# Global burden of 369 diseases and injuries in 204 countries and territories, 1990–2019: a systematic analysis for the Global Burden of Disease Study 2019

**DOI:** 10.1016/S0140-6736(20)30925-9

**Published:** 2020-10-17

**Authors:** GBD 2019 Diseases and Injuries Collaborators, Theo Vos, Stephen S Lim, Cristiana Abbafati, Kaja M Abbas, Mohammad Abbasi, Mitra Abbasifard, Mohsen Abbasi-Kangevari, Hedayat Abbastabar, Foad Abd-Allah, Ahmed Abdelalim, Mohammad Abdollahi, Ibrahim Abdollahpour, Hassan Abolhassani, Victor Aboyans, Elissa M Abrams, Lucas Guimarães Abreu, Michael R M Abrigo, Laith Jamal Abu-Raddad, Abdelrahman I Abushouk, Alyssa Acebedo, Ilana N Ackerman, Maryam Adabi, Abdu A Adamu, Oladimeji M Adebayo, Victor Adekanmbi, Jaimie D Adelson, Olatunji O Adetokunboh, Davoud Adham, Mahdi Afshari, Ashkan Afshin, Emilie E Agardh, Gina Agarwal, Kareha M Agesa, Mohammad Aghaali, Seyed Mohammad Kazem Aghamir, Anurag Agrawal, Tauseef Ahmad, Alireza Ahmadi, Mehdi Ahmadi, Hamid Ahmadieh, Ehsan Ahmadpour, Temesgen Yihunie Akalu, Rufus Olusola Akinyemi, Tomi Akinyemiju, Blessing Akombi, Ziyad Al-Aly, Khurshid Alam, Noore Alam, Samiah Alam, Tahiya Alam, Turki M Alanzi, Samuel B Albertson, Jacqueline Elizabeth Alcalde-Rabanal, Niguse Meles Alema, Muhammad Ali, Saqib Ali, Gianfranco Alicandro, Mehran Alijanzadeh, Cyrus Alinia, Vahid Alipour, Syed Mohamed Aljunid, François Alla, Peter Allebeck, Amir Almasi-Hashiani, Jordi Alonso, Rajaa M Al-Raddadi, Khalid A Altirkawi, Nelson Alvis-Guzman, Nelson J Alvis-Zakzuk, Saeed Amini, Mostafa Amini-Rarani, Arya Aminorroaya, Fatemeh Amiri, Arianna Maever L Amit, Dickson A Amugsi, Gianna Gayle Herrera Amul, Deanna Anderlini, Catalina Liliana Andrei, Tudorel Andrei, Mina Anjomshoa, Fereshteh Ansari, Iman Ansari, Alireza Ansari-Moghaddam, Carl Abelardo T Antonio, Catherine M Antony, Ernoiz Antriyandarti, Davood Anvari, Razique Anwer, Jalal Arabloo, Morteza Arab-Zozani, Aleksandr Y Aravkin, Filippo Ariani, Johan Ärnlöv, Krishna K Aryal, Afsaneh Arzani, Mehran Asadi-Aliabadi, Ali A Asadi-Pooya, Babak Asghari, Charlie Ashbaugh, Desta Debalkie Atnafu, Sachin R Atre, Floriane Ausloos, Marcel Ausloos, Beatriz Paulina Ayala Quintanilla, Getinet Ayano, Martin Amogre Ayanore, Yared Asmare Aynalem, Samad Azari, Ghasem Azarian, Zelalem Nigussie Azene, Ebrahim Babaee, Alaa Badawi, Mojtaba Bagherzadeh, Mohammad Hossein Bakhshaei, Ahad Bakhtiari, Senthilkumar Balakrishnan, Shivanthi Balalla, Shelly Balassyano, Maciej Banach, Palash Chandra Banik, Marlena S Bannick, Agegnehu Bante Bante, Adhanom Gebreegziabher Baraki, Miguel A Barboza, Suzanne Lyn Barker-Collo, Celine M Barthelemy, Lingkan Barua, Akbar Barzegar, Sanjay Basu, Bernhard T Baune, Mohsen Bayati, Gholamreza Bazmandegan, Neeraj Bedi, Ettore Beghi, Yannick Béjot, Aminu K Bello, Rose G Bender, Derrick A Bennett, Fiona B Bennitt, Isabela M Bensenor, Catherine P Benziger, Kidanemaryam Berhe, Eduardo Bernabe, Gregory J Bertolacci, Reshmi Bhageerathy, Neeraj Bhala, Dinesh Bhandari, Pankaj Bhardwaj, Krittika Bhattacharyya, Zulfiqar A Bhutta, Sadia Bibi, Molly H Biehl, Boris Bikbov, Muhammad Shahdaat Bin Sayeed, Antonio Biondi, Binyam Minuye Birihane, Donal Bisanzio, Catherine Bisignano, Raaj Kishore Biswas, Somayeh Bohlouli, Mehdi Bohluli, Srinivasa Rao Rao Bolla, Archith Boloor, Alexandra S Boon-Dooley, Guilherme Borges, Antonio Maria Borzì, Rupert Bourne, Oliver J Brady, Michael Brauer, Carol Brayne, Nicholas J K Breitborde, Hermann Brenner, Paul Svitil Briant, Andrew M Briggs, Nikolay Ivanovich Briko, Gabrielle B Britton, Dana Bryazka, Rachelle Buchbinder, Blair R Bumgarner, Reinhard Busse, Zahid A Butt, Florentino Luciano Caetano dos Santos, Luis LA Alberto Cámera, Ismael R Campos-Nonato, Josip Car, Rosario Cárdenas, Giulia Carreras, Juan J Carrero, Felix Carvalho, Joao Mauricio Castaldelli-Maia, Carlos A Castañeda-Orjuela, Giulio Castelpietra, Chris D Castle, Franz Castro, Ferrán Catalá-López, Kate Causey, Christopher R Cederroth, Kelly M Cercy, Ester Cerin, Joht Singh Chandan, Alex R Chang, Fiona J Charlson, Vijay Kumar Chattu, Sarika Chaturvedi, Odgerel Chimed-Ochir, Ken Lee Chin, Daniel Youngwhan Cho, Hanne Christensen, Dinh-Toi Chu, Michael T Chung, Flavia M Cicuttini, Liliana G Ciobanu, Massimo Cirillo, Emma L Collins, Kelly Compton, Sara Conti, Paolo Angelo Cortesi, Vera Marisa Costa, Ewerton Cousin, Richard G Cowden, Benjamin C Cowie, Elizabeth A Cromwell, Di H Cross, Christopher Stephen Crowe, Jessica A Cruz, Matthew Cunningham, Saad M A Dahlawi, Giovanni Damiani, Lalit Dandona, Rakhi Dandona, Aso Mohammad Darwesh, Ahmad Daryani, Jai K Das, Rajat Das Gupta, José das Neves, Claudio Alberto Dávila-Cervantes, Kairat Davletov, Diego De Leo, Frances E Dean, Nicole K DeCleene, Amanda Deen, Louisa Degenhardt, Robert Paul Dellavalle, Feleke Mekonnen Demeke, Desalegn Getnet Demsie, Edgar Denova-Gutiérrez, Nebiyu Dereje Dereje, Nikolaos Dervenis, Rupak Desai, Assefa Desalew, Getenet Ayalew Dessie, Samath Dhamminda Dharmaratne, Govinda Prasad Dhungana, Mostafa Dianatinasab, Daniel Diaz, Zahra Sadat Dibaji Forooshani, Zachary V Dingels, M Ashworth Dirac, Shirin Djalalinia, Hoa Thi Do, Klara Dokova, Fariba Dorostkar, Chirag P Doshi, Leila Doshmangir, Abdel Douiri, Matthew C Doxey, Tim Robert Driscoll, Susanna J Dunachie, Bruce B Duncan, Andre Rodrigues Duraes, Arielle Wilder Eagan, Mohammad Ebrahimi Kalan, David Edvardsson, Joshua R Ehrlich, Nevine El Nahas, Iman El Sayed, Maha El Tantawi, Iffat Elbarazi, Islam Y Elgendy, Hala Rashad Elhabashy, Shaimaa I El-Jaafary, Iqbal RF Elyazar, Mohammad Hassan Emamian, Sophia Emmons-Bell, Holly E Erskine, Babak Eshrati, Sharareh Eskandarieh, Saman Esmaeilnejad, Firooz Esmaeilzadeh, Alireza Esteghamati, Kara Estep, Arash Etemadi, Atkilt Esaiyas Etisso, Mohammad Farahmand, Anwar Faraj, Mohammad Fareed, Roghiyeh Faridnia, Carla Sofia e Sá Farinha, Andrea Farioli, Andre Faro, Mithila Faruque, Farshad Farzadfar, Nazir Fattahi, Mehdi Fazlzadeh, Valery L Feigin, Rachel Feldman, Seyed-Mohammad Fereshtehnejad, Eduarda Fernandes, Alize J Ferrari, Manuela L Ferreira, Irina Filip, Florian Fischer, James L Fisher, Ryan Fitzgerald, Carsten Flohr, Luisa Sorio Flor, Nataliya A Foigt, Morenike Oluwatoyin Folayan, Lisa M Force, Carla Fornari, Masoud Foroutan, Jack T Fox, Marisa Freitas, Weijia Fu, Takeshi Fukumoto, João M Furtado, Mohamed M Gad, Emmanuela Gakidou, Natalie C Galles, Silvano Gallus, Amiran Gamkrelidze, Alberto L Garcia-Basteiro, William M Gardner, Biniyam Sahiledengle Geberemariyam, Abiyu Mekonnen Gebrehiwot, Ketema Bizuwork Gebremedhin, Assefa Ayalew Ayalew Ayalew Gebreslassie, Anna Gershberg Hayoon, Peter W Gething, Maryam Ghadimi, Keyghobad Ghadiri, Mansour Ghafourifard, Alireza Ghajar, Farhad Ghamari, Ahmad Ghashghaee, Hesam Ghiasvand, Nermin Ghith, Asadollah Gholamian, Syed Amir Gilani, Paramjit Singh Gill, Mojgan Gitimoghaddam, Giorgia Giussani, Srinivas Goli, Ricardo Santiago Gomez, Sameer Vali Gopalani, Giuseppe Gorini, Taren M Gorman, Harrison Chase Gottlich, Houman Goudarzi, Alessandra C Goulart, Bárbara Niegia Garcia Goulart, Ayman Grada, Michal Grivna, Giuseppe Grosso, Mohammed Ibrahim Mohialdeen Gubari, Harish Chander Gugnani, Andre Luiz Sena Guimaraes, Rafael Alves Guimarães, Rashid Abdi Guled, Gaorui Guo, Yuming Guo, Rajeev Gupta, Juanita A Haagsma, Beatrix Haddock, Nima Hafezi-Nejad, Abdul Hafiz, Hailey Hagins, Lydia M Haile, Brian J Hall, Iman Halvaei, Randah R Hamadeh, Kanaan Hamagharib Abdullah, Erin B Hamilton, Chieh Han, Hannah Han, Graeme J Hankey, Josep Maria Haro, James D Harvey, Ahmed I Hasaballah, Amir Hasanzadeh, Maryam Hashemian, Soheil Hassanipour, Hadi Hassankhani, Rasmus J Havmoeller, Roderick J Hay, Simon I Hay, Khezar Hayat, Behnam Heidari, Golnaz Heidari, Reza Heidari-Soureshjani, Delia Hendrie, Hannah J Henrikson, Nathaniel J Henry, Claudiu Herteliu, Fatemeh Heydarpour, Thomas R Hird, Hans W Hoek, Michael K Hole, Ramesh Holla, Praveen Hoogar, H Dean Hosgood, Mehdi Hosseinzadeh, Mihaela Hostiuc, Sorin Hostiuc, Mowafa Househ, Damian G Hoy, Mohamed Hsairi, Vivian Chia-rong Hsieh, Guoqing Hu, Tanvir M Huda, Fernando N Hugo, Chantal K Huynh, Bing-Fang Hwang, Vincent C Iannucci, Segun Emmanuel Ibitoye, Kevin S Ikuta, Olayinka Stephen Ilesanmi, Irena M Ilic, Milena D Ilic, Leeberk Raja Inbaraj, Helen Ippolito, Seyed Sina Naghibi Irvani, M Mofizul Islam, MdMohaimenul Islam, Sheikh Mohammed Shariful Islam, Farhad Islami, Hiroyasu Iso, Rebecca Q Ivers, Chidozie C D Iwu, Ihoghosa Osamuyi Iyamu, Jalil Jaafari, Kathryn H Jacobsen, Farhad Jadidi-Niaragh, Hussain Jafari, Morteza Jafarinia, Deepa Jahagirdar, Mohammad Ali Jahani, Nader Jahanmehr, Mihajlo Jakovljevic, Amir Jalali, Farzad Jalilian, Spencer L James, Hosna Janjani, Manthan Dilipkumar Janodia, Achala Upendra Jayatilleke, Panniyammakal Jeemon, Ensiyeh Jenabi, Ravi Prakash Jha, Vivekanand Jha, John S Ji, Peng Jia, Oommen John, Yetunde O John-Akinola, Catherine Owens Johnson, Sarah Charlotte Johnson, Jost B Jonas, Tamas Joo, Ankur Joshi, Jacek Jerzy Jozwiak, Mikk Jürisson, Ali Kabir, Zubair Kabir, Hamed Kalani, Rizwan Kalani, Leila R Kalankesh, Rohollah Kalhor, Zahra Kamiab, Tanuj Kanchan, Behzad Karami Matin, André Karch, Mohd Anisul Karim, Salah Eddin Karimi, Getachew Mullu Kassa, Nicholas J Kassebaum, Srinivasa Vittal Katikireddi, Norito Kawakami, Gbenga A Kayode, Suzanne H Keddie, Cathleen Keller, Maia Kereselidze, Morteza Abdullatif Khafaie, Nauman Khalid, Maseer Khan, Khaled Khatab, Mona M Khater, Mahalaqua Nazli Khatib, Maryam Khayamzadeh, Mohammad Taghi Khodayari, Roba Khundkar, Neda Kianipour, Christian Kieling, Daniel Kim, Young-Eun Kim, Yun Jin Kim, Ruth W Kimokoti, Adnan Kisa, Sezer Kisa, Katarzyna Kissimova-Skarbek, Mika Kivimäki, Cameron J Kneib, Ann Kristin Skrindo Knudsen, Jonathan M Kocarnik, Tufa Kolola, Jacek A Kopec, Soewarta Kosen, Parvaiz A Koul, Ai Koyanagi, Michael A Kravchenko, Kewal Krishan, Kris J Krohn, Barthelemy Kuate Defo, Burcu Kucuk Bicer, G Anil Kumar, Manasi Kumar, Pushpendra Kumar, Vivek Kumar, Girikumar Kumaresh, Om P Kurmi, Dian Kusuma, Hmwe Hmwe Kyu, Carlo La Vecchia, Ben Lacey, Dharmesh Kumar Lal, Ratilal Lalloo, Jennifer O Lam, Faris Hasan Lami, Iván Landires, Justin J Lang, Van Charles Lansingh, Samantha Leigh Larson, Anders O Larsson, Savita Lasrado, Zohra S Lassi, Kathryn Mei-Ming Lau, Pablo M Lavados, Jeffrey V Lazarus, Jorge R Ledesma, Paul H Lee, Shaun Wen Huey Lee, Kate E LeGrand, James Leigh, Matilde Leonardi, Haley Lescinsky, Janni Leung, Miriam Levi, Sarah Lewington, Shanshan Li, Lee-Ling Lim, Christine Lin, Ro-Ting Lin, Christine Linehan, Shai Linn, Hung-Chun Liu, Shiwei Liu, Zichen Liu, Katharine J Looker, Alan D Lopez, Platon D Lopukhov, Stefan Lorkowski, Paulo A Lotufo, Tim C D Lucas, Alessandra Lugo, Raimundas Lunevicius, Ronan A Lyons, Jianing Ma, Jennifer H MacLachlan, Emilie R Maddison, Ralph Maddison, Fabiana Madotto, Phetole Walter Mahasha, Hue Thi Mai, Azeem Majeed, Venkatesh Maled, Shokofeh Maleki, Reza Malekzadeh, Deborah Carvalho Malta, Abdullah A Mamun, Amir Manafi, Navid Manafi, Helena Manguerra, Borhan Mansouri, Mohammad Ali Mansournia, Ana M Mantilla Herrera, Joemer C Maravilla, Ashley Marks, Francisco Rogerlândio Martins-Melo, Ira Martopullo, Seyedeh Zahra Masoumi, João Massano, Benjamin Ballard Massenburg, Manu Raj Mathur, Pallab K Maulik, Colm McAlinden, John J McGrath, Martin McKee, Man Mohan Mehndiratta, Fereshteh Mehri, Kala M Mehta, Wahengbam Bigyananda Meitei, Peter T N Memiah, Walter Mendoza, Ritesh G Menezes, Endalkachew Worku Mengesha, Meresa Berwo Mengesha, Alibek Mereke, Atte Meretoja, Tuomo J Meretoja, Tomislav Mestrovic, Bartosz Miazgowski, Tomasz Miazgowski, Irmina Maria Michalek, Kebadnew Mulatu Mihretie, Ted R Miller, Edward J Mills, Andreea Mirica, Erkin M Mirrakhimov, Hamed Mirzaei, Maryam Mirzaei, Mehdi Mirzaei-Alavijeh, Awoke Temesgen Misganaw, Prasanna Mithra, Babak Moazen, Masoud Moghadaszadeh, Efat Mohamadi, Dara K Mohammad, Yousef Mohammad, Naser Mohammad Gholi Mezerji, Abdollah Mohammadian-Hafshejani, Noushin Mohammadifard, Reza Mohammadpourhodki, Shafiu Mohammed, Ali H Mokdad, Mariam Molokhia, Natalie C Momen, Lorenzo Monasta, Stefania Mondello, Meghan D Mooney, Mahmood Moosazadeh, Ghobad Moradi, Masoud Moradi, Maziar Moradi-Lakeh, Rahmatollah Moradzadeh, Paula Moraga, Linda Morales, Lidia Morawska, Ilais Moreno Velásquez, Joana Morgado-da-Costa, Shane Douglas Morrison, Jonathan F Mosser, Simin Mouodi, Seyyed Meysam Mousavi, Amin Mousavi Khaneghah, Ulrich Otto Mueller, Sandra B Munro, Moses K Muriithi, Kamarul Imran Musa, Saravanan Muthupandian, Mehdi Naderi, Ahamarshan Jayaraman Nagarajan, Gabriele Nagel, Behshad Naghshtabrizi, Sanjeev Nair, Anita K Nandi, Vinay Nangia, Jobert Richie Nansseu, Vinod C Nayak, Javad Nazari, Ionut Negoi, Ruxandra Irina Negoi, Henok Biresaw Netsere Netsere, Josephine W Ngunjiri, Cuong Tat Nguyen, Jason Nguyen, Michele Nguyen, Minh Nguyen, Emma Nichols, Dabere Nigatu, Yeshambel T Nigatu, Rajan Nikbakhsh, Molly R Nixon, Chukwudi A Nnaji, Shuhei Nomura, Bo Norrving, Jean Jacques Noubiap, Christoph Nowak, Virginia Nunez-Samudio, Adrian Oţoiu, Bogdan Oancea, Christopher M Odell, Felix Akpojene Ogbo, In-Hwan Oh, Emmanuel Wandera Okunga, Morteza Oladnabi, Andrew T Olagunju, Bolajoko Olubukunola Olusanya, Jacob Olusegun Olusanya, Mojisola Morenike Oluwasanu, Ahmed Omar Bali, Muktar Omer Omer, Kanyin L Ong, Obinna E Onwujekwe, Aislyn U Orji, Heather M Orpana, Alberto Ortiz, Samuel M Ostroff, Nikita Otstavnov, Stanislav S Otstavnov, Simon Øverland, Mayowa O Owolabi, Mahesh P A, Jagadish Rao Padubidri, Abhijit P Pakhare, Raffaele Palladino, Adrian Pana, Songhomitra Panda-Jonas, Anamika Pandey, Eun-Kee Park, Priya G Kumari Parmar, Deepak Kumar Pasupula, Sangram Kishor Patel, Angel J Paternina-Caicedo, Ashish Pathak, Mona Pathak, Scott B Patten, George C Patton, Deepak Paudel, Hamidreza Pazoki Toroudi, Amy E Peden, Alyssa Pennini, Veincent Christian Filipino Pepito, Emmanuel K Peprah, Alexandre Pereira, David M Pereira, Norberto Perico, Hai Quang Pham, Michael R Phillips, David M Pigott, Thomas Pilgrim, Tessa M Pilz, Meghdad Pirsaheb, Oleguer Plana-Ripoll, Dietrich Plass, Khem Narayan Pokhrel, Roman V Polibin, Suzanne Polinder, Kevan R Polkinghorne, Maarten J Postma, Hadi Pourjafar, Farshad Pourmalek, Reza Pourmirza Kalhori, Akram Pourshams, Anna Poznańska, Sergio I Prada, V Prakash, Dimas Ria Angga Pribadi, Elisabetta Pupillo, Zahiruddin Quazi Syed, Mohammad Rabiee, Navid Rabiee, Amir Radfar, Ata Rafiee, Alireza Rafiei, Alberto Raggi, Afarin Rahimi-Movaghar, Muhammad Aziz Rahman, Ali Rajabpour-Sanati, Fatemeh Rajati, Kiana Ramezanzadeh, Chhabi Lal Ranabhat, Puja C Rao, Sowmya J Rao, Davide Rasella, Prateek Rastogi, Priya Rathi, David Laith Rawaf, Salman Rawaf, Lal Rawal, Christian Razo, Sofia Boston Redford, Robert C Reiner, Nickolas Reinig, Marissa Bettay Reitsma, Giuseppe Remuzzi, Vishnu Renjith, Andre M N Renzaho, Serge Resnikoff, Nima Rezaei, Mohammad sadegh Rezai, Aziz Rezapour, Phoebe-Anne Rhinehart, Seyed Mohammad Riahi, Antonio Luiz P Ribeiro, Daniel Cury Ribeiro, Daniela Ribeiro, Jennifer Rickard, Nicholas L S Roberts, Shaun Roberts, Stephen R Robinson, Leonardo Roever, Sam Rolfe, Luca Ronfani, Gholamreza Roshandel, Gregory A Roth, Enrico Rubagotti, Susan Fred Rumisha, Siamak Sabour, Perminder S Sachdev, Basema Saddik, Ehsan Sadeghi, Masoumeh Sadeghi, Shahram Saeidi, Sare Safi, Saeid Safiri, Rajesh Sagar, Amirhossein Sahebkar, Mohammad Ali Sahraian, S Mohammad Sajadi, Mohammad Reza Salahshoor, Payman Salamati, Saleh Salehi Zahabi, Hosni Salem, Marwa R Rashad Salem, Hamideh Salimzadeh, Joshua A Salomon, Inbal Salz, Zainab Samad, Abdallah M Samy, Juan Sanabria, Damian Francesco Santomauro, Itamar S Santos, João Vasco Santos, Milena M Santric-Milicevic, Sivan Yegnanarayana Iyer Saraswathy, Rodrigo Sarmiento-Suárez, Nizal Sarrafzadegan, Benn Sartorius, Arash Sarveazad, Brijesh Sathian, Thirunavukkarasu Sathish, Davide Sattin, Alyssa N Sbarra, Lauren E Schaeffer, Silvia Schiavolin, Maria Inês Schmidt, Aletta Elisabeth Schutte, David C Schwebel, Falk Schwendicke, Anbissa Muleta Senbeta, Subramanian Senthilkumaran, Sadaf G Sepanlou, Katya Anne Shackelford, Jamileh Shadid, Saeed Shahabi, Amira A Shaheen, Masood Ali Shaikh, Ali S Shalash, Mehran Shams-Beyranvand, Morteza Shamsizadeh, Mohammed Shannawaz, Kiomars Sharafi, Fablina Sharara, Brittney S Sheena, Abbas Sheikhtaheri, Ranjitha S Shetty, Kenji Shibuya, Wondimeneh Shibabaw Shiferaw, Mika Shigematsu, Jae Il Shin, Rahman Shiri, Reza Shirkoohi, Mark G Shrime, Kerem Shuval, Soraya Siabani, Inga Dora Sigfusdottir, Rannveig Sigurvinsdottir, João Pedro Silva, Kyle E Simpson, Ambrish Singh, Jasvinder A Singh, Eirini Skiadaresi, Søren T Skou Skou, Valentin Yurievich Skryabin, Eugene Sobngwi, Anton Sokhan, Shahin Soltani, Reed J D Sorensen, Joan B Soriano, Muluken Bekele Sorrie, Ireneous N Soyiri, Chandrashekhar T Sreeramareddy, Jeffrey D Stanaway, Benjamin A Stark, Simona Cătălina Ştefan, Caroline Stein, Caitlyn Steiner, Timothy J Steiner, Mark A Stokes, Lars Jacob Stovner, Jacob L Stubbs, Agus Sudaryanto, Mu'awiyyah Babale Sufiyan, Gerhard Sulo, Iyad Sultan, Bryan L Sykes, Dillon O Sylte, Miklós Szócska, Rafael Tabarés-Seisdedos, Karen M Tabb, Santosh Kumar Tadakamadla, Amir Taherkhani, Masih Tajdini, Ken Takahashi, Nuno Taveira, Whitney L Teagle, Hirut Teame, Arash Tehrani-Banihashemi, Berhane Fseha Teklehaimanot, Sonyah Terrason, Zemenu Tadesse Tessema, Kavumpurathu Raman Thankappan, Azalea M Thomson, Hamid Reza Tohidinik, Marcello Tonelli, Roman Topor-Madry, Anna E Torre, Mathilde Touvier, Marcos Roberto Roberto Tovani-Palone, Bach Xuan Tran, Ravensara Travillian, Christopher E Troeger, Thomas Clement Truelsen, Alexander C Tsai, Aristidis Tsatsakis, Lorainne Tudor Car, Stefanos Tyrovolas, Riaz Uddin, Saif Ullah, Eduardo A Undurraga, Bhaskaran Unnikrishnan, Marco Vacante, Alireza Vakilian, Pascual R Valdez, Santosh Varughese, Tommi Juhani Vasankari, Yasser Vasseghian, Narayanaswamy Venketasubramanian, Francesco S Violante, Vasily Vlassov, Stein Emil Vollset, Avina Vongpradith, Ana Vukovic, Rade Vukovic, Yasir Waheed, Madgalene K Walters, Jiayu Wang, Yafeng Wang, Yuan-Pang Wang, Joseph L Ward, Alexandrea Watson, Jingkai Wei, Robert G Weintraub, Daniel J Weiss, Jordan Weiss, Ronny Westerman, Joanna L Whisnant, Harvey A Whiteford, Taweewat Wiangkham, Kirsten E Wiens, Tissa Wijeratne, Lauren B Wilner, Shadrach Wilson, Bogdan Wojtyniak, Charles D A Wolfe, Eve E Wool, Ai-Min Wu, Sarah Wulf Hanson, Han Yong Wunrow, Gelin Xu, Rixing Xu, Simon Yadgir, Seyed Hossein Yahyazadeh Jabbari, Kazumasa Yamagishi, Mousa Yaminfirooz, Yuichiro Yano, Sanni Yaya, Vahid Yazdi-Feyzabadi, Jamal A Yearwood, Tomas Y Yeheyis, Yordanos Gizachew Yeshitila, Paul Yip, Naohiro Yonemoto, Seok-Jun Yoon, Javad Yoosefi Lebni, Mustafa Z Younis, Theodore Patrick Younker, Zabihollah Yousefi, Mahmoud Yousefifard, Taraneh Yousefinezhadi, Abdilahi Yousuf Yousuf, Chuanhua Yu, Hasan Yusefzadeh, Telma Zahirian Moghadam, Leila Zaki, Sojib Bin Zaman, Mohammad Zamani, Maryam Zamanian, Hamed Zandian, Alireza Zangeneh, Mikhail Sergeevich Zastrozhin, Kaleab Alemayehu Zewdie, Yunquan Zhang, Zhi-Jiang Zhang, Jeff T Zhao, Yingxi Zhao, Peng Zheng, Maigeng Zhou, Arash Ziapour, Stephanie R M Zimsen, Mohsen Naghavi, Christopher J L Murray

## Abstract

**Background:**

In an era of shifting global agendas and expanded emphasis on non-communicable diseases and injuries along with communicable diseases, sound evidence on trends by cause at the national level is essential. The Global Burden of Diseases, Injuries, and Risk Factors Study (GBD) provides a systematic scientific assessment of published, publicly available, and contributed data on incidence, prevalence, and mortality for a mutually exclusive and collectively exhaustive list of diseases and injuries.

**Methods:**

GBD estimates incidence, prevalence, mortality, years of life lost (YLLs), years lived with disability (YLDs), and disability-adjusted life-years (DALYs) due to 369 diseases and injuries, for two sexes, and for 204 countries and territories. Input data were extracted from censuses, household surveys, civil registration and vital statistics, disease registries, health service use, air pollution monitors, satellite imaging, disease notifications, and other sources. Cause-specific death rates and cause fractions were calculated using the Cause of Death Ensemble model and spatiotemporal Gaussian process regression. Cause-specific deaths were adjusted to match the total all-cause deaths calculated as part of the GBD population, fertility, and mortality estimates. Deaths were multiplied by standard life expectancy at each age to calculate YLLs. A Bayesian meta-regression modelling tool, DisMod-MR 2.1, was used to ensure consistency between incidence, prevalence, remission, excess mortality, and cause-specific mortality for most causes. Prevalence estimates were multiplied by disability weights for mutually exclusive sequelae of diseases and injuries to calculate YLDs. We considered results in the context of the Socio-demographic Index (SDI), a composite indicator of income per capita, years of schooling, and fertility rate in females younger than 25 years. Uncertainty intervals (UIs) were generated for every metric using the 25th and 975th ordered 1000 draw values of the posterior distribution.

**Findings:**

Global health has steadily improved over the past 30 years as measured by age-standardised DALY rates. After taking into account population growth and ageing, the absolute number of DALYs has remained stable. Since 2010, the pace of decline in global age-standardised DALY rates has accelerated in age groups younger than 50 years compared with the 1990–2010 time period, with the greatest annualised rate of decline occurring in the 0–9-year age group. Six infectious diseases were among the top ten causes of DALYs in children younger than 10 years in 2019: lower respiratory infections (ranked second), diarrhoeal diseases (third), malaria (fifth), meningitis (sixth), whooping cough (ninth), and sexually transmitted infections (which, in this age group, is fully accounted for by congenital syphilis; ranked tenth). In adolescents aged 10–24 years, three injury causes were among the top causes of DALYs: road injuries (ranked first), self-harm (third), and interpersonal violence (fifth). Five of the causes that were in the top ten for ages 10–24 years were also in the top ten in the 25–49-year age group: road injuries (ranked first), HIV/AIDS (second), low back pain (fourth), headache disorders (fifth), and depressive disorders (sixth). In 2019, ischaemic heart disease and stroke were the top-ranked causes of DALYs in both the 50–74-year and 75-years-and-older age groups. Since 1990, there has been a marked shift towards a greater proportion of burden due to YLDs from non-communicable diseases and injuries. In 2019, there were 11 countries where non-communicable disease and injury YLDs constituted more than half of all disease burden. Decreases in age-standardised DALY rates have accelerated over the past decade in countries at the lower end of the SDI range, while improvements have started to stagnate or even reverse in countries with higher SDI.

**Interpretation:**

As disability becomes an increasingly large component of disease burden and a larger component of health expenditure, greater research and development investment is needed to identify new, more effective intervention strategies. With a rapidly ageing global population, the demands on health services to deal with disabling outcomes, which increase with age, will require policy makers to anticipate these changes. The mix of universal and more geographically specific influences on health reinforces the need for regular reporting on population health in detail and by underlying cause to help decision makers to identify success stories of disease control to emulate, as well as opportunities to improve.

**Funding:**

Bill & Melinda Gates Foundation.

**Research in context**

**Evidence before this study**

The Global Burden of Diseases, Injuries, and Risk Factors Study (GBD) 2017 reported on incidence, prevalence, and mortality from 359 diseases and injuries. Information on prevalence and mortality was also analysed in terms of summary measures: years of life lost (YLLs), years lived with disability (YLDs), disability-adjusted life-years (DALYs), and healthy life expectancy. GBD is the only comprehensive assessment providing time trends for a mutually exclusive and collectively exhaustive list of diseases and injuries. For the first time, GBD 2017 also produced internally consistent estimates of population, fertility, mortality, and migration by age, sex, and year for 1950–2017. GBD 2017 also included subnational assessments for 16 countries at administrative level 1 and for local authorities in England.

**Added value of this study**

GBD 2019 updates and expands beyond GBD 2017 in ten ways. (1) The number of countries for which subnational assessments have been undertaken was expanded to include Italy, Nigeria, Pakistan, the Philippines, and Poland. (2) 12 new causes were added to the GBD modelling framework, including pulmonary arterial hypertension, nine new sites of cancer, and two new sites of osteoarthritis (hand and other joints). (3) For each disease, the preferred or reference case definition or measurement method was clearly defined and stored in a database. For both risks and diseases, the statistical relationship between the alternative and reference measurement method was analysed using network meta-regression using only data where two different approaches were measured in the same location–time period. Although statistical cross-walking between alternative and reference definitions and measurement methods has been a feature in all GBD studies, the approach in GBD 2019 was highly standardised and used improved methods across diseases and risks. (4) Some prior distributions used in DisMod-MR, the Bayesian meta-regression tool used to simultaneously estimate incidence, prevalence, remission, excess mortality, and cause-specific mortality, were revised on the basis of simulation studies showing that less informative priors helped to improve the coverage of uncertainty intervals. (5) Redistribution algorithms for sepsis, heart failure, pulmonary embolism, acute kidney injury, hepatic failure, acute respiratory failure, pneumonitis, and five intermediate causes in the central nervous system were revised according to an analysis of 116 million deaths that were attributed to multiple causes. (6) Processing of clinical informatics data on hospital and clinic visits was revised to better take into account differential access across locations to health-care facilities. (7) To enhance the stability of models in the presence of the addition of subnational data in different GBD cycles, we adopted a set of standard locations for the estimation of covariate effects in models. (8) 7333 national and 24 657 subnational vital registration systems, 16 984 published studies, and 1654 household surveys were used in the analysis, including many newly available data sources. (9) Results are presented so as to integrate causes of death, incidence, prevalence, YLDs, YLLs, and DALYs into a comprehensive assessment of each disease and injury. (10) Closer technical coordination with WHO has led to the addition of nine WHO member states to the analysis and revisions of the analytical approach for select diseases.

**Implications of all the available evidence**

GBD 2019 provides the most up-to-date assessment of the descriptive epidemiology of a mutually exclusive and collectively exhaustive list of diseases and injuries for 204 countries and territories from 1990 to 2019. The comprehensive nature of the assessment provides policy-relevant information on the trends of major causes of burden globally, regionally, and by country or territory.

## Introduction

The Global Burden of Diseases, Injuries, and Risk Factors Study (GBD) provides a systematic scientific assessment of published, publicly available, and contributed data on disease and injury incidence, prevalence, and mortality for a mutually exclusive and collectively exhaustive list of diseases and injuries.^1^, ^2^, ^3^ In an era of shifting global agendas and expanded emphasis on non-communicable diseases and injuries along with communicable diseases, sound and up-to-date evidence on trends—both progress and adverse patterns—by cause at the national level is essential to reflect effects of public health policy and medical care delivery.^4^, ^5^, ^6^, ^7^

GBD 2019 provides an opportunity to incorporate newly available datasets, enhance method performance and standardisation, and reflect changes in scientific understanding. Since GBD 2017,^1^, ^2^, ^3^ no comprehensive update of descriptive epidemiology levels and trends has been released, to our knowledge. In this study, we summarise GBD methods and present integrated results on fatal and non-fatal outcomes for the GBD disease and injury hierarchical cause list. GBD 2019 includes estimation of numerous different models for disease and injury outcomes. This Article provides a high-level overview of our findings. Results are presented both broadly and in detail for a selection of diseases, injuries, and impairments in two-page summaries with a standard set of tables and figures.

## Methods

### Overview

The general approach to estimating causes of death and disease incidence and prevalence for GBD 2019 is the same as for GBD 2017.^2^, ^3^ Appendix 1 provides details on the methods used to model each disease and injury. Here, we provide an overview of the methods, with an emphasis on the main methodology changes since GBD 2017.

For each iteration of GBD, the estimates for the whole time series are updated on the basis of addition of new data and change in methods where appropriate. Thus, the GBD 2019 results supersede those from previous rounds of GBD.

GBD 2019 complies with the Guidelines for Accurate and Transparent Health Estimates Reporting (GATHER) statement (appendix 1 section 1.4).^8^ Analyses were completed with Python version 3.6.2, Stata version 13, and R version 3.5.0. Statistical code used for GBD estimation is publicly available online.

### Geographical units, age groups, time periods, and cause levels

GBD 2019 estimated each epidemiological quantity of interest—incidence, prevalence, mortality, years lived with disability (YLDs), years of life lost (YLLs), and disability-adjusted life-years (DALYs)—for 23 age groups; males, females, and both sexes combined; and 204 countries and territories that were grouped into 21 regions and seven super-regions. For GBD 2019, nine countries and territories (Cook Islands, Monaco, San Marino, Nauru, Niue, Palau, Saint Kitts and Nevis, Tokelau, and Tuvalu) were added, such that the GBD location hierarchy now includes all WHO member states. GBD 2019 includes subnational analyses for Italy, Nigeria, Pakistan, the Philippines, and Poland, and 16 countries previously estimated at subnational levels (Brazil, China, Ethiopia, India, Indonesia, Iran, Japan, Kenya, Mexico, New Zealand, Norway, Russia, South Africa, Sweden, the UK, and the USA). All subnational analyses are at the first level of administrative organisation within each country except for New Zealand (by Māori ethnicity), Sweden (by Stockholm and non-Stockholm), the UK (by local government authorities), and the Philippines (by province). In this publication, we present subnational estimates for Brazil, India, Indonesia, Japan, Kenya, Mexico, Sweden, the UK, and the USA; given space constraints, these results are presented in appendix 2. At the most detailed spatial resolution, we generated estimates for 990 locations. The GBD diseases and injuries analytical framework generated estimates for every year from 1990 to 2019.

Diseases and injuries were organised into a levelled cause hierarchy from the three broadest causes of death and disability at Level 1 to the most specific causes at Level 4. Within the three Level 1 causes—communicable, maternal, neonatal, and nutritional diseases; non-communicable diseases; and injuries—there are 22 Level 2 causes, 174 Level 3 causes, and 301 Level 4 causes (including 131 Level 3 causes that are not further disaggregated at Level 4; see appendix 1 sections 3.4 and 4.12 for the full list of causes). 364 total causes are non-fatal and 286 are fatal. For GBD 2019, 12 new causes were added to the modelling framework: pulmonary arterial hypertension, eye cancer, soft tissue and other extraosseous sarcomas, malignant neoplasm of bone and articular cartilage, and neuroblastoma and other peripheral nervous cell tumours at Level 3, and hepatoblastoma, Burkitt lymphoma, other non-Hodgkin lymphoma, retinoblastoma, other eye cancers, and two sites of osteoarthritis (hand and other joints) at Level 4.

### Data

The GBD estimation process is based on identifying multiple relevant data sources for each disease or injury including censuses, household surveys, civil registration and vital statistics, disease registries, health service use, air pollution monitors, satellite imaging, disease notifications, and other sources. Each of these types of data are identified from systematic review of published studies, searches of government and international organisation websites, published reports, primary data sources such as the Demographic and Health Surveys, and contributions of datasets by GBD collaborators. 86 249 sources were used in this analysis, including 19 354 sources reporting deaths, 31 499 reporting incidence, 19 773 reporting prevalence, and 26 631 reporting other metrics. Each newly identified and obtained data source is given a unique identifier by a team of librarians and included in the Global Health Data Exchange (GHDx). The GHDx makes publicly available the metadata for each source included in GBD as well as the data, where allowed by the data provider. Readers can use the GHDx source tool to identify which sources were used for estimating any disease or injury outcome in any given location.

### Data processing

A crucial step in the GBD analytical process is correcting for known bias by redistributing deaths from unspecified codes to more specific disease categories, and by adjusting data with alternative case definitions or measurement methods to the reference method. We highlight several major changes in data processing that in some cases have affected GBD results.

#### Cause of death redistribution

Vital registration with medical certification of cause of death is a crucial resource for the GBD cause of death analysis in many countries. Cause of death data obtained using various revisions of the International Classification of Diseases and Injuries (ICD)^9^ were mapped to the GBD cause list. Many deaths, however, are assigned to causes that cannot be the underlying cause of death (eg, cardiopulmonary failure) or are inadequately specified (eg, injury from undetermined intent). These deaths were reassigned to the most probable underlying causes of death as part of the data processing for GBD. Redistribution algorithms can be divided into three categories: proportionate redistribution, fixed proportion redistribution based on published studies or expert judgment, or statistical algorithms. For GBD 2019, data for 116 million deaths attributed to multiple causes were analysed to produce more empirical redistribution algorithms for sepsis,^10^ heart failure, pulmonary embolism, acute kidney injury, hepatic failure, acute respiratory failure, pneumonitis, and five intermediate causes (hydrocephalus, toxic encephalopathy, compression of brain, encephalopathy, and cerebral oedema) in the central nervous system. To redistribute unspecified injuries, we used a method similar to that of intermediate cause redistribution, using the pattern of the nature of injury codes in the causal chain where the ICD codes X59 (“exposure to unspecified factor”) and Y34 (“unspecified event, undetermined intent”) and GBD injury causes were the underlying cause of death. These new algorithms led to important changes in the causes to which these intermediate outcomes were redistributed. Additionally, data on deaths from diabetes and stroke lack the detail on subtype in many countries; we ran regressions on vital registration data with at least 50% of deaths coded specifically to type 1 or 2 diabetes and ischaemic, haemorrhagic, or subarachnoid stroke to predict deaths by these subtypes when these were coded to unspecified diabetes or stroke.

#### Correcting for non-reference case definitions or measurement methods

In previous cycles of GBD, data reported using alternative case definitions or measurement methods were corrected to the reference definition or measurement method primarily as part of the Bayesian meta-regression models. For example, in DisMod-MR, the population data were simultaneously modelled as a function of country covariates for variation in true rates and as a function of indicator variables capturing alternative measurement methods. To enhance transparency and to standardise and improve methods in GBD 2019, we estimated correction factors for alternative case definitions or measurement methods using network meta-regression, including only data where two methods were assessed in the same location–time period or in the exact same population. This included validation studies where two methods had been compared in populations that were not necessarily random samples of the general population. Details on the correction factors from alternative to reference measurement methods are provided in appendix 1 (section 4.4.2).

#### Clinical informatics

Clinical informatics data include inpatient admissions, outpatient (including general practitioner) visits, and health insurance claims. Several data processing steps were undertaken. Inpatient hospital data with a single diagnosis only were adjusted to account for non-primary diagnoses as well as outpatient care. For each GBD cause that used clinical data, ratios of non-primary to primary diagnosis rates were extracted from claims in the USA, Taiwan (province of China), New Zealand, and the Philippines, as well as USA Healthcare Cost and Utilization Project inpatient data. Ratios of outpatient to inpatient care for each cause were extracted from claims data from the USA and Taiwan (province of China). The log of the ratios for each cause were modelled by age and sex using MR-BRT (Meta-Regression-Bayesian Regularised Trimmed), the Bayesian meta-regression tool. To account for the incomplete health-care access in populations where not every person with a disease or injury would be accounted for in administrative clinical records, we transformed the adjusted admission rates using a scalar derived from the Healthcare Access and Quality Index.^11^ We used this approach to produce adjusted, standardised clinical data inputs. More details are provided in appendix 1 (section 4.3).

### Modelling

For most diseases and injuries, processed data are modelled using standardised tools to generate estimates of each quantity of interest by age, sex, location, and year. There are three main standardised tools: Cause of Death Ensemble model (CODEm), spatiotemporal Gaussian process regression (ST-GPR), and DisMod-MR. Previous publications^2^, ^3^, ^12^ and the appendix provide more details on these general GBD methods. Briefly, CODEm is a highly systematised tool to analyse cause of death data using an ensemble of different modelling methods for rates or cause fractions with varying choices of covariates that perform best with out-of-sample predictive validity testing. DisMod-MR is a Bayesian meta-regression tool that allows evaluation of all available data on incidence, prevalence, remission, and mortality for a disease, enforcing consistency between epidemiological parameters. ST-GPR is a set of regression methods that borrow strength between locations and over time for single metrics of interest, such as risk factor exposure or mortality rates. In addition, for select diseases, particularly for rarer outcomes, alternative modelling strategies have been developed, which are described in appendix 1 (section 3.2).

In GBD 2019, we designated a set of standard locations that included all countries and territories as well as the subnational locations for Brazil, China, India, and the USA. Coefficients of covariates in the three main modelling tools were estimated for these standard locations only—ie, we ignored data from subnational locations other than for Brazil, China, India, and the USA (appendix 1 section 1.1). Using this set of standard locations will prevent changes in regression coefficients from one GBD cycle to the next that are solely due to the addition of new subnational units in the analysis that might have lower quality data or small populations (appendix 1 section 1.1). Changes to CODEm for GBD 2019 included the addition of count models to the model ensemble for rarer causes. We also modified DisMod-MR priors to effectively increase the out-of-sample coverage of uncertainty intervals (UIs) as assessed in simulation testing (appendix 1 section 4.5).

For the cause Alzheimer's disease and other dementias, we changed the method of addressing large variations between locations and over time in the assignment of dementia as the underlying cause of death. Based on a systematic review of published cohort studies, we estimated the relative risk of death in individuals with dementia. We identified the proportion of excess deaths in patients with dementia where dementia is the underlying cause of death as opposed to a correlated risk factor (appendix 1 section 2.6.2). We changed the strategy of modelling deaths for acute hepatitis A, B, C, and E from a natural history model relying on inpatient case fatality rates to CODEm models after predicting type-specific acute hepatitis deaths from vital registration data with specified hepatitis type.

DisMod-MR was used to estimate deaths from three outcomes (dementia, Parkinson's, and atrial fibrillation), and to determine the proportions of deaths by underlying aetiologies of cirrhosis, liver cancer, and chronic kidney disease deaths.

### Socio-demographic Index, annual rate of change, and data presentation

The Socio-demographic Index (SDI) is a composite indicator of a country's lag-distributed income per capita, average years of schooling, and the fertility rate in females under the age of 25 years (appendix 1 section 6).^13^ For changes over time, we present annualised rates of change as the difference in the natural log of the values at the start and end of the time interval divided by the number of years in the interval. We examine the relationship between SDI and the annualised rate of change in age-standardised DALY rates for all causes, apart from HIV/AIDS, natural disasters, and war and conflict, by country or territory, for the time periods 1990–2010 and 2010–19. We deliberately subtracted out DALYs due to HIV/AIDS because their magnitude in some parts of the world would have obscured the trends in all other causes; we also subtracted out DALY rates from natural disasters and war and conflict to avoid trends in disease burden in some countries being dominated by these sudden and dramatic changes. As a measure of the epidemiological transition, we present the ratio of YLDs due to non-communicable diseases and injuries, and due to total burden in DALYs. We present 95% UIs for every metric based on the 25th and 975th ordered values of 1000 draws of the posterior distribution.

### Role of the funding source

The funders of this study had no role in study design, data collection, data analysis, data interpretation, or the writing of the report. The corresponding author had full access to the data in the study and final responsibility for the decision to submit for publication.

## Results

### Global trends

**Figure 1:**
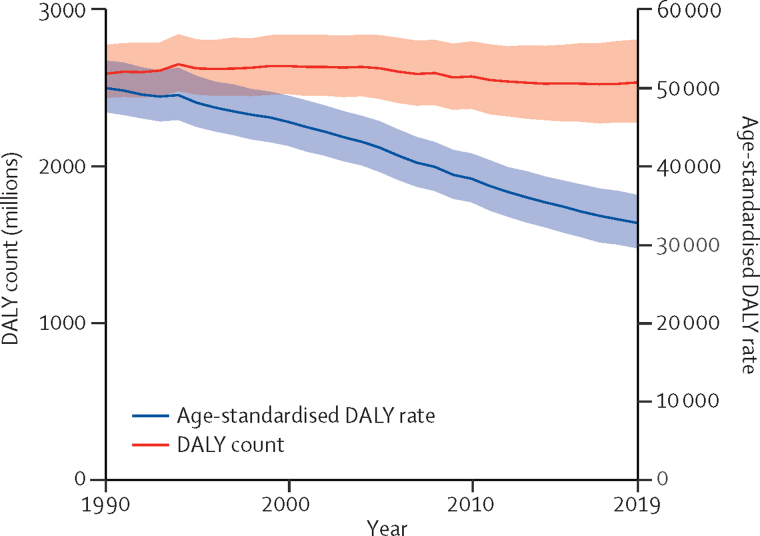
Global DALYs and age-standardised DALY rates, 1990–2019 Shaded sections indicate 95% uncertainty intervals. DALY=disability-adjusted life-year.

Between 1990 and 2019, the number of global DALYs remained almost constant, but once the effects of population growth and ageing were removed by converting counts to age-standardised rates, there were clear improvements in overall health (figure 1). Over the past decade, the pace of decline in global age-standardised DALY rates accelerated in age groups younger than 50 years compared with the 1990–2010 time period (table). The annualised rate of decline was greatest in the 0–9-year age group. In the population aged 50 years and older, the rate of change was slower from 2010 to 2019 compared with the earlier time period.

**Table:** Global DALYs in 2019 and annualised rate of change in DALYs and age-standardised DALY rates over 1990–2010 and 2010–19, by age group and for all ages

	**DALYs 2019**		**Annualised rate of change, 1990–2010**		**Annualised rate of change, 2010–19**	
	Count (millions)	Age-standardised rate (per 100 000)	DALYs	Age-standardised rate	DALYs	Age-standardised rate
0–9 years	531 (458 to 621)	19 125·7 (16 495·1 to 22 382·5)	−2·3% (−2·5 to −2·2)	−2·5% (−2·6 to −2·3)	−3·7% (−4·4 to −2·9)	−4·0% (−4·7 to −3·2)
10–24 years	229 (194 to 270)	12 313·0 (10 399·9 to 14 478·3)	0·2% (0·1 to 0·2)	−0·7% (−0·8 to −0·6)	−1·1% (−1·4 to −0·9)	−1·3% (−1·5 to −1·1)
25–49 years	616 (533 to 709)	22 691·2 (19 613·7 to 26 116·3)	1·4% (1·4 to 1·5)	−0·4% (−0·4 to −0·3)	−0·0% (−0·2 to 0·1)	−1·2% (−1·4 to −1·0)
50–74 years	832 (752 to 919)	28 263·2 (25 527·6 to 31 213·4)	1·3% (1·2 to 1·3)	−1·0% (−1·0 to −0·9)	2·0% (1·8 to 2·1)	−0·9% (−1·1 to −0·8)
≥75 years	329 (308 to 351)	77 320·5 (72 372·5 to 82 440·3)	2·2% (2·2 to 2·2)	−0·9% (−0·9 to −0·9)	2·3% (2·3 to 2·4)	−0·8% (−0·9 to −0·8)
All ages	2540 (2290 to 2810)	32 801·7 (29 535·1 to 36 319·5)	−0·0% (−0·1 to 0·0)	−1·4% (−1·5 to −1·3)	−0·2% (−0·4 to 0·0)	−1·3% (−1·5 to −1·1)

DALY=disability-adjusted life-year.

**Figure 2:**
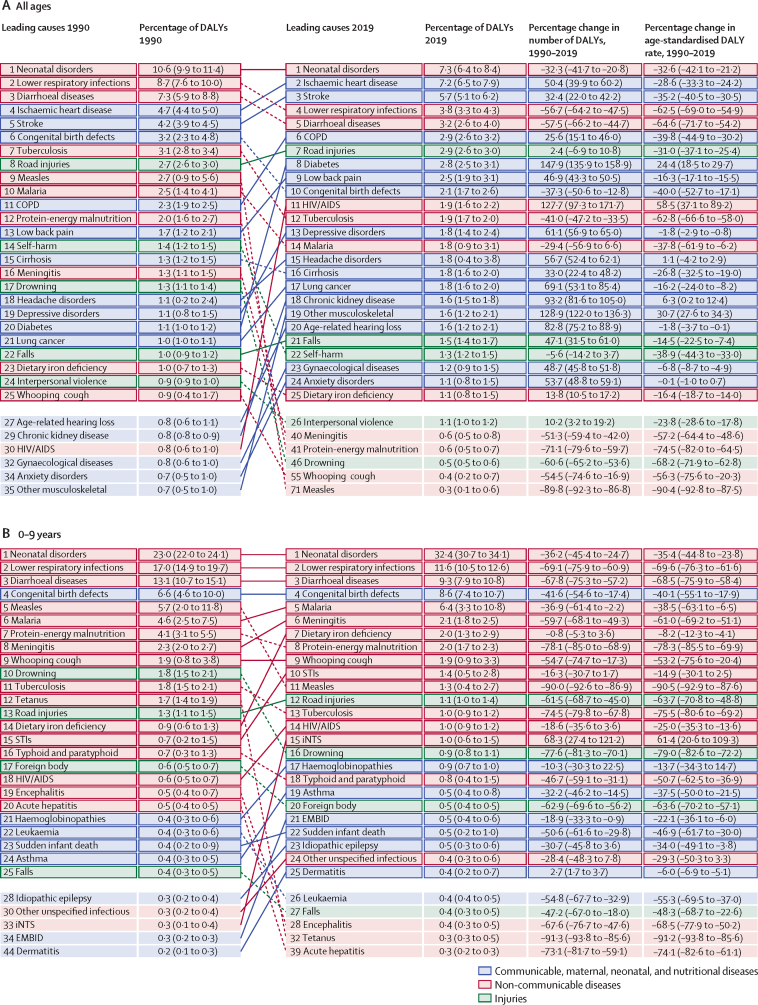

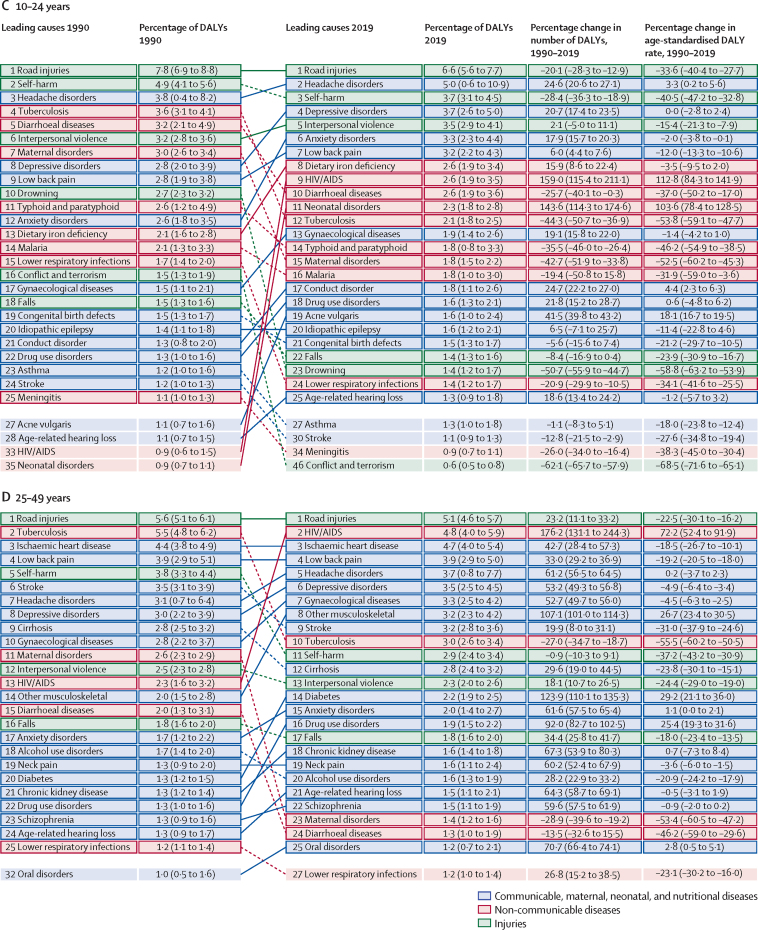

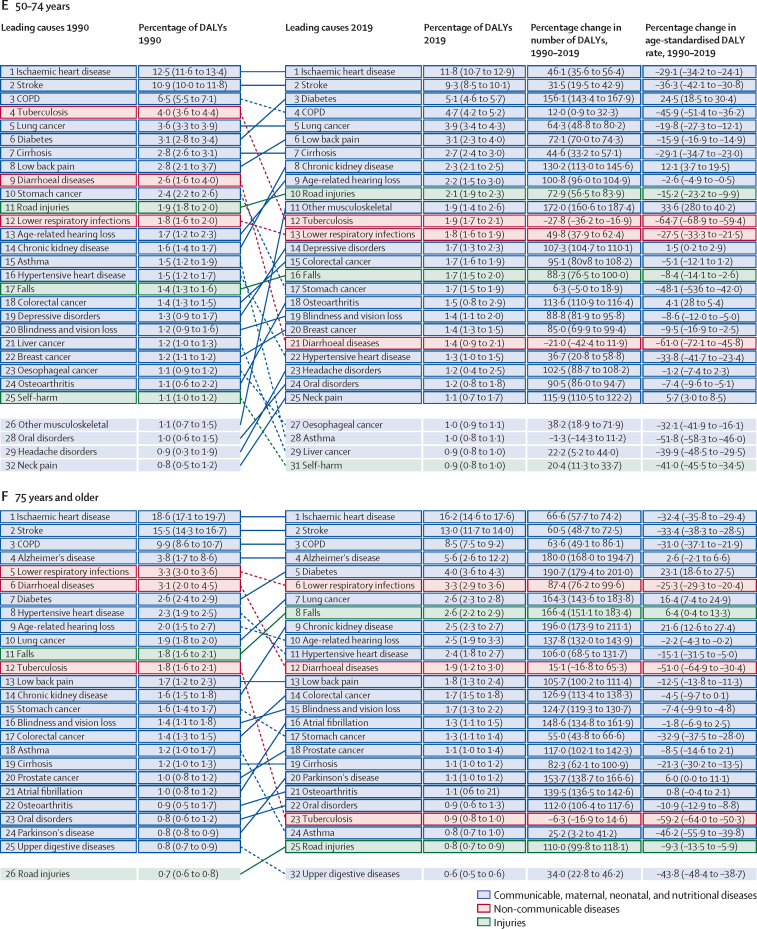
Leading 25 Level 3 causes of global DALYs and percentage of total DALYs (1990 and 2019), and percentage change in number of DALYs and age-standardised DALY rates from 1990 to 2019 for both sexes combined for all ages (A), children younger than 10 years (B), and ages 10–24 years (C), 25–49 years (D), 50–74 years (E), and 75 years and older (F) Causes are connected by lines between time periods; solid lines are increases in rank and dashed lines are decreases. Age-related hearing loss=age-related and other hearing loss. Alzheimer's disease=Alzheimer's disease and other dementias. Atrial fibrillation=atrial fibrillation and flutter. Cirrhosis=cirrhosis and other chronic liver diseases. COPD=chronic obstructive pulmonary disease. EMBID=endocrine, metabolic, blood, and immune disorders. DALY=disability-adjusted life-year. iNTS=invasive non-typhoidal salmonella. Haemoglobinopathies=haemoglobinopathies and haemolytic anaemias. Lung cancer=tracheal, bronchus, and lung cancer. Other musculoskeletal=other musculoskeletal disorders. Other unspecified infectious=other unspecified infectious diseases. Sudden infant death=sudden infant death syndrome. STI=sexually transmitted infections excluding HIV.

These general trends are made up of complex trends for specific diseases and injuries. Overall trends in the number of DALYs across the different age groups between 1990 and 2019 are driven by some key diseases and injuries (figure 2). The ten most important drivers of increasing burden (ie, the causes that had the largest absolute increases in number of DALYs between 1990 and 2019) include six causes that largely affect older adults (ischaemic heart disease, diabetes, stroke, chronic kidney disease, lung cancer, and age-related hearing loss), whereas the other four causes (HIV/AIDS, other musculoskeletal disorders, low back pain, and depressive disorders) are common from teenage years into old age (figure 2). Despite these ten conditions contributing the largest number of additional DALYs over the 30-year period, only HIV/AIDS, other musculoskeletal disorders, and diabetes saw large increases in age-standardised DALY rates, with an increase of 58·5% (95% UI 37·1–89·2) for HIV/AIDS, 30·7% (27·6–34·3) for other musculoskeletal disorders, and 24·4% (18·5–29·7) for diabetes. The burden of HIV/AIDS, however, peaked in 2004 and has dropped substantially after the global scale-up of antiretroviral treatment (ART). The changes in age-standardised rates for chronic kidney disease, age-related hearing loss, and depressive disorders were small (figure 2). Substantial declines in age-standardised rates were seen in ischaemic heart disease (28·6%, 95% UI 24·2–33·3), stroke (35·2%, 30·5–40·5), and lung cancer (16·1%, 8·2–24·0).

The ten most important contributors to declining burden (ie, the causes that had the largest absolute decreases in number of DALYs between 1990 and 2019) include nine that predominantly affect children (lower respiratory infections, diarrhoeal diseases, neonatal disorders, measles, protein-energy malnutrition, congenital birth defects, drowning, tetanus, and malaria), as well as tuberculosis, which largely affects adults. All of these causes with declining burden also had substantial decreases in age-standardised DALY rates, ranging from 32·6% (21·2–42·1) decline for neonatal disorders to 90·4% (87·5–92·8) decline for measles, not just decreases in the absolute number of DALYs due to demographic changes (figure 2A). Although most of the ten leading Level 3 causes of DALYs were the same for both sexes in 2019, road injuries (ranked fourth for males), cirrhosis (ninth), and lung cancer (tenth) were in the top ten for males only, and were replaced by low back pain (ranked sixth for females), gynaecological diseases (ninth), and headache disorders (tenth) for females (appendix 2 figure S5 and tables S2–5, S7, S8, S12, S13, S16). Congenital defects were ranked tenth for both sexes combined in 2019 but did not make the top ten for either sex separately.

The burden for children younger than 10 years declined profoundly between 1990 and 2019, by 57·5% (95% UI 50·3–63·1). Key drivers of this progress included large reductions in major infectious diseases affecting children—namely, lower respiratory infections, diarrhoeal diseases, and meningitis, each of which declined by more than 60% between 1990 and 2019 (figure 2). In 2019, neonatal disorders were the leading cause of burden in this age group, accounting for 32·4% (30·7–34·1) of the group's global DALYs, increasing from 23·0% (22·0–24·1) in 1990. Six infectious diseases were also among the top ten causes of burden in children: lower respiratory infections (ranked second), diarrhoeal diseases (third), malaria (fifth), meningitis (sixth), whooping cough (ninth), and sexually transmitted infections (which were fully accounted for by congenital syphilis in this age group; tenth). Congenital birth defects (ranked fourth) as well as two nutritional disorders—dietary iron deficiency (seventh) and protein-energy malnutrition (eighth)—completed the top ten. The percentage change in age-standardised DALY rates for eight of the ten leading causes was large, ranging from a 35·4% (23·8–44·8) decline for neonatal disorders to 78·3% (69·9–85·5) decline for protein-energy malnutrition over the study period. The decreases for the remaining two top-ten causes, sexually transmitted infections and dietary iron deficiency, were much more modest. Sub-Saharan Africa experienced nearly half of the total DALYs (49·9% [47·6–52·3]) for this age group in 2019.

The change in disease burden in adolescents aged 10–24 years was much more modest (figure 2). DALYs declined by 6·2% (95% UI 2·1–10·5) overall between 1990 and 2019. DALYs for non-communicable diseases increased by 13·1% (9·5–16·3), whereas injuries declined by 24·8% (19·7–29·3) and infectious diseases by 18·7% (13·4–24·0). Three injury causes were among the top ten causes of global DALYs in this age group in 2019: road injuries (ranked first), self-harm (third), and interpersonal violence (fifth; figure 2). Headache disorders, two mental disorders (depression and anxiety), low back pain, dietary iron deficiency, HIV/AIDS, and diarrhoeal disease were the other causes in the top ten for adolescents. Among the top ten causes in this age group, age-standardised DALY rates for road injuries, self-harm, and diarrhoeal diseases decreased by more than a third each between 1990 and 2019. As in the 0–9-year age group, the large increase in burden due to HIV/AIDS in the 10–24-year age group reflects a rapid increase in the first half of the study period followed by a decline after the global scale-up of ART; despite declining in recent years, the HIV/AIDs burden has not yet returned to 1990 levels. The other causes in the top ten showed small or insignificant change (figure 2). The sex differences in the top ten rankings are striking. The three previously mentioned injuries were the top-ranked causes of DALYs among male adolescents (appendix 2 figure S9), whereas headaches, depressive disorders, and anxiety disorders were the top three causes of DALYs among females (appendix 2 figure S10). Maternal disorders, gynaecological disorders, and dietary iron deficiency were also in the top ten causes for females in this relatively young age group (appendix 2 figure S10).

Five causes that were in the top ten for ages 10–24 in 2019 were also in the top ten in the 25–49 age group: road injuries (ranked first), HIV/AIDS (second), low back pain (fourth), headache disorders (fifth), and depressive disorders (sixth; figure 2). Tuberculosis and four non-communicable causes—ischaemic heart disease, gynaecological disorders, other musculoskeletal disorders, and stroke—completed the top ten rankings. There were substantial improvements since 1990 in DALY rates of tuberculosis, road injuries, stroke, and, to a lesser extent, low back pain and ischaemic heart disease. For similar reasons as in the previous age group, HIV/AIDS DALY rates increased substantially. The increase in the residual “other musculoskeletal disorder” category is more difficult to interpret, as it is a collection of several individual diseases. HIV/AIDS, ischaemic heart disease, stroke, and headache disorders appeared in the top-ten rankings for DALYs for both males and females in 2019. Three injury causes (road injuries, self-harm, and interpersonal violence) and cirrhosis ranked prominently among males but not females. Among females, gynaecological disorders, depressive disorders, other musculoskeletal disorders, maternal disorders, and anxiety disorders were top ten causes (appendix 2 figures S9, S10).

In 2019, the ten leading causes of DALYs in age groups 50–74 years and 75 years and older largely overlapped. Ischaemic heart disease and stroke were ranked first and second, respectively, in both age groups. Chronic obstructive pulmonary disease (COPD), diabetes, lung cancer, chronic kidney disease, and age-related hearing loss appeared in the top ten in both age groups. For ages 50–74 years, low back pain, cirrhosis, and road injuries were the remaining top-ten-ranking causes of DALYs, whereas Alzheimer's disease and other dementias, lower respiratory infections, and falls appeared in the top ten for those aged 75 years and older. The most notable changes in top ten causes in these two age groups between 1990 and 2019 were large declines in age-standardised DALY rates for ischaemic heart disease, stroke, COPD, cirrhosis, and road injuries, but increases in DALY rates for diabetes and chronic kidney disease. There was a decline in age-standardised lung cancer rates for ages 50–74 years, but an increase in the oldest age category. The ten leading causes for DALYs by sex in both of these older age groups largely overlapped in 2019. Among 50–74-year-olds, breast cancer, other musculoskeletal disorders, and depressive disorders appeared in the top ten for females only, while road injuries, cirrhosis, and tuberculosis made it into the top ten for males. For the oldest age group, falls and hypertensive heart disease ranked in the top ten among females, but not males; lung cancer and prostate cancer ranked among the top ten in males (appendix 2 figures S9, S10).

### National trends

**Figure 3:**
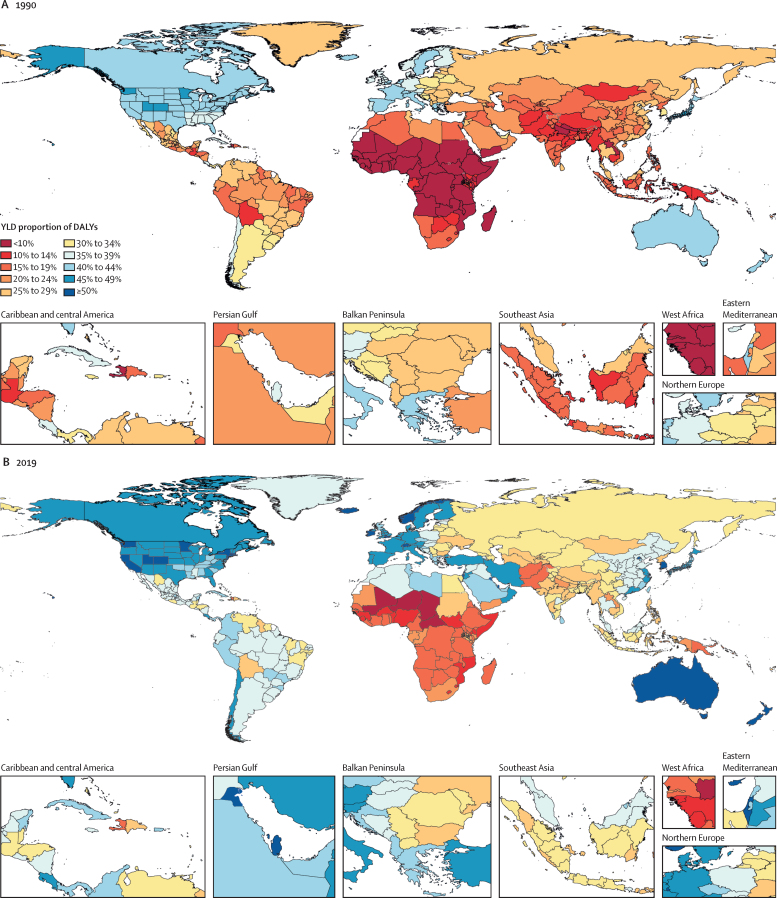
Proportion of total DALYs contributed by injury and non-communicable disease YLDs, by country or territory, 2019 Proportions were rounded to the nearest whole number. DALY=disability-adjusted life-year. YLD=year lived with disability.

Countries and territories vary widely in their stages of the epidemiological transition. With increasing SDI, we expect to see a shift in the burden of disease from communicable, maternal, neonatal, and nutritional diseases towards non-communicable causes. We also expect to see a shift towards a larger fraction of the burden due to YLDs compared with YLLs. These two major trends can be summarised by the percentage of all-cause DALYs made up of non-communicable disease and injury YLDs. Figure 3 shows this proportion across 204 countries and territories in 1990 and 2019. In 2019, this measure of the epidemiological transition ranged from 8·4% (95% UI 6·2–10·9) in Chad to 56·9% (48·7–64·3) in Qatar. The values in 1990 ranged from 3·5% (2·6–4·7) in Niger to 47·5% (37·6–56·0) in Andorra. In 2019, non-communicable and injury YLDs contributed to more than half of all disease burden in 11 countries. All but two countries, Ukraine and Lesotho, had higher ratios in 2019 compared with 1990.

**Figure 4:**
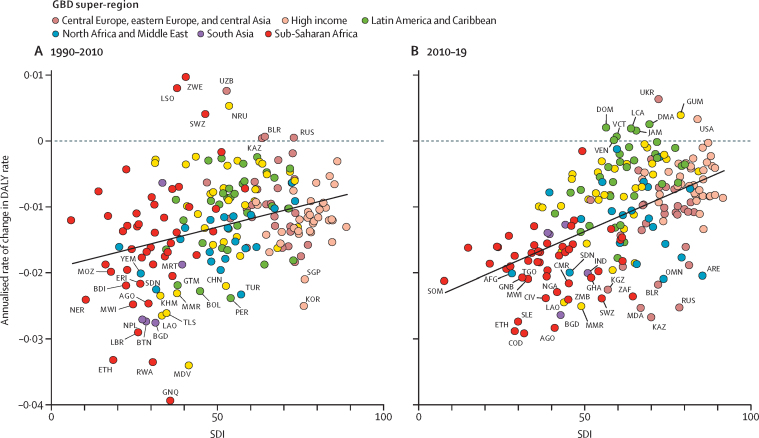
Annualised rate of change in age-standardised DALY rates for all causes excluding HIV/AIDS, natural disasters, and war and conflict, and SDI by country or territory, for 1990–2010 (A) and 2010–19 (B) A simple linear regression line is shown in each figure for the relationship between annualised rate of change and the average SDI value of each country and territory for each time period. AFG=Afghanistan. AGO=Angola. ARE=United Arab Emirates. BDI=Burundi. BGD=Bangladesh. BLR=Belarus. BOL=Bolivia. BTN=Bhutan. CHN=China. CIV=Côte d'Ivoire. CMR=Cameroon. COD=Democratic Republic of the Congo. DALY=disability-adjusted life-year. DMA=Dominica. DOM=Dominican Republic. ERI=Eritrea. ETH=Ethiopia. GHA=Ghana. GNB=Guinea-Bissau. GNQ=Equatorial Guinea. GTM=Guatemala. GUM=Guam. IND=India. JAM=Jamaica. KAZ=Kazakhstan. KHM=Cambodia. KOR=South Korea. KNA=Saint Kitts and Nevis. LAO=Laos. LBR=Liberia. LCA=Saint Lucia. LSO=Lesotho. MDA=Moldova. MDV=Maldives. MMR=Myanmar. MOZ=Mozambique. MRT=Mauritania. MWI=Malawi. NER=Niger. NGA=Nigeria. NPL=Nepal. NRU=Nauru. OMN=Oman. PER=Peru. RUS=Russia. RWA=Rwanda. SDN=Sudan. SGP=Singapore. SLE=Sierra Leone. SOM=Somalia. SWZ=eSwatini. TGO=Togo. TLS=Timor-Leste. TUR=Turkey. UKR=Ukraine. UZB=Uzbekistan. VCT=Saint Vincent and the Grenadines. VEN=Venezuela. YEM=Yemen. ZAF=South Africa. ZWE=Zimbabwe. SDI=Socio-demographic Index.

When comparing the annualised rate of change in age-standardised DALY rates for all causes except HIV/AIDS, natural disasters, and war and conflict between the time periods 1990–2010 and 2010–19 for each country and territory, the rate, as shown by a simple linear regression line, is steeper in the latter time period, suggesting that change has accelerated over the last decade in countries and territories at the lower end of the SDI range (figure 4). Improvements have started to stagnate, or even reverse, in countries with higher SDI, as is the case in Dominica, the Dominican Republic, Guam, Jamaica, Saint Lucia, Saint Vincent and the Grenadines, Ukraine, the USA, and Venezuela. Countries with greater than 2% annual reductions in age-standardised DALY rates over both time periods were Ethiopia, Angola, Burundi, Malawi, Sudan, Myanmar, Laos, and Bangladesh. Four countries from the former Soviet Union—Russia, Belarus, Kazakhstan, and Uzbekistan—experienced increases in age-standardised DALY rates between 1990 and 2010, but recovered in the following decade; Russia, Kazakhstan, and Belarus experienced an estimated annual decline of 2% or greater between 2010 and 2019, and Uzbekistan experienced an estimated 1·5% annual decline. Another former Soviet Union republic, Ukraine, saw modest decline in the 1990 to 2010 period, but a worsening trend in the decade after.

### Cause-specific trends

Two-page cause-specific summaries provide detailed results on mortality, prevalence, incidence, YLLs, YLDs, and DALYs for a selection of diseases, injuries, and impairments in the GBD cause hierarchy. These summaries include 2019 counts, age-standardised rates, and rankings; the fraction of DALYs attributed to risk factors; patterns over time and age; and the relationship between SDI and DALY rates by country or territory. They were written to increase the accessibility to and transparency of GBD estimates for each cause. Summaries for select causes are highlighted in print (pp S2–213); summaries for all diseases, injuries, and impairments can be found online.

## Discussion

### Main findings

Global health has steadily improved over the past 30 years, as measured by changes in age-standardised DALY rates. While health has improved, after accounting for population growth and ageing, the absolute number of DALYs has remained stable. The shift to a much greater number of DALYs occurring at older ages, despite reductions in age-standardised DALY rates, illustrates the importance of understanding how ageing shapes future health needs. Policy makers should remain aware that the number of DALYs represents the burden of disease that the world's health systems must manage.

Although most diseases showed a pattern of stable or slowly changing rates of death and disability over the study period, there are some notable exceptions. Deaths due to drug use disorders have risen sharply over the past decade. In 2019, more than half of all global overdose deaths occurred in the USA. Liberal prescribing of high-dose opioids, inadequate provision of opioid substitution therapy, and the lacing of street drugs with highly potent opioids such as fentanyl are considered major contributors to this public health crisis.^14^, ^15^, ^16^, ^17^ By contrast, a positive, rapid change in disease rates has taken place in Egypt, where close to 80% of the population aged 12 years and older has been screened for hepatitis C, and those with detectable virus are treated with a low-cost treatment regimen.^18^, ^19^ We estimated that the number of cases of chronic hepatitis C has dropped by 65·9% (95% UI 51·1–79·7) since screening and treatment were initiated through regular health services in 2014 and an enhanced national screening programme for the whole population aged 12 years and older was established in 2019.^19^ Egypt had the highest prevalence of chronic hepatitis C in the world, ascribed to iatrogenic infection during treatment campaigns for schistosomiasis in the 1960s and 1970s.^20^, ^21^, ^22^ The sharp decline in chronic infections in Egypt is expected to be reflected in a large decline in deaths from cirrhosis and liver cancer in coming years. Unlike hepatitis B vaccination in children, where the effect of intervention cannot be expected until several decades later, removal of hepatitis C virus in the adult population leads to more immediate health impact.

In children younger than 10 years, the decline in neonatal disorders was slower than for the major infectious diseases, thus increasing neonatal disorders' share of total DALYs. Among injuries in this age group, drowning saw the largest decline in DALYs. The position of congenital syphilis among the top ten causes of DALYs in children is indicative of health system failure. With testing and treatment in the second trimester of pregnancy, this cause could be eliminated.^23^ The main reasons for failure are limited access to health services, the low use of rapid diagnostic tests, the failure of antenatal clinics to screen or treat when a woman is tested positive, and the recent global shortage of benzathine penicillin, the treatment of choice.^24^ Despite the large health gains among children younger than 10 years, considerable burdens still remain in sub-Saharan Africa. Sustaining the global pace of progress will become more challenging as an ever-increasing proportion of the global birth cohort is born in sub-Saharan Africa,^25^ with the highest rates of burden in these age groups. It is encouraging, however, that the largest decreases in DALY rates globally have occurred in sub-Saharan African countries, such as Ethiopia, Angola, Rwanda, and Malawi, although there are others that have seen much less progress.

Among the top ten causes of DALYs in adolescents aged 10–24 years, self-harm had the largest decline (28·4% [95% UI 18·9–36·3]) over the study period. The prevalence of depressive disorders and other mental disorders, which are major underlying causes of self-harm,^26^ did not change, suggesting that the decline in self-harm deaths was largely due to other factors such as better access to mental health services, urbanisation, and a reduction in access to more lethal means of suicide.^27^, ^28^, ^29^, ^30^ The increase in DALY rates of neonatal disorders in this age group is a downside to the large improvements in neonatal survival, causing a greater proportion of the surviving babies to have long-term neurological and sensory deficits.

In the 25–49-year age group, HIV/AIDS was the second leading cause of DALYs in 2019 despite a drop since 2005, when ART became more widely available.^31^ To be on course to end HIV/AIDS as a public health threat by 2030, UNAIDS estimates that a substantial increase in global funding would be required, whereas high-income countries have reduced their funding.^32^ The prominent position of headache disorders in the DALY rankings in the 10–24-year and 25–49-year age groups has received little attention in global health policy debates. While there is no cure for these disorders, there are effective symptomatic and preventive treatments available.^33^ Ischaemic heart disease, stroke, and diabetes were not among the 25 leading causes in the two younger age groups, but emerged as major contributors to burden in the 25–49-year age group and, more prominently, in the older age groups that follow. These diseases share many common risk factors and treatment approaches. The burden in high-income countries has been rapidly declining since the 1980s, but a more recent downturn in this decline over the past 5 years has been noted as an important explanation for the slowdown in life expectancy gains.^34^ Low-income and middle-income countries still have ample opportunity to make greater use of known effective intervention strategies (tobacco control, blood pressure-lowering and cholesterol-lowering treatments, and emergency response and treatment for acute events) that have been so effective in high-income countries.^35^ However, the rising prevalence of diabetes, linked to the almost ubiquitous increase in body-mass index globally,^36^ is mitigating the pathway to reducing the burden of cardiovascular diseases.^37^, ^38^ In the 25–49-year age group, tuberculosis that is not associated with HIV infection ranked among the top ten causes in 2019. There are similar worries about sustained global funding of tuberculosis control as mentioned for HIV/AIDS, let alone having the additional resources and research development effort that would be required to reach WHO's goals to reduce the 2015 levels of tuberculosis deaths and incidence by 90% and 80%, respectively, by the year 2030.^39^, ^40^, ^41^

The prominent rankings of COPD and lung cancer in the 50–74-year and 75-years-and-older age groups emphasise the continuing need for tobacco-control measures and attention to reducing exposure to indoor and outdoor air pollution. Already, low-income and middle-income countries account for 62·6% of the global burden of COPD and lung cancer, and this share is likely to increase sharply over coming decades due to ageing populations and less successful tobacco and air pollution control. The finding that lung cancer DALY rates are declining in the 50–74-year age group but not in those aged 75 years and older is probably due to a cohort effect; this could be encouraging if it reflects a greater response to tobacco control in younger generations that will drive further declines in coming years. Chronic kidney disease is strongly linked to cardiovascular diseases and diabetes, and shares common risks and intervention approaches.^42^ Given its prominent position in the top ten rankings of DALYs in older age groups and the costs associated with end-stage kidney disease treatments, screening and low-cost treatments at earlier stages of chronic kidney disease should be more widely implemented.^43^ Cirrhosis ranked seventh among those aged 50–74 years in 2019. With low-cost treatments available to low-income and middle-income countries, there is an opportunity to eradicate hepatitis C as an underlying cause—a strategy that Egypt is well on the way to achieving in coming years.^19^ Childhood vaccinations for hepatitis B will eventually also reduce cirrhosis (and liver cancer) outcomes, but the full effect will probably not be apparent for years. Alcohol is the third modifiable cause of cirrhosis; there is strong evidence that taxation and regulations can reduce alcohol use to less harmful levels.^44^ Age-related hearing loss is a top ten cause of DALYs in the two older age groups. While some reduction in burden can be achieved by control of loud noises during leisure or occupational activities, most of the burden cannot be prevented through currently known strategies. For a large proportion of the elderly, hearing aids can relieve some of the symptoms and associated social isolation. The quality of hearing aids has improved over the past decade, but low-cost appliances are not readily available in low-income and middle-income countries.^45^

Alzheimer's disease and other dementias, and falls are two causes that appear in the top ten ranking of DALYs only for those aged 75 years and older. The ability to intervene by prevention or treatment for dementia is still limited despite a large research and development effort to identify drugs, but efforts continue.^46^ There is good evidence that a range of modifiable risks (tobacco, physical inactivity, metabolic risks, and hearing loss) contribute to the development of dementia,^47^, ^48^ but little evidence of the effectiveness of interventions addressing these risk factors.^47^, ^49^ Falls in the elderly are common and linked to psychotropic and cardiovascular medications,^50^ cognitive impairment, depression, and general frailty.^51^, ^52^ There is evidence for the effectiveness of multifactorial interventions combining education, exercise, and home safety modification interventions.^53^

The trend towards disability as an increasing share of overall burden has continued. In 11 countries, more than half the burden was from YLDs of NCDs and injuries in 2019. To some extent, the absence of a discernible trend in disability might be an artifact of the poor availability of data on severity, and, therefore, an inability to quantify the effect of health service interventions that modulate severity. The larger issue, however, is that most of the focus of global public health has been on life-saving interventions directed at the main causes of death.^7^, ^54^, ^55^ The large contributors to disability, such as musculoskeletal conditions and mental disorders, are associated with few deaths. As disability becomes an increasingly large component of disease burden and, as importantly, a larger component of health expenditure, a greater research development investment is needed to identify new, and more effective, intervention strategies.^56^, ^57^, ^58^ With a rapidly ageing global population, the demands on health services to deal with disabling outcomes, which increase with age, will require policy makers to anticipate these changes. GBD provides key information on the changes in types of health services in terms of facilities and adequately trained personnel that will be needed.

The finding that health gains in countries at the lower end of the SDI scale have, on average, accelerated over the past decade compared with the two decades before indicates the potential for low-income countries to make a real difference by investing in health. Progress, however, has been uneven. The more recent downturn in reductions in DALY rates in countries and territories with higher SDI is striking and near universal, although an actual reversal into increases of age-standardised DALY rates has only happened in a small number of countries in the Caribbean and the USA. Plausible drivers of this change include obesity, diminishing potential for further reductions in smoking, and improvements in coverage of treatments for high blood pressure and cholesterol to maintain the past declines in cardiovascular mortality.^34^ Inequalities in access to preventive and curative services by lower socioeconomic groups might be a further obstacle to continued improvements in cardiovascular mortality.^59^ The large increase in drug overdose deaths in the USA and the increasing number of deaths from violence in Latin American countries, in addition to the decelerated decline of cardiovascular mortality, are driving the patterns in these locations. The mix of universal and more geographically specific influences on health reinforces the need for regular, detailed reporting on population health by underlying cause to help decision makers to identify success stories of disease control, as well as opportunities to improve and emulate countries that are performing well.

### Limitations

The major limitation of the GBD analysis of the burden of diseases and injuries is the availability of primary data. Where data are not available, the results depend on the out-of-sample predictive validity of the modelling efforts. While improvements to data processing and modelling can lead to incremental improvements in the accuracy of our estimates, fundamental improvements require more and better primary data collection. Even when data are available, they might not have been obtained using the preferred case definition or measurement method. The more explicit identification of the preferred and alternative measurement method for each outcome, and the bias mapping from alternative to reference method undertaken as part of GBD 2019, have led to greater stability in data adjustments. These improvements will also aid in identifying priorities for data collection and in determining preferred case definitions and study methods. Moving to use of standard locations for estimating fixed effects in the models will aid in cycle-to-cycle stability of models. Through the use of standard locations, the addition of more subnational units in a given GBD cycle should not shift the regression model predictions as much as they previously would have. Nevertheless, collinearity between covariates in some of these models might contribute to some instability in fixed effects between cycles. Future work on ensemble models might help to solve the collinearity problem. Of note, because the cause of death models developed using CODEm are an ensemble of all high-performing possible models, they avoid the instability due to collinearity. Although our statistical modelling is designed to capture uncertainty from stochastic variation in input data, age and sex splitting of data, corrections for alternative case definitions or uninformative cause of death codes, other data manipulations, and model choice, it remains a challenge to fully represent the UIs around estimates, particularly in locations with sparse or absent data. This will remain a major focus of GBD by tapping into existing knowledge in other estimation fields as well as our own development of methods.

The shift to adjusting dementia deaths to reflect only those with end-stage disease is conceptually more appealing than the past crude adjustment for the large variation in coding practices. We will, however, need to replicate the methods of determining the share of excess mortality in people with dementia who are in the last stages of the disease and for whom an assignment of dementia as the underlying cause of death is therefore justified. A greater focus in future rounds of GBD will need to be directed to identifying data of treatment effects on severity distributions of the large contributors to YLDs, such as mental, neurological, and musculoskeletal disorders, for which we currently do not distinguish geographical variation in severity. This is of particular importance as these conditions represent an increasing share of total burden. Our effort to improve the consistency between mortality rates, prevalence, and incidence for selected conditions by providing more explicit guidance on excess mortality rates in DisMod-MR has revealed that more attention will be required in future rounds of GBD. After imposing a pattern of excess mortality that follows an expected pattern of lower rates in countries with better health systems, the models might predict prevalence or incidence estimates that are far removed from observed data. The challenge is then to identify whether the inconsistency is due to error in the cause of death estimates, the non-fatal data sources, or a combination of the two. In addition to these general limitations, there are many limitations for each specific modelling exercise reported in this study. Appendix 1 (sections 3.4 and 4.12) provides more insight into some of these issues.

### Future directions

Several method improvements signalled in previous GBD publications have not yet been implemented but remain a priority. For instance, DisMod-AT, a new version of our main non-fatal modelling tool that simultaneously solves for patterns over age and time, is still undergoing testing before it can be implemented in GBD. Methods to make dependent comorbidity corrections computationally feasible, and imposing greater variation in severity distributions based on access to and quality of health care, are also still under development. More generally, imposing GBD principles and methods to the estimation of access to health interventions and the effectiveness thereof, and being able to link those estimates with our future health scenario platform^25^ is a direction we are keen to take. Developing this comprehensively is a large endeavour that will take many years to complete. As this would greatly add value to the policy relevance of GBD, we will also aim to develop less comprehensive methods that will nevertheless allow us to respond to policy makers seeking information on major policy decisions in a more timely fashion.

### Conclusion

Taking into account population growth and shifts in age structure, health continues to improve at the global level. The absolute burden of disease and its associated impact on health systems, however, remain resolutely constant. Some diseases, such as diabetes, are increasing in burden, and more general all-cause DALY stagnation in some high SDI countries points out that further gains are not inevitable. Close monitoring of health trends and careful policy evaluation of the options to counteract adverse trends is required. Leading causes of DALYs, as well as solutions, differ substantially across age groups, highlighting the need to formulate policy for different phases of the life course.

Correspondence to: Prof Christopher J L Murray, Institute for Health Metrics and Evaluation, University of Washington, Seattle, WA 98195, USA cjlm@uw.edu

## Data sharing

To download the data used in these analyses, please visit the Global Health Data Exchange GBD 2019 website.
