# Department of Error

**DOI:** 10.1016/S0140-6736(20)32226-1

**Published:** 2020-11-14

*GBD 2019 Diseases and Injuries Collaborators. Global burden of 369 diseases and injuries in 204 countries and territories, 1990–2019: a systematic analysis for the Global Burden of Disease Study 2019.* Lancet *2020;* 396: *1204–22*—In figure 2 of this Global Health Metrics paper, the colour key has been corrected. This correction has been made to the online version as of Oct 23, 2020.

