# Supplementary material for: Global burden of 369 diseases and injuries in 204 countries and territories, 1990–2019: a systematic analysis for the Global Burden of Disease Study 2019
Source: Lancet. 2020 Oct 17;396(10258):1204–22. doi: 10.1016/S0140-6736(20)30925-9 (PMC7567026; doi:10.1016/S0140-6736(20)30925-9)
Supplement: Supplementary appendix 1 [file mmc1.pdf]

THE LANCET

Supplementary appendix 1

This appendix formed part of the original submission and has been peer reviewed. We post it as supplied by the authors.

Supplement to: GBD 2019 Diseases and Injuries Collaborators. Global burden of 369 diseases and injuries in 204 countries and territories, 1990–2019: a systematic analysis for the Global Burden of Disease Study 2019. *Lancet* 2020; **396:** 1204–22.

Appendix 1: Methods appendix to "Global burden of 369 diseases and injuries in 204 countries and territories, 1990–2019: a systematic analysis for the Global Burden of Disease Study 2019"

This appendix provides further methodological detail for "Global burden of 369 diseases and injuries in 204 countries and territories, 1990–2019: a systematic analysis for the Global Burden of Disease Study 2019."

Portions of this appendix have been reproduced or adapted from Roth et al.,[1] James et al.,[2] Kyu et al.,[3] and Stanaway et al.[4] References are provided for reproduced sections.

Preamble

This appendix provides further methodological detail for "Global burden of 369 diseases and injuries in 204 countries and territories, 1990–2019: a systematic analysis for the Global Burden of Disease Study 2019." This study complies with the Guidelines for Accurate and Transparent Health Estimates Reporting (GATHER) recommendations.[5] It includes detailed tables and information on data to maximize transparency in our estimation processes and provides a comprehensive description of analytical steps. We intend this appendix to be a living document, to be updated with each iteration of the Global Burden of Disease Study (GBD).

Authors' Contributions

Managing the estimation process

Theo Vos, Stephen S Lim, Ashkan Afshin, Tahiya Alam, Charlie Ashbaugh, Celine Barthelemy, Molly Biehl, Michael Brauer, Kelly Compton, Elizabeth Cromwell, Lalit Dandona, Amanda Deen, Mae Dirac, Kara Estep, Alize Ferrai, Nancy Fullman, Christina Fitzmaurice, Lisa Force, Emmanuela Gakidou, Peter Gething, Erin Hamilton, Spencer James, Nicholas Kassebaum, Hmwe Kyu, Alan D Lopez, Ashley Marks, Awoke Misganaw, Ali Mokdad, Meghan Mooney, Jonathan Mosser, Erin Mullany, Molly R Nixon, Puja Rao, Greg Roth, Katya Shackelford, Stein Emil Vollset, Theo Vos, Harvey Whiteford, Eve Wool, Mohsen Naghavi, and Christopher J L Murray.

Writing the first draft of the manuscript

Theo Vos, Stephen S Lim, Catherine Bisignano, Jessica Cruz, Anna Gershberg, Scott Glenn, Gaorui Guo, Vincent Iannucci, Hussain Jafari Hayoon, Cathleen Keller, Varsha Krish, Samantha Larson, Rui Ma, Molly R Nixon, Kyle Simpson, Alexandrea Watson, and Christopher J L Murray.

Providing data or critical feedback on data sources

Theo Vos, Cristiana Abbafati, Mohammad Abbasi, Kanaan Abdullah, Hassan Abolhassani, Lucas Abreu, Michael Abrigo, Laith Abu-Raddad, Abdelrahman Abushouk, Maryam Adabi, Oladimeji Adebayo, Victor Adekanmbi, Jaimie Adelson, Olatunji Adetokunboh, Ashkan Afshin, Gina Agarwal, Mohammad Aghaali, Tomi Akinyemiju, Khurshid Alam, Jacqueline Alcalde-Rabanal, Muhammad Ali, Saqib Ali, Cyrus Alinia, Syed Aljunid, François Alla, Peter Allebeck, Amir Almasi-Hashiani, Nelson Alvis-Guzman, Nelson Alvis-Zakzuk, Saeed Amini, Gianna Gayle Amul, Deanna Anderlini, Tudorel Andrei, Mina Anjomshoa, Fereshteh Ansari, Alireza Ansari-Moghaddam, Davood Anvari, Razique Anwer, Jalal Arabloo, Morteza Arab-Zozani, Filippo Ariani, Johan Ärnlöv, Krishna Aryal, Desta Atnafu, Sachin Atre, Floriane Ausloos, Marcel Ausloos, Beatriz Paulina Ayala Quintanilla, Yared Aynalem, Samad Azari, Zelalem Azene, Ahad Bakhtiari, Senthilkumar Balakrishnan, Maciej Banach, Palash Banik, Miguel Barboza, Akbar Barzegar, Sanjay Basu, Vo Bay, Ettore Beghi, Aminu Bello, Eduardo Bernabe, Reshmi Bhageerathy, Boris Bikbov, Antonio Biondi, Binyam Birihane, Donal Bisanzio, Mahdi Bohluli, Guilherme Borges, Antonio Borzì, Rupert Bourne, Michael Brauer, Carol Brayne, Gabrielle Britton, Dana Bryazka, Luis Cámera, Josip Car, Juan Carrero, Joao Mauricio Castaldelli-Maia, Carlos Castañeda-Orjuela, Chris Castle, Franz Castro, Ferrán Catalá-López, Christopher Cederroth, Alex Chang, Vijay Kumar Chattu, Daniel Cho, Dinh-Toi Chu, Michael Chung, Massimo Cirillo, Paolo Cortesi, Vera Costa, Ewerton Cousin, Richard Cowden, Benjamin Cowie, Matthew Cunningham, Giovanni Damiani, Aso Darwesh, Ahmad Daryani, Rajat Das Gupta, José Das Neves, Kairat Davletov, Nebiyu Dereje, Nikolaos Dervenis, Rupak Desai, Samath Dharmaratne, Govinda Dhungana, Mostafa Dianatinasab, Hoa Do, Klara Dokova, Fariba Dorostkar, Leila Doshmangir, Susanna Dunachie, Bruce Duncan, David Edvardsson, Joshua Ehrlich, Nevine El Nahas, Iman El Sayed, Islam Elgendy, Iqbal Elyazar, Mohammad Hassan Emamian, Holly Erskine, Firooz Esmaeilzadeh, Alireza Esteghamati, Mohammad Farahmand, Anwar Faraj, Mohammad Fareed, Carla Farinha, Andrea Farioli, Andre Faro, Farshad Farzadfar, Nazir Fattahi, Valery Feigin, Seyed-Mohammad Fereshtehnejad, Alize Ferrari, Irina Filip, Carsten Flohr, Morenike Folayan, Lisa Force, Takeshi Fukumoto, João Furtado, Mohamed Gad, Amiran Gamkrelidze, Mansour Ghafourifard, Alireza Ghajar, Farhad Ghamari, Nermin Ghith, Syed Amir Gilani, Giorgia Giussani, Ricardo Gomez, Taren Gorman, Harrison Gottlich, Houman Goudarzi, Alessandra Goulart, Bárbara Goulart, Ayman Grada, Michal Grivna, Giuseppe Grosso, Harish Gugnani, Andre Guimaraes, Yuming Guo, Juanita Haagsma, Beatrix Haddock, Nima Hafezi-Nejad, Lydia Haile, Brian Hall, Kanaan Hamagharib Abdullah, Erin Hamilton, Chieh Han, Hannah Han, Josep Haro, Amir Hasanzadeh, Soheil Hassanipour, Hadi Hassankhani, Behnam Heidari, Reza Heidari-Soureshjani, Claudiu Herteliu, Praveen Hoogar, Mehdi Hosseinzadeh, Sorin Hostiuc, Mowafa Househ, Mohamed Hsairi, Guoqing Hu,

Fernando Hugo, Kevin Ikuta, Helen Ippolito, Seyed Sina Irvani, Md.Mohaimenul Islam, Sheikh Mohammed Shariful Islam, Chidozie Iwu, Kathryn Jacobsen, Morteza Jafarinia, Mohammad Jahani, Nader Jahanmehr, Mihajlo Jakovljevic, Spencer James, Achala Jayatilleke, Panniyammakal Jeemon, John Ji, Oommen John, Catherine Johnson, Sarah Johnson, Jost Jonas, Tamas Joo, Jacek Jozwiak, Mikk Jürisson, Zubair Kabir, Leila Kalankesh, André Karch, Salah Eddin Karimi, Getachew Kassa, Nicholas J Kassebaum, Srinivasa Katikireddi, Gbenga Kayode, Konstantin Kazanjan, Morteza Khafaie, Nauman Khalid, Maseer Khan, Khaled Khatab, Mahalaqua Nazli Khatib, Mohammad Taghi Khodayari, Neda Kianipour, Christian Kieling, Yun Jin Kim, Adnan Kisa, Sezer Kisa, Katarzyna Kissimova-Skarbek, Ann Kristin Skrindo Knudsen, Jonathan Kocarnik, Soewarta Kosen, Michael Kravchenko, Kewal Krishan, Burcu Kucuk Bicer, Manasi Kumar, Pushpendra Kumar, Girikumar Kumaresh, Dian Kusuma, Hmwe Kyu, Dharmesh Lal, Van Lansingh, Savita Lasrado, Kathryn Lau, Jorge Ledesma, Shaun Lee, Kate Legrand, James Leigh, Janni Leung, Shanshan Li, Lee-Ling Lim, Shiwei Liu, Stefan Lorkowski, Jennifer Maclachlan, Fabiana Madotto, Hue Mai, Reza Malekzadeh, Deborah Malta, Abdullah Mamun, Amir Manafi, Navid Manafi, Borhan Mansouri, Mohammad Ali Mansournia, Ana Maria Mantilla Herrera, Joemer Maravilla, Francisco Martins-Melo, Manu Mathur, Colm Mcalinden, Walter Mendoza, Ritesh Menezes, Endalkachew Mengesha, Alibek Mereke, Atte Meretoja, Tomislav Mestrovic, Bartosz Miazgowski, Ted Miller, Andreea Mirica, Erkin Mirrakhimov, Babak Moazen, Masoud Moghadaszadeh, Dara Mohammad, Naser Mohammad Gholi Mezerji, Abdollah Mohammadian-Hafshejani, Reza Mohammadpourhodki, Shafiu Mohammed, Ali Mokdad, Natalie Momen, Lorenzo Monasta, Ghobad Moradi, Masoud Moradi, Maziar Moradi-Lakeh, Ilais Moreno Velásquez, Joana Morgado-Da-Costa, Seyyed Meysam Mousavi, Ulrich Mueller, Kamarul Imran Musa, Ahamarshan Nagarajan, Gabriele Nagel, Sanjeev Nair, Javad Nazari, Ionut Negoi, Ruxandra Irina Negoi, Henok Netsere, Josephine Ngunjiri, Cuong Nguyen, Dabere Nigatu, Rajan Nikbakhsh, Shuhei Nomura, Bo Norrving, Jean Jacques Noubiap, Christoph Nowak, Felix Ogbo, Emmanuel Okunga, Andrew Olagunju, Bolajoko Olusanya, Jacob Olusanya, Kanyin Ong, Obinna Onwujekwe, Heather Orpana, Alberto Ortiz, Adrian Oțoiu, Simon Øverland, Mahesh P A, Jagadish Rao. Padubidri, Raffaele Palladino, Adrian Pana, Songhomitra Panda-Jonas, Deepak Kumar Pasupula, Sangram Patel, Angel Paternina-Caicedo, Ashish Pathak, Scott Patten, Veincent Christian Pepito, Alexandre Pereira, David Pereira, Michael Phillips, David Pigott, Khem Pokhrel, Suzanne Polinder, Kevan Polkinghorne, Maarten Postma, Hadi Pourjafar, Reza Pourmirza Kalhori, Anna Poznańska, Sergio Prada, Dimas Pribadi, Elisabetta Pupillo, Hai Quang Pham, Zahiruddin Quazi Syed, Amir Radfar, Alireza Rafiei, Afarin Rahimi-Movaghar, Ali Rajabpour-Sanati, Kiana Ramezanzadeh, Chhabi Ranabhat, Sowmya J Rao, Priya Rathi, David Laith Rawaf, Salman Rawaf, Lal Rawal, Christian Razo, Nickolas Reinig, Marissa Reitsma, Vishnu Renjith, Andre Renzaho, Seyed Mohammad Riahi, Antonio Luiz Ribeiro, Jennifer Rickard, Nicholas Roberts, Leonardo Roever, Luca Ronfani, Enrico Rubagotti, Basema Saddik, Ehsan Sadeghi, Shahram Saeidi, Saeid Safiri, Rajesh Sagar, S. Mohammad Sajadi, Mohammad Salahshoor, Payman Salamati, Hosni Salem, Inbal Salz, Zainab Samad, Abdallah Samy, Damian Santomauro, Milena Santric-Milicevic, Sivan Saraswathy, Benn Sartorius, Arash Sarveazad, Brijesh Sathian, Alyssa Sbarra, Maria Schmidt, David Schwebel, Sadaf Sepanlou, Masood Shaikh, Mehran Shams-Beyranvand, Morteza Shamsizadeh, Mohammed Shannawaz, Jae Il Shin, Soraya Siabani, Jasvinder Singh, Eirini Skiadaresi, Valentin Skryabin, Joan Soriano, Luisa Sorio Flor, Chandrashekhar Sreeramareddy, Benjamin Stark, Timothy Steiner, Mark Stokes, Lars Stovner, Jacob Stubbs, Agus Sudaryanto, Gerhard Sulo, Dillon Sylte, Miklós Szócska, Rafael Tabarés-Seisdedos, Karen Tabb, Amir Taherkhani, Cuong Tat Nguyen, Nuno Taveira, Hoa Thi Do, Roman Topor-Madry, Mathilde Touvier, Marcos Roberto Tovani-Palone, Bach Tran, Ravensara S Travillian, Christopher Troeger, Thomas Truelsen, Aristidis Tsatsakis, Riaz Uddin, Bhaskaran Unnikrishnan, Marco Vacante, Pascual Valdez, Tommi Vasankari, Yasser Vasseghian, Narayanaswamy Venketasubramanian, Vasily Vlassov, Ana Vukovic, Yasir Waheed, Yafeng Wang, Joseph Ward, Jordan Weiss, Ronny Westerman, Harvey Whiteford, Taweewat Wiangkham, Tissa Wijeratne, Shadrach Wilson, Bogdan Wojtyniak, Charles Wolfe, Ai-Min Wu, Sarah Wulf Hanson, Han Yong Wunrow, Gelin Xu, Mousa

Yaminfirooz, Sanni Yaya, Jamal Yearwood, Naohiro Yonemoto, Seok-Jun Yoon, Mustafa Younis, Theodore Younker, Mahmoud Yousefifard, Abdilahi Yousuf, Mohammad Zamani, Alireza Zangeneh, Mikhail Zastrozhin, Yunquan Zhang, Maigeng Zhou, Arash Ziapour, Stephanie R M Zimsen, Mohsen Naghavi, and Christopher J L Murray.

Developing methods or computational machinery

Theo Vos, Jaimie Adelson, Ashkan Afshin, Kareha Agesa, Saeed Amini, Davood Anvari, Aleksandr Aravkin, Samad Azari, Zelalem Azene, Marlena Bannick, Greg Bertolacci, Reshmi Bhageerathy, Michael Brauer, Paul Briant, Dana Bryazka, Chris Castle, Kate Causey, Kelly Compton, Elizabeth Cromwell, Matthew Cunningham, Ahmad Daryani, Rajat Das Gupta, Mostafa Dianatinasab, Zachary Dingels, M Ashworth Dirac, Matthew Doxey, Sophia Emmons-Bell, Firooz Esmaeilzadeh, Alize Ferrari, Lisa Force, Jack Fox, Natalie C Galles, William Gardner, Farhad Ghamari, Ahmad Ghashghaee, Taren Gorman, Harrison Gottlich, Beatrix Haddock, Lydia Haile, Erin Hamilton, Chieh Han, Hannah Han, Nathaniel Henry, Mowafa Househ, Kevin Ikuta, Spencer James, Catherine Johnson, Sarah Johnson, Nicholas J Kassebaum, Mahalaqua Nazli Khatib, Neda Kianipour, Adnan Kisa, Sezer Kisa, Jonathan Kocarnik, Pushpendra Kumar, Hmwe Kyu, Kathryn Lau, Jorge Ledesma, James Leigh, Haley Lescinsky, Zichen Liu, Emilie Maddison, Amir Manafi, Navid Manafi, Helena Manguerra, Borhan Mansouri, Ira Martopullo, Masoud Moghadaszadeh, Efat Mohamadi, Ali Mokdad, Emma Nichols, Rajan Nikbakhsh, Kanyin Ong, Mona Pathak, Alyssa Pennini, Reza Pourmirza Kalhori, Zahiruddin Quazi Syed, Chhabi Ranabhat, Christian Razo, Marissa Reitsma, Seyed Mohammad Riahi, Nicholas Roberts, Sam Rolfe, Enrico Rubagotti, Mohammad Salahshoor, Zainab Samad, Abdallah Samy, Damian Santomauro, Fablina Sharara, Maral Shayesteh Bonyan, Reed Sorensen, Luisa Sorio Flor, Jeffrey Stanaway, Benjamin Stark, Dillon Sylte, Christopher Troeger, Yasser Vasseghian, Ronny Westerman, Tissa Wijeratne, Lauren Wilner, Shadrach Wilson, Sarah Wulf Hanson, Han Yong Wunrow, Mousa Yaminfirooz, Jamal Yearwood, Seok-Jun Yoon, Theodore Younker, Peng Zheng, Jeff Zhao, Arash Ziapour, Mohsen Naghavi, and Christopher J L Murray.

Providing critical feedback on methods or results

Theo Vos, Cristiana Abbafati, Kaja Abbas, Mitra Abbasifard, Foad Abd-Allah, Ahmed Abdelalim, Ibrahim Abdollahpour, Kanaan Abdullah, Hassan Abolhassani, Victor Aboyans, Lucas Abreu, Michael Abrigo, Laith Abu-Raddad, Abdelrahman Abushouk, Ilana Ackerman, Abdu Adamu, Oladimeji Adebayo, Victor Adekanmbi, Jaimie Adelson, Olatunji Adetokunboh, Mahdi Afshari, Ashkan Afshin, Gina Agarwal, Kareha Agesa, Mohammad Aghaali, Anurag Agrawal, Tauseef Ahmad, Mehdi Ahmadi, Hamid Ahmadieh, Temesgen Akalu, Tomi Akinyemiju, Ziyad Al-Aly, Khurshid Alam, Noore Alam, Samiah Alam, Turki Alanzi, Jacqueline Alcalde-Rabanal, Niguse Alema, Muhammad Ali, Saqib Ali, Gianfranco Alicandro, Mehran Alijanzadeh, Vahid Alipour, Syed Aljunid, Amir Almasi-Hashiani, Rajaa Al-Raddadi, Khalid Altirkawi, Nelson Alvis-Guzman, Nelson Alvis-Zakzuk, Saeed Amini, Arianna Maever Amit, Dickson Amugsi, Gianna Gayle Amul, Deanna Anderlini, Tudorel Andrei, Fereshteh Ansari, Iman Ansari, Alireza Ansari-Moghaddam, Carl Abelardo Antonio, Davood Anvari, Razique Anwer, Jalal Arabloo, Morteza Arab-Zozani, Filippo Ariani, Johan Ärnlöv, Krishna Aryal, Afsaneh Arzani, Mehran Asadi-Aliabadi, Charlie Ashbaugh, Desta Atnafu, Sachin Atre, Floriane Ausloos, Marcel Ausloos, Beatriz Paulina Ayala Quintanilla, Getinet Ayano, Martin Ayanore, Samad Azari, Ghasem Azarian, Zelalem Azene, Ebrahim Babaee, Alaa Badawi, Mojtaba Bagherzadeh, Mohammad Hossein Bakhshaei, Ahad Bakhtiari, Senthilkumar Balakrishnan, Shivanthi Balalla, Maciej Banach, Palash Banik, Agegnehu Bante, Adhanom Baraki, Miguel Barboza, Suzanne Barker-Collo, Lingkan Barua, Sanjay Basu, Bernhard Baune, Vo Bay, Mohsen Bayati, Gholamreza Bazmandegan, Ettore Beghi, Aminu Bello, Rose Bender, Derrick Bennett, Isabela Bensenor, Catherine Benziger, Kidanemaryam Berhe, Eduardo Bernabe, Reshmi Bhageerathy, Dinesh Bhandari, Pankaj Bhardwaj, Krittika Bhattacharya, Zulfiqar Bhutta, Boris Bikbov, Antonio Biondi,

Binyam Birihane, Donal Bisanzio, Mahdi Bohluli, Srinivasa Rao Bolla, Archith Boloor, Guilherme Borges, Antonio Borzì, Rupert Bourne, Oliver Brady, Michael Brauer, Carol Brayne, Nicholas Breitborde, Hermann Brenner, Paul Briant, Andrew Briggs, Nikolay Briko, Gabrielle Britton, Dana Bryazka, Rachelle Buchbinder, Reinhard Busse, Zahid Butt, Florentino Luciano Caetano Dos Santos, Luis Cámera, Ismael Campos-Nonato, Rosario Cárdenas, Joao Mauricio Castaldelli-Maia, Carlos Castañeda-Orjuela, Franz Castro, Ferrán Catalá-López, Kate Causey, Christopher Cederroth, Ester Cerin, Joht Chandan, Vijay Kumar Chattu, Sarika Chaturvedi, Odgerel Chimed-Ochir, Ken Chin, Daniel Cho, Dinh-Toi Chu, Michael Chung, Flavia Cicuttini, Liliana Ciobanu, Massimo Cirillo, Kelly Compton, Paolo Cortesi, Vera Costa, Ewerton Cousin, Richard Cowden, Benjamin Cowie, Elizabeth Cromwell, Di Cross, Matthew Cunningham, Giovanni Damiani, Aso Darwesh, Ahmad Daryani, Jai Das, Rajat Das Gupta, José Das Neves, Claudio Dávila-Cervantes, Kairat Davletov, Diego De Leo, Frances Dean, Robert Dellavalle, Feleke Demeke, Desalegn Demsie, Nebiyu Dereje, Nikolaos Dervenis, Rupak Desai, Mostafa Dianatinasab, Daniel Diaz, Zahra Sadat Dibaji Forooshani, M Ashworth Dirac, Hoa Do, Klara Dokova, Fariba Dorostkar, Chirag Doshi, Leila Doshmangir, Abdel Douiri, Susanna Dunachie, Andre Duraes, Arielle Eagan, Mohammad Ebrahimi Kalan, David Edvardsson, Joshua Ehrlich, Nevine El Nahas, Iman El Sayed, Islam Elgendy, Hala Elhabashy, Iqbal Elyazar, Mohammad Hassan Emamian, Sophia Emmons-Bell, Holly Erskine, Babak Eshrati, Sharareh Eskandarieh, Saman Esmaeilnejad, Firooz Esmaeilzadeh, Alireza Esteghamati, Arash Etemadi, Mohammad Farahmand, Anwar Faraj, Mohammad Fareed, Carla Farinha, Andre Faro, Mithila Faruque, Farshad Farzadfar, Valery Feigin, Seyed-Mohammad Fereshtehnejad, Alize Ferrari, Manuela Ferreira, Irina Filip, Florian Fischer, James Fisher, Carsten Flohr, Nataliya Foigt, Morenike Folayan, Lisa Force, Carla Fornari, Masoud Foroutan, Marisa Freitas, Weijia Fu, Takeshi Fukumoto, Mohamed Gad, Natalie C Galles, Amiran Gamkrelidze, Alberto Garcia-Basteiro, Biniyam Geberemariyam, Ketema Gebremedhin, Maryam Ghadimi, Mansour Ghafourifard, Alireza Ghajar, Ahmad Ghashghaee, Hesam Ghiasvand, Nermin Ghith, Paramjit Gill, Giorgia Giussani, Srinivas Goli, Ricardo Gomez, Sameer Gopalani, Taren Gorman, Harrison Gottlich, Houman Goudarzi, Bárbara Goulart, Ayman Grada, Michal Grivna, Mohammed Gubari, Harish Gugnani, Yuming Guo, Rajeev Gupta, Juanita Haagsma, Beatrix Haddock, Nima Hafezi-Nejad, Lydia Haile, Brian Hall, Randah Hamadeh, Kanaan Hamagharib Abdullah, Chieh Han, Hannah Han, Josep Haro, Amir Hasanzadeh, Maryam Hashemian, Soheil Hassanipour, Hadi Hassankhani, Rasmus Havmoeller, Roderick Hay, Simon I Hay, Khezar Hayat, Behnam Heidari, Golnaz Heidari, Reza Heidari-Soureshjani, Nathaniel Henry, Claudiu Herteliu, Fatemeh Heydarpour, Thomas Hird, Ramesh Holla, Praveen Hoogar, H Dean Hosgood, Mehdi Hosseinzadeh, Mihaela Hostiuc, Mowafa Househ, Vivian Chia-Rong Hsieh, Guoqing Hu, Fernando Hugo, Bing-Fang Hwang, Segun Ibitoye, Kevin Ikuta, Olayinka Ilesanmi, Irena Ilic, Milena Ilic, Leeberk Inbaraj, Seyed Sina Irvani, M Mofizul Islam, Mdmohaimenul Islam, Sheikh Mohammed Shariful Islam, Farhad Islami, Rebecca Ivers, Chidozie Iwu, Ihoghosa Iyamu, Jalil Jaafari, Kathryn Jacobsen, Farhad Jadidi-Niaragh, Morteza Jafarinia, Nader Jahanmehr, Mihajlo Jakovljevic, Amir Jalali, Farzad Jalilian, Spencer James, Manthan Janodia, Panniyammakal Jeemon, Ensiyeh Jenabi, Ravi Jha, Vivekanand Jha, John Ji, Oommen John, Yetunde John-Akinola, Catherine Johnson, Sarah Johnson, Jost Jonas, Tamas Joo, Ankur Joshi, Jacek Jozwiak, Mikk Jürisson, Ali Kabir, Zubair Kabir, Rizwan Kalani, Leila Kalankesh, Rohollah Kalhor, Zahra Kamiab, Tanuj Kanchan, Behzad Karami Matin, André Karch, Mohd Karim, Salah Eddin Karimi, Getachew Kassa, Nicholas J Kassebaum, Srinivasa Katikireddi, Norito Kawakami, Gbenga Kayode, Konstantin Kazanjan, Ali Kazemi Karyani, Morteza Khafaie, Nauman Khalid, Maseer Khan, Khaled Khatab, Mona Khater, Mahalaqua Nazli Khatib, Maryam Khayamzadeh, Mohammad Taghi Khodayari, Roba Khundkar, Neda Kianipour, Christian Kieling, Daniel Kim, Ruth Kimokoti, Adnan Kisa, Sezer Kisa, Katarzyna Kissimova-Skarbek, Mika Kivimäki, Ann Kristin Skrindo Knudsen, Jonathan Kocarnik, Tufa Kolola, Jacek Kopec, Soewarta Kosen, Parvaiz Koul, Ai Koyanagi, Kewal Krishan, Kris Krohn, Burcu Kucuk Bicer, Manasi Kumar, Pushpendra Kumar, Vivek Kumar, Girikumar Kumaresh, Om Kurmi, Dian Kusuma, Hmwe Kyu, Carlo La Vecchia, Dharmesh Lal, Ratilal Lalloo, Faris Lami, Justin Lang, Anders Larsson, Savita Lasrado, Zohra Lassi, Kathryn Lau, Pablo

Lavados, Jorge Ledesma, Paul Lee, Kate Legrand, James Leigh, Matilde Leonardi, Janni Leung, Miriam Levi, Shanshan Li, Lee-Ling Lim, Ro-Ting Lin, Christine Linehan, Shai Linn, Shiwei Liu, Alan D Lopez, Platon Lopukhov, Stefan Lorkowski, Paulo Lotufo, Jennifer Maclachlan, Emilie Maddison, Ralph Maddison, Fabiana Madotto, Phetole Mahasha, Hue Mai, Azeem Majeed, Venkatesh Maled, Shokofeh Maleki, Reza Malekzadeh, Deborah Malta, Abdullah Mamun, Amir Manafi, Navid Manafi, Helena Manguerra, Borhan Mansouri, Mohammad Ali Mansournia, Ana Maria Mantilla Herrera, Joemer Maravilla, Francisco Martins-Melo, João Massano, Benjamin Massenburg, Manu Mathur, Pallab Maulik, Colm Mcalinden, Martin Mckee, Kala Mehta, Wahengbam Bigyananda Meitei, Peter Memiah, Walter Mendoza, Ritesh Menezes, Endalkachew Mengesha, Meresa Mengesha, Atte Meretoja, Tuomo Meretoja, Tomasz Miazgowski, Irmina Maria Michalek, Kebadnew Mihretie, Ted Miller, Edward Mills, Andreea Mirica, Erkin Mirrakhimov, Hamed Mirzaei, Maryam Mirzaei, Mehdi Mirzaei-Alavijeh, Prasanna Mithra, Babak Moazen, Masoud Moghadaszadeh, Dara Mohammad, Yousef Mohammad, Naser Mohammad Gholi Mezerji, Abdollah Mohammadian-Hafshejani, Noushin Mohammadifard, Reza Mohammadpourhodki, Shafiu Mohammed, Ali Mokdad, Natalie Momen, Lorenzo Monasta, Stefania Mondello, Mahmood Moosazadeh, Ghobad Moradi, Maziar Moradi-Lakeh, Rahmatollah Moradzadeh, Linda Morales, Lidia Morawska, Joana Morgado-Da-Costa, Jonathan Mosser, Simin Mouodi, Amin Mousavi Khaneghah, Ulrich Mueller, Moses Muriithi, Kamarul Imran Musa, Saravanan Muthupandian, Mehdi Naderi, Ahamarshan Nagarajan, Gabriele Nagel, Behshad Naghshtabrizi, Sanjeev Nair, Vinay Nangia, Jobert Richie Nansseu, Vinod Nayak, Javad Nazari, Ionut Negoi, Ruxandra Irina Negoi, Henok Netsere, Josephine Ngunjiri, Cuong Nguyen, Emma Nichols, Dabere Nigatu, Yeshambel Nigatu, Rajan Nikbakhsh, Molly R Nixon, Chukwudi Nnaji, Shuhei Nomura, Bo Norrving, Jean Jacques Noubiap, Christoph Nowak, Bogdan Oancea, Felix Ogbo, In-Hwan Oh, Emmanuel Okunga, Andrew Olagunju, Bolajoko Olusanya, Jacob Olusanya, Mojisola Oluwasanu, Muktar Omer, Kanyin Ong, Obinna Onwujekwe, Heather Orpana, Alberto Ortiz, Adrian Oțoiu, Nikita Otstavnov, Stanislav Otstavnov, Simon Øverland, Mayowa Owolabi, Mahesh P A, Jagadish Rao Padubidri, Abhijit Pakhare, Raffaele Palladino, Adrian Pana, Songhomitra Panda-Jonas, Eun-Kee Park, Priyakumari Parmar, Sangram Patel, Angel Paternina-Caicedo, Ashish Pathak, Mona Pathak, Scott Patten, George Patton, Deepak Paudel, Amy Peden, Veincent Christian Pepito, Emmanuel Peprah, Alexandre Pereira, Michael Phillips, David Pigott, Meghdad Pirsaheb, Oleguer Plana-Ripoll, Dietrich Plass, Khem Pokhrel, Roman Polibin, Kevan Polkinghorne, Maarten Postma, Hadi Pourjafar, Farshad Pourmalek, Reza Pourmirza Kalhori, Akram Pourshams, Sergio Prada, V Prakash, Elisabetta Pupillo, Hai Quang Pham, Zahiruddin Quazi Syed, Mohammad Rabiee, Navid Rabiee, Amir Radfar, Ata Rafiee, Alireza Rafiei, Alberto Raggi, Muhammad Aziz Rahman, Ali Rajabpour-Sanati, Fatemeh Rajati, Chhabi Ranabhat, Sowmya J Rao, Prateek Rastogi, Priya Rathi, David Laith Rawaf, Salman Rawaf, Lal Rawal, Christian Razo, Marissa Reitsma, Vishnu Renjith, Andre Renzaho, Serge Resnikoff, Nima Rezaei, Mohammad Sadegh Rezai, Aziz Rezapour, Seyed Mohammad Riahi, Antonio Luiz Ribeiro, Daniel Ribeiro, Daniela Ribeiro, Jennifer Rickard, Nicholas Roberts, Stephen Robinson, Leonardo Roever, Luca Ronfani, Gholamreza Roshandel, Enrico Rubagotti, Siamak Sabour, Perminder Sachdev, Basema Saddik, Ehsan Sadeghi, Shahram Saeidi, Saeid Safiri, Rajesh Sagar, Mohammad Ali Sahraian, Not Available Saifullah, S Mohammad Sajadi, Mohammad Salahshoor, Payman Salamati, Hosni Salem, Marwa Salem, Hamideh Salimzadeh, Inbal Salz, Zainab Samad, Abdallah Samy, Juan Sanabria, Damian Santomauro, Itamar Santos, João Santos, Milena Santric-Milicevic, Sivan Saraswathy, Rodrigo Sarmiento-Suárez, Nizal Sarrafzadegan, Benn Sartorius, Arash Sarveazad, Brijesh Sathian, Thirunavukkarasu Sathish, Davide Sattin, Alyssa Sbarra, Lauren Schaeffer, Silvia Schiavolin, Aletta Schutte, David Schwebel, Falk Schwendicke, Anbissa Senbeta, Subramanian Senthilkumaran, Sadaf Sepanlou, Saeed Shahabi, Amira Shaheen, Masood Shaikh, Ali Shalash, Mehran Shams-Beyranvand, Morteza Shamsizadeh, Mohammed Shannawaz, Kiomars Sharafi, Maral Shayesteh Bonyan, Abbas Sheikhtaheri, Kenji Shibuya, Wondimeneh Shiferaw, Mika Shigematsu, Jae Il Shin, Rahman Shiri, Reza Shirkoohi, Mark Shrime, Kerem Shuval, Soraya Siabani, Kyle Simpson, Ambrish Singh, Jasvinder Singh, Eirini Skiadaresi, Søren Skou, Valentin

Skryabin, Eug.Ne Sobngwi, Shahin Soltani, Reed Sorensen, Joan Soriano, Luisa Sorio Flor, Muluken Sorrie, Ireneous Soyiri, Chandrashekhar Sreeramareddy, Jeffrey Stanaway, Benjamin Stark, Simona Cătălina Ștefan, Mark Stokes, Jacob Stubbs, Agus Sudaryanto, Mu'awiyyah Sufiyan, Gerhard Sulo, Iyad Sultan, Bryan Sykes, Dillon Sylte, Miklós Szócska, Rafael Tabarés-Seisdedos, Karen Tabb, Santosh Tadakamadla, Masih Tajdini, Cuong Tat Nguyen, Nuno Taveira, Arash Tehrani-Banihashemi, Berhane Teklehaimanot, Zemenu Tessema, Kavumpurathu Thankappan, Hoa Thi Do, Hamid Reza Tohidinik, Marcello Tonelli, Mathilde Touvier, Marcos Roberto Tovani-Palone, Bach Tran, Ravensara S Travillian, Christopher Troeger, Thomas Truelsen, Alexander Tsai, Aristidis Tsatsakis, Lorainne Tudor Car, Stefanos Tyrovolas, Riaz Uddin, Bhaskaran Unnikrishnan, Marco Vacante, Alireza Vakilian, Pascual Valdez, Santosh Varughese, Yasser Vasseghian, Narayanaswamy Venketasubramanian, Francesco Violante, Stein Emil Vollset, Ana Vukovic, Rade Vukovic, Yasir Waheed, Yafeng Wang, Yuan-Pang Wang, Joseph Ward, Jingkai Wei, Robert Weintraub, Jordan Weiss, Ronny Westerman, Harvey Whiteford, Taweewat Wiangkham, Kirsten Wiens, Tissa Wijeratne, Shadrach Wilson, Bogdan Wojtyniak, Ai-Min Wu, Sarah Wulf Hanson, Han Yong Wunrow, Gelin Xu, Mousa Yaminfirooz, Yuichiro Yano, Sanni Yaya, Vahid Yazdi-Feyzabadi, Yordanos Yeshitila, Paul Yip, Naohiro Yonemoto, Seok-Jun Yoon, Javad Yoosefi Lebni, Mustafa Younis, Taraneh Yousefinezhadi, Abdilahi Yousuf, Chuanhua Yu, Hasan Yusefzadeh, Telma Zahirian Moghadam, Leila Zaki, Sojib Bin Zaman, Maryam Zamanian, Hamed Zandian, Alireza Zangeneh, Mikhail Zastrozhin, Kaleab Zewdie, Yunquan Zhang, Jeff Zhao, Yingxi Zhao, Maigeng Zhou, Arash Ziapour, Mohsen Naghavi, and Christopher J L Murray.

Drafting the work or revising is critically for important intellectual content

Theo Vos, Cristiana Abbafati, Kaja Abbas, Mohsen Abbasi-Kangevari, Foad Abd-Allah, Ahmed Abdelalim, Kanaan Abdullah, Hassan Abolhassani, Elissa Abrams, Lucas Abreu, Abdelrahman Abushouk, Abdu Adamu, Oladimeji Adebayo, Victor Adekanmbi, Jaimie Adelson, Olatunji Adetokunboh, Davoud Adham, Ashkan Afshin, Gina Agarwal, Mohammad Aghaali, Seyed Mohammad Kazem Aghamir, Temesgen Akalu, Rufus Akinyemi, Tomi Akinyemiju, Blessing Akombi, Khurshid Alam, Noore Alam, Samiah Alam, Jacqueline Alcalde-Rabanal, Niguse Alema, Muhammad Ali, Saqib Ali, Gianfranco Alicandro, Cyrus Alinia, Peter Allebeck, Amir Almasi-Hashiani, Jordi Alonso, Saeed Amini, Mostafa Amini-Rarani, Arya Aminorroaya, Dickson Amugsi, Deanna Anderlini, Mina Anjomshoa, Iman Ansari, Carl Abelardo Antonio, Ernoiz Antriyandarti, Jalal Arabloo, Morteza Arab-Zozani, Johan Ärnlöv, Ali Asadi-Pooya, Babak Asghari, Desta Atnafu, Floriane Ausloos, Marcel Ausloos, Beatriz Paulina Ayala Quintanilla, Martin Ayanore, Yared Aynalem, Alaa Badawi, Mojtaba Bagherzadeh, Mohammad Hossein Bakhshaei, Senthilkumar Balakrishnan, Shivanthi Balalla, Maciej Banach, Suzanne Barker-Collo, Lingkan Barua, Sanjay Basu, Bernhard Baune, Vo Bay, Neeraj Bedi, Yannick Béjot, Aminu Bello, Derrick Bennett, Fiona Bennitt, Isabela Bensenor, Catherine Benziger, Kidanemaryam Berhe, Reshmi Bhageerathy, Dinesh Bhandari, Krittika Bhattacharya, Muhammad Shahdaat Bin Sayeed, Antonio Biondi, Catherine Bisignano, Raaj Kishore Biswas, Srinivasa Rao Bolla, Guilherme Borges, Antonio Borzì, Rupert Bourne, Oliver Brady, Nicholas Breitborde, Hermann Brenner, Andrew Briggs, Nikolay Briko, Gabrielle Britton, Dana Bryazka, Zahid Butt, Florentino Luciano Caetano Dos Santos, Ismael Campos-Nonato, Josip Car, Giulia Carreras, Juan Carrero, Felix Carvalho, Joao Mauricio Castaldelli-Maia, Giulio Castelpietra, Franz Castro, Ferrán Catalá-López, Christopher Cederroth, Ester Cerin, Joht Chandan, Alex Chang, Vijay Kumar Chattu, Sarika Chaturvedi, Ken Chin, Flavia Cicuttini, Liliana Ciobanu, Massimo Cirillo, Sara Conti, Paolo Cortesi, Vera Costa, Ewerton Cousin, Benjamin Cowie, Di Cross, Christopher Crowe, Giovanni Damiani, José Das Neves, Claudio Dávila-Cervantes, Frances Dean, Feleke Demeke, Edgar Denova-Gutiérrez, Nikolaos Dervenis, Rupak Desai, Assefa Desalew, Samath Dharmaratne, Govinda Dhungana, Mostafa Dianatinasab, Daniel Diaz, Zahra Sadat Dibaji Forooshani, M Ashworth Dirac, Hoa Do, Klara Dokova, Leila Doshmangir, Abdel Douiri, Bruce Duncan, Arielle Eagan, David Edvardsson, Joshua Ehrlich, Iman El

Sayed, Maha El Tantawi, Iffat Elbarazi, Islam Elgendy, Hala Elhabashy, Shaimaa El-Jaafary, Mohammad Hassan Emamian, Sharareh Eskandarieh, Saman Esmaeilnejad, Firooz Esmaeilzadeh, Alireza Esteghamati, Arash Etemadi, Roghiyeh Faridnia, Andrea Farioli, Andre Faro, Mithila Faruque, Mehdi Fazlzadeh, Valery Feigin, Seyed-Mohammad Fereshtehnejad, Eduarda Fernandes, Alize Ferrari, Manuela Ferreira, Irina Filip, Florian Fischer, James Fisher, Carsten Flohr, Nataliya Foigt, Morenike Folayan, Carla Fornari, Masoud Foroutan, Marisa Freitas, Weijia Fu, Takeshi Fukumoto, João Furtado, Mohamed Gad, Silvano Gallus, Amiran Gamkrelidze, Alberto Garcia-Basteiro, Biniyam Geberemariyam, Assefa Ayalew Gebreslassie, Maryam Ghadimi, Farhad Ghamari, Ahmad Ghashghaee, Nermin Ghith, Paramjit Gill, Mojgan Gitimoghaddam, Sameer Gopalani, Giuseppe Gorini, Harrison Gottlich, Alessandra Goulart, Bárbara Goulart, Ayman Grada, Michal Grivna, Giuseppe Grosso, Rafael Guimarães, Rajeev Gupta, Beatrix Haddock, Nima Hafezi-Nejad, Abdul Hafiz, Lydia Haile, Brian Hall, Iman Halvaei, Kanaan Hamagharib Abdullah, Erin Hamilton, Hannah Han, Graeme Hankey, Josep Haro, Ahmed Hasaballah, Amir Hasanzadeh, Rasmus Havmoeller, Roderick Hay, Simon I Hay, Khezar Hayat, Golnaz Heidari, Claudiu Herteliu, Thomas Hird, Michael Hole, Ramesh Holla, Praveen Hoogar, Mihaela Hostiuc, Sorin Hostiuc, Mowafa Househ, Guoqing Hu, Fernando Hugo, Segun Ibitoye, Olayinka Ilesanmi, Irena Ilic, Milena Ilic, Helen Ippolito, Seyed Sina Irvani, Sheikh Mohammed Shariful Islam, Farhad Islami, Hiroyasu Iso, Rebecca Ivers, Chidozie Iwu, Ihoghosa Iyamu, Kathryn Jacobsen, Morteza Jafarinia, Mihajlo Jakovljevic, Manthan Janodia, Achala Jayatilleke, Panniyammakal Jeemon, Ensiyeh Jenabi, Ravi Jha, Vivekanand Jha, Catherine Johnson, Jost Jonas, Jacek Jozwiak, Mikk Jürisson, Ali Kabir, Hamed Kalani, André Karch, Mohd Karim, Nicholas J Kassebaum, Srinivasa Katikireddi, Gbenga Kayode, Konstantin Kazanjan, Maseer Khan, Khaled Khatab, Mona Khater, Mahalaqua Nazli Khatib, Roba Khundkar, Christian Kieling, Daniel Kim, Yun Jin Kim, Adnan Kisa, Sezer Kisa, Mika Kivimäki, Cameron Kneib, Ann Kristin Skrindo Knudsen, Jonathan Kocarnik, Tufa Kolola, Parvaiz Koul, Ai Koyanagi, Michael Kravchenko, Kewal Krishan, Kris Krohn, Pushpendra Kumar, Vivek Kumar, Om Kurmi, Dian Kusuma, Hmwe Kyu, Carlo La Vecchia, Ben Lacey, Ratilal Lalloo, Jennifer Lam, Iván Landires, Justin Lang, Anders Larsson, Kathryn Lau, Pablo Lavados, Jeffrey Lazarus, Jorge Ledesma, Shaun Lee, Kate Legrand, James Leigh, Matilde Leonardi, Haley Lescinsky, Janni Leung, Miriam Levi, Sarah Lewington, Lee-Ling Lim, Zichen Liu, Platon Lopukhov, Stefan Lorkowski, Paulo Lotufo, Alessandra Lugo, Jennifer Maclachlan, Hue Mai, Azeem Majeed, Venkatesh Maled, Reza Malekzadeh, Deborah Malta, Abdullah Mamun, Amir Manafi, Navid Manafi, Borhan Mansouri, Mohammad Ali Mansournia, Ana Maria Mantilla Herrera, Francisco Martins-Melo, Seyedeh Zahra Masoumi, João Massano, Benjamin Massenburg, Pallab Maulik, Colm Mcalinden, John Mcgrath, Wahengbam Bigyananda Meitei, Walter Mendoza, Ritesh Menezes, Endalkachew Mengesha, Atte Meretoja, Tuomo Meretoja, Tomislav Mestrovic, Tomasz Miazgowski, Irmina Maria Michalek, Ted Miller, Edward Mills, Prasanna Mithra, Babak Moazen, Masoud Moghadaszadeh, Dara Mohammad, Yousef Mohammad, Abdollah Mohammadian-Hafshejani, Reza Mohammadpourhodki, Shafiu Mohammed, Ali Mokdad, Natalie Momen, Lorenzo Monasta, Stefania Mondello, Maziar Moradi-Lakeh, Paula Moraga, Ilais Moreno Velásquez, Joana Morgado-Da-Costa, Shane Morrison, Jonathan Mosser, Seyyed Meysam Mousavi, Amin Mousavi Khaneghah, Ulrich Mueller, Sandra Munro, Kamarul Imran Musa, Saravanan Muthupandian, Ahamarshan Nagarajan, Gabriele Nagel, Behshad Naghshtabrizi, Sanjeev Nair, Vinay Nangia, Jobert Richie Nansseu, Vinod Nayak, Javad Nazari, Ionut Negoi, Ruxandra Irina Negoi, Josephine Ngunjiri, Cuong Nguyen, Dabere Nigatu, Yeshambel Nigatu, Rajan Nikbakhsh, Molly R Nixon, Bo Norrving, Jean Jacques Noubiap, Christoph Nowak, Virginia Nunez-Samudio, Bogdan Oancea, Felix Ogbo, In-Hwan Oh, Morteza Oladnabi, Andrew Olagunju, Bolajoko Olusanya, Jacob Olusanya, Mojisola Oluwasanu, Muktar Omer, Kanyin Ong, Obinna Onwujekwe, Heather Orpana, Alberto Ortiz, Samuel Ostroff, Adrian Oțoiu, Nikita Otstavnov, Stanislav Otstavnov, Simon Øverland, Mayowa Owolabi, Mahesh P A, Jagadish Rao Padubidri, Raffaele Palladino, Adrian Pana, Songhomitra Panda-Jonas, Ashish Pathak, Mona Pathak, Hamidreza Pazoki Toroudi, Amy Peden, Veincent Christian Pepito, Emmanuel Peprah, Alexandre Pereira, David Pereira, Norberto Perico, Thomas Pilgrim, Oleguer Plana-Ripoll, Dietrich Plass,

Roman Polibin, Suzanne Polinder, Maarten Postma, Farshad Pourmalek, Sergio Prada, Hai Quang Pham, Zahiruddin Quazi Syed, Mohammad Rabiee, Navid Rabiee, Amir Radfar, Alberto Raggi, Muhammad Aziz Rahman, Ali Rajabpour-Sanati, Fatemeh Rajati, Chhabi Ranabhat, Sowmya J Rao, Davide Rasella, Prateek Rastogi, David Laith Rawaf, Salman Rawaf, Lal Rawal, Christian Razo, Marissa Reitsma, Vishnu Renjith, Andre Renzaho, Nima Rezaei, Seyed Mohammad Riahi, Antonio Luiz Ribeiro, Daniel Ribeiro, Daniela Ribeiro, Jennifer Rickard, Nicholas Roberts, Shaun Roberts, Leonardo Roever, Luca Ronfani, Gholamreza Roshandel, Enrico Rubagotti, Siamak Sabour, Perminder Sachdev, Basema Saddik, Masoumeh Sadeghi, Saeid Safiri, Rajesh Sagar, Amirhossein Sahebkar, Mohammad Ali Sahraian, Farkhonde Salehi, Hosni Salem, Marwa Salem, Hamideh Salimzadeh, Inbal Salz, Zainab Samad, Abdallah Samy, Juan Sanabria, Damian Santomauro, Itamar Santos, João Santos, Milena Santric-Milicevic, Rodrigo Sarmiento-Suárez, Arash Sarveazad, Davide Sattin, Lauren Schaeffer, Silvia Schiavolin, Maria Schmidt, Aletta Schutte, David Schwebel, Falk Schwendicke, Sadaf Sepanlou, Saeed Shahabi, Ali Shalash, Mehran Shams-Beyranvand, Fablina Sharara, Maral Shayesteh Bonyan, Ranjitha Shetty, Kenji Shibuya, Wondimeneh Shiferaw, Mika Shigematsu, Reza Shirkoohi, Mark Shrime, Kerem Shuval, Inga Sigfusdottir, Inga Dora Sigfusdottir, João Silva, Kyle Simpson, Jasvinder Singh, Eirini Skiadaresi, Søren Skou, Anton Sokhan, Joan Soriano, Muluken Sorrie, Ireneous Soyiri, Chandrashekhar Sreeramareddy, Jeffrey Stanaway, Benjamin Stark, Simona Cătălina Ştefan, Timothy Steiner, Mark Stokes, Lars Stovner, Jacob Stubbs, Mu'awiyyah Sufiyan, Iyad Sultan, Bryan Sykes, Rafael Tabarés-Seisdedos, Karen Tabb, Santosh Tadakamadla, Cuong Tat Nguyen, Nuno Taveira, Hirut Teame, Hoa Thi Do, Hamid Reza Tohidinik, Marcello Tonelli, Roman Topor-Madry, Anna Torre, Mathilde Touvier, Marcos Roberto Tovani-Palone, Bach Tran, Ravensara S Travillian, Thomas Truelsen, Alexander Tsai, Aristidis Tsatsakis, Stefanos Tyrovolas, Riaz Uddin, Eduardo Undurraga, Bhaskaran Unnikrishnan, Marco Vacante, Sahel Valadan Tahbaz, Tommi Vasankari, Yasser Vasseghian, Narayanaswamy Venketasubramanian, Francesco Violante, Vasily Vlassov, Stein Emil Vollset, Ana Vukovic, Rade Vukovic, Yasir Waheed, Yuan-Pang Wang, Joseph Ward, Robert Weintraub, Ronny Westerman, Harvey Whiteford, Taweewat Wiangkham, Kirsten Wiens, Tissa Wijeratne, Eve Wool, Ai-Min Wu, Han Yong Wunrow, Seyed Hossein Yahyazadeh Jabbari, Kazumasa Yamagishi, Mousa Yaminfirooz, Sanni Yaya, Vahid Yazdi-Feyzabadi, Tomas Yeheyis, Yordanos Yeshitila, Seok-Jun Yoon, Zabihollah Yousefi, Mahmoud Yousefifard, Sojib Bin Zaman, Mohammad Zamani, Maryam Zamanian, Mikhail Zastrozhin, Zhi-Jiang Zhang, Yingxi Zhao, Mohsen Naghavi, and Christopher J L Murray.

Extracting, cleaning, or cataloging data; designing or coding figures and tables

Alyssa Acebedo, Jaimie Adelson, Olatunji Adetokunboh, Kareha Agesa, Sam Albertson, Fatemeh Amiri, Rose Bender, Greg Bertolacci, Alexandra Boon-Dooley, Paul Briant, Dana Bryazka, Chris Castle, Franz Castro, Kate Causey, Jessica Cruz, Matthew Cunningham, Giovanni Damiani, Ahmad Daryani, Nicole DeCleene, Zachary Dingels, M Ashworth Dirac, Matthew Doxey, Iqbal Elyazar, Sophia Emmons-Bell, Holly Erskine, Saman Esmaeilnejad, Firooz Esmaeilzadeh, Rachel Feldman, Alize Ferrari, Jack Fox, Takeshi Fukumoto, Natalie C Galles, William Gardner, Anna Gershberg Hayoon, Taren Gorman, Harrison Gottlich, Gaorui Guo, Beatrix Haddock, Lydia Haile, Chieh Han, Hannah Han, James Harvey, Hannah Henrikson, Fernando Hugo, Vincent Iannucci, Kevin Ikuta, Helen Ippolito, Morteza Jafarinia, Spencer James, Sarah Johnson, Nicholas J Kassebaum, Jonathan Kocarnik, Pushpendra Kumar, Samantha Larson, Kathryn Lau, Jorge Ledesma, Haley Lescinsky, Janni Leung, Christine Lin, Zichen Liu, Jianing Ma, Emilie Maddison, Amir Manafi, Navid Manafi, Helena Manguerra, Borhan Mansouri, Ana Maria Mantilla Herrera, Ira Martopullo, Fereshteh Mehri, Masoud Moghadaszadeh, Ali Mokdad, Javad Nazari, Jason Nguyen, Emma Nichols, Kanyin Ong, Alyssa Pennini, David Pigott, Zahiruddin Quazi Syed, Christian Razo, Nickolas Reinig, Marissa Reitsma, Nima Rezaei, Seyed Mohammad Riahi, Shaun Roberts, Sam Rolfe, Enrico Rubagotti, Hosni Salem, Zainab Samad, Abdallah Samy, Damian Santomauro, Fablina Sharara, Kyle Simpson, Ambrish Singh, Luisa Sorio FLor, Jeffrey Stanaway, Benjamin Stark, Dillon Sylte, Whitney Teagle, Azalea

Thomson, Anna Torre, Christopher Troeger, Yasser Vasseghian, Avina Vongpradith, Alexandrea Watson, Joanna Whisnant, Lauren Wilner, Shadrach Wilson, Sarah Wulf Hanson, Han Yong Wunrow, Mousa Yaminfirooz, Seok-Jun Yoon, Theodore Younker, Mikhail Zastrozhin, Jeff Zhao, Stephanie R M Zimsen, Mohsen Naghavi, and Christopher J L Murray.

Managing the overall research enterprise

Theo Vos, Ashkan Afshin, Peter Allebeck, Charlie Ashbaugh, Celine Barthelemy, Michael Brauer, Kelly Compton, Elizabeth Cromwell, Amanda Deen, M Ashworth Dirac, Kara Estep, Alize Ferrari, Erin Hamilton, Spencer James, Nicholas J Kassebaum, Alan D Lopez, Deborah Malta, Ashley Marks, Ali Mokdad, Molly R Nixon, Christopher Odell, Heather Orpana, Simon Øverland, George Patton, Zainab Samad, Benn Sartorius, Roman Topor-Madry, Stein Emil Vollset, Harvey Whiteford, Eve Wool, Mohsen Naghavi, and Christopher J L Murray.

Table of Contents

Authors' Contributions 3

Managing the estimation process 3

Writing the first draft of the manuscript 3

Providing data or critical feedback on data sources 3

Developing methods or computational machinery 5

Providing critical feedback on methods or results 5

Drafting the work or revising is critically for important intellectual content 8

Extracting, cleaning, or cataloging data; designing or coding figures and tables 10

Managing the overall research enterprise 11

List of appendix figures and tables 14

Appendix figures 14

Appendix tables 14

Section 1: GBD overview 16

Section 1.1 Geographic locations of the analysis 16

Section 1.2: Time period of the analysis 16

Section 1.3: GBD cause list 16

Section 1.4: Statement of GATHER compliance 17

Abbreviations 17

Section 1.5 GBD results overview[1,3] 19

Section 1.6 Data input sources overview[1] 19

Section 1.7 Funding sources 20

Section 2: GBD 2019 Causes of Death database 20

Background 20

Section 2.1: CoD data identification[1] 20

Section 2.2: Verbal autopsy[1] 22

Section 2.3: Standardise input data (step 1)[1] 24

Section 2.4: Map to GBD cause list (step 2)[1] 26

Section 2.5: Age-sex splitting (step 3)[1] 27

Section 2.6: Correction for miscoding of Alzheimer's and other dementias, Parkinson's disease, and atrial fibrillation and flutter (step 4)[1] 29

Section 2.7: Redistribute (Step 5)[1] 31

Section 2.8: HIV/AIDS misclassification correction (step 6)[1] 37

Section 2.9: Scale strata to province (step 7)[1] 38

Section 2.10: Restrictions post-redistribution (step 8)[1] ... 38
Section 2.11: Drop VR country years or mark as non-representative (step 9)[1] ... 39
Section 2.12: Cause aggregation (step 10)[1] ... 39
Section 2.13: Remove shocks and HIV/AIDS maternal adjustments (step 11)[1] ... 39
Section 2.14: Noise reduction (step 12)[1] ... 44
Section 2.15: Cause of death database and outlier identification (step 13)[1] ... 45
Section 2.16: Causes of death data star rating calculation[1] ... 45
Section 3: Causes of death modelling methods ... 48
Section 3.1: CODEm[1] ... 48
Section 3.2: Causes modelled outside of CODEm[1] ... 51
Section 3.3: Central computation[1] ... 55
Section 3.4: CoD cause-specific modelling descriptions ... 57
Section 4:Non-fatal outcome estimation[2] ... 435
Section 4.1:Data sources, identification, and extraction[2] ... 435
Section 4.2:Input data and methods summary[2] ... 436
Section 4.3:Modelling strategy[2] ... 437
Section 4.4:Data adjustment ... 450
Section 4.5:DisMod-MR 2.1 estimation[2] ... 459
Section 4.6:Impairment and underlying cause estimation[2] ... 468
Section 4.7:Severity distribution[2] ... 469
Section 4.8:Disability weights[2] ... 471
Section 4.9:Comorbidity correction (COMO)[2] ... 474
Section 4.10: YLD computation, uncertainty, and residual YLDs[2] ... 476
Section 4.11: Birth prevalence[2] ... 477
Section 4.12: Non-fatal cause-specific modelling descriptions ... 478
Section 5: Estimation process for DALYs[3] ... 1431
Section 5.1: Computing DALYs ... 1431
Section 6: SDI analysis[3] ... 1431
Section 6.1: SDI definition ... 1431
Section 6.2: Development of revised SDI indicator ... 1431
Section 7: References ... 1434
Section 8: Figures and tables ... 1439

List of appendix figures and tables

Appendix figures

Figure S1. Analytical flowchart for the development of the GBD 2019 cause of death database (A) and different strategies used to model different causes (B) and ultimately combine them into a consistent set of cause-specific deaths for each location, age, sex, and year 1439

Figure S2. GBD 2019 Causes of death estimation flowchart by modelling group 1440

Figure S3. Vital registration and verbal autopsy data availability by country, 1980–2018 1441

Figure S4. Percent of vital registration deaths assigned to major garbage codes for all ages and sexes by country, 1980–2018 1442

Figure S5A. Classification of national time series of vital registration and verbal autopsy data 1980–2018 1444

Figure S5B. Classification of national time series of vital registration and verbal autopsy data 2010–2018 1445

Figure S6. Out-of-sample model performance for CODEm models for GBD 2019 and age-standardised cause-specific mortality rate by Level 2 causes 1446

Appendix tables

Table S1. GATHER checklist of information that should be included in reports of global health estimates, with description of compliance and location of information for GBD 2019 1447

Table S2. GBD 2019 cause hierarchy 1450

Table S3. GBD 2019 location hierarchy 1459

Table S4. Total number of site years by cause and source type for 2019 1474

Table S5. List of International Classification of Diseases (ICD) codes mapped to the Global Burden of Disease cause list for causes of death 1476

Table S6. Restrictions on age and sex by cause for GBD 2019 1484

Table S7. Data quality rating from 0 to 5 stars, maximum percent well certified per 5-year interval and percent well certified across time series for 204 countries, 1980-2019. 1493

Table S8. HIV/AIDS-related garbage code redistribution packages 1495

Table S9. Underlying indicators for percent well-certified for data source with maximum percent well certified in each 5-year time interval for 204 countries, 1980-2019 1496

Table S10. CodCorrect cause hierarchy with levels 1505

Table S11. Modelling strategy for individual cause of death models in GBD 2019 1511

Table S12. Percent change before and after CoDCorrect by cause for all ages, both sexes, global, 2019 1519

Table S13. GBD 2019 sequelae, health states, health state lay descriptions, and disability weights 1528

Table S14. GBD 2019 methods of estimating years lived with disability (YLDs) for 34 residual categories 1566

Table S15. List of GBD 2019 non-fatal causes with prevalence at birth 1569

Table S16. CODEm covariates used, level of covariate, and expected direction of covariate by cause, sex, and age 1570

Table S17. CODEm predictive validity results by cause, model type, sex, and age 1757

Table S18. Comparison of GBD 2017 and GBD 2019 covariates and level of covariates used in cause of death modelling 1767

Table S19. Socio-demographic Index R-squared values with lags up to 10 years 1812

Section 1: GBD overview

Section 1.1 Geographic locations of the analysis

We produced estimates for 204 countries and territories that were grouped into 21 regions and seven super-regions (section 8, table S3). The seven super-regions are central Europe, eastern Europe, and central Asia; high income; Latin America and the Caribbean; north Africa and the Middle East; south Asia; southeast Asia, east Asia, and Oceania; and sub-Saharan Africa. For GBD 2019, nine countries and territories (Cook Islands, Monaco, San Marino, Nauru, Niue, Palau, Saint Kitts and Nevis, Tokelau, and Tuvalu) were added, such that the GBD location hierarchy now includes all WHO member states. This round, GBD includes subnational analyses for several new countries and continues to analyse at subnational levels countries that were added in previous cycles. Subnational estimation in GBD 2019 includes five new countries (Italy, Nigeria, Pakistan, the Philippines, and Poland) and 16 countries previously estimated at subnational levels (Brazil, China, Ethiopia, India, Indonesia, Iran, Japan, Kenya, Mexico, New Zealand, Norway, Russia, South Africa, Sweden, the UK, and the USA). All analyses are at the first level of administrative organisation within each country except for New Zealand (by Māori ethnicity), Sweden (by Stockholm and non-Stockholm), the UK (by local government authorities), and the Philippines (by provinces). All subnational estimates for these countries were incorporated into model development and evaluation as part of GBD 2017. To meet data use requirements, in this publication we present subnational estimates for Brazil, India, Indonesia, Japan, Kenya, Mexico, Sweden, the UK, and the USA); given space constraints, these results are presented in appendix 2 instead of the main text. Subnational estimates for China are included in maps but are not reported in appendix tables. Subnational estimates for other countries will be released in separate publications.

For GBD 2019, we have also defined locations as standard locations and non-standard locations. Standard GBD locations are defined as the set of all subnationals belonging to countries where data quality is high and with populations over 200 million, in addition to all other countries. Standard locations include the subnationals for China, India, the USA, and Brazil, but not Indonesia; data for China, India, the USA, and Brazil are also included at the country level. All other countries with subnational estimates are defined as non-standard locations.

Section 1.2: Time period of the analysis

We estimated numbers and rates of incidence, prevalence, years lived with disability (YLDs), and disability-adjusted life-years (DALYs) for the years 1990–2019; we estimated deaths and years of life lost (YLLs) for 1980–2019.

Section 1.3: GBD cause list

The GBD cause and sequelae list is organized hierarchically (see table S2) to accommodate different purposes and needs of various users.

The first two levels aggregate causes into general groupings. At Level 1 there are three cause groups: communicable, maternal, neonatal, and nutritional diseases (Group 1 diseases); non-communicable diseases (Group 2); and injuries (Group 3). These Level 1 aggregates are subdivided at Level 2 of the

hierarchy into 22 cause groupings (eg, neonatal disorders, neurological disorders, and transport injuries). The disaggregation into Levels 3 and 4 contains the finest level of detail for causes captured in GBD 2019. The greatest detail available for some causes, such as anxiety disorders or rheumatoid arthritis, is at Level 3 of the hierarchy, while other specific causes are at Level 4 of the hierarchy with an aggregate category at Level 3 (for example, depressive disorders at Level 3, which encompasses major depressive disorders and dysthymia at Level 4). Sequelae of diseases and injuries are organised at Levels 5 and 6 of the hierarchy. In GBD, sequelae are defined as distinct, mutually exclusive categories of health consequences that can be directly attributed to a cause. For example, both neuropathy and blindness due to diabetic retinopathy are sequelae of diabetes; stroke and ischaemic heart disease are not, as these consequences cannot be categorically ascribed to diabetes in an individual despite good evidence for increased risk of these outcomes. The finest detail for all sequelae estimated in GBD is at Level 6 and is aggregated into summary sequelae categories (Level 5) for causes with large numbers of sequelae. Examples include the grouping of the infectious disease episodes and long-term sequelae of meningitis. For GBD 2019 there are 3473 mutually exclusive and collectively exhaustive sequela, 2063 cause sequelae and 1410 injuries sequelae, and thus our YLD estimates at each level of the hierarchy sum to the total of the level above. Prevalence and incidence aggregation is estimated at the level of individuals who may have more than one sequela or disease and therefore are not additive.

The GBD cause list continues to evolve to reflect the policy relevance, and public health and medical care importance of the causes of major losses of health. The cause and sequelae list expanded based on input from the Scientific Council and GBD collaborator network. For GBD 2019, the causes of death cause list has increased to 286 causes, from the 282 causes in GBD 2017. The non-fatal cause list has expanded from 354 causes in GBD 2017 to 364 causes in GBD 2019. The total number of fatal and non-fatal causes combined for GBD 2019 is 369. As in GBD 2017, we made no estimates for YLDs for just five causes, either because no disability is possible (as is the case with sudden infant death syndrome); because disability may occur rarely but at levels too low for accurate estimation given the data (as for aortic aneurysm); or because the disability is captured by the complicating causes that led to that cause of death (as for indirect maternal deaths, late maternal deaths, and maternal deaths aggravated by HIV/AIDS).

Section 1.4: Statement of GATHER compliance

This study complies with GATHER recommendations.[5] We have documented the steps in our analytical procedures and detailed the data sources used. See table S1 for the GATHER checklist. The GATHER recommendations can be found at the GATHER website under GATHER Statement.

Abbreviations

**Abbreviation**	**Meaning**
5_q0	probability of death from birth to age 5 years
ART	antiretroviral therapy
BTL	basic tabulation list
CDC	United States Centers for Disease Control & Prevention
CoD	causes of death
CODEm	Cause of Death Ensemble modelling

COMO	comorbidity correction
COPD	chronic obstructive pulmonary disease
CSMR	cause-specific mortality rate
CSV	comma-separated values
DALYs	disability-adjusted life-years
DisMod-MR	disease model-Bayesian meta-regression
DSP	disease surveillance points
DW	disability weights
EDU15+	mean education for those 15 years old and older
EMR	excess mortality rate
GATHER	Guidelines for Accurate and Transparent Health Estimates Reporting
GBD	Global Burden of Diseases, Injuries, and Risk Factors Study
GBS	Guillain-Barré syndrome
GHDx	Global Health Data Exchange
HALE	Healthy Life Expectancy
HAQ	Healthcare Access and Quality
HAT	human African trypanosomiasis
HDI	Human Development Index
ICD-	International Classification of Diseases
IFD	in-facility delivery
IHME	Institute for Health Metrics and Evaluation
iNTS	invasive non-typhoidal salmonella
LASSO	least absolute shrinkage and selection operator
LDI	lag-distributed income per capita
LMER	linear mixed effects regression
MAD	median absolute deviation
MCCD	Medical Certification of Causes of Death
MEPS	Medical Expenditure Panel Surveys
MMR	maternal mortality ratio
MR-BRT	meta-regression—Bayesian, regularised, trimmed
NESARC	National Epidemiological Survey on Alcohol and Related Conditions
NSMHWB	Australian National Survey of Mental Health and Wellbeing of Adults
NTDs	neglected tropical diseases
PAF	population attributable fraction
PAHO	Pan American Health Organization
PHMRC	Population Health Metrics Research Consortium
RMSE	root mean square error
SCD	Survey of Causes of Death
SD	Standard deviation
SDI	Socio-demographic Index
SF-12	Short Form 12 questions
SRS	Sample Registration System
ST-GPR	spatiotemporal Gaussian process regression
TFR	total fertility rate
TFU25	total fertility rate for those younger than 25 years old

UI	uncertainty interval
UN	United Nations
UNAIDS	Joint United Nations Programme on HIV and AIDS
USD	US dollars
USSR	Union of Soviet Socialist Republics
VA	verbal autopsy
VR	vital registration
WHO	World Health Organization
YLDs	years lived with disability
YLLs	years of life lost

Section 1.5 GBD results overview[1,3]

Results from GBD 2019 are available through an interactive data downloading tool on the Global Health Data Exchange (GHDx). The GHDx is the world's most comprehensive catalogue of surveys, censuses, vital statistics, and other health-related data. Results are measured in terabytes.

The latest version of the data download tool, available here: http://ghdx.healthdata.org/GBD-results-tool, contains core summary results for GBD 2019. These results include deaths, years of life lost (YLLs), YLDs, disability-adjusted life-years (DALYs), prevalence, incidence, and rate of change. The GHDx includes data for causes, risks, cause-risk attribution, aetiologies, and impairments.

Data above a certain size cannot be viewed online but can be downloaded. Depending on the size of the download, users may need to enter an email address; a download location will be sent to them when the files are prepared.

All GBD 2019 online data visualisations are available at http://vizhub.healthdata.org/GBD-compare, which provides results for all GBD health metrics.

Section 1.6 Data input sources overview[1]

GBD 2019 synthesises a large and growing number of data input sources including surveys, censuses, vital statistics, and other health-related data sources. The data from these sources are used to estimate morbidity; illness, and injury; and attributable risk for 204 countries and territories from 1990 to 2019; mortality deaths are estimated from 1980 to 2019. The input sources are accessible through an interactive citation tool available in the GHDx.

Citations for specific GBD components, causes and risks, and locations can be found through the Data Input Sources Tool in GHDx: http://ghdx.healthdata.org/gbd-2019/data-input-sources. This tool allows users to view and access GHDx records for input sources and export a comma-separated value (CSV) file that includes metadata, citations, and information about where the data were used in GBD. As required by GATHER, additional metadata for input sources are available through the citation tool as well.

Section 1.7 Funding sources

This publication and the research it presents was funded by the Bill & Melinda Gates Foundation; the University of Melbourne; Queensland Department of Health, Australia; the National Health and Medical Research Council, Australia; Public Health England; the Norwegian Institute of Public Health; St. Jude Children's Research Hospital; the Cardiovascular Medical Research and Education Fund; the National Institute on Ageing of the National Institutes of Health (award P30AG047845); and the National Institute of Mental Health of the National Institutes of Health (award R01MH110163). The funders of the study had no role in study design, data collection, data analysis, data interpretation, or writing of the report. All authors had full access to all data in the study and had final responsibility for the decision to submit for publication.

Section 2: GBD 2019 Causes of Death database

Background

All available data on causes of death (CoD) data are standardised and pooled into a single database used to generate cause-specific mortality estimates by age, sex, year, and geography. Appendix figures 1 and 2 show the high-level view of data inputs, analytical steps, and outputs of the CoD analysis frame. Section 2 of this appendix provides details on each step in the development of the CoD database as illustrated in appendix figure 1.

Section 2.1: CoD data identification[1]

Section 2.1.1: Overview of data types

The CoD database contains seven types of data sources (table S4): vital registration (VR), verbal autopsy (VA), cancer registry, police records, sibling history, surveillance, survey/census, and minimally invasive tissue sample (MITS) diagnoses. In countries with complete VR systems, there is no need to use any other data source. Less than half the world's population has deaths captured in a VR system, therefore, for countries with incomplete VR systems, vital statistics for causes of death may be supplemented with other data types (appendix figure 3).

Section 2.1.2: ICD-detail

A majority of the CoD data is VR data obtained from the World Health Organization (WHO) Mortality Database, a compilation of data submitted to the WHO by individual countries. VR is also obtained from country-specific mortality databases operated by official offices. Each cause is coded directly to the most detailed CoD when possible, whereas cause codes in data tabulated by International Classification of Disease (ICD-) are coded to aggregated cause groups. The CoD database contains 2,525 country-years of detailed data from 1980 to 2018, which includes underlying CoD coded with 3–5 digit codes, by country, year, sex, and age groups. Detailed causes are coded to one of the following ICD-detail coding systems: ICD-8, ICD-9, or ICD-10 (table S5). Each coding system has a similar cause hierarchy and cause list that has continually developed over time. ICD-10 is the current standard and the most exhaustive cause list. Within the cause lists, 5-digit codes are truncated to 4-digit codes to condense the lists. Updates to ICD-detail occur biannually as WHO releases new versions or as country

collaborators provide additional data. Updates to data from WHO increasingly include ICD-10 CoD data as it is the most current classification of CoD, while updates to ICD-8 and ICD-9 detailed lists are less common. In the case of overlapping data, preference is given to data from pre-determined country collaborations, which are updated annually.

Section 2.1.3: ICD-tabulations list

The ICD tabulation lists include the ICD-8 List A (ICD-8A), ICD-9 Basic Tabulation List (BTL), ICD-10 Mortality Tabulation, Russia Tabulation, and India Medical Certification of Cause of Death (MCCD). These data sources make up 1096 country-years from 1980 to 2016 in the CoD database. All are condensed versions of the ICD-8, ICD-9 and ICD-10 detail lists with some differences in the format of cause lists depending on the data source. ICD-8A, ICD-9 BTL, and ICD-10 Mortality Tabulation CoD are assigned to subtotal groups (referred to as chapters) and cause groups respective to ICD-detail groups. Additionally, ICD-9 BTL includes ICD-9 detail codes for some cancers and a custom tabulation scheme for the former Union of Soviet Socialist Republics (USSR) countries. The Russia Tabulation lists and India MCCD cause lists each have custom nomenclatures based on ICD-detail cause codes.

Two of the drawbacks in using tabulation lists are discrepancies in the accuracy of death counts and lack of detail due to aggregated cause groups. There are instances where the sum of deaths in chapter subtotals are not equal to the sum of cause groups within the chapter. To account for any missing or duplicate deaths reported within the cause groupings, death counts are systematically adjusted by calculating the differences between subtotals and sub-causes within the cause groups. Any differences are assigned to a remainder cause group. To account for the lack of cause code detail, select cause groups are disaggregated (Step 1.1) to create a complete cause list. Updates to ICD tabulation lists obtained from WHO occur less frequently compared to ICD-detailed lists as more countries are reporting deaths in ICD-detail. In instances of overlapping data, preference is given first to detailed collaborator data, followed by detailed WHO data, then tabulated collaborator data, and finally tabulated WHO data.

Section 2.1.4: China Disease Surveillance Points /China Center for Disease Control and Prevention

The two primary sources of data for China are surveillance data from the China Disease Surveillance Points (DSP) system and VR data collected by the Chinese Center for Disease Control and Prevention (CDC). In the China DSP data, deaths were reported across 145 disease surveillance points used from 1991 to 2003, 161 disease surveillance points from 2004 to 2012, and 605 disease surveillance points from 2013 to 2017. While China DSP with ICD-10 coding is considered sample VR data, it provides national coverage and cause detail. Thus, it receives similar processing and treatment to the China CDC VR from 2008 to 2016. From 2008 to 2017, all of the deaths and CoD information from the DSP system and other system points throughout China were collected and reported via the Mortality Registration and Reporting System, an online reporting system of the Chinese CDC. The deaths in these data are reported at the strata level, a metric that is specific to China. Counties are stratified by urban and rural classification, but definitions of urbanity vary across counties. In Step 7, we use a method developed to scale up deaths from strata level to the province level.

Section 2.1.5: Sample registration system

Sample registration systems are expanding in several countries, and are key sources of data in Indonesia and India. The Sample Registration System (SRS) is a dual-record system wherein a resident part-time enumerator continuously records births and deaths in each household within the sample unit every month. A full-time SRS supervisor thereafter independently collects the vital events along with other related details for each of the preceding six month periods during the calendar year.

Section 2.1.6: India Medical Certification of Cause of Death

The India MCCD has data for the urban parts of the majority of the states and union territories beginning in 1980. Deaths reported in this data source have been medically certified and are considered VR data. The CoD are reported in a tabulation list with a unique numbering scheme that conforms to ICD-9 and ICD-10 detail codes, which must be disaggregated. MCCD is state-split to fill in data gaps (Step 1.2 State Splitting) prior to age-sex splitting. Because SRS is widely considered a more credible assessment of CoD in India, we chose to use MCCD data only in certain cases for modelling with cause of death ensemble modelling (CODEm). We preserved MCCD data in the database for two primary reasons. First, where the three midpoint years of SRS data resulted in the loss of a clear time trend, as was the case for maternal mortality, we chose to preserve MCCD in addition to SRS. Second, MCCD has an advantage over SRS in cases where VA is not a valid instrument for ascertaining CoD, like encephalitis and dengue fever. In these cases, we kept MCCD over SRS.

Section 2.2: Verbal autopsy[1]

Section 2.2.1: Verbal autopsy coded to ICD-10 and other lists

In countries without VR systems, VA studies are a viable data source to inform CoD. Data are obtained by trained interviewers who use a standardised questionnaire to ask relatives about the signs, symptoms, and demographic characteristics of recently deceased family members. CoD is assigned based on the answers to the questionnaires.

VA data are highly heterogeneous: studies use different instruments, different cause lists (from single causes to full ICD cause lists), different methods for assigning CoD, different recall periods, and different age groups. Cultural differences may also affect the interpretation of specific questions. CoD validity must be considered when mapping to a GBD cause. VAs are likely accurate in assigning CoD to road injury or homicide but less accurate for causes requiring medical certification, such as cardiovascular causes. Studies may also occur as stand-alone assessments or as part of an extended network, such as The International Network for the Demographic Evaluation of Populations and their Health (INDEPTH) Network[6]— a continuous surveillance source with several Demographic Surveillance Systems sites that collect data coded to ICD-detail causes.

Section 2.2.2: InterVA-modelled verbal autopsy

InterVA (Interpreting Verbal Autopsy), a set of computer models intended to facilitate interpreting VAs, was found to be non-credible by the Population Health Metrics Research Consortium (PHMRC).[7] As a result, InterVA-modelled VAs are typically excluded from our analysis because of low validations, except for injuries and maternal causes, used to fill gaps and stabilise patterns.

Section 2.2.3: Other data types

Section: 2.2.3.1 Maternal mortality data

In locations with low-quality, or no VR, maternal mortality metrics can be found in surveillance, surveys, census, and sibling history data sources. The best data have death counts due to maternal causes and the total number of deaths for women within the reproductive ages of 10–54 by year. If a data source is missing these components, creating a complete cause list is necessary by using live births and all-cause mortality deaths.[8] Though death counts are the preferred metric, maternal mortality is often measured by using the maternal mortality ratio (MMR), which is easily converted to deaths by using live births. The China Maternal and Child Surveillance data is adjusted by scaling data from the strata to the province level (Step 7).

Section: 2.2.3.2 Surveys and censuses reporting fraction of deaths due to selected injuries

Surveys and censuses are often used in countries with less developed VR systems; in countries with adequate VR, surveys and censuses are supplementary. Much like VAs, the CoD validity is a concern because of lack of medical certification at the time of death. For these data sources, we keep only causes related to maternal mortality and injuries. The remaining causes are accounted for as a remainder of total deaths in the sample size.

Section 2.2.4: Police records

In most countries, police and crime reports are an important source of information for some types of injury deaths, notably road injuries and interpersonal violence. Our police data come from reports on road traffic and crime trends. The police reports used in this analysis were obtained from published studies, national agencies, and institutional surveys such as the United Nations (UN) Crime Trends survey and the UN Office on Drugs and Crime Global Study on Homicides. We assessed whether police reports were likely to be complete and to cover the entire country by comparing police trends with those seen in VR. Data are excluded in instances where police data for road traffic injuries are significantly lower than the VR. Police data that meet our inclusion criteria and provide complete coverage are uploaded to the database for use in road injuries and interpersonal violence deaths estimation.

Section 2.2.5: Population-based cancer registries

Section 2.2.5.1 Cancer registries with incidence

Data on cancer incidence were sought from individual population-based cancer registries as well as from databases that include multiple registries, including Cancer Incidence in Five Continents, NORDCAN, and EUREG. Cancer registries were identified through the membership list of the International Association of Cancer Registries, through the GBD collaborator network, through publications, or through the GHDx. Registries were excluded if they were not representative of the coverage population, if the data were limited to years prior to 1980, if the source did not provide details on the population covered, or if the list of cancer types included was not comprehensive for the age group covered. Beginning in GBD 2019, childhood cancer-specific population-based cancer registry data were sought and included.

Section 2.2.5.2 Cancer registries with incidence and high-quality mortality data

In addition to incidence, some high-quality cancer registries also report cancer mortality data. These data were also extracted and used as inputs to the mortality-to-incidence model.

Section 2.3: Standardise input data (step 1)[1]

The input data to the CoD database are received in various formats and must be standardised to run through central CoD machinery to then upload to the database. Raw data inputs come from data sources such as mortality databases, literature reviews, or reports. Usable data sources must have a clear sample size of the number of deaths in the population and exhaustive cause lists. The complexity of the data cleaning process varies drastically across data sources. For VR microdata with the location, age, sex, year, and ICD-coded cause of every death, very little effort is necessary to standardise it into a consistent structure. Other sources may require weeks of careful review to accurately extract scans of hardcover CoD reports into spreadsheets that can be transformed and standardised.

At this point, data are assigned source identifiers so that they can be linked to the GHDx and cited appropriately. Any aggregate age and sex categories are flagged for age-sex splitting. The methods of cause-of-death assignment and data collection are reviewed to determine which source type to assign; for example, we distinguish sibling history data from surveys with a VA module. Only data at the most detailed level of the GBD location hierarchy are used. Documentation from the source is reviewed to determine if the population is representative of the location or only a subset of the population in that location. Data sources representing a subset of the population are flagged as non-representative; this flag is used by Cause of Death Ensemble modelling (CODEm) to increase the variance associated with such data points.

Finally, diagnostics are reviewed at this stage to avoid sending cleaning errors downstream. We review cause-specific deaths for each demographic group to ensure the data are reasonable. For example, it is unlikely that male breast cancer deaths are higher than female breast cancer deaths or deaths from neonatal causes occur in age groups over one year. All death totals are compared with the sum of cause-specific deaths to ensure the observed deaths are accounted for and sample size is complete.

Section 2.3.1: Disaggregation (step 1.1)

CoD in tabulated VR data are condensed into aggregated groups, some of which can be mapped directly to GBD causes, while other aggregated cause groups are not informative and cannot be mapped to them. To correct for this, aggregated causes were mapped and split onto multiple ICD-8, ICD-9, and ICD-10 detail causes, or targets, based on the ICD groupings within the aggregated causes. ICD-8, ICD-9, and ICD-10 detail codes serve as targets because they are the highest-quality VR data and enable the calculation of proportions used to split the aggregated cause data into detailed causes. The proportions of deaths from nearby countries within the super-region were used to fill in data gaps as they were likely to have similar CoD trends.

We determined the targets based on detail causes missing from the tabulated cause list. For example, in ICD-9 BTL, the tabulated cause list includes a viral diseases group. In the hierarchy of causes, this group is comprised of “measles”, “yellow fever”, “encephalitis”, “hepatitis”, “rabies”, “other infectious diseases”, “garbage code”, and “remainder of viral diseases”. We did not consider this list to be an

exhaustive list of viral diseases based on the range of ICD-detail codes given in the ICD-9 BTL documentation. To make the cause list exhaustive and inclusive of other viral diseases, we split the remainder of the viral diseases group into “other meningitis”, “other infectious diseases”, “herpes”, “dengue”, “other neglected tropical diseases”, and “garbage code”. After a list of targets was determined, the aggregated deaths were disaggregated to the target causes by using ICD-8, ICD-9, and ICD-10 detail proportions generated at the super-region level for the corresponding sex and age groups across all years in the time series. For example, in ICD-9 detail data, 54.8% of deaths in males in Latin America and the Caribbean within the target group for the BTL “remainder of viral diseases” group were designated to “other meningitis.” Thus, 54.8% of deaths in the tabulated group “remainder of viral diseases” were assigned to “other meningitis” for any country within that particular super-region. For any cause and demographic group for which we lacked ICD-detail, global proportions were used.

Section 2.3.2: State splitting (step 1.2)

Two sources for CoD estimation in India are the MCCD report, which reports medically certified deaths from health facilities in mostly urban areas[9], and the SRS, which collects information via VA about one-half of 1% of the total population in India, including both urban and rural areas, from 8853 sampling units as of 2014.[10] For MCCD, missing data impedes estimation of trends at the state level. We used a first-order, log-linear model of the four-way contingency table of deaths by sex, age, state, and year to estimate the missing state-years. We fit the model to all available data for MCCD separately for each cause, including state-specific all-age measurements and age-specific national measurements. From this, we produced estimates for each combination of sex, age, state, and year. We then used these estimates wherever the raw data did not include sex-specific, age-specific, and state-specific death counts.

For MCCD, the model was fit separately for ICD-10-based and ICD-9-based reports by using the tabulated cause list present in the data.

Section 2.3.3: Calculate non-maternal deaths (step 1.3)

In cases when maternal mortality metrics do not include both deaths due to maternal causes and deaths due to non-maternal causes for women of reproductive age, live births and all-cause mortality estimates can be used to calculate deaths. Many studies report maternal deaths as the MMR. MMR is the number of maternal deaths per 100,000 live births and can be used to calculate deaths when it has been derived from primary data and not estimated. Maternal deaths were calculated by using MMR and live births; if live births were missing we substituted live birth estimates and used the following equation:

$$Maternal\ deaths\ = \frac{MMR}{100{,}000} \times Live\ births$$

If a study was non-representative, we extracted sample size and live births from that study. After maternal deaths were calculated, we used the difference from all-cause mortality estimates to determine non-maternal deaths.

A more accurate and data-inclusive method of calculating maternal and non-maternal deaths incorporates coverage and splits deaths for a range of years into individual years. If there were live births in the study, we adjusted the coverage.

$$Coverage = \frac{Live\ births}{GBD\ estimated\ live\ births}$$

After coverage was calculated, totals deaths were scaled to be more representative. This gives a more accurate death count since the envelope assumes representative coverage. We then calculated non-maternal deaths by using all-cause mortality as an all-cause total.

$$Maternal\ envelope\ with\ coverage\ = Maternal\ envelope \times Coverage$$

An additional adjustment can be applied to maternal data spanning over a range of consecutive years, which allows for more data inclusion. The years within specified year ranges are separated into individual years, and total deaths within the year range were split between each individual year by using the fixed proportions of maternal deaths from VR in that particular country. We used only VR data to inform the proportions because it was both high-quality and representative.

Section 2.4: Map to GBD cause list (step 2)[1]

In GBD 2019, we used 439 maps to translate causes found in the input data to the GBD 2019 cause list. This included 31 maps for VR data, 314 for VA data sources, and 98 for other data types. The largest, and most universal, maps used were those for ICD-9 and ICD-10 VR data. The input data causes varied from 3–4 digit ICD codes to custom cause lists with cause names such as “cholera” or “hepatitis”. Our mapping process enabled us to compare these various data sources across demographic groups.

A crucial aspect of enhancing the comparability of data for cause of death is to deal with uninformative, so-called garbage codes. Garbage codes are codes to which deaths were assigned that cannot or should not be considered as the underlying cause of death, for example: heart failure, ill-defined cancer site, senility, ill-defined external causes of injuries, and septicaemia. In GBD 2019, we developed additional maps to translate ICD- codes found in the input data that are non-underlying causes to appropriate target codes based on the levels of the GBD cause list. These garbage codes were mapped to Levels 1–4 of the GBD cause list according to the following criteria:

1. **Level 1** includes all garbage codes for which a Level 1 GBD cause cannot be directly assigned. For example, the underlying causes of “sepsis” or “peritonitis”, if not specified in the data, could be an injury, a non-communicable disease, or a type of communicable disease. In these cases, deaths will be redistributed across all three of these Level 1 causes. In addition, deaths coded to impossible or ill-defined causes of death (including “senility” and “unspecified causes”) fall into this category, as they will be redistributed onto all causes.

2. **Level 2** includes all garbage codes that can be assigned to Level 1 causes in the GBD cause list. This would include deaths coded to “unspecified injuries” (X59), which are redistributed onto all injuries.

3. **Level 3** includes all garbage codes for which we know the Level 2 CoD and can redistribute onto Level 3 causes. This includes deaths coded to causes such as “unspecified cardiovascular disease”, which falls within the Level 2 cause “cardiovascular diseases”, as well as those coded to “unspecified cancer site”, which falls within the Level 2 cause “neoplasms”.

4. **Level 4** includes all garbage codes for underlying causes of death that can be redistributed within a Level 3 cause. This includes garbage codes such as “unspecified stroke” or “unspecified road injuries.”

Section 2.5: Age-sex splitting (step 3)[1]

Different sources, particularly VA studies, report deaths for a wide range of age groups with varying intervals. For the analysis of CoD, we mapped these different age intervals to the GBD standard set of age groups. The approach to undertake this mapping was the same as in the prior GBD studies (GBD 2017, GBD 2016, GBD 2015, GBD 2013, and GBD 2010).

In the process of assembling a consolidated demographic database, we found that the aggregation of age groups is perhaps the strongest source of inconsistency. By convention, such data are reported in broad age groupings such as 0–4, 5–14, and 15–49, or with both sexes together. The issue of comparability between age-sex groups arose when assembling the GBD CoD database. We developed a tool called age-sex splitting that takes aggregated age groupings and the “both sexes combined” grouping and divides them into what their constituent age groups would likely have been if respective cause-specific and country-specific age distributions had been used. The analytical framework for GBD includes three infant age categories: early neonatal (0–6 days), late neonatal (7–27 days), and post-neonatal (28–364 days), and 20 non-infant age categories: 1–4 years, 5–9 years, and so forth proceeding in five-year age groups until the terminal age group of 95 years and older. We treat unknown ages and sexes in the same manner we treated the “all ages combined” age category and “both sexes combined” sex group. Through this process, we were able to directly compare all data sources on even terms.

The approach to age splitting is based on the following formula. The key assumption underlying this formula is that the relative risk of death by age group compared to a reference age group is invariant across populations. Although this assumption is likely violated in specific cases, a strong biologically based pattern of the relative risk of death for a cause by age is observed for most causes. The basic formula is as follows:

$$D_a = R_a N_a \left(\frac{D_a^{a+x}}{\sum_a^{a+x}(R_a N_a)}\right)$$

Where:

D_a= the number of deaths from a cause in age group *a*

R_a= global cause-specific mortality rate of age group *a*

N_a= the country-year-sex-specific population in age group *a*

D_{a}^{a+x}= the number of deaths in the age group *a* to *a+x*

With the assumption of invariant relative risks of death by age with respect to a reference age group, this equation can be used, along with population distribution by age, to split an aggregate number of deaths for the age groups *a* to *a+x* into specific deaths for each age group within the aggregate interval.

$$D_{as} = R_{as}N_{as}\left(\frac{D_{as}^{a+x,s}}{\sum_{a}^{a+x}(R_{as}N_{as})}\right)$$

Where:

D_{as}= the number of deaths from a cause in age group *a,* sex *s*

R_{as}= global cause-specific mortality rate of age group *a,* sex *s*

N_{as}= the country-year-sex-specific population in age group *a* for sex *s*

$D_{a,s}^{a+x,s}$= the number of deaths in the age group *a* to *a+x* for sex *s*

In some cases, deaths are reported for an aggregate age group for both sexes combined. The task in this case is more complicated, but the same principle can be applied. In this case we assumed that the relative risks of death by age and sex are constant.

This equation can be used to split data aggregated by age and sex. The assumption, however, of invariant relative risks across age and sex is a stronger assumption. Fortunately, data pooled across sexes are less common in the published or unpublished CoD data.

The relative risk of death in a particular age group for a given sex is derived from the global distribution of cause-specific mortality rates found in available VR data. Location-years from the following code systems are used, provided they report the requisite age-detail and sex-detail: ICD-7, ICD-8, ICD-9 BTL, ICD-10 tabulated, ICD-9, and ICD-10. Upon compiling these data, we mapped them to GBD causes and aggregated up to cause Level 3. This is the level at which a particular cause is split—that is, any child cause of a Level 3 parent is split by using the age distribution of that parent (so, chronic kidney disease due to diabetes would be split by using the age pattern of chronic kidney disease).

We next adjusted separately for estimated adult and child VR completeness. Location-year-age-sex-cause specific deaths and population were then aggregated across all location-years, to produce cause-specific mortality rates by age and sex. These were used to determine the risk of death at any age relative to any reference age group, as shown in the above equations.

Section 2.5.1: Correct age-sex violations

Occasionally, data sources include deaths by a cause for which medical consensus exists that death is impossible for the sex and age. For example, some number of deaths may be attributed to cervical cancer in males, or to maternal causes in children younger than 10 years. We have constructed a conservative list of age-sex restrictions. When deaths violate these restrictions, we redistribute them

proportionally onto all causes. All restrictions are included in table S5, Restrictions on age and sex by cause for GBD 2019.

Section 2.6: Correction for miscoding of Alzheimer's and other dementias, Parkinson's disease, and atrial fibrillation and flutter (step 4)[1]

Section 2.6.1: Objective

For certain causes of death, mortality rates reported in VR systems are impossible to reconcile with observed trends in disease prevalence and excess mortality. For dementia, Parkinson's disease, and atrial fibrillation and flutter, these disparities can largely be attributed to death certification practices. We sought to address the known bias in CoD data by first identifying the proportion of all deaths that should be assigned to these causes and next determining the GBD causes and garbage groups to which these deaths are being incorrectly assigned.

In past GBD iterations, we estimated Alzheimer's disease and other dementias, Parkinson's disease, and atrial fibrillation and flutter on the basis of longitudinal prevalence and excess-mortality data to help account for changing patterns in death certification and corresponding implausible time trends in many VR sources. This method was first implemented for Alzheimer's disease and other dementias in GBD 2013. We added atrial fibrillation and flutter to the causes modelled in GBD 2015 and Parkinson's disease to the causes modelled in GBD 2016 by using this strategy. All of these causes were processed in CoDCorrect in a manner that was agnostic to the likely targets of misclassification, which inappropriately led to changes in mortality estimates for causes unrelated to these three in GBD 2015. For GBD 2016, we improved this process by completing a literature review to identify the causes of death most closely associated with Parkinson's and Alzheimer's diseases[11–14] and limiting the CoDCorrect adjustments to include only those causes. For GBD 2017, we refined this approach further by using multiple CoD data to determine the GBD causes and garbage codes from which we move deaths as well as the pattern of misclassification.

Section 2.6.2: Correction process

Changes in coding practices for Alzheimer's diseases and other dementias, Parkinson's disease and Atrial fibrillation and flutter, cause results in spatial-temporal mortality trends that are incompatible with prevalence and case-fatality trends. These changes in coding practices are believed to be the result of shifting consensus in cause of death certification, meaning there is a bias in vital registration (VR) data that needs correction. For Parkinson's disease and atrial fibrillation and flutter, we first estimated excess mortality from prevalence and CoD data in countries with the highest ratio of cause-specific mortality to prevalence, which represents the greatest willingness to code to an under-coded cause. Then, using DisMod-MR 2.1 (see Section 4.5), we derived estimates of cause-specific mortality rates from available prevalence surveys as well as the estimates of excess mortality rate, applied across all countries and over time. We divide this value by the all-cause mortality rate to determine the fraction of overall mortality to attribute to each under-coded cause. For dementia, the modelling process was redesigned in 2019 to no longer depend on vital registration data from the highest dementia mortality locations. Instead, we used relative risk data from cohort studies to calculate total number of excess deaths due to dementia, and end-stage disease proportions from linked hospital to death records to subset these deaths to the proportion of excess deaths with end-stage conditions, which we attributed to dementia.

Finally, we used log-linear interpolation to interpolate final estimates of death due to dementia for the entire time series, and saved as a custom CoD model.

To ascertain the causes from which we would move deaths to under-coded causes, we leveraged multiple CoD data from the USA—by looking to the combinations of intermediate and immediate causes (ie, chain causes) present on death certificates with an under-coded cause listed as underlying, and identifying other causes with similar or identical chain causes, we can determine the expected pattern of miscoded deaths.

The first stage in this process is to parse out years we believe coding practices in the USA to be relatively stable. For dementia, this "gold standard" dataset features 2010–2015, for Parkinson's 2005–2015, and for atrial fibrillation and flutter 2014–2015. We then collect all deaths in those years with the under-coded cause listed as underlying and remove any mention of the under-coded cause from the death certificate. Next, for each unique chain, we search the entire time series of data (1980–2015) to identify the distribution of underlying causes that share that chain. The premise here is that if the diagnosis of dementia, Parkinson's, or atrial fibrillation and flutter were missed, the other causes listed on the death certificate would have been the basis for certification. We then reallocate the under-coded deaths by chain based on that alternative underlying cause distribution.

Upon iterating through all unique chains, we are left with a dataset excluding under-coded causes of death, each remaining cause able to be subdivided into correctly coded deaths and deaths that have been recoded from an under-coded cause by the process described (although not all causes are necessarily targeted by the recoding algorithm). The quantity of interest is the ratio of miscoded deaths to total deaths by cause, age, and sex in our counterfactual dataset.

We apply the ratios derived from the multiple cause data to all VR data to determine the local pattern of miscoding. In this way, the method is sensitive to the observed epidemiology of a given place and time. Then, we calculate the deficit in under-coded cause mortality for each location, year, age, and sex by taking the difference in the expected cause fraction based on prevalence and excess mortality compared to the proportion of deaths actually certified by the VR system. Finally, we scale the cause-specific miscoded deaths to match the deficit and then move them accordingly. We assumed that misclassification of actual dementia and Parkinson's deaths in past years occurred only for reported causes of death that might have plausibly been the direct result of dementia or resulted from misdiagnosis of other organic brain diseases based on clinical expert judgement. A similar assumption is used for atrial fibrillation and flutter, for which only cardiovascular causes and ill-defined garbage codes are considered.

Because the deaths being reallocated vary by location-year, we need a mechanism to ensure plausible limits to how many deaths are extracted from each GBD cause and garbage code. To achieve this, we first run the above-mentioned algorithm on all 5-star VR data (see Section 2.16 of this appendix for an explanation of the star data quality rating system). Then, we determine the 95th percentile of the proportion of deaths moved for each GBD cause and garbage code group by age and sex across location-years among these data. Those values are subsequently stored and applied as the limits for deaths moved by this process.

Section 2.7: Redistribute (Step 5)[1]

A crucial aspect of enhancing the comparability of data for CoD is to deal with uninformative, so-called garbage codes. Garbage codes to which deaths were assigned should not be considered as the underlying cause of death—for example: heart failure, ill-defined cancer site, senility, ill-defined external causes of injuries, and septicaemia. The methods for redistributing these garbage-coded deaths were outlined in detail in Naghavi et al,[15] and the underlying algorithm for redistributing deaths assigned to these codes has not changed since GBD 2013.

Section 2.7.1: Redistribute HIV-related garbage codes (step 5.1)

Because of the disparate nature of HIV/AIDS mortality across space and time, dynamic redistribution of HIV/AIDS-related garbage codes was needed (table S6). To inform this redistribution, we generated target proportions for each garbage group by age band (under 1 month, 1–59 months, 5–19 years, 20–49 years, 50–59 years, 60–69 years, 70–79 years, and 80 years and older), five-year time interval, and sex. The garbage groups either target HIV or a remainder target. The allotment of deaths to either of these is based on the regional increase in the mortality rate of all codes in the group relative to the rates seen from 1980 to 1984—an increase greater than 5% is assumed to be HIV/AIDS-related, and the proportion of those deaths exceeding 5% are redistributed to HIV/AIDS. Any increase less than or equal to 5% is then assigned to the remainder target.

Section 2.7.2: Regress garbage codes versus non-garbage codes (step 5.2)

For each redistribution package, we defined the “universe” of data as all deaths coded to either the package’s garbage codes or the package’s redistribution targets for each country, year, age, and sex. We then ran a regression based on the following equation separately for each target group and sex:

$$TG_{crt} = \alpha + \beta_1 Gar_{crt} + \beta_2 Age_{crt} Gar_{crt} + \theta_r Gar_{crt} + \gamma_r + \varepsilon_{ct}$$

Where:

TG_{crt} = percentage of deaths within the given garbage code’s universe that were coded to a given target group, by country

Gar_{crt} = percentage of deaths within the given garbage code’s universe that were coded to a given set of garbage codes

Age_{crt} = age interaction term for the fixed effect on the interaction of garbage and age

α = constant

β_1 = slope coefficient describing the association between Gar_{crt} and TG_{crt}

β_2 = slope coefficient describing the association between the interaction $Age_{crt}Gar_{crt}$ and G_{crt}

γ_r = region-specific random intercept (or super-region if the random effect on region is not significant)

θ_r = region-specific random slope (or super-region if the random effect on region is not significant)

ε_{ct} = standard error, normally distributed and calculated by bootstrapping

This regression was adjusted from GBD 2013 to include fixed effects on the interaction of garbage and age to ensure smooth age patterns. We made this decision after investigating diagnostic visualisations that showed unlikely gaps between proportions assigned to different age groups.

Once proportions were produced for each country, sex, age, and target group, certain adjustments were made to conform our packages to the best medical evidence available. In some cases, we implemented restrictions on the proportions that the regressions could yield. For example, we did not allow any redistribution onto "Chagas disease" outside of Latin America and the Caribbean or "suicide" under the age of 15 years. In other cases, we capped the proportion for some targets to the level that would be produced from proportional redistribution; for example, "haemoglobinopathy" and "haemolytic anaemia" were restricted to the level of proportional redistribution in the redistribution of "left heart failure". Occasionally, further adjustments were made on a case-by-case basis per country, age, sex, and target group to suppress the impact of outliers based on existing epidemiological evidence and expert judgment.

In GBD 2019, we updated the regressions for stroke and diabetes. We dropped the proportion of garbage from the regression formula and ran regression on high-quality, low proportion garbage data (4/5 stars, < 50% GC). We also included all covariates included in the CODEm models for both stroke and diabetes.

Section 2.7.3: Development of an algorithm for redistribution of garbage codes based on multiple CoD data

Multiple CoD data are a form of individual record causes of death data that include an underlying CoD along with other causes in the death chain, including intermediate and immediate causes. By analysing this type of data, we can sometimes find the true underlying CoD in other CoD data where the underlying cause is a garbage code or a mis-assigned CoD.

For GBD 2019, this method was expanded and used in redistribution of the following intermediate causes: sepsis, embolism (pulmonary and arterial), heart failure (left, right, and unspecified), acute kidney injury, hepatic failure, acute respiratory failure, pneumonitis, and unspecified central nervous system disorders. Using multiple CoD records for the USA, Mexico, Brazil, Taiwan (province of China), Italy, and Colombia we identified the fraction of deaths where the underlying cause of death and the intermediate cause was in the causal chain. Using a mixed effect linear regression, we estimated the fraction of intermediate-cause related deaths by underlying GBD cause. These fractions were multiplied by the GBD 2017 CoDCorrect result to calculate the number of deaths intermediate cause-related deaths for each GBD cause. Lastly, we calculated the "intermediate cause fraction", with total intermediate-cause related deaths as the denominator, by age, sex, location, year GBD cause. These fractions were used to redistribute the intermediate-cause-related deaths to a GBD cause. An example

is given below for sepsis where a, s, l, y, c denotes a given age group, sex, location, year, and underlying cause of death:

1. $sepsis\ fraction = \beta_{HAQ\ Index} + \beta_{age\ group} + \beta_{sex} + Y_{cause} + \varepsilon$

2. $sepsis\ deaths_{a,s,l,y,c} = sepsis\ fraction_{a,s,l,y,c} * GBD\ deaths_{a,s,l,y,c}$

3. $total\ sepsis\ deaths_{a,s,l,y} = \sum_c sepsis\ deaths_{a,s,l,y,c}$

4. $fraction\ of\ sepsis\ to\ redistribute_{a,s,l,y} = \frac{sepsis\ deaths_{a,s,l,y,c}}{total\ sepsis\ deaths_{a,s,l,y}}$

To redistribute X59 and Y34 (unspecified injuries) deaths, we used a multi-step approach that utilised the pattern of nature of injury codes in the causal chain in the multiple CoD data. First, we looked at deaths where X59, Y34, and GBD injuries causes were the underlying cause of death and got the pattern of nature of injury codes in the chain. We then derived a cause-specific redistribution proportion based on the probability of a given pattern being coded to X59/Y34 or a GBD injuries cause and summing up these proportions for all patterns. An example below is given for X59:

5. $P_{(pattern_j | UCoD\ X59)} = \frac{\#\ of\ pattern_j\ deaths\ |\ UCoD\ X59}{\sum_{j=0}^{m}(\#\ of\ pattern_j\ deaths\ |UCoD\ X59)}$

6. $P_{(GBD\ injuries\ cause_i | pattern_j)} = \frac{\#\ of\ UCoD\ GBD\ injuries\ cause_i\ deaths\ |\ pattern_j}{\sum_{i=0}^{n}(\#\ of\ UCoD\ GBD\ injuries\ cause_i\ deaths\ |pattern_j)}$

7. $redistribution\ proportion_{GBD\ injuries\ cause_i} = \sum_{j=0}^{m}\left(P\left(pattern_j \middle| UCoDX59\right) * P(GBD\ injuries\ cause_i | pattern_j)\right)$

Where:

$pattern_j$ = a given nature of injury code pattern in the chain of the multiple CoD data

UCoD X59 = a death with X59 coded as the underlying cause of death (UCoD)

UCoD GBD injuries $cause_i$ = a death with a GBD injuries causes coded as the UCoD

We applied these cause-specific redistribution proportions on the data where X59/Y34 were the underlying cause of death to get the number of X59/Y34 deaths “attributable” to each GBD injuries cause. Then, for each GBD injuries cause in the multiple CoD data, we calculated the fraction of redistributed X59/Y34 deaths over the fraction of total injuries death for that cause and modelled this intermediate cause fraction using a mixed effects linear regression similar to the one mentioned above. Like mentioned above, these fractions were then multiplied by GBD 2017 CoDCorrect results, and the cause fractions for X59 and Y34 were calculated by age, sex, location, year, and GBD injuries cause, and then used to redistribute X59 and Y34 deaths to GBD injuries causes.

Additionally, multiple CoD data were used in the correction of the mis-assignment of deaths due to drug

overdoses to unintentional other poisoning. More than 90% of these types of poisonings are due to exposure to narcotics, psychodysleptics, and other drugs, specified or unspecified. More than 97% of these poisonings by substance or drug occurred in ages 15–65 years. These are clearly not cases of accidental ingestion of substances but rather deliberate ingestion and unintentional poisoning. Using multiple CoD records for the USA, Mexico, Brazil, Taiwan (province of China), Italy, Colombia, Australia, and various European countries from 1980 to 2017, we selected all deaths with underlying causes coded to X40–X44 (table A below). Table B shows the combination of other potential causes that can be found in the multiple CoD data for these underlying causes, and table A shows the ICD-10 codes corresponding to these causes. On the basis of Table B, we proportionally redistributed mis-assigned unintentional poisoning deaths to one of these causes. The main assumption behind this algorithm is the predominance of the fatality of some substances when a combination of drugs is considered. Given the combination of different drugs and substances in these codes, opium is the main cause of fatality.[16,17] Other substances, like cocaine, methamphetamine, and alcohol in combination with cannabis are less likely to be dominant in fatality.[18]

For example, if the multiple CoD data show that 40% of deaths include opioid use disorders as an intermediate cause where the underlying cause is X40–X44, the redistribution proportion for opioid use disorders will be exactly 40% due to the dominance of the fatality of opioid use disorders compared to other drugs in the above table. Additionally, in our final results, cannabis and psychoactive and psychedelic drug use disorder deaths were mapped to other drug use disorders.

Table A. ICD-10 codes for substances or drugs used to assign deaths coded to an underlying cause of unintentional poisoning by using multiple CoD data

Accidental poisoning codes	All X40, X41, X42, X43, X44 codes
Opioid Codes	T40.0, T40.1, T40.2, T40.3, T40.4, T40.6, F11.0, F11.1, F11.2, F11.3, F11.4, F11.5, F11.6, F11.7, F11.8, F11.9
Amphetamine Codes	T43.6, F15.0, F15.1, F15.2, F15.3, F15.4, F15.5, F15.6, F15.7, F15.8, F15.9
Cocaine Codes	T40.5, F14.0, F14.1, F14.2, F14.3, F14.4, F14.5, F14.6, F14.7, F14.8, F14.9
Psychoactive and psychedelic drug	T40.8, T40.9, T43.6, F16.0, F16.1, F16.2, F16.3, F16.4, F16.5, F16.6, F16.7, F16.8, F16.9
Alcohol Codes	T51.0, F10.0, F10.1, F10.2, F10.3, F10.4, F10.5, F10.6, F10.7, F10.8, F10.9
Cannabis Codes	T40.7, F12.0, F12.1, F12.2, F12.3, F12.4, F12.5, F12.6, F12.7, F12.8, F12.9

Table B. Multiple cause of death selection algorithm used for redistributing unintentional poisoning causes of death to substance or drug use cause of death

Selection Algorithm						
	Opioids	Cannabis	Cocaine	Amphetamines	Alcohol	Psychoactive and psychedelic drugs
Opioids	Opioids	Opioids	Opioids	Opioids	Opioids	Opioids

Cannabis	Opioids	Cannabis	Cocaine	Amphetamines	Alcohol	Psychoactive and psychedelic drugs
Cocaine	Opioids	Cocaine	Cocaine	Amphetamines + cocaine	Cocaine + alcohol	Cocaine
Amphetamines	Opioids	Amphetamines	Amphetamines + cocaine	Amphetamines	Amphetamines + alcohol	Amphetamines
Alcohol	Opioids	Alcohol	Cocaine + alcohol	Amphetamines + alcohol	Alcohol	Psychoactive and psychedelic drugs
Psychoactive and psychedelic drugs	Opioids	Psychoactive and psychedelic drugs	Cocaine	Amphetamines	Psychoactive and psychedelic drugs	Psychoactive and psychedelic drugs

Multiple CoD data were only available to us for the USA, Mexico, Brazil, Taiwan (province of China), Italy, Colombia, Australia, and various European countries. Because of this limited sample, we applied the result from the multiple CoD analysis from each country to its respective super-region and used global proportions for sub-Saharan Africa. We hope for increased availability of multiple CoD data in future analyses to achieve a more precise distribution for more locations.

Section 2.7.4: Verbal autopsy anaemia adjustment (step 5.3)

To compensate for the over-representative cause fractions from anaemia found in VA studies, we redistributed these deaths based on the causal attribution of severe anaemia from GBD 2015. The proportions were country-year-age-sex specific.

Section 2.7.5: Calculate redistribution uncertainty (step 5.4)

We categorised garbage codes into four levels in order of increasing specificity (see Section 2.4). Some garbage codes are redistributed on all causes (eg, unspecified causes of death) and others are only redistributed onto specific causes (eg, unspecified cancer). Major garbage refers to garbage codes in Levels 1 or 2. Because of the variation in redistribution, estimating uncertainty from garbage redistribution for CODEm modelling was an important goal for GBD 2019.

We assigned redistribution variance to each data point in the CoD database by calculating residual variance from a regression predicting the percentage of garbage coded deaths redistributed to a cause, given the proportion of garbage codes we observed for that location, year, age, sex, cause, and the age standardised relative rate of major garbage codes across all causes. If there is a cause that has greater residual variance, we assume greater redistribution uncertainty.

The two model inputs are the observed percentage of Levels 1, 2, and 3 garbage codes (by cause, age, sex, location, and year) in redistributed CoD data and the percentage of garbage codes in the raw data (calculated as the age standardised mortality rate ratio of major garbage coded deaths to all deaths in the raw data by location, year, and sex). Level 4 garbage codes were excluded from the model to avoid over estimating uncertainty in countries with high percentages of major garbage codes. Additionally, the classification of Level 4 garbage codes is not stable between successive GBD rounds—for example, “unspecified diabetes” was not a garbage code in GBD 2016, and in GBD 2017 was re-classified as a

Level 4 garbage code to permit estimation of diabetes by type. These deaths are still taken into account later in the uncertainty estimation process. The model predicts the percentage of garbage coded deaths redistributed to a cause, given the proportion of garbage codes we observed for that location, year, age, sex, cause, and the age standardised relative rate of major garbage codes across all causes. From this model, we calculate residual variance. It is important to note that the variance here is a measurement of uncertainty of redistribution, not of the level of miscoding in the raw CoD data for a given demographic.

To calculate variance, a dataset was generated that contained percent garbage by location, year, age, sex, and cause, where percent garbage is determined by the equation

$$pct_{garbage} = \frac{deaths_{redistributed} - deaths_{raw}}{deaths_{redistributed}}$$

A mixed-effect linear regression model was then fit to predict the logit percent of deaths from redistribution by age-standardised relative rate of major garbage codes.

$$\begin{aligned} logit\left(pct_{garbage_{ij}}\right) \\ &= \beta_0 + \beta_1 * \log\left(ASR_{majorgarbage_{ij}}\right) + \beta_2 * 15yearage_{ij} + \gamma_{1j} \\ &* \log\left(ASR_{majorgarbage_{ij}}\right) + u_j + e_{ij},\ \ \theta_{\{i\}} \sim N(0, \sigma^2) \end{aligned}$$

Where:

i indexes dataset-location-year-age-sex-cause data points nested within j groups by GBD region

$ASR_{majorgarbage_{ij}}$ is age-standardised relative rate of major garbage

Residual variance, as estimated by the mean absolute deviation, was calculated for each cause, sex, and age.

The next step was to use the residual variance to calculate uncertainty around each data point in the CoD database. First, we calculated the percent garbage of each data point by treating all deaths that could not be directly mapped to a GBD cause as garbage, including Level 4 garbage codes. Percent garbage was calculated as

$$pct_{garbage} = \frac{deaths_{redistributed} - deaths_{corrected}}{deaths_{corrected}}$$

Where:

$deaths_{corrected}$: deaths post misdiagnosis correction (Section 2.6)

$deaths_{redistributed}$: deaths post redistribution (Section 2.7)

Residual variance was matched to each data point and 100 draws were sampled from a normal distribution by using the cause, age, sex, specific residual variance, and mean of 0. The logit transformed

percent garbage was added to each value in the distribution. Each draw was then transformed out of logit space, and the post-redistribution deaths were calculated as

$$deaths = \frac{deaths_{corrected}}{1 - pct\_garbage}$$

Draws of deaths were processed through noise reduction before calculating the final redistribution variance passed to CODEm, which was added to the total data variance. The mean of the draws was not used as the final estimate because it was found that the logit transformation biased the distribution of cause fractions higher. Instead, only point estimates were used.

Section 2.8: HIV/AIDS misclassification correction (step 6)[1]

In many location-years, certain causes of death known to be comorbid with HIV/AIDS (eg, tuberculosis, other infectious diseases) are seen to have age patterns that diverge from those observed in location-years without widespread HIV epidemics and are in fact more reflective of HIV mortality trends. To identify these instances, a global relative age pattern is generated by using all VR deaths in countries with observed HIV prevalence less than 1% by using the following equation

$$RR_{asc} = \frac{R_{asc}}{\bar{x}(R_{65sc}, R_{70sc}, R_{75sc})}$$

Where:

RR_{asc} is the relative death rate for age group *a*, sex *s*, cause *c*;

R_{asc} is the rate for that age group

$\bar{x}(R_{65sc}, R_{70sc}, R_{75sc})$ is the mean of the rates in ages 65–69, 60–74, and 75–79 for that sex and cause.

This is preferable to comparing mortality rates because we are able to isolate divergence in age pattern while accounting for varying levels of overall mortality by fixing death rates to age groups that are unlikely to be confounded by the presence of HIV. Expected deaths for an identified cause were then determined by the equation

$$ED_{lyasc} = \bar{x}\left(R_{ly65sc}, R_{ly70sc}, R_{ly75sc}\right) \times p_{lasc} \times RR_{asc}$$

Where:

ED_{lasc} are deaths for location *l*, year *y*, age group *a*, sex *s*, and cause *c*;

$\bar{x}(R_{l65sc}, R_{l70sc}, R_{l75sc})$ is the mean of the rates for ages 65–69, 60–74, and 75–79 for that location-year-sex-cause;

p_{lasc} is the population for that location-year-age-sex-cause

RR_{asc} is the global standard relative rate determined in the previous step for that age-sex-cause.

The expected deaths remain attributed to that particular cause, while the difference between observed and expected are reallocated to HIV/AIDS.

Section 2.9: Scale strata to province (step 7)[1]

Over time, a higher proportion of deaths have been registered in China through the expansion of the DSP system and provincial/county efforts to increase CoD registration. With the expansion of coverage, it is possible that province aggregates do not accurately represent the population distribution between urban and rural areas in each year. For this reason, we stratified the data preparation by urban and rural status for each county within each province. Stratification was based on the median level of urbanisation across counties within each province as recorded in the 2010 China census. In the provinces of Tibet and Hainan, all counties were placed into one strata based on largely homogeneous urbanisation levels within each province. This yielded a total of 62 analytical province-strata. Macao and Hong Kong were not included in this stratification system as the VR systems there are independent from that on the mainland; no weighting scheme needs to be carried out in these complete VR systems with quality CoD data.

Within each province-strata, a larger proportion of deaths in-hospital might be reported than that of deaths outside of hospital because of the internet hospital reporting system. To avoid bias, we reweighted in-hospital and out-of-hospital deaths based on the age-sex-province-specific fraction of deaths in and out of hospital in the DSP system. DSP data have been used to establish these percentages because in these communities, there is a concerted effort to identify all out-of-hospital deaths. Province-strata death rates are combined to produce overall province death rates by weighting each strata by population in each age-sex-year group. Province death rates are rescaled so that all-cause mortality equals the estimated death rate in each age-sex-year estimated in the life-table analysis. The Bayesian noise reduction algorithm was used to deal with zero counts and small number issues for rare causes.[18]

Section 2.10: Restrictions post-redistribution (step 8)[1]

Some causes of death can only be reliably assigned through an autopsy by a trained physician. For example, a VA would be unlikely to reliably distinguish between ischaemic and haemorrhagic stroke.

This step ensures that the detail of the cause list at this point in the data prep process is reasonable given the detail of the original data source and the methods by which the CoD was assigned. A "bridge map" is applied over a certain set of sources to ensure that these sources do not contain causes that could not reliably be determined by the methods used. These causes, identified to be too detailed, are then aggregated to their parent cause. This correction is applied to ICD-9 detail, ICD-9 BTL, ICD-10 tabulated, ICD-8 detail, ICD-8 A, China DSP (tabulated ICD-9), India MCCD, India SRS, USSR tabulated ICD-9, the Philippine Vital Statistics Reports, Iran ICD-10 VR from the Ministry of Health and Medical Education, and all VA. An example of this would be the aggregation of all sub-types of lower respiratory infection to lower

respiratory infection in ICD-9 BTL.

Section 2.11: Drop VR country years or mark as non-representative (step 9)[1]

Lozano and colleagues[20] describe the negative impact that low-completeness VR data could have on CoD modelling for GBD 2010. In particular, in settings where a data source does not capture all deaths in a population, the cause composition of deaths captured might be different from those that are not. However, a completeness sensitivity test found that low-completeness VR data had little impact on the cause-specific mortality trends at the global level.

For GBD 2019, we investigated the impact of these data at the country and subnational and determined that these data produced unlikely trends in the models affected. Despite the minimal impact on global trends, better models were produced by eliminating or marking as non-representative data with extremely low completeness. VR completeness was estimated as the number of deaths registered divided by the number of deaths estimated in the GBD mortality envelope.

For this round, VR location-years with completeness less than 50% were dropped, while location-years with completeness between 50% and 69% were marked as non-representative.
In addition, any country-year with a number of deaths registered to major garbage codes greater than 50% of the deaths registered was dropped. Major garbage coding refers to garbage codes redistributed across Levels 1 and 2 of the cause hierarchy. When we redistribute garbage codes across Levels 1 and 2 of the cause hierarchy, this is because we do not have enough information to distribute them to more detailed Levels [3 and 4].

Section 2.12: Cause aggregation (step 10)[1]

The cause list is organised in a top-down hierarchical format containing four levels. The first group, or Level 1, sums all causes. Following all-cause mortality are Level 2 causes, which include three broad groupings of causes of deaths: “communicable, maternal, neonatal, and nutritional diseases”; “non-communicable diseases”; and “injuries”. Within those Level 2 groupings are finer levels used for modelling. Level 3, or parent causes, are aggregated; the mortality estimate for a parent cause in the hierarchy represents the sum of the causes under that rubric. Sub-causes within Level 3 causes—Level 4—are more detailed. For example, the parent cause “intestinal infectious diseases” contains the three sub-causes: “typhoid fever”, “paratyphoid fever”, and “other intestinal infectious diseases”. Included in the parent cause estimate are deaths mapped directly to the parent and any Level 4 sub-causes. In data where there was not enough information to assign a Level 4 cause, we aggregated to the Level 3 parent cause. Exceptions to aggregating the Level 4 sub-causes to the parent are instances when certain sub-causes are not present. The United Nations Crime Trends police data only identify homicides, and aggregating homicides to injuries would not accurately represent all injuries.

Section 2.13: Remove shocks and HIV/AIDS maternal adjustments (step 11)[1]

For GBD 2019, CODEm models use an HIV/AIDS- and shock-free envelope. To be comparable, cause fractions must also be HIV/AIDS- and shock-free. Cause fractions were uploaded to the CoD database as the number of deaths due to the cause over an adjusted sample in which the number of deaths due to “HIV/AIDS”, “conflict and terrorism”, “police conflict and executions”, and “exposure to forces of nature”

were removed.

Section 2.13.1: Remove HIV/AIDS and shocks from denominator where cause list includes HIV/AIDS (step 11.1)

The first step to generate HIV- and shock-free cause fractions was to remove any deaths from the sample that were directly coded to "HIV/AIDS", "collective violence and legal intervention", or "exposure to forces of nature". The cause fraction uploaded to the database can be calculated by a simple equation.

$$CF_{l,t,a,x,c} = \frac{D_{l,t,a,x,c}}{D_{l,t,a,x} - D_{l,t,a,x,hiv} - D_{l,t,a,x,war} - D_{l,t,a,x,disaster}}$$

Where:

$CF_{l,t,a,x,c}$ is the cause fraction for a location *l*, year *t*, age *a*, sex *x*, and cause *c*

$D_{l,t,a,x,c}$ is the number of deaths observed for cause *c* in location *l*, year *t*, age *a*, and sex *x*

$D_{l,t,a,x}$ is the total number of deaths due to all causes observed in location *l*, year *t*, age *a*, and sex *x*

$D_{l,t,a,x,hiv}$, $D_{l,t,a,x,war}$, and $D_{l,t,a,x,disaster}$ are the numbers of deaths observed in location *l*, year *t*, age *a*, and sex *x* for causes "HIV/AIDS", "collective violence and legal intervention", and "exposure to forces of nature", respectively

Cause fractions for HIV/AIDS and shock causes were also uploaded to the database for use in separate estimation processes described by Wang et al.[21] In this case, cause fractions followed the standard equation, with variables following the same explanation.

$$CF_{l,t,a,x,c} = \frac{D_{l,t,a,x,c}}{D_{l,t,a,x}}$$

Section 2.13.2: Remove HIV/AIDS deaths from maternal mortality sources (step 11.2)

HIV-free cause fractions were also uploaded for sources on mortality due to maternal causes. In these cases, the sample of all deaths observed in the study is likely to contain some amount of deaths due to HIV/AIDS and shocks, but the sample only includes cause information on maternal deaths. To account for the presence of HIV/AIDS and shocks in the entire sample, we assumed the same proportion of total deaths due to HIV/AIDS by location, age, sex, and year as provided from the estimation of HIV/AIDS and all-cause mortality described by Wang et al.[21]

Maternal mortality studies were only corrected for HIV/AIDS if the sample of total deaths was provided in the data source. Where sources provided only the MMR, we applied the rate to the HIV- and shock-free envelope produced by the analysis described in Wang et al.[21] and thus did not need to adjust cause fractions at this point in the process.

Where a correction was applied, we used the following equation:

$$CF_{l,t,a,x,mat} = \frac{D_{l,t,a,x,maternal}}{D_{l,t,a,x,maternal} + \frac{E[D_{l,t,a,x,hiv\_shock\_free}]}{E[D_{l,t,a,x}]} D_{l,t,a,x,non-maternal}}$$

Where:

$CF_{l,t,a,x,mat}$ is the resulting cause fraction due to maternal causes for the location (l), year (t), age (a), sex (x);

$D_{l,t,a,x,mat}$ is the number of observed deaths in the sample due to maternal causes

$D_{l,t,a,x,non-maternal}$ is the number of observed deaths in the sample due to non-maternal causes

$E[D_{l,t,a,x}]$ is the GBD estimate of all-cause mortality in the location, year, age, and sex

$E[D_{l,t,a,x,hiv\_shock\_free}]$ is the GBD estimate of HIV- and shock-free mortality in the location, year, age, and sex

Section 2.13.3: HIV/AIDS correction of sibling history, census, and survey data (step 11.3)

As described in our analysis from GBD 2013, many studies have failed to find increased mortality in HIV+ pregnant mothers, but those who have advanced HIV are known to have increased baseline mortality. Prior to GBD 2013, we did not distinguish between deaths in HIV+ women that were caused by pregnancy and those for whom the pregnancy was incidental to their death. To more explicitly quantify the contribution of pregnancy to death in HIV+ women, and therefore more accurately estimate the maternal death count, we completed two additional analyses for GBD 2013 and all subsequent GBD analyses. First, we determined the population attributable fraction (PAF) of HIV/AIDS to pregnancy-related death. Second, we determined the proportion of pregnancy-related deaths in HIV+ pregnant mothers that are aggravated by pregnancy and are therefore by definition maternal deaths.

$$PAF = \frac{P(RR-1)}{1+P(RR-1)}$$

Where:

PAF is the population attributable fraction

P denotes the prevalence of HIV in pregnancy

RR is relative risk of mortality in HIV+ vs HIV- pregnant mothers.

To recap our analysis for GBD 2013, we used the paper published by Calvert and Ronsmans[22] to identify sources that could inform Step 1 of our HIV-correction analysis. We independently reviewed each of

the component studies in Calvert and Ronsmans' review and extracted data directly, not from the systematic review paper. We identified only one additional study that was not used in Calvert and Ronsmans' analysis. We have, however, not used all the studies included in that review. Specific details are as follows:

1) Figueroa-Damian et al.[23] was excluded for not including any postpartum deaths at all.

2) In the case of Ryder et al.[24] and Zvandasara et al.[25] we excluded those deaths that occurred more than 12 months after delivery.

3) We excluded the results from Chilongozi et al.[26] from the site that did not include any HIV-patients.

4) Leroy et al.[27] was not in the bibliography. We could not locate it for review so it was excluded.

5) Kourtis et al.[28] was extracted with adjustment of the denominator based on the average number of hospitalisations per delivery in each group.

6) Ticconi et al.[29] was excluded for being both non-representative and including subgroup data from mothers with malaria infection.

A total of 21 sources were included in our analysis of the increased mortality risk of HIV+ versus HIV- women in pregnancy.[30] We performed DerSimonian-Laird random effects meta-analysis to derive a pooled estimate of RR of death during pregnancy given HIV positivity.[31] The pooled effect size was 6·40 (95% uncertainty interval [UI] 3·98–10·29), which was then used to calculate an HIV PAF for each country, age group, and year. To determine the proportion of those HIV-related deaths that were attributable to maternal causes, we performed a second systematic literature review. This time we sought evidence for the excess mortality risk of pregnancy in those women who are already HIV+. Most studies have failed to find such an effect, but most also did not stratify their study population by stage of HIV or ART (antiretroviral therapy) status. Only two studies did this stratification, with a pooled effect size of 1·13 (95% UI 0·73−1·77).[32,33]

An updated literature review to inform the relative risk of mortality in pregnancy in HIV+ versus HIV- women had 14 non-usable sources. We completed this search on May 10, 2019, using the following search strings:

((HIV[Title/Abstract] OR "Acquired Immunodeficiency Syndrome"[Title/Abstract] OR AIDS[Title/Abstract]) AND ("pregnant"[Title/Abstract] OR "pregnancy"[Title/Abstract] OR "postpartum"[Title/Abstract] OR "post partum"[Title/Abstract]) AND ("mortality"[Title/Abstract] OR "death"[Title/Abstract]) NOT "case report" NOT (animals[MeSH] NOT humans[MeSH])

AND (2016/08/15[PDat] : 3000/12/31[PDat]))

Prevalence of HIV in pregnant women was calculated by using the Joint United Nations Programme on HIV and AIDS (UNAIDS) Spectrum model,[34] a compartmental HIV progression model used to generate age-specific incidence, prevalence, and death rates from pre-calculated incidence curves and assumptions about intervention scale-up and local variation in epidemiology. For each location, we used UNAIDS' age-specific ratios of fertility in women living with HIV to fertility in women not living with HIV. In most locations, this ratio is assumed to be greater than one in women aged 15–24 years and less than one and decreasing as age increases beyond 24 years. Since Spectrum assumes fertile ages of 15–49 years, we used the ratio of HIV prevalence in pregnant women to HIV prevalence in the general population at either end of that range to extend estimates to age bands 10–14 years and 50–54 years.

Unlike GBD 2013, when we applied the PAF correction to the envelope of maternal deaths predicted by CODEm, we instead applied country-year-age-group-specific PAF to maternal mortality input data prior to modelling in CODEm. This ensured that both the numerator and denominator of all CF data were internally consistent in their exclusion of background HIV/AIDS mortality. The cause fractions for maternal deaths in sibling history, survey, and census data were therefore adjusted as follows:

$$CF_{l,t,a,x,mat_{adj}} = CF_{l,t,a,x,mat} \times \left(1 - ProP_{hiv_{l,t,a,x}}\right)$$

$$ProP_{hiv_{l,t,a,x}} = PAF_{l,t,a,x,hivpos} \times (1 - rr_{mat})$$

$$CF_{l,t,a,x,mat_{hiv}} = CF_{l,t,a,x,mat} \times ProP_{maternalhiv_{l,t,a,x}}$$

$$ProP_{maternalhiv_{l,t,a,x}} = PAF_{l,t,a,x,hivpos} \times rr_{mat}$$

Where:

$CF_{l,t,a,x,mat}$ = The proportion of deaths due to all maternal causes before HIV/AIDS correction for the location, year, age, and sex.

$CF_{l,t,a,x,mat_{adj}}$= The proportion of deaths due to maternal causes after the adjustment for the location, year, age, and sex.

$CF_{l,t,a,x,mat_{hiv}}$ = The proportion of deaths due to maternal deaths aggravated by HIV/AIDS after the adjustment for the location, year, age, and sex.

$PAF_{l,t,a,x,hivpos}$ = The PAF that describes the percentage of all maternal deaths that were HIV-related for the location, year, age, and sex

$ProP_{hiv_{l,t,a,x}}$ = The proportion of deaths in pregnancy for the location, year, age, and sex that are estimated to be incidental deaths due to HIV/AIDS and therefore not a maternal CoD.

$ProP_{maternalhiv_{l,t,a,x}}$ =The proportion of deaths in pregnancy for the location, year, age, and sex that are estimated to be HIV+ and maternal deaths that are aggravated by HIV/AIDS.

rr_{mat} = 0.13/1.13 = The proportion of HIV/AIDS deaths during pregnancy that were exacerbated by the pregnancy.

Section 2.13.4: HIV/AIDS correction of other maternal mortality data (step 11.4)

Although a specific subset of codes in ICD-10 corresponds to HIV/AIDS deaths aggravated by pregnancy, these codes are sparsely used and unreliable. We therefore adapted the method described to also correct VR and VA sources for the systematic exclusion of HIV-related maternal deaths. This correction was calculated in the same manner, by using the same input data as above, with the only difference being that HIV correction of VR and VA sources resulted in a net increase in the maternal correction factor maternal deaths aggravated by HIV/AIDS are calculated in the following way:

$$CF_{l,t,a,x,mat_{hiv}} = CF_{l,t,a,x,mat} \times ProP_{maternalhiv_{l,t,a,x}}$$

$$ProP_{maternalhiv_{l,t,a,x}} = \frac{PAF_{l,t,a,x,hivpos} \times rr_{mat}}{1 - PAF_{l,t,a,x,hivpos} \times rr_{mat}}$$

Section 2.14: Noise reduction (step 12)[1]

To deal with problems of zero counts in VR, VA, cancer registries, or sibling histories for a given age group in a given year, we use a Bayesian noise-reduction algorithm. For this algorithm, we assume a normal prior and a normal data likelihood. We estimate the normal prior for a given country-series of data by running a Poisson regression to estimate the number of deaths due to each respective cause and sex with dummy variables for age and year. With two notable exceptions (detailed below), these regressions are sex-, cause-, and country-specific, so borrowing strength over age and year is only within a given data type, country, cause, and sex. The variance of the prior, τ^2, is estimated from the Poisson regression, taking into account the variance-covariance matrix of the regression coefficients. For the data variance, we use the Wilson approximation which provides an estimate of σ^2 even in cases with a zero count of cause-specific deaths. The posterior estimate for each data point is

$$Mean = \left(\frac{\tau^2}{\tau^2 + \sigma^2} X + \frac{\sigma^2}{\tau^2 + \sigma^2} \mu\right)$$

$$Variance = \left(\frac{\tau^2 \sigma^2}{\tau^2 + \sigma^2}\right)$$

Where

X is the mean of the data

μ is the mean of the prior.

This approach to noise reduction avoids the problem that zero counts in an *ln* rates model or a logit cause fraction model will be dropped from the regression and lead to upward bias in the estimates. This is particularly important in two settings: high-income countries with small numbers of cause-specific

deaths, and the analysis of sibling history data where for any given age group in any given year the number of deaths reported in the survey that are pregnancy-related or the number of deaths from all causes in that age group may be small.

Regarding the exceptions to the regression, the first is that country-years with populations under 1 million are pooled with the region data to prevent over-dispersion and provide a stronger signal. Additionally, VA data diverge from the above description in two ways. First, all data for a given super-region are pooled together and a study dummy variable is added, allowing for different studies and surveillance sites to borrow strength from one another within a super-region. Second, unless the data are part of a time series (eg, the Matlab Health and Demographic Surveillance System), the regression has no year component.

Section 2.15: Cause of death database and outlier identification (step 13)[1]

Death rates for different causes of death generally have a stable age pattern. In large populations, these patterns will not change very rapidly over time. We can assume a relatively stable pattern in death rates for all causes except for some epidemic diseases and specific types of injuries. Rare causes in large populations and prevalent causes in small populations usually have stochastic patterns. To correct for these stochastic patterns, we implemented a noise-reduction process, explained in Step 12.

In VR data, we infrequently find one or more data points for specific geography/age/sex/year combinations that lie very far from the stable pattern of death rates. In these situations, the model usually ignores the data point(s). If the model fails to ignore these data, dramatic jumps or drops can occur in the death rates. When no logical explanation exists for variation in the death rates to this degree, we regard the data point(s) as outlier(s). The selection of data points to regard as outliers occurs after data have been prepped for modelling, as well as during preliminary reviews of the models.

In non-VR sources, data-collection methods and data quality can vary widely from source to source. Where data points in each age-sex-geography-year are very sparse, extreme data points can have a bad effect on regional estimation. In these situations, we investigate the study's methods and consider lower-quality data points as outliers.

Identifying outliers in the CoD data occurs prior to finalisation of models for each cause. We do not automate the selection of outliers but investigate the source of the offending data as well as reviewing other data sources for the same cause, geography, and year. Ultimately, outliers are identified based on the judgement of the modeller and senior faculty. Outlier decisions are reversible and may be revisited.

Section 2.16: Causes of death data star rating calculation[1]

GBD estimates are most accurate when computed with a full time series of complete VR with a low percentage of garbage codes. For GBD 2016, we developed a simple star-rating system from 0 to 5 to give a picture of the quality of data available in a given country over the full time series used in GBD estimates. Countries improve in the star rating as they increase availability, completeness, and detail of their mortality data and reduce the percentage of deaths coded to ill-defined garbage codes or highly

aggregated causes (table 7, figures 5a and 5b). Underlying indicators for the percent well-certified calculation are listed in table S8.

We assign star ratings to rate the quality of data for any given location year. Two dimensions determine this star rating: (I) the percentage of total deaths determined to be major garbage (such as ill-defined). Causes such as "injuries" or "cancer" will also be included in major garbage percentage because this percentage includes use of highly aggregated causes; and (II) the level of completeness of death registration. These two values were used to create a "percent well-certified" value between 0 and 1, determined as:

$$pct_{wellcertified} = Completeness \times (1 - pct_{majgarbage})$$

The mapping of percent well certified to star rating is as followed:

0 star: $0\% = pct_{wellcertified}$

1 star: $0\% < pct_{wellcertified} < 10\%$

2 star: $10\% <= pct_{wellcertified} < 35\%$

3 star: $35\% <= pct_{wellcertified} < 65\%$

4 star: $65\% <= pct_{wellcertified} < 85\%$

5 star: $pct_{wellcertified} \geq 85\%$

While stars are calculated for each five-year time interval as well as the full time series from 1980 to 2019, stars in the main text are presented for the full time series only.

In the case of VA, all garbage codes are considered ill-defined because redistribution for VA is highly imprecise.

For each VA data source, percent well-certified is

$$pct_{wellcertified} = VerbalAutopsyAdjustment \times (1 - pct_{majgarbage})$$

Where:

$$VerbalAutopsyAdjustment = SubAdj \times RegAdj \times AgeSexCoverage$$

SubAdj is 10% for subnationally representative studies; 100% for nationally representative studies. This adjustment, while arbitrary in its specific value, reflects the bias that can be associated with studies that only cover a potentially non-representative sample of a country's population.

RegAdj is 64% for all VA data sources. This accounts for the inaccuracy of VA in assigning CoD compared to medically verified VR. The specific multiplier 0·64 is based on the chance-corrected concordance of Physician Certified Verbal Autopsy (PCVA) versus medical certification by the Population Health Metrics Research Consortium.[35]

Age-Sex Coverage is the number of deaths estimated in the GBD mortality envelope for the ages and sexes in the study for the country and year divided by the number of deaths estimated in the GBD mortality envelope for the country and year. Studies that only cover children under 5 years or maternal mortality, for example, will be highly discounted by this multiplier.

Once percent well-certified is calculated for each location-year of VR and each VA study-year, we then combine these into one measurement for each five-year time interval and the full time series 1980–2019. For each five-year time interval, we take the maximum percent well-certified. Then for 1980–2019, we take the average of the maximum percentages well-certified for the seven five-year time intervals. Any five-year time interval in which no data were available were given a percent well-certified value of zero.

Prior to GBD 2019, the causes of death team used an all ages, both sex cause fraction to estimate the percentage of garbage coded deaths in a given location year. Thus, the percentage of garbage for a given location year was determined as:

$$CF_G = \frac{D_G}{D}$$

Where:

CF_G represents the cause fraction of percent garbage

D_G represents total garbage coded deaths

D represents the total deaths in a given location/year.

In GBD 2019, we moved to calculating the percentage of garbage coded deaths using an age-standardised cause fraction. The steps for creating these age-standardised cause fractions, in the case of garbage, are as follows:

1. Create both-sex, age-specific cause fractions of garbage for each age group
2. Scale these cause fractions by a set of both-sex age weights, determined by global mortality estimates from 2010 to present. That is, weights for each GBD age group were determined as:

$$W_a = \frac{D_a}{D}$$

Where:

W_a is the weight for given age group "a"

D_a is the total both sex, global deaths from 2010 to present in age group "a"

D is the total both sex, global deaths from 2010 to present across all ages.

3. Sum these weighted cause fractions across all age groups to produce the age-standardised cause fraction

In the case of percent garbage for a given location year, the formula to calculate percent garbage would be given as the sum of the weighted age specific cause fractions across all age groups “a”:

$$CF_G = \Sigma_a(\frac{G_a}{D_a} \times W_a)$$

Where:

G_a represents the total both sex garbage deaths in age group “a”

D_a represents the total both sex deaths in age group “a”

W_a represents the weight generated from mortality estimates for age group “a”

ICD-10 and ICD-9 codes assigned to Level 1 or 2 garbage can be found in table S4.

Section 3: Causes of death modelling methods

Section 3.1: CODEm[1]

Section 3.1.1: Overview of methods

Cause of death ensemble modelling (CODEm) is the framework used to model most cause-specific death rates in the GBD.[36] It relies on four key components:

First, all available data are identified and gathered to be used in the modelling process. Although the data may vary in quality, they all contain some signal of the true epidemiological process.

Second, a diverse set of plausible models are developed to capture well-documented associations in the estimates. Using a wide variety of individual models to create an ensemble predictive model has been shown to outperform techniques using only a single model both in CoD estimation[36] and in more general prediction applications.[37,38]

Third, the out-of-sample predictive validity is assessed for all individual models, which are then ranked for use in the ensemble modelling stage.

Finally, differently weighted combinations of individual models are evaluated to select the ensemble model with the highest out-of-sample predictive validity.

For some causes (eg, lower respiratory infections), evidence exists that the relationship between covariates and death rates might differ between children and adults. Separate models are therefore run for different age ranges, when applicable. Additionally, separate models are developed for countries with extensive, complete, and representative VR for every cause to ensure that uncertainty can better reflect the more complete data in these locations.

In order to ensure the addition of subnational locations are not driving changes in estimates, in GBD 2019, we run a global model that excludes data from non-standard locations; the resulting covariate

betas are then used as priors for the true global model.

In addition to CoD modelling, we also estimate fatal discontinuities. Fatal discontinuities are events that are stochastic in nature, that cannot be modelled because they do not have a predictable time trend. The fatal discontinuities by cause are aggregated by age and sex and added to the estimated number of deaths in CoD modelling for those causes during CoDCorrect. Details on their methods can be found in Section 3.4.

Section 3.1.2: Model pool development

Because many factors may co-vary with any given CoD, a range of plausible statistical models are developed for each cause. In the CODEm framework, four families of statistical models are used: linear mixed effects regression (LMER) models of the natural log of the cause-specific death rate, LMER models of the logit of the cause fraction, spatiotemporal Gaussian process regression (ST-GPR) models of the natural logarithm of the cause-specific death rate, and ST-GPR models of the logit of the cause fraction (see the 2x2 table in Foreman et al).[36] For more on ST-GPR, see section 4.3.3. For each family of models, all plausible relationships between covariates and the response variable are identified. Because all possible combinations of selected covariates are considered for each family of models, multi-collinearity between covariates may produce implausible signs on coefficients or unstable coefficients. Each combination is therefore tested for statistical significance (covariate coefficients must have a coefficient with p-value $< 0{\cdot}05$) and plausibility (the coefficients must have the directions expected on the basis of the literature). Only covariate combinations meeting these criteria are retained. This selection process is run for both cause fractions and death rates, then ST-GPR and LMER-only models are created for each set of covariates. For a detailed explanation of the covariate selection algorithm, see Foreman et al.[36]

Section 3.1.3: Data variance estimation

The families of models that go through ST-GPR described in Section 3.1.2 incorporate information about data variance. The main inputs for a Gaussian process regression (GPR) are a mean function, a covariance function, and data variance for each data point. These inputs are described in detail in Foreman et al.[36] For GBD 2019, we have updated this calculation to incorporate garbage code redistribution uncertainty.

Three components of data variance are now used in CODEm: sampling variance, non-sampling variance, and garbage code redistribution variance. The computation of sampling variance and non-sampling variance has not changed since previous iterations of the GBD and is also described in Foreman et al.[36] Garbage code redistribution variance is computed in the CoD database process described in Section 2.7 of this appendix. Since variance is additive, we calculate total data variance as the sum of sampling variance, non-sampling variance, and redistribution variance. Increased data variance in GPR results in the GPR draws not following the data point as closely.

Section 3.1.4: Testing model pool on 15% sample

The performance of all models (individual and ensemble) is evaluated by means of out-of-sample predictive validity tests. Thirty percent of the data are randomly excluded from the initial model fits.

These individual model fits are evaluated and ranked by using half of the excluded data (15% of the total), then used to construct the ensembles on the basis of their performance. Data are held out from the analysis on the basis of the cause-specific missingness patterns for ages and years across locations. Out-of-sample predictive validity testing is repeated 20 times for each model, which has been shown to produce stable results.[36] These performance tests include the root mean square error (RMSE) for the log of the cause-specific death rate, the direction of the predicted versus actual trend in the data, and the coverage of the predicted 95% UI.

Section 3.1.5: Ensemble development and testing

The component models are weighted on the basis of their predictive validity rank to determine their contribution to the ensemble estimate. The relative weights are determined both by the model ranks and by a parameter ψ, whose value determines how quickly the weights taper off as rank decreases. The distribution of ψ is described in more detail in Foreman et al.[36] A set of ensemble models is then created by using the weights constructed from the combinations of ranks and ψ values. These ensembles are tested by using the predictive validity metrics described in Section 3.1.4 on the remaining 15% of the data, and the ensemble with the best performance in out-of-sample trend and RMSE is chosen as the final model.

Section 3.1.6: Final estimation

Once a weighting scheme has been chosen, 1000 draws are created for the final ensemble, and the number of draws contributed by each model is proportional to its weight. The mean of the draws is used as the final estimate for the CODEm process, and a 95% UI is created from the 0·025 and 0·975 quantiles of the draws. The validity of the UI can be checked via its coverage of the out-of-sample data; ideally, the 95% UI would capture 95% of these data. Higher coverage suggests that the UIs are too large, and lower coverage suggests overfitting.

Section 3.1.7: Selection of causes for which CODEm is used

CODEm is used to model 193 causes, described in detail in Section 3.3. However, it is unsuitable for use in modelling certain causes, including those with very low death counts, those where cause-specific death record availability is inadequate, or those for which there are marked biases or variability for CoD certification over time that cannot be fully accounted for with the current garbage code redistribution algorithms. Criteria for causes where CODEm is not used are discussed in further detail in Section 3.2.

Section 3.1.8: Model-specific covariates

Modellers select covariates to be used in CODEm, but those covariates may not be significant or in the direction specified during the covariate selection step of CODEm and will therefore not be used in the model. These covariates are listed with a '—' for number of draws. Additionally, covariates may be selected by CODEm but only exist in submodels that perform poorly and may end up with zero draws included in the final ensemble. Finally, all other covariates are listed with the number of draws in the final ensemble from submodels that had the covariate.

Section 3.2: Causes modelled outside of CODEm[1]

Section 3.2.1: Overview

A number of causes required alternative modelling strategies to those used for CODEm because they were not compatible with CODEm estimation infrastructure and processes. Such unsuitability included having very low death counts; inadequate availability of cause-specific death records; and marked biases or variability for CoD certification over time that could not be fully accounted for with current garbage code redistribution algorithms. The inclusion of these causes in CODEm often renders its out-of-sample predictive validity testing unstable, but the validity of this type of testing is a key advantage of using CODEm for CoD estimation. Alternately, CODEm simply fails to generate plausible mortality rates in the absence of enough VR or VA data when these causes are included. Because of increased data availability and redistribution algorithm refinements, we were able to incorporate several new causes, which were modelled separately for GBD 2013, into CODEm for this iteration of the GBD study; with each annual update of GBD, we aim to add more causes within the CODEm estimation space. For GBD 2019, we used alternative modelling approaches for these causes, including negative binomial models, natural history models, sub-cause proportion models, and prevalence-based models (table S10).

Section 3.2.2: Negative binomial models

For eight rare causes of death, too few observed deaths were included in the CoD database to produce stable estimates. For these causes, we ran negative binomial regression models, with either a constant or a constant multiplied by the mean assumption for the dispersion parameter, by using reverse step-wise model building. We selected one of the two model dispersion assumptions based on best fit to the data by using the same method as GBD 2013. For GBD 2015, we also tested zero-inflated Poisson models for these rare causes of death but rejected them after finding that they did not substantially affect the mean predictions but instead produced unrealistically large UIs. Descriptions of the modelling process for each of these causes follows in the next sections.

Section 3.2.3: DisMod-MR 2.1

Until GBD 2010, non-fatal estimates were based on a single data source on prevalence, incidence, remission, or a mortality risk selected by the researcher as most relevant to a particular location and time. For GBD 2010, we set a more ambitious goal: to evaluate all available information on a disease that passes a minimum quality standard. That required a different analytical tool that would be able to pool disparate information presented in varying age groupings and from data sources by using different methods. The DisMod-MR 1.0 tool used in GBD 2010 evaluated and pooled all available data, adjusted data for systematic bias associated with methods that varied from the reference, and produced estimates with UIs by world regions. For GBD 2013, the improved DisMod-MR 2.0 had increased computational speed, allowing computations that were consistent between all disease parameters at the country rather than the region level. The hundred-fold increase in speed of DisMod-MR 2.0 was partly due to a more efficient rewrite of the code in C++ but also to changing to a model specification using log rates rather than a negative binomial model used in DisMod-MR 1.0. In cross-validation tests, the log rates specification worked as well as or better than the negative binomial specification.[39] For GBD 2015, the computational engine (DisMod-MR 2.1) remained substantively unchanged, but we re-

wrote the wrapper code that organised the flow of data and settings at each level of the analytical cascade. The sequence of estimation occurred at five levels: global, super-region, region, country, and, where applicable, subnational locations (see flow diagram of DisMod-MR 2.1 cascade that follows). The super-region priors were generated at the global level with mixed-effects, non-linear regression by using all available data; the super-region fit, in turn, informed the region fit and so on down the cascade. The wrapper gave analysts the choice to branch the cascade in terms of time and sex at different levels depending on data density. The default used in most models was to branch by sex after the global fit but to retain all years of data until the lowest level in the cascade. For GBD 2015, we generated fits for the years 1990, 1995, 2000, 2005, 2010, and 2015.

In updating the wrapper, we consolidated the code base into a single language, Python, to make the code more transparent and efficient and to better deal with subnational estimation. The computational engine is limited to three levels of random effects; we differentiated estimates at the super-region, region, and country levels. In GBD 2013, the subnational units of China, Mexico, and the UK were treated as countries, such that a random effect was estimated for every location with contributing data. However, the lack of a hierarchy between country and subnational units meant that the fit to country data contributed as much to the estimation of a subnational unit as the fits for all other countries in the region. We found inconsistency between the country fit and the aggregation of subnational estimates when the country's epidemiology varied from the average of the region. Adding an additional level of random effects required a prohibitively comprehensive rewrite of the underlying DisMod-MR engine. Instead, we added a fifth layer to the cascade, with subnational estimation informed by the country fit and country covariates, plus an adjustment based on the average of the residuals between the subnational unit's available data and its prior. This procedure mimicked the impact of a random effect on estimates between subnationals.

For GBD 2015, we improved how country covariates differentiate non-fatal estimates for diseases with sparse data. The coefficients for country covariates were re-estimated at each level of the cascade. For a given location, country coefficients were calculated by using both data and prior information available for that location. In the absence of data, the coefficient of its parent location was chosen to utilise the predictive power of our covariates in data sparse situations.

For GBD 2017, the DisMod-MR 2.1 tool was used. Updates included estimation of new age groups through the GBD 2017 terminal age group of 95 years and older in addition to the new locations added for the GBD 2017 cycle.

Section 3.2.4: DisMod-MR 2.1 likelihood estimation

Analysts have the choice of using a Gaussian, log-Gaussian, Laplace, or log-Laplace likelihood function in DisMod-MR 2.1. The default log-Gaussian equation for the data likelihood is as follows:

$$-log\left[p\left(y_j|\Phi\right)\right] = \log\left(\sqrt{2\pi}\right) + \log\left(\delta_j + s_j\right) + \frac{1}{2}\left(\frac{\log\left(a_j + \eta_j\right) - \log\left(m_j + \eta_j\right)}{\delta_j + s_j}\right)^2$$

Where:

y_j is a measurement value (ie, data point)

Φ denotes all model random variables

η_j is the offset value, *eta*, for a particular integrand (prevalence, incidence, remission, excess mortality rate, with-condition mortality rate, cause-specific mortality rate, relative risk, or standardised mortality ratio)

a_j is the adjusted measurement for data point j, defined by

$$a_j = e^{(-u_j - c_j)} y_j$$

Where:

u_j is the total area effect (ie, the sum of the random effects at three levels of the cascade: super-region, region, and country)

c_j is the total covariate effect (ie, the mean combined fixed effects for sex, study-level, and country-level covariates), defined by

$$c_j = \sum_{k=0}^{K[I(j)]-1} \beta_{I(j),k} \hat{X}_{k,j}$$

with standard deviation (SD)

$$s_j = \sum_{l=0}^{L[I(j)]-1} \zeta_{I(j),l} \hat{Z}_{k,j}$$

Where:

k denotes the mean value of each data point in relation to a covariate (also called x-covariate)

$I(j)$ denotes a data point for a particular integrand, j

$\beta_{I(j),k}$ is the multiplier of the kth x-covariate for the ith integrand

$\hat{X}_{k,j}$ is the covariate value corresponding to the data point j for covariate k

l denotes the SD of each data point in relation to a covariate (also called z-covariate)

$\zeta_{I(j),k}$ is the multiplier of the lth z-covariate for the ith integrand

δ_j is the SD for adjusted measurement j, defined by

$$\delta_j = log\left[y_j + e^{(-u_j - c_j)}\eta_j + c_j\right] - log\left[y_j + e^{(-u_j - c_j)}\eta_j\right]$$

Where mj denotes the model for the jth measurement, not counting effects or measurement noise and defined by

$$m_j = \frac{1}{B(j) - A(j)} \int_{A(j)}^{B(j)} I_j(\text{a})\ \text{da}$$

Where:

$A(j)$ is the lower bound of the age range for a data point j

$B(j)$ is the upper bound of the age range for a data point j

$I(j)$ denotes the function of age corresponding to the integrand for data point j

The source code for DisMod-MR 2.1 as well as the wrapper code is available at https://github.com/ihmeuw/ihme-modelling/tree/master/gbd_2017/shared_code/central_comp/nonfatal/dismod.

Section 3.2.5: Natural history models

For some causes for which CoD data may be systematically biased either owing to misclassification or because the disease exists in focal communities without VR or VA studies, we have developed natural history models. In natural history models, incidence and case-fatality rates are modelled separately and then combined to produce estimates of cause-specific mortality.

Section 3.2.6: Prevalence-based models

The modelling strategies for atrial fibrillation and flutter are distinct from those used for other causes modelled as natural history models. These models use prevalence estimates and excess mortality rates (EMR) generated through DisMod-MR 2.1 rather than incidence and case-fatality rates.

Section 3.2.7: Sub-cause proportion models

For certain sub-causes for which accurate diagnoses are known to be very difficult, we first modelled the parent cause in the GBD hierarchy with CODEm and then allocated deaths to specific causes by using proportions of the parent cause for each age-sex-location-year for each sub-cause. For these causes, we identified no significant predictors in negative binomial regressions. This approach was taken because the available data on these specific causes may come from sources other than VR, such as end-stage renal disease registries, or may come from too few places to model the death rates directly. Details for each cluster of causes analysed in this way follow.

Section 3.3: Central computation[1]

Section 3.3.1: Imported cases

Imported cases are fatalities that occur in a geographic area where a particular CoD is known to be eradicated in a specific time period or where infection cannot occur. We apply space-time restrictions to these causes in the modelling strategy for that location and time period. However, in some rare cases, deaths from these causes occur outside of restricted locations and time periods. These deaths are referred to as imported cases.

Illustrating this concept, Chagas disease is transmitted by insect vectors that only exist in the Americas. For this reason, Chagas disease is restricted in the models for countries such as Russia. However, someone traveling in Latin America could contract Chagas disease and then die after returning home to Russia. Imported cases accounts for these kinds of deaths.

To calculate these imported cases, we find all cases from the VRs of data-rich countries for any CoD that is otherwise geographically or temporally restricted. We then create a beta distribution from that data point by using the sample size of the VR for that data point and upload these draws as a custom CoD model. This model is then used as an input to CoDCorrect.

Section 3.3.2: CoDCorrect

Section 3.3.2.1 Objective of CoDCorrect

As mentioned in the main text, the CoD models are cause-specific. As such, there is no guarantee that the sum of these models will equal the results of the all-cause mortality estimates or that model results of child causes add up to the parent model results. The CoDCorrect process is used to make the CoD and all-cause mortality estimates internally consistent by using a very simple algorithm.

Section 3.3.2.2 Algorithm and levels

The core algorithm remains the same as it did in GBD 2013. The equation can be written as follows:

$$CD_{lyasjd} = D_{lyasjd}\left(\frac{PD_{lyasjd}}{\sum_{j=1}^{j=k} D_{lyasjd}}\right)$$

Where:

CD_{lyasjd} is the corrected number of deaths for a location l, year y, age a, sex s, cause j, and draw d

PD_{lyasjd} is the parent CoD for a location l, year y, age a, sex s, cause j, and draw d

D_{lyasjd} is the uncorrected number of deaths estimated from a cause-specific model for a l, year y, age a, sex s, cause j, and draw d

The CoDCorrect process starts by rescaling the Level 1 causes to match the all-cause mortality estimates (used for PD_{lyasjd} in the previous equation). Level 2 causes are then rescaled to their corrected parent causes. This process continues until all levels of the hierarchy have been rescaled. Causes and their levels within the CoDCorrect hierarchy can be found in table S9.

Since GBD 2017, HIV has not been included in the CoDCorrect process. To account for this change, Level 1 CoDCorrect causes are rescaled to HIV-deleted mortality estimates that are produced as part of the mortality and HIV estimation process. Results from the GBD version of Spectrum are added to the post-CoDCorrect death estimates with fatal discontinuities and imported cases to generate the full set of death estimates.

Section 3.3.2.3 Diagnostic results of CoDCorrect by cause and location

For more detail on diagnostic results of CodCorrect by cause see table S15.

Section 3.3.3: Years of life lost calculation

Years of life lost (YLLs) owing to premature mortality were computed for 1082 locations and 39 years. First, we used the lowest observed age-specific mortality rates by location and sex across all estimation years from locations with total populations greater than 5 million in 2016 to establish a theoretical minimum risk reference life table.

The YLL is a metric that is computed by multiplying the number of estimated deaths by the standard life expectancy at age of death. The metric therefore highlights premature deaths by applying a larger weight to deaths that occur in younger age groups. We propagated uncertainty from CoDCorrected deaths for all demographics. The core equation can be written as follows:

$$YLL = \sum_{c=1,a=0,s=1}^{\infty} d_{cas}e_a$$

Section 3.3.4: GBD world population age standard

Age-standardised populations in the GBD were calculated by using the GBD world population age standard. For GBD 2013, GBD 2015, and GBD 2016, the age-specific proportional distributions of all national locations from the UN Population Division World Population Prospects 2012 revision for all years from 2010 to 2035 were used to generate a standard population age structure by using the non-weighted mean across all the aforementioned country-years. For GBD 2017, we used the non-weighted mean of 2017 age-specific proportional distributions from the GBD 2017 population estimates for all national locations with a population greater than 5 million people in 2017 to generate an updated standard population age structure.[40] For GBD 2019, we have continued to use this method using GBD 2019 population estimates.[8]

Section 3.4: CoD cause-specific modelling descriptions

GBD 2019 cause of death appendix write-ups in order:

1. HIV/AIDS
2. HIV/AIDs–multidrug-resistant tuberculosis without extensive drug resistance, HIV/AIDS–extensively drug-resistant tuberculosis, and HIV/AIDS–drug-susceptible tuberculosis
3. Sexually transmitted diseases excluding HIV
4. Tuberculosis
5. Multidrug-resistant tuberculosis, extensively drug-resistant tuberculosis, and drug-susceptible tuberculosis
6. Lower respiratory infections
7. Upper respiratory infections
8. Otitis media
9. Diarrhoeal diseases
10. Typhoid fever
11. Paratyphoid fever
12. Invasive non-typhoidal Salmonella (iNTS)
13. Other intestinal infectious diseases
14. Malaria
15. Chagas disease
16. Visceral leishmaniasis
17. African trypanosomiasis
18. Schistosomiasis
19. Cysticercosis
20. Cystic echinococcosis
21. Dengue
22. Yellow fever
23. Rabies
24. Ascariasis
25. Ebola virus disease
26. Zika virus disease
27. Other neglected tropical diseases
28. Meningitis
29. Encephalitis
30. Diphtheria
31. Whooping cough
32. Tetanus
33. Measles
34. Varicella and herpes zoster
35. Acute hepatitis
36. Other unspecified infectious diseases
37. Maternal disorders

38. Neonatal disorders
39. Nutritional deficiencies
40. Neoplasms
41. Cardiovascular diseases
42. Rheumatic heart disease
43. Ischaemic heart disease
44. Stroke
45. Ischaemic stroke
46. Intracerebral haemorrhage
47. Subarachnoid haemorrhage
48. Hypertensive heart disease
49. Non-rheumatic valvular heart disease, non-rheumatic calcific aortic valvular heart disease, non-rheumatic degenerative mitral valvular heart disease, and other non-rheumatic valvular heart diseases
50. Cardiomyopathy and myocarditis
51. Myocarditis
52. Alcoholic cardiomyopathy
53. Other cardiomyopathy
54. Atrial fibrillation and flutter
55. Aortic aneurysm
56. Peripheral artery disease
57. Endocarditis
58. Other cardiovascular and circulatory diseases
59. Chronic respiratory diseases
60. Chronic obstructive pulmonary disease
61. Pneumoconiosis: silicosis, asbestosis, coal worker's pneumoconiosis, and other pneumoconiosis
62. Asthma
63. Interstitial lung disease and pulmonary sarcoidosis
64. Other chronic respiratory diseases
65. Digestive diseases
66. Cirrhosis and other chronic liver diseases
67. Upper digestive system diseases
68. Peptic ulcer disease
69. Gastritis and duodenitis
70. Appendicitis
71. Paralytic ileus and intestinal obstruction
72. Inguinal, femoral, and abdominal hernia
73. Inflammatory bowel disease
74. Vascular intestinal disorders
75. Gallbladder and biliary diseases
76. Pancreatitis
77. Other digestive diseases

78. Alzheimer’s disease and other dementias
79. Parkinson disease
80. Idiopathic epilepsy
81. Multiple sclerosis
82. Motor neuron disease
83. Other neurological disorders
84. Eating disorders
85. Anorexia nervosa
86. Bulimia nervosa
87. Alcohol use disorders
88. Drug use disorders
89. Opioid use disorders
90. Cocaine use disorders
91. Amphetamine use disorders
92. Other drug use disorders
93. Diabetes mellitus
94. Chronic kidney disease
95. Acute glomerulonephritis
96. Skin and subcutaneous diseases
97. Bacterial skin diseases
98. Cellulitis
99. Pyoderma
100. Decubitus ulcer
101. Other skin and subcutaneous diseases
102. Musculoskeletal disorders
103. Rheumatoid arthritis
104. Other musculoskeletal disorders
105. Congenital birth defects
106. Urinary diseases and male infertility
107. Urinary tract infection and interstitial nephritis
108. Urolithiasis
109. Other urinary diseases
110. Gynaecological diseases
111. Haemoglobinopathies and haemolytic anaemias
112. Endocrine, metabolic, blood, and immune disorders
113. Sudden infant death syndrome
114. Injuries
115. Fatal discontinuities

HIV/AIDS

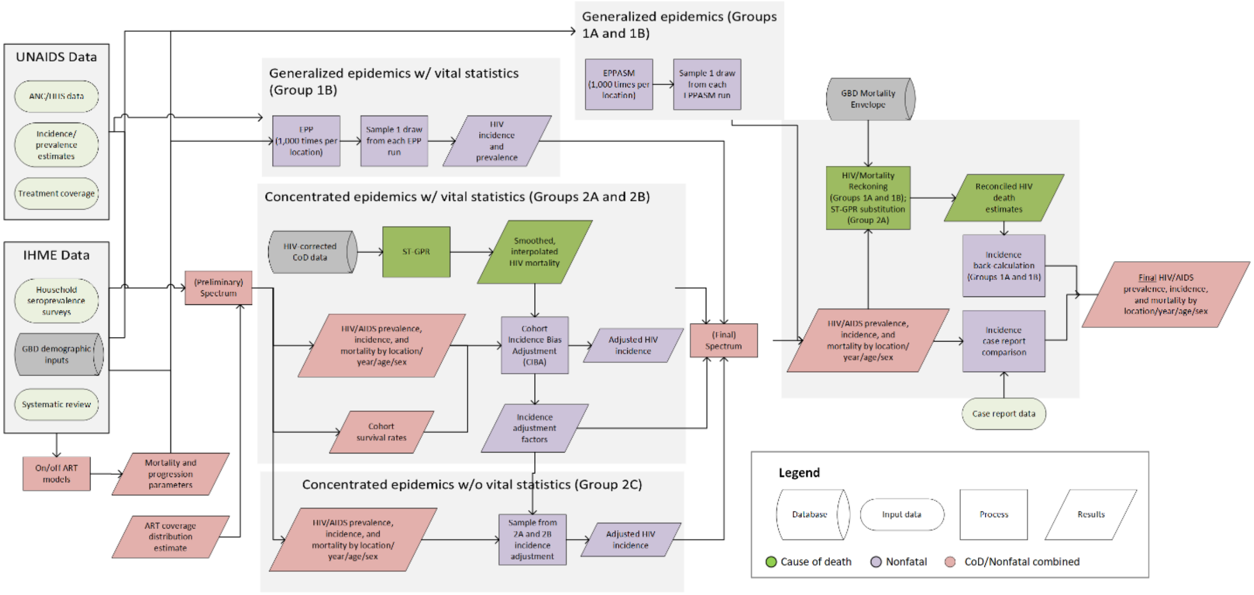

Case definition

Infection with the human immunodeficiency virus (HIV) causes influenza-like symptoms during the acute period following infection and can lead to acquired immunodeficiency syndrome (AIDS) if untreated. HIV attacks the immune system of its host, leaving infected individuals more susceptible to opportunistic infections like tuberculosis. Although there are two different subtypes of HIV, HIV-1 and HIV-2, no distinction is made in our estimation process or presentation of results. For HIV, ICD 10 codes are B20-B24, C46-C469, D84.9; ICD 9 codes are 042-044, 112-118 (after 1980), 130 (after 1980), 136.3-136.8 (after 1980), 176.0-176.9 (after 1980), 279 (after 1980); and ICD9 BTL codes are B184-B185.

Input data

Household seroprevalence surveys
Geographically representative HIV seroprevalence survey results were used as inputs to the model for countries with generalised HIV epidemics where available.

GBD demographic inputs
Location-specific population, fertility, migration and HIV-free survival rates from GBD 2019 were used as inputs in modelling all locations.

Data from countries
The files compiled by UNAIDS for their HIV/AIDS estimation process were our main source of data for producing estimates of HIV burden. Spectrum files are often built by within-country experts with the support of UNAIDS, which publishes estimates annually on behalf of countries and only shares their Spectrum files when permission is granted. The files contain the HIV-specific information which is needed to run the Estimation and Projection Package (EPP) model and the Estimation and Projection Package Age Sex Model (EPPASM).

Spectrum and EPPASM require the following input data: AIDS mortality among people living with HIV with and without ART, CD4 progression among people living with HIV not on ART, ART coverage among adults and children, cotrimoxazole coverage among children, coverage of breastfeeding among women living with HIV, prevention of mother-to-child transmission coverage, and CD4 thresholds for treatment eligibility. EPPASM additionally uses HIV prevalence data from surveillance sites and representative surveys. In contrast to Spectrum and EPPASM, EPP fits a simpler model to HIV prevalence data from surveillance sites and representative surveys only. Antenatal care (ANC), incidence, prevalence, and treatment coverage data from UNAIDS were used in modelling for all locations. We extracted all of these data from the proprietary format used by UNAIDS.

We did not have country UNAIDS files for 40 locations, many of them countries with small populations and/or low HIV prevalence. In those places, we generated regional averages of all needed inputs. This enabled us to run Spectrum for every GBD location.

Vital registration data
We used all available sources of vital registration and sample registration data from the GBD Causes of Death database after garbage code redistribution and HIV/AIDS mis-coding correction, except in Group 1A countries as described below.[1, 2] There are two different cause of death data sources for HIV/AIDS in China: the Disease Surveillance Point (DSP) system and the Notifiable Infectious Disease Reporting (NIDR) system. Both systems are administered by the Chinese Center for Disease Control and Prevention, but the reported number of deaths due to HIV is significantly lower in DSP. Therefore, we have used the provincial-level ratio of deaths due to HIV/AIDS from NIDR to those from DSP, choosing the larger ratio between years 2013 and 2014, and scaled the reported deaths in the DSP system, which is in turn used in the spatiotemporal Gaussian process regression (ST-GPR).

On-ART literature data
Data were identified by using search terms “HIV,” “mortality,” and “antiretroviral therapy” in PubMed searches across the literature. To be included, studies must include only HIV-positive people who receive antiretroviral therapy (ART) but who were ART-naïve prior to the study. In addition, studies must report either a duration-specific (time since initiation of ART) mortality proportion or a hazard ratio across age or sex, and must not include children.

For duration-specific survival data, studies must report uncertainty on mortality estimates or provide stratum-specific sample sizes and must include duration-specific data to allow for calculation of 0-6, 7-12, or 13-24 month conditional mortality. In addition, studies must either report separate mortality and loss-to-follow-up (LTFU) curves, be corrected for LTFU using vital registration data or double sampling, or be conducted in a high-income setting. Finally, studies must report the percentage of participants who are male and the median age of participants.

Hazard ratio data for ages or sexes can only be used if the hazard ratios are controlled for other variables of interest (age, sex, and CD4 category). In GBD 2013, we identified 102 papers for extraction. For GBD 2015, we included 13 additional studies informing the duration-specific mortality estimation process and 26 studies informing the age and sex hazard ratio estimation process (some studies were used and counted in both). We also added one study to our LTFU analysis. For GBD 2016, we included 12 additional studies informing the duration-specific mortality estimation process and 11 studies informing the age and sex hazard ratio estimation process (some studies were used and counted in both). For GBD 2017, we included 17 additional studies informing the duration-specific mortality estimation process and 13 studies informing the age and sex hazard ratio estimation process (some studies were used and

counted in both). We also included two new studies in our LTFU analysis. For GBD 2019, we did not update the systematic review or add cohort studies.

Off-ART literature data
In GBD 2013, we systematically reviewed the literature on mortality without ART to characterise uncertainty in the progression and death rates. We searched terms related to pre-ART or ART-naive survival since seroconversion.[3] After screening, we identified 13 cohort studies that included the cohorts used by UNAIDS, from which we extracted survival at each one-year point after infection. Screening for additional, recently published studies in GBD 2015, GBD 2016 and GBD 2017 identified no new cohort studies for inclusion in this analysis. We did not search for new studies in GBD 2019.

Severity splits and disability weights
The basis of the GBD disability weight survey assessments are lay descriptions of sequelae highlighting major functional consequences and symptoms. The lay descriptions and disability weights for HIV/AIDS severity levels are shown below.

**Severity level**	**Lay description**	**DW (95% CI)**
Symptomatic HIV	Has weight loss, fatigue, and frequent infections.	0.274 (0.184–0.377)
AIDS with antiretroviral treatment	Has occasional fevers and infections. The person takes daily medication that sometimes causes diarrhoea.	0.078 (0.052–0.111)
AIDS without antiretroviral treatment	Has severe weight loss, weakness, fatigue, cough and fever, and frequent infections, skin rashes, and diarrhoea.	0.582 (0.406–0.743)

Modelling strategy

We continued to estimate on-ART and off-ART mortality by CD4 count as in GBD 2017, which is described below. However, in GBD 2019, our burden estimation strategy for HIV incidence, prevalence, and mortality diverged from GBD 2017. We continued to use the Spectrum program rewritten in Python for GBD 2013 to facilitate faster and more flexible execution necessary for our more intensive computational needs for Group 2 countries. For India, we used EPP and Spectrum, as in GBD 2017. However, we used EPPASM exclusively for the remaining Group 1 countries. Both EPP and EPPASM are open-source computer programmes in R written by Jeffrey Eaton.[4,5]

On-ART
First, we corrected reported probabilities of death for loss to follow-up using an approach developed by Verguet and colleagues.[6] Verguet and colleagues used tracing and follow-up studies to empirically estimate the relationship between death in LTFU and the rate of LTFU.

To create estimates of age-specific hazard ratios, we synthesised hazard ratio data in five broad age groups: 15-25, 25-35, 35-45, 45-55, 55-100, and modelled the data using DisMod-MR 2.1.

To create estimates of sex-specific hazard ratios, we use the *metan* function in Stata to create estimates of relative risks separately by region, using female age groups as the reference group.
The age and sex hazard ratios were applied to the study-level mortality rates, accounting for the distribution of ages and sexes in the mortality data. We then subtracted HIV-free mortality from the model life table process to calculate study-level age-sex HIV-specific mortality.

We used DisMod-MR 2.1 to synthesise the age-sex-split study-level data into estimates of conditional probability of death over initial CD4 count.[3] We modelled the data separately by duration, age, sex, and region and added a fixed effect on whether the study was conducted prior to 2002. We estimate mortality for each region in its own DisMod model based on data from the IeDEA cohort collaboration,[7] and include a covariate for year as mortality among the LFTU has been found to decline in recent years.[8] Finally, we replaced our on-ART mortality rates with those estimated off treatment if they were higher.

Off-ART
Following UNAIDS assumptions, no-ART mortality is modelled as shown in the figure below.[3]

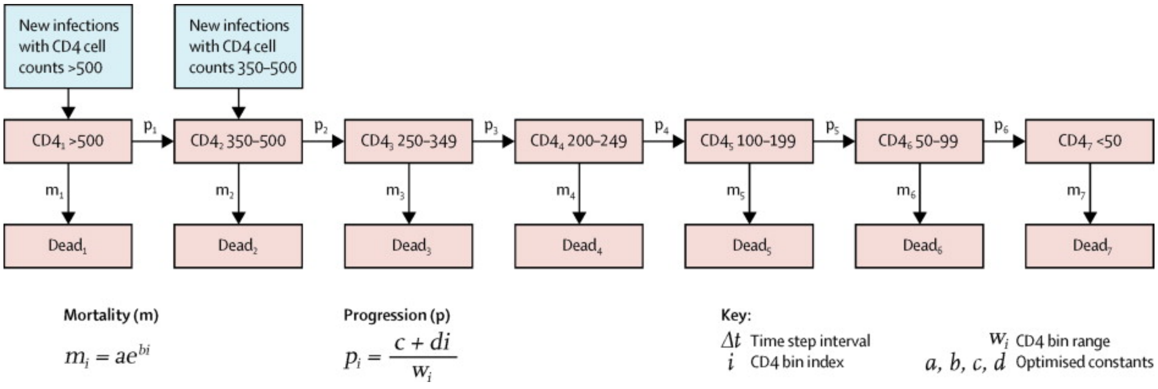

The death and progression rates between CD4 categories vary by age according to four age groups: 15–24 years, 25–34 years, 35–44 years, and 45 years or older. We modelled the logit of the conditional probability of death between years in these studies using the following formula:

$$logit\ (m_{ijk}) = \beta_0 + \sum_{i=1}^{4} \beta_{1i}\, a_i + \sum_{j=1}^{12} \beta_{2j} t_j + u_k + \varepsilon_{ijk}$$

In the formula, m is conditional probability of death from year t_j to t_{j+1}, a_i is an indicator variable for age group at seroconversion (15–24 years, 25–34 years, 35–44 years, and 45 years or older), t_j is an indicator variable of year since seroconversion, and u_K is a study-level random effect.

By sampling the variance-covariance matrix of the regression coefficients and the study-level random effect, we generated 1,000 survival curves for each age group that capture the systematic variation in survival across the available studies. For each of the 1,000 survival curves, we used a framework modelled after the UNAIDS optimisation framework in which we find a set of progression and death rates that minimises the sum of the squared errors for the fit to the survival curve.[9, 10]

We estimate mortality for each region in its own DisMod model based on data from the IeDEA cohort collaboration,[9] and include a covariate for year as mortality among the LFTU has been found to decline in recent years.[10] Finally, in cases where on-ART rates were higher, we replaced our estimated on-ART mortality rates by rates off ART to account for progression to lower CD4 categories. This ensured individuals would not experience higher mortality when they entered treatment in Spectrum or EPPASM.

GBD 2019 burden estimation overview

We used three different components to derive year-, age- and sex-specific estimates of HIV incidence, prevalence, and mortality depending on locations' availability of data and extent of HIV burden, as described below:

1. EPPASM was used to estimate incidence, prevalence, and mortality that are consistent with serosurveillance data from antenatal care clinics and/or prevalence surveys.

2. EPP was used to estimate age- and sex-aggregate incidence and prevalence trajectories that are consistent with serosurveillance data from antenatal care clinics and/or prevalence surveys in India subnational locations.

3. Spectrum is a compartmental HIV progression model used to generate age-sex-specific incidence, prevalence, and death rates from input incidence and prevalence curves and assumptions about intervention scale-up and local variation in epidemiology. This model was used in conjunction with EPP for India, and for all Group 2 countries.

Changes for GBD 2019

EPPASM

For GBD 2019, we modified the UNAIDS version of EPP-ASM both to improve the fit to data and to generate paediatric estimates. We built a paediatric module in EPP-ASM that mirrored the recent developments to the paediatric module in Spectrum.[11] This child module included CD4 progression and CD4-specific mortality rates taken from a model fit to survival data from IeDEA and child initiation of ART based on ART distribution data from IeDEA. Perinatal and breastfeeding transmission was calculated as a function of prevalence among pregnant women and PMTCT programme data. We were thus able to utilise EPP-ASM to produce HIV incidence, prevalence, and mortality estimates for all ages. Additionally, we improved fit to prevalence data through allowing flexibility in the age distribution of incidence over time. We parameterised the ratio of incidence among ages 15-24:25+ as a constant before year 2000 and a linear regression thereafter. This allowed for the shifts in the age distribution of incidence observed over the course of the HIV epidemic to be reflected in our results. Finally, we utilised GBD demographic inputs and substituted in our own assumptions about HIV progression rates and on/off-ART mortality.

To incorporate uncertainty in our demographic and progression parameters, we run EPP-ASM with separate draws of CD4 progression, on- and off-ART mortality rates, fertility, and HIV-free mortality. This process produced 1000 posterior distributions for each of the locations that make up Group 1A. For every location in the group, we sampled one draw from each of the sets of EPP-ASM results in order to create a final distribution. By sampling one draw from each set, we ensured that the distribution of

mortality parameters dictating the relationship between incidence and prevalence aligned with those used in the GBD demographics estimates.

ANC bias adjustment

For GBD 2019, we also implemented a new approach to address selection bias resulting from temporal and geographical variation in ANC reporting. The ANC data which EPPASM uses cannot be assumed as representative of HIV prevalence in the full population. This is especially the case when there are minimal or no nationally representative prevalence surveys to anchor estimates, as in the early epidemic.[12]

EPPASM has embedded approaches to adjust for the bias associated with using prevalence among ANC-site-attending pregnant women to estimate prevalence among the both-sexes population. For the bias between pregnant women and the national both-sexes population, it makes assumptions around the difference in total fertility rate among HIV positive and HIV negative women, and the difference in prevalence between men and women. For the bias associated with the data coming from ANC sites, the specification of the likelihood of observed ANC data includes random intercepts for each clinic. The random intercepts allow each site's baseline prevalence to vary randomly around the overall mean prevalence. In other words, factors that could drive differences between sites' HIV prevalence levels are "adjusted" for.

However, the embedded approach does not explicitly account for the fact that the location of the clinic in space may also drive its HIV prevalence level. For example, we might expect rural sites to be more correlated than urban sites. Thus, to further adjust for this bias, we used an offset term that represents the difference in the prevalence among the national, both-sexes population and the prevalence among the female, pregnant population associated with an ANC site location. The offset term was derived for each location as the difference between the adjusted prevalence in a given site-year and the adjusted national prevalence in that year. These estimates are adjusted for covariates that are thought to influence prevalence, for example, access to health-care facilities, malaria incidence, and male circumcision.

Thus, our final strategy for estimating the likelihood of the observed ANC data was:

$$\boldsymbol{W_{st} = \varphi^{-1}(\rho_t) + \vartheta_{st} + u_s + e_{st}}$$

$$\boldsymbol{e_{st} \sim N(0, \sigma_{st}^2)}$$

$$\boldsymbol{u_s \sim N(0, \sigma_s^2)}$$

Where:

W_{st} = the probit transformed prevalence at site *s* and time *t*
ρ_t= The national prevalence adjusted to represent prevalence among pregnant women from the model simulation
ϑ_{st} = The offset term representing the difference between the adjusted prevalence in a given site-year and the adjusted national prevalence in that year
φ^{-1} = probit transformation
e_{st} = Site-specific error term
u_s = Site-specific intercept

Spectrum

For GBD 2013, we created an exact replica of Spectrum in Python. This enabled us to run thousands of iterations of the model at once on our computing cluster and allowed for more flexible input data structures. Additionally, we scaled all input values by a uniformly sampled factor between 0.9 and 1.1 to generate estimates with realistic ranges of uncertainty. For example, if treatment retention rates across CD4 categories were 0.906, 0.759, 0.787, 0.795, 0.785, 0.756, 0.813, and 0.700, we multiplied each number by an array of equivalent size that contained factors ranging from 0.9 to 1.1. At each draw, the array would contain different, randomly selected factors in the same range. Further, we previously improved our sex-specific modelling strategy in Spectrum by sex-splitting incidence based on a model fit to the sex ratio of prevalence observed in countries with representative surveys and updated the Spectrum paediatric module to reflect changes made by UNAIDS.[11] Our child module was revised to include CD4 progression and CD4-specific mortality rates taken from a model fit to survival data from IeDEA. Finally, we updated child initiation of ART to include data on ART distribution from IeDEA. These changes were retained in GBD 2019.

ART coverage distribution

Spectrum determines the number of people initiating ART treatment across each CD4 category based on eligibility criteria, and the number of expected deaths and untreated people. In other words, groups with a large proportion of people living with HIV and high numbers of expected deaths initiated the most individuals into treatment.

We improved the basis for this distribution using survey microdata and country-level wealth information. Three relevant surveys were available: Uganda AIS 2011 and Kenya AIS 2007 and 2012. These surveys conducted CD4 count measurements and include a question regarding the amount of time that an individual receiving ART had been enrolled in treatment. Survey data provide cross-sectional CD4 count information; however, the Spectrum modelling framework tracks individuals by categorical CD4 count at the initiation of treatment. In order to cross-walk the cross-sectional survey data into estimates of CD4 count at treatment initiation, we built a model using relevant cohort data which tracked changes in CD4 count after initiation of treatment to translate an individual's current CD4 count and duration on treatment into CD4 count at initiation of treatment. The functional form for changes in CD4 count as a function of duration on treatment was a natural spline on duration with knots at 3, 12, 24, and 36 months, and an interaction between initial CD4 count and duration.

After cross-walking, we predicted the probability of being on treatment as a function of individual income (measured through an asset-based index), stratified by CD4 count, age, and sex. The results of this prediction were translated into country-specific age-sex-year-CD4 count probabilities of coverage using a conversion factor between individual income and lag-distributed GDP per capita. We used stochastic frontier analysis to constrain the maximum possible coverage for a given degree of income and CD4 count.

Predicted probabilities of coverage were input to Spectrum to inform the distribution, and not the overall level, of ART treatment by CD4 count. Within Spectrum, the probabilities of coverage are converted to counts of expected individuals on treatment in each CD4 count group. These are scaled to the distribution across CD4 count groups to match the input data on the number of people on ART coming from UNAIDS country files. In cases where the predicted number of individuals initiating treatment exceeds the total number of untreated individuals in a CD4 count group, we reallocate treatment evenly to other CD4 count groups.

Countries with seroprevalence surveys and antenatal clinic data (Groups 1A and 1B)

We identified 50 countries – as well as subnational locations in India, Kenya, Ethiopia, Nigeria and South Africa – with at least 0.5% adult HIV prevalence and at least one geographically representative HIV seroprevalence survey or available antenatal care clinic (ANC) data. For all locations except India we used a version of EPPASM, and for India we used a version of EPP. Both were written in R and C++ by Jeffrey Eaton. The version of EPP and EPPASM used in GBD 2019 was updated to incorporate the new ANC bias adjustment. Further we added a paediatric module in EPPASM which was a replicate of the paediatric model embedded in Spectrum.

EPP and EPPASM rely on the parameter estimation via the IMIS procedure, described in Raftery and Bao.[13] Two optimisation methods have been introduced. The main algorithm is Broyden–Fletcher–Goldfarb–Shanno (BFGS) optimisation. If BFGS fails, Nelder-Mead optimum is used instead.[14-16] To incorporate uncertainty in our mortality and progression parameters, we run EPP with separate draws of each of these parameters. Then, for every location, we have 1000 linked draws of adult incidence and prevalence and the exact mortality and progression parameters that generated those draws. For EPP locations (India), we then ran these results, along with the previously described demographic and HIV-specific inputs, through Spectrum to produce location-, year-, age-, and sex-specific estimates of HIV incidence, prevalence, and mortality.

The HIV/mortality reckoning process is intended as a method of reconciling separate estimates of HIV mortality (and its resulting effect on estimates of HIV-free and all-cause mortality) in Group 1 countries by averaging estimates of HIV mortality from the model life table process and our modelled estimates. Additional details on the reckoning can be found elsewhere.[17]

Since EPP-ASM produces HIV incidence, prevalence, and deaths that are consistent with one another over time, the reckoning process results in death numbers that are no longer consistent with the incidence and prevalence produced in Spectrum. In order to recreate this consistency, we recalculated incidence for all Group 1 locations using reckoned deaths and prevalence produced by EPP-ASM. The updated incidence is calculated by aggregating counts of new infections, HIV deaths from EPP-ASM, and HIV deaths after reckoning at the year-sex level. The difference between reckoned HIV deaths and HIV deaths from EPP-ASM is added to EPP-ASM incidence, and we calculate the ratio between updated incidence and EPP-ASM incidence. Age-specific counts of new infections are then scaled by their corresponding sex-year ratios.

Countries with vital registration data (All of Group 2A, 2B and India)
Vital registration is one of the highest-quality sources of data on HIV burden in many countries, so generating estimates that are consistent with these data with necessary adjustment to account for any potential underreporting is critical. We identified 121 countries – as well as 632 subnational locations from China, Japan, Indonesia, India, Mexico, Sweden, Philippines, Poland, Italy, the United Kingdom, Ukraine, Russia, New Zealand, Iran, Norway and the United States – with usable points of vital registration data, verbal autopsy (VA) data, or sample registration system (SRS) data. In India, Vietnam and Indonesia, we used SRS and VA data, respectively, as input mortality for CIBA. For India we extracted the resulting age-sex distribution of incidence but scaled the level to match the adult incidence rate estimated from EPP for each state.

We imputed missing years of data to generate a complete time series for HIV from the estimated start year of the epidemic using ST-GPR. We analysed mortality trends using ST-GPR starting in 1981, the year that HIV was first identified in the USA.[18] For ST-GPR, we adjusted the lambda (time weight) and GPR scale according to the completeness of vital registration data, with 4- and 5-star quality VR using

parameters designed to follow the data more closely. We produced separate splines by country/age group, up to the peak year of death rate. We then ran a linear regression with fixed effects on region, age, and sex. Following this, we ran space-time residual smoothing, in which time, age, and space weights are used to inform smoothing of the residuals between datapoints and the linear regression estimate. From this process, we generated space-time estimates with the applied weights, along with the median absolute deviation (MAD) of the space-time estimates from the data. The MAD was calculated at various levels of the geographical hierarchy (eg, subnational and national), and was added into the data variance term. The data variance and space-time estimates were then analysed using Gaussian process regression to return a final estimate of mortality along with uncertainty.

Although Spectrum produces HIV mortality estimates that are within the realm of possibility in most countries using the incidence curves provided in the UNAIDS country files, it is a deterministic model that has not yet been integrated into an optimisable framework. Therefore, in order to "fit" it to vital registration data, we need to adjust input incidence.

To improve the fit of this process, in GBD 2015, we restructured Spectrum to track cohorts by year of HIV infection. With this version of Spectrum we can output, among many other metrics, HIV deaths by year, age, sex, and infection cohort. This enables us to adjust incidence to fit to death much more precisely and without making any rigid assumptions about the time from HIV infection to HIV death.

We have incorporated these improvements into a cohort incidence bias adjustment (CIBA) process. First, we ran Spectrum normally to produce 1000 draws of incidence, prevalence, and mortality. Then, by year, age, and sex, we took the ratio of VR deaths to Spectrum deaths to quantify the amount of bias in Spectrum. Using draw-level duration data from the new version of Spectrum, for every year-, age-, and sex-specific infection cohort, we calculated the share of all HIV deaths observed over the course of the projection period in that cohort that would occur in each year after the year of infection. For example, projecting from 1970 through 2019, we identified the cohort of men infected in 1992 at the age of 16, calculated the total number of HIV deaths in that cohort in all subsequent years through the end of 2019, and divided the annual number of deaths by that total. This showed us the distribution of deaths among that cohort over the projection period. In the most extreme case (infections in 2018), we could only produce one point of that distribution (2019), so that single value is exactly 1·0; 100% of the deaths observed in that cohort occurred in 2019.

We then used these distributions of death to weigh the ratio of VR deaths to Spectrum deaths, meaning that ratios in the years where we expect the largest share of deaths were weighed most heavily. We then multiplied the initial size of that cohort from the normal run of Spectrum by the sum of the combined ratios to get a new estimate of new cases in that year/age/sex combination. We can write this method mathematically in the following way:

$$r_t = \frac{VR_t}{D_t}$$

$$\rho_t^{t-i} = \frac{d_t^{t-i}}{\sum_{k=t-i+1}^{n} d_k^{t-i}}$$

$$\alpha^{t-i} = \sum_{k=t-i+1}^{n} r_k * \rho_k^{t-i}$$

$$n_{\text{adjusted}}^{t-i} = \alpha^{t-i} * n^{t-i}$$

VR_t is the number of HIV/AIDS deaths in year t from ST-GPR, and D_t is the number of HIV/AIDS deaths from the first run of Spectrum. In the second equation, d_t^{t-i} is the number of HIV/AIDS deaths among members of infection cohort $t - i$ in year t, with $i \geq 1$, from the new, duration-tracking version of Spectrum, and n is final year of the projection. Therefore, ρ_t^{t-i} is the share of observed deaths in cohort $t - i$ that we expect to occur in year t. It follows that α^{t-i} is the weighted adjustment ratio described above, which we multiply by the estimated initial size of infection cohort $t - i$ as calculated in the first-stage Spectrum run to get the adjusted number of new cases, $n_{\text{adjusted}}^{t-i}$. This process is run separately for every sex, single age, and draw.

CIBA allows ratios in each year after a given infection year to influence the final adjustment to incidence. The size of that influence is determined by the relative importance of that year in the cohort-year's distribution of deaths over time. The result is a new set of 1000 draws of incidence and a set of 1000 ratios of post-adjustment incidence to pre-adjustment incidence. We perform this adjustment using mean durations from the new version of Spectrum in order to try to shift the mean of the regular distribution of deaths.

Finally, to produce location-, year-, age-, and sex-specific estimates of HIV incidence, prevalence, and mortality, we ran the new estimates of incidence and all previously input data through Spectrum.

Countries without survey data and vital registration data (Group 2C)
40 countries had neither geographically representative seroprevalence surveys nor reliable vital registration systems. To produce estimates of HIV burden in these countries, we assumed that Spectrum is similarly biased as in other Group 2 countries within the same super-region. This involved running Spectrum, adjusting incidence using 1000 adjustment ratios randomly sampled from CIBA results from the same super-region, and rerunning Spectrum using the new draws of adjusted incidence. As above, the estimates of incidence, prevalence, and mortality were incorporated into the rest of the machinery via the reckoning process.

References

1. Global, regional, and national age–sex specific all-cause and cause-specific mortality for 240 causes of death, 1990–2013: a systematic analysis for the Global Burden of Disease Study 2013. The Lancet 2015; 385: 117–71.

2. Birnbaum JK, Murray CJ, Lozano R. Exposing misclassified HIV/AIDS deaths in South Africa. Bull World Health Organ 2011; 89: 278–85.

3. Murray CJL, Ortblad KF, Guinovart C, et al. Global, regional, and national incidence and mortality for HIV, tuberculosis, and malaria during 1990–2013: a systematic analysis for the Global Burden of Disease Study 2013. The Lancet 2014; 384: 1005–70.

4. jeffeaton/epp. GitHub. https://github.com/jeffeaton/epp (accessed July 1, 2019).

5. mrc-ide/eppasm. GitHub. https://github.com/mrc-ide/eppasm (accessed July 1, 2019).

6. Verguet S, Lim SS, Murray CJL, Gakidou E, Salomon JA. Incorporating Loss to Follow-up in Estimates of Survival Among HIV-Infected Individuals in Sub-Saharan Africa Enrolled in Antiretroviral Therapy Programs. J Infect Dis 2013; 207: 72–9.

7. Anderegg N, Johnson LF, Zaniewski E, et al. All-cause mortality in HIV-positive adults starting combination antiretroviral therapy: correcting for loss to follow-up. AIDS 2017; 31 Suppl 1: S31-40.

8. Zürcher K, Mooser A, Anderegg N, et al. Outcomes of HIV-positive patients lost to follow-up in African treatment programmes. Trop Med Int Health 2017; 22: 375-387.

9. Ghys PD, Zaba B, Prins M. Survival and mortality of people infected with HIV in low and middle income countries: results from the extended ALPHA network. AIDS Lond Engl 2007; 21 Suppl 6: S1–4.

10. Hallett TB, Zaba B, Todd J, et al., ALPHA Network. Estimating incidence from prevalence in generalised HIV epidemics: methods and validation. PLoS Med 2008; 5: e80.

11. Mahy M, Penazzato M, Ciaranello A, et al. Improving estimates of children living with HIV from the Spectrum AIDS Impact Model. Aids 2017;31: S13–S22

12. Ng M, Gakidou E, Murray CJL, Lim S. A comparison of missing data procedures for addressing selection bias in HIV sentinel surveillance data. Population Health Metrics 2013; 11: 12.

13. Raftery AE, Bao L. Estimating and Projecting Trends in HIV/AIDS Generalized Epidemics Using Incremental Mixture Importance Sampling. Biometrics 2010; 66: 1162–73.

14. Nelder JA, Mead R. A simplex algorithm for function minimization. Comput J 1965;7:308-13.

15. Nash JC. Compact numerical methods for computers. Linear algebra and function minimization. 2nd edn. Bristol, England: Adam Hilger, 1990.

16. Byrd RH, Lu P, Nocedal J, et al. A limited memory algorithm for bound constrained optimization. SIAM J Sci Comput 1995;16:1190-208.

17. Wang H, Murray CJL, Carter A, He F. Global, regional, and national under-5 mortality, adult mortality, age-specific mortality, and life expectancy, 1970-2016: a systematic analysis for the Global Burden of Disease Study 2016. The Lancet 2017; 390: 1151-1210.

18. CDC. Pneumocystis Pneumonia --- Los Angeles. MMWR Wkly. 1981; published online June 5. http://www.cdc.gov/mmwr/preview/mmwrhtml/june_5.htm (accessed April 21, 2016).

HIV/AIDS – multidrug-resistant tuberculosis without extensive drug resistance, HIV/AIDS – extensively drug-resistant tuberculosis, and HIV/AIDS – drug-susceptible tuberculosis

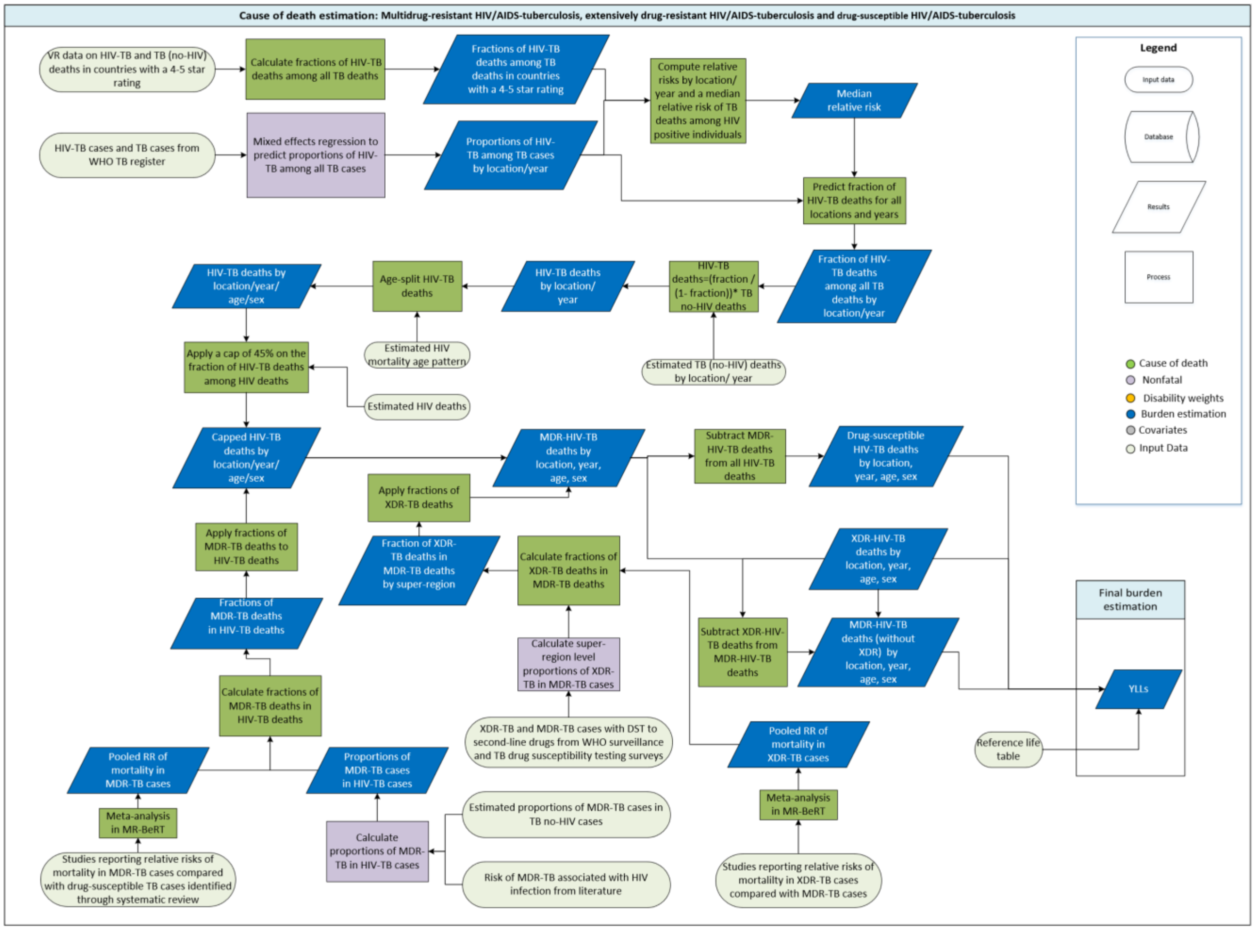

Input data

Input data for HIV/AIDS-tuberculosis (HIV-TB) mortality estimation include (i) 438 site-years of vital registration data from countries with a four- or five-star rating where cause of death data for directly coded HIV-TB and tuberculosis (TB) were available, and (ii) the number of TB cases (new and re-treatment) recorded as HIV-positive and the number of TB cases (new and re-treatment) with an HIV test result recorded in the TB register from the World Health Organization (WHO). We excluded data from countries with ten HIV-TB deaths or less. We also excluded data that were largely conflicting with the majority of data for other years from the same country.

Input data for estimation of multidrug-resistant and extensively drug-resistant HIV-TB include: (i) the number of drug-resistant cases by type (multidrug-resistant tuberculosis [MDR-TB], extensively drug-resistant tuberculosis [XDR-TB], all TB cases with a drug sensitivity testing [DST] result for isoniazid and rifampicin, and MDR-TB cases with DST for second-line drugs) from routine surveillance and surveys reported to WHO. Additional input data include relative risks of mortality in MDR-TB cases compared with drug-susceptible TB cases, and relative risks of mortality in XDR-TB cases compared with MDR-TB

cases reported by studies identified through our systematic review, and the risk of MDR-TB associated with HIV infection from the literature.[1]

Prisma Diagram of MDR-TB mortality relative risk in GBD2019

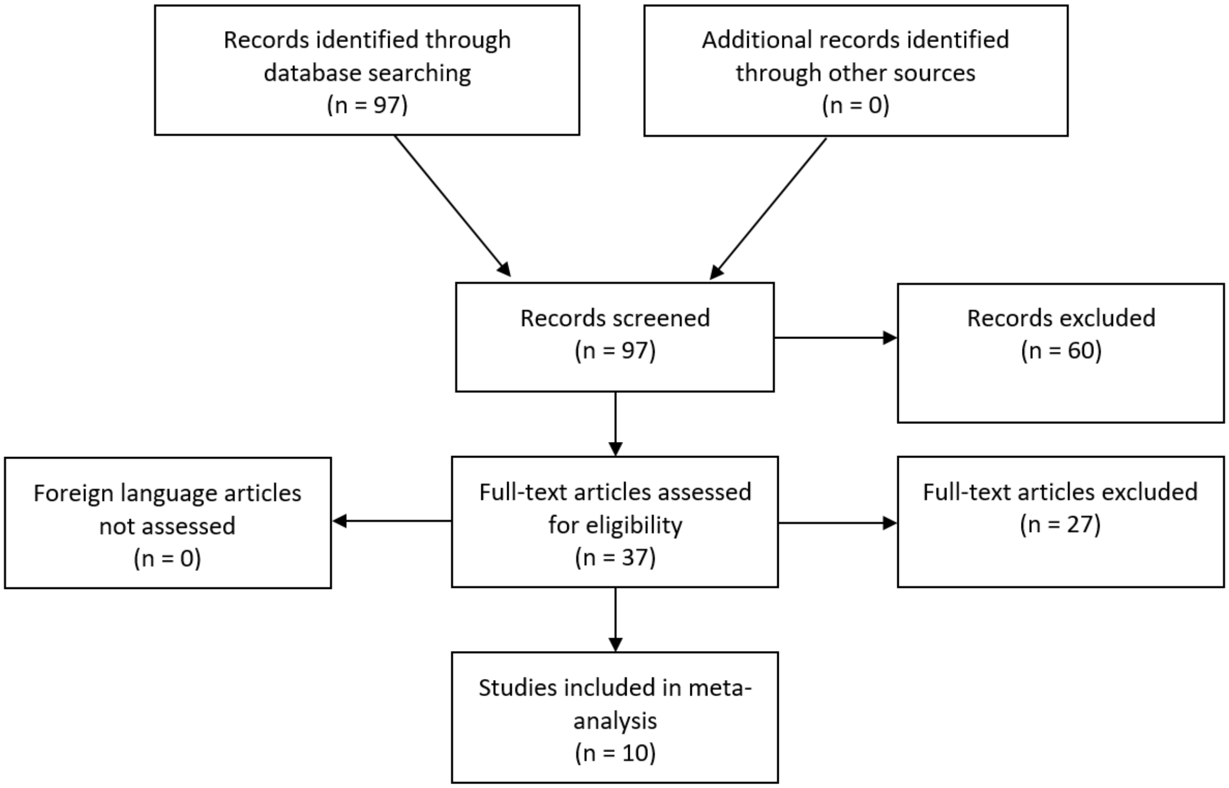

Modelling strategy

To determine TB deaths in HIV-positive individuals, we first computed the fraction of HIV-TB deaths among all TB deaths using vital registration data from countries with a four- or five-star rating. We also calculated the proportion of TB cases that are HIV-positive (ie, number of TB cases recorded as HIV-positive/number of TB cases with an HIV test result recorded in the WHO TB register). We used these proportions as input data for a mixed effects regression to predict the proportions of HIV-TB cases among all TB cases for all locations and years using an adult HIV death rate covariate. We estimated the fraction of HIV-TB deaths among all TB deaths in each location and year $(D_{c,y})$, defined by

$$D_{c,y} = \frac{P_{c,y}RR}{P_{c,y}RR + 1 - P_{c,y}}$$

where $P_{c,y}$ is the proportion of HIV-TB cases among all TB cases and *RR* is the relative risk of TB deaths in HIV positive individuals, defined by:

$$RR = \frac{D_{c,y}P_{c,y} - D_{c,y}}{D_{c,y}P_{c,y} - P_{c,y}}$$

We took the median relative risk (RR) from each calculation. We then applied the median RR and the predicted proportions of HIV-TB cases among all TB cases to get the fractions of HIV-TB deaths among all TB deaths for all locations and years. Location-year-specific HIV-TB deaths were then calculated using the following equation:

$$Deaths_{HIV-TB} = \frac{D_{c,y}}{1 - D_{c,y}} Deaths_{TB}$$

where $Deaths_{TB}$ is location-year specific deaths from the CODEm TB no-HIV model. Finally, we applied the age-sex pattern of the HIV mortality estimates to these HIV-TB deaths to generate location-year-age-sex-specific HIV-TB deaths. As the HIV-TB deaths were estimated based on the fraction of HIV-TB deaths among all TB deaths, the total number of HIV-TB deaths could exceed the total number of HIV deaths in some locations. To avoid this, we applied a cap of 45% on the fraction of HIV-TB deaths among HIV deaths, based on a review by Cox and colleagues, 2010,[2] and a systematic review and meta-analysis by Ford and colleagues, 2016.[3]

To split HIV-TB into HIV-MDR-TB and HIV-drug-susceptible-TB, we first calculated the proportion of HIV-MDR-TB among all HIV-TB cases ($P_{MDR-HIVc,y,a,s}$) for each location, year, age, and sex using the following formula:

$$P_{MDR-HIVc,y,a,s} = P_{MDRnoHIVc,y,a,s} RR_{HIV}$$

where $P_{MDRnoHIVc,y,a,s}$ is the estimated proportion of MDR-TB among HIV-negative TB cases for each location, year, age, and sex (see MDR-TB modelling strategy for the detail) and RR_{HIV} is the relative risk of MDR-TB associated with HIV infection.

We then computed the fraction of HIV-MDR-TB deaths among all HIV-TB deaths ($D_{MDR-HIVc,y,a,s}$) using the following formula:

$$D_{MDR-HIVc,y,a,s} = \frac{P_{MDR-HIVc,y,a,s} RR_{MDR}}{P_{MDR-HIVc,y,a,s} RR_{MDR} + 1 - P_{MDR-HIVc,y,a,s}}$$

where RR_{MDR} is the pooled relative risk of mortality in MDR-TB cases compared with drug-susceptible TB cases. In GBD 2019, the pooled relative risk was derived from a meta-analysis in the meta-regression with Bayesian priors, regularization, and trimming (MR-BRT) model. After derivation of the pooled relative risk, we then applied the predicted HIV-MDR-TB death fractions to all HIV-TB death estimates to generate HIV-MDR-TB deaths by location, year, age, and sex. Next, we subtracted HIV-MDR-TB deaths from all HIV-TB deaths at the 1000 draw level to generate drug-susceptible HIV-TB deaths by location, year, age, and sex.

To separate out HIV-XDR-TB from HIV-MDR-TB, we aggregated the XDR-TB cases and MDR-TB cases (with DST for second-line drugs) up to the super-region level and calculated the super-region-level proportions of XDR-TB among MDR-TB cases. Next, we computed the super-region-specific fraction of XDR-TB deaths among all MDR-TB deaths (D_{XDRsr}) using the following formula:

$$D_{XDRsr} = \frac{P_{XDRsr}RR_{XDR}}{P_{XDRsr}RR_{XDR} + 1 - P_{XDRsr}}$$

where P_{XDRsr} is the proportion of XDR-TB among MDR-TB cases for each super-region, and RR_{XDR} is the pooled relative risk of mortality in XDR-TB cases compared with MDR-TB cases. Similar to the pooled relative risk for MDR-TB, the derivation of the pooled relative risk of mortality in XDR-TB was computed with a meta-analysis in the MR-BRT model for GBD 2019. The fractions were then applied to MDR-TB deaths in corresponding countries within the super-regions to produce XDR-TB deaths by location, age, and sex for the most recent year of estimation. We linearly extrapolated XDR-TB mortality rates back, assuming the mortality rates were zero in 1992, one year before 1993 when XDR-TB was first recorded in USA surveillance data.[4] Finally, we subtracted HIV-XDR-TB deaths from HIV-MDR-TB deaths to generate HIV-MDR-TB (without extensive drug resistance) deaths by location, year, age, and sex.

References

1. Mesfin YM, Hailemariam D, Biadglign S, Kibret KT. Association between HIV/AIDS and multi-drug resistance tuberculosis: a systematic review and meta-analysis. PLoS One. 2014;9(1):e82235.
2. Cox JA, Lukande RL, Lucas S, Nelson AM, Van Marck E, Colebunders R. Autopsy causes of death in HIV-positive individuals in sub-Saharan Africa and correlation with clinical diagnoses. *AIDS Rev* 2010; **12**: 183–94.
3. Ford N, Matteelli A, Shubber Z, *et al.* TB as a cause of hospitalization and in-hospital mortality among people living with HIV worldwide: a systematic review and meta-analysis. *J Int AIDS Soc* 2016; **19**: 20714.
4. Centers for Disease Control and Prevention (CDC). Extensively Drug-Resistant Tuberculosis --- United States, 1993—2006. MMWR. 2007; 56(11);250-253

Sexually Transmitted Infections Excluding HIV

Total, chlamydia, gonorrhea, syphilis, and other

Flowchart

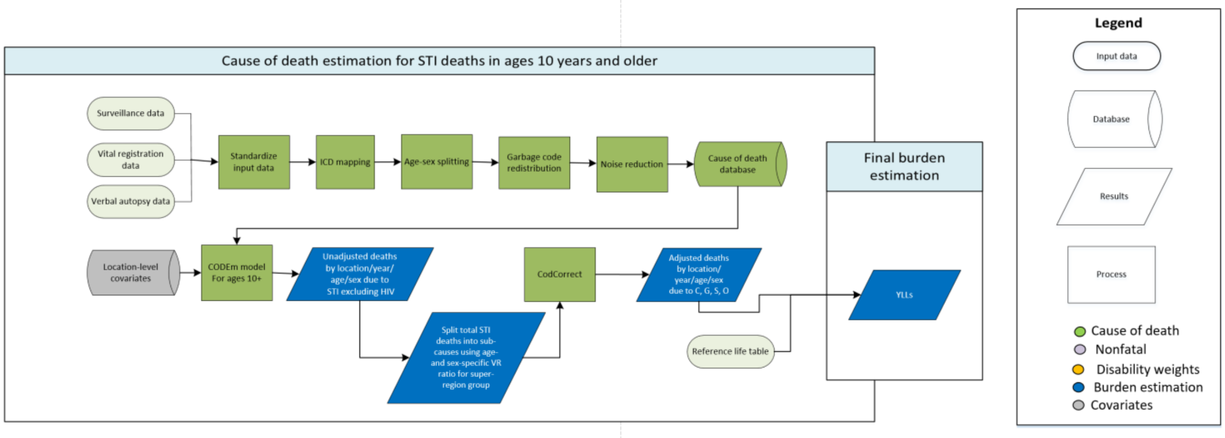

Input Data – Adult STIs

Total adult deaths due to STI excluding HIV were modeled in aggregate for males and females 10 years and older using centrally processed vital registration, verbal autopsy, and surveillance data from the cause of death (COD) database. These data included deaths from all geographies and coding systems for syphilis, chlamydial infection, gonococcal infection, and other STIs excluding HIV. Data were excluded if they violated well-established patterns for age, time or space. Data were also excluded for locations where sparse data, small numbers, and data processing combined to produce implausible cause fractions.

To produce estimates of deaths specifically due to syphilis, chlamydial infection, gonococcal infection and other STIs, estimates from the total model were divided according to proportions that were estimated from all available cause-specific vital registration data.

Modelling strategy – Adult STIs

We completed data-rich (DR) and global CODEm models for ages 10 years and over for males and females separately. Ten covariates were entered for possible selection in each CODEm model, including 1) prevalence of positive syphilis serology; 2) coverage of one antenatal care (ANC) visit; 3) coverage of four or more ANC visits; 4) age-specific fertility rate; 5) total fertility rate; 6) maternal care & immunization (a covariate based on a principal components analysis of ANC, in-facility delivery, skilled birth attendance, and vaccine coverage); 7) health care access and quality index (HAQI), 8) lag-distributed income (LDI); 9) years of education per capita; and 10) abortion legality (a categorical rating of abortion laws that range from 1 (always illegal) to 7 (always legal on demand)).

Table 1: Covariates used in STI mortality modelling

Level	Covariate	Direction
1	Syphilis prevalence	+
2	Abortion legality	-
	Age-specific fertility rate	+
	Education (years per capita)	-
	Total fertility rate	+
	Maternal Care & Immunization	-
	Health care access and quality index	-
3	Antenatal care coverage, 1+ visits	-
	Antenatal care coverage, 4+ visits	-
	Lag-distributed income	-

The CODEm model for STI was split into the sub-causes using vital registration (VR) data from the COD database. Trichomoniasis and HSV-2 were assumed not to cause mortality. Chlamydia was further assumed not to cause death in males. Therefore, for males the STI CODEm model was split into deaths due to syphilis, gonorrhea or other STI. For females, the STI CODEm model was split into deaths due to syphilis, gonorrhea, chlamydia, or other STI.

In GBD 2017, cause-specific VR data were summed by age group and sex, then scaled to the total STI death model in order to calculate proportions for each specific infection. These proportions were then applied to all locations. In GBD 2019, to account for geographic variation in proportions, cause-specific VR data were summed by age group, sex, and super-region, then scaled to the total. Unfortunately, the COD database had very sparse data on STI cause of death in sub-Saharan Africa and North Africa & the Middle East, which resulted in implausible proportions estimated for these super-regions. As a result, the decision was made to calculate cause-specific proportions by age, sex, and two super-region groups.

Table 2: Super-Region Groups for STI sub-cause proportions

Super-Region Group	Super-Regions Included
ALMA	• *Southeast Asia, East Asia & Oceania*<br>• *Latin America & Caribbean*<br>• *North Africa & Middle East*<br>• *South Asia*<br>• *Sub-Saharan Africa*
CECAH	• *Central Europe, Eastern Europe, and Central Asia*<br>• *High-Income*

Males: Adult STI proportional split

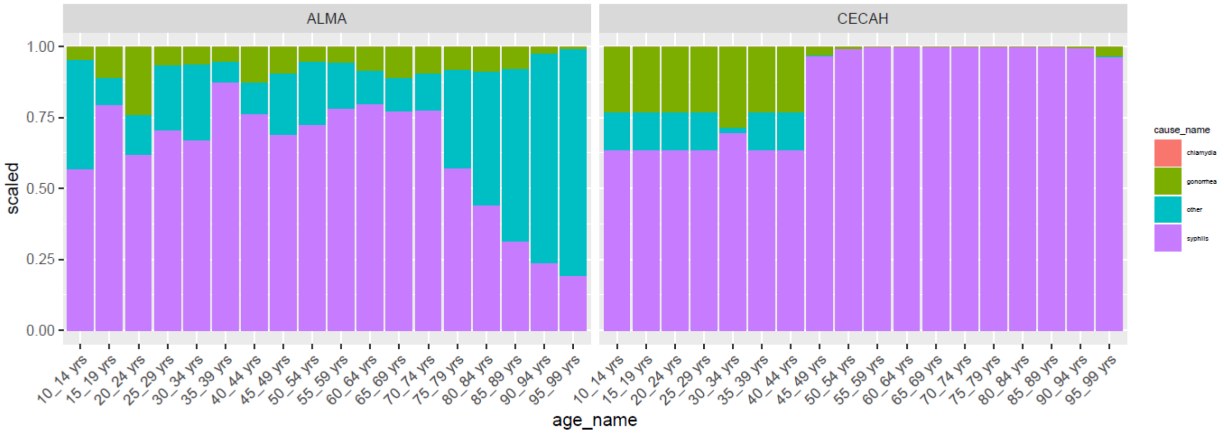

Females: Adult STI proportional split

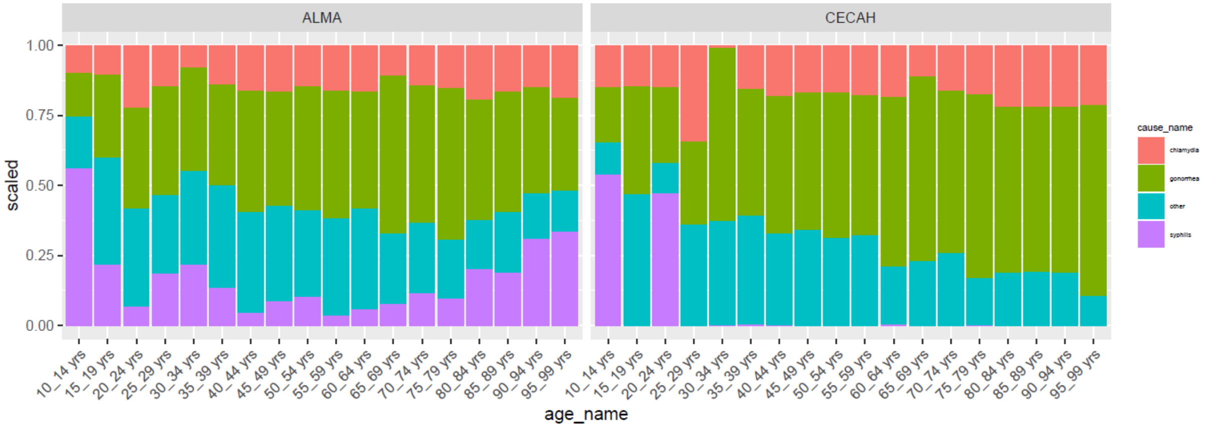

Congenital Syphilis

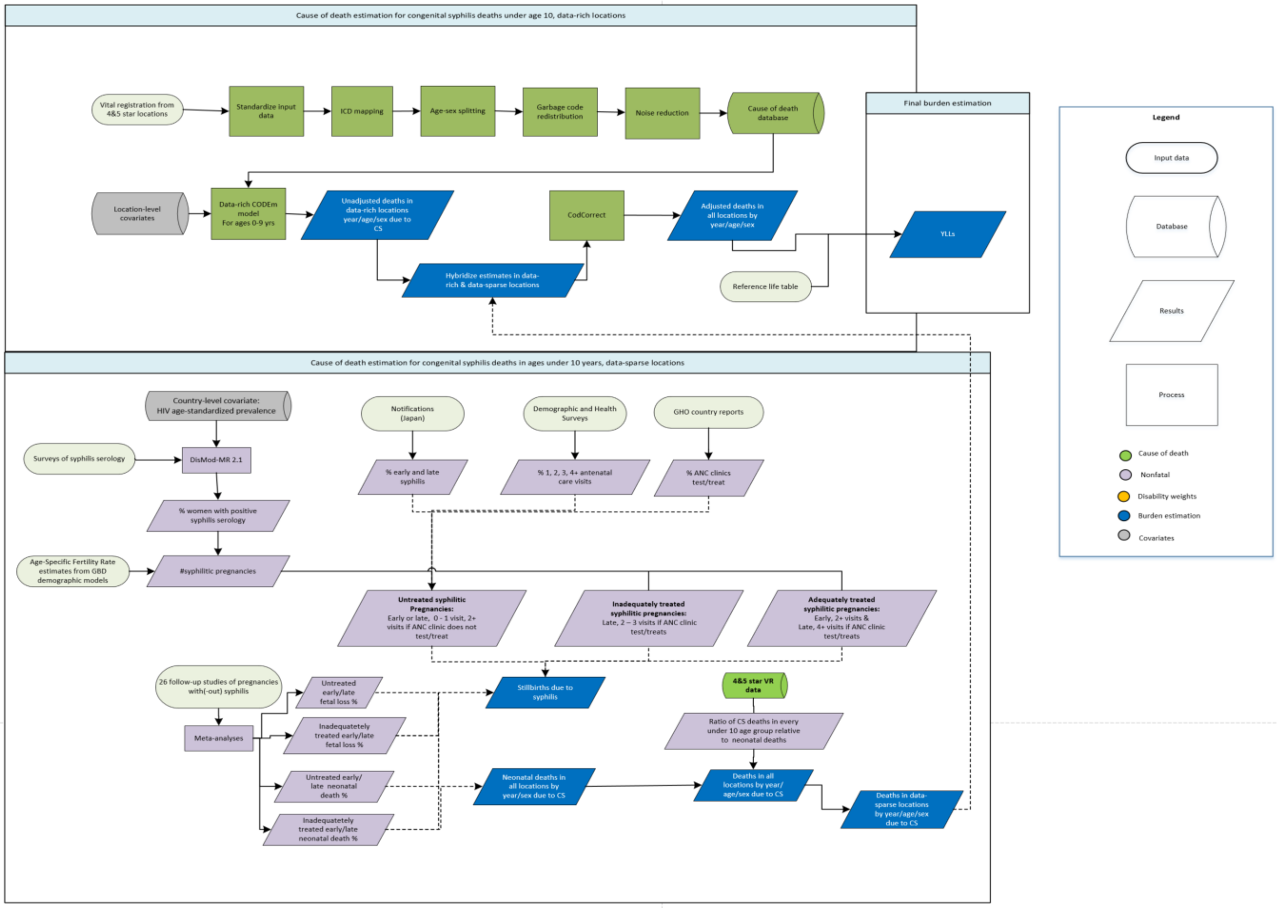

Congenital syphilis arises from the transmission of syphilis from mother to child, in the womb or during childbirth. We model deaths due to congenital syphilis for males and females aged 0 to 9 years. Of all STIs excluding HIV, only syphilis is regarded as causing deaths in children under 10 years. In GBD 2017, congenital syphilis deaths were estimated in all locations with a natural history model. However, we found that our natural history model exceeded the number of deaths recorded by countries with high quality vital registration (VR) and a record of investment into the eradication of congenital syphilis. To produce more plausible estimates based on data considered to be highly complete and reliable, we decided that congenital syphilis deaths in data-rich countries would be estimated in a CODEm model. We continue to use the natural history model to produce estimates for countries with no or lesser quality VR (data-sparse). Outputs for data-sparse countries produced in the natural history model are combined with outputs for data-rich countries produced in the CODem model, then passed on to the CodCorrect process as a hybrid model and included in final GBD estimates of mortality due to congenital syphilis. In the sections below, the input data and the modelling strategy for each method are described.

Input data – Congenital Syphilis

CODEm

Deaths due to congenital syphilis in data-rich countries were modeled using centrally processed vital registration data from the cause of death (COD) database.

Natural History

Five different inputs were used to model the natural history of congenital syphilis. Inputs were drawn from both data-rich and data-sparse locations, and the model produced outputs for all location-years. Only the outputs for data-sparse location-years were passed on to the hybrid model that went into CodCorrect, and subsequently included in final GBD estimates of mortality due to congenital syphilis. Our first inputs were estimates of positive syphilis serology in women of reproductive age pulled from our nonfatal Dismod model of syphilis seroprevalence. A more detailed description of these estimates can be found in the nonfatal methods appendix for STIs. Our second inputs were age-specific fertility rates estimated in the GBD 2019 demographic analyses. Third, we used GBD estimates of the number of antenatal care (ANC) visits per pregnant woman. Fourth, we used published data from the Global Health Observatory on the proportion of ANC clinics that test for syphilis and the proportion of women testing positive who receive treatment. Fifth, we used cohort studies on the risk of fetal loss and neonatal death in syphilitic women. In GBD 2017, 11 studies were collected through recommendations from our GBD collaborator network. In GBD 2019, we conducted a systematic review of congenital syphilis. The search string below was run on April 4th, 2019 through Pubmed. It returned 1,675 articles. After title/abstract review, 442 articles remained for full text screening. Of these, 165 were deemed eligible for data extraction. 15 of these articles were combined with the 11 studies from GBD 2017 and included in a meta-analysis of excess neonatal death and fetal loss.

(syphilis[tiab] OR "treponema pallidum"[tiab]) AND ((pregnan[tiab] OR fetal[tiab] OR foetal[tiab] or fetus*[tiab] OR foetus*[tiab] OR neonat*[tiab] OR infan*[tiab] OR newborn*[tiab] OR congenital[tiab]) OR ((vertical*[tiab] OR maternal[tiab] OR mother[tiab] OR fetomaternal[tiab]) AND transmi*[tiab])) AND (outcomes[tiab] OR sequela*[tiab] OR manifestation*[tiab] OR morbidity*[tiab] OR diagnos*[tiab] OR hutchinson*[tiab])*

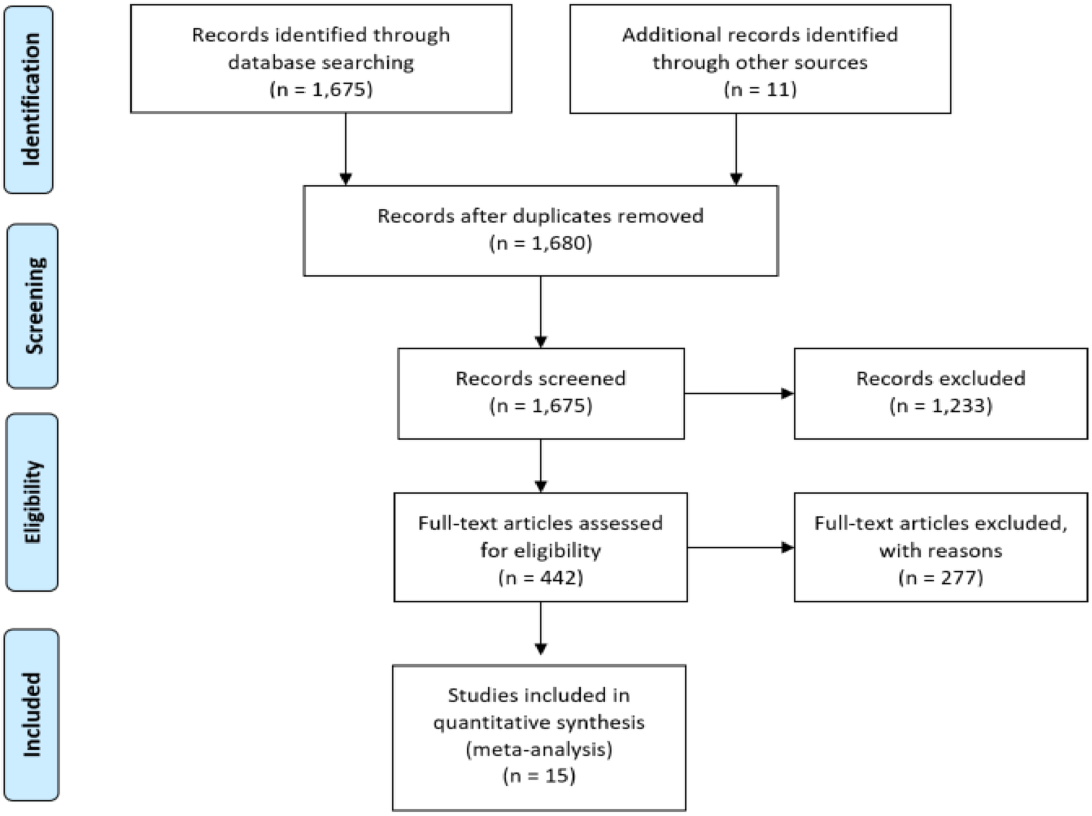

Modelling strategy – Congenital Syphilis

CODEm

We completed a data-rich CODEm model for ages 0-9 years for males and females separately. Ten covariates were entered for possible selection in each CODEm model, including 1) female age-standardized prevalence of positive syphilis serology; 2) coverage of one antenatal care (ANC) visit; 3) coverage of four or more ANC visits; 4) maternal care & immunization (a covariate based on a principal components analysis of ANC, in-facility delivery, skilled birth attendance, and vaccine coverage); 5) abortion legality, an index that includes a categorical rating of abortion laws that range from 1 (always illegal) to 7 (always legal on demand); 6) age-specific fertility rate (ASFR); 7) total fertility rate (TFR), 8) years of education per capita; 9) health care access and quality index (HAQI); and 10) lag-distributed income (LDI).

Table 3: Covariates used in congenital syphilis data-rich CODEm model

Level	Covariate	Direction
1	Syphilis prevalence	+
	Antenatal care coverage, 1+ visits	-
	Antenatal care coverage, 4+ visits	-
	Maternal Care & Immunization	-
2	Abortion Legality	-
	Age-specific Fertility Rate	+
	Total Fertility Rate	+
3	Years of education	-
	Health care access & quality	-
	Lag-distributed income	-

Natural History

Our natural history model for congenital syphilis mortality begins with the estimation of pregnancies that are at risk of vertical transmission. To calculate this, we multiply the prevalence of positive syphilis serology in women of child-bearing age by age-specific fertility rates.

Next, we incorporate 5 separate measures that allow us to estimate the number of fetal and neonatal deaths in children of infected mothers. These are: 1) the proportion of antenatal (ANC) clinics that both test and treat for syphilis, 2) the number of times that a mother visits an ANC clinic during pregnancy, 3) the stage of disease in infected mothers, 4) excess risk of stillbirth and neonatal death in syphilitic pregnancies by treatment status and stage, and 5) ratios of syphilis death for every age group up to 10 years of age, relative to neonatal deaths.

1) ANC testing and treatment data are obtained from 132 countries via the Global Health Observatory. The first of these measures is the proportion of ANC attendees that are tested for syphilis at their first visit. The second is the proportion of infected women that receive treatment if they test positive for syphilis. These data are entered into a ST-GPR model to estimate these measures for all year-age-location combinations with socio-demographic index (SDI) as a covariate.

2) The distribution of the number of skilled antenatal care visits during pregnancy are produced by internal GBD analyses of maternal health. There are 3 categories: 1 visit, 2-3 visits, and 4+ visits.
3) Detailed notification data from Japan on the stage of syphilis infection in pregnant women diagnosed during antenatal screening.
4) The excess risk of stillbirth and neonatal death in syphilitic vs non-syphilitic pregnancies as estimated in a meta-analysis described below.

5) 4&5 star vital registration data on deaths from congenital syphilis for males and females in every age group up to 10 years (early neonatal, late neonatal, post neonatal, 1-4 years, 5-9 years). Using this data, we calculate a ratio of deaths for every age-group relative to neonatal deaths.

Measures 1-4 are used to estimate total fetal loss and neonatal death from congenital syphilis. The 5th measure allows us to disaggregate neonatal deaths into early and late neonatal groups, and estimate the number of deaths in infected infants that survive the neonatal stage.

Delving into the methods behind measure 4, the excess risk of fetal loss and neonatal death for syphilitic mothers relative to non-syphilitic mothers were estimated using a meta-analysis of 26 studies. Risks were calculated detailed by treatment status of the mother. The time period that studies were conducted in had great variance, so we accounted for the higher risk of adverse pregnancy outcomes in the past by subtracting rates of these outcomes among healthy mothers from the rates among syphilitic mothers from the same study. Forest plots of the estimated risks are below. Values of mortality from women of unknown treatment status were excluded from the analysis.

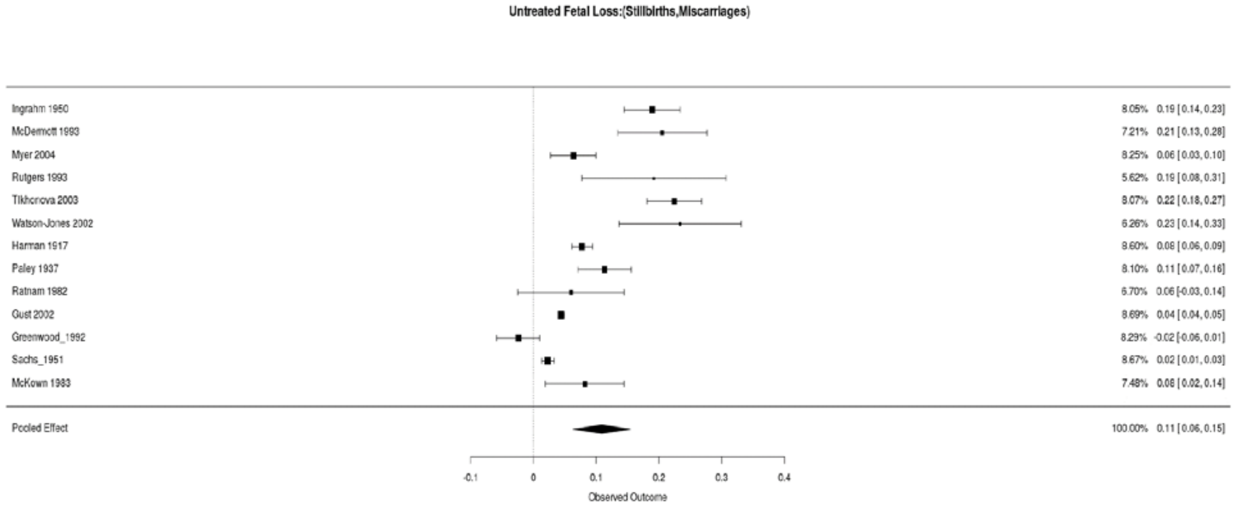

Inadequately Treated Fetal Loss:(Stillbirths,Miscarriages)

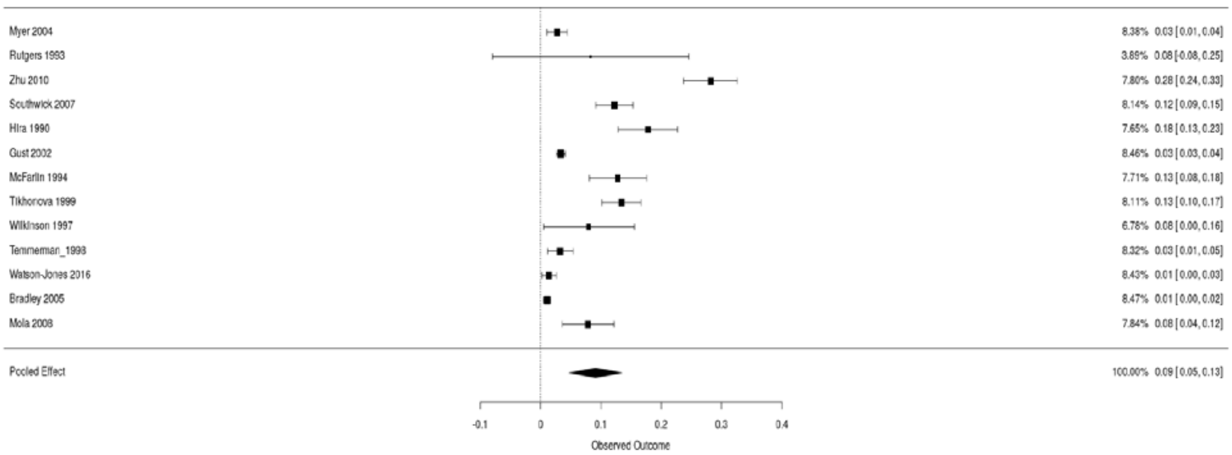

Untreated Neonatal Deaths

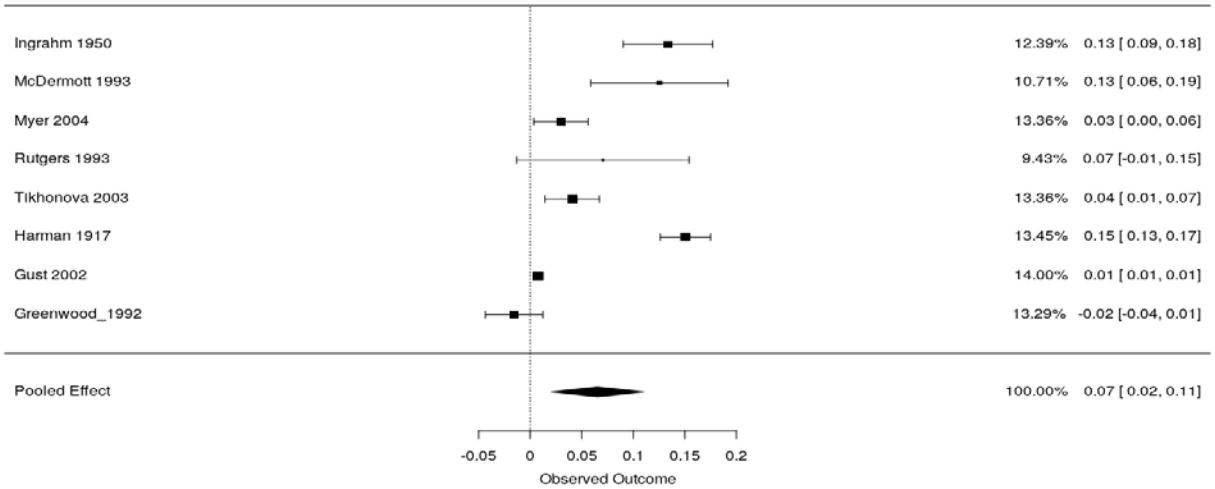

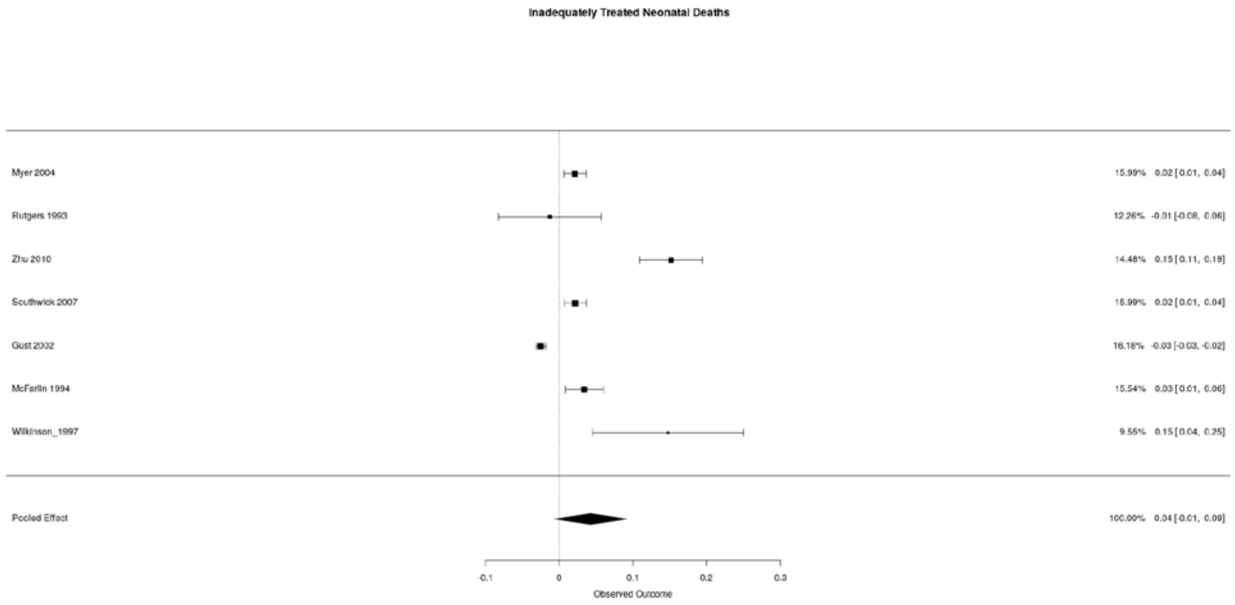

No excess mortality or fetal loss was assumed for adequately treated cases of maternal syphilis. A comparison of the neonatal mortality rates between adequately treated women and uninfected women showed a smaller proportion of babies from adequately treated women died than babies from uninfected women.

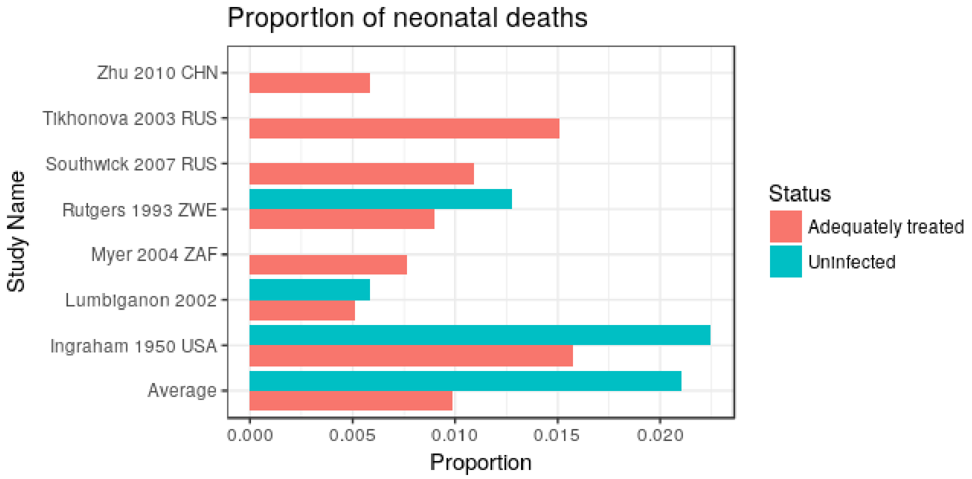

To combine these measures and obtain the final numbers of death:

We adjusted syphilitic pregnancies for the excess risk of stillbirth to estimate the number of stillbirths attributable to congenital syphilis. We then subtracted the stillbirths from the pregnancies at risk to estimate the number of live births to syphilitic mothers.

We then multiplied the live births in syphilitic mothers by the proportions of mothers attending antenatal clinics at least 1, 2, or 4 times during pregnancy, the probability of attending a clinic that tests and treats, and the proportions of early and late syphilis in pregnant women. This gave us the number of

live births that stemmed from mothers with untreated status, inadequately treated status, or adequately treated status. These three groups are estimated because treatment status impacts the risk of fetal loss and neonatal death. The recommendation throughout literature is that individuals with early syphilis infection require 1 dose of penicillin to be adequately treated, while those with late syphilis infection are recommended 3 doses of penicillin for adequate treatment. We assume that women need to attend an ANC clinic at least two times – once to undergo syphilis testing, and a second time to receive test results and get treatment. Thus, for those with early infection, 0-1 anc visits indicate untreated status, and 2 or more visits indicate adequately treated status. For those with late infection, 0-1 visits indicate untreated status, 2-3 visits indicate inadequately treated status, and 4+ visits indicate adequately treatment status.

After the number of women in each treatment group is calculated, we multiply each category by the risk of fetal loss or neonatal death specific to each treatment category. This produces the number of stillbirths in mothers at each treatment stage, and the number of neonatal deaths in infants born alive to mothers at each treatment stage.

Finally, we distribute neonatal deaths across early and late neonatal age groups, and estimate the number of deaths for the post-neonatal, 1-4 year & 5-9 year age groups. In GBD 2017, ratios for each age group relative to neonatal deaths were calculated using vital registration (VR) data from all location-years. However, this produced implausible differences between males and females in the estimated ratios. To solve this, in GBD 2019, only 4 and 5-star VR data were used to calculate ratios of deaths for every age group relative to neonatal deaths. (A further explanation of the star rating system can be found in the appendix.) We multiply the ratios calculated from high-quality VR data by our estimated number of neonatal deaths..

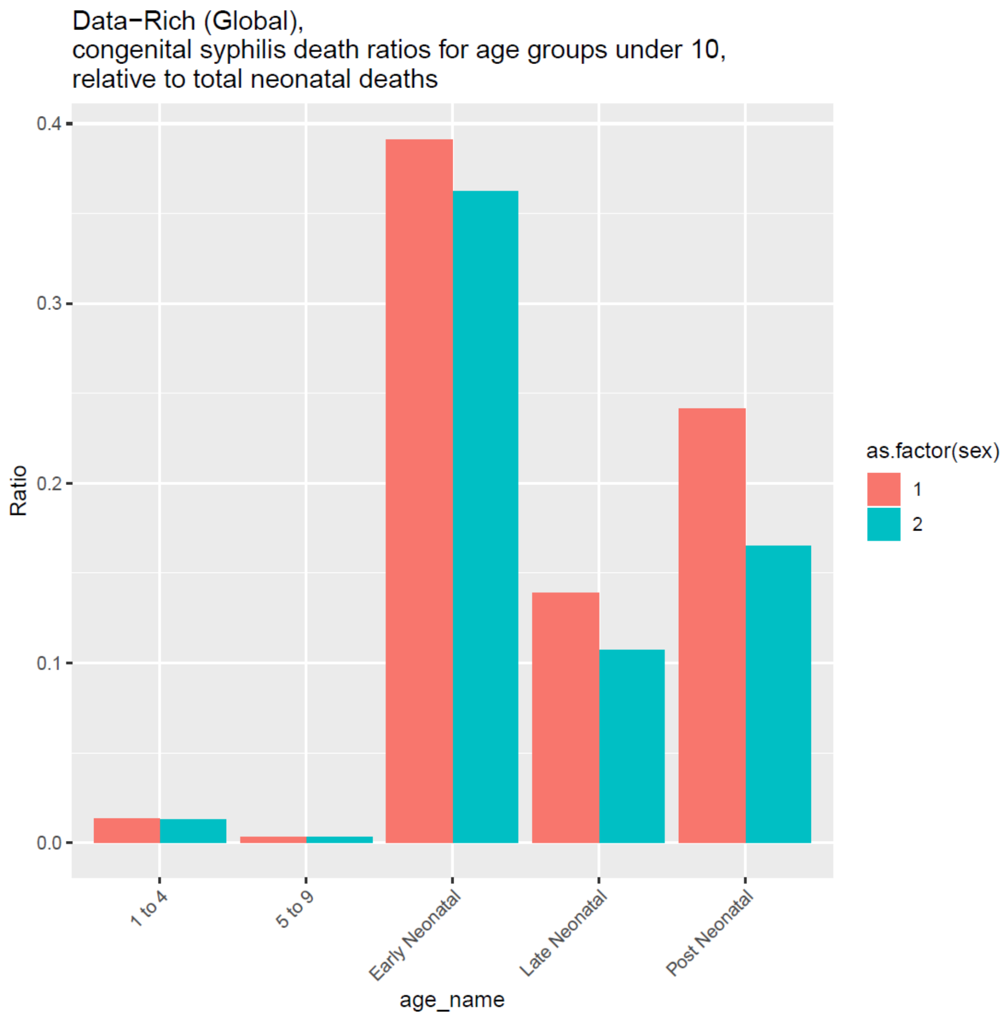

Subsequently, the sex and age-specific congenital syphilis deaths estimated in the natural history model for data-sparse location-years were hybridized with the deaths estimated in the CODEm model for data-rich locations, and the hybrid model results were uploaded to the causes of death database and entered into the CoDCorrect process.

Tuberculosis

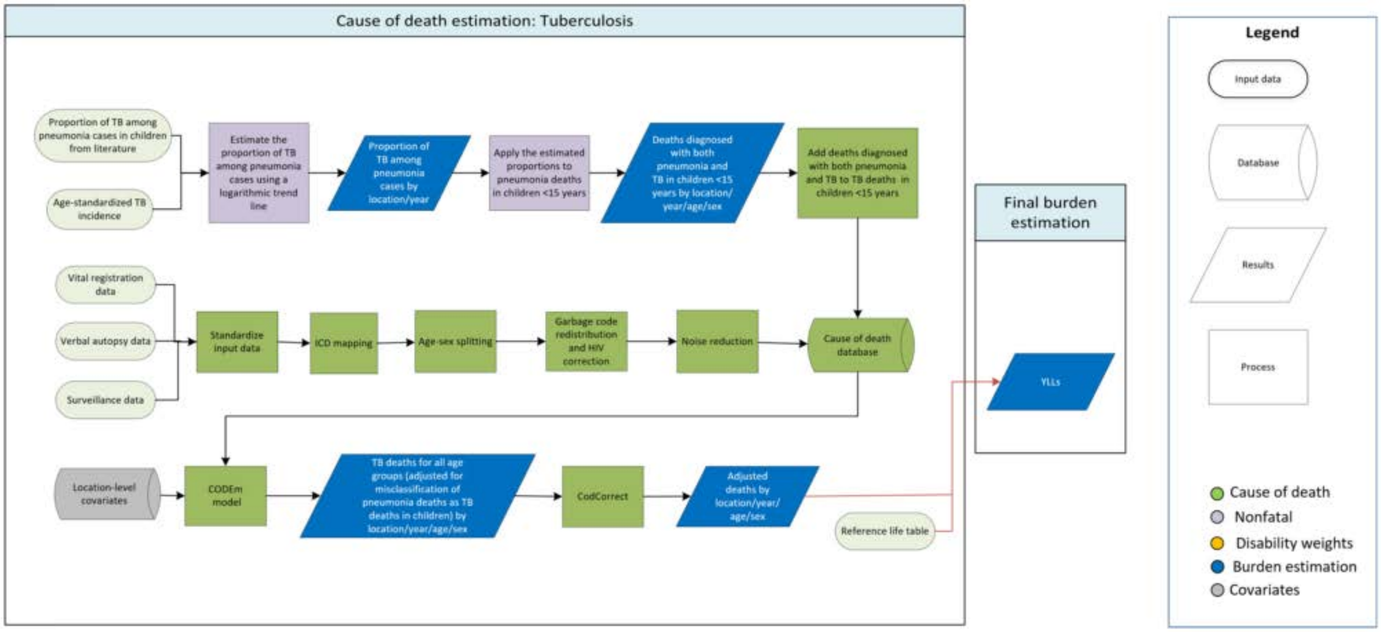

Input data

Input data for modelling tuberculosis (TB) mortality among HIV-negative individuals include vital registration, verbal autopsy, and surveillance data. Vital registration data were adjusted for garbage coding (including ill-defined codes and the use of intermediate causes) following GBD algorithms and misclassified HIV deaths (ie, HIV deaths being assigned to other underlying causes of death such as tuberculosis or diarrhoea because of stigma or misdiagnosis).

Verbal autopsy data in countries with age-standardised HIV prevalence greater than 5% were removed because of a high probability of misclassification, as verbal autopsy studies have poor validity in distinguishing HIV deaths from HIV-TB deaths.

Modelling strategy

A general CODEm modelling strategy was used. In GBD 2019, we made a small change with regard to the alcohol litres per capita covariate where we exchanged it for an all-age and both-sex equivalent that aligns better with the covariate framework for CODEm. We continued to use the TB strain prevalence-weighted transmission risk and cigarettes per capita covariate that were introduced in GBD 2017. Other location-level covariates included in the CODEm model were the same as in previous GBD cycles: adult underweight proportion, alcohol (litres per capita), diabetes (fasting plasma glucose mmol/L), education (years per capita), Healthcare Access and Quality Index, lag-distributed income, indoor air pollution, outdoor air pollution, population density, prevalence of active tuberculosis, prevalence of latent tuberculosis infection, smoking prevalence, Socio-demographic Index, and a summary exposure variable reflecting the average exposure to all of the risk factors.

Covariate table

	Covariate	Direction
Level 1	TB prevalence<br>Latent TB infection prevalence<br>SEV scalar<br>Litres of alcohol consumed per capita<br>Smoking prevalence<br>Cigarettes per capita<br>Fasting plasma glucose<br>TB strain prevalence-weighted transmission risk	+<br>+<br>+<br>+<br>+<br>+<br>+<br>+
Level 2	HAQ Index<br>Adult underweight proportion<br>Indoor air pollution<br>Outdoor air pollution<br>Population density	-<br>+<br>+<br>+<br>+
Level 3	Log LDI<br>Education (years per capita)<br>Socio-demographic Index (SDI)	-<br>-<br>-

Correcting for a potential misclassification of tuberculosis deaths as pneumonia deaths in children

Since GBD 2017, we have addressed the potential for misclassification of TB deaths as pneumonia deaths among children in locations with high TB burden. First, we estimated the proportion of tuberculosis among pneumonia cases as a function of age-standardised TB incidence using data from eight clinical studies[2,3,4,5,6,7,8,9] reporting the proportion of pneumonia cases that had tuberculosis (or the data to calculate them) and the age-standardised TB incidence estimates. We used a logarithmic trend line to fit these data. In GBD 2019, we applied the estimated proportions to pneumonia deaths reported in data among children younger than 15 years to compute the number of deaths diagnosed with both pneumonia and TB, which were then added to child TB data. Following this correction in our input data, the CODEm model was run to provide location-year-age-sex specific estimates. This is a departure from GBD 2017, where the estimated proportions were applied after CODEm. Finally, the CODEm estimates were adjusted using CoDCorrect, which ensures that the number of deaths from each cause add up to all-cause mortality deaths for a given year.

References

1. Graham SM, Sismanidis C, Menzies HJ, Marais BJ, Detjen AK, Black RE. Importance of tuberculosis control to address child survival. *Lancet* 2014; **383**(9928): 1605-7.
2. Adegbola RA, Falade AG, Sam BE, et al. The etiology of pneumonia in malnourished and well-nourished Gambian children. Pediatr Infect Dis J 1994; 13: 975–82.

3. Chisti MJ, Graham SM, Duke T, et al. A prospective study of the prevalence of tuberculosis and bacteraemia in Bangladeshi children with severe malnutrition and pneumonia including an evaluation of Xpert MTB/RIF assay. PloS One 2014; 9: e93776.
4. Madhi SA, Petersen K, Madhi A, Khoosal M, Klugman KP. Increased disease burden and antibiotic resistance of bacteria causing severe community-acquired lower respiratory tract infections in human immunodeficiency virus type 1-infected children. Clin Infect Dis 2000; 31: 170–76.
5. McNally LM, Jeena PM, Gajee K, et al. Effect of age, polymicrobial disease, and maternal HIV status on treatment response and cause of severe pneumonia in South African children: a prospective descriptive study. Lancet 2007; 369: 1440–51.
6. Moore DP, Klugman KP, Madhi SA. Role of Streptococcus pneumoniae in hospitalisation for acute community-acquired pneumonia associated with culture-confirmed Mycobacterium tuberculosis in children: a pneumococcal conjugate vaccine probe study. Pediatr Infect Dis J 2010; 29: 1099–104.
7. Nantongo JM, Wobudeya E, Mupere E, et al. High incidence of pulmonary tuberculosis in children admitted with severe pneumonia in Uganda. BMC Pediatr 2013; 13: 16.
8. Zar HJ, Hanslo D, Tannenbaum E, et al. Aetiology and outcome of pneumonia in human immunodeficiency virus-infected children hospitalized in South Africa. Acta Paediatr 2001; 90: 119–25.
9. Moore DP, Higdon MM, Hammitt LL, Prosperi C, DeLuca AN, Da Silva P, Baillie VL, Adrian PV, Mudau A, Deloria Knoll M, Feikin DR. The incremental value of repeated induced sputum and gastric aspirate samples for the diagnosis of pulmonary tuberculosis in young children with acute community-acquired pneumonia. Clinical Infectious Diseases. 2017 May 27;64(suppl_3):S309-16.

TB strain prevalence-weighted transmission risk covariate

In GBD 2017, we incorporated a TB covariate that incorporated data on the global distribution of TB strains and the relative risk of transmission associated with those strains. We continued the use of this covariate in GBD 2019. For this covariate, we defined TB strains according to the seven phylogenetic lineages of the *Mycobacterium tuberculosis* complex (MTBC) identified by S. Gagneaux and colleagues.[1] We determined the global distribution of these strains using a systematic review of human TB molecular epidemiology studies from 1990 to 2017 in PubMed and Scopus, as described in greater detail elsewhere.[2] All studies that used population-based sampling methods or collected isolates from all culture-positive TB cases in a given location and time period were included. All genotypes that could be converted to phylogenetic lineages were extracted, including genotypes determined by spoligotyping, MIRU-VNTR typing, and PCR or whole-genome sequencing. Studies of sub-populations, such as prison populations or drug-resistant cases only, were excluded. In total, 206 studies representing 85 countries and over 200,000 bacterial isolates were included. In GBD 2019, the systematic review was updated, which yielded an additional 18 studies published between 2017 and 2019. A map of these strains highlighted the widespread global distribution of Euro-American Lineage 4 strains and East Asian Lineage 2 strains, and the geographical restriction of Lineage 5 and 6 strains to West Africa. Thirty of these studies also reported transmission chains associated with bacterial genotypes, as defined by genetic clustering.[3]

We used spatiotemporal Gaussian process regression (ST-GPR) to model the distribution of each strain in each GBD location across all ages and sexes, as described in greater detail elsewhere.[4] The covariates tested in each model included HIV age-standardised prevalence, population density, and a custom-made human movement covariate. The human movement covariate took into account (1) immigration and emigration patterns[5] and (2) airplane passenger flow[6] to and from each country. In the ST-GPR models we assumed strong correlation and smoothing over both space and time. We then used a random-effects meta-analysis to determine the relative risk (RR) of transmission associated with each strain, as defined by genetic clustering. We used the most widespread strains, Euro-American Lineage 4 strains, as the reference group. We found that East Asian Lineage 2 strains were associated with increased risk of transmission overall (relative risk [95% CI] = 1.24 [1.07, 1.45]), while West African Lineage 5 and 6 strains were associated with reduced transmission (relative risk [95% CI] = 0.61 [0.43, 0.86]). We used the following formula to calculate a TB strain prevalence-weighted risk of transmission based on these estimates:

$$\sum_{i=1}^{n} Pr_i RR_i$$

i=TB strain; Pr=proportion; RR=relative risk

References

1. Comas I, Coscolla M, Luo T, *et al.* Out-of-Africa migration and Neolithic coexpansion of *Mycobacterium tuberculosis* with modern humans. *Nat Genet* 2013; **45**: 1176–82.
2. Wiens KE, Woyczynski LP, Ledesma JR, *et al*. Global variation in bacterial strains that cause tuberculosis disease: a systematic review and meta-analysis. *BMC Medicine 2018*; 16:196.
3. Dheda K, Gumbo T, Maartens G, *et al.* The epidemiology, pathogenesis, transmission, diagnosis, and management of multidrug-resistant, extensively drug-resistant, and incurable tuberculosis. *Lancet Respir Med* 2017; **5**: 291–360.
4. Manuscript in preparation.
5. United Nations Population Division. United Nations Trends in International Migrant Stock: The 2015 Revision. New York City, United States: United Nations Population Division, 2015.
6. Huang Z, Wu X, Garcia AJ, *et al*. An open-access modeled passenger flow matrix for the global air network in 2010. *PLoS ONE* **8(5)**: e64317.

Multidrug-resistant tuberculosis, extensively drug-resistant tuberculosis, and drug-susceptible tuberculosis

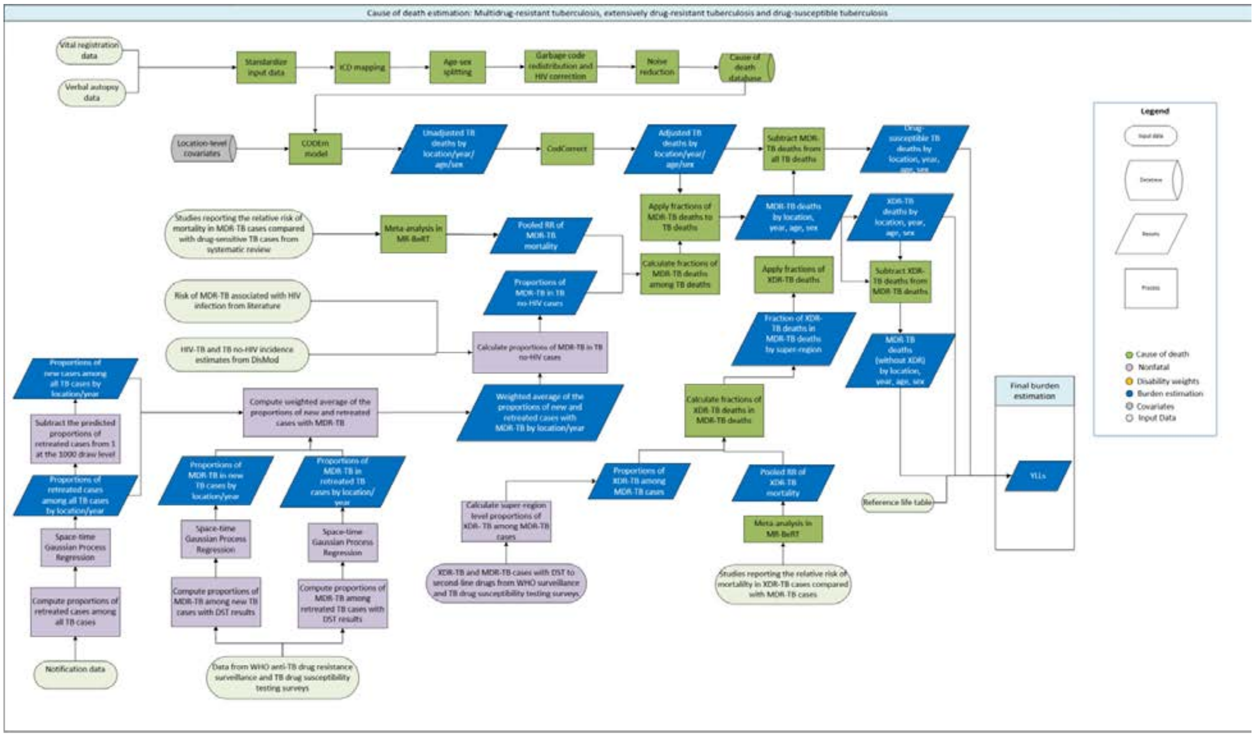

Input data

Input data include: (i) the number of drug-resistant cases by type (multidrug-resistant tuberculosis [MDR-TB], extensively drug-resistant tuberculosis [XDR-TB], all TB cases with a drug-susceptible testing [DST] result for isoniazid and rifampicin, and MDR-TB cases with DST for second-line drugs) from routine surveillance and surveys reported to the World Health Organization, (ii) data from studies (identified through our systematic review) reporting on the relative risk of death in MDR-TB cases compared with non-MDR TB (drug-susceptible TB) cases, and the relative risk of death in XDR-TB cases compared with MDR-TB cases, and (iii) the risk of MDR-TB associated with HIV infection from the literature.[1]

PRISMA diagram of MDR-TB mortality relative risk in GBD2019

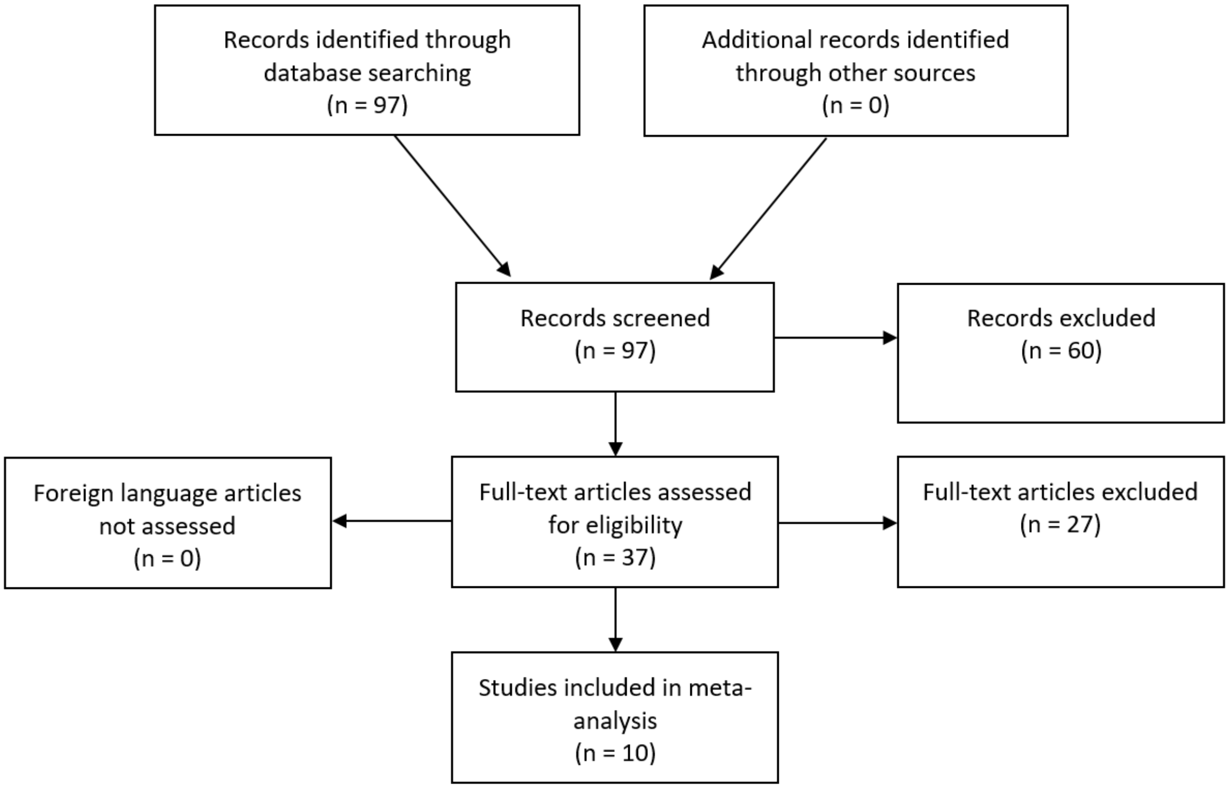

Modelling strategy

We conducted a systematic review and meta-analysis of studies reporting the relative risk of death in MDR-TB cases compared with drug-susceptible TB cases. We ran spatiotemporal Gaussian process regressions to predict the proportions of new TB cases with MDR-TB, proportions of retreated TB cases with MDR-TB, and proportions of retreated cases among all TB cases for all locations and years. We also calculated the proportions of new TB cases among all TB cases. We then computed the weighted average of the proportions of new and retreated cases with MDR-TB at the 1000-draw level. We then used the weighted average proportions of MDR-TB, along with the HIV-TB and TB no-HIV incidence estimates (from our modelling of non-fatal TB), and the relative risk of MDR-TB associated with HIV infection from the literature[1] to compute the proportions of MDR-TB cases among HIV-negative TB cases $\left(P_{MDRnoHIVc,y,a,s}\right)$ by location, year, age, and sex using the following formula:

$$P_{MDRnoHIVc,y,a,s} = \frac{MDR_{c,y}}{\left(1 + \left(RR_{HIV}\frac{HIVTB_{c,y,a,s}}{TBnoHIV_{c,y,a,s}}\right)\right) TBnoHIV_{c,y,a,s}}$$

where $MDR_{c,y}$ is the number of all MDR-TB cases among HIV-positive and HIV-negative individuals by location and year, RR_{HIV} is the relative risk of MDR-TB associated with HIV infection, $HIVTB_{c,y,a,s}$ is the number of HIV-TB incident cases by location, year, age, and sex, and $TBnoHIV_{c,y,a,s}$ is the number of TB no-HIV incident cases by location, year, age, and sex.

We then computed the fraction of MDR-TB deaths among all HIV-negative TB deaths ($D_{MDRnoHIVc,y,a,s}$) using the following formula:

$$D_{MDRnoHIVc,y,a,s} = \frac{P_{MDRnoHIVc,y,a,s} RR_{MDR}}{P_{MDRnoHIVc,y,a,s} RR_{MDR} + 1 - P_{MDRnoHIVc,y,a,s}}$$

where RR_{MDR} is the relative risk of death in MDR-TB cases compared with drug-susceptible TB cases. In GBD 2019, the pooled relative risk was derived from a meta-analysis in the meta-regression with Bayesian priors, regularization, and trimming (MR-BRT) model. After derivation of the pooled relative risk, we then applied the predicted HIV-MDR-TB death fractions to all HIV-TB death estimates to generate HIV-MDR-TB deaths by location, year, age, and sex. Next, we subtracted MDR-TB deaths from all TB deaths to generate drug-susceptible TB deaths by location, year, age, and sex.

To separate out XDR-TB from MDR-TB, we aggregated the XDR-TB cases and MDR-TB cases (with DST for second-line drugs) up to the super-region level and calculated the super-region-level proportions of XDR-TB among MDR-TB cases. Next, we computed the super-region-specific fractions of XDR-TB deaths among all MDR-TB deaths (D_{XDRsr}) using the following formula:

$$D_{XDRsr} = \frac{P_{XDRsr} RR_{XDR}}{P_{XDRsr} RR_{XDR} + 1 - P_{XDRsr}}$$

where P_{XDRsr} is the proportion of XDR-TB among MDR-TB cases for each super-region, and RR_{XDR} is the pooled relative risk of mortality in XDR-TB cases compared with MDR-TB cases. Similar to the pooled relative risk for MDR-TB, the derivation of the pooled relative risk of mortality in XDR-TB was computed with a meta-analysis in the MR-BRT model for GBD 2019. These fractions were then applied to MDR-TB deaths in corresponding countries within the super-regions to produce XDR-TB deaths by location, age, and sex for the most recent year of estimation. We linearly extrapolated XDR-TB mortality rates back, assuming the mortality rates were zero in 1992, one year before 1993 when XDR-TB was first recorded in USA surveillance data.[2] Finally, we subtracted XDR-TB deaths from MDR-TB deaths to generate MDR-TB (without extensive drug resistance) deaths by location, year, age, and sex.

References

1. Mesfin YM, Hailemariam D, Biadglign S, Kibret KT. Association between HIV/AIDS and multi-drug resistance tuberculosis: a systematic review and meta-analysis. PLoS One. 2014;9(1):e82235.
2. Centers for Disease Control and Prevention (CDC). Extensively Drug-Resistant Tuberculosis --- United States, 1993—2006. MMWR. 2007; 56(11);250-253

Lower respiratory infections

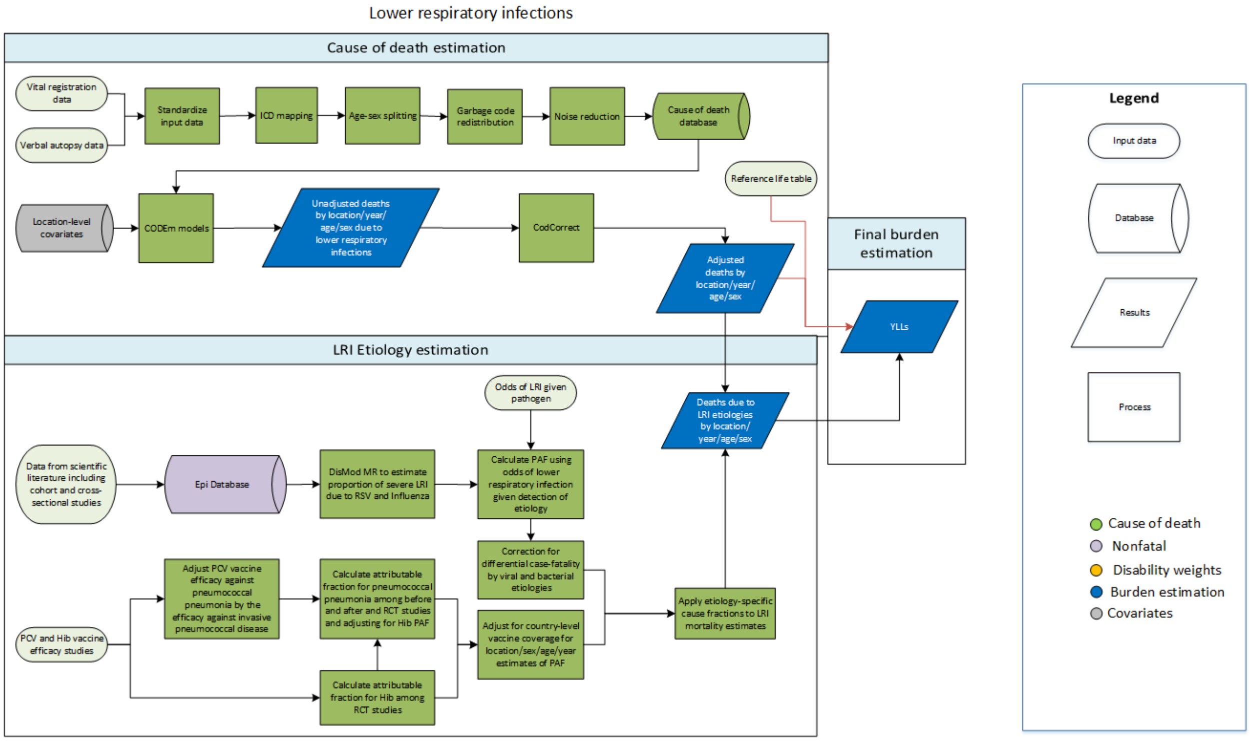

Input data

Cause of death

Lower respiratory infection (LRI) mortality was estimated in CODEm. We estimated LRI mortality separately for males and females and for children under 5 years and older than 5 years. We used all available data from vital registration systems, surveillance systems, and verbal autopsy. We checked for and excluded outliers from our data by country or region. We also excluded ICD9-coded mortality data in Sri Lanka (1982, 1987–1992), ICD9-coded neonatal mortality data in Guatemala (1980, 1981, 1984, 2000–2004), and medically coded cause of death data and Civil Registration System data in many Indian states (1986–2013).

Aetiologies

We updated our systematic review of scientific literature for the proportion of LRI that tested positive for influenza and respiratory syncytial virus (RSV) to include all data from GBD 2017 and from studies published between August 1, 2018 and February 7, 2019. We performed the search using PubMed and the following search string:

*((“lower respiratory”[title] OR pneumonia[title]) AND (**2018/08/01[PDat] : 2019/2/7[PDat]**) AND ((incidence OR prevalence OR epidemiology) OR (etiolog*[title/abstract] OR influenza[title/abstract] OR “respiratory syncytial virus”[title/abstract])) AND Humans[MeSH Terms]) NOT(autoimmune[title/abstract] OR COPD [title/abstract] OR “cystic fibrosis”[title/abstract] OR **Review[ptyp]**)*

Inclusion criteria were studies that had a sample size of at least 100, studies that were at least one year in duration, and studies describing lower respiratory infections, pneumonia, or bronchiolitis as the case definition. During our literature review we identified 121 studies, of which two met our inclusion criteria and were extracted. We excluded studies that described pandemic H1N1 influenza solely and studies that used influenza-like illness as the case definition. An age pattern based on age-specific data was estimated and then used to split data where the age range was more than 25 years.

We also conducted a systematic literature review of studies on the *Haemophilus influenzae* type B (Hib) vaccine and pneumococcal conjugate vaccine (PCV) effectiveness studies against x-ray-confirmed pneumonia and against pneumococcal and Hib disease until May 2017. This review was not updated for GBD 2019. For PCV studies, we extracted, if available, the distribution of pneumococcal pneumonia serotypes and the serotypes included in the PCV used in the study. We excluded observational and case-control studies due to implausibly high vaccine efficacy estimates. Hib trial data were exclusively from children under 5 years, so we did not include the effect of Hib on ages over 5 years. PCV trial data are also frequently limited to younger populations. To understand the contribution of pneumococcal pneumonia in older populations, we also included PCV efficacy studies that used before-after approaches.

Modelling strategy

Cause of death. LRI fatal modelling occurs using CODEm. Because of starkly different patterns, LRI CODEm models include under-5 years and 5–95+ years. Like all models of mortality in GBD, LRI mortality models are single-cause, requiring in effect that the sum of all mortality models must be equal to the all-cause mortality envelope. We correct LRI mortality estimates, and other causes of mortality, by rescaling them according to the uncertainty around the cause-specific mortality rate. This process is called CoDCorrect and is essential to ensure internal consistency among causes of death.

Table 1. Covariates used in LRI mortality modelling. Table 1A is for children under 5 and Table 1B shows the covariates used for ages 5–95+. The *Level* is the associated strength of relationship between the covariate and LRI mortality, ranked from 1 (proximally related) to 3 (distally related). *Direction* is the direction of the association between the covariate and LRI mortality.

Table 1A. Covariates used in under 5 years model

Level	Covariate	Direction
1	Childhood stunting summary exposure value (SEV)	+
	Childhood underweight SEV	+
	Childhood wasting SEV	+
	Indoor air pollution	+
	LRI SEV	+
	Antibiotics for LRI	-
	Hib vaccine coverage	-
	PCV coverage	-
	Vitamin A deficiency	+
2	Secondhand smoking prevalence	+
	Zinc deficiency	+
	DTP3 vaccine coverage	-
	Healthcare Access and Quality Index	-

	Ambient particulate matter SEV	+
	Household air pollution	+
	Outdoor air pollution ($PM_{2.5}$)	+
	Handwashing SEV	+
3	Sanitation SEV	+
	Population density > 1000/km^2	+
	Population density < 150/km^2	+
	Maternal education	-
	Socio-demographic Index	-

Table 1B. Covariates used in 5-95+ years model

Level	Covariate	Direction
1	Indoor air pollution	+
	LRI SEV	+
	Outdoor air pollution	+
	Secondhand smoking prevalence	+
	Smoking prevalence	+
2	DTP3 vaccine coverage	-
	Adult underweight	+
	Healthcare Access and Quality Index	-
	PCV coverage	-
	Handwashing access	+
3	Education years per capita	-
	Lag distributed income per capita	-
	Socio-demographic Index	-
	Sanitation SEV	+

Aetiologies

We estimated LRI aetiologies separately from overall LRI mortality using two distinct counterfactual modelling strategies to estimate population attributable fractions (PAFs), described in detail below. The PAF represents the relative reduction in LRI mortality if there was no exposure to a given aetiology. As LRIs can be caused by multiple pathogens and the pathogens may co-infect, PAFs can overlap and are not scaled to sum to 100%. Separate strategies were used for viral (influenza and RSV) and bacterial (*Streptococcus pneumoniae* and Hib) aetiologies. We did not attribute aetiologies to neonatal pneumonia deaths due to a dearth of reliable data in this age group. We calculated uncertainty of our PAF estimates from 1,000 draws of each parameter using normal distributions in log space.

Influenza and RSV. We calculated the PAF from the proportion of severe LRI cases positive for influenza and RSV. We assumed that hospitalised LRI cases are a proxy of severe cases. We used the following formula to estimate the PAF:[1]

$$PAF = Proportion\ (modelled) * (1 - \frac{1}{OR})$$

Where *Proportion* is the proportion of LRI cases that test positive for influenza or RSV and *OR* is the odds ratio of LRI given the presence of the pathogen. There are two published estimates of the odds ratios of influenza and RSV. One is based on detection in children younger than 5 years and the second is based on adults over 65 years. We applied the separate odds ratios for those age groups and log-linearly interpolated values between those ages to determine odds ratios for ages between those groups.[2, 3]

We modelled the proportion data using the meta-regression tool DisMod-MR to estimate the proportion of LRI cases that are positive for influenza and RSV, separately, by location/year/age/sex. To make disparate data types directly comparable such as the diagnostic technique (detection by PCR served as our reference), studies that investigated RSV or influenza exclusively (multi-pathogen studies were our reference), and studies from inpatient populations (community-based sample populations was our reference), we performed a meta-regression of the ratios of the reference to non-reference definitions. These meta-regression results were used to adjust the mean and variance of nonreference data. The value for the ratio of community to inpatient LRI was used as a scalar in our final estimate of fatal attributable fractions because we assumed that the frequency of influenza or RSV in hospitalised episodes of LRI represented the frequency in fatal LRI.

As the case-fatality of viral causes of pneumonia is lower than for bacterial causes, we adjusted for differential case-fatality by determining the aetiological fractions for mortality attributable to RSV and influenza (**Table 2**). We measured the aetiological fractions by applying a relative case-fatality adjustment based on in-hospital case-fatality, which we coded to specific pneumonia aetiologies. Hospital admissions data of this type were limited to data from Austria, Brazil, Chile, China, Ecuador, Italy, Kenya, Mexico, New Zealand, the Philippines, Portugal, and the United States. We generated the pooled estimate of the case-fatality differential between bacterial (pneumococcus, Hib) and viral aetiologies (RSV, influenza) using DisMod-MR to determine an age pattern for this ratio. Therefore, the final attributable fraction for fatal LRI was:

$$Fatal\ PAF = Proportion * \left(1 - \frac{1}{OR}\right) * Inpatient\ scalar * Case\ fatality\ scalar$$

Pneumococcal pneumonia and Hib. For *Streptococcus pneumoniae* (pneumococcal pneumonia) and Hib, we calculated the PAF using a vaccine probe design.[4,5] The ratio of vaccine effectiveness against nonspecific pneumonia to pathogen-specific disease represents the fraction of pneumonia cases attributable to each pathogen.

To estimate the PAF for Hib and pneumococcal pneumonia, we calculated the ratio of vaccine effectiveness against nonspecific pneumonia to pathogen-specific pneumonia (equations 1 and 3). We estimated a study-level estimate of the PAF from a meta-analysis of these ratios. To estimate the PAF for Hib, we only used randomised controlled trials because of implausibly high values of vaccine efficacy in case-control studies. To estimate the PAF for pneumococcal pneumonia, we included RCTs and before and after vaccine introduction longitudinal studies.

We adjusted the study-level PAF estimate by vaccine coverage and expected vaccine performance to estimate country- and year-specific PAF values. For pneumococcal pneumonia, we adjusted the PAF by the final Hib PAF estimate and by vaccine serotype coverage. Finally, we used an age distribution of the PAF modelled in DisMod to determine the PAF by age. Because of an absence of data describing vaccine

efficacy against Hib in children older than 2 years, we did not attribute Hib to episodes of LRI in ages 5 years and older.

We used a vaccine probe design to estimate the PAF for pneumococcal pneumonia and Hib by first calculating the ratio of vaccine effectiveness against nonspecific pneumonia to pathogen-specific pneumonia at the study level (equations 1 and 2).[4–6] We then adjusted this estimate by vaccine coverage and expected vaccine performance to estimate country- and year-specific PAF values (equations 3 and 4).

$$1)\quad HibPAF_{Base} = \frac{VE_{Pneumonia}}{VE_{Hib}}$$

$$2)\quad PneumoPAF_{Base} = \frac{VE_{Pneumonia} * (1 - PAF_{Hib} * VE_{Hib\ Optimal})}{VE_{Streptococcus} * Cov_{Serotype}}$$

$$3)\quad PAF_{Hib} = PAF_{Base} * \frac{(1 - Cov_{Hib} * VE_{Hib\ Optimal})}{(1 - PAF_{Base} * Cov_{Hib} * VE_{Hib\ Optimal})}$$

$$4)\quad PAF_{Pneumo} = \frac{PAF_{Base} * (1 - Cov_{PCV} * VE_{PCV\ Optimal})}{(1 - PAF_{Hib} * Cov_{Hib} * VE_{Hib\ Optimal}) * \left(1 - \frac{PAF_{Base} Cov_{PCV} * VE_{PCV\ Optimal}}{(1 - PAF_{Hib} * Cov_{Hib} * VE_{Hib\ Optimal})}\right)}$$

Where $VE_{Pneumonia}$ is the vaccine efficacy against nonspecific pneumonia, VE_{Hib} is the vaccine efficacy against invasive Hib disease, $VE_{Streptococcus}$ is the vaccine efficacy against serotype-specific pneumococcal pneumonia, $Cov_{serotype}$ is the serotype-specific vaccine coverage for PCV,[7] $VE_{Hib\ Optimal}$ is the Hib effectiveness in the community (0.8),[8] PAF_{Hib} is the final PAF for Hib, Cov_{PCV} is the PCV coverage, Cov_{Hib} is the Hib coverage by country, and $VE_{PCV\ Optimal}$ is the vaccine effectiveness in the community (0.8).[9]

For Hib, we assumed that the vaccine efficacy against invasive Hib disease is the same against Hib pneumonia. For pneumococcal pneumonia, a recent study in adults[10] found that the vaccine efficacy against invasive pneumococcal disease may be significantly higher than against pneumococcal pneumonia. We used this ratio to adjust estimates of vaccine efficacy against invasive pneumococcal disease from other studies. However, recognising that the study is unique in that it uses a urine antigen test among adults, we added uncertainty around our adjustment using a wide uniform distribution (median 0.65, 0.3–1.0). This has increased the estimates of pneumococcal pneumonia mortality in a meaningful way.

Table 2: **The median values for the ratio of viral to bacterial pneumonia case-fatality ratio by age is shown.** These estimates are modelled using hospital-based, ICD-coded admissions and mortality for aetiology-specified pneumonia. Values in parentheses represent 95% uncertainty interval.

Age group	Ratio
Early neonatal	0.59 (0.36–0.84)
Late neonatal	0.58 (0.37–0.84)
Post neonatal	0.58 (0.41–0.77)
1 to 4	0.69 (0.64–0.74)
5 to 9	0.85 (0.77–0.93)
10 to 14	0.84 (0.79–0.89)
15 to 19	0.83 (0.78–0.87)
20 to 24	0.82 (0.77–0.87)
25 to 29	0.82 (0.78–0.86)
30 to 34	0.82 (0.79–0.85)
35 to 39	0.82 (0.8–0.85)
40 to 44	0.82 (0.8-0.85)
45 to 49	0.82 (0.8–0.85)
50 to 54	0.82 (0.79–0.85)
55 to 59	0.82 (0.79–0.86)
60 to 64	0.82 (0.79–0.86)
65 to 69	0.82 (0.8–0.85)
70 to 74	0.82 (0.79–0.85)
75 to 79	0.82 (0.78–0.85)
80 to 84	0.83 (0.8–0.87)
85 to 89	0.86 (0.83–0.89)
90 to 94	0.89 (0.85–0.93)
95 to 99	0.92 (0.86–0.97)

Changes from GBD 2017

The main changes from GBD 2017 involved methods used in determining the attributable fractions for influenza and RSV. For GBD 2019, we applied a consistent and reproducible approach to estimating the ratio of reference to nonreference data. For example, we found the ratio of the proportion of LRI that tested positive for RSV among community episodes and divided that by the proportion positive in inpatient populations.

$$\frac{Proportion_{Community}}{Proportion_{Inpatient}}$$

This value was the input in a meta-regression to find the mean relative difference in those values. This scalar was used to adjust all inpatient data to the *expected* value if it used a community sample instead. The approach described here was used to make inpatient, non-PCR, and single etiology studies more similar to our reference definitions.

The second main change implemented in GBD 2019 was the differential odds ratios by age. Previously, we used a single study of the odds ratio of influenza and RSV for children younger than 5 and applied that to all ages. With a recently published article on the odds for these pathogens in adults over 65 years, we were able to have different values by age.

References

1 Miettinen OS. Proportion of disease caused or prevented by a given exposure, trait or intervention. *Am J Epidemiol* 1974; **99**: 325–32.

2 Shi T, McLean K, Campbell H, Nair H. Aetiological role of common respiratory viruses in acute lower respiratory infections in children under five years: A systematic review and meta-analysis. *J Glob Health* 2015; **5**: 10408.

3 Shi T, Arnott A, Semogas I, Falsey AR, Openshaw P, Wedzicha JA, Campbell H, Nair H, RESCEU Investigators. The etiological role of common respiratory viruses in acute respiratory infections in older adults: a systematic review and meta-analysis. J Infect Dis. 2019 Mar 8. doi: 10.1093/infdis/jiy662

4 Feikin DR, Scott JAG, Gessner BD. Use of vaccines as probes to define disease burden. *Lancet Lond Engl* 2014; **383**: 1762–70.

5 O'Brien KL, Wolfson LJ, Watt JP, *et al.* Burden of disease caused by Streptococcus pneumoniae in children younger than 5 years: global estimates. *Lancet Lond Engl* 2009; **374**: 893–902.

6 Watt JP, Wolfson LJ, O'Brien KL, *et al.* Burden of disease caused by Haemophilus influenzae type b in children younger than 5 years: global estimates. *Lancet Lond Engl* 2009; **374**: 903–11.

7 Johnson HL, Deloria-Knoll M, Levine OS, *et al.* Systematic evaluation of serotypes causing invasive pneumococcal disease among children under five: the pneumococcal global serotype project. *PLoS Med* 2010; **7**. DOI:10.1371/journal.pmed.1000348.

8 Swingler G, Fransman D, Hussey G. Conjugate vaccines for preventing Haemophilus influenzae type B infections. *Cochrane Database Syst Rev* 2007; : CD001729.

9 Lucero MG, Dulalia VE, Nillos LT, *et al.* Pneumococcal conjugate vaccines for preventing vaccine-type invasive pneumococcal disease and X-ray defined pneumonia in children less than two years of age. *Cochrane Database Syst Rev* 2009; : CD004977.

10 Bonten MJM, Huijts SM, Bolkenbaas M, *et al.* Polysaccharide conjugate vaccine against pneumococcal pneumonia in adults. *N Engl J Med* 2015; **372**: 1114–25.

Upper respiratory infections

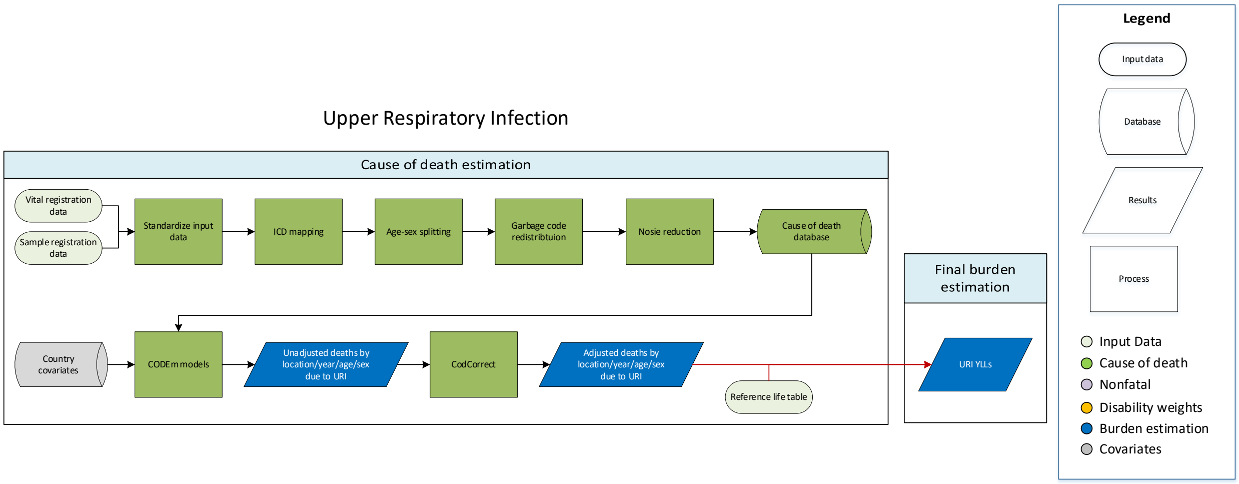

Input data and methodological summary for upper respiratory infections

Input data

Vital registration and surveillance data from the cause of death (CoD) database were used. Outliers were identified by systematic examination of datapoints. Datapoints that violated well-established age or time trends, were inconsistent with other country- or region-specific points, or that resulted in extremely high or low mortality rates were determined to be outliers.

Modelling strategy

A generic CODEm approach was used to estimate mortality due to upper respiratory infections (URI) in GBD 2019. In GBD 2016, mortality from URI was modelled using a negative binomial regression. It was determined that a negative binomial regression was an appropriate approach for estimating URI due to a small number of deaths due to URI in the CoD database. However, due to changes in how we redistribute cause of death codes, more deaths were attributed to URI in the CoD database, and thus it was determined that a generic CODEm approach was feasible for estimating URI mortality in GBD 2017. The covariates used are displayed below. We have made no substantive changes to the modelling strategy in 2019.

Level	Covariate	Direction
1	Smoking prevalence	+
2	Indoor pollution	+
	Outdoor pollution ($PM_{2.5}$)	+
	Healthcare Access and Quality Index	-
3	Socio-demographic Index	-
	Lag distributed income	-
	Education (years per capita)	-

Otitis media

Flowchart

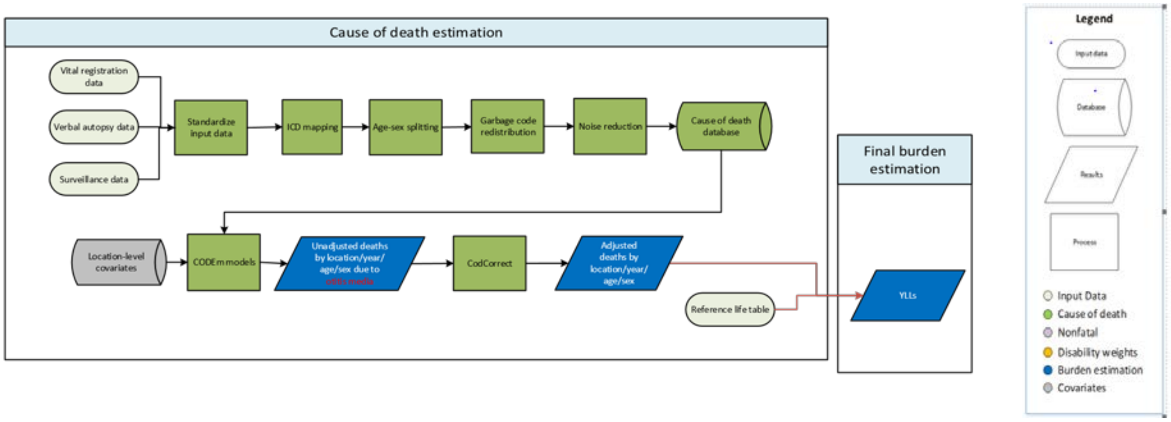

Input data and methodological summary for otitis media

Input data

Vital registration, verbal autopsy, and surveillance data were used. Outliers were identified by systematic examination of datapoints. Datapoints that violated well-established age or time trends, were inconsistent with other country- or region-specific points, or that resulted in extremely high or low mortality rates were determined to be outliers.

Modelling strategy

A general CODEm modelling strategy was used. There were no substantive changes from GBD 2017 in terms of modelling strategy. The covariates used are displayed below.

Table 1. Covariates used in otitis media mortality modelling

Level	Covariate	Direction
1	Otitis summary exposure value (SEV)	+
	Smoking prevalence	+
2	Indoor pollution	+
	Healthcare Access and Quality Index	-
	Outdoor pollution ($PM_{2.5}$)	+
3	Socio-demographic Index (SDI)	-
	Log-transformed lag distributed income	-
	Education (years per capita)	-

Diarrhoeal diseases

Flowchart

Diarrheal diseases

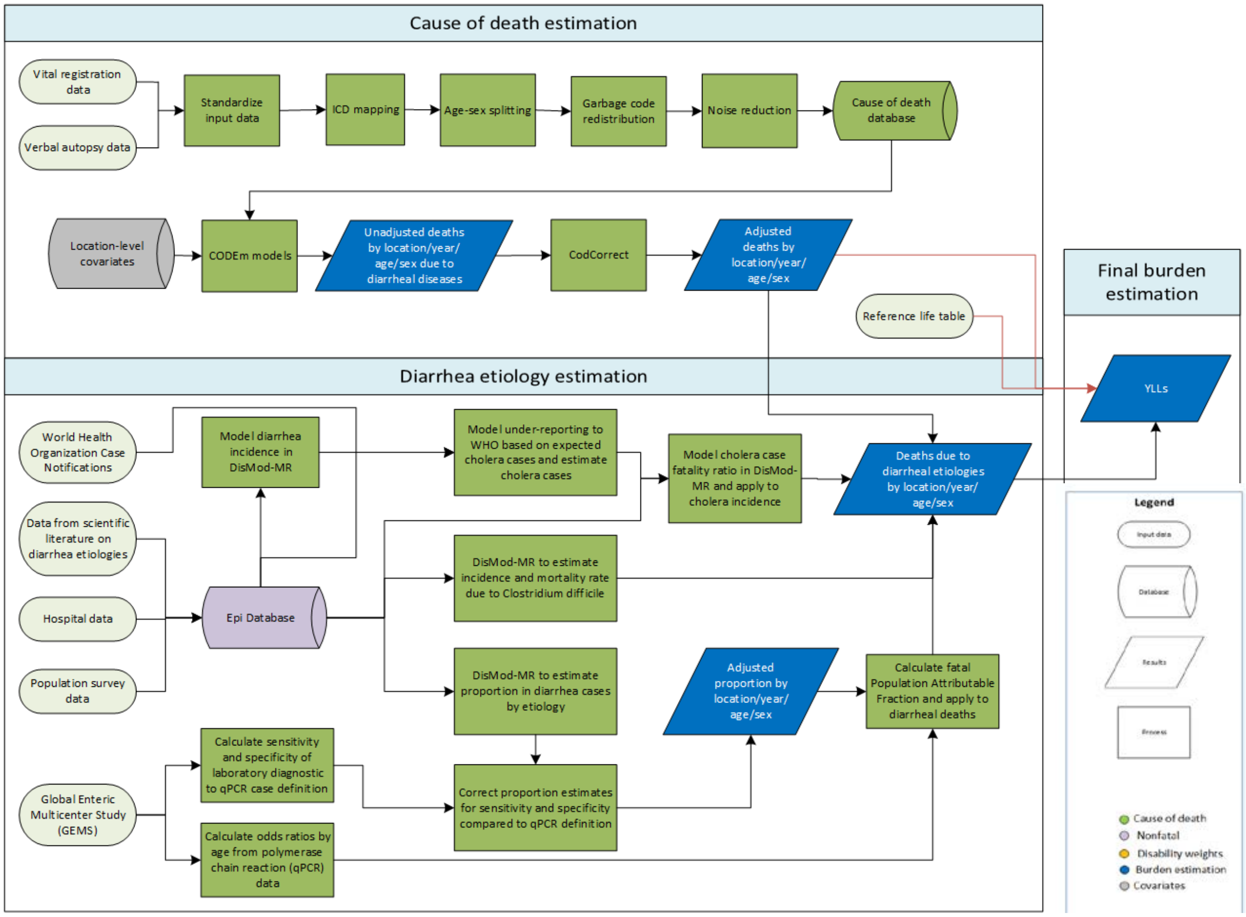

Diarrhoeal diseases are a cause of death in GBD. We also estimated the attributable deaths from 13 diarrhoeal aetiologies using an independent modelling strategy. These pathways are shown in the flowchart above and will both be described in this report.

Input data

Cause of death. We used all available data from vital registration systems, surveillance systems, and verbal autopsy. Data points that violated well-established age or time trends were determined to be outliers. We also excluded early neonatal mortality data in the Philippines (1994–1998), India Civil Registration System data, and medically certified cause of death (MCCD) data in all states (1986–2013).

Aetiologies. The second type of data describes diarrhoea aetiologies. There are 13 aetiologies in GBD 2019 for diarrhoea: adenovirus, *aeromonas, campylobacter, vibrio cholerae, clostridium difficile, cryptosporidium*, *entamoeba histolytica,* typical enteropathogenic *E. coli* (typical EPEC), heat-stable toxin producing enterotoxigenic *E. coli* (ST-ETEC), norovirus, rotavirus, non-

typhoidal salmonella, and shigella. We extracted data on all aetiologies except *C. difficile* from scientific literature that reported the proportion of diarrhoea cases that tested positive for each pathogen. We completed a systematic literature review covering the time period May 2018 to February 2019 for diarrhoea prevalence, incidence, and all diarrhoea aetiologies. Inclusion criteria included diarrhoea as the case definition, studies with a sample size of at least 100, and studies with at least one year of follow up. We excluded studies that reported on diarrhoeal outbreaks exclusively and those that used acute gastroenteritis with or without diarrhoea.

We searched articles using a PubMed search term that combined nonspecific and aetiology-specific diarrhoea in February 2019 using the following search string:

(diarrhoea[title/abstract] OR diarrhea[title/abstract]) AND (2018/07/30:2019/2/7[PDat]) AND Humans[MeSH Terms] AND (incidence[title/abstract] OR prevalence[title/abstract] OR epidemiology[title/abstract] OR salmonella[title/abstract] OR aeromona[title/abstract] OR shigell*[title/abstract] OR enteropathogenic[title/abstract] OR enterotoxigenic[title/abstract] OR campylobacter[title/abstract] OR amoebiasis[title/abstract] OR entamoeb*[title/abstract] OR cryptosporid*[title/abstract] OR rotavirus[title/abstract] OR norovirus[title/abstract] OR adenovirus[title/abstract] OR etiology[title/abstract]) NOT (appendicitis[title/abstract] OR esophag*[title/abstract] OR surger*[title/abstract] OR gastritis[title/abstract] OR liver[title/abstract] OR case report[title] OR case-report[title] OR therapy[title] OR treatment[title] Crohn[title/abstract] OR "inflammatory bowel"[title/abstract] OR irritable[title/abstract] OR travel*[title] OR Outbreak[title] OR Review[ptyp] OR vomiting[title/abstract).*

We identified 82 studies, of which three met our inclusion criteria. We extracted data for location, sex, year, and age.

We used the Global Enteric Multicenter Study (GEMS), a seven-site, case-control study of moderate-to-severe diarrhoea in children under 5 years,[1] and the MAL-ED study,[2] a multi-site birth cohort, to calculate odds ratios for the diarrhoeal pathogens. We analysed raw data for a systematic reanalysis, representative of the distribution of cases and controls by age and site that were tested for the presence of pathogen using quantitative polymerase chain reaction (qPCR).[3]

Data that did not use qPCR for detection were adjusted for sensitivity and specificity prior to modelling in order to standardize data regardless of detection method. Adjusting these data prior to modelling allowed us to adjust only data that did not use qPCR, as well as better control for values at extreme bounds, and capture uncertainty in modelling.

Modelling strategy

Cause of death. Diarrhoeal disease mortality was estimated in the Cause of Death Ensemble modelling platform (CODEm). We estimated diarrhoea mortality separately for males and

females and for children under 5 years and older than 5 years. We used country-level covariates to inform our CODEm models (**Table 1**).

Table 1. The covariates used in diarrhoea mortality modelling. Table 1A shows the covariates used in the 0–4 years model, and Table 2B shows the covariates used in the 5–95+ years model. The *Level* represents the strength of the association between the covariate and diarrhoea mortality from 1 (proximally related) to 3 (distally related). The *Direction* indicates the positive or negative association between the covariate and diarrhoea mortality.

Table 1A. The covariates used in the 0–4 years model

Level	Covariate	Direction
1	Oral rehydration solution treatment	-
	Safe sanitation access	-
	Safe water access	-
	Rotavirus vaccine	-
2	Vitamin A deficiency	+
	Zinc deficiency	+
	Zinc treatment for diarrhoea	-
3	Handwashing access	-
	Lag distributed income (LDI) per capita	-
	Maternal education years	-
	Healthcare Access and Quality Index	-
	Socio-demographic Index (SDI)	-

Table 1B. The covariates used in the 5–95+ years model.

Level	Covariate	Direction
1	Diarrhoea summary exposure value (SEV)	+
	Unsafe sanitation SEV	+
	Unsafe water SEV	+
	Sanitation access	-
	Improved water source access	-
2	Healthcare Access and Quality Index	-
	Rotavirus vaccine coverage	-
3	Education years per capita	-
	LDI per capita	-
	Adult underweight	+
	SDI	-
	Oral rehydration accesss	-
	Population density less than 150/km^2	+
	Population density greater than 1000/km^2	+

Aetiologies. We estimated diarrhoeal disease aetiologies independently from overall diarrhoea mortality using a counterfactual strategy for enteric adenovirus, *aeromonas*, *entamoeba histolytica* (amoebiasis), *campylobacter*, *cryptosporidium*, typical EPEC, enterotoxigenic *Escherichia coli* (ETEC), norovirus, non-typhoidal salmonella infections, rotavirus, and shigella. *Vibrio cholerae* and *C. difficile* were modelled separately.

Diarrhoeal aetiologies are attributed to diarrhoeal deaths using a counterfactual approach. We calculated a population attributable fraction (PAF) from the proportion of severe diarrhoea cases that are positive for each aetiology. The PAF represents the relative reduction in diarrhoea mortality if there was no exposure to a given aetiology. As diarrhoea can be caused by multiple pathogens and the pathogens may co-infect, PAFs can overlap and are not scaled to sum to 100%. We calculated the PAF from the proportion of severe diarrhoea cases that are positive for each aetiology. We assumed that hospitalised diarrhoea cases are a proxy of severe and fatal cases. We used the following formula to estimate PAF:[4]

$$PAF = Proportion * (1 - \frac{1}{OR})$$

Where *Proportion* is the proportion of diarrhoea cases positive for an aetiology and *OR* is the odds ratio of diarrhoea given the presence of the pathogen.

We dichotomised the continuous qPCR test result using the value of the cycle threshold (Ct) that most accurately discriminated between cases and controls. The Ct values range from 0 to 35 cycles representing the relative concentration of the target gene in the stool sample. A low value indicates a higher concentration of the pathogen while a value of 35 indicates the absence of the target in the sample. We used the lower Ct value when we had multiple Ct values for the cutpoint. The case definition for each pathogen is a Ct value that is below the established cutoff point.

We used a mixed effects conditional logistic regression model to calculate the odds ratio for under 1 year and 1–4 years old for each of our pathogens. The stool samples from cases and controls in GEMS were used exclusively to calculate these odds ratios as we assumed that the association between pathogens and moderate-to-severe diarrhoea is a proxy for fatal outcomes. The odds ratio for 1–4 years was applied to all GBD age groups over 5 years. There were three pathogen-age odds ratios that were not statistically significant: aeromonas and amoebiasis in under 1 year and campylobacter in 1–4 years. The mean value of the odds ratio was above 1 in all three cases, so we transformed the odds ratios for these three exceptions only in log space such that exponentiated values could not be below 1. The transformation was:

Odds ratio = exp(log(OR) – 1)) + 1

We modelled the proportion data using the Bayesian meta-regression tool DisMod-MR to estimate the proportion of positive diarrhoea cases for each separate aetiology by location/year/age/sex and to adjust for the covariates. We used the estimated sensitivity and specificity of the original laboratory diagnostic test results from the pooled GEMS and MAL-ED qPCR stool samples compared to the qPCR test result to adjust our proportion before we modelled the proportions:[5]

$$Proportion_{True} = \frac{(Proportion_{Observed} + Specificity - 1)}{(Sensitivity + Specificity - 1)}$$

We used this correction to account for the fact that the proportions we used are based on a new test that is not consistent with the laboratory-based case definition (qPCR versus GEMS conventional laboratory testing for pathogens).[6] Because differences in the type of PCR used in the original (nonreference qPCR diagnostic) between GEMS and MAL-ED in detecting norovirus, we combined the sensitivity and specificity results for norovirus such that 50% of the draws were coming from GEMS test results exclusively and 50% of the draws were coming from MAL-ED test results exclusively. Additionally, because the original laboratory diagnostic technique used for *campylobacter* in MAL-ED was one not commonly used, we only used GEMS to determine the sensitivity and specificity of bacterial culture compared to qPCR in detecting *campylobacter.*[7]

Our literature review extracted the proportion of any EPEC without differentiating between typical (tEPEC) and atypical (aEPEC). In order to be consistent with the odds ratios that we obtained, we adjusted our proportion estimates of any EPEC to typical EPEC only. This adjustment was informed by a subset of our literature review that reported both atypical and typical EPEC. We estimated a ratio by super-region of tEPEC to any EPEC and adjusted our proportion estimates accordingly. We found that the majority of EPEC diarrhoea cases were positive for atypical EPEC, consistent with other published work.[8] We applied the same approach to differentiate between heat-stable toxin (ST) and heat labile toxin producing (LT) ETEC. For the first time, GBD 2019 split these serotypes so that estimates in GBD 2019 represent the diarrhoeal disease burden attributable to ST-ETEC. This was based on work showing that ST-ETEC was much more pathogenic than LT-ETEC. As our proportion data were extracted for any ETEC, we determined a proportion of all ETEC that produced ST from the GEMS and MAL-ED studies and applied that ratio to our input data so that they represented ST-ETEC only. We re-estimated the sensitivity and specificity values as well as the odds ratios for our new definition of ST-ETEC.

For *vibrio cholerae* (cholera), we used the literature review to estimate the expected number of cholera cases for each country-year using the incidence of diarrhea (estimated using DisMod-MR) and the proportion of diarrhoea cases that are positive for cholera. We assigned cholera PAF using odds ratios from the qPCR results to estimate a number of cholera-attributable cases. We compared this expected number of cholera cases to the number reported to the World Health Organization at the country-year level.[9] We modelled the underreporting fraction to correct the cholera case notification data for all countries using health system access and the diarrhoea SEV scalar to predict total cholera cases. We used the age-specific proportion of positive cholera samples in DisMod-MR and our incidence estimates to predict the number of cholera cases for each age/sex/year/location. Finally, we modelled the case fatality ratio of cholera using DisMod-MR and to estimate the number of cholera deaths.

For *C. difficile*, we modelled incidence and mortality in DisMod-MR for each age, sex, year, location. DisMod-MR is a Bayesian meta-regression tool that uses spatiotemporal information

as priors to estimate prevalence, incidence, remission, and mortality for *C. difficile* infection. DisMod-MR uses a compartmental model to relate prevalence, incidence, remission, and mortality. We set remission in our model to 1 month.

For rotavirus, we made a change to the process of estimating attributable fraction to explicitly account for rotavirus vaccine efficacy in GBD 2019. The impact of the rotavirus vaccine is dependent on modelled vaccine coverage for a location-year and on the rotavirus vaccine efficacy (VE). There are numerous studies that demonstrate a difference in VE by location.[10] We determined that SDI was the best predictor of rotavirus VE, and we used a meta-regression with this covariate to predict the rotavirus VE by location where the VE was higher in areas with larger SDI values and followed a logit-linear distribution.

For GBD 2019, we explicitly incorporated the results from our analysis of VE to produce more robust estimates of the proportion of diarrhoea that has rotavirus over time and space. We assumed that the impact of the vaccine can be represented as one minus the product of the estimated vaccine coverage and VE.

$$Vaccine\ impact = 1 - vaccine\ coverage * vaccine\ efficacy$$

Both of these values vary in time and space but not by age. To avoid discontinuities in our model, we adjusted the input proportion data to remove the impact of the rotavirus vaccine by dividing the observed proportion by the vaccine impact.

$$Rotavirus\ proportion_{Adjusted} = \frac{Rotavirus\ proportion}{1 - Cov_{RotaV} * VE_{Modeled}}$$

The result is the modelled proportion of diarrhoea positive for rotavirus in the absence of the vaccine. This modelled value is then multiplied by the impact of the rotavirus vaccine to determine the estimated proportion of diarrhoea positive for rotavirus in the presence of the vaccine. Our modified attributable fraction is then:

$$DisModPAF = Modeled\ Proportion\ (from\ DisMod)\ * \left(1 - \frac{1}{OR}\right)$$

The last step is to account for the expected impact of the rotavirus vaccine. We do this using the equation below:

$$PAF_{Rota} = DisModPAF * \frac{(1 - Cov_{RotaV} * VE_{Modeled})}{(1 - DisModPAF * Cov_{RotaV} * VE_{Modeled})}$$

Where the final attributable fraction for rotavirus is the product of the PAF estimated in DisMod-MR and the expected reduction in that PAF given modelled vaccine coverage and modelled VE by location-year, and this value is only applied to children 28 days to 5 years old. The product of the rotavirus attributable fraction and the number of deaths or cases of diarrhoea is the number of deaths and cases caused by rotavirus.

References

1 Kotloff KL, Nataro JP, Blackwelder WC, *et al.* Burden and aetiology of diarrhoeal disease in infants and young children in developing countries (the Global Enteric Multicenter Study, GEMS): a prospective, case-control study. *Lancet Lond Engl* 2013; **382**: 209–22.

2 Platts-Mills J, Liu J, Rogawski E. Aetiology, burden and clinical characteristics of diarrhoea in children in low-resource settings using quantitative molecular diagnostics: results from the MAL-ED cohort study. *Lancet Glob Health* 2018; : Accepted.

3 Liu J, Gratz J, Amour C, *et al.* A laboratory-developed TaqMan Array Card for simultaneous detection of 19 enteropathogens. *J Clin Microbiol* 2013; **51**: 472–80.

4 Miettinen OS. Proportion of disease caused or prevented by a given exposure, trait or intervention. *Am J Epidemiol* 1974; **99**: 325–32.

5 Reiczigel J, Földi J, Ozsvári L. Exact confidence limits for prevalence of a disease with an imperfect diagnostic test. *Epidemiol Infect* 2010; **138**: 1674–8.

6 Platts-Mills JA, Operario DJ, Houpt ER. Molecular diagnosis of diarrhea: current status and future potential. *Curr Infect Dis Rep* 2012; **14**: 41–6.

7 Platts-Mills JA, Liu J, Gratz J, *et al.* Detection of Campylobacter in stool and determination of significance by culture, enzyme immunoassay, and PCR in developing countries. *J Clin Microbiol* 2014; **52**: 1074–80.

8 Ochoa TJ, Barletta F, Contreras C, Mercado E. New insights into the epidemiology of enteropathogenic Escherichia coli infection. *Trans R Soc Trop Med Hyg* 2008; **102**: 852–6.

9 World Health Organization. Global Health Observatory data repository: Cholera. 2016. http://apps.who.int/gho/data/node.main.174?lang=en (accessed Aug 25, 2016).

10 Lamberti LM, Ashraf S, Walker CLF, Black RE. A Systematic Review of the Effect of Rotavirus Vaccination on Diarrhea Outcomes Among Children Younger Than 5 Years. *Pediatr Infect Dis J* 2016; **35**: 992–8.

Typhoid fever

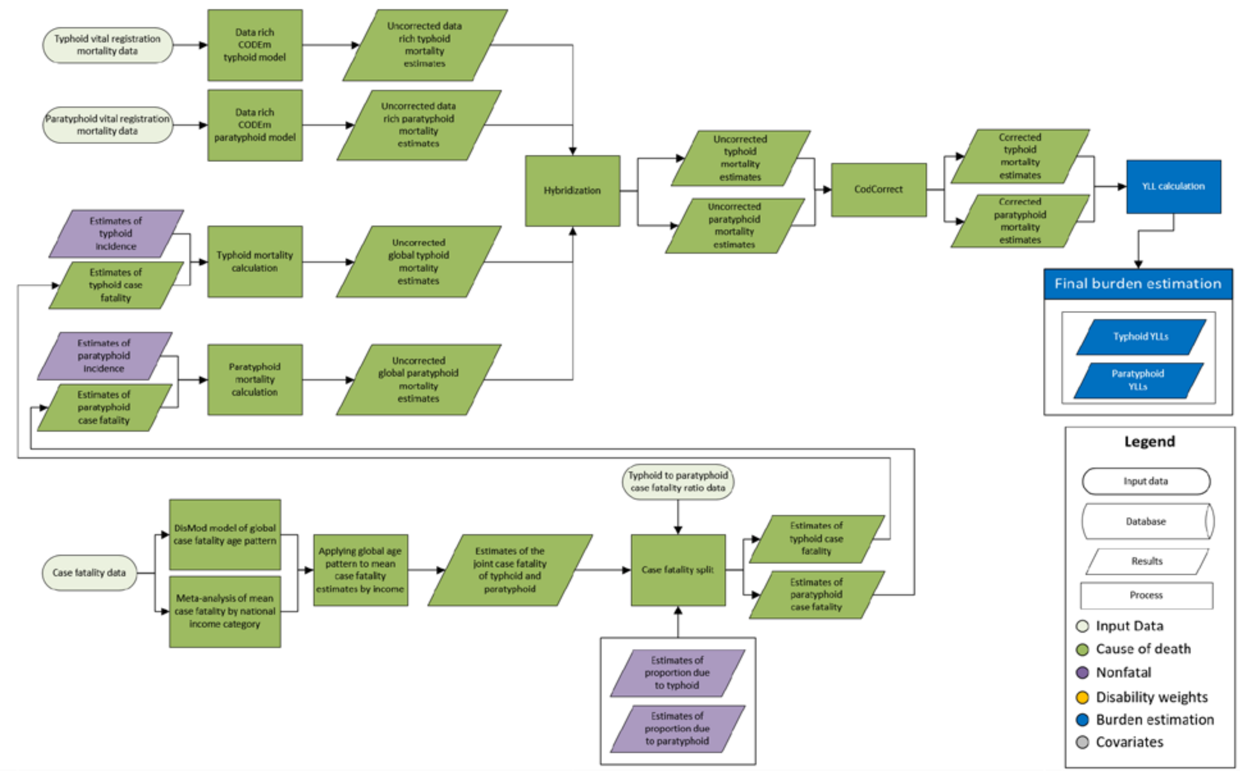

Input data

Our CODEm model used all available data in the cause of death database from data-rich countries. No data were outliered for this cause. For the natural history model, our incidence dataset included a combination of data from prospective cohort studies and national surveillance systems. Similarly, data on proportions due to typhoid and paratyphoid included a combination of prospective cohort studies and national surveillance systems. Case fatality data were from national surveillance systems and hospital databases.

Modelling strategy

We model typhoid deaths using a hybrid modelling strategy with two components: 1) for data-rich locations we estimate typhoid mortality using a CODEm model of CoD data; and 2) in all other locations (ie, not data-rich) we use a natural history model in which we derive deaths as the product of cases and case fatality.

The CODEm model included six covariates:

Level	Covariate	Direction
1	Sanitation (proportion with access)	-
	Improved water source (proportion of the population with access)	-
	Proportion of the population living in the Indian Ocean monsoon belt	+
	SEV unsafe water	+

	SEV unsafe sanitation	+
2	Healthcare Access and Quality Index	-

For the natural history model, we first model total incidence of typhoid and paratyphoid combined. Second, we model the proportion of this total due to typhoid and the proportion due to paratyphoid. Third, we estimate case fatality by age and national income category for typhoid and paratyphoid combined. Fourth, we use data on the relative fatality of typhoid and paratyphoid to split the joint case fatality estimates into typhoid- and paratyphoid-specific case fatality estimates. Finally, we estimate cause-specific mortality rates as the product of incidence and case fatality.

Total incidence was modelled using DisMod-MR 2.1 using the proportion of the population with access to clean water, and the proportion of the population living in the Indian Ocean monsoon belt as covariates. We performed a crosswalk using a study-level covariate indicating sources that were based on passive versus active surveillance, with active surveillance as the reference. This adjusts for incomplete case capture by passive surveillance. Incidence data were inflated to account for poor diagnostic sensitivity, based on a meta-analysis of the sensitivity of blood culture, the most common diagnostic used for typhoid. Similarly, we used two DisMod models to estimate aetiologic proportions: one for the proportion of total incidence due to typhoid, and one for the proportion due to paratyphoid.

Case fatality data were too limited to allow for a complete DisMod model, or to allow for varying estimates by time and space. We had sufficient data, however, to estimate case fatality by age and by three categories of national income. We used DisMod to extract a global age-pattern in case fatality, and meta-regression to estimate the mean case fatality by income category. Finally, we estimated the relative risk of death from typhoid relative to paratyphoid based on data from Chinese surveillance and used that relative risk to estimate case fatality separately for typhoid and paratyphoid, by age and income.

Finally, we estimated typhoid mortality as the product of total incidence, the proportion of the total due to typhoid, and case fatality for typhoid. We propagated uncertainty through every step of the modelling process by pulling 1,000 draws from the distribution of each model component (eg, incidence, proportion due to typhoid, overall case fatality, case fatality age pattern, relative fatalness of typhoid versus paratyphoid), and performing all calculations at the draw level.

We have made no substantive changes to our natural history modelling strategy between GBD 2017 and 2019.

Paratyphoid fever

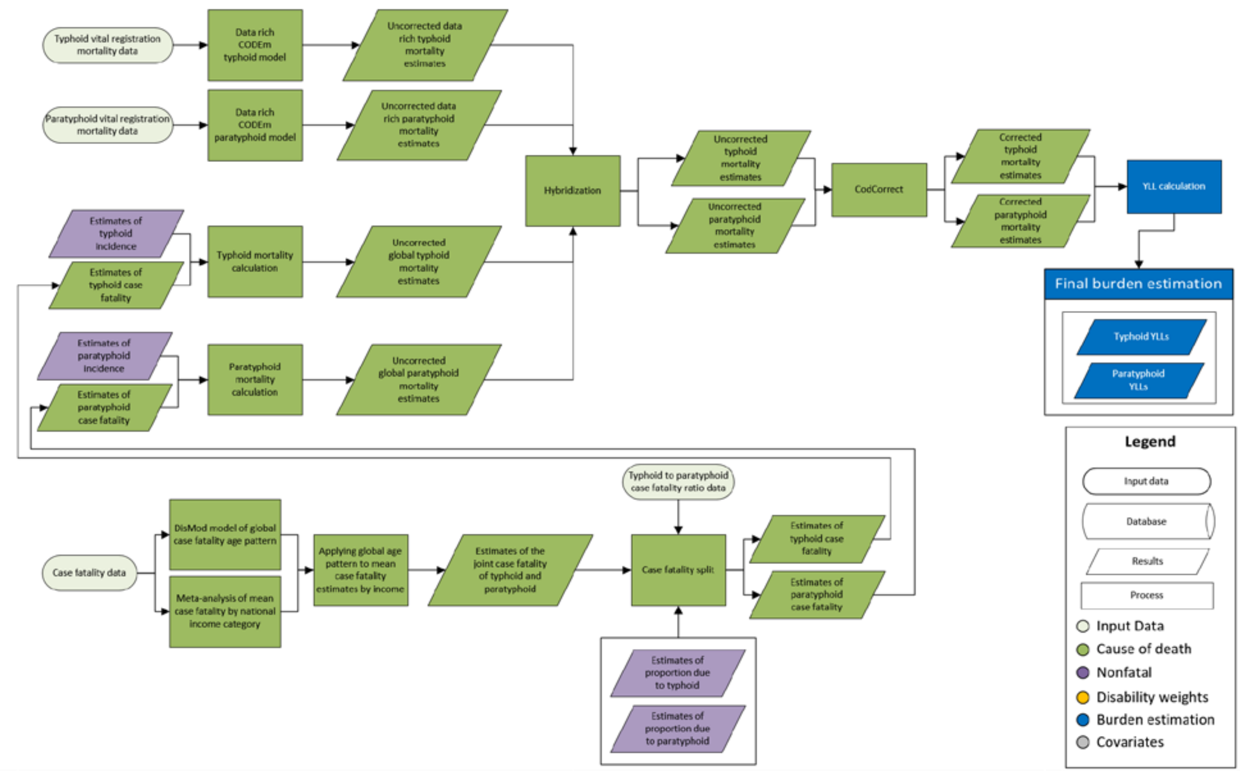

Input data

Our CODEm model used all available data in the cause of death database from data-rich countries. No data were outliered for this cause. For the natural history model, our incidence dataset included a combination of data from prospective cohort studies and national surveillance systems. Similarly, data on proportions due to typhoid and paratyphoid included a combination of prospective cohort studies and national surveillance systems. Case fatality data were from national surveillance systems and hospital databases.

Modelling strategy

We model paratyphoid deaths using a hybrid modelling strategy with two components: 1) for data-rich locations we estimate paratyphoid mortality using a CODEm model of CoD data; and 2) in all other locations (ie, not data-rich) we use a natural history model in which we derive deaths as the product of cases and case fatality.

The CODEm model included six covariates:

Level	Covariate	Direction
1	Sanitation (proportion with access)	-
	Improved water source (proportion of the population with access)	-
	Proportion of the population living in the Indian Ocean monsoon belt	+
	SEV unsafe water	+

	SEV unsafe sanitation	+
2	Healthcare Access and Quality Index	-

For the natural history model, we first model total incidence of typhoid and paratyphoid combined. Second, we model the proportion of this total due to typhoid and the proportion due to paratyphoid. Third, we estimate case fatality by age and national income category for typhoid and paratyphoid combined. Fourth, we use data on the relative fatality of typhoid and paratyphoid to split the joint case fatality estimates into typhoid- and paratyphoid-specific case fatality estimates. Finally, we estimate cause-specific mortality rates as the product of incidence and case fatality.

Total incidence was modelled using DisMod-MR 2.1, using the proportion of the population with access to clean water, and the proportion of the population living in the Indian Ocean monsoon belt as covariates. We performed a crosswalk using a study-level covariate indicating sources that were based on passive versus active surveillance, with active surveillance as the reference. This adjusts for incomplete case capture by passive surveillance. Incidence data were inflated to account for poor diagnostic sensitivity, based on a meta-analysis of the sensitivity of blood culture, the most common diagnostic used for typhoid and paratyphoid. Similarly, we used two DisMod models to estimate aetiologic proportions: one for the proportion of total incidence due to typhoid, and one for the proportion due to paratyphoid.

Case fatality data were too limited to allow for a complete DisMod model, or to allow for varying estimates by time and space. We had sufficient data, however, to estimate case fatality by age and by three categories of national income. We used DisMod to extract a global age-pattern in case fatality, and meta-regression to estimate the mean case fatality by income category. Finally, we estimated the relative risk of death from typhoid relative to paratyphoid based on data from Chinese surveillance and used that relative risk to estimate case fatality separately for typhoid and paratyphoid, by age and income.

Finally, we estimated paratyphoid mortality as the product of total incidence, the proportion of the total due to paratyphoid, and case fatality for paratyphoid. We propagated uncertainty through every step of the modelling process by pulling 1,000 draws from the distribution of each model component (eg, incidence, proportion due to paratyphoid, overall case fatality, case fatality age pattern, relative fatalness of typhoid versus paratyphoid), and performing all calculations at the draw level.

We have made no substantive changes to our natural history modelling strategy between GBD 2017 and 2019.

Invasive non-typhoidal salmonella (iNTS)

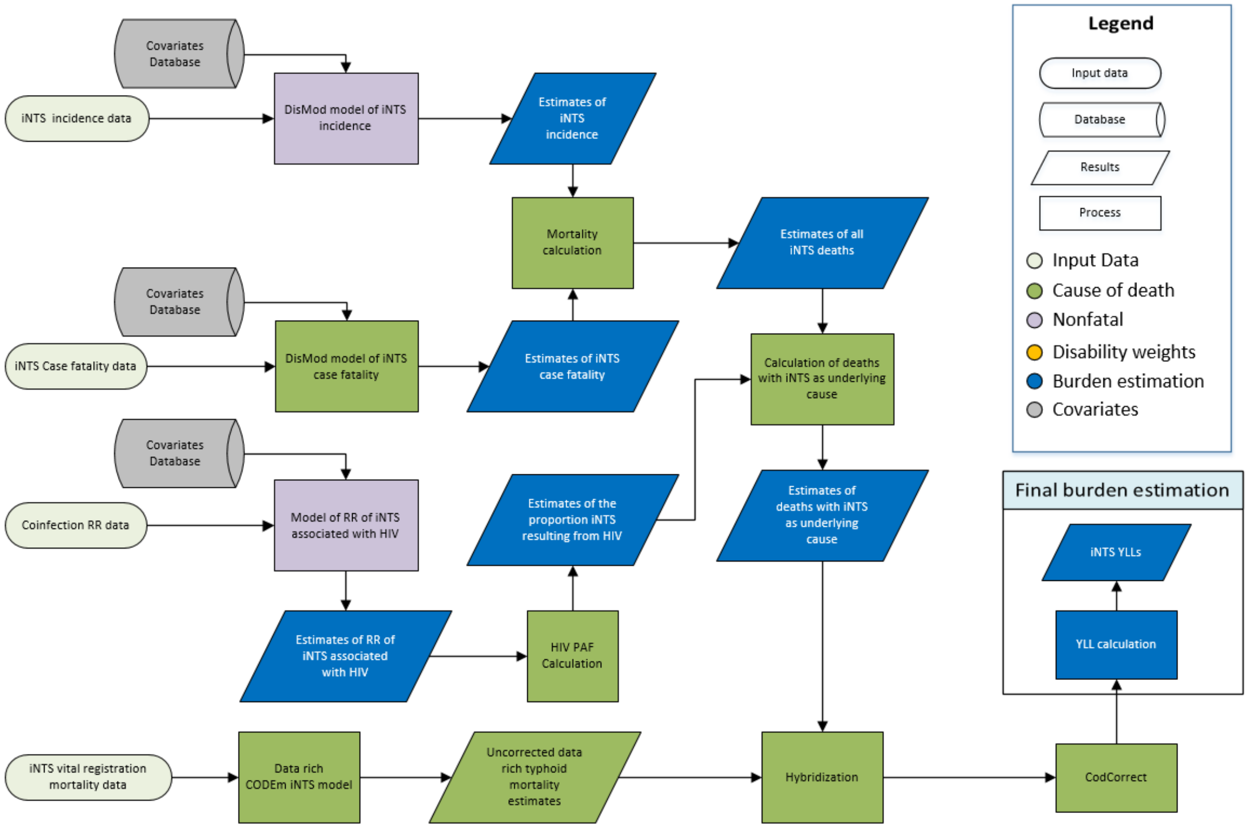

Input data

Our CODEm model used all available data in the cause of death database from data-rich countries. No data were outliered for this cause. Incidence estimates for the natural history model are modelled using an incidence dataset based principally on prospective cohort studies and facility-based surveillance. Similarly, data on case fatality and co-infection come from prospective cohort studies and facility-based surveillance.

Modelling strategy

We model iNTS deaths using a hybrid modelling strategy with two components: 1) for data-rich locations we estimate iNTS mortality using a CODEm model of CoD data; and 2) in all other locations (ie, not data-rich) we use a natural history model in which we derive deaths as the product of cases and case fatality.

The CODEm model included three covariates:

Level	Covariate	Direction
1	SEV unsafe water	+
	Malaria incidence adjusted for antimalarial coverage and drug effectiveness	+
	HIV mortality rate	+

For the natural history model, we estimate iNTS deaths as the product of cases and case fatality. Incidence was modelled with DisMod-MR 2.1, using the HIV mortality rate, malaria incidence adjusted for antimalarial coverage and drug effectiveness, and the summary exposure value (SEV), unsafe water, as covariates. We estimated the relative risk of iNTS comparing people with HIV to those without using a negative binomial model with log-age and log of the summary exposure value (SEV) for water as predictors. We used the resulting relative risk estimates and HIV prevalence estimates to calculate the proportion of iNTS that was attributable to HIV in each location, year, age, and sex. Using these proportions, we divided iNTS cases into those that were attributable to HIV and those that were not. We modelled case fatality by age and Socio-demographic Index (SDI) separately for those with and without HIV using a generalised additive model, parameterising age with P-splines, and estimated mortality as the product of incidence and case fatality. Where iNTS occurs among those with HIV, we assume that iNTS is an opportunistic infection and that HIV is therefore the underlying cause of death. We therefore estimate deaths with iNTS as the underlying cause as total iNTS deaths times the proportion of cases not attributable to HIV.

The hybrid approach is a new for GBD 2019, as estimates for GBD 2017 were based on a natural history model for all locations. For countries that are not data-rich, we have made no substantive changes to our natural history modelling strategy between GBD 2017 and 2019.

Other intestinal infectious diseases

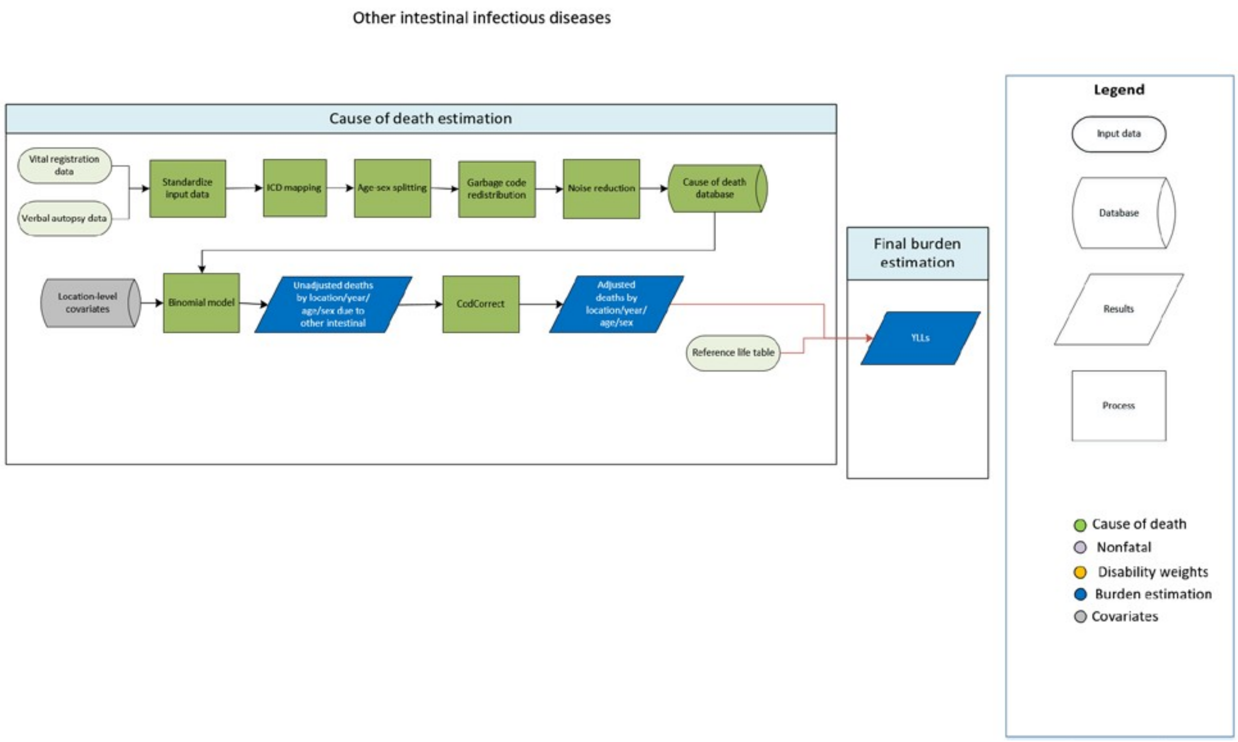

Input data

We modelled other intestinal infectious disease mortality using all available data in the cause of death (CoD) database. Data points were outliered if they reported an improbable number of deaths or if their inclusion in the model yielded distorted trends. In some cases, multiple data sources for the same location differed dramatically both in their quality and reported other intestinal infectious disease mortality (eg, a verbal autopsy and vital registration source). In these cases the lower-quality data source was outliered.

Modelling strategy

We modelled other intestinal infectious disease mortality using a custom binomial model of all data in the CoD database. The custom model was used because of very small death counts. We used the number of cause-specific deaths as the outcome, with the all-cause mortality envelope as the exposure term. We included the square root of Socio-demographic Index, age group, and sex as covariates, and included a random effect on region.

We have made no substantive changes to the modelling strategy in 2019.

Malaria

Flowchart

Malaria cause of death estimation – case fatality rate (CFR) approach

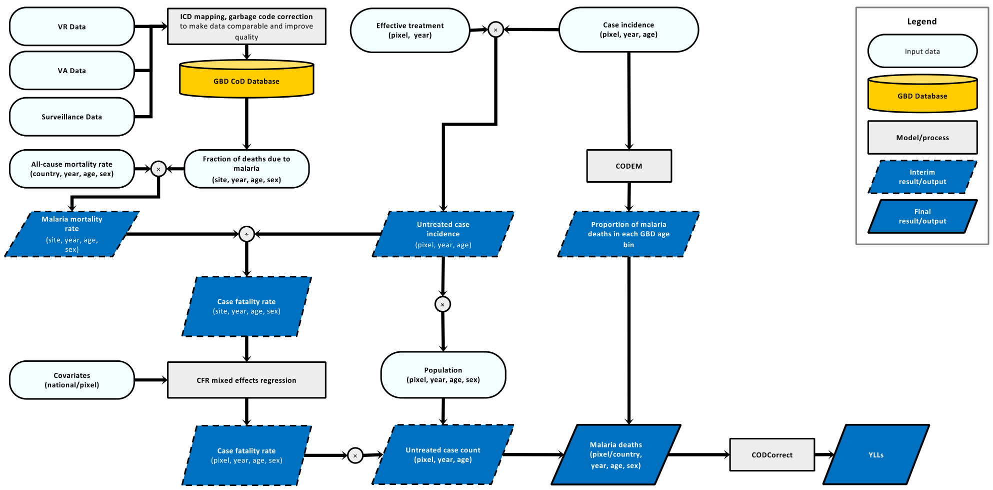

Malaria cause of death estimation - *P. vivax* only countries

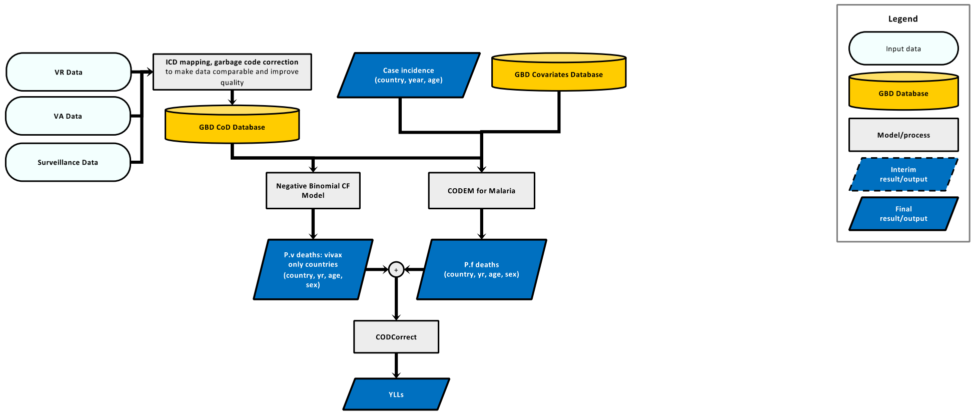

Input data and methodological summary for malaria

Overview

Variability in the presence of *Plasmodium falciparum* necessitated distinct approaches for estimating malaria mortality. In countries with *Plasmodium falciparum*, which is responsible for the vast majority of malaria deaths and for which there is cause of death evidence available, this species was considered the only cause of malaria deaths. For these countries we applied a model that generates a geographically heterogeneous case fatality rate (CFR) grid, which we then intersect with untreated incidence to determine the number of deaths. Pixel-level death totals are then aggregated to the admin-level to produce the GBD estimates. In countries where the only species present was *Plasmodium vivax*, a very simplistic model was used instead to attribute some nominal number of deaths to malaria.

Input data

The cause of death (CoD) data included vital registration, verbal autopsy, and surveillance data from the GBD database. For the CFR model, we only used CoD data (mostly verbal autopsy) where we have been able to successfully geo-reference the site (i.e., find associated geographic coordinates). Systematic literature reviews for malaria were not conducted. Our outlier criteria excluded data points that (i) were implausibly high or low relative to global or regional patterns, (ii) substantially conflicted with established age or temporal patterns, or (iii) significantly conflicted with other data sources conducted from the same locations or locations with similar characteristics (i.e., local Socio-demographic Index).

Modelling strategy

For most GBD causes, epidemiologic measures may be used as covariates in a traditional CODEm approach, if at all. To estimate the fatal burden of *P. falciparum* malaria in Africa, we used epidemiological measures in our estimation process directly. The Malaria Atlas Project (MAP) at the University of Oxford has generated updated spatiotemporal “cubes” estimating clinical incidence (rates and case counts) for each 5x5 km pixel, by year, from 1980 to 2019. MAP has also generated an equivalent spatiotemporal prediction of effective treatment with an antimalarial drug (combining treatment seeking, the fraction of malaria cases receiving different classes of antimalarial, and the estimated country-year-specific efficacy of each antimalarial class though time). This estimated effective treatment rate was combined with the incidence rate cube to derive a third cube estimating the incidence of untreated cases at the pixel level.

For each site-year for which CoD malaria cause fraction data were available we (i) estimated a site-year-specific malaria mortality rate as the product of malaria cause fraction and all-cause mortality rate (with the latter drawn from national-level values); (ii) divided the malaria mortality rate by the site-year-specific estimate of untreated malaria incidence rate (drawn from the MAP cube) to estimate a site-year-specific case fatality rate (CFR) among untreated malaria cases. These derived site-year-specific CFR values were then used in a geostatistical model to estimate pixel-year CFR for each 5x5 km grid cell. The response variable for this model was logit all-ages CFR (for untreated cases), and Gaussian likelihood was used. The model included a separate intercept for each IHME super-region. Similarly, each continent was given its own smooth temporal effect (random walk of order 2). There was no global intercept or global temporal term as some continents had many data points while Africa in particular had very few. The fixed effect covariates used were travel time to cities, proportion of adults, proportion of infants, log country-year all-cause mortality, and sickle cell anemia rate (proportion of heterozygotes).

Finally, a sample location random effect was included (and not used in prediction) to account for sampling biases between sites.

Pixel-year predictions of CFR were then multiplied by the untreated incidence rate rasters from the MAP cube to yield pixel-year mortality rate estimates, which were then multiplied by pixel-year population to derive pixel-year malaria death counts. Pixel-level results were then aggregated to yield the GBD national and subnational death estimates. By applying this logic over a set of raster realizations, we created a distribution of results from which we obtained measures of uncertainty.

To age-spilt the deaths we relied on the age-specific death ratios that emerged from a separate CODEm modelling strategy. This strategy was carried out in four parts: males <5 years, males >5 years, females <5 years, and females >5 years. The resulting predicted age-patterns were used to distribute the country-year mortality estimates proportionally into the 23 GBD age bins. The covariates used in CODEm were:

Level	Covariate	Direction
1	*Pf*-only incidence	1
1	Effective antimalarial treatment	-1

For countries where the exclusive strain of malaria was *P. vivax*, deaths were estimated using a zero-inflated negative binomial mixed model where the outcome is study deaths. The model included as fixed effect the logarithm of mortality rate, age, and sex. Locations were included as random effects.

The results from the *P. falciparum* and *P. vivax* models were collated, uploaded in CODEm and marked as best model in order to incorporate the estimation in the CodCorrect algorithm.

References

Bhatt, S. et al. The effect of malaria control on Plasmodium falciparum in Africa between 2000 and 2015. Nature (2015).

Gething, P. W. et al. Mapping Plasmodium falciparum Mortality in Africa between 1990 and 2015. New England Journal of Medicine 375, 2435-2445 (2016).

Weiss, D. J. *et al.* Mapping the global prevalence, incidence, and mortality of *Plasmodium falciparum*, 2000-17: a spatial and temporal modelling study. *The Lancet*, doi:10.1016/S0140-6736(19)31097-9 (2019).

Chagas disease

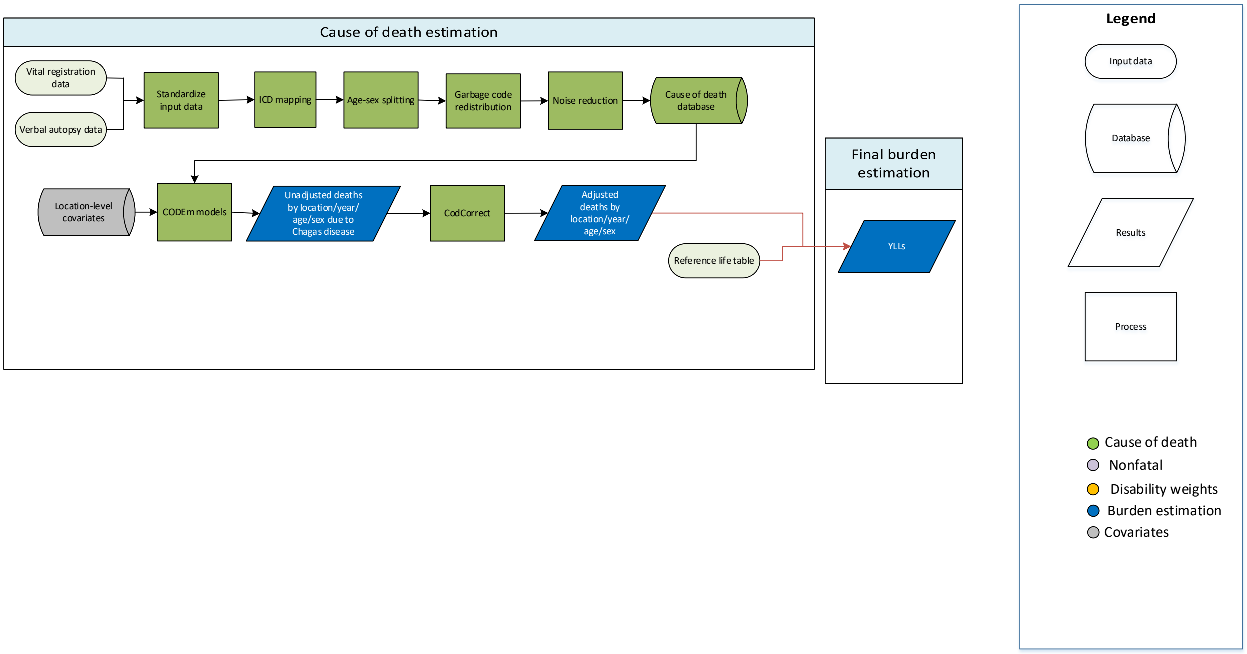

Input data

We modelled Chagas mortality using all available data in the cause of death database. No data were outliered for this cause.

Modelling strategy

We modelled Chagas mortality using a CODEm model of all Chagas-endemic countries of Latin America using all data in the CoD database. Estimates of Chagas mortality in endemic countries were drawn from the CODEm model. Estimates of mortality in countries without known endemic transmission were added as imported cases if reported through vital registration systems.

The CODEm models included three covariates:

Level	Covariate	Direction
1	Chagas prevalence	+
2	Healthcare Access and Quality Index	-
	Socio-demographic Index	-

We have made no substantive changes in the modelling strategy from GBD 2017 to GBD 2019.

Visceral leishmaniasis

Visceral leishmaniasis – GBD2019

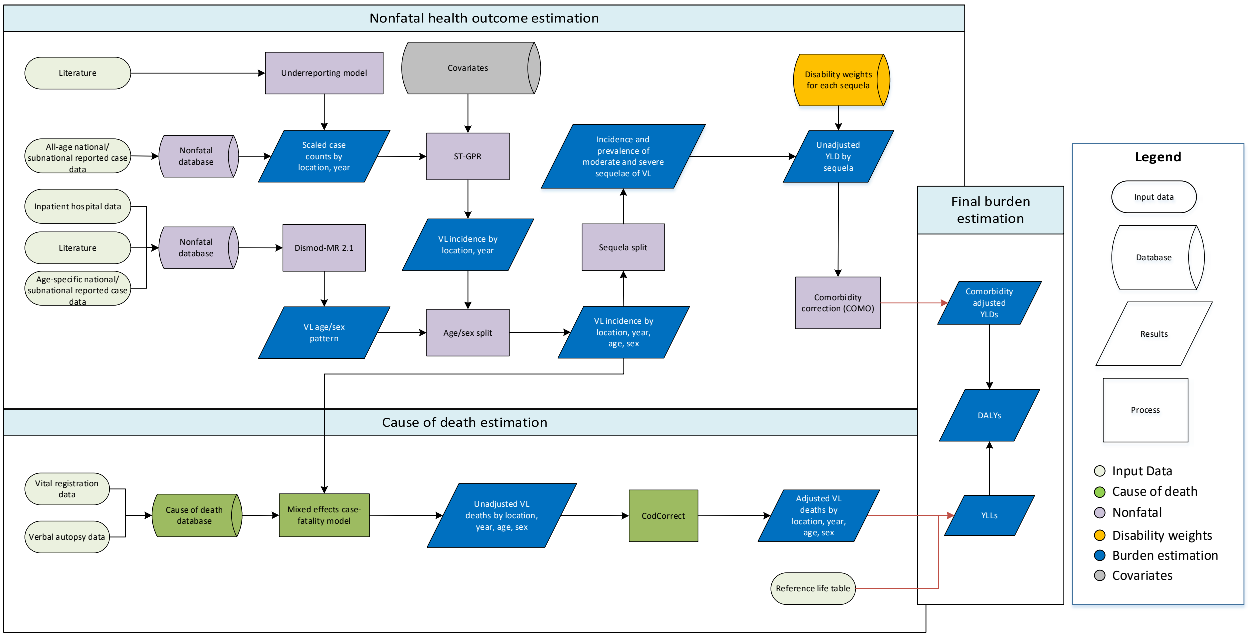

Visceral leishmaniasis (VL) is the most serious manifestation of disease caused by the *Leishmania* parasite, transmitted through the bite of phlebotomine sandflies. Those infected typically present with fever, weight loss, anaemia, leukopenia, thrombocytopenia, and enlargement of the spleen and liver. If left untreated, it can be fatal. Transmission varies by geographical region, with a variety of reservoir hosts implicated, and different vector species associated, maintaining both zoonotic and anthroponotic transmission cycles. The ICD9 code related to visceral leishmaniasis is 085.0, and the ICD10 code is B55.0.

Description of general methodology

The fatal estimation process for visceral leishmaniasis is built from incident case notification data representative of the GBD geographical location, which is adjusted for underreporting. The upscaled all-age, both-sex case counts are modelled using spatiotemporal Gaussian process regression (ST-GPR) in order to impute for missing location-year combinations as well as to account for further biases and inaccuracies in reporting. Datasets that disaggregate VL cases by age and sex are modelled using DisMod-MR 2.1 to produce a global age-sex split which is applied to the all-age, both-sex envelope estimates resulting from ST-GPR. The mean incidence estimates are compared with estimated death counts to generate a case-fatality rate model that is subsequently used to estimate deaths for each age, sex, location, year.

Input data – case notification time series

Current estimation for the all-age, both-sex incidence envelope is based upon location-representative information rather than site-specific epidemiological measures due to the absence of global foci maps allowing for upscaling of geographically precise information. The primary data resource therefore is the case notification time-series reported by National Control Programs and Ministries of Health to the World Health Organization. This is supplemented by systematic literature review (last updated for GBD

2015) to identify alternate sources of data for years missing information. For countries with subnational estimates, in-country collaborators have compiled information for respective programmes or identified key resources.

Input data – underreporting assessments

It is recognised that case notification series record only a subset of the true cases present. A review was undertaken to identify articles that compared reported cases with alternate measures to estimate the degree of underreporting. The following search strings were used: ‘leish* AND under*’; ‘active passive leish*’. Inclusion criteria were broad to maximise spatiotemporal coverage in potential estimates – any report that compared reported statistics with some notion of “truth” (whether capture-recapture, active surveillance, etc.) were extracted. Values for both cutaneous and visceral leishmaniasis were included. For GBD 2019, nine articles were included, summarised in Table 1.

Input data – mortality

Deaths were extracted from a variety of sources, ranging from vital registration (VR) records, to verbal autopsy (VA) assessments. Deaths assigned to visceral leishmaniasis were processed following central cause of death processing, outlined elsewhere.

Citation	GBD location	Time period	Pathogen	Method synopsis	Proportion of “true” cases reported
Yadon *et al.* 2001 “Assessment of Leishmaniasis notification system in Santiago del Estero, Argentina, 1990-1993” (Yadón et al. 2001)	Argentina	1990–1993	CL	Capture-recapture methods were used to evaluate four reporting sources.	94/210
Sesma *et al.* 1997 “Leishmaniasis in Navarra: a review of activities” (Sesma and Barricarte 1997)	Spain	1990–1997	CL, VL	Comparison of active searching within the region with reporting via Epidemiological Surveillance System	8/21
Maia-Elkhoury *et al.* 2007 “Analysis of visceral leishmaniasis reports by the capture-recapture method” (Maia-Elkhoury et al. 2007)	Brazil	2002–2003	VL	Comparison of three notification systems for completeness	5896/10691
Gkolfinopoulou *et al.* 2013 “Epidemiology of human leishmaniasis in Greece, 1981-2011” (Gkolfinopoulou et al. 2013)	Greece	2004–2009	VL	Comparing number of cases identified at national reference laboratory with mandatory notification system.	260/361
Singh *et al.* 2010 “Estimation of under-reporting of Visceral Leishmaniasis cases in Bihar India” (V. P. Singh et al. 2010)	Bihar, India	2006	VL	Comparison of actual reported number of cases with estimates age-sex-stratified incidence proportions for a cohort of 31,324 persons	34/177
Hirve *et al.* 2010 “Effectiveness and feasibility of active and passive case detection in the Visceral Leishmaniasis Elimination Initiative in India, Bangladesh, and Nepal” (Hirve et al. 2010)	Bihar, India<br>Nepal<br>Bangladesh	2008	VL	Comparing active case detection evaluations (conducting via house-to-house screening) with passive case detection systems	111/130<br>119/127<br>18/25<br>20/32
Faraj *et al.* 2016 “Effectiveness and cost of insecticide-treated bed nets and indoor residual spraying for the control of cutaneous leishmaniasis: A cluster-randomized control trial in Morocco” (Faraj et al. 2016)	Morocco	2008–2013	CL	Comparison of incidence of new CL cases by both active and passive case detection	409/670

Das *et al.* 2014 “Active and passive case detection strategies for the control of leishmaniasis in Bangladesh” (Das et al. 2014)	Bangladesh	2010–2011	VL	Comparing two districts’ estimates [identified in the paper as being directly comparable] of cases, one via active case detection, the other via passive case detection. Active case detection was via community education and outreach workers targeting households	756/1087
Rahman *et al.* 2015 “Performance of Kala-azar surveillance in Gaffargaon subdistrict of Mymensingh, Bangladesh” (Rahman et al. 2015)	Bangladesh	2010–2011	VL	Comparison of cases reported to the local health complex versus active search for kala-azar cases	29/58
Eid *et al.* 2017 “Assessment of a Leishmaniasis reporting system in tropical Bolivia using the capture-recapture method” (Eid et al. 2017)	Bolivia	2013–2014	CL	Active surveillance during medical campaigns were compared to registered cases reported by the National Program of Leishmaniasis Control	23/86.4

Table 1: Metadata for underreporting scalars used in GBD 2019. For each record, a citation, GBD location of relevance, year, pathogen, brief summary of methods, and output values used in modelling are listed.

Input data – age/sex-split data

Where possible, information disaggregating location-level statistics by age and sex was extracted.

Method – geographical restrictions

There are strong climatic and biogeographic constraints on the geographical distribution of VL resulting in a focal rather than cosmopolitan global distribution. As a result, it is necessary to identify locations burdened by the disease through space and time as distinct from countries were VL is absent. Tags were assigned to each location-year based upon the outcome of a search of IHME databases, as well as location-specific searches of PubMed. Each location-year is tagged as follows:

- Present – where a specific citation of either an autochthonous laboratory-confirmed case (ie, a case with PCR, serological, or parasitological diagnosis), reported case (ie, a case noted as VL, but with no supporting diagnostic), or supporting evidence (ie, confirmed infection in animal reservoirs or sandfly vectors)
- Protocol Present – for a given location-year, where no specific citation is used, but is present for another year in the same location, it is assumed that VL is present given that eradication of the pathogen has not been achieved
- Absent – where PubMed location-specific searches returned zero relevant results, in locations scoring -25 or lower as evaluated by Pigott and colleagues (2014) [the threshold for "absence" in that study (Pigott et al. 2014)], locations were tagged as Absent
- Protocol Absent – as with Absent, locations with zero relevant PubMed results, but with greater than -25 as evaluated by Pigott and colleagues (2014), were tagged as Protocol Absent (Pigott et al. 2014)

We did not make estimates for locations that were tagged Absent or Protocol Absent.

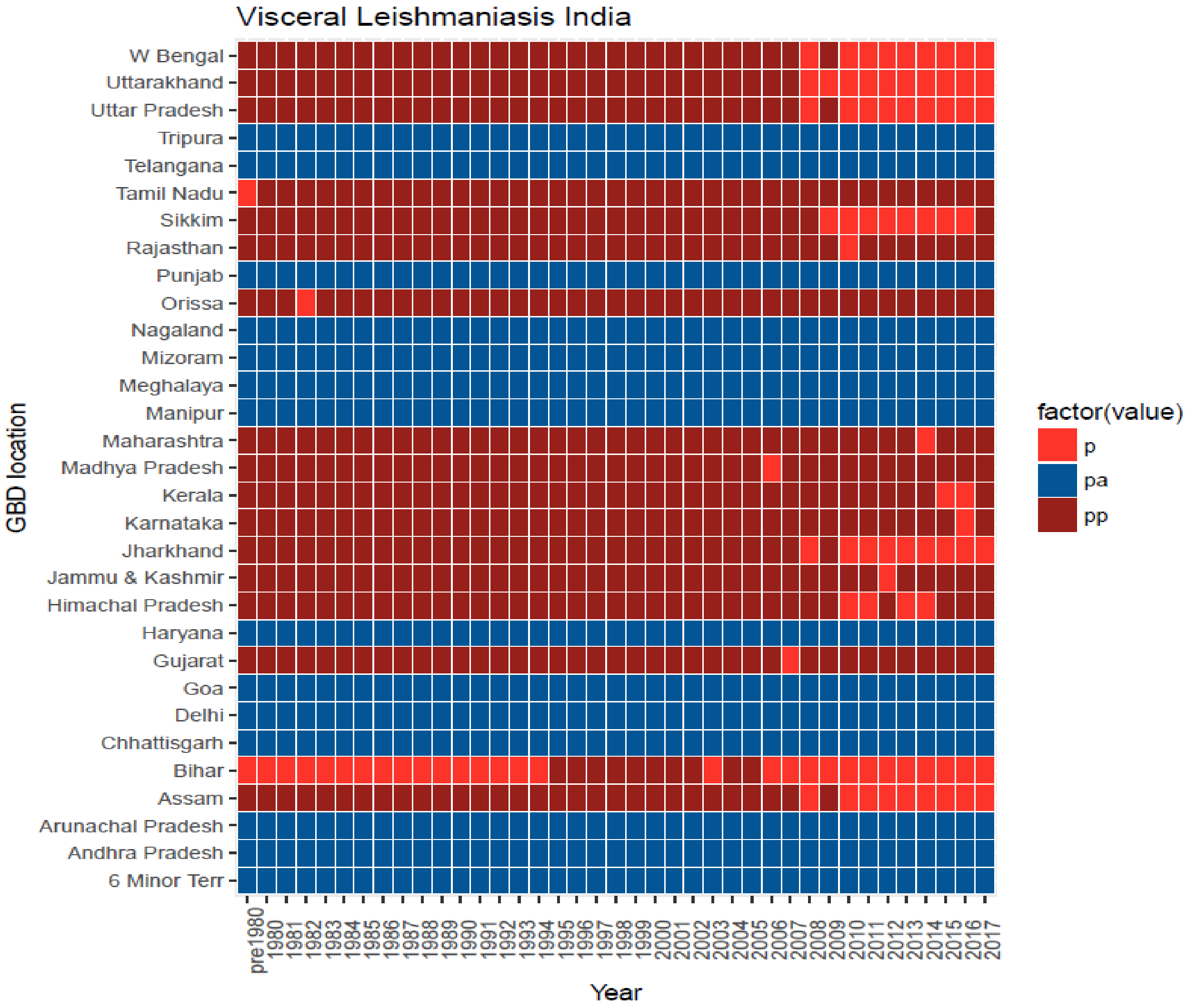

Figure 1: Visceral Leishmaniasis geographical restrictions for Indian subnationals. Locations tagged as present are coloured in red (denoted as p), yellow represents protocol presence (denoted as pp), and dark blue represents protocol absence (denoted as pa).

Full time series of maps and tables, with relevant GHDx NIDs, are available upon request from gbdsec@uw.edu.

Method – underreporting modelling and scaled case counts

Underreporting scalars were modelled as a generalised linear model estimating the proportion of true cases captured by reporting systems: a value of 1 therefore represents all actual cases of leishmaniasis being reported through notification systems. The specific model is as follows:

$$\frac{reported\ cases}{\text{"true" cases}} = Pathogen + Year + Sociodemographic\ Index$$

To account for potential biases inherently present based upon differing survey methods or location-specific confounders, 1000 models were run, with each model randomly dropping all data from a specific location, and then one additional datapoint from the remaining dataset. Similarly, for estimates

that spanned multiple years, for each model one of the years within the range of possible years was randomly assigned.

To generate scaled case counts, for each of the 1000 models a random number was generated, using a normal distribution with mean being that of the mean estimated scalar bounded by the upper and lower confidence interval. With these 1000 scalars, 1000 scaled case counts were calculated and summarised for modelling within ST-GPR.

Method – ST-GPR

Using existing IHME tools, the summarised values were modelled using ST-GPR to produce a complete time series of estimates for each location-year tagged "Present" or "Protocol Present". In short, ST-GPR attempts to model non-linear trends utilising a Gaussian process to fit a trend, rather than a definitive functional form. The following model specifications were used:

$$Incidence = \ Health\ Access\ and\ Quality\ Index + Sociodemographic\ Index + (1|level\ 1) + (1|level\ 2) + (1|level\ 3)$$

where levels 1, 2, and 3, referring to GBD location hierarchies, were treated as random effects. The following hyperparameters were used: st-lambda = 0.4, st-omega = 1, st-zeta = 0.01, gpr-scale = 10. The coefficients can be found in the table below.

Table 2: ST-GPR model coefficients.

Covariate	Beta coefficient, logit (95% CI)	Standard error	Exponentiated beta (95% CI)
Socio-demographic Index	-8.455	1.276	$2.12 * 10^{-4}$ ($1.74 * 10^{-5}$ – $2.60 * 10^{-3}$)
Healthcare Access and Quality Index	-0.006	0.012	0.99 (0.97 – 1.02)

Method – DisMod MR-2.1

DisMod MR-2.1 was used to generate an age-sex curve to disaggregate all-age, both-sex incidence data. DisMod is an integrated meta-regression framework that allows for multiple datasets to be integrated into a singular analysis regardless of age-binning, sources, and geographies. As a consequence, a variety of differently aggregated information can be evaluated to generate a consensus output. From this model, the global fit was used.

Method – YLL estimation

Deaths were modelled using a mixed effect model parameterising case-fatality rate, with data derived from taking attributed-death data and dividing it by the mean predicted incident cases.

$$Case\ Fatality\ Rate \sim Age + Sex + (Age|Super\ Region\ /\ Region) + (Sex|Super\ Region)$$

Only data from countries defined as present or protocol present were used, as these represent locations that are generalisable to all endemic regions for VL. The deaths in non-endemic countries, while not used in the case-fatality rate model, are subsequently added back into the death envelope as-is by

central computation. For African and European countries as well as South Sudan from 1990–1994, we assumed custom case-fatality rates as described below.

Case-fatality rate estimates had high uncertainty in some geographies. In general, female mean case-fatality rates were higher than male case-fatality rates. Typically an all-age estimate of 10% case-fatality rate is discussed when looking at visceral leishmaniasis (Alvar et al. 2012).

Changes from GBD 2017

A number of changes to the methodology were implemented for GBD 2019:

Underreporting model – considerable changes were undertaken in GBD 2017 for underreporting. Rather than using a single scalar, taken from expert opinion (Alvar and colleagues 2012), applied across the entire time series, a model was developed, parameterised by real data, allowing for spatiotemporal variation in estimates. These variable scalars were then applied to their relevant location-year case count values. In GBD 2019, we maintained this model while outliering three articles due to concerns of their representativeness for other locations as the proportion of cases detected was less than 15%.

Case-fatality rate – We assumed a custom case-fatality rate for African and European countries as well as South Sudan between the years of 1990 and 1994. These assumptions were more consistent with external literature of visceral leishmaniasis case-fatality rates. For African case-fatality rates, 1000 draws were taken from a uniform distribution between 0.10 and 0.30 (Alvar and colleagues 2012, Martins-Melo and colleagues 2014). For European case-fatality rates, including endemic Italian subnationals, we drew 1000 draws from a uniform distribution between 0.06 and 0.10 (Martins-Melo and colleagues 2014), and we assumed a 0.69 case fatality rate for South Sudan between the years of 1990 and 1994, based on data reported during the VL epidemic from the late 1980s to 1994 (Seamen and colleagues 1996).

Results specific to visceral leishmaniasis model

The aim here is to provide insights in some of the sub-models that are involved in the VL estimation process that are not published as part of the GBD capstones or readily available via the supplemental materials. For further questions, please direct to gbdsec@uw.edu.

<u>Underreporting</u>

Coefficients
Pathogen: 0.39 (-0.06 to 1.06) (where pathogen order is CL, VL)
Year: 0.06 (0.01 to 0.11)
SDI: 0.64 (-1.44 to 1.20)

<u>Age- and sex-specific trends in incidence rate</u>

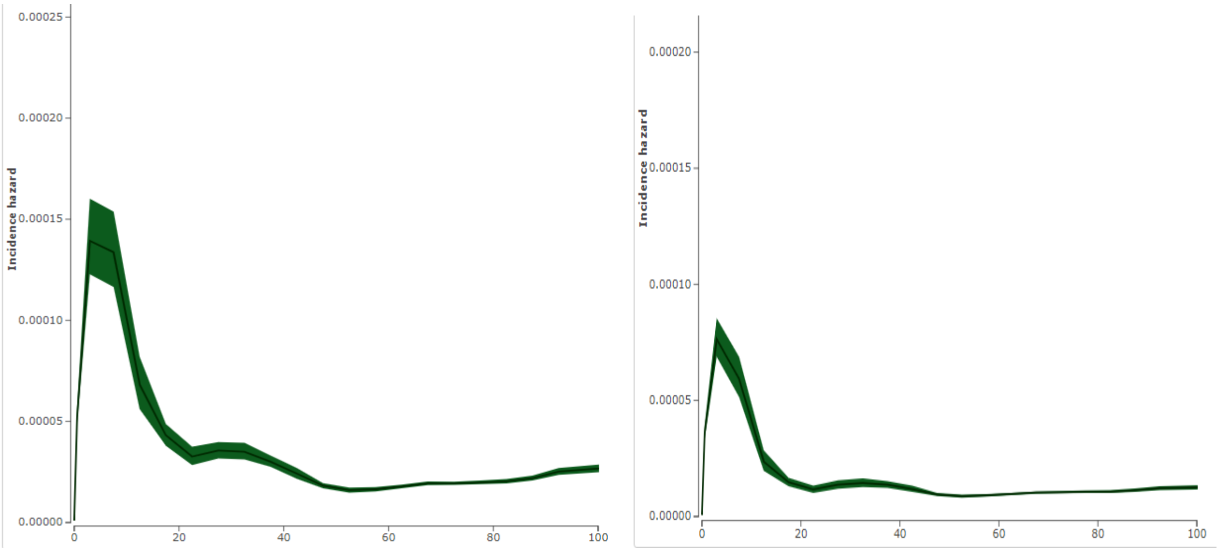

Figure 4: Global age-specific incidence estimates for males (left) and females (right) for the year 2010. Incidence is on the y-axis (rate per total population), and age in years on the x-axis. Screenshot from EpiViz.

Figure 4 shows the age-specific variation in incidence rates, differentiated by sex. When considered as a global aggregate, we see that reported incidence rates for males are approximately double those of females, with highest rates observed in younger age groupings. In adults, levels are comparatively flat, but there is an uptick in older age groups.

<u>ST-GPR</u>

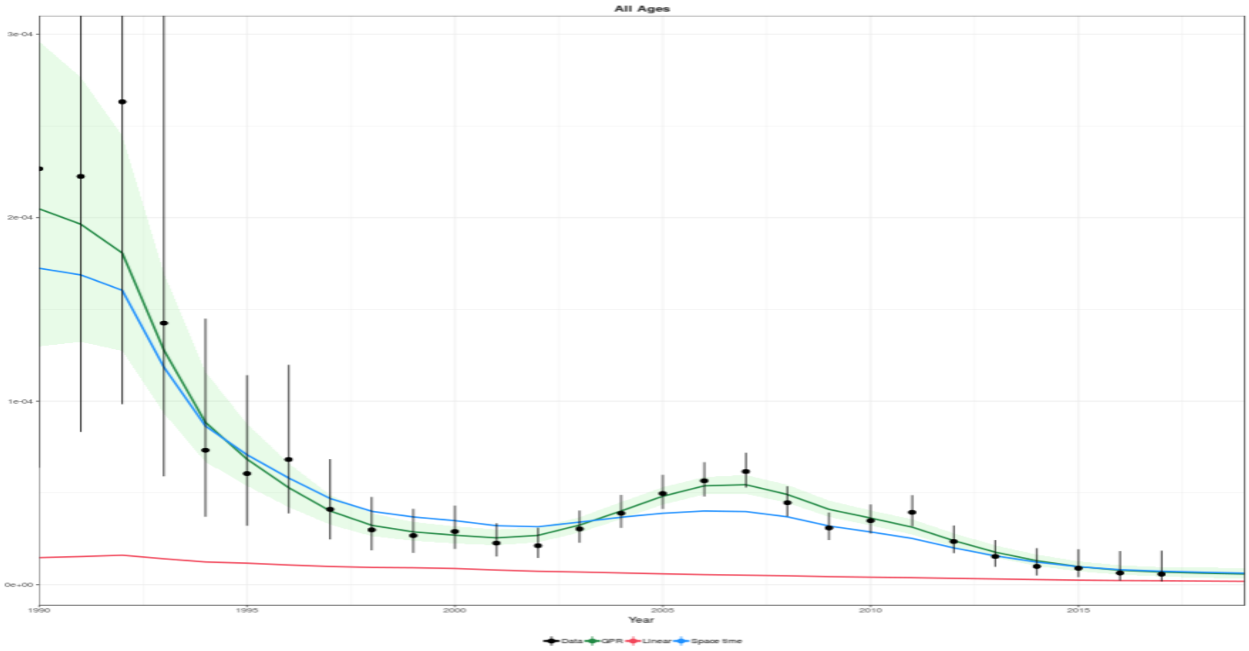

Figure 5: ST-GPR estimates for India (all-age, both sex) for years 1990–2019. Black dots represent input datapoints (post processing for underreporting) with the black lines indicating variance. The green line represents the mean GPR estimated value, with uncertainty shown by the green polygon. The blue line

indicates the space-time component of the ST-GPR; the red indicates the linear regression component derived from global data. Transparent black dots represent data from other locations in the GBD region.

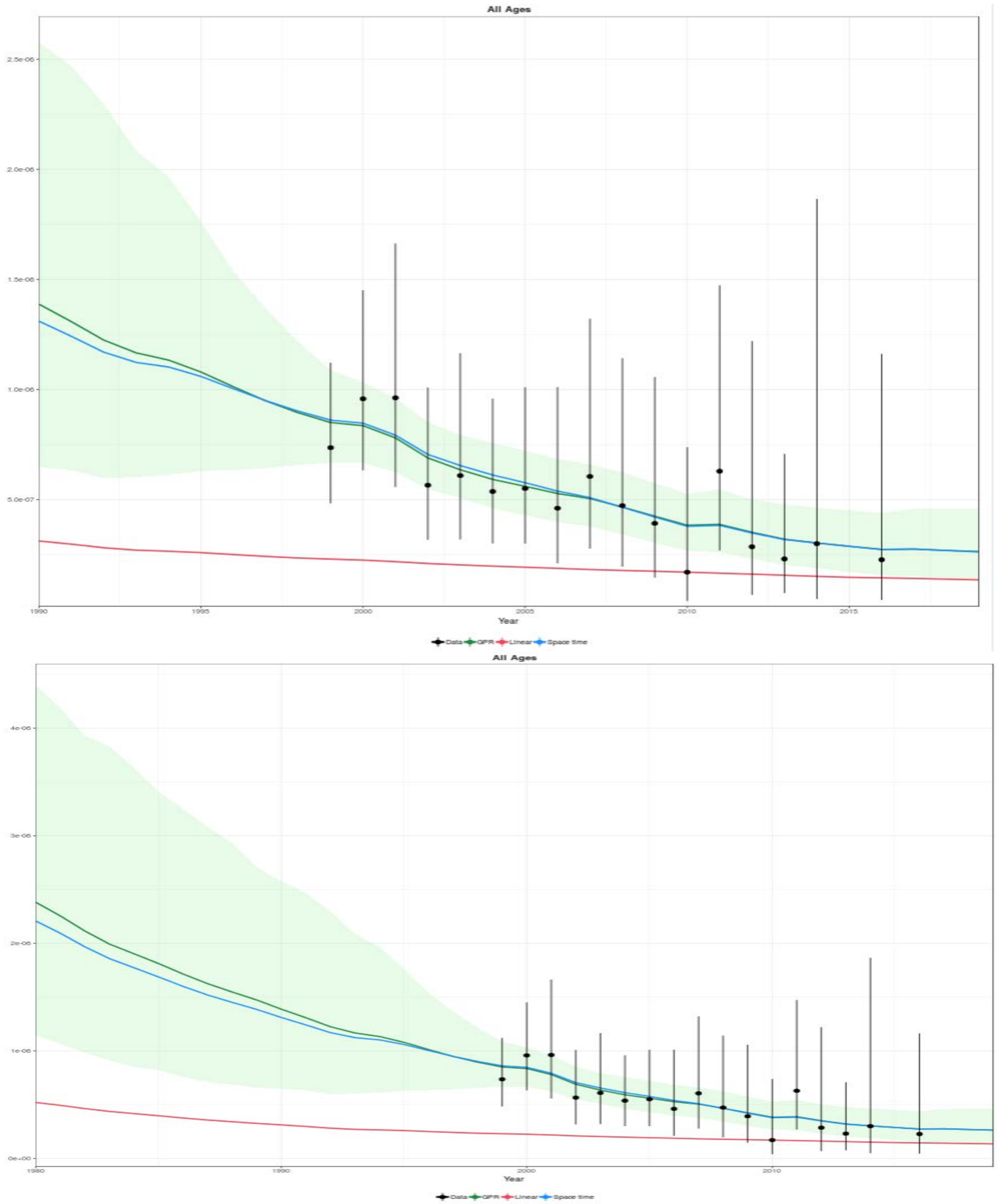

Figure 6: ST-GPR estimates for France (all-age, both sex) for years 1990–2019. Colouration and symbols are as stated in caption for Figure 5.

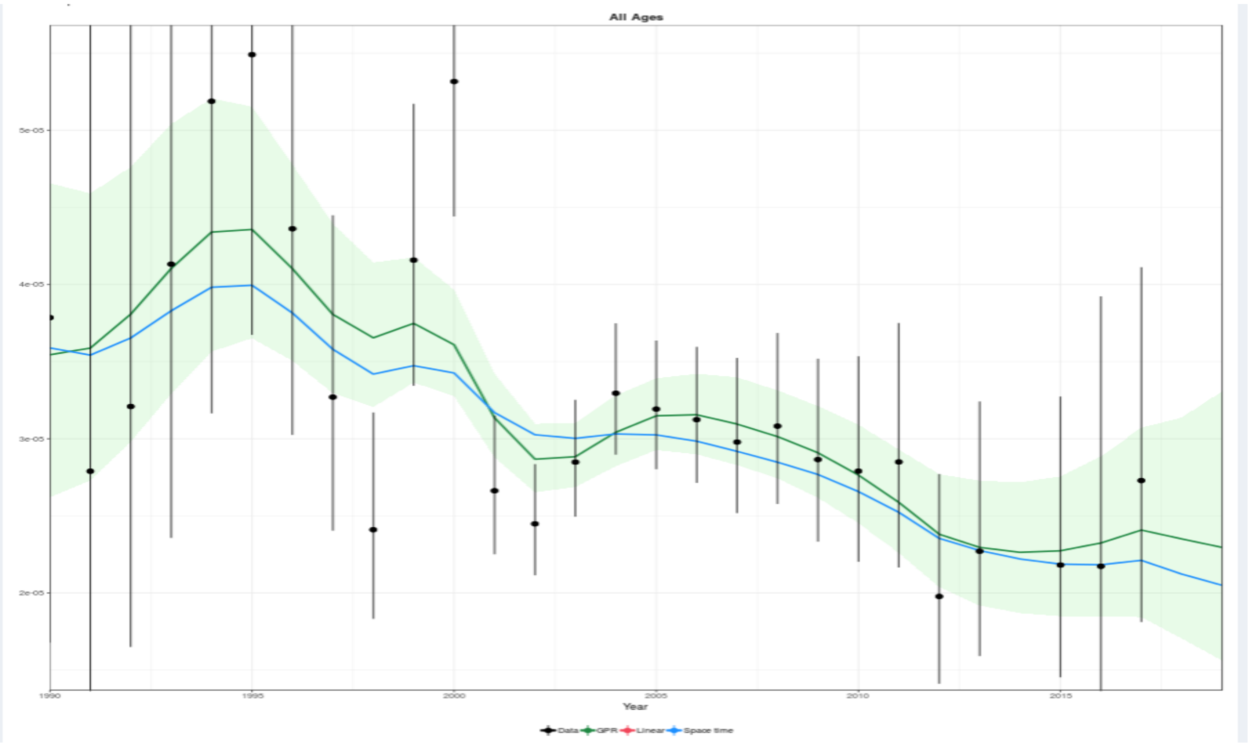

Figure 7: ST-GPR estimates for Brazil (all-age, both sex) for years 1990–2019. Colouration and symbols are as stated in caption for Figure 5.

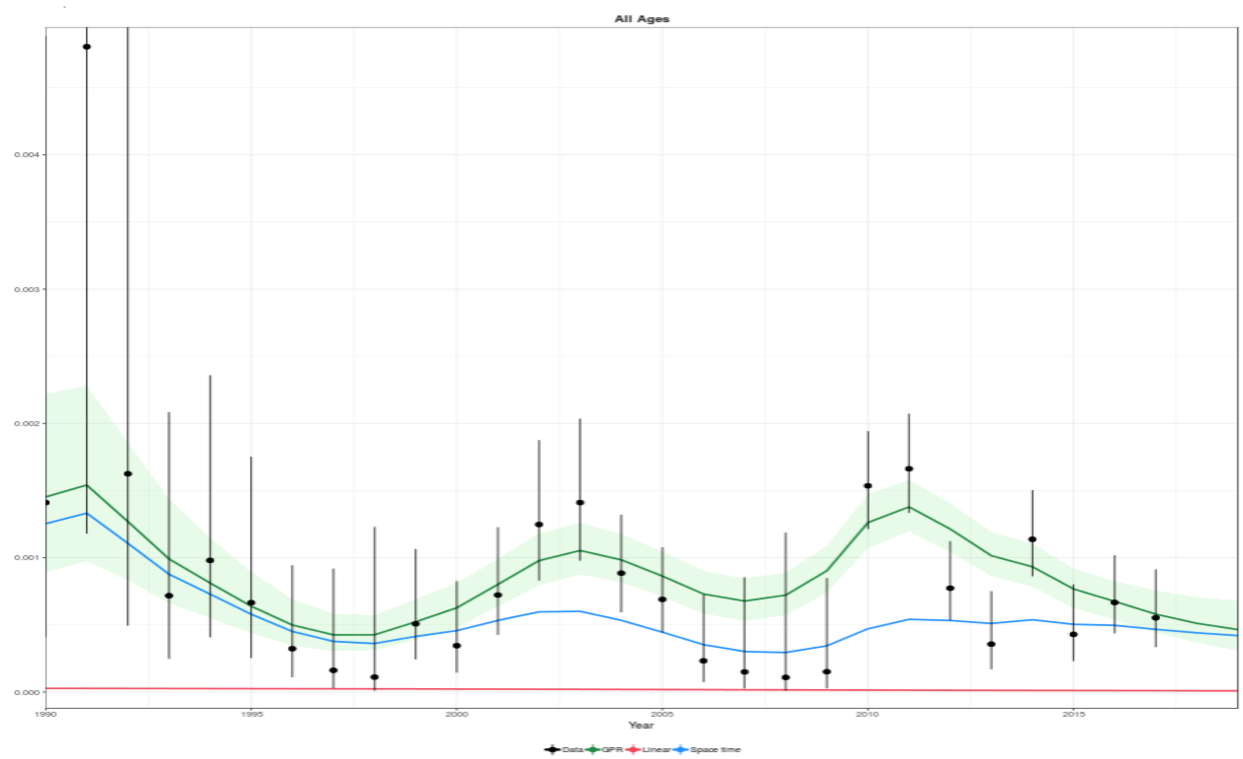

Figure 8: ST-GPR estimates for South Sudan (all-age, both sex) for years 1990–2019. Colouration and symbols are as stated in caption for Figure 5.

Limitations

As with any modelling process, a number of limitations are known, which will be the focus of additional effort in upcoming GBD cycles and engagement with collaborators. Given the focus on location-representative estimates, the existing model is based upon national case counts. This excludes a large resource of published literature and grey literature focused on site-specific surveillance or surveys. In the next cycle of GBD, there is a need to identify an independent resource to aid in quantifying the population at risk.

Age-sex patterns are highly reflective of the countries from which data are obtained. Importantly, there is a large skew in information coming from Brazil. This information has potential biases due to the nature of the data inputs (notification and hospital data) and the corresponding age-sex variation in health-seeking behaviours which may not be generalisable to other settings.

References

Alvar, Jorge, Iván D Vélez, Caryn Bern, Mercé Herrero, Philippe Desjeux, Jorge Cano, Jean Jannin, and Margriet den Boer. 2012. "Leishmaniasis Worldwide and Global Estimates of Its Incidence." *PLoS One* 7 (5): e35671.

Copeland, H W, B A Arana, and T R Navin. 1990. "Comparison of Active and Passive Case Detection of Cutaneous Leishmaniasis in Guatemala." *Am. J. Trop. Med. Hyg.* 43 (3): 257–259.

Das, A K, A D Harries, S G Hinderaker, R Zachariah, B Ahmed, G N Shah, M A Khogali, G I Das, E M Ahmed, and K Ritmeijer. 2014. "Active and Passive Case Detection Strategies for the Control of Leishmaniasis in Bangladesh." *Public Health Action* 4 (1): 15–21.

Eid, Daniel, Miguel Guzman-Rivero, Ernesto Rojas, Isabel Goicolea, Anna-Karin Hurtig, Daniel Illanes, and Miguel San Sebastian. 2017. "Assessment of a Leishmaniasis Reporting System in Tropical Bolivia Using the Capture-Recapture Method," October, tpmd170308.

Faraj, Chafika, Joshua Yukich, El Bachir Adlaoui, Rachid Wahabi, Abraham Peter Mnzava, Mustapha Kaddaf, Abderrahmane Laamrani El Idrissi, Btissam Ameur, and Immo Kleinschmidt. 2016. "Effectiveness and Cost of Insecticide-Treated Bed Nets and Indoor Residual Spraying for the Control of Cutaneous Leishmaniasis: A Cluster-Randomized Control Trial in Morocco." *Am. J. Trop. Med. Hyg.* 94 (3): 679–685.

Gkolfinopoulou, K, N Bitsolas, S Patrinos, L Veneti, A Marka, G Dougas, D Pervanidou, et al. 2013. "Epidemiology of Human Leishmaniasis in Greece, 1981-2011." *Euro Surveill.* 18 (29): 20532.

Hirve, S, S P Singh, N Kumar, M R Banjara, P Das, S Sundar, S Rijal, et al. 2010. "Effectiveness and Feasibility of Active and Passive Case Detection in the Visceral Leishmaniasis Elimination Initiative in India, Bangladesh, and Nepal." *Am. J. Trop. Med. Hyg.* 83 (3): 507–511.

Maia-Elkhoury, Ana Nilce Silveira, Eduardo Hage Carmo, Marcia Leite Sousa-Gomes, and Eduardo Mota. 2007. "[Analysis of visceral leishmaniasis reports by the capture-recapture method]." *Rev. Saude Publica* 41 (6): 931–937.

Martins-Melo, Francisco Rogerlândio, Mauricélia da Silveira Lima, Alberto Novaes Ramos, Carlos Henrique Alencar, and Jorg Heukelbach. 2014. "Mortality and Case Fatality Due to Visceral Leishmaniasis

in Brazil: A Nationwide Analysis of Epidemiology, Trends and Spatial Patterns.” PLoS ONE 9, no. 4. https://doi.org/10.1371/journal.pone.0093770.

Pigott, David M, Samir Bhatt, Nick Golding, Kirsten A Duda, Katherine E Battle, Oliver J Brady, Jane P Messina, et al. 2014. “Global Distribution Maps of the Leishmaniases.” *Elife* 3 (January): e02851.

Rahman, Kazi Mizanur, Indira V M Samarawickrema, David Harley, Anna Olsen, Colin D Butler, Shariful Amin Sumon, Subrata Kumar Biswas, Stephen P Luby, and Adrian C Sleigh. 2015. “Performance of Kala-Azar Surveillance in Gaffargaon Subdistrict of Mymensingh, Bangladesh.” Edited by Carlos Franco-Paredes. *PLoS Negl. Trop. Dis.* 9 (4): e0003531.

Seaman, J., A. J. Mercer, and E. Sondorp.1996. “The Epidemic of Visceral Leishmaniasis in Western Upper Nile, Southern Sudan: Course and Impact from 1984 to 1994.” International Journal of Epidemiology 25, no. 4 : 862–71. https://doi.org/10.1093/ije/25.4.862.

Sesma, B, and A Barricarte. 1997. “[Leishmaniasis in Navarra: review of activities].” *An. Sist. Sanit. Navar.* 20 (2): 209–216.

Singh, S P, D C S Reddy, M Rai, and S Sundar. 2006. “Serious Underreporting of Visceral Leishmaniasis through Passive Case Reporting in Bihar, India.” *Trop. Med. Int. Health* 11 (6): 899–905.

Singh, V P, A Ranjan, R K Topno, R B Verma, N A Siddique, V N Ravidas, N Kumar, K Pandey, and P Das. 2010. “Estimation of Under-Reporting of Visceral Leishmaniasis Cases in Bihar, India.” *Am. J. Trop. Med. Hyg.* 82 (1): 9–11.

Yadón, Z E, M A Quigley, C R Davies, L C Rodrigues, and E L Segura. 2001. “Assessment of Leishmaniasis Notification System in Santiago Del Estero, Argentina, 1990-1993.” *Am. J. Trop. Med. Hyg.* 65 (1): 27–30.

Human African trypanosomiasis (HAT)

Flowchart

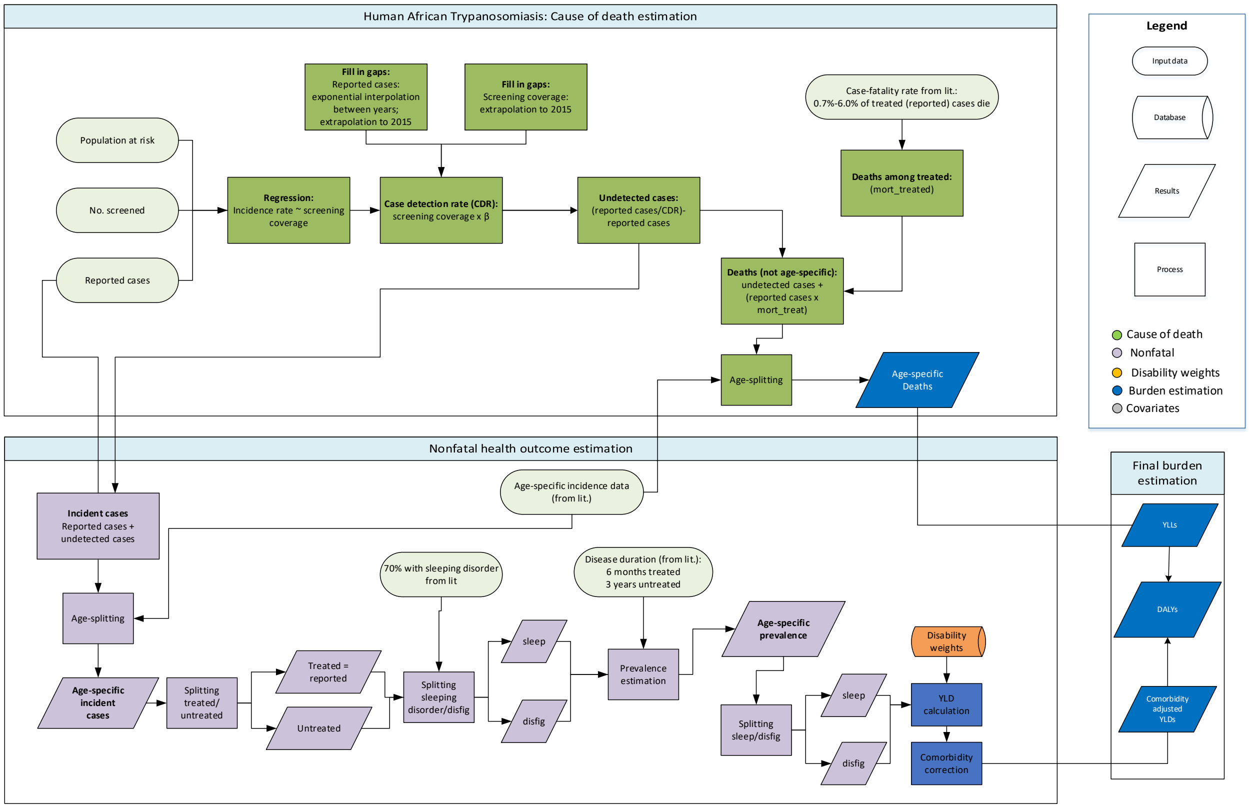

Input data & methodological summary

Case definition

Human African trypanosomiasis (HAT), also known as sleeping sickness, is a vector-borne disease which is transmitted by the bite of the tsetse fly. It is caused by the parasite *Trypanosoma brucei* with two subspecies, namely *T.b. rhodesience* (makes up less than 5% of total HAT cases) and *T.b. gambiense*. Cases are diagnosed through laboratory methods which rest on finding the parasite in body fluid or tissue by microscopy. In highly endemic or epidemic areas where the likelihood of false positives in serological tests is deemed lower, a seropositive individual is considered affected even in the absence of parasitological confirmation. The ICD-10 codes for HAT are B56.0, B56.1 and B56.9.

Input data

Model inputs

Data sources for GBD 2019:

1) Annual case totals 1980–2018: National-level annual case totals from 1990–2018 were obtained from WHO's publicly available dataset, available here: http://apps.who.int/gho/data/node.main.A1635?lang=en

   Subnational data:

   Kenya: Deaths due to HAT were attributed to Busia county. Identification of subnational locations for Kenyan case data were obtained via studies published in the peer-reviewed literature[1] and review of maps published from via the WHO HAT Atlas: http://www.who.int/entity/trypanosomiasis_african/country/Kenya_whole_0014.jpg?ua=1

   Nigeria: Review of historical data on the distribution of HAT indicated that cases have been reported from Delta State. All Nigeria estimates were then applied to that location.

2) Age/sex data: Data on the age and sex distribution of HAT cases were extracted from the peer-reviewed literature via a systematic review of sources identified in PubMed using the following search string:

   ((African trypanosomiasis[Title/Abstract] AND (incidence[Title/Abstract] OR burden[Title/Abstract] OR prevalence[Title/Abstract] OR community[Title/Abstract])) AND ("1990"[Date – Publication] : "2017"[Date – Publication]))

   This yielded 219 studies of which only three met the inclusion criteria and were extracted. The inclusion criteria were:

   1. Studies representative of the national population
   2. Population-based studies
   3. Studies with primary data on incidence
   4. Studies of human African trypanosomiasis (excluded studies on animal African trypanosomiasis)

3) Population at risk estimates 1980–2015: population at risk estimates from GBD 2010 ArcGIS analysis using geocoded case notifications for 2000 to 2009[2] and population Count Grid estimates from Gridded Population of the World 3.

4) Screening coverage: Data on active versus passive screening coverage were obtained from a Weekly Epidemiological Report[3] identifying the population screened from 1997 to 2004 at the national level.

5) Geographical restrictions: Data file of all GBD locations, defining location as either endemic or non-endemic for HAT. Estimates are not produced for non-endemic countries, nor are they generated for countries with a history of HAT transmission but no data reported by WHO from 1990–2018.

Modelling strategy

Geographical restrictions
For countries historically considered endemic for HAT, but which have no reported case data or estimate of the population at risk, estimates are not produced. These countries include Botswana, Ethiopia, Guinea-Bissau, and Rwanda.

Among countries where population at risk data are available, if no cases were reported to WHO, we assume the incidence of HAT is zero for those years and generate model estimates accordingly.

Modelling steps
The cause of death model for HAT is implemented as follows:

1. The incidence of reported HAT cases among the population at risk was calculated as the total number of reported cases divided by the population at risk estimates generated by the GBD working group for the period 1980–2015. Population at risk estimates for 2016–2017 were generated by assuming an annual 2% rate of population growth.

2. To estimate the number of cases that were likely undetected by country and year, a multilevel mixed-effects linear regression of log-transformed incidence rate (ratio of reported HAT cases to population at risk) on log-transformed screening coverage[3] (ratio of number screened for HAT to population at risk), with country random effects, was performed. Gaps were then filled using interpolation between years and extrapolation from 2018 to 2019 for reported cases. This model generates a beta-coefficient which is used to estimate the case detection rate (see step 4).

   For country-years in which no screening coverage data were reported:
   - Among countries with data reported, 1997–2004, the proportion of the at-risk population screened from 1997 was used retrospectively for the period 1980–1996 and the screening coverage from 2004 was carried forward from 2005 to 2019.
   - For countries with no screening data reported, the mean screening coverage for the region was used to impute a value over time.

3. To construct an estimate of total deaths, we first assume that all detected cases receive treatment, and that mortality among the treated occurs for a small proportion of cases. Deaths among detected cases are estimated by generating 1,000 draws of mortality among treated cases, assuming that between 0.7% and 6.0% of all reported (and therefore assumed to have received treatment) cases die.[4-6]

4. We then assume that all undetected cases experience mortality. This is estimated via generation of 1,000 draws of the case detection rate (CDR), given the expected screening coverage from the regression (in step 2). Undetected deaths were then estimated as the difference between the ratio of reported cases to CDR and reported cases (reported cases/CDR – reported cases).

5. Estimates of death were obtained by adding the deaths among treated cases to the total number of undetected cases. Without information on sex-specific incidence or deaths, death rates between both sexes were equal.

6. Finally, an age-pattern was applied to the mortality estimates using the incidence studies from Sudan[7], DRC[8], and Uganda[9]. The age-pattern in GBD 2019 employed a cubic spline to account for the higher risk of infection among working-age adults.

References

1. Rutto JJ, Osano O, Thuranira EG, Kurgat RK, Odenyo VA. Socio-economic and cultural determinants of human african trypanosomiasis at the Kenya - Uganda transboundary. *PLoS Negl Trop Dis* 2013; **7**(4): e2186.
2. Simarro PP, Cecchi G, Paone M, et al. The Atlas of human African trypanosomiasis: a contribution to global mapping of neglected tropical diseases. *Int J Health Geogr* 2010; **9**: 57.
3. World Health O. Human African trypanosomiasis (sleeping sickness): epidemiological update. *Weekly epidemiological record* 2006; **February 24**(8): 69-80.
4. Kato CD, Nanteza A, Mugasa C, Edyelu A, Matovu E, Alibu VP. Clinical profiles, disease outcome and co-morbidities among T. b. rhodesiense sleeping sickness patients in Uganda. *PLoS One* 2015; **10**(2): e0118370.
5. Balasegaram M, Harris S, Checchi F, Hamel C, Karunakara U. Treatment outcomes and risk factors for relapse in patients with early-stage human African trypanosomiasis (HAT) in the Republic of the Congo. *Bull World Health Organ* 2006; **84**(10): 777-82.
6. Odiit M, Kansiime F, Enyaru JC. Duration of symptoms and case fatality of sleeping sickness caused by Trypanosoma brucei rhodesiense in Tororo, Uganda. *East Afr Med J* 1997; **74**(12): 792-5.
7. Moore A, Richer M, Enrile M, Losio E, Roberts J, Levy D. Resurgence of sleeping sickness in Tambura County, Sudan. *Am J Trop Med Hyg* 1999; **61**(2): 315-8.
8. Lutumba P, Makieya E, Shaw A, Meheus F, Boelaert M. Human African trypanosomiasis in a rural community, Democratic Republic of Congo. *Emerg Infect Dis* 2007; **13**(2): 248-54.
9. Fevre EM, Odiit M, Coleman PG, Woolhouse ME, Welburn SC. Estimating the burden of rhodesiense sleeping sickness during an outbreak in Serere, eastern Uganda. *BMC Public Health* 2008; **8**: 96.

Schistosomiasis

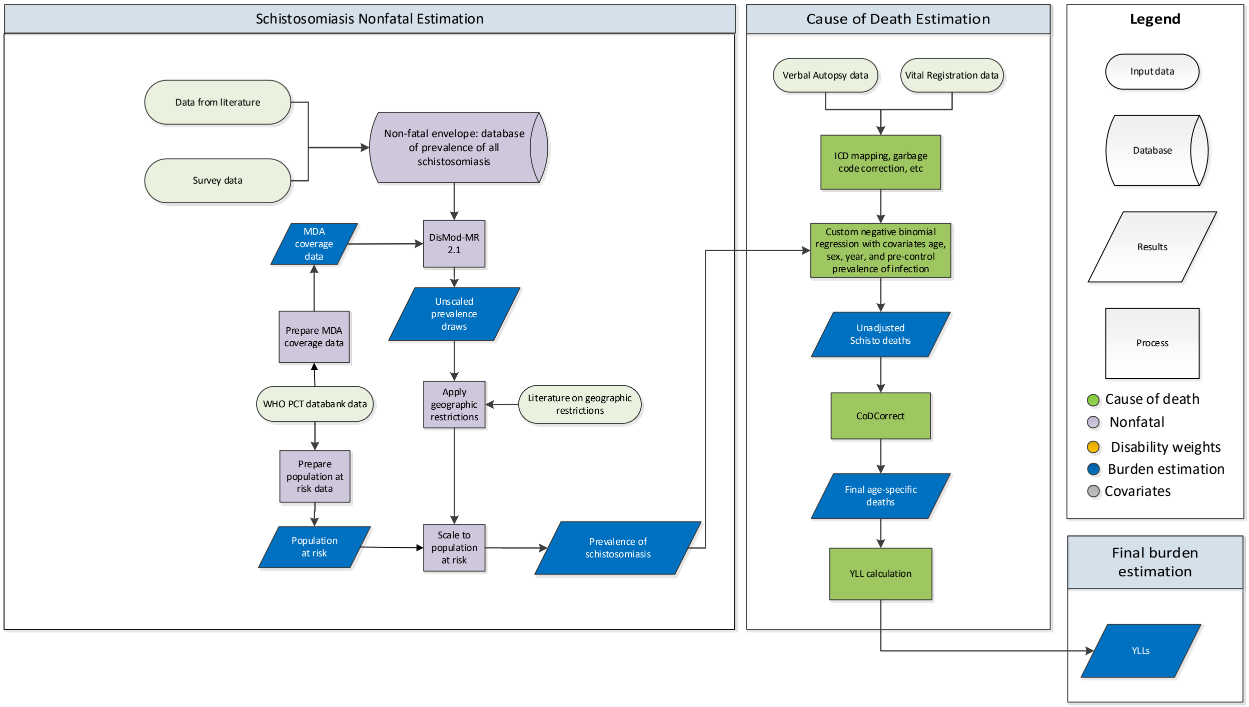

Input data

To estimate mortality due to schistosomiasis, data on deaths and prevalence of infection were used. The prevalence data were prepared this year for GBD 2019, and further information on prevalence data is available in the non-fatal write-up for this cause. Country-year-age-sex-specific verbal autopsy and vital registration data were used in the mortality model.

Geographical restrictions

We conducted a literature review to determine the geographical extent of the disease and classify locations based on whether the disease is absent or present in each year. Locations that were geographically restricted in any given year did not have estimates made for them but could have imported cases attributed to them at a later stage. Evidence of absence or presence was not available for every location for each year, and so assumptions were made for missing years by taking into consideration the epidemiological characteristics of the disease. If evidence indicated disease presence for two non-consecutive years, we assumed presence for all years between the two. If evidence indicated disease absence for two non-consecutive years, we assumed absence for all years between the two. If evidence indicated a change in status (ie, from absent to present, or present to absent) between two non-consecutive years, then we conducted targeted searches to ascertain the relevant year of introduction or elimination for that location. In the cases where presence or absence information was missing for the start or end years of our study interval (1990–2019) without evidence of any introduction or elimination events within the interval, we applied the status of the first and last presence/absence observations respectively to all years between the interval bound and the observation year. For schistosomiasis, we used a combination of Chitsulo and colleagues' *The global*

status of schistosomiasis and its control (1) and WHO's *Preventative chemotherapy in human helminthiasis* (2) report as a baseline. Where country-level endemicity statuses conflicted between the two sources, we searched PubMed and Google Scholar for country- and subnational-specific endemicity status. Our search yielded 22 sources that were used to develop our annual geographical restriction map for schistosomiasis.

Modelling strategy

To estimate deaths due to schistosomiasis, a negative binomial regression model of country-year-age-sex-specific deaths on natural log-transformed age-standardized schistosomiasis infection prevalence with a 15-year lag was used. The negative binomial regression was selected due to its suitability for modelling count data. In addition, there are relatively low numbers of deaths attributable to schistosomiasis. Indicator variables for endemic Brazil subnationals and South Africa subnationals were used to allow the model to follow data in those areas. A multivariate normal distribution using the mean and variance-covariance matrix from the model was used to generate 1000 draws of deaths due to schistosomiasis.

Models were evaluated by assessing the AIC and plotting the predicted deaths against time, age, and sex. In addition, the Cause of Death visualisation tool was used to evaluate time trends across locations, age, and sex. A map of the global distribution of schistosomiasis across age groups was also used to assess the changes in death rates over time. The final model was selected based on how well the estimated numbers fit the input data and how plausible the predicted distribution of disease was over time and with age.

References

(1) Chitsulo, L., Engels, D., Montresor, A., & Savioli, L. (2000). The global status of schistosomiasis and its control. Acta Tropica, 77(1), 41-51. doi:10.1016/s0001-706x(00)00122-4
(2) World Health Organization (2006). Preventive chemotherapy in human helminthiasis: coordinated use of anthelminthic drugs in control interventions : a manual for health professionals and programme managers.

Cysticercosis

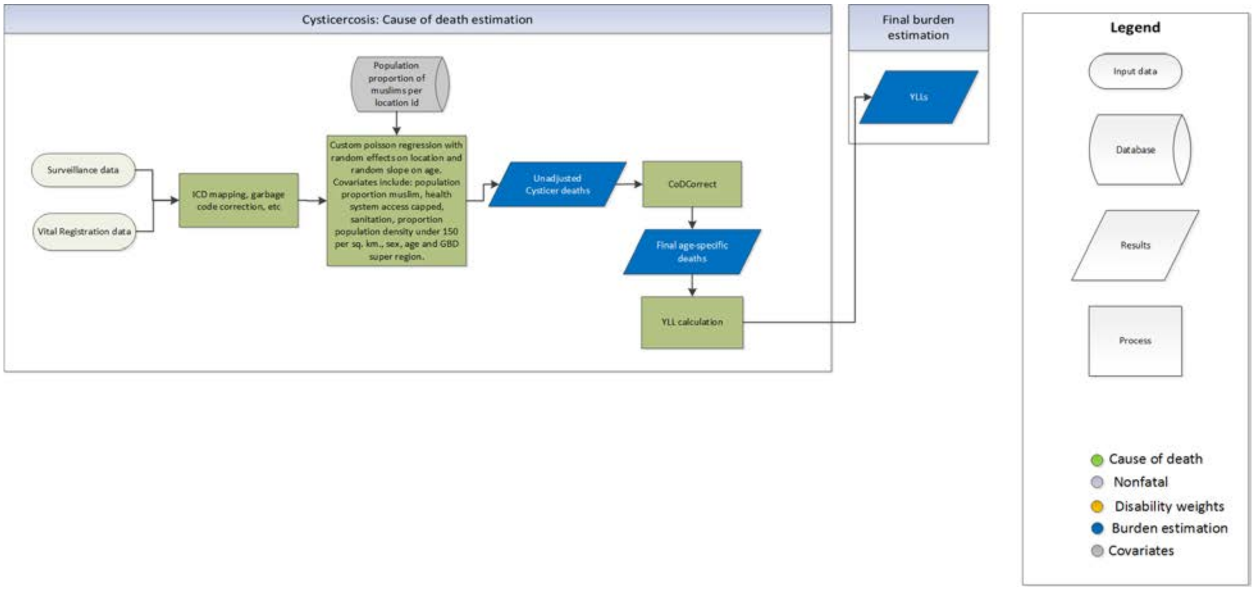

Input data

The model for mortality due to cysticercosis relied on vital registration and surveillance data from endemic countries. In addition, we used data from the Pew Research Center on percentage of population that is Muslim by country. The primary covariates adjusted for in the model were proportion of the population that is Muslim, health system access capped, proportion of the population with access to sanitation, proportion of the country with population density under 150 people per square kilometer, sex, age, and GBD super-region.

Geographical restrictions

We conducted a literature review to determine the geographical extent of the disease and classify locations based on whether the disease is absent or present in each year. Locations that were geographically restricted in any given year did not have estimates made for them but could have imported cases attributed to them at a later stage. Of note, we did not attempt a complete systematic review, since a single high-quality source could offer sufficient evidence of presence. Evidence of absence or presence was not available for every location for each year, and so assumptions were made for missing years by taking into consideration the epidemiological characteristics of the disease. If evidence indicated disease presence for two non-consecutive years, we assumed presence for all years between the two. If evidence indicated disease absence for two non-consecutive years, we assumed absence for all years between the two. If evidence indicated a change in status (ie, from absent to present, or present to absent) between two non-consecutive years, then we conducted targeted searches to ascertain the relevant year of introduction or elimination for that location. In the cases where presence or absence information was missing for the start or end years of our study interval (1990–2016) without evidence of any introduction or elimination events within the interval, we applied the status of the first and last presence/absence observations respectively to all years between the interval bound and the observation year. For cysticercosis, we performed targeted searches to classify

location-years in PubMed and Google Scholar. Our map was populated by 21 peer-reviewed articles and meta-analyses and WHO reports.

Modelling strategy

Globally, deaths due to cysticercosis are relatively low. Therefore, a Poisson model was used to model cysticercosis deaths due to its suitability for count data. This model choice was validated by tests for overdispersion. Random effects were used on location with random slopes on age by location. A multivariate normal distribution using the mean and variance-covariance matrix from the model was used to generate 1,000 draws of deaths due to cysticercosis.

Estimates for new subnational locations were also added in GBD 2019. Since the Pew Research Center only has data on proportion of Muslims by country, we applied the national proportions to subnational locations. We understand that this does not account for sometimes large expected differences in proportions of Muslims within a country, but were limited by data availability.

Cystic echinococcosis

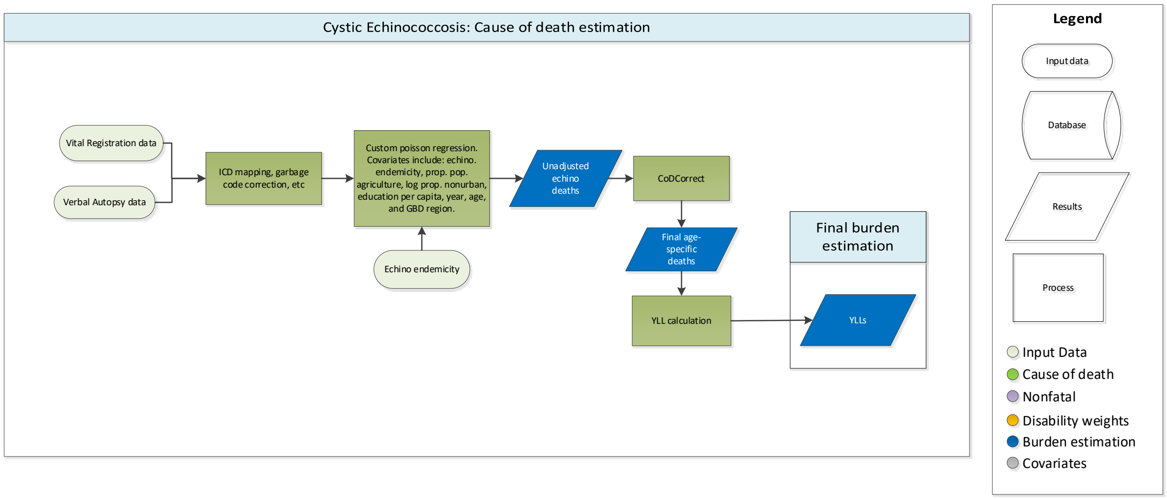

Input data

Geographical restrictions

We conducted a literature review to determine the geographical extent of the disease and classify locations based on whether the disease is absent or present in each year. Locations that were geographically restricted in any given year did not have estimates made for them but could have imported cases attributed to them at a later stage. Of note, we did not attempt a complete systematic review, since a single high-quality source could offer sufficient evidence of presence. Evidence of absence or presence was not available for every location for each year, and so assumptions were made for missing years by taking into consideration the epidemiological characteristics of the disease. If evidence indicated disease presence at a given point in time, we assumed presence for all years. If evidence indicated disease absence, we assumed absence for all years. If evidence indicated a change in status (ie, from absent to present, or present to absent) between two non-consecutive years, then we conducted targeted searches to ascertain the relevant year of introduction or elimination for that location. In the cases where presence or absence information was missing from the start or end years of our study interval (1990–2019) without evidence of any introduction or elimination events within the interval, we applied the status of the first and last presence/absence observations respectively to all years between the interval bound and the observation year. For cystic echinococcosis (CE), we reviewed all references pertaining to CE in Global Distribution of Alveolar and Cystic Echinococcosis by Deplazes and colleagues and supplemented with targeted searches to classify location-years in PubMed and the GHDx.

Data sources

Mortality due to cystic echinococcosis was modelled using vital registration data and covariates. The Mortality and Cause of Death team provided country-year-age-sex-specific vital registration. Of note, the ICD codes mapped to cystic echinococcosis are:

Table 1: ICD-9 codes mapped to CE

ICD code	ICD name
**122**	Echinococcosis
**122.0**	*Echinococcus granulosus* infection of liver
**122.1**	*Echinococcus granulosus* infection of lung
**122.2**	*Echinococcus granulosus* infection of thyroid
**122.3**	*Echinococcus granulosus* infection, other
**122.4**	*Echinococcus granulosus* infection, unspecified
**122.8**	Echinococcosis unspecified, of liver
**122.9**	Echinococcosis other and unspecified

Table 2: ICD-10 codes mapped to CE

ICD code	ICD name
**B67.0**	*Echinococcus granulosus* infection of liver
**B67.1**	*Echinococcus granulosus* infection of lung
**B67.2**	*Echinococcus granulosus* infection of bone
**B67.3**	*Echinococcus granulosus* infection, other and multiple sites
**B67.31**	*Echinococcus granulosus* infection, thyroid gland
**B67.32**	*Echinococcus granulosus* infection, multiple sites
**B67.39**	*Echinococcus granulosus* infection, other sites
**B67.4**	*Echinococcus granulosus* infection, unspecified
**B67.8**	Echinococcosis, unspecified, of liver
**B67.9**	Echinococcosis, other and unspecified
**B67.90**	Echinococcosis, unspecified
**B67.99**	Other echinococcosis

Due to the scarcity of hospital data, especially in endemic areas, we incorporated covariates to drive global distribution of deaths in the model.

We created a categorical cystic echinococcosis endemicity covariate based on expert opinion and an endemicity map published by WHO [1]. We assigned GBD locations to one of four categories: probable absence, rare and/or sporadic transmission, suspected and/or confirmed transmission, and high endemic areas.

We based further selection of covariates on a meta-analysis of potential risk factors associated with cystic echinococcosis [2]. According to the meta-analysis, statistically significant potential risk factors include living in rural endemic areas, slaughtering, feeding dogs with viscera, and low income. Hence, we also included two other covariates: the proportion of the population participating in agricultural activities and the log of proportion non-urban.

Modelling strategy

We implemented a Poisson regression model to estimate deaths due to cystic echinococcosis. The Poisson regression was selected due to its suitability for modelling count data that are not over-dispersed. Covariates for the model, including echinococcosis endemicity, log of proportion non-urban, proportion of the population participating in agricultural activities, and education (years per capita), were incorporated into the model to influence the global trend due to paucity of data. Random effects were used on location with random slopes on age by location. A multivariate normal distribution using the mean and variance-covariance matrix from the model was used to generate 1,000 draws of deaths due to cystic echinococcosis. The final model was selected based on how well the estimated numbers fit the input data and how plausible the predicted distribution of disease was over time and with age.

References

1. World Health Organization (2010). Global Echinococcosis Granulosus and Cystic Echinococcosis (hydatisosis) Worldwide 2009.

2. Possenti A, Manzano-Román R, Sánchez-Ovejero C, et al. Potential Risk Factors Associated with Human Cystic Echinococcosis: Systematic Review and Meta-analysis. Flisser A, ed. *PLoS Neglected Tropical Diseases*. 2016;10(11):e0005114. doi:10.1371/journal.pntd.0005114.

Dengue

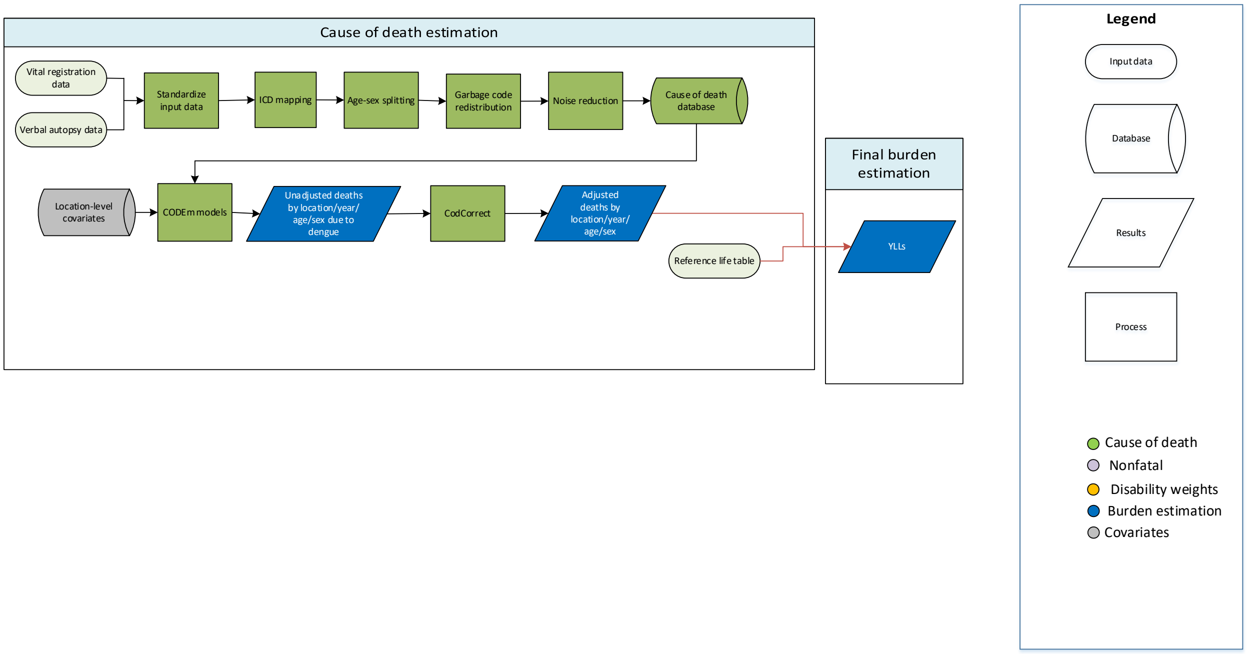

Input data

We modelled dengue mortality using all available data in the cause of death database. Data points were outliered if they reported an improbably low number of dengue deaths (eg, zero dengue deaths in a hyper-endemic country) or an improbably high number of dengue deaths.

Modelling strategy

We modelled dengue mortality using three-model hybrid approach: 1) a global CODEm model of all locations, using all data in the CoD database; 2) a CODEm model restricted to data-rich countries; and 3) estimates of mortality from imported cases in non-endemic, data-rich countries. Where dengue deaths were reported in non-endemic data-rich countries, we produced non-zero estimates by drawing from a beta distribution based on number of reported deaths and the underlying sample size. Estimates of dengue mortality in endemic data-rich countries were drawn from the data-rich CODEm model. Finally, estimates in other endemic countries were drawn from the global CODEm model.

We use county-level covarites to inform our model. The *Level* is the associated strength of relationship between the covariate and LRI mortality, ranked from 1 (proximally related) to 3 (distally related). The direction is the forced direction of the association between the covariate and dengue mortality (Table 1).

Table 1. CODEm model covariates and directions

Level	Covariate	Direction
1	Population density (over 1000 ppl/sqkm, proportion)	+
	Population weighted probability of dengue transmission	+
2	Health system access	-
	Latitude under 15 (proportion)	+
	Elevation under 100m (proportion)	+
	Rainfall quintile 4 (proportion)	+
	Rainfall quintile 5 (proportion)	+
	Dengue outbreaks (binary)	+
3	Education (years per capita)	-
	LDI (1$ per capita)	-

While we've made no substantive changes to the modelling strategy since GBD 2017, we have updated the geographic restrictions that determine whether a location is considered non-endemic (and, therefore, will have estimates based on the imported case model) in a given year. We derived our geographical restrictions for 2010 from Brady and colleagues(1). We have also refreshed our literature review to determine locations and years in which dengue was introduced or eliminated, to allow for time-varying geographical restrictions.

References

1. Brady OJ, Gething PW, Bhatt S, Messina JP, Brownstein JS, Hoen AG, et al. Refining the Global Spatial Limits of Dengue Virus Transmission by Evidence-Based Consensus. PLoS Negl Trop Dis. 2012 Aug 7;6(8):e1760.

2. Al ST et. Autochthonous Dengue Fever, Tokyo, Japan, 2014 - Volume 21, Number 3—March 2015 - Emerging Infectious Disease journal - CDC. [cited 2017 Apr 28]; Available from: https://wwwnc.cdc.gov/eid/article/21/3/14-1662_article

3. Guzman MG, Kouri G. Dengue and dengue hemorrhagic fever in the Americas: lessons and challenges. J Clin Virol. 2003 May;27(1):1–13.

4. Boshell J, Groot H, Gacharna M, Márquez G, González M, Gaitán MO, et al. Dengue en Colombia. Biomédica. 1986;6(3–4):101–6.

5. Effler PV, Pang L, Kitsutani P, Vorndam V, Nakata M, Ayers T, et al. Dengue Fever, Hawaii, 2001–2002. Emerg Infect Dis. 2005 May;11(5):742–9.

6. McBride WJH. Dengue fever: is it endemic in Australia? Intern Med J. 2010 Apr 1;40(4):247–9.

7. Kay BH, Barker-Hudson P, Stallman ND, Wiemers MA, Marks EN, Holt PJ, et al. Dengue fever. Reappearance in northern Queensland after 26 years. Med J Aust. 1984 Mar 3;140(5):264–8.

8. Al GA et. Dengue Reemergence in Argentina - Volume 5, Number 4—August 1999 - Emerging Infectious Disease journal - CDC. [cited 2017 Apr 28]; Available from: https://wwwnc.cdc.gov/eid/article/5/4/99-0424_article

9. Ramos MM, Mohammed H, Zielinski-Gutierrez E, Hayden MH, Lopez JLR, Fournier M, et al. Epidemic Dengue and Dengue Hemorrhagic Fever at the Texas–Mexico Border: Results of a Household-based Seroepidemiologic Survey, December 2005. Am J Trop Med Hyg. 2008 Mar 1;78(3):364–9.

10. Luo L, Liang H, Hu Y, Liu W, Wang Y, Jing Q, et al. Epidemiological, virological, and entomological characteristics of dengue from 1978 to 2009 in Guangzhou, China. J Vector Ecol. 2012 Jun 1;37(1):230–40.

11. Murray KO, Rodriguez LF, Herrington E, Kharat V, Vasilakis N, Walker C, et al. Identification of Dengue Fever Cases in Houston, Texas, with Evidence of Autochthonous Transmission Between 2003 and 2005. Vector Borne Zoonotic Dis. 2013 Dec 1;13(12):835–45.

12. Locally Acquired Dengue --- Key West, Florida, 2009--2010 [Internet]. [cited 2017 Apr 28]. Available from: https://www.cdc.gov/mmwr/preview/mmwrhtml/mm5919a1.htm

13. Bouri N, Sell TK, Franco C, Adalja AA, Henderson DA, Hynes NA. Return of Epidemic Dengue in the United States: Implications for the Public Health Practitioner. Public Health Rep. 2012;127(3):259–66.

14. Wilder-Smith A, Quam M, Sessions O, Rocklov J, Liu-Helmersson J, Franco L, et al. The 2012 dengue outbreak in Madeira: exploring the origins. 2014 [cited 2017 Apr 28]; Available from: https://dr.ntu.edu.sg/handle/10220/19685

15. Brathwaite Dick O, San Martín JL, Montoya RH, del Diego J, Zambrano B, Dayan GH. The History of Dengue Outbreaks in the Americas. Am J Trop Med Hyg. 2012 Oct 3;87(4):584–93.

Yellow fever

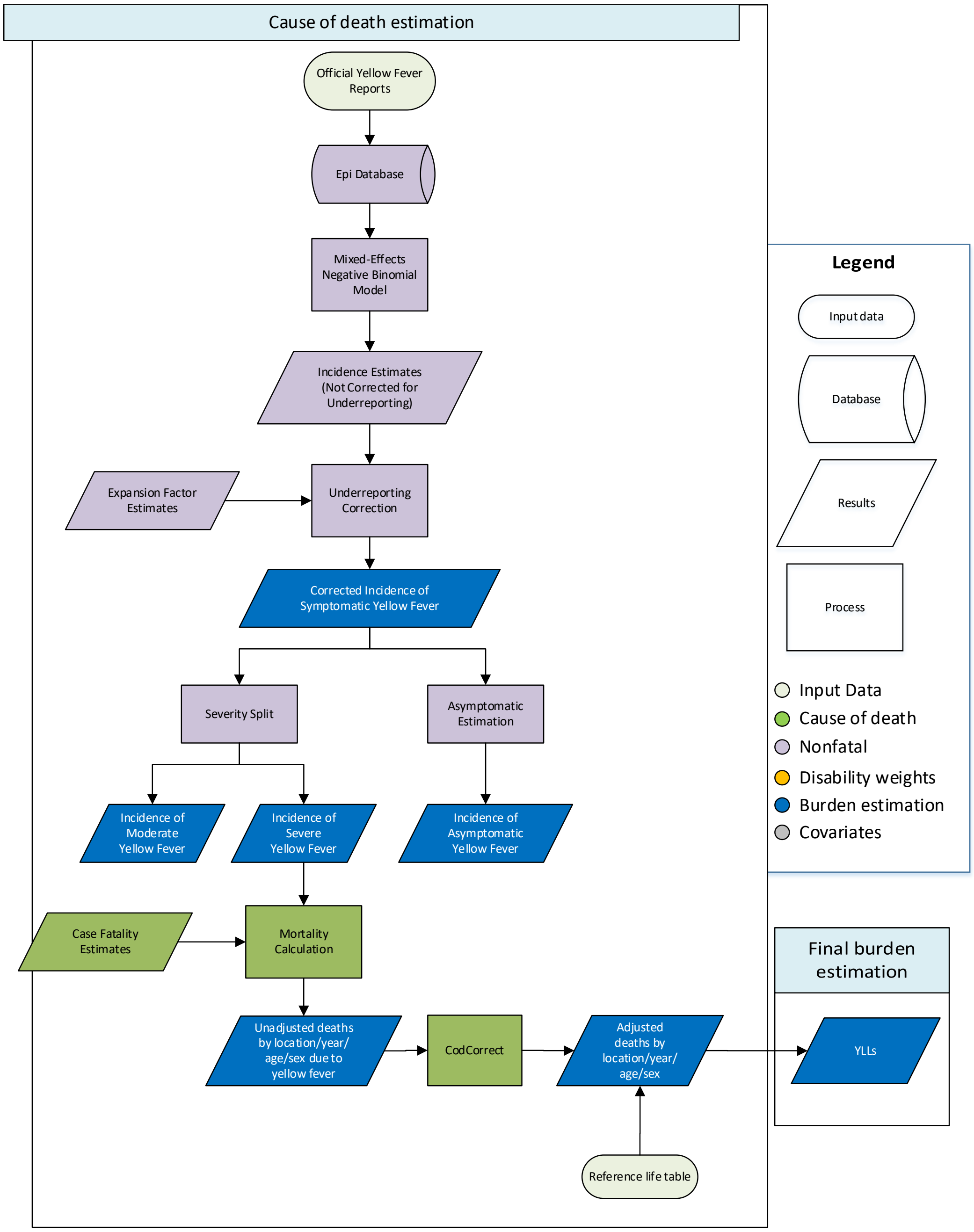

Input data

Incident case data come from official case reports filed with the World Health Organization. Data on case fatality come from published studies of yellow fever fatality. Data on deaths in non-endemic countries are restricted to only vital registration data.

Modelling strategy

We model yellow fever deaths using a hybrid approach. For countries in which yellow fever is endemic, we use a natural history approach in which we estimate deaths as the product of cases and case fatality. For non-endemic countries, we allow for deaths among imported cases where we have vital registration data indicating yellow fever deaths. That is, we assume no yellow fever deaths in non-endemic countries; however, where yellow fever deaths are reported in vital registration data, we accept those as true imported yellow fever deaths.

We model reported cases using a mixed-effects negative binomial model, with fixed effects for year and socio-demographic index, and random effects for super-region, region, and country. We assume that yellow fever cases are underreported and that this underreporting mirrors that of dengue (a disease for which we have better data on underreporting). With that, we estimate symptomatic cases as the product of our base case estimates and dengue expansion factors (ie, the factor by which you must multiply reported cases to derive true cases). Based on published estimates, we assume that 27% of symptomatic cases will be severe.[1]

We performed a meta-analyses of case fatality using data from published studies of yellow fever fatality. Studies tend to report deaths among those with severe infection (eg, hospitalised cases), rather than among all cases. We assume that no deaths occur with asymptomatic infection or among those with only moderate symptoms. With that, we estimate deaths as the product of severe cases and case fatality. We accept deaths reported in vital registration data as true imported deaths. We have made no substantive changes to the modelling strategy for GBD 2019 with the exception of adjusting total death estimates to account for the high case burden observed in the 2017-2018 outbreak in Brazil. We used reported deaths from Brazilian vital registration data from 2017 to derive an age and sex distribution of these deaths, simulated uncertainty for case totals from a Poisson distribution to inflate modeled death estimates to account for this outbreak.

Reference

1 Johansson MA, Vasconcelos PFC, Staples JE. The whole iceberg: estimating the incidence of yellow fever virus infection from the number of severe cases. *Trans R Soc Trop Med Hyg* 2014; 108: 482–7.

Rabies

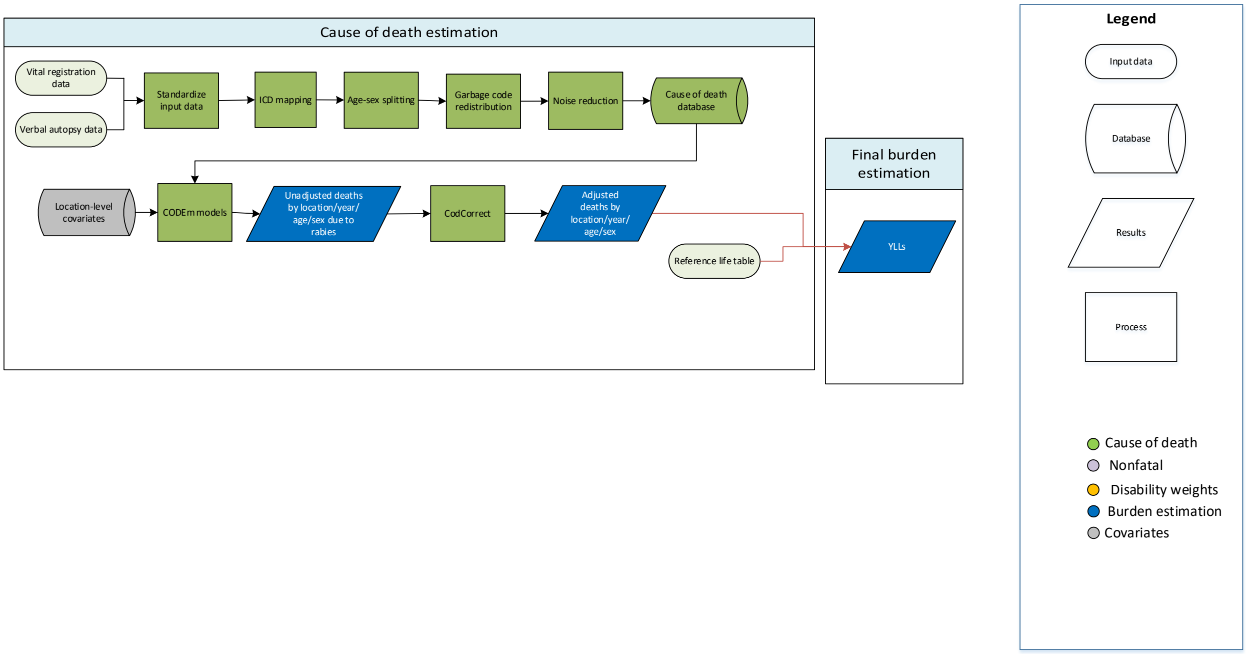

Input data

We modelled rabies mortality using all available data in the cause of death database. Data points were outliered if they reported an improbable number of rabies deaths (eg, zero rabies deaths in a hyper-endemic country) or if their inclusion in the model yielded distorted trends. In some cases, multiple data sources for the same location differed dramatically both in their quality and reported rabies mortality (eg, a verbal autopsy and vital registration source). In these cases the lower-quality data source was outliered.

Modelling strategy

We modelled rabies mortality using a two-model hybrid approach: 1) a global CODEm model of all locations, using all data in the CoD database; and 2) a CODEm model restricted to data-rich countries. The CODEm models included nine covariates:

Level	Covariate	Direction
1	Antenatal care coverage (4 visits)	-
	Health system access	-
	In-facility delivery coverage	-
2	Healthcare access and quality index	-
	Skilled birth attendance coverage	-
	Health system access (capped)	-
3	Population density, 500-1000 per km^2	+
	Population density, <150 per km^2	+
	Socio-demographic Index	-

We have made no substantive changes to the modelling strategy in GBD 2019.

Ascariasis

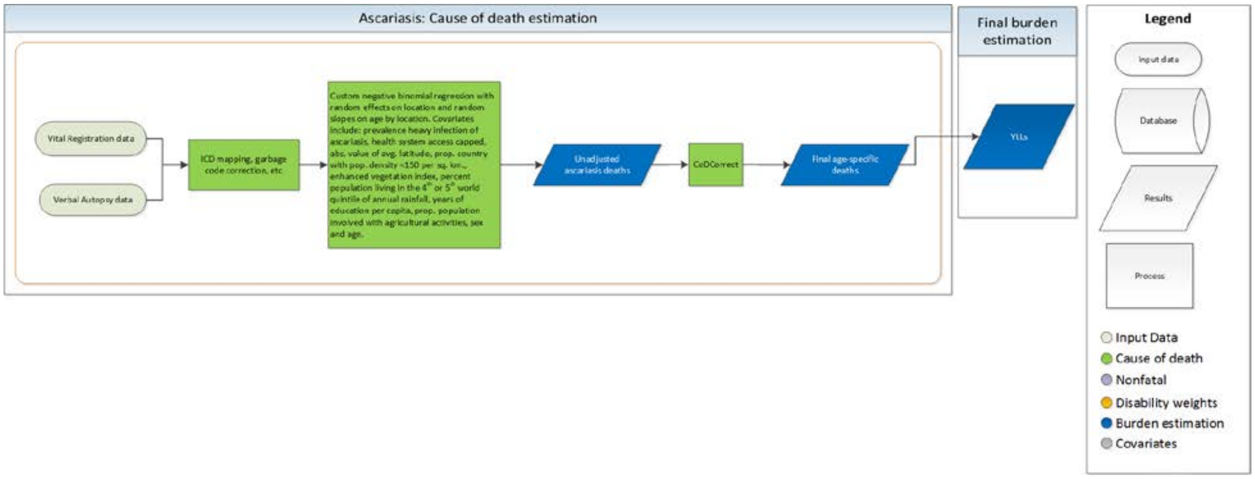

Input data

To estimate mortality due to ascariasis, country-year-age-sex-specific verbal autopsy and vital registration data were used. Covariates used include prevalence of heavy infection of ascariasis, the absolute value of average latitude, the proportion of the country with population density under 150 people per square kilometer, enhanced vegetation index, percentage of the population living in the fourth or fifth world quintile of annual rainfall, number of years of education per capita, proportion of the population involved with agricultural activities, age, and sex.

Geographical restrictions

We conducted a literature review to determine the geographical extent of the disease and classify locations based on whether the disease is absent or present in each year. Locations that were geographically restricted in any given year did not have estimates made for them but could have imported cases attributed to them at a later stage. Of note, we did not attempt a complete systematic review, since a single high-quality source could offer sufficient evidence of presence. Evidence of absence or presence was not available for every location for each year and so assumptions were made for missing years by taking into consideration the epidemiological characteristics of the disease. If evidence indicated disease presence for two non-consecutive years, we assumed presence for all years between the two. If evidence indicated disease absence for two non-consecutive years, we assumed absence for all years between the two. If evidence indicated a change in status (ie, from absent to present, or present to absent) between two non-consecutive years, then we conducted targeted searches to ascertain the relevant year of introduction or elimination for that location. In the cases where presence or absence information was missing for the start or end years of our study interval (1990–2019) without evidence of any introduction or elimination events within the interval, we applied the status of the first and last presence/absence observations respectively to all years between the interval bound and the observation year. Our search was done in conjunction with the title/abstract screening portion of a systematic literature review for prevalence data. The search strings and yield can be viewed in the table below for each of the databases queried.

Database	Search string	Yield
PubMed	(Ascariasis[Title/Abstract] OR Ascaris[Title/Abstract] OR "A. lumbricoides"[Title/Abstract] OR Ascaris[MeSH] OR Trichuris[Title/Abstract] OR Trichuriasis[Title/Abstract] OR "Whip Worm"[Title/Abstract] OR "T. trichura"[Title/Abstract] OR Trichuris[MeSH] OR Hookworm[Title/Abstract] OR "A. duodenale"[Title/Abstract] OR "Ancylostoma duodenale"[Title/Abstract] OR ancylostomiasis[Title/Abstract] OR "N. americanus"[Title/Abstract] OR "Necator americanus"[Title/Abstract] OR necatoriasis[Title/Abstract] OR Ancylostoma [MeSH] OR Necator[MeSH]) AND (prevalence[Title/Abstract] OR incidence[Title/Abstract] OR epidemiology[Title/Abstract] OR surveillance[Title/Abstract]) NOT(Animals[MeSH] NOT Humans[MeSH])	2376
Web of Science	(Ascariasis OR Ascaris OR A. lumbricoides OR Trichuris OR Trichuriasis OR Whip Worm OR T. trichura OR Hookworm OR A. duodenale OR Ancylostoma duodenale OR anclyostomiasis OR N. americanus OR Necator americanus OR necatoriasis) AND TOPIC:(prevalence OR incidence OR epidemiology OR surveillance) NOTTOPIC: ((Animals NOT Humans))<br>Timespan: 1980-2016. Indexes: SCI-EXPANDED, SSCI, A&HCI, ESCI.	2266
SCOPUS	TITLE-ABS_KEY (ascariasis OR ascaris OR a. lumbricoides OR trichuris OR trichuriasis OR whip worm OR t. trichura OR hookworm OR a. duodenale OR ancylostoma duodenale OR anclyostomiasis OR n. americanus OR necator americanus OR necatoriasis) AND PUBYEAR>1979	29

These papers were used to classify location-years for all locations and years present in the literature. Additionally, systematic literature reviews, meta-analyses, national health statistics publications, and collaborator input were used to classify location-years not present in the literature review wherever possible.

Modelling strategy

A negative binomial model was used to estimate deaths from ascariasis with random intercepts for locations and random slopes for age groups by location. A multivariate normal distribution using the mean and variance-covariance matrix from the model was used to generate 1,000 draws of deaths due to ascariasis. The final model was selected based on how well the estimated number fit the input data and how plausible the predicted distribution of disease was over time and with age.

Ebola virus disease

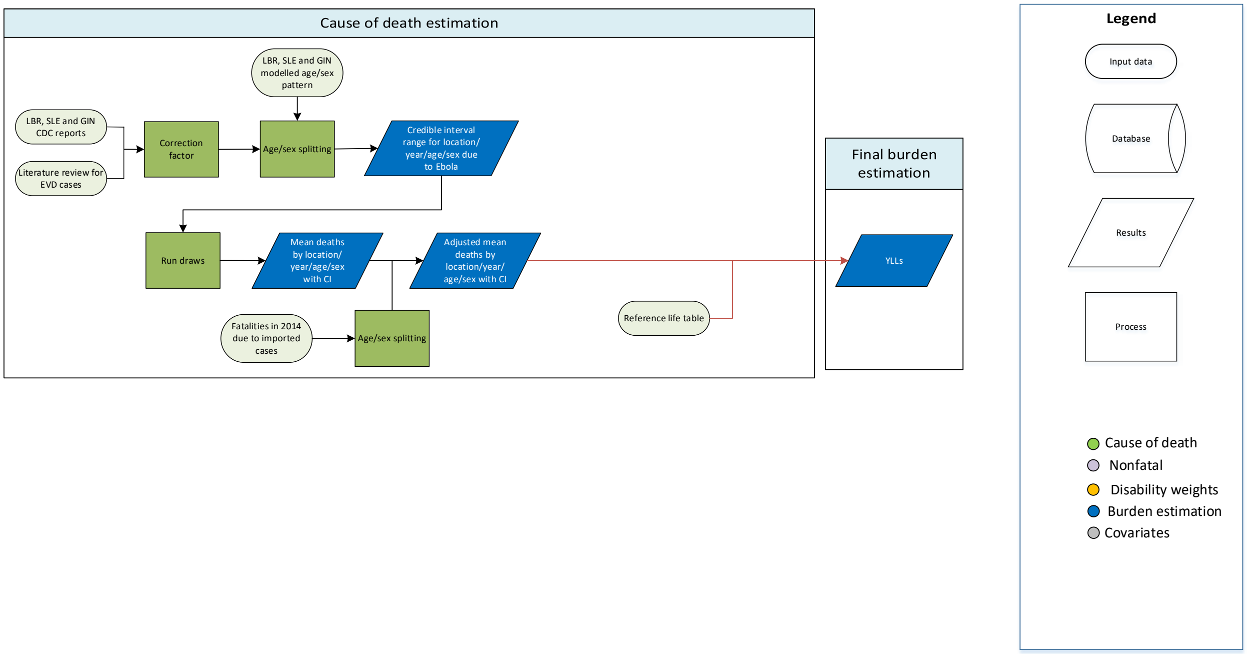

Input data

The input data for deaths due to Ebola virus disease (EVD) came in two forms: (i) total case reports for the West African outbreak from 2013 to 2016 provided by the Centers for Disease Control (CDC) focused specifically on the three worst-affected countries (Liberia, Guinea, and Sierra Leone) and (ii) literature searches for reported deaths due to EVD not captured by the West African dataset. In order to capture the small number of fatalities that occurred in countries outside of the core three mentioned above, WHO Situation Reports were consulted. Fatalities were reported in the USA (specifically Texas), Mali, and Nigeria, [3] and these deaths occurred in 2014. Additional age and sex information could only be obtained for the death that occurred in the USA.

Using a previous review of historical outbreaks,[4,5] original articles describing the progression of historical outbreaks were reviewed. This resulted in datasets describing each outbreak with variable degrees of detail – some fully describing the age and sex breakdown of all deaths [eg, Rosello and colleagues[6]] and others simply providing the final total. Only confirmed or probable deaths were included; suspected EVD deaths were omitted. Outbreaks that spanned multiple years, in the absence of sufficient data providing an accurate breakdown, were split between the years by evenly assigning a uniform number of deaths to each month of the outbreak's duration.

These data were supplemented with WHO External Situation reports detailing the 2018 Democratic Republic of Congo Equateur province outbreak[7] as well as the ongoing 2018–2019 Democratic Republic

of Congo outbreak[8], including reported Ugandan cases[9]. The case totals for the ongoing outbreak were last updated November, 26th, 2019, and more information may be available since submission.

A full tabulation of death metadata availability is found in Table 1.

Outbreak	Number of deaths	Sex metadata	Age metadata	Year metadata
Côte d'Ivoire 1994	No deaths	N/A	N/A	N/A
Gabon 1994/1995	Georges 1999	Imputed	Imputed	Georges 1999
Democratic Republic of the Congo 1995	Rosello 2015	Rosello 2015	Rosello 2015 [94.5% coverage]	Rosello 2015
Gabon 1996	Milleliri 2004	Imputed	Imputed	Milleliri 2004
Gabon 1996/1997	Milleliri 2004	Imputed	Imputed	Imputed
Uganda 2000/2001	Okware 2002	Imputed	Imputed	Imputed
Congo 2002/2003	Kuhn 2008	Imputed	Imputed	Imputed
Congo 2003	Boumandouki 2005	Imputed	Imputed	Boumandouki 2005
South Sudan 2004	WHO 2004	WHO 2004	WHO 2004 [42.86% coverage]	WHO 2004
Congo 2005	Nkoghe 2011	Nkoghe 2011	Nkoghe 2011	Nkoghe 2011
Democratic Republic of the Congo 2007	Rosello 2015	Rosello 2015	Rosello 2015	Rosello 2015
Uganda 2007	Wamala 2010	Wamala 2010	Imputed	Wamala 2010
Democratic Republic of the Congo 2008	Rosello 2015	Rosello 2015	Rosello 2015	Rosello 2015
Uganda 2011	Shoemaker 2012	Shoemaker 2012	Shoemaker 2012	Shoemaker 2012
Democratic Republic of the Congo 2012	Rosello 2015	Rosello 2015	Rosello 2015	Rosello 2015
Uganda 2012	Albarino 2013	Imputed	Imputed	Albarino 2013
Uganda 2012/2013	Albarino 2013	Imputed	Imputed	Imputed
West Africa 2013/2015	WHO/CDC	Imputed	Imputed	WHO/CDC
Democratic Republic of the Congo 2014	Rosello 2015	Rosello 2015	Rosello 2015	Rosello 2015
Democratic Republic of the Congo, Equateur province 2018	WHO 2018	WHO 2018	WHO 2018	WHO 2018

Democratic Republic of the Congo, Kivu 2018/2019 (ongoing)	WHO 2018, WHO 2019	WHO 2018, WHO 2019	WHO 2018, WHO 2019	WHO 2018, WHO 2019

Modelling strategy

Data on deaths resulting from imported cases from 2014 were used as specific count data as it was assumed to be an accurate representation of the cases and outbreaks in these countries, all of which were on high alert for importation of cases.[10,11]

The other input data were processed prior to inclusion in GBD to account for any potential underreporting of deaths. A meta-analysis of existing underreporting studies from the literature was performed, using a random effects model with a DerSimonian-Laird estimator. A variety of sources were included, capturing a number of different estimation processes, all identified by literature review. The figure below shows the different effect sizes of the different studies,[12–18] as well as the resulting GBD 2016 correction factor, with the GBD 2015 correction factor for reference. The correction factor ranged from 1.4580 to 2.5475, with a mean of 2.0027. For GBD 2019 the GBD 2016 factor was used.

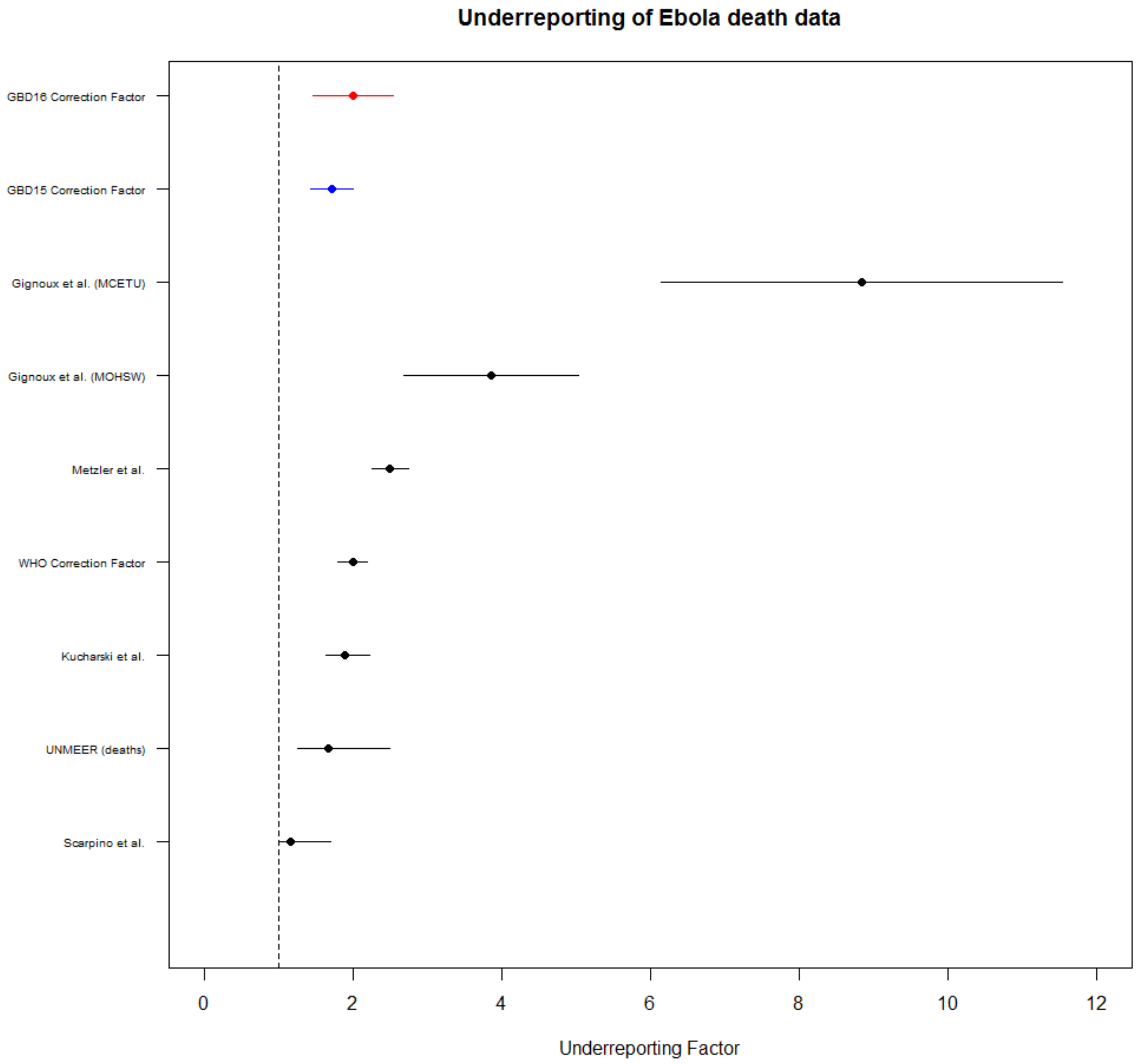

In order to capture this potential variation, all input data were multiplied by the lower and upper limit of this estimated correction factor; these numbers then provided the lower and upper bounds from which draw values were taken. For outbreaks where no data were supplied for age and/or sex, the pattern observed in the age- and sex-specific case data was used to apportion these total values.

One thousand draws were taken from a normal distribution fitted between these lower and upper bound values, which generated mean estimates stratified by age, sex, location, and year along with credible intervals for these numbers. These estimates were then adjusted by including the count data for imported cases from 2014.

Data on Ebola outbreaks prior to 2014 are sparse, and as a result many values derived from the West African outbreak were assumed to be valid for historical outbreaks as well. This may mask significant differences in the distribution of cases by age and sex that exist between these outbreaks, some of which were caused by different species of *Ebolavirus*. In order to minimise this problem, we chose to

implement a data-driven approach – for those outbreaks where sufficiently detailed historical data could be obtained, these were used in preference to any assumed age/sex breakdown.

References

1 Agua-Agum J, Ariyarajah A, Aylward B, *et al.* West African Ebola Epidemic after One Year — Slowing but Not Yet under Control. *N Engl J Med* 2015; **372**: 584–7.

2 Ebola Virus Disease in West Africa - The First 9 Months of the Epidemic and Forward Projections. *N Engl J Med* 2014; **371**: 1481–95.

3 World Health Organization. Ebola Situation Reports. 2016. Interview (accessed March 14, 2016).

4 Pigott DM, Golding N, Mylne A, *et al.* Mapping the zoonotic niche of Ebola virus disease in Africa. *Elife* 2014; **3**: e04395.

5 Mylne A, Brady OJ, Huang Z, *et al.* A comprehensive database of the geographic spread of past human Ebola outbreaks. *Sci Data* 2014; **1**: 140042.

6 Maganga GD, Kapetshi J, Berthet N, *et al.* Ebola virus disease in the Democratic Republic of Congo. *N Engl J Med* 2014; **371**: 2083–91.

7 World Health Organization (WHO). WHO Ebola Situation Report 2018 - Number 17. 2018.

8 World Health Organization (WHO). WHO Ebola Situation Report 2019 - Number 45. 2019.

9 World Health Organization (WHO). WHO Ebola Situation Report 2019 - Number 51. 2019.

10 Rosello A, Mossoko M, Flasche S, *et al.* Ebola virus disease in the Democratic Republic of the Congo, 1976-2014. *Elife* 2015; **4**. DOI:10.7554/eLife.09015.

11 Fasina FO, Shittu A, Lazarus D, *et al.* Transmission dynamics and control of Ebola virus disease outbreak in Nigeria, July to September 2014. *Euro Surveill* 2014; **19**: 20920.

12 Althaus CL, Low N, Musa EO, Shuaib F, Gsteiger S. Ebola virus disease outbreak in Nigeria: Transmission dynamics and rapid control. *Epidemics* 2015; **11**: 80–4.

13 Gignoux E, Idowu R, Bawo L, *et al.* Use of Capture-Recapture to Estimate Underreporting of Ebola Virus Disease, Montserrado County, Liberia. *Emerg Infect Dis* 2015; **21**: 2265–7.

14 Meltzer MI, Atkins CY, Santibanez S, *et al.* Estimating the future number of cases in the Ebola epidemic--Liberia and Sierra Leone, 2014-2015. *MMWR Suppl* 2014; **63**: 1–14.

15 Scarpino S V, Iamarino A, Wells C, *et al.* Epidemiological and viral genomic sequence analysis of the 2014 ebola outbreak reveals clustered transmission. *Clin Infect Dis* 2015; **60**: 1079–82.

16 Kucharski AJ, Camacho A, Flasche S, Glover RE, Edmunds WJ, Funk S. Measuring the impact of Ebola control measures in Sierra Leone. *Proc Natl Acad Sci U S A* 2015; **112**: 14366–71.

17 UNMEER. Sierra Leone: Ebola emergency Weekly Situation Report No. 7. 2014 https://www.humanitarianresponse.info/system/files/documents/files/UNMEER_NERC_SitRep_07Dec.pdf.

18 Enserink M. How many Ebola cases are there really? | Science | AAAS. 2014.

http://www.sciencemag.org/news/2014/10/how-many-ebola-cases-are-there-really (accessed Jan 28, 2017).

Zika virus disease

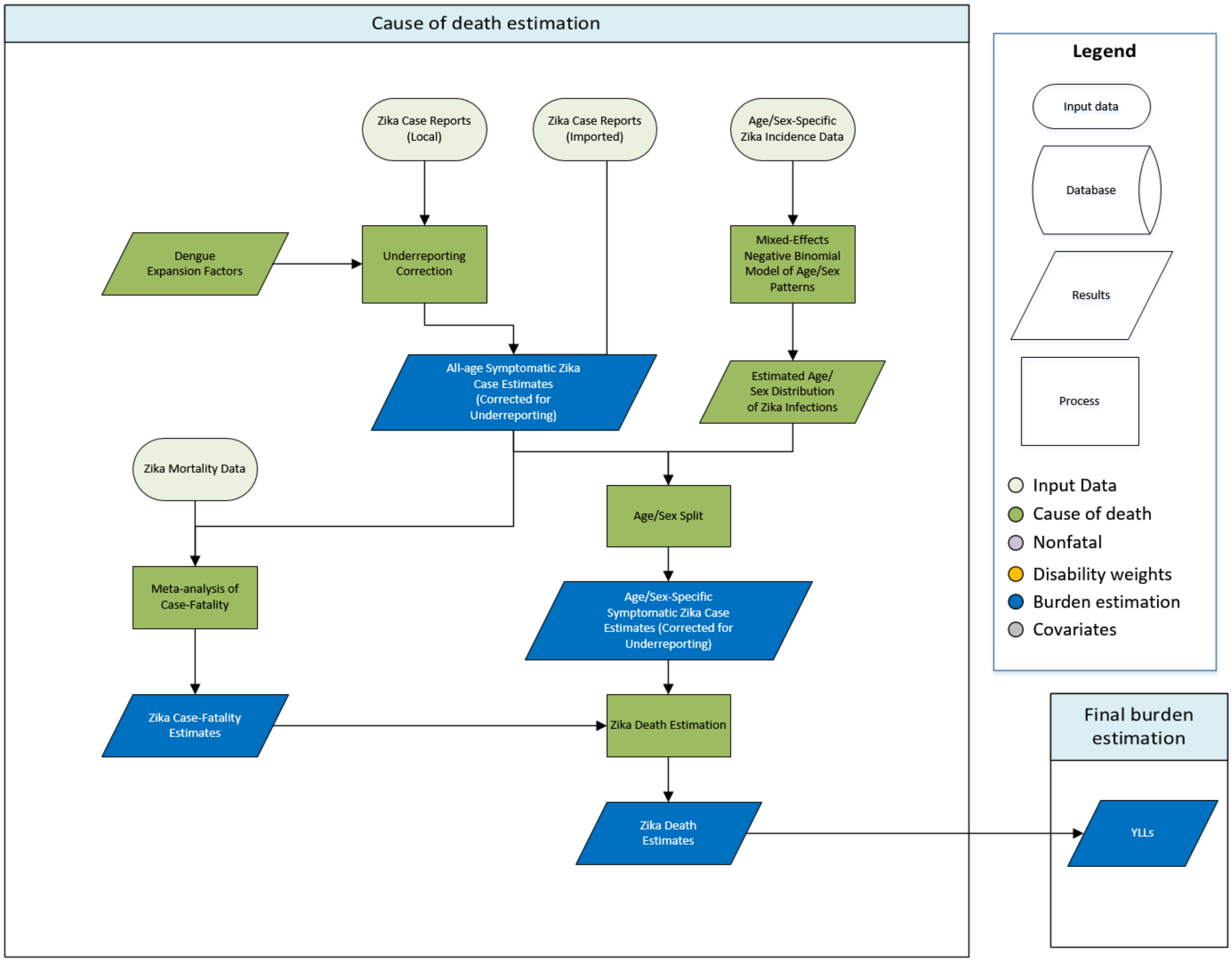

Input data

Case data and death data come from official reports, primarily from PAHO, in which deaths attributed to Zika virus infection were reported for the period 2015–2018. Overall, a total of 22 deaths were reported in Brazil, Suriname, and Puerto Rico during this period. Of these cases, the majority were among adult males.

Modelling strategy

We model Zika deaths using mixed effects negative binomial regression model, with the log of total Zika incidence (all-age and all-sex as estimated by the non-fatal model) and year as covariates, including a random effect for location. This model was used to generate an estimate of total deaths due to Zika. Data on the age and sex distribution was used to generate the proportion of deaths by age and sex, with uncertainty simulated around those proportions applied to the split the total deaths estimated by the model into age- and sex-specific deaths.

Other neglected tropical diseases (NTDs)

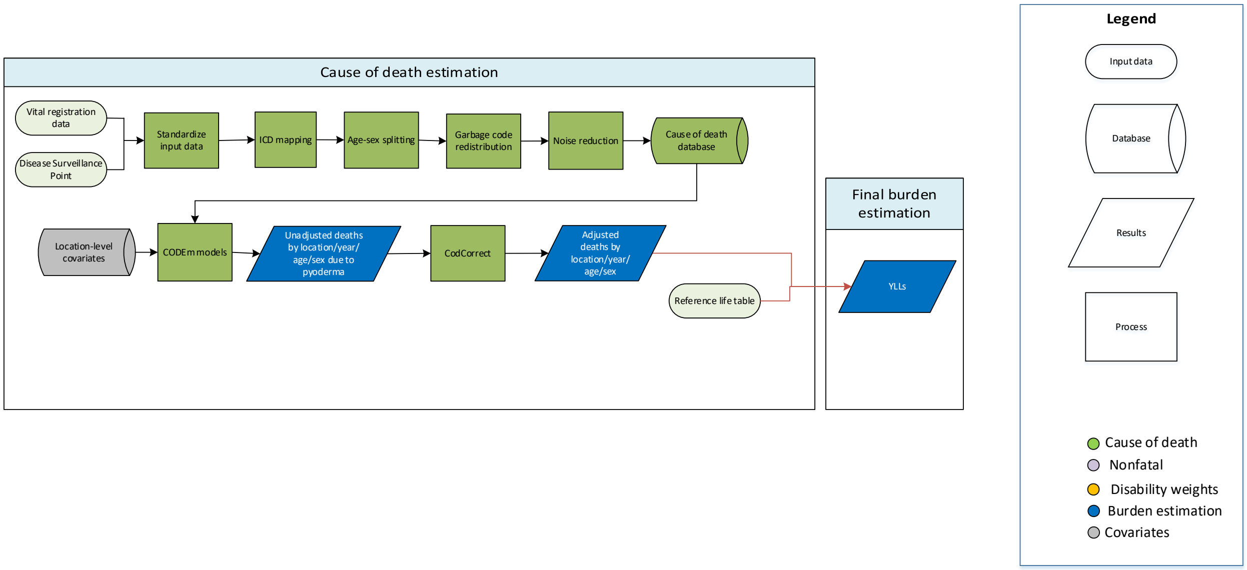

There are many diverse types of neglected tropical diseases, which are encompassed by the following ICD 10 codes:

A68 Relapsing fevers

A68.0 Louse-borne relapsing fever

A68.1 Tick-borne relapsing fever

A68.9 Relapsing fever, unspecified

A69.2 Lyme disease

A69.20 Lyme disease, unspecified

A69.21 Meningitis due to Lyme disease

A69.22 Other neurologic disorders in Lyme disease

A69.23 Arthritis due to Lyme disease

A69.29 Other conditions associated with Lyme disease

A69.5 There is not this code in ICD10 site, but we have this in mortality data

A69.8 Other specified spirochetal infections

A69.9 Spirochetal infection, unspecified

A75 Typhus fever

A75.0 Epidemic louse-borne typhus fever due to Rickettsia prowazekii

A75.1 Recrudescent typhus [Brill's disease]

A75.2 Typhus fever due to Rickettsia typhi

A75.3 Typhus fever due to Rickettsia tsutsugamushi

A75.9 Typhus fever, unspecified

A77 Spotted fever [tick-borne rickettsioses]

A77.0 Spotted fever due to Rickettsia rickettsii

A77.1 Spotted fever due to Rickettsia conorii

A77.2 Spotted fever due to Rickettsia siberica

A77.3 Spotted fever due to Rickettsia australis

A77.4 Ehrlichiosis

A77.40 Ehrlichiosis, unspecified

A77.41 Ehrlichiosis chafeensis [E. chafeensis]

A77.49 Other ehrlichiosis

A77.8 Other spotted fevers

A77.9 Spotted fever, unspecified

A78 Q fever

A79 Other rickettsioses

A79.0 Trench fever

A79.1 Rickettsialpox due to Rickettsia akari

A79.8 Other specified rickettsioses

A79.81 Rickettsiosis due to Ehrlichia sennetsu

A79.89 Other specified rickettsioses

A79.9 Rickettsiosis, unspecified

A92 Other mosquito-borne viral fevers

A92.0 Chikungunya virus disease

A92.1 O'nyong-nyong fever

A92.2 Venezuelan equine fever

A92.3 West Nile virus infection

A92.30 West Nile virus infection, unspecified

A92.31 West Nile virus infection with encephalitis

A92.32 West Nile virus infection with other neurologic manifestation

A92.39 West Nile virus infection with other complications

A92.4 Rift Valley fever

A92.8 Other specified mosquito-borne viral fevers

A92.9 Mosquito-borne viral fever, unspecified

A93 Other arthropod-borne viral fevers, not elsewhere classified

A93.0 Oropouche virus disease

A93.1 Sandfly fever

A93.2 Colorado tick fever

A93.8 Other specified arthropod-borne viral fevers

A94 Unspecified arthropod-borne viral fever

A94.0 Unspecified arthropod-borne viral fever

A96 Arenaviral hemorrhagic fever

A96.0 Junin hemorrhagic fever

A96.1 Machupo hemorrhagic fever

A96.2 Lassa fever

A96.8 Other arenaviral hemorrhagic fevers

A96.9 Arenaviral hemorrhagic fever, unspecified

A98 Other viral hemorrhagic fevers, not elsewhere classified

A98.0 Crimean-Congo hemorrhagic fever

A98.1 Omsk hemorrhagic fever

A98.2 Kyasanur Forest disease

A98.3 Marburg virus disease

A98.5 Hemorrhagic fever with renal syndrome

A98.8 Other specified viral hemorrhagic fevers

B33.0 Epidemic myalgia

B33.1 Ross River disease

B60 Other protozoal diseases, not elsewhere classified

B60.0 Babesiosis

B60.1 Acanthamebiasis

B60.10 Acanthamebiasis, unspecified

B60.11 Meningoencephalitis due to Acanthamoeba (culbertsoni)

B60.12 Conjunctivitis due to Acanthamoeba

B60.13 Keratoconjunctivitis due to Acanthamoeba

B60.19 Other acanthamebic disease

B60.2 Naegleriasis

B60.8 Other specified protozoal diseases

B67.5 Echinococcus multilocularis infection of liver

B67.6 Echinococcus multilocularis infection, other and multiple sites

B67.61 Echinococcus multilocularis infection, multiple sites

B67.69 Echinococcus multilocularis infection, other sites

B67.7 Echinococcus multilocularis infection, unspecified

B70 Diphyllobothriasis and sparganosis

B70.0 Diphyllobothriasis

B70.1 Sparganosis

B71 Other cestode infections

B71.0 Hymenolepiasis

B71.1 Dipylidiasis

B71.8 Other specified cestode infections

B71.9 Cestode infection, unspecified

B74.3 Loiasis

B74.4 Mansonelliasis

B74.8 Other filariases

B74.9 Filariasis, unspecified

B75 Trichinellosis

B83 Other helminthiases

B83.0 Visceral larva migrans

B83.1 Gnathostomiasis

B83.2 Angiostrongyliasis due to Parastrongylus cantonensis

B83.3 Syngamiasis

B83.4 Internal hirudiniasis

B83.8 Other specified helminthiases

P37.1 Congenital toxoplasmosis

Input data

We modelled other neglected tropical disease mortality using all available data in the cause of death database. Data points were outliered if they reported an improbable number of deaths or if their inclusion in the model yielded distorted trends.

Modelling strategy

We modelled other neglected tropical disease mortality using a two-model hybrid approach: 1) a global CODEm model of all locations, using all data in the CoD database; and 2) a CODEm model restricted to data-rich countries.

We have made no substantive changes in the modelling strategy for other neglected tropical diseases from GBD 2017.

Level	Covariate	Direction
1	Healthcare Access and Quality Index	−
	Proportion of the population living between 0 and 15 degrees latitude	+
2	Proportion of the population living in the 5th quintile of rainfall	+
	Sanitation	−
3	Education (years per capita)	−
	Lag-distributed income (per capita)	−
	Socio-demographic Index	−

Meningitis

Flowchart

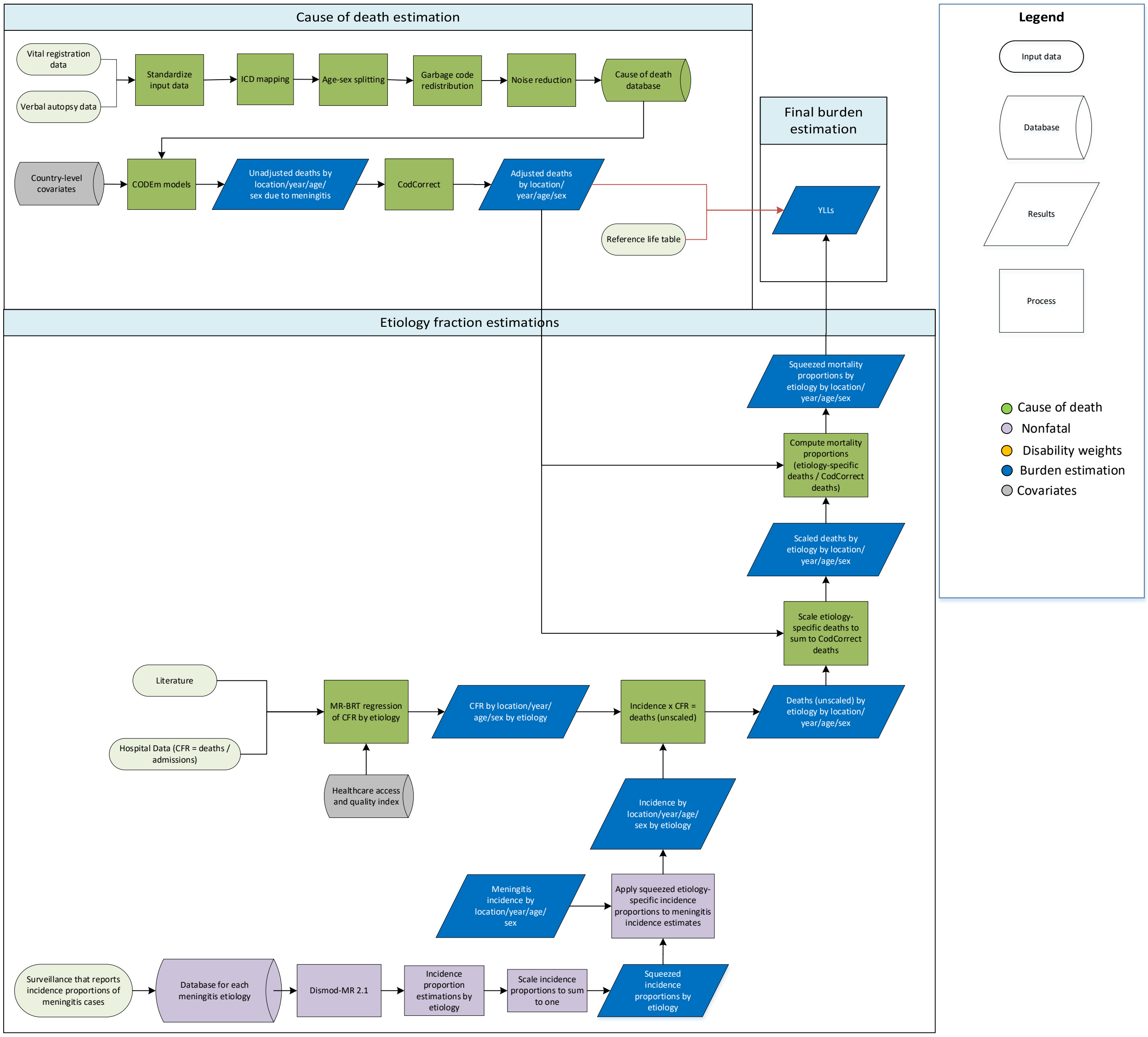

Input data and methodological summary for meningitis

Input data

Input data for the overall meningitis model came from the cause of death database, which includes vital registration (VR) and verbal autopsy (VA) data. We outliered data in instances where garbage code redistribution and noise reduction, in combination with small sample sizes, resulted in unreasonable cause fractions when compared to regional, super-regional, and global rates, and data that violated well-established time or age trends. Outliering methods were consistent across both VR and VA data.

Modelling strategy

We modelled deaths due to all meningitis with two CODEm models, separately for each sex and two age categories – under 5 and 5 years and above. The mortality trends differ substantially between children and adults, and there are a significant number of data sources that only have data for children under 5. The two models used the same covariates (with the exception of the covariate for underweight, which is age-specific) and otherwise standard CODEm parameters. The final sex-specific models for deaths due to all meningitis were a hybridised model of separate global and data-rich models for males and females.

Mortality estimates for each of the three aetiologies of bacterial meningitis – meningococcal, pneumococcal, *H. influenzae* type B – were derived from aetiology-specific incidence and case fatality rate (CFR) estimates. First, incident cases of bacterial meningitis were split into four aetiologies (pneumococcal, meningococcal, *H. influenzae* type B, and other bacterial meningitis) using four proportion models run in DisMod-MR 2.1. Input data for these models were from published studies reporting incidence proportions for each aetiology. Within each location, year, age group, and sex, we squeezed the proportions to ensure that they summed to 100% at the draw level. We applied a Hib3 vaccine coverage for the *H. influenzae* type B proportion model, the proportion of the population living in the meningitis belt covariate, the proportion of the population living in areas covered by the MenAfriVac initiative (meningitis meningococcal type A) to the meningococcal proportion model, and a PCV3 coverage covariate to the pneumococcal meningitis model. We also estimated pathogen-specific CFRs as a function of healthcare access and quality using MR-BRT. Input data for this model included inpatient-hospital data as well as data from published studies reporting CFRs.

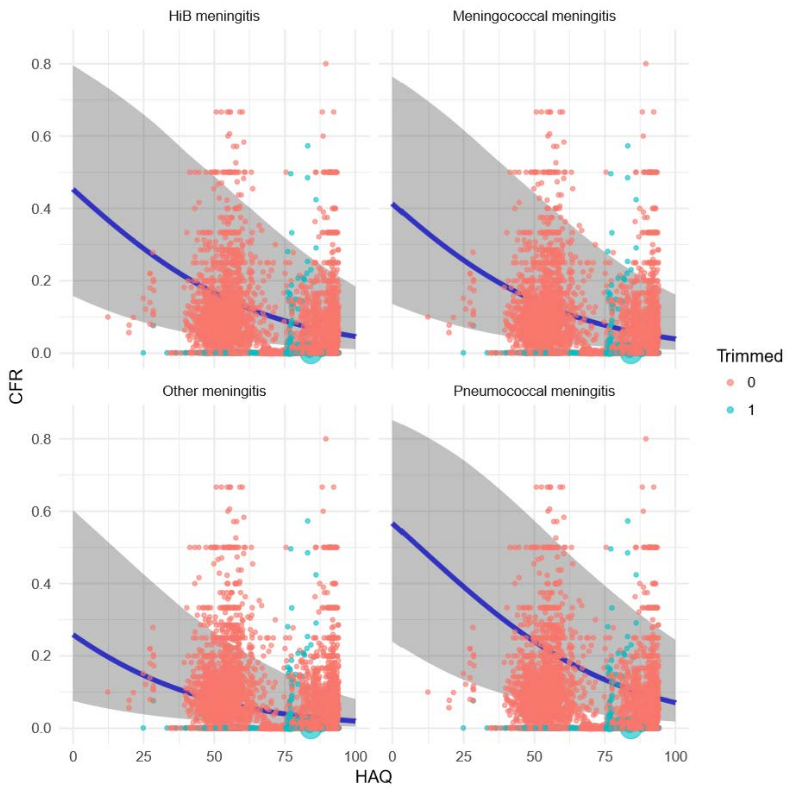

Figure 1 Regression of aetiology-specific CFR as a function of healthcare access and quality. Size of points is proportional to inverse variance.

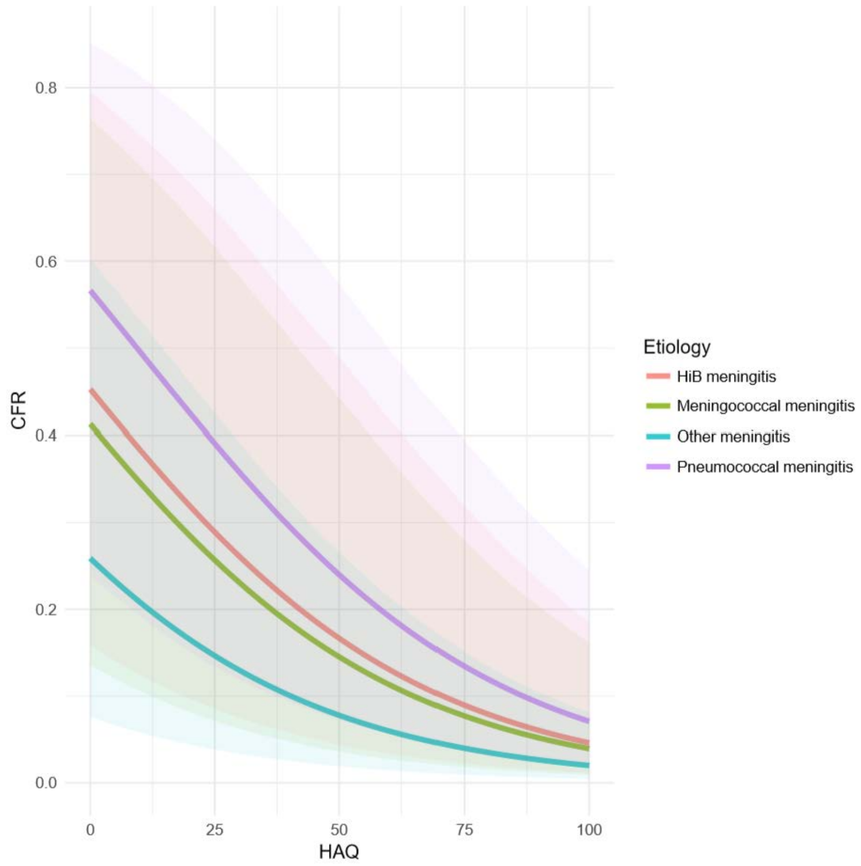

Figure 2 Same regression as figure 1, but now showing all of the aetiologies together

The aetiology-specific deaths were then squeezed to the total number meningitis deaths after CoDCorrect and meningococcal shocks deaths were included at the draw level.

Table 1. Covariates used in meningitis mortality modelling (0–4 years, 5–95+ years)

Covariate Name	Level	Direction
Meningitis belt (proportion of population in belt)	1	+
MenAfriVac coverage	1	-
H. influenzae type B proportion covered	1	-
PCV3 coverage proportion	1	-
Age- and sex-specific summary exposure value (SEV) for child underweight	2	+
Logit-transformed water (proportion with access)	2	-
Maternal care and immunization	2	-
Healthcare Access and Quality Index	2	-
Log-transformed lag distributed income	3	-
Sanitation (proportion with access)	3	-
Maternal education (years per capita)	3	-
Socio-demographic Index	3	-

References

(1) Centers for Disease Control (CDC). CDC health information for international travel 2016: the yellow book. New York City, United States: Oxford University Press, USA, 2016.

Encephalitis

Flowchart

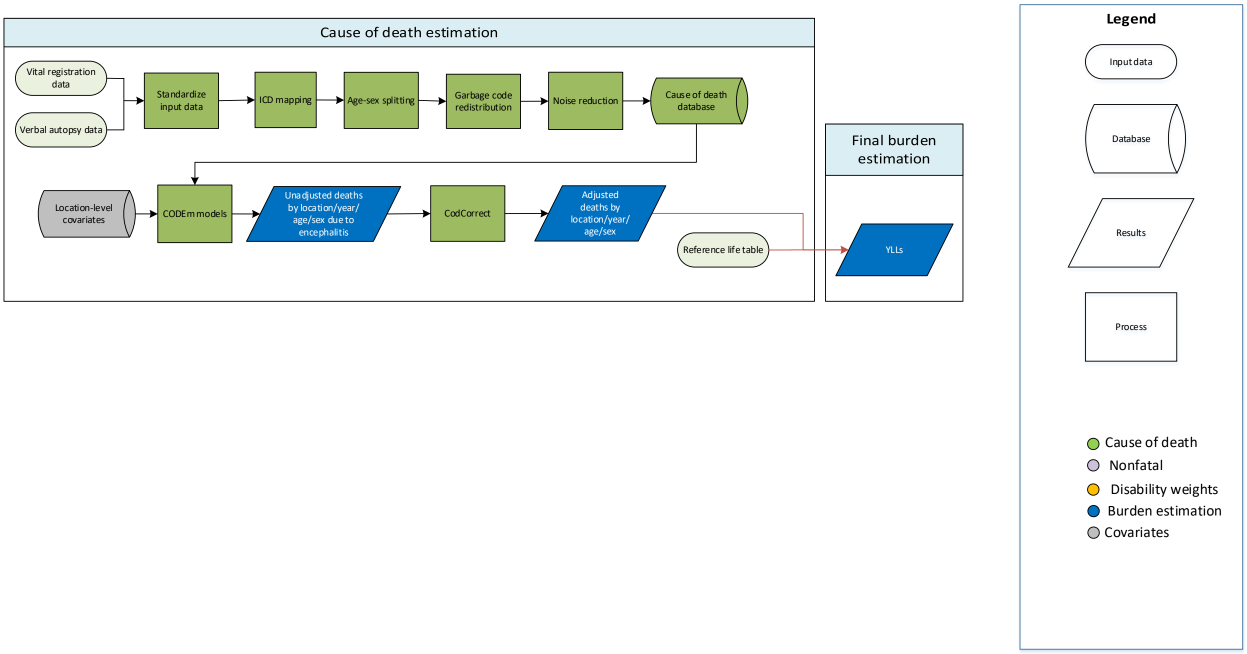

Input data and methodological summary for encephalitis

Input data

For GBD 2019, vital registration and verbal autopsy data were used to model this cause. We outliered data in instances where garbage code redistribution and noise reduction, in combination with small sample sizes, resulted in unreasonable cause fractions when compared to regional, super-regional, and global rates, and data that violated well-established time or age trends. Outliering methods were consistent across both vital registration and verbal autopsy data.

Modelling strategy

We modelled deaths due to encephalitis with a standard CODEm model using the cause of death database and location-level covariates as inputs. We hybridised separate global and data-rich models to acquire unadjusted results, which were adjusted using CodCorrect to reach final years of life lost due to encephalitis.

We previously used two separate age models for encephalitis, 0–5 years and 5–95. Starting in GBD 2015, we modelled encephalitis using the full age range in one model. Another significant change was the addition of the Japanese encephalitis covariate, which is a binary covariate indicating if the location is known to be endemic for Japanese encephalitis. The covariate was modelled according to data from the Centers for Disease Control and Prevention (CDC).[1] For GBD 2017, we updated the Japanese encephalitis covariate to include regions of Russia that are included as endemic regions in the CDC report. We also added the DTP3 coverage covariate to the model. A full list of covariate inputs in the published model can be found below. Covariates were weighted and selected based on the ensemble model process.

Table 1. Covariates used in encephalitis mortality modelling

Level	Covariate	Direction
1	Japanese encephalitis binary	+
	Age- and sex-specific summary exposure value (SEV) for child underweight	+
2	Log-transformed lag distributed income	-
	Healthcare Access and Quality Index	-
	Maternal care and immunization	-
3	Squared proportion of in-facility deliveries	-
	Socio-demographic Index	-
	Logit-transformed sanitation (proportion with access)	-
	Logit-transformed water (proportion with access)	-
	DTP3 coverage	-
	Maternal education (years per capita)	-

References

(1) Centers for Disease Control (CDC). CDC health information for international travel 2016: the yellow book. New York City, United States: Oxford University Press, USA, 2016.

Diphtheria

Flowchart

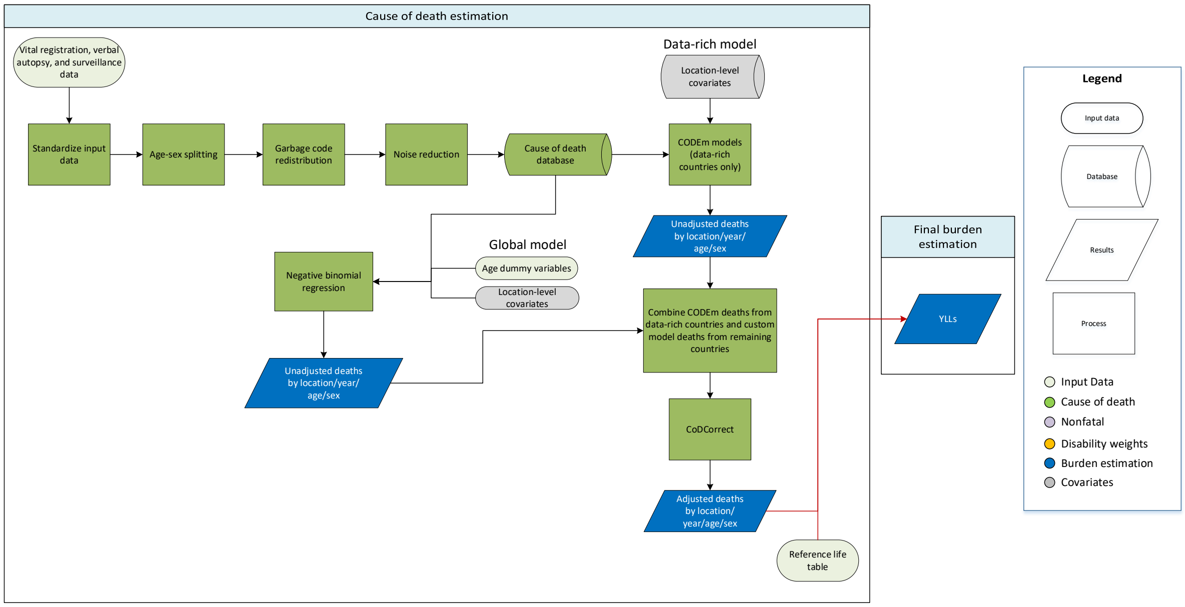

Input data

Diphtheria cause of death (COD) data for GBD 2019 included vital registration, verbal autopsy, and surveillance sources from all locations as available. We excluded COD data if they were highly incongruent with other available data from the same location or locations with similar sociodemographic characteristics.

Modelling strategy

We used two distinct methods to estimate diphtheria mortality for different countries based on the quality of vital registration data available. We used a counts-based Cause of Death Ensemble modeling strategy (CODEm) for countries with well-defined vital registration (ie, "data-rich" countries), and for remaining countries a custom count negative binomial regression model. Each approach is further described in more detail below.

1. Data-rich countries

We used CODEm counts models rather than standard rate-space CODEm models, as the models in count space had lower out-of-sample root mean squared error (RMSE) than those in rate-space. For data-rich locations, we used the covariates outlined in Table 1 to inform CODEm predictions. New covariates in the GBD 2019 models were age- and sex-specific summary exposure values (SEV) for child wasting to replace the wasting proportion covariate; Healthcare Access and Quality (HAQ) Index and Socio-

demographic Index (SDI) were used to capture the effect of the maternal care and immunisation (MCI) covariate used in prior GBD cycles.

Table 1. Covariates. Summary of covariates used in the data-rich diphtheria cause of death model

Level	Covariate	Direction
1	Diphtheria-tetanus-pertussis third-dose vaccination coverage (DTP3)	-
	Healthcare Access and Quality (HAQ) Index	-
	Age- and sex-specific SEV for child wasting	+
3	Lag-distributed income (LDI)	-
	Socio-demographic Index (SDI)	-
	Mean years of education per capita	-

2. *Custom count model*

Our custom counts mortality model for all non-data-rich locations also used COD data as available by location. We excluded data with extremely high cause fractions (ie, greater than the 99th percentile of all diphtheria cause fractions). Using a negative binomial regression with a log link, cause fractions representing the number of deaths due to diphtheria as a proportion of the all-cause mortality envelope were regressed using five-year rolling diphtheria-pertussis-tetanus third-dose (DPT3) vaccine coverage as a covariate, with dummy variables for each GBD age group as predictors:

$$Y_{ij} = \beta_0 + \beta_1\, DTP3_{ij} + \beta_a\, age_a + e_{ij}\,,$$

where Y_{ij} is the log-transformed cause fraction (counts of deaths with an offset of the total number of deaths); β_0 is the fixed-effect intercept; β_1 is the fixed-effects slope on vaccine coverage; β_a is the fixed-effects slope on age_a, the dummy variable for each GBD age group in the estimation; e_{ij} is the residual; i is the year; and j is the location. In past GBD cycles, estimates of routine DTP3 coverage among infants in the modeled year were used as the routine immunization input into this model rather than the average DTP3 coverage over the previous five years.

Uncertainty was estimated by predicting 1000 draws based on the variance-covariance matrix, and a random sample of the dispersion parameter from a gamma distribution. Results were summarised as the mean of all draws and an associated 95% uncertainty interval (the 2.5th and 97.5th quantile of all draws).

Pertussis (whooping cough)

Flowchart

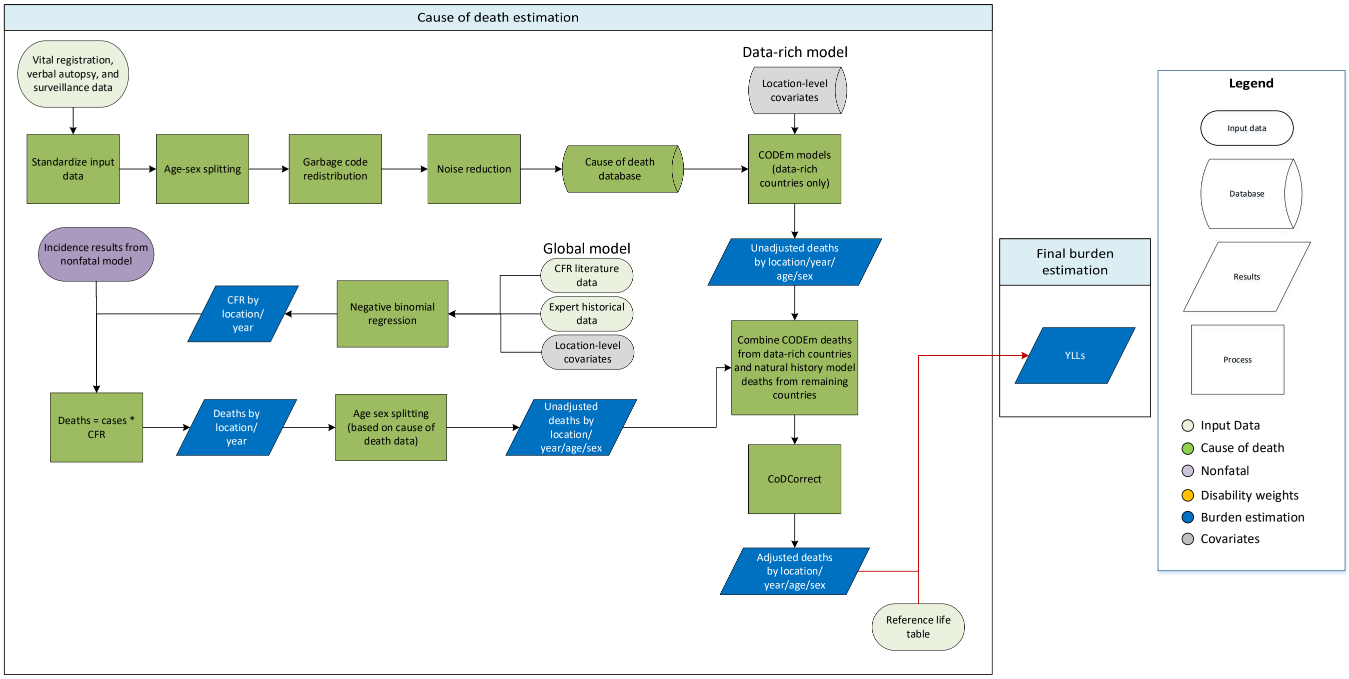

Modelling strategy overview

The GBD 2019 pertussis mortality estimates were generated one of two ways depending on the quality of available vital registration data for the country. For countries with well-defined vital registration (ie, "data-rich" countries), we used a Cause of Death Ensemble model (CODEm). For the remaining countries, we leveraged a natural history model approach, drawing from preceding non-fatal case estimates. For all countries, we made estimates for all age groups between post-neonatal and 59 years.

1. Data-rich countries

For data-rich countries modeled in CODEm, we used the covariates listed in Table 1 to inform predictions. New this cycle, the maternal care and immunisation (MCI) covariate was removed in favor of using measures of health access and quality (HAQ) and sociodemographic index (SDI) to predict. In addition, age- and sex-specific summary exposure values (SEV) for child underweight were added to the model to replace the malnutrition proportion covariate used in prior GBD cycles.

Table 1. Covariates. Summary of covariates used in the data-rich pertussis cause of death model

Level	Covariate	Direction
1	Diphtheria-tetanus-pertussis third-dose vaccination coverage (DTP3)	-
	Age- and sex-specific SEV for child underweight	+
	Healthcare Access and Quality (HAQ) Index	-

3	Lag-distributed income (LDI)	-
	Socio-demographic Index (SDI)	-
	Mean years of education per capita	-

2. *Natural history model*

The pertussis natural history model uses GBD estimates of non-fatal pertussis cases and an intermediate, custom model of pertussis case fatality rate (CFR) to produce estimates in non-data-rich locations where pertussis mortality data are sparse. As described in the non-fatal pertussis modelling text, case notifications informing the pertussis non-fatal model come from the World Health Organization (WHO) Joint Reporting Form (JRF) and historical documentation of pertussis cases and vaccination from the UK. The pertussis CFR data are compiled through systematic reviews of the literature. This systematic review was not updated for GBD 2019.

With the available pertussis CFR input data, we make location- and year-specific estimates using a negative binomial model with the Healthcare Access and Quality (HAQ) Index as a covariate:

$$Y_{ij} = \beta_0 + \beta_1 HAQ_{ij} + u_j + e_{ij} \,,$$

Pertussis log-transformed incidence – modelled independently – is generated from a mixed effects linear regression model predicting pertussis cases as a function of vaccination coverage. Combining these estimates of incidence for every estimated location and year with location-/year-specific estimates of pertussis CFR, pertussis deaths were calculated as:

$$deaths = incidence * CFR \,.$$

This calculation was replicated at the draw level 1000 times in order to produce estimates of total deaths by location and year and associated uncertainty. These draw-level estimates were age- and sex-split using an age-sex distribution based on global-level age- and sex-specific patterns found in the cause of death data, then summarised as the mean of the draws and a 95% uncertainty interval (the 2.5th and 97.5th quantile of all draws).

Tetanus

Flowchart

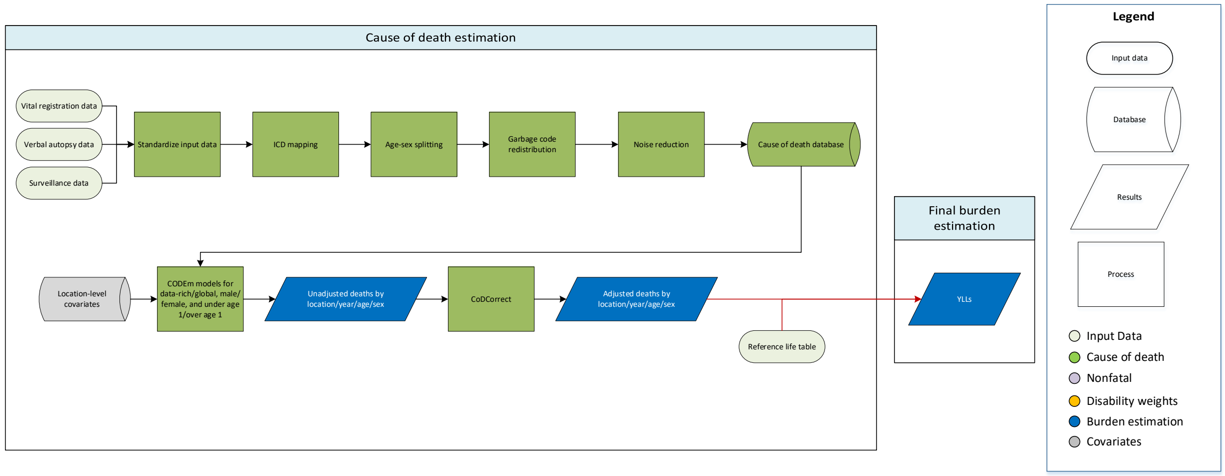

Input data

Tetanus cause of death (COD) data for GBD 2019 included vital registration, verbal autopsy, and surveillance sources from all locations as available. We excluded prepared COD data if they were highly incongruent with other available data from the same location or locations of similar sociodemographic characteristics.

Modelling strategy

We used a Cause of Death Ensemble modelling approach (CODEm) to compute age-, sex-, location-, and year-specific estimates. Given the relative rarity of tetanus mortality, we modelled directly in count-space. These models in count space had lower out-of-sample root mean squared error (RMSE) than rate-space models, and thus were frequently the top models selected in the ensemble.

Separate, sex-specific models were run for neonatal tetanus (under-1-year age groups) and all other tetanus (1 year to 95+ age groups). We also stratified models by vital registration data quality, running both “data-rich” and global models for each age- and sex-specific group. Following model completion, the data-rich and global model outputs were combined to produce a single set of estimates for all locations by sex and age (under-1 and over-1 age groups).

Table 1a lists the covariates used in the data-rich and global under-1 models, and table 1b the covariates in the over-1 model. In both the under-1 and over-1 models, Healthcare Access and Quality (HAQ) Index and Socio-demographic Index (SDI) were used to capture the effect of the maternal care and immunisation (MCI) covariate used in prior GBD cycles.

Table 1a. Covariates. Summary of covariates used in the under-1 tetanus cause of death model

Level	Covariate	Direction
1	Diphtheria-tetanus-pertussis third-dose vaccination coverage (DTP3)	-
	Tetanus toxoid coverage	-
2	In-facility deliveries (proportion)	-
	Skilled birth attendance (proportion)	-
	Healthcare Access and Quality (HAQ) Index	-
3	Lag-distributed income (LDI)	-
	Socio-demographic Index (SDI)	-
	Mean years of education per capita	-

Table 1b. Covariates. Summary of covariates used in the over-1 tetanus cause of death model

Level	Covariate	Direction
1	Diphtheria-tetanus-pertussis third-dose vaccination coverage (DTP3)	-
2	Healthcare Access and Quality (HAQ) Index	-
3	Sanitation access (proportion)	-
	Lag-distributed income (LDI)	-
	Socio-demographic Index (SDI)	-
	Mean years of education per capita	-

Measles

Model flowchart

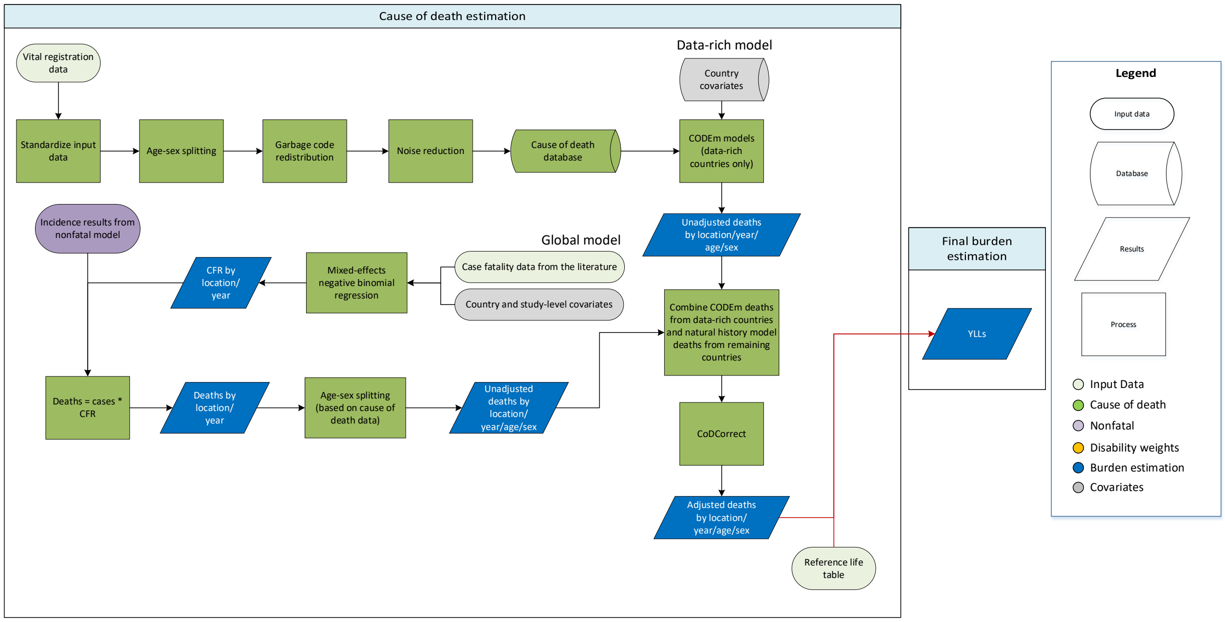

Modelling strategy overview

The GBD 2019 measles mortality estimates were generated in one of two ways depending on the quality of available vital registration data for the country. For countries with well-defined vital registration (ie, "data-rich" countries), we used a Cause of Death Ensemble model (CODEm). For the remaining countries, we leveraged a natural history model approach, drawing from preceding non-fatal case estimates. For all countries, we made estimates for all age groups between post-neonatal and 59 years.

Data-rich countries

For data-rich countries modeled in CODEm, we used the covariates listed in Table 1 to inform predictions. New this cycle, the Healthcare Access and Quality (HAQ) Index and Socio-demographic Index (SDI) covariates were used to capture the effect of the maternal care and immunisation (MCI) covariate used in prior GBD cycles.

Table 1. Covariates. Summary of covariates used in the data-rich measles cause of death model

Level	Covariate	Direction
1	Measles-containing vaccination dose one (MCV1)	-
2	Healthcare Access and Quality (HAQ) Index	-
3	Socio-demographic index (SDI)	-
	Mean years of education per capita	-

Natural history model

A natural history model is used to estimate measles mortality in non-data-rich locations where mortality data are sparse. GBD estimates of non-fatal measles cases are combined with estimates of measles case-fatality rate (CFR) generated by an intermediate, custom CFR model to produce this output. As described in the non-fatal measles modelling methods text, case notifications informing the measles non-fatal model come from the World Health Organization (WHO) Joint Reporting Form (JRF) and additional case notification sources identified by collaborators (eg, Japan and USA subnational measles surveillance data). The measles CFR data are compiled through systematic reviews of the literature, and this search was updated in GBD 2019. This search was conducted in PubMed using the following search string: *((((measles[MeSH Terms] OR measles) AND (mortality[MeSH Terms] OR mortality OR "case fatality rate" OR "case fatality ratio" OR "case fatality"))) AND ("2016"[Date - Publication] : "2019"[Date - Publication]))*.

With the available measles CFR input data, we make location- and year-specific death estimates using a negative binomial model with Socio-demographic Index (SDI) as a country-level covariate, additionally accounting for three indicators (hospital-based or not; outbreak or not; and rural or urban/mixed) as study-level covariates, with country random effects:

$$Y_{ij} = \beta_0 + \beta_1 SDI_{ij} + \beta_2 hospital_{ij} + \beta_3 outbreak_{ij} + \beta_4 rural_{ij} + u_j + e_{ij}$$

where Y_{ij} is the number of deaths (using measles cases as the offset term); β_0 is the fixed-effect intercept; β_1, β_2, β_3, and β_4 are the fixed-effects slopes on the Socio-demographic Index (SDI) and hospital, outbreak, and rurality study-level covariates; u_j is country-level random effects; e_{ij} is the residual; *i* is the year; and *j* is the location. Uncertainty was estimated by taking 1000 iterations of the predictions based on the variance-covariance matrix and uncertainty in country random effects.

Measles log-transformed incidence – modelled independently – is generated from a mixed effects linear regression model predicting measles cases as a function of vaccination coverage (rolling means of MCV1 and MCV2 over the preceding five years, and five-year lagged SIA coverage) given WHO case notification data from countries in the high-income, central Europe/eastern Europe/central Asia, and Latin America and Caribbean super-regions. Combining these estimates of incidence for every estimated location-year with location- and year-specific estimates of measles CFR, measles deaths were calculated as:

$$deaths = incidence * CFR$$

This calculation was replicated at the draw level 1000 times, producing draw-level estimates of total measles deaths for each location and year, which were then split by age and sex using an age-sex distribution based on global-level age- and sex-specific patterns found in the cause of death data. All draw-level estimates were then summarised as the mean of the draws along with a 95% uncertainty interval (the 2.5th and 97.5th quantile of all draws).

Varicella

Flowchart

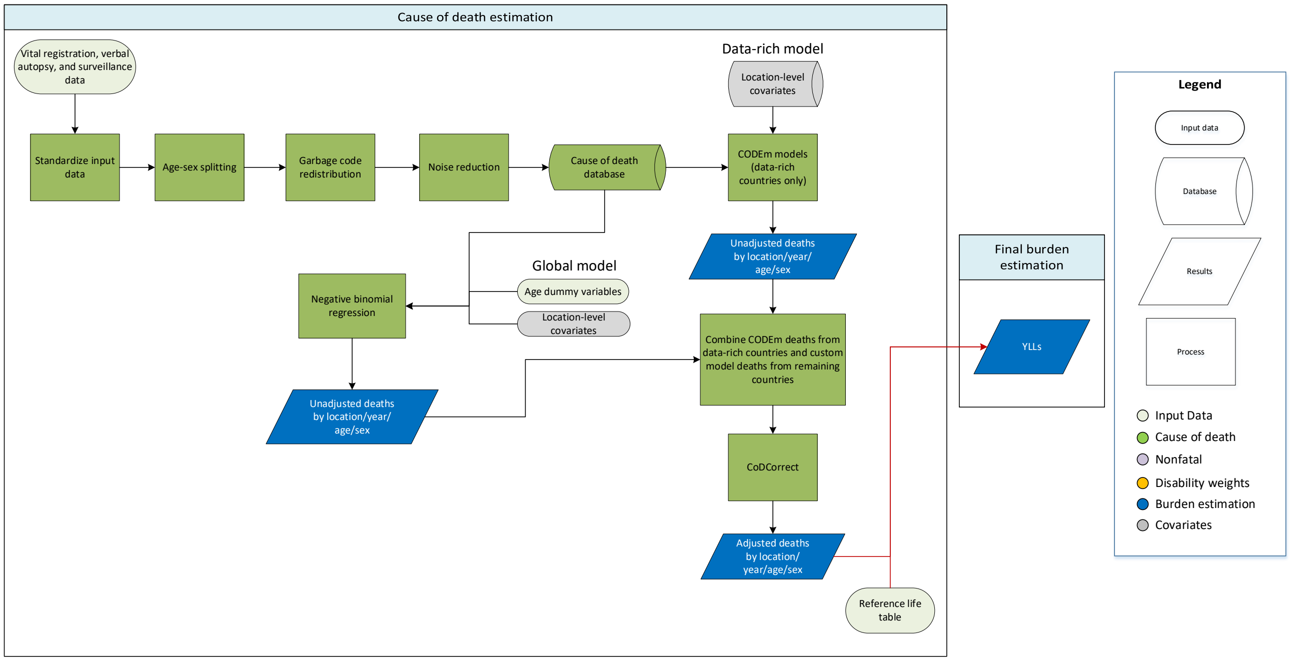

Input data

Varicella cause of death (COD) data for GBD 2019 included vital registration, verbal autopsy, and surveillance sources from all locations as available. We excluded COD data if they were highly incongruent with other available data from the same location or locations of similar sociodemographic characteristics.

Modelling strategy overview

We used two distinct methods to estimate varicella mortality based on the quality of vital registration data available for each country. We used a counts-based Cause of Death Ensemble modelling strategy (CODEm) for countries with well-defined vital registration (ie, "data-rich" countries), and for remaining countries a custom count negative binomial regression model. Each approach is further described in more detail below.

1. Data-rich countries

For data-rich countries, the covariates listed in Table 1 were used to inform CODEm predictions. New this cycle, all covariates were assigned prediction directions enforced during compilation of the ensemble. The Healthcare Access and Quality (HAQ) Index, Socio-demographic Index (SDI) and lag-distributed income (LDI) covariates were all reviewed and assigned a negative directional influence; the maternal care and immunisation (MCI) covariate was removed in favor of using HAQ and SDI to predict. In addition, age- and sex-specific summary exposure values (SEV) for child underweight were added to the model to replace the malnutrition proportion covariate used in prior GBD cycles. Age- and sex-

specific summary exposure values (SEV) for child wasting, mean years of education per capita, sanitation access proportion, and percentage population density over 1000 people per square kilometer covariates were also added to the model this GBD cycle, improving overall root mean square error (RMSE).

Table 1. Covariates. Summary of covariates used in the data-rich varicella cause of death model

Level	Covariate	Direction
1	Healthcare Access and Quality (HAQ) Index	-
	Age- and sex-specific SEV for child underweight	+
	Age- and sex-specific SEV for child wasting	+
3	Lag-distributed income (LDI)	-
	Mean years of education per capita	-
	Sanitation access (proportion)	-
	Population density over 1000 people per square kilometer (proportion)	+
	Socio-demographic Index (SDI)	-

2. Custom count model

Our custom counts mortality model for all non-data-rich locations also used COD data as available by location, and we used a negative binomial regression to model varicella mortality. We modelled counts of deaths due to varicella using the Healthcare Access and Quality (HAQ) Index and age dummy variables with the offset set to the location- year- age- and sex-specific populations:

$$Y_{ij} = \beta_0 + \beta_1 \, HAQ_{ij} + age_{a\,ij} + e_{ij},$$

where Y_{ij} is the log-transformed number of varicella deaths offset by population size; β_0 is the fixed-effect intercept; β_1 is the fixed-effects slope on location- and year-specific HAQ_{ij}; $age_{a\,ij}$ is a dummy variable for each GBD age group in the estimation; e_{ij} is the residual; i is the year; and j is the location. Uncertainty was estimated by taking 1000 samples of the predictions based on the variance-covariance matrix and a random sample of the dispersion parameter from a gamma distribution.

Acute Hepatitis

Flowchart

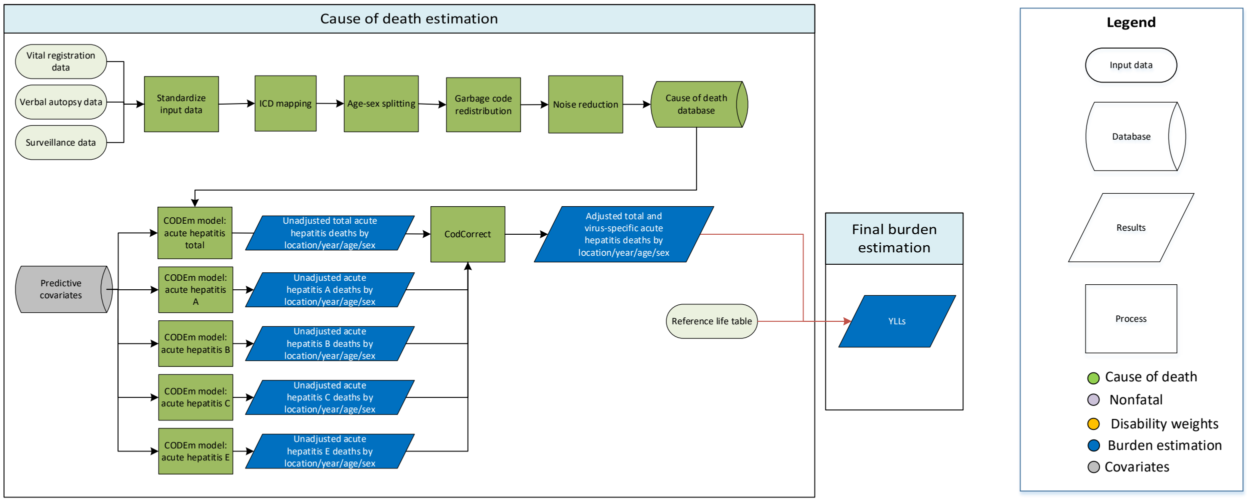

Input Data and Methodological Summary for Acute Hepatitis

"Acute hepatitis" in GBD methodology refers to acute viral hepatitis caused by the hepatitis A, B, C or E viruses.

Input data

We modelled acute hepatitis mortality using vital registration, surveillance, and verbal autopsy data from the cause of death database. We investigated the subset of our data from vital registration systems that allow recording multiple diagnostic codes as causes of death (underlying, intermediate, etc) and found that where a code for acute viral hepatitis was assigned as the underlying cause of death, ICD codes for chronic liver disease often appeared in the cause of death chain. This investigation revealed that hepatitis B unspecified deaths had a combination of cirrhosis or chronic liver disease in the underlying causes while some did not. As such, hepatitis B unspecified deaths were redistributed to mostly cirrhosis and other chronic liver diseases and a small proportion to acute hepatitis B, unlike in GBD2019 where all hepatitis B unspecified deaths were redistributed to cirrhosis deaths. The remaining acute viral hepatitis deaths were included in the database for total acute hepatitis; those that specified virus type (A, B, C, or E) were also assigned to separate databases by viral type. Unspecified acute viral hepatitis deaths were included in the database for total acute hepatitis and distributed proportionately to the databases for acute hepatitis due to hepatitis A, B, C and E. Additionally, acute delta infections of hepatitis B carrier deaths were mapped to acute hepatitis B.

Data points were marked as outliers and excluded if they reported an improbable number of acute hepatitis deaths. In some cases, multiple data sources for the same location differed dramatically both in their quality and reported acute hepatitis mortality (eg, a verbal autopsy and vital registration source). In these cases, the lower-quality data source was excluded.

Modeling strategy

The models used to estimate acute hepatitis mortality employed the GBD's standard approach of running two models - 1) a global CODEm model of all locations, using all data in the CoD database; and 2) a

CODEm model restricted to data-rich countries – and hybridizing the results. (See appendix section on CODEm method for details.)

We modeled acute hepatitis deaths encompassing all hepatitis virus types (A, B, C, and E) in a parent CODEm model and also modeled acute hepatitis A, B, C, and E in separate CODEm models. The virus-specific acute hepatitis deaths were then rescaled to fit within the envelope defined by the parent acute hepatitis CODEm model through the CoDCorrect process.

This modeling strategy was a substantive change from GBD2017. In that round, we developed a parent acute hepatitis mortality model using CODEm and all acute viral hepatitis deaths in the CoD database, similar to now. The deaths due to hepatitis A, B, C and E, however, were estimated in four separate natural history models that used incidence estimates from the nonfatal hepatitis A, B, C, and E models and case fatality ratios from hospital data. These virus-specific natural history models were then rescaled to fit the distribution of the parent model. The older approach relied on the assumption that case fatality ratios in hospital data could be applied to all acute hepatitis cases in the community.

The following are the covariates included in each model. Some covariates were changed in GBD 2019. We introduced a new covariate of injection drug use in the parent model and changed all-age seroprevalence covariates to age-standardized seroprevalence covariates.

Covariates used in parent acute hepatitis mortality modelling

Level	Covariate	Direction
1	SEV scalar age standardized hepatitis	+
	Seroprevalence (HBsAg) age standardized	+
	Seroprevalence (anti-HCV) age standardized	+
	Seroprevalence (anti-HAV) age standardized	+
	Seroprevalence (anti-HEV) age standardized	+
2	Health care access and quality index	-
	SEV unsafe sanitation	+
	SEV unsafe water	+
	Socio-demographic Index	-
	Hep B vaccine coverage proportion, aged through time	-
	Injection drug use proportion by age	+
3	Education (years per capita)	-
	Lag distributed income (LDI) (ln transformation)	-

Covariates used in acute hepatitis A mortality modelling

Level	Covariate	Direction
1	SEV scalar (hepatitis)	+
	Seroprevalence (anti-HAV) age standardized	+
2	Health care access and quality index	-
	SEV unsafe sanitation	+
	SEV unsafe water	+

	Socio-demographic Index	-
3	Education (years per capita)	-
	Lag distributed income (LDI) (ln transformation)	-

Covariates used in acute hepatitis B mortality modelling

Level	Covariate	Direction
1	SEV scalar (hepatitis)	+
	Seroprevalence (HBsAg) age standardized	+
2	Health care access and quality index	-
	Socio-demographic Index	-
	Hep B vaccine coverage proportion, aged through time	-
	Injection drug use proportion by age	+
3	Education (years per capita)	-
	Lag distributed income (LDI) (ln transformation)	-

Covariates used in acute hepatitis C mortality modelling

Level	Covariate	Direction
1	SEV scalar (hepatitis)	+
	Seroprevalence (anti-HCV) age standardized	+
2	Health care access and quality index	-
	Socio-demographic Index	-
	Injection drug use proportion by age	+
3	Education (years per capita)	-
	Lag distributed income (LDI) (ln transformation)	-

Covariates used in acute hepatitis E mortality modelling

Level	Covariate	Direction
1	SEV scalar (hepatitis)	+
	Seroprevalence (anti-HEV) age standardized	+
2	Health care access and quality index	-
	SEV unsafe sanitation	+
	SEV unsafe water	+
	Socio-demographic Index	-
3	Education (years per capita)	-
	Lag distributed income (LDI) (ln transformation)	-

Other unspecified infectious diseases

Flowchart

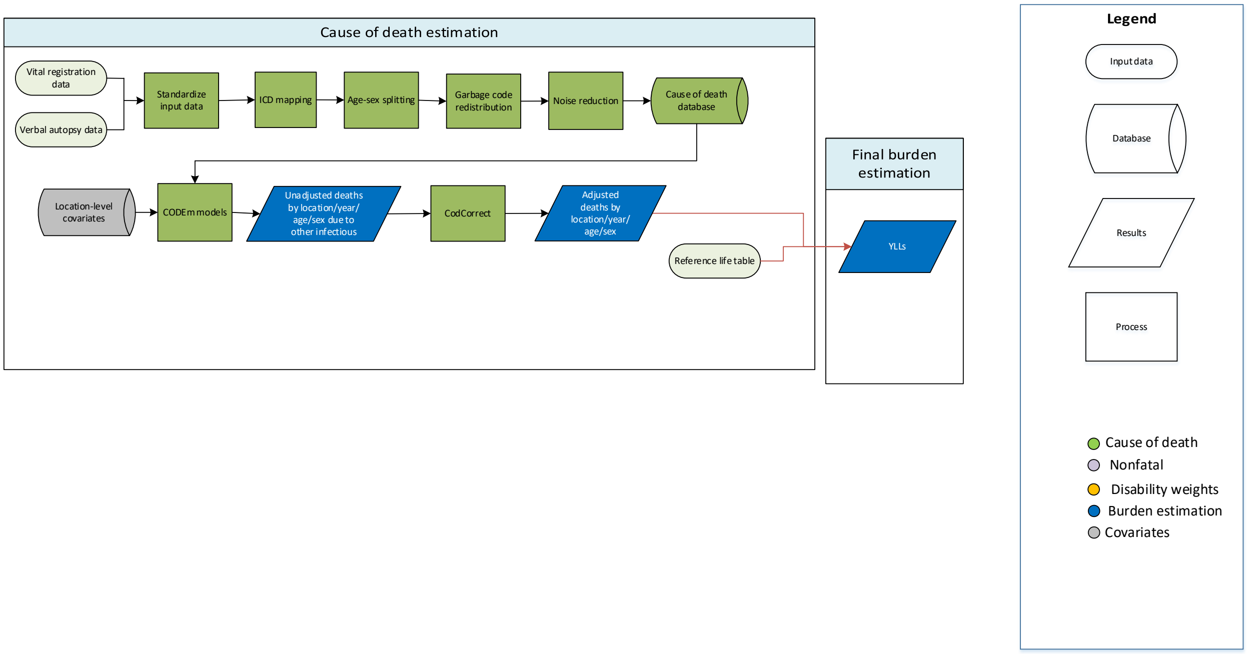

Input data and methodological summary for other unspecified infectious diseases

Input data

We modelled other infectious disease mortality using all available data in the cause of death database. Datapoints were outliered if they reported an improbable number of deaths or if their inclusion in the model yielded distorted trends.

Modelling strategy

We modelled other unspecified infectious disease mortality using a two-model hybrid approach: 1) a global CODEm model of all locations, using all data in the CoD database; and 2) a CODEm model restricted to data-rich countries. We have made no substantive changes to the modelling strategy since GBD 2017.

Table 1. Covariates used in other unspecified infectious diseases mortality modelling

Covariate name	Level	Direction
ANC proportion	3	-
DPT3 coverage	1	-
Sanitation proportion	2	-
Clear water proportion	2	-
Socio-demographic Index	3	-
Healthcare Access and Quality Index	2	-

Maternal disorders

Flowchart

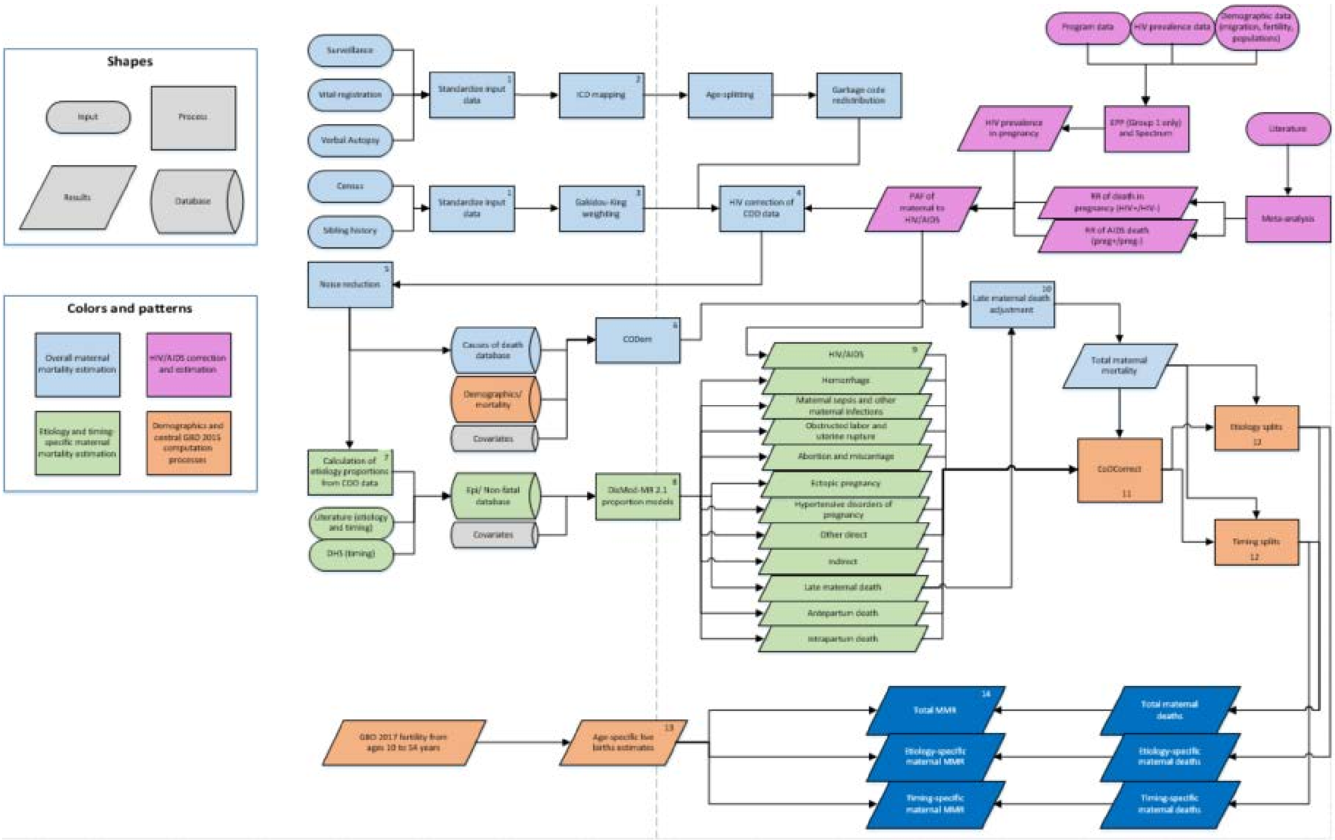

Input data

CODEm models of overall maternal mortality were informed by centrally prepped data stored in the cause of death (COD) database. All data were corrected for incidental HIV deaths by combining estimated HIV prevalence in pregnancy with relative risk (RR) of mortality during pregnancy for HIV-positive women to calculate a population attributable fraction (PAFs) that was then divided between incidental and maternal deaths based on RR of death in HIV-positive women during pregnancy. Incidental HIV deaths were removed from sibling history and census data, while maternal HIV deaths were added to vital registration, verbal autopsy, and surveillance data. This process is described in more detail in the appendix section on HIV/AIDS estimation.

For cause-specific maternal mortality, we used data from the COD database, other data sources and reports from the Global Health Data Exchange, and data from published studies identified through the search below. All data from all geographies were reviewed in CODEm models. Outliers were identified as those data where age patterns or temporal patterns were inconsistent with neighbouring age groups or locations or where sparse data were predicting implausible overall temporal or age patterns for a given location.

Our systematic literature review for maternal disorders is completed annually and encompasses all aspects of maternal disorder burden estimation including overall maternal mortality, cause-specific

maternal mortality, incidence of pregnancy complications by type, relative risk of mortality in pregnancy in HIV-positive versus HIV-negative women, and relative risk of mortality in HIV-positive women who are pregnant versus non-pregnant. We completed this search May 10, 2019, using the following search string:

> (((("Postpartum Hemorrhage" OR "Uterine Hemorrhage") OR (maternal[Title/Abstract] OR pregnan*[Title/Abstract] OR mothers) AND (haemorrhag*[Title/Abstract] OR hemorrhag*[Title/Abstract]) NOT "case report"[All fields]) OR (("induced abortion" OR "Therapeutic abortion" OR "legal Abortion" OR "medical abortion" OR "miscarriage" OR "Abortion, Induced"[Mesh] OR "Abortion, Therapeutic"[Mesh] OR "Abortion, Legal"[Mesh] OR "ectopic Pregnancy") NOT ("case report"[Title/Abstract] OR "birth defect"[Title/Abstract] OR congenital[Title/Abstract])) OR ("obstructed labour" OR "obstructed labor" OR "labour dystocia" OR "labor dystocia" OR dystocia OR "cephalopelvic disproportion" OR "cephalo-pelvic disproportion") OR (("obstetric fistula" OR "vesicovaginal fistula") OR "rectovaginal fistula") OR (("Puerperal Infection"[Mesh] OR "Puerperal Infection" OR ((maternal[Title/Abstract] OR pregnan*[Title/Abstract]) AND (Sepsis OR infection[Title/Abstract]))) NOT "case report") OR ((pre-eclampsia[Title/Abstract] OR preeclampsia[Title/Abstract] OR eclampsia[Title/Abstract] OR Pre-Eclampsia[Mesh] OR Eclampsia[Mesh] OR "Hypertension, Pregnancy-Induced"[Mesh] OR "pregnancy induced hypertension"[Title/Abstract] OR "gestational hypertension"[Title/Abstract] OR "Hypertensive disorders of pregnancy"[Title/Abstract]) NOT ("case report" OR "kidney donor"[Title/Abstract] OR "kidney donors"[Title/Abstract] OR polymorphism*[Title/Abstract] OR endotheli*[Title/Abstract]))) OR((("maternal mortality"[Title/Abstract] OR "maternal death"[Title/Abstract] OR "maternal deaths"[Title/Abstract] OR "MM"[Title/Abstract] OR "confidential enquiry"[Title/Abstract] OR "confidential inquiry"[Title/Abstract] OR ((obstetric[Title/Abstract] OR pregnan*[Title/Abstract]) AND (etiology[Title/Abstract] OR cause[Title/Abstract] OR pattern[Title/Abstract]) AND (death[Title/Abstract] OR mortality[Title/Abstract]))) NOT (fetal[Title/Abstract] OR newborn*[Title/Abstract] OR neonatal[Title/Abstract] OR "case report" [Title/Abstract] OR "case study" [Title/Abstract] OR pathogenesis[Title/Abstract] OR thromboprophylaxis[Title/Abstract])) OR ((("maternal mortality"[Title/Abstract] OR "maternal death"[Title/Abstract] OR "maternal deaths"[Title/Abstract] OR "MMR"[Title/Abstract]) AND ("Afghanistan"[Title/Abstract] OR "Albania"[Title/Abstract] OR "Algeria"[Title/Abstract] OR "Andorra"[Title/Abstract] OR "Angola"[Title/Abstract] OR "Antigua and Barbuda"[Title/Abstract] OR "Argentina"[Title/Abstract] OR "Armenia"[Title/Abstract] OR "Azerbaijan"[Title/Abstract] OR "Bahrain"[Title/Abstract] OR "Bangladesh"[Title/Abstract] OR "Barbados"[Title/Abstract] OR "Belarus"[Title/Abstract] OR "Belize"[Title/Abstract] OR "Benin"[Title/Abstract] OR "Bhutan"[Title/Abstract] OR "Bolivia"[Title/Abstract] OR "Bosnia and Herzegovina"[Title/Abstract] OR "Botswana"[Title/Abstract] OR "Brazil"[Title/Abstract] OR "Brunei"[Title/Abstract] OR "Bulgaria"[Title/Abstract] OR "Burkina Faso"[Title/Abstract] OR "Burundi"[Title/Abstract] OR "Cambodia"[Title/Abstract] OR "Cameroon"[Title/Abstract] OR "Cape Verde"[Title/Abstract] OR "Central African Republic"[Title/Abstract] OR "Chad"[Title/Abstract] OR "China"[Title/Abstract] OR "Colombia"[Title/Abstract] OR "Comoros"[Title/Abstract] OR "Congo"[Title/Abstract] OR "Costa Rica"[Title/Abstract] OR "Croatia"[Title/Abstract] OR "Cuba"[Title/Abstract] OR "Cyprus"[Title/Abstract] OR "Côte d'Ivoire"[Title/Abstract] OR "Democratic Republic of the Congo"[Title/Abstract] OR "Djibouti"[Title/Abstract] OR "Dominica"[Title/Abstract] OR "Dominican Republic"[Title/Abstract] OR "Ecuador"[Title/Abstract] OR "Egypt"[Title/Abstract] OR "El Salvador"[Title/Abstract] OR "Equatorial Guinea"[Title/Abstract] OR "Eritrea"[Title/Abstract] OR "Ethiopia"[Title/Abstract] OR "Federated States of Micronesia"[Title/Abstract] OR "Fiji"[Title/Abstract] OR "Gabon"[Title/Abstract] OR "Georgia"[Title/Abstract] OR "Ghana"[Title/Abstract] OR "Grenada"[Title/Abstract] OR "Guatemala"[Title/Abstract] OR "Guinea"[Title/Abstract] OR "Guinea-Bissau"[Title/Abstract] OR "Guyana"[Title/Abstract] OR "Haiti"[Title/Abstract] OR "Honduras"[Title/Abstract] OR "India"[Title/Abstract] OR "Indonesia"[Title/Abstract] OR "Iran"[Title/Abstract] OR "Iraq"[Title/Abstract] OR "Jamaica"[Title/Abstract] OR "Jordan"[Title/Abstract] OR "Kazakhstan"[Title/Abstract] OR "Kenya"[Title/Abstract] OR "Kiribati"[Title/Abstract] OR "Kuwait"[Title/Abstract] OR "Kyrgyzstan"[Title/Abstract] OR "Laos"[Title/Abstract] OR "Latvia"[Title/Abstract] OR "Lebanon"[Title/Abstract] OR "Lesotho"[Title/Abstract] OR "Liberia"[Title/Abstract] OR "Libya"[Title/Abstract] OR "Lithuania"[Title/Abstract] OR "Macedonia"[Title/Abstract] OR "Madagascar"[Title/Abstract] OR "Malawi"[Title/Abstract] OR "Malaysia"[Title/Abstract] OR "Maldives"[Title/Abstract] OR "Mali"[Title/Abstract] OR "Malta"[Title/Abstract] OR "Marshall Islands"[Title/Abstract] OR "Mauritania"[Title/Abstract] OR "Mauritius"[Title/Abstract] OR "Moldova"[Title/Abstract] OR "Mongolia"[Title/Abstract] OR "Montenegro"[Title/Abstract] OR "Morocco"[Title/Abstract] OR "Mozambique"[Title/Abstract] OR "Myanmar"[Title/Abstract] OR "Namibia"[Title/Abstract] OR

"Nepal"[Title/Abstract] OR "Nicaragua"[Title/Abstract] OR "Niger"[Title/Abstract] OR "Nigeria"[Title/Abstract] OR "North Korea"[Title/Abstract] OR "Oman"[Title/Abstract] OR "Pakistan"[Title/Abstract] OR "Palestine"[Title/Abstract] OR "Panama"[Title/Abstract] OR "Papua New Guinea"[Title/Abstract] OR "Paraguay"[Title/Abstract] OR "Peru"[Title/Abstract] OR "Philippines"[Title/Abstract] OR "Qatar"[Title/Abstract] OR "Romania"[Title/Abstract] OR "Russia"[Title/Abstract] OR "Rwanda"[Title/Abstract] OR "Saint Lucia"[Title/Abstract] OR "Saint Vincent and the Grenadines"[Title/Abstract] OR "Samoa"[Title/Abstract] OR "Saudi Arabia"[Title/Abstract] OR "Senegal"[Title/Abstract] OR "Serbia"[Title/Abstract] OR "Seychelles"[Title/Abstract] OR "Sierra Leone"[Title/Abstract] OR "Singapore"[Title/Abstract] OR "Solomon Islands"[Title/Abstract] OR "Somalia"[Title/Abstract] OR "South Africa"[Title/Abstract] OR "South Sudan"[Title/Abstract] OR "Sri Lanka"[Title/Abstract] OR "Sudan"[Title/Abstract] OR "Suriname"[Title/Abstract] OR "Swaziland"[Title/Abstract] OR "Syria"[Title/Abstract] OR "São Tomé and Principe"[Title/Abstract] OR "Taiwan"[Title/Abstract] OR "Tajikistan"[Title/Abstract] OR "Tanzania"[Title/Abstract] OR "Thailand"[Title/Abstract] OR "The Bahamas"[Title/Abstract] OR "The Gambia"[Title/Abstract] OR "Timor-Leste"[Title/Abstract] OR "Togo"[Title/Abstract] OR "Tonga"[Title/Abstract] OR "Trinidad and Tobago"[Title/Abstract] OR "Tunisia"[Title/Abstract] OR "Turkmenistan"[Title/Abstract] OR "Uganda"[Title/Abstract] OR "Ukraine"[Title/Abstract] OR "United Arab Emirates"[Title/Abstract] OR "Uruguay"[Title/Abstract] OR "Uzbekistan"[Title/Abstract] OR "Vanuatu"[Title/Abstract] OR "Venezuela"[Title/Abstract] OR "Vietnam"[Title/Abstract] OR "Yemen"[Title/Abstract] OR "Zambia"[Title/Abstract] OR "Zimbabwe"[Title/Abstract])) NOT ("demographic and health survey"[Title/Abstract] OR "demographic and health surveys "[Title/Abstract] OR DHS[Title/Abstract] OR "reproductive health survey"[Title/Abstract] OR "reproductive health surveys"[Title/Abstract] OR RHS[Title/Abstract])) OR ((HIV[Title/Abstract] OR "Acquired Immunodeficiency Syndrome"[Title/Abstract] OR AIDS[Title/Abstract]) AND (pregnan*[Title/Abstract] OR "postpartum"[Title/Abstract] OR "post partum"[Title/Abstract]) AND ("mortality"[Title/Abstract] OR "death"[Title/Abstract]) NOT "case report")) AND (2017/07/01[PDat] : 3000[PDat]) NOT (animals[MeSH] NOT humans[MeSH]))

A total of 12 964 literature sources were reviewed for their title and abstract. Of the 272 sources selected for full text review, 81 were extracted to inform maternal disorder models (fatal and non-fatal). There were no new sources extracted for maternal deaths aggravated by HIV. All cause-specific maternal mortality data were extracted as maternal mortality ratio (MMR; cause-specific deaths per live birth). All cause-specific COD data, along with any sources that reported cause-specific maternal deaths in cause fraction or population rate terms, were converted to MMR using all-cause mortality, population, and age-specific fertility results estimated in GBD 2019.

One exception was late maternal death, where only raw, unprocessed COD data were included from the COD database, and only for the subset of locations where the proportion of late maternal deaths coded in VR exceeded the lowest published rate from a comprehensive study.[1] Our assumption is that any location that has never reported a late maternal death in its VR does not capture any late maternal deaths. These data were supplemented with late maternal death data, all of which was extracted and prepped as proportion of the total. for the subset of locations where they were reliably coded in raw VR. All cause-specific MMR and proportion (late only) data were uploaded to the non-fatal database.

Modelling strategy

Overall maternal mortality

Overall maternal mortality was estimated with CODEm. Covariates included in this model, their level, and directionality are show in the table below:

Table 1: Covariates used in CODEm models of overall maternal mortality

Level	Covariate	Direction
Level 1	Age-specific fertility rate	+
	Total fertility rate (log-transformed)	+
	Maternal education (years per capita)	–
	In-facility delivery (proportion)	–
	Skilled birth attendance (proportion)	–
	Neonatal mortality ratio (log-transformed)	+
	Age-specific HIV mortality in females 10-54 (log-transformed)	+
Level 2	Antenatal care 1-visit coverage (proportion)	-
	Antenatal care 4-visits coverage (proportion)	-
	Age-standardised wasting (weight-for-height) summary exposure value (SEV)	+
	Age-standardised stunting (height-for-age) SEV	+
	Healthcare Access and Quality Index	-
	Age- and sex-specific SEV for high body-mass index (BMI)	+
	Age- and sex-specific SEV for high blood pressure (SBP)	+
	Underweight women of reproductive age	+
Level 3	Socio-demographic Index	-
	Mortality shock (cumulative rate in last 10 years)	+
	LDI (log-transformed)	-
	Hospital beds (per 1,000 population)	–

Cause-specific maternal mortality

We used spatiotemporal Gaussian process regression (ST-GPR) to estimate MMRs for each of the eight maternal subcauses. This modeling strategy requires data to be in standard GBD age groups. To achieve this, we used the global age pattern of the COD data for each cause and applied it to all data that were not in the standard GBD age groups. ST-GPR also requires variance for each datapoint. In order to compute variance, we ran a Lowess regression on the data by year and used the variance of the residuals resulting from the difference between the data and the predicted values.

The first step in the past has been a mixed-effects ordinary least squares regression of the quantity of interest and a specified set of location-level covariates. For GBD 2019 we revised this first step to instead be informed by an ensemble of regressions where weighting of each component model was based on out-of-sample coverage prediction performance. This approach allowed us to test a larger number of covariates and also specify the directionality of relationships between location-level covariates and the outcome of interest. Country covariates were specific for each subcause model, as shown in the table below:

Table 2: Covariates used in generation of ensemble stage 1 predictions of cause-specific maternal mortality ST-GPR models

Maternal subcause	Country-level covariates	Direction
Maternal haemorrhage	In-facility delivery (proportion)<br>Skilled birth attendance (proportion)<br>Age- and sex-specific SEV for unsafe sanitation<br>Neonatal mortality ratio (log-transformed)<br>Maternal education<br>Healthcare Access and Quality Index	-<br>-<br>+<br>+<br>-<br>-
Maternal hypertensive disorders	Age- and sex-specific SEV for fasting plasma glucose (FPG)<br>Age- and sex-specific SEV for high body-mass index (BMI)<br>Age- and sex-specific SEV for high blood pressure (SBP)<br>Neonatal mortality ratio (log-transformed)<br>Hospital beds (per 1000 population)<br>Antenatal care 1-visit coverage (proportion)<br>Antenatal care 4-visits coverage (proportion)<br>Healthcare Access and Quality Index	+<br>+<br>+<br>+<br>-<br>-<br>-<br>-
Obstructed labour and uterine rupture	In-facility delivery (proportion)<br>Skilled birth attendance (proportion)<br>Underweight women of reproductive age<br>Neonatal mortality ratio (log-transformed)<br>Hospital beds (per 1000 population)<br>Age-standardised wasting (weight-for-height) SEV<br>Age-standardised stunting (height-for-age) SEV	-<br>-<br>+<br>+<br>-<br>+<br>+
Abortion and miscarriage	Abortion legality<br>Antenatal care 1-visit coverage (proportion)<br>Antenatal care 4-visits coverage (proportion)<br>Hospital beds (per 1,000 population)<br>Maternal education<br>Healthcare Access and Quality Index	-<br>-<br>-<br>-<br>-<br>-
Ectopic pregnancy	Abortion legality<br>Pelvic inflammatory disease age-standardised prevalence<br>Antenatal care 1-visit coverage (proportion)<br>Antenatal care 4-visits coverage (proportion)<br>Hospital beds (per 1,000 population)<br>Maternal education<br>Healthcare Access and Quality Index	-<br>+<br>-<br>-<br>-<br>-<br>-
Maternal sepsis and other maternal infections	In-facility delivery (proportion)<br>Skilled birth attendance (proportion)<br>Age- and sex-specific SEV for unsafe sanitation<br>Age- and sex-specific SEV for fasting plasma glucose (FPG)<br>Antenatal care 1-visit coverage (proportion)<br>Antenatal care 4-visits coverage (proportion)<br>LDI (log-transformed)<br>Healthcare Access and Quality Index	-<br>-<br>+<br>+<br>-<br>-<br>-<br>-
Other maternal deaths	In-facility delivery (proportion)<br>Skilled birth attendance (proportion)<br>Antenatal care 1-visit coverage (proportion)<br>Antenatal care 4-visits coverage (proportion)	-<br>-<br>-<br>-

	LDI (log-transformed)<br>Age- and sex-specific SEV for high body-mass index (BMI)<br>Maternal education<br>Healthcare Access and Quality Index	-<br>+<br>-<br>-
Indirect maternal deaths	In-facility delivery (proportion)<br>Skilled birth attendance (proportion)<br>Antenatal care 1-visit coverage (proportion)<br>Antenatal care 4-visits coverage (proportion)<br>LDI (log-transformed)<br>Age- and sex-specific SEV for high body-mass index (BMI)<br>Maternal education<br>Healthcare Access and Quality Index	-<br>-<br>-<br>-<br>-<br>+<br>-<br>-

Late maternal death and model processing

Aetiology-specific estimates were derived by scaling the results from the ST-GPR subcause-specific models scaled in relation to each other to equal one and then multiplying them by the total maternal deaths, corrected for late maternal deaths, for that age group, location, and year. A single parameter proportion model was run in Dismod-MR 2.1 for late maternal deaths using the data described above. The proportions coming for the VR data sources were taken before any of the central data processing. We used the Healthcare Access and Quality Index as a country-level covariate for the model.

Neonatal disorders

Flowchart

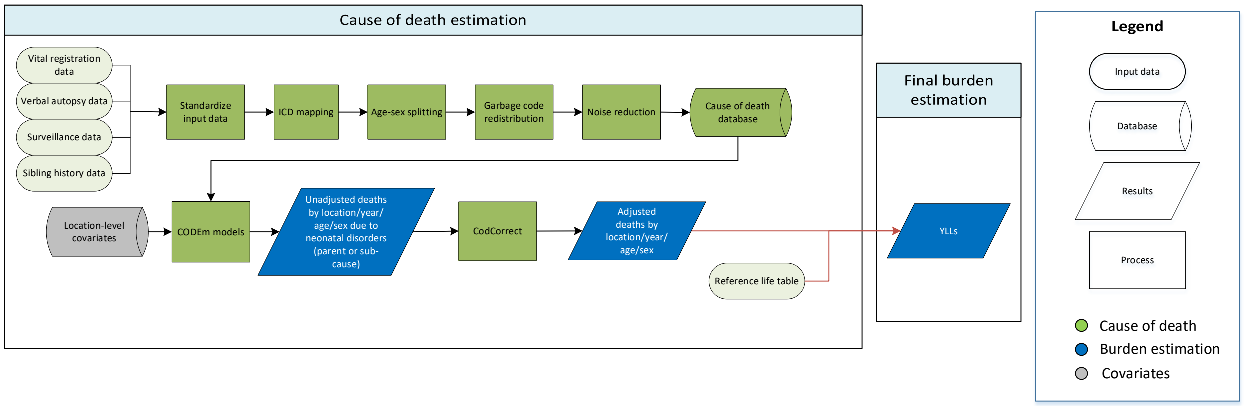

Input Data and Methodological Summary for Neonatal Disorders

Mortality for five causes are modeled within "neonatal disorders": neonatal preterm birth complications, neonatal encephalopathy due to birth asphyxia and trauma, neonatal sepsis and other neonatal infections, hemolytic disease and other neonatal jaundice, and other neonatal disorders. An overall neonatal disorders "parent" envelope is also estimated, to which all neonatal causes are squeezed.

Input data

Vital registration and surveillance were the majority of data sources used for GBD 2019 to estimate number of deaths from each condition. In Indian states, only verbal autopsy were used to inform estimates. Only deaths among males and females under age 5 were modelled, in four separate age groups: early neonatal period, late neonatal period, post-neonatal period, and 1-4 years. Data points were selected as outliers if they were implausibly high, low, or significantly conflicted with established age or temporal patterns. A significant new data source in GBD 2019 is Child Health and Mortality Prevention Surveillance (CHAMPS) in Bangladesh, Kenya, Mozambique, South Africa and Mali.

Modeling strategy

The standard CODEm modelling approach was used to model each of the neonatal conditions. Varying levels of data quality and coding issues may have affected our results. Validation studies suggest that verbal autopsy methods tend to be less accurate for cause of death ascertainment in the neonatal age groups.[1-4] Thus, for GBD 2019, except for the Indian states, the majority of verbal autopsy data were excluded. All neonatal causes used the following pool of covariates in covariate selection:

Table 1. Covariates used in neonatal disorders mortality modelling

Level	Covariate	Direction
1	Maternal care and immunization	-
	Age-standardized SEV for Ambient particulate matter	+
	Age-standardized SEV for Household air pollution	+
	Age-standardized SEV for Short gestation	+
	Age-standardized SEV for Low birth weight	+
	Age-standardized SEV for Smoking	+

2	Proportion of the population with at least 12 years of education, maternal	-
	Proportion of the population with at least 6 years of education, maternal	-
	Live Births 35+ (proportion)	+
	Socio-demographic Index	-
	Healthcare access and quality index	-
3	Antenatal Care (1 visit) Coverage (proportion)	-
	Antenatal Care (4 visits) Coverage (proportion)	-
	In-Facility Delivery (proportion)	-
	LDI (I$ per capita)	-
	Skilled Birth Attendance (proportion)	-
	Total Fertility Rate	+

References

1. Anker M, Black RE, Coldham C, *et al.* A Standard Verbal Autopsy Method for Investigating Causes of Death in Infants and Children. Geneva, Switzerland: World Health Organization Department of Communicable Disease Surveillance and Response; The Johns Hopkins School of Hygiene and Public Health; The London School of Hygiene and Tropical Medicine, 1999.

2. Kalter HD, Gray RH, Black RE, Gultiano SA. Validation of postmortem interviews to ascertain selected causes of death in children. *Int J Epidemiol* 1990; **19**: 380–6.

3. Quigley MA, Armstrong Schellenberg JR, Snow RW. Algorithms for verbal autopsies: a validation study in Kenyan children. *Bull World Health Organ* 1996; **74**: 147–54.

4. Snow RW, Armstrong JR, Forster D, *et al.* Childhood deaths in Africa: uses and limitations of verbal autopsies. *The Lancet* 1992; **340**: 351–5.

Nutritional deficiencies: *Parent nutritional deficiencies, protein-energy malnutrition, and other nutritional deficiencies*

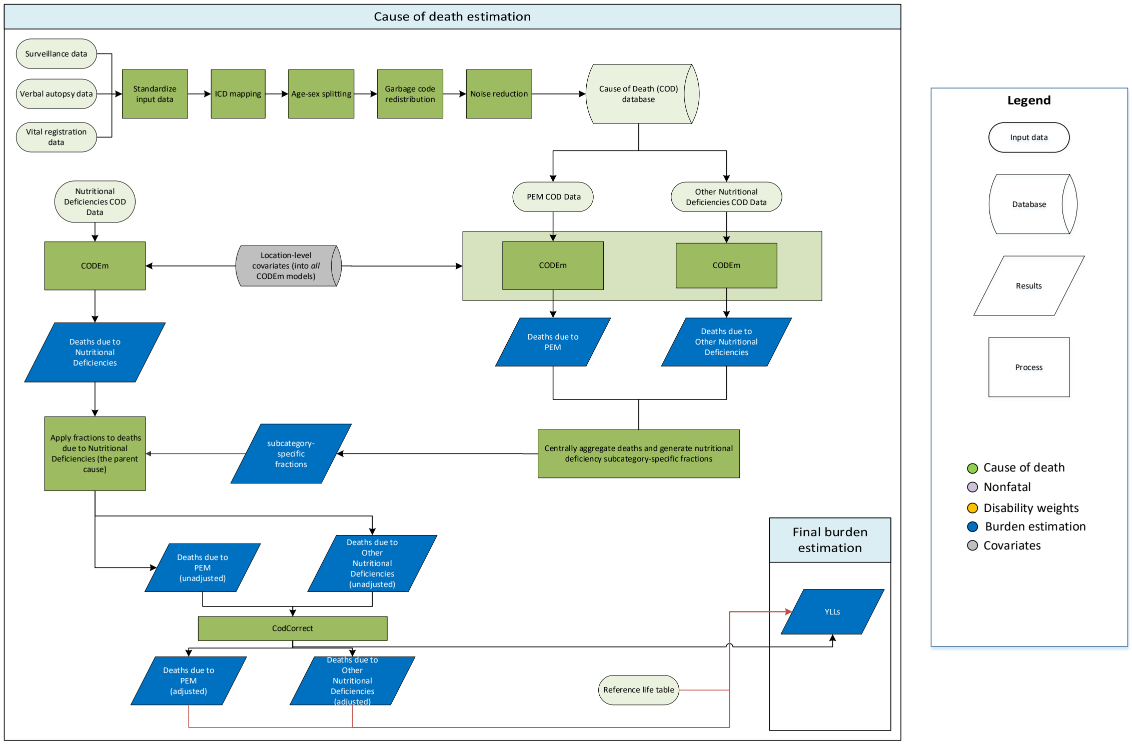

Input data and methodological summary for nutritional deficiencies

Input data

Vital registration (VR), verbal autopsy (VA), and surveillance data were used to model deaths due to nutritional deficiencies. We outliered data that were largely conflicting with the majority of data from other studies conducted either in the same countries or different countries (with similar socio-demographic characteristics) in the same region. ICD codes, which can be interpreted as case definitions, for each of the nutritional deficiencies are listed in Table 1 below.

Table 1. ICD-10 codes included in the nutritional deficiency models

GBD cause	ICD-10 code
Protein-energy malnutrition	E40-E46.9 (Kwashiorkor, marasmus, specified and unspecified protein-calorie malnutrition)
Other nutritional deficiencies	D51-D52.0 (vitamin B12 deficiency anaemia and folate deficiency anaemia)
Other nutritional deficiencies	D52.8-D53.9 (other nutritional anaemias)
Other nutritional deficiencies	D64.3 (other sideroblastic anaemias)

Other nutritional deficiencies	E51-E61.9 (thiamine, niacin, other B group vitamins, ascorbic acid, vitamin D, other vitamin, dietary calcium, dietary selenium, dietary zinc, and other nutrient element deficiencies)
Other nutritional deficiencies	E63-E64.0 (other nutritional deficiencies and sequelae of protein-calorie malnutrition)
Other nutritional deficiencies	E64.2-E64.9 (sequelae of vitamin C deficiency, rickets, other nutritional deficiencies, and unspecified nutritional deficiencies)
Other nutritional deficiencies	M12.1-M12.19 (Kashin-Beck disease)
Garbage code	D50, D50.0 and D50.9 (unspecified anaemia)

Modelling strategy

We estimated mortality for the nutritional deficiencies in two steps. CODEm was first used to generate mortality estimates for total nutritional deficiencies. The sub-categories of nutritional deficiencies, protein-energy malnutrition and other nutritional deficiencies, were modelled individually. Protein-energy malnutrition was modelled separately for age groups under 5 and over 5 so that the data trends and patterns in children under 5 were accurately captured. Estimates from the two nutritional sub-categories were then scaled at the 1000 draw level in CODCorrect to match that for total nutritional deficiencies.

Data and data processing methods were updated centrally by the cause of death team for GBD 2019. Of these changes, the VA data processing updates that resulted in lower VA input data, general noise reduction around VR data, and a decrease in the population envelope for children under 5 had the biggest impact on the nutritional deficiencies models. Additionally, the new methodology for the dementia misdiagnosis correction decreased the estimates of deaths attributed to dementia, therefore decreasing the number of deaths redistributed from nutritional deficiencies and increasing data estimates in the oldest ages for nutritional deficiencies. Apart from putting a definitive direction on every covariate, our team made no updates to the modelling strategy for fatal nutritional deficiency models this cycle. The CODEm covariates (including level and direction) used for each of the models are listed in the table below.

Table 2. Covariates used in mortality modelling

Nutritional deficiencies (overall)		
**Level**	**Covariate**	**Direction**
1	Age-standardised prevalence of severe anaemia	+
	Age-standardised SEV for child underweight	+
	Age-standardised SEV for child wasting	+
	Proportion of households using iodised salt	-
	Total kcal per person per day availability	-
2	Population living in the 1st world quintile (least) of annual rainfall	+
	Population living in the 2nd world quintile (2nd least) of annual rainfall	+
	Unsafe sanitation SEV	+
	Unsafe water SEV	+

	Log-transformed diarrhoeal diseases SEV	+
	Mortality rate due to war shocks	+
	Healthcare Access and Quality Index	-
	Age and sex-specific SEV for alcohol use	+
	Maternal care and immunisation	-
3	Education (years per capita)	-
	Lag-distributed income per capita	-
	Socio-demographic Index	-
	Maternal education (years per capita)	-
**Protein-energy malnutrition**		
**Level**	**Covariate**	**Direction**
1	Age-standardised prevalence of severe anaemia	+
	Total kcal per person per day availability	–
	Age-standardised SEV for child wasting	+
2	Population living in the 1st world quintile (least) of annual rainfall	+
	Population living in the 2nd world quintile (2nd least) of annual rainfall	+
	Unsafe sanitation SEV	+
	Unsafe water SEV	+
	Log-transformed diarrhoeal diseases SEV	+
	Mortality rate due to war shocks	+
	Healthcare Access and Quality Index	-
	Age and sex-specific SEV for alcohol use	+
	Maternal care and immunisation	-
3	Antenatal care (4 visits) coverage proportion	-
	Education (years per capita)	–
	Lag-distributed income per capita	–
	Socio-demographic Index	–
**Other nutritional deficiencies**		
**Level**	**Covariate**	**Direction**
1	Age-standardised prevalence of severe anaemia	+
	Total kcal per person per day availability	-
	Age-standardised SEV for child underweight	+
2	Population living in the 1st world quintile (least) of annual rainfall	+
	Population living in the 2nd world quintile (2nd least) of annual rainfall	+
	Unsafe sanitation SEV	+
	Unsafe water SEV	+
	Log-transformed diarrhoeal diseases SEV	+
	Mortality rate due to war shocks	+
	Healthcare Access and Quality Index	-
	Age and sex-specific SEV for alcohol use	+
	Maternal care and immunisation	-
3	Education (years per capita)	–
	Lag-distributed income per capita	–
	Socio-demographic Index	–

Cancers

Input data and methodological summary for all cancers except for non-melanoma skin cancer

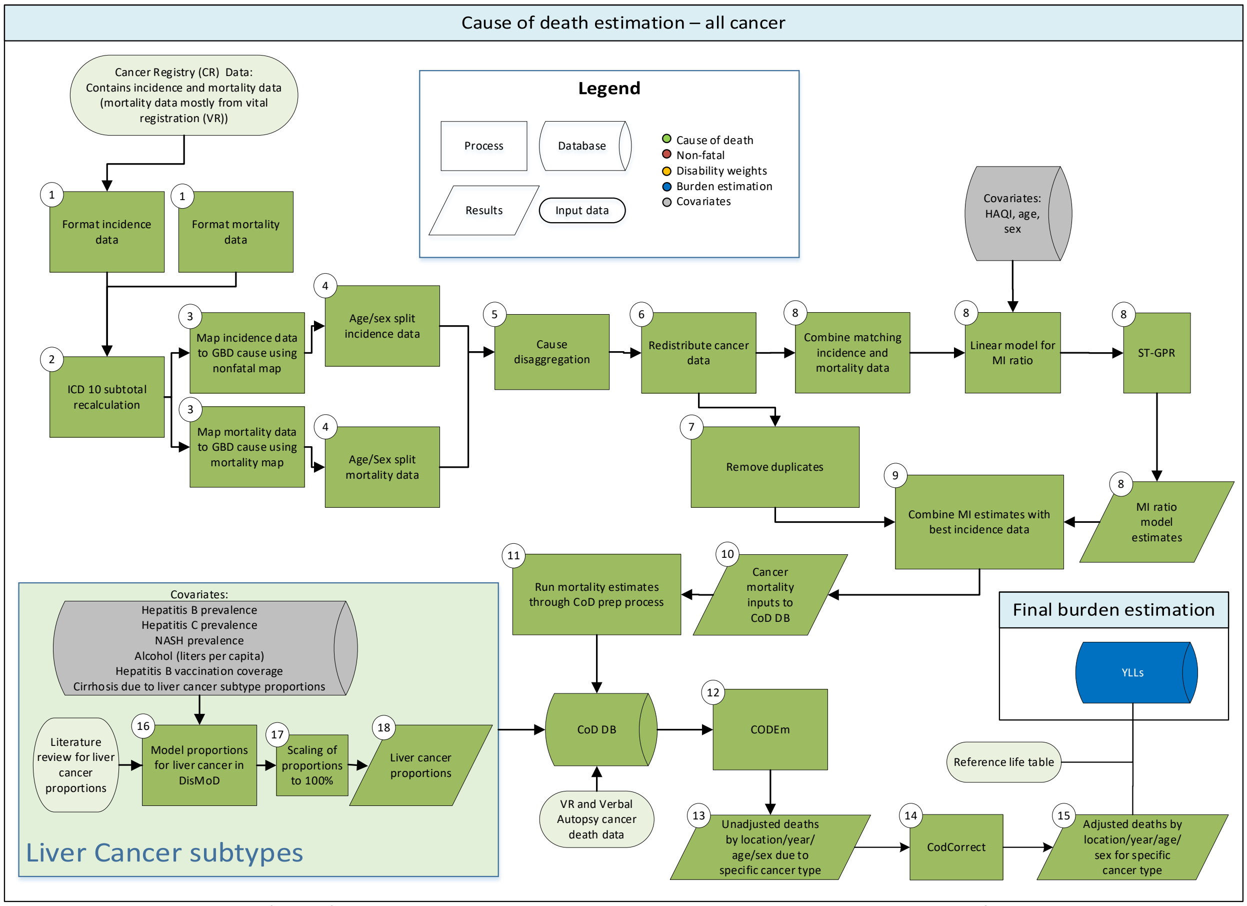

Abbreviations: ICD: International classification of diseases; DB: database, ST-GPR: Space-time smoothing, Gaussian process regression, COD: Causes of death

Data

The cause of death (COD) database contains multiple sources of cancer mortality data. These sources include vital registration, verbal autopsy, and cancer registry data. The cancer registry mortality estimates that are uploaded into the COD database stem from cancer registry incidence data that have been transformed to mortality estimates through the use of mortality-to-incidence ratios (MIR).

Data-seeking processes

Cancer mortality data in the cause of death database other than cancer registry data

Sources for cancer mortality data other than cancer registry data are described in the COD database description (Appendix Section 2.2).

Cancer registry data

Cancer registry data were used from publicly available sources or provided by collaborators. We used all data from GBD 2017 and added registry data from Argentina, Australia, Austria, Bermuda, Canada, Chile, China, Colombia, Germany, Netherlands, Switzerland, United Kingdom, Uruguay, and Yemen.

Inclusion and exclusion criteria

Only population-based cancer registries were included, and only those that included all cancers (no specialty registries), data for all age groups (except for paediatric cancer registries), and data for both sexes. Pathology-based cancer registries were included if they had a defined population. Hospital-based cancer registries were excluded.

Cancer registry data were excluded from either the final incidence data input or the MI model input if a more detailed source (eg, providing more detailed age or diagnostic groups) was available for the same population. Preference was given to registries with national coverage over those with only local coverage, except those from countries where the GBD study provides subnational estimates.

Data were excluded if the coverage population was unknown.

Bias of categories of input data

Cancer registry data can be biased in multiple ways. A high proportion of ill-defined cancer cases in the registry data requires redistribution of these cases to other cancers, which introduces a potential for bias. Changes between coding systems can lead to artificial differences in disease estimates; however, we adjust for this bias by mapping the different coding systems to the GBD causes. Underreporting of cancers that require advanced diagnostic techniques (eg, leukaemia, brain, pancreatic, and liver cancer) can be an issue in cancer registries from low-income countries. On the other hand, misclassification of metastatic sites as primary cancer can lead to overestimation of cancer sites that are common sites for metastases, like the brain or liver. Since many cancer registries are located in urban areas, the representativeness of the registry for the general population can also be problematic. The accuracy of mortality data reported in cancer registries usually depends on the quality of the vital registration system. If the vital registration system is incomplete or of poor quality, the mortality-to-incidence ratio can be biased to lower ratios.

Data for liver cancer aetiology splits

To find the proportion of liver cancer cases due to the five aetiology groups included in GBD (1. Liver cancer due to hepatitis B, 2. Liver cancer due to hepatitis C, 3. Liver cancer due to alcohol, 4. Liver cancer due to non-alcoholic steatohepatitis (NASH), 5. Liver cancer due to other causes), a systematic literature search was performed in PubMed on 10/24/2016 using the following search string: “("liver neoplasms"[All Fields] OR "HCC"[All Fields] OR "liver cancer"[All Fields] OR "Carcinoma, Hepatocellular"[Mesh]) AND (("hepatitis B"[All Fields] OR "Hepatitis B"[Mesh] OR "Hepatitis B virus"[Mesh] OR "Hepatitis B Antibodies"[Mesh] OR "Hepatitis B Antigens"[Mesh]) OR ("hepatitis C"[All Fields] OR "Hepatitis C"[Mesh] OR "hepatitis C antibodies"[MESH] OR "Hepatitis C Antigens"[Mesh] OR "Hepacivirus"[Mesh]) OR ("alcohol"[All Fields] OR "Alcohol Drinking"[Mesh] OR "Alcohol-Related Disorders"[Mesh] OR "Alcoholism"[Mesh] OR "Alcohol-Induced Disorders"[Mesh])) NOT (animals[MeSH] NOT humans[MeSH])”. Also, studies not found through this search but included in the meta-analysis by de Martel and colleagues were included.[10] We also included the study by Hong and colleagues after the authors provided us with additional data on the overlap in risk factors.[11]

Studies were included if the study population was representative of liver cancer for the respective location. For each study, the proportions of liver cancer due to the five specific risk factors were calculated. Cases were considered to be due to NASH when the manuscript explicitly listed the aetiology to be NASH or non-alcoholic fatty liver disease (NAFLD). Cases where the aetiology was listed as “cryptogenic”, “idiopathic”, or “unknown” were included within the “other causes” category. In

manuscripts where the aetiology for a case was not known but major categories could not be ruled out (for example, the study tested for hepatitis B and C, but did not assess alcohol use), these cases were excluded from the numerator of the study (in other words, did not contribute to the proportion of any aetiology). Remaining risk factors were included under a combined "other" group (for example, haemochromatosis, autoimmune hepatitis, Wilson's disease, etc.). If multiple risk factors were reported for an individual patient, these were apportioned proportionally to the individual risk factors. These estimated proportions are then used to split the overall liver cancer estimates into estimates for their respective aetiologies.

Methods

Steps of analysis and data transformation processes

Cancer registry data went through multiple processing steps before integration with the COD database. First, the original data were transformed into standardised files, which included standardisation of format, categorisation, and registry names (#1 in flowchart).

Second, some cancer registries report individual codes as well as aggregated totals (eg, C18, C19, and C20 are reported individually, but the aggregated group of C18-C20 [colorectal cancer] is also reported in the registry data). The data-processing step "subtotal recalculation" (#2 in flowchart) verifies these totals and subtracts the values of any individual codes from the aggregates.

In the third step (#3 in the flowchart), cancer registry incidence data and cancer registry mortality data are mapped to GBD causes. A different map is used for incidence data and for mortality data because of the assumption that there are no deaths for certain cancers. One example is basal-cell carcinoma of the skin. In the cancer registry incidence data, basal-cell carcinoma is mapped to "non-melanoma skin cancer (basal-cell carcinoma)". However, if basal-cell skin cancer is recorded in the cancer registry mortality data, the deaths are instead mapped to "non-melanoma skin cancer (squamous-cell carcinoma)" under the assumption that they were indeed squamous-cell skin cancers that had been misclassified as basal-cell skin cancers. Other examples are benign or in situ neoplasms. Benign or in situ neoplasms found in the cancer registry incidence dataset were simply dropped from that dataset. The same neoplasms reported in a cancer registry mortality dataset were mapped to the respective invasive cancer (eg, melanoma in situ in the cancer registry incidence dataset was dropped from the dataset; melanoma in situ in the cancer registry mortality dataset was mapped to melanoma).

In the fourth data-processing step (#4 in the flowchart) cancer registry data were standardised to the GBD age groups. Age-specific incidence rates were generated using all datasets that include microdata, and datasets that report age groups up to 95+ years of age, while age-specific mortality rates were generated from the CoD data through a method described in Appendix section 2.5. Age-specific proportions were then generated by applying the age-specific rates to a given registry population that required age-splitting to produce the expected number of cases/deaths for that registry by age. The expected number of cases/deaths for each sex, age, and cancer were then normalised to 1, creating final, age-specific proportions. These proportions were then applied to the total number of cases/deaths by sex and cancer to get the age-specific number of cases/deaths.

In the rare case that the cancer registry only contained data for both sexes combined, the now-age-specific cases/deaths were split and reassigned to separate sexes using the same weights that are used for the age-splitting process. Starting from the expected number of deaths, proportions were generated by sex for each age (eg, if for ages 15 to 19 years old there are six expected deaths for males and four expected deaths for females, then 60% of the combined-sex deaths for ages 15-19 years would be assigned to males and the remaining 40% would be assigned to females).

In the fifth step (#5 in the flowchart) data for cause entries that are aggregates of GBD causes were redistributed. Examples of these aggregated causes include some registries reporting ICD10 codes C00-C14 together as, "lip, oral cavity, and pharyngeal cancer." These groups were broken down into sub-causes that could be mapped to single GBD causes. In this example, those include lip and oral cavity cancer (C00-C08), nasopharyngeal cancer (C11), cancer of other parts of the pharynx (C09-C10, C12-C13), and "Malignant neoplasm of other and ill-defined sites in the lip, oral cavity, and pharynx" (C14). To redistribute the data, weights were created using the same "rate-applied-to-population" method employed in age-sex splitting (see step four above). For the undefined code (C14 in the example) an "average all cancer" weight was used, which was generated by adding all cases from SEER/NORDCAN/CI5 and dividing the total by the combined population. Then, proportions were generated by sub-cause for each aggregate cause as in the sex-splitting example above (see step four). The total number of cases from the aggregated group (C00-C14) was then recalculated for each subgroup and the undefined code (C14). C14 was then redistributed as a "garbage code" in step six. Distinct proportions were used for C44 (non-melanoma skin cancer) and C46 (Kaposi's sarcoma). Non-melanoma skin cancer processing is described under section "Input data and methodological summary for non-melanoma skin cancer (squamous-cell carcinoma)." C46 entries were redistributed as "other cancer" and HIV using proportions described in Appendix Section 2.

In the sixth step (#6 in the flowchart) unspecified codes ("garbage codes") were redistributed. Redistribution of cancer registry incidence and mortality data mirrored the process of the redistribution used in the cause of death database (Appendix Section 2.7).

In the seventh step (#7 in the flowchart) duplicate or redundant sources were removed from the processed cancer registry dataset. Duplicate sources were present if, for example, the cancer registry was part of the CI5 database but we also had data from the registry directly. Redundancies occurred and were removed as described in "Inclusion and Exclusion Criteria," where more detailed data were available, or when national registry data could replace regionally representative data. From here, two parallel selection processes were run to generate input data for the MI models and to generate incidence for final mortality estimation. When creating the final incidence input, higher priority was given to registry data from the most standardised source; whereas for the MI model input, only sources that reported both incidence and mortality were used.

In the eighth step (#8 in the flowchart) the processed incidence and mortality data from cancer registries were matched by cancer, age, sex, year, and location to generate MI ratios. These MI ratios were used as input for a three-step modelling approach using ST-GPR, with HAQ Index as a covariate in the linear step mixed effects model using a logit link function. Predictions were made without the random effects. The ST-GPR model has three main hyper-parameters that control for smoothing across time, age, and geography, which were adjusted for GBD 2019. The time adjustment parameter lambda

(λ) aims to borrow strength from neighbouring time points (ie, the exposure in this year is highly correlated with exposure in the previous year but less so further back in time). Lambda was lowered from 2 to 0.05, reducing the weight of more distant years. The age adjustment parameter omega (ω) borrows strength from data in neighbouring age groups and was set to 0.5 (unchanged). The space adjustment parameter zeta (ξ) aims to borrow strength across the hierarchy of geographical locations.[12] Zeta was lowered from 0.95 to 0.01, reducing the weight of more distant geographical data. For the remaining parameters in the Gaussian process regression, we lowered the amplitude from 2 to 1 (reducing fluctuation from the mean function) and reduced the scale value from 15 to 10 (reducing the time distance over which points are correlated). These model specification changes generally led to less smoothing of the data compared to GBD 2017 models.

Data-cleaning steps were similar as for GBD 2017. For each cancer, MI ratios from locations in HAQ quintiles 1-4 were dropped if they were below the median of MI ratios from locations in HAQ quintile 5. We also dropped MI ratios from locations in HAQ quintiles 1-4 if the MI ratios were above the third quartile + 1.5 * IQR (inter-quartile range). We dropped all MIR that were based on less than 15 (this was 25 in 2017) cases to avoid noise due to small numbers, except for mesothelioma and acute myeloid leukaemia, where we dropped MIR that were based on less than ten cases because of lower data availability for these two cancers. We also aggregated incidence and mortality to the youngest five-year age bin where SEER reported at least 50 cases from 1990 to 2015, to avoid unstable MIR predictions in young age groups on too few datapoints. The MIR in the minimum age-bin was used to backfill the MIR down to the lowest age group estimated for that cancer.

Since MI ratios can be above 1, especially in older age groups and cancers with low cure rates, we used the 95th percentile (by age group) of the cleaned dataset (detailed above) to cap the MIR input data. This "upper cap" was used to allow MIR over 1 but to constrain the MIR to a maximum level. To run the logit model, the input data were divided by the upper caps to get data from 0 to 1. Model predictions from ST-GPR were then rescaled back by multiplying them by the upper caps.
To constrain the MIRs at the lower end, we used the fifth percentile of the cancer and age-specific cleaned MIR input data to replace all model predictions with this lower cap.

Final MI ratios were matched with the cancer registry incidence dataset in the ninth step (#9 in the flowchart) to generate mortality estimates (Incidence * Mortality/Incidence = Mortality) (#10 in the flowchart). These mortality estimates are then smoothed by a Bayesian noise-reduction algorithm (to deal with problems with zero counts, as also applied to the VR and VA data) and uploaded into the COD database (#11 in the flowchart). Cancer-specific mortality modelling then followed the general CODEm process.

Liver cancer aetiology split models

The proportion data found through the systematic literature review were used as input for five separate DisMod-MR 2.1 models to determine the proportion of liver cancers due to the five subgroups for all locations, both sexes, all years, and all age groups (step #16 in the flowchart). For GBD 2019 we used MR-BRT to split sex-combined input data into sex-specific proportion data. For liver cancer due to hepatitis C and hepatitis B, a prior value of 0 was set between age 0 and 0.01. For liver cancer due to alcohol, a prior value of 0 was set for ages 0 to 5 years. For liver cancer due to hepatitis C, hepatitis C (IgG) seroprevalence was used as a covariate, forcing a positive relationship between the hepatitis C

seroprevalence covariate and the outcome of liver cancer due to hepatitis C proportion. For liver cancer due to hepatitis B, seroprevalence of HBsAg was used as a covariate as well as the population coverage of three-dose Hepatitis B vaccination, forcing a negative relationship between vaccination and the outcome of liver cancer due to hepatitis B proportion. For liver cancer due to alcohol, alcohol (litres per capita) was used as a covariate as well as a covariate for proportion of alcohol abstainers, forcing a negative relationship between the proportion of alcohol abstainers and the outcome of liver cancer due to alcohol proportion. For liver cancer due to NASH, NASH/NAFLD prevalence was used as a covariate as well as a covariate for obesity prevalence and mean body-mass index (BMI), forcing a positive relationship between these covariates and the outcome of liver cancer due to NASH proportion. All covariates used were modelled independently. To ensure consistency between cirrhosis and liver cancer estimates and to take advantage of the data for the respective other related cause (eg, liver cancer due to hepatitis C and the related cause cirrhosis due to hepatitis C), we generated covariates from the liver cancer proportion models that were subsequently used in separate cirrhosis aetiology proportion models. We then created covariates from the cirrhosis aetiology proportion models and used those in final liver cancer aetiology models.

Since the proportion models are run independently of each other, the final proportion models were scaled to sum to 100% within each age, sex, year, and location, by dividing each proportion by the sum of the five (step # 17). For the liver cancer subtype mortality estimates, we multiplied the parent cause "liver cancer" by the corresponding scaled proportions (step # 18). Single cause estimates were adjusted to fit into the separately modelled all-cause mortality envelope in the GBD-wide CoDCorrect process.

Results

Interpretation of results

Cancer mortality estimates for GBD 2019 can differ from the GBD 2017 results for multiple reasons. Updated cancer mortality data were added from vital registration system data, verbal autopsy studies, and cancer registry incidence data. Previously some deaths mapped to liver cancer contained deaths from liver metastases rather than primary liver cancer; for GBD 2019, these deaths were instead mapped as garbage codes and redistributed. The mortality-to-incidence ratio estimation was updated with lower case inclusion criteria and different model hyperparameters compared to GBD 2017, leading to more training data and less smoothing across time and geography. Covariates used in CODEm models were updated for GBD 2019. This included removing or replacing covariates that had been updated by other GBD teams (most of the dietary covariates), assigning a direction of association prior to all covariates (previously covariates such as income and Socio-demographic Index had been allowed to have agnostic direction priors), and changing the minimum age ranges for which the models estimated mortality. Compared to GBD 2017, large differences in the incidence and prevalence estimates for the benign and in-situ neoplasms is due to changes in how the clinical informatics data are processed for these causes. These data are now adjusted for HAQ Index and corrected for outpatient encounters, which should capture significantly more of these cases than before (since that relied on hospital admissions).

The other group producing country-level cancer mortality estimates is the International Agency for Research on Cancer (IARC) with their GLOBOCAN database. Significantly different methods between the GBD study and GLOBOCAN can lead to differences in results. Whereas estimates in GLOBOCAN are based on the assumption that there are "In theory, […] as many methods as countries,"[13] the cancer

estimation process for the GBD study follows a coherent, well-documented method for all cancers, which allows cross-validation of models as well as determination of uncertainty. Another major difference is the ability in the GBD study to adjust single cause estimates to the all-cause mortality, which is being determined independently. This also allows us to adjust individual causes of death to the all-cause mortality envelope, which permits us to correct for the underdiagnosis of cancer in countries with inadequate diagnostic resources. Redistribution of a fraction of undefined causes of death to certain cancers is another methodological advantage the GBD study has over GLOBOCAN, and estimates for cancer mortality can therefore differ substantially in countries with a large proportion of undefined causes of deaths in their vital registration data or a large proportion of undefined cancer cases in their cancer registry data.

Limitations

There are certain limitations to consider when interpreting the GBD mortality cancer estimates. First, even though every effort is made to include the most recently available data for each country, data-seeking resources are not limitless and new data cannot always be accessed as soon as they are made available. It is therefore possible that the GBD study does not include all available data sources for cancer incidence or cancer mortality. Second, different redistribution methods can potentially change the cancer estimates substantially if the data sources used for the estimated location contain a large number of undefined causes; however, neglecting to account for these undefined deaths would likely introduce an even greater bias in the disease estimates. Third, using mortality-to-incidence ratios to transform cancer registry incidence data to mortality estimates requires accurate MIR. For GBD 2019 we have made further changes to the MIR estimation, but the method remains sensitive to underdiagnosis of cancer cases or under-ascertainment of cancer deaths. However, given that the majority of data used for the cancer mortality estimation come from vital registration data and not cancer registry data, this is not a major limitation.

Non-melanoma skin cancer (squamous-cell carcinoma)

Data

Data-seeking processes

Since squamous-cell carcinomas are only very infrequently recorded by cancer registries, only vital registration system data were used as input for the squamous-cell carcinoma mortality modelling.

Inclusion and exclusion criteria

Inclusion and exclusion criteria followed the same methods as described for the vital registration data sources (Appendix Section 2).

Bias of categories of input data

The potential biases of the input data are the same as for other cancers (see above).

Methods

Overall methodological process

Vital registration system data were used as input to model deaths due to squamous-cell skin cancer.

Steps of analysis and data transformation processes

Since mortality estimates for non-melanoma skin cancer are only produced for squamous-cell carcinoma

under the assumption that basal-cell carcinoma causes almost no deaths, all mortalities reported as "C44" or "173" were mapped to the "squamous-cell carcinoma" GBD cause.

Model selection

The modelling strategy for non-melanoma skin cancer (squamous-cell carcinoma) followed the general CODEm process.

Model performance and sensitivity

The modelling performance and sensitivity for non-melanoma skin cancer (squamous-cell carcinoma) mirrored that of the general CODEm process.

Uncertainty intervals

Uncertainty was determined using standard CODEm methodology.

Results

Interpretation of results

Non-melanoma skin cancer mortality estimates are not available from other sources. GLOBOCAN, for example, does not report deaths due to non-melanoma skin cancer. Even though the data availability for non-melanoma skin cancer is poor, the fact that it is the most common incident cancer, with rates expected to rise, makes it a necessity to include the disease in the GBD framework.

Limitations

Cancer registry data for non-melanoma skin cancer incidence have to be interpreted with caution due to a substantial amount of underreporting or rules that only the first non-melanoma skin cancer has to be registered. Many cancer registries therefore do not include non-melanoma skin cancers at all. However, the information if registries capture NMSC or not is not consistently available. Therefore, no cancer registry data were used to estimate deaths due to squamous-cell carcinoma of the skin. For vital registration data, we make the assumption that there are no deaths due to basal-cell non-melanoma skin cancer, and therefore all deaths attributed to basal-cell carcinoma were included instead as squamous-cell carcinoma.

Covariates by cancer:

Lip and oral cavity cancer

Level	Covariate	Direction
1	*Litres of alcohol consumed per capita*	+
	Cumulative cigarettes (10 years)	+
	Cumulative cigarettes (20 years)	+
	Tobacco (cigarettes per capita)	+
	Log-transformed SEV scalar: Mouth Cancer	+
2	*Age- and sex-specific SEV for high red meat*	+
	Age- and sex-specific SEV for low vegetables	+
	Age- and sex-specific SEV for low fruit	+
	Healthcare Access and Quality Index	–
3	*Education (years per capita)*	–
	LDI (I$ per capita)	+
	Socio-demographic Index	+

Nasopharynx cancer

Level	Covariate	Direction
1	*Litres of alcohol consumed per capita*	+
	Cumulative cigarettes (10 years)	+
	Cumulative cigarettes (20 years)	+
	Tobacco (cigarettes per capita)	+
	Log-transformed SEV scalar: Nasopharynx Cancer	+
2	*Age- and sex-specific SEV for low vegetables*	+
	Population density (over 1000 ppl/sqkm, proportion)	+
	Healthcare Access and Quality Index	–
3	*Education (years per capita)*	–
	Age- and sex-specific SEV for low fruit	+
	LDI (I$ per capita)	-
	Socio-demographic Index	+

Oesophageal cancer

Level	Covariate	Direction
1	*Litres of alcohol consumed per capita*	+
	Log-transformed age-standardised SEV scalar: Oesophageal Cancer	+
	Mean BMI	+
	Smoking prevalence	+
2	*Indoor air pollution (all cooking fuels)*	+
	Tobacco (cigarettes per captia)	+
	Age- and sex-specific SEV for low vegetables	+
	Age- and sex-specific SEV for low fruit	+
	Healthcare Access and Quality Index	–
3	*Education (years per capita)*	–
	Sanitation (proportion with access)	–
	Improved water source (proportion with access)	–
	LDI (I$ per capita)	+
	Socio-demographic Index	+

Other pharynx cancer

Level	Covariate	Direction
1	*Litres of alcohol consumed per capita*	+
	Smoking prevalence	+
	Log-transformed SEV scalar: Other Pharynx Cancer	+
2	*Cumulative cigarettes (5 years)*	+
	Age- and sex-specific SEV for low fruit	+
	Age- and sex-specific SEV for low vegetables	+
	Population density (over 1000 ppl/sqkm, proportion)	+
	Population density (under 150 ppl/sqkm, proportion)	+
	Healthcare Access and Quality Index	–
3	*Education (years per capita)*	–
	LDI (I$ per capita)	+
	Socio-demographic Index	+

Stomach cancer

Level	Covariate	Direction
1	*Diet high in sodium*	+
	Tobacco (cigarettes per capita)	+
	Log-transformed SEV scalar: Stomach Cancer	+
	Log-transformed SEV scalar: Stomach Cancer	+
2	*Cumulative cigarettes (20 years)*	+
	Age- and sex-specific SEV for unsafe water	+
	Age- and sex-specific SEV for unsafe sanitation	+
	Mean BMI	+
	Sanitation (proportion with access)	–
	Improved water source (proportion with access)	–
	Healthcare Access and Quality Index	–
3	*Education (years per capita)*	–
	Age- and sex-specific SEV for low fruits	+
	Age- and sex-specific SEV for low vegetables	+
	LDI (I$ per capita)	+
	Socio-demographic Index	–

Testicular cancer

Level	Covariate	Direction
2	*Cumulative cigarettes (5 years)*	+
	Cumulative cigarettes (10 years)	+
	Cumulative cigarettes (15 years)	+
	Cumulative cigarettes (20 years)	+
	Tobacco (cigarettes per capita)	+
	Smoking prevalence	+
	Age- and sex-specific SEV for low fruits	+
	Age- and sex-specific SEV for low vegetables	+
	Healthcare Access and Quality Index	–
3	*Education (years per capita)*	–
	LDI (I$ per capita)	+
	Socio-demographic Index	+

Liver Cancer

Level	*Covariate*	*Direction*
1	*Litres of alcohol consumed per capita*	+
	HIV age-standardised prevalence	+
	Hepatitis B seroprevalence (HBsAg) age-standardised	+
	Hepatitis C seroprevalence (anti-HCV) age-standardised	+
	Log-transformed SEV scalar: Liver Cancer	+
2	*Hepatitis B 3-dose coverage (proportion)*	–
	Hepatitis B vaccine coverage (proportion), aged through time	–
	Intravenous drug use (age-standardised proportion)	+
	Cumulative cigarettes (20 years)	+
	Mean BMI	+
	Tobacco (cigarettes per capita)	+
	Healthcare Access and Quality Index	–
	Diabetes fasting plasma glucose (mmol/L), age-standardised 25+	+

Liver cancer (continued)

Level	*Covariate*	*Direction*
3	*Education (years per capita)*	–
	Age- and sex-specific SEV for high red meat	+
	LDI (I$ per capita)	–
	Socio-demographic Index	–

Gallbladder and biliary tract cancer

Level	*Covariate*	*Direction*
1	*Log-transformed SEV scalar: Gallbladder Cancer*	+
	Mean BMI	+
2	*Litres of alcohol consumed per capita*	+
	Cumulative cigarettes (5 years)	+
	Cumulative cigarettes (10 years)	+
	Smoking prevalence	+
	Tobacco (cigarettes per capita)	+
	Age- and sex-specific SEV for low fruit	+
	Age- and sex-specific SEV for low vegetables	+
	Diabetes age-atandardised prevalence (proportion)	+
	Healthcare Access and Quality Index	–
3	*Education (years per capita)*	–
	LDI (I$ per capita)	+
	Socio-demographic Index	-

Pancreatic cancer

Level	Covariate	Direction
1	*Cumulative cigarettes (10 years)*	+
	Cumulative cigarettes (20 years)	+
	Tobacco (cigarettes per capita)	+
	Log-transformed SEV scalar: Pancreas Cancer	+
	Mean BMI	+
2	*Age- and sex-specific SEV for high red meat*	+
	Litres of alcohol consumed per capita	+
	Age- and sex-specific SEV for low vegetables	+
	Energy unadjusted (kcal)	+
	Diabetes fasting plasma glucose (mmol/L), age-standardised 25+	+
	Diabetes age-standardised prevalence (proportion)	+
	Healthcare Access and Quality Index	–
3	*Education (years per capita)*	–
	Age- and sex-specific SEV for low fruit	+
	LDI (I$ per capita)	+
	Socio-demographic Index	+

Larynx cancer

Level	Covariate	Direction
1	*Litres of alcohol consumed per capita*	+
	Log-transformed SEV scalar: Larynx Cancer	+
2	*Smoking prevalence*	+
	Asbestos consumption (metric tons per year per capita)	+
	Age- and sex-specific SEV for low vegetables	+
	Cumulative cigarettes (10 years)	+
	Cumulative cigarettes (20 years)	+
	Population density (over 1000 ppl/sqkm, proportion)	+
	Healthcare Access and Quality Index	–
3	*Age- and sex-specific SEV for low fruit*	+
	LDI (I$ per capita)	+
	Socio-demographic Index	+

Tracheal, bronchus, and lung cancer

Level	Covariate	Direction
1	*Asbestos consumption (metric tons per year per capita)*	+
	Smoking prevalence	+
	Secondhand smoke	+
	Log-transformed SEV scalar: Lung Cancer	+
	Log-transformed age-standardised SEV scalar: Lung Cancer	+
2	*Indoor air pollution (all cooking fuels)*	+
	Cumulative cigarettes (10 years)	+
	Cumulative cigarettes (20 years)	+
	Outdoor air pollution ($PM_{2.5}$)	+
	Residential radon	+
	Diabetes fasting plasma glucose (mmol/L), age-standardised 25+	+
	Healthcare Access and Quality Index	–
3	*Education (years per capita)*	–
	LDI (I$ per capita)	+
	Socio-demographic Index	+

Malignant skin melanoma

Level	Covariate	Direction
1	Litres of alcohol consumed per capita	+
2	Latitude under 15 (proportion)	–
	Latitude 15 to 30 (proportion)	–
	Latitude 30 to 45 (proportion)	–
	Latitude over 45 (proportion)	–
	Healthcare Access and Quality Index	–
3	Education (years per capita)	–
	LDI (I$ per capita)	–
	Socio-demographic Index	+

Non-melanoma skin cancer

Level	Covariate	Direction
1	Cumulative cigarettes (5 years)	+
	Cumulative cigarettes (10 years)	+
	Cumulative cigarettes (15 years)	+
	Smoking prevalence	+
2	Average latitude	–
	Healthcare Access and Quality Index	–
3	Education (years per capita)	–
	LDI (I$ per capita)	–
	Socio-demographic Index	+

Breast cancer

Level	Covariate	Direction
1	Litres of alcohol consumed per capita	+
	Mean BMI	+
	Log-transformed SEV scalar: Breast Cancer	+
2	Age-specific fertility rate	–
	Total fertility rate	–
	Age- and sex-specific SEV for low fruit	+
	Age- and sex-specific SEV for low vegetables	+
	Cumulative cigarettes (10 years)	+
	Cumulative cigarettes (20 years)	+
	Smoking prevalence	+
	Diabetes fasting plasma glucose (mmol/L), age-standardised 25+	+
	Healthcare Access and Quality Index	–
3	LDI (I$ per capita)	–
	Socio-demographic Index	+

Cervical cancer

Level	Covariate	Direction
1	Cumulative cigarettes (5 years)	+
	HIV age-standardised prevalence	+
2	Age-specific fertility rate	+
	Total fertility rate	+
	Smoking prevalence	+
	Age- and sex-specific SEV for low fruit	+
	Age- and sex-specific SEV for low vegetables	+
	Healthcare Access and Quality Index	–
3	Education (years per capita)	–
	LDI (I$ per capita)	–
	Socio-demographic Index	–

Uterine cancer

Level	Covariate	Direction
1	Log-transformed SEV scalar: Uterus Cancer	+
	Mean BMI	+
2	Cumulative cigarettes (5 years)	+
	Cumulative cigarettes (10 years)	+
	Smoking prevalence	+
	Tobacco (cigarettes per capita)	+
	Diabetes age-standardized prevalence (proportion)	+
	Total fertility rate	–
	Age- and sex-specific SEV for low fruit	+
	Age- and sex-specific SEV for low vegetables	+
	Healthcare Access and Quality Index	–
3	Education (years per capita)	–
	LDI (I$ per capita)	+
	Socio-demographic Index	+

Prostate cancer

Level	Covariate	Direction
1	Log-transformed SEV scalar: Prostate Cancer	+
2	Smoking prevalence	+
	Healthcare Access and Quality Index	–
3	Education (years per capita)	–
	LDI (I$ per capita)	–
	Socio-demographic Index	+

Kidney cancer

Level	Covariate	Direction
1	*Cumulative cigarettes (5 years)*	+
	Cumulative cigarettes (10 years)	+
	Cumulative cigarettes (15 years)	+
	Mean BMI	+
	Log-transformed SEV scalar: Kidney Cancer	+
2	*Litres of alcohol consumed per capita*	+
	Diabetes age-standardised prevalence (proportion)	+
	Systolic blood pressure (mmHg)	+
	Smoking prevalence	+
	Healthcare Access and Quality Index	–
3	*Education (years per capita)*	–
	LDI (I$ per capita)	+
	Socio-demographic Index	+

Bladder cancer

Level	Covariate	Direction
1	*Schistosomiasis prevalence (proportion)*	+
	Cumulative cigarettes (10 years)	+
	Smoking prevalence	+
	Log-transformed SEV scalar: Bladder Cancer	+
2	*Litres of alcohol consumed per capita*	+
	Diabetes fasting plasma glucose (mmol/L), age-standardised 25+	+
	Age- and sex-specific SEV for low vegetables	+
	Healthcare Access and Quality Index	–
3	*Age- and sex-specific SEV for low fruits*	+
	LDI (I$ per capita)	+
	Socio-demographic Index	+

Brain and nervous system cancer

Level	Covariate	Direction
1	*Litres of alcohol consumed per capita*	+
	Cumulative cigarettes (10 years)	+
	Smoking prevalence	+
2	*Cholesterol (total, mean per capita)*	+
	Systolic blood pressure (mmHg)	+
	Age- and sex-specific SEV for high red meat	+
	Age- and sex-specific SEV for low vegetables	+
	Age- and sex-specific SEV for low fruit	+
	Healthcare Access and Quality Index	–
3	*Education (years per capita)*	–
	LDI (I$ per capita)	+
	Socio-demographic Index	+

Thyroid cancer

Level	Covariate	Direction
1	*Litres of alcohol consumed per capita*	+
	Log-transformed SEV scalar: Thyroid Cancer	+
2	*Age- and sex-specific SEV for low vegetables*	+
	Age- and sex-specific SEV for high red meat	+
	Tobacco (cigarettes per capita)	+
	Mean BMI	+
	Healthcare Access and Quality Index	–
3	*Education (years per capita)*	–
	Sanitation (proportion with access)	–
	Improved water source (proportion with access)	–
	Age- and sex-specific SEV for low fruits	+
	LDI (I$ per capita)	+
	Socio-demographic Index	+

Mesothelioma

Level	Covariate	Direction
1	*Asbestos consumption (metric tons per year per capita)*	+
	Cumulative cigarettes (5 years)	+
	Log-transformed SEV scalar: Mesothelioma	+
	Log-transformed age-standardized SEV scalar: Mesothelioma	+
	Smoking prevalence	+
2	*Gold production (binary)*	+
	Indoor air pollution (all cooking fuels)	+
	Population density (over 1000 ppl/sqkm, proportion)	+
	Healthcare Access and Quality Index	–
3	*Education (years per capita)*	–
	LDI (I$ per capita)	–
	Socio-demographic Index	+

Hodgkin lymphoma

Level	Covariate	Direction
2	*Healthcare Access and Quality Index*	–
3	*Education (years per capita)*	–
	LDI (I$ per capita)	–
	Socio-demographic Index	–

Non-Hodgkin lymphoma

Level	Covariate	Direction
2	Cumulative cigarettes (5 years)	+
	Cumulative cigarettes (10 years)	+
	Cumulative cigarettes (15 years)	+
	Cumulative cigarettes (20 years)	+
	Litres of alcohol consumed per capita	+
	Smoking prevalence	+
	Mean BMI	+
	Healthcare Access and Quality Index	–
3	Total fertility rate	–
	LDI (I$ per capita)	+
	Socio-demographic Index	+

Multiple myeloma

Level	Covariate	Direction
1	Litres of alcohol consumed per capita	+
	Smoking prevalence	+
	Tobacco (cigarettes per capita)	+
2	Age- and sex-specific SEV for low vegetables	+
	Age- and sex-specific SEV for low fruits	+
	Age- and sex-specific SEV for high red meat	+
	Mean BMI	+
	Sanitation (proportion with access)	–
	Improved water source (proportion with access)	–
	Healthcare Access and Quality Index	–
3	Education (years per capita)	–
	LDI (I$ per capita)	+
	Socio-demographic Index	+

Leukaemia

Level	Covariate	Direction
1	Log-transformed age-standardised SEV scalar: Leukaemia	+
	Log-transformed SEV scalar: Leukaemia	+
2	Litres of alcohol consumed per capita	+
	Mean BMI	+
	Cumulative cigarettes (10 years)	+
	Cumulative cigarettes (20 years)	+
	Tobacco (cigarettes per capita)	+
	Healthcare Access and Quality Index	–
3	Education (years per capita)	–
	LDI (I$ per capita)	+
	Socio-demographic Index	–

Myelodysplastic, myeloproliferative, other haemopoietic neoplasms

Level	*Covariate*	*Direction*
1	*Log-transformed age-standardised SEV scalar: Leukaemia*	+
	Log-transformed SEV scalar: Leukaemia	+
2	*Litres of alcohol consumed per capita*	+
	Cumulative cigarettes (5 years)	+
	Cumulative cigarettes (10 years)	+
	Cumulative cigarettes (15 years)	+
	Cumulative cigarettes (20 years)	+
	Smoking prevalence	+
	Tobacco (cigarettes per capita)	+
	Healthcare Access and Quality Index	–
3	*Education (years per capita)*	–
	LDI (I$ per capita)	+
	Socio-demographic Index	+

Other malignant cancers

Level	*Covariate*	*Direction*
1	*Smoking prevalence*	+
	Tobacco (cigarettes per capita)	+
2	*Age- and sex-specific SEV for low vegetables*	+
	Age- and sex-specific SEV for low fruits	+
	Age- and sex-specific SEV for low nuts and seeds	+
	PUFA adjusted (percent)	–
	Healthcare Access and Quality Index	–
3	*Education (years per capita)*	–
	LDI (I$ per capita)	+
	Socio-demographic Index	+

Other neoplasms

Level	*Covariate*	*Direction*
2	*Healthcare Access and Quality Index*	–
3	*Education (years per capita)*	–
	LDI (I$ per capita)	+
	Socio-demographic Index	–

Colon and rectum cancer

Level	Covariate	Direction
1	Mean BMI	+
	Tobacco (cigarettes per capita)	+
	Total physical activity (MET-min/week), age-specific	–
	Log-transformed SEV scalar: Colorectal Cancer	+
	Age- and sex-specific SEV for high red meat	+
2	Litres of alcohol consumed per capita	+
	PUFA adjusted (percent)	–
	Age- and sex-specific SEV for low vegetables	+
	Age- and sex-specific SEV for low fibre	+
	Age- and sex-specific SEV for low calcium	+
	Cumulative cigarettes (5 years)	+
	Diabetes fasting plasma glucose (mmol/L), age-standardised 25+	+
3	Education (years per capita)	–
	Age- and sex-specific SEV for low milk	+
	Age- and sex-specific SEV for low fruit	+
	Age- and sex-specific SEV for low nuts and seeds	+
	Healthcare Access and Quality Index	–
	LDI (I$ per capita)	+
	Socio-demographic Index	+

Ovarian cancer

Level	Covariate	Direction
1	Litres of alcohol consumed per capita	+
	Cumulative cigarettes (10 years)	+
	Cumulative cigarettes (20 years)	+
	Contraception (modern) prevalence (proportion)	–
	Log-transformed SEV scalar: Ovary Cancer	+
2	Asbestos consumption (metric tons per year per capita)	+
	Smoking prevalence	+
	Total fertility rate	–
	Energy unadjusted (kcal)	+
	Mean BMI	+
	Diabetes age-standardized prevalence (proportion)	+
	Healthcare Access and Quality Index	`–
3	Education (years per capita)	–
	Age- and sex-specific SEV for low fruits	+
	Age- and sex-specific SEV for low vegetables	+
	LDI (I$ per capita)	–
	Socio-demographic Index	+

References

1 Waterhouse J, Muir C, Shanmugaratnam K, Powell J. Cancer Incidence in Five Continents IV. Lyon: IARC, 1982.

2 Curado M, Edwards B, Shin H, *et al.* Cancer Incidence in Five Continents IX. Lyon: IARC, 2007 http://www.iarc.fr/en/publications/pdfs-online/epi/sp160/CI5vol9-A.pdf.

3 Muir C, Mack T, Powell J, Whelan S. Cancer Incidence in Five Continents V. Lyon: IARC, 1987.

4 Parkin D, Muir C, Whelan S, Gao Y, Ferlay J, Powell J. Cancer Incidence in Five Continents VI. Lyon: IARC, 1992.

5 Parkin D, Whelan S, Ferlay J, Raymond L, Young J. Cancer Incidence in Five Continents VII. Lyon: IARC, 1997.

6 Parkin D, Whelan S, Ferlay J, Teppo L, Thomas D. Cancer Incidence in Five Continents VIII. Lyon: IARC, 2002.

7 Forman D, Bray F, Brewster D, *et al.* Cancer Incidence in Five Continents X. 2013. http://ci5.iarc.fr.

8 Engholm G, Ferlay J, Christensen N, *et al.* NORDCAN: Cancer Incidence, Mortality, Prevalence and Survival in the Nordic Countries, Version 7.3 Association of the Nordic Cancer Registries. Danish Cancer Society. 2016; published online Aug 7. http://www.ancr.nu.

9 Steliarova-Foucher E, O'Callaghan M, Ferlay J, Masuyer E, Forman D, Comber H, Bray F. European Cancer Observatory: Cancer Incidence, Mortality, Prevalence and Survival in Europe. Version 1.0 European Network of Cancer Registries, International Agency for Research on Cancer. 2012; published online Sept. http://eco.iarc.fr.

10 de Martel C, Maucort-Boulch D, Plummer M, Franceschi S. World-wide relative contribution of hepatitis B and C viruses in hepatocellular carcinoma. *Hepatology* 2015; **62**: 1190–200.

11 Hong TP, Gow P, Fink M, *et al.* Novel population-based study finding higher than reported hepatocellular carcinoma incidence suggests an updated approach is needed. *Hepatology* 2016; **63**: 1205–12.

12 GBD 2015 Risk Factors Collaborators. Global, regional, and national comparative risk assessment of 79 behavioural, environmental and occupational, and metabolic risks or clusters of risks, 1990–2015: a systematic analysis for the Global Burden of Disease Study 2015. *The Lancet* 2016; **388**: 1659–724.

13 International Agency for Research on Cancer, World Health Organization. GLOBOCAN estimated cancer incidence, mortality, and prevalence worldwide in 2012. Lyon, France: IARC, 2014 http://globocan.iarc.fr/Default.aspx (accessed April 19, 2016).

14 Karagas MR, Greenberg ER, Spencer SK, Stukel TA, Mott LA. Increase in incidence rates of basal cell and squamous cell skin cancer in New Hampshire, USA. New Hampshire Skin Cancer Study Group. *Int J Cancer* 1999; **81**: 555–9.

Cardiovascular Diseases

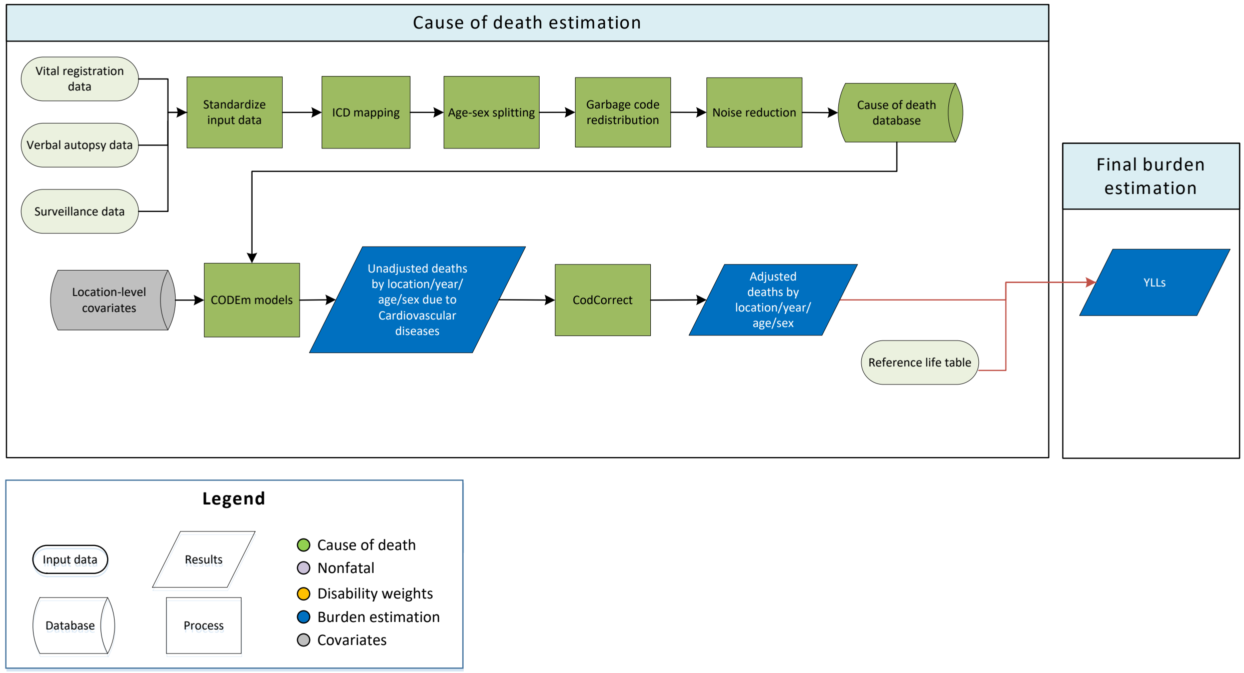

Input data

Vital registration and verbal autopsy data were used to model the parent cardiovascular envelope. We outliered non-representative subnational verbal autopsies from a number of Indian states and verbal autopsy data in Nepal and Papua New Guinea that were implausible in terms of time and age trends. We also outliered verbal autopsy data sources that were implausibly low in all age groups and ICD8 and ICD9BTL data points that were inconsistent with the rest of the data and created implausible time trends.

Modelling strategy

We used a standard CODEm approach to model deaths from cardiovascular diseases. The covariates included in the ensemble modelling process are listed in the table below. For GBD 2019, adjusted dietary covariates for consumption of fruits, omega-3 fatty acids, vegetables, nuts and seeds, and polyunsaturated fatty acids were replaced with the summary exposure value scalars for diet low in each of these factors. The direction for each dietary covariate was changed from -1 to 1 to as our *a priori* assumption is that low levels of intake of these dietary factors are associated with increasing mortality risk from cardiovascular disease. In addition, the dietary covariate for whole grains (kcal/capita, adjusted) the covariate for socio-demographic index as exploratory analyses indicated that these covariates were not predictive of the outcome. The summary exposure value scalar for CVD was dropped as this covariate was not produced for Level 2 causes in GBD 2019. Apart from these changes to the covariates, there are no other substantive changes from the approach used in GBD 2017.

Table: Selected covariates for CODEm models, cardiovascular diseases

Covariate	Transformation	Level	Direction
Cholesterol (total, mean per capita)	None	1	1
Smoking prevalence	None	1	1
Systolic blood pressure (mmHg)	None	1	1
Mean BMI	None	2	1
Elevation over 1500m (proportion)	None	2	-1
Fasting plasma glucose (mmol/L)	None	2	1
Outdoor pollution ($PM_{2.5}$)	None	2	1
Indoor air pollution (all fuel types)	None	2	1
Healthcare access and quality index	None	2	-1
Lag distributed income per capita (I$)	Log	3	-1
Summary exposure value, omega-3 fatty acids	None	3	1
Summary exposure value, fruits	None	3	1
Summary exposure value, vegetables	None	3	1
Summary exposure value, Nuts and seeds	None	3	1
Pulses/legumes (kcal/capita, unadjusted)	None	3	-1
Summary exposure value, PUFA adjusted (percent)	None	3	1
Alcohol (litres per capita)	None	3	1
Trans fatty acid	None	3	1

Rheumatic heart disease

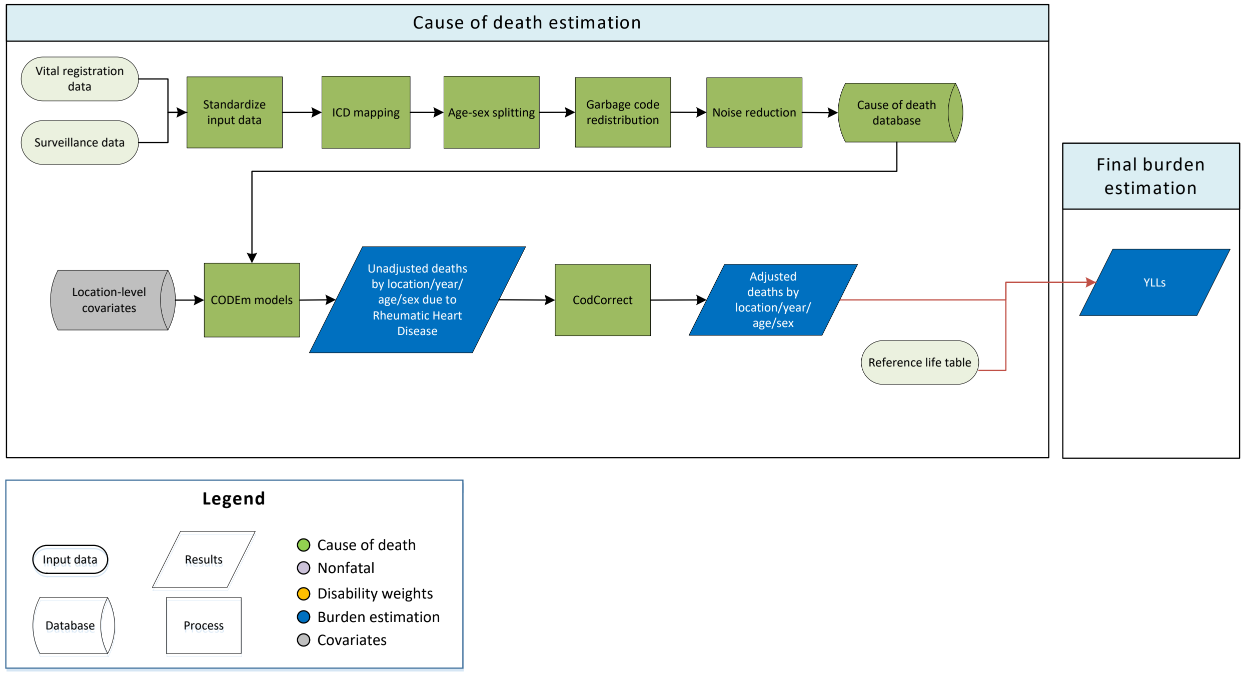

Input data

Vital registration and surveillance data were used to model rheumatic heart disease. We outliered ICD8 and ICD9 BTL datapoints which were inconsistent with the rest of the data and created implausible time trends. We also outliered datapoints which were too high after the redistribution process in a number of age groups. In addition, we outliered verbal autopsy datapoints in Nepal and Pakistan which created an implausibly low cause fraction.

Modelling strategy

We used a standard CODEm approach to model deaths from rheumatic heart disease. There have been no substantive changes from the approach used in GBD 2017, including any covariate changes.

Table 1: Selected covariates for CODEm models, rheumatic heart disease

Level	Covariate	Transformation	Direction
1	Rheumatic heat disease summary exposure value scalar	None	1
1	Improved water (proportion)	None	-1
1	Malnutrition	None	1
1	Sanitation (proportion with access)	None	-1
2	Healthcare access and quality index	None	-1
3	Lag distributed income per capita (I$)	Log	-1
3	Socio-demographic Index	None	-1
3	Education (years per capita)	None	-1

Ischaemic Heart Disease

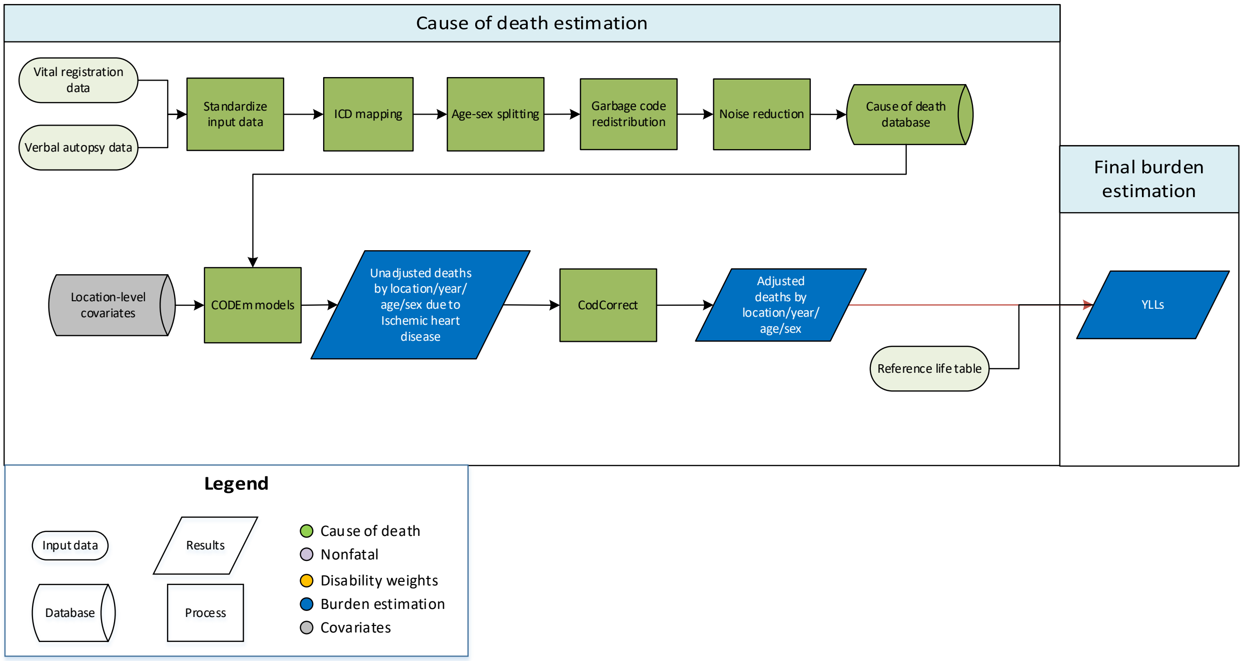

Input data

Vital registration and verbal autopsy data were used to model ischaemic heart disease. We outliered verbal autopsy data in countries and subnational locations where high-quality vital registration data were also available. We also outliered non-representative subnational verbal autopsy data points, ICD8 and ICD9BTL data points which were inconsistent with the rest of the data and created implausible time trends, and data in a number of Indian states identified by experts as poor-quality.

Modelling strategy

We used a standard CODEm approach to model deaths from ischemic heart disease. For GBD 2019, adjusted dietary covariates for consumption of fruits, omega-3 fatty acids, vegetables, nuts and seeds, and polyunsaturated fatty acids were replaced with the summary exposure value scalars for diet low in each of these factors. The direction for each dietary covariate was changed from -1 to 1 to as our *a priori* assumption is that low levels of intake of these dietary factors are associated with increasing mortality risk from ischaemic heart disease. We changed the direction of the alcohol variable from 0 to 1 to reflect our *a priori* hypothesis about the expected direction of the association between this risk factor and mortality risk of ischaemic heart disease. In addition, we changed the level of the covariate for trans fatty acid from 1 to 3. Besides these covariate changes, there are no other substantive changes from the approach used in GBD 2017.

Table: Selected covariates for CODEm models, ischaemic heart disease

Covariate	Transformation	Level	Direction
Summary exposure value, IHD	None	1	1
Cholesterol (total, mean per capita)	None	1	1
Smoking prevalence	None	1	1
Systolic blood pressure (mmHg)	None	1	1
Mean BMI	None	2	1
Elevation over 1500m (proportion)	None	2	-1
Fasting plasma glucose	None	2	1
Outdoor pollution ($PM_{2.5}$)	None	2	1
Indoor air pollution	None	2	1
Healthcare access and quality index	None	2	-1
Lag distributed income per capita (I$)	Log	3	-1
Summary exposure value, omega-3	None	3	1
Summary exposure value, fruits	None	3	1
Summary exposure value, vegetables	None	3	1
Summary exposure value, nuts and seeds	None	3	1
Pulses/legumes (kcal/capita, unadjusted)	None	3	-1
Summary exposure value, PUFA (percent, adjusted)	None	3	1
Alcohol (litres per capita)	None	3	1
Trans fatty acid	None	3	1

Stroke

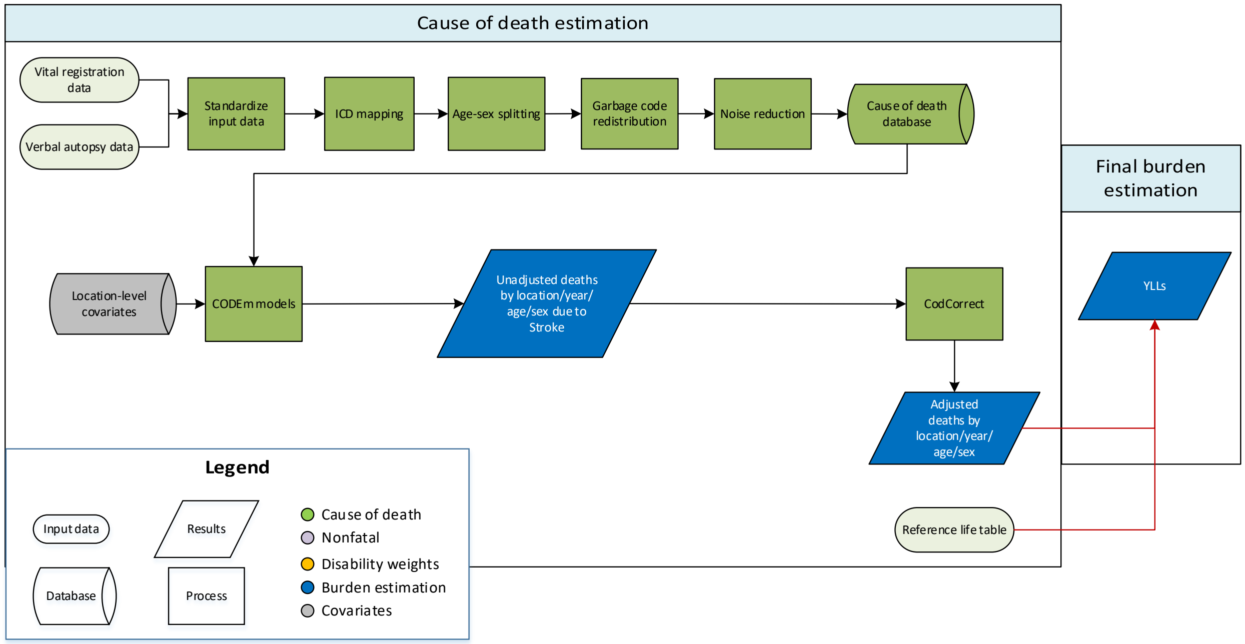

Input data

Verbal autopsy and vital registration data were used to model cerebrovascular disease (stroke). We reassigned deaths from verbal autopsy reports for cerebrovascular disease to the parent cardiovascular disease for both sexes for those under 20 years of age. We outliered non-representative subnational verbal autopsy datapoints. We also outliered ICD8, ICD9BTL, and tabulated ICD10 datapoints which were inconsistent with the rest of the data and created implausible time trends. Datapoints from sources which were implausibly low in all age groups and data points that were causing the regional estimates to be improbably high were outliered.

Modelling strategy

We used a standard CODEm approach to model deaths from stroke. The covariates included in the ensemble modelling process are listed in the table below. For GBD 2019, adjusted dietary covariates for consumption of fruits, omega-3 fatty acids, vegetables, nuts and seeds, and polyunsaturated fatty acids (PUFA) were replaced with the summary exposure value scalars for diet low in each of these factors. The direction for each dietary covariate was changed from -1 to 1 to as our a priori assumption is that low levels of intake of these dietary factors are associated with increasing mortality risk from stroke. We dropped the dietary covariate for whole grains (kcal/capita, adjusted) and the socio-demographic index covariate as exploratory analyses indicated that these variables were not predictive of stroke mortality. In addition, we changed the direction of the alcohol consumption covariate from 0 to 1 to reflect the expected direction of the association for this risk factor with stroke mortality. Apart from these covariate changes, there are no substantive changes from the approach used in GBD 2017.

Table: Selected covariates for CODEm models, stroke

Covariate	Transformation	Level	Direction
Summary exposure variable, stroke	None	1	1
Cholesterol (total, mean per capita)	None	1	1
Smoking prevalence	None	1	1
Systolic blood pressure (mmHg)	None	1	1
Mean BMI	None	2	1
Elevation over 1,500m (proportion)	None	2	-1
Fasting plasma glucose	None	2	1
Outdoor pollution ($PM_{2.5}$)	None	2	1
Indoor air pollution	None	2	1
Healthcare Access and Quality Index	None	2	-1
Lag distributed income per capita (I$)	Log	3	-1
Summary exposure value, omega-3	None	3	1
Summary exposure value, fruits	None	3	1
Summary exposure value, vegetables	None	3	1
Summary exposure value, nuts and seeds	None	3	1
Pulses/legumes (kcal/capita, unadjusted)	None	3	-1
Summary exposure value, PUFA adjusted (percent)	None	3	1
Alcohol (litres per capita)	None	3	1
Trans fatty acid	None	3	1

Ischaemic Stroke

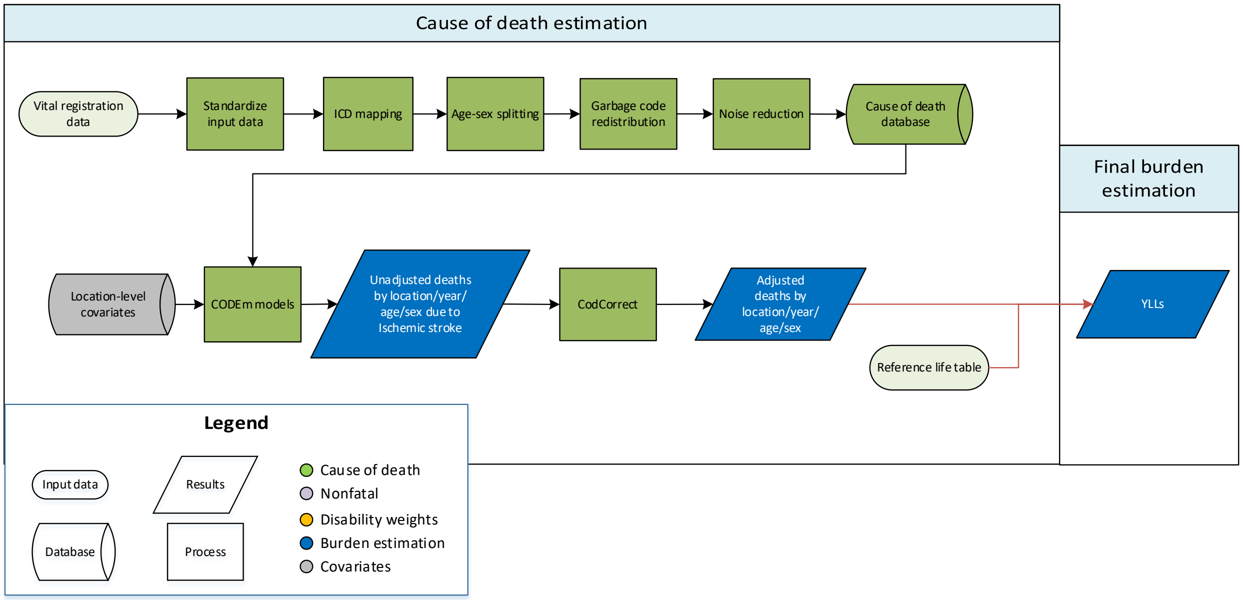

Input data

Vital registration data were used to model deaths from ischaemic stroke. We outliered ICD8 data points which were inconsistent with the rest of the data and created implausible time trends. We also outliered ICD10 data points in The Republic of Tajikistan due to unstable and implausible estimates in similar age groups.

Modelling strategy

We used a standard CODEm approach to model deaths from ischemic stroke. For GBD 2019, adjusted dietary covariates for consumption of fruits, omega-3 fatty acids, vegetables, nuts and seeds, and polyunsaturated fatty acids were replaced with the summary exposure value scalars for diet low in each of these factors. The direction for each dietary covariate was changed from -1 to 1 to as our *a priori* assumption is that low levels of intake of these dietary factors are associated with increasing mortality risk from ischaemic stroke. In addition, the dietary covariate for whole grains (kcal/capita, adjusted) and the socio-demographic index covariate were dropped as exploratory analyses indicated that the covariates were not predictive of the outcome. In addition, we changed the direction of the alcohol variable from 0 to 1 to reflect our *a priori* hypothesis about the expected direction of the association between this risk factor and mortality risk of ischaemic stroke. We also changed the level of the trans fatty acid covariate from 1 to 3. Besides these covariate changes, there are no other substantive changes from the approach used in GBD 2017.

Table: Selected covariates for CODEm models, ischaemic stroke

Covariate	Transformation	Level	Direction
Summary exposure value, ischaemic stroke	None	1	1
Cholesterol (total, mean per capita)	None	1	1
Smoking prevalence	None	1	1
Systolic blood pressure (mmHg)	None	1	1
Mean BMI	None	2	1
Elevation over 1500m (proportion)	None	2	-1
Fasting plasma glucose	None	2	1
Outdoor pollution ($PM_{2.5}$)	None	2	1
Indoor air pollution	None	2	1
Healthcare access and quality index	None	2	-1
Lag distributed income per capita (I$)	Log	3	-1
Summary exposure value, omega-3	None	3	1
Summary exposure value, fruits	None	3	1
Summary exposure value, vegetables	None	3	1
Summary exposure value, nuts and seeds	None	3	1
Pulses/legumes (kcal/capita, unadjusted)	None	3	-1
Summary exposure value PUFA adjusted	None	3	1
Alcohol (litres per capita)	None	3	1
Trans fatty acid	None	3	1

Intracerebral haemorrhage

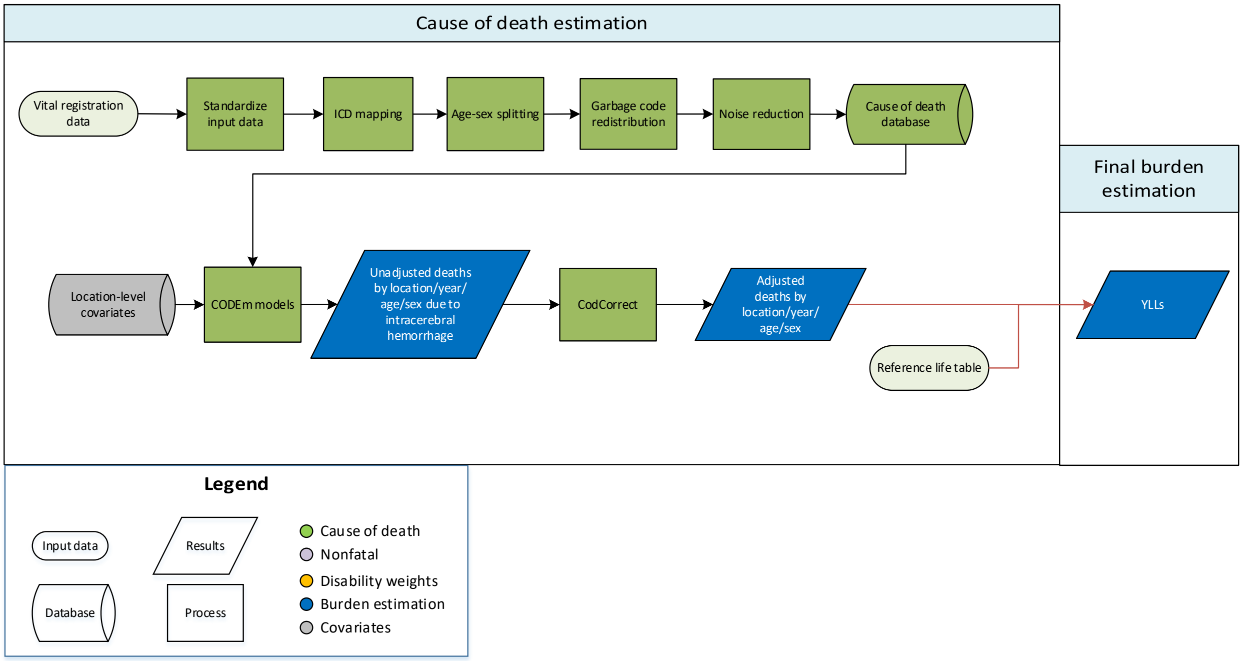

Input data

Vital registration data were used to model intracerebral haemorrhage. We outliered ICD8 data points which were inconsistent with the rest of the data and created implausible time trends. In addition, we outliered vital registration data points in certain countries in Latin American countries due to implausibly high values at the oldest age groups resulting in inconsistencies in time trends.

Modelling strategy

We used a standard CODEm approach to model deaths from intracerebral haemorrhage. For GBD 2019, adjusted dietary covariates for consumption of fruits, omega-3 fatty acids, vegetables, nuts and seeds, and polyunsaturated fatty acids were replaced with the summary exposure value scalars for diet low in each of these factors. The direction for each dietary covariate was changed from -1 to 1 to as our *a priori* assumption is that low levels of intake of these dietary factors are associated with increasing mortality risk from intracerebral haemorrhage. In addition, the dietary covariate for whole grains (kcal/capita, adjusted) and the social demographic index covariate were dropped as exploratory analyses indicated that these covariates were not predictive of the mortality risk from intracerebral haemorrhage. We changed the direction of the covariate for alcohol from 0 to 1 due to our *a priori* hypothesis about the direction of the association for this covariate. We also changed the level of the cholesterol covariate from 1 to 3 and the direction from 0 to -1 to reflect the mixed and inconclusive evidence regarding cholesterol levels and risk of intracerebral haemorrhage. In addition, we changed the level of the trans fatty acid from covariate from 1 to 3 in accordance with the expected importance of this risk factor on mortality from intracerebral haemorrhage. Besides these covariate changes, there are no other substantive changes from the approach used in GBD 2017.

Table: Selected covariates for CODEm models, intracerebral haemorrhage

Covariate	Transformation	Level	Direction
Summary exposure variable, ICH	None	1	1
Smoking prevalence	None	1	1
Systolic blood pressure (mmHg)	None	1	1
Mean BMI	None	2	1
Elevation over 1500m (proportion)	None	2	-1
Fasting plasma glucose	None	2	1
Outdoor pollution ($PM_{2.5}$)	None	2	1
Indoor air pollution	None	2	1
Healthcare access and quality index	None	2	-1
Lag distributed income per capita (I$)	Log	3	-1
Summary exposure value omega-3	None	3	1
Summary exposure value fruits	None	3	1
Summary exposure value vegetables	None	3	1
Summary exposure value nuts and seeds	None	3	1
Pulses/legumes (kcal/capita, un-adjusted)	None	3	-1
Summary exposure value PUFA	None	3	1
Cholesterol (total, mean per capita)	None	3	-1
Alcohol (litres per capita)	None	3	1
Trans fatty acid	None	3	1

Subarachnoid haemorrhage

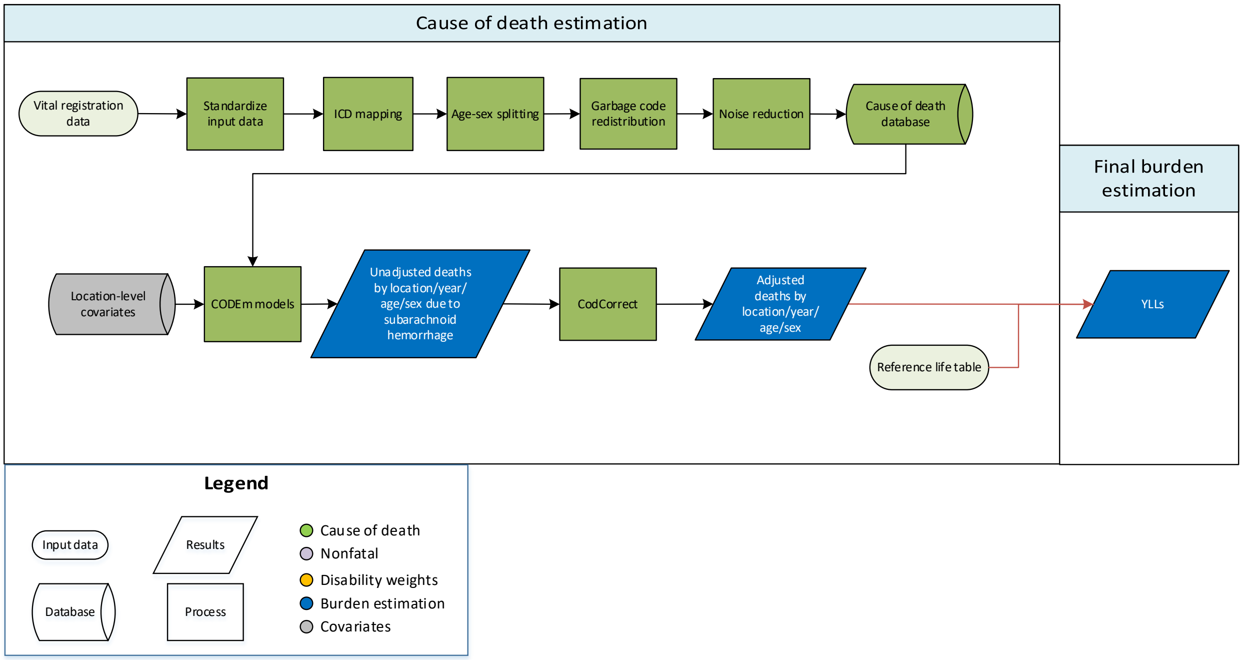

Input data

Vital registration data were used to model subarachnoid haemorrhage. We outliered ICD8 datapoints which were inconsistent with the rest of the data and created implausible time trends. In addition, we outliered vital registration data in Tibet that was implausibly high for all years and age groups.

Modelling strategy

We used a standard CODEm approach to model deaths from subarachnoid haemorrhage. The covariates chosen for inclusion in the ensemble modelling process are listed in the table below. For GBD 2019, we dropped the Socio-demographic Index covariate as exploratory analyses indicated that it was not predictive of the outcome. We also changed the direction of the alcohol covariate from 0 to 1 to reflect the expected direction of the association of this risk factor with mortality risk. Apart from these changes to the covariates, there are no substantive changes from the approach used in GBD 2017.

Table: Selected covariates for CODEm models, subarachnoid haemorrhage

Level	Covariate	Transformation	Direction
1	Smoking prevalence	None	1
1	Systolic blood pressure (mmHg)	None	1
2	Healthcare access and quality index	None	-1
3	Lag distributed income per capita (I$)	Log	-1
3	Alcohol (litres per capita)	None	1

Hypertensive Heart Disease

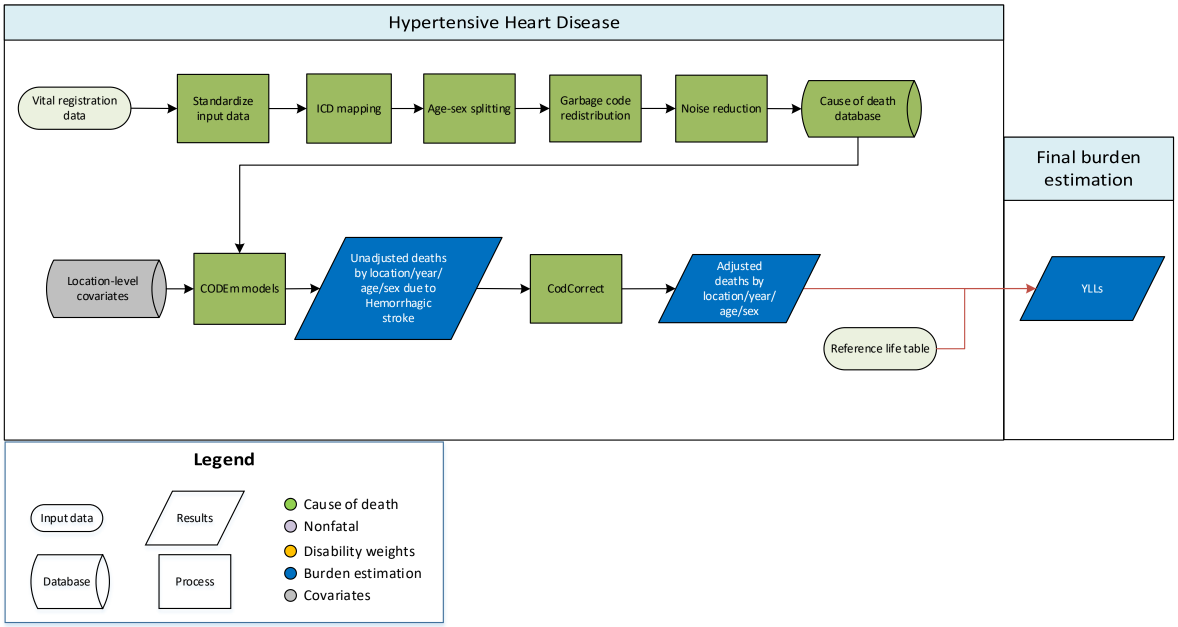

Input data

Vital registration data were used to model cause-specific mortalty for hypertensive heart disease. We outliered ICD9BTL data points, which were inconsistent with the rest of the data and created implausible time trends. In addition, we outliered vital registration data from Grenada in 2017 for being implausibly low across all age groups.

Modelling strategy

We used a standard CODEm approach to model deaths from hypertensive heart disease. For GBD 2019, adjusted dietary covariates for consumption of fruits, omega-3 fatty acids, vegetables, nuts and seeds, and polyunsaturated fatty acids were replaced with the summary exposure value scalars for diet low in each of these factors. The direction for each dietary covariate was changed from -1 to 1 to as our *a priori* assumption is that low levels of intake of these dietary factors are associated with increasing mortality risk from hypertensive heart disease. We also changed the direction of the covariates for alcohol and socio-demographic index from 0 to 1 to reflect the expected direction of these covariates with mortality risk. Apart from these covariate updates, there are no other substantive changes from the approach used in GBD 2017.

Table: Selected covariates for CODEm models, hypertensive heart disease

Covariate	Transformation	Level	Direction
Systolic blood pressure (mmHg)	None	1	1
Cholesterol (total, mean per capita)	None	2	1
Smoking prevalence	None	2	1
Mean BMI	None	2	1
Healthcare access and quality index	None	2	-1
Lag distributed income per capita (I$)	Log	3	-1
Socio-demographic Index	None	3	1
Alcohol (litres per capita)	None	3	1
Summary exposure value, omega-3	None	3	1
Summary exposure value, fruits	None	3	1
Summary exposure value, nuts and seeds	None	3	1
Summary exposure value, PUFA	None	3	1
Summary exposure value, vegetables	None	3	1
Pulses/legumes (kcal/capita, unadjusted)	None	3	-1
Trans fatty acid (percent)	None	3	1

Non-rheumatic valvular heart disease
Non-rheumatic calcific aortic valve disease
Non-rheumatic degenerative mitral valve disease
Other non-rheumatic valvular heart diseases

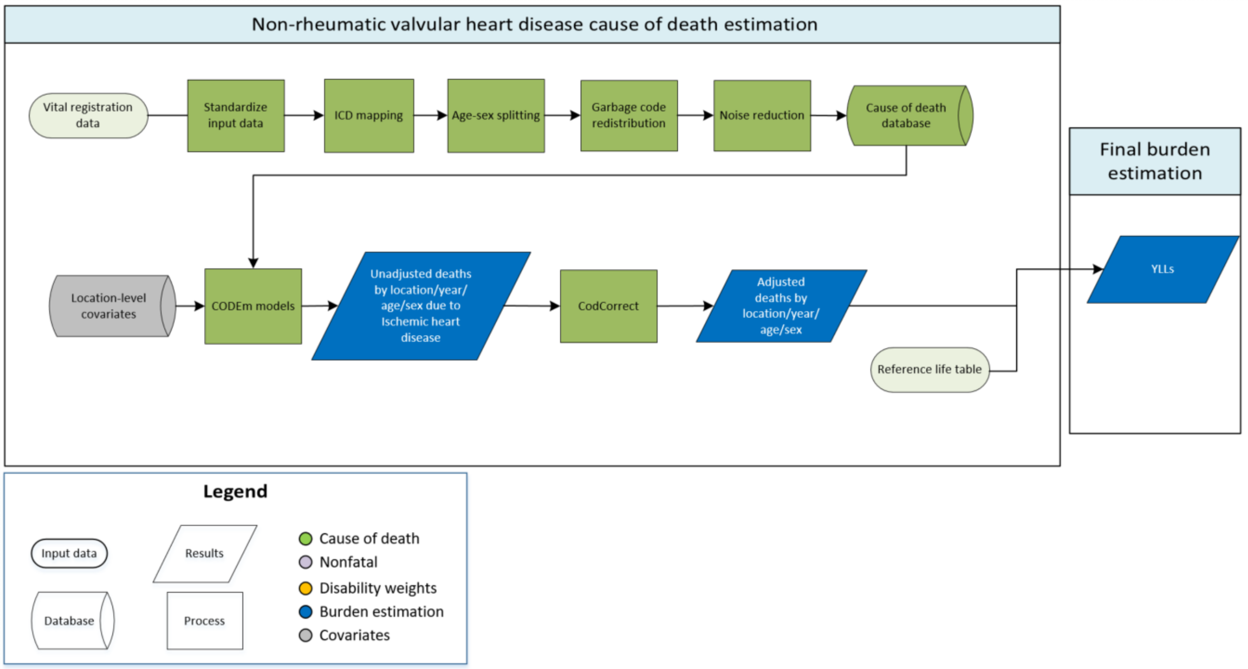

Input data
Vital registration data were used to model non-rheumatic valvular heart disease, non-rheumatic calcific valve disease, non-rheumatic degenerative mitral valve disease, and other non-rheumatic valve diseases. We outliered ICD8, ICD9BTL, and tabulated ICD10 datapoints which were inconsistent with the rest of the data and created implausible time trends. Datapoints from sources which were implausibly low in all age groups and datapoints that were causing the regional estimates to be improbably high were outliered.

Modelling strategy
We used a standard CODEm approach to model deaths from non-rheumatic valvular heart disease, non-rheumatic calcific valve disease, non-rheumatic degenerative mitral valve disease, and other non-rheumatic valvular diseases. The covariates used in the GBD 2019 models, along with their transformations, importance levels, and imposed directions are reported by cause in the tables below. For non-rheumatic valvular heart disease and non-rheumatic calcific aortic valve disease, we added the appropriate summary exposure value, setting both the direction and level to 1. We changed the direction of the Socio-demographic Index covariate from 0 to 1; this change affected the non-rheumatic valve disease, non-rheumatic calcific aortic valve disease, and non-rheumatic degenerative mitral valve disease models. We also changed the direction of the alcohol consumption variable from 0 to 1; this update affected the non-rheumatic valvular heart disease and calcific aortic valve disease models. All covariates for the other non-rheumatic valvular heart disease model were changed. In GBD 2017, we

had included only the summary exposure value for cardiovascular diseases in the model. For GBD 2019, we updated the model to include the summary exposure value for non-rheumatic valvular heart disease (level 1, direction 1), Healthcare Access and Quality Index (level 1, direction -1), and Socio-demographic Index (level 2, direction -1).

Table 1: Selected covariates for CODEm models, non-rheumatic valvular heart disease

Level	Covariate	Transformation	Direction
1	Smoking prevalence	None	1
1	Summary exposure value, non-rheumatic valve disease	None	1
1	Systolic blood pressure (mmHg)	None	1
2	Cholesterol (total, mean per capita)	None	1
2	Mean BMI	None	1
2	Healthcare Access and Quality Index	None	-1
3	Lag distributed income per capita (I$)	Log	-1
3	Socio-demographic Index	None	1
3	Alcohol (litres per capita)	None	1

Table 2: Selected covariates for CODEm models, non-rheumatic calcific aortic valve disease

Level	Covariate	Transformation	Direction
1	Smoking prevalence	None	1
1	Summary exposure value, non-rheumatic calcific aortic valve disease	None	1
1	Systolic blood pressure (mmHg)	None	1
2	Cholesterol (total, mean per capita)	None	1
2	Mean BMI	None	1
2	Fasting plasma glucose	None	1
2	Healthcare Access and Quality Index	None	-1
3	Lag distributed income per capita (I$)	Log	-1
3	Socio-demographic Index	None	1
3	Alcohol (litres per capita)	None	1

Table 3: Selected covariates for CODEm models, non-rheumatic degenerative mitral valve disease

Level	Covariate	Transformation	Direction
1	Healthcare Access and Quality Index	None	-1
1	Lag distributed income per capita (I$)	Log	1
1	Socio-demographic Index	None	1

Table 4: Selected covariates for CODEm models, other non-rheumatic valvular heart diseases

Level	Covariate	Transformation	Direction
1	Summary exposure value, non-rheumatic valve disease	None	1
1	Healthcare Access and Quality Index	None	-1
2	Socio-demographic Index	None	-1

Cardiomyopathy and Myocarditis

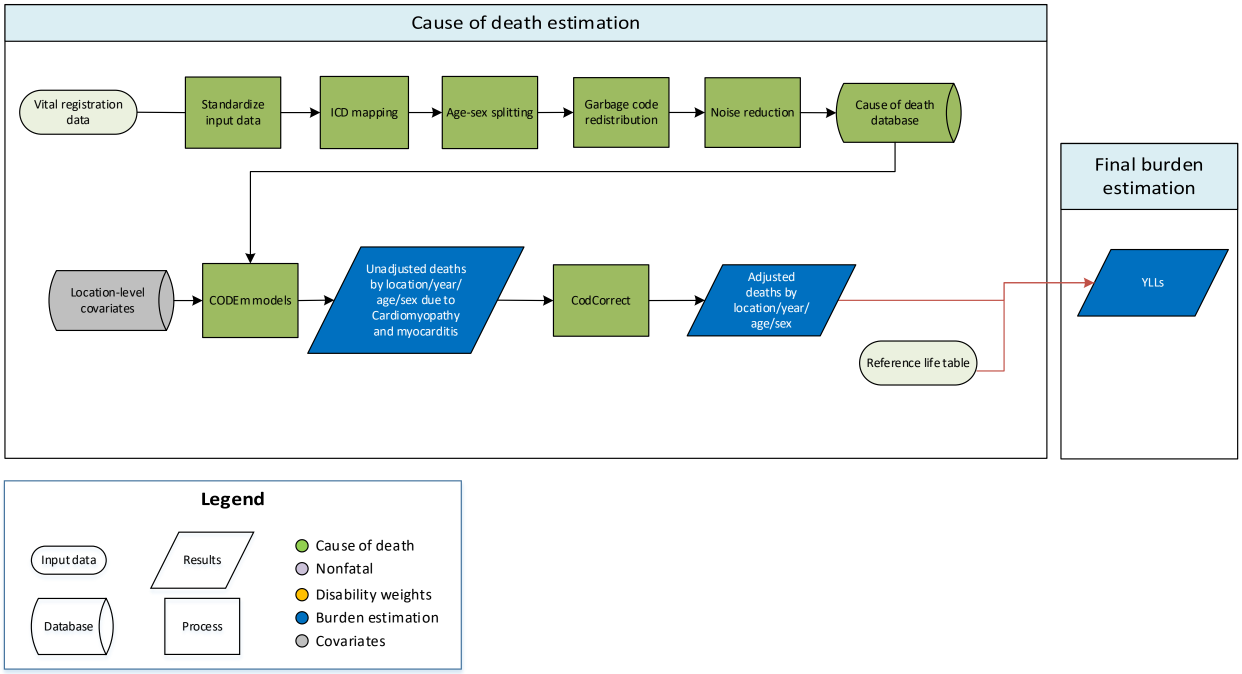

Input data

Vital registration data were used to model deaths due to cardiomyopathy and myocarditis. We outliered data points in Central Asia, Central Europe, and Eastern Europe due to implausibly high values which we attributed to variation in local coding practices. We also outliered ICD8 and ICD9BTL data points in countries where they were discontinuous with other data in the time series or were implausibly high or low. Additionally, we outliered ICD10 data points in Grenada that were improbably low and causing inconsistencies in the time pattern.

Modelling strategy

We used a standard CODEm approach to model deaths from cardiomyopathy and myocarditis. The covariates selected for inclusion in the CODEm modelling process can be found in the table below. A select few changes were made to the covariates as compared with GBD 2017. We dropped the alcohol (litres per capita) covariate as exploratory analyses indicated that it was not predictive of the outcome. We also changed the directions of the socio-demographic index covariate and lag distributed income (per capita) covariate from 0 to -1 to reflect our *a priori* hypotheses about the relationships of these covariates with mortality risk from cardiomyopathy and myocarditis. Aside from these covariate changes, there have been no substantive changes to the modelling strategy since GBD 2017.

Table: Selected covariates for CODEm models, cardiomyopathy and myocarditis

Covariate	Transformation	Level	Direction
Summary exposure value, CMP	none	1	1
Mean systolic blood pressure (mmHg)	none	1	1
Smoking prevalence	none	1	1
Mean BMI (kg/m^2)	None	2	1
Healthcare access and quality index	none	2	-1
Lag distributed income per capita (I$)	log	3	-1
Socio-demographic Index	none	3	-1

Myocarditis

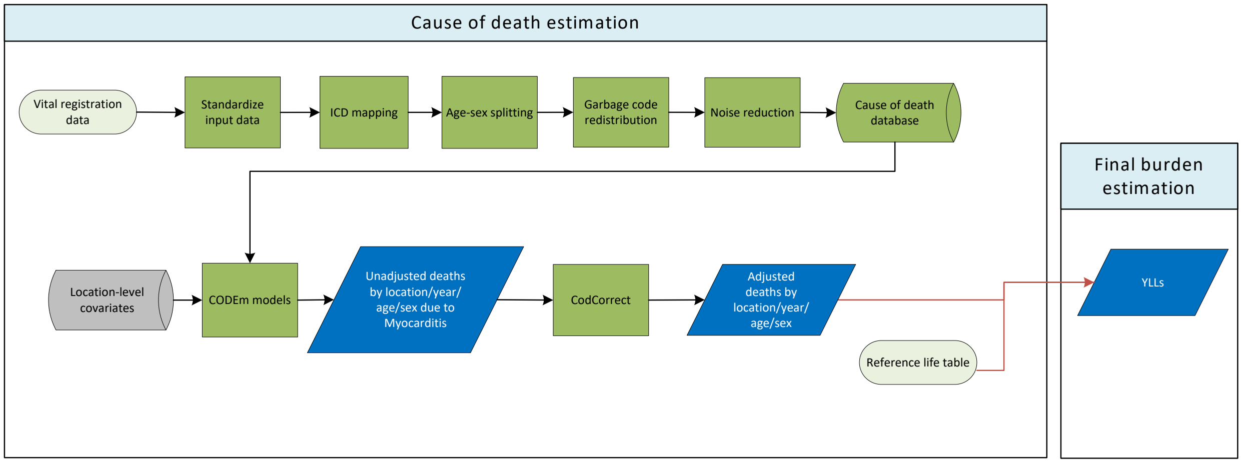

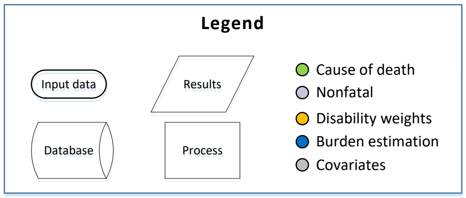

Input data

Vital registration data were used to model deaths due to myocarditis.

Modelling strategy

We used a standard CODEm approach to model deaths from myocarditis. The covariates selected for evaluation in the CODEm ensemble modelling process can be found in the table below. We changed the direction on the lag distributed income per capita and socio-demographic index covariates from 0 for both to -1 and 1, respectively, to reflect our *a priori* hypotheses regarding these associations. Aside from these changes, there have been no substantive changes to the modelling strategy since GBD 2017.

Table: Selected covariates for CODEm models, myocarditis

Covariate	Transformation	Level	Direction
Summary exposure variable, CMP	none	1	1
Systolic blood pressure (mm Hg)	none	1	1
Healthcare access and quality index	none	2	-1
Lag distributed income per capita (I$)	log	3	-1
Socio-demographic Index	none	3	1

Alcoholic Cardiomyopathy

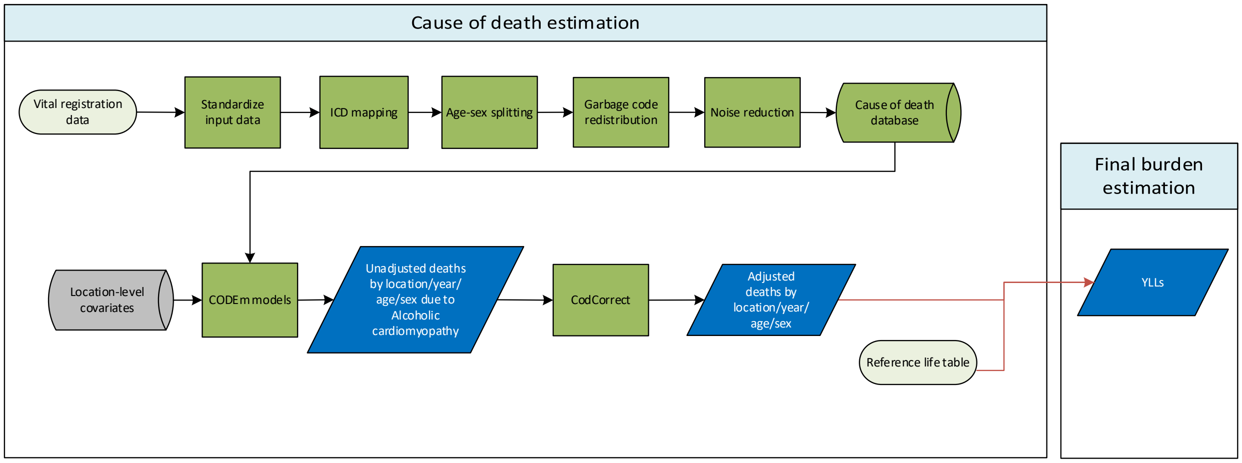

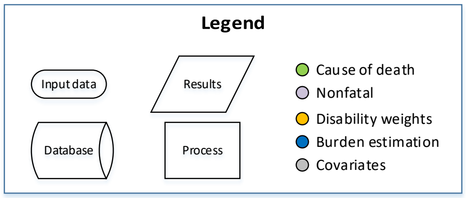

Input data

Vital registration data were used to model deaths due to alcoholic cardiomyopathy. We outliered ICD9 data points in Cyprus that were implausibly high and discontinuous with the rest of the time series. We also dropped ICD9BTL data points in locations in Central and Eastern Europe where we were unable to disaggregate them appropriately. Additionally, we outliered tabulated ICD10 data points in locations where unreliable estimates caused an abrupt inconsistency with detailed ICD10 data.

Modelling strategy

We used a standard CODEm approach to model deaths from alcoholic cardiomyopathy. The covariates selected for inclusion in the CODEm modelling process can be found in the table below. For GBD 2019, we dropped the covariate on socio-demographic index as exploratory analyses indicated that it was not predictive of the outcome. Additionally, we changed the direction of the lag distributed income per capita covariate from 0 to -1 to reflect our *a priori* hypothesis about the expected relationship between this covariate and deaths from alcoholic cardiomyopathy. Aside from these covariate changes, there have been no substantive changes from the approach used in GBD 2017.

Table: Selected covariates for CODEm models, alcoholic cardiomyopathy

Covariate	Transformation	Level	Direction
Summary exposure value, CMP	none	1	1
Smoking prevalence	none	1	1
Alcohol (litres per capita)	none	1	1
Healthcare access and quality index	none	2	-1
Lag distributed income per capita (I$)	log	3	-1

Other cardiomyopathy

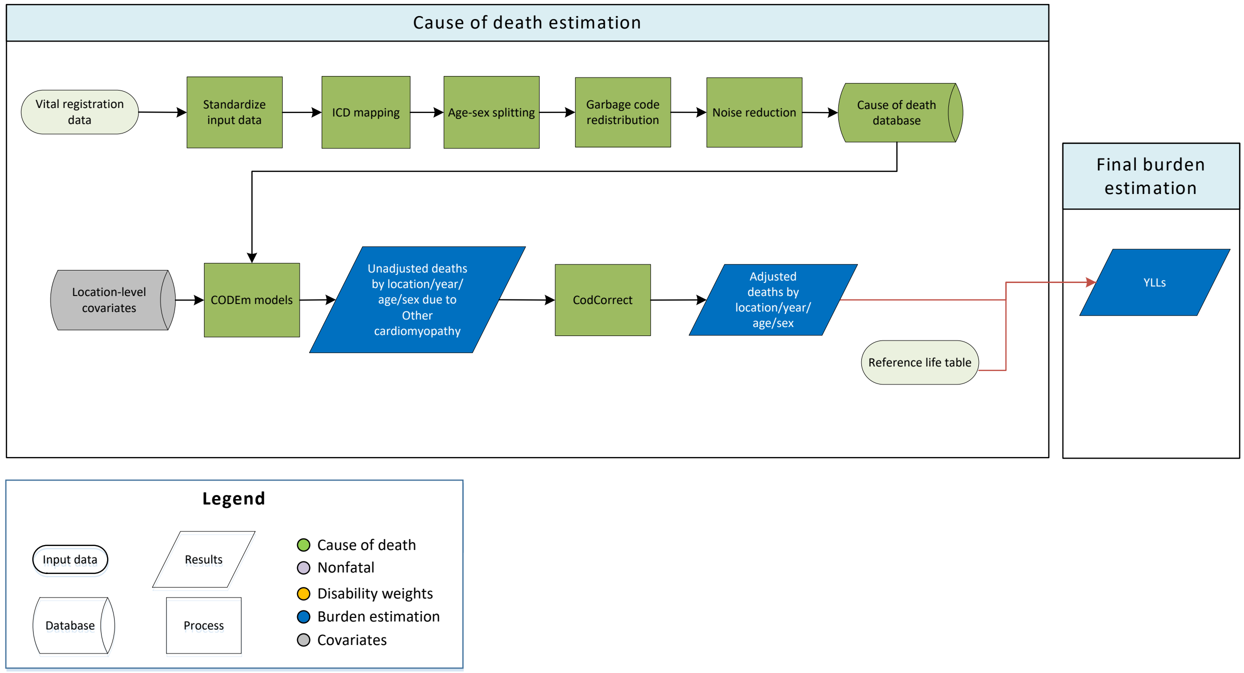

Input data

Vital registration data were used to model deaths due to other cardiomyopathy. We outliered datapoints in Central Asia and Central and Eastern Europe due to implausibly high values which we attributed to variation in local coding practices after review with experts.

Modelling strategy

We used a standard CODEm approach to model deaths from other cardiomyopathy. The covariates selected for inclusion in the CODEm modelling process can be found in the table below. We changed the directions of the Socio-demographic Index and lag distributed income per capita covariates from 0 for both to 1 and -1, respectively. Aside from these covariate changes, there have been no substantive changes to the modelling process since GBD 2017.

Table: Selected covariates for CODEm models, other cardiomyopathy

Level	Covariate	Transformation	Direction
1	Summary exposure variable, CMP	none	1
1	Systolic blood pressure (mmHg)	none	1
1	Smoking prevalence	none	1
2	Body mass index (kg/m^2)	none	1
2	Healthcare Access and Quality Index	none	-1
3	Lag distributed income per capita (I$)	log	-1
3	Socio-demographic Index	none	1

Atrial Fibrillation and Flutter

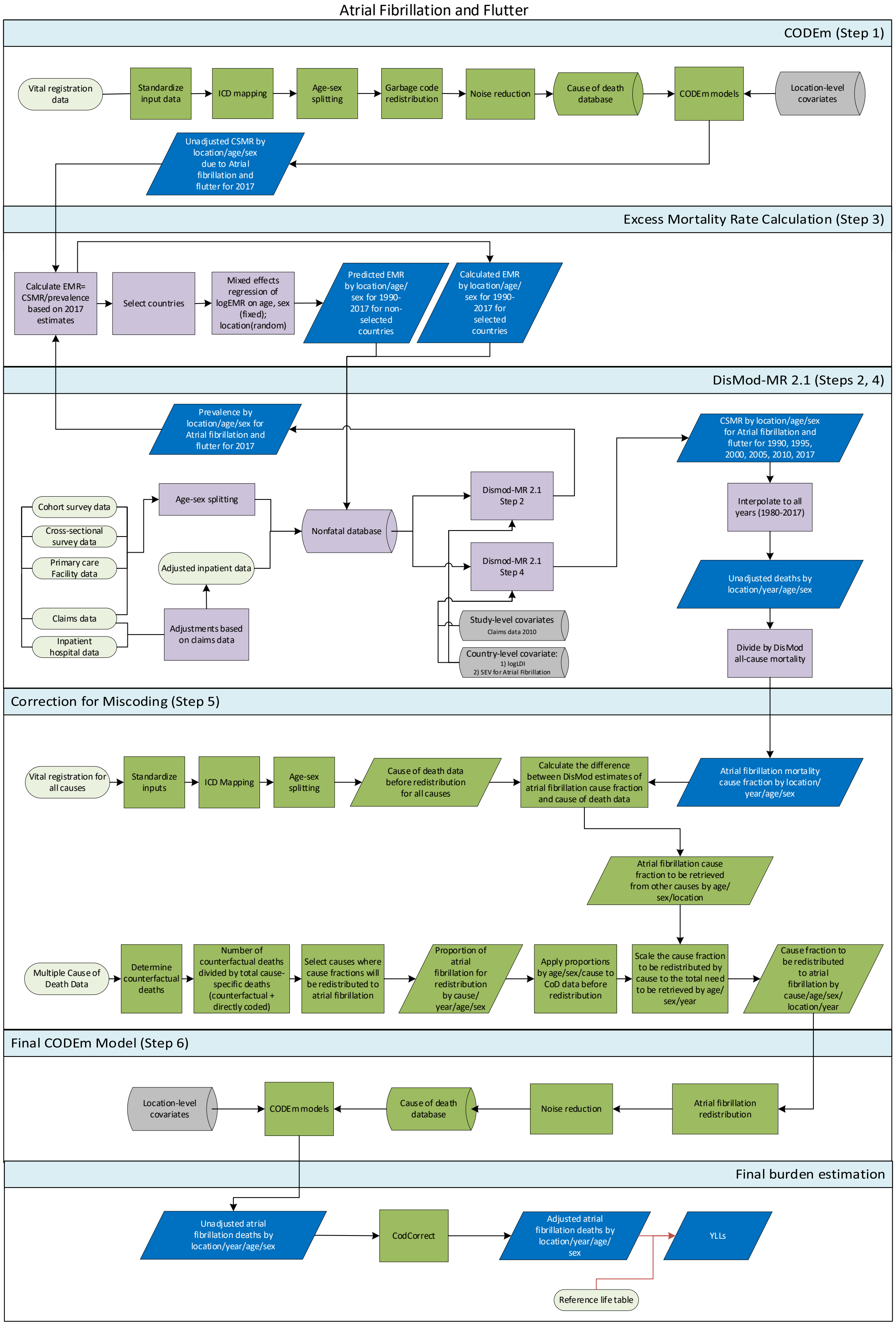

Input data

Vital registration (VR) data: We outliered ICD8 and ICD9 data points that were discontinuous from other data in the time series and created an unlikely time trend. We also outliered data points that were implausibly low in multiple age groups.

Modelling strategy

In order to address changes in coding practices for atrial fibrillation, we used an integrated approach that combined DisMod-MR 2.1 and CODEm models to estimate deaths from atrial fibrillation and flutter. This approach allowed us to adjust estimates to more accurately reflect the number of deaths for which atrial fibrillation was the true underlying cause of death. Due to the restrictions of the decomposition analysis implemented for GBD 2019, we utilized the CSMR from the final GBD 2017 DisMod-MR 2.1 model to inform the misdiagnosis correction described below.

The modelling steps are illustrated in the above flowchart. Covariates included in both the DisMod-MR 2.1 and CODEm models can be found in the table below. In Step 1, we estimated deaths for atrial fibrillation using a standard CODEm approach. In Step 2, we estimated prevalence rates in DisMod-MR 2.1 using data from published reports of cross-sectional and cohort surveys, as well as primary care facility data. We also used claims data covering inpatient and outpatient visits for the United States along with inpatient hospital data from 163 locations in 15 countries. Inpatient hospital data were adjusted using age- and sex-specific information for: 1) readmission within one year; 2) primary diagnosis code to secondary codes; and, 3) the ratio of inpatient to outpatient visits. We set priors of no remission and no excess mortality prior to age 30.

In Step 3, we calculated the excess mortality rate (EMR) for 2017 (defined as the cause-specific mortality rate (CSMR) estimated from CODEm divided by the prevalence rate from DisMod-MR 2.1). We then selected 17 countries based on four conditions: 1) ranking of 4 or 5 stars on the newly developed system for assessing the quality of VR data; 2) prevalence data available from the literature were included in the DisMod-MR 2.1 estimation; 3) prevalence rate ≥ 0.005; and, 4) CSMR ≥ 0.00002. Using information from these countries as input data, we ran a linear mixed-effects regression of logEMR on sex, age, and location. Sex and age were treated as fixed effects for the regression, while location was considered a random effect. We then predicted age- and sex-specific EMR using the results of this regression for all non-selected countries. Countries included in the regression were assigned their directly calculated values. These EMR data points were assigned to the time period 1990–2017 and uploaded into the nonfatal database in order to be used in modelling.

In Step 4, we reran DisMod-MR 2.1 including the EMR estimated in Step 3 as input data using the same priors as in Step 2 to obtain CSMR estimates from DisMod-MR 2.1 that are consistent with the available data for incidence and prevalence. As DisMod-MR 2.1 only generates estimates for six years (1990, 1995, 2000, 2005, 2010, 2017), we interpolated using a log-linear approach for 1990–2017. Estimates for 1980–1990 were generated via regression on the entire time series, using sociodemographic index as a predictor.

In Step 5, the CSMR estimates were divided by the all-cause mortality estimates used in DisMod-MR 2.1 to calculate the cause fraction for atrial fibrillation and flutter. We then calculated the difference between the cause fraction estimated by DisMod-MR 2.1 and the cause fraction in the VR data generated by the Cause of Death data preparation process. This yielded the cause fraction that would need to be retrieved from other causes via the process described in Section 2.6: Correction for miscoding of Alzheimer's and other dementias and Parkinson's disease. After this correction process, the cause fraction data are processed through the standard redistribution and noise reduction processes.

In Step 6, these adjusted cause fraction data are then used as inputs for a final CODEm model, using the covariates described below. The results from the CODEm model are processed through CoDCorrect; these post-CoDCorrected results are the final estimates for cause-specific mortality for atrial fibrillation and flutter.

Modelling strategy

We used a standard CODEm approach to model deaths from ischemic heart disease. For GBD 2019, adjusted dietary covariates for consumption of fruits, omega-3 fatty acids, vegetables, nuts and seeds, and polyunsaturated fatty acids were replaced with the summary exposure value scalars for diet low in each of these factors. The direction for each dietary covariate was changed from -1 to 1 to as our *a priori* assumption is that low levels of intake of these dietary factors are associated with increasing mortality risk from ischaemic heart disease. We changed the direction of the alcohol variable from 0 to 1 to reflect our *a priori* hypothesis about the expected direction of the association between this risk factor and mortality risk of ischaemic heart disease. In addition, we changed the level of the covariate for trans fatty acid from 1 to 3. Besides these covariate changes, there are no other substantive changes from the approach used in GBD 2017.

For GBD 2019, adjusted dietary covariates for consumption of fruits, omega-3 fatty acids, vegetables, nuts and seeds, and polyunsaturated fatty acids were replaced with the summary exposure value scalars for diet low in each of these factors. The direction for each dietary covariate was changed from -1 to 1 to as our *a priori* assumption is that low levels of intake of these dietary factors are associated with increasing mortality risk from atrial fibrillation. In addition, the dietary covariate for whole grains (kcal/capita, adjusted) was dropped as exploratory analyses indicated that it was not associated with mortality risk. The direction for the alcohol and socio-demographic index covariates was changed from 0 to 1 to reflect our *a priori* hypotheses about the expected directions of the associations between these covariates and mortality risk of atrial fibrillation. Besides these covariate changes, there are no other substantive changes from the approach used in GBD 2017.

CODEm Covariates, atrial fibrillation and flutter

Covariate	Transformation	Level	Direction
Summary exposure variable, atrial fibrillation	None	1	1
Smoking prevalence	None	1	1
Systolic blood pressure (mmHg)	None	1	1
Mean BMI	None	2	1
Fasting plasma glucose	None	2	1
Healthcare Access and Quality Index	None	2	-1
Cholesterol (total, mean per capita)	None	2	1
Lag distributed income per capita (I$)	Log	3	-1
Socio-demographic Index	None	3	1
Summary exposure value, omega-3	None	3	1
Summary exposure value, fruits	None	3	1
Summary exposure value, vegetables	None	3	1
Summary exposure value, nuts and seeds	None	3	1
Pulses/legumes (kcal/capita, unadjusted)	None	3	-1
Summary exposure value, PUFA	None	3	1
Alcohol (litres per capita)	None	3	1
Trans fatty acid	None	3	1

DisMod-MR 2.1 Covariates – Step 2

Covariate	Parameter	Beta	Exponentiated beta
All MarketScan, year 2010	Prevalence	-0.077 (-0.099 to -0.051)	0.93 (0.91 to 0.95)
SEV scalar: Atrial fibrillation	Prevalence	0.75 (0.75 to 0.75)	2.12 (2.12 to 2.12)
Healthcare access and quality index	Excess mortality rate	-0.11 (-0.13 to -0.088)	0.90 (0.88 to 0.92)

DisMod-MR 2.1 Covariates – Step 4

Covariate	Parameter	Beta	Exponentiated beta
All MarketScan, year 2010	Prevalence	0.017 (-0.013 to 0.040)	1.02 (0.99 to 1.04)
SEV scalar: Atrial fibrillation	Prevalence	0.75 (0.75 to 0.75)	2.12 (2.12 to 2.12)
LDI (I$ per capita)	Excess mortality rate	-0.1 (-0.1 to -0.1)	0.90 (0.90 to 0.90)

Aortic Aneurysm

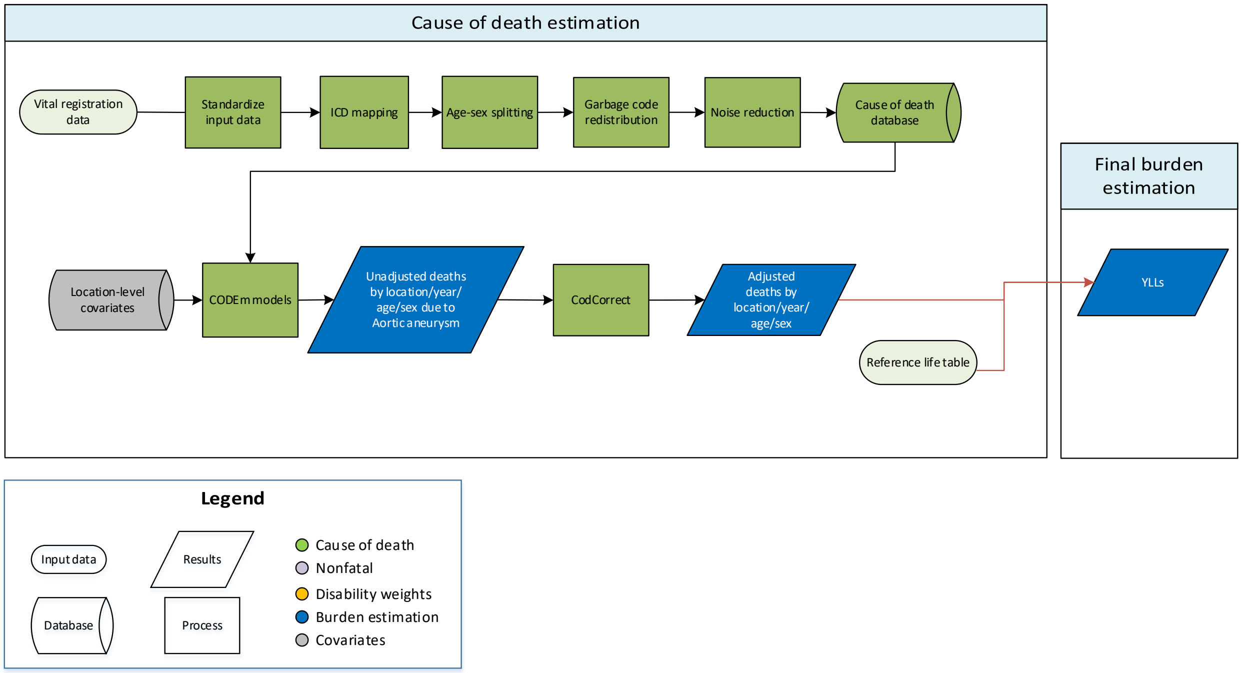

Input data

Vital registration data were used to model cause-specific mortality for aortic aneurysm. We outliered data in Oman as they were improbably high in comparison with the rest of the region. We also outliered ICD8 data that were discontinuous with the rest of the time series and created implausible time trends. In addition, we outliered a subset of vital registration data points in Latin America due to implausibly high values at the oldest age groups that resulted in inconsistencies in time trends.

Modelling strategy

We used a standard CODEm approach to model deaths from aortic aneurysm. The covariates selected for inclusion in the CODEm modelling process can be found in the table below. For GBD 2019, adjusted dietary covariates for consumption of fruits, omega-3 fatty acids, vegetables, nuts and seeds, and polyunsaturated fatty acids were replaced with the summary exposure value scalars for diet low in each of these factors. The direction for each dietary covariate was changed from -1 to 1 to as our *a priori* assumption is that low levels of intake of these dietary factors are associated with increasing mortality risk from aortic aneurysm. We also changed the direction of the covariates for alcohol consumption and the socio-demographic index from 0 to 1. Besides these covariate changes, there are no other substantive changes from the approach used in GBD 2017.

Table: Selected covariates for CODEm models, aortic aneurysm

Covariate	Transformation	Level	Direction
Summary exposure variable, aortic aneurysm	None	1	1
Cholesterol (total, mean per capita)	None	1	1
Cumulative cigarettes (10 yrs)	None	1	1
Systolic blood pressure (mmHg)	None	1	1
Mean BMI	None	2	1
Healthcare access and quality index	None	2	-1
Lag distributed income per capita (I$)	Log	3	-1
Socio-demographic Index	None	3	1
Summary exposure value omega-3	None	3	1
Summary exposure value fruits	None	3	1
Summary exposure value vegetables	None	3	1
Summary exposure value nuts and seeds	None	3	1
Pulses/legumes (kcal/capita, un-adjusted)	None	3	-1
Summary exposure value PUFA	None	3	1
Alcohol (litres per capita)	None	3	1

Peripheral artery disease

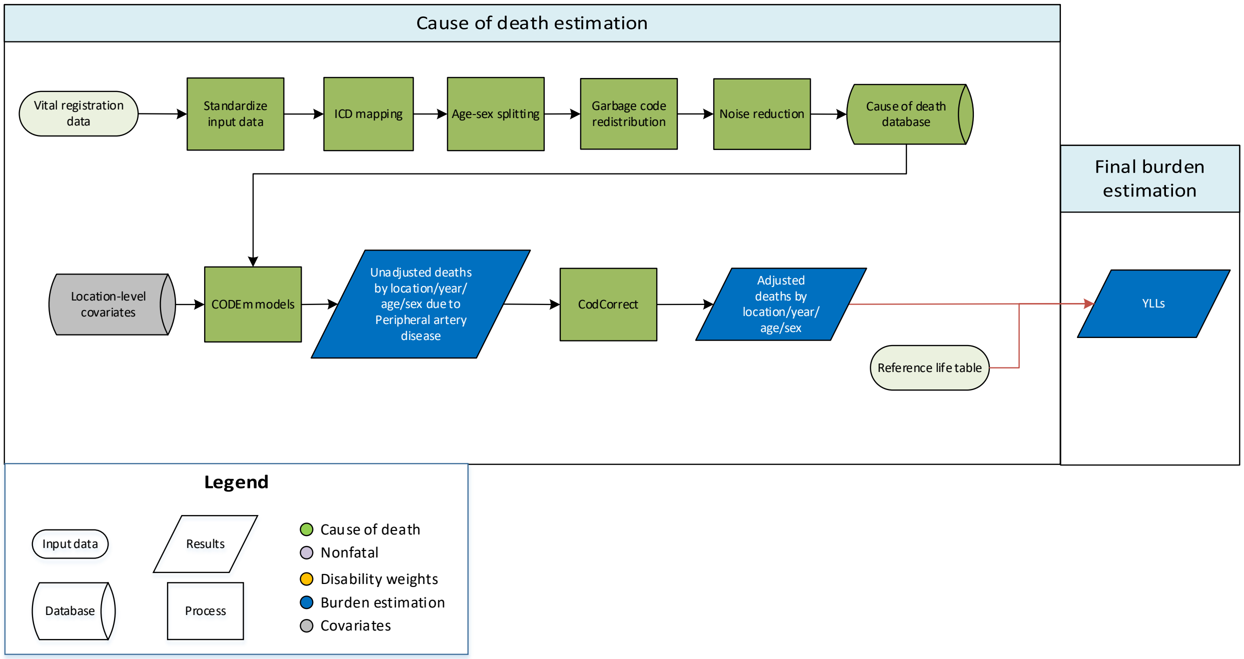

Input data

Vital registration data were used to model peripheral artery disease. We outliered all datapoints with less than 1 death in Egypt per expert review.

Modelling strategy

We used a standard CODEm approach to model deaths from peripheral artery disease. For GBD 2019, adjusted dietary covariates for consumption of fruits, omega-3 fatty acids, vegetables, nuts and seeds, and polyunsaturated fatty acids were replaced with the summary exposure value scalars for diet low in each of these factors. The direction for each dietary covariate was changed from -1 to 1 to as our a priori assumption is that low levels of intake of these dietary factors are associated with increasing mortality risk from peripheral arterial disease. In addition, we dropped the dietary covariates for whole grains (kcal/capita, adjusted) and trans fatty acid (percent). We changed the direction of the alcohol and the Socio-demographic Index covariates from 0 to 1 to reflect the expected direction of the association for these risk factors with mortality risk. Apart from these changes, there are no substantive changes from the approach used in GBD 2017.

Table: Selected covariates for CODEm models, peripheral artery disease

Level	Covariate	Transformation	Direction
1	Summary exposure variable, PAD	None	1
1	Systolic blood pressure (mmHg)	None	1
1	Cholesterol (total, mean per capita)	None	1
1	Smoking prevalence	None	1
2	Mean body mass index (kg/m^2)	None	1
2	Healthcare Access and Quality Index	None	-1
2	Diabetes fasting plasma glucose (mmol/L)	None	1
3	Lag distributed income per capita (I$)	Log	-1
3	Socio-demographic Index	None	1
3	Summary exposure value, omega-3	None	1
3	Summary exposure value, fruits	None	1
3	Summary exposure value, vegetables	None	1
3	Summary exposure value, nuts and seeds	None	1
3	Pulses/legumes (kcal/capita, unadjusted)	None	-1
3	Summary exposure value, polyunsaturated fatty acids	None	1
3	Alcohol (litres per capita)	None	1

Endocarditis

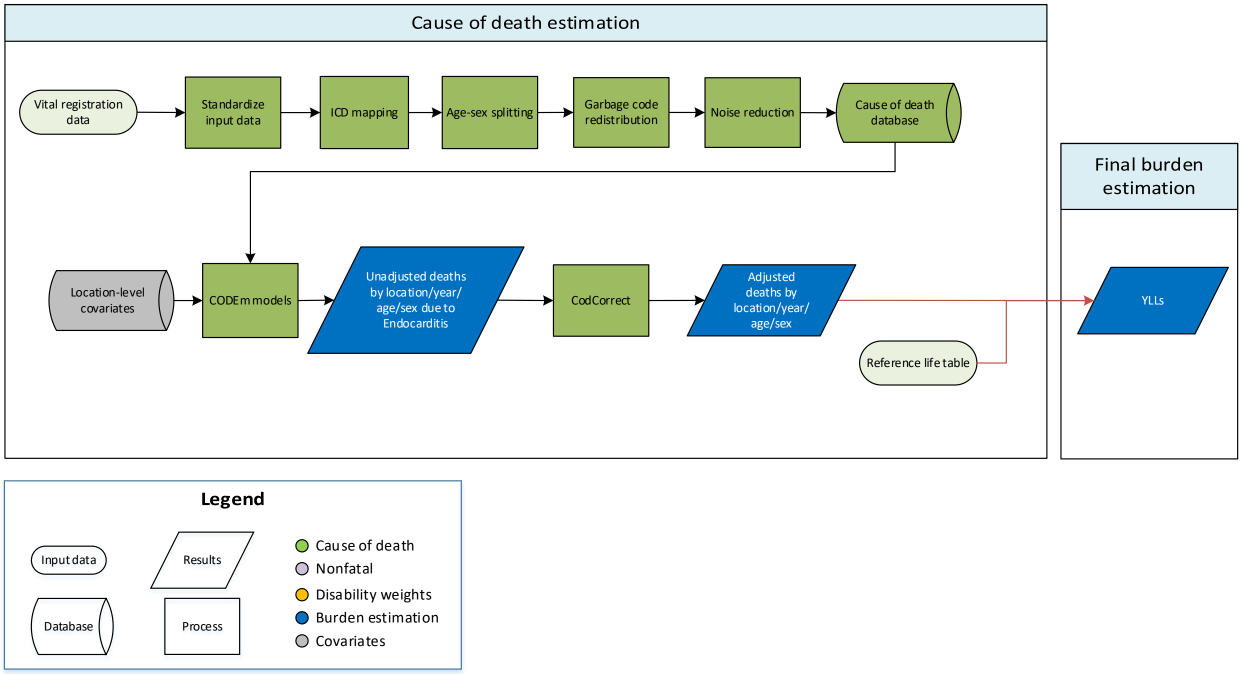

Input data

Vital registration data were used to model endocarditis. We outliered data in Mozambique as these were non-representative for sub-Saharan Africa and were causing regional estimates to be implausibly low. We also outliered ICD8 data that were discontinuous from the rest of the data series and created an implausible time trend.

Modelling strategy

We used a standard CODEm approach to model deaths from endocarditis. Covariates selected for inclusion in the CODEm ensemble modelling process are listed in the table below. For GBD 2019, the same covariates as GBD 2017 were used. We changed the level of the healthcare access and quality index covariate from 1 to 2 for consistency with our *a priori* hypothesis about the relative impact of the covariate on mortality from endocarditis. We also changed the direction of the socio-demographic index covariate from 0 to -1. Apart from these updates to the covariates, there have been no substantive changes from the approach used in GBD 2016.

Table: Selected covariates for CODEm models, endocarditis

Covariate	Transformation	Level	Direction
Summary exposure value, endocarditis	None	1	1
Improved water (proportion)	None	1	-1
Sanitation (proportion with access)	None	1	-1
Healthcare access and quality index	None	2	-1
Lag distributed income per capita (I$)	Log	3	-1
Socio-demographic Index	None	3	-1

Other cardiovascular and circulatory diseases

Flowchart

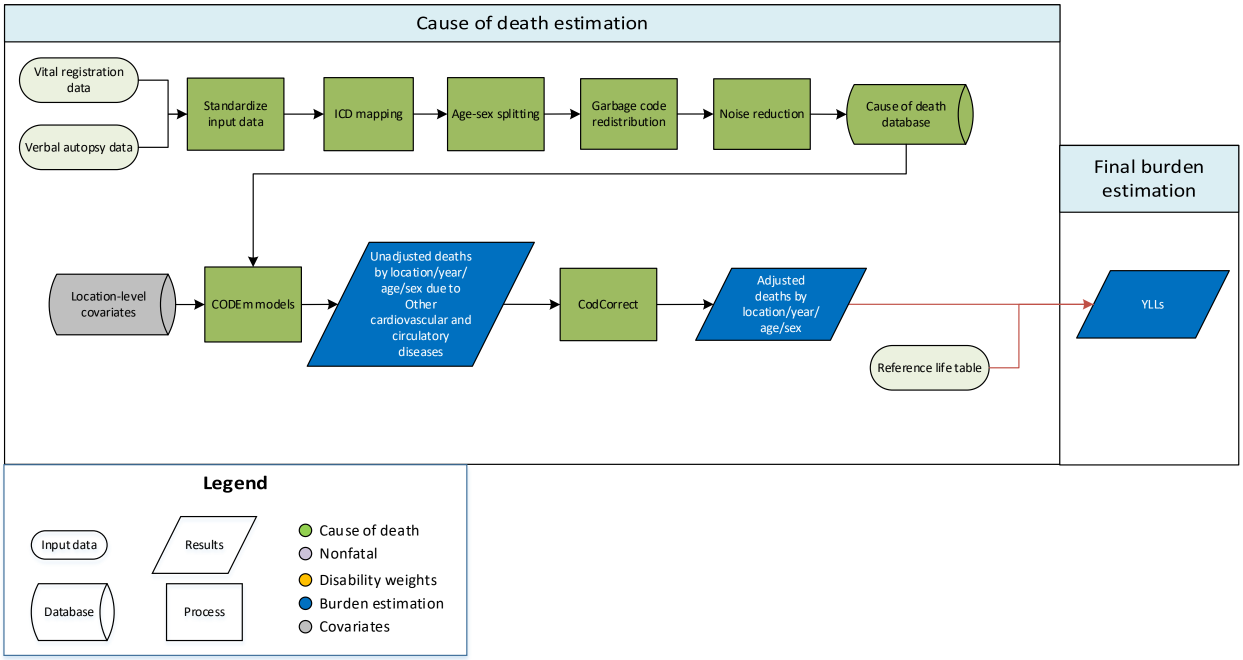

Input data and methodological summary

Input data

Vital registration and verbal autopsy data were used to model other cardiovascular and circulatory diseases. We outliered ICD8 and ICD9 BTL datapoints that were inconsistent with the rest of the data and created implausible time trends. We also outliered ICD8 datapoints which were not nationally representative.

Modelling strategy

We used a standard CODEm approach to model deaths from other circulatory and cardiovascular diseases. Covariates selected for inclusion in the ensemble model are listed in the table below. For GBD 2019, multiple cause of death data were used to redistribute deaths originally coded to heart failure. This strategy is detailed elsewhere in the appendix. Additionally, we specified a positive direction on the alcohol consumption covariate, and a negative direction on the Socio-demographic Index covariate; previously both had a direction of 0. There were no other substantial methodological changes from GBD 2017.

Table: Selected covariates for CODEm models, cardiovascular diseases

Covariate	Transformation	Level	Direction
Summary exposure value, other CVD	None	1	1
Cholesterol (total, mean per capita)	None	1	1
Smoking prevalence	None	1	1
Systolic blood pressure (mmHg)	None	1	1
Mean BMI	None	2	1
Elevation over 1500m (proportion)	None	2	-1
Fasting plasma glucose (mmol/L)	None	2	1
Indoor air pollution (all fuel types)	None	2	1
Outdoor air pollution ($PM_{2.5}$)	None	2	1
Healthcare Access and Quality Index	None	2	-1
Lag distributed income per capita (I$)	Log	3	-1
Socio-demographic Index	None	3	-1
Omega-3 (kcal/capita, adjusted)	Log	3	-1
Fruits (kcal/capita, adjusted)	None	3	-1
Vegetables (kcal/capita, adjusted)	None	3	-1
Nuts and seeds (kcal/capita, adjusted)	None	3	-1
Pulses/legumes (kcal/capita, adjusted)	None	3	-1
PUFA adjusted (percent)	None	3	-1
Alcohol (litres per capita)	None	3	1

Chronic Respiratory Diseases

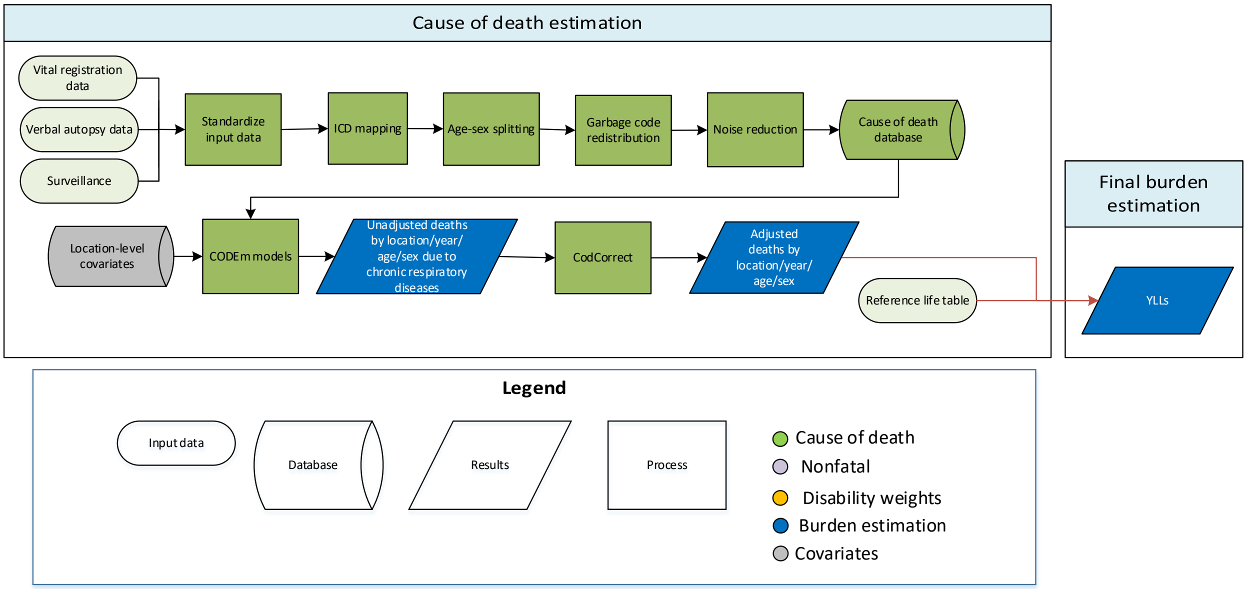

Input data

Sources used to estimate chronic respiratory disease mortality included vital registration, verbal autopsy, and surveillance data from China. Our outlier criteria excluded data points that (1) were implausibly high or low, (2) substantially conflicted with established age or temporal patterns, or (3) significantly conflicted with other data sources conducted from the same locations or locations with similar characteristics (ie, Socio-demographic Index).

Modelling strategy

The standard CODEm modelling approach was applied to estimate deaths due to chronic respiratory diseases. Separate models were conducted for male and female mortality, and the age range for both models was 1 to 95+ years.

Key Changes from GBD 2017

- We added estimates for the following new locations: Monaco, San Marino, Cook Islands, Nauru, Niue, Palau, Tokelau, Tuvalu, Monaco, San Marino, St Kitts and Nevis
- We added subnational location data for the following: Italy, Poland, Pakistan, the Philippines, and Nigeria
- We excluded all MCCD (the very incomplete hospital death data largely from urban areas) and all SCD (earlier verbal autopsy data using lesser quality instruments and analysis) from India, based on discussions with GBD India collaborators. Thus, the estimates are driven by the more recent higher quality SRS verbal autopsy data and covariates.
- Healthcare quality and access index covariate changed to a level 2 covariate from level 1.
- Smoking prevalence and indoor air pollution both moved to a level 1 covariate from level 2.
- We removed the covariate SEV for chronic respiratory disease.
- The SDI covariate was allowed to take a positive or negative direction in GBD 2017, but was specified to only be selected if a negative association was detected in GBD 2019.

The following covariates were used for GBD 2019:

Level	Covariate	Direction
1	indoor air pollution (all cooking fuels)	+
	cumulative cigarettes (10 years)	+
	cumulative cigarettes (5 years)	+
	smoking prevalence	+
2	healthcare quality and access index	-
	outdoor air pollution ($PM_{2.5}$)	+
	population above 1500m elevation (proportion)	+
3	LDI (I$ per capita)	-
	education (years per capita)	-
	socio-demographic index	-
	population between 500 and 1,500m elevation (proportion)	+
	population density over 1,000 people/kilometer^2 (proportion)	+

Chronic respiratory diseases served as a "parent" to the following causes:

- chronic obstructive pulmonary disease
- pneumoconiosis (silicosis, asbestosis, coal worker's pneumoconiosis, other pneumoconiosis)
- asthma
- interstitial lung disease and pulmonary sarcoidosis
- other chronic respiratory diseases

The unadjusted death estimates for all these "child" causes are summed and fit to the distribution of deaths estimated for the "parent" during the CODCorrect adjustment process. This results in deaths recorded using non-specific coding systems, such as verbal autopsy, being included in the parent model and redistributed to the child models proportionately. This approach assumes that deaths reported in non-specific data-sources have the same underlying distribution of specific causes as deaths reported in more specific data-sources.

Covariate Influences:

The following plots show the influence of each covariate on the four CODEm models (male global, male data rich, female global, and female data rich). A positive standardized beta (to the right) means that the covariate was associated with increased death. A negative standardized beta (to the left) means the covariate was associated with decreased death.

Male Global

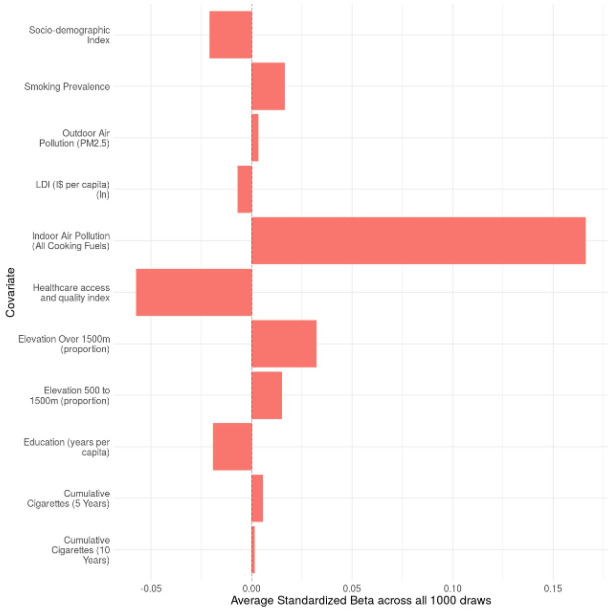

Male Data Rich

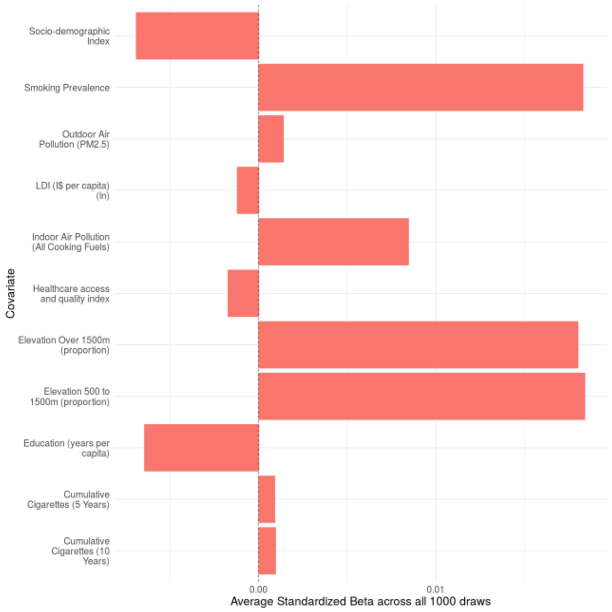

Female Global

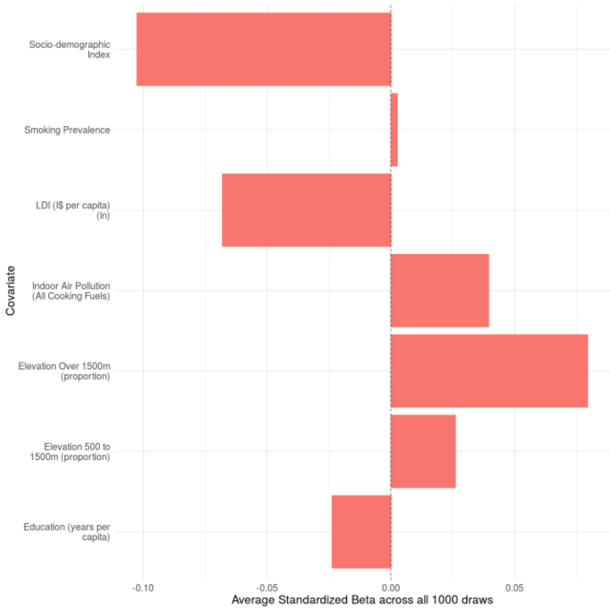

Female Data Rich

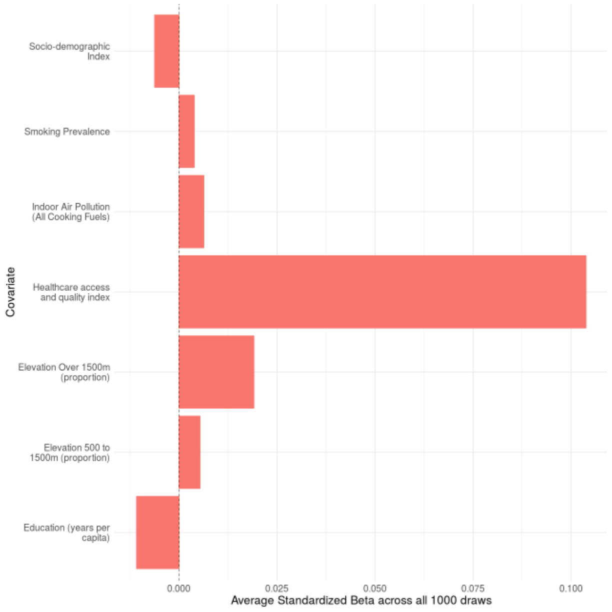

Chronic Obstructive Pulmonary Disease

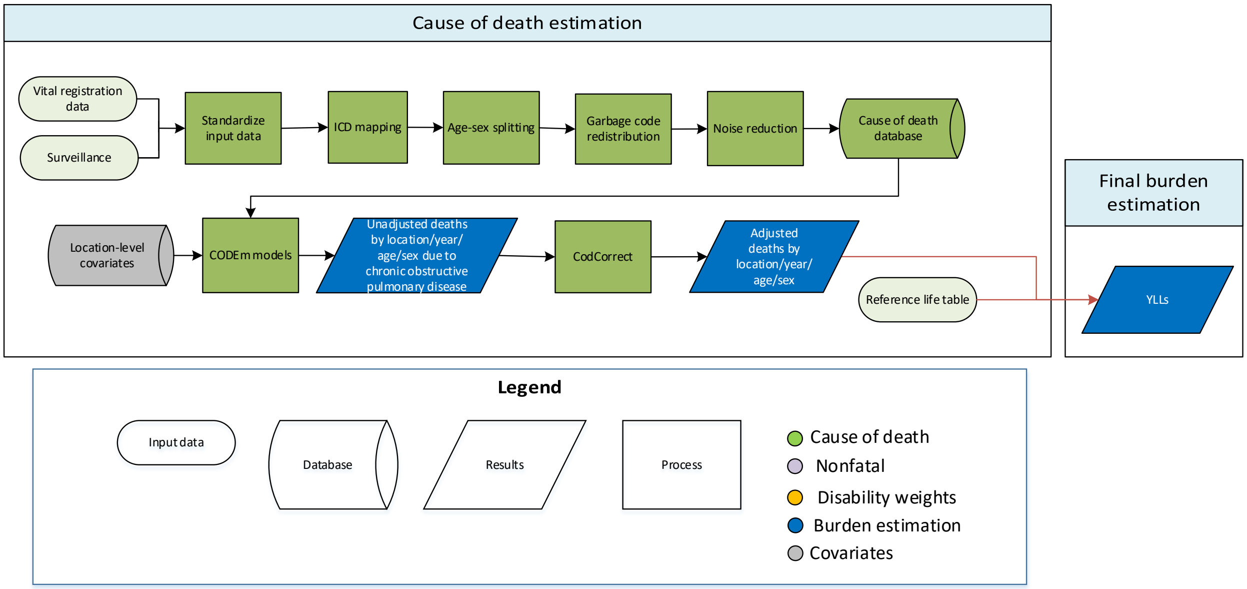

Input data

Data used to estimate chronic obstructive pulmonary disease (COPD) mortality included vital registration and surveillance data from the cause of death (COD) database. Verbal autopsy data were not included and were instead mapped to an overall chronic respiratory disease model. Our outlier criteria excluded data points that (1) were implausibly high or low, (2) substantially conflicted with established age or temporal patterns, or (3) substantially conflicted with other data sources conducted from the same locations or locations with similar characteristics (ie, Socio-demographic Index).

Modelling strategy

The standard CODEm modelling approach (as described in the relevant appendix section) was applied to estimate deaths due to COPD. Separate models were conducted for male and female mortality, and the age range for both models was 1-95+ years.

Key Changes from GBD 2017

- We added estimates for the following new locations: Monaco, San Marino, Cook Islands, Nauru, Niue, Palau, Tokelau, Tuvalu, Monaco, San Marino, St Kitts and Nevis
- We added subnational location data for the following: Italy, Poland, Pakistan, the Philippines, and Nigeria
- We added a covariate for total number of cigarettes smoked in the past 20 years, by age group. We also replaced the covariate for log income per capita with 10-year lagged income per capita.
- Outdoor air pollution covariate was moved to level 1.

The following covariates were used for GBD 2019:

Level	Covariate	Direction
1	log-transformed SEV scalar: COPD	+
	cumulative cigarettes (10 years)	+
	cumulative cigarettes (5 years)	+
	cumulative cigarettes (20 years)	+
	elevation over 1,500m (proportion)	+
	outdoor air pollution ($PM_{2.5}$)	+
2	smoking prevalence	+
	indoor air pollution (all cooking fuels)	+
	healthcare access and quality index	-
3	socio-demographic index	-
	lagged 10 year income per capita (I$ per capita)	-
	education (years per capita)	-

Chronic obstructive pulmonary disease is a “child’ disease that is fit into an overall “parent” chronic respiratory disease model. The unadjusted death estimates from COPD are summed alongside other ”child” causes (asthma, interstitial lung disease and pulmonary sarcoidosis, and pneumoconiosis) and fit to the distribution of deaths in an overall chronic respiratory disease “parent” model as part of the CODCorrect adjustment process. This results in deaths recorded using non-specific coding systems, such as verbal autopsy, being included in the parent model and redistributed to the child models proportionately.

Covariate Influences:

The following plots show the influence of each covariate on the four CODEm models (male global, male data rich, female global, and female data rich). A positive standardized beta (to the right) means that the covariate was associated with increased death. A negative standardized beta (to the left) means the covariate was associated with decreased death.

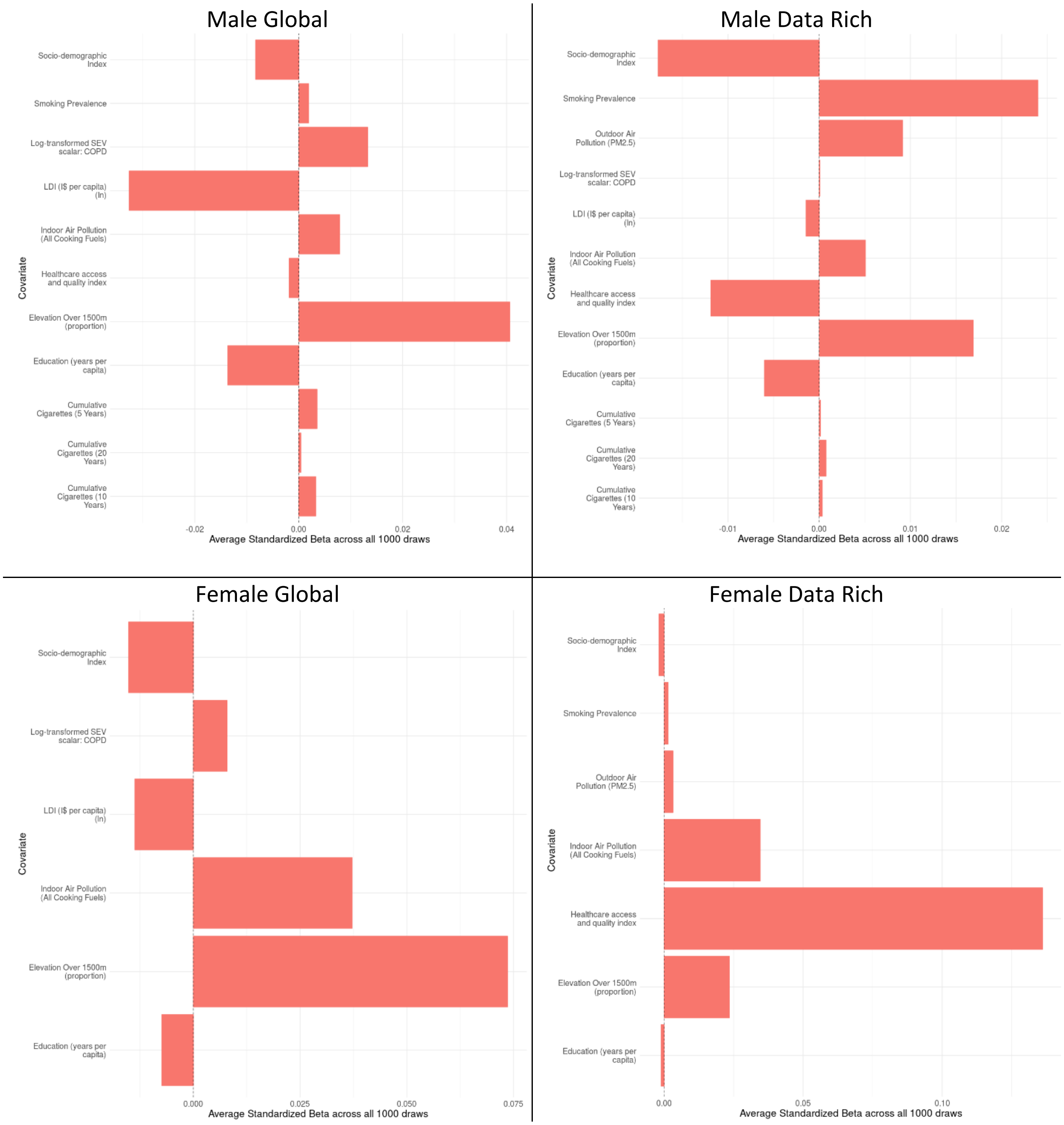

Pneumoconiosis Diseases: Silicosis, Asbestosis, Coal Worker's Pneumoconiosis, and Other Pneumoconiosis

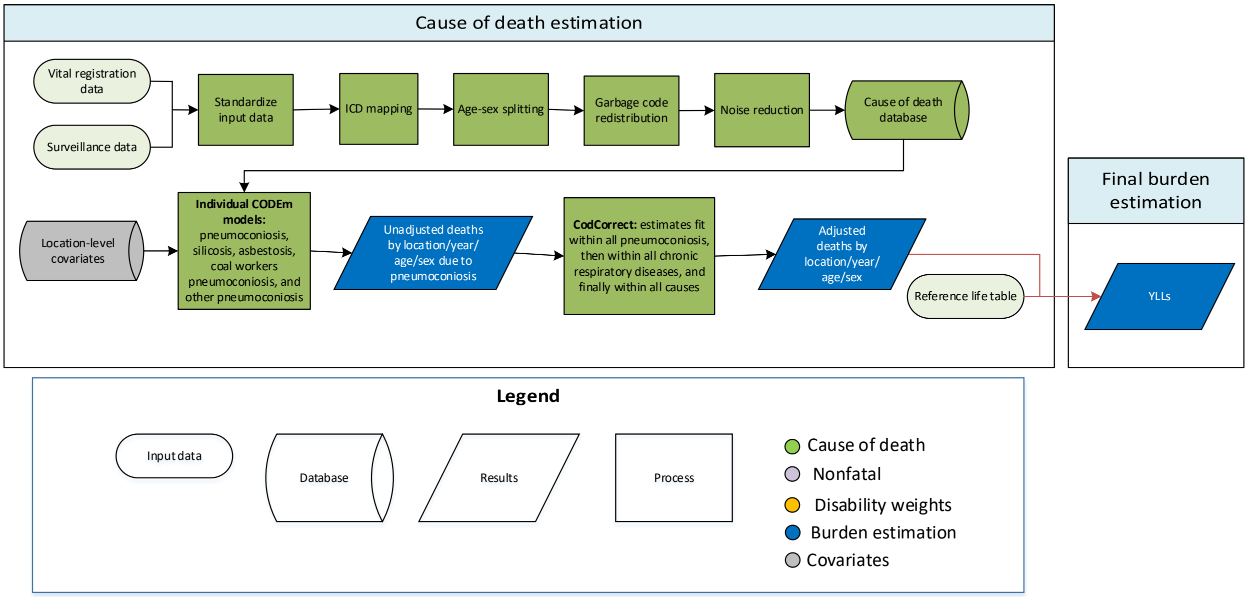

Input data

Data used to estimate pneumoconiosis mortality included vital registration and China mortality surveillance data from the cause of death (COD) database. Our outlier criteria excluded data points that (1) were implausibly high or low, (2) substantially conflicted with established age or temporal patterns, or (3) substantially conflicted with other data sources conducted from the same locations or locations with similar characteristics (ie, socio-demographic index).

Modelling strategy

The standard CODEm modelling approach was applied to estimate deaths due to pneumoconiosis diseases. Separate models were conducted for male and female mortality, and the age range for both models was 15–95+ years. The mortality estimates from pneumoconiosis disease models were ultimately fit into the chronic respiratory envelope, which is the parent cause for pneumoconiosis disease. The pneumoconiosis model serves as an envelope or "parent" model for silicosis, asbestosis, coal worker's pneumoconiosis, and other pneumoconiosis. In CoDCorrect, estimates for each of these "child" models are first fit within all pneumoconiosis, then within all chronic respiratory disease, before being fit to the all-cause mortality envelope.

Key Changes from GBD 2017

- We added estimates for the following new locations: Monaco, San Marino, Cook Islands, Nauru, Niue, Palau, Tokelau, Tuvalu, Monaco, San Marino, St Kitts and Nevis
- We added subnational location data for the following: Italy, Poland, Pakistan, the Philippines, and Nigeria
- We switched the covariate from log income per capita to a 10-year lagged income per capita and removed the elevation covariates that were previously in GBD 2017.

- We added back SEV scalars that were previously dropped in GBD 2017. These are SEVs for occupational asbestos, beryllium, and silica.

The following table indicates covariates used in the pneumoconiosis models, their level, and direction:

**Level**	**Covariate**	**Direction**
1	asbestos consumption per capita*	+
	coal production per capita*	+
	gold production per capita*	+
	age- and sex-specific SEV for occupational asbestos	+
	age- and sex-specific SEV for occupational beryllium	+
	age- and sex-specific SEV for occupational silica	+
2	smoking prevalence	+
	indoor air pollution (all cooking fuels)	+
	cumulative cigarettes (5 years)	+
	healthcare access and quality index	-
3	LDI (I$ per capita)	-
	education (years per capita)	-
	socio-demographic index	-

* asbestos, coal, and gold covariates are each only used in a subset of the pneumoconiosis models, as follows: all three are included in the parent all pneumoconiosis model, asbestos consumption is included in the asbestosis model, coal production is included in the coal worker's pneumoconiosis model, and gold production is included in the silicosis model.

Covariate Influences:

The following plots show the influence of each covariate on the four CODEm models (male global, male data rich, female global, and female data rich). A positive standardized beta (to the right) means that the covariate was associated with increased death. A negative standardized beta (to the left) means the covariate was associated with decreased death.

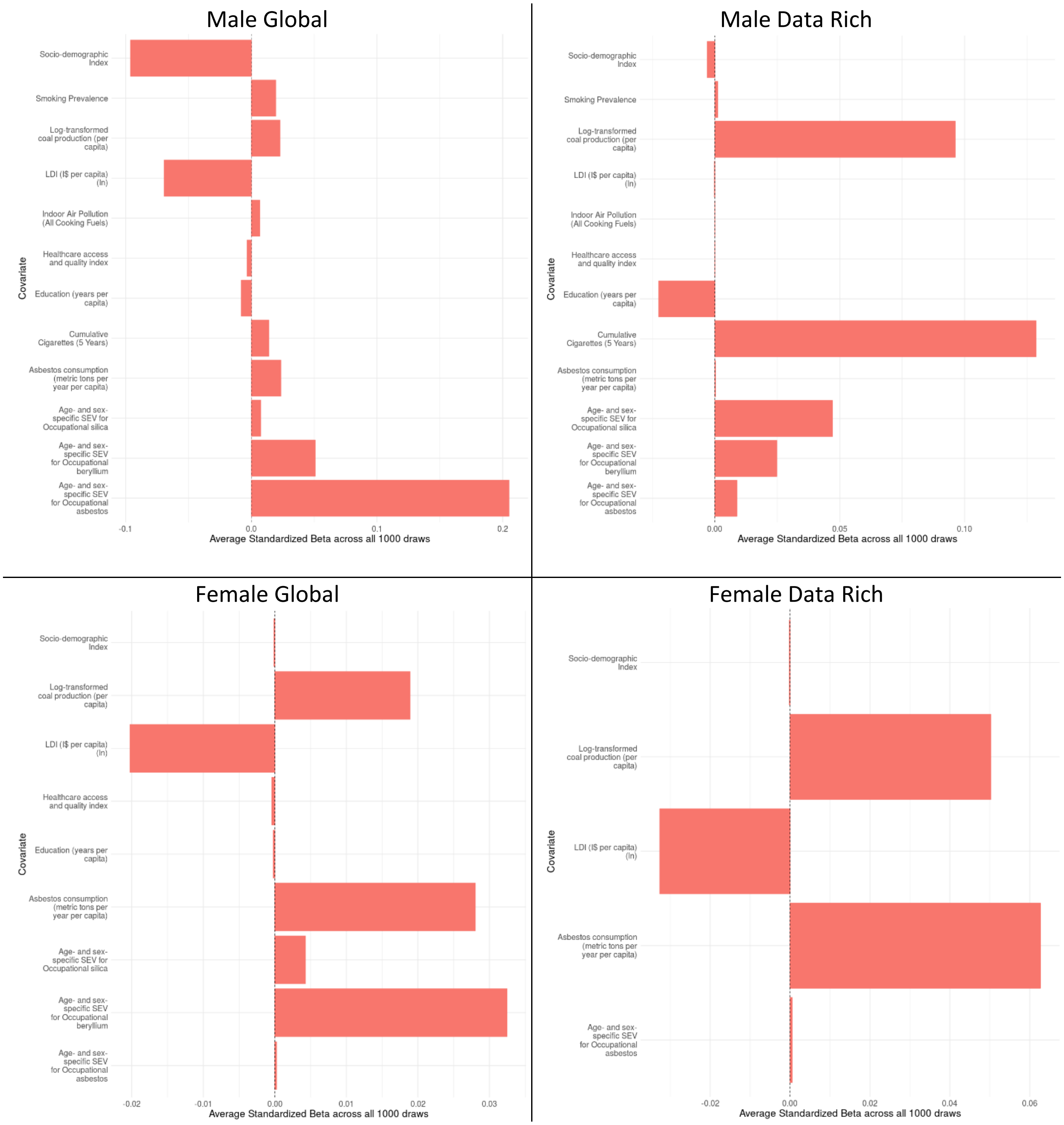

Asthma

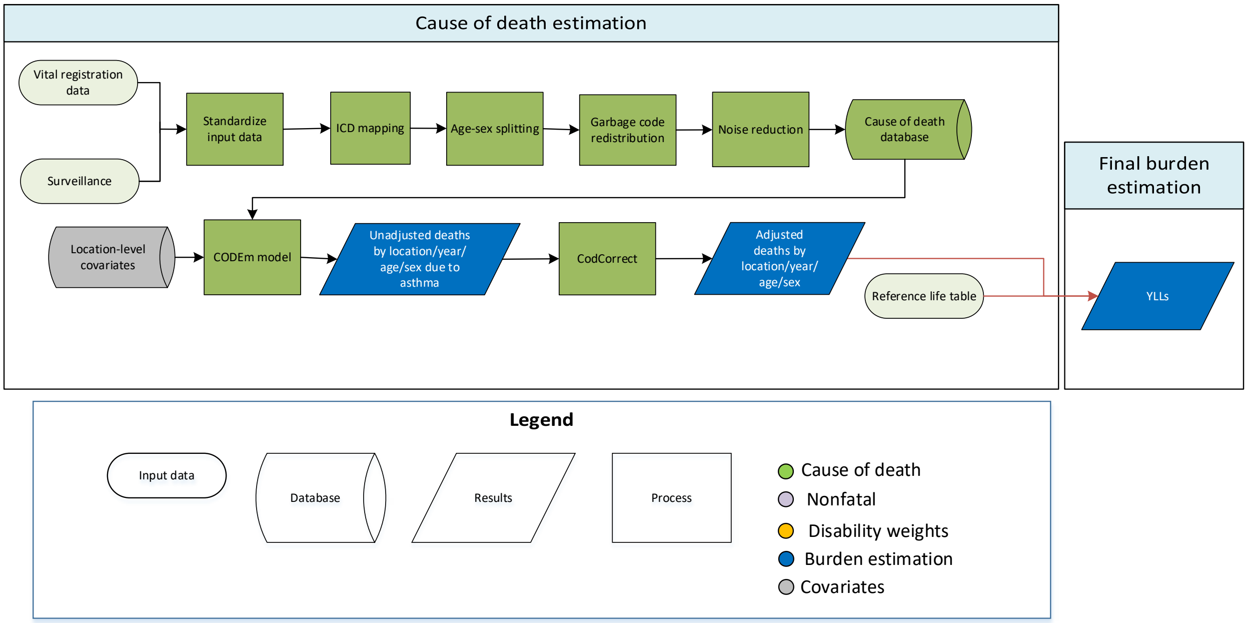

Input data

Data used to estimate asthma mortality included vital registration and surveillance data from the cause of death (COD) database. Verbal autopsy data were not included and were instead mapped to an overall chronic respiratory model. Our outlier criteria excluded data points that (1) were implausibly high or low relative to global or regional patterns, (2) substantially conflicted with established age or temporal patterns, or (3) significantly conflicted with other data sources conducted from the same locations or locations with similar characteristics (ie, Socio-demographic Index).

Modelling strategy

The standard CODEm modelling approach was applied to estimate deaths due to asthma. Separate models were conducted for male and female mortality, and the age range for both models was 1–95+ years.

Key Changes from GBD 2017

- We added estimates for the following new locations: Monaco, San Marino, Cook Islands, Nauru, Niue, Palau, Tokelau, Tuvalu, Monaco, San Marino, St Kitts and Nevis
- We added subnational location data for the following: Italy, Poland, Pakistan, the Philippines, and Nigeria
- We switched the covariate from log income per capita to a 10-year lagged income per capita.

The following table has the full list of covariates used in GBD 2019.

Level	Covariate	Direction
1	log-transformed SEV scalar: asthma	+
	cumulative cigarettes (10 years)	+
	cumulative cigarettes (5 years)	+
	healthcare access and quality index	-
2	smoking prevalence	+
	indoor air pollution (all cooking fuels)	+
	outdoor air pollution ($PM_{2.5}$)	+
3	lagged 10 year LDI (I$ per capita)	-
	education (years per capita)	-
	socio-demographic index	-

Asthma is a “child’ disease that is fit into an overall chronic respiratory disease model. In CODCorrect, the unadjusted death estimates for asthma are combined with those for chronic obstructive pulmonary disease, interstitial lung disease and pulmonary sarcoidosis, pneumoconiosis, and other chronic respiratory diseases and fit to the distribution of deaths in an overall chronic respiratory disease “parent” model. This results in deaths recorded using non-specific coding systems, such as verbal autopsy, being included in the parent model and redistributed to the child models proportionately.

Covariate Influences:

The following plots show the influence of each covariate on the four CODEm models (male global, male data rich, female global, and female data rich). A positive standardized beta (to the right) means that the covariate was associated with increased death. A negative standardized beta (to the left) means the covariate was associated with decreased death.

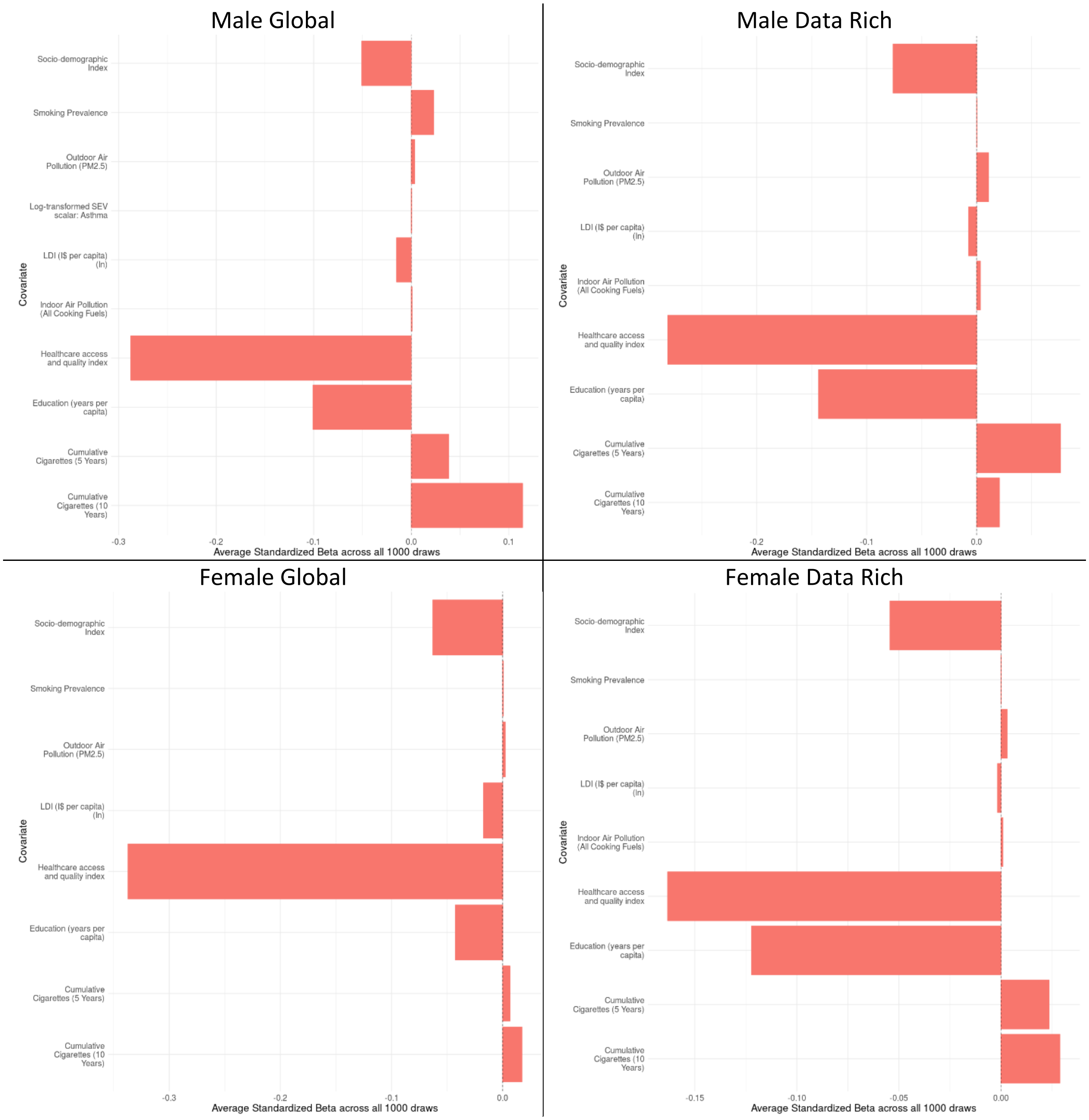

Interstitial Lung Disease and Pulmonary Sarcoidosis

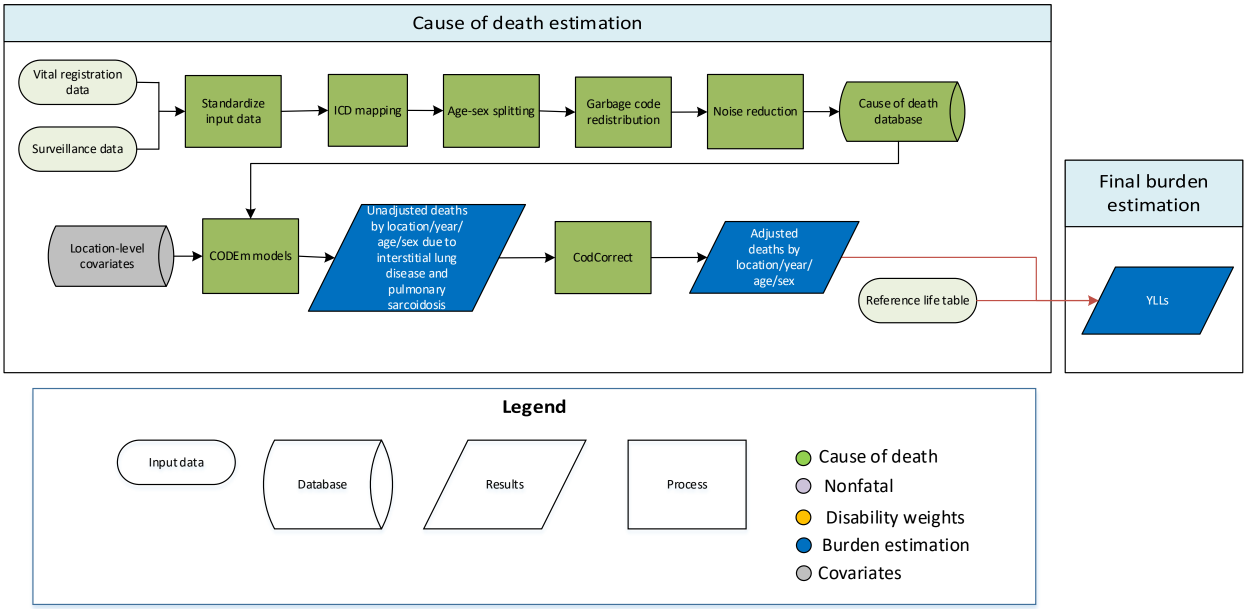

Input data

Data used to estimate interstitial lung disease and pulmonary sarcoidosis mortality included vital registration and surveillance data from the cause of death (COD) database. Our outlier criteria excluded data points that (1) were implausibly high or low, (2) substantially conflicted with established age or temporal patterns, or (3) substantially conflicted with other data sources conducted from the same locations or locations with similar characteristics (ie, Socio-demographic Index).

Modelling strategy

The standard CODEm modelling approach was applied to estimate deaths due to interstitial lung disease and pulmonary sarcoidosis. Separate models were conducted for male and female mortality, and the age range for both models was 1–95+ years.

Key Changes from GBD 2017

- We added estimates for the following new locations: Monaco, San Marino, Cook Islands, Nauru, Niue, Palau, Tokelau, Tuvalu, Monaco, San Marino, St Kitts and Nevis
- We added subnational location data for the following: Italy, Poland, Pakistan, the Philippines, and Nigeria
- We removed the covariate for the population density and added a covariate for the proportion of employed population working in professional occupations.
- The direction for the socio-demographic index covariate was changed from no direction to a negative in 2019.

The following covariates were used for GBD 2019:

Level	Covariate	Direction
1	log-transformed SEV scalar: interstitial lung disease	+
	smoking prevalence	+
	cumulative cigarettes (5 years)	+
	occupational professionals	-
2	elevation over 1,500m (proportion)	+
	elevation between 500 and 1,500m (proportion)	+
	indoor air pollution (all cooking fuels)	+
	outdoor air pollution ($PM_{2.5}$)	+
	healthcare access and quality index	-
3	log LDI (I$ per capita)	-
	education (years per capita)	-
	socio-demographic index	-

Interstitial lung disease and pulmonary sarcoidosis is a “child’ disease that is fit into an overall chronic respiratory disease model. The unadjusted death estimates from interstitial lung disease and pulmonary sarcoidosis are summed alongside other “child” causes (chronic obstructive pulmonary disease, asthma, and pneumoconiosis) and fit to the distribution of deaths in an overall chronic respiratory disease “parent” model as part of the CODCorrect adjustment process. This results in deaths recorded using non-specific coding systems, such as verbal autopsy, being included in the parent model and redistributed to the child models proportionately.

Covariate Influences:

The following plots show the influence of each covariate on the four CODEm models (male global, male data rich, female global, and female data rich). A positive standardized beta (to the right) means that the covariate was associated with increased death. A negative standardized beta (to the left) means the covariate was associated with decreased death.

Male Global

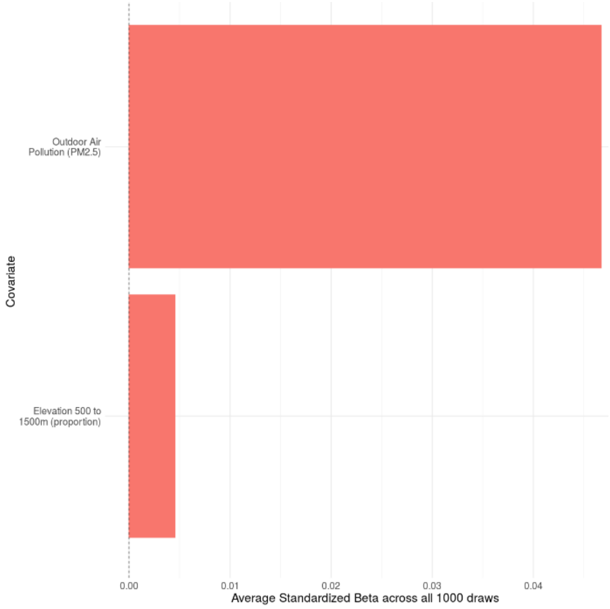

Male Data Rich

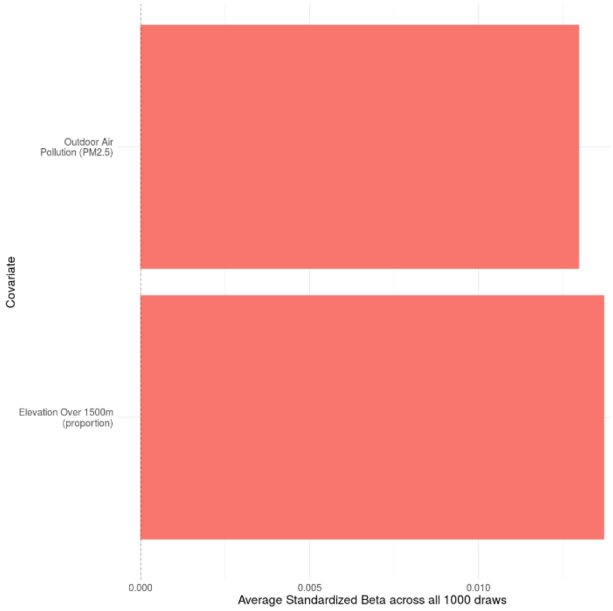

Female Global

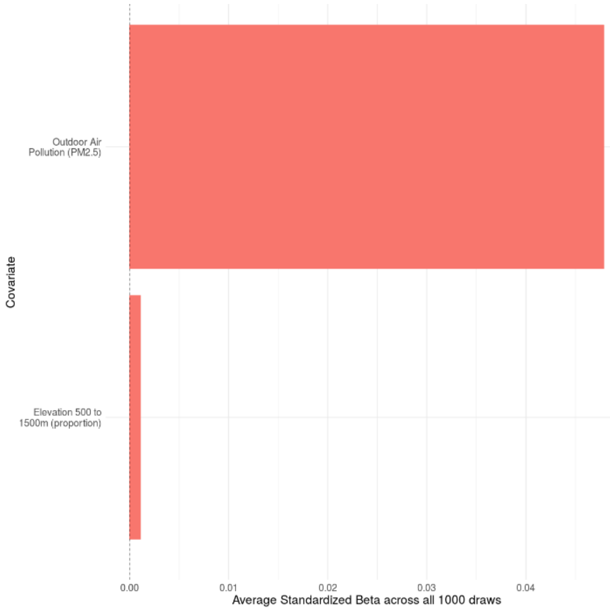

Female Data Rich

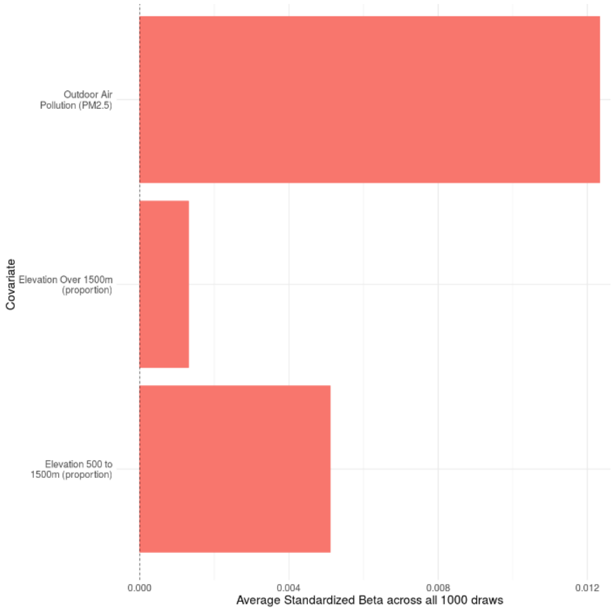

Other Chronic Respiratory Diseases

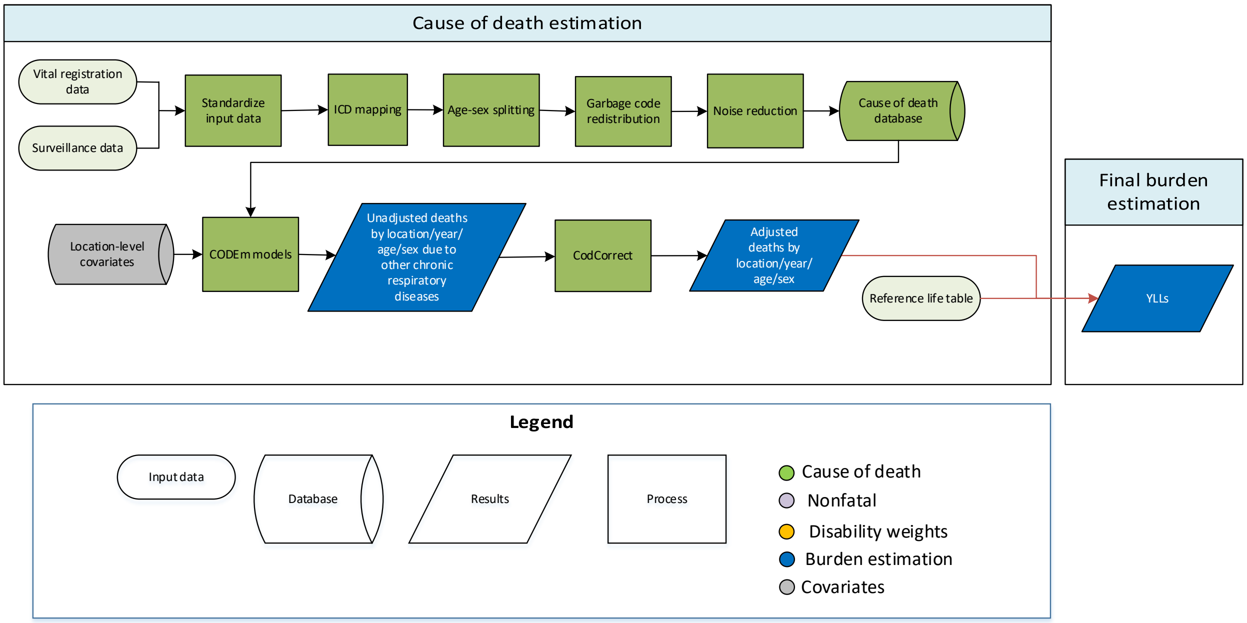

Input data

Data used to estimate other chronic respiratory diseases included vital registration and surveillance data from the cause of death (COD) database. Our outlier criteria excluded data points that (1) were implausibly high or low, (2) substantially conflicted with established age or temporal patterns, or (3) substantially conflicted with other data sources conducted from the same locations or locations with similar characteristics (ie, Socio-demographic Index).

Modelling strategy

The standard CODEm modelling approach was applied to estimate deaths due to other chronic respiratory diseases. Separate models were conducted for male and female mortality, and the age range for both models was 1 year to 95+ years.

Key Changes from GBD 2017

- We removed the log transformed SEV and changed log income per capita into a 10 year-lagged income per capita.

The following covariates were used for GBD 2019:

Level	Covariate	Direction
1	smoking prevalence	+
	cumulative cigarettes (5 years)	+
	indoor air pollution (all cooking fuels)	+

	outdoor air pollution ($PM_{2.5}$)	+
2	elevation over 1,500m (proportion)	+
	elevation between 500 and 1,500m (proportion)	+
	population density over 1,000 ppl/km^2 (proportion)	+
	healthcare access and quality index	-
3	LDI (I$ per capita)	-
	education (years per capita)	-
	socio-demographic Index	-

Other chronic respiratory is a "child' cause that is fit into an overall chronic respiratory disease model. The unadjusted death estimates from Other chronic respiratory are summed alongside unadjusted estimates for other "child" causes (chronic obstructive pulmonary disease, interstitial lung disease and pulmonary sarcoidosis, pneumoconiosis and asthma) and fit to the distribution of deaths in an overall chronic respiratory disease "parent" model. This results in deaths recorded using non-specific coding systems, such as verbal autopsy, being included in the parent model and redistributed to the child models proportionately.

Covariate Influences:

The following plots show the influence of each covariate on the four CODEm models (male global, male data rich, female global, and female data rich). A positive standardized beta (to the right) means that the covariate was associated with increased death. A negative standardized beta (to the left) means the covariate was associated with decreased death.

Male Global

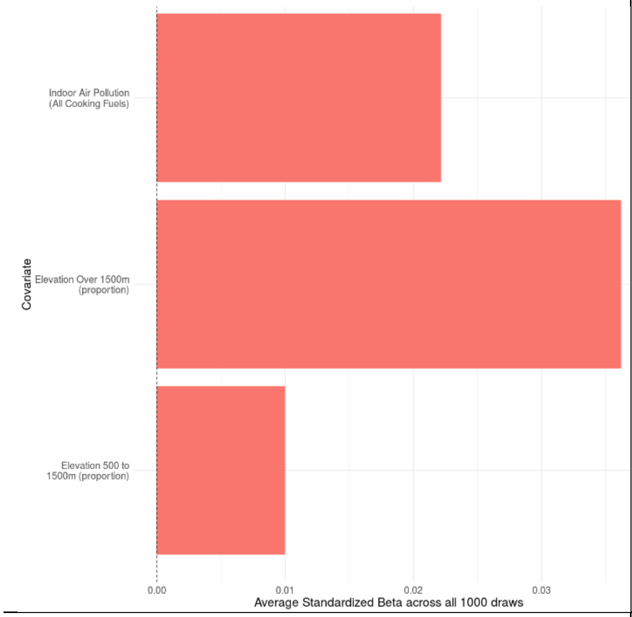

Male Data Rich

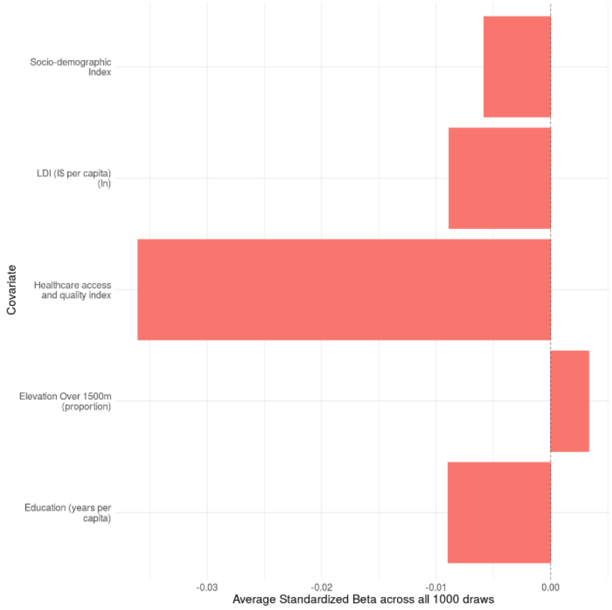

Female Global

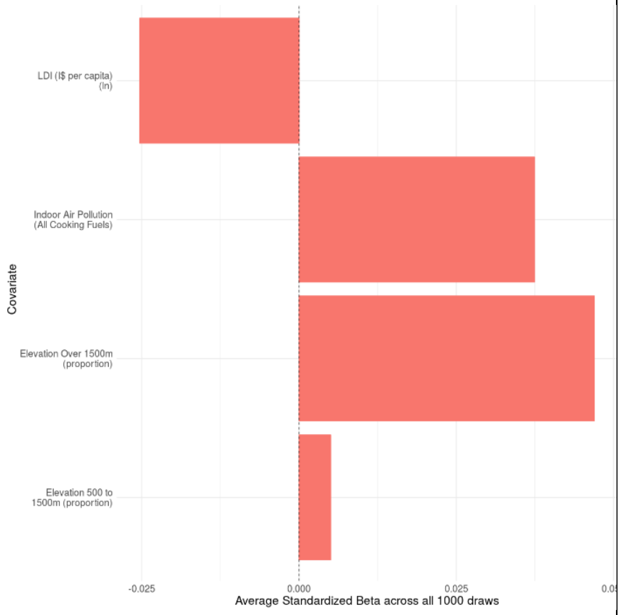

Female Data Rich

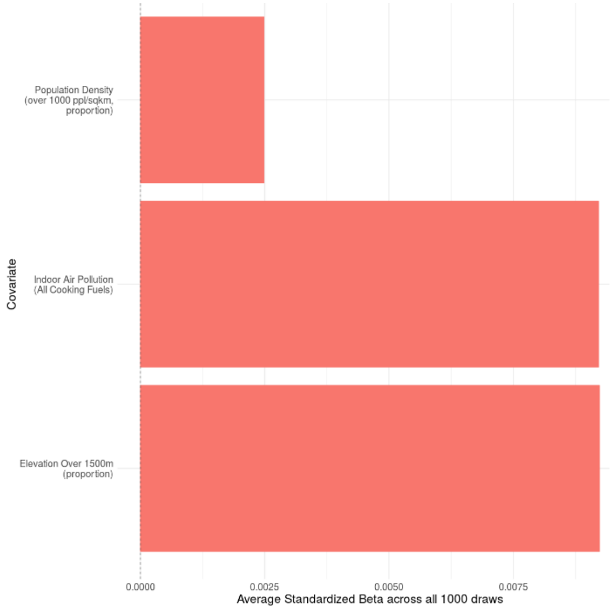

Digestive diseases

Flowchart

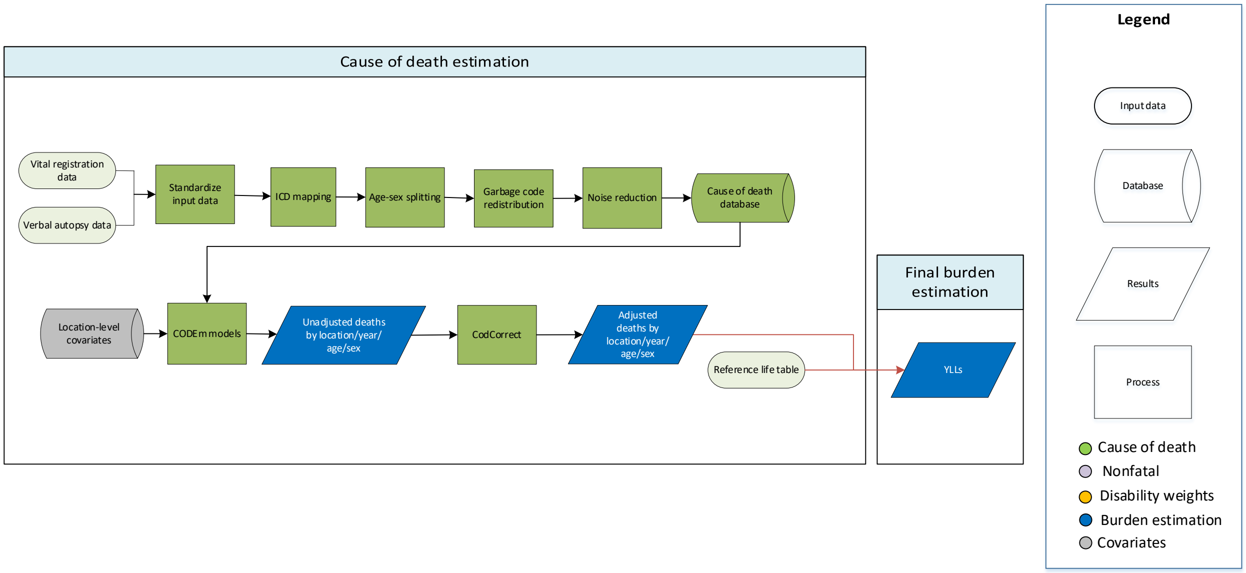

Input data

Data used to estimate mortality of digestive diseases consisted of vital registration data and verbal autopsy data from the cause of death (COD) database. The data in digestive diseases consisted of aggregated data from all other specific digestive diseases (peptic ulcer disease, gastritis and duodenitis, gallbladder and biliary diseases, pancreatitis, cirrhosis and other chronic liver diseases, inguinal, femoral and abdominal hernias, inflammatory bowel disease, vascular intestinal disorders, paralytic ileus and intestinal obstruction), as well as unique data points from deaths reported with a set of non-specific digestive disease codes.

We marked data as outliers and excluded them in instances where garbage code redistribution and noise reduction, in combination with small sample sizes, resulted in unreasonable cause fractions. We also marked as outliers those data that violated well-established time or age trends. Methods for selecting outliers were consistent across both vital registration and verbal autopsy data.

Modelling strategy

The estimation strategy used for fatal digestive diseases is largely similar to methods used in GBD 2017. A standard CODEm model with location-level covariates was used to model deaths due to digestive diseases (see appendix section on CODEm method for details). Separate models were conducted for male and female mortality, and age-restrictions for death estimations included 0 days for lower bound and 95+ for upper bound. We hybridised separate global and data-rich models to acquire unadjusted results, which we finalised and adjusted using CodCorrect to reach final YLLs due to digestive diseases.

Key changes from GBD 2017

- We added estimates for the following new locations: Monaco, San Marino, Cook Islands, Palau, and Saint Kitts and Nevis.

- We added subnational location data for the following: Italy, Poland, Pakistan, and the Philippines.
- We replaced adjusted dietary covariates with age-sex-specific scaled exposure variable covariates with a direction of 1.
- We newly added the red meat consumption and smoking prevalence covariates. The direction of the Socio-demographic Index covariate also changed from 0 to -1 in GBD 2019.

The following table has the full list of covariates used for fatal digestive diseases.

Table 1. Covariates used in digestive diseases mortality modelling

Level	Covariate	Direction
1	Sanitation (proportion with access)	-
	Cumulative cigarettes (10 years)	+
	Cumulative cigarettes (5 years)	+
	Smoking prevalence	+
	Alcohol (litres per capita)	+
2	Mean BMI	+
	Age-sex-specific scaled exposure variable for low fruit consumption	+
	Age-sex-specific scaled exposure variable for low vegetable consumption	+
	Age-sex-specific scaled exposure variable for high red meat consumption	+
	Healthcare Access and Quality Index	-
3	Socio-demographic Index	-
	Education (year per capita)	-
	Log LDI ($I per capita)	-

Adjustment in CodCorrect included fitting unadjusted death estimates for all other specific and non-specific digestive diseases to overall digestive disease deaths, which was, then, adjusted with all other causes to sum to all-cause counts of death.

Cirrhosis

Flowchart

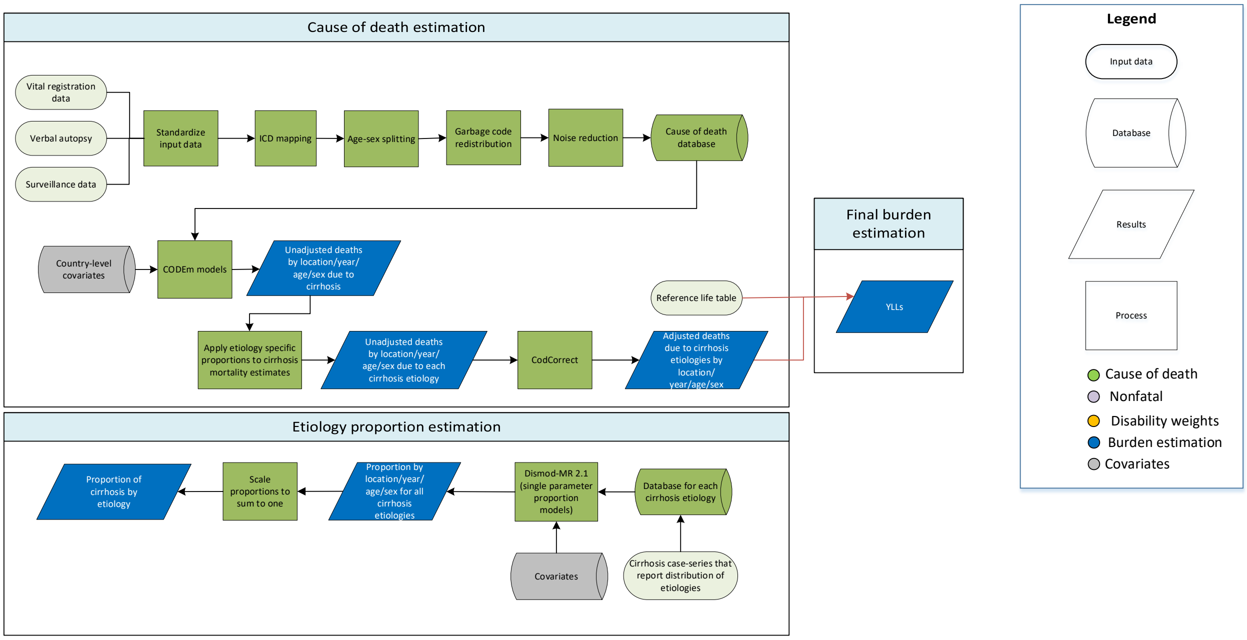

Input Data and Methodological Summary for Cirrhosis

Input data

We modelled cirrhosis mortality using vital registration and verbal autopsy data in the cause of death database. See the appendix section on causes of death data preparation for detailed description of this database. We marked data as outliers and excluded them in instances where garbage code redistribution and noise reduction, in combination with small sample sizes, resulted in unreasonable cause fractions or unreasonable time, age, or spatial trends.

Additionally, we use data from cirrhosis case-series that report the proportion of cirrhosis cases attributed to alcohol, hepatitis B, hepatitis C, NASH and other causes. See the nonfatal methods appendix on cirrhosis estimation for the details of this database. In GBD 2019 12 new case-series studies from GBD collaborators were added.

Modeling strategy

We modelled total cirrhosis mortality using a standard CODEm approach, restricting to ages 1 to 95+. Predictive covariates entered for selection in this CODEm model are shown in the table below. Proportions of cirrhosis due to alcohol, cirrhosis due to hepatitis B, cirrhosis due to hepatitis C, cirrhosis due to other causes, and cirrhosis due to NASH/NAFLD were modeled using DisMod-MR 2.1. Proportions from the five aetiology models were then rescaled to sum to one (at the draw level) and used to split the total cirrhosis mortality estimates from CODEm. The summary of DisMod model covariates are listed below.

Covariates used in CODem model for Cirrhosis and other chronic liver diseases (parent)

Level	Covariate	Direction
1	Liters of alcohol per capita	+
	Seroprevalence (HBsAg) age standardized	+
	Seroprevalence (anti-HCV) age standardized	+
	Hepatitis B vaccine coverage proportion, aged through time	-
2	Mean BMI	+
	Healthcare access and quality index	-
	Diabetes prevalence age standardized	+
	Schistosomiasis prevalence	+
	Intravenous drug use	+
3	Education (years per capita)	-
	Lag distributed income (LDI) (ln transformation)	-
	Socio-demographic index	-

Covariates used in the Proportion of cirrhosis due to hepatitis B DisMod-MR meta-regression model

Covariate	Exponentiated beta<br>(95% Uncertainty Interval)
Seroprevalence (HBsAg) age standardized	2.37 (1.88 — 2.70)
Proportion of liver cancer due to hepatitis B (age-standardised)	1.59 (1.17 — 2.16)
Hepatitis B 3-dose coverage (proportion), lagged 10 years	0.50 (0.45 — 0.55)
Proportion of cirrhosis due to alcohol	0.88 (0.70 — 0.99)
Proportion of cirrhosis due to hepatitis C	0.41 (0.37 — 0.50)
Proportion of cirrhosis due to other causes	0.93 (0.82 — 1.00)
Proportion of cirrhosis due to NASH	0.69 (0.45 — 0.98)

Covariates used in the Proportion of cirrhosis due to hepatitis C DisMod-MR meta-regression model

Covariate	Exponentiated beta<br>(95% Uncertainty Interval)
Seroprevalence (anti-HCV) age standardized	1.72 (1.07 — 2.59)
Proportion of liver cancer due to hepatitis C (Age Standardized)	1.81 (1.14 — 2.62)
Proportion of cirrhosis due to alcohol	0.44 (0.37 — 0.60)
Proportion of cirrhosis due to hepatitis B	0.64 (0.40 — 0.96)
Proportion of cirrhosis due to other causes	0.90 (0.76 — 1.00)
Proportion of cirrhosis due to NASH	0.58 (0.38 — 0.91)

Covariates used in the Proportion of cirrhosis due to alcohol DisMod-MR meta-regression model

Covariate	Exponentiated beta<br>(95% Uncertainty Interval)
Liters of alcohol consumed per capita	1.02 (1.00 — 1.04)
Alcohol abstainer proportion, age-standardized	0.90 (0.76 — 1.00)
Proportion of liver cancer due to alcohol (Age Standardized)	1.40 (1.02 — 2.21)
Proportion of cirrhosis due to hepatitis B	0.83 (0.63 — 0.99)

Proportion of cirrhosis due to hepatitis C	0.43 (0.37 — 0.60)
Proportion of cirrhosis due to other causes	0.68 (0.45 — 0.95)
Proportion of cirrhosis due to NASH	0.65 (0.42 — 0.96)

Covariates used in the Proportion of cirrhosis due to other causes DisMod-MR meta-regression model

Covariate	Exponentiated beta (95% Uncertainty Interval)
Proportion of liver cancer due to other causes (Age Standardized)	1.59 (1.05 — 2.56)
Proportion of cirrhosis due to hepatitis B	0.59 (0.39 — 0.91)
Proportion of cirrhosis due to hepatitis C	0.92 (0.78 — 1.0)
Proportion of cirrhosis due to alcohol	0.41 (0.37 — 0.50)
Proportion of cirrhosis due to NASH	0.64 (0.42 — 0.94)

Covariates used in the Proportion of cirrhosis due to NASH DisMod-MR meta-regression model

Covariate	Exponentiated beta (95% Uncertainty Interval)
Mean BMI	1.00 (1.00 — 1.01)
Prevalence of obesity	1.16 (1.01 — 1.50)
NAFLD/NASH prevalence	2.20 (1.07 — 5.08)
Proportion of liver cancer due to NASH (Age Standardized)	3.88 (1.59 — 7.13)
Proportion of cirrhosis due to hepatitis B	0.48 (0.37 — 0.78)
Proportion of cirrhosis due to hepatitis C	0.88 (0.70 — 0.99)
Proportion of cirrhosis due to alcohol	0.43 (0.37 — 0.56)
Proportion of cirrhosis due to other causes	0.73 (0.53 — 0.96)

Compared to GBD 2017, modeling the proportions of cirrhosis due to NASH vs "other causes" changed in GBD 2019. Epidemiological studies and hepatologists have indicated that cryptogenic cases of cirrhosis may be un-identified cases of cirrhosis due to NASH. In GBD 2017, when a cirrhosis case-series identified all of our aetiologies of interest as well as cryptogenic cirrhosis, cryptogenic cases were extracted as "other causes", but when a case-series did not explicitly identify NASH, cases reported as "cryptogenic" were extracted as NASH. In GBD 2019 we analyzed case-series studies that reported both NASH and cryptogenic cases, modeling the proportion due to NASH (out of NASH plus cryptogenic) in MR-BRT. We then identified the case-series in our database that reported cryptogenic, but not NASH, as an aetiology of cirrhosis, and extracted a proportion due to NASH and a proportion due to other causes based on the proportion modeled in MR-BRT.

Proportion of cryptogenic cases in studies that did not specify NASH believed to be NASH, as modeled in MR-BRT

Data input	Beta Coefficient, Logit (95% CI)	Gamma
Proportion of cryptogenic cases out of cryptogenic cases plus NASH cases reported in the same study	0.624 (-0.659 – 1.887)	0.567

Upper Digestive Diseases

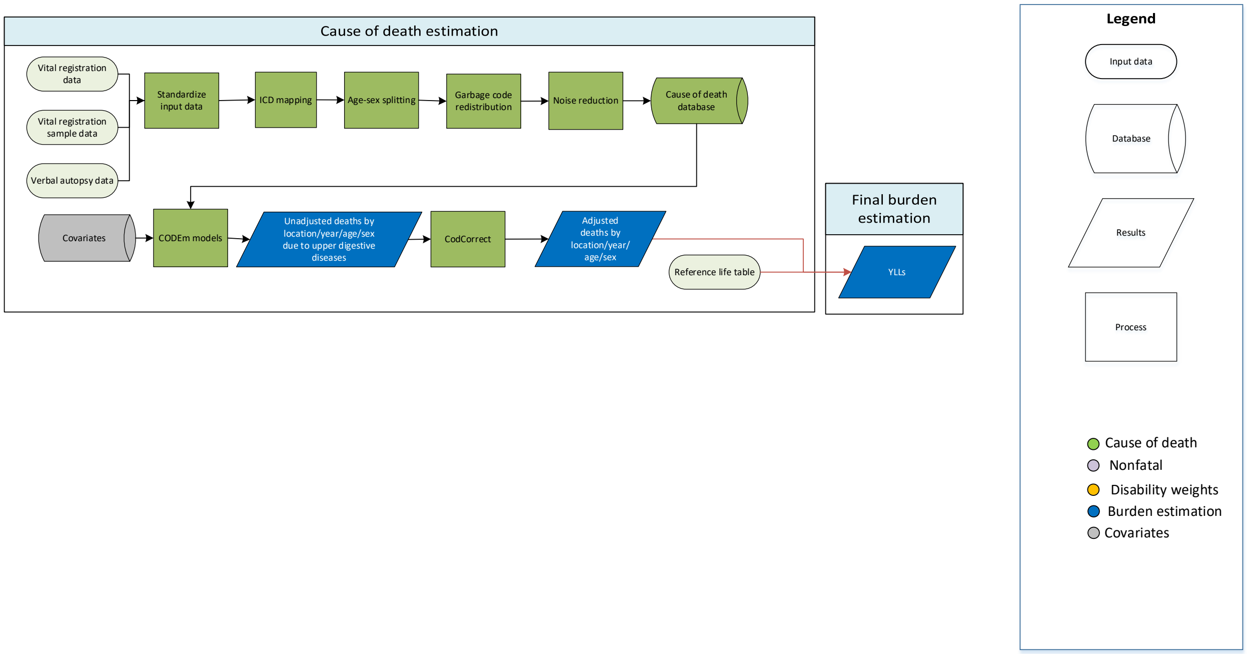

Input data

Data used to estimate mortality due to upper digestive diseases consisted of vital registration data, vital registration sample data, and verbal autopsy data from the cause of death (COD) database. Upper digestive disease data aggregate deaths due to peptic ulcer disease and gastritis and duodenitis, which are also modelled separately. For sources of data that were considered too low-quality to definitively assign peptic ulcer or gastritis deaths to one of these two causes, data were included only in the upper digestive disease dataset.

We marked data as outliers and excluded them in instances where garbage code redistribution and noise reduction, in combination with small sample sizes, resulted in unreasonable cause fractions or unreasonable time, age, or spatial trends; data from Tibet and Kiribati were excluded for these reasons. In situations where unreasonable temporal and spatial trends were observed at transitions between data sources, higher-quality data-sources were retained and lower-quality sources were excluded; this affected subnational locations in India, where vital registration data biased toward in-hospital deaths were available for urban locations only (MCCD), whereas high-quality verbal autopsy data with representative sampling were available for both urban and rural locations.

Modelling strategy

We modelled deaths due to upper digestive diseases with a standard CODEm model. The model followed standard parameters, with the exception that the start age of the model was 1 year and the linear floor rate was lowered to 0.0001 in order to better capture low data.

Covariates entered into CODEm were the same in GBD 2019 as GBD 2017, with the following exceptions: covariates related to water and sanitation were promoted from level 2 to level 1, the alcohol covariate was demoted from level 1 to level 2, maternal education was replaced by a general education covariate,

and the adjusted vegetable covariate was replaced by an unadjusted vegetable covariate and forced to take a negative direction (or not be selected). A complete list is provided in the table below.

Covariate	Level	Direction
Sanitation, proportion with access	1	-1
Scaled exposure variable for unsafe water source	1	1
Smoking prevalence	1	1
Cumulative cigarettes (10 years)	1	1
Cumulative cigarettes (5 years)	1	1
Litres of alcohol consumed per capita	2	1
Vegetables (grams, unadjusted)	2	-1
Healthcare access and quality index	2	-1
Lag distributed income (per capita)	3	-1
Education (years per capita)	3	-1
Socio-demographic Index	3	-1

Adjustment in CoDCorrect included fitting estimates for peptic ulcer disease and gastritis and duodenitis to all upper digestive disease deaths first before the adjustment with all other cause to sum to all-cause counts of death.

Peptic Ulcer Disease

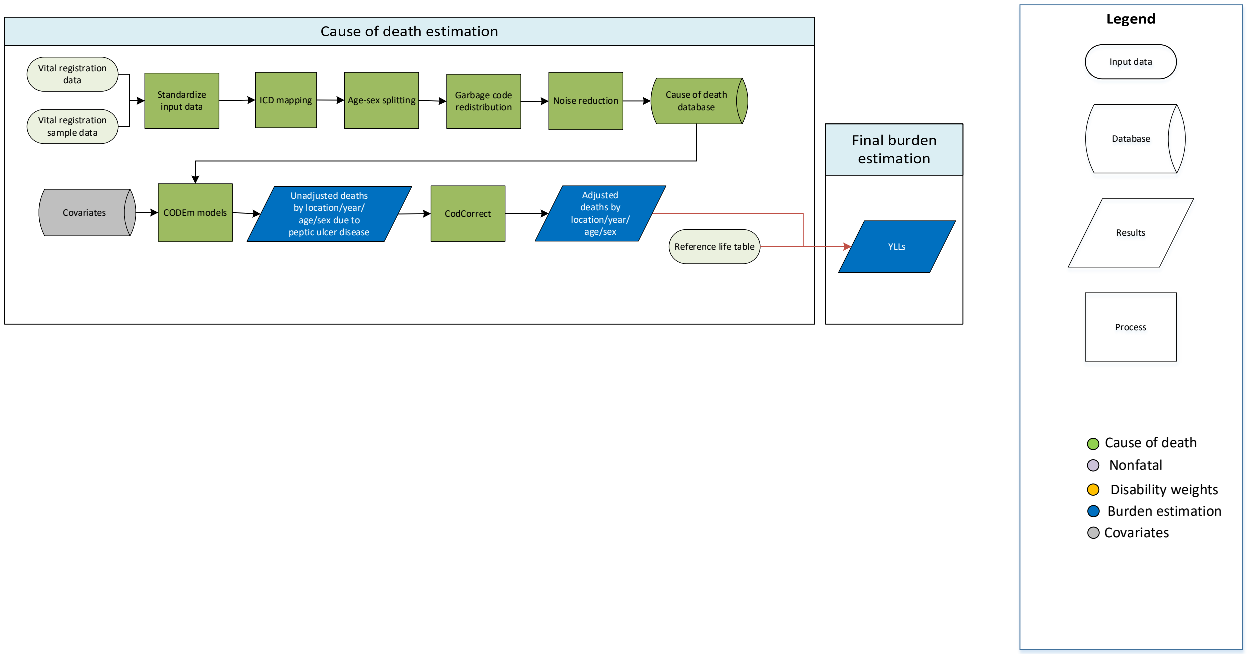

Input data

Data used to estimate unadjusted mortality of peptic ulcer disease consisted of vital registration data and vital registration sample data from those sources in the cause of death (COD) database that use ICD9 or ICD10 codes and report un-tabulated (individual) deaths. We marked data as outliers and excluded them in instances where garbage code redistribution and noise reduction, in combination with small sample sizes, resulted in unreasonable cause fractions or unreasonable time, age, or spatial trends; data from Tibet, Fiji, Kiribati, Palestine, Stockholm, and Mozambique were excluded for these reasons. In situations where unreasonable temporal and spatial trends were observed at transitions between data sources, higher-quality data-sources were retained and lower-quality sources were excluded; this affected Kazakhstan, at the transition between ICD9-BTL and ICD10 coding, and subnational locations in India, where vital registration data biased toward in-hospital deaths (MCCD) were available for urban locations only.

Modelling strategy

We modelled deaths due to peptic ulcer disease with a standard CODEm model. The model followed standard parameters, with the exception that the start age of the model was 1 year instead of 0 and the linear floor rate was lowered to 0.0001 in order to better capture low data.

Covariates entered into CODEm were the same in GBD 2019 as GBD 2017, with the following exceptions: covariates related to water and sanitation were promoted from level 2 to level 1, the alcohol covariate was demoted from level 1 to level 2, maternal education was replaced by a general education covariate, and the adjusted vegetable covariate was replaced by an unadjusted vegetable covariate and forced to take a negative direction (or not be selected). A complete list is provided in the table below.

Covariate	Level	Direction
Sanitation, proportion with access	1	-1
Scaled exposure variable for unsafe water source	1	1

Smoking prevalence	1	1
Cumulative cigarettes (10 years)	1	1
Cumulative cigarettes (5 years)	1	1
Litres of alcohol consumed per capita	2	1
Vegetables (grams, unadjusted)	2	-1
Healthcare access and quality index	2	-1
Lag distributed income (per capita)	3	-1
Education (years per capita)	3	-1
Socio-demographic Index	3	-1

In CoDCorrect estimates for peptic ulcer disease and gastritis and duodenitis were first adjusted to sum to all upper digestive disease deaths, and then to sum to all-cause mortality with all other causes.

Gastritis and Duodenitis

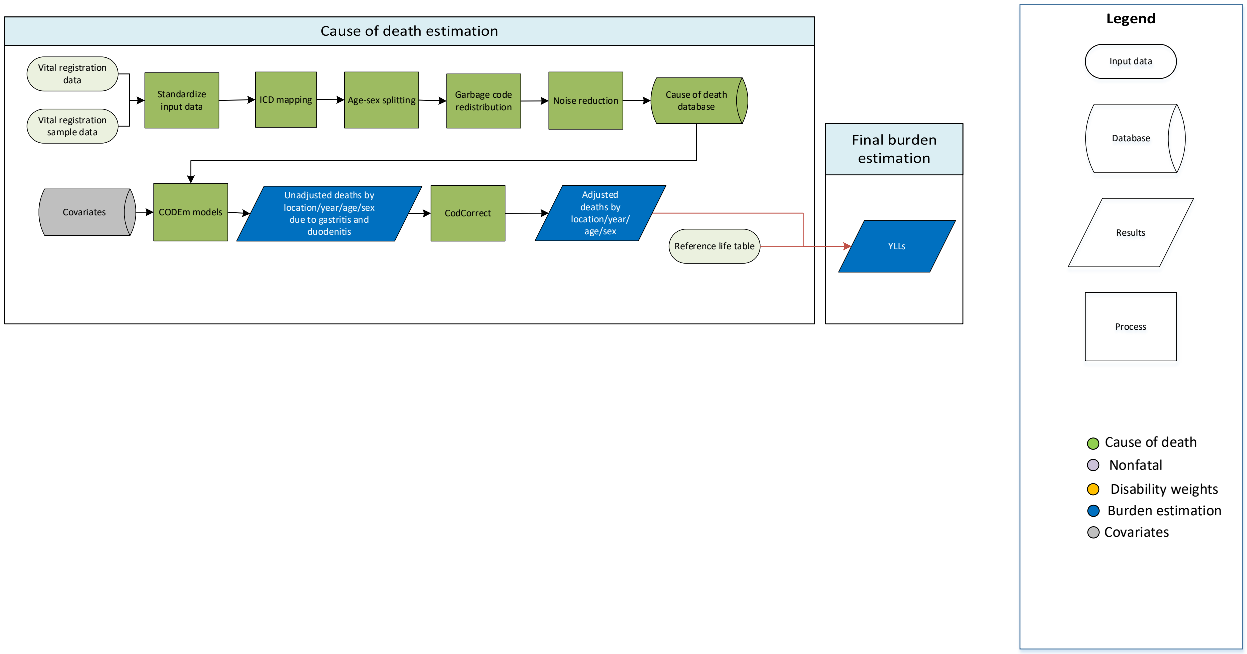

Input data

Data used to estimate unadjusted mortality of gastritis and duodenitis consisted of vital registration data and vital registration sample data from those sources in the cause of death (COD) database that use ICD9 or ICD10 codes and report un-tabulated (individual) deaths. We marked data as outliers and excluded them in instances where garbage code redistribution and noise reduction, in combination with small sample sizes, resulted in unreasonable cause fractions or unreasonable time, age, or spatial trends; data from Tibet, Yunnan, Ghana, Qatar, Kiribati, Bahrain, Palestine, and Grenada were excluded for these reasons. In situations where unreasonable temporal and spatial trends were observed at transitions between data sources, higher-quality data-sources were retained and lower-quality sources were excluded; this affected subnational locations in India, where vital registration data biased toward in-hospital deaths (MCCD) were available for urban locations only. We also excluded data for young-adult age-groups in South African subnational locations where adjustments for mis-coded HIV deaths were inadequate.

Modelling strategy

We modelled deaths due to gastritis and duodenitis with a standard CODEm model. The model followed standard parameters, with the exception that the start age of the model was 1 year instead of 0 and the linear floor rate was lowered to 0.00001 in order to better capture low data.

Covariates entered into CODEm were the same in GBD 2019 as GBD 2017, with the following exceptions: covariates related to water and sanitation were promoted from level 2 to level 1, the alcohol and smoking-related covariates were demoted from level 1 to level 2, and the adjusted vegetable covariate was replaced by an unadjusted vegetable covariate and forced to take a negative direction (or not be selected). A complete list is provided in the table below.

Covariate	Level	Direction
Sanitation, proportion with access	1	-1
Scaled exposure variable for unsafe water source	1	1
Smoking prevalence	2	1
Cumulative cigarettes (10 years)	2	1
Cumulative cigarettes (5 years)	2	1
Litres of alcohol consumed per capita	2	1
Vegetables (grams, unadjusted)	2	-1
Healthcare access and quality index	2	-1
Lag distributed income (per capita)	3	-1
Education (years per capita)	3	-1
Socio-demographic Index	3	-1

In CoDCorrect estimates for peptic ulcer disease and gastritis and duodenitis were first adjusted to sum to all upper digestive disease deaths and then to sum to all-cause mortality with all other causes.

Appendicitis

Flowchart

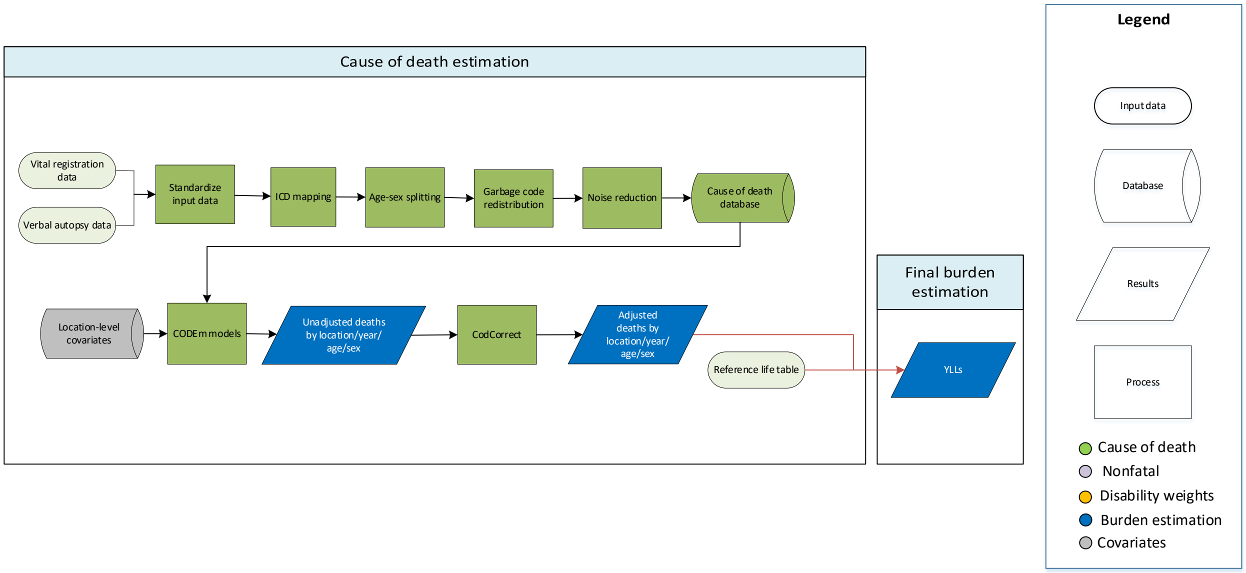

Input data

Data used to estimate mortality of appendicitis consisted of vital registration and verbal autopsy data from the cause of death (COD) database. Outliers were identified if data violated well-established time or age trends. We also excluded data in instances where garbage code redistribution and noise reduction, in combination with small sample sizes, resulted in unreasonable cause fractions.

Modelling strategy

The estimation strategy used for fatal appendicitis is largely similar to methods used in GBD 2017. A standard CODEm model with location-level covariates was used to model deaths due to appendicitis with age restrictions for death estimations of 1 year for lower bound and 95+ for upper bound (see appendix section on CODEm method for details). Separate models were conducted for male and female mortality. We hybridised separate global and data-rich models to acquire unadjusted results, which we finalised and adjusted using CodCorrect to reach final YLLs due to appendicitis.

Key changes from GBD 2017

- We added estimates for the following new locations: Monaco, San Marino, Cook Islands, and Saint Kitts and Nevis.
- We added subnational location data for the following: Italy, Poland, Pakistan, the Philippines, and Nigeria.
- We excluded the maternal care and immunisation (MCI) covariate because it is redundant with the Healthcare Access and Quality Index covariate that was pre-existing in the model. The MCI covariate is often used as a proxy for health system access measured through clinic accessibility, attendance, and immunisation status.
- We replaced adjusted dietary covariates with age-sex specific scaled exposure variable covariates with a direction of 1.
- We changed the direction of Socio-demographic Index covariate from 0 to -1.

The following table has the full list of covariates used for appendicitis.

Table 1. Covariates used in appendicitis mortality modelling

Level	Covariate	Direction
2	Age-sex-specific scaled exposure variable for low fruit consumption	+
	Age-sex-specific scaled exposure variable for low vegetable consumption	+
	Healthcare Access and Quality Index	-
3	Socio-demographic Index	-
	Education (years per capita)	-
	Log LDI ($I per capita)	-

Paralytic ileus and intestinal obstruction

Flowchart

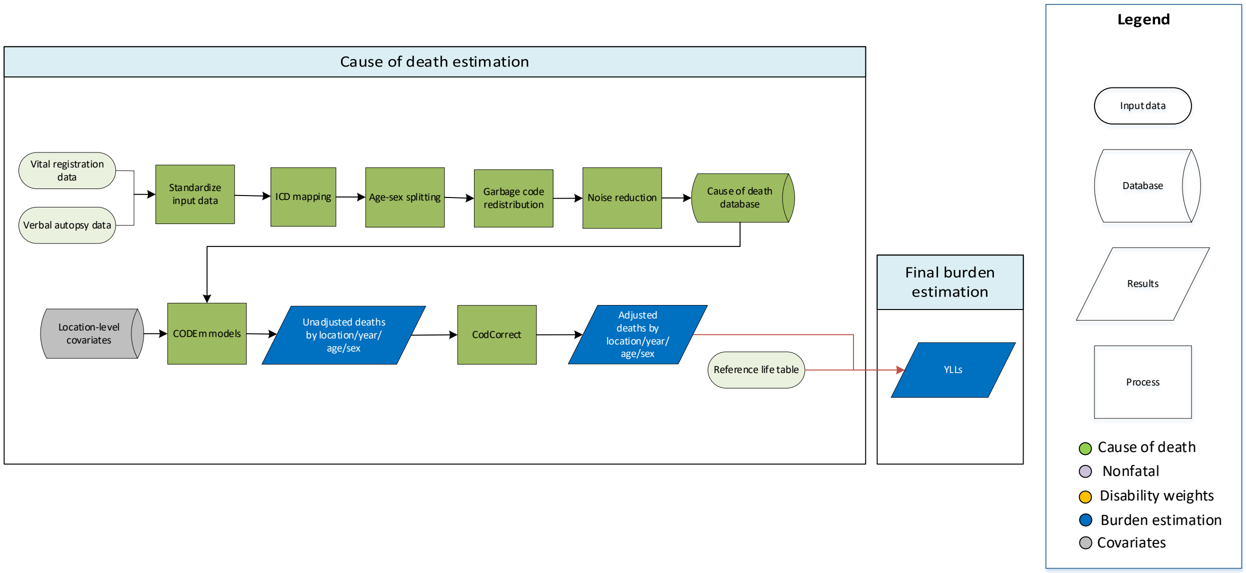

Input data

Data used to estimate mortality of paralytic ileus and intestinal obstruction consisted of vital registration and verbal autopsy data from the cause of death (COD) database. Outliers were identified by systematic examination of datapoints for all location-years. We excluded all VA data in children under the age of 1 because it is not possible to accurately diagnose paralytic ileus or intestinal obstruction in this age group using verbal autopsy methods. We also excluded data that violated well-established time or age trends; and data in instances where garbage code redistribution and noise reduction, in combination with small sample sizes, resulted in unreasonable cause fractions.

Modelling strategy

The estimation strategy used for fatal paralytic ileus and intestinal obstruction is largely similar to methods used in GBD 2017. A standard CODEm model with location-level covariates was used to model deaths due to paralytic ileus and intestinal obstruction with age restrictions for death estimations of 1 year for lower bound and 95+ for upper bound (see appendix section 3.1 details). Separate models were conducted for male and female mortality. We hybridised separate global and data-rich models to acquire unadjusted results, which we finalised and adjusted using CodCorrect to reach final YLLs due to paralytic ileus and intestinal obstruction.

Key changes from GBD 2017

- We added estimates for the following new locations: Monaco, San Marino, Cook Islands, and Saint Kitts and Nevis.
- We added subnational location data for the following: Italy, Poland, Pakistan, the Philippines, and Nigeria.
- We excluded the maternal care and immunisation (MCI) covariate because it is redundant with the Healthcare Access and Quality Index covariate that was pre-existing in the model. The MCI

covariate is often used as a proxy for health system access measured through clinic accessibility, attendance, and immunisation status.

- We replaced adjusted dietary covariates with age-sex-specific scaled exposure variable covariates with a direction of 1.
- We changed the direction of the Socio-demographic Index covariate from 0 to -1.

The following table has the full list of covariates used for paralytic ileus and intestinal obstruction.

Table 1. Covariates used in paralytic ileus and intestinal obstruction mortality modelling

Level	Covariate	Direction
2	Age-sex-specific scaled exposure variable for low fruit consumption	+
	Age-sex-specific scaled exposure variable for low vegetable consumption	+
	Healthcare Access and Quality Index	-
3	Socio-demographic Index	-
	Education (years per capita)	-
	Log LDI ($I per capita)	-

Inguinal, femoral, and abdominal hernia

Flowchart

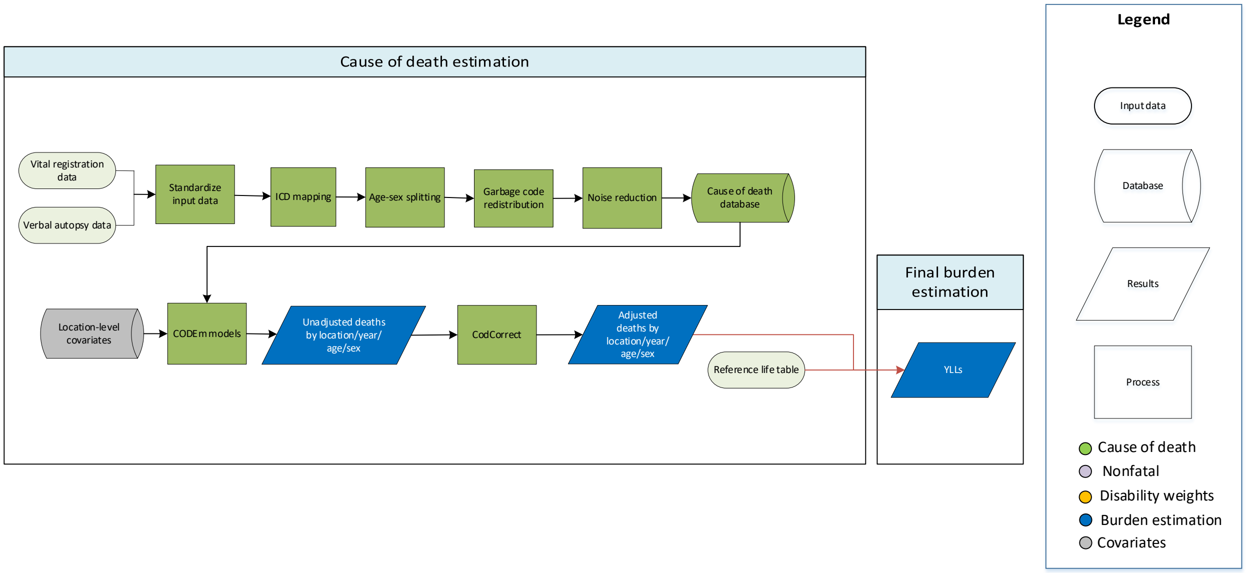

Input data

Data used to estimate mortality of inguinal, femoral, and abdominal hernia consisted of vital registration and verbal autopsy data from the cause of death (COD) database. Outliers were identified by systematic examination of datapoints for all location-years. Data that violated well-established time or age trends were marked as outliers and excluded. Data were also marked as outliers in instances where garbage code redistribution and noise reduction, in combination with small sample sizes, resulted in unreasonable cause fractions. Methods for assigning outlier status were consistent across both vital registration and verbal autopsy data.

Modelling strategy

The estimation strategy used for fatal inguinal, femoral, and abdominal hernia is largely similar to methods used in GBD 2017. A standard CODEm model with location-level covariates was used to model deaths due to inguinal, femoral, and abdominal hernia (see appendix section 3.1 for details). Separate models were conducted for male and female mortality. We hybridised separate global and data-rich models to acquire unadjusted results, which we finalised and adjusted using CodCorrect to reach final YLLs due to inguinal, femoral, and abdominal hernia.

Key changes from GBD 2017

- We added estimates for the following new locations: Monaco, San Marino, and Saint Kitts and Nevis.
- We added subnational location data for the following: Italy, Poland, Pakistan, and the Philippines.
- We excluded ICD9_BTL data sources from both male and female models because they were producing implausibly high estimates compared to ICD9_detail and ICD10_detail data sources.
- We changed the lower bound of age-restrictions for death estimations from 1 year to 0 days. The upper bound remained the same at 95+ years.

- We changed the direction of the Socio-demographic Index covariate from 0 to -1.

The following table has the full list of covariates used for fatal inguinal, femoral, and abdominal hernia.

Table 1. Covariates used in inguinal, femoral, and abdominal hernia mortality modelling

Level	Covariate	Direction
1	BMI (mean)	-
	Cumulative cigarettes (10 years)	+
	Cumulative cigarettes (5 years)	+
	Smoking prevalence	+
2	Healthcare Access and Quality Index	-
3	Socio-demographic Index	-
	Education (years per capita)	-
	Log LDI ($I per capita)	-

Inflammatory bowel disease

Flowchart

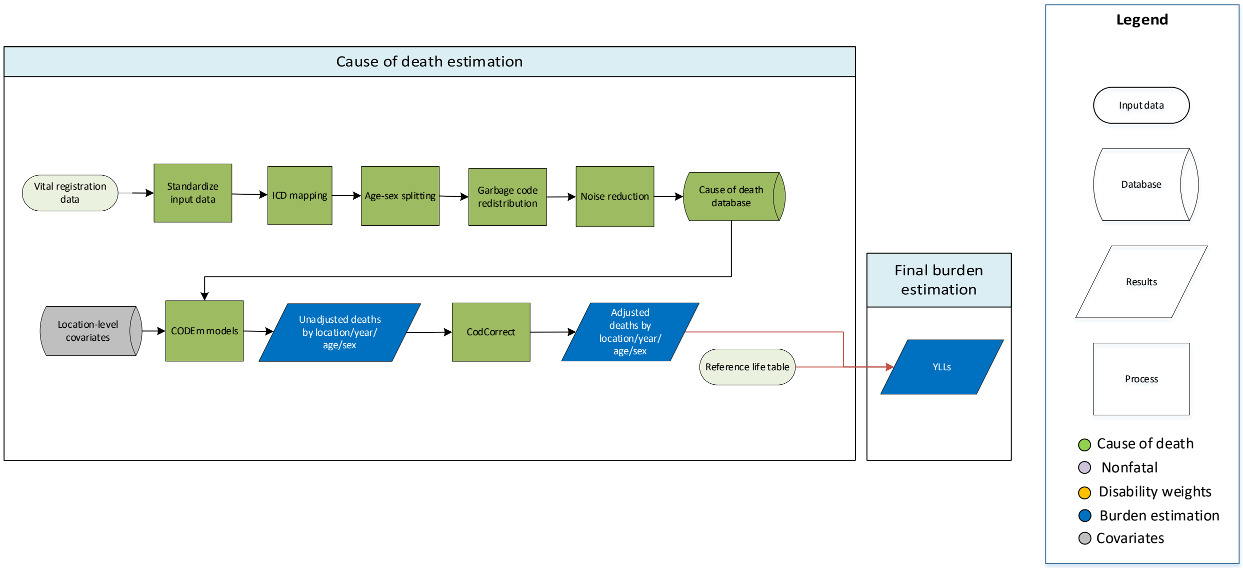

Input data

Data used to estimate mortality of inflammatory bowel disease consisted of vital registration data from the cause of death (COD) database. Outliers were identified by systematic examination of datapoints for all location-years. Data were excluded if they violated well-established time or age trends, and data in instances where garbage code redistribution and noise reduction, in combination with small sample sizes, resulted in unreasonable cause fractions.

Modelling strategy

The estimation strategy used for fatal inflammatory bowel disease is largely similar to methods used in GBD 2017. A standard CODEm model with location-level covariates was used to model deaths due to inflammatory bowel disease with age restrictions for death estimations of 1 year for lower bound and 95+ for upper bound (see appendix section 3.1 for details). Separate models were conducted for male and female mortality. We hybridised separate global and data-rich models to acquire unadjusted results, which we finalised and adjusted using CodCorrect to reach final YLLs due to inflammatory bowel disease.

Key changes from GBD 2017

- We added estimates for the following new locations: Monaco, San Marino, Palau, and Saint Kitts and Nevis.
- We added subnational location data for the following: Italy, Poland, and the Philippines.
- We changed the direction of Socio-demographic Index and lag-distributed income covariates from 0 to 1.
- We replaced adjusted dietary covariates with age-sex-specific scaled exposure variable covariates with a direction of 1.

The following table has the full list of covariates used for inflammatory bowel disease.

Table 1. Covariates used in inflammatory bowel disease mortality modelling

Level	Covariate	Direction
1	Age-sex-specific scaled exposure variable for low polyunsaturated fatty acids consumption	+
	Age-sex-specific scaled exposure variable for low fruit consumption	+
	Age-sex-specific scaled exposure variable for low vegetable consumption	+
	Age-sex-specific scaled exposure variable for high red meat consumption	+
2	Healthcare Access and Quality Index	-
	Latitude 15 to 30 (proportion)	-
	Latitude 30 to 45 (proportion)	+
	Latitude 45 plus (proportion)	+
3	Socio-demographic Index	+
	Education (years per capita)	-
	Log LDI ($I per capita)	+

Vascular intestinal disorders

Flowchart

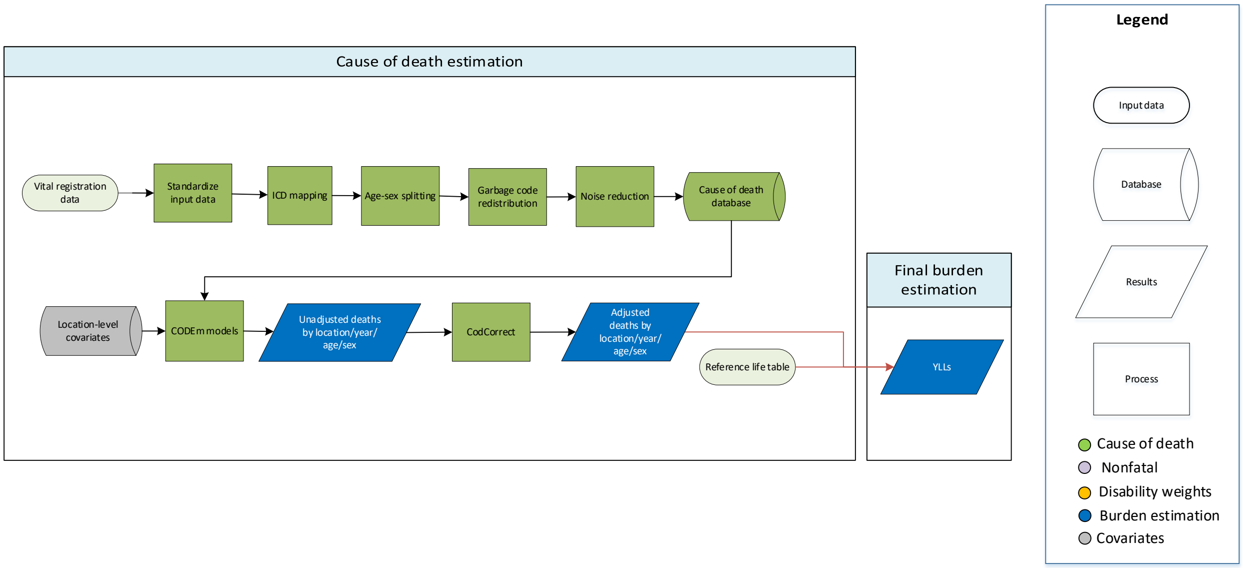

Input data

Data used to estimate mortality of vascular intestinal disorders consisted of vital registration data from the cause of death (COD) database. Outliers were identified by systematic examination of datapoints for all location-years, as well as data that violated well-established time or age trends; and data in instances where garbage code redistribution and noise reduction, in combination with small sample sizes, resulted in unreasonable cause fractions.

Modelling strategy

The estimation strategy used for fatal vascular intestinal disorders is largely similar to methods used in GBD 2017. A standard CODEm model with location-level covariates was used to model deaths due to vascular intestinal disorders with age restrictions for death estimations of 1 year for lower bound and 95+ for upper bound (see appendix section 3.1 for details). Separate models were conducted for male and female mortality. We hybridised separate global and data-rich models to acquire unadjusted results, which we finalised and adjusted using CodCorrect to reach final YLLs due to vascular intestinal disorders.

Key changes from GBD 2017

- We added estimates for the following new locations: Monaco, San Marino, Palau, and Saint Kitts and Nevis.
- We added subnational location data for the following: Italy, Poland, and the Philippines.
- We updated the list of covariates to mimic the covariates that were used in fatal ischaemic heart disease. The newly included covariates were mean BMI, smoking prevalence, pulses/legumes (kcal/capita, adjusted), and other dietary covariates, such as consumption of nuts, fish, and food with high trans-unsaturated fatty acids.
- We excluded the covariates related to diabetes and proportion of the population living between latitude of 30 and 45 absolute degrees.
- We changed the direction of the Socio-demographic Index covariate from 0 to -1

- We changed the level of the alcohol consumption covariate from 2 to 3.

The following table has the full list of covariates used for vascular intestinal disorders.

Table 1. Covariates used in vascular intestinal disorders mortality modelling

Level	Covariate	Direction
1	Fasting plasma glucose	+
	Cholesterol (total, mean per capita)	+
	Systolic blood pressures (mmHg)	+
2	BMI (mean)	+
	Smoking prevalence	+
	Healthcare Access and Quality Index	-
3	Socio-demographic Index	-
	Education (yeard per capita)	-
	Log LDI ($I per capita)	-
	Pulses/legumes (kcal/capita, adjusted)	-
	Age-sex-specific scaled exposure variable for low fruit consumption	+
	Age-sex-specific scaled exposure variable for low vegetable consumption	+
	Age-sex-specific scaled exposure variable for high red meat consumption	+
	Age-sex-specific scaled exposure variable for low fish consumption	+
	Age-sex-specific scaled exposure variable for low nut consumption	+
	Consumption of high trans-unsaturated fatty acids	+
	Alcohol (litres per capita)	+

Gallbladder and biliary diseases

Flowchart

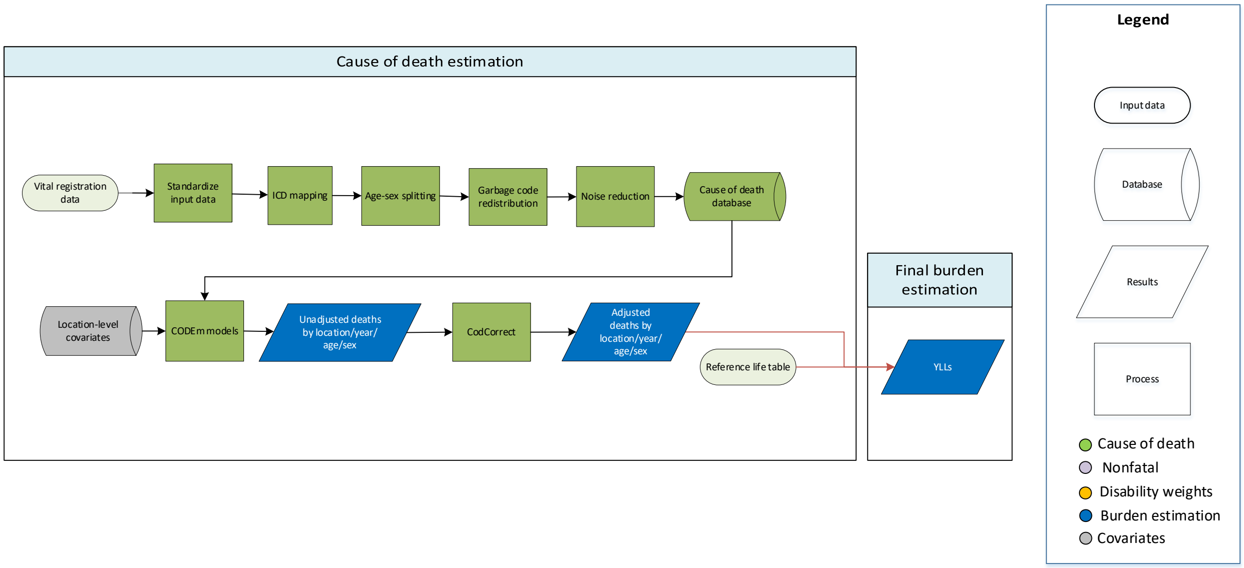

Input data

Data used to estimate mortality of gallbladder and biliary diseases consisted of vital registration data from the cause of death (COD) database. Outliers were identified by systematic examination of datapoints for all location-years. Specifically, we marked data as outliers in instances where garbage code redistribution and noise reduction, in combination with small sample sizes, resulted in unreasonable cause fractions. We also marked as outliers those data that violated well-established time or age trends.

Modelling strategy

The estimation strategy used for fatal gallbladder and biliary diseases is largely similar to methods used in GBD 2017. A standard CODEm model with location-level covariates was used to model deaths due to gallbladder and biliary diseases with age-restrictions for death estimations of 1 year for lower bound and 95+ years for upper bound (see appendix section on CODEm method for details). Separate models were conducted for male and female mortality. We then hybridised separate global and data-rich models to acquire unadjusted results, which we finalised and adjusted using CodCorrect to reach final YLLs due to gallbladder and biliary diseases.

Key changes from GBD 2017

- We added estimates for the following new locations: Monaco, San Marino, Palau, and Saint Kitts and Nevis.
- We added subnational location data for the following: Italy and Poland.
- We changed the direction of Socio-demographic Index and lag-distributed income covariates from 0 to -1 in GBD 2019.
- We replaced adjusted dietary covariates with age-sex-specific scaled exposure variable covariates with a direction of 1.

The following table has the full list of covariates used for fatal gallbladder and biliary diseases.

Table 1. Covariates used in gallbladder and biliary diseases mortality modelling

Level	Covariate	Direction
1	Age-sex-specific scaled exposure variable for low polyunsaturated fatty acids	+
	BMI (mean)	+
2	Alcohol (litres per capita)	+
	Healthcare Access and Quality Index	-
	Age-sex-specific scaled exposure variable for high red meat consumption	+
	Population over 65 (proportion)	+
3	Socio-demographic Index	-
	Education (years per capita)	-
	Log LDI ($I per capita)	-

Pancreatitis

Flowchart

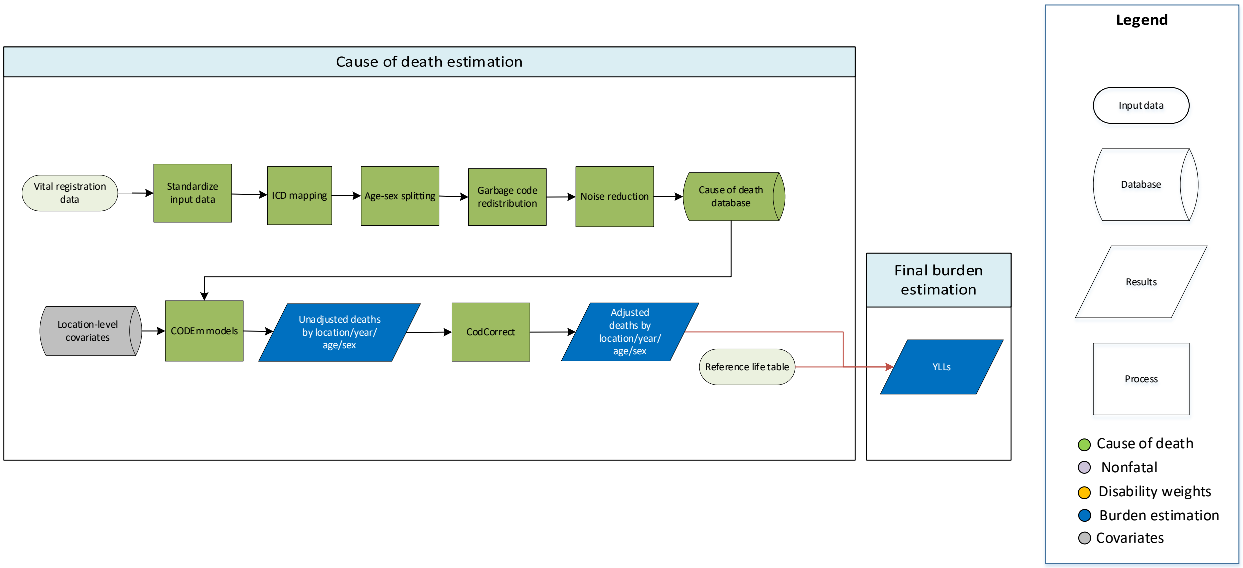

Input data

Data used to estimate mortality of pancreatitis consisted of vital registration data from the cause of death (COD) database. Outliers were identified by systematic examination of datapoints for all location-years. Data were excluded if they violated well-established time or age trends and in instances where garbage code redistribution and noise reduction, in combination with small sample sizes, resulted in unreasonable cause fractions.

Modelling strategy

The estimation strategy used for fatal pancreatitis is largely similar to methods used in GBD 2017. A standard CODEm model with location-level covariates was used to model deaths due to pancreatitis with age restrictions for death estimations of 1 year for lower bound and 95+ for upper bound (See appendix section 3.1 for details). Separate models were conducted for male and female mortality. We hybridised separate global and data-rich models to acquire unadjusted results, which we finalised and adjusted using CodCorrect to reach final YLLs due to pancreatitis.

Key changes from GBD 2017

- We added estimates for the following new locations: Monaco, San Marino, Palau, and Saint Kitts and Nevis.
- We added subnational location data for the following: Italy, Poland, and the Philippines.
- We changed the direction of Socio-demographic Index and lag-distributed income covariates from 0 to -1.

The following table has the full list of covariates used for pancreatitis.

Table 1. Covariates used in pancreatitis mortality modelling

Level	Covariate	Direction
1	Log-transformed scaled exposure variable for pancreatitis	+
	Alcohol (litres per capita)	+
2	Healthcare Access and Quality Index	-
	BMI (mean)	+
3	Socio-demographic Index	-
	Education (years per capita)	-
	Log LDI ($I per capita)	-

Other digestive diseases

Flowchart

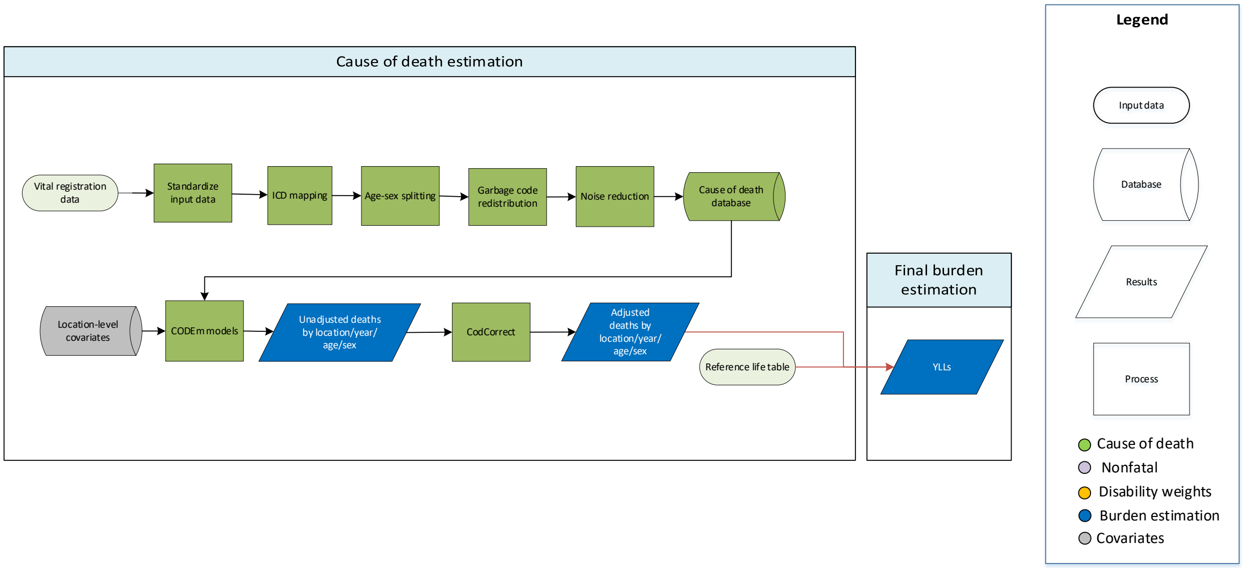

Input data

Data used to estimate mortality of other digestive diseases consisted of vital registration data from the cause of death (COD) database. The data in other digestive diseases consist of unique datapoints from deaths reported with a set of non-specific digestive disease codes (see appendix section on ICD mapping for details). We marked data as outliers in instances where garbage code redistribution and noise reduction, in combination with small sample sizes, resulted in unreasonable cause fractions. We also marked as outliers those data that violated well-established time or age trends.

Modelling strategy

The estimation strategy used for fatal other digestive diseases is largely similar to methods used in GBD 2017. A standard CODEm model with location-level covariates was used to model deaths due to other digestive diseases with age restrictions for death estimations of 1 year for lower bound and 95+ for upper bound (see appendix section 3.1 for details). Separate models were conducted for male and female mortality. We hybridised separate global and data-rich models to acquire unadjusted results, which we finalised and adjusted using CodCorrect to reach final YLLs due to other digestive diseases.

Key changes from GBD 2017

- We added estimates for the following new locations: Monaco, San Marino, Cook Islands, Palau, and Saint Kitts and Nevis.
- We added subnational location data for the following: Italy, Poland, and the Philippines.
- We replaced adjusted dietary covariates with age-sex-specific scaled exposure variable covariates with a direction of 1.
- We changed the direction of the Socio-demographic Index covariate from 0 to -1.

The following table has the full list of covariates used for other digestive diseases.

Table 1. Covariates used in other digestive diseases mortality modelling

Level	Covariate	Direction
1	Cumulative cigarettes (10 years)	+
	Cumulative cigarettes (5 years)	+
	Smoking prevalence	+
	Alcohol (litres per capita)	+
2	Diabetes age-standardised prevalence (proportion)	+
	BMI (mean)	+
	Sanitation (proportion with access)	-
	Improved water source (proportion with access)	-
	Age-sex-specific scaled exposure variable for low polyunsaturated fatty acids consumption	+
	Age-sex-specific scaled exposure variable for low fruit consumption	+
	Age-sex-specific scaled exposure variable for low vegetable consumption	+
	Age-sex-specific scaled exposure variable for high red meat consumption	+
	Healthcare Access and Quality Index	-
3	Socio-demographic Index	-
	Education (years per capita)	-
	Log LDI ($I per capita)	-

Alzheimer's disease and other dementias

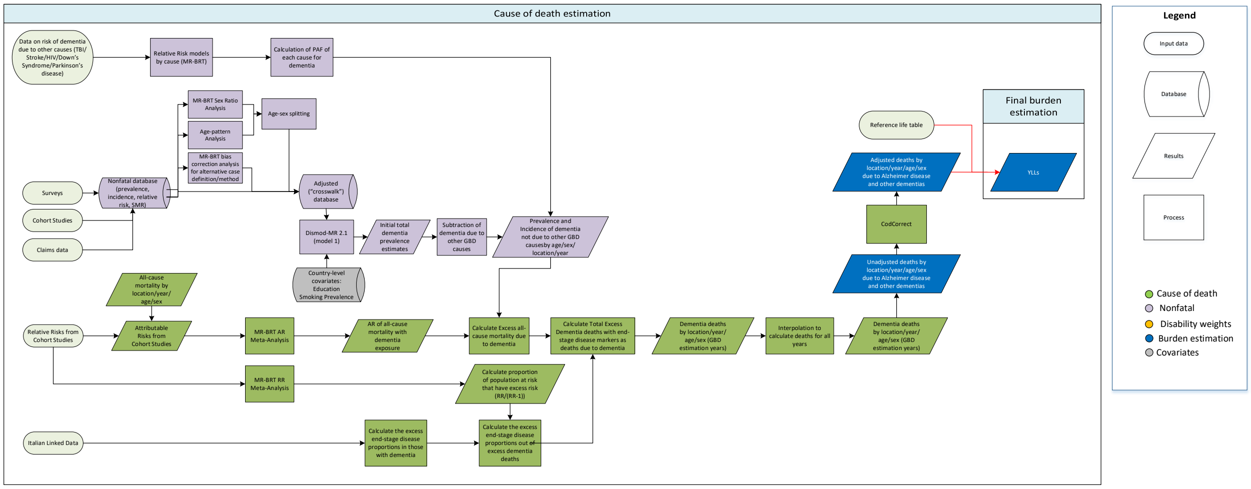

Input data

In GBD 2019, fatal modeling was redesigned to remove reliance on vital registration data (described in more detail in the "Modelling strategy" section). Instead, two new source types were extracted:

(1) Literature on the relative risk of all-cause mortality given the exposure of dementia. Relative risk sources were identified through a systematic review using search terms[2] in PubMed. This yielded 4470 total hits, of which 34 studies were marked for extraction. Overall, the data were heterogeneous and varied in the exposure category measured (all dementia, Alzheimer's disease, cognitive impairment) and in the different factors controlled for in analyses.

(2) Linked vital registration and hospitalisation data. We used mortality records linked to inpatient records, covering all deaths from 2003 to 2017 in the Emilia-Romagna region of Italy.

Table 1: Results of systematic review on all-cause excess mortality with dementia

N		60
Region name (%)	East Asia	4 (6.7)
	Eastern sub-Saharan Africa	2 (3.3)
	High-income Asia Pacific	4 (6.7)
	High-income North America	22 (36.7)
	North Africa and Middle East	1 (1.7)
	Tropical Latin America	1 (1.7)
	Western Europe	26 (43.3)
Exposure (%)	Alzheimer's disease	11 (18.3)
	cognitive impairment	10 (16.7)
	other dementia	35 (58.3)
	vascular dementia	4 (6.7)

Conducted in clinical setting (%)	Clinical setting	10 (16.7)
	Population representative	50 (83.3)
Controlled for education (%)	Controlled	32 (53.3)
	No control	28 (46.7)
Controlled for basic CVD info (%)	Controlled	33 (55.0)
	No control	27 (45.0)
Extensive CVD control (%)	Controlled	15 (25.0)
	No control	45 (75.0)
Controlled for smoking and alcohol (%)	Controlled	11 (18.3)
	No control	49 (81.7)
Controlled for factors in causal pathway (%)	Controlled	13 (21.7)
	No control	47 (78.3)

Modelling strategy

Overview

Dementia mortality rates have increased more than five-fold since 1980 in high-quality vital registration systems such as in the USA and Scandinavia. We have not seen an equivalent increase in prevalence and incidence data sources. If at all, there has been a modest decline in incidence and prevalence of dementia in studies in the UK and the USA.[1,2] Also, the greater than 20-fold variation in mortality rates of dementia between countries is much greater than the four-fold difference in prevalence and incidence between countries. As it is unlikely that case fatality from dementia has dramatically increased over the time period and that it would differ by a very large margin between countries, the hypothesis is that certifying and coding practices have changed over time and at a different pace between countries. To avoid spurious large trends over time in the fatal component of the burden of dementia, we decided for GBD 2013 to make dementia mortality rates consistent with the most recent rates relative to prevalence of countries that are most likely to certify or code dementia as an underlying cause of death. This approach was applied again for GBD 2017 with some modifications. For GBD 2019, the fatal modelling process was redesigned to avoid the need for using estimates only from the highest dementia mortality locations. This was accomplished with an attributable risk model based on a systematic review of cohort studies and relative risk data, and end-stage disease proportions from linked hospital and death records. The modelling process is described below.

Modelling steps

Relative risk data

First, using relative risk data extracted from studies identified by systematic review, we calculated attributable risk and the GBD estimate of all-cause mortality rate for a given study location and time, using the following formula:

[1] Akushevich I, Kravchenko J, Ukraintseva S, Arbeev K, Yashin AI. Time trends of incidence of age-associated diseases in the US elderly population: Medicare-based analysis. *Age and ageing*. 2013 Jul 1;42(4):494-500.

[2] Matthews FE, Arthur A, Barnes LE, Bond J, Jagger C, Robinson L, Brayne C, Medical Research Council Cognitive Function and Ageing Collaboration. A two-decade comparison of prevalence of dementia in individuals aged 65 years and older from three geographical areas of England: results of the Cognitive Function and Ageing Study I and II. *The Lancet*. 2013 Nov 1;382(9902):1405-12.

$$Attributable\ Risk = (Relative\ Risk - 1) * All\text{-}Cause\ Mortality$$

We then conducted a meta-analysis on the attributable risk data, using covariates for age, sex, exposure category (all dementia, Alzheimer's disease, cognitive impairment), whether the study was conducted in a clinical sample, and categories indicating different types of variables that were controlled for in the component studies (educational attainment, cardiovascular disease comorbidities, smoking and alcohol consumption, and daily activities or residence in a nursing home). Relative risks were estimated using a second Bayesian bias-reduction meta-regression model and the same studies identified through systematic review. Regression results for relative risk and attributable risk analyses are displayed below.

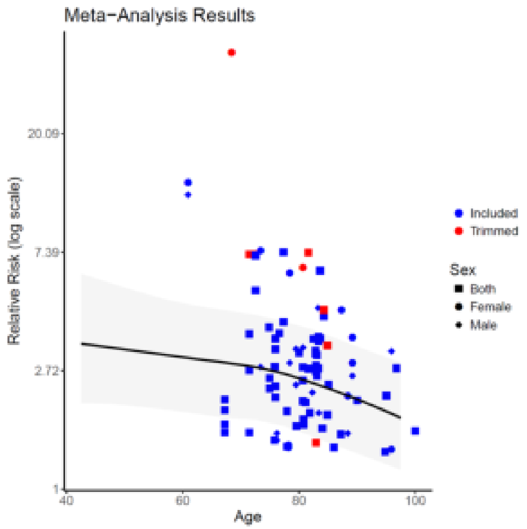

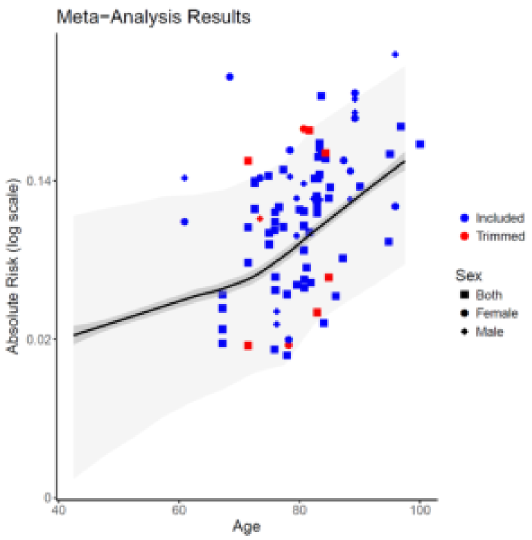

Meta-regression results were used to calculate the total number of excess deaths due to dementia as the product of our prevalence estimates (post-adjustment for dementia caused by other GBD diseases) and our estimates of attributable risk. See the non-fatal write-up on dementia for details on prevalence calculations.

Linked data

The excess deaths calculated through the multiplication of attributable risk and prevalence represent the total number of excess deaths due to having dementia, which likely includes deaths due to other conditions, such as cardiovascular diseases, that are more common in those with dementia as compared to the general population due to common underlying risk factors such as blood pressure, smoking, and lower educational attainment. In order to subset this total number of excess dementia deaths to calculate the number of deaths that were caused by dementia, we completed an analysis of linked clinical and mortality data. We used mortality records linked to inpatient records, covering all deaths from 2003 to 2017 in the Emilia-Romagna region of Italy. Using these data, we looked for markers of severe, end-stage disease in the clinical records up to one year before death.

To select these markers, for each ICD code that appeared in the data we calculated the difference in the proportion of individuals who died with dementia and had a record of each code in the year before death and the proportion of individuals who died without dementia and had a record of the same code in the year before death. We reviewed the 150 codes with the highest difference and selected codes

that indicated end-stage disease, excluding codes for conditions such as cardiovascular disease. Codes for decubitus ulcer, malnutrition, sepsis, pneumonia, urinary tract infections, falling from bed, senility, dehydration, sodium imbalance, muscular wasting, bronchitis, dysphagia, hip fracture, and bedridden status were used as indicators of severe disease.

In order to determine the proportion of excess deaths that were caused by dementia, we calculated the proportion of dementia deaths that had clinical markers of end-stage disease in the year before death, above and beyond the occurrence of end-stage disease markers in those who died without dementia. The subtraction of the proportions with end-stage disease markers in those without dementia from the proportions in those with dementia represents the proportion of individuals who are assumed to have died with severe, end-stage dementia out of total deaths in those with dementia.

Figure 2: Percent of individuals who died with and without dementia with end stage conditions in the last year before death

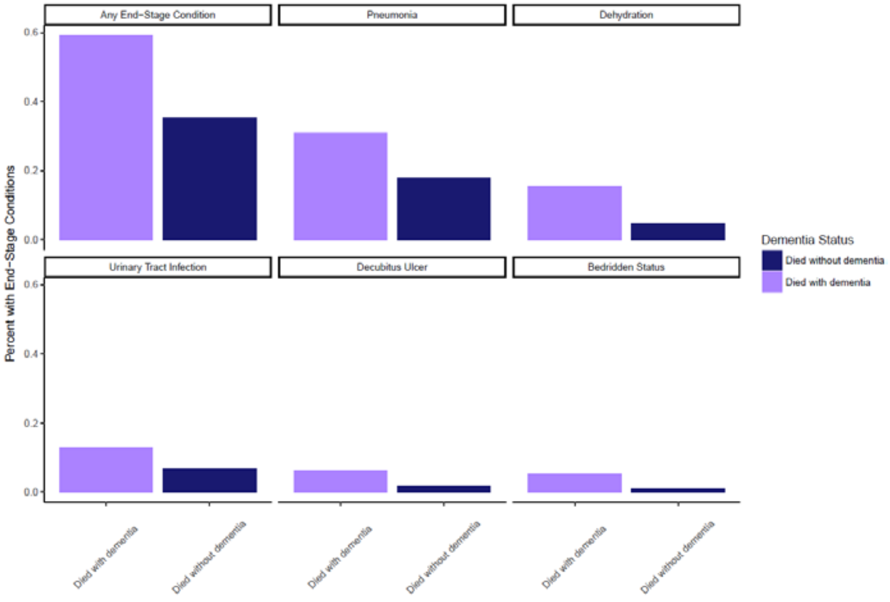

Calculation of deaths due to dementia

In order to apply these estimates to the total excess deaths we then adjusted these proportions to calculate the proportion of individuals who died with severe, end-stage dementia out of excess dementia deaths using the formula:

$$\frac{Died\ with\ Severe\ Disease}{Excess\ Dementia\ Deaths} = \frac{Died\ with\ Severe\ Disease}{Total\ Dementia\ Deaths} * \frac{Relative\ Risk}{Relative\ Risk - 1}$$

We then calculated the number of deaths due to dementia as the product of total excess dementia deaths and the proportion of those who died with severe disease out of excess dementia deaths. These final estimates of deaths due to dementia were then used to adjust data on causes of death from all other causes in vital registration systems.

Interpolation for all years

Finally, we used log-linear interpolation to interpolate these results (limited to 1990, 1995, 2000, 2005, 2010, 2015, 2017, 2019) to create estimates for the entire time series from 1980 to 2019. Socio-demographic Index was used as a covariate to extrapolate back to the year 1980.

Parkinson's Disease

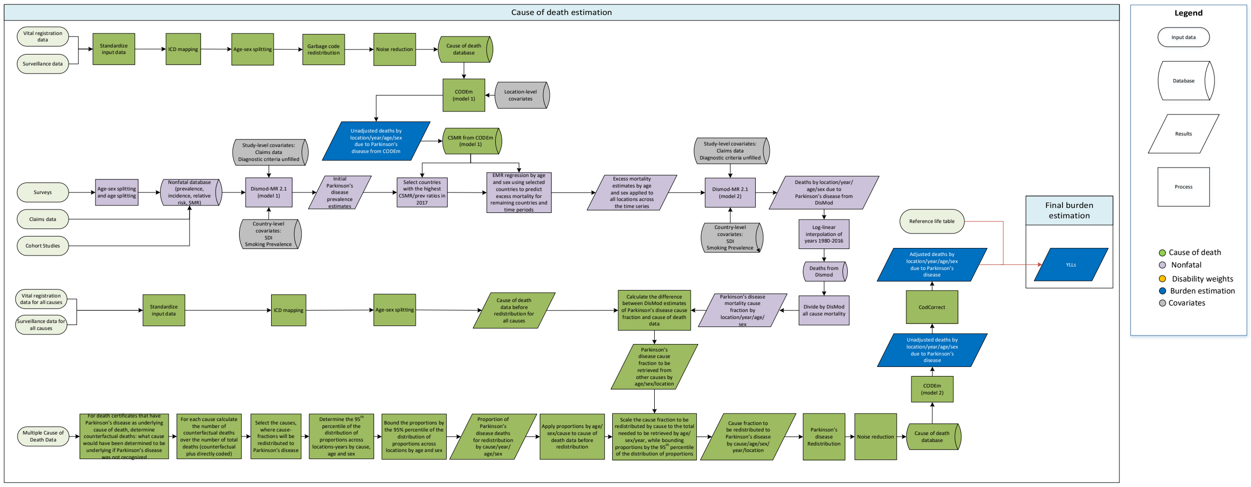

Input Data

In GBD 2017, data used to estimate deaths due to Parkinson's disease included mortality data from vital registration systems and prevalence data from surveys and claims sources.

An updated systematic review was conducted from September 2015 to August 2017, and search terms[1] were set to capture studies for Parkinson's disease. Inclusion criteria comprised studies that reported prevalence, incidence, remission rate, excess mortality rate, relative risk of mortality, standardized mortality ratio, or with-condition mortality rate. Studies with no clearly defined sample or that drew from specific clinic/patient organizations were excluded. We also added US claims data for 2011 and 2012-2015. No further prevalence or incidence data were added in GBD 2019.

Modelling Strategy

Overview

Parkinson's disease mortality rates have more than doubled since 1980 in high-quality vital registration systems such as in the US, Canada, Australia, France, Germany, the United Kingdom and Finland, while other European countries like the Netherlands, Sweden, and Norway have not seen such increases over time. We have not seen an equivalent increase in prevalence and incidence data sources. Additionally, the greater than 15-fold variation in mortality rates of Parkinson's disease between countries is much greater the three-fold difference in prevalence and incidence between high-income countries. As it is unlikely that case fatality from Parkinson's disease has dramatically increased over the time period and that it would differ by a very large margin between countries, the hypothesis is that certifying and coding practices have changed over time and at a different pace between countries. For GBD 2016, we decided to employ a modelling strategy which we have previously used to model mortality from

[1] (Parkinson disease[Title/Abstract] OR Parkinson's disease[Title/Abstract]) AND (epidemiology[Title/Abstract] OR prevalence[Title/Abstract] OR incidence[Title/Abstract]) AND ("2015/09/31"[PDAT] : "2017/08/23"[PDAT])

Alzheimer disease and other dementias, which avoids spurious large trends over time in the fatal component of the burden of Parkinson's disease by making Parkinson's mortality rates consistent with the rates observed in 2016, relative to prevalence in countries that are most likely to certify or code Parkinson's disease as an underlying cause of death. For GBD 2017, we again employed this strategy.

Modelling steps

Fatal modeling for Parkinson's Disease is described in the following steps. The initial steps were not re-run in GBD 2019, and so the Multiple Cause of Death (MCOD) Parkinson's disease inputs were identical to those used in the GBD 2017 capstone.

First, we ran a CODEm model for Parkinson's disease and extracted the mortality rates by age, sex, and geography. The covariates used in this intermediary model are displayed below; some have a direction of 0 because this model was run early in the GBD 2019 cycle. The final Parkinson's model has a negative or positive direction specified for all covariates (see final table).

Level	Covariate	Direction
1	Cumulative cigarette consumption (10 years)	-
2	Absolute latitude	+
	Cholesterol (total, mean per capita)	+
	Sanitation (proportion with access)	0
	Improved water source (proportion with access)	0
	Fruit consumption adjusted (g)	-
	Healthcare access and quality index	-
3	Education (years per capita)	-
	Socio-demographic index	+
	Lag distributed income	0

Second, we ran a DisMod-MR 2.1 model with all data on incidence, prevalence, and mortality risk (RR, SMR, or with-condition mortality rates) and a setting of zero remission and extracted prevalence by age, sex, and geography. Studies where the case definition of two of the four cardinal symptoms of Parkinson's disease was not filled were crosswalked to studies using the reference case definition. No random effects were used in the model in order to prevent spurious inflation of regional differences due to differences in measurement and measurement error.

Third, we selected the seven countries (France, England, the United States, the Netherlands, Finland, Scotland, and Wales) with the highest cause-specific mortality rate (from step 1) to prevalence (from step 2) ratio in 2017, which also had an age-standardised prevalence rate greater than 0.0005, and a population greater than 1 million.

Fourth, we used a linear effects regression with dummies on age group and sex to predict excess mortality (i.e., the ratio of cause-specific mortality rate and prevalence) by age and sex, the results of which are found in the tables below.

Table: Fixed effect coefficients of EMR regression. Outcome: ln(EMR)

Independent variables	Coef	Std. error	P value	95% Confidence Interval	
Male	0.288	0.036	0.000	0.218	0.358

Age 40-59	-3.25	0.076	0.000	-3.399	-3.101
Age 60-64	-2.557	0.076	0.000	-2.706	-2.407
Age 65-69	-2.021	0.076	0.000	-2.17	-1.871
Age 70-74	-1.42	0.076	0.000	-1.57	-1.271
Age 75- 80	-0.898	0.076	0.000	-1.047	-0.749
Age 80-84	-0.502	0.076	0.000	-0.651	-0.352
Age 85-89	-0.248	0.076	0.001	-0.397	-0.099
Age 90-94	-0.047	0.076	0.537	-0.196	0.102
Constant	-2.357	0.057	0.000	-2.469	-2.246

Table: Predicted EMR values by age and sex (95% CI)

	**Male**	**Female**
Age 40-59	0.005 (0.004 - 0.005)	0.004 (0.003 - 0.004)
Age 60-64	0.01 (0.009 - 0.011)	0.007 (0.007 - 0.008)
Age 65-69	0.017 (0.015 - 0.019)	0.013 (0.011 - 0.014)
Age 70-74	0.031 (0.027 - 0.034)	0.023 (0.02 - 0.025)
Age 75- 80	0.051 (0.046 - 0.057)	0.039 (0.035 - 0.043)
Age 80-84	0.076 (0.068 - 0.085)	0.058 (0.052 - 0.064)
Age 85-89	0.099 (0.089 - 0.111)	0.074 (0.066 - 0.083)
Age 90-94	0.12 (0.108 - 0.135)	0.09 (0.081 - 0.1)
Age 95+	0.126 (0.113 - 0.142)	0.095 (0.085 - 0.106)

Fifth, these estimates were added to a second DisMod-MR 2.1 model as pertaining to the full 1990–2017 estimation period. For the countries included in the regression, we allowed them to retain their original EMR values when the age-standardized EMR for a country was higher than the age-standardized EMR prediction generated from the regression. These countries retained their age- and sex-specific ratios and entered those also as pertaining to the full 1990–2017 estimation period. Smoking prevalence was used as a country-level covariate. We excluded data for standardized mortality ratio, with-condition mortality rate, and relative risk as we wanted to estimate cause-specific mortality rates that were consistent with the level of excess mortality from the seven chosen countries in 2017.

Sixth, we took the predictions of cause-specific mortality by age, sex, geography, and year that DisMod-MR 2.1 calculated as being consistent with the data on incidence, prevalence, and the priors on excess mortality from step five. Because DisMod-MR 2.1 produces estimates in five-year intervals only, we expanded the time series by log-linear interpolation; values for 1980-1990 were generated using a regression on the entire time series with Socio-demographic index included as a predictor. We divided this cause-specific mortality by the all-cause mortality used in DisMod to calculate the Parkinson's disease cause-fraction based on prevalence data and the excess mortality derived from countries most likely to code to Parkinson's disease as a cause of death.

Seventh, we calculated the difference between this cause-fraction derived from DisMod and the cause-fraction derived from the cause of death data prep process before redistribution in order to get the amount of cause fraction that needed to be retrieved from other causes through the Parkinson's disease redistribution process.

Eighth, in order to calculate where these Parkinson's disease deaths should be retrieved from, we analysed multiple cause of death (MCOD) data. We only used data from the US, and asserted that the data from 2010-2015, during which the increases in coding to Parkinson's disease as a cause of death leveled off, is the reference data.

Ninth, for deaths where Parkinson's disease is the underlying cause of death in the years 2010-2015, we calculated what the underlying cause of death would have been in the counterfactual scenario in which Parkinson's disease had not been recognized. In order to calculate this counterfactual, we examined the causes listed in part one of the chain of the death certificate. For each death certificate chain we looked across the entire dataset from 1980-2015 and determine what the distribution of underlying causes of death was in individuals with that particular death certificate chain. Then, we assigned the counterfactual deaths proportionally to the causes that are listed as underlying in these death certificates. If, over the time period, there were less than 1000 death certificates that had exactly the same death certificate chain, then we included all death certificate chains that had those same causes, but which could additionally include other causes in the chain as well. To assign counterfactual deaths for these chains, we further subsetted the data to death certificate chains where any of the causes in the original death certificate chain were listed as underlying, determined the distribution of underlying causes of death among just this subset, and then assigned counterfactual deaths proportionally in the same manner.

Tenth, once we determined the counterfactual causes of death stemming from all Parkinson's disease deaths from 2010-2015, we calculated the proportion of deaths by cause that should be Parkinson's disease deaths according to the reference data by taking the counterfactual deaths for each cause and dividing by the sum of the counterfactual deaths for that cause plus the directly coded deaths for that cause.

Eleventh, we applied the proportions to cause of death data in cause fraction space and scaled the cause fractions to the total mortality cause fraction to be retrieved based on the DisMod model. We set caps on the percent of deaths that were moved by age, sex and cause. The caps were determined by finding the 95th percentile of the percentages of deaths moved in each age-sex-cause category across all 5-star VR locations. The COD data is then processed using general redistribution strategies and noise reduction.

Finally, the data derived from this process was used in a final CODEm model, using the same covariates as the original CODEm model. These covariates were adjusted for this model in GBD 2019 so that every covariate had a specified directionality (see table below), and with some adjustments for level. These results were then adjusted through CodCorrect and become the final cause of death estimates for Parkinson's disease.

Level	Covariate	Direction
1	Cumulative cigarette consumption (10 years)	-
	Fruit consumption adjusted (g)	-
2	Absolute latitude	+
	Cholesterol (total, mean per capita)	+
	Sanitation (proportion with access)	+
	Improved water source (proportion with access)	+
	Healthcare access and quality index	-

3	Education (years per capita)	-
	Socio-demographic index	+
	Lag distributed income	+

The following plots show the influence of each covariate on the four CODEm models (male global, male data rich, female global, and female data rich). A positive standardized beta (to the right) means that the covariate was associated with increased death. A negative standardized beta (to the left) means the covariate was associated with decreased death.

**Male, global**	**Male, data rich**
**Female, global**	**Female, data rich**

Idiopathic Epilepsy

Flowchart

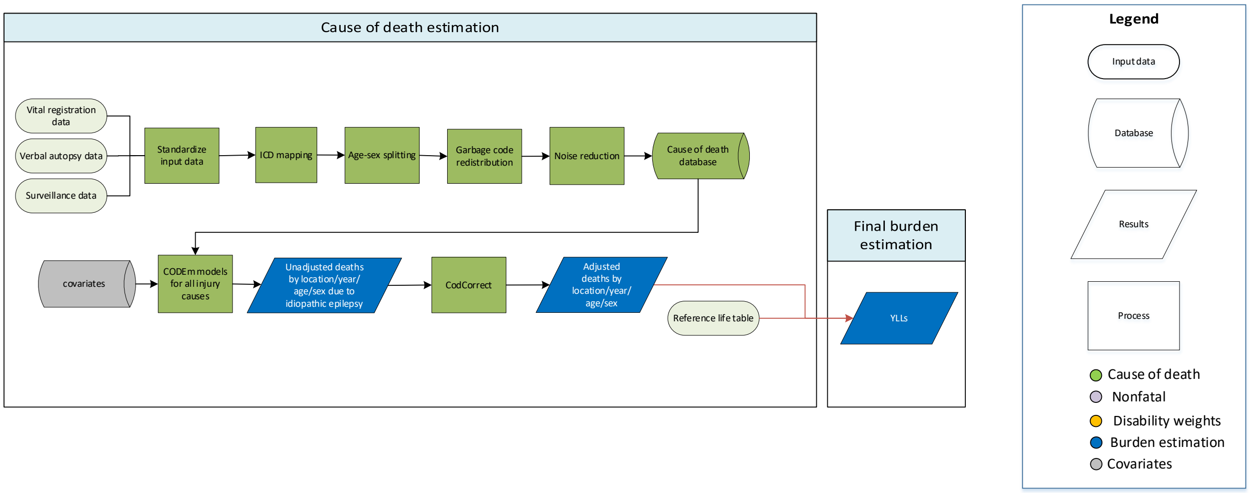

Input Data and Methodological Summary for Idiopathic Epilepsy

Input data

Data used to estimate epilepsy mortality included vital registration (VR), verbal autopsy, and China mortality surveillance data from the cause of death (COD) database. Our outlier criteria were to exclude data points that were (1) implausibly high or low relative to global or regional patterns, (2) substantially conflicted with established age or temporal patterns, or (3) substantially conflicted with other data sources based from the same locations or locations with similar characteristics (i.e., socio-demographic index).

Modeling strategy

The standard CODEm modelling approach (detailed in a appendix section 3.1) was used to estimate deaths due to idiopathic epilepsy. Separate models were conducted for male and female mortality, and the age range for both models was 28 days – 95+ years. Changes to these models relative to GBD 2017, and the complete list of covariates used in GBD 2019 are displayed below. Unadjusted death estimates were adjusted using CoDCorrect to produce final estimates of YLLs.

Key Changes from GBD 2017

- Introduction of subnational location data for Italy, Poland, Pakistan, the Philippines, and Nigeria.
- Introduction of the following new locations: Monaco, San Marino, Cook Islands, Nauru, Niue, Palau, Tokelau, Tuvalu, Monaco, San Marino, St Kitts, and Nevis.
- Changes in covariate choices. A covariate for pig meat consumption (kcal per capita) used in GBD 2017 was not modeled for use in CODEm in GBD 2019. All other covariates remained from GBD2017 (see Table 1).

Table 1. Covariates used in Idiopathic Epilepsy mortality modelling

Level	Covariate	Direction
1	Pigs (per capita)	+
	SEV scalar: epilepsy	+
	Mean systolic blood pressure (mmHg)	+
2	Health access and quality index	-
	Mean body mass index	+
	Mean serum total cholesterol (mmol/L)	+
3	Cumulative cigarettes (10 years)	+
	Cumulative cigarettes (5 years)	+
	Education (years per capita)	-
	Log LDI (per capita)	-
	Socio-demographic Index	-

The following plots show the influence of each covariate on the four CODEm models (male global, male data rich, female global, and female data rich). A positive standardized beta (to the right) means that the covariate was associated with increased death. A negative standardized beta (to the left) means the covariate was associated with decreased death.

Male, global

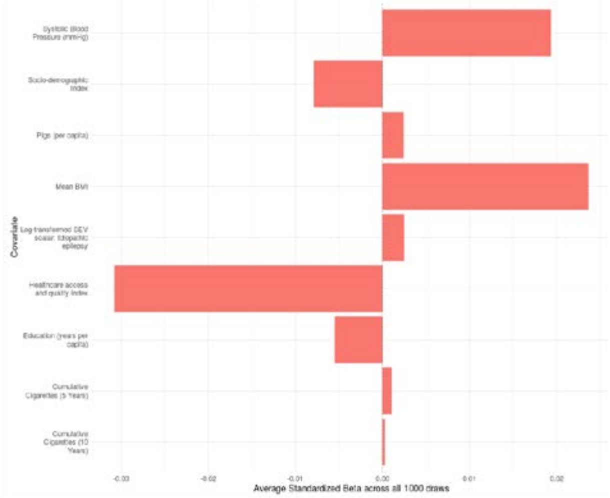

Male, data rich

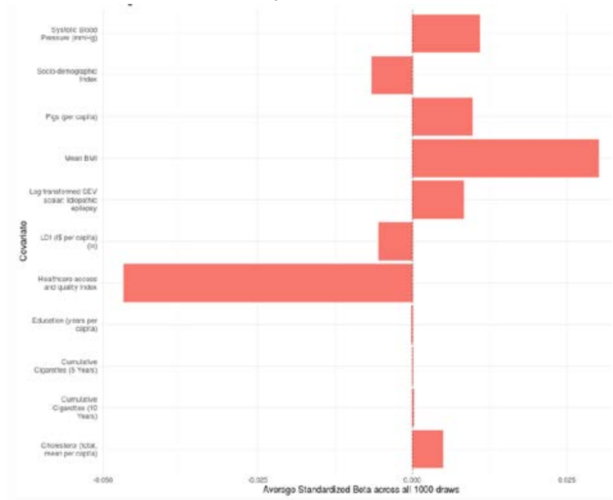

Female, global	Female, data rich
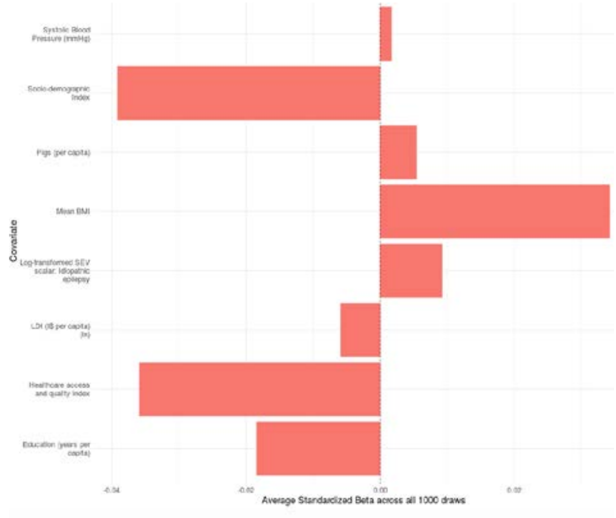	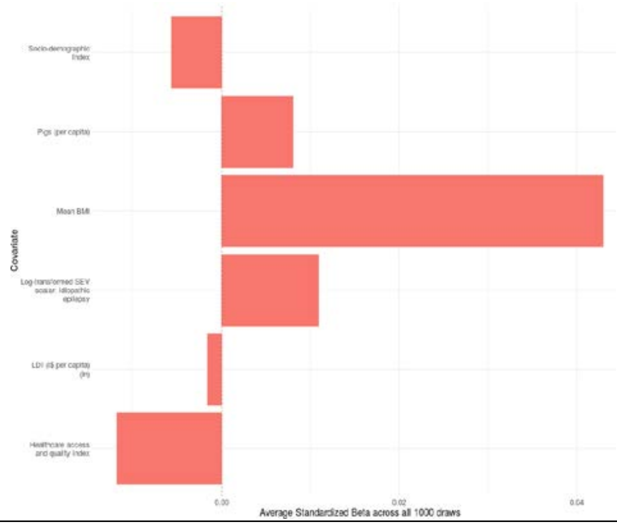

Multiple Sclerosis

Flowchart

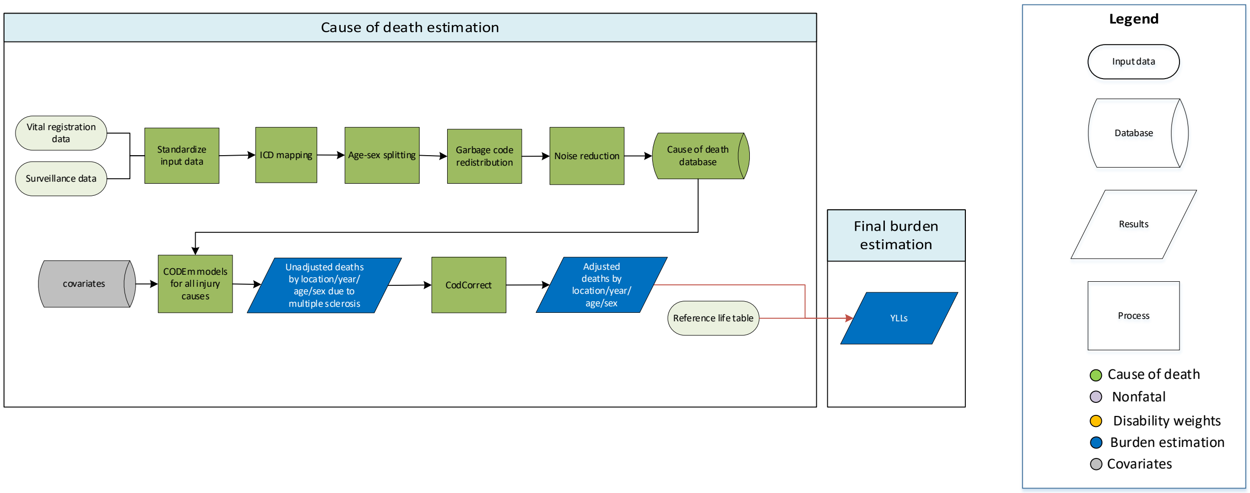

Input Data and Methodological Summary for Multiple Sclerosis

Input data

Data used to estimate multiple sclerosis included vital registration and surveillance data from the cause of death (COD) database. Our outlier criteria were to exclude data points that (1) were implausibly high or low, (2) substantially conflicted with established age or temporal patterns, or (3) substantially conflicted with other data sources conducted from the same locations or locations with similar characteristics (i.e., Socio-demographic Index). In particular, where data-processing could not resolve discrepancies between different coding systems for the same location over time, one system was selected as more reliable and the other was excluded. In particular, this affected Kazakhstan, where the conversion from ICD9-BTL tabulated vital registration data (for years1981-2003) to ICD10-coded data (for year 2013 onwards) led to an implausible 5-fold increase between 1980 and 2017 and 2017 estimates more than two-fold greater than anywhere else in the world. The ICD10-coded data were excluded.

Modeling strategy

The standard CODEm modelling approach (detailed in a appendix section 3.1) was used to estimate deaths due to multiple sclerosis. Separate models were conducted for male and female mortality, and the age range for both models was 5-95+ years (differing from previous years where the age range was 20-95+ years). The linear floor was set to 0.0001. Key changes from GBD 2017 and the full list of covariates used in GBD 2019 are displayed below. Unadjusted death estimates were adjusted using CoDCorrect to produce final estimates of YLLs.

Key Changes from GBD 2017

- Changes to Garbage Code redistribution and Noise Reduction (as detailed in the appendix section on Cause of Death data preparation)

- Introduction of subnational location data for Italy, Poland, Pakistan, the Philippines, and Nigeria.
- Introduction of the following new locations: Monaco, San Marino, Cook Islands, Nauru, Niue, Palau, Tokelau, Tuvalu, Monaco, San Marino, St Kitts, and Nevis.

Table 1. Covariates used in Multiple Sclerosis mortality modelling

Level	Covariate	Direction
1	Absolute value of average latitude	+
2	Mean serum total cholesterol (mmol/L)	+
	Health care access and quality index	-
3	Cumulative cigarettes (10 years)	+
	Cumulative cigarettes (5 years)	+
	Education (years per capita)	-
	Log-transformed LDI (per capita)	-
	Smoking prevalence	+
	Socio-demographic Index	+

The following plots show the influence of each covariate on the four CODEm models (male global, male data rich, female global, and female data rich). A positive standardized beta (to the right) means that the covariate was associated with increased death. A negative standardized beta (to the left) means the covariate was associated with decreased death.

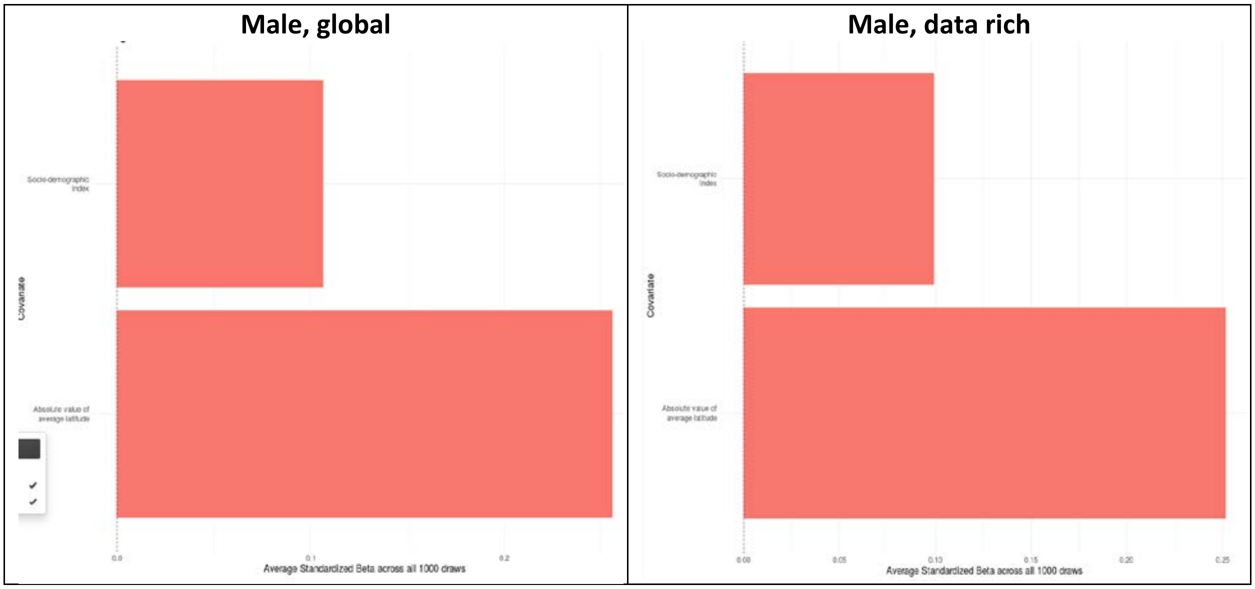

Female, global

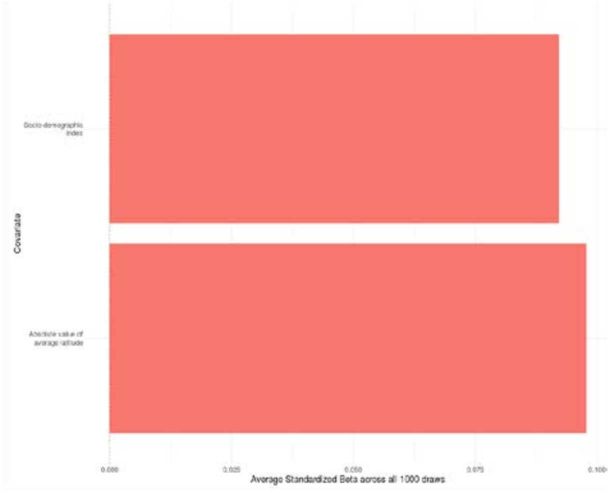

Female, data rich

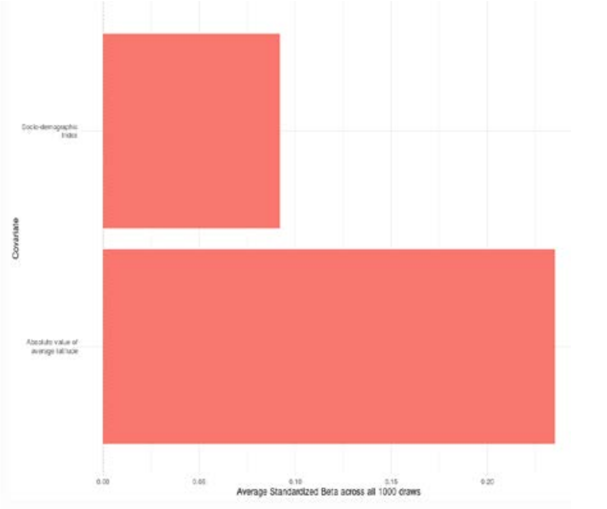

Motor Neuron Disease

Flowchart

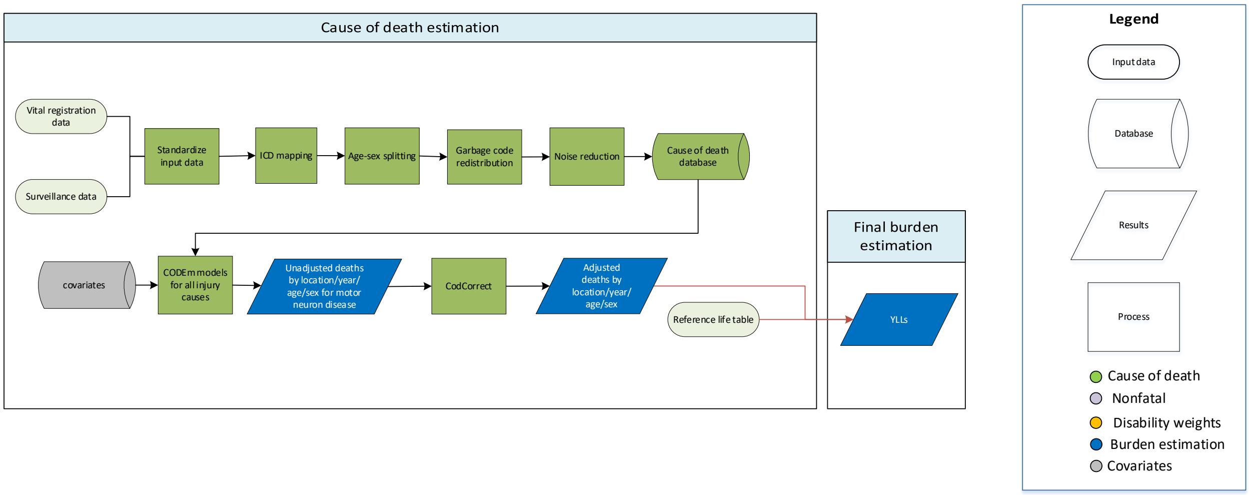

Input Data and Methodological Summary for Motor Neuron Disease

Input data

Data used to estimate Motor Neuron Disease included vital registration and surveillance data from the cause of death (COD) database. Our outlier criteria were to exclude data points that (1) were implausibly high or low, (2) substantially conflicted with established age or temporal patterns, or (3) substantially conflicted with other data sources from the same locations or locations with similar characteristics (i.e., Socio-demographic Index). In GBD 2019, this affected Kazakhstan where ICD9-BTL tabulated vital registration data were available for 1991-2003 and ICD10-coded vital registration were available for 2013 onwards. The raw ICD9-BTL data for 1991 were 14-fold higher than raw ICD9-BTL (1992-2003) and ICD-10 (2013 onwards) causing an implausible time pattern via noise reduction data processing methods for ICD9-BTL data. For that reason, the ICD9-BTL data were excluded and the ICD-10 data retained.

Modeling strategy

The standard CODEm modelling approach (described appendix section 3.1) was used to estimate deaths due to multiple sclerosis. Separate models were conducted for male and female mortality, and the age range for both models was 0-days to 95+ years. Unadjusted death estimates were adjusted using CoDCorrect to produce final estimates of YLLs.

Key Changes from GBD 2017

- Changes to Garbage Code redistribution and Noise Reduction (as detailed in the appendix section on Cause of Death data preparation)
- Introduction of subnational location data for Italy, Poland, Pakistan, the Philippines, and Nigeria.
- Introduction of the following new locations: Monaco, San Marino, Cook Islands, Nauru, Niue, Palau, Tokelau, Tuvalu, Monaco, San Marino, St Kitts, and Nevis.

Table 1. Covariates used in Motor Neuron Disease mortality modelling

Level	Covariate	Direction
	Mean total body mass index (kg/m^2)	-
	Mean serum total cholesterol (mmol/L)	-
	Absolute value of average latitude	+
	Mean diabetes fasting plasma glucose (mmol/L)	+
	Fruit consumption (grams per day adjusted)	-
	Socio-demographic Index	+
	Health care access and quality index	-
2	Population-weighted mean temperature	-
	Sanitation (proportion with access)	+
	Improved water source (proportion with access)	-
3	Education (years per capita)	+
	Log-transformed LDI (per capita)	+

"The following plots show the influence of each covariate on the four CODEm models (male global, male data rich, female global, and female data rich). A positive standardized beta (to the right) means that the covariate was associated with increased death. A negative standardized beta (to the left) means the covariate was associated with decreased death.

Male, global

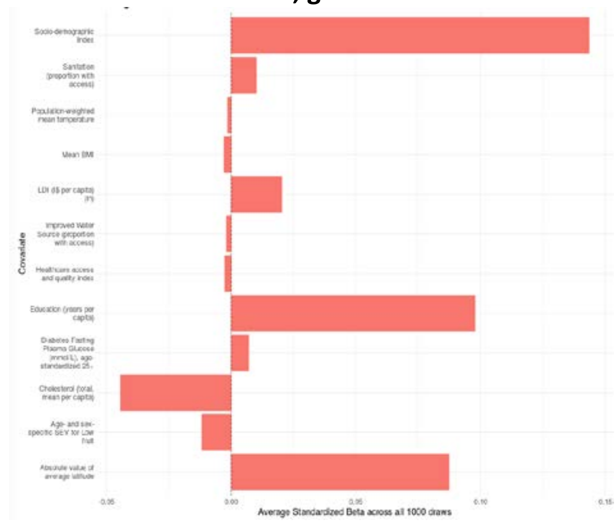

Male, data rich

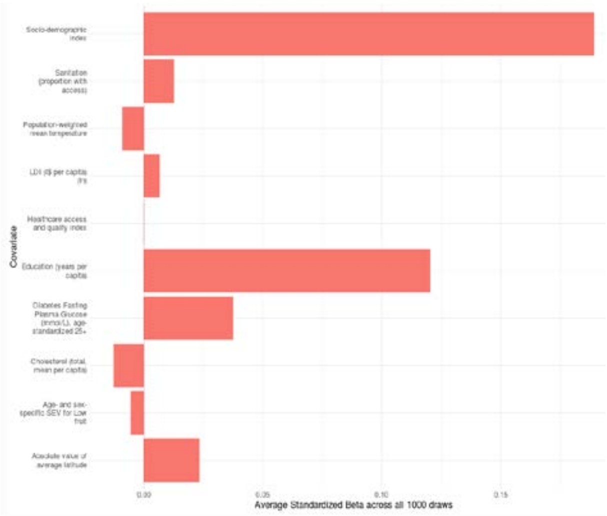

Female, global

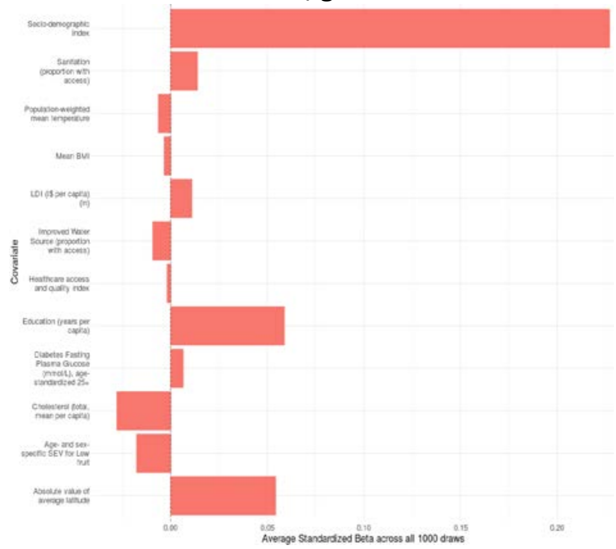

Female, data rich

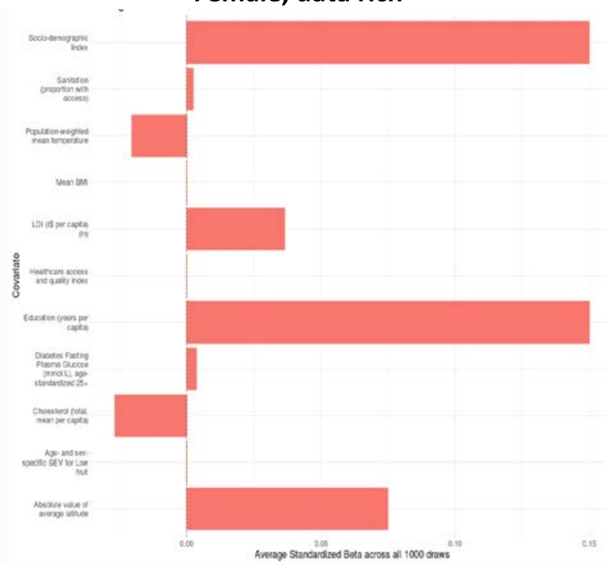

Other Neurological Disorders

Flowchart

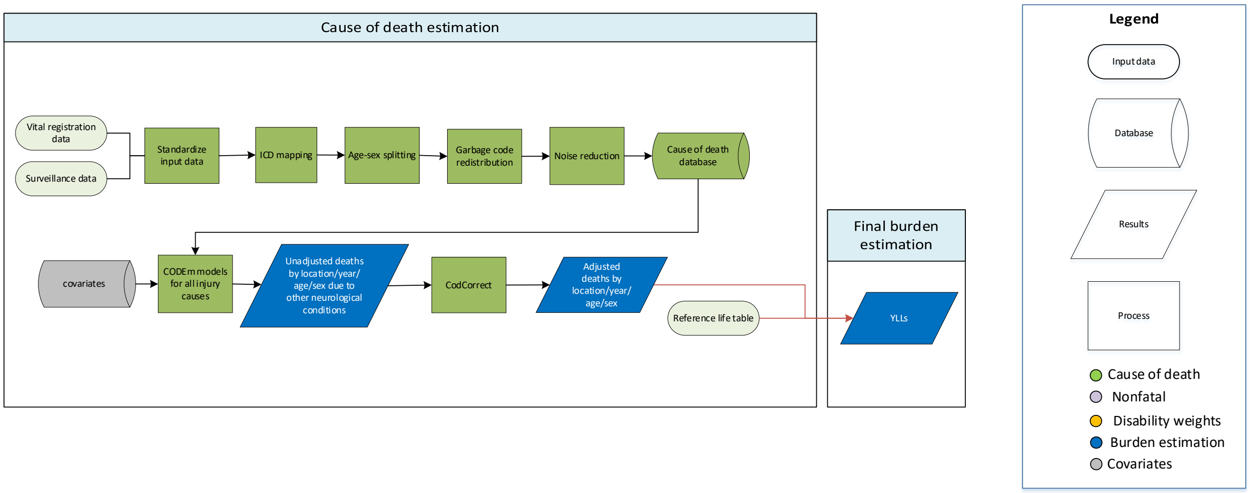

Input Data and Methodological Summary for Other Neurological Disorders

Input data

Data used to estimate other neurological disorders included vital registration and surveillance data from the cause of death (COD) database. Our outlier criteria were to exclude data points that (1) were implausibly high or low, (2) substantially conflicted with established age or temporal patterns, or (3) significantly conflicted with other data sources conducted from the same locations or locations with similar characteristics (i.e., Socio-demographic Index). In particular,

- Data excluded as outliers in GBD2017 continued to be excluded in GBD2019
- ICD-10 data were available for Kazakhstan for 2013 onwards, but were marked as outliers as the raw data were 10-fold greater than the previously modelled mean.
- Similarly, Brunei data from 2016 were marked as outliers because they were more than three-fold higher than the median for countries in the high-income Asia Pacific countries. These high values were evident in the raw data for 2011-2014 years. Raw data for Brunei for 2015 and 2016, in contrast, were similar to other regions in High Income Asia Pacific, but in the process of noise reduction, data for Brunei 2015 onwards were adjusted to the high values from 2011-2014.

Modeling strategy

The standard CODEm modelling approach (as described in appendix section 3.1) was used to estimate deaths due to multiple sclerosis. Separate models were conducted for male and female mortality, and the age range for both models was 28-days to 95+ years. Changes from GBD 2017 and the full list of covariates used in GBD 2019 are displayed below. Unadjusted death estimates were adjusted using CoDCorrect to produce final estimates of YLLs.

Key Changes from GBD 2017

- Changes to Garbage Code redistribution and Noise Reduction (as detailed in the appendix section on Cause of Death data preparation)
- Introduction of subnational location data for Italy, Poland, Pakistan, the Philippines, and Nigeria.
- Introduction of the following new locations: Monaco, San Marino, Cook Islands, Nauru, Niue, Palau, Tokelau, Tuvalu, Monaco, San Marino, St Kitts, and Nevis.
- Changes in covariate choices. Alcohol consumption and per capita pig meat consumption (kcal per capita) were not used in GBD 2019, but all other covariates remained from GBD2017 (see Table 1). Note that age-, and sex-specific adjusted covariates for red meat and fruit consumption, SEV for underweight children and pigs per capita were utilized this year.

Table 1. Covariates used in Other Neurological Disorders mortality modelling

Level	Covariate	Direction
1	Mean total body mass index	+
	Mean serum total cholesterol (mmol/L)	+
	Mean systolic blood pressure (mm/Hg)	+
	Pigs per capita	+
	Underweight proportion under 2 standard deviations	+
	Red meat consumption adjusted	+
2	Population density over 1,000 per square kilometer pct	+
	Health care access and quality index	-
	Fruit consumption (grams per day adjusted)	-
3	Cumulative cigarettes (10 years)	+
	Cumulative cigarettes (5 years)	+
	Education (years per capita)	-
	Log-transformed LDI (per capita)	-
	Smoking prevalence	+
	Socio-demographic Index	+

The following plots show the influence of each covariate on the four CODEm models (male global, male data rich, female global, and female data rich). A positive standardized beta (to the right) means that the covariate was associated with increased death. A negative standardized beta (to the left) means the covariate was associated with decreased death.

Male, global

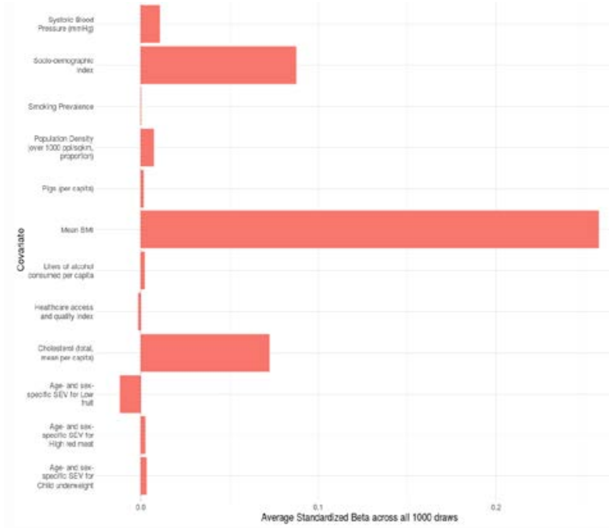

Male, data rich

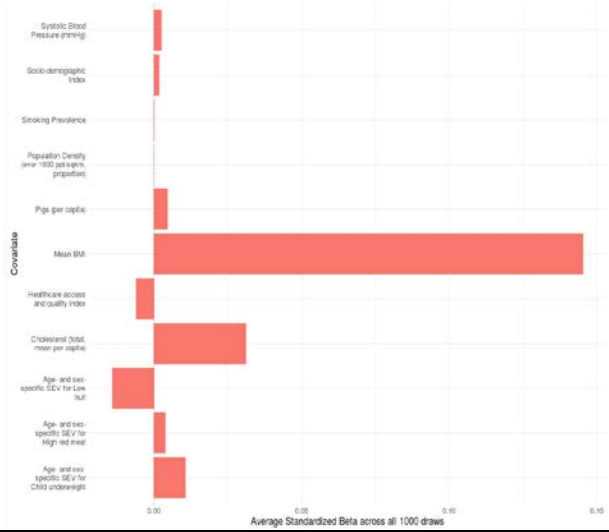

Female, global

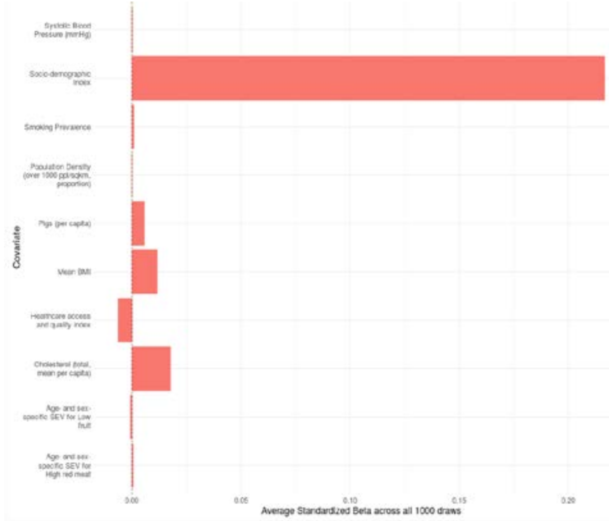

Female, data rich

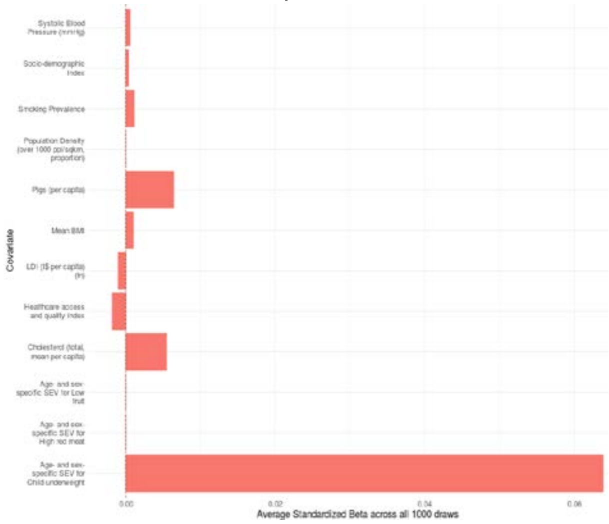

Eating Disorders

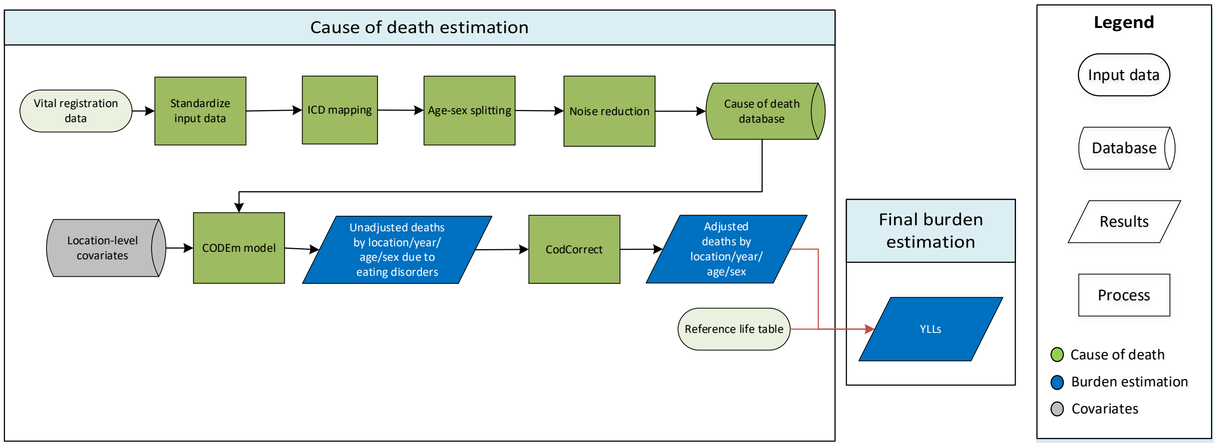

Input data

Data used to estimate eating disorders mortality included vital registration data from the cause of death (COD) database. No garbage codes were redistributed to eating disorders given previous issues with dehydration deaths in low- and middle-income countries causing unfeasible results.

Modelling strategy

Eating disorders were modelled using standard CODEm modelling approach and encompassing the two child models of anorexia nervosa and bulimia nervosa. Age was restricted to deaths occurring between 5 and 49 years of age based on expert advice and patterns of prevalence seen in the non-fatal models of anorexia nervosa and bulimia nervosa. Several covariates were applied to this model and are listed in the table below, along with the direction in which they were applied.

Level	Covariate	Direction
1	education (years per capita)	+
	log LDI (I$ per capita)	+
	Age- and sex-specific SEV for child underweight	-
	sanitation (proportion with access)	+
	maternal education (years per capita)	+
2	healthcare access and quality index	-
3	Socio-demographic Index	+

In GBD 2013, eating disorders were modelled as a negative binomial model using a custom approach. This approach was changed in GBD 2015, with eating disorders being modelled as a standard CODEm model, as no obvious benefit was seen from using the custom modelling approach. GBD 2016 utilised the same approach as GBD 2015 with the only difference being the inclusion of covariates. For GBD

2017, garbage codes were no longer redistributed to eating disorders given the impact of these codes on the feasibility of the geographical distribution. For example, while only a relatively small proportion of dehydration garbage code deaths were redistributed to eating disorders, this added a comparatively large number of deaths to eating disorders, particularly in regions with higher rates of infectious diseases, and they were redistributed equally between males and females despite the prevalence of eating disorders known to be up to ten times higher in females. As such, a decision was made to no longer redistribute garbage codes to eating disorders.

Anorexia Nervosa

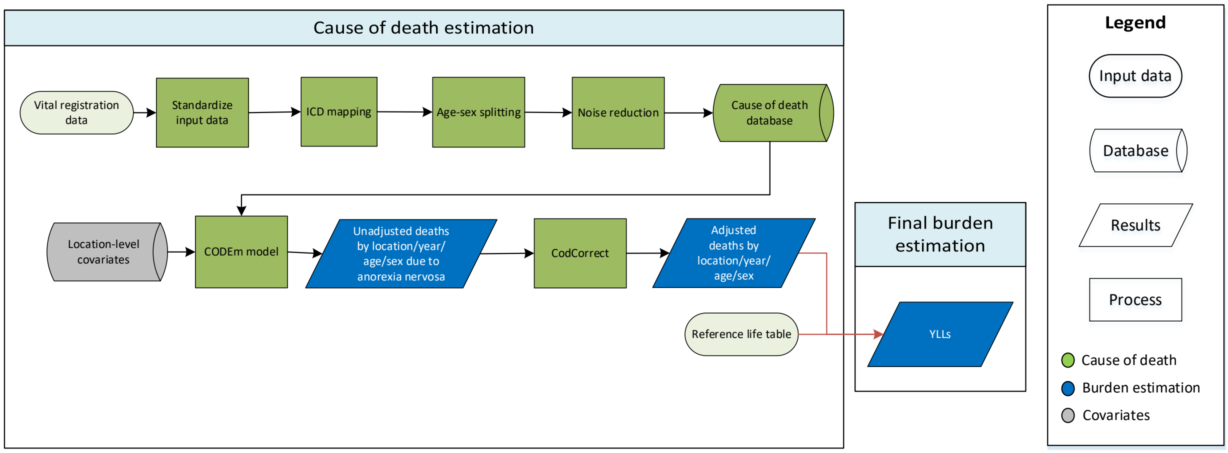

Input data

Data used to estimate anorexia nervosa mortality included centrally prepped vital registration data from the cause of death (COD) database. No garbage codes were redistributed to anorexia nervosa given previous issues with dehydration deaths in low- and middle-income countries causing unfeasible results.

Modelling strategy

Anorexia nervosa was modelled using the standard CODEm approach and came under the eating disorders parent model. Age was restricted to deaths occurring between 5 and 49 years based on expert advice and patterns of prevalence seen in the non-fatal model. Several covariates were applied to this model and are listed in the table below, along with the direction in which they were applied.

Level	Covariate	Direction
1	education (years per capita)	+
	log LDI (I$ per capita)	+
	Age- and sex-specific SEV for child underweight	-
	sanitation (proportion with access)	+
	maternal education (years per capita)	+
2	healthcare access and quality index	-
3	Socio-demographic Index	+

In GBD 2013, anorexia nervosa deaths were extrapolated from the eating disorders model, which was modelled through a negative binomial approach. This approach was changed in GBD 2015, with anorexia nervosa deaths being modelled through a standard CODEm approach under the overarching eating disorders model, as there was no benefit observed from applying the custom approach. GBD 2016 utilised the same approach as GBD 2015 with the only difference being the inclusion of covariates. For

GBD 2017, garbage codes were no longer redistributed to anorexia nervosa given the impact of these codes on the feasibility of the geographical distribution. For example, while only a relatively small proportion of dehydration garbage code deaths were redistributed to anorexia nervosa, this added a comparatively large number of deaths to anorexia nervosa, particularly in regions with higher rates of infectious diseases, and were redistributed equally between males and females despite the prevalence of anorexia nervosa known to be up to ten times higher in females. As such, a decision was made to no longer redistribute garbage codes to anorexia nervosa.

Bulimia Nervosa

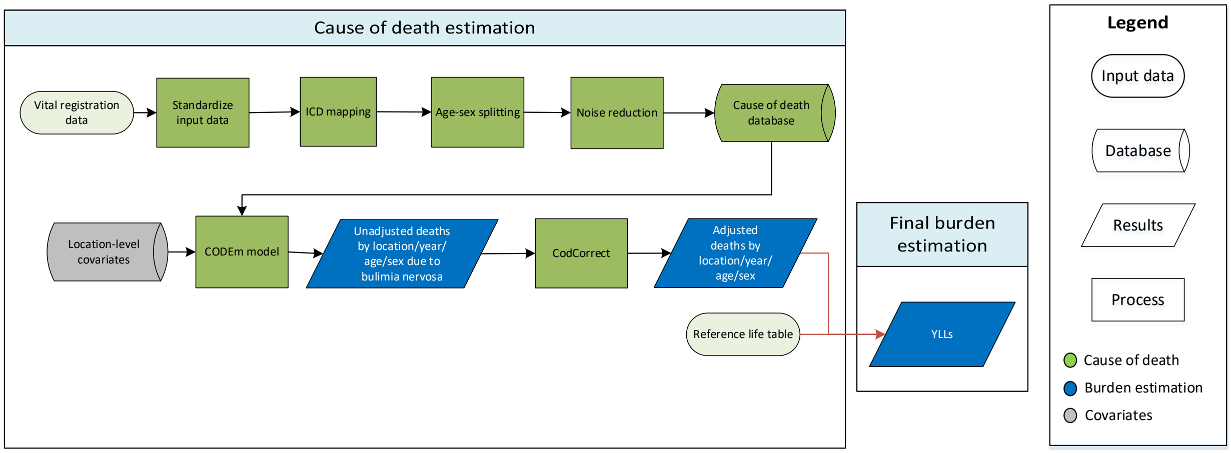

Input data

Data used to estimate bulimia nervosa mortality included centrally prepped vital registration data from the cause of death (COD) database. No garbage codes were redistributed to bulimia nervosa given previous issues with deaths in low- and middle-income countries causing unfeasible results.

Modelling strategy

Bulimia nervosa was modelled using the standard CODEm approach and comes under the eating disorders parent model. Age was restricted to deaths occurring between 5 and 49 years based on expert advice and patterns of prevalence seen in the non-fatal model. Several covariates were applied to this model and are listed in the table below, along with the direction in which they were applied.

Level	Covariate	Direction
1	education (years per capita)	+
	log LDI (I$ per capita)	+
	Age- and sex-specific SEV for child underweight	-
	sanitation (proportion with access)	+
	maternal education (years per capita)	+
2	healthcare access and quality index	-
3	Socio-demographic Index	+

In GBD 2013, bulimia nervosa was not modelled as a distinct cause of death. Any deaths due to bulimia nervosa were attributed to the eating disorders model. We changed this approach in GBD 2015, recognising bulimia nervosa as an individual cause of death, and therefore modelled it as a standard CODEm model under the overarching eating disorders model. This decision was based on observing

deaths due to bulimia nervosa in high-quality vital registration data, such as data from the USA. These data also include eating disorders not otherwise specified. GBD 2016 utilised the same approach as GBD 2015 with the only difference being the inclusion of covariates. For GBD 2017, garbage codes were no longer redistributed to bulimia nervosa given the impact of these codes on the feasibility of the geographical distribution.

Alcohol use disorders

Flowchart

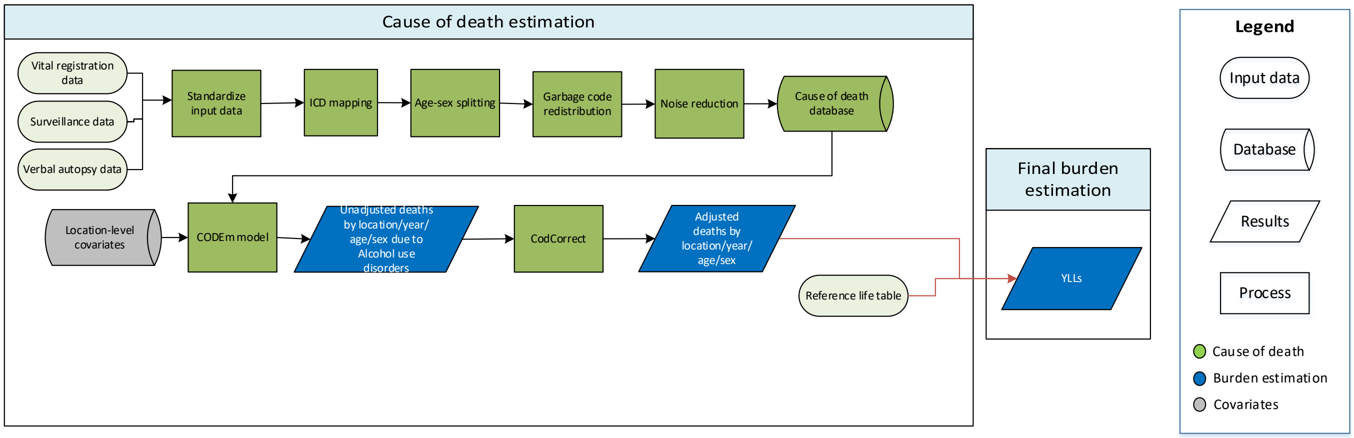

Input data and methodological summary for alcohol use disorders

Input data

All data were from vital registration, China surveillance, and verbal autopsy sources. Some data were outliered from countries with sparse yet heterogeneous data if they created implausible fluctuations in deaths and regional patterns. As an example, Medical Certification of Cause of Death data from India were excluded for alcohol use disorders due to the extremely low estimates. All data came from the following ICD 10 codes: E24.4, F10, G31.2, G62.1, G72.1, P04.3, Q86.0, R78.0, X45, X65, Y15.

Modelling strategy

Cause of death modelling for alcohol use disorders followed the general CODEm strategy. There were no substantial, model-specific changes from GBD 2017. Model covariate inclusion was based on empirical evidence and expert feedback, which resulted in a set of model covariates that reflected alcohol consumption, smoking, education, health system access, domestic income, and Socio-demographic Index (SDI).

Table 1: Covariates used in alcohol use disorders mortality model

Level	Covariate	Direction
1	Alcohol consumption (litres per capita)	+
	Alcohol binge drinking	+
	Alcohol consumption, age-standardised, in grams per day	+
	Alcohol drinker proportion, age-standardised	+
2	Cumulative cigarettes (10 years)	+

	Smoking prevalence	+
	Healthcare Access and Quality Index	-
3	Log LDI (I$ per capita)	-
	Education (years per capita)	-
	Socio-demographic Index	-

In GBD 2019, ICD codes for a number of garbage codes, including shock and cardiac arrest, alcoholic hepatic failure, and unspecified heart failure, were redistributed to alcohol use disorders using an algorithm devised from analysing national registry data from several countries and expert feedback.

Drug Use Disorders

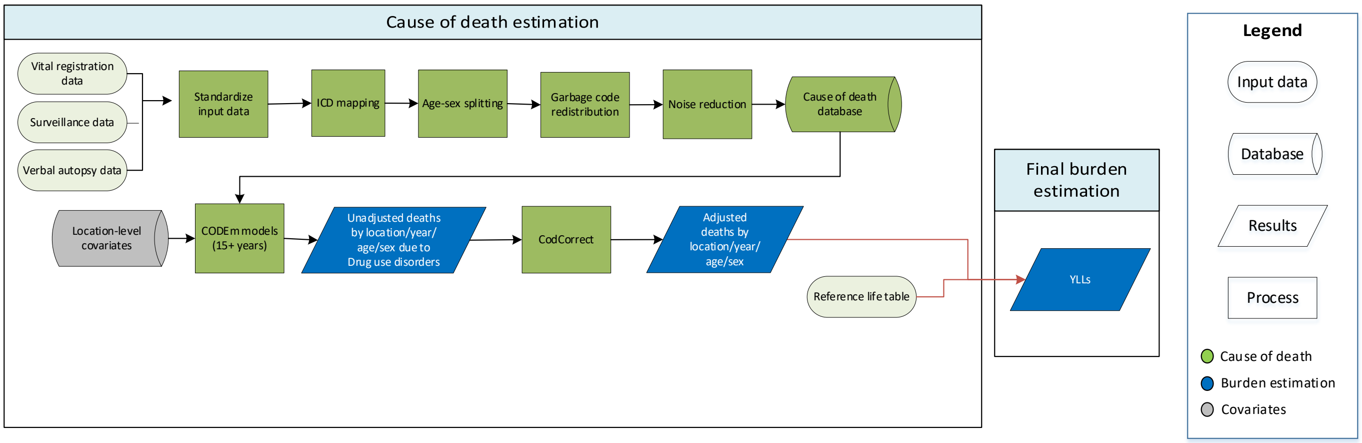

Input data

All data were from vital registration, verbal autopsy, and surveillance sources. Data from countries with sparse yet heterogeneous data were excluded as the data exaggerated fluctuations in deaths and gave implausible regional patterns. Excluded data were typically from low-income countries. Notably, a considerable amount of Medical Certification of Cause of Death (MCCD) data from India were excluded for drug use disorders. Specifically, it was decided to remove the MCCD ICD-9 data, as a specific garbage redistribution package was not available for that time series. Additionally, it was decided to remove MCCD-ICD10 data from the Northeastern states of Meghalaya, Mizoram, Nagaland, and Manipur (where the much lower values in MCCD compared to SRS removed the expected higher death rates there) and also from the four states of Punjab, Uttarakhand, Jharkand, and Karnataka (where the raw data showed almost no deaths from drug use disorders).

Redistribution of garbage codes remains a major challenge in estimating global drug deaths. Garbage codes most relevant to drug use disorders include ICD codes for accidental poisonings (X40-44 and X49), exposure to unspecified factors (X59), and external causes of undetermined intent (Y34). As in past rounds, we have used multiple cause of death (MCOD) records to inform redistribution packages. This year, we added new data from Colombia, Italy, and Taiwan, in addition to data used in GBD 2017 from USA, Australia, Mexico, and Brazil. Drug-specific redistribution follows an algorithm based on the fatality of different substances when considering a combination of drugs (Table 1).

Table 1. Algorithm for the selection and assignment of a substance or drug use cause of death for deaths coded to an underlying cause of unintentional poisoning using multiple cause of death data

selection algorithm						
Other cause / Other cause	Opioids	Cannabis	Cocaine	Amphetamines	alcohol	Psychoactive and psychedelic drug
Opioids	Opioids	Opioids	Opioids	Opioids	Opioids	Opioids
Cannabis	Opioids	Cannabis	Cocaine	Amphetamines	alcohol	Psychoactive and psychedelic drug
Cocaine	Opioids	Cocaine	Cocaine	Amphetamines	Cocaine	Cocaine
Amphetamines	Opioids	Amphetamines	Amphetamines	Amphetamines	Amphetamines	Amphetamines
alcohol	Opioids	alcohol	Cocaine	Amphetamines	alcohol	Psychoactive and psychedelic drug
Psychoactive and psychedelic drug	Opioids	Psychoactive and psychedelic drug	Cocaine	Amphetamines	Psychoactive and psychedelic drug	Psychoactive and psychedelic drug

The addition of new MCOD data, along with new data processing methods, resulted in a significant decrease in garbage code deaths redistributed to drug use disorders from Y34. This resulted in decreases in drug deaths mainly in lower- and middle-income countries where Y34 is commonly used. The changes resulted in implausibly low drug deaths in Philippines, Thailand, Malaysia, Iraq, and South Africa, given what is known about drug use in these countries based on survey data. While the MCOD data analysis is improved in GBD 2019 by greater geographic coverage, notably the places where it performed poorly are in geographically distinct areas with no MCOD coverage. As a result, we removed data from these countries and allowed the model to follow covariates. In future rounds, additional modelling to more accurately predict redistribution in regions with no MCOD coverage could help to alleviate this issue.

Other notable changes between GBD 2017 and GBD 2019 include excluding deaths coded to tobacco dependence (F17.2) from drug use disorders, as well as assigning a larger proportion of deaths coded as undetermined intent poisoning by psychoactive drugs (Y12) to drug deaths rather than suicide. The magnitude of the impact of these changes depended on location-specific coding practices. We also utilized European death data that has single combinations of E and N codes to inform age- and sex-specific drug-specific redistributions for Europe, resulting in a lower proportion of deaths assigned to other drug use disorders and a higher proportion of deaths assigned to opioid use disorders.

Additionally, we identified several ICD codes from the F19 chapter that were previously mapped to “other drug use disorders” but should instead be mapped to the parent “drug use disorders” category, informing the level in order to allow other more definitive codes to determine the drug-specific splits. This change resulted in a lower proportion of drug deaths in the other drug category, and generally a higher proportion of deaths in the opioid use disorder category.

Modelling strategy

Cause of death modelling for drug use disorders follows the general CODEm strategy. Level 1 covariates include intravenous drug use prevalence and opioid consumption per million inhabitants per day. The

latter covariate is derived from data from the International Narcotics Control Board (INCB), which measures *"defined daily doses for statistical purposes" (S-DDD),* and is considered an approximate measure to rank consumption in different countries.

Due to the extremely small number of drug deaths being recorded, drug models are restricted to ages 15 and above. To capture drug deaths among ages under 15, deaths recorded in vital registration for ages less than 15 were directly added during post-processing steps, rather than being modeled. As a rule, in GBD2019 we no longer specified covariates with a 'zero' direction and therefore changed the direction of the log LDI, education and SDI covariates to be positive.

Table 2. Covariates used in drug use disorders CODEm model

Level	Covariate	Direction
1	Intravenous drug use age-standardised	+
	Intravenous drug use age-specific	+
	Opioid standard doses per million per day (10-year lag)	+
2	cumulative cigarettes (10 years)	+
	cumulative cigarettes (5 years)	+
	opium cultivation bin	+
	smoking prevalence	+
	healthcare access and quality index	-
3	log LDI (I$ per capita)	+
	education (years per capita)	+
	Socio-demographic Index	+

The drug use model is the parent model of all other drug use causes (ie, amphetamine, cocaine, opioid, and other drug). It forms an envelope into which all four individual drug use models are scaled during the CoDCorrect process.

Covariate Influences:

The following plots show the influence of each covariate on the four CODEm models (male global, male data rich, female global, and female data rich). A positive standardized beta (to the right) means that the covariate was associated with increased death. A negative standardized beta (to the left) means the covariate was associated with decreased death.

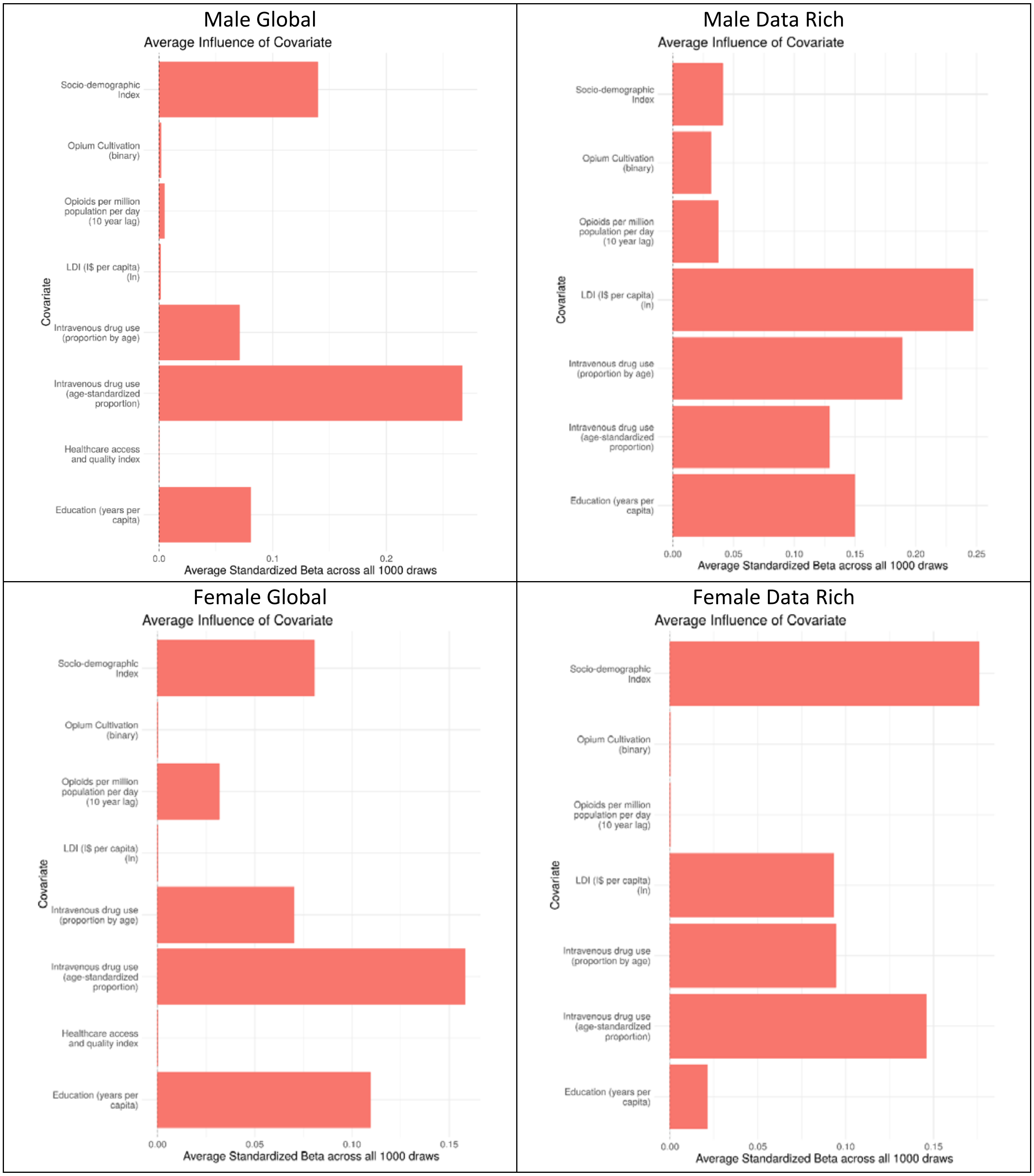

Opioid Use Disorders

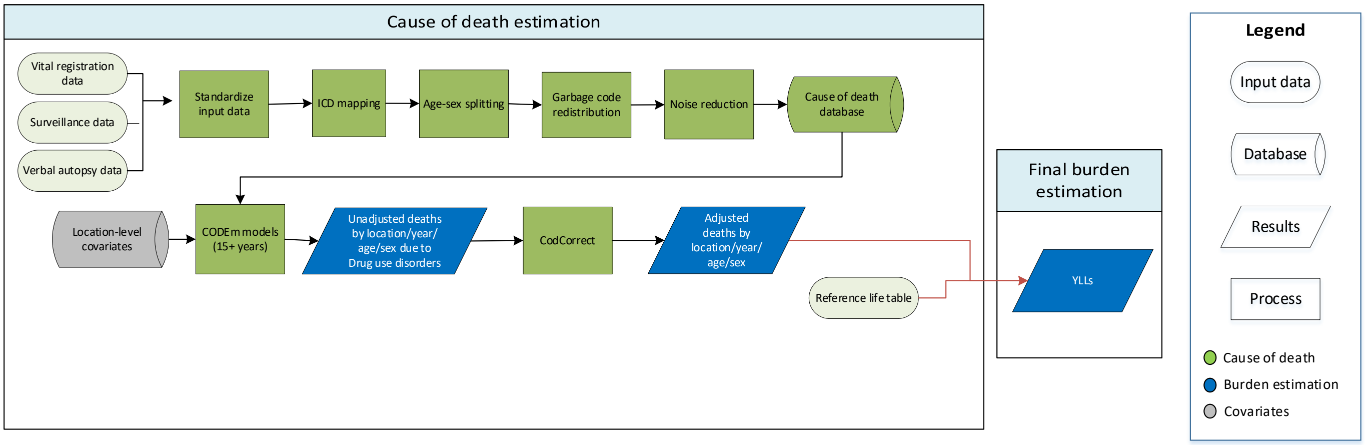

Input data

All input data were from vital registration and surveillance sources. Data from countries with sparse yet heterogeneous data were excluded as the data exaggerated fluctuations in deaths and gave implausible regional patterns. Excluded data were typically from low- and middle-income countries. The locations for which there was the most data included North America, Australia, Western Europe, and parts of Latin America.

A full description of changes to coding and redistribution are described in the appendix section focusing on aggregate drug use disorders. Globally, estimated deaths due to opioid use disorders decreased compared to GBD 2017, mainly due to decreases resulting from the new Y34 redistribution package. These changes mainly impacted lower- and middle-income countries where the Y34 code is commonly used. In high-income countries, deaths due to opioid use disorders increased compared to GBD 2017. These changes were the result of improved drug-specific redistribution in Europe, which assigned a greater proportion of drug deaths to opioid use disorders compared and a smaller proportion of deaths to other drug use disorders, as well as improved redistribution of Y12, which assigned a greater proportion of poisoning deaths of undetermined intent to drug use disorders rather than suicide.

Modelling strategy

Cause of death modelling for opioid use disorders followed the general CODEm strategy. Several covariates are particularly important for the opioid use disorder models to be able to capture the rapid increases in opioid use disorder deaths recently observed in the United States. These include intravenous drug use prevalence from the model used to estimate exposure for the drug use as a risk analyses, and opioid consumption per million inhabitants per day. The latter covariate was derived from data from the International Narcotics Control Board (INCB) which measures *“defined daily doses for statistical purposes” (S-DDD),* which translates all different opioids of different types and dosages into comparable units to quantify consumption in different countries. As a rule, in GBD2019 we no longer specified covariates with a ‘zero’ direction and therefore changed the direction of the log LDI, education and SDI covariates to be positive.

Table 1: Covariates used in opioid use CODEm model

Level	Covariate	Direction
1	Intravenous drug use age-standardised	+
	Intravenous drug use age-specific	+
	Opioid standard doses per million per day (10-year lag)	+
2	cumulative cigarettes (10 years)	+
	cumulative cigarettes (5 years)	+
	opium cultivation bin	+
	smoking prevalence	+
	healthcare access and quality index	-
3	log LDI (I$ per capita)	+
	education (years per capita)	+
	Socio-demographic Index	+

Covariate Influences:

The following plots show the influence of each covariate on the four CODEm models (male global, male data rich, female global, and female data rich). A positive standardized beta (to the right) means that the covariate was associated with increased death. A negative standardized beta (to the left) means the covariate was associated with decreased death.

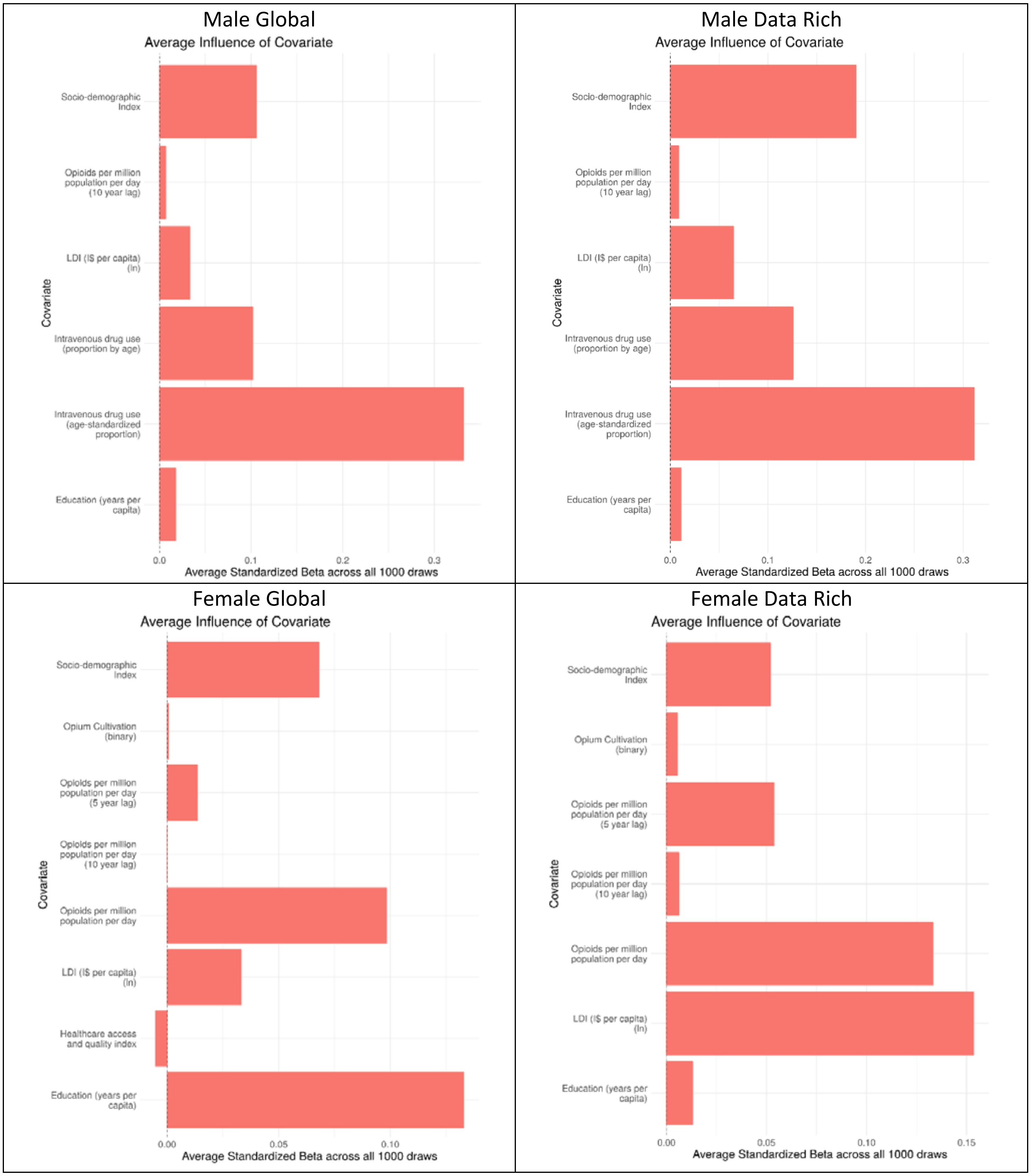

Cocaine Use Disorder

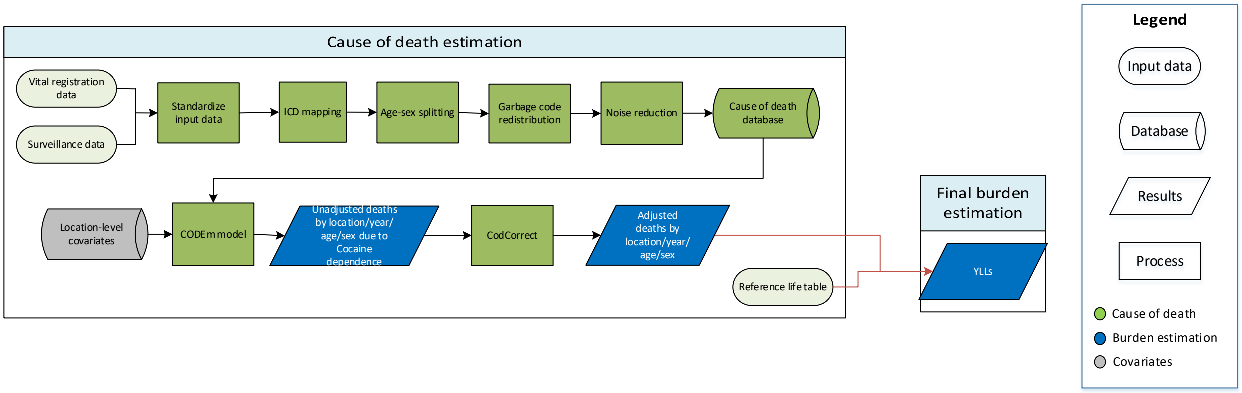

Input data

All data were from vital registration and surveillance sources. Data from countries with sparse yet heterogeneous data were excluded as the data exaggerated fluctuations in deaths and gave implausible regional patterns. Excluded data were typically from low- and middle-income countries. A full description of changes to coding and redistribution are described in the appendix section focusing on aggregate drug use disorders. Overall, estimated deaths due to cocaine use disorders increased compared to GBD 2017, as a result of additional data added in GBD 2019 to inform drug-specific redistribution, particularly the new MCOD data from Colombia.

Modelling strategy

Cause of death modelling for cocaine use followed the general CODEm strategy. There were no substantial changes from GBD 2017. Model covariate inclusion was based on empirical evidence and expert feedback, which resulted in a set of model covariates that reflected alcohol consumption, smoking, education, health system access, income per capita, and Socio-demographic Index (SDI) (Table 1). As a rule, in GBD2019 we no longer specified covariates with a 'zero' direction and therefore changed the direction of the log LDI, education and SDI covariates to be positive.

Table 1: Covariates used in cocaine use CODEm model

Level	Covariate	Direction
1	alcohol (litres per capita)	+
	current drinking prevalence	+
	Intravenous drug use age-standardised	+
	Intravenous drug use age-specific	+
	cumulative cigarettes (10 years)	+
	cumulative cigarettes (5 years)	+
	cigarettes per capita	+
	smoking prevalence	+
2	healthcare access and quality index	-
3	log LDI (I$ per capita)	+
	education (years per capita)	+
	Socio-demographic Index	+

Covariate Influences:

The following plots show the influence of each covariate on the four CODEm models (male global, male data rich, female global, and female data rich). A positive standardized beta (to the right) means that the covariate was associated with increased death. A negative standardized beta (to the left) means the covariate was associated with decreased death.

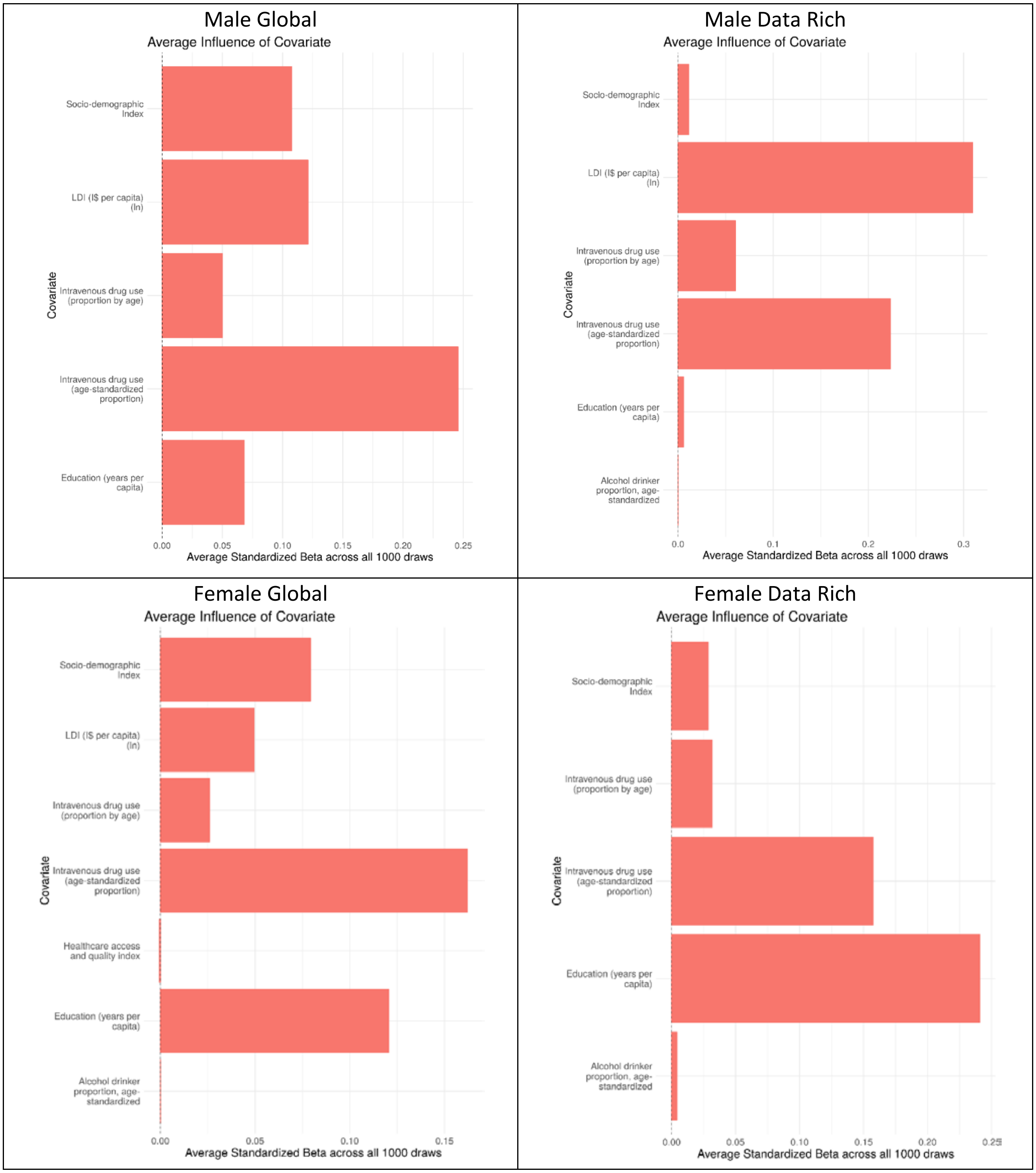

Amphetamine Use Disorder

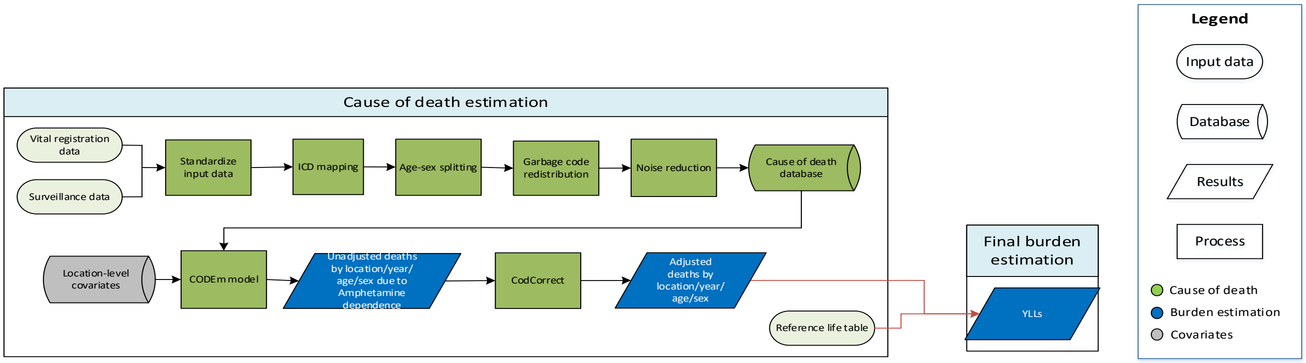

Input data

All data were from vital registration and surveillance sources. Data from countries with sparse yet heterogeneous data were excluded as the data exaggerated fluctuations in deaths and gave implausible regional patterns. Excluded data were typically from lower-income countries. A full description of changes to coding and redistribution are described in the appendix section focusing on aggregate drug use disorders. Overall, estimated deaths due to amphetamine use disorders increased compared to GBD 2017, as a result of additional data added in GBD 2019 to inform drug-specific redistribution.

Modelling strategy

Cause of death modelling for amphetamine use followed the general CODEm strategy. There were no substantial changes from GBD 2017. Model covariate inclusion was based on empirical evidence and expert feedback, which resulted in a set of model covariates that reflected alcohol consumption, smoking, education, health system access, domestic income, and Socio-demographic Index (SDI) (Table 1). As a rule, in GBD2019 we no longer specified covariates with a 'zero' direction and therefore changed the direction of the log LDI, education and SDI covariates to be positive.

Table 1: Covariates used in amphetamine use CODEm model

Level	Covariate	Direction
1	alcohol (litres per capita)	+
	current drinking prevalence	+
	Intravenous drug use age-standardised	+
	Intravenous drug use age-specific	+
	cumulative cigarettes (10 years)	+
	cumulative cigarettes (5 years)	+
	cigarettes per capita	+
	smoking prevalence	+
2	healthcare access and quality index	-
3	log LDI (I$ per capita)	+
	education (years per capita)	+
	Socio-demographic Index	+

Covariate Influences:

The following plots show the influence of each covariate on the four CODEm models (male global, male data rich, female global, and female data rich). A positive standardized beta (to the right) means that the covariate was associated with increased death. A negative standardized beta (to the left) means the covariate was associated with decreased death.

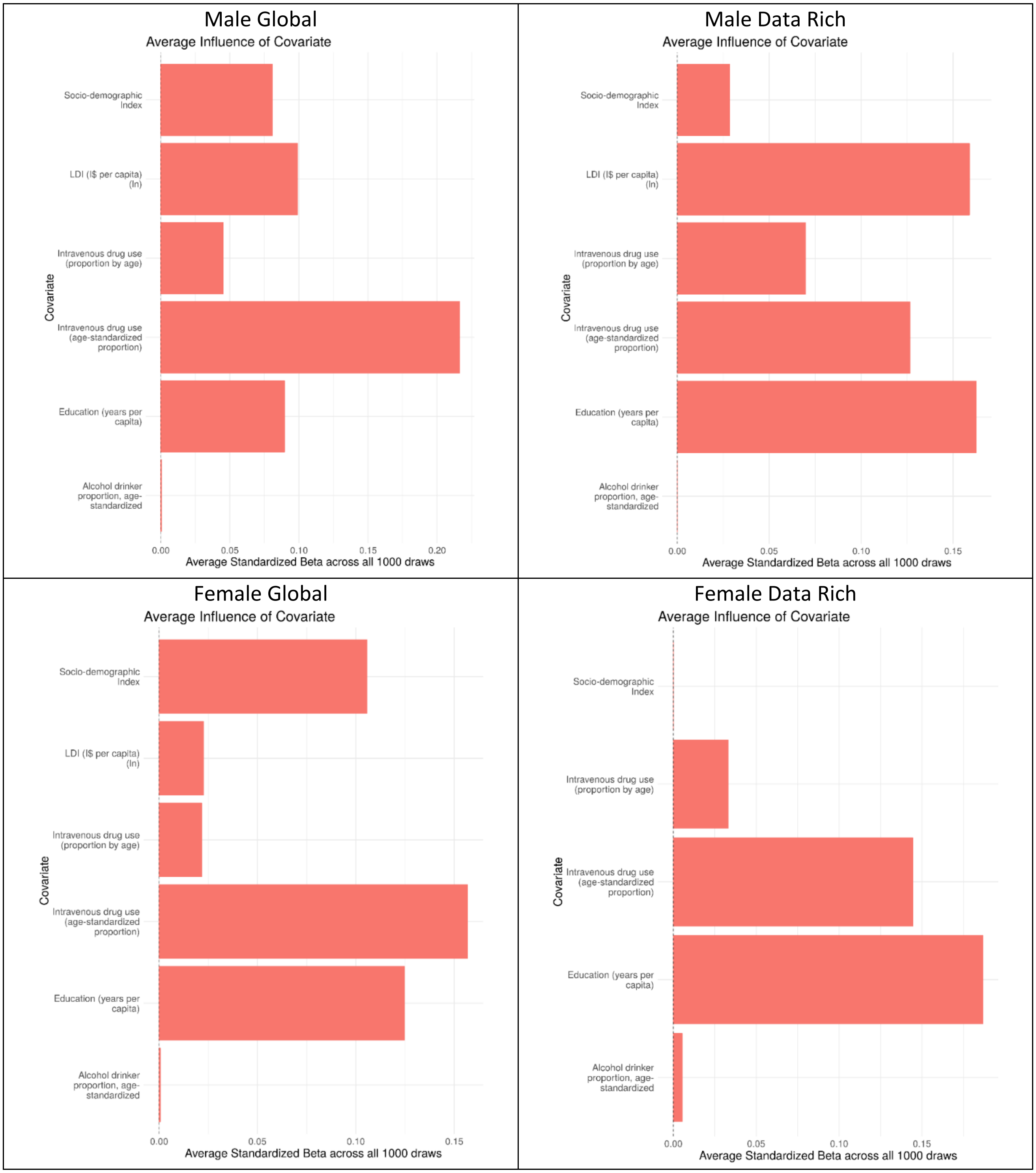

Other Drug Use Disorders

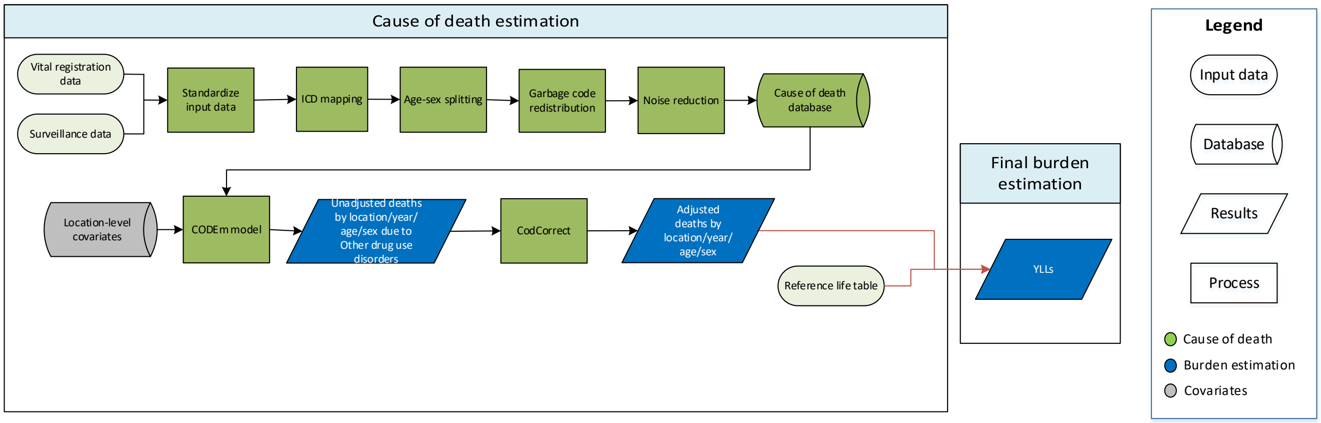

Input data

All data were from vital registration and surveillance sources. Data from countries with sparse yet heterogeneous data were excluded as the data exaggerated fluctuations in deaths and gave implausible regional patterns. Excluded data were typically from lower-income countries. A full description of changes to coding and redistribution are described in the appendix section focusing on aggregate drug use disorders. Overall, estimated deaths due to other drug use disorders decreased compared to GBD 2017, as a result of coding changes that both decreased the total envelope of drug use disorder deaths as well as changes that decreased the proportion of drug deaths that were categorized as other drug use disorder deaths.

Modelling strategy

Cause of death modelling for other drug use followed the general CODEm strategy. There were no substantial changes from GBD 2017. Model covariate inclusion was based on empirical evidence and expert feedback, which resulted in a set of model covariates that reflected alcohol consumption, smoking, education, health system access, domestic income, and Socio-demographic Index (SDI) (Table 1). As a rule, in GBD2019 we no longer specified covariates with a 'zero' direction and therefore changed the direction of the log LDI, education and SDI covariates to be positive.

Table 1: Covariates used in other drug use CODEm model

Level	Covariate	Direction
1	alcohol (litres per capita)	+
	current drinking prevalence	+
	Intravenous drug use age-standardised	+
	Intravenous drug use age-specific	+
	cumulative cigarettes (10 years)	+
	cumulative cigarettes (5 years)	+
	cigarettes per capita	+
	smoking prevalence	+
2	healthcare access and quality index	-
3	log LDI (I$ per capita)	+
	education (years per capita)	+
	Socio-demographic Index	+

Covariate Influences:

The following plots show the influence of each covariate on the four CODEm models (male global, male data rich, female global, and female data rich). A positive standardized beta (to the right) means that the covariate was associated with increased death. A negative standardized beta (to the left) means the covariate was associated with decreased death.

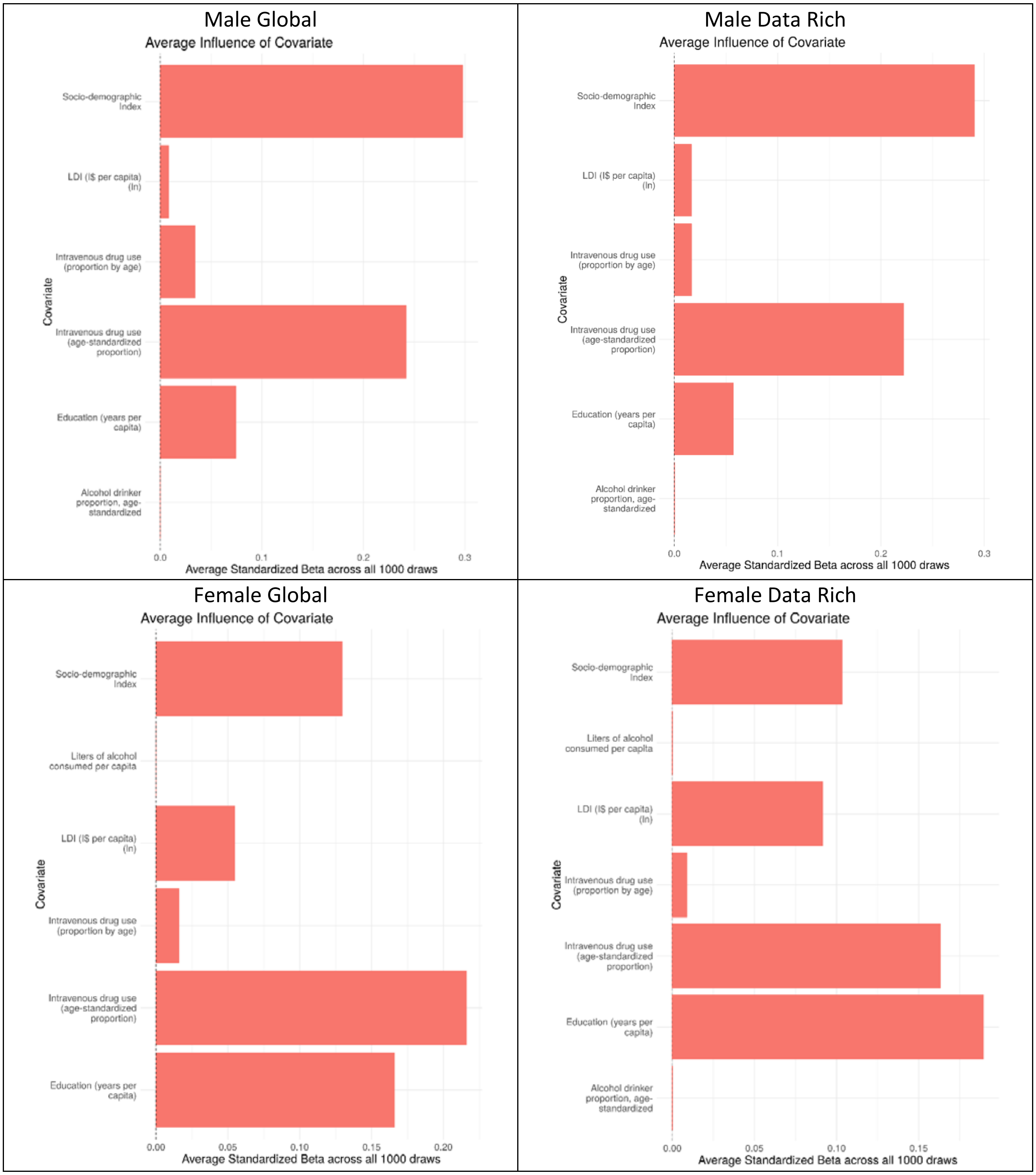

Diabetes Mellitus

Diabetes mellitus mortality was estimated for overall diabetes mellitus, diabetes mellitus type 1, and diabetes mellitus type 2 in GBD 2019.

Overall Diabetes Mellitus

Flowchart

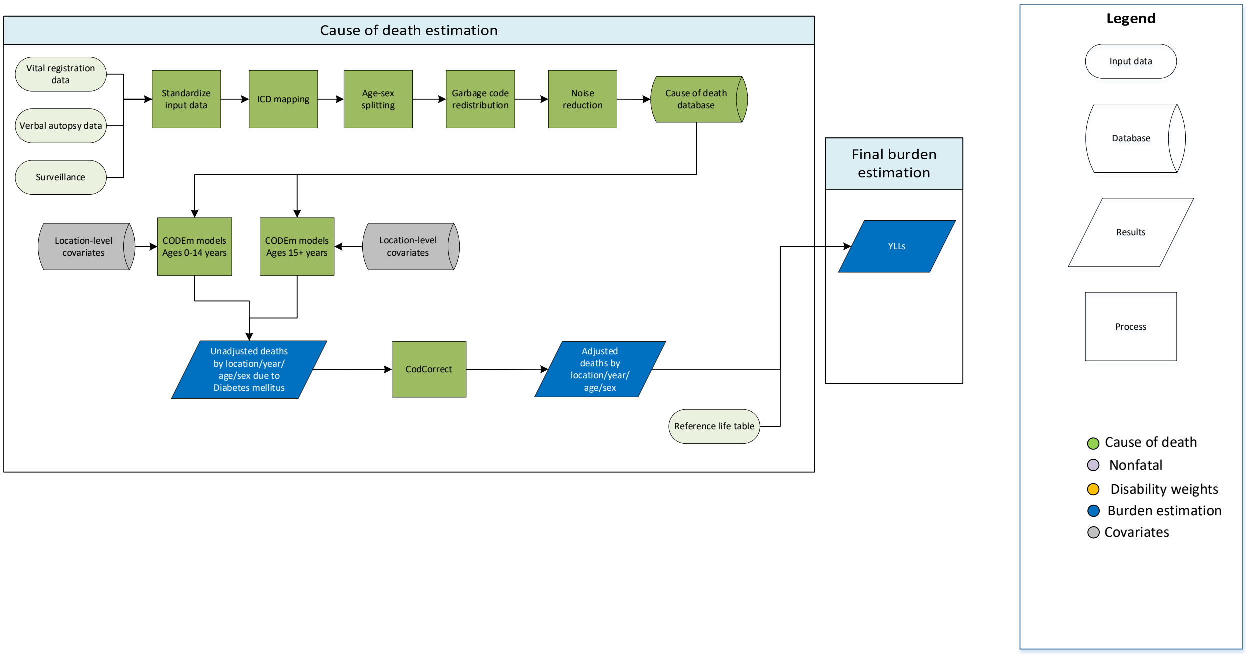

Input Data and Methodological Summary for diabetes mellitus

Input data

Overall diabetes mellitus mortality was estimated using deaths directly attributed to diabetes mellitus. We used verbal autopsy and vital registration data as inputs into the model.

Verbal autopsy data: We outliered data points from sources where there were zero deaths estimated in an age group as this was not realistic for deaths due to diabetes and we determined that these data sources were unreliable.

Vital registration data: We outliered all data from the India Medical Certification of Cause of Death report since the source of the data was unreliable according to expert opinion. We also outliered ICD9BTL data points that were inconsistent with the rest of the data series and created unlikely time trends.

Modelling strategy

The Cause of Death Ensemble model (CODEm) was used for deaths due to diabetes mellitus estimation.

In the overall diabetes mellitus model, we used two models to estimate overall diabetes deaths with different age restrictions. This is because deaths in younger age groups are almost exclusively due to type 1 diabetes, while deaths in older ages are primarily due to type 2 diabetes. This allowed us to select predictive covariates that are specific to the pathophysiology of diabetes type 1 and type 2. We set the younger age model from 0-14 years and the older age model from 15-95+ years. We determined the age threshold based on evidence of the onset age of diabetes type 2 occurring at younger ages.

Covariate selection

The following table lists the covariates included in the model. This requires that the covariate selected for the model must have the directional relationship with diabetes mellitus deaths. In GBD 2019, we made 2 updates. First, we changed 4 covariates to reflect the most current covariate available, proportion underweight to age-standardised underweight (weight-for-age) summary exposure variable, proportion stunting to age-standardised stunting (height-for-age) summary exposure variable, energy-adjusted grams of fruits to age- and sex-specific summary exposure variable for low fruit, and energy-adjusted grams of vegetables to age- and sex-specific summary exposure variable for low vegetables. Second, we selected a direction on covariates that we did not set a direction in previous GBD. We determined the direction based on the strength of the evidence.

Model	Level	Covariate	Direction
0-14 years	1	Healthcare access and quality index	-
	3	Education years per capita	-
	2	Age-standardised fertility rate	+
	2	Latitude	+
	2	Age-standardised underweight (weight-for-age) summary exposure variable	-
	2	Percentage of births occurring in women >35 years old	+
	2	Percentage of births occurring in women >40 years old	+
	3	Socio-demographic Index	-
	2	Age-standardised stunting (height-for-age) summary exposure variable	-
	2	Mean birth weight	-
15 + model	1	Age-standardised mean fasting plasma glucose (mmol/L)	+
	1	Age-standardised prevalence of diabetes	+
	3	Education years per capita	-
	3	Lag-distributed income per capita	+
	1	Mean BMI	+
	2	Mean cholesterol	+
	2	Mean systolic blood pressure	+
	1	Prevalence of obesity	+
	2	Age- and sex-specific summary exposure variable for low fruit	-
	2	Energy-adjusted grams of sugar	+

Model	Level	Covariate	Direction
	2	Age- and sex-specific summary exposure variable for low vegetables	-
	3	Healthcare access and quality index	-
	2	Age- and sex-specific summary exposure variable for alcohol use	+

Covariate Influences:

The following plots show the influence of each covariate on the four CODEm models (male global, male data rich, female global, and female data rich). A positive standardized beta (to the right) means that the covariate was associated with increased death. A negative standardized beta (to the left) means the covariate was associated with decreased death.

Female 0-14 model

Data rich

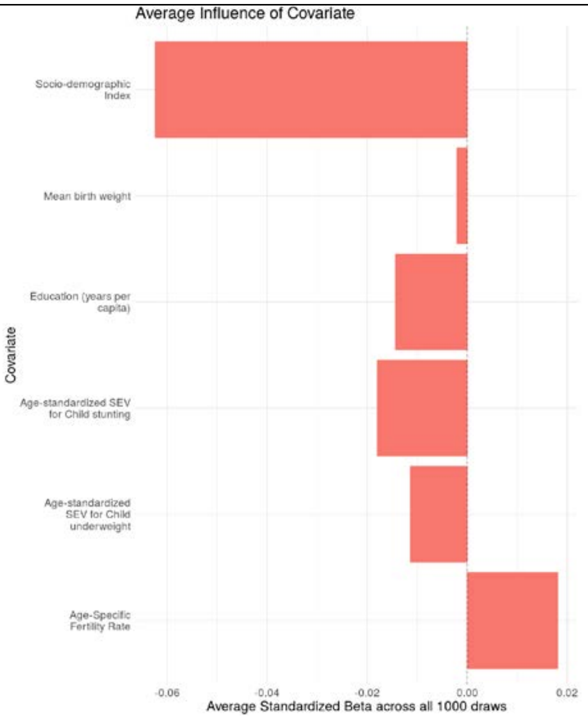

Global

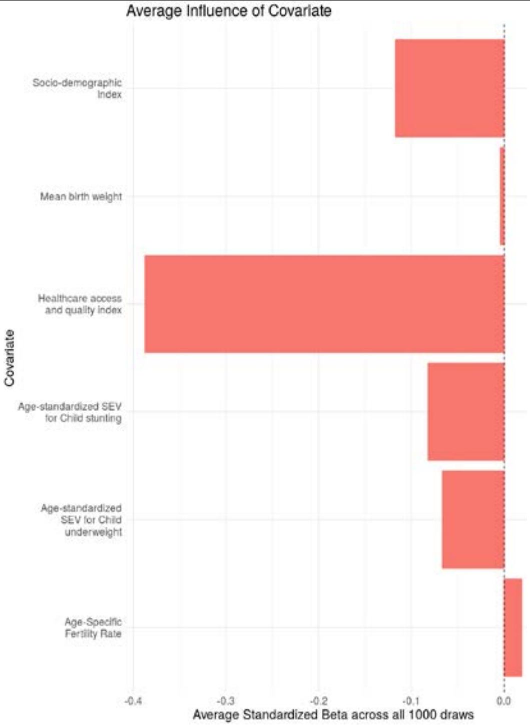

<table>
<tr><th></th><th>Data rich</th><th>Global</th></tr>
<tr><td>Male
0-14
model</td><td>
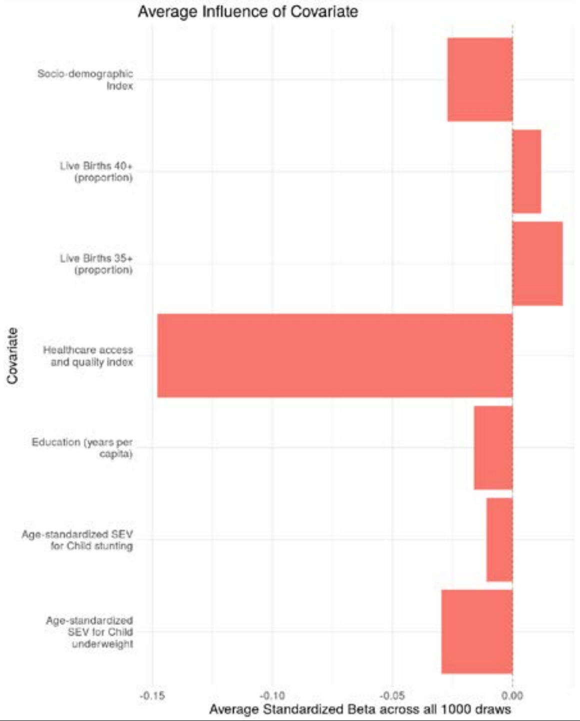

</td><td>
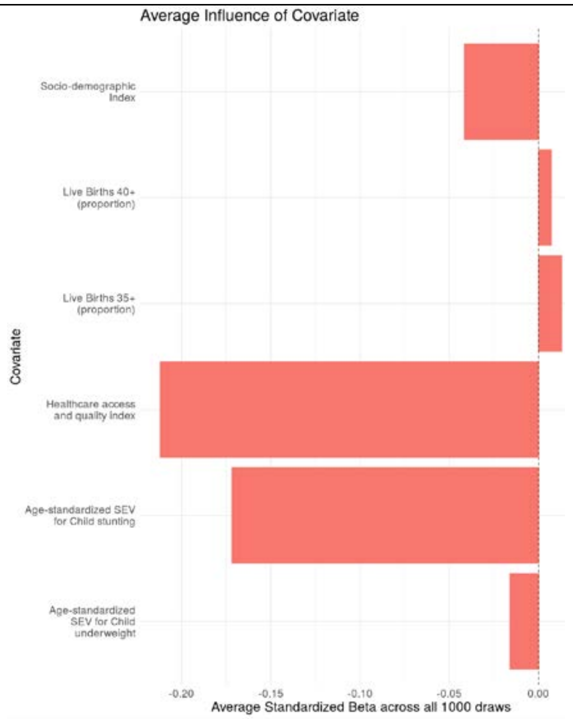

</td></tr>
<tr><td>Female
15+
model</td><td>
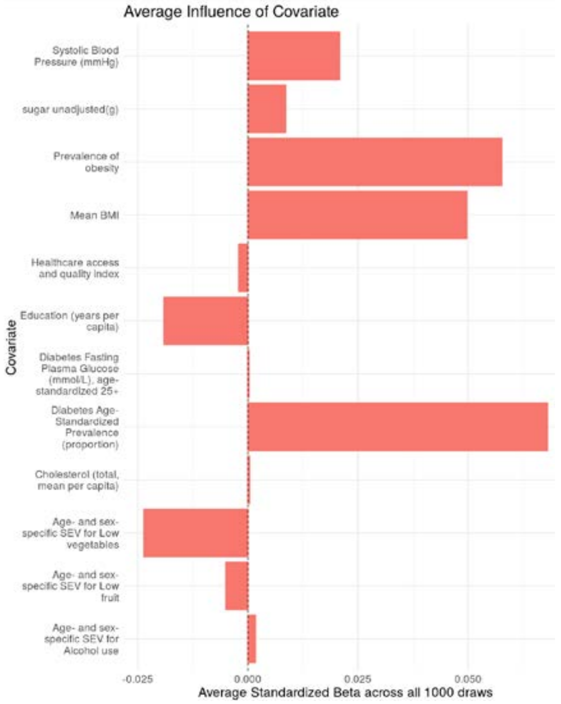

</td><td>
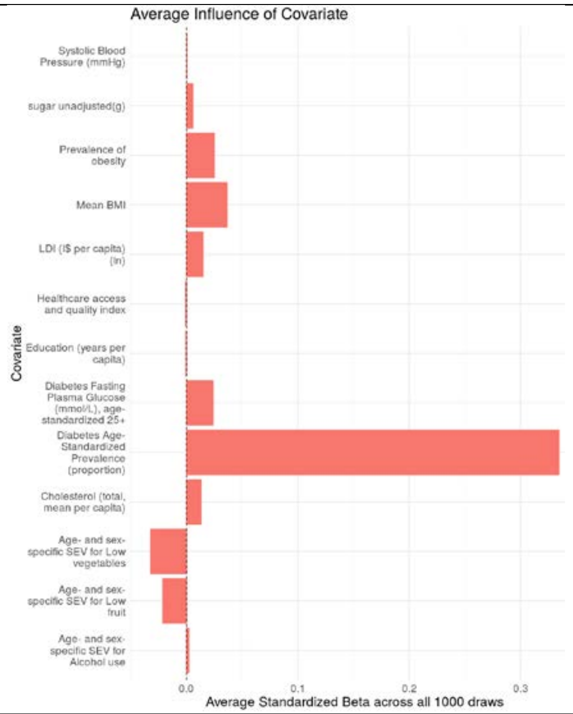

</td></tr>
</table>

Male 15+ model

Data rich

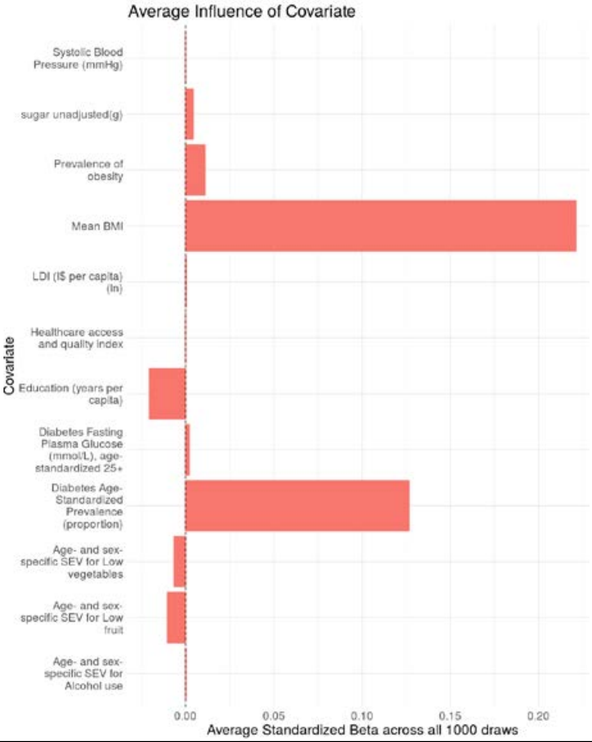

Global

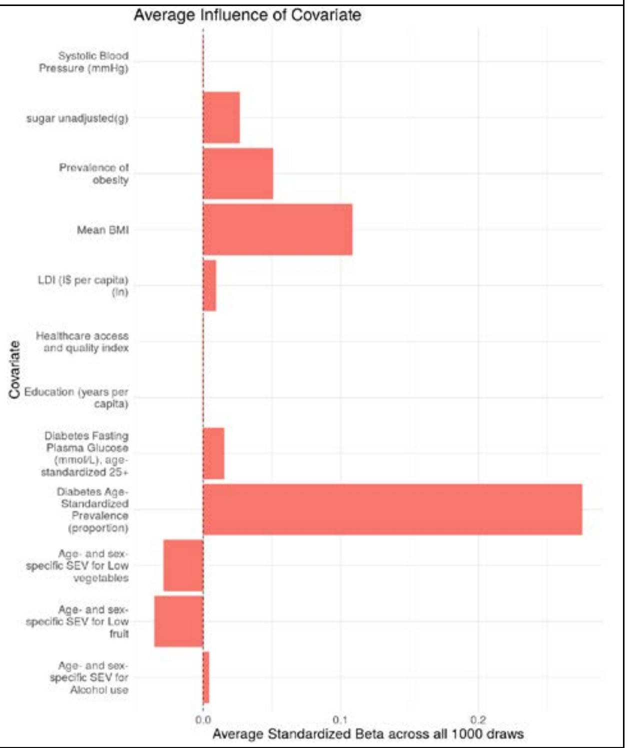

Diabetes mellitus Type 1 and Type 2

Flowchart

Diabetes mellitus Type 1

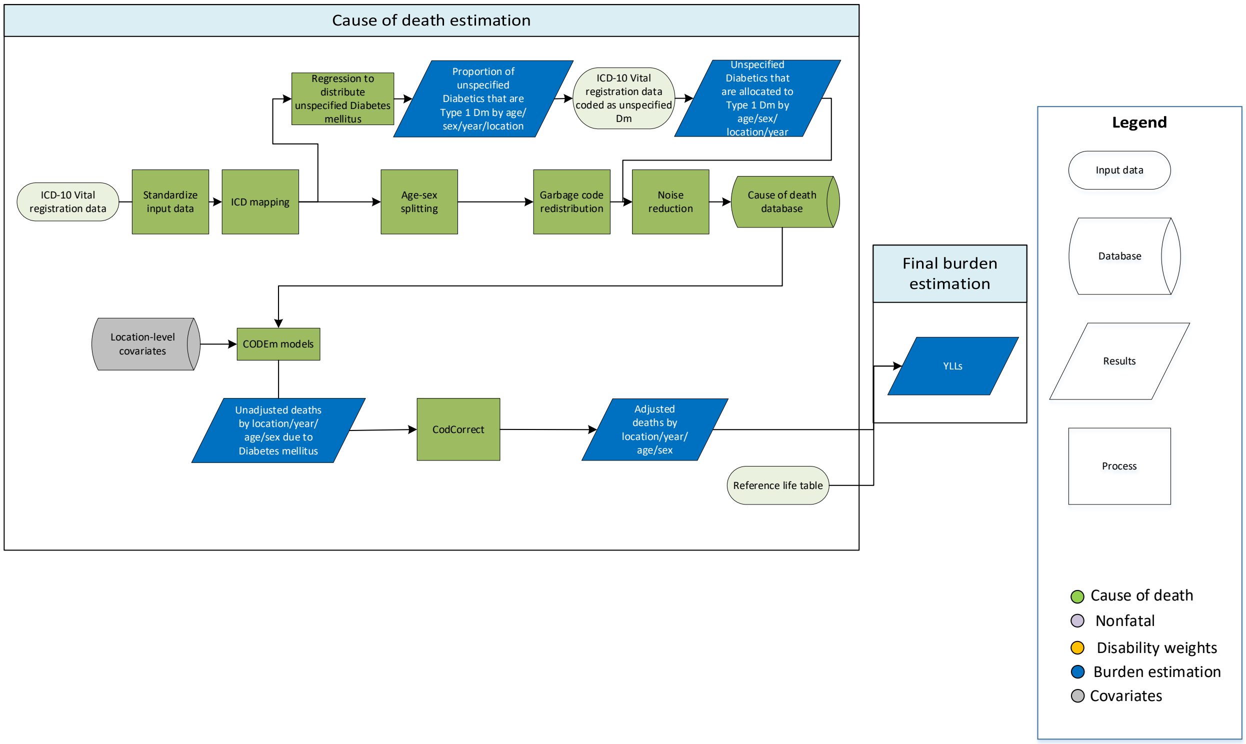

Diabetes mellitus Type 2

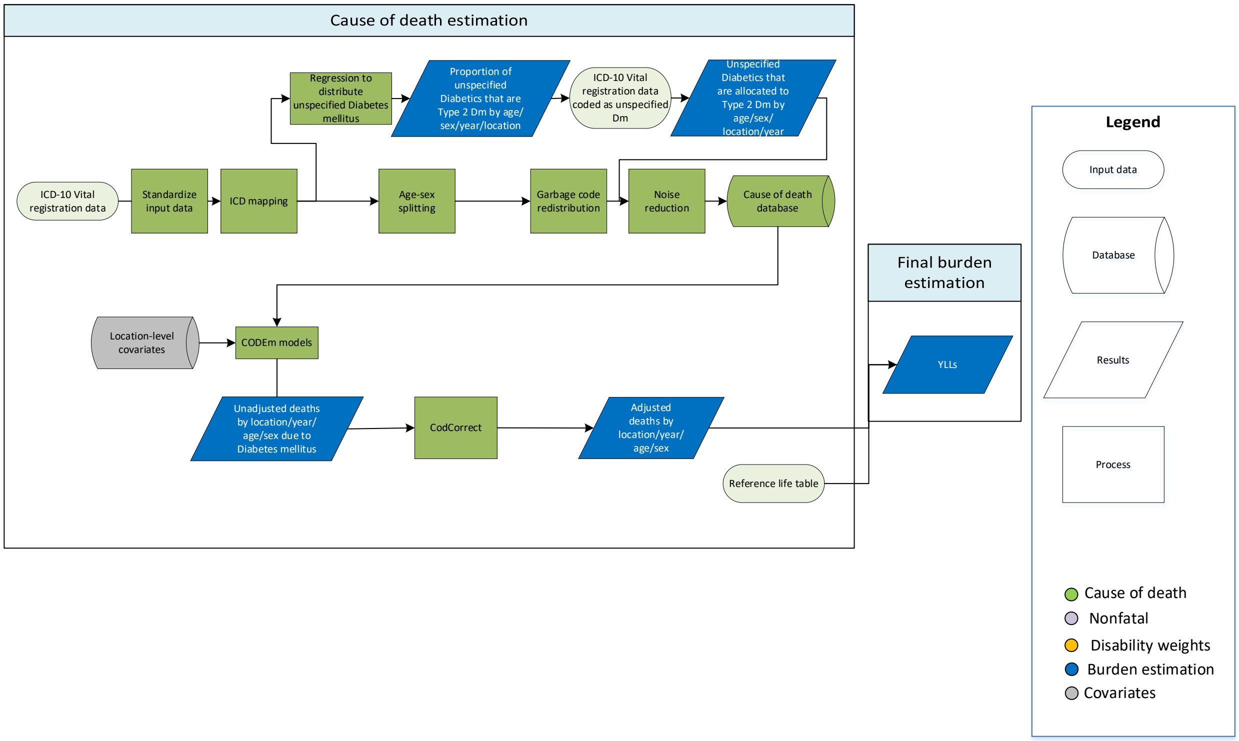

Input Data and Methodological Summary for Type 1 and Type 2 diabetes mellitus

Input data

Type-specific diabetes mellitus mortality was estimated using deaths from vital registration sources in ICD-10 codes only. Diabetes type-specific information was not available in ICD-9 codes or deaths determined by verbal autopsy.

Modelling strategy

The Cause of Death Ensemble model (CODEm) was used for deaths due to diabetes mellitus estimation.

Deaths in younger age groups are almost exclusively due to type 1 diabetes, while deaths in older ages are primarily due to type 2 diabetes. To account for this age pattern, we set the age range of the diabetes type 1 model to 0-95+ years and the age range of the diabetes type 2 model to 15-95+ years. We used the same covariates in the diabetes type 1 model and diabetes type 2 model as the 0-14 year and 15-95+ year in the overall diabetes models, respectively.

There were two unique data manipulation steps that occurred in order to prepare the data as part of the modelling process.

1. We assumed that all deaths <15 years were due to type 1 regardless of the ICD-10 code assigned to the death. We imposed 100% attribution of diabetes mellitus deaths in <15 years to type 1 diabetes mellitus.
2. ICD-10 diabetes data were reported as type 1, type 2, or unspecified. We developed a regression to estimate the fraction of unspecified diabetes mellitus that was type 1 and type 2. We only used data from 703 country-years to inform the regression. This is because these country-years had more than 50% of the deaths typed to type 1 or type 2 AND at least 70% of type-specific deaths in people >25 years were coded to type 2. Since there was a separate regression to estimate the proportion of type 1 diabetes mellitus and type 2 diabetes mellitus, we scaled the predicted proportions to one. These scaled proportions were then applied to number of deaths coded to unspecified diabetes in each location, year, sex where ICD-10 data was reported.

<u>Regression equation</u>

Type 1:

$$logit\left(\frac{number\ type\ 1\ DM}{number\ total\ DM}\right) \sim logit\left(\frac{number\ unspecified\ DM}{number\ total\ DM}\right) + \beta_1 age\ group + \beta_2 age\text{-}st\ prev\ obesity * age\ group + age\text{-}st\ prev\ obesity$$

Type 2:

$$logit\left(\frac{number\ type\ 2\ DM}{number\ total\ DM}\right) \sim logit\left(\frac{number\ unspecified\ DM}{number\ total\ DM}\right) + \beta_1 age\ group + \beta_2 age\text{-}st\ prev\ obesity * age\ group + age\text{-}st\ prev\ obesity$$

Covariate selection

The following are the covariates included in the model. We selected the same covariates for the type 1 diabetes model as the 0-14 year diabetes model and the type 2 diabetes model as the 15-95+ year diabetes model. In GBD 2019, we made 2 updates. First, we changed 4 covariates to reflect the most current covariate available, proportion underweight to age-standardised underweight (weight-for-age) summary exposure variable, proportion stunting to age-standardised stunting (height-for-age) summary exposure variable, energy-adjusted grams of fruits to age- and sex-specific summary exposure variable for low fruit, and energy-adjusted grams of vegetables to age- and sex-specific summary exposure variable for low vegetables. Second, we selected a direction on covariates that we did not set a direction in previous GBD. We determined the direction based on the strength of the evidence.

Model	Level	Covariate	Direction
Type 1	1	Healthcare access and quality index	-
	3	Education years per capita	-
	2	Age-standardised fertility rate	+
	2	Latitude	+
	2	Age-standardised underweight (weight-for-age) summary exposure variable	-
	2	Percentage of births occurring in women >35 years old	+
	2	Percentage of births occurring in women >40 years old	+
	3	Socio-demographic Index	-
	2	Age-standardised stunting (height-for-age) summary exposure variable	-
	2	Mean birth weight	-
Type 2	1	Age-standardised mean fasting plasma glucose (mmol/L)	+
	1	Age-standardised prevalence of diabetes	+
	3	Education years per capita	-
	3	Lag-distributed income per capita	+
	1	Mean BMI	+
	2	Mean cholesterol	+
	2	Mean systolic blood pressure	+
	1	Prevalence of obesity	+
	2	Age- and sex-specific summary exposure variable for low fruit	-
	2	Energy-adjusted grams of sugar	+
	2	Age- and sex-specific summary exposure variable for low vegetables	-
	3	Healthcare access and quality index	-
	2	Age- and sex-specific summary exposure variable for alcohol use	+

Covariate Influences:

The following plots show the influence of each covariate on the four CODEm models (male global, male data rich, female global, and female data rich). A positive standardized beta (to the right) means that the covariate was associated with increased death. A negative standardized beta (to the left) means the covariate was associated with decreased death.

Type 1 diabetes

	Data rich	Global
Male	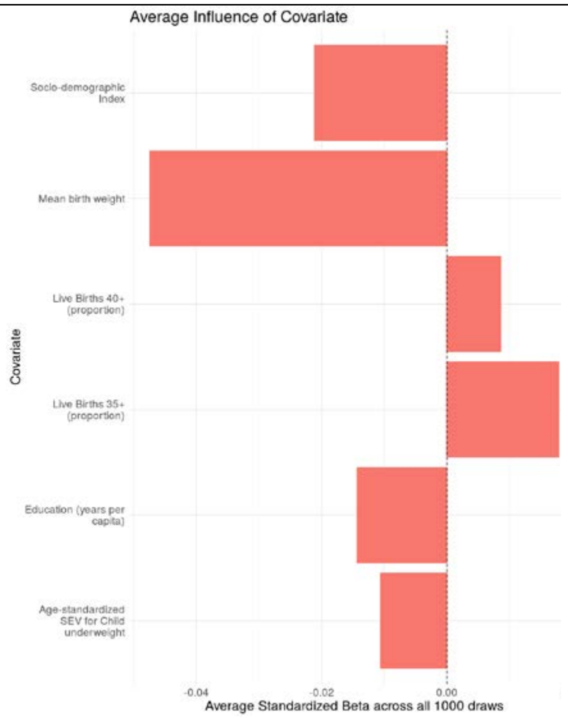	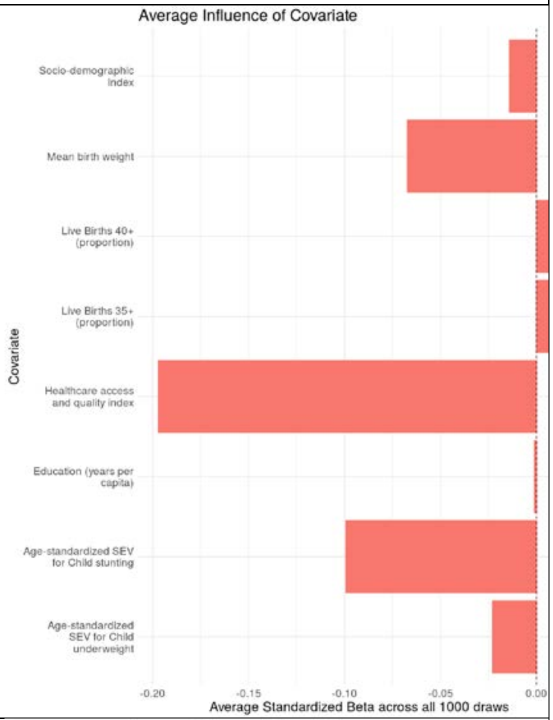
Female	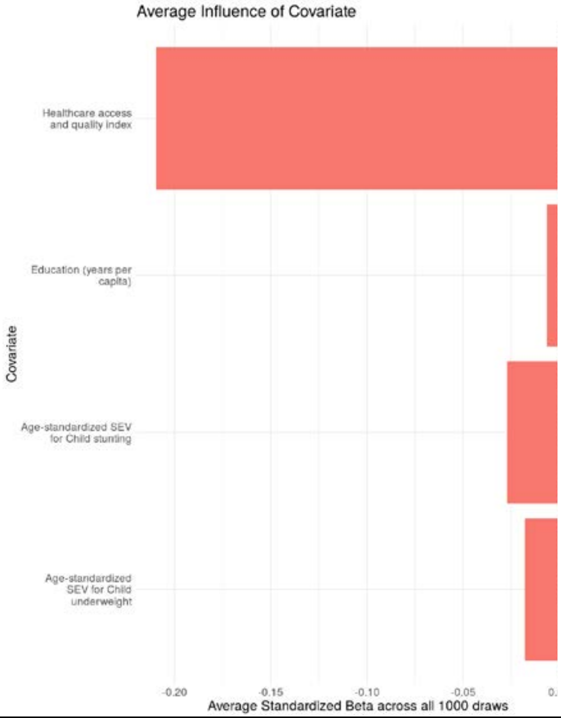	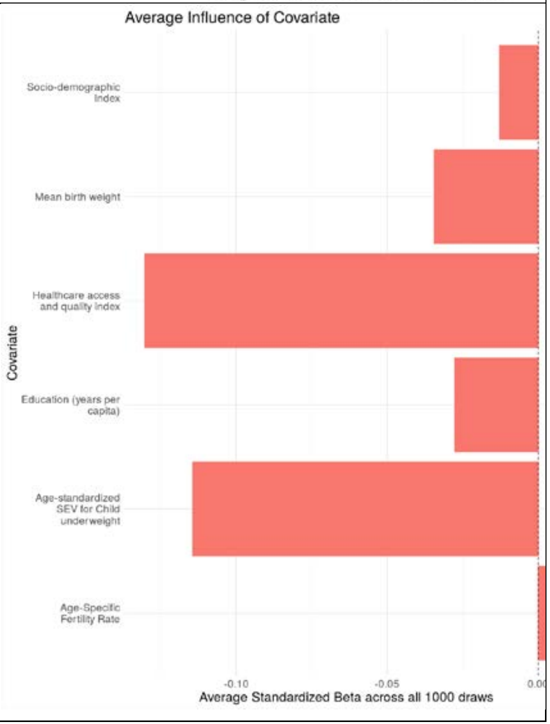

Type 2 diabetes

	Data rich	Global

Male

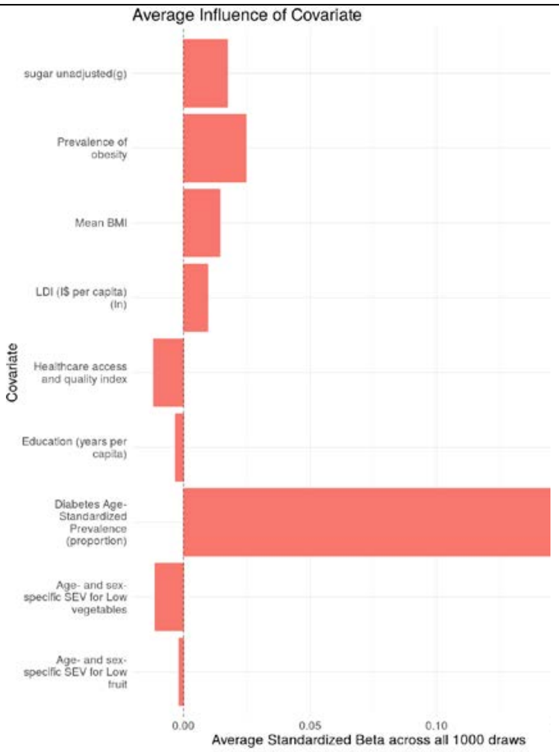

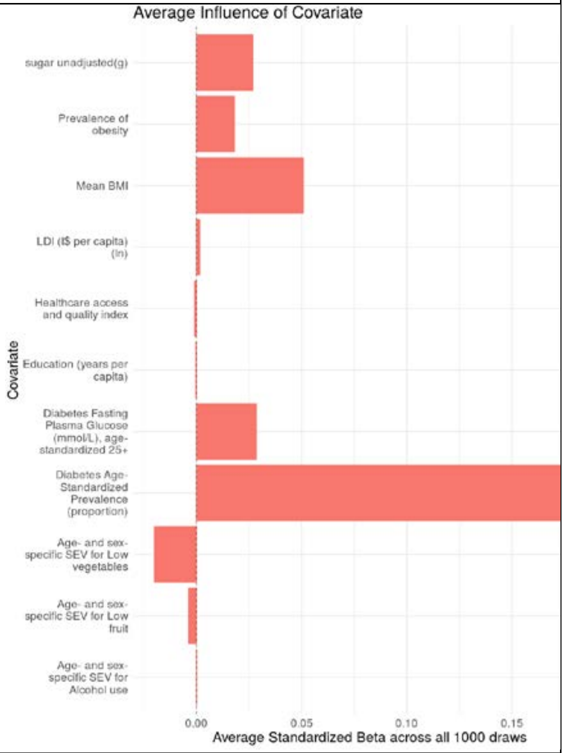

Female

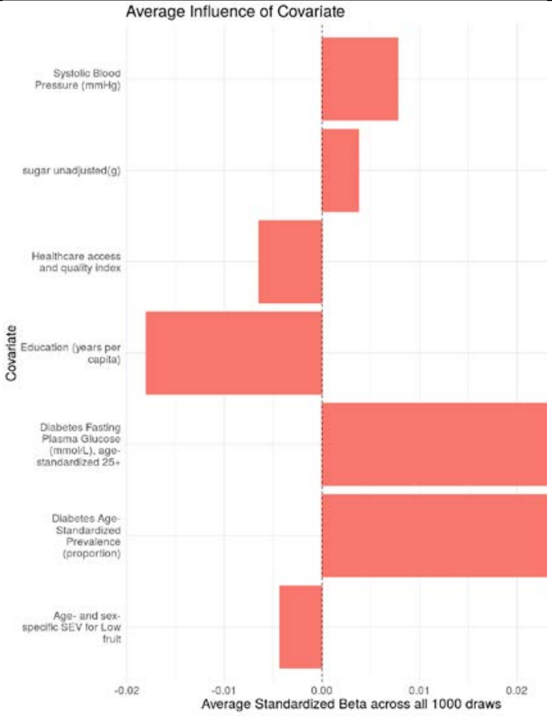

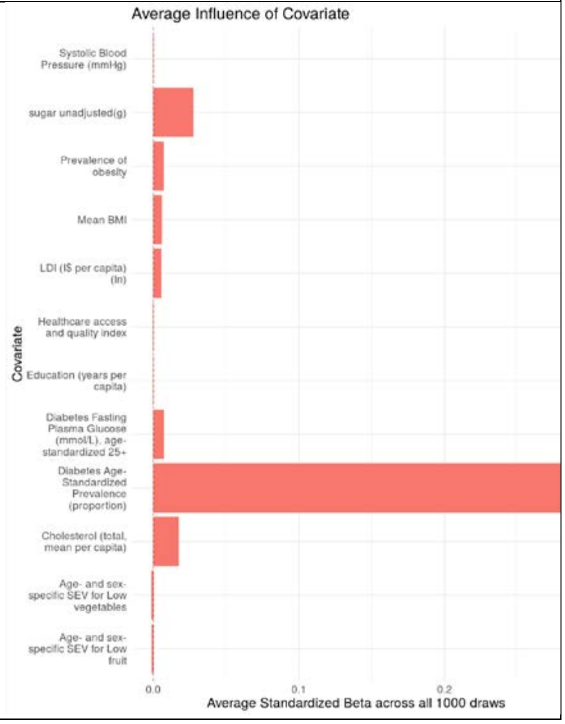

Chronic Kidney Disease

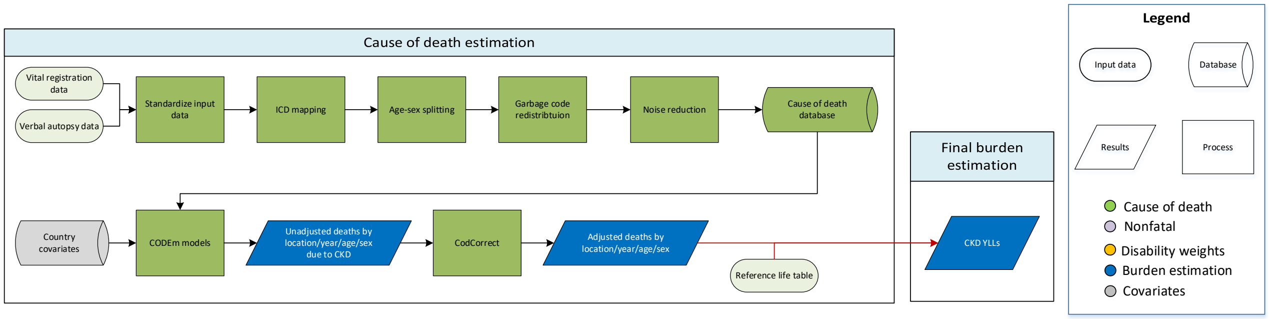

Input data

Vital registration and verbal autopsy data were used to model mortality due to chronic kidney disease. Data were standardised and mapped according to the GBD causes of death ICD mapping method. These data were then age-sex split, and appropriate redistribution of garbage code data was performed. Data points that violated well-established age or time trends or that resulted in extremely high or low cause fractions were marked as outliers and excluded.

Modelling strategy

The estimation strategy used for fatal chronic kidney disease is largely similar to methods used in GBD 2017. A standard CODEm model with location-level covariates was used to model deaths due to chronic kidney disease.

Key Changes from GBD 2017

- We removed the following covariates: whole grains per capita, animal fat per capita, and log lagged 10-year income per capita. We added lagged 10-year income per capita.
- Specified that CODem could only select covariates if the relationship detected between the covariate and mortality was in the direction known or suspected based on prior studies. This resulted the following changes: 1) SDI specified as having a negative association - previously not specified; 2) Red meat consumption specified with a positive association - previously not specified

The full list of covariates used in the GBD 2019 model are displayed below.

Level	Covariate	Direction
1	Diabetes fasting plasma glucose (mmol/L)	+
	Diabetes age-standardised prevalence (proportion)	+
	Mean systolic blood pressure (mmHg)	+
	Mean BMI	+
	Healthcare access and quality index	−
2	Mean cholesterol	+
	Total Calories available per capita per day	+
	Red meat unadjusted (kcal per capita)	+
3	Socio-demographic Index	−
	Education (years per capita)	−
	LDI (I$ per capita)	−

Covariate Influences:

The following plots show the influence of each covariate on the four CODEm models (male global, male data rich, female global, and female data rich). A positive standardized beta (to the right) means that the covariate was associated with increased death. A negative standardized beta (to the left) means the covariate was associated with decreased death.

Male Global

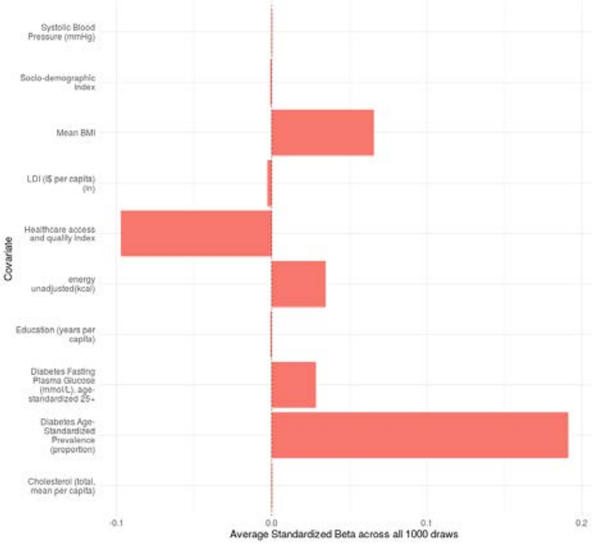

Male Data Rich

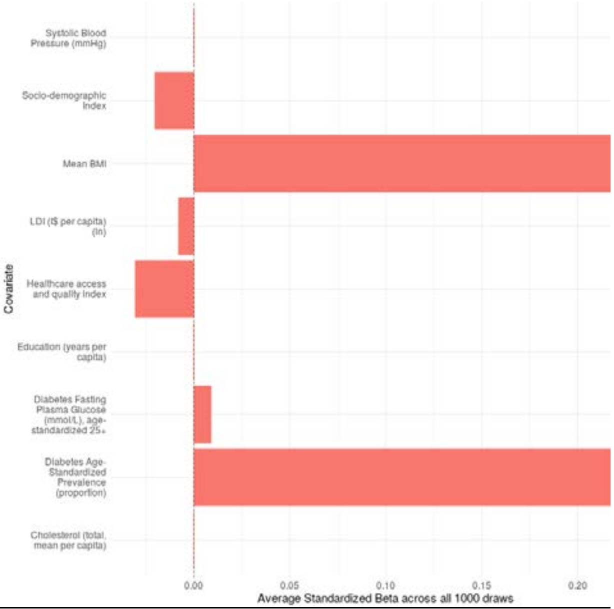

Female Global

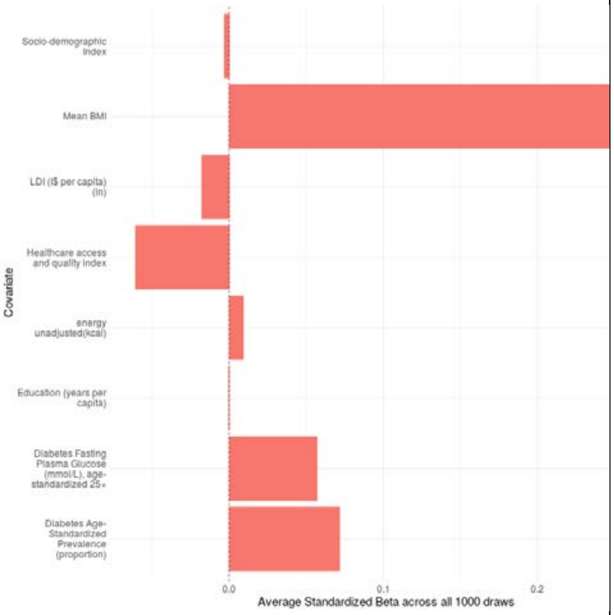

Female Data Rich

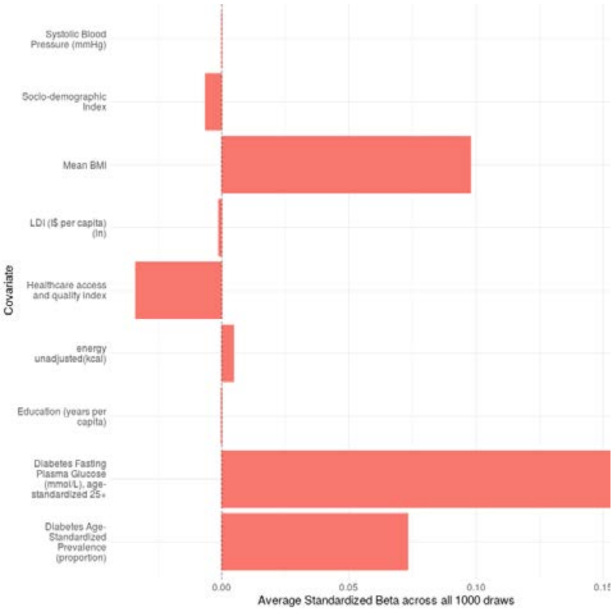

Chronic Kidney Disease subtypes

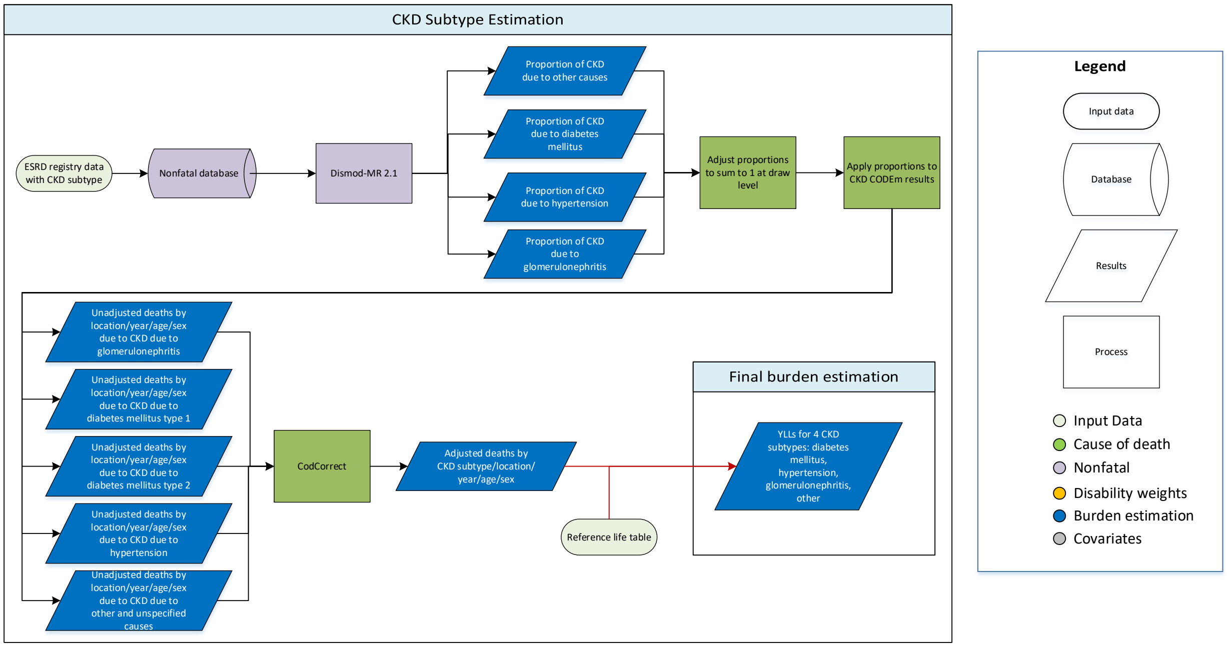

Input data

We estimated deaths due to five subtypes of chronic kidney disease: diabetes mellitus (DM) type 1, diabetes mellitus (DM) type 2, hypertension, glomerulonephritis, and other causes. Deaths due to congenital kidney anomalies (cystic kidney disease and reflux hydronephrosis) were included in the latter category. Data from end-stage renal disease registries were used to estimate proportion of CKD mortality attributable to each CKD subtype. Age-specific data on the proportion of ESRD by subtype was available from the United States, Australia, New Zealand, Nigeria, and Russia.

Vital registration (VR) data were excluded from subtype-specific estimates, as etiology coding in VR sources was considered to be of highly variable quality between countries.

Modelling strategy

We utilized data primarily from end-stage kidney registries that included CKD aetiologies to model CKD-death aetiology proportions.

Data for CKD due to overall DM were more widely available than data by type of DM. In order to make use of all available data, we modelled the proportion of CKD due to overall DM, DM type 1, and DM type 2. We ran DisMod-MR 2.1 models including diabetes prevalence and mean systolic blood pressure as country-level covariates to obtain estimates of proportions for each subtype by location, year, age, and sex. Proportion of CKD due to DM type 1 and DM type 2 were then scaled to sum to the proportion of overall DM at the gender, age, and country-matched level. The results from all subtype-specific models were adjusted so that estimates across the subtypes equaled 1 at each of 1,000 draws. These adjusted proportions were applied to the parent CKD CODEm model to obtain type-specific estimates of CKD mortality.

Model	Covariate	Value	Exponentiated
CKD proportion YLD due to diabetes mellitus	Diabetes age-standardised prevalence	0.49 (0.36–0.61)	1.63 (1.44–1.84)
CKD proportion YLD due to hypertension	Mean systolic blood pressure	0.30 (0.010–1.05)	1.35 (1.01–2.86)

Acute glomerulonephritis

Flowchart

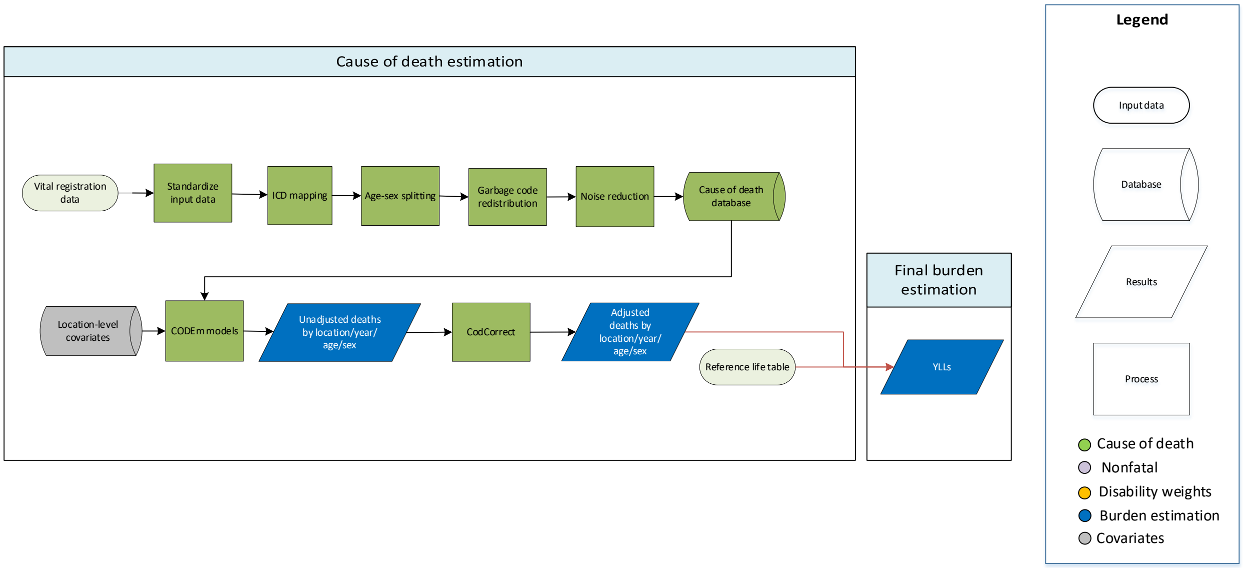

Input data

Data used to estimate mortality of acute glomerulonephritis consisted of vital registration data from the cause of death (COD) database. Outliers were identified by systematic examination of datapoints for all location-years. Specifically, we marked data as outliers in instances where garbage code redistribution and noise reduction, in combination with small sample sizes, resulted in unreasonable cause fractions. We also marked as outliers those data that violated well-established time or age trends.

Modelling strategy

The estimation strategy used for fatal acute glomerulonephritis is largely similar to methods used in GBD 2017. A standard CODEm model with location-level covariates was used to model deaths due to acute glomerulonephritis (see appendix section 3.1 for details). Separate models were conducted for male and female mortality, and age-restrictions for death estimations included 28 days for lower bound and 95+ for upper bound. We hybridised separate global and data-rich models to acquire unadjusted results, which we finalised and adjusted using CodCorrect to reach final YLLs due to acute glomerulonephritis.

Key changes from GBD 2017

- We added estimates for the following new locations: Monaco, San Marino, Palau, San Marino, Saint Kitts and Nevis.
- We added subnational location data for the following: Italy, Poland, and the Philippines.
- We changed the direction of the Socio-demographic Index covariate from 0 to -1.

The following table has the full list of covariates used for fatal acute glomerulonephritis.

Table 1. Covariates used in acute glomerulonephritis mortality modelling

Level	Covariate	Direction
2	Age-standardised prevalence of diabetes	+
	Mean systolic blood pressure (mmHg)	+
	Sanitation (proportion with access)	-
	Improved water sources (proportion with access)	-
	Healthcare Access and Quality Index	-
3	Socio-demographic Index	-
	Education (years per capita)	-
	Log LDI ($I per capita)	-

Skin and subcutaneous diseases

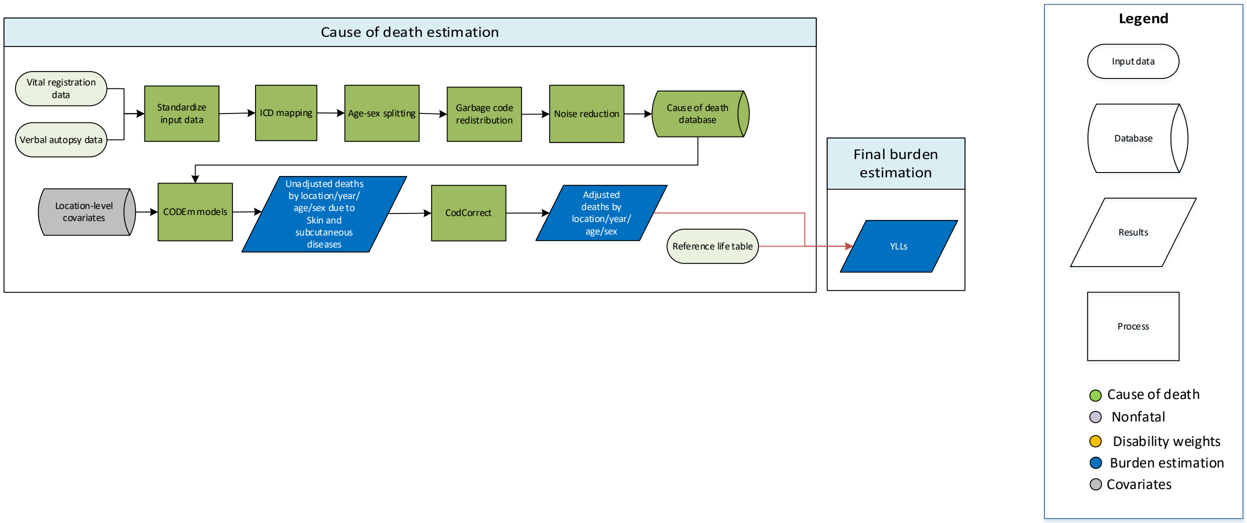

Input data

Data used to estimate mortality of skin and subcutaneous diseases consisted of vital registration data and verbal autopsy data from the cause of death (COD) database. We marked data as outliers in instances where garbage code redistribution and noise reduction – in combination with small sample sizes – resulted in unreasonable cause fractions, as well as data that violated well-established time or age trends. The data in skin and subcutaneous diseases consist of aggregated data from all other specific skin diseases (cellulitis, pyoderma, decubitus ulcer) as well as unique datapoints from unspecified codes of skin and subcutaneous disease.

Modelling strategy

We modelled deaths due to skin and subcutaneous diseases with a standard CODEm model using the cause of death database and location-level covariates as inputs. The model followed standard parameters, with the exception that the start age of the model was 28 days instead of 0. We hybridised separate global and data-rich models to acquire unadjusted results, which we finalised and adjusted using CoDCorrect to reach final years of life lost (YLLs) due to skin and subcutaneous diseases. In GBD 2019 we added these covariates to the model:

- Prevalence of overweight and obesity
- Diabetes fasting plasma glucose (mmol/L), by age

Table 1. Covariates used in skin and subcutaneous disease mortality modelling

Level	Covariate	Direction
1	Summary exposure value (SEV) scalar for unsafe sanitation*	+
	Prevalence of overweight and obesity*	+
	Healthcare Access and Quality Index*	-
	Diabetes fasting plasma glucose (mmol/L), by age*	+
	Improved water source (proportion with access)*	-
2	Alcohol (litres per capita)	+
	Cumulative cigarettes (5 years)	+
	Cumulative cigarettes (10 years)	+
	Smoking prevalence	+
3	Education (years per capita)*	-
	Lag distributed income (per capita)*	-
	Socio-demographic Index*	-

*Selected by CODEm

Bacterial skin diseases

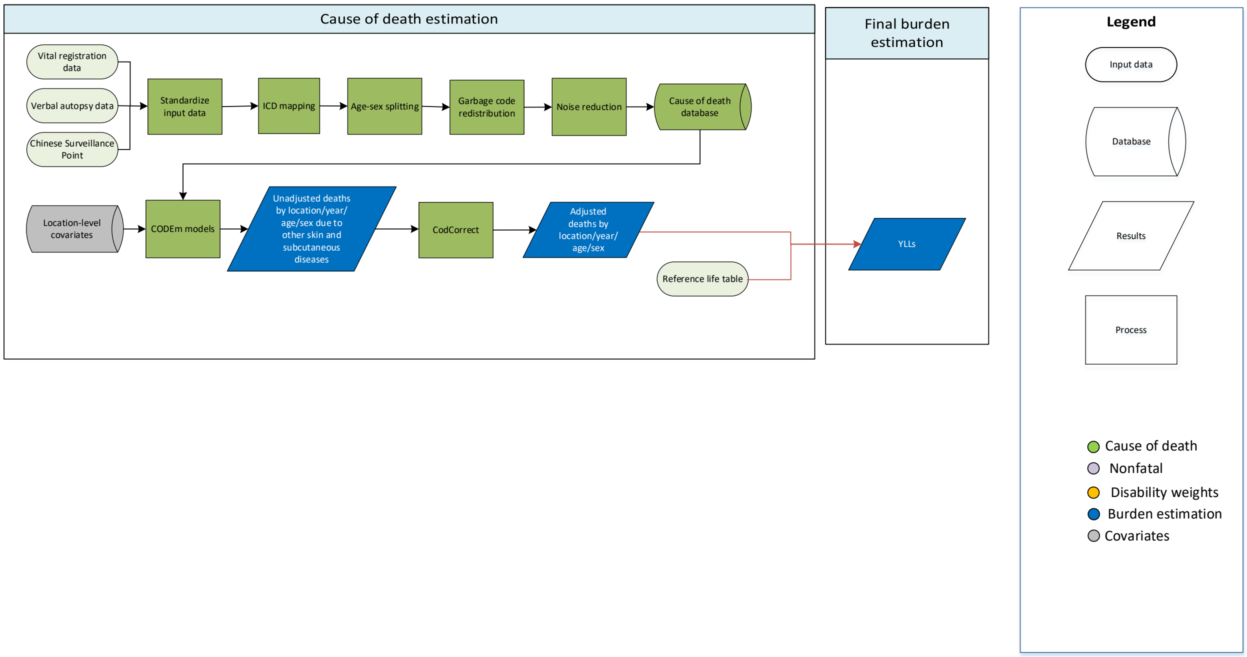

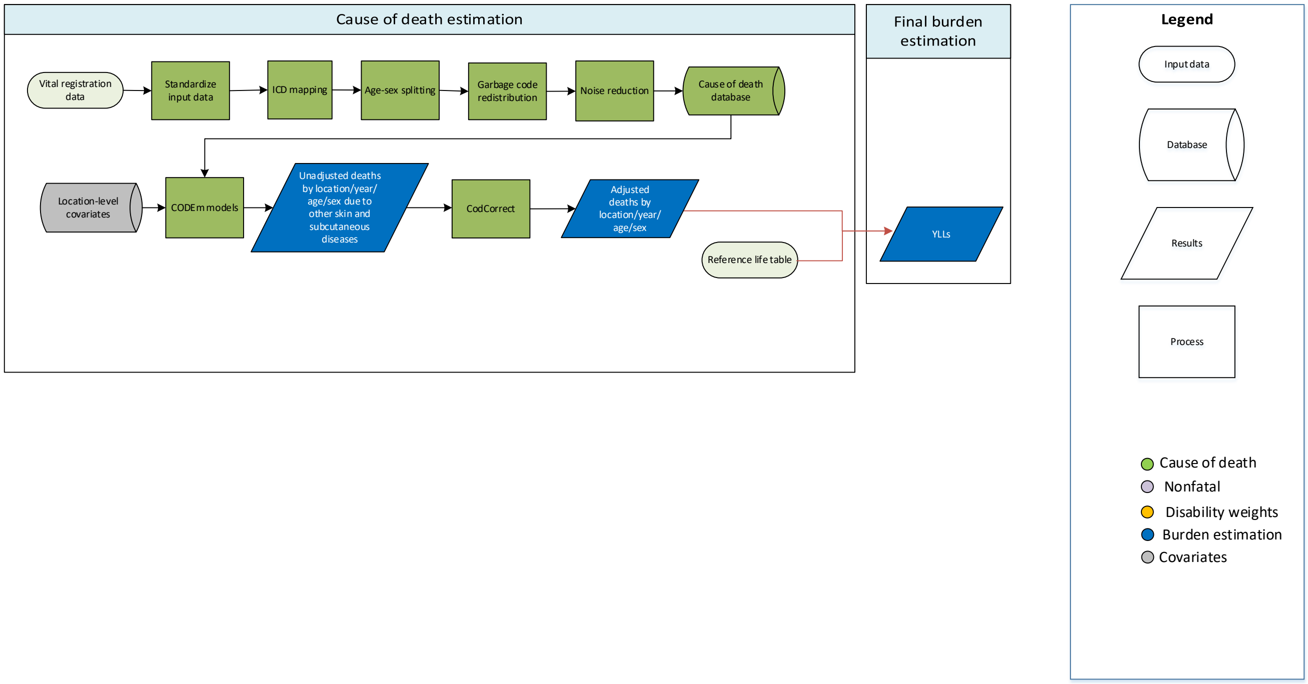

Input data

Data used to estimate bacterial diseases consisted of vital registration, verbal autopsy, and Chinese disease surveillance point (DSP) data from the cause of death (COD) database. Outlier criteria excluded data points that were implausibly high or low relative to global or regional patterns and data from countries with small populations.

Modelling strategy

This is a parent model of pyoderma and cellulitis. The standard CODEm modelling approach was used to estimate deaths due to bacterial skin diseases. CODEm parameters were a combination of those from pyoderma and cellulitis. In GBD 2019 we added these covariates to the model:

- Prevalence of overweight and obesity
- Diabetes fasting plasma glucose (mmol/L), by age

There were no significant changes in the modelling process between GBD 2017 and GBD 2019.

Table 1. Covariates used in bacterial skin mortality modelling

Level	Covariate	Direction
1	Summary exposure value (SEV) scalar for unsafe sanitation*	+
	Prevalence of overweight and obesity*	+
	Healthcare Access and Quality Index*	-
	Diabetes fasting plasma glucose (mmol/L), by age*	+
	Improved water source (proportion with access)*	-
2	Alcohol (litres per capita)	+
	Cumulative cigarettes (5 years)	+
	Cumulative cigarettes (10 years)	+
	Smoking prevalence	+
3	Education (years per capita)*	-
	Lag distributed income (per capita)	-
	Socio-demographic Index*	-

*Selected by CODEm

Cellulitis

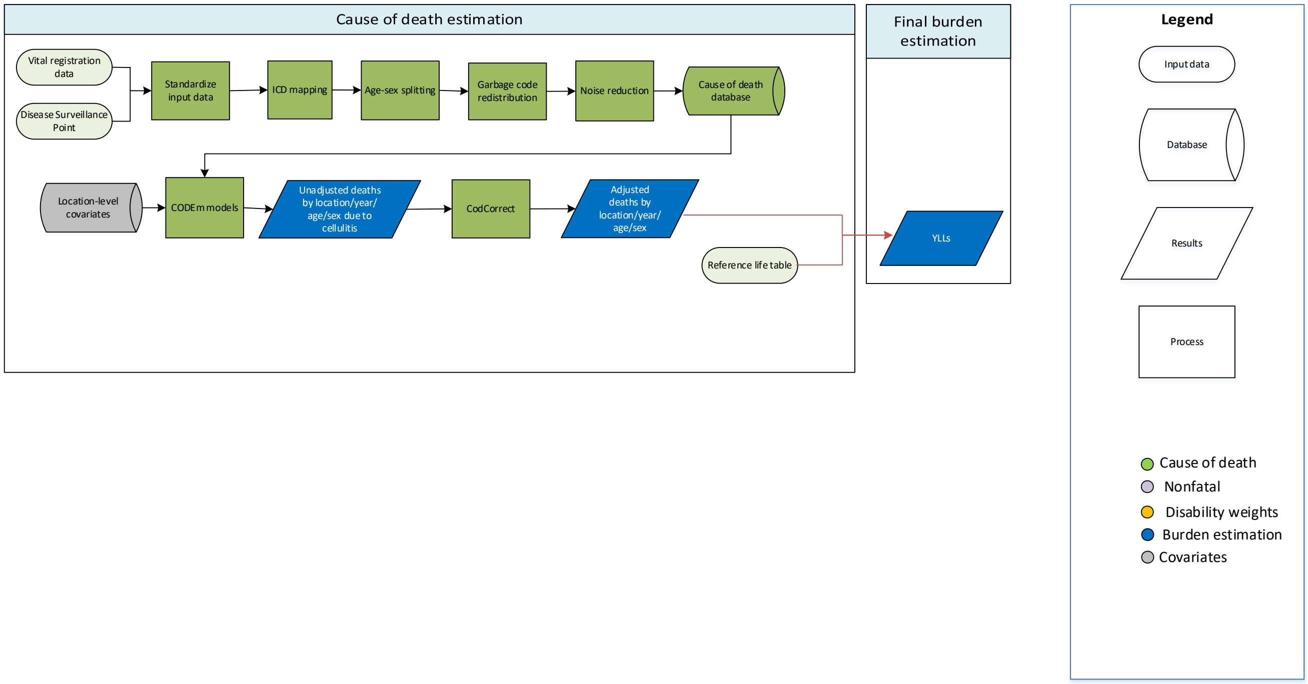

Input data

Data used to estimate cellulitis mortality consisted of vital registration and Chinese disease surveillance point (DSP) data from the cause of death (COD) database. Outlier criteria excluded data points that were implausibly high or low relative to global or regional patterns and data from countries with small populations.

Modelling strategy

We modelled deaths due to cellulitis with a standard CODEm model using the cause of death database and location-level covariates as inputs. The model followed standard parameters. We hybridised separate global and data-rich models to acquire unadjusted results, which we finalised and adjusted using CodCorrect to reach final years of life lost (YLLs) due to cellulitis. In GBD 2019 we added these covariates to the model:

- Prevalence of overweight and obesity
- Diabetes fasting plasma glucose (mmol/L), by age

There were no significant changes in the modelling process between GBD 2017 and GBD 2019.

Table 1. Covariates used in Cellulitis mortality modelling

Level	Covariate	Direction
1	Healthcare Access and Quality Index*	-
	Diabetes fasting plasma glucose (mmol/L), by age*	+
	Prevalence of overweight and obesity*	+
2	Lag distributed income (per capita)	-
3	Education (years per capita)	-

*Selected by CODEm

Pyoderma

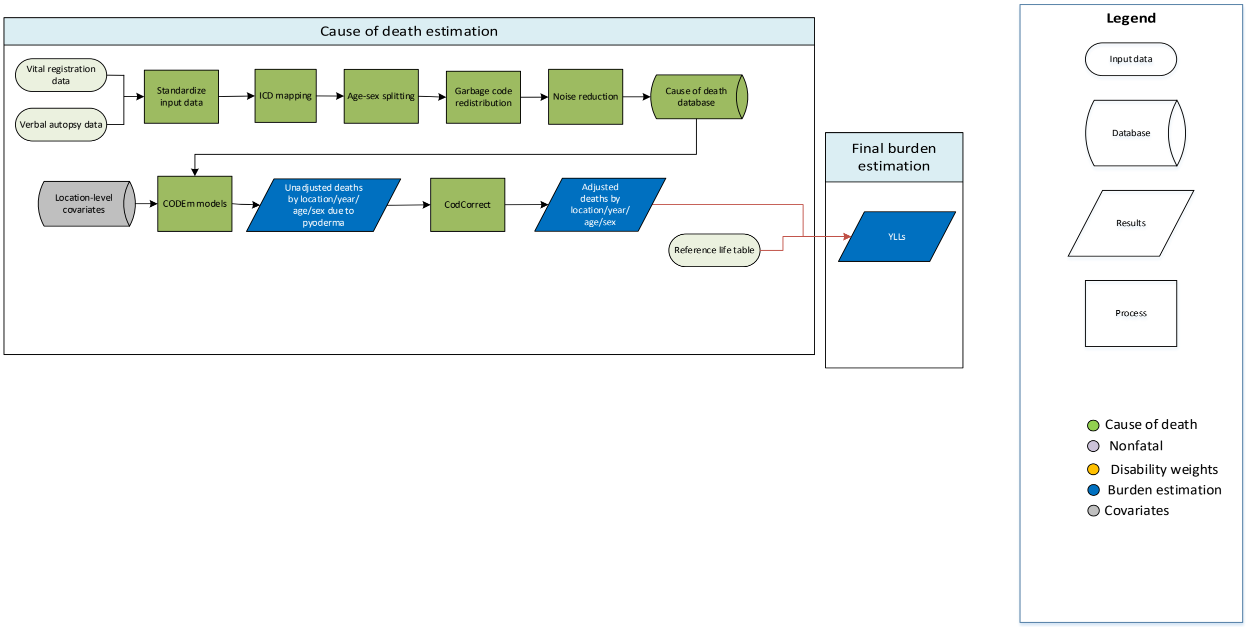

Input data

Data used to estimate pyoderma mortality included centrally prepped vital registration and verbal autopsy data from the cause of death (COD) database. Outlier criteria excluded data points that were implausibly high or low relative to global or regional patterns and data from countries with small populations.

Modelling strategy

We modelled deaths due to pyoderma with a standard CODEm model using the COD database and location-level covariates as inputs. The model followed standard parameters. We hybridised separate global and data-rich models to acquire unadjusted results, which we finalised and adjusted using CodCorrect to reach final years of life lost due to pyoderma. In GBD 2019 we added these covariates to the model:

- The prevalence of overweight and obesity
- Diabetes fasting plasma glucose (mmol/L), by age

There were no significant changes in the modelling process between GBD 2017 and GBD 2019.

Table 1. Covariates used in pyoderma mortality modelling

Level	Covariate	Direction
1	Improved water source (proportion with access)	-
	Prevalence of overweight and obesity*	+
	Healthcare Access and Quality Index*	-
	Diabetes fasting plasma glucose (mmol/L), by age*	+
	Unsafe sanitation (summary exposure value)*	+
2	Alcohol (litres per capita)	+
	Cumulative cigarettes (10 years)	+
	Cumulative cigarettes (5 years)	+
	Smoking prevalence	+
3	Lag distributed income (per capita)*	-
	Education (years per capita)	-
	Socio-demographic Index	-

*Selected by CODEm

Decubitus ulcer

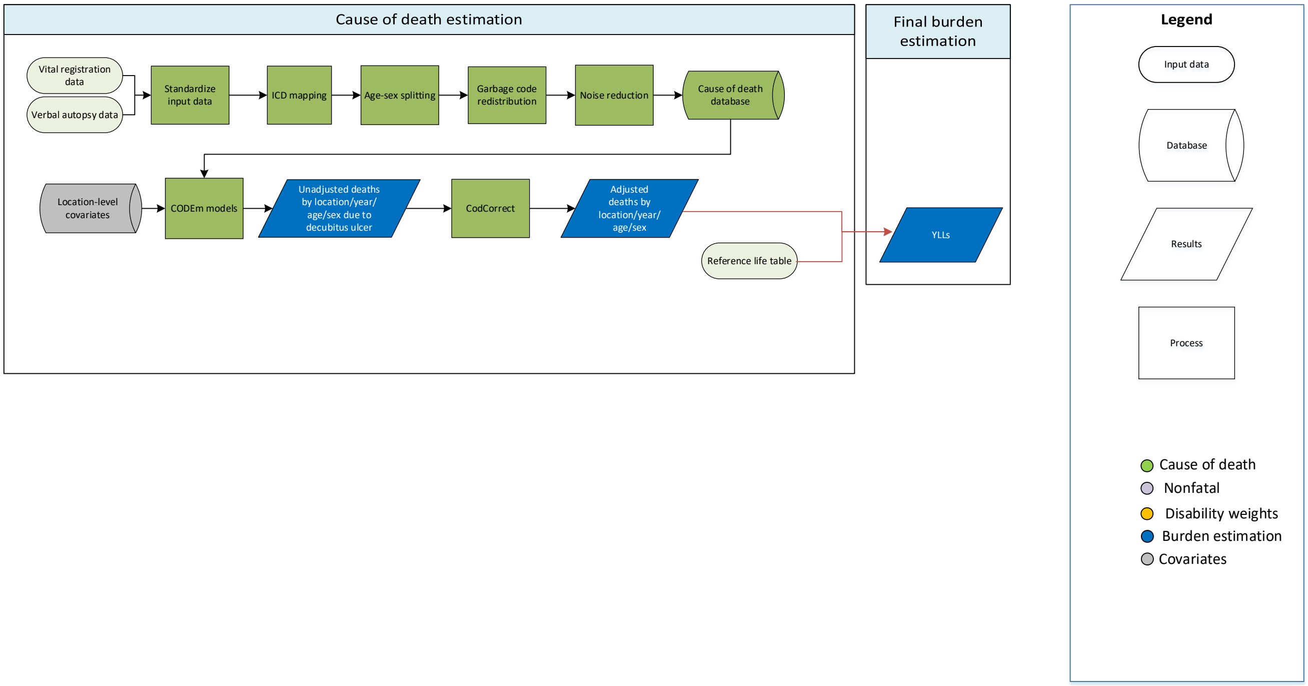

Input data

Data used to estimate decubitus ulcer mortality consisted of vital registration sources and verbal autopsy sources from the cause of death (COD) database. Outlier criteria excluded datapoints that were implausibly high or low relative to global or regional patterns and data from countries with small populations.

Modelling strategy

We modelled deaths due to decubitus ulcer with a standard CODEm model using the cause of death database and location-level covariates as inputs. The model followed standard parameters. We hybridised separate global and data-rich models to acquire unadjusted results, which we finalised and adjusted using CodCorrect to reach final years of life lost (YLLs) due to decubitus ulcer. Decubitus ulcer death estimates were also corrected for misclassification of Alzheimer's and Parkinson's disease deaths. In GBD 2019 we added the prevalence of overweight and obesity and diabetes fasting plasma glucose (mmol/L) by age covariates to the model.

There were no significant changes in the modelling process between GBD 2017 and GBD 2019.

Table 1. Covariates used in decubitus ulcer mortality modelling

Level	Covariate	Direction
1	Alcohol (litres per capita)*	+
	Prevalence of overweight and obesity	+
	Diabetes fasting plasma glucose (mmol/L), by age*	+
	Improved water source (proportion with access)	-
	Healthcare Access and Quality Index*	–
	Cumulative cigarettes (5 years)	+

2	Cumulative cigarettes (10 years)*	+
	Smoking prevalence*	+
3	Education (years per capita)*	-
	Summary exposure variable (SEV) scalar for unsafe sanitation	+
	Socio-demographic Index*	-
	Lag distributed income (per capita)	-

*Selected by CODEm

Other skin and subcutaneous diseases

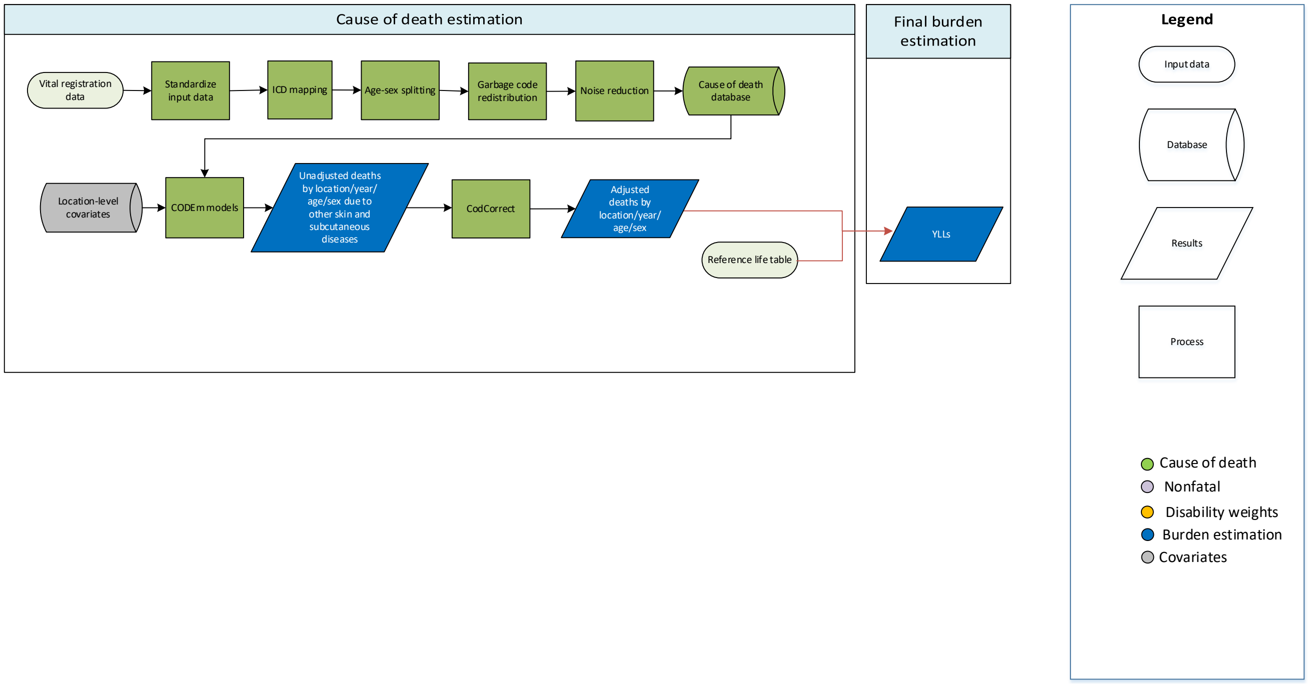

Input data

Data used to estimate mortality due to other skin and subcutaneous diseases consisted of vital registration data from the cause of death (COD) database. We outliered data in instances where garbage code redistribution and noise reduction, in combination with small sample sizes, resulted in unreasonable cause fractions. We also outlliered data that violated well-established time or age trends.

Modelling strategy

We modelled deaths due to other skin and subcutaneous diseases with a standard CODEm model using the COD database and location-level covariates as inputs. The model followed standard parameters. We hybridised separate global and data-rich models to acquire unadjusted results, which we finalised and adjusted using CodCorrect to reach final years of life lost due to other skin diseases. In GBD 2019 we added these covariates to the model:

- The prevalence of overweight and obesity
- Diabetes fasting plasma glucose (mmol/L), by age

There were no significant changes in the modelling process between GBD 2017 and GBD 2019.

Table 1. Covariates used in other skin and subcutaneous diseases mortality modelling

Level	Covariate	Direction
1	Age-standardised summary exposure value (SEV) for child underweight	+
	Improved water source (proportion with access)*	-
	SEV)scalar for unsafe sanitation	+
	Diabetes fasting plasma glucose (mmol/L), by age*	+
	Healthcare Access and Quality Index*	-
	Prevalence of overweight and obesity*	+
2	Smoking prevalence*	+
	Alcohol (litres per capita)	+
	Cumulative cigarettes (5 years)	+
	Cumulative cigarettes (10 years)*	+
3	Education (years per capita)*	-
	Lag distributed income (per capita)*	-
	Socio-demographic Index*	-

*Selected by CODEm

Musculoskeletal Disorders

Flowchart

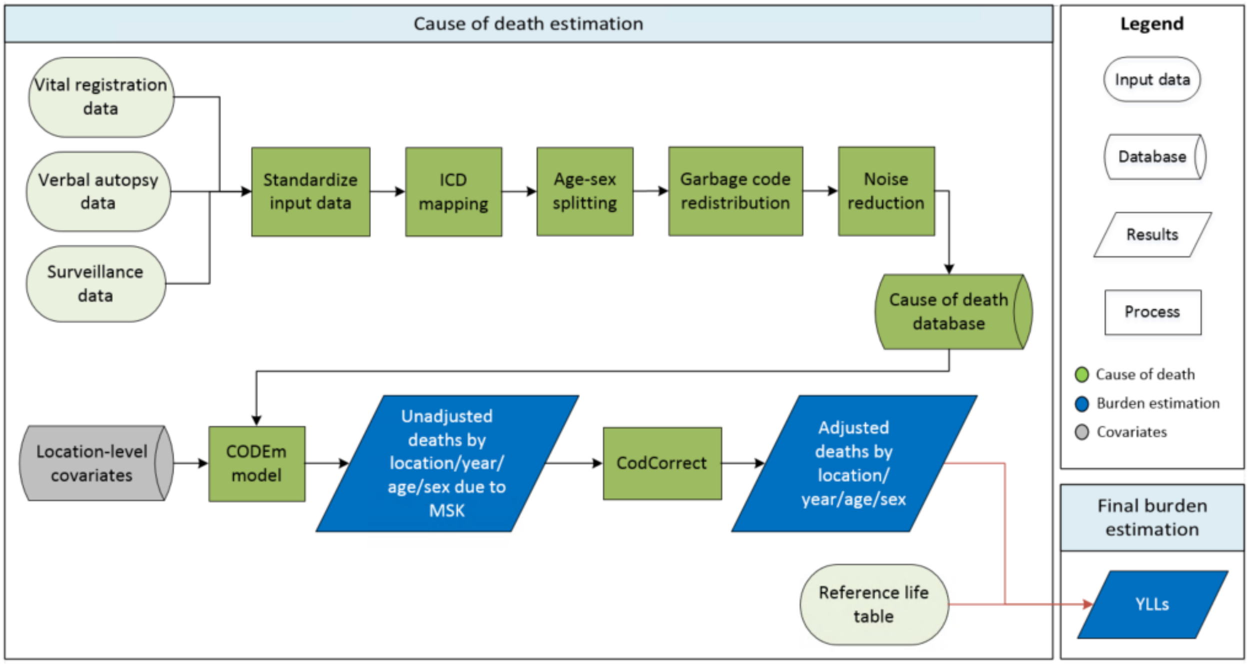

Input Data and Methodological Summary for MSK

Input data

Data used to estimate mortality from musculoskeletal disorders (MSK) included vital registration (VR) and China disease surveillance point data from the cause of death (COD) database. Our outlier criteria excluded (1) data points that were implausibly high or low relative to global or regional patterns, (2) substantially conflicted with established age or temporal patterns, or (3) significantly conflicted with other data sources based from the same locations or locations with similar characteristics (ie, Socio-demographic Index), and (4) from verbal autopsy sources due to the inability of verbal autopsy to accurately capture most musculoskeletal conditions.

Based on these criteria, in GBD 2017 we excluded VA data from Bangladesh, Vietnam, South Africa, Burkina Faso, Ghana, and all countries in Eastern sub-Saharan Africa, including Ethiopia, Kenya, Tanzania, Mozambique, and Zambia, as VA tools have poor validity in identifying MSK deaths. In India, the number of deaths from new Sample Registration System (SRS) data in urban parts of states was substantially higher than the number of deaths from Medical Certification of Cause of Death (MCCD) data. In rural India, the SRS data are the only source. We have outliered the MCCD data to make the models follow the SRS data. This does lead to higher estimates in India compared to other parts of the world. However, as SRS is also the only exception made to the exclusion criteria of no verbal autopsy data and estimates remained implausibly high in some subnational locations in GBD 2019, SRS data were outliered in the urban and rural states of Madhya Pradesh, Uttar Pradesh, Chhattisgarh, Odisha,

Arunachal Pradesh, Punjab, Rajasthan, and Mizoram. For Indonesia, we excluded verbal autopsy data from the national surveillance system for a few states with high estimates based on small numbers, ie, Kalimantan Selatan and Kalimantan Timur in males, and Maluku in females. Recent years of data from Kazakhstan (2013–2016) were outliered as they presented a discontinuity with previous years, which has been ascribed to the country's attempt to reduce deaths due to CVD leading to an increase of deaths. All data from Saint Kitts and Nevis and Philippines subnationals were outliered because a small number of nonzero estimates caused these locations to have the highest prevalence globally. ICD9-BTL data from Latin American countries (Ecuador, Costa Rica, El Salvador, Guatemala, Honduras, Nicaragua, Panama, Venezuela, Antigua and Barbuda, the Bahamas, Barbados, Belize, Bermuda, Cuba, Dominica, Grenada, Guyana, Jamaica, Saint Lucia, Saint Vincent and Grenadines, Suriname, and Trinidad and Tobago) were outliered. The data from these countries provided in ICD9-detail or ICD10 were kept in the analysis.

Modeling strategy

The standard CODEm modelling approach was applied to estimate deaths due to musculoskeletal disorders. We applied mostly the same covariates used in GBD 2017, with a few changes. Otherwise, there were no changes from the GBD 2017 modelling strategy. The CODEm model for musculoskeletal disorders is limited by a lack of strong predictive covariates. Many are selected as a proxy for Socio-demographic Index (SDI), as many musculoskeletal disorders are auto-immune conditions which tend to have increasing prevalence with SDI. Covariates are shown in the following table

Table 1. Covariates used in [insert cause name] mortality modelling

Level	Covariate	Direction
1	Mean BMI	+
	Vegetables (g), unadjusted	+
	Alcohol consumption (litres per capita)	+
2	Cumulative cigarettes (10 years)	+
	Cumulative cigarettes (5 years)	+
	Education (years per capita)	+
	Log-transformed LDI: lag-distributed income ($ per capita)	+
	Mean cholesterol	+
	Smoking prevalence	+
	Healthcare access and quality index	-
3	SDI: Socio-demographic Index	+

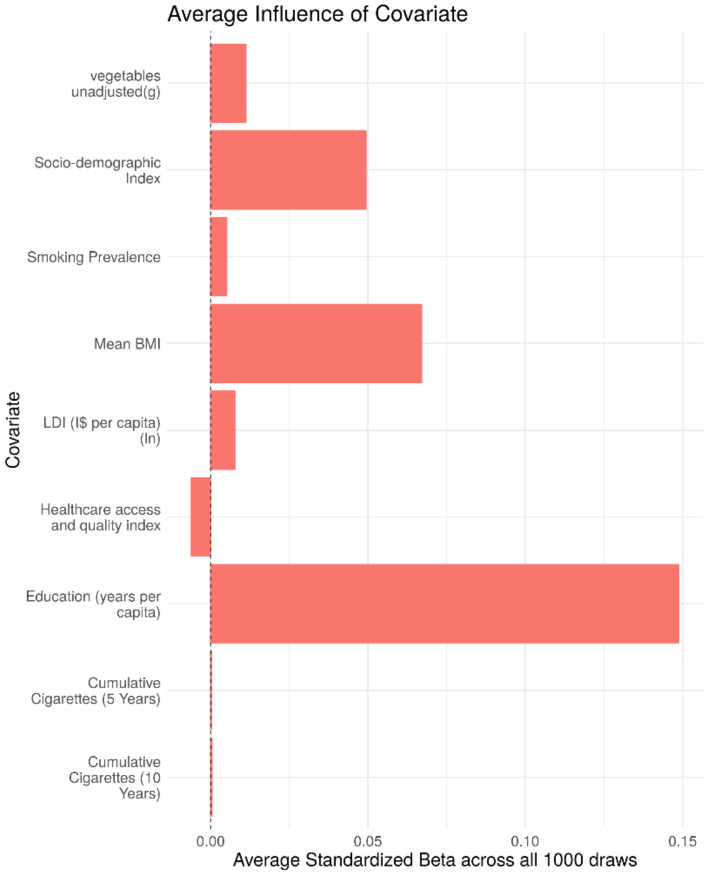

Male Global Model

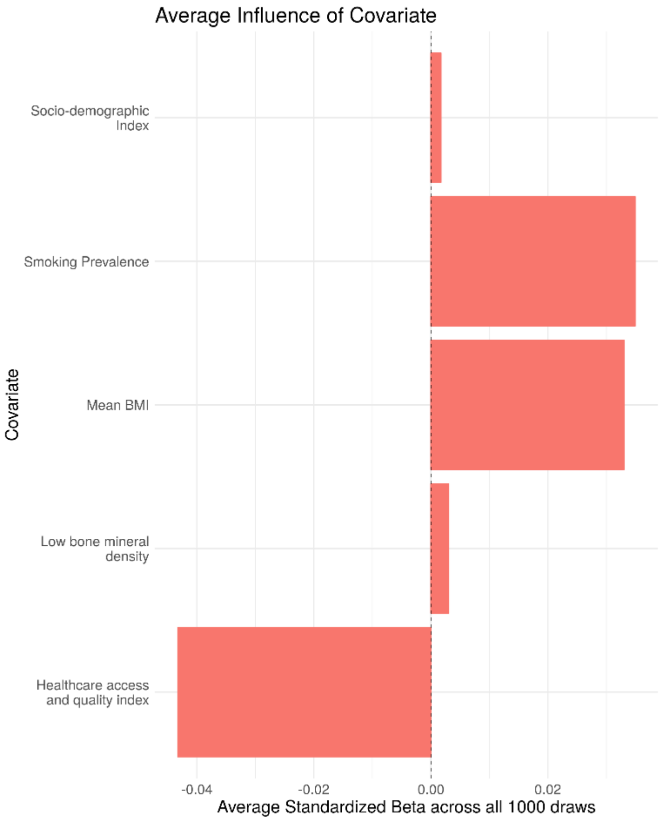

Male Data Rich Model

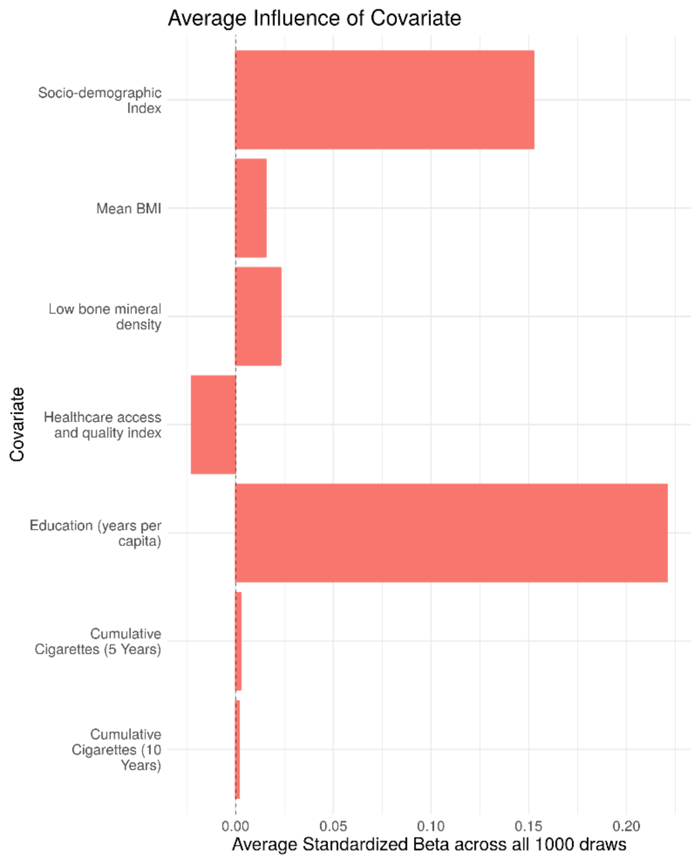

Female Global Model

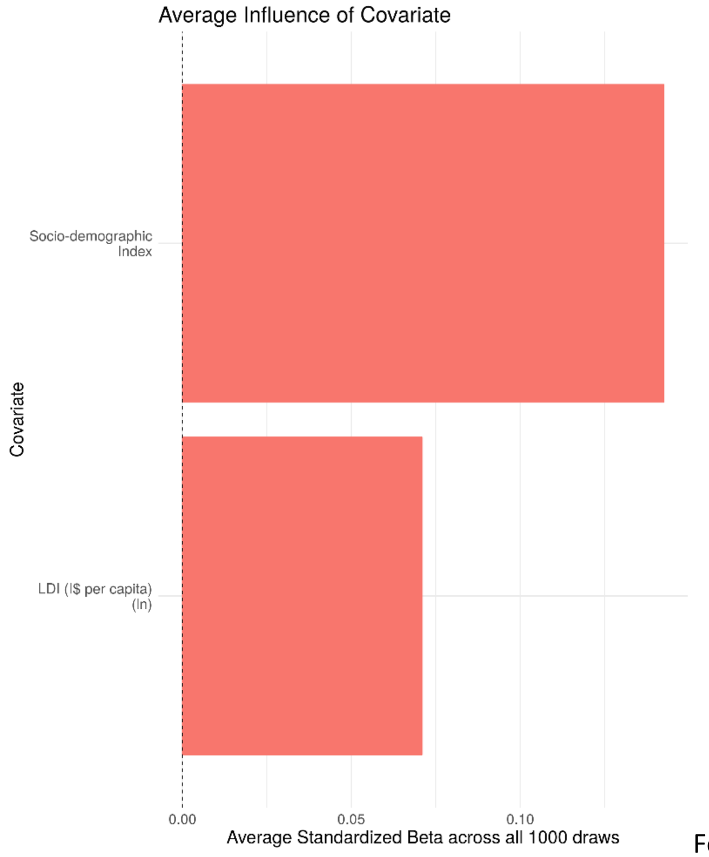

Female Data Rich Model

Rheumatoid Arthritis

Flowchart

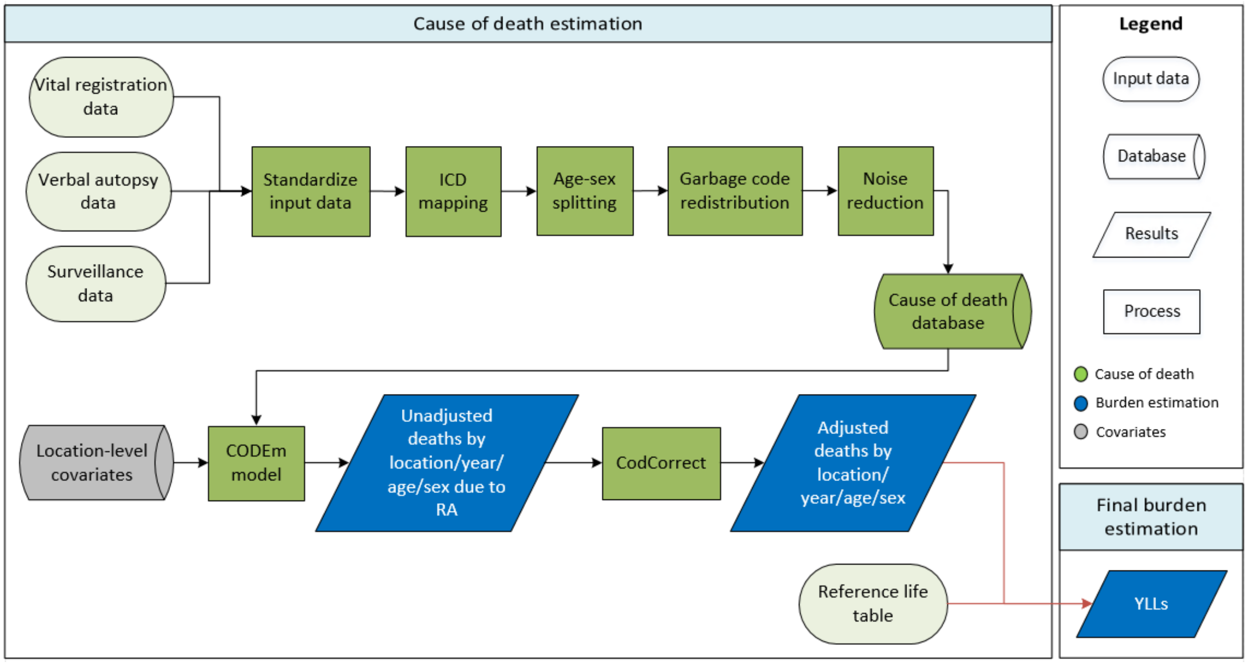

Input Data and Methodological Summary for Rheumatoid Arthritis

Input data

Data used to estimate rheumatoid arthritis mortality included vital registration, and China disease surveillance data from the cause of death database. Our outlier criteria were to exclude data points that were (1) implausibly high or low relative to global or regional patterns, (2) substantially conflicted with established age or temporal patterns, or (3) significantly conflicted with other data sources based from the same locations or locations with similar characteristics (ie, Socio-demographic Index), and (4) from verbal autopsy sources due to the inability of verbal autopsy to accurately capture most musculoskeletal conditions.

Based on these criteria, we excluded a few data points from China. For males, we outliered data points from all sources in Tibet and data points from China disease surveillance in 1991 in all states, as these led to disproportionately high estimates. For females, we outliered Tibet data points from all sources up to 2007 and China disease surveillance data points in several southern states, ie, Guangxi, Hainan, and Yunnan. In addition, as the vital registration data in Limpopo for both males and females in 2003 and before are implausibly higher than the other provinces in South Africa, we outliered this data source and kept the data for 2004–2016 in the analysis. Also, as the vital registration data of mid-age males in Greenland are unrealistically high and much higher than, eg, in Canada and Denmark, the data for males age 45 and above were outliered. Recent years of data from Kazakhstan (2013–2016) were outliered as they presented a discontinuity with previous years, which has been ascribed to the country's attempt to

reduce deaths due to CVD leading to an increase of deaths from all other causes including rheumatoid arthritis. All data from Saint Kitts and Nevis and Philippines subnationals were outliered because a small number of nonzero estimates caused these locations to have the highest prevalences globally. Lastly, we outliered ICD9-BTL data from Latin American countries (Ecuador, Costa Rica, El Salvador, Guatemala, Honduras, Nicaragua, Panama, Venezuela, Antigua and Barbuda, the Bahamas, Barbados, Belize, Bermuda, Cuba, Dominica, Grenada, Guyana, Jamaica, Saint Lucia, Saint Vincent and Grenadines, Suriname, and Trinidad and Tobago). The data from these countries in the years that used ICD10 were kept in the analysis.

Modeling strategy

The standard CODEm modelling approach was applied to estimate deaths due to rheumatoid arthritis. We mostly applied the same covariates used in GBD 2017, with a few changes such as including the milk, unadjusted covariate in lieu of the deprecated milk, adjusted covariate. Otherwise, there were no changes from the GBD 2017 modelling strategy. The CODEm model for rheumatoid arthritis is limited by a lack of strong predictive covariates. Many are selected as a proxy for Socio-demographic Index (SDI), as auto-immune conditions are expected to increase with SDI. All the covariates are shown in the following table.

Table 1. Covariates used in rheumatoid arthritis mortality modelling

Level	Covariate	Direction
1	Cumulative cigarettes (10 years)	+
	Cumulative cigarettes (5 years)	+
	Smoking prevalence	+
	Milk (g), unadjusted	-
	Healthcare access and quality index	-
	Alcohol consumption (litres per capita)	+
2	Mean BMI	+
	Mean cholesterol	+
3	Education (years per capita)	+
	Log-transformed LDI: lag-distributed income ($ per capita)	+
	SDI: Socio-demographic Index	+

Covariate Influences:

The following plots show the influence of each covariate on the four CODEm models (male global, male data rich, female global, and female data rich). A positive standardized beta (to the right) means that the covariate was associated with increased death. A negative standardized beta (to the left) means the covariate was associated with decreased death.

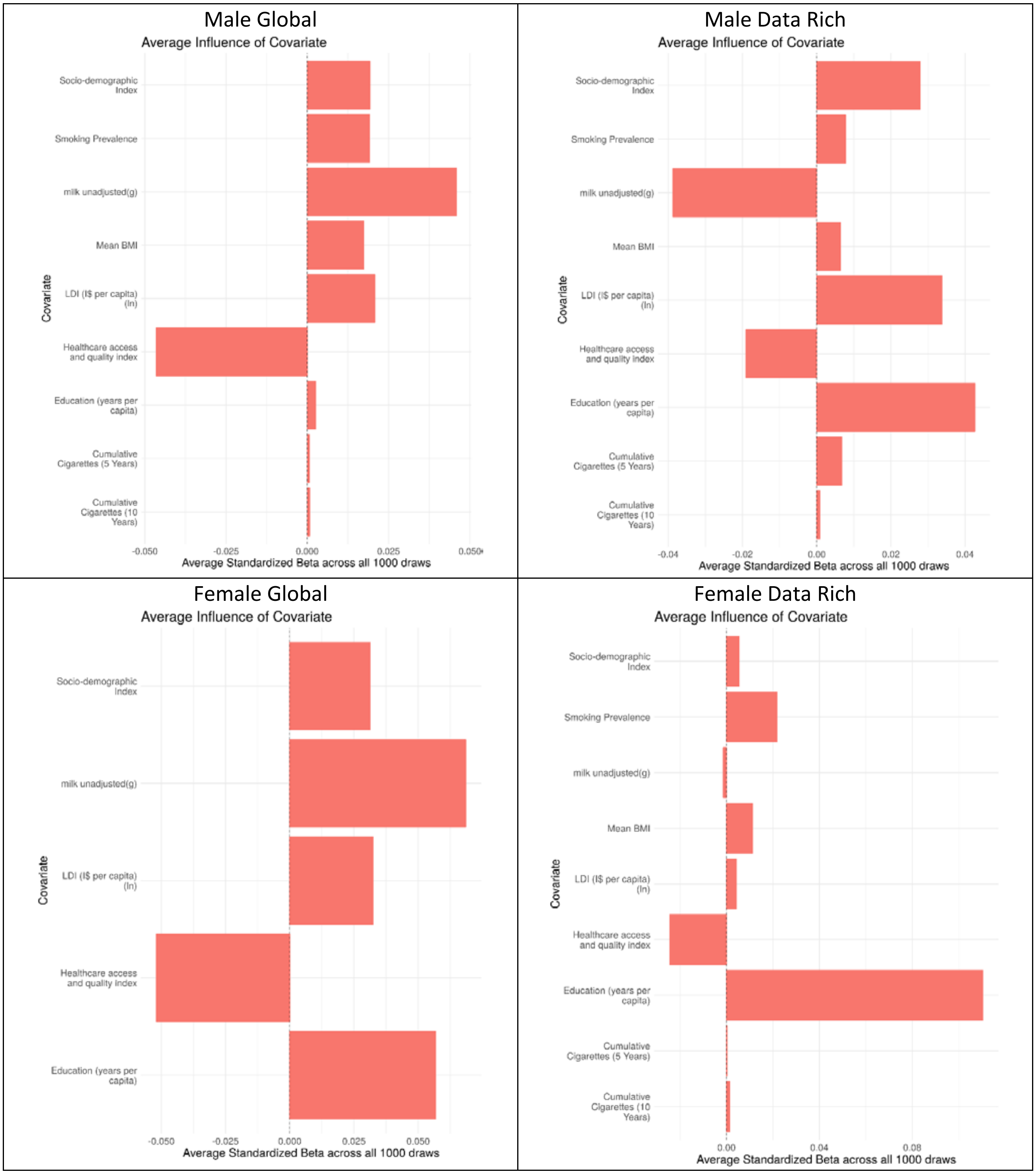

Other Musculoskeletal Disorders

Flowchart

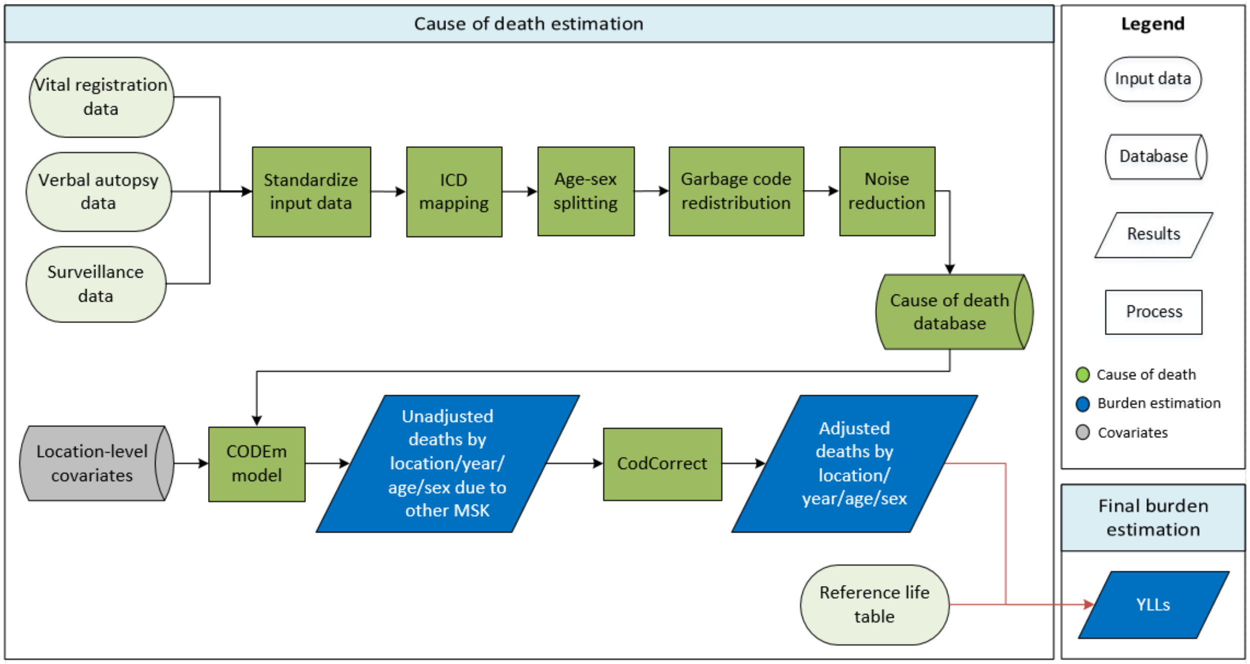

Input Data and Methodological Summary for Other Musculoskeletal Disorders

Input data

Data used to estimate mortality of other musculoskeletal disorders (MSK) included vital registration and China disease surveillance point data from the cause of death database. Our outlier criteria excluded data points that were (1) implausibly high or low relative to global or regional patterns, (2) substantially conflicted with established age or temporal patterns, (3) significantly conflicted with other data sources based from the same locations or locations with similar characteristics (ie, sociodemographic index), or (4) from verbal autopsy sources due to the inability of verbal autopsy to accurately capture most musculoskeletal conditions.

In all ICD-10 coded deaths globally, 60% of deaths in this category were coded to autoimmune disorders (like systemic lupus erythematosus and systemic sclerosis), 21% to osteoporosis, 7% to pyogenic arthritis, and 4% to spinal deformities.

Recent years of data from Kazakhstan (2013–2016) were outliered as they presented a discontinuity with previous years, which has been ascribed to the country's attempt to reduce deaths from CVD leading to an increase of deaths from all other causes, including other MSK. All data from Saint Kitts and Nevis and Philippines subnationals were outliered because a small number of nonzero estimates caused these locations to have the highest prevalences globally. We also outliered all ICD-9 BTL data in Latin American countries (Ecuador, Costa Rica, El Salvador, Guatemala, Honduras, Nicaragua, Panama, Venezuela, Antigua and Barbuda, the Bahamas, Barbados, Belize, Bermuda, Cuba, Dominica, Grenada, Guyana, Jamaica, Saint Lucia, Saint Vincent and Grenadines, Suriname, and Trinidad and Tobago). The data from these countries in the years that used ICD9-detail or ICD10 were kept in the analysis.

Modeling strategy

The standard CODEm modelling approach was applied to estimate deaths due to other musculoskeletal disorders. We applied the same covariates used in GBD 2017 and there were no major changes from the GBD 2017 modelling strategy. The CODEm model for other musculoskeletal disorders is limited by a lack of strong predictive covariates. Many are selected as a proxy for Socio-demographic Index (SDI), as many other musculoskeletal disorders are auto-immune conditions whose prevalence is expected to increase with SDI. Covariates are shown in the following table.

Table 1. Covariates used in other MSK mortality modelling

Level	Covariate	Direction
1	Mean BMI	+
	Vegetables (g), unadjusted	-
	Alcohol consumption (litres per capita)	+
2	Cumulative cigarettes (10 years)	+
	Cumulative cigarettes (5 years)	+
	Education (years per capita)	+
	Log-transformed LDI: lag-distributed income ($ per capita)	+
	Mean cholesterol	+
	Smoking prevalence	+
	Healthcare access and quality index	-
3	SDI: Socio-demographic Index	+

Covariate Influences:

The following plots show the influence of each covariate on the four CODEm models (male global, male data rich, female global, and female data rich). A positive standardized beta (to the right) means that the covariate was associated with increased death. A negative standardized beta (to the left) means the covariate was associated with decreased death.

Male Global

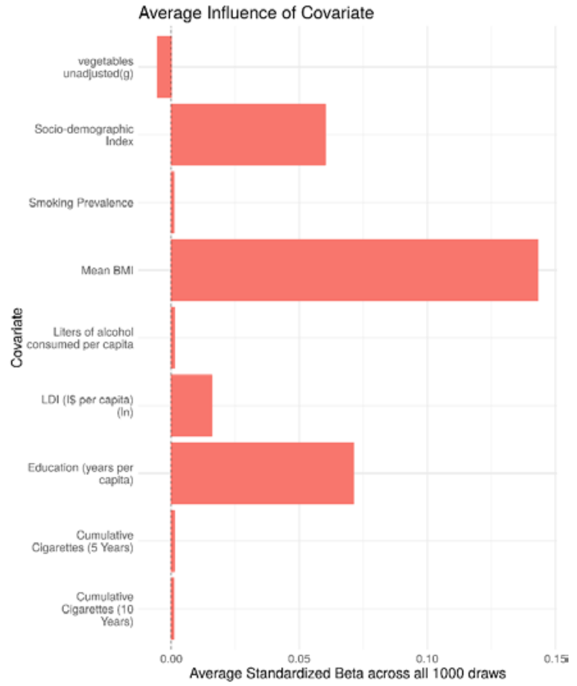

Male Data Rich

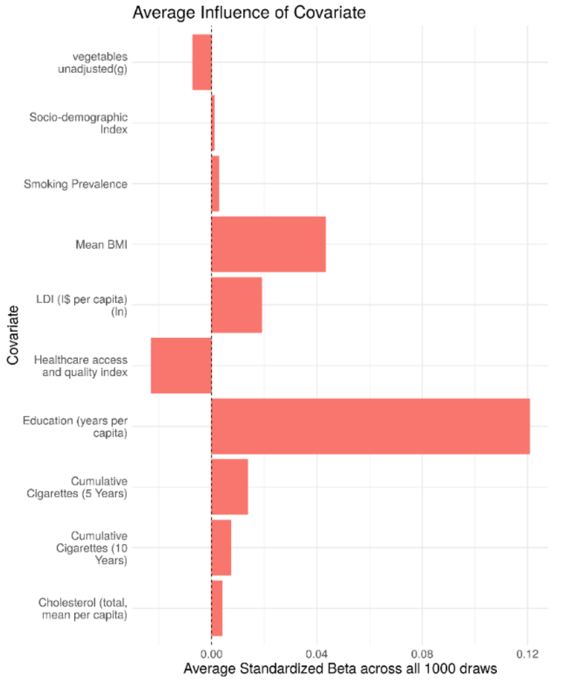

Female Global

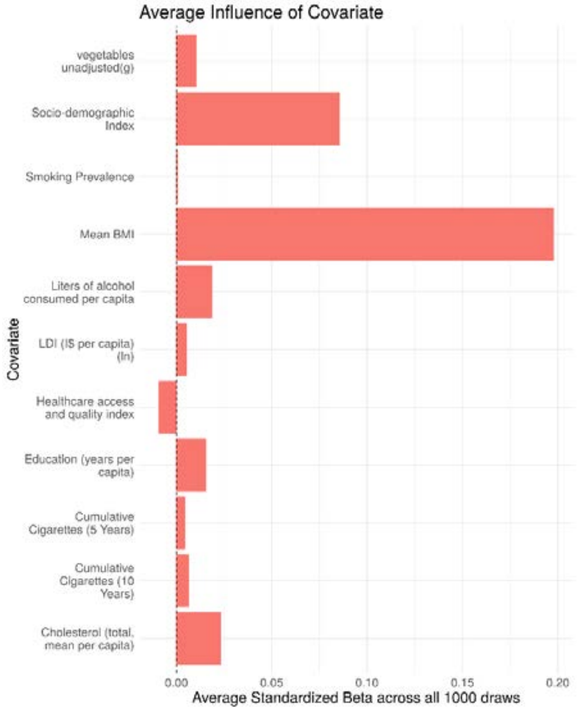

Female Data Rich

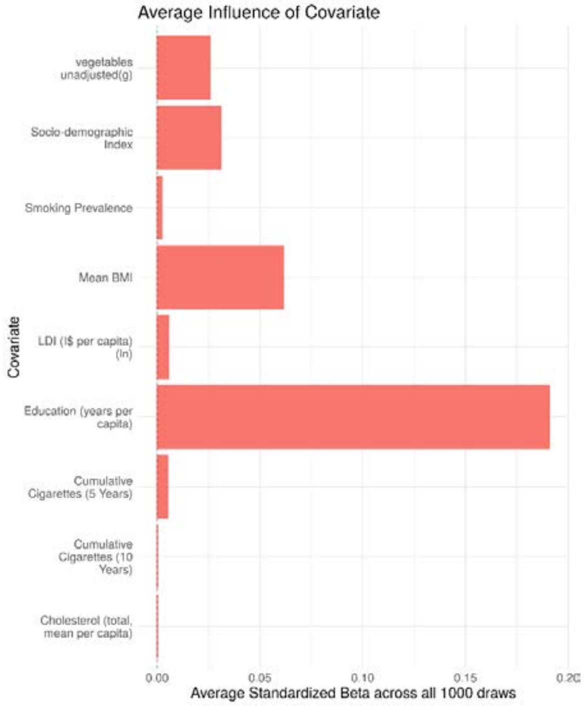

Congenital birth defects: neural tube defects, congenital heart anomalies, orofacial clefts, Down syndrome, Turner syndrome, Klinefelter syndrome, other chromosomal disorders, congenital musculoskeletal anomalies, urogenital congenital anomalies, digestive congenital anomalies, and other congenital birth defects.

Flowchart

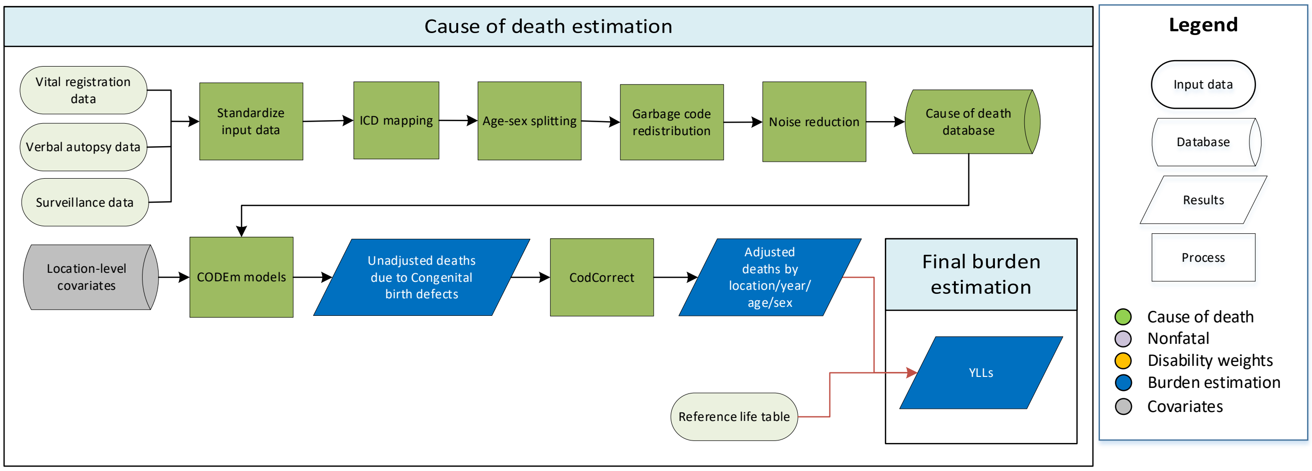

Input data

For GBD 2019, input data for estimating mortality due to congenital anomalies was centrally extracted, processed, and stored in cause of death (CoD) database. Vital registration (VR) was the dominant data type, followed by verbal autopsy (VA) and surveillance. Those CoD data sources that specified the subcause of birth defect were included in estimation of both the parent congenital anomalies model as well as in subtype-specific models.

For GBD 2019, data exclusions were limited. The majority of VA data were outliered in those over 5 years old as the age patterns were unreliable and led to poor model performance in the under-5 age groups. We also excluded some data sources from the parent model where only a subset of subcauses were specified (e.g., congenital heart disease, neural tube defects, and other congenital anomalies) and the sum of the subcauses clearly represented systematic underreporting of one of the subcauses. Systematic underreporting was suspected when sex- and age-specific rates were more than an order of magnitude lower than neighbouring or comparable locations. Data sources for those locations were still included by default for subcause specific models because underreporting of the total was not assumed to necessarily be associated with underreporting of all of the component conditions.

Modelling strategy

All types of congenital anomalies were estimated using cause of death ensemble modelling (CODEm) for GBD 2019, as was done for previous iterations of the GBD study. Specific causes included neural tube

defects, congenital heart anomalies, orofacial clefts, Down syndrome, other chromosomal anomalies, congenital musculoskeletal anomalies, urogenital congenital anomalies, digestive congenital anomalies, and other congenital birth defects. We assumed no mortality from either Klinefelter syndrome or Turner syndrome, for which we model nonfatal outcomes only. For GBD 2019, we modelled congenital anomalies as a cause of death for ages 0–69 years only, assuming that all mortality from congenital conditions occurs before age 70 years of age.

For GBD 2016, we added three new causes to the congenital anomalies: congenital musculoskeletal and limb anomalies; urogenital congenital anomalies; and digestive congenital anomalies. We made no additions to the causes of congenital anomalies for GBD 2017 or 2019.

Table 1: Covariates tested for CODEm model of overall congenital birth defects

Covariate	Transformation	Level	Direction
Maternal alcohol consumption during pregnancy (proportion)	None	1	+
In-facility delivery (proportion)	None	1	-
Live births 35+ (proportion)	None	1	+
Folic acid unadjusted (ug)	None	1	-
Folic acid fortification index	None	1	-
Birth prevalence of congenital heart disease	None	1	+
Birth prevalence of chromosomal anomalies	None	1	+
Legality of abortion	None	2	-
Antenatal care (1 visit) coverage (proportion)	None	2	-
Age-standardised summary exposure value (SEV) of smoking	None	2	+
Antenatal care (4 visits) coverage (proportion)	None	2	-
Healthcare Access and Quality Index	None	2	-
Maternal education (years per capita)	None	3	-
Alcohol (litres per capita)	None	3	+
Age-standardised SEV of low fruits	None	3	+
Outdoor air pollution (PM2.5)	None	3	+
Age-standardised SEV of household air pollution	None	3	+
Socio-demographic Index	None	3	-
Age-standardised SEV of low vegetables	None	3	+

Table 2: Covariates tested for CODEm model of neural tube defects

Covariate	Transformation	Level	Direction
In-facility delivery (proportion)	None	1	-
Folic acid unadjusted (ug)	None	1	-
Folic acid fortification index	None	1	-
Healthcare Access and Quality Index	None	2	-
Antenatal care (1 visit) coverage (proportion)	None	2	-
Antenatal care (4 visits) coverage (proportion)	None	2	-
Age-standardised SEV of smoking	None	2	+
Age-standardised SEV of low fruits	None	3	+
Age-standardised SEV of low vegetables	None	3	+
Maternal education (years per capita)	None	3	-
Socio-demographic Index	None	3	-
Legality of abortion	None	2	-
Maternal alcohol consumption during pregnancy (proportion)	None	3	+
Age-standardised SEV of household air pollution	None	3	+

Age-standardised SEV of fasting plasma glucose	None	3	+
Litres of alcohol consumed per capita	None	3	+

Table 3: Covariates selected for CODEm model of congenital heart anomalies

Covariate	Transformation	Level	Direction
Maternal alcohol consumption during pregnancy (proportion)	None	1	+
Birth prevalence of congenital heart disease	None	1	+
Socio-demographic Index	Log	2	-
Age-standardised SEV of smoking	None	2	+
Age-standardised SEV of diabetes	None	2	+
Healthcare Access and Quality Index	None	2	-
Legality of abortion	None	2	-
Antenatal care (1 visit) coverage (proportion)	None	2	-
In-facility delivery (proportion)	None	2	-
Maternal education (years per capita)	None	3	-
Alcohol (litres per capita)	None	3	+
Antenatal care (4 visits) coverage (proportion)	None	3	-
Skilled birth attendance (proportion)	None	3	-
Live births 35+ (proportion)	None	3	+

Table 4: Covariates selected for CODEm model of cleft lip and cleft palate

Covariate	Transformation	Level	Direction
Socio-demographic Index	None	1	-
Folic acid fortification index	None	1	-
Age-standardised SEV of diabetes	None	2	+
Maternal alcohol consumption during pregnancy (proportion)	None	2	+
Healthcare Access and Quality Index	None	2	-
Legality of abortion	None	2	-
Skilled birth attendance (proportion)	None	2	-
Age-standardised SEV of smoking	None	2	+
Age-standardised SEV of household air pollution	None	3	+
Age-standardised SEV of low vegetables	None	3	+
Alcohol (litres per capita)	None	3	+
Antenatal care (4 visits) coverage (proportion)	None	3	-
Maternal education (years per capita)	None	3	-
Age-standardised SEV of low fruits	None	3	+
Antenatal care (1 visit) coverage (proportion)	None	3	-

Table 5: Covariates selected for CODEm model of Down syndrome

Covariate	Transformation	Level	Direction
Live births 35+ (proportion)	None	1	+
Legality of abortion	None	1	-
Live births 40+ (proportion)	None	1	+
Birth prevalence of chromosomal anomalies	None	1	+
Socio-demographic Index	None	2	-
In-facility delivery (proportion)	None	2	-
Healthcare Access and Quality Index	None	2	-
Maternal alcohol consumption during pregnancy (proportion)	None	3	+
Antenatal care (1 visit) coverage (proportion)	None	3	-
Maternal education (years per capita)	None	3	-

Age-standardised SEV of household air pollution	None	3	+
Antenatal care (4 visits) coverage (proportion)	None	3	-
Age-standardised SEV of low vegetables	None	3	-
Age-standardised SEV of smoking	None	3	+
Litres of alcohol consumed per capita	None	3	+

Table 6: Covariates selected for CODEm model of other chromosomal abnormalities

Covariate	Transformation	Level	Direction
Live births 35+ (proportion)	None	1	+
Live births 40+ (proportion)	None	1	+
Legality of abortion	None	1	-
Lag distributed income (LDI) (I$ per capita)	Log	2	-
Healthcare Access and Quality Index	None	2	-
Antenatal care (4 visits) coverage (proportion)	None	2	-
Antenatal care (1 visit) coverage (proportion)	None	2	-
In-facility delivery (proportion)	None	2	-
Maternal alcohol consumption during pregnancy (proportion)	None	2	+
Socio-demographic Index	None	3	-
Alcohol (litres per capita)	None	3	+
Age-standardised SEV of smoking	None	3	+
Age-standardised SEV of household air pollution	None	3	+
Maternal education (years per capita)	None	3	-
Skilled birth attendance (proportion)	None	3	-

Table 7: Covariates selected for CODEm model of congenital musculoskeletal and limb anomalies

Covariate	Transformation	Level	Direction
Maternal alcohol consumption during pregnancy (proportion)	None	1	+
Legality of abortion	None	1	-
In-facility delivery (proportion)	None	2	-
Ag-standardised SEV of diabetes	None	2	+
Socio-demographic Index	None	2	-
Healthcare Access and Quality Index	None	2	-
Age-standardised SEV of household air pollution	None	2	+
Age-standardised SEV of smoking	None	2	+
Antenatal care (4 visits) coverage (proportion)	None	3	-
Alcohol (litres per capita)	None	3	+
Age-standardised SEV of low fruits	None	3	+
Age-standardised SEV of low vegetables	None	3	+
Maternal education (years per capita)	None	3	-
Antenatal care (1 visit) coverage (proportion)	None	3	-
LDI per capita	Log	3	-

Table 8: Covariates selected for CODEm model of urogenital congenital anomalies

Covariate	Transformation	Level	Direction
Age-standardised SEV of smoking	None	1	+
Maternal alcohol consumption during pregnancy (proportion)	None	1	+
Healthcare Access and Quality Index	None	2	-
Diabetes age-standardised prevalence (proportion)	None	2	+
Socio-demographic Index	None	2	-

Age-standardised SEV of outdoor air pollution	None	2	+
In-facility delivery (proportion)	None	2	-
Age-standardised SEV of household air pollution	None	2	+
Antenatal care (1 visit) coverage (proportion)	None	3	-
Alcohol (litres per capita)	None	3	+
Maternal education (years per capita)	None	3	-
LDI (I$ per capita)	Log	3	-
Antenatal care (4 visits) coverage (proportion)	None	3	-

Table 9: Covariates selected for CODEm model of digestive congenital anomalies

Covariate	Transformation	Level	Direction
Maternal alcohol consumption during pregnancy (proportion)	None	1	+
Age-standardised SEV of smoking	None	1	+
Age-standardised SEV of household air pollution	None	2	+
Diabetes age-standardised prevalence (proportion)	None	2	+
Age-standardised SEV of diabetes	None	2	+
Socio-demographic Index	None	2	-
Age-standardised SEV of obesity	None	2	+
In-facility delivery (proportion)	None	2	-
Healthcare Access and Quality Index	None	2	-
Alcohol (litres per capita)	None	3	+
Maternal education (years per capita)	None	3	-
Age-standardised SEV of low vegetables	None	3	+
Antenatal care (1 visit) coverage (proportion)	None	3	-
Antenatal care (4 visits) coverage (proportion)	None	3	-
Age-standardised SEV of low fruits	None	3	+
LDI (I$ per capita)	Log	3	-
MCI	None	3	-

Table 10: Covariates selected for CODEm model of other congenital birth defects

Covariate	Transformation	Level	Direction
Maternal alcohol consumption during pregnancy (proportion)	None	1	+
Live births 35+ (proportion)	None	1	+
Maternal education (years per capita)	None	2	-
Legality of abortion	None	2	-
In-facility delivery (proportion)	None	2	-
Age-standardised SEV of household air pollution	None	2	+
Healthcare Access and Quality Index	None	2	-
Antenatal care (1 visit) coverage (proportion)	None	3	-
Age-standardised SEV of diabetes	None	3	+
LDI (I$ per capita)	Log	3	-
Socio-demographic Index	None	3	-
Antenatal care (4 visits) coverage (proportion)	None	3	-
Alcohol (litres per capita)	None	3	+

Urinary diseases and male infertility

Flowchart

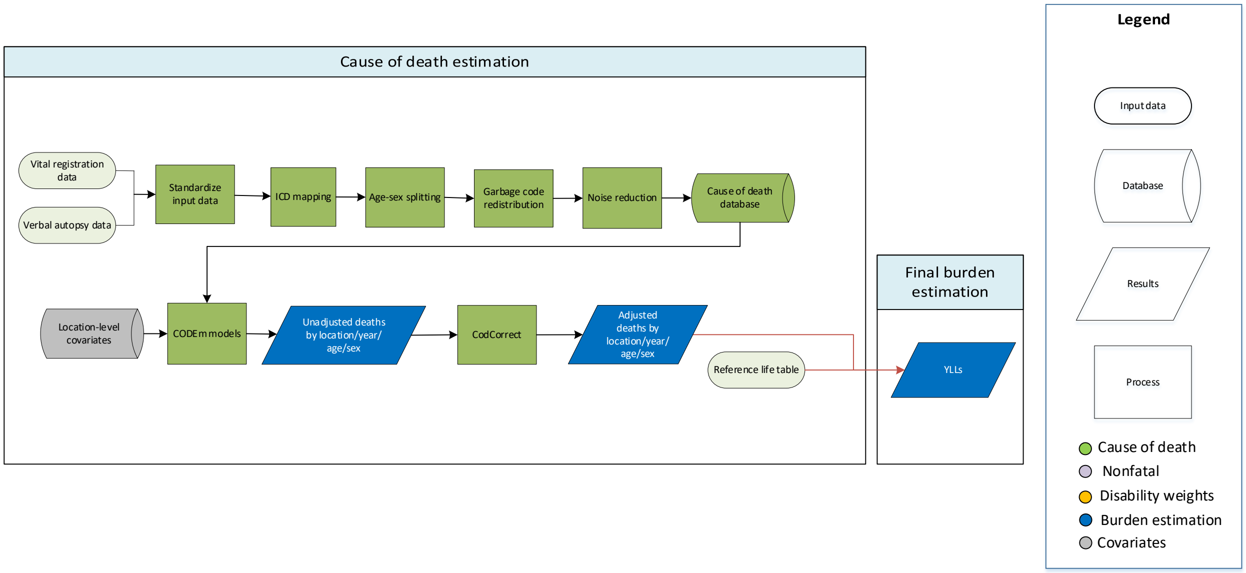

Input data

Data used to estimate mortality of urinary diseases and male infertility consisted of vital registration data and verbal autopsy data from the cause of death (COD) database. The data in urinary diseases consist of aggregated data from all other specific urinary diseases (i.e., urolithiasis, urinary tract infections), as well as unique datapoints from deaths reported with a set of non-specific urinary disease codes (i.e. renal osteodystrophy, bladder-neck obstruction). We marked data as outliers in instances where garbage code redistribution and noise reduction, in combination with small sample sizes, resulted in unreasonable cause fractions. We also marked as outliers those data that violated well-established time or age trends. Methods for selecting outliers were consistent across both vital registration and verbal autopsy data.

Modelling strategy

The estimation strategy used for fatal urinary diseases and male infertility is largely similar to methods used in GBD 2017. A standard CODEm model with location-level covariates was used to model deaths due to urinary diseases and male infertility with age-restrictions for death estimation of 0 days for lower bound and 95+ for upper bound (see appendix section 3.1 for details). We hybridised separate global and data-rich models to acquire unadjusted results, which we finalised and adjusted using CodCorrect to reach final YLLs due to urinary diseases and male infertility.

Key changes from GBD 2017

- We added estimates for the following new locations: Monaco, San Marino, Cook Islands, Palau, and Saint Kitts and Nevis.
- We added subnational location data for the following: Italy, Poland, Pakistan, and the Philippines.
- We excluded the Level 2 latitude-related covariates. Instead, we added the Level 2 temperature (90th percentile) covariate.

- We newly included the sanitation (proportion with access) covariate with a direction of 1.
- We changed the direction of the Socio-demographic Index covariate from 0 to -1

The following table has the full list of covariates used for fatal urinary diseases and male infertility.

Table 1. Covariates used in urinary diseases and male infertility mortality modelling

Level	Covariate	Direction
2	Temperature (90th percentile)	+
	Sanitation (proportion with access)	+
	Mean BMI	+
	Healthcare Access and Quality Index	-
3	Socio-demographic Index	-
	Education (years per capita)	-
	Log LDI ($I per capita)	-

Adjustment in CodCorrect included fitting unadjusted death estimates for all other specific and non-specific urinary diseases to overall urinary disease deaths, which was, then, adjusted with all other causes to sum to all-cause counts of death.

Urinary tract infection

Flowchart

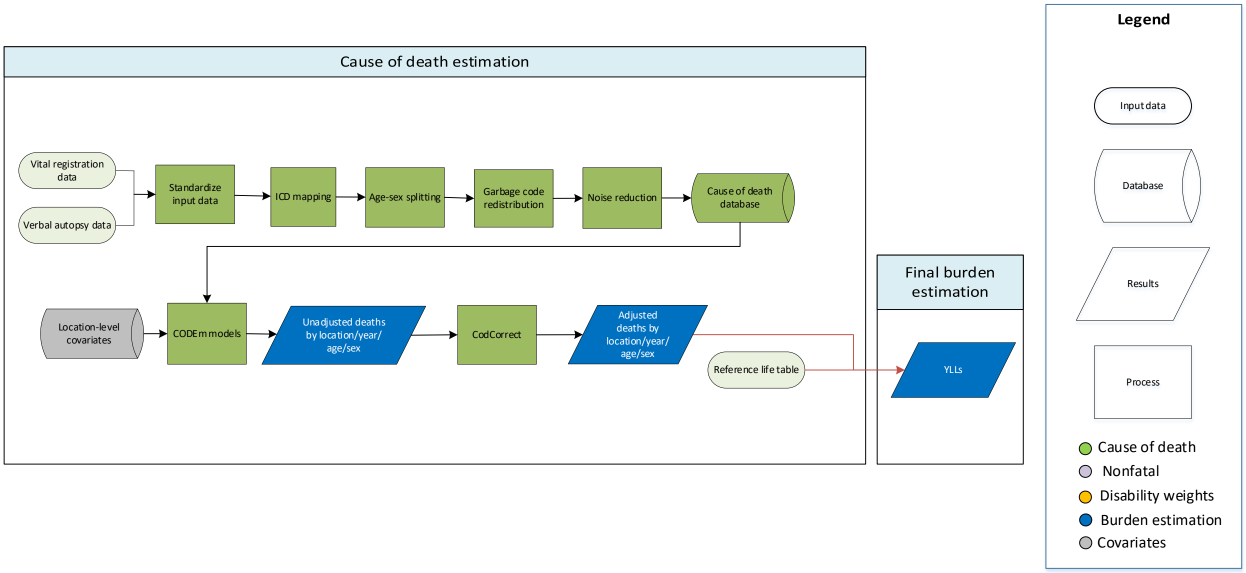

Input data

Data used to estimate mortality of urinary tract infection consisted of vital registration data and verbal autopsy data from the cause of death (COD) database. There was an ICD mapping change in GBD 2019 (see appendix section 2.2.1 for details). ICD codes related to irradiation cystitis N30.4, N30.40, and N30.41 were excluded, and N13.6 pyonephrosis was newly added in GBD 2019.

Outliers were identified by systematic examination of datapoints for all location-years. Specifically, we marked data as outliers in instances where garbage code redistribution and noise reduction, in combination with small sample sizes, resulted in unreasonable cause fractions. We also marked as outliers those data that violated well-established time or age trends. Methods for selecting outliers were consistent across both vital registration and verbal autopsy data.

Modelling strategy

The estimation strategy used for fatal urinary tract infection is largely similar to methods used in GBD 2017. A standard CODEm model with location-level covariates was used to model deaths due to urinary tract infection with age-restrictions for death estimation of 0 days for lower bound and 95+ for upper bound (see appendix section on CODEm method for details). Separate models were conducted for male and female mortality. We then hybridised separate global and data-rich models to acquire unadjusted results, which we finalised and adjusted using CodCorrect to reach final YLLs due to urinary tract infection.

Key changes from GBD 2017

- We added estimates for the following new locations: Monaco, San Marino, Palau, and Saint Kitts and Nevis.

- We added subnational location data for the following: Italy, Poland, Pakistan, and the Philippines.
- We changed the direction of the Socio-demographic Index covariate from 0 to -1.

The following table has the full list of covariates used for fatal urinary tract infection.

Table 1. Covariates used in urinary tract infection mortality modelling

Level	Covariate	Direction
1	Sanitation (proportion with access)	+
2	Education (years per capita)	-
	Log LDI ($I per capita)	-
	Healthcare Access and Quality Index	-
3	Socio-demographic Index	-

Urolithiasis

Flowchart

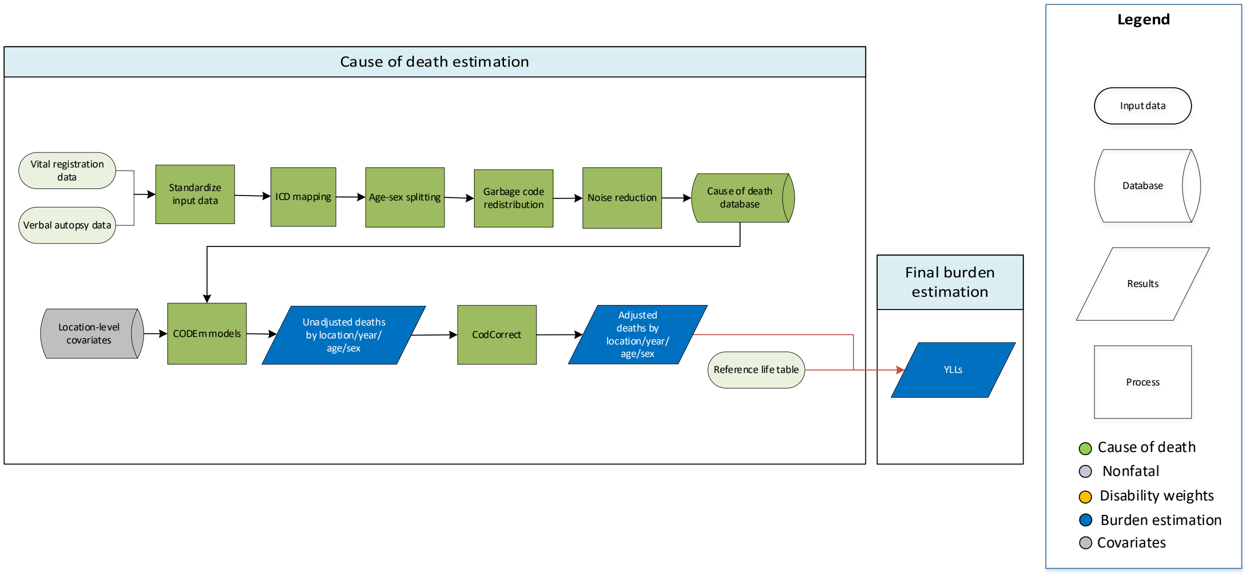

Input data

Data used to estimate mortality of urolithiasis consisted of vital registration data and verbal autopsy data from the cause of death (COD) database. Outliers were identified by systematic examination of datapoints for all location-years. Specifically, we marked data as outliers in instances where garbage code redistribution and noise reduction, in combination with small sample sizes, resulted in unreasonable cause fractions. We also marked as outliers those data that violated well-established time or age trends. Methods for selecting outliers were consistent across both vital registration and verbal autopsy data.

Modelling strategy

The estimation strategy used for fatal urolithiasis is largely similar to methods used in GBD 2017. A standard CODEm model with location-level covariates was used to model deaths due to urolithiasis (see appendix section 3.1 for details). Separate models were conducted for male and female mortality. We then hybridised separate global and data-rich models to acquire unadjusted results, which we finalised and adjusted using CodCorrect to reach final YLLs due to urolithiasis.

Key changes from GBD 2017

- We added estimates for the following new locations: Monaco, San Marino, Palau, and Saint Kitts and Nevis.
- We added subnational location data for the following: Italy, Poland, Pakistan, and the Philippines.
- We changed the lower bound of age-restriction for death estimations from 5 years to 1 year for lower bound. The upper bound of age-restriction remained the same at 95+.
- We changed the direction of the Socio-demographic Index covariate from 0 to -1 in GBD 2019.
- We replaced adjusted dietary covariates with unadjusted dietary covariates.

The following table has the full list of covariates used for fatal urolithiasis.

Table 1. Covariates used in urolithiasis mortality modelling

Level	Covariate	Direction
1	Temperature (90th percentile)	+
	Red meat consumption (unadjusted, kcal per capita)	+
2	Fruit consumption (unadjusted, kcal per capita)	-
	Vegetable consumption (unadjusted, kcal per capita)	-
	Healthcare Access and Quality Index	-
3	Socio-demographic Index	-
	Education (years per capita)	-
	Log LDI ($I per capita)	-

Other urinary diseases

Flowchart

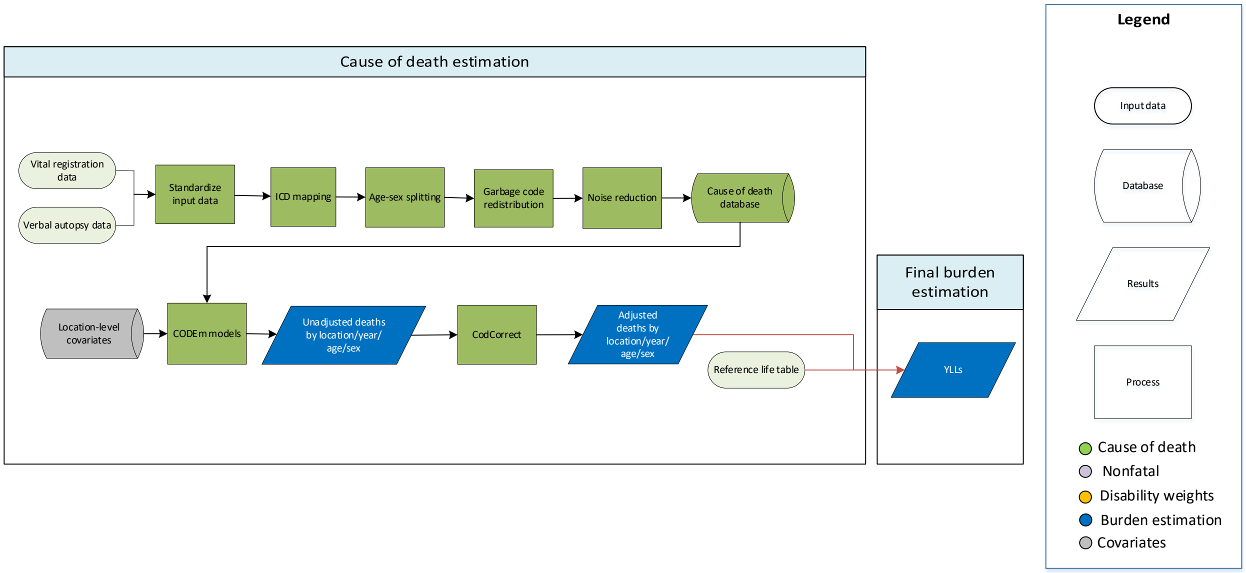

Input data

Data used to estimate mortality of other urinary diseases consisted of vital registration and verbal autopsy data from the cause of death (COD) database. The data in other urinary diseases consist of unique datapoints from deaths reported with a set of non-specific urinary disease codes (see appendix section 2.2.1 for details). Outliers were identified by systematic examination of datapoints for all location-years. Datapoints that violated well-established age or time trends or that resulted in extremely high or low cause fractions were determined to be outliers.

Modelling strategy

The estimation strategy used for other urinary diseases is largely similar to methods used in GBD 2017. A standard CODEm model with location-level covariates was used to model deaths due to other urinary diseases (see appendix section 3.1 for details). Age-restrictions for death estimations secondary to other urinary diseases included 0 days for lower bound, 95+ for upper bound. Separate models were conducted for male and female mortality. We hybridised separate global and data-rich models to acquire unadjusted results, which we finalised and adjusted using CodCorrect to reach final YLLs due to other urinary diseases.

Key changes from GBD 2017

- We added estimates for the following new locations: Monaco, San Marino, Palau, and Saint Kitts and Nevis.
- We added subnational location data for the following: Italy, Poland, the Philippines.
- We changed the direction of the Socio-demographic Index covariate from 0 to -1, and that of education and lag-distributed income covariates from 1 to -1.
- We changed the level of education and lag-distributed income covariates in the female models from 1 to 2.

The following table has the full list of covariates used for other urinary diseases.

Table 1. Covariates used in other urinary diseases mortality modelling

Level	Covariate	Direction
1	Mean BMI	+
2	Education (years per capita)	-
	Log LDI ($I per capita)	-
	Healthcare Access and Quality Index	-
3	Socio-demographic Index	-

Gynaecological diseases

Flowchart

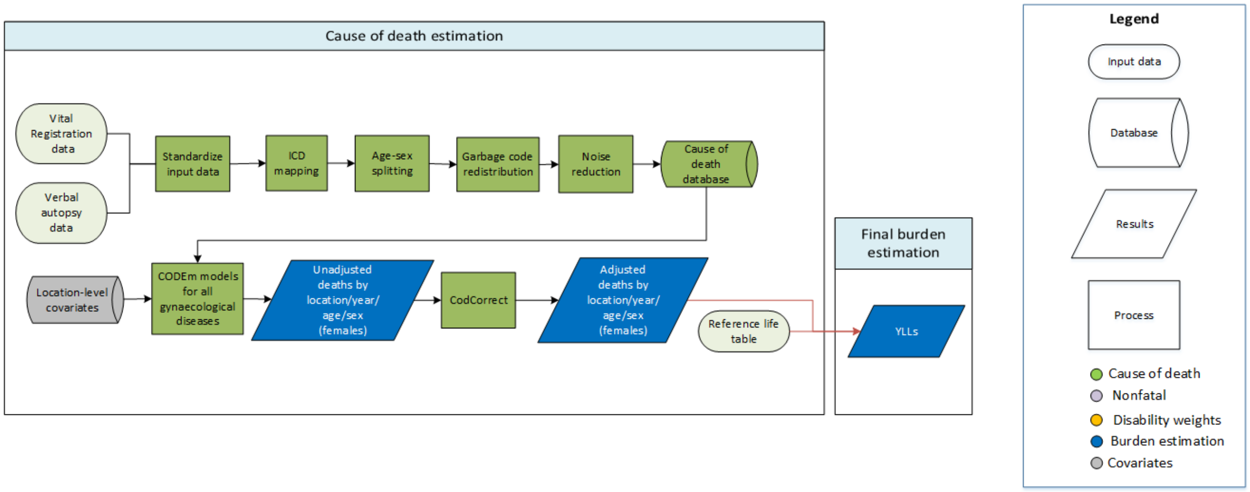

Input Data and Methodological Summary for gynaecological diseases

Input data

For GBD 2019, vital registration and verbal autopsy data were used to estimate deaths for each of the four fatal gynaecological conditions, which include uterine fibroids, endometriosis, genital prolapse, and other gynaecological conditions such as inflammatory diseases of the cervix and uterus and non-inflammatory disorders of the ovary, among others. ICD9 and ICD10 codes for each are listed in table 1. These causes are sex-specific to women, therefore, we only model deaths among women. Data points were selected as outliers if they were implausibly high, low, or significantly conflicted with established age or temporal patterns. For GBD 2019 we had to outlier most of the non-data rich countries such as Bangladesh, Afghanistan, Mongolia, Republic of Palao, among others, to address inconsistent geographical patterns and inconsistencies between the estimated mortality due to all gynaecological diseases and each of the individual causes.

Table 1. ICD10 and ICD9 codes used for gyneacological diseases

Cause	ICD10 code	ICD9 code
Uterine Fibroids	D25-D26.9, D28.2	218-219.9, 236.0
Endometriosis	N80-N80.9	617-617.9
Genital Prolapse	N81-N81.9	618-618.9
Other Gyneacological Disorders*	N72, N75 – N77.8	613-619, 620-629.81

*Other gyneacological disorders include inflammatory disease of cervix uteri, diseases of Bartholin's gland, other inflammation of vagina and vulva, vulvovaginal ulceration and inflammation in diseases classified elsewhere and non-inflammatory disorders of ovary, fallopian tube and broad ligament.

Modeling strategy

For GBD 2019, we estimated mortality due to all gynaecological diseases as well as each of the sub-categories using CODEm. As in GBD 2017, we reassigned deaths due to leiomyomas and other benign uterine tumors to uterine fibroids and we assumed no deaths from premenstrual syndrome and primary infertility, which we model as nonfatal outcomes only. For GBD 2019, following consultation with the GBD Scientific Council, polycystic ovarian syndrome (PCOs) was also no longer considered as a cause of death due to its low lethality and the lack of evidence around the physiopathology and biological mechanism's through which PCOs can be considered a direct cause of death[1]. All gynaecological causes used the pool of covariates shown in table 2. The primary limitations of our estimation is data availability and the lack of evidence of predictors of these conditions.

Table 2. Covariates used in gynaecological diseases mortality modelling

Level	Covariate	Direction
1	Age and sex specific SEV for smoking	-1
2	Percentage of births in women over 35 years	1
	Skilled birth attendance proportion	-1
	Total fertility rate	1
	Healthcare access and quality index	-1
	Health system access capped	-1
3	Education, years per capita	-1
	Lag-distributed income per capita	-1
	Socio-demographic index	-1

References

1. Zhou Y, Wang X, Jiang Y, et al. Association between polycystic ovary syndrome and the risk of stroke and all-cause mortality: insights from a meta-analysis. Gynecol Endocrinol 2017; 33: 904–10.

Haemoglobinopathies and haemolytic anaemias

This write-up covers the following sub-causes: sickle cell disorders, thalassaemias, glucose-6-phosphate dehydrogenase (G6PD) deficiency, and other haemoglobinopathies and haemolytic anaemias

Flowchart

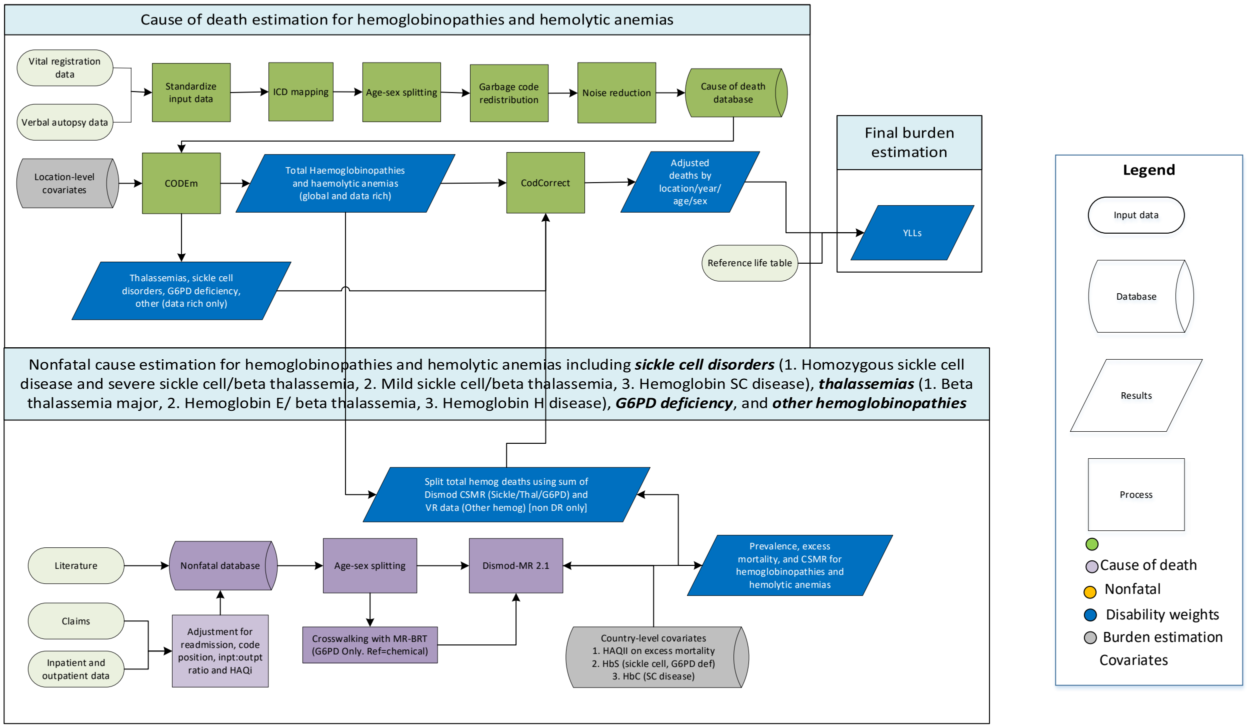

Input data and methodological summary

For GBD 2019, our approach was as follows: Cause of death Ensemble modelling (CODEm) models were developed for all of haemoglobinopathies and haemolytic anaemias combined across all age groups and years. CODEm models were run separately for males and females; one model was run for all locations (global) and a separate for all "data-rich" locations and described elsewhere. For subcauses of haemoglobinopathies and haemolytic anaemias, we changed our approach in GBD 2019. Previously, we summed and scaled prevalence times excess mortality rate (ie, cause-specific mortality rate) results from DisMod-MR 2.1 models of each of thalassaemias, sickle cell disorders, and G6PD deficiency to split the total deaths to component causes for all demographic groups. This approach was retained for non-data-rich locations, but for the data-rich locations we instead ran another set of male and female CODEm models for each of the four subcauses of haemoglobinopathies and haemolytic anaemias.

Input data to CODEm models was centrally processed along with all other specific causes of death and stored in the cause of death (COD) database. Data processing steps are described elsewhere. It should be noted that updates to garbage code redistribution algorithms in GBD 2019 had substantial impact on the CODEm input data in some location-year-age-sex combinations. Outliers were identified as those data where age patterns or temporal patterns were inconsistent with neighbouring age groups or locations or where sparse data were predicting implausible overall temporal or age patterns for a given location. Covariates used in each of the CODEm models, along with their level and direction, are shown in the table below. Most notably, prevalence of hemoglobin S trait and hemoglobin C trait, as estimated

by the Malaria Atlas Project, were added as covariates to the total CODEm model and the subcause models for sickle cell disorders. Other haemoglobinopathies and haemolytic anaemias has several covariates unique to it, reflecting the risk factors for aplastic anaemias that constitute a large proportion of this cause category.

Table 1. Covariates used in <u>haemoglobinopathies and haemolytic anaemias</u> CODEm models (data-rich and global models)

Level	Covariate	Direction	Cause
1	Sickle S trait from Malaria Atlas Project	+	Total (squared), sickle (linear)
	Sickle C trait from Malaria Atlas Project	+	Total (squared), sickle (linear)
	Lysenko 1 (holoendemic) proportion	+	Total, sickle, thal
	Haemoglobinopathies prevalence * excess mortality	+	All
	Sickle cell and thalassaemias prevalence * excess mortality	+	All
	SEV – Leukaemia	+	Other
	SEV – WaSH (water)	+	Other
	SEV – WaSH (sanitation)	+	Other
2	Maternal care and immunisation (MCI)	-	Total, sickle
	Healthcare Access and Quality Index	-	All
	SEV – drugs/alcohol (age-standardised)	+	Other
	SEV – high BMI (age-specific)	+	Other
3	Lag-distributed income (LN-transformed)	-	All
	Population proportion (0-15 latitude)	+	Total, sickle, thal, G6PD
	Population proportion (15-30 latitude)	+	Total, sickle, thal, G6PD
	Population proportion (30-45 latitude)	-	Total, sickle, thal, G6PD
	Population proportion (45+ latitude)	-	Total, sickle, thal, G6PD
	Education (years per capita)	-	Total, other
	Education (proportion w 6+ years schooling)	-	Sickle, thal, G6PD, other
	Education (proportion w 12+ years schooling)	-	Sickle, thal, G6PD, other
	Socio-demographic Index	-	All

***Level refers to a categorical assessment of the strength of mechanistic relationship between the covariate and mortality (1 = more likely; 3 = less likely); direction refers to the direction of the relationship (1 = positive correlation; -1 = negative correlation).**

As mentioned above, DisMod-MR 2.1 was used to estimate sickle cell disorders, thalassaemias, and G6PD deficiency age- and sex-specific prevalence and mortality for each location and year in the GBD. More details on this modelling process, including input data processing, are described in the corresponding non-fatal appendix section. Briefly, each datum for sickle cell disease models was used for one of three mutually exclusive conditions: 1) homozygous sickle cell disease and severe sickle cell/beta thalassaemia, 2) mild sickle cell/beta thalassaemia, or 3) hemoglobin SC disease. We similarly extracted data for thalassaemias using three mutually exclusive disease states: 1) beta thalassaemia major, 2) haemoglobin E/beta thalassaemia, and 3) haemoglobin H disease. G6PD deficiency was estimated as a single model. Cause-specific mortality rates for other haemoglobinopathies and haemolytic anaemias, lacking more specific data, was assumed to be geographically uniform, but did vary by age and sex; the levels and trends were informed by analysis of VR data from the COD database.

Case definitions for each of the types of thalassemias and sickle cell were based on genotype. G6PD deficiency is an X-linked recessive genetic disease, and our reference definition was based on quantitative decline in G6PD activity reagent (ie, chemical) testing. Three sources of data were used for DisMod-MR 2.1 models: literature (generally from community prevalence surveys, birth screening, and cohort studies), claims data, and ICD-9 & ICD-10 hospital discharge data that were adjusted for ICD code position, readmission, inpatient-to-outpatient ratio, and location-specific Healthcare Access and Quality Index. We added data from select geographies identified by GBD collaborators for GBD 2019. Of note, there were no hospital data available for haemoglobin E/beta-thalassaemia, haemoglobin H disease, or G6PD deficiency. Our last comprehensive literature review was completed in GBD 2016, where we identified data on prevalence, excess mortality rate, or with-condition mortality rate. Age-specific survival probabilities from cohort studies were converted to corresponding with-condition mortality rates.

The primary limitation of our estimation is data availability, especially in the locations thought to have the highest burden. We elected a hybrid approach of CODEm and DisMod-MR 2.1 to improve the quality of estimates in data-poor locations, but in most of these location data are still relatively sparse for non-fatal models, which leads to relatively large uncertainty. Further adding to the uncertainty is the fact that the mechanism of death in many with haemoglobinopathies is due to infectious agents such as malaria, lower respiratory infections, and diarrhoea, or due to cardiovascular diseases such as ischaemic heart disease or stroke, and are associated with increased risk of death during pregnancy. In locations with poor diagnostic capabilities and high infectious burden, it is thus very plausible that mortality due to haemoglobinopathies may be even higher. Secondly, our specification of seven distinct entities for DisMod-MR 2.1 models does not align perfectly with the cause categories in the central COD prep, which limits the extent to which CSMR data from the COD database can inform non-fatal models.

Endocrine, metabolic, blood, and immune disorders

Flowchart

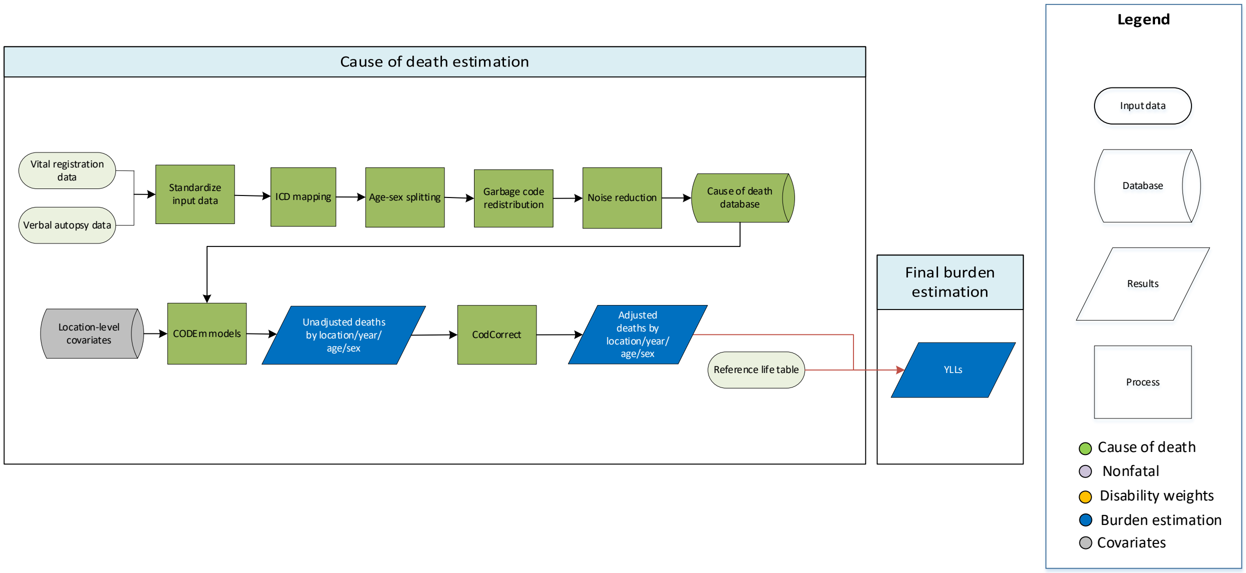

Input data

Vital registration and verbal autopsy data from the cause of death (COD) database were used to model mortality due to endocrine, metabolic, blood, and immune disorders. Relative to GBD 2017, in GBD 2019 we re-mapped codes for a small number of secondary endocrine, immune, or metabolic disorders to their underlying causes (ICD codes D70.2, D89.3, and E24.4).

Outliers were identified by systematic examination of datapoints for all location-years. Datapoints that violated well-established age or time trends or that resulted in extremely high or low cause fractions were determined to be outliers. Methods for selecting outliers were consistent across both vital registration and verbal autopsy data.

Modelling strategy

The estimation strategy used for fatal endocrine, blood, metabolic, and immune disorders is largely similar to methods used in GBD 2017. A standard CODEm model with location-level covariates was used to model deaths due to endocrine, blood, metabolic, and immune disorders (see appendix section 3.1 for details). Separate models were conducted for male and female mortality, and age-restrictions for death estimations to digestive diseases included 0 days for lower bound and 95+ for upper bound. We hybridised separate global and data-rich models to acquire unadjusted results, which we finalised and adjusted using CodCorrect to reach final YLLs due to endocrine, blood, metabolic, and immune disorders.

Key changes from GBD 2017

- We added estimates for the following new locations: Monaco, San Marino, Cook Islands, and Saint Kitts and Nevis.
- We added subnational location data for the following: Italy, Poland, Pakistan, and the Philippines.

- We changed the direction of the Socio-demographic Index covariate from 0 to -1.

The following table has the full list of covariates used for fatal endocrine, blood, metabolic, and immune disorders.

Table 1. Covariates used in endocrine, blood, metabolic, and immune disorders mortality modelling

Level	Covariate	Direction
1	Mean BMI	+
2	Mean cholesterol	+
	Alcohol (liters per capita)	+
	Healthcare Access and Quality Index	-
3	Socio-demographic Index	-
	Education (year per capita)	-
	Log LDI ($I per capita)	-

Sudden infant death syndrome (SIDS)

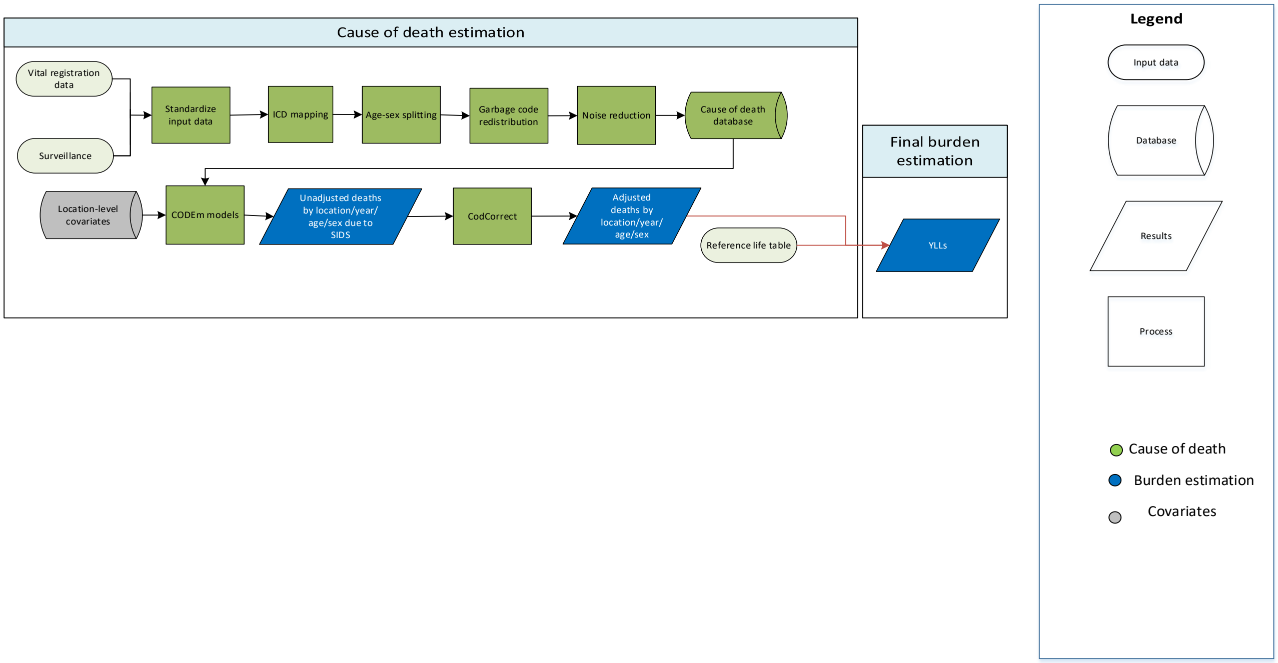

Input data

Vital registration data were used to estimate deaths due to sudden infant SIDS. Datapoints were selected as outliers if they met the following criteria: (1) implausibly high values relative to country time trends or global or regional patterns, based on the assumption that there are not "outbreaks" of SIDS, or (2) substantial conflict with established age or temporal patterns. In addition, for GBD 2017, all deaths assigned to SIDS outside of 4- and 5-star countries were reassigned to neonatal disorders. SIDS can only be ascertained as a cause of death by autopsy, which is unlikely to have been used outside of 4- and 5-star countries. All deaths coded to SIDs in verbal autopsy data were mapped to neonatal disorders.

Modelling strategy

The standard CODEm modelling approach was applied to estimate deaths due to SIDS. We ran CODEm models for ages 7–27 days and 28–364 days because we believe that deaths assigned to SIDS in other age groups are mis-assigned, and these are therefore treated as garbage codes. Surveillance data and verbal autopsy data were not used as inputs to this model because these sources do not use data collection methods that can accurately diagnose deaths due to SIDS.

Notable differences between the GBD 2013 and GBD 2015 strategy included updates across the board to smoking-related covariates, total fertility rate, and Socio-demographic Index covariates. The addition of American Samoa to the Oceania region was also of note, as well as the shift to including more ICD detail codes in the input data for some countries that previously reported only aggregated codes. There were no significant changes in strategy from GBD 2015 to GBD 2017.

Covariates in GBD 2017 are shown in the following table.

Level	Covariate	Direction
1	Tobacco (cigarettes per capita)	+
	In-facility delivery (proportion)	-
2	Underweight (proportion <2SD weight for age, <5 years)	+
	Skilled birth attendance (proportion)	-
3	Lag distributed income (I$ per capita)	0
	Education (years per capita)	-
	Total fertility rate	+
	Socio-demographic Index	0

Covariates in GBD 2019 are shown in the following table.

			Data rich		Global	
Level	Covariate	Direction	Acceptance in males	Acceptance in females	Acceptance in males	Acceptance in females
1	Tobacco (cigarettes per capita)	+	Y	Y	Y	Y
	In-facility delivery (proportion)	-	N	N	N	N
2	Maternal care and immunisation	+	N	N	N	N
	Skilled birth attendance (proportion)	-	N	N	N	N
	Healthcare Access and Quality Index	-	N	N	N	N
3	Lag distributed income (I$ per capita)	+	Y	Y	Y	Y
	Education (years per capita)	-	N	N	N	N
	Total fertility rate	+	N	N	N	N
	Socio-demographic Index	+	Y	Y	Y	Y

Injuries

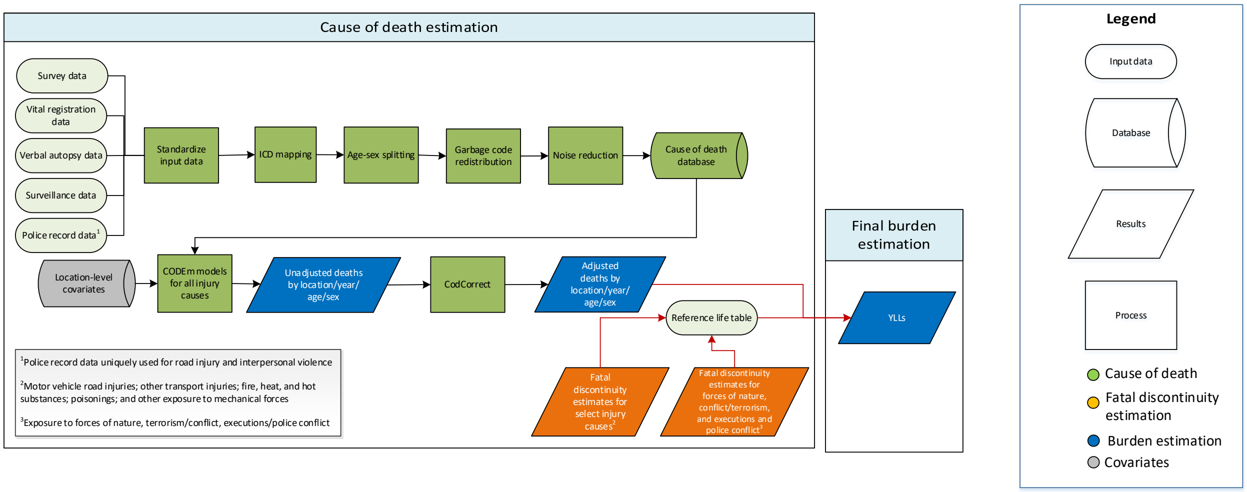

Input data

In GBD 2017, we estimated injury mortality from vital registration, verbal autopsy, mortality surveillance, censuses, surveys, and police record data. Police and crime reports were data sources uniquely used for the estimation of deaths from road traffic injury and interpersonal violence. The police data were collected from published studies, national agencies, and institutional surveys such as the United Nations Crime Trends Survey and the WHO Global Status Report on Road Safety Survey. For countries with vital registration data we did not use police records, except if the recorded number of road injury and interpersonal violence deaths from police records exceeded that in the vital registration.

Infrequently, data points were marked as outliers. Outlier criteria excluded data points that (1) were implausibly high or low relative to global or regional patterns, (2) substantially conflicted with established age or temporal patterns, or (3) significantly conflicted with other data sources conducted from the same locations or locations with similar characteristics (ie, Socio-demographic Index).

Modelling strategy

Overview

In GBD 2019, the standard CODEm modelling approach was applied to estimate deaths due to all causes of injury, excluding “Exposure to forces of nature,” and “Conflict and terrorism”. These causes were modelled solely outside of the CODEm process as fatal discontinuities estimation; this process is detailed further in the section on fatal discontinuities estimation in the appendix.

Fatal discontinuity was estimated for ten injury causes also modeled in CODEm. These causes included “Other transport injuries”, “Fire, heat, and hot substances”, “Poisoning by other means”, “Other exposure to mechanical forces”, “Non-venomous animal contact”, “Environmental heat and cold exposure”, “Physical violence by firearm”, “Physical violence by sharp object”, “Physical violence by other means”, “Executions and police conflict”. Final fatal discontinuity estimations for these causes were merged with CODEm results post-CoDCorrect to produce final cause of death results.

Refer to the table at the end of this section for a complete list of the cause-of-injury categories, modelling strategies, and covariate changes from GBD 2017.

GBD injury codes and categories

The International Classification of Diseases (ICD) was used to classify injuries. In GBD, injury incidence and death are defined as ICD-9 codes E000-E999 and ICD-10 chapters V to Y. There is one exception: deaths and cases of alcohol poisoning and drug overdoses are classified under drug and alcohol use disorders. In GBD 2019, injury causes were organized into 30 mutually exclusive and collectively exhaustive external cause-of-injury categories.

Preparation of data

The preparation of cause of death data includes age splitting, age-sex splitting, smoothing, and outlier detection. These steps are described in detail by Naghavi et al and Lozano et al.[1,2,3] The concept of "garbage codes" and redistribution of these codes was proposed in GBD 1990.[4] Garbage codes are causes of death that should not be identified as specific underlying causes of death but have been entered as the underlying cause of death on death certificates. A classic example of these types of codes in injuries chapters are "Exposure to unspecified factor" (X59 in ICD-10 and E887 in ICD-9) and all undetermined intent codes (Y10-Y34 in ICD-10 and E980-E988 in ICD-9). Other examples of garbage codes in injuries are the coding of an injury death to intermediate codes like septicemia or peritonitis or as an ill-defined and unknown cause of mortality (R99). Approximately 2% of total deaths in countries with vital registration data are assigned to these three injury garbage code categories.

Splitting into sublevel causes

In countries with non-detail ICD code data, cause-of-injury categories were proportionally split into sublevel cause-of-injury categories. The sublevel cause-of-injury causes were created in the CoDCorrect process. One of the countries with non-detail ICD code data is South Africa, and in GBD 2013 the proportions of sublevel cause-of-injury were based on vital registration data. For GBD iterations of 2015, 2016, 2017, and 2019, the proportions were based on post-mortem investigation of injury deaths as described in the paper by Matzopoulos et al. 2015.[5]

Limitations and model assumptions

We added police data for road injuries and interpersonal violence to help predict level and age patterns in countries with sparse or absent cause of death data even though we know from countries with near-complete vital registration data that police records tend to underestimate the true level of deaths. However, we applied police data estimates in instances where reported deaths were higher than vital registration numbers.

During GBD 2019, the input data for the US was reviewed for completeness, and we determined that the US National Vital Statistics System (NVSS) systematically underreports deaths due to police violence by about 50% every year. In order to quantify this bias, we ran a network meta-regression on NVSS data with direct comparisons by state and year to Mapping Police Violence (MPV), an alternate open-source database that we believe more accurately captures deaths due to police violence, and indirect comparisons to an additional source, Fatal Encounters (FE). The regression included a fixed effect on state to capture different underreporting rates across states, but assumed that underreporting rates are constant across age, sex, and year. Additionally, since MPV does not attempt to capture police killed by civilians and neither MPV nor FE attempt to capture executions, death counts from the FBI's Law

Enforcement Officers Killed and Assaulted database and the Death Penalty Information Center (DPIC) were added to these data sources in order to conform them to the GBD definition of executions and police conflict. We then used the underreporting rates estimated by the network meta-regression to scale the CODCorrect estimates for executions and police conflict in the United States upwards to a more accurate level. To maintain consistency with the all-cause mortality envelope, the deaths added to executions and police conflict were also removed proportionally from interpersonal violence and its relevant sub-causes. Record linkage between NVSS and open-source databases has shown that interpersonal violence is the most common underlying cause of death listed on death certificates for mis-assigned police violence deaths.[6]

Covariates

The following covariates were included.

**Transport Injuries**		
Level	Covariate	Direction
1	BAC law professional drivers (quartile)	1
1	BAC law general population (quartile)	1
1	BAC law youth drivers (quartile)	1
1	Liters of alcohol consumed per capita	1
1	Speed limit law rural (quartile)	1
1	Speed limit law urban (quartile)	1
1	Vehicles - 2 wheels fraction (proportion)	1
1	Vehicles - 2+4 wheels (per capita)	1
2	Education (years per capita)	-1
2	Healthcare access and quality index	-1
2	LDI (I$ per capita)	-1
2	Population 15 to 30 (proportion)	1
2	Population Density (300-500 ppl/sqkm, proportion)	1
2	Population Density (500-1000 ppl/sqkm, proportion)	1
2	Population-weighted mean temperature	1
2	Socio-demographic Index	-1
3	Rainfall Quintile 5 (proportion)	1
**Road injuries**		
Level	Covariate	Direction
1[a]	BAC law professional drivers (quartile)	1
1[a]	BAC law general population (quartile)	1
1[a]	BAC law youth drivers (quartile)	1
1	Liters of alcohol consumed per capita	1
1	Log-transformed SEV scalar: Road Inj	1
1[a]	Speed limit law rural (quartile)	1
1[a]	Speed limit law urban (quartile)	1
1	Vehicles - 2 wheels (per capita)	1

1	Vehicles - 2 wheels fraction (proportion)	1
1	Vehicles - 2+4 wheels (per capita)	1
1	Vehicles - 4 wheels (per capita)	1
2[b]	Education (years per capita)	-1
2	Healthcare access and quality index	-1
2[b]	LDI (I$ per capita)	-1
2	Population 15 to 30 (proportion)	1
2	Population Density (300-500 ppl/sqkm, proportion)	1
2	Population Density (500-1000 ppl/sqkm, proportion)	1
2	Population-weighted mean temperature	1
2[b]	Socio-demographic Index	-1
3	Rainfall Quintile 5 (proportion)	1
**Pedestrian road injuries**		
Level	Covariate	Direction
1	BAC law professional drivers (quartile)	1
1	BAC law general population (quartile)	1
1	BAC law youth drivers (quartile)	1
1	Liters of alcohol consumed per capita	1
1	Log-transformed SEV scalar: Pedest	1
1	Speed limit law rural (quartile)	1
1	Speed limit law urban (quartile)	1
1	Vehicles - 2 wheels fraction (proportion)	1
1	Vehicles - 2+4 wheels (per capita)	1
2	Education (years per capita)	-1
2	Healthcare access and quality index	-1
2	LDI (I$ per capita)	-1
2	Population 15 to 30 (proportion)	1
2	Population Density (300-500 ppl/sqkm, proportion)	1
2	Population Density (500-1000 ppl/sqkm, proportion)	1
2[c]	Population-weighted mean temperature	1
2	Socio-demographic Index	-1
3	Rainfall Quintile 5 (proportion)	1
**Pedestrian road injuries**		
Level	Covariate	Direction
1	BAC law professional drivers (quartile)	1
1	BAC law general population (quartile)	1
1	BAC law youth drivers (quartile)	1
1	Liters of alcohol consumed per capita	1
1	Log-transformed SEV scalar: Cyclist	1
1	Speed limit law rural (quartile)	1

1	Speed limit law urban (quartile)	1
1	Vehicles - 2 wheels fraction (proportion)	1
1	Vehicles - 2+4 wheels (per capita)	1
2	Education (years per capita)	-1
2	Healthcare access and quality index	-1
2	LDI (I$ per capita)	-1
2	Population 15 to 30 (proportion)	1
2	Population Density (300-500 ppl/sqkm, proportion)	1
2	Population Density (500-1000 ppl/sqkm, proportion)	1
2	Population-weighted mean temperature	1
2	Socio-demographic Index	-1
3	Rainfall Quintile 5 (proportion)	1
**Motorcyclist road injuries**		
Level	Covariate	Direction
1	BAC law professional drivers (quartile)	1
1	BAC law general population (quartile)	1
1	BAC law youth drivers (quartile)	1
1	Liters of alcohol consumed per capita	1
1	Log-transformed SEV scalar: Mot Cyc	1
1	Speed limit law rural (quartile)	1
1	Speed limit law urban (quartile)	1
1[d]	Vehicles - 2 wheels fraction (proportion)	1
2	Education (years per capita)	-1
2	Healthcare access and quality index	-1
2	LDI (I$ per capita)	-1
2	Population 15 to 30 (proportion)	1
2	Population Density (300-500 ppl/sqkm, proportion)	1
2	Population Density (500-1000 ppl/sqkm, proportion)	1
2	Population-weighted mean temperature	1
2	Socio-demographic Index	-1
3	Rainfall Quintile 5 (proportion)	1
**Motor vehicle road injuries**		
Level	Covariate	Direction
1	BAC law professional drivers (quartile)	1
1	BAC law general population (quartile)	1
1	BAC law youth drivers (quartile)	1
1	Liters of alcohol consumed per capita	1
1	Log-transformed SEV scalar: Mot Veh	1
1	Speed limit law rural (quartile)	1
1	Speed limit law urban (quartile)	1

1	Vehicles - 4 wheels (per capita)	1
2	Education (years per capita)	-1
2	Healthcare access and quality index	-1
2	LDI (I$ per capita)	-1
2	Population 15 to 30 (proportion)	1
2	Population Density (300-500 ppl/sqkm, proportion)	1
2	Population Density (500-1000 ppl/sqkm, proportion)	1
2	Population-weighted mean temperature	1
2	Socio-demographic Index	-1
3	Rainfall Quintile 5 (proportion)	1
**Other road injuries**		
Level	Covariate	Direction
1	BAC law professional drivers (quartile)	1
1	BAC law general population (quartile)	1
1	BAC law youth drivers (quartile)	1
1	Liters of alcohol consumed per capita	1
1	Log-transformed SEV scalar: Oth Road	1
1	Speed limit law rural (quartile)	1
1	Speed limit law urban (quartile)	1
1	Vehicles - 2 wheels fraction (proportion)	1
1	Vehicles - 2+4 wheels (per capita)	1
2	Education (years per capita)	-1
2	Healthcare access and quality index	-1
2	LDI (I$ per capita)	-1
2	Population 15 to 30 (proportion)	1
2	Population-weighted mean temperature	1
3	Rainfall Quintile 5 (proportion)	1
3[e]	Socio-demographic Index	-1
**Other transport injuries**		
Level	Covariate	Direction
1	BAC law professional drivers (quartile)	1
1	BAC law general population (quartile)	1
1	BAC law youth drivers (quartile)	1
1	Liters of alcohol consumed per capita	1
1	Log-transformed SEV scalar: Oth Trans	1
1	Speed limit law rural (quartile)	1
1	Speed limit law urban (quartile)	1
1[f]	Vehicles - 2 wheels fraction (proportion)	1
1	Vehicles - 2+4 wheels (per capita)	1
2	Education (years per capita)	-1
2	Healthcare access and quality index	-1

2	LDI (I$ per capita)	-1
2	Population 15 to 30 (proportion)	1
2	Population Density (300-500 ppl/sqkm, proportion)	1
2	Population Density (500-1000 ppl/sqkm, proportion)	1
2	Population-weighted mean temperature	1
2	Socio-demographic Index	-1
3	Rainfall Quintile 5 (proportion)	1
**Falls**		
Level	Covariate	Direction
1	Education (years per capita)	-1
1	Liters of alcohol consumed per capita	1
1	Log-transformed SEV scalar: Falls	1
2	Healthcare access and quality index	-1
2	Population-weighted mean temperature	-1
3	Elevation Over 1500m (proportion)	1
3	LDI (I$ per capita)	-1
3	Socio-demographic Index	-1
**Drowning**		
Level	Covariate	Direction
1	Coastal Population within 10km (proportion)	1
1	Landlocked Nation (binary)	-1
1	Log-transformed SEV scalar: Drown	1
1	Population-weighted mean temperature	1
1	Rainfall Quintile 1 (proportion)	-1
1	Rainfall Quintile 5 (proportion)	1
2	Elevation Under 100m (proportion)	1
3	Education (years per capita)	-1
3	LDI (I$ per capita)	-1
3	Socio-demographic Index	-1
**Fire, heat, and hot substances**		
Level	Covariate	Direction
1	Log-transformed SEV scalar: Fire	1
1	Population-weighted mean temperature	1
2	Healthcare access and quality index	-1
2	Indoor Air Pollution (All Cooking Fuels)	1
2	Population Density (over 1000 ppl/sqkm, proportion)	1
2	Tobacco (cigarettes per capita)	1
3	Education (years per capita)	-1
3	LDI (I$ per capita)	-1
3	Socio-demographic Index	-1

**Poisonings**		
Level	Covariate	Direction
1	Log-transformed SEV scalar: Poison	1
1	Opium Cultivation (binary)	1
1	Population-weighted mean temperature	1
2	Healthcare access and quality index	-1
2	Population Density (over 1000 ppl/sqkm, proportion)	-1
2	Population Density (under 150 ppl/sqkm, proportion)	1
3	Education (years per capita)	-1
3	LDI (I$ per capita)	-1
3	Socio-demographic Index	-1
**Poisoning by carbon monoxide**		
Level	Covariate	Direction
1	Log-transformed SEV scalar: Inj Pois CO	1
2	Population-weighted mean temperature	-1
3	Education (years per capita)	-1
3	Healthcare access and quality index	-1
3	LDI (I$ per capita)	-1
3	Socio-demographic Index	-1
**Poisoning by other means**		
Level	Covariate	Direction
1	Log-transformed SEV scalar: Inj Pois Oth	1
1	Population-weighted mean temperature	1
3	Education (years per capita)	-1
3	Healthcare access and quality index	-1
3	LDI (I$ per capita)	-1
3	Socio-demographic Index	-1
**Exposure to mechanical forces**		
Level	Covariate	Direction
1	Population-weighted mean temperature	1
2	Healthcare access and quality index	-1
2	Population Density (over 1000 ppl/sqkm, proportion)	-1
2	Population Density (under 150 ppl/sqkm, proportion)	1
3	Education (years per capita)	-1
3	LDI (I$ per capita)	-1
3	Socio-demographic Index	-1
**Unintentional firearm injuries**		
Level	Covariate	Direction
1	Log-transformed SEV scalar: Mech Gun	1
1	Population-weighted mean temperature	1

2	Healthcare access and quality index	-1
3	Education (years per capita)	-1
3	LDI (I$ per capita)	-1
3	Population Density (over 1000 ppl/sqkm, proportion)	-1
3	Population Density (under 150 ppl/sqkm, proportion)	1
3	Socio-demographic Index	-1
**Other exposure to mechanical forces**		
Level	Covariate	Direction
1	Log-transformed SEV scalar: Oth Mech	1
1	Population-weighted mean temperature	1
2	Healthcare access and quality index	-1
2	Population Density (over 1000 ppl/sqkm, proportion)	-1
2	Population Density (under 150 ppl/sqkm, proportion)	1
3	Education (years per capita)	-1
3	LDI (I$ per capita)	-1
3	Socio-demographic Index	-1
**Adverse effects of medical treatment**		
Level	Covariate	Direction
1	Education (years per capita)	-1
1[g]	Liters of alcohol consumed per capita	1
1	Population-weighted mean temperature	1
2	Healthcare access and quality index	-1
3	LDI (I$ per capita)	1
3	Socio-demographic Index	-1
**Environmental heat and cold exposure**		
Level	Covariate	Direction
2	Healthcare access and quality index	-1
3	90th percentile climatic temperature in the given country-year.	1
3	Education (years per capita)	-1
3	Elevation 500 to 1500m (proportion)	1
3	Elevation Over 1500m (proportion)	1
3	LDI (I$ per capita)	-1
3	Population Density (150-300 ppl/sqkm, proportion)	-1
3	Population-weighted mean temperature	1
3	Rainfall (Quintiles 4-5)	1
3	Sanitation (proportion with access)	-1
3	Socio-demographic Index	-1
**Animal contact**		

Level	Covariate	Direction
1	Liters of alcohol consumed per capita	1
1	Log-transformed SEV scalar: Animal	1
1	Population-weighted mean temperature	1
2	Healthcare access and quality index	-1
2	Population 15 to 30 (proportion)	1
3	Education (years per capita)	-1
3	Elevation Over 1500m (proportion)	-1
3	Elevation Under 100m (proportion)	1
3	LDI (I$ per capita)	-1
3	Population Density (over 1000 ppl/sqkm, proportion)	-1
3	Population Density (under 150 ppl/sqkm, proportion)	1
3	Socio-demographic Index	-1
**Venomous animal contact**		
Level	Covariate	Direction
1	Liters of alcohol consumed per capita	1
1	Log-transformed SEV scalar: Venom	1
1	Absolute value of average latitude	-1
1	Liters of alcohol consumed per capita	1
1	Mean number of venomous snake species	1
1	Proportion of population vulnerable to snake species	1
1	Population-weighted mean temperature	1
1	Rainfall population-weighted (mm/yr)	1
1	Proportion of population involved in agricultural activities	1
1	Sahel Region of Africa (binary)	1
1	Urbanicity	-1
2	Healthcare access and quality index	-1
3	Education (years per capita)	-1
3	Elevation Over 1500m (proportion)	-1
3	Elevation Under 100m (proportion)	-1
3	LDI (I$ per capita)	-1
3	Population Density (over 1000 ppl/sqkm, proportion)	-1
3	Population Density (under 150 ppl/sqkm, proportion)	1
3	Socio-demographic Index	-1
**Non-venomous animal contact**		
Level	Covariate	Direction
1[k]	Elevation Over 1500m (proportion)	-1
1[k]	Elevation Under 100m (proportion)	1

1	Liters of alcohol consumed per capita	1
1	Log-transformed SEV scalar: Non Ven	1
1	Population-weighted mean temperature	1
2[l]	Healthcare access and quality index	-1
3	Education (years per capita)	-1
3[m]	Elevation Over 1500m (proportion)	-1
3[m]	Elevation Under 100m (proportion)	1
3	LDI (I$ per capita)	-1
3[m]	Population Density (over 1000 ppl/sqkm, proportion)	-1
3[m]	Population Density (under 150 ppl/sqkm, proportion)	1
3	Socio-demographic Index	-1
**Foreign body**		
Level	Covariate	Direction
1	Education (years per capita)	1
1	Indoor Air Pollution (All Cooking Fuels)	1
1	LDI (I$ per capita)	1
1	Liters of alcohol consumed per capita	1
1	Population Over 65 (proportion)	1
1	Population-weighted mean temperature	1
2	Healthcare access and quality index	-1
3	Socio-demographic Index	-1
**Pulmonary aspiration and foreign body in airway**		
Level	Covariate	Direction
1[n]	Education (years per capita)	-1
1	Liters of alcohol consumed per capita	1
1	Log-transformed SEV scalar: F Body Asp	1
1	Population-weighted mean temperature	1
2[o]	Alcohol binge drinker proportion, age-standardized	1
2	Healthcare access and quality index	-1
2	Mean BMI	1
3	LDI (I$ per capita)	-1
3	Socio-demographic Index	-1
**Foreign body in other body part**		
Level	Covariate	Direction
1	Liters of alcohol consumed per capita	1
1	Log-transformed SEV scalar: Oth F Body	1
1	Population-weighted mean temperature	1
2	Healthcare access and quality index	-1
3	Education (years per capita)	-1
3	LDI (I$ per capita)	-1

3	Socio-demographic Index	-1
**Other unintentional injuries**		
Level	Covariate	Direction
1	Liters of alcohol consumed per capita	1
1	Log-transformed SEV scalar: Oth Unint	1
1	Population-weighted mean temperature	1
1	Vehicles - 2 wheels (per capita)	1
1	Vehicles - 4 wheels (per capita)	1
2	Healthcare access and quality index	-1
3	Education (years per capita)	-1
3	LDI (I$ per capita)	-1
3	Population Density (over 1000 ppl/sqkm, proportion)	-1
3	Population Density (under 150 ppl/sqkm, proportion)	1
3	Socio-demographic Index	-1
**Self-harm**		
Level	Covariate	Direction
1	12-month non-partner sexual violence	1
1	Liters of alcohol consumed per capita	1
1[h]	Log-transformed SEV scalar: Self Harm	1
1	Major depressive disorder	1
1[i]	Muslim Religion (proportion of population)	1
1	Population-weighted mean temperature	1
2	Healthcare access and quality index	-1
2	Population Density (150-300 ppl/sqkm, proportion)	1
2	Population Density (300-500 ppl/sqkm, proportion)	-1
2	Population Density (500-1000 ppl/sqkm, proportion)	-1
2	Population Density (over 1000 ppl/sqkm, proportion)	-1
2	Population Density (under 150 ppl/sqkm, proportion)	1
3	Education (years per capita)	-1
3	LDI (I$ per capita)	-1
3	Socio-demographic Index	-1
**Self-harm by firearm**		
Level	Covariate	Direction
1	12-month non-partner sexual violence	1
1	Liters of alcohol consumed per capita	1
1	Log-transformed SEV scalar: Self Harm	1

1	Major depressive disorder	1
1	Population-weighted mean temperature	1
2	Healthcare access and quality index	-1
2	Population Density (150-300 ppl/sqkm, proportion)	1
2	Population Density (300-500 ppl/sqkm, proportion)	-1
2	Population Density (500-1000 ppl/sqkm, proportion)	-1
2	Population Density (over 1000 ppl/sqkm, proportion)	-1
2	Population Density (under 150 ppl/sqkm, proportion)	1
3	Education (years per capita)	-1
3	LDI (I$ per capita)	-1
3	Socio-demographic Index	-1
**Self-harm by other specified means**		
Level	Covariate	Direction
1	12-month non-partner sexual violence	1
1	Liters of alcohol consumed per capita	1
1	Log-transformed SEV scalar: Self Harm	1
1	Major depressive disorder	1
1	Population-weighted mean temperature	1
2	Healthcare access and quality index	-1
2	Population Density (150-300 ppl/sqkm, proportion)	1
2	Population Density (300-500 ppl/sqkm, proportion)	-1
2	Population Density (500-1000 ppl/sqkm, proportion)	-1
2	Population Density (over 1000 ppl/sqkm, proportion)	-1
2	Population Density (under 150 ppl/sqkm, proportion)	1
3	Education (years per capita)	-1
3	LDI (I$ per capita)	-1
3	Socio-demographic Index	-1
**Interpersonal violence**		
Level	Covariate	Direction
1	Education Relative Inequality (Gini)	1
1	Liters of alcohol consumed per capita	1
1	Log-transformed SEV scalar: Violence	1
1	Population 15 to 30 males (proportion)	1
1	Population-weighted mean temperature	1
2	Healthcare access and quality index	-1

2	Opium Cultivation (binary)	1
2	Population Density (over 1000 ppl/sqkm, proportion)	1
3	Education (years per capita)	-1
3	LDI (I$ per capita)	-1
3	Socio-demographic Index	-1
**Assault by firearm**		
Level	Covariate	Direction
1	Education Relative Inequality (Gini)	1
1	Liters of alcohol consumed per capita	1
1	Log-transformed SEV scalar: Viol Gun	1
1	Population 15 to 30 males (proportion)	1
1	Population-weighted mean temperature	1
2	Healthcare access and quality index	-1
2	Opium Cultivation (binary)	1
2	Population Density (over 1000 ppl/sqkm, proportion)	1
3	Education (years per capita)	-1
3	LDI (I$ per capita)	-1
3	Socio-demographic Index	-1
**Assault by sharp object**		
Level	Covariate	Direction
1	Education Relative Inequality (Gini)	1
1	Liters of alcohol consumed per capita	1
1	Log-transformed SEV scalar: Viol Knife	1
1	Population 15 to 30 males (proportion)	1
1[j]	Population-weighted mean temperature	1
2	Healthcare access and quality index	-1
2	Opium Cultivation (binary)	1
2	Population Density (over 1000 ppl/sqkm, proportion)	1
3	Education (years per capita)	-1
3	LDI (I$ per capita)	-1
3	Socio-demographic Index	-1
**Assault by other means**		
Level	Covariate	Direction
1	Education Relative Inequality (Gini)	1
1	Liters of alcohol consumed per capita	1
1	Log-transformed SEV scalar: Oth Viol	1
1	Population 15 to 30 males (proportion)	1
1	Population-weighted mean temperature	1
2	Healthcare access and quality index	-1
2	Opium Cultivation (binary)	1

2	Population Density (over 1000 ppl/sqkm, proportion)	1
3	Education (years per capita)	-1
3	LDI (I$ per capita)	-1
3	Socio-demographic Index	-1

a: Used at level 1 in female models, level 2 in males
b: Used at level 3 in global models, level 2 in data-rich models
c: Used at level 1 in male data-rich model. Level 2 in other three models.
d: Only used in Female global model
e: Used at level 2 in male global model, level 3 for the other three models
f: Not used in female global model
g: Only used in female global model
h: Only used in female models
i: Used at level 2 in male global mode, used at level 1 in male data-rich model. Not used in female model.
j: Used at level 2 in female, global model and level 1 for all others
k: Only used in male global model
l: Used at level 3 in male global model
m: Used at level 2 in male global model
n: Used at level 3 in the female global model
o: Only used in the female global model

Table – Injury Cause List			
**ID**	**Cause**	**Modelling Strategy**	**Covariate changes from GBD 2017**
1	Transport injuries	CODEm	Additions: Population-weighted mean temperature; Quartile on the strictness of blood-alcohol content laws of professional, general, and youth drivers; Quartile on the strictness of speed limit laws in rural and urban places; Proportion of population aged 15 to 30
1.1	Road injuries	CODEm	Additions: Population-weighted mean temperature; Quartile on the strictness of blood-alcohol content laws of professional, general, and youth drivers; Quartile on the strictness of speed limit laws in rural and urban places; Proportion of population aged 15 to 30
1.1.1	Pedestrian road injuries	CODEm	Additions: Population-weighted mean temperature; Quartile on the strictness of blood-alcohol content laws of professional, general, and youth drivers; Quartile on the strictness of speed limit laws in rural and urban places; Proportion of population aged 15 to 30
1.1.2	Cyclist road injuries	CODEm	Additions: Population-weighted mean temperature; Quartile on the strictness of

Table – Injury Cause List			
ID	Cause	Modelling Strategy	Covariate changes from GBD 2017
			blood-alcohol content laws of professional, general, and youth drivers; Quartile on the strictness of speed limit laws in rural and urban places; Proportion of population aged 15 to 30
1.1.3	Motorcyclist road injuries	CODEm	Additions: Population-weighted mean temperature; Quartile on the strictness of blood-alcohol content laws of professional, general, and youth drivers; Quartile on the strictness of speed limit laws in rural and urban places; Proportion of population aged 15 to 30
1.1.4	Motor vehicle road injuries	CODEm	Additions: Population-weighted mean temperature; Quartile on the strictness of blood-alcohol content laws of professional, general, and youth drivers; Quartile on the strictness of speed limit laws in rural and urban places; Proportion of population aged 15 to 30
1.1.5	Other road injuries	CODEm	Additions: Population-weighted mean temperature; Quartile on the strictness of blood-alcohol content laws of professional, general, and youth drivers; Quartile on the

Table – Injury Cause List			
**ID**	**Cause**	**Modelling Strategy**	**Covariate changes from GBD 2017**
			strictness of speed limit laws in rural and urban places; Proportion of population aged 15 to 30
1.2	Other transport injuries	CODEm and fatal discontinuity estimation	Additions: Population-weighted mean temperature; Quartile on the strictness of blood-alcohol content laws of professional, general, and youth drivers; Quartile on the strictness of speed limit laws in rural and urban places; Proportion of population aged 15 to 30<br><br>Dropped: Education (years per capita)
2	Unintentional injuries	Not modeled at parent cause level	
2.1	Falls	CODEm	Added: Population-weighted mean temperature; education in years per capita
2.2	Drowning	CODEm	Added: Population-weighted mean temperature
2.3	Fire, heat, and hot substances	CODEm and fatal discontinuity estimation	Added: Population-weighted mean temperature
2.4	Poisonings	CODEm	Added: Population-weighted mean temperature
2.4.1	Poisoning by carbon monoxide	CODEm	Added: Population-weighted mean temperature; summary exposure value of risk factors for poisoning by carbon monoxide, log-transformed
2.4.2	Poisoning by other means	CODEm and fatal discontinuity estimation	Added: Population-weighted mean

Table – Injury Cause List			
**ID**	**Cause**	**Modelling Strategy**	**Covariate changes from GBD 2017**
			temperature; Summary exposure value of risk factors for poisoning by other means, log-transformed
2.5	Exposure to mechanical forces	CODEm	Added: Population-weighted mean temperature
2.5.1	Unintentional firearm injuries	CODEm	Added: Population-weighted mean temperature
2.5.2	Other exposure to mechanical forces	CODEm and fatal discontinuity estimation	Added: Population-weighted mean temperature
2.6	Adverse effects of medical treatment	CODEm	Added: Alcohol liters per capita; population-weighted mean temperature; education (years per capita)
2.7	Animal contact	CODEm	Added: Population-weighted mean temperature
2.7.1	Venomous animal contact	CODEm	Added: Population-weighted mean temperature
2.7.2	Non-venomous animal contact	CODEm and fatal discontinuity estimation	Added: Population-weighted mean temperature
2.8	Foreign body	CODEm	Added: Population-weighted mean temperature<br>Dropped: Population of people living at greater than 1500 meters (proportion); Population density over 1,000 per square kilometer (proportion); Population density under 150 per square kilometer (proportion); Population of people living under 100 meters elevation (proportion)
2.8.1	Pulmonary aspiration and foreign body in airway	CODEm	Added: Population-weighted mean temperature;

Table – Injury Cause List			
**ID**	**Cause**	**Modelling Strategy**	**Covariate changes from GBD 2017**
			education (years per capita)
2.8.2	Foreign body in other body part	CODEm	Added: Population-weighted mean temperature<br>Dropped: Population of people living at greater than 1500 meters (proportion); Population density over 1,000 per square kilometer (proportion); Population density under 150 per square kilometer (proportion); Population of people living under 100 meters elevation (proportion)
2.9	Environmental exposure to heat and cold	CODEm and fatal discontinuity estimation	
2.10	Exposure to forces of nature	Fatal discontinuity estimation	
2.11	Other unintentional injuries	CODEm and fatal discontinuity estimation	Added: Population-weighted mean temperature<br>Dropped: Population living at over 1,500 meters elevation (proportion); Population living under 100 meters elevation (proportion)
3	Self-harm and interpersonal violence	Not modeled at parent cause level	
3.1	Self-harm	CODEm	Population-weighted mean temperature; 12-month non-partner sexual violence
3.1.1	Self-harm by firearm	CODEm	Population-weighted mean temperature; 12-month non-partner sexual violence
3.1.2	Self-harm by other specified means	CODEm	Population-weighted mean temperature; 12-

Table – Injury Cause List			
ID	Cause	Modelling Strategy	Covariate changes from GBD 2017
			month non-partner sexual violence
3.2	Interpersonal violence	CODEm	Population-weighted mean temperature; Education relative inequality (Gini); Proportion of population males 15 to 30 years old
3.2.1	Physical violence by firearm	CODEm and fatal discontinuity estimation	Population-weighted mean temperature; Education relative inequality (Gini); Proportion of population males 15 to 30 years old
3.2.2	Physical violence by sharp object	CODEm and fatal discontinuity estimation	Population-weighted mean temperature; Education relative inequality (Gini); Proportion of population males 15 to 30 years old
3.2.3	Physical violence by other means	CODEm and fatal discontinuity estimation	Population-weighted mean temperature; Education relative inequality (Gini); Proportion of population males 15 to 30 years old
3.3	Conflict and terrorism	Fatal discontinuity estimation	
3.4	Executions and police conflict	CODEm and fatal discontinuity estimation	Population-weighted mean temperature; Proportion of population males 15 to 30 years old

References

1 Lozano R, Naghavi M, Foreman K, *et al.* Global and regional mortality from 235 causes of death for 20 age groups in 1990 and 2010: a systematic analysis for the Global Burden of Disease Study 2010. *The Lancet* 2012; **380**: 2095–128.

2 Global, regional, and national age–sex specific all-cause and cause-specific mortality for 240 causes of death, 1990–2013: a systematic analysis for the Global Burden of Disease Study 2013. *The Lancet* 2015; **385**: 117–71.

3 Global, regional, and national life expectancy, all-cause mortality, and cause-specific mortality for 249 causes of death, 1980-2015: a systematic analysis for the Global Burden of Disease Study 2015. The Lancet 2016; **388**: 1459-1544.

4 Murray CJL, Lopez AD, Harvard School of Public Health, World Health Organization, World Bank. The global burden of disease: a comprehensive assessment of mortality and disability from diseases, injuries, and risk factors in 1990 and projected to 2020. Cambridge, MA: Published by the Harvard School of Public Health on behalf of the World Health Organization and the World Bank : Distributed by Harvard University Press, 1996.

5 Matzopoulos R, Prinsloo M, Wyk VP, Gwebushe N, Mathews S, et al. Injury-related mortality in South Africa: a retrospective descriptive study of postmortem investigations. *Bull World Health Organ* 2015; **93**: 303–13.

6 Feldman JM, Gruskin S, Coull BA, Krieger N (2017) Quantifying underreporting of law-enforcement-related deaths in United States vital statistics and news-media-based data sources: A capture–recapture analysis. PLOS Medicine 14(10): e1002399.

Fatal Discontinuities

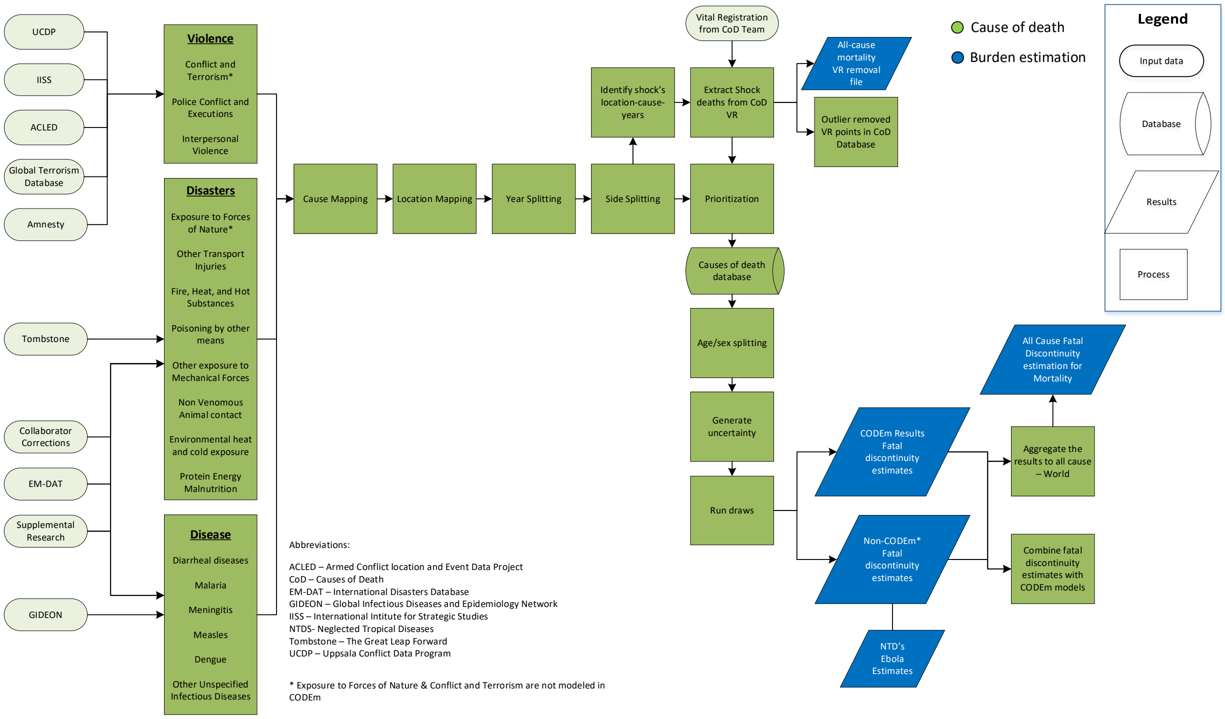

Fatal Discontinuities are defined as events that are stochastic in nature and cannot be modelled because they do not have a predictable time trend. Some causes have both fatal discontinuities, as well as a continuous background mortality that has a smooth time trend and can be modelled, these include, Police violence and Executions, Interpersonal Violence, Other Transport Injuries, Fire Heat and Hot Substances, Poisoning by other Means, Other exposure to Mechanical Forces, Non-Venomous Animal Contact, Environmental Heat and Cold Exposure, Protein Energy Malnutrition, Diarrheal Disease, Malaria, Meningitis, Measles, Dengue, And Other Unspecified Infectious Disease. Causes without a continuous background mortality are exclusively estimated using the fatal discontinuity method, are Conflict and Terrorism, and Exposure to Forces of Nature. Any other causes are not captured in Fatal Discontinuities.

Input data

Overall

Input data for fatal discontinuities are compiled from a range of sources, including country vital registration (VR) data; international databases that capture several cause-specific fatal discontinuities; and supplemental data in the presence of known issues with data quality or representativeness, or time lags in reporting. A Twitter scrape was used in place of a systematic literature review as a way to identify supplemental input data for missing fatal discontinuities. Below more detail is provided on the different input data sources by sub-causes of fatal discontinuities.

Only Discontinuity (Non-CODEm)

For causes that are not modelled in CODEm, all of the deaths captured in VR are considered to be fatal discontinuities. Deaths that are extracted from cause specific VR are then subtracted from the all-cause VR data used in the all-cause mortality estimation process.

Conflict and Terrorism

In GBD 2019, War is defined as *"a state of armed conflict between states, governments, societies and paramilitary groups. It is generally characterized by extreme violence, aggression, destruction, and mortality and the use of regular or irregular military forces."* and Terrorism is defined as *"The unlawful use or threatened use of force or violence against individuals or property in an attempt to coerce or intimidate governments or societies to achieve political, religious or ideological objectives"*. Data for conflict and terrorism came from the Uppsala Conflict Data Program (UCDP), International Institute for Strategic Studies (IISS), Armed Conflict Location & Event Data Project (ACLED), Global Terrorism Database (GTD) and Vital Registration (VR) and other supplemental data sources. Causes were assigned for each event using the source's cause coding and any description from the notes available.

Data source name	Date accessed	Years of data downloaded	Type of data included
**Uppsala Conflict Data Program[1]**			
Georeferenced Event Dataset, Version 19.1	6/10/2019	1989-2018	UCDP battles, non-state, and one-sided conflict deaths with the most disaggregated location information available
PRIO Battles Deaths Dataset, Version 3.1	1/16/2018	1946-2008	Armed conflict (civil wars, etc.)
**International Institute for Strategic Studies**			
Armed Conflict Dataset	11/17/2016	1997-2016	Insurgency, Inter-state, Intra-state conflict deaths
**Robert S. Strauss Center For International Security And Law**			
Armed Conflict Location and Event Dataset (ACLED)	2/5/2019	1997-2019	Actions of opposition groups, governments, and militias in selected locations in Africa, Asia, and the Middle East specifying the exact location and date of battle events, transfers of military control, headquarter establishment, civilian violence, and rioting
**University of Maryland, Global Terrorism Database**			
Global Terrorism Database (GTD)	6/10/2019	1970-2017	Attacks aimed at attaining political, economic, religious, or social goal, includes evidence of intention to coerce, action was outside precepts of International Humanitarian Law.
**University of Chicago, Chicago Project on Security and Threats**			
Suicide Attack Database (CPOST SAD)	11/26/2018	1982-2018	Attacks in which an attacker kills him/herself in a deliberate attempt to kill others, includes only attacks perpetrated by non-state actors
**Amnesty International**			
Amnesty	6/20/2019	1991-2018	Police conflict and executions

Four major conflicts were identified that were not represented in these databases: 1997 civil conflict in Albania[4]; 1971 genocide in Bangladesh[5]; 1972 genocide in Burundi[6]; and 1993 genocide in Burundi[6]. In these cases, literature sources were used to account for these fatal discontinuities.

Exposure to forces of nature

In GBD 2019, Exposure to forces of nature is defined as *"A force which is beyond human control"* The Centre for Research on the Epidemiology of Disasters' International Disaster Database (EM-DAT[7]) served as the primary non-VR source of fatal discontinuities due to exposure to forces of nature (i.e., natural disasters, Lightning, Earthquake, Volcanic Eruption, Avalanche, Storms, and Floods). Data from EM-DAT were last accessed June 20, 2019. Supplemental online research was conducted for events where EM-DAT and VR were not up-to-date.

Partial Discontinuity (CODEm)

For causes modelled in CODEm that have fatal discontinuities hiding in the time trend, a process was established to avoid duplication of fatal discontinuity deaths in CODEm and the fatal discontinuity estimates. First, location-cause-years were identified through outside non-VR sources. If these location-cause-years also had VR death estimates that were greater than the average of the immediate surrounding years, the difference between the identified year and the average of the surrounding years was included in the relevant cause for the fatal discontinuities database. The extracted deaths for all fatal discontinuity causes from VR are then subtracted from the all-cause VR data used in the all-cause mortality estimation process.

Executions and Police Conflict

In GBD 2019, *Executions and Police Conflict is* defined as *"The lawful use or threatened use of force or violence against individual or group of people or property in an attempt to achieve political or socioeconomic objectives for a state."* Data for Executions and Police Conflict mainly came from Amnesty International but other sources such as UCDP, ACLED, and VR that reported deaths due to legal intervention were also cause mapped to executions and police conflict.

Homicide

In GBD 2019, Homicide is defined as *"The use of violence against an individual or group of people in an attempt to achieve nonpolitical, religious or ideological objectives."* Data for Homicide comes from VR, IISS, GED, ACLED and other supplements. Events are mapped to Homicide where the notes found in the raw data indicate gang violence. Deaths from IISS, GED, and ACLED were then split among three homicide sub-types; physical violence by firearms, physical violence by sharp object, and physical violence by other means, based on the rates calculated from VR by country if available, and by region if country VR was unavailable.

Protein-Energy Malnutrition (PEM)

Protein-energy malnutrition is defined as *"A lack of dietary protein and/or energy"* and covers famines as well as severe droughts. The Primary source for PEM, other than VR, is EM-DAT. Supplemental online research was conducted for events where EM-DAT and VR were not up-to-date. The Tombstone report was used to estimate deaths attributed to the Famine during the Great Leap Forward in China in the 1960's.[8]

Other Injury Causes

Other injury causes include other transport injuries (e.g., plane, train, and boat accidents); poisonings; fire, heat, and hot substances; and other exposure to mechanical forces (e.g., building collapse). The primary data source other than VR for these events is EM-DAT. Supplemental online research was conducted for events where EM-DAT and VR were not up-to-date.

Meningococcal meningitis and other diseases

In GBD 2019, fatal discontinuities due to a subset of infectious diseases were estimated, including, meningococcal meningitis (or meningococcal infection), diarrheal disease caused by cholera, Dengue, and Malaria. These infectious diseases were first included on the fatal discontinuity cause list for GBD 2016 because (1) their current modelling strategies with the Cause of Death Ensemble model (CODEm) does not optimally capture the potentially highly variable – or epidemic – mortality levels and trends characteristic of these two causes; and (2) they can contribute to significant total fatalities in a given location-year. Other infectious diseases for which the latter is true – high death rates in the presence of an outbreak or epidemic – are currently modelled with alternative cause of death methods (eg, natural history models for measles and yellow fever), which allow for greater variation year-over-year if or when outbreaks occur.

The Global Infectious Diseases and Epidemiology Network (GIDEON) and EM-DAT served as the primary data sources for collating cholera and meningococcal meningitis or meningococcal infection death reports.[9,10] For any year that cholera or meningococcal meningitis deaths were recorded in a country or territory covered by the GBD, reported deaths were directly extracted from 1950 to 2019. If GIDEON or EMDAT had reporting gaps in cholera or meningococcal meningitis deaths, and the World Health Organization (WHO) reports had coverage for those years, the WHO reports were used. For the Yemen Cholera outbreak in 2016 and 2017, estimates from local collaborators were used in the absence of other data sources.

Location Mapping

Every event in the fatal discontinuities database was mapped to a GBD location using a four step process that includes the following steps in succession: Manual Mapping, String Matching, GPS Overlay, and Geocoding. If an event was manually mapped, the location was assigned without the use of any other

map types. In manual mapping, events are manually assigned to locations by matching the location provided in the raw data to a GBD location. During string matching, an event's location strings are directly compared to the GBD ASCII location names. During GPS Overlay, events that have GPS coordinates provided are overlaid onto a map of GBD locations. If the event is placed over a GBD most-detailed location the event is assigned to that location. During geocoding, the event's location string is entered into Open Street Maps that returns GPS coordinates. These coordinates are processed using GPS Overlay to return GBD locations. This hierarchy provides results where the results of Manual mappings are considered the most reliable, followed successively by string matching, GPS coordinates, and then geocoding.

Side Splitting

Many fatal discontinuities, such as war, have deaths that are reported across multiple locations. In these instances, deaths are split the population from both locations, unless estimates by side are provided. If the resulting locations are at the most detailed level according to GBD no further splitting is needed. If a location is not most detailed the deaths are distributed among the child locations by population.

Prioritization

Choosing between multiple sources for same event (Prioritization)
Where multiple sources reported shock deaths for the same location-year-cause, a cause-specific prioritization scheme was followed that reflected the available detail in the cause-specific datasets. For example, the Georeferenced Event Dataset from UCDP was prioritized above all other non-VR sources because it included detail on how deaths were distributed between multiple actors and locations in each conflict event. In most cases, VR from 4- or 5-star locations was used where available. In some cases, VR from 4- or 5-star locations was not chosen if there were well-known data quality issues or discrepancies in the cause of death data reporting related to a particular event (e.g., supplemental death data for Louisiana was used for Hurricane Katrina because of established data reporting issues).

Age Sex Splitting

All compiled data was ran through the causes of death age-sex splitting process, except for where we had strong supplemental information on the age distribution of specific, large events, such as United States mortality in the Vietnam War and Iranian mortality from the Iran-Iraq conflict in the early 1980s.

Assigning Uncertainty and Generating Draws

Uncertainty analysis

Uncertainty intervals for deaths due to conflict and terrorism were generated using UCDP high and low death estimates, except in the case of Iraq 2003-2016.During this time period deaths due to conflict and terrorism in Iraq were estimated using a combination of supplemental sources. The source found with the lowest number of deaths, Iraq Body Count[2], was used as the lower bound of the uncertainty interval from 2003 to 2016. Estimates from the Iraq Mortality Study by Hagopian et al[3] from 2003 to 2006, the deadliest years of the war, were used to scale deaths to generate the upper uncertainty interval limits using the following formula:

$$deaths_{GBD\ 2017,\ high} = deaths_{IBC} \cdot \overline{\left[\frac{deaths_{IMS}}{deaths_{IBC}}\right]}_{2003-2006}$$

GBD 2019 used the average ratio between IMS and IBC reported deaths between 2003 and 2006, multiplied by the number of deaths reported by the IBC. This high estimate was carried forward through 2017 under the assumption that the Iraq Body Count similarly undercounts the number of deaths due to the ongoing civil war in Iraq. The final, best estimate for conflict and terrorism deaths in Iraq from 2003 to 2016 is the midpoint of the high and low estimates given above.

In cases where low and high estimates were not included in the available data, the regional average uncertainty interval was applied to the available death estimate across all fatal discontinuity causes.

A log-normal distribution was assumed, using mean death rates and standard error based on high and low estimates. In the case that standard error was less than 10e-8, the draws were set equal to the mean rate. 1,000 draws were sampled from this log-normal distribution. These 1,000 draws were then converted back to count space and used for final calculations of means and uncertainty intervals.

Changes from GBD 2017

In GBD 2019, all events were assigned a unique identifier that is derived from the source's internal tracking system. This unique identifier is consistent over time and improved versioning of changes made during cause and location mapping.

In GBD 2017, the location matching process only retained location detail from one phase of location mapping at a time. In GBD 2019, each location mapping phase retains the detail that was provided by the previous phases. For instance, if string matching provides national location information, the following phases will only map subnational locations that correspond with that national location.

In past GBD rounds, if an event spanned multiple years, and no detail on the distribution of deaths across years was provided in the raw data, deaths were split evenly across the time span. In GBD 2019, months are used when distributing deaths over time, to improve accuracy. Year distributions are calculated by taking the months of a year an event occurred over and divided by 12. These weights are

then normalized to sum to one. For example, an event that started in September and lasted until June, the weight for year 1 would be $\frac{4}{12}$ and the year for weight 2 would be $\frac{6}{12}$. The fractions are then multiplied by the inverse of the sum of both fractions so that they sum to 1 and can be used to distribute deaths.

References

1 UCDP/PRIO Armed Conflict Dataset Codebook. Uppsala Conflict Data Program (UCDP); Centre for the Study of Civil Wars, International Peace Research Institute, Oslo (PRIO), 2013.

2 Iraq Body Count. https://www.iraqbodycount.org/database/ (accessed May 8, 2017).

3 Hagopian A, Flaxman AD, Takaro TK, *et al.* Mortality in Iraq Associated with the 2003–2011 War and Occupation: Findings from a National Cluster Sample Survey by the University Collaborative Iraq Mortality Study. *PLOS Medicine* 2013; **10**: e1001533.

4 Jarvis C. The Rise and Fall of Albania's Pyramid Schemes. *F&D* 2000; **37**. http://www.imf.org/external/pubs/ft/fandd/2000/03/jarvis.htm.

5 Obermeyer Z, Murray CJL, Gakidou E. Fifty years of violent war deaths from Vietnam to Bosnia: analysis of data from the world health survey programme. *BMJ* 2008; **336**: 1482–6.

6 Milton L. Rwanda, 1994: International incompetence produces genocide. 1994 https://ezproxy.uwc.edu/login?url=http://search.proquest.com/docview/234405747?accountid=42411.

7 Centre for Research on the Epidemiology of Disasters (CRED). EM-DAT: The OFDA/CRED International Disaster Database. Brussels, Belgium: Catholic University of Leuven

8 Jisheng Y, Friedman E, Guo J, Mosher S. Tombstone: The Great Chinese Famine, 1958-1962. New York: Farrar, Straus and Giroux (Macmillan), 2012.

9 Inc GI, Berger DS. Cholera: Global Status: 2017 edition. GIDEON Informatics Inc, 2017.

10 Inc GI, Berger DS. Bacterial Meningitis: Global Status: 2017 edition. GIDEON Informatics Inc, 2017.

Section 4: Non-fatal outcome estimation[2]

The GBD 2019 non-fatal estimation process describes the steps necessary to estimate incidence, prevalence, and YLDs for disease and injury sequelae in GBD 2019. Conceptually, the estimation effort is divided into eight major components: (1) compiling data sources through data identification and extraction; (2) data adjustment; (3) estimation of prevalence and incidence by cause and sequelae by using DisMod-MR 2.1 or alternative modelling strategies for selected cause groups; (4) estimation by impairment; (5) severity distributions; (6) incorporation of disability weights (DWs); (7) comorbidity adjustment; and (8) the estimation of YLDs by sequelae and causes. Section 4.12 contains additional detail specific to each non-fatal disease, impairment, and injury, and their sequelae. Non-fatal modelling strategies vary significantly between causes.

Section 4.1: Data sources, identification, and extraction[2]

Section 4.1.1: Systematic reviews

For GBD 2019, updated systematic reviews were conducted for 49 causes. Over 123,925 studies were screened for inclusion, and over 1250 articles were newly incorporated into GBD 2019 non-fatal models. For other disease sequelae, only a small fraction of the existing data appears in the published literature, and other sources predominate, such as survey data, disease registers, notification data, or hospital inpatient data. As was done in past rounds of GBD, data were systematically screened from household surveys archived in the GHDx (http://ghdx.healthdata.org/), including Demographic and Health Surveys, Multiple Indicator Cluster Surveys, Living Standards Measurement Surveys, and Reproductive Health Surveys. Other national health surveys were identified on the basis of survey series that had yielded usable data for past rounds of GBD, sources suggested to us by in-country collaborators, and surveys identified in major multinational survey data catalogues such as the International Household Survey Network and the WHO Central Data Catalog, as well as through country Ministry of Health and Central Statistical Office websites. Case notifications reported to the WHO were updated through 2019. Citations for all data sources used for non-fatal estimation in GBD 2019 are provided in searchable form through a web tool (http://ghdx.healthdata.org/). A description of the search terms used for cause-specific systematic reviews are detailed by cause in Section 4.12.

Section 4.1.2: Survey data preparation

For GBD 2019, survey data for which we have access to the unit record data constitute a substantial part of the underlying data used in the estimation process. During extraction, we concentrated on demographic variables (eg, location, sex, age), survey design variables (eg, sampling strategy and sampling weights), and the variables used to define the population estimate (eg, prevalence or a proportion) and a measure of uncertainty (standard error, confidence interval or sample size, and number of cases).

Section 4.1.3: Disease registries

For GBD 2019 non-fatal estimation, disease registries were an important source for a select number of conditions such as cancers, end-stage renal disease, and congenital disorders.

Registry data is particularly key in the estimation of neoplasms when we consider the increasing attention to non-communicable diseases, particularly cancers, in low and middle-income areas of the world. The GHDx source tool (http://ghdx.healthdata.org/data-type/disease-registry) provides a comprehensive list of registry data used in GBD estimation processes.

Section 4.1.4: Estimation of hospital envelope

Figure A. Overview process of estimation of hospital envelope.

This process utilises administrative data, reported tabulations, and survey microdata to estimate the rates of inpatient admissions per capita for every location and demographic group in the GBD hierarchy.

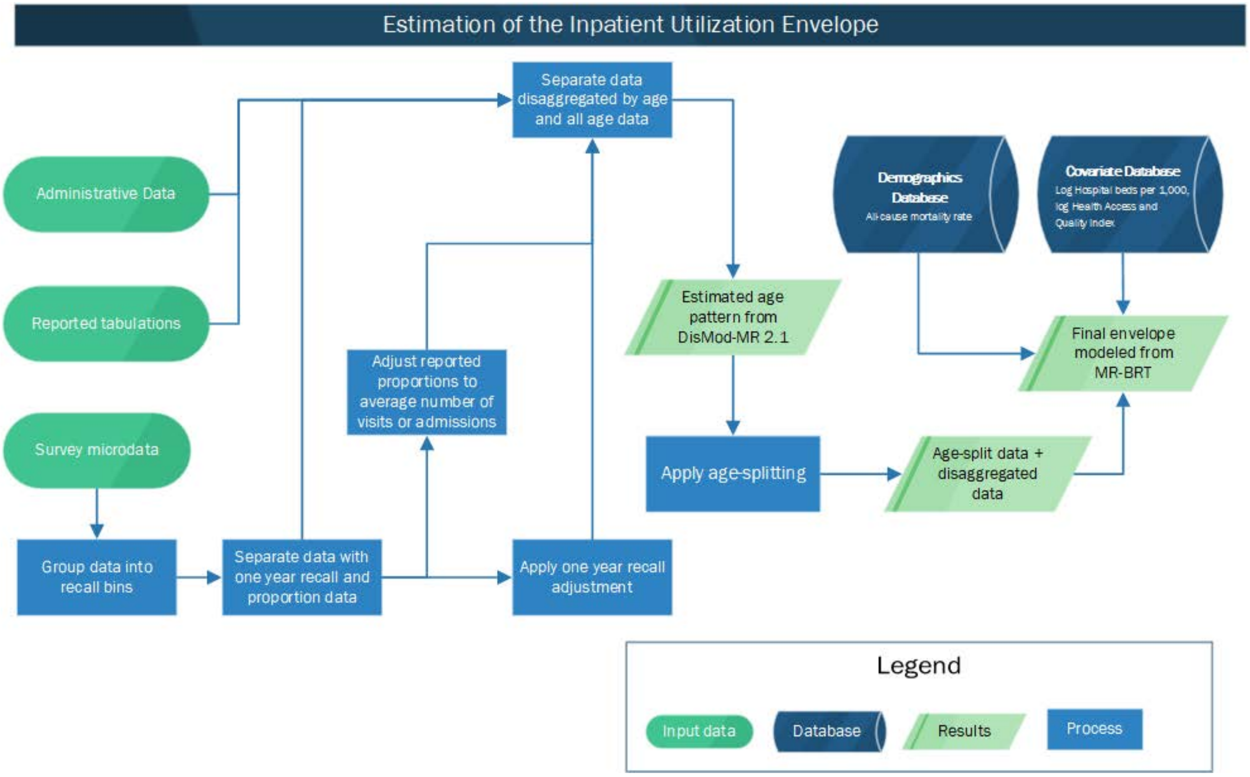

Section 4.2: Input data and methods summary[2]

Section 4.2.1: Case definition

We defined a hospital admission as admission into a formal health care facility for an overnight stay. However, we excluded admissions to long-term care facilities (>120 days), nursing care facilities, and facilities staffed by traditional or spiritual healers.

Section 4.2.2: Input data

We searched the GHDx for population surveys, administrative records, and censuses from January 1990 to September 2017. We applied the following keyword filters: “Health care use” OR “Length of stay” AND “Hospitals” OR “Health care services”. We applied no language restrictions to our search and required all returned records to contain either microdata or tabulated reports. We searched the returned records’ metadata for measures of inpatient care. For inclusion, we required all measures to be

nationally or subnationally representative. Additionally, we consulted with experts and GBD collaborators to gather data sources that were not within the GHDx.

To estimate inpatient admission rates for newborns, we input estimates of the in-facility delivery (IFD) rates for every subnational and national location at 5-year intervals starting at 1990 and including the most recent 2019 estimate. IFD was estimated by using an ST-GPR model based on population-representative surveys and administrative data. We accepted data sources from 28,646 location-years (1413 from administrative records and 27,233 from population surveys).

Section 4.3: Modelling strategy[2]

Section 4.3.1: Data adjustment

We classified each of the accepted data sources into four data types: (1) proportion of survey respondents who were admitted into the hospital in the last 30 days; (2) proportion of survey respondents who were admitted to the hospital in the last year; (3) average number of admissions (utilisation rate) reported by survey respondents in the last year; and (4) average number of visits reported by annual administrative records. We assigned measures reported by annual administrative records as our reference group because these data types were free from recall bias and most closely matched our case definition. From data sources for which microdata were available, we extracted and binned the data based on gender and age groups of less than 1 year, 1–4 years, 4–9 years, 10–14 years, and similar increments of years up to 95 years and older.

We crosswalked each of the three non-reference (survey) data types to the reference (administrative record) data type through the use of penalised spline regressions to account for non-systematic differences between the data types. For each non-reference data type and each sex, we looked for overlap between the non-reference data type and the reference data type based on location, year, age group, and sex. With the overlapping data, we calculated the ratio of the point estimate from the reference data type, μ_{ref}, to the non-reference data type, μ_s. We fit these ratios with a penalised spline regression equation

$$\ln\left(\frac{\mu_{ref,i}}{\mu_{s,i}}\right) = h(age_i) + \varepsilon_i \ (1)$$

Where:

i denotes a given matched observation

$h(age_i)$ represents a basis function that estimated a cross-validated, penalised spline over the population weighted mean age of the age group

ε represents the residual

In the figures that follow, for each non-reference data type, we plot the ratio of μ_{ref} and μ_s across age and by sex and the predictions from the penalised spline regressions.

Figure B. Global age-sex specific crosswalks to equate each non-reference data type to the reference data type.

For each non-reference data type and each sex, we plotted the ratio of reference data points to non-reference data points, which were matched based on location, age group, year, and sex. Using a penalized spline regression, we estimated the crosswalk between each non-reference data type and the reference type. We plotted the crosswalk and the associated prediction error in the following figures:

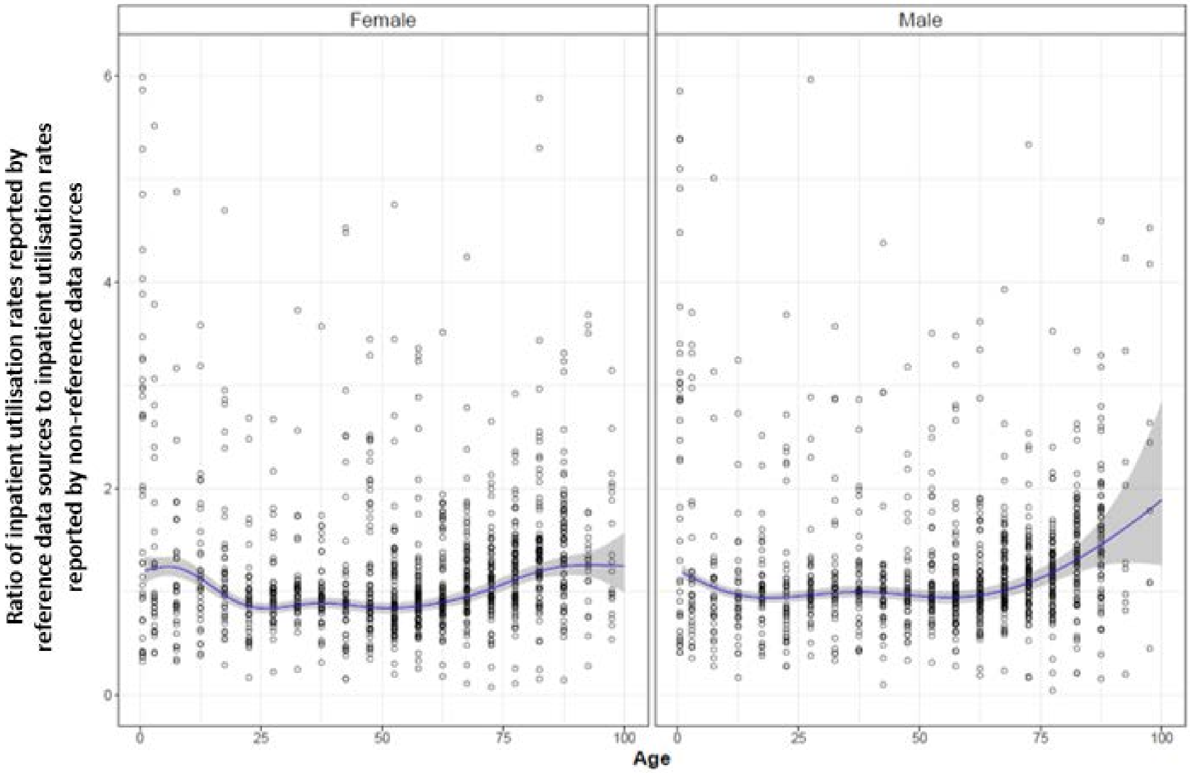

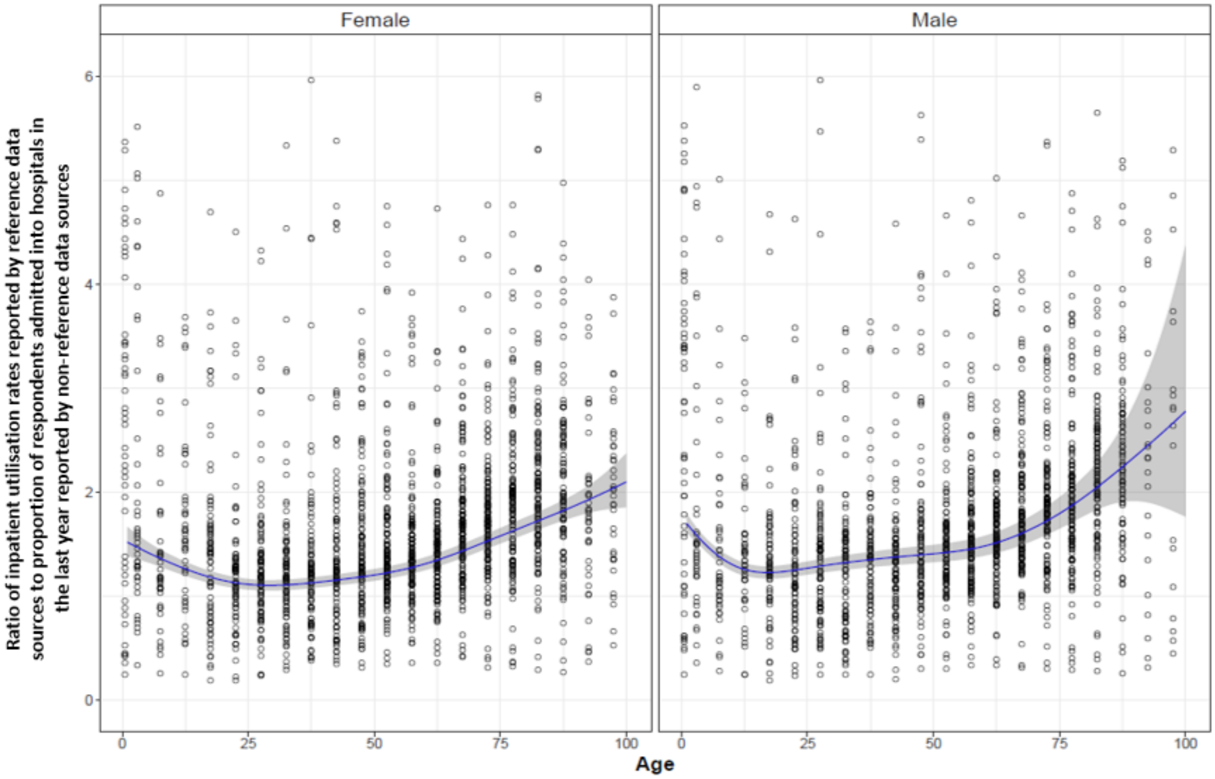
Female
Male
Ratio of inpatient utilisation rates reported by reference data sources to proportion of respondents admitted into hospitals in the last year reported by non-reference data sources
Age

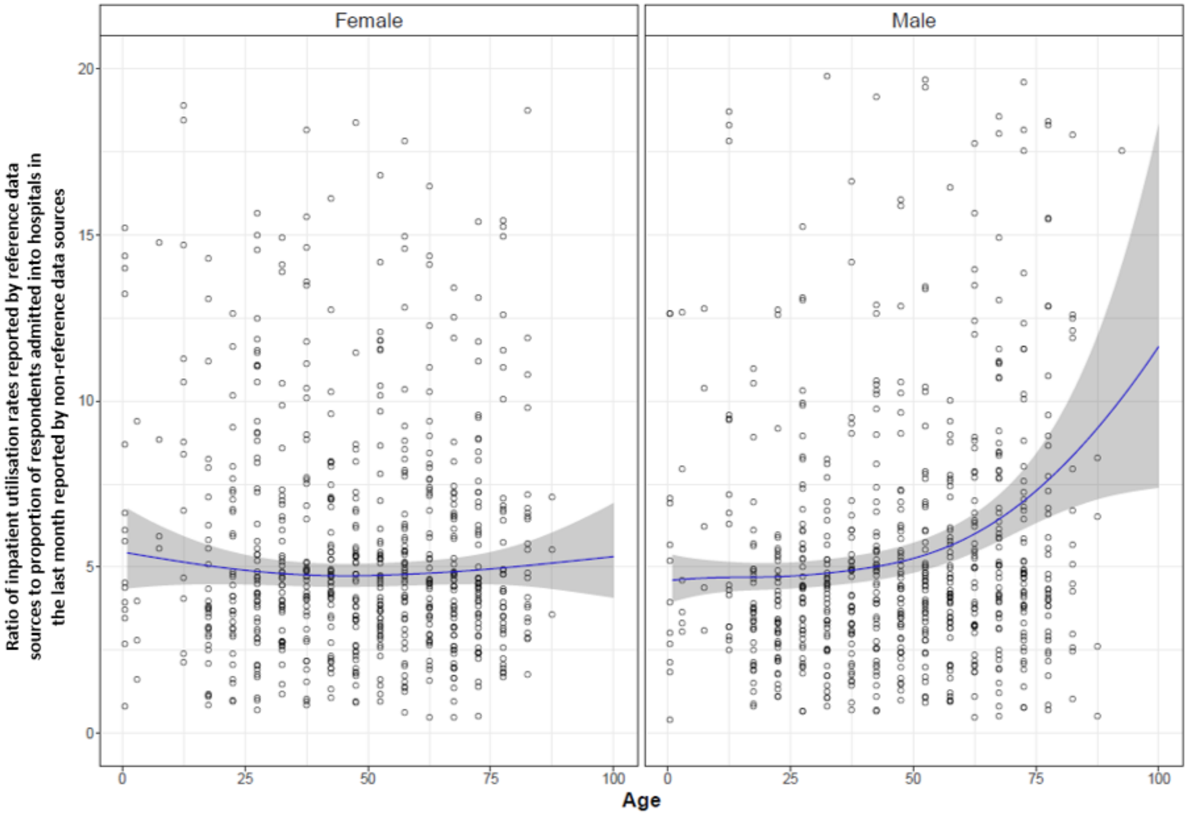
Female
Male
Ratio of inpatient utilisation rates reported by reference data sources to proportion of respondents admitted into hospitals in the last month reported by non-reference data sources
Age

To crosswalk non-reference data types to reference data types, we multiplied non-reference data types by the exponentiated predictions from respective penalised spline regressions. Uncertainty from the adjustments was accounted for by the equation

$$se_a = \sqrt{se_m^2 \cdot se_s^2 + se_m^2 \cdot \mu_s^2 + se_s^2 \cdot \mu_m^2} \ (2)$$

Where:

se_a is the standard error of the adjusted non-reference data point

se_m is the standard error of the exponentiated crosswalk prediction

se_s is the standard error of the non-reference data point

μ_s is the mean of the non-reference data point

μ_m is the exponentiated crosswalk prediction from the penalised spline regression

Section 4.3.2: Age-sex splitting

Before modelling, we ran a Dismod-MR 2.1 model with data disaggregated by age to estimate countries' age-pattern and then applied the estimated age-pattern to split aggregated all-age data into the age groups that are necessary 5-year age groups encouraged by ST-GPR. This procedure was done by calculating a constant, k, which was the ratio of the aggregated all-age data point, $\mu_{all\ age}$, to the all-age estimated utilisation rate from the DisMod-MR 2.1 model, $\widehat{\mu_d}$

$$k = \frac{\mu_{all\ age}}{\widehat{\mu_d}} \ (3)$$

The constant, k, was then multiplied by age-specific utilisation rates from the DisMod-MR 2.1 model. The uncertainty from the data and the age-pattern were propagated by following Equation 2. The split data were then incorporated into the final DisMod-MR 2.1 model.

Section 4.3.3: Spatiotemporal Gaussian process regression (ST-GPR) modelling[4]

The input data were modelled by using ST-GPR to allow for smoothing over age, time, and location in locations that were missing complete datasets.

The flowchart showing the analytic steps can be found elsewhere.[41] The approach is a stochastic modelling technique that is designed to detect signals amidst noisy data. It also serves as a powerful tool for interpolating non-linear trends.[42,43] Unlike classical linear models that assume that the trend underlying data follows a definitive functional form, GPR assumes that the specific trend of interest follows a Gaussian process, which is defined by a mean function $m(\cdot)$ and a covariance function $Cov(\cdot)$. For example, let $p_{c,a,s,t}$ be the prevalence, in normal, log, or logit space, observed in country c, for age group a, and sex s at time t:

$$(p_{c,a,s,t}) = g_{c,a,s}(t) + \epsilon_{c,a,s,t}$$

where

$$\epsilon_{c,a,s,t} \sim Normal(0, \sigma_p^2),$$

$$g_{c,a,s}(t) \sim GP\left(m_{c,a,s}(t), Cov\left(g_{c,a,s}(t)\right)\right).$$

The derivation of the mean and covariance functions, $m_{c,a,s}(t)$ and $Cov\left(g_{c,a,s}(t)\right)$, along with a more detailed description of the error variance (σ_p^2), is described below.

Section 4.3.3.1: Estimating mean functions

We estimated mean functions by using a two-step approach. To be more specific, $m_{c,a,s}(t)$ can be expressed, depending on the prevalence transformation, as:

$$log(p_{c,a,s}(t)) = X_{c,a,s}\beta + h(r_{c,a,s,t})$$

$$logit(p_{c,a,s}(t)) = X_{c,a,s}\beta + h(r_{c,a,s,t})$$

$$p_{c,a,s}(t) = X_{c,a,s}\beta + h(r_{c,a,s,t})$$

where $X\beta$ is the summation of the components of a hierarchical mixed-effects linear regression, including the intercept and the product of covariates with their corresponding fixed-effect coefficients. Some models were run as hierarchical mixed-effects linear regressions with random effects on the levels of the location hierarchy. For most mixed-effects models, random effects were only used in the fit, not in the prediction. The second part of the equation, $h(r_{c,a,s,t})$, is a smoothing function for the residuals, $r_{c,a,s,t}$, derived from the linear model.[44] Cause-specific methods details can be found in appendix sections 3.4 and 4.12.

Although the linear component captures general trends over time, much of the data variability may still not be adequately accounted for. To address this, we fit a locally weighted polynomial regression (locally estimated scatterplot smoothing, or LOESS) function $h(r_{c,a,s,t})$ to systematically estimate this residual variability by borrowing strength across time, age, and space patterns (the spatiotemporal component of ST-GPR).[45,46] The time adjustment parameter, defined by λ , aims to borrow strength from neighboring time points (ie, the prevalence in this year is highly correlated with prevalence in the previous year but less so further back in time). The age-adjustment parameter, defined by ω, borrows strength from data in neighboring age groups. The space-adjustment parameter, defined by ξ, aims to borrow strength across the hierarchy of geographical locations. The spatial and temporal weights are combined into a single space-time weight to allow the amount of spatial weight given to a particular point $r_{c,a,s,t}$ to fluctuate given the data availability at each time *t* and location-level *l* in the location hierarchy.

Let $w_{c,a,s,t}$ be the final weight assigned to observation $r_{c,a,s,t}$ with reference to a focal observation r_{c_0,a_0,s_0,t_0}. We first generated a temporal weight $t.w_{c,a,s,t}$ for smoothing over time, which was based on the scaled distance along the time dimension of the two observations[46]:

$$t.w_{c,a,s,t} = \frac{1}{e^{\lambda|t-t_0|}}$$

Next, we generated a spatial weight to smooth over geography. Specifically, we defined a geospatial relationship by categorizing data based on the GBD location hierarchy (table S3). *zeta* acts as a scalar on a given datapoint given its proximity to the target location:

$$t.w_{c,a,s,t} = \zeta^{|c-c_0|}$$

For example, estimating a country, would use the following weighting scheme:

- Country data: ζ^0 = 1
- Regional data not from the country being estimated: ζ^1
- Data from other regions in the same super region: ξ^2
- Global data from other super regions: ζ^3

Under the spatial weighting specification, typical values of ζ range from [0.001, 0.2], where ζ can be interpreted as the amount to downweight regional datapoints compared to country datapoints for a given estimating country. For example, for a given datapoint $r_{c,a,s,t}$ and ζ = 0.01, a datapoint not within country *c* but within the same region *r* as $r_{c,a,s,t}$ would be assigned $\frac{1}{100}$ the weight of a datapoint within the country.

The spatial and temporal weights were then multiplied and summed across each level of the location hierarchy and normalised for each time period *t* . This procedure allowed the space-time weight to implicitly take into account the amount of data available at the country vs. region vs. super-region level and attribute spatial weight accordingly.

Given a normalisation constant,

$$K_i = \sum_{c \epsilon C} s.w_{c,t} * t.w_{c.t} + \sum_{c \epsilon R} s.w_{c,t} * t.w_{c.t} + \sum_{c \epsilon SR} s.w_{c,t} * t.w_{c.t}$$

the final space-time weight would then equal

$$w'_{c,a,s,t} = \frac{s.w_{c,t} * t.w_{c,t}}{K_i}$$

Finally, we calculated the weight $w''_{c,a,s,t}$ to smooth over age, which is based on a distance along the age dimension of two observations. For a point between the age a of the observation $r_{c,a,s,t}$ and a focal observation r_{c_0,a_0,s_0,t_0}, the weight is defined as follows:

$$w''_{c,a,s,t} = \frac{1}{e^{\omega|a-a_0|}}$$

The final weights were then computed by simply multiplying the space-time weights and age weights and normalising so all weights for a given time period *t* sum to 1. A full derivation of weights for each category, assuming the location being estimated was a country, follows:

1) If the observation $r_{c,t}$ belongs to the same country c_0 of the focal observation r_{c_0,t_0}:

$$w_{c,a,s,t} = \frac{(w'_{c,a,s,t}w''_{c,a,s,t})}{\sum_{c=c_0}(w'_{c,a,s,t}w''_{c,a,s,t})} \qquad \forall c = c_0$$

2) If the observation $r_{c,t}$ belongs to a different country than the focal observation r_{c_0,t_0}, but both belong to the same region *R*:

$$w_{c,a,s,t} = \frac{(w'_{c,a,s,t}w''_{c,a,s,t})}{\sum_{c\neq c_0}(w'_{c,a,s,t}w''_{c,a,s,t})} \qquad \forall c \neq c_0 \cap R[c] = R[c_0]$$

3) If the observation $r_{c,t}$ belongs to the same super region *SR* but to both a different country c_0 and a different region $R[c_0]$ than the focal observation r_{c_0,t_0}:

$$w_{c,a,s,t} = \frac{(w'_{c,a,s,t}w''_{c,a,s,t})}{\sum_{c\neq c_0}(w'_{c,a,s,t}w''_{c,a,s,t})} \qquad \forall c \neq c_0 \cap R[c] \neq R[c_0] \cap SR[c] = SR[c_0]$$

4) If the observation $r_{c,t}$ is from a different super region than the focal observation r_{c_0,t_0}(Ie, all other data currently not receiving a weight):

$$w_{c,a,s,t} = \frac{(w'_{c,a,s,t}w''_{c,a,s,t})}{\sum_{c\neq c_0}(w'_{c,a,s,t}w''_{c,a,s,t})} \qquad \forall c \neq c_0 \cap R[c] \neq R[c_0] \cap SR[c] \neq SR[c_0]$$

Observations could be downweighted by a factor of 0.1, usually because they were not geographically representative at the unit of estimation. Details of reasons for downweighting can be found in cause-specific modeling summaries. The final weights were then normalised such that the sum of weights across age, time, and geographic hierarchy for a reference group was 1.

Section 4.3.3.2: Estimating error variance

σ_p^2 represents the error variance in normal or transformed space including the sampling variance of the estimates and prediction error from any crosswalks performed. First, variance was systematically imputed if the data extraction did not include any measure of uncertainty. When some sample sizes for data were available, missing sample sizes were imputed as the 5th percentile of available sample sizes. Missing variances were then calculated as $\sigma_p^2 = \frac{p*(1-p)}{n}$ for proportions or were predicted from the mean by using a regression for continuous values. When sample sizes were entirely missing and could not be imputed, the 95th percentile of available variances at the most granular geographic level (ie, first country, then region, etc.) were used to impute missing variances. For proportions where *p*n* or *(1-p)*n* is <20, variance was replaced by using the Wilson Interval Score method.

Next, if prevalence was modelled as a log transformation, the error variance was transformed into log-space by using the delta method approximation as follows:

$$\sigma_p^2 \cong \frac{\sigma_{p'}^2}{p_{c,a,s,t}^2}$$

where $\sigma^2_{p'}$ represents the error variance in normal space. If prevalence was modelled as a logit transformation, the error variance was transformed into logit-space by using the delta method approximation as follows:

$$\sigma_p^2 \cong \frac{\sigma^2_{p'}}{(p_{c,a,s,t} * (1 - p_{c,a,s,t}))^2}$$

Finally, prior to GPR, an approximation of non-sampling variance was added to the error variance. Calculations of non-sampling variance were done on normal-space variances. Non-sampling variance was calculated as the variance of inverse-variance weighted residuals from the space-time estimate at a given location-level hierarchy. If there were <10 data points at a given level of the location hierarchy, the non-sampling variance was replaced with that of the next highest geography level with >10 data points.

Section 4.3.3.3: Estimating the covariance function

The final input into GPR is the covariance function, which defines the shape and distribution of the trends. Here, we have chosen the Matern-Euclidian covariance function, which offers the flexibility to model a wide spectrum of trends with varying degrees of smoothness. The function is defined as follows:

$$M(t, t') = \sigma^2 \frac{2^{1-v}}{\Gamma(v)} \left(\frac{d(t, t')\sqrt{2v}}{l}\right)^v K_v \left(\frac{d(t, t')\sqrt{2v}}{l}\right)$$

where $d(\cdot)$is a distance function; σ^2, v, l, and K_v are hyperparameters of the covariance function—specifically σ^2 is the marginal variance, v is the smoothness parameter that defines the differentiability of the function, l is the length scale, which roughly defines the distance between which two points become uncorrelated, and K_v is the Bessel function. We approximated σ^2 by taking the normalised median absolute deviation $MADN(r'_c)$ of the difference, which is the normalised absolute deviation of the difference of the first-stage linear regression estimate from the second-stage spatiotemporal smoothing step for each country. We then took the mean of these country-level MADN estimates for all countries with 10+ country-years of data to ensure that differences between first- and second-stage estimates had sufficient data to truly convey meaningful information on model uncertainty. We used the parameter specification $v = 2$ for all models. The scale parameter l used for each cause is reported in appendix sections 3.4 and 4.12.

Section 4.3.3.4: Prediction using GPR

We integrated over $g_{c,t}(t_*)$ to predict a full time series for country c, age *a*, sex *s*, and prediction time t_*as follows:

$$p_{c,a,s}(t_*) \sim N\left(m_{c,a,s,t}(t_*), \sigma_p^2 I + Cov\left(g_{c,a,s,t}(t_*)\right)\right)$$

Random draws of 1000 samples were obtained from the distributions above for every country for a given indicator. The final estimated mean for each country was the mean of the draws. In addition, 95% UIs were calculated by taking the 2.5 and 97.5 percentile of the sample distribution. The linear modelling process was implemented by using the lmer4 package in R, and the ST-GPR analysis was implemented through the PyMC2 package in Python.

Section 4.3.3.5: Subnational scaling and aggregation

To ensure internal consistency of the estimates between countries and their respective subnational locations, national estimates were either created by population-weighted aggregation or subnational estimates were adjusted by population-weighted scaling to the national estimates, depending on the data coverage of a given country compared to that of its subnational locations. For example, if data coverage was better at the national level than at its corresponding subnational locations for a given country and cause across age, sex, and time, estimates were rescaled to be consistent with the national level. Conversely, if data coverage was better at the subnational level, estimates for its parent country were generated through population-weighted aggregation of subnational estimates.

Estimates can also be scaled within logit space. Scaling in logit space ensures that subnational estimates of proportion models do not exceed one after being rescaled to the national estimate.

Section 4.3.3.6: Example: ST-GPR hospital bed estimation

To further help explain variation in geographies with little to no data, we used the covariates of the natural log of hospital beds per 1000 and the HAQ Index for every location. Hospital beds per 1000 was estimated by using ST-GPR on data sourced from the World Bank. Coefficients for the covariates are presented in the table that follows.

Table B. Estimated coefficients of the hospital envelope model.

**Covariate**	**Sex**	**Coefficient (95% UI)**	**Exponentiated Coefficient**
Log hospital beds per 1000	Male	0.41<br>(0.36 to 0.45)	1.50<br>(1.44 to 1.57)
	Female	0.41<br>(0.37 to 0.45)	1.50<br>(1.45 to 1.56)
HAQ Index	Male	0.029<br>(0.027 to 0.030)	1.029<br>(1.027 to 1.030)
	Female	0.028<br>(0.026 to 0.029)	1.028<br>(1.027 to 1.029)
All-cause mortality	Male	2.14<br>(2.11 to 2.17)	8.49<br>(8.25 to 8.73)
	Female	2.33<br>(2.30 to 2.36)	10.24<br>(9.93 to 10.55)

Section 4.3.4: Claims, inpatient hospital, and outpatient data

Claims, inpatient hospital, and outpatient data played a key role in the process of estimating many non-fatal causes in GBD 2019. All sources of administrative clinical data were aggregated and processed together for all causes of disease that included this type of data in their estimates. Data sources were heterogeneous in granularity, comprehensiveness, and level of detail, and the methods described below were used to transform data to be comparable and complete across locations, ages, sexes, and years, and causes.

Section 4.3.4.1 Claims data

For GBD 2019, we accessed aggregate data derived from the Truven database of USA private health insurance and subset of public insurance schemes of Medicaid and Medicare for the years 2000, 2010-2016. The population covered in each year was 3.3 million in 2000, 40.4 million in 2010, 44.4 million in 2011, 40.8 million in 2012, 42.2 million in 2013, 36.4 million in 2014, 22.6 million in 2015, and 22.4 million in 2016. For each of these individuals, information on every health service encounter was collected and all episodes of care were linked to individuals by unique identifiers. Outpatient claims could have up to four diagnoses while inpatient claims had up to 15 diagnoses. Data from Taiwan (province of China), the Philippines, Poland, Russia, and Singapore were also incorporated as claims data. We mapped ICD diagnoses in each source to GBD causes. GBD conditions were extracted as "prevalence" or "incidence" depending on cause duration and based on the specification of the research team responsible for the cause. In a given year, for each individual in the claims data, a prevalent case was defined as any mention in any diagnostic field associated with any claim, including inpatient and outpatient encounters. To reduce noise from spurious coding practices, an additional requirement is placed on prevalence in outpatient claims whereby a minimum of two claims must be filed in a calendar year to count as a prevalent case. An incident case was defined the same way but assumed that claims within a condition-specific duration were the same case. In this way, an individual could have multiple incident cases in a given year, but double-counting of cases with multiple claims from a single illness episode was avoided.

Figure C. GBD 2019 Claims Data Processing

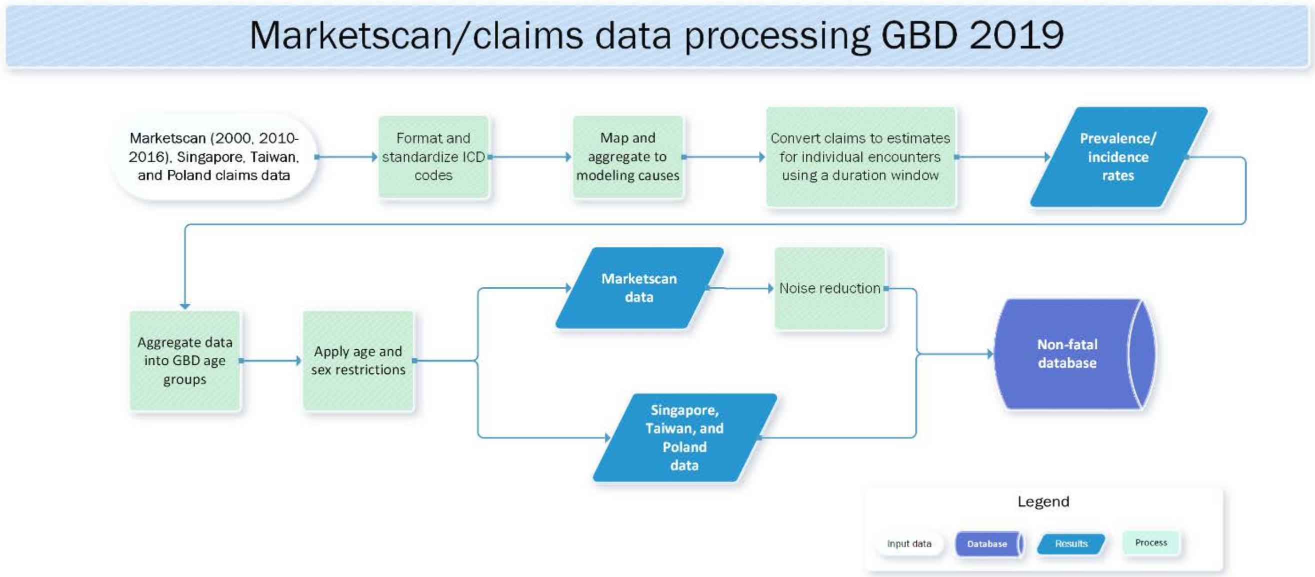

Section 4.3.4.2 Inpatient hospital admissions

Inpatient hospital data were extracted from 4401 location-years in 45 countries. ICD coding was standardised across sources and versions of ICD. Counts of admissions with a primary diagnosis of each cause were extracted from all sources and modelled through the inpatient hospital process. Secondary diagnostic detail was included in estimation through corrections as described below. A case of any cause of disease was defined as an overnight inpatient admission with a primary diagnosis of that cause.

For GBD 2015, our use of hospital data in non-fatal disease estimation was limited by the challenge of accessing accurate information on coverage populations for any given data source. Section 4.1.4 of the appendix describes the modelling strategy that was developed for the hospital utilisation envelope, an estimate of admission per capita in each location. In GBD 2016, we used the hospital utilisation envelope in place of information on coverage population. We calculated age-specific and sex-specific cause fractions in each inpatient hospital data source and multiplied these fractions by the hospital utilisation envelope to produce incidence or prevalence rates. In GBD 2017, we used the same approach except the hospital envelope was measured in ST-GPR to accommodate admissions data reflecting newborns being delivered in facilities. In GBD 2019, we updated the modelling framework to the hospital utilisation envelope, adding all-cause mortality as a covariate and improving the space-time smoothing to more accurately fit locations with and without data.

We performed three adjustments on inpatient hospital data to synthesise all inpatient sources to the same definition of care and to account for cases that were not captured in some inpatient sources depending on data availability. Data were first adjusted to account for multiple admissions for a single case of disease. It was then adjusted to account for cases of any cause that were non-primary reasons for admission. Finally, admissions were scaled by the ratio of outpatient cases observed for any inpatient case of disease to account for additional cases that did not warrant an inpatient admission. Combined with the uncorrected version (with no scalar applied), this process resulted in four stages of incidence and prevalence estimates from inpatient hospital data: (1) (un-corrected) inpatient admissions by episode, primary diagnosis; (2) inpatient admissions by individual, primary diagnosis only; (3) inpatient hospital admissions, accounting for all diagnoses; and (4) an estimate of inpatient admissions and outpatient visits by individual, accounting for all diagnoses. Estimate 4 was applied to all causes except those where outpatient care or non-primary diagnosis was not expected based on the nature of the disease. Adjustment ratios were calculated using all clinical inpatient sources that had patient-level data and primary and non-primary diagnoses. Sources of this data include Marketscan and Taiwan (province of China) claims data as described above; claims and inpatient data from Singapore, the Philippines, Ecuador, and New Zealand; and the HCUP SID database spanning years 2003–2008. Only Marketscan and Taiwan (province of China) claims data included a link between inpatient and outpatient care to be used in the fourth estimate described. Ratios from these sources were modelled over age and sex using a mixed-effects model in MR-BRT for each cause. If data for any ratio did not exist for the youngest or oldest age groups, we assumed a uniform tail on the model from the nearest age group with data. All models were conducted in log-space in order to bound the model to be greater than one for any age, sex, and cause. We used the following equations for each of the three scalars:

1) Correction to account for multiple admissions, which gives us inpatient admissions by individual, primary diagnosis only
    a. $inpatient_{admin}^{1^{\circ}} * \left(\frac{inpatient_{indiv}^{1^{\circ}}}{inpatient_{admin}^{1^{\circ}}}\right) = inpatient_{indiv}^{1^{\circ}}$
2) Correction to adjust for non-primary diagnoses, which gives us inpatient admissions by individual, all diagnoses
    a. $inpatient_{admin}^{1^{\circ}} * \left(\frac{inpatient_{indiv}^{all}}{inpatient_{admin}^{1^{\circ}}}\right) = inpatient_{indiv}^{all}$

3) Correction to account for inpatient and outpatient care, which gives us inpatient admissions and outpatient visits by individual for all diagnoses
    a. $inpatient_{admission}^{1°} * \left(\frac{inpatient_{indiv}^{all} \cup outpatient_{indiv}^{all}}{inpatient_{admissions}^{1°}}\right) = inpatient|outpatient_{indiv}^{all}$

Determination of maternal causes used separate cause-fractions and a different scalar calculated from a maternal hospital admissions rate instead of the hospital envelope, and the equation

$$\left(\frac{events}{\#\ of\ total\ hospital\ visits}\right) * \left(\frac{hospital\ visits}{live\ births}\right) * \left(\frac{births}{population}\right)$$

Determination of injuries used a separate correction factor from those described above which adjusted data that was only E-coded by data that contained E-codes and N-codes (nature of injury codes) with the following equation

$$\frac{1}{\frac{E\text{-}code\ primary\ dx}{E\text{-}code\ any\ dx + N\text{-}code\ any\ dx}}$$

A final adjustment was applied to each of the above estimates. The HAQ Index was used to account for differences in access and quality of health care across time and space. The HAQ Index adjustment was applied by dividing the above estimates by a scalar ranging from 0 to 100, where 0 represents the first percentile of observed access and quality and 100 the 99th percentile.

Figure D. GBD 2019 Inpatient Hospital Data Processing

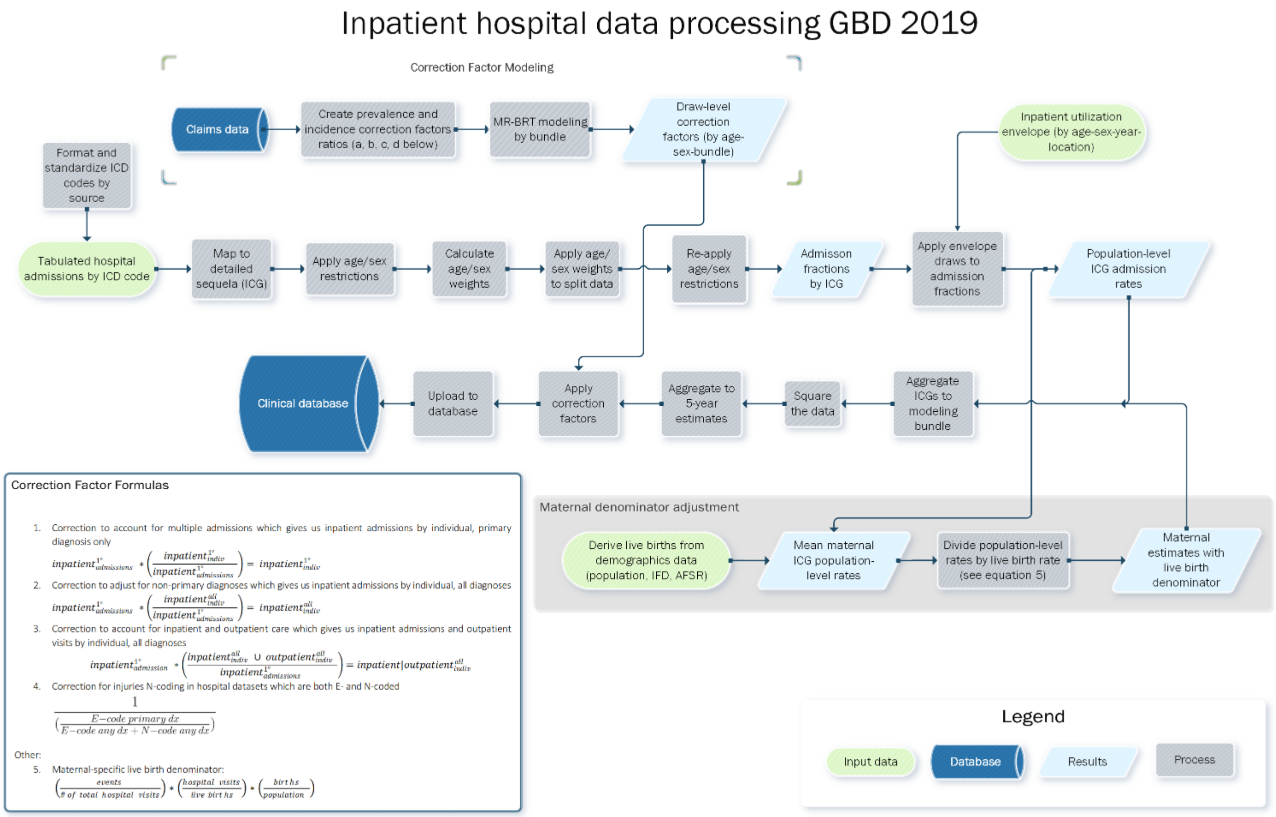

Section 4.3.4.3 Outpatient encounter data

Outpatient encounter data were available from the USA and Sweden for 109 location-years. No changes were made in the processing of outpatient data from GBD 2017, except for updates to the ICD mappings to GBD cause.

As with the inpatient hospital data, a scalar was calculated by using Marketscan claims data to adjust for multiple visits per individual within one year (for prevalent conditions) and within a cause-specific duration (for incident causes).

Figure E. GBD 2019 Outpatient data extraction process

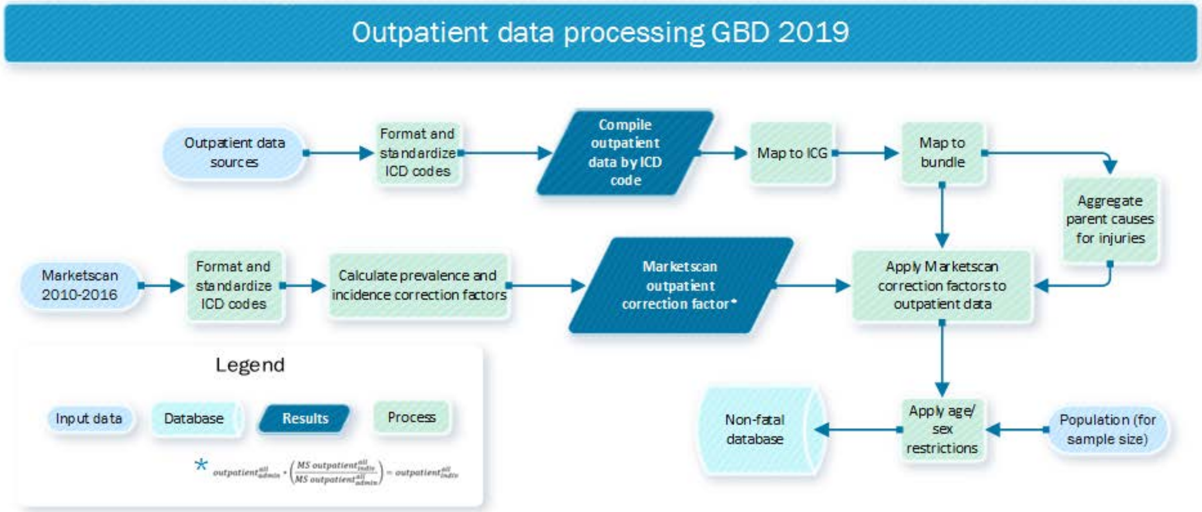

Section 4.3.5: Case notifications

Case notifications, active screening, intervention coverage studies, and surveillance contributed to estimates of infectious diseases. If data were available, we extracted it from survey and administrative microdata; otherwise, data were extracted from published literature and reports. For many infectious diseases and neglected tropical diseases (NTDs), we used of cases for which notification was made by countries to the WHO and other global monitoring entities. The causes for which we used WHO case notification data included tuberculosis, measles, yellow fever, rabies, dengue, cholera, whooping cough, human African trypanosomiasis (HAT), meningitis, all sexually transmitted infections, and other infectious diseases and NTDs, such as Ebola.

Section 4.4: Data adjustment

Section 4.4.1: MR-BRT and Fitting Procedures

This section details the statistical models underlying MR-BRT, and fitting procedure used to obtain estimates. Further details on models and algorithms can be found in the technical report.[47]

The MR-BRT program is a set of wrappers customized for global health problems that use the open source mixed effects package `LimeTr` (https://github.com/zhengp0/limetr). We describe the basic functionality in the sections below.

Section 4.4.1.1 Mixed-Effects Model

We consider the following nonlinear mixed effects model:

$$\boldsymbol{y}_i = \mathbf{F}_i(\beta) + \mathbf{Z}_i \boldsymbol{u}_i + \boldsymbol{\epsilon}_i$$

$$\boldsymbol{u}_i \sim N(\mathbf{0}, \boldsymbol{\Gamma}),\ \boldsymbol{\Gamma} = \mathrm{diag}(\gamma), \qquad \boldsymbol{\epsilon}_i \sim N(\mathbf{0}, \boldsymbol{\Lambda}), \tag{1}$$

where $y_i \in \mathbb{R}^{n_i}$ is the vector of observations from the ith study, $\boldsymbol{\epsilon}_i \in \mathbb{R}^{n_i}$ are measurement errors with given covariance $\boldsymbol{\Lambda}$, $\boldsymbol{u}_i \in \mathbb{R}^{k_\gamma}$ are independent random effects, and $\mathbf{Z}_i \in \mathbb{R}^{n_i \times k_\gamma}$is a linear map, and β are regression coefficients. The models F_i may be nonlinear.

To fit (β, γ) we solve the marginal likelihood problem:

$$\min_{\beta,\gamma}\ f(\beta,\gamma) \coloneqq \sum_{i=1}^{m} \frac{1}{2}\left(y_i - \mathbf{F}_i(\beta)\right)^\top \left(\mathbf{Z}_i \boldsymbol{\Gamma} \mathbf{Z}_\mathrm{i}^\top + \boldsymbol{\Lambda}_{\boldsymbol{i}}\right)^{-1} \left(\boldsymbol{y}_i - \mathbf{F}_i(\beta)\right) + \frac{1}{2} ln\left|\mathbf{Z}_i \boldsymbol{\Gamma} \mathbf{Z}_\mathrm{i}^\top + \boldsymbol{\Lambda}_i\right|. \tag{2}$$

When the model is linear, we can write:

$$\mathbf{F}_i(\beta) = \boldsymbol{X}\beta. \tag{3}$$

Linear models are very common in cross-walks, and for network analysis, which is detailed below.

Section 4.4.1.2. Network Analysis

Network analysis is a special case of the linear model (3) that is used to compare multiple treatment effects. To explain the coding we use a running example with four treatments A, B, C, D.

For simplicity assume A is this reference treatment. We then have the following coding.

$$AB \to B - A: \quad [1 \quad 0 \quad 0]$$

$$AC \to C - A: \quad [0 \quad 1 \quad 0]$$

$$AD \to D - A: \quad [0 \quad 0 \quad 1].$$

We see from this simple example that the design matrix under the basic network assumption is always full rank, since a subset of rows forms the identity matrix.

Comparisons that do not include the reference can be computed. For example,

$$BC \to C - B = (C - A) - (B - A)$$

$$= [0 \quad 1 \quad 0] - [1 \quad 0 \quad 0]$$

$$= [-1 \quad 1 \quad 0]$$

Using this simple algebra, we quickly obtain the remaining codings.

$$BC \to C - B: \quad [-1 \quad 1 \quad 0]$$

$$BD \to D - B: \quad [-1 \quad 0 \quad 1]$$

$$CD \rightarrow D - C: \quad [0 \quad -1 \quad 1]$$

Each row of the design matrix $\boldsymbol{X}$ is coded according to the comparison.

When doing network analysis, the design matrix $\boldsymbol{X}$ does not include the intercept term (**1** column).

Section 4.4.1.3. Constraints and Priors

The ML estimate (2) can be extended to incorporate nonlinear inequality constraints

$$\mathbf{C}(\boldsymbol{\theta}) \leq c,$$

where $\boldsymbol{\theta} = (\beta, \gamma)$. Constraints play a key role for polynomial splines.

It is also essential to allow priors on parameters of interest. We assume that priors are given by a functional form

$$\boldsymbol{\theta} \sim \exp(-\rho(\boldsymbol{\theta}))$$

The likelihood problem is then augmented by adding the term $\rho(\boldsymbol{\theta})$ to the ML objective. The function ρ may be nonlinear and nonconvex, but we assume it is smooth.

Section 4.4.1.4. Trimming outliers

Least trimmed squares (LTS) is a robust estimator[48,49] for the standard regression problem. Given the problem

$$\min_{\beta} \sum_{i=1}^{n} \frac{1}{2}\,(y_i - \langle \boldsymbol{X}_i, \beta \rangle)^2, \tag{4}$$

the LTS estimator minimizes the sum of *smallest h* residuals rather than all residuals. These estimators were initially introduced to develop linear regression estimators that have a high breakdown point (in this case 50%) and good statistical efficiency (in this case $n^{-1/2}$). Breakdown refers to the percentage of outlying points which can be added to a dataset before the resulting M-estimator can change in an unbounded way. Here, outliers can affect both the outcomes and training data (features).

LTS estimators are robust against outliers, and arbitrarily large deviations that are trimmed do not affect the final $\hat{\beta}$.

Rather than writing the objective in terms of order statistics, it is far simpler to extend the likelihood using an auxiliary variable $\boldsymbol{W}$:

$$\min_{\beta, \mathbf{W}} \sum_{i=1}^{n} w_i \left(\frac{1}{2}\,(y_i - \langle \mathbf{X}_i, \beta \rangle)\right)^2 \quad \text{s.t.} \quad \mathbf{1}^\top \boldsymbol{W} = h, \quad \mathbf{0} \leq \boldsymbol{W} \leq \mathbf{1}. \tag{5}$$

The set

$$\Delta_h \coloneqq \{\mathbf{W} : \mathbf{1}^\top \mathbf{W} = h, \quad \mathbf{0} \leq \mathbf{W} \leq \mathbf{1}\} \tag{6}$$

is known as the *capped simplex*, since it is the intersection of the h-simplex with the unit box.[48] For a fixed β, the optimal solution of (5) with respect to $\mathbf{W}$ assigns weight 1 to each of the smallest h residuals, and 0 to the rest. Problem (5) is solved *jointly* in $(\beta, \mathbf{W})$, simultaneously finding the regression

estimate and classifying the observations into inliers and outliers. This joint strategy makes LTS different from post-hoc analysis, where a model is fit first with all data, and then outliers are detected using that estimate.

To explain how trimming enters the marginal likelihood problem, we focus on a single group term from the ML likelihood (2):

$$\left(\frac{1}{2}(\boldsymbol{y}_i - \mathbf{F}_{i(\beta)})^\top (\mathbf{Z}_i\boldsymbol{\Gamma}^{-1}\mathbf{Z}_i^\top + \boldsymbol{\Lambda}_i)^{-1}(\boldsymbol{y}_i - \mathbf{F}_i(\beta)) + \frac{1}{2}\ln|\mathbf{Z}_i\boldsymbol{\Gamma}^{-1}\mathbf{Z}_i^\top + \boldsymbol{\Lambda}_i|\right)$$

We introduce auxiliary variables $\mathbf{W}_i \in \mathbb{R}^{n_i}$, and define

$$\boldsymbol{r}_i := y_i - \mathbf{F}_i(\beta), \quad \mathbf{W}_i := \mathrm{diag}(\mathbf{W}_i), \quad \sqrt{\mathbf{W}_i} := \mathrm{diag}(\sqrt{\mathbf{W}_i}).$$

We now form the objective

$$\tfrac{1}{2}\boldsymbol{r}_I^\top \sqrt{\mathbf{W}_i}\left(\sqrt{\mathbf{W}_i}\mathbf{Z}_i\boldsymbol{\Gamma}^{-1}\mathbf{Z}_i^\top\sqrt{\mathbf{W}_i} + \boldsymbol{\Lambda}_i^{\odot\mathbf{W}_i}\right)^{-1}\sqrt{\mathbf{W}_i}\,\boldsymbol{r}_i + \tfrac{1}{2}\ln\left|\sqrt{\mathbf{W}_i}\,\mathbf{Z}_i\boldsymbol{\Gamma}^{-1}\mathbf{Z}_i^\top\sqrt{\mathbf{W}_i} + \boldsymbol{\Lambda}_i^{\odot\mathbf{W}_i}\right|, \quad (7)$$

where $\odot$ denotes the elementwise power operation:

$$\boldsymbol{\Lambda}_i^{\odot\mathbf{W}_i} := \begin{bmatrix} (\lambda_{1j})^{w_{i1}} & 0 & \cdots & 0 \\ 0 & \ddots & \ddots & \vdots \\ 0 & \cdots & 0 & (\lambda_{in_i})^{w_{in_i}} \end{bmatrix} \quad (8)$$

When $w_{ij} = 1$, we recover the contribution of the ijth observation to the original likelihood. As $w_{ij} \downarrow 0$, the ijth contribution to the residual is correctly eliminated by $\sqrt{w_{ij}} \downarrow 0$. The jth row and column of $\sqrt{\mathbf{W}_i}\mathbf{Z}_i\boldsymbol{\Gamma}^{-1}\mathbf{Z}_i^\top\sqrt{\mathbf{W}_i}$ both go to 0, while the jth entry of $\boldsymbol{\Lambda}_i^{\odot\mathbf{W}_i}$ goes to 1, which effectively removes all impact of the jth point on the covariance matrix.

For full details and analysis, please see the technical report.[47]

Section 4.4.1.5. Final Estimator

Putting together the trimmed ML with priors and constraints, we arrive at the following estimator.

$$\begin{aligned} \min_{\beta,\gamma,\mathbf{W}} f(\beta,\gamma,\mathbf{W}) := \sum_{i=1}^{m} \frac{1}{2} r_i^\top \sqrt{\mathbf{W}_i}\left(\sqrt{\mathbf{W}_i}\,\mathbf{Z}_i\boldsymbol{\Gamma}^{-1}\mathbf{Z}_i^\top\sqrt{\mathbf{W}_i} + \boldsymbol{\Lambda}_i^{\odot\mathbf{W}_i}\right)^{-1} \\ \sqrt{\mathbf{W}_i}\, r_i + \tfrac{1}{2}\ln\left|\sqrt{\mathbf{W}_i}\,\mathbf{Z}_i\boldsymbol{\Gamma}^{-1}\mathbf{Z}_i^\top\sqrt{\mathbf{W}_i} + \boldsymbol{\Lambda}_i^{\odot\mathbf{W}_i}\right| + \rho(\beta,\gamma,\boldsymbol{\Lambda}) \quad (9) \\ \text{s.t.}\ \ \boldsymbol{r}_i = \boldsymbol{y}_i - \mathbf{F}_i(\beta),\quad \mathbf{1}^\top\mathbf{W} = h,\quad 0 \le \mathbf{W} \le 1,\quad \boldsymbol{C}\begin{pmatrix}\beta\\\gamma\end{pmatrix} \le c\,. \end{aligned}$$

The fit is obtained using iterative optimization techniques. Problem (9) is nonlinear and non-smooth, and the optimization is implemented in the `LimeTR` package[3] (https://github.com/zhengp0), and relies on the IPopt interior point method.[50]

Section 4.4.1.6. Nonlinear Dose-Response Curves with Constrained Splines

In this section we discuss spline models for dose-response relationships. General background on splines and spline regression are available elsewhere.[51,52]

Section 4.4.1.6.1. B-splines and bases

A spline basis is a set of piecewise polynomial functions with designated degree and domain. If we denote polynomial order by p, and the number of knots by k, we need $p + k$ basis elements s_j^p, which can be generated recursively as illustrated in Figure A.

Figure A. Recursive generation of b-spline basis elements (orders 0, 1, 2)

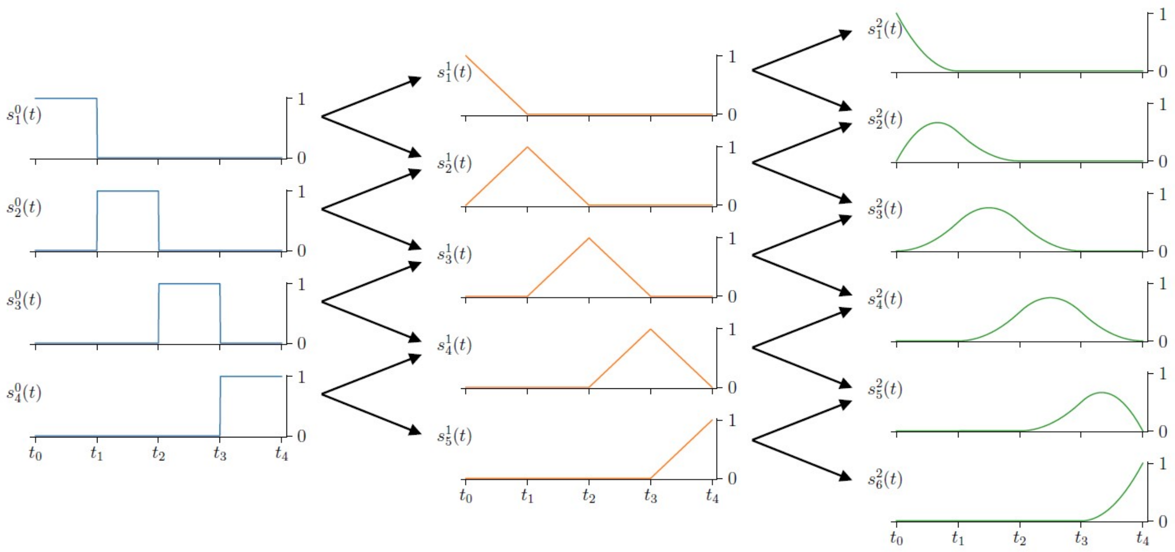

.

Given such a basis, we can represent any dose-response relationship as the linear combination of the spline basis elements, with coefficients $\beta \in \mathbb{R}^{\mathrm{p+k}}$:

$$f(t) = \sum_{j=1}^{p+k} \beta_j^p s_j^p(t). \tag{10}$$

These coefficients are then inferred as part of the general estimator (9) as discussed in the previous section. An explicit representation of (11) is obtained by building a design matrix $\mathbf{X}$. Given a set of t values at which we have data, the jth column of $\mathbf{X}$ is given by the expression

$$\mathbf{X}_{\cdot,j} = \begin{bmatrix} s_j^p(t_0) \\ \vdots \\ s_j^p(t_k) \end{bmatrix}. \tag{11}$$

The model for direct observations data coming from (11) can now be written compactly as

$$\boldsymbol{y} = \mathbf{X}\beta + \mathbf{Z}_i \boldsymbol{u}_i + \boldsymbol{\epsilon}_i \,,$$

which is a special case of the main problem class (1).

Section 4.4.1.6.2. Shape constraints

We can impose shape constraints such as monotonicity, concavity, and convexity on splines. Constraints on splines have been developed in the past through reformulation techniques.[53] The development in this section uses explicit constraints instead.

Monotonicity. Spline monotonicity across the domain of interest follows from monotonicity of the spline coefficients.[51] Given coefficients

$$\beta = \begin{bmatrix} \beta_1 \\ \vdots \\ \beta_n \end{bmatrix},$$

the curve $f(t)$ in (11) is monotonically non-decreasing when

$$\alpha_1 \leq \alpha_2 \leq \cdots \leq \alpha_n$$

and monotonically non-increasing if

$$\alpha_1 \geq \alpha_2 \geq \cdots \geq \alpha_n.$$

The relationship $\alpha_1 \leq \alpha_2$ can be written as $\alpha_1 - \alpha_2 \leq 0$. Stacking these inequality constraints for each pair (α_i, α_{i+1}) we can write all constraints simultaneously as

$$\underbrace{\begin{bmatrix} 1 & -1 & 0 & \cdots & 0 \\ 0 & 1 & -1 & \cdots & 0 \\ \ddots & \ddots & \ddots & \ddots & \vdots \\ 0 & \cdots & \cdots & 1 & -1 \end{bmatrix}}_{\mathbf{C}} \begin{bmatrix} \alpha_1 \\ \alpha_2 \\ \alpha_3 \\ \vdots \\ \alpha_n \end{bmatrix} \leq \begin{bmatrix} 0 \\ 0 \\ \vdots \\ 0 \end{bmatrix}.$$

These linear constraints are a special case of the general estimator (9) that allows $\mathbf{C}(\beta) \leq c_\beta$.

Convexity and Concavity. For any twice continuously differentiable function: $f : \mathbb{R} \to \mathbb{R}$, convexity and concavity are captured by the signs of the second derivative. Specifically, f is convex if $f''(t) \geq 0$ is everywhere, and concave if $f''(t) \leq 0$ everywhere. We can compute $f''(t)$ for each interval, and impose linear inequality constraints on these expressions.

Enforcing linear tails. For large consumption with little data, we need the capability to ensure that the last segment of the spline is linear, with slopes that match the adjacent segment at the knot. The estimated spline is then a best fit to the data, subject to this specification. Priors on the tails can also be provided.

Figure B. Spline extrapolation. Left: linear extrapolation. Right: nonlinear extrapolation.

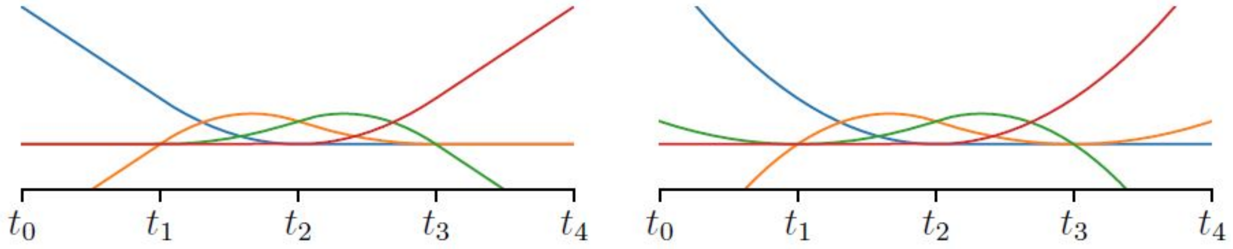

In general, using linear head and/or tail pieces to extrapolate outside the original domain or interpolate in the data sparse region is far more stable that using higher order polynomials, see figure B. The figure shows symmetric linear tail modifications, but for the analyses in the paper we only impose a right linear tail shape constraint.

Section 4.4.1.6.3. Posterior Variance Estimation

To obtain posterior uncertainty, we use a parametric bootstrap.[54] Once we solve (9) to obtain estimates $\hat{\beta}$ and $\hat{\gamma}$, we have a model distribution of the errors (1):

$$\boldsymbol{y}_i = \mathbf{F}_i(\hat{\beta}) + \mathbf{Z}_i \boldsymbol{u}_i + \boldsymbol{\epsilon}_i$$

We sample datasets from this distribution to generate full data sets $\{\mathbf{Y}\}^j$, for $j = 1, \ldots, N$. For each dataset $\mathbf{Y}^j$, we then re-solve the fitting problem (9) to obtain estimates $\hat{\beta}^j$ and $\hat{\gamma}^j$, and the set $\{\hat{\beta}^j, \hat{\gamma}^j\}$ over all j allows us to estimate any posterior statistic we need.

In particular, the posterior set of dose-response curves is given by

$$\{f(t)^j + u_0^j\}$$

where $f(t)^j$ is the curve obtained by using the re-fit value $\hat{\beta}^j$, and u_0^j is a sample from $N(0, \hat{\gamma}_0^j)$, the associated unexplained heterogeneity parameter.

Section 4.4.2: Bias adjustment for alternative case definitions and study methods

In GBD 2019, we decided to do all our adjustments of non-fatal and risk exposure data to deal with alternative case definitions or study methods prior to entering data into our main analytical tools of DisMod-MR 2.1 and ST-GPR. This decision also included the adjustment of data presented for both sexes to a male and female equivalent. The starting point was to explicitly state the reference case definition and study method and identify alternative definitions and study characteristics that fall within our inclusion criteria.

We compiled data from both within-study comparisons (ie, data that used alternative and reference definitions in the same population) and between-study comparisons (ie, data that used an alternative definition in one population and a reference definition in another population that overlap in location, time, age, and sex) of different case definitions. For between-study comparisons, we allowed a maximum calendar year difference between studies of five years. Where validation studies (ie, those carried out at the introduction of a new set of diagnostic criteria comparing to previous criteria) were available, we extracted data on the comparison of alternative to reference. For quantities of interest with multiple alternative definitions/methods we also look for pairs comparing two alternatives. In a network analysis, if A is the reference and B and C are two alternatives, a comparison of A vs B and B vs C provides an indirect comparison of the alternative C against the reference A.

We pooled either the logit difference between alternative and reference or the natural log of the ratio of alternative to reference. From simulations we found that the two methods provide almost identical results for quantities that after adjustment do not exceed a value of 0.5 (eg, prevalence or proportion). The logit difference method much better dealt with higher values and avoided prevalence or proportions to exceed one. If the values of either the reference or alternative were zero, we aggregated

values across age groups until both values had non-zero observations. We used the delta method to compute the standard error of the reference and alternative measures in logit space. The standard error of the logit difference was computed as the square root of the sum of the variances of each data point in a pair.

Section 4.4.2.1 Age-sex splitting

Age-sex splitting was commonly applied to literature data reported by age or sex but not by age and sex. For GBD 2019, we split all data reported in age groups with a width greater than 20 years, and we did so by using age patterns from available survey microdata or regional patterns derived from an initial run of the main modelling tool, DisMod-MR 2.1.

Section 4.4.2.2 Data analysis

We used a network random effects meta-regression in meta-regression—Bayesian, regularised, trimmed (MR-BRT). In a network analysis, if A is the reference and B and C are two alternatives, a comparison of A vs B and B vs C provides an indirect comparison of the alternative C against the reference A. To implement the network we included dummy variables with a particular structure. This was implemented as follows, where A is the reference definition/method:

- Create *k* dummy variables where *k* are all definitions/methods other than A (eg, *k* = B, C)
- Code dummy *k* as
  - 1 if the first term of the logit difference is *k*;
  - -1 if *k* is second term of the logit difference;
  - 0 otherwise

For example:

Study	Comparison	DummyB	DummyC
1	logit(B)-logit(A)	1	0
2	logit(B)-logit(A)	1	0
3	logit(C)-logit(A)	0	1
4	logit(C)-logit(A)	0	1
5	logit(C)-logit(B)	-1	1
6	logit(C)-logit(B)	-1	1

The coding structure outlined above in step 1 assumes that all case definitions are mutually exclusive. In some cases, however, individual case definitions are a function of different components

or dimensions. For example, case definitions may vary by the type of symptoms that a respondent experiences as well as the recall period over which those symptoms are experienced. In the presence of sparse data, it may be difficult to find both direct and indirect comparisons of all individual case definitions. In these case, an alternative approach is to assume different dimensions of case definitions have a multiplicative effect. In other words, the effect of recall period has the same relative effect across different categories of symptoms reported by respondents. To implement this coding scheme:

- Create *k* dummy variable columns for each case definition dimension
- For each dummy variable k:
    - Add 1 if *k* is a component of the first term in the logit difference
    - Subtract 1 if *k* is a component of the second term in the logit difference

In MR-BRT, we ran random effects meta-regression of the logit difference (or log ratio) with all the *k* dummy variables as covariates, omitting the intercept in the meta-regression. We used a study_id variable for the unique identifier of the reference and alternative studies (or alternative1 to alternative2). The coefficients on the *k* dummy variables represent the pooled logit difference of the *k* alternative definition to the reference taking into account evidence from both direct and indirect comparisons. In the example above, the coefficient on DummyA is the pooled logit difference of B minus A; the coefficient on DummyB is the pooled logit difference of C minus A. The standard error of the pooled logit difference incorporating the between study variance was calculated as:

$$se(\text{logit}(difference_k)) = \sqrt{var_k + \gamma^2}$$

Where:

$se(\text{logit}(difference_k)$ is the standard error of the pooled logit difference of alternative *k* to the reference

var_k is the variance of the coefficient on dummy variable *k*

γ^2 is the between-study variance

If both between and within study pairs were available, we examined whether there was a systematic difference between these. If there was a significant difference, we made judgement call as to whether within-study or between study data comparisons were most appropriate. In general, this was the within-study data, however, there were important measurement or conceptual reasons for choosing between-study data. For example, for crosswalks between self-reported height and weight compared to measured height and weight, between-study comparisons may be preferable if respondents knew they would be measured and, therefore, were less likely to misreport their height and weight.

We also examined whether there were systematic differences in the adjustments by key demographics (age, sex, geographic location, year) and other potential factors that may lead to variation in crosswalks. This could only be done at present in a direct comparison model and not in a network. We did this when there was a strong rationale, eg, biological plausibility, for variation by such characteristics.

After obtaining the pooled logit difference or log ratio estimates, we predicted adjustments based on the statistical model, including uncertainty in the adjustment and sampling error of each data point. For non-significant logit differences or log ratios we still applied the adjustments if there was a conceptual reason to believe that the alternative definition is biased. This expands the variance of these alternative definition data points.

Interpreting the coefficients of a logit difference model is not so straightforward as the adjustment to alternative data points is dependent of its value. For instance, the figure below on the left, shows the MR-BRT fit using a spline function by age to the logit differences of all overlapping pairs. The graph on the right indicates the adjustment by age for a hypothetical data point of 5%. The larger logit difference at younger ages, and to a lesser extent older ages, leads to a greater downward (in this case) adjustment of the 5% data point than at the mid age range.

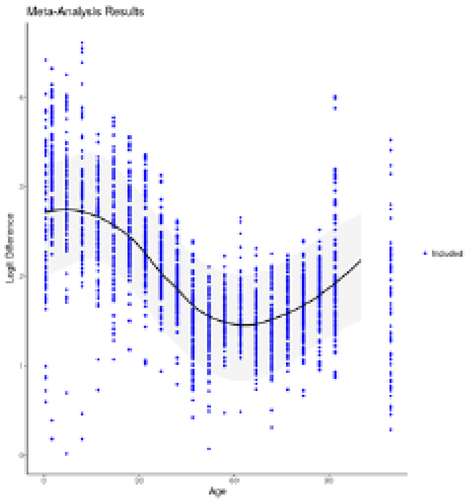

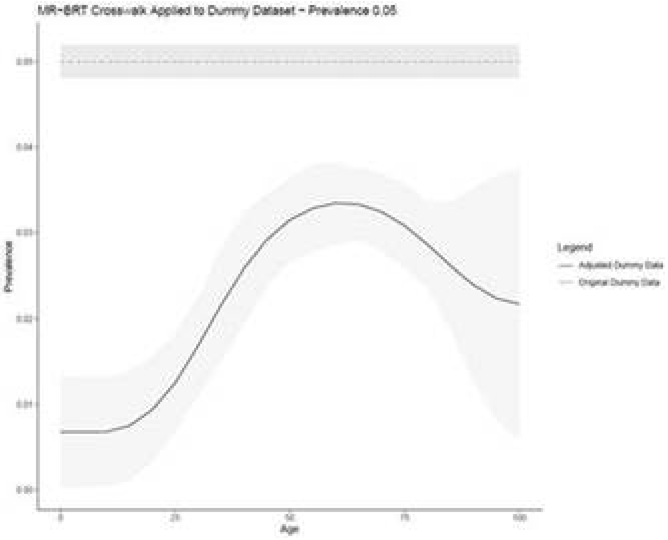

Section 4.5: DisMod-MR 2.1 estimation[2]

Section 4.5.1: Estimation of sequelae and causes

The most extensively used estimation method is the Bayesian meta-regression method DisMod-MR 2.1. For some causes such as HIV/AIDS or measles, disease-specific natural history models have been used for which the underlying three state model in DisMod-MR 2.1 (susceptible, cases, dead) is insufficient to capture the complexity of a disease process. For some diseases with a range of sequelae differentiated by severity, such as COPD or diabetes mellitus, DisMod-MR 2.1 was used to meta-analyse the data on overall prevalence with separate DisMod-MR 2.1 models of the proportions of cases with different severity levels or sequelae. Likewise, DisMod-MR 2.1 was used to meta-analyse data on the proportions of liver cancer and cirrhosis due to underlying aetiologies such as hepatitis B, hepatitis C, and alcohol use.

Section 4.5.2: DisMod-MR 2.1 description

Until GBD 2010, non-fatal estimates in burden of disease assessments were based on a single data source on prevalence, incidence, remission, or a mortality risk selected by the researcher as most relevant to a particular location and time. For GBD 2010, we set a more ambitious goal: to evaluate all

available information on a disease that passes a minimum quality standard. That required a different analytical tool that would be able to pool disparate information presented for varying age groupings and from data sources by using different methods. The DisMod-MR 1.0 tool used in GBD 2010 evaluated and pooled all available data, adjusted data for systematic bias associated with methods that varied from the reference, and produced estimates by world regions with UIs by using Bayesian statistical methods. For GBD 2013, the improved DisMod-MR 2.0 increased computational speed, which allowed computations to be consistent between all disease parameters at the country rather than the region level. The hundred-fold increase in speed of DisMod-MR 2.0 was partly due to a more efficient rewrite of the code in C++ but also to changing to a model specification by using log rates rather than a negative binomial model used in DisMod-MR 1.0. In cross-validation tests, the log rates specification worked as well or better than the negative binomial specification.[39] The sequence of estimation occurs at five levels: global, super-region, region, country and, where applicable, subnational location. The super-region priors are generated at the global level with mixed-effects, nonlinear regression by using all available data; the super-region fit, in turn, informs the region fit, and so on down the cascade. The wrapper gives analysts the choice to branch the cascade in terms of time and sex at different levels depending on data density. The default used in most models is to branch by sex after the global fit but to retain all years of data until the lowest level in the cascade is reached.

The computational engine is limited to three levels of random effects; we differentiate estimates at the super-region, region and country level. In GBD 2013, the subnational units of China, the UK and Mexico were treated as "countries" to enable a random effect to be estimated for every location with contributing data. However, the lack of a hierarchy between country and subnational units meant that the fit to country data contributed as much to the estimation of a subnational unit as the fits for all other countries in the region. We found inconsistency between the country fit and the aggregation of subnational estimates when the country's epidemiology varied from the average of the region. Adding an additional level of random effects required a prohibitively comprehensive rewrite of the underlying DisMod-MR engine. Instead, we added a fifth layer to the cascade, with subnational estimation informed by the country fit and country covariates, plus an adjustment based on the average of the residuals between the subnational location's available data and its prior. This technique mimicked the impact of a random effect on estimates between subnationals.

In GBD 2015, we also improved how country covariates differentiate non-fatal estimates for diseases with sparse data. The coefficients for country covariates are re-estimated at each level of the cascade. For a given location, country coefficients are calculated by using both data and prior information available for that location. In the absence of data, the coefficient of its parent location is used to utilise the predictive power of our covariates in data-sparse situations.

For GBD 2016, the computational engine (DisMod-MR 2.1) remained substantively unchanged from GBD 2015. We changed the prediction year set to generate fits for the years 1990, 1995, 2000, 2005, 2010, and 2016. We updated the age prediction sets to include age groups 80–84 years, 85–89 years, 90–94 years, and 95 years and older to comply with changes across all functional areas of the GBD. We also expanded the set of locations where subnational units are modelled; the set now includes Brazil, China, England, India, Indonesia, Japan, Kenya, Mexico, South Africa, Sweden, and the US.

In GBD 2017, we continued to use DisMod-MR 2.1 because no substantial changes were made. Updates to computation include extending the terminal prediction year to 2017 and additional subnational units in Ethiopia, Iran, New Zealand, Norway, and Russia. Saudi Arabia was also modelled only at the national level in 2017.

In GBD 2019, no substantial changes were made to DisMod-MR 2.1 but we made more substantial changes to how we use the tool. First, we added the year 2019 as an additional year of estimation. Second, we also included the option again to have random effects on cause-specific mortality rates (CSMR) and EMR This functionality had been dropped a couple of GBD rounds earlier. Third, as we did all our adjustments for alternative case definition and study methods as well as adjustments to both sex data points prior to entering data into DisMod-MR 2.1, we no longer used the functionality in DisMod-MR 2.1 to estimate coefficients for study covariates.

Fourth, based on simulation testing we found that coverage improved and errors reduced when passing down priors with a wider setting of minimum coefficient of variation (which determines the uncertainty around priors and hence how 'informative' the priors are) than had generally been used in past GBD iterations. We settled on a default value of 0.8 where in the past values of 0.4 or less had been more commonly used. We made some exceptions for high prevalent conditions where a lower minimum coefficient of variation (CV) setting achieved the task of making priors less informative but not completely uninformative.

We carried out simulation testing using DisMod-MR 2.1 based on an internally consistent set of 15,601 data points for prevalence, incidence, excess mortality, CSMR, and remission. The dataset was generated by the simulation capability of the DisMod-AT tool that is under development. We aimed to test what level of minimum CV would create the best fit based on the following three performance statistics:

(1) Coverage, ie, the proportion of data point mean values that fall between the 2.5th and 97.5th percentile of the draws of the fit values;
(2) Root mean square error: the square root of the mean of the squares of the difference between data point mean values and the mean fit value; and
(3) Bias: the difference between the mean fit value and the data point mean value.

We created different datasets culling the initial complete set with values at every age, sex, and location to more realistic data sparsity scenarios for analysis.

A first strategy was to randomly reduce the dataset to 10%, 5%, 2.5%, 1%, and 0.5% of the original data points. Initial results indicated little variation between the data samples culled to 10%, 5%, 2.5%, and 1%. The 0.5% culled dataset was an exception with markedly worse performance statistics, particularly with regard to bias and RMSE as illustrated in figure 1. We conducted further studies using the datasets culled to 10%, 5%, and 0.5%.

Figure 1. Performance statistics for randomly culled datasets

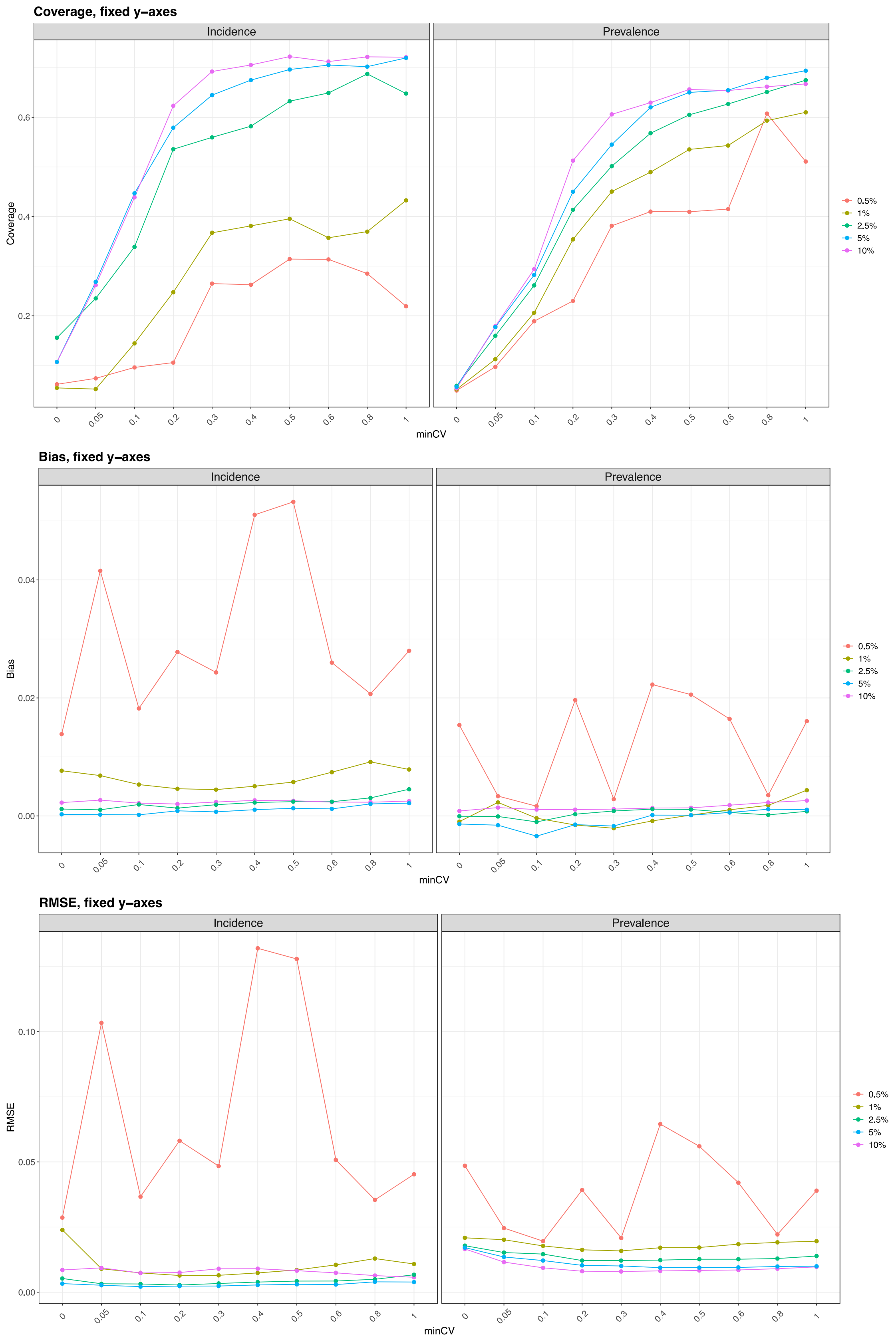

The second strategy was to compare randomly culled dataset for 10%, 5%, and 0.5% with datasets culled to the same percentages, but differentially by SDI, such that we culled all the data in sub-Saharan Africa and for the other super-regions based on the probability diminishing with increasing SDI. This pattern of differential data coverage by SDI is commonly observed in datasets used for modelling. The plots shown in figure 2, generally also show diminished performance for this more realistic scenario of differential sparseness by location based on SDI.

Figure 2. Performance statistics comparing randomly and differentially culled datasets.

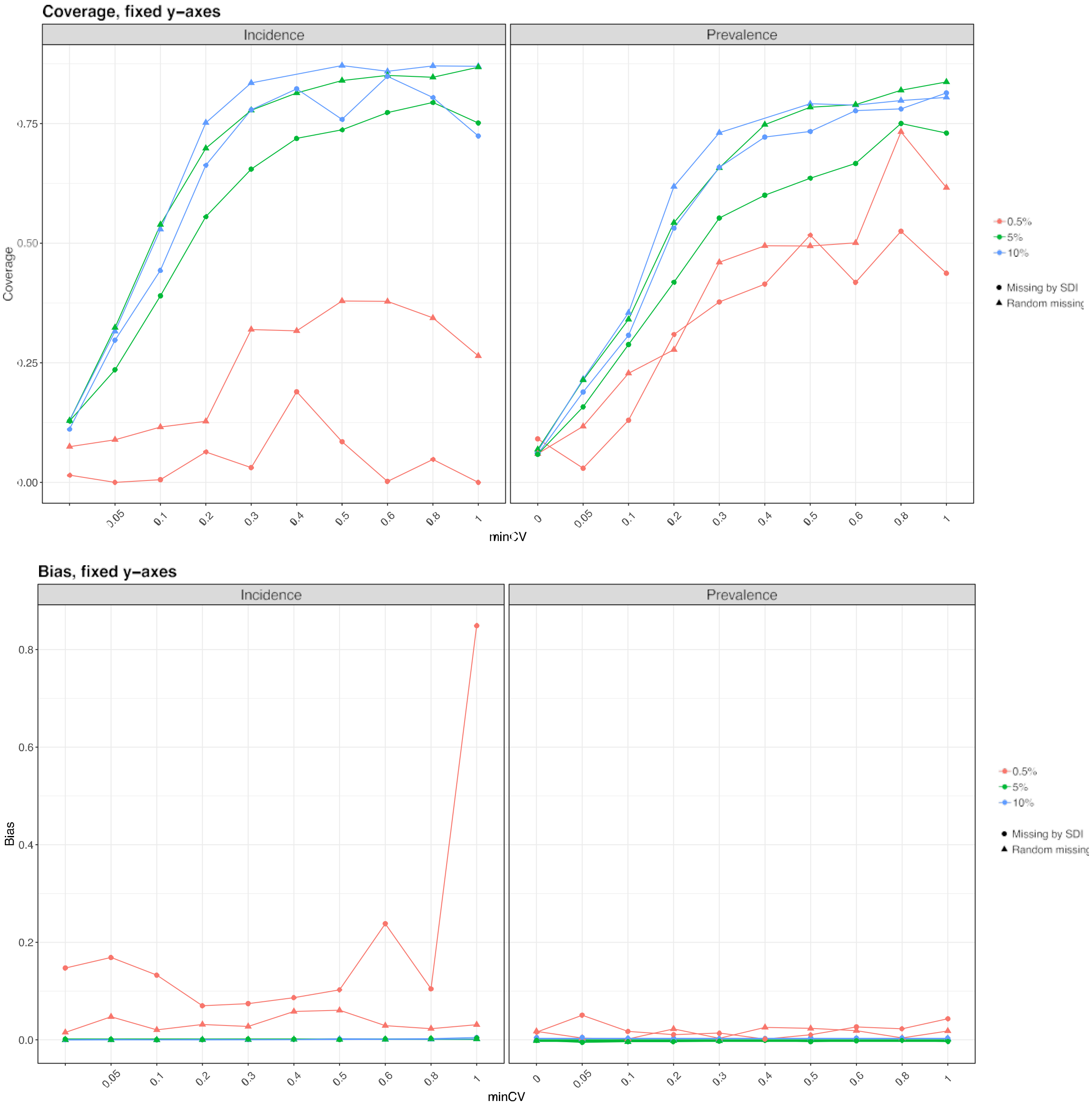

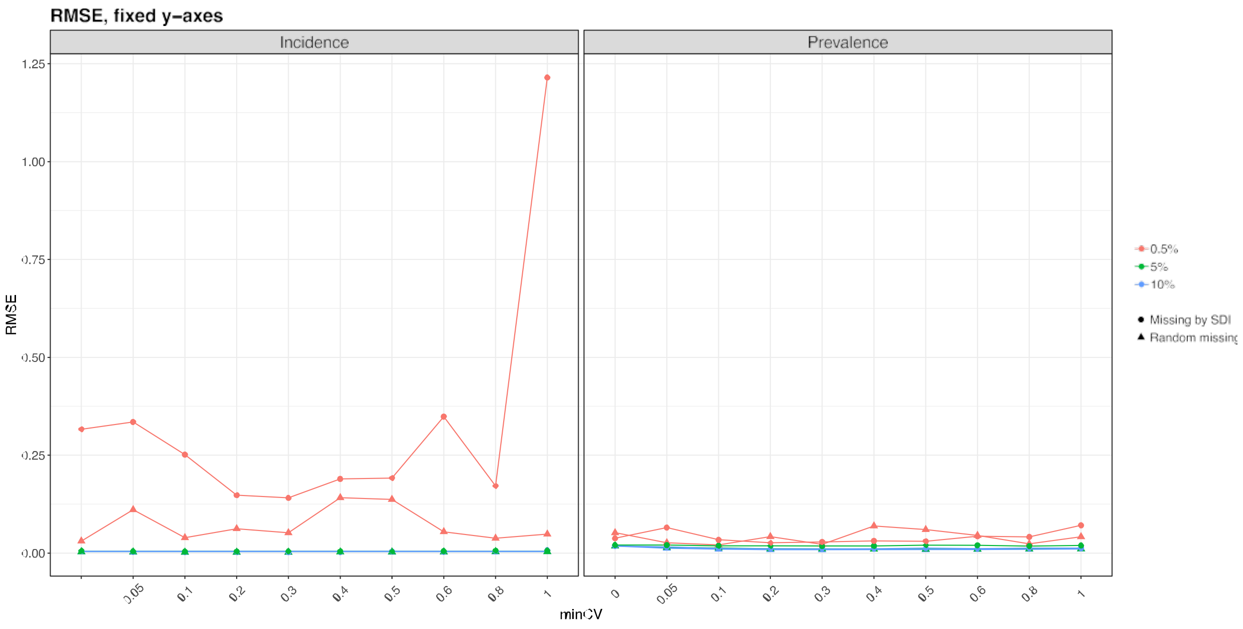

A third strategy was to apply a further distinction of complete culling of either prevalence and CSMR, or incidence data points, using the 10% randomly culled or 10% differentially culled datasets as comparators. In these scenarios, we found that the coverage statistic starts to level off at a value of 0.8 for minimum CV. All three metrics are much worse for datasets with incidence data culled. Performance statistics for this strategy are shown in figure 3.

Figure 3. Performance statistics comparing datasets with specific measures held out vs. randomly or differentially culled datasets.

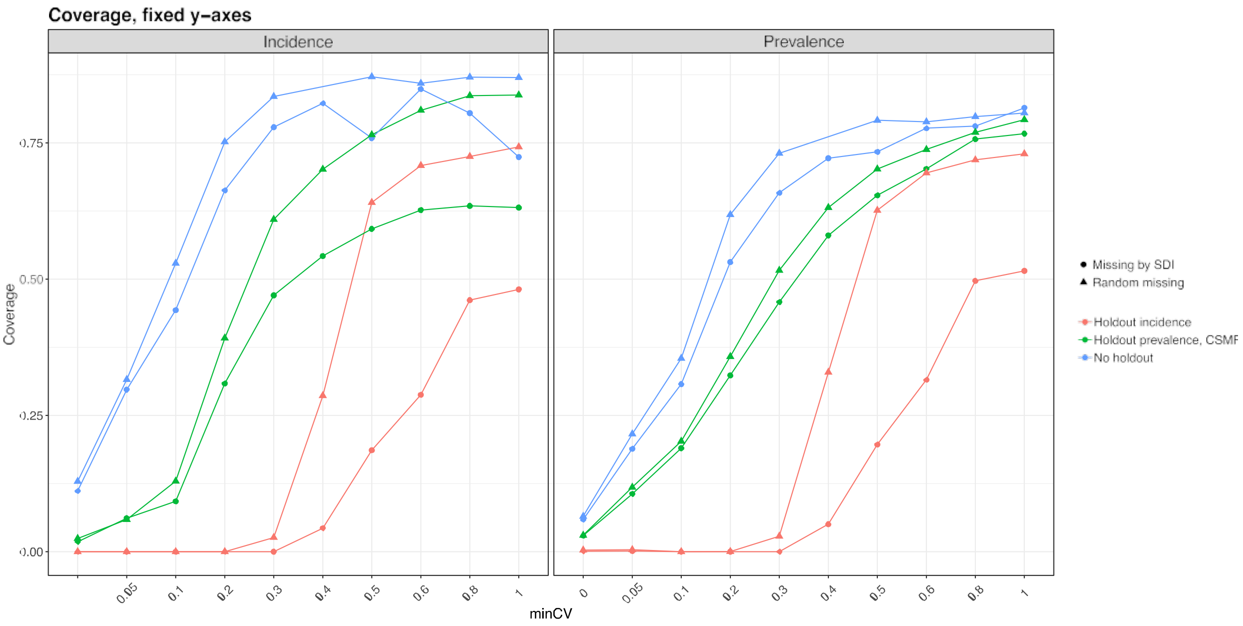

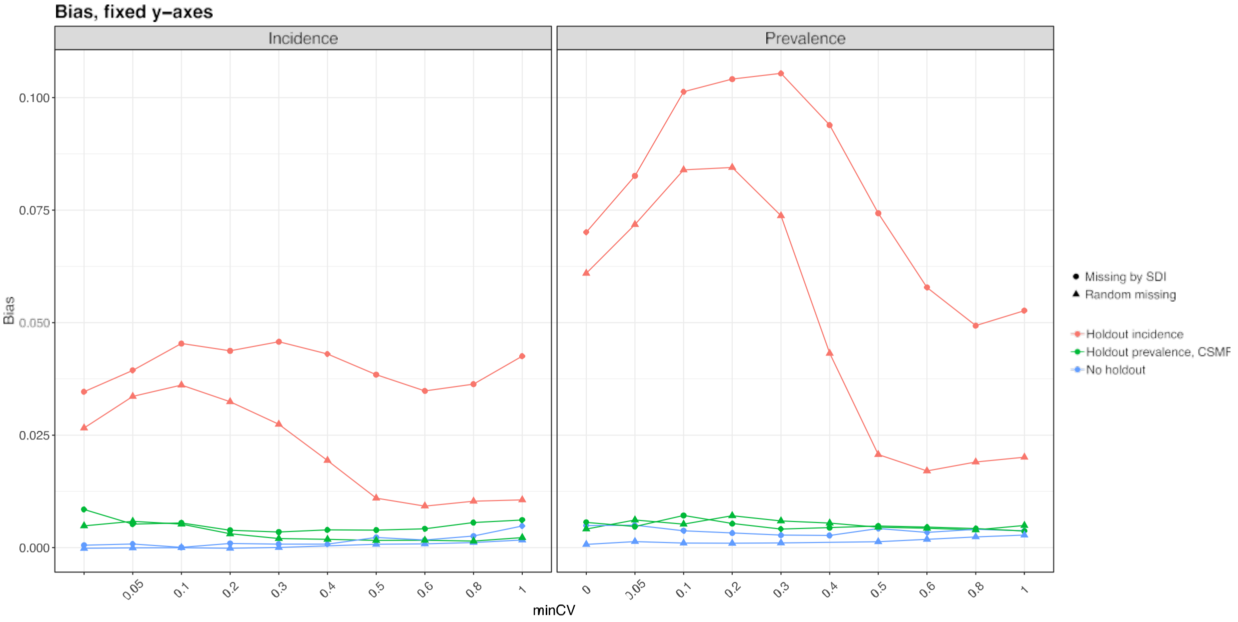

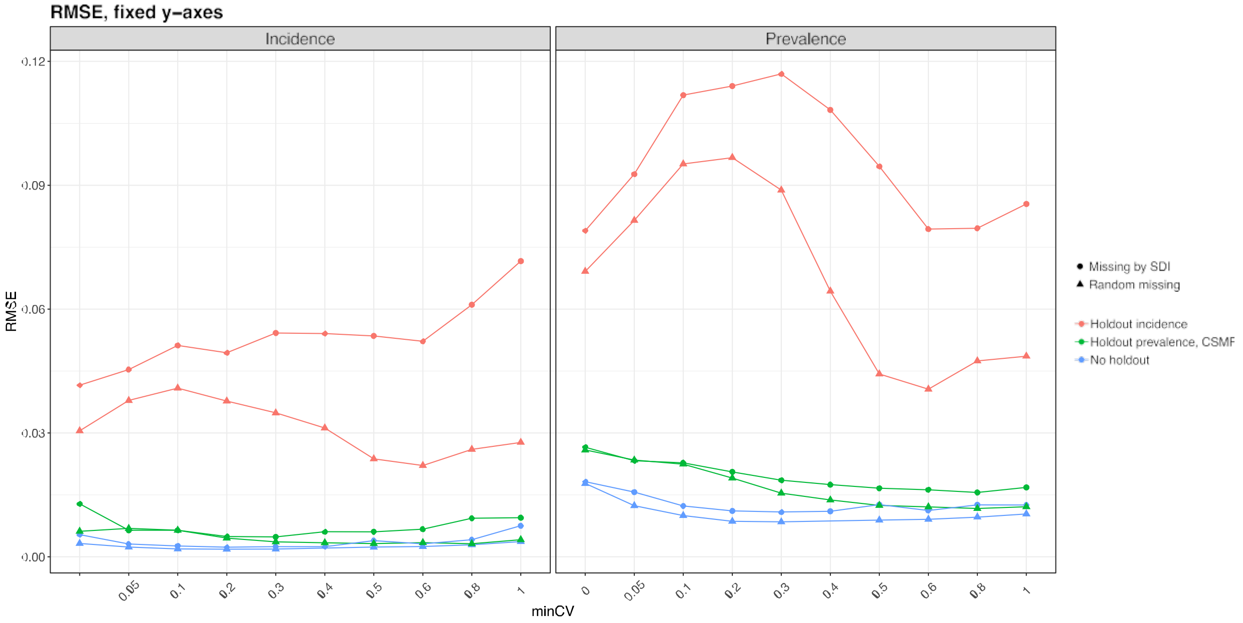

Fifth, we changed our approach to estimating excess mortality rates, the key link in the model between cause-specific mortality rates (CSMR) and incidence and prevalence. In the past two GBD rounds we calculated priors on excess mortality and entered these as data points by matching sex-specific prevalence data with an age width of 20 or less with the corresponding CSMR for the same location and year. For stability sake, we excluded calculation of EMR for prevalence data points of less than 1 in a million. EMR is simply calculated as CSMR divided by prevalence. As with previous GBD years, for diseases with an average duration of less than a year (as indicated by a setting of remission greater than one), we ran an initial global model to get an equivalent prevalence and used the following formula to calculate EMR:

$$EMR = \frac{CSMR * (remission + (ACMR - CSMR) + EMR\_pred)}{incidence}$$

where,

ASMR is the all-cause mortality rate

EMR_pred is the EMR fit from an initial global DisMod model

Despite using the log of LDI or the HAQ Index as a covariate with a prior that the coefficient had to be negative, we found many disease models with an implausible distribution of mortality to prevalence (or incidence) ratios implying lower case fatality in locations with lower HAQ Index than in countries with higher HAQ Index. This likely signals an inconsistency between fatal and non-fatal data inputs. For GBD 2019, we decided to run regressions on EMR data (calculated as described above) first using MR-BRT with HAQ Index as a predictor. In general, we tend to think that CSMR estimates are more robust than non-fatal data because of much greater data availability and a lesser task in adjusting cause death data for garbage coding than the complex task of adjusting non-fatal data sources for alternative case definitions and study methods. To indicate that we would reduce the random effects on EMR and the minimum coefficient of variation for priors on EMR being created at each next level down the cascade. However, there were exceptions. For drug use disorders, the risk of overdose deaths is less a function of a country's quality of health services but driven more by the availability of harm reduction strategies such as opioid substitution therapy and the availability of highly potent opioids such as fentanyl, which have been an important contributor to the large increase in overdose deaths in the USA in the last decade. We settled on a model for opioid use disorder with wider random effects and higher minimum coefficient of variation to give less emphasis on CSMR when enforcing consistency with prevalence data. In a next round, we will work to find covariates that are more relevant to drug overdose deaths such as a grading of harm reduction strategies by country and over time. In the case of COPD, we noted that following the data on CSMR and EMR led to large increases in prevalence estimates in east Asia, Oceania and, to a lesser extent, south Asia. In the oldest age groups, prevalence estimates would be higher than the prevalence data for these locations and reach a level of close to 80% in the oldest age groups. In these locations, we will pay attention to how garbage codes are being redistributed onto COPD in the next round of GBD.

Section 4.5.3: DisMod-MR 2.1 likelihood estimation

Analysts have the choice of using a Gaussian, log-Gaussian, Laplace, or Log-Laplace likelihood function in DisMod-MR 2.1. The default log-Gaussian equation for the data likelihood is

$$-log\left[p\left(y_j|\Phi\right)\right] = \log\left(\sqrt{2\pi}\right) + \log\left(\delta_j + s_j\right) + \frac{1}{2}\left(\frac{\log\left(a_j + \eta_j\right) - \log\left(m_j + \eta_j\right)}{\delta_j + s_j}\right)^2$$

Where,

y_j is a "measurement value" (ie, data point)

Φ denotes all model random variables

η_j is the offset value, *eta*, for a particular "integrand" (prevalence, incidence, remission, excess mortality rate, with-condition mortality rate, cause-specific mortality rate, relative risk, or standardised mortality ratio)

a_j is the adjusted measurement for data point j, defined by

$$a_j = e^{(-u_j - c_j)} y_j$$

Where:

u_j is the total "area effect" (ie, the sum of the random effects at three levels of the cascade: super-region, region and country) and

c_j is the total covariate effect (ie, the mean combined fixed effects for sex, study level, and country level covariates), defined by

$$c_j = \sum_{k=0}^{K[I(j)]-1} \beta_{I(j),k} \hat{X}_{k,j}$$

with SD

$$s_j = \sum_{l=0}^{L[I(j)]-1} \zeta_{I(j),l} \hat{Z}_{k,j}$$

Where:

k denotes the mean value of each data point in relation to a covariate (also called x-covariate)

I(j) denotes a data point for a particular integrand, *j*

$\beta_{I(j),k}$ is the multiplier of the kth x-covariate for the ith integrand

$\hat{X}_{k,j}$ is the covariate value corresponding to the data point *j* for covariate *k*;

l denotes the SD of each data point in relation to a covariate (also called z-covariate)

$\zeta_{I(j),k}$ is the multiplier of the lth z-covariate for the ith integrand

δ_j is the SD for adjusted measurement *j*, defined by:

$$\delta_j = log\left[y_j + e^{(-u_j - c_j)} \eta_j + c_j\right] - log\left[y_j + e^{(-u_j - c_j)} \eta_j\right]$$

Where:

m_j denotes the model for the jth measurement, not counting effects or measurement noise, and defined by:

$$m_j = \frac{1}{B(j) - A(j)} \int_{A(j)}^{B(j)} I_j(\text{a}) \text{ da}$$

Where:

A(j) is the lower bound of the age range for a data point

B(j) is the upper bound of the age range for a data point

I_j denotes the function of age corresponding to the integrand for data point *j*

Section 4.6: Impairment and underlying cause estimation[2]

For GBD 2019, as in GBD 2017 and GBD 2016, we estimated the country-age-sex-year prevalence of nine impairments. Impairments in GBD are conditions or specific domains of functional health loss that are spread across many GBD causes as sequelae and for which there are better data to estimate the occurrence of the overall impairment than for each sequela based on the underlying cause. These impairments included anaemia, epilepsy, hearing loss, heart failure, intellectual disability, infertility, vision loss, Guillain-Barré syndrome, and pelvic inflammatory disease. Overall impairment prevalence was estimated by using DisMod-MR 2.1. We constrained cause-specific estimates of impairments, as in the 19 causes of blindness, to sum to the total prevalence estimated for that impairment. Anaemia, epilepsy, hearing loss, heart failure, and intellectual disability were estimated at different levels of severity. Estimates were made separately for primary infertility (those unable to conceive), secondary infertility (those having trouble conceiving again), and whether the impairment affected men and/or women. In the case of epilepsy, we determined the proportions with idiopathic and secondary epilepsy as well as the proportions with severe and less severe epilepsy by using mixed effects regressions. The sparse data for the proportion of seizure-free, treated epilepsy were pooled in a random effects meta-analysis. DisMod-MR 2.1 models produced country-, age-, sex-, and year-specific severity levels of hearing loss and vision loss. Because of limited information on the severity levels of intellectual disability, we assumed a similar distribution of severity globally based on random effects meta-analysis of IQ-specific data for the overall impairment. This assumption was supplemented by cause-specific severity distributions for chromosomal causes and iodine deficiency; the severity of intellectual disability included in the long-term sequelae of causes including neonatal disorders, meningitis, encephalitis, neonatal tetanus, and malaria was estimated in combined health states of multiple impairments such as motor impairment, blindness, and/or seizures.[55] We changed the name of the intellectual disability impairment to specify that estimates reflect cases arising during the developmental period, which we have defined as ages under 20 years. The severity of heart failure was derived from our Medical Expenditure Panel Surveys (MEPS) analysis and therefore was not specific for country, year, age, or sex.

A detailed description of the methods of each impairment can be found at the end of Section 4.12 of this appendix.

Section 4.6.1: Impairment squeeze

For impairments like epilepsy, intellectual disability, and blindness, mentioned above in Step 4, we often have better information regarding the total prevalence of the impairment rather than the prevalence of said impairment due to its various causes. For example, we have more data and a better idea of the total number of blind individuals (which we refer to herein as the blindness “envelope”) in the world than we do the number of individuals who are blind due to a specific cause like retinopathy of prematurity or

cataract. We achieve this consistency by either squeezing or inflating the individual sequela prevalence values so that their sums fit into each appropriate envelope. Blindness, epilepsy, and/or intellectual disability appear in various combinations with motor impairment levels as sequelae for a number of neonatal disorders and infectious diseases like malaria and neonatal tetanus ("Moderate motor impairment with blindness and epilepsy due to neonatal tetanus", for example). This presents an extra challenge because any squeeze or inflation of one of the impairments making up a sequela affects the others.

We set some rules on how to do these adjustments sequentially. First, when the envelope of an impairment is smaller than the sum of all contributing causes, we redistribute the excess prevalent cases of combined impairment sequelae onto the sequelae that only have motor impairment (at a mild, moderate, or severe level) within the same cause grouping. Second, we apply the adjustments in a particular order such that we always fit at least one of the envelopes exactly where the other one or two envelopes may be exceeded by some amount. We first enforce a fit to the epilepsy impairment envelope, then intellectual disability, and last, blindness. Thus, the epilepsy envelope always matches exactly, whereas the intellectual disability and blindness envelopes may occasionally be exceeded on a draw-by-draw basis.

Section 4.7: Severity distribution[2]

Sequelae were defined in terms of severity for 169 causes. We generally followed the same approach for estimating the distribution of severity we used in GBD 2017. In cases in which severity was related to a particular impairment, such as mild, moderate, and severe heart failure due to ischaemic heart disease or the newly added cause of pulmonary arterial hypertension, the analysis was driven by impairment estimation methods. Severity levels for causes such as chronic kidney disease, epilepsy and COPD were modelled using DisMod-MR 2.1 or ST-GPR, whereas we performed meta-analyses to estimate the allocation of severity for causes such as rheumatoid arthritis, and multiple sclerosis. For dementia, we changed from using meta-analysis of three age categories to a more flexible model in MR-BRT using a spline on age. That allowed us to increase the number of studies informing severity from 7 to 67. For gallbladder and biliary diseases, we performed a meta-analysis of six community-based studies of the proportion of cases of gallbladder disease identified by ultrasonography who are symptomatic. In previous rounds, inpatient admission for gall bladder and biliary disease as a primary diagnosis were taken to represent symptomatic cases. For the new cancer sites included in GBD 2019, we used the same strategy as for all other cancer sites. For the newly added sites of osteoarthritis of the hand and sites other than hip or knee, we assumed the same severity distribution as for osteoarthritis of the knee.

For many causes, we continue to have inadequate data on severity from surveys or the epidemiological literature. For those diseases, we made use of three population surveys: the MEPS 2000–2014, the [US] National Epidemiological Survey on Alcohol and Related Conditions (NESARC) 2000–2001 and 2004–2005, and the Australian National Survey of Mental Health and Wellbeing of Adults (NSMHWB) 1997.[56–58] Each dataset contained individual-level measurements of functional health status made by using the 12-Item Short Form Health Survey (SF-12) as well as diagnostic information on the causes affecting each individual.

To use the data collected by measuring the distribution of severity with the SF-12, the individual SF-12 summary scores were mapped to an equivalent DW. A convenience sample of respondents was asked to complete SF-12 for the hypothetical individual living in a health state described by using a selection of 60 of the 235 health states with their lay descriptions from the GBD DW surveys reflecting the full range of severity. Each of these health states has a measured DW associated with it on a zero to one scale. We collected 1980 usable responses in total. To deal with heterogeneity in responses, we excluded from the statistical analysis responses that were more than two median absolute deviations from the median for each health state. After correcting for outliers, the rank order correlation between SF-12 scores for the hypothetical individuals in each health state characterised by the lay description with the measured DW was -0.815. The health states served as random effect groups such that the composite score would be equal to the intercept plus the random effect estimated for that health state, or

$$DW_i = \alpha + U_{health\ state}$$

The final relationship between SF-12 score and DW is depicted in figure A:

Figure A. SF-12 composite scores and disability weights for 60 health states with fitted loess regression

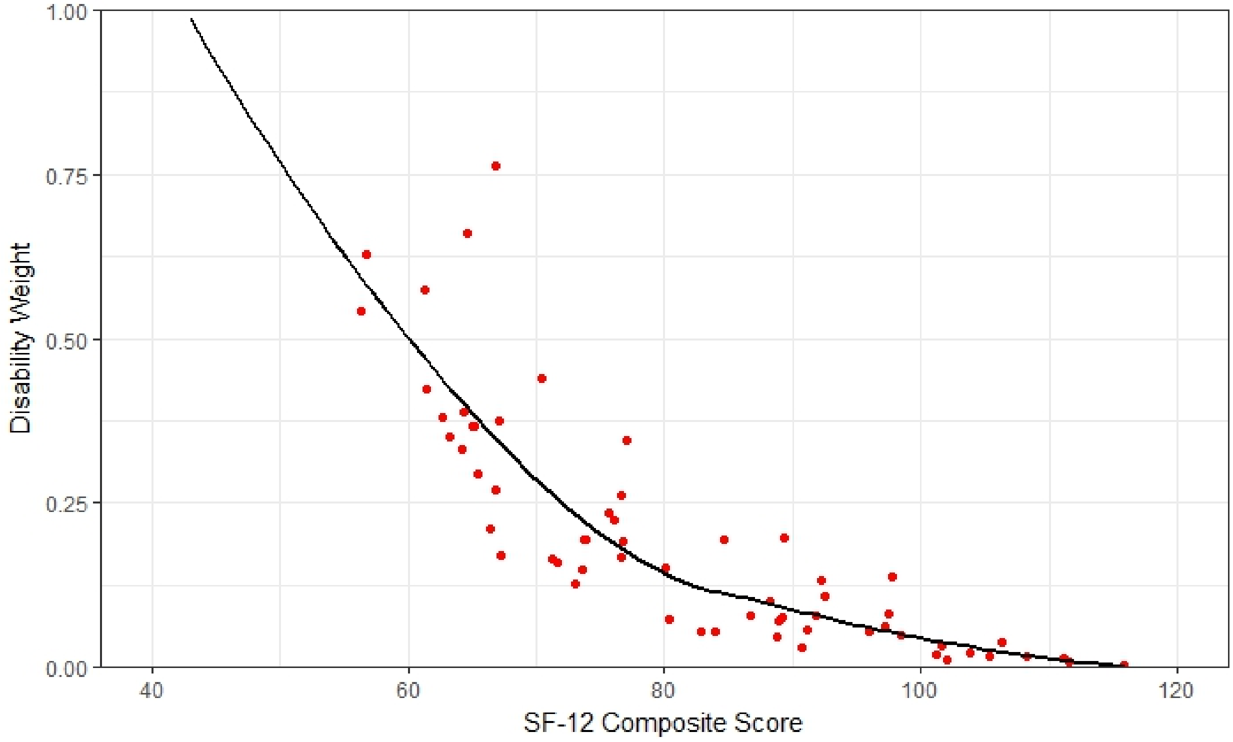

To generate a smooth mapping from SF-12 combined scores to the GBD DW space, we used locally estimated scatterplot smoothing regression on the random effects for each health state. Because DWs are defined in the range from 0 to 1, we truncated the function at a combined SF-12 score of 116.36 (any combined score above this level was set to 0) and truncated the function at 42.7 so that any combined score less than that value was set to 1. All SF-12 survey data were thus transformed into DW space.

The second stage of the analysis was to build models predicting the transformed SF-12 scores as a function of the number of causes suffered by each individual. First, variable selection was performed by using least absolute shrinkage and selection operator (LASSO) regression to penalize the regression coefficients of highly correlated causes. The tuning parameter, λ, controls the strength of the least-squares penalty. When λ=0, LASSO regression returns the same results as ordinary least-squares regression. Higher values of λ impose a stronger penalty and constrain a greater number of model parameters to 0. A ten-fold cross-validation was used to find the value of the λ that minimized the mean cross-validated error. This process resulted in a λ value of 0.0013 and eliminated 10 causes from the analysis. Transformed SF-12 scores into the DW scale for the remaining 190 causes were then modelled for each measure *m* of each individual *i* over *n* total causes in the survey as follows:

$$logit(DW)_{im} = \beta_0 + \beta_1 Condition1_{im} + \cdots + \beta_n Condition\ n_{im}$$

This equation effectively assumes that comorbid causes act to change SF-12 scores in a multiplicative fashion rather than an additive fashion.

To estimate the comorbidity-corrected effect of each cause (ie, in isolation) on total disability, we compared the predicted DW without the cause of interest (counterfactual DW) with the predicted DW including the cause of interest. Following the multiplicative comorbidity equation, the joint effect can be written

$$Condition\ specificDW = 1 - \frac{1 - predictedDW_m}{1 - counterfactualDW_m}$$

The mean of this cause-specific effect over all observations is the population marginal effect of a cause.

Using the model above, we estimate a counterfactual DW – the total individual DW excluding the effect of the cause of interest. We compared the observed distribution of functional health status with this counterfactual distribution to determine the marginal effect of the cause of interest. In other words, we estimated the health state for each individual and for each cause as the cumulative individual weight minus the effects of all comorbid causes.

$$Health\ state\ DW = 1 - \frac{1 - individual\ cumulativeDW_m}{1 - counterfactualDW_m}$$

The estimation strategy for health state-specific severity distributions for which there are multiple severity categories involved binning individuals' weights into severity cut-offs (eg, mild, moderate, and severe) for which DWs were derived. These bins were defined by using results from the GBD Disability Weights Studies[59] for causes that had multiple health states defined. Cut-offs were taken as the midpoints between levels of health state and cases distributed into severity bins accordingly. Cases were considered asymptomatic if the counterfactual weight was equal to or greater than the individual cumulative weight.

Section 4.8: Disability weights[2]

To compute YLDs for a particular health outcome in a given population, the number of people living with that outcome is multiplied by a DW that represents the magnitude of health loss associated with the

outcome. DWs are measured on a scale from 0 to 1; 0 implies a state equivalent to full health, and 1, a state equivalent to death.

DWs used in GBD studies before GBD 2010 have been criticized for the method used (ie, person trade-off), the small elite panel of international public health experts who determined the weights, and the lack of consistency over time as the GBD cause list expanded and additional DWs from a study in the Netherlands[60] were added or others were derived by ad-hoc methods.

Section 4.8.1: GBD 2010 disability weights measurement study

For GBD 2010, a primary data collection effort focused on measuring health loss rather than welfare loss by using a standardised approach of simple comparison questions directed to the general public across diverse communities.

Multi-country household surveys were conducted between Oct 28, 2009 and June 23, 2010 in five countries (Bangladesh, Indonesia, Peru, Tanzania, and the USA) selected to provide diversity across culture, language, and socioeconomic status.

Personal face-to-face computer-assisted interviews were conducted for all household surveys except for the survey in the US, which was conducted by computer-assisted telephone interview. Households were randomly selected by using a multistage stratified sampling design for which the probability of selection was proportional to the population size. In all cases, samples were designed to be representative of a given geographical area and, in the USA, to provide national representation.

For every contacted household, an adult respondent age 18 years or older was randomly selected by the survey program by means of the Kish approach. For face-to-face interviews, as many as three visits were made to selected households to establish contact. When a respondent was identified, as many as three return visits were made to do the survey at a time when the respondent was available. For the US telephone surveys, repeated calls were made up to seven times.

A web-based survey was posted at a dedicated URL between July 26, 2010 and May 16, 2011. The survey was initially available in English and subsequently available in Spanish and Mandarin. Recruitment of respondents occurred through several channels, such as news items and editorials in scientific journals, announcements at scientific meetings, postings on websites of institutions participating in the GBD, and social networking and communication mobilisation channels as well as direct contact with individuals and groups with known global health interests by tapping into the professional networks of the study investigators and their colleagues. Participants in the web-based survey were required to be ages 18 or older. Household surveys obtained oral informed consent from all participants; written informed consent was obtained from participants in the web survey. Ethical review board approval was obtained from each household survey site and the University of Washington, Seattle, WA.

Standardised survey instruments were developed to obtain comparative assessments of the full array of disease and injury sequelae, parsimoniously captured in 220 unique health states. Lay descriptions of health states formed the basis for all comparisons. These descriptions used simple, non-clinical vocabulary that emphasised the major functional consequences and symptoms associated with each

health state. Development of these descriptions involved an iterative process of detailed consultation with experts participating in the GBD 2010 study; the goals was to capture the most relevant details of each health state while avoiding ambiguity and ensuring consistency. When possible, health states were grounded in standard clinical classifications systems. For example, the Canadian Cardiovascular Society grading scale was referenced for descriptions of stages of angina,[61] and the New York Heart Association functional classification was referenced for severity of heart failure.[62] Pilot testing indicated that the lay descriptions in face-to-face interviews should not exceed 30 words.

A paired comparison question formed the basis of all surveys. The questions in the survey were framed with the following statement, "A person's health may limit how well parts of his body or mind work. As a result, some people are not able to do all of the things in life that others may do, and some people are more severely limited than others. I am going to ask you a series of questions about different health problems. In each question, I will describe two different people…" Descriptions of two hypothetical people, each with a particular health state, were presented to respondents who were then asked which person they regarded as healthier. Health pairs in all surveys were selected by a randomizing computer algorithm. In the five household surveys, paired comparisons were presented for a subset of 108 health states pertaining to chronic conditions. The framing of chronic and acute conditions is different as they were presented as causing life-long or temporary health loss. We chose to only field health states that could be framed as lasting a lifetime in the household surveys as we hypothesized that presenting differently framed comparisons would be difficult to convey in face-to-face interviews. In the web survey, we considered this more feasible because respondents could read and refer to the framing of the question for each pair-wise comparison. All 220 health states were thus evaluated in the web survey.

In addition, the web survey included questions relating to population health and health programs specifically—such as "Imagine two different health programs. The first program prevented 1000 people from getting an illness that causes rapid death. The second program prevented 2000 people from getting an illness that is not fatal but causes lifelong health problems resulting in moderate to severe disability. Which program would you say produced the greater overall health benefits?" This information was used to anchor the results from the pair-wise comparisons on the 0–1 DW scale.

Section 4.8.2: GBD 2013 European disability weights measurement study

The GBD 2010 DWs were critically dependent on the ways that outcomes were described to survey respondents. Descriptions for health states were designed to balance validity and parsimony, and this approach necessarily meant that some details of different health states had to be omitted. Because lay descriptions were developed collaboratively through individual expert groups organised around a particular set of health issues, some amount of variability in language and detail inevitably occurred. Criticisms and suggestions for improvement came from a number of commentators on the GBD 2010 DWs measurement study.[63–65]

GBD 2013 expanded the list of disease and injury causes and sequelae mapped to 235 unique health states. Additional data for the European Disability Weights Measurement Study were collected between September 23, 2013 and November 11, 2013 in Hungary, Italy, the Netherlands, and Sweden. The

initiation of these surveys was connected to a project sponsored by the European Centre for Disease Prevention and Control (the Burden of Communicable Diseases in Europe project).[66] The four selected countries were chosen to be representative of the four regions of Europe (east, south, middle, and north) in terms of age, sex, and education of the respondents. Respondents were recruited from standing internet panels in each country on the basis of quota sampling with reference to age, sex, and education in such a way as to maintain the population representativeness of these characteristics. Eligible participants were 18–65 years old and were preselected in the Netherlands, where the age, sex, and education of respondents were already known, or in the other three countries, invited to participate via a web-link and then selected on the basis of their individual characteristics.

The protocol for the European DWs measurement study followed the protocol that was developed and implemented in the GBD 2010 DWs measurement study. Lay descriptions for some health states that lacked mention of an important symptom or for which consistency of wording across different levels of severity had been noted were reworded. The European DWs measurement study included 255 health states, of which 183 were used in the analyses of GBD 2013. Those 183 consisted of 135 of the 220 health states that were included in the European DWs measurement study with unmodified lay descriptions and 30 from GBD 2010 for which alternative lay descriptions were included. DWs were estimated for additional sequelae that were incorporated into GBD 2013 but had not been included in GBD 2010.

Finding high correlation in resulting DW values between the country surveys and the web survey, we analysed the results of all surveys together. We ran probit regression analyses on the answers to the pair-wise comparison questions by using dummies for each health state with a value of 1 for the first state in a pair, –1 for the second state in a pair, and 0 for all states other than the pair. This method formalizes the intuition that if two health states in a pair produce similar health loss, the answers are likely to be evenly split; a pair of health states with very different health loss get many more responses favouring one over the other. The statistical methods infer the distances between values attached to different health states based on the frequencies of responses to the paired comparisons.

A second analytic step is needed to anchor the resulting estimates onto the 0–1 DWs scale. We anchored results from the probit regression analysis onto the 0–1 scale by using population health equivalence data from the GBD 2010 web survey by using a linear regression of the probit coefficients from the analysis of paired comparisons on the logit-transformed DW estimates derived from interval regression of the population health equivalence responses. Using numerical integration, we then estimated mean values for DWs on the natural 0–1 scale. Uncertainty was estimated by bootstrapping with 1000 samples.

A complete listing of the lay descriptions and values for the 440 health states (including combined health states) used in GBD 2019 is provided in table S12.

Section 4.9: Comorbidity correction (COMO)[2]

The final stage in the estimation of YLDs is a micro-simulation, which adjusts for comorbidity. We refer to this micro-simulation process as “COMO” (for comorbidity correction). For GBD 2019, we estimated the co-occurrence of different diseases by simulating 40,000 individuals in each location-age-sex-year

combination as exposed to the independent probability of having any of the sequelae included in GBD 2019 based on disease prevalence. We tested the contribution of dependent and independent comorbidity in the US MEPS data and found that independent comorbidity was the dominant factor even though well-known examples of dependent comorbidity exist, such as clustering of conditions like diabetes and stroke or anxiety and alcohol use disorders. Age was the main predictor of comorbidity such that age-specific micro-simulations accommodated most of the required comorbidity correction.[67]

The two components necessary for the computation of YLDs, prevalence of each disease sequelae and DWs, are the two inputs into COMO. The prevalence values are primarily produced by using DisMod-MR 2.1. The DWs have been described earlier in this appendix.

The micro-simulation, as performed for each age-sex-location-year, can best be represented as a four-step process. First, simulants are exposed to independent probabilities of having each sequela, where the probability is equal to the prevalence estimate. For each simulant, the probability of having a disease sequela is equal to the estimated prevalence from that draw from the uncertainty distribution. Each simulant is determined to have or not have the disease sequelae based on a draw from a binomial distribution. From this simulation, simulants end up having from no to multiple disease sequelae. Second, the DW for each simulant is estimated on the basis of the disease sequelae that they have acquired. The formula for the cumulative DW for a simulant is one minus the multiplicative sum of one minus each DW present

$$Simulant\ DW_l = 1 - \prod_{k=i}^{j}(1 - DW_k)$$

Where:

DW_k is the DW for the kth disease sequela that the simulant *l* has acquired.

Once the simulant DW is computed, the DW attributable to each sequela for the simulant is calculated by using the following formula:

$$ADW_{lk} = \frac{DW_k}{\sum_{k=i}^{k=j} DW_k} * Simulant\ DW_l$$

Where:

ADW_{lk} is the attributable DW for disease sequela *k* in simulant *l*

DW_k is the DW for disease sequela *k*

Simulant DW_l is the DW for simulant *l* from the combination of all sequelae that they have acquired.

This formula apportions the overall simulant DW to each condition in proportion to the DW of each condition in isolation.

Finally, YLDs per capita in an age-sex-country-year are computed by taking the sum of the attributable DWs for a disease sequela across simulants.

$$YLD\ Rate_k = \frac{\sum_{l=1}^{n} ADW_{lk}}{n}$$

The actual number of YLDs from disease sequela *k* in an age-sex-location-year is then computed as the YLD rate *k* times the appropriate age-sex-location-year population.

By repeating the simulation process for each age-sex-country-year 1000 times, the uncertainty in the prevalence of each disease sequela and the DW is propagated into the final comorbidity corrected YLD results. We selected 40,000 simulants for each age-sex-location-year group on the basis of simulation testing, which has shown that results are stable for YLDs at this number of simulants even in the younger age groups when prevalence is relatively low. Mean results for YLDs that reflect 40 million simulants (40,000 simulants multiplied by 1000 iterations to capture uncertainty) are very stable in each age-sex-location-year. For any given location-year-age-sex group, sequelae with a prevalence of less than one in 20,000 were excluded from the micro-simulation.

Section 4.10: YLD computation, uncertainty, and residual YLDs[2]

For GBD 2019, we computed YLDs by sequela as prevalence multiplied by the DW for the health state associated with that sequela. The uncertainty ranges reported around YLDs incorporate uncertainty in prevalence and uncertainty in the DW. To do this, we take the 1000 samples of comorbidity-corrected YLDs and 1000 samples of the DW to generate 1000 samples of the YLD distribution. We assume no correlation in the uncertainty in prevalence and DWs. The 95% uncertainty interval is reported as the 25th and 975th values of the distribution. UIs for YLDs at different points in time (1990, 1995, 2000, 2005, 2010, and 2016) for a given disease or sequela are correlated because of the shared uncertainty in the DW. For this reason, changes in YLDs over time can be significant even if the UIs of the two estimates of YLDs largely overlap because significance is determined by the uncertainty around the prevalence estimates.

Section 4.10.1: Residual YLDs

Despite expanding our list of causes and sequelae in successive GBD iterations, many diseases remain for which we do not explicitly estimate disease prevalence and YLDs. Less common diseases and their sequelae were included in 35 residual categories (table S13). For 22 of these residual categories, epidemiological data on incidence or prevalence were available, so these were modelled accordingly. For 13 residual categories, epidemiological data on incidence and prevalence were not available, but sufficient CoD data allowed for CoD estimates. For these residual categories, we estimated YLDs by multiplying the residual YLL estimates by the ratio of YLDs to YLLs from the estimates Level 3 causes in the same disease category that were explicitly modelled. This scaling was done for each country-sex-year. This approach made the simplifying assumption that the residual diseases caused disability proportionate to the ratio of disability to mortality in explicitly modelled diseases. We did not include causes with large disability but no or little mortality in estimating these ratios. For example, we estimated the YLDs from other neurological disorders from the YLD to YLL ratios for dementia, multiple sclerosis, and Parkinson's disease but did not include the YLDs from headaches and epilepsy in the ratio.

Section 4.11: Birth prevalence[2]

A number of conditions are present at birth, and quantifying them is important in fully describing the epidemiology of diseases within populations. These include many conditions included in the GBD cause group of neonatal disorders, infections that are transmitted from mother to child either transplacentally or during birth, and congenital birth defects arising either *de novo* or from maternal exposures. Although these conditions were included in the underlying models informing previous GBD iterations, we developed a system for reporting them for the first time in GBD 2017; a list of these causes is reported in table S14.

Mathematically (ie, in the models), conditions present at birth are equivalent to "birth prevalence." However, we report these as "incidence" in recognition of the way that GBD defines incidence as a new case of a disease or injury entering the population. To process these results for publication in GBD, we used a three-step process. First, the number of cases at birth was calculated as birth prevalence rate multiplied by number of live births for each location, sex, and year. Second, the number of cases present at birth were summed with incident cases during the early neonatal period (calculated as the 0-to-6-days incidence rate times the 0-to-6-days population), and the early neonatal incidence rate was recalculated by re-dividing by the 0-to-6-days population. Third, incidence rates for aggregate age groups were re-calculated by using the revised incidence figures for the early neonatal period.

Causes included in reporting are all of those for which birth prevalence has been estimated in GBD 2019 as part of existing modelling processes. Although extensive, this list should not be considered exhaustive of all of the conditions that can be present at birth. Future efforts in GBD will focus on identifying and comprehensively including all conditions present at birth, including revision of model frameworks as necessary. These efforts will also be facilitated by continuing improvements in the resolution of epidemiologic estimates of disease burden during pregnancy. These efforts are also expected to facilitate subsequent analyses derived from GBD that evaluate how maternal interventions, including pregnancy surveillance, can influence patterns of neonatal, infant, and child health.

Section 4.12: Non-fatal cause-specific modelling descriptions

GBD 2019 non-fatal appendix write-ups in order:

1. HIV/AIDS
2. Sexually transmitted infections excluding HIV
3. Tuberculosis
4. Lower respiratory infections
5. Upper respiratory infections
6. Otitis media
7. Diarrhoeal diseases
8. Typhoid and paratyphoid
9. Invasive non-typhoidal Salmonella (iNTS)
10. Other intestinal infectious diseases
11. Malaria
12. Chagas disease
13. Visceral leishmaniasis
14. Cutaneous and mucocutaneous leishmaniasis
15. African trypanosomiasis
16. Schistosomiasis
17. Cysticercosis
18. Cystic echinococcosis
19. Lymphatic filariasis
20. Onchocerciasis
21. Dengue
22. Yellow fever
23. Rabies
24. Ascariasis
25. Trichuriasis
26. Hookworm disease
27. Food-borne trematodiases

28. Leprosy
29. Ebola virus disease
30. Zika virus disease
31. Guinea worm disease
32. Other neglected tropical diseases
33. Meningitis
34. Encephalitis
35. Diphtheria
36. Whooping cough (pertussis)
37. Tetanus
38. Measles
39. Varicella and herpes zoster
40. Acute hepatitis
41. Other unspecified infectious diseases
42. Maternal disorders
43. Neonatal preterm birth
44. Nutritional deficiencies
45. Neoplasms
46. Rheumatic heart disease
47. Ischaemic heart disease
48. Stroke
49. Non-rheumatic valvular heart disease
50. Myocarditis
51. Atrial fibrillation and flutter
52. Peripheral artery disease
53. Endocarditis
54. Other cardiovascular and circulatory diseases
55. Chronic obstructive pulmonary disease
56. Pneumoconiosis

57. Asthma

58. Interstitial lung disease and pulmonary sarcoidosis

59. Other chronic respiratory diseases

60. Cirrhosis and other chronic liver diseases

61. NAFLD

62. Peptic ulcer disease

63. Gastritis and duodenitis

64. Gastro-oesophageal reflux disease

65. Appendicitis

66. Paralytic ileus and intestinal obstruction

67. Inguinal, femoral, and abdominal hernia

68. Inflammatory bowel disease

69. Vascular intestinal disorders

70. Gallbladder and biliary diseases

71. Pancreatitis

72. Other digestive diseases

73. Alzheimer's disease and other dementias

74. Parkinson's disease

75. Multiple sclerosis

76. Motor neuron disease

77. Headache disorders

78. Other neurological disorders

79. Schizophrenia

80. Major depressive disorder

81. Dysthymia

82. Bipolar disorder

83. Anxiety disorders

84. Anorexia nervosa

85. Bulimia nervosa

86. Autism spectrum disorders

87. Attention-deficit/hyperactivity disorder

88. Conduct disorder

89. Other mental disorders

90. Alcohol use disorders

91. Alcohol use disorders (fetal)

92. Opioid use disorders

93. Cocaine use disorders

94. Amphetamine use disorders

95. Cannabis use disorders

96. Other drug use disorders

97. Diabetes mellitus

98. Chronic kidney disease

99. Acute glomerulonephritis

100. Dermatitis

101. Psoriasis

102. Cellulitis

103. Pyoderma

104. Scabies

105. Fungal skin diseases

106. Viral skin diseases

107. Acne vulgaris

108. Alopecia areata

109. Pruritus

110. Urticaria

111. Decubitus ulcer

112. Other skin and subcutaneous diseases

113. Other sense organ diseases

114. Rheumatoid arthritis

115. Osteoarthritis

116. Low back pain

117. Neck pain

118. Gout

119. Other musculoskeletal disorders

120. Congenital birth defects

121. Urinary tract infection and interstitial nephritis

122. Urolithiasis

123. Benign prostatic hyperplasia

124. Other urinary diseases

125. Gynaecological diseases

126. Haemoglobinopathies and haemolytic anaemias

127. Endocrine, metabolic, blood, and immune disorders

128. Oral disorders

129. Injuries

130. Sexual violence

131. Anaemia

132. Epilepsy

133. Guillain-Barré syndrome

134. Hearing loss

135. Heart failure

136. Infertility

137. Developmental intellectual disability

138. Pelvic inflammatory disease

139. Blindness and vision impairment

140. Fistula

HIV/AIDS

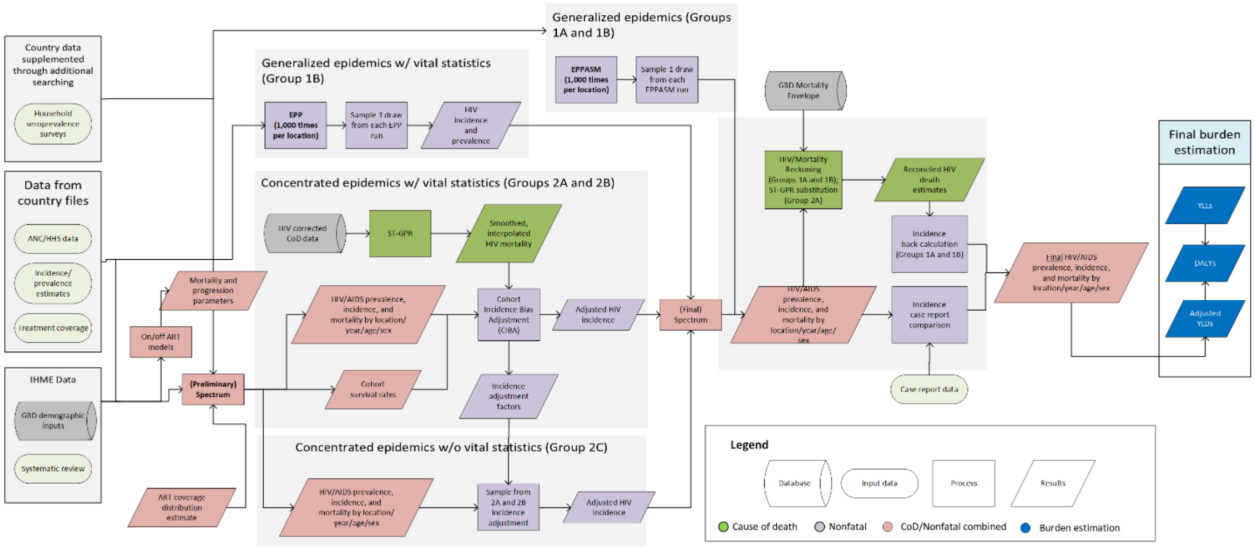

Case definition

Infection with the human immunodeficiency virus (HIV) causes influenza-like symptoms during the acute period following infection and can lead to acquired immunodeficiency syndrome (AIDS) if untreated. HIV attacks the immune system of its host, leaving infected individuals more susceptible to opportunistic infections like tuberculosis. Although there are two different subtypes of HIV, HIV-1 and HIV-2, no distinction is made in our estimation process or presentation of results. For HIV, ICD 10 codes are B20-B24, C46-C469, D84.9; ICD 9 codes are 042-044, 112-118 (after 1980), 130 (after 1980), 136.3-136.8 (after 1980), 176.0-176.9 (after 1980), 279 (after 1980); and ICD9 BTL codes are B184-B185.

Input data

Case reports

We used case reports from countries believed to have high quality data for case notifications, mainly countries in our high-income super region and with 4 or 5-star vital registration data (Group 2A, as described below). These reports were extracted from country-level reports.

Household seroprevalence surveys

Geographically representative HIV seroprevalence survey results were used as inputs to the model for countries with generalised HIV epidemics where available.

GBD demographic inputs

Location-specific population, fertility, migration and HIV-free survival rates from GBD 2019 were used as inputs in modelling all locations.

Data from countries

The files compiled by UNAIDS for their HIV/AIDS estimation process were our main source of data for producing estimates of HIV burden. Spectrum files are often built by within-country experts with the support of UNAIDS, who publishes estimates annually on behalf of countries and only shares their Spectrum files when permission is granted. The files contain the HIV-specific information which is

needed to run the Spectrum, the Estimation and Projection Package (EPP) model and the Estimation and Projection Package Age Sex Model (EPPASM).

Spectrum and EPPASM require the following input data: AIDS mortality among people living with HIV with and without ART, CD4 progression among people living with HIV not on ART, ART coverage among adults and children, Cotrimoxazole coverage among children, coverage of breastfeeding among women living with HIV, prevention of mother-to-child transmission coverage, and CD4 thresholds for treatment eligibility. EPPASM additionally uses HIV prevalence data from surveillance sites and representative surveys. In contrast to Spectrum and EPPASM, EPP fits a simpler adult-only model to HIV prevalence data from surveillance sites and representative surveys. Antenatal care (ANC), incidence, prevalence, and treatment coverage data from UNAIDS were used in modelling for all locations. We extracted all of these data from the proprietary format used by UNAIDS.

We did not have country UNAIDS files for 40 locations, many of them countries with small populations and/or low HIV prevalence. In those places, we generated regional averages of all needed inputs. This enabled us to run Spectrum for every GBD location.

Vital registration data

We used all available sources of vital registration and sample registration data from the GBD Causes of Death database after garbage code redistribution and HIV/AIDS mis-coding correction, except in Group 1A countries as described below.[1, 2] There are two different cause of death data sources for HIV/AIDS in China: the Disease Surveillance Point (DSP) system and the Notifiable Infectious Disease Reporting (NIDR) system. Both systems are administered by the Chinese Center for Disease Control and Prevention, but the reported number of deaths due to HIV is significantly lower in DSP. Therefore, we have used the provincial-level ratio of deaths due to HIV/AIDS from NIDR to those from DSP, choosing the larger ratio between years 2013 and 2014, and scaled the reported deaths in the DSP system, which is in turn used in the spatiotemporal Gaussian process regression (ST-GPR).

On-ART literature data

Data were identified by using search terms "HIV," "mortality," and "antiretroviral therapy" in PubMed searches across the literature. To be included, studies must include only HIV-positive people who receive antiretroviral therapy (ART) but who were ART-naïve prior to the study. In addition, studies must report either a duration-specific (time since initiation of ART) mortality proportion or a hazard ratio across age or sex, and must not include children.

For duration-specific survival data, studies must report uncertainty on mortality estimates or provide stratum-specific sample sizes and must include duration-specific data to allow for calculation of 0-6, 7-12, or 13-24 month conditional mortality. In addition, studies must either report separate mortality and loss-to-follow-up (LTFU) curves, be corrected for LTFU using vital registration data or double sampling, or be conducted in a high-income setting. Finally, studies must report the percent of participants who are male and the median age of participants.

Hazard ratio data for ages or sexes can only be used if the hazard ratios are controlled for other variables of interest (age, sex, and CD4 category). In GBD 2013, we identified 102 papers for extraction. For GBD 2015, we included 13 additional studies informing the duration-specific mortality estimation process and 26 studies informing the age and sex hazard ratio estimation process (some studies were used and counted in both). We also added one study to our LTFU analysis. For GBD 2016, we included 12 additional studies informing the duration-specific mortality estimation process and 11 studies informing the age and sex hazard ratio estimation process (some studies were used and counted in both). For GBD

2017, we included 17 additional studies informing the duration-specific mortality estimation process and 13 studies informing the age and sex hazard ratio estimation process (some studies were used and counted in both). We also included two new studies in our LTFU analysis. For GBD 2019, we did not update the systematic review or add cohort studies.

Off-ART literature data

In GBD 2013, we systematically reviewed the literature on mortality without ART to characterise uncertainty in the progression and death rates. We searched terms related to pre-ART or ART-naive survival since seroconversion.[3] After screening, we identified 13 cohort studies that included the cohorts used by UNAIDS, from which we extracted survival at each one-year point after infection. Screening for additional, recently published studies in GBD 2015, GBD 2016 and GBD 2017 identified no new cohort studies for inclusion in this analysis. We did not search for new studies in GBD 2019.

Severity splits and disability weights

The basis of the GBD disability weight survey assessments are lay descriptions of sequelae highlighting major functional consequences and symptoms. The lay descriptions and disability weights for HIV/AIDS severity levels are shown below.

**Severity level**	**Lay description**	**DW (95% CI)**
Symptomatic HIV	Has weight loss, fatigue, and frequent infections.	0.274 (0.184–0.377)
AIDS with antiretroviral treatment	Has occasional fevers and infections. The person takes daily medication that sometimes causes diarrhoea.	0.078 (0.052–0.111)
AIDS without antiretroviral treatment	Has severe weight loss, weakness, fatigue, cough and fever, and frequent infections, skin rashes, and diarrhoea.	0.582 (0.406–0.743)

Modelling strategy

We continued to estimate on-ART and off-ART mortality by CD4 count as in GBD 2017, which is described below. However, in GBD 2019, our burden estimation strategy for HIV incidence, prevalence, and mortality diverged from GBD 2017. We continued to use the Spectrum program rewritten in Python for GBD 2013 to facilitate faster and more flexible execution necessary for our more intensive computational needs for Group 2 countries. For India, we used EPP and Spectrum, as in GBD 2017. However, we used EPPASM exclusively for the remaining Group 1 countries. Both EPP and EPPASM are open-source computer programmes in R written by Jeffrey Eaton.[4,5]

On-ART

First, we corrected reported probabilities of death for loss to follow-up using an approach developed by Verguet and colleagues.[6] Verguet and colleagues used tracing and follow-up studies to empirically estimate the relationship between death in LTFU and the rate of LTFU.

To create estimates of age-specific hazard ratios, we synthesised hazard ratio data in five broad age groups: 15-25, 25-35, 35-45, 45-55, 55-100, and modelled the data using DisMod-MR 2.1.

To create estimates of sex-specific hazard ratios, we use the *metan* function in Stata to create estimates of relative risks separately by region, using female age groups as the reference group.

The age and sex hazard ratios were applied to the study-level mortality rates, accounting for the distribution of ages and sexes in the mortality data. We then subtracted HIV-free mortality from the model life table process to calculate study-level age-sex HIV-specific mortality.

We used DisMod-MR 2.1 to synthesise the age-sex-split study-level data into estimates of conditional probability of death over initial CD4 count.[3] We modelled the data separately by duration, age, sex, and region and added a fixed effect on whether the study was conducted prior to 2002. We estimate mortality for each region in its own DisMod model based on data from the IeDEA cohort collaboration,[7] and include a covariate for year as mortality among the LFTU has been found to decline in recent years.[8] Finally, we replaced our on-ART mortality rates with those estimated off treatment if they were higher.

Off-ART

Following UNAIDS assumptions, no-ART mortality is modelled as shown in the figure below.[3]

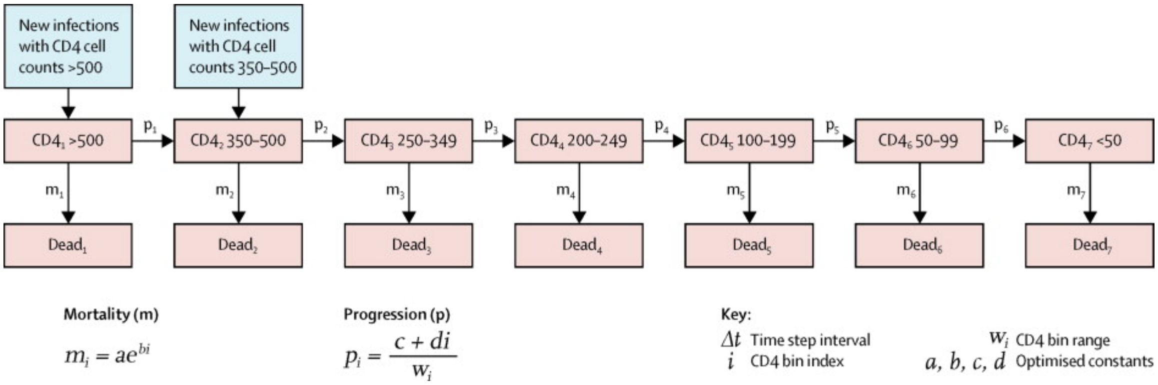

The death and progression rates between CD4 categories vary by age according to four age groups: 15–24 years, 25–34 years, 35–44 years, and 45 years or older. We modelled the logit of the conditional probability of death between years in these studies using the following formula:

$$logit\ (m_{ijk}) = \beta_0 + \sum_{i=1}^{4} \beta_{1i}\, a_i + \sum_{j=1}^{12} \beta_{2j} t_j + u_k + \varepsilon_{ijk}$$

In the formula, m is conditional probability of death from year t_j to t_{j+1}, a_i is an indicator variable for age group at seroconversion (15–24 years, 25–34 years, 35–44 years, and 45 years or older), t_j is an indicator variable of year since seroconversion, and u_K is a study-level random effect.

By sampling the variance-covariance matrix of the regression coefficients and the study-level random effect, we generated 1,000 survival curves for each age group that capture the systematic variation in survival across the available studies. For each of the 1,000 survival curves, we used a framework

modelled after the UNAIDS optimisation framework in which we find a set of progression and death rates that minimises the sum of the squared errors for the fit to the survival curve.[9, 10]

GBD 2019 Burden estimation overview

We used three different components to derive year-, age- and sex-specific estimates of HIV incidence, prevalence, and mortality depending on locations' availability of data and extent of HIV burden. As described below:

1. EPPASM was used to estimate incidence, prevalence and mortality that are consistent with serosurveillance data from antenatal care clinics and/or prevalence surveys.

2. EPP was used to estimate age- and sex- aggregate incidence and prevalence trajectories that are consistent with serosurveillance data from antenatal care clinics and/or prevalence surveys in India subnational locations.

3. Spectrum is compartmental HIV progression model used to generate age-sex-specific incidence, prevalence, and death rates from input incidence and prevalence curves and assumptions about intervention scale-up and local variation in epidemiology. This model was used in conjunction with EPP for India, and for all Group 2 countries.

Changes for GBD 2019

EPPASM

For GBD 2019, we modified the UNAIDS version of EPPASM both to improve the fit to data and to generate pediatric estimates. We built a pediatric module in EPPASM that mirrored the recent developments to the pediatric module in Spectrum.[11] This child module included CD4 progression and CD4-specific mortality rates taken from a model fit to survival data from IeDEA and child initiation of ART based on ART distribution data from IeDEA. Perinatal and breastfeeding transmission was calculated as a function of prevalence among pregnant women and PMTCT program data. We were thus able to utilize EPP-ASM to produce HIV incidence, prevalence, and mortality estimates for all ages. Additionally, we improved fit to prevalence data through allowing flexibility in the age distribution of incidence over time. We parameterized the ratio of incidence among ages 15-24:25+ as a constant before year 2000 and a linear regression thereafter. This allowed for the shifts in the age distribution of incidence observed over the course of the HIV epidemic to be reflected in our results. Finally, we utilized GBD demographic inputs and substituted in our own assumptions about HIV progression rates and on/off ART mortality.

To incorporate uncertainty in our demographic and progression parameters, we run EPP-ASM with separate draws of CD4 progression, on- and off-ART mortality rates, fertility, and HIV-free mortality. This process produced 1,000 posterior distributions for each of the locations that make up Group 1A. For every location in the group, we sampled one draw from each of the sets of EPP-ASM results in order to create a final distribution. By sampling one draw from each set, we ensured that the distribution of mortality parameters dictating the relationship between incidence and prevalence aligned with those used in the GBD demographics estimates.

ANC Bias Adjustment

For GBD 2019, we also implemented a new approach to address selection bias resulting from temporal and geographic variation in ANC reporting. The ANC data which EPPASM uses cannot be assumed as representative of HIV prevalence in the full population. This is especially the case when there are minimal or no nationally representative prevalence surveys to anchor estimates, as in the early epidemic.[12]

EPPASM has embedded approaches to adjust for the bias associated with using prevalence among ANC-site attending pregnant women to estimate prevalence among the both-sexes population. For the bias between pregnant women and the national both sexes population, it makes assumptions around the difference in total fertility rate among HIV positive and HIV negative women, and the difference in prevalence between men and women. For the bias associated with the data coming from ANC sites, the specification of the likelihood of observed ANC data includes random intercepts for each clinic. The random intercepts allow each site's baseline prevalence to vary randomly around the overall mean prevalence. In other words, factors that could drive differences between sites' HIV prevalence levels are 'adjusted' for.

However, the embedded approach does not explicitly account for the fact that the location of the clinic in space may also drive its HIV prevalence level. For example, we might expect rural sites to be more correlated than urban sites. Thus, to further adjust for this bias, we used an offset term that represents the difference in the prevalence among the national, both sexes population and the prevalence among the female, pregnant population associated with an ANC site location. The offset term was derived for each location as the difference between the adjusted prevalence in a given site-year and the adjusted national prevalence in that year. These estimates are adjusted for covariates that are thought to influence prevalence, for example, access to health facilities, malaria incidence and male circumcision.

Thus our final strategy for estimating the likelihood of the observed ANC data was:

$$\boldsymbol{W_{st} = \varphi^{-1}(\rho_t) + \vartheta_{st} + u_s + e_{st}}$$

$$\boldsymbol{e_{st} \sim N(0, \sigma^2_{st})}$$

$$\boldsymbol{u_s \sim N(0, \sigma^2_s)}$$

Where:

W_{st} = the probit transformed prevalence at site *s* and time *t*
ρ_t= The national prevalence adjusted to represent prevalence among pregnant women from the model simulation
ϑ_{st} = The offset term representing the difference between the adjusted prevalence in a given site-year and the adjusted national prevalence in that year
φ^{-1} = probit transformation
e_{st} = Site-specific error term
u_s = Site specific intercept

Spectrum

For GBD 2013, we created an exact replica of Spectrum in Python. This enabled us to run thousands of iterations of the model at once on our computing cluster and allowed for more flexible input data structures. Additionally, we scaled all input values by a uniformly sampled factor between 0.9 and 1.1 to generate estimates with realistic ranges of uncertainty. For example, if treatment retention rates across CD4 categories were 0.906, 0.759, 0.787, 0.795, 0.785, 0.756, 0.813, and 0.700, we multiplied each

number by an array of equivalent size that contained factors ranging from .9 and 1.1. At each draw, the array would contain different, randomly selected factors in the same range. Further, we previously improved our sex-specific modelling strategy in Spectrum by sex-splitting incidence based on a model fit to the sex ratio of prevalence observed in countries with representative surveys and updated the Spectrum pediatric module to reflect changes made by UNAIDS.[11] Our child module was revised to include CD4 progression and CD4-specific mortality rates taken from a model fit to survival data from IeDEA. Finally, we updated child initiation of ART to include data on ART distribution from IeDEA. These changes were retained in GBD 2019.

ART coverage distribution

Spectrum determines the number of people initiating ART treatment across each CD4 category based on eligibility criteria, and the number of expected deaths and untreated people. In other words, groups with a large proportion of PLHIV and high numbers of expected deaths initiated the most individuals into treatment.

We improved the basis for this distribution using survey microdata and country-level wealth information. Three relevant surveys were available: Uganda AIS 2011 and Kenya AIS 2007 and 2012. These surveys conducted CD4 count measurements and include a question regarding the amount of time that an individual receiving ART had been enrolled in treatment. Survey data provide cross-sectional CD4 count information; however, the Spectrum modelling framework tracks individuals by categorical CD4 count at the initiation of treatment. In order to cross-walk the cross-sectional survey data into estimates of CD4 count at treatment initiation, we built a model using relevant cohort data which tracked changes in CD4 count after initiation of treatment to translate an individual's current CD4 count and duration on treatment into CD4 count at initiation of treatment. The functional form for changes in CD4 count as a function of duration on treatment was a natural spline on duration with knots at 3, 12, 24 and 36 months, and an interaction between initial CD4 count and duration.

After cross-walking, we predicted the probability of being on treatment as a function of individual income (measured through an asset-based index), stratified by CD4 count, age, and sex. The results of this prediction were translated into country-specific age-sex-year-CD4 count probabilities of coverage using a conversion factor between individual income and lagged distributed GDP per capita. We used stochastic frontier analysis to constrain the maximum possible coverage for a given degree of income and CD4 count.

Predicted probabilities of coverage were input to Spectrum to inform the distribution, and not the overall level, of ART treatment by CD4 count. Within Spectrum, the probabilities of coverage are converted to counts of expected individuals on treatment in each CD4 count group. These are scaled to the distribution across CD4 count groups to match the input data on the number of people on ART coming from UNAIDS country files. In cases where the predicted number of individuals initiating treatment exceeds the total number of untreated individuals in a CD4 count group, we reallocate treatment evenly to other CD4 count groups.

Countries with seroprevalence surveys and antenatal clinic data (Groups 1A and 1B)

We identified 50 countries – as well as subnational locations in India, Kenya, Ethiopia, Nigeria and South Africa – with at least 0.5% adult HIV prevalence and at least one geographically representative HIV seroprevalence survey or available antenatal care clinic (ANC) data. For all locations except India we used a version of EPPASM, and for India we used a version of EPP. Both were written in R and C++ by Jeffrey Eaton. The version of EPP and EPPASM used in GBD 2019 was updated to incorporate the new

ANC bias adjustment. Further we added a pediatric module in EPPASM which was a replicate of the pediatric model embedded in Spectrum.

EPP and EPPASM rely on the parameter estimation via the IMIS procedure, described in Raftery and Bao.[13] Two optimisation methods have been introduced. The main algorithm is Broyden–Fletcher–Goldfarb–Shanno (BFGS) optimisation. If BFGS fails, Nelder-Mead optimum is used instead.[14-16] To incorporate uncertainty in our mortality and progression parameters, we run EPP with separate draws of each of these parameters. Then, for every location, we have 1000 linked draws of adult incidence and prevalence and the exact mortality and progression parameters that generated those draws. For EPP locations (India), we then ran these results, along with the previously described demographic and HIV-specific inputs, through Spectrum to produce location-, year-, age-, and sex-specific estimates of HIV incidence, prevalence, and mortality.

The HIV/mortality reckoning process is intended as a method of reconciling separate estimates of HIV mortality (and its resulting effect on estimates of HIV-free and all-cause mortality) in Group 1 countries by averaging estimates of HIV mortality from the model life table process and our modelled estimates. Additional details on the reckoning can be found elsewhere.[17]

Since EPPASM produces HIV incidence, prevalence, and deaths that are consistent with one another over time, the reckoning process results in death numbers that are no longer consistent with the incidence and prevalence produced in Spectrum. In order to recreate this consistency, we recalculated incidence for all Group 1 locations using reckoned deaths and prevalence produced by EPP-ASM. The updated incidence is calculated by aggregating counts of new infections, HIV deaths from EPP-ASM, and HIV deaths after reckoning at the year-sex level. The difference between reckoned HIV deaths and HIV deaths from EPPASM is added to EPPASM incidence, and we calculate the ratio between updated incidence and EPPASM incidence. Age-specific counts of new infections are then scaled by their corresponding sex-year ratios.

Countries with vital registration data (All of Group 2A, 2B and India)

Vital registration is one of the highest-quality sources of data on HIV burden in many countries, so generating estimates that are consistent with these data with necessary adjustment to account for any potential underreporting is critical. We identified 121 countries – as well as 632 subnational locations from China, Japan, Indonesia, India, Mexico, Sweden, Philippines, Poland, Italy, the United Kingdom, Ukraine, Russia, New Zealand, Iran, Norway and the United States – with usable points of vital registration data, verbal autopsy (VA) data, or sample registration system (SRS) data. In India, Vietnam and Indonesia, we used SRS and VA data, respectively, as input mortality for CIBA. For India we extracted the resulting age-sex distribution of incidence but scaled the level to match the adult incidence rate estimated from EPP for each state.

We imputed missing years of data to generate a complete time series for HIV from the estimated start year of the epidemic using ST-GPR. We analysed mortality trends using ST-GPR starting in 1981, the year that HIV was first identified in the United States.[18] For ST-GPR, we adjusted the lambda (time weight) and GPR scale according to the completeness of vital registration data, with 4- and 5-star quality VR using parameters designed to follow the data more closely. We produced separate splines by country/age group, up to the peak year of death rate. We then ran a linear regression with fixed effects on region, age, and sex. Following this, we ran space-time residual smoothing, in which time, age, and space weights are used to inform smoothing of the residuals between data points and the linear regression estimate. From this process, we generated space-time estimates with the applied weights,

along with the median absolute deviation (MAD) of the space-time estimates from the data. The MAD was calculated at various levels of the geographic hierarchy (eg, subnational and national), and was added into the data variance term. The data variance and space-time estimates were then analysed using Gaussian process regression to return a final estimate of mortality along with uncertainty.

Although Spectrum produces HIV mortality estimates that are within the realm of possibility in most countries using the incidence curves provided in the UNAIDS country files, it is a deterministic model that has not yet been integrated into an optimisable framework. Therefore, in order to "fit" it to vital registration data, we need to adjust input incidence.

To improve the fit of this process, in GBD 2015, we restructured Spectrum to track cohorts by year of HIV infection. With this version of Spectrum we can output, among many other metrics, HIV deaths by year, age, sex, and infection cohort. This enables us to adjust incidence to fit to death much more precisely and without making any rigid assumptions about the time from HIV infection to HIV death.

We have incorporated these improvements into a cohort incidence bias adjustment (CIBA) process. First, we ran Spectrum normally to produce 1,000 draws of incidence, prevalence, and mortality. Then, by year, age, and sex, we took the ratio of VR deaths to Spectrum deaths to quantify the amount of bias in Spectrum. Using draw-level duration data from the new version of Spectrum, for every year-, age-, and sex-specific infection cohort, we calculated the share of all HIV deaths observed over the course of the projection period in that cohort that would occur in each year after the year of infection. For example, projecting from 1970 through 2019, we identified the cohort of men infected in 1992 at the age of 16, calculated the total number of HIV deaths in that cohort in all subsequent years through the end of 2019, and divided the annual number of deaths by that total. This showed us the distribution of deaths among that cohort over the projection period. In the most extreme case (infections in 2018), we could only produce one point of that distribution (2019), so that single value is exactly 1·0; 100% of the deaths observed in that cohort occurred in 2019.

We then used these distributions of death to weigh the ratio of VR deaths to Spectrum deaths, meaning that ratios in the years where we expect the largest share of deaths were weighed most heavily. We then multiplied the initial size of that cohort from the normal run of Spectrum by the sum of the combined ratios to get a new estimate of new cases in that year/age/sex combination. We can write this method mathematically in the following way:

$$r_t = \frac{VR_t}{D_t}$$

$$\rho_t^{t-i} = \frac{d_t^{t-i}}{\sum_{k=t-i+1}^{n} d_k^{t-i}}$$

$$\alpha^{t-i} = \sum_{k=t-i+1}^{n} r_k * \rho_k^{t-i}$$

$$n_{\text{adjusted}}^{t-i} = \alpha^{t-i} * n^{t-i}$$

VR_t is the number of HIV/AIDS deaths in year t from ST-GPR, and D_t is the number of HIV/AIDS deaths from the first run of Spectrum. In the second equation, d_t^{t-i} is the number of HIV/AIDS deaths among members of infection cohort $t-i$ in year t, with $i \geq 1$, from the new, duration-tracking version of Spectrum, and n is final year of the projection. Therefore, ρ_t^{t-i} is the share of observed deaths in cohort $t-i$ that we expect to occur in year t. It follows that α^{t-i} is the weighted adjustment ratio described above, which we multiply by the estimated initial size of infection cohort $t-i$ as calculated in the first-

stage Spectrum run to get the adjusted number of new cases, $n_{\text{adjusted}}^{t-i}$. This process is run separately for every sex, single-age, and draw.

CIBA allows ratios in each year after a given infection year to influence the final adjustment to incidence. The size of that influence is determined by the relative importance of that year in the cohort-year's distribution of deaths over time. The result is a new set of 1,000 draws of incidence and a set of 1,000 ratios of post-adjustment incidence to pre-adjustment incidence. We perform this adjustment using mean durations from the new version of Spectrum in order to try to shift the mean of the regular distribution of deaths.

To produce final location-, year-, age-, and sex-specific estimates of HIV incidence, prevalence, and mortality, we ran the new estimates of incidence and all previously input data through Spectrum.

For countries with high quality case reports data we then took an additional step of scaling Spectrum incidence to the case reports. We assumed a five-year lag to diagnosis, meaning, for example, that case reports from 2008 were assumed to be incident cases in year 2003. We applied the scalar from the first year of case reports data to years prior to case reports data. For years after the five-year lag on the most recent case reports data, we applied the same scalar from the last year with case reports data, resulting in an adjustment on the full incidence time series. Importantly, we only scaled upwards. In years where the case reports reported lower incident cases than the Spectrum estimates, we did not scale the incidence.

Countries without survey data and vital registration data (Group 2C)
40 countries had neither geographically representative seroprevalence surveys nor reliable vital registration systems. To produce estimates of HIV burden in these countries, we assumed that Spectrum is similarly biased as in other Group 2 countries within the same super-region. This involved running Spectrum, adjusting incidence using 1,000 adjustment ratios randomly sampled from CIBA results from the same super-region, and rerunning Spectrum using the new draws of adjusted incidence. As above, the estimates of incidence, prevalence, and mortality were incorporated into the rest of the machinery via the reckoning process.

Measure	Total Sources	Countries with data
All measures	6390	193
Prevalence	107	45
Incidence	1092	70
Cause-specific mortality rate	3960	164
Proportion	1231	152

References

1. Global, regional, and national age–sex specific all-cause and cause-specific mortality for 240 causes of death, 1990–2013: a systematic analysis for the Global Burden of Disease Study 2013. The Lancet 2015; 385: 117–71.

2. Birnbaum JK, Murray CJ, Lozano R. Exposing misclassified HIV/AIDS deaths in South Africa. Bull World Health Organ 2011; 89: 278–85.

3. Murray CJL, Ortblad KF, Guinovart C, et al. Global, regional, and national incidence and mortality for HIV, tuberculosis, and malaria during 1990–2013: a systematic analysis for the Global Burden of Disease Study 2013. The Lancet 2014; 384: 1005–70.

4. jeffeaton/epp. GitHub. https://github.com/jeffeaton/epp (accessed July 1, 2019).

5. mrc-ide/eppasm. GitHub. https://github.com/mrc-ide/eppasm (accessed July 1, 2019).

6. Verguet S, Lim SS, Murray CJL, Gakidou E, Salomon JA. Incorporating Loss to Follow-up in Estimates of Survival Among HIV-Infected Individuals in Sub-Saharan Africa Enrolled in Antiretroviral Therapy Programs. J Infect Dis 2013; 207: 72–9.

7. Anderegg N, Johnson LF, Zaniewski E, et al. All-cause mortality in HIV-positive adults starting combination antiretroviral therapy: correcting for loss to follow-up. AIDS 2017; 31 Suppl 1: S31-40.

8. Zürcher K, Mooser A, Anderegg N, et al. Outcomes of HIV-positive patients lost to follow-up in African treatment programmes. Trop Med Int Health 2017; 22: 375-387.

9. Ghys PD, Zaba B, Prins M. Survival and mortality of people infected with HIV in low and middle income countries: results from the extended ALPHA network. AIDS Lond Engl 2007; 21 Suppl 6: S1–4.

10. Hallett TB, Zaba B, Todd J, et al., ALPHA Network. Estimating incidence from prevalence in generalised HIV epidemics: methods and validation. PLoS Med 2008; 5: e80.

11. Mahy M, Penazzato M, Ciaranello A, et al. Improving estimates of children living with HIV from the Spectrum AIDS Impact Model. Aids 2017;31: S13–S22

12. Ng M, Gakidou E, Murray CJL, Lim S. A comparison of missing data procedures for addressing selection bias in HIV sentinel surveillance data. Population Health Metrics 2013; 11: 12.

13. Raftery AE, Bao L. Estimating and Projecting Trends in HIV/AIDS Generalized Epidemics Using Incremental Mixture Importance Sampling. Biometrics 2010; 66: 1162–73.

14. Nelder JA, Mead R. A simplex algorithm for function minimization. Comput J 1965;7:308-13.

15. Nash JC. Compact numerical methods for computers. Linear algebra and function minimization. 2nd edn. Bristol, England: Adam Hilger, 1990.

16. Byrd RH, Lu P, Nocedal J, et al. A limited memory algorithm for bound constrained optimization. SIAM J Sci Comput 1995;16:1190-208.

17. Wang H, Murray CJL, Carter A, He F. Global, regional, and national under-5 mortality, adult mortality, age-specific mortality, and life expectancy, 1970-2016: a systematic analysis for the Global Burden of Disease Study 2016. The Lancet 2017; 390: 1151-1210.

18. CDC. Pneumocystis Pneumonia --- Los Angeles. MMWR Wkly. 1981; published online June 5. http://www.cdc.gov/mmwr/preview/mmwrhtml/june_5.htm (accessed April 21, 2016).

Sexually transmitted infections (STIs), excluding HIV:
Chlamydia, gonorrhea, trichomoniasis, genital herpes due to HSV-2, syphilis, and other STIs

Flowcharts

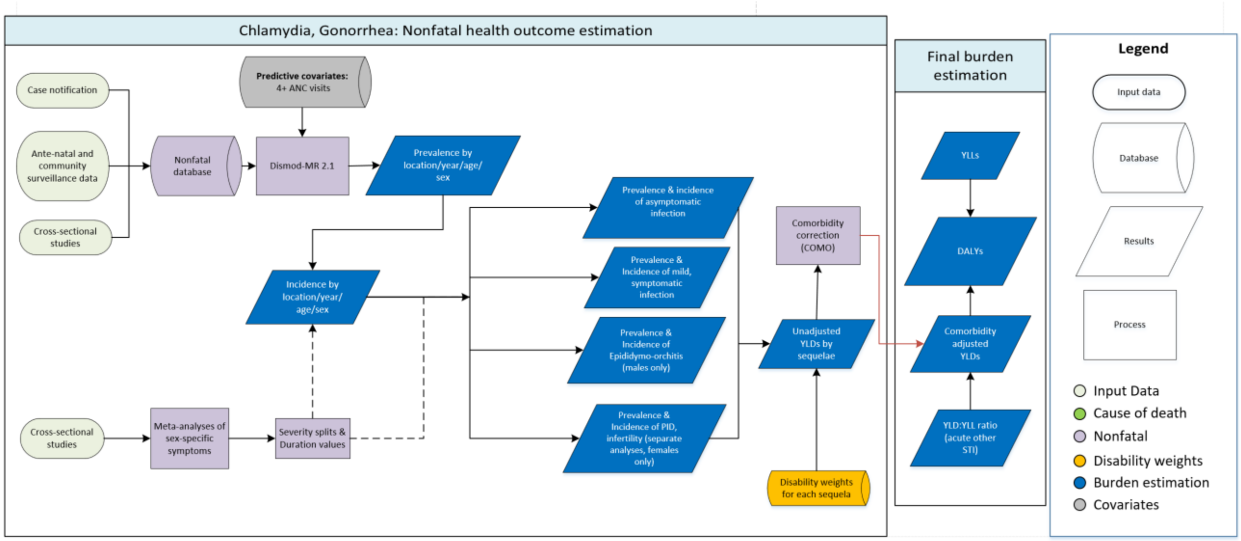

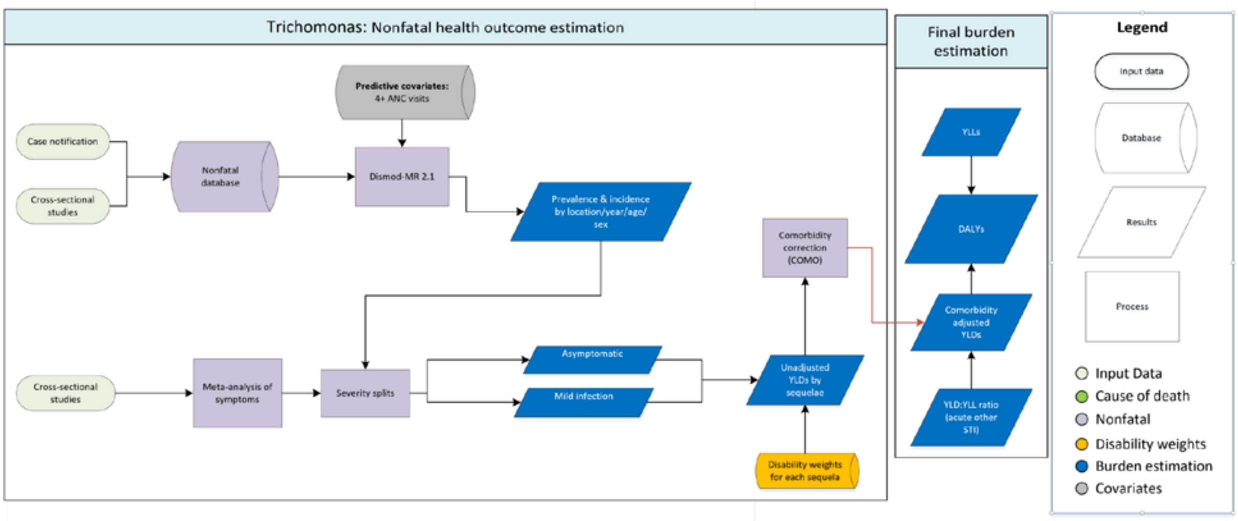

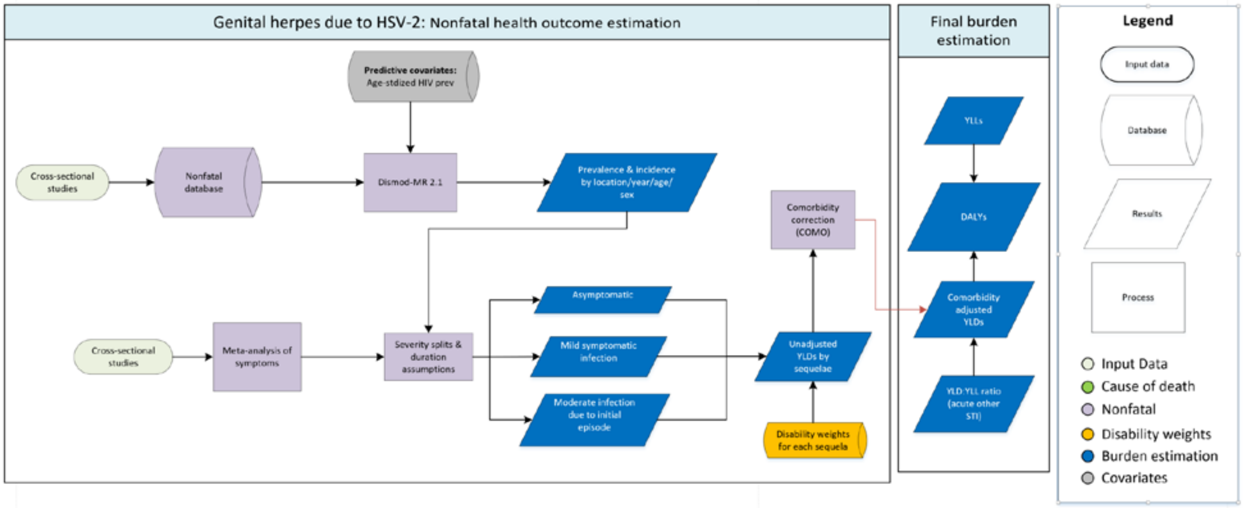

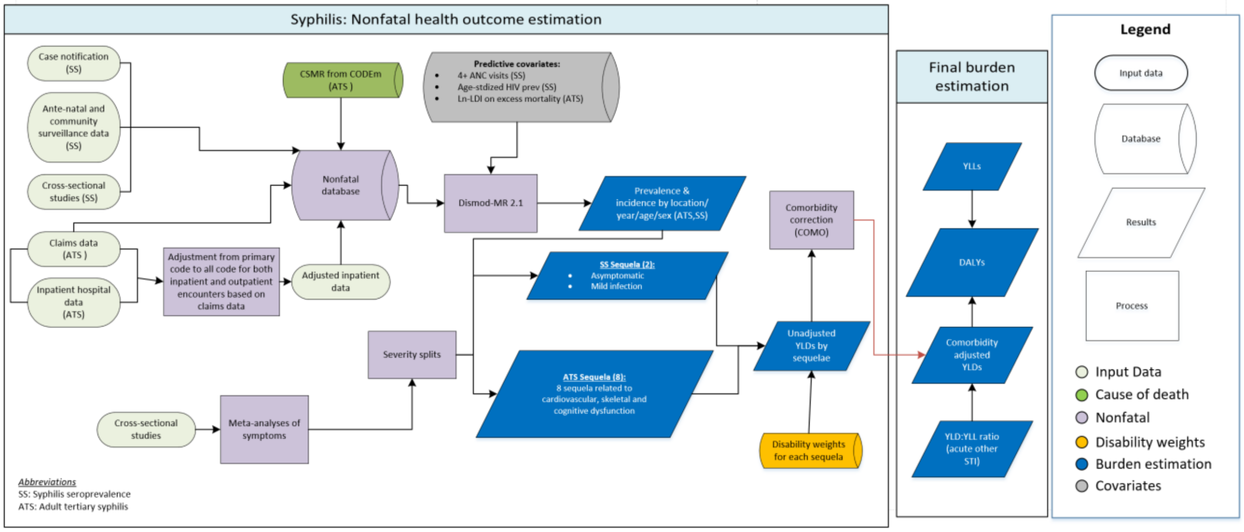

Input data and methodological summary

Case definition

For GBD 2019, we estimated the prevalence, incidence, and YLDs of genital and reproductive tract infection with several sexually transmitted infections (STIs): *Chlamydia trachomatis*, *Neisseria gonorrhoea*, *Trichomonas vaginalis*, *Treponema pallidum* (syphilis), and HSV-2. Syphilis was estimated in two separate models, an adult seroprevalence model, from which we estimated the occurrence of early (primary, secondary, and early latent), sexually acquired syphilis, and a separate model of adult tertiary syphilis. The seroprevalence model also served as a covariate in other estimation processes in GBD; see separate appendix sections on estimation of fatal burden of STI for details. The nonfatal burden of congenital syphilis was not estimated. Case definitions for all of these infections were based on laboratory findings (see below for details), except late syphilis, which was ascertained from administrative data using ICD-9 093-095 and ICD-10A52 and I98.0.

Input data

Prevalence and incidence data sources

Systematic literature reviews for STIs were completed on April 17, 2015 for GBD 2015. These were done for chlamydia, gonorrhoea, trichomonas, genital herpes, and syphilis. Three related search strings were used as many studies report on multiple infections. With the exception of the syphilis literature review, which was first conducted in GBD 2015, these were the same search strings and strategies that were previously employed in systematic reviews for GBD 2013.

462 initial hits; 54 sources selected from full text review for data extraction: (((chlamydia[Title/Abstract] OR chlamydia tracomatis[Title/Abstract] OR trachoma[Title/Abstract]) AND prevalence[Title/Abstract]) AND ('2013'[Date - Publication] : '2015'[Date - Publication])) /// ((gonorrhea[Title/Abstract] OR Neisseria[Title/Abstract] OR gonococcal[Title/Abstract]) AND prevalence[Title/Abstract]) AND ("2013"[PDAT] : "2015"[PDAT]) /// ((trichomonal[Title/Abstract] OR trichomonas[Title/Abstract]) AND prevalence[Title/Abstract]) AND ('2013'[PDAT] : '2015'[PDAT])

1265 initial hits; 178 sources selected from full text review for data extraction: ("syphilis"[MeSH] OR "Treponema pallidum"[MeSH]) NOT "Yaws"[MeSH] AND "prevalence"[MeSH] AND "1990"[PDAT] : "2015"[PDAT] AND "humans"[MeSH] **///** ("syphilis"[MeSH] OR "Treponema pallidum"[Mesh]) NOT "Yaws"[MeSH] AND ("incidence"[MeSH]) AND ("1990"[PDAT] : "2015"[PDAT]) AND "humans"[MeSH])

13 initial hits; 1 selected from full text review for data extraction: herpes"[Title/Abstract] OR "Herpesvirus 2, Human"[Mesh]) AND ("Prevalence"[Title/Abstract] OR "Incidence"[Title/Abstract] AND ("2015"[PDAT] : "2015"[PDAT])

For all STIs excluding genital herpes, we supplemented our datasets with manual search of national ministry of health websites, antenatal clinic surveillance reports, data from the GBD collaborator network and case-notification data from locations where centralised reporting is mandatory. The genital herpes dataset was only supplemented by sources from the GBD collaborator network.

Table 1: Data Inputs for Gonococcal Infection morbidity modelling by parameter

Measure	Total Sources	Countries with data
Prevalence	138	64
Incidence	561	53
Proportion	13	6

Table 2: Data Inputs for Chlamydial Infection morbidity modelling by parameter

Measure	Total Sources	Countries with data
Prevalence	269	94
Incidence	1030	52
Proportion	19	9

Table 3: Data Inputs for Trichomoniasis morbidity modelling by parameter

Measure	Total Sources	Countries with data
Prevalence	136	56

Incidence	2	1

Table 4: Data Inputs for Syphilis morbidity modelling by parameter

Measure	Total Sources	Countries with data
Prevalence	923	161
Incidence	657	44

Table 5: Data Inputs for Genital Herpes morbidity modelling by parameter

Measure	Total Sources	Countries with data
Prevalence	314	77
Incidence	42	19

Prevalence and incidence data processing

In order to sex-split data sources reported for both sexes combined, sources reporting for each sex separately were matched by age and location for each STI. Log ratios between the prevalence of each STI in females and the prevalence of each STI in males were input into MR-BRT to estimate an adjustment factor. An adjustment factor to split both sex data points into sex-specific data points was calculated for each STI, as pooled values across all ages and geographies. The log adjustment factor for both sex-data points was 0.09 (-0.03, 0.51) for chlamydia, 0.34 (-0.63, 1.25) for gonorrhea, 1.4 (0.53, 3.49) for trichomoniasis, -0.54 (-1.63, 0.52) for syphilis, and 0.46 (-0.09, 1.05) for genital HSV-2.

To be included, a study had to report on laboratory-confirmed diagnosis of an STI. For chlamydia, gonorrhea, and trichomoniasis, the reference case definition was diagnosis with a nucleic acid amplification test (NAAT). Data from high-quality sources using any other diagnostic test were considered for inclusion. For these data collected with alternative methods, we estimated an adjustment factor in MR-BRT by running a meta-regression on the log ratios of the prevalence of infection diagnosed with an alternative test to prevalence of infection diagnosed with a NAAT. In order to estimate these log ratios, we searched for validation studies that compared the sensitivity of alternative tests to the reference, DNA-based test for each respective STI. Thus, we could quantitatively adjust data collected with alternative tests to the level expected had the reference test been used.

Table 6: MR-BRT Crosswalk Adjustment Factors for Chlamydial infection

Data input	Reference or alternative case definition	Gamma	Beta Coefficient, Log (95% CI)	Adjustment factor*
Nucleic Acid Amplification Test	Ref	0.068	---	---
Culture Diagnostic	Alt		-0.53 (-0.77, -0.31)	0.59 (0.46, 0.73)
Other Diagnostic	Alt		-0.78 (-1.03, -0.53)	0.46 (0.36, 0.59)

**Adjustment factor is the transformed Beta coefficient in normal space, and can be interpreted as the factor by which the alternative case definition is adjusted to reflect what it would have been if measured as the reference.*

Table 7: MR-BRT Crosswalk Adjustment Factors for Gonococcal infection

Data input	Reference or alternative case definition	Gamma	Beta Coefficient, Log (95% CI)	Adjustment factor*
Nucleic Acid Amplification Test	Ref	0.97	---	---
Culture Diagnostic	Alt		-1.02 (-3.099, 1.053)	0.36 (0.04, 2.87)

**Adjustment factor is the transformed Beta coefficient in normal space, and can be interpreted as the factor by which the alternative case definition is adjusted to reflect what we expect the measurement would have been if measured with reference methods.*

Table 8: MR-BRT Crosswalk Adjustment Factors for Trichomoniasis infection

Data input	Reference or alternative case definition	Gamma	Beta Coefficient, Log (95% CI)	Adjustment factor*
Nucleic Acid Amplification Test	Ref	0.16	---	---
Culture Diagnostic	Alt		-0.23 (-0.61, 0.11)	0.79 (0.54, 1.12)
Other Diagnostic	Alt		-0.58 (-0.99, -0.22)	0.56 (0.37, 0.80)

**Adjustment factor is the transformed Beta coefficient in normal space, and can be interpreted as the factor by which the alternative case definition is adjusted to reflect what we expect the measurement would have been if measured with reference methods.*

For syphilis infection, the reference case definition was diagnosis with both a treponemal and non-treponemal serologic test. The alternative case definitions were diagnosis with only a treponemal test, or diagnosis with only a non-treponemal test. To adjust data collected with alternative methods, we ran a meta-regression in MR-BRT. In this instance, we estimated log ratios by matching sources by age, sex, and location to find comparisons between data collected with alternative case definitions and data collected with the reference case definition. Additionally, we adjusted populations of blood donors to the level of syphilis expected in the general population by using matched sources as inputs to MR-BRT.

Table 9: MR-BRT Crosswalk Adjustment Factors for Syphilis infection

Data input	Reference or alternative case definition	Gamma	Beta Coefficient, Log (95% CI)	Adjustment factor*
Both treponemal & nontreponemal Diagnostic Tests	Ref	0	---	---
Treponemal Diagnostic	Alt		0.44 (0.15, 0.74)	1.55 (1.16, 2.09)
Nontreponemal Diagnostic	Alt		0.21 (0.01, 0.40)	1.23 (1.01, 1.49)
General Population	Ref		---	---
Blood Donors	Alt		-0.20 (-0.72, 0.33)	0.82 (0.48, 1.39)

**Adjustment factor is the transformed Beta coefficient in normal space, and can be interpreted as the factor by which the alternative case definition is adjusted to reflect what we expect the measurement would have been if measured with reference methods.*

Adult Tertiary Syphilis is defined by clinical syndrome, rather than acquisition of an infectious agent, and it was modeled using data from claims and hospital discharges as prepared by the GBD Clinical Informatics team and described in detail in a separate section of this Appendix.

In GBD 2019, claims data linked multiple inpatient and outpatient claims to a single individual; prevalent cases were extracted if an individual had at least one inpatient or two outpatient encounters with an appropriate ICD code as any diagnosis within a one-year duration. Data from hospital discharges were adjusted using correction factors from claims, converting encounters to estimates of cases, correcting for most locations providing only primary diagnostic codes, and estimating outpatient cases from inpatient cases.

For adult tertiary syphilis, claims data from the United States were adjusted to inpatient hospital data prior to analysis in DisMod. A priori, we believed that claims data reflected a certain level of selection bias due to commercial insurance, while inpatient hospital data was more reflective of the general population. The adjustment factor was estimated as a single pooled value across all ages. It was modelled in MR-BRT as a meta-regression of log-transformed ratios between US claims data sources and inpatient data sources. Ratios were formed between sources matched by age and location.

Table 10: MR-BRT Crosswalk Adjustment Factors for Adult tertiary syphilis

Data input	Reference or alternative case definition	Gamma	Beta Coefficient, Log (95% CI)	Adjustment factor*
Inpatient Data	Ref	0	---	---
US Claims (Marketscan)	Alt		1.02 (0.90, 1.14)	2.77 (2.46, 3.12)

**Adjustment factor is the transformed Beta coefficient in normal space, and can be interpreted as the factor by which the alternative case definition is adjusted to reflect what we expect the measurement would have been if measured with reference methods.*

For genital herpes, neither validation studies nor matched studies could be found to estimate adjustment factors, so any sources that did not use nucleic acid amplification tests for HSV-2 were excluded. However, adjustments were made for non-representative populations. Adjustment factors were calculated in MR-BRT for populations of blood donors and pregnant women. The log-ratios that were inputs to MR-BRT were estimated from matched comparisons by age, sex, and location using all data in the genital herpes database.

Table 11: MR-BRT Crosswalk Adjustment Factors for Genital herpes

Data input	Reference or alternative case definition	Gamma	Beta Coefficient, Log (95% CI)	Adjustment factor*

General Population	Ref	0.35	---	---
Population of pregnant women	Alt		-0.24 (-0.97, 0.46)	0.78 (0.37, 1.58)
Population of blood donors	Alt		0.64 (-0.13, 1.39)	1.89 (0.88, 4.01)

Adjustment factor is the transformed Beta coefficient in normal space, and can be interpreted as the factor by which the alternative case definition is adjusted to reflect what we expect the measurement would have been if measured with reference methods.

For all STIs, sources were excluded if the sample population was drawn exclusively from a high-risk group (eg, HIV-positive, men who have sex with men [MSM], or sex workers). Additionally, for sources reported for age groups spanning more than 15 years, these data points were disaggregated by imposing an age pattern from the respective GBD 2017 model. The exception was trichomoniasis. For this cause, broad age groups were disaggregated by imposing the age pattern from a preliminary GBD 2019 model run only with age-specific data points.

Due to difficulty in reconciling differences between prevalence and incidence sources, likely due to underreporting in surveillance data, incidence data were ignored for all STIs.

Remission inputs

Remission inputs for each STI excluding genital herpes were estimated from disease duration ranges calculated as follows. Duration ranges were calculated using a sum of the duration of untreated and treated disease, weighted by the percent of individuals that are symptomatic and the probability of receiving treatment if symptomatic with the formula below.

$$\textbf{Duration} = (\%\,Symptomatic)(Prob_{Rx})(Duration_{Rx}) + (1 - \%\,Symptomatic)(Duration_{\,not\,Rx}) + (\%\,Symptomatic)(1 - Prob_{Rx})\,(Duration_{\,not\,Rx})$$

The durations and probabilities of symptoms used in this formula were taken from GBD 2000 and WHO 2005, and were largely expert-driven. The probability of treatment if symptomatic was modeled using the Healthcare Access and Quality (HAQ) index to compute this probability for each location and year.

For syphilis, durations per stage (primary, secondary, latent, and tertiary) were calculated individually and summed along with the average seroreversion by stage, weighing by the proportion of cases remaining at each stage and including the time it would take to serorevert after adequate treatment.

Remission inputs were not modeled for genital HSV-2 infection.

Modelling strategy

We estimated the nonfatal burden of STIs in three parts.

First, we estimated the incidence and prevalence of trichomoniasis, genital herpes, syphilis (adult seroprevalence and adult tertiary), and pelvic inflammatory disease (PID); each in separate models in DisMod-MR 2.1. We estimated the prevalence of chlamydia and gonorrhea, also in separate models in Dismod. The incidence of chlamydia and gonorrhea were estimated in a custom process outside of Dismod, as is described in the post-processing section below. Specific modelling considerations in DisMod

for each of these entities are also described below, except PID, which is described in detail in a separate section of this Appendix.

Second, we split cases of each STI into asymptomatic and symptomatic health states, based on assumptions about probability and duration of symptoms. This included estimating the proportion of gonorrhea and chlamydia cases that experienced epididymo-orchitis. The subset of gonorrhea and chlamydia cases that experienced PID was determined by separately estimating the incidence and prevalence of PID and the proportion of those cases due to each etiology, then deducting PID cases from the overall chlamydia and gonorrhea occurrence described here.

Third, we found the ratio of YLDs to YLLs for all specified STIs (excluding other STI) and then applied that ratio to other STI YLLs.

DisMod models

Gonococcal infection

The inputs to the gonococcal infection model were prevalence data from cross-sectional studies and modeled remission rates as described above.

Incidence was restricted to occur only between ages 10 and 69. EMR was set to have a maximum value of 0.0001. The proportion of pregnant women estimated to experience four visits to antenatal care clinics (ANC4) was used as a covariate to help predict prevalence.

Table 12: Predictive Covariates, Gonorrhoea

Predictive covariate	Parameter	Beta (95% UI)	Exponentiated beta
Antenatal Care (4 visits) Coverage (proportion)	prevalence	-0.057 (-0.097 to -0.0096)	0.95 (0.91–0.99)

Chlamydial infection

The inputs to the chlamydial infection model were prevalence data from cross-sectional studies and modeled remission rates as described above.

Incidence was restricted to occur only between ages 10 and 69. EMR was set to have a maximum value of 0.0001. The proportion of pregnant women estimated to experience four visits to antenatal care clinics (ANC4) was used as a covariate to help predict prevalence.

Table 13: Predictive Covariates, Chlamydia

Predictive covariate	Parameter	Beta (95% UI)	Exponentiated beta
Antenatal Care (4 visits) Coverage (proportion)	prevalence	-0.07 (-0.098, -0.029)	0.93 (0.91–0.97)

Trichomoniasis infection

The primary inputs to the trichomoniasis model were prevalence data from cross-sectional studies and modeled remission rates as described above.

Incidence was restricted to occur only between ages 10 and 69. EMR was set to have a maximum value of 0.0001. The proportion of pregnant women estimated to experience four visits to antenatal care clinics (ANC4) was used as a covariate to help predict prevalence.

Table 14: Predictive Covariates, Trichomoniasis

Predictive covariate	Parameter	beta	Exponentiated beta
Antenatal care (4 visits) coverage (proportion)	prevalence	-0.083 (-0.099, -0.052)	0.92 (0.91 - 0.95)

Genital herpes infection due to HSV-2

Prevalence data from cross-sectional studies were the primary input.

Genital herpes estimation assumed mortality is zero and remission is a small value (0–0.02) to account for a subset of herpes-infected patients who experience seroreversion. Incidence was restricted to occur between ages 10 and 79. A predictive covariate for age-standardised HIV prevalence was used to guide estimates in geographies with sparse data in recognition of the strong relationship between HSV-2 and HIV transmission.

Table 15: Predictive Covariates, Genital Herpes

Predictive covariate	Parameter	beta	Exponentiated beta
HIV age-standardised prevalence	Prevalence	0.96 (0.87–1.00)	2.60 (2.38–2.71)

Syphilis infection

The primary inputs to the adult seroprevalence model were prevalence data from cross-sectional studies and ANC clinic reports, and modeled remission rates as described above. Implausibly high data from Argentina and the Solomon Islands previously included were marked as outliers and excluded in GBD 2019.

Incidence was restricted to occur only between ages 10 and 69. The age range was restricted from 10 to 64 years. HIV age-standardised prevalence was applied as a predictive covariate on prevalence.

Table 16: Predictive Covariates, Syphilis infection

Predictive covariate	Parameter	beta	Exponentiated beta
HIV age-standardised prevalence	prevalence	0.052 (0.00067–0.19)	1.05 (1.00–1.21)

Adult tertiary syphilis

Inputs for this model included prevalence data from hospital discharge and claims data, as described above, and cause-specific mortality rate (CSMR) estimates for syphilis from the GBD causes of death analysis. Each prevalence datum was paired with a CSMR estimate to calculate an excess mortality rate (EMR) input datum, as well.

Incidence was restricted to not occur until age 15. Excess mortality rate was capped at 0.1, which equates to minimum duration of five years. Remission was set to zero.

Natural log of lag-distributed income (LN-LDI) was used as a predictive covariate on EMR.

Table 17: Predictive covariates, Adult Tertiary Syphilis

Country-level covariate	Parameter	beta	Exponentiated beta
LDI (I$ per capita)	excess mortality rate	-0.5 (-0.5 to -0.49)	0.61 (0.61 – 0.61)

Pelvic inflammatory disease due to chlamydia & gonorrhea

We modelled the prevalence, incidence, remission, case fatality and excess mortality rate from pelvic inflammatory disease (PID) and PID-induced primary and secondary infertility. Briefly, we used discharge and claims data to estimate total PID incidence and prevalence using DisMod-MR 2.1. We use proportions from published PID case-series to run separate DisMod models of the proportion of PID due to each underlying etiology (chlamydia, gonorrhoea, and other STIs) and then split the results of the PID model according to these proportions. PID-induced primary and secondary infertility were then modeled assuming only a fixed subset of incident PID cases specific to each etiology develop infertility and that there is no remission in these cases. These estimation processes are described in detail in separate sections of this Appendix.

Sequela of specified STIs

Gonococcal and chlamydial infection outcomes

Gonococcal and chlamydial infections in females are split into asymptomatic cases, symptomatic cases with mild infection, and cases that go on to develop pelvic inflammatory disease. In males, gonococcal and chlamydial infections are split into asymptomatic cases, symptomatic cases with mild infection, and cases that go on to develop epididymo-orchitis (EO).

For females, 0.34 (95% UI 0.306–0.374) of gonococcal prevalence and incidence, and 0.17 (0.153–0.187) of chlamydia prevalence and incidence were estimated to be symptomatic and the remainder were considered asymptomatic. The prevalence of PID due to gonorrhea and PID due to chlamydia were estimated in a separate process. Briefly, cases of PID were assigned to moderate disease and severe disease and deducted from the prevalent symptomatic cases of gonorrhea and chlamydia. A proportion of PID cases were assumed to go on to infertility. Further details on infertility due to chlamydia & gonorrhea, as well as PID due to chlamydia & gonorrhea, are described in separate sections of this Appendix.

For males, 0.5875 (0.5288–0.6463) of gonococcal prevalence and incidence, and 0.505 (0.4545–0.5555) of chlamydia prevalence and incidence were estimated to be symptomatic and the remainder were considered asymptomatic. A proportion of all male incident cases were assumed to progress to epididymo-orchitis. The proportion of incident cases that developed epididymo-orchitis was assumed to differ by specific pathogen (gonorrhea *versus* chlamydia) and with better healthcare access, and healthcare access was assumed to correspond to high-quality vital registration systems. Thus, GBD locations with long time-series of high quality vital registration data were labeled as “developed”, while all others were marked as “developing”. The proportion of incident cases thought to experience epididymo-orchitis in locations considered “developed” was 0.03 (0.015–0.045) for gonorrhoea and 0.02 (0.01–0.03) for chlamydia. The proportion of incident cases thought to experience epididymo-orchitis in "developing” locations was 0.0975 (0.0483–0.143) for gonorrhoea and 0.0625 (0.0325–0.0975) for chlamydia.

In GBD 2019, we found that the number of YLDs due to male chlamydial & gonococcal infection (particularly those attributable to epididymo-orchitis), exceeded the number of YLDs due to female chlamydial & gonococcal infection (particularly those attributable to PID). Given the epidemiology of PID and of epididymo-orchitis, this was deemed to be implausible. We determined that the incidence of gonorrhea and chlamydia estimated by Dismod was implausibly high. This particularly impacted the epididymo-orchitis estimation process, which stemmed from the incident cases of chlamydia and gonorrhea in males. Thus, we abandoned results of incidence estimated in the full compartmental DisMod model for gonorrhea and chlamydia, and instead optimized the fit of prevalence estimates to prevalence data inputs. We then estimated incidence in a custom process outside of DisMod. To estimate incidence, we divided prevalence estimates from Dismod by the sum of the multiplied duration and proportion value for each sequela. We assumed a duration of 3 weeks for epididymo-orchitis, a duration of 1 week for mild, symptomatic, infection, and a duration of 1 year for asymptomatic infection.

Estimation of female incidence:

$$1)\ prevalence_{female} = prevalence_{asymptomatic} + prevalence_{mild}$$

$$2)\ prevalence_{female} = (proportion_{asymptomatic} * duration_{asymptomatic} * \ incidence_{female}) + (proportion_{mild} * duration_{mild} * \ incidence_{female})$$

$$3)\ incidence_{female} = \frac{prevalence_{female}}{(proportion_{asymptomatic} * duration_{asymptomatic}) + (proportion_{mild} * duration_{mild})}$$

Estimation of male incidence:

$$1)\ prevalence_{male} = prevalence_{asymptomatic} + prevalence_{mild} + prevalence_{EO}$$

$$2)\ prevalence_{male} = \left(proportion_{asymptomatic} * duration_{asymptomatic} * \ incidence_{male}\right) + (proportion_{mild} * duration_{mild} * \ incidence_{male}) + (proportion_{EO} * duration_{EO} * incidence_{male})$$

$$3)\ incidence_{male} = \frac{prevalence_{male}}{\left(proportion_{asymptomatic} * duration_{asymptomatic}\right) + (proportion_{mild} * duration_{mild}) + (proportion_{EO} * duration_{EO})}$$

After we procured estimates of male and female incidence, we estimated the incidence of each sequela by applying the proportion of asymptomatic, symptomatic, and for males, epididymo-orchitis, to incidence. We estimated the prevalence of each sequela by multiplying incident cases for each sequela by the assumed duration for each sequela. The prevalence and incidence of PID induced infertility and PID due to chlamydia and gonorrhea are described in other sections of this Appendix.

Trichomoniasis infection outcomes

For trichomoniasis, 0.067 (0.063 – 0.073) of males were assumed to be symptomatic, and assigned a health state of mild, acute infectious disease. For females, 0.34 (0.306–0.374) were assumed symptomatic and assigned a health state of mild, acute infectious disease. For each sex, the remaining proportion was assumed to be asymptomatic.

HSV-2 genital infection outcomes

A systematic literature review revealed a few studies that informed our estimation that 0.175 (0.10–0.25) of initial herpes cases have symptoms of moderate, acute infectious disease lasting 3 (2–4) weeks and 0.189 of prevalent cases have 6 (5–7) recurrent episodes per year each lasting 2 (1–3) weeks.

Syphilis outcomes

Our review of literature indicated that 0.043 (0.014–0.073) of primary, secondary, and early latent syphilis infections (from our adult seroprevalence model) are assumed to be symptomatic and assigned a health state of mild, acute, infectious disease. The remainder were considered asymptomatic. For adult tertiary syphilis, there are eight sequelae, including asymptomatic.

Table 18: Adult Tertiary Syphilis Proportions

Sequela name	Proportion (95% UI) - Males	Proportion (95% UI) - Females
Asymptomatic	0.3932 (0.338 – 0.448)	0.689 (0.652 – 0.727)
Cardiovascular complications	0.0999 (0.0662 – 0.1337)	0.058 (0.0391 – 0.0769)
Neurological problems	0.0193(0.0038 – 0.0348)	0.034 (0.0196 – 0.0492)
Neurological problems & cardiovascular complications	0.0845 (0.0532 – 0.1158)	0.004 (0.0 – 0.0091)
Severe disfigurement	0.1283 (0.0906 – 0.1659)	0.1853 (0.1538 – 0.2168)
Severe disfigurement & cardiovascular complications	0.1475 (0.1076 – 0.1874)	0.0171 (0.0066 – 0.0276)
Severe disfigurement & neurological problems	0.0931 (0.0604 – 0.1258)	0.0107 (0.0024 – 0.019)
Severe disfigurement, neurological problems, & cardiovascular	0.0341 (0.0136 – 0.0545)	0.000856 (0.0 – 0.0032)

Indirect YLD estimation for other sexually transmitted infections

To calculate YLDs due to acute infection with other STI, we calculated the YLD to YLL ratio for all STI (excluding other STI) and then applied that same ratio to other STI YLLs. YLDs were also estimated to other STI as a result of the proportion of PID and PID-induced infertility that was not due to gonorrhoea or chlamydia.

Flowchart

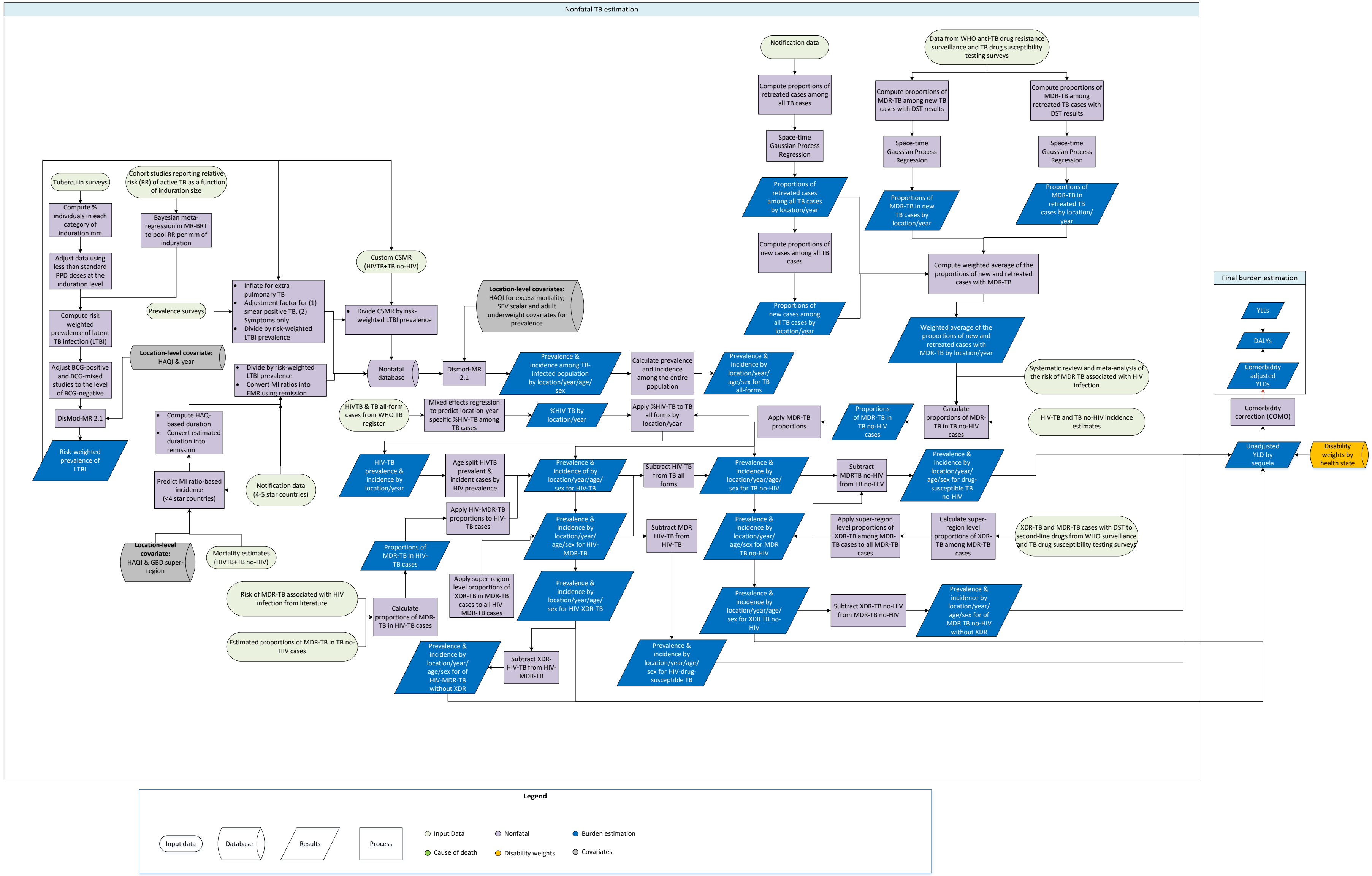

Nonfatal TB estimation
Notification data
Data from WHO anti-TB drug resistance surveillance and TB drug susceptibility testing surveys
Compute proportions of retreated cases among all TB cases
Compute proportions of MDR-TB among new TB cases with DST results
Compute proportions of MDR-TB among retreated TB cases with DST results
Space-time Gaussian Process Regression
Proportions of retreated cases among all TB cases by location/year
Proportions of MDR-TB in new TB cases by location/year
Proportions of MDR-TB in retreated TB cases by location/year
Compute proportions of new cases among all TB cases
Compute weighted average of the proportions of new and retreated cases with MDR-TB
Proportions of new cases among all TB cases by location/year
Weighted average of the proportions of new and retreated cases with MDR-TB by location/year
Tuberculin surveys
Cohort studies reporting relative risk (RR) of active TB as a function of induration size
Compute % individuals in each category of induration mm
Bayesian meta-regression in MR-BRT to pool RR per mm of induration
Adjust data using less than standard PPD doses at the induration level
Custom CSMR (HIVTB+TB no-HIV)
Prevalence surveys
Inflate for extra-pulmonary TB
Adjustment factor for (1) smear positive TB, (2) Symptoms only
Divide by risk-weighted LTBI prevalence
Divide CSMR by risk-weighted LTBI prevalence
Location-level covariates: HAQI for excess mortality; SEV scalar and adult underweight covariates for prevalence
Compute risk weighted prevalence of latent TB infection (LTBI)
Location-level covariate: HAQI & year
Adjust BCG-positive and BCG-mixed studies to the level of BCG-negative
Divide by risk-weighted LTBI prevalence
Convert MI ratios into EMR using remission
Nonfatal database
Dismod-MR 2.1
Prevalence & incidence among TB-infected population by location/year/age/sex
Calculate prevalence and incidence among the entire population
Prevalence & incidence by location/year/age/sex for TB all-forms
Systematic review and meta-analysis of the risk of MDR TB associated with HIV infection
DisMod-MR 2.1
Compute HAQ-based duration
Convert estimated duration into remission
HIVTB & TB all-form cases from WHO TB register
Mixed effects regression to predict location-year specific %HIV-TB among TB cases
%HIV-TB by location/year
Apply %HIV-TB to TB all forms by location/year
Apply MDR-TB proportions
Proportions of MDR-TB in TB no-HIV cases
Calculate proportions of MDR-TB in TB no-HIV cases
HIV-TB and TB no-HIV incidence estimates
Risk-weighted prevalence of LTBI
Predict MI ratio-based incidence (<4 star countries)
Notification data (4-5 star countries)
HIV-TB prevalence & incidence by location/year
Age split HIVTB prevalent & incident cases by HIV prevalence
Prevalence & incidence of by location/year/age/sex for HIV-TB
Subtract HIV-TB from TB all forms
Prevalence & incidence by location/year/age/sex for TB no-HIV
Subtract MDRTB no-HIV from TB no-HIV
Prevalence & incidence by location/year/age/sex for drug-susceptible TB no-HIV
Location-level covariate: HAQI & GBD super-region
Mortality estimates (HIVTB+TB no-HIV)
Apply HIV-MDR-TB proportions to HIV-TB cases
Proportions of MDR-TB in HIV-TB cases
Prevalence & incidence by location/year/age/sex for HIV-MDR-TB
Subtract MDR HIV-TB from HIV-TB
Prevalence & incidence by location/year/age/sex for MDR TB no-HIV
Apply super-region level proportions of XDR-TB among MDR-TB cases to all MDR-TB cases
Calculate super-region level proportions of XDR-TB among MDR-TB cases
XDR-TB and MDR-TB cases with DST to second-line drugs from WHO surveillance and TB drug susceptibility testing surveys
Risk of MDR-TB associated with HIV infection from literature
Calculate proportions of MDR-TB in HIV-TB cases
Apply super-region level proportions of XDR-TB in MDR-TB cases to all HIV-MDR-TB cases
Prevalence & incidence by location/year/age/sex for HIV-XDR-TB
Prevalence & incidence by location/year/age/sex for XDR TB no-HIV
Subtract XDR-TB no-HIV from MDR-TB no-HIV
Prevalence & incidence by location/year/age/sex for of MDR TB no-HIV without XDR
Estimated proportions of MDR-TB in TB no-HIV cases
Prevalence & incidence by location/year/age/sex for of HIV-MDR-TB without XDR
Subtract XDR-HIV-TB from HIV-MDR-TB
Prevalence & incidence by location/year/age/sex for HIV-drug-susceptible TB
Final burden estimation
YLLs
DALYs
Comorbidity adjusted YLDs
Comorbidity correction (COMO)
Unadjusted YLD by sequela
Disability weights by health state
Legend
Input data
Database
Results
Process
Input Data
Nonfatal
Burden estimation
Cause of death
Disability weights
Covariates

Case Definition

Tuberculosis (TB) is an infectious disease caused by *Mycobacterium tuberculosis*. The case definition includes all forms of TB, including pulmonary TB and extrapulmonary TB, which are bacteriologically confirmed or clinically diagnosed. For TB, the ICD 10 codes are A10-A19.9, B90-B90.9, K67.3, K93.0, M49.0, P37.0, and ICD 9 codes are 010-019.9, 137-137.9, 138.0, 138.9, 139.9, 320.4, 730.4-730.6. For HIV-TB, the ICD 10 code is B20.0.

Latent TB infection is defined as an infection with *Mycobacterium tuberculosis*, without any symptoms or signs of active TB disease.

We separately estimated the incidence and prevalence of multidrug-resistant tuberculosis and extensively drug-resistant tuberculosis by HIV status. The case definitions are shown below.

(1) Multidrug-resistant TB without extensive drug resistance: a form of TB (among HIV-negative individuals) that is resistant to the two most effective first-line anti-tuberculosis drugs (isoniazid and rifampicin), but is not resistant to any fluoroquinolone and any second-line injectable drugs (amikacin, kanamycin, or capreomycin).

(2) Extensively drug-resistant TB: a form of TB (among HIV-negative individuals) that is resistant to isoniazid and rifampicin, plus any fluoroquinolone and any second-line injectable drugs.

(3) Drug-susceptible TB: TB (among HIV-negative individuals) that is susceptible to isoniazid and rifampicin.

(4) HIV/AIDS - Multidrug-resistant TB without extensive drug resistance: a form of TB (among HIV-positive individuals) that is resistant to the two most effective first-line anti-tuberculosis drugs (isoniazid and rifampicin), but is not resistant to any fluoroquinolone and any second-line injectable drugs (amikacin, kanamycin, or capreomycin).

(5) HIV/AIDS - Extensively drug-resistant TB: a form of TB (among HIV-positive individuals) that is resistant to isoniazid and rifampicin, plus any fluoroquinolone and any second-line injectable drugs.

(6) HIV/AIDS - Drug-susceptible TB: TB (among HIV-positive individuals) that is susceptible to isoniazid and rifampicin.

Input data

Model inputs

Input data for TB include annual case notifications, data from prevalence surveys, and estimated cause-specific mortality rates (CSMR) of TB among HIV-positive and HIV-negative individuals. For latent TB infection (LTBI), input date include: (1) population-based tuberculin surveys, and (2) cohort studies examining the risk of developing active TB disease as a function of induration size. An updated systematic review was done for GBD 2019. The search terms, number of studies identified, and number of studies included are shown in the table below.

Outcome	Search Terms	Total number of studies identified	Number of studies included
Tuberculosis	Pubmed: ("tuberculosis"[MeSH] OR tuberculosis[Title/Abstract]) OR TB[Title/Abstract] OR Mycobacterium tuberculosis[Title/Abstract] AND prevalence[Title/Abstract] AND ("2016/08/01"[PDAT] : "2017/09/15[PDAT]) NOT (animals[MESH] NOT humans[MESH])	997	2
LTBI (tuberculin surveys)	Pubmed: ("tuberculin survey"[tiab] OR (("risk"[MeSH Terms] OR "risk"[tiab] OR "risk of"[tiab]) AND ("tuberculosis"[MeSH Terms] OR "tuberculosis"[tiab] OR "tuberculous"[tiab]) AND ("infection"[MeSH Terms] OR "infection"[tiab])) OR (("risk"[MeSH Terms] OR "risk"[tiab] OR "risk of"[tiab]) AND TB[tiab] AND ("infection"[MeSH Terms] OR "infection"[tiab])) OR "latent tuberculosis infection"[tiab] OR "latent TB infection"[tiab] OR "latent tuberculosis"[MESH]) AND ("survey"[tiab] OR "surveys"[tiab]) NOT (animals[MESH] NOT humans[MESH]) ("2016/08/01"[PDAT] : "2017/09/07"[PDAT])<br><br>Google Scholar: ("tuberculin survey" OR "risk of tuberculous infection" OR "risk of tuberculosis infection" OR "risk of TB infection" OR "latent tuberculosis infection" OR "latent TB infection") AND "survey". (01-01-2016 to 09-08-2017).	42	0
LTBI (cohort studies)	Pubmed: ("tuberculin"[tiab] OR ("tuberculin"[tiab] AND "positive"[tiab]) OR "Mantoux"[tiab] OR ("Mantoux"[tiab] AND "positive"[tiab]) OR "induration"[tiab]) AND (active[tiab] AND ("tuberculosis"[MeSH] OR "tuberculosis"[tiab])) AND ("risk"[MeSH] OR "risk"[tiab]) AND ("prospective"[tiab] OR "follow up"[tiab] OR "longitudinal"[tiab]) NOT (animals[MESH] NOT humans[MESH]) ("2016/08/01"[PDAT] : "2017/09/21"[PDAT])	955	12

Input data for multidrug-resistant TB (MDR-TB) and extensively drug-resistant TB (XDR-TB) include: (i) the number of MDR-TB cases, XDR-TB cases, new and retreated TB cases with a drug sensitivity testing (DST) result for isoniazid and rifampicin, and MDR-TB cases with DST for second-line drugs from routine surveillance and surveys reported to the World Health Organization, and (ii) the risk of MDR-TB associated with HIV infection from the literature.[1]

PRISMA Diagram of TB All Forms Prevalence in GBD2019

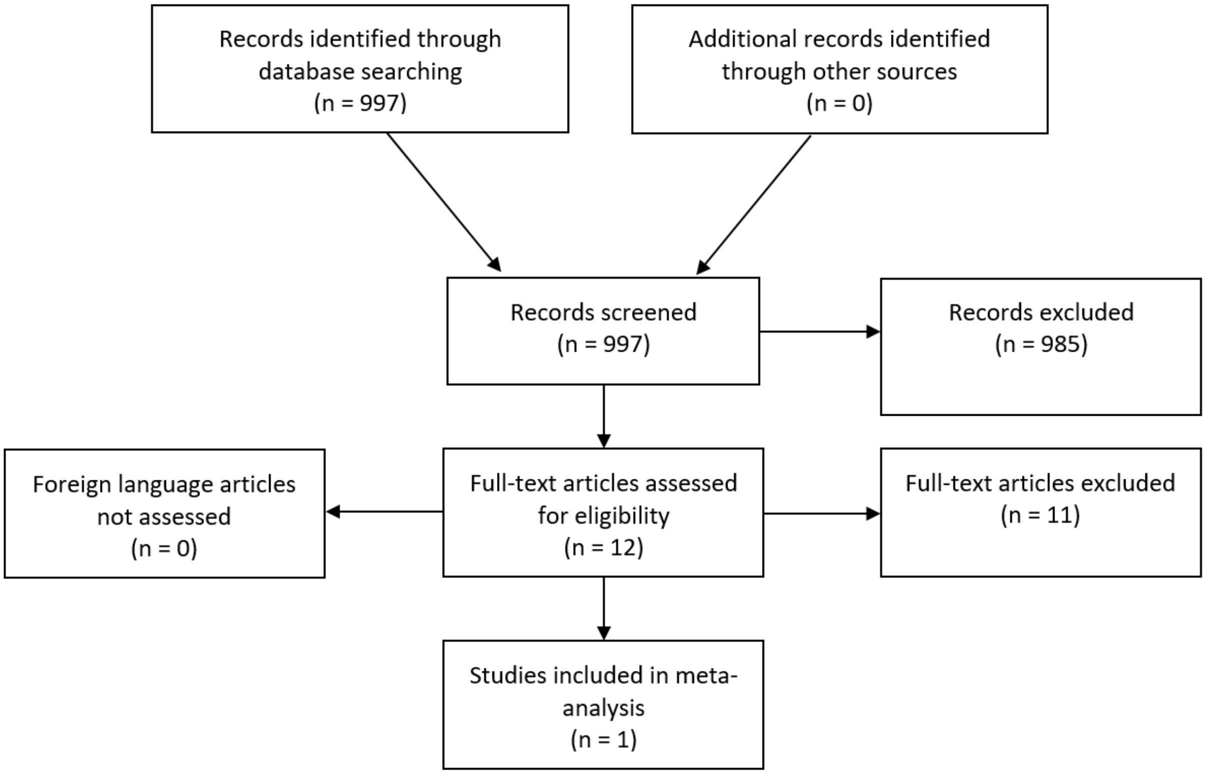

Prisma Diagram of Latent Tuberculosis Infectious in GBD2019

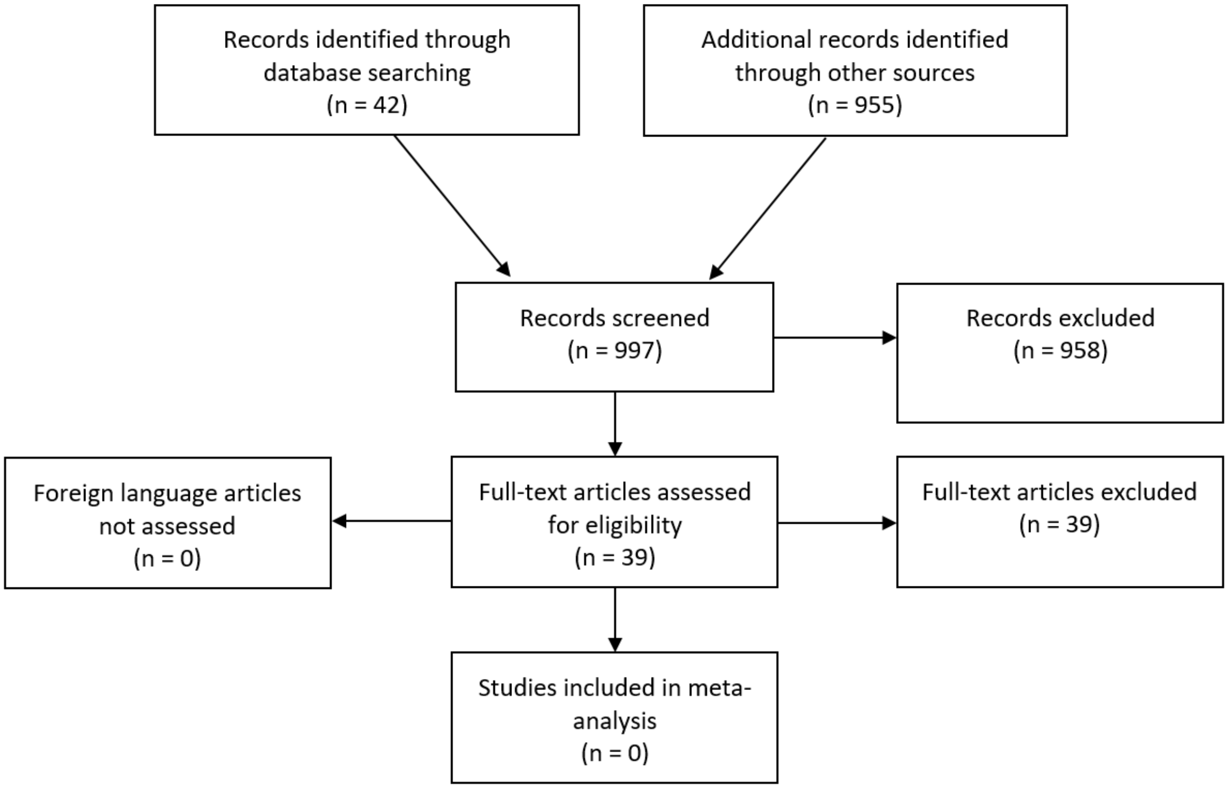

Modelling Strategy

Overview

Our TB modelling strategy has not changed substantially from GBD 2017, but we made refinements to our modeling approach: we used the Meta-Regression with Bayesian Priors, Regularization, and Trimming (MR-BRT) model as the primary analytical engine to predict MI ratios instead of a mixed-effects regression, and we used modeled excess mortality rate (EMR) as input in DisMod. First, we estimated risk-weighted prevalence of LTBI by location, year, age, and sex using data from population-based tuberculin surveys and cohort studies reporting the risk of developing active TB disease as a function of induration size. Next, we divided the inputs on prevalence (from surveys in low- and middle-income countries), incidence (notification data from countries with a four- or five-star rating, and estimated incidence for countries with a less than four-star rating), and cause-specific mortality rate (CSMR) by the risk-weighted LTBI prevalence in order to model TB among those at risk in each country. Next we run MR-BRT (with GBD super region fixed effects) using MI ratios (logit transformed) from locations with a 4- or 5-star rating on causes of death with HAQ index as a covariate anchoring the lower end of the HAQ index scale with a data point from the Bangalore study[2] reporting that 49.2% of 126 untreated new pulmonary TB cases were dead at the end of the five-year follow up period, to predict age-sex specific MI ratios for all locations and years. We then estimated age-sex-specific incidence using the predicted MI ratios and CSMR estimates. Finally, we modeled remission as a function of the HAQ index and used estimated remission to convert MI ratios into excess mortality rates (EMR).

We used DisMod-MR 2.1, the GBD Bayesian meta-regression tool to generate consistent trends in all parameters. We then multiplied the DisMod-MR 2.1 outputs by the risk-weighted prevalence of LTBI to get population-level estimates of incidence and prevalence. Because the outputs from DisMod-MR 2.1 are for all forms of TB, we split them into MDR-TB and XDR-TB by HIV status. To do so, we estimated the proportions of TB cases with MDR-TB for all locations and years, using data from notifications and survey data. We then estimated the proportions of MDR-TB among HIV-negative individuals and MDR-TB among HIV-positive individuals based on the risk of MDR-TB associated with HIV infection from a meta-analysis[1]. To split MDR-TB into MDR-TB with and without extensive drug resistance, we pooled the limited notification and survey data on the proportion of MDR-TB cases with extensive drug resistance by super-region, and applied these proportions to MDR-TB cases among HIV-negative and HIV-positive individuals, respectively.

Modelling risk-weighted latent TB infection prevalence

Input data for modelling risk-weighted LTBI prevalence were from two sources: (i) population-based tuberculin skin test (TST) surveys, and (ii) cohort studies examining the risk of developing active TB disease as a function of induration size. First, we extracted the prevalence of tuberculin skin testing results by induration size using the most detailed induration categories reported by studies. Second, from cohort studies reporting on the relative risk of developing active TB disease as a function of induration size. In GBD 2019, we pooled the risk of developing active TB by induration size in millimeters using MR-BRT to allow for integration over binned data. Third, we multiplied the LTBI prevalence by induration in millimeters ranging from 0-20+ with the relative risk of developing active TB at each induration size, and summed them up to derive risk-weighted LTBI prevalence for each age group.

Available evidence[3] suggests that people with very advanced HIV infection (CD4 counts <200 cells/mm^3) may have a false-negative TST (0mm induration) due to profound immune suppression, but still have very high risk for TB. For those who are HIV-positive, but with higher CD4 counts, the risk for active TB increases with greater induration size as in HIV-negative individuals (ie, the shape of the tuberculin response curve is similar to that for the general population). To take into account the false-negative TST response in HIV cases with profound immune suppression, we first computed the proportion of HIV-positive individuals with CD4 counts <200 cells/mm for the 0 mm induration group using our HIV prevalence estimates for that particular category. We then multiplied that proportion by the relative risk of developing active TB disease in the 0 mm induration group compared with the 20+ mm induration group among HIV-positive individuals. The relative risk was computed using data from a prospective, multicenter cohort study of HIV-positive people in the United States.[3]

Additional evidence[4] indicates that lower doses of PPD (e.g. 1 TU RT23) in a tuberculin skin test yields smaller reactions compared to the standard dose (2 TU RT23; 5 TU PPD-S). In GBD 2019, we adjusted for this bias by collating data from studies that report the difference in reactivity between the standard dose and smaller doses in the same population. We used the reported mean difference from two studies[4,5] in the MR-BRT model to derive a pooled difference. We then added this pooled difference to every reported induration category from studies using lower doses of PPD to adjust the data to the level of the standard dose. In GBD 2019 we also utilized the MR-BRT model to derive adjustment factors for studies where the entire sample is BCG-positive and for studies where BCG status is mixed. The table below contains adjustment factors for BCG status in GBD 2019:

Table 1: MR-BRT Crosswalk Adjustment Factors for Latent Tuberculosis Infection

Reference or alternative case definition	Gamma	Beta Coefficient, Logit (95% CI)	Adjustment factor*
BCG Negative	0.36	---	---
BCG Mixed		0.11 (-0.03 to 0.24)	0.53
BCG Positive		0.42 (0.40 to 0.45)	0.60

Adjustment factor is the transformed Beta coefficient in normal space, and can be interpreted as the factor by which the alternative case definition is adjusted to reflect what it would have been if measured as the reference. The adjustment occurred in logit space where the difference was taken to adjust the data to the level of the reference.

Using the risk-weighted LTBI prevalence (adjusted for a false-negative TST among people with advanced HIV infection, for non-standard PPD doses, and for BCG status) as input data, we ran a DisMod-MR 2.1 model with the HAQ index covariate to help inform variation over year and geography, with priors that at higher HAQ index values, LTBI prevalence decreases. To stabilize temporal trends we included a covariate for year with priors such that LTBI prevalence decreases over time.

Modelling TB incidence

Incidence inputs were from two different sources: (1) incidence from notification data for countries with a four- or five-star rating on their cause of death data[6] as a proxy for the quality of health-related administrative data systems, and (2) estimated incidence for countries with a less than four-star rating. We used the age- and sex-specific notifications (all new and relapse cases combined) in our analysis. Prior to 2013, notification data were available by case type (new pulmonary smear-positive, new pulmonary

smear-negative, and new extra-pulmonary) and there were missing age data, especially for younger age groups in some countries. We imputed the missing age groups for the three forms of TB notifications. Smear-positive age-specific notifications were inflated with the proportion smear-unknown and relapsed cases only reported at the country-year level. Some countries reported only pulmonary smear-positive cases for selected years. Missing smear-negative and extrapulmonary cases were predicted from the adjusted smear-positive cases using a seemingly unrelated regression. All three types of notifications were added together to represent TB-all-form incidence for countries with a four- or five-star rating.

To generate incidence estimates for locations with a less than four-star rating, we implemented the MR-BRT model with age and sex dummies and super-region fixed effects, using MI ratios (logit transformed) from locations with a 4- or 5-star rating on causes of death as input data with HAQ index as a covariate anchoring the lower end of the HAQ index scale with a data point from a cohort study in the 1960s[2] reporting that 49.2% of 126 untreated new pulmonary TB cases were dead at the end of the five-year follow-up period, in order to predict age-sex-specific MI ratios for all locations and years. We then used the MI ratios and cause-specific mortality estimates to compute the incidence input for DisMod-MR 2.1 for locations with a less than four-star rating. In locations where MI ratio based incidence was lower than notification-based incidence, we dropped the MI ratio based incidence and allowed DisMod to estimate incidence by triangulating between mortality, prevalence, excess mortality, and remission. For comparisons between MI ratio based incidence and notification based incidence, we used the year 2010 and assumed a similar proportional difference across all other years. Finally, we computed the age-sex-specific incidence of TB among the latent TB-infected population, using TB incidence as the numerator and our estimated risk-weighted latent TB infection prevalence as the denominator.

Modelling TB prevalence

Data from prevalence surveys reporting on pulmonary smear-positive TB and bacteriologically positive TB were included. Because incidence data are for all forms of TB, we adjusted prevalence surveys to account for extrapulmonary cases. We ran a spatiotemporal Gaussian process regression to predict location-year-age-sex-specific proportions of extrapulmonary TB among all TB cases using data on the three forms of TB from the incidence data above. We then computed the extrapulmonary inflation factor as *1+(proportion of extrapulmonary TB /(1- proportion of extrapulmonary TB))*, and applied it to data from prevalence surveys.

In GBD 2019, we used the MR-BRT model to derive adjustment factors for studies where the case definition was smear-positive TB rather than bacteriologically positive TB (reference). For the adjustment, we identified all prevalence surveys that provided comparisons of smear-positive TB and bacteriologically positive TB from the same sample. Overall, 16 prevalence surveys from Cambodia, China, Ethiopia, Gambia, India, Myanmar, South Korea, Philippines, Rwanda, and Vietnam were included as inputs in the MR-BRT model. The model also contained covariates for sex and age to reflect gradients across demographics. In GBD 2019 we also computed an adjustment factor to adjust studies that used symptoms only as a screening method compared to studies using both symptoms and chest X-ray during screening (reference). To derive the adjustment factor, we ran a MR-BRT model where data from six studies[7,8,9,10,11,12] comparing prevalence between using symptoms only as opposed to symptoms and chest X-ray in the same population as input. The adjustment factors are in the table below.

Finally, we computed the prevalence of TB among the TB-infected population, using TB prevalence as the numerator and our estimated risk-weighted LTBI prevalence as the denominator. We included two location-level covariates, namely, age-standardised adult underweight prevalence and log-transformed age-standardised Summary Exposure Variable (SEV) scalar for TB (a summary variable of the exposure levels of TB risk factors weighted by relative risk) to help inform variation of TB prevalence over year and geography.

Table 2: MR-BRT Crosswalk Adjustment Factors for Tuberculosis Prevalence

Reference or alternative case definition	Gamma	Beta Coefficient, Log (95% CI)	Adjustment factor*
Bacteriologically positive	0.17	---	---
Smear positive		-0.39 (-0.58 to -0.22)	0.67
Symptoms and chest X-ray	0.01	---	---
Symptoms only		-0.38 (-0.50 to -0.25)	0.68

Adjustment factor is the transformed Beta coefficient in normal space, and can be interpreted as the factor by which the alternative case definition is adjusted to reflect what it would have been if measured as the reference.

Modelling TB remission and excess mortality

In GBD 2019 we computed TB duration based on a systematic review of studies during the pre-chemotherapy era finding that duration from onset to cure or death is 3 years.[13] To anchor the lowest end of TB duration we assumed a duration of 6 months based on treatment regiments. We then linearly interpolated between 6 months and 3 years across the HAQ index to compute TB duration for every country-year. We converted duration into remission by taking the inverse (e.g. Remission = 1/duration). Using HAQ-based remission and estimated MI ratios, we computed excess mortality rate (EMR) with the following computation: EMR = MI*Remission (formula derived from Prevalence=Incidence*Duration)

DisMod-MR 2.1

For each location, we included the following as input in the DisMod model: case notifications for locations with a four- or five-star rating, predicted MI-ratio-based incidence for locations with a less than four-star rating, prevalence survey data where available, predicted excess mortality estimates, HAQ-based remission, and CSMR (TB and HIV-TB combined) by age and sex.

The output from the DisMod model was for all forms of TB in TB-infected populations, including both HIV-negative and HIV-positive individuals. We computed the incidence and prevalence of TB among the entire population, by multiplying the prevalence of LTBI with the DisMod model estimates. Betas and exponentiated values from the DisMod model are shown in the table below.

Covariate	Parameter	Beta (95% CI)	Exponentiated beta (95% CI)
Sex (male)	Prevalence	0.23 (0.19 to 0.26)	1.26 (1.21 to 1.30)
Sex (male)	Incidence	0.35 (0.35 to 0.35)	1.42 (1.42 to 1.42)
Age-standardised proportion adult underweight	Prevalence	2.08 (1.77 to 2.38)	7.97 (5.90 to 10.86)
Age-standardised SEV scalar (log-transformed)	Prevalence	0.75 (0.75 to 0.76)	2.12 (2.12–2.14)

HIV-TB incidence and prevalence

To distinguish HIV-TB from all forms of TB, we first estimated the proportions of HIV-TB cases among all TB cases using data on the number of TB cases recorded as HIV-positive and the number of TB cases with an HIV test result recorded in the WHO TB notifications register. We ran a mixed effects regression using the adult HIV death rate as a covariate to predict location-year-specific HIV-TB proportions, which were then applied to TB incident and prevalent cases from DisMod, to generate HIV-TB incident and prevalent cases by location and year. These cases were then age-sex split based on the age-sex pattern of estimated HIV prevalence by location-year to generate location-year-age-sex-specific HIV-TB incident and prevalent cases.

Multidrug-resistant TB, extensively drug-resistant TB, and drug-susceptible TB

We ran spatiotemporal Gaussian process regressions to predict the proportions of new TB cases with MDR-TB, proportions of retreated TB cases with MDR-TB, and proportions of retreated cases among all TB cases for all locations and years. We calculated the proportions of new TB cases among all TB cases as *1- estimated proportions of retreated cases*. Next, we computed the weighted average of the proportions of new and retreated cases with MDR-TB at the 1000 draw level. We then used the weighted average proportions of MDR-TB, along with the HIV-TB and TB no-HIV incidence estimates, and the relative risk of MDR-TB associated with HIV infection from the literature[1] to compute the proportions of MDR-TB cases among HIV-negative TB cases $\left(PnoHIV_{c,y,a,s}\right)$ by location, year, age, and sex using the following formula:

$$PnoHIV_{c,y,a,s} = \frac{MDR_{c,y}}{\left(1 + \left(RR\,\frac{HIVTB_{c,y,a,s}}{TBnoHIV_{c,y,a,s}}\right)\right) TBnoHIV_{c,y,a,s}}$$

where $MDR_{c,y}$ is the number of all MDR-TB cases among HIV-positive and HIV-negative individuals by location and year, *RR* is the relative risk of MDR-TB associated with HIV infection, $HIVTB_{c,y,a,s}$ is the number of HIV-TB incident cases by location, year, age, and sex, and $TBnoHIV_{c,y,a,s}$ is the number of TB no-HIV incident cases by location, year, age, and sex.

We then applied the predicted proportions of MDR-TB cases among HIV-negative TB cases to our predicted HIV-negative TB incident and prevalent cases to generate MDR-TB incident and prevalent cases by location, year, age, and sex. Next, we subtracted MDR-TB cases from all HIV-negative TB cases to generate drug-susceptible TB cases by location, year, age, and sex. To distinguish XDR-TB from MDR-TB, we aggregated the XDR-TB cases and MDR-TB cases (with drug sensitivity testing for second-line drugs) up to the super-region level and calculated the super-region-level proportions of XDR-TB among MDR-TB cases, which were then applied to MDR-TB cases in corresponding countries within the super-regions to produce XDR-TB cases by location, year, age, and sex. We linearly extrapolated XDR-TB prevalence and incidence back assuming the rates were zero in 1992, one year before 1993 when XDR-TB was first recorded in USA surveillance data.[14] Finally, we subtracted XDR-TB cases from MDR-TB cases to generate MDR-TB (without XDR) cases by location, year, age, and sex.
`

HIV/AIDS - Multidrug-resistant TB, HIV/AIDS - extensively drug-resistant TB, and HIV/AIDS - drug-susceptible TB

To split HIV-TB into HIV-MDR-TB and HIV-drug-susceptible-TB, we first calculated the proportions of HIV-MDR-TB among all HIV-TB cases ($PHIV_{c,y,a,s}$) for each location, year, age, and sex using the following formula:

$$PHIV_{c,y,a,s} = PnoHIV_{c,y,a,s}RR$$

where $PnoHIV_{c,y,a,s}$ is the proportions of MDR-TB among all HIV-negative TB cases for each location, year, age, and sex and RR is the relative risk of MDR-TB associated with HIV infection. We then applied the predicted proportions of MDR-TB cases among HIV-TB cases to our estimated HIV-TB incident and prevalent cases to generate HIV-MDR-TB incident and prevalent cases by location, year, age, and sex. Next, we subtracted HIV-MDR-TB cases from all HIV-TB cases to generate HIV-drug-susceptible-TB cases by location, year, age, and sex. To separate out HIV-XDR-TB from HIV-MDR-TB, we applied the super-region level proportions of XDR-TB among MDR-TB cases, to HIV-MDR-TB cases in corresponding countries within the super-regions to produce HIV-XDR-TB cases by location, year, age, and sex. We linearly extrapolated HIV-XDR-TB prevalence and incidence back assuming the rates were zero in 1992, one year before 1993 when XDR-TB was first recorded in USA surveillance data.[14] Finally, we subtracted HIV-XDR-TB cases from HIV-MDR-TB cases to generate HIV-MDR-TB (without extensive drug resistance) cases by location, year, age, and sex.

New MDR-TB and XDR-TB cases among retreated cases by HIV status

Because we split TB incidence (new and relapse cases combined) by drug-resistance type, the above estimation did not capture new MDR-TB and XDR-TB cases arising from retreated TB cases other than relapse cases. We therefore separately estimated new MDR-TB and XDR-TB cases arising from retreated TB cases and added them to the incident cases estimated above. To do so, we first ran a spatiotemporal Gaussian process regression using notification data and HAQ index as a covariate to predict the proportion of retreated cases (excluding relapse cases) among all TB patients for all locations and years. Next, we computed retreated cases as *(retreated proportion*estimated incident cases)/(1-retreated proportion)*. We then computed the total number of TB cases by summing estimated incident cases and retreated cases. Similar to our estimation for MDR-TB and XDR-TB among TB incident cases by HIV status, we estimated MDR-TB and XDR-TB cases among all TB cases (incident cases and retreated cases combined) by HIV status. Finally, the number of retreated cases with MDR-TB was computed by subtracting MDR-TB among TB incident cases from MDR-TB among all TB cases (incident cases and retreated cases combined), separately for HIV-positive and HIV-negative individuals. Similarly, the number of retreated cases with XDR-TB was computed by subtracting XDR-TB among TB incident cases from XDR-TB among all TB cases, separately for HIV-positive and HIV-negative individuals. All computations were done at the 1000-draw level.

Disability weights

The lay descriptions and disability weights for severity levels derived from the GBD disability weights study are shown below.

Health state name	Lay description	Disability Weights (95% CI)
Tuberculosis, not HIV infected	has a persistent cough and fever, is short of breath, feels weak, and has lost a lot of weight	0.333 (0.224–0.454)
Tuberculosis, HIV infected	has a persistent cough and fever, shortness of breath, night sweats, weakness and fatigue and severe weight loss	0.408 (0.274–0.549)

For drug-susceptible TB, MDR-TB without extensive drug resistance, and XDR-TB, we used the same disability weight [0.333 (0.224–0.454)] as in non-HIV-infected TB. For HIV-drug-susceptible-TB, HIV-MDR-TB without extensive drug resistance, and HIV-XDR-TB, we used the same disability weight [0.408 (0.274–0.549))] as in HIV-infected TB.

Source Counts

Data	Measure	Total sources	Countries with data
Tuberculosis	All measures	4048	194
	Prevalence	144	52
	Incidence	624	78
	Relative risk	34	25
	Proportion	3577	193
Latent tuberculosis infection	All measures	139	54
	Prevalence	105	43
	Relative risk	34	24
Proportion of HIV-TB among all TB cases	All measures	1231	151
	Proportion	1231	151
MDR-TB and MDR-HIV-TB proportions	All measures	4413	192
	Proportion	4413	192
XDR-TB and XDR-HIV-TB proportions	All measures	85	83
	Proportion	85	83

References

1. Mesfin YM, Hailemariam D, Biadglign S, Kibret KT. Association between HIV/AIDS and multi-drug resistance tuberculosis: a systematic review and meta-analysis. *PLoS One*. 2014 Jan 8;9(1):e82235.
2. Institute NT. Tuberculosis in a rural population of South India: a five-year epidemiological study. *Bulletin of the World Health Organization*. 1974;51(5):473.
3. Markowitz N, Hansen NI, Hopewell PC, Glassroth J, Kvale PA, Mangura BT, Wilcosky TC, Wallace JM, Rosen MJ, Reichman LB. Incidence of tuberculosis in the United States among HIV-infected persons. *Annals of internal medicine*. 1997 Jan 15;126(2):123-32.
4. Chadha VK, Jagannath PS, Nagaraj AV, Prasad DN, Anantha A. A comparative study of tuberculin reactions to 1 TU and 2 TU of PPD-RT23. *Indian Journal of Tuberculosis*. 2000;47(15):15-20.
5. Chadha VK, Jagannath PS, Vaidyanathan PS, Jagota P. PPD RT23 for tuberculin surveys in India. *International Journal of Tuberculosis and Lung Disease*. 2003;7(2):172-179.

6. GBD 2017 Mortality and Causes of Death Collaborators. Global, regional, and national age-sex specific mortality and life expectancy, 1950–2017: a systematic analysis for the Global Burden of Disease Study 2017. *The Lancet* (under review)
7. Gothi GD, Narayan R, Nair S, Chakraborty A, Srikantaramu N. Estimation of prevalence of bacillary on the bases if of chest X-ray and/or symptomatic screening. *Indian Journal of Tuberculosis*. 1976;64(8):1150-1159.
8. Chadha VK, Kumar P, Anjinappa SM, Singh S, Narasimhaiah S, et al. Prevalence of Pulmonary Tuberculosis among Adults in a Rural Sub-District of South India. *PLoS ONE* 2012;7(8): e42625.
9. Datta M, Radhamani MP, Sadacharam K, Selvaraj R, Satyanarayana Rao DL Nagabushana Rao RS, Gopalan BN, Prabhakar R. Survey for tuberculosis in a tribal population in North Arcot District. *International Journal of Tuberculosis and Lung Disease*. 2001;5(2):240-249.
10. Datta M, Gopi PG, Appeqgowda BN, Bhima Rao KR, Gopalan BN. *Indian Journal of Tuberculosis*. 2000;47:147-154.
11. Gopi PG, Subramani R, Sadacharam K, Narayanan R. Yield of pulmonary tuberculosis cases by employing two screening methods in a community survey. *International Journal of Tuberculosis and Lung Disease*. 2006;10(3):343-345.
12. Revised National Tuberculosis Control Program (India). Tuberculosis Survey in Gujarat, Gujarat, 2011-2012. [Unpublished].
13. Tiemersma EW, Van der Werf MJ, Borgdorff MW, Williams BG, Nagelkerke N. Natural History of Tuberculosis: Duration and Fatality of Untreated Pulmonary Tuberculosis in HIV Negative Patients: A Systematic Review. *PLoS ONE*. 2011;6(4): e17601.
14. Centers for Disease Control and Prevention (CDC). Extensively Drug-Resistant Tuberculosis --- United States, 1993—2006. *MMWR*. 2007; 56(11);250-253.

Lower respiratory infections (LRI)

Flowchart

Lower respiratory infections

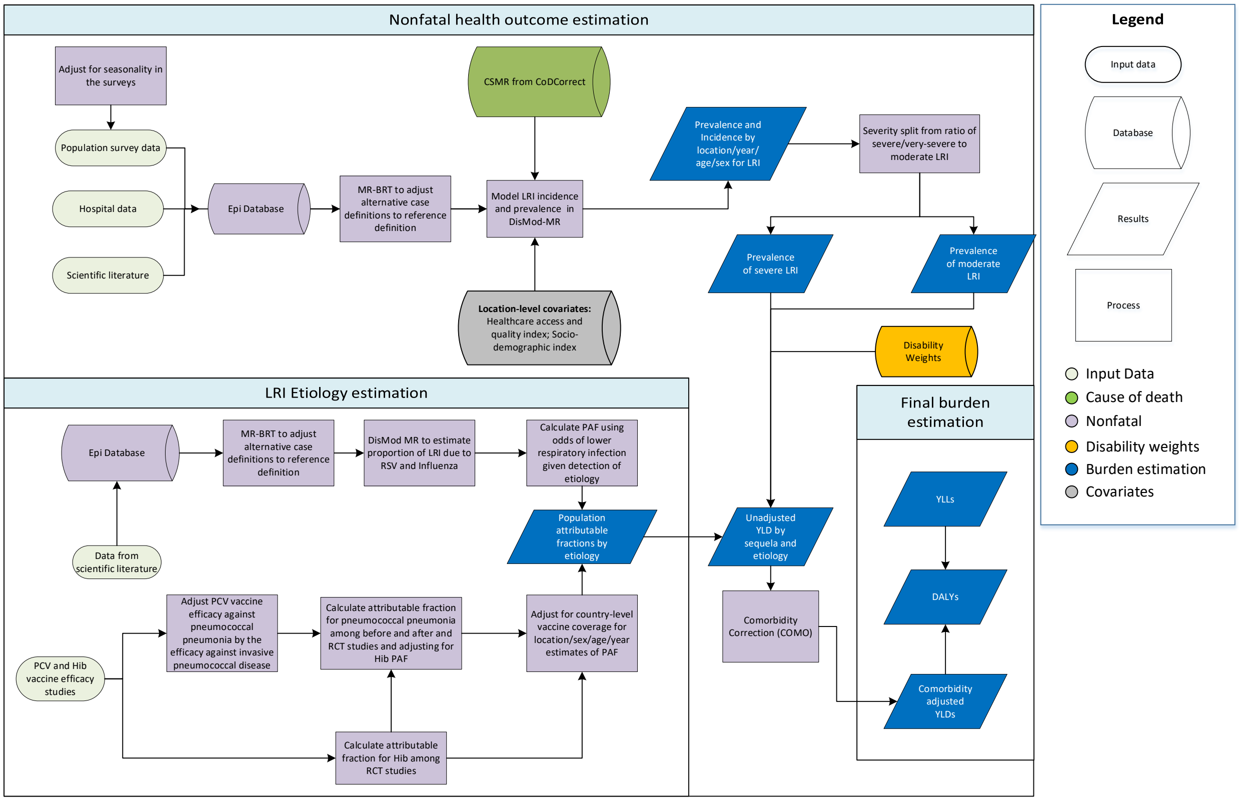

Case definition

We used clinician-diagnosed pneumonia or bronchiolitis as our case definition for lower respiratory infections (LRI). We included ICD9 codes 073.0-073.6, 079.82, 466-469, 480-489, 513.0, and 770.0 and ICD10 codes A48.1, J09-J22, J85.1, P23-P23.9, and U04. LRI etiologies are modeled separately from overall LRI incidence and prevalence. The etiologies include influenza, respiratory syncytial virus, *Streptococcus pneumoniae,* and *Haemophilus influenzae* type b and are episodes of LRI where the etiology is the causal pathogen in the infection.

Input data

Model inputs

Input data included all data used in GBD 2017 and new data identified in our updated systematic review, newly acquired surveys, and new claims and inpatient data. We used two primary types of input data for lower respiratory infections. The first is lower respiratory infection incidence and prevalence data. These data come from a systematic literature review, hospital inpatient and outpatient data, claims data from the US, and population-representative surveys. The second type of data is on the aetiologies of LRI.

Influenza and respiratory syncytial virus (RSV) population attributable fractions were informed by a systematic literature review of the proportion of LRI cases that are positive for each pathogen. *Haemophilus influenzae* type B (Hib) and *Streptococcus pneumoniae* (pneumococcal pneumonia) are informed by a systematic review of vaccine efficacy and effectiveness.

This search string below looks for the incidence and prevalence of LRI cases, and the etiology proportion for influenza and RSV.

((“lower respiratory”[title] OR pneumonia[title]) AND (2018/08/01[PDat] : 2019/2/7[PDat]) AND ((incidence OR prevalence OR epidemiology) OR (etiolog[title/abstract] OR influenza[title/abstract] OR “respiratory syncytial virus”[title/abstract])) AND Humans[MeSH Terms]) NOT(autoimmune[title/abstract] OR COPD [title/abstract] OR “cystic fibrosis”[title/abstract] OR Review[ptyp]) NOT (animals[MeSH] NOT humans[MeSH])*

Our inclusion criteria were studies that had a sample size of at least 100, were at least one year in duration, and included lower respiratory infections, pneumonia, or bronchiolitis in the case definition.

We identified 121 studies, of which 2 met our inclusion criteria and were extracted. We excluded studies that described pandemic H1N1 influenza solely and studies that used influenza-like illness as the case definition. We assigned an age range based on the prevalence-weighted mean age of LRI in the appropriate year/sex/location if the ages of the study participants were not reported.

Figure 1. Lower Respiratory Infection systematic review flowchart

PRISMA Flow Diagram

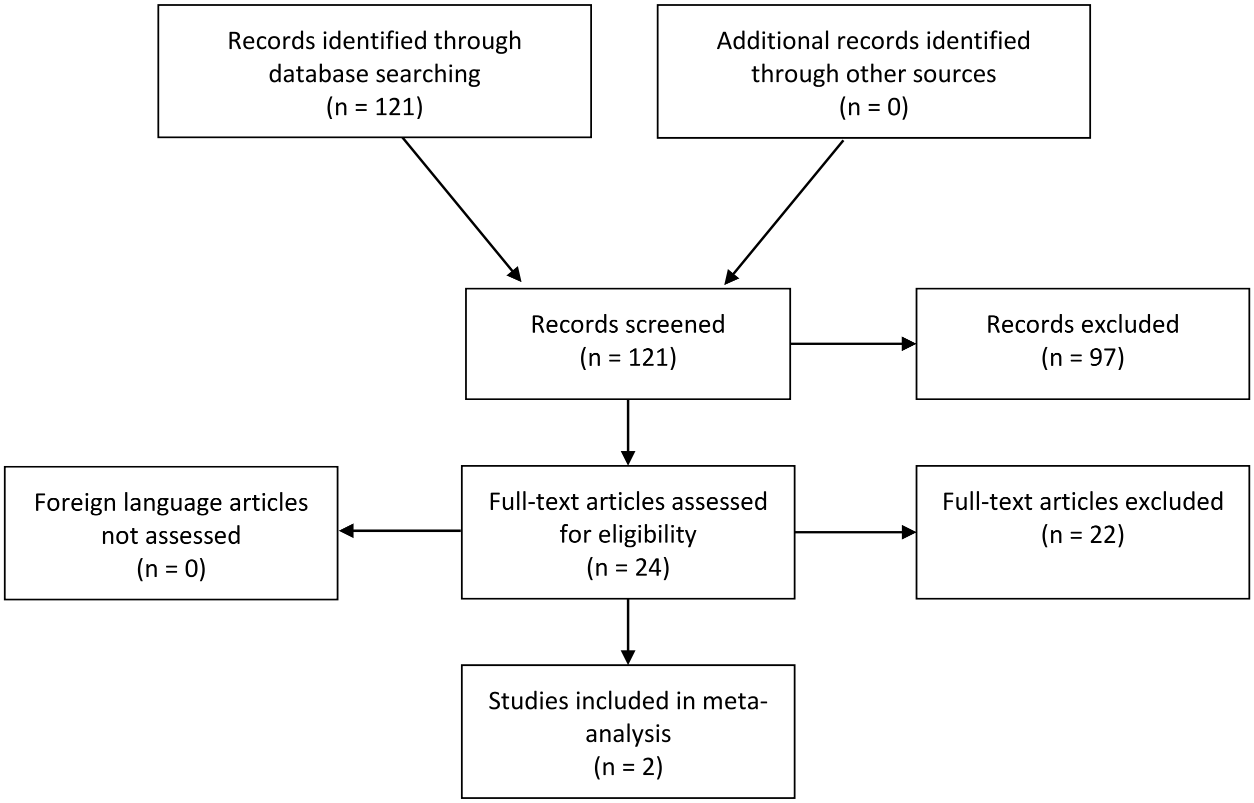

We conducted a systematic literature review of studies on the Hib vaccine and PCV effectiveness studies against X-ray-confirmed pneumonia and against pneumococcal and Hib disease until May 2017. For PCV studies, we extracted, if available, the distribution of pneumococcal pneumonia serotypes and the serotypes included in the PCV used in the study. No new studies were identified for GBD 2019. For Hib, we excluded observational and case-control studies due to implausibly high vaccine efficacy estimates. Hib trial data were exclusively from children <5 years so we did not model Hib in ages over 5 years. PCV trial data are also frequently limited to younger age populations. To understand the contribution of pneumococcal pneumonia in older populations, we also included PCV efficacy studies that used before-after approaches.

PRISMA Flow Diagram

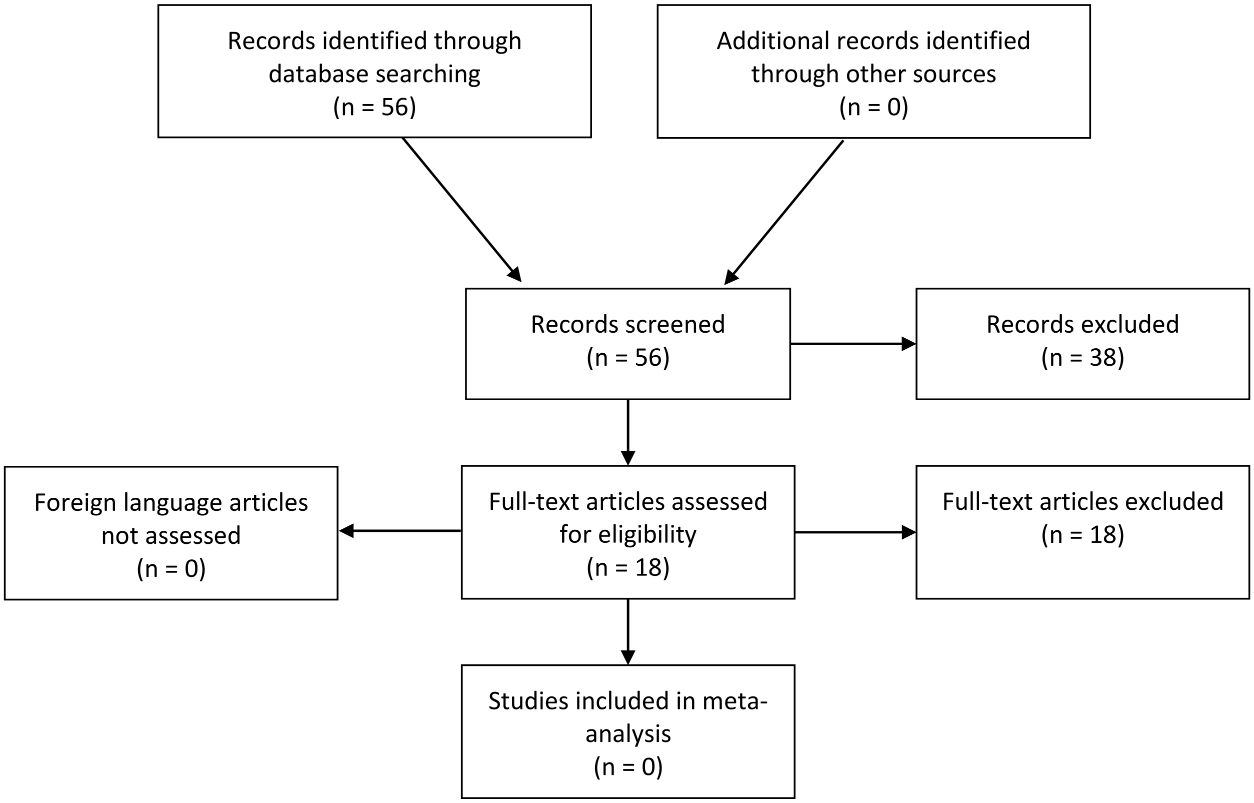

These new sources were added to studies and sources identified in previous rounds of the GBD, resulting in 1283 total unique sources for lower respiratory infection, representing data from 162 countries **(table 1)**.

Table 1. Unique source counts for lower respiratory infections by measure

Measure	Total sources	Countries with data
All measures	1152	162
Prevalence	918	154
Proportion	253	75

To estimate the non-fatal burden of LRI, we also used self-reported prevalence of LRI symptoms from population-representative surveys, such as the Demographic and Health Survey and the Multiple Indicator Cluster Survey. When possible, we extracted survey data by one-year age group and by sex. We converted these data from two-week period prevalence to point prevalence. The equation for this adjustment is

$$1)\quad Point\ Prevalence = \frac{Period\ Prevalence * Duration}{(Recall\ Period + Duration - 1)}$$

We accepted four survey definitions for the prevalence of symptoms of LRI: 1) Cough with difficulty breathing with the symptoms in the chest with a fever was our gold standard but we also accepted 2) Cough with difficulty breathing with the symptoms in the chest *without* fever, 3) Cough with difficulty breathing with fever, and 4) Cough with difficulty breathing *without* fever. To make these definitions comparable, we identified the surveys that met the best case definition (definition 1). Within these surveys, we calculated the ratio of the prevalence of the best case definition to the prevalence of the alternate definitions. This ratio was used as the dependent variable in a meta-regression. The results from that meta-regression were used to adjust the prevalence and uncertainty for all the surveys that reported alternate case definitions **(Table 1)**.

Table 1. Survey crosswalk coefficients

Data Input	Reference or alternative case definition	Gamma	Crosswalk covariate	Beta Coefficient, Logit (95% UI)
Cough, with difficulty breathing and fever	ref	--	--	--
Survey, chest without fever	alt	0.18	intercept	-0.5 (-0.85, -0.15)
Survey, difficulty breath without fever	alt	0.55	intercept	-0.78 (-1.87, 0.31)
Survey, difficulty breathing with fever	alt	0.23	intercept	-0.6 (-1.04, -0.15)

Survey data were adjusted for seasonality. An inclusion criterion for scientific literature is a study duration longer than one year to avoid bias in the seasonal timing of LRI. Surveys are frequently conducted over several months. To account for seasonal variation in LRI symptom prevalence, we fit a generalised additive model with a forced periodicity for each GBD region. The model is mixed-effects with random effects on each country. The model accounts for the year of the survey and the case definition used. The percent difference between the monthly model fit LRI prevalence and the mean fitted LRI prevalence is a scalar to adjust survey data by month and geography.

In addition to survey data, hospital inpatient, outpatient data, and US claims data were included in the LRI modelling. These data are adjusted prior to modelling for multiple admissions, multiple diagnoses, and for outpatient claims. To make the data more consistent in the modelling process, we converted all incidence data to prevalence. We found the ratio of the prevalence of LRI in hospitalisation records to the prevalence of LRI in our case definition (clinician-diagnosed pneumonia or bronchiolitis) for locations that contained data on both these prevalence values. We then regressed this ratio in a meta-regression to predict the adjustment factor for hospitalisation data to make them compatible with the reference case definition for our modelling. This meta-regression considered the Socio-demographic Index (SDI) as a predictor of this ratio for inpatient data, assuming that location-years with higher values of SDI are

more likely to have access to healthcare, making this ratio smaller in those location-years **(Table 2)**. Similarly, age was considered a predictor for hospital-based studies, and data was adjusted accordingly using age midpoint **(Table 3)**.

Table 2. Crosswalk coefficient, clinical inpatient to reference definition

Data Input	Reference or alternative case definition	Gamma	Crosswalk covariate	Beta Coefficient, Logit (95% UI)
clinician-diagnosed pneumonia or bronchiolitis	ref		--	--
Clinical, inpatient	alt	1.49	sdi_0	2.77 (-0.37, 5.92)
Clinical, inpatient	alt		sdi_1	4.82 (3.77, 5.87)
Clinical, inpatient	alt		sdi_2	1.25 (0.22, 2.29)
Clinical, inpatient	alt		sdi_3	0.47 (0.04, 0.9)

Table 3. Crosswalk coefficient, hospital-based studies to reference definition

Data Input	Reference or alternative case definition	Gamma	Covariate	Beta Coefficient, Logit (95% UI)
clinician-diagnosed pneumonia or bronchiolitis	ref		--	--
Literature, hospital-based	alt	0.3	age_mid_0	1.06 (0.03, 2.08)
Literature, hospital-based	alt		age_mid_1	1.98 (-0.16, 4.12)
Literature, hospital-based	alt		age_mid_2	1.31 (0.38, 2.25)
Literature, hospital-based	alt		age_mid_3	0.95 (0.56, 1.34)

Claims data for GBD 2019 include Marketscan (US), and data from Taiwan, Poland, and Russia. Marketscan data are retrieved by IHME's the Clinical Informatics Team. As with inpatient clinical data, these data are converted first to prevalence, then compared to the reference definition for LRI using a meta-regression model **(Table 4)**. Taiwan claims data were dropped as there were no reference data to match with and because the values there were systematically different from those in the United States.

Table 4. Claims to reference crosswalk coefficients

Data Input	Reference or alternative case definition	Gamma	Crosswalk covariate	Beta Coefficient, Logit (95% UI)
Claims, marketscan	Alt	0.39	intercept	-0.87 (-1.67, -0.067)

We performed a systematic review of the duration of symptoms of LRI. We sought consistency with our case definition of LRI and defined our duration as the time between the onset of symptoms to the

resolution of increased work of breathing. Although crucial, there were very limited data on spatial, temporal, or age-specific duration, which may vary based on severity, aetiology, and treatment. We identified 485 titles from PubMed and extracted six studies which were used in a meta-analysis (mean duration 7.79 days, 6.2–9.64 days). We used this as the duration of LRI in our conversions from period to point prevalence and for the conversion between incidence and prevalence.

Severity splits

The distribution of moderate (85%) and severe (15%) lower respiratory infections is determined by a meta-analysis of the ratio of severe to all LRI from studies that report the incidence of moderate and severe lower respiratory infections.

We used the health states of acute infectious disease episode, moderate and severe, with the lay descriptions and disability weight values shown in table below:

Table 5: Severity Splits

Severity level	Lay description	DW (95% CI)
Moderate	Has a fever and aches and feels weak which causes some difficulty with daily activities.	0.051 (0.032–0.074)
Severe	Has a high fever and pain and feels very weak, which causes great difficulty with daily activities.	0.133 (0.088–0.19)

Modelling strategy

The non-fatal lower respiratory infection burden is modelled in model-MR, a Bayesian meta-regression modelling framework. DisMod-MR produces estimates of the incidence, prevalence, and remission of LRI for each age, sex, geographic location, and year. We defined the time to recovery as an average of 10 days (5-15 days), which corresponds with a remission 36.5. The models are informed by country-level covariates (**Table 6**).

Table 6. Model covariates

Study covariate	Type	Parameter	Exponentiated beta (95% Uncertainty Interval)
Socio-demographic Index	Country-level	Prevalence	0.14 (0.14 – 0.14)
Healthcare access and quality index	Country-level	Excess mortality	0.38 (0.15 – 1.00)

Aetiologies

We estimated LRI etiologies separately from overall LRI mortality using two distinct counterfactual modeling strategies to estimate population attributable fractions (PAFs), described in detail below. The PAF represents the relative reduction in LRI mortality if there was no exposure to a given etiology. As LRIs can be caused by multiple pathogens and the pathogens may co-infect, PAFs can overlap and may add up to more than 100%. Separate strategies were used for viral- influenza and respiratory syncytial virus (RSV)- and bacterial- *Streptococcus pneumoniae* and *Haemophilus influenzae* type B- etiologies. We did not attribute etiologies to neonatal LRI deaths due to a dearth of reliable data in this age group. We calculated uncertainty of our PAF estimates from 1,000 draws of each parameter using normal distributions in log space.

Influenza and RSV. We calculated the population attributable fraction (PAF) from the proportion of severe LRI cases positive for influenza and RSV. We used the following formula to estimate PAF[2]:

$$PAF = Proportion\ (modeled) * (1 - \frac{1}{OR})$$

Where *Proportion* is the proportion of LRI cases that test positive for influenza or RSV and *OR* is the odds ratio of LRI given the presence of the pathogen. There are two published estimates of the odds ratios of influenza and RSV. One is based on detection in children younger than 5 years[3] and the second is based on adults over 65 years[4]. We applied the separate odds ratios for those age groups and log-linearly interpolated values between those ages to determine odds ratios for ages between those groups.

We modelled the proportion data using the meta-regression tool DisMod-MR to estimate the proportion of LRI cases that are positive for influenza and RSV, separately, by location/year/age/sex. To make disparate data types directly comparable such as the diagnostic technique (detection by PCR served as our reference), studies that investigated RSV or influenza exclusively (multi-pathogen studies were our reference), and studies from inpatient populations (community-based sample populations was our reference), we performed a meta-regression of the ratios of the reference to non-reference definitions. These meta-regression results were used to adjust the mean and variance of non-reference data **(Table 7)**.

Table 7. Influenza and RSV crosswalk coefficients for lab diagnostic adjustments

Etiology	Data Input	Reference or alternative case definition	Gamma	Crosswalk covariate	Beta Coefficient, Logit (95% CI)
Influenza	PCR diagnostic resting	ref	0.68	--	--
Influenza	Literature, ELISA diagnostic testing	alt		intercept	1.09 (-0.31, 2.5)
Influenza	Community-based samples	ref	0.42	--	--
Influenza	Clinical, inpatient	alt		intercept	0.32 (-0.58, 1.23)
RSV	PCR diagnostic resting	ref	0.69	--	--
RSV	Literature, ELISA diagnostic testing	alt		intercept	0.73 (-0.69, 2.16)

RSV	Community-based samples	ref	0.58	--	--
RSV	Clinical, inpatient	alt		intercept	-0.86 (-2.07, 0.35)

Pneumococcal pneumonia and Hib. For *Streptococcus pneumoniae* (pneumococcal pneumonia) and *Haemophilus influenzae* type B (Hib), we calculated the population attributable fraction using a vaccine probe design.[5,6] The ratio of vaccine effectiveness against nonspecific pneumonia to pathogen-specific disease represents the fraction of pneumonia cases attributable to each pathogen.

To estimate the PAF for Hib and pneumococcal pneumonia, we calculated the ratio of vaccine effectiveness against nonspecific pneumonia to pathogen-specific pneumonia (Equations 1 and 3). We estimated a study-level estimate of PAF from a meta-analysis of these ratios. To estimate the PAF for Hib, we only used randomised controlled trials because of implausibly high values of vaccine efficacy in case-control studies. To estimate the PAF for pneumococcal pneumonia, we included RCTs and before and after vaccine introduction longitudinal studies.

We adjusted the study-level PAF estimate by vaccine coverage and expected vaccine performance to estimate country- and year-specific PAF values. For pneumococcal pneumonia, we adjusted the PAF by the final Hib PAF estimate and by vaccine serotype coverage. Finally, we used an age distribution of PAF modelled in DisMod to determine the PAF by age. Because of an absence of data describing vaccine efficacy against Hib in children older than two years, we did not attribute Hib to episodes of LRI in ages five years and older.

We used a vaccine probe design to estimate the PAF for pneumococcal pneumonia and (Hib) by first calculating the ratio of vaccine effectiveness against nonspecific pneumonia to pathogen-specific pneumonia at the study level (Equations 1 and 2).[5–7] We then adjusted this estimate by vaccine coverage and expected vaccine performance to estimate country- and year-specific PAF values (Equations 3 and 4).

$$1)\quad HibPAF_{Base} = \frac{VE_{Pneumonia}}{VE_{Hib}}$$

$$2)\quad PneumoPAF_{Base} = \frac{VE_{Pneumonia} * (1 - PAF_{Hib} * VE_{Hib\ Optimal})}{VE_{Streptococcus} * Cov_{Serotype}}$$

$$3)\quad PAF_{Hib} = PAF_{Base} * \frac{(1 - Cov_{Hib} * VE_{Hib\ Optimal})}{(1 - PAF_{Base} * Cov_{Hib} * VE_{Hib\ Optimal})}$$

$$4)\quad PAF_{Pneumo} = \frac{PAF_{Base} * (1 - Cov_{PCV} * VE_{PCV\ Optimal})}{(1 - PAF_{Hib} * Cov_{Hib} * VE_{Hib\ Optimal}) * \left(1 - \frac{PAF_{Base} Cov_{PCV} * VE_{PCV\ Optimal}}{(1 - PAF_{Hib} * Cov_{Hib} * VE_{Hib\ Optimal})}\right)}$$

Where $VE_{Pneumonia}$ is the vaccine efficacy against nonspecific pneumonia, VE_{Hib} is the vaccine efficacy against invasive Hib disease, $VE_{Streptococcus}$ is the vaccine efficacy against serotype-specific pneumococcal pneumonia, $Cov_{serotype}$ is the serotype-specific vaccine coverage for PCV,[8] $VE_{Hib\ Optimal}$ is the Hib effectiveness in the community (0.8),[9] PAF_{Hib} is the final PAF for Hib, Cov_{PCV} is the PCV coverage, Cov_{Hib} is the Hib coverage by country, and $VE_{PCV\ Optimal}$ is the vaccine effectiveness in the community (0.8).[10]

For Hib, we assumed that the vaccine efficacy against invasive Hib disease is the same against Hib pneumonia. For pneumococcal pneumonia, a recent study in adults[11] found that the vaccine efficacy against invasive pneumococcal disease may be significantly higher than against pneumococcal pneumonia. We used this ratio to adjust estimates of vaccine efficacy against invasive pneumococcal disease from other studies. However, recognizing that the study is unique in that it uses a urine antigen test among adults, we added uncertainty around our adjustment using a wide uniform distribution (median 0.65, 0.3-1.0).

Changes from GBD 2017

There is one key methodological change from GBD 2017. All data adjustments in GBD 2019 occur before modeling using a standardized approach. Data adjustments for non-fatal LRI include survey prevalence, inpatient clinical prevalence, and clinical claims prevalence. All of these data sources are adjusted to be comparable with our reference definition using a meta-regression model where the dependent variable is the ratio of non-reference to reference data in studies or location-years that have overlap in the definitions. The result is sometimes large changes in the adjustment factors compared to GBD 2017. We believe that this represents an improvement in our methodology because it standardizes these adjustments, accounts for between and within study variance, and explicitly creates these ratios using data within studies or location-years.

References

1 World Health Organization: Department of Child and Adolescent Health and Development. Handbook Integrated Management of Childhood Illness. 2005.

2 Miettinen OS. Proportion of disease caused or prevented by a given exposure, trait or intervention. *Am J Epidemiol* 1974; **99**: 325–32.

3 Shi T, McLean K, Campbell H, Nair H. Aetiological role of common respiratory viruses in acute lower respiratory infections in children under five years: A systematic review and meta-analysis. *J Glob Health* 2015; **5**: 10408.

4 Shi T, Arnott A, Semogas I, Falsey AR, Openshaw P, Wedzicha JA, Campbell H, Nair H, RESCEU Investigators. The etiological role of common respiratory viruses in acute respiratory infections in older adults: a systematic review and meta-analysis. J Infect Dis. 2019 Mar 8. doi: 10.1093/infdis/jiy662.

5 Feikin DR, Scott JAG, Gessner BD. Use of vaccines as probes to define disease burden. *Lancet Lond Engl* 2014; **383**: 1762–70.

6 O'Brien KL, Wolfson LJ, Watt JP, *et al.* Burden of disease caused by Streptococcus pneumoniae in children younger than 5 years: global estimates. *Lancet Lond Engl* 2009; **374**: 893–902.

7 Watt JP, Wolfson LJ, O'Brien KL, *et al.* Burden of disease caused by Haemophilus influenzae type b in children younger than 5 years: global estimates. *Lancet Lond Engl* 2009; **374**: 903–11.

8 Johnson HL, Deloria-Knoll M, Levine OS, *et al.* Systematic evaluation of serotypes causing invasive pneumococcal disease among children under five: the pneumococcal global serotype project. *PLoS Med* 2010; **7**. DOI:10.1371/journal.pmed.1000348.

9 Swingler G, Fransman D, Hussey G. Conjugate vaccines for preventing Haemophilus influenzae type B infections. *Cochrane Database Syst Rev* 2007; : CD001729.

10 Lucero MG, Dulalia VE, Nillos LT, *et al.* Pneumococcal conjugate vaccines for preventing vaccine-type invasive pneumococcal disease and X-ray defined pneumonia in children less than two years of age. *Cochrane Database Syst Rev* 2009; : CD004977.

11 Bonten MJM, Huijts SM, Bolkenbaas M, *et al.* Polysaccharide conjugate vaccine against pneumococcal pneumonia in adults. *N Engl J Med* 2015; **372**: 1114–25.

Upper respiratory infections

Flowchart

Upper respiratory infections (URI)

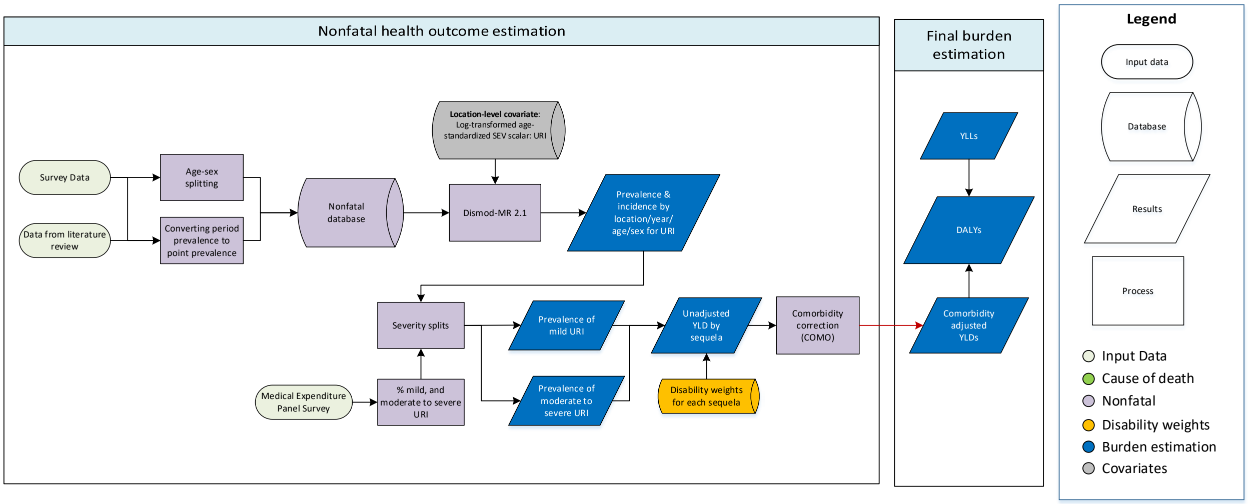

Case Definition

Upper respiratory infections (URI) include cough, acute nasopharyngitis, sinusitis, pharyngitis, tonsillitis, laryngitis/tracheitis, epiglottitis, rhinitis, rhinosinusitis, rhinopharyngitis, supraglottitis, and the common cold. For URI, ICD 10 codes are J00-J02, J02.8-J03, J03.8-J06.9, J36, J36.0, `and ICD 9 codes are 460-465.9, 475-475.9, 476.9.

Input data

Model Inputs

For GBD 2019, a systematic review of URI was conducted using the following PubMed search string:

((upper respiratory infection[Title/Abstract] or rhinitis[Title/Abstract] or rhinitis[MeSH] or rhinosinusitis[Title/Abstract] or sinusitis[Title/Abstract] or sinusitis[MeSH] or nasopharyngitis[Title/Abstract] or rhinopharyngitis[Title/Abstract] or common cold[Title/Abstract] or common cold[MeSH] or pharyngitis[Title/Abstract] or pharyngitis[MeSH] or tonsillitis[Title/Abstract] or epiglottitis[Title/Abstract] or supraglottitis[Title/Abstract] or supraglottitis[MeSH] or laryngitis[Title/Abstract] or laryngitis[MeSH] or laryngotracheitis[Title/Abstract] or tracheitis[Title/Abstract] or tracheitis[MeSH]) AND (prevalence[Title/Abstract] OR incidence[Title/Abstract] OR remission[Title/Abstract] OR duration[Title/Abstract]) NOT (allergies or allergy or allergic rhinitis or asthma) AND (2018/02/11[PDAT] : 2019/02/07[PDAT])) NOT (animals[MeSH] NOT humans[MeSH])

The exclusion criteria for both systematic reviews were:

1. Studies that were not population-based, eg, hospital or clinic-based studies
2. Studies that did not provide primary data on epidemiological parameters, eg, a commentary piece

3. Studies with a sample size of less than 150
4. Reviews

We identified 691 studies via PubMed, of which only one met the above inclusion criteria. Given the low yield of the most recent systematic review, we will prioritise adding data from national surveys as opposed to journal articles in future rounds, given that we expect comprehensive, national surveys to be more likely to estimate the burden of URI.

PRISMA Flow Diagram

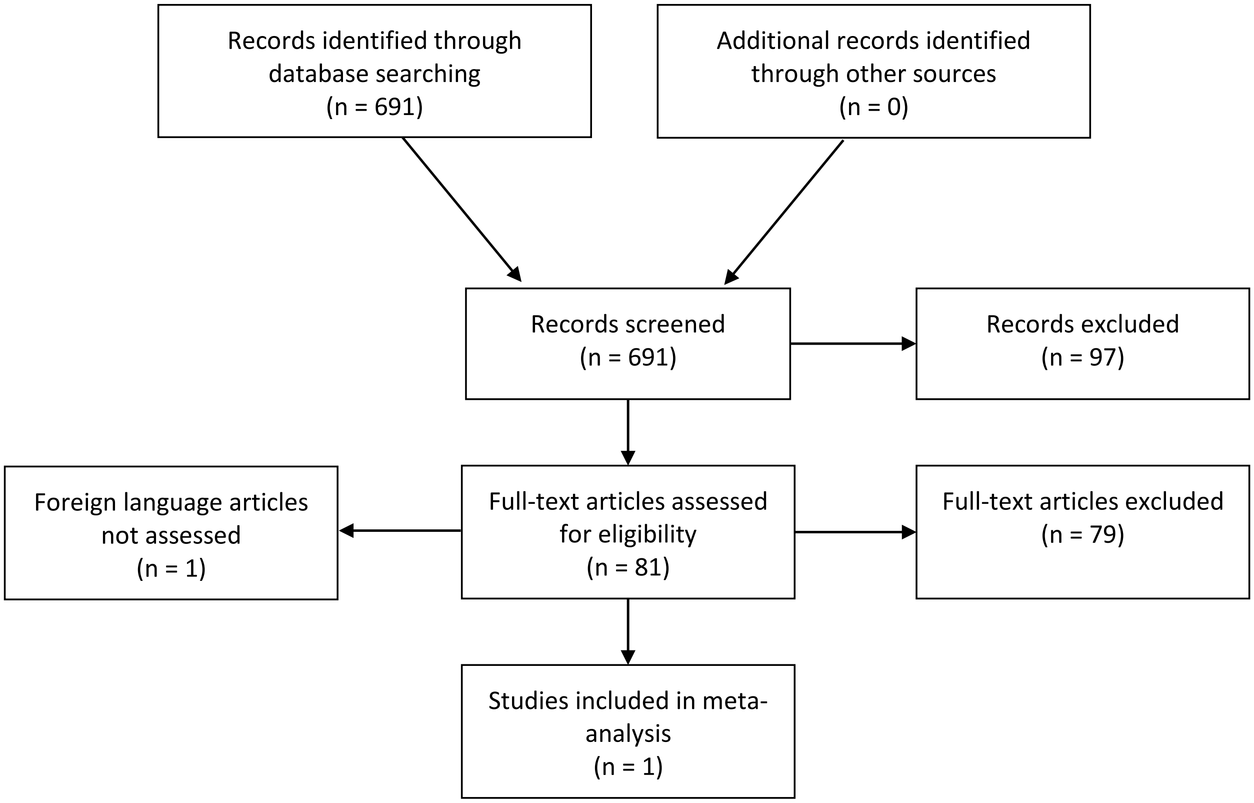

Additionally, data from nationally representative surveys including United States National Health Interview Surveys and Demographic and Health Surveys were included.

Newly identified data sources were added to sources and studies identified in previous rounds of the GBD, resulting in a total of 241 unique data sources from 74 countries (**Table 1).**

Table 1. Unique data sources for upper respiratory infections by measure

Measure	Total sources	Countries with data
All measures	241	74
Prevalence	223	74
Incidence	3	1
Proportion	15	1

Severity Splits

The table below shows the severity distributions based on the data from Medical Expenditure Panel Surveys where we categorised “acute nasopharyngitis or acute URI multi sites/nos” as mild URI and “acute sinusitis, acute pharyngitis, acute tonsillitis, and acute laryngitis/tracheitis and epiglottitis” as moderate URI.

Table 2. URI severity split proportions

Mild URI Proportion	Moderate URI Proportion
.56 (.43 - .68)	.44 (.32 - .57)

The lay descriptions and disability weights for severity levels derived from the GBD disability weights study are shown below.

Table 3. Severity split disability weights

Severity level	Lay description		DW (95% CI)
Mild upper respiratory infections	has a low fever and mild discomfort , but no difficulty with daily activities.		0.006 (0.002–0.012)
Moderate/severe upper respiratory infections	has a fever and aches, and feels weak, which causes some difficulty with daily activities.		0.051 (0.032–0.074)

Modelling Strategy

URI was modeled using a standard DisMod MR 2.1 model. We used secondhand smoke as the location-level covariate in the model. Betas and exponentiated values are shown in the table below:

Table 4. URI DisMod covariates

Covariate	Parameter	beta	Exponentiated beta
Secondhand smoke	Prevalence	0.095 (-0.027 — 0.23)	1.10 (0.97 — 1.26)
Sex	Prevalence	0.0026 (-0.016 — 0.022)	1.00 (0.98 — 1.02)

Otitis media

Flowchart

Otitis media (OM)

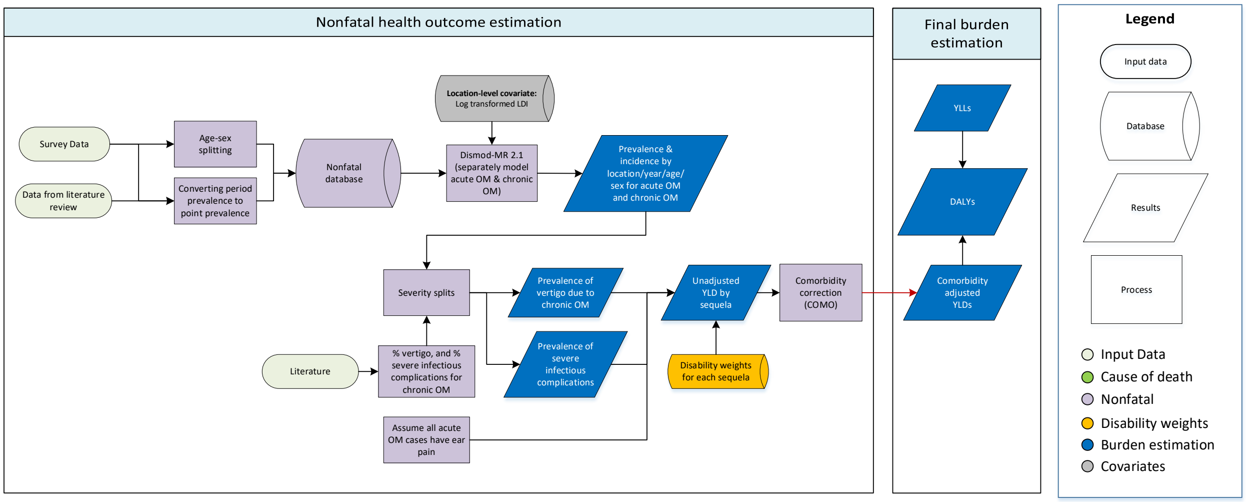

Case definition

Otitis media is an infection of the middle ear space. We included acute otitis media, chronic otitis media, and hearing loss due to chronic otitis media in the GBD non-fatal outcome modelling. Hearing loss due to chronic otitis media estimation is included in the hearing loss report provided separately. The ICD 10 codes are H65-H75.83, and ICD 9 codes are 381-384.9.

Input data

Model Inputs

A systematic review of the prevalence of otitis media was conducted for GBD 2013. The PubMed search terms were: (((otitis media[Title/Abstract] AND (incidence[Title/Abstract] OR prevalence[Title/Abstract])) AND ("2009"[Date – Publication] : "2013"[Date – Publication])).

The exclusion criteria were:

1. Studies that were not population-based, eg, hospital or clinic-based studies
2. Studies that did not provide primary data on epidemiological parameters, eg, commentaries
3. Studies with a sample size of less than 150
4. Reviews
5. Case series

Updates to systematic reviews are performed on an ongoing schedule across all GBD causes, and an update for otitis media will be performed in the next round.

In addition, data from the United States Medical Expenditure Panel Surveys and Australia National Health Surveys were included. The addition of US claims data in the acute otitis model was one main change for GBD 2017.

Table 1: Source Counts

Measure	Total sources	Countries with data
All measures	83	27
Prevalence	30	19
Incidence	50	10
Remission	5	4

Severity splits

We assume that all acute otitis media cases would experience ear pain. The severity distributions for chronic otitis media based on the study by Lin and colleagues (2009) were as follows: (i) vertigo (2.9%, 95% CI: 2.4–3.6%), and (ii) severe infectious complications (0.05%, 95% CI: 0.01–0.2%). We assumed that all chronic otitis media cases experience either mild or moderate hearing loss. The lay descriptions and disability weights for severity levels derived from the GBD disability weights study are shown below.

Table 2. Severity distribution, details on the severity levels for otitis media in GBD 2019 and the associated disability weight (DW) with that severity.

Severity level	Lay description	DW (95% CI)
Acute otitis media	Has an ear-ache that causes some difficulty with daily activities.	0.013 (0.007 to 0.024)
Severe infectious complications due to chronic otitis media	Has an ear-ache that causes some difficulty with daily activities.	0.013 (0.007 to 0.024)
Mild hearing loss due to chronic otitis media	Has great difficulty hearing and understanding another person talking in a noisy place (for example, on an urban street).	0.01 (0.004 to 0.019)
Moderate hearing loss due to chronic otitis media	Is unable to hear and understand another person talking in a noisy place (for example, on an urban street), and has difficulty hearing another person talking even in a quiet place or on the phone.	0.027 (0.015 to 0.042)
Mild hearing loss with ringing due to chronic otitis media	Has great difficulty hearing and understanding another person talking in a noisy place (for example, on an urban street), and sometimes has annoying ringing in the ears.	0.021 (0.012 to 0.036)
Moderate hearing loss with ringing due to chronic otitis media	Is unable to hear and understand another person talking in a noisy place (for example, on an urban	0.074 (0.049 to 0.107)

	street), and has difficulty hearing another person talking even in a quiet place or on the phone, and has annoying ringing in the ears for more than 5 minutes at a time, almost everyday.	
Vertigo with mild hearing loss due to chronic otitis media	*	0.122<br>(0.079 to 0.17)
Vertigo with mild hearing loss and ringing due to chronic otitis media	*	0.132<br>(0.086 to 0.184)
Vertigo with moderate hearing loss due to chronic otitis media	*	0.137<br>(0.089 to 0.189)
Vertigo with moderate hearing loss and ringing due to chronic otitis media	*	0.179<br>(0.12 to 0.247)

* See the hearing loss report for the lay descriptions and disability weights for different severity levels.

Modelling Strategy

We modelled acute and chronic otitis media as separate non-fatal health outcomes using DisMod-MR 2.1. Log-transformed LDI covariate was used as a location-level covariate to model chronic otitis media.

Table 3. Summary of covariates used in the acute otitis media DisMod-MR model

Covariate	Type	Parameter	Exponentiated beta (95% CI)
Sex	Study-level	Prevalence	0.98 (0.81 — 1.19)
Sex	Study-level	Incidence	0.80 (0.79 — 0.80)

Table 4. Summary of covariates used in the chronic otitis media DisMod-MR model

Covariate	Type	Parameter	Exponentiated beta (95% CI)
Log LDI	Country-level	Prevalence	0.63 (0.61 – 0.67)
Sex	Study-level	Prevalence	1.35 (1.12 — 1.62)
Sex	Study-level	Incidence	1.16 (0.43 — 2.82)

Reference

Lin, Y. S., Lin, L. C., Lee, F. P., & Lee, K. J. (2009). The prevalence of chronic otitis media and its complication rates in teenagers and adult patients. *Otolaryngology-Head and Neck Surgery*, *140*(2), 165-170.

Diarrhoeal diseases

Flowchart

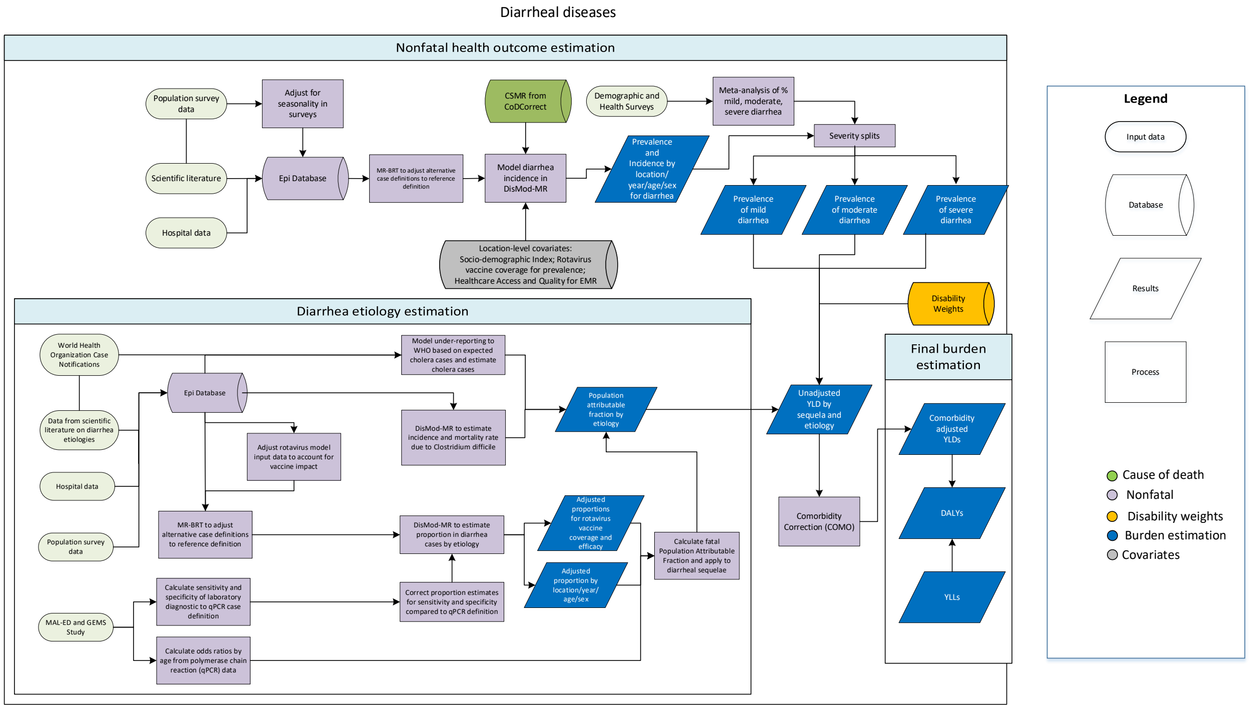

Case definition

We defined diarrhoeal disease episodes as three or more loose stools in a 24-hour period. In the diarrhoea models, self-reported prevalence is the reference category for all data adjustments. Hospital input data use ICD9 codes 001-009.9 and ICD10 codes A00-A09. We excluded gastroenteritis as a case definition as this is often syndromic (vomiting or diarrhea).

Input data

Model inputs

We used two main types of data in the diarrhoea non-fatal burden estimation and the attribution of diarrhoeal aetiologies. Moreover, we included all data sources used in GBD 2017 and conducted new reviews of scientific literature, surveys, and hospitalisation data.

The first type of data is the incidence and prevalence of diarrhoea in community and hospital settings. Hospital data and healthcare utilisation data were identified using the ICD9 codes 001-009.9 and ICD10 codes A00-A09. These data are adjusted prior to modelling for multiple admissions, multiple diagnoses, and for outpatient claims. The outpatient adjustment is informed by claims data in the US, Taiwan, and the Philippines, and estimates that the number of community cases given inpatient data. To be consistent with the survey data, hospital and health care data were transformed from incidence to prevalence using the following equation:

$$Prevalence = Incidence * \frac{duration(days)}{365}$$

The second type of data are from population-representative surveys, such as the Demographic and Health Surveys and the Multiple Indicator Cluster Surveys. We converted the prevalence of maternal-reported two-week period from surveys to point prevalence in one-year age groups using this equation:

$$Point\ Prevalence = Period\ Prevalence * \frac{Duration}{(Recall\ Period + Duration - 1)}$$

Where the mean duration was the duration in days, an average of 4.3 (4.2–4.4) in both equations.[1]

Survey data were adjusted for seasonality. An inclusion criterion for scientific literature is a study duration longer than 1 year to avoid bias in the seasonal timing of diarrhea. Surveys are frequently conducted over several months. To account for seasonal variation in diarrhea prevalence, we fit a mixed-effects generalized additive model for each GBD region with a forced periodicity and a random intercept by country. The ratio between the monthly model fit diarrhea prevalence and the mean fitted diarrheal prevalence is a scalar to adjust survey data by month and geography.

Aetiologies

The second type of data describes diarrhoea aetiologies. We extracted data on all aetiologies except *C. difficile* from scientific literature that reported the proportion of diarrhoea cases that tested positive for each pathogen. We completed a systematic literature review covering the time period May 2018 to February 2019 for diarrhoea prevalence, incidence, and all diarrhoea aetiologies. Inclusion criteria included diarrhoea as the case definition, studies with a sample size of at least 100, and studies with at least one year of follow-up. We excluded studies that reported on diarrhoeal outbreaks exclusively and those that used acute gastroenteritis with or without diarrhoea.

We searched articles using a PubMed search term that combined non-specific and aetiology-specific diarrhoea in February 2019 using the following search string:

(diarrhoea[title/abstract] OR diarrhea[title/abstract]) AND (2018/07/30:2019/2/7[PDat]) AND Humans[MeSH Terms] AND (incidence[title/abstract] OR prevalence[title/abstract] OR epidemiology[title/abstract] OR salmonella[title/abstract] OR aeromona[title/abstract] OR shigell*[title/abstract] OR enteropathogenic[title/abstract] OR enterotoxigenic[title/abstract] OR campylobacter[title/abstract] OR amoebiasis[title/abstract] OR entamoeb*[title/abstract] OR cryptosporid*[title/abstract] OR rotavirus[title/abstract] OR norovirus[title/abstract] OR adenovirus[title/abstract] OR etiology[title/abstract]) NOT (appendicitis[title/abstract] OR esophag*[title/abstract] OR surger*[title/abstract] OR gastritis[title/abstract] OR liver[title/abstract] OR case report[title] OR case-report[title] OR therapy[title] OR treatment[title] Crohn[title/abstract] OR "inflammatory bowel"[title/abstract] OR irritable[title/abstract] OR travel*[title] OR Outbreak[title] OR Review[ptyp] OR vomiting[title/abstract) NOT (animals[MeSH] NOT humans[MeSH])*

We identified 82 studies, of which three met our inclusion criteria. We extracted data for location, sex, year, and age.

Figure 1. Diarrheal disease etiology systematic review flowchart

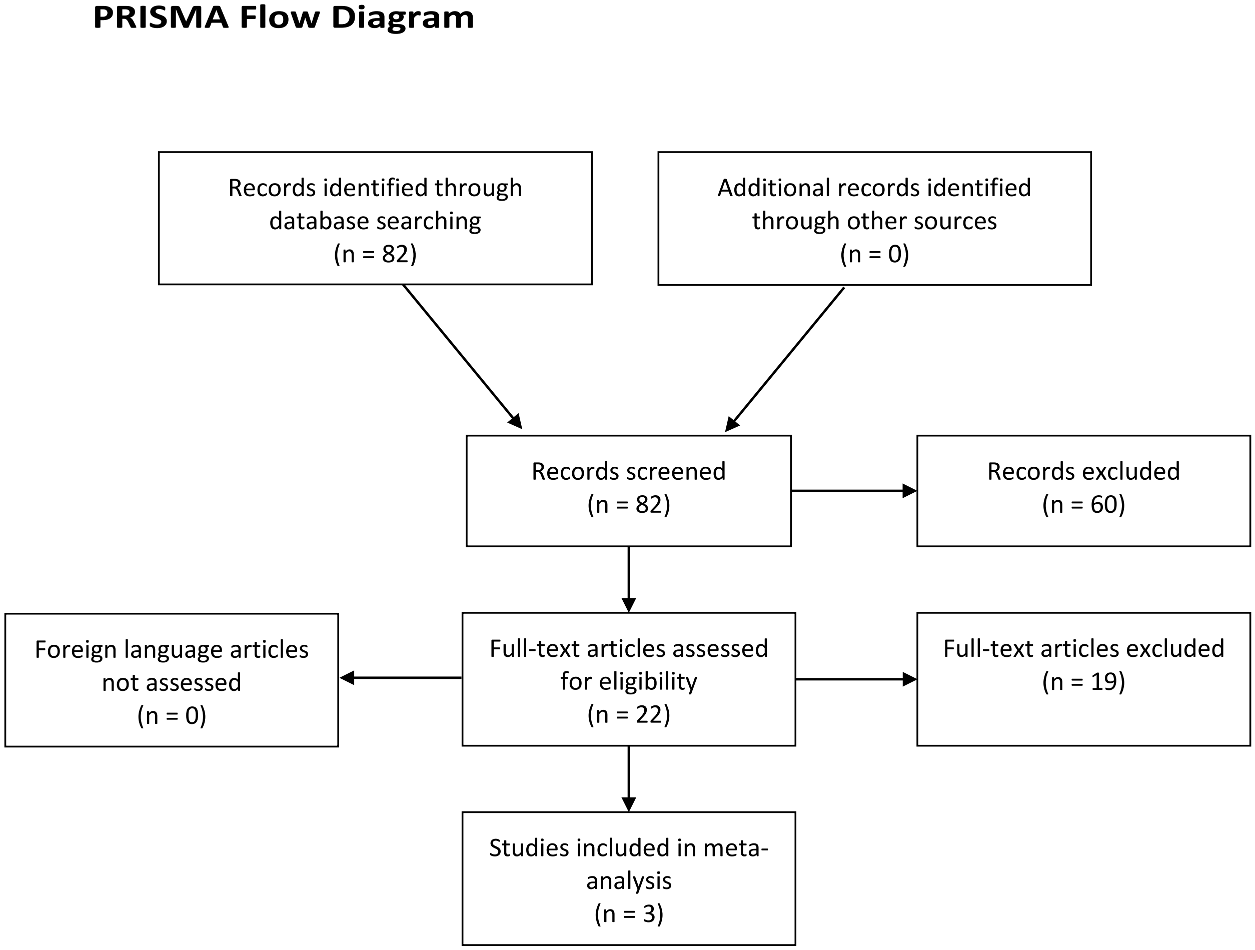

Similiarly, we used the following search string to supplement incidence data on *C. difficile:*

"clostridium difficile" AND diarrhea[title/abstract] AND (epidemiolog OR incidence OR prevalence) AND (("2017/06/05"[PDat] : "2019/2/7"[PDat])) NOT (animals[MeSH] NOT humans[MeSH])*

We identified 185 studies, of which five met our inclusion criteria. We extracted data points for location, sex, year, and age.

Figure 2. C. difficile systematic review flowchart

PRISMA Flow Diagram

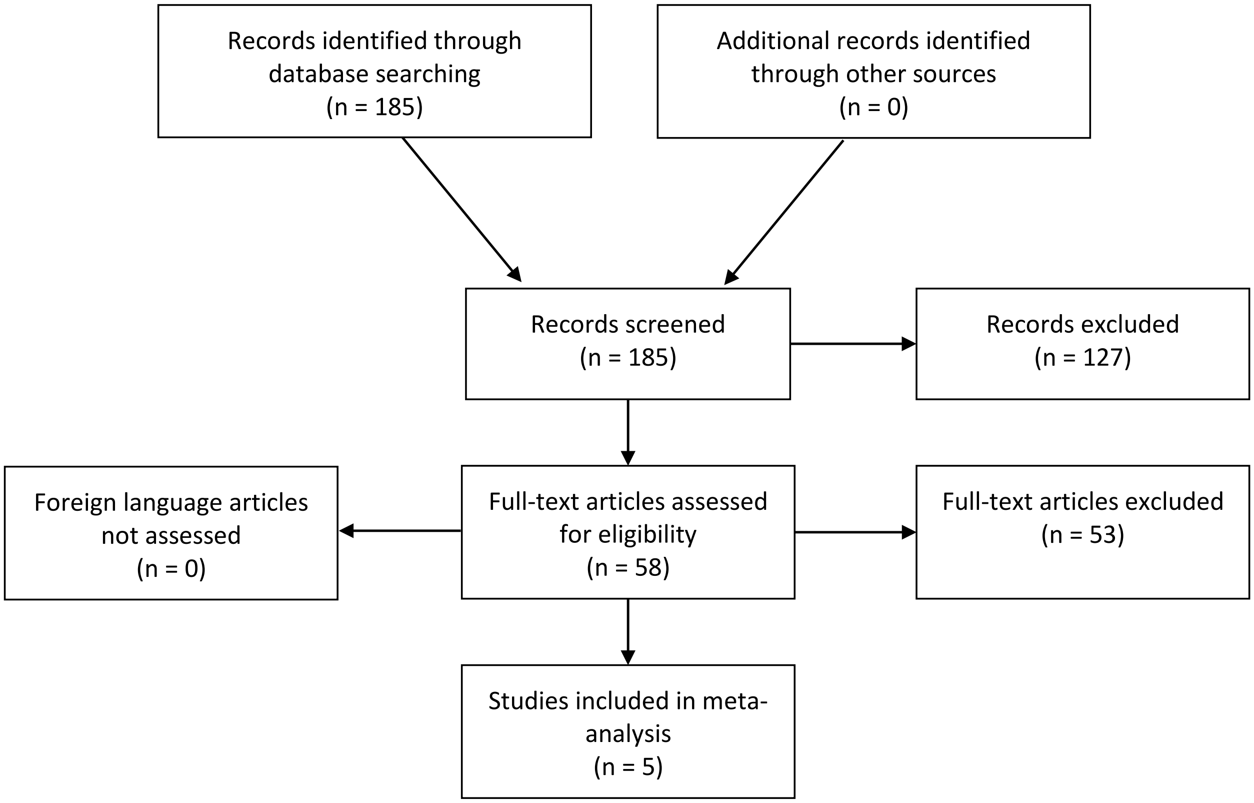

Additionally, we searched specifically for data sources detailing rotavirus coverage and vaccine efficacy using the following search string:

(((rotavirus[title/abstract] AND vaccine[title/abstract] AND (efficacy[title/abstract] OR effectiveness[title/abstract]) AND (2018/06/21[PDAT] : 2019/2/7[PDAT]) AND "humans"[MeSH Terms]))) NOT Review[Publication Type] NOT (animals[MeSH] NOT humans[MeSH])

We identified 603 studies via PubMed and additional 119 studies through manual reference search. Of the 722 studies identified, 56 met our inclusion criteria.

Figure 3. Rotavirus vaccine efficacy systematic review flowchart

PRISMA Flow Diagram

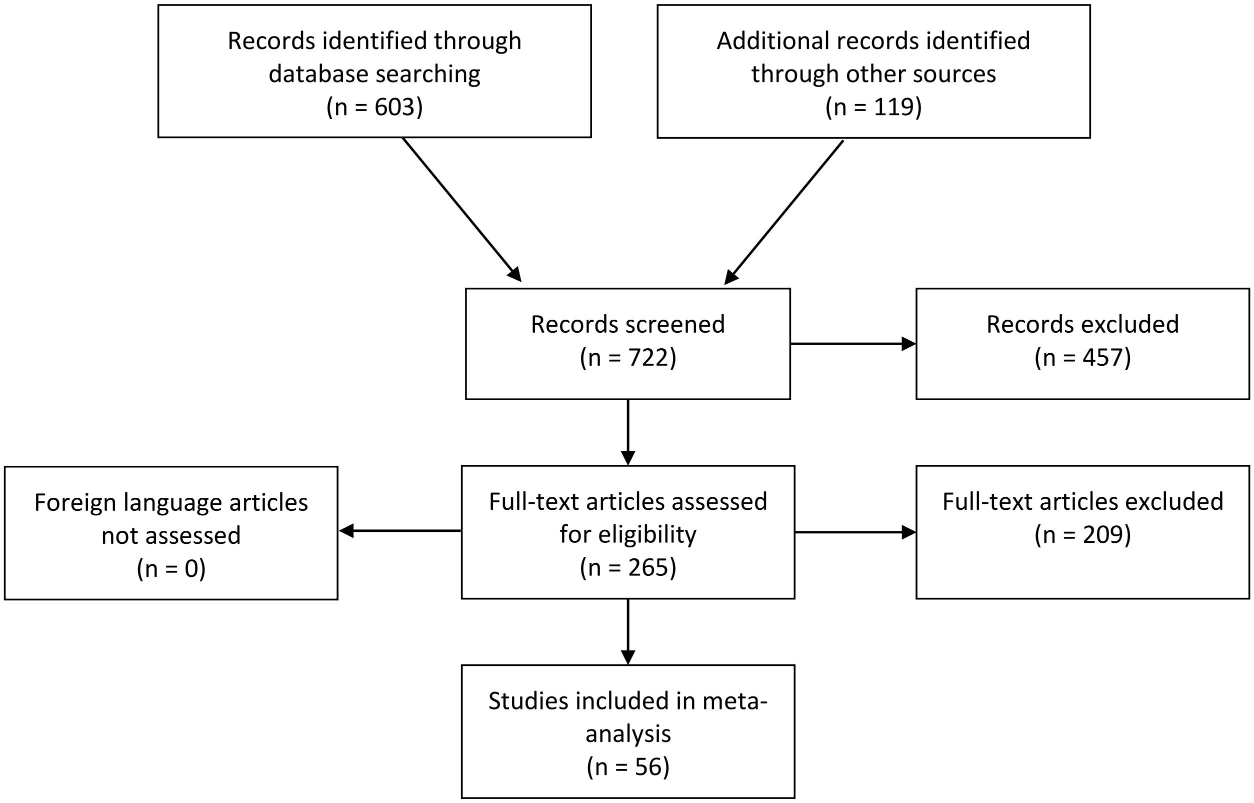

For the data that describe proportion of episodes positive for a given pathogen, we assigned an age range based on the prevalence-weighted mean age of diarrhea in the appropriate year/sex/location if the age of the study participants was not reported.

We used the Global Enteric Multicenter Study (GEMS), a seven-site, case-control study of moderate-to-severe diarrhoea in children under 5 years,[2] and the MAL-ED study,[3] a multi-site birth cohort, to calculate odds ratios for the diarrhoeal pathogens. We analysed raw data for a systematic reanalysis, representative of the distribution of cases and controls by age and site that were tested for the presence of pathogen using quantitative polymerase chain reaction (qPCR).[4]

Data that did not use qPCR for detection were adjusted for sensitivity and specificity prior to modelling in order to standardize data regardless of detection method. Adjusting these data prior to modelling allowed us to adjust only data that did not use qPCR, as well as better control for values at extreme bounds, and capture uncertainty in modelling.

Newly-identified sources were added to studies and sources identified in previous rounds of the GBD, resulting in 3768 total unique sources for diarrheal diseases, representing data from 182 countries (table 1).

Table 1. Unique sources counts for diarrheal diseases by measure

Measure	Total sources	Countries with data
All measures	3299	183
Prevalence	1188	171
Incidence	224	26
Proportion	2111	160
Continuous	1	0
Other	14	0

Data crosswalks

One of the GBD core principles is to use all available data to inform our estimates. In order to account for differences between studies, we conducted a meta-regression of the ratio of reference to non-reference data means using the Meta-Regression Bayesian Regularized Trimmed (MR-BRT) tool. MR-BRT is new innovation for GBD 2019, and uses within study comparisons when possible to crosswalk alternative and reference case definitions/methods by estimating coefficients on study covariates. When possible, crosswalks were based on data matched within studies on age, sex, and location are used. When not possible, ratios between alternative and reference case definitions/methods were based on data matched between studies, nearby in age, year, with exact matches on sex and location. We adjusted inpatient clinical data, clinical claims data, incidence of hospitalized diarrhea, and incidence of medically-attended diarrhea up to the level of self-reported data (our reference case definition) (table 2). Additionally, age was shown to be a predictor of this adjustment for claims data. To accommodate any non-linear association between age and the crosswalk ratios, we incorporated splines on age midpoint as shown in table 2.

Table 2. Diarrhoeal disease crosswalk coefficients

<table>
<tr><th>Data Input</th><th>Reference or alternative case definition</th><th>Gamma</th><th>Crosswalk covariate</th><th>Beta Coefficient, Logit (95% UI)</th></tr>
<tr><td>self-reported diarrhea</td><td>ref</td><td>--</td><td>--</td><td>--</td></tr>
<tr><td>Clinical, inpatient</td><td>alt</td><td>1.50</td><td>intercept</td><td>6.7 (3.76, 9.64)</td></tr>
<tr><td>Claims, marketscan</td><td>alt</td><td rowspan="5">0</td><td>age_mid_0</td><td>3.84 (3.26, 4.41)</td></tr>
<tr><td>Claims, marketscan</td><td>alt</td><td>age_mid_1</td><td>4.09 (3.67, 4.5)</td></tr>
<tr><td>Claims, marketscan</td><td>alt</td><td>age_mid_2</td><td>4.78 (4.54, 5.01)</td></tr>
<tr><td>Claims, marketscan</td><td>alt</td><td>age_mid_3</td><td>4.92 (4.67, 5.17)</td></tr>
<tr><td>Claims, marketscan</td><td>alt</td><td>age_mid_4</td><td>4.06 (3.76, 4.36)</td></tr>
<tr><td>Literature, inpatient</td><td>alt</td><td>1.8</td><td>intercept</td><td>3.02 (-1.07, 7.11)</td></tr>
<tr><td>Literature, hospital-based</td><td>alt</td><td>0.16</td><td>intercept</td><td>0.29 (-0.1, 0.69)</td></tr>
</table>

Age-sex splits

Data were age and sex split based on population and a modeled age-curve generated using age-specific data as inputs in MR-BRT in order to better estimate the distribution of non-age specific data.

Severity split inputs

Diarrhoeal diseases have three severity levels: mild, moderate, and severe (**Table 3**). The proportion of diarrhoea cases that are assigned to each comes from a systematic review of diarrhoea severity.[1] Mild cases are the proportion of diarrhoea cases that did not seek medical care (64.8%); moderate cases are the proportion that sought medical care but did not have severe dehydration or bloody stool (28.9%); and severe cases are the proportion that sought medical care with severe dehydration or bloody stool (6.9%). These proportions are based on the frequency of dehydration and bloody stool among community-based studies reported in the systematic review.

Table 3. Severity splits, details on the severity levels for diarrhoea in GBD 2019 and the associated disability weight (DW) with that severity.

Severity level	Lay description	Disability weight (95% CI)	Proportion
Mild	Has diarrhea defined as 3 or more loose stools in a 24-hour period with no dehydration	0.074 (0.049-0.104)	64.8%
Moderate	Has diarrhea defined as 3 or more loose stools in a 24-hour period with painful cramps and feeling thirsty and any dehydration	0.188 (0.125-0.264)	28.9%
Severe	Has diarrhea defined as 3 or more loose stools in a 24-hour period with painful cramps and is very thirsty or feels nauseated or tired and/or severely dehydrated	0.247 (0.164-0.348)	6.9%

Modelling strategy

Diarrhoea incidence and prevalence

The non-fatal diarrhoeal disease burden is modelled in DisMod-MR 2.1, a Bayesian meta-regression modelling framework. DisMod-MR produces estimates of the incidence, prevalence, and remission of diarrhoea for each age, sex, geographic location, and year. We defined remission, or the time to recovery, as five days average. The reference category for our input data is community-based diarrhoea episodes such as data from population-representative surveys or community cohorts. As described in the data crosswalks section above, input data that are from a different population, such as hospital inpatient groups, are adjusted before modeling by determining a meta-regression ratio of non-reference to reference data values, so that they are consistent with the reference category.

Country-level covariates are used to inform the model (**Table 4**). In previous rounds, priors on excess mortality rate (EMR) were estimated in DisMod by matching prevalence data points with their

corresponding CSMR values within the same age, sex, year, location (by dividing CSMR by prevalence). For short duration conditions (remission>1), the corresponding prevalence was derived by running an initial model and then applying the same CSMR/prevalence method. However, for many causes, DisMod estimated a rather unrealistic pattern of EMR compared to an expected pattern of decreasing EMR with greater access to quality health care. Such unexpected patterns often signal inconsistencies between CSMR estimates and the measures of prevalence and/or incidence. In effort to provide greater guidance to DisMod on the expected pattern of EMR, EMR data generated in the previous round were modeled using the MR-BRT approach by age and sex with a prior on healthcare access and quality index (HAQi) having a negative coefficient. Results from MR-BRT were then predicted for each location year, sex and for ages 0, 10, 20 ….100. We included HAQi as a country-level covariate to inform EMR with a mean and standard deviation produced from MR-BRT. However, even without this setting DisMod would tend to estimate a coefficient that was consistent with the MR-BRT analysis.

Table 4. Covariates. Summary of covariates used in the diarrhoea DisMod-MR meta-regression model

Covariate	Type	Parameter	Exponentiated beta (95% Uncertainty Interval)
Socio-demographic Index	Country-level	Prevalence	0.14 (0.14-0.14)
Rotavirus vaccine coverage	Country-level	Prevalence	1.00 (1.00-1.00)
Healthcare access and quality index	Country-level	Excess mortality	0.95 (0.95-0.95)

Aetiologies

We estimated diarrhoeal disease aetiologies independently from overall diarrhoea envelope using a counterfactual strategy for enteric adenovirus, *aeromonas*, *entamoeba histolytica* (amoebiasis), *campylobacter*, *cryptosporidium*, typical EPEC, enterotoxigenic *Escherichia coli* (ETEC), norovirus, non-typhoidal salmonella infections, rotavirus, and shigella. *Vibrio cholerae* and *C. difficile* were modelled separately (**Table 5)**.

Table 5. Inpatient to community crosswalk coefficients for diarrhoeal disease etiologies, not including *Vibrio cholerae* or *C. difficile*

Etiology	Data Input	Reference or alternative case definition	Gamma	Crosswalk covariate	Beta Coefficient, Logit (95% UI)
All	Community-based samples	ref	--	--	--
adenovirus	Hospital-based samples	alt	0.65	intercept	-0.29 (-0.53, -0.04)
aeromonas	Hospital-based samples	alt	0.64	intercept	0.38 (0.16, 0.6)

amoebiasis	Hospital-based samples	alt	0.45	intercept	0.35 (-0.61, 1.3)
campylobacter	Hospital-based samples	alt	0.31	intercept	-0.12 (-0.76, 0.52)
cryptosporidium	Hospital-based samples	alt	0.74	intercept	-0.1 (-1.63, 1.44)
Epec	Hospital-based samples	alt	0.1	intercept	-0.05 (-0.34, 0.24)
Etec	Hospital-based samples	alt	0.28	intercept	0.12 (-0.47, 0.72)
norovirus	Hospital-based samples	alt	0.37	intercept	-0.16 (-0.92, 0.6)
rotavirus	Hospital-based samples	alt	0.46	intercept	-0.78 (-1.7, 0.14)
salmonella	Hospital-based samples	alt	0.48	intercept	-0.63 (-1.64, 0.37)
shigellosis	Hospital-based samples	alt	0.38	intercept	0.05 (-0.74, 0.84)

Diarrhoeal aetiologies are attributed to diarrhoeal deaths using a counterfactual approach. We calculated a population attributable fraction (PAF) from the proportion of severe diarrhoea cases that are positive for each aetiology. The PAF represents the relative reduction in diarrhoea mortality if there was no exposure to a given aetiology. As diarrhoea can be caused by multiple pathogens and the pathogens may co-infect, PAFs can overlap and are not scaled to sum to 100%. We calculated the PAF from the proportion of severe diarrhoea cases that are positive for each aetiology. We assumed that hospitalised diarrhoea cases are a proxy of severe and fatal cases. We used the following formula to estimate PAF:[5]

$$PAF = Proportion * (1 - \frac{1}{OR})$$

Where *Proportion* is the proportion of diarrhoea cases positive for an aetiology and *OR* is the odds ratio of diarrhoea given the presence of the pathogen.

We dichotomised the continuous qPCR test result using the value of the cycle threshold (Ct) that most accurately discriminated between cases and controls. The Ct values range from 0 to 35 cycles representing the relative concentration of the target gene in the stool sample. A low value indicates a higher concentration of the pathogen while a value of 35 indicates the absence of the target in the sample. We used the lower Ct value when we had multiple Ct values for the cut-point. The case definition for each pathogen is a Ct value that is below the established cutoff point **(Table 6)**.

Table 6. Single to multi-pathogen study crosswalk coefficients for diarrhoeal disease etiologies, not including *Vibrio cholerae* or *C. difficile*

Etiology	Data Input	Reference or alternative case definition	Gamma	Crosswalk covariate	Beta Coefficient, Logit (95% UI)
all	Multi-pathogen studies	ref	--	--	--
adenovirus	Single pathogen	alt	0.65	intercept	-0.32 (-1.65, 1)
aeromonas	Single pathogen	alt	0.64	intercept	-0.69 (-1.99, 0.62)
amoebiasis	Single pathogen	alt	0.85	intercept	-0.6 (-2.31, 1.11)
campylobacter	Single pathogen	alt	0.45	intercept	0 (-0.07, 0.07)
cryptosporidium	Single pathogen	alt	0.54	intercept	-0.11 (-1.2, 0.98)
epec	Single pathogen	alt	0.55	intercept	-0.32 (-1.5, 0.86)
etec	Single pathogen	alt	0.32	intercept	-0.02 (-0.67, 0.63)
norovirus	Single pathogen	alt	0.68	intercept	-0.31 (-1.65, 1.02)
rotavirus	Single pathogen	alt	0.88	intercept	-0.52 (-2.24, 1.2)
salmonella	Single pathogen	alt	0.89	intercept	-0.37 (-2.14, 1.4)
shigellosis	Single pathogen	alt	0.51	intercept	-0.3 (-1.31, 0.72)

We used a mixed effects conditional logistic regression model to calculate the odds ratio for under 1 year and 1–4 years old for each of our pathogens. The stool samples from cases and controls in GEMS were used exclusively to calculate these odds ratios as we assumed that the association between pathogens and moderate-to-severe diarrhoea is a proxy for fatal outcomes. The odds ratio for 1–4 years was applied to all GBD age groups over 5 years. There were three pathogen-age odds ratios that were not statistically significant: aeromonas and amoebiasis in under 1 year and campylobacter in 1–4 years. The mean value of the odds ratio was above 1 in all three cases, so we transformed the odds ratios for

these three exceptions only in log space such that exponentiated values could not be below 1. The transformation was:

$$Odds\ ratio = exp(log(OR) - 1)) + 1$$

We modelled the proportion data using the Bayesian meta-regression tool DisMod-MR to estimate the proportion of positive diarrhoea cases for each separate aetiology by location/year/age/sex and to adjust for the covariates. We used the estimated sensitivity and specificity of the original laboratory diagnostic test results from the pooled GEMS and MAL-ED qPCR stool samples compared to the qPCR test result to adjust our proportion before we modelled the proportions:[6]

$$Proportion_{True} = \frac{(Proportion_{Observed} + Specificity - 1)}{(Sensitivity + Specificity - 1)}$$

We used this correction to account for the fact that the proportions we used are based on a new test that is not consistent with the laboratory-based case definition (qPCR versus GEMS conventional laboratory testing for pathogens).[7] Because differences in the type of PCR used in the original (nonreference qPCR diagnostic) between GEMS and MAL-ED in detecting norovirus, we combined the sensitivity and specificity results for norovirus such that 50% of the draws were coming from GEMS test results exclusively and 50% of the draws were coming from MAL-ED test results exclusively. Additionally, because the original laboratory diagnostic technique used for *campylobacter* in MAL-ED was one not commonly used, we only used GEMS to determine the sensitivity and specificity of bacterial culture compared to qPCR in detecting *campylobacter.*[8]

Our literature review extracted the proportion of any EPEC without differentiating between typical (tEPEC) and atypical (aEPEC). In order to be consistent with the odds ratios that we obtained, we adjusted our proportion estimates of any EPEC to typical EPEC only. This adjustment was informed by a subset of our literature review that reported both atypical and typical EPEC. We estimated a ratio by super-region of tEPEC to any EPEC and adjusted our proportion estimates accordingly. We found that the majority of EPEC diarrhoea cases were positive for atypical EPEC, consistent with other published work.[9] We applied the same approach to differentiate between heat-stable toxin (ST) and heat labile toxin producing (LT) ETEC. For the first time, GBD 2019 split these serotypes so that estimates in GBD 2019 represent the diarrhoeal disease burden attributable to ST-ETEC. This was based on work showing that ST-ETEC was much more pathogenic than LT-ETEC. As our proportion data were extracted for any ETEC, we determined a proportion of all ETEC that produced ST from the GEMS and MAL-ED studies and applied that ratio to our input data so that they represented ST-ETEC only. We re-estimated the sensitivity and specificity values as well as the odds ratios for our new definition of ST-ETEC.

For *vibrio cholerae* (cholera), we used the literature review to estimate the expected number of cholera cases for each country-year using the incidence of diarrhea (estimated using DisMod-MR) and the proportion of diarrhoea cases that are positive for cholera. We assigned cholera PAF using odds ratios from the qPCR results to estimate a number of cholera-attributable cases. We compared this expected number of cholera cases to the number reported to the World Health Organization at the country-year level.[10] We modelled the underreporting fraction to correct the cholera case notification data for all countries using health system access and the diarrhoea SEV scalar to predict total cholera cases. We

used the age-specific proportion of positive cholera samples in DisMod-MR and our incidence estimates to predict the number of cholera cases for each age/sex/year/location. Finally, we modelled the case fatality ratio of cholera using DisMod-MR and to estimate the number of cholera deaths.

For *C. difficile*, we modelled incidence and mortality in DisMod-MR for each age, sex, year, location. DisMod-MR uses a compartmental model to relate prevalence, incidence, remission, and mortality. We set remission in our model to 1 month. Additionally, age was found to be a predictor for both inpatient and claims data. As with diarrhoeal diseases overall, we used multiple splines on age-midpoint to accommodate any non-linear association between the crosswalk ratios and age.

and these sources were adjusted accordingly using splines on multiple age midpoints **(Table 7)**.

Table 7. Crosswalk coefficients for *C. difficile*

Data Input	Reference or alternative case definition	Gamma	Crosswalk covariate	Beta Coefficient, Logit (95% UI)
Clinical, inpatient	alt	0.97	age_mid_0	1.01 (-2.36, 4.37)
Clinical, inpatient	alt		age_mid_1	0.73 (-2.32, 3.79)
Clinical, inpatient	alt		age_mid_2	0.71 (-1.12, 2.55)
Clinical, inpatient	alt		age_mid_3	-1.96 (-4.15, 0.23)
Clinical, inpatient	alt		age_mid_4	-2.29 (-3.49, -1.08)
Claims, marketscan	alt	1.17	age_mid_0	0.03 (-2.66, 2.71)
Claims, marketscan	alt		age_mid_1	0.45 (-0.36, 1.26)
Claims, marketscan	alt		age_mid_2	-0.45 (-1.23, 0.33)
Claims, marketscan	alt		age_mid_3	0.45 (-0.33, 1.23)
Claims, marketscan	alt		age_mid_4	-0.41 (-1.19, 0.37)

For rotavirus, we made a change to the process of estimating attributable fraction to explicitly account for rotavirus vaccine efficacy in GBD 2019. The impact of the rotavirus vaccine is dependent on modelled vaccine coverage for a location-year and on the rotavirus vaccine efficacy (VE). There are numerous studies that demonstrate a difference in VE by national income and development.[11] We also determined via LASSO (least absolute shrinkage and selection operator) that Socio-demographic Index (SDI) was the best predictor of rotavirus VE. We used a meta-regression with SDI as covariate to predict the rotavirus VE by location and year.

For GBD 2019, we explicitly incorporated the results from our analysis of VE to produce more robust estimates of the proportion of diarrhoea that has rotavirus over time and space. We assumed that the impact of the vaccine can be represented as one minus the product of the estimated vaccine coverage and VE.

$$Vaccine\ impact = 1 - vaccine\ coverage * vaccine\ efficacy$$

Both of these values vary in time and space but not by age. To avoid discontinuities in our model, we adjusted the input proportion data to remove the impact of the rotavirus vaccine by dividing the observed proportion by the vaccine impact.

$$Rotavirus\ proportion_{Adjusted} = \frac{Rotavirus\ proportion}{1 - Cov_{RotaV} * VE_{Modeled}}$$

The result is the modelled proportion of diarrhoea positive for rotavirus in the absence of the vaccine. This modelled value is then multiplied by the impact of the rotavirus vaccine to determine the estimated proportion of diarrhoea positive for rotavirus in the presence of the vaccine. Our modified attributable fraction is then:

$$DisModPAF = Modeled\ Proportion\ (from\ DisMod)\ * \left(1 - \frac{1}{OR}\right)$$

The last step is to account for the expected impact of the rotavirus vaccine. We do this using the equation below:

$$PAF_{Rota} = DisModPAF * \frac{(1 - Cov_{RotaV} * VE_{Modeled})}{(1 - DisModPAF * Cov_{RotaV} * VE_{Modeled})}$$

Where the final attributable fraction for rotavirus is the product of the PAF estimated in DisMod-MR and the expected reduction in that PAF given modelled vaccine coverage and modelled VE by location-year, and this value is only applied to children 28 days to 5 years old. The product of the rotavirus attributable fraction and the number of deaths or cases of diarrhoea is the number of deaths and cases caused by rotavirus.

References

1 Lamberti LM, Fischer Walker CL, Black RE. Systematic review of diarrhea duration and severity in children and adults in low- and middle-income countries. *BMC Public Health* 2012; **12**: 276.

2 Kotloff KL, Nataro JP, Blackwelder WC, *et al.* Burden and aetiology of diarrhoeal disease in infants and young children in developing countries (the Global Enteric Multicenter Study, GEMS): a prospective, case-control study. *Lancet Lond Engl* 2013; **382**: 209–22.

3 Platts-Mills J, Liu J, Rogawski E. Aetiology, burden and clinical characteristics of diarrhoea in children in low-resource settings using quantitative molecular diagnostics: results from the MAL-ED cohort study. *Lancet Glob Health* 2018; : Accepted.

4 Liu J, Gratz J, Amour C, *et al.* A laboratory-developed TaqMan Array Card for simultaneous detection of 19 enteropathogens. *J Clin Microbiol* 2013; **51**: 472–80.

5 Miettinen OS. Proportion of disease caused or prevented by a given exposure, trait or intervention. *Am J Epidemiol* 1974; **99**: 325–32.

6 Reiczigel J, Földi J, Ozsvári L. Exact confidence limits for prevalence of a disease with an imperfect diagnostic test. *Epidemiol Infect* 2010; **138**: 1674–8.

7 Platts-Mills JA, Operario DJ, Houpt ER. Molecular diagnosis of diarrhea: current status and future potential. *Curr Infect Dis Rep* 2012; **14**: 41–6.

8 Platts-Mills JA, Liu J, Gratz J, *et al.* Detection of Campylobacter in stool and determination of significance by culture, enzyme immunoassay, and PCR in developing countries. *J Clin Microbiol* 2014; **52**: 1074–80.

9 Ochoa TJ, Barletta F, Contreras C, Mercado E. New insights into the epidemiology of enteropathogenic Escherichia coli infection. *Trans R Soc Trop Med Hyg* 2008; **102**: 852–6.

10 World Health Organization. Global Health Observatory data repository: Cholera. 2016. http://apps.who.int/gho/data/node.main.174?lang=en (accessed Aug 25, 2016).

11 Lamberti LM, Ashraf S, Walker CLF, Black RE. A Systematic Review of the Effect of Rotavirus Vaccination on Diarrhea Outcomes Among Children Younger Than 5 Years. *Pediatr Infect Dis J* 2016; **35**: 992–8.

Typhoid and paratyphoid fevers

Flowchart

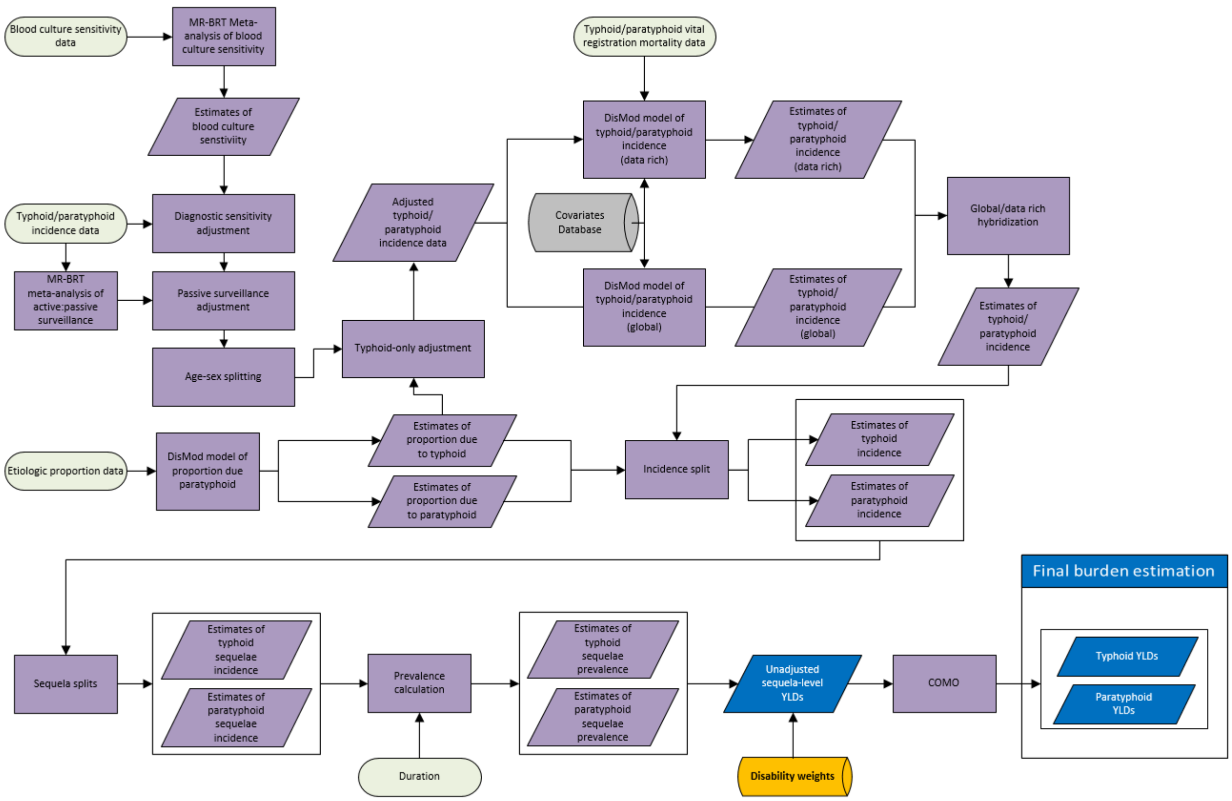

Case definition

Typhoid and paratyphoid are acute bacterial infections that most commonly cause febrile illness and gastrointestinal symptoms. Severe cases are associated with intestinal bleeding and perforation, altered mental state and, in some cases, death. We define a confirmed case as one for which there has been a positive blood culture test for either *Salmonella enterica typhi* or *paratyphi*. Diagnostic criteria do not typically accompany national surveillance reports; however, with blood culture being the standard diagnostic, we treat reported cases as confirmed. Given the poor sensitivity of blood culture, however, we estimated case definition as simply febrile illness resulting from an infection with *Salmonella enterica typhi* or *paratyphi*. This is effectively a counterfactual definition in which we attempt to estimate the number of true infections regardless of test result. These causes include all ICD-10 codes under the heading A01 (Typhoid and paratyphoid fevers).

Input data

Model inputs

Our incidence dataset included a combination of data from prospective cohort studies and national surveillance systems. Similarly, data on proportions due to typhoid and paratyphoid included a combination of prospective cohort studies and national surveillance systems.

Updates to systematic reviews are performed on an ongoing schedule across all GBD causes; an update for typhoid and paratyphoid fevers will be performed in the next one to two iterations. While no systematic update was conducted, we did incorporate new data that were provided by collaborators, and re-extracted all incidence data to ensure consistency and accuracy, and to extract additional meta-data about the source studies.

Table 1: Data inputs for typhoid and paratyphoid fever

Measure	Total sources	Countries with data
All measures	205	33
Incidence	179	26
Proportion	78	23

Severity splits

For GBD 2019, we derived severity splits based on a published review of enteric fever outcomes from (Azmatullah A, Qamar FN, Thaver D, et al. 2005).

Paratyphoid is split into four sequelae: mild (28.5% [15.6–44.2]), moderate (52.25% [27.2–77.7]), severe (14.25% [8.2–21.8]), and abdominal pain and distention (5.0% [2.8–7.6]):

Table 2: Severity distribution for paratyphoid fever

Sequela	Description	Disability weight
Mild	Has a low fever and mild discomfort, but no difficulty with daily activities.	0.006 (0.002–0.012)
Moderate	Has a fever and aches, and feels weak, which causes some difficulty with daily activities.	0.051 (0.032–0.074)
Severe	Has a high fever and pain, and feels very weak, which causes great difficulty with daily activities.	0.133 (0.088–0.19)
Abdominal pain & distention due to paratyphoid	Has pain in the belly and feels nauseated. The person has difficulties with daily activities.	0.114 (0.078–0.159)

Similarly, typhoid is split into four sequelae: moderate (35.0% [26.0–44.3]), severe (47.75% [38.0–57.4]), severe abdominal pain and distention (17.0% [10.0–25.7]), and intestinal bleeding (0.25% [0–2.0]):

Table 3: Severity distribution for typhoid fever

Sequela	Description	Disability weight
Moderate	Has a fever and aches, and feels weak, which causes some difficulty with daily activities.	0.051 (0.032–0.074)
Severe	Has a high fever and pain, and feels very weak, which causes great difficulty with daily activities.	0.133 (0.088–0.19)

Gastrointestinal bleeding	Vomits blood and feels nauseated.	0.325 (0.209–0.462)
Abdominal pain and distention (includes intestinal perforation)	Has severe pain in the belly and feels nauseated. The person is anxious and unable to carry out daily activities.	0.324 (0.22–0.442)

Modelling strategy

We first model total incidence of typhoid and paratyphoid combined. Second, we model the proportion of this total due to typhoid and the proportion due to paratyphoid. Finally, we split the case estimates into sequelae representing different major symptoms and levels of severity.

Before modelling, we applied four adjustments to the incidence data: 1) diagnostic sensitivity adjustment, 2) passive surveillance adjustment, 3) typhoid-only adjustment, and 4) age/sex splits. Incidence data were inflated to account for poor diagnostic sensitivity, based on an internal meta-analysis of the sensitivity of blood culture, the most common diagnostic used for typhoid. We updated our meta-analysis of blood culture sensitivity for GBD 2019 to use MR-BRT, resulting in an increase in our estimates of diagnostic sensitivity from 54.9% (38.5 - 71.3) to 60.3% (50.3 – 68.8). We performed a crosswalk adjusts for incomplete case capture data from passive versus active surveillance, with active surveillance as the reference. Whereas this was previously done using a study-level covariate in DisMod, we used a MR-BRT model and adjusted the data before modelling in GBD 2019. In reviewing our incidence data, we noted some studies that only tested for and reported typhoid, and did not include paratyphoid. As a new adjustment for GBD 2019, we used estimates from our etiologic proportion models to adjust these typhoid-only sources and calculated an adjusted joint incidence by dividing the typhoid-only incidence by the estimated proportion due to typhoid. We performed this calculation using posterior simulation with 1,000 draws to propagate uncertainty from both the incidence data and the proportion estimate. Finally, where incidence data were reported for both sexes combined or for age categories spanning more than 25 years, we produced data points that were age and sex-specific based on a MR-BRT model of sex ratios, and a DisMod model of age patterns.

Total incidence was modelled using DisMod-MR, using the summary exposure values (SEV) for unsafe water, and the proportion of the population living in the Indian Ocean monsoon belt as covariates. Similarly, we used a DisMod model to estimate aetiologic proportions: whereas for GBD 2017 we used two models (one for the proportion of total incidence due to typhoid, and one for the proportion due to paratyphoid), for GBD 2019 we switched to a single model of the proportion due to paratyphoid. We made this change because previous aetiologic proportion models failed to capture the high proportion of enteric fever due to *Salmonella* Typhi in sub-Saharan Africa. Regarding proportion models, DisMod performs better with proportions that are near-zero, than with proportions that are near-one. By changing our approach to model only the proportion due to *Salmonella* Paratyphi we were able to better capture these proportions.

Typhoid cases are split between four sequelae: moderate typhoid fever, severe typhoid fever, severe typhoid fever with intestinal bleeding, and typhoid fever with abdominal complications. Paratyphoid

cases are split between four sequelae: mild paratyphoid fever, moderate paratyphoid fever, severe paratyphoid fever, and paratyphoid fever with abdominal complications.

Changes from GBD 2017 to GBD 2019

We updated our incidence data processing methods for GBD 2019. We have used a new MR-BRT based meta-analysis for the diagnostic sensitivity adjustment. We have changed our methods for adjusting data from passive surveillance to now be based on a MR-BRT model, rather than an in-DisMod crosswalk. Finally, we've added two new adjustments: first we've age/sex split data points that covered either both sexes or wide age spans; and, second, we've adjusted data from studies of only typhoid to account for missed cases of paratyphoid.

Since GBD 2013 we have modelled the incidence of typhoid and paratyphoid jointly and split the two based on DisMod models of etiologic proportions. Previous etiologic proportion models failed to capture the high proportion of enteric fever due to *Salmonella* Typhi in sub-Saharan Africa. Regarding proportion models, DisMod performs better with proportions that are near-zero, than with proportions that are near-one. By changing our approach to model the proportion due to *Salmonella* Paratyphi we were able to better capture these proportions.

Invasive non-typhoidal salmonella (iNTS)

Flowchart

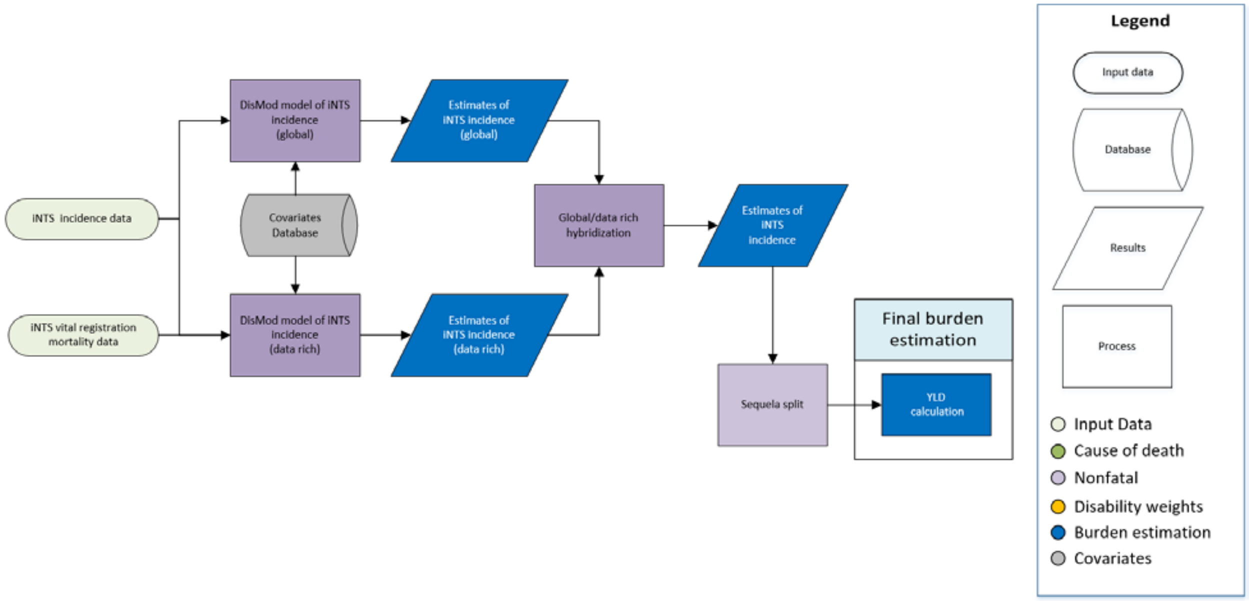

Case definition

Non-typhoidal salmonella infections are typically associated with diarrhoea. When these bacteria invade a typically sterile site like blood, they produce invasive non-typhoidal salmonella (iNTS) disease. Whereas non-typhoidal salmonella infections typically produce diarrhoeal illness, iNTS is typically febrile and can manifest in diverse symptoms that vary with severity and the exact site of the infection. Blood culture is the standard diagnostic for iNTS, and has good sensitivity and specificity. We thus define a case of iNTS as any blood-culture-confirmed non-typhoidal salmonella infection.

Input data

Model inputs

We conducted a systematic review for studies of iNTS incidence for GBD 2017, including sources that provided iNTS incidence rates derived from either active surveillance or, more commonly, hospital- or clinic-based surveillance with adjustments for health care utilisation. Studies of special populations (eg, people living with HIV/AIDS) were excluded. In total, we found 34 sources meeting our inclusion criteria. Updates to systematic reviews are performed on an ongoing schedule across all GBD causes; an update for iNTS was not performed for GBD 2019 and will be performed in the next one to two iterations.

Table 1: Data inputs for invasive non-typhoidal salmonella

Measure	Total sources	Countries with data
All measures	34	26
Incidence	34	26

Severity splits

Given the typical severity of iNTS and the breadth of potential symptoms and manifestations, we assign all cases to the severe acute infectious disease episode health state, with a disability weight of 0.133 (0.088–0.19)

Table 2: Severity distribution for invasive non-typhoidal salmonella

Sequela	Description	Disability weight
Severe acute infectious disease episode	Has a high fever and pain, and feels very weak, which causes great difficulty with daily activities.	0.133 (0.088–0.19)

Modelling strategy

We modelled incidence using two DisMod models: 1) a model that includes only incidence data, used to produce estimates for moderate and high burden regions; and 2) a model that includes additional incidence estimates derived from vital registration data from data rich counties, used to produce estimates for low burden regions. Both DisMod models used HIV mortality rate, malaria incidence adjusted for antimalarial coverage and drug effectiveness, and the summary exposure values (SEV) for sanitation as country-level covariates. We used no study-level covariates in the models.

We estimated prevalence as the product of incidence times duration. We estimated the duration of iNTS based on duration parameters reported in the scientific literature, with reported duration parameters including mean, median, range, standard deviation, and interquartile range. Because studies differed in how they reported duration, we were unable to use a simple meta-analysis approach. To leverage information on duration from all studies, we used approximate Bayesian computation (ABC). ABC employs a simple grid search in which we assumed that iNTS duration, in days, follows a negative binomial distribution with a one-day offset such that the resulting distribution had a minimum possible value of one-day. We used a random negative binomial generator that took three inputs: the length of the randomly generated vector, N, the number of trials, n, and the probability of success in each trial, p. We trialed combinations of values of n and p using a simple grid search. For each combination, and for each duration data point, we generated 10,000 vectors from an offset random negative binomial distribution, where the length of each vector equaled the sample size of the study. Thus, each vector represented a random realization of a possible distribution of durations for a given study. We estimated deviations between these realizations and the corresponding input data using an empirical cumulative distribution, and selected the best combination of values for n and p based on the root mean squared error. We estimated a mean duration of 7 days (95% CI: 1–24).

Changes from GBD 2017 to GBD 2019

Our approach of using incidence estimates based on vital registration data, and hybrid DisMod models are new for GBD 2019. Whereas for GBD 2017 we estimated duration based on the

duration of severe typhoid fever, for GBD 2019 we implemented the ABC model to estimate iNTS duration.

Other intestinal infectious diseases

In addition to the intestinal infectious diseases described above, there are many diverse types of intestinal infectious diseases. Because these intestinal infectious diseases are diverse in their underlying causes and risk factors as well as in their associated health outcomes, modelling them together in a DisMod-MR model would not produce reliable estimates of prevalence or excess mortality. Instead, we calculated the YLDs caused by intestinal infectious diseases directly using a YLD/YLL ratio.

We calculated the ratio of YLDs to YLLs across the specified intestinal infectious diseases for which nonfatal outcomes were modelled, using YLL estimates from the GBD 2019 cause of death (CoD) analysis. We then multiplied this YLD/YLL ratio by the YLL estimates for other intestinal infectious diseases from the GBD 2019 CoD analysis, providing us with an estimate of the YLDs associated with other intestinal infectious diseases.

Malaria

Flowchart

Malaria non-fatal outcomes (parasite rate and case incidence) in Sub-Saharan Africa

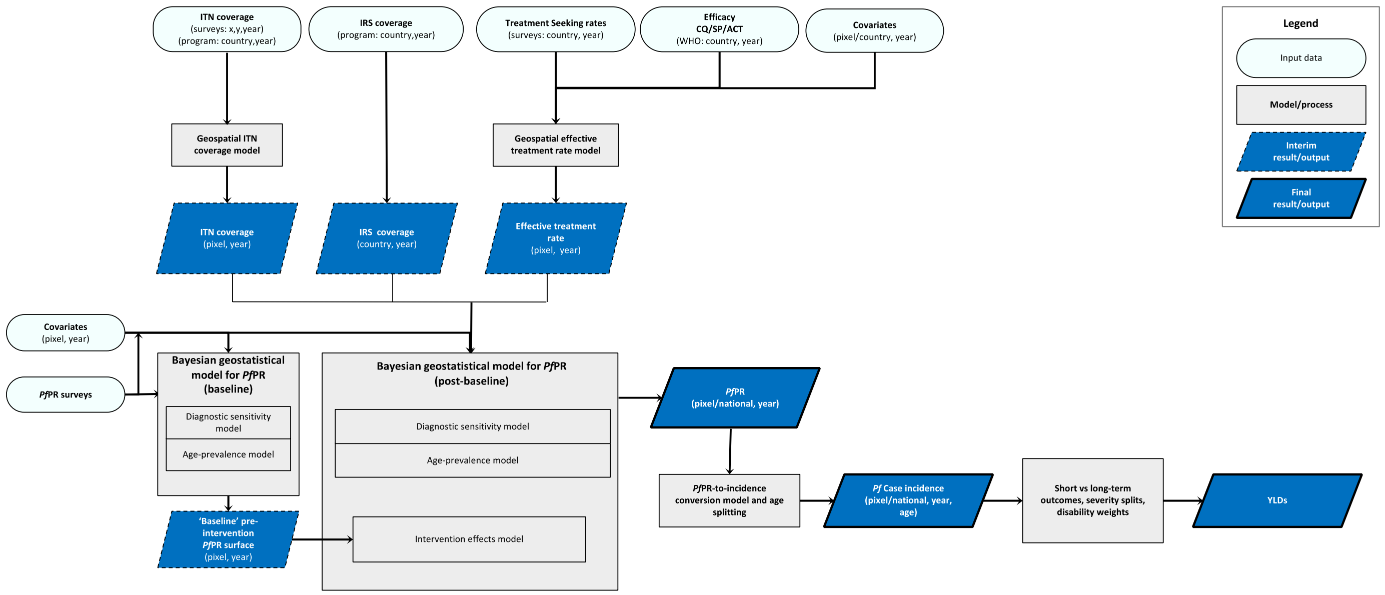

Malaria non-fatal outcomes (parasite rate and case incidence) outside Sub-Saharan Africa

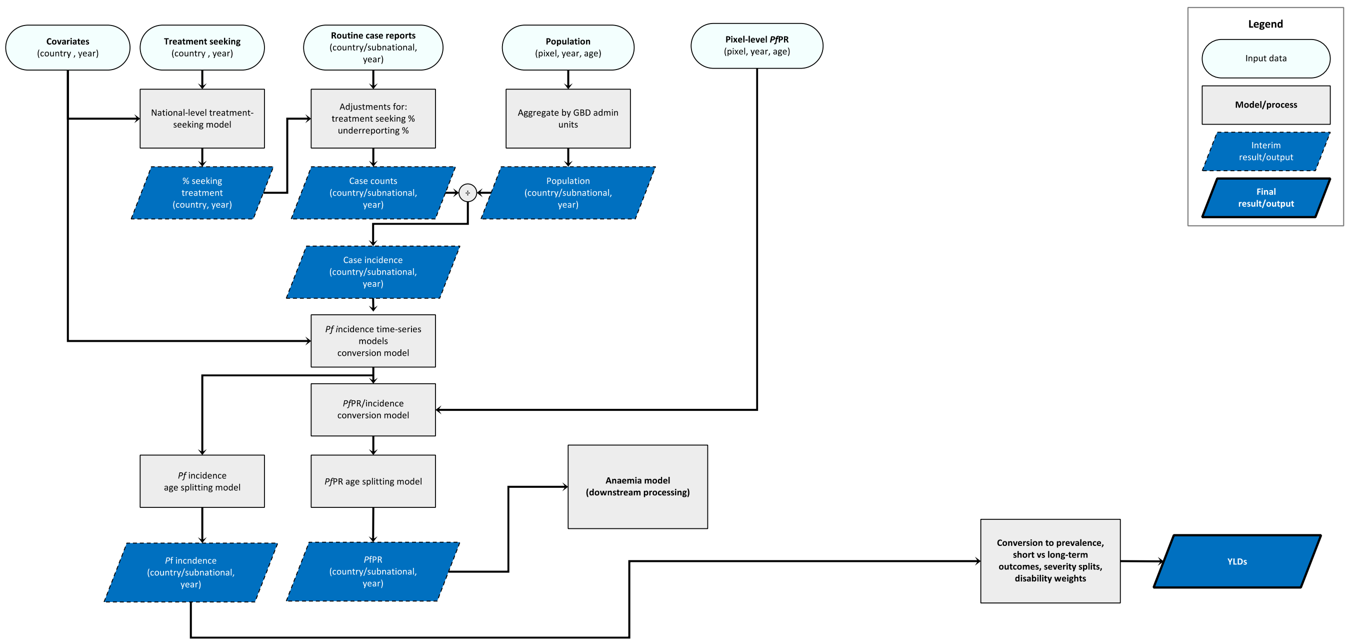

Case definition

Malaria is an acute parasitic mosquito-borne disease. An individual with uncomplicated malaria experiences one to two weeks of persistent fever, chills/shivering, sweating, joint pains, and headache. The individual will likely be lethargic and feverish, causing loss of daily function during the attack. Individuals with an untreated *P. falciparum* infection may develop severe malaria, which includes the symptoms of uncomplicated malaria but may also involve swelling, difficulty breathing, unconsciousness, and potentially death. Microscopy is considered the gold-standard diagnostic approach for the purposes of GBD. The relevant ICD-10 codes are B50-B54.

Data input

Primary data inputs were:

(i) Routine malaria case reports from national routine surveillance systems. These were obtained at the national level from the WHO World Malaria Report and at the subnational administrative level, wherever possible, via an exhaustive search of published and grey literature sources along with online data portals hosted by national ministries of health. Each retained record consisted of an annual count of malaria cases along with a distinction between confirmed and unconfirmed diagnoses, and differentiation by malaria parasite species.

(ii) Cross-sectional, geolocated, and community-representative observations of infection prevalence for *Plasmodium falciparum* (referred to hereafter as *P. falciparum* parasite rate, *Pf*PR).

These malaria epidemiological metrics were augmented in the modelling by:

(iii) Malaria Atlas Project (MAP) modelled estimates of malaria control intervention population coverage (ITNs, IRS, and effective treatment with an antimalarial drug) resolved to 5 km x 5 km pixel-year level (for sub-Saharan Africa) and country-year level (outside sub-Saharan Africa).

(iv) A large suite of environmental, sociodemographic, and economic covariates resolved to 5 km x 5 km pixel-year level (for sub-Saharan Africa) and country-year level (outside sub-Saharan Africa).

Table 1: Data Inputs for malaria morbidity modelling by parameter.

Measure	Total sources	Countries with data
All measures	6928	105
Prevalence	1616	85
Incidence	4089	104
Proportion	4304	104
Other	1118	51

Modelling strategy

The suitability, availability, and quality of *Pf*PR and routine case reporting data, as well as detailed intervention coverage information, differ markedly inside versus outside sub-Saharan Africa. As such, we developed separate modelling strategies for countries inside sub-Saharan Africa versus those outside. The exceptions were Algeria, Botswana, Cabo Verde, Comoros, Djibouti, Egypt, Eritrea, Ethiopia, Mauritania, Mauritius, Morocco, Namibia, Sao Tome and Principe, Senegal, South Africa, and Swaziland. Despite being part of Africa, these countries exhibit epidemiological trends and have data availability/quality more akin to non-African settings.

*Pf*PR and case incidence modelling: Africa

Modelling was conducted in the following steps:

(i) The large assembly of geolocated *Pf*PR surveys maintained by MAP was used in a Bayesian spatiotemporal geostatistical model to predict *Pf*PR for every pixel-year in sub-Saharan Africa, representing an update to earlier work (Bhatt et al Nature, Gething et al NEJM). The model considered (i) *Pf*PR survey participant age ranges and diagnostic type; (ii) coverage of ITNs, IRS, and effective antimalarial drug coverage, and how these metrics changed through time at each date and prediction location; (iii) environmental conditions at each date and prediction location (including density of vegetation, temperature, humidity, rainfall, elevation, and proximity to populated areas). The outcome was a predicted space-time "cube" of *Pf*PR, standardized to the 2-10 age range, for each year 1980–2017.

(ii) The *Pf*PR cube was then converted into an equivalent cube of the predicted incidence rate of clinical malaria. This conversion was achieved using an established model (Cameron et al Nature Communications) and provided estimates stratified first into three broad age bins (0-5; 5-15; <15) and then into the final 23 GBD 2017 age bins.

*Pf*PR and case incidence modelling: Outside Africa

Malaria endemic countries outside Africa tend to have less *Pf*PR data than those inside, in part because prevalence is generally lower. Furthermore, *Pf*PR surveys are rare in areas of lower prevalence and thuse this metric becomes an inefficient way to measure malaria risk. In contrast, routine surveillance systems outside Africa are generally stronger, meaning that reports of malaria cases from health systems are more reliable and provide some insight into the total malaria burden in the community. Modelling outside Africa was carried out in the following steps:

(i) National and subnational case reports were first subject to adjustments to identify and minimize bias. Bias in reported case numbers arises from various sources. First, a fraction of cases in the community will fail to seek treatment or will attend a private or informal health care provider that will not provide a record of that case to the routine surveillance system. We adjusted for these factors by modelling the fraction of cases seeking care from different provider categories based on data from nationally representative cross-sectional household surveys (primarily from the Demographic and Health Survey (DHS) program and the Multiple Indicator Cluster Survey program). Another factor for which we must adjust is cases reaching formal clinics that may not be subject to a confirmatory diagnostic test. We adjusted for this by assuming the fraction of unconfirmed cases that were truly malaria would equal the fraction of positives among all those tested. A final factor we adjust for is incomplete data as many routine surveillance systems fail to capture all case reports, with facilities/regions

missing from the national totals in a given year. We adjusted for this based on reporting completeness statistics published nationally by WHO.

(ii) These adjusted routine case reports were georeferenced using digitized administrative boundary data using a spatial database of such boundaries collated and maintained by MAP.

(iii) Each case report was converted into an estimate of clinical incidence rate by dividing it by the estimated population in each unit, with the latter quantity derived by combing high-resolution gridded population data and the aforementioned administrative boundaries.

(iv) Bayesian time-series models were then applied to the case reports for each country to impute incidence rates for years with missing data. The results from this analysis, in conjunction with the adjusted case reports, constitute the incidence values delivered for GBD 2017.

(v) The incidence rate for each country-year was then converted to an inferred *Pf*PR value using the same model described earlier (Cameron et al). This allowed us to utilize these polygon-level surveillance data and the *Pf*PR point-level data (where present) within the same modelling framework.

(vi) The combined *Pf*PR survey point data and (pseudo) *Pf*PR administrative unit data were then used in a Bayesian spatiotemporal geostatistical model to predict *Pf*PR at pixel-year level across all countries. As for the Africa model, *Pf*PR was standardized by age and diagnostic type and informed by a wide suite of covariates. An additional mechanism was developed to allow polygon (i.e., administrative unit) and point (i.e., survey) data to be used jointly to infer the predicted space-time surfaces.

(vii) The predicted *Pf*PR cube was then adjusted to ensure that, after conversion to pixel-level incidence, the incidence counts per country-year would precisely match the incidence results from step (iv). The summarized *Pf*PR values (i.e., population-weighted and tallied for each country-year) from the adjusted *Pf*PR cube constitute the *Pf*PR values delivered for GBD 2017.

Total malaria cases by country, year, sex

The pixel-level predictions of clinical incidence rate (both inside and outside Africa) were combined with high-resolution gridded population data to estimate total cases per pixel-year. These were then aggregated to GBD national/subnational areas. Inside sub-Saharan Africa, for countries endemic for *P. vivax* and *P. falciparum*, we calculated the number of cases due to *P. vivax* by applying the fraction of *P. vivax* and *P. falciparum* obtained from WHO and a literature review. Outside sub-Saharan Africa we followed the identical procedure for *P. vivax* and *P. falciparum.* Final age-splitting was accomplished using age-versus-incidence rate relationships gleaned from the paper by Cameron and colleagues (2014).

Determining YLDs for malaria

As in GBD 2017, we use a two-step process for determining malaria severity. For acute cases, severity splits for mild, moderate, and severe malaria were produced by analysis of MEPS data. These sequelae and their associated disability weights are presented below.

Table 1. Severity level, lay description, and DW

Severity level	Lay description	DW (95% CI)
Mild	Has a low fever and mild discomfort but no difficulty with daily activities.	0.006 (0.002–0.012)

Moderate	Has a fever and aches and feels weak, which causes some difficulty with daily activities.	0.051 (0.032–0.074)
Severe	Has a high fever and pain and feels very weak, which causes great difficulty with daily activities.	0.133 (0.088–0.19)

To determine long-term neurological burden due to malaria, we use the work by Roca-Felter and colleagues (2008) that examined the number of uncomplicated cases that led to longer-term impairment. Analytically, this means multiplying incidence estimates (described in the section above for persons under 20 by 0.00029 (0.000077–0.00057). This adjusted case estimate is then combined with excess mortality rates derived from all-cause mortality and standardized mortality ratios for neonatal encephalopathy (NE) in a DisMod model to produce prevalence estimates of long-term sequelae for all estimation years. Implicit in this process is an assumption that the disability and trend of impairment due to severe malaria follow NE. The subsequent severity splitting follows NE as well.

To determine the burden of acute (short-term) malaria, the incidence estimation results are combined and converted to prevalence by matching each draw with a draw of duration of clinical illness. Consistent with GBD 2017, we use a uniform distribution between 14 and 28 days for duration.

References

Bhatt, S. et al. The effect of malaria control on Plasmodium falciparum in Africa between 2000 and 2015. Nature (2015).

Cameron, E., et al. Defining the relationship between infection prevalence and clinical incidence of Plasmodium falciparum malaria. *Nature Communications* 6:8170 (2015).

Gething, P. W. et al. Mapping Plasmodium falciparum Mortality in Africa between 1990 and 2015. New England Journal of Medicine 375, 2435-2445 (2016).

Weiss, D. J. *et al.* Mapping the global prevalence, incidence, and mortality of *Plasmodium falciparum*, 2000-17: a spatial and temporal modelling study. *The Lancet*, doi:10.1016/S0140-6736(19)31097-9 (2019).

Chagas disease

Flowchart

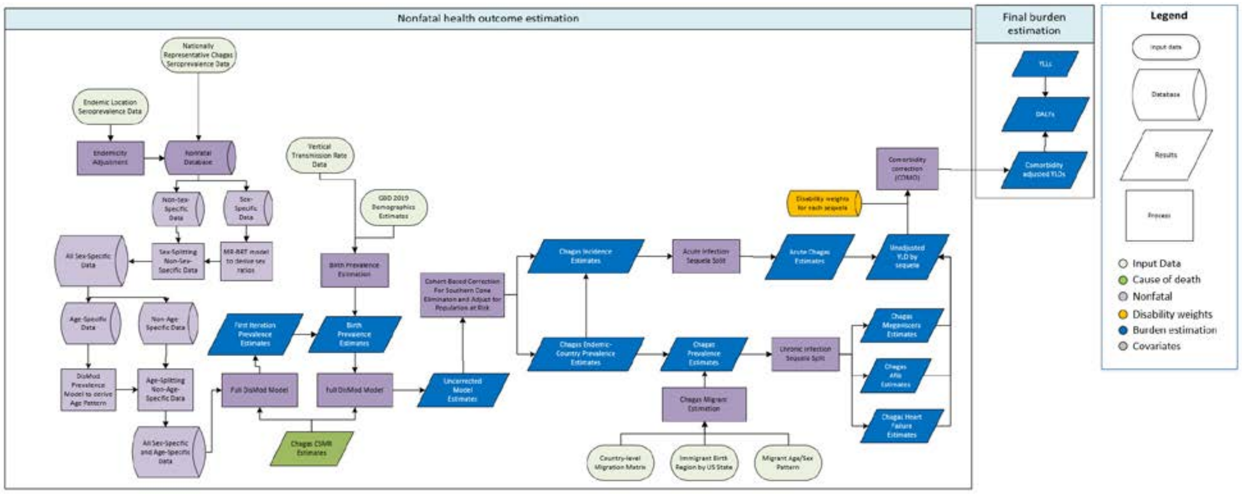

Case definition

Chagas disease is defined by infection with the protozoa *Trypanosoma cruzi,* which is transmitted by *Triatominae* insect vectors (most common), blood transfusion, organ transplant, and congenital transmission. It includes an acute phase corresponding with the time of infection, and is typically asymptomatic. Chronic infection may be latent (ie, asymptomatic), or result in cardiovascular or digestive sequelae. It includes all ICD-10 codes under the heading B57 (Chagas disease), with codes B57.0-B75.1 corresponding to the acute phase, B57.2 corresponding to chronic cardiovascular sequelae, and B57.3 corresponding to chronic digestive sequelae.

Input data

Model inputs

Table 1: Source Counts

Measure	Total sources	Countries with data
All measures	84	21
Prevalence	81	20
Proportion	3	1
Population	1	1

For GBD 2019 estimation, we used seroprevalence data to model Chagas prevalence. We used a MR-BRT model with our sex-specific data to derive an estimate of the ratio of the male prevalence of Chagas disease to female prevalence of Chagas disease to split non-sex-specific data. Then, a DisMod-MR 2.1

Bayesian meta-regression model using the age-specific input data was run to derive an age pattern to apply to split the all-age data.

Table 2: MR-BRT Crosswalk Adjustment Factors for Chagas Disease

Data input	Reference or alternative case definition	Gamma	Beta Coefficient, Log (95% CI)	Adjustment factor*
Female data	Ref	0.37	---	---
Male data	Alt		0.07 (-0.65, 0.79)	1.07

**Adjustment factor is the transformed Beta coefficient in normal space, and can be interpreted as the factor by which the alternative case definition is adjusted to reflect what it would have been if measured as the reference.*

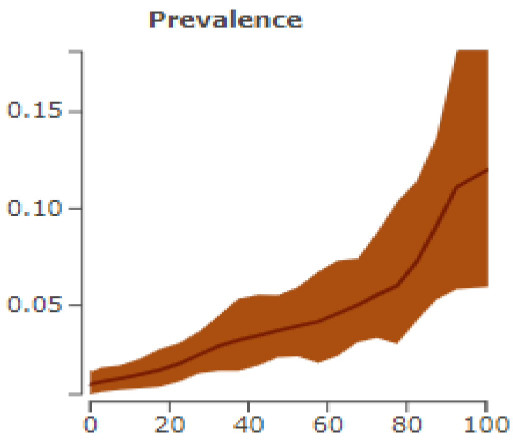

Figure 1: Latin America-specific age-pattern for Chagas disease used to split all-age data into age-specific data points for further modeling.

We also use CSMR estimates in the modelling process, which will be addressed in further detail below.

Modelling strategy

We modelled Chagas disease using a full DisMod-MR 2.1 Bayesian meta-regression model incorporating seroprevalence data, as above, and CSMR estimates. We assume no remission. We eliminate all new infections, except those via vertical transmission, in Chile and Uruguay for years after the interruption of vector-based transmission (Abad-Franch F, Diotaiuti L, Gurgel-Gonçalves R, Gürtler RE. Certifying the interruption of Chagas disease transmission by native vectors: cui bono? Mem Inst Oswaldo Cruz 2013;108:251–4.; Coura JR. Chagas disease: control, elimination and eradication. Is it possible? Mem Inst Oswaldo Cruz 2013;108:962–7.). We then adjust these estimates for population at-risk as estimated by the Pan-American Health Organization in 2005 (Pan American Health Organization (PAHO), World Health Organization (WHO). Quantitative Estimation of Chagas in the Americas). For non-endemic countries, we estimate the prevalence of imported chronic infections based on migration. For each non-endemic country, we estimate the total number of people infected with Chagas as the sum of the number of

immigrants from each endemic country multiplied by the corresponding prevalence of Chagas in that endemic country.

We estimate five sequelae: symptomatic acute infection from incidence; and megaviscera, heart failure, atrial fibrillation, and chronic asymptomatic infection from prevalence. We assume that 5% of acute infections will be symptomatic (Teixeira AR, Nitz N, Guimaro MC, Gomes C, Santos-Buch CA. Chagas disease. Postgrad Med J 2006;82:788–98.). The proportion of chronic infections resulting in a given sequela varies by sex and age: the prevalence of megaviscera among those infected with Chagas ranges from 0% in children to nearly 10% among older adults (Coura JR, Naranjo MA, Willcox HP. Chagas' disease in the Brazilian Amazon: II. A serological survey. Rev Inst Med Trop São Paulo 1995; 37:103–7.); the prevalence of atrial fibrillation attributable to Chagas ranges from 0% among children to approximately 10% in men over 80 years of age (Ribeiro AL, Marcolino MS, Prineas RJ, Lima-Costa MF. Electrocardiographic abnormalities in elderly Chagas disease patients: 10-year follow-up of the Bambuí Cohort Study of Aging. J Am Heart Assoc 2014;3:e000632.); and the prevalence of heart failure attributable to Chagas among those who are infected ranges from 0% among young children, to a maximum of 23% among men over 80 years of age (Sabino EC, Ribeiro AL, Salemi VM, et al., for the National Heart, Lung, and Blood Institute Retrovirus Epidemiology Donor Study-II (REDS-II), International Component. Ten-year incidence of Chagas cardiomyopathy among asymptomatic Trypanosoma cruzi-seropositive former blood donors. Circulation 2013;127:1105–15.).

Severity splits and disability weights

The table below illustrates the sequelae, lay descriptions, and DWs for Chagas disease.

Table 3. Sequelae, lay description and DWs

Sequelae	Description	Disability Weight
Atrial fibrillation and flutter due to Chagas disease	Has periods of rapid and irregular heartbeats and occasional fainting.	0.224 (0.151–0.312)
Mild heart failure due to Chagas disease	Is short of breath and easily tires with moderate physical activity, such as walking uphill or more than a quarter-mile on level ground. The person feels comfortable at rest or during activities requiring less effort.	0.041 (0.026–0.062)
Moderate heart failure due to Chagas disease	Is short of breath and easily tires with minimal physical activity, such as walking only a short distance. The person feels comfortable at rest but avoids moderate activity.	0.072 (0.047–0.103)
Severe heart failure due to Chagas disease	Is short of breath and feels tired when at rest. The person avoids any physical activity, for fear of worsening the breathing problems.	0.179 (0.122–0.251)

Mild chronic digestive disease due to Chagas disease	Has some pain in the belly that causes nausea but does not interfere with daily activities.	0.011 (0.005–0.021)
Moderate chronic digestive disease due to Chagas disease	Has pain in the belly and feels nauseated. The person has difficulties with daily activities.	0.114 (0.078–0.159)
Acute Chagas disease	Has a fever and aches, and feels weak, which causes some difficulty with daily activities.	0.051 (0.032–0.074)
Asymptomatic Chagas disease	Latent Chagas infection (ie, chronic infection with no apparent symptoms)	NA

Changes from GBD 2017 to GBD 2019

Data reported as either both sex and/or by age groups broader than 25 years were disaggregated using a sex ratio estimated by MR-BRT and age-splitting using a Latin America-specific age-pattern derived from a DisMod-MR 2.1 Bayesian meta-regression model.

Visceral leishmaniasis

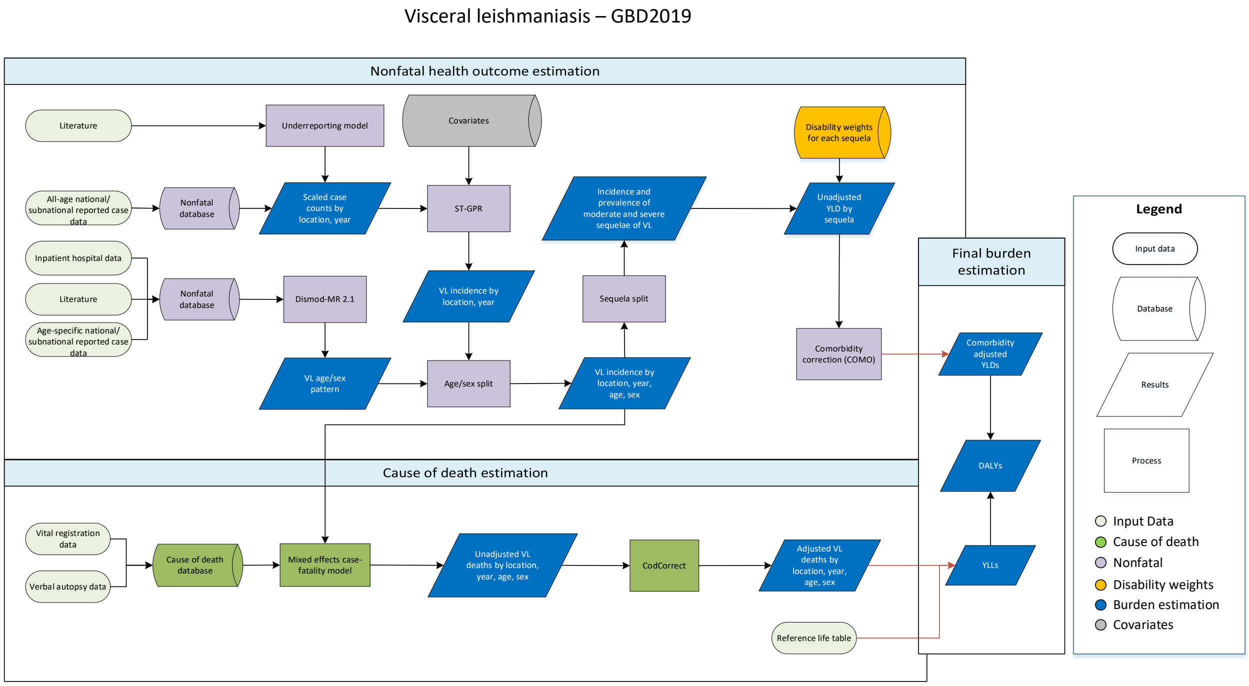

Visceral leishmaniasis (VL) is the most serious manifestation of disease caused by the *Leishmania* parasite, transmitted through the bite of phlebotomine sandflies. Those infected typically present with fever, weight loss, anaemia, leukopenia, thrombocytopenia, and enlargement of the spleen and liver. If left untreated, it can be fatal. Transmission varies by geographic region, with a variety of reservoir hosts implicated, and different vector species associated, maintaining both zoonotic and anthroponotic transmission cycles. The ICD9 code related to visceral leishmaniasis is 085.0, and the ICD10 code is B55.0.

Description of general methodology

The fatal estimation process for visceral leishmaniasis is built from incident case notification data representative of the GBD geographic location, which is adjusted for underreporting. The upscaled all-age, both-sex case counts are modelled using spatiotemporal Gaussian process regression (ST-GPR) in order to impute for missing location-year combinations as well as to account for further biases and inaccuracies in reporting. Datasets that disaggregate VL cases by age and sex are modelled using DisMod MR-2.1 to produce a global age-sex split which is applied to the all-age, both-sex envelope estimates resulting from ST-GPR. The mean incidence estimates are compared with estimated death counts to generate a case-fatality rate model that is subsequently used to estimate deaths for each age, sex, location, year.

Input Data – Case Notification time series

Table 1: Source Counts

Measure	Total sources	Countries with data
All measures	1098	71
Incidence	1079	71
Proportion	20	17

Current estimation for the all-age, both-sex incidence envelope is based upon location-representative information rather than site-specific epidemiological measures due to the absence of global foci maps allowing for upscaling of geographically precise information. The primary data resource therefore is the case notification time-series reported by National Control Programs and Ministries of Health to the World Health Organization. This is supplemented by systematic literature review (last updated for GBD 2015) to identify alternate sources of data for years missing information. For countries with subnational estimates, in-country collaborators have compiled information for respective programs, or identified key resources. Notifications from 1,151 location-years were available.

Input Data – Underreporting assessments

It is recognised that case notification series record only a subset of the true cases present. A review was undertaken to identify articles that compared reported cases with alternate measures to estimate the degree of underreporting. The following search strings were used: 'leish* AND under*'; 'active passive leish*'. Inclusion criteria were broad to maximise spatiotemporal coverage in potential estimates – any report that compared reported statistics with some notion of "truth" (whether capture-recapture, active surveillance, etc.) were extracted. Values for both cutaneous and visceral leishmaniasis were included. For GBD 2019, 9 articles were included, summarised in Table 2.

Table 2: Metadata for underreporting scalars used in GBD 2019. For each record, a citation, GBD location of relevance, year, pathogen, brief summary of methods, and output values used in modelling are listed.

Citation	GBD location	Time period	Pathogen	Method synopsis	Proportion of "true" cases reported
Yadon *et al.* 2001 "Assessment of Leishmaniasis notification system in Santiago del Estero, Argentina, 1990-1993" (Yadón et al. 2001)	Argentina	1990–1993	CL	Capture-recapture methods were used to evaluate four reporting sources.	94/210
Sesma *et al.* 1997 "Leishmaniasis in Navarra: a review of activities" (Sesma and Barricarte 1997)	Spain	1990–1997	CL, VL	Comparison of active searching within the region with reporting via Epidemiological Surveillance System	8/21
Maia-Elkhoury *et al.* 2007 "Analysis of visceral leishmaniasis reports by the capture-recapture method" (Maia-Elkhoury et al. 2007)	Brazil	2002–2003	VL	Comparison of three notification systems for completeness	5896/10691
Gkolfinopoulou *et al.* 2013 "Epidemiology of human leishmaniasis in Greece, 1981-2011" (Gkolfinopoulou et al. 2013)	Greece	2004–2009	VL	Comparing number of cases identified at national reference laboratory with mandatory notification system.	260/361
Singh *et al.* 2010 "Estimation of under-reporting of Visceral Leishmaniasis cases in Bihar India" (V. P. Singh et al. 2010)	Bihar, India	2006	VL	Comparison of actual reported number of cases with estimates age-sex stratified incidence proportions for a cohort of 31,324 persons	34/177
Hirve *et al.* 2010 "Effectiveness and feasibility of active and passive case detection in the Visceral Leishmaniasis Elimination Initiative in India, Bangladesh, and Nepal" (Hirve et al. 2010)	Bihar, India<br>Nepal<br>Bangladesh	2008	VL	Comparing active case detection evaluations (conducting via house-to-house screening) with passive case detection systems	111/130<br>119/127<br>18/25<br>20/32

Faraj *et al.* 2016 “Effectiveness and cost of insecticide-treated bed nets and indoor residual spraying for the control of cutaneous leishmaniasis: A cluster-randomized control trial in Morocco” (Faraj et al. 2016)	Morocco	2008–2013	CL	Comparison of incidence of new CL cases by both active and passive case detection	409/670
Das *et al.* 2014 “Active and passive case detection strategies for the control of leishmaniasis in Bangladesh” (Das et al. 2014)	Bangladesh	2010–2011	VL	Comparing two districts’ estimates [identified in the paper as being directly comparable] of cases, one via active case detection, the other via passive case detection. Active case detection was via community education and outreach workers targeting households	756/1087
Rahman *et al.* 2015 “Performance of Kala-azar surveillance in Gaffargaon subdistrict of Mymensingh, Bangladesh” (Rahman et al. 2015)	Bangladesh	2010–2011	VL	Comparison of cases reported to the local health complex versus active search for kala-azar cases	29/58
Eid *et al.* 2017 “Assessment of a Leishmaniasis reporting system in tropical Bolivia using the capture-recapture method” (Eid et al. 2017)	Bolivia	2013–2014	CL	Active surveillance during medical campaigns were compared to registered cases reported by the National Program of Leishmaniasis Control	23/86.4

Input data – age/sex-split data

Where possible, information disaggregating location-level statistics by age and sex were extracted.

Method – geographic restrictions

There are strong climatic and biogeographic constraints on the geographic distribution of VL resulting in a focal rather than cosmopolitan global distribution. As a result, it is necessary to identify locations burdened by the disease through space and time as distinct from countries were VL is absent. Tags were assigned to each location-year based upon the outcome of a search of IHME databases, as well as location-specific searches of PubMed. Each location-year is tagged as follows:

- Present – where a specific citation of either an autochthonous laboratory-confirmed case (ie, a case with PCR, serological, or parasitological diagnosis), reported case (ie, a case noted as VL, but with no supporting diagnostic), or supporting evidence (ie, confirmed infection in animal reservoirs or sandfly vectors)
- Protocol Present – for a given location-year, where no specific citation is used, but is present for another year in the same location, it is assumed that VL is present given that eradication of the pathogen has not been achieved
- Absent – where PubMed location-specific searches returned zero relevant results, in locations scoring -25 or lower as evaluated by Pigott *et al.* (2014) [the threshold for "absence" in that study (Pigott et al. 2014)], locations were tagged as Absent
- Protocol Absent – as with Absent, locations with zero relevant PubMed results, but with greater than -25 as evaluated by Pigott *et al.* (2014), were tagged as Protocol Absent (Pigott et al. 2014)

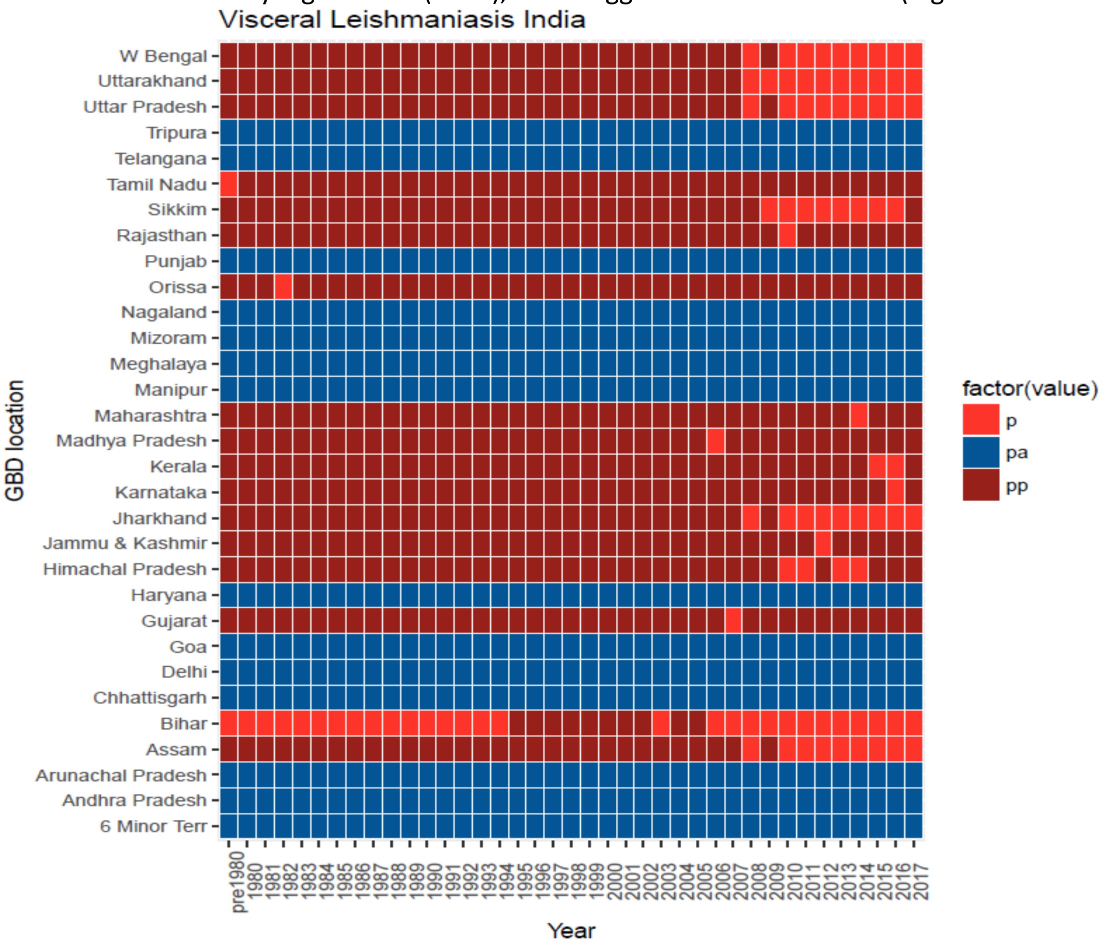

Figure 1: Visceral Leishmaniasis geographical restrictions for Indian subnationals. Locations tagged as present are coloured in red (denoted as p), yellow represents protocol presence (denoted as pp), and dark blue represents protocol absence (denoted as pa).

Full time series of maps and tables, with relevant GHDx NIDs, are available upon request from gbdsec@uw.edu.

Method – underreporting modelling and scaled case counts

Underreporting scalars were modelled as a generalised linear model estimating the proportion of true cases captured by reporting systems: a value of 1 therefore represents all actual cases of leishmaniasis being reported through notification systems. The specific models is as follows:

$$\frac{reported\ cases}{\text{"true" cases}} = Pathogen + Year + Sociodemographic\ Index$$

To account for potential biases inherently present based upon differing survey methods or location-specific confounders, 1,000 models were run, with each model randomly dropping all data from a specific location, and then one additional data point from the remaining dataset. Similarly, for estimates that spanned multiple years, for each model one of the years within the range of possible years was randomly assigned.
To generate scaled case counts, for each of the 1,000 models a random number was generated, using a normal distribution with mean being that of the mean estimated scalar bounded by the upper and lower confidence interval. With these 1,000 scalars, 1,000 scaled case counts were calculated and summarised for modelling within ST-GPR.

Method – ST-GPR

Using existing IHME tools, the summarised values were modelled using ST-GPR to produce a complete time series of estimates for each location-year tagged “Present” or “Protocol Present”. In short, ST-GPR attempts to model non-linear trends utilising a Gaussian process to fit a trend, rather than a definitive functional form. The following model specifications were used:

$$\begin{aligned} Incidence = \ & Healthcare\ Access\ and\ Quality\ Index + Sociodemographic\ Index + (1|level\ 1) \\ & + (1|level\ 2) + (1|level\ 3) \end{aligned}$$

where levels 1, 2, and 3, referring to GBD location hierarchies, treated as random effects. The following hyperparameters were used: st-lambda = 0.4, st-omega = 1, st-zeta = 0.01, gpr-scale = 10. The coefficnets can be found in the table below.

Table 3: ST-GPR Model coefficients.

Covariate	Beta Coefficient, Logit (95% CI)	Standard Error	Exponentiated beta (95% CI)
Socio-demographic Index	-8.455	1.276	2.12 * 10^{-4} (1.74 * 10^{-5} – 2.60 * 10^{-3})
Health Access and Quality Index	-0.006	0.012	0.99 (0.97 – 1.02)

Method – DisMod MR-2.1

DisMod MR-2.1 was used to generate an age-sex curve to disaggregate all-age, both-sex incidence data. DisMod is an integrated meta-regression framework that allows for multiple datasets to be integrated into a singular analysis regardless of age-binning, sources, and geographies. As a consequence, a variety of differently aggregated information can be evaluated to generate a consensus output. From this model, the global fit was used.

Method – YLD estimation (incorporating duration and disability weighting) / COMO

Following standard GBD estimation protocols, incidence estimates were used to calculate disease prevalence (by multiplication with duration), disaggregated by disease sequelae. In total, two health states are assigned to visceral leishmaniasis, "moderate visceral leishmaniasis" and "severe visceral leishmaniasis" [Table 4]. Duration values were taken from Murray et *al.* (2005).

Table 4: Sequelae and associated metadata. For the sequelae used in GBD 2019, the lay descriptor health state, disability weight, and duration are listed.

Sequela	Health state lay description	Disability weight	Duration
Moderate visceral leishmaniasis	Infectious disease, acute episode, moderate "has a fever and aches, and feels weak, which causes some difficulty in daily activities"	0.051 (0.032–0.074)	2.5 months
Severe visceral leishmaniasis	Infectious disease, acute episode, severe "has a high fever and pain, and feels very weak, which causes great difficulty with daily activities"	0.133 (0.088–0.19)	15 days

Central processing is used to generate the final estimates, including co-morbidity simulations.

Changes from GBD 2017

A number of changes to the methodology were implemented for GBD 2019:

The under-reporting model was fit with an updated dataset in which three articles were outliered due to concerns of their representativeness for other locations as the proportion of cases detected was less than 15%.

References

Alvar, Jorge, Iván D Vélez, Caryn Bern, Mercé Herrero, Philippe Desjeux, Jorge Cano, Jean Jannin, and Margriet den Boer. 2012. “Leishmaniasis Worldwide and Global Estimates of Its Incidence.” *PLoS One* 7 (5): e35671.

Copeland, H W, B A Arana, and T R Navin. 1990. “Comparison of Active and Passive Case Detection of Cutaneous Leishmaniasis in Guatemala.” *Am. J. Trop. Med. Hyg.* 43 (3): 257–259.

Das, A K, A D Harries, S G Hinderaker, R Zachariah, B Ahmed, G N Shah, M A Khogali, G I Das, E M Ahmed, and K Ritmeijer. 2014. “Active and Passive Case Detection Strategies for the Control of Leishmaniasis in Bangladesh.” *Public Health Action* 4 (1): 15–21.

Eid, Daniel, Miguel Guzman-Rivero, Ernesto Rojas, Isabel Goicolea, Anna-Karin Hurtig, Daniel Illanes, and Miguel San Sebastian. 2017. “Assessment of a Leishmaniasis Reporting System in Tropical Bolivia Using the Capture-Recapture Method,” October, tpmd170308.

Faraj, Chafika, Joshua Yukich, El Bachir Adlaoui, Rachid Wahabi, Abraham Peter Mnzava, Mustapha Kaddaf, Abderrahmane Laamrani El Idrissi, Btissam Ameur, and Immo Kleinschmidt. 2016. “Effectiveness and Cost of Insecticide-Treated Bed Nets and Indoor Residual Spraying for the Control of Cutaneous Leishmaniasis: A Cluster-Randomized Control Trial in Morocco.” *Am. J. Trop. Med. Hyg.* 94 (3): 679–685.

Gkolfinopoulou, K, N Bitsolas, S Patrinos, L Veneti, A Marka, G Dougas, D Pervanidou, et al. 2013. “Epidemiology of Human Leishmaniasis in Greece, 1981-2011.” *Euro Surveill.* 18 (29): 20532.

Hirve, S, S P Singh, N Kumar, M R Banjara, P Das, S Sundar, S Rijal, et al. 2010. “Effectiveness and Feasibility of Active and Passive Case Detection in the Visceral Leishmaniasis Elimination Initiative in India, Bangladesh, and Nepal.” *Am. J. Trop. Med. Hyg.* 83 (3): 507–511.

Maia-Elkhoury, Ana Nilce Silveira, Eduardo Hage Carmo, Marcia Leite Sousa-Gomes, and Eduardo Mota. 2007. “[Analysis of visceral leishmaniasis reports by the capture-recapture method].” *Rev. Saude Publica* 41 (6): 931–937.

Pigott, David M, Samir Bhatt, Nick Golding, Kirsten A Duda, Katherine E Battle, Oliver J Brady, Jane P Messina, et al. 2014. “Global Distribution Maps of the Leishmaniases.” *Elife* 3 (January): e02851.

Rahman, Kazi Mizanur, Indira V M Samarawickrema, David Harley, Anna Olsen, Colin D Butler, Shariful Amin Sumon, Subrata Kumar Biswas, Stephen P Luby, and Adrian C Sleigh. 2015. “Performance of Kala-Azar Surveillance in Gaffargaon Subdistrict of Mymensingh, Bangladesh.” Edited by Carlos Franco-Paredes. *PLoS Negl. Trop. Dis.* 9 (4): e0003531.

Sesma, B, and A Barricarte. 1997. “[Leishmaniasis in Navarra: review of activities].” *An. Sist. Sanit. Navar.* 20 (2): 209–216.

Singh, S P, D C S Reddy, M Rai, and S Sundar. 2006. “Serious Underreporting of Visceral Leishmaniasis through Passive Case Reporting in Bihar, India.” *Trop. Med. Int. Health* 11 (6): 899–905.

Singh, V P, A Ranjan, R K Topno, R B Verma, N A Siddique, V N Ravidas, N Kumar, K Pandey, and P Das. 2010. “Estimation of Under-Reporting of Visceral Leishmaniasis Cases in Bihar, India.” *Am. J. Trop. Med. Hyg.* 82 (1): 9–11.

Yadón, Z E, M A Quigley, C R Davies, L C Rodrigues, and E L Segura. 2001. “Assessment of Leishmaniasis Notification System in Santiago Del Estero, Argentina, 1990-1993.” *Am. J. Trop. Med. Hyg.* 65 (1): 27–30.

Cutaneous leishmaniasis

Cutaneous & Mucocutaneous Leishmaniasis

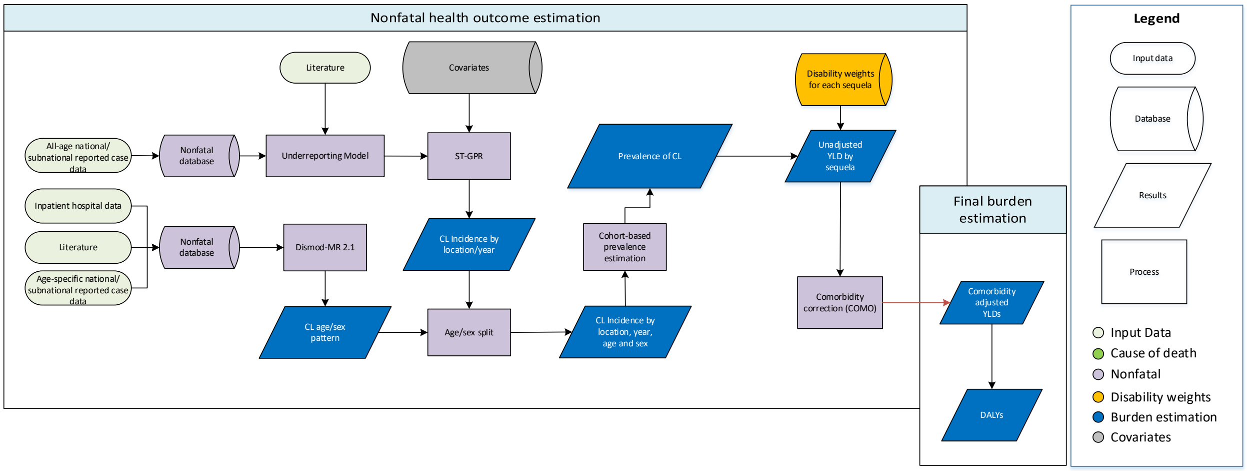

Description of general methodology

The non-fatal estimation process for cutaneous leishmaniasis is built from incident case notification data representative of the GBD geographic location, which are adjusted for underreporting. The upscaled all-age, both sex, case counts are modelled using spatiotemporal Gaussian process regression (ST-GPR) in order to impute for missing location-year combinations as well as to account for further biases and inaccuracies in reporting. Datasets that disaggregate CL cases by age and sex are modelled using DisMod to produce a global age-sex split which is applied to the all-age, both-sex envelope estimates resulting from ST-GPR. These incidence estimates are used to derive prevalence measures, as well as compute the resulting years lived with disability values.

Input Data – Case Notification time series

Table 1: Source Counts

Measure	Total sources	Countries with data
All measures	1056	72
Incidence	1056	72

Current estimation for the all-age, both-sex, incidence envelope is based upon location-representative information rather than site-specific epidemiological measures due to the absence of global foci maps allowing for upscaling of geographically precise information. The primary data resource therefore is the case notification time-series reported by National Control Programs and Ministries of Health to the World Health Organization. This is supplemented by systematic literature review (last updated for GBD 2015) to identify alternate sources of data for years missing information. For countries with subnational estimates, in-country collaborators have compiled information for respective programs, or identified key resources,

again supplemented by literature reviews. Where possible, information disaggregating location-level statistics by age and sex were extracted.

Method – Geographic restrictions

There are strong climatic and biogeographic constraints on the geographic distribution of CL resulting in a focal, rather than cosmopolitan global distribution. As a result, it is necessary to identify locations burdened by the disease through space and time as distinct from countries where CL is absent. Tags were assigned to each location-year based upon the outcome of a search of IHME databases, as well as location-specific searches of PubMed. Each location-year is tagged as follows:

- Present – where a specific citation of either an autochthonous laboratory-confirmed case (ie, a case with PCR, serological, or parasitological diagnosis), reported case (ie, a case noted as CL, but with no supporting diagnostic), or supporting evidence (ie, confirmed infection in animal reservoirs or sandfly vectors)
- Protocol Present – for a given location-year, where no specific citation is used, but is present for another year in the same location, it is assumed that CL is present given that eradication of the pathogen has not been achieved
- Absent – where PubMed location-specific searches returned zero relevant results, in locations scoring -25 or lower as evaluated by Pigott and colleagues (2014) [the threshold for "absence" in that study], locations were tagged as Absent
- Protocol Absent – as with Absent, locations with zero relevant PubMed results, but with greater than -25 as evaluated by Pigott and colleagues (2014), were tagged as Protocol Absent

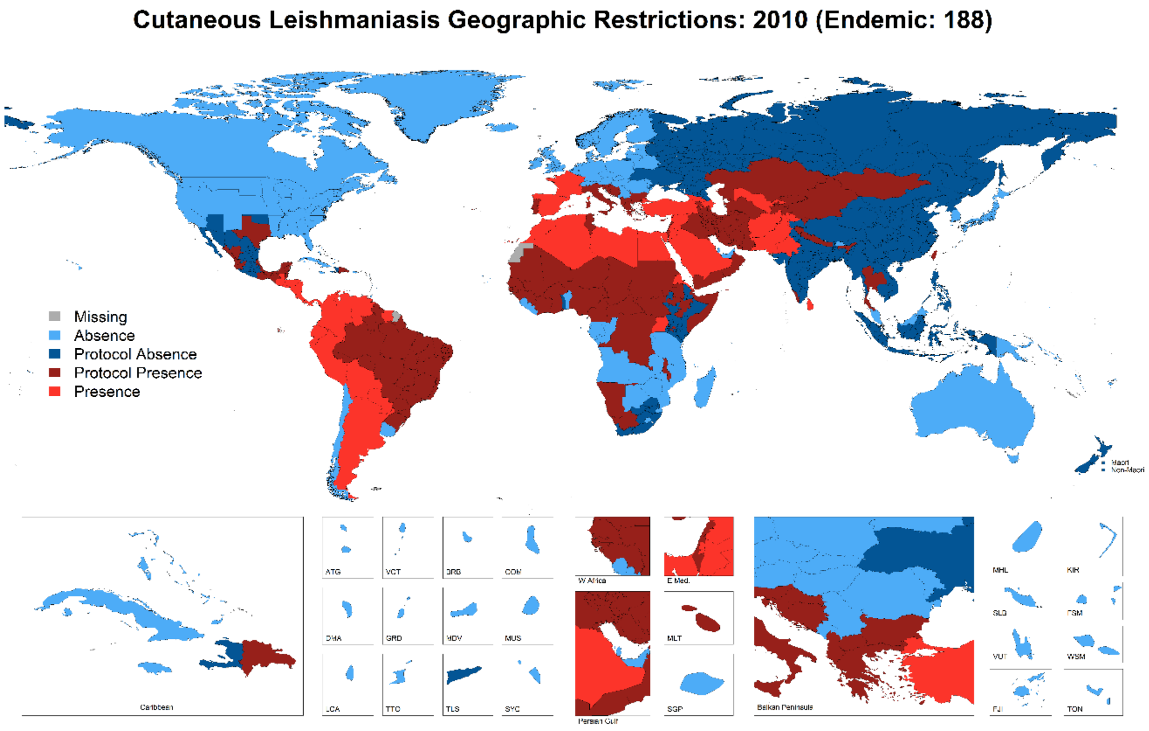

Figure 1: Cutaneous Leishmaniasis geographic restrictions for the year 2010. GBD locations tagged as present are coloured in red, dark red represents protocol presence, dark blue represents protocol absence, and absence is represented by light blue. Locations missing tags are presented in grey.

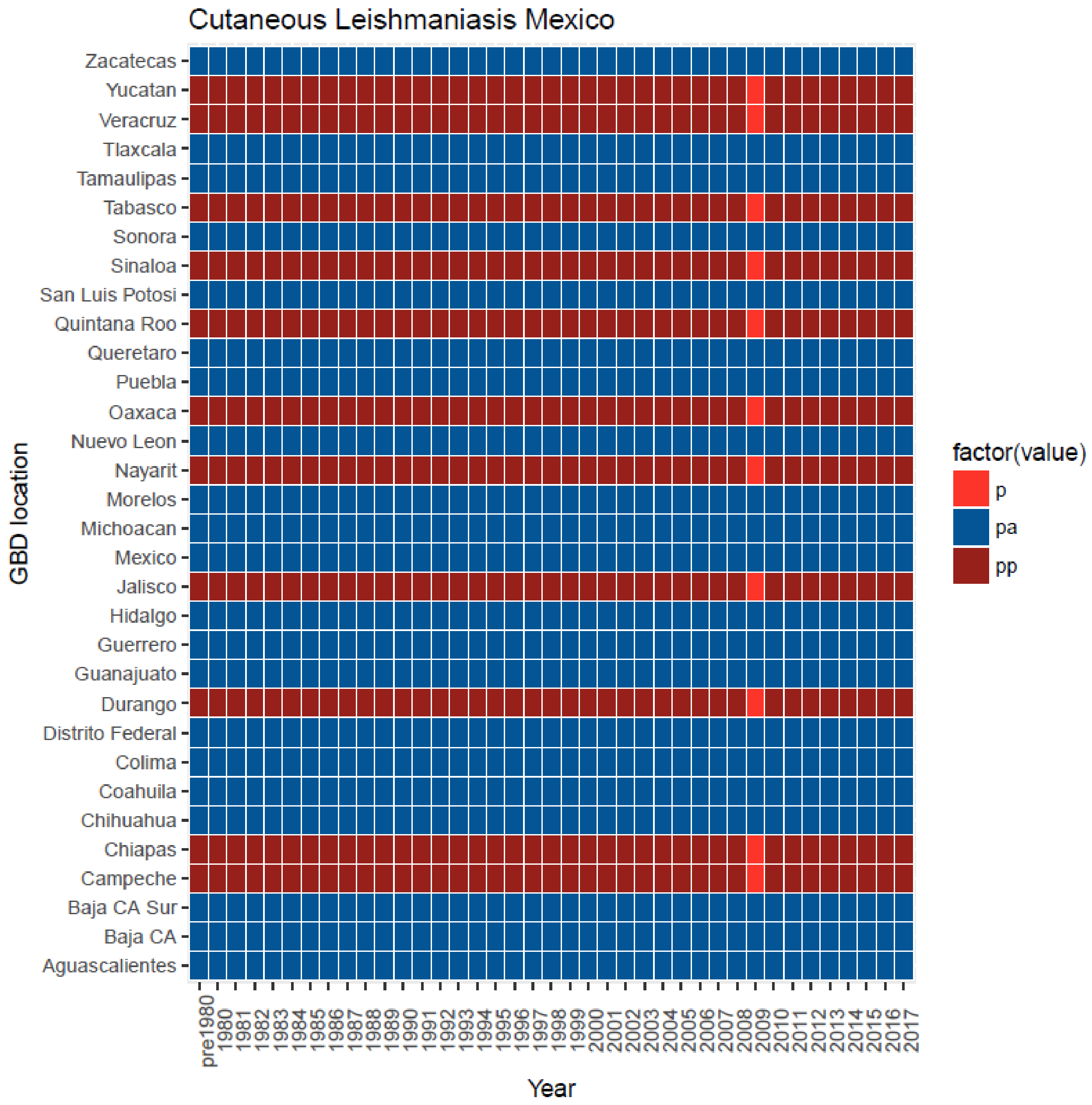

Figure 2: Cutaneous Leishmaniasis geographic restrictions for Mexican subnationals. Locations tagged as present are coloured in red (denoted as p), dark red represents protocol presence (denoted as pp), and dark blue represents protocol absence (denoted as pa).

Full time series of maps and tables, with relevant GHDx NIDs are available upon request from gbdsec@uw.edu.

Method – ST-GPR

Using existing IHME tools, the summarised values were modelled using ST-GPR to produce a complete time series of estimates for each location-year tagged “Present” or “Protocol Present”. In short, ST-GPR attempts to model non-linear trends utilizing a Gaussian process to fit a trend, rather than a definitive

functional form. Case count data were translated into estimates of true case counts by using underreporting scalars as identified by Alvar et al. (2012).

Method – DisMod

DisMod was used to generate an age-sex curve to disaggregate all-age, both-sex, incidence data. DisMod is an integrated meta-regression framework that allows for multiple datasets to be integrated into a singular analysis regardless of age-binning, sources, and geographies. As a consequence, a variety of differently aggregated information can be evaluated to generate a consensus output. From this model, the global fit was used.

Method – YLD estimation (incorporating duration and disability weighting) / COMO

Following standard GBD estimation protocols, incidence estimates were used to calculate disease prevalence (by multiplication with duration), disaggregated by disease sequelae. One health state is assigned to Cutaneous Leishmaniasis, [Table 2]. Duration value of initial acute infection was set to six months (Reithinger et al. 2007). Prevalence of long-term sequelae was based upon the proportion of cases that would result in facial scarring. The average proportion of sores that occurred on the face was calculated based upon a sample-weighted average of the proportion from four studies conducted in North Africa/Middle East. This proportion was 0.476. Of these people, only those who did not have appropriate access to health care were assigned long-term sequelae, estimated via the Healthcare Access and Quality Index. CL incidence, multiplied by proportion of people with facial sores, times the proportion of people without adequate health care access in each location-year, was used to obtain incidence of people with long-term sequelae, with cohorts streamed through time.

Sequela	Health state lay description	Disability weight	Duration
Cutaneous and mucocutaneous leishmaniasis	"has a slight, visible physical deformity that others notice, which causes some worry and discomfort"	0.011 (0.005–0.021)	6 months (46.7% * HAQ Index) Lifelong

Table 2: Sequelae and associated metadata. For the sequelae used in GBD 2019, the lay descriptor health state, disability weight, and duration are listed.

Central processing is used to generate the final estimates, including co-morbidity simulations.

Changes from GBD 2017

There were no substantive changes from the GBD 2017 methodology.

Limitations

As with any modelling process, a number of limitations are known, which will be the focus of additional effort in upcoming GBD cycles and engagement with collaborators. Given the focus on location-representative estimates, the existing model is focused on national case counts. This excludes a large resource of published literature and grey literature focused on site-specific surveillance or surveys. While some pathogens have integrated subnational approaches as a building block for national estimates (eg, schistosomiasis) this has yet to be implemented for cutaneous leishmaniasis. Regardless of contribution

to the global incidence model, these data can be used to inform age-sex splits, as well as a variety of other key parameters, particularly duration parameters, which are currently lacking uncertainty.

References

Alvar et al. (2012) Leishmaniasis Worldwide and Global Estimates of Its Incidence. PLoS One 7(5):e35671

Pigott et al. (2014) Global Distribution Maps of the Leishmaniases. eLife 3:e02851

Reithinger et al. (2007) Cutaneous Leishmaniasis. Lancet Infect Dis 7(9):581-96

Human African Trypanosomiasis (HAT)

Flowchart

African trypanosomiasis

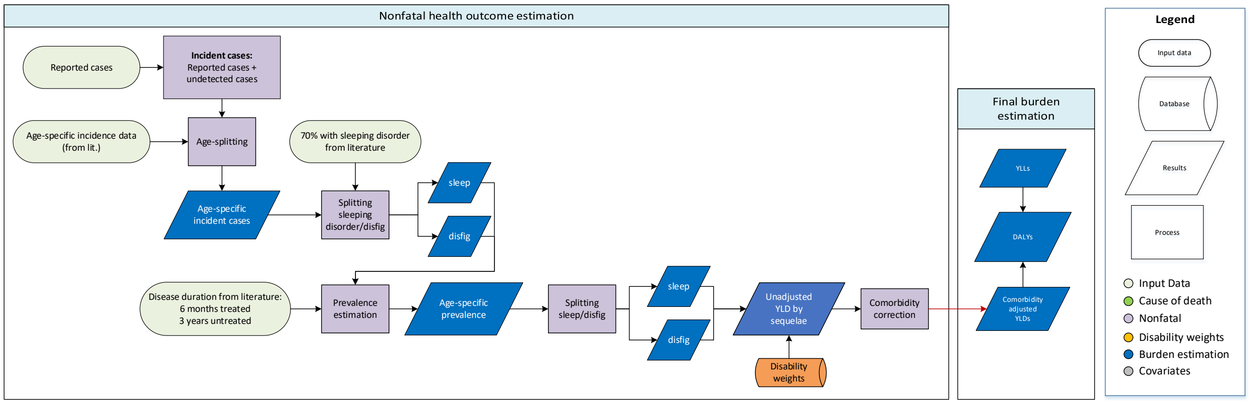

Input Data & Methodological Summary

Case Definition

Human African trypanosomiasis (HAT), also known as sleeping sickness, is a vector-borne disease which is transmitted by the bite of the tsetse fly. It is caused by the parasite *Trypanosoma brucei* with two subspecies, namely *T.b. rhodesience* (makes up less than 5% of total HAT cases) and *T.b. gambiense*. Cases are diagnosed through laboratory methods which rest on finding the parasite in body fluid or tissue by microscopy. In highly endemic or epidemic areas where the likelihood of false positives in serological tests is deemed lower, a seropositive individual is considered affected even in the absence of parasitological confirmation. The ICD-10 codes for HAT are B56.0, B56.1 and B56.9.

Input data

Model inputs

Data sources for GBD 2019:

1) Annual case totals 1980–2018: National-level annual case totals from 1990–2018 were obtained from the publicly available data via WHO, available here: http://apps.who.int/gho/data/node.main.A1635?lang=en

Subnational data:

Kenya: Kenyan subnational estimates are attributed to Busia County. Identification of subnational locations for Kenyan case data were obtained via studies published in the peer-reviewed literature[1] and review of maps published from via the WHO HAT Atlas[2]: http://www.who.int/entity/trypanosomiasis_african/country/Kenya_whole_0014.jpg?ua=1.

Nigeria: Nigeria subnational estimates were assigned by review of historical case data, identifying Delta State as the only subnational location reporting HAT disease.

2) Age/sex data: Data on the age and sex distribution of HAT cases were extracted from the peer-reviewed literature via a systematic review of sources identified in PubMed using the following search string:

((African trypanosomiasis[Title/Abstract] AND (incidence[Title/Abstract] OR burden[Title/Abstract] OR prevalence[Title/Abstract] OR community[Title/Abstract])) AND (“1990”[Date – Publication] : “2017”[Date – Publication]))

This yielded 219 studies, of which only three met the inclusion criteria and were extracted[3-5]. The inclusion criteria were:

1. Studies representative of the national population
2. Population-based studies
3. Studies with primary data on incidence
4. Studies of human African trypanosomiasis (excluded studies on animal African trypanosomiasis)

3) Population at risk estimates 1980–2019: population at risk estimates from GBD 2010 ArcGIS analysis using geocoded case notifications for 2000 to 2009[2] and population Count Grid estimates from Gridded Population of the World.

4) Screening coverage: Data on active versus passive screening coverage were obtained from a Weekly Epidemiological Report[6] identifying the population screened from 1997 to 2004 at the national level.

5) Geographic restrictions: Data file of all GBD locations, defining location as either endemic or non-endemic for HAT. Estimates are not produced for non-endemic countries, nor are they generated for countries with a history of HAT transmission but no data reported by WHO from 1990 to 2018.

Table 1 presents the total number of data sources used in this model.

Table 1. Total data source counts

Measure	Total sources	Countries with data
All measures	2944	35
Prevalence	1	1
Incidence	959	33
Proportion	1044	29
Population	940	29

Modelling strategy

Geographic restrictions

For countries historically considered endemic for HAT, but which have no reported case data or estimate of the population at risk, estimates are not produced. These countries include Botswana, Ethiopia, Guinea-Bissau, and Rwanda.

Among countries where population at-risk data are available, if no cases were reported to WHO, we assume the incidence of HAT is zero for those years and generate model estimates accordingly.

Modelling steps

Non-fatal estimates for HAT were generated as follows:

1. The incidence of reported HAT cases among the population at-risk was calculated as the total number of reported cases divided by the population at-risk estimates generated by the GBD working group for the period 1980–2015. Population at-risk estimates for 2016–2017 were generated by assuming an annual 2% rate of population growth.

2. To estimate the number of cases that were likely undetected by country and year, a multi-level mixed-effects linear regression of log-transformed incidence rate (ratio of reported HAT cases to population at risk) on log-transformed screening coverage (ratio of number screened for HAT to population at risk), with country random effects, was performed. Gaps were then filled using interpolation between years and extrapolation from 2018 to 2019 for reported cases. This model generates a beta-coefficient which is used to estimate the case detection rate (see step 4).

   For country-years in which no screening coverage data were reported:
   - Among countries with data reported, 1997–2004, the proportion of the at-risk population screened from 1997 was used retrospectively for the period 1980–1996 and the screening coverage from 2004 was carried forward from 2005–2019.
   - For countries with no screening data reported, the mean screening coverage for the region was used to impute a value over time.

3. Assuming the same proportion in treated (reported) and untreated (undetected) cases, the incidence estimates were then split into the two sequelae, skin disfigurement and sleeping disorder. This was done by generating 1,000 draws of the splitting proportion for the sequelae (70%–74% with sleeping disorder) based on a study that reported presence of symptoms at admission of patients in treatment centers[7]. Draws were generated from a beta distribution with alpha parameter = 1884 and beta parameter = 649.

4. To compute prevalence of HAT, 1,000 draws of total duration of symptoms in untreated cases were generated from a normal distribution with mean = [ln(3) – 0.5 * sigma^2], and standard deviation = sigma, where sigma = [ln(4.39)-ln(1.92))/(invnormal(0.975)*2)]: these parameters were based on a study of *T.b. gambiense*[7] which estimated an average duration of three years to untreated cases. An estimated duration of six months was applied to cases that received treatment, based on findings from a paper about *T.b. rhodesiense* in Uganda[8].

5. Prevalence was then estimated from the incident cases before applying age pattern. Prevalence of treated and untreated cases were summed up, assuming that untreated cases have been prevalent up to their death for a certain duration[9]. For untreated cases, it was assumed that half the duration is spent with sleeping disorder (severe motor and cognitive impairment) and disfigurement[7]. Treated (ie, reported) cases are assumed to have been prevalent for 0.5 years, and for the fraction of treated cases that present with sleeping disorder, it was assumed that this is present for half the total duration and that the rest of the duration is spent suffering from disfiguring skin disease. Among reported cases assumed to be detected prior to stage 2 infection, we do not attribute any of the duration of morbidity to sleeping disorder.

6. Finally, an age-pattern was applied to the prevalence estimates using the incidence studies from Sudan[5], DRC[3], and Uganda[4]. The age-pattern in GBD 2019 employed a cubic spline to account for the higher risk of infection among working-age adults.

Severity splits/sequelae

The basis of the GBD disability weight (DW) survey assessments are lay descriptions of sequelae highlighting major functional consequences and symptoms. The lay descriptions and disability weights for HAT sequelae due to HAT are shown below in Table 2.

Table 2. Health states for human African trypanosomiasis

Sequela	Lay description	DW (95% CI)
Skin disfigurement, level 1	has a slight, visible physical deformity that is sometimes sore or itchy. Others notice the deformity, which causes some worry and discomfort.	0.027 (0.015–0.042)
Motor plus cognitive impairments, severe	cannot move around without help, and cannot lift or hold objects, get dressed or sit upright. The person also has very low intelligence, speaks few words, and needs constant supervision and help with all daily activities	0.542 (0.37–0.702)

References

1. Rutto JJ, Osano O, Thuranira EG, Kurgat RK, Odenyo VA. Socio-economic and cultural determinants of human african trypanosomiasis at the Kenya - Uganda transboundary. *PLoS Negl Trop Dis* 2013; **7**(4): e2186.

2. Simarro PP, Cecchi G, Paone M, et al. The Atlas of human African trypanosomiasis: a contribution to global mapping of neglected tropical diseases. *Int J Health Geogr* 2010; **9**: 57.

3. Lutumba P, Makieya E, Shaw A, Meheus F, Boelaert M. Human African trypanosomiasis in a rural community, Democratic Republic of Congo. *Emerg Infect Dis* 2007; **13**(2): 248-54.

4. Fevre EM, Odiit M, Coleman PG, Woolhouse ME, Welburn SC. Estimating the burden of rhodesiense sleeping sickness during an outbreak in Serere, eastern Uganda. *BMC Public Health* 2008; **8**: 96.

5. Moore A, Richer M, Enrile M, Losio E, Roberts J, Levy D. Resurgence of sleeping sickness in Tambura County, Sudan. *Am J Trop Med Hyg* 1999; **61**(2): 315-8.

6. World Health Organization. Human African trypanosomiasis (sleeping sickness): epidemiological update. *Weekly epidemiological record* 2006; **February 24**(8): 69-80.

7. Blum J, Schmid C, Burri C. Clinical aspects of 2541 patients with second stage human African trypanosomiasis. *Acta Trop* 2006; **97**(1): 55-64.

8. Odiit M, Kansiime F, Enyaru JC. Duration of symptoms and case fatality of sleeping sickness caused by Trypanosoma brucei rhodesiense in Tororo, Uganda. *East Afr Med J* 1997; **74**(12): 792-5.

9. Checchi F, Filipe JA, Haydon DT, Chandramohan D, Chappuis F. Estimates of the duration of the early and late stage of gambiense sleeping sickness. *BMC Infect Dis* 2008; **8**: 16.

Schistosomiasis

Flowchart

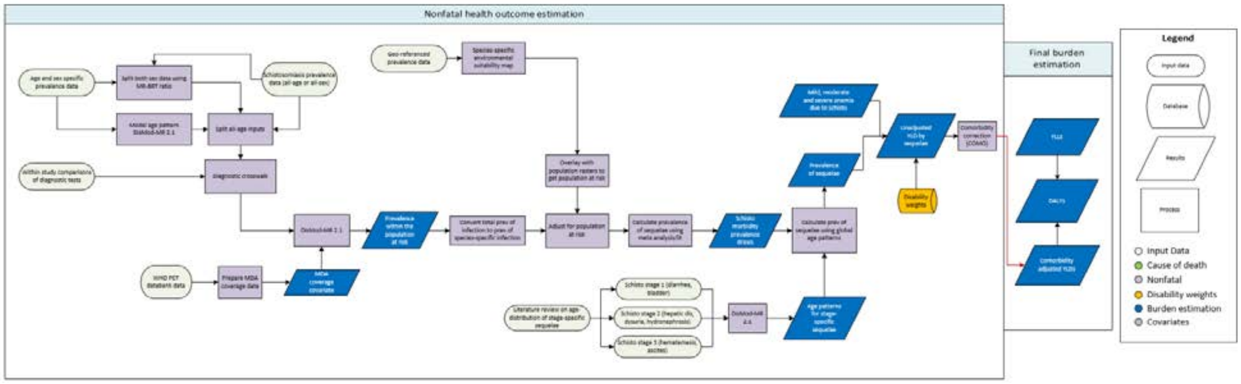

Case definition

Schistosomiasis, also known as bilharzia or "snail fever," is a helminth disease caused by infection with five species of the parasite *Schistosoma,* namely, *S mansoni, S japonicum, S haematobium, S mekongi, and S intercalatum*. It is considered a neglected tropical disease (NTD). The first three species cause the most infection and the last two rarely cause disease. Diagnosis is made by microscopic exam of stool or urine for parasite eggs. For less advanced infections, serologic techniques are used. The ICD-10 codes for schistosomiasis are B65-B65.9.

Input data

Model inputs

To model non-fatal outcomes due to schistosomiasis, we conducted a systematic literature review, extracting prevalence data from 1980 to 2016 for the five species of schistosomiasis listed above. The search string used in the systematic review is (schistosom*[Title/Abstract] OR bilharzia*[Title/Abstract] OR "snail fever"[Title/Abstract]) AND ("1990"[Date - Publication] : "3000"[Date - Publication]) AND (epidemiolog* OR inciden* OR prevalen* OR seroprevalen*) NOT (animals[mesh] NOT humans[mesh]). Additionally, we used data obtained through the Expanded Special Project for the Elimination of Neglected Tropical Diseases (ESPEN) data portal (maintained by WHO AFRO) and data compiled by the Global Atlas of Helminth Infections (GAHI), which includes grey literature and unpublished data. Site-specific prevalence data is aggregated by GBD location and year.

Table 1 presents the total source counts used to produce burden estimates of schistosomiasis.

Table 1. Total data source counts

Measure	Total sources	Countries with data
All measures	524	173
Prevalence	450	171
Proportion	81	23

Mass drug administration data

Mass drug administration data were extracted from the WHO PCT Databank [1].

Severity splits/sequelae

Table 2 shows the list of clinical sequelae (including mild, moderate, and severe anaemia) due to schistosomiasis, their lay descriptions, and the associated disease stages and disability weights. Using literature [1], a list of eight possible clinical sequelae and anaemia sequelae were defined (mild infection, mild diarrhoea, haematemesis (vomiting blood), hepatomegaly, ascites (buildup of fluid in the peritoneal cavity), dysuria (painful urination), bladder pathology, hydronephrosis (swelling of kidney due to buildup of urine in the kidney), mild anaemia, moderate anaemia, and severe anaemia).

Table 2. Clinical sequela, lay descriptions, disease stages, and DWs

Clinical sequela	Lay description	Disease stage	Disability weights (DWs)
Mild infection	has a low fever and mild discomfort , but no difficulty with daily activities	1	0.006 (0.002–0.012)
Mild diarrhoea		1	0.056
Hepatomegaly	has some pain in the belly that causes nausea but does not interfere with daily activities	2	0.011 (0.005–0.021)
Dysuria	has some pain in the belly that causes nausea but does not interfere with daily activities	2	0.011 (0.005–0.021)
Hydronephrosis	has some pain in the belly that causes nausea but does not interfere with daily activities	2	0.011 (0.005–0.021)
Haematemesis	vomits blood and feels nauseated	3	0.325 (0.209–0.463)
Ascites	has pain in the belly and feels nauseated. The person has difficulties with daily activities	3	0.114 (0.078–0.159)
Bladder pathology	has some pain in the belly that causes nausea but does not interfere with daily activities	3	0.011 (0.005–0.021)
Mild anaemia	feels slightly tired and weak at times, but this does not interfere with normal daily activities	NA	0.004 (0.001–0.008)
Moderate anaemia	feels moderate fatigue, weakness, and shortness of breath after exercise, making daily activities more difficult	NA	0.052 (0.034–0.076)
Severe anaemia	feels very weak, tired, and short of breath, and has problems with activities that require physical effort or deep concentration	NA	0.149 (0.101–0.210)

Data processing

Schistosomiasis prevalence data reported for both sexes was first split into sex-specific inputs using a sex-ratio estimated by MR-BRT. All age data were then split into five-year age groups by using a global age pattern obtained via Dismod, illustrated in Figure 1.

Figure 1. Global age pattern of schistosomiasis prevalence produced by Dismod.

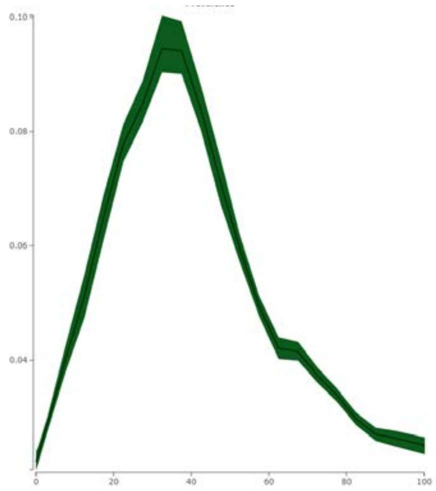

In GBD 2019, we updated our method for diagnostic adjustment to account for species-specific diagnostic tests, generating an adjustment for *S. haematobium*, *S. mansoni* and *S. japonicum* separately. For *S. mansoni*, we identified 90 within study comparisons including at least two of the following diagnostic methods : Kato-Katz (1, 2 or 3 stool smears); ELISA; CCA; formol-ether concentration; sedimentation and PCR.   At total of 56 diagnostic comparisons were identified for *S. haematobium*: CCA; urine filtration, dipstick tests, centrifugation and sedimentation. 37 comparisons were identified for japonicum, including Kato-Katz, IHA, hatch test, and ELISA. The reference categories by species were defined as Kato-Katz for *S. mansoni*, urine filtration for *S. haematobium* and PCR for *S. japonicum* (adjustment factors presented in Tables 3-5).

Table 3: MR-BRT Crosswalk Adjustment Factors for *S. mansoni*

Data input	Reference or alternative case definition	Gamma	Beta Coefficient, Logit (95% CI)	Adjustment factor*
Kato-Katz 3 sample	Ref	0.701	---	---
Kato-Katz 2 sample	Alt		-0.423 (-0.6, -.24)	1.53
Kato-Katz 1 sample	Alt		0.495 (0.26,0.72)	0.61
CCA	Alt		2.306 (1.77, 2.84)	0.10
Sedimentation	Alt		1.636 (1.44, 1.82)	0.19
Formol-ether	Alt		-0.36 (-1.18, 0.45)	1.44
PCR	Alt		-0.011 (-0.57, 0.55)	1.01
ELISA	Alt		1.122 (1.01, 1.23)	0.33

**Adjustment factor is the transformed Beta coefficient in normal space, and can be interpreted as the factor by which the alternative case definition is adjusted to reflect what it would have been if measured as the reference.*

Table 4: MR-BRT Crosswalk Adjustment Factors for *S. haematobium*

Data input	Reference or alternative case definition	Gamma	Beta Coefficient, Logit (95% CI)	Adjustment factor*
Urine filtration	Ref	0.80	---	---
CCA	Alt		2.42 (1.88, 2.95)	11.24
Dipstick	Alt		-0.21 (-0.4, 0.07)	0.81
PCR	Alt		-0.07 (-1.7, 1.6)	0.94
Centrifugation	Alt		-0.13 (-0.78, 0.53)	0.88
Sedimentation	Alt		-0.56 (-1.7, 0.60)	0.57

**Adjustment factor is the transformed Beta coefficient in normal space, and can be interpreted as the factor by which the alternative case definition is adjusted to reflect what it would have been if measured as the reference.*

Table 5: MR-BRT Crosswalk Adjustment Factors for *S. japonicum*

Data input	Reference or alternative case definition	Gamma	Beta Coefficient, Logit (95% CI)	Adjustment factor*
IHA	Ref	0.506	---	---
ELISA	Alt		0.94 (0.49, 1.4)	2.57
Hatch test	Alt		-1.54 (-1.89, -1.15)	0.21
Kato-Katz 1 sample	Alt		-1.50 (-1.73, -1.2)	0.22
Kato-Katz 2 sample	Alt		-1.21 (-1.6, -0.82)	0.30
Kato-Katz 3 sample	Alt		-1.40 (-2.1, -0.64)	0.24

**Adjustment factor is the transformed Beta coefficient in normal space, and can be interpreted as the factor by which the alternative case definition is adjusted to reflect what it would have been if measured as the reference.*

Modelling strategy

The morbidity model for schistosomiasis involved a multi-step process. First, we ran a single-parameter prevalence model in DisMod-MR 2.1 using the prevalence data after adjusting for age, sex and diagnostic. We make the assumption that all of our data are measured within a population at risk – therefore, the estimates from the DisMod model represent prevalence estimates among the population at risk for schistosomiasis. Additionally, we included the MDA treatment data from WHO as a country-level covariate in the DisMod model (Table 6).

Table 6. Dismod Model Covariates

Covariate	Type	Parameter	Exponentiated beta
Socio-demographic Index	Country-level	Prevalence	0.76 (0.65, 0.92)
MDA treatments	Country-level	Prevalence	0.61 (0.59, 0.64)

Second, we ran three separate ecological niche maps for the three major species of schistosomiasis (*S mansoni, S haematobium,* and *S japonicum*) using a boosted regression tree and all geolocated data that

were extracted from both the literature review and the GAHI database. The output was 1,000 maps (representing 1,000 draws) for each of the three species representing the suitability for schistosomiasis to exist in each 5x5 km square. Then, we extracted population at risk by optimising the area under the curve for each of the 1,000 maps for each of the three species, overlaid the three species maps over one another, and extracted 1,000 draws of proportion of the population at risk for schistosomiasis at the GBD location level.

To avoid over-estimation of prevalence using the population at risk raster in urban areas in Brazil and China, we masked out urban areas. In China we used year-specific masks based off of published literature on county-specific elimination of schistosomiasis, allowing the geographic restrictions to be implemented at a more detailed level where information is available (5).

We then scaled the prevalence estimates to the population at risk estimates from the ecological niche map to get age/sex/location/year all-schistosomiasis prevalence envelopes. 4) We ran a generalised linear model to get species-specific proportional prevalence on data from literature that reported both *S. haematobium* and *S. mansoni* infection, and 5) literature-informed parameters (a, b, c) for translating infection (x) to morbidity (y): $y = (a + bx^c)/(1 + bx^c) – a$ [2-4]. We used the species-specific conversion factors calculated in step (4) to split the all-schistosomiasis envelope into species-specific schistosomiasis. We then used the parameters determined in step (5) to translate infection into morbidity to get age/sex/year/location-specific prevalence of sequelae. The burden of anaemia due to schistosomiasis was estimated (see anaemia documentation for details).

Model evaluation was done by separately assessing the fit of the single-parameter DisMod models and checking the final estimates produced after age-sex splits. Plots of time trends of prevalence across locations and age were used to evaluate the results. In addition, maps of the global distribution of total schistosomiasis prevalence and prevalence of sequelae due to schistosomiasis were also assessed across time.

References

1. World Health Organization (WHO). WHO PCT Databank - Schistosomiasis. Geneva, Switzerland: World Health Organization (WHO).
2. van der Werf MJ, de Vlas SJ, Brooker S, et al. Quantification of clinical morbidity associated with schistosome infection in sub-Saharan Africa. Acta Trop. 2003;86(2-3):125-39
3. van der Werf MJ, de Vlas SJ, Looman CW, Nagelkerke NJ, Habbema JD, Engels D. Associating community prevalence of Schistosoma mansoni infection with prevalence of signs and symptoms. Acta Trop. 2002;82(2):127-37
4. van der Werf MJ, de Vlas SJ. Diagnosis of urinary schistosomiasis: A novel approach to compare bladder pathology measured by ultrasound and three methods for hematuria detection. Am. J. Trop. Med. Hyg. 2004;82:98-106
5. Zhou, Xiao-Nong & Bergquist, Robert & Leonardo, Lydia & Olveda, Remigio. (2018). Schistosomiasis: The Disease and its Control.

Cysticercosis

Flowchart

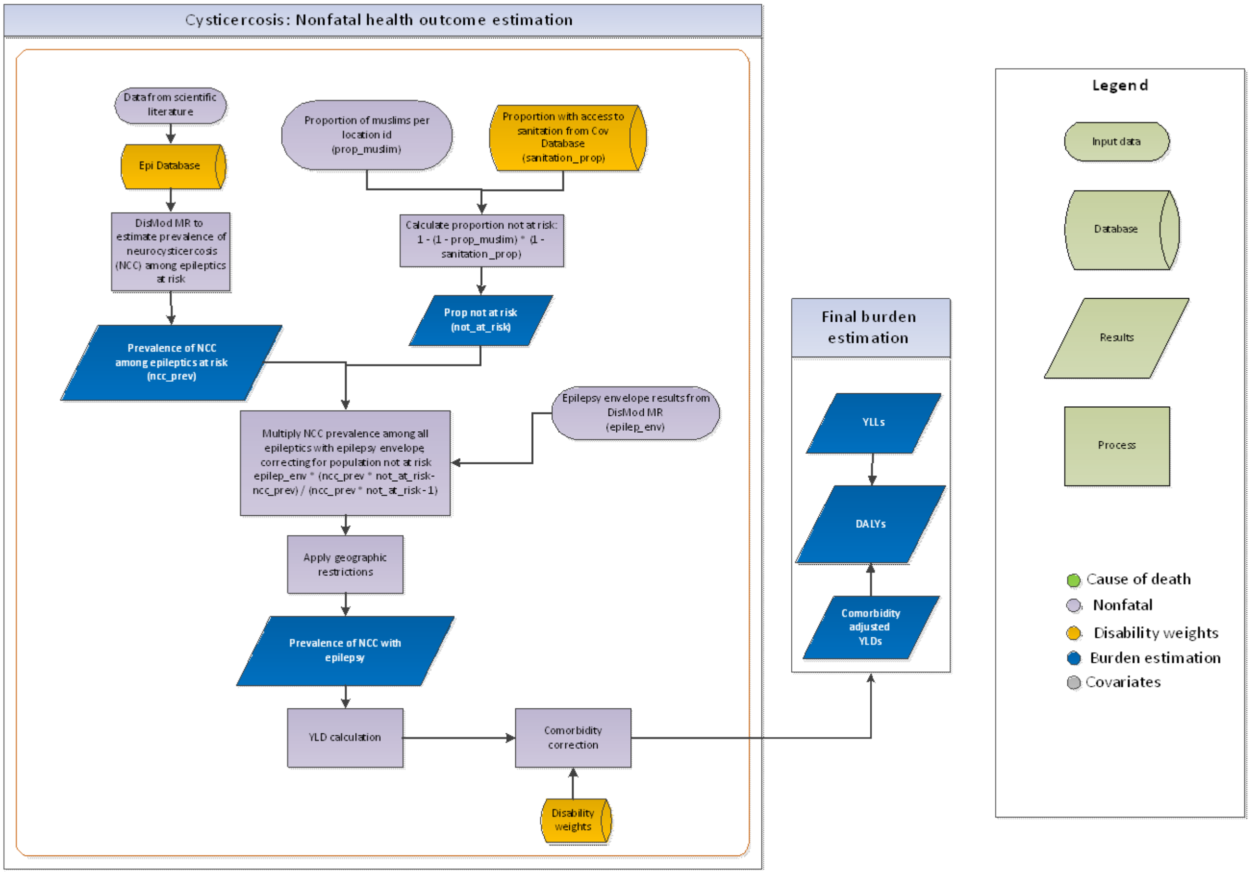

Input Data & Methodological Summary

Case Definition

Cysticercosis, or neurocysticercosis (NCC), is a parasitic disease caused by the pig tapeworm *Taenia solium.* It is transmitted via ingestion of eggs or gravid proglottids shed by a human or non-human host with an intestinal infection of the same helminth known as Taeniasis. In rare cases, auto-infection is also possible among people with intestinal infections. Diagnosis is made by magnetic resonance imaging (MRI) or computerized tomography (CT) brain scans to identify cysts. The ICD-10 codes for cysticercosis are B69-B69.9.

Input data

Systematic literature review

The nonfatal estimation for cysticercosis focused on estimating prevalence of NCC among epileptics at risk as well as the prevalence of NCC with epilepsy. A systematic review of literature was conducted in PubMed for GBD 2015 using the following search string:

> ("cysticercosis"[Title/Abstract] OR "neurocysticercosis"[Title/Abstract] OR "cysticerciasis"[Title/Abstract] OR "Taenia solium"[Title/Abstract]) AND ("1990"[Date – Publication] : "2015"[Date – Publication]) AND (epidemiology OR prevalence)).

This yielded 1,038 studies, of which 166 were included during the title/abstract screening. Following the full-text screening, 17 studies were included and extracted – studies were excluded because of one or more of the following reasons:

1. study not in epileptics
2. study not population-based
3. study does not have primary data on prevalence of NCC among epileptics at risk
4. study not in humans (some studies were on cysticercosis in pigs)
5. study on comorbidities with NCC (other than epilepsy)
6. study on sub-population, eg, patients with neurological disorders
7. review study

Table 1 presents a summary of source counts for this model.

Table 1. Total data source counts

Measure	Total sources	Countries with data
All measures	30	16
Prevalence	30	16

Data processing

Input data were classified as either probable or definite diagnosis. We extracted 16 within-study comparisons to crosswalk the data using definite diagnosis as a reference using MR-BRT (Table 2).

Table 2. MR-BRT Crosswalk Adjustment Factors

Data input	Reference or alternative case definition	Gamma	Beta Coefficient, Log (95% CI)	Adjustment factor*
Definite	Ref	0.62	---	---
Probable	Alt		0.59 (0.22, 0.96)	0.55

**Adjustment factor is the transformed Beta coefficient in normal space, and can be interpreted as the factor by which the alternative case definition is adjusted to reflect what it would have been if measured as the reference.*

Covariates

Data were ascertained from the PEW Research Center [1] on the proportion of the population that is Muslim and incorporated as a continuous covariate with a range between 0 and 1.

Epilepsy envelope

The modelling process incorporates 1,000 draws of epilepsy envelope prevalence from the GBD 2019 epilepsy DisMod-MR model – details on this modelling process can be found elsewhere.

Modelling strategy

DisMod-MR was used to model the prevalence of NCC among epileptics at risk. In the model, pigs raised in extensive agricultural systems per capita, SDI, and religion (binary, >50% Muslim) were used as country-level covariates (Table 3).

Table 3. DisMod model covariates

Covariate	Type	Parameter	Exponentiated beta
Religion (binary, > 50% Muslim)	Country-level	Prevalence	0.22 (0.15, 0.37)
Socio-demographic Index	Country-level	Prevalence	0.14 (0.14, 0.16)
Pigs raised in extensive agricultural systems per capita	Country-level	Prevalence	3.27 (1.40, 6.83)

After running DisMod, we adjusted the fraction of people with epilepsy attributable to cysticercosis in endemic countries for the population at risk based on the proportion of the population without access to sanitation and the proportion of the population that is Muslim. The following is the computation for estimating NCC prevalence among epileptics at risk:

$$Prevalence_{NCC\ prevalence} = Prevalence_{epilepsy} * \frac{NM - N}{NM - 1}$$

Where prevalence = prevalence of all-cause epilepsy in total population, N = proportion of NCC among epileptics at risk (non-Muslims without access to sanitation), and M = proportion of population not at risk of contracting NCC. It was assumed that the prevalence of epilepsy due to causes other than NCC is the same regardless of whether a population is at risk or not. It was also assumed that Muslims and non-Muslims have equal access to sanitation. Geographic restrictions were applied to set prevalence to zero in non-endemic locations.

Model evaluation was done by separately assessing the fit of the DisMod-MR model and checking the estimates produced after estimating prevalence of NCC with epilepsy. Plots of time trends of prevalence across locations and age were used to evaluate the results. In addition, maps of the global distribution of prevalence of NCC among epileptics at risk and prevalence of NCC with epilepsy were also assessed across time.

Several changes were made compared to the GBD 2017 modelling strategy. First, we made slight changes to model parameters in DisMod-MR to improve model fit. Second, we incorporated two new

covariates (ie, pigs raised in extensive agricultural systems per capita, SDI) to better inform the model. Lastly, we updated geographic restrictions and updated proportion of population with Muslim data by imputing subnational locations with national proportions due to a lack of data at the subnational level.

References:

1. “Table: Muslim Population by Country Pew Research Center, Washington, D.C.” (July 7, 2017). http://www.pewforum.org/2011/01/27/table-muslim-population-by-country/

Cystic Echinococcosis

Flowchart

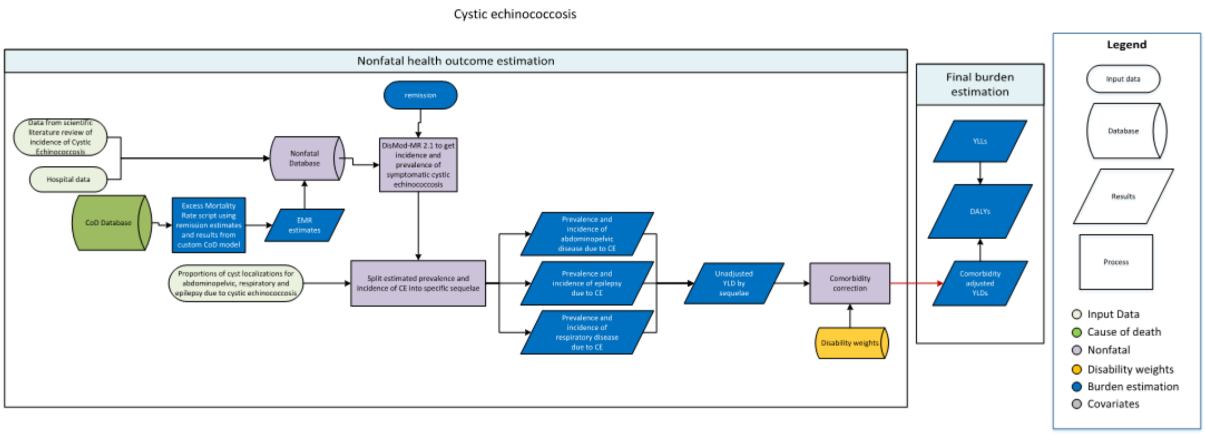

Input Data & Methodological Summary

Case definition

Cystic echinococcosis is a parasitic disease caused by infection with the *Echinococcus granulosis* tapeworm. It is a natural parasite of canines, with sheep being the most common intermediate host in the two-stage lifecycle, but can be spread to humans through ingestion of soil, water, or food contaminated with the fecal matter of an infected dog containing infective eggs. Diagnosis is made by clinical findings, imaging, serology, and tissue pathology. The ICD-9 and ICD-10 codes for echinococcosis are 122.0-122.9 and B67-B67.9, respectively.

Input data

Table 1: Source Counts

Measure	Total sources	Countries with data
All measures	286	196
Incidence	285	61
Proportion	1	196

Systematic Literature Review

The non-fatal estimation for cystic echinococcosis (CE) focused on estimating incidence and prevalence of CE and its sequelae. A systematic review of literature was conducted in PubMed for GBD 2015 using the following search string:

> ("echinococcosis"[Title/Abstract] OR "hydatid disease"[Title/Abstract] OR "hydatidosis"[Title/Abstract] OR "echinococcal disease"[Title/Abstract] OR "Echinococcus granulosus infection"[Title/Abstract]) AND (“1990”[Date – Publication] : “2015”[Date – Publication]) AND (epidemiology OR incidence OR prevalence).

This yielded 1,619 studies of which 279 were included during the title/abstract screening. Following the full-text screening, 77 studies (32 incidence, 43 prevalence, and 2 both) were included and extracted – studies were excluded because of one or more of the following reasons:

1. study not population-based
2. study does not have primary data on prevalence and/or incidence
3. study not in humans
4. study on sub-populations
5. review study

Since we were interested in modelling symptomatic CE cases, we only used data on incidence of patients diagnosed by imaging techniques (mainly ultrasonography). Therefore, we excluded prevalence data, which were mostly from serological studies. Data from these extracted studies were combined with data from studies extracted during GBD 2013.

Hospital data

Hospital data prepared by the GBD team were used as additional input into our models. These data were adjusted to account for multiple hospital episodes of a single case and non-primary diagnoses.

Geographic restrictions

We conducted a literature review to determine the geographic extent of the disease and classify locations based on whether the disease is absent or present in each year. Locations that were geographically restricted in any given year did not have estimates made. Of note, we did not attempt a complete systematic review, since a single high-quality source could offer sufficient evidence of presence. Evidence of absence or presence was not available for every location for each year, and so assumptions were made for missing years by taking into consideration the epidemiological characteristics of the disease.

If evidence indicated disease presence for two non-consecutive years, we assumed presence for all years between the two. If evidence indicated disease absence for two non-consecutive years, we assumed absence for all years between the two. If evidence indicated a change in status (ie, from absent to present, or present to absent) between two non-consecutive years, then we conducted targeted searches to ascertain the relevant year of introduction or elimination for that location. In the cases where presence or absence information was missing for the start or end years of our study interval (1990–2019) without evidence of any introduction or elimination events within the interval, we applied the status of the first and last presence/absence observations respectively to all years between the interval bound and the observation year. For cystic echinococcosis, we performed targeted searches to classify location-years in PubMed and Google Scholar. Geographic restrictions were populated by reviewing sources referenced by Deplazes and colleagues along with ad hoc searches in PubMed for evidence of active transmission of cystic echinococcosis in respective countries [1].

Sequelae due to cystic echinococcosis

The table below shows the sequelae due to echinococcosis and their associated disability weights.

Table 2. Sequelae, lay descriptions, and disability weights (DWs)

Sequela	Lay description	DW (95% CI)
Chronic respiratory disease	"has cough and shortness of breath after heavy physical activity, but is able to walk long distances and climb stairs."	0.019 (0.011–0.033)
Abdominal problems	"has pain in the belly and feels nauseated. The person has difficulties with daily activities."	0.114 (0.078–0.159)
Epilepsy	(Combined DW)	NA

Modelling strategy

The morbidity model for cystic echinococcosis involved a multi-step process. First, DisMod-MR was used to model incidence and prevalence of symptomatic cystic echinococcosis using incidence data from systematic reviews in GBD 2013 and 2015 and hospital data, excess mortality rate estimates, and an assumed remission of 0.15–0.25 per case per year (duration 2–6.7 years, average 5 years). Estimates of excess mortality rate were obtained by pulling death estimates from our CoD model. The following steps were followed to estimate excess mortality rate: 1) create custom age groups for CE deaths with uncertainty; 2) calculate CSMR as CSMR=deaths/population at the 1,000 draw level – calculate mean CSMR, uncertainty interval, and standard error; and 3) calculate EMR as EMR=CSMR/(prevalence), where prevalence = (incidence*5) – standard error of EMR was calculated taking into consideration the standard errors of both prevalence and CSMR. Geographic restrictions were applied to set incidence and prevalence to zero in location-years where the disease was not endemic. These computations provided 655 site-years of EMR data.

Table 3. DisMod model covariates

Covariate	Type	Parameter	Exponentiated beta
Sex	Study-level	Incidence	0.66 (0.63–0.70)
Urbanicity	Country-level	Incidence	1.00 (0.98–1.00)
Echinococcosis endemicity	Country-level	Incidence	6.03 (5.75–6.37)
Proportion of population involved in agricultural activities	Country-level	Incidence	1.00 (1.00–1.00)
Sex	Study-level	Excess mortality rate	1.63 (1.56–1.70)

After producing all-case prevalence draws, 1,000 draws of proportions for abdominal, respiratory, and epileptic symptoms among echinococcosis cases adding up to 1 were generated. Uncertainty in the splitting proportions was captured by drawing them from a Dirichlet distribution, informed by published data on cysts localization [2]. On average, the proportions of abdominal, respiratory, and epileptic symptoms due to echinococcosis were 0.5, 0.47, and 0.03, respectively. These proportions were used to split the prevalence and incidence from DisMod into the three sequelae.

Model evaluation was done by separately assessing the fit of the DisMod MR model and checking the estimates produced after estimating incidence and prevalence of sequelae due to cystic echinococcosis. Plots of time trends of incidence and prevalence across locations and age were used to evaluate the results. In addition, maps of the global distribution of incidence and prevalence were assessed across time.

Changes from GBD 2017 to GBD 2019

We have made no substantive changes in the modeling strategy from GBD 2017.

References

1. Deplazes P, Rinaldi L, Alvarez Rojas CA, Torgerson PR, Harandi MF, Romig T, Antolova D, Schrurer JM, Lahmar S, Cringoli G, Magambo J, Thompson RC, Jenkins EJ. Global Distribution of Alveolar and Cystic Echinococcosis. Advanced Parasitology. 2017. 95: 315-493.
2. Raether W, Hänel H. Epidemiology, clinical manifestations and diagnosis of zoonotic cestode infections: an update. Parasitology Research. 2003. 91:412-438.

Lymphatic Filariasis

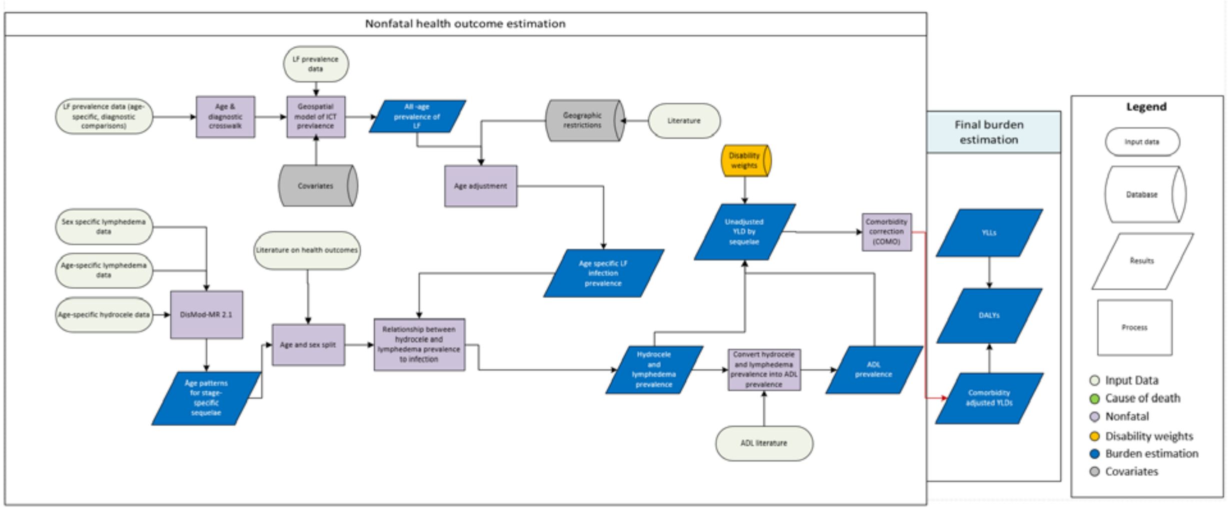

Input Data and Methodological Summary

Case Definition

Lymphatic filariasis (LF) is a neglected tropical disease in which threadlike nematodes invade the lymphatic system. The worms responsible – *Wuchereria bancrofti*, *Brugia malayi,* and *Brugia timori* – are spread from human to human via mosquitoes. The most prominent clinical manifestations of LF are lymphoedema (a swelling of the legs, also known in its more extreme manifestation as elephantiasis) and hydrocele (a collection of fluid in the sac around the testicles).

Input data

A systematic review of literature for GBD 2016 in the PubMed database was done on October 14, 2016, for prevalence and incidence data using the search (Lymphatic filariasis AND prevalence) OR (Lymphatic filariasis AND (prevalence OR incidence OR "mass drug administration" OR MDA OR coverage)) OR (Lymphoedema, hydrocele) OR (Transmission Assessment Survey (TAS)) OR (Lymphatic filariasis AND mapping). This literature review was updated again in May 2019. Additional data on LF infection prevalence collected under the Global Programme for the Elimination of Lymphatic Filariasis were obtained through the Expanded Special Project for Elimination of Neglected Tropical Diseases and the World Health Organization.

Table 1. Total data source counts

Measure	Total sources	Countries with data
All measures	561	43
Prevalence	561	43

Modelling strategy

We first model the prevalence of LF infection represented by ICT using a geospatial model to generate an estimate of all-age prevalence. We then relate the prevalence of LF infection to the prevalence of hydrocele and lymphoedema, and ADL.

Model of LF infection prevalence

Covariates

The geospatial model relied on covariates at the 5 × 5-km grid-cell resolution to represent environmental factors associated with LF transmission, including elevation, precipitation, vegetation, and temperature, as well as socioeconomic measures potentially associated with vector-borne disease burden. Geospatial estimates of population coverage with insecticide-treated bednets (ITN), indoor residual spraying and LF MDA (of any drug regimen) were included to account for interventions known to reduce transmission, and malaria (*Plasmodium falciparum* and *Plasmodium vivax*) prevalence and incidence were included as proxies for exposure to vector-borne disease. VIF analysis was performed to identify the set of covariates for modeling. The final analyses included a total of 22 covariates for Africa, 20 covariates for Asia, and 17 covariates for Hispaniola.

Age & diagnostic adjustment

In order to derive a global estimate of LF infection using data reported across different age and diagnostic categories, reflecting all-age infection prevalence, we used age and diagnostic crosswalk models to adjust the input data prior to the main modelling analysis. Due to the introduction and rapid adoption of ICT card tests in the mid-2000s and their higher sensitivity, data derived from identification of MF by blood microscopy were first adjusted to be comparable with ICT prevalence estimates. Prevalence measured in a single age group (typically adults in baseline surveys or children in TAS) were adjusted to reflect all-age prevalence. We identified peer-reviewed published surveys that reported prevalence in at least two age groups in the same study population. The non-linear age-dependent relationship between MF and ICT prevalence was then calculated using surveys that reported both measures by fitting a logistic regression model with a basis spline on the ratio of ICT to MF prevalence by age. The age crosswalk model was similarly structured and was fit using surveys reporting ICT prevalence for multiple age groups.

Geostatistical analysis

Bayesian geostatistical modelswere fit separately for each of the following modelling regions based on a review of LF endemicity: (1) Africa and Yemen, including Madagascar, São Tomé and Príncipe, and Comoros; (2) South and Southeast Asia; and (3) the island of Hispaniola. We first employed an ensemble method to select covariates, capture possible non-linear effects, and account for the complex interactions among them. For each modelling region, we fit three sub-models to predict prevalence of LF for geo-referenced data points, with cross validation: generalised additive models (GAM), generalised boosted models (GBM), and lasso regression. All sub-models included country-level fixed effects. We modelled LF infection prevalence using a spatially- and temporally-explicit generalised linear mixed effects model *via* integrated nested Laplace approximation (INLA). The spatiotemporal variation beyond that described by the included covariates was modelled as a Gaussian process with covariance as a Kronecker product of the spatial and temporal error processes. Spatial covariance was modelled using a Matérn function, and the temporal covariance was modelled using a

first- or second-order autoregressive function. Predictions were generated using the in-sample sub-model predictions as covariates and summarising 1 000 samples from the posterior distribution as the mean; 95% uncertainty intervals (UIs) were generated from the 2·5th percentile and 97·5th percentile. This model was fit in R-INLA using stochastic partial differential equations (SPDE) to model the spatiotemporal processes.

Model validation was performed using spatially stratified five-fold out-of-sample cross validation, with examination of mean bias, mean absolute error, total error variance (root-mean-square error, RMSE), 95% data coverage within prediction intervals, and correlations of observed to predicted values. Geostatistical methods were not practical for estimating the prevalence of LF infection for the following locations due to small area (<25 km^2), missing covariate data, or limited geo-referenced data: American Samoa, Brazil, Cook Islands, Fiji, French Polynesia, Guyana, Kiribati, Maldives, Marshall Islands, New Caledonia, Niue, Palau, Samoa, Tonga, Tuvalu, Vanuatu, and Wallis and Futuna. Instead, Bayesian time series models for endemic IUs were fit to estimate annual national prevalence (Appendix Section 5·6). We masked all final model outputs for which land cover was classified as “barren or sparsely vegetated” on the basis of 2013 MODIS satellite data (the most recent year available), as well as areas in which total population density was less than ten individuals per 5 × 5-km grid cell in 2015.

To estimate of the number of infected individuals from the 5 × 5-km model predictions, the total number of cases per country was calculated first by multiplying grid-cell-level prevalence by the grid-cell-level population estimate produced by WorldPop, then aggregating those case estimates to national boundaries by draw. The mean total cases infected was calculated across the 1 000 draws of case totals and the UI was constructed from the 2·5th and 97·5th percentile. WHO regional totals were produced by aggregating up to regional boundaries, also by draw. Mean case estimates from the non-MBG locations were produced by applying the model-predicted national prevalence (mean, 2·5th and 97·5th percentile values) to the national population estimates produced for the Global Burden of Disease study) or other sources for the relevant IU populations.

Lymphoedema and hydrocele modeling

For lymphoedema and hydrocele, we reviewed published studies on the prevalence of hydrocele or lymphoedema, as well as program monitoring data for which LF infection and hydrocele or lymphoedema prevalence were reported in the same study population. We first adjusted data on lymphoedema reported in both males and females to be sex specific. We do not model the prevalence of hydrocele in females. We then adjusted any all-age lymphoedema and hydrocele data to be age-specific according to 5-year age groups using age patterns modeled from age-specific data in DisMod-MR 2.1. Two separate disability models were implemented, one for lymphoedema and one for hydrocele – the process essentially the same. The community-level prevalence reported in studies for which hydrocele or lymphoedema were also reported was used as a covariate (adjusted to represent ICT prevalence) to predict prevalence of hydrocele and lymphoedema. The age-specific national estimates of ICT prevalence estimated by the geospatial model were then used to predict national hydrocele and lymphoedema prevalence. Overall prevalence of LF infection was predicted accounting for the impact of MDA on prevalence – we further restricted countries at least five years post-elimination from the estimates.

ADL prevalence estimates

After prevalence of lymphoedema and hydrocele were estimated, we assumed the following for prevalent lymphoedema cases: 95% experience a total of 4 episodes per year, with an average duration of 7 days. For prevalent hydrocele, we assume: 70% of cases experience a total of two episodes per year, with an average duration of 7 days.

Table 2. Sequela and lay description

Sequela	Lay description	DW (95% CI)
Lymphoedema	Has swollen legs with hard and thick skin, which causes difficulty in moving around	0.109 (0.073, 0.154)
Hydrocele	Has swelling and tenderness in the testicles and pain during urination	0.128 (0.086, 0.18)
Acute adenolymphangitis due to lymphatic filariasis	Has a fever and aches and feels weak, which causes some difficulty with daily activities	0.051 (0.032, 0.074)

Changes from GBD 2017 to GBD 2019

Use of a geospatial model to predict LF infection prevalence enables us to better account for the focal distribution of disease.

Onchocerciasis

Flowchart

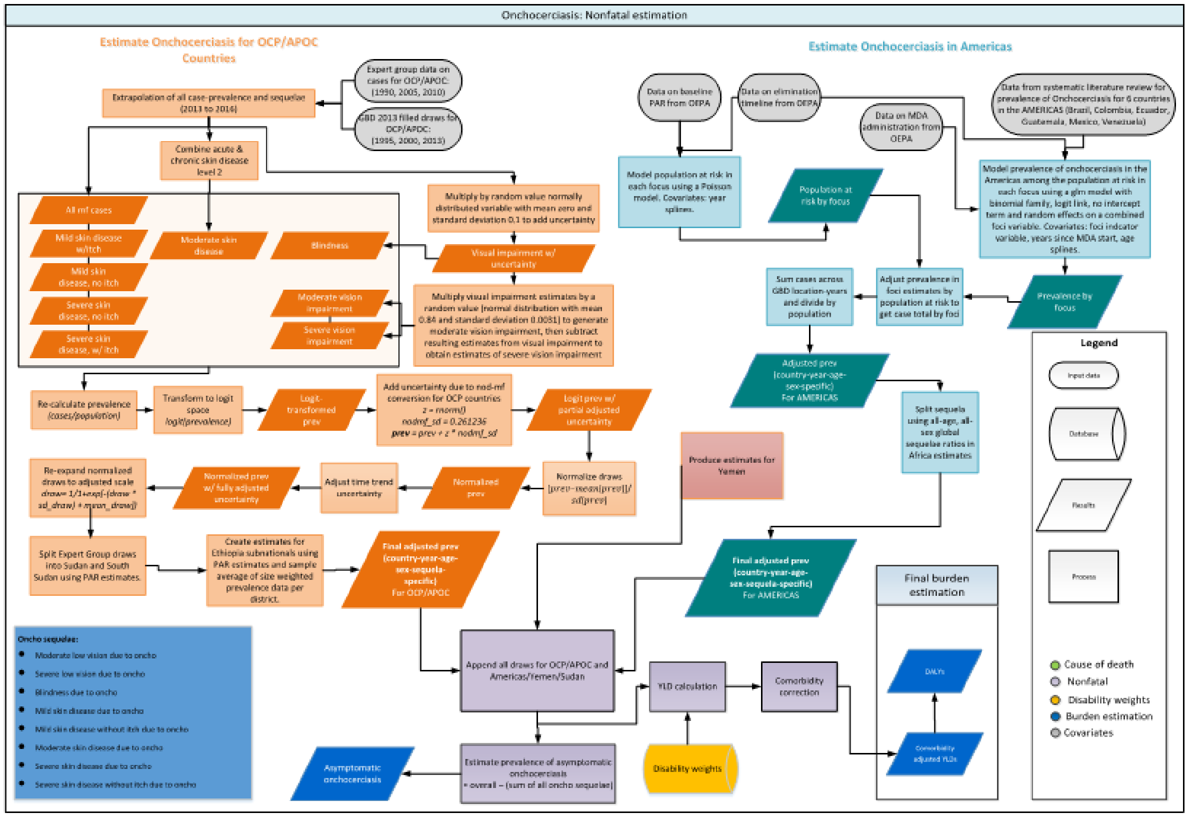

Input data & methodological summary

Case definition

Onchocerciasis, also known as river blindness, is a parasitic disease caused by *Onchocerca volvulus*. It is transmitted via the bite of one of several species of *Simulium* blackflies that have historically bred in fast-moving freshwater rivers and tributaries throughout sub-Saharan Africa, Central America, and South America. Diagnosis can be made by skin snip biopsy to identify larvae, surgical removal of nodules and exam for adult worms, slit lamp exam of anterior part of the eye where larvae or lesions caused by them are visible, and antibody tests (mostly useful to visitors to areas with parasites). The ICD-10 code for onchocerciasis is B73.

Input data

Table 1: Source Counts

Measure	Total sources	Countries with data
All measures	351	32
Prevalence	345	32
Population	6	6

Model inputs

Prevalence data prepared by the GBD 2010 expert group (EG) was used for modelling the nonfatal outcomes resulting from onchocerciasis in Africa. This included 1,000 draws of infection and morbidity (visual impairment, blindness, and skin conditions) cases with confidence intervals categorised by country, age, and sex for years 1990, 1995, 2000, 2005, and 2010. Details about the materials and methods used by the EG to generate these draws can be found elsewhere [1-5]. These data represented all African countries included in the African Programme for Onchocerciasis Control (APOC) and the Onchocerciasis Control Programme (OCP) for which initial Rapid Epidemiological Mapping of Onchocerciasis (REMO) assessments demonstrated a need for Community-Directed Treatment with Ivermectin (CDTI) (defined as having a prevalence of skin nodules greater than 20%). Four countries – Rwanda, Mozambique, Kenya, and Gabon – were designated as hypo-endemic countries after initial REMO assessments and not included due to sparsity of cases and paucity of data. Estimates for Sudan from GBD 2010 were reassigned to South Sudan in GBD 2013 after its independence in 2011 since REMO assessments indicated that the vast majority of cases occurred in that area of the former Sudan. The tables below show the countries included in each program and the number of corresponding GBD locations they represent.

	**APOC Countries**	**OCP Countries**
Countries included	Angola, Burundi, Cameroon, Central African Republic, Chad, Congo, Democratic Republic of Congo, Ethiopia, Equatorial Guinea, Liberia, Malawi, Nigeria, South Sudan, Tanzania, and Uganda	Benin, Burkina Faso, Côte d'Ivoire, Ghana, Guinea Bissau, Guinea, Mali, Niger, Senegal, Sierra Leone, and Togo
Hypo-endemic countries not included	Rwanda, Mozambique, Kenya, Gabon, Sudan	
GBD countries & subnationals provided by EG	15	11
GBD world regions	3	1

Prevalence data for modelling non-fatal outcomes resulting from onchocerciasis in the Americas was extracted via a systematic literature review. Web of Science, Scopus, and PubMed were searched with the following search strings:

**Database**	**Search string**	**Yield**
PubMed	(oncho*[Title/Abstract] OR "river blindness"[Title/Abstract] OR "O. volvulus"[Title/Abstract] OR "robles disease"[Title/Abstract] OR "blinding filariasis"[Title/Abstract] OR "coast erysipelas"[Title/Abstract] OR “sowda” [Title/Abstract] OR “nodding syndrome”[Title/Abstract]) AND (“1980”[Date – Publication] : “2016”[Date – Publication]) AND (epidemiology[Title/Abstract] OR prevalence[Title/Abstract] OR incidence[Title/Abstract] OR surveillance[Title/Abstract] OR”MDA”[Title/Abstract] OR “Mass Drug Administration”[Title/Abstract] OR “Community-directed treatment with ivermectin”[Title/Abstract] OR “CDTI”[Title/Abstract] OR “mass treatment”[Title/Abstract] OR “multiple ivermectin treatments”[Title/Abstract] OR “monthly doses of	986

	ivermectin"[Title/Abstract] OR "large scale treatment"[Title/Abstract] OR REMO[Title/Abstract] OR "Rapid epidemiological mapping of onchocerciasis"[Title/Abstract] OR APOC[Title/Abstract] OR "African Programme for Onchocerciasis Control"[Title/Abstract] OR OCP[Title/Abstract] OR "Onchocerciasis Control Programme"[Title/Abstract]) NOT(Animals[MeSH] NOT Humans[MeSH])	
Web of Science	TS=(oncho* OR "river blindness" OR "O. volvulus" OR "robles disease" OR "blinding filariasis" OR "coast erysipelas" OR sowda OR "nodding syndrome") AND TS=(epidemiology OR prevalence OR incidence OR surveillance OR MDA OR "Mass Drug Administration" OR "Community-directed treatment with ivermectin" OR CDTI OR "mass treatment" OR "multiple ivermectin treatments" OR "monthly doses of ivermectin" OR "large scale treatment" OR REMO OR "Rapid epidemiological mapping of onchocerciasis" OR APOC OR "African Programme for Onchocerciasis Control" OR OCP OR "Onchocerciasis Control Programme") NOT TS=((Animals NOT Humans))	1,144
SCOPUS	(TITLE-ABS-KEY(oncho* OR "river blindness" OR "O. volvulus" OR "robles disease" OR "blinding filariasis" OR "coast erysipelas")) AND TITLE-ABS-KEY(epidemiology OR prevalence OR incidence OR surveillance OR MDA OR "Mass Drug Administration" OR "Community-directed treatment with ivermectin" OR CDTI OR "mass treatment" OR "multiple ivermectin treatments" OR "monthly doses of ivermectin" OR "large scale treatment" OR REMO OR "Rapid epidemiological mapping of onchocerciasis" OR APOC OR "African Programme for Onchocerciasis Control" OR OCP OR "Onchocerciasis Control Programme") AND NOT KEY(Animals NOT Humans) AND PUBYEAR > 1979	2,000

This yielded 4,130 results in total, which was reduced to 2,502 after removing duplicates. The title and abstracts were screened for inclusion or exclusion with the following criteria:

Exclusion criteria:

- Pre-1980
- Non-original source
- Non-representative population
    - Vulnerable populations (eg, slum-dwellers, prisoners, orphans, high-risk jobs, etc.)
    - Hospital-based samples (including saved stool samples)
    - Non-native peoples (eg, migrants, expats, nomads, etc.)
    - Immunosuppression/illness (eg, HIV, TB, CA, RA, asthma, malaria, handicap, etc.)
- Non-human population
- Does not meet case definition
- Case-control study

Sixty-one articles were identified for full text screening and extraction from the historically endemic American countries: Guatemala, Brazil, Ecuador, Venezuela, Mexico, and Colombia.

Severity splits/sequelae

The table below shows the list of common clinical manifestations of onchocerciasis and the sequelae to which they have been mapped along with the lay description and the associated disability weight (DW) of each sequela.

**Clinical manifestation**	**Sequela name**	**Lay description**	**DW**
Uveitis; Punctate keratitis; Optic neuritis; Torpid Iritis; Onchochorioretinitis	Moderate vision impairment	"has vision problems that make it difficult to recognize faces or objects across a room"	0.031 (0.019–0.049)
Sclerosing keratitis; Optic neuropathy; Optic atrophy; Choroidoretinopathy; Cataracts	Severe vision impairment	"has severe vision loss, which causes difficulty in daily activities, some emotional impact (for example worry), and some difficulty going outside the home without assistance"	0.184 (0.125–0.258)
Blindness	Blindness	"is completely blind, which causes great difficulty in some daily activities, worry and anxiety, and great difficulty going outside the home without assistance"	0.187 (0.124–0.260)
Acute papular onchodermatitis; Onchocercomata (subcutaneous nodules)	Mild skin disease	"has a slight, visible physical deformity that is sometimes sore or itchy. Others notice the deformity, which causes some worry and discomfort"	0.027 (0.015–0.042)
Chronic papular onchodermatitis; Lichenified onchodermatitis ("sowda"); Lymphadenopathy	Mild skin disease without itch	"has a slight, visible physical deformity that others notice, which causes some worry and discomfort"	0.011 (0.005–0.021)
Skin atrophy; Depigmentation ("leopard skin")	Moderate skin disease	"has a visible physical deformity that is sore and itchy. Other people stare and comment, which causes the person to worry. The person has trouble sleeping and concentrating"	0.188 (0.124–0.267)
Hanging groin; Lymphoedema	Severe skin disease without itch	"has an obvious physical deformity that makes others uncomfortable, which causes the person to avoid social contact, feel worried, sleep poorly, and think about suicide"	0.405 (0.275–0.546)
	Asymptomatic onchocerciasis	NA	NA

Modelling strategy

The nonfatal modelling for onchocerciasis included six major steps. In the first step, GBD 2010 prevalence was exponentially extrapolated to obtain GBD 2019 estimates. Acute skin disease level 2 and

chronic skin disease level 2 were summed to create the moderate skin disease sequela. Uncertainty was quantified and provided by the EG for all estimates except those of visual impairment and blindness. In these cases, for each of the OCP draws the number of cases were multiplied by a random value (the exponent of a normally distributed variable with mean zero and standard deviation 0.1) in order to add uncertainty. Within each draw, the same randomly drawn value was applied to all country-year-age-sex estimates. Visual impairment was then split into moderate and severe vision impairment by first multiplying the visual impairment estimates by a random value (from a normal distribution with mean 0.84 and standard deviation 0.0031) to generate moderate vision impairment, and then subtracting the resulting estimates from visual impairment to obtain estimates of severe vision impairment. Prevalence of sequelae was calculated by dividing the cases by the population.

The second step in modelling morbidity due to onchocerciasis was the adjustment of uncertainty in the conversion of nodule prevalence to microfilaria (mf) prevalence and in the effects of mass drug administration (MDA). To adjust for uncertainty in translation of nodule prevalence to mf prevalence, the final OCP draws from the first step were logit transformed and uncertainty was added from a random value drawn from a normal distribution to the transformed estimates. The resulting estimates were then normalised and scaled using estimates published elsewhere [1]. To adjust for uncertainty due to MDA, the year when MDA with ivermectin started was set according to the table below.

**Country**	**MDA start year**
Angola, Burundi, South Sudan	2005
Congo, Ethiopia, DRC	2001
Cameroon, Central African Republic, Equatorial Guinea, Liberia, Nigeria, Uganda	1999
Chad, Niger, Tanzania	1998
Malawi	1997
All others	1990

The uncertainty in the time trend was then multiplied by the normalised prevalence estimates and the final prevalence was obtained by re-expanding the scaled normalised draws and adjusting the scale back from logit scale.

Third, since EG draws were provided before the independence of South Sudan in 2011, Sudan estimates from the EG were partitioned between Sudan and South Sudan. Population at risk (PAR) estimates pre- and post-Abu Hamed foci elimination in 2015 in Sudan were used to proportionally split cases between the two countries [2]. REMO maps showing definite needs for community-directed treatment with ivermectin (CTDI) were digitised and overlaid with population per pixel rasters to produce estimates of PAR pre-Abu Hamed elimination. Post-Abu Hamed elimination in 2015, REMO maps were edited to remove the foci as a definite CDTI areas and estimates were reproduced.

In the fourth step, prevalence in the Ethiopia subnationals was estimated separately and appended to the Africa model. Subnational draws were split proportionally based on sample size weighted prevalence from prevalence data, using population at risk estimates derived from digitising a map of onchocerciasis endemic districts in 2015 from Meribo and colleagues to convert into case space [3]. A proportion of cases falling into each subnational was then used to split national case numbers provided by EG draws into each subnational.

In the fifth step, prevalence of onchocerciasis in Yemen was modelled separately and combined with the Africa model. Due to limited data, this was done utilising one data point from the Ministry of Health published in 1991 only accounting for population change [22]. Furthermore, the global age-sex trend was imposed to produce age-sex-specific estimates. The clinical manifestation of Yemeni onchocerciasis is different from other regions, notably the atypical and most severe cutaneous manifestation known as sowda [23]. Therefore, all cases of onchocerciasis are being mapped to mild skin disease due to onchocerciasis without itch.

In the sixth step, prevalence of onchocerciasis in the Americas was modelled separately and combined with the Africa and Yemen models. For the GBD estimation period, onchocerciasis is known to have occurred in six countries of Central and Southern America: Mexico, Guatemala, Colombia, Ecuador, Brazil and Venezuela. The epidemiology of onchocerciasis is very different in these countries than in Africa because it has only occurred in relatively small, well defined foci. These foci have been mapped and thoroughly monitored since the early 1990s with the formation of the Onchocerciasis Elimination Program of the Americas (OEPA) and all of the prevalence surveys conducted are only representative of these areas. Additionally, certain foci are geographically continuous across national boundaries. Therefore, we modelled onchocerciasis in these countries at the focus level among the population at risk in each focus instead of at the national level.

Population at risk for each focus was modelled using data from OEPA on baseline population at risk [6] and data from OEPA and peer-reviewed studies on dates of elimination in each focus [6-19]. This was done with a Poisson model using year splines as a covariate, and 1,000 draws of the population at risk were drawn from the predicted mean and standard error. The prevalence of disease among the population at risk was subsequently modelled using a generalised linear model with a binomial family, logit link, no intercept term, and random effects on a combined-foci variable created by grouping foci by geographic contiguity and nearness when data were sparse. Covariates included an indicator term on the foci, the number of years since MDA began, and splines on age. One thousand draws of prevalence were calculated from 1,000 draws of beta values from the variance-covariance matrix and adjusted by the estimated population at risk in each focus-year to determine the number of cases. The cases were then summed by GBD geography and year and divided by national population to find the national prevalence. While the model predicted case values very close to zero in the countries where elimination has occurred, these were overwritten to zero values for all years after certified elimination. The ratio of global all-age, all-sex prevalence of each sequela to the all-cases prevalence from the Africa estimates was applied to all-cases prevalence from the Americas to calculate prevalence of each sequelae.

Lastly, to estimate the prevalence of asymptomatic onchocerciasis, the prevalence of morbidity (vision loss, blindness and skin conditions) was subtracted from the overall onchocerciasis prevalence. Moderate vision impairment, severe vision impairment, and blindness estimates were each multiplied by a factor of 8/33 before subtraction to account for cases that have concurring symptoms.

Changes from GBD 2017 to GBD 2019

We have made no substantive changes in the modeling strategy from GBD 2017.

References

1. Zouré HG, Noma M, Tekle AH, Amazigo UV, Diggle PJ, Giorgi E, Remme JH. The geographic distribution of onchocerciasis in the 20 participating countries of the African Programme for Onchocerciasis Control: (2) pre-control endemicity levels and estimated number infected. Parasites & Vectors. 2014. 7-326.
2. Zarroung IM, Hashim K, ElMubark WA, Shumo ZA, Salih KA, ElNojomi NA, Awad HA, Aziz N, Katabarwa M, Hassan HK, Unnasch TR, Machenzie CD, Richards F, Higazi TB. The First Confirmed Elimination of an Onchocerciasis Focus in Africa: Abu Hamed, Sudan. The American Journal of Tropical Medicine and Hygeine. 2016. 95(5):1037-1040.
3. Meribo K, Kebede B, Mekasha Feleke S, Mengistu B, Mulugeta A, Sileshi M, Samuel A, Deribe K, Tadesse Z. Review of Ethiopian Onchocerciasis Elimination Program. Ethiopian Medical Journa. 2017. 55(Suppl 1): 55-63.
4. Coffeng L, Stolk W, Hoerauf A, Habbema D, Bakker R, Hopkins A, de Vlas S. Elimination of African onchocerciasis: modeling the impact of increasing the frequency of ivermectin mass treatment. PLoS One. 2014. 9(12):e115886.
5. Coffeng LE, Stolk WA, Zouré HG, Veerman JL, Agblewonu KB, Murdoch ME, Noma M, Fobi G, Richardus JH, Bundy DA, Habbema D, de Vlas SJ, Amazigo UV. African Programme For Onchocerciasis Control 1995-2015: model-estimated health impact and cost. PLoS Negl Trop Dis. 2013; 7(1): e2032.
6. Murdoch ME, Asuzu MC, Hagan M, Makunde WH, Ngoumou P, Ogbuagu KF, Okello D, Ozoh G, Remme J. Onchocerciasis: the clinical and epidemiological burden of skin disease in Africa. Ann Trop Med Parasitol. 2002; 96(3): 283-296.
7. Brieger WR, Awedoba AK, Eneanya CI, Hagan M, Ogbuagu KF, Okello DO, Ososanya OO, Ovuga EB, Noma M, Kale OO, Burnham GM, Remme JH. The effects of ivermectin on onchocercal skin disease and severe itching: results of a multicentre trial. Trop Med Int Health. 1998; 3(12): 951-61.
8. México. http://www.oepa.net/Mexico.htm (accessed July 7, 2017).
9. Guatemala. http://www.oepa.net/guatemala.html (accessed July 7, 2017).
10. Venezuela. http://www.oepa.net/venezuela.html (accessed July 7, 2017).
11. Colombia. http://www.oepa.net/colombia.html (accessed July 7, 2017).
12. Ecuador. http://www.oepa.net/ecuador.html (accessed July 7, 2017).
13. Rodríguez-Pérez MA, Unnasch TR, Domínguez-Vázquez A, *et al.* Lack of Active Onchocerca volvulus Transmission in the Northern Chiapas Focus of Mexico. *The American Journal of Tropical Medicine and Hygiene* 2010; **83**: 15–20.
14. Rodríguez-Pérez MA, Domínguez-Vázquez A, Unnasch TR, *et al.* Interruption of Transmission of Onchocerca volvulus in the Southern Chiapas Focus, México. *PLOS Neglected Tropical Diseases* 2013; **7**: e2133.
15. Rodríguez-Pérez MA, Unnasch TR, Domínguez-Vázquez A, *et al.* Interruption of Transmission of Onchocerca volvulus in the Oaxaca Focus, Mexico. *The American Journal of Tropical Medicine and Hygiene* 2010; **83**: 21–7.
16. Cruz-Ortiz N, Gonzalez RJ, Lindblade KA, *et al.* Elimination of Onchocerca volvulus Transmission in the Huehuetenango Focus of Guatemala. Journal of Parasitology Research. 2012. https://www.hindawi.com/journals/jpr/2012/638429/abs/ (accessed July 7, 2017).
17. Jr FR, Rizzo N, Espinoza CED, *et al.* One Hundred Years After Its Discovery in Guatemala by Rodolfo Robles, Onchocerca volvulus Transmission Has Been Eliminated from the Central Endemic Zone. *The American Journal of Tropical Medicine and Hygiene* 2015; **93**: 1295–304.

18. Gonzalez RJ, Cruz-Ortiz N, Rizzo N, *et al.* Successful interruption of transmission of Onchocerca volvulus in the Escuintla-Guatemala focus, Guatemala. *PLoS Negl Trop Dis* 2009; **3**: e404.
19. Lindblade KA, Arana B, Zea-Flores G, *et al.* Elimination of Onchocercia volvulus transmission in the Santa Rosa focus of Guatemala. *Am J Trop Med Hyg* 2007; **77**: 334–41.
20. Convit J, Schuler H, Borges R, *et al.* Interruption of Onchocerca volvulus transmission in Northern Venezuela. *Parasites & Vectors* 2013; **6**: 289.
21. WHO | WHO declares Ecuador free of onchocerciasis (river blindness). WHO. http://www.who.int/neglected_diseases/ecuador_free_from_onchocerciasis/en/ (accessed July 7, 2017).
22. Onchocerciasis and its control: report of a WHO Expert Committee on Onchocerciasis Control. WHO. http://apps.who.int/iris/bitstream/handle/10665/37346/WHO_TRS_852.pdf;jsessionid=023018C4198968F3E918A2EA8334432C?sequence=1
23. Al-Kubati A, Mackenzie CD, Boakye D, Al-Qubati Y, Al-Samie A, Awad IE, Thylefors B, Hopkins A. Onchocerciasis in Yemen: moving forward towards an elimination program. *International Health*. March 2018; 10(1): i89–i96.

Dengue

Flowchart

Dengue – GBD 2019

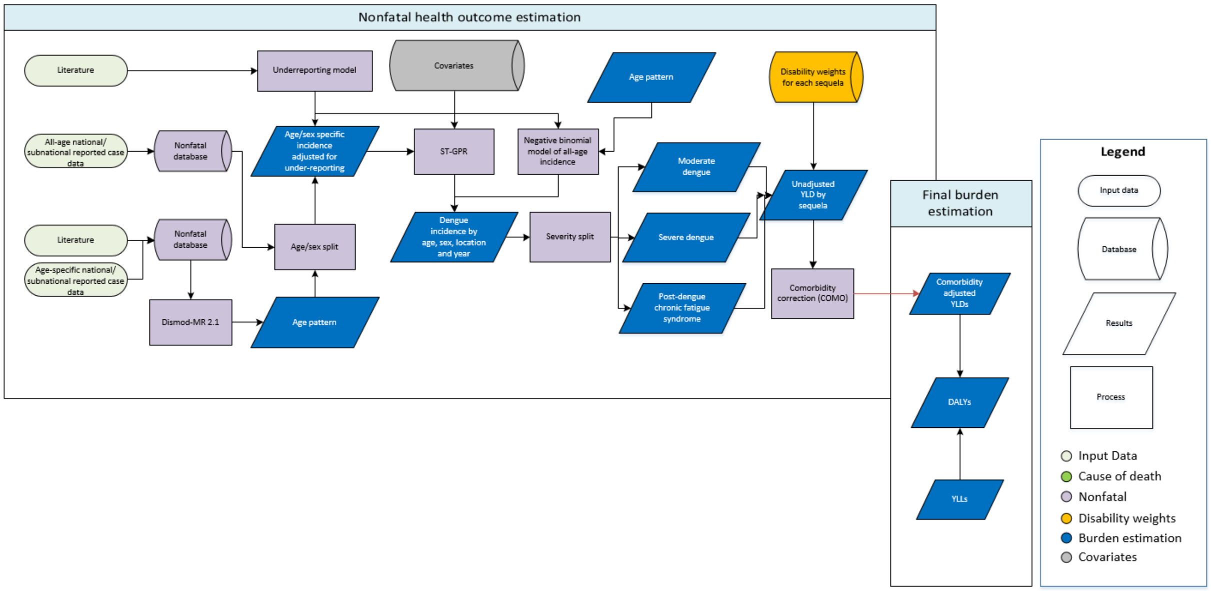

Case definition

Dengue is mosquito-borne viral infection that causes febrile illness and, in severe cases, jaundice, haemorrhage, and death. It includes all ICD-10 codes under the heading A90 (Dengue fever [classical dengue]) and A91 (Dengue haemorrhagic fever).

Input data

Model inputs

For GBD 2019, we modelled dengue incidence based on reported cases. In GBD 2019, data-seeking updates targeted specific geographies (India, Indonesia, Pakistan, Brazil and China) for subnational case details, along with years updates for years 2016 – 2018. Age specific data were collated separately to enable disaggregation of all-age and both-sex case data into age and sex-specific inputs prior to modeling. A systematic literature review was conducted to identify studies that compared incidence of dengue among passive and active case detection systems to estimate a correction factor to adjust for under-reporting. Scientific literature sources were used for assumptions related to severity.

Table 1 presents the total number of data sources used in the non-fatal estimation.

Table 1. Total data source counts

Measure	Total sources
All measures	1980
Incidence	1964
Duration	2
Proportion	1
Continuous	17

Modelling strategy

To model incidence of clinical dengue disease, we first adjusted all-age, all-sex national case notification data. First, all-sex national case notification data were sex split according to the ratio of males : females derived using MR-BRT. The sex ratio estimate was derived from 1,492 matched comparisons, with males having a higher incidence (Table 2).

Table 2. Ratio of males: females estimated using MR-BRT

Data input	Reference or alternative case definition	Gamma	Beta Coefficient, Log (variance)	Adjustment factor
Intercept	Females (ref)	0.14	0.0121 (0.0018)	1.012195

We then used a total of 3,945 age-specific data inputs to derive an age pattern disease using Dismod. All-age data were then split into five-year age groups using super-region age patterns, visualized in Figure 1.

Figure 1. Super-region age patterns used for splitting all-age case notification data.

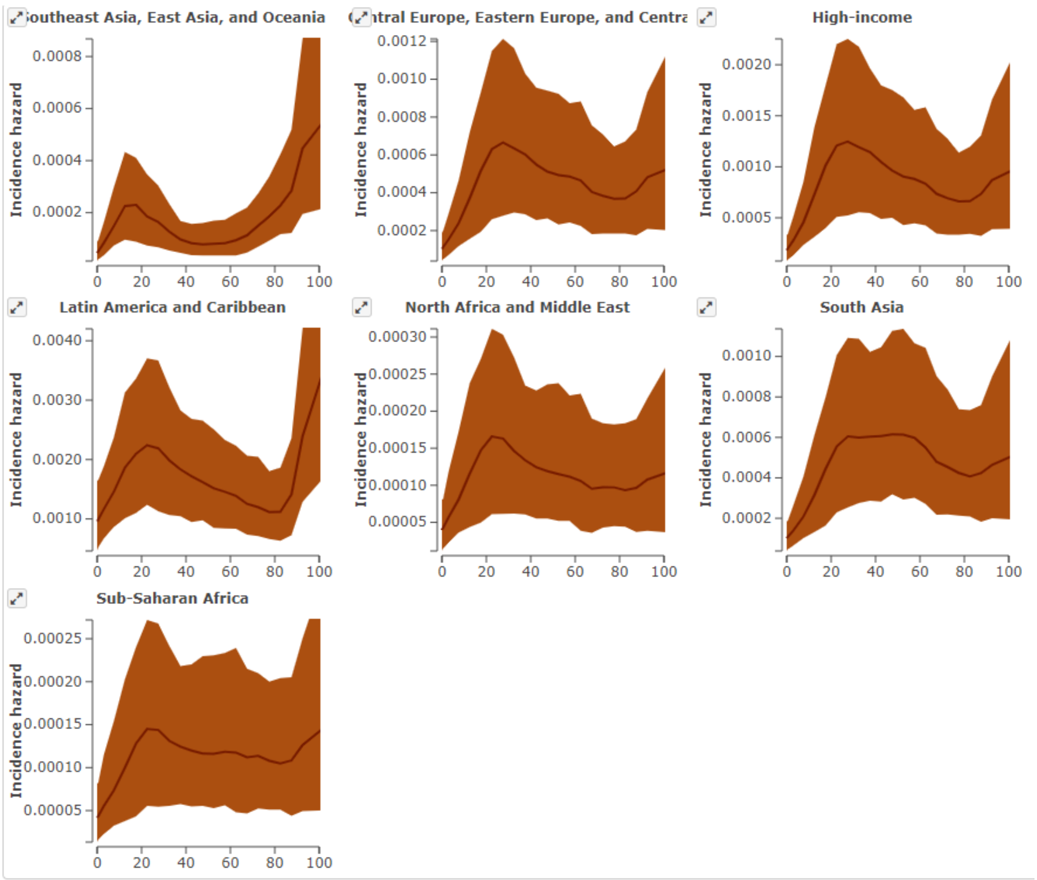

Correction for under-reporting

Since dengue disease is often under-reported due to health system capacity or misdiagnosed as other febrile illnesses, we conducted a systematic literature review to identify sources that compared incidence rates reported via active versus passive surveillance.

We searched PubMed for dengue underreporting with the following search terms (without date restrictions) on 24 May 2019:

> (("active"[Title/Abstract] AND "passive"[Title/Abstract]) OR "case detection"[Text Word] OR "under reporting"[Text Word] OR "coverage"[Text Word]) AND dengue[MeSH Terms]

Figure 2. PRISMA Chart for systematic review for under-reporting of dengue

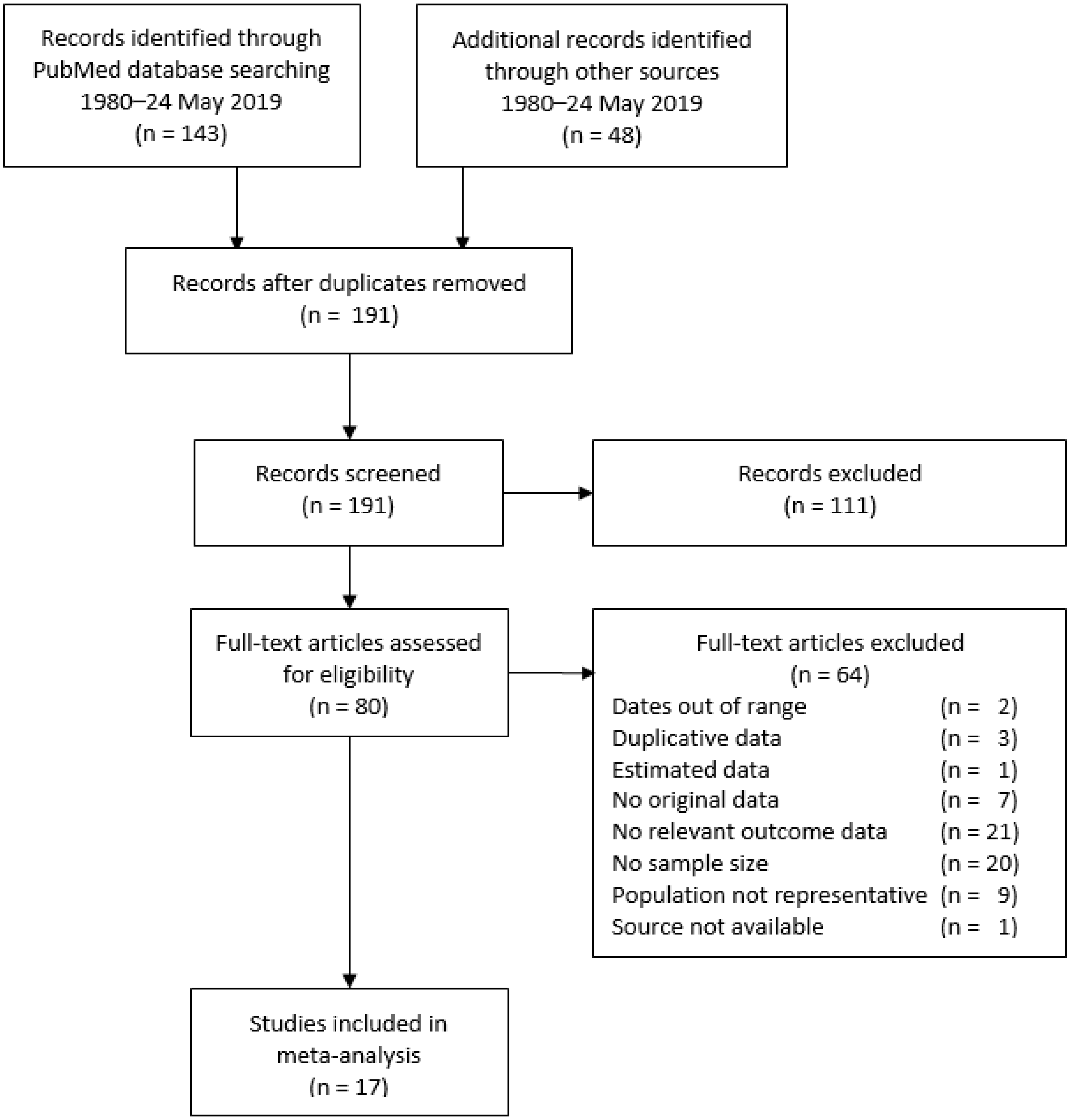

The search returned 143 results (see Figure 2), published between 1982 and 2019. We added 4 sources previously extracted, and 46 more discovered by other means (generally from reference lists of meta-analyses or other sources with composite results). In screening titles and abstracts, we excluded 111 sources. The remaining 80 were subject to full-text screening for extraction. Of these, 64 were excluded as not meeting extraction criteria; 17 sources were extracted. We identified a total of 34 comparisons to generate an adjustment factor to correct for under-reporting. The under-reporting adjustment factors

were estimated using MR-BRT and included SDI and reported incidence rate, trimming 10% of the input data. The uncertainty from the MR-BRT meta-regression was applied to the age and sex-specific adjustment. Table 3 presents the correction factors for under-reporting.

Table 3: MR-BRT Crosswalk Adjustment Factors for under-reporting due to dengue

Data input	Gamma	Beta Coefficient*, Log (95% CI)	Adjustment factor
Intercept	0.798	-3.1 (-5.5, -0.76))	23.1
HAQI (>53)		.66 (-1.5, 2.8)	11.9
Incidence per capita			
0.001 – 0.002		0.94 (-1.4, 3.3)	9.0
0.002 – 0.003		-0.03 (-0.7, 3.9)	24.1
0.003 – 0.004		1.62 (-.72, 3.9)	4.5
>0.004		0.74 (-1.5, 3.1)	10.9

**Coefficients reflect passive v. active (e.g. negative coefficient on the intercept illustrates how passive surveillance under-reports relative to active case detection).*

Once the data were adjusted for under-reporting, a hybrid approach was used to generate incidence estimates using two models: (1) a space-time Gaussian process regression (ST/GPR) and a (2) negative binomial regression using fixed effects to model all-incidence. These two models were hybridized (500 draws from each approach were combined to generate 1,000 draws of incidence).

ST-GPR

The ST/GPR model for incidence included the settings listed in Table 4. The covariates used were the population-weighted probability of dengue infection, GBD-location level cause-specific mortality rate (csmr), population density and HAQI. ST/GPR was used to model incidence, excluding inputs for which zero cases were reported (under the assumption that in dengue-endemic settings zero reported cases would be implausible).

Table 4. ST/GPR Model settings

**Parameter**	**Value**
Lambda	0.5
Omega	1
Zeta	.01
Scale	1
Amplitude	1

Initial model testing showed that inclusion of data from the 2009 Cabo Verde dengue outbreak resulted in implausibly high values for West African locations, largely due to the limited number of data inputs for this modeling region (34 total inputs). The model was run again excluding Cabo Verde data to estimate incidence for West Africa. Estimates of dengue disease incidence were generated for 1990, 1995, 2000, 2005, 2010, 2015, 2017 and 2019.

Negative binomial regression

A negative binomial regression was implemented with the csmr and population-weighted probability of dengue transmission as predictors to model total incidence of dengue disease. Input data were adjusted for under-reporting using the MR-BRT method described above. The fixed effects from this model were used to generate estimates of all-age, both sex incidence which were then disaggregated by age and sex using an overall age pattern derived from the same age-specific data inputs used to develop regional age patterns in Dismod. This age pattern was modeled using a negative binomial regression with cubic spline variables for age group.

Severity splits and disability weights

The resulting incidence estimates were then split into moderate (94.5%) and severe (5.5%) sequelae, based on the proportion of reported cases that were severe. Prevalence of moderate dengue was calculated assuming a duration of 6 days and prevalence of severe dengue estimated using an assumption of duration of 14 days. We assume that 8.4% of symptomatic infections will produce post-acute chronic fatigue lasting an average of six months (Teixeira L de AS, Lopes JSM, Martins AG da C, Campos FAB, Miranzi S de SC, Nascentes GAN. Persistence of dengue symptoms in patients in Uberaba, Minas Gerais State, Brazil. *Cad Saúde Pública* 2010; **26**: 624–30.). Disability weights are presented in Table 5.

Table 5. Severity distribution.

Severity level	Lay description	DW (95% CI)
Moderate	Has a fever and aches, and feels weak, which causes some difficulty with daily activities.	0.051 (0.032, 0.074)
Severe	Has a high fever and pain, and feels very weak, which causes great difficulty with daily activities.	0.133 (0.088-0.19)
Post-dengue chronic fatigue syndrome	Is always tired and easily upset. The person feels pain all over the body and is depressed.	0.219 (0.148-0.308)

Yellow Fever

Flowchart

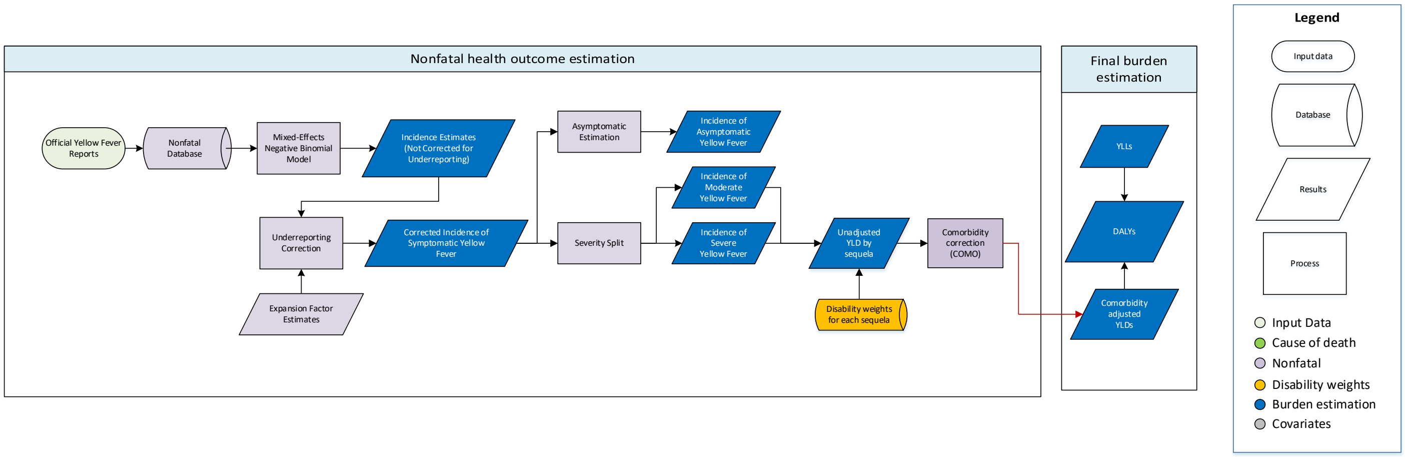

Case definition

Yellow fever is mosquito-borne viral infection that causes febrile illness and, in severe cases, jaundice, haemorrhage, and death. It is considered a neglected tropical disease (NTD). It includes all ICD-10 codes under the heading A95 (yellow fever).

Input data

Model inputs

Case data for the yellow fever estimate process comes from official case reports filed with the World Health Organization. Table 1 presents the total sources used in the analysis.

Table 1. Total data source counts

Measure	Total sources	Countries with data
All measures	2762	195
Incidence	2761	195
Cause-specific mortality rate	4	3
Case fatality rate	6	4
Proportion	4	4

Severity splits

Yellow fever is split into three levels of severity: moderate (33% [13–52]), severe (12% [5–26]), and asymptomatic (55% [37–74]). Table 2 below illustrates this breakdown.

Table 2. Sequela, description, and disability weight (DW)

Sequela	Description	Disability weight (DW)
Moderate	Has a fever and aches, and feels weak, which causes some difficulty with daily activities.	0.051 (0.032–0.074)
Severe	Has a high fever and pain, and feels very weak, which causes great difficulty with daily activities.	0.133 (0.088–0.19)
Asymptomatic	Infection with no apparent illness.	NA

Modelling strategy

We modelled reported cases of yellow fever using a mixed-effects negative binomial model, with fixed effects for year (centered on 2004) and socio-demographic index and random effects for super-region, region, and country. We use GBD population estimates for the location level as the offset. We assume that yellow fever cases are underreported, and that this underreporting mirrors that for dengue (a disease for which we have better data on underreporting). With that, we estimate symptomatic cases as the product of our base case estimates and dengue expansion factors (ie, the factor by which you must multiply reported cases to derive true cases). Expansion factors are applied to the all-age modeled incidence prior to splitting incidence by age and sex. Data that are age and sex-specific are used to generate an age and sex-specific incidence pattern via a negative binomial regression with fixed effects for sex and age group (with cubic splines). Based on published estimates from Johansson and colleagues (2014), we assume that 27% of symptomatic cases will be severe.

Changes from GBD 2017

We have made no substantive changes in the modelling strategy for endemic countries from GBD 2017 to GBD 2019.

Rabies

Flowchart

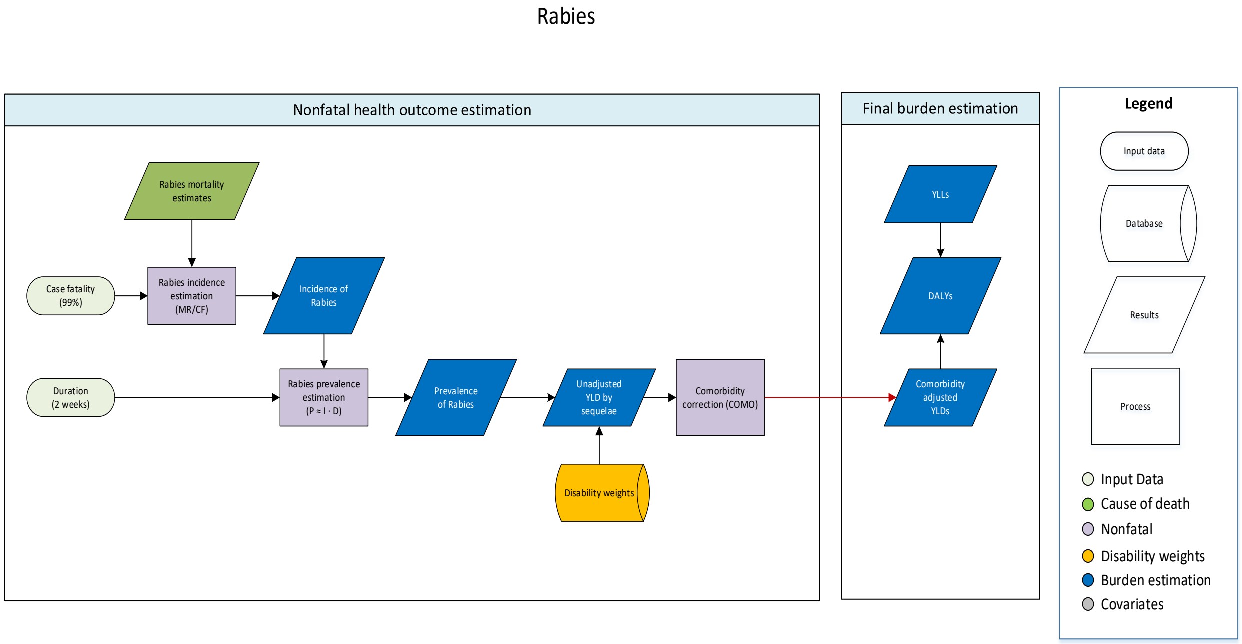

Input data and methodological summary

Case definition

Rabies is a fatal viral infection transmitted by animal bites. Without prophylactic vaccination the disease is almost universally fatal. The disease has a long incubation period (1-3 months), and early intervention with prophylactic vaccination is nearly 100% effective in preventing symptomatic disease. It is considered a neglected tropical disease (NTD). We model symptomatic infections, not including those infections in which intervention prevented the onset of symptomatic disease, corresponding to the ICD10 code A82.

Input data

Model inputs

As we derive our estimate of cases from our estimate of deaths, no incidence data are used in the model. For GBD 2019, we modelled rabies mortality using all available data in the cause of death database. Data points were outliered if they reported an improbable number of rabies deaths (eg, zero rabies deaths in a hyperendemic country) or if their inclusion in the model yielded distorted trends. In some cases, multiple data sources for the same location differed dramatically both in their quality and reported rabies mortality (eg, a verbal autopsy and vital registration source). In these cases, the lower-quality data source was outliered.

Modelling strategy

We derive estimates of the number of symptomatic rabies infections (ie, those not averted through prophylactic vaccination) based on rabies mortality estimates, assuming 99% case fatality. All cases are assumed to be severe.

We modelled rabies mortality using a two-model hybrid approach 1) a global CODEm model of all locations, using all data in the CoD database; and 2) a CODEm model restricted to data-rich countries.

Sequela description and DW

There is only one sequela and associated disability weight for rabies, which is severe. The lay description is included in the table below.

Table 1. Sequela, description, and DW

Sequela	Description	Disability Weight (95% CI)
Severe	Has a high fever and pain, and feels very weak, which causes great difficulty with daily activities.	0.133 (0.088–0.19)

Changes from GBD 2017 to GBD 2019

We have made no substantive changes in the modeling strategy from GBD 2017.

Ascariasis

Flowchart

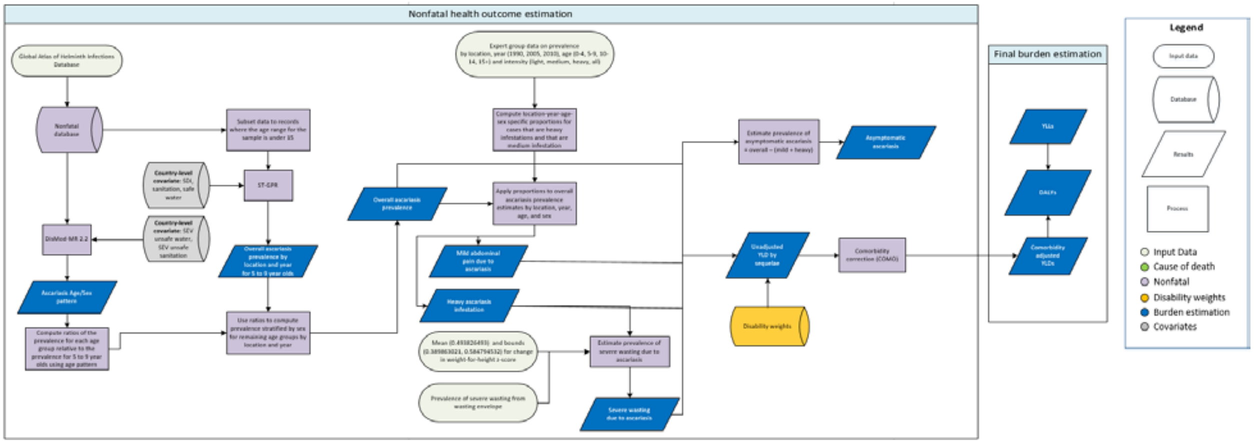

Input data and methodological summary

Case definition

Ascariasis is a helminthic disease caused by the parasitic roundworm *Ascaris lumbricoides*. It is one of the three intestinal nematode infections (INI), or soil-transmitted helminthiasis (STH), that are modelled in GBD. Diagnosis is made by examination of stool by microscope or PCR, with or without concentration procedures. The ICD-10 codes for ascariasis are B77-B77.9.

Input data

Table 1: Source Counts

Measure	Total sources	Countries with data
All measures	166	140
Prevalence	165	83
Proportion	1	134

Global Atlas of Helminth Infections Data

Input data for this model were primarily compiled from the Global Atlas of Helminth Infections (GAHI) database and the Expanded Special Project for the Elimination of Neglected Tropical Diseases (ESPEN). The GAHI and ESPEN databases include surveys and studies conducted to measure the prevalence of STH [1]. Each record in the database contained metadata (ie, location, year, age range, sex) of each study sample and the prevalence of ascariasis in that sample. We excluded data points where the age range of the sample was unknown and retained only those surveys where the Kato-Katz diagnostic was used.

We supplemented the GAHI and ESPEN data with survey-data collected in a literature review performed by Children Without Worms, including countries outside of Sub-Saharan Africa. Additionally, a 2001-2004 China sub-national survey was incorporated to better inform our China estimates.

Geographic restrictions

We conducted a literature review to determine the geographic extent of the disease and classify locations based on whether the disease is absent or present in each year. Locations that were geographically restricted in any given year did not have estimates made for them. Of note, we did not attempt a complete systematic review, since a single high-quality source could offer sufficient evidence of presence. Evidence of absence or presence was not available for every location for each year, and so assumptions were made for missing years by taking into consideration the epidemiological characteristics of the disease.

If evidence indicated disease presence for two non-consecutive years, we assumed presence for all years between the two. If evidence indicated disease absence for two non-consecutive years, we assumed absence for all years between the two. If evidence indicated a change in status (ie, from absent to present, or present to absent) between two non-consecutive years, than we conducted targeted searches to ascertain the relevant year of introduction or elimination for that location. In the cases where presence or absence information was missing for the start or end years of our study interval (1990–2019) without evidence of any introduction or elimination events within the interval, we applied the status of the first and last presence/absence observations, respectively, to all years between the interval bound and the observation year. Our search was done in conjunction with the title/abstract screening portion of a systematic literature review for prevalence data. The search strings and yield can be viewed in the table below for each of the databases queried.

Table 2. Geographic restriction search strings

Database	Search String	Yield
PubMed	(Ascariasis[Title/Abstract] OR Ascaris[Title/Abstract] OR "A. lumbricoides"[Title/Abstract] OR Ascaris[MeSH] OR Trichuris[Title/Abstract] OR Trichuriasis[Title/Abstract] OR "Whip Worm"[Title/Abstract] OR "T. trichura"[Title/Abstract] OR Trichuris[MeSH] OR Hookworm[Title/Abstract] OR "A. duodenale"[Title/Abstract] OR "Ancylostoma duodenale"[Title/Abstract] OR ancylostomiasis[Title/Abstract] OR "N. americanus"[Title/Abstract] OR "Necator americanus"[Title/Abstract] OR necatoriasis[Title/Abstract] OR Ancylostoma [MeSH] OR Necator[MeSH]) AND (prevalence[Title/Abstract] OR incidence[Title/Abstract] OR epidemiology[Title/Abstract] OR surveillance[Title/Abstract]) NOT(Animals[MeSH] NOT Humans[MeSH])	2,376
Web of Science	(Ascariasis OR Ascaris OR A. lumbricoides OR Trichuris OR Trichuriasis OR Whip Worm OR T. trichura OR Hookworm OR A. duodenale OR Ancylostoma duodenale OR anclyostomiasis OR N. americanus OR Necator americanus OR necatoriasis) AND TOPIC:(prevalence OR incidence OR epidemiology OR surveillance) NOTTOPIC: ((Animals NOT Humans))<br>Timespan: 1980-2016. Indexes: SCI-EXPANDED, SSCI, A&HCI, ESCI.	2,266
SCOPUS	TITLE-ABS_KEY (ascariasis OR ascaris OR a. lumbricoides OR trichuris OR trichuriasis OR whip worm OR t. trichura OR hookworm OR a. duodenale OR	29

	ancylostoma duodenale OR anclyostomiasis OR n. americanus OR necator americanus OR necatoriasis) AND PUBYEAR>1979	

These papers were used to classify location-years for all locations and years present in the literature. We only utilised papers that are explicitly concerned with ascariasis. Additionally, systematic literature reviews, meta-analyses, national health statistics publications, and collaborator input were used to classify location-years not present in the literature review wherever possible.

Health states/sequelae

The table below shows the list of sequelae due to ascariasis and the associated disability weights (DW). Prevalence of medium infection and heavy infection were mapped to *mild abdominopelvic problems* and *heavy infestation of ascariasiss,* respectively. Light infection or asymptomatic was not attributed any disability. To inform the wasting model, 1,000 draws of severe wasting prevalence among children under 5 years were ascertained from GBD 2019 estimates – the methods used to generate estimates of wasting prevalence are detailed elsewhere (part of risk factors documentation) [2].

Table 3. Sequelae, lay descriptions, and disability weights (DWs)

Sequela	Lay description	DW
Mild abdominopelvic problems	"has some pain in the belly that causes nausea but does not interfere with daily activities"	0.011 (0.005–0.021)
Heavy infestation	"has cramping pain and a bloated feeling in the belly"	0.027 (0.015–0.043)
Severe wasting	"is extremely skinny and has no energy"	0.128 (0.082–0.183)
Asymptomatic ascariasis	N/A	N/A

Modelling strategy

DisMod-MR 2.1

In the estimation of overall morbidity due to ascariasis, we implemented a three-stage modelling framework. The first stage of the modelling process was using DisMod-MR 2.1 to generate a global age-sex curve to disaggregate all-age, both-sex prevalence data. DisModis an integrated meta-regression framework that allows for multiple datasets to be used within a singular analysis regardless of age-binning, sources, and geographies. As a result, a variety of differently aggregated information can be evaluated to generate a consensus output. Our final model contained all processed GAHI data as input and was informed by two country-level covariates (ie, all risk factors SEV for unsafe water, and all risk factors SEV for unsafe sanitation). From this model, the global fits were used.

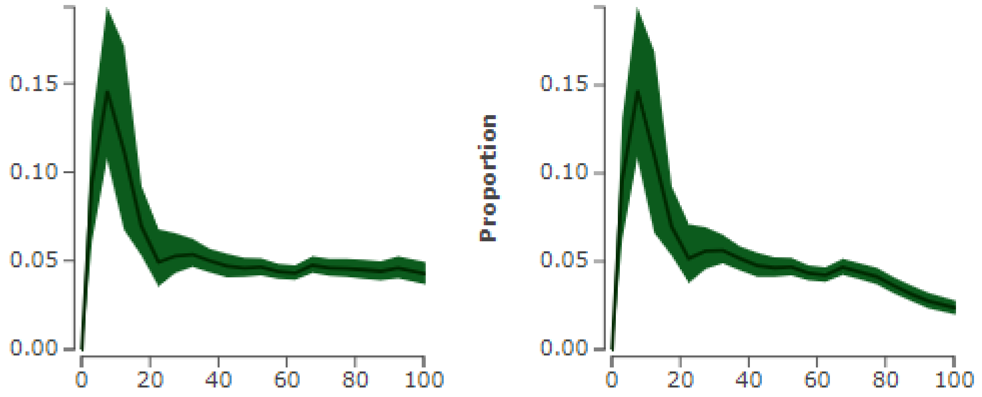

Figure 1: Global age-specific proportion estimates for males (left) and females (right) for the year 2019. Proportion (prevalence) is on the Y-axis, and age in years on the X-axis. Screenshot from EpiViz tool.

Figure 1 shows the age-specific variation in the proportion of prevalence, differentiated by sex. When considered as a global aggregate, we see that reported male and female prevalence are very similar. We use the age-specific proportions to adjust the output of the ST/GPR to predict prevalence in adults ages 15 and older.

ST-GPR

After obtaining a global age-sex pattern from DisMod, we utilise a spatiotemporal Gaussian process regression (ST-GPR) to generate a complete time series of estimates for each location where there are no geographic restrictions. ST-GPR attempts to model non-linear trends utilising a Gaussian process to fit a trend. The following model specifications were used:

$$Prevalence = Proportion\ Safe\ Water + Sociodemographic\ Index + Proportion\ Improved\ Sanitation + (1|level\ 2) + (1|level\ 3)$$

Where Levels 2 and 3 refer to GBD location hierarchies, or random effects for region and location. Notably, the covariates for the model were Sociodemographic Index, proportion of improved sanitation, and safe water or proportion of population with access to improved water sources. Improved water sources are defined by the Joint Monitoring Program. The following hyperparameters were used: st-lambda = 0.25, st-omega =2, st-zeta = 0.01, gpr-scale = 15. We selected these hyperparameters as they provided more weight to country-level data rather than region-level data when estimating the prevalence for a given location-year. In other words, these hyperparameters ensure that the Gaussian process regressions follow country-specific data rather than region-specific data when estimating a time series for a location.

It is important to note that we did not use all processed GAHI data for the ST-GPR model. We opted to run a child-only model because the bulk of our data is among adolescents and there is more granular age information that we can leverage during modelling processes. More specifically, any data points that had age bins between 0 and 15 years were assigned to the 5 to 9 age group. We selected all data with age bins between 0 and 15 because they fall within the peak in prevalence across all age groups; this is where a majority of data are, and this provides sufficient statistical power for our model.

Table 4. ST-GPR model covariates

Covariate	Beta Coefficient, Log	Standard Error	Exponentiated beta (95% CI)
Improved Water	-2.642	1.044	0.071 (0.009 – 0.551)
Improved Sanitation	3.332	0.735	27.994 (6.629 – 118.226)
Socio-demographic Index	-8.131	1.739	$2.94 * 10^{-4}$ ($9.738 * 10^{-6}$ – $8.892 * 10^{-3}$)

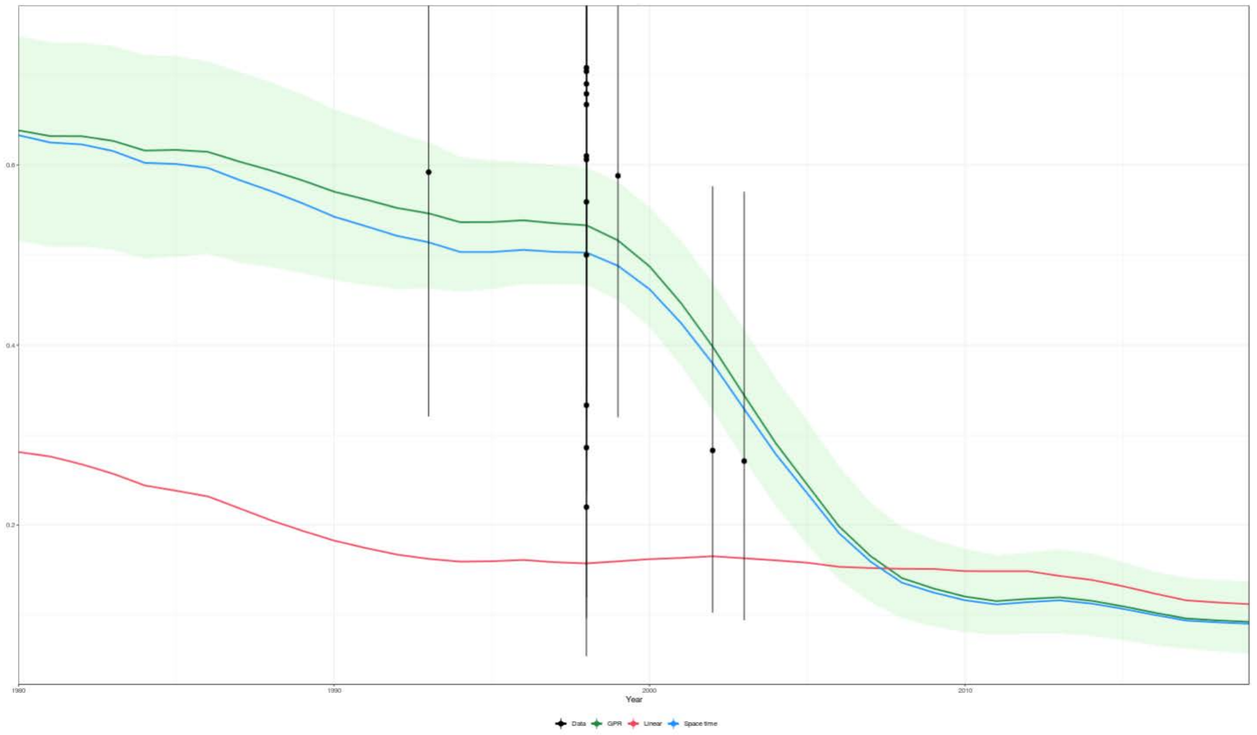

Figure 2: ST-GPR estimates for Cameroon (0- to 15-year-olds, both sex) for years 1990–2019. Black dots represent input data points, with the black lines indicating variance. The green line represents the mean GPR estimated values, with uncertainty shown by the green polygon. The blue line indicates the space-time component of the ST-GPR; the red line indicates the linear regression component derived from global data. Transparent black dots represent data from other locations in the GBD region (Western sub-Saharan Africa).

Figure 2 displays the time trends as computed by ST-GPR. For the most part, locations looked similar to Cameroon, where we see consistent declines in prevalence throughout time.

Imputations

The final stage of the overall prevalence modelling process is to impute the remaining age groups by borrowing information from the ST-GPR time series for 5- to 9-year-olds and the DisMod global age-sex pattern. First, we assign each age group a ratio of how much larger or smaller the prevalence is compared to the prevalence for 5- to 9-year-olds using the DisMod global age-sex pattern. More specifically, the following is the computation for each age group:

$$Ratio = \frac{prevalence_{[age\ start] to\ [age\ end]}}{prevalence_{5\ to\ 9}}$$

We opted not to use the age-sex curves by location or region, because DisMod performed better at disaggregating our heterogeneous data at the global level. With a ratio for every age group by sex, we multiplied the ratio by the ST-GPR location-year estimates to impute estimates for the remaining age groups.

Health states/sequelae

Following computations of location-year-age-sex-specific prevalence of ascariasis, we leverage information from the 2010 EG data to conduct sequelae splits. The 2010 EG data provided estimates for heavy infestation, mild abdominopelvic problems, and asymptomatic ascariasis by location and for 1990, 2005, and 2010. These three values add up to all cases of ascariasis. Thus, for heavy infestation and mild abdominopelvic problems, we computed the proportion of cases that belong to our sequelae of interest over all cases of ascariasis. More specifically, the following is the computation by heavy infestation and mild abdominopelvic problems:

$$Proportion_{sequelae} = \frac{prevalence_{sequelae}}{prevalence_{all\ cases}}$$

This calculation was done for every location, year, and age group available. Because the EG data only had four age groups (0-4, 5-9, 10-14, 15+ years), we applied the 15+ age group proportion for all remaining age groups. In addition, for 1995 and 2000 we applied the 1990 proportions, and for 2017 and 2019 we applied the 2010 proportions. Using these location-year-age-specific proportions, we multiplied the total ascariasis estimates to compute heavy infestation and mild abdominopelvic prevalence. To estimate the prevalence of asymptomatic ascariasis, prevalence of mild and heavy infestation was subtracted from the overall ascariasis prevalence.

The final step in the modelling process was to estimate the prevalence of severe wasting due to ascariasis in age groups 28–364 days and 1–4 years. This was done separately using 1,000 draws of prevalence of heavy infestation due to ascariasis and the wasting envelope prevalence. The initial step in determining prevalence of severe wasting due to ascariasis was generating 1,000 draws of change in weight-for-height z-score per heavy prevalent case from a random normal distribution with mean = 0.493826493 and standard deviation = 0.04972834 (calculated from upper and lower bounds of the mean estimate). The mean, upper, and lower bounds were based on a published article [2]. The prevalence of severe wasting due to ascariasis was then obtained as a function of change in weight-for-height z-score. The following are the computations:

$$Prevalence_{wasting\ due\ to\ ascariasis} = wasting - \Phi(\Phi^{-1}(\text{wasting}) - \text{z score} * \text{heavy infestation})$$

Where Φ is the standard normal cumulative distribution function and Φ^{-1} is the inverse standard normal cumulative distribution function.

Changes from GBD 2017

We have made no substantive changes in the modeling strategy from GBD 2017.

Limitations

As we attempt to improve the modelling processes for ascariasis, we recognise that there are several limitations. We only include studies where Kato-Katz was used to identify infected individuals. Future updates to the model will include a systematic review for within-study comparisons of diagnostic performance to facilitate a crosswalk model.

A secondary limitation to our data is that several included studies are not considered to be nationally representative, and therefore at a location level, the data are highly heterogeneous (Figure 3). Numerous studies within the database were conducted in districts or townships, and in some cases the studies were done in known areas where prevalence is high.

Furthermore, we made a large assumption that the global age-sex distributions were applicable to all locations. While we believe that prevalence should peak among adolescents and slowly decline afterward, there is likely variation across regions and locations. Given that our data are either among children or all-age, it is very difficult to build an age trend at granular location levels. Thus, we allowed DisMod to disaggregate our heterogeneous data in an effort to provide sensible age-sex curves.

We believe that more work needs to be done to improve our sequelae split methods. Since the EG data do not provide all estimation years and age groups, several assumptions had to be made. Thus, we will explore conducting literature searches to provide novel data points for sequelae estimations.

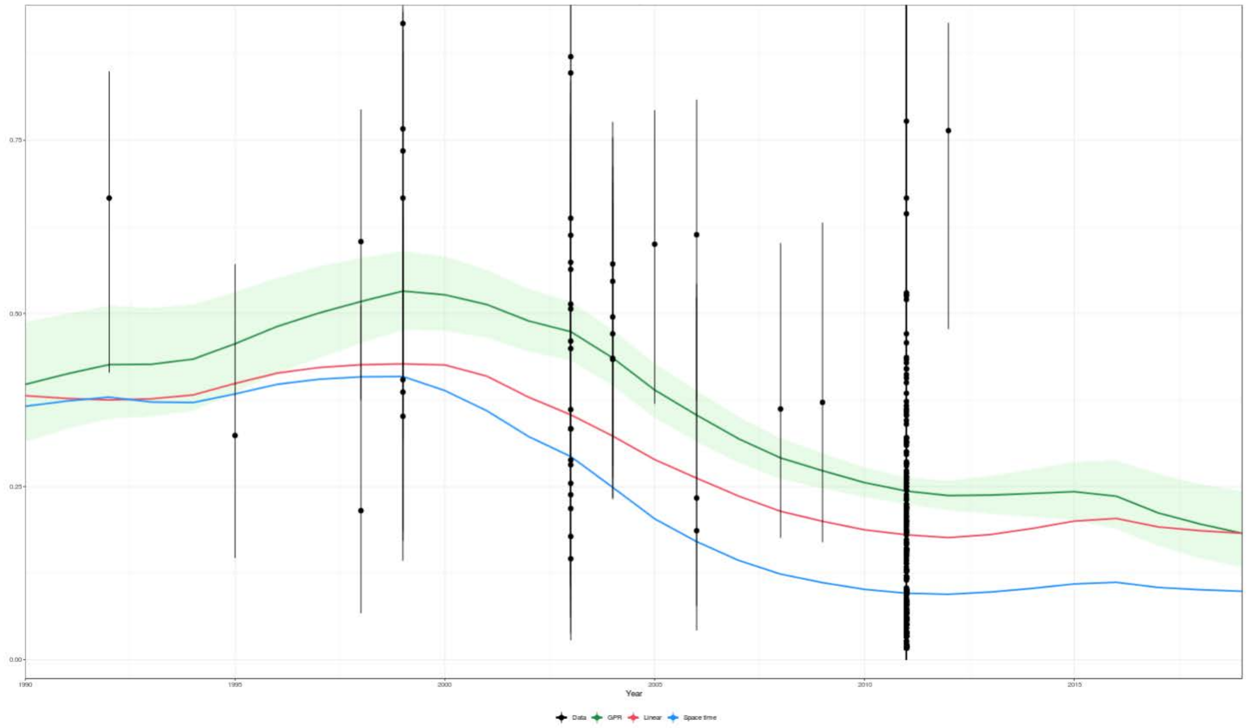

Figure 3: ST-GPR estimates for Nigeria (0 to 15 year olds, both sex) for years 1990–2019. Coloration and symbols are as stated in caption for Figure 2.

Figure 3 shows the time trend for Nigeria as computed by ST-GPR. For some locations, we estimate this fluctuating time trend which is a function of the heterogeneity in our input data.

References

1. London School of Hygiene and Tropical Medicine. Global Atlas of Helminth Infections – Soil Transmitted Helminths. London, United Kingdom: London School of Hygiene and Tropical Medicine.
2. Hall A, Hewitt G, Tuffrey V, de Silva N. A review and meta-analysis of the impact of intestinal worms on child growth and nutrition. *Maternal and Child Nutrition*. 2008. 4. 118-236.

Trichuriasis

Flowchart

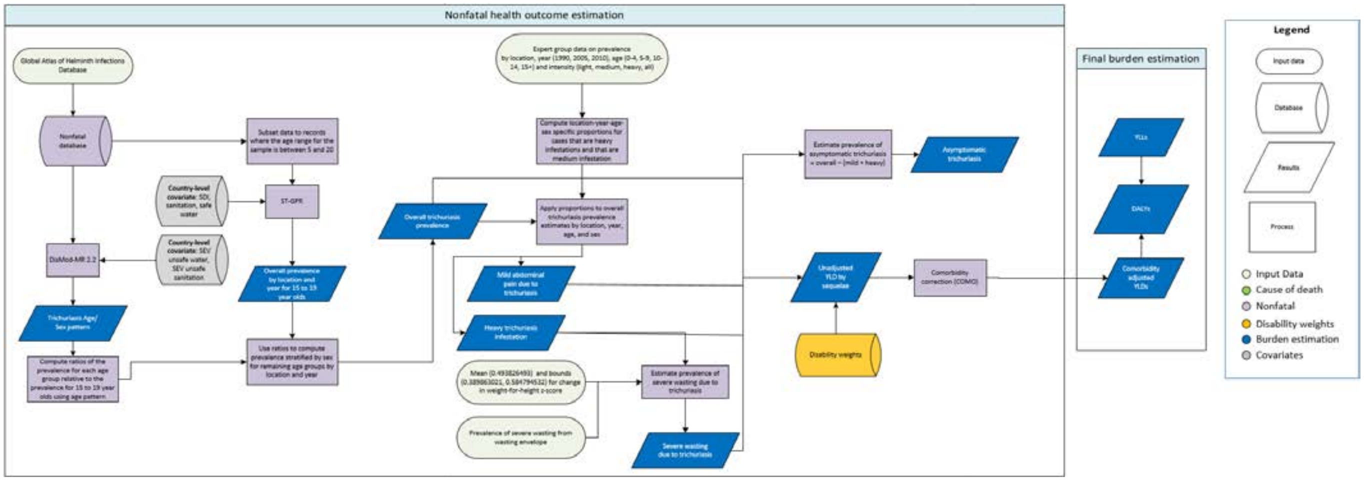

Input data and methodological summary

Case definition

Trichuriasis is a helminth diseases caused by the parasitic whipworm *Trichuris trichiura*. It is one of the three intestinal nematode infections (INI), or soil-transmitted helminthiasis (STH), that we model in GBD. Diagnosis is made by examination of stool by microscope or PCR, with or without concentration procedures. The ICD-10 code for trichuriasis is B79.

Input data

Table 1: Source Counts

Measure	Total sources	Countries with data
All measures	156	140
Prevalence	155	82
Proportion	1	134

Global Atlas of Helminth Infections Data

Input data for this model were primarily compiled from the Global Atlas of Helminth Infections (GAHI) database and the Expanded Special Project for the Elimination of Neglected Tropical Diseases (ESPEN). The GAHI and ESPEN databases include surveys and studies conducted to measure the prevalence of STH [1]. Each record in the database contained metadata (ie, location, year, age range, sex) of each study sample and the prevalence of trichuriasis in that sample. We excluded data points where the age range of the sample was unknown and retained only those surveys where the Kato-Katz diagnostic was used.

We supplemented the GAHI data with survey-data collected in a literature review performed by Children Without Worms, including countries outside of Sub-Saharan Africa. Additionally, a 2001-2004 China sub-national survey was incorporated to better inform our China estimates.

Geographic restrictions

We conducted a literature review to determine the geographic extent of the disease and classify locations based on whether the disease is absent or present in each year. Locations that were geographically restricted in any given year did not have estimates made for them. Of note, we did not attempt a complete systematic review, since a single high-quality source could offer sufficient evidence of presence. Evidence of absence or presence was not available for every location for each year, and so assumptions were made for missing years by taking into consideration the epidemiological characteristics of the disease.

If evidence indicated disease presence for two non-consecutive years, we assumed presence for all years between the two. If evidence indicated disease absence for two non-consecutive years, we assumed absence for all years between the two. If evidence indicated a change in status (ie, from absent to present, or present to absent) between two non-consecutive years, then we conducted targeted searches to ascertain the relevant year of introduction or elimination for that location. In the cases where presence or absence information was missing for the start or end years of our study interval (1990–2019) without evidence of any introduction or elimination events within the interval, we applied the status of the first and last presence/absence observations, respectively, to all years between the interval bound and the observation year. Our search was done in conjunction with the title/abstract screening portion of a systematic literature review for prevalence data. The search strings and yield can be viewed in the table below for each of the databases queried.

Table 2. Geographic restriction search strings

Database	Search String	Yield
PubMed	(Ascariasis[Title/Abstract] OR Ascaris[Title/Abstract] OR "A. lumbricoides"[Title/Abstract] OR Ascaris[MeSH] OR Trichuris[Title/Abstract] OR Trichuriasis[Title/Abstract] OR "Whip Worm"[Title/Abstract] OR "T. trichura"[Title/Abstract] OR Trichuris[MeSH] OR Hookworm[Title/Abstract] OR "A. duodenale"[Title/Abstract] OR "Ancylostoma duodenale"[Title/Abstract] OR ancylostomiasis[Title/Abstract] OR "N. americanus"[Title/Abstract] OR "Necator americanus"[Title/Abstract] OR necatoriasis[Title/Abstract] OR Ancylostoma [MeSH] OR Necator[MeSH]) AND (prevalence[Title/Abstract] OR incidence[Title/Abstract] OR epidemiology[Title/Abstract] OR surveillance[Title/Abstract]) NOT(Animals[MeSH] NOT Humans[MeSH])	2,376
Web of Science	(Ascariasis OR Ascaris OR A. lumbricoides OR Trichuris OR Trichuriasis OR Whip Worm OR T. trichura OR Hookworm OR A. duodenale OR Ancylostoma duodenale OR anclyostomiasis OR N. americanus OR Necator americanus OR necatoriasis) AND TOPIC:(prevalence OR incidence OR epidemiology OR surveillance) NOTTOPIC: ((Animals NOT Humans))<br>Timespan: 1980-2016. Indexes: SCI-EXPANDED, SSCI, A&HCI, ESCI.	2,266
SCOPUS	TITLE-ABS_KEY (ascariasis OR ascaris OR a. lumbricoides OR trichuris OR trichuriasis OR whip worm OR t. trichura OR hookworm OR a. duodenale OR ancylostoma duodenale OR anclyostomiasis OR n. americanus OR necator americanus OR necatoriasis) AND PUBYEAR>1979	29

These papers were used to classify location-years for all locations and years present in the literature. We only utilised papers that are explicitly concerned with trichuriasis. Additionally, systematic literature reviews, meta-analyses, national health statistics publications, and collaborator input were used to classify location-years not present in the literature review wherever possible.

Health states/sequelae

The table below shows the list of sequelae due to trichuriasis and the associated disability weights (DW). Prevalence of medium infection and heavy infection were mapped to *mild abdominopelvic problems* and *heavy infestation of trichuriasis,* respectively. Light infection was not attributed any disability. To inform the wasting model, 1,000 draws of severe wasting prevalence among children under 5 years were ascertained from GBD 2019 estimates – the methods used to generate estimates of wasting prevalence are detailed elsewhere (part of risk factors documentation) [2].

Table 3. Sequelae, lay description, and disability weights (DWs)

Sequela	Lay description	DW (95% CI)
Mild abdominopelvic problems	"has some pain in the belly that causes nausea but does not interfere with daily activities"	0.011 (0.005–0.021)
Heavy infestation	"has cramping pain and a bloated feeling in the belly"	0.027 (0.015–0.044)
Severe wasting	"is extremely skinny and has no energy"	0.128 (0.082–0.183)
Asymptomatic trichuriasis	N/A	N/A

Modelling strategy

DisMod-MR 2.1

In the estimation of overall morbidity due to trichuriasis, we implemented a three-stage modelling framework. The first stage of the modelling process was using DisMod-MR 2.1 to generate a global age-sex curve to disaggregate all-age, both-sex prevalence data. DisMod is an integrated meta-regression framework that allows for multiple datasets to be used within a singular analysis regardless of age-binning, sources, and geographies. As a result, a variety of differently aggregated information can be evaluated to generate a consensus output. Our final model contained all processed GAHI data as input and was informed by two country-level covariates (ie, all risk factors SEV for unsafe water, and all risk factors SEV for unsafe sanitation). From this model, the global fits were used.

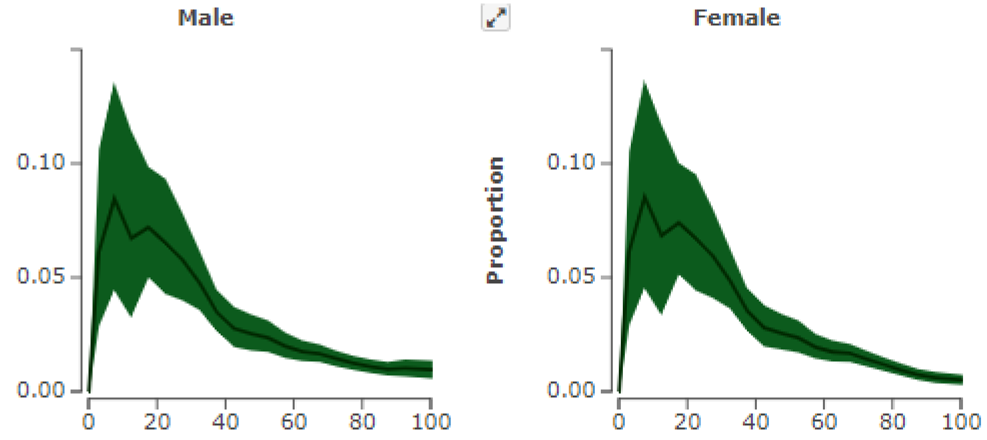

Figure 1: Global age-specific prevalence estimates for males (left) and females (right) for the year 2019. Proportion (prevalence) is on the Y-axis, and age in years on the X-axis. Screenshot from EpiViz tool.

Figure 1 shows the age-specific variation in prevalence rates, differentiated by sex. When considered as a global aggregate, we see that reported male and female prevalence are very similar. This is mostly a function of data used for modelling mainly being reported for both sexes. The highest prevalence rates are among young adults and then decline among adults.

ST-GPR

After obtaining a global age-sex pattern from DisMod, we utilise a spatiotemporal Gaussian process regression (ST-GPR) to generate a complete time series of estimates for each location where there are no geographic restrictions. ST-GPR attempts to model non-linear trends utilising a Gaussian process to fit a trend. The following model specifications were used:

$$Prevalence = Proportion\ Sanitation + Proportion\ Safe\ Water + Proportion\ STH\ MDA + (1 | level\ 2) + (1 | level\ 3)$$

Where Levels 2 and 3 refer to GBD location hierarchies, or random effects for region and location. Notably, the covariates for the model were sanitation or proportion of population with access to improved toilet types, proportion of MDA (mass-drug administration) coverage, and safe water or proportion of population with access to improved water sources. Improved toilet types and improved water sources are defined by the Joint Monitoring Programme. The following hyperparameters were used: st-lambda = 0.25, st-omega =2, st-zeta = 0.01, gpr-scale = 15. We selected these hyperparameters as they provided more weight to country-level data rather than region-level data when estimating the prevalence for a given location-year. In other words, these hyperparameters ensure that the Gaussian process regressions follow country-specific data rather than region-specific data when estimating a time series for a location.

It is important to note that we only model prevalence among ages 5 – 19 years using the ST-GPR model. We opted to run an adolescent-only model because the bulk of our data are among children and there is more granular age information that we can leverage during modelling processes. More specifically, any data points that had age bins between 5 and 20 years were assigned to the 15 to 19 age group. We selected all data with age bins between 5 and 20 because it falls right below the peak in prevalence

across all age groups, this is where a majority of data are, and it provides sufficient statistical power for our model.

Table 4. ST-GPR model covariates

Covariate	Beta Coefficient, Log	Standard Error	Exponentiated beta (95% CI)
Improved Water	-0.158	**0.594**	0.854 (0.183 – 4.00)
WHO STH MDA Coverage	-0.0006	0.001	1.00 (1.00 – 1.00)
Sanitation	**-0.826**	0.594	0.438 (0.127 – 1.402)

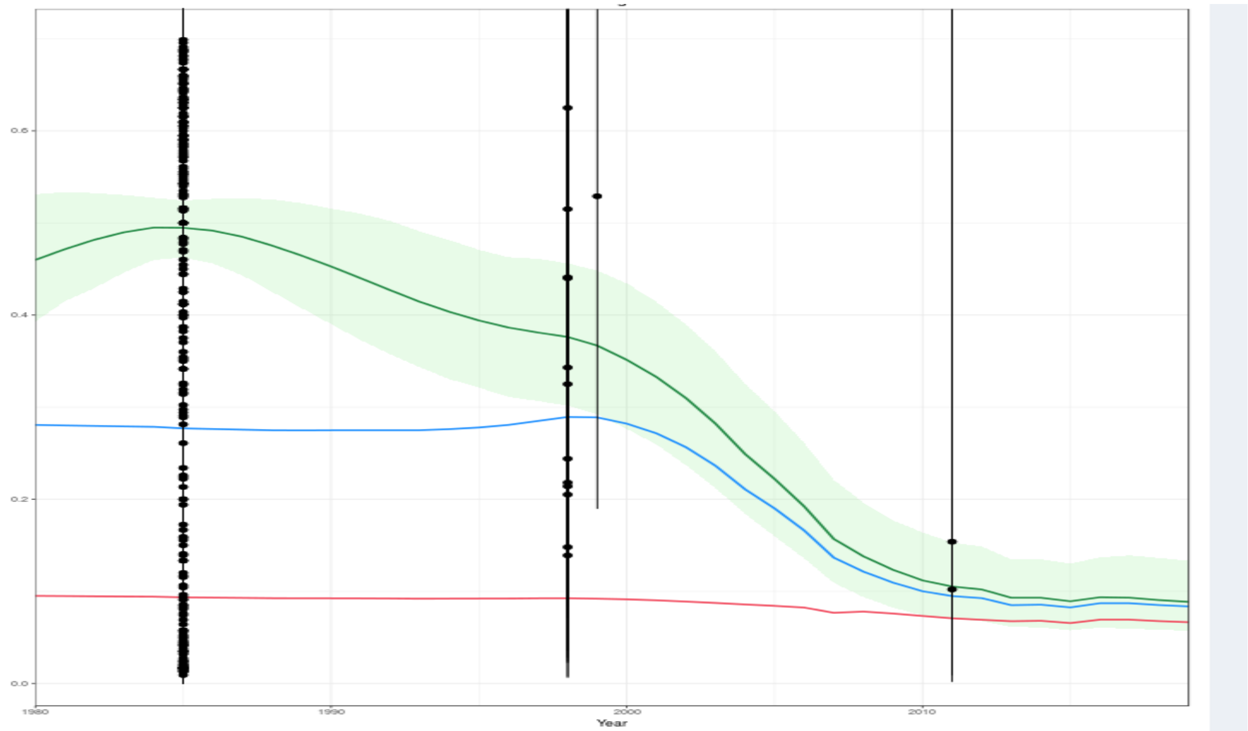

Figure 2: ST-GPR estimates for Cameroon (5- to 20-year-olds, both sexes) for years 1990–2019. Black dots represent input data points, with the black lines indicating variance. The green line represents the mean GPR estimated values, with uncertainty shown by the green polygon. The blue line indicates the space-time component of the ST-GPR; the red line indicates the linear regression component derived from global data. Transparent black dots represent data from other locations in the GBD region (Western sub-Saharan Africa).

Figure 2 displays the time trends as computed by ST-GPR. For the most part, locations looked similar to Cameroon, where we see consistent declines in prevalence throughout time.

Imputation

The final stage of the overall prevalence modelling process is to impute the remaining age groups by borrowing information from the ST-GPR time series for 15- to 19-year-olds and the DisMod global age-sex pattern. First, we assign each age group a ratio of how much larger or smaller the prevalence is compared to the prevalence for 15- to 19-year-olds using the DisMod global age-sex pattern. More specifically, the following is the computation for each age group:

$$Ratio = \frac{prevalence_{[age\ start] to\ [age\ end]}}{prevalence_{15\ to\ 19}}$$

We opted not to use the age-sex curves by location or region, because DisMod performed better at disaggregating our heterogeneous data at the global level. With a ratio for every age group by sex, we multiplied the ratio by the ST-GPR location-year estimates to impute estimates for the remaining age groups.

Health states/sequelae

Following computations of location-year-age-sex-specific prevalence of trichuriasis, we leverage information from the 2010 EG data to conduct sequelae splits. The 2010 EG data provided estimates for heavy infestation, mild abdominopelvic problems, and asymptomatic trichuriasis by location and for 1990, 2005, and 2010. These three values add up to all cases of trichuriasis. Thus, for heavy infestation and mild abdominopelvic problems, we computed the proportion of cases that belong to our sequelae of interest over all cases of trichuriasis. More specifically, the following is the computation by heavy infestation and mild abdominopelvic problems:

$$Proportion_{sequelae} = \frac{prevalence_{sequelae}}{prevalence_{all\ cases}}$$

This calculation was done for every location, year, and age group available. Because the EG data only had four age groups (0-4, 5-9, 10-14, 15+ years), we applied the 15+ age group proportion for all remaining age groups. In addition, for 1995 and 2000 we applied the 1990 proportions, and for 2017 and 2019 we applied the 2010 proportions. Using these location-year-age-specific proportions, we multiplied the total trichuriasis estimates to compute heavy infestation and mild abdominopelvic prevalence. To estimate the prevalence of asymptomatic trichuriasis, prevalence of mild and heavy infestation was subtracted from the overall trichuriasis prevalence.

The final step in the modelling process was to estimate the prevalence of severe wasting due to trichuriasis in age groups 28–364 days and 1–4 years. This was done separately using 1,000 draws of prevalence of heavy infestation due to trichuriasis and the wasting envelope prevalence. The initial step in determining prevalence of severe wasting due to trichuriasis was generating 1,000 draws of change in weight-for-height z-score per heavy prevalent case from a random normal distribution with mean = 0.493826493 and standard deviation = 0.04972834 (calculated from upper and lower bounds of the mean estimate). The mean, upper, and lower bounds were based on a published article [2]. The prevalence of severe wasting due to trichuriasis was then obtained as a function of change in weight-for-height z-score. The following are the computations:

$$Prevalence_{wasting\ due\ to\ trichuriasis} = wasting - \Phi(\Phi^{-1}(\text{wasting}) - \text{z score} * \text{heavy infestation})$$

Where Φ is the standard normal cumulative distribution function and Φ^{-1} is the inverse standard normal cumulative distribution function.

Changes from GBD 2017

The major change from GBD 2017 was in specifying new covariates for the ST-GPR global prevalence model, specifically in removing socio-demographic index due to collinearity with sanitation and adding the WHO STH MDA covariate.

Limitations

As we attempt to improve the modelling processes for trichuriasis, we recognise that there are several limitations. We only include studies where Kato-Katz was used to identify infected individuals. Future updates to the model will include a systematic review for within-study comparisons of diagnostic performance to facilitate a crosswalk model.

A secondary limitation to our data is that several included studies are not considered to be nationally representative, and therefore at a location level, the data are highly heterogeneous (Figure 3). Numerous studies within the database were conducted in districts or townships, and in some cases the studies were done in known areas where prevalence is high.

Furthermore, we made a large assumption that the global age-sex distributions were applicable to all locations. While we believe that prevalence should peak among young adults and slowly decline afterward, there is likely variation across regions and locations. Given that our data are either among adolescents or all-age, it is very difficult to build an age trend at granular location levels. Thus, we allowed DisMod to disaggregate our heterogeneous data in an effort to provide sensible age-sex curves.

We believe that more work needs to be done to improve our sequelae split methods. Since the EG data do not provide all estimation years and age groups, several assumptions had to be made. Thus, we will explore conducting literature searches to provide novel data points for sequelae estimations.

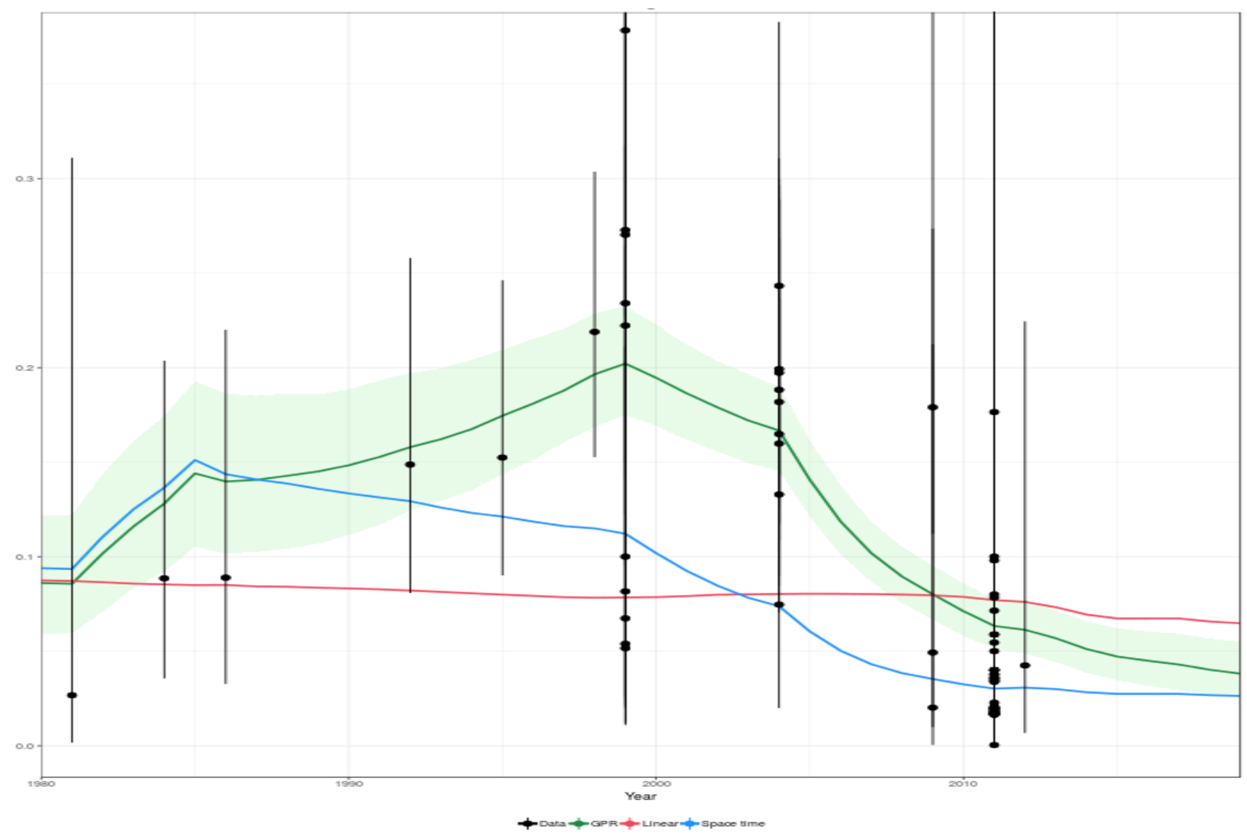

Figure 3: ST-GPR estimates for Nigeria (5- to 20-year-olds, both sexes) for years 1990–2019. Coloration and symbols are as stated in caption for Figure 2.

Figure 3 shows the time trend for Nigeria as computed by ST-GPR. For some locations, we estimate this fluctuating time trend which is a function of the heterogeneity in our input data.

References

1. London School of Hygiene and Tropical Medicine. Global Atlas of Helminth Infections – Soil Transmitted Helminths. London, United Kingdom: London School of Hygiene and Tropical Medicine.
2. Hall A, Hewitt G, Tuffrey V, de Silva N. A review and meta-analysis of the impact of intestinal worms on child growth and nutrition. *Maternal and Child Nutrition*. 2008. 4. 118-236.

Hookworm Disease

Flowchart

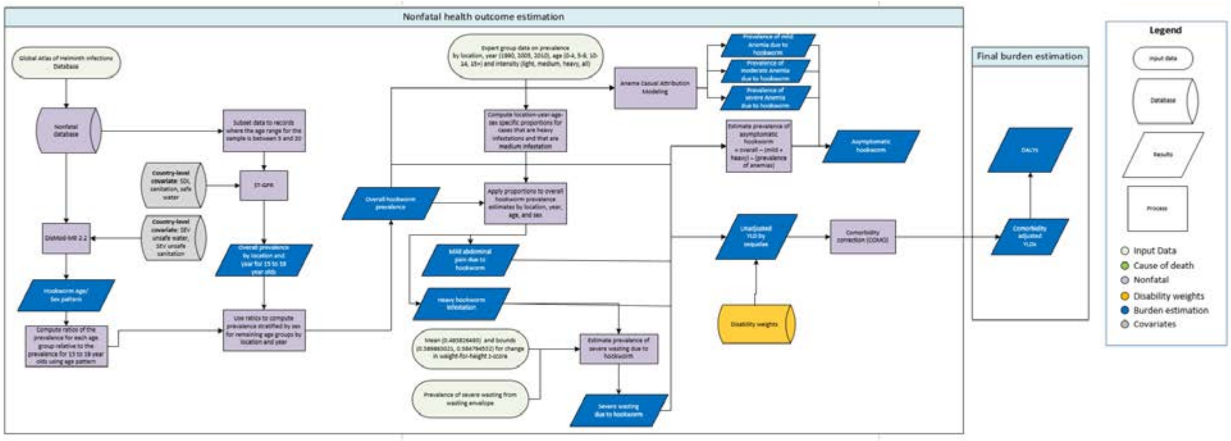

Input data and methodological summary

Case Definition

Hookworm disease is a helminthic disease caused by intestinal parasites in the roundworm group, *Ancylostoma duodenale* and *Necator americanus*. It is one of the three intestinal nematode infections (INI), or soil-transmitted helminthiasis (STH), that we model in GBD. Diagnosis is made by examination of stool by microscope or PCR, with or without concentration procedures. The ICD-10 codes for hookworm disease are B76-B76.9.

Input data

Table 1: Source Counts

Measure	Total sources	Countries with data
All measures	168	140
Prevalence	167	80
Proportion	1	134

Global Atlas of Helminth Infections and ESPEN Data Sources

Input data for this model were primarily compiled from the Global Atlas of Helminth Infections (GAHI) database and the Expanded Special Project for the Elimination of Neglected Tropical Diseases (ESPEN). The GAHI and ESPEN databases include surveys and studies conducted to measure the prevalence of STH [1]. Each record in the database contained metadata (ie, location, year, age range, sex) of each study sample and the prevalence of hookworm in that sample. We excluded data points where the age range of the sample was unknown and retained only those surveys where the Kato-Katz diagnostic was used.

We supplemented the GAHI data with survey-data collected in a literature review performed by Children Without Worms, including countries outside of Sub-Saharan Africa. Additionally, a 2001-2004 China sub-national survey was incorporated to better inform our China estimates

Geographic Restrictions

We conducted a literature review to determine the geographic extent of the disease and classify locations based on whether the disease is absent or present in each year. Locations that were geographically restricted in any given year did not have estimates made for them. Of note, we did not attempt a complete systematic review, since a single high-quality source could offer sufficient evidence of presence. Evidence of absence or presence was not available for every location for each year, and so assumptions were made for missing years by taking into consideration the epidemiological characteristics of the disease.

If evidence indicated disease presence for two non-consecutive years, we assumed presence for all years between the two. If evidence indicated disease absence for two non-consecutive years, we assumed absence for all years between the two. If evidence indicated a change in status (ie, from absent to present, or present to absent) between two non-consecutive years, then we conducted targeted searches to ascertain the relevant year of introduction or elimination for that location. In the cases where presence or absence information was missing for the start or end years of our study interval (1990–2017) without evidence of any introduction or elimination events within the interval, we applied the status of the first and last presence/absence observations, respectively, to all years between the interval bound and the observation year. Our search was done in conjunction with the title/abstract screening portion of a systematic literature review for prevalence data. The search strings and yield can be viewed in the table below for each of the databases queried.

Table 2. Geographic Restriction Search Strings

Database	Search String	Yield
PubMed	(Ascariasis[Title/Abstract] OR Ascaris[Title/Abstract] OR "A. lumbricoides"[Title/Abstract] OR Ascaris[MeSH] OR Trichuris[Title/Abstract] OR Trichuriasis[Title/Abstract] OR "Whip Worm"[Title/Abstract] OR "T. trichura"[Title/Abstract] OR Trichuris[MeSH] OR Hookworm[Title/Abstract] OR "A. duodenale"[Title/Abstract] OR "Ancylostoma duodenale"[Title/Abstract] OR ancylostomiasis[Title/Abstract] OR "N. americanus"[Title/Abstract] OR "Necator americanus"[Title/Abstract] OR necatoriasis[Title/Abstract] OR Ancylostoma [MeSH] OR Necator[MeSH]) AND (prevalence[Title/Abstract] OR incidence[Title/Abstract] OR epidemiology[Title/Abstract] OR surveillance[Title/Abstract]) NOT(Animals[MeSH] NOT Humans[MeSH])	2,376
Web of Science	(Ascariasis OR Ascaris OR A. lumbricoides OR Trichuris OR Trichuriasis OR Whip Worm OR T. trichura OR Hookworm OR A. duodenale OR Ancylostoma duodenale OR anclyostomiasis OR N. americanus OR Necator americanus OR necatoriasis) AND TOPIC:(prevalence OR incidence OR epidemiology OR surveillance) NOTTOPIC: ((Animals NOT Humans))<br>Timespan: 1980-2016. Indexes: SCI-EXPANDED, SSCI, A&HCI, ESCI.	2,266
SCOPUS	TITLE-ABS_KEY (ascariasis OR ascaris OR a. lumbricoides OR trichuris OR trichuriasis OR whip worm OR t. trichura OR hookworm OR a. duodenale OR	29

	ancylostoma duodenale OR anclyostomiasis OR n. americanus OR necator americanus OR necatoriasis) AND PUBYEAR>1979	

These papers were used to classify location-years for all locations and years present in the literature. We only utilised papers that are explicitly concerned with hookworm. Additionally, systematic literature reviews, meta-analyses, national health statistics publications and collaborator input were used to classify location-years not present in the literature review wherever possible.

Health states/sequelae

The table below shows the list of sequelae due to hookworm and the associated disability weights (DW). Prevalence of medium infection and heavy infection were mapped to *mild abdominopelvic problems* and *heavy infestation of hookworm,* respectively. Light infection was not attributed any disability. To inform the wasting model, 1,000 draws of severe wasting prevalence among children under 5 years were ascertained from GBD 2017 estimates – the methods used to generate estimates of wasting prevalence are detailed elsewhere (part of risk factors documentation) [2].

Table 3. Sequelae, lay descriptions, and disability weights (DWs)

Sequela	Lay description	DW
Mild abdominopelvic problems	"has some pain in the belly that causes nausea but does not interfere with daily activities"	0.011 (0.005–0.021)
Heavy infestation	"has cramping pain and a bloated feeling in the belly"	0.027 (0.015–0.044)
Severe wasting	"is extremely skinny and has no energy"	0.128 (0.082–0.183)
Asymptomatic hookworm disease	NA	NA
Mild anaemia	"feels slightly tired and weak at times, but this does not interfere with normal daily activities"	0.004 (0.001–0.008)
Moderate anaemia	"feels moderate fatigue, weakness, and shortness of breath after exercise, making daily activities more difficult"	0.052 (0.034–0.076)
Severe anaemia	"feels very weak, tired and short of breath, and has problems with activities that require physical effort or deep concentration"	0.149 (0.101–0.210)

Modelling strategy

DisMod-MR 2.1

In the estimation of overall morbidity due to hookworm, we implemented a three-stage modelling framework. The first stage of the modelling process was using DisMod-MR 2.1 to generate a global age-sex curve to disaggregate all-age, both-sex prevalence data. DisMod is an integrated meta-regression framework that allows for multiple datasets to be used within a singular analysis regardless of age-binning, sources, and geographies. As a result, a variety of differently aggregated information can be evaluated to generate a consensus output. Our final model contained all processed GAHI data as input

and was informed by two country-level covariates (ie, all risk factors SEV for unsafe water, and all risk factors SEV for unsafe sanitation). From this model, the global fits were used.

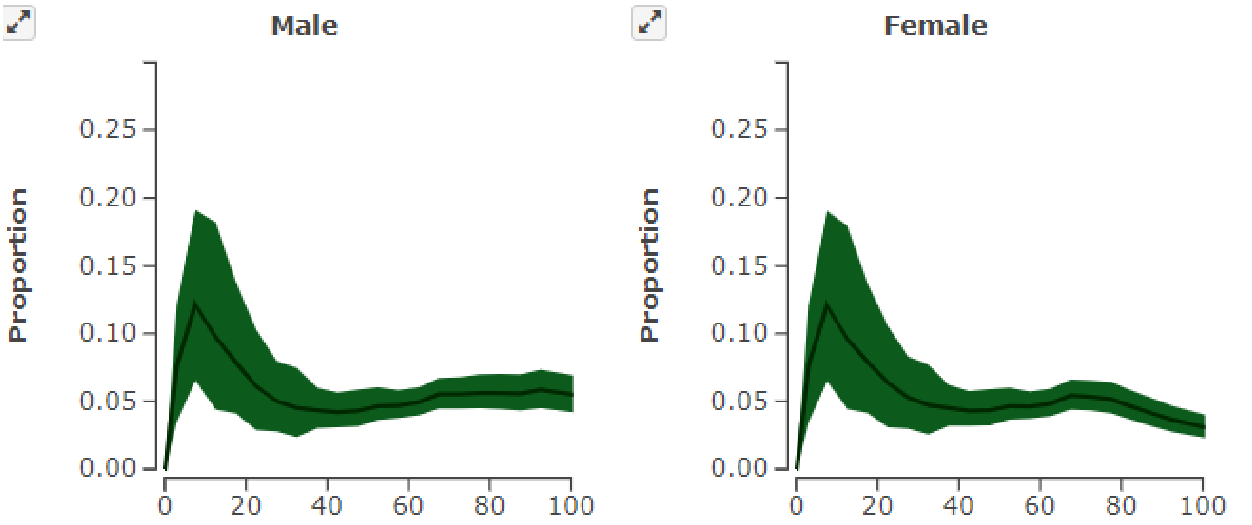

Figure 1: Global age-specific prevalence estimates for males (left) and females (right) for the year 2010. Proportion (prevalence) is on the Y-axis, and age in years on the X-axis. Screenshot from EpiViz tool.

Figure 1 shows the age-specific variation in prevalence rates, differentiated by sex. When considered as a global aggregate, we see that reported male and female prevalence are very similar. This is mostly a function of data used for modelling mainly being reported for both sexes. Prevalence peaks among young adults, followed by a decline and then stabilising during adulthood. These age-sex curves are similar to what has been reported in the literature [3, 4].

ST-GPR

After obtaining a global age-sex pattern from DisMod, we utilise a spatiotemporal Gaussian process regression (ST-GPR) to generate a complete time series of estimates for each location where there are no geographic restrictions. ST-GPR attempts to model non-linear trends utilising a Gaussian process to fit a trend. The following model specifications were used:

$$Prevalence = Proportion\ Sanitation + Proportion\ STH\ MDA\ Coverage + Proportion\ Water + (1|level\ 2) + (1|level\ 3)$$

Where levels 2 and 3 refer to GBD location hierarchies, or random effects for region and location. Notably, the covariates for the model were sanitation or proportion of population with access to improved toilet types, proportion of MDA (mass-drug administration) coverage, and safe water or proportion of population with access to improved water sources. Improved toilet types and improved water sources are defined by the Joint Monitoring Programme. The following hyperparameters were used: st-lambda = 0.25, st-omega =2, st-zeta = 0.01, gpr-scale = 15. We selected these hyperparameters as they provided more weight to country-level data rather than region-level data when estimating the prevalence for a given location-year. In other words, these hyperparameters ensure that the Gaussian process regressions follow country-specific data rather than region-specific data when estimating a time series for a location.

It is important to note that we did not use all processed GAHI data for the ST-GPR model. We opted to run an adolescent-only model because the bulk of our data are among children and there is more granular age information that we can leverage during modelling processes. More specifically, any data points that had age bins between 5 and 20 years were assigned to the 15 to 19 age group. We selected all data with age bins between 5 and 20 because this falls right below the peak in prevalence across all age groups, this is where a majority of data are, and it provides sufficient statistical power for our model.

Table 4. ST-GPR model covariates

Covariate	Beta Coefficient, Log (95% CI)	Standard Error	Exponentiated beta (95% CI)
Improved Water	-2.437(-3.849 - -1.026)	0.720	0.09 (0.02 – 0.36)
WHO STH MDA Coverage	0.003 (0.001 – 0.005)	0.001	1.00 (1.00 – 1.00)
Sanitation	-3.297 (-4.410 - -2.184)	0.568	0.99 (0.96 – 1.02)

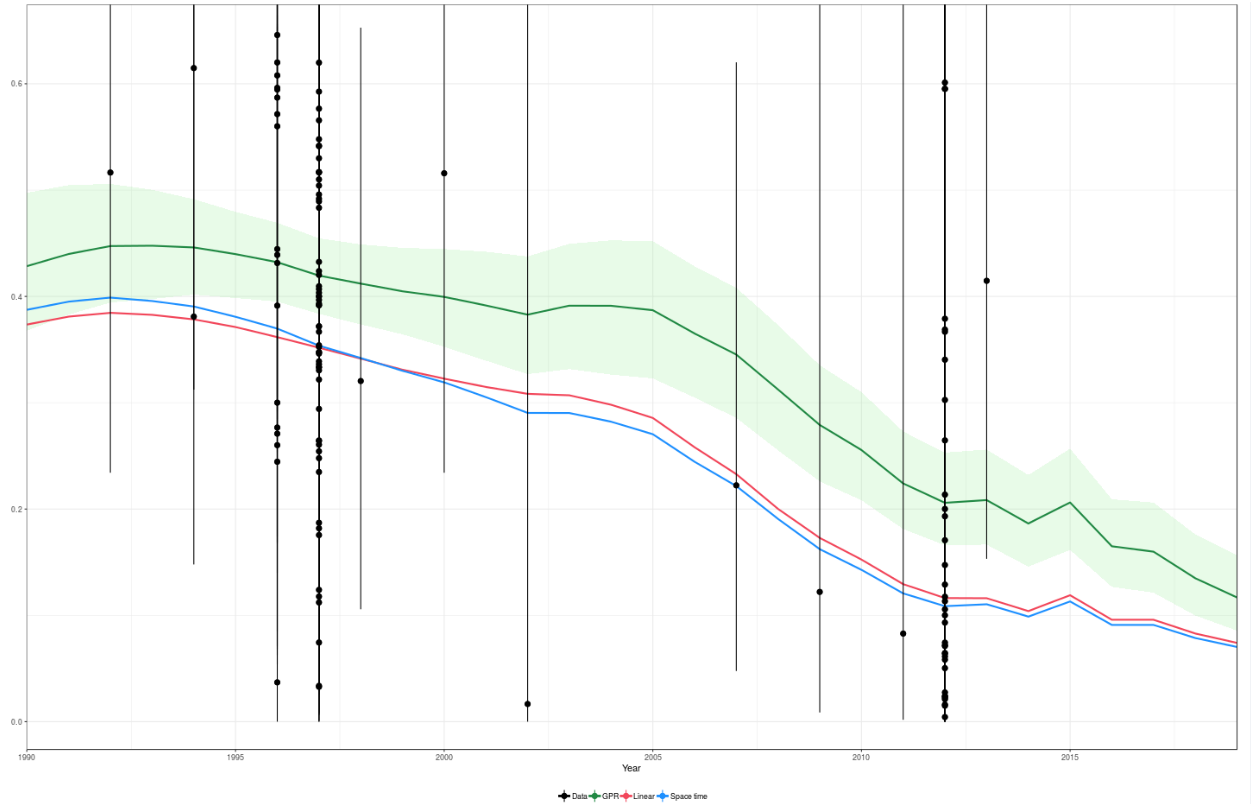

Figure 2: ST-GPR estimates for Tanzania (5- to 20-year-olds, both sexes) for years 1990–2019. Black dots represent input data points, with the black lines indicating variance. The green line represents the mean GPR estimated values, with uncertainty shown by the green polygon. The blue line indicates the space-time component of the ST-GPR; the red line indicates the linear regression component derived from

global data. Transparent black dots represent data from other locations in the GBD region (Western sub-Saharan Africa).

Figure 2 displays the time trends as computed by ST-GPR. For the most part, locations looked similar to Tanzania, where we see steady declines in prevalence throughout time.

Imputation

The final stage of the overall prevalence modelling process is to impute the remaining age groups by borrowing information from the ST-GPR time series for 15- to 19-year-olds and the DisMod global age-sex pattern. First, we assign each age group a ratio of how much larger or smaller the prevalence is compared to the prevalence for 15- to 19-year-olds using the DisMod global age-sex pattern. More specifically, the following is the computation for each age group:

$$Ratio = \frac{prevalence_{[age\ start] to\ [age\ end]}}{prevalence_{15\ to\ 19}}$$

We opted not to use the age-sex curves by location or region, because DisMod performed better at disaggregating our heterogeneous data at the global level. With a ratio for every age group by sex, we multiplied the ratio by the ST-GPR location-year estimates to impute estimates for the remaining age groups.

Health states/sequelae

Following computations of location-year-age-sex-specific prevalence of hookworm, we leverage information from the 2010 EG data to conduct sequelae splits. The 2010 EG data provided estimates for heavy infestation, mild abdominopelvic problems, and asymptomatic hookworm by location and for 1990, 2005, and 2010. These three values add up to all cases of hookworm. Thus, for heavy infestation and mild abdominopelvic problems, we computed the proportion of cases that belong to our sequelae of interest over all cases of hookworm. More specifically, the following is the computation by heavy infestation and mild abdominopelvic problems:

$$Proportion_{sequelae} = \frac{prevalence_{sequelae}}{prevalence_{all\ cases}}$$

This calculation was done for every location, year, and age group available. Because the EG data only had four age groups (0-4, 5-9, 10-14, 15+ years), we applied the 15+ age group proportion for all remaining age groups. In addition, for 1995 and 2000 we applied the 1990 proportions, and for 2017 we applied the 2010 proportions. Using these location-year-age specific proportions, we multiplied the total hookworm estimates to compute heavy infestation and mild abdominopelvic prevalence. To estimate the prevalence of asymptomatic hookworm, prevalence of mild and heavy infestation was subtracted from the overall hookworm prevalence.

The final step in the modelling process was to estimate the prevalence of severe wasting due to hookworm in age groups 28–364 days and 1–4 years. This was done separately using 1,000 draws of prevalence of heavy infestation due to hookworm and the wasting envelope prevalence. The initial step in determining prevalence of severe wasting due to hookworm was generating 1,000 draws of change in weight-for-height z-score per heavy prevalent case from a random normal distribution with mean = 0.493826493 and standard deviation = 0.04972834 (calculated from upper and lower bounds of the mean estimate). The mean, upper, and lower bounds were based on a published article [2]. The

prevalence of severe wasting due to hookworm was then obtained as a function of change in weight-for-height z-score. The following are the computations:

$$Prevalence_{wasting\ due\ to\ hookworm} = wasting - \Phi(\Phi^{-1}(\text{wasting}) - \text{z score} * \text{heavy infestation})$$

Where Φ is the standard normal cumulative distribution function and Φ^{-1} is the inverse standard normal cumulative distribution function. Finally, the age- and sex-specific anemia prevalence for hookworm was analysed as part of overall anemia causal attribution for GBD 2019. The details of the anemia analysis are described separately in the "Anemia Impairment" section. Briefly, after estimating total anemia, a series of counterfactual distributions are generated based on the age- and sex-specific prevalence of each anaemia-causing condition and the quantitative effect that the condition has on haemglobin concentration in the blood, a so-called "haemoglobin shift," that was derived by meta-analyzing cohort studies, observational studies, or trials comparing the haemotologic status of those with as compared to without the disease. Due to limited data on haemologbin shift, all were assumed to be invariant over age, sex, location, and year.

Changes from GBD 2017

The major change from GBD 2017 was in specifying new covariates for the ST-GPR global prevalence model, specifically in removing socio-demographic index due to collinearity concerns and adding the WHO STH MDA covariate.

Limitations

As we attempt to improve the modelling processes for hookworm, we recognise that there are several limitations. We only include studies where Kato-Katz was used to identify infected individuals. Future updates to the model will include a systematic review for within-study comparisons of diagnostic performance to facilitate a crosswalk model.

A secondary limitation to our data is that several included studies are not considered to be nationally representative, and therefore at a location level, the data are highly heterogeneous (Figure 3). Numerous studies within the database were conducted in districts or townships, and in some cases the studies were done in known areas where prevalence is high.

Furthermore, we made a large assumption that the global age-sex distributions were applicable to all locations. While we believe that prevalence should peak among young adults and slowly decline afterward, there is likely variation across regions and locations. Given that our data are either among adolescents or all-age, it is very difficult to build an age trend at granular location levels. Thus, we allowed DisMod to disaggregate our heterogeneous data in an effort to provide sensible age-sex curves.

We believe that more work needs to be done to improve our sequelae split methods. Since the EG data do not provide all estimation years and age groups, several assumptions had to be made. Thus, we will explore conducting literature searches to provide novel data points for sequelae estimations.

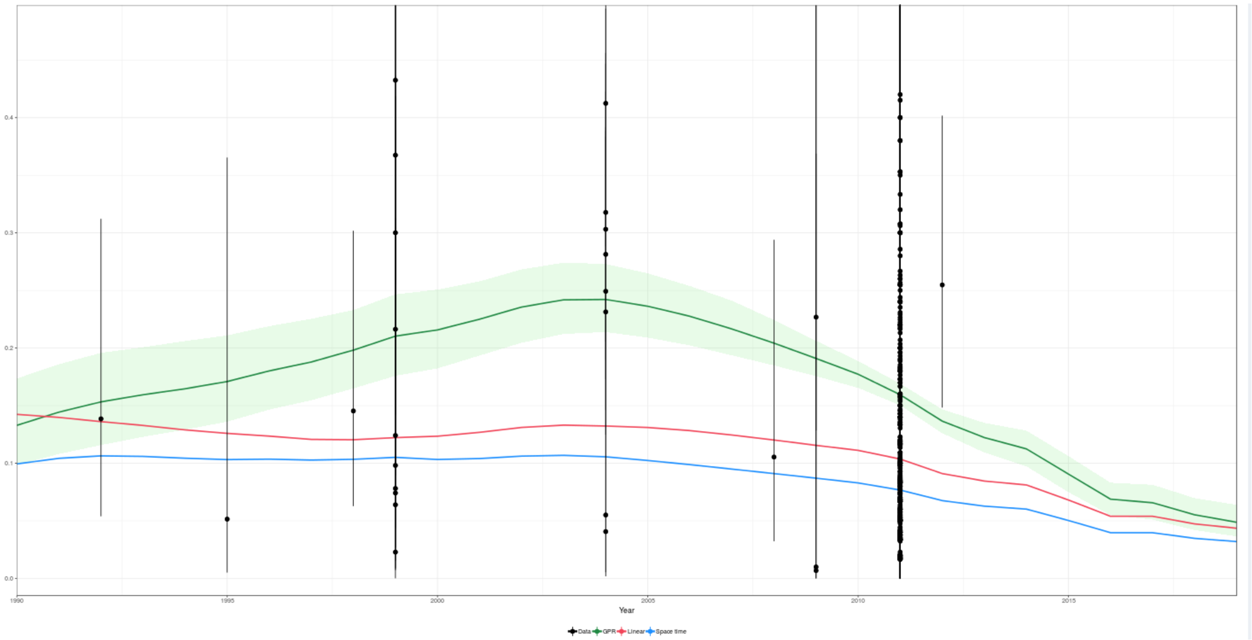

Figure 3: ST-GPR estimates for Nigeria (5- to 20-year-olds, both sexes) for years 1990–2019. Colouration and symbols are as stated in caption for Figure 2.

Figure 3 shows the time trend for Nigeria as computed by ST-GPR. For some locations, we estimate this fluctuating time trend, which is a function of the heterogeneity in our input data.

References

1. London School of Hygiene and Tropical Medicine. Global Atlas of Helminth Infections – Soil Transmitted Helminths. London, United Kingdom: London School of Hygiene and Tropical Medicine.
2. Hall A, Hewitt G, Tuffrey V, de Silva N. A review and meta-analysis of the impact of intestinal worms on child growth and nutrition. *Maternal and Child Nutrition*. 2008. 4. 118-236.
3. Riess H, Clowes P, Kroidl, Kowuor D, Nsojo A, Mangu C, Schule S, Mansmann U, Geldmacher C, Mhina S, Maboko L, Hoelscher M, Saathoff E. Hookworm Infection and Environmental Factors in Mbeya Region, Tanzania: A Cross-Sectional, Population-Based Study. *PLoS Neglected Tropical Diseases*. 2013. 7. e2408.
4. Pullan R, Kabatereine N, Quinnell R, Brooker S. Spatial and Genetic Epidemiology of Hookworm in a Rural Community in Uganda. *PLoS Neglected Tropical Diseases*. 2010. 4. e713.

Foodborne Trematodiases

Clonorchiasis

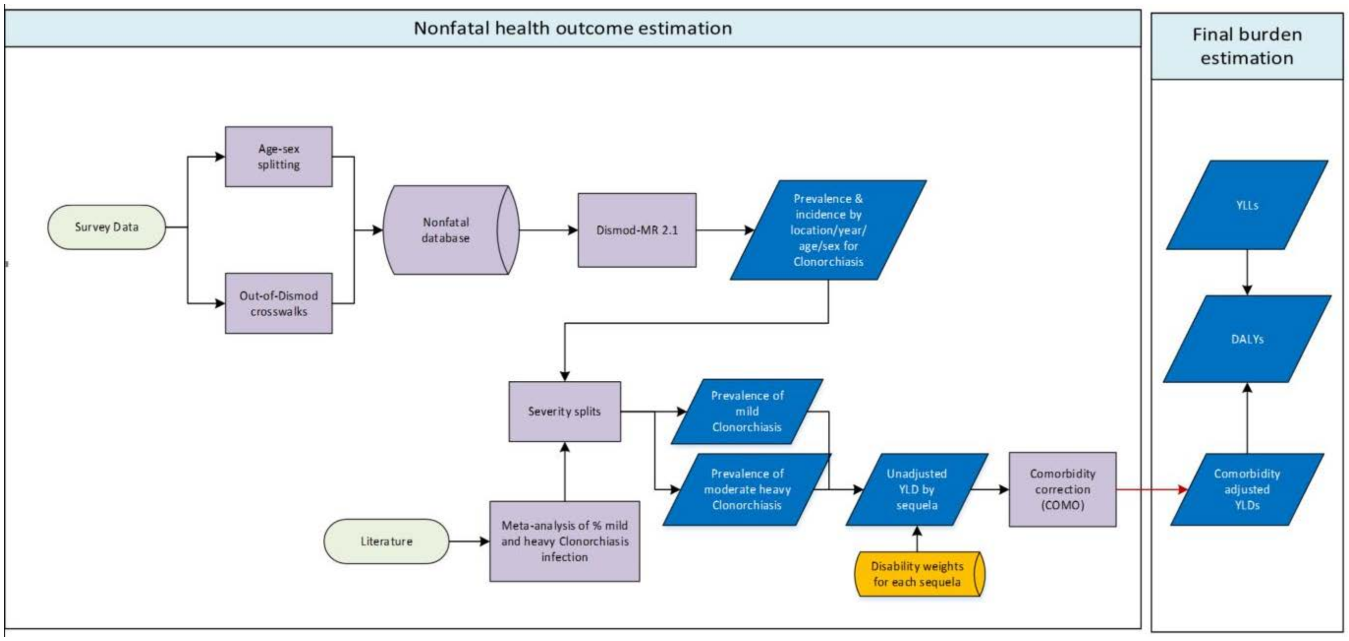

Fascioliasis

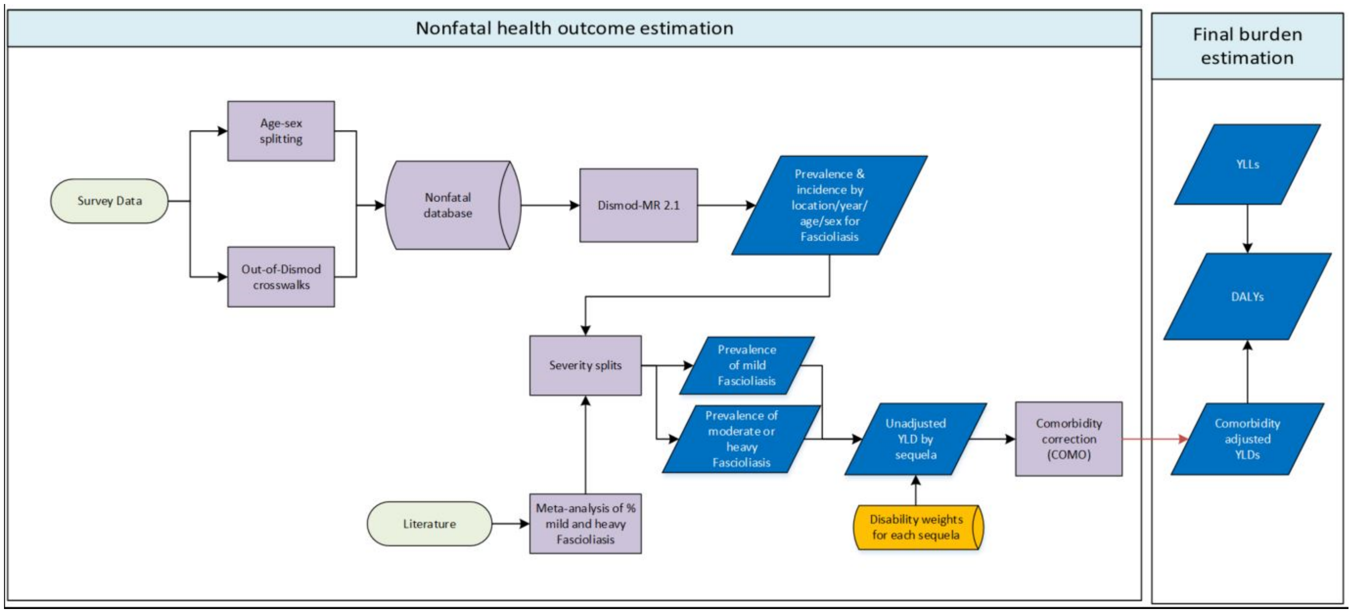

Intestinal Fluke

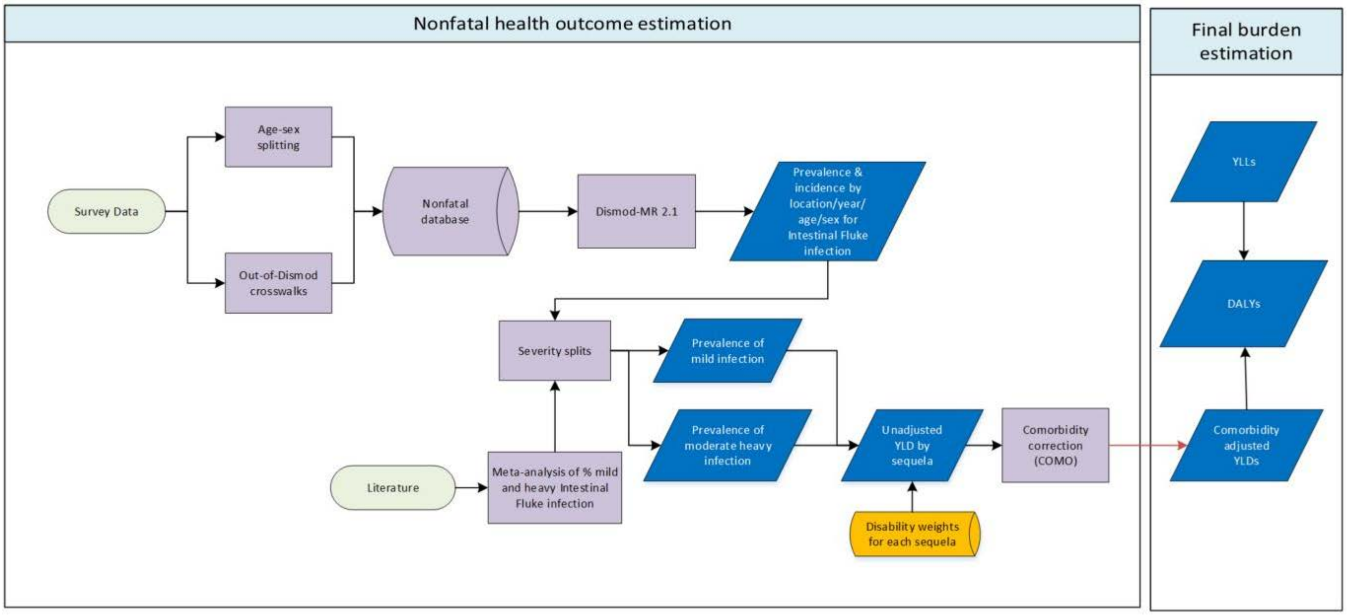

Opisthorchiasis

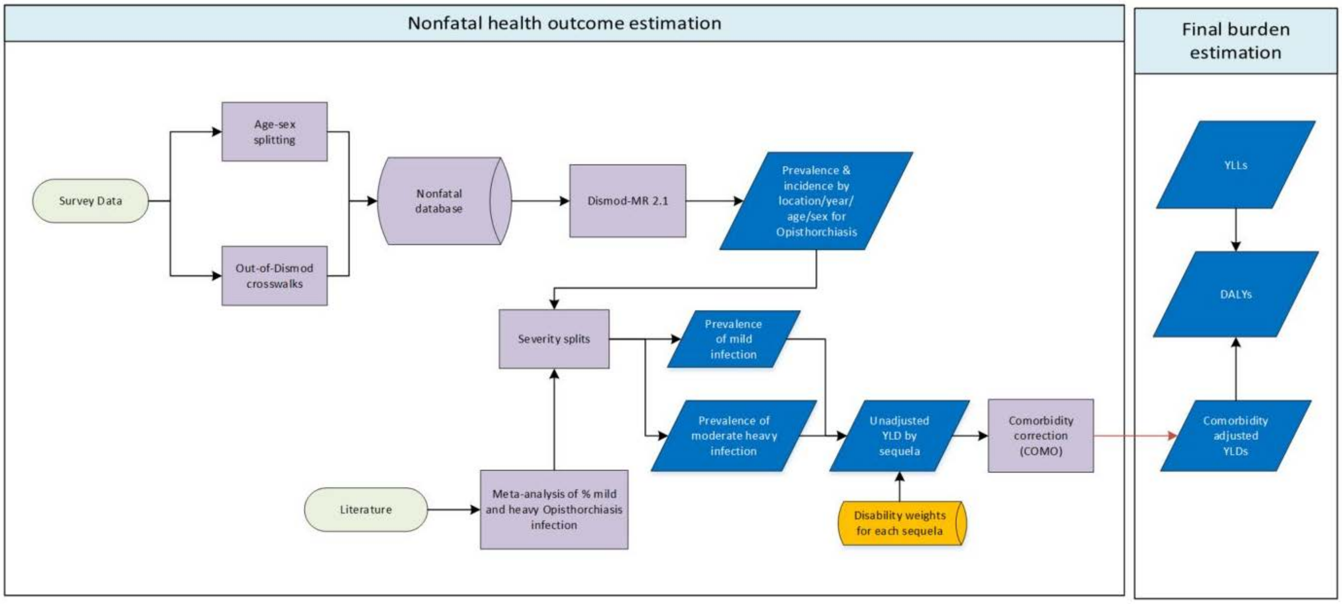

Paragonimiasis

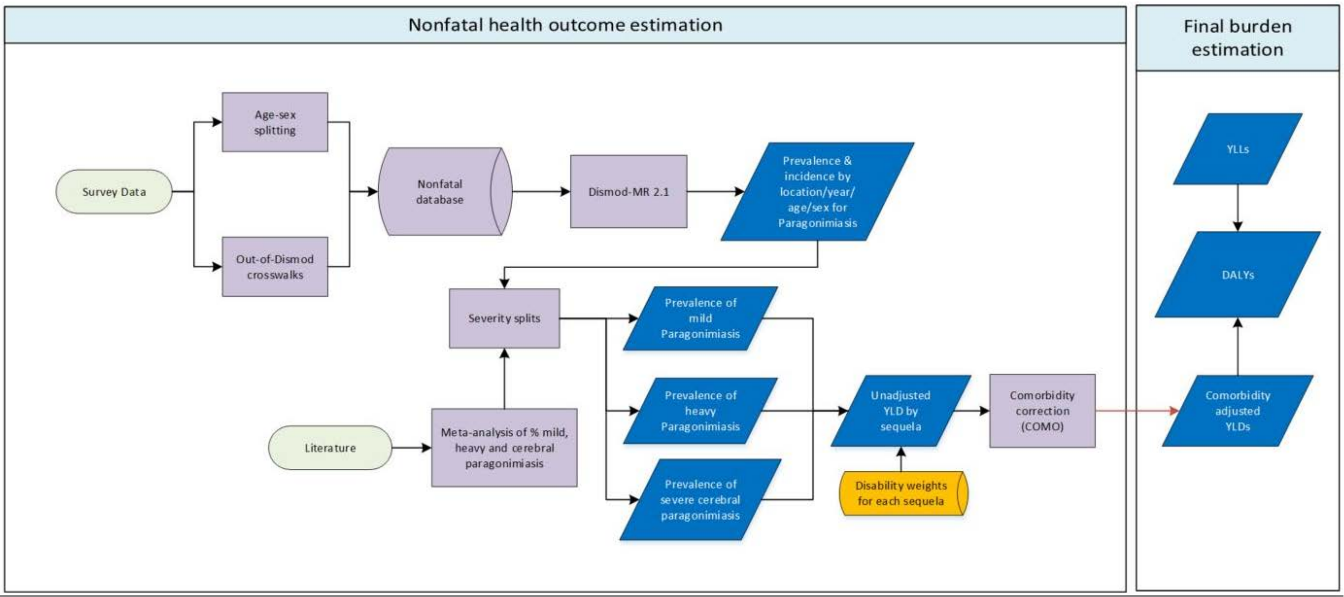

Input Data & Methodological Summary

Case definition

Human foodborne trematodiases (FBT) is defined as the infection with parasitic worms of the class trematoda, which are also known as flukes. Trematodes are transmitted via contaminated food, and infection is highly related to food habits. Definitive hosts, including humans, become infected when ingesting viable metacercariae by consuming contaminated aquatic products (eg, watercress). In the ICD-10, FBT are listed under code B66 [1].

FBT is subdivided into six types of FBT (see Table 1):

- Clonorchiasis
- Fascioliasis
- Intestinal fluke
- Opisthorchiasis
- Paragonimiasis (normal and cerebral infections)

Table 1. Subtypes of FBT

	Species of FBT	Also known as:	Carcinogen
1	Chlonorchiasis	(Chinese) Liver fluke	Associated with choliangiocarcinoma
2	Opisthorchiasis<br><br>(*O viverrini* & *O felineus*)	Liver fluke	Associated with choliangiocarcinoma<br><br>(*O viverrini*)
3	Fascioliasis	Liver fluke	No available evidence

4	Intenstinal fluke	Liver fluke	No available evidence
5	Paragonimiasis	Lung fluke	

Thresholds for heavy infection and duration by species of FBT

The majority of people infected with FBTs are asymptomatic. When symptoms do occur, they are often non-specific. Among the clinical symptomatic group, severity is associated with worm burden, typically measured by fecal egg counts, and the duration of infection. The thresholds for heavy infection and duration by species of FBT are shown in Table 2. The clinical presentation of FBT depends on the target organs (liver, lung, or intestines). Clonorchiasis and opisthorchiasis patients may suffer from loss of appetite, fullness, indigestion, diarrhoea, pain in the right upper quadrant, lassitude, weight loss, ascites, and oedema.[2, 3] Cholangitis, obstructive jaundice, intra-abdominal mass, cholecystitis, and gallbladder or intrahepatic stones may occur as complications.[3, 4]

Table 2. Thresholds for heavy infection and duration by species of FBT

	Species of FBT	Case thresholds for heavy infection	Duration
1	Chlonorchiasis	10,000 eggs per g of feces	lifelong
2	Opisthorchiasis	10,000 eggs per g of feces	lifelong
3	Fascioliasis	1,000 eggs per g of faces	lifelong
4	Intenstinal fluke	1,000 eggs per g of faces	lifelong
5	Paragonimiasis	100 eggs per 5 ml sputum	lifelong
6	Cerebral paragonimiasis	Any infection of the brain with flukes and/or eggs of *Paragonimus* spp.	lifelong

Input data

Table 3: Source Counts

Measure	Total sources
All measures	57
Prevalence	56
Proportion	1

Model inputs

For GBD 2010, the data came from the expert group and is the result of their analysis. The expert group analysis used the results of a systematic literature review performed by Furst and colleagues as a starting point for the analysis.[5] Furst and colleagues searched PubMed, WHOLIS, FAOBIB, Embase, CAB Abstracts, Literatura Latino Americana e do Caribe em Ciências de Saùde (LILACS), ISI Web of Science, BIOSIS preview, Science Direct, African Journals OnLine (AJOL), and the System for Information on Grey Literature in Europe (SIGLE), period Jan 1, 1980, to Dec 31, 2008. The initial number of studies identified through the literature review was ~34,000 references. The literature review included extracted data from 181 studies. For GBD 2013 and GBD 2015, the search strategy was replicated to capture epidemiological studies published between 2008 and 2015.

Input data for the assessment of the total national number of infected people

Only studies that used countrywide surveys to estimate the national prevalence rates were included (or for China, province-wide surveys). Reason for choosing only national studies is that FBT shows a highly focal spatial distribution and local cross-sectional surveys would profoundly under- or overestimate true national prevalences. We decided not to model national and subnational together and get a coefficient on subnational, because there is not a one-fits-all relationship across the world. Infection is highly related to food habits, and there are highly varying differences between national and subnational prevalence rates. The final GBD 2016 dataset contained 29 prevalence studies from 17 countries. We used raw data from the selected studies as input for DisMod.

Prevalence of intestinal fluke infection

Intestinal fluke is different from the other types of FBT, because there are several pathogens that fall under intestinal fluke infection. It can be caused by pathogens, such as *Metagonimus* spp., *Echinostoma* spp., and *Neodiplostomatidae*.[6] When assessing the prevalence of intestinal fluke infection, we added the identified prevalence for each parasite species in order to obtain the overall prevalence of intestinal fluke infections. This approach may lead to a certain overestimation of the true prevalence, because people may be co-infected with more than one intestinal fluke species. There is no sufficient evidence about the proportion of co-infections, but the resulting overestimation of the true prevalence may be more than offset by the assumptions made in our previous modelling approach and the many challenges in generating the underlying epidemiological parameters (eg, diagnostic inaccuracy in the detection of infections with the more than 50 intestinal fluke species). Also of note: the transmission source of intestinal fluke infections are species-specific and therefore vary. For instance, *Fasciolopsis buski* is usually transmitted by eating raw water plants with the infective parasite stage attached to the water plants, whereas *Neodiplostomatidae* are transmitted by eating undercooked and infested frogs, snakes, and tadpoles. Because of these different transmission pathways, the rate of co-infection might in fact be smaller than expected.

Input data to differentiate between asymptomatic and heavy infections

We estimated the proportion of heavily infected among all infected in all available national and regional cross-sectional surveys. It is expected that heavy infection increases with age and there are data available on heavy infection by age group. We therefore decided to include age-dependent rates of heavy infection for clonorchiasis, opisthorchiasis, and intenstinal fluke infection. For (cerebral) paragonimiasis and fascioliasis there were not sufficient age-dependent data on high intensity FBT infection.

Data Pre-Processing

We used a MR-BRT model with our sex-specific data to derive an estimate of the ratio of the male prevalence of all-species FBT infection to female prevalence of all-species FBT infection to split non-sex-specific data. Then, a DisMod-MR 2.1 Bayesian meta-regression model using the age-specific input data was run to derive an age pattern to apply to split the all-age data.

Table 4: MR-BRT Crosswalk Adjustment Factors for all-species FBT Infection

Data input	Reference or alternative case definition	Gamma	Beta Coefficient, Log (95% CI)	Adjustment factor*
Female data	Ref	0.82	---	---
Male data	Alt		0.48 (-1.16 – 2.12)	1.62

**Adjustment factor is the transformed Beta coefficient in normal space, and can be interpreted as the factor by which the alternative case definition is adjusted to reflect what it would have been if measured as the reference.*

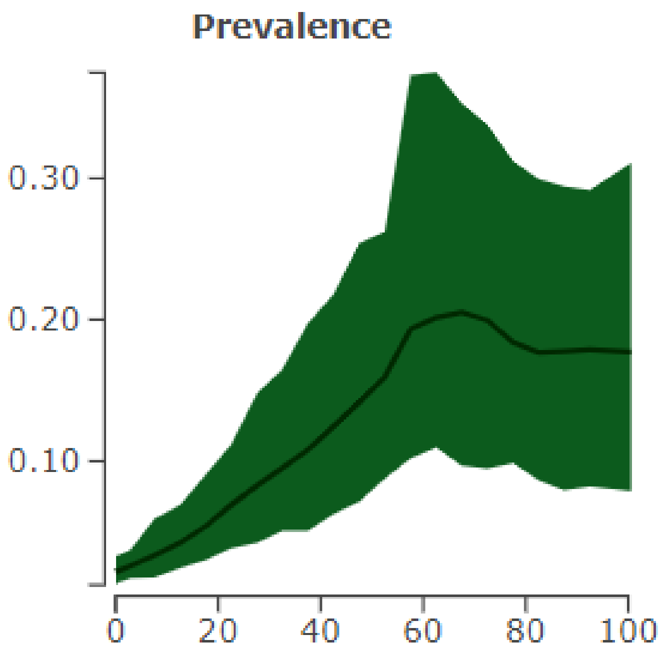

Figure 1: Global age-pattern for all-species FBT infection used to split all-age data into age-specific data points for further modeling.

Modelling strategy

We used a three-step process for the disease modelling of FBT. In the first step we used DisMod-MR 2.0 to estimate the prevalence of FBT by age, sex, year, and country. In the second we differentiated between asymptomatic and heavy infections. MetaXL (a meta-analysis add-in for Microsoft Excel) was used to estimate

the proportion of heavily infected among all infected by age group for clonorchiasis, opisthorchiasis, and intenstinal fluke infection (see Table 4 and 5). These proportions were used to estimate the prevalence of heavy FBT infection. The third step consisted of deselecting countries that have no autochtonous case reports of FBTs.

Table 5. Percentage of high-intensity infection by age group and type of FBT (based on eight FBT prevalence studies)

Age category	Clonorchiasis			Opisthorchiasis			Intestinal fluke infection		
	Mean	Low	High	Mean	Low	High	Mean	Low	High
0-9	30%	17%	44%	10%	0%	29%	8%	3%	14%
10-19	15%	0%	43%	15%	0%	69%	11%	8%	14%
20-29	18%	10%	29%	16%	0%	52%	18%	15%	21%
30-39	17%	5%	34%	21%	0%	56%	22%	17%	28%
40-49	22%	13%	32%	28%	1%	68%	22%	13%	32%
50-59	18%	0%	49%	29%	0%	75%	17%	9%	28%
60+	32%	18%	47%	25%	0%	64%	15%	8%	23%

Table 6. Percentage of high-intensity infection by type of FBT (based on four FBT prevalence studies)

Type of FBT	Mean	Low	High
Paragonimiasis	23%	0%	59%
Fascioliasis	19%	3%	41%

Cerebral paragonimiasis

It was assumed that 0.8% of paragonimiasis cases have cerebral involvement. This proportion was used to estimate the prevalence of cerebral paragonimiasis. This proportion is based on one study. The data are from Oh SJ. The rate of cerebral involvement in paragonimiasis: an epidemiologic study. *Jpn J Parasitol* 1969;18:211-14. The study was performed in Paju, South Korea. This is an area with 6,738 inhabitants, and according to the survey, it was estimated that 29.6% of all individuals would react to intradermal test (= an immunological reaction indicating previous or current contact with the parasite). 25% of all "positive reactors" may have eggs in their sputum (= active infection with the parasite currently present in the human host). If these rates are applied to the community as a whole, the number of patients with active paragonimiasis would be at least 498 (=6,738*0.296*0.250). Furthermore, four cases of cerebral paragonimiasis were found in this community. Therefore, four out of 498 individuals with active paragonimus infection suffered from cerebral infection (=0.80%; 95% confidence interval 0.019%–1.587%).

Severity splits and disability weights

For GBD 2016, FBT was not split into health states with different severities. The table below shows the GBD 2016 disability weights that were used to calculate the burden of FBT in YLDs.

Table 7. Disability weights that were used to calculate FBT YLDs

Sequelae	Severity description	Health state name	Disability weight
Asymptomatic clonorchiasis	Clonorchiasis, currently without symptoms	N/A	0.000 (0.000–0.000)
Heavy clonorchiasis	Abdominal pain and nausea reported as moderate	Abdominopelvic problem, moderate	0.114 (0.078–0.159)
Asymptomatic opisthorchiasis	Opisthorchiasis, currently without symptoms	N/A	0.000 (0.000–0.000)
Heavy opisthorchiasis	Abdominal pain and nausea reported as moderate	Abdominopelvic problem, moderate	0.114 (0.078–0.159)
Asymptomatic fascioliasis	Fascioliasis, currently without symptoms	N/A	0.000 (0.000–0.000)
Heavy fascioliasis	Abdominal pain and nausea reported as moderate	Abdominopelvic problem, moderate	0.114 (0.078–0.159)
Asymptomatic intestinal fluke infection	Intestinal fluke infection, currently without symptoms	N/A	0.000 (0.000–0.000)
Heavy intestinal fluke infection	Abdominal pain and nausea reported as moderate	Abdominopelvic problem, moderate	0.114 (0.078–0.159)
Asymptomatic paragonimiasis	Paragonimiasis, currently without symptoms	N/A	0.000 (0.000–0.000)
Heavy paragonimiasis	Cough, fever, and weight loss	Tuberculosis, not HIV-infected	0.333 (0.224–0.454)
Cerebral paragonimiasis	Epilepsy due to cerebral paragonimiasis	Epilepsy, less severe (seizures < once per month)	0.263 (0.173–0.367)
		Epilepsy, severe (seizures >= once per month	0.552 (0.375–0.710)

Note. N/A: not applicable

Changes from GBD 2017 to GBD 2019

A major change between GBD 2017 and GBD 2019 was in implementing our data pre-processing sex and age splitting methods as described above.

References

1. WHO. *International Statistical Classification of Diseases and Related Health Problems. 10th Revision. Version for 2007.* 2007 [cited 2009 October 14, 2009]; Available from: http://apps.who.int/classifications/apps/icd/icd10online/.
2. Rim, H.J., *Clonorchiasis: an update.* J Helminthol, 2005. **79**(3): p. 269-81.
3. Pungpak, S., et al., *Clinical features in severe opisthorchiasis viverrini.* Southeast Asian J Trop Med Public Health, 1985. **16**(3): p. 405-9.

4. Rim, H.J., *The current pathobiology and chemotherapy of clonorchiasis.* Korean J Parasitol, 1986. **24**(Suppl.): p. 1-141.
5. Furst, T., J. Keiser, and J. Utzinger, *Global burden of human food-borne trematodiasis: a systematic review and meta-analysis.* Lancet Infect Dis, 2012. **12**(3): p. 210-21.
6. Furst, T., et al., *Manifestation, diagnosis, and management of foodborne trematodiasis.* BMJ, 2012. **344**: p. e4093.

Leprosy

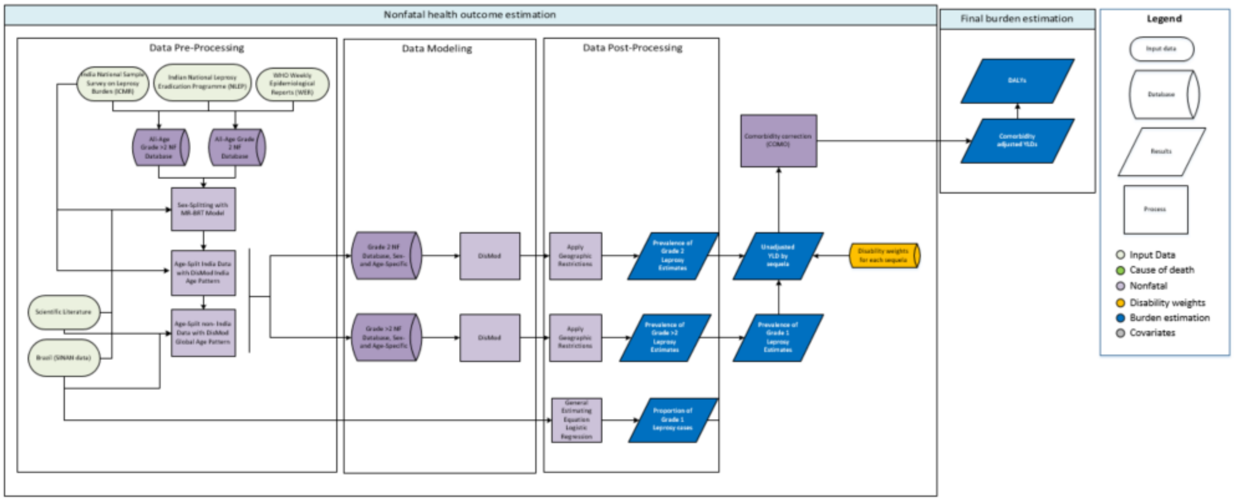

Input Data and Methodological Summary

Case definition

Leprosy is a chronic bacterial infection caused by *Mycobacterium leprae*, primarily affecting the nervous system, skin, respiratory tract, and eyes. Transmission is facilitated through contact with fluid from the nose and mouth of an infected individual. The ICD-10 code for Leprosy is A30.9.

Input data

Description of general methodology

The non-fatal estimation process for Leprosy begins with nationally case notification data, available published by the World Health Organization or ministries of health. The analysis is implemented in three steps: (1) data pre-processing, (2) data modeling, and post-processing, including applying geographic restrictions and (3) quantification of sequela.

Input Data

Table 1: Source Counts

Measure	Total sources	Countries with data
All measures	1684	172
Prevalence	692	121
Incidence	1636	172

There were five distinct data sources used to estimate Leprosy prevalence by grade-classification:

(i) WHO Weekly Epidemiological Record (WER) reports) disaggregated by Grade 2 and less than Grade 2 disability from 2000 to 2017. Data from 1990-2000 was not disaggregated by grade and we hope to split it to use in future cycles.
(ii) Indian National Leprosy Eradication Programme (NLEP) subnational incidence data was used from 2010-2017.
(iii) The 2010 – 2011 India National Sample Survey on Leprosy Burden (ICMR) prevalence data was used to in the subnational India prevalence estimation as well as to inform sex- and age-models.
(iv) Brazilian SINAN data informed the sex- and age-models as well as the severity split model to disaggregate less than Grade 2 estimates into Grade 1 and Grade 0 estimates. These data were not used in the main prevalence models due to concerns that hospital-based reporting might over-represent prevalence at the subnational- and national-level.
(v) Associated scientific literature was used to inform the sex- and age-model.

First, data reported in both sexes were split into male and female prevalence inputs. Sex-specific data inputs were used to estimate the ratio of males to females in MR-BRT (see Table 2). To sex-split our non-sex-specific data points, we use a MR-BRT model to derive a ratio of male Leprosy prevalence to female Leprosy prevalence (using SINAN, ICMR, and scientific literature data). The adjustment factor corresponded to nearly twice the amount of prevalence of Leprosy in males as opposed to females and is consistent with published gender disparity in Leprosy cases[1-3].

Table 2: MR-BRT Crosswalk Adjustment Factors for Leprosy

Data input	Reference or alternative case definition	Gamma	Beta Coefficient, Log (95% CI)	Adjustment factor*
Female data	Ref	00.400	---	---
Male data	Alt		0.73 (-0.14 – 1.56)	2.07

*Adjustment factor is the transformed Beta coefficient in normal space, and can be interpreted as the factor by which the alternative case definition is adjusted to reflect what it would have been if measured as the reference.

We then split all-age case data into age-specific observations using two age patterns derived by a DisMod Bayesian Meta-Regression model, one specific for India (derived using ICMR and Indian scientific literature) and another Global age pattern for non-India locations (derived using SINAN and non-Indian scientific literature). Two age patterns were developed (one for India, one global) using single-parameter incidence models, using DisMod.

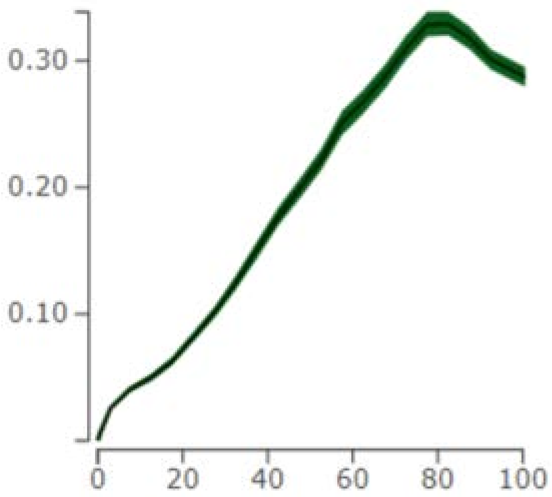

Figure 1a: Global age-pattern for Leprosy used to split non-India all-age data into age-specific data points for further modeling.

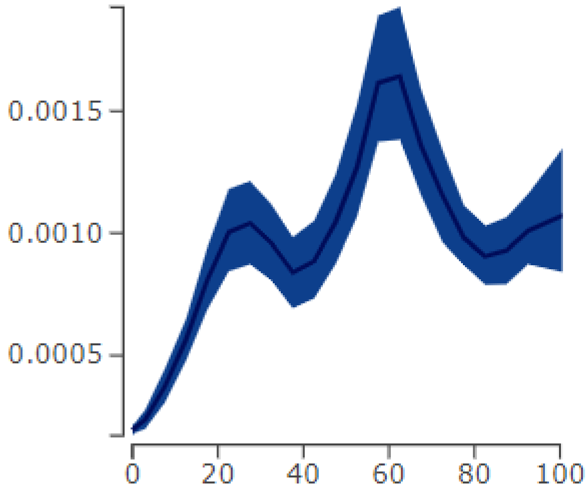

Figure 1b: India age-pattern for Leprosy used to split India all-age data into age-specific data points for further modeling.

Modeling Strategy

We used a compartmental model to derive prevalence of Leprosy from incident case reports. Since reported case data were grade-specific, we implemented two models, one for the prevalence of Grade 2 and a second for Grade <2 cases. For Grade <2 Leprosy model, we assumed no incident cases among children less than 15 years old and a remission of 0.5 to account for broad spectrum of disability associated with Grade 1 and the availability of treatment. For the Grade 2 model, we also assumed no

incident cases occurred among children less than 15 years old and no remission. since Grade 2 Leprosy consists of permanent disfigurement or disability.

Lastly, estimates of Grade <2 leprosy were disaggregated into Grade 1 and Grade 0 estimates using age- and sex-specific proportions reported by Brazil via logistic regression using a general estimating equation to account for repeated measures among the subjects in that cohort.

Table 3a. Covariates. Summary of covariates used in the Leprosy DisMod-MR less than Grade 2 meta-regression model

Covariate	Type	Parameter	Exponentiated beta (95% Uncertainty Interval)
Healthcare access and quality index	Log-transformed	Incidence	0.17 (0.16 — 0.18)

Table 3b. Covariates. Summary of covariates used in the Leprosy DisMod-MR Grade 2 meta-regression model

Covariate	Type	Parameter	Exponentiated beta (95% Uncertainty Interval)
Socio-demographic Index	Log-transformed	Prevalence	0.011 (0.0068 — 0.065)
Healthcare access and quality index	Log-transformed	Prevalence	0.0069 (0.0067 — 0.0072)

Geographic restrictions were applied to generate zero estimates in countries for which transmission is not considered endemic. We do not account for imported cases of Leprosy.

Table 4. Severity distribution, details on the severity levels for Leprosy in GBD 2019 and the associated disability weight (DW) with that severity.

Severity level	Lay description	DW (95% CI)
Disfigurement level 1 due to Leprosy	Has a slight, visible physical deformity that others notice, which causes some worry and discomfort.	0.011 (0.005 – 0.021)
Disfigurement level 2 due to Leprosy	Has a visible physical deformity that causes others to stare and comment. As a result, the person is worried and has trouble sleeping and concentrating.	0.067 (0.044 – 0.100)

Changes from GBD 2017 to GBD 2019

The Leprosy model was extensively revised for GBD 2019. There were substantial changes in data, modeling, and processing approaches.

Data: National case notification data were updated and formed the core input data for the models. Additionally, NLEP and ICMR data were added to improve India subnational estimates.

Data Processing: MR-BRT was used to sex-split the both-sex data and separate DisMod models were used to derive a global and India-specific age pattern to disaggregate all-age data prior to modeling. In prior versions of GBD, we modeled all Leprosy prevalence and then used Brazil data to determine global proportional splits between grades 2 and 1. In GBD 2019, we use the Grade 2 reported data available.

Model: In GBD 2017, WER Leprosy data was used as both an envelope and a basis for modeling prevalent disability cohorts. This cycle we changed this approach to model the grade-classification-specific incidence data and with assumptions regarding remission. We hope in future cycles to incorporate more remission information to better account for the cohort of prevalent cases over time.

References

1 Kumar, Rajendra, Pratap Singhasivanon, Jeevan Bahadur Sherchand, Punkae Mahaisavariya, Jaranit Kaewkungwal, Somchai Peerapakorn, and Krisada Mahotarn. “Gender Difference in Socio-Epidemiological Factors for Leprosy in the Most Hyper-Endemic District of Nepal.” Nepal Medical College Journal: NMCJ 6, no. 2 (December 2004): 98–105.

2 Peters, E. S., and A. L. Eshiet. “Male-Female (Sex) Differences in Leprosy Patients in South Eastern Nigeria: Females Present Late for Diagnosis and Treatment and Have Higher Rates of Deformity.” Leprosy Review 73, no. 3 (September 2002): 262–67.

3 Ramos, José M, Miguel Martínez-Martín, Francisco Reyes, Deriba Lemma, Isabel Belinchón, and Félix Gutiérrez. “Gender Differential on Characteristics and Outcome of Leprosy Patients Admitted to a Long-Term Care Rural Hospital in South-Eastern Ethiopia.” International Journal for Equity in Health 11 (October 4, 2012): 56. https://doi.org/10.1186/1475-9276-11-56.

Ebola virus disease

Flowchart

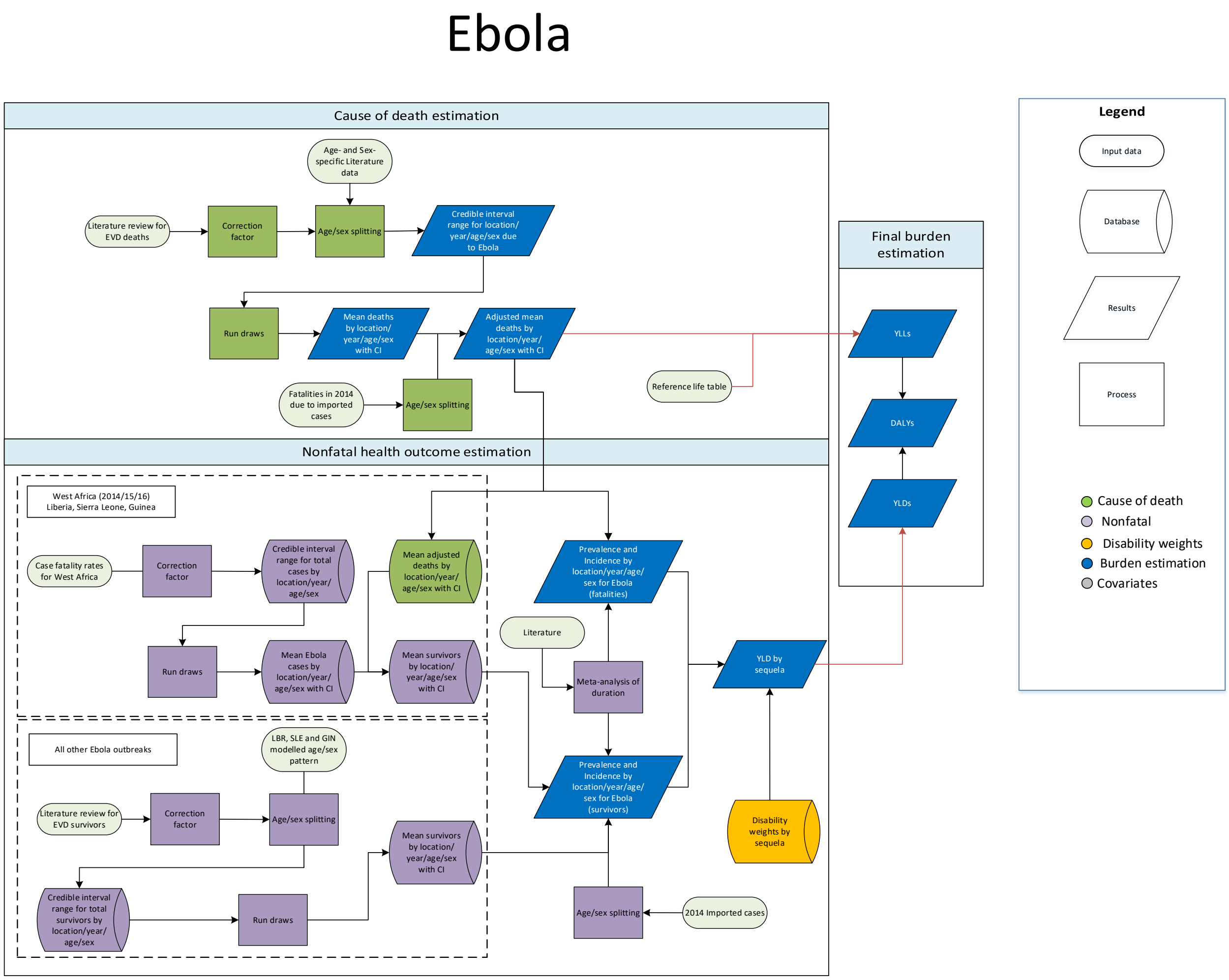

Input data and methodological summary

Background and case definition

Ebola virus is a relatively rare viral pathogen linked with high case fatality rates in both humans and non-human primates. The disease is zoonotic, and while bats have been implicated as reservoirs, definitive host species are yet to be identified. Once a human becomes infected after viral transmission from animal sources either directly or indirectly, secondary human-to-human transmission is possible, primarily through exchange of infectious bodily fluids and secretions. Clinical cases typically present initially as a febrile illness, similar to a number of different pathogens, which can be subsequently followed by haemorrhagic complications and death. Historically there have been a number of outbreaks, usually no more than a few hundred cases, typically constrained to one country, focused in Central Africa. The West African outbreak, however, which started in Guinea in 2013, claimed more lives than all previous outbreaks combined, and spread across the region seeding additional outbreaks. There is an ICD code for

Ebola, A98.4, but no data used in the modelling reference that coding (ie, all the data are from literature extractions). Data for Ebola virus disease were only included if the case was identified as either "probable" or "confirmed" as per WHO definitions [http://www.who.int/csr/resources/publications/ebola/ebola-case-definition-contact-en.pdf]. A confirmed case is any suspected or probable case with a positive laboratory result through either detection of virus RNA via reverse transcriptase-polymerase chain reaction, or by detection of IgM antibodies directed against Ebola. A probable case is any suspected case evaluated by a clinician or any deceased suspected case with an epidemiological link to a confirmed case.

Input data

Table 1: Source Counts

Measure	Total sources
All measures	50
Causes of death	18
Duration	6
Continuous	1
Population	42

Model inputs

Two distinct sequelae were assigned to Ebola virus disease (EVD) to be incorporated into the YLD estimation process: (i) sequela associated with the initial symptomatic phase of the infection (associated with all cases of Ebola virus disease) and (ii) sequela characterising the long-term post-EVD consequences of infection. As such, data were required both to ascertain the number of deaths as well as those surviving from each outbreak.

Data on fatal cases were inherited from the GBD 2017 mortality estimation process and were converted into incidence of cases of Ebola (with fatal outcomes) by cross-referencing locational annualised population estimates.

In order to calculate the numbers of survivors from each outbreak, two data sources were referenced, one based upon modelled estimates of the main three countries in the West African Ebola outbreak (namely Sierra Leone, Liberia, and Guinea), supplemented by WHO Situation Reports covering the clusters of 2016 cases and literature references covering all other subsequent outbreaks.

Age-sex patterns derived from the age- and sex-specific input data were applied to total envelope estimates as reported by WHO and CDC. Raw number of survivors were estimated by subtracting total deaths as reported by WHO and CDC from total cases.

For all other outbreaks, numbers of survivors were directly evaluated based upon numbers published in a previous review[1,2] and consulting original documents describing these outbreaks. This initial review was also updated to include the outbreak that occurred in the Democratic Republic of the Congo (DRC) in

2014[3], cases in 2016 and 2017, the 2018 DRC Equateur province outbreak[4], and the ongoing 2018-2019 DRC outbreak[5], including cases in Uganda[6]. The case totals for the ongoing outbreak were last updated July 23rd and more information may be available since submission. This resulted in datasets describing each outbreak with variable degrees of detail: some fully describing the age and sex breakdown of all survivors [eg, Rosello et al.[7]] and others simply providing the final total. Only confirmed or probable cases were included as per the case definition. Outbreaks that spanned multiple years, in the absence of sufficient data providing an accurate breakdown, were apportioned between the years by evenly assigning a uniform number of survivors to each month of the outbreak's duration. An additional search was conducted to identify imported cases from the West African outbreak during 2014 and 2015.

Table 2. Sequelae and disability weights (DWs) associated with Ebola

Sequelae	Description	Disability weight
Infectious disease, acute episode, severe	Has a high fever and pain and feels very weak, which causes great difficulty with daily activities	0.133 (0.088–0.19)
Infectious disease, post-acute consequences (fatigue, emotional lability, insomnia)	Is always tired and easily upset. The person feels pain all over the body and is depressed	0.219 (0.148–0.308)

It was not possible to create bespoke disability weights for the more specific sequelae often associated with Ebola virus disease (eg, haemorrhaging or ocular complications in survivors), so existing disability weights were co-opted. General high fevers and weakness characterise the majority of presenting cases[8] with long-term complications generally related to weakness and arthralgia.[9]

Modelling strategy

Data on cases (both survivors and fatalities) resulting from imported cases from 2014 and 2015 were used as specific count data as it was assumed to be an accurate representation of the cases and outbreaks in these countries, all of which were on high alert for importation of cases.[10,11]

The other input data were processed prior to inclusion in GBD to account for any potential underreporting of deaths. A meta-analysis of existing underreporting studies from the literature was performed, using a random effects model with a DerSimonian-Laird estimator. A variety of sources were included, capturing a number of different estimation processes, all identified by literature review. The figure below shows the different effect sizes of the different studies, as well as the resulting GBD 2016 (used in GBD 2017) correction factor, with the GBD 2015 correction factor for reference. The correction factor ranged from 1.5147 to 2.5720 with a mean of 2.0433.

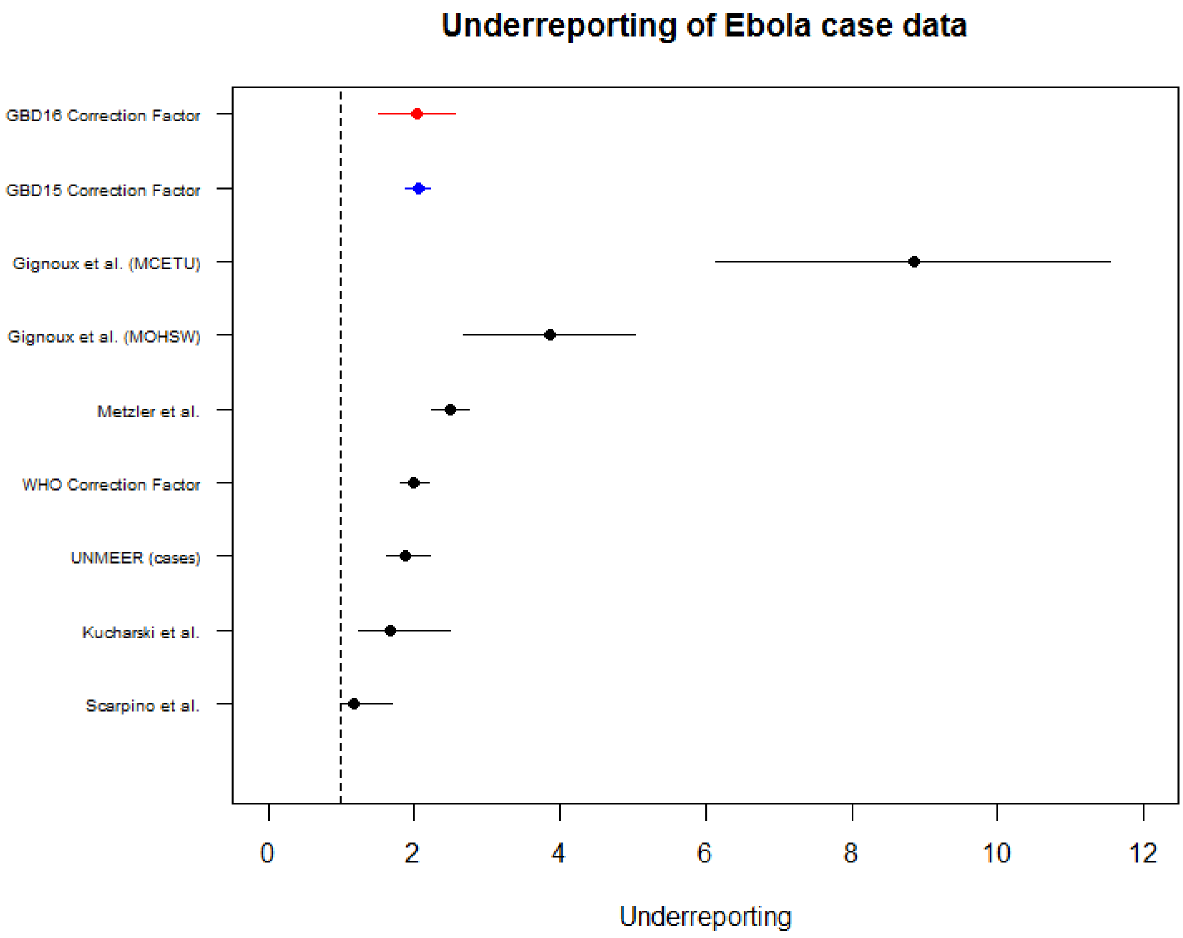

In order to capture this potential variation, all input data were multiplied by the lower and upper limit of this estimated correction factor; these numbers then provided the lower and upper bounds from which draw values were taken. For outbreaks where no data were supplied for age and/or sex, the pattern observed in the West African outbreak (for which there were the most comprehensive data) was used to apportion these total values.

One thousand draws were taken from a normal distribution fitted between these lower and upper bound values, which generated mean estimates stratified by age, sex, location, and year along with credible intervals for these numbers. For the West African outbreak, this generated total case numbers, from which the estimated number of deaths was subtracted in order to provide an estimate for the total number of survivors. For all other outbreaks, this data processing directly estimated the total number of survivors from each outbreak. These count data were converted into prevalence estimates by cross-referencing estimates of population size.

In order to estimate the duration of the sequelae categories, previous modelled assessments of the West African outbreak were consulted.[1,2] The duration of initial infection for patients was calculated as the total time period between onset of symptoms to death or to discharge from hospital (8.2 days [7.9–8.4] and 15.1 [14.6–15.6], respectively). These time periods were assumed to be appropriate for

characterising all other outbreaks. This time period was then assigned a disability weight corresponding to "infectious disease, acute episode, severe."

For long-term sequelae estimation, the proportion of survivors still suffering post-acute consequences was modelled using an exponential function with proportions of survivors still reporting poor health states (derived from a number of survivor studies[12,16–23]) reported over different time periods. The average duration of post-Ebola sequelae was then calculated as 0.9042 years (0.3673–1.4268).

The final combination of YLDs associated with prevalent initial onset of disease and prevalent post-EVD consequences was then calculated to provide an overall YLD estimate stratified by age, sex, location, and year. Estimates were provided for the years 1990, 1995, 2000, 2005, 2010, 2015, 2017, and 2019 as per non-fatal GBD estimation protocols.

Potential limitations

Data on Ebola outbreaks prior to 2014 are sparse, and as a result many values derived from the West African outbreak were assumed to be valid for historical outbreaks as well. This may mask significant differences that exist between these outbreaks, some of which were caused by different species of Ebola virus. In order to minimize this problem, we chose to implement a data-driven approach – for those outbreaks where sufficiently detailed historical data could be obtained, this was used in preference to any assumed age/sex breakdown.

Haemorrhagic manifestations are currently not considered as an explicit health state for disability weighting, and as a result, the current classification (of infectious disease, acute episode, severe) may be an underestimate. In contrast, the post-Ebola disease sequelae disability weighting may overestimate this burden, particularly when applied over a long period of time. In both instances, however, these disability weightings represent the most relevant linkages in the absence of bespoke values being generated.

Due to so few historical survivors of Ebola virus disease, only a handful of studies have tracked the long-term sequelae among cohorts of survivors beyond a two-year period. Given the large number of survivors from the West African outbreak, it is likely that future parameterization of this component will become much better data-driven. The current log-linear regression model extends for a period of 20 years and therefore could prove to be an overestimate of duration. In addition, ocular manifestations are not currently considered within the sequelae envelope – future iterations will consider health states identified by ongoing cohort analyses of Ebola survivors. Comments from collaborators in previous cycles have highlighted ocular conditions for inclusion; however, definitive evidence of a linkage with Ebola remains inconclusive. A study (conducted in West Africa) comparing Ebola survivors with background prevalence rates of many of the symptoms reported in survivors (eg, uveitis), suggested no difference in rates of these ophthalmic complications[25]. Understanding which of the many observed clinical outcomes in patients are caused by the virus, as opposed to incidentally co-morbid, is a necessary prerequisite for inclusion in the GBD.

References

1 Pigott DM, Golding N, Mylne A, *et al.* Mapping the zoonotic niche of Ebola virus disease in Africa. *Elife* 2014; **3**: e04395.

2 Mylne A, Brady OJ, Huang Z, *et al.* A comprehensive database of the geographic spread of past

human Ebola outbreaks. *Sci Data* 2014; **1**: 140042.

3 Maganga GD, Kapetshi J, Berthet N, *et al.* Ebola virus disease in the Democratic Republic of Congo. *N Engl J Med* 2014; **371**: 2083–91.

4 World Health Organization (WHO). WHO Ebola Situation Report 2018 - Number 17. 2018.

5 World Health Organization (WHO). WHO Ebola Situation Report 2019 - Number 45. 2019.

6 World Health Organization (WHO). WHO Ebola Situation Report 2019 - Number 51. 2019.

7 Rosello A, Mossoko M, Flasche S, *et al.* Ebola virus disease in the Democratic Republic of the Congo, 1976-2014. *Elife* 2015; **4**. DOI:10.7554/eLife.09015.

8 Schieffelin JS, Shaffer JG, Goba A, *et al.* Clinical Illness and Outcomes in Patients with Ebola in Sierra Leone. *N Engl J Med* 2014; **371**: 2092–100.

9 Tiffany A, Vetter P, Mattia J, *et al.* Ebola Virus Disease Complications as Experienced by Survivors in Sierra Leone. *Clin Infect Dis* 2016; **62**: 1360–6.

10 Fasina FO, Shittu A, Lazarus D, *et al.* Transmission dynamics and control of Ebola virus disease outbreak in Nigeria, July to September 2014. *Euro Surveill* 2014; **19**: 20920.

11 Althaus CL, Low N, Musa EO, Shuaib F, Gsteiger S. Ebola virus disease outbreak in Nigeria: Transmission dynamics and rapid control. *Epidemics* 2015; **11**: 80–4.

12 UNMEER. Sierra Leone: Ebola emergency Weekly Situation Report No. 7. 2014 https://www.humanitarianresponse.info/system/files/documents/files/UNMEER_NERC_SitRep_07Dec.pdf.

13 Clark D V, Kibuuka H, Millard M, *et al.* Long-term sequelae after Ebola virus disease in Bundibugyo, Uganda: a retrospective cohort study. *Lancet Infect Dis* 2015; **15**: 905–12.

14 Qureshi AI, Chughtai M, Loua TO, *et al.* Study of Ebola Virus Disease Survivors in Guinea. *Clin Infect Dis* 2015; **61**: 1035–42.

15 Rowe AK, Bertolli J, Khan AS, *et al.* Clinical, virologic, and immunologic follow-up of convalescent Ebola hemorrhagic fever patients and their household contacts, Kikwit, Democratic Republic of the Congo. Commission de Lutte contre les Epidémies à Kikwit. *J Infect Dis* 1999; **179 Suppl**: S28-35.

16 Bwaka MA, Bonnet MJ, Calain P, *et al.* Ebola hemorrhagic fever in Kikwit, Democratic Republic of the Congo: clinical observations in 103 patients. *J Infect Dis* 1999; **179 Suppl**: S1-7.

17 Mohammed H, Vandy AO, Stretch R, *et al.* Sequelae and Other Conditions in Ebola Virus Disease Survivors, Sierra Leone, 2015. *Emerg Infect Dis* 2017; **23**: 66–73.

18 Nanyonga M, Saidu J, Ramsay A, Shindo N, Bausch DG. Sequelae of Ebola Virus Disease, Kenema District, Sierra Leone. *Clin Infect Dis* 2016; **62**: 125–6.

19 Mattia JG, Vandy MJ, Chang JC, *et al.* Early clinical sequelae of Ebola virus disease in Sierra Leone: a cross-sectional study. *Lancet Infect Dis* 2016; **16**: 331–8.

20 Epstein L, Wong KK, Kallen AJ, Uyeki TM. Post-Ebola Signs and Symptoms in U.S. Survivors. *N Engl J Med* 2015; **373**: 2484–6.

21 Etard J-F, Sow MS, Leroy S, *et al.* Multidisciplinary assessment of post-Ebola sequelae in Guinea (Postebogui): an observational cohort study. *Lancet Infect Dis* 2017. DOI:10.1016/S1473-3099(16)30516-3.

22 Scott JT, Sesay FR, Massaquoi TA, Idriss BR, Sahr F, Semple MG. Post-Ebola Syndrome, Sierra Leone. *Emerg Infect Dis* 2016; **22**: 641–6.

23 Steptoe, PJ, Scott JT, Baxter, JM, *et al.* Novel retinal lesion in Ebola survivors, Sierra Leone, 2016. *Emerg Infect Dis* 2017; **23**: 1102-9

Zika

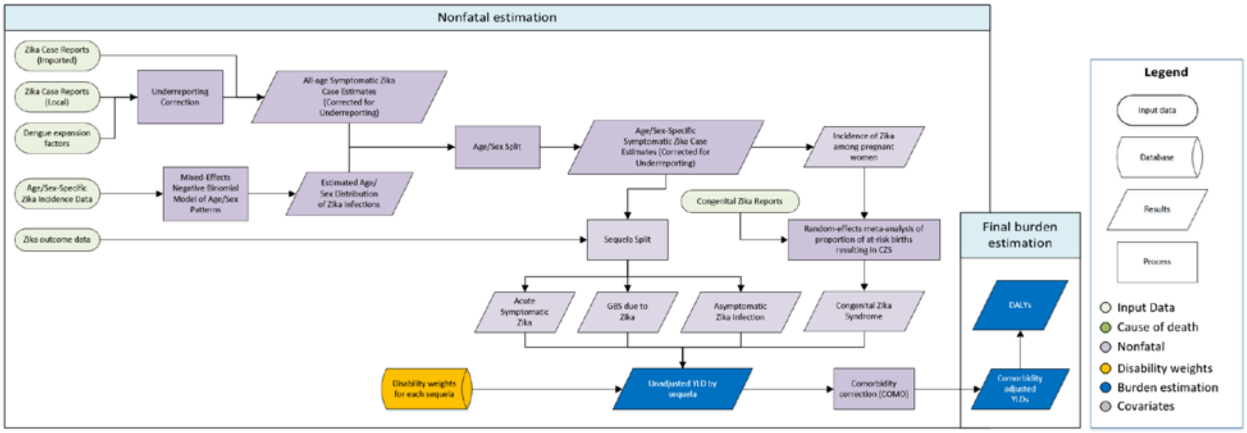

Input data

Data on cases of acute Zika and Congenital Zika Syndrome (CZS) come from official reports, primarily from the Pan American Health Organization (PAHO).

Table 1 presents the total number of source counts included in the analysis.

Table 1. Total data source counts

Measure	Total sources	Countries with data
All measures	407	149
Incidence	399	149
Cause-specific mortality rate	5	3
Proportion	15	10

Modelling strategy

We estimate the all-age incidence of symptomatic Zika as the product of reported Zika cases and country-specific expansion factors that adjust for underreporting. Those expansion factors are derived from our dengue model, and the methods used for their estimation are detailed in the dengue model documentation and by Stanaway and colleagues.(1) A subset of incidence data were age/sex-specific, and we used a mixed-effects negative binomial model with cubic splines on age and interaction terms with sex to estimate the age/sex distribution of cases. We then split total incidence based on the age/sex-distribution model to estimate the incidence of symptomatic Zika by location, year, age, and sex.

We conducted a meta-analysis of three studies(2–4) to estimate the proportion of all Zika infections that are symptomatic. We estimate that 41% of Zika infections are symptomatic (14–68%), with 59% being asymptomatic. We then estimated incidence of asymptomatic infections as

$$I_{asymp} = \frac{I_{symp}}{Pr_{symp}} - I_{symp}$$

Where I_{asymp} is the incidence of asymptomatic infections, I_{symp} is the incidence of symptomatic Zika, and Pr_{symp} is the proportion of infections that are symptomatic (ie, 41%).

We assume that the incidence of Zika among pregnant women equals the incidence of Zika among all women, within a given location, year, and age group. We then estimate the number of pregnant women infected with Zika as the product of incidence of Zika and the number of pregnant women in every location, year, and age group. Finally, we used an intercept only, mixed-effects Poisson regression model, with random effects on location and year, the number of at-risk births as the exposure term, and the number of reported CZS cases as the outcome to estimate proportion of at-risk births (ie, those in which the mother was infected with Zika during pregnancy) resulting in CZS.

References

1. Stanaway JD, Shepard DS, Undurraga EA, Halasa YA, Coffeng LE, Brady OJ, et al. The global burden of dengue: an analysis from the Global Burden of Disease Study 2013. Lancet Infect Dis [Internet]. 2016 Feb [cited 2016 May 23]; Available from: http://linkinghub.elsevier.com/retrieve/pii/S1473309916000268

2. Gallian P, Cabié A, Richard P, Paturel L, Charrel RN, Pastorino B, et al. Zika virus in asymptomatic blood donors in Martinique. Blood. 2017 Jan 12;129(2):263–6.

3. Duffy MR, Chen T-H, Hancock WT, Powers AM, Kool JL, Lanciotti RS, et al. Zika virus outbreak on Yap Island, Federated States of Micronesia. N Engl J Med. 2009 Jun 11;360(24):2536–43.

4. Aubry M, Teissier A, Huart M, Merceron S, Vanhomwegen J, Roche C, et al. Zika Virus Seroprevalence, French Polynesia, 2014–2015. Emerg Infect Dis. 2017 Apr;23(4):669–72.

Dracunculiasis (Guinea worm)

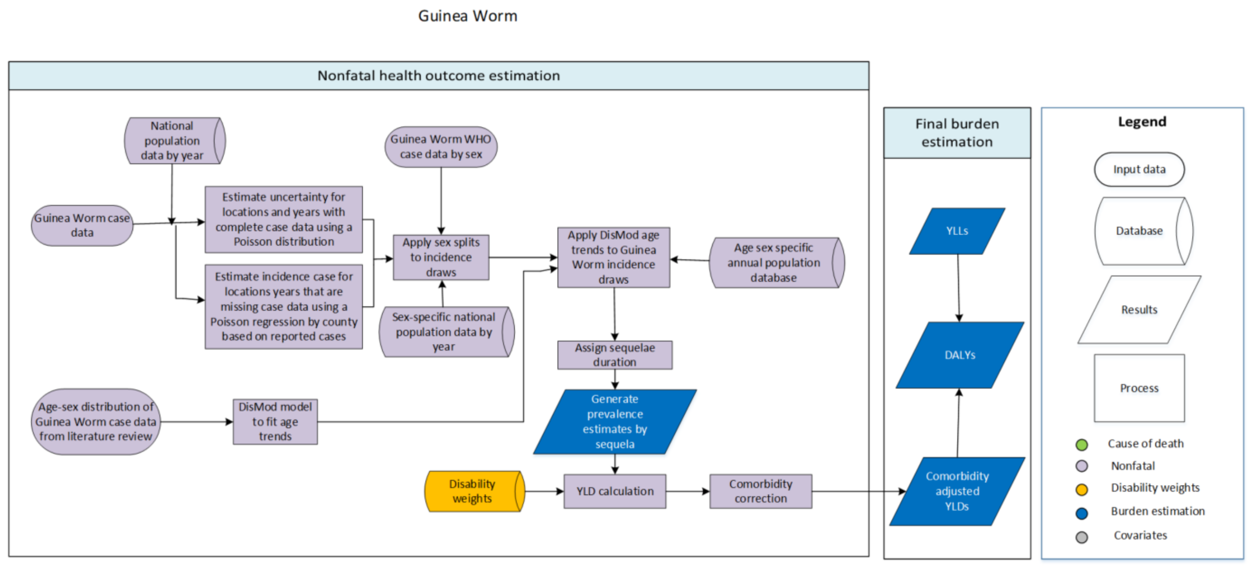

Background

Guinea-worm disease is caused by the parasitic worm *Dracunculus medinensis*. The transmission cycle begins when Guinea worm larvae are released in stagnant water (e.g., ponds, lakes, open wells) where they are ingested by freshwater copepods (small crustaceans sometimes called water fleas) of the genus *Cyclops* [1]. When a person consumes water containing *Cyclops*, the copepods are dissolved by gastric acids and intestinal enzymes and the larvae are released. Larvae then migrate through the intestinal wall and travel to the connective tissues. The larvae mature and mate 60–90 days after infection; shortly thereafter, the male dies and the pregnant female worm continues to move through the victim's connective tissues. Approximately 10–14 months post-infection, the adult worm creates a painful burning blister on the skin that develops and enlarges over several days, usually from the feet or lower limbs. Blister formation may be preceded by a slight fever, itchy rash, nausea, vomiting, and diarrhoea. To relieve the pain associated with the worm's emergence, infected persons immerse the infected part of their body in local stagnant water sources, such as ponds. Upon entering the water, the female worm will expel her larvae and the cycle can begin again [1-4].

The global campaign to eradicate Guinea worm began in 1980, when the US Centers for Disease Control and Prevention (CDC) suggested that Guinea worm eradication would be an ideal indicator of the success of the International Drinking Water Supply and Sanitation Decade of 1981–1990; in 1981, Guinea worm eradication was adopted as a sub-goal of this United Nations advocacy effort [1, 5]. In 1986, the World Health Assembly adopted a resolution to eliminate Guinea worm disease, and since then, the Carter Center has led a coalition that includes ministries of health of endemic countries, CDC, the World Health Organization (WHO), the United Nations Children's Fund (UNICEF), thousands of village volunteers, and supervisory staff supported by numerous donors [5].

To break the cycle of transmission, ministries of health in endemic countries implement a suite of interventions: case detection and containment, provision of safe water sources, distribution of filter cloths and pipe filters, water source treatment with Abate® (a larvacide), and health education.

By design, the Guinea worm eradication programmatic infrastructure covers the entire at-risk population in endemic countries. Since case containment[6] is a key intervention designed to not only interrupt transmission but also monitor progress toward eradication, incident cases of Guinea worm disease are nationally representative. To implement case containment as an intervention, all cases of Guinea worm disease are identified. Containment is defined as detection within 24 hours of the worm's emergence; the patient did not contaminate any water source; the patient received proper wound care and health education on not entering any water source; a supervisor verified the case as dracunculiasis within seven days; and Abate® is used if there is any uncertainty about contamination of water sources or known contamination of water sources [7]. Case reporting occurs at the village level on a monthly basis; case data are then aggregated within the national Guinea Worm Eradication Program and reported to WHO. In settings where annual case reports are low (suggesting no transmission) or transmission has been interrupted, cash rewards are promoted to enhance surveillance activities.

Input Data & Methodological Summary

Case Definition

A Guinea worm case is defined as an individual with Guinea worm disease. A person is counted as a case only once in a calendar year, ie, when the first Guinea worm emerged from that person, although an individual may have more than one worm emerge at a time and/or more than one worm emerge during the year. These cases are confirmed through the Guinea worm eradication program infrastructure by clinical exam and verification by local supervisors. All specimens from case-patients are sent to the CDC for laboratory evaluation and confirmation [7].

Input data

Model inputs

Geographic restrictions

Only the following countries were identified as guinea-worm endemic as of 1990[8]: Benin, Burkina Faso, Cameroon, Central African Republic, Chad, Cote d'Ivoire, Ethiopia, Ghana, India, Kenya, Mali, Mauritania, Niger, Nigeria, Pakistan, Senegal, Sudan, South Sudan, Togo, Uganda, and Yemen[8]. Any country not reporting Guinea worm as of 1990 is not included in the GBD model.

Geographic restrictions by year were also implemented to account for the period post-transmission to reflect the accomplishments of the Guinea worm eradication campaign. Geographic restriction for countries that were endemic in 1990 was defined based on data reported post-interruption of transmission. In the GBD analysis, Guinea worm disease was no longer modelled for the year that followed the last reported case (imported or indigenous) provided that the subsequent years through 2018 also had no case reports. To ensure that cases were attributed to burden in the country in which the case was detected, both indigenous and imported cases were included. For example, Kenya reported its last (imported) case in 2005, and as no other cases were reported through 2018, incidence from 2006

onward is zero. For Chad, a country that had years during which no cases were reported, the model covers the entire period 1990–2019.

Data sources

1) Case data by geography, by year
2) Literature review of age/sex distribution
3) Literature review for sequelae (type, duration, and proportion)

Case data: Annual case data were reported by WHO in the Weekly Epidemiological Record for the period 1990–2018. For years or geographies for which WER reports were not published, the following sources were also used to extract case counts:

1) CDC's MMWR reports
2) 1990–1999 total country reports from Hopkins *et al*[8]
3) India subnational estimates: India MOH report (1984–1999)
4) The Carter Center's Guinea worm wrap-up: disaggregation of case totals for Sudan and South Sudan pre-2011 (independence) to ensure case totals from 1990–2010 are consistent with current national boundaries; 2019 provisional case data.

The number of cases annually was compared to official total numbers published in WER 2016 to ensure accuracy of data entry.

Table 1 presents the total number of data sources used to generate burden estimates.

Table 1. Total data source counts

Measure	Total sources	Countries with data
All measures	436	21
Prevalence	7	4
Incidence	429	21

Subnational data

India: Subnational data for India were obtained from the Ministry of Health for the period 1984–1999; cases were reported by year and state: http://www.ncdc.gov.in/index2.asp?slid=329&sublinkid=216.

Kenya: Subnational data from Kenya were requested from the MOH but not obtained. To split cases by subnational unit, the Carter Center Guinea Worm Wrap-Up was reviewed to identify districts with endemic villages. A national survey conducted 1993/1994 found cases in Turkana and West Pokot counties, but case totals were not reported by county. Indigenous transmission was interrupted in 1995, with imported cases reported until 2005. WER reports from 1999 to 2006 document that all imported cases from 1998 to 2005 occurred in Turkana County. All cases in Kenya are currently analysed in GBD as occurring in Turkana County as we are unable to disaggregate the data.

Accounting for possible under-reporting

Once national eradication programs were initiated, national case searches were conducted to improve the accuracy of national case estimates. These searches were designed to enumerate prevalent Guinea worm disease cases and identify endemic villages to direct intervention and surveillance activities. For the majority of years included in the GBD analysis, the total number of Guinea worm cases reported is equivalent to a national census, as all cases are identified and reported. Nevertheless, not all endemic countries were able to initiate full national surveillance as of 1990.

The model does not account for the possibility that cases occurred in communities that were not included in routine surveillance or did not achieve 100% reporting coverage over time. However, any cases that may have been undetected would likely not have been a significant increase over annual totals given the comprehensive nature of Guinea worm disease surveillance activities. Nevertheless, there are years for which the annual case data is inconsistent with preceding/following annual case totals and could not be accounted for in our model. For example, Niger reported 500 cases in 1992, despite reporting 32,829 cases in 1991 and 25,346 cases in 1993. In those instances, the following data points were identified as outliers and excluded from analysis as follows:

Table 2. List of reported case data outliered in the analysis to account for possible under-reporting

Country	Year	Reported Cases
Central African Republic	1996	9
Central African Republic	1997	5
Ethiopia	1992	303
Kenya (Turkana County)	1990	6
Uganda	1990	4,704
Uganda*	1992	126,369
Benin	1991	4,006
Benin	1992	4,315
Chad	1992	156
Cote d'Ivoire	1990	1,360
Mali	1990	884
Mauritania	1992	1,557
Niger	1992	500
Senegal	1990	38
Togo	1990	3,042
Togo	1991	5,118
South Sudan*	1996	116,844
Sudan	1994	132

*For these two data points, we do not dispute that over 100,000 cases of Guinea worm likely occurred. However, given the amount of missing data in the early time series for these two countries, inclusion of these resulted in implausibly high case predictions (over 1 million cases in Uganda in 1990 and over 1.5 million for South Sudan from 1990 to 1995).

Age/sex distribution

Generally, the risk of Guinea worm infection varies according to sex- or age-specific differences in access to safe drinking water. A study in Ethiopia found women were more likely to experience Guinea worm disease than men; in India, men experienced greater risk of infection [1]. Exposure to unsafe water sources varies largely on mobility patterns and type of water sources: communities in which infected water is carried in for consumption are more likely to see more Guinea worm disease in children and older adults [9]. Once interventions to control the spread of Guinea worm infection are implemented, the age and sex distribution likely changes to reflect variation in coverage and uptake of eradication interventions, such as larvacide of water sources and case-containment rates; age/sex case data are currently not available.

The evidence base available to describe risk of infection by age is as follows:

1) Studies from Nigeria:
    a. Adeyeba *et al* [10]: Guinea worm disease not common among children <1 year of age; increase in risk by age
    b. Kale *et al* [11]: More boys ages 5-9 years than girls were infected (11.9% v. 6.8%); Women ages 20-29 years had higher prevalence of infection than men (13.4% v. 4.7%); Overall, the prevalence in both men and women was highest in ages 10-14 years and 30 years or older.
    c. Greenwood *et al* [12]: The mean age of male cases was 25.8 years (95% CI: 23.9, 27.7) and 26.9 years for females (95% CI: 23.7, 30.1).
2) Other countries:
    a. Sudan [13]: No significant age trend among lower-endemicity villages; higher-endemicity villages (n=4) had higher prevalence in children and older adults. This study attributes the difference in age trends to community-level water source.
    b. Ghana [14]: The trend in age of first infection reported was similar for males and females, with more females experiencing first infection between 15 and 19 years and males between 20 and 24 years of age. The proportion of men with Guinea worm disease was much higher than among women 25-54 years of age. Adults >15 years of age were more likely to be infected than children.

The evidence base available to describe the risk of infection by gender is as follows:

1) Studies from Nigeria:
    a. Adeyeba *et al* [10]: No difference among males and females.
    b. Kale *et al* [11]: No overall gender difference comparing total males infected to total females infected, although gender differences for certain age groups (see notes above).
    c. Greenwood *et al* [12]: Two-thirds of cases reported among 47 villages from 1971 to 1974 were male.

WHO Weekly Epidemiological Record (WER) age reports: Age and sex data were reported by country for 2009 onward; these data capture the age distribution for Chad, Ethiopia, Ghana, Mali, and South Sudan. We excluded these data as the age/sex distribution is only described for children <15 years or adults, which does not permit fitting an age trend across multiple categories.

WER sex-specific data: Sex-specific differences in the burden of Guinea worm disease could reflect differing levels of access to eradication program interventions, in addition to risk factors associated with local transmission dynamics. Since the data reported from 2009 to 2015 are the only available nationally representative data, we used the overall sex difference to generate sex-specific incidence and prevalence, with females experiencing a slightly higher risk (53%) compared to males (47%):

Table 3. WHO Weekly Epidemiological Record total worm burden by gender, by year

Year	Female	Male	Total	% Fem	% Male
**2009**	1699	1490	3189	53%	47%
**2010**	976	821	1797	54%	46%
**2011**	524	534	1058	50%	50%
**2012**	273	269	542	50%	50%
**2013**	79	69	148	53%	47%
**2014**	63	63	126	50%	50%
**2015**	9	13	22	41%	59%
**Total**	3623	3259	6882	53%	47%

There is limited evidence to suggest that risk varies jointly by sex and age; however, evidence for this modification also suggests that such age- and sex-specific risks may vary by endemic community within a given geography (in some settings, women at higher risk, in others men, but not for all age strata). Without additional data sources in which cases are disaggregated by age and sex, this joint relationship is not modelled.

To model age-specific variation, we used data from seven studies with age-specific case data to generate an age-trend in a DisMod model. We further assumed no Guinea worm disease occurred in infants less than 1 year of age.

Severity splits/sequelae

Sequelae associated with Guinea worm relate to the wound at the site of the worm's emergence, which can include abscesses and chronic ulcerations. Joint and tissue damage can occur, as well as secondary infection in connective tissues [15]. During the worm's emergence, which takes approximately one month to exit the body, the ulcer is painful and itchy [1]. The wound is subject to secondary infection and scarring. Possible long-term consequences of Guinea worm infection include arthritis or other permanent damage to connective tissues; however, data on this are limited. In the Greenwood study, 41.7% of all cases experienced infection at the site of emergence, and the annual proportion of cases with definite arthritis ranged from 1.6% to 7.3% of all cases.

While an individual experiences Guinea worm disease, they are generally unable to work and have limited mobility at the time prior and during emergence and in the subsequent period in which they are healing. Although most worms emerge in the feet and lower legs, there are reports of worms exiting at other sites [15], which could cause other disability not accounted for here. A study in Nigeria found that 98% of worms emerged in the lower limbs[16]. The Greenwood study also observed that 88.4% emerged in the lower limbs. Therefore, for the purposes of estimating the burden of Guinea worm disease in GBD, all disability associated with Guinea worm disease is attributed to lower limb conditions, pain, and lack of mobility. Due to limited data, we cannot account for differential disability based on number of worms emerging at the same time.

The following evidence base was reviewed to determine the proportion of cases attributed to each sequela, as well as duration of sequelae.

Duration of disability and type of disability:

Studies from Nigeria:

1) Adeyeba *et al* [10]: 93.4% incapacitated for an average of 26 days.
2) Smith *et al* [17]: Average disability duration 12.7 weeks; 58% unable to leave the home for a mean duration of 4.2 weeks; duration of disability greater among those older than 50 years compared to those younger than 50 years.
3) Okoye *et al* [16]: 21% of cases were totally incapacitated due to their infection (not permanently disabled).
4) Kate *et al* [11]: A survey of 17 villages from 1971 to 1975 found that duration of disability was approximately 100 days.
5) Greenwood *et al* [12]: Weekly visits to 47 villages from 1971 to 1974 reported mean duration of illness ranging from 4.2 weeks to 7.2 weeks. 17.4% of cases had an active infection which persisted for 10 weeks or more.

Other countries:

6) Benin [18]: From two villages in highly endemic areas, estimated 39-59 days of disability experienced after worm emergence.
7) Ghana [19]: 28.2% experienced pain 12-18 months post-emergence; 5% unable to carry out at least one daily activity, 0.5% permanently impaired (ligament damage to thumb).
8) Ghana [14]: Complete disability experienced among males with Guinea worm disease lasted approximately 5 weeks among those untreated. Among cases provided supportive care (wound management), the duration of disability was 2.5 weeks.

For all cases, we assume each experiences pain and disfigurement (level 2), and musculoskeletal problems, lower limb (moderate) for a period of one month, followed by two months of pain and disfigurement (mild). We then assume that 30% of all cases will then experience disfigurement level 1 with itch/pain for an additional nine months (approximately a year of disability) to account for longer-term disability associated with recovery.

Table 4. Sequela associated with Guinea worm disease in the Global Burden of Disease study

Sequela	Lay description	DW (95% CI)
Disfigurement, level 2, with itch/pain	Has a visible physical deformity that is sore and itchy. Other people stare and comment, which causes the person to worry. The person has trouble sleeping and concentrating.	0.188 (0.125–0.267)
Disfigurement, level 1, with itch/pain	Has a slight, visible physical deformity that is sometimes sore or itchy. Others notice the deformity, which causes some worry and discomfort.	0.027 (0.015–0.042)
Musculoskeletal problems, lower limbs, moderate	Has moderate pain in the leg, which makes the person limp, and causes some difficulty walking, standing, lifting and carrying heavy things, getting up and down and sleeping.	0.079 (0.054–0.11)

Modelling strategy

Total incidence

The incidence of Guinea worm disease is modelled in GBD using two approaches: for years and locations for which case data were reported, 1,000 draws of incidence were estimated using a beta distribution of cases and total population minus cases. For years and locations for which case data were missing (largely the early 1990s) a Poisson regression of all case data was implemented per country, using the total population as the offset. The predicted incidence and standard error were used to generate a random distribution of 1,000 incidence draws. Incidence is multiplied by duration of sequelae to calculate prevalence. Country-level incidence predictions are shown in the following figures.

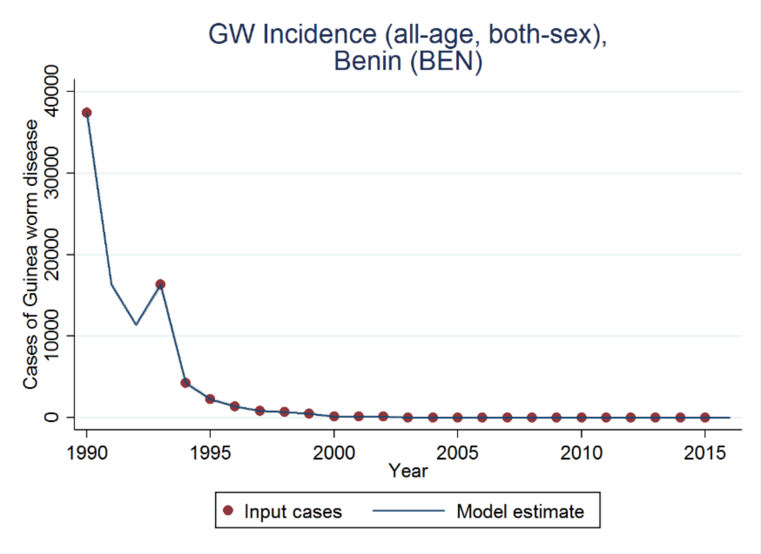
GW Incidence (all-age, both-sex),
Benin (BEN)
Cases of Guinea worm disease
0
10000
20000
30000
40000
1990
1995
2000
2005
2010
2015
Year
Input cases
Model estimate

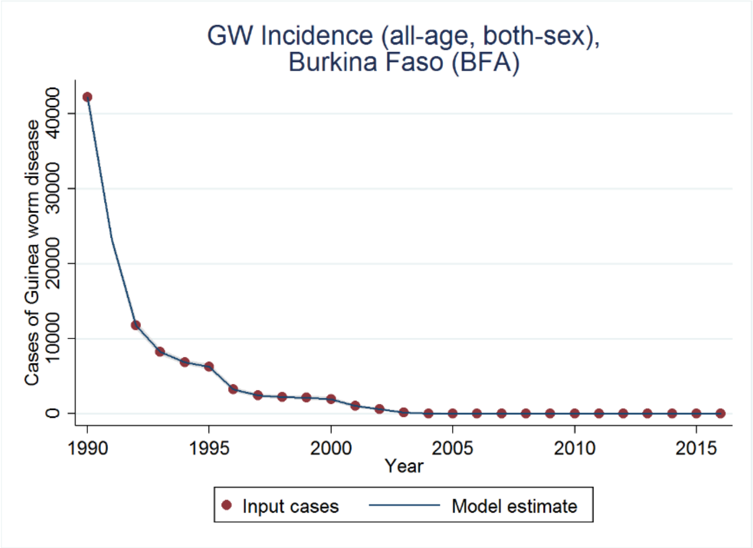
GW Incidence (all-age, both-sex),
Burkina Faso (BFA)
Cases of Guinea worm disease
0
10000
20000
30000
40000
1990
1995
2000
2005
2010
2015
Year
Input cases
Model estimate

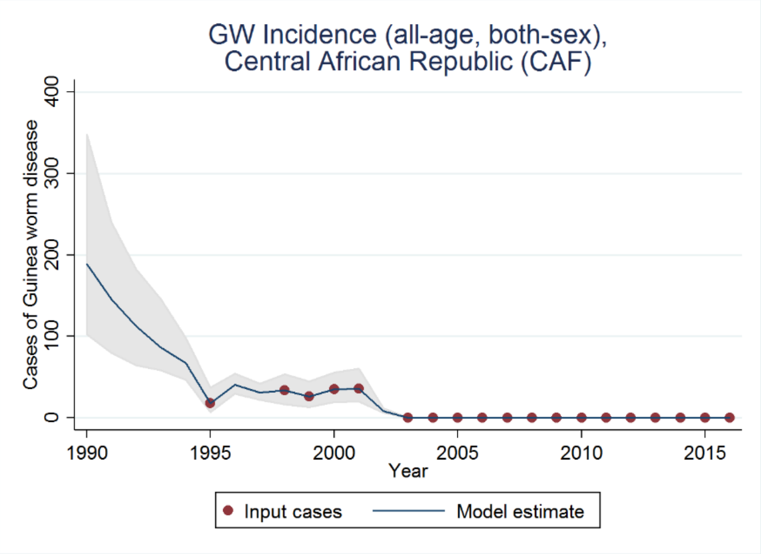
GW Incidence (all-age, both-sex),
Central African Republic (CAF)
Cases of Guinea worm disease
0
100
200
300
400
1990
1995
2000
2005
2010
2015
Year
Input cases
Model estimate

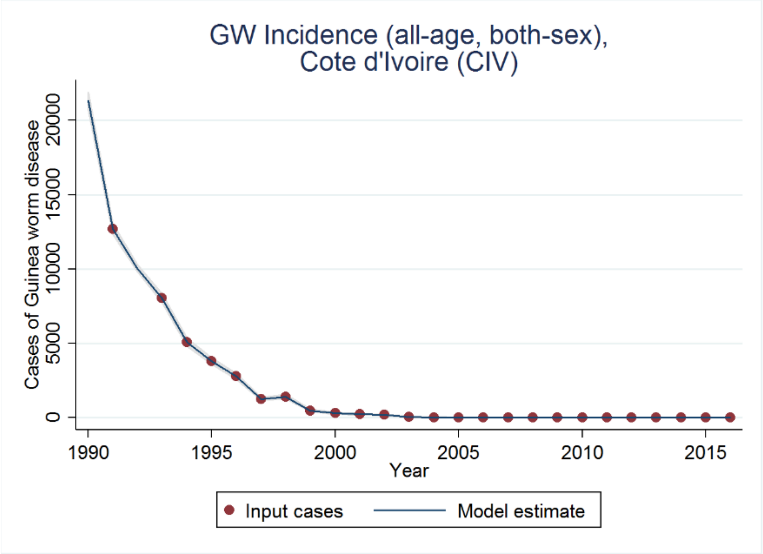
GW Incidence (all-age, both-sex),
Cote d'Ivoire (CIV)
Cases of Guinea worm disease
0
5000
10000
15000
20000
1990
1995
2000
2005
2010
2015
Year
Input cases
Model estimate

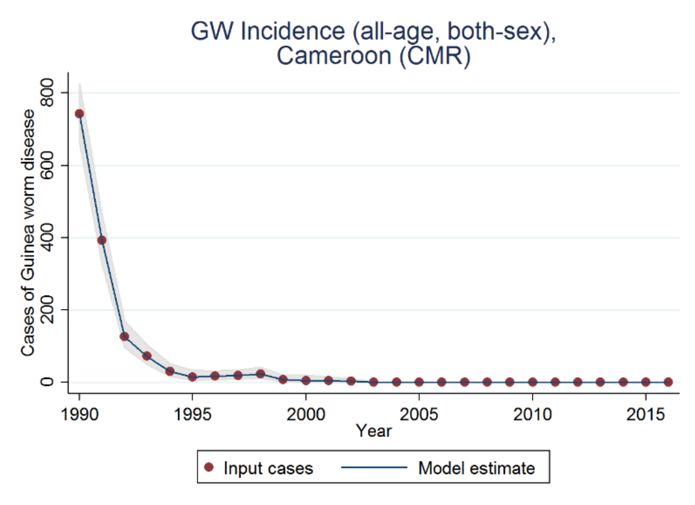
GW Incidence (all-age, both-sex),
Cameroon (CMR)
Cases of Guinea worm disease
0
200
400
600
800
1990
1995
2000
2005
2010
2015
Year
Input cases
Model estimate

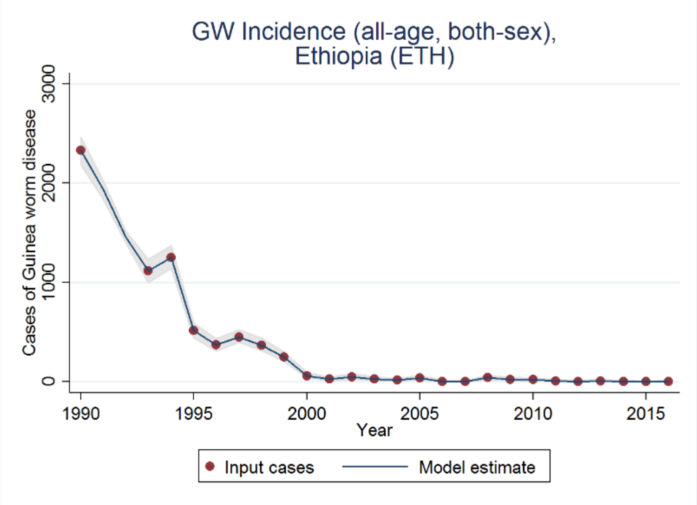
GW Incidence (all-age, both-sex),
Ethiopia (ETH)
Cases of Guinea worm disease
0
1000
2000
3000
1990
1995
2000
2005
2010
2015
Year
Input cases
Model estimate

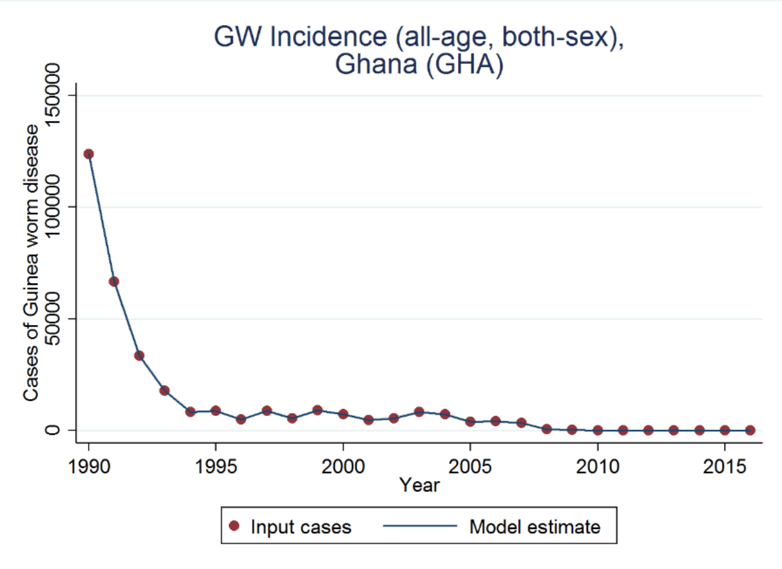
GW Incidence (all-age, both-sex),
Ghana (GHA)
Cases of Guinea worm disease
0
50000
100000
150000
1990
1995
2000
2005
2010
2015
Year
Input cases
Model estimate

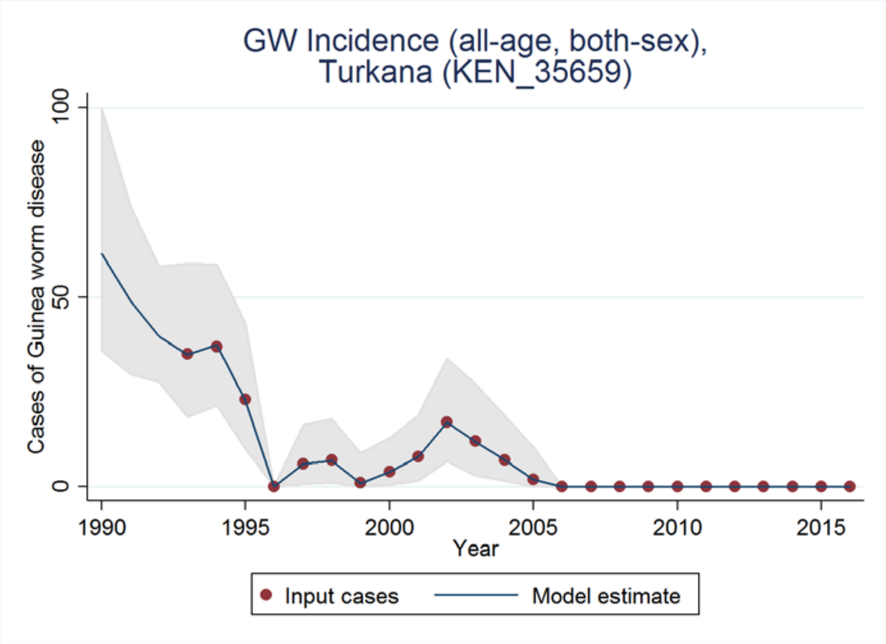
GW Incidence (all-age, both-sex),
Turkana (KEN_35659)
Cases of Guinea worm disease
0
50
100
1990
1995
2000
2005
2010
2015
Year
Input cases
Model estimate

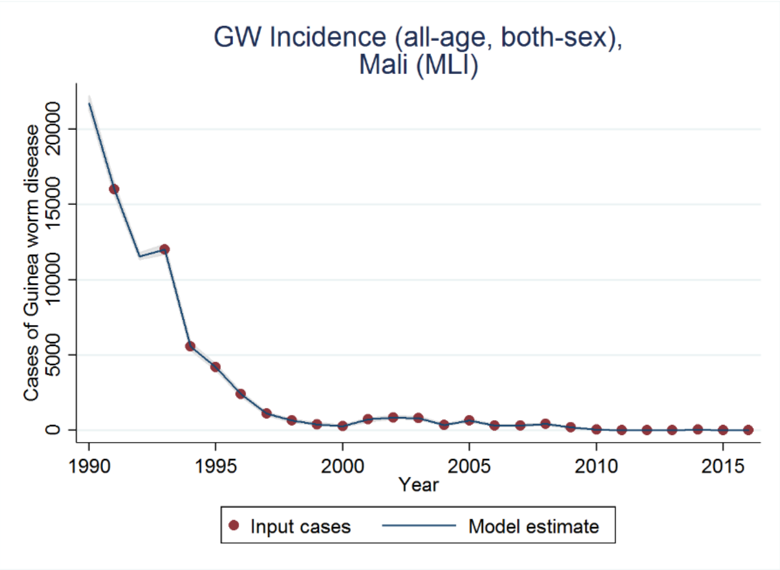
GW Incidence (all-age, both-sex),
Mali (MLI)
Cases of Guinea worm disease
0
5000
10000
15000
20000
1990
1995
2000
2005
2010
2015
Year
Input cases
Model estimate

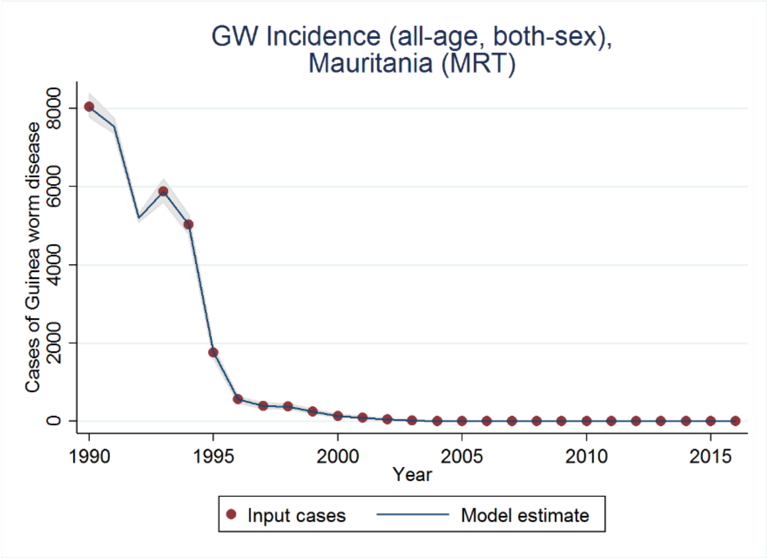
GW Incidence (all-age, both-sex),
Mauritania (MRT)
Cases of Guinea worm disease
0
2000
4000
6000
8000
1990
1995
2000
2005
2010
2015
Year
Input cases
Model estimate

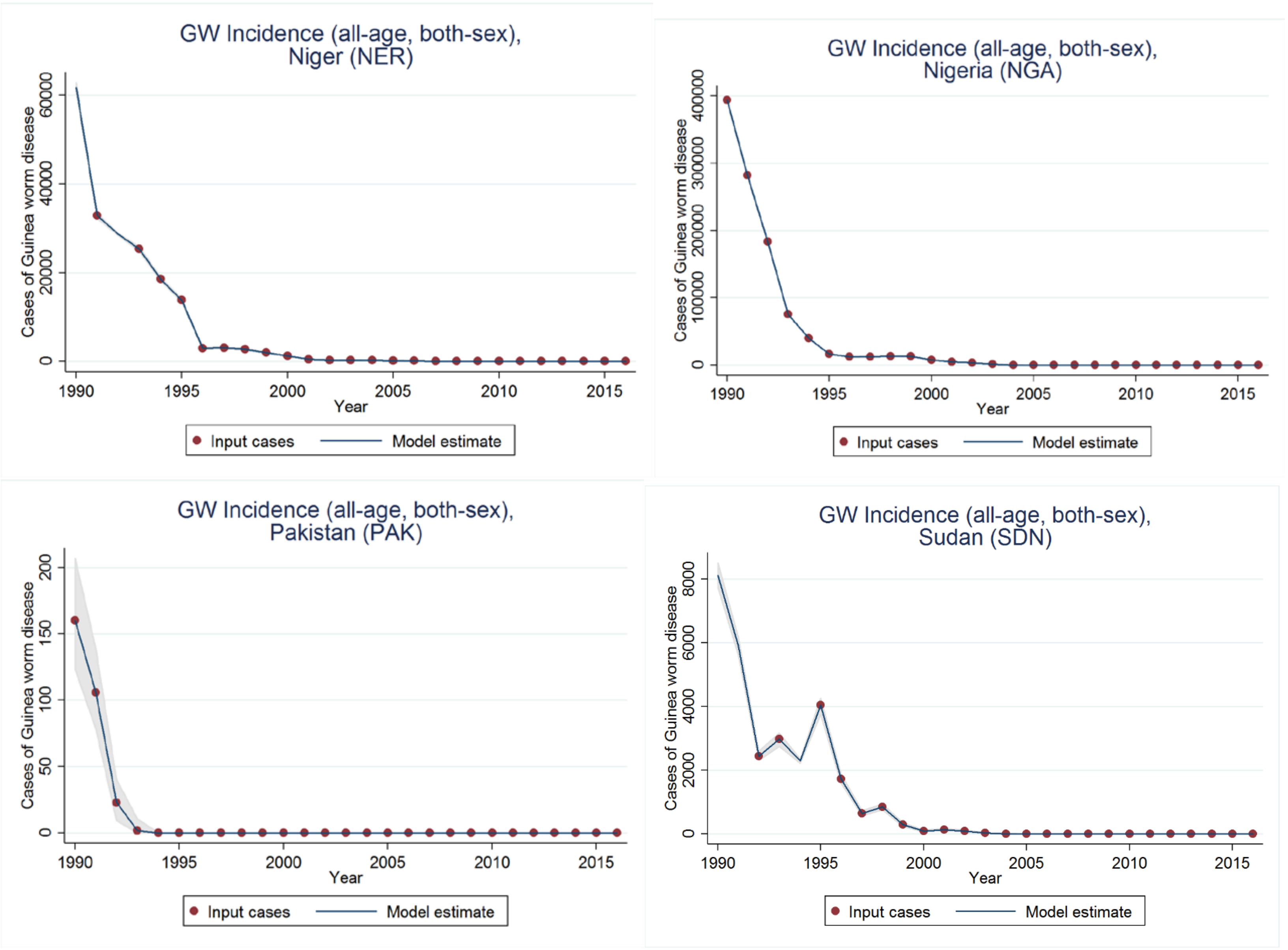
GW Incidence (all-age, both-sex), Niger (NER)
Cases of Guinea worm disease
0
20000
40000
60000
1990
1995
2000
2005
2010
2015
Year
Input cases
Model estimate
GW Incidence (all-age, both-sex), Nigeria (NGA)
Cases of Guinea worm disease
0
100000
200000
300000
400000
1990
1995
2000
2005
2010
2015
Year
Input cases
Model estimate
GW Incidence (all-age, both-sex), Pakistan (PAK)
Cases of Guinea worm disease
0
50
100
150
200
1990
1995
2000
2005
2010
2015
Year
Input cases
Model estimate
GW Incidence (all-age, both-sex), Sudan (SDN)
Cases of Guinea worm disease
0
2000
4000
6000
8000
1990
1995
2000
2005
2010
2015
Year
Input cases
Model estimate

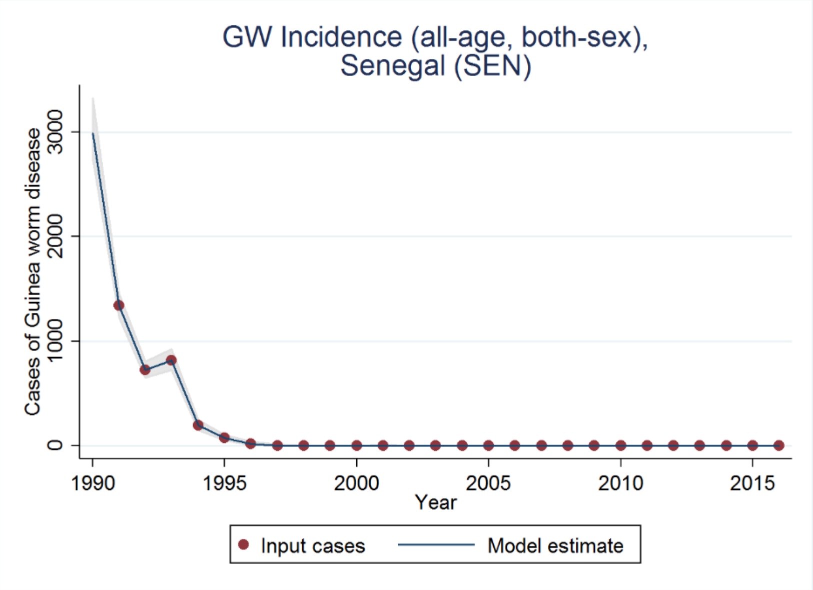

GW Incidence (all-age, both-sex),
Senegal (SEN)
Cases of Guinea worm disease
0
1000
2000
3000
1990
1995
2000
2005
2010
2015
Year
Input cases
Model estimate

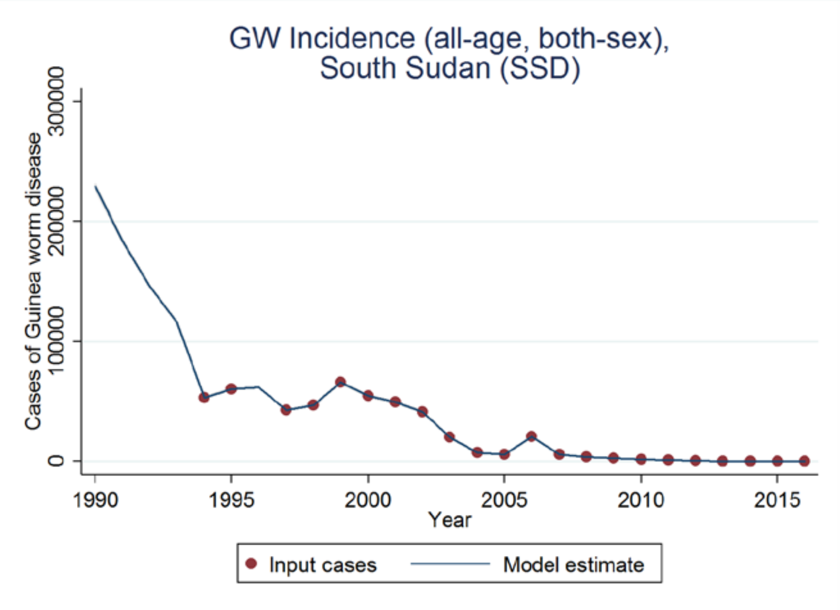

GW Incidence (all-age, both-sex),
South Sudan (SSD)
Cases of Guinea worm disease
0
100000
200000
300000
1990
1995
2000
2005
2010
2015
Year
Input cases
Model estimate

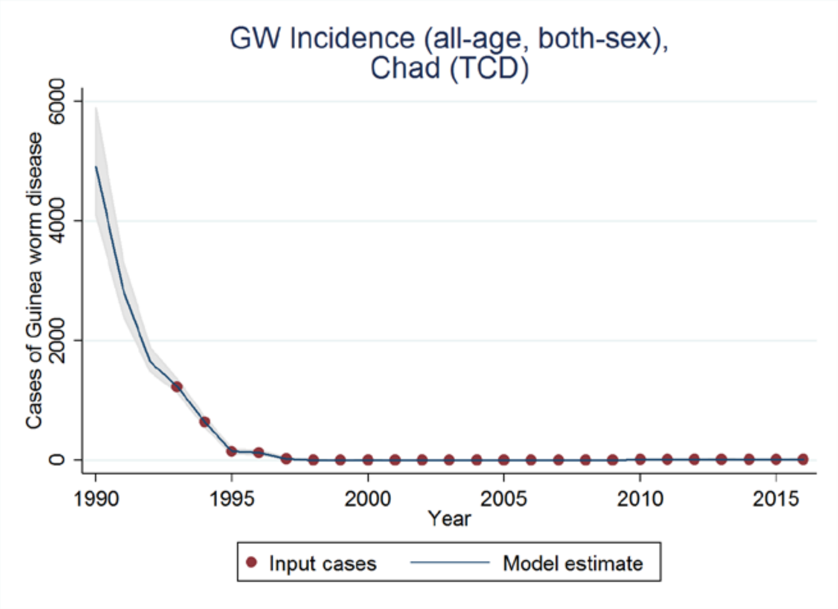

GW Incidence (all-age, both-sex),
Chad (TCD)
Cases of Guinea worm disease
0
2000
4000
6000
1990
1995
2000
2005
2010
2015
Year
Input cases
Model estimate

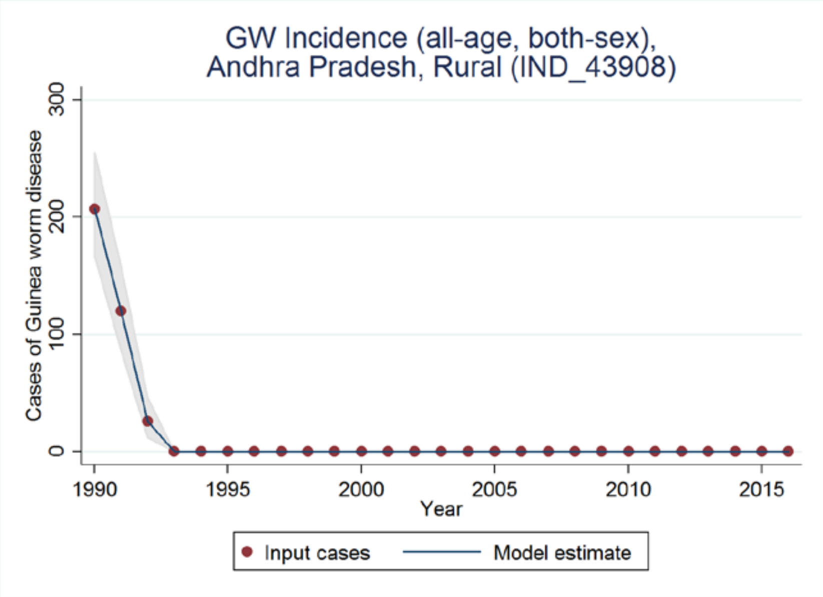

GW Incidence (all-age, both-sex),
Andhra Pradesh, Rural (IND_43908)
Cases of Guinea worm disease
0
100
200
300
1990
1995
2000
2005
2010
2015
Year
Input cases
Model estimate

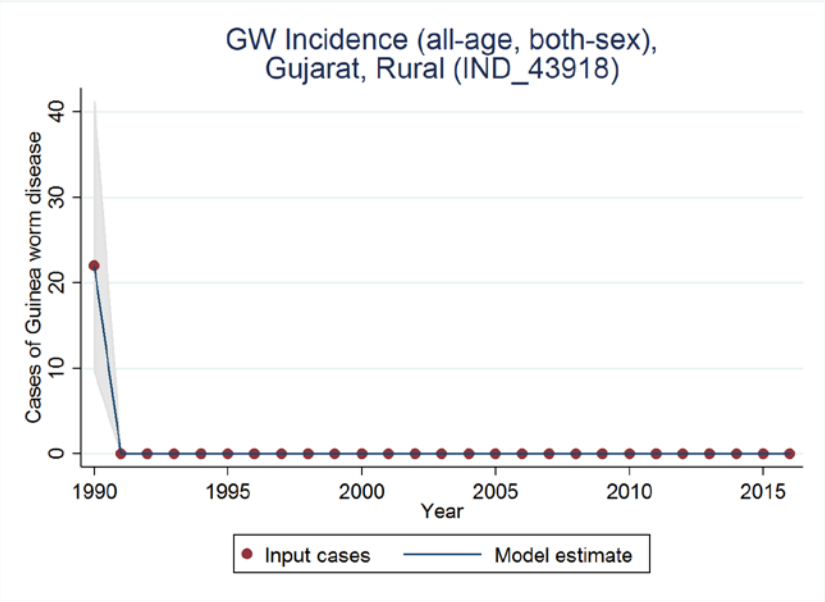
GW Incidence (all-age, both-sex),
Gujarat, Rural (IND_43918)
Cases of Guinea worm disease
0
10
20
30
40
1990
1995
2000
2005
2010
2015
Year
Input cases
Model estimate

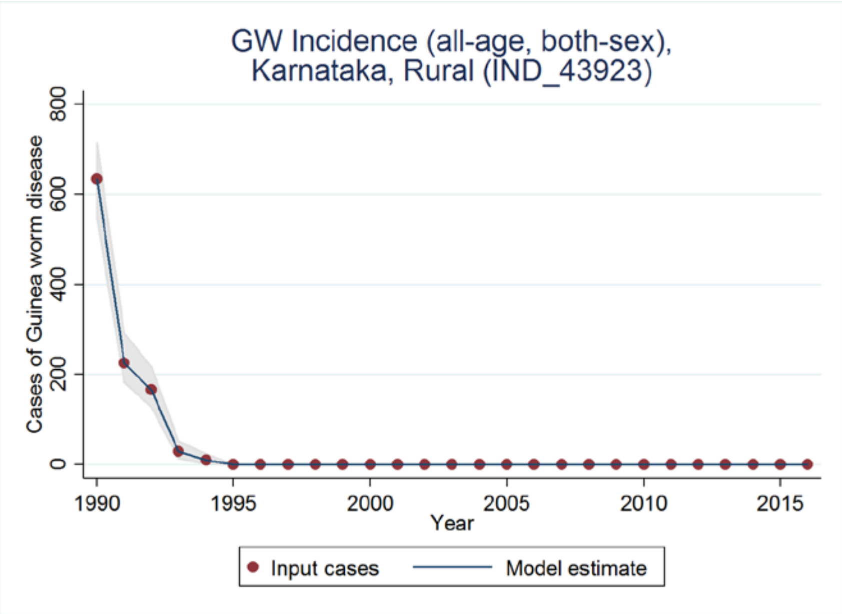
GW Incidence (all-age, both-sex),
Karnataka, Rural (IND_43923)
Cases of Guinea worm disease
0
200
400
600
800
1990
1995
2000
2005
2010
2015
Year
Input cases
Model estimate

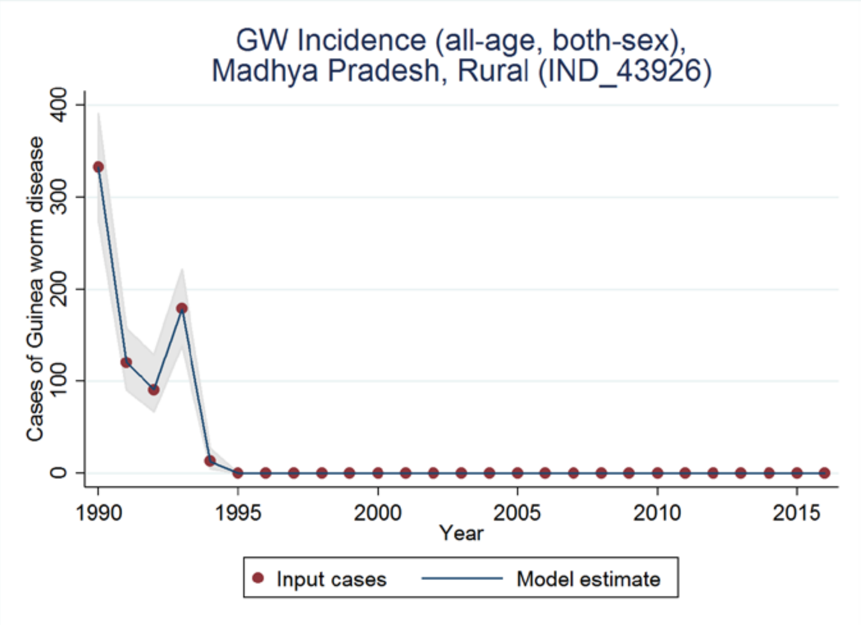
GW Incidence (all-age, both-sex),
Madhya Pradesh, Rural (IND_43926)
Cases of Guinea worm disease
0
100
200
300
400
1990
1995
2000
2005
2010
2015
Year
Input cases
Model estimate

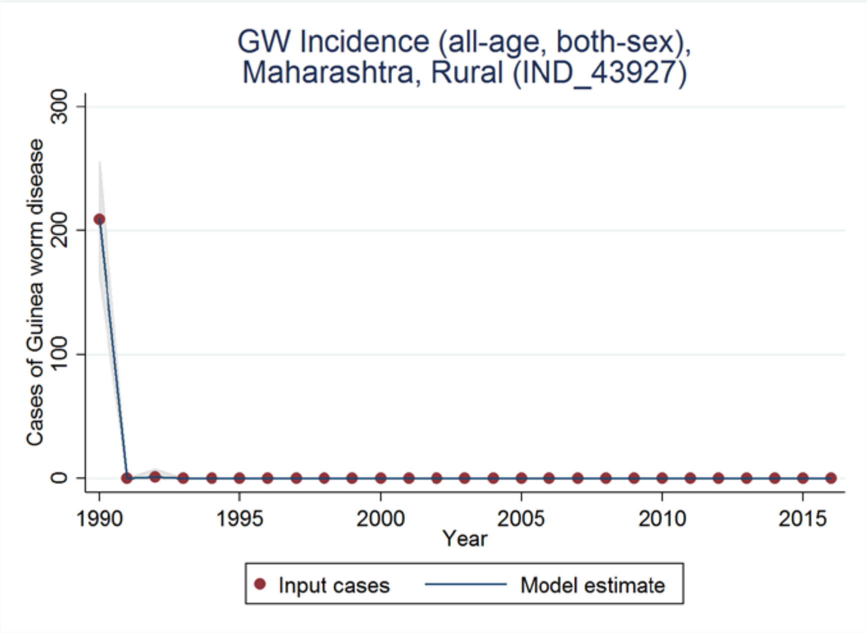
GW Incidence (all-age, both-sex),
Maharashtra, Rural (IND_43927)
Cases of Guinea worm disease
0
100
200
300
1990
1995
2000
2005
2010
2015
Year
Input cases
Model estimate

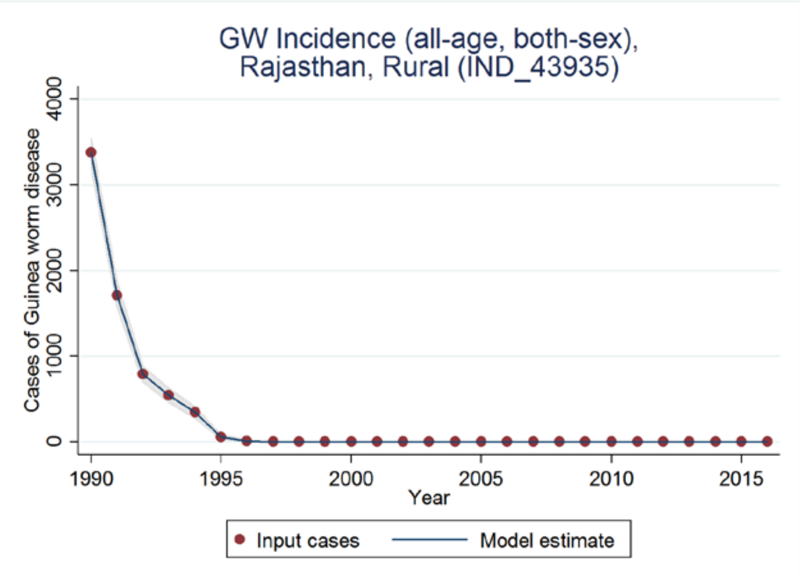
GW Incidence (all-age, both-sex),
Rajasthan, Rural (IND_43935)
Cases of Guinea worm disease
0
1000
2000
3000
4000
1990
1995
2000
2005
2010
2015
Year
Input cases
Model estimate

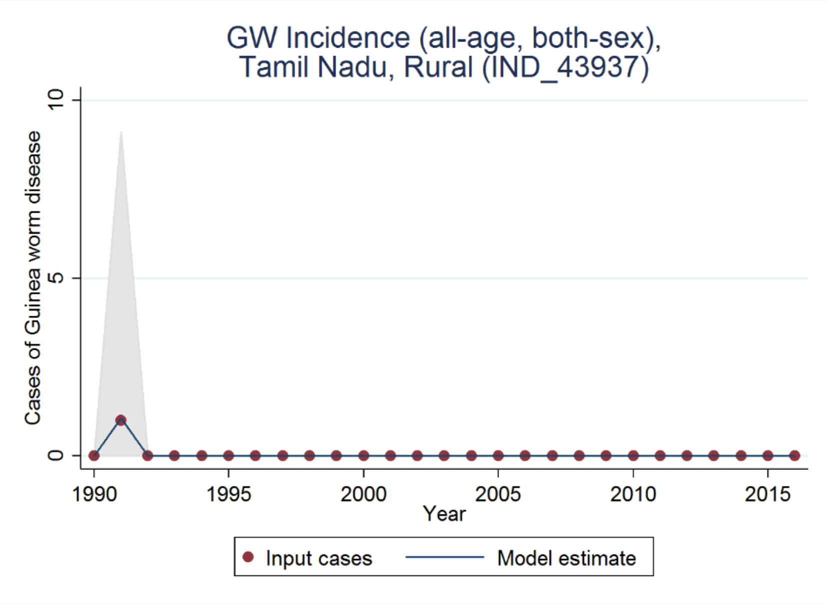
GW Incidence (all-age, both-sex),
Tamil Nadu, Rural (IND_43937)
Cases of Guinea worm disease
0
5
10
1990
1995
2000
2005
2010
2015
Year
Input cases
Model estimate

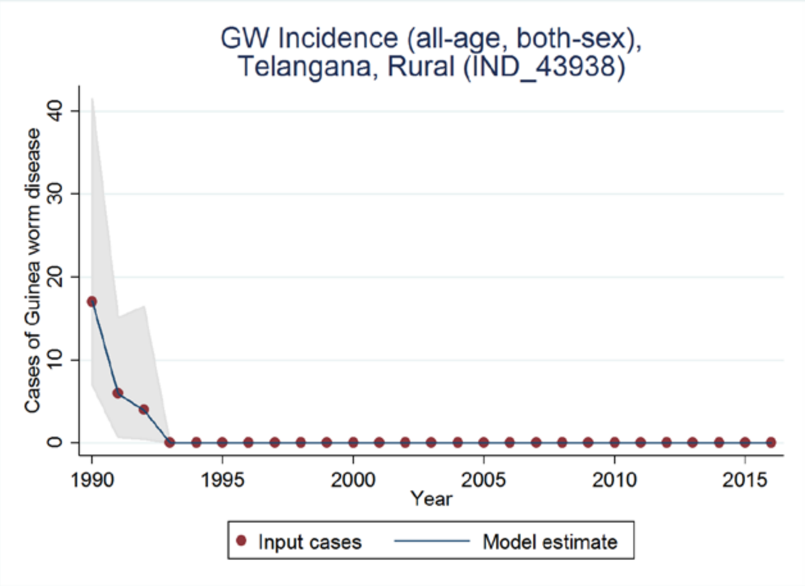
GW Incidence (all-age, both-sex),
Telangana, Rural (IND_43938)
Cases of Guinea worm disease
0
10
20
30
40
1990
1995
2000
2005
2010
2015
Year
Input cases
Model estimate

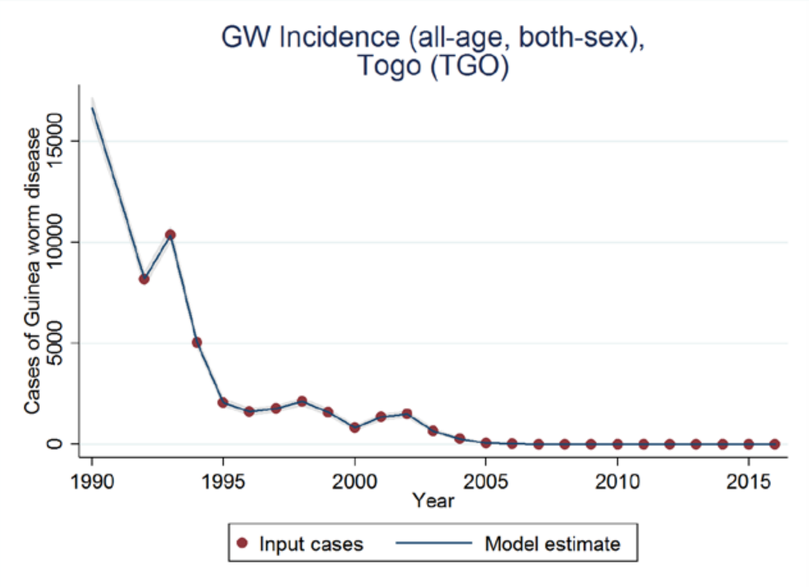
GW Incidence (all-age, both-sex),
Togo (TGO)
Cases of Guinea worm disease
0
5000
10000
15000
1990
1995
2000
2005
2010
2015
Year
Input cases
Model estimate

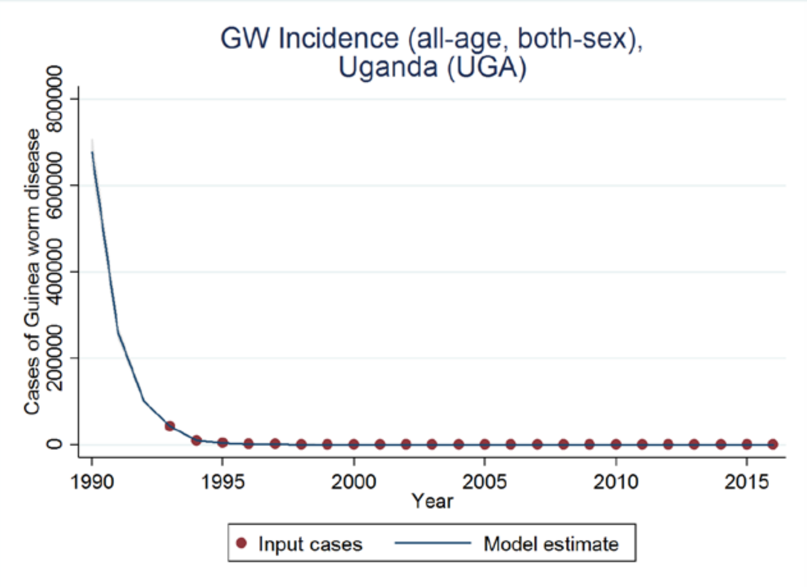
GW Incidence (all-age, both-sex),
Uganda (UGA)
Cases of Guinea worm disease
0
200000
400000
600000
800000
1990
1995
2000
2005
2010
2015
Year
Input cases
Model estimate

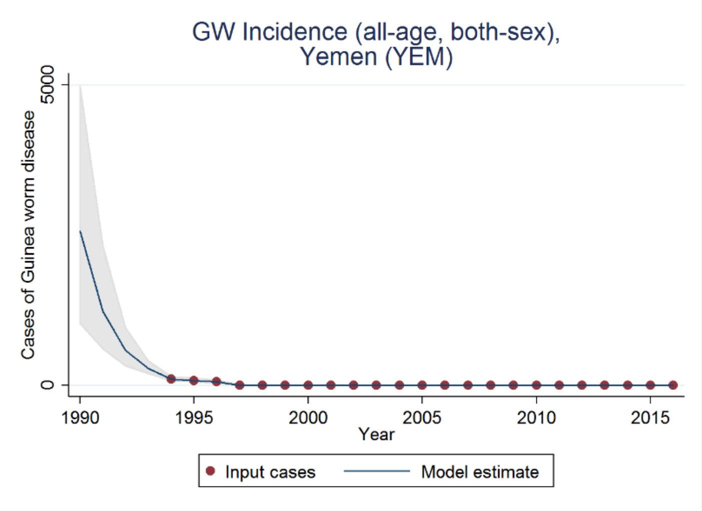
GW Incidence (all-age, both-sex),
Yemen (YEM)
Cases of Guinea worm disease
0
5000
1990
1995
2000
2005
2010
2015
Year
Input cases
Model estimate

Figure 1. Overall comparison of model versus reported cases (excluding outliers)

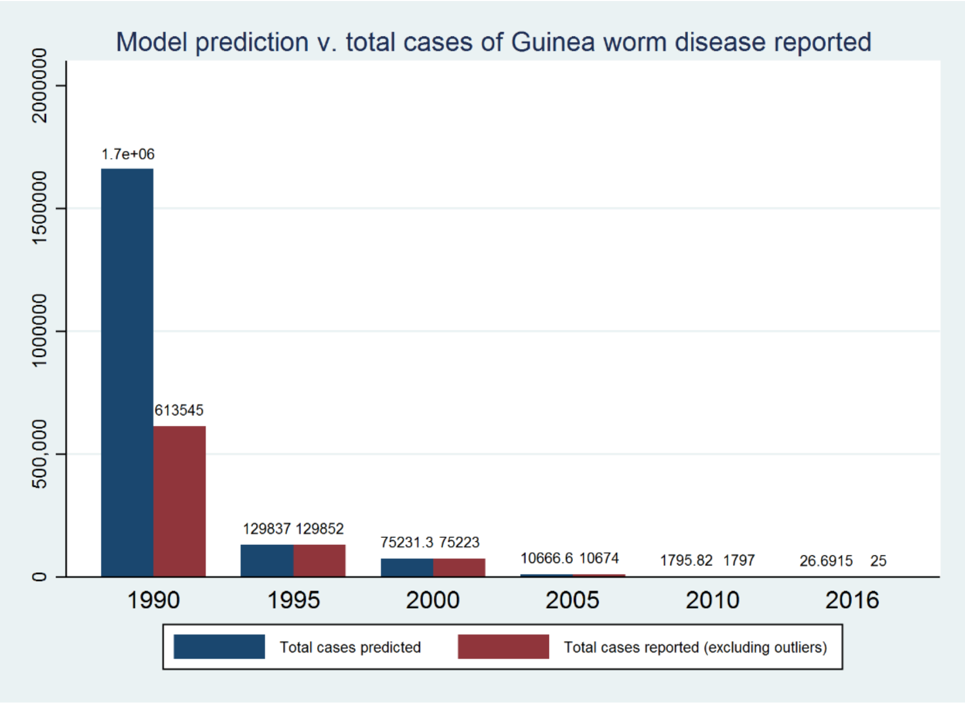

Sex-specific incidence

To account for the proportion of cases in females compared to males (53% to 47%), the incidence draws were multiplied by the sex proportion and the total population (to estimate number of cases by sex), then divided by the sex-specific total population for that year to calculate sex-specific incidence.

Age-specific incidence

In order to generate age-specific incidence, a literature search was conducted to identify national and subnational data sources in which age-specific prevalence was reported. The only nationally representative data available were WER reports from 2009 onward; however, age was only reported as less than 15 years of age or older than 15 years of age. In order to generate a trend over the life course, eight subnational data sources were identified. The prevalence of Guinea worm disease was extracted by age category reported in the original paper. An age trend was then fit using DisMod 2.0, with the following model settings:

Age mesh points: 0 0.01 5 10 15 20 25 30 35 40 45 50 55 60 65 70 75 80 85 90 1000

Drill year: 2000; Drill location: Global; no birth prevalence; 30 year time window

The age data were used to generate one single-age trend that we assumed applied to all geographies and all estimation periods from 1990 to 2019.

Figure 2. Age-specific prevalence model generated by DisMod

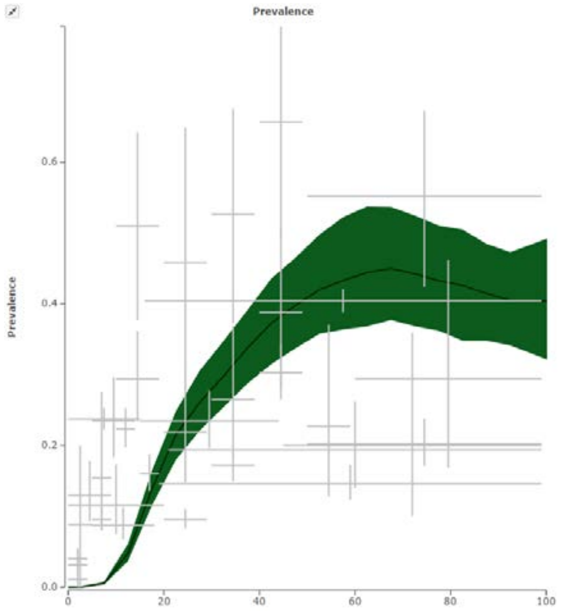

To apply this age prevalence curve to the sex-split incidence draws, 1,000 draws of output were downloaded from DisMod and applied to the incidence data as follows:

j indexes the age strata

i indexes the draw (1 to 1,000)

sex cases draw is the total number of cases for the sex stratum (all ages)

$$age\ cases_j = \ DisMod\ Draw_{i,j} * age\ population_j$$

$$age\ incidence\ draw_i = \frac{age\ cases_j \left(\frac{sex\ cases\ draw_i}{total\ cases}\right)}{age\ population_j}$$

Under the assumption that Guinea worm disease occurs approximately one year post-infection, incidence among children aged less than 1 year was set to zero.

Sequelae splits

Prevalence of the sequelae listed in Table 4 was calculated by multiplying the age- and sex-specific incidence draw by the duration of the health state (in years).

1) Guinea worm pain associated with worm emergence (Level 2): all cases, 1 month
2) Guinea worm pain associated with worm emergence (Level 1): all cases, 2 months plus 30% of cases for an additional 9 months
3) Lower limb musculoskeletal problems: all cases, 1 month

References

1. Cairncross S, Muller R, Zagaria N. Dracunculiasis (Guinea worm disease) and the eradication initiative. Clin Microbiol Rev. 2002;15(2):223-46.
2. Biswas G, Sankara DP, Agua-Agum J, Maiga A. Dracunculiasis (guinea worm disease): eradication without a drug or a vaccine. Philos Trans R Soc Lond B Biol Sci. 2013;368(1623):20120146.
3. Ruiz-Tiben E, Hopkins DR. Dracunculiasis (Guinea worm disease) eradication. Adv Parasitol. 2006;61:275-309.
4. Greenaway C. Dracunculiasis (guinea worm disease). CMAJ. 2004;170(4):495-500.
5. Hopkins DR, Ruiz-Tiben E, Downs P, Withers PC, Jr., Roy S. Dracunculiasis eradication: neglected no longer. Am J Trop Med Hyg. 2008;79(4):474-9.
6. Kappus KD, Hopkins DR, Ruiz-Tiben E, Imtiaz R, Andersen J, Azam M, et al. A strategy to speed the eradication of dracunculiasis. World Health Forum. 1991;12(2):220-5.
7. Prevention CfDCa. Guinea worm wrap-up Atlanta, GA: WHO Collborating center for Research, Training and Eradication of Dracunculiasis, CDC; 2015.
8. Hopkins DR, Ruiz-Tiben E, Diallo N, Withers PC, Jr., Maguire JH. Dracunculiasis eradication: and now, Sudan. Am J Trop Med Hyg. 2002;67(4):415-22.
9. Watts SJ, Brieger WR, Yacoob M. Guinea worm: an in-depth study of what happens to mothers, families and communities. Soc Sci Med. 1989;29(9):1043-9.
10. Adeyeba OA, Kale OO. Epidemiology of dracunculiasis and its socio-economic impact in a village in south-west Nigeria. West Afr J Med. 1991;10(3-4):208-15.
11. Kale OO. The clinico-epidemiological profile of guinea worm in the Ibadan district of Nigeria. Am J Trop Med Hyg. 1977;26(2):208-14.
12. Greenwood B, Greenwood A, Bradley A. Guinea worm infection in northern Nigeria: reflections on a disease approaching eradication. Trop Med Int Health. 2017.
13. Tayeh A, Cairncross S. The impact of dracunculiasis on the nutritional status of children in South Kordofan, Sudan. Ann Trop Paediatr. 1996;16(3):221-6.
14. Belcher DW, Wurapa FK, Ward WB, Lourie IM. Guinea worm in southern Ghana: its epidemiology and impact on agricultural productivity. Am J Trop Med Hyg. 1975;24(2):243-9.
15. Muller R. Guinea worm disease: epidemiology, control, and treatment. Bull World Health Organ. 1979;57(5):683-9.
16. Okoye SN, Onwuliri CO, Anosike JC. A survey of predilection sites and degree of disability associated with guineaworm (Dracunculus medinensis). Int J Parasitol. 1995;25(9):1127-9.
17. Smith GS, Blum D, Huttly SR, Okeke N, Kirkwood BR, Feachem RG. Disability from dracunculiasis: effect on mobility. Ann Trop Med Parasitol. 1989;83(2):151-8.
18. Chippaux JP, Banzou A, Agbede K. [Social and economic impact of dracunculosis: a longitudinal study carried out in 2 villages in Benin]. Bull World Health Organ. 1992;70(1):73-8.
19. Hours M, Cairncross S. Long-term disability due to guinea worm disease. Trans R Soc Trop Med Hyg. 1994;88(5):559-60.

Other neglected tropical diseases

In addition to the neglected tropical diseases described above, there are many diverse types of neglected tropical diseases, which are encompassed by the following ICD 10 codes:

A68 Relapsing fevers

A68.0 Louse-borne relapsing fever

A68.1 Tick-borne relapsing fever

A68.9 Relapsing fever, unspecified

A69.2 Lyme disease

A69.20 Lyme disease, unspecified

A69.21 Meningitis due to Lyme disease

A69.22 Other neurologic disorders in Lyme disease

A69.23 Arthritis due to Lyme disease

A69.29 Other conditions associated with Lyme disease

A69.5 There is not this code in ICD10 site, but we have this in mortality data

A69.8 Other specified spirochetal infections

A69.9 Spirochetal infection, unspecified

A75 Typhus fever

A75.0 Epidemic louse-borne typhus fever due to Rickettsia prowazekii

A75.1 Recrudescent typhus [Brill's disease]

A75.2 Typhus fever due to Rickettsia typhi

A75.3 Typhus fever due to Rickettsia tsutsugamushi

A75.9 Typhus fever, unspecified

A77 Spotted fever [tick-borne rickettsioses]

A77.0 Spotted fever due to Rickettsia rickettsii

A77.1 Spotted fever due to Rickettsia conorii

A77.2 Spotted fever due to Rickettsia siberica

A77.3 Spotted fever due to Rickettsia australis

A77.4 Ehrlichiosis

A77.40 Ehrlichiosis, unspecified

A77.41 Ehrlichiosis chafeensis [E. chafeensis]

A77.49 Other ehrlichiosis

A77.8 Other spotted fevers

A77.9 Spotted fever, unspecified

A78 Q fever

A79 Other rickettsioses

A79.0 Trench fever

A79.1 Rickettsialpox due to Rickettsia akari

A79.8 Other specified rickettsioses

A79.81 Rickettsiosis due to Ehrlichia sennetsu

A79.89 Other specified rickettsioses

A79.9 Rickettsiosis, unspecified

A92 Other mosquito-borne viral fevers

A92.0 Chikungunya virus disease

A92.1 O'nyong-nyong fever

A92.2 Venezuelan equine fever

A92.3 West Nile virus infection

A92.30 West Nile virus infection, unspecified

A92.31 West Nile virus infection with encephalitis

A92.32 West Nile virus infection with other neurologic manifestation

A92.39 West Nile virus infection with other complications

A92.4 Rift Valley fever

A92.8 Other specified mosquito-borne viral fevers

A92.9 Mosquito-borne viral fever, unspecified

A93 Other arthropod-borne viral fevers, not elsewhere classified

A93.0 Oropouche virus disease

A93.1 Sandfly fever

A93.2 Colorado tick fever

A93.8 Other specified arthropod-borne viral fevers

A94 Unspecified arthropod-borne viral fever

A94.0 Unspecified arthropod-borne viral fever

A96 Arenaviral hemorrhagic fever

A96.0 Junin hemorrhagic fever

A96.1 Machupo hemorrhagic fever

A96.2 Lassa fever

A96.8 Other arenaviral hemorrhagic fevers

A96.9 Arenaviral hemorrhagic fever, unspecified

A98 Other viral hemorrhagic fevers, not elsewhere classified

A98.0 Crimean-Congo hemorrhagic fever

A98.1 Omsk hemorrhagic fever

A98.2 Kyasanur Forest disease

A98.3 Marburg virus disease

A98.5 Hemorrhagic fever with renal syndrome

A98.8 Other specified viral hemorrhagic fevers

B33.0 Epidemic myalgia

B33.1 Ross River disease

B60 Other protozoal diseases, not elsewhere classified

B60.0 Babesiosis

B60.1 Acanthamebiasis

B60.10 Acanthamebiasis, unspecified

B60.11 Meningoencephalitis due to Acanthamoeba (culbertsoni)

B60.12 Conjunctivitis due to Acanthamoeba

B60.13 Keratoconjunctivitis due to Acanthamoeba

B60.19 Other acanthamebic disease

B60.2 Naegleriasis

B60.8 Other specified protozoal diseases

B67.5 Echinococcus multilocularis infection of liver

B67.6 Echinococcus multilocularis infection, other and multiple sites

B67.61 Echinococcus multilocularis infection, multiple sites

B67.69 Echinococcus multilocularis infection, other sites

B67.7 Echinococcus multilocularis infection, unspecified

B70 Diphyllobothriasis and sparganosis

B70.0 Diphyllobothriasis

B70.1 Sparganosis

B71 Other cestode infections

B71.0 Hymenolepiasis

B71.1 Dipylidiasis

B71.8 Other specified cestode infections

B71.9 Cestode infection, unspecified

B74.3 Loiasis

B74.4 Mansonelliasis

B74.8 Other filariases

B74.9 Filariasis, unspecified

B75 Trichinellosis

B83 Other helminthiases

B83.0 Visceral larva migrans

B83.1 Gnathostomiasis

B83.2 Angiostrongyliasis due to Parastrongylus cantonensis

B83.3 Syngamiasis

B83.4 Internal hirudiniasis

B83.8 Other specified helminthiases

P37.1 Congenital toxoplasmosis

Because these neglected tropical diseases are diverse in their underlying causes and risk factors as well as in their associated health outcomes, modelling them together in a DisMod-MR model would not produce reliable estimates of prevalence or excess mortality. Instead, we calculated the YLDs caused by neglected tropical diseases directly using a YLD/YLL ratio.

We calculated the ratio of YLDs to YLLs across the specified neglected tropical diseases for which non-fatal outcomes were modelled, using YLL estimates from the GBD 2019 cause of death (CoD) analysis. We

then multiplied this YLD/YLL ratio by the YLL estimates for other neglected tropical diseases from the GBD 2019 CoD analysis, providing us with an estimate of the YLDs associated with other neglected tropical diseases. Table 1 presents the total number of data sources from the Cause of Death Database that are used to produce burden estimates for this cause.

Meningitis

Flowchart

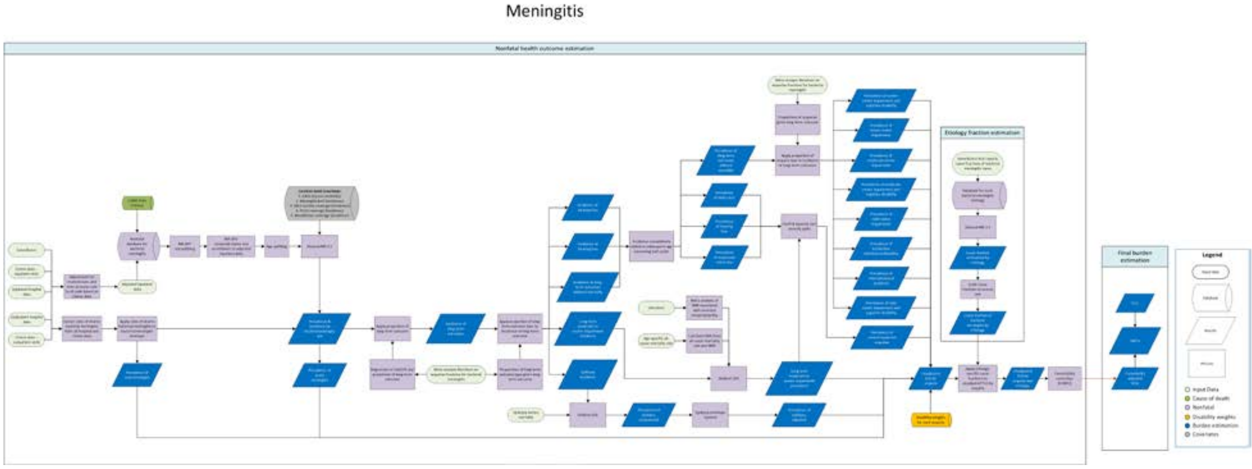

Case definition

Meningitis is a disease caused by inflammation of the meninges, the protective membrane surrounding the brain and spinal cord, and is typically caused by an infection in the cerebrospinal fluid. Symptoms include headache, fever, stiff neck, and sometimes seizures. Included in the GBD modelling were cases meeting ICD-10 diagnostic criteria for meningitis due to bacteria or viruses (A39-A39.9, A87-A87.9, and G00.0-G00.8). In GBD 2019, meningitis encompasses viral meningitis and four bacterial aetiologies: pneumococcal, *Haemophilus influenzae* type B (HiB), meningococcal, and other bacterial meningitis.

Input data

Model inputs

In the GBD 2010 study, a systematic review of literature was conducted to capture studies of incidence and excess mortality rate for all bacterial meningitis cases. For each of the four aetiologies, literature included excess mortality rate, incidence, proportion, remission, and standardised mortality ratio. The inclusion criteria stipulated that: (1) the publication year must be between 1980 and 2010; (2) “caseness” was based on diagnoses by antigen test, blood test, cerebrospinal fluid test, polymerase chain reaction test, or latex agglutination test; (3) sufficient information must be provided on study method and sample characteristics to assess the quality of the study; and (4) study samples must be representative of the general population. No limitation was set on the language of publication. For GBD 2013, the search strategy was replicated to capture epidemiological studies published between 2010 and 2013. The search strategy was repeated in 2015 only to capture excess mortality. For GBD 2019, the search strategy was again replicated to capture epidemiological studies published between 2015 and 2019. The PubMed search terms were: ("meningitis"[MeSH Terms] OR "meningitis"[Title/Abstract]) AND ("incidence"[Title/Abstract] OR "incidence"[MeSH Terms]) AND (2015[Date – Publication] : 3000[Date – Publication]) NOT ("animals"[MeSH Terms] NOT "humans"[MeSH Terms])

Additional sources we included in the acute bacterial meningitis model were surveillance data, inpatient-only hospital data and USA claims data from 2000, 2010, and 2012, 2015, primary diagnosis and inpatient only. Sequelae and severity splits were informed by a meta-analysis, Edmond and colleagues (1), while an internal meta-analysis informed mortality estimates for long-term moderate to severe impairments.

For GBD 2019, a systematic review of literature was conducted to capture studies for case-fatality ratio for the four bacterial aetiologies: pneumococcal, *Haemophilus influenzae* type B (HiB), meningococcal, other bacterial meningitis. The PubMed search terms were: ("meningitis"[MeSH Terms] OR "meningitis"[Title/Abstract]) AND ("case fatality rate"[Title/Abstract] OR "mortality"[MeSH Terms] OR "mortality"[Title/Abstract] OR "fatality"[Title/Abstract]) NOT ("animals"[MeSH Terms] NOT "humans"[MeSH Terms]) AND (1990[DP] : 3000[DP]) AND ("Meningitis, Haemophilus"[MeSH Terms] OR "Haemophilus"[Title/Abstract] OR "Meningitis, Pneumococcal"[MeSH Terms] OR "Pneumococcal"[Title/Abstract] OR "Meningitis, Meningococcal"[MeSH Terms] OR "Meningococcal"[Title/Abstract] OR "Meningitis, Viral"[MeSH Terms] OR "Viral"[Title/Abstract] OR "Streptococcus agalactiae"[MeSH Terms] OR "Streptococcus agalactiae"[Title/Abstract]).

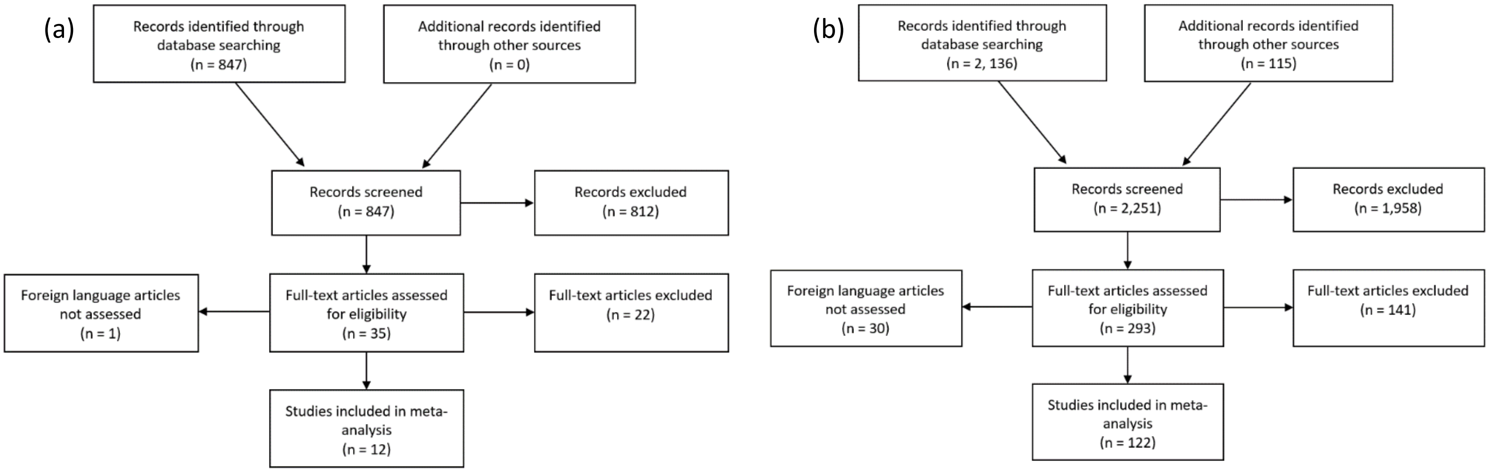

Figure 1 PRISMA diagram for meningitis 2019 systematic review for (a) incidence, and (b) case fatality rate.

Table 1: Source Counts

Measure	Total sources	Countries with data
All measures	925	108
Incidence	349	68
Excess mortality rate	52	38
Case fatality rate	545	100
Proportion	57	39

Data were outliered or excluded if we found them unreasonable when compared to regional, super-regional, and global rates.

Bias corrections

Hospital data were flagged with a covariate for inpatient hospital data and was used as the reference category. Claims data were flagged with year-specific covariates. Both claims and surveillance data were crosswalked up to the reference category.

To inform the Marketscan crosswalk we used 1470 paired observations from Arizona, Colorado, Iowa, Maryland, New York, Washington, and Wisconsin. To inform the Marketscan data from 2000, we used 626 paired observations from Alaska, Arizona, Arkansas, California, Colorado, Florida, Iowa, Maryland, Michigan, Nevada, New Jersey, New York, North Carolina, Washington, and Wisconsin. To inform the surveillance data crosswalk, we used 1809 paired observations from 34 locations in High Income North America, Europe, and Latin America.

Table 2a: MR-BRT Crosswalk Adjustment Factors for Meningitis Marketscan claims data

Data input	Reference or alternative case definition	Gamma	Basis function on age midpoint	B-spline Coefficient, Logit (95% CI)
Inpatient (CF2)	Ref			---
Marketscan claims	Alt	0.0	age_mid_0	1.29 (1.11, 1.46)
			age_mid_1	3.57 (3.31, 3.83)
			age_mid_2	0.482 (0.0404, 0.923)
			age_mid_3	1.53 (1.23, 1.83)
			age_mid_4	2.72 (2.58, 2.86)

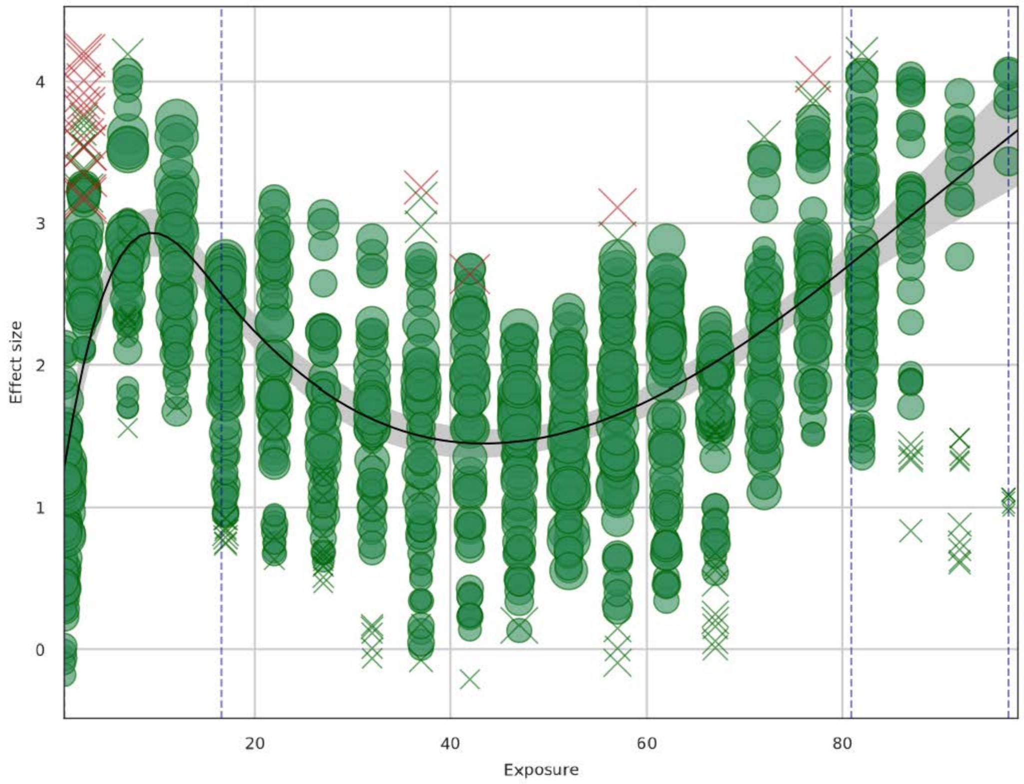

Figure 2a Cubic spline on age midpoint for Marketscan claims crosswalk (exposure is age midpoint, effect size is the adjustment factor in logit space). Circles are data used in the regression, crosses are trimmed data.

Table 2b: MR-BRT Crosswalk Adjustment Factors for Meningitis Marketscan 2000 claims data

Data input	Reference or alternative case definition	Gamma	Basis function on age midpoint	B-spline Coefficient, Logit (95% CI)
Inpatient (CF2)	Ref			---
Marketscan 2000 claims	Alt	0.30	age_mid_0	1.97 (0.688, 3.24)
			age_mid_1	2.6 (-0.0164, 5.22)
			age_mid_2	0.694 (-3.32, 4.71)
			age_mid_3	0.208 (-1.94, 2.36)
			age_mid_4	1.66 (0.94, 2.37)

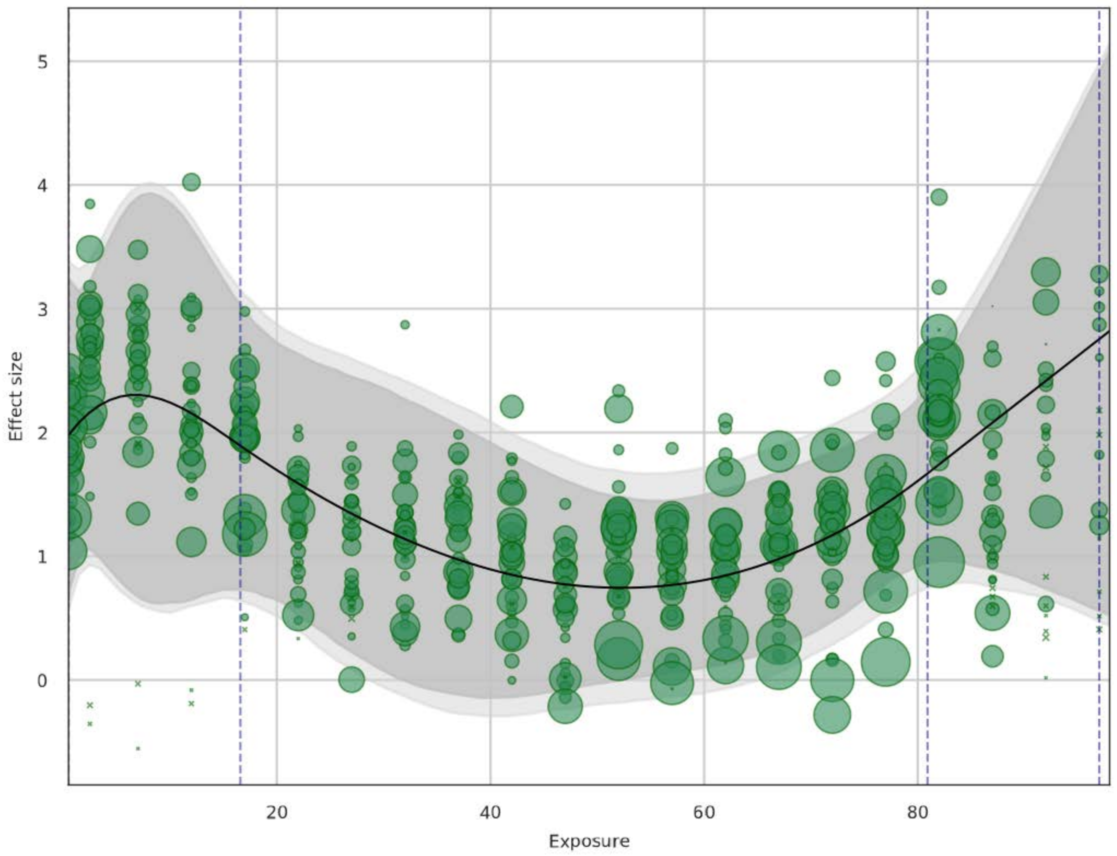

Figure 1b Cubic spline on age midpoint for Marketscan 2000 claims crosswalk (exposure is age midpoint, effect size is the adjustment factor in logit space). Circles are data used in the regression, crosses are trimmed data.

Table 2c: MR-BRT Crosswalk Adjustment Factors for Meningitis surveillance data

Data input	Reference or alternative case definition	Gamma	Covariate	Beta Coefficient, Logit (95% CI)	Adjustment factor*
Surveillance	Alt	0.54	HAQi	0.00285 (-0.00296, 0.00866)	0.5007125

**Adjustment factor is the transformed Beta coefficient in normal space, and can be interpreted as the factor by which the alternative case definition is adjusted to reflect what it would have been if measured as the reference.*

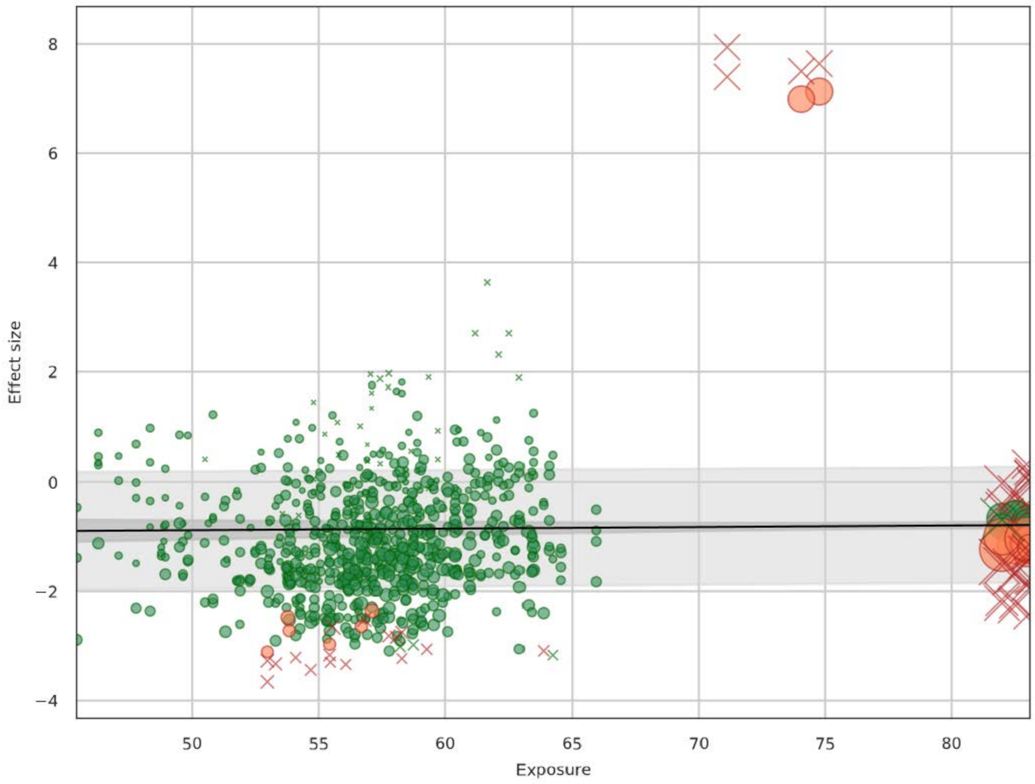

Figure 2c Regression on healthcare access and quality index (exposure is healthcare access and quality index, effect size is the difference between alternative and reference in logit space). Circles are data used in the regression, crosses are trimmed data.

Disability weights

The basis of the GBD disability weight survey assessments are lay descriptions of sequelae highlighting major functional consequences and symptoms. The lay descriptions and disability weights for sequelae associated with each aetiology are shown below.

Table 3. Severity distribution, details on the severity levels for meningitis in GBD 2019 and the associated disability weight (DW) with that severity.

Severity split	Lay description	DW (95% CI)
Acute meningitis	This person has a high fever and pain, and feels very weak, which causes great difficulty with daily activities.	0.133 (0.088–0.19)
Acute viral meningitis	This person has a high fever and pain, and feels very weak, which causes great difficulty with daily activities.	0.133 (0.088–0.19)

Mild behaviour problems	This person is hyperactive and has difficulty concentrating, remembering things, and completing tasks.	0.045 (0.028–0.066)
Mild hearing loss	This person has great difficulty hearing and understanding another person talking in a noisy place (for example, on an urban street).	0.01 (0.004–0.019)
Mild hearing loss with ringing	This person has great difficulty hearing and understanding another person talking in a noisy place (for example, on an urban street), and sometimes has annoying ringing in the ears.	0.021 (0.012–0.036)
Moderate hearing loss	This person is unable to hear and understand another person talking in a noisy place (for example, on an urban street), and has difficulty hearing another person talking even in a quiet place or on the phone.	0.027 (0.015–0.042)
Moderate hearing loss with ringing	This person is unable to hear and understand another person talking in a noisy place (for example, on an urban street), and has difficulty hearing another person talking even in a quiet place or on the phone, and has annoying ringing in the ears for more than 5 minutes at a time, almost every day.	0.074 (0.048–0.107)
Moderately severe hearing loss	(custom DW from hearing loss impairment envelope)	
Moderately severe hearing loss with ringing	(custom DW from hearing loss impairment envelope)	
Severe hearing loss	This person is unable to hear and understand another person talking, even in a quiet place, and unable to take part in a phone conversation. Difficulties with communicating and relating to others cause emotional impact at times (for example worry or depression).	0.158 (0.105–0.227)
Profound hearing loss	This person is unable to hear and understand another person talking, even in a quiet place, is unable to take part in a phone conversation, and has great difficulty hearing anything in any other situation. Difficulties with communicating and relating to others often cause worry, depression, or loneliness.	0.204 (0.134–0.288)
Complete hearing loss	This person cannot hear at all in any situation, including even the loudest sounds, and cannot communicate verbally or use a phone. Difficulties with communicating and relating to others often cause worry, depression, or loneliness.	0.215 (0.144–0.307)
Severe hearing loss with ringing	This person is unable to hear and understand another person talking, even in a quiet place, is unable to take part in a phone conversation, and has annoying ringing in the ears for more than 5 minutes	0.261 (0.175–0.36)

	at a time, almost every day. Difficulties with communicating and relating to others cause emotional impact at times (for example worry or depression).	
Profound hearing loss with ringing	This person is unable to hear and understand another person, even in a quiet place, is unable to take part in a phone conversation, has great difficulty hearing anything in any other situation, and has annoying ringing in the ears for more than 5 minutes at a time, several times a day. Difficulties with communicating and relating to others often cause worry, depression, or loneliness.	0.277 (0.182–0.387)
Complete hearing loss with ringing	This person cannot hear at all in any situation, including even the loudest sounds, and cannot communicate verbally or use a phone, and has very annoying ringing in the ears for more than half of the day. Difficulties with communicating and relating to others often cause worry, depression, or loneliness.	0.316 (0.212–0.435)
Moderate motor impairment	This person has some difficulty in moving around, and difficulty in lifting and holding objects, dressing and sitting upright, but is able to walk without help.	0.061 (0.04–0.089)
Moderate motor plus cognitive impairments	This person has some difficulty in moving around, holding objects, dressing and sitting upright, but can walk without help. This person has low intelligence and is slow in learning to speak and to do simple tasks.	0.203 (0.134–0.29)
Long-term mild motor impairment	This person has some difficulty in moving around but is able to walk without help.	0.01 (0.005–0.02)
Borderline intellectual disability	This person is slow in learning at school. As an adult, the person has some difficulty doing complex or unfamiliar tasks but otherwise functions independently.	0.011 (0.005–0.02)
Severe motor impairment	This person is unable to move around without help, and is not able to lift or hold objects, get dressed or sit upright.	0.402 (0.268–0.545)
Epilepsy	(combined DW)	NA
Blindness	Is completely blind, which causes great difficulty in some daily activities, worry and anxiety, and great difficulty going outside the home without assistance.	0.187 (0.124–0.26)
Mild intellectual disability	This person has low intelligence and is slow in learning at school. As an adult, the person can live independently, but often needs help to raise children and can only work at simple supervised jobs.	0.043 (0.026–0.065)
Monocular distance vision loss	This person is blind in one eye and has difficulty judging distances.	0.017 (0.009–0.029)

Mild motor plus cognitive impairments	This person has some difficulty in moving around but is able to walk without help. The person is slow in learning at school. As an adult, the person has some difficulty doing complex or unfamiliar tasks but otherwise functions independently.	0.031 (0.018–0.05)
Severe motor plus cognitive impairments	This person cannot move around without help, and cannot lift or hold objects, get dressed or sit upright. The person also has very low intelligence, speaks few words, and needs constant supervision and help with all daily activities.	0.542 (0.37–0.702)
Moderate vision impairment	The person has vision problems that make it difficult to recognize faces or objects across a room.	0.031 (0.019 to 0.049)
Severe vision impairment	The person has severe vision loss, which causes difficulty in daily activities, some emotional impact (for example worry), and some difficulty going outside the home without assistance.	0.184 (0.125 to 0.258)

Modelling strategy

Non-fatal outcomes were modelled using a combination of custom models, DisMod-MR 2.1, and in GBD 2017, we added the use of an ordinary differential equations solver (ODE) for more timely and accurate estimates. First, the overall incidence and prevalence of bacterial meningitis were modelled to estimate the short-term morbidity due to acute infection. This DisMod model had a set duration (1/remission) of four weeks with a range ±2 weeks. We also imposed caps on excess mortality for neonates and elders based on the highest excess mortality estimates from GBD 2019. We used the function in DisMod-MR 2.1 to pull in cause-specific mortality rate (CSMR) data from our CODEm and CODcorrect analyses. We calculated excess mortality rate to estimate priors by dividing CSMR by prevalence, calculated from remission and incidence. To help inform trends where we lack data, we applied a country-level covariate for proportion of the population at the subnational and country levels that lives within the meningitis belt in sub-Saharan Africa (2). In GBD 2017 we added country-level covariates for coverage of Hib3 vaccine and the MenAfriVac vaccine initiative to the parent meningitis model. In GBD 2019, we added a country-level covariate for coverage of PCV3. We also outliered incidence input data points with zero cases that were pulling down final estimates. Betas and exponentiated values (which can be interpreted as an odds ratio) are shown in the tables below country-level covariates.

Table 4a. Covariates. Summary of covariates used in the meningitis DisMod-MR meta-regression model

Covariate	Type	Parameter	Exponentiated beta (95% Uncertainty Interval)
Hib3 vaccine coverage	Country-level	Incidence	0.67 (0.65, 0.70)
PCV3 coverage	Country-level	Incidence	0.76 (0.75, 0.78)
Meningitis belt	Country-level	Incidence	7.28 (7.05, 7.39)
MenAfriVac initiative	Country-level	Incidence	0.14 (0.14, 0.14)
Healthcare Access and Quality index	Country-level	Excess mortality	0.998 (0.993, 0.999)

Incidence of bacterial meningitis was split into four aetiologies (pneumococcal, meningococcal, *H influenza* type B, and other bacterial meningitis) using four proportion models run in DisMod-MR 2.1; input data for these models were from published studies reporting incidence proportions for each etiology. Within each location, year, age group, and sex, we squeezed the proportions to ensure that they summed to 100% at the draw level. We applied a Hib3 vaccine coverage for the *H influenzae* type B proportion model, the proportion of the population living in the meningitis belt covariate and the proportion of the population living in areas covered by the MenAfriVac initiative (meningitis meningococcal type A) to the meningococcal proportion model, and a PCV3 coverage covariate to the pneumococcal meningitis model.

Table 4b. Covariates. Summary of covariates used in the etiology incidence proportion DisMod-MR meta-regression models

Covariate	Etiology	Parameter	Exponentiated beta (95% Uncertainty Interval)
Hib3 vaccine coverage	Hib	Proportion	0.25 (0.18, 0.35)
Meningitis belt (proportion of population)	Meningococcal	Proportion	2.06 (1.06, 4.23)
MenAfriVac coverage	Meningococcal	Proportion	0.57 (0.31,1.08)
PCV3 vaccine coverage	Pneumococcal	Proportion	0.83 (0.61, 0.99)

Data for viral meningitis were only available from hospitals or USA claims data, and not from population studies, so incidence and prevalence of viral meningitis were extrapolated from bacterial meningitis incidence by applying age- and sex-specific ratios between bacterial and viral cases from a combination of hospital data and USA claims data. In addition to short-term sequelae as a result of acute bacterial and viral meningitis, we also modelled the long-term outcomes from bacterial meningitis infection. In GBD 2017, we moved to produce both prevalence and incidence estimates of the viral meningitis outcome.

Sequelae splits

We first split the long-term sequelae among survivors of acute infection. We calculated the acute-phase survivors by applying the excess mortality (estimated by the acute meningitis DisMod model) to incidence, excess mortality was converted to case fatality rate by $e^{(\text{-excess mortality x 1/(excess mortality + remission)})}$. The survivors were then subject for long-term sequelae by applying the post-discharge proportions of health consequences calculated by a meta-analysis by Edmond and colleagues (1). We calculated the ratio of acute meningitis survivors that experience major long-term impairments for all aetiologies, and the ratio of minor impairments to major impairments for pneumococcal meningitis versus all other aetiologies (because pneumococcal meningitis showed significantly higher risk of morbidity than other aetiologies). This ratio was based off a regression of log-transformed GDP and ratio values from Edmonds and colleagues – this was different from GBD 2015, which used GNI. The regression is shown below:

$$y = -0.33590\ln(GDP) + 1.15230$$

We used these two ratios to calculate the proportions of survivors who contract a long-term minor impairment and those who contract a long-term major impairment. The proportion with major impairments were further split (again using pooled proportions from Edmond and colleagues) into specific major impairments, which were grouped into vision loss, hearing loss, moderate-to-severe cognitive impairments, and epilepsy.

The calculated incidence of long-term sequelae was then converted to prevalence by two different approaches. For the sequelae not associated with excess mortality, which were vision loss, hearing loss, intellectual disability, motor impairment, and behavioural problems, the incidence of each age was cumulatively added up to the subsequent age (assuming half-cycle) to construct prevalence at each age. If the sequela is associated with excess mortality (epilepsy and moderate-to-severe cognitive impairments), the calculated incidence was used as in input to the ODE solver together with the corresponding mortality parameters (excess mortality data from the epilepsy envelope DisMod model, and standardised mortality ratio data from a neonatal encephalopathy meta-analysis, converted to excess mortality using all-cause mortality estimates) to estimate the prevalence. Vision loss, hearing loss, and epilepsy estimates were squeezed and severity split centrally.

References

(1) Edmond, K. *et al.* Global and regional risk of disabling sequelae from bacterial meningitis: a systematic review and meta-analysis. *The Lancet Infectious Diseases* **10,** 317–328 (2010).

(2) Centers for Disease Control (CDC). CDC health information for international travel 2016: the yellow book. New York City, United States: Oxford University Press, USA, 2016.

Encephalitis

Flowchart

Case definition

Encephalitis is a disease caused by an acute inflammation of the brain. Symptoms of encephalitis can include flu-like symptoms like headache, fever, drowsiness, and fatigue, and at times, seizures, hallucinations, or stroke. Included in the GBD modelling were cases meeting ICD-10 diagnostic criteria for encephalitis (A83-A86.4, B94.1, F07.1, G04-G05.8).

Input data

Model inputs

In the GBD 2015 study, a systematic review of literature was conducted to capture studies of incidence, excess mortality rate, remission, and standardized mortality ratio for encephalitis. These data sources included hospital data and literature. The inclusion criteria stipulated that: (1) the publication year must be between 1980 and 2013; (2) sufficient information must be provided on study method and sample characteristics to assess the quality of the study; and (3) study samples must be representative of the general population. No limitation was set on the language of publication.

We did perform an updated systematic literature review for GBD 2019 to capture studies of incidence through the present year. The PubMed search terms were: ("encephalitis"[MeSH Terms] OR "encephalitis"[Title/Abstract] OR motor cognitive impairments[Title/Abstract]) AND ("incidence"[Title/Abstract] OR "incidence"[MeSH Terms]) AND (2015[Date – Publication] : 3000[Date –

Publication]) NOT ("animals"[MeSH Terms] NOT "humans"[MeSH Terms])

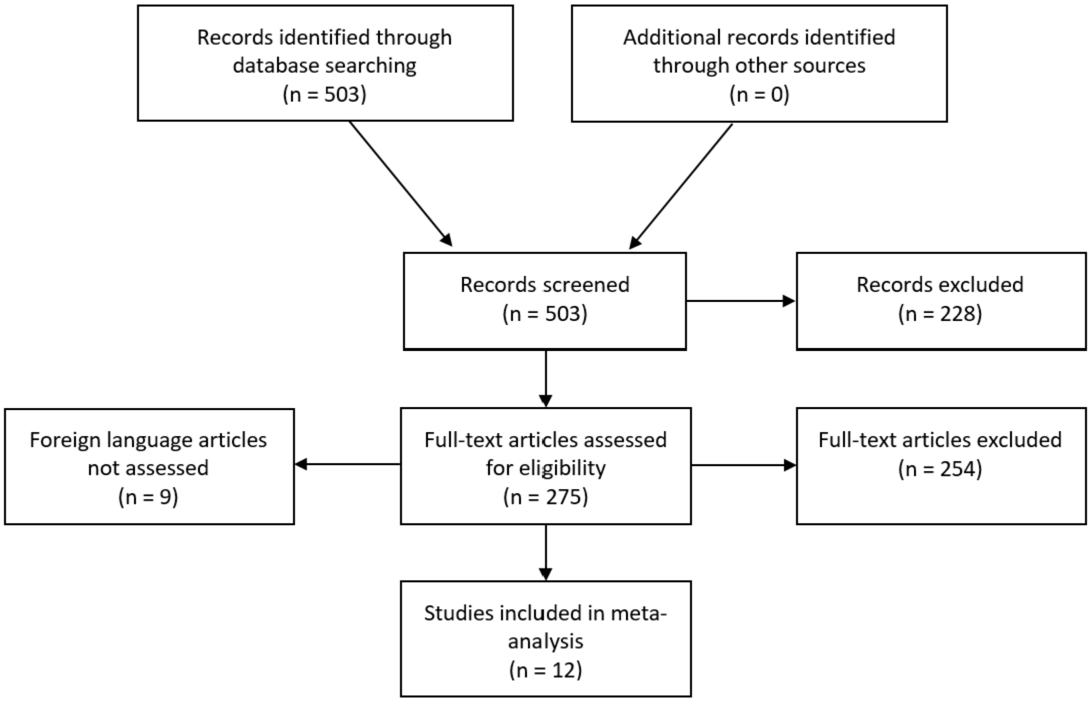

Figure 1 PRISMA diagram for encephalitis 2019 systematic review

Additional sources we included were inpatient hospital data and USA claims data from 2000, 2010, 2012, and 2015, primary diagnosis and inpatient only. Sequelae and severity splits were informed by a meta-analysis, Edmond and colleagues(1), while an internal meta-analysis informed mortality estimates for long-term moderate-to-severe impairments.

Table 1: Source Counts

Measure	Total sources	Countries with data
All measures	329	53
Incidence	329	53

Data were outliered or excluded if we found they differed significantly when compared to regional, super-regional, and global rates.

Bias corrections

Hospital data were flagged with a covariate for inpatient hospital data and was used as the reference category. Claims data were flagged with year-specific covariates. Surveillance data were flagged with covariates specific to the type of surveillance (e.g., active vs. passive and sentinel-based vs. population-based). Both claims and surveillance data were crosswalked up to the reference category.

To inform the Marketscan crosswalk we used 1470 paired observations from Arizona, Colorado, Iowa, Maryland, New York, Washington, and Wisconsin. To inform the Marketscan data from 2000, we used 628 paired observations from Alaska, Arizona, Arkansas, California, Colorado, Florida, Iowa, Maryland, Michigan, Nevada, New Jersey, New York, North Carolina, Washington, and Wisconsin. To inform the

surveillance data crosswalk, we used 3858 paired observations from 2016 locations in high-income North America, Europe, and East Asia.

Table 2a: MR-BRT Crosswalk Adjustment Factors for Encephalitis Marketscan claims data

Data input	Reference or alternative case definition	Gamma	Basis function on age midpoint	B-spline Coefficient, Logit (95% CI)
Inpatient (CF2)	Ref			---
Marketscan claims	Alt	0.00	age_mid_0	3.15 (2.76, 3.53)
			age_mid_1	4.1 (3.86, 4.33)
			age_mid_2	1.62 (1.24, 2.01)
			age_mid_3	3.03 (2.77, 3.3)
			age_mid_4	2.37 (2.24, 2.51)

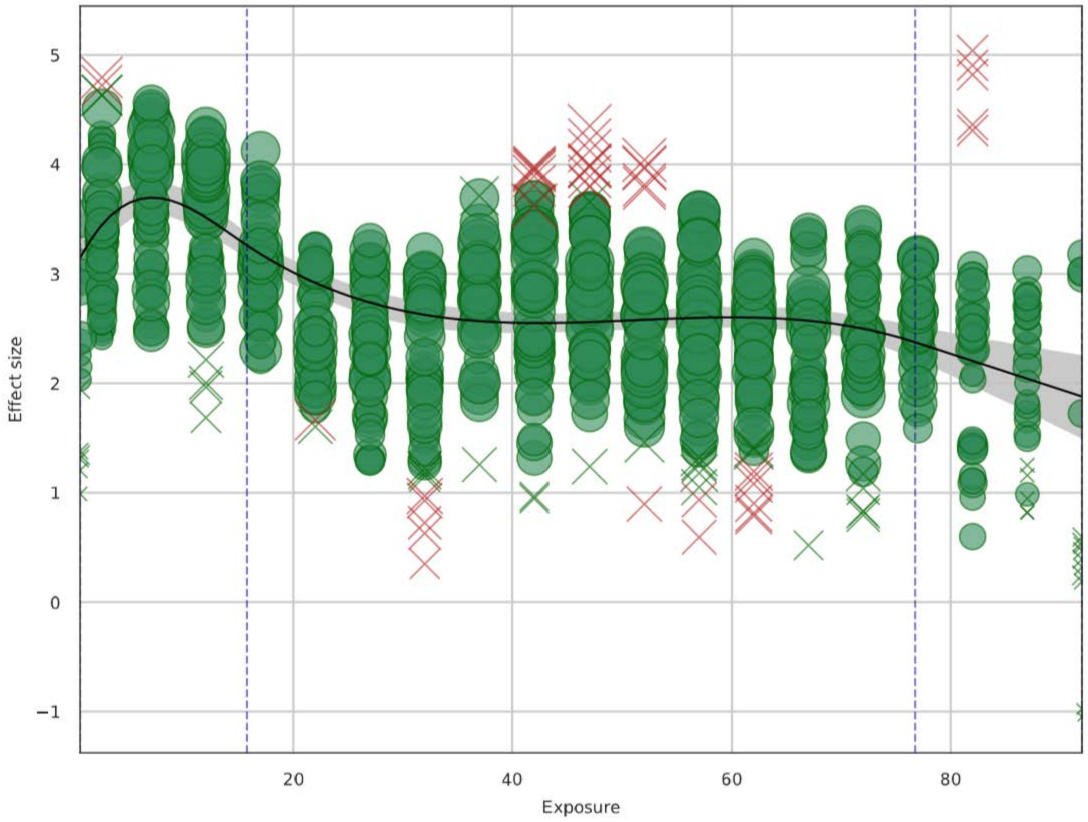

Figure 2a Cubic spline on age midpoint for Marketscan claims crosswalk (exposure is age midpoint, effect size is the adjustment factor in logit space)

Table 2b: MR-BRT Crosswalk Adjustment Factors for Encephalitis Marketscan 2000 claims data

Data input	Reference or alternative case definition	Gamma	Basis function on age midpoint	B-spline Coefficient, Logit (95% CI)
Inpatient (CF2)	Ref			---
Marketscan 2000 claims	Alt	0.00	age_mid_0	3.56 (1.2, 5.93)
			age_mid_1	3.73 (2.46, 4.99)
			age_mid_2	1.99 (0.247, 3.73)
			age_mid_3	2.55 (1.34, 3.76)
			age_mid_4	1.82 (1.22, 2.42)

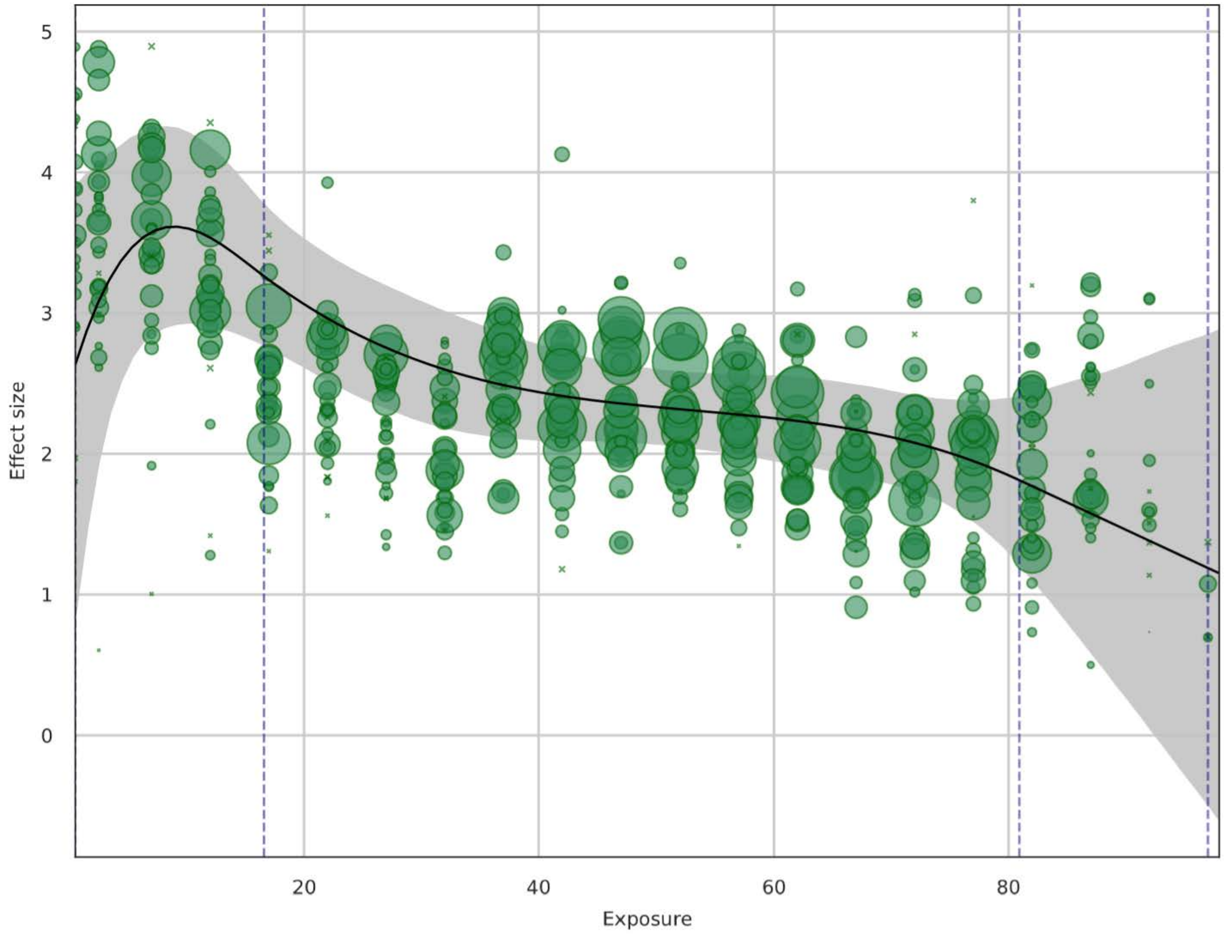

Figure 2b Cubic spline on age midpoint for Marketscan 2000 claims crosswalk (exposure is age midpoint, effect size is the adjustment factor in logit space)

Table 2c: MR-BRT Crosswalk Adjustment Factors for Encephalitis surveillance data

Data input	Reference or alternative case definition	Gamma	Beta Coefficient, Logit (95% CI)	Adjustment factor*
Inpatient(CF2)	Ref	--	--	--
Surveillance	Alt	0.77	-4.00 (-4.05, -3.94)	0.01807403

**Adjustment factor is the transformed Beta coefficient in normal space, and can be interpreted as the factor by which the alternative case definition is adjusted to reflect what it would have been if measured as the reference.*

Modelling strategy

Non-fatal outcomes were modelled using a combination of custom models and DisMod-MR 2.1. First, the overall incidence and prevalence of encephalitis were modelled to estimate the short-term morbidity due to acute infection. This DisMod model had a set duration (1/remission) of three weeks. We also imposed caps on excess mortality for ages 10–50. USA claims data were grouped into year-specific covariates based on quality, and were crosswalked to the reference data, which we extracted from literature and inpatient hospital data. We used the function in DisMod-MR 2.1 to pull in cause-specific mortality rate (CSMR) data from our CODEm and CODcorrect analyses and match with incidence data points for the same geography. We calculated excess mortality rate to estimate priors for EMR by dividing CSMR by prevalence, calculated from remission and incidence. To help inform trends where we lack data, we applied a binary country-level covariate at the subnational and country level that indicates if the location is in a Japanese Encephalitis endemic area (2). We also applied a lag-distributed income covariate to excess mortality. Betas and exponentiated values (which can be interpreted as an odds ratio) are shown in the tables below for study-level covariates and country-level covariates. In GBD 2019 we updated the Japanese Encephalitis covariate to include all Philippine subnationals and all Pakistan subnationals. We outliered incidence input data points with zero cases that were dragging down final estimates. We also improved our time efficiency and estimation accuracy by using an ordinary differential equations solver (ODE solver) in place of traditional DisMod-MR.

Table 3. Covariates. Summary of covariates used in the encephalitis DisMod-MR meta-regression model

Covariate	Type	Parameter	Exponentiated beta (95% Uncertainty Interval)
Japanese Encephalitis endemic area	Country-level covariate	Incidence	1.10 (1.10, 1.11)
LDI (log transformed)	Country-level covariate	Excess mortality	1.00 (1.00, 1.00)

In addition to short-term sequelae as a result of acute encephalitis, we also modelled the long-term outcomes from encephalitis.

Sequelae splits

We first split the long-term sequelae among survivors of acute infection. We calculated the acute phase survivors by applying the excess mortality (calculated by the acute encephalitis DisMod model) to the

incidence of each etiology (excess mortality was converted to case fatality rate by $e^{(-\text{excess mortality} \times 1/(\text{excess mortality} + \text{remission}))}$. The survivors were then subject to long-term sequelae by applying the post-discharge proportions of health consequences calculated by a meta-analysis by Edmond and colleagues (2). We calculated the ratio of acute encephalitis survivors that result in a major long-term impairment, and the ratio of minor impairments to major impairments, based off a regression of log-transformed GDP and ratio values from Edmond and colleagues. This regression was done differently from last year, which previously used GNI. The regression is shown below:

$$y = -0.33590 \ln(GDP) + 1.15230$$

We assumed a similar pattern of health outcomes for encephalitis infection survivors as with other bacterial meningitis survivors (except hearing loss, as we could not find evidence of hearing loss as a consequence of encephalitis infection). We used these two ratios to calculate the proportions of survivors who contract a long-term minor impairment and those who contract a long-term major impairment. The proportion with major impairments were further split (again using pooled proportions from Edmond and colleagues) into specific major impairments, which were grouped into vision loss, moderate to severe cognitive impairments, and epilepsy.

The calculated incidence of long-term sequelae was then converted to prevalence by two different approaches. For the sequelae not associated with excess mortality, which were vision loss, intellectual disability, motor impairment, and behavioural problems, the incidence of each age was cumulatively added up to the subsequent age (assuming half-cycle) to construct prevalence at each age. If the sequela is associated with excess mortality (epilepsy and moderate-to-severe cognitive impairments), the calculated incidence was used as an input the ODE solver, together with the corresponding mortality parameters (excess mortality data from the epilepsy envelope DisMod model, and standardised mortality ratio data from a neonatal encephalopathy meta-analysis, converted to excess mortality using all-cause mortality estimates) to estimate the prevalence. Vision loss and epilepsy estimates were squeezed and severity split centrally.

Disability weights

The basis of the GBD disability weight survey assessments is lay descriptions of sequelae highlighting major functional consequences and symptoms. The lay descriptions and disability weights for sequelae associated with encephalitis are shown below.

Table 4. Severity distribution, details on the severity levels for encephalitis in GBD 2019 and the associated disability weight (DW) with that severity.

Severity split	Lay description	DW (95% CI)
Mild behaviour problems	This person is hyperactive and has difficulty concentrating, remembering things, and completing tasks.	0.045 (0.028–0.066)
Moderate motor impairment	This person has some difficulty in moving around, and difficulty in lifting and holding objects, dressing and sitting upright, but is able to walk without help.	0.061 (0.04–0.089)
Moderate motor plus cognitive impairments	This person has some difficulty in moving around, holding objects, dressing and sitting upright, but can walk without help. This person has low intelligence	0.203 (0.134–0.29)

	and is slow in learning to speak and to do simple tasks.	
Long-term mild motor impairment	This person has some difficulty in moving around but is able to walk without help.	0.01 (0.005–0.02)
Borderline intellectual disability	This person is slow in learning at school. As an adult, the person has some difficulty doing complex or unfamiliar tasks but otherwise functions independently.	0.011 (0.005–0.02)
Severe motor impairment	This person is unable to move around without help, and is not able to lift or hold objects, get dressed, or sit upright.	0.402 (0.268–0.545)
Epilepsy	(combined DW)	NA
Blindness	Is completely blind, which causes great difficulty in some daily activities, worry and anxiety, and great difficulty going outside the home without assistance.	0.187 (0.124–0.26)
Acute encephalitis	This person has a high fever and pain, and feels very weak, which causes great difficulty with daily activities.	0.133 (0.088–0.19)
Mild intellectual disability	This person has low intelligence and is slow in learning at school. As an adult, the person can live independently but often needs help to raise children and can only work at simple supervised jobs.	0.043 (0.026–0.065)
Monocular distance vision loss	This person is blind in one eye and has difficulty judging distances.	0.017 (0.009–0.029)
Mild motor plus cognitive impairments	This person has some difficulty in moving around but is able to walk without help. The person is slow in learning at school. As an adult, the person has some difficulty doing complex or unfamiliar tasks but otherwise functions independently.	0.031 (0.018–0.05)
Severe motor plus cognitive impairments	This person cannot move around without help, and cannot lift or hold objects, get dressed or sit upright. The person also has very low intelligence, speaks few words, and needs constant supervision and help with all daily activities.	0.542 (0.37–0.702)
Moderate vision impairment due to encephalitis	This person has vision problems that make it difficult to recognize faces or objects across a room.	0.031 (0.019 to 0.049)
Severe vision impairment due to encephalitis	This person has severe vision loss, which causes difficulty in daily activities, some emotional impact (for example worry), and some difficulty going outside the home without assistance.	0.184 (0.125 to 0.258)

References

(1) Edmond, K. *et al.* Global and regional risk of disabling sequelae from bacterial meningitis: a systematic review and meta-analysis. *The Lancet Infectious Diseases* **10,** 317–328 (2010).

(2) Centers for Disease Control (CDC). CDC health information for international travel 2016: the yellow book. New York City, United States: Oxford University Press, USA, 2016.

Diphtheria

Model flowchart

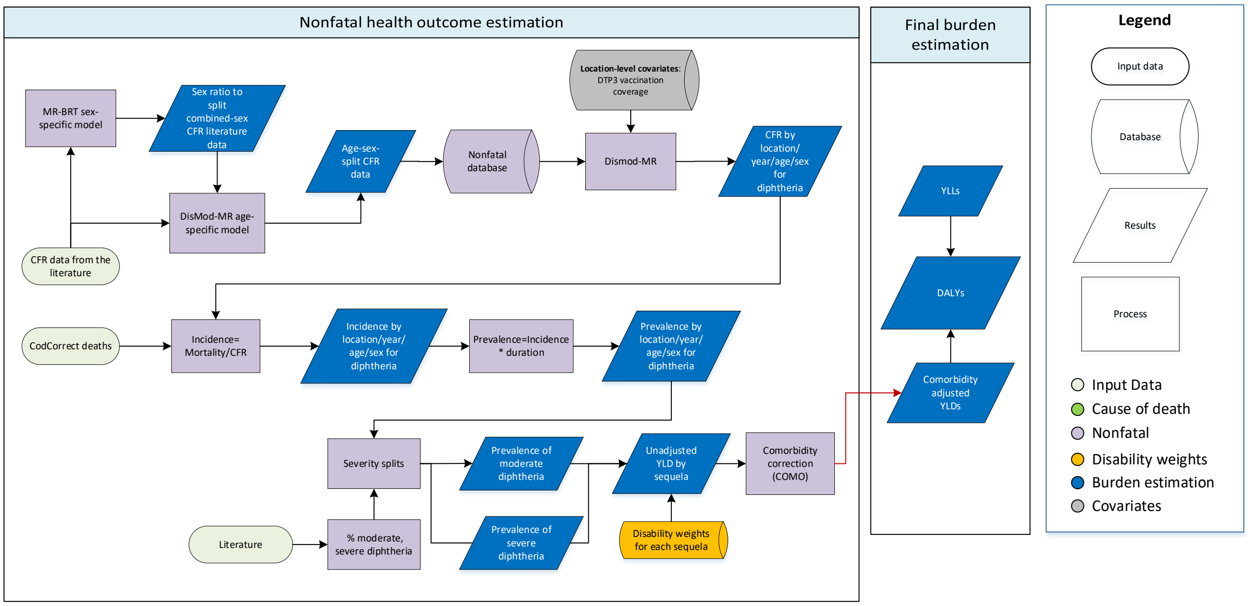

Case definition

Diphtheria is a bacterial infection caused by *Corynebacterium diphtheriae*. For diphtheria, ICD 10 codes are A36-A36.9, Z22.2, Z23.6, and ICD9 codes are 032-032.9, V02.4, V03.5, and V74.3.

Input data

Model inputs

The nonfatal diphtheria model has two primary inputs. The first is literature data obtained from systematic reviews of diphtheria case fatality ratio (CFR). The second is GBD mortality estimates of diphtheria, calculated per country by either Cause of Death Ensemble modeling (CODEm) or a negative binomial regression modelling method.

The diphtheria CFR systematic review was updated in GBD 2019. New data were added to existing sources from systematic reviews completed in prior GBD cycles, the most recent of which took place in GBD 2016. In PubMed, the search terms used were: *((((diphtheria[MeSH Terms] OR diphtheria) AND (mortality[MeSH Terms] OR mortality OR "case fatality rate" OR "case fatality ratio" OR "case fatality"))) AND ("2016"[Date - Publication] : "2019"[Date - Publication]))*. Data were not included if they were excluded if they were missing information about diphtheria cases and deaths or referred to diphtheria outbreaks in camps of refugees, internally displaced people, or ethnic minority groups. Table 1 summarizes the literature-extracted nonfatal input data used in the diphtheria model.

Table 1. Input data counts for the diphtheria nonfatal model

Measure	Total sources
All measures	30
Duration	4
Proportion	30

Input data processing

All extracted diphtheria CFR data that was not sex- and age-specific (i.e. the data that was reflective of both sexes combined and/or age ranges greater than a 20-year start and end difference) were split into sex- and age-specific groups prior to use in modelling. Scant age- and sex-specific diphtheria CFR data is currently available, which precludes the estimation of location- or year-specific age and sex patterns. Instead, global sex ratios and age patterns were generated using all available sex- and age-specific diphtheria CFR data. These were then used to split all non-age- or sex-specific CFR data prior to inclusion in the final CFR model while propagating uncertainty from the splitting process.

The ratios used to make the sex splits were calculated using MR-BRT, the meta-regression, Bayesian tool developed for GBD 2019. Few diphtheria CFR data sources matching inclusion criteria had sufficient, paired sex information to create a standard male to female ratio. To supplement these sources, paired, sex-specific, non-0 CFRs from hospital claims data from the Philippines and nine Brazil states were used only during generation of the ratio. The sex adjustment factor calculated for use in GBD 2019 modeling was 1.31 (Table 2). The adjustment factor that was calculated during modeling in GBD 2017 was 1.10 (0.47 to 2.41). The more robust MR-BRT based approach with 10% trimming and new input data sources used in GBD 2019 suggest slightly larger differences in diphtheria CFR between males versus females than was previously estimated in GBD 2017.

Table 2: MR-BRT Sex-splitting Adjustment Factor for diphtheria CFR

Data input	Reference or alternative case definition	Beta Coefficient, Log (95% CI)	Adjustment factor*
Sex	N/A	0.269 (-0.123 to 0.686)	1.31

**Adjustment factor is the transformed Beta coefficient in normal space, and can be interpreted as the factor by which the alternative case definition is adjusted to reflect what it would have been if measured as the reference.*

For diphtheria CFR data representing an age range wider than 20 years, the extracted CFR values were split proportionally to follow a global age pattern generated from available age-specific diphtheria CFR data available. To generate this global age pattern, diphtheria CFR data representing age groups less than 20 years in width were used to fit a DisMod-MR model with the GBD health access and quality index (HAQI) as a location-level covariate. Then, the final global age pattern output – produced by DisMod in five-year age-bins from early neonatal to 95+ age groups – was used to split the death counts in the remaining data sources.

Severity split & disability weights

Our estimated, nonfatal diphtheria cases are split by severity following distributions summarized from literature reviews. Seventy percent of cases (95% CI:66.5-73.5%) are presumed moderate, and the

remaining 30% (95% CI: 26.5-33.5%) severe. Table 3 provides severity level descriptions in addition to these weights.

Table 3. Severity distribution, details on the severity levels for diphtheria in GBD 2019 and the associated disability weight (DW) with that severity.

Severity level	Lay description	DW (95% CI)
Moderate diphtheria	Has a fever and aches, and feels weak, which causes some difficulty with daily activities.	0.051 (0.032-0.074)
Severe diphtheria	Has a high fever and pain, and feels very weak, which causes great difficulty with daily activities.	0.133 (0.088-0.19)

Modeling strategy

We utilized DisMod-MR to produce location-, year-, age-, and sex-specific diphtheria CFR estimates from our available sex- and age-specific input data. In the model, we used the healthcare access and quality (HAQ) index as a location-level covariate, enforcing a directional prior so locations with increasing HAQ are predicted to have a reduced diphtheria CFR. This directional prior is a new addition in GBD 2019 and drives differences in CFR estimates in comparison to GBD 2017; in particular, we observed a decreased regional CFR in Southeast Asia, East Asia, and Oceania, and an increased regional CFR in South Asia. As a result, CFR model estimates now better reflect the expected relationship between HAQ and CFR across geographies and years, particularly in data-sparse locations.

Table 4 displays the raw and exponentiated magnitudes of covariate influence, which can be interpreted as odds ratios. Additionally, in this GBD cycle, DisMod model parameters were adjusted to decrease the influence of hierarchical priors in the DisMod geographic cascade. These adjustments allow the model to more closely track CFR data in locations where data is present and tend to result in broader uncertainty in CFR estimates for locations where no data is available. In most locations, the net effect of the new age- and sex-splitting approach and adjustments to these DisMod settings resulted in increased CFR estimates compared to GBD 2017.

Incidence was calculated as mortality rate divided by case fatality ratio. The diphtheria mortality rate was produced in GBD 2019, modeled using CODEm or a negative binomial regression and data from the cause of death database with the five-year rolling mean DTP3 coverage covariate and age dummy variables as key predictors (see diphtheria in cause of death appendix). Then, prevalence was calculated as the product of incidence and diphtheria case duration (mean of 27.5 days, based on a meta-analysis of duration data from the literature). These calculations were completed in 1000-draw space to encompass and propagate uncertainty throughout the modeling process. Draw-level estimates were then summarized as means of draws and 95% uncertainty intervals (2.5th and 97.5th quantiles of all draws).

Table 4. Covariates. Summary of covariates used in the diphtheria CFR DisMod-MR meta-regression model

Covariate	Type	Parameter	Exponentiated beta (95% Uncertainty Interval)
Healthcare access and quality (HAQ) index	Country-level	Case fatality ratio	0.86 (0.69 –0.99)

We made no additional substantive changes in the modeling strategy from GBD 2017.

Pertussis (whooping cough)

Flowchart

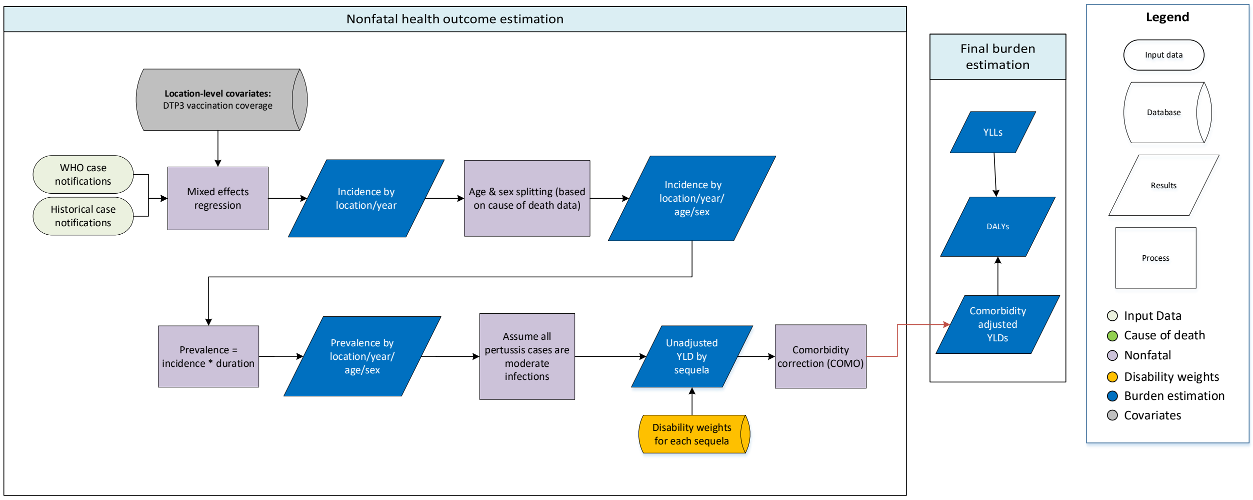

Case definition

Pertussis (whooping cough), is a contagious respiratory disease caused by the bacterium *Bordetella pertussis*. For pertussis, ICD 10 codes are A37-A37.91, Z23.7, and ICD 9 codes are 033-033.9, 484.3, V03.6.

Input data

Model inputs

To estimate pertussis incidence and prevalence rates, our primary input data are the pertussis case notifications annually released by the World Health Organization (WHO) through the Joint Reporting Form (JRF). Historical case notifications and vaccination coverage for the United Kingdom back to 1940 were also included to better inform the natural history model. Table 1 contains counts of all nonfatal input data used in the pertussis model.

Table 1. Input data counts for the pertussis nonfatal model

Measure	Total sources
All measures	7273
Incidence	7272
Duration	1

Severity splits

Each estimated pertussis case was assumed a moderate episode of acute infectious disease, given associated symptoms. The lay description and disability weight derived from the GBD Disability Weights study are shown in Table 2.

Table 2. Severity splits, lay descriptions, and disability weights

Severity level	Lay description	DW (95% CI)
Moderate	Has a fever and aches and feels weak, which causes some difficulty with daily activities.	0.051 (0.032-0.074)

Modeling strategy

As in GBD 2017, we use a mixed-effects linear regression model to make a prediction of pertussis cases for every estimated location. Along with the case notification input data, we use GBD 2019 estimates of diphtheria-tetanus-pertussis third-dose (DTP3) vaccine coverage as a predictor in the model. In past GBD cycles, estimates of DTP3 coverage among infants in the modeled year were used as the primary covariate for this linear regression. In GBD 2019, we now use a lagged mean of DTP3 coverage calculated over a rolling, five-year interval in order to capture population-level vaccine-derived immunity among under-5-year olds, including coverage both in the current year and in recent years. This model also includes location-specific random effects to capture variation in reported pertussis incidence not explained by DTP3 coverage:

$$Y_{ij} = \beta_0 + \beta_1\ (1\text{-}DTP3_{ij}) + u_j + e_{ij}\,,$$

where Y_{ij} is the log-transformed incidence rate (in cases per 100,000 persons using WHO case notifications and GBD populations); β_0 is the fixed effect intercept; β_1 is the fixed effects slope on the log-transformed proportion of unvaccinated individuals (using the rolling mean of DTP3 coverage over the past five years); u_j is the country random effect; e_{ij} is the residual; i is the year; and j is the location.

As in GBD 2017, to adjust for underreporting in case notifications we used the random effect of Switzerland – the location with the largest random effect and known to have a robust pertussis monitoring system – when predicting from the model for all locations. This approach, which has also been used in previous GBD cycles, implies an attack rate assumed stable across unvaccinated populations. With the addition of updated case notification data in this GBD cycle, the random effect of Switzerland increased compared to GBD 2017. This result implies a higher degree of underreporting in other countries as compared to Switzerland than was estimated in GBD 2017, and incidence increased in most locations as a result. From this model, 1000 predictions of incidence were generated using the estimated variance-covariance matrix in order to capture uncertainty.

The results of this model were used to predict prevalence and incidence rates. Prevalence rate was the product of cases and duration, assuming average case duration of fifty days, divided by GBD-estimated populations. Incidence rate was the result of predicted cases divided by GBD-estimated populations. All draw-level results were summarized as means of draws and 95% uncertainty intervals (the 2.5th and 97.5th quantiles of all draws).

Tetanus

Flowchart

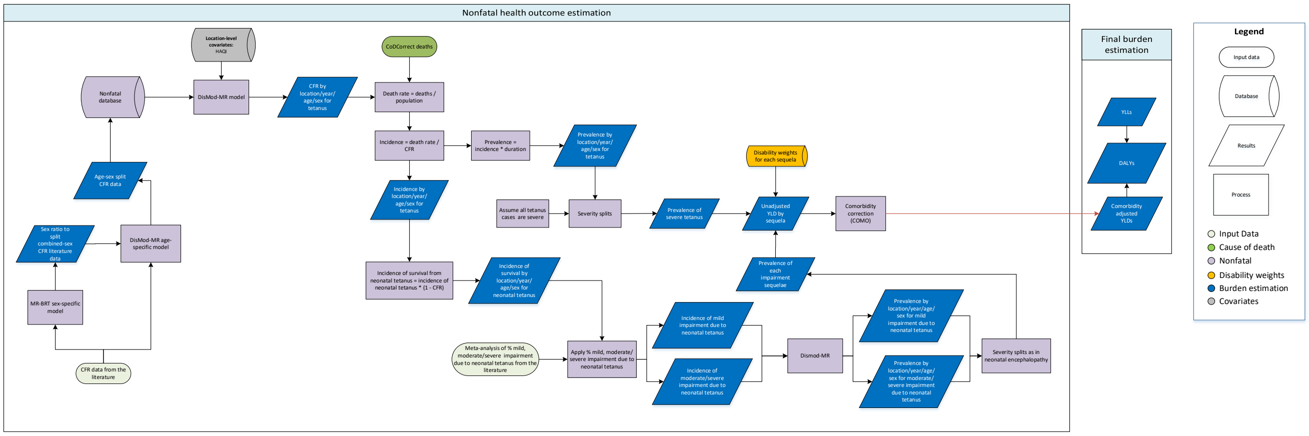

Input Data and Methodological Summary for Tetanus

Case definition

Tetanus is a serious bacterial disease caused by the bacterium *Clostridium tetani.* For tetanus, the ICD 10 codes are A33-A35.0, Z23.5, and ICD 9 codes are 037-037.9,771.3,V03.7.

Input data

Model inputs

The tetanus nonfatal model requires case fatality ratio (CFR) data obtained from systematic reviews of the literature, and the mortality rate outputs from the GBD 2019 tetanus mortality model.

A new systematic review of tetanus CFR literature was not completed in GBD 2019. The last systematic review occurred in GBD 2016, using the following search string in PubMed: *(tetanus[Title/Abstract]) AND (case fatality[Title/Abstract]) AND ("2013"[Date - Publication]: "2016"[Date - Publication])*. As new literature on the topic is published, this systematic review will be updated in subsequent GBD cycles. Table 1 summarizes the literature-extracted nonfatal input data used in the tetanus model.

Table 1. Input data counts for the tetanus nonfatal model

Measure	Total sources
All measures	98
Duration	6
Proportion	92

Input data processing

All extracted tetanus CFR data that was not sex- and age-specific (i.e. the data that was reflective of both sexes combined and/or age ranges greater than a 20-year start and end difference) were split into sex- and age-specific groups prior to use in modelling.

Because scant age- and sex-specific tetanus CFR data is currently available, location or year-specific age and sex patterns could not be estimated. Instead, global sex ratios and age patterns were generated using all available sex- and age-specific tetanus CFR data; these ratios were then used to split all non-age- or sex-specific data prior to inclusion in the model while propagating uncertainty from the splitting process.

The ratios used to make the sex splits were calculated using MR-BRT, the meta-regression, Bayesian tool developed for GBD 2019. The sex adjustment factor calculated for use in GBD 2019 modeling was 0.96 (0.79 to 1.15) (Table 2). The adjustment factor that was calculated during modeling in GBD 2017 was 0.93 (0.72 to 1.20), and we observe similar sex distributions in nonfatal tetanus burden using this MR-BRT approach.

Table 2: MR-BRT Sex-splitting Adjustment Factor for tetanus CFR

Data input	Reference or alternative case definition	Beta Coefficient, Log (95% CI)	Adjustment factor*
Sex	N/A	-0.045 (-0.233 to 0.142)	0.96

Adjustment factor is the transformed Beta coefficient in normal space, and can be interpreted as the factor by which the alternative case definition is adjusted to reflect what it would have been if measured as the reference.

For tetanus CFR data with ages greater than a range of 20 years, the extracted CFR values were split proportionally to follow a global age pattern generated using all available age-specific tetanus CFR data. To generate the global age pattern for tetanus CFR, all available age-specific tetanus CFR data (i.e. CFR data representing an age group less than 20 years in width) was used to fit a DisMod-MR model with the GBD health access and quality index (HAQI) as a location-level covariate. Then, the final global age pattern output – produced by DisMod in five-year age-bins from early neonatal to 95+ age groups – was used to split the death counts in the remaining data sources.

Severity splits and disability weights

All of the tetanus cases estimated are assumed to be severe, acute infections. Table 3 presents our lay description of severe tetanus in addition to the disability weight applied. For neonatal tetanus impairments, our distribution matches the distribution of neonatal encephalopathy.

Table 3. Severity distribution, details on the severity levels for tetanus in GBD 2019 and the associated disability weight (DW) with that severity.

Severity level	Lay description	DW (95% CI)
Severe	Has a high fever and pain, and feels very weak, which causes great difficulty with daily activities.	0.133 (0.088-0.19)

Modeling strategy

We utilized DisMod-MR to produce location-, year-, age-, and sex-specific tetanus CFR estimates from sex- and age-specific input data, following the age- and sex-splitting process described above. In the model, we used the healthcare access and quality (HAQ) index as a location-level covariate, enforcing a directional prior so locations with increasing HAQ are predicted to have a reduced tetanus CFR. This directional prior is a new addition to the model in GBD 2019. As a result, CFR model estimates now better reflect the expected relationship between HAQ and CFR across geographies and years, particularly in data-sparse locations. Table 4 displays the raw and exponentiated magnitude of covariate influence, which can be interpreted as odds ratios. Additionally, in this GBD cycle, DisMod model parameters were adjusted to decrease the influence of hierarchical priors in the DisMod geographic cascade. These adjustments allow the model to more closely track CFR data in locations where data is present and tend to result in broader uncertainty in CFR estimates for locations where no data is available. In most locations, the net effect of the new age- and sex-splitting approach and adjustments to these DisMod settings resulted in increased CFR estimates compared to GBD 2017.

Incidence rates were then calculated using estimates of tetanus CFR and GBD 2019 tetanus mortality estimates. In GBD 2019, tetanus mortality rates are produced using CODEm separately for all combinations of children under one year of age and those ages one to eighty, data-rich and non-data-rich countries, and for males and females. Using these results, incidence was calculated as the quotient of mortality rate by CFR. From tetanus incidence and tetanus case duration sourced from a prior literature review, tetanus prevalence was computed. These calculations were completed at the draw level for each of 1000 draws, then summarized using the mean of draws and a 95% uncertainty interval (the 2.5th and 97.5th quantile of all draws).

Table 4. Covariates. Summary of covariates used in the tetanus CFR DisMod-MR meta-regression model.

Covariate	Type	Parameter	Exponentiated beta (95% CI)
Healthcare access and quality (HAQ) index	Country-level	Case fatality ratio	0.85 (0.75 — 0.97)

To estimate mild and moderate impairment due to neonatal tetanus, we first computed the incidence of survival from neonatal tetanus as:

$$incidence\ of\ survival = incidence * (1 - CFR) .$$

To appropriately proportion impairments as either mild or moderate-to-severe, we leveraged a systematic review of this proportion in cases in the literature. We applied these splits to the incidence of survival to calculate the incidence of survival from neonatal tetanus with mild impairment and with moderate-to-severe impairment. These estimates were each then used as input data sets for separate DisMod-MR models, which in turn produced draw-level estimates of the prevalence of mild or moderate-to-severe impairment due to neonatal tetanus for all ages, sexes, years, and locations.

Measles

Flowchart

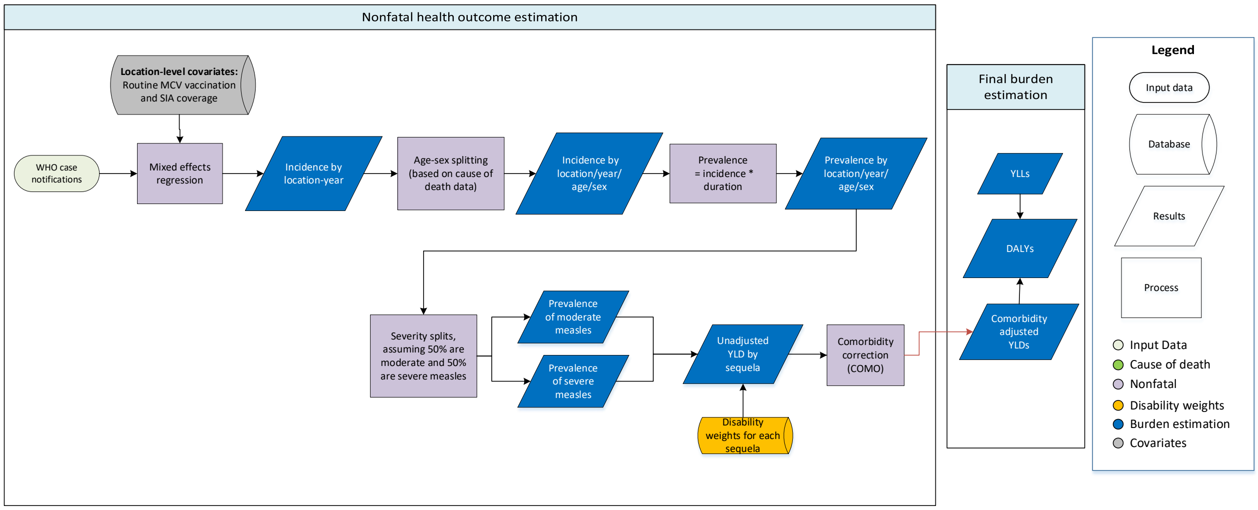

Case definition

Measles is a contagious infection caused by the measles virus. Symptoms include cough, runny nose, fever, conjunctivitis, and red, blotchy skin. For measles, ICD 10 codes are B05-B05.9, Z24.4, and ICD 9 codes are 055-055.9, 484.0, V04.2, V73.2.

Input data

Model inputs

The custom measles incidence model primarily leverages the relationship between direct reports of measles case notifications annually released by the World Health Organization (WHO) in the Joint Reporting Form (JRF), modeled estimates of measles-containing-vaccine (MCV) vaccination coverage proportions for doses 1 and 2, and supplementary immunization campaign (SIA) coverage to produce global estimates of measles cases. We supplement the national, JRF-reported case notifications with subnational case notifications from national health agencies in United States and Japan when complete and publicly available. In total for GBD 2019, we included complete case notifications through December 31, 2017, adding in supplemental notifications from 2018 and 2019 where available. For high-income, Central Europe/Eastern Europe/Central Asia and Latin America and Caribbean super-regions, modeled estimates of measles incidence are replaced directly by reported case notifications after the model is fit, assuming complete reporting in these locations. To better capture global measles outbreaks in 2019, we also used annualized, reported case notifications as available from 2019 in outbreak locations where the estimates produced by the custom incidence model were lower than suggested by available outbreak data. Table 1 contains counts of all nonfatal input data used in the measles model.

Table 1. Input data counts for the measles nonfatal model

**Measure**	**Total sources**
All measures	7903
Incidence	7901
Duration	1
Proportion	1

Severity splits

We assume 50% of measles cases were acute episodes of moderate infectious disease and 50% were acute episodes of severe infectious disease. The lay descriptions and disability weights for measles severity levels derived from the GBD Disability Weights study are shown in Table 2.

Table 2. Severity distribution, details on the severity levels for measles in GBD 2019 and the associated disability weight (DW) with that severity.

**Severity level**	**Lay description**	**DW (95% CI)**
Moderate	Has a fever and aches, and feels weak, which causes some difficulty with daily activities.	0.051 (0.032-0.074)
Severe	Has a high fever and pain, and feels very weak, which causes great difficulty with daily activities.	0.133 (0.088-0.19)

Modeling strategy

The general modelling approach used for GBD 2019 is similar to that used in GBD 2017. First, we make estimates of measles cases (i.e. direct counts) in every location, using a mixed-effects linear regression model and the case notification inputs. This model uses measles case notifications as the dependent variable with GBD 2019 estimates of five-year rolling lagged routine measles vaccination rates (first- and second-dose measles-containing vaccines) and coverage of supplementary immunization activities (SIAs) as predictors. In past GBD cycles, estimates of routine MCV coverage among infants in the modeled year were used as the routine immunization input into this model. In GBD 2019, we now use rolling means of MCV coverage calculated over the preceding five-year interval in order to better capture population-level vaccine-derived immunity among under-5-year olds. This approach now incorporates coverage both in the current year and in recent years.

In more detail, log-transformed incidence rates were regressed on the log of the proportion unvaccinated with first- and second-dose measles-containing vaccine (calculated using five-year rolling mean coverage), and additional SIA coverage lagged by one, two, three, four, and five years, with super-region, region, and country-level random effects:

$$Y_{ij} = \beta_0 + \beta_1 \,(1\text{-}MCV1_{ij}) + \beta_2 \,(1\text{-}MCV2_{ij}) + \beta_{a3}\, SIA_{aij} + u_j + e_{ij}\,,$$

In the equation above, Y_{ij} is the natural log of measles incidence rate per 100,000 people; β_0 is the fixed-effect intercept; β_1 is the fixed-effects slope on the log-transformed proportion unvaccinated with first-dose measles vaccine (calculated using rolling mean coverage over the preceding five years); β_2 is the fixed-effects slope on the log-transformed proportion unvaccinated with second-dose measles vaccine coverage (similarly calculated using rolling mean 5-year coverage); β_{a3} is the fixed-effects slope on supplementary measles immunization campaign coverage (administered doses over the target population

of all under-15s) lagged by a=1-5 years; u_j is the location-level random effects; e_{ij} is the residual; i is the year; and j is the location. We also assume a universal 95% attack rate in the absence of vaccination by generating a standard random effect consistent with this assumption, then applying that random effect in all years and locations when generating predictions from the model. From the fitted model, 1000 incidence predictions (draws) were generated for all ages, sexes, locations, and years using the estimated variance-covariance matrix.

These both-sex / all-age measles case estimates for every location were split into age- and sex-specific cases counts by utilizing age-sex distributions obtained from cause of death modeling in CODEm. Prevalence rates were then calculated by multiplying case predictions at the draw level by an average case duration of ten days and dividing by GBD-estimated population in each location; incidence rates were computed by draw by dividing estimated cases by population in each location. All draw-level results were then summarized by the mean of the draws with 95% uncertainty intervals (2.5th and 97.5th quantiles of all draws).

Varicella (chickenpox) and herpes zoster

Flowchart

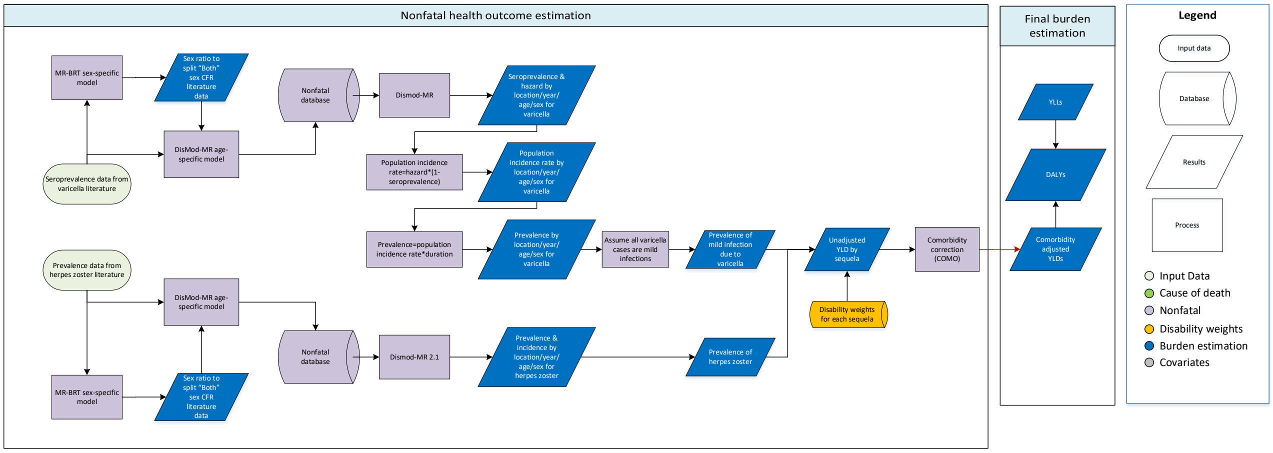

Case definition

Varicella (also known as chickenpox) is an acute infectious disease caused by primary infection of the varicella-zoster virus. Herpes zoster (also known as shingles) is caused by the reactivation of the same virus that causes varicella in adults. For varicella and herpes zoster, the ICD 10 codes are B01-B02.9, P35.8, Z20.820, and ICD 9 codes are 052-053.9, V01.71, V01.79, V05.4.

Input data

Model inputs

The varicella nonfatal models require varicella seroprevalence literature reports to produce estimates of chickenpox, and herpes zoster incidence literature reports to produce estimates of herpes zoster. The last systematic reviews of these topics were conducted in GBD 2016 using the following queries: *(varicella[Title/Abstract] AND seroprevalence[Title/Abstract]) AND (incidence[Title/Abstract] OR prevalence[Title/Abstract]) NOT (herpes zoster[Title/Abstract] OR shingles[Title/Abstract]) AND ("2013"[Date - Publication] : "2016"[Date - Publication])*; and *((herpes zoster[Title/Abstract] OR shingles[Title/Abstract]) AND (incidence[Title/Abstract)) NOT (varicella[Title/Abstract] OR chicken pox[Title/Abstract]) AND ("2013"[Date - Publication] : "2016"[Date - Publication])*.

We excluded studies that were: (1) not population-based, e.g., hospital or clinic-based studies; (2) did not provide primary data on epidemiological parameters, e.g., commentaries; (3) review articles; (4) case series; or (5) self-reported cases. Table 1 contains counts of all nonfatal input data used in the varicella and herpes zoster models.

Table 1. Input data counts for the varicella and herpes zoster nonfatal models

Measure	Total sources
All measures	124
Prevalence	61
Incidence	60
Remission	2
Duration	1

Input data processing

All extracted varicella seroprevalence and herpes zoster incidence data that was not sex- and age-specific (i.e. the data that was reflective of both sexes combined and/or age ranges greater than a 20-year start and end difference) were split into sex- and age-specific groups prior to use in modelling. Because scant age- and sex-specific on varicella seroprevalence and herpes zoster incidence are available, global sex ratios and age patterns were generated as described below and used to split non age- or sex-specific data while propagating uncertainty.

The ratios used to make the sex splits were calculated using MR-BRT, the meta-regression, Bayesian tool developed for GBD 2019. The sex adjustment factor calculated for use in GBD 2019 modeling for varicella seroprevalence was 0.97, and 0.94 for herpes zoster incidence (Tables 2a, 2b). The adjustment factors that were calculated during modeling in GBD 2017 were 0.91 and 0.92, respectively.

Table 2a: MR-BRT Sex-splitting Adjustment Factor for varicella seroprevalence

Data input	Reference or alternative case definition	Beta Coefficient, Log (95% CI)	Adjustment factor*
Sex	N/A	-0.027 (-0.071 to 0.018)	0.97

**Adjustment factor is the transformed Beta coefficient in normal space, and can be interpreted as the factor by which the alternative case definition is adjusted to reflect what it would have been if measured as the reference.*

Table 2b: MR-BRT Sex-splitting Adjustment Factor for herpes zoster incidence

Data input	Reference or alternative case definition	Beta Coefficient, Log (95% CI)	Adjustment factor*
Sex	N/A	-0.064 (-0.349 to 0.231)	0.94

**Adjustment factor is the transformed Beta coefficient in normal space, and can be interpreted as the factor by which the alternative case definition is adjusted to reflect what it would have been if measured as the reference.*

For both datasets, data representing an age group that spanned more than 20 years were split proportionally to follow a global age pattern that was generated using available age-specific data in DisMod. To estimate the global age pattern for herpes zoster incidence and varicella seroprevalence, all data representing an age group of less than 20 years in width were used to fit in separate DisMod-MR models. Then, the final global age pattern output – produced by DisMod in five-year age-bins from early neonatal to 95+ age groups – was used to split data from the remaining non-age-specific data sources.

Severity splits & disability weights

We assume all varicella cases are mild episodes of acute infectious disease, and herpes zoster is treated as a sequela. The lay descriptions and corresponding disability weights are presented in Table 3.

Table 3. Severity distribution, details on the severity levels for varicella-related nonfatal burden in GBD 2019 and the associated disability weight (DW) with that severity.

Severity level	Lay description	DW (95% CI)
Mild acute infectious disease	Has a low fever and mild discomfort but no difficulty with daily activities.	0.006 (0.002-0.012)
Herpes zoster	Has a blistering skin rash that causes pain, with some burning and itching.	0.058 (0.035-0.09)

Modeling strategy

The modeling of varicella (chickenpox) requires an intermediate model of varicella seroprevalence. Using the sex- and age-split varicella seroprevalence data, a DisMod-MR model was run to produce an estimate for every location and year, using HAQI as a covariate (Table 4). Model parameters are constrained so that there is zero remission and no excess mortality. Using the incidence hazard and prevalence outputs of the seroprevalence model, incidence rate is calculated as expanded below:

$$incidence\ rate\ =\ hazard\ *(1-prevalence)$$

Then, we calculate varicella prevalence as below, assuming a mean case duration of seven days:

$$prevalence = incidence\ rate * duration$$

Herpes zoster morbidity – modeled separately – uses the age- and sex-split herpes zoster incidence data directly in a DisMod model. There are no covariates used in the DisMod model. Like varicella, we assume that there is no excess mortality associated with herpes zoster.

In both models, the DisMod model parameters were newly adjusted in GBD 2019 to decrease the influence of hierarchical priors in the DisMod geographic cascade. These adjustments allow the model to more closely track available data in locations where data is present, and tend to result in broader uncertainty in resultant seroprevalence or incidence estimates, respectively, for locations where no data is available. In most locations, the net effect of the new age- and sex-splitting approach and adjustments to these DisMod settings resulted in increases in our final varicella seroprevalence estimates (e.g. Sub-Saharan Africa and Central Europe and Eastern Europe, & Central Asia) and decreases in our final herpes zoster incidence estimates (e.g. Southeast Asia, East Asia, & Oceania and high-income locations) while better following available data, reflecting uncertainty, and following the age and sex patterns present in age- and sex-specific data.

Table 4. Covariates. Summary of covariates used in the varicella seroprevalence DisMod-MR meta-regression model

Covariate	Type	Parameter	Exponentiated beta (95% CI)
Healthcare access and quality (HAQ) index	Country-level	Case fatality ratio	0.60 (0.37 — 0.97)

Acute Hepatitis: A, B, C, and E

Acute Hepatitis A

Flowchart

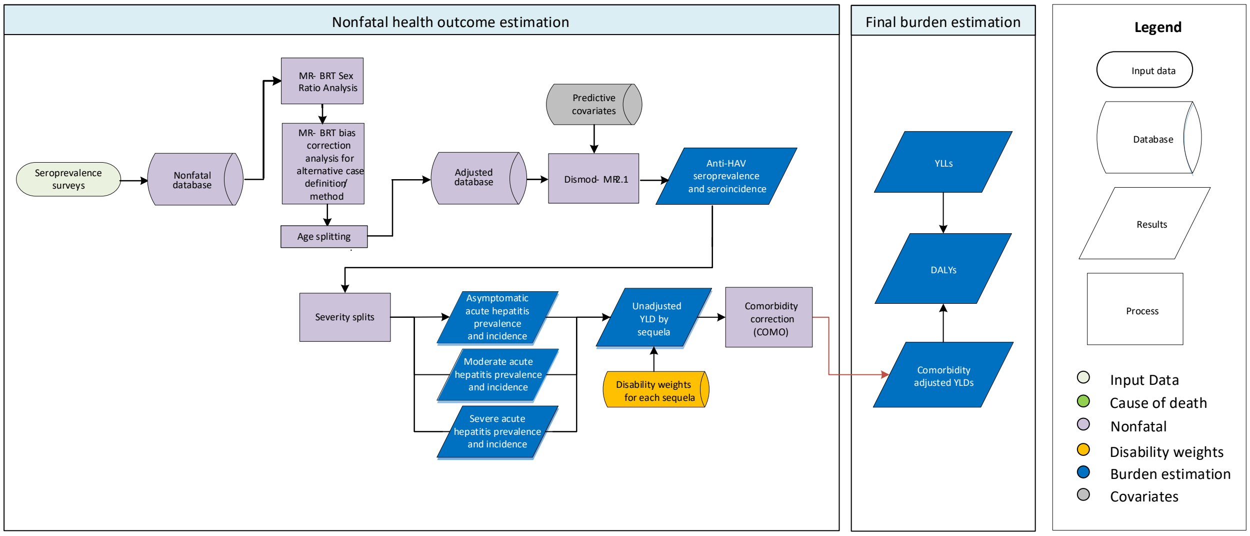

Input Data and Methodological Summary for Hepatitis A

Case definition

We define acute hepatitis A as an infection with the hepatitis A virus resulting in anti-HAV IgG seroconversion, regardless of symptoms.

Input data

Model inputs

We use anti-hepatitis A virus (HAV) seroprevalence data from population-based studies and surveys to estimate seroprevalence and seroincidence. The last systematic review was performed as part of GBD 2013. Additional data sources provided by collaborators were included in GBD2019.

Data inputs for anti-HAV seroprevalence modelling

Measure	Total sources	Countries with data
Prevalence	472	117

Modelling strategy

We model the seroprevalence of anti-hepatitis A virus IgG using a DisMod-MR 2.1 model. (See appendix section on DisMod method for details.) Remission and excess mortality value priors of zero were used, and an incidence value prior range between 0 and 0.5 was used. Given its reasonably stable force of infection among susceptible people across age groups, we derive incidence from the prevalence estimates using the following formula:

$$incid = \frac{-\ln(1 - prev)}{\mathrm{age_{mid}}} * (1 - prev)$$

In GBD 2019, we changed the method used to sex split data points. Previously studies that reported on “both” sex data points were split inside DisMod MR 2.1 using the sex covariate’s fixed-effect coefficient. However, this round we modeled the ratio of female/male prevalence in MR-BRT using the sex-ratios calculated directly from the studies that reported on both sexes separately and 10% trimming of the calculated ratios. (See appendix section on MR-BRT method for details.) Then, for studies only reporting prevalence for both sexes, we used GBD estimated population to estimate male prevalence as:

$$prev_{male} = prev_{both} * \frac{pop_{both}}{(pop_{male} + ratio * pop_{female})}$$

And then calculated female prevalence:

$$prev_{female} = ratio * prev_{male}$$

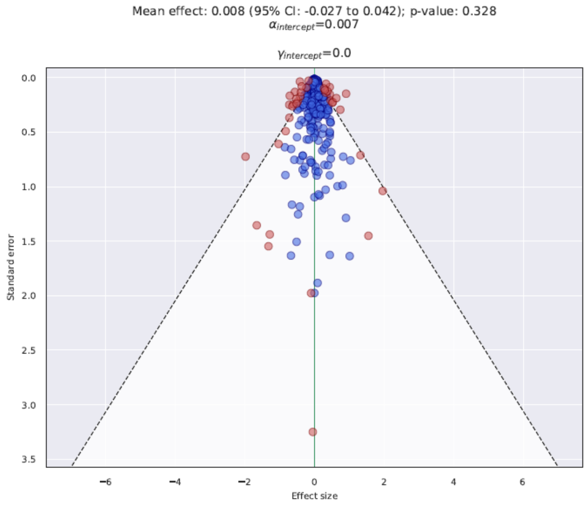

Figure: Estimated sex ratio; blue data points are included and red data points are excluded

In GBD 2017, we also split data points where the age range was greater than 25 years. This method was continued in GBD2019, in which the age pattern from the GBD2017 model was applied to large age range data to split into more granular age groups.

For GBD 2019, adjustment factors for all study-level covariates were determined using matched data (by year, age, sex, location) for reference and alternative case definitions in a logit difference network meta-regression. Study-level covariates included studies that were not population representative, such as blood donors and pregnant women. Furthermore, this round we added an adjustment to studies that included vaccinated participants. In GBD2017, we assumed that anti-HAV IgG only indicated past infection. We ignored the fact that someone could test positive for anti-HAV IgG because of vaccination. However, this meant that our results could be overestimating cases of acute hepatitis A, particularly in countries and years where there has been routine vaccination against HAV. In order to account for this, we crosswalked studies that study participants to studies that explicitly excluded individuals that had been vaccinated to approximate seroprevalence only in the unvaccinated population. Additionally, predictive covariates were included in the DisMod model to inform estimates for location-years with little or no primary data. The following tables provide an overview of the adjustment factors and predictive covariates used in the anti-HAV seroprevalence DisMod MR-2.1 model.

Summary of country-level covariates used in the anti-HAV seroprevalence DisMod-MR 2.1 model

Covariate	Parameter	Exponentiated beta (95% Uncertainty Interval)
Log-transformed age-standardized SEV scalar: Diarrhea	Prevalence	1.28 (1.26 — 1.31)

MR-BRT Crosswalk Factors for anti-HAV seroprevalence non representative populations

Data input	Reference or alternative case definition	Gamma	Beta Coefficient, Logit (95% CI)
General population	Ref	0.87	---
Blood donors	Alt		0.85 (-0.95 – 2.58)
Pregnant women	Alt		1.31 (-1.18 – 3.80)

MR-BRT Crosswalk Factors for anti-HAV seroprevalence vaccination status

Data input	Reference or alternative case definition	Gamma	Beta Coefficient, Logit (95% CI)
Unvaccinated study population	Ref	1.01	---
Study population included both vaccinated and unvaccinated individuals	Alt		0.59 (-1.41 – 2.61)

Severity splits & disability weights

The table below illustrates the sequelae associated with acute hepatitis A, as well as the lay descriptions and associated disability weights.

Severity distributions and disability weights

Sequela	Description	Disability Weight
Moderate	Has a fever and aches, and feels weak, which causes some difficulty with daily activities.	0.051 (0.032–0.074)
Severe	Has a high fever and pain, and feels very weak, which causes great difficulty with daily activities.	0.133 (0.088–0.19)
Asymptomatic	Infection with no apparent illness	NA

We calculate acute symptomatic infections by multiplying incidence of acute infection by the probability of acute symptomatic infection. The probability of symptomatic infection comes from Armstrong and

Bell[1] and is shown in the figure below (where probability of symptomatic infection is represented as "probability of jaundice") [1]. The probability increases with age from ~1% in the first year of life to ~85% in adulthood. The probability function is:

$$Prob\ (symptomatic) = 0.852 * (1 - \ e^{-0.01244 * age^{1.903}})$$

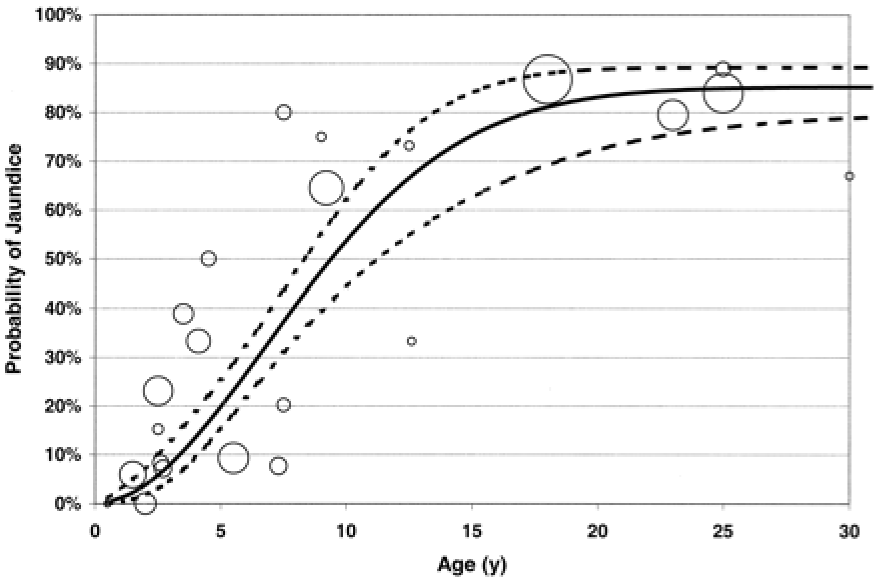

The remainder of acute infections are assumed to be asymptomatic.

We then base severity splits for moderate and severe on expert opinion that the probability of severe infection follows a beta distribution with mean 0.6% (the below table reports percentiles of this distribution.) We assume the rest of symptomatic infections are moderate.

Percentiles of the probability distribution of severe acute hepatitis A

0 percentile	25 percentile	50 percentile	75 percentile	100 percentile
0.0024	0.0054	0.006	0.007	0.01

Acute Hepatitis B

Flowchart

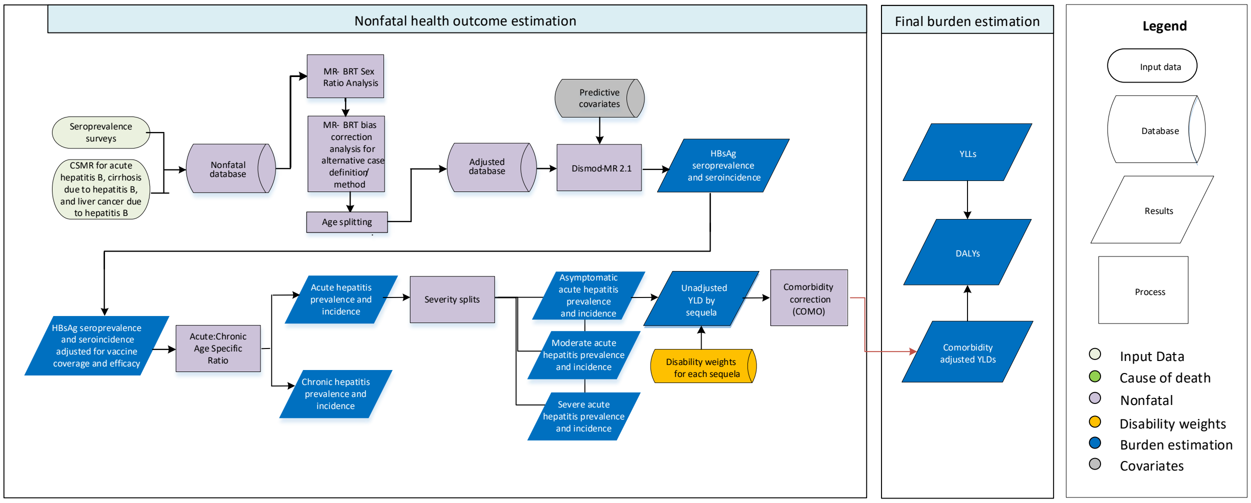

Input Data and Methodological Summary for Hepatitis B

Case definition

We define acute hepatitis B as the period corresponding to initial infection with the hepatitis B virus, regardless of symptoms.

Input data

Model inputs

We use hepatitis B surface antigen (HBsAg) seroprevalence data from population-based studies and surveys. The last systematic review conducted by IHME was performed as part of GBD 2013.. This round we began to align our sources with those used by the London School of Tropical Medicine and WHO. Sources were screened from the appendix of the Schweitzer 2015 systematic review[2] and added where data matched our inclusion criteria. Given the length of the citation list, we prioritized data time periods and geographies that were data-scarce in previous rounds of GBD or have particularly dynamic hepatitis B epidemiology. New sources were added this round for Sub-Saharan Africa, Australasia, Andean South America, Eastern Europe, and High Income North America. The remainder of the appropriate sources from this systematic review will be incorporated in future rounds of GBD.

We also use cause-specific mortality rate (CSMR) estimates for acute hepatitis B, cirrhosis and other chronic liver diseases due to hepatitis B, and liver cancer due to hepatitis B from the GBD Causes of Death modelling process.

We used estimates of vaccination coverage of hepatitis B 3 dose vaccine from the GBD Vaccine Team and efficacy of 0.95 to construct location and country specific reductions of seroprevalence and seroincidence estimates from a DisMod model. This team uses a combination of country-reported administrative data, such as MICS and DHS data, and survey data to inform their estimates of hepatitis B vaccine coverage.

Data inputs for HBsAg seroprevalence modelling

Measure	Total sources	Countries with data
Prevalence	468	108

Modelling strategy

We modeled HBsAg seroprevalence using a multi-step approach. First, we create a “counterfactural” HBsAg seroprevalence model, using only data from unvaccinated populations in a full DisMod-MR 2.1 model to obtain estimates of what the incidence and prevalence of chronic carriage would be in a steady-state without vaccine intervention. Next, we modify those results using estimates of hepatitis B vaccine coverage and efficacy to obtain estimates of the true incidence and prevalence of chronic hepatitis B carriage. Finally, we use natural history studies to infer what the total incidence of acute hepatitis B was from the incidence of chronic carriage. These processes are described in more detail below.

In GBD 2019, we changed the method used to sex split data points. Previously studies that reported on “both” sex data points were split inside DisMod MR 2.1 using the sex covariate’s fixed-effect coefficient. However, this round we modeled the ratio of female/male prevalence in MR-BRT using the sex-ratios calculated directly from the studies that reported on both sexes separately and 10% trimming of the calculated ratios. (See appendix section on MR-BRT method for details.) Then, for studies only reporting prevalence for both sexes, we used GBD estimated population to estimate male prevalence as:

$$prev_{male} = prev_{both} * \frac{pop_{both}}{(pop_{male} + ratio * pop_{female})}$$

And then calculated female prevalence:

$$prev_{female} = ratio * prev_{male}$$

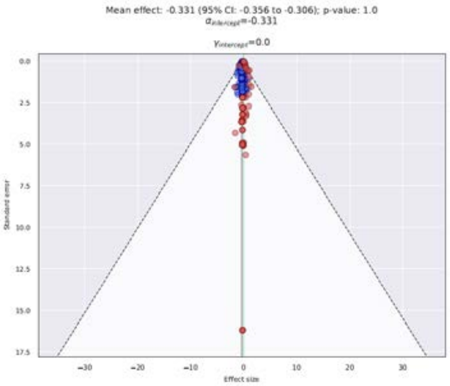

Figure: Estimated sex ratio in MR-BRT; blue data points are included and red data points are excluded

In GBD 2017, we split data points where the age range was greater than 25 years. This method was continued in GBD2019, in which the age pattern from the GBD2017 model was applied to large age range data to split into more granular age groups.

For GBD 2019, adjustment factors for all study-level covariates were determined using matched data (by year, age, sex, location) for reference and alternative case definitions in a logit difference network meta-regression. Study-level covariates included studies that were not population representative, such as blood

donors and pregnant women. Predictive covariates were included in the DisMod model to inform global patterns. The following tables provide an overview of the study-level and predictive covariates used in the anti-HAV seroprevalence DisMod MR-2.1 model.

Summary of predictive covariates used in the HBsAg seroprevalence DisMod-MR 2.1 model

Covariate	Parameter	Exponentiated beta (95% Uncertainty Interval)
Log-transformed age-standardized SEV scalar: Hep B	Prevalence	1.13 (1.00 — 1.43)
Socio-demographic Index	Prevalence	0.14 (0.14 — 0.14)
Healthcare access and quality index	Excess mortality rate	1.00 (1.00 — 1.00)

MR-BRT Crosswalk Factors for HBsAg seroprevalence non representative populations

Data input	Reference or alternative case definition	Gamma	Beta Coefficient, Logit (95% CI)
General population	Ref	0.72	---
Blood donors	Alt		-0.53 (-1.94 – 0.81)
Pregnant women	Alt		-0.86 (-2.44 – 0.65)

As mentioned above, in GBD2019, we employed a counterfactual DisMod-MR model using only data from unvaccinated populations. In previous rounds, we used a DisMod-MR model of hepatitis B surface antigen positivity that employed all available data for vaccinated or unvaccinated populations. This older model tended to follow the data from unvaccinated populations, and poorly fit prevalence data from vaccinated populations at younger ages. Thus, for GBD 2019, we marked seroprevalence data from vaccinated populations as outliers and did not use them in the DisMod model, effectively producing a "counter-factual" model of HBsAg seroprevalence in the absence of vaccination programs. We excluded studies in which participants were exposed to vaccination by using the ages of study participants and years of the study to determine possible years of birth. A study was excluded if all or at least 50% of a normal distribution of study participants were born after the location specific year of vaccine introduction. Data collected from vaccinated populations were retained in the database to verify that subsequent modeling steps adequately accounted for the effect of vaccine programs.

After the completion of the counter-factual DisMod model, a post-hoc adjustment was performed to modify estimates of HBsAg seropositivity based on vaccine coverage and efficacy. The proportion of coverage by location and year were multiplied by efficacy of vaccine to get the proportion of the population effectively covered by the HBV vaccine. Then these results were subtracted from the HBsAg seroprevalence DisMod estimates to get estimates of incidence and prevalence.

An example of the DisMod MR 2.1 model and data (included in grey and excluded in red) for Taiwan is pictured in Figure A. Figure B shows the results after the post-hoc adjustment where the estimates are adjusted by vaccine coverage and efficacy. Note that the adjusted results closely match the seroprevalence data points in red, which were not used in any modeling step and are presented only to validate the final outputs. These excluded data points are from age groups that had been exposed to vaccination.

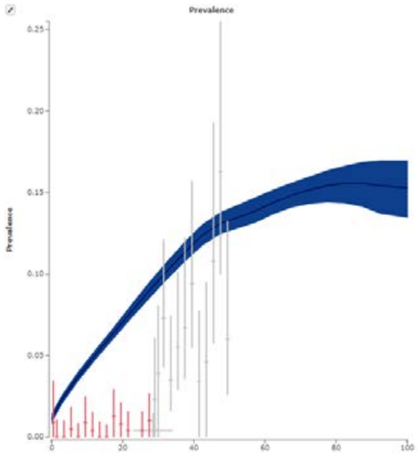

Figure A

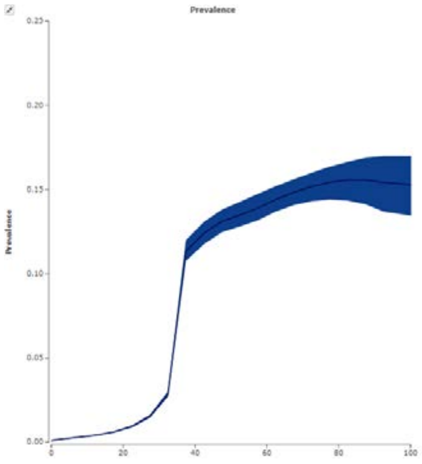

Figure B

These final estimates of HBsAg seroprevalence serve as inputs to models for several entities, as described in the methods appendix sections on the estimation of the fatal and nonfatal burden of cirrhosis and other chronic liver diseases and liver cancer. The remainder of this section only discusses how HBsAg seroprevalence estimates are used to estimate acute hepatitis B infection.

The incidence obtained from the DisMod model of HBsAg seroprevalence is regarded as the incidence of chronic carriage. This is converted to the total incidence of hepatitis B infection by dividing age-specific estimates of the incidence of chronic carriage by age-specific estimates of the probability of infection resulting in carriage based on Edmunds and colleagues[3]:

$$P(carrier \mid age \leq 6\,months) = 0.885$$

$$P(carrier \mid 6\,months \leq age < 25\,years) = e^{-0.645 \times age^{0.455}}$$

$$P(carrier \mid age \geq 25\,years) = e^{-0.645 \times 25^{0.455}} = 0.061$$

We then split symptomatic cases into moderate (73%) and severe (27%) based on data from McMahon and colleagues[4]. We then assigned the moderate and severe cases the following health states and disability weights.

Severity distributions and disability weights

Sequela	Description	Disability Weight
Moderate	Has a fever and aches, and feels weak, which causes some difficulty with daily activities.	0.051 (0.032–0.074)
Severe	Has a high fever and pain, and feels very weak, which causes great difficulty with daily activities.	0.133 (0.088–0.19)
Asymptomatic	Infection with no apparent illness.	NA

Acute Hepatitis C

Flow Chart

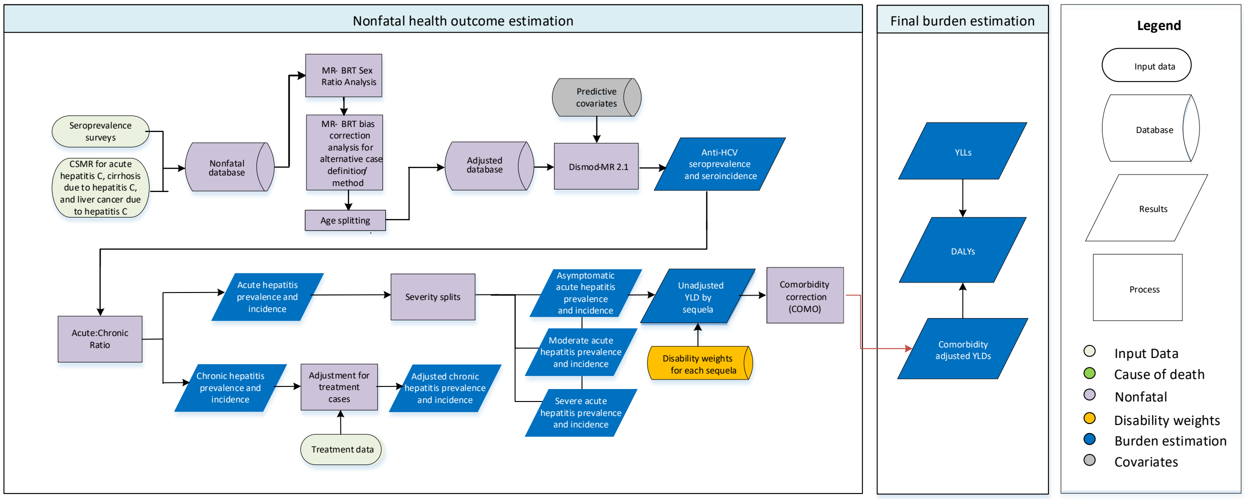

Input Data and Methodological Summary for Hepatitis C

Case definition

We define acute hepatitis C as the period corresponding to initial infection with the hepatitis C virus, resulting in anti-HCV IgG seroconversion, regardless of symptoms.

Input data

To estimate morbidity for hepatitis C, we use anti-HCV seroprevalence data from population-based studies and surveys to estimate incidence and prevalence of hepatitis C infection. The last systematic review performed by IHME was part of GBD 2013. This round we augmented our database with sources collated by the Center for Disease Analysis. Sources were taken from the appendix of the systematic review by Blach 2016[5]. We included all sources in this appendix except 40 sources that could not be located.

We also use cause-specific mortality rate (CSMR) estimates for acute hepatitis C, cirrhosis and other chronic liver diseases due to hepatitis C, and liver cancer due to hepatitis C from the GBD Causes of Death modelling process as inputs in our DisMod compartmental model.

Additionally, we use hepatitis C treatment data from Egypt, Japan, and Australia to perform reductions in our estimates of chronic hepatitis C. These data report on demographics where available and treatment type, which relates to the efficacy of the intervention.

Data inputs for acute hepatitis C modelling by parameter

Measure	Total sources	Countries with data
All Measures	332	98
Prevalence	300	98
Proportion	32	3

Modelling strategy

In GBD 2019, we changed the method used to sex split data points. Previously studies that reported on "both" sex data points were split inside DisMod MR 2.1 using the sex covariate's fixed-effect coefficient. However, this round we modeled the ratio of female/male prevalence in MR-BRT using the sex-ratios calculated directly from the studies that reported on both sexes separately and 10% trimming of the calculated ratios. (See appendix section on MR-BRT method for details.) Then, for studies only reporting prevalence for both sexes, we used GBD estimated population to estimate male prevalence as:

$$prev_{male} = prev_{both} * \frac{pop_{both}}{(pop_{male} + ratio * pop_{female})}$$

And then calculated female prevalence:

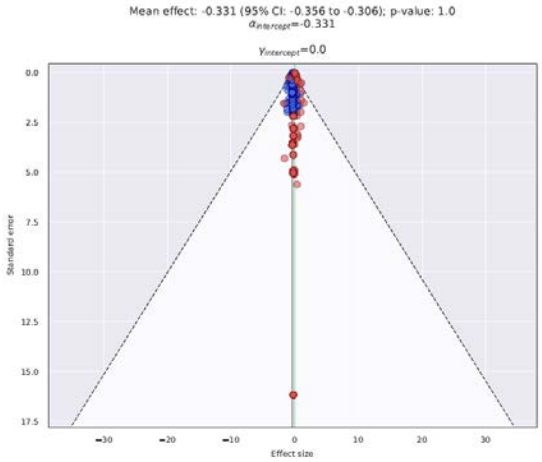

Estimated sex ratio in MR-BRT; blue data points are included and red data points are excluded

In GBD 2017, we split data points where the age range was greater than 25 years. This method was continued in GBD2019, in which the age pattern from the GBD2017 model was applied to large age range data to split into more granular age groups.

For GBD 2019, adjustment factors for all study-level covariates were determined using matched data (by year, age, sex, location) for reference and alternative case definitions in a logit difference meta-regression. Study-level covariates included studies that were not population representative, such as blood donors. Because of lack of overlapping matches, no adjustment factor could be estimated for pregnant women. As a result, data sources reporting on pregnant women were outliered. We used predictive covariates to help inform estimates where data were sparse or absent. The following tables provide an overview of the study-level and predictive covariates used in the anti-HCV seroprevalence DisMod MR-2.1 model.

Summary of covariates used in the anti-HCV seroprevalence DisMod-MR 2.1 model

Covariate	Parameter	Exponentiated beta (95% Uncertainty Interval)
Log-transformed age-standardized SEV scalar: Hep C	Prevalence	2.47 (2.46 — 2.50)
Socio-demographic Index	Prevalence	0.14 (0.14 — 0.14)

LDI ($ per capita)	Excess mortality rate	0.96 (0.94 – 0.98)

MR-BRT Crosswalk Factors for anti-HCV seroprevalence non representative populations

Data input	Reference or alternative case definition	Gamma	Beta Coefficient, Logit (95% CI)
General population<br>Blood donors	Ref<br>Alt	0.74	---<br>-0.55 (-1.92 – 0.88)

To estimate burden due to acute hepatitis C, incident infections estimated from the DisMod model were divided into asymptomatic (75%), moderate (24%), and severe (1%) states based on expert opinion and assigned the following health states and disability weights.

Severity distributions and disability weights

Sequela	Description	Disability Weight
Moderate	Has a fever and aches, and feels weak, which causes some difficulty with daily activities.	0.051 (0.032–0.074)
Severe	Has a high fever and pain, and feels very weak, which causes great difficulty with daily activities.	0.133 (0.088–0.19)
Asymptomatic	Infection with no apparent illness.	NA

Beyond estimating burden due to acute hepatitis C, the DisMod model of hepatitis C infection was used to estimate prevalence of chronic infection, which serves as an input to multiple estimation processes described in separate sections of this appendix (fatal and non-fatal burden of cirrhosis and other liver disease and liver cancer). We estimate chronic infections from total incident infections by multiplying incidence as estimated by DisMod-MR by the probability an incident infection will be chronic. In previous rounds of the GBD a single study by Guadagnino and colleagues 1997 [6] was used to convert estimates from incident infection to chronic. In GBD2019, we conducted a meta-analysis in MR-BRT using 42 studies that reported on the prevalence of anti-HCV antibody and HCV-RNA to produce a pooled estimate of proportion viraemic among the seropositive. This was used to correct outputs of our model of anti-HCV seropositivity to estimate viraemia. We examined the estimated coefficient based on super-region, particularly looking to see if there is a difference in the ratio of anti-HCV to HCV RNA positivity in Sub-Saharan Africa as suggested by expert collaborators. However, no significant difference was identified and we used the same conversion factor globally. Below is a graph of the pooled estimated logit difference and logit difference and standard error of input studies.

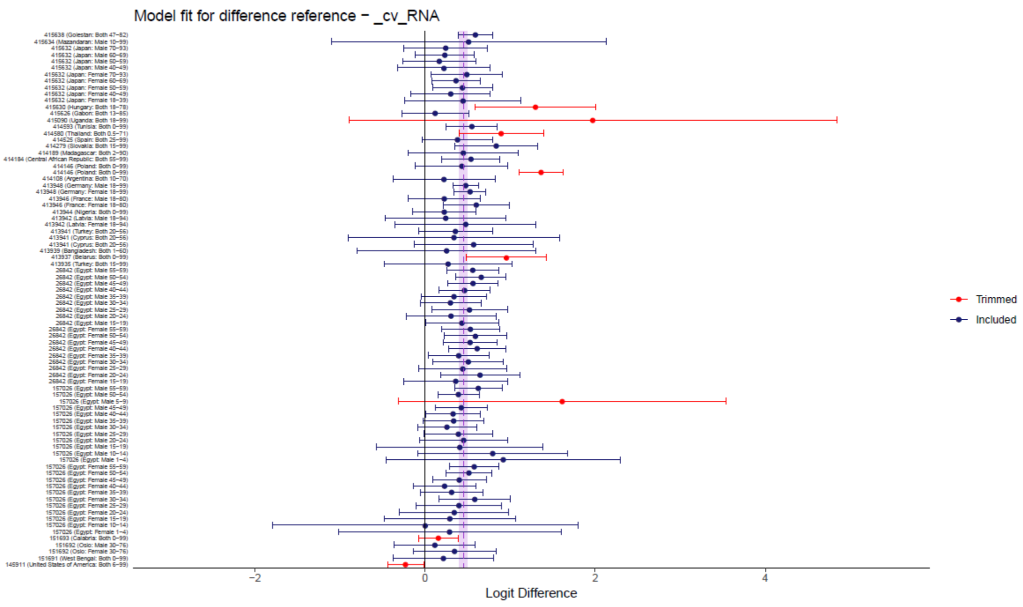

In GBD2019, we included information on treatment effects for countries where national treatment data were available. Estimates of chronic hepatitis in Egypt, Japan, and Australia were adjusted to account for virus-clearing treatment by subtracting the number of individuals treated multiplied by the efficacy of treatment. Based on expert opinion, pegylated interferon and direct acting antivirals (DAA) treatments were considered to have efficacy of 70% and 95%, respectively. We estimated the cumulative effect of treatment effects from year to year as the reduction in cases of chronic infection. As data on treatment volumes becomes available in other locations, we will perform a similar reduction in those countries in future rounds.

Acute Hepatitis E

Flowchart

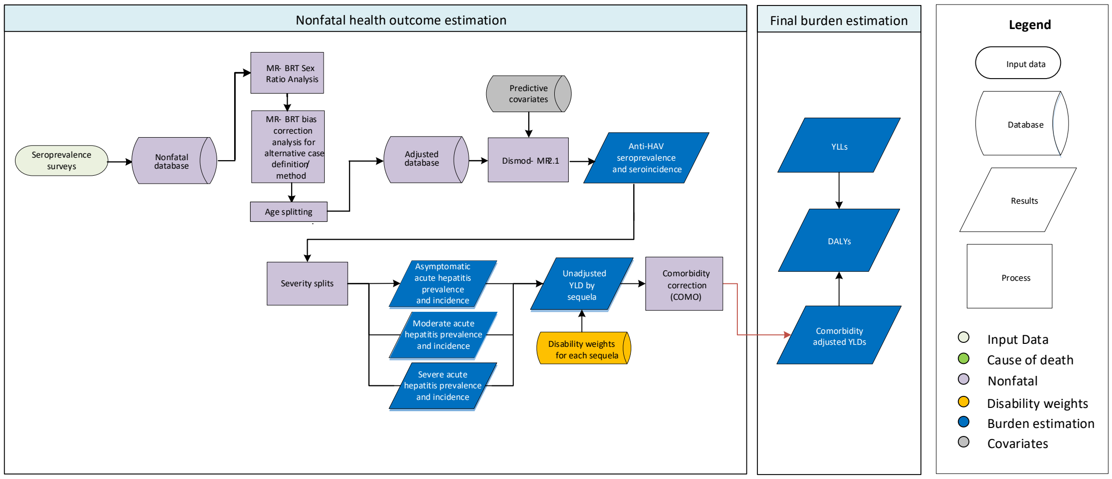

Input Data and Methodological Summary for Hepatitis E

Case definition

We define acute hepatitis E as an infection with the hepatitis E virus resulting in anti-HEV IgG seroconversion, regardless of symptoms.

Input data

We use anti-HEV seroprevalence data from population-based studies and surveys to estimate incidence of infection. The last systematic review was performed as part of GBD 2013.

Data inputs for anti-HEV seroprevalence modelling

Measure	Total sources	Countries with data
Prevalence	81	44

Modelling Strategy

We model the incidence of hepatitis E using a full DisMod-MR 2.1 model of anti-HEV seroprevalence, assuming no remission.

In GBD 2019, we changed the method used to sex split data points. Previously studies that reported on “both” sex data points were split inside DisMod MR 2.1 using the sex covariate’s fixed-effect coefficient. However, this round we modeled the ratio of female/male prevalence in MR-BRT using the sex-ratios calculated directly from the studies that reported on both sexes separately and 10% trimming of the calculated ratios. (See appendix section on MR-BRT method for details.) Then, for studies only reporting prevalence for both sexes, we used GBD estimated population to estimate male prevalence as:

$$prev_{male} = prev_{both} * \frac{pop_{both}}{(pop_{male} + ratio * pop_{female})}$$

And then calculated female prevalence:

$$prev_{female} = ratio * prev_{male}$$

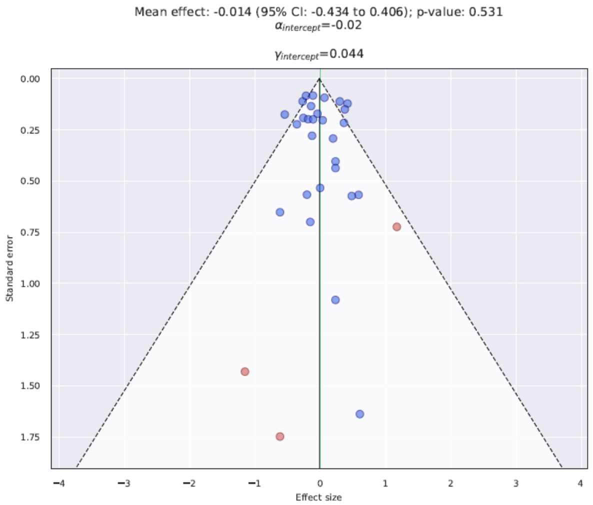

Figure: Estimated sex ratio in MR-BRT; blue data points are included and red data points are excluded

In GBD 2017, we split data points where the age range was greater than 25 years. This method was continued in GBD2019, in which the age pattern from the GBD2017 model was applied to large age range data to split into more granular age groups.

For GBD 2019, adjustment factors for all study-level covariates were determined using matched data (by year, age, sex, location) for reference and alternative case definitions in a logit difference meta-regression. Study-level covariates included studies that were not population representative, such as blood donors. There were insufficient matched studies of anti-HEV seroprevalence in alternative and reference populations from the same year-age-sex-location combinations to estimate an adjustment factor in MR-BRT. Thus, we combined matched pairs of studies of anti-HEV and matched pairs of studies of anti-HAV, to estimate an adjustment factor for all viral hepatitis with fecal-oral transmission, and applied these adjustments to anti-HEV data collected by non-reference methods. Because of lack of overlapping matches, no adjustment factor could be estimated for pregnant women. As a result, data sources from pregnant samples were outliered.

We employed predictive covariates in the DisMod MR-2.1 model to improve estimates in location-years with little or no data. The following tables provide an overview of the study-level and predictive covariates used in the anti-HEV seroprevalence model.

Summary of covariates used in the anti-HEV seroprevalence DisMod-MR 2.1 model

Covariate	Parameter	Exponentiated beta (95% Uncertainty Interval)
Proportion of the population living in the classic monsoon region (low-income countries)	Prevalence	1.19 (1.01 — 1.56)
Log-transformed SEV scalar: Diarrhea	Prevalence	1.07 (1.01 – 1.14)

MR-BRT Crosswalk Factors for anti-HEV seroprevalence non representative populations

Data input	Reference or alternative case definition	Gamma	Beta Coefficient, Logit (95% CI)

General population	Ref	0.88	---
Blood donors	Alt		0.90 (-0.84 – 2.66)

Based on information published by Rein and colleagues [7], we assume that the probability of symptomatic infection increases with age from ~1% in the first year of life to ~60% in adulthood.

The table below illustrates the sequelae associated with acute hepatitis E, along with their descriptions and disability weights.

Severity distributions and disability weights

Sequela	Description	Disability Weight
Moderate	Has a fever and aches, and feels weak, which causes some difficulty with daily activities.	0.051 (0.032–0.074)
Severe	Has a high fever and pain, and feels very weak, which causes great difficulty with daily activities.	0.133 (0.088–0.19)
Asymptomatic	Infection with no apparent illness.	NA

References

1. Armstrong GL, Bell BP. Hepatitis A Virus Infections in the United States: Model-Based Estimates and Implications for Childhood Immunization. Pediatrics. 2002 May 1;109(5):839–45.
2. Schweitzer, A., Horn, J., Mikolajczyk, R. T., Krause, G., & Ott, J. J. Estimations of worldwide prevalence of chronic hepatitis B virus infection: a systematic review of data published between 1965 and 2013. Lancet. 2015; 386(10003), 1546–1555
3. Edmunds WJ, Medley GF, Nokes DJ, Hall AJ, Whittle HC. The influence of age on the development of the hepatitis B carrier state. Proc Biol Sci. 1993 Aug 23;253(1337):197–201.
4. McMahon BJ, Alward WL, Hall DB, Heyward WL, Bender TR, Francis DP, et al. Acute hepatitis B virus infection: relation of age to the clinical expression of disease and subsequent development of the carrier state. J Infect Dis. 1985 Apr;151(4):599–603).
5. Blach S et al. Global prevalence and genotype distribution of hepatitis C virus infection in 2015: a modelling study. The Lancet Gastroenterology & Hepatology. 2017; 2(3):161-176.
6. Guadagnino, Vincenzo, et al. "Prevalence, risk factors, and genotype distribution of hepatitis C virus infection in the general population: a community-based survey in southern Italy." Hepatology 26.4 (1997): 1006-1011.
7. Rein DB, Stevens GA, Theaker J, Wittenborn JS, Wiersma ST. The global burden of hepatitis E virus genotypes 1 and 2 in 2005. Hepatology. 2012 Apr 1;55(4):988–97.

Other unspecified infectious diseases

Flowchart

Anemia Impairment Estimation

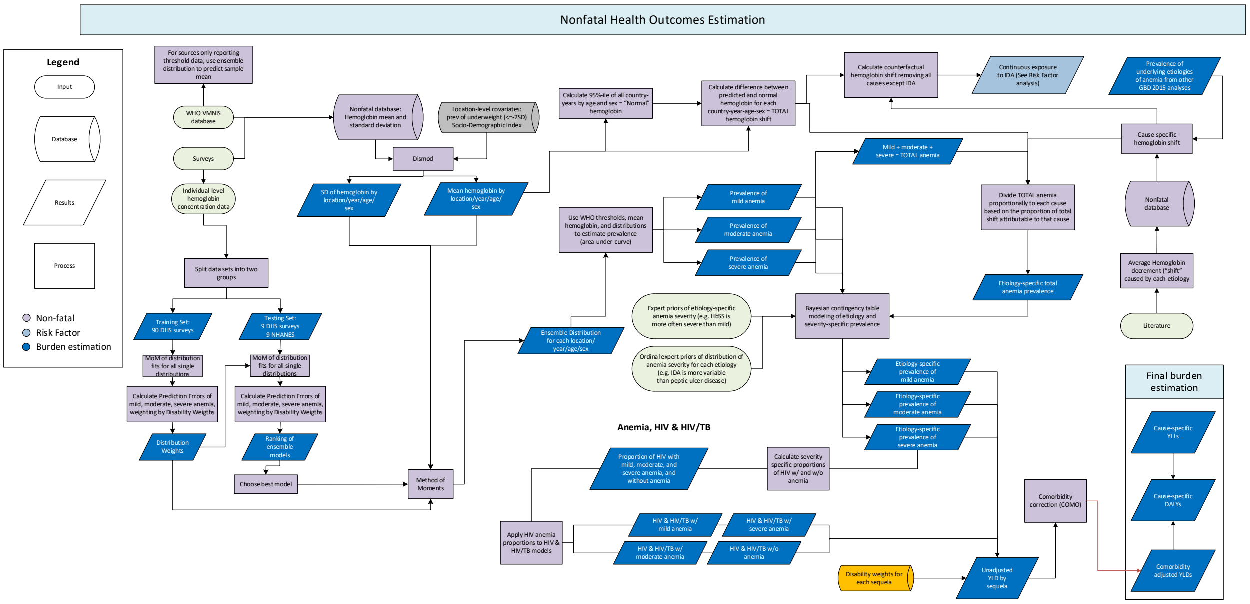

Input data and Methodological Summary for Other Unspecified Infectious Diseases

For GBD 2019, we estimate other unspecified infectious diseases using the residual anemia impairment envelope based on a fixed proportion of redistribution. The resulting models of Mild anemia due to other infectious diseases, Moderate anemia due to other infectious diseases, and Severe anemia due to other infectious diseases go into our central computation to generate YLDs based on our prevalence values.

Causes for which allocation of residual anemia envelope was based on fixed proportion redistribution methods*:

Iron-deficiency anemia (IDA)

Other infectious diseases

Other neglected tropical diseases

Other endocrine, nutrition, blood and immune disorders

Other hemoglobinopathies and hemolytic anemias

* A minimum of 10% of all anemia was assigned to residual categories based on analysis of NHANES-III data from the United States

References

1. Kassebaum NJ. The Global Burden of Anemia. *Hematology/Oncology Clinics* 2016; **30**: 247–308.

2. Kassebaum NJ, Jasrasaria R, Naghavi M, *et al.* A systematic analysis of global anemia burden from 1990 to 2010. *Blood* 2014; **123**: 615–24.

Maternal disorders

Maternal disorders nonfatal estimation includes disability due to seven of ten maternal subcauses, including 1) Abortion and miscarriage; 2) Ectopic pregnancy; 3) Obstructed labour and uterine rupture; 4) Maternal haemorrhage; 5) Maternal sepsis and other maternal infections; 6) Maternal hypertensive disorders; and 7) Other [direct] maternal disorders. Indirect maternal disorders, late maternal deaths, and maternal deaths aggravated by HIV/AIDS did not have any estimated disability based on the premise that it is captured in the respective underlying causes.

Flowchart

Maternal disorders: 1) Abortion and miscarriage; 2) Obstructed labor and uterine rupture; 3) Maternal hemorrhage; 4) Maternal sepsis and other maternal infections; 5) Maternal hypertensive disorders; 6) Ectopic pregnancy 7) Other maternal disorders

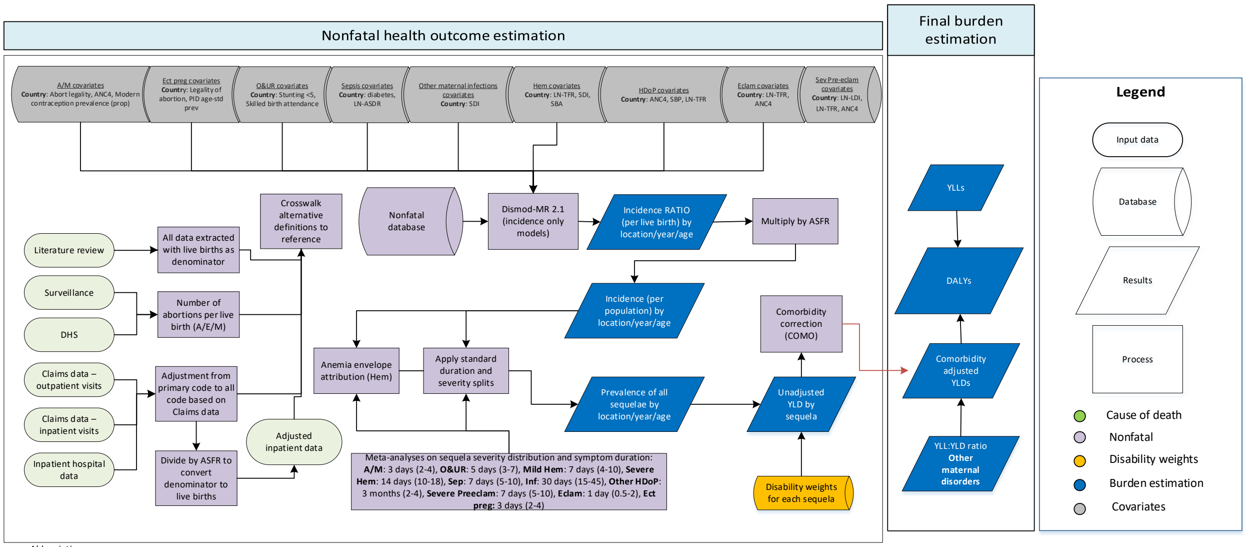

Abbreviations

A/M: Abortion and miscarriage; Ect Preg: Ectopic pregnancy; O&UR: Obstructed labor and uterine rupture; Hem: Maternal hemorrhage; Sep: Maternal sepsis; Inf: Other maternal infections; HDoP: Maternal hypertensive disorders; Eclam: Eclampsia; Sev pre-eclam: Severe pre-eclampsia; ASFR: Age-specific fertility rate; Stunting <5: Stunting (proportion <2SD height for age, <5 years); MS: Marketscan; ANC4: coverage of 4 visits of antenatal care; SDI: Socio-Demographic Index; lit: Literature data; inpt: Inpatient data; not repr: Not representative; LN: Natural log; TFR: Total fertility rate; LDI: Lag-distributed income per capita; SBA: Skilled birth attendance (proportion), IFD: In facility delivery

Input data and methodological summary

Case definition

Maternal disorders are those complications occurring during pregnancy, childbirth, and the postpartum period. Nine different statistical models were completed for GBD 2019 across six of the maternal subcauses. These included, by GBD cause:

1) Abortion is defined as elective or medically-indicated termination of pregnancy at any gestational age and miscarriage is defined as spontaneous loss of pregnancy before 24 weeks of gestation with complications requiring medical care.
2) Ectopic pregnancy is defined as any pregnancy occurring outside of the uterus.
3) Obstructed labour and uterine rupture –
    a. Acute event includes failure to progress (no advance of the presenting part of the fetus despite strong uterine contractions), cephalopelvic disproportion (foetal size that is too large for maternal pelvic dimensions), non-vertex foetal positioning during labour (any foetal position besides head down during labour; excludes non-vertex positioning during antepartum period), and uterine rupture during labour (non-surgical breakdown of uterine wall during labour and delivery). Perineal lacerations without any of the above conditions are excluded from the case definition.

   b. Fistula is defined as an abnormal connection between either vagina and large intestine (rectovaginal fistula) or between vagina bladder (vesicovaginal fistula). Fistula YLDs are included in YLDs for obstructed labour; estimation is described in a separate appendix section on "Fistula – impairment."
4) Maternal haemorrhage (including placental disorders) – includes both postpartum (>500 ml for vaginal delivery and >1,000 ml for cesarean delivery) and antepartum haemorrhage vaginal bleeding from any cause at or beyond 20 weeks of gestation and prior to onset of labour). This also includes placental disorders with haemorrhage regardless of blood volume lost or timing of bleeding event. Placental disorders without haemorrhage are included with other [direct] maternal disorders.
5) Maternal sepsis and other maternal infections –
   a. Maternal sepsis is defined as a temperature <36°C or >38°C and clinical signs of shock including systolic blood pressure <90 mmHg and tachycardia >120 bpm
   b. Other maternal infections are defined as any maternal infections excluding HIV, sexually-transmitted infections, or are not believed to have epidemiologic relationship with pregnancy. Examples include urinary tract infections, mastitis, candidiasis, and bacterial vaginosis during pregnancy.
6) Hypertensive disorders of pregnancy – overall category defined as having blood pressure (BP) >140/90 based on multiple measurements in persons who were not hypertensive prior to pregnancy. This category includes several subcategories
   a. Severe pre-eclampsia is defined by severe hypertension (>160/100), proteinuria (>=0.3 g/l), and additional signs of end organ damage (liver: low platelets, elevated liver enzymes, coagulation issues; kidney: elevated creatinine; CNS: headaches or visual disturbances) and includes hypertension elevated liver low platelets (HELLP) syndrome.
   b. Eclampsia is defined as hypertension +/- proteinuria and seizures.
   c. Other hypertensive disorders of pregnancy include gestational hypertension (>140/90 without proteinuria or other symptoms) and pre-eclampsia (hypertension [>140/90] and proteinuria without signs of end-organ damage).
7) Other [direct] maternal disorders include a variety of different obstetric complications. The most common of these in ICD-10 coded vital registration sources in terms of number of deaths include O88 (obstetric embolism), O26 (Maternal care for other conditions predominantly related to pregnancy), O90 (Complications of the puerperium, not elsewhere classified), O75 (Other complications of labor and delivery, not elsewhere classified), C58 (Malignant neoplasm of placenta), and O36 (Maternal care for other fetal problems).

We estimated YLDs for other [direct] maternal disorders YLD-to-YLL ratio approach where the ratio of YLD:YLL were pooled for all the causes in the list above and multiplied by the YLL for other [direct] maternal disorders. For other subcauses of maternal disorders, including late maternal death, indirect maternal disorders, and maternal death complicated by HIV/AIDS, we did not estimate any nonfatal burden based on the premise that the associated disability is captured in the respective causes.

Input data

Systematic literature reviews have been completed annually since GBD 2010 and use a consolidated search string for all components of maternal burden estimation. These were updated on May 10, 2019, using the search string below.

(((("Postpartum Hemorrhage" OR "Uterine Hemorrhage") OR (maternal[Title/Abstract] OR pregnan*[Title/Abstract] OR mothers) AND (haemorrhag*[Title/Abstract] OR hemorrhag*[Title/Abstract]) NOT "case report"[All fields]) OR (("induced abortion" OR "Therapeutic abortion" OR "legal Abortion" OR "medical abortion" OR "miscarriage" OR "Abortion,

Induced"[Mesh] OR "Abortion, Therapeutic"[Mesh] OR "Abortion, Legal"[Mesh] OR "ectopic Pregnancy") NOT ("case report"[Title/Abstract] OR "birth defect"[Title/Abstract] OR congenital[Title/Abstract])) OR ("obstructed labour" OR "obstructed labor" OR "labour dystocia" OR "labor dystocia" OR dystocia OR "cephalopelvic disproportion" OR "cephalo-pelvic disproportion") OR (("obstetric fistula" OR "vesicovaginal fistula") OR "rectovaginal fistula") OR (("Puerperal Infection"[Mesh] OR "Puerperal Infection" OR ((maternal[Title/Abstract] OR pregnan*[Title/Abstract]) AND (Sepsis OR infection[Title/Abstract]))) NOT "case report") OR ((pre-eclampsia[Title/Abstract] OR preeclampsia[Title/Abstract] OR eclampsia[Title/Abstract] OR Pre-Eclampsia[Mesh] OR Eclampsia[Mesh] OR "Hypertension, Pregnancy-Induced"[Mesh] OR "pregnancy induced hypertension"[Title/Abstract] OR "gestational hypertension"[Title/Abstract] OR "Hypertensive disorders of pregnancy"[Title/Abstract]) NOT ("case report" OR "kidney donor"[Title/Abstract] OR "kidney donors"[Title/Abstract] OR polymorphism*[Title/Abstract] OR endotheli*[Title/Abstract]))) OR((("maternal mortality"[Title/Abstract] OR "maternal death"[Title/Abstract] OR "maternal deaths"[Title/Abstract] OR "MM"[Title/Abstract] OR "confidential enquiry"[Title/Abstract] OR "confidential inquiry"[Title/Abstract] OR ((obstetric[Title/Abstract] OR pregnan*[Title/Abstract]) AND (etiology[Title/Abstract] OR cause[Title/Abstract] OR pattern[Title/Abstract]) AND (death[Title/Abstract] OR mortality[Title/Abstract])))) NOT (fetal[Title/Abstract] OR newborn*[Title/Abstract] OR neonatal[Title/Abstract] OR "case report" [Title/Abstract] OR "case study" [Title/Abstract] OR pathogenesis[Title/Abstract] OR thromboprophylaxis[Title/Abstract])) OR ((("maternal mortality"[Title/Abstract] OR "maternal death"[Title/Abstract] OR "maternal deaths"[Title/Abstract] OR "MMR"[Title/Abstract]) AND ("Afghanistan"[Title/Abstract] OR "Albania"[Title/Abstract] OR "Algeria"[Title/Abstract] OR "Andorra"[Title/Abstract] OR "Angola"[Title/Abstract] OR "Antigua and Barbuda"[Title/Abstract] OR "Argentina"[Title/Abstract] OR "Armenia"[Title/Abstract] OR "Azerbaijan"[Title/Abstract] OR "Bahrain"[Title/Abstract] OR "Bangladesh"[Title/Abstract] OR "Barbados"[Title/Abstract] OR "Belarus"[Title/Abstract] OR "Belize"[Title/Abstract] OR "Benin"[Title/Abstract] OR "Bhutan"[Title/Abstract] OR "Bolivia"[Title/Abstract] OR "Bosnia and Herzegovina"[Title/Abstract] OR "Botswana"[Title/Abstract] OR "Brazil"[Title/Abstract] OR "Brunei"[Title/Abstract] OR "Bulgaria"[Title/Abstract] OR "Burkina Faso"[Title/Abstract] OR "Burundi"[Title/Abstract] OR "Cambodia"[Title/Abstract] OR "Cameroon"[Title/Abstract] OR "Cape Verde"[Title/Abstract] OR "Central African Republic"[Title/Abstract] OR "Chad"[Title/Abstract] OR "China"[Title/Abstract] OR "Colombia"[Title/Abstract] OR "Comoros"[Title/Abstract] OR "Congo"[Title/Abstract] OR "Costa Rica"[Title/Abstract] OR "Croatia"[Title/Abstract] OR "Cuba"[Title/Abstract] OR "Cyprus"[Title/Abstract] OR "Côte d'Ivoire"[Title/Abstract] OR "Democratic Republic of the Congo"[Title/Abstract] OR "Djibouti"[Title/Abstract] OR "Dominica"[Title/Abstract] OR "Dominican Republic"[Title/Abstract] OR "Ecuador"[Title/Abstract] OR "Egypt"[Title/Abstract] OR "El Salvador"[Title/Abstract] OR "Equatorial Guinea"[Title/Abstract] OR "Eritrea"[Title/Abstract] OR "Ethiopia"[Title/Abstract] OR "Federated States of Micronesia"[Title/Abstract] OR "Fiji"[Title/Abstract] OR "Gabon"[Title/Abstract] OR "Georgia"[Title/Abstract] OR "Ghana"[Title/Abstract] OR "Grenada"[Title/Abstract] OR "Guatemala"[Title/Abstract] OR "Guinea"[Title/Abstract] OR "Guinea-Bissau"[Title/Abstract] OR "Guyana"[Title/Abstract] OR "Haiti"[Title/Abstract] OR "Honduras"[Title/Abstract] OR "India"[Title/Abstract] OR "Indonesia"[Title/Abstract] OR "Iran"[Title/Abstract] OR "Iraq"[Title/Abstract] OR "Jamaica"[Title/Abstract] OR "Jordan"[Title/Abstract] OR "Kazakhstan"[Title/Abstract] OR "Kenya"[Title/Abstract] OR "Kiribati"[Title/Abstract] OR "Kuwait"[Title/Abstract] OR "Kyrgyzstan"[Title/Abstract] OR "Laos"[Title/Abstract] OR "Latvia"[Title/Abstract] OR "Lebanon"[Title/Abstract] OR "Lesotho"[Title/Abstract] OR "Liberia"[Title/Abstract] OR "Libya"[Title/Abstract] OR "Lithuania"[Title/Abstract] OR "Macedonia"[Title/Abstract] OR "Madagascar"[Title/Abstract] OR "Malawi"[Title/Abstract] OR "Malaysia"[Title/Abstract] OR "Maldives"[Title/Abstract] OR "Mali"[Title/Abstract] OR "Malta"[Title/Abstract] OR "Marshall Islands"[Title/Abstract] OR "Mauritania"[Title/Abstract] OR "Mauritius"[Title/Abstract] OR "Moldova"[Title/Abstract] OR "Mongolia"[Title/Abstract] OR "Montenegro"[Title/Abstract] OR "Morocco"[Title/Abstract] OR "Mozambique"[Title/Abstract] OR "Myanmar"[Title/Abstract] OR "Namibia"[Title/Abstract] OR "Nepal"[Title/Abstract] OR "Nicaragua"[Title/Abstract] OR "Niger"[Title/Abstract] OR "Nigeria"[Title/Abstract] OR "North Korea"[Title/Abstract] OR "Oman"[Title/Abstract] OR "Pakistan"[Title/Abstract] OR "Palestine"[Title/Abstract] OR "Panama"[Title/Abstract] OR "Papua New Guinea"[Title/Abstract] OR "Paraguay"[Title/Abstract] OR "Peru"[Title/Abstract] OR "Philippines"[Title/Abstract] OR "Qatar"[Title/Abstract] OR "Romania"[Title/Abstract] OR "Russia"[Title/Abstract] OR "Rwanda"[Title/Abstract] OR "Saint Lucia"[Title/Abstract] OR "Saint Vincent and the Grenadines"[Title/Abstract] OR "Samoa"[Title/Abstract] OR "Saudi Arabia"[Title/Abstract] OR "Senegal"[Title/Abstract] OR "Serbia"[Title/Abstract] OR "Seychelles"[Title/Abstract] OR "Sierra Leone"[Title/Abstract] OR "Singapore"[Title/Abstract] OR "Solomon Islands"[Title/Abstract] OR "Somalia"[Title/Abstract] OR "South Africa"[Title/Abstract] OR "South Sudan"[Title/Abstract] OR "Sri Lanka"[Title/Abstract] OR "Sudan"[Title/Abstract] OR "Suriname"[Title/Abstract] OR "Swaziland"[Title/Abstract] OR "Syria"[Title/Abstract] OR "São Tomé and Principe"[Title/Abstract] OR "Taiwan"[Title/Abstract] OR "Tajikistan"[Title/Abstract] OR "Tanzania"[Title/Abstract] OR "Thailand"[Title/Abstract] OR "The Bahamas"[Title/Abstract] OR "The Gambia"[Title/Abstract] OR "Timor-Leste"[Title/Abstract] OR "Togo"[Title/Abstract] OR "Tonga"[Title/Abstract] OR "Trinidad and Tobago"[Title/Abstract] OR "Tunisia"[Title/Abstract] OR "Turkmenistan"[Title/Abstract] OR "Uganda"[Title/Abstract] OR "Ukraine"[Title/Abstract] OR "United Arab

Emirates"[Title/Abstract] OR "Uruguay"[Title/Abstract] OR "Uzbekistan"[Title/Abstract] OR "Vanuatu"[Title/Abstract] OR "Venezuela"[Title/Abstract] OR "Vietnam"[Title/Abstract] OR "Yemen"[Title/Abstract] OR "Zambia"[Title/Abstract] OR "Zimbabwe"[Title/Abstract])) NOT ("demographic and health survey"[Title/Abstract] OR "demographic and health surveys "[Title/Abstract] OR DHS[Title/Abstract] OR "reproductive health survey"[Title/Abstract] OR "reproductive health surveys"[Title/Abstract] OR RHS[Title/Abstract])) OR ((HIV[Title/Abstract] OR "Acquired Immunodeficiency Syndrome"[Title/Abstract] OR AIDS[Title/Abstract]) AND (pregnan*[Title/Abstract] OR "postpartum"[Title/Abstract] OR "post partum"[Title/Abstract]) AND ("mortality"[Title/Abstract] OR "death"[Title/Abstract]) NOT "case report")) AND (2017/07/01[PDat] : 3000[PDat]) NOT (animals[MeSH] NOT humans[MeSH]))

PRISMA 2009 Flow Diagram

From: Moher D, Liberati A, Tetzlaff J, Altman DG, The PRISMA Group (2009). *P*referred *R*eporting *I*tems for *S*ystematic Reviews and *M*eta-*A*nalyses: The PRISMA Statement. PLoS Med 6(7): e1000097. doi:10.1371/journal.pmed1000097

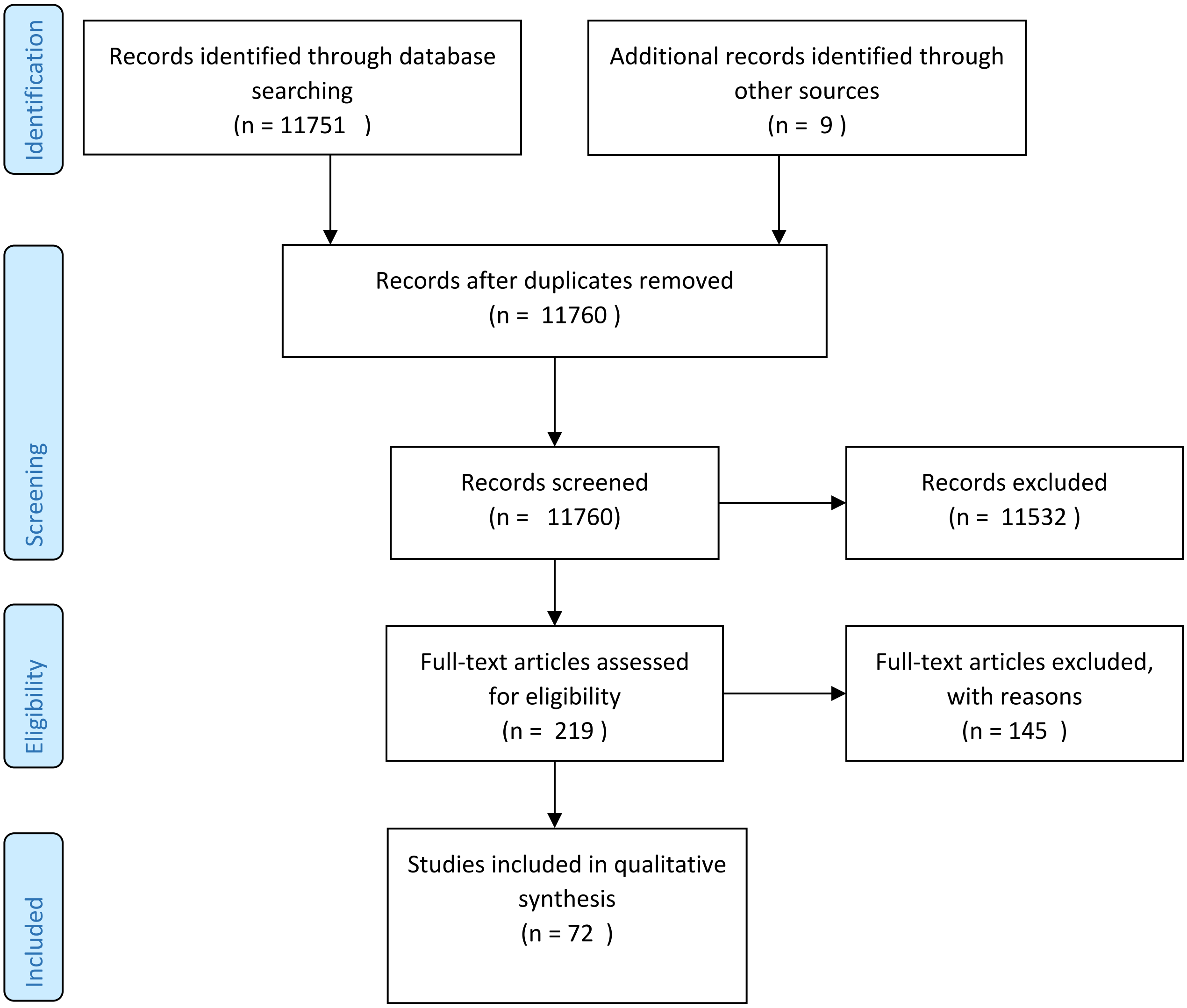

This search produced 12964 hits for title and abstract review. Of these 272 were selected for full-text review and 81 were extracted for inclusion in the models.

In addition, we searched ministry of health websites for pregnancy complication data and used Confidential Enquiry and other sources used in our maternal mortality analyses when they presented data on pregnancy complications. We also performed snowball searches for abortion reporting and surveillance data systems, finding multiple such systems throughout high-income countries and several geographies in Central and Eastern Europe. We found 9 new surveillance sources this year. The table below summarizes the number of sources used in each model by cause:

Table 1. Data sources used in estimation of nonfatal pregnancy complications

Cause/Impairment Name	Measure	Total sources	Countries with data
Maternal hemorrhage	All measures	463	84
Maternal hemorrhage	Incidence	463	84
Maternal sepsis and other maternal infections	All measures	388	75
Maternal sepsis and other maternal infections	Incidence	388	75
Maternal hypertensive disorders	All measures	523	104
Maternal hypertensive disorders	Incidence	523	104
Maternal obstructed labor and uterine rupture	All measures	295	64
Maternal obstructed labor and uterine rupture	Prevalence	33	26
Maternal obstructed labor and uterine rupture	Incidence	249	46
Maternal obstructed labor and uterine rupture	Other	14	6
Ectopic pregnancy	All measures	313	55
Ectopic pregnancy	Incidence	313	55
Maternal abortion and miscarriage	All measures	593	59
Maternal abortion and miscarriage	Incidence	593	59

Inpatient and outpatient data were used, as were claims data from Taiwan and Singapore as well as MarketScan in the United States. These data were extracted and processed as described in the appendix section on clinical informatics data, including use of primary-to-any inpatient ratio to correct for under-reporting of pregnancy complications in hospital datasets that rely only on primary discharge codes, and inpatient-to-outpatient ratio. Processing of clinical administrative data (i.e. hospital and claims) were based on ICD-9 and ICD-10 codes as listed in the table below. The extraction and processing of hospital and claims data is described separately. We only used inpatient data, corrected for location-year-specific HAQI value for all models, with four exceptions – Hypertensive disorders of pregnancy (total), abortion and miscarriage, ectopic pregnancy, and other maternal infections.

All data were either extracted as incidence ratio (number of events / live birth) or, if data were only available with population as the denominator, they were converted to incidence ratio using GBD 2019 age-specific fertility rate (number of live births / population). The reason is that most literature and surveillance data are expressed in terms of number of events per livebirth rather than per population. Hospital and claims data, which were centrally processed for all GBD 2019 causes to have population as the denominator, were transformed to have livebirths as the denominator by dividing by age-specific fertility rate (ASFR; live births per population). All data were extracted in standard fashion, and were uploaded and stored on a centralised SQL database.

Data processing

Previously we derived empirical age patterns and performed all crosswalks in DisMod-MR 2.1. Our data processing approach changed for GBD 2019 such that all of this occurred prior to DisMod-MR 2.1 modeling. The first step of data processing was age-sex splitting. For any datum that did not entirely fit within a GBD age group or was for both sexes combined, the observation was split to be multiple age-specific and sex-specific data points based on the age and sex pattern predicted by GBD 2017 DisMod-MR 2.1 models. It is our intention to update this age-sex splitting with each cycle of GBD.

The second step was crosswalking all data from alternate to reference definitions. For all other models, we adjusted data to the reference category for each cause by age using Meta-Regression - Bayesian, Regularized, Trimmed (MR-BRT), a meta-analytic tool developed for GBD 2019. In accordance with GBD 2019 principles for data processing, to make data comparable, we began by evaluating the number of observations of each alternate definition that matched with a corresponding observation from the reference definition. We excluded some alternative definitions from this process, e.g. studies reporting chronic hypertension and studies reporting severe diagnoses of maternal disorders except for sepsis and eclampsia. The standard error of the ratio was calculated using the delta method. The details of each of the crosswalks are described below. All data sources that only reported event rates for severe maternal morbidity or “near miss” were excluded as a reliable crosswalk model could not be developed.

Abortion and miscarriage

Surveillance data are the reference category for abortion and miscarriage. Claims and inpatient in the US data had similar levels so we created a binary covariate to distinguish US clinical data from the rest of clinical data and crosswalked all of them to the surveillance data by age. The crosswalk changes direction after age 45.

Figure 1. Clinical to surveillance for abortion and miscarriage

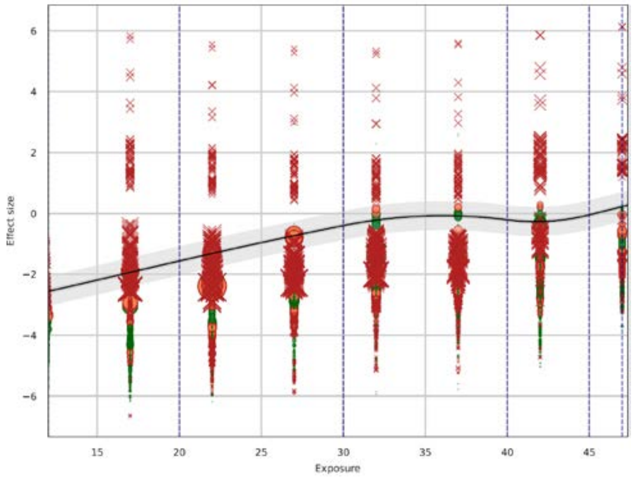

Ectopic pregnancy

We used outpatient data for ectopic pregnancy. Claims data were the reference category. We crosswalked outpatient hospital data to claims by age. The age-pattern is not significant until 35. For the older ages, the ratio of hospital to claims decreases with age.

Figure 1. Outpatient data to claims data for ectopic pregnancy

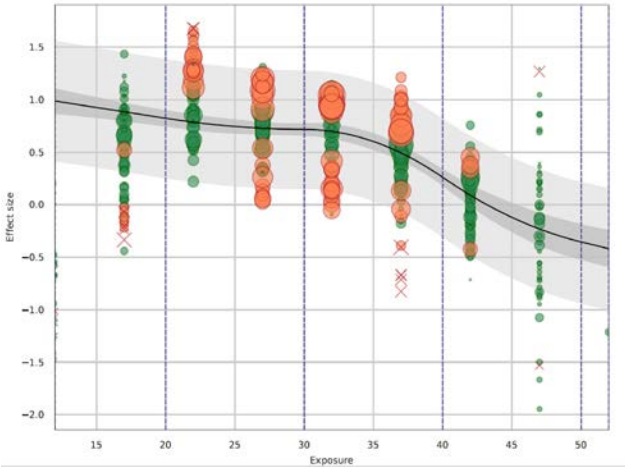

Obstructed labour and uterine rupture

For obstructed labour, we adjusted the clinical data of obstructed labor by using a ratio of the number of cases of the ICD-9 code 664 ICD-10 codes of O70.x. These codes capture cases of perineal laceration which are not included in our case definition of obstructed labour.

Maternal haemorrhage

For maternal haemorrhage, the reference is all cases of maternal haemorrhage including post-partum bleeding $\geq$ 500ml in vaginal births and $\geq$ 1000ml in caesarean sections and any amount of bleeding prior to birth. All data sources that reported only on antepartum haemorrhage (APH) or postpartum haemorrhage (PPH) were crosswalked to total haemorrhage by age. The age-specific crosswalk was retained for consistency across all maternal pregnancy complications even though it was not significant in this case. We included only within-study matches for this crosswalk.

Figure 3. PPH to all haemorrhage

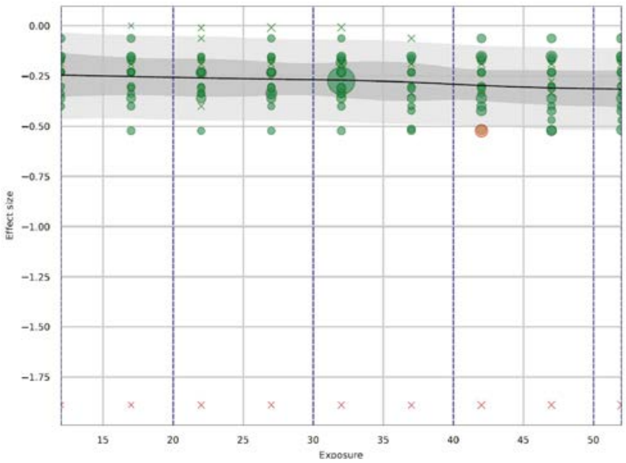

Figure 4. APH to all haemorrhage

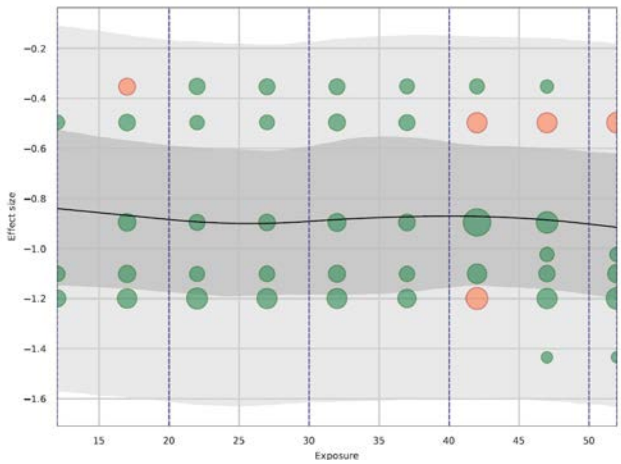

Puerperal sepsis

Puerperal sepsis cases reported in literature studies were the reference category. We crosswalked claims data to inpatient data by age. After this adjustment we crosswalked all of the clinical data to the

literature data by age. The age pattern for the claims to inpatient crosswalk was significant with an increase with age until age 40. The age pattern of clinical to literature was slightly decreasing with age.

Figure 5. Claims to inpatient hospital

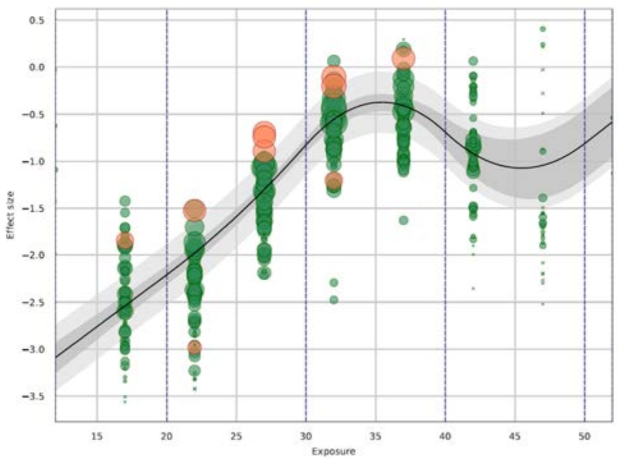

Figure 6. Clinical to lit. for puerperal sepsis

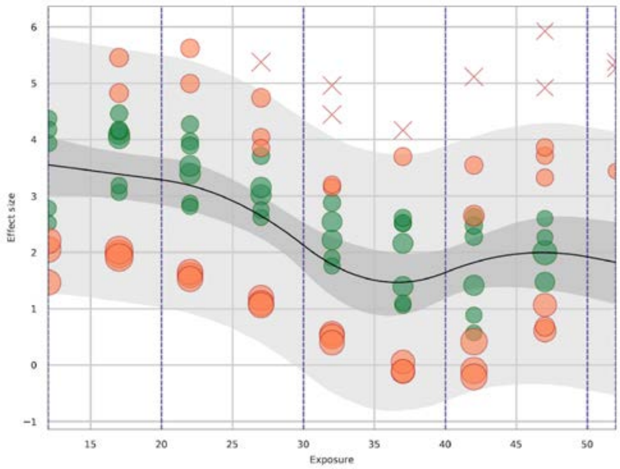

Other maternal infections

Inpatient hospital data were the reference for other maternal infections. We crosswalked claims data to inpatient hospital data by age. The age pattern shows a steep increase in the ratio from ages 10 to 35.

Figure 7. Claims to inpatient hospital data for other maternal infections

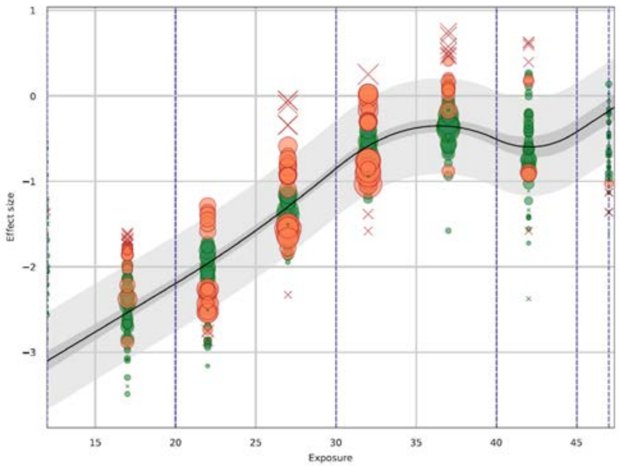

Hypertensive disorders of pregnancy

For the overall hypertensive disorders of pregnancy (HDoP), any sources that reported only on pre-eclampsia (PE) or pregnancy induced hypertension (PIH) were crosswalked to total HDoP. This crosswalk was again completed using only within study matches and in an age-specific manner, although the age pattern was not significant.

Figure 8. PE to all HDoP

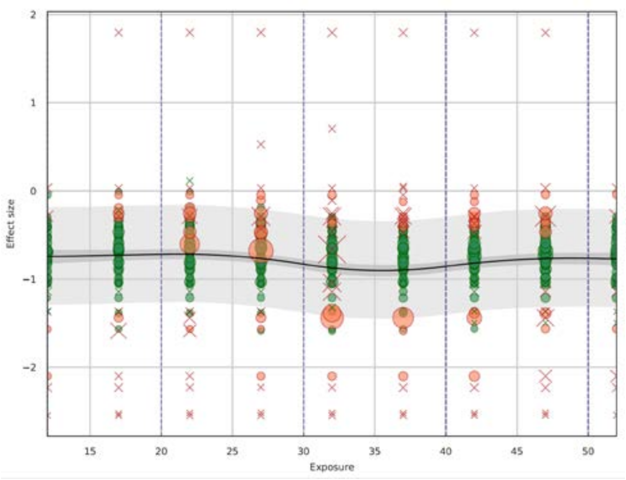

Figure 9. PIH to all HDoP

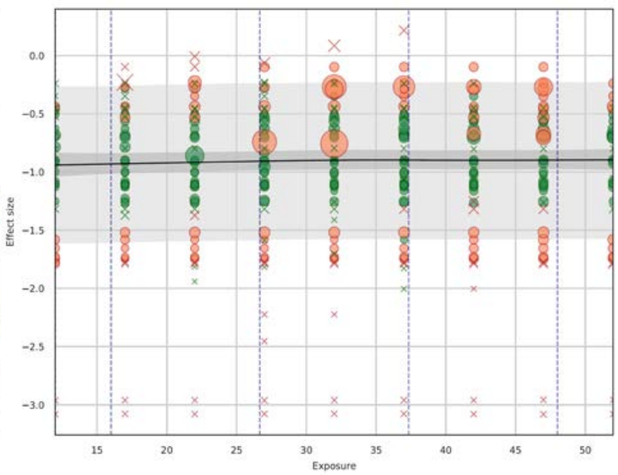

Severe pre-eclampsia

We crosswaked claims data to inpatient hospital data for severe pre-eclampsia. The crosswalk had a significant age pattern with a slight increase in the ratio of claims to inpatient data with age (mostly from 10 to 35).

Figure 10. Claims to inpatient data for severe pre-eclampsia

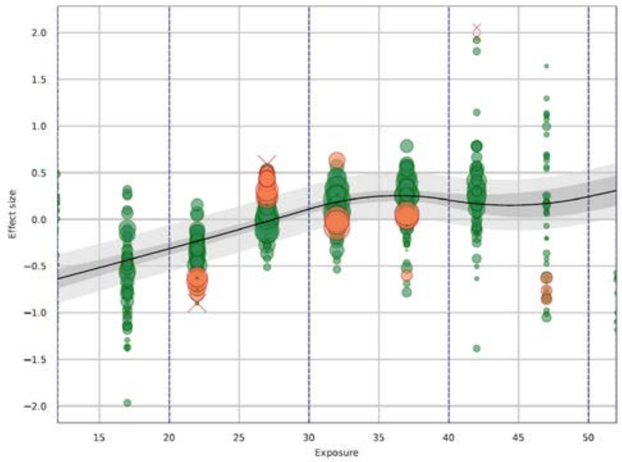

Eclampsia

For eclampsia we considered the cases reported in literature as the reference. We adjusted claims data to inpatient hospital data and then adjusted all of the clinical data to the literature data. These crosswalks were age-specific. Both crosswalks had significant and opposite age patterns and directions. The claims to inpatient ratio decreases with age whereas the clinical to literature crosswalk increases with age.

Figure 11. Claims data to inpatient hospital data

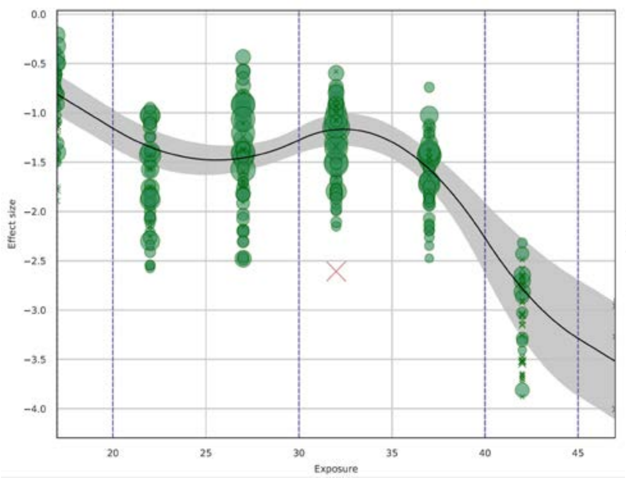

Figure 12. Clinical to lit. data for eclampsia

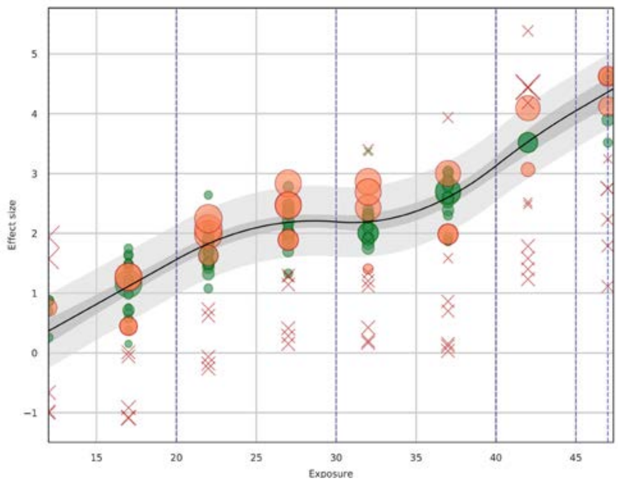

Modelling strategy

We estimated the incidence ratio of each category of pregnancy complications using DisMod-MR 2.1, with the exception of other maternal disorders, which we estimated using a YLD-to-YLL ratio approach used in multiple causes across GBD 2019.

We used the datasets described above to estimate incidence ratio for each age-sex-location-year in the GBD 2019 location hierarchy using DisMod-MR 2.1. A series of country covariates were chosen to help drive the magnitude of estimates in areas of sparse or absent data. We included the respective log transformed maternal mortality ratio (MMR) for each maternal disorder that was estimated in GBD 2017 as a country level covariate for almost every model. Puerperal sepsis and ectopic pregnancy used the log transformed age standardized death rate (LN-ASDR) as a covariate, instead of MMR. No specific age or slope priors were used. All models were run with a time window of five years. The quantitative results of country-level covariates for each condition are shown below.

Abortion and miscarriage

Covariate Name	Type	Measure	Beta value	Exponentiated value
Legality of Abortion	Country covariate	Incidence	0.017 (0.016 - 0.018)	1.02 (1.02 - 1.02)
Contraception (Modern) Prevalence (proportion)	Country covariate	Incidence	-0.0012 (-0.0029 - -0.000071)	1.00 (1.00 - 1.00)

Ectopic pregnancy

Covariate Name	Type	Measure	Beta value	Exponentiated value
Ectopic pregnancy	lnASDR	Incidence	0.00656 (0.00055 - 0.01431)	1.01 (1.00 - 1.01)
Legality of Abortion	Country covariate	Incidence	-0.00058 (-0.00158 - -0.00003)	1.00 (1.00 - 1.00)
Pelvic inflammatory disease age-standardized prevalence	Country covariate	Incidence	0.51087 (0.08438 - 0.93981)	1.67 (1.09 - 2.56)

Maternal haemorrhage

Covariate Name	Type	Measure	Beta value	Exponentiated value
Skilled Birth Attendance (proportion)	Country covariate	Incidence	-0.01151 (-0.03090 - -0.00060)	0.99 (0.97 - 1.00)
Socio-demographic Index	Country covariate	Incidence	-0.10502 (-0.11390 - -0.10020)	0.90 (0.89 - 0.90)
MMR due toe maternal hemorrhage	Country covariate	Incidence	0.98611 (0.00967 - 1.93866)	2.68 (1.01 - 6.95)

Hypertensive disorders of pregnancy

Covariate Name	Type	Measure	Beta value	Exponentiated value
Antenatal Care (4 visits) Coverage (proportion)	Country covariate	Incidence	-0.00004 (-0.00009 - 0.00000)	1.00 (1.00 - 1.00)
MMR due to maternal hypertensive disorders	Country covariate	Incidence	1.01152 (0.02075 - 1.99978)	2.75 (1.02 - 7.39)
Age-standardized SEV for High blood pressure	Country covariate	Incidence	0.00014 (0.00013 - 0.00015)	1.00 (1.00 - 1.00)
Age-standardized SEV for High body-mass index	Country covariate	Incidence	1.99901 (1.99600 - 2.00000)	7.38 (7.36 - 7.39)

Eclampsia

Covariate Name	Type	Measure	Beta value	Exponentiated value
Antenatal Care (4 visits) Coverage (proportion)	Country covariate	Incidence	-0.00004 (-0.00009 - 0.00000)	1.00 (1.00 - 1.00)
MMR due to maternal hypertensive disorders	Country covariate	Incidence	1.01152 (0.02075 - 1.99978)	2.75 (1.02 - 7.39)
Age-standardized SEV for High blood pressure	Country covariate	Incidence	0.00014 (0.00013 - 0.00015)	1.00 (1.00 - 1.00)
Age-standardized SEV for High body-mass index	Country covariate	Incidence	1.99901 (1.99600 - 2.00000)	7.38 (7.36 - 7.39)

Severe pre-eclampsia

Covariate Name	Type	Measure	Beta value	Exponentiated value
Antenatal Care (4 visits) Coverage (proportion)	Country covariate	Incidence	-0.00736 (-0.02277 - -0.0003)	0.99 (0.98 - 1.00)
MMR due to maternal hypertensive disorders	Country covariate	Incidence	0.99911 (0.03094 - 1.9681)	2.72 (1.03 - 7.16)
Age-standardized SEV for High body-mass index	Country covariate	Incidence	1.98172 (1.94600 - 1.9990)	7.26 (7.00 - 7.38)

Obstructed labour and uterine rupture

Covariate Name	Type	Measure	Beta value	Exponentiated value
Skilled Birth Attendance (proportion)	Country covariate	Incidence	-0.00373 (-0.01011 - -0.00016)	1.00 (0.99 - 1.00)
Age-standardized SEV for Child stunting	Country covariate	Incidence	0.06696 (0.00219 - 0.18971)	1.07 (1.00 - 1.21)
MMR due to obstructed labor	Country covariate	Incidence	0.99704 (0.01045 - 1.95593)	2.71 (1.01 - 7.07)

Maternal sepsis

Covariate Name	Type	Measure	Beta value	Exponentiated value
Maternal sepsis and other maternal infections	lnASDR	Incidence	0.05640 (0.02232 - 0.08984)	1.06 (1.02 - 1.09)
Diabetes Age-Standardized Prevalence (proportion)	Country covariate	Incidence	1.77585 (1.27300 - 1.99300)	5.91 (3.57 - 7.34)

Other maternal infections:

Covariate Name	Type	Measure	Beta value	Exponentiated value
Socio-demographic Index	Country covariate	Incidence	-0.01038 (-0.03228 - -0.00031)	0.99 (0.97 - 1.00)
Log-transformed age-standardized SEV scalar: HIV	Country covariate	Incidence	0.09656 (0.05602 - 0.13931)	1.10 (1.06 - 1.15)
MMR due to sepsis and other maternal infections	Country covariate	Incidence	0.99307 (0.00000 - 1.97634)	2.70 (1.00 - 7.22)

Severity splits and post-model processing to estimate incidence and prevalence rates

After completion of DisMod-MR 2.1 models, all age-specific ratios were then converted to incidence rates by multiplying by ASFR and then to prevalence rates by applying a global assumed duration of disability for each type of pregnancy complications.

Maternal haemorrhage was split between moderate (500 to <1000 ml blood loss) and severe ($\geq$1000 ml blood loss) on the basis of a meta-analysis of 19 studies[1]. Data on the average duration of acute symptoms were not available so, after consultation with clinician collaborators, we assigned a duration of seven days (+/-3) for moderate haemorrhage and 14 days (+/- 4) for severe haemorrhage. The age- and sex-specific anemia prevalence for maternal haemorrhage was also analysed as part of overall anemia causal attribution for GBD 2019. The details of the anemia analysis are described separately in the “Anemia Impairment” section. Briefly, after estimating total anemia, a series of counterfactual distributions are generated based on the age- and sex-specific prevalence of each anaemia-causing condition and the quantitative effect that the condition has on haemglobin concentration in the blood, a so-called “haemoglobin shift,” that was derived by meta-analyzing cohort studies, observational studies, or trials comparing the haemotologic status of those with as compared to without the disease. Due to limited data on haemologbin shift, all were assumed to be invariant over age, sex, location, and year.

For abortion and miscarriage, prevalence was calculated assuming incident cases have acute disability that persist for an average of three days (+/-1). The same was calculated for ectopic pregnancy. Obstructed labour was assigned a duration of five days (+/-2). Again, these determinations were based on clinical expert determination as we could not identify any data to inform this.

Hypertensive disorders of pregnancy (HDoP) was estimated in three models. The duration of severe pre-eclampsia was assigned to be 7 days (+/-2) and other HDoP was assigned a duration of three months (2-4). Eclampsia was a separate model, assigned a duration of one day (+/-1). The disability weight for eclampsia and severe pre-eclampsia is estimated as a combination of the disability weights hypertensive disorders of pregnancy and the respective specific condition. A large number of those with severe pre-eclampsia go on to have long-term sequelae of the condition[2], as do those with eclampsia[3,4]. We estimate these long-term sequelae by using the prevalence results of severe pre-eclampsia and eclampsia as input data for 2 full-compartment DisMod-MR 2.1 models. Sixty-two percent (57% - 67%) of the severe pre-eclampsia cases are estimated to be long-term sequela. For eclampsia we estimate that 6.5% (6.1% - 6.9%) of the cases continue on to long-term sequela in data-rich locations, whereas 11% (10.8% to 12%) in not data-rich.

Maternal sepsis and other maternal infections were also estimated separately. Maternal sepsis was assigned a duration of five days (+/-2) and, based on the same data identified in our review of pelvic inflammatory disease (PID; described separately), 9% (7.7% - 10%) of incident cases of puerperal sepsis were estimated to continue on to have secondary infertility due to maternal sepsis. We apply this proportion to the incidence results of puerperal sepsis and use them as input data for a full-compartment DisMod-MR 2.1 model. Other maternal infections were assigned a wide potential duration of 15 to 45 days (mean 30).

The sequelae, health states, lay descriptions and disability weights for each maternal disorder are listed in table 3. We assigned abdominopelvic pain of varying severity to approximate the disability from maternal hemorrhage, obstructed labour, ectopic pregnancy, and abortion and miscarriage. We used

two health states to estimate the disability weight due to eclampsia (moderate abdominal pain and severe epilepsy). Tension-type headaches and mild motor plus cognitive impairment were used for severe pre-eclampsia. When two or more health states were combined for one sequela we calculated the disability weight as described in YLD calculation section of this paper.

Table 2: Health states and disability weights for each of the nonfatal maternal disorders

Sequela	Healthstate name	Health state description	Disability weight
Maternal hemorrhage (< 1L blood lost)	Abdominopelvic problem, moderate	Has pain in the belly and feels nauseous. The person has difficulties with daily activities.	0.114 (0.078-0.159)
Maternal hemorrhage (> 1L blood lost)	Abdominopelvic problem, severe	Has severe pain in the belly and feels nauseous. The person is anxious and unable to carry out daily activities.	0.324 (0.22-0.442)
Mild anemia due to maternal hemorrhage	Anemia, mild	Feels slightly tired and weak at times, but this does not interfere with normal daily activities.	0.004 (0.001-0.008)
Moderate anemia due to maternal hemorrhage	Anemia, moderate	Feels moderate fatigue, weakness, and shortness of breath after exercise, making daily activities more difficult.	0.052 (0.034-0.076)
Severe anemia due to maternal hemorrhage	Anemia, severe	Feels very weak, tired and short of breath, and has problems with activities that require physical effort or deep concentration.	0.149 (0.101-0.209)
Severe pre-eclampsia	Moderate abdominal pain, tension-type headaches, mild motor plus cognitive impairment	Has pain in the belly and feels nauseous. The person has difficulties with daily activities. Has a moderate headache that also affects the neck, which causes difficulty in daily activities. Has some difficulty in moving around but is able to walk without help. The person is slow in learning at school. As an adult, the person has some difficulty doing complex or unfamiliar tasks but otherwise functions independently.	0.174 (0.120 – 0.239)
Eclampsia	Moderate abdominal pain and severe epilepsy	Has pain in the belly and feels nauseous. The person has difficulties with daily activities. Has sudden seizures with violent muscle contractions and stiffness, loss of consciousness, and loss of urine or bowel control. Between seizures the person has memory loss and difficulty concentrating.	0.602 (0.427 – 0.753)
Long term sequelae of severe pre-eclampsia	Tension-type headaches, mild motor plus cognitive impairment	Has a moderate headache that also affects the neck, which causes difficulty in daily activities. Has some difficulty in moving around but is able to walk without help. The person is slow in learning at school. As an adult, the person has some difficulty doing complex or unfamiliar tasks but otherwise functions independently.	0.067 (0.041 – 0.103)
Long term sequelae of eclampsia	Tension-type headaches, mild motor plus cognitive impairment	Has a moderate headache that also affects the neck, which causes difficulty in daily activities. Has some difficulty in moving around but is able to walk without help. The person is slow in learning at school. As an adult, the person has some difficulty doing complex or unfamiliar tasks but otherwise functions independently.	0.067 (0.041 – 0.103)
Other hypertensive disorders of pregnancy	Generic uncomplicated disease: worry and daily medication	Has a chronic disease that requires medication every day and causes some worry but minimal interference with daily activities.	0.049 (0.031-0.072)
Puerperal sepsis	Infectious disease, acute episode, severe	Has a high fever and pain, and feels very weak, which causes great difficulty with daily activities.	0.133 (0.088-0.19)

Infertility due to puerperal sepsis	Infertility, secondary	Has at least one child, and wants to have more children. The person has a fertile partner, but the couple cannot conceive.	0.005 (0.002-0.011)
Other maternal infections	Infectious disease, acute episode, moderate	Has a fever and aches, and feels weak, which causes some difficulty with daily activities.	0.051 (0.032-0.074)
Obstructed labor, acute event	Abdominopelvic problem, severe	Has severe pain in the belly and feels nauseous. The person is anxious and unable to carry out daily activities.	0.324 (0.22-0.442)
Rectovaginal fistula	Rectovaginal fistula	Has an abnormal opening between her vagina and rectum causing flatulence and feces to escape through the vagina. The person gets infections in her vagina, and has pain when urinating.	0.501 (0.339-0.657)
Vesicovaginal fistula	Vesicovaginal fistula	Has an abnormal opening between the bladder and the vagina, which makes her unable to control urinating. The woman is anxious and depressed.	0.342 (0.227-0.478)
Maternal abortive outcome	Abdominopelvic problem, moderate	Has pain in the belly and feels nauseous. The person has difficulties with daily activities.	0.114 (0.078-0.159)
Ectopic Pregnancy	Abdominopelvic problem, moderate	Has pain in the belly and feels nauseous. The person has difficulties with daily activities.	0.114 (0.159-0.078)

Uncertainty and model selection

For all maternal disorders, uncertainty bounds include uncertainty due to input data, crosswalks from non-reference definitions, uncertainty in numerical solutions (posteriors) of each DisMod-MR 2.1 model, duration of symptoms, and proportion of all persons with each type of symptom.

In consultation with GBD researchers and collaborators, final models were selected on a combination of qualitative and quantitative goodness of fit to input data, plausibility of geographic and temporal trends, consistency of age pattern, and, when available, comparison with other published studies on the epidemiology of pregnancy complications. Directionality, magnitude, and plausibility of study-level and country-level covariates were also considered in the process of model development. Of note, due to the nature of statistical modelling, final results do not always cover the values reported in input data.

References

1 Sloan N, Durocher J, Aldrich T, Blum J, Winikoff B. What measured blood loss tells us about postpartum bleeding: a systematic review. *BJOG* 2010; 117: 788–800.

2 Roes EM, Raijmakers MT, Schoonenberg M, Wanner N, Peters WH, Steegers EA. Physical well-being in women with a history of severe preeclampsia. *J Matern Fetal Neonatal Med* 2005; 18: 39–45.

3 Okanloma KA, Moodley J. Neurological complications associated with the pre-eclampsia/eclampsia syndrome. *Int J Gynaecol Obstet* 2000; 71: 223–5.

4 Usta IM, Sibai BM. Emergent management of puerperal eclampsia. *Obstet Gynecol Clin North Am* 1995; 22: 315–35.

Neonatal disorders

Morbidity due to neonatal disorders is modelled as five individual causes: neonatal preterm birth complications, neonatal encephalopathy due to birth asphyxia and trauma, neonatal sepsis and other neonatal infections, hemolytic disease and other neonatal jaundice, and other neonatal disorders. Each cause is modeled separately due to differences in data availability and pathology, though many input data types and modeling approaches are shared across the causes. The process for each cause is documented below.

Neonatal preterm birth complications

Flowchart

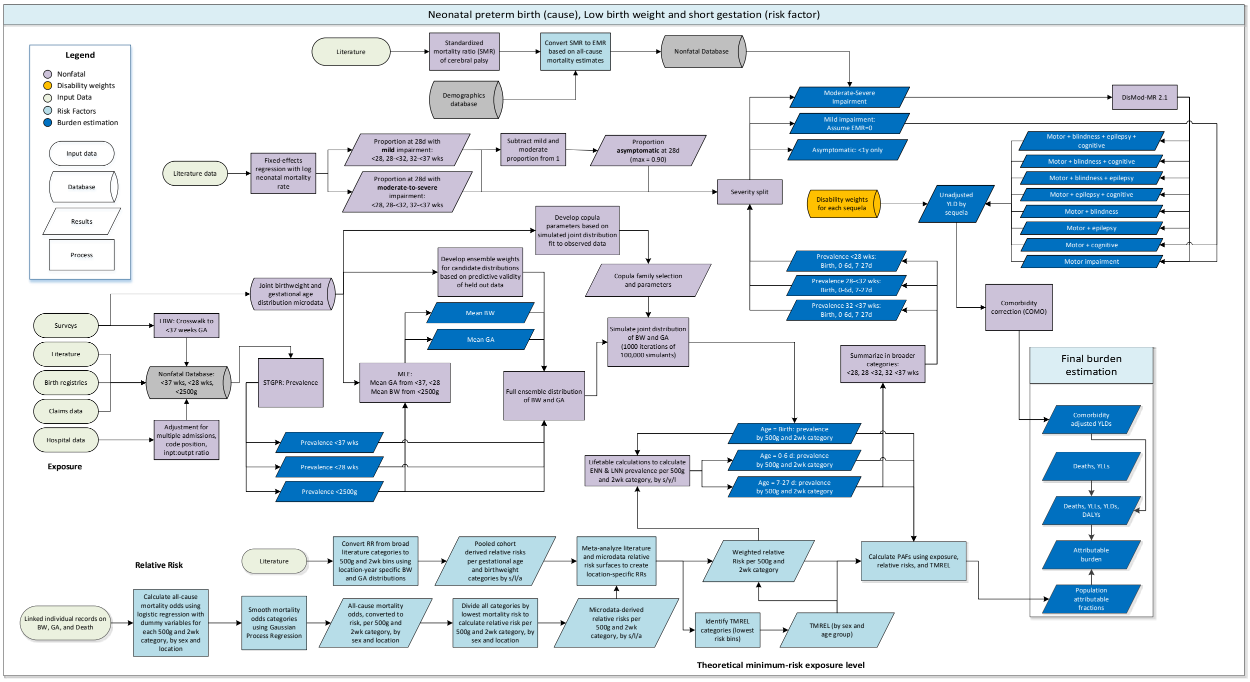

Case definition

Preterm birth is defined as live birth before 37 completed weeks of gestation. Three categories of preterm birth, based on WHO definitions of prematurity, are presented in GBD estimates: extremely preterm birth (<28 weeks), very preterm birth (28 to <32 weeks), and moderate-to-late preterm birth (32 to <37 weeks).

Modelling Strategy

We model the nonfatal burden of neonatal preterm birth in five main steps (Table 1). To estimate nonfatal health burden due to neonatal preterm birth, the distribution of gestational age at birth is modeled for every location/year/sex. Models of all-cause mortality rates by gestational age are used to estimate the gestational age distribution of surviving neonates from birth until 28 days (Step 1). The proportion of extremely preterm, very preterm, and moderate-to-late preterm neonates who experience long-term impairment are modeled in three severity categories: no impairment (asymptomatic cases), mild impairment, and moderate-to-severe impairment (Step 2). The impairment proportions are applied to estimates of all survivors of preterm birth from birth to 95+ years (the

terminal age group in modeled GBD) in order to estimate the prevalence of impairment due to preterm birth by severity category, at all ages. Disability due to asymptomatic preterm birth is estimated until the first year of life, after which no impairment is assumed. Mild and moderate-to-severe impairment is assumed to persist until death, with all excess mortality due to preterm birth attributed to moderate-to-severe impairment (Step 3). Mild and moderate-to-severe impairment are further split into estimates of sequela (Step 4) and then disability weights are applied (Step 5).

Table 1. Analytic steps in estimation of YLDs due to preterm birth

Step	Summary of Modeling Strategy
1	A. Model gestational age distributions for all locations/years/sexes at birth<br>B. Model all-cause mortality rates by gestational age<br>C. Model gestational age distribution of surviving neonates for all l/y/s from birth to 28 days, using all-cause mortality rates by gestational age
2	Model proportion of neonates born preterm who will go on to experience mild, moderate-to-severe, or no long-term impairment, by gestational age category
3	Model all survivors of preterm birth, by severity category, at all ages
4	Model sequela due to preterm birth
5	Apply disability weights to each sequela to calculate YLDs

The strategy to model gestational age distributions from birth until 28 days is the same for both the estimation of nonfatal health burden due to preterm birth, described in this appendix, and the estimation of the exposure due to the risk factors "Low birth weight and short gestation" (LBWSG). Estimates of nonfatal health burden due to preterm birth require only the modeled gestational age distributions as inputs; however, LBWSG exposure requires the joint distribution of gestational age and birth weight. Because the nonfatal burden due to preterm birth and LBWSG exposure share the same process, the joint estimation of gestational age and birth weight distributions is described in this appendix, even though only gestational age distributions are used in this analysis.

Table 2. Input Data – Neonatal preterm birth

Measure	Total sources	Countries with data
All measures	1609	160
Proportion	1609	160

Step 1: Model gestational age distributions from birth to 28 days

Input data

Estimates of prevalence of extremely preterm birth and prevalence of preterm birth are modeled using data from clinical data, vital registration, and surveys. Only inpatient and insurance claims data were included from clinical informatics datasets; outpatient data was excluded because it was more likely to capture repeated visits by the same child rather than unique visits. Clinical data processing is described separately.

The preterm birth (<37 weeks) model was informed by low birth weight (<2500 grams) data. Low birth weight data are more readily available than preterm birth data, especially in low- and middle-income countries. In DHS surveys where additional covariates were available, missingness in the birth weight

data was imputed using multiple imputation through the R Package Amelia. Low birth weight data was crosswalked to preterm data and used to inform the preterm model (see Data Processing for more information).

Literature review

Before GBD 2016, available preterm data was sourced by a technical working group. In GBD 2016 and GBD 2017, we conducted systematic reviews to identify additional sources beyond the data already used in the models. The PubMed database was searched using the following search string:

((("Infant, Premature"[Mesh] OR ("infant"[All Fields] AND "premature"[All Fields]) OR "premature infant"[All Fields] OR ("preterm"[All Fields] AND "infant"[All Fields]) OR "preterm infant"[All Fields] OR ("infant, newborn"[MeSH Terms] OR ("infant"[All Fields] AND "newborn"[All Fields]) OR "newborn infant"[All Fields] OR ("newborn"[All Fields] AND "infant"[All Fields])) AND (premature[All Fields] OR preterm[All Fields]) OR "premature birth"[MeSH Terms] OR ("premature"[All Fields] AND "birth"[All Fields]) OR "premature birth"[All Fields] OR ("preterm"[All Fields] AND "birth"[All Fields]) OR "preterm birth"[All Fields]) ((("Infant, Premature"[Mesh] OR ("infant"[All Fields] AND "premature"[All Fields]) OR "premature infant"[All Fields] OR ("preterm"[All Fields] AND "infant"[All Fields]) OR "preterm infant"[All Fields] OR ("infant, newborn"[MeSH Terms] OR ("infant"[All Fields] AND "newborn"[All Fields]) OR "newborn infant"[All Fields] OR ("newborn"[All Fields] AND "infant"[All Fields])) AND (premature[All Fields] OR preterm[All Fields]) OR "premature birth"[MeSH Terms] OR ("premature"[All Fields] AND "birth"[All Fields]) OR "premature birth"[All Fields] OR ("preterm"[All Fields] AND "birth"[All Fields]) OR "preterm birth"[All Fields]) AND ("1985"[PDAT] : "3000"[PDAT]) AND "humans"[MeSH Terms].

The exclusion criteria were: Studies that did not provide primary data on epidemiological parameters, non-representative studies (eg, only high-risk pregnancies), and reviews. Table 2 shows the search hits, number of full-texts reviewed, and number of extracted sources.

Table 3. Preterm search hits, full-text review, extracted sources

Search	Hits	Full-text Review	Extracted	Search date
GBD 2017	16174	2200	154	6/6/2017

Data Processing

Starting in GBD 2019, as was the case with all other non-fatal analyses, we applied empirical age and sex-ratios from previous GBD 2019 Decomposition 1 models to disaggregate observations that did not entirely fit in one GBD age category or sex. Ratios were determined by dividing the result for a specific age and sex by the result for the aggregate age and sex specified in a given observation. It is our intention to update this splitting process annually.

Low birth weight (<2500 grams) data was extracted from literature, vital registration systems, and surveys. DHS survey data were observed to have high missingness; to correct for the missingness, birth weight was imputed using the Amelia package in R. Birth weight was predicted using standard Amelia imputation methods from the following variables also in the DHS surveys: urbanicity, sex, birthweight recorded on card, birth order, maternal education, paternal education, child age, child weight, child height, mother's age at birth, mother's weight, shared toilet facility, and household water treated.

"Crosswalking", or the process of reducing non-random bias by adjusting non-standard data to the likely value had the data been "gold-standard", was used to process data in the extremely preterm (<28

weeks) and preterm (<37 weeks) models. All preterm crosswalks were done using Meta Regression – Regularized, Bayesian, Trimmed (MR-BRT). Insurance claims data in extremely preterm (<28 weeks) data was adjusted to vital registration data. Insurance claims data and inpatient data were also adjusted to vital registration in preterm (<37 weeks) conditions. The crosswalk for inpatient data had a spline on the prevalence of inpatient data. Once all claims & inpatient preterm (<37 weeks) data was adjusted, low birth weight data was crosswalked to post-claims and inpatient preterm (<37 weeks) data. If low birth weight data in countries that were 1) categorized as "data-rich" locations in cause-of-death modeling or had at least 10 consecutive years of vital registration data recording gestational age and 2) had both preterm birth and low birth weight data, crosswalked low birth weight data was outliered so that the model was informed only by the gestational age data.

Table 4. MR-BRT VR-Insurance Claims Crosswalk Adjustment Factor for Extremely Preterm Birth (<28 weeks of gestation)

Data input	Reference or alternative case definition	Gamma	Beta Coefficient, Log (95% CI)	Adjustment factor*
Vital registration	Reference	0.00	---	---
Insurance Claims	Alt		-0.651 (-0.602, -0.699)	0.521 (0.500, 0.548)

**Adjustment factor is the transformed Beta coefficient in normal space, and can be interpreted as the factor by which the alternative case definition is adjusted to reflect what it would have been if measured as the reference.*

Table 5. MR-BRT VR-Insurance Claims Crosswalk Adjustment Factor for Preterm Birth (<37 weeks of gestation)

Data input	Reference or alternative case definition	Gamma	Beta Coefficient, Log (95% CI)	Adjustment factor*
Vital registration	Reference	0.16	---	---
Insurance Claims	Alt		-0.728 (-0.705, -0.752)	0.483 (0.471, 0.494)

**Adjustment factor is the transformed Beta coefficient in normal space, and can be interpreted as the factor by which the alternative case definition is adjusted to reflect what it would have been if measured as the reference.*

Figure 1: MR-BRT Clinical Inpatient Data Crosswalk with Spline on Prevalence of Preterm Birth

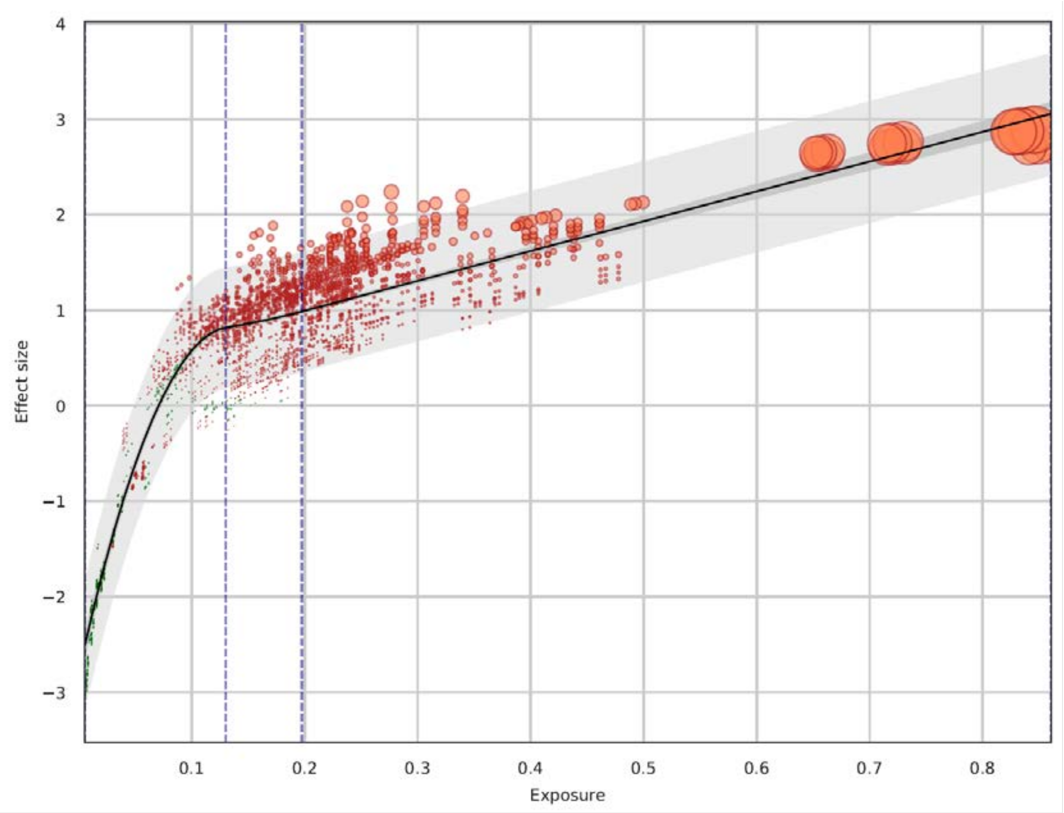

Table 6. MR-BRT Preterm birth-Low birth weight Crosswalk Adjustment Factor for Neonatal Preterm Birth (<37 weeks of gestation)

Data input	Reference or alternative case definition	Gamma	Beta Coefficient, Log (95% CI)	Adjustment factor*
Preterm birth	Reference	0.41	---	---
Low birth weight	Alt		-0.0974 (-0.0807, -0.1161)	0.907 (0.890, 0.922)

**Adjustment factor is the transformed Beta coefficient in normal space, and can be interpreted as the factor by which the alternative case definition is adjusted to reflect what it would have been if measured as the reference.*

Modelling Strategy

Step 1A: Model univariate birth weight and gestational age distributions at birth, by l/y/s

Microdata is the ideal data source for modelling distributions; however, microdata is not widely available for birth weight and is more scarce for gestational age. Categorical prevalence data is more readily available from a wider range of locations and years for low birth weight (<2500g), extremely preterm (<28 weeks of gestation), and preterm birth (<37 weeks of gestation). Because categorical

prevalence has wider availability than microdata, we use prevalence data to assist in modelling birth weight and gestational age ensemble distributions.

Ensemble distribution models can be constructed with three pieces of information: mean of the distribution, variance of the distribution, and the weights of the distributions being ensemble. To model mean and variance for all l/y/s for birth weight and gestational age, we first used Spatio-temporal Gaussian Process Regression (STGPR) models to model prevalence of low birth weight, extremely preterm, and preterm birth for all l/y/s at birth. To model mean birth weight for all l/y/s, OLS linear regression was used to regress mean birth weight on log-transformed low birth weight prevalence. This model was then used to predict mean birth weight for all l/y/s, using the prevalence of low birth weight (<2500 grams) modelled for all l/y/s in STGPR. Similarly, to model gestational age mean for all l/y/s, OLS linear regression model was used to regress mean gestational age on log-transformed preterm prevalence. Mean gestational age for all l/y/s was predicted using the preterm birth (<37 weeks) estimated modelled in STGPR.

Global ensemble weights for gestational age were derived by using a 3 million sample of all available gestational age and birth weight microdata in Table 6 to select the ensemble weights. The two distribution families that received the highest weights were the Weibull (43%) and log-logistic (21%) distributions. Global ensemble weights for birth weight were derived using a 3 million sample of all available microdata in Table 6, in addition to birth weight microdata available primarily through the DHS and MICS surveys. The four distribution families that received the highest weights were the mirror gamma (31%), log-logistic (19%), normal (10%), and mirror gumbel (10%) distributions.

For each l/y/s, given the mean and ensemble weights, the variance was optimized to minimze error on the prevalence of preterm birth (<37 weeks) for the gestational age distribution and prevalence of low birth weight (<2500 grams) for the birth weight distribution.

Step 1B: Model joint birth weight and gestational age distributions at birth, by l/y/s

In order to model the joint distribution of gestational age and birth weight from separate distributions, information was needed about the correlation between the two distributions. Distributions of gestational age and birth weight are not independent; the Spearman correlation for each country where joint microdata was available (Table 6), pooling across all years of data available, ranged from 0.25-0.49. The overall Spearman correlation was 0.38, pooling across all countries in the dataset.

Table 7. Summary of Data Inputs

Location	Years of data	Total births*	Format of data	Spearman correlation	Used in Ensemble Weight Selection	Used in Copula Parameter Selection	Used in Relative Risk Models
BRA	2016	2,854,380	Microdata	0.37	Yes	Yes	No
ECU	2003-2015	2,473,039	Microdata	0.34	Yes	Yes	No
ESP	1990-2014	8,537,220	Microdata	0.42	Yes	Yes	No
JPN	1995-2015	23,644,506	Tabulations	0.41	No	No	Yes
MEX	2008-2012	10,256,117	Microdata	0.35	Yes	Yes	No
NOR	1990-2014	1,489,210	Microdata	0.44	Yes	Yes	Yes
NZL	1990-2016	1,600,501	Microdata	0.25	Yes	Yes	Yes
SGP	1993-2015	972,775	Tabulations	0.41	No	No	Yes
TWN	1998-2002	1,331,760	Tabulations	0.38	No	No	Yes

URY	1996-2014	698,622	Microdata	0.49	Yes	Yes	No
USA	1990-2014	81,929,879	Microdata	0.38	Yes	Yes	Yes

** Pooled across all year and sexes, excluding data missing year of birth, gestational age, or birth weight*

Joint distributions between the birth weight and gestational age marginal distributions were modeled with copulae. The Copula and VineCopula packages in R were used to select the optimal copula family and copula parameters to model the joint distribution, using joint microdata from the country-years in Table 6. The copula family selected from the microdata was "Survival BB8", with theta parameter set to 1.75 and delta parameter set to 1.

The joint distribution of birth weight and gestational age per location-year-sex was modelled using the global copula family and parameters selected and the location-year-sex gestational age and birth weight distributions. The joint distribution was simulated 100 times to capture uncertainty. Each simulation consisted of 10,000 simulated joint birth weight and gestational age data points. Each joint distribution was divided into 500g by 2wk bins to match the categorical bins of the relative risk surface. Birth prevalence was then calculated for each 500g by 2wk bin.

Step 1C: Model joint distributions from birth to the end of the neonatal period, by l/y/s

Early neonatal prevalence and late neonatal prevalence was estimated using life table approaches for each 500g & 2wk bin. Using the all-cause early neonatal mortality rate for each location-year-sex, births per location-year-sex-bin, and the relative risks for each location-year-sex-bin in the early neonatal period, the all-cause early neonatal mortality rate was calculated for each location-year-sex-bin. The early neonatal mortality rate per bin was used to calculate the number of survivors at 7 days and prevalence in the early neonatal period. Using the same process, the all-cause late neonatal mortality rate for each location-year-sex was paired with the number of survivors at 7 days and late neonatal relative risks per bin to calculate late neonatal prevalence and survivors at 28 days.

Step 2: Model impairment proportions

Using mild impairment proportion and moderate-to-severe impairment proportion data, we ran a single mixed-effects linear regression model, regressing on HAQI and with a dummy variable on each gestational age and proportion type, to generate country-year-sex-specific estimates of both parameters for each gestational age (Figure 2). The remainder of 1 – (mild proportion + moderate-severe proportion) was assigned to asymptomatic proportion, by gestational age. The maximum sum of the mild and moderate-severe proportions was capped at 90%.

Figure 2: Preterm birth mild, moderate-severe impairment regression on HAQI (log), by gestational age

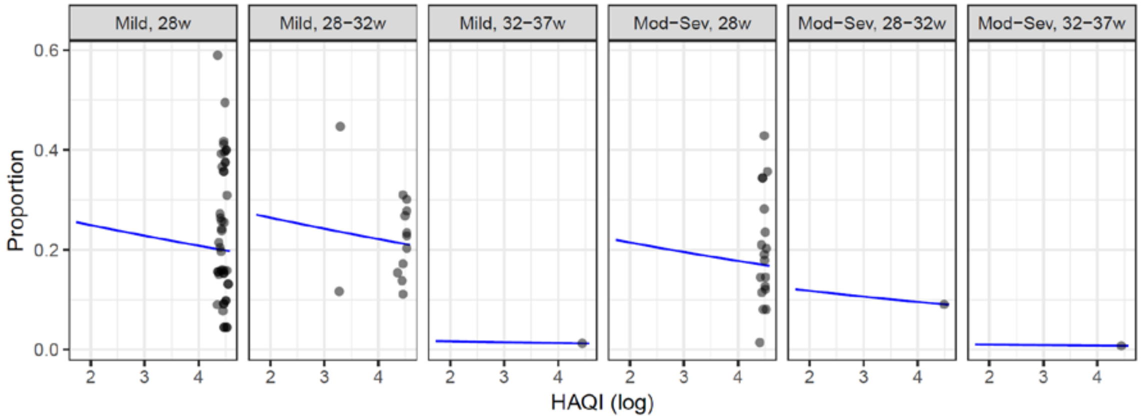

Step 3: Model long-term impairment at all ages

Asymptomatic, mild, and moderate-severe impairment proportions at 28 days, modeled in Step 2, were applied to prevalence at 28 days. Prevalence of survivors of extremely preterm birth, very preterm birth, and moderate-to-late preterm birth to 28 days was estimated in the modeling step described in Step 1C. Asymptomatic prevalence was assumed to be the same from birth to one year as at 28 days. Asymptomatic prevalence was set to 0 after one year, as no burden is assumed after the first year of life. Mild prevalence was assumed to be the same at all GBD age groups as at 28 days. This was both a pragmatic decision in terms of reducing complexity of subsequent modeling steps, but also reflects a lack of data and therefore an assumption of no excess mortality among those born preterm who develop mild impairment.

The sum of asymptomatic and mild impairment in the early and late neonatal periods was subtracted from the neonatal preterm birth envelope estimates for each gestational age in the early and late neonatal periods, respectively, in order to estimate moderate-severe impairment. For moderate/severe impairment, moderate-severe prevalence calculated at birth, early neonatal, and late neonatal periods were combined with excess mortality estimates derived from the standard mortality ratios (SMR) of cerebral palsy and used as inputs into a second DisMod-MR model. SMR was converted to EMR by multiplying age-specific mortality by age-specific standardized mortality ratios - 1. For this model, remission and incidence were also set to zero.

Step 4: Split into sequela

Asymptomatic cases were by definition assigned no disability weight and therefore no YLDs. Mild impairment and moderate-severe impairment due to neonatal preterm birth are split into the sequelae listed in Table 7. The proportion for mild sequelae were split equally between motor and motor plus cognitive impairment. The proportions for each moderate/severe sequelae were extracted from a study by Badawi et al and are listed in Table 7. The proportions were the same across gestational age categories.

Prematurity was additionally assessed to be a cause of vision loss via development of retinopathy of prematurity. The proportion of infants born with prematurity and surviving to the end of the neonatal period who go onto develop retinopathy of prematurity is applied to prevalence of preterm birth at 28 days. Proportional splits were estimated by regressing proportion of ROP among preterm infants on

natural log-transformed neonatal mortality rate from 55 studies in 19 countries. The prevalence of infants with ROP is then split into five vision sequelae of varying severity: asymptomatic, mild, moderate, severe, and complete vision loss (blindness). The proportional splits of retinopathy of prematurity by severity are also listed in Table 7 and are the same across gestational age categories.

Table 8. Proportion of each sequelae by neonatal preterm birth

Sequelae of neonatal preterm birth	Proportion
Mild motor impairment	0.25
Mild motor plus cognitive impairment	0.25
Moderate Motor only	0.17
Moderate Motor impairment + Epilepsy	0.10
Moderate Motor impairment + Blindness	0.02
Moderate Motor impairment + Blindness + Epilepsy	0.01
Moderate Motor impairment + Blindness + Cognitive impairment	0.03
Moderate Motor impairment + Epilepsy + Cognitive impairment	0.18
Moderate Motor impairment + Blindness + Epilepsy + Cognitive impairment	0.02
Severe Motor only	0.15
Severe Motor impairment + Epilepsy	0.03
Severe Motor impairment + Blindness	0.01
Severe Motor impairment + Blindness + Epilepsy	0.003
Severe Motor impairment + Blindness + Cognitive impairment	0.04
Severe Motor impairment + Epilepsy + Cognitive impairment	0.22
Severe Motor impairment + Blindness + Epilepsy + Cognitive impairment	0.02
Mild Retinopathy of Prematurity	0.07
Moderate Retinopathy of Prematurity	0.19
Severe Retinopathy of Prematurity	0.13
Retinopathy of Prematurity with Blindness	0.26

Step 5: Use disability weights to calculate YLDs

Each sequela is associated with a health state, which is used to calculate YLDs. The disability weights for all the health states of all the neonatal disorders are listed in the table below. Some health states are combined using a multiplicative approach to calculate the disability of certain sequelae.

Table 9. Disability weights and lay descriptions by health state

Health State	Description	Disability Weight
Motor impairment, mild	Has some difficulty in moving around but is able to walk without help	0.01 (0.005-0.019)
Motor impairment, moderate	Has some difficulty in moving around, and difficulty in lifting and holding objects, dressing and sitting upright, but is able to walk without help	0.061 (0.040-0.089)

Motor impairment, severe	Is unable to move around without help, and is not able to lift or hold objects, get dressed or sit upright	0.402 (0.268-0.545)
Motor plus cognitive impairments, mild	Has some difficulty moving around but is able to walk without help. The person is slow in learning at school. As an adult, the person has some difficulty doing complex or unfamiliar tasks but otherwise functions independently	0.031 (0.018-0.050)
Motor plus cognitive impairments, moderate	Has some difficulty in moving around, holding objects, dressing and sitting upright, but can walk without help. The person has low intelligence and is slow in learning to speak and to do simple tasks.	0.203 (0.134-0.290)
Motor plus cognitive impairments, severe	Cannot move around without help, and cannot lift or hold objects, get dressed or sit upright. The person also has very low intelligence, speaks few words, and needs constant supervision and help with all daily activities.	0.542 (0.374-0.702)
Distance vision blindness	Is completely blind, which causes great difficulty in some daily activities, worry and anxiety, and great difficulty going outside the home without assistance.	0.187 (0.124-0.260)
Epilepsy, less severe (seizures < once per month)	Has sudden seizures two to five times a year, with violent muscle contractions and stiffness, loss of consciousness, and loss of urine or bowel control.	0.263 (0.173-0.367)
Epilepsy, severe (seizures >= once per month)	Has sudden seizures one or more times each month, with violent muscle contractions and stiffness, loss of consciousness, and loss of urine or bowel control. Between seizures the person has memory loss and difficulty concentrating.	0.552 (0.375-0.71)
Abdominopelvic problem, severe (proxy for EHB without kernicterus)	Has severe pain in the belly and feels nauseous. The person is anxious and unable to carry out daily activities	0.324 (0.220-0.442)

Neonatal encephalopathy due to birth asphyxia and trauma

Flowchart

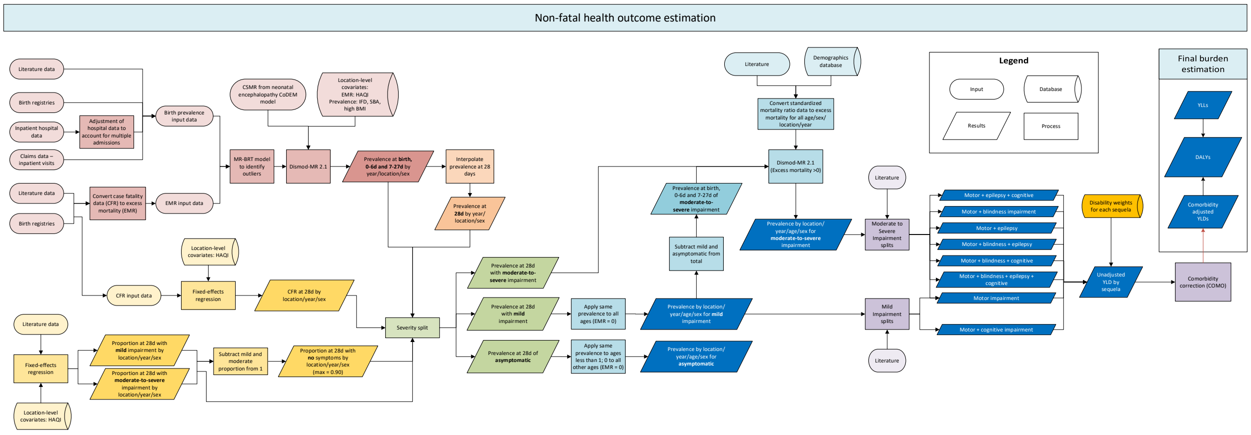

Case definition

Neonatal encephalopathy (NE) due to birth asphyxia and birth trauma is defined in the GBD 2019 nonfatal analyses as injury to the brain in the first few moments or days of life in an infant born at term. This is a change from GDB 2017 when all cases of birth trauma were included in the case definition of NE. We made the change to reflect data source limitations, namely that clinical administrative datasets inconsistently code trauma that is not associated with brain injury. NE is often used interchangeably with the term hypoxic-ischemic encephalopathy (HIE), but the terms are not strictly synonymous because it is believed that only a subset of NE cases are actually triggered by a hypoxic or ischemic event. NE has multiple aetiologies and is defined by its symptoms – abnormal neurological function, including reduced level of consciousness, seizures, depression of tone and reflexes, or difficulty maintaining respiration.

Modeling strategy

Modelling the nonfatal burden of neonatal encephalopathy occurs in five main steps.

Table 10. Analytic steps in estimation of YLDs due to neonatal encephalopathy due to birth asphyxia and trauma

Step	Summary of modeling strategy
1	Model NE prevalence envelope at birth, early neonatal period, late neonatal period, and at exactly 28 days using DisMod-MR 2.1
2	Model case fatality ratio and asymptomatic, mild, and moderate-severe impairment proportions at 28 days using mixed effect regressions, then split prevalence at 28 days by severity of impairment
3	Model impairment prevalence at younger and older ages based on 28 day impairment prevalence
4	Split mild and moderate/severe impairment prevalence into sequelae
5	Apply disability weights to each sequela to calculate YLDs

Table 11. Input Data – Neonatal encephalopathy due to birth asphyxia and trauma

Measure	Total sources	Countries with data
All measures	349	60
Prevalence	301	55
Excess mortality rate	36	24
Proportion	50	26

Step 1: Estimate NE prevalence envelope at birth, early neonatal, and late neonatal periods

DisMod-MR 2.1 was used to model an envelope of neonatal encephalopathy prevalence at birth, early neonatal, and late neonatal periods for all locations, years, and sexes estimated in GBD. Two types of input data inform the model: prevalence data and case fatality ratio (CFR) data.

Input data and data processing

Prevalence

Data on prevalence of neonatal encephalopathy at birth were sourced from literature and clinical informatics data.

A systematic review for NE was last completed for GBD 2015. The PubMed database was searched using the following search string:

> ((("infant"[Title/Abstract] OR "newborn"[Title/Abstract] OR "newborn infant"[Title/Abstract]) AND ("encephalopathy"[Title/Abstract] OR "neonatal encephalopathy"[Title/Abstract] OR "perinatal asphyxia"[Title/Abstract] OR "asphyxia neonatorum"[Title/Abstract] OR "newborn encephalopathy"[Title/Abstract] OR "hypoxic ischaemic encephalopathy"[Title/Abstract] OR ("birth trauma"[Title/Abstract] AND "birth asphyxia"[Title/Abstract]))) AND ("2012"[PDAT] : "3000"[PDAT]) AND "humans"[MeSH Terms])

The exclusion criteria were: Studies that did not provide primary data on epidemiological parameters, non-representative studies (eg, only high-risk pregnancies), and reviews. Sixty studies were extracted.

Clinical informatics data (hospital and claims) formed the bulk of the input data for the NE envelope model. Only inpatient data were included from these datasets, because we believe it is more representative of the true prevalence of neonatal encephalopathy than outpatient data. Infants with neonatal encephalopathy in the countries from which hospital data were available are almost sure to be admitted to the hospital, whereas outpatient data are more likely to capture repeated visits by the same child as they grow. Only inpatient data has been used since GBD 2015. Clinical data processing methods are described separately.

NE cause mapping for GBD 2019 was changed in two ways to address extreme heterogeneity in input data. First, we standardized data processing to be the same across all sources of clinical informatics data (namely hospital and claims data). GBD 2017 hospital data included only discharges with one of four ICD-10 codes: P20 (intrauterine hypoxia), P21 (birth asphyxia), P24 (neonatal aspiration syndromes), and P91 (hypoxic ischaemic encephalopathy, unspecified), while claims data sources included several additional codes representing many types of probable birth injury. These codes are listed in the table below and do not necessarily correspond with brain injury, which is part of our case definition of NE. In GBD 2017 we addressed this inconsistency in clinical data by applying a study-level covariate in DisMod-MR 2.1 to crosswalk claims data to the combined reference category of hospital and literature data. In GBD 2019, we standardized the codes included in claims data to match the codes included in hospital data, eliminating the need for this crosswalk. This approach standardized the clinical data, but we still

observed substantial heterogeneity between clinical and literature data. Investigation of the root cause of the heterogeneity led to a second change: exclusion of those with a solitary discharge diagnosis of P20 (intrauterine hypoxia) from being counted as cases of NE.

Both of these changes technically create a mismatch between GBD mapping of ICD codes for NE for non-fatal versus mortality analyses, but we believe this is likely a more accurate representation of how the codes are used. For neonates who die with any of the codes listed in the table below certified as the underlying cause of death, it is a relatively safe assumption that the neonate experienced birth trauma, and likely brain injury, leading to their death. The same assumption of brain injury cannot be made when the same codes are used on neonates who survive. P20 in particular is recommended for recording fetal distress, a common indication for urgent or emergent cesarean section, and a large proportion of such neonates will receive care and therefore not experience brain damage or develop NE. These changes in clinical mapping and processing eliminated the need for a crosswalk, but also had the consequence of limiting the size of the dataset because not all sources contained the necessary level of detail to make a distinction. Significant heterogeneity in NE data from clinical sources remains and is a priority research area going forward in GBD.

Table 12. ICD Codes Mapped to NE claims data in GBD 2017 that were not included in GBD 2019

Code	Name
P02	Newborn (suspected to be) affected by complications of placenta, cord and membranes
P03	Newborn (suspected to be) affected by other complications of labor and delivery
P10	Intracranial laceration and hemorrhage due to birth injury
P11	Other birth injuries to central nervous system
P12	Birth injury to scalp
P13	Birth injury to skeleton
P14	Birth injury to peripheral nervous system
P15	Other birth injuries
P20	Intrauterine hypoxia
P90	Convulsions of newborn

Starting in GBD 2019, as was the case with all other non-fatal analyses, we applied empirical age and sex-ratios from previous DisMod-MR 2.1 models to disaggregate observations that did not entirely fit in one GBD age category or sex. Ratios were determined by dividing the result for a specific age and sex by the result for the aggregate age and sex specified in a given observation. It is our intention to update this splitting process annually.

Lastly, because of significant residual heterogeneity in input data, especially from clinical administrative sources, we used MR-BRT model to identify outliers in the prevalence data, running a cubic spline with healthcare access and quality index (HAQI) as a covariate and fixed effects on sex and age group, trimming 40% of data. All trimmed data were marked as outliers in the model.

Figure 3. NE prevalence data with spline on HAQI

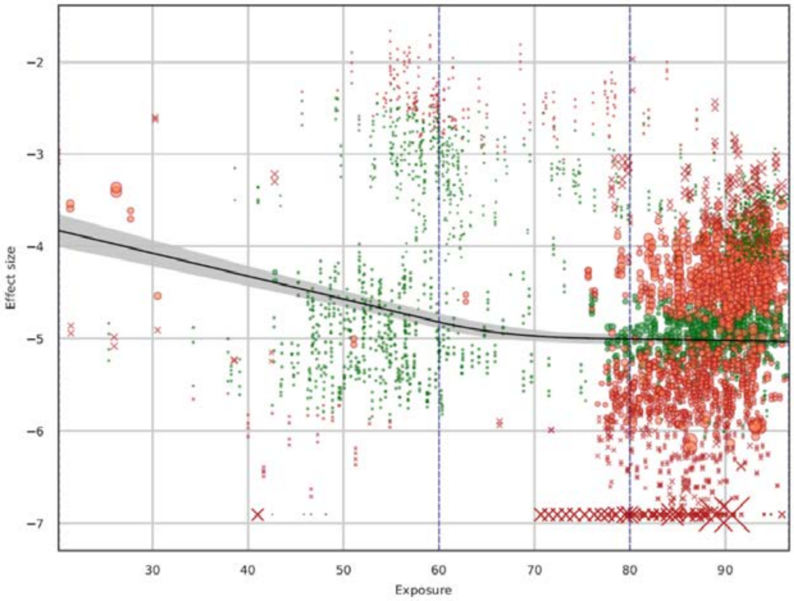

Case Fatality Ratio

Case fatality ratio (CFR) data were extracted from literature as the proportion of deaths in the neonatal period (<28 days of life) amongst cases of NE. A separate literature review was not conducted to identify CFR data, but it was extracted whenever identified from the search described above. In order to enter this CFR data into DisMod-MR 2.1, CFR is transformed into an excess mortality rate (EMR) using the formula

$$EMR = -\frac{\ln(1 - CFR)}{\frac{days\ of\ observation\ period}{365}}$$

This is analogous to the transformation of cumulative incidence (proportion) to an incidence rate (person-year denominator). The denominator in this equation is the number of days in the observation period for the data point – for example, data that followed newborns with neonatal encephalopathy for one year would have a denominator of 1.

Modeling strategy

A DisMod-MR 2.1 model estimated prevalence at birth, and early and late neonatal age groups. Remission and incidence are both set to zero, as no one can develop encephalopathy after birth, and no one can cease to have been born with encephalopathy after the fact. Three country-level covariates informed prevalence estimates: in-facility delivery, skilled birth attendance, and age-standardized SEV for high body-mass index (proxy for maternal body anthropometric status). The latter was changed in GBD 2019; previously we used categorical prevalence of BMI <18.5 in women of reproductive age as a covariate. EMR was informed by the location-level covariate HAQI, a change from GBD 2017 when natural log-transformed lag-distributed income per capita (LN-LDI) was used as a covariate on EMR. The beta values from the DisMod-MR 2.1 model for each location-level covariate are shown in the table below.

Table 13. Summary of covariates used to model prevalence of neonatal encephalopathy at birth and in the neonatal period

Covariate	Measure	Transform	Exponentiated beta (95% UI)
In-facility delivery (proportion)	Prevalence	None	0.95 (0.83 — 1.00)
Skilled birth attendance (proportion)	Prevalence	None	0.92 (0.73 — 1.00)
Age-standardized summary exposure value for High body-mass index	Prevalence	None	3.14 (1.36 — 8.30)
Healthcare access and quality index	Excess mortality rate	None	0.97 (0.96 — 0.97)

A second change in GBD 2019 was inclusion of cause-specific mortality rate (CSMR) results into DisMod-MR 2.1 models of NE, taking advantage of a feature (described in the DisMod-MR 2.1 description in this appendix) that when CSMR is incorporated into DisMod-MR 2.1 models, each CSMR data is paired with corresponding prevalence values matched for specific age group, year, location, and sex. After pairing, an implied EMR datum is generated by dividing CSMR by prevalence. The EMR and CSMR data also therefore inform the model. Utilization of this approach was made possible by the age-sex splitting of prevalence data that occurred prior to modeling – otherwise there would have been no matches. This improved internal consistency of COD and nonfatal estimates. We added a prior of monotonically-decreasing EMR with increasing age.

After estimating prevalence at birth, early neonatal, and late neonatal age groups, prevalence at 28 days was estimated by linearly extrapolating early neonatal and late neonatal prevalence. Prevalence at 28 days is not an age group that is reported in GBD, but it is required for modelling since the proportional severity splits from literature, which determine prevalence of asymptomatic, mild, and moderate-severe impairment, are based on prevalence at 28 days (the end of the neonatal period).

Step 2: Model impairment proportions and case fatality ratio at 28 days, then split prevalence at 28 days by severity of impairment

Infants who survive neonatal encephalopathy may go on to experience long-term disability or impairment. We categorized impairment for neonatal encephalopathy into three severities: asymptomatic, mild, and moderate to severe impairment.

Input Data

Data on the proportion of cases of neonatal encephalopathy that go on to develop mild impairment and moderate-to-severe impairment were extracted from a systematic literature review that was last completed in GBD 2013 and updated in GBD 2015. The same search string described above was used to identify impairment data.

Modeling strategy

To model proportion of mild impairment and moderate-severe impairment, we ran a mixed-effect linear regression on mild impairment and moderate-severe impairment proportion data, using a dummy variable to represent the type of impairment, and HAQI as a predictor. Moderate-severe impairment was the reference category.

With this method, it was possible for the modeled proportion of mild impairment and proportion of moderate-severe impairment to sum to a value greater than one. To address this, we checked the sum of the two values in any of the 1,000 iterations of the uncertainty analysis, and if greater than 0.9, proportionately rescaled both estimates to sum to 0.9 (we picked 0.9 rather than 1 to allow at least

some probability of a child having no impairment). The remainder of 1 – (mild proportion + moderate-severe proportion) was assigned to asymptomatic proportion.

We ran another mixed-effect linear regression on case fatality ratio data, using HAQI as a predictor, to generate location-year-sex-specific estimates of CFR. Prevalence at 28 days was then multiplied by 1 - CFR to determine the number of survivors. The number of survivors was then divided into asymptomatic, mild, and moderate-severe categories by multiplying the number of survivors by the impairment proportions.

Asymptomatic prevalence is extended to other ages based on the assumption that prevalence at 28 days is the same as at early neonatal, late neonatal, and post-neonatal, and that there is no burden and therefore no prevalence after 1 year. Mild prevalence is extended to other ages based on the assumption that prevalence at 28 days is the same as the prevalence at all other ages because there is no excess mortality and no remission among those born with mild neonatal encephalopathy (e.g. no one can develop the disease after birth, no one dies from it, and no one recovers from it, so the number of cases is constant across age).

Step 3: Model impairment prevalence at other ages based on 28 day impairment prevalence

Input Data

Standardized mortality ratios (SMR) of cerebral palsy are used as input data to model the prevalence of moderate-to-severe impairment for ages greater than the neonatal period. Cerebral palsy is used because it has essentially the same symptoms as moderate-to-severe long-term impairment. This data is used across all four neonatal causes. The same data is also used by other causes on the GBD. A meta-analysis was run for a 0-19 age group and a 20-99 age group, and the SMR values were converted to EMR for use in DisMod-MR 2.1 using the formula:

*EMR = (location-sex-age-specific all-cause mortality) * (age-specific SMR – 1)*

Modelling Strategy

To estimate the prevalence of moderate-severe impairment at other ages, we needed to account for excess mortality. Because there is excess mortality, the number of cases of moderate-severe impairment declines with age. The sum of asymptomatic and mild impairment in the early and late neonatal periods was subtracted from the NE envelope estimates (Step 1) in the early and late neonatal periods in order to estimate moderate-severe impairment. This reflects the assumption that all deaths in the early and late neonatal period were among those with moderate-severe impairment, and all newborns born with asymptomatic or mild NE did not experience excess mortality.

To model moderate-severe prevalence, a DisMod-MR 2.1 model was run on the moderate-severe prevalence estimates (e.g. prevalence at birth, early neonatal period, late neonatal period, and 28 days), and on excess mortality estimates derived from the standard mortality ratios (SMR) of cerebral palsy. Remission and incidence were set to zero. The input dataset was entirely complete as every location had an input datum for 28-day prevalence as well as specific values for EMR at every age-location-sex-year so no location-level covariates or priors were specified in the running of the model.

Step 4: Split mild and moderate-to-severe prevalence into sequelae

The mild impairment estimates are split into two sequelae, and the moderate-to-severe impairment estimates are split into 14 sequelae:

Table 14. Health states by severity

Health State	Mild	Moderate	Severe
Motor only	X	X	X
Motor + Cognitive	X		
Motor + Epilepsy		X	X
Motor + Blindness		X	X
Motor + Blindness + Epilepsy		X	X
Motor + Blindness + Cognitive		X	X
Motor + Epilepsy + Cognitive		X	X
Motor + Blindness + Epilepsy + Cognitive		X	X

The mild sequelae were derived by splitting the mild prevalence equally. The proportions for each moderate/severe sequelae were extracted from a study by Badawi et al[1] and are listed in the table below in descending order. This data was also used to split impairments into sequelae across the other neonatal causes.

Table 15. Proportion of each sequelae of moderate/severe neonatal encephalopathy

Sequelae of moderate/severe neonatal encephalopathy	Proportion
Severe motor plus cognitive impairment with epilepsy	0.216
Moderate motor plus cognitive impairment with epilepsy	0.183
Moderate motor impairment	0.173
Severe motor impairment	0.152
Moderate motor impairment with epilepsy	0.100
Severe motor plus cognitive impairment with blindness	0.038
Severe motor impairment with epilepsy	0.033
Moderate motor plus cognitive impairment with blindness	0.032
Severe motor plus cognitive impairment with blindness and epilepsy	0.020
Moderate motor impairment with blindness	0.018
Moderate motor plus cognitive impairment with blindness and epilepsy	0.017
Moderate motor impairment with blindness and epilepsy	0.009
Severe motor impairment with blindness	0.006
Severe motor impairment with blindness and epilepsy	0.003

Step 5: Use disability weights to calculate YLDs

Each sequela is associated with a health state, which is used to calculate YLDs. The health states used for NE are largely the same as the health states for other neonatal causes (see Table 8. Disability weights and lay descriptions by health state for list). Some health states were combined to calculate the burden of certain sequela.

Haemolytic disease and other neonatal jaundice

Flowchart

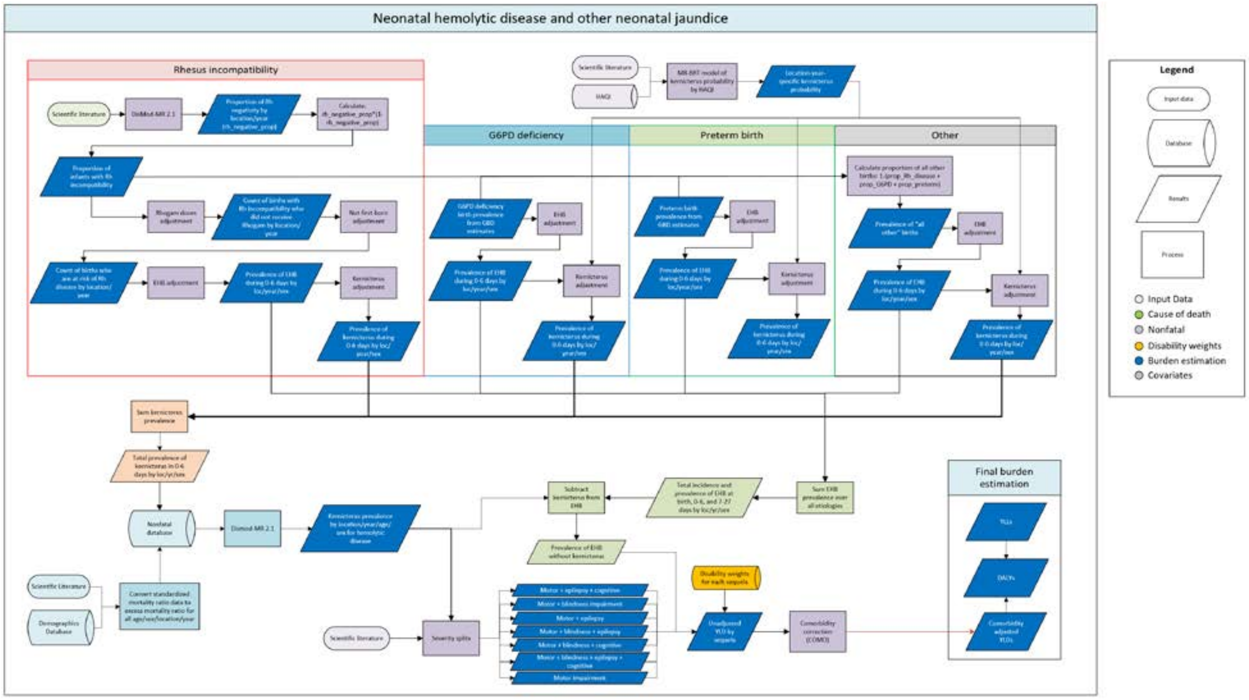

Case definition

Haemolytic disease of the newborn and other neonatal jaundice refers to several aetiologies by which an infant develops extreme hyperbilirubinemia (EHB) and can then go on to develop kernicterus. We define jaundice as serum bilirubin >5 mg/dl and EHB as >25 mg/dl in the neonatal period. Kernicterus is defined as bilirubin-induced brain injury following an EHB episode and is a clinical diagnosis. GBD estimates are limited to incidence, prevalence, and YLDs due to EHB and kernicterus. We classify EHB that does not progress to kernicterus as mild impairment and kernicterus as moderate/severe impairment. The aetiologies that inform our estimates for EHB and kernicterus are Rhesus (Rh) disease, preterm birth, glucose-6-phosphate dehydrogenase deficiency (G6PD), and other causes.

Modelling strategy

Modelling the nonfatal burden of hemolytic disease occurs in seven main steps.

Table 16. Analytic steps in estimation of YLDs due to hemolytic disease and other neonatal jaundice

Step	Summary of modeling strategy
1	Estimate prevalence of EHB due to Rh disease using DisMod-MR 2.1
2	Estimate prevalence of EHB due to G6PD deficiency, preterm birth complications, and other causes
3	Estimate prevalence of kernicterus due to each etiology
4	Estimate prevalence of kernicterus (moderate/severe impairment) starting at age 7 days using DisMod-MR 2.1

5	Calculate EHB without kernicterus (mild impairment) as prevalence of EHB minus prevalence of EHB with kernicterus
6	Split moderate/severe impairment prevalence into sequelae
7	Apply disability weights to each sequela to calculate YLDs

Table 17. Input Data – Hemolytic disease and other neonatal jaundice

Measure	Total sources	Countries with data
All measures	307	147
Prevalence	56	50
Incidence	1	1
Proportion	250	143

Step 1: EHB due to Rh Disease

Birth prevalence of EHB due to Rh disease is estimated using the following equation:

*EHB Prevalence = Rh negative prevalence * (1 – Rh negative prevalence) * (2010 Rhogam doses / 2010 Rh incompatible babies) * (not-firstborn prevalence) * 0.15*

The inputs and analytic approach that inform each component of the equation are described below.

Input Data

Birth prevalence data

Rh negativity prevalence was extracted from literature based on the following search, first completed as a systematic review for GBD 2010. For GBD 2019, the systematic review was updated to include years since GBD 2010. The PubMed database was searched using the search string below on February 7, 2019 and returned 466 results. 39 were screened for full-text review, and 8 were extracted. The exclusion criteria were: Studies that did not provide primary data on epidemiological parameters, non-representative studies (eg, only high-risk pregnancies), and reviews.

((newborn[Title/Abstract] OR neonat*[Title/Abstract]) AND (haemolytic[Title/Abstract] OR hemolytic[Title/Abstract] OR hyperbilirubin*[Title/Abstract] OR jaundice[Title/Abstract] OR "glucose-6"[Title/Abstract] OR G6PD[Title/Abstract] OR EHB[Title/Abstract] OR phototherapy[Title/Abstract] OR "ABO incompatibility"[Title/Abstract] OR "RH incompatibility"[Title/Abstract] OR "rh blood group system"[Title/Abstract] OR Rhesus[Title/Abstract] OR "erythroblastosis fetalis"[Title/Abstract] OR kernicterus[Title/Abstract]) AND (prevalen*[Title/Abstract] OR inciden*[Title/Abstract] OR mortality[Title/Abstract] OR severity[Title/Abstract] OR "long term"[Title/Abstract])) AND (2015/05/01[PDAT] : 3000[PDAT]) NOT "Case Reports"[PT]

PRISMA flow diagram (for GBD2019 extraction update)

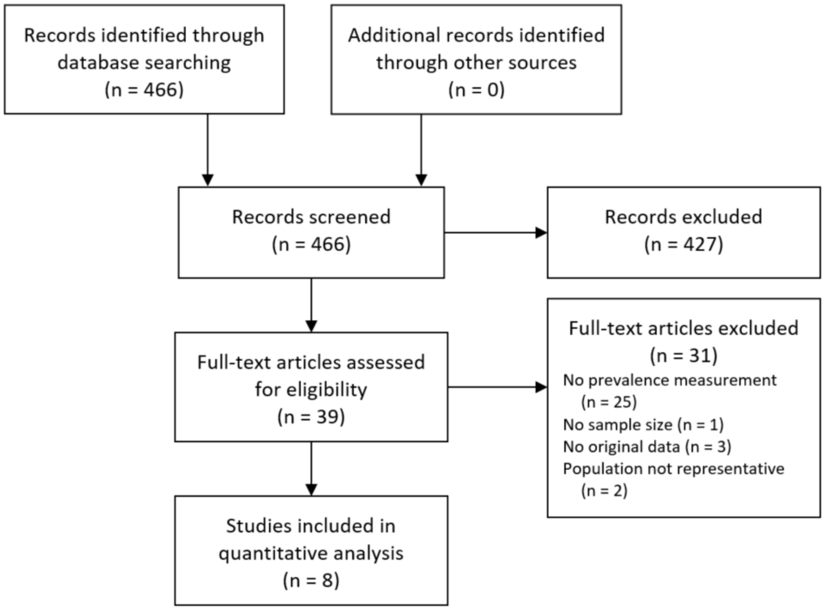

US claims data and hospital data were not included in the haemolytic disease modelling process because they are not coded separately by aetiology. We are working to develop an analytic framework whereby these data could be incorporated into GBD estimates.

Data on Rhogam doses were from market research surveys on Rhogam distribution, and prevalence of not-firstborn children was extracted from the Demographic and Health Survey series for multiple countries.

EHB proportion

The 0.15 multiplier used in the EHB prevalence formula was also from literature[2] and was used to represent the proportion of babies at risk for Rh disease who go on to develop EHB. We do not have corresponding information on the proportion of babies at risk for Rh disease who only develop jaundice (and not EHB), which prevents our being able to estimate overall jaundice.

Modelling Strategy

We began with data on the prevalence of Rh negativity in the population, the number of Rhogam (Rh0 immune globulin) doses distributed to countries in 2010, and the proportion of children who are not firstborn. A single-parameter DisMod-MR 2.1 model was run on Rh negativity prevalence, and a mixed effect regression on birth order greater than one to generate estimates of these values for every location-year. We made the assumptions that Rh negativity did not vary by age, the proportion of Rhogam doses to Rh-incompatible children stayed constant over time, and that countries with NMR<5 had complete Rhogam coverage, based on similar assumptions made in the literature.[2] These quantities were then plugged into the overall equation (repeated below) to calculate EHB prevalence:

*EHB Prevalence = Rh negative prevalence * (1 – Rh negative prevalence) * (2010 Rhogam doses / 2010 Rh incompatible babies) * (not-firstborn prevalence) * 0.15*

Step 2: EHB due to G6PD deficiency, neonatal preterm birth, and other causes

Input data

The data used to estimate EHB due to non-Rh disease were prevalence of neonatal preterm birth, prevalence of G6PD deficiency, and the proportion of cases who develop EHB. The GBD 2019 estimation of neonatal preterm birth is described above and that of G6PD deficiency is described in the appendix section on "Haemoglobinopathies and haemolytic anaemias." The proportion who develop EHB were derived from Bhutani 2013.[2] The etiology-specific EHB proportions are listed in the table below.

Table 18. Proportion of cases of G6PD, preterm birth, and other causes that develop EHB

Etiology	EHB proportion	95% CI
G6PD deficiency	0.0013	(0.00085, 0.002)
Neonatal preterm birth	0.00045	(0.00029, 0.0007)
Other	0.00038	(0.00033, 0.00163)

Modeling strategy

To model the prevalence of EHB due to G6PD deficiency, preterm, and other causes, we started with birth prevalence results for these three conditions. Birth prevalence estimates for G6PD deficiency and neonatal preterm birth came from the corresponding GBD 2019 models of those two conditions. The birth prevalence of other causes was based on the assumption that all babies who don't have any of the three modelled conditions (Rh, G6PD deficiency, and preterm birth) still have some probability of developing EHB. We therefore summed the birth prevalence of Rh disease, G6PD deficiency, and preterm births (as calculated in previous steps), and subtracted this from 1 to get the birth prevalence of all other causes:

$$other\_birth\_prev = 1 - (rh\_birth\_prev + g6pd\_birth\_prev + preterm\_birth\_prev)$$

We calculated prevalence of EHB by multiplying each birth prevalence estimate by the aetiology-specific scalar from the table above, representing the proportion of children who are expected to develop EHB.

Step 3: Estimating Kernicterus Prevalence

Input data

Data on the probability of kernicterus was extracted from literature based on the following search, first completed as a systematic review for GBD 2019. This search was also designed to identify data on probability of EHB and prevalence of neonatal jaundice as a whole. The PubMed database was searched using the search string below on April 25, 2019 and returned 2,212 results. 151 were screened for full-text review, and 36 were extracted.

((newborn[Title/Abstract] OR neonat*[Title/Abstract]) AND (haemolytic[Title/Abstract] OR hemolytic[Title/Abstract] OR hyperbilirubin*[Title/Abstract] OR jaundice[Title/Abstract] OR icter*[Title/Abstract] OR "exchange transfusion"[Title/Abstract] OR "acute bilirubin encephalopathy" [Title/Abstract] OR EHB[Title/Abstract] OR phototherapy[Title/Abstract] OR kernicterus[Title/Abstract]) AND (prevalen*[Title/Abstract] OR inciden*[Title/Abstract] OR mortality[Title/Abstract] OR severity[Title/Abstract] OR "long term"[Title/Abstract])) AND (1980[PDAT] : 3000[PDAT]) NOT "Case Reports"[PT]

We included data in our model of kernicterus probability if the total serum bilirubin level in study participants was directly specified or could be reasonably inferred, and if the outcome matched our case definition of kernicterus (bilirubin-induced brain dysfunction). The exclusion criteria were: Studies that did not provide primary data on epidemiological parameters, non-representative studies (eg, only high-risk pregnancies), and reviews.

PRISMA flow diagram

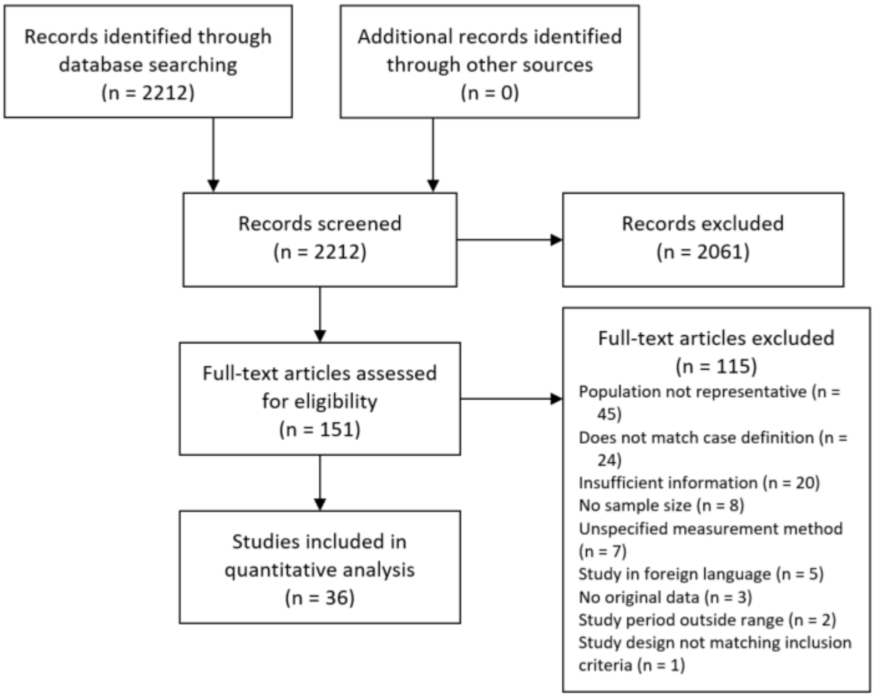

Modeling strategy

In GBD 2017, kernicterus prevalence was calculated with the same approach used to calculate EHB prevalence – in this case by multiplying EHB prevalence by literature-derived scalars representing the proportion of EHB cases that develop kernicterus. Starting in GBD 2019, we instead modeled kernicterus probability as a function of HAQI and initial total serum bilirubin level (TSB), and generated location-year-specific kernicterus proportions. These proportions were used to calculate kernicterus from non-Rh EHB. However, we continued to use a pooled value from literature of 0.072 (0.038, 0.112)[3–5] for proportion of cases of EHB due to Rh disease who develop kernicterus.

To go into more detail about the modeling approach to estimate these new location-year-specific kernicterus proportions, we used all extracted data to develop a monotonic cubic spline model in MR-BRT, with 10% trimming and covariates for HAQI and TSB as shown in the figure below. We used the probability of kernicterus when initial TSB is 25 mg/dL from this model to represent the probability of kernicterus among those with EHB, pairing with location-year specific HAQI values.

Figure 4: Predicted kernicterus proportion for total serum bilirubin levels as a function of HAQI as predicted by MR-BRT

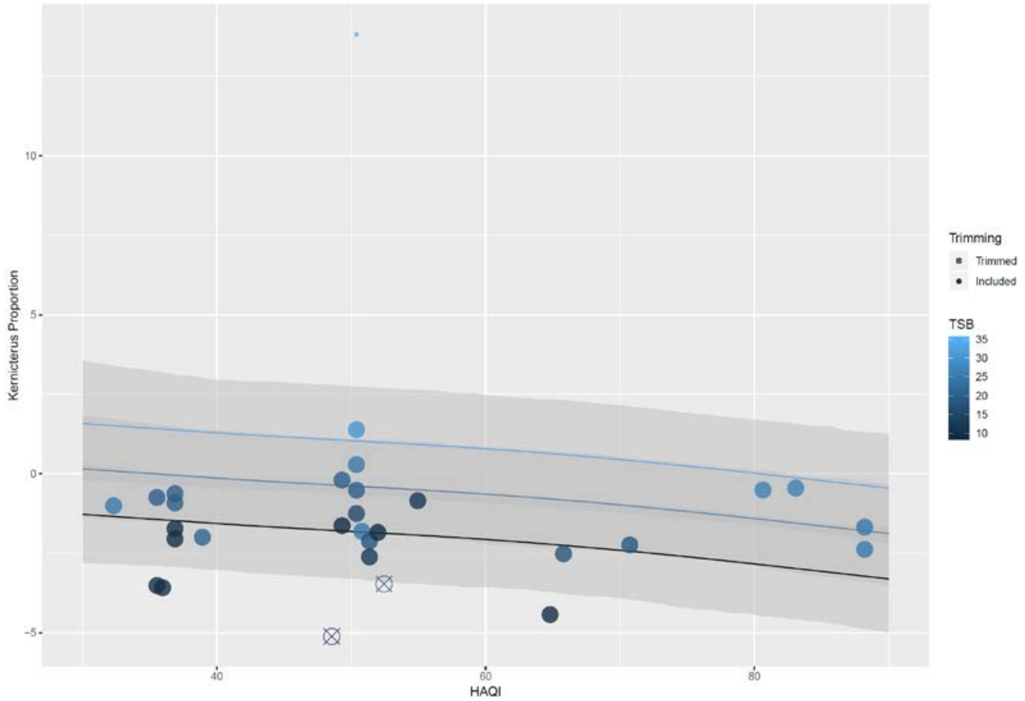

Finally, we calculated total kernicterus prevalence across etiologies in the 0-6 day period by summing kernicterus prevalence from Rh disease, G6PD, and other causes. Kernicterus prevalence due to preterm birth complications was excluded because we assumed that all disability due to preterm birth complications was already captured in our preterm models, and therefore should not be counted twice. Thus, total prevalence of kernicterus is represented as the following equation:

Kernicterus prevalence Total = (kernicterus prevalence Rh disease) + (kernicterus prevalence G6PD) + (kernicterus prevalence Other)

Step 4: Kernicterus Prevalence at Older Ages (Moderate/Severe Impairment)

Input Data

Standardized mortality ratios of cerebral palsy were used as input data to model the prevalence of kernicterus for ages greater than the neonatal period. Cerebral palsy is used because it has essentially the same symptoms as moderate-to-severe long-term impairment. This data is used across all four neonatal causes. The same data is also used by other causes on the GBD. See **Table 15. Geographic representation of SMR of cerebral palsy data** for the geographic coverage of the SMR data. A meta-analysis was run for a 0-19 age group and a 20-99 age group, and the SMR values were converted to EMR for use in DisMod-MR 2.1 using the formula:

*EMR = (location-sex-age-specific all-cause mortality) * (age-specific SMR – 1)*

Modeling Strategy

To model moderate-severe (kernicterus) prevalence at older ages, a DisMod-MR 2.1 model was run on the existing moderate-severe prevalence estimate (e.g. prevalence in the early neonatal period), and on excess mortality estimates derived from the standard mortality ratios (SMR) of cerebral palsy. Remission and incidence were set to zero. The input dataset was entirely complete as every location had an input

datum for early neonatal prevalence as well as specific values for EMR at every age-location-sex-year, so no location-level covariates or priors were specified in the running of the model.

Step 5: EHB Without Kernicterus (Mild Impairment)

We represent mild impairment as impairment due to having EHB alone (no progression to kernicterus). To estimate this, we summed EHB prevalence across all four etiologies, and then subtracted the summed kernicterus prevalence across the three etiologies (excluding preterm). This was estimated for the 0-6 and 7-27 day age groups. Prevalence of EHB without kernicterus from the post-neonatal period onward was assumed to be zero.

Step 6: Split into Health States

The kernicterus estimates were split into 14 sequelae corresponding to moderate and severe disability, and the EHB without kernicterus estimate was associated with one sequela with mild disability.

Table 19. Health states of hemolytic disease and other neonatal jaundice by severity

Health State	Mild	Moderate	Severe
Motor only		X	X
Motor + Cognitive			
Motor + Epilepsy		X	X
Motor + Blindness		X	X
Motor + Blindness + Epilepsy		X	X
Motor + Blindness + Cognitive		X	X
Motor + Epilepsy + Cognitive		X	X
Motor + Blindness + Epilepsy + Cognitive		X	X
Extreme hyperbilirubinemia due to hemolytic disease and other neonatal jaundice, without kernicterus	X		

The proportions for each moderate/severe sequelae were extracted from a study by Badawi et al.[1] This data was also used to split impairments into sequelae across the other neonatal causes.

Process 7: Use disability weights to calculate YLDs

Each sequela was associated with a health state, which was used to calculate YLDs. The health states used for neonatal hemolytic disease are the same as the health states for other neonatal causes (see **Table 8. Disability weights and lay descriptions by health state** for list). Some health states were combined to calculate the burden of certain sequela.

Neonatal sepsis and other neonatal infections

Flowchart

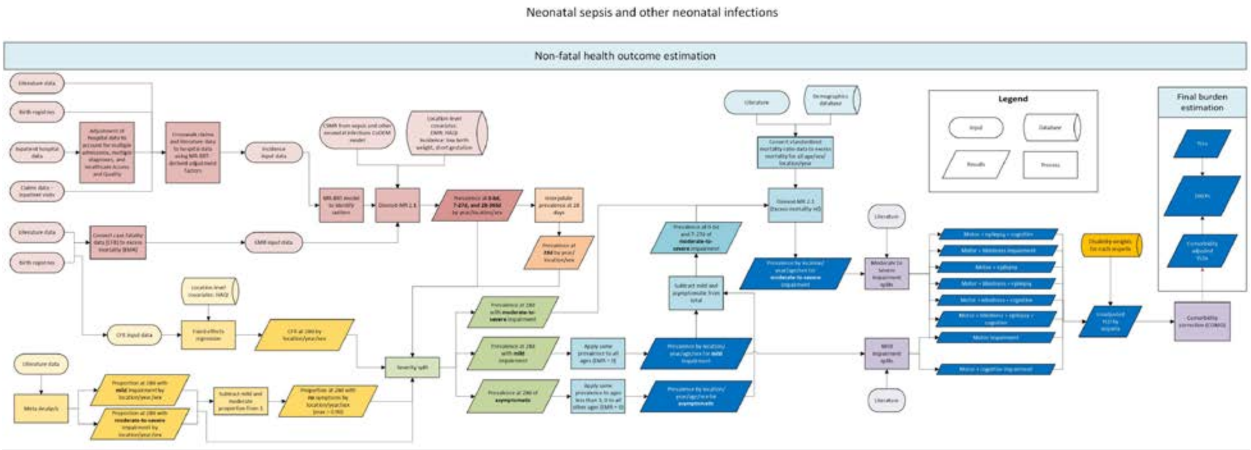

Case definition

Neonatal sepsis and other neonatal infections are infections during the neonatal period that advance to a systemic bloodstream infection (sepsis) and infections that occur during the neonatal period that are not already modeled separately in the GBD.

Modelling Strategy

Modelling the nonfatal burden of neonatal sepsis and other neonatal infections occurs in five main steps:

Table 20. Analytic steps in estimation of YLDs due to neonatal sepsis and other neonatal infections

Step	Summary of modeling strategy
1	Model neonatal sepsis prevalence envelope at the early neonatal period, the late neonatal period, and the post-neonatal period using DisMod-MR 2.1
2	Model case fatality ratio and meta-analyze asymptomatic, mild, and moderate-severe impairment proportions at 28 days, then split prevalence at 28 days by severity of impairment
3	Model impairment prevalence at younger and older ages based on 28 day impairment prevalence
4	Split mild and moderate/severe impairment prevalence into sequelae
5	Apply disability weights to each sequela to calculate YLDs

Table 21. Input Data – Neonatal sepsis and other neonatal infections

Measure	Total sources	Countries with data
All measures	340	54
Incidence	323	45
Excess mortality rate	15	15
Proportion	2	3

Step 1: Estimate neonatal sepsis prevalence envelope at early, late, and post-neonatal periods

DisMod-MR 2.1 was used to estimate an envelope of neonatal sepsis prevalence at the early neonatal and late neonatal periods for all locations, years, and sexes estimated in GBD. Two types of input data inform the model: incidence data and case fatality ratio (CFR) data.

Input data

Incidence

We extracted data on prevalence and incidence of neonatal sepsis and other neonatal infections from literature and clinical informatics data. All prevalence data were then converted to incidence before being input to DisMod-MR 2.1

A systematic literature review for neonatal sepsis was last completed for GBD 2015. The PubMed database was searched using the following search string:

> (("infant"[Title/Abstract] OR "newborn"[Title/Abstract] OR "newborn infant"[Title/Abstract]) AND ("neonatal sepsis"[All Fields] OR "neonatal septicaemia"[All Fields] OR "neonatal meningitis"[All Fields] OR "early sepsis"[All Fields] OR "early septicaemia"[All Fields] OR "tetanus"[All Fields] OR "meningitis"[All Fields] OR "sepsis"[All Fields])) AND ("2012"[PDAT] : "3000"[PDAT]) AND "humans"[MeSH Terms]

To be included, published data sources had to report on specific infections, or groups of infections, and provide diagnostic criteria for how cases were identified. The exclusion criteria were: studies that did not provide primary data on epidemiological parameters (e.g. a commentary piece), Non-representative studies (e.g. only high-risk pregnancies, nosocomial infection rates, preterm infants, ICU populations), and review articles. We did not find any studies that reported on all neonatal infections, only sepsis.

Clinical informatics data (hospital and claims) formed the bulk of the input data for the neonatal sepsis envelope model. Only inpatient data were included from these datasets, because we believe it is more representative of the true prevalence of neonatal sepsis than outpatient data; infants with neonatal sepsis in the countries from which hospital data were available are almost sure to be admitted to the hospital, whereas outpatient data are more likely to capture repeated visits by the same child as they grow. Clinical data processing is described separately.

Case Fatality Ratio

Case fatality ratio (CFR) data were extracted from literature sources as the proportion of deaths in the neonatal period (<28 days of life) amongst cases of neonatal sepsis and other neonatal infections. A separate literature review was not conducted to identify CFR data, but it was extracted whenever identified from the incidence data systematic review described above.

Data Processing

Starting in GBD 2019, we applied empirical age and sex-ratios from previous DisMod-MR 2.1 models to disaggregate observations that did not entirely fit in one GBD age category or sex. Ratios were determined by dividing the result for a specific age and sex by the result for the aggregate age and sex specified in a given observation. It is our intention to update this splitting process annually.

In GBD 2017, we applied study-level covariates in DisMod-MR 2.1 to crosswalk claims and literature incidence data to inpatient hospital data (our reference category). Consistent with non-fatal analyses across the GBD, in GBD 2019 we used MR-BRT to estimate these crosswalk adjustment factors and applied them to our data before input to the DisMod-MR 2.1 model. The adjustment factors applied were as follows:

Table 22. MR-BRT Crosswalk Adjustment Factors for Neonatal Sepsis and Other Neonatal Infections

Data input	Reference or alternative case definition	Gamma	Beta Coefficient, Log (95% CI)	Adjustment factor*
Hospital Data	Ref	0.72	---	---
Claims Data	Alt		0.51 (-1.13 – 2.13)	1.66 (0.32 – 8.41)
Literature Data	Alt		-2.69 (-4.41 – -0.98)	0.07 (0.01 – 0.38)

**Adjustment factor is the transformed Beta coefficient in normal space, and can be interpreted as the factor by which the alternative case definition is adjusted to reflect what it would have been if measured as the reference. The adjusted value is calculated as the alternative case definition value divided by this adjustment factor*

Prior to input into DisMod-MR 2.1, CFR data were transformed into excess mortality rate (EMR) using the formula

$$EMR = -\frac{\ln(1 - CFR)}{\frac{days\ of\ observation\ period}{365}}$$

This is analogous to the transformation of cumulative incidence (proportion) to an incidence rate (person-year denominator). The denominator of this equation is the number of days in the observation period for the data point – for example, data that followed newborns with neonatal sepsis for one year would have a denominator of 1.

Modelling Strategy

A DisMod-MR 2.1 model estimated prevalence in early, late, and post-neonatal age groups. Unlike other neonatal cause models using similar modelling strategies (preterm birth and encephalopathy), no birth prevalence was estimated for neonatal sepsis. Incidence was set to 0 after 27 days, as by definition neonatal sepsis must occur within the neonatal period (0-27 days). Two location-level covariates informed incidence estimates: summary exposure value (SEV) for low birth weight and SEV for short gestation. These were the two most-often selected covariates in the CODEm model of neonatal sepsis and other neonatal infections and represent a change from GBD 2017 when SEV for unsafe water and SEV for unsafe sanitation were used. Excess mortality was informed by the location-level Healthcare Access and Quality index covariate which is also a change from GBD 2017 when LN-LDI was used. The beta values from the DisMod-MR 2.1 model for each location-level covariate are shown in the table below.

Table 23. Summary of covariates used to model prevalence of neonatal sepsis and other neonatal infections

Covariate	Measure	Transform	Exponentiated beta (95% UI)
SEV for low birth weight	Incidence	None	2.09 (1.08 – 4.04)
SEV for short gestation	Incidence	None	2.09 (1.10 – 4.05)
Healthcare access and quality index	Excess mortality rate	None	0.95 (0.94 — 0.96)

Starting in GBD 2019, we included cause-specific mortality rate (CSMR) data from GBD cause of death (COD) analyses into our DisMod-MR 2.1 model to inform nonfatal estimates and improve internal consistency between fatal and nonfatal results. When CSMR is incorporated into DisMod-MR 2.1

models, each CSMR data is paired with corresponding incidence values matched for specific age group, year, location, and sex. After pairing, an implied EMR datum is generated using the following formula:

$$EMR = \frac{CSMR * [remission + (ACMR - CSMR) + EMR_{pred}]}{incidence}$$

where *EMR* is excess mortality rate, *CSMR* is cause-specific mortality, *ACMR* is all-cause mortality rate, and EMR_{pred} is the excess mortality fit from the global DisMod model. Utilization of this approach was made possible by the age-sex splitting of incidence data that occurred prior to modeling, as previously there were no matches.

After estimating prevalence in the early, late, and post-neonatal age groups, prevalence at 28 days was estimated by linearly interpolating early, late, and post-neonatal prevalence. Prevalence at 28 days is not an age group that is reported in GBD, but it is required for modelling since the proportional severity splits from literature, which determine asymptomatic, mild, and moderate-severe prevalence, are based on prevalence at 28 days. The post-neonatal age group estimated in this model is dropped and not used in further modelling steps; only the early neonatal, late neonatal, and 28-day prevalence estimates are retained for the envelope.

Step 2: Model impairment proportions and case fatality ratio at 28 days, then split prevalence at 28 days by severity of impairment

Infants who survive neonatal sepsis may go on to experience long-term disability or impairment. We categorized impairment for neonatal sepsis and other neonatal infections into three severities: asymptomatic, mild, and moderate-to-severe impairment.

Input Data

Data on the proportion of cases of neonatal sepsis that go on to develop mild impairment and moderate-to-severe impairment were extracted from a systematic literature review that was last completed in GBD 2013 and updated in GBD 2015. The same search string described above was used to identify impairment data.

Modeling strategy

Using mild impairment proportion and moderate-to-severe impairment proportion data, we ran separate meta-analyses to generate estimates of both parameters. The remainder of 1 – (mild proportion + moderate-severe proportion) was assigned to asymptomatic proportion.

Table 24. Proportion of mild and moderate-to-severe impairment of neonatal sepsis and other neonatal infections at 28 days

Parameter	Estimate (95% UI)
Mild impairment proportion	10.2% (7.2% - 12.9%)
Moderate-to-severe impairment proportion	4.3% (2.5% - 6.0%)

Figure 5. Mild impairment meta-analysis

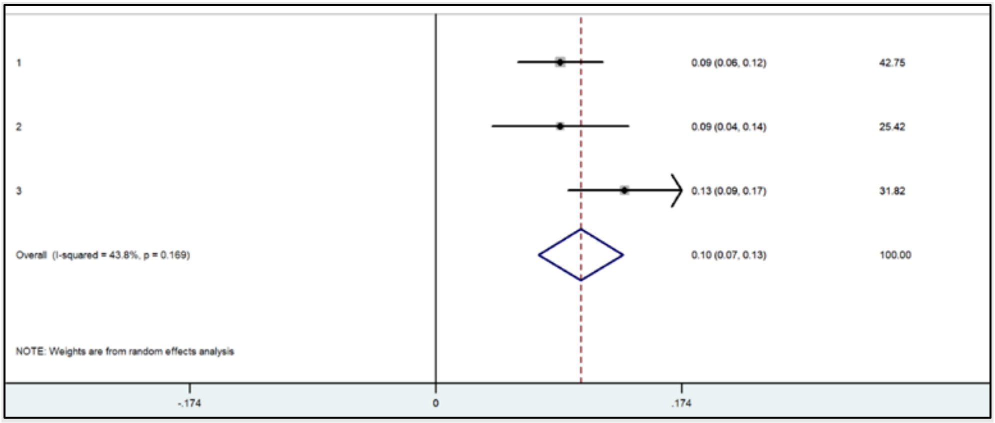

Figure 6. Moderate-to-severe impairment meta-analysis

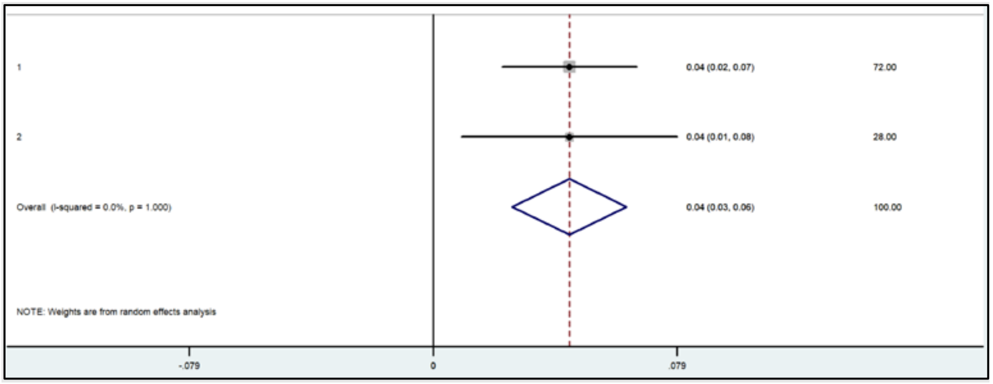

Next, we ran a mixed-effects linear regression of case fatality ratio (CFR) data with healthcare access and quality index (HAQi) as a predictor to generate location-year-sex-specific estimates of CFR. Prevalence at 28 days was then multiplied by 1 - CFR to determine the number of survivors. The number of survivors was then divided into asymptomatic, mild, and moderate-severe categories by multiplying the number of survivors by the impairment proportions.

Asymptomatic prevalence is extended to other ages based on the assumption that prevalence at 28 days is the same as at early neonatal, late neonatal, and post-neonatal, and that there is no burden and therefore no prevalence after one year. Mild prevalence is extended to other ages based on the assumption that prevalence at 28 days is the same as the prevalence at all other ages because there is no excess mortality among those who develop mild neonatal sepsis.

Step 3: Model impairment prevalence at other ages based on 28 day impairment prevalence

Input Data

Standardized mortality ratios (SMR) of cerebral palsy were used as input data to model the prevalence of moderate-to-severe impairment for ages greater than the neonatal period. Cerebral palsy was used because it has essentially the same symptoms as moderate-to-severe long-term impairment. This data was used across all four neonatal causes. A meta-analysis was run for a 0-19 age group and a 20-99 age group, and the SMR values were converted to EMR for use in DisMod-MR 2.1 using the formula:

*EMR = (location-sex-age-specific all-cause mortality) * (age-specific SMR – 1)*

Modelling Strategy

To estimate the prevalence of moderate-severe impairment at other ages, we needed to account for excess mortality. Because there is excess mortality, the number of cases of moderate-severe impairment declines with age. The sum of asymptomatic and mild impairment in the early and late neonatal periods was subtracted from the neonatal sepsis envelope estimates (Step 1) in the early and late neonatal periods in order to estimate moderate-severe impairment. This reflects the assumption that all deaths in the early and late neonatal period were among those with moderate-severe impairment, and all newborns who developed asymptomatic or mild neonatal sepsis did not experience excess mortality.

To model moderate-severe prevalence, a DisMod-MR 2.1 model was run on the moderate-severe prevalence estimates (e.g. prevalence at birth, early neonatal period, late neonatal period, and 28 days), and on excess mortality estimates derived from the standard mortality ratios (SMR) of cerebral palsy. Remission and incidence were set to zero. The input dataset was entirely complete as every location had an input datum for 28-day prevalence as well as specific values for EMR at every age-location-sex-year so no location-level covariates or priors were specified in the model.

Process 4: Splitting mild and moderate-severe impairment prevalence into sequelae

Mild impairment and moderate-severe impairment due to neonatal sepsis and other neonatal infections are split into the following sequelae:

Table 25. Health states by severity

Health State	Mild	Moderate	Severe
Motor only	X	X	X
Motor + Cognitive	X		
Motor + Epilepsy		X	X
Motor + Blindness		X	X
Motor + Blindness + Epilepsy		X	X
Motor + Blindness + Cognitive		X	X
Motor + Epilepsy + Cognitive		X	X
Motor + Blindness + Epilepsy + Cognitive		X	X

To determine the proportion of people within each of these severity levels, one study by Badawi et al[1] informed moderate-to-severe impairment splits, and mild impairments cases were divided equally into both categories.

Step 5: Use disability weights to calculate YLDs

Each sequela is associated with a health state, which is used to calculate YLDs. The health states used for neonatal sepsis and other neonatal infections are the same as the health states for other neonatal causes (see Table 8. Disability weights and lay descriptions by health state for list). Some health states were combined to calculate the burden of certain sequela.

Other neonatal disorders

In addition to the neonatal disorders described above, there are many diverse types of neonatal disorders with a range of severities and associated sequelae. Because these other neonatal disorders are diverse in their underlying causes and risk factors as well as in their associated health outcomes, modelling them together in a DisMod-MR 2.1 model would not produce reliable estimates of prevalence or excess mortality. Instead, we calculated the YLDs caused by other neonatal disorders directly using a YLD/YLL ratio.

We calculated the ratio of YLDs to YLLs across the specified neonatal disorders for which non-fatal outcomes were modelled, using YLL estimates from the GBD 2019 cause of death (CoD) analysis. We then multiplied this YLD/YLL ratio by the YLL estimate for other neonatal disorders from the GBD 2019 CoD analysis, providing us with an estimate of the YLDs associated with other neonatal disorders.

A full list of the ICD codes classified as other neonatal disorders in the mortality analysis are provided below. The codes that made up the largest proportion of deaths were P52: Intracranial nontraumatic hemorrhage of newborn, P29: Cardiovascular disorders originating in the perinatal period, and P00: Newborn (suspected to be) affected by maternal conditions that may be unrelated to present pregnancy.

ICD9 codes:
760, 760.0-760.6, 760.8-760.9, 761, 761.2-761.6, 764, 766, 770, 771, 772, 772.0, 775, 775.0, 775.4-775.9, 776, 776.0-776.5, 776.7-776.9, 777, 777.0-777.4, 777.7-777.9, 778, 779, 779.3, 779.6-779.8

ICD10 codes:
P00, P01, P01.2-01.6, P01.8-01.9, P04, P04.0-04.2, P04.5-04.6, P04.8-04.9, P05, P08, P09, P19, P29, P50, P51, P52, P53, P54, P60, P61, P61.0-61.1, P61.3-61.6, P61.8-61.9, P70, P70.1, P70.3-70.4, P70.8-70.9, P71, P72, P74, P75, P76, P78, P80, P81, P83, P84, P92, P93, P94, P96, P96.3-96.4, P96.8

References

1 Badawi N, Felix JF, Kurinczuk JJ, *et al.* Cerebral palsy following term newborn encephalopathy: a population-based study. *Dev Med Child Neurol* 2005; **47**: 293–8.

2 Bhutani VK, Zipursky A, Blencowe H, *et al.* Neonatal hyperbilirubinemia and Rhesus disease of the newborn: incidence and impairment estimates for 2010 at regional and global levels. *Pediatr Res* 2013; **74 Suppl 1**: 86–100.

3 Walker W. Haemolytic Disease of the Newborn. In: Recent Advances in Paediatrics, 4th edn. London, UK: JA Churchill, 1970.

4 Vaughan VC. Kernicterus in erythroblastosis fetalis. *J Pediatr* 1946; **29**: 462–73.

5 Mollison PL, Cutbush M. Exchange transfusion in haemolytic disease of the newborn. *Lancet* 1948; **2**: 522–7.

Nutritional Deficiencies

Nutritional deficiencies is a parent cause for the nonfatal estimation of the following subcauses:

1. vitamin A deficiency
2. iodine deficiency
3. dietary iron deficiency
4. protein-energy-malnutrition
5. other nutritional deficiencies

Since these 5 subcauses are modeled separately with differences in case definition, input data, strategy, and severity distribution analysis, we present each subcause sequentially.

Vitamin A deficiency

Flowchart

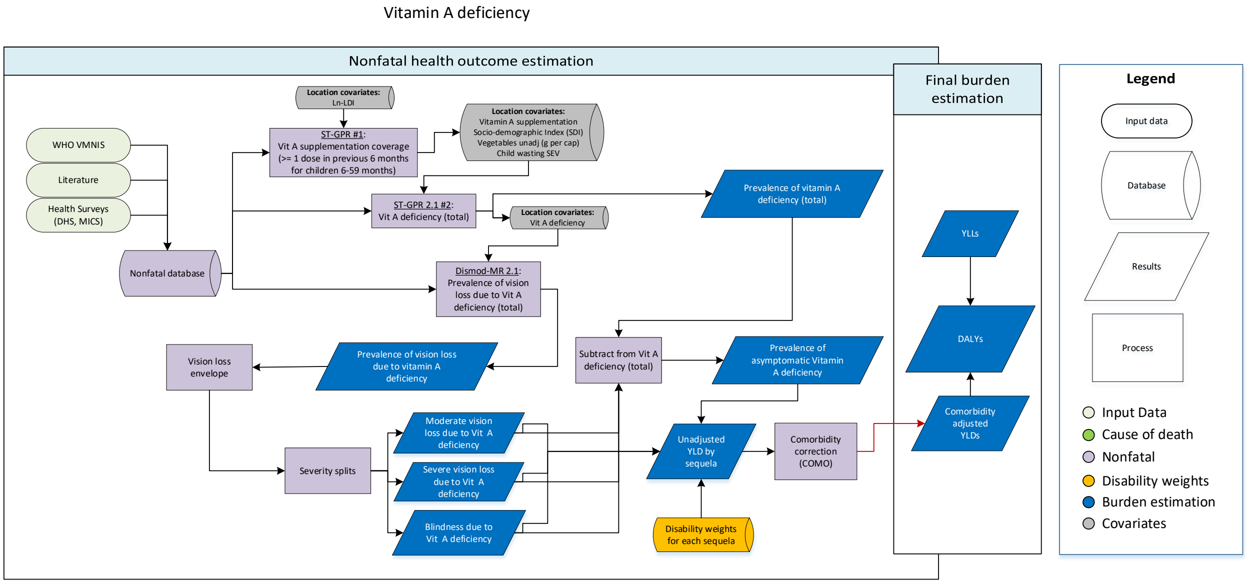

Case definition

The case definition of vitamin A deficiency is the prevalence of serum retinol < 0.7 µmol/L.

In GBD 2019, the assessment of vitamin A deficiency burden involves the quantification of total vitamin A deficiency as well as blindness and vision loss due to vitamin A deficiency, which are associated with corneal ulcerations and corneal scars.

Input data

For GBD 2019, we used data from the WHO Vitamin and Mineral Nutrition Information System, health surveys such as DHS and MICS, and studies identified through literature review for the vitamin A deficiency model. We used data from the UNICEF State of the World's Children database and DHS and MICS surveys for the vitamin A supplementation model, and data from the WHO Vitamin and Mineral Nutrition Information System for the vision loss model. Table 1 provides a summary of data inputs for

vitamin A deficiency modeling**.** A systematic review was last conducted for GBD 2013. The PubMed search terms were: ((vitamin A deficiency[Title/Abstract] AND prevalence[Title/Abstract]) AND (“2009”[Date – Publication] : “2013”[Date – Publication])). Exclusion criteria were:

1. Studies that were not population-based, eg, hospital or clinic-based studies
2. Studies that did not provide primary data on epidemiological parameters, eg, commentaries
3. Review articles
4. Case series
5. Self-reported cases

Table 1: Data Inputs for Vitamin A deficiency morbidity modelling by parameter.

Measure	Total sources	Countries with data
All measures	320	101
Prevalence	46	27
Proportion	274	96

Modeling strategy

The steps of the modelling strategy for GBD 2019 remained consistent with those used in GBD 2017, however several step-specific updates were made. Broadly the strategy consists of three steps, beginning with a model of vitamin A supplementation coverage. The supplementation estimates are then used as a location-level covariate to guide prevalence estimates of overall vitamin A deficiency, which is subsequently used as a location-level covariate to guide prevalence estimates of vision loss due to vitamin A deficiency. The difference between total vitamin A deficiency and vision loss due to vitamin A deficiency is considered asymptomatic. Total vitamin A deficiency was separately considered as a risk factor in the GBD 2019 comparative risk assessment analysis.

To ensure we are using as much information as possible, and therefore maximise the data basis of our estimates, we first model vitamin A supplementation. The case definition for the supplementation model is the proportion individuals who received at least one dose of vitamin A in the previous six months; although the typical metric on which supplementation is tracked is 2+ doses of vitamin A in the previous 12 months for children under 5 years, most existing health surveys do not routinely provide sufficient information to calculate it. In GBD 2019, the supplementation model was moved to ST-GPR to achieve a better time trend that accounts for the introduction of supplementation programs in the late 1990s. Additionally, vitamin A supplementation was previously modeled as an all-age and both-sex indicator with the proportion of children 6-59 months of age who received at least one dose of vitamin A in the previous six months as the case definition. In an effort to capture the effect of supplementation programs on the prevalence of deficiency across age-specific groups, we modeled vitamin A supplementation as an age and sex-specific indicator for GBD 2019 so that high coverage would be restricted to children 6-59 months who are targeted in supplementation campaigns. As in GBD 2017, we used the natural log of lag-distributed income per capita (LN-LDI) as a location-level covariate to inform supplementation estimates where data were absent.

Second, we estimated the age- and sex-specific prevalence of vitamin A deficiency (serum retinol < 0.7 μmol/L). This year we updated the deficiency data processing steps to include a separate sex ratio

model (using MR-BRT) and a separate age pattern model (using DisMod) which were used to split both-sex and all-age data prior to modeling. As with the supplementation model, we moved vitamin A deficiency to ST-GPR to utilize its superior time trends. The age-specific stunting SEV was added as a location-level covariate for the vitamin A deficiency ST-GPR model, alongside the three used last year: sociodemographic index, the availability of retinol activity equivalent (rae) units in foods, and (newly updated) vitamin A supplementation.

Thirdly, the vision loss due to vitamin A deficiency model was run as a single-parameter meta-regression on prevalence in DisMod with vitamin A deficiency prevalence as a location-level covariate. The case definition for vision loss due to vitamin A deficiency is aligned with the WHO Vitamin and Mineral Nutrition Information System database's definition of a corneal scar. In GBD 2019 we modeled the sex ratio for vision loss due to vitamin A deficiency outside of DisMod using MR-BRT and applied this ratio to split both sex data prior to DisMod modeling. Apart from the out-of-dismod sex split, no modeling changes were made for the vision loss model this cycle.

Table 2. Covariates. Summary of covariates used in the vitamin A deficiency models

Vitamin A model	Modeling strategy	Covariate	Type	Parameter
Supplementation	ST-GPR	LDI (I$ per capita)	Country-level	Prevalence
Deficiency	ST-GPR	Vitamin A supplementation	Country-level	Prevalence
	ST-GPR	Vitamin A rae unadjusted (g)	Country-level	Prevalence
	ST-GPR	Stunting SEV	Country-level	Prevalence
	ST-GPR	SDI	Country-level	Prevalence
Vision loss	DisMod-MR	Vitamin A deficiency (age standardized)	Country-level	Prevalence

Our GBD 2019 results include explicit estimates of total vitamin A deficiency, although those without vision loss are assumed to be asymptomatic. Description of how our estimates of total vision loss described above are parsed into moderate vision loss, severe vision loss, and blindness can be found in the modelling description for the "vision loss impairment". Sequelae and corresponding disability weights for each of the health states associated with vitamin A deficiency are shown in Table 3.

Table 3. Severity, lay description, and disability weight (DW)

Sequela	Health state name	Lay description	Disability weight
Moderate vision impairment loss due to vitamin A deficiency	Distance vision, moderate impairment	has vision problems that make it difficult to recognise faces or objects across a room.	0.031 (0.019–0.049)

Severe vision impairment loss due to vitamin A deficiency	Distance vision, severe impairment	has severe vision loss, which causes difficulty in daily activities, some emotional impact (for example worry), and some difficulty going outside the home without assistance.	0.184 (0.125–0.258)
Blindness due to vitamin A deficiency	Distance vision blindness	is completely blind, which causes great difficulty in some daily activities, worry and anxiety, and great difficulty going outside the home without assistance.	0.187 (0.124–0.26)
Asymptomatic vitamin A deficiency	Asymptomatic	--	--

Iodine Deficiency

Flowchart

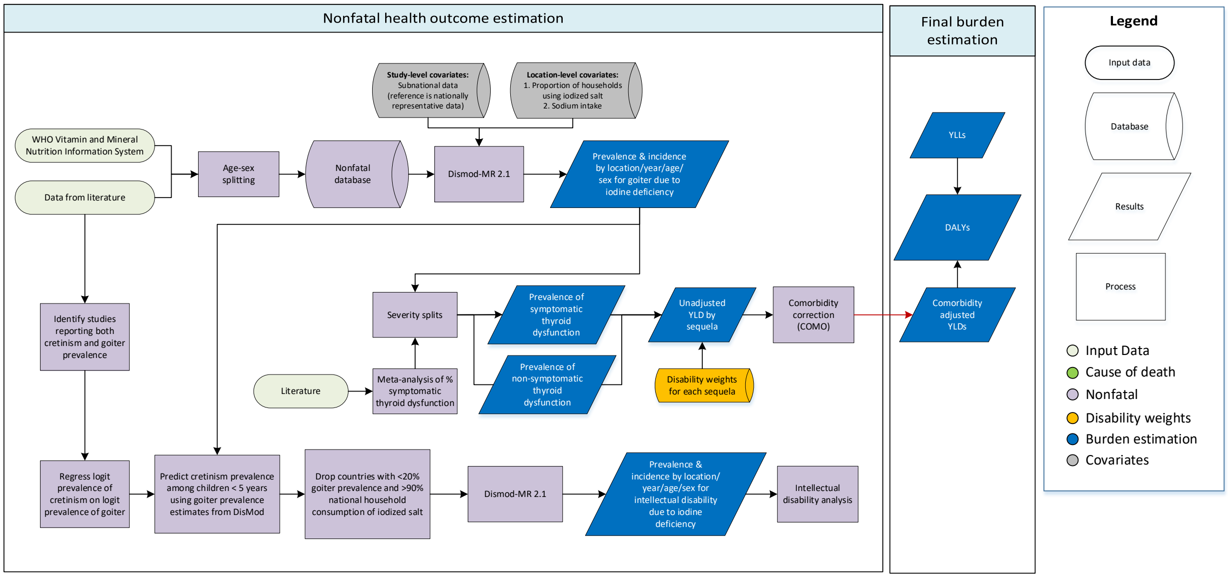

Case definition

Our assessment of the non-fatal burden of iodine deficiency includes estimates of only the subset of iodine deficiency associated with visible goiter (grade 2) and its associated sequelae, including thyroid dysfunction, heart failure, and intellectual disability (historically referred to as "cretinism"). It does not include estimates of sub-clinical iodine deficiency or non-visible goiter (grade 1) induced by iodine deficiency.

Input data

For GBD 2019, data from the WHO Vitamin and Mineral Nutrition Information System and published studies were used for the visible goiter model (Table 1). The extraction and accompanying systematic

review were last conducted for GBD 2013. The PubMed search terms were: ((iodine deficiency[Title/Abstract] AND prevalence[Title/Abstract]) AND (“2009”[Date – Publication] : “2013”[Date – Publication]))

The exclusion criteria were:

1. Studies that were not population-based, eg, hospital or clinic-based studies
2. Studies that did not provide primary data on epidemiological parameters, eg, commentaries
3. Review articles
4. Case series
5. Self-reported cases

Updates to systematic reviews are performed on an ongoing schedule across all GBD causes; an update for iodine deficiency will be performed in the next iteration

Table 1: Data Inputs for iodine deficiency morbidity modelling by parameter.

Measure	Total sources	Countries with data
All measures	207	81
Prevalence	201	78
Relative Risk	5	4
Standardized mortality ratio	1	1

All input data for iodine deficiency is already in our gold-standard case definition (prevalence of visible goiter and prevalence of intellectual disability due to iodine deficiency), so no bias corrections are needed.

Modeling strategy

The iodine deficiency modeling strategy includes iodine deficiency and associated sequelae heart failure, thyroid dysfunction, and intellectual disability. The process is comprised of two models for visible goiter and intellectual disability due to iodine deficiency and severity splits for the other sequela.

For GBD 2019 we changed the strategy for the visible goiter model, estimating the prevalence of grade 2 goiter in a two-step process. We first used all available data to construct an age pattern model that captured the prevalence age-trend in the data, which was used to split data spanning an age range greater than 25 years into narrower age bins. Then we modeled the prevalence of visible goiter using the new age split data. In this model, we introduced several new assumptions: visible goiter incidence can be non-decreasing across age (i.e. we removed a decreasing slope prior), a small amount of remission is possible, and birth prevalence is not possible. These assumptions were based on evidence in the literature showing that the highest levels of visible goiter are in middle aged people and were prompted by observing that the previously strict parameters were limiting the predictive power of the model. We also used proportion of households using iodized salt and sodium intake as country-level covariates, with sodium intake being new for GBD 2019. The coefficients for these covariates are in the table below.

Table 2. Visible goiter covariates. Summary of covariates used in the visible goiter DisMod-MR meta-regression model

Covariate	Type	Parameter	Exponentiated beta (95% UI)
Proportion of households using iodized salt	Country-level	Prevalence	0.0028 (0.0024 – 0.0034)
Sodium intake	Country-level	Prevalence	1.11 (1.08-1.13)

For GBD 2019, no changes were made to the strategy for the intellectual disability model. Consistent with the GBD 2017 approach, we estimated the prevalence of intellectual disability due to iodine deficiency (cretinism) by regressing data points from studies reporting both cretinism and goiter prevalence in the same population. To do so, we first transformed cretinism prevalence and goiter prevalence into logit space, regressed the logit prevalence of cretinism on the logit prevalence of goiter, and predicted for all national locations using the goiter estimates from the DisMod-MR 2.1 model above. We dropped locations with total goiter prevalence less than 20% and locations with household iodised salt consumption greater than 90%. We kept observations in children younger than 5 years and used these data as incidence input in a second DisMod-MR 2.1 to generate location-year-age-sex-specific estimates. This was combined with relative risk (RR) and standardised mortality ratio (SMR) data on intellectual disability identified in the literature review described above. We modeled with zero remission, zero incidence after age 5, and proportion of households using iodized salt as a covariate on incidence (Table 3). We repeated the dropout criteria of total goiter prevalence and iodised salt consumption on the DisMod-MR 2.1 output.

Table 3. Intellectual disability due to iodine deficiency covariates. Summary of covariates used in the intellectual disability due to iodine deficiency DisMod-MR meta-regression model

Covariate	Type	Parameter	Exponentiated beta (95% UI)
Proportion of households using iodized salt	Country-level	Incidence	0.14 (0.14-0.14)

The severity split distribution did not change for GBD 2019. Initial severity proportions are: visible goiter without symptoms of thyroid dysfunction (proportion=0.915, 95% confidence interval (CI): 0.904–0.926); goiter with symptoms of thyroid dysfunction (proportion=0.085, 95% confidence interval (CI): 0.084–0.086). Additionally, we split the intellectual disability due to iodine deficiency model into severe and profound ID using ID proportion assumptions. Everyone with ID is assumed to have thyroid dysfunction, while heart failure is assumed to only occur in people with profound intellectual disability (which we split into mild, moderate and severe heart failure). Heart failure attributable to iodine deficiency was modelled separately, and the methods for this outcome are presented separately in the section for heart failure and its etiologies. Table 4 provides details on the severity states downstream of iodine deficiency.

Table 4. Severity distribution, details on the severity levels for iodine deficiency in GBD 2019 and the associated disability weight (DW) with that severity.

<table>
<tr><th>Sequela</th><th>Health state name</th><th>Lay description</th><th>Disability weight</th></tr>
<tr><td>Visible goiter without symptoms</td><td>Disfigurement, level 1</td><td>has a slight, visible physical deformity that others notice, which causes some worry and discomfort.</td><td>0.011
(0.005–0.021)</td></tr>
<tr><td>Visible goiter with symptoms without intellectual disability or heart failure</td><td>Iodine-deficiency goiter</td><td>has a large mass in the front of the neck. The person sometimes has weakness and fatigue, constipation and weight gain.</td><td>0.199
(0.133–0.276)</td></tr>
<tr><td rowspan="2">Visible goiter with severe intellectual disability due to iodine deficiency</td><td>Intellectual disability / mental retardation, severe</td><td>has very low intelligence and cannot speak more than a few words, needs constant supervision and help with most daily activities, and can do only the simplest tasks.</td><td rowspan="2">0.326
(0.233–0.438)*</td></tr>
<tr><td>Iodine-deficiency goiter</td><td>(see above)</td></tr>
<tr><td rowspan="2">Visible goiter with profound intellectual disability due to iodine deficiency</td><td>Intellectual disability / mental retardation, profound</td><td>has very low intelligence, has almost no language, and does not understand even the most basic requests or instructions. The person requires constant supervision and help for all activities.</td><td rowspan="2">0.358
(0.252–0.475)*</td></tr>
<tr><td>Iodine-deficiency goiter</td><td>(see above)</td></tr>
<tr><td rowspan="3">Visible goiter with profound intellectual disability and mild heart failure due to iodine deficiency</td><td>Intellectual disability / mental retardation, profound</td><td>(see above)</td><td rowspan="3">0.384
(0.276–0.502)*</td></tr>
<tr><td>Iodine-deficiency goiter</td><td>(see above)</td></tr>
<tr><td>Heart failure, mild</td><td>is short of breath and easily tires with moderate physical activity, such as walking uphill or more than a quarter-mile on level ground. The person feels comfortable at rest or during activities requiring less effort.</td></tr>
<tr><td rowspan="3">Visible goiter with profound intellectual disability and moderate heart failure due to iodine deficiency</td><td>Intellectual disability / mental retardation, profound</td><td>(see above)</td><td rowspan="3">0.403
(0.293–0.524)*</td></tr>
<tr><td>Iodine-deficiency goiter</td><td>(see above)</td></tr>
<tr><td>Heart failure, moderate</td><td>is short of breath and easily tires with minimal physical activity, such as walking only a short distance. The person feels comfortable at rest but avoids moderate activity.</td></tr>
<tr><td rowspan="3">Visible goiter with profound intellectual disability with severe heart failure due to iodine deficiency</td><td>Intellectual disability / mental retardation, profound</td><td>(see above)</td><td rowspan="3">0.471
(0.344–0.602)*</td></tr>
<tr><td>Iodine-deficiency goiter</td><td>(see above)</td></tr>
<tr><td>Heart failure, severe</td><td>is short of breath and feels tired when at rest. The person avoids any physical activity, for fear of worsening the breathing problems.</td></tr>
</table>

Dietary Iron Deficiency

Flowchart

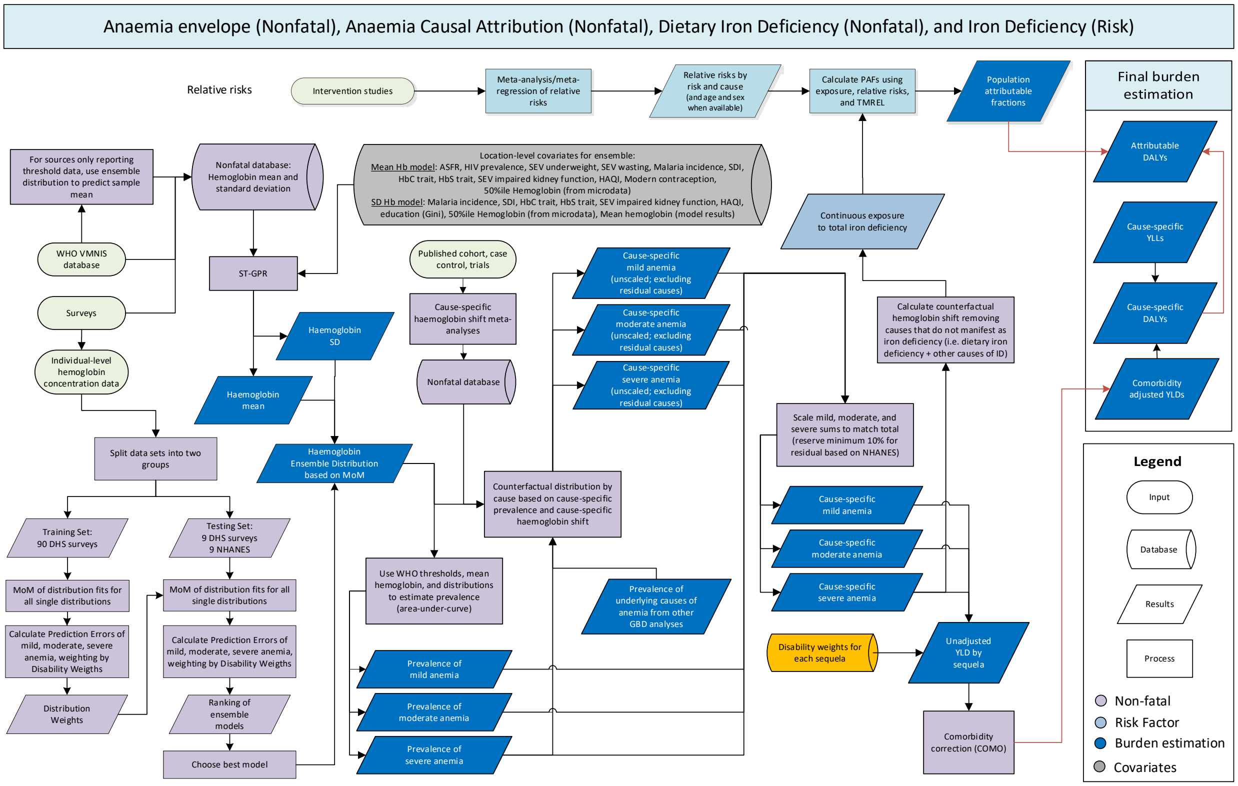

Case definition

Dietary iron deficiency in the GBD cause analysis is defined as inadequate iron to meet the body's needs due to inadequate dietary intake of iron, but not due to other causes of absolute or functional iron deficiency.

Methodological summary

Dietary iron deficiency was quantified as an output of the GBD Anaemia Causal Attribution framework. The GBD anaemia model has two main steps – estimation of the anaemia envelope and causal attribution – both of which inherently impact estimates of iron deficiency. See the methodological description of "Anaemia (Impairment)" for detailed description of the analytic approach and inputs.

Briefly, the first step is estimating anaemia envelope – the prevalence of mild, moderate, and severe anaemia prevalence for each GBD location, age-group, sex, and year. The inputs to the envelope model are mean and standard deviation (SD) of haemoglobin concentration, each of which are modeled in ST-GPR. Individual level data sources are then used to develop a set of ensemble distribution weights using method of moments, which are then paired with mean and SD model results to produce estimates of the entire distribution of haemoglobin for each population group. A population group is a specific geography, sex, age-group, and year combination. The second step is anaemia causal attribution, the approach for which was revised in GBD 2019 to, instead of Bayesian contingency table modeling, generate counterfactual haemoglobin distributions for each cause of anaemia based on the cause-level

prevalence (or incidence, in the case of maternal haemorrhage) estimates from the respective GBD analyses and cause-specific haemoglobin shifts that were determined via meta-analysis for each cause. The counterfactual distribution methods used the same ensemble distribution weights as the overall anaemia envelope because there is inadequate data to guide alternate distributions for each subcause. Mild, moderate, and severe anaemia were assigned to each cause based on the difference between the counterfactual and observed haemoglobin distributions in each population group. The sum of severity-specific prevalence was then summed to match the total, with a minimum residual of 10%,[1,2] and then the remainder was distributed between five GBD causes using fixed proportion redistribution methods: 1) dietary iron deficiency (GBD cause), 2) other haemoglobinopathies and haemolytic anaemias, 3) other infectious diseases, 4) other neglected tropical disease, and 5) endocrine, metabolic, blood, and immune disorders.

It is important to take note of the difference between "dietary iron deficiency" as a GBD cause and "iron deficiency" as a GBD risk. Many GBD causes lead to anaemia that clinically manifests as iron deficiency (or microcytosis), but where inadequate intake is not the underlying problem. Examples include neglected tropical diseases such as hookworm, malaria, and schistosomiasis, gastrointestinal disorders, cirrhosis, maternal haemorrhage, menstural disorders, uterine fibroids, and Vitamin A deficiency. The name "dietary iron deficiency" is intended to differentiate, therefore, between inadequate intake and haemorrhagic or disorders of iron metabolism. Additionally, because we have yet to include 100% of anaemia causes, estimates should be interpreted to also include some acute and chronic haemorrhagic states for which supplementation may be helpful, but poor nutritional intake is not the only underlying problem. Examples include malabsorption syndromes, other micronutrient deficiencies besides Vitamin A deficiency, and injuries with associated acute blood loss anaemia. "Iron deficiency" exposure as estimated for the GBD risk factors analysis, in contrast, includes a combined assessment of the magnitude of haemotologic insult from all causes that manifest as iron deficiency. As mentioned above, our goal is to systematically add all causes of anaemia as specific inputs to GBD Anaemia Causal Attribution, including inadequate iron intake, and eliminate the need for residual attribution.

References

1 Centers for Disease Control and Prevention (CDC). Iron deficiency--United States, 1999-2000. *MMWR Morb Mortal Wkly Rep* 2002; **51**: 897–9.

2 Looker AC, Dallman PR, Carroll MD, Gunter EW, Johnson CL. PRevalence of iron deficiency in the united states. *JAMA* 1997; **277**: 973–6.

3 Murray-Kolb LE, Chen L, Chen P, Shapiro M, Caulfield L. CHERG Iron Report: Maternal Mortality, Child Mortality, Perinatal Mortality, Child Cognition, and Estimates of Prevalence of Anemia due to Iron Deficiency | GHDx. 2013. http://ghdx.healthdata.org/record/cherg-iron-report-maternal-mortality-child-mortality-perinatal-mortality-child-cognition-and (accessed Nov 12, 2019).

Protein-energy malnutrition

Flowchart

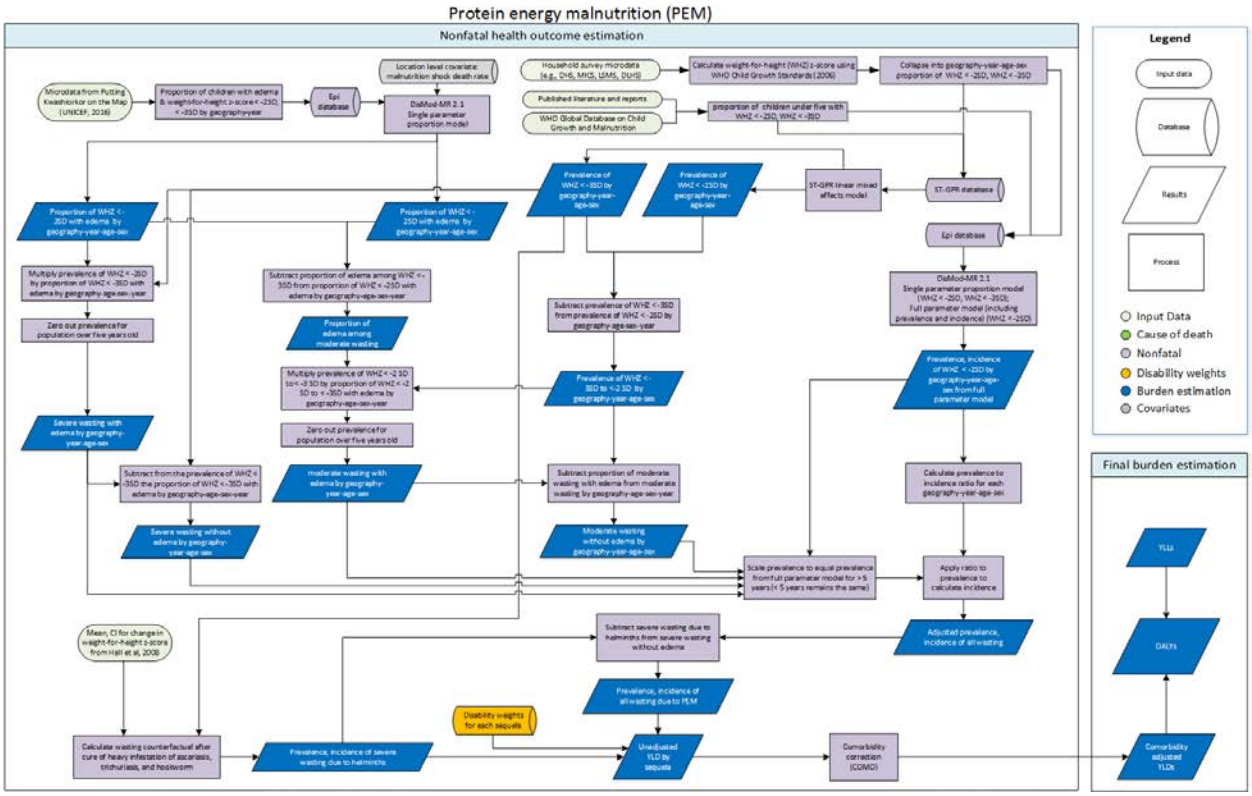

Case Definition

Protein-energy malnutrition (PEM) includes moderate and severe acute malnutrition, commonly referred to as "wasting," and was defined in terms of weight-for-height Z-scores (WHZ) on the WHO 2006 growth standard for children. We quantified non-fatal PEM burden in four mutually exclusive and collectively exhaustive categories, reflecting distinct gradations of disability that can occur: moderate wasting ***without oedema*** (WHZ < -2SD to < -3 SD), moderate wasting ***with oedema*** (WHZ < -2SD to < -3 SD), severe wasting ***without oedema*** (WHZ < -3SD), and severe wasting ***with oedema*** (WHZ < -3SD). The aggregate of categories that include "oedema" can be considered equivalent to the disease state commonly referred to as "kwashiorkor" and severe wasting can likewise be considered equivalent to "marasmus." For PEM, ICD 10 codes are E40-E46.9, E64.0, and ICD 9 codes are 260-263.9.

This classification reflects a moderate shift from GBD 2015, when moderate wasting without oedema was not included in our non-fatal estimates, and by definition is associated with higher prevalence estimates than previously published by GBD. The other GBD 2015 categories – kwashiorkor, marasmus, and severe wasting – have unchanged case definitions, but have been renamed for clarity and consistency. This revised GBD 2016 case definition more closely aligns with other and allows for better application to the international nutrition community's programming and estimates related to non-fatal PEM. This change has been continued into GBD 2019.

Input data & Data Processing

The input data for this model come in two primary streams. First, we used individual-level and tabulated child anthropometry data from health surveys, literature, and national reports, and centralised them to inform the prevalence of WHZ decrement in each category corresponding to our case definitions. For details on estimation of wasting (WHZ <-2 and WHZ <-3) data identification and processing, see the methodological description of "Child Growth Failure" in the GBD 2019 Risk Factors appendix. Second, to inform the proportion of children under 5 years who have signs of organ failure manifested as oedema (ie, kwashiorkor), we used a compiled dataset of surveys conducted using Standardized Monitoring and Assessment of Relief and Transitions (SMART) methods. All data were extracted with the most detailed standard demographic identifiers available, including age, sex, country, year, and subnational location if available. No alternate case identifications were identified for oedema data so no crosswalks were required or performed.

Table 1: Data Inputs for PEM modelling by parameter.

Measure	Total sources	Countries with data
All measures	1687	158
Prevalence	288	92
Proportion	1443	151
Continuous	970	142

Modelling Strategy

We used five parallel models to inform our estimates, all of which produced age-sex-specific results: 1) Prevalence of WHZ <-2 in children under 5 years in ST-GPR, 2) Prevalence of WHZ <-3 in children under 5 years in ST-GPR, 3) Proportion of those with WHZ <-2 who have oedema in under 5 years in DisMod-MR 2.1, 4) Proportion of those with WHZ <-3 who have oedema in under 5 years in DisMod-MR 2.1, and 5) Prevalence, incidence, and excess mortality of WHZ <-2 in all ages in DisMod-MR 2.1.

Using available information from scientific publications, which suggest the mean duration of illness is nine months, and conversations with collaborators and nutrition experts, we applied what we consider a plausible set of remission rate bounds of 0.25–1.25 (# of remitted cases of PEM per person-year of illness) to the final of the five models. These bounds allowed DisMod to mathematically derive an internally consistent solution for incidence, prevalence, remission, excess mortality, and cause-specific mortality using all available data. This could only be done for the aggregate PEM definition (prevalence of WHZ <-2) to ensure that the case definition for prevalence matched that of the mortality results. The incidence-to-prevalence ratio derived from the final model was applied equally across all the categories of non-fatal PEM. Future work in systematically evaluating longitudinal datasets on nutrition and growth failure will allow us to improve the empirical basis for PEM incidence estimates, including improved resolution for the component categories.

For details on estimation of wasting (WHZ <-2 and WHZ <-3) estimation, see the methodological description of "Child Growth Failure" in the GBD 2019 Risk Factors appendix. Location-level covariate effects for each of the three DisMod-MR 2.1 models are shown in the tables below.

Table 2a: Location-level covariate effects for proportion of oedema among total wasting

Measure	Covariate	Beta value	Exponentiated
Proportion	Energy unadjusted (kcal)	-1 (-1 - -1)	0.37 (0.37–0.37)
Proportion	Malnutrition shock log-transformed mortality rate	1 (1 - 1)	2.72 (2.72 – 2.72)

Table 2b. Location-level covariate effects for proportion of oedema among severe wasting

Measure	Covariate	Beta Value	Exponentiated
Proportion	energy unadjusted(kcal)	-1 (-1 - -1)	0.37 (0.37–0.37)
Proportion	Malnutrition shock log-transformed mortality rate	1 (1 - 1)	2.72 (2.72–2.72)

Table 2c. Location-level covariate effects for total wasting (moderate + severe, with and without oedema)

Measure	Covariate	Beta Value	Exponentiated
Prevalence	Sanitation (prop access)	-0.033 (-0.045 — -0.022)	0.97 (0.96 — 0.98)
Prevalence	Socio-demographic Index	-0.025 (-0.089 - -0.00088)	0.98 (0.91 — 1.00)
Prevalence	Malnutrition Shock, log-trans mortality rate	0.00044 (0.000016 — 0.0017)	1.00 (1.00 — 1.00)
Excess mortality rate	Healthcare Access and Quality index	-0.038 (-0.04 - -0.036)	0.96 (0.96 – 0.96)

The results of the first four models were used for children under 5 years. Arithmetic transformations were performed to ensure that the final results fit into the mutually exclusive, collectively exhaustive categories of moderate and severe wasting, with and without oedema. We assumed zero prevalence of oedema in people over 5 years old. The results of the final model were used for all age groups 5 years and older and the proportion of moderate versus severe wasting in each of those age groups was derived from the first set of models.

As a final step, we subtracted a number of cases of PEM where the underlying aetiology is severe worm infestation. See the appendix section on "Neglected Tropical Diseases" for more details of that process. Briefly, because both worms and PEM can cause wasting, we needed to divide out the wasting envelope to attribute wasting to both PEM and worms. We determined the amount of wasting attributable to worms by referencing Hall and colleagues 2008[1] to determine the mean and confidence interval estimates of the z-score shift. We then calculated the counterfactual wasting prevalence given no worms, according to the z-score shift. From this, we calculated the fraction of wasting that is attributable to worms and assigned the remainder of wasting to PEM. We assumed no oedema due to worms and the same prevalence-to-incidence ratio as in each of the other models.

We applied disability weights from the GBD disability weight survey to the prevalence of the above sequelae according to their corresponding health state and severity level. The sequelae, along with their lay descriptions and disability weights for health states derived from the GBD disability weights study, are shown below. We assumed that those with moderate wasting, but no oedema, did not have any direct disability due to this condition.

Table 3. Sequelae, severity, lay description, and DWs

<table>
<tr><th>Sequela</th><th>Health state name</th><th>Lay description</th><th>DW (95% CI)</th></tr>
<tr><td>Moderate wasting without oedema</td><td>Asymptomatic</td><td>--</td><td>--</td></tr>
<tr><td>Moderate wasting with oedema</td><td>Kwashiorkor</td><td>Is very tired and irritable and has diarrhoea.</td><td>0.051 (0.031–0.079)</td></tr>
<tr><td>Severe wasting without oedema</td><td>Severe wasting</td><td>Is extremely skinny and has no energy.</td><td>0.128 (0.082–0.183)</td></tr>
<tr><td rowspan="2">Severe wasting with oedema</td><td rowspan="2">Kwashiorkor + severe wasting</td><td>Is very tired and irritable and has diarrhoea.</td><td>0.051 (0.031–0.079)</td></tr>
<tr><td>Is extremely skinny and has no energy.</td><td>0.128 (0.082–0.183)</td></tr>
</table>

Following the assignment of disability weights to the various sequelae, the resulting years lived with disability (YLDs) go through the comorbidity simulator, which accounts for any comorbidity and corrects accordingly. The final outputs are comorbidity-adjusted YLDs, which are combined with years of life lost (YLLs) for final disability-adjusted life-years (DALYs).

References

1 Hall A, Hewitt G, Tuffrey V, de Silva N. A review and meta-analysis of the impact of intestinal worms on child growth and nutrition. *Matern Child Nutr* 2008; **4 Suppl 1**: 118–236.

Other nutritional deficiencies

Other nutritional deficiencies encompass a wide variety of causes of morbidity, ranging from vitamin deficiencies to other nutritional anaemias. In GBD 2019, as done previously, we treat these causes as a single category, given their relatively limited burden, diversity in underlying causes and risk factors, and data availability. Instead of modelling them in a traditional modelling format, we calculate the YLDs associated with other nutritional deficiencies using a YLD/YLL ratio.

The first input for this non-fatal portion of other nutritional deficiencies burden is the YLL estimates from the GBD 2019 causes of death (CoD) analysis. The causes and their associated ICD-10 codes that constitute other nutritional deficiencies for CoD are listed below. Additionally, CoD includes specific models for protein-energy malnutrition, another nutritional cause of morbidity and mortality; as protein-energy malnutrition has a specific non-fatal model that results in YLDs, we can calculate the YLD/YLL ratio for protein-energy malnutrition. We multiply the YLL estimates for other nutritional deficiencies from CoD by the YLD/YLL ratio for PEM, providing us with an estimate of the YLDs associated with other nutritional deficiencies. There were no changes in modeling strategy for other nutritional deficiencies from GBD 2017.

Table 1. Definitions, ICD-10 codes and descriptions included in the other nutritional deficiencies model

GBD cause	ICD-10 code
Other nutritional deficiencies	D51-D52.0 (vitamin B12 deficiency anaemia and folate deficiency anaemia)
Other nutritional deficiencies	D52.8-D53.9 (other nutritional anaemias)
Other nutritional deficiencies	D64.3 (other sideroblastic anaemias)
Other nutritional deficiencies	E51-E61.9 (thiamine, niacin, other B group vitamins, ascorbic acid, vitamin D, other vitamin, dietary calcium, dietary selenium, dietary zinc, and other nutrient element deficiencies)
Other nutritional deficiencies	E63-E64.0 (other nutritional deficiencies and sequelae of protein-calorie malnutrition)
Other nutritional deficiencies	E64.2-E64.9 (sequelae of vitamin C deficiency, rickets, other nutritional deficiencies, and unspecified nutritional deficiencies)
Other nutritional deficiencies	M12.1-M12.19 (Kaschin-Beck disease)

Neoplasms

The general framework for the GBD 2019 cancer estimation applies to all malignant neoplasms (i.e. cancers) except for: non-melanoma skin cancer (basal cell carcinoma and squamous cell carcinoma); benign and in situ neoplasms (which include intestinal, cervical and uterine, and other benign neoplasms); and myelodysplastic, myeloproliferative, and other hematopoietic neoplasms.

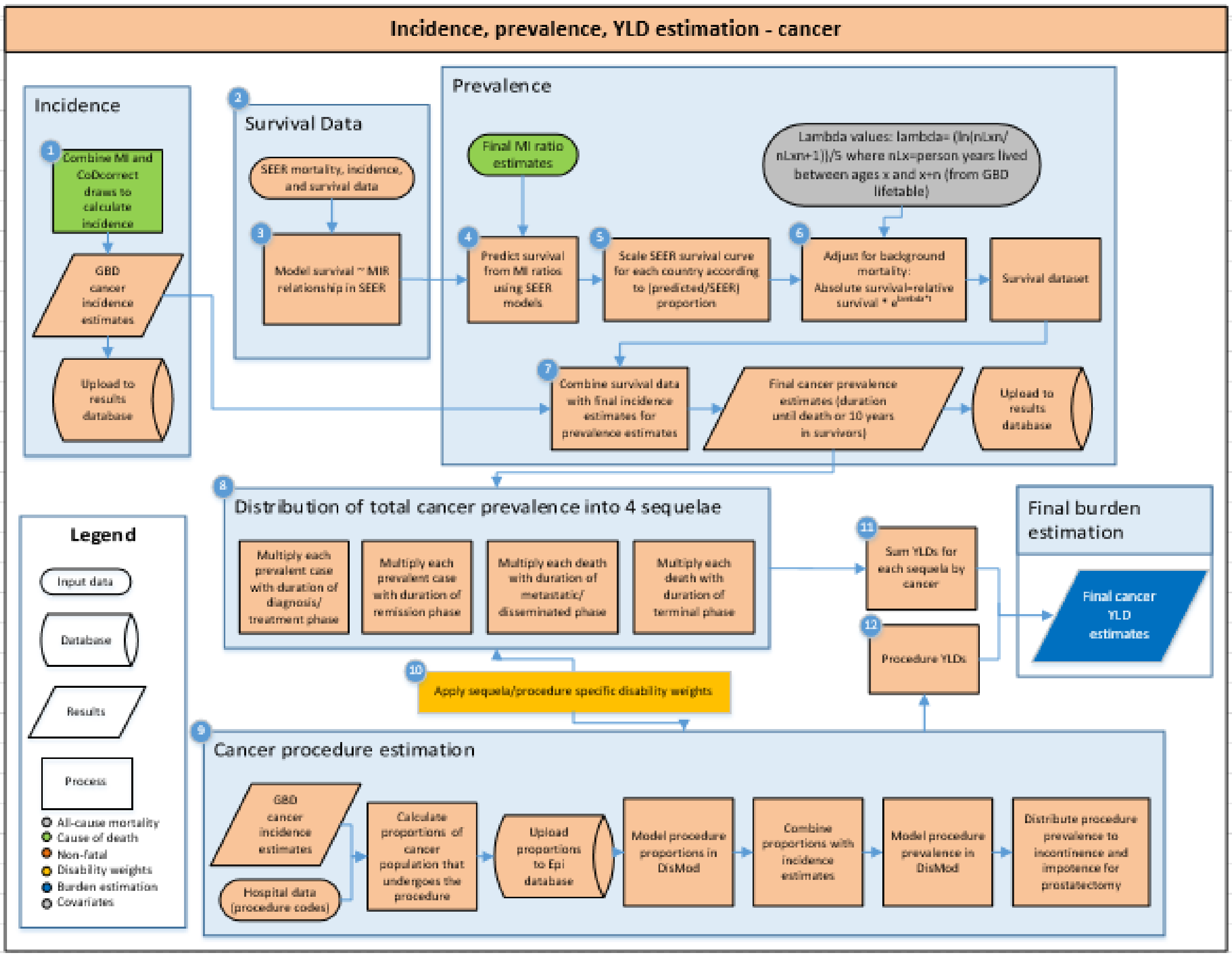

Input data and methodological appendix

Case definition

For GBD 2019, incidence, prevalence, and disability are estimated for all cancers and benign neoplasms as defined in ICD-10 (C00-D49). The associated ICD codes for neoplasms estimated for GBD 2019 are listed in Appendix Table 4. Prevalence for all cancers is estimated for a maximum of 10 years after incidence, as in GBD 2013, GBD 2015, GBD 2016, and GBD 2017. Prevalence extending beyond the 10year period is only estimated for permanent sequelae resulting from five treatment-related surgical procedures (cystectomy, laryngectomy, mastectomy, prostatectomy, and stoma).

To estimate disability for each cancer, total prevalence is split into four sequelae: 1. diagnosis and primary therapy; 2. controlled phase; 3. metastatic phase; and 4. terminal phase. The diagnosis and primary therapy phase is defined as the time from the onset of symptoms to the end of treatment. The controlled phase is defined as the time between finishing primary treatment and the earliest of either: cure (defined as recurrence- and progression-free survival after 10 years); death from another cause; or progression to the metastatic phase. The metastatic phase is defined as the time period of intensive treatment for metastatic disease, as determined for each cancer by SEER (Surveillance, Epidemiology, and End Results Program) averages (Table 1). The terminal phase is defined as the one-month period prior to death. Each of these four sequelae has a separate disability weight, which are the same across cancer types (Table 3: Lay description and disability weights). Because of long-term disability associated with treatment-related procedures, additional disability beyond these four sequelae is estimated for five cancers: breast cancer (disability due to mastectomy), larynx cancer (disability due to laryngectomy), colon and rectum cancer (disability due to stoma), bladder cancer (disability due to incontinence from cystectomy), and prostate cancer (disability due to either incontinence or impotence from prostatectomy).

Input data

Cancer incidence is directly estimated from cancer mortality using mortality to incidence ratios (MIRs). Data sources for cancer mortality are described in detail elsewhere.[1] To estimate the proportion of cancer patients undergoing surgical procedures we used SEER data form 1983 to 2008[2] and Mexico Hospital Data from 2001 to 2009[3]. Data sources used to adjust procedure sequelae will be listed below.

Table 1a. Data Inputs for neoplasms morbidity modelling by parameter.

Cause	Prevalence sources	Incidence sources	Deaths sources	All measures sources
Neoplasms	299	4329	5489	8574
Esophageal cancer	3	3305	5336	7460
Stomach cancer	3	3316	5211	7335
Liver cancer	3	3361	5352	7800
Larynx cancer	3	3311	5236	7325
Tracheal, bronchus, and lung cancer	3	3341	5390	7514
Breast cancer	3	3365	5362	7539
Cervical cancer	3	3303	5193	7312
Uterine cancer	3	3311	5168	7290
Prostate cancer	3	3293	5204	7305

Colon and rectum cancer	3	3357	5354	7523
Lip and oral cavity cancer	3	2909	4656	6786
Nasopharynx cancer	3	3314	4938	7078
Other pharynx cancer	3	3221	4872	6986
Gallbladder and biliary tract cancer	3	3283	4926	7009
Pancreatic cancer	3	3359	4985	7157
Malignant skin melanoma	3	3245	4910	7042
Non-melanoma skin cancer	0	1434	3462	3462
Ovarian cancer	3	3325	4959	7099
Testicular cancer	3	3215	4854	6970
Kidney cancer	3	3209	4897	6991
Bladder cancer	3	2997	4500	6707
Brain and central nervous system cancer	3	3339	5131	7292
Thyroid cancer	3	3355	4985	7151
Mesothelioma	3	1329	2020	3226
Hodgkin lymphoma	3	3318	4975	7114
Non-Hodgkin lymphoma	3	3537	4581	7472
Multiple myeloma	3	3265	4329	6413
Leukemia	3	3539	5107	7531
Other malignant neoplasms	3	3466	5271	7390
Other neoplasms	296	0	2630	2922

Table 1b. Data Inputs for liver cancer subtypes morbidity modelling by parameter.

**Cause**	**Proportion data sources**
Neoplasms	268
Liver cancer due to hepatitis B	267
Liver cancer due to alcohol use	96
Liver cancer due to other causes (internal)	55

Modelling strategy

Estimation of cancer mortality and MIR estimation has been described in the GBD 2019 Mortality and Causes of Death capstone paper. The final GBD cancer mortality estimates are transformed to incidence estimates by using MIRs (which are modeled separately). To summarize the MIR estimation process: incidence and mortality data from cancer registries were matched by cancer, age, sex, year, and location to generate M/I ratios. These MIR data were used to fit cause-specific fixed effect logistic regression models with covariates for sex, categorical age, and the Healthcare-access and quality index (HAQ index) [4]:

$$\text{logit}\left(MI\ ratio_{c,a,s,t}\right) = \alpha + \beta_1 HAQI_{c,t} + \sum_{a}^{A} \beta_2 I_a + \beta_3 I_s + \epsilon_{c,a,s,t}$$

c: country, a: age group, t: time (years); s: sex
HAQI: Healthcare access and quality index
I: indicator variable
$\epsilon_{c,a,s,t}$: error term

These models were then used to obtain MIR estimates for all combinations of GBD age, sex, year, cause, and location. Data points were outliered manually if they clearly influenced the model in an unrealistic way. For example, a data point was marked as an outlier if it created a single-year, single age group spike in model predictions that was inconsistent with the trend suggested by surrounding data points. Results from the final linear model were used as input for space-time smoothing and a Gaussian Process Regression (ST-GPR). The ST-GPR process has been updated for GBD 2019 to utilize more MIR input data (by lessening the inclusion criteria for MIR data from 25 incident cases to 15) and to perform more smoothing across age and time (by adjusting modeling hyperparameters that control the weighting of adjacent data values).

Final MIR estimates at the 1000-draw level were combined with final mortality estimates (also at the 1000-draw level) to generate 1000 draws of incidence estimates (which provides an estimated mean incidence with 95% uncertainty interval). It was assumed that uncertainty in the MIR is independent of uncertainty in the estimated mortality.

After transforming the final GBD cancer mortality estimates to incidence estimates (step 1 in the general cancer flowchart), incidence was combined with annual relative survival estimates from 1 to 10 years (step 7 in the flowchart). Our survival estimation methods were first implemented in GBD 2017 to more directly utilize MIRs to generate yearly cancer relative survival estimates; for GBD 2019 we updated these methods to utilize age-specific rather than all-ages survival curves. Previous reports suggest that the value of (1 – MIR) may serve as a proxy for 5-year relative survival, with the exact correlation varying slightly by cancer type.[5] We used SEER*Stat[6] to obtain mortality, incidence, and relative survival statistics from the 9 SEER registries[7] reporting from 1980-2014 (step 2), by cancer type, sex, 5-year blocks (i.e., 1980-84, 1985-1989, etc.), and 5-year age groups (except combining 80+). For each cancer, we modelled 5-year relative survival with the SEER MIRs. For GBD 2019 we updated this model from the Poisson regression used in GBD 2017 to using a generalized linear model with a quasibinomial family and logit link, weighted by the number of index cases (step 3). To reduce variability due to small samples, we only included MIRs based

on at least 25 incident cases (except for the rarer cancers mesothelioma, nasopharyngeal cancer, and acute myeloid leukemia, where MIRs based on at least 10 cases were included). These models were then applied to the GBD MIR estimates to predict an estimated 5-year survival for each age/sex/year/location (step 4). To prevent unrealistic values, predicted 5-year survival values were winsorized to be between 0% and 100% survival. Unlike GBD 2017, we did not require the estimated survival to be greater than the all-ages worst-case survival scenario from SurvCan and US 1950 survival data[8,9], since age-specific survival could be plausibly lower than for these all-ages scenarios.). To generate yearly survival estimates up to 10 years, for GBD 2019 we downloaded SEER sex- and age-specific annual 1- through 10-year relative survival data from patients diagnosed between 2001 and 2010 (compared to GBD 2017 where we downloaded all-ages survival data from 2004).[10] The proportion of the predicted GBD 5-year survival estimate to the SEER 5-year survival statistic was calculated as a scalar, and then used to generate yearly survival estimates by scaling the 1-10 year SEER curve to the GBD survival predictions under the proportional hazard assumption (step 5). This change from GBD 2017 (where we used SEER all-ages data from 2004 as the scalar and survival curve) impacts prevalence and YLD estimation, generally leading to survival estimates that are higher for younger ages and lower for older ages compared to estimates using the all-ages curve.

To transform relative to absolute survival (adjusting for background mortality), GBD 2019 lifetables were used (step 6 and 7 in the flowchart) to calculate lambda values: lambda= (ln(nLxn/nLxn+1))/5, where nLx=person years lived between ages x and x+n (from GBD lifetable). Absolute survival was then calculated using an exponential survival function (absolute survival = relative survival * $e^{lambda*t}$). Absolute survival is combined with incidence to estimate the prevalence at each year after diagnosis, which is then split into the four sequelae (step 8 in the flowchart).

For the purposes of calculating disability due to cancer, survivors beyond 10 years were considered cured. For this group, the survivor population prevalence was divided into two sequelae (1. diagnosis and primary therapy; 2. controlled phase). For the population that did not survive beyond 10 years, the yearly prevalence was divided into the four sequelae by assigning the fixed durations for each of the diagnosis and primary therapy phase, metastatic phase, and terminal phase, and assigning the remaining prevalence to the controlled phase (step 8 in the flowchart). Duration of these four sequelae remained the same as for GBD 2013, GBD 2015, GBD 2016, and GBD 2017.[11] Table 1 lists the duration of each, along with the sources used to determine their length.

Table 2. Duration of four prevalence sequelae by cancer

	Diagnosis/ Treatment (months)	Remission	Disseminated/metastatic (months)	Note	Terminal (months)
Esophageal cancer	5[12]	Calculated based on remainder of time after attributing	4.6[10]	SEER Summary Stage 1997 (Distant site/node involved) 1995-2000	1 months
Stomach cancer	5.2[12]		3.88[10]	SEER Summary Stage 1997 (Distant site/node involved) 1995-2000	

Liver cancer	4	other sequelae.	2.51[10]	SEER Summary Stage 1997 (Distant site/node involved) 1995-2000
Larynx cancer	5.3[12]		8.84[10]	SEER Stage IVc
Lung cancer	3.3[13]		4.51[10]	SEER Summary Stage 1997 (Distant site/node involved) 1995-2000
Breast cancer	3[13]		17.7[10]	SEER Summary Stage 1997 (Distant site/node involved) 1995-2000
Cervical cancer	4.8[12]		9.21[10]	SEER Summary Stage 1997 (Distant site/node involved) 1995-2000
Uterine cancer	4.6[12]		11.6[10]	SEER Summary Stage 1997 (Distant site/node involved) 1995-2000
Prostate cancer	4[13]		30.35[10]	SEER Summary Stage 1997 (Distant site/node involved) 1995-2000
Colorectal cancer	4[13]		9.69[10]	SEER Summary Stage 1997 (Distant site/node involved) 1995-2000
Oral cancer	5.3[12]		9.33[10]	SEER Stage IVc
Nasopharyngeal cancer	5.3[12]		13.19[10]	SEER Stage IVc
Cancer of other part of pharynx	5.3[12]		7.91[10]	SEER Stage IVc
Gallbladder cancer	4		3.47[10]	SEER Summary Stage 1997 (Distant site/node involved) 1995-2000
Pancreas cancer	4.1[12]		2.54[10]	SEER Summary Stage 1997 (Distant site/node involved) 1995-2000
Melanoma	2.9[14]		7.18[10]	SEER Summary Stage 1997 (Distant site/node involved) 1995-2000
Ovarian cancer	3.2[13]		25.6[10]	SEER Summary Stage 1997 (Distant site/node involved) 1995-2000
Testicular cancer	3.7[12]		19.47[10]	SEER Stage III
Kidney cancer	5.3[12]		5.38[10]	SEER Summary Stage 1997 (Distant site/node involved) 1995-2000

Bladder cancer	5.1[12]		5.8[10]	SEER Summary Stage 1997 (Distant site/node involved) 1995-2000	
Brain cancer	5		6.93[10]	SEER Median age standardized survival all patients, all years	
Thyroid cancer	3		19.39[10]	SEER Stage IVc	
Mesothelioma	4		7.75[10]	SEER Summary Stage 1997 (Distant site/node involved) 1995-2000	
Hodgkin lymphoma	3.7[13]		26[15]		
Non Hodgkin lymphoma	3.7[13]		7.7[15]		
Multiple myeloma	7[12]		36.82[10]	SEER Median age standardized survival all patients, all years	
Leukemia[12]	5		43.67[10]	SEER Median age standardized survival all patients, all years	
ALL	12		7.02[10]	SEER Median age standardized survival all patients, all years	
AML	6		4.6[10]	SEER Median age standardized survival all patients, all years	
CLL	6		48[16]	SEER Median age standardized survival all patients, all years	
CML	6		4.6[10]	SEER Median age standardized survival for AML (patients with CML die in blast crisis, which is treated like AML) all patients, all years	
Leukemia other	6		48[16]	SEER Median age standardized survival all patients, all years	
Other	4.4 (mean of other cancer durations)		15.81[10]	SEER Median age standardized survival all patients, all years	

For cancer-specific procedure sequelae, hospital data were used to estimate the number of cancer patients undergoing mastectomy, laryngectomy, stoma, prostatectomy, and cystectomy (step 9 in the flowchart). These proportions remained the same as in GBD 2013, GBD 2015 GBD 2016, and GBD 2017.[11]

Proportions were generated by dividing the rate of procedures generated from the diagnostic codes in the hospital dataset and the coverage population by the GBD age-, and sex-specific disease incidence rates for that country. Diagnostic codes used are listed in Table 2:

Table 3. Procedure codes used to estimate cancer procedure proportions		
Procedure	Cancer	Procedure code (ICD-9_CM)
Mastectomy	Breast cancer	854, 8541, 8542, 8543, 8544, 8545, 8546, 8547, 8548
Laryngectomy	Larynx cancer	301, 303, 304, 3029
Stoma	Colon and rectum cancer	461, 4610, 4611, 4613, 4862
Cystectomy	Bladder cancer	5771, 5779
Prostatectomy	Prostate	603, 604, 605, 606, 6062

To estimate procedure-related disability for each of these five cancers, the procedure proportions (proportion of each cancer population that undergo these procedures) from hospital data were used as input for a proportion model in Dismod-MR 2.1 to estimate the proportions for all locations, by age, year, and by sex.

Since colostomy or ileostomy procedures are done for reasons other than cancer, a literature review was conducted to determine the proportion of ostomies due to colorectal cancer. Based on the results of the literature review that an average of 58% of ostomies are done for colorectal cancer, the “all cause” colostomy proportions were multiplied by 0.58.[17–19]

The final procedure proportions were applied to the incidence cases of the respective cancers and multiplied with the proportion of the incidence population surviving for 10 years to determine the incident cases of the cancer population that underwent procedures and that survived beyond 10 years. These incident cases were used again as an input for DisMod-MR 2.1, with a remission specification of zero and an excess mortality rate prior of 0 to 0.1, as well as with increasing the age of the population and the year by 10 years to reflect prevalence after that population has survived 10 years. The results from this model are incidence and lifetime prevalent cases of persons with these cancer-related sequelae who have survived beyond 10 years.

Since disability associated with prostatectomy comes from impotence and incontinence, and not from the prostatectomy itself, 18% of the prostatectomy prevalence was assumed to have incontinence and 55% was assumed to have impotence, based on a literature review done for GBD 2013.[20–27] Cases were assigned disability for either impotence or incontinence, but no cases were assigned disability from both.

We assumed that for the population surviving up to 10 years, only the prevalence population being in remission experiences additional disability due to procedures (e.g. a women suffering from metastatic breast cancer do not experience additional disability due to a mastectomy during this phase). To estimate the prevalence of the cancer population in remission during the first 10 years after diagnosis with and without procedure-related disability, we multiplied the prevalence of the population in the remission phase with the proportion of the population undergoing a procedure. This step allowed us to estimate disability during the remission phase for both the population experiencing disability due to the remission phase alone, as well as the population experiencing disability from the remission phase and the additional procedure-related disability.

Lastly, the procedure sequelae prevalence and general sequelae prevalence were multiplied with their respective disability weights (Table 3) to obtain the number of YLDs (steps 11 and 12 in the flowchart). The sum of these YLDs is the final YLD estimate associated with each cancer.

Table 4. Lay description and disability weights

Health state	Lay description	Estimate	Uncertainty interval	
Cancer, diagnosis and primary therapy (cancer_diagnosis)	This person has pain, nausea, fatigue, weight loss and high anxiety.	0.288	0.193	0.399
Cancer, controlled phase (generic_medication)	This person has a chronic disease that requires medication every day and causes some worry but minimal interference with daily activities.	0.049	0.031	0.072
Cancer, metastatic (cancer_metastatic)	This person has severe pain, extreme fatigue, weight loss and high anxiety.	0.451	0.307	0.600
Terminal phase, with medication (cancer_terminal_treat)	This person has lost a lot of weight and regularly uses strong medication to avoid constant pain. The person has no appetite, feels nauseous, and needs to spend most of the day in bed.	0.540	0.377	0.687
Mastectomy (cancer_mastectomy)	This person had one of her breasts removed and sometimes has pain or swelling in the arms.	0.036	0.020	0.057
Stoma (cancer_stoma)	This person has a pouch attached to an opening in the belly to collect and empty stools.	0.095	0.063	0.131
Laryngectomy (speech_problems)	This person has difficulty speaking, and others find it difficult to understand.	0.051	0.032	0.078
Urinary incontinence (incontinence)	This person cannot control urinating.	0.139	0.094	0.198
Impotence (impotence)	This person has difficulty in obtaining or maintaining an erection.	0.017	0.009	0.030

Non-melanoma skin cancer (squamous and basal cell carcinoma)

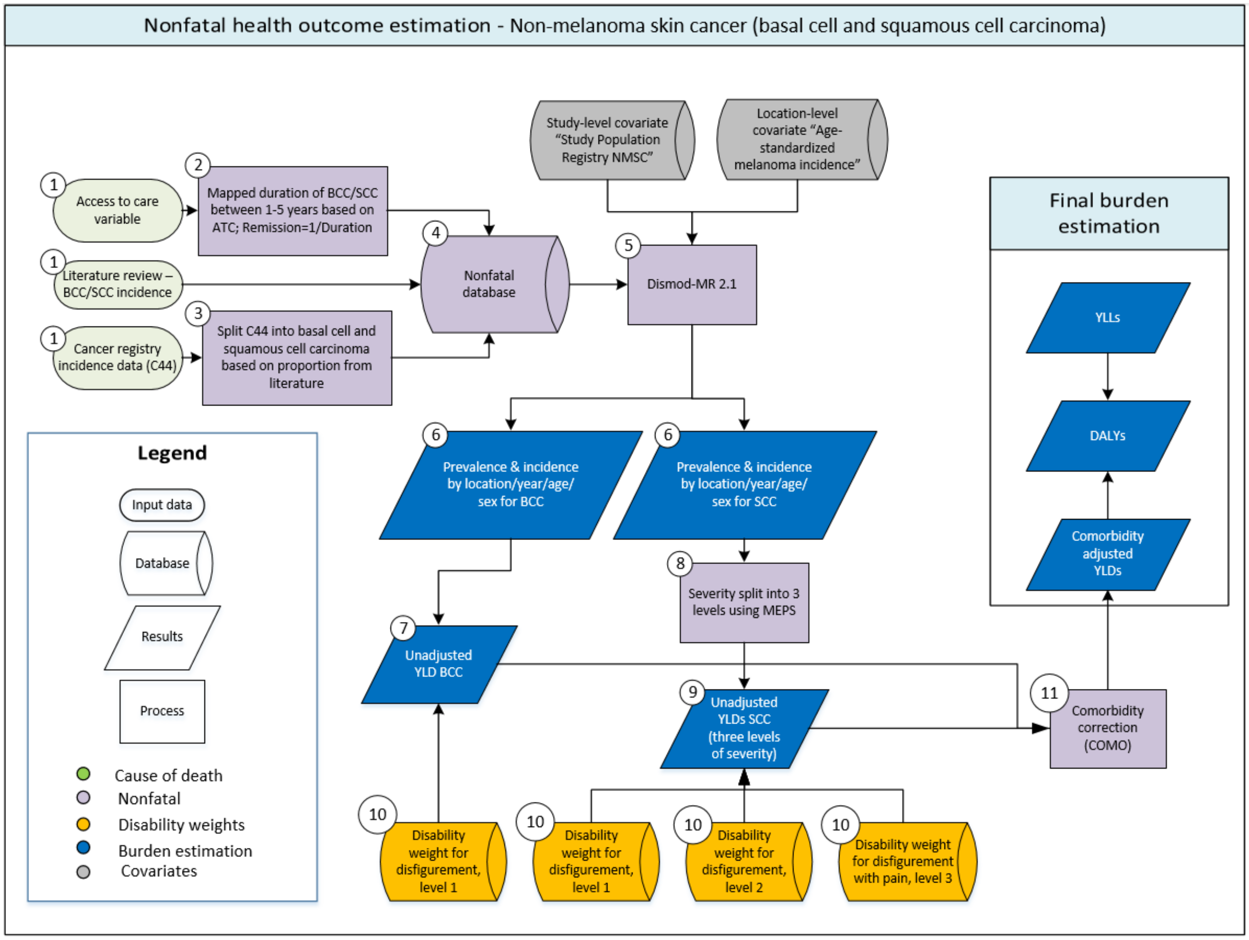

Case definition

Non-melanoma skin cancer (NMSC) is defined as basal cell carcinoma and squamous cell carcinoma. NMSC does not include other types of skin cancer (e.g. melanoma, Merkel cell carcinoma).

Input data

We estimated squamous cell and basal cell skin cancer incidence by using cancer registry as well as primary literature, and clinical informatics data (such as Marketscan) for incidence. Only cancer registries that were listed in CI5 VIII as registering squamous cell carcinoma or basal cell carcinoma, respectively, were included in the analysis. For 2019, the clinical data were adjusted for the healthcare access and quality index of the country, and accounts for outpatient encounters. This is a change from GBD 2017, where these data only included non-primary diagnoses in inpatient admissions. This change led to higher values in the input clinical informatics data compared to last year, as it now includes diagnoses from outpatient procedures that did not require hospital admission (whereas previously these data approximated the rate of inpatient admissions for cases with benign neoplasms who had access to hospitals).

Modelling strategy

For cancer registry data reported at the three digit level (i.e., C44: Other and unspecified malignant neoplasm of skin), proportions from Karagas et al were used to split C44 into squamous cell carcinoma and basal cell carcinoma.[28] The only new data we added compared to GBD 2017 was additional data from hospital and outpatient sources. DisMod-MR 2.1 was used to model incidence and prevalence. Prevalence was calculated as a function of two extreme scenarios (duration 1 versus 5 years). Country, age, sex and year-specific duration was estimated using a country-age-sex-year specific relative access-to-care-score.

The access to care score was based on the melanoma mortality to incidence ratio:

$$Access\ to\ care = 1 - \frac{Age\ standardized\ MIR_{cys} - Age\ standardized\ MIR_{min}}{Age\ standardized\ MIR_{max} - Age\ standardized\ MIR_{min}}$$

c=country; y=year; s=sex; Age-standardized MI ratio$_{min}$=lowest MIR for all countries and years; Age standardized MIR$_{max}$=highest MIR for all countries and years

Remission was calculated as the inverse of the duration estimates and used as additional input for DisMod-MR 2.1.

To reflect differing degrees of disability due to squamous cell carcinoma we used three levels of severity that were derived from MEPS (Medical Expenditure Panel Survey), resulting in proportions of 80% mild, 15% moderate, and 5% severe disfigurement. For basal cell carcinoma, disability severity was split into 60% asymptomatic (without disability) and 40% with mild disfigurement. Prevalence was multiplied by distinct disability weights (Table 4) to generate YLDs.

Table 5. Lay description and disability weights			
Cause	Health state		Estimate (95% Uncertainty Interval)
Cutaneous squamous cell carcinoma, mild	Disfigurement, level 1	has a slight, visible physical deformity that others notice, which causes some worry and discomfort.	0.011 (0.005-0.021)
Cutaneous squamous cell carcinoma, moderate	Disfigurement, level 2	has a visible physical deformity that causes others to stare and comment. As a result, the person is worried and has trouble sleeping and concentrating.	0.067 (0.044-0.096)
Cutaneous squamous cell carcinoma, severe	Disfigurement, level 3, with itch/pain	has an obvious physical deformity that is very painful and itchy. The physical deformity makes others uncomfortable, which causes the person to avoid social contact, feel worried, sleep poorly, and think about suicide.	0.576 (0.401-0.731)
Disfigurement due to basal cell carcinoma	Disfigurement, level 1	has a slight, visible physical deformity that others notice, which causes some worry and discomfort.	0.011 (0.005-0.021)

Myelodysplastic, myeloproliferative, and other hematopoieticneoplasms

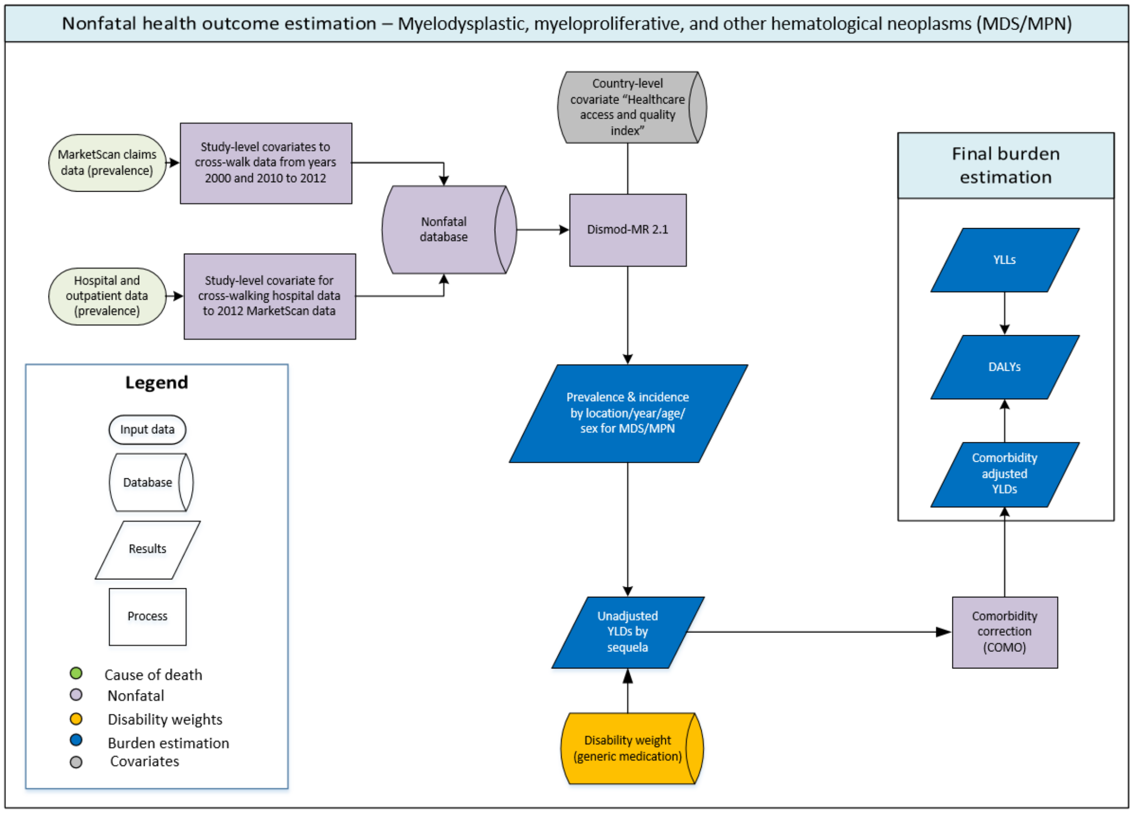

Case definition

Myelodysplastic, myeloproliferative, and other hematopoietic neoplasms (MDS/MPN) comprise a wide variety of diseases and outcomes. These were modelled together as a single group for GBD 2019 (the same as for GBD 2017).

Input data

We estimated MDS/MPN deaths using vital registration data (as outlined above). We did not use cancer registry data for these neoplasms, as it has only been reported within some cancer registries since 2001 and is recognized to be underreported.[29] We estimated MDS/MPN prevalence using MarketScan claims data from the United States in the years 2000, 2010, and 2012, as well as hospital and outpatient data from other health systems worldwide. For 2019, these prevalence data were adjusted for the healthcare access and quality index of the country, and accounts for outpatient encounters. This is a change from GBD 2017, where prevalence only included non-primary diagnoses in inpatient admissions. This change led to a large increase in incidence and prevalence compared to last year, as it now includes diagnoses from outpatient procedures that did not require hospital admission (whereas previously these data approximated the rate of inpatient admissions for cases with benign neoplasms who had access to hospitals).

Modelling strategy

We modelled deaths for all locations and years, by age and by sex, using CODEm. As MDS/MPN can be a precursor to leukemia, our MDS/MPN CODEm model used the same covariate priors as the CODEm model for acute myeloid leukemia.

We modelled the prevalence of these diseases for all combinations of location, age, year, and sex using a prevalence model in Dismod-MR 2.1. For Dismod model specifications, cause-specific mortality rates came from the CODEm model, remission was specified to be zero, and the excess mortality rate was set to be inversely related to the healthcare access and quality index covariate.

While this broad category of hematological neoplasms is heterogeneous in its components' severity or propensity for transformation to leukemia, modelling these components separately was not feasible for 2019. This is an admitted limitation, and an area of desired future improvement as data availability improves. For GBD 2019, the "generic medication" disability weight was assigned for all MDS/MPN cases (see Table 3).

Benign and in situ intestinal neoplasms; Benign and in situ cervical and uterine neoplasms; Other benign and in situ neoplasms

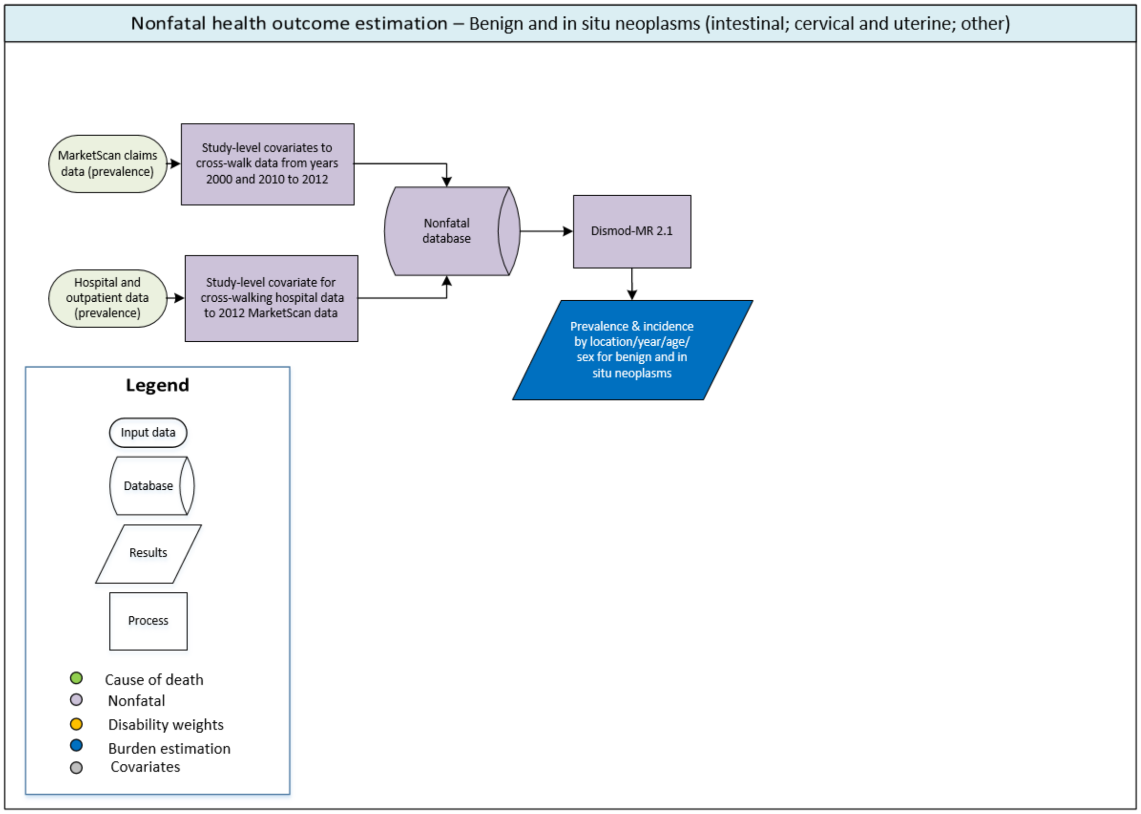

Case definition

For GBD 2019 we estimated three categories of benign and in-situ neoplasms: intestinal neoplasms; cervical and uterine neoplasms; and other benign and in situ neoplasms. Benign and in situ intestinal neoplasms were defined as any non-invasive intestinal growth. Benign and in situ cervical and uterine neoplasms were defined as any non-invasive cervical and uterine growth, except for uterine fibroids. Other benign and in situ neoplasms were defined as any non-invasive neoplasms not covered by other GBD causes.

Input data

To estimate the prevalence of each of these categories for all locations, by age, year, and sex, the prevalence of these neoplasms from hospital data was used as input for a prevalence model in Dismod-MR 2.1. These inputs included MarketScan claims data from the United States in the years 2000, 2010, and 2012, as well as hospital and outpatient data from other health systems worldwide. For GBD 2019, these prevalence data were adjusted for the healthcare access and quality index of the country, and accounts for outpatient encounters. This is a change from GBD 2017, where prevalence only included non-primary diagnoses in inpatient admissions. This change led to a large increase in incidence and prevalence compared to last year, as it now includes diagnoses from outpatient procedures that did not require hospital admission (whereas previously these data approximated the rate of inpatient admissions for cases with benign neoplasms who had access to hospitals).

Modelling strategy

In the Dismod model for benign and in situ intestinal neoplasms, excess mortality rate was specified to be zero, and remission was allowed to vary from 0 to 1. In the Dismod model for benign and in situ cervical and uterine neoplasms, excess mortality rate was specified to be zero, and remission was allowed to vary from 0 to 0.75. In the Dismod model for other benign and in situ neoplasms, excess mortality rate was specified to be zero, and remission was allowed to vary from 0 to 1.

All three of these benign and in-situ neoplasms are by definition benign and localized. As such, no deaths or disability were attributed to their occurrence in GBD 2017.

References

1 GBD 2017 Causes of Death Collaborators. Global, regional, and national age-sex specific mortality for 264 causes of death, 1980-2017: a systematic analysis for the Global Burden of Disease Study 2017. *Lancet Lond Engl*; **submitted**.

2 National Cancer Institute (United States). United States SEER Cancer Data 1973-2010. Bethesda, United States: National Cancer Institute (United States). .

3 Ministry of Health (Mexico). Mexico Ministry of Health Hospital Discharges 2000-2012. Mexico City, México: Ministry of Health (Mexico). .

4 Barber RM, Fullman N, Sorensen RJD, *et al.* Healthcare Access and Quality Index based on mortality from causes amenable to personal health care in 195 countries and territories, 1990–2015: a novel analysis from the Global Burden of Disease Study 2015. *The Lancet* 2017; **390**: 231–66.

5 Asadzadeh Vostakolaei F, Karim-Kos HE, Janssen-Heijnen MLG, Visser O, Verbeek ALM, Kiemeney LALM. The validity of the mortality to incidence ratio as a proxy for site-specific cancer survival. *Eur J Public Health* 2011; **21**: 573–7.

6 SEER*Stat Software. 2014 http://seer.cancer.gov/seerstat/.

7 Surveillance, Epidemiology, and End Results (SEER) Program (www.seer.cancer.gov) SEER*Stat Database: Incidence - SEER 18 Regs Research Data + Hurricane Katrina Impacted Louisiana Cases, Nov 2012 Sub (1973-2010 varying) - Linked To County Attributes - Total U.S., 1969-2011 Counties, National Cancer Institute, DCCPS, Surveillance Research Program, Surveillance Systems Branch, released April 2013, based on the November 2012 submission. .

8 Sankaranarayanan R, Swaminathan R, Lucas E. Cancer survival in Africa, Asia, the Caribbean and Central America (SurvCan). .

9 National Center for Health Statistics, Centers for Disease Control and, Prevention. US Mortality Files. 61-Year Trends in U.S. Cancer Death Rates. http://seer.cancer.gov/archive/csr/1975_2010/results_merged/topic_historical_mort_trends.pdf.

10 SEER Cancer Statistics Review 1975-2011. http://seer.cancer.gov/csr/1975_2011/results_merged/topic_survival_by_year_dx.pdf.

11 Fitzmaurice C, Dicker D, Pain A, *et al.* The Global Burden of Cancer 2013. *JAMA Oncol* 2015; published online May 28. DOI:10.1001/jamaoncol.2015.0735.

12 Neal RD, Din NU, Hamilton W, *et al.* Comparison of cancer diagnostic intervals before and after implementation of NICE guidelines: analysis of data from the UK General Practice Research Database. *Br J Cancer* 2014; **110**: 584–92.

13 Allgar VL, Neal RD. Delays in the diagnosis of six cancers: analysis of data from the National Survey of NHS Patients: Cancer. *Br J Cancer* 2005; **92**: 1959–70.

14 Neal RD, Cannings-John R, Hood K, *et al.* Excision of malignant melanomas in North Wales: effect of location and surgeon on time to diagnosis and quality of excision. *Fam Pract* 2008; **25**: 221–7.

15 Kewalramani T, Nimer SD, Zelenetz AD, *et al.* Progressive disease following autologous transplantation in patients with chemosensitive relapsed or primary refractory Hodgkin’s disease or aggressive non-Hodgkin’s lymphoma. *Bone Marrow Transplant* 2003; **32**: 673–9.

16 Esteban D, Tovar N, Jiménez R, *et al.* Patients with relapsed/refractory chronic lymphocytic leukaemia may benefit from inclusion in clinical trials irrespective of the therapy received: a case-control retrospective analsysis. *Blood Cancer J* 2015; **5**: e356.

17 Canova C, Giorato E, Roveron G, Turrini P, Zanotti R. Validation of a stoma-specific quality of life questionnaire in a sample of patients with colostomy or ileostomy. *Colorectal Dis Off J Assoc Coloproctology G B Irel* 2013; **15**: e692-698.

18 Caricato M, Ausania F, Ripetti V, Bartolozzi F, Campoli G, Coppola R. Retrospective analysis of long-term defunctioning stoma complications after colorectal surgery. *Colorectal Dis Off J Assoc Coloproctology G B Irel* 2007; **9**: 559–61.

19 Erwin-Toth P, Thompson SJ, Davis JS. Factors impacting the quality of life of people with an ostomy in North America: results from the Dialogue Study. *J Wound Ostomy Cont Nurs Off Publ Wound Ostomy Cont Nurses Soc WOCN* 2012; **39**: 417–22; quiz 423–4.

20 Catalona WJ, Carvalhal GF, Mager DE, Smith DS. Potency, continence and complication rates in 1,870 consecutive radical retropubic prostatectomies. *J Urol* 1999; **162**: 433–8.

21 Donnellan SM, Duncan HJ, MacGregor RJ, Russell JM. Prospective assessment of incontinence after radical retropubic prostatectomy: objective and subjective analysis. *Urology* 1997; **49**: 225–30.

22 Eastham JA, Kattan MW, Rogers E, *et al.* Risk factors for urinary incontinence after radical prostatectomy. *J Urol* 1996; **156**: 1707–13.

23 Kundu SD, Roehl KA, Eggener SE, Antenor JAV, Han M, Catalona WJ. Potency, continence and complications in 3,477 consecutive radical retropubic prostatectomies. *J Urol* 2004; **172**: 2227–31.

24 Potosky AL, Davis WW, Hoffman RM, *et al.* Five-Year Outcomes After Prostatectomy or Radiotherapy for Prostate Cancer: The Prostate Cancer Outcomes Study. *JNCI J Natl Cancer Inst* 2004; **96**: 1358–67.

25 Sacco E, Prayer-Galetti T, Pinto F, *et al.* Urinary incontinence after radical prostatectomy: incidence by definition, risk factors and temporal trend in a large series with a long-term follow-up. *BJU Int* 2006; **97**: 1234–41.

26 Stanford JL, Feng Z, Hamilton AS, *et al.* Urinary and sexual function after radical prostatectomy for clinically localized prostate cancer: the Prostate Cancer Outcomes Study. *JAMA* 2000; **283**: 354–60.

27 Walsh PC, Marschke P, Ricker D, Burnett AL. Patient-reported urinary continence and sexual function after anatomic radical prostatectomy. *Urology* 2000; **55**: 58–61.

28 Karagas MR, Greenberg ER, Spencer SK, Stukel TA, Mott LA. Increase in incidence rates of basal cell and squamous cell skin cancer in New Hampshire, USA. New Hampshire Skin Cancer Study Group. *Int J Cancer J Int Cancer* 1999; **81**: 555–9.

29 Cogle CR, Craig BM, Rollison DE, List AF. Incidence of the myelodysplastic syndromes using a novel claims-based algorithm: high number of uncaptured cases by cancer registries. *Blood* 2011; **117**: 7121–5.

Rheumatic Heart Disease

Flowchart

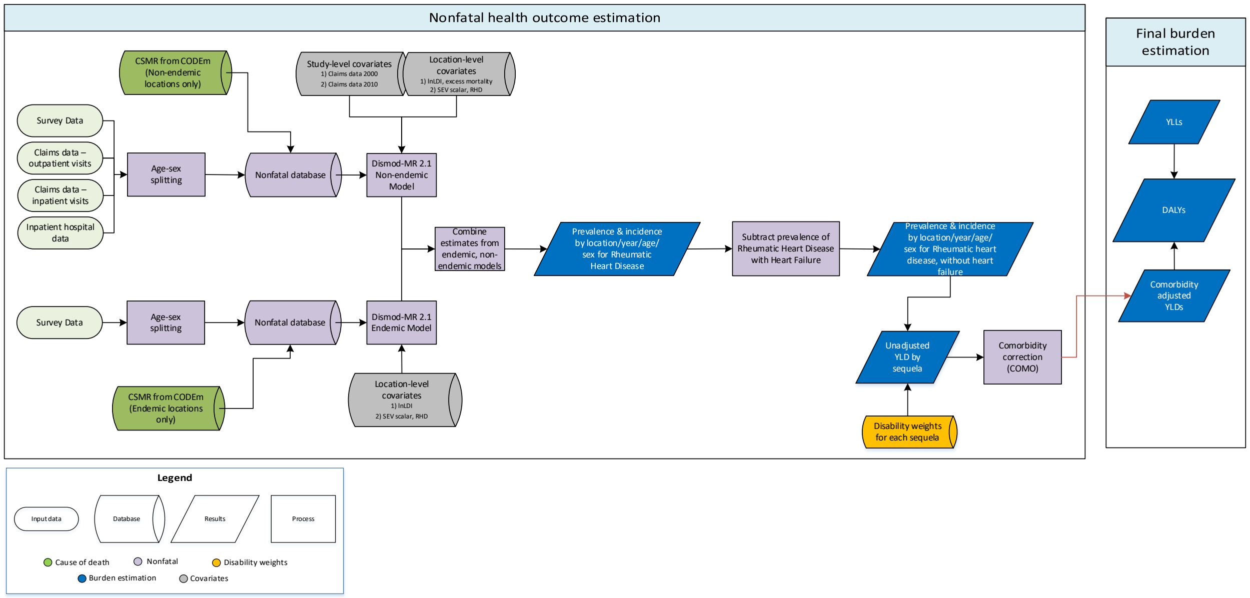

Input data and methodological appendix

Case definition

Rheumatic heart disease (RHD) was defined as a clinical diagnosis by a physician with or without confirmation using echocardiography. This case definition for echocardiographic confirmation of RHD follows the World Heart Federation criteria for echocardiographic diagnosis of rheumatic heart disease (1).

Criterion	Definition
1. Echocardiography	Prevalent rheumatic heart disease based on echocardiographic assessment and clinical confirmation
2. Clinical diagnosis	Prevalent rheumatic heart disease based on physician diagnosis

ICD codes for data included from hospital records can be found elsewhere in the appendix.

Input data

Model inputs

Table 1: Source counts for rheumatic heart disease

Measure	Total sources	Countries with data
All measures	198	58
Prevalence	198	58

Table 1 shows the source counts for rheumatic heart disease. We did not perform a systematic review for GBD 2017. A systematic review was performed for GBD 2013 and updated for GBD 2015. The GBD 2015 search information encompassed the following:

- Search terms: ('rheumatic heart disease' AND epidemiology[MeSH Subheading]) OR ('acute rheumatic fever' AND epidemiology[MeSH Subheading]) OR ('rheumatic fever' AND epidemiology[MeSH Subheading]) OR (RHD AND epidemiology[MeSH Subheading]) OR ('valvular heart disease' AND epidemiology[MeSH Subheading]) OR (((streptococcus OR streptococci) AND heart) AND epidemiology[MeSH Subheading]) OR (heart AND valve AND disease AND epidemiology[MeSH Subheading]) OR ('mitral valve stenosis' AND epidemiology[MeSH Subheading]) OR (('rheumatic heart disease' OR 'rheumatic fever') AND prevalence) OR (('rheumatic heart disease' OR 'rheumatic fever') AND incidence) OR (('rheumatic heart disease' OR 'rheumatic fever') AND ('standardized mortality ratio' OR SMR)) OR ('rheumatic heart disease' OR 'rheumatic fever' AND 'case fatality')
- Dates included in search: 1/1/2013 – 3/16/2015
- Number of initial hits: 2,045
- Number of sources included: 17

These differed from the GBD 2013 search terms:

- (hasabstract[text] AND Humans[Mesh] AND middle age[MeSH])) OR 21) AND ((rheumatic heart disease/epidemiology[Mesh] OR rheumatic heart disease/mortality[Mesh]) AND (prevalence[Title/Abstract] OR incidence[Title/Abstract]) AND ("2010"[Date - Publication] : "3000"[Date - Publication]) AND (hasabstract[text] AND Humans[Mesh] AND middle age[MeSH]))

We did not include any non-literature-based data types other than the hospital and claims data described elsewhere. Prevalence from hospital and claims data sources were included only for the non-endemic country model. Inpatient data were adjusted for multiple visits, non-primary diagnoses, and inpatient to outpatient utilisation ratios. This methodology is detailed elsewhere in the appendix.

Severity splits and disability weights

Severity level	Lay description	DW (95% CI)
Rheumatic heart disease, not including heart failure	Has a chronic disease that requires medication every day and causes some worry but minimal interference with daily activities.	0.049 (0.031–0.072)

Modelling strategy

For GBD 2019 estimation, we ran two models using DisMod-MR – one for non-endemic countries and one for endemic countries. For GBD 2016, we identified locations as endemic if the estimated death rate due to RHD was greater than 0.15 per 100,000 in the 5 to 9 age group, or if that location had an SDI less than 0.6. Beginning in GBD 2017, we identified locations as endemic if the estimated death rate due to RHD was greater than 0.15 per 100,000 in the 10 to 14 age group, or if that location had an SDI less than 0.6. This change in age group was made based on feedback from RHD expert reviewers due to concerns that the death rate in 5 to 9 age group would not capture endemicity in locations where RHD is common only in later age groups. Each location estimated as part of GBD 2019 is listed below as either "Endemic" or "Non-endemic".

Remission

In GBD 2016, we assumed that there was no remission from RHD. Beginning in GBD 2017, we estimated remission in both the endemic and non-endemic DisMod models. This decision was based on two studies[2,3] that observed remission among confirmed RHD cases. We used the equation below to convert reported proportion of remitted individuals in each study to a remission rate, defined as the number of remitted cases divided by the total person-years of disease:

$$remission\ rate = \frac{\log(1 - proportion\ remitted)}{years\ of\ followup}$$

Where *proportion remitted* is the reported proportion of all individuals with RHD at baseline who ended up remitting, and *years of followup* is the mean follow-up time in the study. The relevant values for the two papers and the calculated remission rates are listed in the table below.

Study	Remitted proportion	Mean follow-up time	Calculated remission rate
Beaton et al[2]	0.3	2.4 years	0.14 cases per person-year
Engelman et al[3]	0.1	7.5 years	0.014 cases per person-year

In order to acknowledge the uncertainty in these calculated remission rates and to allow DisMod flexibility in estimating remission, we input 0.2 as the upper bound for remission the remission prior and 0.00 as the lower bound for remission the remission prior. Because the two studies used to estimate remission were done only in children, we applied these remission priors to only those younger than age 20, and setting a remission prior of zero for adults older than age 20.

DisMod models

Non-endemic model: We included hospital data, claims data, and limited literature data on prevalence. We also included CSMR from our mortality estimates of RHD for non-endemic locations only. A prior of no remission was set, and excess mortality was capped at 0.1 for all ages. Coefficients for selected covariates are listed in the table below.

Endemic model: We included prevalence data from surveys published in the literature. As with the high-income model, we included CSMR from our mortality estimates of RHD for endemic locations only. A prior of no remission was set for all ages, and excess mortality was capped at 0.07, the highest observed mean excess mortality rate data point observed in this model. We also set priors of 0 on incidence for ages 0 to 1 and 50 to 100 to account for patterns of incidence in endemic countries. We used lnLDI as a fixed-effect country-level covariate on prevalence and excess mortality, enforcing an inverse relationship for both. The log-transformed, age-standardised SEV scalar was also used as a fixed-effect country-level covariate on prevalence.

We combined estimates from the endemic and non-endemic models, selecting estimates for the locations identified as non-endemic from the non-endemic model and estimates for the locations identified as endemic from the endemic model. Estimates of heart failure due to RHD were then subtracted from the estimates for RHD, giving the overall prevalence of RHD without heart failure. A description of the modelling strategy for heart failure due to RHD can be found in the heart failure appendix. We evaluated models based on comparing estimates with input data as well as estimates from previous rounds of GBD.

The table below shows the country covariates, parameters, betas, and exponentiated betas:

Covariate	Parameter	Beta	Exponentiated beta
Endemic model			
Log-transformed age-standardised SEV scalar: RHD	Prevalence	0.95 (0.76 to 1.17)	2.57 (2.15 to 3.23)
LDI (I$ per capita)	Excess mortality rate	-0.3 (-0.49 to -0.11)	0.74 (0.61 to 0.90)
Non-endemic model			
Log-transformed age-standardised SEV scalar: RHD	Prevalence	0.76 (0.75 to 0.78)	2.14 (2.12 to 2.18)
LDI (I$ per capita)	Excess mortality rate	-0.94 (-0.96 to -0.93)	0.39 (0.38 to 0.40)

Endemic locations: Aceh, Acre, Addis Ababa, Afar, Afghanistan, Alagoas, Albania, Alborz, Algeria, Amapá, Amazonas, American Samoa, Amhara, Andean Latin America, Andhra Pradesh, Andhra Pradesh, Rural, Andhra Pradesh, Urban, Angola, Anhui, Antigua and Barbuda, Ardebil, Argentina, Armenia, Arunachal Pradesh, Arunachal Pradesh, Rural, Assam, Assam, Rural, Assam, Urban, Azerbaijan, Bahia, Bangladesh, Barbados, Baringo, Belize, Bengkulu, Benin, Benishangul-Gumuz, Bhutan, Bihar, Bihar, Rural, Bihar, Urban, Bolivia, Bomet, Botswana, Brazil, Bungoma, Burkina Faso, Burundi, Busia, Cambodia, Cameroon, Cape Verde, Caribbean, Ceará, Central African Republic, Central Asia, Central Europe, Eastern Europe, and Central Asia, Central Kalimantan, Central Sub-Saharan Africa, Chad, Chahar Mahaal and Bakhtiari, Chhattisgarh, Chhattisgarh, Rural, Chhattisgarh, Urban, Chiapas, China, Chongqing, Comoros, Congo, Costa Rica, Cote d'Ivoire, Cuba, Delhi, Delhi, Rural, Delhi, Urban, Democratic Republic of the Congo, Dire Dawa, Distrito Federal, Djibouti, Dominica, Dominican Republic, East Asia, East Azarbayejan, East Nusa Tenggara, Eastern Cape, Eastern Sub-Saharan Africa, Ecuador, Egypt, El Salvador, Elgeyo-Marakwet, Embu, Equatorial Guinea, Eritrea, Espírito Santo, Ethiopia, Fars, Federated States of Micronesia, Fiji, Free State, Gabon, Gambella, Gansu, Garissa, Gauteng, Georgia, Ghana, Gilan, Global, Goa, Goa, Rural, Goa, Urban, Goiás, Golestan, Gorontalo, Grenada, Guam, Guangxi, Guatemala, Guerrero, Guinea, Guinea-Bissau, Guizhou, Gujarat, Gujarat, Rural, Gujarat, Urban, Guyana, Hainan, Haiti, Hamadan, Harari, Haryana, Haryana, Rural, Haryana, Urban, Hebei, Heilongjiang, Henan, Hidalgo, Himachal Pradesh, Himachal Pradesh, Rural, Himachal Pradesh, Urban, HomaBay, Honduras, Hormozgan, Hubei, Hunan, Ilam, India, Inner Mongolia, Iran, Iraq, Isfahan, Isiolo, Jamaica, Jammu and Kashmir, Jammu and Kashmir, Rural, Jammu and Kashmir, Urban, Jharkhand, Jharkhand, Rural, Jharkhand, Urban, Jiangxi, Jilin, Kajiado, Kakamega, Karnataka, Karnataka, Rural, Karnataka, Urban, Kenya, Kerala, Kerala, Rural, Kerala, Urban, Kericho, Kerman, Kermanshah, Khorasan-e-Razavi, Khuzestan, Kiambu, Kilifi, Kiribati, Kirinyaga, Kisii, Kisumu, Kitui, Kohgiluyeh and Boyer-Ahmad, Kurdistan, Kwale, KwaZulu-Natal, Kyrgyzstan, Laikipia, Lamu, Laos, Latin America and Caribbean, Lesotho, Liaoning, Liberia, Libya, Limpopo, Lorestan, Machakos,

Madagascar, Madhya Pradesh, Madhya Pradesh, Rural, Madhya Pradesh, Urban, Maharashtra, Maharashtra, Rural, Maharashtra, Urban, Makueni, Malawi, Malaysia, Maldives, Mali, Maluku, Mandera, Manipur, Manipur, Rural, Manipur, Urban, Maranhão, Markazi, Marsabit, Marshall Islands, Mato Grosso, Mato Grosso do Sul, Mauritania, Mauritius, Mazandaran, Meghalaya, Meghalaya, Rural, Meghalaya, Urban, Meru, Mexico City, Michoacán de Ocampo, Migori, Minas Gerais, Mizoram, Rural, Mombasa, Mongolia, Morocco, Mozambique, Mpumalanga, Murang'a, Myanmar, Nagaland, Nagaland, Rural, Nairobi, Nakuru, Namibia, Nandi, Narok, Nepal, Nicaragua, Niger, Nigeria, Ningxia, North Africa and Middle East, North Africa and Middle East, North Khorasan, North Korea, North Maluku, North-West, Northern Cape, Northern Mariana Islands, Nyamira, Nyandarua, Nyeri, Oaxaca, Oceania, Odisha, Odisha, Rural, Odisha, Urban, Oromia, Pakistan, Palestine, Panama, Papua, Papua New Guinea, Pará, Paraguay, Paraíba, Paraná, Pernambuco, Peru, Philippines, Piaui, Puebla, Punjab, Punjab, Rural, Punjab, Urban, Qazvin, Qinghai, Rajasthan, Rajasthan, Rural, Rajasthan, Urban, Republic of Tuva, Riau Islands, Rio de Janeiro, Rio Grande do Norte, Rio Grande do Sul, Rondônia, Roraima, Rwanda, Saint Lucia, Saint Vincent and the Grenadines, Samburu, Samoa, Santa Catarina, São Paulo, Sao Tome and Principe, Semnan, Senegal, Sergipe, Seychelles, Shaanxi, Shandong, Shanxi, Siaya, Sichuan, Sierra Leone, Sikkim, Sikkim, Rural, Sikkim, Urban, Sistan and Baluchistan, Solomon Islands, Somali, Somalia, South Africa, South Asia, South Asia, South Kalimantan, South Khorasan, South Sudan, Southeast Asia, Southeast Asia, East Asia, and Oceania, Southeast Sulawesi, Southern Nations, Nationalities, and Peoples, Southern Sub-Saharan Africa, Sub-Saharan Africa, Sudan, Suriname, Swaziland, Syria, TaitaTaveta, Tajikistan, Tamil Nadu, Tamil Nadu, Rural, Tamil Nadu, Urban, TanaRiver, Tanzania, Tehran, Telangana, Telangana, Rural, Telangana, Urban, Thailand, TharakaNithi, The Bahamas, The Gambia, Tianjin, Tibet, Tigray, Timor-Leste, Tocantins, Togo, Tonga, TransNzoia, Trinidad and Tobago, Tripura, Tripura, Rural, Tripura, Urban, Tropical Latin America, Turkana, Turkmenistan, Tyumen oblast without autonomous areas, UasinGishu, Uganda, Union Territories other than Delhi, Union Territories other than Delhi, Rural, Union Territories other than Delhi, Urban, United Arab Emirates, Uttar Pradesh, Uttar Pradesh, Rural, Uttar Pradesh, Urban, Uttarakhand, Uttarakhand, Rural, Uttarakhand, Urban, Uzbekistan, Vanuatu, Veracruz de Ignacio de la Llave, Vihiga, Wajir, West Azarbayejan, West Bengal, West Bengal, Rural, West Bengal, Urban, West Kalimantan, West Nusa Tenggara, West Papua, West Sulawesi, West Sumatra, Western Cape, Western Sub-Saharan Africa, WestPokot, Xinjiang, Yemen, Yunnan, Zambia, Zanjan, Zimbabwe

Non-endemic locations: Aguascalientes, Aichi, Akershus, Akita, Alabama, Alaska, Altai kray, Amur oblast, Andorra, Aomori, Arizona, Arkansas, Arkhangelsk oblast without Nenets autonomous district, Arunachal Pradesh, Urban, Astrakhan oblast, Aust-Agder, Australasia, Australia, Austria, Bahrain, Baja California, Baja California Sur, Bali, Bangka-Belitung Islands, Banten, Barking and Dagenham, Barnet, Barnsley, Bath and North East Somerset, Bedford, Beijing, Belarus, Belgium, Belgorod oblast, Bermuda, Bexley, Birmingham, Blackburn with Darwen, Blackpool, Bolton, Bosnia and Herzegovina, Bournemouth, Bracknell Forest, Bradford, Brent, Brighton and Hove, Bristol, City of, Bromley, Brunei, Bryansk oblast, Buckinghamshire, Bulgaria, Bury, Bushehr, Buskerud, Calderdale, California, Cambridgeshire, Camden, Campeche, Canada, Central Bedfordshire, Central Europe, Central Java, Central Latin America, Central Sulawesi, Chechen Republic, Chelyabinsk oblast, Cheshire East, Cheshire West and Chester, Chiba, Chihuahua, Chile, Chukchi autonomous area, Chuvash Republic, Coahuila, Colima, Colombia, Colorado, Connecticut, Cornwall, County Durham, Coventry, Croatia, Croydon, Cumbria, Cyprus, Czech Republic, Darlington, Delaware, Denmark, Derby, Derbyshire, Devon, District of Columbia, Doncaster, Dorset, Dudley, Durango, Ealing, East Java, East Kalimantan, East Midlands, East of England, East Riding of Yorkshire, East Sussex, Eastern

Europe, Ehime, Enfield, England, Essex, Estonia, Finland, Finnmark, Florida, France, Fujian, Fukui, Fukuoka, Fukushima, Gateshead, Georgia, Germany, Gifu, Gloucestershire, Greater London, Greece, Greenland, Greenwich, Guanajuato, Guangdong, Gunma, Hackney, Halton, Hammersmith and Fulham, Hampshire, Haringey, Harrow, Hartlepool, Havering, Hawaii, Hedmark, Herefordshire, County of, Hertfordshire, High-income, High-income Asia Pacific, High-income North America, Hillingdon, Hiroshima, Hokkaidō, Hong Kong Special Administrative Region of China, Hordaland, Hounslow, Hungary, Hyōgo, Ibaraki, Iceland, Idaho, Illinois, Indiana, Indonesia, Iowa, Ireland, Irkutsk oblast, Ishikawa, Isle of Wight, Islington, Israel, Italy, Ivanovo oblast, Iwate, Jakarta, Jalisco, Jambi, Japan, Jewish autonomous oblast, Jiangsu, Jordan, Kabardian-Balkar Republic, Kagawa, Kagoshima, Kaliningrad oblast, Kaluga oblast, Kamchatka kray, Kanagawa, Kansas, Karachaev-Chercassian Republic, Kazakhstan, Kemerovo oblast, Kensington and Chelsea, Kent, Kentucky, Khabarovsk kray, Khanty-Mansi autonomous area, Kingston upon Hull, City of, Kingston upon Thames, Kirklees, Kirov oblast, Knowsley, Kōchi, Komi Republic, Kostroma oblast, Krasnodar kray, Krasnoyarsk kray, Kumamoto, Kurgan oblast, Kursk oblast, Kuwait, Kyōto, Lambeth, Lampung, Lancashire, Latvia, Lebanon, Leeds, Leicester, Leicestershire, Leningrad oblast, Lewisham, Lincolnshire, Lipetzk oblast, Lithuania, Liverpool, Louisiana, Luton, Luxembourg, Macao Special Administrative Region of China, Macedonia, Magadan oblast, Maine, Malta, Manchester, Maryland, Massachusetts, Medway, Merton, Mexico, México, Michigan, Middlesbrough, Mie, Milton Keynes, Minnesota, Mississippi, Missouri, Miyagi, Miyazaki, Mizoram, Mizoram, Urban, Moldova, Montana, Montenegro, Møre og Romsdal, Morelos, Moscow City, Moscow oblast, Murmansk oblast, Nagaland, Urban, Nagano, Nagasaki, Nara, Nayarit, Nebraska, Nenets autonomous district, Netherlands, Nevada, New Hampshire, New Jersey, New Mexico, New York, New Zealand, New Zealand Maori population, New Zealand non-Maori population, Newcastle upon Tyne, Newham, Niigata, Nizhny Novgorod oblast, Nordland, Norfolk, North Carolina, North Dakota, North East England, North East Lincolnshire, North Kalimantan, North Lincolnshire, North Somerset, North Sulawesi, North Sumatra, North Tyneside, North West England, North Yorkshire, Northamptonshire, Northern Ireland, Northumberland, Norway, Nottingham, Nottinghamshire, Novgorod oblast, Novosibirsk oblast, Nuevo León, Ohio, Ōita, Okayama, Okinawa, Oklahoma, Oldham, Oman, Omsk oblast, Oppland, Oregon, Orenburg oblast, Oryol oblast, Ōsaka, Oslo, Østfold, Oxfordshire, Pennsylvania, Penza oblast, Perm kray, Peterborough, Plymouth, Poland, Poole, Portsmouth, Portugal, Primorsky kray, Pskov oblast, Puerto Rico, Qatar, Qom, Querétaro, Quintana Roo, Reading, Redbridge, Redcar and Cleveland, Republic of Adygeya, Republic of Altai, Republic of Bashkortostan, Republic of Buryatia, Republic of Crimea, Republic of Dagestan, Republic of Ingushetia, Republic of Kalmykia, Republic of Karelia, Republic of Khakasia, Republic of Mariy El, Republic of Mordovia, Republic of North Ossetia-Alania, Republic of Sakha (Yakutia), Republic of Tatarstan, Rhode Island, Riau, Richmond upon Thames, Rochdale, Rogaland, Romania, Rostov oblast, Rotherham, Russian Federation, Rutland, Ryazan oblast, Saga, Saitama, Sakhalin oblast, Salford, Samara oblast, San Luis Potosí, Sandwell, Sankt-Petersburg, Saratov oblast, Saudi Arabia, Scotland, Sefton, Serbia, Sevastopol, Shanghai, Sheffield, Shiga, Shimane, Shizuoka, Shropshire, Sinaloa, Singapore, Slough, Slovakia, Slovenia, Smolensk oblast, Sogn og Fjordane, Solihull, Somerset, Sonora, South Carolina, South Dakota, South East England, South Gloucestershire, South Korea, South Sulawesi, South Sumatra, South Tyneside, South West England, Southampton, Southend-on-Sea, Southern Latin America, Southwark, Spain, Sri Lanka, St Helens, Staffordshire, Stavropol kray, Stockholm, Stockport, Stockton-on-Tees, Stoke-on-Trent, Suffolk, Sunderland, Surrey, Sutton, Sverdlovsk oblast, Sweden, Sweden except Stockholm, Swindon, Switzerland, Tabasco, Taiwan, Tamaulipas, Tambov oblast, Tameside, Telemark, Telford and Wrekin, Tennessee, Texas, Thurrock, Tlaxcala, Tochigi, Tokushima, Tōkyō, Tomsk oblast, Torbay, Tottori, Tower Hamlets, Toyama,

Trafford, Troms, Trøndelag, Tula oblast, Tunisia, Turkey, Tver oblast, Udmurt Republic, Ukraine, Ukraine (without Crimea & Sevastopol), Ulyanovsk oblast, United Kingdom, United States, Uruguay, Utah, Venezuela, Vermont, Vest-Agder, Vestfold, Vietnam, Virgin Islands, U.S., Virginia, Vladimir oblast, Volgograd oblast, Vologda oblast, Voronezh oblast, Wakayama, Wakefield, Wales, Walsall, Waltham Forest, Wandsworth, Warrington, Warwickshire, Washington, West Berkshire, West Java, West Midlands, West Sussex, West Virginia, Western Europe, Westminster, Wigan, Wiltshire, Windsor and Maidenhead, Wirral, Wisconsin, Wokingham, Wolverhampton, Worcestershire, Wyoming, Yamagata, Yamaguchi, Yamalo-Nenets autonomous area, Yamanashi, Yaroslavl oblast, Yazd, Yogyakarta, York, Yorkshire and the Humber, Yucatán, Zabaikalsk kray, Zacatecas, Zhejiang

1. Reményi, B. et al. Nat. Rev. Cardiol. 9, 297–309 (2012); published online 28 February 2012
2. Beaton A, Aliku T, Dewyer A, et al. Latent Rheumatic Heart Disease: Identifying the Children at Highest Risk of Unfavorable Outcome. Circulation. 2017;136(23):2233-2244.
3. Engelman D, Wheaton GR, Mataika RL, et al. Screening-detected rheumatic heart disease can progress to severe disease. Heart Asia. 2016;8(2):67-73.

Ischaemic heart disease

Flowchart

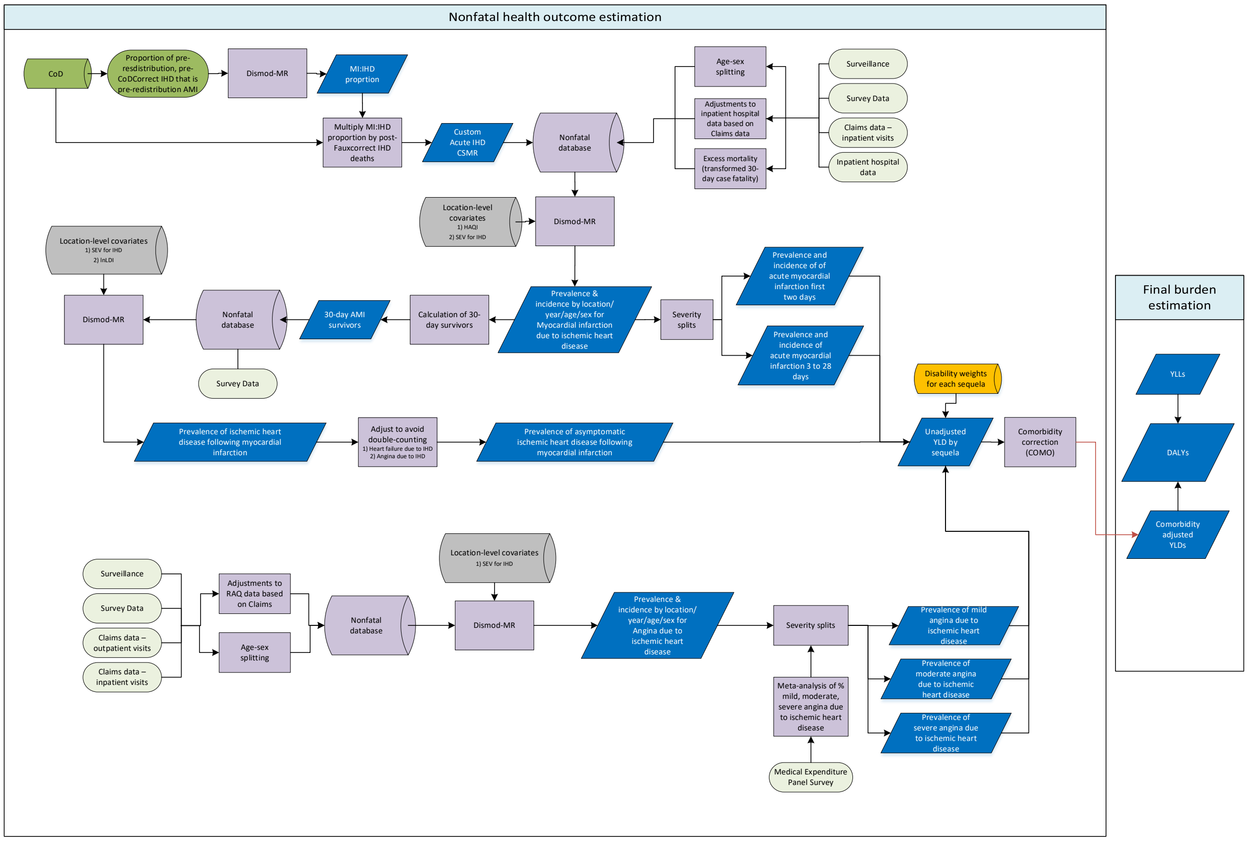

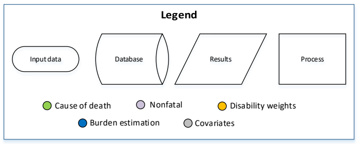

Input data and methodological summary

Case definition

Case definitions:

1) Acute myocardial infarction (MI): Definite and possible MI according to the third universal definition of myocardial infarction:
    a. When there is clinical evidence of myocardial necrosis in a clinical setting consistent with myocardial ischaemia or
    b. Detection of a rise and/or fall of cardiac biomarker values and with at least one of the following: i) symptoms of ischaemia, ii) new or presumed new ST-segment-T wave changes or new left bundle branch block, iii) development of pathological Q waves in the ECG, iv) imaging evidence of new loss of viable myocardium or new regional wall motion abnormality, or v) identification of an intracoronary thrombus by angiography or autopsy.
    c. Sudden (abrupt) unexplained cardiac death, involving cardiac arrest or no evidence of a non-coronary cause of death
    d. Prevalent MI is considered to last from the onset of the event to 28 days after the event and is divided into an acute phase (0–2 days) and subacute (3–28 days).

2) Chronic IHD
    a. Angina; clinically diagnosed stable exertional angina pectoris or definite angina pectoris according to the Rose Angina Questionnaire, physician diagnosis, or taking nitrate medication for the relief of chest pain.
    b. Asymptomatic ischaemic heart disease following myocardial infarction; survival to 28 days following incident MI. The GBD study does not use estimates based on ECG evidence for prior MI, due to its limited specificity and sensitivity (1).

ICD codes used for inclusion of hospital and claims data for MI and angina can be found elsewhere in the appendix.

Input data

The total source counts for non-fatal ischaemic heart disease are shown in the table below by measure.

Table 1: Source counts for all non-fatal ischaemic heart disease models.

Measure	Total sources	Countries with data
All measures	442	84
Prevalence	88	61
Incidence	296	44
Excess mortality rate	90	21
Relative risk	1	1
Standardized mortality ratio	1	1
With-condition mortality rate	4	4
Proportion	16	1

Myocardial infarction

A systematic review was done for myocardial infarction for GBD 2019 in order to update our current database. The search strings used were ((“myocardial infarction”[tiab] AND (incidence OR “case fatality” OR “excess mortality”)) OR (“acute coronary syndrome”[tiab] AND (incidence OR “case fatality” OR “excess mortality”)) OR (angina[tiab] AND (incidence OR prevalence OR “case fatality” OR “excess mortality”))) AND ("2019/01/01"[PDAT] : "2019/12/31"[PDAT]) NOT rat[tiab] NOT mice[tiab] NOT monkey[tiab] NOT pig[tiab] NOT animals[tiab].

The dates of the search were 1/1/2019 – 12/31/2019. 28957 studies were returned, 80 were extracted. The PRISMA diagram for the systematic review is given below. In the diagram, screening refers to reviewing of the title and abstract of an article for relevant information, not screening of the entire article.

PRISMA Diagram

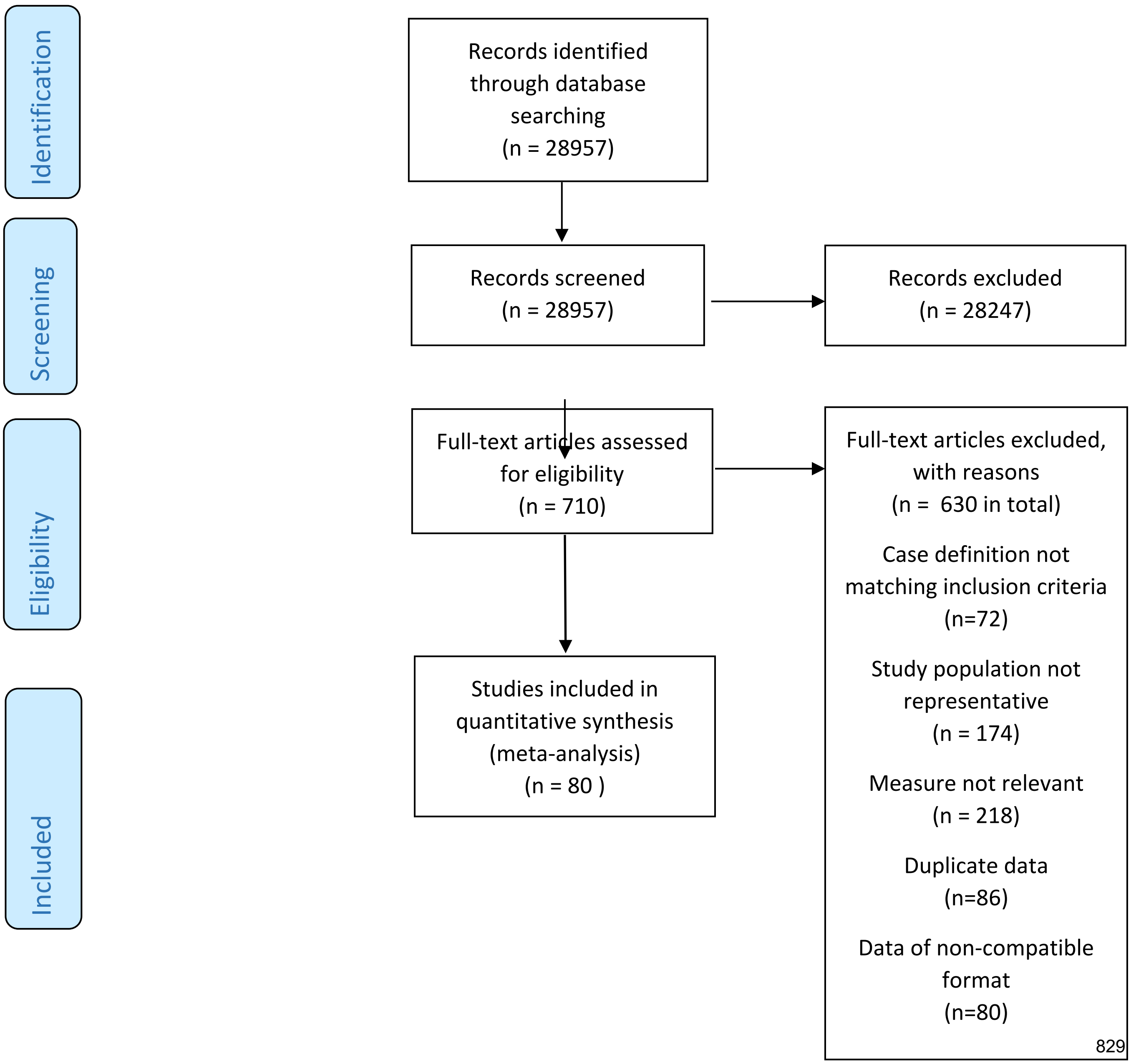

The last systematic review for myocardial infarction was done for GBD 2015. The dates of the search were 1/1/2009 – 2/3/2015. 38,522 studies were returned; 194 were extracted (this number includes extractions that were done for STEMI/NSTEMI models and revascularisation models that are not currently part of the MI modelling process but may be in the future).

A systematic review for myocardial infarction was also done for GBD 2013. The extensive search terms for that review will be provided on request.

Apart from inpatient hospital and inpatient claims data, we did not include any data from sources other than the literature for myocardial infarction. We also split excess mortality data points where the age range was greater than 25 years. Age splitting was based on the global sex-specific age pattern from a Dismod model that only used excess mortality input data from scientific literature with less than a 25-year age range. We excluded incidence data with broad age ranges where it was impossible to obtain more granular data, as these data caused the known age pattern for increased risk of myocardial infarction to be masked in the estimates generated from DisMod.

We crosswalked incidence measurements for myocardial infarction literature data with alternative definitions to agree with our case reference definition using MR-BRT (Meta Regression – Bayesian, Regularized, Trimmed) modeling tool. MR-BRT and the process of data adjustment are discussed elsewhere in the appendix. For myocardial infarction we crosswalked using multiple different covariates: a covariate to capture only first-ever MI, using studies where all events were included as the reference; a covariate to adjust estimates from studies that only included non-fatal cases, using sources that included fatal and non-fatal cases as reference; and a covariate to adjust for studies that did not use troponin measurements in their case diagnosis, using sources that did include troponin measurements in their diagnostic method. The coefficients in Table 2 below can be used to calculate adjustment factors for alternative definitions. The formula for computing adjustment factors is given in equation 1 below. We also included a standardized age variable (age scaled) and a sex variable to the regression to adjust for the possibly of bias.

Equation 1: Calculation of adjustment factors:

$$Estimated\ Reference\ Def = invlogit(logit(Alternative\ Def) - Beta_{Alternative\ Def} - Beta_{Sex} * Sex - Beta_{Age_{scaled}} * Age\ Scaled)$$

Table 2a: MR-BRT Crosswalk Adjustment Factors for Myocardial Infarction

Data input	Measure	Reference or alternative case definition	Gamma	Beta Coefficient, Logit (95% CI)
Any event, fatal and nonfatal events, used troponin	Incidence	Ref	0.27	---
Troponin not used as part of definition	Incidence	Alt		-0.55 (-1.08 - -0.01)
First-ever	Incidence	Alt		-0.59 (-1.21 – 0.03)
Non-fatal	Incidence	Alt		-0.35 (-0.98 – 0.29)
Age scaled	Incidence	Alt		-0.05 (-0.59 – 0.49)
Sex (male)	Incidence	Alt		-0.001 (-0.54 – 0.54)

Asymptomatic ischaemic heart disease following myocardial infarction

No systematic review was performed for Asymptomatic ischaemic heart disease following myocardial infarction in GBD 2019. The primary input for this model are 28-day survivors calculated from the excess mortality estimates for the myocardial infarction model. We included data for excess mortality and standardised mortality ratio to inform the estimates of survival after myocardial infarction.

Angina

A systematic review was not performed for GBD 2019. Updates to systematic reviews are performed on an ongoing schedule across all GBD causes; an update for angina will be performed in the next one to two iterations.

A systematic review for angina was last performed for GBD 2013. The search terms for that are: (Angina Pectoris/epidemiology[Mesh] OR Angina Pectoris/mortality[Mesh]) AND (prevalence[Title/Abstract] OR incidence[Title/Abstract]) AND ("2010"[Date - Publication] : "3000"[Date - Publication])

We included survey data (including NHANES and World Health Study questionnaires) which included the RAQ items. Prevalence of angina was calculated using the standard algorithm to determine whether the RAQ was positive or negative.

We excluded data with broad age ranges where it was impossible to obtain more granular data, as these data caused the known age pattern for increased risk of angina to be masked in the estimates generated from DisMod.

We also included US claims data, but did not include inpatient hospital data from any locations. Stable angina (unstable angina is modeled as part of MI) is expected to be rare in inpatient but common in outpatient data as it is a condition usually managed on an outpatient basis, except for specific surgical interventions. This discrepancy leads to implausible correction factors based on inpatient/outpatient information from claims data (~150X); thus adjusted data cannot be used. Including uncorrected data in the model is likely to lead to incorrect estimates as hospitalisation and procedure rates are likely to vary between geographies based on access to and patterns of care. All outpatient data were excluded as they were implausibly low for all locations when compared with literature and claims data.

We crosswalked prevalence data obtained from survey data using the RAQ using claims data as a reference since the RAQ has been shown to be neither sensitive nor specific. Specifics on the crosswalking process are discussed elsewhere in the appendix. Table 2b shows the coefficients adjustments made to the alternative definition.

Table 2b: MR-BRT Crosswalk Adjustment Factors for Angina

Data input	Measure	Reference or alternative case definition	Gamma	Beta Coefficient, Logit (95% CI)
United States Claims Data	Prevalence	Ref	0.11	---
Rose Angina Questionnaire	Prevalence	Alt		2.21 (1.97 to 2.44)
Age (scaled)	Prevalence	Alt		-0.97 (-1.20 to -0.74)
Sex (male)	Prevalence	Alt		-0.62 (-0.86 to -0.38)

Severity split inputs

Acute myocardial infarction was split into two severity levels by length of time since the event – days 1 and 2 versus days 3 through 28. Disability weights were established for these two severities using the standard approach for GBD 2019.

Asymptomatic ischaemic heart disease following myocardial infarction was all assigned to the asymptomatic severity level. No disability weight is assigned to this level.

Angina was split into asymptomatic, mild, moderate, and severe groups using information from MEPS. Disability weights were established for these severities using the standard approach for GBD 2019.

Acute myocardial infarction

Table 3a. Severity distribution, details on the severity levels for Myocardial Infarction in GBD 2019 and the associated disability weight (DW) with that severity.

Severity level	Lay description	DW (95% CI)
Acute myocardial infarction, days 1-2	Has severe chest pain that becomes worse with any physical activity. The person feels nauseated, short of breath, and very anxious.	0.432 (0.288–0.579)
Acute myocardial infarction, days 3-28	Gets short of breath after heavy physical activity, and tires easily, but has no problems when at rest. The person has to take medication every day and has some anxiety.	0.074 (0.049–0.105)

Asymptomatic ischaemic heart disease following myocardial infarction

Table 3b. Severity distribution, details on the severity levels for Asymptomatic ischaemic heart disease following myocardial infarction in GBD 2019 and the associated disability weight (DW) with that severity.

Severity level	Lay description	DW (95% CI)
Asymptomatic ischaemic heart disease		N/A

Angina pectoris

Table 3c. Severity distribution, details on the severity levels for Angina pectoris in GBD 2019 and the associated disability weight (DW) with that severity.

Severity level	Lay description	DW (95% CI)
Asymptomatic angina		N/A
Mild angina	Has chest pain that occurs with strenuous physical activity, such as running or lifting heavy objects. After a brief rest, the pain goes away.	0.033 (0.02–0.052)
Moderate angina	Has chest pain that occurs with moderate physical activity, such as walking uphill or more than half a kilometer (around a quarter-mile) on level ground. After a brief rest, the pain goes away.	0.08 (0.052–0.113)
Severe angina	Has chest pain that occurs with minimal physical activity, such as walking only a short distance. After a brief rest, the pain goes away. The person avoids most physical activities because of the pain.	0.167 (0.11–0.24)

Modelling strategy

Myocardial infarction

- We first calculated custom cause-specific mortality estimates using cause of death data prior to garbage code redistribution, generating age-sex-country-specific proportions of IHD deaths that were due to MI (acute IHD) versus those due to other causes of IHD (chronic IHD). Estimates of this proportion for all locations were then generated using a DisMod proportion-only model. Due to a high degree of variability in pre-redistribution coding practices by location, we used the global age-, sex-, and year-specific proportions of acute deaths in subsequent calculations. The global proportions were multiplied by post-Fauxcorrect (final GBD 2019 CoD estimates with GBD 2017 scalers) IHD deaths by location to generate CSMR estimates for MI. These data, along with incidence and excess mortality data, informed a DisMod model to estimate the prevalence and incidence of myocardial infarction due to ischaemic heart disease.
- These estimates were split into estimates for days 1-2 and days 3-28 post-event. Disability weights were assigned to each of these two groupings.
- We set a value prior of one month for remission (11/13) from the MI model. We also set a value prior for the maximum excess mortality rate of 10 for all ages. We included the Healthcare Access and Quality (HAQ) Index as a fixed-effect country-level covariate on excess mortality, forcing an inverse relationship.

Table 4a. Covariates. Summary of covariates used in the Myocardial Infarction DisMod-MR meta-regression model

Covariate	Parameter	Beta	Exponentiated beta
Healthcare Access and Quality (HAQ) Index	Excess mortality rate	-0.01 (-0.01 to -0.01)	0.99 (0.99 to 0.99)
Log-transformed age-standardised SEV scalar: IHD	Incidence	0.75 (0.75 to 0.76)	2.12 (2.12 to 2.13)

Asymptomatic ischaemic heart disease

- Excess mortality estimates from the myocardial infarction model were used to generate data of the incidence of surviving 28 days post-event.
- We used these data, along with the estimates of CSMR due to chronic IHD (the other part of the proportion described in step 1) and excess mortality data in a DisMod model to estimate the prevalence of persons with IHD following myocardial infarction. This estimate included subjects with angina and heart failure; a proportion of this prevalence was removed in order to avoid double-counting based on evidence from the literature (2). The result of this step generates estimates of asymptomatic ischaemic heart disease following myocardial infarction.
- We set a value prior of 0 for remission for all ages.
- We also included the log-transformed, age-standardised SEV scalar for IHD as a fixed effect, country-level covariate on prevalence and LDI (I$ per capita) as a fixed-effect country-level covariate on excess mortality, forcing an inverse relationship for LDI.

Table 4b. Covariates. Summary of covariates used in Asymptomatic Ischaemic Heart Disease DisMod-MR meta-regression model

Covariate	Parameter	Beta	Exponentiated beta
LDI (I$ per capita)	Excess mortality rate	-0.28 (-0.45 to -0.13)	0.76 (0.63 to 0.88)
Log-transformed age-standardised SEV scalar: IHD	Incidence	1.00 (0.77 to 1.24)	2.72 (2.15 to 3.47)

Angina

- We used prevalence data from the literature and USA claims databases, along with data on mortality risk to estimate the prevalence and incidence of angina for all locations. Data which used the Rose Angina Questionnaire to determine prevalence of angina was adjusted using MR-BRT as described above.
- The proportion of mild, moderate, and severe angina was determined by the standard approach for severity splitting for GBD 2019.
- We included a value prior of 0 for remission for all ages. We also included a value prior of 1 for excess mortality for all ages.
- We also included the log-transformed, age-standardised SEV scalar for IHD as a fixed effect, country-level covariate on prevalence and LDI (I$ per capita) as a fixed effect, country-level covariate on excess mortality, forcing an inverse relationship LDI.

Table 4c. Covariates. Summary of covariates used in the Angina DisMod-MR meta-regression model

Covariate	Parameter	Beta	Exponentiated beta
Log-transformed age-standardised SEV scalar: IHD	Prevalence	1.09 (1.01 to 1.18)	2.99 (2.74 to 3.27)
LDI (I$ per capita)	Excess mortality rate	-0.54 (-0.99 to -.10)	0.58 (0.37 to 0.90)

There have been no substantive changes in the modelling strategy for myocardial infarction, asymptomatic ischaemic heart disease following myocardial infarction, and angina from GBD 2017.

Ischaemic Stroke, Intracerebral Haemorrhage, and Subarachnoid Haemorrhage

Flowchart

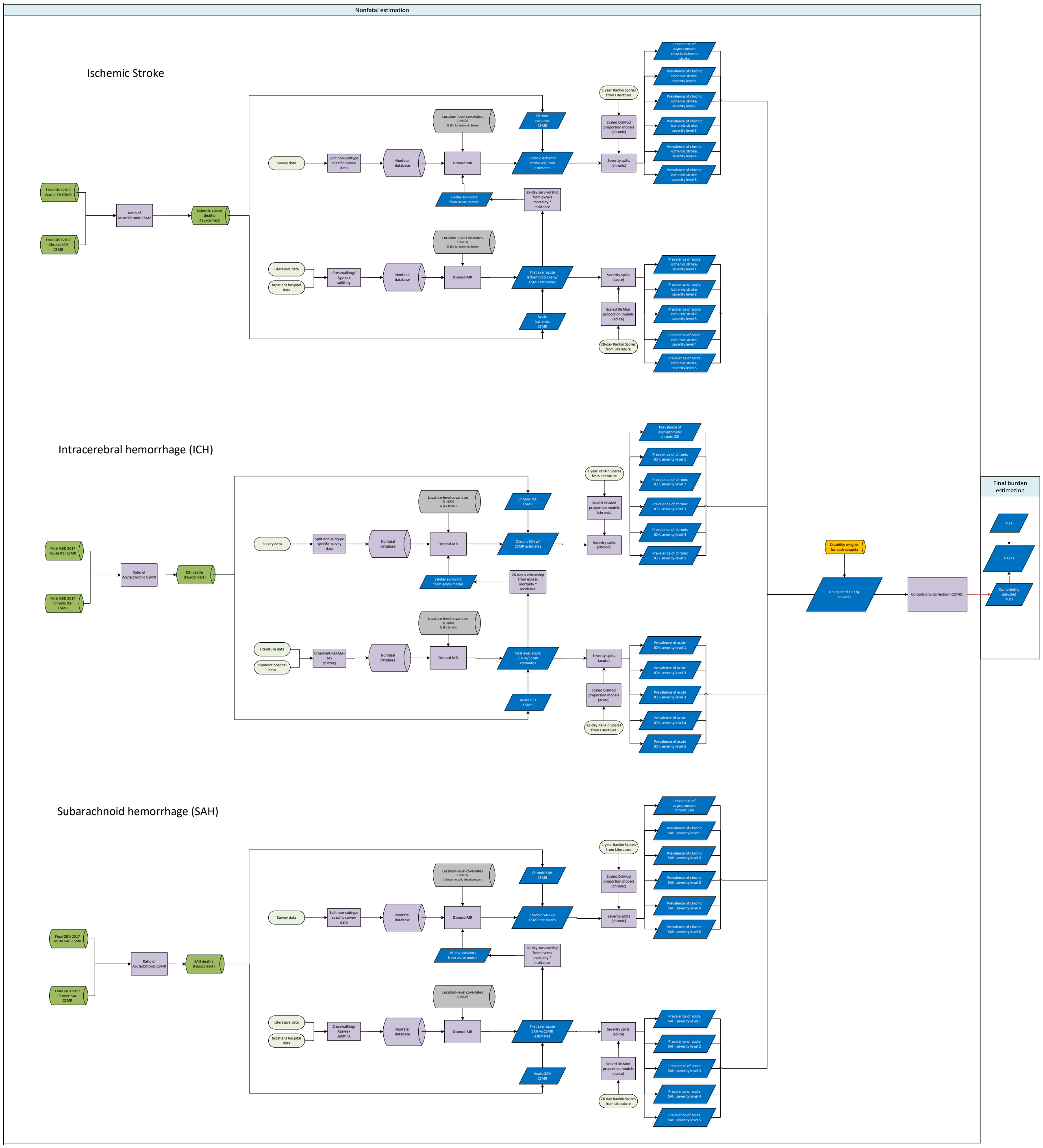

Input data and methodological summary

Case definition

Stroke was defined according to WHO criteria – rapidly developing clinical signs of focal (at times global) disturbance of cerebral function lasting more than 24 hours or leading to death with no apparent cause other than that of vascular origin (1). Data on transient ischaemic attack (TIA) were not included.

Acute stroke: Stroke cases are considered acute from the day of incidence of a first-ever stroke through day 28 following the event.

Chronic stroke: Stroke cases are considered chronic beginning 28 days following the occurrence of an event. Chronic stroke includes the sequelae of an acute stroke AND all recurrent stroke events. GBD 2015 adopts this broader definition of chronic stroke than was used in prior iterations in order to model acute strokes using only first-ever incident events.

Ischaemic stroke: an episode of neurological dysfunction caused by focal cerebral, spinal, or retinal infarction

Intracerebral haemorrhage: a focal collection of blood within the brain parenchyma or ventricular system that is not caused by trauma

Subarachnoid haemorrhage: bleeding into the subarachnoid space (the space between the arachnoid membrane and the pia mater of the brain or spinal cord)

ICD codes used for inclusion of hospital and claims data can be found elsewhere in the appendix.

Input data

Tables 1a, 1b, and 1c display source count information for non-fatal ischaemic stroke, intracerebral haemorrhage, and subarachnoid haemorrhage respectively.

Table 1a: Source counts for ischaemic stroke models.

Measure	Total sources	Countries with data
All measures	523	76
Prevalence	117	24
Incidence	332	62
Excess mortality rate	141	47
Case fatality rate	50	22

Table 1b: Source counts for intracerebral haemorrhage models.

Measure	Total sources	Countries with data
All measures	502	74
Prevalence	117	24
Incidence	322	61
Excess mortality rate	125	41
Case fatality rate	40	18

Table 1c: Source counts for subarachnoid haemorrhage models.

Measure	Total sources	Countries with data
All measures	435	63
Prevalence	117	24
Incidence	260	47
Excess mortality rate	88	28

A systematic review was not performed for GBD 2019. However, a systematic review was performed for GBD 2017. Search terms, dates of search, and databases queried follow:

1) Ischaemic stroke
    a. Google scholar: ("ischemic stroke" OR “cerebral infarction” OR “ischaemic stroke”) AND (incidence OR prevalence OR mortality OR epidemiology). Reviewed first 1000 hits, sorted by relevance
    b. Global Index Medicus search: (tw:("ischemic stroke") OR tw:(“cerebral infarction” OR tw:(“ischaemic stroke”)) AND (tw:(incidence) OR tw:(prevalence) OR tw:(mortality) OR tw:(epidemiology)) AND NOT (tw:(rats) OR tw:(mice) OR tw:(dogs) OR tw:(apes) OR tw:(monkeys)). Dates of search: 01Jan2010 – 31Aug2017
2) Intracerebral haemorrhage
    a. Google scholar: ("hemorrhagic stroke" OR “intracerebral hemorrhage” OR “haemorrhagic stroke” OR “intracerebral haemorrhage”) AND (incidence OR prevalence OR mortality OR epidemiology). Reviewed first 1000 hits, sorted by relevance
    b. GIM search: (tw:("intracerebral hemorrhage") OR tw:(“intracerebral haemorrhage”) OR tw:(“hemorrhagic stroke”) OR tw:(“haemorrhagic stroke”)) AND (tw:(incidence) OR tw:(prevalence) OR tw:(mortality) OR tw:(epidemiology)) AND NOT (tw:(rats) OR tw:(mice) OR tw:(dogs) OR tw:(apes) OR tw:(monkeys)). Dates of search: 01Jan2010 – 31Aug2017
3) Subarachnoid haemorrhage
    a. Google scholar search: ("subarachnoid hemorrhage" OR “subarachnoid haemorrhage”) AND (incidence OR prevalence OR mortality OR epidemiology). Reviewed first 1000 hits, sorted by relevance.
    b. GIM search: (tw:("subarachnoid hemorrhage") OR tw:(“subarachnoid haemorrhage”)) AND (tw:(incidence) OR tw:(prevalence) OR tw:(mortality) OR tw:(epidemiology)) AND NOT (tw:(rats) OR tw:(mice) OR tw:(dogs) OR tw:(apes) OR tw:(monkeys)). Dates of search: 01Jan2010 – 31Aug2017

We included inpatient hospital data, adjusted for readmission and primary to any diagnosis using correction factors estimated from US claims data. We excluded data for locations where the data points were implausibly low (Vietnam, Philippines, India). In addition, we included unpublished stroke registry data for acute ischaemic stroke, acute intracerebral haemorrhage, and acute subarachnoid haemorrhage. We also included survey data for chronic stroke. These surveys were identified based on expert opinion and review of major survey series focused on world health that included questions regarding self-reported history of stroke. For GBD 2019, we split unspecified strokes (ICD-10 I64) into ischaemic stroke, intracerebral haemorrhage, and subarachnoid haemorrhage according to the proportions of subtype-specific coded strokes in the original data. We also split ICD-10 I62 into intracerebral haemorrhage, and subarachnoid haemorrhage using the same approach.

As with many models in GBD, the diversity of data sources available means that we needed to adjust available data to our reference case definition. We thus crosswalked incidence and excess mortality data that did not meet our reference case definitions using MR- BRT, a Bayesian meta-regression tool develop for the GBD. More information on MR-BRT can be found elsewhere in the appendix.

We adjusted data points for first and recurrent strokes combined, using data for first strokes only as reference. For ischaemic stroke and intracerebral haemorrhage, we also adjusted data points that reported all stroke subtypes combined, using as reference studies with subtype-specific information. We also adjusted data which included only persons who survived to hospital admission, using as reference data on both fatal and nonfatal strokes. In addition, we adjusted subtype-specific, inpatient clinical informatics data using subtype-specific literature estimates as a reference. These adjustments can be examined more closely in Table 2. The coefficients in Tables 2a, 2b, and 2c below can be used to calculate adjustment factors for alternative definitions. The formula for computing adjustment factors is given in equation 1 below. We also included a standardized age variable (age scaled) and a sex variable to the crosswalking procedure to adjust for the possibly of bias.

Equation 1: Calculation of adjustment factors:

$$Estimated\ Reference\ Def = invlogit(logit(Alternative\ Def) - Beta_{Alternative\ Def} - Beta_{Sex} * Sex - Beta_{Age_{scaled}} * Age\ Scaled)$$

No data adjustments were necessary for the chronic stroke models.

Table 2a: MR-BRT Crosswalk Adjustment Factors for Ischaemic stroke

	Data input	Measure	Reference or alternative case definition	Gamma	Beta Coefficient, Logit (95% CI)
Ischaemic stroke	First-ever, subtype-specific, fatal and nonfatal events	Incidence	Ref	---	---
Ischaemic stroke	Hospital data	Incidence	Alt	0.97	-0.26 (-2.22 to 1.70)
Ischaemic stroke	Any stroke	Incidence	Alt		0.02 (-1.94 to 1.98)
Ischaemic stroke	Acute first-ever stroke	Incidence	Alt		0.22 (-1.67 to 2.12)
Ischaemic stroke	Inpatient clinical informatics	Incidence	Alt		0.70 (-1.26 to 2.66)
Ischaemic stroke	Sex (male)	Incidence	Alt		0.07 (-1.82 to 1.96)
Ischaemic stroke	Age scaled	Incidence	Alt		0.28 (-1.61 to 2.17)

Table 2b: MR-BRT Crosswalk Adjustment Factors for Intracerebral Haemorrhage

	Data input	Measure	Reference or alternative case definition	Gamma	Beta Coefficient, Logit (95% CI)
Intracerebral Haemorrhage	First-ever, subtype-specific, fatal and nonfatal events	Incidence	Ref	---	---
Intracerebral Haemorrhage	Hospital data	Incidence	Alt	0.50	0.04 (-0.93 to 1.02)
Intracerebral Haemorrhage	Any stroke	Incidence	Alt		1.78 (0.80 to 2.76)
Intracerebral Haemorrhage	Acute first-ever stroke	Incidence	Alt		0.15 (-0.83 to 1.13)
Intracerebral Haemorrhage	Inpatient clinical informatics	Incidence	Alt		1.40 (0.41 to 2.38)
Intracerebral Haemorrhage	Age scaled	Incidence	Alt		0.09 (-0.88 to 1.07)
Intracerebral Haemorrhage	Sex (male)	Incidence	Alt		0.10 (-0.88 to 1.06)

Table 2c: MR-BRT Crosswalk Adjustment Factors for Subarachnoid Haemorrhage

	Data input	Measure	Reference or alternative case definition	Gamma	Beta Coefficient, Logit (95% CI)
Subarachnoid Haemorrhage	First-ever, subtype-specific, fatal and nonfatal events	Incidence	Ref	---	---
Subarachnoid Haemorrhage	Aneurysmal subarachnoid haemorrhage only	Incidence	Alt	0.76	-0.79 (-2.28 to 0.70)
Subarachnoid Haemorrhage	Age scaled	Incidence	Alt		-0.11 (-1.59 to 1.38)
Subarachnoid Haemorrhage	Sex (male)	Incidence	Alt		-0.07 (-1.56 to 1.42)

Severity split inputs

The table below illustrates the severity level, lay description, and disability weights for GBD 2019. In previous iterations of GBD, severity splits for stroke were based on the standard approach described elsewhere (3). For GBD 2016, we undertook a review to identify epidemiologic literature which reported the degree of disability at 28 days (for acute stroke) or one year (for chronic stroke) using the modified Rankin scale (mRS) and the Mini-Mental State Examination (MMSE) or the Montreal Cognitive Assessment (MoCA). The mRS assesses functional capabilities, while the MMSE and MoCA tests provide evaluations of cognitive functioning. We then mapped these measures to the existing GBD categories as indicated below. This approach allowed us to include location-specific information and can be updated as more data on functional or cognitive status become available.

Acute stroke severity splits

Table 3a. Severity distribution, details on the severity levels for Acute Stroke in GBD 2019 and the associated disability weight (DW) with that severity.

Severity level	Lay description	Modified Rankin score	Cognitive status	DW (95% CI)
Stroke, mild	Has some difficulty in moving around and some weakness in one hand, but is able to walk without help.	1	N/A	0.019 (0.01–0.032)
Stroke, moderate	Has some difficulty in moving around, and in using the hands for lifting and holding things, dressing, and grooming.	2, 3	MoCA>=24 or MMSE>=26	0.07 (0.046–0.099)
Stroke, moderate plus cognition problems	Has some difficulty in moving around, in using the hands for lifting and holding things, dressing and grooming, and in speaking. The person is often forgetful and confused.	2, 3	MoCA<24 or MMSE<26	0.316 (0.206–0.437)

Stroke, severe	Is confined to bed or a wheelchair, has difficulty speaking, and depends on others for feeding, toileting, and dressing.	4, 5	MoCA>=24 or MMSE>=26	0.552 (0.377–0.707)
Stroke, severe plus cognition problems	Is confined to bed or a wheelchair, depends on others for feeding, toileting, and dressing, and has difficulty speaking, thinking clearly, and remembering things.		MoCA<24 or MMSE<26	0.588 (0.411–0.744)

Chronic stroke severity splits

Table 3b. Severity distribution, details on the severity levels for Chronic Stroke in GBD 2019 and the associated disability weight (DW) with that severity.

Severity level	Lay description	Modified Rankin score	Cognitive status	DW (95% CI)
Stroke, asymptomatic		0	N/A	N/A
Stroke, long-term consequences, mild	Has some difficulty in moving around and some weakness in one hand, but is able to walk without help.	1	N/A	0.019 (0.01–0.032)
Stroke, long-term consequences, moderate	Has some difficulty in moving around, and in using the hands for lifting and holding things, dressing, and grooming.	2, 3	MoCA>=24 or MMSE>=26	0.07 (0.046–0.099)
Stroke, long-term consequences, moderate plus cognition problems	Has some difficulty in moving around, in using the hands for lifting and holding things, dressing and grooming, and in speaking. The person is often forgetful and confused.	2, 3	MoCA<24 or MMSE<26	0.316 (0.206–0.437)
Stroke, long-term consequences, severe	Is confined to bed or a wheelchair, has difficulty speaking, and depends on others for feeding, toileting, and dressing.	4, 5	MoCA>=24 or MMSE>=26	0.552 (0.377–0.707)
Stroke, long-term consequences, severe plus cognition problems	Is confined to bed or a wheelchair, depends on others for feeding, toileting, and dressing, and has difficulty speaking, thinking clearly, and remembering things.	4, 5	MoCA<24 or MMSE<26	0.588 (0.411–0.744)

Table 4: Data input counts for the estimation process for the custom severity splits.

	Acute proportion	Chronic proportion
**Site-years (total)**	9	16
**Number of countries with data**	6	13
**Number of GBD regions with data (out of 21 regions)**	6	7
**Number of GBD super-regions with data (out of 7 super-regions)**	4	5

We used DisMod-MR, a Bayesian meta-regression tool, to model the six severity levels, with an independent proportion model for each. Reports which grouped mRS scores differently than our mapping (eg, 0-2) were adjusted in DisMod by estimating the association between these alternate groupings and our preferred mappings. These statistical associations were used to adjust data points to the referent category as necessary. The six models were scaled such that the sum of the proportions for all levels equaled 1.

Modelling strategy

The general approach employed for all of the components of the stroke modelling process is detailed in the table below.

- Data points were adjusted from alternative to reference case definitions using estimates from statistical models generated by MR-BRT (discussed elsewhere in the appendix) for the acute models. Coefficients for these crosswalks can be found in Table 2a, 2b, and 2c.

- The GBD summary exposure values (SEV), which are the relative risk-weighted prevalence of exposure, were included as covariates for the ischaemic stroke or intracerebral haemorrhage models as appropriate, and a covariate for country income was used as a country-level covariate for both models (4). Subarachnoid haemorrhage did not included an SEV covariate, but did include a covariate for country income for excess mortality. Coefficients for these covariates can be found in Table 5a, 5b, 5c for fixed effects located below.

- We used the ratio of acute:chronic cause-specific mortality estimated by the final GBD 2017 dismod model estimates to divide GBD 2019 stroke deaths into acute and chronic stroke deaths, using the global average for the proportion of acute:chronic stroke mortality. The acute and chronic models were then run using the same incidence, prevalence, and case fatality data as well as the custom cause-specific mortality rates as input data.

- We ran the first-ever acute subtype-specific models with CSMR as derived from FauxCorrect and epidemiological data as described above using Dismod-MR.

- We then calculated the rate of surviving until 28 days after an acute event for all three subtypes using the modelled estimates of excess mortality and incidence from the acute stroke models.

- Twenty-eight-day survivorship data was uploaded into the chronic subtype-specific with CSMR models. These chronic models also use CSMR as derived from FauxCorrect and epidemiological data as described above. Models were evaluated based on expert opinion, comparison with previous iterations, and model fit.

Table 5a, 5b, 5c below indicate the covariates used by cause in the estimation process, as well as the beta and exponentiated beta values.

Table 5a: Coefficients for covariates used in the acute and chronic ischemic stroke DisMod-MR models

Model	Variable name	Measure	beta	Exponentiated beta
First-ever acute ischaemic stroke with CSMR	Log-transformed age-standardised SEV scalar: Ischaemic stroke	Incidence	0.90 (0.85 to 0.95)	2.46 (2.34 to 2.58)
First-ever acute ischaemic stroke with CSMR	Healthcare access and quality index	Excess mortality rate	-0.035 (-0.035 to -0.035)	0.97 (0.97 to 0.97)
Chronic ischaemic stroke with CSMR	Log-transformed SEV scalar: Ischaemic stroke	Prevalence	0.85 (0.78 to 0.92)	2.34 (2.18 to 2.51)
Chronic ischaemic stroke with CSMR	LDI (I$ per capita)	Excess mortality rate	-0.41 (-0.46 to -0.36)	0.67 (0.63 to 0.70)

Table 5b: Coefficients for covariates used in the acute and chronic intracerebral haemorrhage DisMod-MR models

Model	Variable name	Measure	beta	Exponentiated beta
First-ever acute intracerebral haemorrhage with CSMR	Log-transformed SEV scalar: Intracerebral Haemorrhage	Incidence	0.76 (0.75 to 0.77)	2.13 (2.12 to 2.15)
First-ever acute intracerebral haemorrhage with CSMR	Healthcare access and quality index	Excess mortality rate	-0.07 (-0.07 to -0.069)	0.93 (0.93 to 0.93)
Chronic intracerebral haemorrhage with CSMR	Log-transformed SEV scalar: Intracerebral haemorrhage	Prevalence	0.75 (0.75 to 0.76)	2.12 (2.12 to 2.14)
Chronic intracerebral haemorrhage with CSMR	LDI (I$ per capita)	Excess mortality rate	-0.5 (-0.5 to -0.5)	0.61 (0.61 to 0.61)

Table 5a: Coefficients for covariates used in the acute and chronic subarachnoid DisMod-MR models

Model	Variable name	Measure	beta	Exponentiated beta
First-ever acute subarachnoid haemorrhage with CSMR	LDI (I$ per capita)	Excess mortality rate	-0.3 (-0.49 to -0.11)	0.74 (0.61 to 0.90)

Non-rheumatic valvular heart diseases:

Calcific aortic valve disease

Degenerative mitral valve disease

Other non-rheumatic valve disease

Flowchart: Calcific aortic valve and degenerative mitral valve disease

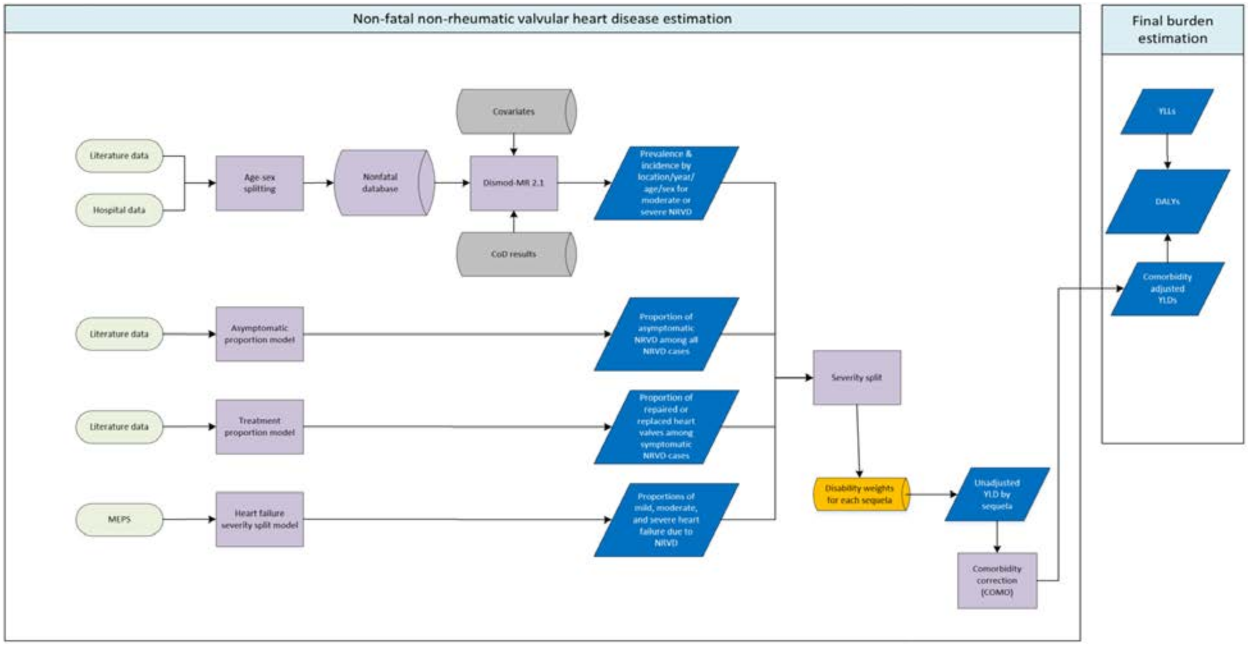

Case definitions

Calcific aortic valve disease

Calcific aortic valve disease was defined as clinical diagnosis of aortic valve stenosis or regurgitation due to progressive calcification of the aortic valve or annulus leading to haemodynamically moderate or severe aortic stenosis or regurgitation. Cases were determined by echocardiography. Calcific aortic valve disease in the GBD did not include aortic valve disease with an aetiology that was congenital, rheumatic, or infectious. Disease due to these aetiologies are modelled in other causes in the GBD. Information on unicuspid or bicuspid valves was generally not available and is often unknown in advanced calcific disease. Therefore, we included cases of unicuspid or bicuspid valves in our case definition if they developed clinically significant aortic stenosis. The criteria for aortic stenosis follow the American Heart Association/American College of Cardiology definition of haemodynamically moderate or severe aortic stenosis and are listed in Table 1. The criteria for aortic regurgitation follow the American Heart Association/American College of Cardiology definition of haemodynamically moderate or severe aortic regurgitation and are listed in Table 2. Mild haemodynamic aortic stenosis or regurgitation was not included in our case definition because mildly abnormal haemodynamic parameters are difficult to differentiate from non-pathological stenosis and/or regurgitation, and are generally not reported in population-based studies.

Table 1: AHA/ACC definitions of aortic stenosis

Maximum jet velocity ≥ 3 m/s
Mean pressure gradient ≥ 20 mmHg

Table 2: AHA/ACC definitions of aortic regurgitation

Central jet mitral regurgitation ≥ 25% of the left ventricular outflow tract
Vena contracta ≥ 0.3 cm
Regurgitant volume ≥ 30 mL/beat
Regurgitant fraction ≥ 30%
Angiography grade ≥ 2+

Degenerative mitral valve disease

Degenerative mitral valve disease was defined as myxomatous degeneration of the mitral valve leading to regurgitation or prolapse. Cases were determined by echocardiography by a physician. Degenerative mitral valve disease did not include mitral valve disease with an aetiology that was congenital, rheumatic, infectious, traumatic, carcinoid, or functional (ie, secondary to left ventricular remodeling due to heart failure from another cause). Mitral valve stenosis was always considered to have a rheumatic aetiology and therefore was not included in the definition of degenerative mitral valve disease. Degenerative mitral valve disease was restricted to persons at or above the age of 15 in order to exclude congenital mitral valve disorders. This age restriction is consistent with other progressive cardiovascular diseases modelled in the GBD. The criteria for mitral regurgitation follow the American Heart Association/American College of Cardiology definition of haemodynamically progressive or severe mitral regurgitation and are listed in Table 3. Mild haemodynamic mitral regurgitation was not included in our case definition because mild mitral valve disease cannot be differentiated from nonpathological regurgitation and is generally not reported in population-based studies.

Table 3: AHA/ACC definitions of mitral regurgitation

Central jet mitral regurgitation > 20% of the left atrium
Vena contracta ≥ 0.7 cm
Regurgitant volume ≥ 60 mL/beat
Regurgitant fraction ≥ 50%
Effective regurgitant orifice ≥ 0.4 cm^2
Angiography grade ≥ 2+

Other non-rheumatic valve disease

Other non-rheumatic valve disease is a residual category that captures non-rheumatic, non-congenital valve disorders of the tricuspid and pulmonary valves. This includes tricuspid regurgitation, tricuspid stenosis, pulmonary regurgitation, and pulmonary stenosis. Other non-rheumatic valve disease did not include tricuspid or pulmonary valve disease with an aetiology that was congenital, rheumatic, infectious, traumatic, carcinoid, or functional (ie, secondary to heart failure due to another cause).

Input data

Data on the prevalence, incidence, treatment, haemodynamic severity, and asymptomatic status were collected from PubMed using the following search strings on 8/21/2017:

Calcific aortic valve disease

("aortic stenosis"[Title/Abstract] OR "aortic regurgitation"[Title/Abstract]) NOT ("Transcatheter Aortic Valve Replacement"[MeSH] OR "Transcatheter aortic valve implantation"[KEYWORD]) AND (epidemiology[MeSH Major Topic] OR epidemiology[Subheading] OR epidemiology[MeSH Terms] OR prevalence[Title/Abstract] OR mortality[Title/Abstract]) NOT (animals[MeSH] NOT humans[MeSH]) AND ("1980/1/01"[PDAT] : "2017/12/31"[PDAT]) NOT Comment[ptyp] NOT Case Reports[ptyp]

Degenerative mitral valve disease

("mitral stenosis"[Title/Abstract] OR "mitral regurgitation"[Title/Abstract]) AND ("epidemiology"[MeSH Major Topic] OR "epidemiology"[Subheading] OR "epidemiology"[MeSH Terms] OR prevalence[Title/Abstract] OR mortality[Title/Abstract]) NOT (animals[MeSH] NOT humans[MeSH]) AND ("1980/1/01"[PDAT] : "2017/12/31"[PDAT]) NOT Comment[ptyp] NOT Case Reports[ptyp]

Other non-rheumatic valve disease

We did not run a literature review for "other non-rheumatic valve diseases" because we did not directly model non-fatal burden due to this cause.

We excluded literature that was not representative, included rheumatic, endocarditic, or congenital heart disease in its case definition, or included haemodynamically mild valve disease in its case definition.

Data on the prevalence of calcific aortic valve and degenerative mitral valve disease were also obtained from inpatient hospital data. These data were adjusted for multiple visits, non-primary diagnoses, and inpatient to outpatient utilisation ratios. Hospital data were excluded below age 30 or if the age-series for a given hospital data source was implausible. Prevalence data from both inpatient and outpatient hospital claims were used in the United States.

For GBD 2019, we used the modeling software Meta-Regression, Baysian Regularized Trimming (MR-BRT) to correct for biases in data types, replacing the in-DisMod crosswalks used in GBD 2017. We used a network meta-analysis to adjust inpatient data, MarketScan data from 2010-2016, and MarketScan data from 2000, which used a different sampling methodology than other years, to literature and inpatient data. Tables 4 and 5 show MR-BRT crosswalk adjustment factors.

MR-BRT was used to split both-sex data points into sex-specific estimates. This methodology is detailed elsewhere in the appendix. We also split data points where the age range was greater than 25 years. Age splitting was based on the global sex-specific age pattern from a Dismod model that only used input data from scientific literature with less than a 25-year age range.

Source counts

	Measure	Total sources	Countries with data
Calcific aortic valve disease	Prevalence	221	35
Calcific aortic valve disease	Case fatality rate	1	1
Degenerative mitral valve disease	Prevalence	198	30
Degenerative mitral valve disease	With-condition mortality rate	1	1
Degenerative mitral valve disease	Case fatality rate	1	1

Table 4: MR-BRT adjustment factors for calcific aortic valve disease

$$Estimated\ Reference\ Def = invlogit(logit(Alternative\ Def) - Beta_{Alternative\ Def} - Beta_{Sex} * Sex - Beta_{Age_{scaled}} * Age\ Scaled)$$

Data input	Reference or alternative case definition	Gamma	Beta Coefficient, Logit (95% CI)	Beta Coefficient, real-space
Literature	Reference	0.07	---	---
Inpatient	Alternate		-1.08 (-1.27, -0.89)	0.25
Marketscan, 2000	Alternate		-0.78 (-0.98, -0.58)	0.31
Marketscan, 2010-2016	Alternate		-0.04 (-0.23, 0.15)	0.49
Age, scaled			0.45 (0.32, 0.59)	0.61
Male			0.06 (-0.08, 0.19)	0.51

Table 5: MR-BRT adjustment factors for degenerative mitral valve disease

$$Estimated\ Reference\ Def = invlogit(logit(Alternative\ Def) - Beta_{Alternative\ Def} - Beta_{Sex} * Sex - Beta_{Age_{scaled}} * Age\ Scaled)$$

Data input	Reference or alternative case definition	Gamma	Beta Coefficient, Logit (95% CI)	Beta Coefficient, real-space
Literature	Reference	0.07	---	---
Inpatient	Alternate		-1.88 (-2.34, -1.43)	0.13
Marketscan, 2000	Alternate		-1.53 (-1.99, -1.06)	0.18
Marketscan, 2010-2016	Alternate		-0.82 (-1.28, -0.37)	0.31

Age, scaled			0.41 (0.03, 0.80)	0.60
Male			0.01 (-0.38, 0.39)	0.50

Modelling strategy

For other non-rheumatic valve diseases, we estimated nonfatal burden using the cause of death heart failure approach. This method is used for most cardiovascular diseases that cause heart failure and is described in detail in the appendix section on heart failure.

In order to estimate non-fatal burden for calcific aortic valve disease and degenerative mitral valve disease, we first determined the sequelae and corresponding health states that result from these conditions. This information, along with the disability weights applied to each health state, are displayed in Table 6.

Table 6: Sequelae, health state lay descriptions, and disability weights

Sequela	Health state name	Health state lay description	Disability weight
Asymptomatic non-rheumatic valve disease	Asymptomatic	--	0
Non-rheumatic valve disease after treatment	Generic uncomplicated disease: worry and daily medication	Has a chronic disease that requires medication every day and causes some worry but minimal interference with daily activities.	0.049 (0.031–0.072)
Mild heart failure due to non-rheumatic valve disease	Heart failure, mild	Is short of breath and easily tires with moderate physical activity, such as walking uphill or more than a quarter-mile on level ground. The person feels comfortable at rest or during activities requiring less effort.	0.041 (0.026–0.062)
Moderate heart failure due to non-rheumatic valve disease	Heart failure, moderate	Is short of breath and easily tires with minimal physical activity, such as walking only a short distance. The person feels comfortable at rest but avoids moderate activity.	0.072 (0.047–0.103)
Severe heart failure due to non-rheumatic valve disease	Heart failure, severe	Is short of breath and feels tired when at rest. The person avoids any physical activity, for fear of worsening the breathing problems.	0.179 (0.122–0.251)

To model the burden due to each of the sequela above, we first modelled the overall prevalence of combined haemodynamically moderate and severe calcific aortic valve disease and degenerative mitral valve disease. We then estimated the proportion of those with prevalent disease who were haemodynamically moderate, assuming that this would approximate the proportion who were asymptomatic. We next estimated the proportion of those with symptomatic disease (ie, those with haemodynamically severe disease) who were treated. The remaining proportion – those with untreated symptomatic disease – was split into four proportions: 1) controlled, medically managed; 2) mild; 3) moderate; and 4) severe heart failure. All proportions were calculated and converted to population prevalence at the draw level, thus propagating uncertainty from each step through to all subsequent steps. Population prevalence for each severity level are necessary in order to accurately calculate the burden for these diseases. Figure 1 visualises this framework. Each of these modelling steps is outlined in greater detail below.

Figure 1: Modelling framework for calcific aortic valve disease and degenerative mitral valve disease

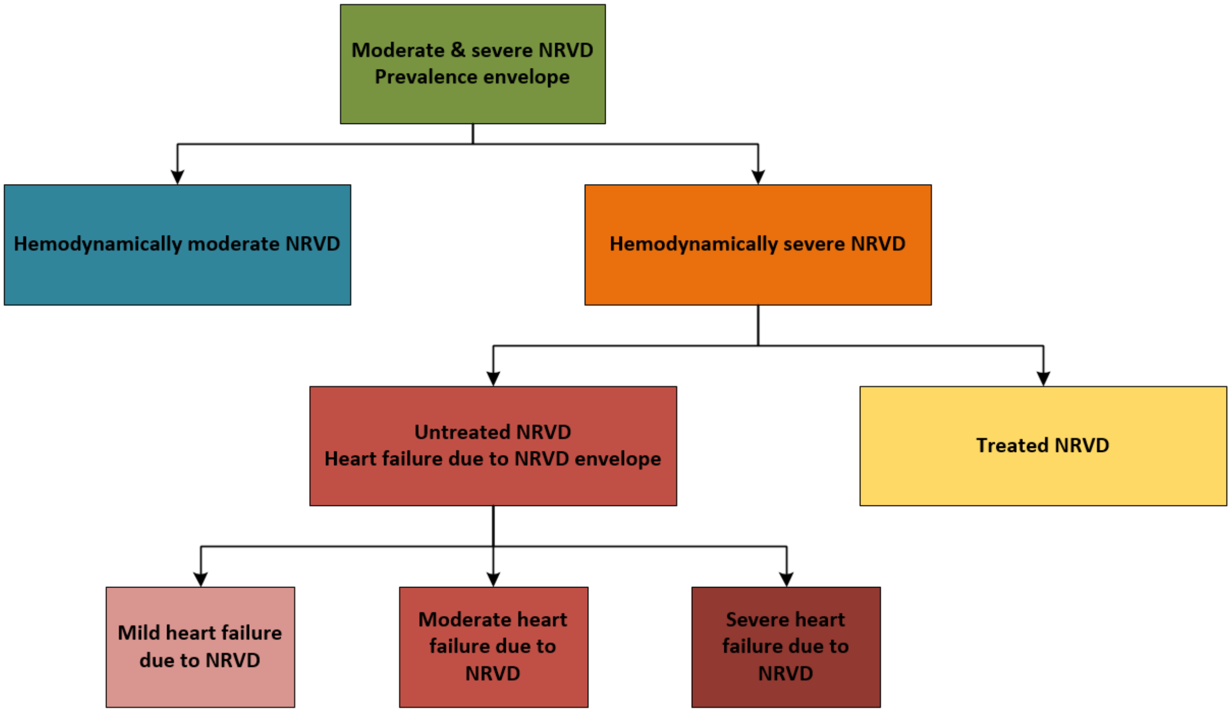

Prevalence envelope

We separately modelled the overall prevalence of calcific aortic valve disease and degenerative mitral valve disease in DisMod-MR 2.1. We used cause-specific mortality rates from the fatal modelling process as inputs. These two models estimate the prevalence of these two valve diseases for each age, sex, location, and year. Covariates included in the DisMod models for prevalence of calcific aortic valve and degenerative mitral valve disease are presented in tables 9 and 10.

Table 9: Covariates and resulting coefficients for calcific aortic valve disease DisMod model

Covariate	Integrand	Coefficients	Exponentiated coefficients
Mean BMI	Prevalence	1.76 (1.74-1.77)	5.79 (5.72-5.88)
Smoking Prevalence	Prevalence	0.0026 (0.000086 to 0.0095)	1.00 (1.00 to 1.01)
HAQ index	Excess mortality rate	-0.079 (-0.082 to -0.077)	0.92 (0.92 to 0.93)

Table 10: Covariates and resulting coefficients for degenerative mitral valve disease DisMod model

Covariate	Integrand	Coefficients	Exponentiated coefficients
HAQ index	Excess mortality rate	-0.073 (-0.18 to –0.005)	0.93 (0.84 to 1.00)

Haemodynamically moderate proportion

We estimated the proportion of individuals with haemodynamically moderate or severe valve disease who were haemodynamically moderate. As mentioned above, we assumed that individuals with haemodynamically moderate disease were asymptomatic. There were a total of five data sources that reported the proportion of individuals who were haemodynamically moderate. Because of the sparsity of data, we modelled the haemodynamically moderate proportion together for both calcific aortic valve disease and degenerative mitral valve disease. We modelled a proportion with uncertainty that varied by age with the following regression:

$$logit(y) = \beta_0 + \beta_1 age + \gamma$$

Where y is the proportion of haemodynamically moderate disease, *age* is the midpoint age for each data point, and γ is a random effect for each data source. The regression coefficients are reported in Table 11.

Table 11: Moderate NRVD regression coefficients

Covariate	Coefficients	Transformed coefficients
Intercept (β_0)	6.6 (4.9 to 8.4)	0.998 (0.992 to 0.999)

Age (β_1)	-0.07 (-0.093 to -0.047)	0.932 (0.911 to 0.954)

The prevalence of those with haemodynamically moderate valve disease and the prevalence of those with haemodynamically severe disease were calculated using the prevalence envelope and the proportion of those with haemodynamically moderate disease for each five-year age group, sex, location, and year.

Treated proportion

We estimated the proportion of individuals who had haemodynamically severe disease who had been treated. Treatment was defined as valve replacement or repair. We assumed that treatment was not performed on any individuals with only haemodynamically moderate disease. The number of data points are reported in Table 10.

Table 12: Data on treated calcific aortic and degenerative mitral valve disease

Input data	Number of data points
**Unique sources**	**23**
**Geography-years**	**35**

These data were all from relatively high-income geographies, yet it is important that we capture the difference in treatment between high- and low-income locations. Because of this challenge, we ran a regression using the Healthcare Access and Quality (HAQ) index predicting the level of treatment and set a prior that the proportion of individuals with a valve replacement or repair was zero where HAQ index was equal to zero. This assumption allowed us to estimate an increasing relationship between HAQ index and proportion treated, where the estimated proportion treated was based on data where HAQ index was high. We used the regression equation:

$$logit(y) = \alpha + \beta_1 * haqi + \beta_2 * age + \beta_3 * severity$$

where y is the proportion of individuals with haemodynamically severe disease who had a valve replacement or repair, $haqi$ is the Healthcare Access and Quality index, age is the midpoint of the age range for a given data point, and $severity$ is an indicator variable to adjust for data points where the denominator of the proportion treated included both haemodynamically moderate and haemodynamically severe individuals. The prevalence of those with treated valve disease and the prevalence of those with untreated haemodynamically severe disease were calculated using the prevalence of haemodynamically severe disease and the proportion of those with treated valve disease. The results of this regression are reported in Table 13 and plotted for three ages in Figure 2.

Table 13: Treated calcific aortic valve and degenerative mitral valve disease regression coefficients

Covariate	Coefficients	Transformed coefficients
Intercept (β_0)	-4.69 (-5.90 to -3.43)	0.009 (0.003 to 0.032)

HAQI (β_1)	0.080 (0.070 to 0.089)	1.083 (1.073 to 1.093)
Age (β_2)	-0.029 (-0.04 to -0.015)	0.971 (0.957 to 0.985)
Severity (β_3)	-0.947 (-1.40 to -0.54)	0.377 (0.246 to 0.578)

Figure 2: Results of treatment model for three ages

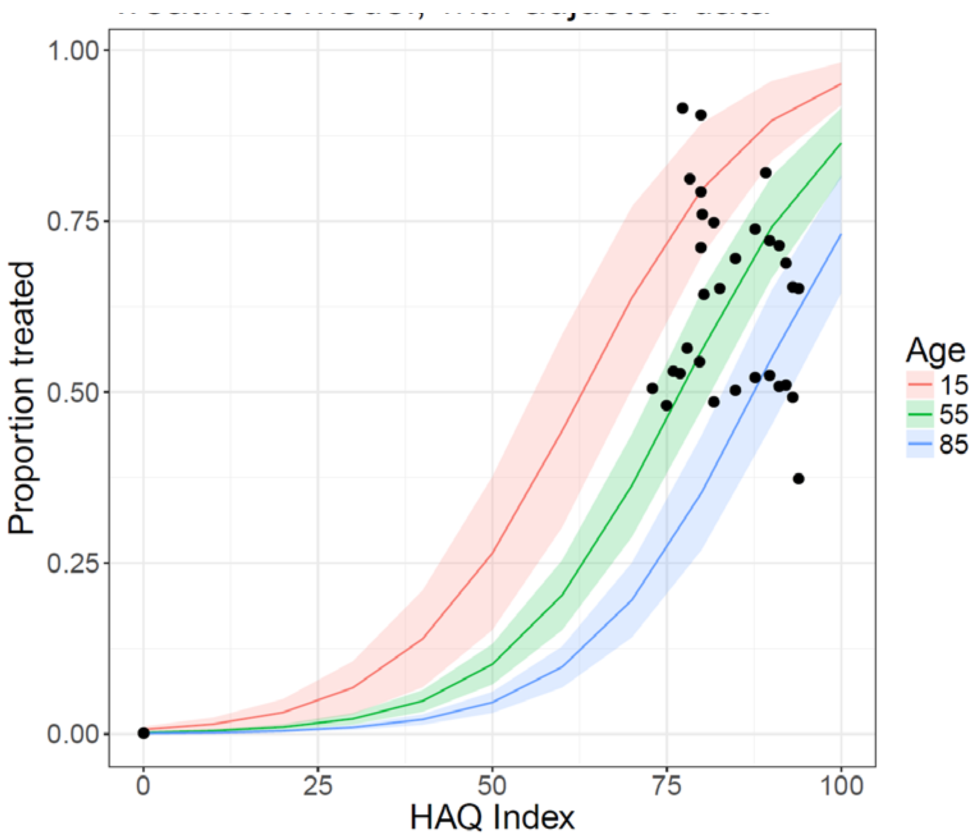

Final burden estimation

The proportions of 1) controlled, medically managed, 2) mild, 3) moderate and 4) severe heart failure due to valve disease were estimated using the approach described in the heart failure section of the appendix. Prevalence for each of these health states was estimated using the prevalence of haemodynamically severe disease and the corresponding proportion for each severity of heart failure. Burden due to each severity of valve disease was estimated by multiplying the prevalence of each severity by the corresponding disability weight.

Acute Myocarditis

Flowchart

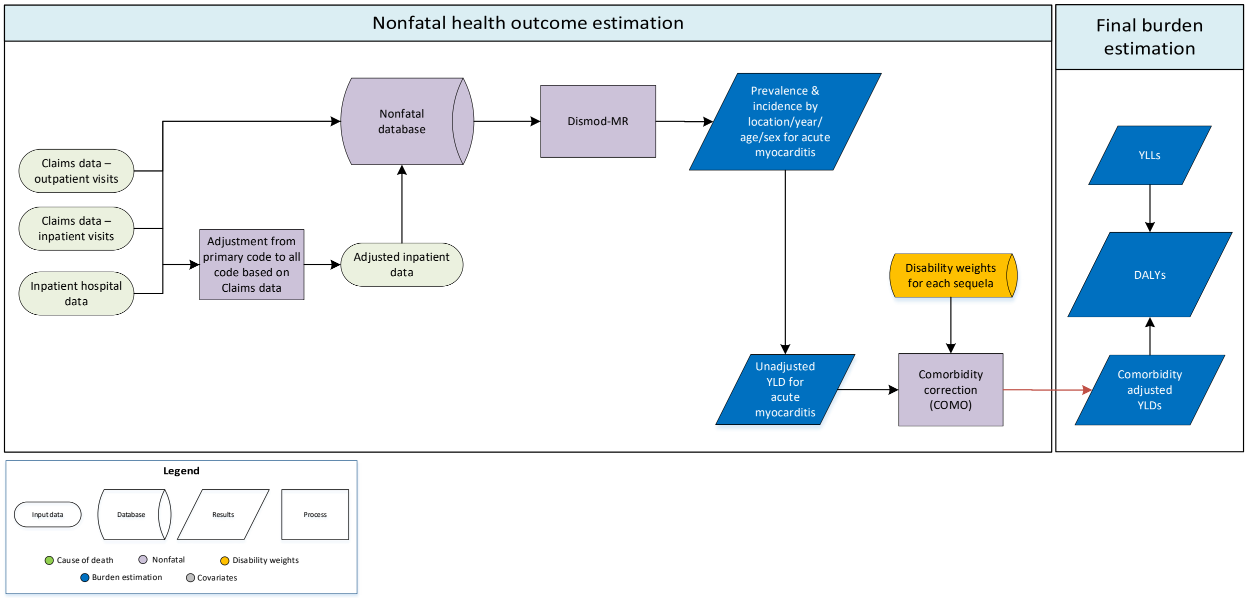

Input data and methodological summary

Case definition

Myocarditis refers to a heterogenous group of diseases with variable clinical and pathological features. Acute myocarditis was defined for GBD as the acute and time-limited symptoms of myocarditis separate from its chronic heart failure-related sequelae. Heart failure due to myocarditis is estimated separately in GBD (see methods for heart failure). Symptoms of acute myocarditis are nonspecific and include a flu-like or gastrointestinal syndrome, followed by anginal-type chest pain, arrhythmias, syncope, or heart failure.

A list of the ICD codes included can be found in elsewhere in the appendix.

Input data

Model inputs

The preferred data sources for acute myocarditis were hospital admission data and other health facility data identifying cases of acute myocarditis. Table 1 shows the source counts for acute myocarditis.

Table 1: Source counts for acute myocarditis

Measure	Total sources	Countries with data
All measures	250	39
Incidence	250	39

A systematic review was performed for GBD 2013 and updated for GBD 2015. A systematic review was not performed for GBD 2019.

The GBD 2015 search terms included: (cardiomyopathy AND epidemiology [MeSH Subheading]) OR (myocarditis AND epidemiology [MeSH Subheading]) OR (cardiomyopathy AND (incidence OR prevalence OR “case fatality”)) OR (myocarditis AND (incidence OR prevalence OR “case fatality”))

- Dates included in search: 1/1/2013 – 3/16/2015
- Number of initial hits: 3,598
- Number of sources included: 0

The GBD 2013 search terms included: (hasabstract[text] AND Humans[Mesh] AND middle age[MeSH])) OR 21) AND ((cardiomyopathy/epidemiology[Mesh] OR cardiomyopathy/mortality[Mesh]) AND (prevalence[Title/Abstract] OR incidence[Title/Abstract]) AND ("2010"[Date - Publication] : "3000"[Date - Publication]) AND (hasabstract[text] AND Humans[Mesh] AND middle age[MeSH]))

We did not include any non-literature-based data, apart from the hospital and claims data described elsewhere. We used inpatient hospital data adjusted for readmission, primary to any diagnosis, and inpatient to outpatient utilisation based on correction factors generated using USA claims data. We excluded all outpatient data, as they were implausibly low when compared with inpatient data from the same locations and with claims data. Inpatient hospital data points that were more than two-fold higher or 0.5-fold lower than the median absolute deviation value for high-income North America, Central Europe, and Western Europe for that age-sex group were excluded.

Severity splits and disability weights

Table 2. Severity distribution, details on the severity levels for Acute Myocarditis in GBD 2019 and the associated disability weight (DW) with that severity.

Severity level	Lay description	DW (95% CI)
Acute myocarditis	Has a fever and aches, and feels weak, which causes some difficulty with daily activities.	0.051 (0.032–0.074)

Modelling strategy

For GBD 2019, we estimated acute myocarditis using a DisMod-MR Bayesian meta-regression model, setting a minimum of 3 and maximum of 5 as value priors on remission to establish an average duration of three months. We set a value prior of 0 for all ages on excess mortality. In GBD 2017, the country-level covariates used included the cardiomyopathy and myocarditis summary exposure variable (SEV) on incidence and the Healthcare Access and Quality index (HAQ Index) on excess mortality. For GBD 2019, The only country level covariate used was Healthcare Access and Quality Index (HAQ Index) on excess mortality.

Table 3 below gives the parameters, betas, and exponentiated betas for study-level and country-level covariates used in the model

Table 3. Covariates. Summary of covariates used in the Acute Myocarditis DisMod-MR meta-regression model

Study covariate	Parameter	beta	Exponentiated beta
Healthcare Access and Quality index	Excess mortality rate	-0.55 (-0.99 to -0.1)	0.58 (0.37 to 0.90)

Aside from the minor covariate change, no other substantive changes were made to the modelling approach for GBD 2017.

Atrial Fibrillation and Flutter

Flowchart

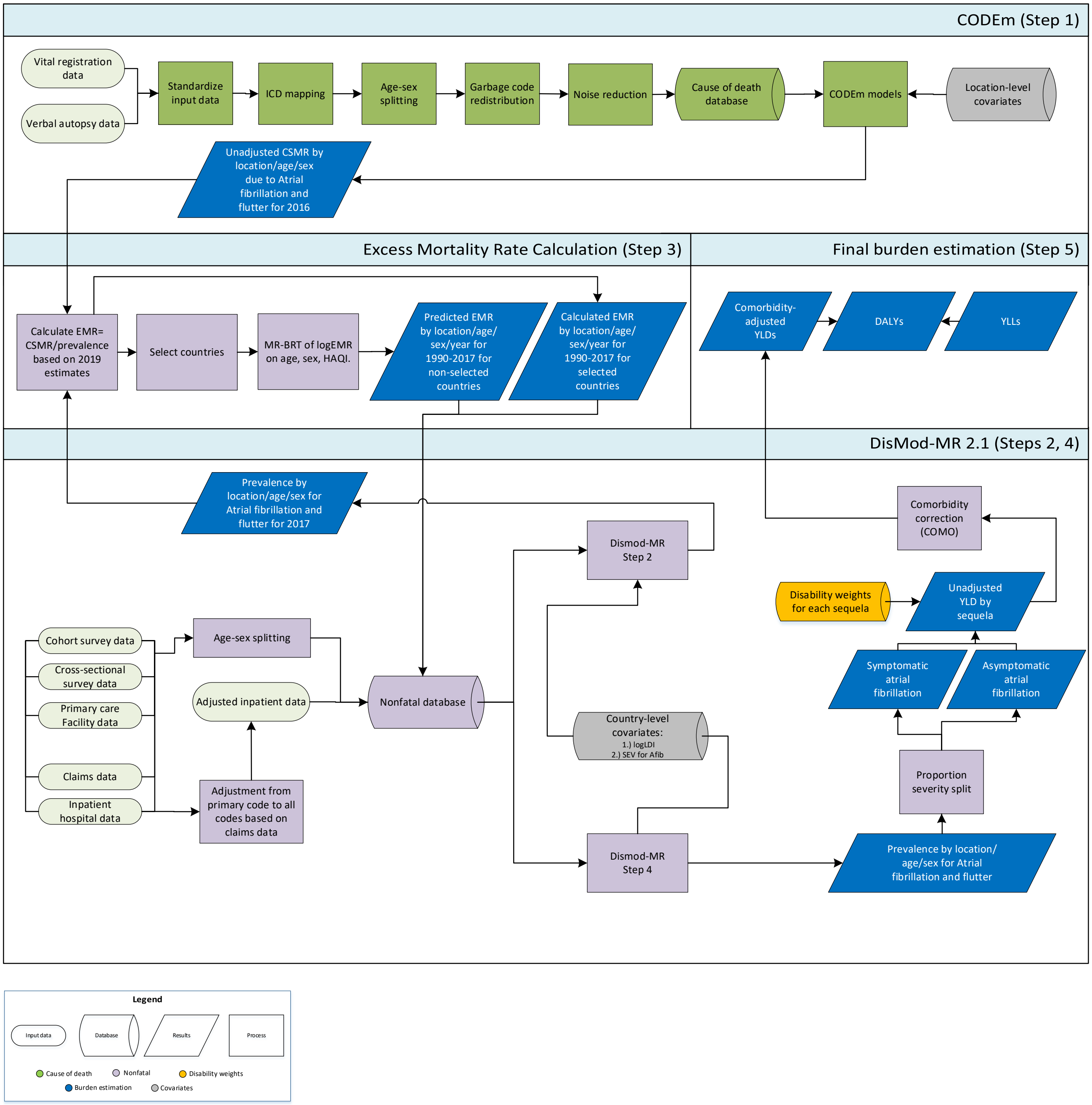

Input data and methodological summary

Case definition

Atrial fibrillation is a supraventricular arrhythmia due to disorganised depolarisation of the atrium. Atrial flutter is a macro-reentrant supraventricular arrhythmia, usually involving the cavo-tricuspid isthmus. Diagnosis requires an ECG demonstrating: 1) irregularly irregular RR intervals (in the absence of complete AV block); 2) no distinct P waves on the surface ECG, and; 3) an atrial cycle length (when visible) that is usually variable and less than 200 milliseconds.

ICD codes used for inclusion of hospital and claims data can be found elsewhere in the appendix.

Input data

Model inputs

Table 1 shows the source counts for atrial fibrillation and flutter in GBD 2019.

Measure	Total sources	Countries with data
All measures	347	51
Prevalence	335	51
Incidence	11	8
Excess mortality rate	4	4
With-condition mortality rate	6	6

We did not perform a systematic review for GBD 2019. A systematic review was performed for GBD 2015 with the following search terms: (“atrial fibrillation” AND epidemiology[MeSH Subheading]) OR (“atrial flutter” AND epidemiology[MeSH Subheading]) OR (“atrial fibrillation” AND (prevalence OR incidence OR “case fatality”)) OR (“atrial flutter” AND (prevalence OR incidence OR “case fatality”)) OR (“heart atrium fibrillation” AND epidemiology[MeSH Subheading]) OR (“heart atrium fibrillation” AND (prevalence OR incidence OR “case fatality”))

The dates of the search were 1/1/2013 – 3/15/2016. There were 5,630 studies returned and, of those, 27 were extracted.

A systematic review was also performed for GBD 2013, with the search terms: (hasabstract[text] AND Humans[Mesh] AND middle age[MeSH])) OR 21) AND ((atrial fibrillation/epidemiology[Mesh] OR atrial fibrillation/mortality[Mesh]) AND (prevalence[Title/Abstract] OR incidence[Title/Abstract]) AND ("2010"[Date - Publication]: "3000"[Date - Publication]) AND (hasabstract[text] AND Humans[Mesh] AND middle age[MeSH]))

Apart from hospital and claims data points on prevalence, no non-literature-based data were included. We included hospital data corrected for readmission, primary to any diagnosis, and inpatient to outpatient utilisation ratios using adjustment factors calculated from US claims data. We excluded hospital data in certain geographies (eg, Philippines, China, India, Mexico, Botswana) where the data were implausibly low. We also excluded all outpatient administrative data as the values for all locations were implausibly low.

We adjusted claims and inpatient hospital data using literature data in which an ECG reading was used as a reference using MR-BRT crosswalking procedures. These procedures are discussed in detail elsewhere in the appendix. Table 2 shows the adjustment factors produced by the crosswalking procedure. The crosswalking coefficients in Table 2 below can be used to calculate adjustment factors for alternative definitions. The formula for computing adjustment factors is given in equation 1 below. We also included a standardized age variable (age scaled) and a sex variable to the crosswalking procedure to adjust for the possibly of bias.

Equation 1: Calculation of adjustment factors:

$$Estimated\ Reference\ Def = invlogit(logit(Alternative\ Def) - Beta_{Alternative\ Def} - Beta_{Sex} * Sex - Beta_{Age_{scaled}} * Age\ Scaled)$$

Table 2: MR-BRT Crosswalk Adjustment Factors for Atrial Fibrillation and Flutter

Data input	Reference or alternative case definition	Gamma	Beta Coefficient, Logit (95% CI)
Literature using ECG reading	Ref	0.99	---
Claims and hospital inpatient data	Alt		-0.29(-2.33 to 1.75)
Age scaled	Alt		-0.04 (-1.98 to 1.89)
Sex (male)	Alt		-0.07 (-2.00 to 1.87)

Severity splits & disability weights

Atrial fibrillation is split into symptomatic and asymptomatic based on standard GBD proportion information. The table below includes lay descriptions and disability weights for the severity levels of atrial fibrillation:

Table 3. Severity distribution, details on the severity levels for Atrial Fibrillation and Flutter in GBD 2019 and the associated disability weight (DW) with that severity.

Severity level	Lay description	DW (95% CI)
Asymptomatic	No symptoms	N/A
Symptomatic	Has periods of rapid and irregular heartbeats and occasional fainting	0.224 (0.151–0.312)

Modelling strategy

In order to address changes in coding practices for atrial fibrillation that resulted in an implausible trend of increasing death-certificate-based mortality rates, we used a prevalence-based modelling approach that combined DisMod-MR and CODEm models to generate estimates for atrial fibrillation and flutter. This approach, first used in GBD 2015, allowed us to generate more accurate estimates, using observed prevalence and incidence rates along with modelled excess mortality rates generated from prevalence and cause-specific mortality estimates.

The modelling steps are illustrated in the above flowchart. Effect sizes for covariates included in both the DisMod-MR 2.1 and CODEm models can be found in the table below.

- In Step 1, we estimated deaths for atrial fibrillation using a standard CODEm approach.

- In Step 2, we estimated prevalence rates in DisMod-MR using data from published reports of cross-sectional and cohort surveys, as well as primary care facility data. We also used claims data covering inpatient and outpatient visits for the United States along with inpatient hospital data from 247 locations in 15 countries. For GBD 2019, inpatient hospital data were adjusted using age- and sex-specific information for: 1) readmission within one year; 2) primary diagnosis code to secondary codes; and, 3) the ratio of inpatient to outpatient visits. These clinical informatics data were then

further adjusted using MR-BRT to account for misclassification compared with reference data. We set priors of no remission and capped excess mortality at 0.4 for all ages. We included the Healthcare Access and Quality (HAQ) index as a country-level, fixed-effect covariate on excess mortality and the log-transformed, age-standardised SEV scalar for atrial fibrillation and flutter as a country-level, fixed-effect covariate on prevalence.

- In Step 3, we calculated the excess mortality rate (EMR) for 2019 (defined as the cause-specific mortality rate [CSMR] estimated from CODEm divided by the prevalence rate from DisMod-MR). We then selected 17 countries based on four conditions: 1) ranking of 4 or 5 stars on the system for assessing the quality of VR data; 2) prevalence data available from the literature were included in the DisMod-MR estimation; 3) prevalence rate ≥ 0.005; and, 4) CSMR ≥ 0.00002. Using information from these countries as input data, we ran a MR-BRT model of logEMR on sex, a cubic spline of age, and HAQI. Specifics on the MR-BRT framework can be found elsewhere in the appendix. We then predicted year-, age- and sex-specific EMR using the results of this regression for all non-selected countries. Countries included in the regression were assigned their directly calculated values. These EMR data points were assigned to the time period 1990–2017 and uploaded into the non-fatal database in order to be used in modelling.

- In Step 4, we re-ran DisMod-MR using the input data described in Step 2 along with the EMR estimated in Step 3. We included Healthcare access and quality index (HAQI) as a fixed-effect, country-level covariate on excess mortality and the log-transformed, age-standardised SEV scalar for atrial fibrillation and flutter as a fixed-effect, country-level covariate on prevalence. We included a value prior of 0 for remission for all ages and set a value prior of 0 for excess mortality for ages 0-30.

The prevalence from the DisMod-MR model in Step 4 was used as the finalised output for upload to COMO and further processing into YLDs and DALYs.

Models were evaluated based on expert opinion, comparison with results from previous rounds of GBD, and model fit.

The tables below include the study covariates, parameters, betas, and exponentiated betas.

Table 4a. Covariates. Summary of covariates used in the Atrial Fibrillation and Flutter step 2 DisMod-MR meta-regression model

Covariate	Parameter	Beta	Exponentiated beta
Log-transformed age-standardised SEV scalar: A Fib	Prevalence	0.75 (0.75 to 0.76)	2.12 (2.12 to 2.13)
Healthcare Access and Quality Index	Excess mortality rate	-0.11 (-0.13 to -0.099)	0.89 (0.88 to 0.91)

Table 4b. Covariates. Summary of covariates used in the Atrial Fibrillation and Flutter step 4 DisMod-MR meta-regression model

Covariate	Parameter	Beta	Exponentiated beta
Log-transformed age-standardised SEV scalar: A Fib	Prevalence	1.15 (1.10 to 1.21)	3.17 (3.01 to 3.34)
Healthcare Access and Quality Index	Excess mortality rate	-0.017 (-0.017 to -0.017)	0.98 (0.98 to 0.98)

No substantive changes were made to the modelling strategy for GBD 2019.

Peripheral arterial disease

Flowchart

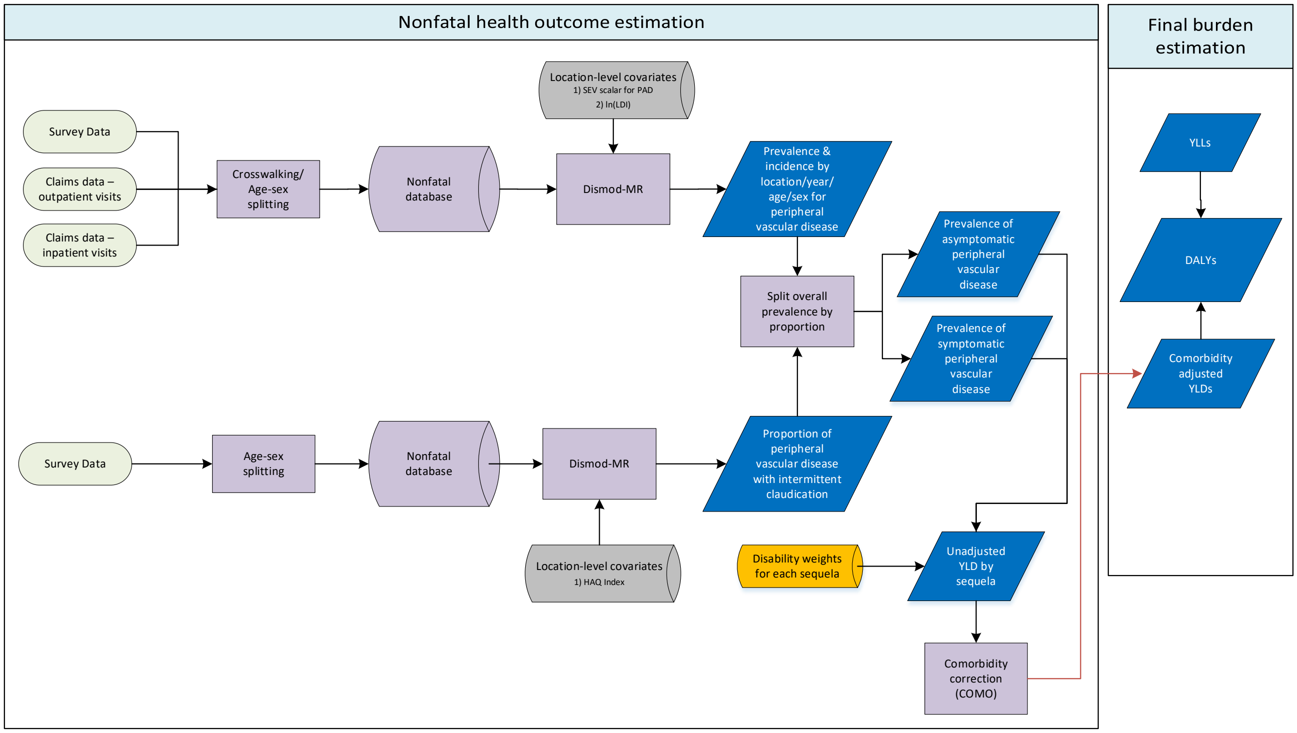

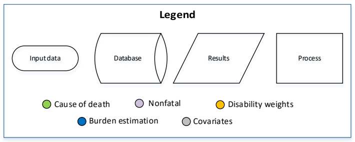

Input data and methodological appendix

Case definition

For GBD 2019, peripheral arterial disease was defined as having an ankle-brachial index (ABI) < 0.9. Intermittent claudication was defined clinically.

Specific ICD codes for claims data included can be found elsewhere in the appendix.

Input data

Model inputs

Table 1: Source counts for peripheral arterial disease

Measure	Total sources	Countries with data
All measures	45	15
Prevalence	37	14
Proportion	11	4

Table 1 shows the source counts for peripheral arterial disease modeling. We did not perform a systematic review for GBD 2019. A systematic review was performed for peripheral arterial disease and intermittent claudication for GBD 2015. The search terms were: ('peripheral vascular disease'[TIAB] AND 'epidemiology'[Subheading]) OR ('peripheral arterial disease'[TIAB] AND 'epidemiology'[Subheading]) OR ('peripheral artery disease'[TIAB] AND 'epidemiology'[Subheading]) OR ('intermittent claudication'[TIAB] AND 'epidemiology'[Subheading]) OR ('ankle-brachial index'[TIAB] AND 'epidemiology'[Subheading]) OR ('ankle brachial index'[TIAB] AND 'epidemiology'[Subheading]) OR ('peripheral artery occlusive disease'[TIAB] AND 'epidemiology'[Subheading]) OR ('peripheral obliterative arteriopathy'[TIAB] AND 'epidemiology'[Subheading]) OR ('peripheral vascular disease'[TIAB] AND 'prevalence'[MeSH Terms]) OR ('peripheral vascular disease'[TIAB] AND 'incidence'[MeSH Terms]) OR ('peripheral vascular disease'[TIAB] AND 'case fatality'[All Fields]) OR ('symptomatic claudication'[TIAB] AND (proportion[All Fields] OR percent[All Fields]))

The search was conducted from 1/1/2013 to 3/16/2015. 1,658 results were returned, of which six were extracted.

A systematic review was also performed for peripheral arterial disease and intermittent claudication for GBD 2013. Search terms can be provided upon request.

Apart from the claims data from the United States, we did not include any non-literature-based data types. We did not use inpatient hospital data, as peripheral arterial disease is expected to be rare in inpatient data but common in outpatient data as it is a condition usually managed on an outpatient basis, except for specific surgical interventions. This discrepancy leads to implausible correction factors based on inpatient/outpatient information from claims data (~150X); thus, adjusted data cannot be used. Including uncorrected data in the model is likely to lead to incorrect estimates as hospitalisation and procedure rates are likely to vary between geographies based on access to and patterns of care.

For GBD 2019 we adjusted prevalence data from claims using the MR-BRT data adjustment procedure described elsewhere the appendix. Our reference data was from literature in which the prevalence of PAD was based on directly-measured ABI values. The coefficients in Table 2 below can be used to calculate adjustment factors for alternative definitions. The formula for computing adjustment factors is given in equation 1 below. We also included a standardized age variable (age scaled) and a sex variable to the crosswalking procedure to adjust for the possibly of bias.

Equation 1: Calculation of adjustment factors:

$$Estimated\ Reference\ Def = invlogit(logit(Alternative\ Def) - Beta_{Alternative\ Def} - Beta_{Sex} * Sex - Beta_{Age_{scaled}} * Age\ Scaled)$$

Table 2: MR-BRT Crosswalk Adjustment Factors for Peripheral Arterial Disease

Data input	Measure	Reference or alternative case definition	Gamma	Beta Coefficient,Logit (95% CI)
Measured ABI less than or equal to 0.90	Prevalence	Ref	0	---
Claims data	Prevalence	Alt		-1.87 (-1.92 to -1.82)
Age scaled	Prevalence	Alt		0.27 (0.23 to 0.31)
Sex (male)	Prevalence	Alt		0.29 (0.22 to 0.36)

Severity splits and disability weights

We used the proportion of intermittent claudication to split the overall prevalence of peripheral arterial disease into symptomatic and asymptomatic peripheral vascular disease. The table below illustrates these values:

Table 3. Severity distribution, details on the severity levels for Peripheral Arterial Disease in GBD 2019 and the associated disability weight (DW) with that severity.

Severity level	Lay description	DW (95% CI)
Asymptomatic	No symptoms	No DW assigned
Symptomatic	Has cramping pains in the legs after walking a medium distance. The pain goes away after a short rest.	0.014 (0.007–0.025)

Modelling strategy

For GBD 2019, we used DisMod MR 2.1 to model the overall prevalence of peripheral arterial disease using prevalence data from literature studies and and crosswalked claims data.

We included the log-transformed, age-standardised SEV scalar for PAD and log-transformed LDI as fixed-effect, country-level covariates. We set value priors of 0 for incidence from ages 0 to 30. We also set a value prior of 0 for remission for all ages. Additionally, we set a value prior of 0 for excess mortality inbetween ages 0 and 30 as well as a value prior between 0 and 0.05 for excess mortality inbetween ages 30 and 100.

The table below illustrate the beta values and and exponentiated beta values for the covariates chosedn for the overall peripheral vascular disease model.

Table 4a. Covariates. Summary of covariates used in the Peripheral Arterial Disease DisMod-MR meta-regression model

Covariate	Parameter	Beta	Exponentiated beta
Log-transformed age-standardised SEV scalar: PAD	Prevalence	1.24 (1.22 to 1.25)	3.46 (3.39 to 3.49)
LDI (I$ per capita)	Excess mortality rate	-0.3 (-0.5 to -0.1)	0.74 (0.61 to 0.90)

We used DisMod MR to model the proportion of peripheral vascular disease with intermittent claudication. We set a value prior of 0 for proportion for ages 0 to 40. We included the Health Access and Quality Index score as a country-level covariate for excess mortality.

The table below illustrate the study covariates, parameters, beta, and exponentiated beta values for the proportion model for intermittent claudication.

Table 4b. Covariates. Summary of covariates used in the Intermittent Claudication DisMod-MR meta-regression model

Covariate	Parameter	Beta	Exponentiated beta
Healthcare Access and Quality index	Proportion	-.0064 (-.014 to -.00066)	0.99 (.99 to 1.00)

To obtain final estimates for the sequelae of interest, we multiplied the prevalence model by the proportion model at the draw level to generate the prevalence of symptomatic and asymptomatic peripheral vascular disease.

Models were evaluated based on expert review, comparisons with estimates from prior rounds of GBD, and assessing model fit.

There have been no substantive changes from GBD 2017 in terms of modelling strategy for peripheral arterial disease.

Acute Endocarditis

Flowchart

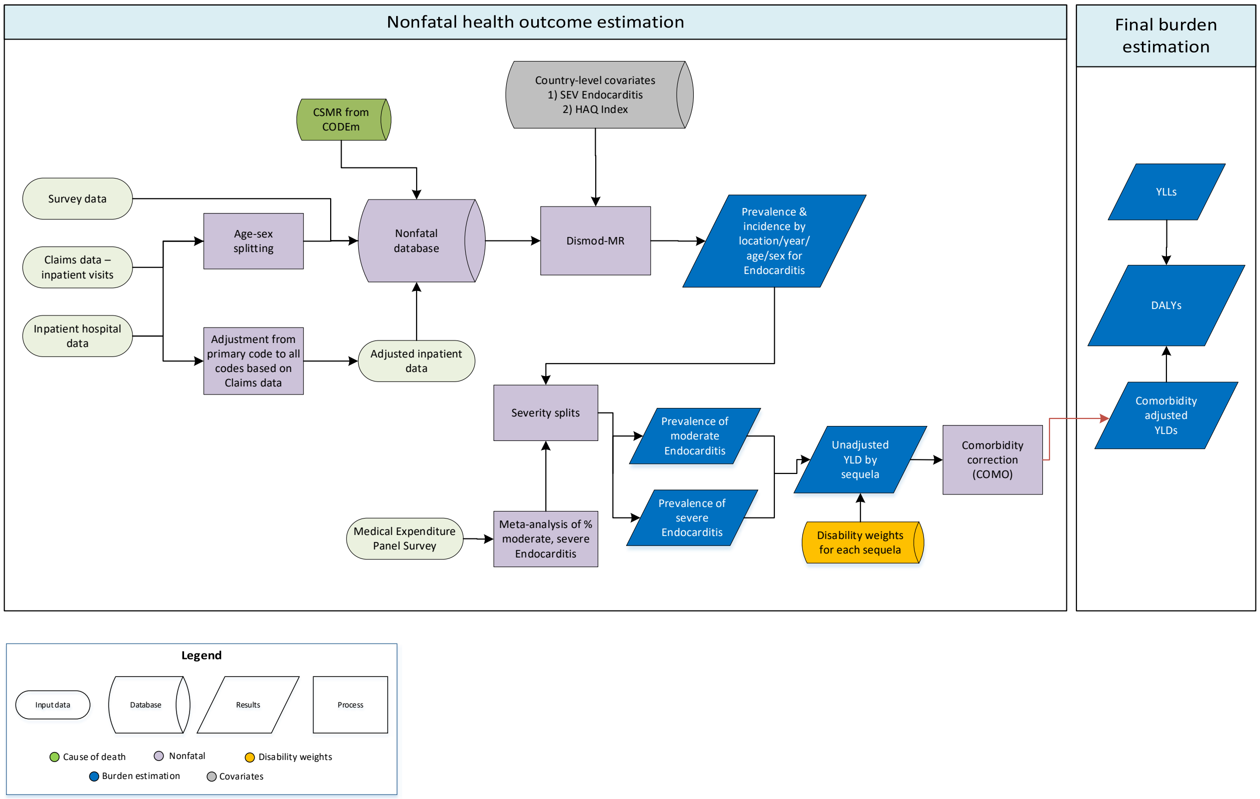

Input data and methodological appendix

Case definition

Our case definition for acute endocarditis was a clinical diagnosis of infective endocarditis. The ICD codes included can be found elsewhere in the appendix.

Input data

Model inputs

Table 1: Source counts for acute endocarditis

Measure	Total sources	Countries with data
All measures	303	41
Incidence	303	41

Table 1 displays the source counts for the non-fatal acute endocarditis model. We did not perform a systematic review for GBD 2019. A systematic review was performed for GBD 2013 and updated for GBD 2015. . The following search terms were used: ((‘endocarditis’[MeSH Terms] OR ‘endocarditis’[All Fields]) AND ‘epidemiology’[Subheading]) OR ((‘endocarditis’[MeSH Terms] OR ‘endocarditis’[All Fields]) AND

((‘epidemiology’[Subheading] OR ‘epidemiology’[All Fields] OR ‘incidence’[All Fields] OR ‘incidence’[MeSH Terms]) OR (‘epidemiology’[Subheading] OR ‘epidemiology’[All Fields] OR ‘prevalence’[All Fields] OR ‘prevalence’[MeSH Terms]) OR ‘case fatality’[All Fields])) OR ((‘endocardium’[MeSH Terms] OR ‘endocardium’[All Fields]) AND inflammation[TIAB] AND ‘epidemiology’[Subheading]) OR ((‘endocardium’[MeSH Terms] OR ‘endocardium’[All Fields]) AND inflammation[TIAB] AND ((‘epidemiology’[Subheading] OR ‘epidemiology’[All Fields] OR ‘incidence’[All Fields] OR ‘incidence’[MeSH Terms]) OR (‘epidemiology’[Subheading] OR ‘epidemiology’[All Fields] OR ‘prevalence’[All Fields] OR ‘prevalence’[MeSH Terms]) OR ‘case fatality’[All Fields]))

- Dates included in search: 1/1/2013 – 3/16/2015
- Number of initial hits: 1,246
- Number of sources included: 6

We did not include any non-literature-based data types, apart from the hospital and claims data described elsewhere. We excluded all outpatient data, as they were implausibly low when compared with inpatient data from the same locations and claims data. We used hospital data corrected for readmission and primary to any diagnosis based on the correction factors generated by the clinical informatics team. We excluded any inpatient hospital data points which were more than two-fold higher or 0.5-fold lower than the median absolute deviation value for high-income North America, Central Europe, and Western Europe for that age-sex group. No data adjustments was done for acute endocarditis in GBD 2019.

Severity split inputs

We used the standard GBD approach, which utilises MEPS data to split overall estimates of endocarditis into moderate and severe categories. The table below includes the severity level, lay descriptions, and DWs associated with acute endocarditis.

Table 2. Severity distribution, details on the severity levels for Acute Endocarditis in GBD 2019 and the associated disability weight (DW) with that severity.

Severity level	Lay description	DW (95% CI)
Moderate	Has a fever and aches, and feels weak, which causes some difficulty with daily activities.	0.051 (0.032–0.074)
Severe	Has a high fever and pain, and feels very weak, which causes great difficulty with daily activities.	0.133 (0.088–0.19)

Modelling strategy

For GBD 2019, we estimated endocarditis using a DisMod-MR Bayesian meta-regression model, setting a minimum of 11 and maximum of 13 as value priors on remission to establish an average duration of one month. For GBD 2019, we outliered cause specific mortality rate data from Mali due to implausibly high estimates. Country-level covariates used included the endocarditis summary exposure variable (SEV) on incidence and Health Access and Quality Index on excess mortality.

We evaluated models by comparing model fits with the data and with results from previous GBD estimation cycles.

The table below gives the parameters, betas, and exponentiated betas for study-level and country-level covariates used in the model

Table 3. Covariates. Summary of covariates used in the Acute Endocarditis DisMod-MR meta-regression model

Covariate	Parameter	Beta	Exponentiated beta (95% Uncertainty Interval)
Health Access and Quality Index	Excess mortality rate	-0.1 (-0.1 to -0.1)	0.90 (0.90 to 0.90)
Log-transformed age-standardised SEV scalar: endocarditis	Incidence	0.78 (0.75 to 0.83)	2.19 (2.12 to 2.30)

No significant changes were made to the modelling strategy from GBD 2017.

Other cardiovascular disease

Flowchart

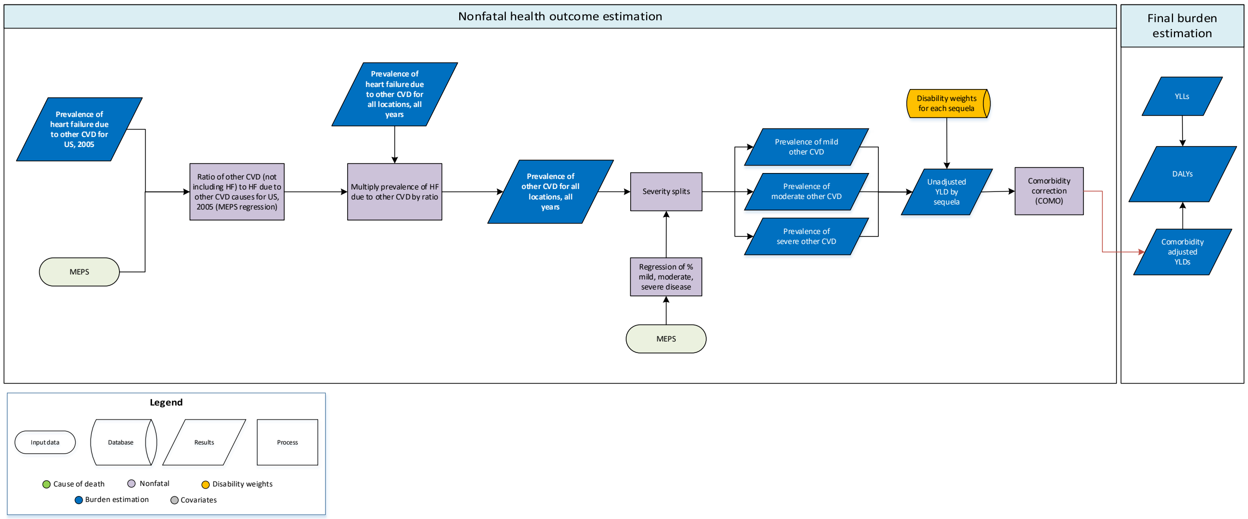

Case definition

Other cardiovascular disease is a residual category resulting from the GBD approach of estimating the total burden of all causes. Prevalence estimates are produced in order to provide YLDs consistent with the estimated YLLs from the death modelling process and to enable the calculation of DALYs.

Conditions included in this cause, based on ICD codes used for both fatal and non-fatal modelling, are Other diseases of pulmonary vessels; Acute pericarditis; Other diseases of pericardium; Pericarditis in diseases classified elsewhere; Paroxysmal tachycardia; Cardiac septal defect, acquired; Rupture of chordae tendineae, not elsewhere classified; Rupture of papillary muscle, not elsewhere classified; Intracardiac thrombosis, not elsewhere classified; Cerebral amyloid angiopathy; Other aneurysm; Other disorders of arteries and arterioles; Diseases of capillaries; Disorders of arteries, arterioles, and capillaries in diseases classified elsewhere; Phlebitis and thrombophlebitis; Portal vein thrombosis; Other venous embolism and thrombosis; Varicose veins of lower extremities; Varicose veins of other sites; Other disorders of veins; Nonspecific lymphadenitis; Other non-infective disorders of lymphatic vessels and lymph nodes; Other disorders of circulatory system in diseases classified elsewhere.

Input data

As this is a residual category, we used data from the Medical Expenditure Panel Survey and modelled estimates from heart failure due to other cardiovascular disease to estimate prevalence of other cardiovascular disease.

Severity split inputs

The table below includes lay descriptions and disability weights for the severity levels of other cardiovascular disease for GBD 2019.

Severity level	Lay description	DW (95% CI)
Asymptomatic		N/A
Mild	Is short of breath and easily tires with moderate physical activity, such as walking uphill or more than a quarter-mile on level ground. The person feels comfortable at rest or during activities requiring less effort.	0.041 (0.026–0.062)
Moderate	Is short of breath and easily tires with minimal physical activity, such as walking only a short distance. The person feels comfortable at rest but avoids moderate activity.	0.072 (0.047–0.103)
Severe	Is short of breath and feels tired when at rest. The person avoids any physical activity, for fear of worsening the breathing problems.	0.179 (0.122–0.251)

Source counts

**Measure**	Total sources	Countries with data
Proportion	19	1

Modelling strategy

To obtain prevalence estimates of other cardiovascular disease, we used MEPS data combined with prevalence estimates of heart failure due to other CVD for the USA in 2005 to estimate the ratio of the prevalence of heart failure due to other CVD causes to the prevalence of other CVD causes. We then applied this ratio to the age-, sex-, and year-specific prevalence estimates for heart failure due to other CVD causes for all locations to generate prevalence estimates of other cardiovascular disease.

No significant changes were made from GBD 2017.

Chronic obstructive pulmonary disease (COPD)

Flowchart

Chronic Obstructive Pulmonary Disease (COPD)

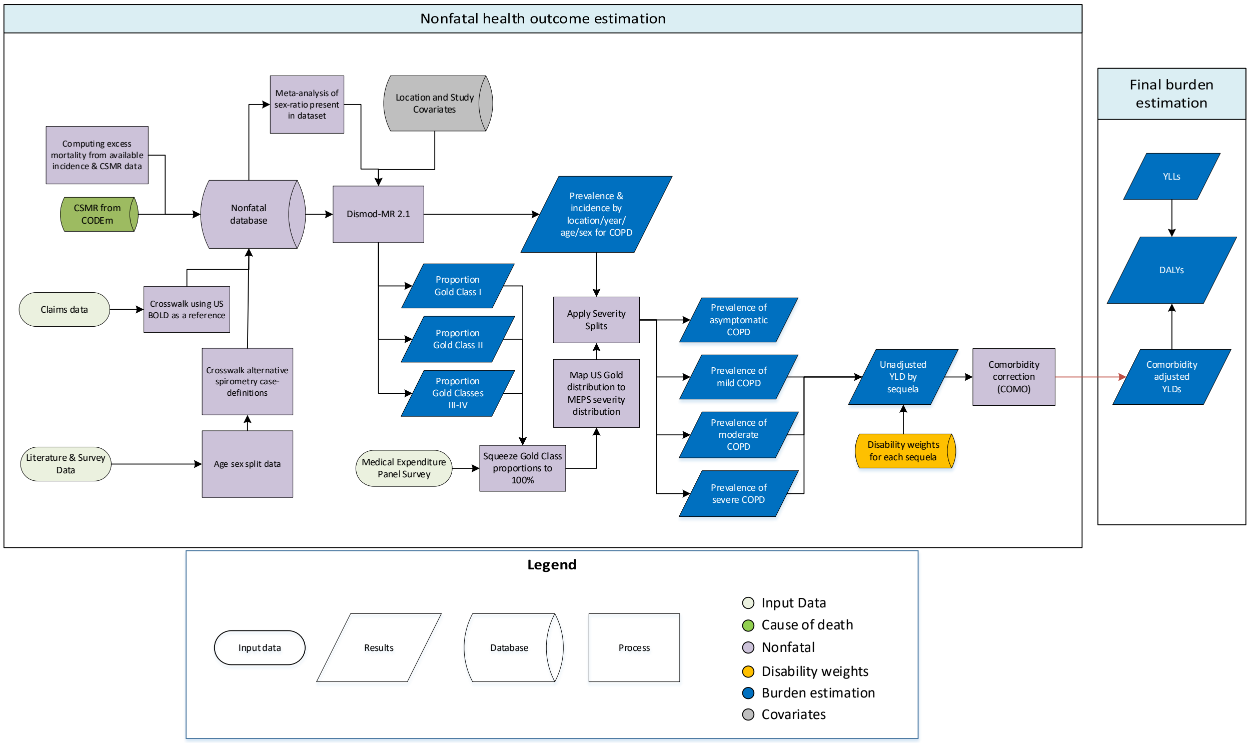

Case definition

COPD is defined as in the Global Initiative for Chronic Obstructive Lung Disease (GOLD) classification: a measurement of <0.7 FEV_1/FVC (one second of forceful exhalation/total forced expiration) on spirometry after bronchodilation. The severity grading of COPD follows this GOLD class definition.

GOLD CLASS	FEV_1 Score
I: Mild	>=80% of normal
II: Moderate	50-79% of normal
IV: Severe	<50% of normal

ICD-10 codes associated with COPD include J41, J42, J43, J44, and J47. The corresponding ICD-9 codes are 491-492, and 496. J40 & 490 (Bronchitis, not specified as acute or chronic) and J47 & 494 (Bronchiectasis) were removed from COPD mapping in GBD 2017.

Alternative case definitions that differ from the GOLD Post-bronchodilation definition are as follows: GOLD Pre-bronchodilation, Lower Limit of Normal (LLN) Post-bronchodilation, LLN Pre-bronchodilation, and European Respiratory Society (ERS) guidelines. These are all different methods of evaluating whether an individual has COPD.

Input data

No systematic review of the literature was completed for GBD 2019; however, for GBD 2016, we updated the systematic review from previous iterations. The full search term was:

(chronic obstructive pulmonary disease[Title/Abstract] AND (prevalence[Title/Abstract] or incidence [Title/Abstract] or mortality [Title/Abstract] or death [Title/Abstract]) AND "Cross-Sectional Studies"[MeSH Terms]) Filters: Publication date from 04/01/2015 to 11/01/2016; Humans

COPD has the following data sources

- Prevalence, incidence, and remission data from literature
- Hospital claims data
- Proportion data of GOLD class severities
- Burden of Obstructive Lung Disease (BOLD) Study data

Prevalence, incidence, and remission data relating to COPD are extracted from literature provided by collaborators or found with a systematic review. All data include spirometry-based measures. Other data come from hospital claims data for nonfatal estimation and vital registrations for cause of death.

GOLD class proportions are extracted from literature when the severity is available. Our models estimate three separate severities:

- Mild COPD: GOLD class I
- Moderate COPD: Gold class II
- Severe COPD: Gold class III & IV

These severities are used in the modelling process to split COPD by severities.

The Burden of Obstructive Lung Disease (BOLD) data is specifically notable because of its use in bias adjustments described in the data processing section.

New data this year include the English Longitudinal Study of Aging (ELSA), and claims data for the United States. Additional information on the claims data collection and pre-corrections are provided elsewhere. Briefly, we determined USA national and state-level estimates of COPD prevalence from a database of individual-level ICD-coded health service encounters. Persons with any inpatient claim or at least two outpatient claims associated with COPD were marked as a prevalent case for that year.

Data Inputs for Chronic Obstructive Pulmonary Disease

Measure	Total sources	Countries with data
All measures	166	57
Prevalence	142	54
Incidence	6	6
Relative risk	2	2
Proportion	36	32

Data Processing

Age-Sex and Sex Split

In some cases, data are reported by only age or only sex, but not both. For example, a study may have included the prevalence of males and females with COPD and then separately reported the prevalence for both sexes in smaller age bins (e.g. age 40-45, 46-50, etc.) that have COPD. In these cases, we perform an age-sex split by utilizing proportions within the study to disaggregate the data.

When data are not disaggregated into male and female categories for a given data source, we instead perform a sex-split on the data by applying sex proportions from other studies that do have male and female specific data. When data are aggregated into age categories larger than 25 years, we split into smaller age bins based on super-regional age patterns in the 2017 COPD model.

Modeled excess mortality data

For GBD 2019, we implemented a new method of modeling excess mortality rate (EMR).

In previous rounds, priors on EMR were estimated in DisMod by matching prevalence data points with their corresponding CSMR values within the same age, sex, year, location (by dividing CSMR by prevalence).

However, for many causes, DisMod estimated a rather unrealistic pattern of EMR compared to an expected pattern of decreasing EMR with greater access to quality health care. Such unexpected patterns often signal inconsistencies between CSMR estimates and the measures of prevalence and/or incidence.

In an effort to provide greater guidance to DisMod on the expected pattern of EMR, EMR data generated in the previous round were used as inputs for modeling in MR-BRT with age, sex, and healthcare access and quality index (HAQi) included as covariates. Results from MR-BRT were then predicted for each location year, sex and for ages 0, 10, 20 ….100.

This method led to improvements in the consistency of EMR relative to health care access. We also included HAQi as a country-level covariate in Dismod to inform EMR with the mean and standard deviation produced from MR-BRT analysis.

Bias Adjustments

In GBD 2019, we improved the bias adjustment methods by utilizing a MR-BRT model outside of DisMod to allow a more direct comparison between different case definitions and/or study designs. In GBD 2017, these adjustments were performed within DisMod.

We made a series of adjustments to data that do not completely match our case definition. Different diagnosis often leads to different estimates of COPD. Similarly, claims data is subject to biases. Claims data are often systemically lower than survey data, probably due to selection bias with regard to socioeconomic status. Adjustments are made to these data to correct these biases.

The adjustment is a logit-transformation method in MR-BRT. The general process is described below:

1. Identify data points with overlapping year, age, sex, and location between reference and alternative definitions.
2. Logit transform overlapping data points of alternative and reference case definitions
3. Convert overlapping data points into a difference in logit space using the following equation: $logit(altnerative) - logit(reference)$
4. Use the delta method to compute standard errors of overlapping data points in logit space, then calculate standard error of logit difference using the following equation: $\sqrt{(variance\ of\ alternative) + (variance\ of\ reference)}$
5. Using MR-BRT, conduct a random effects meta-regression to obtain the pooled logit difference of alternative to reference
6. Apply the pooled logit difference to all data points of alternative case definitions using the following equation:
$$new_{estimate} = inverse.logit((logit(alternative)) - (pooled\ logit\ difference))$$
7. Calculate new standard errors using the delta method, accounting for gamma (between-study heterogeneity)

Data derived from claims from commercial health insurance in the United States were also adjusted using a factor estimated in MR-BRT. Claims data, notably US Marketscan was adjusted in relation to the BOLD study data. In this case, the BOLD data serves as the reference definition while the marketscan data are the alternative definition.

MR-BRT Crosswalk Adjustment Factors

Data input	Status	Gamma	Beta Coefficient, Logit (95% CI)	Adjustment factor*
GOLD Post	Ref	0.25	---	---
GOLD Pre	Alt		0.50 (-0.02 - 1.07)	0.62 (0.49 - 0.74)
ERS	Alt		0.70 (0.11 - 1.31)	0.67 (0.53 - 0.79)
LLN Pre	Alt	0.08	0.10 (0.01 - 0.19)	0.52 (0.50 - 0.55)
LLN Post			-0.34 (-0.50 - -0.19)	0.42 (0.38 -0.45)
BOLD	Ref	.19	---	---
Marketscan	Alt		-1.93 (-2.35 - -1.50)	0.13 (0.08 - 0.18)

**Adjustment factor is the inverse-logit transformed Beta coefficient; <0.5 represents that alternative is adjusted upward; >0.5 represents alternative is adjusted downward*

Modelling strategy

The estimation of COPD burden has two distinct steps.

1. Estimate prevalence and incidence using a DisMod-MR 2.1 model
2. Estimate proportion of COPD severities using GOLD class groupings in DisMod-MR 2.1

After these two steps, the COPD prevalence and incidence is split by age, sex, location for each severity level.

Step 1: Main COPD model – Estimate prevalence and incidence using DisMod-MR 2.1

Model Settings

We set remission to 0 because individuals do not recover once they have COPD. The symptoms are only managed. Incidence ceiling is set at .0002 before age 15 and a ceiling at .0005 before age 30 to avoid a kick-up of estimates in age ranges with few or no primary data.

Each model includes a series of country-level covariates that describe spatiotemporal patterns.

- COPD standardised exposure variables (SEV) aggregates multiple risk factors into a single variable.
- Healthcare Access and Quality (HAQi) index on EMR to capture country-level variation of EMR, assuming a negative coefficient (ie, lower mortality with rising GDP and HAQ). The priors of HAQi came from the EMR MRBRT prediction.
- The proportion of elevation over 1500m was included as a country-level covariate on EMR because of its significance in COPD cause of death models.

Model coefficients for COPD

Model	Variable name	Measure	Beta	Exponentiated
COPD	Elevation over 1500m (proportion)	excess mortality rate	0.60 (0.14 — 0.95)	1.81 (1.15 — 2.58)
COPD	Healthcare access and quality index	excess mortality rate	-0.022 (-0.023 — -0.022)	0.98 (0.98 — 0.98)
COPD	Log age-standardised SEV scalar: COPD	prevalence	0.91 (0.90 — 0.92)	2.47 (2.46 — 2.50)

Step 2: GOLD class models to estimate proportions of severities

The GOLD class models use data from surveys that specified prevalence by GOLD class after expressing the values as a proportion of all COPD cases. For GBD 2016 we used fixed effects from the SEV scalar and the log of lag-distributed income (LDI) per capita to assist estimation. For GBD 2017, we dropped these covariates because they did not produce significant coefficients and also did not use them for GBD 2019. We also restricted random effects to +/-0.5 to control implausible geographical variation.

Severity Splits

The three GOLD class groupings reflect a grading based on a physiological measurement rather than a direct measurement of disease severity. In order to map the epidemiological findings by GOLD class into

the three COPD health states for which we have disability weights (DW), we used the 2001–2011 Medical Expenditure Panel Survey (MEPS) data from the United States. Specifically, we convert the GOLD class designations estimated for the USA in 2005 (the midpoint of MEPS years of analyses) into GBD classifications of asymptomatic, mild, moderate, and severe COPD.

The table below shows the three health states of COPD and the corresponding lay descriptions and disability weights. The graph shows the average proportion by GOLD class (after scaling to 100%) across all ages for USA in 2005. We also show the proportion of MEPS respondents reporting any health service contact in the past year for COPD with a DW value attributable to COPD of 0, mild range (0 to midpoint between DWs for mild and moderate), moderate range (midpoint of DW values mild and moderate to midpoint of DW values for moderate and severe) and severe range (midpoint between DW values moderate and severe or higher). The DW value for COPD was derived from a regression with indicator variables for all health states reported by MEPS respondents and their reported overall level of disability derived from a conversion of 12-Item Short Form Surveys (SF-12) answers to GBD DW values. This analysis gave the severity distribution for each GBD cause reported in MEPS after correcting for any comorbid causes individual respondents reported during a year.

Description of Health States

Health state	Lay description	DW (95% CI)
Mild COPD	This person has cough and shortness of breath after heavy physical activity, but is able to walk long distances and climb stairs.	0.019 (0.011–0.033)
Moderate COPD	This person has cough, wheezing, and shortness of breath, even after light physical activity. The person feels tired and can walk only short distances or climb only a few stairs.	0.225 (0.153–0.31)
Severe COPD	This person has cough, wheezing, and shortness of breath all the time. The person has great difficulty walking even short distances or climbing any stairs, feels tired when at rest, and is anxious.	0.408 (0.273–0.556)

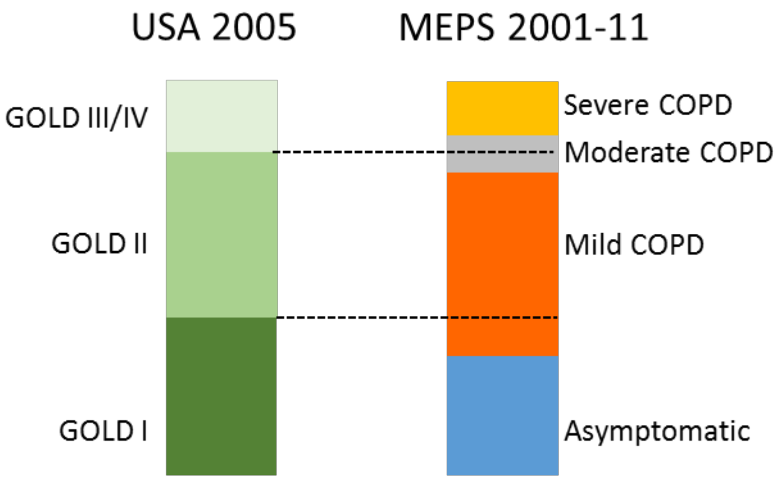

The algorithm to translate GOLD class to COPD DW categories first assigns GOLD III&IV to severe COPD and what remains to moderate. Next, GOLD class I is assigned to the asymptomatic category first and what remains goes to mild COPD. This algorithm is repeated for each age and sex category and for all 1,000 draws from the DisMod models of GOLD classes and the MEPS analyses. We end up with proportions of each of the GOLD class categories that map onto GBD COPD health states with uncertainty bounds determined by the 25th and 975th values of the 1,000 draws. These values are then applied to the estimates of the proportion of cases by GOLD class category, after scaling to 100%, by location, year, age, and sex. This assumes that the relationship between GOLD class and GBD COPD health states in the United States applies everywhere.

Pneumoconiosis

Coal Worker's Pneumoconiosis, Asbestosis, Silicosis, and Other Pneumoconiosis

Flowchart

Pneumoconiosis:
Coal Worker's, Asbestosis, Silicosis, Other

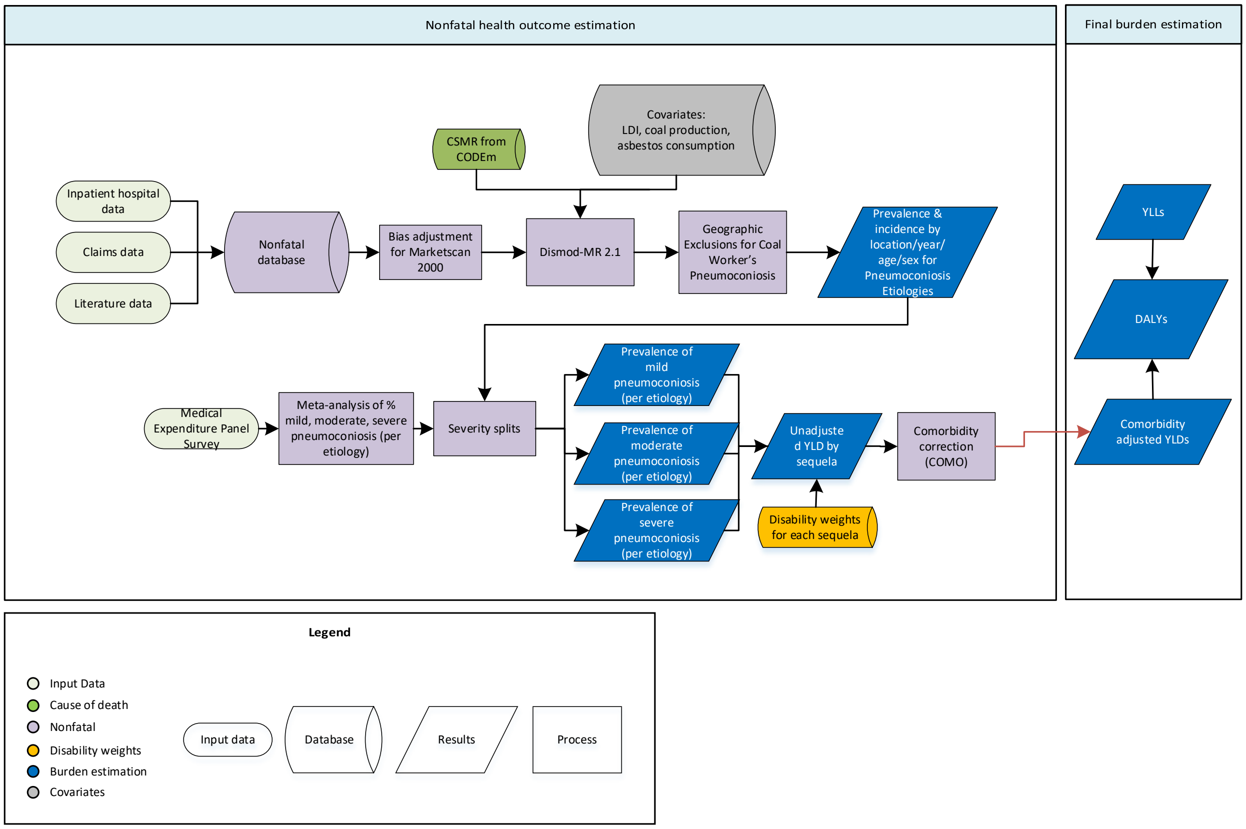

Input data and methodological appendix

Case definition

Pneumoconiosis is a chronic lung disease characterized by lung scarring and other interstitial damage caused by exposure to dust and other containments – usually through occupational exposure. For GBD, we model pneumoconiosis by exposure type: coal, asbestos, silica, and other.

Input data

Data used to make estimates of pneumoconiosis come from two sources: inpatient hospital reports, and hospital claims data. For GBD 2019, new claims data were added for the U.S. for the years 2015 and 2016.

Data Inputs for Pneumoconiosis

Cause/Impairment Name	Measure	Total sources	Countries with data
Pneumoconiosis	All measures	309	44
Pneumoconiosis	Prevalence	294	44
Pneumoconiosis	Proportion	15	1
Asbestosis	All measures	279	37
Asbestosis	Prevalence	279	37
Coal workers pneumoconiosis	All measures	251	35
Coal workers pneumoconiosis	Prevalence	251	35
Other pneumoconiosis	All measures	259	41
Other pneumoconiosis	Prevalence	259	41

Data Processing

Bias Adjustments

In GBD 2019, we improved the bias adjustment methods by utilizing a MR-BRT model outside of DisMod to allow a more direct comparison between different case definitions and/or study designs.

For the pneumoconiosis, adjusted U.S. Marketscan claims data collected in th year 2000 to all other U.S. Marketscan data. To do so, we used the logit difference for data points from reference (non-2000 claims data) and alternative (2000 claims data) matched on age, sex and location as input into MR-BRT.

The adjustment is a logit-transformation method in MR-BRT. The general process is described below:

1. Identify data points with overlapping age, sex, and location between reference and alternative definitions.
2. Logit transform overlapping data points of alternative and reference case definitions
3. Convert overlapping data points into a difference in logit space using the following equation: $logit(altnerative) - logit(reference)$
4. Use the delta method to compute standard errors of overlapping data points in logit space, then calculate standard error of logit difference using the following equation: $\sqrt{(variance\ of\ alternative) + (variance\ of\ reference)}$
5. Using MR-BRT, conduct a random effects meta-regression to obtain the pooled logit difference of alternative to reference
6. Apply the pooled logit difference to all data points of alternative case definitions using the following equation:
$$new_{estimate} = inverse.logit((logit(alternative)) - (pooled\ logit\ difference))$$
7. Calculate new standard errors using the delta method, accounting for gamma (between-study heterogeneity)

The coefficients for bias adjustments are shown below:

MR-BRT Crosswalk Adjustment Factor: Asbestosis

Data input	Status	Gamma	Beta Coefficient, Logit (95% CI)	Adjustment factor*

Marketscan (not 2000)	Ref		---	---
Marketscan 2000	Alt	0.0	-0.25 (-0.36 to -0.15)	0.44

**Adjustment factor is the inverse-logit transformed Beta coefficient; <0.5 represents that alternative is adjusted upward; >0.5 represents alternative is adjusted downward*

MR-BRT Crosswalk Adjustment Factor: Coal Worker's Pneumoconiosis

Data input	Status	Gamma	Beta Coefficient, Logit (95% CI)	Adjustment factor*
Marketscan (not 2000)	Ref		---	---
Marketscan 2000	Alt	0.0	-0.34 (-0.76 to 0.07)	0.42

**Adjustment factor is the inverse-logit transformed Beta coefficient; <0.5 represents that alternative is adjusted upward; >0.5 represents alternative is adjusted downward*

MR-BRT Crosswalk Adjustment Factor: Silicosis

Data input	Status	Gamma	Beta Coefficient, Logit (95% CI)	Adjustment factor*
Marketscan (not 2000)	Ref		---	---
Marketscan 2000	Alt	0.0	-0.48 (-1.91 to 0.96)	0.38

**Adjustment factor is the inverse-logit transformed Beta coefficient; <0.5 represents that alternative is adjusted upward; >0.5 represents alternative is adjusted downward*

MR-BRT Crosswalk Adjustment Factor: Other Pneumoconiosis

Data input	Status	Gamma	Beta Coefficient, Logit (95% CI)	Adjustment factor*
Marketscan (not 2000)	Ref		---	---
Marketscan 2000	Alt	0.0	0.14 (-0.32 to 0.59)	0.53

**Adjustment factor is the inverse-logit transformed Beta coefficient; <0.5 represents that alternative is adjusted upward; >0.5 represents alternative is adjusted downward*

Modeled excess mortality data

As part of iteration of estimates for all pneumoconioses, we tested a new method of modeling excess mortality rate (EMR) that was not used in the final model.

In previous rounds, priors on EMR were estimated in DisMod by matching prevalence data points with their corresponding CSMR values within the same age, sex, year, location (by dividing CSMR by prevalence). For short duration conditions (remission>1), the corresponding prevalence was derived by running an initial model and then applying the same CSMR/prevalence method.

However, for many causes, DisMod estimated a rather unrealistic pattern of EMR compared to an expected pattern of decreasing EMR with greater access to quality health care. Such unexpected patterns often signal inconsistencies between CSMR estimates and the measures of prevalence and/or incidence.

In effort to provide greater guidance to DisMod on the expected pattern of EMR, EMR data generated in the previous round were used as inputs in MR-BRT modeling with age, sex, and healthcare access and quality index (HAQi) included as covariates. Results from MR-BRT were then predicted for each location year, sex and for ages 0, 10, 20 ….100.

While this method led to some improvements in the consistency of EMR relative to health care access, the resulting prevalence estimates were unrealistic. We decided to continue using the DisMod EMR estimations while keeping HAQi as a country-level covariate to inform EMR with a mean and standard deviation produced from MR-BRT.

This method was utilized for all pneumoconiosis: asbestosis, coal worker's pneumoconiosis, silicosis, and other pneumoconiosis.

Modelling strategy

Estimates for the pneumoconioses are produced using a standard DisMod-MR 2.1 approach.

For all aetiologies, we use prior settings of zero remission. Additionally, we assume no incidence and prevalence before the age of 15. We include a predictive covariate on healthcare access and quality. Location random effects are set at -20 to 20 for prevalence and incidence hazard to reflect large location variations.

Covariates on Asbestos, Mesothelioma, and coal production were removed in GBD 2019.

Cause	Measure	Variable name	Beta	Exponentiated
Asbestosis	Prevalence	Asbestos consumption (per capita)	0.47 (0.015–1.70)	1.60 (1.02–5.47)
Asbestosis	Prevalence	Log-transformed age-standardised SEV scalar: Mesothelioma	0.029 (0.000016–0.32)	1.03 (1.00–1.38)
Asbestosis	Excess Mortality	Healthcare access and quality index	-0.025 (-0.025 — -0.024)	0.98 (0.98 — 0.98)
Coal worker's	Prevalence	Coal production (per capita)	0.0017 (-0.00025 to 0.0045)	1.00 (1.00–1.00)
Coal Worker's	Excess Mortality	Healthcare Access and Quality index	-0.07809	0.013502

Severity Split Inputs

Data to inform estimates of the severity gradient due to pneumoconiosis etiologies are derived from previous analyses of the Medical Expenditure Panel Survey (MEPS). The disability weights are shared by all aetiologies.

Severity level	Lay description	DW (95% CI)	Severity Distributions
Asymptomatic			23.0% (20.8 – 25.0)
Mild	Has cough and shortness of breath after heavy physical activity, but is able to walk long distances and climb stairs.	0.019 (0.011–0.033)	34.2% (26.4 – 37.5)
Moderate	Has cough, wheezing, and shortness of breath, even after light physical activity. The person feels tired and can walk only short distances or climb only a few stairs.	0.225 (0.153–0.312)	13.3% (9.7 – 19.4)
Severe	Has cough, wheezing, and shortness of breath all the time. The person has great difficulty walking even short distances or climbing any stairs, feels tired when at rest, and is anxious.	0.408 (0.273–0.556)	29.5 (20.8 – 36.1)

Geographical Exclusions

In GBD 2019, we set estimates for coal worker's pneumoconiosis to zero prevalence for any location with no coal production for all years. This exclusion was applied after running a DisMod model. The assumption here is that coal worker's pneumoconiosis should be near zero in areas where there is no coal production.

Asthma

Flowchart

Asthma

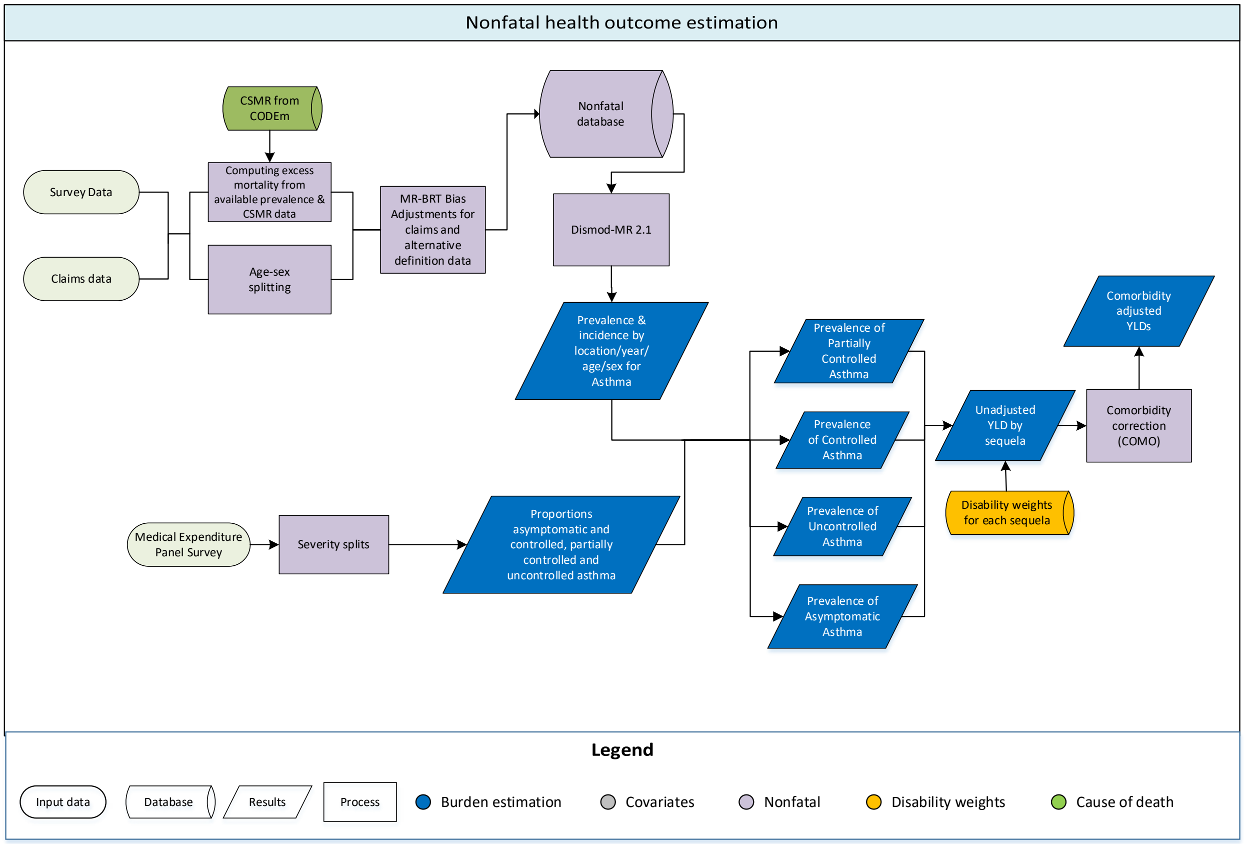

Case definition

Asthma is a chronic lung disease marked by spasms in the bronchi usually resulting from an allergic reaction or hypersensitivity and causing difficulty in breathing. We define asthma as a doctor's diagnosis and wheezing in the past year. The relevant ICD-10 codes are J45 and J46. ICD-9 code is 493.

Alternative case definitions include the following:

- Self-reported Asthma in the past year
- Self-reported Asthma ever
- Only a doctor's diagnosis in the past year
- Only wheezing in the past year

Input data

The last full systemic review of the literature on Asthma was done for GBD 2016. The following search string was used in PubMed and filtered by studies of humans published between January 2012 and November 2016.

(Asthma[Title/Abstract] AND prevalence[Title/Abstract] AND "Cross-Sectional Studies"[MeSH Terms])

Data in literature matching our case definitions were extracted. Those that had definitions outside our alternative case definitions were not included. In addition to claims data used in GBD 2017, we added new USA claims data for the years 2015 and 2016. We also added new data for Wave 7 of the English Longitudinal Study of Ageing (ELSA). Surveys carried out as part of the International Study of Asthma and Allergies in Childhood (ISAAC) collaboration are the most important source of prevalence data in children.

Data Inputs for Asthma

Measure	Total sources	Countries with data
All measures	413	136
Prevalence	374	136
Incidence	11	6
Remission	28	16
Relative risk	5	3
Standardized mortality ratio	1	1
With-condition mortality rate	4	2
Proportion	15	1

Data processing

Age-Sex and Sex Split

In some cases, data are reported by only age or only sex, but not both. For example, a study may have included the prevalence of males and females with Asthma and then separately reported the prevalence of both sexes combined in smaller age bins (e.g. 40-45 years, 46-50, etc.) that have Asthma. In these cases, we perform an age-sex split by utilizing proportions within the study to disaggregate the data.

When data are not disaggregated into male and female categories, we instead perform a sex-split on the data by applying sex proportions from outside studies. The sex split analysis was carried out using MR-BRT and included a cubic spline on age to reflect the higher prevalence of asthma in males at young ages, which then transitions to a higher prevalence of asthma in females during the teenage years.
When data are aggregated into age categories larger than 25 years, we split the data into smaller age bins based on the global age pattern from an initial Dismod model that only included input data with age ranges under 25 years.

Modeled excess mortality data

As part of iteration of estimates for Asthma, we tested a new method of modeling excess mortality rate (EMR) that was not used in the final model.

In previous rounds, priors on EMR were estimated in DisMod by matching prevalence data points with their corresponding CSMR values within the same age, sex, year, location (by dividing CSMR by prevalence).

However, for many causes, DisMod estimated a rather unrealistic pattern of EMR compared to an expected pattern of decreasing EMR with greater access to quality health care. Such unexpected patterns often signal inconsistencies between CSMR estimates and the measures of prevalence and/or incidence.

In effort to provide greater guidance to DisMod on the expected pattern of EMR, EMR data generated in the previous GBD round were used as inputs into aMR-BRT model that includedage, sex and healthcare access and quality index (HAQi) as covariates. Results from MR-BRT were then predicted for each location year, sex and for ages 0, 10, 20 ….100.

While this method led to some improvements in the consistency of EMR relative to health care access, the resulting prevalence estimates were unrealistic. We decided to continue using the DisMod EMR estimations while keeping HAQi as a country-level covariate to inform EMR with a mean and standard deviation produced from MR-BRT.

Bias Adjustments

In GBD 2019, we improved the bias adjustment methods by utilizing a MR-BRT model outside of DisMod to allow a more direct comparison between different case definitions and/or study designs.

We made a series of adjustments to data that don't completely match our case definition, doctor's diagnosis and wheezing in the past year. The estimation of Asthma in a population varies slightly by the case definition used (wheezing and diagnosis, only wheezing, etc). Similarly, claims data is subject to biases. An analysis for GBD 2017 showed that claims data were systemically lower than asthma survey data, probably reflecting selection bias with regard to socioeconomic status. Adjustments are made to these data to correct these biases.

The adjustment is a logit-transformation method in MR-BRT. The general process is described below:

1. Identify data points with overlapping year, age, sex, and location between reference and alternative definitions.
2. Logit transform overlapping data points of alternative and reference case definitions
3. Convert overlapping data points into a difference in logit space using the following equation: $logit(altnerative) - logit(reference)$
4. Use the delta method to compute standard errors of overlapping data points in logit space, then calculate standard error of logit difference using the following equation: $\sqrt{(variance\ of\ alternative) + (variance\ of\ reference)}$
5. Using MR-BRT, conduct a random effects meta-regression to obtain the pooled logit difference of alternative to reference
6. Apply the pooled logit difference to all data points of alternative case definitions using the following equation: $new_{estimate} = inverse.logit((logit(alternative)) - (pooled\ logit\ difference))$
7. Calculate new standard errors using the delta method, accounting for gamma (between-study heterogeneity)

Data derived from claims from commercial health insurance in the United States were also adjusted using a factor estimated in MR-BRT. To account for this, we estimated a MarketScan 2000 coefficient and a separate MarketScan coefficient for the remaining years of Marketscan data, by comparing the national

values in these datasets to national asthma estimates from the USA National Health and Nutrition Examination Survey and National Health Interview Surveys.

The coefficients for bias adjustments are shown:

MR-BRT Crosswalk Adjustment Factors

Data input	Status	Gamma	Beta Coefficient, Logit (95% CI)	Adjustment factor*
Wheezing + Doctor's Diagnosis	Ref	0.26	---	---
Only Wheezing	Alt		1.09 (0.61, 1.59)	0.75 (.65, 0.83)
Only Diagnosis	Alt		0.99 (0.50, 1.48)	0.73 (0.62, 0.82)
Self-reported currently have asthma	Alt		.01 (-0.48, 0.56)	0.50 (0.38, 0.64)
Self-reported ever having asthma	Alt		0.66 (0.11, 1.20)	0.66 (0.53, 0.77)
Marketscan 2000	Alt	0.00	-1.35 (-1.37, -1.33)	0.21 (0.20, 0.21)
Marketscan 2010 - 2016	Alt	0.60	-1.60 (-2.71, -0.43)	.17 (.06, .41)

**Adjustment factor is the inverse-logit transformed Beta coefficient; <0.5 represents that alternative is adjusted upward; >0.5 represents alternative is adjusted downward*

Modelling strategy

We use DisMod-MR 2.1 as the main modelling tool for asthma. Prior settings include a maximum remission of 0.3 (reflecting the upper bound of the highest observed data) and no incidence between the ages of 0 and 0.5 year, as a diagnosis cannot be made in young infants.

Predictive covariates

To assist estimation, particularly in locations with few or no data, we included covariates in our DisMod model that are associated with measures of asthma epidemiology in prior studies and for which estimates of those covariates are available for all GBD year-age-sex-location combinations. Specifically, we use log LDI and the asthma standardised exposure variable (SEV), a scalar that combines exposure of all GBD risks that influence asthma.

We also used HAQi covariate with priors from tests on excess mortality rate in MR-BRT.

Covariate Table	Measure	Beta	Exponentiated
Healthcare Access and Quality Index	EMR	-0.06 (-.062 to -.059)	.94 (.93 to .94)

Log SEV scalar: asthma	prevalence	0.75 (0.75–0.76)	2.13 (2.12–2.14)
Log LDI (I$ per capita)	excess mortality rate	-0.5 (-0.5 to -0.5)	0.61 (0.61–0.61)

Severity split inputs

Lay descriptions and disability weights for the asthma health states are shown in the table below. The distribution between the three health states is derived from an analysis of the USA Medical Expenditure Panel Surveys (MEPS). The methods are described in full in a separate section of this appendix. Briefly, MEPS is an ongoing survey of health service encounters with as its main objective to collect data on health expenditure. Panels are recruited every year and followed up for a period of two years. Diagnostic information provided by respondents on the reasons for any health care contact are coded into three-digit ICD-9 codes by professional coders.

Twice over the two-year follow-up period, respondents are asked to fill in 12-Item Short Form Surveys (SF-12). From convenience samples asking respondents to fill in SF-12 for 60 of the GBD health states, IHME has created a mapping from SF-12 scores to GBD disability weights (DW). We perform a regression with indicator variables for all GBD causes that we can identify from the ICD codes in MEPS to derive for each individual with a diagnosis the amount of disability that can be attributed to that condition after controlling for any comorbid conditions. Anyone with a diagnosis of asthma in whom the disability assigned to asthma is negative or zero we assume is asymptomatic (at the time of asking SF-12 question relating to their health status in the past four weeks). Non-zero values we bin into the three health states assuming a split between these at the midpoint between DW values. The table below gives the proportions in MEPS in each of the health states and an asymptomatic state.

Severity level	Lay description	DW (95% CI)	Severity distribution
Asymptomatic			36.2% (35.0–37.3%)
Controlled	This person has wheezing and cough once a month, which does not cause difficulty with daily activities.	0.015 (0.007–0.026)	19.9% (13.6–27.8%)
Partially controlled	This person has wheezing and cough once a week, which causes some difficulty with daily activities.	0.036 (0.022–0.055)	20.6% (15.1–25.8%)
Uncontrolled	This person has wheezing, cough, and shortness of breath more than twice a week, which causes difficulty with daily activities and sometimes wakes the person at night.	0.133 (0.086–0.192)	23.3% (18.7–30.3%)

Interstitial lung disease and pulmonary sarcoidosis (ILD)

Flowchart

Interstitial Lung Disease and Pulmonary Sarcoidosis (ILD)

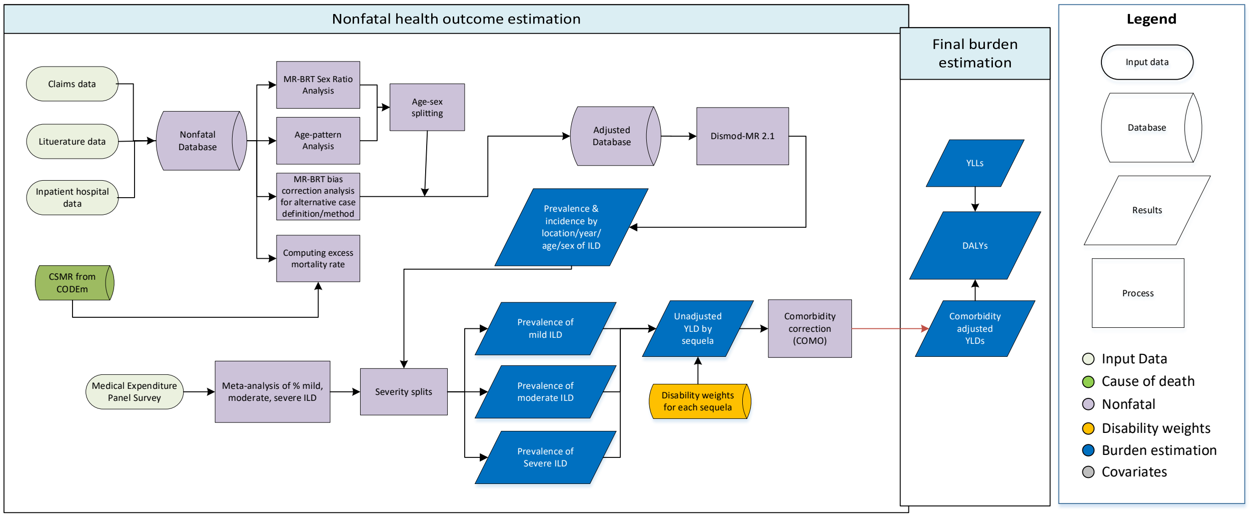

Case definition

Interstitial lung diseases and pulmonary sarcoidosis are a collection of chronic respiratory diseases that impair lung function and oxygen uptake through scarring and/or inflammation. The relevant ICD codes are D86 and J84. For interstitial lung disease, we use the American Thoracic Society as the gold standard definition.

Input data

Model Inputs

No systematic review of the literature was conducted for ILD for this iteration of the Global Burden of Disease. These reviews are done on a rotating basis and updates will be made for a future iteration.

Data used to make estimates of ILD are from three sources. The first is literature data from previous systematic reviews – usually from smaller-scale studies of prevalence or incidence. The second data type is claims data for the United States. The source and preparation of these data is described elsewhere. The third data type is adjusted hospital inpatient records. Because these records only report primary diagnosis, a priori adjustments are made based on location and healthcare access and quality.

Data inputs for interstitial lung disease and pulmonary sarcoidosis

Measure	Total sources	Countries with data
All measures	342	51
Prevalence	306	45
Incidence	27	16
With-condition mortality rate	2	2
Proportion	15	1

Data Processing

Age-Sex and Sex Split

In some cases, data are reported by only age or only sex, but not both. For example, a study may have included the proportion of males and females with ILD and then separately reported the proportion of both sexes in smaller age bins (e.g. age 40-45, 45-50, etc.) that have ILD. In these cases, we perform an age-sex split by utilizing proportions within the study to disaggregate the data.

When no information by sex in a study is present, we instead perform a sex-split on the data by applying separate sex proportions. The sex split analysis was carried out using MR-BRT. When data are aggregated into age categories larger than 25 years, we split the data into smaller age bins based on the global age pattern from an initial DisMod model.

Bias Adjustments

In GBD 2019, we improved the bias adjustment methods by utilizing a MR-BRT model outside of DisMod to allow a more direct comparison between different case definitions and/or study designs.

We made a series of adjustments to data that don't completely match our case definition. Data that only reports IPF or only sarcoidosis tend to vary estimates of ILD in a population. Similarly, claims tends to differ from the population, probably representing selection bias due to socioeconomic status. We make adjustments to these data to reflect these possible variations. The adjustment is a logit-transformation method in MR-BRT. The general process is described below:

1. Identify data points with overlapping year, age, sex, and location between reference and alternative definitions.
2. Logit transform overlapping data points of alternative and reference case definitions
3. Convert overlapping data points into a difference in logit space using the following equation: $logit(altnerative) - logit(reference)$
4. Use the delta method to compute standard errors of overlapping data points in logit space, then calculate standard error of logit difference using the following equation: $\sqrt{(variance\ of\ alternative) + (variance\ of\ reference)}$
5. Using MR-BRT, conduct a random effects meta-regression to obtain the pooled logit difference of alternative to reference
6. Apply the pooled logit difference to all data points of alternative case definitions using the following equation: $new_{estimate} = inverse.logit((logit(alternative)) - (pooled\ logit\ difference))$
7. Calculate new standard errors using the delta method, accounting for gamma (between-study heterogeneity)

MR-BRT Crosswalk Adjustment Factors

Data input	Status	Gamma	Beta Coefficient, Logit (95% CI)	Adjustment factor*
IPF and Sarcoidosis	Ref	0.23	---	---
Only IPF	Alt		-1.46 (-2.09 - -0.79)	0.19 (0.11 - 0.31)
Only Sarcoidosis	Alt		-1.07 (-1.71 - -0.40)	0.26 (0.15—0.40)
Marketscan 2000	Alt	0	-0.31 (-0.32 - -0.29)	0.42 (0.42 - 0.43)

**Adjustment factor is the inverse-logit transformed Beta coefficient; <0.5 represents that alternative is adjusted upward; >0.5 represents alternative is adjusted downward*

Modelling strategy

Estimates for ILD are produced using a standard DisMod-MR 2.1 approach. We use prior settings of zero remission and we constrain the super-region random effects to -0.5 to 0.5 to ensure model stability.

We employed predictive covariates to improve estimation in locations with scarce prevalence data. These were income per capita and the healthcare access and quality index (HAQi). The priors on HAQi were model outputs from the MRBRT modelling on EMR as described in the next section.

Variable name	Measure	Beta	Exponentiated
LDI (I$ per capita)	excess mortality rate	-0.2 (-0.2 to -0.2)	0.82 (0.82–0.82)
Healthcare Access and Quality index	excess mortality rate	-0.014 (-0.014 — -0.014)	0.99 (0.99 — 0.99)

Predicted excess mortality rate with MR-BRT

Similar to other causes, we include estimates of cause-specific mortality rate (CSMR) and Excess Mortality Rate (EMR) as model inputs. In previous rounds, priors on excess mortality rate (EMR) were estimated in DisMod by matching prevalence data points with their corresponding CSMR values within the same age, sex, year, location (by dividing CSMR by prevalence). For short duration conditions (remission>1), the corresponding prevalence was derived by running an initial model and then applying the same CSMR/prevalence method.

However, for many causes, DisMod estimated a rather unrealistic pattern of EMR compared to an expected pattern of decreasing EMR with greater access to quality health care. Such unexpected patterns often signal inconsistencies between CSMR estimates and the measures of prevalence and/or incidence.

To provide greater guidance to DisMod on the expected pattern of EMR, EMR data generated in the previous round were modeled using the MR-BRT approach by age and sex with a prior on healthcare access and quality index (HAQi) having a negative coefficient. Results from MR-BRT were then predicted for each location year, sex and for ages 0, 10, 20 ….100. We included HAQi as a country-level covariate to inform EMR with a mean and standard deviation produced from MR-BRT.

Severity Splits

Data to inform estimates of the severity gradient due to ILD are derived from previously analyses of the Medical Expenditure Panel Survey (MEPS). The table below illustrates the lay descriptions and disability weights associated with different levels of severity of interstitial lung disease.

Severity level	Lay description	DW (95% CI)
Mild	Has cough and shortness of breath after heavy physical activity, but is able to walk long distances and climb stairs.	0.019 (0.011–0.033)
Moderate	Has cough, wheezing, and shortness of breath, even after light physical activity. The person feels tired and can walk only short distances or climb only a few stairs.	0.225 (0.153–0.312)
Severe	Has cough, wheezing, and shortness of breath all the time. The person has great difficulty walking even short distances or climbing any stairs, feels tired when at rest, and is anxious.	0.408 (0.273–0.556)

Other chronic respiratory diseases

In addition to the chronic respiratory diseases described above, there are other types of chronic respiratory diseases with a range of severities and associated sequelae. Because these chronic respiratory diseases are diverse in their underlying causes and risk factors as well as in their associated health outcomes, modelling them together in a DisMod-MR model would not produce reliable estimates of prevalence. Instead, we calculated the YLDs caused by other chronic respiratory diseases directly using a YLD/YLL ratio as a 'place holder'.

We calculated the ratio of YLDs to YLLs across the specified chronic respiratory diseases for which non-fatal outcomes were modelled, using YLL estimates from the GBD 2019 cause of death (CoD) analysis. We then multiplied this YLD/YLL ratio by the YLL estimates for other chronic respiratory diseases.

Cirrhosis

Flowchart

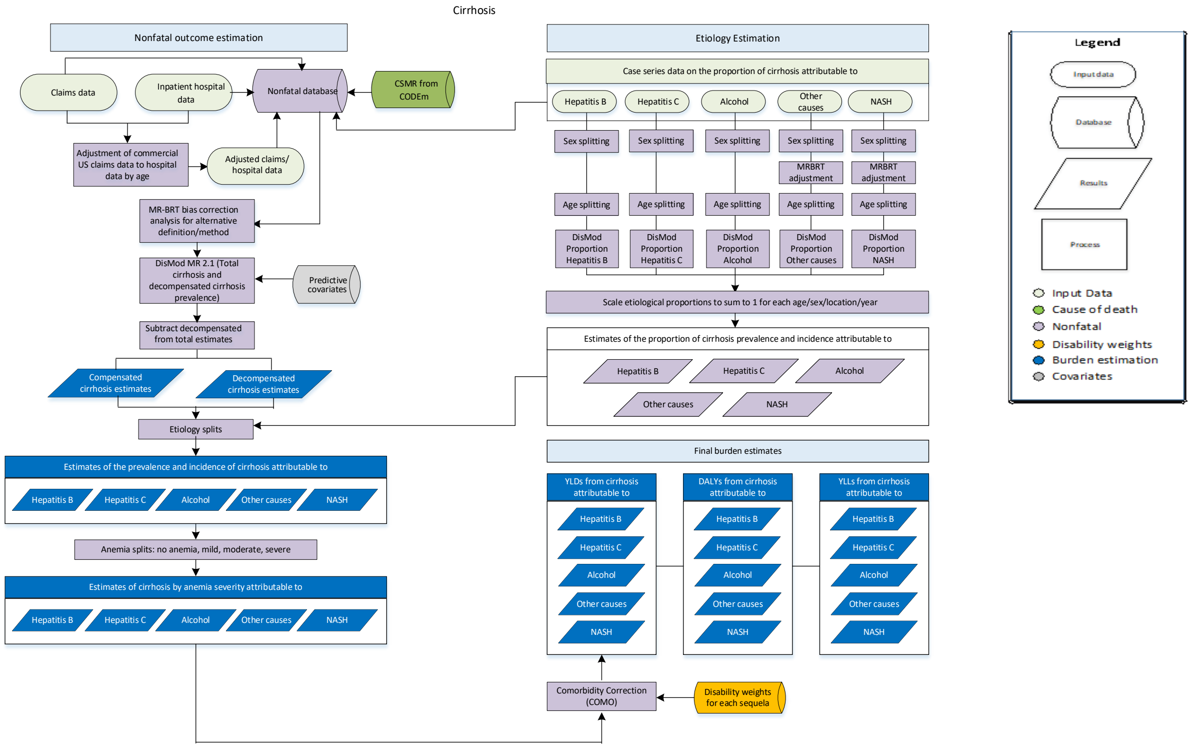

Input Data and Methodological Summary for Cirrhosis

Case definition

Cirrhosis is a chronic liver disease in which there is progressive destruction of functional hepatic cells and replacement with fibrosis (scarring) of the liver. It is most often caused by alcohol use, chronic infection with hepatitis B or C, non-alcoholic steatohepatitis but there is also a residual category of multiple other causes. Early disease is typically asymptomatic as the liver's resilience compensates for cirrhotic damage. Decompensated cirrhosis occurs when the disease progresses beyond the capacity of the liver to compensate for the damage, and is marked by profound symptoms, health loss and typically progresses to death in a few years. ICD10 codes are K70-K77, I85, P78.81.

Input data and processing

We modelled total cirrhosis and decompensated cirrhosis prevalence based on hospital discharge data and claims data. The total cirrhosis model uses claims data for both inpatient and outpatient care and inpatient discharge data adjusted to total cases diagnosed in inpatient and outpatient encounters using a correction factor estimated from claims data. The decompensated model uses claims data only for inpatient care, and hospital discharge data adjusted only to account for readmissions. (See sections of this appendix for details of hospital and claims data processing.)

Data inputs for total cirrhosis and decompensated cirrhosis morbidity modelling

Measure	Total sources	Countries with data
Prevalence	334	48

Additionally, we use case-series data to estimate the proportion of cirrhosis cases attributed to alcohol, hepatitis B, hepatitis C, NASH and other causes. In GBD 2019, we added 12 new case-series from GBD collaborators.

The inclusion criteria for case-series data stipulated that: 1) the publication year was from 1980 onward; 2) the sample was a representative sample of those with decompensated cirrhosis (eg, studies of patients with both HCC and hepatitis were excluded); 3) sufficient information was provided on study method and sample characteristics to assess the quality of the study; 4) hepatitis B and C were confirmed via HBsAg, in the case of hepatitis B, and anti-HCV IgG, in the case of hepatitis C.

Data inputs for cirrhosis and other chronic liver diseases aetiological proportion modelling

Model	Measure	Total sources	Countries with data
Cirrhosis and other chronic liver diseases due to hepatitis B	Proportion	84	34
Cirrhosis and other chronic liver diseases due to hepatitis C	Proportion	85	36
Cirrhosis and other chronic liver diseases due to alcohol	Proportion	54	24
Cirrhosis and other chronic liver diseases due to other causes	Proportion	31	19
Cirrhosis and other chronic liver diseases due to NASH	Proportion	25	16

Data processing

In GBD 2019, we changed the method used to sex split data points. Previously studies that reported on "both" sex data points were split inside DisMod MR 2.1 using the sex covariate's fixed-effect coefficient. However, this round we modeled the ratio of female/male prevalence in MR-BRT using the sex-ratios calculated directly from the studies that reported on both sexes separately and 10% trimming of the calculated ratios. (See appendix section on MR-BRT method for details.) Then, for studies only reporting prevalence for both sexes, we used GBD estimated population to estimate male prevalence as:

$$prev_{male} = prev_{both} * \frac{pop_{both}}{(pop_{male} + ratio * pop_{female})}$$

And then calculated female prevalence:

$$prev_{female} = ratio * prev_{male}$$

The table below lists out the estimated sex ratio by proportion aetiology.

MR-BRT Sex Ratios for cirrhosis etiology proportions

Cirrhosis etiology proportion	Beta Coefficient, Log (95% CI)
Cirrhosis due to hepatitis B	-0.21 (-0.56 – 0.14)
Cirrhosis due to hepatitis C	0.05 (-1.79 – 1.90)

Cirrhosis due to alcohol	0.71 (-1.05 – 2.46)
Cirrhosis due to other causes	0.96 (0.14 – 1.75)
Cirrhosis due to NASH	0.58 (-0.11 – 1.26)

In GBD 2017, we split data points where the age range was greater than 25 years. This method was continued in GBD2019, in which the age pattern from the GBD2017 model was applied to large age range data to split into more granular age groups.

For GBD2019, adjustment factors for all study-level covariates were determined using matched data (by year, age, sex, location) for reference and alternative case definitions in a logit difference meta-regression. Adjustments were made for data from MarketScan, a database of claims data for commercial insurance in the USA, which may be biased because because commercially insured individuals may have differential healthcareseeking behaviors compared to those in the general population. We conducted an analysis in MR-BRT with a spline on age to adjust these commercial claims data to hospital data differentially by age. The analysis was conducted between MarketScan data in 2000 compared to hospital data in 2000, and then all other years of MarketScan data compared to other years of hospital data. The figures below show examples of splines on age for the different adjustments.

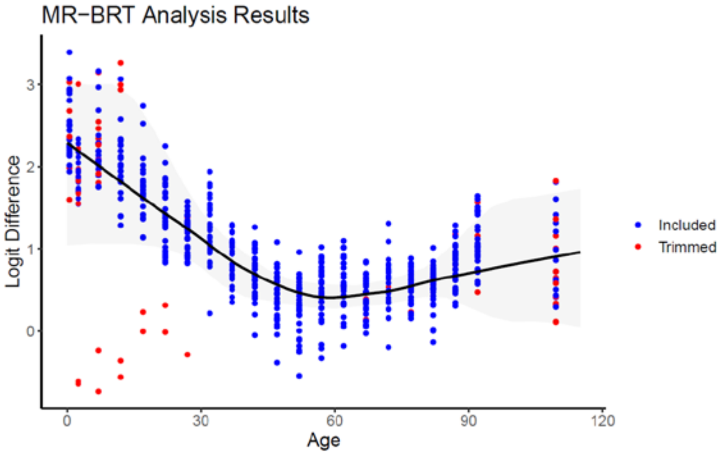

Figure: Spline on age for decompensated cirrhosis, MarketScan years after 2000

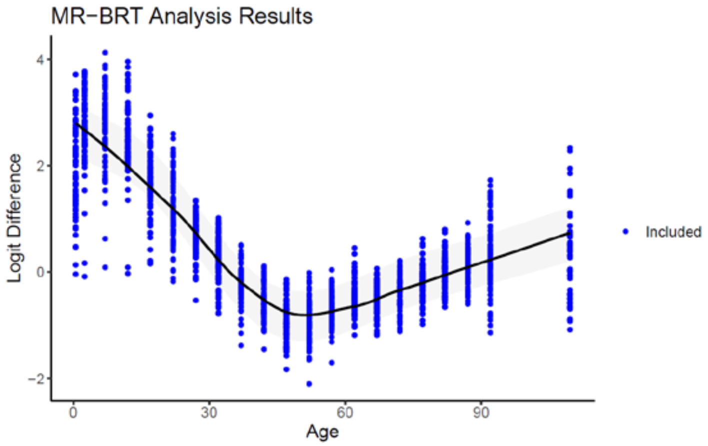

Figure: Spline on age for total cirrhosis, MarketScan years after 2000

Compared to GBD 2017, modeling the proportions of cirrhosis due to NASH vs "other causes" changed in GBD 2019. Epidemiological studies and hepatologists have indicated that cryptogenic cases of cirrhosis may be un-identified cases of cirrhosis due to NASH. In GBD 20sdf17, when a cirrhosis case-series identified all of our etiologies of interest as well as cryptogenic cirrhosis, cryptogenic cases were extracted as "other causes", but when a case-series did not explicitly identify NASH, cases reported as "cryptogenic" were extracted as NASH. In GBD 2019 we analyzed case-series studies that reported both NASH and cryptogenic cases, modeling the proportion due to NASH (out of NASH plus cryptogenic) in MR-BRT. We then identified the case-series in our database that reported cryptogenic, but not NASH, as an aetiology of cirrhosis, and extracted a proportion due to NASH and a proportion due to other causes based on the proportion modeled in MR-BRT.

Adjustment of cryptogenic cases that did not specify NASH to cases of NASH modeled in MR-BRT

Data input	Beta Coefficient, Logit (95% CI)	Gamma
Proportion of cryptogenic cases out of cryptogenic cases plus NASH cases reported in the same study	0.624 (-0.659 – 1.887)	0.567

Modeling strategy

We modelled cirrhosis prevalence using hospital data and EMR, assuming no remission, using DisMod-MR 2.1. The summary of covariates and the exponentiated betas of the total cirrhosis and decompensated cirrhosis DisMoD-MR 2.1 models are listed in tables below. To estimate the prevalence of cirrhosis due to alcohol, hepatitis B, hepatitis C, NASH, and other causes, we developed aetiological proportion models using DisMod-MR 2.1, and used the results of these models to split the parent total cirrhosis and decompensated cirrhosis prevalence estimates.

Total and decompensated cirrhosis models

As stated above, we used excess mortality rate data as inputs into our total and decompensated cirrhosis DisMod models. In previous rounds, priors on excess mortality rate (EMR) were estimated in DisMod by matching prevalence data points with their corresponding CSMR values within the same age, sex, year, location (by dividing CSMR by prevalence). However, for many causes, DisMod estimated a rather unrealistic pattern of EMR compared to an expected pattern of decreasing EMR with greater access to quality health care. Such unexpected patterns often signal inconsistencies between CSMR estimates and the measures of prevalence and/or incidence. In effort to provide greater guidance to DisMod on the expected pattern of EMR, EMR data generated in the previous round were modeled using the MR-BRT approach by age and sex with a prior on healthcare access and quality index (HAQi) having a negative coefficient. Results from MR-BRT were then predicted for each location year, sex and for ages 0, 10, 20 ….100. We included HAQi as a country-level covariate to inform EMR with a mean and standard deviation produced from MR-BRT. However, even without this setting DisMod would tend to estimate a coefficient that was consistent with the MR-BRT analysis.

Summary of covariates used in the total cirrhosis DisMod-MR 2.1 model

Covariate	Parameter	Exponentiated beta (95% Uncertainty Interval)
Hepatitis B Seroprevalence (HBsAg) age standardized	Prevalence	39.30 (29.25 — 50.60
Hepatitis C Seroprevalence (anti-HCV) age standardized	Prevalence	1.34 (1.01 — 2.26)
Liters of alcohol consumed per capita	Prevalence	1.00 (1.00 — 1.00)
Prevalence of obesity	Prevalence	1.05 (1.00 — 1.16)
Healthcare access and quality index	Excess mortality rate	0.98 (0.98 — 0.98)

Summary of covariates used in the decompensated cirrhosis DisMod-MR 2.1 model

Covariate	Parameter	Exponentiated beta (95% Uncertainty Interval)
Hepatitis B Seroprevalence (HBsAg) age standardized	Prevalence	53.28 (51.37 — 54.54)
Hepatitis C Seroprevalence (anti-HCV) age standardized	Prevalence	13.04 (3.49 — 40.45)
Liters of alcohol consumed per capita	Prevalence	1.00 (1.00 — 1.00)
Prevalence of obesity	Prevalence	1.01 (1.00 — 1.02)
Healthcare access and quality index	Excess mortality rate	0.98 (0.98 — 0.98)

Aetological proportion models

To estimate morbidity from cirrhosis due to alcohol, hepatitis B, hepatitis C, NASH, and other causes, we developed aetiological proportion models using DisMod-MR to split the parent cirrhosis morbidity estimates. Proportions from the five aetiology models were then rescaled to sum to one at the draw level and used to split the parent cirrhosis morbidity estimates.Data for aetiologic proportion models are scant, and estimates are strengthened by using predictive covariates. As in previous rounds, we included the prevalence of the precursor states that can give rise to each aetiology of cirrhosis (prevalence of hepatitis B, hepatitis C, alcohol consumption, *etcetera*) and the most recent estimate of the proportion of liver cancer cases due to each aetiology, all with bounds limiting to positive associations. (See liver cancer appendix section for details on estimation of aetiologic proportions for liver cancer.) In GBD 2019, we introduced predictive covariates with specified negative associations for the proportions of cirrhosis due to other aetiologies. The summary of covariates and the exponentiated betas of each aetiological proportion model are listed in tables 8-12.

Summary of covariates used in the proportion of cirrhosis due to hepatitis B DisMod-MR meta-regression model

Covariate	Exponentiated beta (95% Uncertainty Interval)
Hepatitis B Seroprevalence (HBsAg) age standardized	2.37 (1.88 — 2.70)
Proportion of liver cancer due to hepatitis B (age-standardised)	1.59 (1.17 — 2.16)
Hepatitis B 3-dose coverage (proportion), lagged 10 years	0.50 (0.45 — 0.55)
Proportion of cirrhosis due to alcohol	0.88 (0.70 — 0.99)

Proportion of cirrhosis due to hepatitis C	0.41 (0.37 — 0.50)
Proportion of cirrhosis due to other causes	0.93 (0.82 — 1.00)
Proportion of cirrhosis due to NASH	0.69 (0.45 — 0.98)

Summary of covariates used in the Proportion of cirrhosis due to hepatitis C DisMod-MR meta-regression model

Covariate	Exponentiated beta (95% Uncertainty Interval)
Hepatitis C Seroprevalence (anti-HCV) age standardized	1.72 (1.07 — 2.59)
Proportion of liver cancer due to hepatitis C (Age Standardized)	1.81 (1.14 — 2.62)
Proportion of cirrhosis due to alcohol	0.44 (0.37 — 0.60)
Proportion of cirrhosis due to hepatitis B	0.64 (0.40 — 0.96)
Proportion of cirrhosis due to other causes	0.90 (0.76 — 1.00)
Proportion of cirrhosis due to NASH	0.58 (0.38 — 0.91)

Summary of covariates used in the Proportion of cirrhosis due to alcohol DisMod-MR meta-regression model

Covariate	Exponentiated beta (95% Uncertainty Interval)
Liters of alcohol consumed per capita	1.02 (1.00 — 1.04)
Alcohol abstainer proportion, age-standardized	0.90 (0.76 — 1.00)
Proportion of liver cancer due to alcohol (Age Standardized)	1.40 (1.02 — 2.21)
Proportion of cirrhosis due to hepatitis B	0.83 (0.63 — 0.99)
Proportion of cirrhosis due to hepatitis C	0.43 (0.37 — 0.60)
Proportion of cirrhosis due to other causes	0.68 (0.45 — 0.95)
Proportion of cirrhosis due to NASH	0.65 (0.42 — 0.96)

Summary of covariates used in the Proportion of cirrhosis due to other causes DisMod-MR meta-regression model

Covariate	Exponentiated beta (95% Uncertainty Interval)
Proportion of liver cancer due to other causes (Age Standardized)	1.59 (1.05 — 2.56)
Proportion of cirrhosis due to hepatitis B	0.59 (0.39 — 0.91)
Proportion of cirrhosis due to hepatitis C	0.92 (0.78 — 1.0)
Proportion of cirrhosis due to alcohol	0.41 (0.37 — 0.50)
Proportion of cirrhosis due to NASH	0.64 (0.42 — 0.94)

Summary of covariates used in the Proportion of cirrhosis due to NASH DisMod-MR meta-regression model

Covariate	Exponentiated beta (95% Uncertainty Interval)
Mean BMI	1.00 (1.00 — 1.01)
Prevalence of obesity	1.16 (1.01 — 1.50)

NAFLD/NASH prevalence	2.20 (1.07 — 5.08)
Proportion of liver cancer due to NASH (Age Standardized)	3.88 (1.59 — 7.13)
Proportion of cirrhosis due to hepatitis B	0.48 (0.37 — 0.78)
Proportion of cirrhosis due to hepatitis C	0.88 (0.70 — 0.99)
Proportion of cirrhosis due to alcohol	0.43 (0.37 — 0.56)
Proportion of cirrhosis due to other causes	0.73 (0.53 — 0.96)

Sequelae and disability weights

In GBD2019, we estimated the proportion of individuals with decompensated cirrhosis that had different severity levels of anemia: no anemia, mild anemia, moderate anemia, and severe anemia. After estimation of decompensated cirrhosis due to each etiology, we further split estimates to reflect anemia severity state. As such, the disability weight for cirrhosis changed in GBD2019 to be a combined weight to account for disability due to decompensated cirrhosis and the different levels of anemia.

Severity distribution, details on the severity level decompensated cirrhosis for in GBD 2019 and the associated disability weight (DW) with that severity

Health state	Lay description	DW (95% CI)
Decompensated cirrhosis of the liver, no anemia	Has a swollen belly and swollen legs. The person feels weakness, fatigue and loss of appetite.	0.178 (0.113–0.243)
Decompensated cirrhosis of the liver and mild anemia	Feels slightly tired and weak at times, but this does not interfere with normal daily activities.	0.181 (0.116 – 0.246)
Decompensate cirrhosis of the liver and moderate anemia	Feels moderate fatigue, weakness, and shortness of breath after exercise, making daily activities more difficult.	0.220 (0.146 – 0.295)
Decompensated cirrhosis of the liver and severe anemia	Feels very weak, tired and short of breath, and has problems with activities that require physical effort or deep concentration.	0.300 (0.202 – 0.397)

Nonalcoholic fatty liver disease without cirrhosis

Flowchart

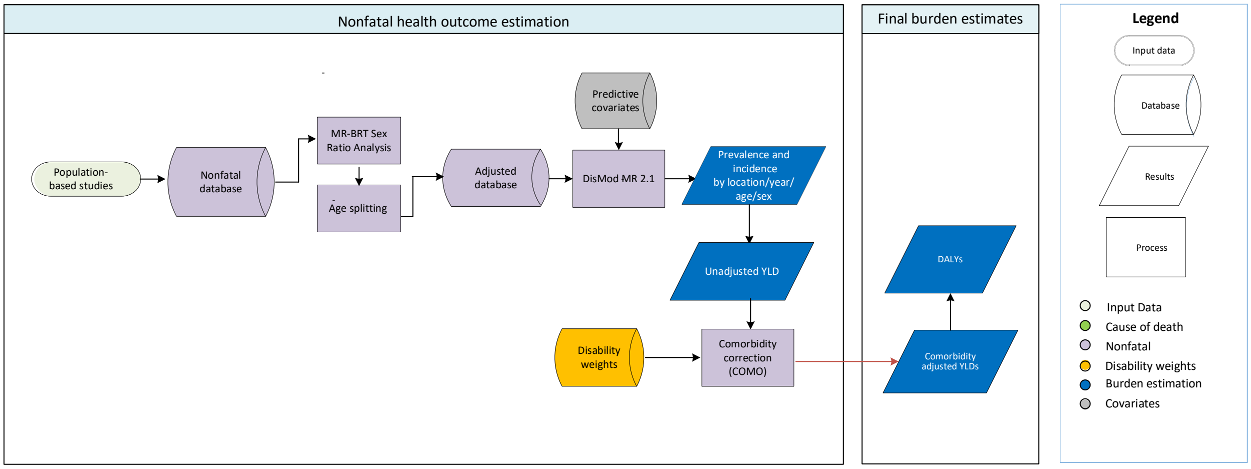

Input Data and Methodological Summary for Non-alcoholic fatty liver disease

Case definition

Non-alcoholic fatty liver disease (NAFLD) is an umbrella term for a range of liver conditions that mimic alcoholic liver disease – both in the appearance of tissue on biopsy and in clinical signs and symptoms – but occur in people who drink little to no alcohol. This range includes non-alcoholic fatty liver, characterized by fat deposition in liver cells, non-alcoholic steatohepatitis, characterized by fat deposition and inflammation, and cirrhosis. Non-alcoholic fatty liver disease without cirrhosis includes all degrees of NAFLD that have not progressed to cirrhosis, although we refer to it simply as "NAFLD" in this appendix section.

Input data

We use population-based studies that report the prevalence of NAFLD. The following inclusion criteria were used:

(1) Sample size greater than 100
(2) Sample representative of general population for location
(3) Sufficient description of methods to assess study quality
(4) Does not exclude comorbidities
(5) NAFLD diagnosed by ultrasound (USS) or other diagnostic imaging modality

The last systematic review was performed for GBD 2017, using the search string below.

("Steatohepatitides"[Title/Abstract]) OR ("NAFLD"[Title/Abstract] OR "NAFL"[Title/Abstract] OR "NASH"[Title/Abstract] OR)) AND ("prevalence"[Title/Abstract] OR "incidence"[Title/Abstract) AND ("1990/01/01"[PDAT] : "2017/07/26"[PDAT]) NOT (animals[MeSH] NOT humans[MeSH]))

Although biopsy provides the gold-standard clinical case definition, this invasive procedure is not typically employed in population-based surveys or screening programs. In consultation with GI experts, we thus chose ultrasound or other imaging study as our reference case diagnostics. We excluded any studies using serum diagnostics or fatty liver indexes and scores to diagnose NAFLD. Studies were excluded if they ascertained cases only among patients with GI distress or in specialty outpatient clinics, or if they excluded patients with comorbidities.

Since the majority of NAFLD cases are asymptomatic, we generally preferred studies with active case-finding methods and did not make use of administrative data from hospitals or claims, which severely underestimate NAFLD prevalence. An exception to this is that we accepted Asian studies pooling data from general checkups, where participation in checkups is high and USS is a part of the checkup regimen (eg, South Korea, Japan, and some parts of China). Data were marked as outliers and excluded if we found they differed substantially when compared to regional, super-regional, and global rates.

Data Processing

In GBD 2019, we changed the method used to sex split data points. Previously studies that reported on "both" sex data points were split inside DisMod MR 2.1 using the sex covariate's fixed-effect coefficient. However, this round we modeled the ratio of female/male prevalence in MR-BRT using the sex-ratios calculated directly from the studies that reported on both sexes separately and 10% trimming of the calculated ratios. (See appendix section on MR-BRT method for details.) Then, for studies only reporting prevalence for both sexes, we used GBD estimated population to estimate male prevalence as:

$$prev_{male} = prev_{both} * \frac{pop_{both}}{(pop_{male} + ratio * pop_{female})}$$

And then calculated female prevalence:

$$prev_{female} = ratio * prev_{male}$$

In GBD 2017, we also split data points where the age range was greater than 25 years. This method was continued in GBD2019, in which the age pattern from the GBD2017 model was applied to large age range data to split into more granular age groups.

Modeling strategy

We have made few changes in the modeling strategy from GBD 2017. We modelled prevalence and incidence of NAFLD using DisMod-MR 2.1. Our prior inputs include zero excess mortality for all ages and zero incidence from age 0-5.

Several factors known to be associated with NAFLD prevalence in prior studies, for which we have prevalence estimates available for all GBD year-age-sex-location combinations, were employed as predictive covariates. Associations between predictive covariates and NAFLD prevalence for year-age-sex-location combinations with NAFLD prevalence data are used to help predict NAFLD prevalence for year-age-sex-location combinations with little or no data. In GBD2019, we added prevalence of obesity and age standardized SEV for high fasting plasma glucose as predictive covariates in the model. A table of predictive covariates and their coefficients is shown below.

Summary of covariates used in the NAFLD DisMod-MR meta-regression model

Covariate	Parameter	Exponentiated beta (95% Uncertainty Interval)
Mean BMI	Prevalence	1.12 (1.08 — 1.17)
Prevalence of obesity	Prevalence	4.21 (1.70 — 7.26)
Age-standardized SEV* for High fasting plasma glucose	Prevalence	2.72 (2.49 — 2.97)

*Estimation of scaled exposure variables (SEVs) is described in a separate appendix section

Studies of NAFLD vary in the level of alcohol consumption they use to define those at risk of NAFLD. We first ran models adjusting different definitions of alcohol exclusion towards the most frequently used definition: 70 grams and 140 grams per week for men and women, respectively. The effect of these adjustments were insignificant and dropped in the final model.

Because many studies excluded individuals with high alcohol consumption from the study sample, prevalence measurements from these studies reflect the prevalence in low- or non-consumers of alcohol, not a general population. Thus, we multiplied location-year-sex-age specific prevalence estimates from the NAFLD DisMod model by the proportion of the general population that consumes < 70g (female) and < 140g (male) of alcohol per week to approximate data for the general population. This proportion is estimated by the alcohol risk factor team and is year, age, sex and location specific. We did not develop a similar post-hoc adjustment for studies that excluded individuals with hepatitis or other forms of chronic liver disease from their samples.

Disability weights

Cases of NAFLD without cirrhosis are asymptomatic and assigned a disability weight of zero.

Peptic Ulcer Disease

Flowchart

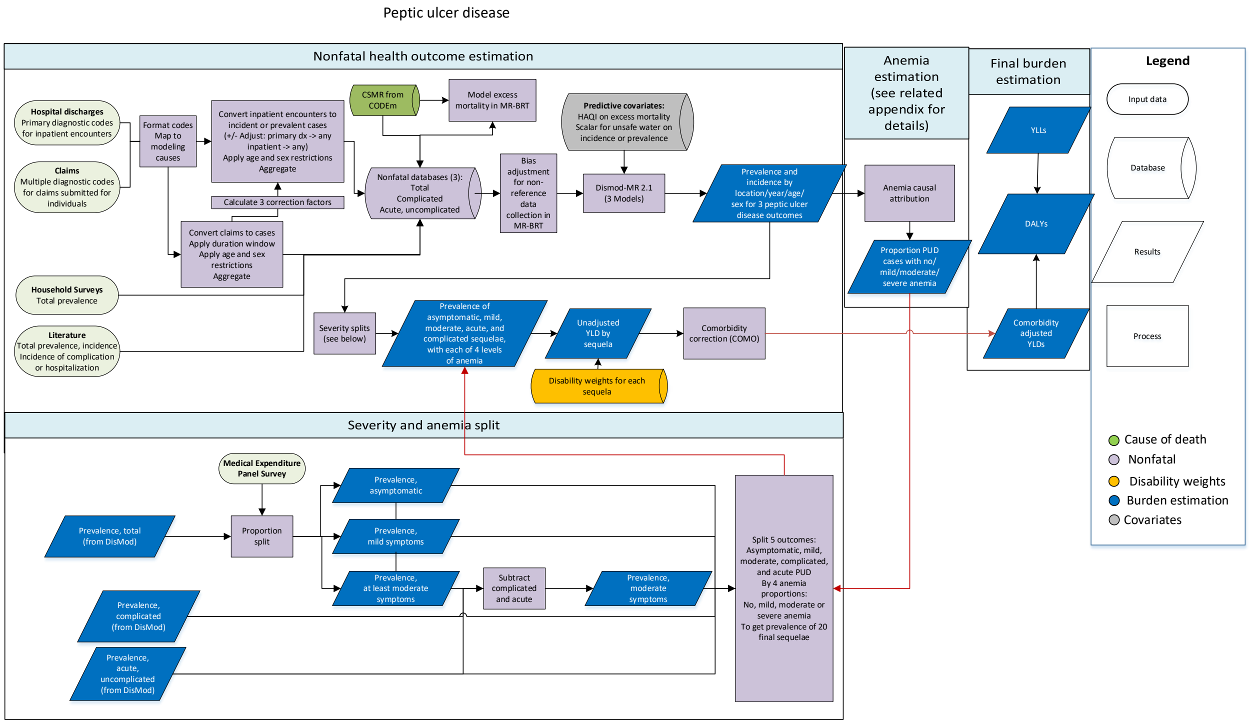

Case definition

Peptic ulcer disease is a digestive disorder defined by defects in the lining of the stomach (gastric ulcers) or the duodenum (duodenal ulcers) that extend through the muscularis mucosa. Diagnosis by endoscopy is considered the gold standard. Peptic ulcers can develop marked abdominal pain acutely or can have a more insidious onset and develop into a chronic disease with asymptomatic and symptomatic periods. Symptomatic periods of peptic ulcer disease are characterised by abdominal pain, bloating, nausea, and early satiety. Regardless of the duration of the disease, acute, life-threatening complications of bleeding, perforation, or gastric outlet obstruction can develop. Chronic gastric ulcer disease predisposes to gastric cancer.

For GBD, cases were defined by diagnostic codes in administrative data. ICD10 codes used to identify cases of peptic ulcer disease are K25, K26, K27, K28, and K31. ICD10 codes for complicated peptic ulcer disease are K25.0-2, K25.4-6, K26.0-2, K26.4-6, K27.0-2, K27.4-6, K28.0-2, and K28.4-6. ICD10 codes for acute peptic ulcer disease without complication are K25.3, K26.3, K27.3 and K28.3. Equivalent ICD9 codes were used where appropriate.

Input data

Data sources

As in previous rounds, our GBD 2019 peptic ulcer disease models relied primarily on data from hospital discharges and medical claims, as obtained and processed by the GBD Clinical Informatics team and described in a separate section of this Appendix. New data added in GBD 2019 included Polish claims, additional years of USA claims (years 2015-2016), and hospital discharges in Mexico, India, New Zealand, Sweden, Georgia, and Ecuador. Notably, we added hospital discharge data from Botswana; southern sub-Saharan Africa previously did not have data. In GBD 2017, data from outpatient encounters from facilities in the United States and Sweden were considered for inclusion in the total peptic ulcer disease database, but these data were inconsistent with established regional trends and age distributions and were excluded; this exclusion was maintained in GBD 2019.

Additional sources of data for peptic ulcer disease included peer-reviewed publications identified via systematic reviews of the literature conducted using recognized search engines (PubMed, Embase) for previous rounds of GBD, most recently, GBD 2016. They also included studies contributed to the Global Health Data Exchange by GBD Network Collaborators and identified by a keyword search. In brief, to be included, studies from all sources needed to:

1) report a standard epidemiologic measure (incidence, prevalence, case fatality ratio, standardized mortality rate, *etcetera*) of peptic ulcer disease or its complications (bleeding, perforation, hospital admission)
2) provide sufficient information on study methods and sample characteristics to assess its quality and make appropriate adjustments
3) use a gold-standard endoscopic case definition, or use a well-defined alternative case-definition that could be adjusted toward a reference standard
4) be conducted in a representative sample of a general population defined only by year, age, sex and location, or be conducted in a representative sample of a well-defined sub-population for which valid adjustments could be made, or ascertain all cases for a defined catchment area for which GBD population estimates are available

As in GBD 2017, the GBD 2019 peptic ulcer disease modeling strategy used three separate categories: total peptic ulcer disease, peptic ulcer disease with complication (such as haemorrhage or perforation), and peptic ulcer disease, acute, without complication (but with sufficient severity and diagnostic uncertainty to require hospitalisation). The total peptic ulcer disease model included data from hospital discharges and claims coded with any peptic ulcer disease ICD code, as well as data from peer-reviewed publications and household surveys. The peptic ulcer disease with complication dataset included hospital discharges and inpatient claims with ICD codes specifying the occurrence of complications, as well as data from peer-reviewed publications. The peptic ulcer disease, uncomplicated, acute dataset included only hospital discharges and inpatient claims with ICD codes specifying that a complication did not occur.

Data inputs for peptic ulcer disease morbidity modelling by parameter

Measure	Total sources	Countries with data
All measures	388	52
Prevalence	355	51
Incidence	322	44
Proportion	15	1

Data extraction and processing

Hospital discharge data provide observations about encounters, generally with only the primary diagnostic code for the encounter. Claims data, on the other hand, link claims for all inpatient and outpatient encounters for a single individual, and provide primary and secondary diagnoses for all encounters. The details of how these data are extracted and processed are described in greater detail elsewhere in this Appendix.

For the total peptic ulcer disease database, an individual was extracted from claims data as a prevalent case if they had any peptic ulcer disease code as any diagnosis in one or more inpatient encounters or two or more outpatient encounters. Hospital discharges were extracted if an appropriate code appeared as a discharge diagnosis. Correction factors from claims data were then applied to the hospital discharges to estimate the number of cases represented by the encounters, adjusting for the fact that some facilities only provide the primary discharge diagnosis, and estimating the number of outpatient cases represented by each inpatient case. For the peptic ulcer disease with complication dataset and the peptic ulcer disease, uncomplicated, acute dataset claims were extracted as incident cases, linking multiple encounters for an individual and assuming multiple encounters within a 60-day window represented a single episode. Discharges with an appropriate ICD code in any diagnostic field were extracted as encounters and adjusted using a correction factor from claims data to estimate the number of incident cases, and another to account for some sources only providing primary diagnoses.

Epidemiologic measurements from peer-reviewed publications were manually extracted and marked with dichotomous variables for non-reference case definitions. Prevalence estimates were extracted from individual-level data from household surveys using questionnaire text, skip-pattern, and weights for complex sampling strategies provided in the documentation from original study investigators.

Pre-modelling bias adjustments

For total peptic ulcer disease, we sought to use a gold-standard case definition of endoscopy without clinical indication, and to develop adjustments for alternative case definitions of endoscopy with clinical indication, diagnostic code in administrative data, and self-reported diagnosis (current or with 12-month recall). Unfortunately, the few (four) endoscopy-based studies in our database were not performed in samples from locations for which we had data with alternative case definitions available. Thus, we dropped the endoscopy-based data and adopted diagnostic code in administrative data as our reference case definition. Two pre-modeling adjustments were made to non-reference data sources: data using self-reported diagnosis and data from a claims database that only covers a commercially insured sub-population. Twenty-six sources used self-reported diagnosis and 18 of these were matched to hospital discharge data, claims data or both. Commercial claims data were available for all 51 USA subnational locations, and matched hospital discharge data covering the general population for one or more years for 24 USA subnational locations. These sets of paired data were used as inputs to a model of the

difference in logit prevalence between alternative and reference data types using a network model in MR-BRT. The estimated mean logit differences were applied to non-reference data types as bias correction prior to modeling in DisMod-MR 2.1 (below).

The process of adjusting for non-reference data using MR-BRT with the logit-transformation method is described below:

1. Identify data points with overlapping year, age, sex, and location between commercial claims or self-report (alternative data collection methods) and hospital discharges (reference data)
2. Logit transform overlapping data points of alternative and reference case definitions
3. Convert overlapping data points into a difference in logit space using the following equation: $logit(altnerative) - logit(reference)$
4. Use the delta method to compute standard errors of overlapping data points in logit space, then calculate standard error of logit difference using the following equation: $\sqrt{(variance\ of\ alternative) + (variance\ of\ reference)}$
5. Using MR-BRT, conduct a random effects meta-regression to obtain the pooled logit difference of alternative to reference
6. Apply the pooled logit difference to all data points of alternative case definitions using the following equation:
$$new_{estimate} = inverse.logit((logit(alternative)) - (pooled\ logit\ difference))$$
7. Calculate new standard errors using the delta method, accounting for gamma (between-study heterogeneity)

The table below shows bias correction factors estimated using MR-BRT.

MR-BRT Crosswalk Adjustment Factors for Total Peptic Ulcer Disease

Data input	Reference or alternative data collection	Gamma	Beta Coefficient, Logit difference (95% CI)	Adjustment factor*
Hospital + non-USA claims	Reference	0.163	---	---
USA claims from year 2000	Alternative		0.00936 (-0.319 to 0.340)	0.50 (0.42 to 0.58)
USA claims from year 2010-2016	Alternative		-0.138 (-0.463 to 0.193)	0.47 (0.39 to 0.55)
Self-reported diagnosis	Alternative		2.37 (2.05- to 2.70)	0.91 (0.89 to 0.94)

**Adjustment factor is the inverse-logit transformed Beta coefficient; <0.5 represents that alternative is adjusted upward; >0.5 represents that alternative is adjusted downward*

For peptic ulcer disease with complication, similar to the total peptic ulcer disease model, we sought to use a gold-standard endoscopic case definition, and to develop adjustments for the alternative case definitions by diagnostic code in administrative data. Unfortunately, there were only five studies that used endoscopy to define peptic ulcer disease with complications our database, and they were not conducted in the same year, age, sex and location as studies with other designs, so these data were dropped, and diagnosis in administrative data was adopted as the reference case definition. Pre-

modelling adjustments were made to data from commercial claims, using an approach similar to that described above for total peptic ulcer disease data.

MR-BRT Crosswalk Adjustment Factors for Peptic Ulcer Disease with Complication

Data input	Reference or alternative data collection	Gamma	Beta Coefficient, Logit difference (95% CI)	Adjustment factor*
Hospital + non-USA claims	Reference	0.118	---	---
USA claims from year 2000	Alternative		0.861 (0.214 to 1.50)	0.70 (0.55 to 0.82)
USA claims from year 2010-2016	Alternative		0.778 (0.511 to 1.03)	0.69 (0.62 to 0.74)

**Adjustment factor is the inverse-logit transformed Beta coefficient; <0.5 represents that alternative is adjusted upward; >0.5 represents that alternative is adjusted downward*

For peptic ulcer disease, uncomplicated, acute, all data were based on diagnostic codes in administrative data. Pre-modelling adjustments were made to data from commercial claims, as described above.

MR-BRT Crosswalk Adjustment Factors for Peptic Ulcer Disease, uncomplicated, acute

Data input	Reference or alternative data collection	Gamma	Beta Coefficient, Logit difference (95% CI)	Adjustment factor*
Hospital + non-USA claims	Reference	0.550	---	---
USA claims from year 2000	Alternative		0.291 (-1.22 to 1.72)	0.57 (0.23 to 0.85)
USA claims from year 2010-2016	Alternative		0.220 (-0.903 to 1.39)	0.55 (0.29 to 0.80)

**Adjustment factor is the inverse-logit transformed Beta coefficient; <0.5 represents that alternative is adjusted upward; >0.5 represents that alternative is adjusted downward*

Outlier identification and exclusion

After adjustment, for each source-location-year-sex combination, age-standardised mean was calculated, and the data series was excluded if this was 0 or was greater than two times the median absolute deviation above or below the median for the database.

Modelling strategy

Total peptic ulcer disease, symptomatic and asymptomatic

The DisMod model for total peptic ulcer disease used prevalence data as described above, cause-specific mortality rate (CSMR) estimates from the GBD causes of death analysis, modeled excess mortality rate inputs, and expert priors for other epidemiologic measures.

In previous rounds, priors on excess mortality rate (EMR) were estimated in DisMod by matching prevalence data points with their corresponding CSMR values within the same age, sex, year, and location (by dividing CSMR by prevalence). With this approach, however, DisMod estimated a rather unrealistic pattern of EMR compared to an expected pattern of decreasing EMR with greater access to quality health care. This highlighted inconsistencies between CSMR estimates and the measures of prevalence and/or incidence in many locations. In effort to provide greater guidance to DisMod on the expected pattern of EMR, EMR data generated in the previous round were modeled using the MR-BRT approach by age and sex with a prior on healthcare access and quality index (HAQi) having a negative coefficient. Results from MR-BRT were then predicted for each location, year, sex, and for ages 0, 10, 20….100 and entered as data inputs for our DisMod model. Additionally, we included HAQi as a country-level covariate in our DisMod model to inform EMR with a mean and standard deviation produced from MR-BRT.

The prior value of remission was bounded from 0.1 to 0.5 (a duration of two to ten years) and the prior value of incidence was that no incidence occurs before age 5. The summary exposure variable (SEV) for access to safe water was applied as a covariate to predict prevalence. Betas and exponentiated values (which can be interpreted as an odds ratio) are shown in the tables below for all predictive covariates in the DisMod model.

DisMod-MR 2.1 Predictive Covariates for Total Peptic Ulcer Disease

Covariate	Parameter	Beta coefficient	Exponentiated beta
Summary exposure variable for unsafe water	Prevalence	1.23 (1.21 to 1.25)	3.41 (3.34 to 3.48)
Healthcare access and quality index	Excess mortality	-0.018 (-0.018 to -0.018)	0.98 (0.98 to 0.98)

Complicated peptic ulcer disease

The DisMod model for complicated peptic ulcer disease included incidence data as described above. The prior value of incidence was set to 0 before age 5, the prior value of excess mortality rate was bounded to 0.1 to 10, and the prior value of remission was bounded to 6 to 13 cases of remission per person-year (disease duration 4 to 8.7 weeks). A covariate for a Healthcare Access and Quality index was applied to excess mortality ratio, and a covariate for the log-transformed age-standardised death rate due to peptic ulcer disease was applied to incidence, but neither of these were found to be predictive.

DisMod-MR 2.1 Predictive Covariates for Peptic Ulcer Disease with Complication

Covariate	Parameter	Beta coefficient	Exponentiated beta
Natural log of age-standardised death rate	Incidence	0.0012 (0.000063 to 0.0030)	1.00 (1.00 to 1.00)
Healthcare access and quality index	Excess mortality	-0.034 (-1.92 to 1.85)	0.97 (0.15 to 6.36)

Acute peptic ulcer disease, without complication

The DisMod model for acute, uncomplicated peptic ulcer disease included incidence data as described above. Incidence was forced to 0 through age 5 years, the range of prior values on excess mortality rate was bounded to 0 to 0.1, and the range of prior values on remission was bounded to 16.5 to 17.5 cases per person-year (duration of approximately three weeks). Covariates were applied for Healthcare Access and Quality index (on excess mortality rate), log-transformed age-standardised death rate due to peptic ulcer disease (on incidence), and unsafe water (on incidence).

DisMod-MR 2.1 Predictive Covariates for Peptic Ulcer Disease, uncomplicated, acute

Covariate	Parameter	Beta coefficient	Exponentiated beta
Natural log of age-standardised death rate	Incidence	0.000074 (0.0000016 to 0.00027)	1.00 (1.00 to 1.00)
Healthcare access and quality index	Excess mortality	-0.49 (-0.98 to -0.022)	0.61 (0.38 to 0.98)

Severity split & disability weight

The basis of the GBD disability weight survey assessments are lay descriptions of health states highlighting major functional consequences and symptoms.

Peptic ulcer disease, with complication, and peptic ulcer disease, uncomplicated, acute, were assigned the following lay descriptions and disability weights.

Severity level	Lay description	DW (95% CI)
Peptic ulcer disease, with complication	This person vomits blood and feels nauseous.	0.325 (0.209–0.462)
Peptic ulcer disease, uncomplicated, acute	This person has severe pain in the belly and feels nauseous. The person is anxious and unable to carry out daily activities.	0.324 (0.220–0.442)

Prevalence draws from the total peptic ulcer disease model were divided into asymptomatic, mild, and at least moderate severity levels using proportions derived from the Medical Expenditure Panel Survey (MEPS). It must be noted that the MEPS analysis uses quality-of-life data from individuals who had a health care encounter for peptic ulcer disease within the preceding 12 months, and were interviewed about their quality of life in the preceding four weeks, so the asymptomatic proportion represents those with diagnosed disease who were asymptomatic in a given period of time, not those always asymptomatic who may have peptic ulcer disease on endoscopy if examined for study or screening purposes. After dividing the total prevalence draws by these three proportions, the complicated and uncomplicated, acute prevalence draws were subtracted from the at least moderate draws. The asymptomatic, mild, and remaining moderate prevalent cases were then assigned the following lay descriptions and disability weights.

Severity level	Lay description	DW (95% CI)

Diagnosed peptic ulcer disease, not in a symptomatic episode	--	0
Mild peptic ulcer disease episode	This person has some pain in the belly that causes nausea but does not interfere with daily activities.	0.011 (0.005–0.021)
Moderate peptic ulcer disease episode	This person has pain in the belly and feels nauseous. The person has difficulties with daily activities.	0.114 (0.080–0.159)

*The numerous sequelae generated from exclusive combinations of anaemia and peptic ulcer disease each contain custom disability weights. More information can be found in the appendix detailing disability weights.

Methods for causal attribution of anaemia due to peptic ulcer can be found elsewhere in the appendix detailing strategies for impairments

Gastritis and duodenitis

Flowchart

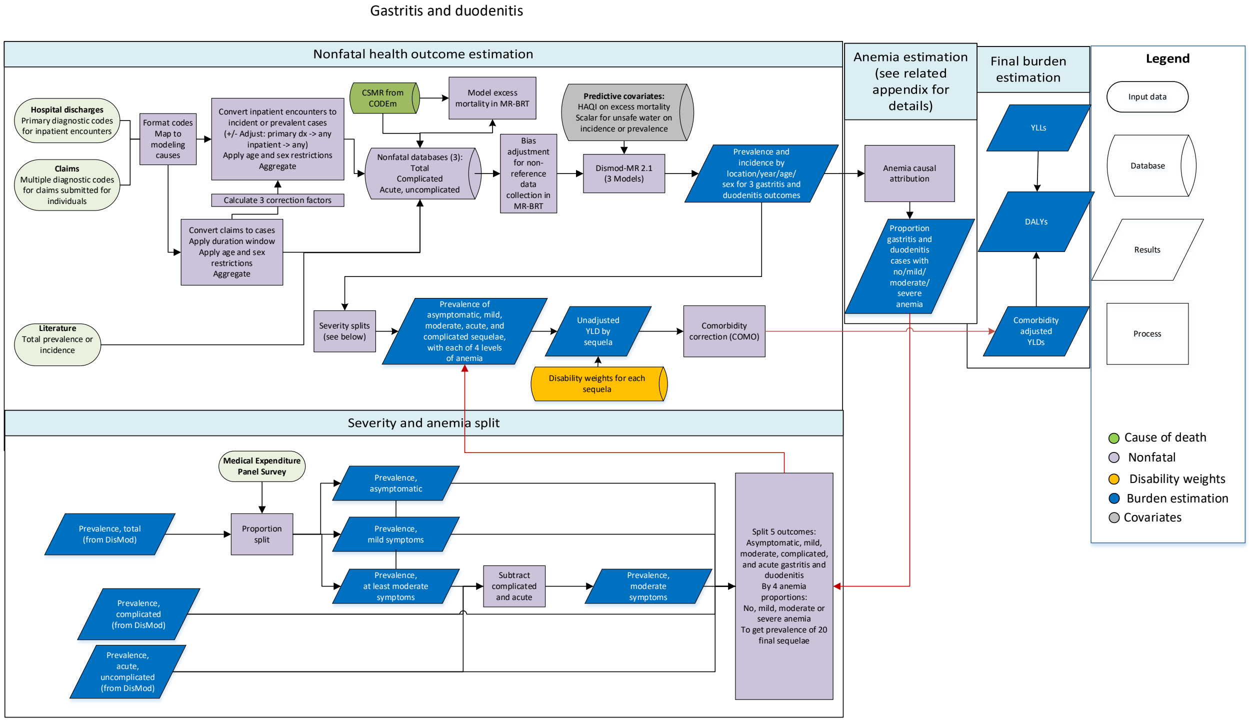

Case definition

Gastritis and duodenitis refer to inflammation of the lining of the stomach and duodenum, respectively, often with damage to epithelial cells lining the gut that is visible via endoscope. Gold standard diagnosis is by biopsy, although a number of biochemical and microbiological tests have good predictive value. This inflammation can acutely produce severe symptoms, or have a subtle onset and evolve into a chronic illness characterised by asymptomatic periods and periods of abdominal pain, bloating, nausea, and early satiety. Complications such as haemorrhage may develop. Chronic gastritis predisposes to gastric cancer.

In GBD 2019, gastritis and duodenitis were defined by diagnostic codes, as described below. The ICD10 code for gastritis and duodenitis is K29. ICD10 codes for complicated gastritis and duodenitis are K29.01, K29.21, K29.31, K29.41, K29.51, K29.61, K29.71, K29.81, K29.91. ICD10 codes for acute gastritis are K29.0, K 29.00, K29.1, K29.2, and K29.20. Equivalent ICD9 codes were used where appropriate.

Input data

Data sources

As in previous rounds, our GBD 2019 gastritis and duodenitis models relied primarily on data from hospital discharges and claims, as obtained and processed by the GBD Clinical Informatics team and described in a separate section of this Appendix. New data added in GBD 2019 included Polish claims, additional years of USA claims (years 2015-2016), and hospital discharges in Mexico, India, New Zealand,

Sweden, Georgia, and Ecuador. Notably, we added hospital discharge data from Botswana; southern sub-Saharan Africa previously did not have data. In GBD 2017, data from outpatient encounters from facilities in the United States and Sweden were considered for inclusion in the total peptic ulcer disease database, but these data violated established regional trends and age distributions and were excluded; this exclusion was maintained in GBD 2019.

Additional sources of data for gastritis and duodenitis included peer-reviewed publications identified via systematic reviews of the literature conducted using recognized search engines (PubMed, Embase) for previous rounds of GBD, most recently, GBD 2016. In brief, to be included, studies from all sources needed to:

1) report a standard epidemiologic measure (incidence, prevalence, case fatality ratio, standardized mortality rate, *etcetera*) of gastritis, duodenitis, or both
2) provide sufficient information on study methods and sample characteristics to assess its quality and make appropriate adjustments
3) use a gold-standard endoscopic case definition, or use a well-defined alternative case-definition that could be adjusted toward a reference standard
4) be conducted in a representative sample of a general population defined only by year, age, sex and location, or be conducted in a representative sample of a well-defined sub-population for which valid adjustments could be made, or ascertain all cases for a defined catchment area for which GBD population estimates are available

As in GBD 2017, the GBD 2019 gastritis and duodenitis modeling strategy used three separate databases: total gastritis and duodenitis, gastritis and duodenitis with complication (such as haemorrhage), and gastritis and duodenitis, acute, without complication (but with sufficient severity and diagnostic uncertainty to require hospitalisation). The total gastritis and duodenitis dataset included data from hospital discharges and claims coded with any gastritis or duodenitis ICD code, as well as data from peer-reviewed publications. The gastritis and duodenitis with complication dataset included hospital discharges and inpatient claims with ICD codes specifying the occurrence of complications. The gastritis and duodenitis, uncomplicated, acute dataset included only hospital discharges and inpatient claims with ICD codes specifying that a complication did not occur.

Data inputs for gastritis and duodenitis morbidity modelling by parameter

Measure	Total sources	Countries with data
All measures	337	45
Prevalence	295	44
Incidence	241	22
Proportion	15	1

Data extraction and processing

Hospital discharge data provide observations about encounters, generally with only the primary diagnostic code for the encounter. Claims data, on the other hand, link claims for all inpatient and outpatient encounters for a single individual, and provide primary and secondary diagnoses for all encounters. The details of how these data are extracted and processed are described in greater detail elsewhere in this Appendix.

For the total gastritis and duodenitis database, an individual was extracted from claims data as a prevalent case if they had any gastritis and duodenitis code as any diagnosis in one or more inpatient encounters or two or more outpatient encounters. Hospital discharges were extracted if an appropriate code appeared as a discharge diagnosis. Correction factors from claims data were then applied to the hospital discharges to estimate the number of cases represented by the encounters, adjusting for the fact that some facilities only provide the primary discharge diagnosis, and estimating the number of outpatient cases represented by each inpatient case. For the gastritis and duodenitis with complication dataset and the gastritis and duodenitis, uncomplicated, acute dataset claims were extracted as incident cases, linking multiple encounters for an individual and assuming multiple encounters within a 60-day window represented a single episode. Discharges with an appropriate ICD code as any diagnosis were extracted as encounters and adjusted using a correction factor from claims data to estimate the number of incident cases, and another to account for some sources only providing primary diagnoses.

Epidemiologic measurements from peer-reviewed publications were manually extracted and marked with dichotomous variables for non-reference case definitions.

For total gastritis and duodenitis, we sought to use a gold-standard case definition of endoscopy without clinical indication, and to develop adjustments for alternative case definitions of endoscopy with clinical indication, serology (pepsinogen), diagnostic code in administrative data, and self-reported diagnosis (current or with 12-month recall). Unfortunately, only a single study in our database used endoscopy to survey for gastritis in a general population selected without regard to symptoms, two used endoscopy performed only in symptomatic persons, eight used serology, and four used self-report; among these, a total of three matches in year, age, sex and location were observed between the studies, and no matches were observed between any of these data types and data from administrative sources. Thus, valid adjustments toward the gold-standard definition could not be estimated, we dropped the endoscopy-based data, and we adopted diagnostic code in administrative data as our reference case definition.

A pre-modeling adjustment was made to account for the fact that claims data from the USA only cover a commercially insured sub-population. Commercial claims data were available for all 51 USA subnational locations, and matched hospital discharge data covering the general population for one or more years for 24 USA subnational locations. These sets of paired data were used as inputs to a model of the difference in logit prevalence between alternative and reference data in MR-BRT. The estimated mean logit differences were applied to non-reference data types as bias correction prior to modeling in DisMod-MR 2.1 (below).

The process of adjusting for non-reference data using MR-BRT with the logit-transformation method is described below:

1. Identify data points with overlapping year, age, sex, and location between commercial claims (alternative data collection method) and hospital discharges (reference data)
2. Logit transform overlapping data points of alternative and reference case definitions
3. Convert overlapping data points into a difference in logit space using the following equation: $logit(altnerative) - logit(reference)$
4. Use the delta method to compute standard errors of overlapping data points in logit space, then calculate standard error of logit difference using the following equation: $\sqrt{(variance\ of\ alternative) + (variance\ of\ reference)}$

5. Using MR-BRT, conduct a random effects meta-regression to obtain the pooled logit difference of alternative to reference
6. Apply the pooled logit difference to all data points of alternative case definitions using the following equation:
$$new_{estimate} = inverse.logit((logit(alternative)) - (pooled\ logit\ difference))$$
7. Calculate new standard errors using the delta method, accounting for gamma (between-study heterogeneity)

The table below shows bias correction factors estimated using MR-BRT.

MR-BRT Crosswalk Adjustment Factors for Total gastritis and duodenitis

Data input	Reference or alternative data collection	Gamma	Beta Coefficient, Logit difference (95% CI)	Adjustment factor*
Hospital + non-USA claims	Reference	0.83	---	---
USA claims from year 2000	Alternative		-0.44 (-2.7 to 1.9)	0.39 (0.066 to 0.87)
USA claims from year 2010-2016	Alternative		-0.030 (-1.7 to 1.7)	0.49 (0.15 to 0.85)

For gastritis and duodenitis with complication, and gastritis and duodenitis, uncomplicated, acute, only administrative data were available. Pre-modelling adjustments were made to data from commercial claims, using an approach similar to that described above for total peptic ulcer disease data.

MR-BRT Crosswalk Adjustment Factors for Gastritis and duodenitis with complication

Data input	Reference or alternative data collection	Gamma	Beta Coefficient, Logit difference (95% CI)	Adjustment factor*
Hospital + non-USA claims	Reference	0.16	---	---
USA claims from year 2000	Alternative		-0.42 (-0.89 to 0.054)	0.40 (0.29 to 0.51)
USA claims from year 2010-2016	Alternative		-0.24 (-0.57 to 0.093)	0.44 (0.36 to 0.52)

MR-BRT Crosswalk Adjustment Factors for Gastritis and duodenitis, uncomplicated, acute

Data input	Reference or alternative data collection	Gamma	Beta Coefficient, Logit difference (95% CI)	Adjustment factor*
Hospital + non-USA claims	Reference	0.21	---	---
USA claims from year 2000	Alternative		0.29 (-0.29 to 0.87)	0.57 (0.43 to 0.71)
USA claims from year 2010-2016	Alternative		-0.072 (-0.51 to 0.36)	0.48 (0.37 to 0.59)

**Adjustment factor is the inverse-logit transformed Beta coefficient; <0.5 represents that alternative is adjusted upward; >0.5 represents that alternative is adjusted downward*

Outlier identification and exclusion

After adjustment, for each source-location-year-sex combination, age-standardised mean was calculated, and the data series was excluded if this was 0 or was greater than two times the median absolute deviation above or below the median for the database.

Modelling strategy

Total gastritis and duodenitis, symptomatic and asymptomatic

The DisMod model for total gastritis and duodenitis used prevalence and incidence data as described above, cause-specific mortality rate (CSMR) estimates from the GBD causes of death analysis, modeled excess mortality rate inputs, and expert priors for other epidemiologic measures.

In previous rounds, priors on excess mortality rate (EMR) were estimated in DisMod by matching prevalence data points with their corresponding CSMR values within the same age, sex, year, and location (by dividing CSMR by prevalence). With this approach, however, DisMod estimated a rather unrealistic pattern of EMR compared to an expected pattern of decreasing EMR with greater access to quality health care. This highlighted inconsistencies between CSMR estimates and the measures of prevalence and/or incidence in many locations. In effort to provide greater guidance to DisMod on the expected pattern of EMR, EMR data generated in the previous round were modeled using the MR-BRT approach by age and sex with a prior on healthcare access and quality index (HAQi) having a negative coefficient. Results from MR-BRT were then predicted for each location, year, sex, and for ages 0, 10, 20….100 and entered as data inputs for our DisMod model. Additionally, we included HAQi as a country-level covariate in our DisMod model to inform EMR with a mean and standard deviation produced from MR-BRT.

Prior value of remission was bounded from 0 to 1 (a minimum duration of one year). Predictive covariates for alcohol consumption and access to safe water were applied to prevalence, which we forced positive with a lower bound of 0 on the priors. Betas and exponentiated values (which can be interpreted as an odds ratio) are shown in the tables below for all predictive covariates in the DisMod model; the covariate for alcohol consumption was not found to be predictive and will be removed in future iterations.

DisMod-MR 2.1 model covariates for Total gastritis and duodenitis

Covariate	Parameter	beta	Exponentiated beta
Liters of alcohol per capita	Prevalence	0.00 (0.00 to 0.00)	1.00 (1.00 to 1.00)
Scaled exposure variable for unsafe water	Prevalence	1.24 (1.23 to 1.25)	3.45 (3.41 to 3.49)
Healthcare access and quality index	Excess mortality	-0.032 (-0.032 to -0.031)	0.97 (0.97 to 0.97)

Complicated gastritis and duodenitis

The DisMod model for complicated gastritis and duodenitis included incidence data as described above. The prior value of incidence was bounded to 0 to 0.3, the prior value of excess mortality rate was bounded to 0.1 to 10, and the prior value of remission was bounded to 6 to 13 cases of remission per person-year (disease duration 4 to 8.7 weeks). A location-level covariate for a Healthcare Access and Quality index was applied to excess mortality ratio, and location-level covariates for the log-transformed age-standardised death rate due to gastritis and duodenitis and unsafe water access were applied to incidence. Random effects for all super-regions except for the High-income super-region were bounded to -0.25 to 0.25.

Betas and exponentiated values (which can be interpreted as odds ratios) are shown in the table below for all covariates. The natural log of the age-standardized death rate was not found to be predictive and will be removed in future iterations.

DisMod-MR 2.1 model covariates for Gastritis and duodenitis with complication

Covariate	Parameter	beta	Exponentiated beta
Natural log of age-standardised death rate	Incidence	0.00 (0.00 to 0.00)	1.00 (1.00 — 1.00)
Scaled exposure variable for unsafe water access	Incidence	0.013 (0.00043 to 0.039)	1.01 (1.00 — 1.04)
Healthcare Access and Quality index	Excess mortality rate	-0.51 (-0.99 to -0.035)	0.60 (0.37 — 0.97)

Acute gastritis and duodenitis, without complication

The DisMod model for acute, uncomplicated gastritis and duodenitis included incidence data as described above. Incidence was forced to 0 through age 5 years, the range of prior values on excess mortality rate was bounded to 0 to 0.1, and the range of prior values on remission was bounded to 6 to 13 cases per person-year. Location-level covariates were applied for log-transformed, lag-distributed income (on excess mortality rate), log-transformed age-standardised death rate due to gastritis and duodenitis (on incidence), and for per capita alcohol consumption (on incidence). Betas and exponentiated values (which can be interpreted as odds ratios) are shown for these covariates in the tables below. The natural log of the age-standardized death rate was not found to be predictive and will be removed in future iterations.

DisMod-MR 2.1 model covariates for Gastritis and duodenitis, uncomplicated, acute

Covariate	Parameter	beta	Exponentiated beta
Log-transformed lag-distributed income	Excess mortality rate	-0.5 (-0.99 to -0.034)	0.61 (0.37 to 0.97)
Natural log of age-standardised death rate	Incidence	0.00 (0.00 to 0.00)	1.00 (1.00 to 1.00)

Severity split & disability weight

The basis of the GBD disability weight survey assessments are lay descriptions of health states highlighting major functional consequences and symptoms.

Prevalence draws from the total gastritis and duodenitis model were divided into asymptomatic, mild, and at least moderate severity levels using proportions derived from the Medical Expenditure Panel Survey (MEPS). It must be noted that the MEPS analysis uses quality-of-life data from individuals who had a health care encounter for gastritis and duodenitis within the preceding 12 months and were interviewed about their quality of life in the preceding four weeks, so the asymptomatic proportion represents those with diagnosed disease who were asymptomatic in a given period of time, not those always asymptomatic who may have gastritis and duodenitis on lab tests or endoscopy if examined for study or screening purposes. After dividing the total prevalence draws by these three proportions, the complicated and uncomplicated acute prevalence draws were subtracted from the at least moderate draws.

The asymptomatic, mild, and remaining moderate prevalent cases were then assigned the following lay descriptions and disability weights.

Severity level	Lay description	DW (95% CI)
Diagnosed gastritis and duodenitis, not in a symptomatic episode	--	0
Mild gastritis and duodenitis episode	This person has some pain in the belly that causes nausea but does not interfere with daily activities.	0.011 (0.005–0.021)
Moderate gastritis and duodenitis episode	This person has pain in the belly and feels nauseous. The person has difficulties with daily activities.	0.114 (0.080–0.159)

Gastritis and duodenitis, with complication, and gastritis and duodenitis, uncomplicated, acute, were then assigned the following lay descriptions and disability weights.

Severity level	Lay description	DW (95% CI)
Gastritis and duodenitis, with complication	This person vomits blood and feels nauseous.	0.325 (0.209–0.462)
Gastritis and duodenitis, acute, uncomplicated	This person has severe pain in the belly and feels nauseous. The person is anxious and unable to carry out daily activities.	0.324 (0.220–0.442)

These five final health states were then combined with health states for anaemia. Methods for causal attribution of anaemia due to gastritis and duodenitis can be found elsewhere in this Appendix.

Gastroesophageal reflux disease

Flowchart

Gastroesophageal reflux disease

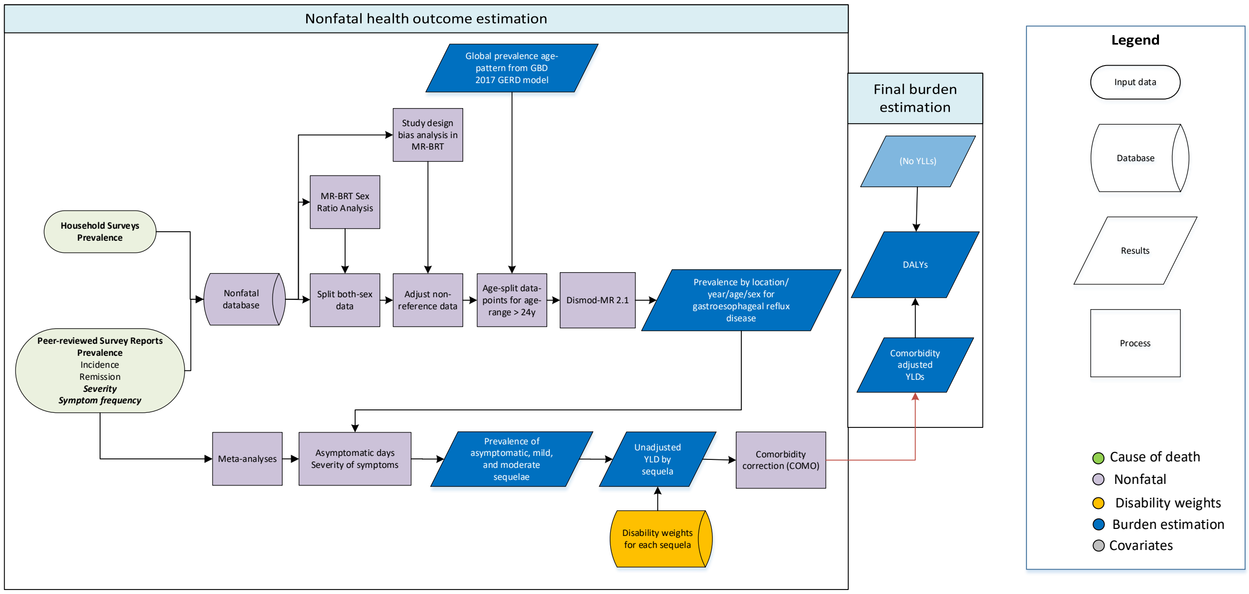

Case definition

Gastroesophageal reflux disease (GERD) is a digestive disorder that develops when the reflux of stomach contents causes troublesome symptoms, complications, or both. The cardinal symptoms of typical GERD are heartburn (a burning feeling behind the breastbone) and regurgitation (the unpleasant sensation of material moving upward from the stomach toward the mouth).

In GBD 2019, the occurrence of heartburn, regurgitation, or both, at least once weekly over a 12-month recall period was adopted as the reference case definition.

Individuals who experience esophageal complications (ulceration, metaplasia, etc.) without symptoms, whose sole symptom of gastroesophageal reflux is chest pain without typical reflux symptoms, or who experience reflux primarily as a trigger or exacerbating factor in respiratory or head and neck diseases (chronic cough, dental erosion, etc.) were not included. This strategy avoids double-counting disability already attributed to other underlying diseases modelled in GBD. Likewise, we regarded newborn reflux as a separate disease, which is modelled elsewhere and excluded from this analysis.

Input data

Data inputs

Data inputs for estimating the prevalence of GERD in GBD 2019 came from a systematic review conducted for GBD 2017. In brief, peer-reviewed publications reporting epidemiologic measures of GERD were identified via a search-string-based review in PubMed, citations of those articles identified by search-string, and suggestions from the GBD Collaborator Network. Two household surveys - the USA National Health Interview Surveys in 2007 and 2012 – were identified from the Global Health Data Exchange as asking participants about the occurrence of typical reflux symptoms, and were also included. In brief, data from all sources had to:

1) report a standard epidemiologic measure (incidence, prevalence, case fatality ratio, standardized mortality rate, *etcetera*) of GERD or provide individual-level data from which one could be calculated
2) provide sufficient information on study methods and sample characteristics to assess its quality and make appropriate adjustments
3) use our reference case-definition, or use a well-defined alternative case-definition that could be adjusted toward our reference standard
4) be conducted in a representative sample of a general population defined only by year, age, sex and location, or be conducted in a representative sample of a well-defined sub-population for which valid adjustments could be made, or ascertain all cases for a defined catchment area for which GBD population estimates are available
5) provide information on uncertainty (sample size, standard deviation, or confidence interval) and follow-up time
6) be written in a language that the modelling team could read (English, French, Portuguese or Spanish)

In our search, all studies reporting incidence or remission of GERD provided insufficient information on person-time of observation and were excluded, so only prevalence data were included. Data from claims data extracted and prepared by the GBD Clinical Informatics team (and described elsewhere in this Appendix) were used to develop adjustments factors for published studies from the search-string-based review that ascertained cases based on diagnostic codes in administrative data, but were not used in the primary analysis of GERD prevalence.

Prevalence measurements from peer-reviewed publications for 112 studies were manually extracted. Prevalence estimates were extracted from individual-level data from two household surveys using questionnaire text, skip-pattern, and weights for complex sampling strategies provided in the documentation from original study investigators. Data were marked with dichotomous variables for non-reference study design features.

Data inputs for GERD morbidity modelling by parameter

Measure	Total sources	Countries with data
Prevalence	110	37

Data processing

For studies that reported prevalence by age for both sexes combined, and prevalence by sex for all ages combined, we calculated the sex-ratio of cases in that study and applied it to the age-specific prevalence measures to estimate age-sex-specific prevalence.

To estimate sex-specific prevalence from studies that reported prevalence only for both sexes combined, we modeled the log sex ratio in MR-BRT using all sex-specific prevalence measurements from all other studies in the database: 0.24 (-0.23 to 0.70) and combined this with the GBD sex-specific population estimates for the relevant age-group. These were applied by calculating male prevalence:

$$prev_{male} = prev_{both} * \frac{pop_{both}}{(pop_{male} + ratio * pop_{female})}$$

and then calculating female prevalence:

$$prev_{female} = ratio * prev_{male}$$

For GERD, 27 studies used our reference case definition. The remaining studies had one or more non-reference study design feature thought to systematically bias prevalence measurements: questionnaire only asked subjects about heartburn, questionnaire only asked subjects about regurgitation, case definition required subjects to have additional symptoms to qualify as having GERD (such as sleep disruption or sour taste in mouth), case definition allowed subjects to qualify as having GERD due to having symptoms other than heartburn and regurgitation, recall period was less than 12 months, case definition required more than weekly symptoms, case definition included those with less than weekly symptoms, case definition used a scoring system that integrated information on number, frequency and duration of symptoms, or cases were identified based on diagnostic code in administrative data. These were modeled as independent effects in a network meta-analysis in MR-BRT, using 82 studies. Adjustments were modeled as difference in logit prevalence between alternative and reference data. The estimated mean logit differences were applied to non-reference data types as bias correction prior to modeling in DisMod-MR 2.1 (below).

The process of adjusting non-reference data using MR-BRT with the logit-transformation method is described below:

1. Mark all data points with dichotomous variables for all study design characteristics to be adjusted
2. Identify data points with overlapping year, age, sex, and location that differ with regard to one or more study design characteristics
3. Logit transform prevalence estimates for all overlapping data points
4. For all pair-wise combinations of overlapping data points, calculate the difference between prevalence estimates in logit space
5. Use the delta method to compute standard errors of overlapping data points in logit space, then calculate standard error of logit difference
6. Using MR-BRT, conduct a random effects meta-regression to estimate the logit difference of alternative to reference study designs, with covariates for each study design variable and no intercept
7. Logit transform the prevalence estimates for all data (not just points that overlap)

8. Transform the logit prevalence of each non-reference data point by subtracting the coefficients from MR-BRT for all applicable study design variables
9. Calculate new standard errors using the delta method, accounting for gamma (between-study heterogeneity)

The table below shows bias correction factors for study design characteristics estimated using MR-BRT.

MR-BRT Crosswalk Adjustment Factors for Total gastritis and duodenitis

Data input	Reference or alternative data collection	Gamma	Beta Coefficient, Logit difference (95% CI)	Adjustment factor*
Heartburn and/or regurgitation at least weekly for 12 months	Reference	0.61	---	---
Only asked about heartburn	Alternative		-0.61 (-2.1 to 0.92)	0.35 (0.11 to 0.72)
Only asked about regurgitation	Alternative		-0.26 (-1.8 to 1.3)	0.43 (0.14 to 0.78)
Required additional symptoms to meet case definition	Alternative		0.25 (-1.3 to 1.8)	0.56 (0.23 to 0.86)
Could meet case definition with other symptom options	Alternative		0.58 (-0.96 to 2.1)	0.64 (0.28 to 0.89)
Shorter recall period	Alternative		0.26 (-1.3 to 1.8)	0.56 (0.22 to 0.86)
Required greater minimum symptom frequency to meet case definition	Alternative		-1.2 (-2.7 to 0.35)	0.23 (0.063 to 0.59)
Had lower symptom frequency requirement to meet case definition	Alternative		0.89 (-0.63 to 2.4)	0.71 (0.35 to 0.92)
Used diagnostic score integrating multiple domains	Alternative		-0.027 (-1.6 to 1.5)	0.49 (0.17 to 0.82)
Diagnostic code in administrative data	Alternative		-1.7 (-3.2, -0.13)	0.16 (0.039 to 0.47)

**Adjustment factor is the inverse-logit transformed Beta coefficient; <0.5 represents that alternative is adjusted upward; >0.5 represents that alternative is adjusted downward*

Data sources that used non-reference study designs were dropped for which valid adjustments could not be developed: sampling of populations defined by profession (4), convenience sampling from waiting-rooms (3), case definition limited to endoscopically confirmed erosive esophagitis (1), and self-reported diagnosis without symptom-based questions (1).

Subsequently, data-points for samples spanning 25 years of age or more were disaggregated by applying the age-pattern observed in the global fit for the GBD 2017 GERD model.

Specific data points from some sources from subnational locations were excluded if relatively high values in young age groups led to overestimation of the entire age range.

Modelling strategy

Compartmental DisMod model

A full compartmental model of GERD epidemiology was developed using DisMod-MR 2.1. Adjusted prevalence data as described above were the inputs. Excess mortality was assumed a priori to be 0, and remission prior was set to 0.2 to 0.5 cases per person-year. Incidence was forced to 0 from birth to age 5 years, and after this age prior was set to 0 to 0.2 cases per person-year. We trialed covariates for mean body-mass index, prevalence of obesity, and per capita alcohol consumption, but these were not predictive, so were removed from the model.

Severity split & disability weight

Throughout the literature, the severity of GERD is often divided into three or four categories, using definitions such as those in the table below. We reviewed the studies in our prevalence database, above, and, if provided, extracted counts of cases of each severity as reported. These cases were then mapped to one of two GBD 2017 GERD severities (also shown in the table below). These categories were mapped to GBD health states, which are associated with disability weights. The basis of the GBD disability weight survey assessments are lay descriptions of health states highlighting major functional consequences and symptoms, also shown below.

Sample mapping of reported GERD severity levels to GBD GERD severity levels

Literature severity levels	GBD severity level	Lay description
Mild: can be ignored	Mild/moderate	Often has a burning sensation in the back of the chest after eating
Moderate: cannot be ignored but does not affect lifestyle	Mild/moderate	Often has a burning sensation in the back of the chest after eating
Severe: affects lifestyle	Severe (abdom_mod)	Has pain in the belly* and feels nauseous. Has difficulty with daily activities.
Very severe: has marked effect on lifestyle	Severe (abdom_mod)	Has pain in the belly* and feels nauseous. Has difficulty with daily activities.

*We acknowledge that gastroesophageal reflux symptoms are felt in the chest, not the belly, but opine that a health state that incorporates other gastrointestinal symptoms and indicates interference with daily activities, such as difficulty eating and sleeping, better represents more severe gastroesophageal reflux disease than a health state that describes only post-prandial heartburn.

The proportion of cases in each of the GBD 2017 GERD severities was then estimated using the metafor package (version 2.0-0) in R (version 3.4). Inputs to this meta-analysis are shown below. In GBD 2017, all studies with severity information for sample of cases defined by at least weekly symptoms were included, whether the defining symptoms were heartburn, regurgitation, either or both and regardless of recall period or duration; thus 15 studies were included. In GBD 2019, we limited the severity meta-analysis to only those studies that used the reference case definition of heartburn and/or regurgitation at least weekly for 12 months, thus only four studies were included.

Meta-analysis of proportion severe/very severe for GERD

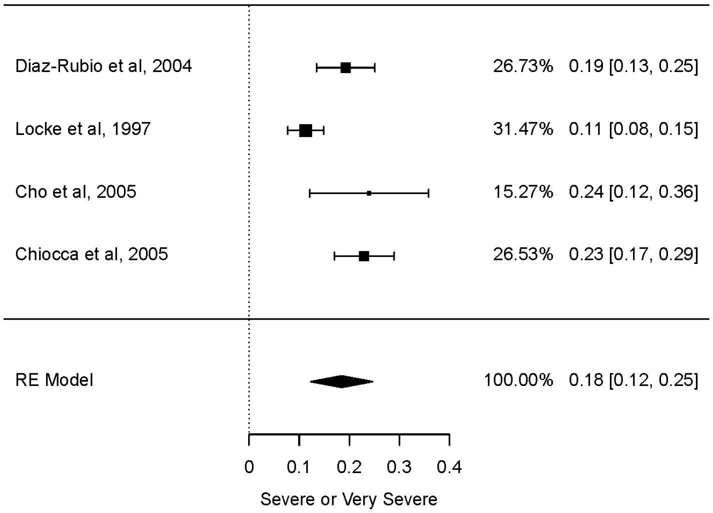

Many studies in the literature also report the frequency of GERD symptoms as the proportions of cases in each of a set of mutually exclusive and collectively exhaustive frequency categories. Examples include: 1-6 days/week and daily; 1 day/week, 2-6 days/week and daily; 1-3 days/week, 4-6 days/week and daily; etc. For each study, for each frequency category, 1,000 proportion draws were generated using a beta distribution with case counts in and out of the frequency category as shape parameters. We then assume that the number of days symptomatic within a category are uniformly distributed. We combine proportion draws and this assumption about mean days symptomatic in each category to produce draws of the mean number of days/week symptomatic across all cases in a study. Means and standard deviations of these draws were combined in a meta-analysis, and final mean and standard deviation were divided by 7 to estimate the proportion of cases symptomatic on a given day, with uncertainty.

Meta-analysis of days/week spent symptomatic for GERD

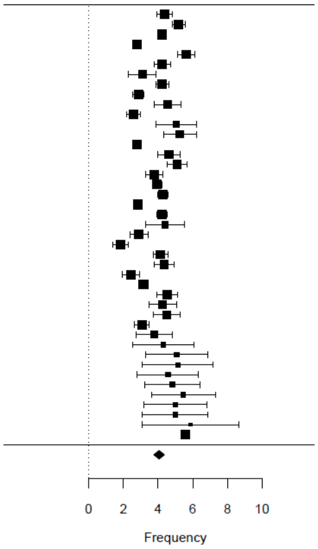

Severity and frequency categories were combined to generate four categories, as shown below.

GBD severity-frequency category	Proportion	Proportion	DW (95% CI)
Mild/moderate GERD, asymptomatic days	0.72 (0.71 – 0.74)	0.42 (0.38 – 0.46)	None
Mild/moderate GERD, symptomatic days		0.58 (0.54 – 0.62)	0.027 (0.015–0.046)
Severe GERD, asymptomatic days	0.28 (0.26 – 0.29)	0.42 (0.38 – 0.46)	None
Severe GERD, symptomatic days		0.58 (0.54 – 0.62)	0.114 (0.080–0.159)

Appendicitis

Flowchart

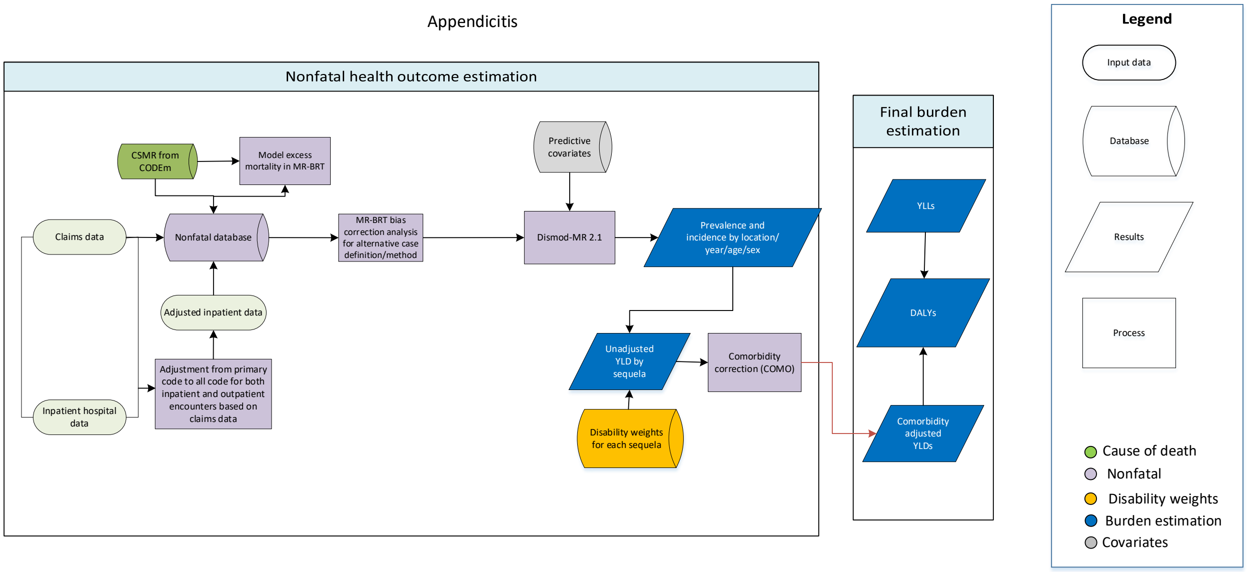

Input Data and Methodological Summary for Appendicitis

Case definition

Appendicitis is an inflammation of the appendix that causes nausea, vomiting, and sharp pain in the right lower abdomen. Appendicitis carries risk of severe complications, including sepsis and death, and is usually treated surgically. ICD-10 codes included are K35-K35.3, K35.8, K35.80, K35.89, K35.9, K36, K36.0, K37, K37.0, K37.9, and K38.3.

Input data and data processing

Input data

Like GBD 2017, the appendicitis model included data from hospital discharges and claims. In GBD 2019, we newly added Poland claims data and additional years of data from USA claims (years 2015-2016) and hospital discharges in Mexico, India, New Zealand, Sweden, Georgia, and Ecuador. Notably, we included hospital data from Botswana; southern sub-Saharan Africa previously did not have data.

Table 1. Data Inputs for appendicitis morbidity modelling by parameter.

Measure	Total sources	Countries with data
Incidence	297	46

Data processing

Hospital discharge data provide observations about encounters, generally with only the primary diagnostic code for the encounter. Claims data, on the other hand, link claims for all inpatient and outpatient encounters for a single individual, and provide primary and secondary diagnoses for all encounters.

In GBD 2017, an individual was extracted from claims data as an incident case if that individual had one or more inpatient encounters with an appropriate ICD code as any diagnosis; readmissions within 28 days were assumed to be for the same episodes of illness. Data from hospital discharges with appropriate ICD codes as primary diagnostic code were, then, adjusted using correction factors derived from inpatient claims data, estimating the number of individuals represented by each encounter.

In GBD 2019, we improved data processing methods to capture cases that were diagnosed and/or treated in an outpatient setting. Specifically, incident cases were extracted from claim data if an individual had at least one inpatient or outpatient encounter with an appropriate ICD code as any diagnosis within one year. Data from hospital discharges were, then, adjusted using correction factors from claims, converting encounters to estimates of cases, accounting for most locations providing only primary diagnostic codes, and estimating outpatient cases from inpatient cases.

The USA claims data from the year 2000 and from the years 2010–2016 were each adjusted to data from hospital discharges outside DisMod using MR-BRT analysis to adjust for selection bias due to commercial insurance.

The table below shows bias correction factors estimated using MR-BRT.

Table 2. MR-BRT Crosswalk Adjustment Factors for Appendicitis

Data input	Reference or alternative data collection	Gamma	Beta Coefficient, Log (95% CI)	Adjustment factor*
Hospital + non-USA claims	Ref	0.06	---	---
USA claims from year 2000	Alt		-0.57 (-0.30, -0.85)	0.56 (0.43, 0.74)
USA claims from year 2010-2016	Alt		-0.06 (-0.20, 0.08)	0.94 (0.82, 1.09)

**Adjustment factor is the transformed Beta coefficient in normal space, and can be interpreted as the factor by which the alternative case definition is adjusted to reflect what it would have been if measured as the reference.*

Data points with an age-standardised incidence rate greater than three median absolute deviations from the median of the age-standardised incidence rate for all inpatient and non-USA claims data were marked as outliers and excluded from analysis.

Severity split & disability weight

The basis of the GBD disability weight survey assessments are lay descriptions of sequelae highlighting major functional consequences and symptoms. The lay descriptions and disability weights for appendicitis are shown below.

Table 3. Severity Distribution, details on the severity levels for appendicitis in GBD 2019 and the associated disability weight (DW) with that severity.

Severity level	Lay description	DW (95% CI)
Severe	This person has severe pain in the belly and feels nauseated. The person is anxious and unable to carry out daily activities.	0.324 (0.219–0.442)

Modeling strategy

Similar to GBD 2017, we ran a DisMod-MR 2.1 model to produce estimates by age, sex, year, and location. Prior settings in the DisMod model included cure after about two weeks (remission set to 25-27) for all age groups. We used the function in DisMod to pull in cause-specific mortality rate (CSMR) data from our CODEm and CODcorrect analyses. The minimum coefficient of variation at the regional, super-regional, and global-level was changed from 0.4 to 0.8 in GBD 2019 to improve model fit against input data.

In previous rounds, priors on excess mortality rate (EMR) were estimated in DisMod by matching prevalence data points with their corresponding CSMR values within the same age, sex, year, and location (by dividing CSMR by prevalence). For short duration conditions (remission >1), the corresponding prevalence was derived by running an initial model and then applying the same CSMR/prevalence method. However, for many causes, DisMod estimated a rather unrealistic pattern of EMR compared to an expected pattern of decreasing EMR with greater access to quality health care. Such unexpected patterns often signal inconsistencies between CSMR estimates and the measures of prevalence and/or incidence. In effort to provide greater guidance to DisMod on the expected pattern of EMR, EMR data generated in the previous round were modeled using the MR-BRT approach by age and sex with a prior on healthcare access and quality index (HAQi) having a negative coefficient. Results from MR-BRT were then predicted for each location, year, sex, and for ages 0, 10, 20….100. We included HAQi as a country-level covariate in our DisMod model to inform EMR with a mean and standard deviation produced from MR-BRT.

The fibre (g per day) consumption covariate was applied as a predictive covariate to incidence. Betas and exponentiated values (which can be interpreted as an odds ratio) of predictive covariates are shown in the table below.

Table 4. Covariates. Summary of covariates used in the appendicitis DisMod-MR meta-regression model

**Covariate**	**Type**	**Parameter**	**Exponentiated beta (95% Uncertainty Interval)**
Fibre unadjusted (g)	Country-level	Incidence	1.00 (0.99, 1.00)
Healthcare access and quality index	Country-level	Excess mortality rate	0.94 (0.94, 0.94)

Paralytic Ileus and Intestinal Obstruction

Flowchart

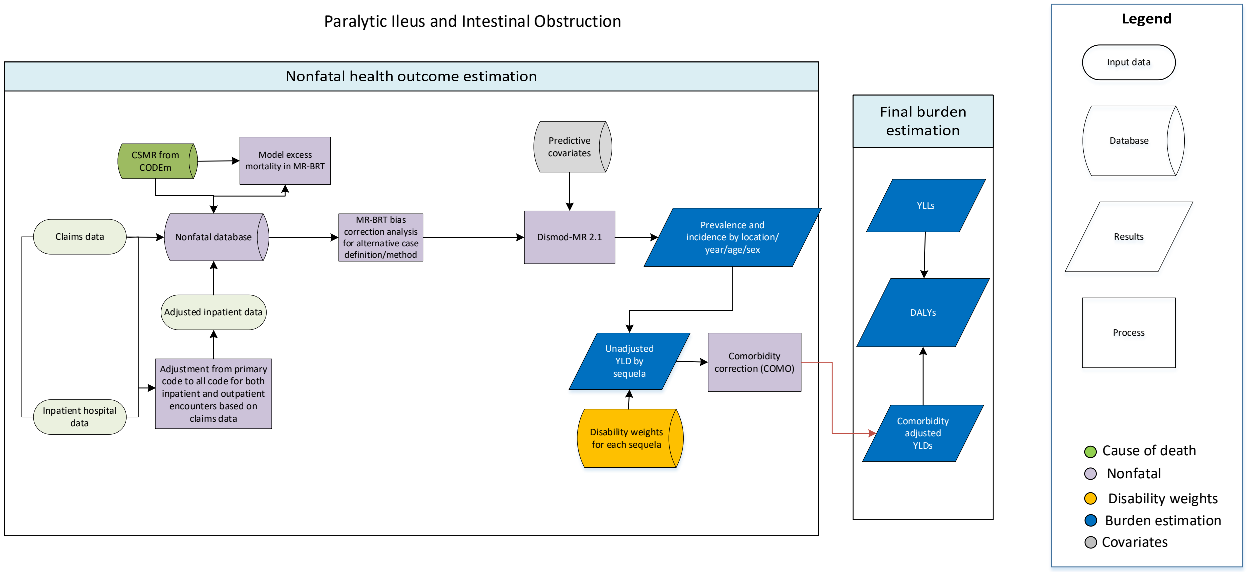

Input Data and Methodological Summary for Paralytic Ileus and Intestinal Obstruction

Case definition

Paralytic ileus and intestinal obstruction is a lack of digestive propulsion caused by failed peristalsis, typically requiring surgery. ICD code for paralytic ileus and intestinal obstruction is K56.

Input data and data processing

Input data

Like GBD 2017, the model included data from hospital discharges and claims. In GBD 2019, we newly added Poland claims data and additional years of data from USA claims (years 2015-2016) and hospital discharges in Mexico, India, New Zealand, Sweden, Georgia, and Ecuador. Notably, we included hospital data from Botswana; southern sub-Saharan Africa previously did not have data.

Table 1. Data Inputs for paralytic ileus and intestinal obstruction morbidity modelling by parameter.

Measure	Total sources	Countries with data
Incidence	297	46

Data processing

Hospital discharge data provide observations about encounters, generally with only the primary diagnostic code for the encounter. Claims data, on the other hand, link claims for all inpatient and

outpatient encounters for a single individual, and provide primary and secondary diagnoses for all encounters.

In GBD 2017, an individual was extracted from claims data as an incident case if that individual had one or more inpatient encounters with an appropriate ICD code as any diagnosis; readmissions within 28 days were assumed to be for the same episodes of illness. Data from hospital discharges with appropriate ICD codes as primary diagnostic code were, then, adjusted using correction factors derived from inpatient claims data, estimating the number of individuals represented by each encounter.

In GBD 2019, we improved data processing methods to capture cases that were diagnosed and/or treated in an outpatient setting. Specifically, incident cases were extracted from claim data if an individual had at least one inpatient or outpatient encounter with an appropriate ICD code as any diagnosis within one year. Data from hospital discharges were, then, adjusted using correction factors from claims, converting encounters to estimates of cases, accounting for most locations providing only primary diagnostic codes, and estimating outpatient cases from inpatient cases.

The USA claims data from the year 2000 and from the years 2010–2016 were each adjusted to data from hospital discharges outside DisMod using MR-BRT analysis to adjust for selection bias due to commercial insurance.

The process of adjusting for non-reference data using MR-BRT with the logit-transformation method is described below:

1. Identify data points with overlapping year, age, sex, and location between claims (alternative case definition) and hospital discharges (reference case definition)
2. Logit transform overlapping data points of alternative and reference case definitions
3. Convert overlapping data points into a difference in logit space using the following equation: $logit(altnerative) - logit(reference)$
4. Use the delta method to compute standard errors of overlapping data points in logit space, then calculate standard error of logit difference using the following equation: $\sqrt{(variance\ of\ alternative) + (variance\ of\ reference)}$
5. Using MR-BRT, conduct a random effects meta-regression to obtain the pooled logit difference of alternative to reference
6. Apply the pooled logit difference to all data points of alternative case definitions using the following equation:
$$new_{estimate} = inverse.logit((logit(alternative)) - (pooled\ logit\ difference))$$
7. Calculate new standard errors using the delta method, accounting for gamma (between-study heterogeneity)

The table below shows bias correction factors estimated using MR-BRT.

Table 2. MR-BRT Crosswalk Adjustment Factors for Paralytic Ileus and Intestinal Obstruction

Data input	Reference or alternative data collection	Gamma	Beta Coefficient, Logit (95% CI)	Adjustment factor*
Hospital + non-USA claims	Ref	0.03	---	---

USA claims from year 2000	Alt		-0.08 (-0.15, -0.01)	0.48 (0.46, 0.50)
USA claims from year 2010-2016	Alt		0.01 (-0.05, 0.07)	0.50 (0.49, 0.52)

**Adjustment factor is the inverse-logit transformed Beta coefficient; <0.5 represents that alternative is adjusted upward; >0.5 represents that alternative is adjusted downward*

Data points with an age-standardised incidence rate greater than two median absolute deviations from the median of the age-standardised incidence rate for all inpatient and non-USA claims data were marked as outliers and excluded from analysis.

Severity split & disability weight

The basis of the GBD disability weight survey assessments are lay descriptions of sequelae highlighting major functional consequences and symptoms. The lay descriptions and disability weights for paralytic ileus and intestinal obstruction are shown below.

Table 3. Severity Distribution, details on the severity levels for paralytic ileus and intestinal obstruction in GBD 2019 and the associated disability weight (DW) with that severity.

Severity level	Lay description	DW (95% CI)
Severe	This person has severe pain in the belly and feels nauseated. The person is anxious and unable to carry out daily activities.	0.324 (0.219–0.442)

Modeling strategy

Similar to GBD 2017, we ran a DisMod-MR 2.1 model to produce estimates by age, sex, year, and location. Prior settings in the DisMod model included bounding remission between 25 and 26 for all age groups (for a duration of approximately two weeks), and the maximum incidence of 0.002 for ages 0 to 5. We used the function in DisMod to pull in cause-specific mortality rate (CSMR) data from our CODEm and CODcorrect analyses. The minimum coefficient of variation at the regional, super-regional, and global-level was changed from 0.4 to 0.8 in GBD 2019 to improve model fit against input data.

In previous rounds, priors on excess mortality rate (EMR) were estimated in DisMod by matching prevalence data points with their corresponding CSMR values within the same age, sex, year, and location (by dividing CSMR by prevalence). For short duration conditions (remission >1), the corresponding prevalence was derived by running an initial model and then applying the same CSMR/prevalence method. However, for many causes, DisMod estimated a rather unrealistic pattern of EMR compared to an expected pattern of decreasing EMR with greater access to quality health care. Such unexpected patterns often signal inconsistencies between CSMR estimates and the measures of prevalence and/or incidence. In effort to provide greater guidance to DisMod on the expected pattern of EMR, EMR data generated in the previous round were modeled using the MR-BRT approach by age and sex with a prior on healthcare access and quality index (HAQi) having a negative coefficient. Results from MR-BRT were then predicted for each location, year, sex, and for ages 0, 10, 20….100. We included HAQi as a predictive covariate to inform EMR with a mean and standard deviation produced from MR-BRT.

The Beta and exponentiated values of this predictive covariate (which can be interpreted as an odds ratio) are shown in the table below.

Table 4. Covariates. Summary of covariates used in the paralytic ileus and intestinal obstruction DisMod-MR meta-regression model

Covariate	Type	Parameter	Exponentiated beta (95% Uncertainty Interval)
Healthcare access and quality index	Country-level	Excess mortality rate	0.97 (0.97, 0.97)

Inguinal, Femoral, and Abdominal Hernia

Flowchart

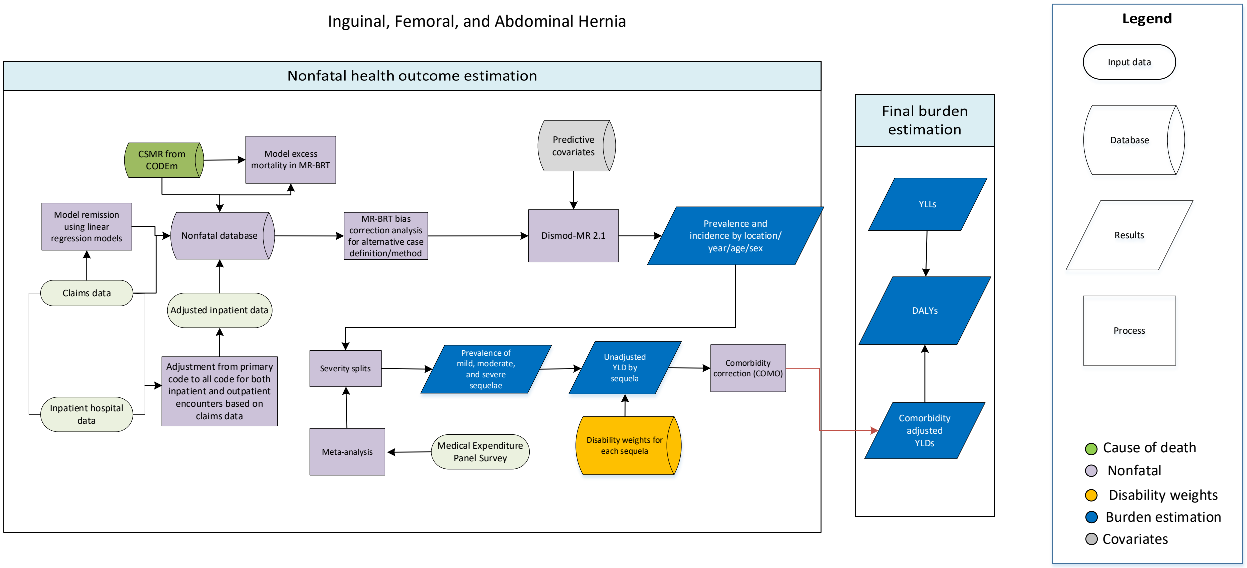

Input Data and Methodological Summary for Inguinal, Femoral, and Abdominal Hernia

Case definition

Hernia refers to when an internal organ protrudes through an opening in the tissue that holds it in place. Inguinal, femoral, and abdominal hernia comprises the disorders in which portions of the digestive tract protrude through defects in the walls of the abdominal cavity. These occasionally lead to life-threatening acute complications, but more commonly are asymptomatic or cause chronic or intermittent pain. Symptomatic hernia is surgically repaired.

ICD10 codes are K40, K41, K42, K44, K45, and K46 and all their 4-digit and 5-digit constituents. The ICD9 codes are 550, 551, 552, 553 and their constituents, with the exceptions of 551.1-3, 552.1-3, and 553.1-3. The procedure codes for hernia repair are 43336-43337, 44050, 49491-49492, 49495-49496, 49500-49501, 49505, 49507, 49525, 49540, 49550, 49553, 49555, 49557, 49560-49561, 49565-49566, 49568, 49570, 49572, 49585, 49587, 49590, 49650-49653, and 54640.

Overall strategy

In GBD 2017, two databases were developed for inguinal, femoral, and abdominal hernia to separately model total (symptomatic + asymptomatic cases) and symptomatic cases. In GBD 2019, the DisMod model for symptomatic cases was dropped, and we only modeled total cases of hernia in DisMod; an updated severity distribution was, then, applied as described below.

Input data and data processing

Input data

Like GBD 2017, the total hernia model included hospital and claims data. In GBD 2019, we newly added Poland claims data and additional years of hospital discharge data from Mexico, India, New Zealand, Sweden, Georgia, and Ecuador, as well as two additional years of USA Marketscan claims data. Most notably, we included hospital discharge data from Botswana; southern sub-Saharan Africa previously did not have data. Encounter data from outpatient facilities used in GBD 2017 were excluded this round because they were highly heterogeneous and inconsistent with other data sources from the same locations.

Table 1. Data Inputs for inguinal, femoral, and abdominal hernia morbidity modelling by parameter.

Measure	Total sources	Countries with data
All measures	312	46
Prevalence	297	46
Proportion	15	1

Data processing

Hospital discharge data provide observations about encounters, generally only with the primary diagnostic code for the encounter. Claims data, on the other hand, link claims for all inpatient and outpatient encounters for a single individual, and provide primary and secondary diagnoses for all encounters. For this reason, hospital discharge data are adjusted to estimate prevalent cases by using standard GBD correction factors derived from claims data: one factor is based on the ratio of inpatient encounters to individuals, one is based on the ratio of primary diagnostic codes to secondary diagnostic codes, and the last is based on the ratio of inpatient encounters to outpatient encounters. These ratios are modeled with additional information on healthcare access and quality index (HAQi) and other factors; the details are provided in a separate appendix section on clinical informatics data preparation. Claims data come predominantly from the USA, and this correction strategy relies on the assumption that the ratio of inpatient to outpatient encounters in an insured population is the same as the ratio in the general population.

For the symptomatic hernia model in GBD 2017, individuals were extracted as cases from claims data if they had an inpatient encounter with a hernia ICD code as any diagnosis. Hospital discharges with hernia as primary diagnosis were corrected with a ratio from claims data to estimate the number of unique individuals represented by this diagnosis. A key assumption of this GBD 2017 modeling strategy was that symptomatic cases of hernia are admitted as inpatients, and patients seen only in the outpatient setting are asymptomatic. Laparoscopic surgery is one of the most common operative procedures to repair symptomatic hernia. Because of relatively low risk of complications and short recovery time, laparoscopic surgery is often done in outpatient settings in some high-income countries,

including the USA. Thus, the GBD 2017 strategy likely underestimated symptomatic cases and overestimated total cases.

In GBD 2019, we extracted prevalent cases of hernia for the total hernia database from claims data in the same manner as in GBD 2017—extracting prevalent cases from claims data if an individual had one inpatient or two outpatient encounters with a hernia ICD code as any diagnosis. However, we developed custom correction factors for hospital discharge data. In GBD 2019, we assumed that in USA claims data, individuals with either an inpatient encounter with a hernia ICD code or an outpatient encounter with both hernia ICD code and procedural code for hernia repair was symptomatic, but that most symptomatic cases of hernia were treated in an inpatient setting in most locations. Consequently, we summed the inpatient and outpatient encounters with procedures in claims data, and estimated the ratio of this sum to all encounters with hernia ICD codes, and applied this ratio to international hospital discharge data to estimate total hernia cases for populations for which individual-level claims data were not available. This resulted in a smaller corrections of hospital discharges to total hernia cases in GBD 2019 than in GBD 2017.

Although better able to capture the relationship between inpatient and outpatient care, USA claims data were regarded as suffering from selection bias due to commercial health insurance status. Thus, total hernia prevalence data extracted from USA claims from the year 2000 and from the years 2010–2016 were ultimately adjusted to total hernia prevalence data from hospital discharges. This was done in MR-BRT using the logit-transformation method is described below:

1. Identify data points with overlapping year, age, sex, and location between claims (alternative case definition) and hospital discharges (reference case definition)
2. Logit transform overlapping data points of alternative and reference case definitions
3. Convert overlapping data points into a difference in logit space using the following equation: $logit(altnerative) - logit(reference)$
4. Use the delta method to compute standard errors of overlapping data points in logit space, then calculate standard error of logit difference using the following equation: $\sqrt{(variance\ of\ alternative) + (variance\ of\ reference)}$
5. Using MR-BRT, conduct a random effects meta-regression to obtain the pooled logit difference of alternative to reference
6. Apply the pooled logit difference to all data points of alternative case definitions using the following equation:
$$new_{estimate} = inverse.logit((logit(alternative)) - (pooled\ logit\ difference))$$
7. Calculate new standard errors using the delta method, accounting for gamma (between-study heterogeneity)

The table below shows bias correction factors of alternative case definitions using MR-BRT.

Table 2. MR-BRT Crosswalk Adjustment Factors for Inguinal, femoral, and abdominal hernia

Data input	Reference or alternative case definition	Gamma	Beta Coefficient, Logit (95% CI)	Adjustment factor*
Hospital + non-USA claims	Ref	1.08	---	---

USA claims from year 2000	Alt		0.36 (-2.65, 3.36)	0.59 (0.07, 0.97)
USA claims from year 2010-2016	Alt		0.62 (-2.37, 3.61)	0.65 (0.09, 0.97)

**Adjustment factor is the inverse-logit transformed Beta coefficient; <0.5 represents that alternative is adjusted upward; >0.5 represents alternative is adjusted downward*

Data points with an age-standardised prevalence greater than two median absolute deviations from the median of the age-standardised prevalence for all inpatient and non-USA claims data were marked as outliers and excluded from analysis.

Severity split & disability weight

The DisMod model of symptomatic hernia used in GBD 2017 was dropped in GBD 2019, and symptom occurrence and severity distribution were estimated from MEPS data using standard GBD methodology. The basis of the GBD disability weight survey assessments are lay descriptions of sequelae highlighting major functional consequences and symptoms. Prevalent cases of symptomatic hernia were divided according to severity distributions derived from data from the Medical Expenditure Panel Survey (MEPS) to assign them to mild, moderate, and severe sequelae. Asymptomatic cases were assigned no disability. The lay descriptions and disability weights for inguinal, abdominal, and femoral hernia are shown below.

Table 3. Severity Distribution, details on the severity levels for Inguinal, femoral, and abdominal hernia in GBD 2019 and the associated disability weight (DW) with that severity.

Severity level	Lay description	DW (95% CI)
Asymptomatic	--	0
Mild	This person has some pain in the belly that causes nausea but does not interfere with daily activities.	0.011 (0.005–0.021)
Moderate	This person has pain in the belly and feels nauseous. The person has difficulties with daily activities.	0.114 (0.080–0.159)
Severe	This person has severe pain in the belly and feels nauseated. The person is anxious and unable to carry out daily activities.	0.324 (0.219–0.442)

Modeling strategy

We ran a DisMod-MR 2.1 model to produce estimates of total inguinal, femoral and abdominal hernia by year, age, sex, and location. Prior settings included in GBD 2019 were bounding excess mortality rate (EMR) from 0 to 0.00002 between ages 0 and 15 and an upper bound of incidence rate at 0.01 between ages 0 and 20. We used the function in DisMod to pull in cause-specific mortality rate (CSMR) data from our CODEm and CODcorrect analyses. The minimum coefficient of variation at the regional, super-regional, and global-level was changed from 0.4 to 0.8 in GBD 2019 to improve model fit against input

data. We also assumed no birth prevalence of hernia to adjust for implausibly high prevalence in younger age groups.

In previous rounds, priors on EMR were estimated in DisMod by matching prevalence data points with their corresponding CSMR values within the same age, sex, year, and location (by dividing CSMR by prevalence). For short duration conditions (remission >1), the corresponding prevalence was derived by running an initial model and then applying the same CSMR/prevalence method. However, for many causes, DisMod estimated a rather unrealistic pattern of EMR compared to an expected pattern of decreasing EMR with greater access to quality health care. Such unexpected patterns often signal inconsistencies between CSMR estimates and the measures of prevalence and/or incidence. In effort to provide greater guidance to DisMod on the expected pattern of EMR, EMR data generated in the previous round were modeled using the MR-BRT approach by age and sex with a prior on healthcare access and quality index (HAQi) having a negative coefficient. Results from MR-BRT were then predicted for each location, year, sex, and for ages 0, 10, 20….100. We included HAQi as a country-level covariate to inform EMR with a mean and standard deviation produced from MR-BRT. However, even without this setting, DisMod would tend to estimate a coefficient that was consistent with the outputs from the MR-BRT analysis.

In GBD 2017, we used remission estimates derived from a single, large study of mean wait times for elective surgical repair in OECD countries conducted by Siciliani and colleagues. To better inform DisMod on the increasing pattern of remission with greater access to quality health care, in GBD 2019 we used remission data from the USA claims, defined as a number of people with a hernia repair procedure code among all people with hernia diagnosis, and regressed against HAQi and sex with an assumption that hernia does not resolve on its own without a surgical repair, so remission is 0 at a theoretical HAQi value of 0. The results from the regression model were then used to predict remission estimates for each location, year, sex and for ages 0, 10, 20…100.

We used smoking prevalence and mean BMI as predictive covariates for prevalence. The HAQi and lag-distributed income (log transformed) covariates were applied to EMR and remission, respectively. Betas and exponentiated values for these predictive covariates (which can be interpreted as an odds ratio) are shown in the table below.

Table 4. Covariates. Summary of predictive covariates used in the total inguinal, femoral, and abdominal hernia DisMod-MR meta-regression model

Covariate	Type	Parameter	Exponentiated beta (95% Uncertainty Interval)
Smoking prevalence	Country-level	Prevalence	3.25<br>(2.95, 3.59)
Mean BMI	Country-level	Prevalence	0.96<br>(0.96, 0.97)
Healthcare access and quality index	Country-level	Excess mortality rate	0.98<br>(0.98, 0.98)
LDI (I$ per capita)	Country-level	Remission	1.65<br>(1.65, 1.65)

Inflammatory Bowel Disease

Flowchart

Inflammatory Bowel Disease

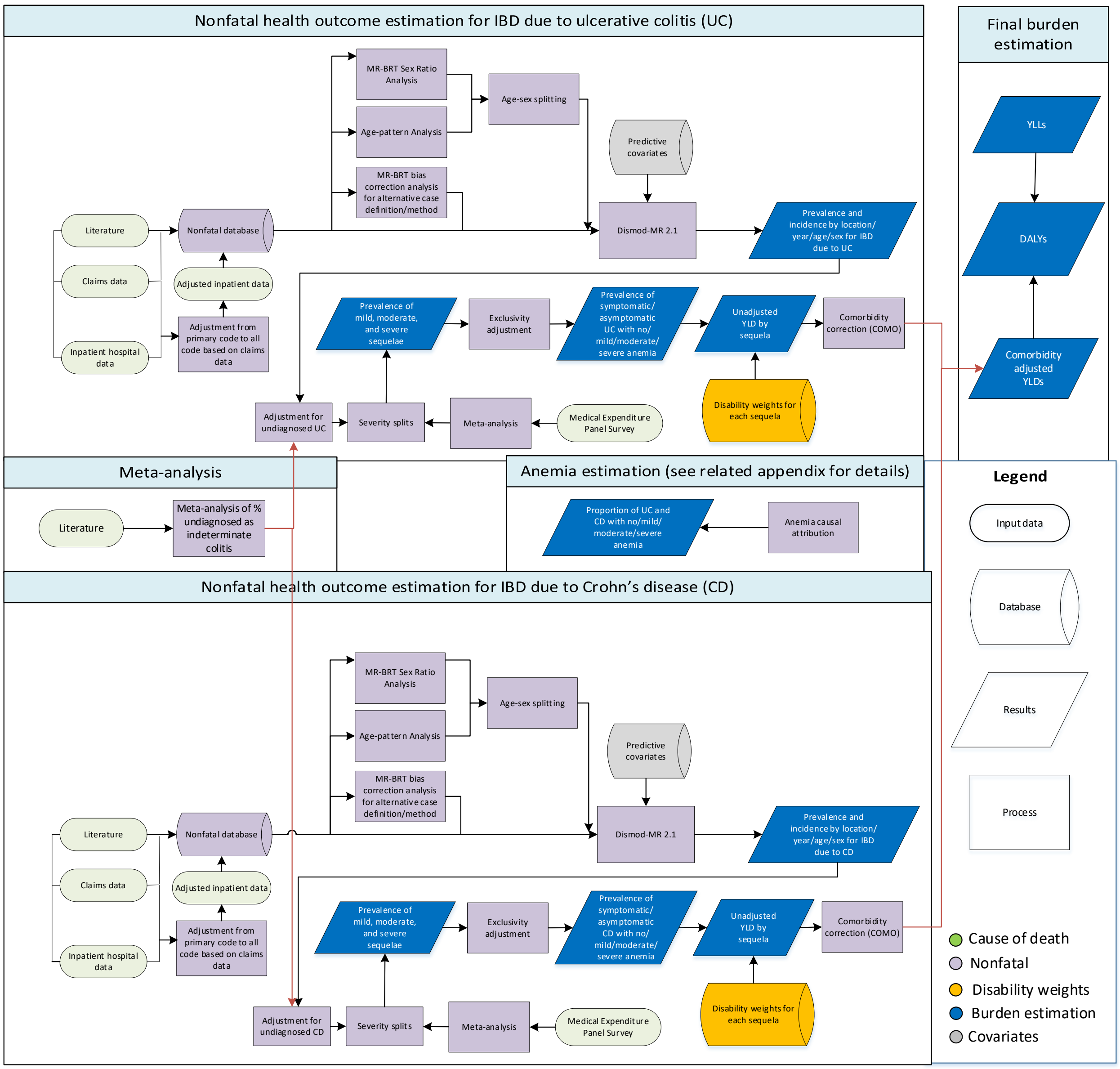

Input Data and Methodological Summary for Inflammatory Bowel Disease

Case definition

Inflammatory bowel disease comprises digestive disorders resulting from non-infectious inflammation of the colon and gastrointestinal tract, predominantly Crohn's disease (inflammation of the small and large intestine) and ulcerative colitis (inflammation of the colon and rectum). These disorders are diagnosed

by endoscopy, imaging studies, or biopsy in a patient with appropriate clinical signs and symptoms. In some cases of inflammatory bowel disease, neither Crohn's disease nor ulcerative colitis can be definitively diagnosed, and a diagnosis of indeterminate colitis is applied, indefinitely, or until definitive features of Crohn's or ulcerative colitis declare themselves.

ICD codes are K50 for Crohn's disease, K51 for ulcerative colitis, and K52 for indeterminate colitis.

Overall strategy

Like in GBD 2017, we utilized two databases for inflammatory bowel disease as inputs to two separate, complete compartmental DisMod models: ulcerative colitis and Crohn's disease.

Input data and data processing

Input data

For GBD 2016, a systematic literature review was conducted to capture studies of prevalence and incidence for all inflammatory bowel diseases. A PubMed search was conducted using the following search string: (("crohn disease"[MeSH Terms] OR ("crohn"[All Fields] AND "disease"[All Fields]) OR "crohn disease"[All Fields] OR ("crohn's"[All Fields] AND "disease"[All Fields]) OR "crohn's disease"[All Fields]) OR ("colitis, ulcerative"[MeSH Terms] OR ("colitis"[All Fields] AND "ulcerative"[All Fields]) OR "ulcerative colitis"[All Fields] OR ("ulcerative"[All Fields] AND "colitis"[All Fields])) OR (Inflammatory[All Fields] AND Bowl[All Fields]) OR (("irritable bowel syndrome"[MeSH Terms] OR ("irritable"[All Fields] AND "bowel"[All Fields] AND "syndrome"[All Fields]) OR "irritable bowel syndrome"[All Fields]) AND ("diarrhoea"[All Fields] OR "diarrhea"[MeSH Terms] OR "diarrhea"[All Fields]))) AND ("epidemiology"[Subheading] OR "epidemiology"[All Fields] OR "epidemiology"[MeSH Terms]) AND (("2016"[PDAT]) NOT (animals[MeSH] NOT humans[MeSH])).

The exclusion criteria were:

1. Studies clearly not representative of a geographically defined general population
2. Studies that did not provide primary data on epidemiological parameters, eg, a commentary piece

For GBD 2019, we added additional data from peer-reviewed publications identified via a systematic review that was conducted by Ng and her colleagues in 2017[1].

In addition to the literature studies, both databases included administrative data that were extracted as prevalence. In GBD 2019, we newly added Poland claims data and additional years of hospital discharge data from Mexico, India, New Zealand, Sweden, Georgia, and Ecuador, as well as two additional years of USA Marketscan claims data. Most notably, we included hospital discharge data from Botswana; southern sub-Saharan Africa previously did not have data. Russia claims data were newly added only to the ulcerative colitis database as it did not provide data for Crohn's disease.

[1]Ng SC, Shi HY, Hamidi N, Underwood FE, Tang W, Benchimol EI, et al. Worldwide incidence and prevalence of inflammatory bowel disease in the 21st century: a systematic review of population-based studies. Lancet. 2018 23;390(10114):2769–78.

In GBD 2017, the databases included data points extracted as case fatality rate, proportion, relative risk, and standardized mortality ratio. However, these data points were scant (17 site-years total) and had limited spatiotemporal coverage. Therefore, these data were excluded from analysis in GBD 2019.

Table 1. Data Inputs for inflammatory bowel disease morbidity modelling by parameter.

Measure	Total sources	Countries with data
All measures	501	80
Prevalence	370	61
Incidence	167	62
Standardized mortality ratio	1	1

Data processing

Claims data link multiple inpatient and outpatient claims to a single individual, whereas hospital data report discharges. In GBD 2019, an individual was extracted as a prevalent case of ulcerative colitis or Crohn's disease if they had at least one inpatient or two outpatient encounters with an appropriate ICD code as any diagnosis. This is in contrast to GBD 2017, when individuals were extracted as a prevalent case if they had at least one outpatient encounter. In both GBD 2019 and GBD 2017, data from hospital discharges were adjusted using correction factors from claims, converting encounters to estimates of cases, accounting for most locations providing only primary diagnostic codes, and estimating outpatient cases from inpatient cases.

In GBD 2019, we improved the bias adjustment methods to allow a more direct comparison between different case definitions and/or study designs. In the past GBD cycles, we adjusted alternative case definitions or study design characteristics to the reference standard by creating binary covariates for these alternative groups, and estimating a fixed effect for these covariates in our DisMod meta-regression modeling process. This amounts to adjusting data using an ecological comparison, and vulnerable to compositional bias; if data from different location-years were collected using different methods or case definitions, true spatiotemporal differences in epidemiology can be erroneously adjusted, and differences truly due to differences in methods can be erroneously estimated as differences in underlying epidemiology. In GBD 2019, we avoided this risk by making pre-modeling bias adjustments and dropping data types that could not be rigorously adjusted. This was done by conducting a meta-regression of the relationship between data points matched on year, age, sex, and location, but differing with regard to one or more case definition and study design characteristic.

For both ulcerative colitis and Crohn's disease models, we decided to use data from literature studies that identified cases through detailed chart review as the reference standard. These studies used a combination of clinical presentation, endoscopic, histological, and radiological and/or biochemical findings to validate a case definition, which we refer to as "stringent criteria" in shorthand. Using the stringent criteria, we would, then, adjust other ICD-code based administrative data without validation (i.e. data from claims and hospital discharges). However, the number of matched pairs between reference and alternative (based on year, age, sex and location) was small and yielded highly uncertain adjustment factors for the alternative case definitions. As a result, we reverted back to using diagnosis of ulcerative colitis or Crohn's disease as indicated by ICD code in a clinical encounter as the reference in GBD 2019. This reference standard included literature studies that ascertained cases using claims or hospital databases without further validation of diagnosis via chart review. The USA claims data from

the year 2000 and from the years 2010–2016 were separately adjusted to the reference to account for selection bias due to commercial insurance.

The table below shows bias correction factors estimated using MR-BRT.

Table 2. MR-BRT Crosswalk Adjustment Factors for Inflammatory Bowel Disease

Ulcerative colitis: Incidence

Data input	Reference or alternative case definition	Gamma	Beta Coefficient, Log (95% CI)	Adjustment factor*
ICD-code based administrative data	Ref	0.02	-	-
Stringent criteria	Alt		-0.09 (0.15, -0.04)	0.91 (0.86, 0.96)

**Adjustment factor is the transformed Beta coefficient in normal space, and can be interpreted as the factor by which the alternative case definition is adjusted to reflect what it would have been if measured as the reference.*

Ulcerative colitis: Prevalence

Data input	Reference or alternative case definition	Gamma	Beta Coefficient, Log (95% CI)	Adjustment factor*
ICD-code based administrative data	Ref	0.13	-	-
USA claims from year 2000	Alt		0.14 (-0.49, 0.78)	1.15 (0.61, 2.18)
USA claims from year 2010-2016	Alt		0.61 (0.32, 0.90)	1.84 (1.37, 2.46)
Stringent criteria	Alt		-0.16 (-0.43, 0.11)	0.85 (0.65, 1.12)

**Adjustment factor is the transformed Beta coefficient in normal space, and can be interpreted as the factor by which the alternative case definition is adjusted to reflect what it would have been if measured as the reference.*

Crohn’s disease: Incidence

Data input	Reference or alternative case definition	Gamma	Beta Coefficient, Log (95% CI)	Adjustment factor*
ICD-code based administrative data	Ref	0.02	-	-
Stringent criteria	Alt		-0.09 (-0.15, -0.04)	0.91 (0.86, 0.96)

**Adjustment factor is the transformed Beta coefficient in normal space, and can be interpreted as the factor by which the alternative case definition is adjusted to reflect what it would have been if measured as the reference.*

Crohn’s disease: Prevalence

Data input	Reference or alternative case definition	Gamma	Beta Coefficient, Log (95% CI)	Adjustment factor*
ICD-code based administrative data	Ref	0.16	-	-
USA claims from year 2000	Alt		-0.08 (-0.61, 0.45)	0.92 (0.54, 1.57)
USA claims from year 2010-2016	Alt		0.34 (-0.05, 0.73)	1.40 (0.95, 2.08)
Stringent criteria	Alt		-0.46 (-0.91, -0.01)	0.63 (0.40, 0.99)

**Adjustment factor is the transformed Beta coefficient in normal space, and can be interpreted as the factor by which the alternative case definition is adjusted to reflect what it would have been if measured as the reference.*

We split data points where the age range was greater than 20 years using the global age pattern informed by the data points with fine age groups (i.e. ages 5-9, 10-14, and 15-20…). We also split data reported for both sexes using the pooled sex-ratio estimated from studies that reported prevalence in males and females separately. The ratios of female to male cases derived from MR-BRT analysis were 0.81 (CI: 0.36, 1.82) and 1.13 (CI: 0.59, 2.16) for ulcerative colitis and Crohn's disease, respectively.

Data points with an age-standardised prevalence greater than three median absolute deviations from the median of the age-standardised prevalence for all inpatient and non-USA claims data were marked as outliers and excluded from analysis. We excluded any data for subnational locations under the age of 20 years that had excessive influence on the estimation of pseudo-random effects and the subnational prior distribution, and led the model to ignore more abundant data in older age-groups; this occurred in some subnational locations in Japan and USA. Russian claims data from the ulcerative colitis model were also marked as outliers because their estimates were too high when compared to regional, super-regional, and global rates.

Severity split & disability weight

The basis of the GBD disability weight survey assessments are lay descriptions of sequelae highlighting major functional consequences and symptoms. For GBD 2019, we used the Medical Expenditure Panel Survey to find the proportion of ulcerative colitis and Crohn's disease asymptomatic versus symptomatic during a given four-week period. The lay descriptions and disability weights for sequelae associated with inflammatory bowel disease are shown below.

Table 3. Severity Distribution, details on the severity levels for inflammatory bowel disease in GBD 2019 and the associated disability weight (DW) with that severity.

Severity split	Lay description	DW (95% CI)
Crohn's disease, currently asymptomatic	--	0
Crohn's disease, symptomatic	This person has cramping abdominal pain, has diarrhoea several times a day, and feels very tired for two months every year. When the person does not have symptoms, there is anxiety about them returning.	0.231 (0.156–0.32)

Ulcerative colitis, currently asymptomatic	--	0
Ulcerative colitis, symptomatic	This person has cramping abdominal pain, has diarrhoea several times a day, and feels very tired for two months every year. When the person does not have symptoms, there is anxiety about them returning.	0.231 (0.156–0.32)

*The numerous sequelae generated from exclusive combinations of anaemia and inflammatory bowel disease each contain custom disability weights. More information can be found in the appendix detailing disability weights

Modeling strategy

The modelling strategy for all inflammatory bowel disease encompasses separate DisMod models for ulcerative colitis and Crohn's disease, which are then adjusted to account for inflammatory bowel disease due to indeterminate colitis.

Non-infective inflammatory bowel disease due to ulcerative colitis, pre-adjustment (for indeterminant colitis)

Similar to GBD 2017, we ran a DisMod-MR 2.1 model to produce estimates by age, sex, year, and country. Prior settings in GBD 2017 included setting remission to 0 for all ages and setting incidence to 0 for ages 0 to 1. In GBD 2019, the prior setting on remission remained the same. But, we set incidence to 0 for ages 0 to 2 and 0.00025 for ages 80 to100. We also set priors on excess mortality rate (EMR) at 0.2 for all ages. The minimum coefficient of variation at the regional, super-regional, and global-level was changed from 0.4 to 0.8 in GBD 2019 to improve model fit against input data. Predictive covariates included socio-demographic index on incidence and healthcare access index on EMR.

Non-infective inflammatory bowel disease due to Crohn's disease, pre-adjustment (for indeterminant colitis)

Similar to GBD 2017, we ran a DisMod-MR 2.1 model to produce estimates by age, sex, year, and country. Prior settings in GBD 2017 included setting remission to 0 for all ages and setting incidence to 0 for ages 0 to 2. In GBD 2019, the prior setting on remission remained the same. But, we set incidence to 0.00025 for ages 80 to100 in addition to 0 for ages 0 to 2. We also set priors on excess mortality rate (EMR) at 0.2 for all ages. The minimum coefficient of variation at the regional, super-regional, and global-level was changed from 0.4 to 0.8 in GBD 2019 to improve model fit against input data. Predictive covariates included socio-demographic index on incidence and healthcare access index on EMR.

Betas and exponentiated values for predictive covariates (which can be interpreted as an odds ratio) are shown in the table below.

Table 4. Covariates. Summary of covariates used in the inflammatory bowel disease DisMod-MR meta-regression model

Ulcerative colitis

Covariate	Type	Parameter	Exponentiated beta (95% Uncertainty Interval)
Socio-demographic index	Country-level	Incidence	7.26 (7.03-7.38)
Healthcare access and quality index	Country-level	Excess mortality rate	0.61 (0.37, 0.98)

Crohn's disease

Covariate	Type	Parameter	Exponentiated beta (95% Uncertainty Interval)
Socio-demographic index	Country-level	Incidence	7.37 (7.32, 7.39)
Healthcare access and quality index	Country-level	Excess mortality rate	0.61 (0.39, 0.96)

Vascular intestinal disorders

Flowchart

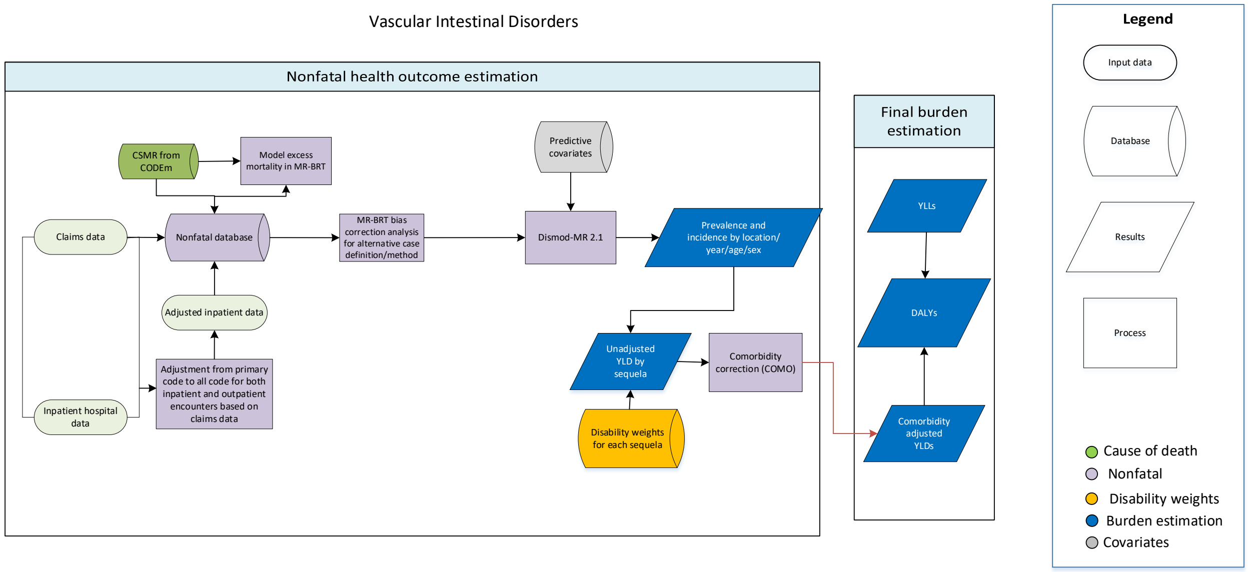

Input Data and Methodological Summary for Vascular Intestinal Disorders

Case definition

Vascular intestinal disorders comprise ischaemic disorders and vascular malformations (ie, angiodysplasias). Ischaemia occurs when there is decreased blood supply to the gastrointestinal tract, causing injury to the bowel, and vascular malformations occur when blood vessels in the bowel grow inappropriately, predisposing to bleeding. Vascular intestinal disorders typically require surgical treatment. The ICD10 code for vascular intestinal disorders is K55; ischaemia and angiodysplasia are only distinguished at the level of 4-digit and 5-digit codes. Equivalent codes for ICD9 are 569.84, 569.85 and 569.86 (for angiodysplasia), and 557 and its 4- and 5-digit constituents (for ischaemia).

Input data and data processing

Input data

Like GBD 2017, the model included data from hospital discharges and claims. In GBD 2019, we newly added Poland claims data and additional years of data from USA claims (years 2015-2016) and hospital discharges in Mexico, India, New Zealand, Sweden, Georgia, and Ecuador. Notably, we included hospital data from Botswana; southern sub-Saharan Africa previously did not have data.

Table 1. Data Inputs for vascular intestinal disorders morbidity modelling by parameter.

Measure	Total sources	Countries with data
Incidence	294	43

Data processing

Hospital discharge data provide observations about encounters, generally with only the primary diagnostic code for the encounter. Claims data, on the other hand, link claims for all inpatient and outpatient encounters for a single individual, and provide primary and secondary diagnoses for all encounters.

In GBD 2017, an individual was extracted from claims data as an incident case if that individual had one or more inpatient encounters with an appropriate ICD code as any diagnosis; readmissions within 28 days were assumed to be for the same episodes of illness. Data from hospital discharges with appropriate ICD codes as primary diagnostic code were, then, adjusted using correction factors derived from inpatient claims data, estimating the number of individuals represented by each encounter, and adjusting the number of individuals with vascular intestinal disorders as primary diagnostic code to the number expected if information on all diagnoses had been provided.

In GBD 2019, however, we improved data processing methods to capture cases that were diagnosed and/or treated in an outpatient setting. Specifically, incident cases were extracted from claim data if an individual had at least one inpatient or outpatient encounter with an appropriate ICD code as any diagnosis within one year. Data from hospital discharges were adjusted using correction factors from claims, converting encounters to estimates of cases, correcting for most locations providing only primary diagnostic codes, and estimating outpatient cases from inpatient cases.

The USA claims data from the year 2000 and from the years 2010–2016 were each adjusted to data from hospital discharges outside DisMod using MR-BRT analysis to adjust for selection bias due to commercial insurance.

The process of adjusting for non-reference data using MR-BRT with the logit-transformation method is described below:

1. Identify data points with overlapping year, age, sex, and location between claims (alternative case definition) and hospital discharges (reference case definition)
2. Logit transform overlapping data points of alternative and reference case definitions
3. Convert overlapping data points into a difference in logit space using the following equation: $logit(altnerative) - logit(reference)$
4. Use the delta method to compute standard errors of overlapping data points in logit space, then calculate standard error of logit difference using the following equation: $\sqrt{(variance\ of\ alternative) + (variance\ of\ reference)}$
5. Using MR-BRT, conduct a random effects meta-regression to obtain the pooled logit difference of alternative to reference
6. Apply the pooled logit difference to all data points of alternative case definitions using the following equation:
$$new_{estimate} = inverse.logit((logit(alternative)) - (pooled\ logit\ difference))$$
7. Calculate new standard errors using the delta method, accounting for gamma (between-study heterogeneity)

The table below shows bias correction factors estimated using MR-BRT.

Table 2. MR-BRT Crosswalk Adjustment Factors for Vascular Intestinal Disorders

Data input	Reference or alternative data collection	Gamma	Beta Coefficient, Logit (95% CI)	Adjustment factor*
Hospital + non-USA claims	Ref	0.05	---	---
USA claims from year 2000	Alt		-0.24 (-0.71, 0.22)	0.44 (0.33, 0.55)
USA claims from year 2010-2016	Alt		0.12 (-0.02, 0.26)	0.53 (0.50, 0.56)

**Adjustment factor is the inverse-logit transformed Beta coefficient; <0.5 represents that alternative is adjusted upward; >0.5 represents that alternative is adjusted downward*

Data points with an age-standardised incidence rate greater than three median absolute deviations from the median of the age-standardised incidence rate for all inpatient and non-USA claims data were marked as outliers and excluded from analysis.

Severity split & disability weight

The basis of the GBD disability weight survey assessments are lay descriptions of sequelae highlighting major functional consequences and symptoms. The lay description and disability weights for vascular intestinal disorders are shown below. All cases are assumed to be severe.

Table 3. Severity Distribution, details on the severity levels for vascular intestinal disorders in GBD 2019 and the associated disability weight (DW) with that severity.

Severity level	Lay description	DW (95% CI)
Severe	This person has severe pain in the belly and feels nauseated. The person is anxious and unable to carry out daily activities.	0.324 (0.219–0.442)

Modeling strategy

Similar to GBD 2017, we ran a DisMod-MR 2.1 model to produce estimates by age, sex, year, and country. Prior settings of the DisMod model included bounding remission between 2 and 12 (a duration from about four weeks to half a year) for all age groups. We used the function in DisMod to pull in cause-specific mortality rate (CSMR) data from our CODEm and CODcorrect analyses. The minimum coefficient of variation at the regional, super-regional, and global-level was changed from 0.4 to 0.8 in GBD 2019 to improve model fit against input data.

In previous rounds, priors on excess mortality rate (EMR) were estimated in DisMod by matching prevalence data points with their corresponding CSMR values within the same age, sex, year, and location (by dividing CSMR by prevalence). For short duration conditions (remission >1), the corresponding prevalence was derived by running an initial model and then applying the same CSMR/prevalence method. However, for many causes, DisMod estimated a rather unrealistic pattern of EMR compared to an expected pattern of decreasing EMR with greater access to quality health care. Such unexpected patterns often signal inconsistencies between CSMR estimates and the measures of

prevalence and/or incidence. In effort to provide greater guidance to DisMod on the expected pattern of EMR, EMR data generated in the previous round were modeled using the MR-BRT approach by age and sex with a prior on healthcare access and quality index (HAQi) having a negative coefficient. Results from MR-BRT were then predicted for each location, year, sex, and for ages 0, 10, 20….100. We included HAQi as a country-level covariate to inform EMR with a mean and standard deviation produced from MR-BRT.

A lag-distributed income covariate (log transformed) and a mean total cholesterol covariates were applied to incidence as predictive covariates. Betas and exponentiated values (which can be interpreted as an odds ratio) are shown in the table below.

Table 4. Covariates. Summary of covariates used in the vascular intestinal disorders DisMod-MR meta-regression model

Covariate	Type	Parameter	Exponentiated beta (95% Uncertainty Interval)
Cholesterol (total, mean per capita)	Country-level	Incidence	1.24 (1.17, 1.33)
LDI (I$ per capita)	Country-level	Incidence	1.21 (1.18, 1.22)
Healthcare access and quality index	Country-level	Excess mortality rate	0.96 (0.96, 0.96)

Gallbladder and biliary diseases

Flowchart

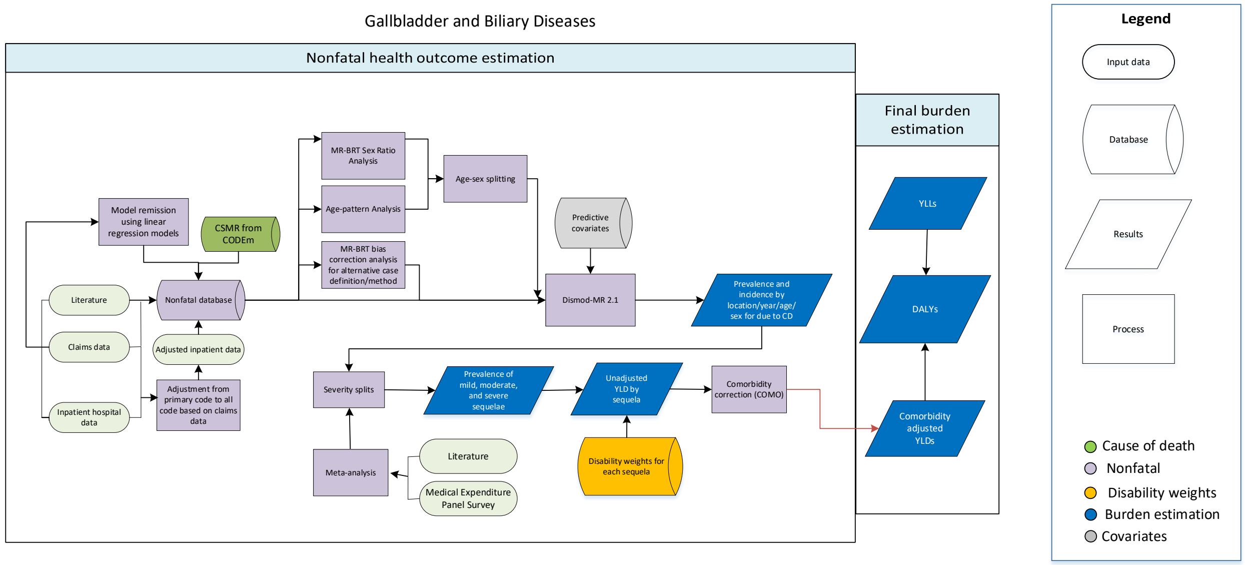

Input Data and Methodological Summary for Gallbladder and Biliary Diseases

Case definition

Gallbladder and biliary diseases include gallstones, cholecystitis, cholangitis, and other diseases of the gallbladder and biliary tract. Gallstones are crystalline masses formed abnormally in the gallbladder or bile ducts from bile pigments, cholesterol, or calcium salts. Gallstones can be asymptomatic and can cause symptomatic episodes of severe abdominal pain, nausea, and vomiting. Cholecystitis is an inflammation of the gallbladder, and cholangitis is an inflammation of the bile duct, both of which can result from obstruction by gallstones and cause severe symptoms. ICD codes for gallstone and biliary diseases included in GBD are K80, K81, K82, and K83. The procedure codes used to identify remission of gallbladder and biliary diseases are 47400-47480, 47490-47544, 47550-47556, 47562-47579, 47600-47715, 47720-47900, 47999-47999.

Overall strategy

In GBD 2017, two databases were created for gallbladder and biliary diseases as inputs to two separate, complete compartmental DisMod models: total (symptomatic + asymptomatic cases) and symptomatic. In GBD 2019, the DisMod model for symptomatic cases was dropped, and we only modeled total cases of gallbladder and biliary diseases in DisMod; an updated severity distribution was, then, applied as described below.

Input data and data processing

Input data

Literature data in the total gallbladder and biliary disease database were drawn from a systematic literature review that was conducted in GBD 2016. The search string used was ((gall bladder disease[Title/Abstract] OR cholecyst*[Title/Abstract] AND prevalence[Title/Abstract] AND ("2010/01/01"[Date - Publication] : "2016/11/01"[Date - Publication])) NOT(animals[MeSH] NOT humans[MeSH])). Studies not representative of the national population (ie, *H. pylori* cohorts, patients presenting with pain), studies without sufficient information on study and sampling methods, and reviews were excluded.

In addition to literature data, input data for the total model included clinical administrative data that were extracted as prevalence. In GBD 2019, we newly added Poland claims data and additional years of hospital discharges data from Mexico, India, New Zealand, Sweden, Georgia, and Ecuador, as well as two additional years of USA Marketscan claims data. Most notably, we included hospital discharges data from Botswana; southern sub-Saharan Africa previously did not have data.

Table 1. Data Inputs for gallbladder and biliary diseases morbidity modelling by parameter.

Measure	Total sources	Countries with data
All measures	335	54
Prevalence	320	54
Proportion	15	1

Data processing

Similar to GBD 2017, claims data link multiple inpatient and outpatient claims to a single individual; prevalent cases were extracted if an individual had at least one inpatient or two outpatient encounters with an appropriate ICD code as any diagnosis, and correction factors were derived to apply to other data sources. Data from hospital discharges were adjusted using correction factors from claims, converting encounters to estimates of cases, correcting for some facilities providing only primary diagnostic codes, and estimating outpatient cases from inpatient cases.

In GBD 2017, the total model utilized ICD-code based clinical administrative data as the reference standard. In GBD 2019, we improved our reference case definition, employing data from literature studies in which general population samples were screened for both symptomatic and asymptomatic cases of gallbladder and biliary diseases using ultrasonography. Claims and hospital discharge data were adjusted toward this new reference standard to account for systematic differences prior to modeling in DisMod. The USA claims data from the year 2000 and from the years 2010–2016 were separately adjusted outside DisMod using MR-BRT analysis to account for selection bias due to commercial insurance.

The process of adjusting for non-reference data using MR-BRT with the logit-transformation method is described below:

1. Identify data points with overlapping year, age, sex, and location between claims (alternative case definition) and hospital discharges (reference case definition)
2. Logit transform overlapping data points of alternative and reference case definitions
3. Convert overlapping data points into a difference in logit space using the following equation: $logit(altnerative) - logit(reference)$
4. Use the delta method to compute standard errors of overlapping data points in logit space, then calculate standard error of logit difference using the following equation: $\sqrt{(variance\ of\ alternative) + (variance\ of\ reference)}$
5. Using MR-BRT, conduct a random effects meta-regression to obtain the pooled logit difference of alternative to reference
6. Apply the pooled logit difference to all data points of alternative case definitions using the following equation:
$$new_{estimate} = inverse.logit((logit(alternative)) - (pooled\ logit\ difference))$$
7. Calculate new standard errors using the delta method, accounting for gamma (between-study heterogeneity)

The table below shows bias correction factors estimated using MR-BRT.

Table 2. MR-BRT Crosswalk Adjustment Factors for Gallbladder and Biliary Diseases

Data input	Reference or alternative case definition	Gamma	Beta Coefficient, logit difference (95% CI)	Adjustment factor (inverse logit)*
Ultrasound-based diagnosis	Ref	0.66	---	---
Hospital + non-USA claims	Alt		-1.88 (-3.42, -0.35)	0.13 (0.03, 0.41)
USA claims from year 2000	Alt		-1.51 (-3.53, 0.51)	0.18 (0.03, 0.62)
USA claims from year 2010-2016	Alt		-1.80 (-3.59, -0.02)	0.14 (0.03, 0.50)

**Adjustment factor is the inverse-logit transformed Beta coefficient; <0.5 represents that alternative is adjusted upward; >0.5 represents that alternative is adjusted downward*

We split data points where the age range was greater than 20 years using the global age pattern informed by the data points with fine age groups (i.e. ages 5-9, 10-14, and 15-20…). We also split data reported for both sexes using the pooled sex-ratio estimated from studies that reported prevalence in males and females separately. The ratio of female to male cases derived from MR-BRT analysis was 2.40 (CI: 1.26, 4.57).

Data points with an age-standardised prevalence greater than two median absolute deviations from the median of the age-standardised prevalence for all inpatient and non-USA claims data were marked as outliers and excluded from analysis.

Severity split & disability weight

The basis of the GBD disability weight survey assessments are lay descriptions of sequelae highlighting major functional consequences and symptoms. In GBD 2019, cases from the total model were divided into asymptomatic and symptomatic groups using proportions found in a review of six literature studies

of the natural history of gallbladder and biliary diseases through the MR-BRT analysis. Symptomatic cases of gallbladder and biliary diseases were, then, divided according to severity distributions derived from data from the Medical Expenditure Panel Survey (MEPS) to assign them to mild, moderate, and severe sequelae. Asymptomatic cases were assigned no disability. The lay descriptions and disability weights for gallbladder and biliary diseases are shown below.

Table 3. Severity Distribution, details on the severity levels for gallbladder and biliary diseases in GBD 2019 and the associated disability weight (DW) with that severity.

Severity level	Lay description	DW (95% CI)
Asymptomatic	--	0
Mild	This person has some pain in the belly that causes nausea but does not interfere with daily activities.	0.011 (0.005–0.021)
Moderate	This person has pain in the belly and feels nauseous. The person has difficulties with daily activities.	0.114 (0.080–0.159)
Severe	This person has severe pain in the belly and feels nauseated. The person is anxious and unable to carry out daily activities.	0.324 (0.219–0.442)

Modeling strategy

We ran a DisMod-MR 2.1 model to produce estimates by age, sex, year, and country. We used the function in DisMod-MR 2.1 to pull in cause-specific mortality rate (CSMR) data from our CODEm and CODcorrect analyses and match with prevalence data points for the same geography. We calculated excess mortality rate (EMR) to estimate priors by dividing CSMR by prevalence. The minimum coefficient of variation at the regional, super-regional, and global-level was changed from 0.4 to 0.8 in GBD 2019 to improve model fit against input data.

In previous rounds, priors on remission were estimated in DisMod by using prevalence, CSMR, and DisMod-estimated EMR. To better inform DisMod on the increasing pattern of remission with greater access to quality health care, in GBD 2019 we used remission data from the USA claims, defined as a number of people with procedure codes among all people with diagnosis of gallbladder and biliary diseases, and regressed against healthcare access and quality index (HAQi) and sex. The results from the regression model were then used to predict remission estimates for each location, year, sex and for ages 0, 10, 20…100.

We applied a lag-distributed income covariate to EMR, log transformed and forced negative with an upper bound of 0 and a lower bound of -1. The Beta and exponentiated values of this predictive covariate (which can be interpreted as an odds ratio) are shown in the table below.

Table 4. Covariates. Summary of covariates used in the gallbladder and biliary diseases DisMod-MR meta-regression model

Covariate	Parameter	Exponentiated beta (95% Uncertainty Interval)
LDI (I$ per capita)	Excess mortality rate	1.00 (1.00, 1.00)

Pancreatitis

Flowchart

Pancreatitis

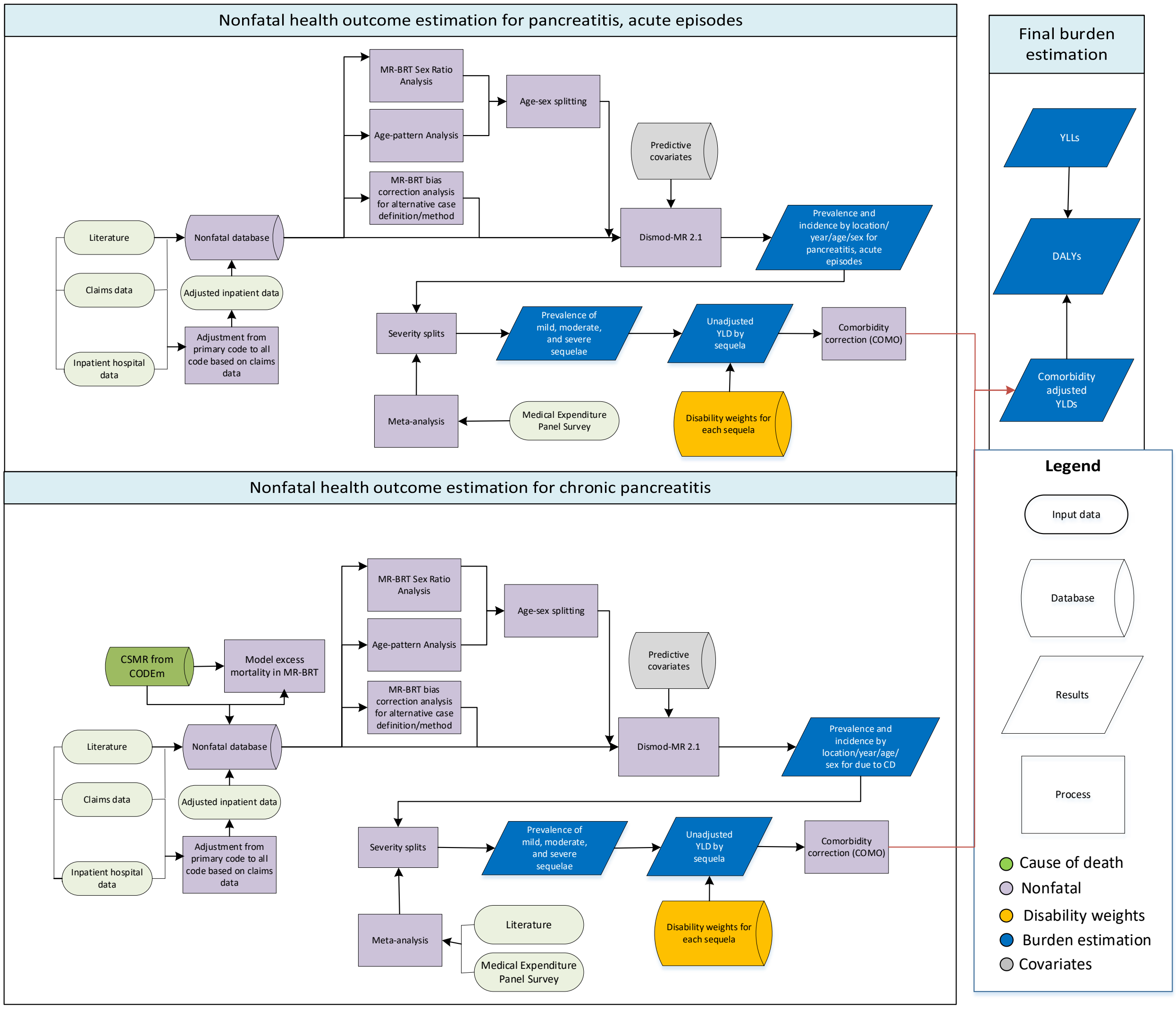

Input Data and Methodological Summary for Chronic Pancreatitis and Pancreatitis, Acute Episodes

Case definition

Pancreatitis is the inflammation of the pancreas. Acute pancreatitis involves active inflammation and injury to the pancreas, resulting in severe upper abdominal pain and nausea, inappropriate release of pancreatic contents, and a systemic inflammatory response with fever, low blood pressure, and, in some cases, failure of one or more organs. Chronic pancreatitis involves permanent damage to the pancreas from longstanding or recurrent inflammation; this produces chronic or episodic abdominal pain and

nausea and ultimately failure of the pancreas to produce and release digestive enzymes and hormones, leading to chronic diarrhea, poor absorption of nutrients from food, and diabetes. Individuals with chronic pancreatitis can have superimposed episodes of acute pancreatitis. In prior rounds of GBD, we modelled acute and chronic pancreatitis together, but in GBD 2017 we developed separate models for these two diseases.

ICD10 codes are K85 for acute and K86 for chronic pancreatitis. ICD9 code 577.0 corresponds to acute pancreatitis, and 577 and the remainder of its four-digit and five-digit constituents refer to chronic or unspecified pancreatitis.

Overall strategy

Like in GBD 2017, two databases were used as inputs to two separate, complete compartmental DisMod models: pancreatitis with acute episodes and chronic pancreatitis.

Input data and data processing

Input data

For GBD 2013, a systematic literature review was conducted to capture studies of prevalence and incidence of pancreatitis throughout the world. This search was updated for GBD 2015 and, again, for GBD 2016. A PubMed search was conducted using the following search terms: Pancreatitis[Title/Abstract] OR "Pancreatitis"[Mesh] OR "Pancreatitis, Acute Necrotizing"[Mesh] OR "Pancreatitis, Chronic"[Mesh]) AND (prevalence[Title/Abstract] OR incidence[Title/Abstract]) AND (“2010/01/01”[Date - Publication] : “2016/11/01”[Date - Publication]) NOT(animals[MeSH] NOT humans[MeSH])) NOT("comment"[Publication Type])

The exclusion criteria were:

1. Studies clearly not representative of the national population (ie, alcoholics or smokers)
2. Studies that did not provide primary data on epidemiological parameters, eg, a commentary piece

Studies were added to the acute database if they measured the incidence of acute pancreatitis as defined by appropriate ICD codes, or by a combination of clinical, biochemical, and radiographic criteria. The acute database included studies that measured incidence of first episode of acute pancreatitis only, and studies that measured incidence of all acute pancreatitis, including recurrent episodes. Studies that included individuals with underlying chronic pancreatitis were excluded from the acute database. Studies were added to the chronic database if they employed appropriate ICD codes or appropriate clinical, biochemical, and radiographic criteria of chronic pancreatitis. Some studies reported incidence of acute and chronic disease separately and data were extracted to both databases, but those few studies that reported only a single measure for both disorders were excluded.

In GBD 2017, the acute database included literature data extracted as prevalence from six countries, including Ireland, Japan, and Poland. These data were excluded from analysis in GBD 2019 because they did not meet the inclusion criteria for the acute database.

In addition to the literature studies, both databases included administrative data that were extracted as incidence for acute and prevalence for chronic. In GBD 2019, we newly added Poland claims data and additional years of hospital discharge data from Mexico, India, New Zealand, Sweden, Georgia, and Ecuador, as well as two additional years of USA Marketscan claims data. Most notably, we included hospital discharge data from Botswana; southern sub-Saharan Africa previously did not have data.

Table 1. Data Inputs for pancreatitis morbidity modelling by parameter.

Measure	Total sources	Countries with data
All measures	358	48
Prevalence	292	41
Incidence	336	47
Proportion	15	1

Data processing

Hospital discharge data provide observations about encounters, generally with only the primary diagnostic code for the encounter. Claims data, on the other hand, link claims for all inpatient and outpatient encounters for a single individual, and provide primary and secondary diagnoses for all encounters.

Similar to GBD 2017, in the acute database, an individual was extracted from claims data as an incident case if that individual had one or more inpatient encounters with an appropriate ICD code as any diagnosis; readmissions within 30 days were assumed to be for the same episodes of illness. Hospital discharges were included only if the primary discharge diagnosis was a code for acute pancreatitis, and incident cases were estimated from number of discharges using a correction factor from claims data.

In the chronic database, claims data linked multiple inpatient and outpatient claims to a single individual; prevalent cases were extracted if an individual had at least one inpatient or two outpatient encounters with a chronic pancreatitis ICD code as any diagnosis. Data from hospital discharges were, then, adjusted using correction factors from claims, converting encounters to estimates of cases, accounting for most locations providing only primary diagnostic codes, and estimating outpatient cases from inpatient cases. Encounter data from outpatient facilities used in GBD 2017 were excluded in GBD 2019 because they were highly heterogeneous and inconsistent with other data sources from the same locations.

In GBD 2019, we improved the bias adjustment methods to allow a more direct comparison between different case definitions and/or study designs. In GBD 2017, we used data from published studies that employed rigorous case definitions as our reference standard for acute pancreatitis and adjusted clinical administrative data toward this reference standard by marking administrative data with binary covariates, and estimating a fixed effect for this covariate in our DisMod meta-regression modeling process. This amounts to adjusting data using an ecological comparison, and vulnerable to compositional bias; if data from different location-years were collected using different methods or case definitions, true spatiotemporal differences in epidemiology can be erroneously adjusted, and differences truly due to differences in methods can be erroneously estimated as differences in underlying epidemiology. In GBD 2019, we avoided this risk by making pre-modeling bias adjustments and dropping data types that could not be rigorously adjusted. This was done by conducting a meta-

regression of the relationship between data points matched on year, age, sex, and location, but differing with regard to one or more study design characteristic.

Like in GBD 2017, we decided to use data from literature studies that identified cases through detailed chart review as the reference standard for the acute pancreatitis model. These studies used a combination of clinical presentation, biochemical, and radiographic findings to validate a case definition, which we refer to as "stringent criteria" in shorthand. Using the stringent criteria, we would, then, adjust other ICD-code based administrative data without validation (i.e. data from claims and hospital discharges). However, the number of matched pairs between reference and alternative (based on year, age, sex and location) was small and yielded highly uncertain adjustment factors for the alternative case definitions. As a result, a new case definition was adopted in GBD 2019: diagnosis of acute pancreatitis as indicated by ICD code in a clinical encounter. Other case definitions and study design characteristics were adjusted toward this new reference standard.

The chronic pancreatitis model used ICD-code based administrative data as the reference standard in GBD 2017 due to scant literature data that were available. In GBD 2019, we attempted to employ the new bias adjustment method for chronic pancreatitis using the more rigorous case definition based on clinical, biochemical, and radiographic findings, but, like in the acute pancreatitis model, we could not find an adequate number of comparison pairs to inform reliable adjustment factors. Therefore, we decided to use the same ICD-based administrative data as the reference standard in GBD 2019, adjusting other case definitions and study design characteristics to this reference standard.

For both acute and chronic pancreatitis models, the USA claims data from the year 2000 and from the years 2010–2016 were each adjusted to the reference to adjust for selection bias due to commercial insurance.

The table below shows bias correction factors estimated using MR-BRT.

Table 2. MR-BRT Crosswalk Adjustment Factors for Pancreatitis

Acute pancreatitis episode: Incidence

Data input	Reference or alternative case definition	Gamma	Beta Coefficient, Log (95% CI)	Adjustment factor*
ICD-code based administrative data	Ref	0.30		
USA claims from year 2000	Alt		-0.18 (-1.12, 0.75)	0.83 (0.33, 2.12)
USA claims from year 2010-2016	Alt		0.19 (-0.44, 0.82)	1.21 (0.65, 2.26)
Stringent criteria	Alt		-0.22 (-1.05, 0.60)	0.80 (0.35, 1.82)

**Adjustment factor is the transformed Beta coefficient in normal space, and can be interpreted as the factor by which the alternative case definition is adjusted to reflect what it would have been if measured as the reference.*

Chronic pancreatitis: Incidence

Data input	Reference or alternative case definition	Gamma	Beta Coefficient, Log (95% CI)	Adjustment factor*
ICD-code based administrative data	Ref	0.61		
Stringent criteria	Alt		-0.66 (-2.14, 0.82)	0.52 (0.12, 2.28)

**Adjustment factor is the transformed Beta coefficient in normal space, and can be interpreted as the factor by which the alternative case definition is adjusted to reflect what it would have been if measured as the reference.*

<u>Chronic pancreatitis: Prevalence</u>

Data input	Reference or alternative case definition	Gamma	Beta Coefficient, Log (95% CI)	Adjustment factor*
ICD-code based administrative data	Ref	0.18		
USA claims from year 2000	Alt		-0.89 (-1.83, 0.05)	0.41 (0.16, 1.05)
USA claims from year 2010-2016	Alt		0.10 (-0.35, 0.55)	1.11 (0.70, 1.73)
Stringent criteria	Alt		0.09 (-2.74, 2.93)	1.10 (0.06, 18.79)

**Adjustment factor is the transformed Beta coefficient in normal space, and can be interpreted as the factor by which the alternative case definition is adjusted to reflect what it would have been if measured as the reference.*

We split data points where the age range was greater than 20 years using the global age pattern informed by the data points with fine age groups (i.e. ages 5-9, 10-14, and 15-20…). We also split data reported for both sexes using the pooled sex-ratio estimated from studies that reported prevalence in males and females separately. The ratios of female to male cases derived from MR-BRT analysis were 0.81 (CI: 0.54, 1.20) and 0.66 (CI: 0.36, 1.22) for acute and chronic pancreatitis, respectively.

Data points with an age-standardised prevalence greater than three median absolute deviations from the median of the age-standardised prevalence for all inpatient and non-USA claims data were marked as outliers and excluded from analysis. Data from Nepal, Turkey, and the Philippians were also marked as outliers in the chronic pancreatitis model because their estimates were unreasonably low or high when compared to regional, super-regional, and global rates.

Severity split & disability weight

The basis of the GBD disability weight survey assessments are lay descriptions of sequelae highlighting major functional consequences and symptoms. The lay descriptions and disability weights for pancreatitis are shown below. All prevalent cases from the pancreatitis, acute episode model were assigned a single, combined disability weight for severe abdominal pain and severe infectious disease symptoms. Prevalent cases from the chronic pancreatitis disease model were divided into symptomatic and asymptomatic groups using proportions found in a review of published studies of the natural history

of chronic pancreatitis. The symptomatic group was divided into mild, moderate, and severe groups using proportions from the Medical Expenditure Panel Survey (MEPS).

Table 3. Severity Distribution, details on the severity levels for pancreatitis in GBD 2019 and the associated disability weight (DW) with that severity.

Severity split	Lay description	DW (95% CI)
Acute pancreatitis episodes	This person has severe pain in the belly and feels nauseated. The person has high fevers, pain and feels very weak. This causes great difficulty with daily activities.	*Combined DW: 0.324 (0.220–0.442) 0.133 (0.088–0.190)
Asymptomatic chronic pancreatitis	--	0
Mild chronic pancreatitis	This person has some pain in the belly that causes nausea but does not interfere with daily activities.	0.011 (0.005–0.021)
Moderate chronic pancreatitis	This person has pain in the belly and feels nauseous. The person has difficulties with daily activities.	0.114 (0.080–0.159)
Severe chronic pancreatitis	This person has severe pain in the belly and feels nauseated. The person is anxious and unable to carry out daily activities.	0.324 (0.219–0.442)

*Acute pancreatitis episodes have a custom disability weight combining abdominal pain and infectious disease. More information can be found in the appendix detailing disability weights.

Modeling strategy

Acute pancreatitis episodes

Similar to GBD 2017, we ran a DisMod-MR 2.1 model to produce estimates by age, sex, year, and country, and no other significant modeling changes were made in GBD 2019. The prior value of remission was bounded from 8 to 9 (a duration from about six weeks) for all ages. The minimum coefficient of variation at the regional, super-regional, and global-level was changed from 0.4 to 0.8 in GBD 2019 to improve model fit against input data. Predictive covariates included were per capita alcohol consumption on incidence and healthcare access and quality index on excess mortality rate (EMR).

Chronic Pancreatitis

Similar to GBD 2017, we ran a DisMod-MR 2.1 model to produce estimates by age, sex, year, and country. The value prior on remission was set to 0. We used the function in DisMod to pull in cause-specific mortality rate (CSMR) data from our CODEm and CODcorrect analyses. The minimum coefficient of variation at the regional, super-regional, and global-level was changed from 0.4 to 0.8 in GBD 2019 to improve model fit against input data. Predictive covariates included a log-transformed age-standardised SEV scalar covariate for pancreatitis on prevalence, and healthcare access and quality index on EMR.

In previous rounds, priors on excess mortality rate (EMR) were estimated in DisMod by matching prevalence data points with their corresponding CSMR values within the same age, sex, year, and

location (by dividing CSMR by prevalence). For short duration conditions (remission >1), the corresponding prevalence was derived by running an initial model and then applying the same CSMR/prevalence method. However, for many causes, DisMod estimated a rather unrealistic pattern of EMR compared to an expected pattern of decreasing EMR with greater access to quality health care. Such unexpected patterns often signal inconsistencies between CSMR estimates and the measures of prevalence and/or incidence. In effort to provide greater guidance to DisMod on the expected pattern of EMR, EMR data generated in the previous round were modeled using the MR-BRT approach by age and sex with a prior on healthcare access and quality index (HAQi) having a negative coefficient. Results from MR-BRT were then predicted for each location, year, sex, and for ages 0, 10, 20….100. We included HAQi as a country-level covariate in our DisMod model to inform EMR with a mean and standard deviation produced from MR-BRT.

Betas and exponentiated values of predictive covariates (which can be interpreted as an odds ratio) are shown in the table below.

Table 4. Covariates. Summary of covariates used in the pancreatitis DisMod-MR meta-regression model

<u>Acute pancreatitis episodes</u>

Covariate	Type	Parameter	Exponentiated beta (95% Uncertainty Interval)
Alcohol (litres per capita)	Country-level	Incidence	1.00<br>(1.00, 1.00)
Healthcare access and quality index	Country-level	Excess mortality rate	0.98<br>(0.15, 7.31)

<u>Chronic pancreatitis</u>

Covariate	Type	Parameter	Exponentiated beta (95% Uncertainty Interval)
Log-transformed age-standardised scaled exposure variable for pancreatitis risk factors	Country-level	Prevalence	2.51<br>(2.43, 2.60)
Healthcare access and quality index	Country-level	Excess mortality rate	0.98<br>(0.98, 0.98)

Other digestive diseases

In addition to the digestive diseases described above, there are other types of digestive diseases with a range of severities and associated sequelae. Because these digestive diseases are diverse in their underlying causes and risk factors as well as in their associated health outcomes, modelling them together in a DisMod-MR model would not produce reliable estimates of prevalence. Instead, we calculated the YLDs caused by other digestive diseases directly using a YLD/YLL ratio as a 'place holder'.

We calculated the ratio of YLDs to YLLs across the specified digestive diseases for which non-fatal outcomes were modelled, using YLL estimates from the GBD 2019 cause of death (CoD) analysis. We then multiplied this YLD/YLL ratio by the YLL estimates for other digestive diseases.

Alzheimer's disease and other dementias

Flowchart

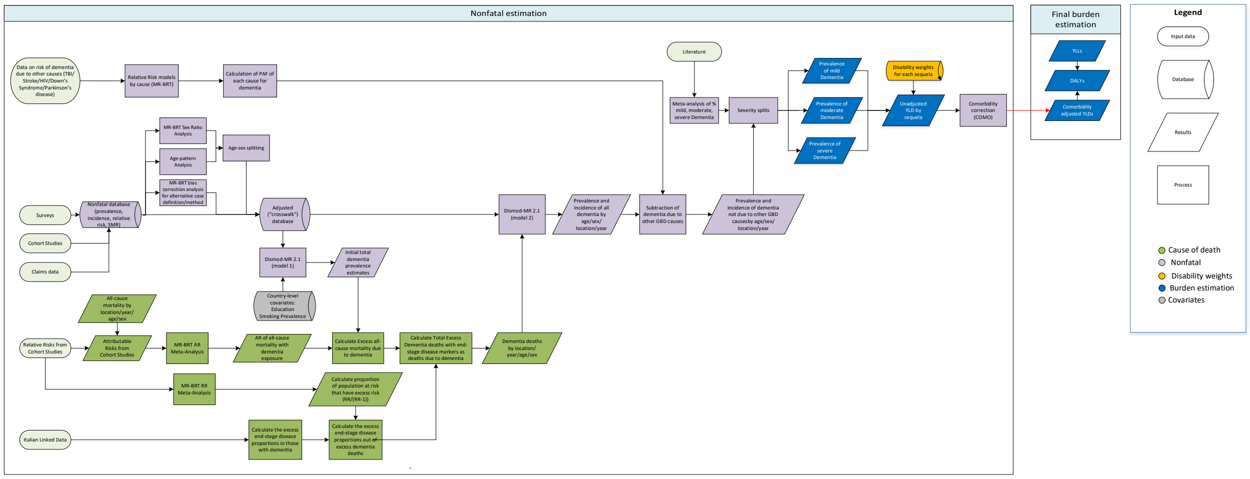

Input data and methodological summary

Case definition

Dementia is a progressive, degenerative, and chronic neurological disorder typified by memory impairment and other neurological dysfunctions. For the purposes of GBD 2019, we use the Diagnostic and Statistical Manual of Mental Disorders III, IV or V, or ICD case definitions as the reference. The DSM-IV definition is:

- Multiple cognitive deficits manifested by both memory impairment and one of the following: aphasia, apraxia, agnosia, disturbance in executive functioning
- Must cause significant impairment in occupational functioning and represent a significant decline.
- Course is characterized by gradual onset and continuing cognitive decline
- Cognitive deficits are not due to other psychiatric conditions
- Deficits do not occur exclusively during the course of a delirium

A wide array of diagnostic and screening instruments exists, including Clinical Dementia Rating scale (CDR), Mini Mental State Examination (MMSE), and the Geriatric Mental State (GMS). For severity rating purposes we use the CDR as the reference. The relevant ICD-10 codes for dementia are F00, F01, F02, F03, G30, and G31. The ICD-9 codes are 290, 291.2, 291.8, 294 and 331.

Unlike most causes in the Global Burden of Disease project, dementia mortality and morbidity estimates are modelled jointly. This is because of marked discrepancies between prevalence data and cause of death data. Specifically, prevalence data suggest little to no variation over time (eg, 1990–2019), whereas age-standardised mortality rates in vital registrations in high-income countries have increased multiple times over this same period. Additionally, prevalence variation between countries is much smaller than the variation in death rates assigned to dementia in vital registration. We attribute these discrepancies to changing coding practices rather than epidemiological change.

Because of this joint procedure, descriptions of the mortality estimation process are included where relevant.

Input data

Model inputs

To inform our estimates of burden due to dementia, we use mortality data from relative risk studies and linked hospital to mortality data, as well as prevalence data from surveys and administrative data such as claims sources.

Item Response Theory for prevalence prediction

The prevalence models for dementia are data sparse, and there are not many surveys done in low income settings. However, there are a larger body of surveys that collect data on cognitive tests and functional limitations which are the two main components of a DSM or ICD diagnosis. Predictions of dementia prevalence using information from these questions would allow for expanded data coverage and additional information in locations where there are currently no data guiding estimates.

Generating these predictions requires calibrating a model to samples that have information about both functional limitations, cognition and adjudicated dementia diagnoses. However, making comparisons across surveys can be difficult, as each survey asks a different set of questions about cognition and limitations, although there is some overlap. This overlap allows for the use of item response theory methods for the harmonization of these scales. Once the scales are harmonized the subsamples can be utilized to create a model for the prediction of prevalence.

In GBD 2019, data from the ADAMS and HRS surveys were extracted and used for Item Response Theory modeling to estimate prevalence. HRS is a nationally representative survey in the US, which has data on cognition and functional limitations. ADAMS is a subsample of HRS that includes much more detailed neuropsychological testing and adjudicated dementia diagnoses. ADAMS includes almost all questions in HRS plus additional questions as well.

Excluding incidence

Since 2016, we have made the decision to exclude incidence data, because in locations with high quality cohort data on prevalence and incidence, the two are not compatible (incidence data implies a higher prevalence than what is reported). Because dementia has a slow, insidious onset and prevalence is easier to measure, we trust prevalence data more and rely on this, excluding incidence data from DisMod.

Severity splits

Methods to determine severity splits for dementia were redesigned in GBD 2019. A new systematic review was conducted to collect information on the proportion of individuals in each dementia severity class out of the population of all individuals with dementia. There are a variety of commonly-used methods for severity rating; for the purposes of GBD 2019, we took the Clinical Dementia Rating (CDR) scale as our reference definition for severity classification, along with a doctor-given diagnosis according to DSM III, IV, V or ICD case definitions as our reference definition for dementia.

However, as a neurodegenerative disorder with a wide range of categories in which symptoms manifest, there are an abundance of classification tools which discern between severity levels along different criteria. We accepted severities classified by:

- Clinical dementia rating sum-of-boxes (CSR-SB)
- Blessed test of information, memory, and concentration (BIMC)
- Global deterioration scale (GDS)
- Geriatric Mental State Examination (GMS)
- CAMDEX
- DSM-III-R
- Karasawa's

We excluded any studies which classified dementia severity according to scales that only evaluated cognitive function and memory, excluding activities of daily living (ADLs). The most prominent such scale is MMSE.

The following search string was used:

((dementia[MeSH Terms] OR dementia[Title] OR Alzheimer disease[Title]) AND (severity[Title/Abstract] OR CDR[Title/Abstract] OR Clinical Dementia Rating Scale[Title/Abstract]) AND (Severity of illness index[MeSH] OR diagnosis[sh] OR Cross-Sectional Studies[MeSH])) AND ("1950/01/01"[Date - Publication] : "2100/02/25"[Date - Publication]) NOT (animals[MeSH] NOT humans[MeSH]))

Prisma diagram of dementia severity split systematic review

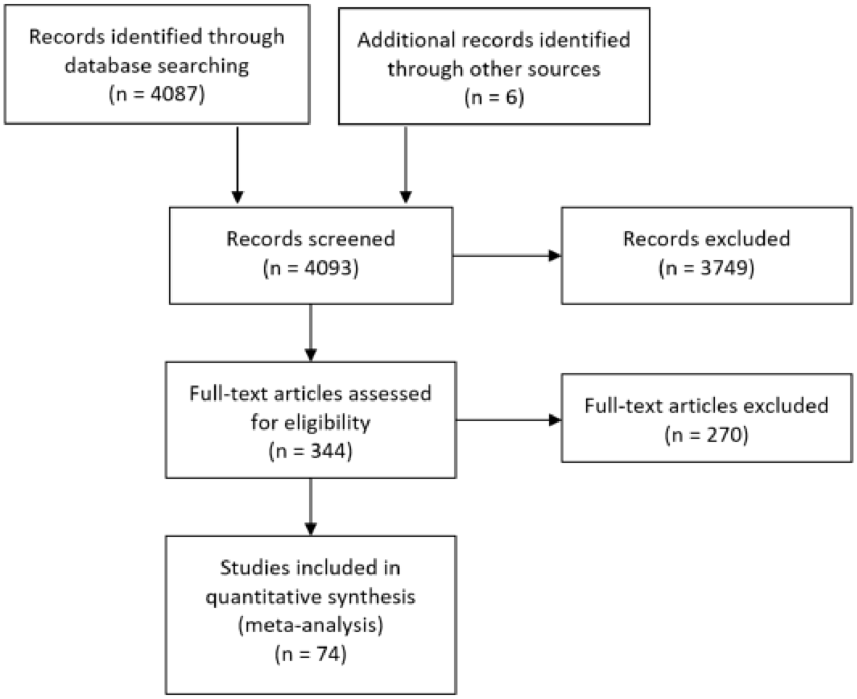

This yielded 4087 total hits, of which 338 passed initial title/abstract screening. After full-text screening, 68 sources met screening criteria and were extracted, along with one source identified through the bibliographies of other sources, and five additional sources used in GBD 2017 for other purposes. A total of 74 sources were extracted and informed the severity split, as compared to the 11 sources used in GBD 2017.

The severity split analysis was conducted using a MR-BRT meta-regression instead of being analyzed as binned meta-analyses as in GBD 2017.
We multiplied estimations of prevalence (country-year-sex-age-specific) by the fractions of mild, moderate, and severe dementia and estimated 95% uncertainty intervals at the 1,000-draw level. The severity distributions over age for each sex are visualized below, followed by a table describing each severity.

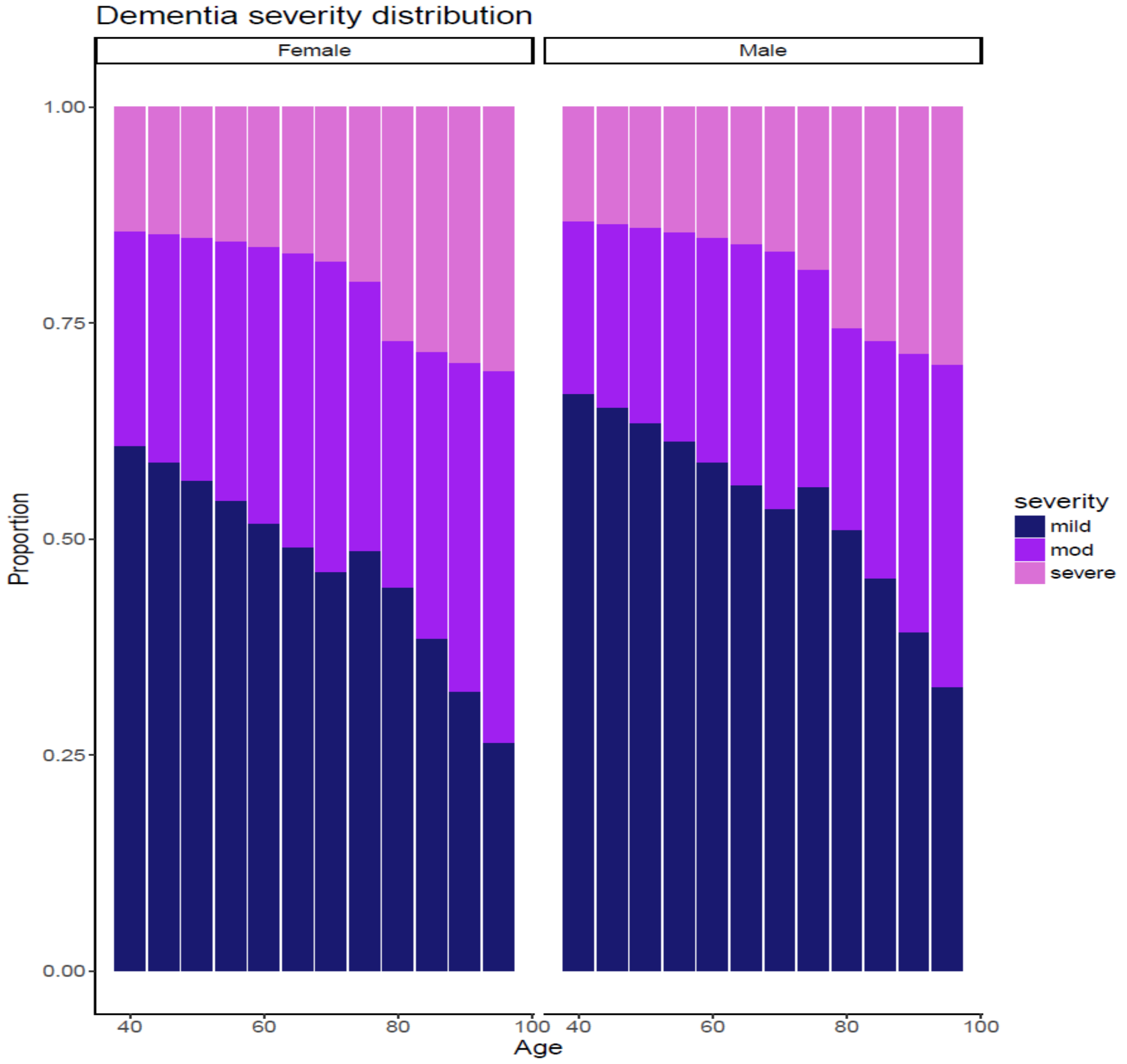

Figure 1 Severity ratios for each 5-year age bin, by sex.

Table of dementia severity levels.

Severity level	Lay description
Mild	The person has some trouble remembering recent events and finds it hard to concentrate and make decisions and plans. They may have slight to moderate difficulty engaging in community affairs, complicated hobbies, and intellectual interests.
Moderate	The person retains highly learned material, but has severe memory problems, is disoriented with respect to time and sometimes place. They are severely impaired in their ability to handle problems and make social judgements. They require assistance with daily activities, and only retain simple chores and hobbies.
Severe	The person has complete memory loss, no longer recognizes close family members, and requires help with all daily activities, including personal care.

Relative risk due to other causes

While the DSM definition excludes dementia cases, where the syndrome is caused by other psychiatric disorders, it does not exclude dementia cases caused by other diseases, not included in DSM. This includes, stroke, Parkinson's disease, Down's syndrome and traumatic brain injury (TBI), which are found elsewhere in the GBD cause list. To prevent double counting of prevalent cases, both under dementia and each of these other causes, we adjusted our dementia prevalence to exclude cases caused by these other conditions. To do so, in GBD 2019 we used data from the Aging, Demographics and Memory study (ADAMS), to estimate the relative risk of getting dementia for each condition included in the ADAMS dataset (stroke, Parkinson's disease, TBI). We then conducted more extensive systematic reviews on all five of these conditions to model each separately. Relative risk models were run using MR-BRT, and population attributable fractions (PAF) for each condition were calculated with the following equation, where exposure is defined as the prevalence of condition:

$$PAF = \frac{exposure * (RR - 1)}{[exposure * (RR - 1)] + 1}$$

Finally, attributable burden was calculated as the PAF multiplied by total burden (i.e. dementia incidence/prevalence).

A summary of each systematic review is displayed in the table below.

	**Stroke**	**Parkinson's disease**	**Down's Syndrome**	**TBI**
	Recent meta-analysis (2018) [46 sources], plus PubMed review for more recent articles			Three recent systematic reviews (2016, 2016, 2019), cross checked and collated all sources [71 total]
**Data Type**	Relative Risks	Proportions and Relative Risks	Proportions	Relative Risks

**Review Hits**	504	1475	355	
**Accepted During Title/Abstract Screening**	79	135	102	
**Accepted During Full Text**	35 (33 from systematic review and 2 from PubMed search)	56	26	45

The total source count used in GBD 2019 modeling is listed in the table below:

Measure	Total sources	Countries with data
All measures	529	56
Prevalence	262	48
Incidence	80	24
Relative risk	83	17
Proportion	97	34
Other	34	17

Modelling strategy

First, prevalence data was sex split, crosswalked and age split. Studies with age and sex detail separately were split into age- and sex-specific data points. Data specified as "both" sex data were split into male- and female-specific data points using MR-BRT to get a model ratio of female/male prevalence and then using the following equations:
Male prevalence:

$$prev_{male} = prev_{both} * \frac{pop_{both}}{(pop_{male} + ratio * pop_{female})}$$

Female prevalence:

$$prev_{female} = ratio * prev_{male}$$

We also split data points where the age range was greater than 25 years using the global age pattern.

Dementia studies are heterogeneous. Even with a smaller number of definitions (DSM/ICD), there are a large number of different ways to diagnose dementia. For example, out of 272 sources used in GBD 2017, there were 263 different methods of diagnosing dementia (overlap was among those who used 10/66 protocol or AGECAT algorithm). Most use a two-step procedure, where you screen using a cognitive test and then only fully evaluate those that fall below a certain pre-defined threshold. We

controlled for methods differences by crosswalking alternative case definitions to reference. Study covariates are based on broad categories determined after going through the diagnostic heterogeneity and there are some added for specific criteria that we know are biased. The same study-level covariates were used in 2019 as in 2017 with the addition of Item Response Theory HRS predictions. Crosswalking was carried out using a logit difference network meta-regression analysis. U.S. Marketscan were separately crosswalked to standardize the claims data relative to existing literature data.

MR-BRT Crosswalk Adjustment Factors for Dementia (Network Analysis)

Data input	Reference or alternative case definition	Gamma	Beta Coefficient, Logit (95% CI)	Adjustment factor*
DSM or ICD case definition	Ref	0.34	---	---
Clinical records diagnosis criteria	Alt		-0.05 (-0.72 – 0.61)	0.51
Algorithm diagnosis criteria (AGECAT)	Alt		0.08 (-0.59 – 0.74)	0.50
U.S. Marketscan	Alt		-0.95 (-1.61 – -0.28)	0.50
NIA-AA diagnosis criteria	Alt		0.51 (-0.16 – 1.17)	0.53
10/66 algorithm diagnosis criteria	Alt		0.97 (0.30 – 1.64)	0.50
GP records used for diagnosis	Alt		-1.21 (-1.88 – -0.54)	

A separate analysis was conducted to crosswalk Marketscan claims data (excluding Marketscan year 2000) to non-claims data using a spline on age. The plot below shows the model fit over different ages (gamma = 0.07).

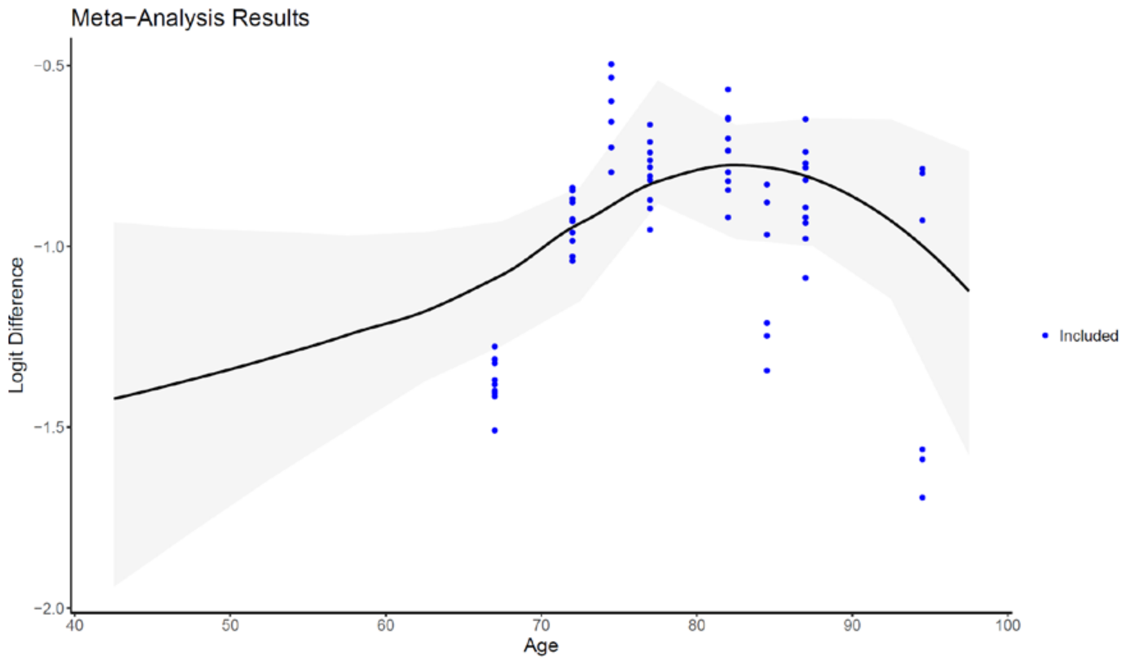

Two country-level covariates were included in the initial Dismod model. Age-standardised education was used as a proxy for general brain health/use that may be protective of dementia – specifically Alzheimer's disease. Smoking prevalence (age-standardised, both sexes) was also used as a covariate to guide estimates, as the literature has shown a positive relationship between smoking and dementia.

Note that two Dismod models were run with prevalence inputs – the first uses adjusted prevalence data (Dismod Model 1 in flowchart), which accounts for dementia caused by other diseases. The second uses unadjusted dementia (Dismod Model 2 in flowchart) which accounts for all dementia regardless of cause (this is the dementia impairment envelope). The tables below summarize country-level covariates used in each of these Dismod model.

Covariates. Summary of covariates used in the Parkinson's Disease DisMod-MR meta-regression model (adjusted prevalence, Model 1)

Covariate	Type	Parameter	Exponentiated beta (95% Uncertainty Interval)
Smoking prevalence (age-standardized)	Prevalence	TBD – asking Emma	
Healthcare access and quality index	Excess mortality rate		

Covariates. Summary of covariates used in the Parkinson's Disease DisMod-MR meta-regression model (unadjusted prevalence, Model 2)

Covariate	Type	Parameter	Exponentiated beta (95% Uncertainty Interval)

Smoking prevalence (age-standardized)	Prevalence	0.005	1.00 (1.00-1.01)
Healthcare access and quality index	Excess mortality rate	-0.08	0.92 (0.92 – 0.92)

As mentioned previously, the estimation of morbidity due to dementia occurs in conjunction with the mortality estimation. **Additional details on this process can be found in the COD capstone appendix.**

We pull the cause-specific mortality results from final fatal estimates into a final DisMod model (Model 2), with the same settings as the models previous. To prevent double counting of prevalent cases, both under dementia and under other causes that can lead to dementia, we adjusted our dementia prevalence to exclude cases caused by these other conditions, which include stroke, Parkinson's disease, traumatic brain injury and Down's Syndrome. To do so, we used data from the Aging, Demographics and Memory study (ADAMS) and new systematic reviews, to estimate the relative risk of getting dementia for each condition included in the ADAMS dataset (stroke, Parkinson's disease, TBI). We first fit logistic regression models predicting the outcome of dementia given each exposure, with an additional covariate on age.

We then used these models to predict the probability of dementia given each exposure at various ages and divided the probability of having dementia by the probability of not having dementia at each age to calculate relative risks. After calculating age specific relative risks, we used these data and estimates of dementia prevalence from our DisMod-MR 2.1 model to calculate the population attributable fractions (PAFs) for each cause and age using the formula:

$$PAF = \frac{prevalence * (RR - 1)}{prevalence * (RR - 1) + 1}$$

Finally, we multiplied the PAF by the total prevalence to get the amount of dementia prevalence that can be attributed to each cause and subtracted this from the total prevalence to get the prevalence of dementia that is not due to other GBD causes.

Parkinson's Disease

Flowchart

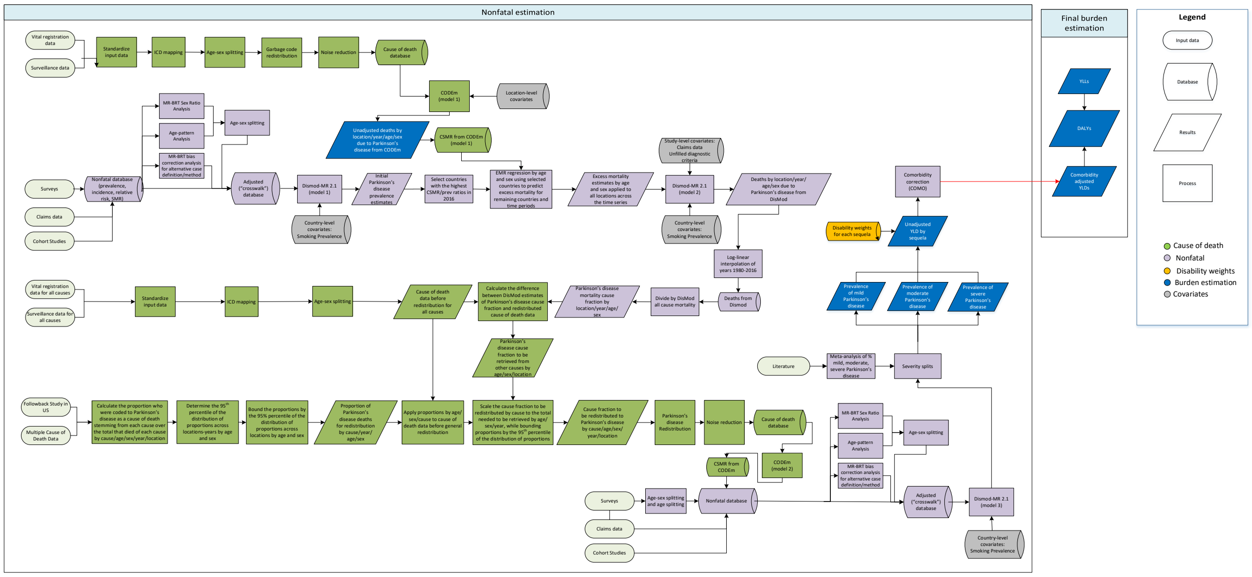

Case definition

Parkinson's disease is a chronic, degenerative, and progressive neurological condition typified by the loss of motor mobility and control – most notably tremors. The corresponding ICD-10 codes are G20, G21, and G22. Our case definition for GBD is the presence of at least two of the four primary symptoms: (1) tremors/trembling, (2) bradykinesia, (3) stiffness of limbs and torso, and (4) posture instability.

Unlike most causes in the Global Burden of Disease project, Parkinson's disease mortality and morbidity estimates are modelled jointly. This is because of marked discrepancies between prevalence data and cause of death data. Specifically, prevalence data suggest little to no variation over time (eg, 1990–2017) whereas age-standardised mortality rates in vital registrations in high-income countries have increased multiple times over this same period. Additionally, prevalence variation between countries is much smaller than the variation in death rates assigned to Parkinson's disease in vital registration. We attribute these discrepancies to changing coding practices rather than epidemiological change.

Because of this joint procedure, descriptions of the mortality estimation process are included where relevant, but see the Parkinson's disease fatal write up for more details.

Input data

Model inputs

To inform our estimates of burden due to Parkinson's disease, we use mortality data from vital registration systems, as well as prevalence data from surveys and administrative data such as claims sources.

An updated systematic review was conducted from September 2015 to August 2017, and the search terms were set to capture studies for Parkinson's disease.[1] This search term resulted in 660 initial hits with 20 sources marked for extraction. Studies with no clearly defined sample or that drew from specific clinic/patient organizations were excluded.

Studies using non-representative populations are excluded from modeling. Certain studies have been outliered on a case-by-case basis due to subsequent review and exclusion due to inappropriateness of the study design, or case ascertainment that conflict with existing gold-standard data – where possible. We exclude claims data from the year 2000 because these data are systematically lower than other years. As of GBD 2017, a prevalent case is identified from claims data where an individual has one inpatient visit, two outpatient visits, or one outpatient and one inpatient visit (arguing that a single mention of a code for PD in an individual could be a provisional diagnosis prior to confirmation). This decreased prevalence estimates for the United States because previously an individual with any inpatient or outpatient visit in a given year counted as a case.

The total source count used for modeling in GBD 2019 is listed in the table below:

Measure	Total sources	Countries with data
All measures	186	45
Prevalence	120	42
Incidence	45	22
Relative risk	1	1
Standardized mortality ratio	6	6
With-condition mortality rate	1	1
Proportion	34	14

Modelling strategy

Studies with age and sex detail separately were split into age- and sex-specific data points. Standard GBD sex splitting methods were used for studies with only "both" sex data points: we modeled the ratio of female/male prevalence in MR-BRT and then calculated male prevalence:

$$prev_{male} = prev_{both} * \frac{pop_{both}}{(pop_{male} + ratio * pop_{female})}$$

And then calculated female prevalence:

$$prev_{female} = ratio * prev_{male}$$

We also split data points where the age range was greater than 25 years. In GBD 2017, age splitting was based on the age pattern from the United States, where we had the most detail by age. In GBD 2019, age splitting was based on the global age pattern from a Dismod model that only used input data with less

[1] (Parkinson disease[Title/Abstract] OR Parkinson's disease[Title/Abstract]) AND (epidemiology[Title/Abstract] OR prevalence[Title/Abstract] OR incidence[Title/Abstract]) AND ("2015/09/31"[PDAT] : "2017/08/23"[PDAT])

than a 25-year age range. Data are location split if they are at country level and cover a number of subnationals (or are UK data).

For GBD 2019, adjustment factors for all study-level covariates were determined using matched data (by year, age, sex, location) for reference and alternative case definitions in a logit difference network meta-regression. Study-level covariates included studies that were not population representative, excluded nursing homes from their estimates, followed UKPD Brain Bank diagnosis criteria, followed MDS diagnosis criteria, or did not explicitly define diagnosis criteria. Country covariates are used to inform global patterns. Cause-specific mortality results from the final fatal Parkinson's disease model is pulled into the final non-fatal DisMod model. The following tables provide an overview of the study-level and country covariates used in the Parkinson's disease DisMod MR-2.1 model.

MR-BRT Crosswalk Adjustment Factors for Parkinson's Disease

Data input	Reference or alternative case definition	Gamma	Beta Coefficient, Logit (95% CI)	Adjustment factor*
2 of 4 diagnostic criteria	Ref	0.48	---	---
Not population representative	Alt		0.03 (-0.95 – 1.04)	0.51
Excluded nursing homes	Alt		0.01 (-0.95 – 0.95)	0.50
UKPD Brain Bank criteria	Alt		0.01 (-1.46 – 0.47)	0.50
MDS criteria	Alt		0.14 (-0.83 – 1.54)	0.53
No explicit criteria	Alt		0.01 (=0.56 – 1.37)	0.50

Covariates. Summary of covariates used in the Parkinson's Disease DisMod-MR meta-regression model

Covariate	Type	Parameter	Exponentiated beta (95% Uncertainty Interval)
Smoking prevalence (age-standardized)	Prevalence	-1.15	0.32 (0.28 – 0.36)
Healthcare access and quality index	Excess mortality rate	-0.025	0.98 (0.97 – 0.98)

Severity splits

As in GBD 2013, we use Hoehn and Yahr stages to determine severity. However, for GBD 2017 onward, the cutpoints were updated in order to more accurately correspond with the lay descriptions of severities. Specifically, a Hoehn and Yahr stage 4 now corresponds to a designation of severe, where before it was classified as moderate.

Severity	Stage

Mild	≤2.0
Moderate	2.5-3.5
Severe	≥4

The following figures show the results of the meta-analysis on Hoehn and Yahr stages.

Figure 1. Percentage of mild cases of Parkinson's disease in population-based studies

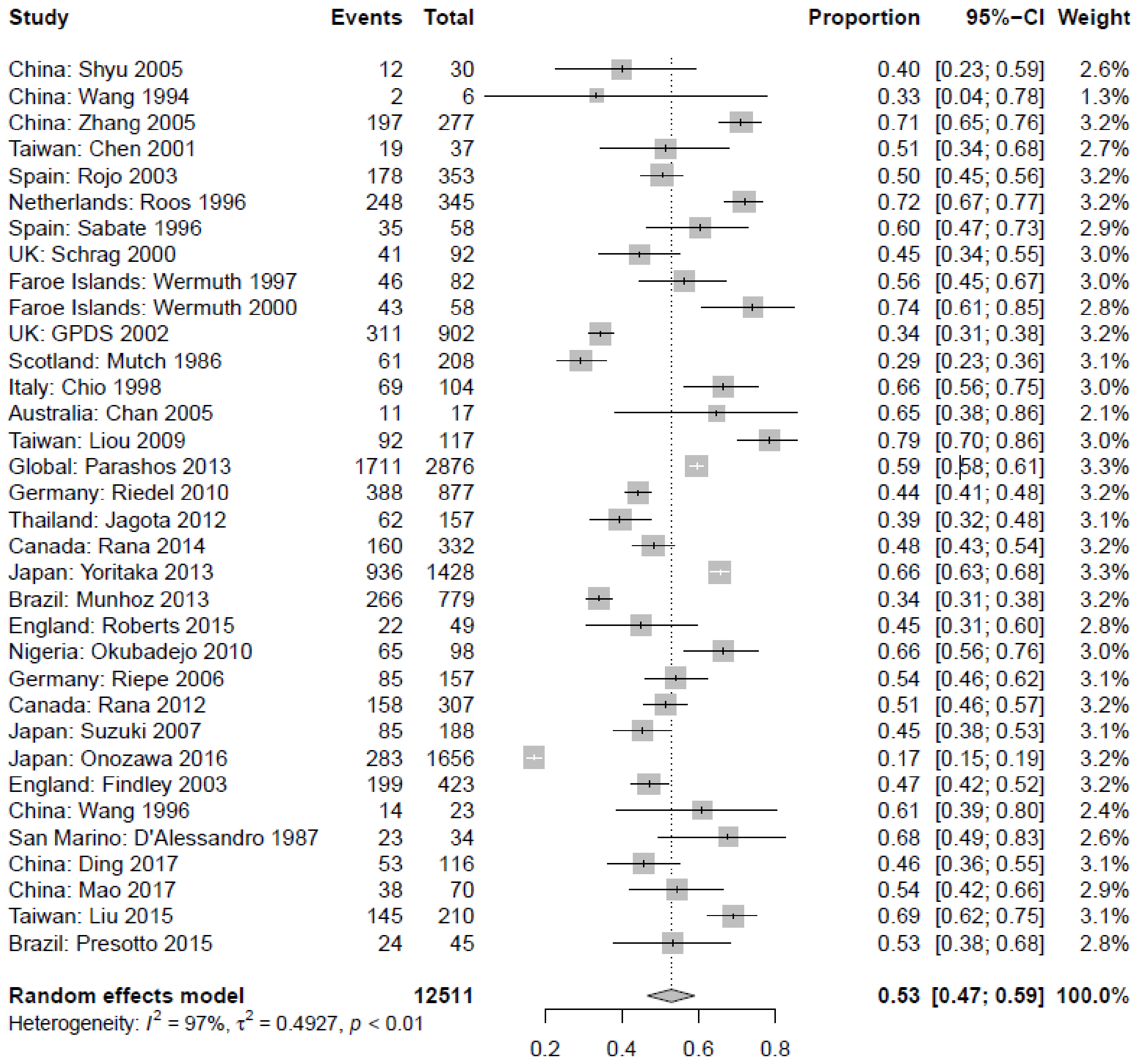

Figure 2. Percentage of moderate cases of Parkinson's disease in population-based studies

Study	Events	Total	Proportion	95%−CI	Weight
China: Shyu 2005	15	30	0.50	[0.31; 0.69]	2.4%
China: Wang 1994	2	6	0.33	[0.04; 0.78]	0.9%
China: Zhang 2005	51	277	0.18	[0.14; 0.23]	3.3%
Taiwan: Chen 2001	12	37	0.32	[0.18; 0.50]	2.5%
Spain: Rojo 2003	144	353	0.41	[0.36; 0.46]	3.5%
Netherlands: Roos 1996	94	345	0.27	[0.23; 0.32]	3.4%
Spain: Sabate 1996	12	58	0.21	[0.11; 0.33]	2.6%
UK: Schrag 2000	45	92	0.49	[0.38; 0.60]	3.1%
Faroe Islands: Wermuth 1997	16	82	0.20	[0.12; 0.30]	2.8%
Faroe Islands: Wermuth 2000	3	58	0.05	[0.01; 0.14]	1.6%
UK: GPDS 2002	444	902	0.49	[0.46; 0.53]	3.6%
Scotland: Mutch 1986	52	208	0.25	[0.19; 0.31]	3.3%
Italy: Chio 1998	15	104	0.14	[0.08; 0.23]	2.8%
Australia: Chan 2005	5	17	0.29	[0.10; 0.56]	1.8%
Taiwan: Liou 2009	18	117	0.15	[0.09; 0.23]	2.9%
Global: Parashos 2013	929	2876	0.32	[0.31; 0.34]	3.6%
Germany: Riedel 2010	339	877	0.39	[0.35; 0.42]	3.6%
Thailand: Jagota 2012	84	157	0.54	[0.45; 0.61]	3.3%
Canada: Rana 2014	121	332	0.36	[0.31; 0.42]	3.5%
Japan: Yoritaka 2013	414	1428	0.29	[0.27; 0.31]	3.6%
Brazil: Munhoz 2013	379	779	0.49	[0.45; 0.52]	3.6%
England: Roberts 2015	23	49	0.47	[0.33; 0.62]	2.8%
Nigeria: Okubadejo 2010	29	98	0.30	[0.21; 0.40]	3.1%
Germany: Riepe 2006	55	157	0.35	[0.28; 0.43]	3.3%
Canada: Rana 2012	105	307	0.34	[0.29; 0.40]	3.4%
Japan: Suzuki 2007	89	188	0.47	[0.40; 0.55]	3.4%
Japan: Onozawa 2016	959	1656	0.58	[0.55; 0.60]	3.6%
England: Findley 2003	120	423	0.28	[0.24; 0.33]	3.5%
China: Wang 1996	3	23	0.13	[0.03; 0.34]	1.5%
San Marino: D'Alessandro 1987	4	34	0.12	[0.03; 0.27]	1.8%
China: Ding 2017	57	116	0.49	[0.40; 0.59]	3.2%
China: Mao 2017	23	70	0.33	[0.22; 0.45]	2.9%
Taiwan: Liu 2015	44	210	0.21	[0.16; 0.27]	3.3%
Brazil: Presotto 2015	21	45	0.47	[0.32; 0.62]	2.7%
**Random effects model**		**12511**	**0.33**	**[0.29; 0.37]**	**100.0%**

Heterogeneity: $I^2 = 95\%$, $\tau^2 = 0.2645$, $p < 0.01$

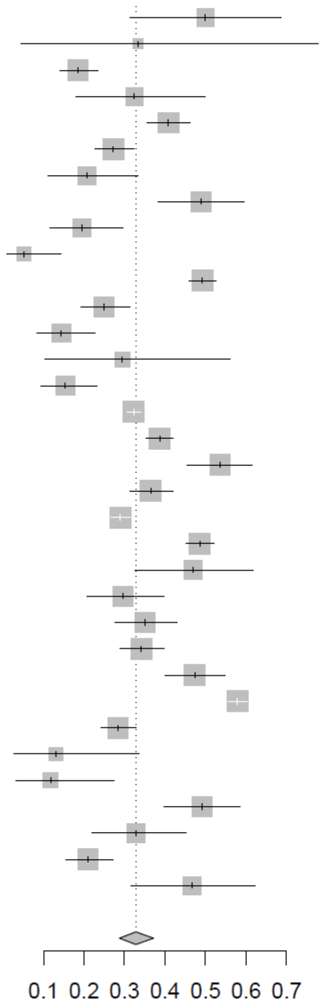

Figure 3. Percentage of severe cases of Parkinson's disease in population-based studies

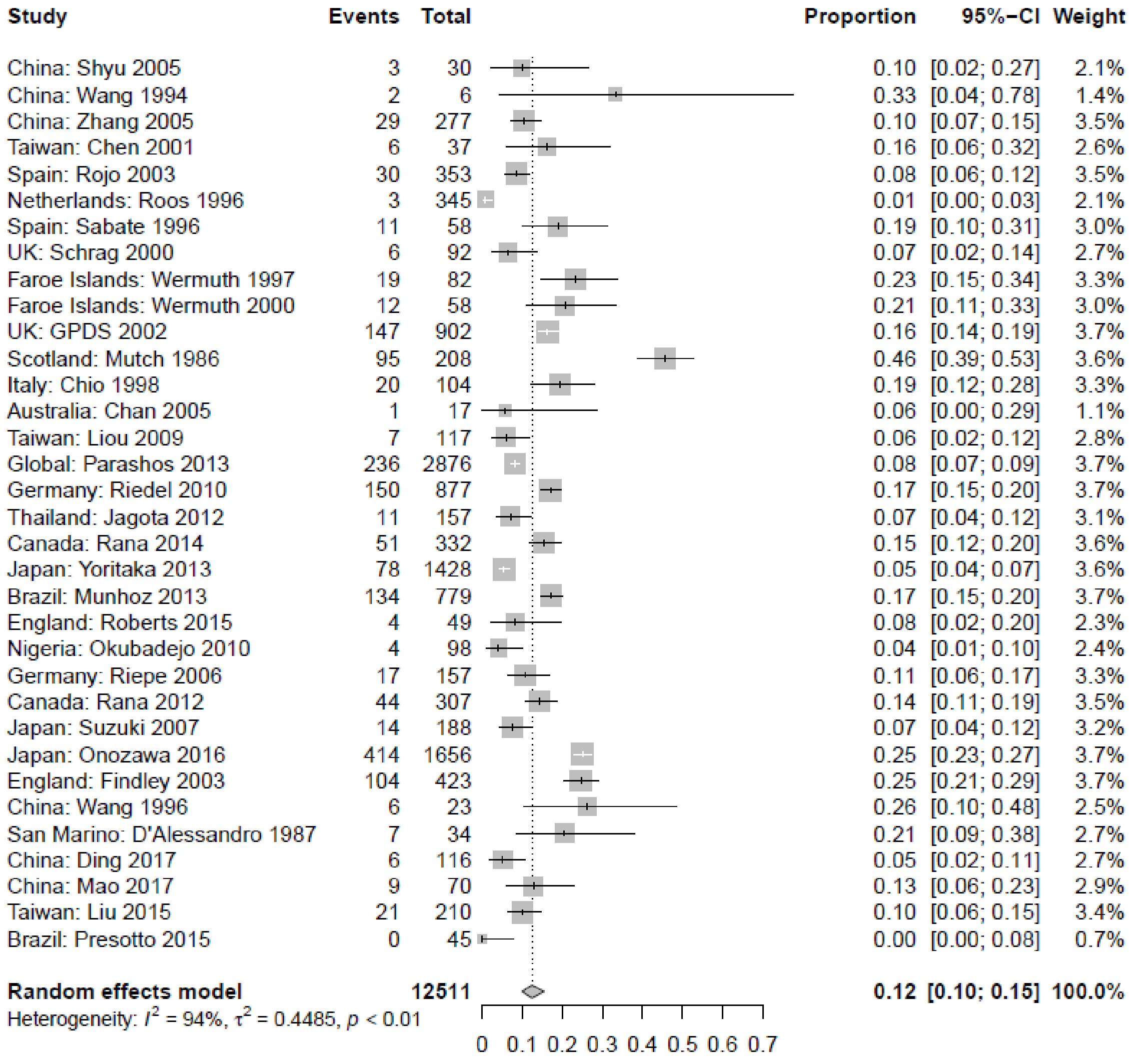

Severity estimates were generated by multiplying estimates of prevalence (country-year-sex-age-specific) by the fractions of mild, moderate, and severe PD, and 95% confidence intervals were estimated by taking 1,000 draws.

The following table provides the lay description and disability weights associated with Parkinson's disease.

Severity level	Lay description	DW (95% CI)
Mild	Has mild tremors and moves a little slowly, but is able to walk and do daily activities without assistance.	0.01 (0.005–0.019)
Moderate	Has moderate tremors and moves slowly, which causes some difficulty in walking and daily activities. The person has some trouble swallowing, talking, sleeping, and remembering things.	0.267 (0.181–0.372)
Severe	Has severe tremors and moves very slowly, which causes great difficulty in walking and daily activities. The person falls easily and has a lot of difficulty talking, swallowing, sleeping, and remembering things.	0.575 (0.396–0.73)

Multiple sclerosis (MS)

Flowchart

Multiple sclerosis

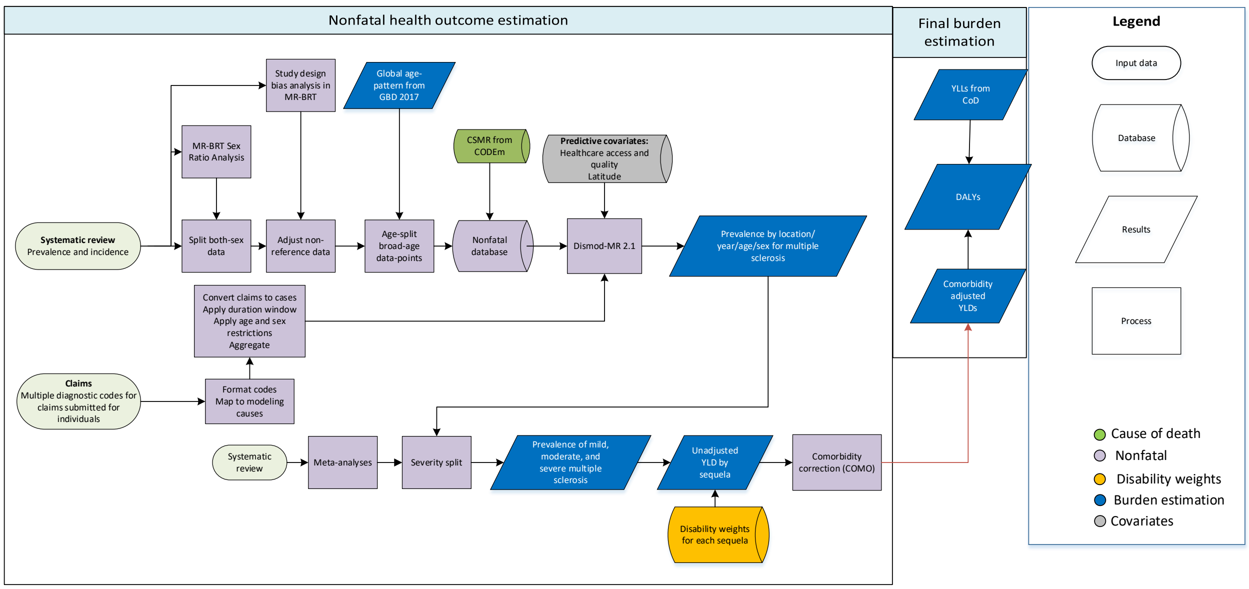

Input data and methodological summary

Case definition

Multiple sclerosis is a chronic, degenerative, and progressive neurological condition typified by the damaging of the myelin sheaths. McDonald's criteria for diagnosis are considered the contemporary gold standard. For GBD 2019, as for previous rounds, diagnosis by McDonald's criteria, other published criteria (such as Poser, Schumacher, or McAllen criteria), and clinical neurological exam are all treated as reference. The ICD-10 code for MS is G35.

Input data and processing

The data underpinning estimates of burden due to MS are generally of two types. The first are representative, population-based, cross-sectional or longitudinal studies reported in peer-reviewed journals and identified via a search-string-based review, last updated for GBD 2017 and described in previous reports. Estimates of epidemiologic measures (prevalence, incidence, *etcetera*) were manually extracted from these publications. The second type are claims data as obtained and processed by the GBD Clinical Informatics team and described in a separate section of this Appendix. New data added in GBD 2019 included Polish claims, additional years of USA claims (years 2015-2016). These data link claims for all inpatient and outpatient encounters for a single individual, and provide primary and secondary diagnoses for all encounters. An individual was extracted from claims data as a prevalent case if they had any peptic ulcer disease code as any diagnosis in one or more inpatient encounters or two or more outpatient encounters.

The total number of sources used for modeling in GBD 2019 are listed in the table below:

Measure	Total sources	Countries with data
All measures	251	53
Prevalence	208	46
Incidence	86	24
Proportion	29	20

For studies that reported epidemiologic measures (generally prevalence or incidence) by age for both sexes combined, and also by sex for all ages combined, we calculated the sex-ratio of cases in that study and applied it to the age-specific measures to estimate age-sex-specific measures.

To estimate sex-specific measures from studies that reported only for both sexes combined, we modeled the log sex ratio in MR-BRT using all sex-specific measurements from all other studies in the database and combined these with the GBD sex-specific population estimates for the relevant age-group. For prevalence, this estimate was 0.63 (0.069 to 1.2); for incidence this estimate was 0.86 (0.53 to 1.2). These were applied by calculating male prevalence:

$$prev_{male} = prev_{both} * \frac{pop_{both}}{(pop_{male} + ratio * pop_{female})}$$

and then calculating female prevalence:

$$prev_{female} = ratio * prev_{male}$$

(Equivalent equations were used for incidence.)

A pre-modelling bias adjustment was then made to data from USA claims in the year 2000 - a dataset that only covers a small commercially insured sub-population. This adjustment was modeled as difference in logit prevalence between USA claims data and reference data matched on year, age, sex and location. The estimated mean logit differences were applied to the USA claims data for 2000 prior to modeling in DisMod-MR 2.1 (below).

The process of adjusting for non-reference data using MR-BRT with the logit-transformation method is described below:

1. Identify data points with overlapping year, age, sex, and location between claims (alternative case definition) and other (reference case definition)
2. Logit transform overlapping data points of alternative and reference case definitions
3. Convert overlapping data points into a difference in logit space using the following equation: $logit(altnerative) - logit(reference)$
4. Use the delta method to compute standard errors of overlapping data points in logit space, then calculate standard error of logit difference using the following equation: $\sqrt{(variance\ of\ alternative) + (variance\ of\ reference)}$
5. Using MR-BRT, conduct a random effects meta-regression to obtain the pooled logit difference of alternative to reference
6. Apply the pooled logit difference to all data points of alternative case definitions using the following equation:

$$new_{estimate} = inverse.logit((logit(alternative)) - (pooled\ logit\ difference))$$

7. Calculate new standard errors using the delta method, accounting for gamma (between-study heterogeneity)

The table below shows bias correction factors.

MR-BRT Crosswalk Adjustment Factors for Multiple sclerosis

Data input	Reference or alternative data	Gamma	Beta Coefficient, Logit difference (95% CI)	Adjustment factor*
McDonald's diagnostic criteria<br>OR<br>Other published diagnostic criteria<br>OR<br>Clinical neuro exam<br>OR<br>Claims for location-years other than USA 2000	Reference	0.32	---	---
Data from USA claims in 2000	Alternative		-0.57 (-1.79 to 0.62)	0.36 (0.14 to 0.65)

**Adjustment factor is the inverse-logit transformed Beta coefficient; <0.5 represents that alternative is adjusted upward; >0.5 represents that alternative is adjusted downward*

Subsequently, data-points for samples spanning 25 years of age or more were disaggregated by applying the age-pattern observed in the global fit for the GBD 2017 model.

After extraction and processing, some studies were marked as outliers and excluded on a case-by-case basis if they were inconsistent with established regional or temporal trends or if concerns about study quality were identified during extraction and processing.

Modelling strategy

Compartmental model

We used DisMod 2.1 as the main analytical tool for the MS estimation process. Inputs included prevalence and incidence data, as described above, as well as the cause-specific mortality rate (CSMR) estimated in the GBD causes of death analysis, and excess mortality rate (EMR) obtained by dividing CSMR by prevalence data-points. Prior settings included zero remission for all ages, no incidence or excess mortality for persons under 5 years old, and incidence limited to less than 0.000005 after the age of 60 years. We also constrained the super-region random effects for prevalence, incidence, and excess mortality to -1 and 1 for all locations except Greenland, United States, and Canada, where location random effects for incidence were constrained to -4, 2 and 2, respectively.

We employed the following covariates to improve model predictions:

Covariate	Measure	Beta coeff (95% CI)	Exponentiated
Absolute value of average latitude	prevalence	0.041 (0.037 to 0.042)	1.04 (1.04 to 1.04)

Absolute value of average latitude	incidence	0.041 (0.036 to 0.045)	1.04 (1.04 to 1.05)
Healthcare Access and Quality index	excess mortality rate	-0.027 (-0.037 to -0.022)	0.97 (0.96 to 0.98)

As described in the literature, extreme latitude is associated with higher prevalence and incidence of MS, although the pathway to explain the association is not understood. Our operationalisation of latitude is created by a population-weighted average of latitude by country and taking the absolute value. The underlying population distribution rasters are part of the Gridded Population of the World dataset.

Although there are no known cures for MS, we expect disease management to differ globally – largely as a function of available resources. To capture this, we use the Healthcare Access and Quality index covariate to capture this relationship in the estimation of excess mortality.

Severity splits

As we have done since GBD 2013, we used Kurtzke's Expanded Disability Status Scale (EDSS) to determine severity splits for MS. The EDSS scores corresponding to each severity are as follows:

Asymptomatic: EDSS = 0
Mild: 0 < EDSS ≤ 3.5
Moderate: 3.5 < EDSS ≤ 6.5
Severe: 6.5 < EDSS ≤ 9.5

The table below illustrates severity levels, lay descriptions, and DWs.

Severity level	Lay description	DW (95% CI)
Asymptomatic	-	0 (0-0)
Mild	Has mild loss of feeling in one hand, is a little unsteady while walking, has slight loss of vision in one eye, and often needs to urinate urgently.	0.183 (0.124–0.253)
Moderate	Needs help walking, has difficulty with writing and arm coordination, has loss of vision in one eye and cannot control urinating.	0.463 (0.313–0.613)
Severe	Has slurred speech and difficulty swallowing. The person has weak arms and hands, very limited and stiff leg movement, has loss of vision in both eyes and cannot control urinating.	0.719 (0.534–0.858)

Because not all sources had information on the number of cases with EDSS stage 0, instead reporting on a mild category, we implemented a two-step meta-analysis strategy. First, we subsetted the studies to those that reported on the number of cases with EDSS stage 0, and did meta-analyses on the proportion of asymptomatic and mild cases. Then, we conducted meta-analyses on the full dataset to get the

proportion mild, moderate, and severe, and we squeezed the asymptomatic and mild categories from the previous meta-analyses into the mild category established by the meta-analysis on the full dataset.

The following figures provide the result of the first meta-analysis on the asymptomatic and mild categories.

Figure 1. Asymptomatic cases of MS

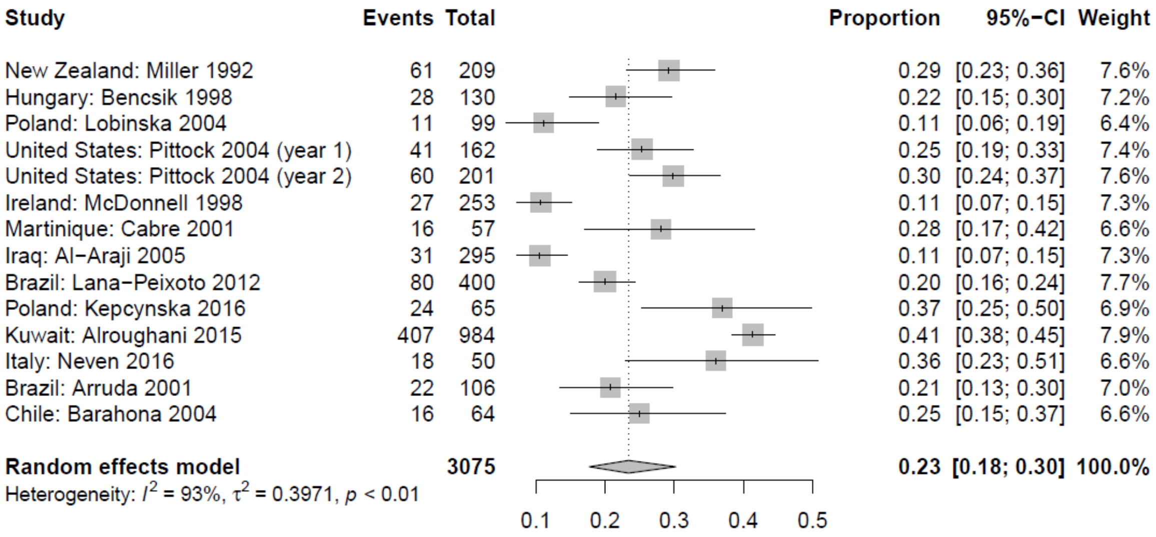

Figure 2. Mild cases of MS

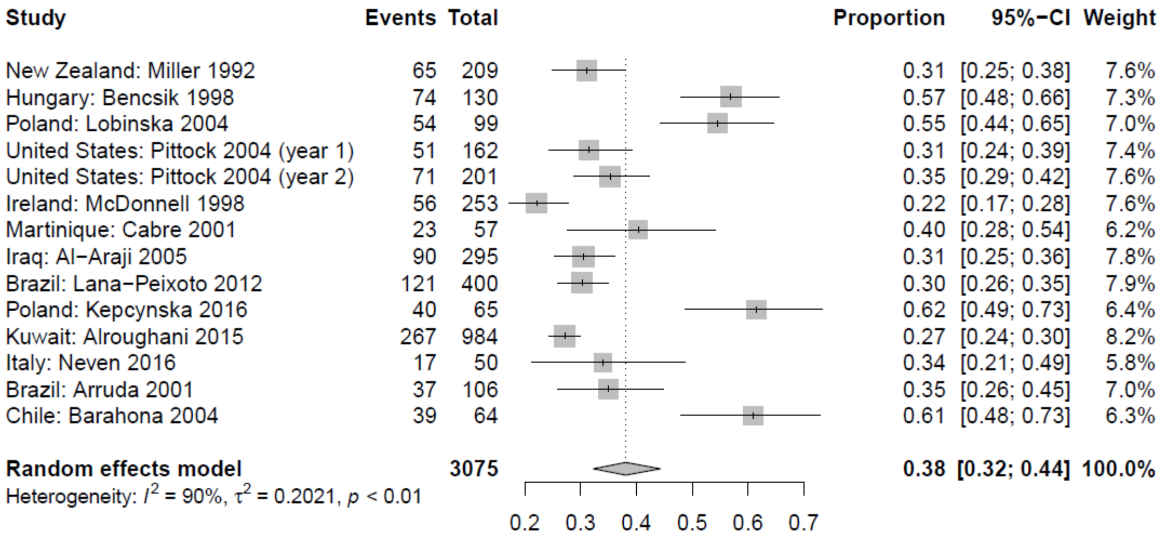

The following figures provide the result of the second meta-analysis on the mild, moderate, and severe categories.

Figure 3. Mild cases of MS (including both asymptomatic and mild categories)

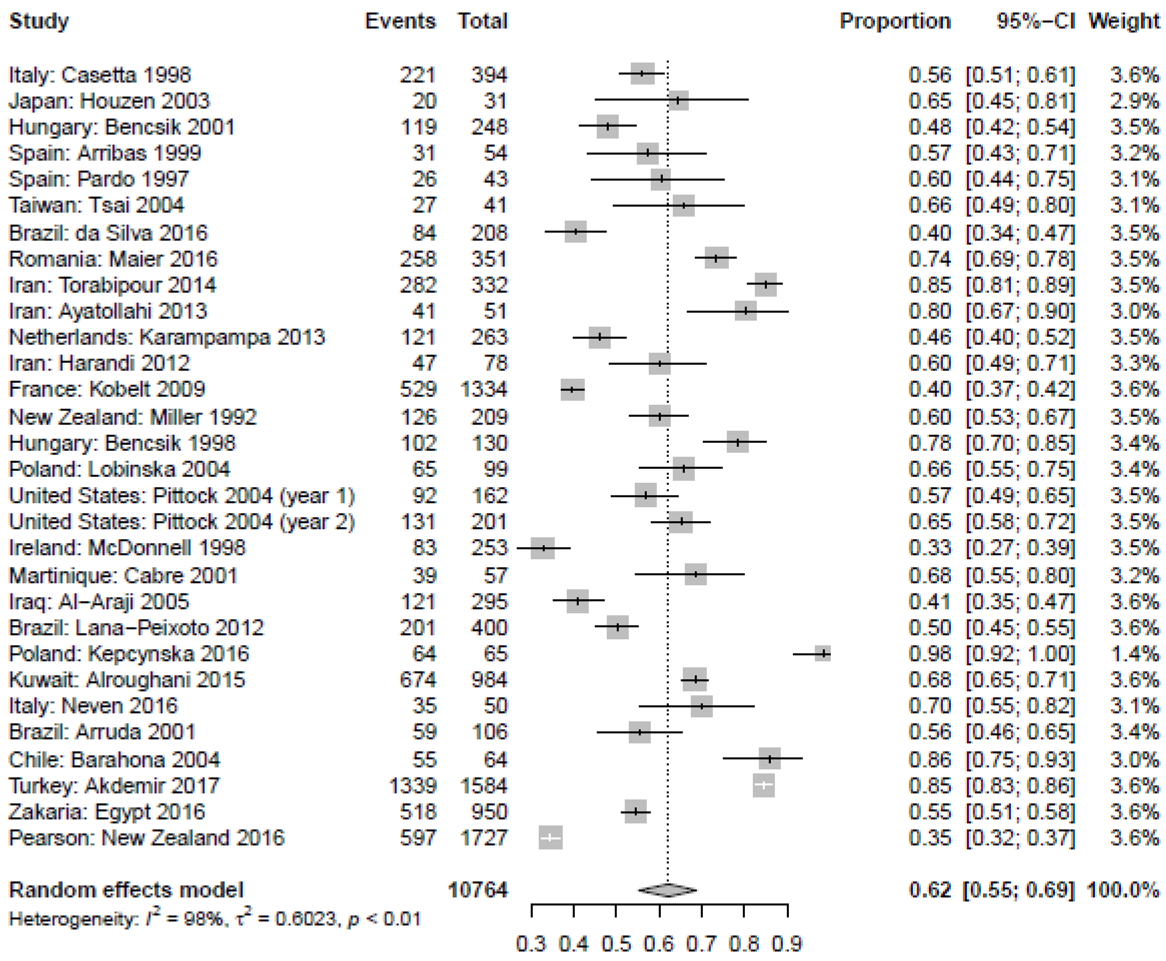

Figure 4. Moderate cases of MS

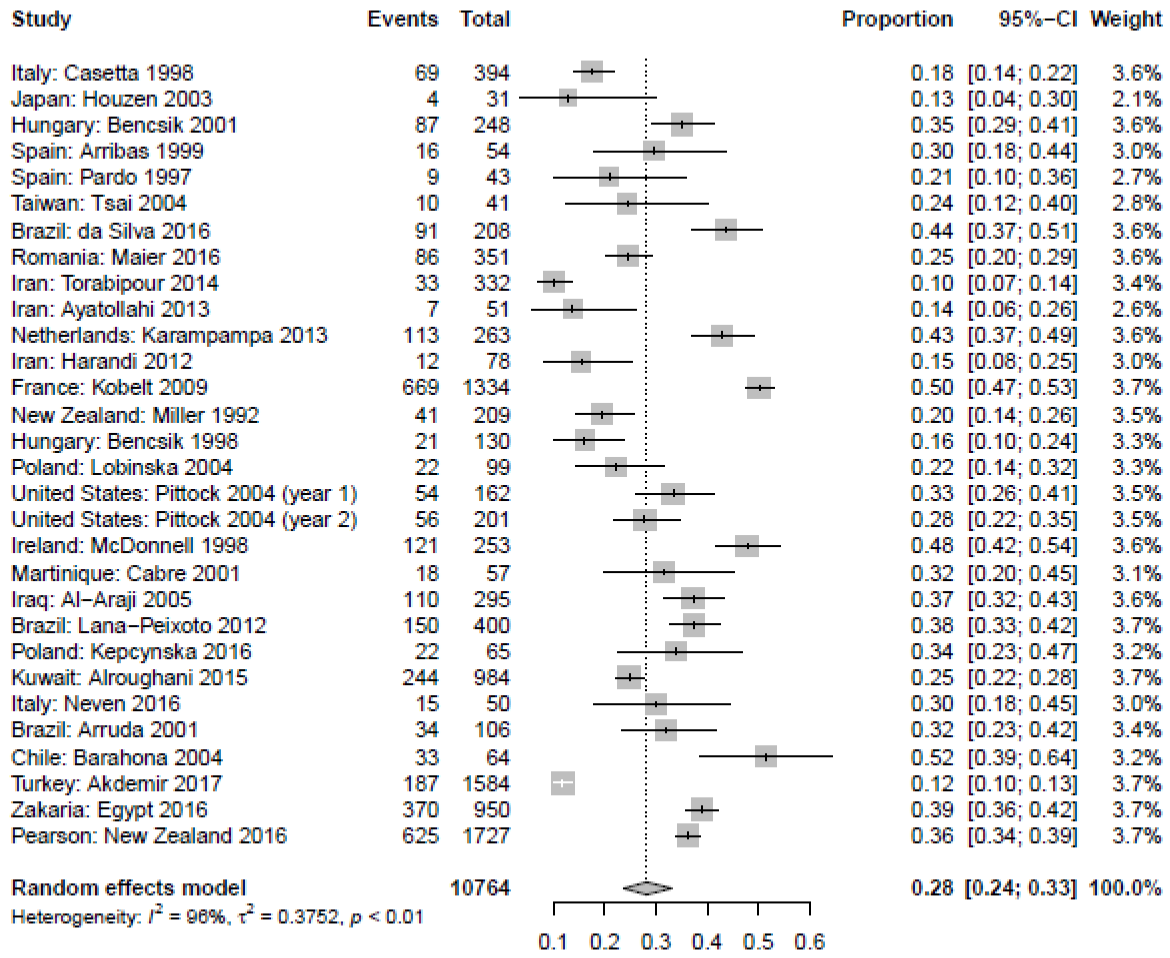

Figure 5. Severe cases of MS

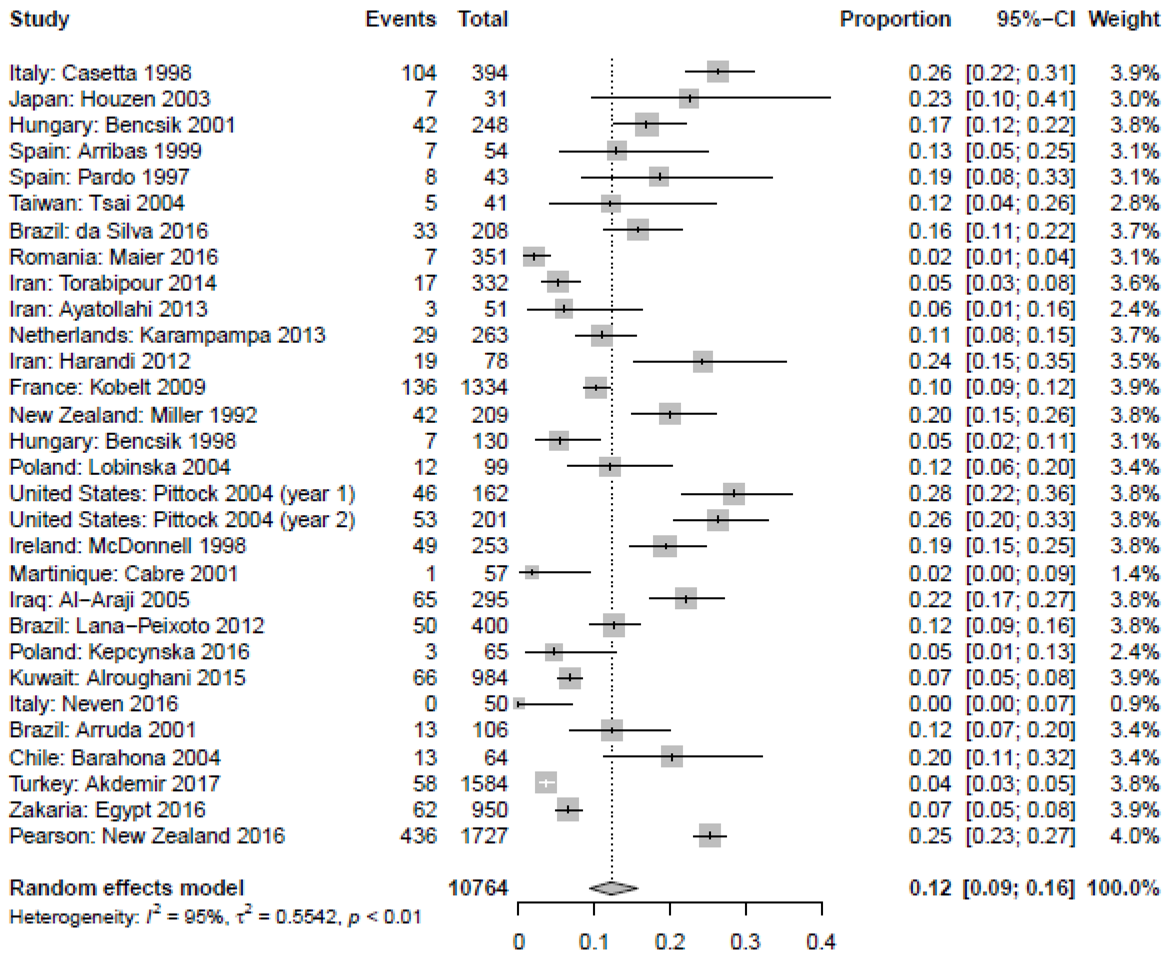

Motor neuron diseases

Flowchart

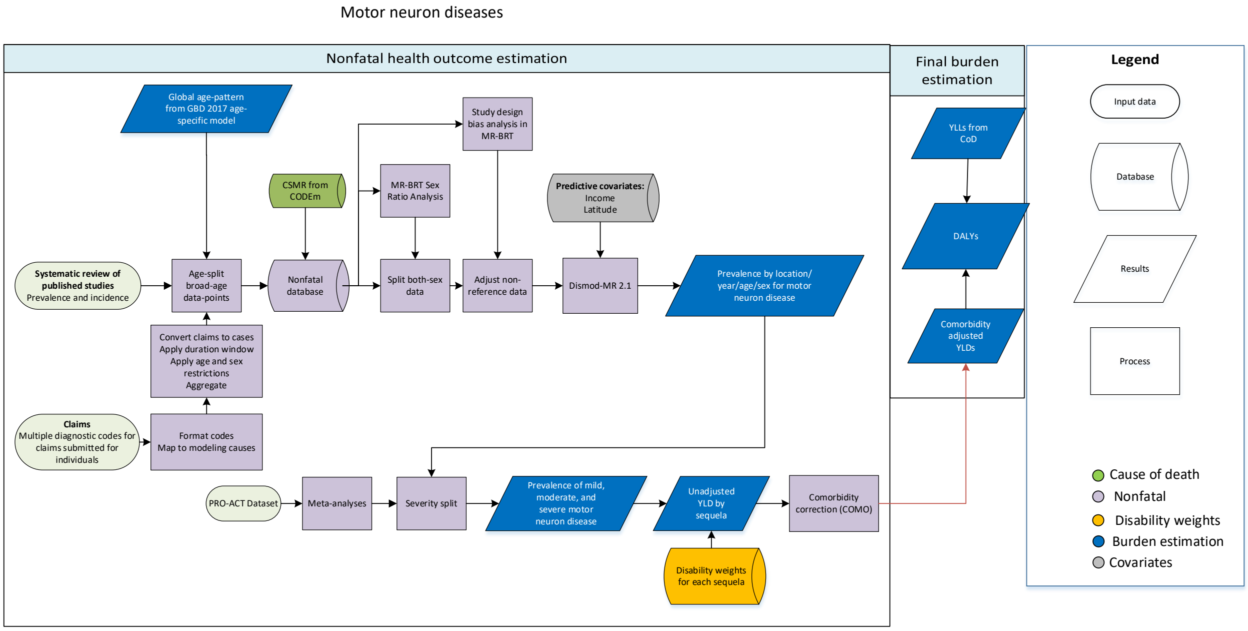

Case definition

Motor neuron diseases (MND) are a set of chronic, degenerative, and progressive neurological conditions typified by the destruction of motor neurons and the subsequent deterioration of voluntary muscle activity. The most common MND is amyotrophic lateral sclerosis (ALS). The El Escorial Criteria are the gold standard diagnostic criteria. The ICD-10 code corresponding to motor neuron diseases is G12.

Input data and data processing

A full systematic review was last conducted for GBD 2015 and will be updated in a future round of GBD. The following search string guided our search, which resulted in 3,146 hits with 58 sources meeting extraction criteria: (1) the study is a representative population-based study with well-defined sample, (2) reports on prevalence, incidence, remission, excess mortality, relative risk of mortality, standardised mortality ratio, or with-condition mortality rate for motor neuron diseases in aggregate or a specified motor neuron disease.

(('motor neuron disease'[MeSH Terms] OR ('motor'[All Fields] AND 'neuron'[All Fields] AND 'disease'[All Fields]) OR 'motor neuron disease'[All Fields] OR ('motor'[All Fields] AND 'neuron'[All Fields] AND 'diseases'[All Fields]) OR 'motor neuron diseases'[All Fields]) OR ('amyotrophic lateral sclerosis'[MeSH Terms] OR ('amyotrophic'[All Fields] AND 'lateral'[All Fields] AND 'sclerosis'[All Fields]) OR 'amyotrophic lateral sclerosis'[All Fields]) OR ALS[All Fields] OR ('motor neuron disease'[MeSH Terms] OR ('motor'[All Fields] AND 'neuron'[All Fields] AND 'disease'[All Fields]) OR 'motor neuron disease'[All Fields] OR ('primary'[All Fields] AND 'lateral'[All Fields] AND 'sclerosis'[All Fields]) OR 'primary lateral sclerosis'[All Fields]) OR ('Politics Life Sci'[Journal] OR 'pls'[All Fields]) OR ('muscular atrophy, spinal'[MeSH Terms] OR

('muscular'[All Fields] AND 'atrophy'[All Fields] AND 'spinal'[All Fields]) OR 'spinal muscular atrophy'[All Fields] OR ('progressive'[All Fields] AND 'muscular'[All Fields] AND 'atrophy'[All Fields]) OR 'progressive muscular atrophy'[All Fields]) OR PBP[All Fields] OR ('pseudobulbar palsy'[MeSH Terms] OR ('pseudobulbar'[All Fields] AND 'palsy'[All Fields]) OR 'pseudobulbar palsy'[All Fields])) AND (('epidemiology'[Subheading] OR 'epidemiology'[All Fields] OR 'epidemiology'[MeSH Terms]) OR population-based[All Fields])

Data from the systematic review were manually extracted for GBD 2015. For GBD 2017, data-points referring to broad age-groups were split according to the age-pattern estimated for that datum's location in a preliminary model that used only age-specific data. For GBD 2019, all previously extracted studies were reviewed and assigned a design variable to indicate if the case definition was limited to ALS only or encompassed all MND.

Beyond data from the systematic review, as in previous rounds of GBD, we made use of claims data as obtained and processed by the GBD Clinical Informatics team and described in a separate section of this Appendix. These data link claims for all inpatient and outpatient encounters for a single individual, and provide primary and secondary diagnoses for all encounters. An individual was extracted from claims data as a prevalent case if they had any MND code as any diagnosis in one or more inpatient encounters or two or more outpatient encounters. New data added in GBD 2019 included Polish claims and additional years of USA claims (years 2015-2016).

Total sources used for modeling in GBD 2019 are listed in the table below:

Measure	Total sources	Countries with data
All measures	73	18
Prevalence	24	1
Incidence	48	18
Proportion	1	1

In GBD 2019, all sex-specific data were used to estimate a pooled sex-ratio using MR-BRT. This ratio was combined with sex-specific population estimates for the year-age-location combinations corresponding to each data point reported for both sexes combined, to estimate sex-specific data-points prior to modeling. These were applied by calculating male prevalence:

$$prev_{male} = prev_{both} * \frac{pop_{both}}{(pop_{male} + ratio * pop_{female})}$$

and then calculating female prevalence:

$$prev_{female} = ratio * prev_{male}$$

(Or the equivalent equations for incidence or other epidemiologic measure.)

Two pre-modeling adjustments were then made adjust for systematic biases in some data sources: data reporting on ALS only and data from USA claims in the year 2000 (a database that only covers a small commercially insured sub-population). Two studies of ALS only were found to be closely matched in year, age, sex and time with three studies of MND more broadly, and the log-ratios for all matched pairs

were entered into an MR-BRT meta-analysis. Commercial claims data from the USA in 2000 were matched to USA claims data from later years with more complete coverage of the population, and these log-ratios were entered into a separate MR-BRT model.

MR-BRT Crosswalk Adjustment Factors

Data input	Reference or alternative case definition	Beta Coefficient, Log (95% CI)	Adjustment factor*
Surveys of all MND using combined clinical, imaging, electrophysiology and imaging criteria<br>OR<br>Claims data from location-years other than USA 2000	Ref	---	---
USA claims from year 2000	Alt	-0.026 (-1.2 to 1.1)	0.97 (0.31 to 3.1)
Surveys limited to ALS only	Alt	-0.13 (-0.23 to -0.029)	0.88 (0.79 to 0.97)

**Adjustment factor is the transformed Beta coefficient in normal space, and can be interpreted as the factor by which the alternative case definition is adjusted to reflect what it would have been if measured as the reference.*

After extraction and processing, some studies were marked as outliers and excluded on a case-by-case basis if they were inconsistent with established regional or temporal trends or if concerns about study quality were identified during extraction and processing.

Modelling strategy

We use DisMod 2.1 as the main analytical tool for MND estimation. Inputs included prevalence and incidence data, as described above, as well as the cause-specific mortality rate (CSMR) estimated in the GBD causes of death analysis, and excess mortality rate (EMR) obtained by dividing CSMR by prevalence data-points. Prior settings are limited to 0 remission at all ages and maximum incidence of 0.0004. We also constrain the super-region random effects for prevalence and incidence to -0.5 and 0.5 to account for spurious inflation of regional differences.

We employed the following covariates to improve model predictions:

Covariate	Measure	Beta coeff (95% CI)	Exponentiated
Absolute value of average latitude	Prevalence	0.032 (0.031 to 0.033)	1.03 (1.03 to 1.03)
LDI (I$ per capita)	Excess mortality rate	-0.5 (-0.5 to -0.5)	0.61 (0.61 to 0.61)

Although there are no known cures for MND, we expect disease management to differ globally – largely as a function of available resources. To capture this, we use the natural log of lagged distributed income per capita as a proxy to capture this relationship in the estimation of excess mortality.

As described in the literature, extreme latitude may be associated with higher prevalence and incidence of motor neuron disease, although the pathway to explain the association is not understood. Our

operationalisation of latitude is created by a population-weighted average of latitude by country and taking the absolute value. The underlying population distribution rasters are part of the Gridded Population of the World dataset.

Severity splits

To calculate severity and disability due to MND we analysed a dataset from Pooled Resource Open-access ALS Clinical Trials (PRO-ACT). This dataset contains the largest ALS clinical trials dataset, with a total of 8,635 ALS patient records from multiple completed clinical trials. Among these, we conducted the final analysis with n=4838 (56%) of the patients with complete ALS Function Rating Score (ALSFRS) with average follow-up time of 184 days (min: -22, max: 648), in which 2,999 (62%) received experimental (medication) treatments and 1,301 (27%) received placebo (in these trials, the medications tested were found to be no better than placebo with respect to their effects on ALS progressions).

The ALSFRS is an instrument for evaluating the functional status of patients with amyotrophic lateral sclerosis. It can be used to monitor functional changes in a patient over time. It measures (1) speech, (2) salivation, (3) swallowing, (4) handwriting, (5) cutting food and handling utensils (with or without gastrostomy), (6) dressing and hygiene, (7) turning in bed and adjusting bed clothes, (8) walking, (9) climbing stairs, and (10) breathing. Each task is rated on a 5-point scale from 0 = can't do, to 4 = normal ability. Individual item scores are summed to produce a reported total score of between 0 and 40 (worst to best). ALSFRS has been revised to ALSFRS-R, which includes 12 questions (ALSFRS Q10 changes to (10) Dyspnea, (11) Orthopnea, and (12) Respiratory insufficiency), with individual item scores summed to a score between 0 and 48.

In order to eliminate any bias from the treatment effects on the ALSFRS, only the first observation at the time of trial is selected. If the first observation is missing at the time of trial (or prior), the next non-missing observation is selected to be included in the final analysis.

We subsequently mapped ALSFRS scores into GBD severities, and sequelae into different combinations of speech problems, chronic obstructive pulmonary disease, and motor impairment using the following logic:

Motor impairment

The ALSFRS assess motor function of the legs through questions on walking (Q8) and stair climbing (Q9).

Combined score	Severity level
8	None
5-7	Mild
2-4	Moderate
0-1	Severe

The ALSFRS also assesses motor impairment through questions on handwriting (Q4), cutting food and handling utensils (Q5), and dressing and hygiene (Q6).

Combined score	Severity level
12	None
9-11	Mild

3-8	Moderate
0-2	Severe

After determining case severity on these two separate metrics, we aggregate by taking the most severe ranking (eg, severe + mild = a severe case).

Respiratory problems:

Question 10 of the ALSFRS describes breathing difficulty as a function of MND.

ALSFRS score	Description	Severity level
4	Normal	None
3	Shortness of breath with minimal exertion	Mild
2	Shortness of breath at rest	Moderate
0-1	Intermittent ventilator assistance required/ventilator-dependent	Severe

Speech problems

Speech impairment due to MND is derived from ALSFRS question 1, which describes speech impediments. A score of 4 on this question denotes no impairment, while all other values suggest some impairment.

Creating sequelae

After determining the severity status of each case for the three symptom umbrellas, we subsequently estimated the relative proportion of each combination of symptom class and their respective severities. Those without any symptoms (eg, no severity) were categorised as having worry about the diagnosis for disability estimation. The following table displays the various sequelae and their associated proportions.

Sequela	Proportion (Mean)	Proportion (Lower)	Proportion (Upper)
Mild motor impairment, mild respiratory problems and speech problems due to motor neuron disease	0.01779	0.01658	0.01909
Mild motor impairment, moderate respiratory problems and speech problems due to motor neuron disease	0.00270	0.00225	0.00324
Mild motor impairment, severe respiratory problems and speech problems due to motor neuron disease	0.00082	0.00059	0.00113
Mild motor impairment, and speech problems due to motor neuron disease	0.02052	0.01922	0.02190
Moderate motor impairment, mild respiratory problems and speech problems due to motor neuron disease	0.03377	0.03210	0.03552
Moderate motor impairment, moderate respiratory problems and speech problems due to motor neuron disease	0.00715	0.00640	0.00799
Moderate motor impairment, severe respiratory problems and speech problems due to motor neuron disease	0.00286	0.00240	0.00342

Moderate motor impairment, and speech problems due to motor neuron disease	0.03041	0.02883	0.03208
Severe motor impairment, mild respiratory problems and speech problems due to motor neuron disease	0.05242	0.05035	0.05457
Severe motor impairment, moderate respiratory problems and speech problems due to motor neuron disease	0.02247	0.02111	0.02392
Severe motor impairment, severe respiratory problems and speech problems due to motor neuron disease	0.01365	0.01259	0.01479
Severe motor impairment and speech problems due to motor neuron disease	0.04765	0.04567	0.04970
Mild respiratory problems and speech problems due to motor neuron disease	0.01157	0.01060	0.01263
Moderate respiratory problems and speech problems due to motor neuron disease	0.00142	0.00111	0.00182
Severe respiratory problems and speech problems due to motor neuron disease	0.00023	0.00013	0.00043
Speech problems due to motor neuron disease	0.02457	0.02315	0.02608
Mild motor impairment and mild respiratory problems due to motor neuron disease	0.02245	0.02109	0.02389
Mild motor impairment and moderate respiratory problems due to motor neuron disease	0.00275	0.00230	0.00329
Mild motor impairment and severe respiratory problems due to motor neuron disease	0.00068	0.00047	0.00097
Mild motor impairment due to motor neuron disease	0.10388	0.10103	0.10681
Moderate motor impairment and mild respiratory problems due to motor neuron disease	0.06744	0.06511	0.06985
Moderate motor impairment and moderate respiratory problems due to motor neuron disease	0.01302	0.01199	0.01413
Moderate motor impairment and severe respiratory problems due to motor neuron disease	0.00412	0.00356	0.00477
Moderate motor impairment due to motor neuron disease	0.20136	0.19760	0.20518
Severe motor impairment and mild respiratory problems due to motor neuron disease	0.06902	0.06666	0.07146
Severe motor impairment and moderate respiratory problems due to motor neuron disease	0.02000	0.01872	0.02137
Severe motor impairment and severe respiratory problems due to motor neuron disease	0.01062	0.00969	0.01163
Severe motor impairment due to motor neuron disease	0.15037	0.14702	0.15378
Mild respiratory problems due to motor neuron disease	0.00643	0.00571	0.00723
Moderate respiratory problems due to motor neuron disease	0.00044	0.00028	0.00069
Severe respiratory problems due to motor neuron disease	0.00005	0.00001	0.00017
Asymptomatic, but worry about diagnosis due to motor neuron disease	0.03738	0.03562	0.03921

To determine disability due to these sequelae, we use the standard multiplicative aggregation formula as described in the main text. The following table provides description and disability weight assigned to the sequelae as appropriate.

Symptom group	Severity level	Lay description	DW (95%)
Respiratory problems	Asymptomatic		
Respiratory problems	Mild	Has cough and shortness of breath after heavy physical activity, but is able to walk long distances and climb stairs.	0.019 (0.011–0.033)
Respiratory problems	Moderate	Has cough, wheezing, and shortness of breath, even after light physical activity. The person feels tired and can walk only short distances or climb only a few stairs.	0.225 (0.153–0.31)
Respiratory problems	Severe	Has cough, wheezing, and shortness of breath all the time. The person has great difficulty walking even short distances or climbing any stairs, feels tired when at rest, and is anxious.	0.408 (0.273–0.556)
Motor impairment	Asymptomatic		
Motor impairment	Mild	Has some difficulty in moving around but is able to walk without help.	0.01 (0.005–0.019)
Motor impairment	Moderate	Has some difficulty in moving around and difficulty in lifting and holding objects, dressing, and sitting upright, but is able to walk without help.	0.061 (0.04–0.089)
Motor impairment	Severe	Is unable to move around without help, and is not able to lift or hold objects, get dressed, or sit upright.	0.402 (0.268–0.545)
Speech problems	No		
Speech problems	Yes	Has difficulty speaking, and others find it difficult to understand.	0.051 (0.032–0.078)
Asymptomatic, but worry	Yes	Has a disease diagnosis that causes some worry but minimal interference with daily activities.	0.012 (0.006–0.023)

Headaches

Flowchart

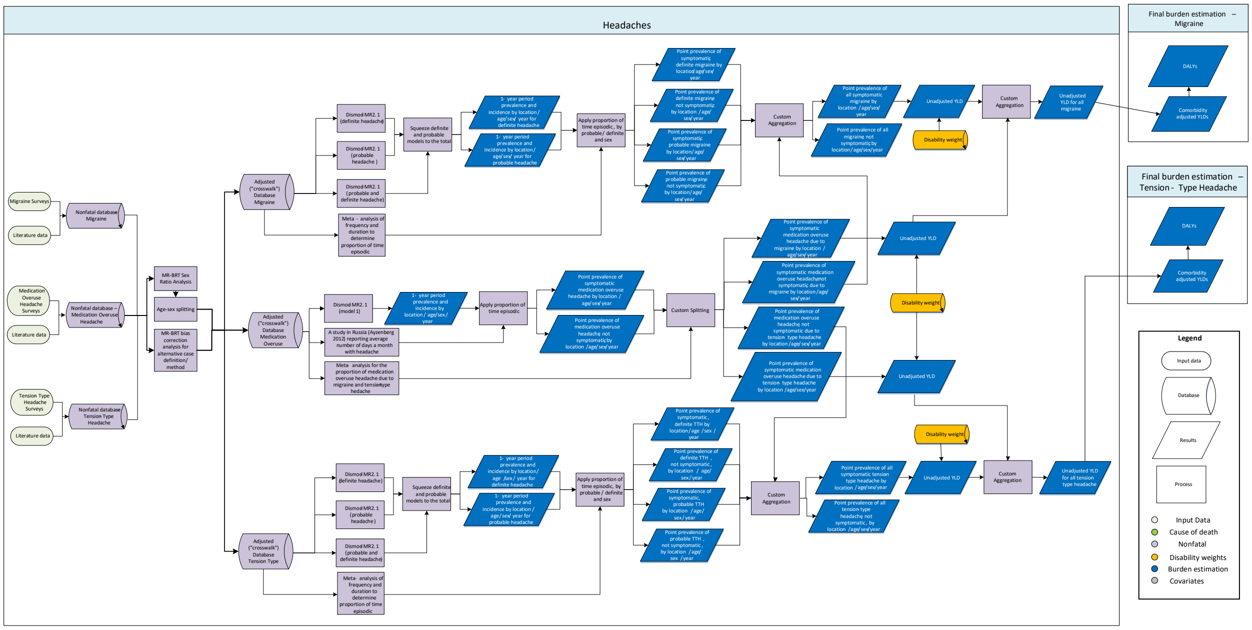

Input Data and Methodological Summary for Headaches

Case definition

Migraine

Migraine is a disabling primary headache disorder, typically characterised by recurrent moderate or severe unilateral pulsatile headaches. The two major types are migraine without aura and migraine with aura (transient neurological symptoms). In GBD, we do not distinguish between migraine with and without aura as most epidemiological studies report on overall migraine only. The reference diagnostic criteria for migraine are from the International Classification of Headache Disorders (ICHD)-3, which describe five criteria:

1. At least five attacks fulfilling criteria 2-5
2. Headache attacks lasting 4-72 hr (untreated or unsuccessfully treated)
3. Headache has at least two of the following four characteristics:
    a. Unilateral location
    b. Pulsating quality
    c. Moderate or severe pain intensity
    d. Aggravation by or causing avoidance of routine physical activity
4. During headache at least one of the following:
    a. Nausea and/or vomiting
    b. Photophobia and phonophobia
5. Not better accounted for by another ICHD-3 diagnosis

Definite migraine is headache that satisfies all the criteria outlined above, while probable migraine satisfies all of the above criteria except one. Studies that have looked at the reasons for cases with probable headache not fulfilling criteria definite diagnosis have suggested that most often it is the duration criterion that is left unfilled.[1,2,3,4,5] Before GBD 2017 we did not distinguish between probable and definite migraine. Since GBD 2017 we accounted for the varying case definitions used by different sources.

Tension-type headache

Tension-type headache (TTH) is characterised by a dull, non-pulsatile, diffuse, band-like (or vice-like) pain of mild to moderate intensity in the head or neck. The reference diagnostic criteria for tension-type headache are from the ICHD-3, which describe five criteria:

1. At least 10 attacks fulfilling criteria 2-5
2. Lasing from 30 minutes to 7 days
3. At least two of the following four characteristics:
    a. Bilateral location
    b. Pressing or tightening (non-pulsating) quality
    c. Mild or moderate intensity
    d. Not aggravated by routine physical activity such as walking or climbing stairs
4. Both of the following:
    a. No nausea or vomiting
    b. No more than one of photophobia or phonophobia
5. Not better accounted for by another ICHD-3 diagnosis

Definite tension-type headache is headache that satisfies all criteria outlined above, while probable tension-type headache satisfies all of the above criteria except one. Before GBD 2017 we did not distinguish between probable and definite tension-type headache. Since GBD 2017 we have accounted for varying case definitions used by different sources.

Medication overuse headache

Both migraine and tension-type headache can give rise to medication overuse headache (MOH), with the following International Classification of Headache Disorders (ICHD-3) diagnostic criteria:

1. Headache occurring ≥15 days/month in a patient with a pre-existing headache disorder
2. Regular overuse for >3 months of one or more drugs that can be taken for acute and/or symptomatic treatment of headache
3. Not better accounted for by another ICHD-3 diagnosis.

[1] Kim B-K, Chung YK, Kim J-M, Lee K-S, Chu MK. Prevalence, clinical characteristics and disability of migraine and probable migraine: A nationwide population-based survey in Korea. *Cephalalgia* 2013; **33**: 1106–16.

[2] Lantéri-Minet M, Valade D, Géraud G, Chautard M, Lucas C. Migraine and probable migraine – results of FRAMIG 3, a French nationwide survey carried out according to the 2004 IHS classification. *Cephalalgia*; **25**: 1146–58.

[3] Pfaffenrath V, Fendrich K, Vennemann M, *et al.* Regional variations in the prevalence of migraine and tension-type headache applying the new IHS criteria: the German DMKG Headache Study. *Cephalalgia*; **29**: 48–57.

[4] Rasmussen BK, Jensen R, Olesen J. A Population-Based Analysis of the Diagnostic Criteria of the International Headache Society. *Cephalalgia* 1991; **11**: 129–34.

[5] Fendrich K, Vennemann M, Pfaffenrath V, *et al.* Headache Prevalence Among Adolescents — The German DMKG Headache Study. *Cephalalgia* 2007; **27**: 347–54.

ICHD-3 explicitly states that, when a person fulfils criteria for both migraine and MOH, both diagnoses should be given. However, our GBD headache collaborators, Steiner and Stovner, say that in survey practice, a screening question on chronic headache is used first, followed by questions to determine if medication overuse is present. This means the diagnoses of migraine and MOH become mutually exclusive (obviating any potential problem of double-counting).

Input data

Migraine

We last conducted a systematic review of migraine for GBD 2017, which covered papers published through September 2017. The search string for this review was ((((("migraine disorders"[MeSH Terms] OR migraine[All Fields]) AND ((prevalence[Title/Abstract] OR incidence[Title/Abstract] OR remission[Title/Abstract] OR epidemiology[Title/Abstract]))))))).

Inclusion criteria of the systematic reviews were:

- Representative, population-based surveys
- Reporting of prevalence of migraine headache

In GBD 2017 we decided to exclude medical claims data as the adjustment needed make the claims data comparable to population representative surveys was unstable.

Tension-type headache

We last conducted a systematic review of TTH for GBD 2017, which covered papers published through September 2017. The search string for this review was ((((("headache"[MeSH Terms]) OR ("headache"[Title/Abstract] AND "tension"[Title/Abstract])) AND ("epidemiology"[Title/Abstract] OR "prevalence"[Title/Abstract] OR "incidence"[Title/Abstract] OR "remission"[Title/Abstract])))).

Inclusion criteria of the systematic reviews were:

- Representative, population-based surveys
- Reporting of prevalence of TTH headache

In GDB 2017 we decided to exclude medical claims data, as the adjustment needed make the claims data comparable to population representative surveys was unstable.

Medication overuse headache

We last conducted a systematic review of MOH for GBD 2017, which covered papers published through September 2017. The search string for this review was (("headache"[MeSH Terms] OR "headache"[Title/Abstract]) AND ("pharmaceutical preparations"[MeSH Terms] OR "pharmaceutical preparations"[Title/Abstract] OR "medication"[Title/Abstract]) AND ("epidemiology"[Title/Abstract] OR "prevalence"[Title/Abstract] OR "incidence"[Title/Abstract] OR "remission"[Title/Abstract])).

Inclusion criteria of the systematic reviews were:

- Representative, population-based surveys
- Reporting of prevalence of MOH headache

Table 1: Data inputs

Cause Name	Measure	Total sources	Countries with data
Tension-type headache	All measures	64	36
Tension-type headache	Prevalence	64	36
Headache disorders	All measures	153	52
Headache disorders	Prevalence	143	49
Headache disorders	Incidence	4	4
Headache disorders	Remission	7	5
Headache disorders	Proportion	1	19

Age and sex splitting

Reported estimates of prevalence were split by age and sex where possible. First, if studies reported prevalence for broad age groups by sex (eg, prevalence in 15- to 65-year-old males and females separately), and also by specific age groups but for both sexes combined (eg, prevalence in 15- to 30-year-olds, then in 31- to 65-year-olds, for males and females combined), age-specific estimates were split by sex using the reported sex ratio and bounds of uncertainty. Second, prevalence data for both sexes that could not be split using a within-study ratio were split using a sex ratio derived from a meta-analysis of existing sex-specific data using MR-BRT. The female to male ratio was 1.90 (1.85 to 1.96). Finally, after the application of bias adjustments, where studies reported estimates across age groups spanning 25 years or more, these were split into five-year age groups using the prevalence age pattern estimated by the best DisMod-MR 2.1 for each headache type from GBD 2017.

Data adjustment (Bias adjustments)

We used a list of binary adjustment criteria which are a modified version of quality indicators of epidemiological studies on headache (Steiner TJ, Stovner LJ et al [2013]. Improving quality in population surveys of headache prevalence, burden, and cost: key methodological considerations. J Headache Pain, 14: 87) and shown in the table below.

Table 2: Study Covariates

Study covariate	Notation	
	Less desirable (1)	Reference (zero)
Other than one-year recall period	Point prevalence	One-year prevalence
Not representative	Selected population	General population or community-based sample from whole country OR general population or community-based sample from defined region within a country, or school-based (for children)
Low-quality sampling method	Not stated OR no (or failed) attempt to secure representativeness	Total defined population, or random sample corrected for population demographics OR random sample uncorrected for population demographics

Poor response	Not stated, or <70%	70–100%
Low-quality survey method and type of interviewer	Not stated OR self-administered (unsupervised) questionnaire OR telephone or face-to-face interview by untrained or unspecified interviewer(s)	Face-to-face interview with headache expert or trained interviewer
Low-quality validation of diagnostic instrument	Instrument not specified or not validated OR validated, but sensitivity and/or specificity <70% OR validated only in screen-positive sub-sample, or in clinic or unspecified sample, but sensitivity and specificity ≥70%	Validated in target population or similar, and sensitivity and specificity ≥70%, or all diagnoses made in face-to-face or telephone interviews by headache expert
Low-quality diagnostic criteria	Not stated OR stated, other than ICHD OR ICHD (or reasonable modification)	ICHD (or reasonable modification)

We also adjusted data reported in studies that were conducted in a school setting. Studies based on lifetime recall of headaches were not included because of the concern of recall bias. For migraine and tension-type headache, we additionally marked studies where the type of headache (probable/definite) was not explicitly mentioned in the report but was determined based on the diagnostic criteria stated.

The mean and standard error for the coefficients were calculated using the MR-BRT adjustment method. All study covariates were initially evaluated independently for each of the three types of headache. However, covariate values varied not only in magnitude but in direction across the three headache types. Because we assume that the same study covariate should adjust data at least in the same direction for all headache types, the final study covariates were evaluated taking all migraine, tension-type, and medication overuse headache data into account. Studies conducting in a school setting remained in the models but were no longer adjusted in this round of the GBD, as we were unable to find matches to inform a reliable crosswalk. The school setting covariate should be re-tested in a future round of the GBD in which new data has been added to better inform the adjustment factor. These studies were not excluded because the headache models are relatively data sparse. Betas and inverse-logit values for these covariates are shown in the table below:

Table 3: MR-BRT Crosswalk Adjustment Factors for Headaches

Data input	Reference or alternative case definition	Gamma	Beta Coefficient, Logit (95% CI)	Adjustment factor*
Other than one-year recall	Alt	1.20	-0.89 (-0.97 to -0.80)	0.30 (0.28 to 0.31)
Not representative	Alt		-0.39 (-0.45 to -0.33)	0.40 (0.39 to 0.42)
Low-quality sampling method	Alt		0.73 (0.66 to 0.79)	0.67 (0.66 to 0.69)

Poor response	Alt		-0.45 (-0.53 to -0.36)	0.40 (0.37 to 0.41)
Low-quality survey method	Alt		-0.22 (-0.31 to -0.13)	0.45 (0.42 to 0.47)
Low-quality diagnostic instrument	Alt		0.15 (0.13 to 0.19)	0.54 (0.53 to 0.55)
Low-quality diagnostic criteria	Alt		-0.37 (-0.43 to -0.32)	0.41 (0.39 to 0.42)
Headache type assumed	Alt		0.37 (0.33 to 0.42)	0.59 (0.58 to 0.60)

**Adjustment factor is the transformed Beta coefficient in normal space, and can be interpreted as the factor by which the alternative case definition is adjusted to reflect what it would have been if measured as the reference.*

Modeling strategy

As in GBD 2017, standard DisMod settings across all headache models include setting excess mortality to 0, and assuming that there was no incidence or prevalence before the age of 5 years.

Migraine

We made no substantive changes in the modeling strategy of migraine from GBD 2017. As in the last round, we ran separate DisMod models for definite migraine, probable migraine, and the total migraine category and set an upper bound on remission of 0.1 across all models. After running the separate models, we then scaled the results of probable and definite headache to the total headache envelope to ensure consistency.

Because some data sources, especially earlier data from before ICHD became the standard (the initial criteria were published in 1988), largely report on definite migraine, we also adjusted studies that reported only on definite migraine to the total migraine category in order to better inform that model. All data that reported on both definite and total migraine were used in regression models by sex in order to derive an age- and sex-specific adjustment. The adjustment is shown in the graphs below.

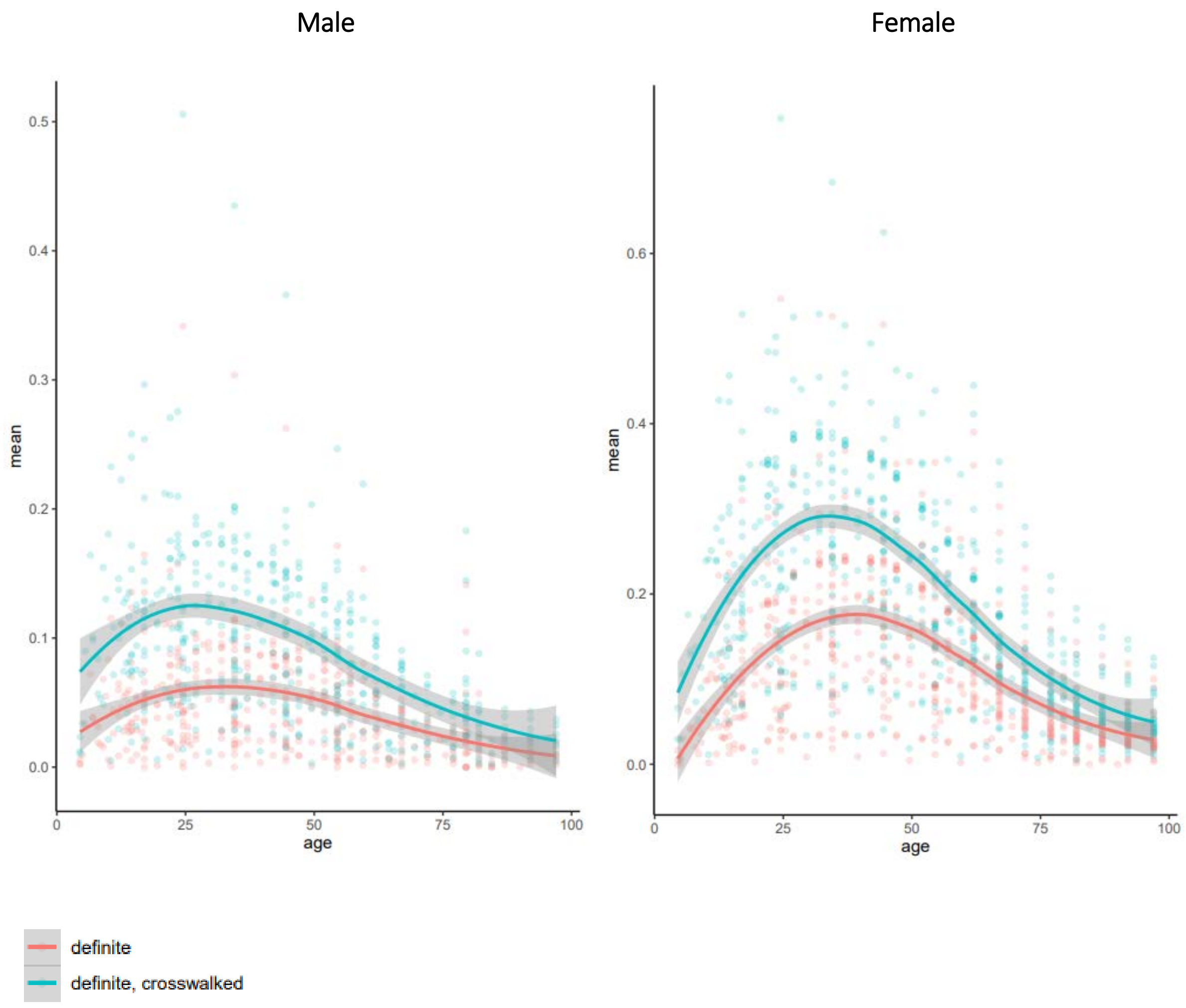

In GBD 2017, to determine the proportion of time over a year spent with migraine headache ("time symptomatic"), we performed a meta-analysis on the frequency and duration of definite headache and total headache combined using the "metafor" package in R. There were not enough data available to obtain reliable estimates on the frequency and duration of probable headache from the literature. As the proportion of time symptomatic for the total migraine category is the weighted average of time symptomatic for definite and probable migraine, weighted by the prevalence of each headache type, the proportion of time symptomatic for probable migraine was calculated as shown below.

$$Time\ Symp_{Probable} = \frac{Time\ Symp_{Total}\ -\ Prevalence_{Definite} * Time\ \ Symp_{Definite}}{Prevalence_{Probable}}$$

For GBD 2019, we used new multi-country survey unit-record data from 19 countries in the Lift the Burden survey series provided by our collaborators on the time symptomatic of various headache types. This source provided greater granularity of time symptomatic data, as we had used summary measures from survey reports instead of microdata in the past. This source also provided data on probable, definite, and total migraine, eliminating the need to back calculate time symptomatic for probable migraine. Using the MR-BRT regression method, we calculated the proportion of time symptomatic is 0.093 for definite migraine and 0.066 for probable migraine.

Tension-type headache

In GBD 2017, we ran a single model for total tension-type headache. For this round of the GBD, we replicated the modeling process for migraine headache and ran separate DisMod models for definite TTH, probable TTH, and the total TTH category, setting an upper bound on remission of 0.5 across all models. After running the separate models, we then scaled the results of probable and definite headache to the total headache envelope to ensure consistency.

Because some data sources, especially earlier data from before ICHD became the standard (the initial criteria were published in 1988), largely report on definite TTH, we also adjusted studies that reported only on definite TTH to the total TTH category in order to better inform that model. Initially, all data that reported on both definite and total TTH were used in regression models by sex in order to derive an age- and sex-specific adjustment. These sex-specific models resulted in an implausible age pattern for females such that the age-pattern of the age-split data points was the inverse of the original data. Consequently, we ran a regression model to derive an age-specific adjustment that was applied to both sexes. The adjustment is shown in the graphs below.

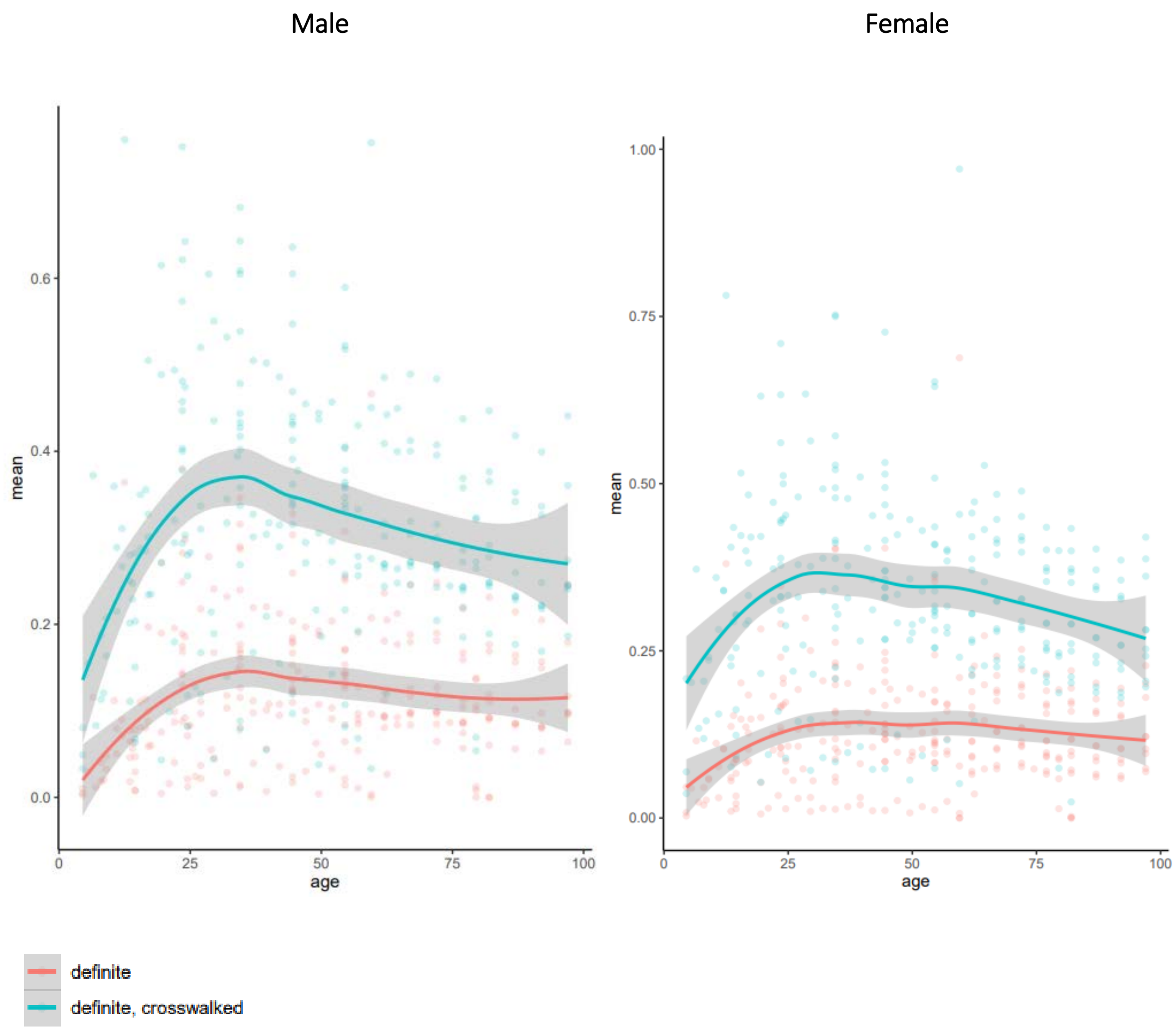

In GBD 2017, a single value derived from a meta-analysis of seven studies on the frequency of the total tension-type headache category was applied to the total TTH model. For GBD 2019 we used the results from the same meta-analysis of Lift the Burden unit-record data on the time symptomatic of headache, which also reported estimates for probable, definite, and total TTH. Using MR-BRT, we calculated the proportion of time symptomatic is 0.029 for definite TTH and 0.0.021 for probable TTH.

Medication overuse headache

Prior settings in the DisMod model included an upper bound on remission of 0.4. In GDB 2017, to determine the proportion of time over a year spent with medication overuse headache, we meta-analysed the two available studies on frequency and used the one available study on duration. The result of the meta-analysis on frequency gave an estimate of 250.83 attacks per year, and the available source on duration estimated an average duration of 18.59 hours. From this data we estimated that the proportion of time symptomatic for medication overuse headache was 0.532. We made no substantive changes in the modeling strategy from GBD 2017.

Medication overuse headache split

As medication overuse headache can develop from migraine or tension-type headache, we split medication overuse into sequelae of both primary headache disorders. Based on a 2017 meta-analysis of three sources, 73.2% (63.7–81.0) of medication-overuse headache is assigned to medication overuse headache due to tension-type headache. The forest plot is shown below.

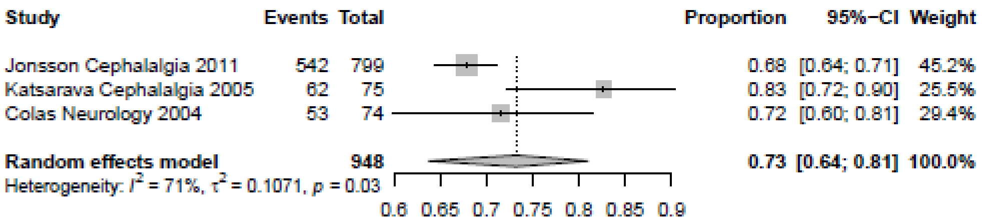

Other neurological disorders

In addition to the neurological disorders described above, there are many diverse types of neurological disorders with a range of severities and associated sequelae. Because these neurological disorders are diverse in their underlying causes and risk factors as well as in their associated health outcomes, modelling them together in a DisMod-MR model would not produce reliable estimates of prevalence or excess mortality. Instead, we calculated the YLDs caused by neurological disorders directly using a YLD/YLL ratio.

We calculated the ratio of YLDs to YLLs across the specified neurological disorders for which non-fatal outcomes were modelled, using YLL estimates from the GBD 2019 cause of death (CoD) analysis. We then multiplied this YLD/YLL ratio by the YLL estimates for other neurological disorders from the GBD 2019 CoD analysis, providing us with an estimate of the YLDs associated with other neurological disorders.

Schizophrenia

Flowchart

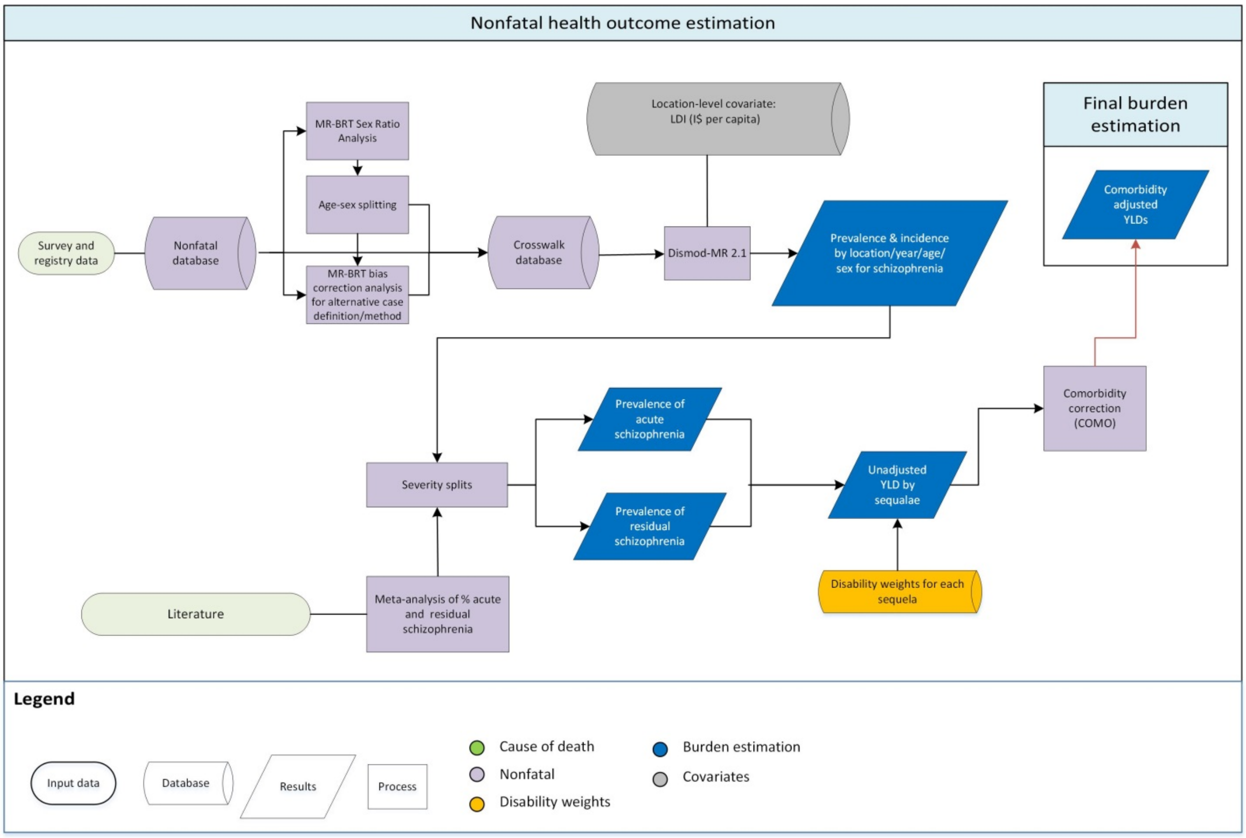

Input Data and Methodological Summary for Schizophrenia

Case definition

Schizophrenia is a chronic psychotic disorder which involves the experience of positive symptoms (e.g., delusions, hallucinations, thought disorder) and negative symptoms (e.g., flat affect, loss of interest, and emotional withdrawal). Included in the GBD disease modelling were cases meeting the Diagnostic and Statistical Manual of Mental Disorders (DSM) or the International Classification of Diseases (ICD) diagnostic criteria for schizophrenia (DSM-IV-TR: 295.10-295.30, 295.60, 295.90; ICD 10: F20)[1, 2.]. Diagnostic criteria are:

A. Two (or more) of the following, each present for a significant portion of time during a one-month period (or less if successfully treated): i) delusions, ii) hallucinations, iii) disorganised speech, iv) grossly disorganised or catatonic behavior, v) negative symptoms

B. Social/occupational dysfunction

C. Continuous signs of the disturbance persist for at least 6 months

D. Exclusions must be met for schizo-affective and mood disorders, substance and general medical conditions, and a relationship to a pervasive development disorder

Input data

The epidemiological systematic review for schizophrenia was conducted in three stages involving electronic searches of the peer-reviewed literature (i.e., PsycInfo, Embase, and PubMed), the grey literature, and expert consultation. For mental disorders, we update our GBD electronic database searches on a two year rolling basis. A systematic review update for schizophrenia was conducted for GBD 2017 [3] , with the next

literature update due for the next round of GBD. The grey literature, and expert consultation was conducted for GBD 2019 and produced new data sources. Consultation with GBD collaborators allowed us to include a large number of studies from Iranian journals which are typically not indexed in the electronic databases searched.

The GBD inclusion criteria stipulated that: (1) the publication year must be from 1980 onward; (2) "caseness" must be based on clinical threshold as established by the DSM or ICD; (3) sufficient information must be provided on study method and sample characteristics to assess the quality of the study; and (4) study samples must be representative of the general population (i.e., inpatient or pharmacological treatment samples, case studies, veterans, or refugee samples were excluded). No limitation was set on the language of publication. Table 1 below summarizes data inputs by parameter for schizophrenia.

Table 1: Data Inputs for schizophrenia morbidity modelling by parameter.

Measure	Total sources
All measures	203
Prevalence	142
Incidence	16
Remission	8
Relative risk	9
Standardized mortality ratio	34
With-condition mortality rate	5
Proportion	1

Age and sex splitting

The extracted data, where possible, underwent three types of age and sex splitting processes:

1. Estimates were further split by sex and age based on the data that was available. For instance, if studies reported prevalence for broad age groups by sex (e.g., prevalence in 15 to 65 year old males and females separately), and also by specific age groups but for both sexes combined (e.g., prevalence in 15- to 30-year-olds, then in 31- to 65-year-olds, for males and females combined); age-specific estimates were split by sex using the reported sex ratio and bounds of uncertainty.
2. A Meta-Regression with Bayesian priors, Regularization, and Trimming (MR-BRT) analysis was used to split the remaining both-sex estimates in the dataset. For each parameter, sex specific estimates were matched by location, age, year and a MR-BRT regression analysis was used to estimate pooled sex ratios and bounds of uncertainty. These were then used to split the both sex estimates in the dataset. The male: female prevalence ratio estimated was 1.17 (95% uncertainty interval [UI]: 0.60 – 1.75).
3. Studies reporting prevalence estimates across age groups spanning 25 years or more, were split into five-year age groups using the prevalence age pattern estimated by DisMod-MR 2.1. The DisMod-MR model used to estimate the age pattern did not contain any previously age split data.

Bias corrections / Crosswalks

Estimates with known and significant biases are typically adjusted / crosswalked prior to DisMod-MR 2.1. For schizophrenia, tested adjustments (e.g., the difference between 12-month vs point prevalence, or between registry- and community-based samples) failed to demonstrate significance, resulting in a model without the inclusion of adjustments.

Severity splits and disability weights

The GBD disability weight survey assessments are lay descriptions of sequelae highlighting major functional consequences and symptoms. The lay descriptions and disability weights for schizophrenia severity levels are shown in Table 2. Severity splits used in GBD 2019 were consistent with those used in GBD 2017 for schizophrenia. Information on the distribution of acute and residual states of schizophrenia was obtained from a separate systematic review of the literature[4] .Meta-XL (a Microsoft Excel add-in for meta-analysis) was used to pool estimates across all studies to calculate the overall proportion of schizophrenia cases in each health state acute 63% (29% – 91%) and residual state 37% (9% – 71%).

Table 2. Severity distribution for Schizophrenia in GBD 2019 and the associated disability weight (DW) with that severity.

Severity level	Lay description	DW (95% UI)
acute state	Hears and sees things that are not real and is afraid, confused, and sometimes violent. The person has great difficulty with communication and daily activities, and sometimes wants to harm or kill himself (or herself).	0.778(0.606 – 0.9)
residual state	Hears and sees things that are not real and has trouble communicating. The person can be forgetful, has difficulty with daily activities, and thinks about hurting himself (or herself).	0.588(0.411 – 0.754)

Modeling strategy

After the above data processes were applied, DisMod MR 2.1 was used to model the epidemiological data for Schizophrenia. The DisMod-MR modeling strategy for schizophrenia followed the standard GBD 2019 decomposition structure. At each decomposition step, we compared the new model against the GBD 2017 best model and the best model from the previous step. All substantial changes between models were explored and explained. Adjustments to model priors or the dataset were made where appropriate. Where outliers were identified in the data, we re-assessed the study's methodology and quality before a decision was made to exclude or include the data.

Data across all epidemiological parameters were initially included in the modelling process. We assumed no incidence before age 10 and after age 80. This minimum age of onset was corroborated with expert feedback and existing literature on schizophrenia. Remission was also restricted to a maximum of 0.04 as guided by data available in the dataset.

Location-level covariates were used to inform the estimation of prevalence in locations with no available data. For schizophrenia, one location-level covariate, lag distributed income (LDI), was used. This covariate represents a moving average of gross domestic product (GDP) over time. LDI was applied to excess mortality data with a negative relationship assumed. Table 3 below illustrates the covariate, parameter, beta and exponentiated beta values for the model.

Table 3. Summary of covariates used in the Schizophrenia DisMod-MR meta-regression model

Covariate	Type	Parameter	Exponentiated beta (95% UI)
LDI	Location-level	Excess mortality rate	0.58 (0.37 – 0.90)

Changes between GBD 2017 and GBD 2019

There were three main changes in the GBD 2019 modelling strategy compared to GBD 2017:

1. In GBD 2019 we updated the age splitting by regional pattern methodology by increasing the age threshold for splitting to 25 years (in GBD 2017 it was 20 years). This meant that there were fewer

estimates eligible for age splitting in this way. Previous age split estimates were on average lower than the global mean leading to an upward shift in prevalence in locations which now had fewer age-split estimates informing prevalence estimation.

4. In GBD 2017 sex ratios were estimated by DisMod-MR as part of the prevalence modelling. In GBD 2019 we conducted a MR-BRT analysis instead. The prevalence male: female ratio remained relative consistent from 1.02 (0.96 – 1.08) in GBD 2017 to 1.17 (0.60 – 1.75) in GBD 2019.
2. In GBD 2019 we included new epidemiological data from 22 locations which further informed the DisMod-MR model.

While we continue to improve on the data and methods used to estimate the burden of mental disorders, some challenges need to be acknowledged. Firstly, we still have a large number of locations with no high-quality raw data available. Secondly, it is difficult to quantify and remove all variation due to measurement error in our prevalence estimates. Whilst we have improved the methodology used to account for known sources of bias (e.g., survey methods or case definitions), we still have very few data points to inform such adjustments. Additionally, there is a paucity of research on the risk factors of mental disorders which can be used as predictive covariates in our epidemiological models.

References

1. American Psychiatric Association. Diagnostic and Statistical Manual of Mental Disorders (DSM-IV-TR). Fourth Edition, Text Revision ed. Washington DC: American Psychiatric Association; 2000.
2. World Health Organization. The ICD-10 Classification of Mental and Behavioural Disorders. Clinical descriptions and diagnostic guidelines. Geneva: World Health Organization; 1992.
3. Charlson FJ, Ferrari AJ, Santomauro DF, Diminic S, Stockings E, Scott JG, et al. Global epidemiology and burden of schizophrenia: findings from the global burden of disease study 2016. Schizophrenia bulletin. 2018;44(6):1195-203.
4. Ferrari AJ, Saha S, McGrath JJ, Norman R, Baxter AJ, Vos T, et al. Health states for schizophrenia and bipolar disorder within the Global Burden of Disease 2010 Study. Population health metrics. 2012;10(1):16.

Major Depressive Disorder

Flowchart

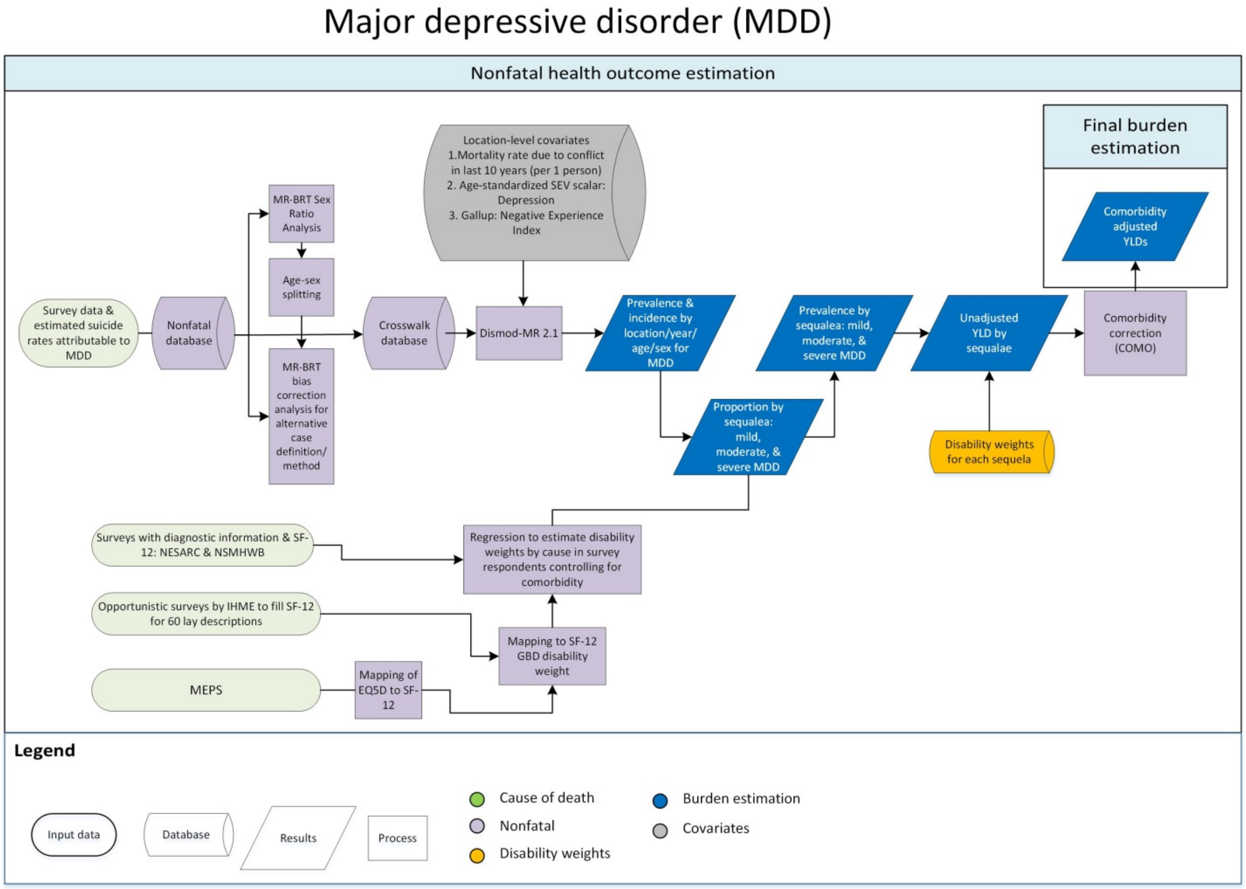

Input Data and Methodological Summary for major depressive disorder

Case Definition

Major depressive disorder (MDD) is an episodic mood disorder involving the experience of one or more major depressive episode(s). Included in the GBD disease modelling were cases meeting diagnostic criteria for MDD according to the Diagnostic and Statistical Manual of Mental Disorders (DSM) or the equivalent diagnosis of recurrent depression in the International Classification of Diseases (ICD).[1,2] These were identified by the following codes: DSM-IV-TR: 296.21–24, 296.31–34; ICD-10: F32.0–9, F33.0–9; excluding those cases due to a general medical condition or substance induced cases.[1,2]

According to DSM-IV-TR criteria, MDD involves the presence of at least one major depressive episode, which is the experience of either depressed mood or loss of interest/pleasure, for most of every day, for at least two weeks. This must represent a change from the person's baseline and impaired functioning observed across social, occupational, and educational domains.

- In addition to one of the two symptoms above, four out of the following nine criteria must also be met to make a diagnosis: change in eating, appetite, or weight
- excessive sleeping or insomnia

- agitated or slow motor activity
- fatigue
- feeling worthless or inappropriately guilty
- trouble concentrating; and
- repeated thoughts about death

MDD was modelled as an episodic disorder with the average length of a major depressive episode (i.e., duration) specified. This method has been discussed in greater detail in previous publications[3,4]

Input data

For mental disorders, we update our GBD electronic database searches on a two-year rolling basis. In GBD 2019 a systematic literature review update was conducted to identify new epidemiological studies on MDD published between September 2016 and December 2018. We included studies reporting the prevalence, incidence, remission, duration, and/or excess mortality associated with MDD. The search was conducted in three stages involving electronic searches of the peer-reviewed literature (i.e., PsycInfo, Embase, and PubMed), the grey literature, and expert consultation. The MDD systematic review was conducted in conjunction with the search for bipolar disorder and dysthymia as these disorders are frequently grouped together in publications. The following search terms were used to develop search strings across all databases searched: ‘dysthymia’, ‘bipolar’, ‘manic’, ‘mania’, ‘mood disorder’, ‘depressive disorder’, ‘bipolar disorder’, ‘dysthymic disorder’, and ‘prevalence’, ‘mortality’, ‘death’, ‘incidence’, ‘recurrence’, ‘remission’, ‘duration’, ‘epidemiology’.

The search generated 18,023 records (after duplicates were removed) across the three electronic databases. The title/abstract screening reduced the number of relevant records to 247 studies, of which 59 met criteria for inclusion for MDD. An additional 39 studies were identified and extracted through a grey literature search. Consultation with GBD collaborators allowed us to include 26 studies from Iranian journals which are typically not indexed in the electronic databases searched. Overall, in GBD 2019 we added 124 new studies into the major depressive disorders dataset.

The GBD inclusion criteria stipulated that: (1) the publication year must be from 1980 onward; (2) “caseness” must be based on clinical threshold as established by the DSM or ICD; (3) sufficient information must be provided on study method and sample characteristics to assess the quality of the study; and (4) study samples must be representative of the general population (i.e., inpatient or pharmacological treatment samples, case studies, veterans, or refugee samples were excluded). No limitation was set on the language of publication. Methods used for this systematic review have been reported in greater detail elsewhere[3]. Table 1 below summarizes data inputs by parameter for major depressive disorders.

Table 1: Data Inputs for Major depressive disorder morbidity modelling by parameter.

Measure	Total sources	Countries with data
All measures	517	111
Prevalence	492	111
Incidence	2	2
Relative risk	20	13
Standardized mortality ratio	2	1
Proportion	2	1

Age and sex splitting

The extracted data underwent three types of age and sex splitting processes:

1. Where possible, estimates were further split by sex and age based on the data that was available. For instance, if studies reported prevalence for broad age groups by sex (e.g., prevalence in 15 to 65 year old males and females separately), and also by specific age groups but for both sexes combined (e.g., prevalence in 15- to 30-year-olds, then in 31- to 65-year-olds, for males and females combined); age-specific estimates were split by sex using the reported sex ratio and bounds of uncertainty.

2. A Meta-Regression with Bayesian priors, Regularization, and Trimming (MR-BRT) analysis was used to split the remaining both-sex estimates in the dataset. For each parameter, sex-specific estimates were matched by location, mid-age, year and a MR-BRT network meta-analysis was used to estimate pooled sex ratios. Given evidence to suggest that the sex ratio in depression varies with age[5-7], we also tested for an age interaction in the model. We found that the sex difference in MDD decreased significantly with age i.e., prevalence in males (compared to females) increased significantly with increasing age. The global estimated all-age male: female prevalence ratio was 0.52 (95% uncertainty interval [UI]: 0.26 – 0.77) while Figure 1 shows the estimated male: female prevalence ratio by age. Age-specific sex ratios were used to split both sex estimates in the dataset.

Figure 1. Sex ratios by age for major depressive disorders

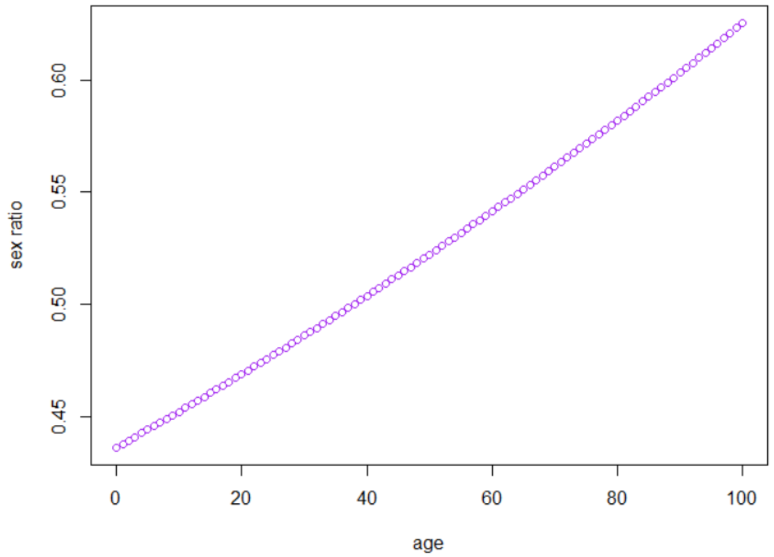

3. Studies reporting prevalence estimates across age groups spanning 25 years or more, were split into five-year age groups using the prevalence age pattern estimated by DisMod-MR 2.1. The DisMod-MR model used to estimate the age pattern did not contain any previously age split data.

Bias corrections / Crosswalks

Estimates with known biases were adjusted / crosswalked accordingly prior to DisMod-MR 2.1. For each crosswalk of interest, pairs of the reference and the alternative estimates were matched by age, sex, location and year. This was done for both within (where possible) and between study pairs. These pairs were then used as inputs in a MR-BRT network meta-analysis. The MR-BRT analysis produced a pooled ratio between the reference estimates and alternative estimates, which was used to adjust all alternative estimates in the dataset. Four adjustment ratios were used for MDD:

1. A past year recall ratio adjusted all data points derived from past year prevalence toward the level they would have been if the study had captured point/past-month prevalence. The latter prevalence period is less affected by recall bias.
2. A symptom scale ratio adjusted all data points derived using a symptom scale toward the level they would have been if the scale had strictly adhered to DSM or ICD thresholds for MDD.
3. A World Health Survey ratio adjusted all World Health Survey data downwards towards the level they would have been had the study strictly adhered to DSM or ICD thresholds for MDD. The World Health Surveys are surveys conducted by the World Health Organization in close to 70 countries. While these surveys capture useful information on the prevalence of depression, they make use of a symptom scale which does not fully meet DSM and ICD criteria for MDD. This adjustment works essentially in the same way as the previous symptom scale adjustment.
4. A lay interviewer ratio was used to adjust all prevalence estimates derived from trained lay-interviewers towards the level they would have been if the estimate was derived from clinically trained interviewers (i.e. psychologist or psychiatrist). We consider interviews conducted by

clinicians to be more sensitive to detecting cases of MDD, particularly in locations where western-based mental health case definitions and instruments are yet to be fully validated.

See Table 2 for adjustment factors used for MDD.

Table 2: MR-BRT Crosswalk Adjustment Factors for MDD

Data input	Reference or alternative case definition	Beta Coefficient, Log (95% UI)	Adjustment factor* (95% UI)	Gamma
Population Survey	Reference: Past month/point prevalence, from a diagnostic tool, administered by a clinician			0.43
Population Survey	Alternative: Past year prevalence	0.69 (-0.20 – 1.57)	1.99 (0.82 – 4.79)	
Population Survey	Alternative: Symptom scale	1.00 (0.10 – 1.88)	2.71 (1.11 – 6.56)	
Population Survey	Alternative: World Health Survey	0.68 (-0.22 – 1.57)	1.98 (0.80 – 4.83)	
Population Survey	Alternative: Lay-interviewer	-0.22 (-1.08 – 0.65)	0.79 (0.34 – 1.91)	

**Adjustment factor is the transformed Beta coefficient in normal space and can be interpreted as the factor by which the alternative case definition is adjusted to reflect what it would have been if measured as the reference.*

UIs incorporate Gamma which represents the between study variance across all input data in the model. This added uncertainty widens the UIs for crosswalks with significant fixed effects.

Attributable suicide estimates

Given that MDD is an established risk factor for suicide,[8] we supplemented the available data on excess mortality with estimated suicide rates (by age, sex, year, and location) attributable to MDD. These were estimated using GBD's comparative risk assessment methodology whereby the current health status was compared with a theoretical-minimum-risk exposure defined as the counterfactual status of the absence of MDD in the population. Population attributable fractions (PAFs) were estimated using this established formula:

$$PAF = \frac{p\ (RR-1)}{p\ (RR-1)+1}$$

P referred to the exposure distribution, which in this case was the DisMod-MR 2.1 prevalence rates of MDD by age, sex, location and year. RR referred to the pooled relative-risk of suicide due to MDD obtained from an existing systematic review and meta-analysis[8]. Age, sex, year, and location-specific PAFs were multiplied by their corresponding GBD suicide rate to estimate the proportion of suicide cases attributable to MDD. These were entered as cause-specific mortality rates in our epidemiological model for MDD.

Severity splits and disability weights

The GBD disability weight survey assessments are lay descriptions of sequelae highlighting major functional consequences and symptoms. The lay descriptions and disability weights for MDD severity levels are shown in Table 3. To determine the proportion of people with MDD within each of the severity levels, the US National Epidemiological Survey on Alcohol and Related Conditions (NESARC, conducted in two waves from 2001 to 2002 and 2004 to 2005)[9] and the Australian National Survey of Mental Health and Wellbeing of Adults (NSMHWB, conducted in 1997)[10] were used. The proportion of MDD cases falling within each severity level were as follows: asymptomatic 13% (10% – 17%), mild 59% (49% – 69%), moderate 17% (13% – 22%), and severe 10% (3% – 20%).

Table 3. Severity distribution for MDD in GBD 2019 and the associated disability weight (DW) with that severity.

Severity level	Lay description	DW (95% UI)
Mild	Feels persistent sadness and has lost interest in usual activities. The person sometimes sleeps badly, feels tired, or has trouble concentrating but still manages to function in daily life with extra effort.	0.145 (0.099 – 0.209)
Moderate	Has constant sadness and has lost interest in usual activities. The person has some difficulty in daily life, sleeps badly, has trouble concentrating, and sometimes thinks about harming himself (or herself).	0.396 (0.267 – 0.531)
Severe	Has overwhelming, constant sadness and cannot function in daily life. The person sometimes loses touch with reality and wants to harm or kill himself (or herself).	0.658 (0.477 – 0.807)

Modelling Strategy

After the above data processes were applied, DisMod MR 2.1 was used to model the epidemiological data for MDD. The DisMod-MR modeling strategy for MDD followed the standard GBD 2019 decomposition structure. At each decomposition step, we compared the new model against the GBD 2017 best model and the best model from the previous step. All substantial changes between models were explored and explained. Adjustments to model priors or the dataset were made where appropriate. Where outliers were identified in the data, we re-assessed the study's methodology and quality before a decision was made to exclude or include the data.

Data across all epidemiological parameters were initially included in the modelling process. However, given that the few incidence data points available typically excluded cases of MDD at baseline, new major depressive episodes in people with previous episodes were not counted and incidence was underestimated. For this reason, we chose to exclude all raw incidence data in the final model and instead allowed Dismod-MR to calculate incidence based on data from other parameters. We assumed no incidence and prevalence before age 3. This minimum age of onset was corroborated with expert feedback and existing MDD literature[3]. An average remission rate for a major depressive episode of 1.45 (1.3–1.6) was used. This was derived from the four longitudinal studies[11-14] fitting a lognormal curve with least squared differences to data on the proportion of incident cases still fulfilling the case definition for major depression at intervals over a one-year period. As data were only available for a follow-up of one year, a decision had to be made about the maximum allowable duration of an episode. Setting this at 40 years, the average duration implied by the lognormal fit was 0.65 (0.59–0.70) of a year[15].

The following location-level covariates were used to inform the estimation of prevalence in locations with no available data:

1. The mean war mortality rate in the previous 10 years: This covariate identified, for each GBD location, the mean mortality rate in the previous ten years due to war and terrorism. It was used given the existing evidence to show a positive association between conflict status and the prevalence of MDD[16,17].
2. An age-standardised SEV scalar: This made use of the fraction of MDD burden caused by its relevant risk factors combined to inform the estimation of prevalence. Intimate partner violence and childhood sexual violence are the two established risk factors of MDD for which attributable burden is estimated in GBD studies.
3. A Gallup negative experience index: The Gallup initiative conducts comprehensive and comparable national surveys across a wide range of countries worldwide[18]. This index measured respondents' past day experiences of physical pain, worry, sadness, stress and anger. The Gallup covariate was included as a means to test for a correlation between negative emotions at a location level and MDD prevalence. Data from the Gallup negative experience index was modelled using the Spatio-temporal Gaussian process regression (STGPR) to produce estimates for all years and locations required by DisMod-MR. The log of the modelled output was used as the covariate in DisMod-MR due to skewedness of the data. The relationship detected was in theexpected direction (i.e. the higher the negative emotion, the higher the prevalence rate) although the association with MDD prevalence was marginally positive.

A summary of covariates and exponentiated values for MDD are shown in Table 4.

Table 4. Summary of covariates used in the MDD DisMod-MR meta-regression model

Covariate	Type	Parameter	Exponentiated beta (95% UI)
Mean war mortality rate in the previous 10 years	Location-level	Prevalence	1.63 (1.07 – 2.53)
Log-transformed age-standardized SEV scalar: Depression	Location-level	Prevalence	3.27 (2.97 – 3.48)
Gallup: Negative experience index	Location-level	Prevalence	1.01 (1.00 — 1.04)

Changes between GBD 2017 and GBD 2019

There were six main changes in the GBD 2019 modelling strategy compared to GBD 2017:

1. In GBD 2019 we updated the age splitting by regional pattern methodology by increasing the age threshold for splitting to 25 years (in GBD 2017 it was 20 years). This meant that there were fewer estimates eligible for age splitting in this way. This impacted on the prevalence for some locations which now had fewer age-split estimates informing prevalence estimation.
2. In GBD 2017 bias corrections and sex ratios were estimated by DisMod-MR as part of the prevalence modelling. In GBD 2019 we conducted a MR-BRT analysis to accommodate for study heterogeneity and estimated pooled ratios with 95% UIs as previously discussed. Ratios estimated between 2017 and 2019 were largely consistent, although the UIs derived by MR-BRT tended to

be larger. MR-BRT UIs incorporate Gamma which represents the between study variance across all input data in the model. This added uncertainty widens the UIs for crosswalks with significant fixed effects.

   a. The prevalence male: female ratio was 0.61 (0.60 – 0.63) in GBD 2017 compared to 0.52 (0.26 – 0.77) in GBD 2019.
   b. The adjustment ratio for past year estimates was 1.96 (1.88 – 2.05) in GBD 2017 compared to 1.99 (0.82 – 4.79) in GBD 2019.
   c. The adjustment ratio for symptom scale estimates was 2.98 (2.82 – 3.15) in GBD 2017 compared to 2.71 (1.11 – 6.56) in GBD 2019, leading to slight overall increase in the adjusted prevalence.
   d. The adjustment factor World Health Survey was 2.31 (2.15 – 2.51) in GBD 2017 compared to 1.98 (0.80 – 4.83) in GBD 2019, leading to a slight overall increase in adjusted prevalence.

3. The GBD 2017 model included an adjustment ratio (as a study level covariate within DisMod-MR) for estimates derived from school surveys. This adjustment was excluded in GBD 2019. The school survey adjustment was used in GBD 2017 based on the premise that school surveys might not be representative of the general population, especially in less developed parts of the world. Estimates derived from school surveys were adjusted downwards by 1.54 (1.36 – 1.75) towards the level of estimates from general household surveys. Part of the new GBD 2019 MR-BRT approach was to assess the availability of data for a given study-level covariate to produce robust matched pairs. We were only able to produce a small number of matched pairs for this covariate, primarily from high income countries which would not be representative of other locations. After further review of the literature and discussion with a number of experts in the area, it became apparent that there was insufficient evidence to fully support the direction and magnitude of the GBD 2017 covariate. It also appeared that bias between school surveys and household samples (and the extent to which the latter would be the gold standard) would vary by location. Until more data becomes available to clarify the above, we have excluded this adjustment from the dataset, accepting both types of surveys. The removal of this adjustment from GBD 2019 meant that prevalence derived from student surveys were no longer being adjusted downwards to the extent they were in GBD 2017.
4. In GBD 2017, a study level covariate was used to adjust prevalence estimates from diagnostic interviews in East Asia, Southeast Asia, and Asia Pacific high-income using a ratio based on a study in China. Phillips and collaborators[19] made use of clinicians as opposed to lay interviewers and reported that the prevalence of MDD in China was 2.07% while the prevalence of mood disorders not otherwise specified (NOS) was 2.06%. Of the 808 individuals diagnosed with mood disorders NOS, 467 (58%) met criteria for minor depression (defined by DSM-IV-TR as two to four of nine symptoms of depression lasting for ≥2 weeks). There is evidence to suggest that these reported cases of minor depression are likely misdiagnosed cases of MDD as DSM/ICD diagnostic criteria are not sensitive to cross-cultural presentations of MDD in Asia[19-22]. Based on this, a ratio of MDD + minor depression: MDD only (1.53, 1.45 – 1.63) was derived from data presented by Phillips and collaborators and used to adjust prevalence estimates from Asia in the model. The aim of this adjustment was not to capture sub-syndromal depression but instead, to pick up on diagnoses of MDD where there is evidence to suggest that the use of Western-based criteria has underestimated prevalence. Given that this is an argument that can be made for other parts of the world, and the original GBD 2017 crosswalk was derived from data from only one Chinese study which could not be incorporated within MR-BRT analyses, we replaced the GBD 2017 covariate with an overall adjustment to prevalence estimates derived from lay interviews. The GBD 2017 ratio translated to an upward adjustment of 0.65 which was a slightly larger adjustment

to that estimated for lay interviews in GBD 2019 (see table 2). The removal of this adjustment from GBD 2019 meant that prevalence data from East Asia, Southeast Asia, and Asia Pacific high-income using were no longer being adjusted upwards to the extent they were in GBD 2017; and prevalence derived from lay interviews from other parts of the world were being adjust upwards for the first time.

5. In GBD 2019 we tested a third location level covariate, Gallup: negative experience index, although the association with MDD prevalence was marginally positive.
6. In GBD 2019 we included new epidemiological data from 80 locations which further informed the DisMod-MR model.

While we continue to improve on the data and methods used to estimate the burden of mental disorders, some challenges need to be acknowledged. Firstly, we still have a large number of locations with no high-quality raw data available. Secondly, it is difficult to quantify and remove all variation due to measurement error in our epidemiological estimates. Whilst we have improved the methodology used to account for known sources of bias, in some case, we still have very few data points to inform these adjustments. Thirdly, there is a paucity of research on the risk factors of mental disorders which can be used as predictive covariates in our epidemiological models.

References

1. American Psychiatric Association. Diagnostic and Statistical Manual of Mental Disorders (DSM). Washington: American Psychiatric Association, 1952.
2. World Health Organization. The ICD-10 Classification of Mental and Behavioural Disorders. Clinical descriptions and diagnostic guidelines. Geneva: World Health Organization; 1992.
3. Ferrari AJ, Charlson FJ, Norman RE, et al. Burden of depressive disorders by country, sex, age, and year: findings from the global burden of disease study 2010. *PLoS Med* 2013; **10**(11): e1001547.
4. Ferrari AJ, Charlson FJ, Norman RE, et al. The epidemiological modelling of major depressive disorder: application for the Global Burden of Disease Study 2010. *PloS one* 2013; **8**(7).
5. Salk RH, Hyde JS, Abramson LY. Gender differences in depression in representative national samples: meta-analyses of diagnoses and symptoms. *Psychological bulletin* 2017; **143**(8): 783.
6. Patten SB, Williams JV, Lavorato DH, Wang JL, Bulloch AG, Sajobi T. The association between major depression prevalence and sex becomes weaker with age. *Social psychiatry and psychiatric epidemiology* 2016; **51**(2): 203-10.
7. Patten SB, Wang JL, Williams JV, et al. Descriptive epidemiology of major depression in Canada. *The Canadian Journal of Psychiatry* 2006; **51**(2): 84-90.
8. Ferrari AJ, Norman RE, Freedman G, et al. The burden attributable to mental and substance use disorders as risk factors for suicide: findings from the Global Burden of Disease Study 2010. *PLoS One* 2014; **9**(4): e91936.
9. Introduction to the National Epidemiologic Survey on Alcohol and Related Conditions [http://pubs.niaaa.nih.gov/publications/arh29-2/74-78.htm]. Access date 1 December 2014.
10. Australian Bureau of Statistics. National Survey of Mental Health and Wellbeing of Adults 1997. Canberra: Australian Bureau of Statistics.
11. Kendler KS, Walters EE, Kessler RC. The prediction of length of major depressive episodes: results from an epidemiological sample of female twins. *Psychol Med* 1997; **27**(1): 107-17.
12. Lewinsohn PM, Clarke GN, Seeley JR, Rohde P. Major depression in community adolescents: age at onset, episode duration, and time to recurrence. *J Am Acad Child Adolesc Psychiatry* 1994; **33**(6): 809-18.

13. Spijker J, de Graaf R, Bijl RV, Beekman AT, Ormel J, Nolen WA. Duration of major depressive episodes in the general population: results from The Netherlands Mental Health Survey and Incidence Study (NEMESIS). *Br J Psychiatry* 2002; **181**: 208-13.
14. McLeod JD, Kessler RC, Landis KR. Speed of recovery from major depressive episodes in a community sample of married men and women. *J Abnorm Psychol* 1992; **101**(2): 277-86.
15. Vos T, Haby MM, Barendregt JJ, Kruijshaar M, Corry J, Andrews G. The burden of major depression avoidable by longer-term treatment strategies. *Archives of General Psychiatry* 2004; **61**(11): 1097-103.
16. Karam E, Bou GM. Psychosocial consequences of war among civilian populations. *Current Opinion in Psychiatry* 2013; **16**(413–419).
17. Steel Z, Chey T, Silove D, Marnane C, Bryant RA, van Ommeren M. Association of torture and other potentially traumatic events with mental health outcomes among populations exposed to mass conflict and displacement: a systematic review and meta-analysis. *JAMA* 2009; **302**(5): 537-49.
18. Gallup G. The Gallup Poll: Public Opinion 2003: Rowman & Littlefield; 2004.
19. Phillips MR, Zhang J, Shi Q, et al. Prevalence, treatment, and associated disability of mental disorders in four provinces in China during 2001–05: an epidemiological survey. *The Lancet* 2009; **373**: 2041–53.
20. Bromet E, Andrade LH, Hwang I, et al. Cross-national epidemiology of DSM-IV major depressive episode. *BMC medicine* 2011; **9**: 90.
21. Wang PS, Aguilar-Gaxiola S, Alonso J, et al. Use of mental health services for anxiety, mood, and substance disorders in 17 countries in the WHO world mental health surveys. *The Lancet* 2007; **370**(9590): 841-50.
22. Simon GE, Goldberg D, Von Korff M, Ustun T. Understanding cross-national differences in depression prevalence. *Psychological Medicine* 2002; **32**(4): 585–94.

Dysthymia

Flowchart

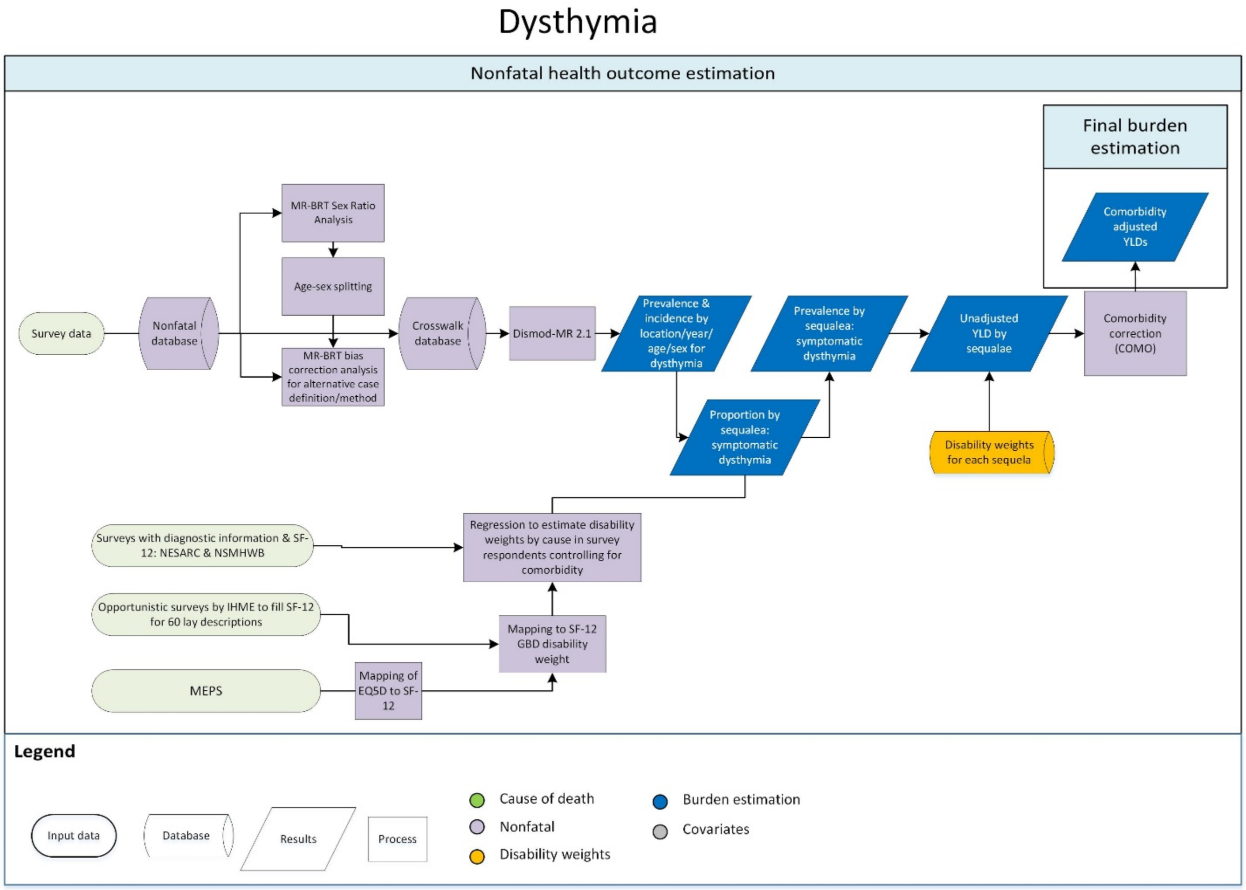

Input Data and Methodological Summary for Dysthymia

Case Definition

Dysthymia is a mood disorder consisting of chronic depression, demonstrating less severe but longer-lasting symptoms than major depressive disorder. Included in GBD disease modelling were cases meeting diagnostic criteria for dysthymia according to the Diagnostic and Statistical Manual of Mental Disorders (DSM), or the equivalent diagnosis in the International Classification of Diseases (ICD). These were identified by the following codes: DSM-IV-TR: 300.4, ICD-10: F34.1; excluding those cases due to a general medical condition or substance-induced cases[1,2].

According to DSM-IV TR criteria, dysthymia involves the experience of chronically depressed mood for most of the day, more days than not, for at least two years (or at least one year in children and adolescents). During this period, at least two of the following symptoms must also be experienced:

- poor appetite or overeating;
- insomnia or hypersomnia;
- low energy or fatigue;
- low self-esteem;

- poor concentration or indecisiveness; and
- feelings of hopelessness

Input data

For mental disorders, we update our GBD electronic database searches on a two year rolling basis. In GBD 2019 a systematic literature review update was conducted to identify new epidemiological studies on dysthymia published between September 2016 and December 2018. We included studies reporting the prevalence, incidence, remission, duration, and/or excess mortality associated with dysthymia. The search was conducted in three stages involving electronic searches of the peer-reviewed literature (i.e., PsycInfo, Embase, and PubMed), the grey literature, and expert consultation. The dysthymia systematic review was conducted in conjunction with the search for bipolar disorder and major depressive disorder as these disorders are frequently grouped together in publications. The following search terms were used to develop search strings across all databases searched: 'dysthymia', 'bipolar', 'manic', 'mania', 'mood disorder', 'depressive disorder', 'bipolar disorder', 'dysthymic disorder', and 'prevalence', 'mortality', 'death', 'incidence', 'recurrence', 'remission', 'duration', 'epidemiology'.

The search generated 18,023 records (after duplicates were removed) across the three electronic databases. The title/abstract screening reduced the number of relevant records to 247 studies, of which 6 met criteria for inclusion for dysthymia. An additional 13 studies were identified and extracted through a grey literature search. Consultation with GBD collaborators allowed us to include 6 studies from Iranian journals which are typically not indexed in the electronic databases searched. Overall, in GBD 2019 we added 25 new studies into the dysthymia dataset.

The GBD inclusion criteria stipulated that (1) the publication year must be from 1980 onward; (2) "caseness" must be based on clinical threshold as established by the DSM or ICD; (3) sufficient information must be provided on study method and sample characteristics to assess the quality of the study; and (4) study samples must be representative of the general population (i.e., inpatient or pharmacological treatment samples, case studies, veterans, or refugee samples were excluded). No limitation was set on the language of publication. Methods used for this systematic review have been reported in greater detail elsewhere[3,4]. Table 1 below summarizes data inputs by parameter for Dysthymia.

Table 1: Data Inputs for dysthymia morbidity modelling by parameter.

Measure	Total sources	Countries with data
All measures	107	37
Prevalence	104	37
Incidence	1	1
Remission	2	2
Proportion	1	1

Age and sex splitting

The extracted data underwent three types of age and sex splitting processes:

1. Where possible, estimates were further split by sex and age based on the data that was available. For instance, if studies reported prevalence for broad age groups by sex (e.g., prevalence in 15 to 65 year old males and females separately), and also by specific age groups but for both sexes combined (e.g., prevalence in 15- to 30-year-olds, then in 31- to 65-year-olds, for males and females combined); age-specific estimates were split by sex using the reported sex ratio and bounds of uncertainty.
2. A Meta-Regression with Bayesian priors, Regularization, and Trimming (MR-BRT) analysis was used to split the remaining both-sex estimates in the dataset. For each parameter, sex-specific estimates were matched by location, mid-age, year and a MR-BRT network meta-analysis was used to estimate pooled sex ratios. Given evidence to suggest that the sex ratio in depressive disorders varies with age[5-7], we also tested for an age interaction in the model. We found that the sex difference in dysthymia decreased significantly with age i.e., prevalence in males (compared to females) increased significantly with increasing age. The estimated all-age male: female prevalence ratio was 0.66 (95% uncertainty interval [UI]: 0.50 – 0.83) while Figure 1 shows the estimated male: female prevalence ratio by age. Age-specific sex ratios were used to split both sex estimates in the dataset.

Figure 1. Sex ratios by age for dysthymia

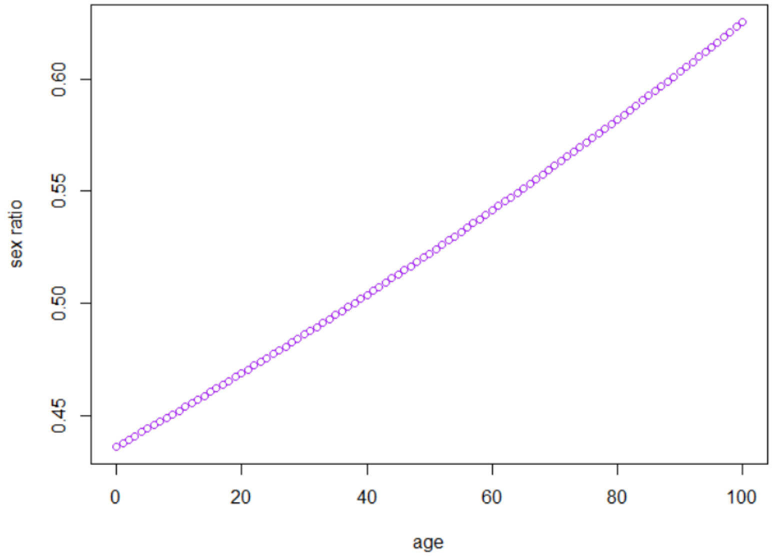

3. Studies reporting prevalence estimates across age groups spanning 25 years or more, were split into five-year age groups using the prevalence age pattern estimated by DisMod-MR 2.1. The DisMod-MR model used to estimate the age pattern did not contain any previously age split data

Bias corrections / Crosswalks

Estimates with known biases were adjusted / crosswalked accordingly prior to DisMod-MR 2.1. For each crosswalk of interest, pairs of the reference and the alternative estimates were matched by age, sex, location and year. This was done for both within (where possible) and between study pairs. These pairs were then used as inputs in a MR-BRT network meta-analysis. The MR-BRT analysis produced a pooled ratio between the reference estimates and alternative estimates, which was used to adjust all alternative estimates in the dataset. For dysthymia a lay interviewer ratio (see Table 2) was used to

adjust all prevalence estimates derived from trained lay-interviewers towards the level they would have been if the estimate was derived from clinically trained interviewers (i.e. psychologist or psychiatrist). We consider interviews conducted by clinicians to be more sensitive to detecting cases of dysthymia, particularly in locations where predominantly westernized mental health case definitions and instruments are yet to be fully validated.

Table 2: MR-BRT Crosswalk Adjustment Factors for Dysthymia

Data input	Reference or alternative case definition	Beta Coefficient, Log (95% UI)	Adjustment factor* (95% UI)	Gamma
Population Survey	Reference: clinical diagnosis			0.43
Population Survey	Alternative: lay-interviewer	-0.22<br>(-1.08 – 0.68)	0.80<br>(0.34 – 1.97)	

**Adjustment factor is the transformed Beta coefficient in normal space and can be interpreted as the factor by which the alternative case definition is adjusted to reflect what it would have been if measured as the reference.*

UIs incorporate Gamma which represents the between study variance across all input data in the model. This added uncertainty widens the UIs for crosswalks with significant fixed effects.

Severity splits and disability weights

The basis of the GBD disability weight survey assessments are lay descriptions of sequelae highlighting major functional consequences and symptoms. The lay description and disability weight for a symptomatic state of dysthymia is shown in Table 3. Given the milder and more stable presentation of dysthymia, it was assigned the same disability weight as that for mild major depressive disorder. To determine the proportion of people with symptomatic and asymptomatic dysthymia, the US National Epidemiological Survey on Alcohol and Related Conditions (NESARC, conducted in two waves from 2001 to 2002 and 2004 to 2005)[8] and the Australian National Survey of Mental Health and Wellbeing of Adults (NSMHWB, conducted in 1997)[9] were used. The proportion of dysthymia cases falling within each severity level were as follows: asymptomatic 29% (23% – 36%), and symptomatic 71% (64% – 77%).

Table 3. Severity distribution for dysthymia in GBD 2019 and the associated disability weight (DW) with that severity.

Severity level	Lay description	DW (95% UI)
Symptomatic dysthymia	Feels persistent sadness and has lost interest in usual activities. The person sometimes sleeps badly, feels tired, or has trouble concentrating but still manages to function in daily life with extra effort.	0.145 (0.099 – 0.209)

Modelling Strategy

After the above data processes were applied, DisMod MR 2.1 was used to model the epidemiological data for dysthymia. The DisMod-MR modeling strategy for dysthymia followed the standard GBD 2019 decomposition structure. At each decomposition step, we compared the new model against the GBD 2017 best model and the best model from the previous step. All substantial changes between models were explored and explained. Adjustments to model priors or the dataset were made where

appropriate. Where outliers were identified in the data, we re-assessed the study's methodology and quality before a decision was made to exclude or include the data.

Data across all epidemiological parameters were initially included in the modelling process. The incidence studies reported estimates which were very low relative to the prevalence data. As prevalence studies contributed much greater world coverage than incidence studies, we excluded the incidence data, relying instead on data from the other parameters. We assumed no incidence and prevalence before age 3. This minimum age of onset was corroborated with expert feedback and was consistent with the available data. Excess-mortality was set to 0 as there is no epidemiological evidence to suggest that dysthymia is associated with a statistically significant risk of mortality[3,4].

Changes between GBD 2017 and GBD 2019

There were three main changes in the GBD 2019 modelling strategy compared to GBD 2017:

1. In GBD 2019 we updated the age splitting by regional pattern methodology by increasing the age threshold for splitting to 25 years (in GBD 2017 it was 20 years). This meant that there were fewer estimates eligible for age splitting in this way. This impacted on the prevalence for some locations which now had fewer age-split estimates informing prevalence estimation.
2. In GBD 2017 bias corrections and sex ratios were estimated by DisMod-MR as part of the prevalence modelling. In GBD 2019 we conducted a MR-BRT analysis to accommodate for study heterogeneity and estimated pooled ratios with 95% UIs as previously discussed. The lay interviewer adjustment was largely consistent, although the UIs derived by MR-BRT tended to be larger. MR-BRT UIs incorporate Gamma which represents the between study variance across all input data in the model. This added uncertainty widens the UIs for crosswalks with significant fixed effects.
    a. The prevalence male: female ratio was 0.61 (0.55 – 0.66) in GBD 2017 compared to 0.66 (0.50 – 0.83) in GBD 2019
    b. The adjustment ratio for lay-interviewer estimates was 0.69 (0.60 – 0.78) in GBD 2017 compared to 0.80 (0.34 – 1.97) in GBD 2019, leading to an overall decrease in the adjusted prevalence.
3. In GBD 2019 we included new epidemiological data from 24 locations which further informed the DisMod-MR model.

While we continue to improve on the data and methods used to estimate the burden of mental disorders, some challenges need to be acknowledged. Firstly, we still have a large number of locations with no high-quality raw data available. Secondly, it is difficult to quantify and remove all variation due to measurement error in our epidemiological estimates. Whilst we have improved the methodology used to account for known sources of bias, in some case, we still have very few data points to inform these adjustments. Thirdly, there is a paucity of research on the risk factors of mental disorders which can be used as predictive covariates in our epidemiological models.

References

1. American Psychiatric Association. Diagnostic and Statistical Manual of Mental Disorders (DSM). Washington: American Psychiatric Association, 1952.
2. World Health Organization. The ICD-10 Classification of Mental and Behavioural Disorders. Clinical descriptions and diagnostic guidelines. Geneva: World Health Organization; 1992.

3. Charlson FJ, Ferrari AJ, Flaxman AD, Whiteford HA. The epidemiological modelling of dysthymia: application for the Global Burden of Disease Study 2010. *J Affect Disord* 2013; **151**(1): 111-20.
4. Ferrari AJ, Charlson FJ, Norman RE, et al. Burden of depressive disorders by country, sex, age, and year: findings from the global burden of disease study 2010. *PLoS Med* 2013; **10**(11): e1001547.
5. Patten SB, Wang JL, Williams JV, et al. Descriptive epidemiology of major depression in Canada. *The Canadian Journal of Psychiatry* 2006; **51**(2): 84-90.
6. Patten SB, Williams JV, Lavorato DH, Wang JL, Bulloch AG, Sajobi T. The association between major depression prevalence and sex becomes weaker with age. *Social psychiatry and psychiatric epidemiology* 2016; **51**(2): 203-10.
7. Salk RH, Hyde JS, Abramson LY. Gender differences in depression in representative national samples: meta-analyses of diagnoses and symptoms. *Psychological bulletin* 2017; **143**(8): 783.
8. Introduction to the National Epidemiologic Survey on Alcohol and Related Conditions [http://pubs.niaaa.nih.gov/publications/arh29-2/74-78.htm]. Access date 1 December 2014.
9. Australian Bureau of Statistics. National Survey of Mental Health and Wellbeing of Adults 1997. Canberra: Australian Bureau of Statistics.

Bipolar Disorder

Flowchart

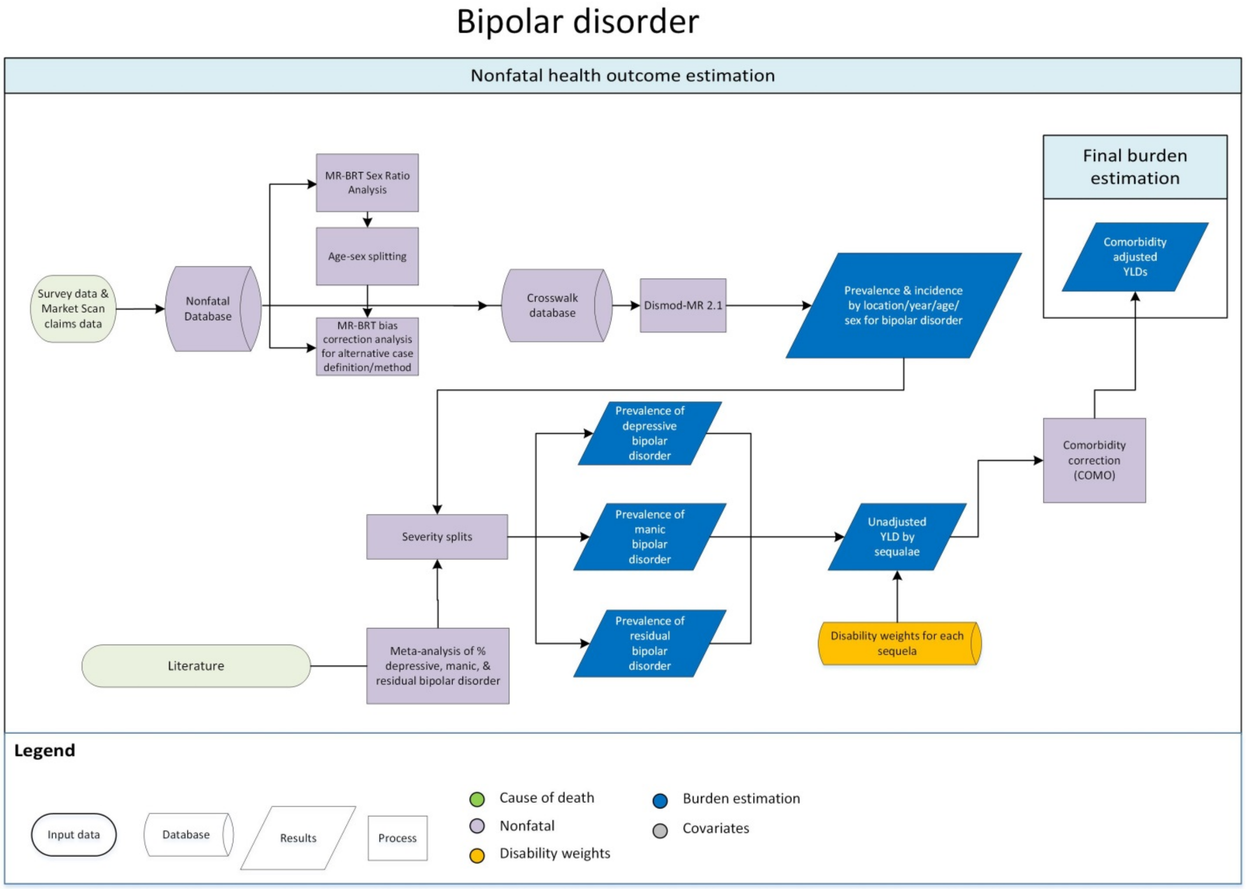

Input Data and Methodological Summary for Bipolar disorder

Case definition

Bipolar disorder is a serious mood disorder with little or no complete remission. Included in GBD disease modelling were cases meeting diagnostic criteria for bipolar disorder according to the Diagnostic and Statistical Manual of Mental Disorders (DSM), or the equivalent diagnosis in the International Classification of Diseases (ICD)[1,2]. These are identified by the following codes: DSM-IV-TR: 296.0–296.7, 296.89, 301.13; ICD-10: F30.0-F30.9, F31.0–F31.6, F31.8–F31.9, F34.0. Excluded were bipolar disorder due to a general medical condition or substance-induced cases. A diagnosis of bipolar disorder involves the experience of one or more manic, hypomanic, and/or major depressive episodes.

According to DSM-IV-TR, a manic episode involves the experience of elevated, expansive, or irritable mood lasting for at least one week. During this period, at least three (or four if mood is only irritable) of the following symptoms must also be experienced: i) inflated self-esteem or grandiosity, ii) decreased need for sleep, iii) more talkative, iv) flight of ideas or experience that thoughts are racing, v) distractibility, vi) increase in goal-directed activity, and vii) excessive involvement in pleasurable activities with high potential for painful consequences.

A hypomanic episode involves the experience of elevated, expansive, or irritable mood lasting for at least four days. During this period, at least three (or four if mood is only irritable) of the symptoms previously listed for a manic episode must also be experienced.

A major depressive episode involves the experience of depressed mood almost all day, every day, for at least two weeks. A total of five out of nine criteria must be met to make a diagnosis and at least one of the five criteria should either be "depressed mood" for most of every day or "loss of interest in nearly all activities" for most of every day. The other seven criteria are: i) change in eating, appetite, or weight, ii) excessive sleeping or insomnia, iii) agitated or slow motor activity, iv) fatigue, v) feeling worthless or inappropriately guilty, vi) trouble concentrating, and vii) repeated thoughts about death.

Different subtypes of bipolar disorder can be diagnosed depending on the combination of symptoms experienced. Bipolar I is characterised by at least one manic episode, which can also alternate with a major depressive episode. Bipolar II is characterised by hypomanic episodes alternating with major depressive episodes. Cyclothymia is characterised by subsyndromal hypomanic and major depressive episode. Bipolar disorder not otherwise specified is characterised by clinically significant symptoms of bipolar disorder which do not meet criteria for the other diagnoses[2,3]. In GBD 2019 we estimated burden for the entire spectrum of bipolar disorder simultaneously, rather than individually for each subtype of the disorder. At a minimum, epidemiological studies needed to report on bipolar I and bipolar II to be included in analyses.

Input data

For mental disorders, we update our GBD electronic database searches on a two-year rolling basis. In GBD 2019 a systematic literature review update was conducted to identify new epidemiological studies on bipolar disorder published between September 2016 and December 2018. We included studies reporting the prevalence, incidence, remission, duration, and/or excess mortality associated with bipolar disorder. The search was conducted in three stages involving electronic searches of the peer-reviewed literature (i.e., PsycInfo, Embase, and PubMed), the grey literature, and expert consultation. The bipolar disorder systematic review was conducted in conjunction with the search for major depressive disorder and dysthymia as these disorders are frequently grouped together in publications. The following search terms were used to develop search strings across all databases searched: 'dysthymia', 'bipolar', 'manic', 'mania', 'mood disorder', 'depressive disorder', 'bipolar disorder', 'dysthymic disorder', and 'prevalence', 'mortality', 'death', 'incidence', 'recurrence', 'remission', 'duration', 'epidemiology'.

The search generated 18,023 records (after duplicates were removed) across the three electronic databases. The title abstract screening reduced the number of relevant records to 247 studies, of which 10 met criteria for inclusion. An additional 10 studies were identified and extracted through grey literature search and consultations with experts. A separate search was also conducted to identify studies (n=39) reporting on the lifetime prevalence of bipolar disorder from 1980 onward which were previously excluded from the GBD dataset. Overall, in GBD 2019, we added 59 new studies into the bipolar dataset.

The GBD inclusion criteria stipulated that: (1) the publication year must be from 1980 onward; (2) "caseness" must be based on clinical threshold as established by the DSM or ICD; (3) sufficient information must be provided on study method and sample characteristics to assess the quality of the study; and (4) study samples must be representative of the general population (i.e., inpatient or pharmacological treatment samples, case studies, veterans, or refugee samples were excluded). No

limitation was set on the language of publication. Table 1 below summarizes data inputs by parameter for bipolar disorders.

Table 1: Data Inputs for bipolar disorders morbidity modelling by parameter.

Measure	Total sources	Countries with data
All measures	153	47
Prevalence	113	41
Incidence	2	2
Relative risk	3	2
Standardized mortality ratio	12	8
Proportion	27	14

As previously explained, we estimated the burden for the entire spectrum of bipolar disorder rather than individually for each subtype of the disorder. Combined estimates of all subtypes of bipolar disorder were required. Studies reporting separate estimates for bipolar I, bipolar II, cyclothymia, and/or bipolar not otherwise specified were accepted if sufficient information was available to sum the disorder-specific estimates.

Age and sex splitting

The extracted data underwent three types of age and sex splitting processes:

1. Where possible, estimates were further split by sex and age based on the data that was available. For instance, if studies reported prevalence for broad age groups by sex (e.g., prevalence in 15 to 65 year old males and females separately), and also by specific age groups but for both sexes combined (e.g., prevalence in 15- to 30-year-olds, then in 31- to 65-year-olds, for males and females combined); age-specific estimates were split by sex using the reported sex ratio and bounds of uncertainty.
2. A Meta-Regression with Bayesian priors, Regularization, and Trimming (MR-BRT) analysis was used to split the remaining both-sex estimates in the dataset. For each parameter, sex specific estimates were matched by location, age, year and a MR-BRT network meta-analysis was used to estimate pooled sex ratios and bounds of uncertainty. These were then used to split the both sex estimates in the dataset. The male: female prevalence ratio estimated for prevalence estimates was 0.82 (95% uncertainty interval [UI]: 0.42 – 1.22).
3. Studies reporting prevalence estimates across age groups spanning 25 years or more, were split into five-year age groups using the prevalence age pattern estimated by DisMod-MR 2.1. The DisMod-MR model used to estimate the age pattern did not contain any previously age split data.

Bias corrections / Crosswalks

Estimates with known biases were adjusted / crosswalked accordingly prior to DisMod-MR 2.1. For each crosswalk of interest, pairs of the reference and the alternative estimates were matched by age, sex, location and year. This was done for both within (where possible) and between study pairs. Pairs were also made between the different alternative estimates. The ratios between these estimates were then used as inputs in a MR-BRT network meta-analysis. This analysis produced pooled ratios between the

reference estimates and alternative estimates, which were used to adjust all alternative estimates in the dataset. Two adjustment ratios were used for bipolar disorder.

1. A point/past-month recall ratio adjusted point/past-month prevalence estimates toward the level they would have been if the study had captured 12-month prevalence. We set 12-month prevalence as the desirable level due to the episodic nature of bipolar disorder. Estimates of point prevalence surveying symptoms experienced in the past 30 days or less may fail to diagnose cases of bipolar disorder in a residual state, thereby underestimating prevalence.
2. A lifetime recall ratio adjusted all data points derived from lifetime prevalence towards the level they would have been if the study had captured 12-month prevalence. Lifetime estimates were included as they are useful to capture potentially missed cases in the residual state.

See Table 2 below for adjustment factors (betas and exponentiated betas, which can be interpreted as odds ratios) used for bipolar disorder.

Table 2: MR-BRT Crosswalk Adjustment Factors for Bipolar disorder

Data input	Reference or alternative case definition	Beta Coefficient, Log (95% UI)	Adjustment factor* (95% UI)	Gamma
Population Survey	Reference: past year or 12-month prevalence of bipolar disorder			0.23
Population Survey	Alternative: point or past-month prevalence	0.45<br>(-0.02 – 0.92)	1.57<br>(0.98 – 2.50)	
Population Survey	Alternative: lifetime prevalence	-0.37<br>(-0.85 – 0.10)	0.69<br>(0.43 – 1.11)	

**Adjustment factor is the transformed Beta coefficient in normal space and can be interpreted as the factor by which the alternative case definition is adjusted to reflect what it would have been if measured as the reference.*

UIs incorporate Gamma which represents the between study variance across all input data in the model. This added uncertainty widens UIs for crosswalks with significant fixed effects.

MarketScan data

We made use of United States (US) MarketScan data in our prevalence dataset. These were prevalence data for bipolar disorder derived from claims information in a database of private and public insurance schemes. Given the sparseness of the bipolar disorder prevalence dataset, this allowed us to incorporate detailed prevalence estimates by state, sex, and age in our modelling. Evaluation of the age-pattern of MarketScan data revealed that it was consistent to what can be observed in population-representative survey estimates; however, given that this data source only captures a subset of the population, the actual levels of prevalence, and the sex difference in prevalence, were not comparable and had to be adjusted accordingly.

We compared each year of MarketScan estimates against corresponding prevalence data from the National Comorbidity Survey Replication (NCS-R), a survey representative of the general US population. The resulting prevalence ratios were used to adjust all MarketScan estimates before they were entered into the bipolar disorder model. The NCS-R: MarketScan ratios are presented in Table 3 below.

Table 3. MarketScan adjustment factors

MarketScan year	Males (95% UI)	Females (95% UI)
2000	3.39 (2.22-4.57)	2.56 (1.74-3.38)
2010	2.17 (1.42-2.92)	1.51 (1.02-1.99)
2011	2.10 (1.38-2.83)	1.49 (1.01-1.97)
2012	2.11 (1.38-2.83)	1.45 (0.99-1.92)
2013	2.09 (1.37-2.82)	1.46 (0.99-1.92)
2014	2.05 (1.34-2.75)	1.37 (0.93-1.81)

Severity splits and disability weights

The GBD disability weight survey assessments are lay descriptions of sequelae highlighting major functional consequences and symptoms. The lay descriptions and disability weights for bipolar disorder severity levels are shown in Table 4. Information on the distribution of manic, depressive, and residual states of bipolar disorder was obtained from a systematic review of the literature[4] capturing data published between 1980 and 2012, and an update we conducted for GBD 2019 capturing data up to February 2018.

Overall, 26 studies provided information on the proportion of bipolar disorder cases in a manic, depressive and residual state. A MR-BRT analysis was used to explore between study heterogeneity and to estimate the pooled proportion of cases falling within each bipolar health state. Two covariates were used in the analysis. The first was a sampling type covariate where the reference was population representative data or data from surveys of in- and out-patients combined. Alternatives for this covariate included data from inpatient only samples, and outpatient only samples. The second covariate was for bipolar subtypes where the reference was surveys screening for overall bipolar disorder (i.e., bipolar I, bipolar II and/or bipolar NOS) and the alternative included studies that reported data for bipolar I only (n=6). An income covariate was tested (i.e., studies representative of high-income countries [n= 21] vs non- high income [n=5]) but it was not statistically significant and was not included in the final analysis. The proportion of bipolar disorder cases falling within each state were as follows: manic 18.7% (9.1% – 30.7%), depressive 31.7% (15.6% – 48.1%), and residual 49.5% (24.9% – 74.1%).

Table 4. Severity distribution for bipolar disorders in GBD 2019 and the associated disability weight (DW) with that severity.

Severity level	Lay description	DW (95% UI)
Manic	Is hyperactive, hears and believes things that are not real, and engages in impulsive and aggressive behavior that endanger the person and others.	0.492 (0.341 – 0.646)
Depressive*	Has constant sadness and has lost interest in usual activities. The person has some difficulty in daily life, sleeps badly, has trouble concentrating, and sometimes thinks about harming himself (or herself).	0.396 (0.267 – 0.531)
Residual	Has mild mood swings, irritability, and some difficulty with daily activities.	0.032 (0.018 – 0.051)

*Note. *Equivalent to the disability weight estimated for moderate major depressive disorder*

Modeling strategy

After the above data processes were applied, DisMod MR 2.1 was used to model the epidemiological data for bipolar disorder. The DisMod-MR modeling strategy for bipolar disorder followed the standard GBD 2019 decomposition structure. At each decomposition step, we compared the new model against the GBD 2017 best model and the best model from the previous step. All substantial changes between models were explored and explained. Adjustments to model priors or the dataset were made where appropriate. Where outliers were identified in the data, we re-assessed the study's methodology and quality before a decision was made to exclude or include the data.

Data across all epidemiological parameters were initially included in the modelling process. The two studies on incidence reported 0% and 0.1% incidence of bipolar disorder and were low relative to the prevalence data. They were excluded from the final model where incidence was estimated using data from other parameters. We assumed no incidence and prevalence before age 10. Remission was set to a maximum of 0.05 in agreement with literature and expert advice suggesting no or very little complete remission from bipolar disorder[5,6].

Changes between GBD 2017 and GBD 2019

There were five main changes in the GBD 2019 modelling strategy compared to GBD 2017:

1. In GBD 2019 we updated the age splitting by regional pattern methodology by increasing the age threshold for splitting to 25 years (in GBD 2017 it was 20 years). This meant that there were fewer estimates eligible for age splitting in this way. This impacted on the prevalence for some locations which now had fewer age-split estimates informing prevalence estimation.
2. In GBD 2017 bias corrections and sex ratios were estimated by DisMod-MR as part of the prevalence modelling. In GBD 2019 we conducted a MR-BRT analysis to accommodate for study heterogeneity and estimated pooled ratios with 95% UIs as previously discussed. Ratios estimated between 2017 and 2019 were largely consistent, although the UIs derived by MR-BRT tended to be larger. MR-BRT UIs incorporate Gamma which represents the between study variance across all input data in the model. This added uncertainty widens the UIs for crosswalks with significant fixed effects. For example:
    a. The prevalence male: female ratio was 0.89 (0.88 – 0.90) in GBD 2017 compared to 0.82 (0.42 – 1.22) in GBD 2019.
    b. The adjustment ratio for point/past month estimates slightly increased from 0.42 (0.31 – 0.54) in GBD 2017 to 0.69 (0.61 – 0.78) in GBD 2019, leading to a slight overall decrease in unadjusted prevalence.
3. In GBD 2019 we included epidemiological data reporting lifetime prevalence of bipolar disorder into the database. Lifetime estimates have been included for bipolar disorder due to their episodic nature, chronicity and lack of remission. As previously discussed, an adjustment ratio was used to adjust lifetime estimates down to the level they would be if they were reported under the gold standard recall period (i.e., past 12 month).
4. In GBD 2019 we updated the severity distribution of bipolar health states. In GBD 2017 the proportion of bipolar disorder cases in each health state were 21% (12% – 33%) of cases in a manic state, 23% (10% – 39%) in a depressive state and, 52% (28% – 77%) in the residual state. In GBD 2019 these proportions changed to manic 19% (9% – 31%), depressive 32% (16% – 47%), or residual 50% (25% – 74%) health state.

5. In GBD 2019 we included new epidemiological data from 13 locations (Argentina, Germany, Hunan, Iran, Japan, Mexico, Kenya, New Zealand, Portugal, Saudi Arabia, Spain, China, United States) which further informed the DisMod-MR model.

While we continue to improve on the data and methods used to estimate the burden of mental disorders, some challenges need to be acknowledged. Firstly, we still have a large number of locations with no high-quality raw data available. Secondly, it is difficult to quantify and remove all variation due to measurement error in our epidemiological estimates. Whilst we have improved the methodology used to account for known sources of bias, in some case, we still have very few data points to inform these adjustments. Thirdly, there is a paucity of research on the risk factors of mental disorders which can be used as predictive covariates in our epidemiological models.

References

1. American Psychiatric Association. Diagnostic and Statistical Manual of Mental Disorders (DSM-IV-TR). Fourth Edition, Text Revision ed. Washington DC: American Psychiatric Association; 2000.

2. World Health Organization. The ICD-10 Classification of Mental and Behavioural Disorders. Clinical descriptions and diagnostic guidelines. Geneva: World Health Organization; 1992.

3. American Psychiatric Association. Diagnostic and Statistical Manual of Mental Disorders (DSM). Washington: American Psychiatric Association, 1952.

4. Ferrari AJ, Saha S, McGrath JJ, et al. Health states for schizophrenia and bipolar disorder within the Global Burden of Disease 2010 Study. *Population health metrics* 2012; **10**(1): 16.

5. Angst J, Sellaro R. Historical perspectives and natural history of bipolar disorder. *Biological Psychiatry* 2000; **48**(6): 445–57.

6. Colom F, Vieta E. The road to DSM-V. Bipolar disorder episode and course specifiers. *Psychopathology* 2009; **42**(4): 209–18.

Anorexia nervosa

Flowchart

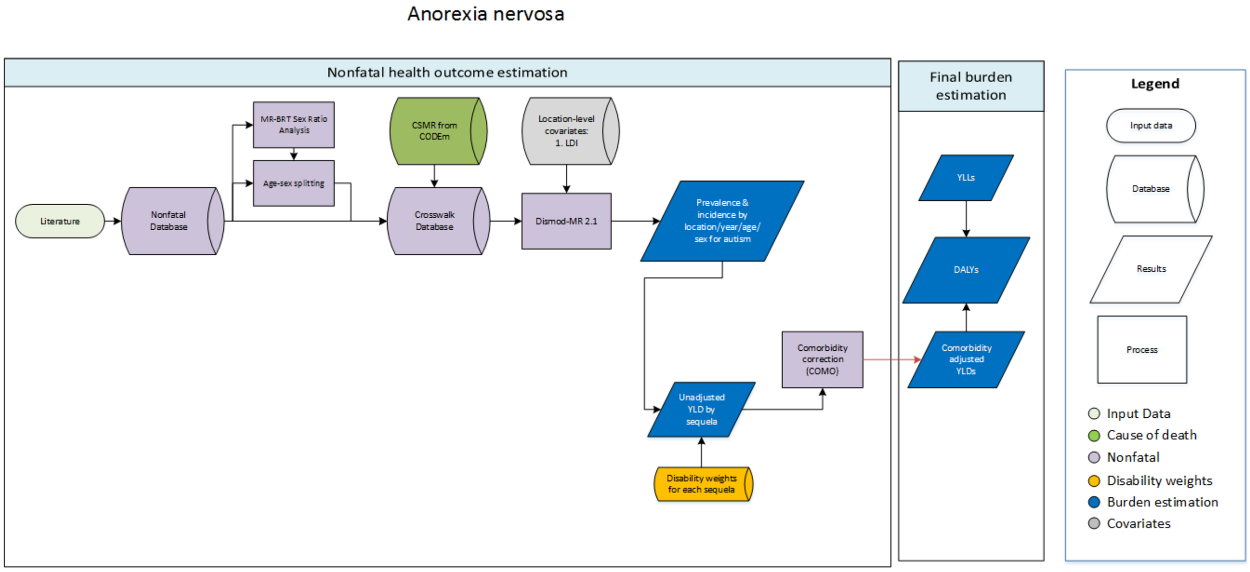

Input Data and Methodological Summary for Anorexia Nervosa

Case definition

According to the Diagnostic and Statistical Manual of Mental Disorders fourth edition, text revision (DSM-IV-TR),[1] anorexia nervosa (AN) is an eating disorder characterised by:

a) Refusal to maintain body weight at or above a minimally normal weight for age and height (eg, weight loss leading to maintenance of body weight less than 85% of that expected; or failure to make expected weight gain during period of growth, leading to body weight less than 85% of that expected).
b) Intense fear of gaining weight or becoming fat, even though underweight (expanded to include any behaviour that interferes with weight gain in DSM-5[2]).
c) Disturbance in the way in which one's body weight or shape is experienced, undue influence of body weight or shape on self-evaluation, or denial of the seriousness of the current low body weight.
d) In postmenarcheal females, amenorrhoea, ie, the absence of at least three consecutive menstrual cycles (this criterion was removed in DSM-5[2]).

Included in GBD were cases meeting diagnostic criteria according to DSM[1] or the International Classification of Diseases (ICD).[3] These were identified by the following codes: 307.1 (DSM-IV-TR) and F50.0-50.1 (ICD-10). Different versions of DSM (DSM-III, DSM-III-R, DSM-IV, DSM-IV-TR, and DSM-5) and ICD (ICD-9 and ICD-10) were accepted.

Input data

Systematic literature reviews were conducted to capture studies reporting the prevalence, incidence, remission, and excess mortality of AN. These were conducted in three stages involving electronic searches of the peer-reviewed literature (i.e., PsycInfo, Embase, and PubMed), the grey literature, and expert consultation. For mental disorders, we update our GBD electronic database searches on a two year rolling basis. A systematic review update for AN was conducted for GBD 2017, with the next

literature update due for the next round of GBD. A grey literature search and expert consultation was conducted for GBD 2019 and produced 8 new data sources.

The GBD inclusion criteria stipulated that: (1) the publication year must be from 1980 onward; (2) "caseness" must be based on clinical threshold as established by the DSM or ICD; (3) sufficient information must be provided on study method and sample characteristics to assess the quality of the study; and (4) study samples must be representative of the general population (i.e., inpatient or pharmacological treatment samples, case studies, veterans, or refugee samples were excluded). No limitation was set on the language of publication. Methods used for this systematic review have been reported in greater detail elsewhere.[6] Table 1 below summarizes data inputs by parameter for anorexia nervosa.

Table 1: Data Inputs for anorexia nervosa morbidity modelling by parameter.

Measure	Total sources	Countries with data
All measures	106	32
Prevalence	65	27
Incidence	6	6
Remission	21	11
Standardized mortality ratio	17	7

Age and sex splitting

The extracted data underwent three types of age and sex splitting processes:

1. Where possible, estimates were further split by sex and age based on the data that was available. For instance, if studies reported prevalence for broad age groups by sex (e.g., prevalence in 15 to 65 year old males and females separately), and also by specific age groups but for both sexes combined (e.g., prevalence in 15- to 30-year-olds, then in 31- to 65-year-olds, for males and females combined); age-specific estimates were split by sex using the reported sex ratio and bounds of uncertainty.
1. A Meta-Regression with Bayesian priors, Regularization, and Trimming (MR-BRT) analysis was used to split the remaining both-sex estimates in the dataset. For each parameter, sex specific estimates were matched by location, age, year and a MR-BRT network meta-analysis was used to estimate pooled sex ratios and bounds of uncertainty. These were then used to split the both sex estimates in the dataset. The male: female prevalence ratio estimated was 0.24 (95% uncertainty interval [UI]: 0.05 – 0.43).
2. Studies reporting prevalence estimates across age groups spanning 25 years or more, were split into five-year age groups using the prevalence age pattern estimated by DisMod-MR 2.1. The DisMod-MR model used to estimate the age pattern did not contain any previously age split data.

Bias corrections / Crosswalks

We tested for a number of potential sources for bias in prevalence between studies (e.g., use of ICD vs. DSM criteria, past year vs. point recall). However none of the crosswalks had a statistically significant impact on prevalence and so no bias corrections were applied to these estimates.

Disability weight

The GBD disability weight survey assessments are lay descriptions of sequelae highlighting major functional consequences and symptoms. No severity splits were applied to AN. The lay description and disability weight for AN are shown in Table 2.

Table 2: Health state and disability weight for anorexia nervosa

Lay description	DW (95% UI)
Feels an overwhelming need to starve and exercises excessively to lose weight. The person is very thin, weak, and anxious.	0.224 (0.150–0.312)

Modelling strategy

After the above data processes were applied, DisMod MR 2.1 was used to model the epidemiological data for AN. The DisMod-MR modeling strategy for AN followed the standard GBD 2019 decomposition structure. At each decomposition step, we compared the new model against the GBD 2017 best model and the best model from the previous step. All substantial changes between models were explored and explained. Adjustments to model priors or the dataset were made where appropriate. Where outliers were identified in the data, we re-assessed the study's methodology and quality before a decision was made to exclude or include the data.

We assumed no incidence prior to age 5 or from 50 years onward. These settings are in line with those placed on the corresponding cause of death model for AN. A cap of 0.6 was placed on remission in order to obtain a more plausible fit of the model. We used the function in DisMod-MR to pull in cause-specific mortality rate (CSMR) data from our CODEm and CoDCorrect analyses. As such, other mortality data (standardised mortality ratios and relative risks) were excluded. We also used these CSMR data to estimate priors on excess mortality rates (EMR) by matching them with prevalence data points for the same geography and study year and dividing CSMR by prevalence. A country-level covariate, lagged distributed income (LDI), was included. This covariate represents a moving average of gross domestic product (GDP) over time. The limits placed on this covariate meant that prevalence was assumed to increase with rising GDP. LDI was also applied to excess mortality data in order to better inform regional distribution. A summary of location-level covariates and exponentiated values for AN are shown in Table 3.

Table 3: Location-level covariates used for anorexia nervosa

Covariate	Parameter	beta	Exponentiated beta
LDI ($ per capita)	Prevalence	0.39 (0.23 — 0.49)	1.48 (1.26 — 1.64)
LDI ($ per capita)	Excess mortality	-0.23 (-0.42 — -0.11)	0.79 (0.66 — 0.90)

Changes between GBD 2017 and GBD 2019

There were two main changes in the GBD 2019 modelling strategy compared to GBD 2017:

1. In GBD 2017 the sex ratio was estimated by DisMod MR 2.1 as part of the prevalence modelling. In GBD 2019 we made use of MR-BRT to run a nested meta-regression analysis on the within-study sex ratios to estimate a pooled sex ratio with 95% uncertainty intervals as previously discussed. Compared to GBD 2017, prevalence male: female ratio increased slightly from 0.21 (0.14 — 0.40) to 0.24 (0.05-0.43).
2. In GBD 2019 we included 8 new epidemiological data sources from 12 locations (Austria, China, East Azarbayejan in Iran, Fars in Iran, Iran, Khorasan-e-Razavi in Iran, Rio Grande do Sul in Brazil, Isfahan in Iran, Saudi Arabia, Switzerland, Tehran in Iran, and United States of America). Three of these studies were from locations where we had no data previously (East Azarbayejan in Iran, Fars in Iran, Iran, Khorasan-e-Razavi in Iran, Isfahan in Iran, Saudi Arabia, and Tehran in Iran).

While we continue to improve on the data and methods used to estimate the burden of mental disorders, some challenges need to be acknowledged. Firstly, we still have a large number of locations with no high-quality raw data available. Secondly, it is difficult to quantify and remove all variation due to measurement error in our epidemiological estimates. Whilst we have improved the methodology used to account for known sources of bias, in some case, we still have very few data points to inform these adjustments. Thirdly, there is a paucity of research on the risk factors of mental disorders which can be used as predictive covariates in our epidemiological models.

References

1. American Psychiatric Association. Diagnostic and Statistical Manual of Mental Disorders (DSM-IV-TR). 4th, Text Revision ed. Washington DC: American Psychiatric Association; 2000.
2. American Psychiatric Association. Diagnostic and Statistical Manual of Mental Disorders, Fifth Edition. Arlington, VA: American Psychiatric Association; 2013.
3. World Health Organisation. ICD-10 Classification of Mental and Behavioural Disorders: Clinical descriptions and diagnostic guidelines. Geneva: World Health Organisation; 1992.
4. Whiteford HA, Degenhardt L, Rehm J, et al. Global burden of disease attributable to mental and substance use disorders: findings from the Global Burden of Disease Study 2010. *The Lancet* 2013; **382**(9904): 1575-86.

Anxiety Disorders

Flowchart

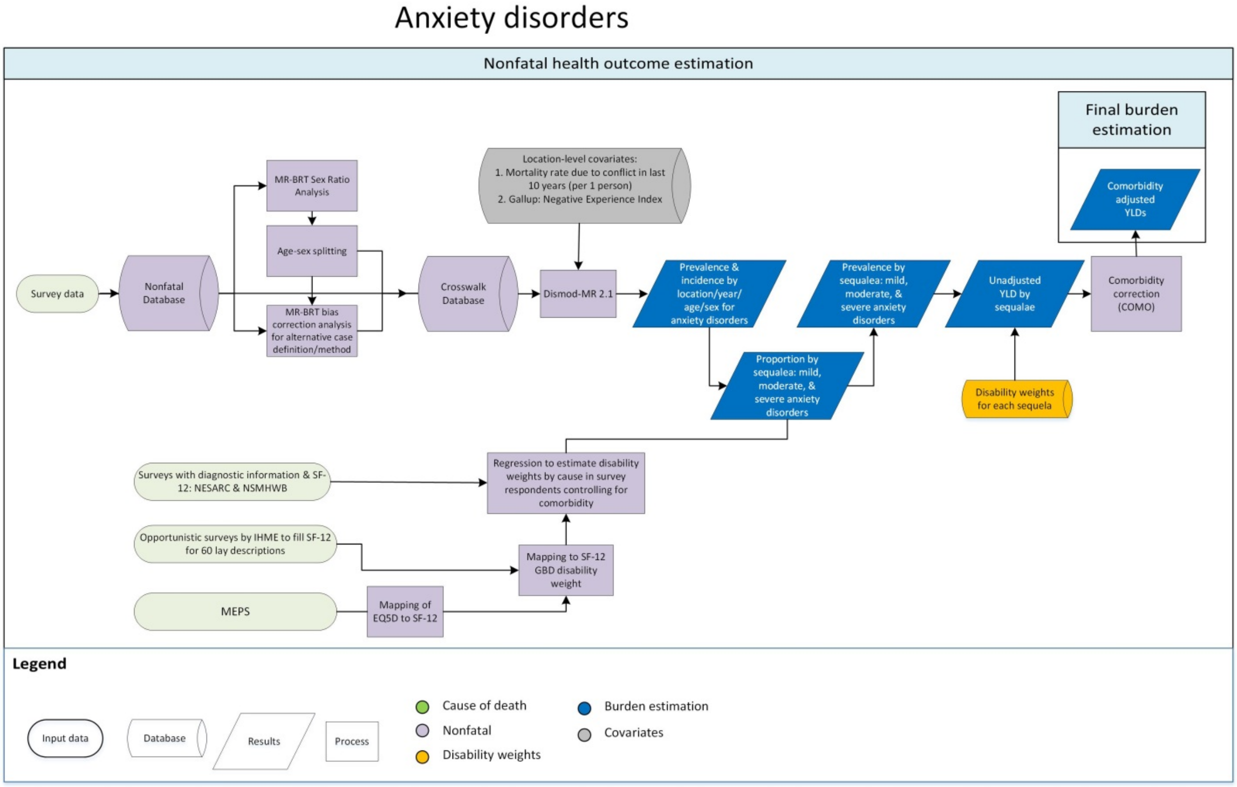

Input Data and Methodological Summary for Anxiety disorders

Case definition

Anxiety disorders are characterised by experiences of intense fear and distress, typically in combination with other physiological symptoms. We aimed to capture all cases of anxiety disorders reaching diagnostic threshold defined by the Diagnostic and Statistical Manual of Mental Disorders (DSM) or the World Health Organization (WHO) International Classification of Diseases (ICD)[1, 2]. Specific anxiety disorders included were: panic disorder, agoraphobia, specific phobia, social phobia, obsessive-compulsive disorder (OCD), posttraumatic stress disorder (PTSD), generalized anxiety disorder (GAD) including overanxious disorder in childhood, separation anxiety disorder (SAD), and anxiety disorder 'not otherwise specified' (NOS). These were identified by the following codes: DSM-IV-TR: 300.0-300.3, 208.3, 309.21, 309.81; ICD-10: F40-42, F43.0, F43.1, F93.0-93.2, F93.8. Excluded were anxiety disorders due to a general medical condition and substance-induced anxiety disorder.

Anxiety disorders were modelled as a single cause for "any" anxiety disorder to avoid the double-counting of individuals meeting criteria for more than one anxiety disorder. Epidemiological estimates reporting an outcome for "any" or "total" anxiety disorders were included in analyses, if they reported on at least three anxiety disorders. This has been further explained in previous publications[3, 4]

Input data

For mental disorders, we update our GBD electronic database searches on a two-year rolling basis. In GBD 2019 a systematic literature review update was conducted to update new epidemiological studies on anxiety disorders published between September 2016 and December 2018. We included studies reporting the prevalence, incidence, remission, duration, and/or excess mortality associated with anxiety disorders. The search was conducted in three stages involving electronic searches of the peer-

reviewed literature (i.e., using PsycInfo, Embase, and PubMed), the grey literature, and expert consultation. The following search terms were used to develop search strings across all databases searched: *'panic disorder', 'agoraphobia', 'social phobia', 'generalised anxiety disorder', 'obsessive compulsive disorder', 'post-traumatic stress disorder', 'anxiety disorder', 'OCD', 'GAD', 'PTSD' and 'epidemiology', 'incidence', 'prevalence', 'mortality', 'remission', 'duration'.*

The search generated 6325 records (after duplicates were removed) across the three electronic databases. The title/abstract screening reduced the number of relevant records to 208 studies, of which 32 studies met criteria for inclusion. An additional 9 studies were identified and extracted through a grey literature search and consultations with experts. Overall, in GBD 2019 we added 41 new studies into the anxiety dataset.

The GBD inclusion criteria stipulated that: (1) the publication year must be from 1980 onward; (2) "caseness" must be based on clinical threshold as established by the DSM or ICD; (3) sufficient information must be provided on study method and sample characteristics to assess the quality of the study; (4) a minimum of 3 (or 2 if occurring during childhood) anxiety disorder subtypes must be included within the overall estimate; and (5) study sample must be representative of the general population (i.e., inpatient or pharmacological treatment samples, case studies, veterans, or refugee samples were excluded). No limitation was set on the language of publications. Methods used in previous systematic reviews have been reported in greater detail elsewhere [3, 4]. Table 1 below summarizes data inputs by parameter for anxiety disorders.

Table 1: Data Inputs for Anxiety disorders morbidity modelling by parameter.

Measure	Total sources	Countries with data
All measures	219	59
Prevalence	199	58
Incidence	1	1
Remission	3	3
Standardized mortality ratio	1	1
Proportion	15	1

Age and sex splitting

The extracted data underwent three types of age and sex splitting processes:

1. Where possible, estimates were further split by sex and age based on the data that was available. For instance, if studies reported prevalence for broad age groups by sex (e.g., prevalence in 15 to 65 year old males and females separately), and also by specific age groups but for both sexes combined (e.g., prevalence in 15- to 30-year-olds, then in 31- to 65-year-olds, for males and females combined); age-specific estimates were split by sex using the reported sex ratio and bounds of uncertainty.
2. A Meta-Regression with Bayesian priors, Regularization, and Trimming (MR-BRT) analysis was used to split the remaining both-sex estimates in the dataset. For each parameter, sex specific estimates were matched by location, age, year and a MR-BRT network meta-analysis was used to estimate pooled sex ratios and bounds of uncertainty. These were then used to split the both sex estimates in the dataset. The male: female prevalence ratio estimated was 0.55 (95% uncertainty interval [UI]: 0.38 – 0.72).
3. Studies reporting prevalence estimates across age groups spanning 25 years or more were split into five-year age groups using the prevalence age pattern estimated by DisMod-MR 2.1.

The DisMod-MR model used to estimate the age pattern did not contain any previously age split data.

Bias corrections / Crosswalks

Estimates with known biases were adjusted / crosswalked accordingly prior to DisMod-MR 2.1. For each crosswalk of interest, pairs of the reference and the alternative estimates were matched by age, sex, location and year. This was done for both within (where possible) and between study pairs. These pairs were then used as inputs in a MR-BRT network meta-analysis. The MR-BRT analysis produced a pooled ratio between the reference estimates and alternative estimates, which was used to adjust all alternative estimates in the dataset. For anxiety disorders, a past year recall ratio was used to adjust all past year recall estimates towards the level they would have been if the estimate had capture point/past-month prevalence. The latter prevalence period is less affected by recall bias. See Table 2 for adjustment factors used for anxiety disorders.

Table 2: MR-BRT Crosswalk Adjustment Factors for Anxiety disorders.

Data input	Reference or alternative case definition	Beta Coefficient, Log (95% UI)	Adjustment factor* (95% UI)	Gamma
Population Survey	Reference: past month or point prevalence			0.23
Population Survey	Alternative: past year prevalence	0.46<br>(0.01 – 0.91)	1.58<br>(0.99 – 2.41)	

**Adjustment factor is the transformed Beta coefficient in normal space and can be interpreted as the factor by which the alternative case definition is adjusted to reflect what it would have been if measured as the reference.*

UIs incorporate Gamma which represents the between study variance across all input data in the model. This added uncertainty widens the UIs for crosswalks with significant fixed effects.

Severity splits and disability weights

The GBD disability weight survey assessments are lay descriptions of sequelae highlighting major functional consequences and symptoms. The lay descriptions and disability weights for anxiety disorder severity levels are shown in Table 3. To determine the proportion of people with anxiety disorders within each of the severity levels we used data from The United States' Medical Expenditure Panel Survey (MEPS, conducted in annual waves since 1996)[5], the US National Epidemiological Survey on Alcohol and Related Conditions (NESARC, conducted in two waves from 2001–2002 and 2004–2005)[6], and the Australian National Survey of Mental Health and Wellbeing of Adults (NSMHWB, conducted in 1997)[7]. The proportion of anxiety disorder cases falling within each level of severity was: asymptomatic 28.8% (27.5% – 30.1%), mild 39.3% (34.2% – 44.2%), moderate 19.1% (15.8% – 22.7%) and severe 12.7% (9.2% – 16.7%).

Table 3. Severity distribution for Anxiety disorders in GBD 2019 and the associated disability weight (DW) with that severity.

Severity level	Lay description	DW (95% UI)
Mild	Feels mildly anxious and worried, which makes it slightly difficult to concentrate, remember things, and sleep. The person tires easily but is able to perform daily activities.	0.03 (0.018 – 0.046)
Moderate	Feels anxious and worried, which makes it difficult to concentrate, remember things, and sleep. The person tires easily and finds it difficult to perform daily activities.	0.133 (0.091 – 0.186)
Severe	Constantly feels very anxious and worried, which makes it difficult to concentrate, remember things, and sleep. The person has lost pleasure in life and thinks about suicide.	0.523 (0.362 – 0.677)

Modeling strategy

After the above data processes were applied, DisMod MR 2.1 was used to model the epidemiological data for anxiety disorders. The DisMod-MR modeling strategy for anxiety disorders followed the standard GBD 2019 decomposition structure. At each decomposition step, we compared the new model against the GBD 2017 best model and the best model from the previous step. All substantial changes between models were explored and explained. Adjustments to model priors or the dataset were made where appropriate. Where outliers were identified in the data, we re-assessed the study's methodology and quality before a decision was made to exclude or include the data.

Data across all epidemiological parameters were initially included in the modelling process. The incidence studies reported estimates which were very low relative to the prevalence data. As prevalence studies contributed much greater world coverage than incidence studies, we excluded the incidence data, relying instead on data from the other parameters. We assumed no incidence and prevalence before age 2 and after age 95. This minimum age of onset was corroborated with expert feedback and existing literature on anxiety disorders. Remission was set to a maximum of 0.2, consistent with the data points available.

The following location-level covariates were used to inform the estimation of prevalence in locations with no available data:

1. The mean war mortality rate in the previous 10 years. This covariate identified, for each GBD location, the mean mortality rate due to war and terrorism. It was used given existing evidence that shows a positive association between conflict status and the prevalence for anxiety disorders[8, 9].
2. The Gallup negative experience index. The Gallup initiative conducts comprehensive and comparable national surveys across a wide range of countries worldwide[10]. This index measured respondents' past day experiences of physical pain, worry, sadness, stress and anger. The Gallup covariate was included as a means to test for a correlation between negative emotions at a location level and anxiety disorder prevalence. Data from the Gallup negative experience index was modelled using the Spatio-temporal Gaussian process regression (STGPR) to produce estimates for all years and locations required by DisMod-MR. The log of the modelled output was used as the covariate in DisMod-MR due to skewedness of the data. The relationship detected was as expected, where the higher the negative emotion, the higher the prevalence rate detected.

A summary of covariates and exponentiated values for anxiety disorders are shown in Table 4.

Table 4. Summary of covariates used in the Anxiety disorders DisMod-MR meta-regression model

Covariate	Type	Parameter	Exponentiated beta (95% UI)
Mean war mortality rate in the previous 10 years	Location-level	Prevalence	1.65 (1.07 — 2.54)
Gallup: Negative experience index	Location-level	Prevalence	2.48 (1.80 — 3.61)

Changes between GBD 2017 and GBD 2019

There were five main changes in the GBD 2019 modelling strategy compared to GBD 2017:

1. In GBD 2019 we updated the age splitting by regional pattern methodology by increasing the age threshold for splitting to 25 years (in GBD 2017 it was 20 years). This meant that there were fewer estimates eligible for age splitting in this way. This impacted on the prevalence for some locations which now had fewer age-split estimates informing prevalence estimation.
2. In GBD 2017 bias corrections and sex ratios were estimated by DisMod-MR as part of the prevalence modelling. In GBD 2019 we conducted a MR-BRT analysis to accommodate for study heterogeneity and estimated pooled ratios with 95% UIs as previously discussed. Ratios estimated between 2017 and 2019 were largely consistent, although the UIs derived by MR-BRT tended to be larger. MR-BRT UIs incorporate Gamma which represents the between study variance across all input data in the model. This added uncertainty widens the UIs for crosswalks with significant fixed effects. For example:
    a. The male: female ratio was 0.54 (0.52 – 0.56) in GBD 2017 compared to 0.55 (0.38 – 0.72) in GBD 2019.
    b. The adjustment ratio for past year estimates was 1.48 (1.41 – 1.56) in GBD 2017 compared to 1.58 (0.99 – 2.41) in GBD 2019, leading to a slight overall decrease in adjusted prevalence.
3. The GBD 2017 model included an adjustment ratio (as a study level covariate within DisMod-MR) for estimates derived from school surveys. This covariate/adjustment was excluded in GBD 2019. The school survey adjustment was used in GBD 2017 based on the premise that school surveys might not be representative of the general population, especially in less developed parts of the world. Estimates derived from school surveys were adjusted downwards by 1.54 (1.36 – 1.75) towards the level of estimates from general household surveys. Part of the new GBD 2019 MR-BRT approach was to assess the availability of data for a given study-level covariate to produce robust matched pairs. We were only able to produce a small number of matched pairs for this covariate, primarily from high income countries which would not be representative of other locations. After further review of the literature and discussion with a number of experts in the area, it became apparent that there was insufficient evidence to fully support the direction and magnitude of the GBD 2017 covariate. It also appeared that bias between school surveys and household samples (and the extent to which the latter would be the gold standard) would vary considerably by location. Until more data becomes available to clarify the above, we have excluded this adjustment from the dataset, accepting both types of surveys. The removal of this adjustment from GBD 2019 meant that prevalence derived from student surveys were no longer being adjusted downwards to the extent they were in GBD 2017.
4. In GBD 2019 we included a second location level covariate, Gallup: negative experience index, to further improve the predictive power of the model. The Gallup covariate was significant at 2.48 (1.80 – 3.61). Resulting changes in prevalence by location were in the expected direction.
5. In GBD 2019 we included new epidemiological data from 18 locations (Argentina, Australia, Austria, Denmark, Finland, France, Germany, Lithuania, Mexico, Netherlands, Ningxia,

Portugal, Brazil, Spain, Switzerland, Taiwan, Iran, and United States) which further informed the DisMod-MR model. Some of these studies were from locations where we had no data previously (e.g., Argentina, Portugal, Iran)

While we continue to improve on the data and methods used to estimate the burden of mental disorders, some challenges need to be acknowledged. Firstly, our case definition for anxiety disorder will need to be revised to better capture changes to latest DSM/ICD criteria. Epidemiological estimates reporting an outcome for "any" or "total" anxiety disorders were included in GBD 2019, if they reported on at least three anxiety disorders. Future iterations of GBD will revisit the unique contribution of specific anxiety disorders. Secondly, we still have a large number of locations with no high-quality raw data available. Thirdly, it is difficult to quantify and remove all variation due to measurement error in our epidemiological estimates. Whilst we have improved the methodology used to account for known sources of bias, in some case, we still have very few data points to inform these adjustments. Fourthly, there is a paucity of research on the risk factors of mental disorders which can be used as predictive covariates in our epidemiological models.

References

1. American Psychiatric Association, *Diagnostic and Statistical Manual of Mental Disorders (DSM-IV-TR)*. Fourth Edition, Text Revision ed. 2000, Washington DC: American Psychiatric Association.
2. World Health Organization, *The ICD-10 Classification of Mental and Behavioural Disorders. Clinical descriptions and diagnostic guidelines.* 1992, World Health Organization: Geneva.
3. Baxter, A.J., et al., *Global prevalence of anxiety disorders: a systematic review and meta-regression.* Psychological Medicine, 2013. **43**(05): p. 897-910.
4. Baxter, A.J., et al., *The global burden of anxiety disorders in 2010.* Psychological Medicine, 2014. **44**(11): p. 2363-2374.
5. Health, U.D.o. and H. Services, *Agency for Healthcare Research and Quality, United States. Preventive services task force."Screening and treatment for major depressive disorder in children and adolescents: recommendation statement; March 2009*. 2010.
6. Introduction to the National Epidemiologic Survey on Alcohol and Related Conditions [http://pubs.niaaa.nih.gov/publications/arh29-2/74-78.htm]. Access date 1 December 2014.
7. Australian Bureau of Statistics, *National Survey of Mental Health and Wellbeing of Adults 1997. Canberra: Australian Bureau of Statistics*.
8. Karam, E. and G.M. Bou, *Psychosocial consequences of war among civilian populations.* Current Opinion in Psychiatry 2013. **16**(413–419).
9. Steel, Z., et al., *Association of torture and other potentially traumatic events with mental health outcomes among populations exposed to mass conflict and displacement: a systematic review and meta-analysis.* JAMA, 2009. **302**(5): p. 537-49.
10. Gallup, G., *The Gallup Poll: Public Opinion 2003*. 2004: Rowman & Littlefield.

Bulimia nervosa

Flowchart

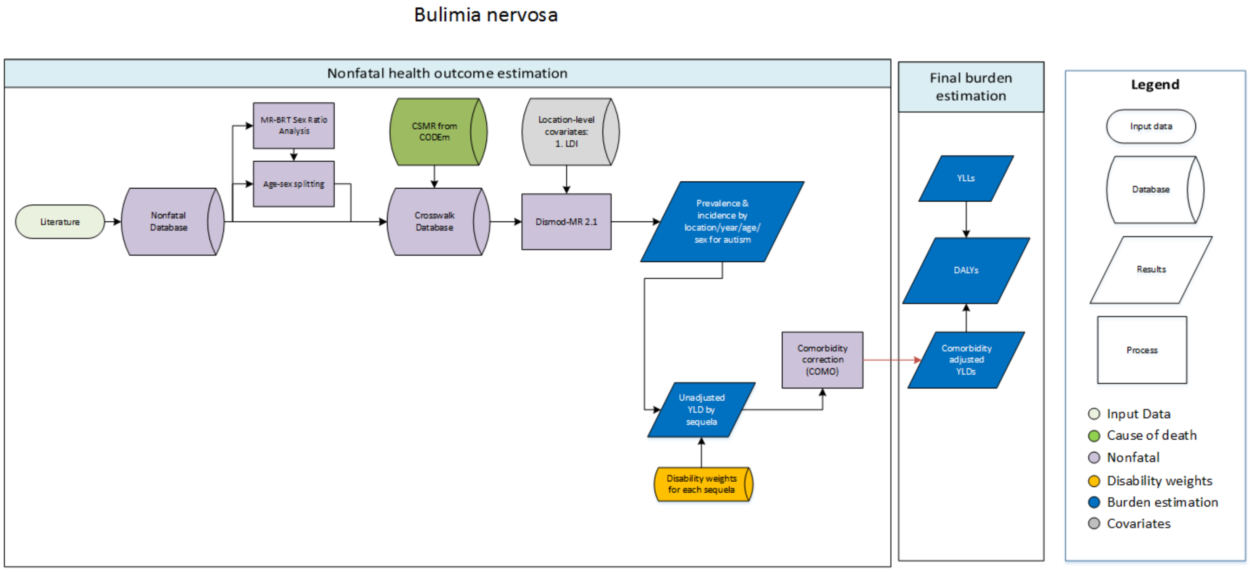

Input Data and Methodological Summary for Bulimia Nervosa

Case definition

According to the Diagnostic and Statistical Manual of Mental Disorders fourth edition, text revision (DSM-IV-TR),[1] bulimia nervosa (BN) is an eating disorder characterised by:

a) Recurrent episodes of binge eating. An episode of binge eating is characterised by both of the following:
   1) eating, in a discrete period of time (e.g., within any two-hour period), an amount of food that is definitely larger than most people would eat during a similar period of time and under similar circumstances
   2) a sense of lack of control over eating during the episode (e.g., a feeling that one cannot stop eating or control what or how much one is eating)

b) Recurrent inappropriate compensatory behavior in order to prevent weight gain, such as self-induced vomiting; misuse of laxatives, diuretics, enemas, or other medications; fasting; or excessive exercise.

c) The binge eating and inappropriate compensatory behaviors both occur, on average, at least twice a week for three months (changed to once a week for three months in DSM-5[2]).

d) Self-evaluation is unduly influenced by body shape and weight.

e) The disturbance does not occur exclusively during episodes of anorexia nervosa.

Included in GBD were cases meeting diagnostic criteria according to DSM[1] or the International Classification of Diseases (ICD).[3] These were identified by the following codes: 307.51 (DSM-IV-TR) and F50.2 (ICD-10). Different versions of DSM (DSM-III, DSM-III-R, DSM-IV, DSM-IV-TR, and DSM-5) and ICD (ICD-9 and ICD-10) were accepted.

Input data

Systematic literature reviews were conducted to capture studies reporting the prevalence, incidence, remission, and excess mortality of BN. These were conducted in three stages involving electronic

searches of the peer-reviewed literature (i.e., PsycInfo, Embase, and PubMed), the grey literature, and expert consultation. For mental disorders, we update our GBD electronic database searches on a two year rolling basis. A systematic review update for BN was conducted for GBD 2017, with the next literature update due for the next round of GBD. A grey literature search and expert consultation was conducted for GBD 2019 and produced new data sources for inclusion.

The GBD inclusion criteria stipulated that: (1) the publication year must be from 1980 onward; (2) "caseness" must be based on clinical threshold as established by the DSM or ICD; (3) sufficient information must be provided on study method and sample characteristics to assess the quality of the study; and (4) study samples must be representative of the general population (i.e., inpatient or pharmacological treatment samples, case studies, veterans, or refugee samples were excluded). No limitation was set on the language of publication. Methods used for this systematic review have been reported in greater detail elsewhere[6]. Table 1 below summarizes data inputs by parameter for bulimia nervosa.

Table 1: Data Inputs for bulimia nervosa morbidity modelling by parameter.

Measure	Total sources	Countries with data
All measures	86	34
Prevalence	66	31
Incidence	4	4
Remission	10	6
Standardized mortality ratio	6	4

Age and sex splitting

The extracted data underwent three types of age and sex splitting processes:

1. Where possible, estimates were further split by sex and age based on the data that was available. For instance, if studies reported prevalence for broad age groups by sex (e.g., prevalence in 15 to 65 year old males and females separately), and also by specific age groups but for both sexes combined (e.g., prevalence in 15- to 30-year-olds, then in 31- to 65-year-olds, for males and females combined); age-specific estimates were split by sex using the reported sex ratio and bounds of uncertainty.
2. A Meta-Regression with Bayesian priors, Regularization, and Trimming (MR-BRT) analysis was used to split the remaining both-sex estimates in the dataset. For each parameter, sex specific estimates were matched by location, age, year and a MR-BRT network meta-analysis was used to estimate pooled sex ratios and bounds of uncertainty. These were then used to split the both sex estimates in the dataset. The male: female prevalence ratio estimated was 0.37 (95% uncertainty interval [UI]: 0.26-0.47).
3. Studies reporting prevalence estimates across age groups spanning 25 years or more, were split into five-year age groups using the prevalence age pattern estimated by DisMod-MR 2.1. The DisMod-MR model used to estimate the age pattern did not contain any previously age split data.

Bias corrections / Crosswalks

We tested for a number of potential sources for bias in prevalence between studies (e.g., use of ICD criteria vs. DSM criteria). However none of the crosswalks had a statistically significant impact on prevalence and so no bias corrections were applied to these estimates.

Disability weight

The GBD disability weight survey assessments are lay descriptions of sequelae highlighting major functional consequences and symptoms. No severity splits were applied to BN. The lay description and disability weight for BN are shown in Table 2 below.

Table 2: Health state and disability weight for bulimia nervosa

Lay description	DW (95% UI)
Has uncontrolled overeating followed by guilt, starving, and vomiting to lose weight.	0.223 (0.149 – 0.311)

Modelling strategy

After the above data processes were applied, DisMod MR 2.1 was used to model the epidemiological data for BN. The DisMod-MR modeling strategy for BN followed the standard GBD 2019 decomposition structure. At each decomposition step, we compared the new model against the GBD 2017 best model and the best model from the previous step. All substantial changes between models were explored and explained. Adjustments to model priors or the dataset were made where appropriate. Where outliers were identified in the data, we re-assessed the study's methodology and quality before a decision was made to exclude or include the data.

We assumed no incidence prior to 10 years of age or onward from 40 years of age. We used the function in DisMod-MR 2.1 to pull in cause-specific mortality rate (CSMR) data from our CODEm and CODcorrect analyses. As such, other mortality data (standardised mortality ratios and relative risks) were excluded. We also used CSMR data to estimate priors on excess mortality rates (EMR) by matching them with prevalence data points for the same geography and study year and dividing CSMR by prevalence. A country-level covariate, lagged distributed income (LDI), was also included. This covariate represents a moving average of gross domestic product (GDP) over time. The limits placed on this covariate meant that prevalence was assumed to increase with rising GDP. LDI was also applied to excess mortality data in order to better inform regional distribution. A summary of location-level covariates and exponentiated values for BN are shown in Table 3.

Table 3: Location-level covariates used for bulimia nervosa

Covariate	Parameter	beta	Exponentiated beta
LDI ($ per capita)	Prevalence	0.43 (0.32 — 0.50)	1.54 (1.38 — 1.64)
LDI ($ per capita)	Excess mortality	-0.29 (-0.46 — -0.13)	0.75 (0.63 — 0.88)

Changes between GBD 2017 and GBD 2019

There were three main changes in the GBD 2019 modelling strategy compared to GBD 2017:

1. In GBD 2017 the sex ratio was estimated by DisMod MR 2.1 as part of the prevalence modelling. In GBD 2019 we made use of MR-BRT to run a nested meta-regression analysis on the within-study sex ratios to estimate a pooled sex ratio with 95% UIs as previously discussed. Compared to GBD 2017, prevalence male: female ratio increased from 0.26 (0.18 — 0.37) to 0.37 (0.26 — 0.47).
2. We removed the study-level covariate for studies using ICD criteria as there was no significant difference between these studies and studies using DSM criteria.
3. In GBD 2019 we included new epidemiological data sources from 6 locations (Austria, China, Iran, Saudi Arabia, Switzerland, and United States of America).

While we continue to improve on the data and methods used to estimate the burden of mental disorders, some challenges need to be acknowledged. Firstly, we still have a large number of locations with no high-quality raw data available. Secondly, it is difficult to quantify and remove all variation due to measurement error in our epidemiological estimates. Whilst we have improved the methodology used to account for known sources of bias, in some case, we still have very few data points to inform these adjustments. Thirdly, there is a paucity of research on the risk factors of mental disorders which can be used as predictive covariates in our epidemiological models.

References

1. American Psychiatric Association. Diagnostic and Statistical Manual of Mental Disorders (DSM-IV-TR). 4th, Text Revision ed. Washington DC: American Psychiatric Association; 2000.
2. American Psychiatric Association. Diagnostic and Statistical Manual of Mental Disorders, Fifth Edition. Arlington, VA: American Psychiatric Association; 2013.
3. World Health Organisation. ICD-10 Classification of Mental and Behavioural Disorders: Clinical descriptions and diagnostic guidelines. Geneva: World Health Organisation; 1992.
4. Whiteford HA, Degenhardt L, Rehm J, et al. Global burden of disease attributable to mental and substance use disorders: findings from the Global Burden of Disease Study 2010. *The Lancet* 2013; **382**(9904): 1575-86.

Autism spectrum disorders

Flowchart

Autism Spectrum Disorders

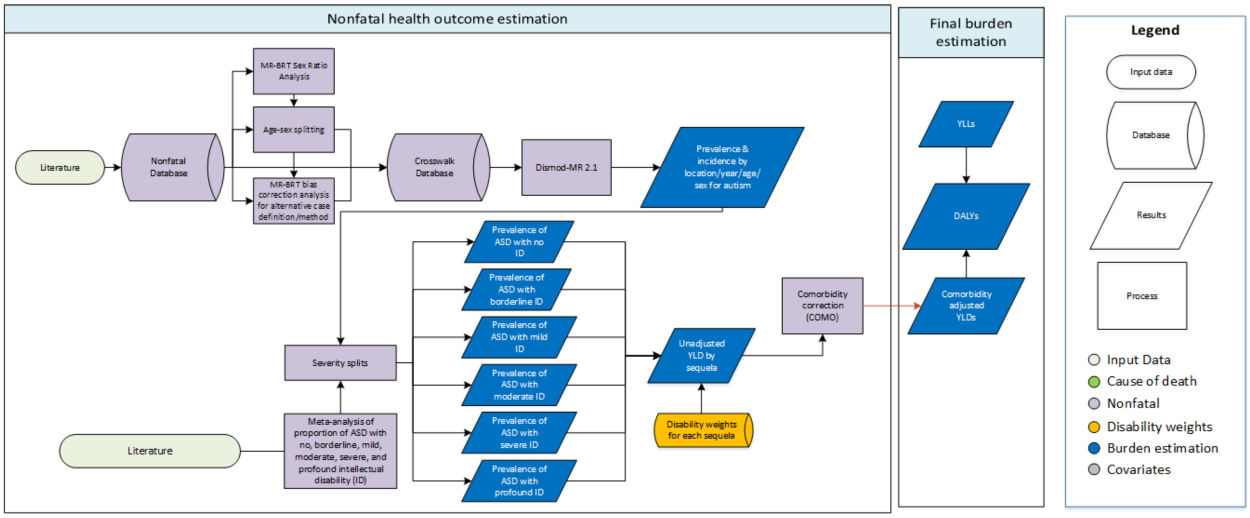

Input Data and Methodological Summary for Autism Spectrum Disorders

Case definition

Autism spectrum disorders (ASD; also known as pervasive developmental disorders) are a group of neurodevelopmental disorders with onset occurring in early childhood. ASD is characterised by pervasive impairment in several areas of development, including social interaction and communication skills, along with restricted and repetitive patterns of behaviours and/or interests.

ASD was an umbrella for five sub-disorders according to the Diagnostic and Statistical Manual of Mental Disorders fourth edition, text revision[2] (DSM-IV-TR): Autistic disorder (299.00), Pervasive Developmental Disorder, Pervasive Developmental Disorder Not Otherwise Specified (299.80), Rett's disorder (299.8), Asperger's Disorder (299.8) and Childhood Disintegrative Disorder (299.10). ASD is still an umbrella for eight sub-disorders according to the International Statistical Classification of Diseases and Related Health Problems 10th Revision[1] (ICD10): Childhood autism (F84.0), Atypical autism (F84.1), Rett syndrome (F84.2), Other childhood disintegrative disorder (F84.3), Overactive disorder associated with mental retardation and stereotyped movements (F84.4), Asperger syndrome (F84.5), Other pervasive developmental disorders (F84.8), and Pervasive disorder unspecified (F84.9). However, it has been amalgamated into a single disorder in the Diagnostic and Statistical Manual for Mental Disorders 5th edition[3] (DSM-5). A diagnosis of ASD according to the DSM-5[3] requires the following criteria to be met:

Persistent deficits in social communication and social interaction across multiple contexts, as manifested by all of the following, currently or by history:

1. *Deficits in social-emotional reciprocity, ranging, for example, from abnormal social approach and failure of normal back-and-forth conversation; to reduced sharing of interests, emotions, or affect; to failure to initiate or respond to social interactions.*
2. *Deficits in nonverbal communicative behaviors used for social interaction, ranging, for example, from poorly integrated verbal and nonverbal communication; to abnormalities in*

eye contact and body language or deficits in understanding and use of gestures; to a total lack of facial expressions and nonverbal communication.

3. *Deficits in developing, maintaining, and understanding relationships, ranging, for example, from difficulties adjusting behavior to suit various social contexts; to difficulties in sharing imaginative play or in making friends; to absence of interest in peers.*

Restricted, repetitive patterns of behaviour, interests, or activities, as manifested by at least two of the following, currently or by history:

1. *Stereotyped or repetitive motor movements, use of objects, or speech (eg, simple motor stereotypies, lining up toys or flipping objects, echolalia, idiosyncratic phrases).*
2. *Insistence on sameness, inflexible adherence to routines, or ritualised patterns of verbal or nonverbal behavior (eg, extreme distress at small changes, difficulties with transitions, rigid thinking patterns, greeting rituals, need to take same route or eat same food every day).*
3. *Highly restricted, fixated interests that are abnormal in intensity or focus (eg, strong attachment to or preoccupation with unusual objects, excessively circumscribed or perseverative interests).*
4. *Hyper- or hyporeactivity to sensory input or unusual interest in sensory aspects of the environment (eg, apparent indifference to pain/temperature, adverse response to specific sounds or textures, excessive smelling or touching of objects, visual fascination with lights or movement).*

The symptoms must be present in the early developmental period, cause clinically significant impairment, and not be better explained by intellectual impairment or global developmental delay.

Input data

The epidemiological systematic review for ASD was conducted in three stages involving electronic searches of the peer-reviewed literature (i.e., PsycInfo, Embase, and PubMed), the grey literature, and expert consultation. For mental disorders, we update our GBD electronic database searches on a two-year rolling basis. A new systematic review for ASD was conducted for GBD 2017, with the next electronic literature update due for the next round of GBD. The grey literature search, and expert consultation was conducted for GBD 2019 and produced an additional four studies.

The GBD inclusion criteria stipulated that: (1) the diagnostic criteria must be from 1980 onward; (2) "caseness" must be based on clinical threshold as established by the DSM, ICD, Chinese Classification of Mental Disorders (CCMD), or diagnosed by a clinician using established tools; (3) sufficient information must be provided on study method and sample characteristics to assess the quality of the study; and (4) study samples must be representative of the general population (i.e., case studies, veterans, or refugee samples were excluded). No limitation was set on the language of publication. Due to insufficient data on ASD, estimates of the prevalence of the DSM-IV-TR sub-disorder Autistic disorder (299.00), ICD-10 Childhood autism (F84.0), and their DSM-III, DSM-II-R, DSM-IV, ICD9, and CCMD equivalents were also included with an adjustment so that they reflected what these estimates would be if the data represented ASD. Table 1 below summarizes data inputs by parameter for Autism spectrum disorders.

Table 1: Data Inputs for Autism spectrum disorders morbidity modelling by parameter.

Measure	Total sources	Countries with data
All measures	167	34
Prevalence	164	34
Standardized mortality ratio	3	2

Age and sex splitting

The extracted data underwent three types of age and sex splitting processes:

1. Where possible, estimates were further split by sex and age based on the data that was available. For instance, if studies reported prevalence for broad age groups by sex (e.g., prevalence in 15 to 65 year old males and females separately), and also by specific age groups but for both sexes combined (e.g., prevalence in 15- to 30-year-olds, then in 31- to 65-year-olds, for males and females combined); age-specific estimates were split by sex using the reported sex ratio and bounds of uncertainty
2. A Meta-Regression with Bayesian priors, Regularization, and Trimming (MR-BRT) analysis was used to split the remaining both-sex estimates in the dataset. For each parameter, sex specific estimates were matched by location, age, year and a MR-BRT network meta-analysis was used to estimate pooled sex ratios and bounds of uncertainty. These were then used to split the both sex estimates in the dataset. The male: female prevalence ratio was 4.39 (95% uncertainty interval [UI]: 3.36 – 5.41).
3. Studies reporting prevalence estimates across age groups spanning 25 years or more, were split into five-year age groups using the prevalence age pattern estimated by DisMod-MR 2.1. The DisMod-MR model used to estimate the age pattern did not contain any previously age split data.

Bias corrections / Crosswalks

Estimates with known biases were adjusted / crosswalked accordingly prior to DisMod-MR 2.1. Within the ASD epidemiological dataset, within and between study estimates were paired by age, sex, location, and year, between the reference and alternative estimates. Pairs were also made between the different alternative estimates. The ratios between these estimates were then used as inputs in a MR-BRT network meta-analysis. This analysis produced pooled ratios between the reference estimates and alternative estimates. These ratios (see Table 2) were used to adjust all alternative estimates in the dataset. ASD had 4 alternative definitions to crosswalk:

1. Estimates of autism (rather than of ASD).
2. General population survey without additional case-finding – These are studies that conduct household or school surveys but do not conduct additional active case-finding (such as reviewing special education records) to find cases likely to be missed by survey methodology.
3. Record report – These are studies where prevalence of ASD is estimated from diagnoses within a clinical or educational registry where no population screening procedure is in place.
4. Review of record notes – These are studies where researchers review notes of high-risk populations from one or more data sources records (e.g., clinical/education records) and determine prevalence based on notes without confirming the diagnosis via clinical evaluation.

Table 2: MR-BRT Crosswalk Adjustment Factors for ASD

Data input	Reference or alternative case definition	Beta Coefficient, Log (95% UI)	Adjustment factor* (95% UI)	Gamma
Population survey	Reference: Estimate represents ASD from general population surveys, with additional case finding or total population screening			0.29
Population survey	Alternative: Estimate represents autism (rather than ASD)	-0.93 (-1.49 – -0.36)	0.40 (0.23 – 0.70)	
Population survey	Alternative: General population survey without additional case finding	-0.29 (-0.91 – 0.33)	0.75 (0.40 – 1.39)	
Registry	Alternative: Record report	-0.17 (-0.74 – 0.41)	0.85 (0.48 – 1.50)	
Surveillance	Alternative: Review of record notes	0.22 (-0.40 – 0.83)	1.24 (0.67 – 2.30)	

**Adjustment factor is the transformed Beta coefficient in normal space, and can be interpreted as the factor by which the alternative case definition is adjusted to reflect what it would have been if measured as the reference.*

UIs incorporate Gamma which represents the between study variance across all input data in the model. This added uncertainty widens the UIs for crosswalks with significant fixed effects.

Severity splits and disability weights

ASD is one of the causes that contributes to the intellectual disability (ID) envelope. As such, a gradation of ASD by level of severity was needed. Meta-analyses were conducted using data from 19 studies that used gold-standard sampling methodology and reported information on the IQ level of those with ASD in order to calculate the severity splits by six sequelae: ASD with 1) no ID, 2) borderline ID, 3) mild ID, 4) moderate ID, 5) severe ID, and 6) profound ID.

The disability weights for each sequela of ASD were calculated using the disability weights for the health states Autism, Asperger's syndrome & other ASD, borderline ID, mild ID, moderate ID, severe ID, and profound ID. These disability weights and their lay descriptions are presented in the table below.

Table 3: Health states and disability weights used to estimate sequela-specific disability weights for ASD.

Health state	Lay description	DW (95% UI)
Autism	Has severe problems interacting with others and difficulty understanding simple questions or directions. The person has great difficulty with basic daily activities and becomes distressed by any change in routine.	0.262 (0.176 – 0.365)
Asperger's syndrome & other ASDs	Has difficulty interacting with other people and is slow to understand or respond to questions. The person is often preoccupied with one thing and has some difficulty with basic daily activities.	0.104 (0.071 – 0.147)
ID, borderline	Is slow in learning at school. As an adult, the person has some difficulty doing complex or unfamiliar tasks but otherwise functions independently.	0.011 (0.005 – 0.020)

ID, mild	Has low intelligence and is slow in learning at school. As an adult, the person can live independently, but often needs help to raise children and can only work at simple supervised jobs.	0.043 (0.026 – 0.064)
ID, moderate	Has low intelligence, and is slow in learning to speak and to do even simple tasks. As an adult, the person requires a lot of support to live independently and raise children. The person can only work at the simplest supervised jobs.	0.100 (0.066 – 0.142)
ID, severe	Has very low intelligence and cannot speak more than a few words, needs constant supervision and help with most daily activities, and can do only the simplest tasks.	0.160 (0.107 – 0.226)
ID, profound	Has very low intelligence, has almost no language, and does not understand even the most basic requests or instructions. The person requires constant supervision and help for all activities.	0.200 (0.133 – 0.283)

To estimate the disability weights for each sequela of ASD, the following steps were conducted, with each step pulling 1,000 draws of each input:

1. A pooled disability weight for ASD was estimated:
$$DW_{ASD} = DW_{Autism} \times P_{Autism} + DW_{Asperger} \times (1 - P_{Autism})$$
Where *DW* is disability weight and *P* is the proportion of ASD cases estimated to meet DSM-IV criteria for the autism subtype.
2. The disability weight for ASD without ID was estimated:
$$DW_{ASD\ no\ ID} = \frac{DW_{ASD} - \sum_{k=Bord.ID}^{Prof.ID}(P_k \times DW_k)}{P_{ASD\ no\ ID} + \sum_{k=Bord.ID}^{Prof.ID}(P_k \times (1 - DW_k))}$$
Where *DW* is disability weight and *P* is the severity proportion estimated from the meta-analysis.
3. The disability weight for ASD and each remaining level of ID was estimated:
$$DW_{ASD+ID} = 1 - (1 - DW_{ASD\ no\ ID}) \times (1 - DW_{ID})$$

The severity proportions from the meta-analysis used in the above process and the resulting disability weights for each sequela are presented in table 4 below.

Table 4: MR-BRT Crosswalk Adjustment Factors for ASD

Sequela	Severity proportion (95% UI)	DW (95% UI)
ASD without ID	0.428 (0.369 – 0.491)	0.143 (0.094 – 0.202)
ASD with borderline ID	0.187 (0.144 – 0.236)	0.152 (0.103 – 0.212)
ASD with mild ID	0.180 (0.134 – 0.231)	0.179 (0.125 – 0.245)
ASD with moderate ID	0.133 (0.094 – 0.177)	0.228 (0.160 – 0.310)
ASD with severe ID	0.057 (0.034 – 0.091)	0.279 (0.195 – 0.378)
ASD with profound ID	0.014 (0.006 – 0.025)	0.313 (0.215 – 0.422)

Modelling strategy

After the above data processes were applied, DisMod MR 2.1 was used to model the epidemiological data for ASD. The DisMod-MR modeling strategy for ASD followed the standard GBD 2019 decomposition structure. At each decomposition step, we compared the new model against the GBD 2017 best model and the best model from the previous step. All substantial changes between models were explored and explained. Adjustments to model priors or the dataset were made where appropriate. Where outliers were identified in the data, we re-assessed the study's methodology and quality before a decision was made to exclude or include the data.

We assumed all incidence of ASD occurred at birth. Remission was set to 0 after expert consultation revealed we would not expect remission for ASD.

Changes between GBD 2017 and GBD 2019

There were three main changes in the GBD 2019 modelling strategy compared to GBD 2017:

1. In GBD 2017 the sex ratio was estimated by DisMod MR 2.1 as part of the prevalence modelling. In GBD 2019 we made use of MR-BRT to run a nested meta-regression analysis on the within-study sex ratios to estimate a pooled sex ratio with 95% UI as previously discussed. The prevalence male : female sex ratio was 4.03 (3.47 – 4.69) in GBD 2017 compared to 4.39 (3.36 – 5.41) in GBD 2019.
2. In GBD 2019 we made use of MR-BRT to run a nested network meta-regression to estimate adjustments to alternative data prior to running DisMod MR 2.1. Ratios estimated between 2017 and 2019 were largely consistent, although the UIs derived by MR-BRT tended to be larger. MR-BRT UIs incorporate Gamma which represents the between study variance across all input data in the model. This added uncertainty widens the UIs for crosswalks with significant fixed effects.
    a. The adjustment ratio for autism to ASD estimates was 0.43 (0.35 – 0.51) in GBD 2017 vs 0.40 (0.23 – 0.70) in GBD 2019
    b. The adjustment ratio for general population survey without additional case finding estimates was 0.87 (0.70 – 1.11) in GBD 2017 vs 0.75 (0.40 – 1.39) in GBD 2019
    c. The adjustment ratio for record report estimates was 0.71 (0.71 – 0.71) in GBD 2017 vs 0.85 (0.48 – 1.50) in GBD 2019

d. The adjustment ratio for review of record notes estimates was 1.48 (1.23 – 1.78) in GBD 2017 vs 1.24 (0.67 – 2.30) in GBD 2019

3. In GBD 2019 we included new epidemiological data from 4 locations (Sweden, Lithuania, Tehran in Iran, and Rio Grande do Sul in Brazil).

While we continue to improve on the data and methods used to estimate the burden of mental disorders, some challenges need to be acknowledged. Firstly, we still have a large number of locations with no high-quality raw data available. Secondly, it is difficult to quantify and remove all variation due to measurement error in our epidemiological estimates. Whilst we have attempted to account for known sources of bias, in some case we still have very few data points to inform these adjustments and to explore other interactions/ bias adjustments. For example there is not enough data to explore the interaction between record report estimates and time or healthcare access quality. This could potentially inflate prevalence in locations with good healthcare access quality where the majority of ASD cases are diagnosed, and underestimate prevalence in locations where healthcare access quality is poor and the majority of ASD cases are missed. We also did not explore interactions between the estimated sex ratio and case detection method which may lead to a change in the sex ratio for ASD. Thirdly, there is a paucity of research on the risk factors of mental disorders which can be used as predictive covariates in our epidemiological models.

References

1. World Health Organisation. ICD-10 Classification of Mental and Behavioural Disorders: Clinical descriptions and diagnostic guidelines. Geneva: World Health Organisation; 1992.

2. American Psychiatric Association. Diagnostic and Statistical Manual of Mental Disorders (DSM-IV-TR). 4th, Text Revision ed. Washington DC: American Psychiatric Association; 2000.

3. American Psychiatric Association. Diagnostic and statistical manual of mental disorders : DSM-5. Arlington, VA: American Psychiatric Publishing; 2013.

4. Baxter AJ, Brugha TS, Erskine HE, Scheurer RW, Vos T, Scott JG. The epidemiology and global burden of autism spectrum disorders. *Psychological Medicine* 2014; **45**(3): 601-13.

Attention-deficit/hyperactivity disorder

Flowchart

Attention-deficit/hyperactivity disorder

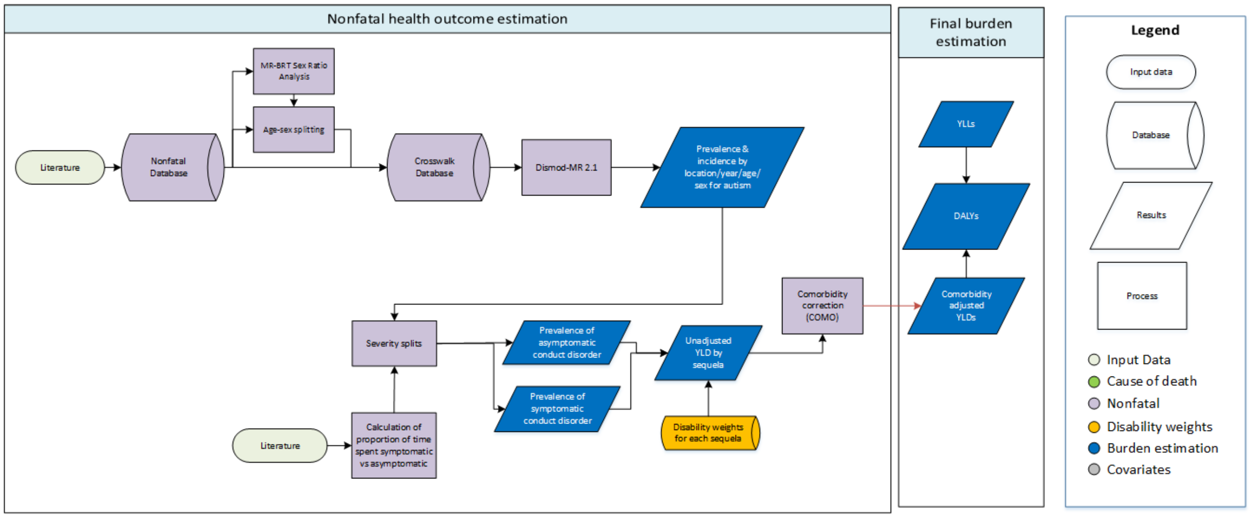

Input Data and Methodological Summary for Attention-deficit/hyperactivity disorder

Case definition

Attention-deficit/hyperactivity disorder (ADHD) is an externalising disorder characterised by persistent inattention and/or hyperactivity-impulsivity. As per criteria set by the Diagnostic and Statistical Manual of Mental Disorders Fourth Edition, Text Revision (DSM-IV-TR)[1], diagnosis requires six or more symptoms of inattention or hyperactivity-impulsivity to have persisted for at least six months in two or more settings causing significant impairment to functioning, with at least some impairing symptoms being present prior to 7 years of age (12 years of age in DSM-5[2]). Recognised symptoms include:

Inattention:

- often fails to give close attention to details or makes careless mistakes in schoolwork, work, or other activities
- often has difficulty sustaining attention in tasks or play activities
- often does not seem to listen when spoken to directly
- often does not follow through on instructions and fails to finish schoolwork, chores, or duties in the workplace (not due to oppositional behavior or failure to understand instructions)
- often has difficulty organising tasks and activities
- often avoids, dislikes, or is reluctant to engage in tasks that require sustained mental effort (such as schoolwork or homework)
- often loses things necessary for tasks or activities (eg, toys, school assignments, pencils, books, or tools)
- is often easily distracted by extraneous stimuli
- is often forgetful in daily activities

Hyperactivity:

- often fidgets with hands or feet or squirms in seat
- often leaves seat in classroom or in other situations in which remaining seated is expected

- often runs about or climbs excessively in situations in which it is inappropriate (in adolescents or adults, may be limited to subjective feelings of restlessness)
- often has difficulty playing or engaging in leisure activities quietly
- is often “on the go” or often acts as if “driven by a motor”
- often talks excessively

Impulsivity:

- often blurts out answers before questions have been completed
- often has difficulty awaiting turn
- often interrupts or intrudes on others (eg, butts into conversations or games)

Included in GBD were cases meeting diagnostic criteria according to DSM[1] or the International Classification of Diseases (ICD)[3] (called “hyperkinetic disorder” in ICD). These were identified by the following codes: 314.0, 314.01 (DSM-IV-TR) and F90 (ICD-10). Different versions of DSM (DSM-III, DSM-III-R, DSM-IV, DSM-IV-TR, and DSM-5) and ICD (ICD-9 and ICD-10) were accepted.

Input data

For mental disorders, we update our GBD electronic database searches on a two year rolling basis. In GBD 2019 a systematic literature review update was conducted to identify new epidemiological studies on ADHD published between September 2016 and December 2018. We included studies reporting the prevalence, remission, incidence, duration, and/or excess mortality associated with ADHD. The systematic review of the literature for ADHD was conducted in conjunction with conduct disorder as they are childhood behavioural disorders and are often reported together. The search was conducted in three stages involving electronic searches of the peer-reviewed literature (i.e., using PsycInfo, Embase, and PubMed), the grey literature, and expert consultation. The following search terms were used to develop search strings across all databases searched: ‘attention’, ‘disorder’, ‘hyperactive’, ‘hyperkinetic’, ‘adhd’, ‘conduct disorder’, ‘disruptive’, ‘externalising’ and ‘prevalence’, ‘mortality’, ‘death’, ‘incidence’, ‘remission’, ‘duration’, ‘remit’, ‘epidemiology’.

The search generated 3135 records (after duplicates were removed) across the three electronic databases. The title/abstract screening reduced the number of relevant records to 224 studies, of which 22 studies met criteria for inclusion for ADHD. An additional 4 studies were identified through a grey literature search and consultations with experts. Overall, in GBD 2019 we added 26 new studies into the ADHD dataset.

The GBD inclusion criteria stipulated that: (1) the publication year must be from 1980 onward; (2) “caseness” must be based on clinical threshold as established by the DSM or ICD; (3) sufficient information must be provided on study method and sample characteristics to assess the quality of the study; (4) study sample must be representative of the general population (i.e., inpatient or pharmacological treatment samples, case studies, veterans, or refugee samples were excluded). No limitation was set on the language of publications. Methods used in previous systematic reviews have been reported in greater detail elsewhere.[4]Table 1 below summarizes data inputs by parameter for ADHD.

Table 1: Data Inputs for Attention-deficit/hyperactivity disorder morbidity modelling by parameter.

Measure	Total sources
All measures	188
Prevalence	172
Incidence	2
Remission	14
Standardized mortality ratio	2
Proportion	1

Age and sex splitting

The extracted data underwent two types of age and sex splitting processes:

1. Where possible, estimates were further split by sex and age based on the data that was available. For instance, if studies reported prevalence for broad age groups by sex (e.g., prevalence in 15 to 65 year old males and females separately), and also by specific age groups but for both sexes combined (e.g., prevalence in 15- to 30-year-olds, then in 31- to 65-year-olds, for males and females combined); age-specific estimates were split by sex using the reported sex ratio and bounds of uncertainty.
2. A Meta-Regression with Bayesian priors, Regularization, and Trimming (MR-BRT) analysis was used to split the remaining both-sex estimates in the dataset. For each parameter, sex specific estimates were matched by location, age, year and a MR-BRT network meta-analysis was used to estimate pooled sex ratios and bounds of uncertainty. These were then used to split the both sex estimates in the dataset. The male: female prevalence ratio estimated was 2.52 (95% uncertainty interval [UI]: 0.57 – 4.46).

Bias corrections / Crosswalks

No crosswalks were applied to the estimates for ADHD. The reasons for this are discussed in the *Changes between GBD 2017 and GBD 2019* section.

Severity splits and disability weight

The GBD disability weight survey assessments are lay descriptions of sequelae highlighting major functional consequences and symptoms. The lay description and disability weight for ADHD is shown in Table 2. A severity split for the proportion of time spent symptomatic versus asymptomatic was based on data from the Great Smoky Mountains Study which assessed the levels of disability found in children and adolescents with mental disorders.[5] Of those with ADHD, 48% reported disability while 20% of individuals with no diagnosis reported disability at the time of survey. Using these as estimates of the proportion of time with disability in the "average case," the proportion of disability in children without a diagnosis was subtracted from the proportion with disability for ADHD, giving an adjusted proportion of 28%. Detailed descriptions of this methodology have been published elsewhere.[6]

Table 2. Lay description for ADHD in GBD 2019 and the associated disability weight (DW).

**Lay description**	**DW (95% UI)**
Is hyperactive and has difficulty concentrating, remembering things, and completing tasks	0.045 (0.028–0.066)

Modelling strategy

After the above data processes were applied, DisMod MR 2.1 was used to model the epidemiological data for ADHD. The DisMod-MR modeling strategy for ADHD followed the standard GBD 2019 decomposition structure. At each decomposition step, we compared the new model against the GBD 2017 best model and the best model from the previous step. All substantial changes between models were explored and explained. Adjustments to model priors or the dataset were made where appropriate. Where outliers were identified in the data, we re-assessed the study's methodology and quality before a decision was made to exclude or include the data.

Data across all epidemiological parameters were initially included in the modelling process. We assumed no incidence prior to 3 years of age or onward from 12 years of age. The minimum age of onset was set in consultation with experts and based on current literature, while the upper age limit on incidence was set in line with the latest DSM-5 criteria. Remission was set to zero prior to 12 years, in line with the restriction on incidence. Excess mortality was set to zero given only three estimates were found for this parameter and there was insufficient data to suggest an elevated risk of mortality in those with ADHD. For ADHD, there are no country–level covariates used to inform the estimation of prevalence in locations with no available data.

Changes between GBD 2017 and GBD 2019

There were four main changes to the GBD 2019 modelling strategy compared to GBD 2017:

1. In GBD 2017 sex ratios were estimated by DisMod-MR as part of the prevalence modelling. In GBD 2019 we conducted a MR-BRT analysis instead. The prevalence male: female ratio was 2.50 (2.11 – 2.91) in GBD 2017 compared to 2.52 (0.57 – 4.46) in GBD 2019.
2. The GBD 2017 model includes three prevalence study-level covariates with downward adjustments to the data that was were not used in GBD 2019;
    a. A covariate adjusting prevalence from small community samples towards the level of nationally representative samples. As only a small number of studies in the dataset were flagged as having alternative data points on this covariate, for which comparable reference data points were not available, a review of their eligibility for inclusion was conducted instead for GBD 2019.
    b. Two covariates adjusting for estimates which did not require agreement between survey informants (e.g. parent and child), and/or did not require impairment for diagnosis. The change in standard GBD methodology between GBD 2017 and GBD 2019 meant that all covariates were required to be based on either within-study or between-study pairs. No within-study pairs were available for these two covariates. Upon consultation with experts, it was a) unclear whether there was systematic bias between these types of survey methodologies, and b) determined that the use of between-study pairs would not be feasible given other variability in methodology between studies that would further impact the ratios e.g. instrument, informant, reporting, age. It was therefore decided that the best approach would be to omit these covariates rather than

attempt to apply crosswalks with known issues that would significantly impact their interpretability.

3. In GBD 2019 we included new epidemiological data from 18 locations (Argentina, Australia, Austria, Brazil, China, Colombia, Cyprus, Finland, United Kingdom, India, Iran, Lebanon, Nigeria, Saudi Arabia, South Korea, Spain, Taiwan, and United States).

While we continue to improve on the data and methods used in GBD, some challenges need to be acknowledged. Firstly, we still have a large number of locations with no high quality raw data available for some disorders. Secondly, it is difficult to quantify and remove all variations due to measurement error in our prevalence estimates. Whilst we have improved the methodology used to account for known sources of bias (e.g., survey methods or case definitions), we still have very few data points to inform such adjustments. Additionally, there is a paucity of research on the risk factors of mental disorders which can be used as predictive covariates in our epidemiological models.

References

1. American Psychiatric Association. Diagnostic and Statistical Manual of Mental Disorders (DSM-IV-TR). 4th, Text Revision ed. Washington DC: American Psychiatric Association; 2000.
2. American Psychiatric Association. Diagnostic and Statistical Manual of Mental Disorders, Fifth Edition. Arlington, VA: American Psychiatric Association; 2013.
3. World Health Organisation. ICD-10 Classification of Mental and Behavioural Disorders: Clinical descriptions and diagnostic guidelines. Geneva: World Health Organisation; 1992.
4. Erskine HE, Ferrari AJ, Nelson P, et al. Research Review: Epidemiological modelling of attention-deficit/hyperactivity disorder and conduct disorder for the Global Burden of Disease Study 2010. *Journal of Child Psychology and Psychiatry* 2013; **54**(12): 1263-74.
5. Ezpeleta L, Keeler G, Erkanli A, Costello EJ, Angold A. Epidemiology of Psychiatric Disability in Childhood and Adolescence. *J Child Psychol Psychiatry* 2001; **42**(7): 901-14.
6. Erskine HE, Ferrari AJ, Polanczyk GV, et al. The global burden of conduct disorder and attention-deficit/hyperactivity disorder in 2010. *J Child Psychol Psychiatry* 2014; **55**(4): 328-36.

Conduct disorder

Flowchart

Conduct disorder

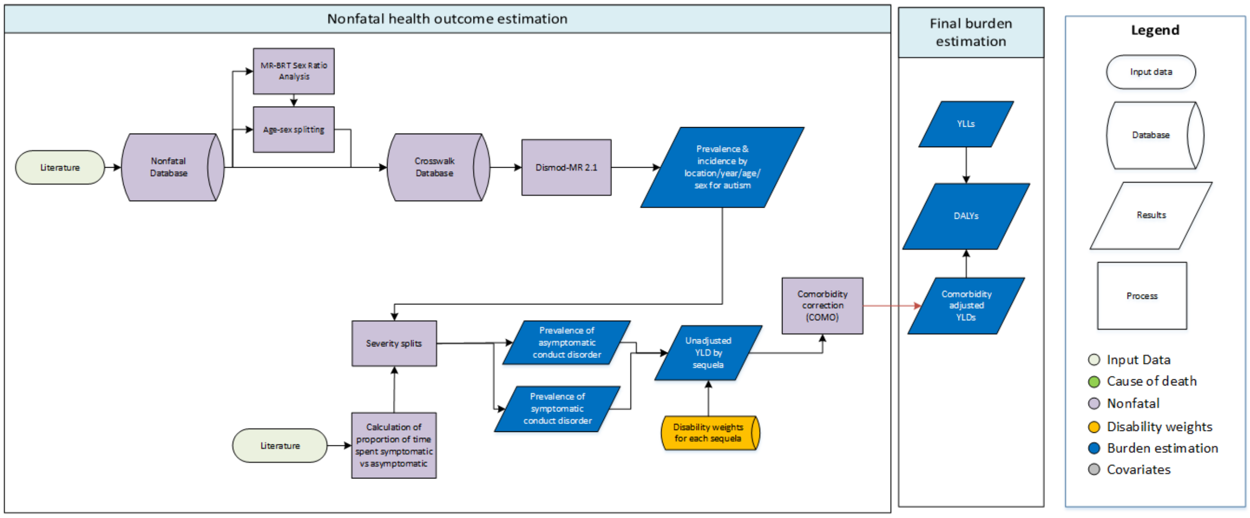

Input Data and Methodological Summary for Conduct Disorder

Case definition

Conduct disorder (CD) is an externalising behaviour disorder characterised by a pattern of antisocial behavior that violates the basic rights of others or major age-appropriate societal norms. As per criteria set by the Diagnostic and Statistical Manual of Mental Disorders fourth edition, text revision (DSM-IV-TR),[1] diagnosis requires three or more of the following symptoms to be present in the past 12 months (with at least one present in the last six months) and cause significant impairment in functioning. Symptoms include:

Aggression to people and animals

- often bullies, threatens, or intimidates others
- often initiates physical fights
- has used a weapon that can cause serious physical harm to others (eg, a bat, brick, broken bottle, knife, gun)
- has been physically cruel to people
- has been physically cruel to animals
- has stolen while confronting a victim (eg, mugging, purse snatching, extortion, armed robbery)
- has forced someone into sexual activity

Destruction of property

- has deliberately engaged in fire setting with the intention of causing serious damage
- has deliberately destroyed others' property (other than by fire setting)

Deceitfulness or theft

- has broken into someone else's house, building, or car
- often lies to obtain goods or favors or to avoid obligations (i.e., "cons" others)

- has stolen items of nontrivial value without confronting a victim (eg, shoplifting, but without breaking and entering; forgery)

Serious violations of rules

- often stays out at night despite parental prohibitions, beginning before age 13 years
- has run away from home overnight at least twice while living in parental or parental surrogate home (or once without returning for a lengthy period)
- is often truant from school, beginning before age 13 years

CD is considered a disorder of childhood but can be diagnosed in adults who display such behaviors yet do not meet the criteria for antisocial personality disorder. However, there are almost no studies measuring adult CD as existing studies in this area tend to measure adult antisocial behavior rather than adult CD.[2] As such, only childhood CD (i.e., cases prior to 18 years of age) was modelled in GBD.

Included in GBD were cases meeting diagnostic criteria according to DSM[1] or the International Classification of Diseases (ICD).[3] These were identified by the following codes: 312.81-312.89 (DSM-IV-TR) and F91 (ICD-10). Different versions of DSM (DSM-III, DSM-III-R, DSM-IV, DSM-IV-TR, and DSM-5) and ICD (ICD-9 and ICD-10) were accepted.

Input data

For mental disorders, we update our GBD electronic database searches on a two year rolling basis. In GBD 2019 a systematic literature review update was conducted to identify new epidemiological studies on CD published between September 2016 and December 2018. We included studies reporting the prevalence, remission, incidence, duration, and/or excess mortality associated with CD. The systematic review of the literature for CD was conducted in conjunction with attention-deficit/hyperactivity disorder as they are childhood behavioural disorders and are often reported together. The search was conducted in three stages involving electronic searches of the peer-reviewed literature (i.e., using PsycInfo, Embase, and PubMed), the grey literature, and expert consultation. The following search terms were used to develop search strings across all databases searched: 'attention', 'disorder', 'hyperactive', 'hyperkinetic', 'adhd', 'conduct disorder', 'disruptive', 'externalising' and 'prevalence', 'mortality', 'death', 'incidence', 'remission', 'duration', 'remit', 'epidemiology'.

The search generated 3135 records (after duplicates were removed) across the three electronic databases. The title/abstract screening reduced the number of relevant records to 224 studies, of which 10 studies met criteria for inclusion for CD. An additional 4 studies were identified through a grey literature search and consultations with experts. Overall, in GBD 2019 we added 14 new studies into the conduct disorders dataset.

The GBD inclusion criteria stipulated that: (1) the publication year must be from 1980 onward; (2) "caseness" must be based on clinical threshold as established by the DSM or ICD; (3) sufficient information must be provided on study method and sample characteristics to assess the quality of the study; (4) study sample must be representative of the general population (i.e., inpatient or pharmacological treatment samples, case studies, veterans, or refugee samples were excluded). No limitation was set on the language of publications. Methods used in previous systematic reviews have been reported in greater detail elsewhere.[2] Table 1 below summarizes data inputs by parameter for conduct disorders.

Table 1: Data Inputs for conduct disorders morbidity modelling by parameter.

Measure	Total sources
All measures	53
Prevalence	49
Incidence	1
Remission	1
Standardized mortality ratio	1
Proportion	1

Age and sex splitting

The extracted data underwent two types of age and sex splitting processes:

1. Where possible, estimates were further split by sex and age based on the data that was available. For instance, if studies reported prevalence for broad age groups by sex (e.g., prevalence in 15 to 65 year old males and females separately), and also by specific age groups but for both sexes combined (e.g., prevalence in 15- to 30-year-olds, then in 31- to 65-year-olds, for males and females combined); age-specific estimates were split by sex using the reported sex ratio and bounds of uncertainty.
2. A Meta-Regression with Bayesian priors, Regularization, and Trimming (MR-BRT) analysis was used to split the remaining both-sex estimates in the dataset. For each parameter, sex specific estimates were matched by location, age, year and a MR-BRT network meta-analysis was used to estimate pooled sex ratios and bounds of uncertainty. The male: female ratio estimated was 2.31 (95% uncertainty interval [UI]: 0.73 – 3.88).

Bias corrections / Crosswalks

No crosswalks were applied to the estimates for CD. The reasons for this are discussed in the *Changes between GBD 2017 and GBD 2019* section.

Severity splits and disability weight

The GBD disability weight survey assessments are lay descriptions of sequelae highlighting major functional consequences and symptoms. The lay description and disability weight for CD is shown in Table 2. A severity split for the proportion of time spent symptomatic versus asymptomatic was based on data from the Great Smoky Mountains Study which assessed the levels of disability found in children and adolescents with mental disorders.[4] Of those with CD, 72% reported disability while 20% of individuals with no diagnosis reported disability at the time of survey. Using these as estimates of the proportion of time with disability in the “average case,” the proportion of disability in children without a diagnosis was subtracted from the proportion with disability for CD, giving an adjusted proportion of 52%. Detailed descriptions of this methodology have been published elsewhere.[5] The lay description and disability weight for CD is shown in the table below.

Table 2. Lay description for conduct disorder in GBD 2019 and the associated disability weight (DW).

Lay description	DW (95% UI)
Has frequent behaviour problems, which are sometimes violent. The person often has difficulty interacting with other people and feels irritable	0.241 (0.159–0.341)

Modelling strategy

After the above data processes were applied, DisMod MR 2.1 was used to model the epidemiological data for CD. The DisMod-MR modeling strategy for CD followed the standard GBD 2019 decomposition structure. At each decomposition step, we compared the new model against the GBD 2017 best model and the best model from the previous step. All substantial changes between models were explored and explained. Adjustments to model priors or the dataset were made where appropriate. Where outliers were identified in the data, we re-assessed the study's methodology and quality before a decision was made to exclude or include the data.

Data across all epidemiological parameters were initially included in the modelling process. We assumed no incidence or prevalence prior to 5 years of age or after 18 years of age. The minimum age of onset was set in consultation with experts while the upper age limit was set in line with DSM criteria. Excess mortality was set to zero given the absence of data demonstrating an association between CD and an increased risk of death. Remission and incidence were capped between ages 4 and 17 years in order to gain more plausible output.

Changes between GBD 2017 and GBD 2019

There were three main changes in the GBD 2019 modelling strategy compared to GBD 2017:

1. In GBD 2017 sex ratios were estimated by DisMod-MR as part of the prevalence modelling. In GBD 2019 we conducted a MR-BRT analysis instead. The prevalence male: female ratio was 2.09 (1.65 – 2.65) in GBD 2017 compared to 2.31 (0.73 – 3.88) in GBD 2019.
2. The GBD 2017 model included estimates that represented the prevalence of both CD and oppositional defiant disorder, which was adjusted via a study-level covariate within DisMod-MR. This covariate/adjustment was excluded in GBD 2019. The vast majority of these studies reported the prevalence of CD and oppositional defiant disorder separately and so these estimates were replaced with the prevalence for CD only. This meant the exclusion of 7 studies which did not report the prevalence of CD only.
3. In GBD 2019 we included new epidemiological data from 13 locations (Argentina, Australia, Austria, Colombia, Denmark, England, Hunan, Kaduna, Rio Grande do Sul, Saudi Arabia, Tehran, Uganda, and United States).

While we continue to improve on the data and methods used to estimate the burden of mental disorders, some challenges need to be acknowledged. Firstly, we still have a large number of locations with no high quality raw data available. Secondly, it is difficult to quantify and remove all variation due to measurement error in our epidemiological estimates. Whilst we have improved the methodology used to account for known sources of bias, in some case, we still have very few data points to inform these adjustments. Thirdly, there is a paucity of research on the risk factors of mental disorders which can be used as predictive covariates in our epidemiological models.

References

1. American Psychiatric Association. Diagnostic and Statistical Manual of Mental Disorders (DSM-IV-TR). 4th, Text Revision ed. Washington DC: American Psychiatric Association; 2000.
2. Erskine HE, Ferrari AJ, Nelson P, et al. Research Review: Epidemiological modelling of attention-deficit/hyperactivity disorder and conduct disorder for the Global Burden of Disease Study 2010. *Journal of Child Psychology and Psychiatry* 2013; **54**(12): 1263-74.
3. World Health Organisation. ICD-10 Classification of Mental and Behavioural Disorders: Clinical descriptions and diagnostic guidelines. Geneva: World Health Organisation; 1992.
4. Ezpeleta L, Keeler G, Erkanli A, Costello EJ, Angold A. Epidemiology of Psychiatric Disability in Childhood and Adolescence. *J Child Psychol Psychiatry* 2001; **42**(7): 901-14.
5. Erskine HE, Ferrari AJ, Polanczyk GV, et al. The global burden of conduct disorder and attention-deficit/hyperactivity disorder in 2010. *Journal of Child Psychology and Psychiatry* 2014; **55**(4): 328-36.

Other mental disorders

Flowchart

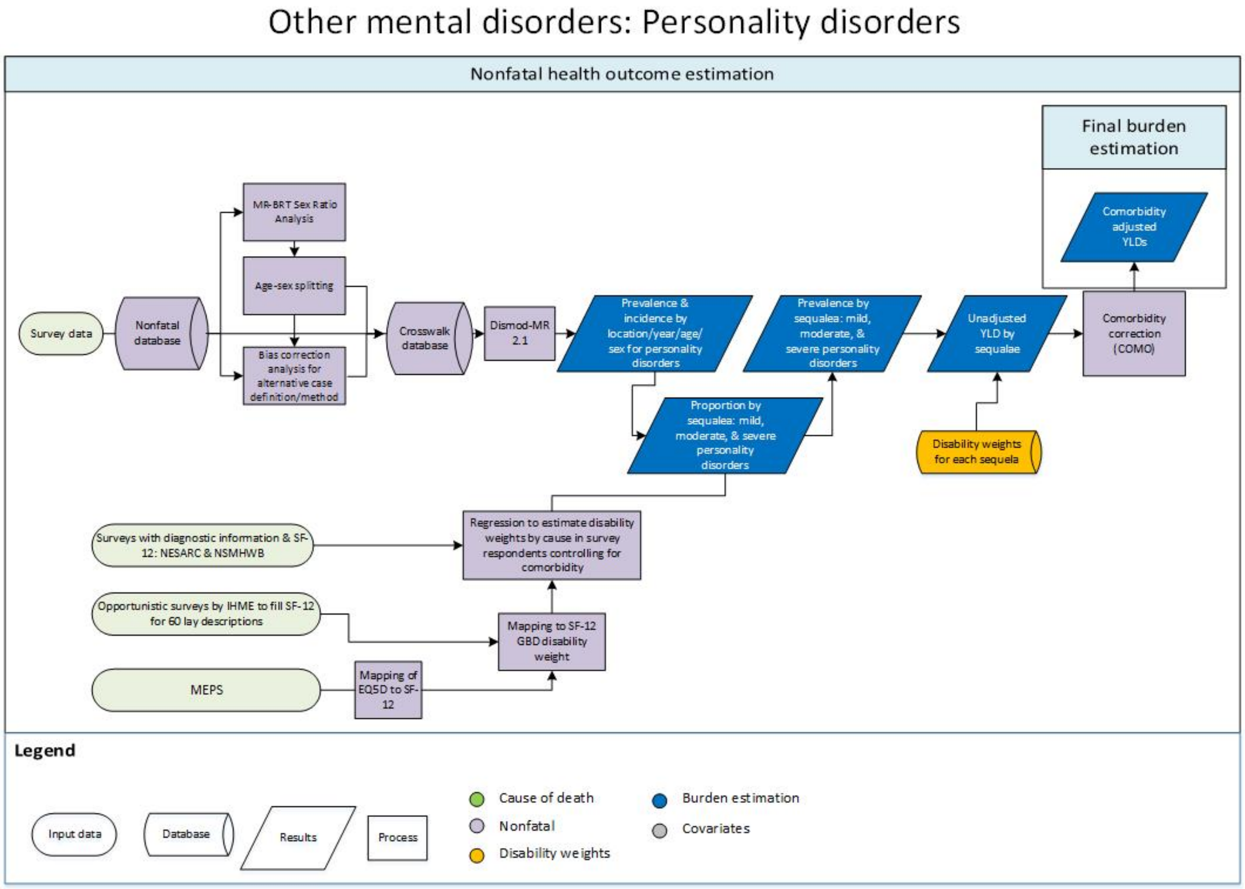

Input Data and Methodological Summary for Anxiety disorders

Case definition

In addition to the individual mental disorders for which we estimate burden, we also estimate the non-fatal burden attributable to a residual cause of "other mental disorders." This is made up of an aggregate group of personality disorders. Personality disorders are characterised by pervasive, inflexible and maladaptive patterns of behaviour and inner experience which are markedly different from what is considered to be acceptable in the individual's culture. These disorders tend to be chronic and are associated with significant distress or disability. Included in GBD 2019 were cases meeting diagnostic criteria for personality disorders according to the Diagnostic and Statistical Manual of Mental Disorders (DSM-IV-TR: 300.3, 301.0; 301.2, 301.22, 301.5–301.9), or the equivalent diagnosis in the International Classification of Diseases (ICD-10: F60)[1,2].The aggregated group of DSM personality disorders used in GBD 2019 captured any of the following;

- Paranoid personality disorder
- Schizoid personality disorder
- Schizotypal personality disorder
- Antisocial personality disorder
- Borderline personality disorder

- Histrionic personality disorder
- Narcissistic personality disorder
- Avoidant personality disorder
- Dependent personality disorder
- Obsessive-compulsive personality disorder
- Personality disorder not otherwise specified

Input data

Prevalence estimates for the above personality disorders were obtained from the US National Epidemiological Survey on Alcohol and Related Conditions (NESARC, conducted in two waves from 2001–2002 and 2004–2005)[3] and the Australian National Survey of Mental Health and Wellbeing of Adults (NSMHWB, conducted in 1997)[4]. Given that personality disorders often co-occur with other mental and substance use disorders, an adjustment for comorbidity is important so as not to overestimate the overall burden attributable to mental and substance use disorders. Participants meeting criteria for any type of personality disorders from the NESARC and NSMHWB surveys were counted as a prevalent case only if they did not simultaneously meet criteria for another mental and substance use disorder featured in GBD 2019. Table 1 below summarizes data inputs by parameter for other mental disorders.

Table 1: Data Inputs for other mental disorders morbidity modelling by parameter.

Measure	Total sources	Countries with data
All measures	3	2
Prevalence	3	2

Bias corrections / Crosswalks

Estimates with known biases were adjusted / crosswalked accordingly prior to DisMod-MR 2.1. A NESARC: NSMHWB prevalence ratio of 2.04 (95% uncertainty interval [UI]: 1.82 – 2.34) was used to adjust all data points derived from NESARC toward the level of data points from the NSMHWB. The latter survey was made up of a more representative list of personality disorders and produced estimates along the levels of what we would expect for personality disorders. As this ratio was informed by only two data sources it was estimated outside of the Meta-Regression with Bayesian priors, Regularization, and Trimming (MR-BRT) analysis typically used for bias correction in GBD 2019.

Severity splits and disability weights

The basis of the GBD disability weight survey assessments are lay descriptions of sequelae highlighting major functional consequences and symptoms. The lay descriptions and disability weights applied to the personality disorders within this residual group are shown below and were those estimated for anxiety disorders (See Table 2). To determine the proportion of people with personality disorders within each of the severity levels, the NSMHWB survey was used to estimate the proportion of cases asymptomatic (30%, 28% – 32%), mild (41%, 33% – 47%), moderate (15%, 11% – 20%) and severe (14%, 10% – 18%).

Table 2. Severity distribution for other mental disorders in GBD 2019 and the associated disability weight (DW) with that severity

Severity level	Lay description	DW (95% UI)
Mild	Feels mildly anxious and worried, which makes it slightly difficult to concentrate, remember things, and sleep. The person tires easily but is able to perform daily activities.	0.03 (0.018 – 0.046)
Moderate	Feels anxious and worried, which makes it difficult to concentrate, remember things, and sleep. The person tires easily and finds it difficult to perform daily activities.	0.133 (0.091 – 0.186)
Severe	Constantly feels very anxious and worried, which makes it difficult to concentrate, remember things, and sleep. The person has lost pleasure in life and thinks about suicide.	0.523 (0.362 – 0.677)

Modelling Strategy

After the above data processes were applied, DisMod MR 2.1 was used to model the epidemiological data for personality disorders. The DisMod-MR modeling strategy followed the standard GBD 2019 decomposition structure. At each decomposition step, we compared the new model against the GBD 2017 best model and the best model from the previous step. All substantial changes between models were explored and explained. Adjustments to model priors or the dataset were made where appropriate. Where outliers were identified in the data, we re-assessed the study's methodology and quality before a decision was made to exclude or include the data.

As we only had prevalence data available, a number of expert priors were used in order to run a full-parameter model. We assumed no incidence and prevalence before age 14. This minimum age of onset was corroborated with expert feedback and DSM criteria highlighting the fact that personality disorders typically become recognizable during adolescence and early adulthood. Remission was set to a maximum of 0.01, given that these are understood to be chronic disorders with little or no complete remission. Excess mortality was set to 0 in this model, in the absence of mortality data required for DisMod-MR 2.1 modelling purposes. Given the sparsity of data, we applied a restriction on location random-effects of -0.1 to 0.1 to further guide prevalence estimation.

Changes between GBD 2017 and GBD 2019

There were two main changes in the GBD 2019 modelling strategy compared to GBD 2017:

1. In GBD 2017 bias corrections were estimated by DisMod-MR as part of the prevalence modelling. In GBD 2019 we estimated the NESARC: NSMHWB prevalence ratio outside of the DisMod-MR. The ratio changed from 1.92 (1.42 – 2.67) in GBD 2017 to 2.04 (1.82 – 2.34) in GBD 2019, leading to slight overall decrease in the adjusted prevalence.
2. In this model, global prevalence was exclusively estimated using prevalence estimates from two surveys from the United States and Australia where we had unit record data available to estimate the prevalence of personality disorders, excluding those not simultaneously meeting criteria for another mental or substance use disorder. The sparsity of data leads to modelled prevalence estimates with large uncertainty bounds, which are sensitive to model re-runs and small changes to model settings. We are currently undertaking a literature review of population-survey data on the epidemiology of personality disorders across low-, middle-, and high-income countries with the aim of providing more robust and globally representative burden estimates for personality disorders in future GBD studies.

References

1. American Psychiatric Association. Diagnostic and Statistical Manual of Mental Disorders (DSM). Washington: American Psychiatric Association, 1952.
2. World Health Organization. The ICD-10 Classification of Mental and Behavioural Disorders. Clinical descriptions and diagnostic guidelines. Geneva: World Health Organization; 1992.
3. Introduction to the National Epidemiologic Survey on Alcohol and Related Conditions [http://pubs.niaaa.nih.gov/publications/arh29-2/74-78.htm]. Access date 1 December 2014.
4. Australian Bureau of Statistics. National Survey of Mental Health and Wellbeing of Adults 1997. Canberra: Australian Bureau of Statistics.

Alcohol use disorders

Flowchart

Alcohol use disorders

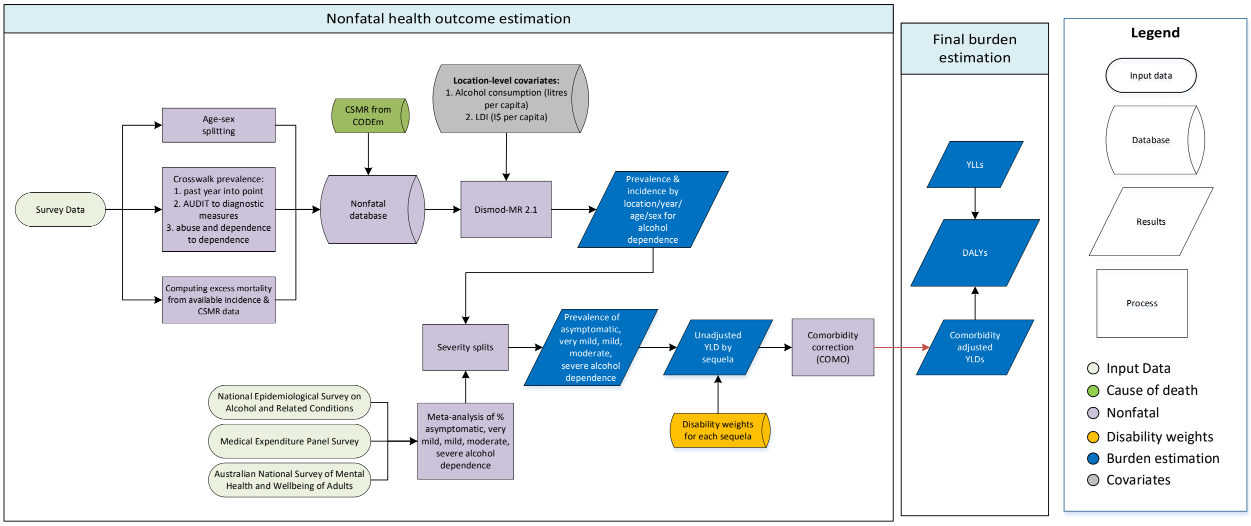

Case definition

Alcohol dependence is a substance-related disorder involving a dysfunctional pattern of alcohol use. According to the Diagnostic and Statistical Manual of Mental Disorders (DSM-IV) criteria for alcohol dependence, at least three out of seven of the following criteria must be manifested during a 12-month period:

- Tolerance
- Withdrawal symptoms or clinically defined alcohol withdrawal syndrome
- Use in larger amounts or for longer periods than intended
- Persistent desire or unsuccessful efforts to cut down on alcohol use
- Time is spent obtaining alcohol or recovering from effects
- Social, occupational, and recreational pursuits are given up or reduced because of alcohol use
- Use is continued despite knowledge of alcohol-related harm (physical or psychological)

The DSM-IV codes for alcohol dependence is 303.90, and the corresponding International Classification of Diseases (ICD-10) codes are F10.1 and F10.2.[1,2]

Input data

Model inputs

In GBD 2013 and GBD 2016, systematic reviews of literature were conducted to capture studies of prevalence, incidence, remission, duration, and excess mortality associated with alcohol dependence. In summary, the search was conducted in three stages involving searches of the peer-reviewed literature (via Medline, Embase, and PubMed), the grey literature, and expert consultation. Updates to systematic reviews are performed on an ongoing schedule across all GBD causes and an update for alcohol dependence will be performed in the next one to two iterations.

The inclusion criteria stipulated that (1) "caseness" must be based on clinical threshold as established by the DSM and ICD; (2) sufficient information must be provided on study method and sample characteristics to assess the quality of the study; and (3) study samples must be representative of the general population (ie, inpatient or pharmacological treatment samples [accepted for estimates of mortality], case studies, and veterans or refugee samples were excluded).

Table 1: Data Inputs for alcohol dependence morbidity modelling by parameter.

	Total Sources	Countries with data
All measures	459	59
Prevalence	395	58
Incidence	3	3
Remission	3	3
Relative risk	7	3
Standardized mortality ratio	34	13
Proportion	15	1
Other	7	4

Prevalence estimates were split by age and sex where necessary. First, studies that reported prevalence for both sexes were split using a global sex ratio estimated using MR-BRT. Second, where studies reported estimates across age groups spanning 20 years or more, these were split into five-year age groups using the global age pattern estimated by DisMod-MR 2.1.

Table 2: MR-BRT Sex Splitting Adjustment Factors for alcohol dependence

Data input	Gamma	Beta Coefficient, Log (95% CI)	Adjustment factor*
Female: Male	0.33	-0.69 (-1.35, -0.04)	0.50
Age < 20		0.12 (0.07, 0.18)	1.13

**Adjustment factor is the transformed Beta coefficient in normal space, and can be interpreted as the factor by which the alternative case definition is adjusted to reflect the ratio by which both-sex data points were split.*

Bias Correction

Due to insufficient data on alcohol dependence in some regions, three crosswalks were performed using MR-BRT to allow for the inclusion of data that did not meet our reference definitions in the epidemiological modelling of alcohol dependence. The first crosswalk converted estimates of alcohol use disorders (alcohol abuse + alcohol dependence) to reflect what they would be if the data represented estimates of alcohol dependence. Similarly, the second crosswalk was performed using MR-BRT to adjust past-year prevalence estimates of alcohol dependence toward the level they would have been had the study measured point prevalence, as the latter is less susceptible to recall bias. The third crosswalk adjusted estimates of prevalence according to the Alcohol Use Disorder Identification Test (AUDIT) to what they would be had prevalence been determined based on diagnostic measures. For this final crosswalk, a systemic review was performed to identify AUDIT validation studies using the following search string:

```
(("audit"[tiab] AND "alcohol"[tiab]) OR "alcohol use disorders identification test"[tiab]) AND
("validation"[tiab] or "validity"[tiab]) NOT (animals[MeSH] NOT humans[MeSH])
```

Out of 303 total studies screened, 38 studies were found to report prevalence of alcohol dependence according to the AUDIT as well as according to physician diagnosis, or reported specificity and sensitivity to allow for the calculation of prevalence. These studies were used to generate crosswalk parameters using MR-BRT. All three crosswalks utilized a logit difference model, which has been described elsewhere. Briefly, alternative definition data points were logit transformed, and the MR-BRT beta was subtracted from them, after which they were transformed back into normal space.

Table 3: MR-BRT Crosswalk Adjustment Factors for alcohol dependence

Data input	Reference or alternative case definition	Gamma	Beta Coefficient, Logit (95% CI)
Point prevalence	Ref	0.68	---
Past- year prevalence	Alt		0.81 (-0.58 – 2.14)
Prevalence according to diagnostic measures	Ref	0.76	---
Prevalence according to AUDIT	Alt		1.09 (-0.40 – 2.63)
Alcohol dependence prevalence	Ref	0.57	---
Alcohol dependence and abuse prevalence	Alt		1.04 (-0.03 – 2.19)

Severity split inputs and disability weights

The basis of the GBD disability weight survey assessments are lay descriptions of sequelae highlighting major functional consequences and symptoms. The lay descriptions and disability weights for alcohol dependence severity levels are shown below.

Table 4. Severity distribution, details on the severity levels for alcohol dependence in GBD 2019 and the associated disability weight (DW) with that severity.

Severity level	Lay description	DW (95% CI)
Very mild	Drinks alcohol daily and has difficulty controlling the urge to drink. When sober, the person functions normally.	0.123 (0.082–0.177)
Mild	Drinks a lot of alcohol and sometimes has difficulty controlling the urge to drink. While intoxicated, the person has difficulty performing daily activities.	0.235 (0.16–0.327)
Moderate	Drinks a lot, gets drunk almost every week and has great difficulty controlling the urge to drink. Drinking and recovering cause great difficulty in daily activities, sleep loss, and fatigue.	0.373 (0.248–0.508)
Severe	Gets drunk almost every day and is unable to control the urge to drink. Drinking and recovering replace most daily activities. The person has difficulty thinking, remembering and communicating, and feels constant pain and fatigue.	0.57 (0.396–0.732)

**asymptomatic cases carried no disability weight*

Severity splits used in GBD 2019 were consistent with those used in GBD 2017. The United States' Medical Expenditure Panel Survey (MEPS, conducted in annual waves since 1996)[3], the US National Epidemiological Survey on Alcohol and Related Conditions (NESARC, conducted in two waves from 2001–2002 and 2004–2005)[4], and the Australian National Survey of Mental Health and Wellbeing of Adults (NSMHWB, conducted in 1997)[5] were used to estimate the proportion of alcohol dependence cases in the asymptomatic 40.9% (38.4%–43.3%); very mild 46.9% (43.7%–50.0%); mild 4.0% (1.8%–5.8%); moderate 3.4% (2.3%–4.5%); and severe 4.8% (3.0%–7.0%) disease categories.

Modelling strategy

We have made no substantive changes in the modeling strategy from GBD 2017. The GBD 2019 epidemiological modelling strategy for alcohol dependence made use of DisMod-MR 2.1 to estimate prevalence by age, sex, year, and location. Standardised mortality ratio and relative risk data were excluded in the modelling process. Instead we pulled in cause-specific mortality rate (CSMR) data from our CODEm and CODcorrect analyses and matched it with prevalence data points for the same geography and study year to estimate priors on excess mortality rates (by dividing CSMR by prevalence). We assumed no incidence and mortality before age 10. An upper limit of 0.6 was placed on remission (in line with data from the US National Epidemiological Survey on Alcohol and Related Conditions (NESARC) as well as a declining trend with age to restrict DisMod-MR 2.1 from straying too far from the data inputs.

Two country-level covariates were included in the DisMod-MR 2.1 model. The LDI covariate represents a moving average of gross domestic product (GDP) over time. LDI was also applied to excess mortality data with a negative relationship assumed. Alcohol consumption was also represented by a covariate representing this in terms of liters of alcohol per capita.

Table 4. Covariates. Summary of covariates used in the alcohol dependence DisMod-MR meta-regression model

Covariate	Type	Parameter	Exponentiated beta (95% Uncertainty Interval)
Liters of alcohol consumed per capita	Country	Prevalence	1.03 (1.00 – 1.07)
LDI (I$ per capita)	Country	Excess mortality rate	0.90 (0.90 – 0.90)

References

1. American Psychiatric Association. Diagnostic and Statistical Manual of Mental Disorders (DSM-IV-TR). 4th, Text Revision ed. Washington DC: American Psychiatric Association; 2000.
2. World Health Organization. The ICD-10 Classification of Mental and Behavioural Disorders. Clinical descriptions and diagnostic guidelines. Geneva: World Health Organization; 1992.
3. Agency for Healthcare Research and Quality. United States Medical Expenditure Panel Survey. Rockville, United States: Agency for Healthcare Research and Quality.
4. Introduction to the National Epidemiologic Survey on Alcohol and Related Conditions [http://pubs.niaaa.nih.gov/publications/arh29-2/74-78.htm]. Access date 1 December 2014.
5. Australian Bureau of Statistics. National Survey of Mental Health and Wellbeing of Adults 1997. Canberra: Australian Bureau of Statistics.

Fetal alcohol syndrome

Flowchart

Fetal alcohol syndrome (FAS)

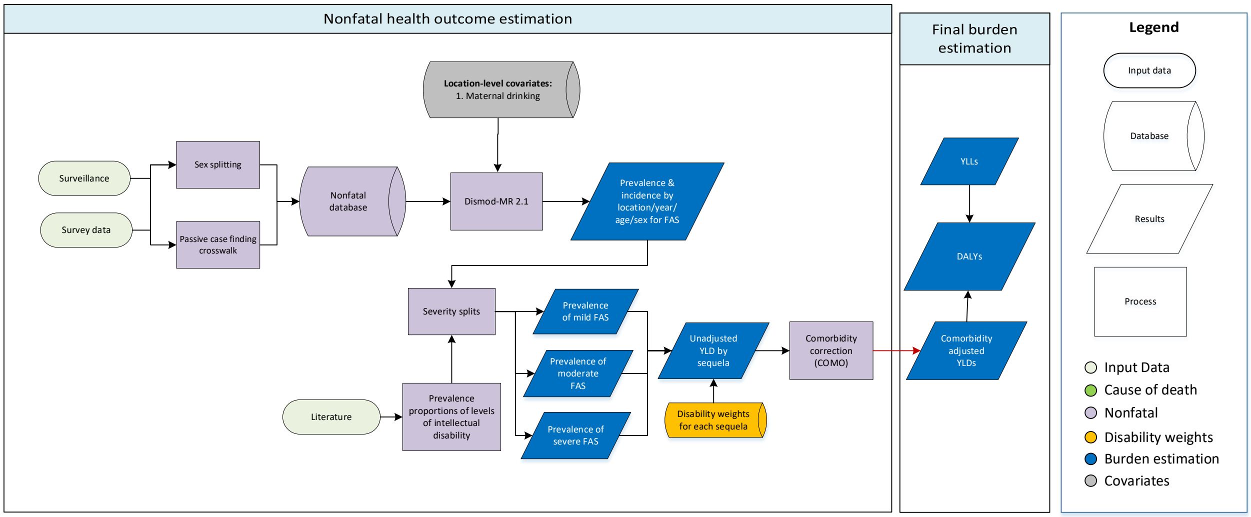

Input data and methodological summary

Case definition

Fetal alcohol syndrome (FAS; ICD-10: Q86.0) is a disorder caused by maternal drinking during pregnancy and is the most severe form of fetal alcohol spectrum disorder (FASD). In GBD, only FAS cases were included in the model. Other manifestations of FASD including partial fetal alcohol syndrome, alcohol-related neurodevelopmental disorder, and alcohol-related birth defects were not included. FAS is characterised by maternal alcohol exposure which results in certain patterns of facial anomalies such as short palpebral fissures and abnormalities in the premaxillary zone (eg, flat upper lip, flattened philtrum, and flat midface), growth retardation (eg, decelerating weight over time not due to nutrition), and central nervous system neurodevelopmental abnormalities (eg, decreased cranial size at birth) in the offspring.[1] Cases were defined according to diagnostic guidelines set by the USA Institute of Medicine, the British Paediatric Association, and other recognised bodies in the area.

Input data

Model inputs

A series of systematic literature reviews were conducted to capture studies reporting the prevalence, incidence, remission, and excess mortality of FAS. The reviews incorporated searches of peer-reviewed literature via electronic databases and consultation with experts. In order for a study to be included, it must use recognised classifications of FAS (eg, the USA Institute of Medicine) and provide sufficient details on study methodology and sample characteristics to determine study quality. No limitation was

set on the language of publication. Data from the European Surveillance of Congenital Anomalies (EUROCAT) were also included and updated where relevant. This methodology was utilised in GBD 2015. Updates to systematic reviews are performed on an ongoing schedule across all GBD causes, and an update for FAS will be performed in the next one to two iterations.

Data reported for both sexes were split using a global sex ratio estimated using MR-BRT.

Table 1: MR-BRT Sex Splitting Adjustment Factors for fetal alcohol syndrome

Data input	Gamma	Beta Coefficient, Log (95% CI)	Adjustment factor*
Female: Male	0	-0.28 (-0.67, 0.11)	0.76

Adjustment factor is the transformed Beta coefficient in normal space, and can be interpreted as the factor by which the alternative case definition is adjusted to reflect the ratio by which both-sex data points were split.

Bias Correction

Prevalence data collected using both passive and active case-finding methodologies was included in this model. As passive case finding methods are likely to underestimate the true prevalence of fetal alcohol syndrome, a crosswalk was applied to increase the uncertainty around those data points. The expected difference in reported prevalence was modeled using MR-BRT. To adjust the passive-case data, a logit difference model was used in which the beta coefficient was subtracted from the logit transformed prevalence data, the inverse logit of which was used in the model. Table 2 summarises the MR-BRT crosswalk coefficients.

Table 2: MR-BRT Crosswalk Adjustment Factors for fetal alcohol syndrome

Data input	Reference or alternative case definition	Gamma	Beta Coefficient, Logit (95% CI)
Active case finding	Ref	1.87	---
Passive case finding	Alt		-0.03 (-3.60, 3.51)

Severity split inputs and disability weights

There were no data available which gave prevalence of FAS by severity. As such, severity splits for FAS were calculated by matching FAS severity to categories of IQ in children for which prevalence data are available. Severe FAS was matched to an IQ of less than 50, moderate FAS to an IQ of 50 to 69, mild FAS to an IQ of 74 to 84, and asymptomatic FAS to an IQ of 85 or higher. Prevalence data for these IQ levels were then used to calculate severity splits for FAS.

Table 3. Severity distribution, details on the severity levels for fetal alcohol syndrome in GBD 2019 and the associated disability weight (DW) with that severity.

Severity level	Lay description	DW (95% CI)
Mild	Is a little slow in developing physically and mentally, which causes some difficulty in learning but no other difficulties in daily activities.	0.016 (0.008–0.03)
Moderate	Is slow in developing physically and mentally, which causes some difficulty in daily activities.	0.056 (0.035–0.083)
Severe	Is very slow in developing physically and mentally, which causes great difficulty in daily activities.	0.179 (0.119–0.257)

Modelling strategy

We have made no substantive changes in the modeling strategy from GBD 2017. Prevalence was set to begin from birth. Incidence was set to zero given cases cannot manifest after birth (despite the fact they may not be diagnosed immediately at birth). Remission was also set to zero. Estimates from known high-drinking populations (eg, indigenous populations) were not considered representative of the general population and were excluded. A country-level covariate was included representing the log proportion of pregnant women who drink during their pregnancy, estimated from a meta-analysis.[2] The table below illustrates the covariate, parameter, beta and exponentiated beta values for the model.

Table 4. Covariates. Summary of covariates used in the fetal alcohol syndrome DisMod-MR meta-regression model

**Covariate**	**Type**	**Parameter**	**Exponentiated beta (95% Uncertainty Interval)**
Maternal drinking	Country	Prevalence	1.09 (1.00 — 1.29)

References

1. Stratton K, Howe C, Battaglia F, editors. Fetal alcohol syndrome. Diagnosis, epidemiology, prevention, and treatment. Washington DC: National Academy Press; 1996.
2. Popova S, Lange S, Probst C, Gmel G, Rehm J. Estimation of national, regional, and global prevalence of alcohol use during pregnancy and fetal alcohol syndrome: a systematic review and meta-analysis. *The Lancet Global Health* 2017.

Opioid use disorders

Flowchart

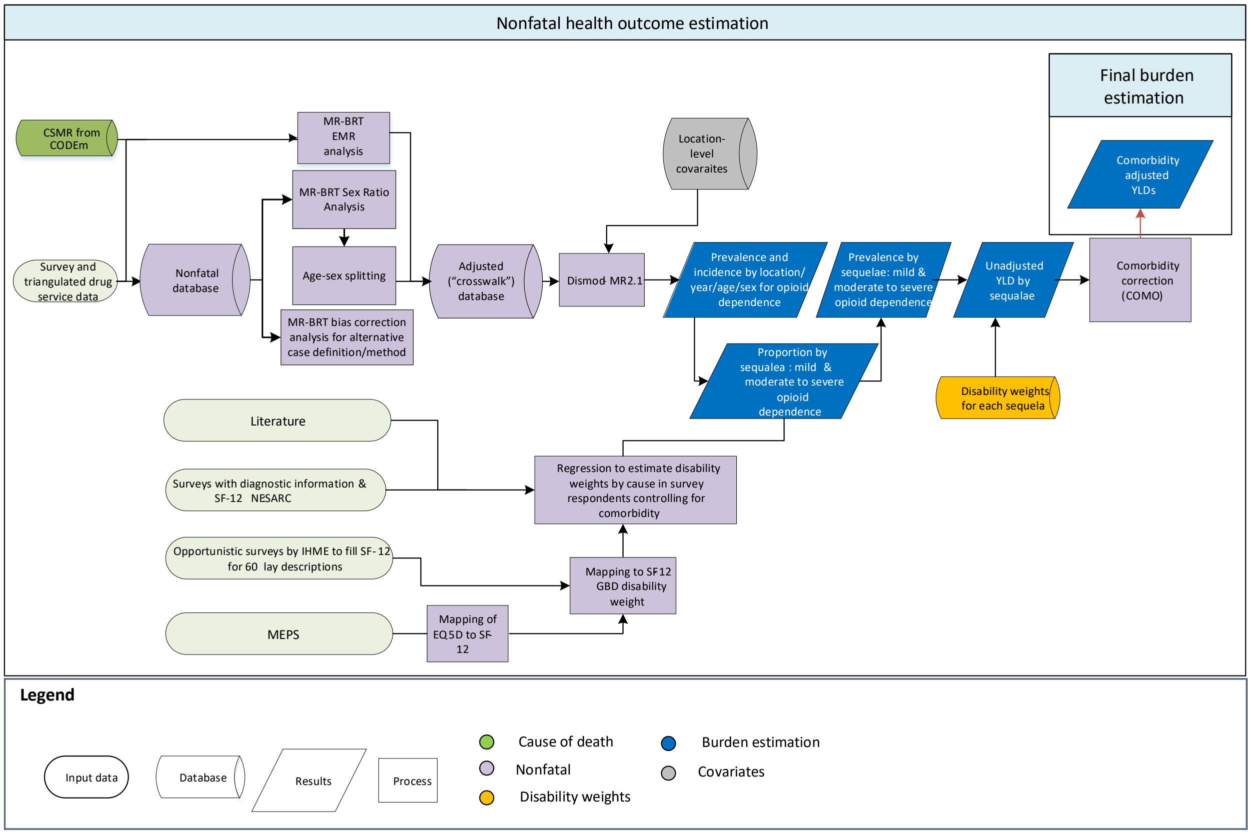

Input Data and Methodological Summary for Opioid Use Disorders

Case definition

Opioid dependence is a substance-related disorder involving a dysfunctional pattern of opioid use. Included in the GBD disease modelling were cases meeting the Diagnostic and Statistical Manual of Mental Disorders (DSM-IV-TR) or the International Classification of Diseases (ICD-10) diagnostic criteria for opioid dependence (DSM: 304.00; ICD: F11.2), excluding those cases due to a general medical condition.[1,2] According to DSM-IV TR criteria, dependence involves a maladaptive pattern of substance use leading to clinically significant impairment or distress. At least three of the following symptoms must be experienced within the same 12-month period:

- Tolerance, characterised by either
  - a need for increased amounts of the substance to achieve intoxication; or
  - markedly diminished effect with continued use of the same amount of the substance;
- Withdrawal, characterised by either
  - Withdrawal symptoms characteristic to dependence; or

- the same (or similar) substance is taken to avoid withdrawal symptoms;
- Substance taken in progressively larger amounts or for longer period;
- Persistent desire or unsuccessful efforts to reduce substance use;
- Disproportionate time dedicated to obtaining the substance;
- Other important activities are given up because of the substance use; and
- Substance use is continued despite knowledge of physical or psychological problems occurring as a result of the substance.

Input data

For GBD 2010, a systematic review of the literature was conducted to capture studies of prevalence, incidence, duration, and excess mortality associated with opioid use disorders. In summary, the search was conducted in three stages involving electronic searches of the peer-reviewed literature (via PsycInfo, Embase, and PubMed), the grey literature, and expert consultation. The agreed-upon approach for mental and substance use disorders was to conduct electronic database searches on a rolling basis. All three stages of GBD 2010's literature review were repeated for GBD 2013 and GBD 2016. For GBD 2017, literature updates focused on data sources captured within the Global Heath Data Exchange (http://ghdx.healthdata.org/).

Additionally, two targeted systematic reviews were conducted in GBD 2017 to further supplement the dataset. The first review captured studies reporting on the epidemiology of opioid use disorders within Maori versus non-Maori populations (as opposed to New Zealand more broadly), given the inclusion of these two sub-groups. The second review searched for studies on the epidemiology of opioid use disorders in China using primarily the China National Knowledge Infrastructure database. The focus was to search for studies published in Chinese journals that would not typically be captured in mainstream databases such as PsycInfo, Embase, and PubMed.

The inclusion criteria stipulated that 1) the publication year must be from 1980 onward; 2) "caseness" must be based on clinical threshold as established by the DSM or ICD; 3) sufficient information must be provided on study method and sample characteristics to assess the quality of the study; and (4) study samples must be representative of the general population (ie, inpatient or pharmacological treatment samples, case studies, veterans or refugee samples were excluded). No limitation was set on the language of publication. Methods used for this systematic review have been reported in greater detail elsewhere.[3,4]

Table 1: Data Inputs for Opioid Use Disorders Morbidity Modeling by Parameter

Measure	Total sources	Countries with data
All measures	162	33
Prevalence	113	31
Remission	8	6

Standardized mortality ratio	1	1
With-condition mortality rate	39	16
Proportion	1	0

Age and sex splitting

Reported estimates of prevalence were split by age and sex where possible. First, if studies reported prevalence for broad age groups by sex (eg, prevalence in 15- to 65-year-old males and females separately), and also by specific age groups for both sexes combined (e.g., prevalence in 15- to 30-year-olds, then in 31- to 65-year-olds, for males and females combined), age-specific estimates were split by sex using the reported sex ratio and bounds of uncertainty. Second, prevalence data for both sexes that could not be split using a within-study ratio were split using a sex ratio derived from a meta-analysis of existing sex-specific data using MR-BRT. The female to male ratio was 0.61 (0.51 to 0.73) for ages 20 and above, and 1.12 (0.92 to 1.35) for ages below 20. Finally, after the application of bias adjustments, where studies reported estimates across age groups spanning 25 years or more, these were split into five-year age groups using the prevalence age pattern estimated by DisMod-MR 2.1 on all data prior to age-splitting.

Data adjustment

The prevalence dataset included data points of both use and dependence estimated using "direct" or "indirect" survey methods. "Direct" methods of measuring opioid dependence predominantly involve surveys of the general population that ask if respondents use or are dependent on opioids. Surveys tend to underestimate the prevalence of the most harmful and stigmatised forms of illicit drug use in ways that probably vary between countries and cultures.[5] "Indirect" methods are considered superior but they use different sources of data to indirectly estimate the total number of drug users (methods include "multiplier methods," back-projection and capture-recapture methods) that are often poorly documented. Due to the lack of data available on opioid dependence from indirect methods (considered to be the gold standard for GBD purposes), estimates of use and/or estimates from direct survey methods were also included in the modelling. We marked studies reporting on the prevalence of opioid dependence obtained via direct methods and derived an adjustment factor using MR-BRT. The beta and exponentiated value for this covariate is shown in the table below:

Table 2: MR-BRT Crosswalk Adjustment Factors for Opioid Use Disorder

Data input	Reference or alternative case definition	Gamma	Beta Coefficient, Logit (95% CI)
Opioid dependence – indirect method	Ref	0.24	---

Opioid dependence – direct method	Alt		-1.05 (-1.56 to -0.55)

Modeling strategy

Prior settings in DisMod included assuming no incidence and excess mortality before age 15. This minimum age of onset was corroborated with expert feedback and existing literature on opioid dependence. We also assumed no incidence after age 64 as supported by data from various sources including the European Monitoring Centre for Drugs and Drug Addiction.[6] An upper limit of 0.2 was placed on remission consistent with limits in the dataset. These settings were retained for GBD 2019.

As in GBD 2017, age-standardised prevalence of intravenous drug use and log-transformed estimates of defined daily doses for statistical purposes (SDDD; consumption per day per million population) of prescribed opioid analgesics were included as country-level covariates. SDDD were modelled in GBD 2017 via spatiotemporal Gaussian process regression (ST-GPR) using data supplied by the International Narcotics Control Board (INCB). Subnational estimates for the USA were estimated by crosswalking national estimates with the state/national ratios of opioid prescriptions per 100 persons supplied by the Centers for Disease Control and Prevention.

In previous rounds, priors on excess mortality rate (EMR) were estimated in DisMod by matching prevalence data points with their corresponding CSMR values within the same age, sex, year, location (by dividing CSMR by prevalence). However, for many causes, DisMod estimated a rather unrealistic pattern of EMR compared to an expected pattern of decreasing EMR with greater access to quality health care. Such unexpected patterns often signal inconsistencies between CSMR estimates and the measures of prevalence and/or incidence. In effort to provide greater guidance to DisMod on the expected pattern of EMR, EMR data generated in the previous round were modeled using the MR-BRT approach by age and sex with a prior on healthcare access and quality index (HAQi) having a negative coefficient. Results from MR-BRT were then predicted for each location year, sex and for ages 0, 10, 20 ….100. We included HAQi as a country-level covariate to inform EMR with a mean and standard deviation produced from MR-BRT.

For opioid use disorder, the MR-BRT analysis for paired prevalence and CSMR data did not find any effect of HAQi under the condition of a negative prior. As such, across high and low HAQi locations predicted EMR was the same, following the EMR trend of the High-income and Central Europe, Eastern Europe, and Central Asia super regions where the majority of data comes from. It did lead to estimates of prevalence following those of cause of deaths estimates more closely. However, estimates in Afghanistan and Iran, two countries in the otherwise low-prevalence North Africa and the Middle East Super Region with some of the highest prevalence input data in the world, were constrained significantly. These two locations have low values of the intravenous drug use and prescription opioid covariates, resulting in country priors that were far lower than their prevalence data.

Intravenous drug use was also included as a country-level covariate on EMR with bounds set between 0 and 2.

Table 3. Covariates. Summary of covariates used in the opioid use disorders DisMod-MR meta-regression model

Covariate	Parameter	Beta, log (95% Uncertainty Interval)	Exponentiated beta (95% Uncertainty Interval)
Intravenous drug use (age-standardised proportion)	Prevalence	0.32 (0.12 to 0.49)	1.38 (1.13 to 1.63)
Opioids per million population per day (10-year lag)	Prevalence	0.50 (0.50 to 0.50)	1.65 (1.65 to 1.65)
Intravenous drug use (age-standardised proportion)	Excess mortality rate	0.29 (0.010 to 0.80)	1.34 (1.01 to 2.21)

Note, a bound was set on the coefficient for opioids per million per day in an effort to make the model follow the high prevalence data in Iran and Afghanistan more closely.

Severity and Disability

The basis of the GBD disability weight survey assessments are lay descriptions of health states highlighting major functional consequences and symptoms. The lay descriptions and disability weights for opioid dependence severity levels are shown below.

Table 4. Severity distribution, details on the severity levels for opioid use disorders in GBD 2019 and the associated disability weight (DW) with that severity.

Severity level	Lay description	DW (95% CI)
Mild	Uses heroin (or methadone) daily and has difficulty controlling the habit. When not using, the person functions normally.	0.335 (0.221–0.473)
Moderate to severe	Uses heroin daily and has difficulty controlling the habit. When the effects wear off, the person feels severe nausea, agitation, vomiting, and fever. The person has a lot of difficulty in daily activities.	0.697 (0.510–0.843)

The proportion of people with opioid dependence within each of the severity levels was determined based on available data from US National Epidemiological Survey on Alcohol and Related Conditions (NESARC), conducted in two waves from 2001–2002 and 2004–2005,[7] and the Comorbidity and Trauma study conducted in 2005-2008.[8] NESARC is a direct household survey. As such, it is expected to underestimate moderate to severe cases of drug dependence. The estimated distribution of opioid dependent cases by severity were asymptomatic (16%, 13%–19%), mild (37%, 20%–55%), and moderate/severe (47%, 29%–64%).

References

1. American Psychiatric Association. Diagnostic and Statistical Manual of Mental Disorders (DSM-IV-TR). 4th, Text Revision ed Washington DC: American Psychiatric Association; 2000.
2. World Health Organization. The ICD-10 Classification of Mental and Behavioural Disorders. Clinical descriptions and diagnostic guidelines Geneva: World Health Organization; 1992.
3. Degenhardt L, Bucello C, Calabria B, Nelson P, Roberts A, Hall W, et al. What data are available on the extent of illicit drug use and dependence globally? Results of four systematic reviews. Drug and alcohol dependence. 2011.
4. Calabria B, Degenhardt L, Briegleb C, Vos T, Hall W, Lynskey M, et al. Systematic review of prospective studies investigating "remission" from amphetamine, cannabis, cocaine or opioid dependence. Addictive Behaviors. 2010.
5. Shand FL, Degenhardt L, Slade T, Nelson EC. Sex differences amongst dependent heroin users: Histories, clinical characteristics and predictors of other substance dependence. Addictive behaviors. 2011; 36(1): p. 27-36.
6. European Montioring Centre for Drugs and Drug Addiction. Lisbon, Portugal; 2014.
7. Grant BF, Dawson DA. National Institute on Alcohol Abuse and Alcoholism. Alcohol Health & Research World. 2006; 29(2): p. 74.
8. Shand FL, Slade T, Degenhardt L, Baillie A, Nelson EC. Opioid dependence latent structure: two classes with differing severity? Addiction. 2011; 106(3): p. 590-8.
9.

Cocaine use disorders

Flowchart

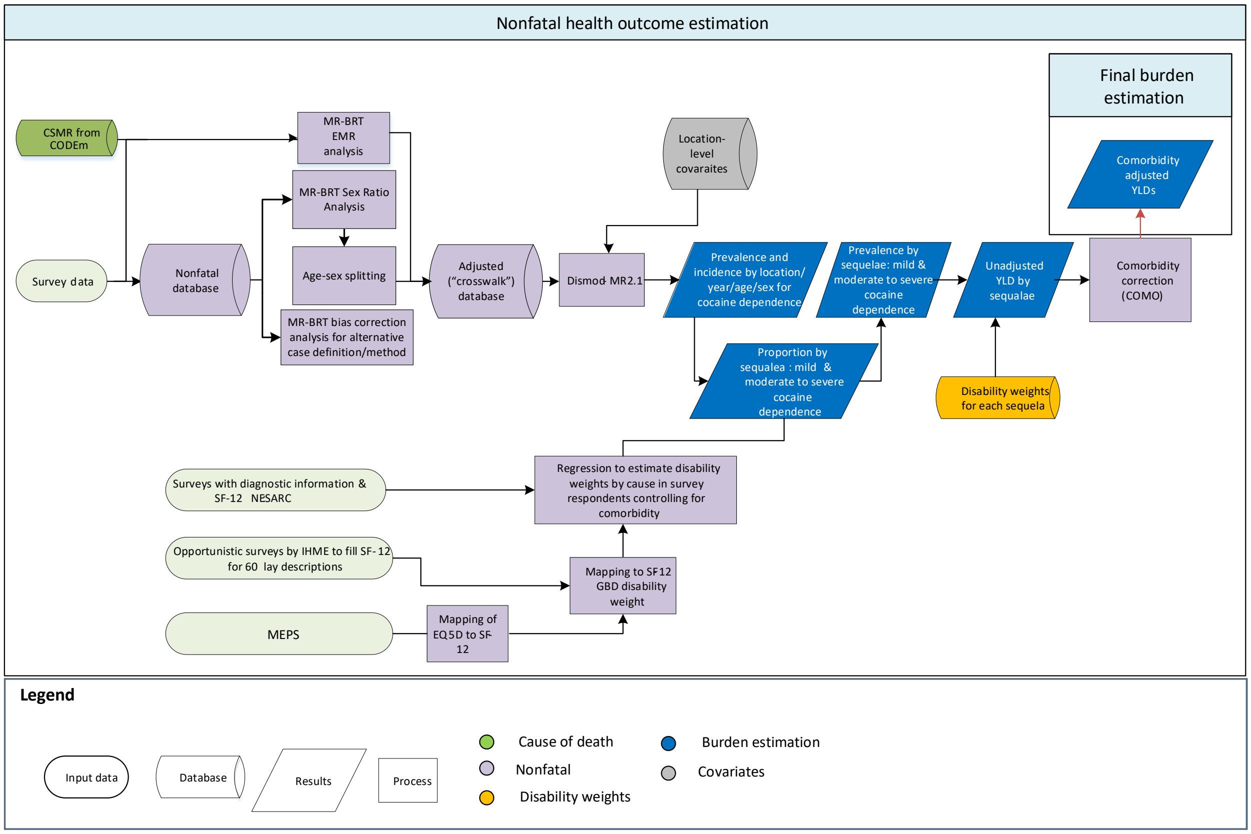

Input Data and Methodological Summary for Cocaine Use Disorders

Case definition

Cocaine dependence is a substance-related disorder involving a dysfunctional pattern of cocaine use. Included in the GBD disease modelling were cases meeting the Diagnostic and Statistical Manual of Mental Disorders (DSM-IV-TR) or the International Classification of Diseases (ICD-10) diagnostic criteria for cocaine dependence (DSM: 304.20; ICD: F14.2), excluding those cases due to a general medical condition.[1,2] According to DSM-IV TR criteria, dependence involves a maladaptive pattern of substance use leading to clinically significant impairment or distress. At least three of the following symptoms must be experienced within the same 12-month period:

- Tolerance, characterised by either
  - a need for increased amounts of the substance to achieve intoxication; or
  - markedly diminished effect with continued use of the same amount of the substance;
- Withdrawal, characterised by either
  - Withdrawal symptoms characteristic to dependence; or

- the same (or similar) substance is taken to avoid withdrawal symptoms;
- Substance taken in progressively larger amounts or for a longer period;
- Persistent desire or unsuccessful efforts to reduce substance use;
- Disproportionate time dedicated to obtaining the substance;
- Other important activities are given up because of the substance use; and
- Substance use is continued despite knowledge of physical or psychological problems occurring as a result of the substance.

Input data

For GBD 2010, a systematic review of the literature was conducted in to capture studies of prevalence, incidence, remission, and excess mortality associated with cocaine dependence. In summary, the search was conducted in three stages involving searches of the peer-reviewed literature (via Medline, Embase, and PubMed), the grey literature, and expert consultation. The agreed-upon approach for mental and substance use disorders was to conduct electronic database searches on a rolling basis. All three stages of GBD 2010's literature review were repeated for GBD 2013 to capture additional data published up to 2013. For GBD 2015, stages 2 and 3 of the literature review were updated, and in GBD 2016, the peer-reviewed database search (stage 1) was conducted via Medline, Embase, and Psycinfo to capture studies published from 2013 to 2016. GBD 2017 included additional sources identified by GBD experts and microdata where available. Additionally, in GBD 2017, two targeted systematic reviews were conducted to further supplement the dataset. The first review captured studies within Maori versus non-Maori populations (as opposed to New Zealand more broadly), given the inclusion of these two sub-groups in GBD 2017. The second review utilised the China National Knowledge Infrastructure database to find studies that would not typically be captured in PubMed, Embase, and PsycINFO.

The inclusion criteria stipulated that 1) the publication year must be from 1980 onward; 2) "caseness" must be based on clinical threshold as established by the DSM or ICD; 3) sufficient information must be provided on study method and sample characteristics to assess the quality of the study; and (4) study samples must be representative of the general population (ie, inpatient or pharmacological treatment samples, case studies, veterans or refugee samples were excluded). No limitation was set on the language of publication. Methods used for this systematic review have been reported in greater detail elsewhere.[3,4]

Table 1: Data Inputs for Cocaine Use Disorders Morbidity Modeling by Parameter

Measure	Total sources	Countries with data
All measures	365	68
Prevalence	353	68
Remission	3	2
Relative Risk	2	2

Standardized mortality ratio	3	3
With-condition mortality rate	3	2
Proportion	2	1

Age and sex splitting

Reported estimates of prevalence were split by age and sex where possible. First, if studies reported prevalence for broad age groups by sex (eg, prevalence in 15- to 65-year-old males and females separately), and also by specific age groups for both sexes combined (eg, prevalence in 15- to 30-year-olds, then in 31- to 65-year-olds, for males and females combined), age-specific estimates were split by sex using the reported sex ratio and bounds of uncertainty. Second, prevalence data for both sexes that could not be split using a within-study ratio were split using a sex ratio derived from a meta-analysis of existing sex-specific data using MR-BRT. The female to male ratio was 0.50 (0.39 to 0.66) for ages 20 and above, and 0.68 (0.51 to 0.89) for ages below 20. Finally, after the application of bias adjustments, where studies reported estimates across age groups spanning 25 years or more, these were split into five-year age groups using the super-region-specific prevalence age pattern estimated by DisMod-MR 2.1 on all data prior to age-splitting.

Data adjustment

Due to insufficient data in the optimal case definition of cocaine dependence, the prevalence dataset included data points of both use and dependence estimated using "direct" or "indirect" survey methods. "Direct" methods of measuring cocaine dependence predominantly involve surveys of the general population that ask if respondents use or are dependent on cocaine. Surveys tend to underestimate the prevalence of the most harmful and stigmatised forms of illicit drug use in ways that probably vary between countries and cultures.[5] "Indirect" methods are considered superior; they use different sources of data to indirectly estimate the total number of drug users (methods include "multiplier methods," back-projection and capture-recapture methods). Due to the lack of data available on cocaine dependence from indirect survey methods (considered to be the gold standard for GBD purposes), estimates of use and/or estimates from direct survey methods were also included in the modelling. We marked studies reporting on the prevalence of cocaine dependence obtained via direct methods as well as those reporting on the prevalence of cocaine use obtained via direct methods and derived adjustment factors using MR-BRT. Due to limited overlapping data and roughly similar patterns of use, we combined amphetamine and cocaine data to derive a single adjustment factor. Betas coefficients, in logit space are shown in the table below:

Table 2: MR-BRT Crosswalk Adjustment Factors for Cocaine and Amphetamine Use Disorders

Data input	Reference or alternative case definition	Gamma	Beta Coefficient, Logit (95% CI)

Cocaine dependence – indirect method	Ref	0.62	---
Cocaine use – indirect method	Alt		1.07 (-0.11 to 2.35)
Cocaine dependence – direct method	Alt		-0.54 (-1.73 to 0.76)
Cocaine use – direct method	Alt		0.54 (-0.65 to 1.81)

Subsequently, we adjusted for recall period to adjust from one-year recall to point-prevalence, again using combined cocaine and amphetamine data. Beta coefficients from MR-BRT are shown in the table below:

Table 3: MR-BRT Crosswalk Adjustment Factors for Cocaine and Amphetamine Use Disorders

Data input	Reference or alternative case definition	Gamma	Beta Coefficient, Logit (95% CI)
Cocaine dependence point prevalence	Ref	0	---
Cocaine dependence 1-year recall	Alt		0.71 (0.63 to 0.79)

Modeling strategy

Prior settings in DisMod included assuming no incidence, remission, and excess mortality before age 15, and an upper limit of 0.2 on remission. The minimum age of onset was corroborated with expert feedback and existing literature from various sources including the European Monitoring Centre for Drugs and Drug Addiction.[6] These settings were retained for GBD 2019.

As in GBD 2017, LDI was included as a country covariate on EMR with bounds set at -0.5 and -0.1.

In previous rounds, priors on excess mortality rate (EMR) were estimated in DisMod by matching prevalence data points with their corresponding CSMR values within the same age, sex, year, location (by dividing CSMR by prevalence). For short duration conditions (remission>1), the corresponding prevalence was derived by running an initial model and then applying the same CSMR/prevalence method. However, for many causes, DisMod estimated a rather unrealistic pattern of EMR compared to an expected pattern of decreasing EMR with greater access to quality health care. Such unexpected

patterns often signal inconsistencies between CSMR estimates and the measures of prevalence and/or incidence. In effort to provide greater guidance to DisMod on the expected pattern of EMR, EMR data generated in the previous round were modeled using the MR-BRT approach by age and sex with a prior on healthcare access and quality index (HAQi) having a negative coefficient. Results from MR-BRT were then predicted for each location year, sex and for ages 0, 10, 20 ….100. We included HAQi as a country-level covariate to inform EMR with a mean and standard deviation produced from MR-BRT.

After testing the modeled EMR approach, the final cocaine use disorder model excludes the predicted data for the EMR parameter in favor of last year's DisMod EMR calculation strategy. This is because the MR-BRT analysis for paired cocaine use disorder prevalence and CSMR data did not find any effect of HAQi under the condition of a negative prior. As such, across high and low HAQi locations predicted EMR remained the same, following the low EMR of the High-income super region where there are the majority of data. This resulted in much lower EMR, and consequently higher prevalence that ignored input data, than in prior rounds in super regions such as Central Europe, Eastern Europe, and Central Asia; Sub-Saharan Africa; South Asia; and Southeast Asia, East Asia, and Oceania.

Table 4. Covariates. Summary of covariates used in the cocaine use disorders DisMod-MR meta-regression model

Covariate	Parameter	Beta, log (95% Uncertainty Interval)	Exponentiated beta (95% Uncertainty Interval)
LDI ($ per capita)	Excess mortality rate	-0.1 (-0.1 to -0.1)	0.90 (0.90 to 0.90)

Severity and Disability

The basis of the GBD disability weight survey assessments are lay descriptions of sequelae highlighting major functional consequences and symptoms. The lay descriptions and disability weights for cocaine dependence severity levels are shown below.

Table 5. Severity distribution, details on the severity levels for cocaine use disorders in GBD 2019 and the associated disability weight (DW) with that severity.

Severity level	Lay description	DW (95% CI)
Mild	Uses cocaine at least once a week and has some difficulty controlling the habit. When not using, the person functions normally.	0.116 (0.074–0.165)
Moderate to severe	Uses cocaine and has difficulty controlling the habit. The person sometimes has mood swings, anxiety, paranoia, hallucinations and sleep problems, and has some difficulty in daily activities.	0.479 (0.324–0.634)

The proportion of people with cocaine dependence within each of the severity levels were determined based on available data from US National Epidemiological Survey on Alcohol and Related Conditions (NESARC), conducted in two waves from 2001 to 2002 and 2004 to 2005.[7] NESARC is a direct household survey. As such, it is expected to underestimate moderate to severe cases of drug dependence. The estimated distribution of cocaine dependent cases by severity were asymptomatic (50%, 37%–64%), mild (25%, 18%–33%), and moderate/severe (25%, 17%–33%).

References

1. American Psychiatric Association. Diagnostic and Statistical Manual of Mental Disorders (DSM-IV-TR). 4th, Text Revision ed Washington DC: American Psychiatric Association. 2000.
2. World Health Organization. The ICD-10 Classification of Mental and Behavioural Disorders. Clinical descriptions and diagnostic guidelines Geneva: World Health Organization. 1992.
3. Degenhardt L, Bucello C, Calabria B, Nelson P, Roberts A, Hall W, et al. What data are available on the extent of illicit drug use and dependence globally? Results of four systematic reviews. Drug and alcohol dependence. 2011.
4. Calabria B, Degenhardt L, Briegleb C, Vos T, Hall W, Lynskey M, et al. Systematic review of prospective studies investigating "remission" from amphetamine, cannabis, cocaine or opioid dependence. Addictive Behaviors. 2010.
5. Reuter P, Trautmann F. A Report on Global Illicit Drugs Markets 1998-2007. Utrecht. 2009.
6. European Montioring Centre for Drugs and Drug Addiction. Lisbon, Portugal 2014.
7. Grant BF, Dawson DA. National Institute on Alcohol Abuse and Alcoholism. Alcohol Health & Research World. 2006; 29(2): p. 74.

Amphetamine use disorders

Flowchart

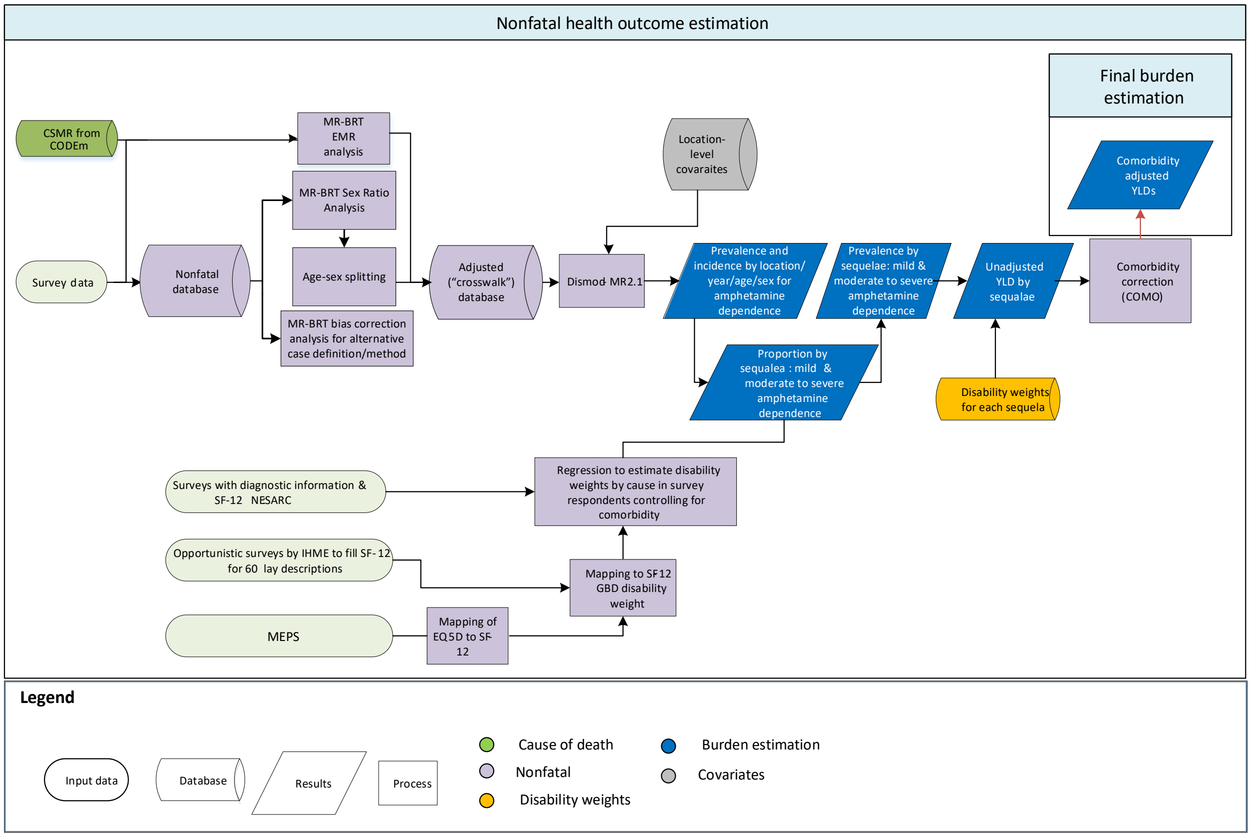

Input Data and Methodological Summary for Amphetamine Use Disorders

Case definition

Amphetamine dependence is a substance-related disorder involving a dysfunctional pattern of amphetamine use. Included in the GBD disease modelling were cases meeting the Diagnostic and Statistical Manual of Mental Disorders (DSM-IV-TR) or the International Classification of Diseases (ICD-10) diagnostic criteria for amphetamine dependence (DSM: 304.40; ICD: F15.2), excluding those cases due to a general medical condition.[1,2] According to DSM-IV TR criteria, dependence involves a maladaptive pattern of substance use, leading to clinically significant impairment or distress. At least three of the following symptoms must be experienced within the same 12-month period:

- Tolerance, characterised by either
    - a need for increased amounts of the substance to achieve intoxication; or
    - markedly diminished effect with continued use of the same amount of the substance;
- Withdrawal, characterised by either
    - Withdrawal symptoms characteristic to dependence; or

- the same (or similar) substance is taken to avoid withdrawal symptoms;
- Substance taken in progressively larger amounts or for longer periods;
- Persistent desire or unsuccessful efforts to reduce substance use;
- Disproportionate time dedicated to obtaining the substance;
- Other important activities are given up because of the substance use; and
- Substance use is continued despite knowledge of physical or psychological problems occurring as a result of the substance.

Input data

For GBD 2010, a systematic review of the literature was conducted to capture studies of prevalence, incidence, remission, and excess mortality associated with amphetamine dependence. In summary, the search was conducted in three stages involving searches of the peer-reviewed literature (via Medline, Embase, and PubMed), the grey literature, and expert consultation. The agreed-upon approach for mental and substance use disorders was to conduct electronic database searches on a rolling basis. All three stages of GBD 2010's literature review were repeated for GBD 2013 to capture additional data published up to 2013. For GBD 2015, stages 2 and 3 of the literature review were updated and in GBD 2016, the peer-reviewed database search (stage 1) was conducted via Medline, Embase, and Psycinfo to capture studies published from 2013 to 2016. GBD 2017 included additional sources identified by GBD experts and microdata where available. Additionally, in GBD 2017, two targeted systematic reviews were conducted to further supplement the dataset. The first review captured studies within Maori versus non-Maori populations (as opposed to New Zealand more broadly), given the inclusion of these two sub-groups in GBD 2017. The second review utilised the China National Knowledge Infrastructure database to find studies that would not typically be captured in PubMed, Embase, and PsycINFO.

The inclusion criteria stipulated that: 1) the publication year must be from 1980 onward; 2) "caseness" must be based on clinical threshold as established by the DSM or ICD; 3) sufficient information must be provided on study method and sample characteristics to assess the quality of the study; and (4) study samples must be representative of the general population (ie, inpatient or pharmacological treatment samples, case studies, veterans, or refugee samples were excluded). No limitation was set on the language of publication. Methods used for this systematic review have been reported in greater detail elsewhere.[3,4]

Table 1: Data Inputs for Amphetamine Use Disorders Morbidity Modeling by Parameter

Measure	Total sources	Countries with data
All measures	323	58
Prevalence	316	58
Remission	1	1
Relative Risk	1	1

With-condition mortality rate	5	4

Age and sex splitting

Reported estimates of prevalence were split by age and sex where possible. First, if studies reported prevalence for broad age groups by sex (eg, prevalence in 15- to 65-year-old males and females separately), and also by specific age groups for both sexes combined (eg, prevalence in 15- to 30-year-olds, then in 31- to 65-year-olds, for males and females combined), age-specific estimates were split by sex using the reported sex ratio and bounds of uncertainty. Second, prevalence data for both sexes that could not be split using a within-study ratio were split using a sex ratio derived from a meta-analysis of existing sex-specific data using MR-BRT. The female to male ratio was 0.63 (0.44 to 0.92) for adults age 20 and older, and 0.60 (0.42 to 0.87) for youth under age 20. Finally, after the application of bias adjustments, where studies reported estimates across age groups spanning 20 years or more, these were split into five-year age groups using the super-region-specific prevalence age pattern estimated by DisMod-MR 2.1 on all data prior to age-splitting.

Data adjustment

Due to insufficient data in the optimal case definition of amphetamine dependence, the prevalence dataset included data points of both use and dependence estimated using "direct" or "indirect" survey methods. "Direct" methods of measuring amphetamine dependence predominantly involve surveys of the general population that ask if respondents use or are dependent on amphetamine. Surveys tend to underestimate the prevalence of the most harmful and stigmatised forms of illicit drug use in ways that probably vary between countries and cultures.[5] "Indirect" methods are considered superior; they use different sources of data to indirectly estimate the total number of drug users (methods include "multiplier methods," back-projection and capture-recapture methods). Due to the lack of data available on amphetamine dependence from indirect survey methods (considered to be the gold standard for GBD purposes), estimates of use and/or estimates from direct survey methods were also included in the modelling. We marked studies reporting on the prevalence of amphetamine dependence obtained via direct methods as well as those reporting on the prevalence of amphetamine use obtained via direct methods and derived adjustment factors using MR-BRT. Due to limited overlapping data and roughly similar patterns of use, we combined amphetamine and cocaine data to derive a single adjustment factor. Betas coefficients, in logit space are shown in the table below:

Table 2: MR-BRT Crosswalk Adjustment Factors for Amphetamine and Cocaine Use Disorders

Data input	Reference or alternative case definition	Gamma	Beta Coefficient, Logit (95% CI)
Amphetamine dependence – indirect method	Ref	0.62	---
Amphetamine use – indirect method	Alt		1.07 (-0.11 to 2.35)

Amphetamine dependence – direct method	Alt		-0.54 (-1.73 to 0.76)
Amphetamine use – direct method	Alt		0.54 (-0.65 to 1.81)

Subsequently, we adjusted for recall period to adjust from one-year recall to point-prevalence, again using combined cocaine and amphetamine data. Beta coefficients from MR-BRT are shown in the table below:

Table 3: MR-BRT Crosswalk Adjustment Factors for Amphetamine and Cocaine Use Disorders

Data input	Reference or alternative case definition	Gamma	Beta Coefficient, Logit (95% CI)
Amphetamine dependence point prevalence	Ref	0	---
Amphetamine dependence 1-year recall	Alt		0.71 (0.63 to 0.79)

Modeling strategy

Prior settings in DisMod included assuming no incidence, remission, and excess mortality before age 15, and an upper limit of 0.35 on remission. The minimum age of onset was corroborated with expert feedback and existing literature from various sources including the European Monitoring Centre for Drugs and Drug Addiction.[6] These settings were retained for GBD 2019.

As in GBD 2017, LDI was included as a country covariate on EMR with bounds set at -1 and -0.1.

In previous rounds, priors on excess mortality rate (EMR) were estimated in DisMod by matching prevalence data points with their corresponding CSMR values within the same age, sex, year, location (by dividing CSMR by prevalence. However, for many causes, DisMod estimated a rather unrealistic pattern of EMR compared to an expected pattern of decreasing EMR with greater access to quality health care. Such unexpected patterns often signal inconsistencies between CSMR estimates and the measures of prevalence and/or incidence. In effort to provide greater guidance to DisMod on the expected pattern of EMR, EMR data generated in the previous round were modeled using the MR-BRT approach by age and sex with a prior on healthcare access and quality index (HAQi) having a negative coefficient. Results from MR-BRT were then predicted for each location year, sex and for ages 0, 10, 20 ….100. We included HAQi as a country-level covariate to inform EMR with a mean and standard deviation produced from MR-BRT.

After testing the modeled EMR approach, the final amphetamine use disorder model excludes the predicted data for the EMR parameter in favor of last year's DisMod EMR calculation strategy. This is because the MR-BRT analysis for paired amphetamine use disorder prevalence and CSMR data did not find any effect of HAQi under the condition of a negative prior. As such, across high and low HAQi locations EMR remained the same and followed the low EMR of the High-income super region where there are the majority of data. This resulted in much lower EMR, and consequently higher prevalence that ignored input data, than in prior rounds, particularly in the Central Europe, Eastern Europe, and Central Asia and Latin America and the Caribbean super regions.

Table 4. Covariates. Summary of covariates used in the amphetamine use disorders DisMod-MR meta-regression model

Covariate	Parameter	Beta, log (95% Uncertainty Interval)	Exponentiated beta (95% Uncertainty Interval)
LDI ($ per capita)	Excess mortality rate	-0.1 (-0.1 to -0.1)	0.90 (0.90 to 0.90)

Severity and Disability

The basis of the GBD disability weight survey assessments are lay descriptions of sequelae highlighting major functional consequences and symptoms. The lay descriptions and disability weights for amphetamine dependence severity levels are shown below.

Table 5. Severity distribution, details on the severity levels for amphetamine use disorders in GBD 2019 and the associated disability weight (DW) with that severity.

Severity level	Lay description	DW (95% CI)
Mild	Uses stimulants (drugs) at least once a week and has some difficulty controlling the habit. When not using, the person functions normally.	0.079 (0.051–0.114)
Moderate to severe	Uses stimulants (drugs) and has difficulty controlling the habit. The person sometimes has depression, hallucinations, and mood swings, and has difficulty in daily activities.	0.486 (0.329–0.637)

The proportion of people with amphetamine dependence within each of the severity levels was determined based on available data from US National Epidemiological Survey on Alcohol and Related Conditions (NESARC), conducted in two waves from 2001 to 2002 and 2004 to 2005.[7] NESARC is a direct household survey. As such, it is expected to underestimate moderate to severe cases of drug dependence. The estimated distribution of amphetamine dependent cases by severity were asymptomatic (55%, 40%–71%), mild (19%, 12%–27%), and moderate/severe (26%, 16%–35%).

References

1. American Psychiatric Association. Diagnostic and Statistical Manual of Mental Disorders (DSM-IV-TR). 4th, Text Revision ed Washington DC: American Psychiatric Association. 2000.
2. World Health Organization. The ICD-10 Classification of Mental and Behavioural Disorders. Clinical descriptions and diagnostic guidelines Geneva: World Health Organization. 1992.
3. Degenhardt L, Bucello C, Calabria B, Nelson P, Roberts A, Hall W, et al. What data are available on the extent of illicit drug use and dependence globally? Results of four systematic reviews. Drug and alcohol dependence. 2011.
4. Calabria B, Degenhardt L, Briegleb C, Vos T, Hall W, Lynskey M, et al. Systematic review of prospective studies investigating "remission" from amphetamine, cannabis, cocaine or opioid dependence. Addictive Behaviors. 2010.
5. Reuter P, Trautmann F. A Report on Global Illicit Drugs Markets 1998-2007. Utrecht. 2009.
6. European Montioring Centre for Drugs and Drug Addiction. Lisbon, Portugal 2014.
7. Grant BF, Dawson DA. National Institute on Alcohol Abuse and Alcoholism. Alcohol Health & Research World. 2006; 29(2): p. 74.

Cannabis use disorders

Flowchart

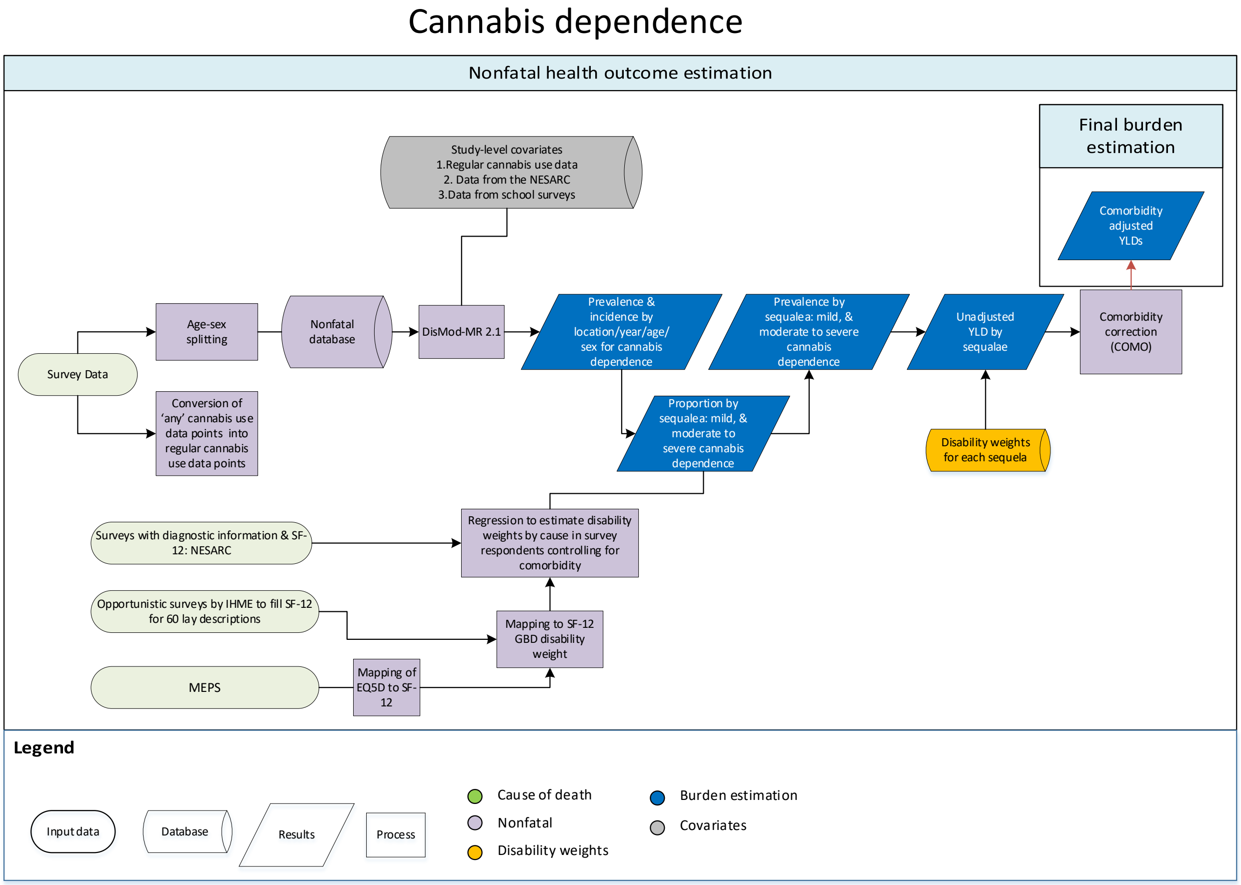

Input Data and Methodological Summary for Cannabis Use Disorders

Case Definition

Cannabis dependence is a substance-related disorder involving a dysfunctional pattern of cannabis use. Included in GBD disease modelling were cases meeting diagnostic criteria for cannabis dependence according to the Diagnostic and Statistical Manual of Mental Disorders (DSM), or the equivalent diagnosis in the International Classification of Diseases (ICD). These were identified by the following codes: DSM:304.30, ICD:F12.2; excluding those cases due to a general medical condition.[1,2]

According to DSM-IV-TR criteria, cannabis dependence involves a maladaptive pattern of cannabis use, leading to clinically significant impairment or distress. At least three of the following symptoms must be experienced within the same 12-month period:

- Tolerance, characterised by either

- a need for increased amounts of the substance to achieve intoxication; or
    - markedly diminished effect with continued use of the same amount of the substance;
- Withdrawal, characterised by either
    - Withdrawal symptoms characteristic to cannabis dependence; or
    - the same (or similar) substance is taken to avoid withdrawal symptoms;
- substance taken in progressively larger amounts or for longer periods;
- persistent desire or unsuccessful efforts to reduce substance use;
- disproportionate time dedicated to obtaining the substance;
- other important activities are given up because of the substance use; and
- substance use is continued despite knowledge of physical or psychological problems occurring as a result of the substance.

Input data

For GBD 2010, a systematic review of the literature was conducted to capture studies of prevalence, incidence, duration, and excess mortality associated with cannabis dependence. In summary, the search was conducted in three stages involving electronic searches of the peer-reviewed literature (via PsycInfo, Embase and PubMed), the grey literature and, expert consultation. The agreed-upon approach for mental and substance use disorders was to conduct electronic database searches on a rolling basis. All three stages of GBD 2010's literature review were repeated for GBD 2013 and GBD 2016. In GBD 2017, stages two and three of the literature review were conducted. Additionally, two targeted systematic reviews were conducted to further supplement the cannabis dependence dataset. The first review captured studies reporting on the epidemiology of cannabis dependence within Maori versus non-Maori populations (as opposed to New Zealand more broadly), given the inclusion of these two sub-groups in GBD 2017. The second review searched for studies on the epidemiology of cannabis dependence in China using primarily the China National Knowledge Infrastructure database. The focus was to search for studies published in Chinese journals that would not typically be captured in mainstream databases such as PsycInfo, Embase, and PubMed.

The inclusion criteria stipulated that: (1) the publication year must be from 1980 onward; (2) "caseness" must be based on clinical threshold as established by the DSM or ICD; (3) sufficient information must be provided on study method and sample characteristics to assess the quality of the study; and (4) study samples must be representative of the general population (ie, inpatient or pharmacological treatment samples, case studies, veterans or refugee samples were excluded). No limitation was set on the language of publication. Methods used for this systematic review have been reported in greater detail elsewhere.[3-6]

Table 1: Data Inputs for Cannabis Use Disorders Morbidity Modeling by Parameter

Measure	Total sources	Countries with data
All measures	806	121

Prevalence	802	121
Remission	3	3
Proportion	2	1

Age and sex splitting

Reported estimates of prevalence were split by age and sex where possible. First, if studies reported prevalence for broad age groups by sex (eg, prevalence in 15- to 65-year-old males and females separately), and also by specific age groups for both sexes combined (eg, prevalence in 15- to 30-year-olds, then in 31- to 65-year-olds, for males and females combined), age-specific estimates were split by sex using the reported sex ratio and bounds of uncertainty. Second, prevalence data for both sexes that could not be split using a within-study ratio were split using a sex ratio derived from a meta-analysis of existing sex-specific data using MR-BRT. The female to male ratio was 0.49 (0.33 to 0.70) for ages 20 and above, and 0.61 (0.42 to 0.88) for ages below 20. Finally, after the application of bias adjustments, where studies reported estimates across age groups spanning 25 years or more, these were split into five-year age groups using the super-region-specific prevalence age pattern estimated by DisMod-MR 2.1 on all data prior to age-splitting.

Data adjustment

Due to insufficient data in the optimal case definition of cannabis dependence, the prevalence dataset included data points originally reporting any cannabis use, regular (ie. weekly) cannabis use, and cannabis dependence. Adjusting any cannabis use and regular cannabis use to cannabis dependence involved a two-step process. In the first stage, estimates of any cannabis use were converted to estimates of regular cannabis use. In GBD 2019 we retained the GBD 2017 adjustment coefficient for this first stage. Briefly a ratio of any use to regular use was calculated by comparing similar regular use and any use estimates in the dataset. To allow for meaningful comparisons, paired regular use and use estimates needed to be similar in terms of the country they were from, year, age group, sex, and prevalence type. Once a dataset was set up with paired regular use and use estimates, MetaXL (a meta-analysis add-in for Microsoft Excel) was used to estimate a ratio of use: regular use whereby use estimates were found to be 2.9 (2.5–3.3) times higher than regular use estimates. This ratio was used to adjust all use estimates in the dataset downward, toward the level they would have been had the study reported regular cannabis use.

In GBD 2019 we focused on updating the second stage of the adjustment, in which regular use estimates were converted to cannabis dependence estimates, using a logit-difference coefficient calculated using MR-BRT. In this second stage we also adjusted for bias in school-based surveys compared to household surveys among youth. We found an age pattern to the relationship between regular use and dependence, and therefore ran separate models for youth (under age 25) and adults (over age 25). A network analysis allowing for both direct and indirect comparisons was preferred for adjusting youth data for the two study-level covariates (regular use and school-based surveys), therefore two separate MR-BRT models were run on cannabis data, one on adults and one on youth. Compared to GBD 2017,

adjustments calculated using a logit-difference approach in MR-BRT resulted in slightly higher post-adjustment prevalence estimates among both youth and adults.

In GBD 2017 a study-level covariate was used to adjust NESARC data upwards based on expert advice. The rationale behind this adjustment was that the NESARC sampling strategy was biased towards less severe cases of drug use disorders. In GBD 2019, we found that there were no direct comparisons between NESARC and non-NESARC data. Given the lack of direct comparisons and the relatively small number of data points coming from NESARC compared to other surveys in the United States, we decided to exclude NESARC data in GBD 2019.

Table 2: MR-BRT Crosswalk Adjustment Factors for Cannabis Use Disorder, Youth

Data input	Reference or alternative case definition	Gamma	Beta Coefficient, Logit (95% CI)
Cannabis dependence, household-based	Ref	0.32	---
Cannabis dependence, school-based	Alt		0.33 (-0.30 to 0.94)
Cannabis regular use, household-based	Alt		0.73 (0.12 to 1.34)
Cannabis regular use, school-based	Alt		1.08 (0.44 to 1.70)

Table 3: MR-BRT Crosswalk Adjustment Factors for Cannabis Use Disorder, Adults

Data input	Reference or alternative case definition	Gamma	Beta Coefficient, Logit (95% CI)
Cannabis dependence	Ref	0.28	---
Cannabis regular use	Alt		1.31 (0.77 to 1.86)

Modelling Strategy

Prior settings in DisMod included assuming no incidence before age 13. This minimum age of onset was corroborated with expert feedback and existing literature on cannabis dependence. We also assumed no incidence after age 70 as supported by data from various sources including the European Monitoring

Centre for Drugs and Drug Addiction.[7] An upper limit of 0.25 was placed on remission consistent with limits in the dataset. These settings were retained for GBD 2019. In GBD 2019, as in GBD 2017, no country-level covariates were used in predictions.

Severity and Disability

The basis of the GBD disability weight survey assessments are lay descriptions of health states highlighting major functional consequences and symptoms. The lay descriptions and disability weights for cannabis dependence severity levels are shown below.

Table 4. Severity distribution, details on the severity levels for cannabis use disorders in GBD 2019 and the associated disability weight (DW) with that severity.

Severity level	Lay description	DW (95% CI)
Mild	Uses marijuana at least once a week and has some difficulty controlling the habit. When not using, the person functions normally.	0.039 (0.024–0.06)
Moderate to severe	Uses marijuana daily and has difficulty controlling the habit. The person sometimes has mood swings, anxiety, and hallucinations, and has some difficulty in daily activities.	0.266 (0.178–0.364)

The US National Epidemiological Survey on Alcohol and Related Conditions (NESARC, conducted in two waves from 2001 to 2002 and 2004 to 2005)[8] was used to estimate the proportion of cannabis dependence cases asymptomatic (58%, 51%–63%), mild (36%, 31%–42%) and moderate to severe (6%, 4%–8%). NESARC is a direct household survey. As such, it is expected to underestimate moderate to severe cases of drug dependence, however, there are very few sources of usable drug severity data.

References

1. American Psychiatric Association. Diagnostic and Statistical Manual of Mental Disorders (DSM-IV-TR). 4th, Text Revision ed. Washington DC: American Psychiatric Association; 2000.
2. World Health Organization. The ICD-10 Classification of Mental and Behavioural Disorders. Clinical descriptions and diagnostic guidelines. Geneva: World Health Organization; 1992.
3. Calabria B, Degenhardt L, Briegleb C, et al. Systematic review of prospective studies investigating "remission" from amphetamine, cannabis, cocaine or opioid dependence. Addictive Behaviors 2010; 35(8): 741-9.
4. Calabria B, Degenhardt L, Hall W, Lynskey M. Does cannabis use increase the risk of death? Systematic review of epidemiological evidence on adverse effects of cannabis use. Drug Alcohol Rev 2010; 29(3): 318-30.
5. Calabria B, Degenhardt L, Nelson P, et al. What do we know about the extent of cannabis use and dependence? Results of a global systematic review. Sydney: National Drug and Alcohol Research Centre, University of NSW, 2010.

6. Degenhardt L, Ferrari AJ, Calabria B, et al. The global epidemiology and contribution of cannabis use and dependence to the global burden of disease: results from the GBD 2010 study. PloS one 2013; 8(10): e76635.
7. Introduction to the National Epidemiologic Survey on Alcohol and Related Conditions [http://pubs.niaaa.nih.gov/publications/arh29-2/74-78.htm]. Access date 1 December 2014.
8. European Montioring Centre for Drugs and Drug Addiction. Lisbon, Portugal; 2014.

Other drug use disorders

Flowchart

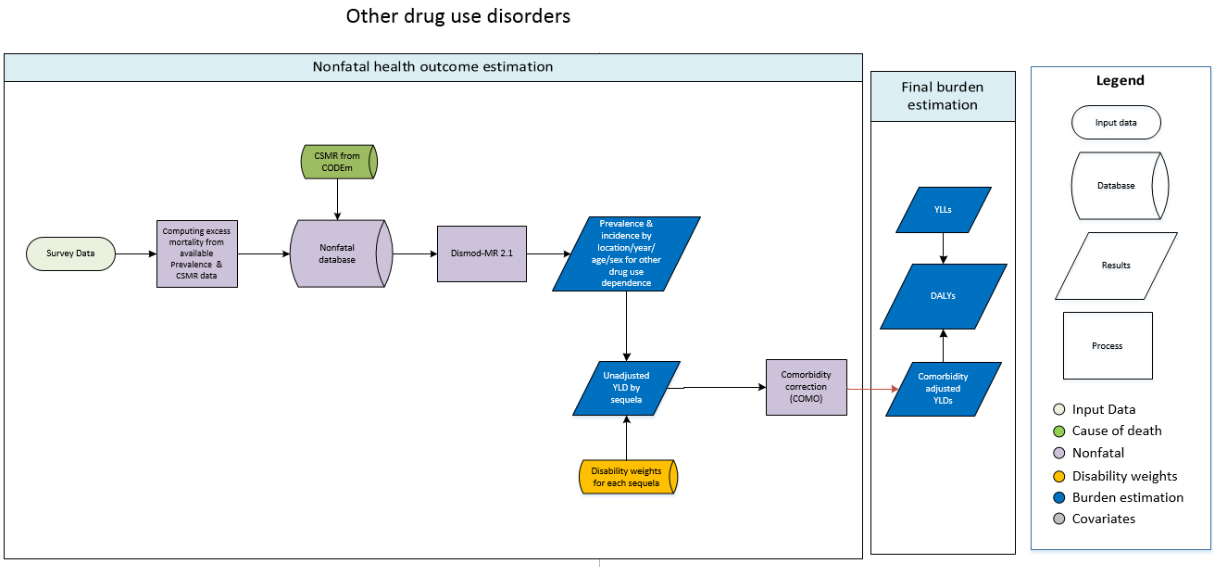

Input Data and Methodological Summary for Other Drug Use Disorders

Case definition

In addition to the four drug use disorders for which we specifically estimate non-fatal burden (opioid, cocaine, amphetamine, and cannabis dependence), we also estimate the burden attributable to a residual cause of "other drug use disorders." This is made up of an aggregate group of other forms of drug dependence. Included in the GBD disease modelling were cases meeting the Diagnostic and Statistical Manual of Mental Disorders (DSM-IV-TR)[1] or the International Classification of Diseases (ICD-10)[2] diagnostic criteria for:

- Hallucinogen dependence
- Inhalant or solvent dependence
- Sedative dependence
- Tranquiliser dependence
- Other medicines, drugs, substance dependence

According to DSM-IV TR criteria, dependence involves a maladaptive pattern of substance use, leading to clinically significant impairment or distress. At least three of the following symptoms must be experienced within the same 12-month period:

- Tolerance, characterised by either
    - a need for increased amounts of the substance to achieve intoxication; or

  - markedly diminished effect with continued use of the same amount of the substance;
- Withdrawal, characterised by either
  - Withdrawal symptoms characteristic to dependence; or
  - the same (or similar) substance is taken to avoid withdrawal symptoms;
- Substance taken in progressively larger amounts or for longer periods;
- Persistent desire or unsuccessful efforts to reduce substance use;
- Disproportionate time dedicated to obtaining the substance;
- Other important activities are given up because of the substance use; and
- Substance use is continued despite knowledge of physical or psychological problems occurring as a result of the substance.

Input data

Prevalence estimates were obtained from the Australian National Survey of Mental Health and Wellbeing (NSMHWB) conducted in 1997[3],and the US National Epidemiological Survey on Alcohol and Related Conditions (NESARC), conducted in two waves in 2001–2002[4] and 2004–2005.[5] Given that other forms of drug dependence often co-occur with the four types of drug dependence for which we estimate non-fatal burden (opioid, cocaine, amphetamine, and cannabis dependence), an adjustment for co-morbidity is important so as not to overestimate the overall burden attributable to drug dependence. Participants meeting criteria for any other form of drug dependence from each of the surveys used were counted as a prevalent case only if they did not simultaneously meet criteria for opioid, cocaine, amphetamine, or cannabis dependence.

Table 1: Data Inputs for Other Drug Use Disorders Morbidity Modeling by Parameter

Measure	Total sources	Countries with data
All measures	4	2
Prevalence	4	2

Modelling Strategy

The GBD 2019 epidemiological modelling strategy made use of DisMod-MR 2.1. A number of additional expert priors were used in order to run a full parameter model. We assumed no incidence before age 14, a maximum of 0.0004 on incidence from the age of 60 years onward, and a maximum remission of 0.2. These priors were corroborated with expert feedback and existing literature on drug use disorders including the European Monitoring Centre for Drugs and Drug Addiction.[6] Finally, cause-specific mortality rates (CSMR) from the GBD 2019 cause of death model for other drug use disorders were included as data-points in the DisMod-MR model.

Severity and Disability

The basis of the GBD disability weight survey assessments are lay descriptions of sequelae highlighting major functional consequences and symptoms. The average disability weight estimated for cocaine and amphetamine dependence was applied to all cases in this residual group of other drug use disorders. The cocaine and amphetamine lay descriptions and disability weights are shown below.

Table 2. Severity distribution, details on the severity levels for amphetamine use and cocaine use disorders in GBD 2019 and the associated disability weight (DW) with that severity.

Severity level	Lay description	DW (95% CI)
**Amphetamine dependence**		
Mild	Uses stimulants (drugs) at least once a week and has some difficulty controlling the habit. When not using, the person functions normally.	0.079 (0.051–0.114)
Moderate to severe	Uses stimulants (drugs) and has difficulty controlling the habit. The person sometimes has depression, hallucinations, and mood swings, and has difficulty in daily activities.	0.486 (0.329–0.637)
**Cocaine dependence**		
Mild	Uses cocaine at least once a week and has some difficulty controlling the habit. When not using, the person functions normally.	0.116 (0.074–0.165)
Moderate to severe	Uses cocaine and has difficulty controlling the habit. The person sometimes has mood swings, anxiety, paranoia, hallucinations, and sleep problems, and has some difficulty in daily activities.	0.479 (0.324–0.634)

References

1. American Psychiatric Association. Diagnostic and Statistical Manual of Mental Disorders (DSM-IV-TR). 4th, Text Revision ed. Washington DC: 2000.

2. World Health Organization. The ICD-10 Classification of Mental and Behavioural Disorders. Clinical descriptions and diagnostic guidelines. Geneva: 1992.

3. Australia Bureau of Statistics. Australia National Survey of Mental Health and Wellbeing 1997. Canberra: 1997.

4. National Institute on Alcohol Abuse and Alcoholism (NIAAA), National Institutes of Health (NIH). United States National Epidemiologic Survey on Alcohol and Related Conditions 2001-2002. 2002.

5. National Institute on Alcohol Abuse and Alcoholism (NIAAA), National Institutes of Health (NIH). United States National Epidemiologic Survey on Alcohol and Related Conditions 2004-2005. 2005.

6. European Monitoring Centre for Drugs and Drug Addiction. Lisbon, Portugal: 2014.

Diabetes Mellitus

Diabetes Mellitus prevalence is estimated for overall Diabetes Mellitus, Diabetes Mellitus Type 1, and Diabetes Mellitus Type 2 in GBD 2019.

Flowchart

Calculating prevalence of diabetes mellitus

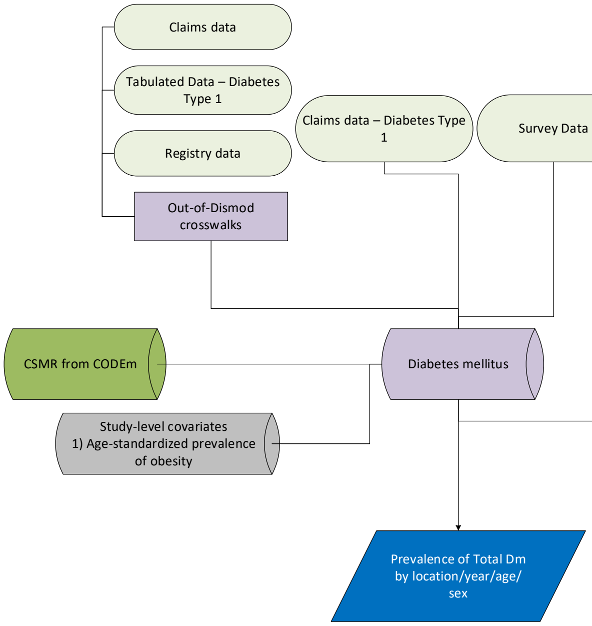

Calculating prevalence of type 1 diabetes mellitus

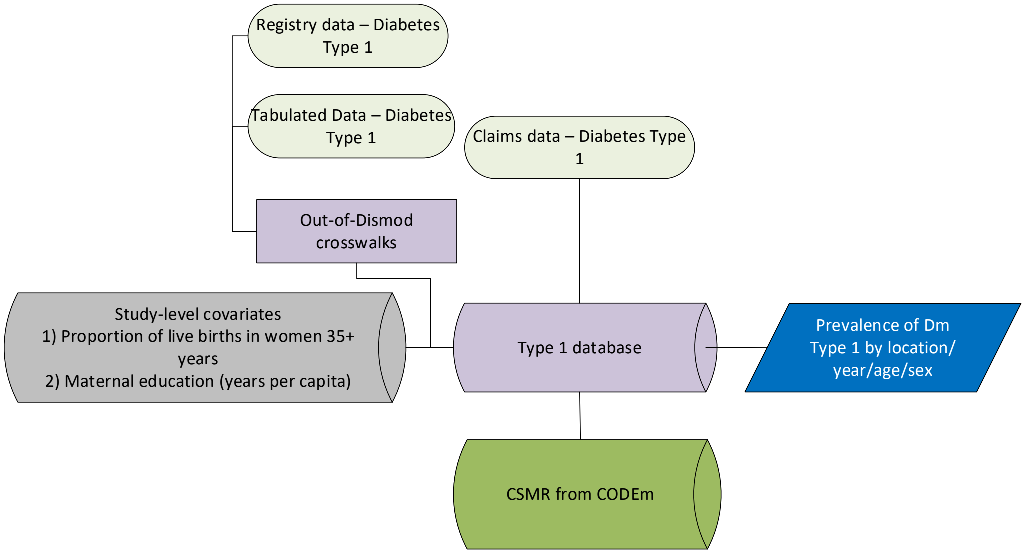

Calculating prevalence of type 2 diabetes mellitus

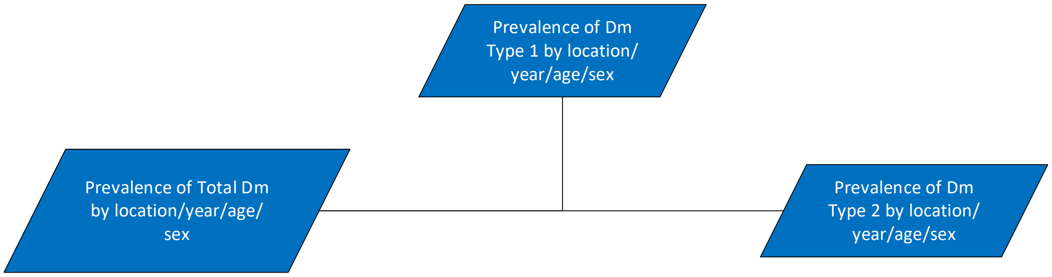

Calculating type-specific diabetic sequeale

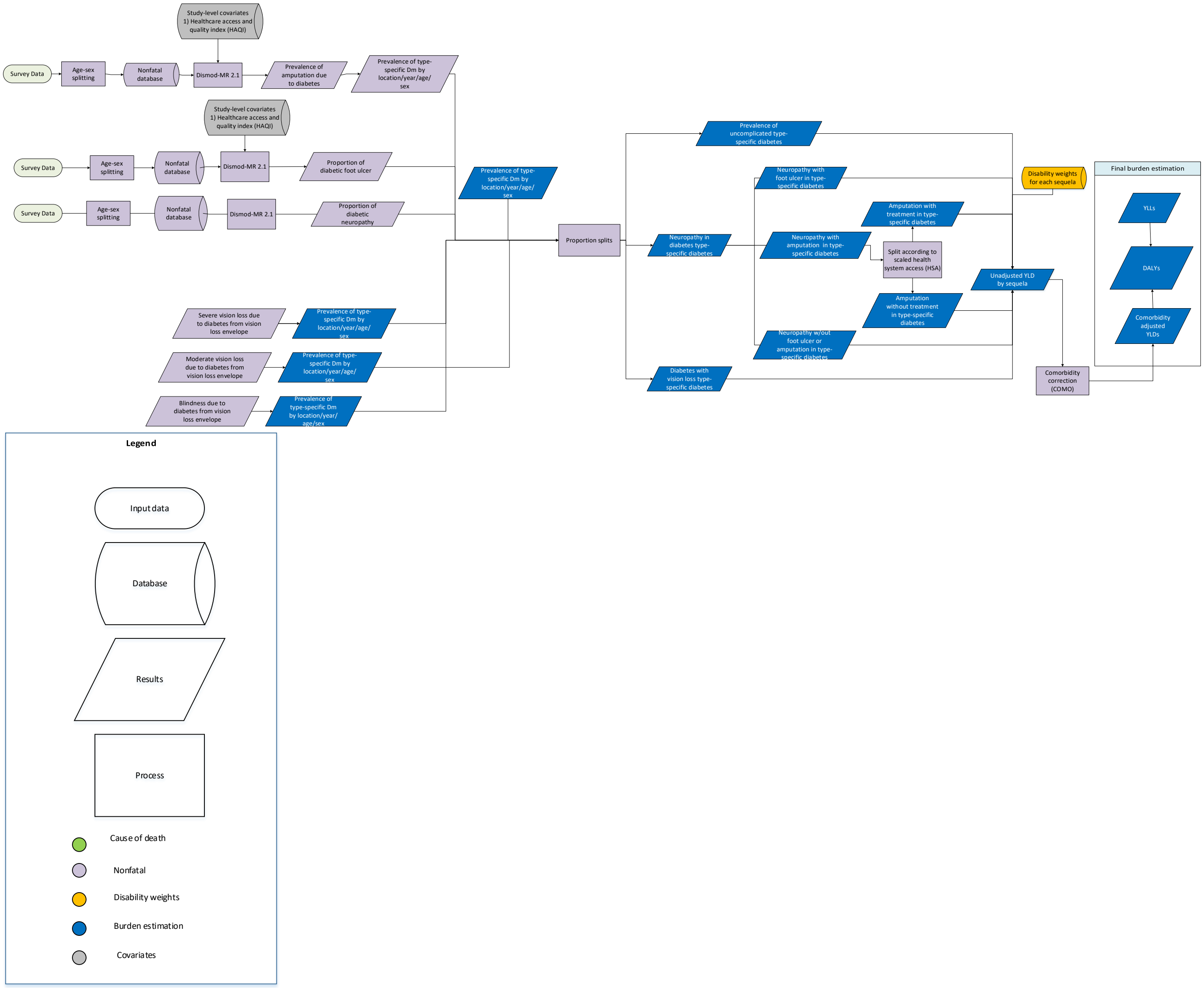

Case definition

The case definitions and diagnostic criteria are presented in the table below.

Overall Diabetes Mellitus

Criterion	Definition
1. Diabetes Mellitus parent	Fasting plasma glucose (FPG) ≥ 126 mg/dL (7 mmol/L), or reporting to be on treatment with drugs or insulin for diabetes, or persons <15 years who are diagnosed by physicians and identified through a diabetic registry or hospital records

Diabetes Mellitus Type 1

Criterion	Definition
1. Diabetes Mellitus Type 1	Cases of type 1 DM diagnosed by physicians and identified through a diabetic registry or hospital records
2. Uncomplicated Diabetes Mellitus Type 1	Cases of type 1 DM that do not have any of the following complications: neuropathy, foot ulcer, leg amputation, or vision loss
3. Diabetic neuropathy among Diabetes Mellitus Type 1	Cases of type 1 DM that experience diagnosable neuropathy
4. Diabetic foot due to neuropathy among Diabetes Mellitus Type 1	Cases of type 1 DM that currently have a foot ulcer
5. Diabetic neuropathy and amputation with treatment among Diabetes Mellitus Type 1	Cases of type 1 DM that have had a leg amputation above or below the knee, with treatment consisting of a prosthetic limb
6. Diabetic neuropathy and amputation without treatment among Diabetes Mellitus Type 1	Cases of type 1 DM that have had a leg amputation above or below the knee, with no prosthetic limb
7. Moderate vision impairment due to Diabetes Mellitus Type 1	Cases of type 1 DM that have moderate vision loss due to diabetic retinopathy
8. Severe vision impairment due to Diabetes Mellitus Type 1	Cases of type 1 DM that have severe vision loss due to diabetic retinopathy
9. Blindness due to Diabetes Mellitus Type 1	Cases of type 1 DM that have blindness due to diabetic retinopathy

Diabetes Mellitus Type 2

Criterion	Definition
1. Diabetes Mellitus Type 2 parent	Fasting plasma glucose (FPG) ≥ 126 mg/dL (7 mmol/L) or reporting to be on drug or insulin treatment for type 2 diabetes
2. Uncomplicated Diabetes Mellitus Type 2	Cases of DM Type 2 that do not have any of the following complications: neuropathy, foot ulcer, leg amputation, or vision loss
3. Diabetic neuropathy among Diabetes Mellitus Type 2	Cases of DM Type 2 that experience diagnosable neuropathy

4. Diabetic foot due to neuropathy among Diabetes Mellitus Type 2	Cases of DM Type 2 that currently have a foot ulcer
5. Diabetic neuropathy and amputation with treatment among Diabetes Mellitus Type 2	Cases of DM Type 2 that have had a leg amputation above or below the knee, with treatment consisting of a prosthetic limb
6. Diabetic neuropathy and amputation without treatment among Diabetes Mellitus Type 2	Cases of DM Type 2 that have had a leg amputation above or below the knee, with no prosthetic limb
7. Moderate vision impairment due to Diabetes Mellitus Type 2	Cases of DM Type 2 that have moderate vision loss due to diabetic retinopathy
8. Severe vision impairment due to Diabetes Mellitus Type 2	Cases of DM Type 2 that have severe vision loss due to diabetic retinopathy
9. Blindness due to Diabetes Mellitus Type 2	Cases of DM Type 2 that have blindness due to diabetic retinopathy

Diabetes Mellitus, Diabetes Mellitus Type 1, Diabetes Mellitus Type 2:

Data seeking

1. A systematic review of the literature was done for GBD 2019 with the following search terms:

Diabetes Mellitus search string: (diabetes[TI] AND (prevalence[TIAB] OR incidence[TIAB])) OR ('Diabetes Mellitus'[MeSH Terms] AND 'epidemiology'[MeSH Terms]) OR (diabetes[TI] AND 'epidemiology'[MeSH Terms]) NOT gestational[All Fields] NOT ('neoplasms'[MeSH Terms] OR 'neoplasms'[All Fields] OR 'cancer'[All Fields]) NOT ('mice'[MeSH Terms] OR 'mice'[All Fields]) NOT ('schizophrenia'[MeSH Terms] OR 'schizophrenia'[All Fields]) NOT ('emigrants and immigrants'[MeSH Terms] OR ('emigrants'[All Fields] AND 'immigrants'[All Fields]) OR 'emigrants and immigrants'[All Fields] OR 'immigrants'[All Fields]) NOT ('pregnancy'[MeSH Terms] OR 'pregnancy'[All Fields] OR 'gestation'[All Fields]) NOT ('rats'[MeSH Terms] OR 'rats'[All Fields] OR 'rat'[All Fields]) NOT ('kidney'[MeSH Terms] OR 'kidney'[All Fields]) NOT renal[All Fields] NOT ('vitamins'[Pharmacological Action] OR 'vitamins'[MeSH Terms] OR 'vitamins'[All Fields] OR 'vitamin'[All Fields])

And

FPG search string: ((“glucose”[Mesh] OR “hyperglycemia”[Mesh] OR “prediabetic state”[Mesh]) AND "Geographic Locations"[Mesh] NOT "United States"[Mesh]) AND ("humans"[Mesh] AND "adult"[MeSH]) AND ("Data Collection"[Mesh] OR "Health Services Research"[Mesh] OR "Population Surveillance"[Mesh] OR "Vital statistics"[Mesh] OR "Population"[Mesh] OR "Epidemiology"[Mesh] OR surve*[TiAb]) NOT Comment[ptyp] NOT Case Reports[ptyp]) NOT "hospital"[TiAb]

Search date: October 17, 2018

The search took place for the following dates: 10/15/2017-10/16/2018. The number of studies returned was 717, and the number of studies extracted was 21.

2. We systematically searched the Global Health Data Exchange (GHDx) for multi-country survey programs, national surveys, and longitudinal studies that were tagged with either fasting plasma glucose (FPG) or Diabetes Mellitus.

3. To capture any remaining sources not identified in the GHDx or in PubMed, we looked to other leaders in the field to ensure our datasets were as comprehensive as possible. These included data sources used by other research groups that report on the global burden of diabetes[3,4], microdata from not-yet published national studies, and publications that were not captured in the PubMed search string.

Figure 1: PRISMA diagram of data sources used in GBD 2019 Diabetes Mellitus model

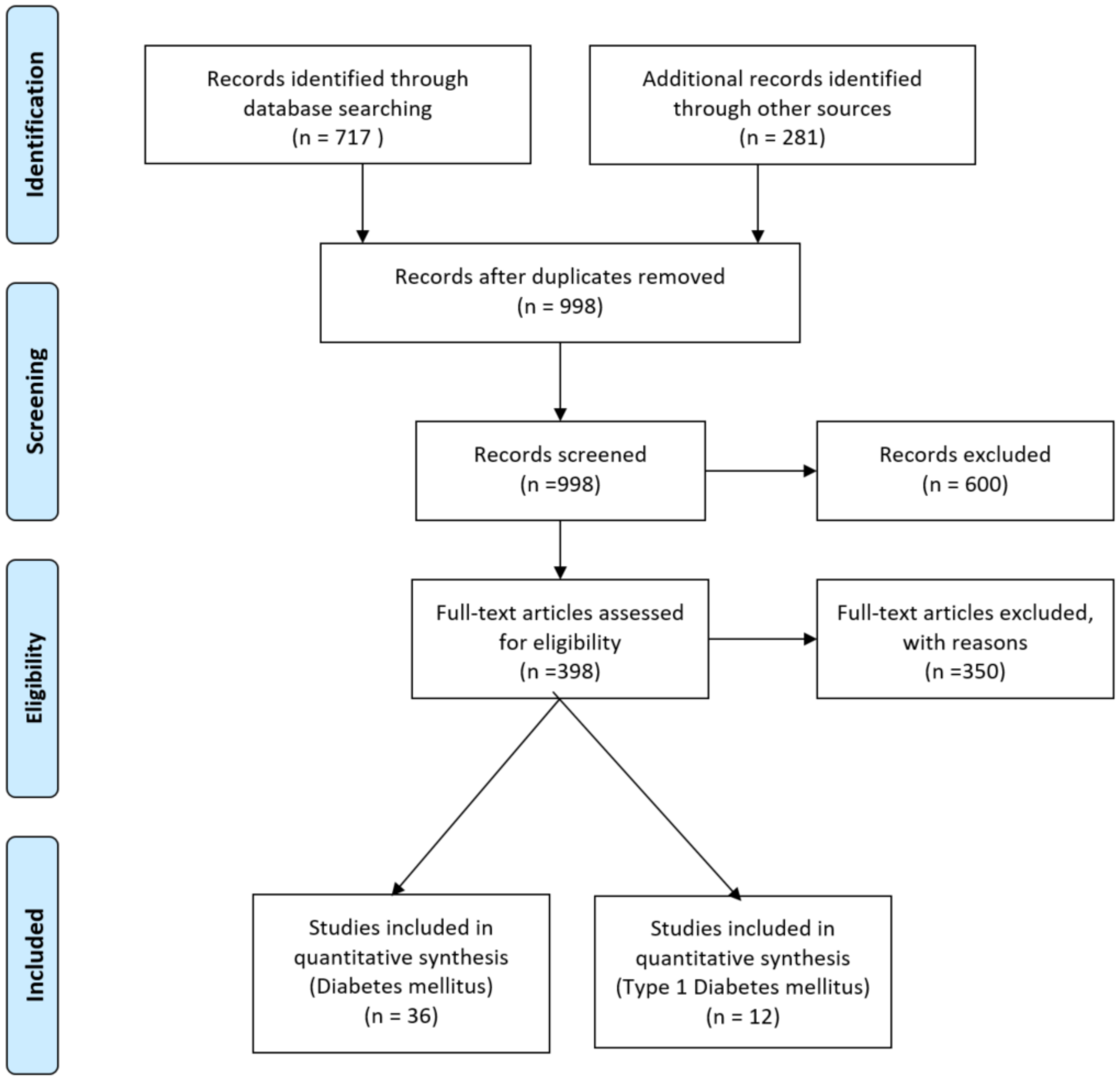

Source counts

Diabetes mellitus

Measure	Total sources	Countries with data
All measures	1289	171
Incidence	214	77
Prevalence	1020	155
Proportion	75	42
Relative risk	1	1
Standardized mortality ratio	5	4
With-condition mortality rate	6	5

Type 1 diabetes mellitus

Measure	Total sources	Countries with data
All measures	193	74
Incidence	163	73
Prevalence	32	14
Standardized mortality ratio	4	4

Data inputs

Overall Diabetes Mellitus

Purpose:

To incorporate all available data related to population-representative estimates of diabetes, we accepted other measures of blood sugar (glycated hemoglobin A1c, oral glucose tolerance test, post prandial glucose test) to define diabetes and mean fasting plasma glucose (FPG) in a population when data on diabetes was not available as data inputs.

Data:

1. Data inputs came from 4 type of sources:
    - Estimates of diabetes in a representative population
    - Estimates of mean FPG in a representative population
    - Individual-level data of fasting plasma glucose measured from surveys
    - Insurance data, claims, from United States and Taiwan

When a study reported both mean fasting plasma glucose (FPG) and prevalence of diabetes, we used the prevalence of diabetes. Where possible, individual-level data from a cohort superseded any data described in a study. Individual-level data was collapsed and aggregated to produce estimates for each age group, sex, location, and year a survey is conducted.

2. Covariates

We used prevalence of obesity per location.

Diabetes Type 1

Purpose:

To incorporate all available data related to population-representative estimates of diabetes Type 1, we accepted data that reported Diabetes Type 1, juvenile-onset Diabetes, and insulin-dependent Diabetes.

Data:

Data inputs comes from 2 types of sources:

- Estimates of Type 1 Diabetes Mellitus in a representative population
- Diabetic registries

Diabetes Type 2

Only 20% of Diabetes Mellitus estimates are available by Type. Furthermore, while the sources report Type 2 Diabetes Mellitus, the diagnostic criteria in the methodological sections are not sufficiently specific. Thus, we calculated estimates of Diabetes Mellitus Type 2 by subtracting the estimates of Diabetes Mellitus Type 1 from estimates Overall Diabetes Mellitus for each age, sex, and location from 1990 to 2019.

Data processing

Overall Diabetes Mellitus

We performed several processing steps to the data in order to address sampling and measurement inconsistencies that will ensure the data are comparable across data sources and between high fasting plasma glucose modeling efforts.

1. *Small sample size:* Estimates in a sex and age group with a sample size <30 persons were considered a small sample size. In order to avoid small sample size problems that may bias estimates, data were collapsed into the next age group in the same study until the sample size reached at least 30 persons. The intent of collapsing the data is to preserve as much granularity between age groups as possible, which determined whether the collapse occurred with a younger or older age group. If the entire study sample consisted of <30 persons and did not include a population-weight, the study was excluded from the modeling process. The estimates were re-calculated if case count and sample size were available or the population-weighted estimate was calculated when only sample size was available.

2. *Mean FPG processing:* We used an ensemble distribution to estimate the prevalence of diabetes based on mean FPG in locations where data on prevalence of diabetes were not available. Essentially, we constructed a distribution based on unit-level data available in 31 different countries. Then we predicted out the prevalence of diabetes by age and sex. This provides the conversion of mean FPG to prevalence of diabetes defined as FPG >126 mg/dL (7 mmol/L). Because this definition is not consistent with our reference case definition (which also includes those on treatment), we then apply an adjustment to adjust these data points to the reference case definition. For information on how these adjustments are made, please see the section, Age splitting and bias adjustments.

3. *Age splitting and bias adjustments*

Reported estimates of prevalence were split by age and sex where possible. First, if studies reported prevalence for broad age groups by sex, and also by specific age groups but for both sexes combined, age-specific estimates were split by sex using the sex ratio from within the study. Second, input data reporting prevalence for both sexes that could not be split using a within-study ratio were split using a sex ratio derived from a meta-analysis of existing sex-specific data using MR-BRT. The female to male ratio for diabetes was 0.85 (0.61-1.09). Finally, after the application of bias adjustments, where studies reported estimates across age groups spanning 25 years or more, these were split into five-year age groups using the prevalence age pattern estimated by DisMod-MR 2.1 in a model that contained the subset of data with age range less than 25 years.

Figure 2: Age pattern used to split data with age range >25 years

a. Female b. Male

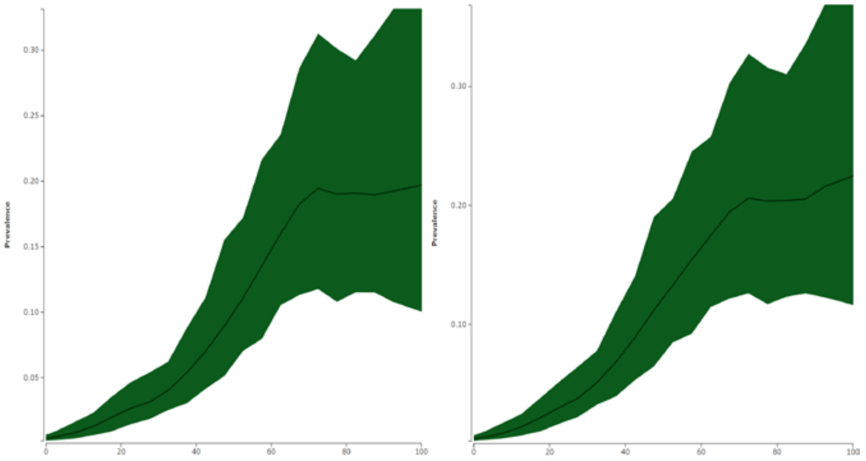

In GBD 2019, we improved the bias adjustment methods to allow a more direct comparison between different case definitions and/or study designs. In GBD 2017, we constructed ratios between alternative case definitions and the reference case definition using data from surveys that measured glucose level based on different glucose tests on a single person. For insurance data we allowed Dismod to estimate the adjustment. In GBD 2019, we constructed ratios between alternative case definitions and the reference case definition using data from surveys that measured glucose level based on different glucose tests on a single person or between survey and the insurance claims data. However, we assume that claims data in persons <15 years are Type 1 diabetes and that 100% of diabetics are captured in this age group. Thus, we only adjust the claims data in persons >15 years. We used MR-BRT analysis to adjust for bias due to commercial insurance or use of alternative case definitions. We performed this analysis in logit-space due to the high prevalence of diabetes (from simulations we learned that for prevalence greater than 50% the log ratio method is biased).

The process of adjusting for non-reference data using MR-BRT with the logit-transformation method is described below:

1. Identify data points with overlapping year, age, sex, and location between alternative case definition and reference case definition
2. Logit transform overlapping data points of alternative and reference case definitions
3. Convert overlapping data points into a difference in logit space using the following equation: logit(alternative)-logit(reference)
4. Use the delta method to compute standard errors of overlapping data points in logit space, then calculate standard error of logit difference using the following equation: √((variance of alternative)+(variance of reference))
5. Using MR-BRT, conduct a random effects meta-regression to obtain the pooled logit difference of alternative to reference
6. Apply the pooled logit difference to all data points of alternative case definitions using the following equation:
   New estimate = inverse.logit((logit(alternative))-(pooled logit difference))
7. Calculate new standard errors using the delta method, accounting for gamma (between-study heterogeneity)

Table: Adjustment factors for alternative case definitions, from MR-BRT analysis

Alternative case definition	# comparisons	Beta coefficient	Lower	Upper
HbA1c > 6.5%	241	0.74	0.33	1.66
HbA1c > 6.4% or Tx	242	1.06	0.57	1.96
HbA1c > 6%	243	2.01	0.65	6.20
HbA1c > 6.5% or Tx	242	0.92	0.52	1.63
FPG > 100 mg/dl (5.6 mmol/L) or Tx	462	4.98	2.89	8.58
FPG > 100 mg/dl (5.6 mmol/L)	462	4.72	2.76	8.08
FPG > 110 mg/dl (6.1 mmol/L) or Tx	462	1.99	1.55	2.54
FPG > 110 mg/dl (6.1 mmol/L)	462	1.8	1.31	2.47
FPG > 115 mg/dl (6.4 mmol/L) or Tx	462	1.46	1.25	1.70
FPG > 120 mg/dl (6.7 mmol/L)	461	0.997	0.77	1.29
FPG > 121 mg/dl (6.7 mmol/L)	461	0.96	0.77	1.20
FPG > 126 mg/dl (7 mmol/L)	460	0.78	0.60	1.02
FPG > 140 mg/dl (7.8 mmol/L) or Tx	456	0.76	0.62	0.93
FPG > 144 mg/dl (8 mmol/L) or Tx	454	0.72	0.57	0.91
OGTT > 180 mg/dl (10 mmol/L) or Tx	120	2.28	1.57	3.30
OGTT > 200 mg/dl (11.1 mmol/L)	120	1.5	1.04	2.15
OGTT > 200 mg/dl (11.1 mmol/L) or Tx	120	1.5	1.04	2.18
FPG > 110 mg/dl (6.1 mmol/L) or OGTT > 200 mg/dl (11.1 mmol/L)	120	4.92	2.94	8.24
FPG > 126 mg/dl (7 mmol/L) or OGTT > 200 mg/dl (11.1 mmol/L)	120	1.85	1.49	2.30

FPG > 126 mg/dl (7 mmol/L) or OGTT > 200 mg/dl (11.1 mmol/L) or Tx	120	1.86	1.49	2.33
FPG > 126 mg/dl (7 mmol/L) or OGTT > 220 mg/dl (12.2 mmol/L)	120	1.44	1.22	1.70
FPG > 144 mg/dl (8 mmol/L) or OGTT > 200 mg/dl (11.1 mmol/L) or Tx	120	1.53	1.06	2.22
FPG > 140 mg/dl (7.8 mmol/L) or OGTT > 200 mg/dl (11.1 mmol/L) or Tx	120	1.54	1.06	2.24
FPG > 140 mg/dl (7.8 mmol/L) or OGTT > 200 mg/dl (11.1 mmol/L)	120	1.53	1.06	2.22
FPG > 126 mg/dl (7 mmol/L) or OGTT > 200 mg/dl (11.1 mmol/L) or HbA1c > 6.1%	77	3.67	1.35	10.00
US claims	140	0.54	0.40	0.73
Taiwan claims	12	1.16	0.53	2.53

Diabetes Type 1

Based on assumption that claims data in persons <15 years are type 1 diabetes and that 100% of diabetics are captured in this age group, we make no adjustments to data in these ages. Claims data are reported as prevalence.

There are a number of different sources and ascertainment methods that were used to identify type 1 diabetics. The majority of data that are reported in the literature are from a diabetic registry, hospital discharge data review, physician interview, or insulin use. We assumed that there is no systematic bias between these sources and consider sources identified through these methods as reference. For the other sources that use alternative ascertainment techniques (eg., pharmacy reports, diabetic camps, school reports), there was not sufficient amount of data to perform an analysis on each individual type, and the model had relatively few data points in locations where these approaches were used. So we collapsed all alternative sources and treated the estimates from these sources as defined as an alternative case definition.

Table: Adjustment factors for alternative case definitions, from MR-BRT analysis

**Alternative case definition**	**Beta coefficient**	**Lower**	**Upper**
Ascertainment through pharmacy, schools, diabetic camps	0.9	0.80	1.10

Modelling Strategy

Overall Diabetes Mellitus

For GBD 2019, we estimated the overall prevalence of diabetes using DisMod MR-2.1, a Bayesian metaregression. DisMod-MR produces estimates of the prevalence of diabetes for each age, sex,

geographic location, and year. We used data that reported prevalence and incidence, for Diabetes Mellitus.

Model parameters and estimates

- We set a value prior of 0 for remission for ages 0 to 14
- We set a value prior of a maximum value of 0.01 for remission for ages 15 to 100
- We set a value prior of a maximum value of 0.15 for excess mortality for all ages
- We set a value prior of 0 for incidence for ages 0 to 1
- We set a value prior of a maximum value of 0.1 for incidence for ages 1 to 100

Country covariate	Parameter	beta	Exponentiated beta
Age-standardized prevalence of obesity	Prevalence	0.66 (0.56- 0.76)	1.93 (1.75-2.13)
Year	Prevalence	0.030 (0.029 – 0.031)	1.03 (1.03 – 1.03)

Diabetes Mellitus Type 1

For GBD 2019, we estimated the overall prevalence of diabetes using DisMod MR-2.1, a Bayesian metaregression. We used data that reported incidence, standardized mortality ratio, and prevalence data in claims data for persons <15 years for Diabetes Mellitus Type 1. We decided to not include reported type 1 diabetes prevalence in non-claims sources because we found that their estimates of prevalence and incidence were inconsistent. We decided to trust the incidence data and thus, had to exclude the prevalence data from the model. Similarly, we did not include prevalence of diabetes type 1 in people >15 years from claims sources, because of poor reporting on type of diabetes.

Model parameters and estimates

- We set a value prior of 0 for remission for all ages

Country covariate	Parameter	beta	Exponentiated beta
Proportion of live births in women 35+ years	Incidence	2.60 (2.34-2.88)	13.42 (10.34 – 17.78)
Maternal education (years per capita)	Incidence	0.091 (0.083 – 0.10)	1.10 (1.09 – 1.11)

Diabetes Type 2

Only 20% of Diabetes Mellitus estimates are available by Type. Furthermore, while the sources report Type 2 Diabetes Mellitus, the diagnostic criteria in the methodological sections are not sufficiently specific. Thus, we calculated estimates of Diabetes Mellitus Type 2 by subtracting the estimated prevalence of Diabetes Mellitus Type 1 from estimated prevalence of Overall Diabetes Mellitus for each age, sex, and location from 1990 to 2019.

Outcomes

<u>Data seeking</u>

Amputation due to Diabetes Mellitus

A systematic review of the literature was performed for GBD 2017 with the following search terms:

(‘Diabetes Mellitus’[MeSH Terms] OR (‘diabetes’[All Fields] AND ‘mellitus’[All Fields]) OR ‘Diabetes Mellitus’[All Fields]) AND ‘amputation’[All Fields] AND (proportion OR prevalence OR incidence) NOT gestational NOT cancer NOT mice NOT schizophrenia NOT immigrants NOT gestation NOT rat NOT kidney NOT renal NOT vitamin

- Dates of search: 12/31/16-10/17/2017
- Number of studies returned: 16
- Number of studies extracted: 1

Diabetic neuropathy

A systematic review of the literature was performed for GBD 2017 with the following search terms:

(“Diabetes Mellitus”[MeSH Terms] OR (“diabetes”[All Fields] AND “mellitus”[All Fields]) OR “Diabetes Mellitus”[All Fields]) AND neuropathy[All Fields] AND (proportion OR prevalence OR incidence) NOT gestational NOT cancer NOT mice NOT schizophrenia NOT immigrants NOT gestation NOT rat NOT kidney NOT renal NOT vitamin

- Dates: 12/31/16-10/17/2017
- Number of studies returned: 170
- Number of studies extracted: 1

Diabetic foot ulcer

A systematic review of the literature was performed for GBD 2017 with the following search terms:

((("Diabetes Mellitus"[MeSH Terms] OR ("diabetes"[All Fields] AND "mellitus"[All Fields]) OR "Diabetes Mellitus"[All Fields] OR "diabetes"[All Fields]) AND ("foot"[MeSH Terms] OR "foot"[All Fields]) AND ("ulcer"[MeSH Terms] OR "ulcer"[All Fields])) NOT ("neoplasms"[MeSH Terms] OR "neoplasms"[All Fields] OR "cancer"[All Fields]) NOT ("mice"[MeSH Terms] OR "mice"[All Fields]) NOT ("emigrants and immigrants"[MeSH Terms] OR ("emigrants"[All Fields] AND "immigrants"[All Fields]) OR "emigrants and immigrants"[All Fields] OR "immigrants"[All Fields]) NOT ("pregnancy"[MeSH Terms] OR "pregnancy"[All Fields] OR "gestation"[All Fields]) NOT ("vitamins"[Pharmacological Action] OR "vitamins"[MeSH Terms] OR "vitamins"[All Fields] OR "vitamin"[All Fields]) NOT renal[All Fields] NOT ("kidney"[MeSH Terms] OR "kidney"[All Fields]) AND (proportion[All Fields] OR "incidence"[All Fields] OR "prevalence"[All Fields]) NOT ("schizophrenia"[MeSH Terms] OR "schizophrenia"[All Fields]) NOT ("rats"[MeSH Terms] OR "rats"[All Fields] OR "rat"[All Fields]))

- Dates: 12/31/16-10/17/2017
- Number of studies returned: 48
- Number of studies extracted: 0

Modeling strategy

For GBD 2019, we estimated amputation due to Diabetes Mellitus, diabetic neuropathy, and diabetic foot for Diabetes Mellitus Type 1 and Diabetes Mellitus Type 2 using DisMod MR-2.1. DisMod-MR produces estimates of the prevalence of diabetes for each age, sex, geographic location, and year. We then multiply all proportion draws from neuropathy/foot/amputation models by the parent diabetes model so that all estimates are in the same population-space.

We ensure that the sum of the prevalence for neuropathy due to Diabetes mellitus, moderate vision loss due to Diabetes mellitus, severe vision loss due to Diabetes mellitus, and blindness due to Diabetes mellitus does not exceed 90% of the prevalence of all Diabetes mellitus. If the sum exceeds 90% then we rescale the individual outcomes to 90%. We do not directly model vision loss. These estimates are derived as part of the vision loss impairment analyses based on data ascribing vision loss to underlying causes in population based surveys. The diabetes process takes these estimates into account when estimating uncomplicated Diabetes mellitus, amputation due to Diabetes Mellitus, diabetic neuropathy, and diabetic foot for Diabetes Mellitus Type 1 and Diabetes Mellitus Type 2.

We perform the same check to ensure that the prevalence of amputation due to diabetes mellitus and prevalence of foot ulcer due to diabetes mellitus does not exceed 90% of the prevalence of neuropathy due to diabetes mellitus. This treats foot ulcer and amputation as mutually exclusive categories by assuming a patient will not have both simultaneously.

From here, we calculate uncomplicated diabetes as the remainder of diabetes cases exclusive of neuropathy and vision loss. In addition, we estimate the prevalence of amputation due to diabetes is split into with and without treatment using scaled health systems access (HSA) values. For diabetic amputation, we calculated a distribution of treated versus untreated amputation, defined as receiving a prosthesis or not. We first rescaled the IHME estimates to be between 0 and 0.9, under the assumption that 10% of amputees will not receive a prosthetic, even in high income countries. We based this assumption on the retrospective study by Moore et al, which found that about 80% of patients following major lower extremity amputation were fitted with prostheses in the authors' institutions from 1978 to 1986 in the USA. We then performed a population-weighted average of this country-specific value to obtain a proxy for the proportion of amputees that receive a prosthetic by super region. Because these are rough estimates based on large assumptions, we applied confidence intervals of +/- 50% of the value to reflect our uncertainty.

Model parameters and estimates

In GBD 2019, we reviewed all input data and sources. We found that nearly all sources reported estimates in age ranges that exceed 50 years. We identified a single study for each outcome that reported estimates in age range <25 years. We applied this age pattern to the remaining datapoints.

Amputation due to diabetes

- We set a value prior of 0 for incidence for ages 0 to 15
- We set a value prior of 0 for remission for all ages

Diabetic neuropathy

- We set a value prior on the proportion of 0 from ages 0 to 1

Diabetic foot ulcer

- We set a value prior on the proportion of 0 from ages 0 to 10.

Severity distributions

We determined the disability weights for each sequela from the GBD disability weight survey. The table below illustrates the severity levels, lay descriptions, and associated disability weights applicable for outcomes related to Diabetes Mellitus Type 1 and Diabetes Mellitus Type 2:

Severity level	Lay description	DW (95% CI)
Uncomplicated Diabetes Mellitus	Has a chronic disease that requires medication every day and causes some worry, but minimal interference with daily activities	0.049 (0.031 – 0.072)
Diabetic neuropathy	Has pain, tingling, and numbness in the arms, legs, hands, and feet. The person sometimes gets cramps and muscle weakness.	0.133 (0.089 – 0.187)
Diabetic neuropathy with diabetic foot	Has a sore on the foot that is swollen and causes some difficulty in walking.	[a]
Diabetic neuropathy with treated amputation	Has lost part of one leg, leaving pain and tingling in the stump. The person has an artificial leg that helps in moving around.	[a]
Diabetic neuropathy with untreated amputation	Has lost part of one leg, leaving pain and tingling in the stump. The person does not have an artificial leg, has frequent sores, and uses crutches.	[a]
Moderate vision loss due to Diabetes Mellitus	Has vision problems that make it difficult to recognize faces or objects across a room.	0.031 (0.019 – 0.049)

Severe vision loss due to Diabetes Mellitus	Has severe vision loss, which causes difficulty in daily activities, some emotional impact (for example worry), and some difficulty going outside the home without assistance.	0.184 (0.125 – 0.259)
Blindness due to Diabetes Mellitus	Is completely blind, which causes great difficulty in some daily activities, worry and anxiety, and great difficulty going outside the home without assistance.	0.187 (0.124 – 0.26)

[a] *The disability weights are produced from a combination of two health states: neuropathy and diabetic foot/amputation*

Comparison to other published estimates

We identified two groups who also make global estimates of diabetes, the International diabetes federation (IDF) and the NCD Risk Factor Collaboration (NCD-RisC). The International diabetes federation publishes annual updates to their estimates, with the most recent estimates published in the 9th atlas (https://www.diabetesatlas.org/en/) and NCD-Risc published estimates in the paper *Worldwide trends in diabetes since 1980: a pooled analysis of 751 population-based studies with 4·4 million participants.*

Below is a table comparing the global number of diabetes reported by GBD 2019, IDF 9th Atlas, and NCD-Risc for the closest years that align with 1990, 2010, and 2019.

Organization	Source	1990	2010	2019
IHME	GBD 2019	159 million	331 million	460 million
International diabetes federation	IDF 9th Atlas	151 million (2000)	285 million (2009)	463 million (2019)
NCD-Risk collaboration	Figure 7	148 million	350 million	422 million (2014)

There are several methodological and analytical differences between each groups approach which explains differences in the number of cases. The table below summarizes the main differences.

Organization	Age	Case definition	Analysis

IHME	All ages	FPG ≥ 7 mmol/L (126 mg/dL)	Bayesian hierarchical meta-regression
International diabetes federation	20-79 years	FPG ≥ 7 mmol/L (126 mg/dL) or OGTT ≥ 11.1 mmol/L (200 mg/dL) or HbA1c ≥ 6.5% or random plasma glucose ≥ 11.1 mmol/L (200 mg/dL) or self-report diabetes status	Generalised linear regression mode
NCD-Risk collaboration*	≥18 years	FPG ≥ 7 mmol/L (126 mg/dL) or self-reported diabetes	Bayesian hierarchical model

References

Moore TJ, Barron J, Hutchinson F 3rd, Golden C, Ellis C, Humphries D. **Prosthetic usage following major lower extremity amputation.** *Clin Orthop Relat Res.* 1989 Jan;(238):219-24.

Chronic Kidney Disease

Flowchart

Chronic Kidney Disease (CKD)

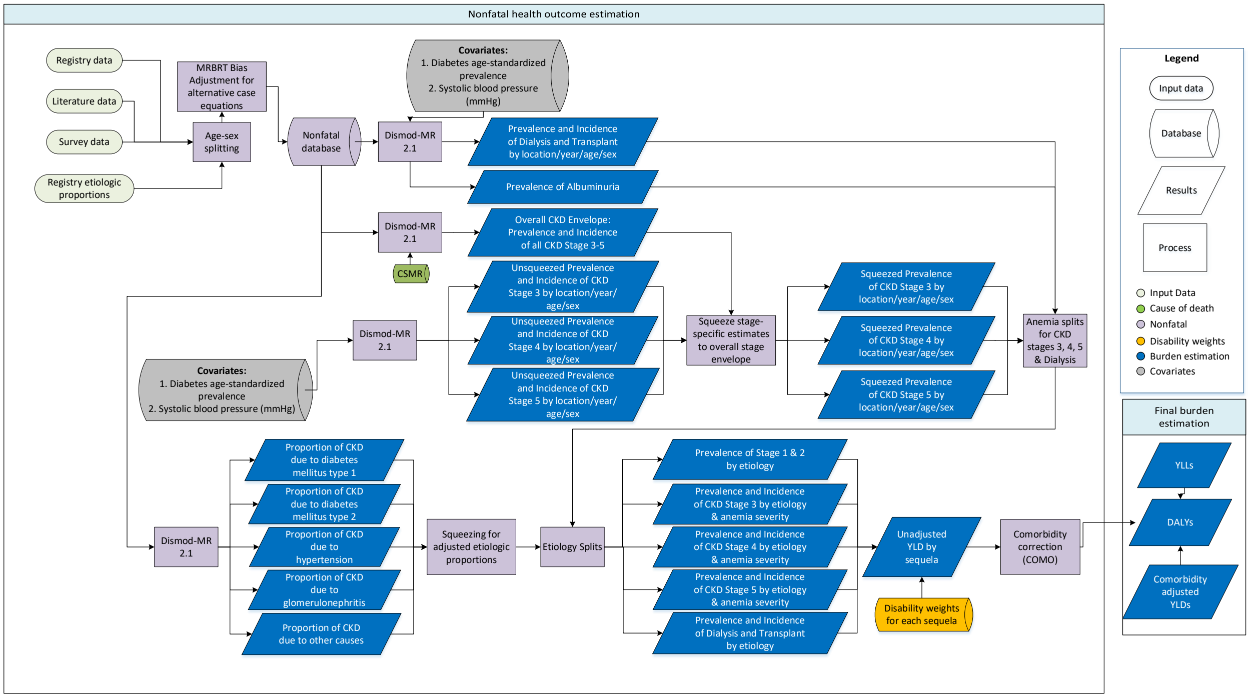

Case definition

Chronic kidney disease (CKD) is defined as a permanent loss of kidney function as indicated by estimated glomerular filtration rate (eGFR) and urinary albumin to creatinine ratio (ACR). The GBD study considers six stages of CKD as defined by degree of loss of kidney function or receipt of kidney replacement therapy: CKD stages 1&2 (eGFR > 60ml/min/1.73m^2 and ACR > 30 mg/g), CKD Stage 3 (eGFR 30-60ml/min/1.73m^2), CKD Stage 4 (eGFR 15-30ml/min/1.73m^2), CKD Stage 5 (eGFR <15ml/min/1.73m^2, not on kidney replacement therapy), maintenance dialysis, and kidney transplantation.[1] The ICD-10 codes associated with CKD include N18.1-N18.9.

Input data

Model inputs

The first systematic review of the prevalence of CKD throughout the world was conducted for GBD 2010. This search was updated for GBD 2013, GBD 2015, and GBD 2016 and GBD 2017. This literature search was repeated using PubMed search terms ((((("chronic kidney disease"[Title/Abstract]) AND prevalen*[Title/Abstract]) AND ("1980/1/1"[Date - Publication] : "3000"[Date - Publication])) NOT ((animals[MeSH] NOT humans[MeSH])))).

The exclusion criteria were:

1. Studies clearly not representative of the national population
2. Studies that did not provide primary data on epidemiological parameters, eg, a commentary piece
3. Studies of a specific aetiology of CKD only

This literature search was augmented by identification of population-based surveys that measured kidney function. For maintenance dialysis and kidney transplantation, data were largely obtained from kidney registry reports.

Data inputs for chronic kidney disease

Measure	Total sources	Countries with data
All measures	1646	122
Prevalence	1204	120
Incidence	1072	90
Excess mortality rate	67	13
With-condition mortality rate	4	4
Proportion	367	55

Data Processing

Age-Sex and Sex Split

In some cases, data are reported by only age or only sex, but not both. For example, a study may have included the proportion of males and females with stage 3 CKD and then separately reported the proportion of both sexes by smaller age bins (e.g. 40 – 44, 45 – 49) that have stage 3 CKD. In these cases, we perform an age-sex split by utilizing proportions within the study to disaggregate the data.

When no information by sex in a study, we instead perform a sex-split on the data by applying separate sex proportions. In order to obtain an appropriate age-pattern with which to age-split input data, we first ran a DisMod-MR 2.1 model containing only age-specific data. We then used age-pattern by super-region from this model to age-split dialysis input data, thereby allowing for variation in the age-pattern by location. After age-splitting, we ran a model on all processed data, including age-split data and age-specific data, to obtain final estimates of dialysis incidence and prevalence by location, year, age, and sex. For dialysis, remission data for dialysis were calculated as the ratio of the incidence of kidney transplantation to prevalence of dialysis at the gender-, age-, and country-matched level.

Modeled excess mortality data

For the Stage 3-5 CKD, we implemented a new method of modeling excess mortality rate (EMR).

In previous rounds, priors on EMR were estimated in DisMod by matching prevalence data points with their corresponding CSMR values within the same age, sex, year, location (estimating EMR by dividing CSMR by prevalence).

However, for many causes, DisMod estimated a rather unrealistic pattern of EMR compared to an expected pattern of decreasing EMR with greater access to quality health care. Such unexpected patterns often signal inconsistencies between CSMR estimates and the measures of prevalence and/or incidence.

In effort to provide greater guidance to DisMod on the expected pattern of EMR, EMR data generated in the previous round were modeled using the MR-BRT approach by age and sex with a prior on healthcare access and quality index (HAQi) having a negative coefficient. Results from MR-BRT were then predicted for each location year, sex and for ages 0, 10, 20 ….100.

We also included HAQi as a country-level covariate to inform EMR with a mean and standard deviation produced from MR-BRT.

Bias Adjustments

In GBD 2019, we improved the bias adjustment methods by utilizing a MR-BRT model outside of DisMod to allow a more direct comparison between different case definitions and/or study designs. In GBD 2017, these adjustments were performed within DisMod.

Glomerular filtration rate (GFR) can be estimated using a variety of equations that lead to different prevalence estimates. Our CKD reference equation is the CKD-Epi Creatinine equation. We also included data estimated with the Modification of Diet in Renal Disease (MDRD) and the Cockcroft-Gault (CG) equation. For children, the Schwartz equation was used as the reference.

We adjusted data using MDRD and CG equations through a MRBRT model to account for different estimates that result from these different equations. The adjustment is a logit-transformation method in MR-BRT. The general process is described below:

1. Identify data points with overlapping year, age, sex, and location between reference and alternative definitions.
2. Logit transform overlapping data points of alternative and reference case definitions
3. Convert overlapping data points into a difference in logit space using the following equation: $logit(altnerative) - logit(reference)$
4. Use the delta method to compute standard errors of overlapping data points in logit space, then calculate standard error of logit difference using the following equation: $\sqrt{(variance\ of\ alternative) + (variance\ of\ reference)}$
5. Using MR-BRT, conduct a random effects meta-regression to obtain the pooled logit difference of alternative to reference
6. Apply the pooled logit difference to all data points of alternative case definitions using the following equation:
$$new_{estimate} = inverse.logit((logit(alternative)) - (pooled\ logit\ difference))$$
7. Calculate new standard errors using the delta method, accounting for gamma (between-study heterogeneity)

This table shows the adjustment factors used to adjust the data:

MR-BRT Crosswalk Adjustment Factors

Data input	Status	*Gamma*	*Beta Coefficient, Logit (95% CI)*	*Adjustment factor**
CKD-EPI	Ref	---	---	---
Stage 3 CG	Alt	*0.25*	0.24 (-0.28 - 0.76)	0.56 (0.43 - 0.68)

Stage 3 MDRD	Alt	*0.03*	0.49<br>(0.34 - 0.64)	0.62<br>(0.58 -0.66)
Stage 4 CG	Alt	*0*	0.09<br>(-0.05 - 0.24)	0.52<br>(0.49 - 0.56)
Stage 4 MDRD	Alt	*0*	-0.07<br>(-0.19 - 0.04)	0.48<br>(0.45 - 0.51)
Stage 5 CG	Alt	*0*	-0.18<br>(-0.45 - 0.09)	0.45<br>(0.39 - 0.52)
Stage 5 MDRD	Alt	*0*	-0.06<br>(-0.28 - 0.18)	0.49<br>(0.43- 0.54)
Stage 3-5 CG	Alt	*0.26*	0.23<br>(-0.29 - 0.75)	0.56<br>(0.43 - 0.68)
Stage 3-5 MDRD	Alt	*0.03*	0.47<br>(0.32 - 0.62)	0.62<br>(0.58—0.65)

**Adjustment factor is the inverse-logit transformed Beta coefficient; <0.5 represents that alternative is adjusted upward; >0.5 represents alternative is adjusted downward*

Modelling strategy

CKD Stage Models

We run a separate DisMod-MR 2.1 model to produce estimates by age, sex, year, and country for each stage of CKD, along with an aggregate CKD Stage III-V model. Each separate CKD Stage model was then rescaled to the aggregate CKD model for every age, sex, year, and country. This was done in order to enforce more consistency in the prevalence and incidence between stage models.

Progression of CKD

To account for the progression of individuals from stage 3 to 4 and 5, we back-calculated remission from later stages of CKD. This was done by calculating the ratio of the incidence of the next stage with the prevalence of the previous stage.

$$remission_s = \frac{incidence_{\ s}}{prevalence_{s-1}}, where\ s\ is\ stage$$

Our assumption is that individuals progress through CKD and do not immediately end up in stage V or needing a transplant.

Furthermore, remission was set to 0 for stage V and the excess mortality parameter was used to account for progression to end-stage kidney disease and mortality due to CKD stage 5 (even though 'technically' this is not correct for those who go onto dialysis, this was a decision to facilitate modeling). Bounds on excess mortality were informed using a meta-analysis of survival analyses of individuals with untreated CKD stage 5.

Model Covariates

A description of covariates and coefficients included in each model can be found in the table below:

	Country-level covariate	Measure	Values	

CKD stage 3-5	diabetes age-standardised	prevalence	0.0045 (0.00012 — 0.015)	1.00 (1.00 — 1.01)
	mean systolic blood pressure	prevalence	0.58 (0.11 — 1.09)	1.79 (1.11 — 2.97)
	healthcare access and quality index	excess mortality	-0.04 (-0.041 — -0.039)	0.96 (0.96 — 0.96)
CKD stage 3	diabetes age-standardised	prevalence	0.017 (0.00067 — 0.055)	1.02 (1.00 — 1.06)
	mean systolic blood pressure	prevalence	0.14 (0.0030 — 0.44)	1.15 (1.00 — 1.55)
CKD stage 4	diabetes age-standardised	prevalence	0.40 (0.039 — 0.86)	1.49 (1.04 — 2.36)
	mean systolic blood pressure	prevalence	0.47 (0.068 — 0.91)	1.60 (1.07 — 2.47)
CKD stage 5	diabetes age-standardised	prevalence	0.69 (0.59 — 0.81)	2.00 (1.80 — 2.24)
	mean systolic blood pressure	prevalence	1.40 (1.22 — 1.50)	4.05 (3.38 — 4.46)

We also added socio-demographic index as a predictive covariate on incidence for transplant. Individuals in lower SDI countries do not have as much access to transplants as those in higher SDI countries. Betas and exponentiated values for SDI are as follows:

	Study covariate	Parameter	beta	Exponentiated beta
ESRD Transplant	Socio-demographic Index	Incidence	1.78 (1.13–2.00)	5.91 (3.08–7.38)

CKD aetiology proportion models

CKD aetiology proportion models

To model aetiology proportions of CKD, we utilized two separate types of data.

The first are data from end-stage kidney registries used to estimate the proportion of each aetiology for those on dialysis or with kidney transplants.

The second data come from the Geisinger Health System in Pennsylvania. These data contain age-sex-stage-specific aetiology proportions that allowed differential aetiologic composition of CKD across stages for disease progression. These data were used for Stages 1&2, Stage 3, Stage 4, and Stage 5 CKD. For each individual with CKD, we scanned their history of recorded ICD codes to identify ICD codes for primary kidney diseases. We used this information to map individuals to GBD aetiologies by stage of CKD; individuals with CKD but with no history of a primary kidney disease ICD code were classified as having CKD of unknown aetiology. We ran a multinomial logistic regression including sex and a non-linear term for age to predict the probability of each aetiology by age and sex for each stage of CKD (1&2, 3, and 4/5 combined). For each stage, aetiology, age, and sex, we converted this probability into the proportion of

CKD due to the given aetiology, and applied these proportions to the prevalence of CKD for the same stage, age, and sex category to estimate the prevalence of each stage of CKD by aetiology, age, and sex. The ICD to GBD aetiology map utilised in this analysis is as follows:

CKD Aetiology	ICD 9 Codes	ICD 10 Codes
Type 1 diabetes	250.41, 250.43	E10.2, E10.21, E10.22, E10.29
Type 2 diabetes	250.40, 250.42	E11.2, E11.21, E11.22, E11.29
Glomerulonephritis	581, 581.0, 581.1, 581.2, 581.3, 581.8, 581.81, 581.89, 581.9, 582, 582.0, 582.1, 582.2, 582.4, 582.8, 582.81, 582.89, 582.9, 583, 583.0, 583.1, 583.2, 583.4, 583.6, 583.7, 583.8, 583.81, 583.89, 583.9	N02, N02.0, N02.1, N02.2, N02.3, N02.4, N02.5, N02.6, N02.7, N02.8, N02.9, N03, N03.0, N03.1, N03.2, N03.3, N03.4, N03.5, N03.6, N03.7, N03.8, N03.9, N04, N04.0, N04.1, N04.2, N04.3, N04.4, N04.5, N04.6, N04.7, N04.8, N04.9, N05, N05.0, N05.1, N05.2, N05.3, N05.4, N05.5, N05.6, N05.7, N05.8, N05.9, N06, N06.0, N06.1, N06.2, N06.3, N06.4, N06.5, N06.6, N06.7, N06.8, N06.9
Hypertension	403, 403.0, 403.00, 403.01, 403.1, 403.10, 403.11, 403.6, 403.9, 403.90, 403.91, 404, 404.0, 404.00, 404.01, 404.02, 404.03, 404.1, 404.10, 404.11, 404.12, 404.13, 404.9, 404.90, 404.91, 404.92, 404.93	I12, I12.0, I12.1, I12.2, I12.9, I13, I13.0, I13.1, I13.10, I13.11, I13.2, I13.9
Other	589, 589.0, 589.1, 589.9, 753.0, 753.1, 753.10, 753.11, 753.12, 753.13, 753.14, 753.15, 753.16, 753.17, 753.19, 753.2, 753.20, 753.21, 753.22, 753.23, 753.29, 753.3, 283.11, 710.0, 753.0, 753.21, 753.22, 753.29	N07, N07.0, N07.1, N07.2, N07.3, N07.4, N07.5, N07.6, N07.7, N07.8, N07.9, N08, N08.0, N08.1, N08.2, N08.3, N08.4, N08.5, N08.8, N15.0, Q61, Q61.0, Q61.00, Q61.01, Q61.02, Q61.1, Q61.11, Q61.19, Q61.2, Q61.3, Q61.4, Q61.5, Q61.8, Q61.9, Q62, Q62.0, Q62.1, Q62.10, Q62.11, Q62.12, Q62.2, Q62.3, Q62.31, Q62.32, Q62.39, Q62.4, Q62.5, Q62.6, Q62.60, Q62.61, Q62.62, Q62.63, Q62.69, Q62.7, Q62.8, D59.3, M31.31, M32.14, M32.15, N11.9, N13.70, N13.8, Q60.2, Q63.8, N14.0, N14.1, N14.3, N25.89, N26.9, N28.0

In order to maintain consistency between GBD estimates of type 1 diabetes prevalence estimates and CKD due to type 1 diabetes prevalence estimates and generalize the results of the Geisinger analysis to all locations, we performed a location-specific correction for the proportion of CKD due to type 1 and type 2 diabetes. Type 1 diabetes makes up a larger proportion of total diabetes in the United States than it does

in other locations. For each diabetic subtype (e) for a given location (l), age (a), and sex (g) the ratio of subtype-specific diabetes prevalence to total diabetes prevalence (r) was calculated as:

$$r_{e,l,a,g} = \frac{prevalence_{e,l,a,g}}{prevalence_{dm1,l,a,g} + prevalence_{dm2,l,a,g}}$$

This ratio is used to adjust the proportion of CKD due to a given diabetic subtype (p) for a given CKD stage (s), l, a, and g by scaling the predicted proportion of CKD due to that subtype (k) by the ratio of total DM due to e in l to the ratio of total DM due to e in the United States (USA).

$$p_{s,e,l,a,g} = k_{s,a,g} \times \frac{r_{e,l,a,s}}{r_{e,USA,a,s}}$$

The stage-specific approach utilised to estimate the prevalenceof CKD stages Is limited by the use of data from a single geographic region.

A change in GBD 2019 was forcing all CKD due to diabetes to be type 1 diabetes under the age of 20.

For end-stage kidney disease on dialysis and end-stage kidney disease after transplant, we ran DisMod-MR 2.1 models to obtain estimates of proportions for each subtype by location, year, age, and sex. Data for CKD due to overall DM were more widely available than data by type of DM. Models for the proportion of CKD due to hypertension and diabetes included covariates for mean systolic blood pressure and the age-standardised prevalence of diabetes, respectively. Coefficient values from these models are as follows:

Model	Covariate	Value	Exponentiated
CKD proportion due to diabetes mellitus	Diabetes age-standardised prevalence	0.72 (0.66–0.78)	2.05 (1.93–2.18)
CKD proportion due to hypertension	Mean systolic blood pressure	0.034 (0.00076–0.12)	1.03 (1.00–1.13)

In order to make use of all available data, we modelled the proportion of CKD due to overall DM, DM type 1, and DM type 2. Proportion of CKD due to DM type 1 and DM type 2 were then scaled to sum to the proportion of overall DM at the gender, age, and country-matched level. The results from all subtype-specific models were adjusted so that estimates across the subtypes equaled 1 at each of 1,000 draws. These adjusted proportions were applied to the DisMod models for dialysis and transplant to obtain estimates of each of these entities by aetiology.

Anemia Causal Attribution

The age- and sex-specific anemia prevalence for CKD was analysed as part of overall anemia causal attribution for GBD 2019. The details of the anemia analysis are described separately in the "Anemia Impairment" section. Briefly, after estimating total anemia, a series of counterfactual distributions were generated based on the age- and sex-specific prevalence of each anaemia-causing condition and the quantitative effect that the condition has on haemglobin concentration in the blood, a so-called

“haemoglobin shift,” that was derived by meta-analyzing cohort studies, observational studies, or trials comparing the haemotologic status of those with as compared to without the disease. Due to limited data on haemologbin shift, all were assumed to be invariant over age, sex, location, and year.

Severity splits and disability weights

Estimates of prevalence and incidence are split using CKD aetiology proportion models, resulting in CKD estimates by stage and aetiology. Then a portion of each aetiology split for CKD stages III, IV, and V is attributed a disability weight associated with mild, moderate, or severe anaemia.[2]

Severity level	Lay description	Disability weight (95% CI)
Albuminuria	Asymptomatic	--
CKD stage III without anaemia	Asymptomatic	--
CKD stage III with mild anaemia	Feels slightly tired and weak at times, but this does not interfere with normal daily activities.	0.004 (0.001–0.008)
CKD stage III with moderate anaemia	Feels moderate fatigue, weakness, and shortness of breath after exercise, making daily activities more difficult.	0.052 (0.034–0.076)
CKD stage III with severe anaemia	Feels very weak, tired, and short of breath, and has problems with activities that require physical effort or deep concentration.	0.149 (0.101–0.21)
CKD stage IV without anaemia	Tires easily, has nausea, reduced appetite, and difficulty sleeping.	0.104 (0.07–0.147)
CKD stage IV with mild anaemia		0.108 (0.072–0.151)
CKD stage IV with moderate anaemia		0.15 (0.103–0.207)
CKD stage IV with severe anaemia		0.237 (0.165–0.324)
CKD stage V without anaemia	Has lost a lot of weight and has constant pain. The person has no appetite, feels nauseated, and needs to spend most of the day in bed.	0.569 (0.389–0.727)
CKD stage V with mild anaemia		0.570 (0.391–0.727)
CKD stage V with moderate anaemia		0.591 (0.414–0.743)
CKD stage V with severe anaemia		0.631 (0.456–0.782)
End-stage kidney disease, on dialysis	Is tired and has itching, cramps, headache, joint pains, and shortness of breath. The person needs intensive medical care every other day lasting about half a day.	0.571 (0.397–0.725)
End-stage kidney disease, with kidney transplant	Sometimes feels tired and down, and has some difficulty with daily activities.	0.024 (0.014–0.039)

Note: the DWs for CKD 4 and 5 stages with anemia are derived from a multiplicative function combining the CKD stage DW and the corresponding severity of anemia DW

Acute Glomerulonephritis

Flowchart

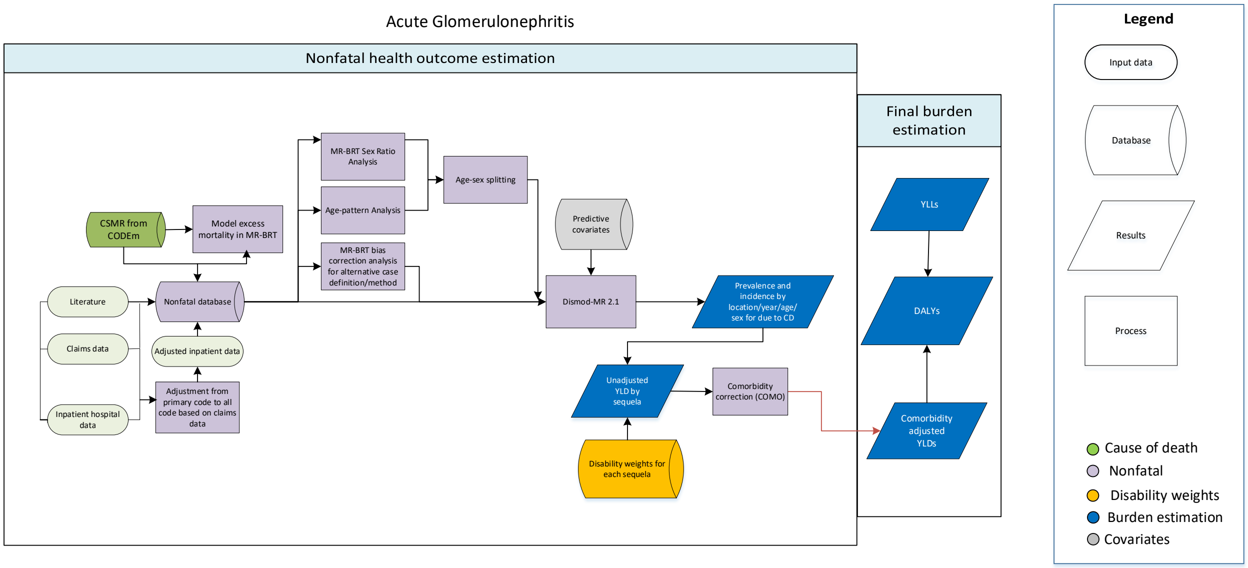

Input Data and Methodological Summary for Acute Glomerulonephritis

Case definition

Acute glomerulonephritis (AG) is an acute episode of glomerular injury accompanied by inflammation, generally presenting with haematuria, oedema, hypertension, and acute kidney injury. ICD codes for AG include N00, N00.0, N00.1, N00.2, N00.3, N00.4, N00.5, N00.6, N00.7, N00.8, N00.9, N01, N01.1, N01.2, N01.3, N01.4, N01.5, N01.6, N01.7, N01.8, and N01.9.

In GBD 2017, our reference case definition for AG was limited to post-infectious AG; data-sources that included other etiologies of AG were adjusted to this reference standard. In GBD 2019, the reference case definition was based on ICD diagnosis in administrative data, and thus was not specific to a single etiology.

Input data and data processing

Input data

A systematic literature review was first conducted in 2010 and, again, in 2013, extracting a total of fourteen articles. These data, however, were too scant and provided too little geographic coverage for robust model, thus the model also included data from hospital discharges and claims.

In addition to claims and hospital discharge data used in GBD 2017, we newly added Poland claims data and additional years of data from USA claims (years 2015-2016) and hospital discharges in Mexico, India, New Zealand, Sweden, Georgia, and Ecuador in GBD 2019. Notably, we included hospital data from Botswana; southern sub-Saharan Africa previously did not have data.

Table 1. Data Inputs for acute glomerulonephritis morbidity modelling by parameter.

Measure	Total sources	Countries with data
Incidence	285	40

Data processing

Claims data link multiple inpatient and outpatient encounters to a single individual. In GBD 2017, individuals were extracted as incident cases if they had one or more inpatient encounters with an appropriate ICD code as any diagnosis; repeat encounters within 90 days were assumed to be readmissions for the same episode of illness. Data from hospital discharges with appropriate ICD codes as primary diagnostic code were, then, adjusted using correction factors derived from inpatient claims data, estimating the number of individuals represented by all encounters and adjusting the number of individuals with AG as primary diagnostic code to the number expected if information on all diagnoses had been provided.

In GBD 2019, we improved data processing methods to capture cases that were diagnosed and/or treated in an outpatient setting. Specifically, incident cases were extracted from claims data if an individual had at least one inpatient or outpatient encounter with an appropriate ICD code as any diagnosis; repeat encounters within 90 days, regardless of setting, were assumed to represent care for the same episode. Data from hospital discharges were adjusted using correction factors from claims, converting encounters to estimates of cases, correcting for most locations providing only primary diagnostic codes, and estimating outpatient cases from inpatient cases.

In addition to the improved case ascertainment of AG, the methods for bias adjustment were updated in GBD 2019 to allow a more direct comparison between different case definitions and/or study designs. In the past GBD cycles, we used data from published studies that employed rigorous case definitions for post-infectious AG as our reference standard, and adjusted clinical administrative data toward this reference standard by marking administrative data with binary covariates, and estimating a fixed effect for this covariate in our DisMod meta-regression modeling process. This amounts to adjusting data using an ecological comparison, and vulnerable to compositional bias; if data from different location-years were collected using different methods or case definitions, true spatiotemporal differences in epidemiology can be erroneously adjusted, and differences truly due to differences in methods can be erroneously estimated as differences in underlying epidemiology. In GBD 2019, we avoided this risk by making pre-modeling bias adjustments and dropping data types that could not be rigorously adjusted. This was done by conducting a meta-regression of the relationship between data points matched with regard to year, age, sex, and location, but differing with regard to one or more study design characteristic. Data from studies that ascertained cases of post-infectious AG based on serological, histological, and/or imaging findings were scarce, and we were not able to find overlapping data points from administrative data sources to estimate adjustment factors. As a result, these data were excluded and a new case definition was adopted: diagnosis of AG of any etiology as indicated by ICD code in a clinical encounter.

The USA claims data from the year 2000 and from the years 2010–2016 shared a case definition with data from hospital discharges, but were adjusted outside DisMod using MR-BRT to compensate for selection bias due to commercial insurance status. The table below shows these bias correction factors. Beta coefficients and adjustment factors incorporate study heterogeneity (gamma).

Table 2. MR-BRT Crosswalk Adjustment Factors for Acute Glomerulonephritis

Data input	Reference or alternative data collection	Gamma	Beta Coefficient, Log (95% CI)	Adjustment factor*
Hospital + non-USA claims	Ref	0.33	---	---
USA claims from year 2000	Alt		1.83 (-0.11, 3.77)	6.21 (0.89, 43.18)
USA claims from year 2010-2016	Alt		1.83 (0.96, 2.70)	6.23 (2.61, 14.89)

**Adjustment factor is the transformed Beta coefficient in normal space, and can be interpreted as the factor by which the alternative case definition is adjusted to reflect what it would have been if measured as the reference.*

Data points with an age-standardised incidence rate greater than 1.5 median absolute deviations from the median of the age-standardised incidence rate for all inpatient and non-USA claims data were marked as outliers and excluded from analysis. Hospital discharge data from Latvia, Meghalaya, Jordan, Qatar, Iran, Turkey, and Georgia, and claims data from Poland were also marked as outliers because their estimates were implausibly high when compared to regional, super-regional, and global rates.

Severity split & disability weight

The basis of the GBD disability weight assessment is lay descriptions of sequelae highlighting major functional consequences and symptoms. Disability weighting (DW) for AG associates with systemic symptoms of fever, aches, weakness, and some difficulty with daily activities. The lay description and disability weight for acute glomerulonephritis are shown below.

Table 3. Severity Distribution, details on the severity levels for Acute Glomerulonephritis in GBD 2019 and the associated disability weight (DW) with that severity.

Severity level	Lay description	DW (95% CI)
Moderate	Has a fever and aches, and feels weak, which causes some difficulty with daily activities.	0.051 (0.032, 0.074)

Modeling strategy

Similar to GBD 2017, we ran a DisMod-MR 2.1 model to produce estimates by age, sex, year, and country. Prior settings in the DisMod model included setting remission of three to four weeks. It was assumed that no one was born with AG. We used the function in DisMod-MR 2.1 to pull in cause-specific mortality rate (CSMR) data from our CODEm and CODcorrect analyses. The minimum coefficient of variation at the regional, super-regional, and global-level was changed from 0.4 to 0.8 in GBD 2019 to improve model fit against input data.

In previous rounds, priors on excess mortality rate (EMR) were estimated in DisMod by matching prevalence data points with their corresponding CSMR values within the same age, sex, year, and location (by dividing CSMR by prevalence). For short duration conditions (remission >1), the corresponding prevalence was derived by running an initial model and then applying the same CSMR/prevalence method. However, for many causes, DisMod estimated a rather unrealistic pattern of EMR compared to an expected pattern of decreasing EMR with greater access to quality health care. Such unexpected patterns often signal inconsistencies between CSMR estimates and the measures of prevalence and/or incidence. In effort to provide greater guidance to DisMod on the expected pattern of EMR, EMR data generated in the previous round were modeled using the MR-BRT approach by age and sex with a prior on healthcare access and quality index (HAQi) having a negative coefficient. Results from MR-BRT were then predicted for each location, year, sex, and for ages 0, 10, 20….100. We included HAQi as a country-level covariate to inform EMR with a mean and standard deviation produced from MR-BRT.

The Beta and exponentiated values of this predictive covariate (which can be interpreted as an odds ratio) are shown in the table below.

Table 4. Covariates. Summary of covariates used in the acute glomerulonephritis DisMod-MR meta-regression model

Covariate	Type	Parameter	Exponentiated beta (95% Uncertainty Interval)
Healthcare access and quality index	Country-level	Excess mortality rate	0.97 (0.97, 0.97)

Dermatitis

Flowcharts for Atopic dermatitis, Contact dermatitis, & Seborrheic dermatitis

Atopic dermatitis

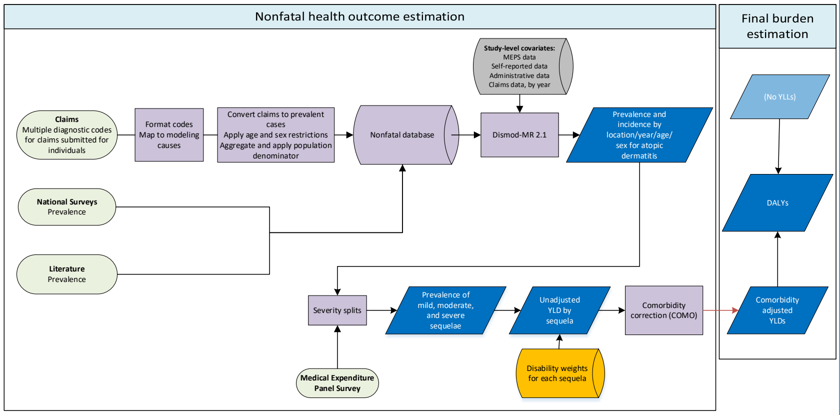

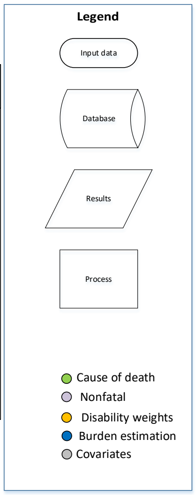

Contact dermatitis

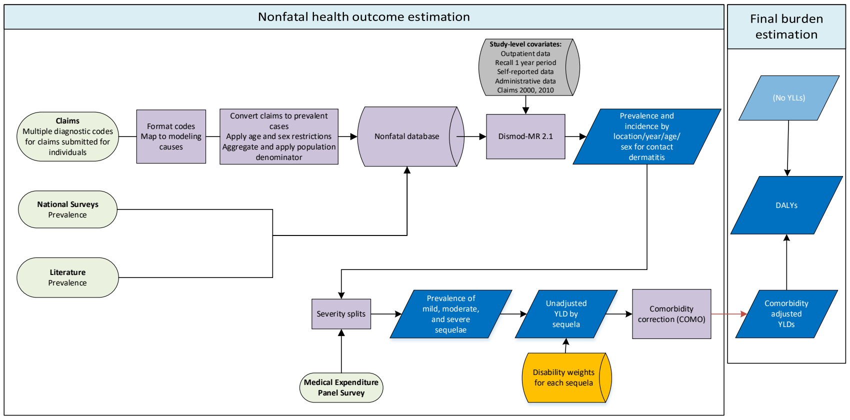

Seborrheic dermatitis

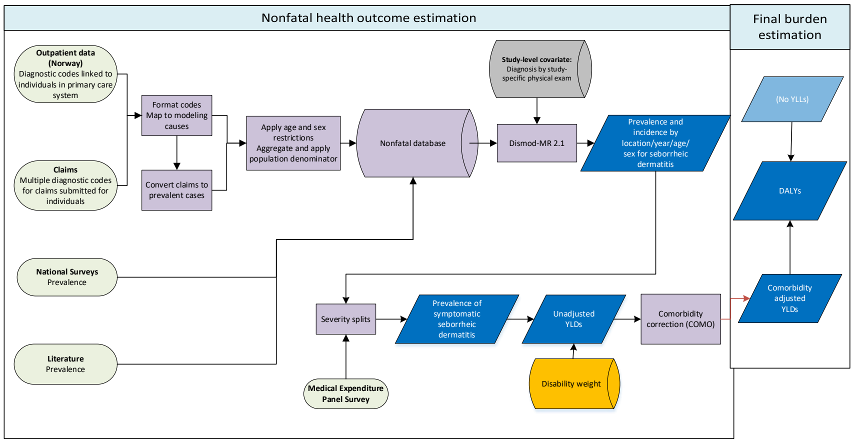

Case definition

Dermatitis, or eczema, refers to inflammation of the dermal layer of the skin, with disruption of the epidermal barrier. This inflammation leads to rashes that are commonly red, scaly, or flaky. Atopic dermatitis is a relapsing dermatitis associated with elevated serum immunoglobulin E and some degree of immune dysregulation; it can be localised or widespread and is commonly characterised by itching that can be extreme (ICD-10: L20). Contact dermatitis is a localised dermatitis caused by direct contact with allergens or irritants; it can be asymptomatic or characterised by itching, stinging or pain (ICD: 10: L22-26). Seborrhoeic dermatitis is a dermatitis affecting the sebaceous-gland-rich areas of skin, and may be itchy (ICD-10: L21). We estimate burden separately for atopic dermatitis, contact dermatitis, and seborrhoeic dermatitis in order to accommodate differences in the epidemiology and burden between these subtypes.

Input data

Model inputs

Data for dermatitis came from scientific literature and claims submitted for individuals to USA commercial insurance. The seborrheic and contact dermatitis model additionally incorporated data from a claims database in Russia, and the atopic dermatitis model incorporated claims data from Poland. A literature review was conducted in GBD 2016 for studies of the incidence and prevalence of dermatitis, the details of which are described in the appendix to GBD 2016, and the results of this review were used in GBD 2019. Inpatient data were regarded as inappropriate for this chronic, non-fatal condition that is primarily cared for in non-acute settings. Data from the Medical Expenditure Panel Survey (MEPS) in the United States in 2000–2009 (2) were included to inform the age pattern of the prevalence output. Data from the NHANES study and the NHIS study (both from the USA) were not extracted, as questions regarding dermatitis were too broad (ie, asked whether a respondent had experienced eczema or any other rash). The data for dermatitis were expanded based on recommendations of research articles and reviews by the skin expert group.

Data from outpatient encounters in the United States and Sweden were considered for inclusion but were found to violate established age patterns and regional trends and were excluded. Additional data were marked as outliers and excluded if we found them unreasonable when compared to regional, super-regional, and global rates. See descriptions of individual modelling approaches for more information.

Cause/Impairment Name	Measure	Total sources	Countries with data
Dermatitis	All measures	341	114
Dermatitis	Prevalence	338	114
Dermatitis	Incidence	2	2
Dermatitis	Proportion	15	1
Atopic dermatitis	All measures	313	113
Atopic dermatitis	Prevalence	313	113
Atopic dermatitis	Incidence	1	1

Contact dermatitis	All measures	66	17
Contact dermatitis	Prevalence	63	17
Contact dermatitis	Incidence	1	1
Contact dermatitis	Proportion	15	1
Seborrhoeic dermatitis	All measures	70	23
Seborrhoeic dermatitis	Prevalence	67	23
Seborrhoeic dermatitis	Incidence	1	1
Seborrhoeic dermatitis	Proportion	15	1

Table 1: MR-BRT Crosswalk Adjustment Factors for dermatitis

Cause	Data input	Reference or alternative case definition	Gamma	Beta Coefficient, Logit (95% CI)	Adjustment factor
Atopic Dermatitis	Literature with physical exam and USA marketscan	Reference	1.03	---	---
	Administrative data	Alternative		-1.04 (-3.07 to 0.98)	0.26
	MEPS	Alternative		-0.56 (-2.59 to 1.47)	0.36
	No physical exam	Alternative		0.25 (-1.78 to 2.28)	0.56
	USA marketscan 2000	Alternative		-1.78 (-3.81 to 0.25)	0.14
Contact Dermatitis	Literature with physical exam and USA marketscan	Reference	0.29	--	--
	No physical exam	Alternative		0.40 (-0.19 to 1.00)	0.60
	MEPS	Alternative		-0.72 (-1.30 to -0.14)	0.33
	Recall 1 year	Alternative		0.40 (-0.19 to 1.00)	0.60
	USA marketscan 2000	Alternative		-0.14 (-0.71 to 0.44)	0.47
Seborrhoeic dermatitis	Literature with physical exam and USA marketscan	Reference		--	--

	RA diagnosis from administrative data	Alternative	0.30	0.42 (-0.23 to 1.07)	0.60
	ICPC	Alternative		-2.97 (-3.60 to -2.35)	0.05
	MEPS	Alternative		-2.69 (-3.29 to -2.10)	0.06
	USA marketscan 2000	Alternative		-0.56 (-1.16 to 0.04)	0.36

Severity splits and disability weights

The basis of the GBD disability weight survey assessments are lay descriptions of sequelae highlighting major functional consequences and symptoms. Severity was split into three levels of disfigurement with pain/itch. See below for a lay descriptions of the severity levels.

Table 2. Severity distribution, details on the severity levels for dermatitis in GBD 2019 and the associated disability weight (DW) with that severity.

Sequela	Severity level	Lay description	DW (95% CI)
Mild atopic dermatitis	Disfigurement, level 1 with itch/pain	The person has a slight, visible physical deformity that is sometimes sore or itchy. Others notice the deformity, which causes some worry and discomfort.	0.027 (0.015–0.042)
Moderate atopic dermatitis	Disfigurement, level 2, with itch/pain	The person has a visible physical deformity that is sore and itchy. Other people stare and comment, which causes the person to worry. The person has trouble sleeping and concentrating.	0.188 (0.124–0.267)
Severe atopic dermatitis	Disfigurement, level 3, with itch/pain	The person has an obvious physical deformity that is very painful and itchy. The physical deformity makes others uncomfortable, which causes the person to avoid social contact, feel worried, sleep poorly, and think about suicide.	0.576 (0.401–0.731)

Mild contact dermatitis	Disfigurement, level 1 with itch/pain	The person has a slight, visible physical deformity that is sometimes sore or itchy. Others notice the deformity, which causes some worry and discomfort.	0.027 (0.015–0.042)
Moderate contact dermatitis	Disfigurement, level 2, with itch/pain	The person has a visible physical deformity that is sore and itchy. Other people stare and comment, which causes the person to worry. The person has trouble sleeping and concentrating.	0.188 (0.124–0.267)
Symptomatic seborrhoeic dermatitis	Disfigurement, level 1 with itch/pain	The person has a slight, visible physical deformity that is sometimes sore or itchy. Others notice the deformity, which causes some worry and discomfort.	0.027 (0.015–0.042)

Modelling strategy

DisMod-MR 2.1 was used to estimate prevalence by age, sex, year, and geography (subnational, country, region, super-region) for atopic dermatitis, contact dermatitis, and seborrhoeic dermatitis. Separate models were run for each cause.

Model parameters

Atopic dermatitis

Since our available data mostly contained information on prevalence, we specified additional expert priors to further inform analyses. The prior value on excess mortality was set to zero, and the prior value on remission was bounded to 0-0.2 (equivalent to five years to life time duration). In GBD 2019, we replaced our within-DisMod crosswalks with crosswalked completed using the MR-BRT modeling tool. We adjusted administrative data, along with data from the Medical Expenditure Panel Survey, USA marketscan 2000 data, and data that were not based on physical exams toward the level of other data points, which were more representative of the general population. To improve regional and global estimates, the minimum coefficient of variation was set at 0.4 and location random effects for Paraguay, Sweden, and England were restricted to [-0.25, 0.25], [-0.25, 0.25], and [-0.5, 0.5], respectively. A time window of ten years was used to determine which data points were used for a particular year of fit.

Contact dermatitis

Similar to atopic dermatitis, mostly prevalence data were available for contact dermatitis. Per expert advice, the remission parameter was set from 0.1 to 4, excess mortality was set to zero, and incidence was set to zero prior to age 6. In GBD 2019, we replaced our within-DisMod crosswalks with crosswalked completed using the MR-BRT modeling tool. We adjusted data with a recall period of 1 year, along with data from the Medical Expenditure Panel Survey, USA marketscan 2000 data, and data that were not based on physical exams toward the level of other data points, which were more representative of the general population. In order to improve model

estimates, location random effects were added for all super regions [-0.25, 0.25]. A time window of 25 years was used to determine which data points were used for a particular year of fit.

Seborrhoeic dermatitis:

As with contact dermatitis, the available data were mostly prevalence estimates. Per expert advice, settings were placed on incidence as follows: 0-4 years = 0-0.1, and 60-100 = 0-0.01. Excess mortality was set to zero while a setting of 0.1-12 was placed on remission, implying a duration of one month to ten years. In GBD 2019, we replaced our within-DisMod crosswalks with crosswalked completed using the MR-BRT modeling tool. We adjusted RA diagnosis from administrative data, along with data from the Medical Expenditure Panel Survey, USA marketscan 2000 data, and Norway outpatient data toward the level of other data points, which were more representative of the general population.

Psoriasis

Flowchart

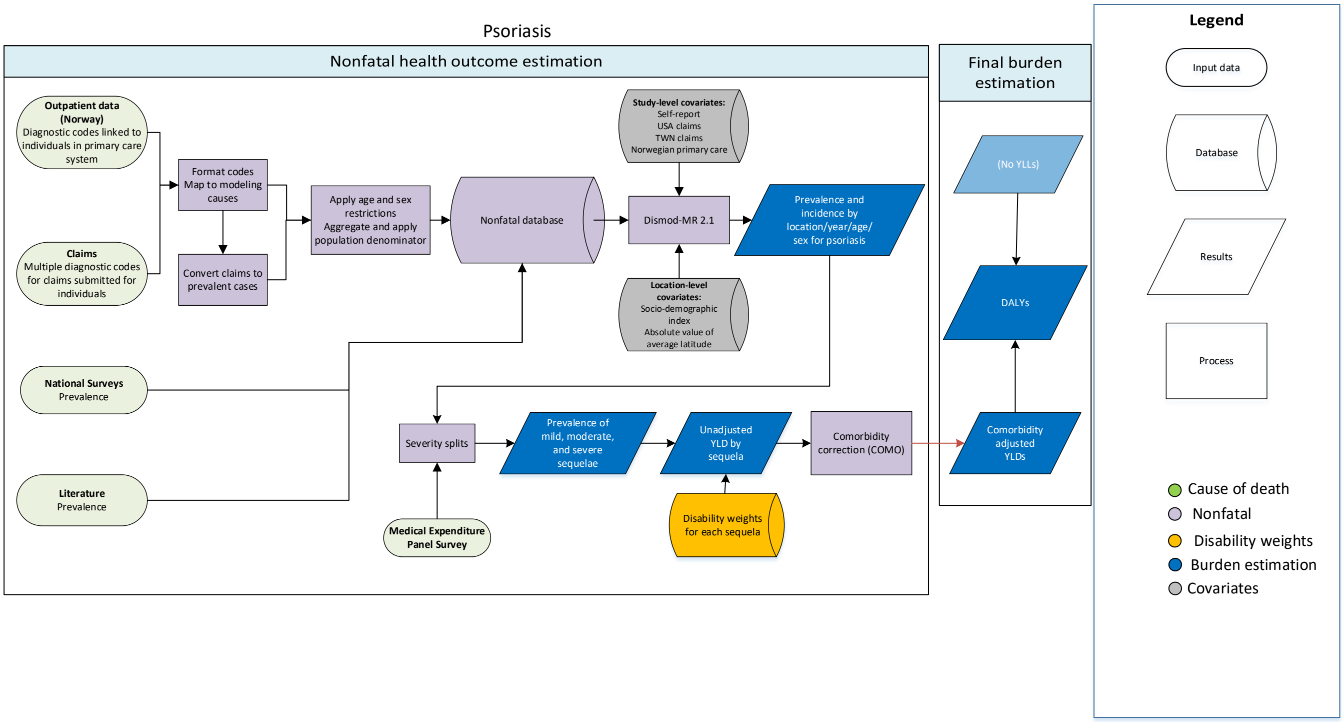

Case definition

Psoriasis is an autoimmune disease characterized by areas of raised, red skin with silvery scales, which may be itchy (ICD-10: L40, L41). It is an immune-mediated disease that involves inflammation and excess growth and abnormal behavior of certain skin cells.

Input data

Model inputs

The data for the psoriasis model come from scientific literature and several large, national surveys, claims data from the United States, Taiwan, Russia, and Poland.

The literature used has been described in greater detail in previous GBD appendices. In brief, in the GBD 2010 study, a systematic review of the literature was conducted using PubMed and Google Scholar to capture epidemiological data for psoriasis. In GBD 2013, the 2010 search strategy was replicated to capture studies from 2012 to 2014, and it was repeated again in GBD 2016 to capture studies through October 1, 2016. The inclusion criteria stipulated that studies (1) must provide data on the incidence or prevalence of psoriasis; (2) must use samples representative of the general population (ie, samples derived from the experimental arm of clinical trials or based in dermatology clinics were excluded); (3) must use a sample size larger than 100; and (4) must provide sufficient information on study method and sample characteristics to assess the quality of the study.

Surveys used include the Medical Expenditure Panel Survey (MEPS) in the United States for 2000–2009, the Australian National Health Survey 1995–1996, 2001, 2004–2005, 2007–2008, and the USA National Health and Nutrition Examination Survey (NHANES) in 2002 and 2005.

Claims data from the United States, Taiwan, Poland, and Russia link claims for multiple inpatient and outpatient encounters to a single individual. An individual was extracted as a prevalent case if they had one or more inpatient or outpatient encounter with a psoriasis ICD code as any encounter diagnosis.

Data from outpatient encounters from facilities in the United States and Sweden were considered for inclusion in the psoriasis database, but these data violated established regional trends and age distributions and were excluded. Data were further considered for exclusion if relatively high values in young age groups led to overestimation of subnational pseudo-random effects and poor model fit, or if we found them unreasonable when compared to regional, super-regional, and global rates, but no data for these models met these criteria for exclusion.

The tables below show the number of studies included in GBD 2019, as well as the number of countries and GBD world regions represented.

Cause/Impairment Name	Measure	Total sources	Countries with data
Psoriasis	All measures	132	31
Psoriasis	Prevalence	123	31
Psoriasis	Incidence	8	4
Psoriasis	Proportion	15	1

Table 1: MR-BRT Crosswalk Adjustment Factors for psoriasis

Data input	Reference or alternative case definition	Gamma	Beta Coefficient, Logit (95% CI)	Adjustment factor
Literature with physical exam	Reference	0.63	---	---
No Physical Exam	Alternative		-0.12 (-1.36 to 1.12)	0.47
USA Marketscan 2000	Alternative		-1.23 (-2.50 to -0.01)	0.22
USA Marketscan 2010-2016	Alternative		-0.82 (-2.06 to 0.43)	0.31
RA diagnosis from administrative data	Alternative		-0.87 (-2.12 to 0.37)	0.29

Severity splits and disability weights

The basis of the GBD disability weight survey assessments are lay descriptions of sequelae highlighting major functional consequences and symptoms. As was the case in GBD 2017, disability weights used were for disfigurement with itch/pain, levels 1, 2, and 3.

Table 2. Severity distribution, details on the severity levels for psoriasis in GBD 2019 and the associated disability weight (DW) with that severity.

Sequela	Severity level	Lay description	DW (95% CI)
Mild psoriasis	Disfigurement, level 1 with itch/pain	The individual has a slight, visible physical deformity that is sometimes sore or itchy. Others notice the deformity, which causes some worry and discomfort.	0.027 (0.015–0.042)
Moderate psoriasis	Disfigurement, level 2, with itch/pain	The individual has a visible physical deformity that is sore and itchy. Other people stare and comment, which causes the person to worry. The person has trouble sleeping and concentrating.	0.188 (0.124–0.267)
Severe psoriasis	Disfigurement, level 3, with itch/pain	The individual has an obvious physical deformity that is very painful and itchy. The physical deformity makes others uncomfortable, which causes the person to avoid social contact, feel worried, sleep poorly, and think about suicide.	0.576 (0.401–0.731)

Modelling strategy

DisMod-MR 2.1, a Bayesian meta-regression tool, was used to estimate prevalence by age, sex, year, and geography (subnational [select countries], country, region, super-region) for psoriasis.

Psoriasis was modelled with remission set between 0.05 and 0.15, implying a duration between 6.6 and 20 years. This was in line with the available epidemiological data, expert opinion, and previous GBD work. Excess mortality was assumed to be zero. The datasets for psoriasis were sufficiently large to make use of a relatively short time window of ten years to determine which data points were used for a particular year of fit. Socio-demographic Index and absolute value of average latitude were used as location-level covariates to guide estimates for countries with few or no data.

In GBD 2019, we replaced our within-DisMod crosswalks with crosswalked completed using the MR-BRT modeling tool. We adjusted USA marketscan data, along with RA diagnosis from administrative data toward the level of other prevalence data points, which were more representative of the general population. In addition, sociodemographic index and absolute value of average latitude were used as country-level covariates to guide estimates for countries with few or no data.

Table 3. Covariates. Summary of covariates used in the psoriasis DisMod-MR meta-regression model

Covariate	Type	Parameter	Exponentiated beta (95% Uncertainty Interval)
Socio-demographic Index	Country-level	Prevalence	0.19 (0.17 — 0.20)
Absolute value of average latitude	Country-level	Prevalence	1.01 (1.01 — 1.01)

Cellulitis

Flowchart

Cellulitis

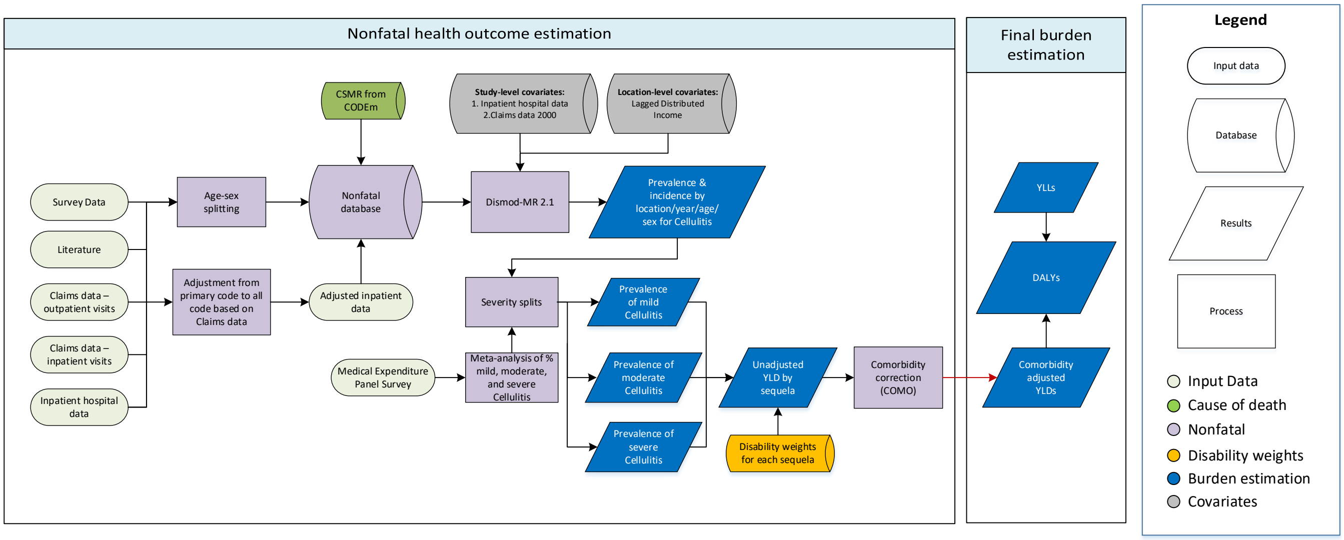

Case definition

Cellulitis was included in the GBD 2019 cause group of skin and subcutaneous conditions. Cellulitis is a skin disease marked by a bacterial infection that affects and spreads through the skin and soft tissues. Symptoms of cellulitis include pain, tenderness, and reddening in the affected area, fever, chills, and lymphadenopathy (ICD-10: L03) (1).

Input data

Model inputs

In the GBD 2010 study, a systematic review of the literature was conducted using PubMed and Google Scholar to capture epidemiological data for cellulitis. Due to lack of published data on the epidemiology of cellulitis, the literature search also included relevant incidence data from national inpatient or outpatient records in Europe, North America, and Latin America. When years in the national data from the hospital records overlapped, inpatient and outpatient data were summed together in an effort to better estimate the population incidence of cellulitis. The final dataset also includes USA claims data, Taiwan claims data, Poland claims data, hospital inpatient data and cause-specific mortality rates for cellulitis estimated by CODEm.

The inclusion criteria stipulated that studies (1) must be published between 1980 and 2012; (2) must provide data on the incidence or prevalence of cellulitis; (3) must use samples representative of the general population (ie, samples derived from the experimental arm of clinical trials or based in dermatology clinics were excluded); (4) must use a sample size larger than 100; and (5) must provide sufficient information on study method and sample characteristics to assess the quality of the study. For GBD 2013, the GBD 2010 search strategy was replicated to capture epidemiological studies published between 2012 and 2013. Data were outliered or excluded if we found them unreasonable when compared to regional, super-regional, and global rates.

Cause/Impairment Name	Measure	Total sources	Countries with data
Cellulitis	All measures	311	44
Cellulitis	Incidence	296	44
Cellulitis	Proportion	15	1

Table 1: MR-BRT Crosswalk Adjustment Factors for cellulitis

Data input	Reference or alternative case definition	Gamma	Beta Coefficient, Logit (95% CI)	Adjustment factor
Literature with physical exam	Reference	0.58	---	---
USA Marketscan 2000	Alternative		0.28 (-1.03 to 1.60)	0.57
USA Marketscan 2010	Alternative		-0.02 (-1.33 to 1.30)	0.50
USA Marketscan 2011	Alternative		-0.03 (-1.35 to 1.28)	0.50
USA Marketscan 2012	Alternative		-0.02 (-1.34 to 1.29)	0.49
USA Marketscan 2013	Alternative		-0.09 (-1.40 to 1.23)	0.48
USA Marketscan 2014	Alternative		-0.06 (-1.38 to 1.25)	0.48
Taiwan claims	Alternative		1.86 (0.55 to 3.17)	0.87
Inpatient data	Alternative		0.16 (-1.15 to 1.48)	0.54

Severity splits

The basis of the GBD disability weight survey assessments are lay descriptions of sequelae highlighting major functional consequences and symptoms. Severity splits for cellulitis were calculated via the Medical Expenditure Panel Survey (MEPS) regression and outlined in the table below.

Table 2. Severity distribution, details on the severity levels for cellulitis in GBD 2019 and the associated disability weight (DW) with that severity.

Sequela	Severity level	Lay description	DW (95% CI)
Mild cellulitis	Infectious disease, acute episode, mild	This person has a low fever and mild discomfort,	0.006 (0.002–0.012)

		but no difficulty with daily activities.	
Moderate cellulitis	Infectious disease, acute episode, moderate	This person has a fever and aches, and feels weak, which causes some difficulty with daily activities.	0.051 (0.032–0.074)
Severe cellulitis	Infectious disease, acute episode, severe	This person has a high fever and pain, and feels very weak, which causes great difficulty with daily activities.	0.133 (0.088–0.19)

Modelling strategy

DisMod-MR 2.1, a Bayesian meta-regression tool, was used to estimate cellulitis prevalence by age, sex, year, and geography (subnational [select countries], country, region, super-region). Cellulitis was modelled with remission set between 12 and 30, implying a duration of 12 days to one month. This was in line with the available epidemiological data, expert opinion, and previous GBD work. The cellulitis dataset was sufficiently large to make use of a relatively short time window of five years to determine which data points were used for a particular year of fit.

In GBD 2019, we replaced our within-DisMod crosswalks with crosswalked completed using the MR-BRT modeling tool. We adjusted inpatient data, along with USA claims data, and Taiwan claims data toward the level of other incidence data points, which were more representative of the general population. In addition, log-transformed lagged distributed income (LDI) was used as a country-level covariate to guide estimates for locations with few or no data. LDI was restricted to a range of -0.5 to -0.1. We restricted location random effects to (-0.5, 0.5) across all 7 GBD super-regions.

In previous rounds, priors on excess mortality rate (EMR) were estimated in DisMod by matching prevalence data points with their corresponding CSMR values within the same age, sex, year, location (by dividing CSMR by prevalence). For short duration conditions (remission>1), the corresponding prevalence was derived by running an initial model and then applying the same CSMR/prevalence method. However, for many causes, DisMod estimated a rather unrealistic pattern of EMR compared to an expected pattern of decreasing EMR with greater access to quality health care. Such unexpected patterns often signal inconsistencies between CSMR estimates and the measures of prevalence and/or incidence. In effort to provide greater guidance to DisMod on the expected pattern of EMR, EMR data generated in the previous round were modeled using the MR-BRT approach by age and sex with a prior on healthcare access and quality index (HAQi) having a negative coefficient. Results from MR-BRT were then predicted for each location year, sex and for ages 0, 10, 20 ….100.

The table below indicates the covariates, parameters, and exponentiated beta values used in GBD 2019.

Table 3. Covariates. Summary of covariates used in the cellulitis DisMod-MR meta-regression model

Covariate	Type	Parameter	Exponentiated beta (95% Uncertainty Interval)
LDI (I$ per capita)	Country-level	Excess Mortality Rate	0.61 (0.61 to 0.61)

Pyoderma

Flowchart

Impetigo

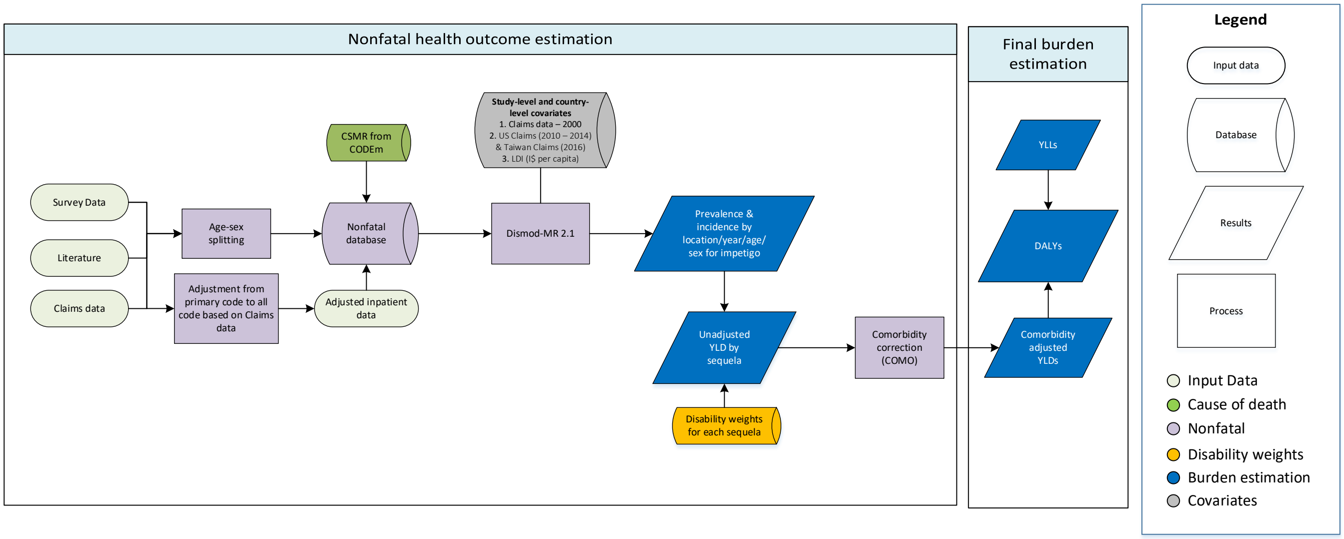

Abscess and Other Bacterial Skin Infections

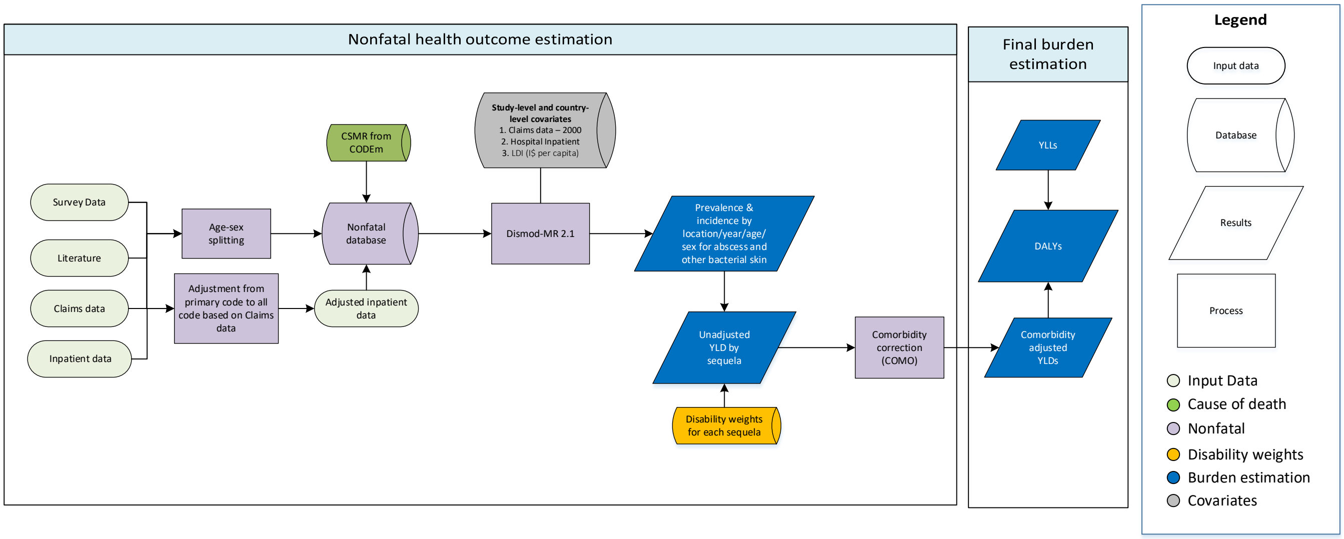

Case definition

Pyoderma refers to any skin disease that is pyogenic, ie, involves the development of pus. These include superficial bacterial conditions such as impetigo, furuncles, ulcers, and abscesses. In line with GBD 2017, for GBD 2019, pyoderma was modelled as two separate groups: impetigo, and abscess and other bacterial skin diseases. Impetigo is a highly contagious bacterial skin infection often characterized by red sores, which eventually leak pus or fluid (ICD-10: L01). An abscess is a collection of pus that builds up within the tissue of the body, with carbuncles and furuncles being examples of specific types of abscess. The abscess and other bacterial skin diseases group included all bacterial skin diseases except impetigo (ICD-10: L00, L02, L04, L05, L08).

Input data

Model inputs

For both impetigo and abscess and other bacterial skin diseases in GBD 2010, a literature review was conducted using PubMed and Google Scholar. The inclusion criteria were studies which were published between 1980 and 2010 and provided data on relevant disease incidence or prevalence. Exclusion criteria were studies with no incidence or prevalence data provided, not community- or population-based, outside of year range, sample size smaller than 100, experimental arm of clinical trial, papers that provided estimates rather than data, and studies that were based in dermatology clinics. For GBD 2016, the GBD 2013 search strategy was replicated to capture epidemiological studies published between 2014 and 2016. Hospital inpatient data were used as model inputs for abscesses and other bacterial skin diseases, but were omitted for impetigo, as the adjustment factor from primary diagnoses codes to all diagnoses codes were found to be implausible.

Cause/Impairment Name	Measure	Total sources	Countries with data
Pyoderma	All measures	301	52
Pyoderma	Prevalence	14	12
Pyoderma	Incidence	289	43

Table 1: MR-BRT Crosswalk Adjustment Factors for pyoderma

Cause	Data input	Reference or alternative case definition	Gamma	Beta Coefficient, Logit (95% CI)	Adjustment factor
Impetigo	Literature with physical exam and claims, USA marketscan, Taiwan claims	Reference	0.32	---	---
	USA Marketscan 2000	Alternative		-0.04 (-0.94 to 0.86)	0.49
	Literature with physical exam	Reference		---	---
	USA Marketscan 2000	Alternative		-0.05 (-0.18 to 0.09)	0.49

Abscess and other bacterial skin	USA Marketscan 2010	Alternative	0.07	0.0017 (-0.14 to 0.14)	0.50
	USA Marketscan 2011	Alternative		0.05 (-0.083 to 0.19)	0.51
	USA Marketscan 2012	Alternative		0.11 (-0.02 to - 0.25)	0.53
	USA Marketscan 2013	Alternative		0.10 (-0.04 to 0.24)	0.52
	USA Marketscan 2014	Alternative		0.21 (0.07 to 0.34)	0.55
	Taiwan claims	Alternative		-1.16 (-1.30 to - 1.03)	0.23
	Inpatient data	Alternative		-1.4 (-1.54 to - 1.27)	0.20

Severity splits and disability weights

Information on the distribution of cases of impetigo and abscess and other bacterial skin diseases, asymptomatic, and within disfigurement levels 1 and 2, were obtained from the Medical Expenditures Panel Survey. The symptomatic cases were assigned the disability weight of a mild acute infectious disease case.

Table 2. Severity distribution, details on the severity levels for pyoderma in GBD 2019 and the associated disability weight (DW) with that severity.

Sequela	Severity level	Lay description	DW (95% CI)
Impetigo	Infectious disease, acute episode, mild	The person has a low fever and mild discomfort, but no difficulty with daily activities.	0.006 (0.002–0.012)
Abscesses and other bacterial skin diseases	Infectious disease, acute episode, mild	The person has a low fever and mild discomfort, but no difficulty with daily activities.	0.006 (0.002–0.012)

Modelling strategy

DisMod-MR 2.1 was used to estimate prevalence by age, sex, year, and geography (country, region, super-region) for impetigo and abscess and other bacterial skin diseases. Separate models were run for each disease.

In previous rounds, priors on excess mortality rate (EMR) were estimated in DisMod by matching prevalence data points with their corresponding CSMR values within the same age, sex, year, location (by dividing CSMR by prevalence). For short duration conditions (remission>1), the corresponding prevalence was derived by running an initial model and then applying the same CSMR/prevalence method. However, for many causes, DisMod estimated a rather unrealistic pattern of EMR compared to an expected pattern of decreasing EMR with greater access to quality health care. Such unexpected patterns often signal inconsistencies between CSMR estimates and the measures of prevalence and/or incidence. In effort to provide greater guidance to DisMod on the expected pattern of EMR, EMR data generated in the previous round were modeled using the MR-BRT approach by age and sex with a prior on healthcare access and quality index (HAQi) having a negative coefficient. Results from MR-BRT were then predicted for each location year, sex and for ages 0, 10, 20 ….100. This approach was used for both impetigo and abscess and other bacterial skin diseases.

Impetigo: Per expert advice, we assumed a remission of 17 to 20, equating to a duration between approximately two and three weeks. A value prior was also placed on incidence, restricting the range between zero and one. In GBD 2019, we replaced our within-DisMod crosswalks with crosswalked completed using the MR-BRT modeling tool. We adjusted USA marketscan data from 2000 toward the level of other data points, which were more representative of the general population. A country-level covariate, log transformed lagged distributed income (I$ per capita), which represents a moving average of gross domestic product (GDP) over time, was also included to inform prevalence and excess mortality estimates. We also used the cause-specific mortality rates for pyoderma estimated using CODEm. We used a time window of five years to determine which data points were used for a particular year of fit.

Abscess and other bacterial skin diseases: Per expert advice, a remission setting of 17 to 30 was applied, which equated to a duration of two to six weeks. In GBD 2019, we replaced our within-DisMod crosswalks with crosswalked completed using the MR-BRT modeling tool. We adjusted USA marketscan data from 2000, inpatient data, and Taiwan claims data toward the level of other data points which were more representative of the general population. We also used the cause-specific mortality rates for pyoderma estimated using CODEm. In addition, we used a log transformed lagged distributed income (I$ per capita) country covariate on excess mortality. We used a time window of five years to determine which data points were used for a particular year of fit and limited random effects to (-0.5, 0.5) for certain GBD regions and super-regions (South Asia, Central Asia, Latin America & Caribbean, North Africa & Middle East, and high-income) to improve model estimates.

Table 3. Covariates. Summary of covariates used in the pyoderma DisMod-MR meta-regression models

Cause	Covariate	Type	Parameter	Exponentiated beta (95% Uncertainty Interval)
Impetigo	LDI (I$ per capita)	Country-level	Prevalence	7.18 (7.06 to 7.32)
	LDI (I$ per capita)	Country-level	Excess Mortality Rate	0.49 (0.48 to 0.50)
Abscess and other bacterial skin diseases	LDI (I$ per capita)	Country-level	Excess Mortality Rate	0.60 (0.58 to 0.61)

Scabies

Flowchart

Scabies

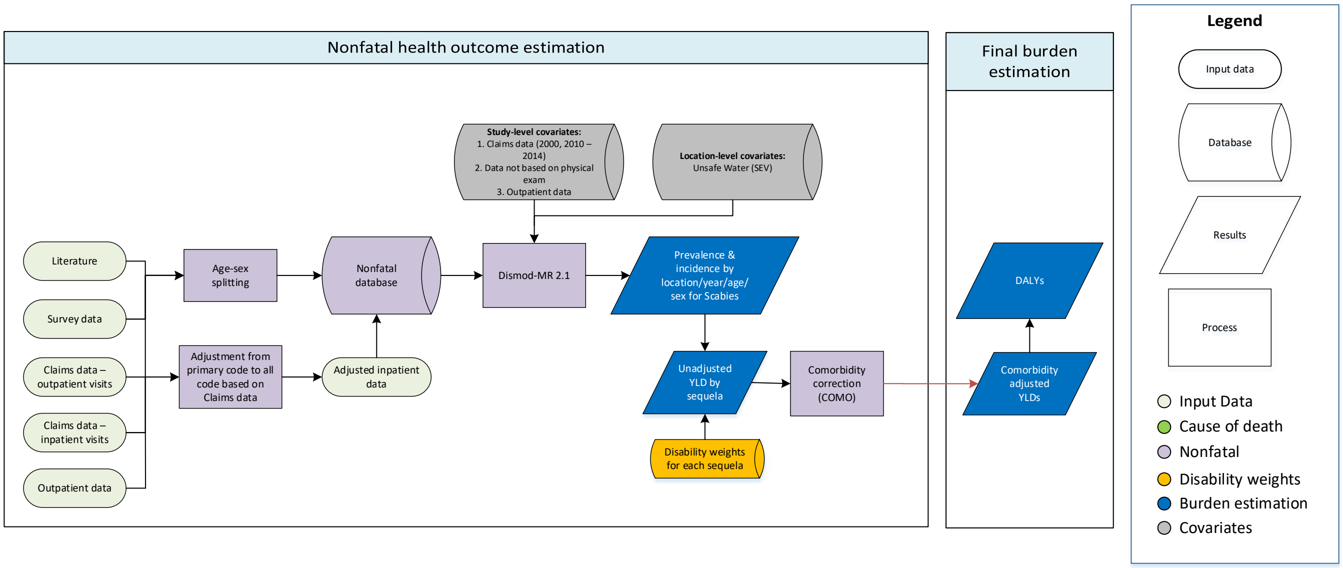

Case definition

Scabies was included in the GBD 2019 cause group of skin and subcutaneous conditions. According to the International Classification of Diseases (ICD-10), scabies is a skin disease caused by the microscopic mite *Sarcoptes scabiei*. The main symptom is an itchy, pimple-like rash (ICD-10: B86) (1).

Input data

Model inputs

In the GBD 2010 study, a systematic review of the literature was conducted using PubMed and Google Scholar to capture epidemiological data for scabies. The inclusion criteria stipulated that studies (1) must be published between 1980 and 2012; (2) must provide data on the incidence or prevalence of scabies; (3) must use samples representative of the general population (ie, samples derived from the experimental arm of clinical trials or based in dermatology clinics were excluded); (4) must use a sample size larger than 100; and (5) must provide sufficient information on study method and sample characteristics to assess the quality of the study. For GBD 2013, the GBD 2010 search strategy was replicated to capture epidemiological studies published between 2011 and 2013. Therefore, we updated the systematic review through October 6, 2016, for GBD 2016. Additionally, USA claims data from 2000 and 2010 through 2016 and outpatient data were included in GBD 2019. Data were outliered or excluded if we found them unreasonable when compared to regional, super-regional, and global rates.

Cause/Impairment Name	Measure	Total sources	Countries with data
Scabies	All measures	164	39
Scabies	Prevalence	144	36
Scabies	Incidence	36	5

Table 1: MR-BRT Crosswalk Adjustment Factors for scabies

Data input	Reference or alternative case definition	Gamma	Beta Coefficient, Logit (95% CI)	Adjustment factor
Literature with physical exam and claims	Reference	3.36	---	---
USA Marketscan 2000	Alternative		1.21 (-8.86 to 11.28)	0.77
No physical exam	Alternative		3.09 (-4.52 to 10.71)	0.96
Outpatient data	Alternative		0.27 (-7.35 to 7.89)	0.57

Scabies was assigned the disability weight for disfigurement level 1. The disability weights used for GBD 2017 were also used for GBD 2019.

Table 2. Severity distribution, details on the severity levels for scabies in GBD 2019 and the associated disability weight (DW) with that severity.

Severity level	Lay description	DW (95% CI)
Disfigurement, level 1 with itch/pain	The individual has a slight, visible physical deformity that is sometimes sore or itchy. Others notice the deformity, which causes some worry and discomfort.	0.027 (0.015–0.042)

Modelling strategy

DisMod-MR 2.1, a Bayesian meta-regression tool, was used to estimate scabies prevalence by age, sex, year, and geography (subnational [select countries], country, region, super-region).

Scabies was modelled with remission set between 2.5 and 3.5, implying four to five months of duration, and excess mortality was assumed to be zero. This was in line with the available epidemiological data, expert opinion, and previous GBD work.

The datasets for scabies were sufficiently large to make use of a relatively short time window of five years to determine which data points were used for a particular year of fit. Additionally, to improve estimation across all regions, we restricted location random effects to (-0.25, 0.25) in Cambodia, Mali, Nepal, Fiji, Timor-Leste, Vanuatu, the Oceania, Southeast Asia, and East Asia GBD regions, and the corresponding super-region. We also restricted the random effect in Kenya (0, 0.5).

In GBD 2019, we replaced our within-DisMod crosswalks with crosswalked completed using the MR-BRT modeling tool. We adjusted outpatient data, along with data that were not based on physical exams, and USA marketscan 2000 data toward the level of other prevalence data points, which were more representative of the general population. In addition, sociodemographic index, sugar consumption, and the Healthcare Access and Quality index were used as country-level covariates to guide estimates for countries with few or no data. In addition, we used the unsafe water SEV (summary exposure value) as a location-level covariate and set the minimum coefficient of variation at 0.4.

The table below indicates the covariates, parameters, and exponentiated beta values used in GBD 2019.

Table 3. Covariates. Summary of covariates used in the acne vulgaris DisMod-MR meta-regression model

Covariate	Type	Parameter	Exponentiated beta (95% Uncertainty Interval)
Unsafe water (SEV)	Country-level	Prevalence	1.12 (1.01 to 1.28)

Fungal skin diseases

Flowchart

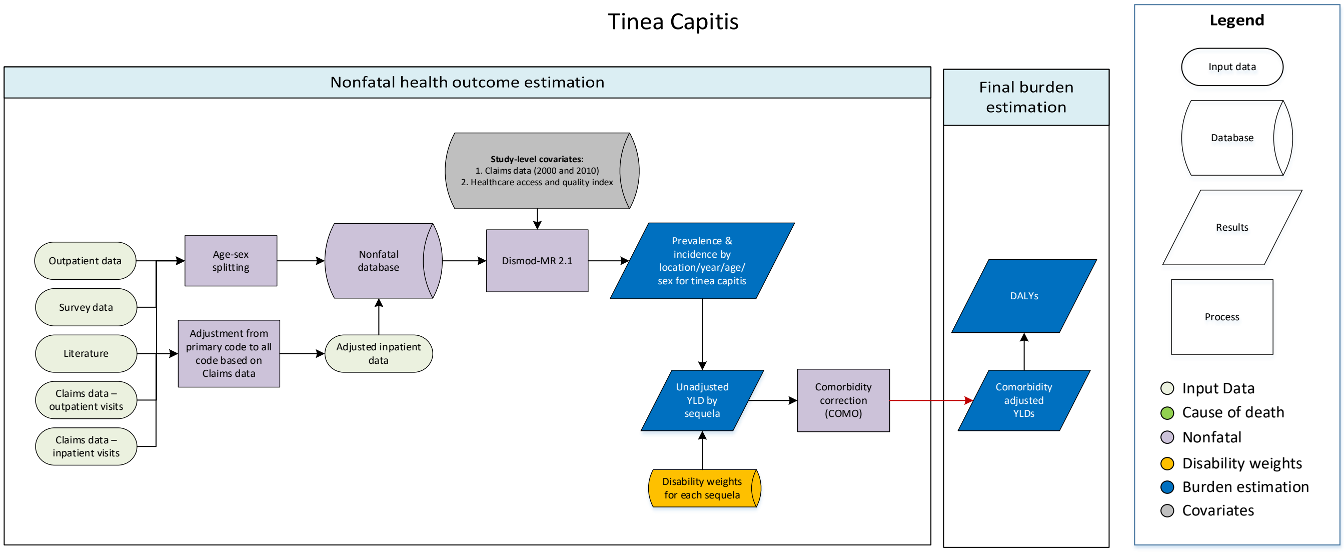

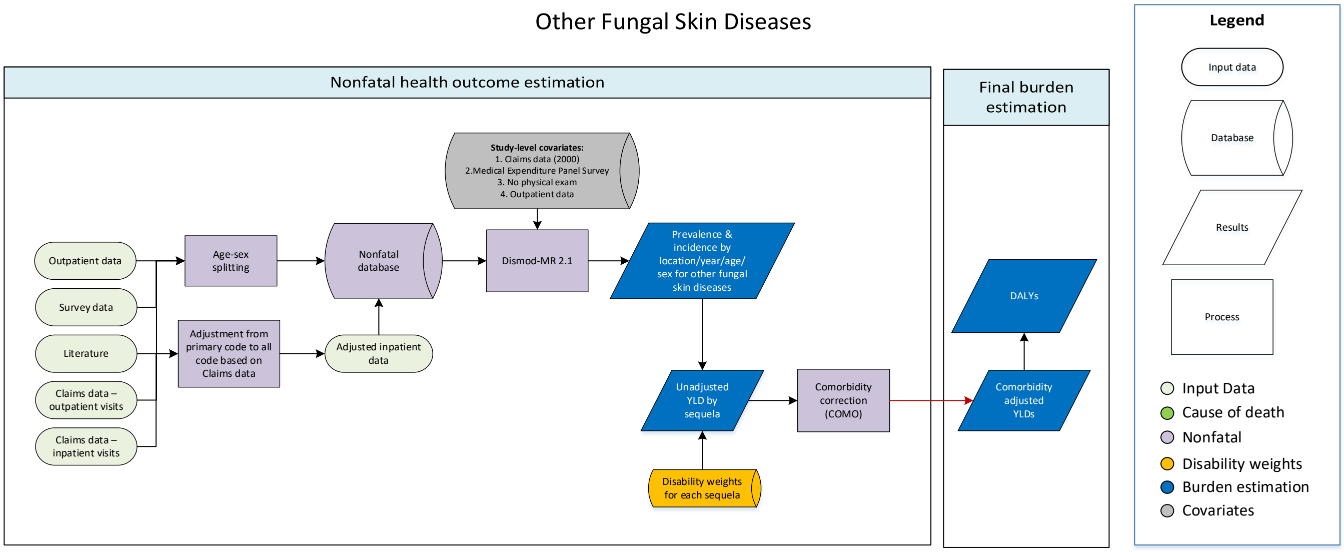

Case definition

Fungal diseases were included in the GBD 2019 cause group of skin and subcutaneous conditions and consisted of tinea capitis and a residual group of "any" other fungal disease. Similar to GBD 2017, tinea capitis was modelled separately from the other fungal skin diseases. This was done to better accommodate differences in burden between tinea capitis and other subtypes of fungal skin diseases.

Tinea capitis is a fungal infection of the scalp and associated hair. It is characterised by the appearance of thickened scaly swellings or as expanding raised red rings (ringworm), mainly caused by species of *Microsporum*, *Trichophyton,* and *Epidermophyton* (ICD-10: B35.0) (1).

The residual group of "any" other fungal skin disease included any fungal skin disease that was specifically not tinea capitis or onychomycosis (ie, fungal nail infection). The ICD-10 (1) list of other fungal skin diseases includes tinea manuum (ICD-10: B35.2), or hand ringworm; tinea pedis (ICD-10: B35.3), or athlete's foot; tinea corporis (ICD-10:B35.4), or ringworm of the body; tinea imbricata (ICD-10:B35.5), a superficial fungal infection limited to parts of Asia and Central America; tinea cruris (ICD-10:B35.6), also known as dhobi itch, groin ringworm, or jock itch. In GBD 2016, we added dermatophytosis (ICD-10:B35.9).

Input data

Model inputs

For GBD 2010, a systematic review of the literature using PubMed and Google Scholar was conducted to capture epidemiological data for fungal skin diseases. The literature search also included any relevant data from the Medical Expenditure Panel Survey (MEPS) in the United States in 2000–2009. The inclusion criteria stipulated that studies (1) must be published between 1980 and 2012; (2) must provide data on the incidence or prevalence of fungal skin diseases; (3) must use samples representative of the general population (ie, samples derived from the experimental arm of clinical trials or based in dermatology clinics were excluded); (4) must use a sample size larger than 100; and (5) must provide sufficient information on study method and sample characteristics to assess the quality of the study. For GBD 2013, the GBD 2010 search strategy was replicated to capture epidemiological studies published between 2012 and 2013. For GBD 2017, the GBD 2010 search strategy was replicated in PubMed to capture epidemiological studies published between 2013 and 2017. Data were outliered or excluded if we found them unreasonable when compared to regional, super-regional, and global rates.

In addition, data from USA claims for 2000 and 2010 through 2016 by state were included for both tinea capitis and other fungal skin diseases, and Poland claims data and USA outpatient data were included for tinea capitis. For tinea capitis, we compared the rates in the outpatient data from Norway, Sweden, Canada, and the USA and found implausibly large differences with the rates from the claims data.

Cause/Impairment Name	Measure	Total sources	Countries with data
Fungal skin diseases	All measures	134	31
Fungal skin diseases	Prevalence	134	31

Table 1: MR-BRT Crosswalk Adjustment Factors for fungal skin diseases

Cause	Data input	Reference or alternative case definition	Gamma	Beta Coefficient, Logit (95% CI)	Adjustment factor
Tinea capitis	Literature with physical exam and USA marketscan	Reference	0.24	---	---
	Outpatient	Alternative		2.04 (1.47 to 2.61)	0.88
Other fungal skin diseases	Literature with physical exam and USA marketscan	Reference	0.13	---	---
	MEPS	Alternative		-0.93 (-1.19 to -0.67)	0.28
	USA Marketscan 2000	Alternative		0.02 (-0.24 to 0.28)	0.51
	No physical exam	Alternative		0.34 (0.06 to 0.62)	0.58

Severity splits

The basis of the GBD disability weight survey assessments are lay descriptions of sequelae highlighting major functional consequences and symptoms. The same disability weight was used for both tinea capitis and other fungal skin diseases.

Table 2. Severity distribution, details on the severity levels for fungal skin diseases in GBD 2019 and the associated disability weight (DW) with that severity.

Severity level	Lay description	DW (95% CI)
Infectious disease, acute episode, mild	The person has a low fever and mild discomfort but no difficulty with daily activities.	0.006 (0.002–0.012)

Modelling strategy

DisMod-MR 2.1, a Bayesian meta-regression tool, was used to estimate tinea capitis and other fungal skin diseases prevalence by age, sex, year, and geography (subnational, country, region, super-region). Separate models were run for tinea capitis and other fungal skin diseases.

Tinea capitis. To help inform the distribution of tinea capitis across the lifespan, excess mortality was set at zero, remission was set at 0.5 to 4, and incidence was set at 0 to 0.02 between 20 and 100 years. This was in agreement with the available prevalence data and expert advice. We made use of a relatively long time window of 20 years to determine which data points were used for a particular year of fit. This means that for the year 2000, for instance, DisMod-MR 2.1 incorporated all data points ranging from 1980 to present to estimate prevalence. In GBD 2019, we replaced our within-DisMod crosswalks with crosswalked completed using the MR-BRT modeling tool. We adjusted USA outpatient data toward the level of other prevalence data points, which

were more representative of the general population. We limited random effects for sub-Saharan Africa (-1,1), North Africa and the Middle East (-1, 1), Southeast Asia, East Asia, and Oceania (-1, 1), and Western Europe (-0.1, 1) to improve model estimates. In addition, sociodemographic index and the Healthcare Access and Quality index were used as country-level covariates to guide estimates for countries with few or no data.

Other fungal skin diseases. The modelling strategy was similar to that for tinea capitis, with remission set between 0.33 and 4. In GBD 2019, we replaced our within-DisMod crosswalks with crosswalked completed using the MR-BRT modeling tool. We adjusted Medical Expenditure Panel Survey (MEPS) data points, USA marketscan data from 2000, and literature data that was not based on a physical exam toward the level of other prevalence data points, which were more representative of the general population. We limited random effects for Nigeria (-0.5, 0.5) and Ethiopia (-0.5, 0.5) to improve model estimates.

The table below indicates the covariates, parameters, and exponentiated beta values used in GBD 2019.

Table 3. Covariates. Summary of covariates used in the fungal skin diseases DisMod-MR meta-regression models

Cause	Covariate	Type	Parameter	Exponentiated beta (95% Uncertainty Interval)
Tinea capitis	Socio-demographic Index	Country-level	Prevalence	6.91 (6.69 to 7.16)
	Healthcare access and quality index	Country-level	Prevalence	0.97 (0.97 to 0.97)

Viral skin diseases

Flowchart

Viral Skin Diseases

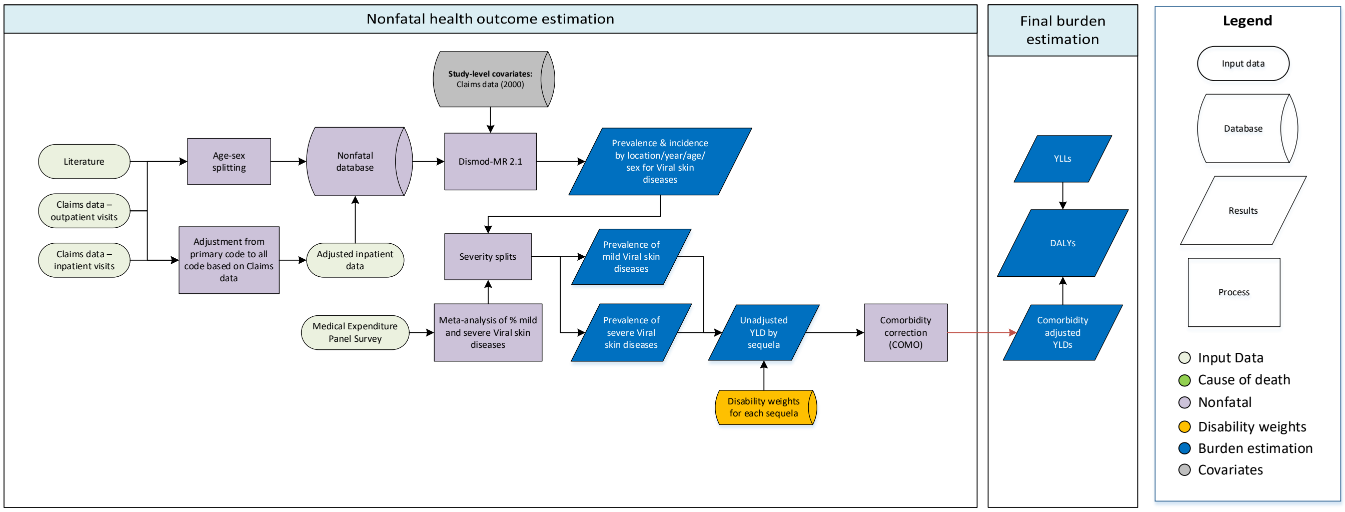

Case definition

Viral skin diseases consist of viral warts and molluscum contagiosum. Viral warts are raised growths on the surface of the skin caused by an infection with the human papillomavirus (ICD-10: B07). Molluscum contagiosum is a viral infection of the skin or occasionally of the mucous membranes characterised by the appearance of waxy, dome-shaped nodules. It is caused by a DNA poxvirus called the molluscum contagiosum virus (ICD-10: B08.1) (1). In GBD 2019, we modelled viral warts and molluscum contagiosum separately in order to better accommodate differences in burden between the subtypes of viral skin diseases.

Input data

Model inputs

In the GBD 2010 study, a systematic review of the literature was conducted using PubMed and Google Scholar to capture epidemiological data for viral skin diseases. Due to lack of published data on the epidemiology of viral skin diseases, the literature search also included relevant incidence data from national inpatient or outpatient records in the USA. The inclusion criteria stipulated that studies (1) must be published between 1980 and 2012; (2) must provide data on the incidence or prevalence of viral warts or molluscum contagiosum; (3) must use samples representative of the general population (ie, samples derived from the experimental arm of clinical trials or based in dermatology clinics were excluded); (4) must use a sample size larger than 100; and (5) must provide sufficient information on study method and sample characteristics to assess the quality of the study. For GBD 2013, the GBD 2010 search strategy was replicated to capture epidemiological studies published between 2012 and 2013. For GBD 2017, the GBD 2010 search strategy was replicated to capture epidemiological studies published between 2013 and 2017. Data were outliered or excluded if we found them unreasonable when compared to regional, super-regional, and global rates.

Cause/Impairment Name	Measure	Total sources	Countries with data
Viral skin diseases	All measures	67	35
Viral skin diseases	Prevalence	58	33
Viral skin diseases	Incidence	10	7

Data from USA claims for 2000 and 2010 through 2016 by state were included in GBD 2019, where appropriate. See descriptions of individual modelling approaches for more information.

Table 1: MR-BRT Crosswalk Adjustment Factors for viral skin diseases

Cause	Data input	Reference or alternative case definition	Gamma	Beta Coefficient, Logit (95% CI)	Adjustment factor
Viral Warts	Literature with physical exam	Reference	0.03	---	---
	USA Marketscan 2000	Alternative		-0.78 (-0.86 to -0.71)	0.31
	USA Marketscan 2010 -2016	Alternative		-0.74 (-0.80 to -0.67)	0.32
Molluscum Contagiosum	Literature with physical exam and claims	Reference	0.51	--	--
	USA Marketscan 2000	Alternative		-0.78 (-2.19 to 0.64)	0.32

Severity splits

In GBD 2019, cases of both disorders were allocated a distribution between mild acute infectious disease and disfigurement level 2. The severity splits and disability weights used in GBD 2017 were also applied in GBD 2019.

Table 2. Sequela and disability weight

Sequela	Severity level	Lay description	DW (95% CI)
Mild viral warts	Infectious disease, acute episode, mild	The person has a low fever and mild discomfort, but no difficulty with daily activities.	0.006 (0.002–0.012)

Severe viral warts	Disfigurement, level 2	The person has a visible physical deformity that causes others to stare and comment. As a result, the person is worried and has trouble sleeping and concentrating.	0.067 (0.044–0.096)
Mild molluscum contagiosum	Infectious disease, acute episode, mild	The person has a low fever and mild discomfort but no difficulty with daily activities.	0.006 (0.002–0.012)
Severe molluscum contagiosum	Disfigurement, level 2	The person has a visible physical deformity that causes others to stare and comment. As a result, the person is worried and has trouble sleeping and concentrating.	0.067 (0.044–0.096)

Modelling strategy

For GBD 2017, DisMod-MR 2.1 was used to estimate prevalence, by age, sex, year, and geography (subnational, country, region, super-region) for viral warts and molluscum contagiosum. Separate models were run for each disease, as illustrated throughout this cause write-up.

Viral warts. Viral warts were modelled with excess mortality set to 0 and remission set between 0.25 and 2, implying a duration of 0.5 to 4 years. This was in line with the levels of prevalence and incidence data, as well as expert opinion. A number of additional settings were used to ensure that DisMod-MR 2.1 sufficiently followed available data points. Incidence was restricted to a maximum of 0.1, and we made use of a relatively long time window of 25 years to determine which data points were used for a particular year of fit. We limited the prevalence random effects for Andean Latin America (-0.2, 0.2) and Central Europe, Eastern Europe, and Central Asia (-1, 1) in order to improve model fit. In GBD 2019, we replaced our within-DisMod crosswalks with crosswalked completed using the MR-BRT modeling tool. We adjusted USA marketscan data toward the level of other prevalence data points, which were more representative of the general population.

Molluscum contagiosum. As available data only contained information on prevalence and incidence, we specified additional expert priors to further inform analyses. Molluscum contagiosum was modelled with excess mortality set to 0 and remission set between 0.5 and 2, implying a duration of 0.5 to 2 years. This was in line with the available epidemiological data, expert opinion, and previous GBD work. We used a time window of 25 years to determine which data points to include for a particular year of fit. Due to data heterogeneity, we restricted the

location random effects to between -0.5 and 0.5 for select GBD regions and super-regions (Southern Latin America, sub-Saharan Africa, high-income, South Asia, and Southeast Asia, East Asia, and Oceania, North Africa and the Middle East, and Central Europe, Eastern Europe, and Central Asia). In GBD 2019, we replaced our within-DisMod crosswalks with crosswalked completed using the MR-BRT modeling tool. We adjusted USA marketscan data 2000 toward the level of other prevalence data points, which were more representative of the general population.

Acne vulgaris

Flowchart

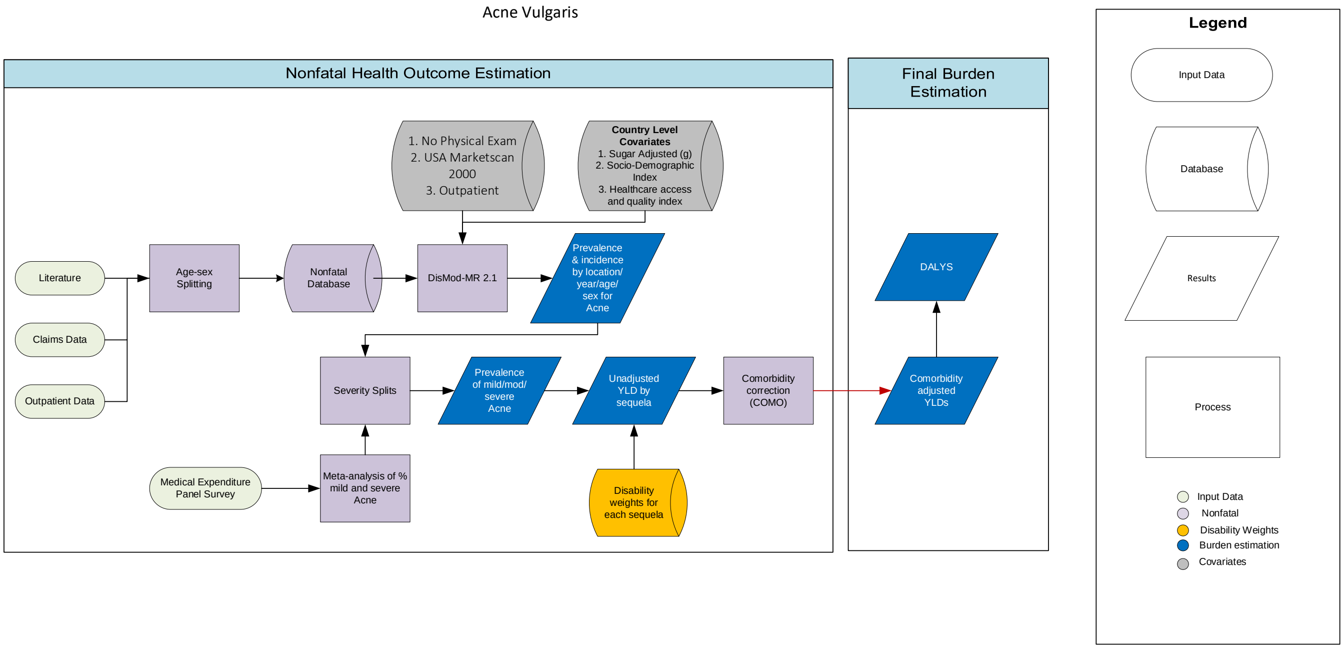

Case definitions

Acne vulgaris was included in the GBD 2019 cause group of skin and subcutaneous conditions. Acne vulgaris (or acne) is a chronic inflammatory disease of the pilosebaceous unit associated with an increase in sebum secretion. Included in the GBD 2019 modelling were cases meeting ICD-10 diagnostic criteria for acne vulgaris (ICD-10: L70, excluding L70.4).

Input data

Model inputs

In the GBD 2010 study, a systematic review of the literature was conducted using PubMed and Google Scholar to capture epidemiological data for acne vulgaris. The inclusion criteria stipulated that studies (1) must be published between 1980 and 2012; (2) must provide data on the incidence or prevalence of acne vulgaris; (3) must use samples representative of the general population (ie, samples derived from the experimental arm of clinical trials or based in dermatology clinics were excluded); (4) must use a sample size larger than 100; and (5) must provide sufficient information on study method and sample characteristics to assess the quality of the study. For GBD 2016, the GBD 2010 search strategy was replicated in PubMed to capture epidemiological studies published between 2013 and 2016. An additional literature search was carried out for GBD 2017 for USA data to better inform the DisMod crosswalk from USA claims data to literature data and capture any studies missed in previous literature searches. This literature search also replicated the GBD 2010 search strategy and captured studies published between 1980 and 2017.

USA claims data from 2000 and 2010 through 2016 are included in this model, along with Poland claims data from 2015-2017, Taiwan claims data from 2016, and outpatient data from both Norway. USA outpatient data were not used due to implausibly high adjusted values.

Cause/Impairment Name	Measure	Total sources	Countries with data
Acne vulgaris	All measures	105	34
Acne vulgaris	Prevalence	90	34
Acne vulgaris	Proportion	15	1

Table 1: MR-BRT Crosswalk Adjustment Factors for acne vulgaris

Data input	Reference or alternative case definition	Gamma	Beta Coefficient, Logit (95% CI)	Adjustment factor
Literature with physical exam and claims	Reference	0.35	---	---
No Physical Exam	Alternative		1.47 (0.78 to 2.17)	0.81
USA Marketscan 2000	Alternative		-0.13 (-0.81 to 0.56)	0.47
Outpatient	Alternative		-2.49 (-3.19 to -1.79)	0.08

Table 2. Severity distribution, details on the severity levels for acne vulgaris in GBD 2019 and the associated disability weight (DW) with that severity.

Sequela	Severity level	Lay description	DW (95% CI)
Mild acne vulgaris	Disfigurement, level 1	The individual has a slight, visible physical deformity that others notice, which causes some worry and discomfort.	0.011 (0.005–0.021)
Moderate acne vulgaris	Disfigurement, level 2	The individual has a visible physical deformity that causes others to stare and comment. As a result, the person is worried and has trouble sleeping and concentrating.	0.067 (0.044–0.096)
Severe acne vulgaris	Disfigurement, level 3	The individual has an obvious physical deformity that makes others uncomfortable, which causes the person to avoid social contact, feel	0.405 (0.275–0.546)

		worried, sleep poorly, and think about suicide.	

Severity splits

The table above illustrates the severity level, lay description, and disability weight for acne. In GBD 2016, we added two additional severity levels – disfigurement 2 and disfigurement 3. The disability weight of each severity of acne was applied across 40% of the total prevalence cases to account for biases in outpatient utilisation. The remaining 60% of prevalence cases were considered mild cases (disfigurement level 1). These proportions were generated using the ratio of patients seeking care captured from claims data, to all individuals captured in literature surveying the general population.

For GBD 2017, we performed a meta-analysis of five literature studies that gave the proportion of people seeking care for acne to replace the estimate from claims data. This was done to get a more geographically diverse estimate of care-seeking acne behavior. The disability weight of mild was applied to those who did not seek care and a small fraction of those seeking care and moderate and severe were applied to those seeking care.

Modelling strategy

DisMod-MR 2.1 was used to estimate prevalence by age, sex, year, and country for acne vulgaris.

Since our available data only contained information on prevalence, we specified additional expert priors to further inform analyses. We assumed zero excess mortality and remission from 0.38 to 0.6, implying a duration of approximately two to three years. This was in line with the available epidemiological data, expert opinion, and previous GBD work. A value prior of zero was set for incidence between the ages of 0 and 6, and 61 and 100. We used a time window of five years to determine which data points were used for a particular year of fit.

In GBD 2019, we replaced our within-DisMod crosswalks with crosswalked completed using the MR-BRT modeling tool. We adjusted outpatient data, along with data that were not based on physical exams toward the level of other prevalence data points, which were more representative of the general population. In addition, sociodemographic index, sugar consumption, and the Healthcare Access and Quality index were used as country-level covariates to guide estimates for countries with few or no data.

The table below indicates the covariates, parameters, and exponentiated beta values used in GBD 2019.

Table 3. Covariates. Summary of covariates used in the acne vulgaris DisMod-MR meta-regression model

Covariate	Type	Parameter	Exponentiated beta (95% Uncertainty Interval)
Socio-demographic Index	Country-level	Prevalence	2.65 (2.60 to 2.71)
Sugar Unadjusted(g)	Country-level	Prevalence	1.00 (1.00 to 1.00)
Healthcare access and quality index	Country-level	Prevalence	1.00 (1.00 to 1.00)

Alopecia areata

Flowchart

Alopecia Areata

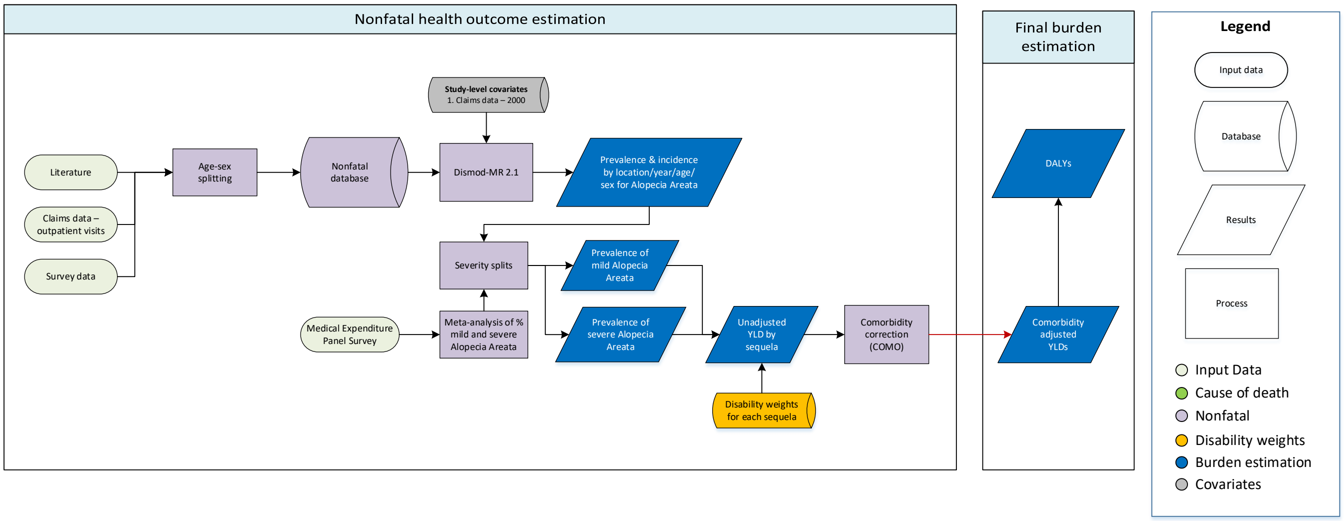

Case definition

Alopecia areata was included in the GBD 2019 cause group of skin and subcutaneous conditions. Alopecia areata is an autoimmune disease that results in hair loss on the scalp and other parts of the body. Included in the GBD disease modelling were cases meeting ICD-10 diagnostic criteria for alopecia (ICD-10: L63).

Input data

Model inputs

In the GBD 2016 study, a systematic review of the literature was conducted using PubMed to expand the GBD dataset (1980–2014) with new epidemiological data for Alopecia areata between 2014 and 2016. The inclusion criteria stipulated that studies (1) must provide data on the incidence or prevalence of alopecia areata; (2) must use samples representative of the general population (ie, samples derived from the experimental arm of clinical trials or based in dermatology clinics were excluded); (3) must use a sample size larger than 100; and (4) must provide sufficient information on study method and sample characteristics to assess the quality of the study. USA claims data from 2000 and 2010 through 2016 are included in this model, along with Taiwan claims data from 2016.

Cause/Impairment Name	Measure	Total sources	Countries with data
Alopecia areata	All measures	55	16
Alopecia areata	Prevalence	38	16
Alopecia areata	Incidence	2	1
Alopecia areata	Proportion	15	1

Table 1: MR-BRT Crosswalk Adjustment Factors for alopecia areata

Data input	Reference or alternative case definition	Gamma	Beta Coefficient, Logit (95% CI)	Adjustment factor
Literature with physical exam and claims	Reference	0.12	---	---
USA Marketscan 2000	Alternative		-0.70 (-0.95 to -0.44)	0.33

Severity splits & disability weights

The table below illustrates the sequelae, severity level, lay description, and disability weights associated with Alopecia areata.

Table 2. Severity distribution, details on the severity levels for alopecia in GBD 2019 and the associated disability weight (DW) with that severity.

Sequela	Severity level	Lay description	DW (95% CI)
Mild alopecia areata	Disfigurement, level 1	The individual has a slight, visible physical deformity that others notice, which causes some worry and discomfort.	0.011 (0.005–0.021)

Severe alopecia areata	Disfigurement, level 2	The individual has a visible physical deformity that causes others to stare and comment. As a result, the person is worried and has trouble sleeping and concentrating.	0.067 (0.044–0.096)

Modelling strategy

DisMod-MR 2.1 was used to estimate prevalence by age, sex, year, and country for alopecia areata. We assumed zero excess mortality and remission priors implying a minimum duration of seven months. This was in line with the available epidemiological data, expert opinion, and previous GBD work. We used a time window of 20 years to determine which data points were used for a particular year of fit.

In GBD 2019, we replaced our within-DisMod crosswalks with crosswalked completed using the MR-BRT modeling tool. We adjusted USA marketscan 2000 data toward the level of other prevalence data points, which were more representative of the general population. To improve estimation across all regions, the minimum global coefficient of variation was set at 0.1. In addition, significant sex differences were observed in the USA claims data, resulting in a higher prevalence in females compared to males, likely due to more females seeking health consultations for alopecia areata compared to males. To minimise this effect, we set the sex covariate to zero, but this had minimal impact on the global estimates.

Pruritus

Flowchart

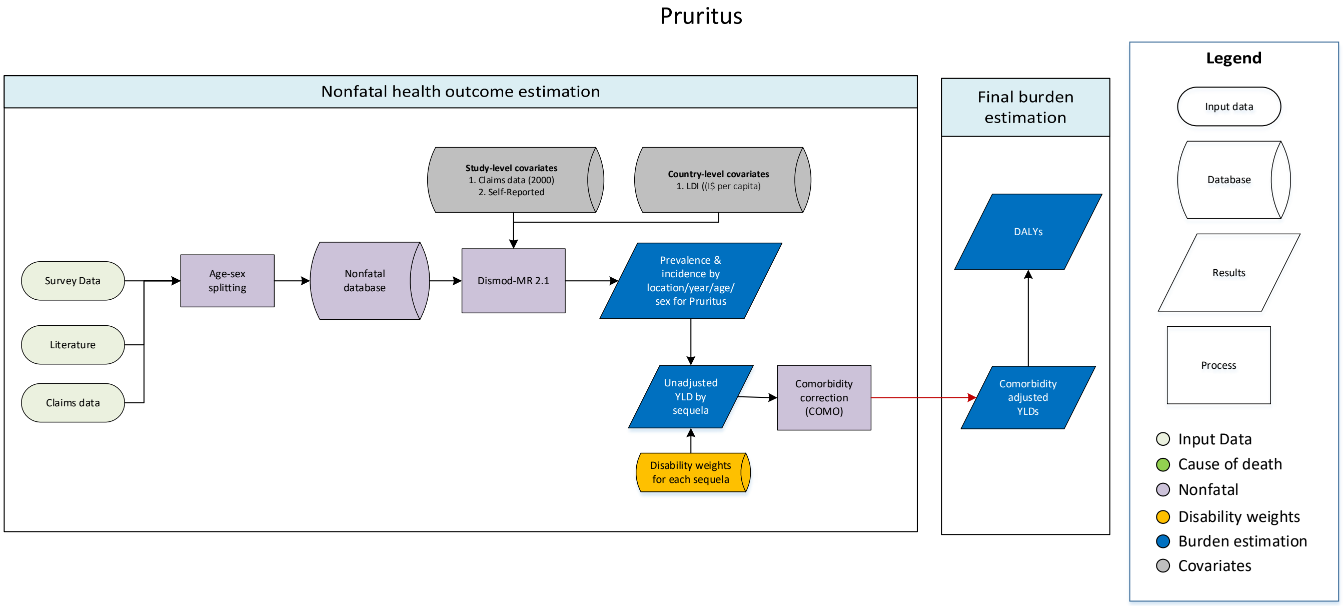

Case definition

Pruritus was included in the GBD 2019 cause group of skin and subcutaneous conditions. Pruritus (or itching) can be a symptom of a condition or disease. Included in the GBD disease modelling were cases meeting ICD-10 diagnostic criteria for pruritus (ICD-10: L29).

Input data

Model inputs

In the GBD 2010 study, a systematic review of the literature was conducted using PubMed and Google Scholar to capture epidemiological data for pruritus. The inclusion criteria stipulated that studies (1) must be published between 1980 and 2012; (2) must provide data on the incidence or prevalence of pruritus; (3) must use samples representative of the general population (ie, samples derived from the experimental arm of clinical trials or based in dermatology clinics were excluded); (4) must use a sample size larger than 100; and (5) must provide sufficient information on study method and sample characteristics to assess the quality of the study. For GBD 2016, the GBD 2010 search strategy was replicated in PubMed to capture epidemiological studies published between 2013 and 2016. Additionally, USA claims data from 2000 and 2010 through 2016 were included.

Cause/Impairment Name	Measure	Total sources	Countries with data
Pruritus	All measures	37	16
Pruritus	Prevalence	37	16

Table 1: MR-BRT Crosswalk Adjustment Factors for pruritus

Data input	Reference or alternative case definition	Gamma	Beta Coefficient, Logit (95% CI)	Adjustment factor
Literature with physical exam and claims	Reference		---	---
No Physical Exam	Alternative	1.46	1.55 (-1.65 to 4.76)	0.83
USA Marketscan 2000	Alternative		-0.74 (-4.80 to 3.31)	0.32

Severity splits

The table below illustrates the severity level, lay description, and disability weight for pruritus.

Table 2. Severity distribution, details on the severity levels for pruritus in GBD 2019 and the associated disability weight (DW) with that severity.

Sequela	Severity level	Lay description	DW (95% CI)
Pruritus	Disfigurement, level 1	The individual has a slight, visible physical deformity that others notice, which causes some worry and discomfort.	0.011 (0.005–0.021)

Modelling strategy

DisMod-MR 2.1 was used to estimate prevalence by age, sex, year, and country for pruritus.

Per expert advice, remission was set from 0.2 to 1, implying a duration of three months to one years. We used a time window of 25 years to determine which data points were used for a particular year of fit.
In GBD 2019, we replaced our within-DisMod crosswalks with crosswalked completed using the MR-BRT modeling tool. We adjusted USA marketscan 2000 data, along with data that were not based on physical exams toward the level of other prevalence data points, which were more representative of the general population. A country-level covariate, log transformed lagged distributed income (I$ per capita), which represents a moving average of gross

domestic product (GDP) over time, was also included to inform prevalence estimates. Additionally, the data in this model were extremely heterogeneous. Therefore, the random effects were constrained to (-0.2, 0.2).

The table below indicates the covariates, parameters, and exponentiated beta values used in GBD 2019.

Table 3. Covariates. Summary of covariates used in the pruritus DisMod-MR meta-regression model

Covariate	Type	Parameter	Exponentiated beta (95% Uncertainty Interval)
LDI (I$ per capita)	Country-level	Prevalence	1.10 (0.91 to 1.26)

Urticaria

Flowchart

Urticaria

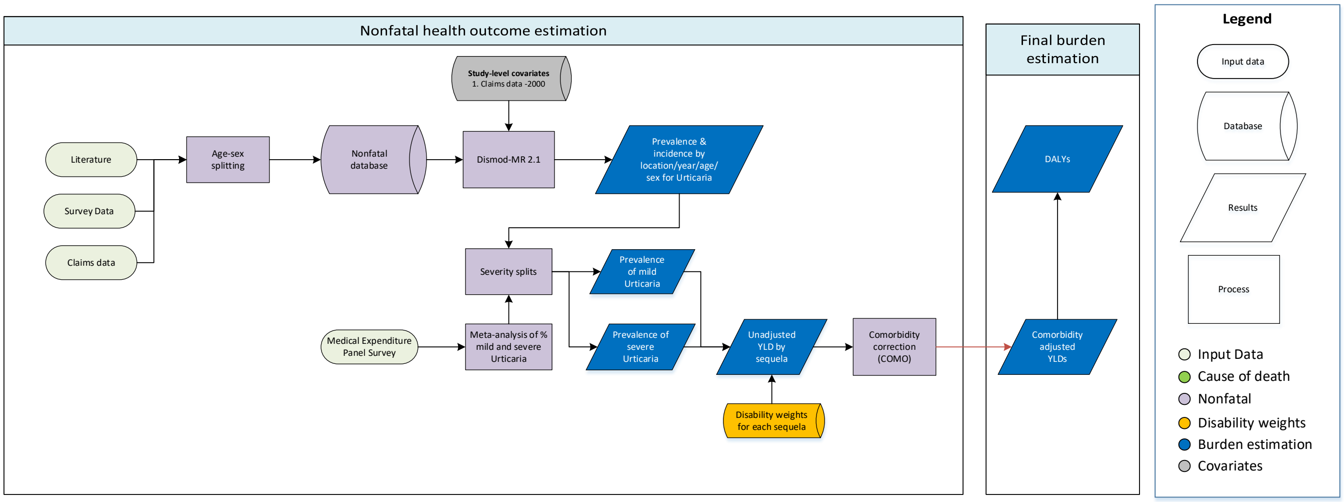

Case definition

Urticaria was included in the GBD 2019 cause group of skin and subcutaneous conditions. Urticaria (hives) refers to a skin reaction that causes itchy, raised bumps. Included in the GBD disease modelling were cases meeting ICD-10 diagnostic criteria for urticaria (ICD-10: L50).

Input data

Model inputs

In the GBD 2010 study, a systematic review of the literature was conducted using PubMed and Google Scholar to capture epidemiological data for urticaria. The inclusion criteria stipulated that studies (1) must be published between 1980 and 2012; (2) must provide data on the incidence or prevalence of urticaria; (3) must use samples representative of the general population (ie, samples derived from the experimental arm of clinical trials or based in dermatology clinics were excluded); (4) must use a sample size larger than 100; and (5) must provide sufficient information on study method and sample characteristics to assess the quality of the study.

For GBD 2016, the GBD 2010 search strategy was replicated to capture epidemiological studies published between 2013 and 2016. Additionally, USA claims data from 2000 and 2010 through 2014 were included in the data used for GBD 2017.

The table below illustrates the data inputs used in GBD 2019 by number of studies, geographic location, and prevalence/incidence.

Cause/Impairment Name	Measure	Total sources	Countries with data
Urticaria	All measures	60	23
Urticaria	Prevalence	45	23
Urticaria	Incidence	1	1
Urticaria	Proportion	15	1

Table 1: MR-BRT Crosswalk Adjustment Factors for urticaria

Data input	Reference or alternative case definition	Gamma	Beta Coefficient, Logit (95% CI)	Adjustment factor
Literature with physical exam and claims	Reference	1.46	---	---
No Physical Exam	Alternative		1.55 (-1.65 to 4.76)	0.83
USA Marketscan 2000	Alternative		-0.74 (-4.80 to 3.31)	0.32

Severity splits & disability weights

The table below illustrates the severity level, lay description, and disability weight for urticaria.

Table 2. Severity distribution, details on the severity levels for urticarial in GBD 2019 and the associated disability weight (DW) with that severity.

Sequela	Severity level	Lay description	DW (95% CI)
Mild urticaria	Disfigurement, level 1 with itch/pain	The person has a slight, visible physical deformity that is sometimes sore or itchy. Others notice the deformity, which causes some worry and discomfort.	0.027 (0.015–0.042)
Severe urticaria	Disfigurement, level 2, with itch/pain	The person has a visible physical deformity that is sore and itchy. Other people stare and comment, which causes the person to worry. The person has trouble	0.188 (0.124–0.267)

		sleeping and concentrating.	

Modelling strategy

DisMod-MR 2.1 was used to estimate prevalence by age, sex, year, and country for urticaria.

The available data were mainly composed of prevalence estimates with a few incidence data points. For GBD 2017, we made both prevalence and incidence estimates. We used a time window set to 25 years. We set excess mortality to zero and remission between 0.5 to 2, implying a duration between ½ and ⅔ years. In addition, location random effects were constrained to (-0.3, 0.3). In GBD 2019, we replaced our within-DisMod crosswalks with crosswalked completed using the MR-BRT modeling tool. We adjusted USA marketscan 2000 data, along with data that were not based on physical exams toward the level of other prevalence data points, which were more representative of the general population. Specific data points were outliered if they were overestimates or underestimates in comparison to country, regional, and global patterns.

Decubitus ulcer

Flowchart

Decubitus Ulcer

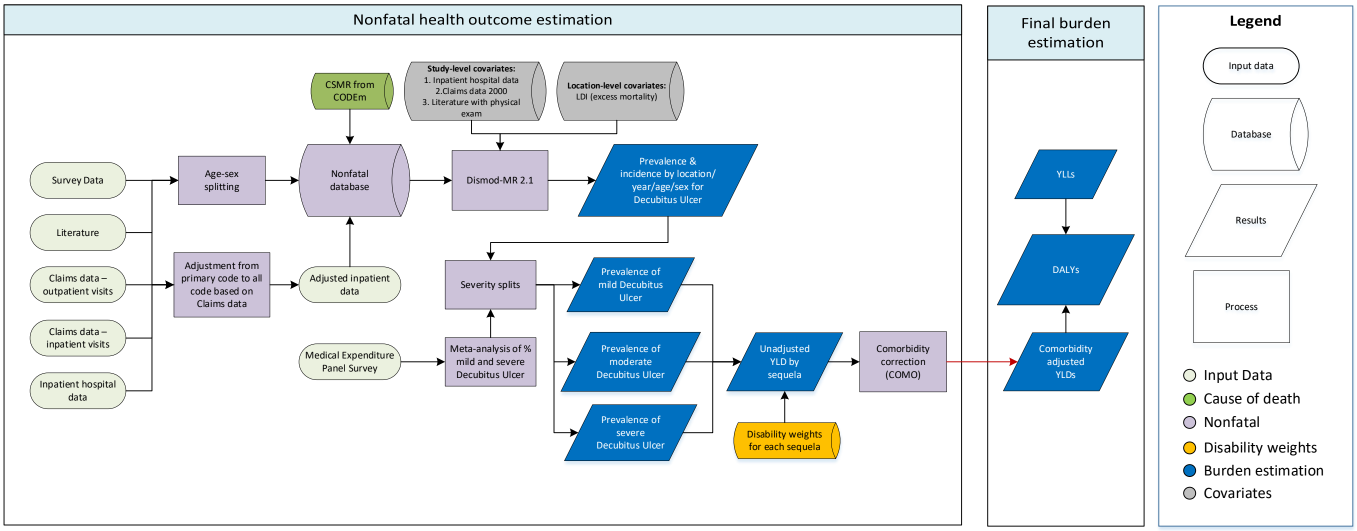

Case definition

Decubitus ulcer was included in the GBD 2019 cause group of skin and subcutaneous conditions. Decubitus ulcer, also known as pressure ulcer/sore, is an injury to the skin and underlying tissue resulting from an obstruction of blood flow due to pressure on the skin. Included in the GBD modelling were cases meeting ICD-10 criteria for decubitus ulcer (ICD-10: L89) (1).

Input data

Model inputs

In the GBD 2010 study, a systematic review of the literature was conducted using PubMed and Google Scholar to capture epidemiological data for decubitus ulcer. The inclusion criteria stipulated that studies (1) must be published between 1980 and 2012; (2) must provide data on the incidence or prevalence of decubitus ulcer; (3) must use samples representative of the general population (ie, samples derived from the experimental arm of clinical trials or based in dermatology clinics were excluded); (4) must use a sample size larger than 100; and (5) must provide sufficient information on study method and sample characteristics to assess the quality of the study. The data from literature were sparse but contained both prevalence and incidence estimates. For GBD 2013, the GBD 2010 search strategy was replicated to capture epidemiological studies published between 2012 and 2013. The available data were from high-income countries. Hospital inpatient, USA claims data from 2000 and 2010 through 2016, Taiwan claims data for 2016, and Poland claims data for 2015-2017 were also used for GBD 2019.

The final dataset also included cause-specific mortality rates for decubitus ulcer estimated by CODEm. Data were outliered or excluded if we found them unreasonable when compared to regional, super-regional, and global rates.

Cause/Impairment Name	Measure	Total sources	Countries with data
Decubitus ulcer	All measures	310	43
Decubitus ulcer	Incidence	295	43
Decubitus ulcer	Proportion	15	1

Table 1: MR-BRT Crosswalk Adjustment Factors for decubitus ulcer

Data input	Reference or alternative case definition	Gamma	Beta Coefficient, Logit (95% CI)	Adjustment factor
Literature with physical exam	Reference	0.11	---	---
USA Marketscan 2000	Alternative		-0.28 (-0.53 to -0.04)	0.43
Inpatient data	Alternative		-0.09 (-0.31 to 0.13)	0.48

Severity splits

In line with GBD 2017, decubitus ulcer was assigned the disability weight, disfigurement with itch/pain, levels 1, 2, and 3.

Table 2. Severity distribution, details on the severity levels for decubitus ulcer in GBD 2019 and the associated disability weight (DW) with that severity.

Sequela	Severity level	Lay description	DW (95% CI)
Mild decubitus ulcer	Disfigurement, level 1 with itch/pain	The person has a slight, visible physical deformity that is sometimes sore or itchy. Others notice the deformity, which causes some worry and discomfort.	0.027 (0.015–0.042)

Moderate decubitus ulcer	Disfigurement, level 2, with itch/pain	The person has a visible physical deformity that is sore and itchy. Other people stare and comment, which causes the person to worry. The person has trouble sleeping and concentrating.	0.188 (0.124–0.267)
Severe decubitus ulcer	Disfigurement, level 3, with itch/pain	The person has an obvious physical deformity that is very painful and itchy. The physical deformity makes others uncomfortable, which causes the person to avoid social contact, feel worried, sleep poorly, and think about suicide.	0.576 (0.401–0.731)

Modelling strategy

DisMod-MR 2.1 was used to estimate prevalence by age, sex, year, and geography (subnational, country, region, super-region) for decubitus ulcer. Per expert advice, remission was set from 3 to 4, implying a duration of three to four months. This was based on the assumption that remission does not change with treatment. These values were also in line with the available epidemiological data, expert opinion, and previous GBD work. The decubitus ulcer dataset was sufficiently large to make use of a relatively short time window of five years to determine which data points were used for a particular year of fit.

In GBD 2019, we replaced our within-DisMod crosswalks with crosswalked completed using the MR-BRT modeling tool. We adjusted inpatient data, along with USA marketscan data 2000 and inpatient data toward the level of other incidence data points, which were more representative of the general population. In addition, log-transformed lagged distributed income (LDI) was used as a country-level covariate to guide estimates for locations with few or no data. LDI was restricted to a range of -0.5 to -0.1. We restricted location random effects to (-0.5, 0.5) across all 7 GBD super-regions.

In previous rounds, priors on excess mortality rate (EMR) were estimated in DisMod by matching prevalence data points with their corresponding CSMR values within the same age, sex, year, location (by dividing CSMR by prevalence). For short duration conditions (remission>1), the corresponding prevalence was derived by running an initial model and then applying the same CSMR/prevalence method. However, for many causes, DisMod estimated a rather unrealistic pattern of EMR compared to an expected pattern of decreasing EMR with greater access to quality health care. Such unexpected patterns often signal inconsistencies between CSMR estimates and the measures of prevalence and/or incidence. In effort to provide greater guidance to DisMod on the expected pattern of EMR, EMR data generated in the previous round were modeled using the MR-BRT approach by age and sex with a prior on healthcare access and quality index (HAQi) having a negative coefficient. Results from MR-BRT were then predicted for each location year, sex and for ages 0, 10, 20 ….100.

The table below indicates the covariates, parameters, and exponentiated beta values used in GBD 2019.

Table 3. Covariates. Summary of covariates used in the cellulitis DisMod-MR meta-regression model

Covariate	Type	Parameter	Exponentiated beta (95% Uncertainty Interval)
LDI (I$ per capita)	Country-level	Excess Mortality Rate	0.61 (0.61 to 0.61)

Other skin and subcutaneous diseases

Flowchart

Other Skin and Subcutaneous Diseases (OSSD)

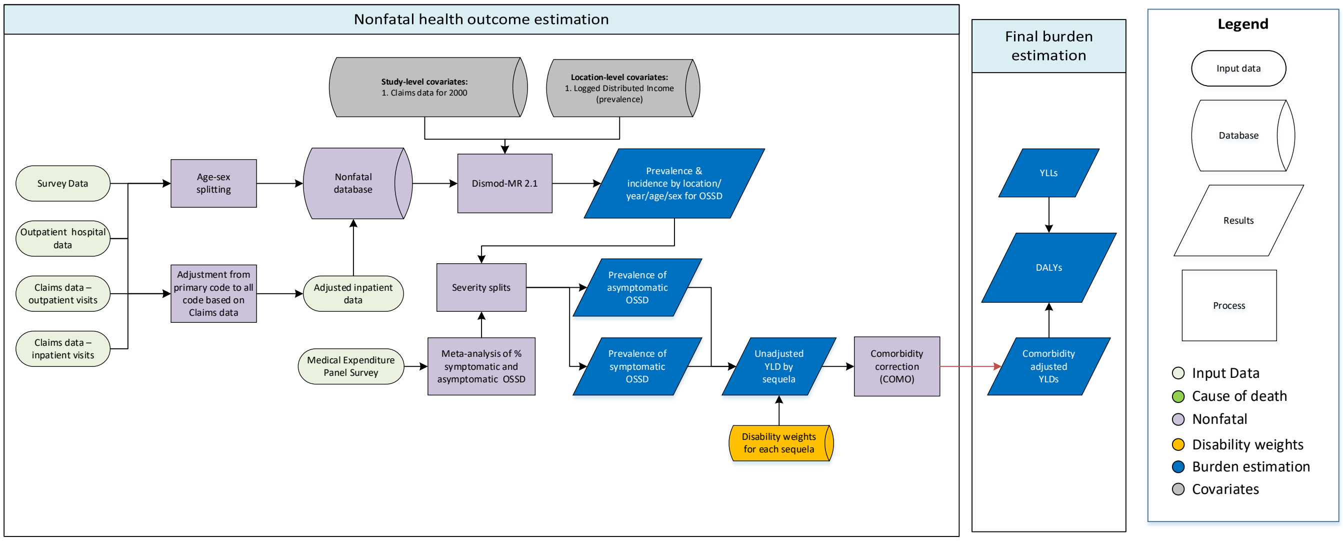

Case definition

The other skin and subcutaneous diseases category encompassed a large group of skin conditions not captured in the other skin categories. We included cases meeting the following ICD-10 diagnostic criteria: other viral infections characterized by skin and mucous membrane lesions, not elsewhere classified (B08), unspecified viral infection characterized by skin and mucous membrane lesions (B09), pediculosis and phthiriasis (B85), myiasis (B87), other infestations (B88), sarcoidosis of skin (D86.3), porphyria cutanea tarda (E80.1), other and unspecified porphyria (E80.2), pemphigus (L10), other acantholytic disorders (L11), pemphigoid (L12), other bullous disorders (L13), bullous disorders in diseases classified elsewhere (L14), lichen simplex chronicus and prurigo (L28), pityriasis rosea (L42), lichen planus (L43), other papulosquamous disorders (L44), papulosquamous disorders in diseases classified elsewhere (L45), exfoliation due to erythematous conditions according to extent of body surface involved (L49), erythema multiforme (L51), erythema nodosum (L52), other erythematous conditions (L53), erythema in diseases classified elsewhere (L54), other acute skin changes due to ultraviolet radiation (L56), skin changes due to chronic exposure to nonionising radiation (L57), other disorders of skin and subcutaneous tissue related to radiation (L59), nail disorders (L60), nail disorders in diseases classified elsewhere (L62), androgenic alopecia (L64), other nonscarring hair loss (L65), cicatricial alopecia [scarring hair loss] (L66), hair color and hair shaft abnormalities (L67), hypertrichosis (L68), rosacea (L71), follicular cysts of skin and subcutaneous tissue (L72), other follicular disorders (L73), eccrine sweat disorders (L74), apocrine sweat disorders (L75), vitiligo (L80), other disorders of pigmentation (L81), seborrhoeic keratosis (L82), acanthosis nigricans (L83), corns and callosities (L84), other epidermal thickening (L85), keratoderma in diseases classified elsewhere (L86), transepidermal elimination disorders (L87), atrophic disorders of skin (L90), hypertrophic disorders of skin (L91),

granulomatous disorders of skin and subcutaneous tissue (L92), other localised connective tissue disorders (L94), vasculitis limited to skin, not elsewhere classified (L95), and other disorders of skin and subcutaneous tissue in diseases classified elsewhere (L99).

Input data

Model inputs

In the GBD 2010 study, a systematic review of the literature was conducted using PubMed and Google Scholar to capture epidemiological data for skin diseases not captured in the other skin categories. The inclusion criteria stipulated that studies (1) must be published between 1980 and 2012; (2) must provide data on the incidence or prevalence; (3) must use samples representative of the general population (ie, samples derived from the experimental arm of clinical trials or based in dermatology clinics were excluded); (4) must use a sample size larger than 100; and (5) must provide sufficient information on study method and sample characteristics to assess the quality of the study. For GBD 2013, the GBD 2010 search strategy was replicated to capture epidemiological studies published between 2012 and 2013. Data from USA claims for 2000 and 2010 through 2016 by US state and Taiwan claims data for 2016 were included in GBD 2019 as well. Data were outliered or excluded if we found them unreasonable when compared to regional, super-regional, and global rates.

Cause/Impairment Name	Measure	Total sources	Countries with data
Other skin and subcutaneous diseases	All measures	35	3
Other skin and subcutaneous diseases	Prevalence	20	3
Other skin and subcutaneous diseases	Proportion	15	1

Table 1: MR-BRT Crosswalk Adjustment Factors for other skin

Data input	Reference or alternative case definition	Gamma	Beta Coefficient, Logit (95% CI)	Adjustment factor
USA Marketscan 2010-2016, outpatient data	Reference	0.32	---	---
USA Marketscan 2000	Alternative		-0.23 (-1.11 to 0.65)	0.44

Severity split & disability weight

Skin and other subcutaneous diseases were assigned the disability weight for disfigurement level 1.

Table 2. Severity distribution, details on the severity levels for other skin in GBD 2019 and the associated disability weight (DW) with that severity.

Sequela	Severity level	Lay description	DW (95% CI)

Asymptomatic other skin and subcutaneous diseases	Asymptomatic		0
Symptomatic other skin and subcutaneous diseases	Disfigurement, level 1	The person has a slight, visible physical deformity that others notice, which causes some worry and discomfort.	0.011 (0.005–0.021)

Modelling strategy

DisMod-MR 2.1, a Bayesian meta-regression tool, was used to estimate prevalence by age, sex, year, and geography (subnational, country, region, super-region) for skin and other subcutaneous diseases.

We assumed remission of one, implying a duration of 12 months. Similar to GBD 2017, we used a time window of 25 years to determine which data points were used for a particular year of fit.

In GBD 2019, we replaced our within-DisMod crosswalks with crosswalked completed using the MR-BRT modeling tool. We adjusted USA marketscan 2000 data toward the level of other prevalence data points, which were more representative of the general population. In addition, log-transformed lagged distributed income (LDI) was used as a country-level covariate to guide estimates for locations with few or no data.

Table 3. Covariates. Summary of covariates used in the other skin DisMod-MR meta-regression model

Covariate	Type	Parameter	Exponentiated beta (95% Uncertainty Interval)
LDI (I$ per capita)	Country-level	Prevalence	1.10 (0.93 to 1.23)

Other sense organ diseases

Flowchart

Other sense organ diseases

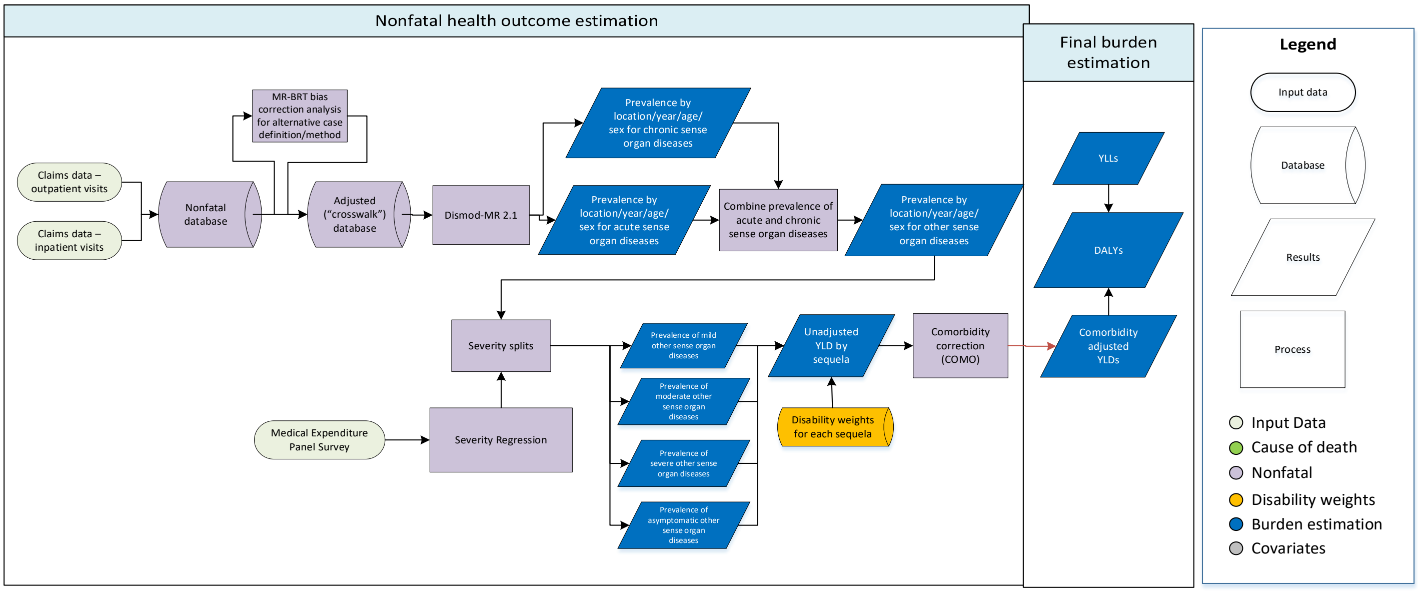

Case definition

Other sense organ disease is a residual cause capturing both acute and chronic conditions that do not map to other causes, but lead to non-trivial morbidity. These include the following ICD-9 codes: 077, 360, 364, 370-77, 379, 380, 386, and 388, which encompass a plethora of eye and ear disorders and conditions.

077	Other diseases of conjunctiva due to viruses and chlamydiae
360	Disorders of the globe
364	Disorders of iris and ciliary body
370-77	Keratitis, corneal opacity and other disorders of cornea, disorders of conjunctiva, inflammation of eyelids, other disorders of eyelids, disorders of the lacrimal system, disorders of the orbit, disorders of optic nerve and visual pathways
379	Other disorders of eye
380	Disorders of external ear
386	Vertiginous syndromes and other disorders of vestibular system
388	Other disorders of ear

Input data

Model inputs

For GBD 2019, we used claims data from the US and Poland to model other sense organ diseases, since these conditions would not appear in inpatient hospital data. ICD-9 codes were assigned at the five-digit level to either acute or chronic conditions as listed elsewhere in the appendix table of all ICD codes.

In GBD 2013, other sense organ disease estimates were based on MEPS self-reported claims data. We transitioned to use of claims data (U.S. Marketscan) in GBD 2015-2019, as this more accurately captures the occurrence of these conditions, which is reflected in higher estimates in GBD 2015 than 2013. In GBD 2016-2019 we began to separately model acute and chronic conditions. In GBD 2019, we added more years of U.S. Marketscan data and a new data source, Poland claims data.

The total source count used in GBD 2019 modeling is listed in the table below:

Measure	Total sources	Countries with data
All measures	44	4
Prevalence	29	4
Incidence	29	4
Proportion	15	1

Modelling strategy

For GBD 2019, data were extracted separately for the chronic and acute conditions included in other sense organ diseases. Chronic data were extracted as prevalence, and acute data as incidence. We then ran two separate DisMod-MR 2.1 models. The chronic model, with prevalence data, was run as a prevalence-only model. The acute model was run as a full model with incidence data, assuming zero excess mortality and duration of one week (remission = 52). In both models, to correct for systematically lower data from 2000 MarketScan claims, we used a study-level covariate to crosswalk the 2000 data. Since the only data sources are from the United States and Poland, we did not use any country-level covariates in this model.

MR-BRT Crosswalk Adjustment Factors for acute other sense organ diseases

Data input	Reference or alternative case definition	Gamma	Beta Coefficient, Log/Logit (95% CI)	Adjustment factor*
U.S. Marketscan (2010 onward)	Reference	0.18	---	---
U.S. Marketscan 2000	Alternative		-0.42 (-0.78 - -0.07)	0.40

**Adjustment factor is the transformed Beta coefficient in normal space, and can be interpreted as the factor by which the alternative case definition is adjusted to reflect what it would have been if measured as the reference.*

MR-BRT Crosswalk Adjustment Factors for chronic other sense organ diseases

Data input	Reference or alternative case definition	Gamma	Beta Coefficient, Logit (95% CI)	Adjustment factor*
U.S. Marketscan (2010 onward)	Reference	0.18	---	---
U.S. Marketscan 2000	Alternative		-0.54 (-0.90 - -0.19)	0.37

Adjustment factor is the transformed Beta coefficient in normal space, and can be interpreted as the factor by which the alternative case definition is adjusted to reflect what it would have been if measured as the reference.

We then aggregated chronic and acute prevalence outputs, resulting in the prevalence of other sense organ diseases by country, age, year, and sex.

Severity splits & disability weights

The basis of the GBD disability weight survey assessments are lay descriptions of sequelae highlighting major functional consequences and symptoms. Severity splits for other sense organ diseases were calculated via the Medical Expenditure Panel Survey (MEPS) regression and outlined in the table below.

Severity distributions are listed in the table below, and provide details on the severity levels for other sense organ diseases in GBD 2019 and the associated disability weight (DW) with that severity.

<u>Chronic:</u>

Severity	Proportion	Health state	Disability weight
Severe (vertigo)	0.21 (0.15–0.28)	Has short spells of dizziness and loss of balance; between spells the person is worried the spells will occur again	0.113 (0.078–0.159)
Mild (disfigurement)	0.37 (0.30–0.42)	This person has slight physical deformity which causes some worry and discomfort	0.011 (0.005–0.021)
Asymptomatic	0.42 (0.41–0.44)	Asymptomatic	N/A

<u>Acute</u>

Severity	Proportion	Health state	Disability weight
Mild (mild infectious disease)	0.30 (0.23–0.37)	This person has low fever and mild discomfort but no difficulty with daily activities	0.006 (0.002–0.012)
Moderate (moderate infectious disease)	0.25 (0.18–0.32)	Has a fever and aches, and feels weak, which causes some difficulty with daily activities	0.05 (0.033–0.073)
Asymptomatic	0.45 (0.43–0.46)	Asymptomatic	N/A

Rheumatoid Arthritis

Flowchart

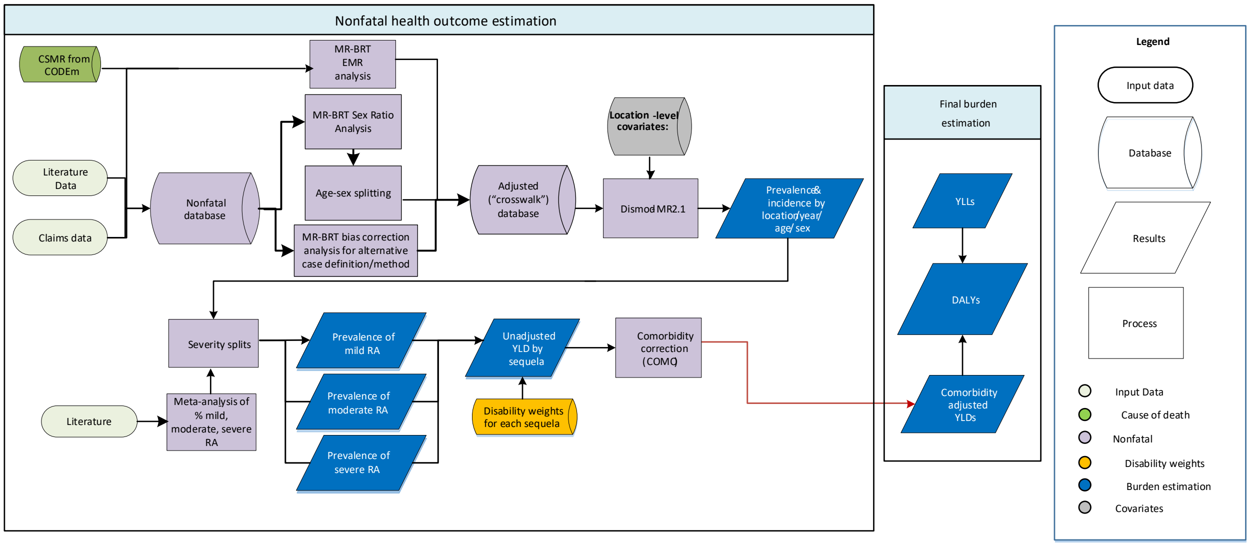

Input Data and Methodological Summary for Rheumatoid Arthritis

Case definition

Rheumatoid arthritis (RA) is a systemic autoimmune disorder that causes pain, swelling, and deformation of the joints and may be accompanied by systemic symptoms. While RA is known to affect internal organs in addition to the joints, these extra-articular effects are currently not quantified in GBD. The reference case definition for rheumatoid arthritis is based on the 1987 criteria by the American College of Rheumatology (ACR 1987) which stipulate seven diagnostic criteria, of which four need to be satisfied for a diagnosis and the first 4 of which need to have been present for at least six weeks:

1. Morning stiffness
2. Arthritis of 3 or more joint areas
3. Arthritis of hand joints
4. Symmetric arthritis
5. Rheumatoid nodules
6. Serum rheumatoid factor
7. Radiographic changes

For RA, ICD-10 codes are M05, M06, and M08, and ICD-9 codes are 714.0–714.9.

Input data

For GBD 2010, a systematic review of the prevalence of RA throughout the world was conducted. Ovid MEDLINE, EMBASE, CINAHL, CAB abstracts, WHOLIS, and SIGLE databases were searched using the

following search terms: (rheumatoid arthritis OR rheumatic disease* OR rheumatism) AND (prevalen* OR inciden* OR cross-sectional OR cross sectional OR epidemiol* OR survey OR population-based OR population based OR population study OR population sample OR cohort OR follow-up OR follow up OR longitudinal OR regist* OR data collection). Opportunistically, we added scientific literatures and population surveys encountered for GBD 2015 and GBD 2016. The most recent PubMed search was conducted in GBD 2017 using the following search terms: ("Arthritis, Rheumatoid"[Mesh] AND ("Prevalence"[Mesh] OR "Incidence"[Mesh])) NOT (Meta-Analysis[ptyp] OR Letter[ptyp] OR Editorial[ptyp] OR Case Reports[ptyp] OR Review[ptyp] OR Controlled Clinical Trial[ptyp]) AND ("2013/01/01"[PDAT] : "2018/1/10"[PDAT]. An age and sex split were applied to extracted data.

The exclusion criteria were:

1. Studies clearly not representative of the national population
2. Studies that were not population-based, eg, hospital or clinic-based studies
3. Studies that did not provide primary data on epidemiological parameters, eg, a commentary piece
4. Studies of a specific type of RA, eg, seropositive RA
5. Studies with a sample size of less than 150
6. Reviews

Opportunistically, additional studies encountered during data review were added for GBD 2019. In addition, data from USA claims data for 2000, 2010–2012, and 2014-2016 by state and Taiwan claims for 2016 were included. We decided not to use hospital inpatient data as we considered they would not be representative of true prevalence and that variation between countries in the proportion of true prevalent cases captured in hospital inpatient data systems would likely vary more than can be captured by a single crosswalk. We compared the rates of RA in the outpatient data from Norway, Sweden, Canada, and the USA and found implausibly large differences with the rates from the claims data. The USA outpatient rates were half the value of the claims data and those for the other countries much lower still. For those reasons we decided not to use the outpatient data.

Table 1: Data Inputs for Rheumatoid Arthritis

Measure	Total sources	Countries with data
All measures	123	45
Prevalence	92	42
Incidence	25	14
Relative risk	1	1
Standardized mortality ratio	12	5
With-condition mortality rate	3	3

Age and sex splitting

Reported estimates of prevalence were split by age and sex where possible. First, if studies reported prevalence for broad age groups by sex (eg, prevalence in 15- to 65-year-old males and females separately), and also by specific age groups for both sexes combined (eg, prevalence in 15- to 30-year-olds, then in 31- to 65-year-olds, for males and females combined), age-specific estimates were split by

sex using the reported sex ratio and bounds of uncertainty. Second, prevalence data for both sexes that could not be split using a within-study ratio were split using a sex ratio derived from a meta-analysis of existing sex-specific data using MR-BRT. The female to male ratio was 2.60 (2.58 to 2.62). Finally, after the application of bias adjustments, where studies reported estimates across age groups spanning 25 years or more, these were split into five-year age groups using the prevalence age pattern estimated by DisMod-MR 2.1 in GBD 2017.

Data adjustment

We used a single study covariate for studies using diagnostic criteria that did not match our reference case definition based on ACR 1987 criteria. We added an additional covariate for claims data in the USA from the year 2000. We treat claims data from the USA from 2010 onward and Taiwan as reference case definition data; rarely would cases of RA not intersect with the health system in the USA and Taiwan. Betas and exponentiated values (which can be interpreted as an odds ratio) for these two covariates are shown in the table below:

Table 2: MR-BRT Crosswalk Adjustment Factors for Rheumatoid Arthritis

Data input	Reference or alternative case definition	Gamma	Beta Coefficient, Log (95% CI)	Adjustment factor*
ACR 1987	Ref	0.38	---	---
RA criteria other than RA 1987	Alt		0.13 (-0.14 to 0.41)	1.14 (0.87 to 1.50)
USA claims data – 2000	Alt		0.54 (-0.25 to 1.34)	1.72 (0.78 to 3.83)

**Adjustment factor is the transformed Beta coefficient in normal space, and can be interpreted as the factor by which the alternative case definition is adjusted to reflect what it would have been if measured as the reference.*

After adjusting data for case definition, we outliered data that with a median absolute deviation of 2 or more above the mean to cull data that were implausibly high.

Modeling strategy

Prior settings in the DisMod model included setting remission to 0 – 0.02 for ages up to 65 and 0 – 0.05 for ages 65+. It was assumed that there was no incidence or prevalence of RA before the age of 5 years. These settings were retained for GBD 2019. We continued to include the Mean BMI country covariate with bounds set at 0 and 1 and increased the coefficient of variation from 0.4 at the Global, Super Region, and Region priors to 0.8 to allow the model to better follow the data. The time window for fit was increased from 10 to 25 years to optimize temporal smoothing.

In previous rounds, priors on excess mortality rate (EMR) were estimated in DisMod by matching prevalence data points with their corresponding CSMR values within the same age, sex, year, location (by dividing CSMR by prevalence). For short duration conditions (remission>1), the corresponding prevalence was derived by running an initial model and then applying the same CSMR/prevalence method. However, for many causes, DisMod estimated a rather unrealistic pattern of EMR compared to an expected pattern of decreasing EMR with greater access to quality health care. Such unexpected patterns often signal inconsistencies between CSMR estimates and the measures of prevalence and/or

incidence. In effort to provide greater guidance to DisMod on the expected pattern of EMR, EMR data generated in the previous round were modeled using the MR-BRT approach by age and sex with a prior on healthcare access and quality index (HAQi) having a negative coefficient. Results from MR-BRT were then predicted for each location year, sex and for ages 0, 10, 20 ….100. We included HAQi as a country-level covariate to inform EMR with a mean and standard deviation produced from MR-BRT. However, even without this setting DisMod would tend to estimate a coefficient that was consistent with the MR-BRT analysis.

Table 3. Covariates. Summary of covariates used in the rheumatoid arthritis DisMod-MR meta-regression model

Covariate	Type	Parameter	Exponentiated beta (95% Uncertainty Interval)
Healthcare access and quality index	Country-level	Excess mortality rate	0.98 (0.98 to 0.98)
Mean BMI	Country-level	Prevalence	1.12 (1.11 to 1.13)

Severity and Disability

The basis of the GBD disability weight survey assessments are lay descriptions of health states highlighting major functional consequences and symptoms. The lay descriptions and disability weights for RA severity levels are shown below.

Table 4. Severity distribution, details on the severity levels for rheumatoid arthritis in GBD 2019 and the associated disability weight (DW) with that severity.

Severity level	Lay description	DW (95% CI)
Mild	This person has moderate pain and stiffness in the arms and hands which causes difficulty lifting, carrying, and holding things, and trouble sleeping because of the pain.	0.117 (0.080–0.163)
Moderate	This person has pain and deformity in most joints, causing difficulty moving around, getting up and down, and using the hands for lifting and carrying. The person often feels fatigue.	0.317 (0.216–0.440)
Severe	This person has severe, constant pain, and deformity in most joints, causing difficulty moving around, getting up and down, eating, dressing, lifting, carrying, and using the hands. The person often feels sadness, anxiety, and extreme fatigue.	0.581 (0.403–0.739)

To determine the proportion of people with RA within each of the severity levels, seven studies from three regions provided information on the severity of RA. Severity was classified according to Health Assessment Questionnaire scores, with the cutoff for each severity level: <1 mild; 1-1.875 moderate; and ≥2 severe. Estimates were across studies. We used a random effects meta-analysis model. The pooled percentages were mild 48.8% (37.9 – 59.6), moderate 37.6% (29.3 – 46.2), and severe 12.2% (7.8

– 17.4). After streaming out 1,000 draws assuming a binomial distribution, percentages were scaled to sum to 1 at each draw.

Figure 1. Severity distribution meta-analysis, details on the studies included in the meta-analysis calculating the proportion of mild RA.

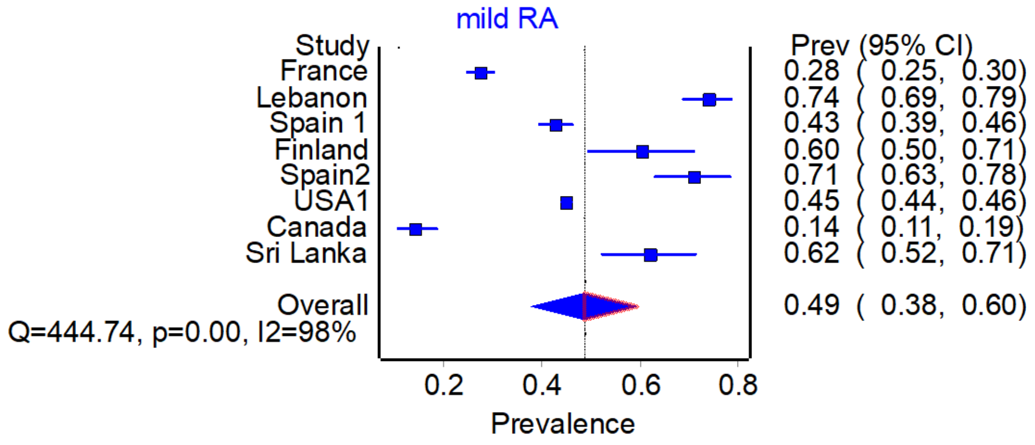

Figure 2. Severity distribution meta-analysis, details on the studies included in the meta-analysis calculating the proportion of moderate RA.

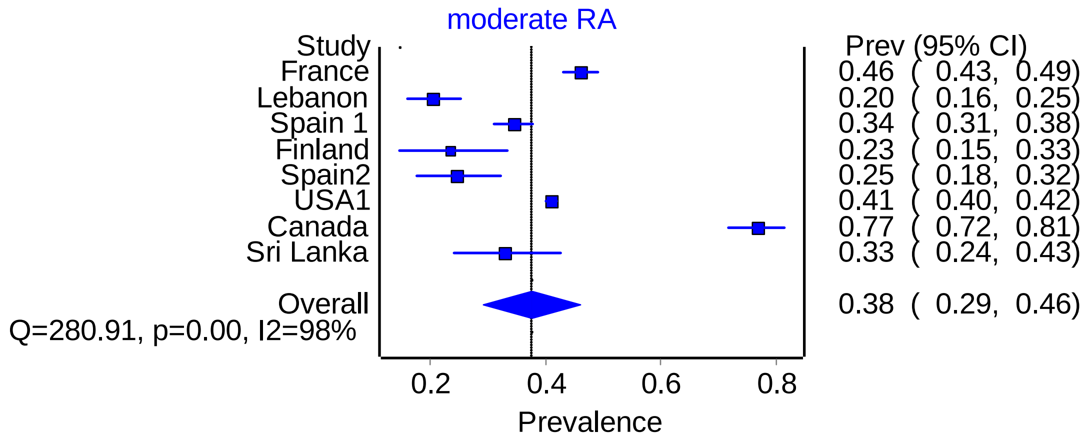

Figure 3. Severity distribution meta-analysis, details on the studies included in the meta-analysis calculating the proportion of severe RA.

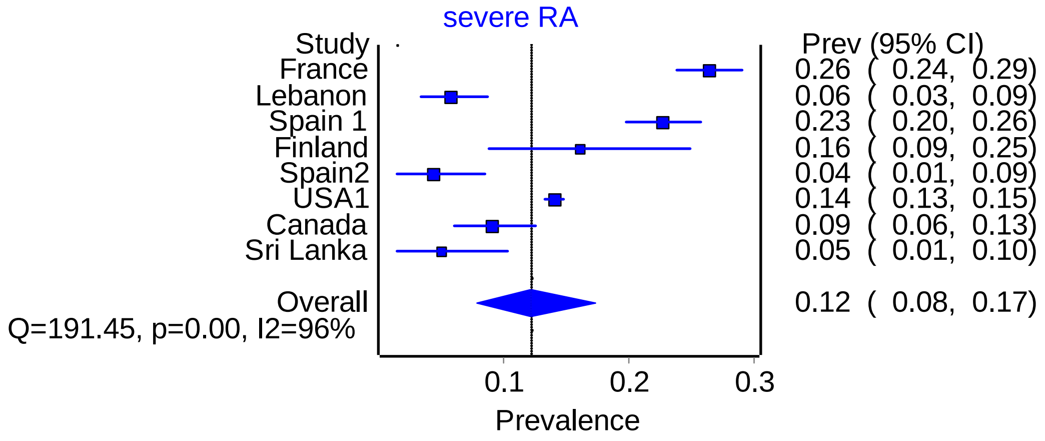

Osteoarthritis

Flowchart

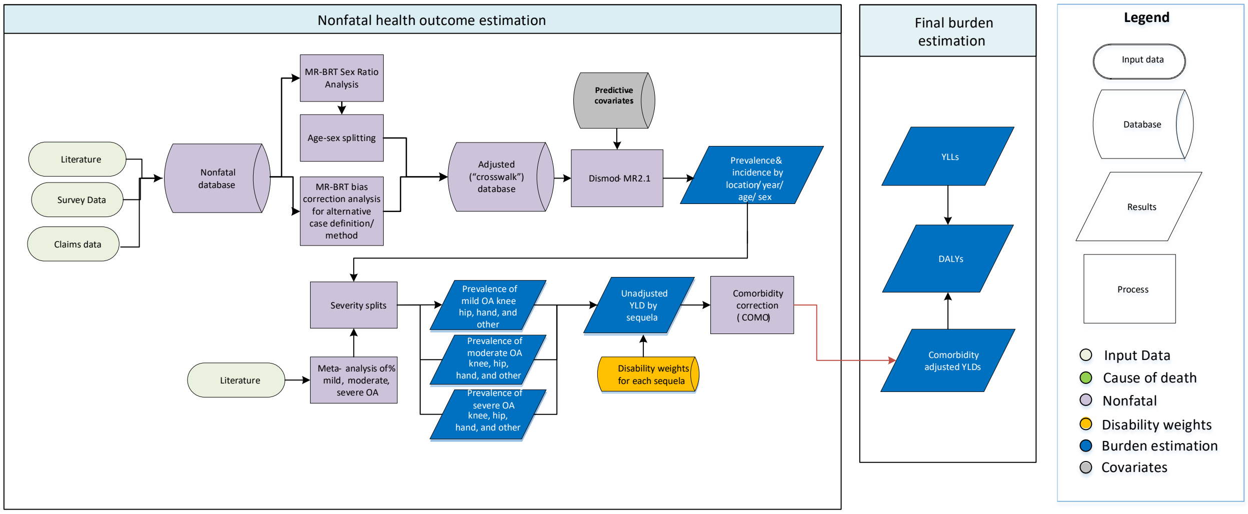

Input Data and Methodological Summary for Osteoarthritis

Case definition

OA is the most common form of arthritis, involving chronic inflammation, breakdown, and structural changes of whole joints. For the purposes of OA estimates for this GBD study, hip, knee, hand, and other sites were reviewed. The hip, knee, and hand are the common sites of OA. OA in the larger joints, such as the hip and knee, are considered to produce the greatest disability. Failure of these joints can lead to need for joint replacement surgery, if available, and thus contributes to a significant proportion of the high direct health care costs attributable to arthritis. OA of the spine is also common; however, it was considered that any symptoms and disability related to the cervical and/or lumbar spine would be captured in the estimates of low back pain and neck pain.

The osteoarthritis (OA) reference case definition is symptomatic osteoarthritis radiologically confirmed as Kellgren-Lawrence grade 2-4. Prior to GBD 2019, we only estimated OA of the hip and knee. For GBD 2019, two new sites of OA were added, OA of the hand, with the same reference criteria present in any single hand joint type, and OA other, with the same reference criteria present in any joint other than those of the hand, hip, knee, or spine. Grade 2 symptomatic requires one defined osteophyte in the affected joint and pain for at least one month out of the last 12. Grade 3-4 symptomatic requires osteophytes and joint space narrowing in the affected joint with deformity also present for grade 4, and pain for at least one month out of the last 12 months.

ICD-10 codes for OA of the hip, knee, hand, and other are M16, M17, M18, and M19, respectively. The ICD-9 code for OA is 715, without specific codes for various sites.

Input data

The most recent systematic review for OA hip and OA knee was conducted in 2017 for studies published between 2013 - 2017. A systematic review of the prevalence, incidence, and mortality was performed on MEDLINE, EMBASE, CINAHL, CAB Abstracts, WHO Library (WHOLIS) and OpenSIGLE. For prevalence and incidence, the following search terms were used: (osteoarth* OR gonarthr*) AND (prevalen* OR inciden* OR cross-sectional OR cross sectional OR epidemiol* OR survey OR population-based OR population based OR population study OR population sample OR cohort OR follow-up OR follow up OR longitudinal OR regist*) AND (list of names of all GBD countries).

Exclusion criteria were:

1. Sub-populations clearly not representative of the national population
2. Not a population-based study
3. Low sample size (less than 150)
4. Review rather than original studies

We identified 1,864 articles and extracted data from 26. These studies were from 19 locations: Australia, Brazil, Canada, China, Ecuador, Egypt, India, Iran, United Kingdom, France, Japan, United States, Mongolia, Portugal, Spain, Mexico, Turkey, Venezuela, and Vietnam.

Figure 1: PRISMA diagram of osteoarthritis systematic review from 2013–2017

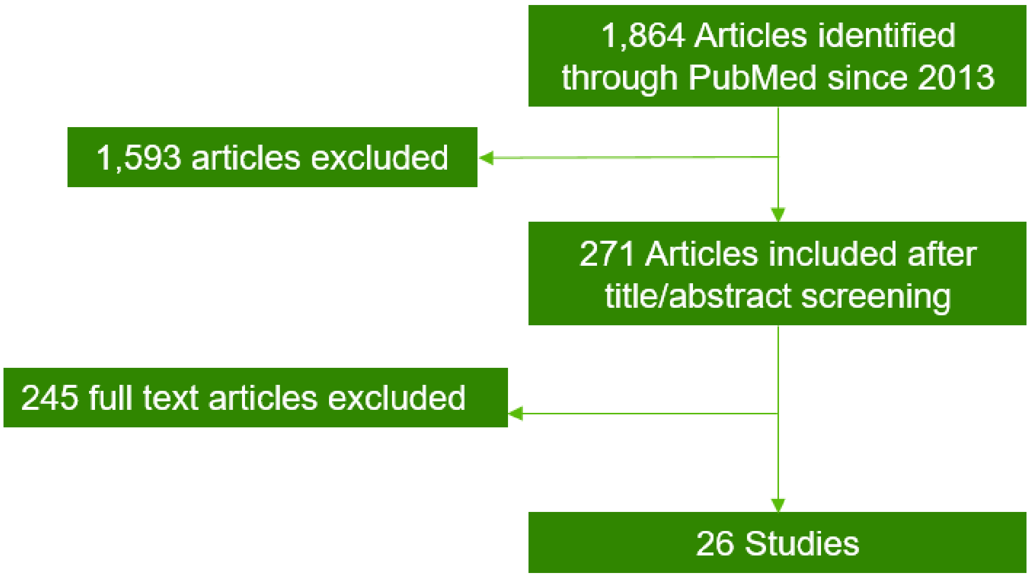

All existing sources used in the hip and knee models were re-reviewed for mention of prevalence and incidence of OA hand or OA other specifically. In order to gather more input data on prevalence for the new OA hand and OA other models, a broad systematic review was also conducted in 2019 specifically for data on these sites. A PubMed search was conducted for studies published between 1980 and 2019 using the following search terms: (("osteoarthritis” AND ("epidemiology" OR "prevalence")) AND "humans") AND ("population" OR "population groups" OR ("population" AND "groups")).

Figure 2: PRISMA diagram of osteoarthritis systematic review from 1980–2019

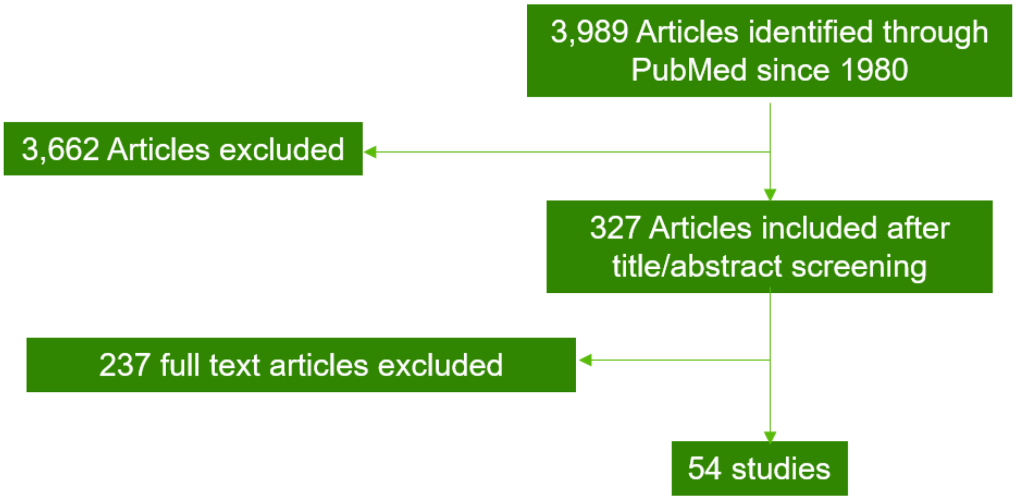

As in past rounds of the GBD, we decided not to use hospital inpatient data as we considered it would not be representative of true prevalence, and that variation between countries in the proportion of true prevalent cases captured in hospital inpatient data system would likely vary more than can be captured by a single crosswalk in DisMod-MR 2.1. Data from USA claims data for 2000 and 2010–2016 by state and Taiwan claims data from 2016 were included. There were very few sources identified through data re-review and systematic review for OA other, with minimal overlap in reported site. As a result, US claims data constituted the only data input source for this model.

The total source counts used for modeling in GBD 2019 are listed below:

Cause	Measure	Total sources	Countries with data
Osteoarthritis hip	All measures	59	23
	Prevalence	52	23
	Incidence	5	3
	Relative risk	1	1
	Standardized mortality ratio	1	1
Osteoarthritis knee	All measures	73	26
	Prevalence	69	25
	Incidence	5	4
Osteoarthritis hand	All measures	88	40
	Prevalence	87	40
	Incidence	1	1
Osteoarthritis other	All measures	12	1
	Prevalence	12	1

Age and sex splitting

Reported estimates of prevalence were split by age and sex where possible. First, if studies reported prevalence for broad age groups by sex (eg, prevalence in 15- to 65-year-old males and females separately), and also by specific age groups but for both sexes combined (eg, prevalence in 15- to 30-year-olds, then in 31- to 65-year-olds, for males and females combined), age-specific estimates were split by sex using the reported sex ratio and bounds of uncertainty. Second, input data reporting prevalence of OA for both sexes that could not be split using a within-study ratio were split using a sex ratio derived from a meta-analysis of existing sex-specific data for each type of OA using MR-BRT. The female to male ratio was 1.10 (1.09 to 1.12) for the hip, 1.44 (1.43 to 1.45) for the knee, and 2.36 (2.33 to 2.38) for the hand. There weren't any both sex input data for OA other. Finally, after the application of bias adjustments, where studies on OA hip and OA knee reported estimates across age groups spanning 25 years or more, these were split into five-year age groups using the prevalence age pattern estimated by DisMod-MR 2.1 for each type of OA in GBD 2017. Remaining wide age bin data for OA hand were split into five-year age groups using the prevalence age pattern of the USA claims input data. There weren't any wide age bin input data for OA other.

Data adjustment

For OA hip and OA knee, we marked studies that reported on X-rays only, self-reported OA with pain, or self-reported OA with no information on pain. Other studies identified cases of osteoarthritis through a review of medical charts. We assumed that these cases were diagnosed by X-ray with pain present. We added three additional covariates for claims data in the USA from the year 2000 and from 2010 onward and for Taiwan claims data. For all these alternative case definitions we derived adjustment factors using MR-BRT. Claims data from Taiwan were excluded from the model, as we did not have data on the reference case definition from Taiwan to inform a reliable adjustment. Betas and exponentiated values (which can be interpreted as an odds ratio) for these two covariates are shown in the table below:

Table 1: MR-BRT Crosswalk Adjustment Factors for OA Hip

Data input	Reference or alternative case definition	Gamma	Beta Coefficient, Log (95% CI)	Adjustment factor*
Radiography with pain	Ref	0.26	---	---
Radiography only	Alt		1.09 (0.89 to 1.28)	2.96 (2.44 to 3.6)
Self-reported OA with pain	Alt		1.32 (1.15 to 1.48)	3.73 (3.16 to 4.39)
Self-reported OA, no mention of pain	Alt		1.60 (1.18 to 2.01)	4.94 (3.26 to 7.49)
USA Claims data – 2000	Alt		-2.50 (-2.96 to -2.01)	0.082 (0.052 to 0.13)
USA Claims data – 2010–2016	Alt		-2.03 (-2.08 to -1.97)	0.13 (0.12 to 0.14)

**Adjustment factor is the transformed Beta coefficient in normal space, and can be interpreted as the factor by which the alternative case definition is adjusted to reflect what it would have been if measured as the reference.*

Table 2: MR-BRT Crosswalk Adjustment Factors for OA Knee

Data input	Reference or alternative case definition	Gamma	Beta Coefficient, Log (95% CI)	Adjustment factor*
Radiography with pain	Ref	0.38	---	---
Radiography only	Alt		0.21 (0.14 to 0.27)	1.23 91.15 to 1.32)
Self-reported OA with pain	Alt		0.063 (-0.027 to 0.15)	1.065 (0.97 to 1.17)
Self-reported OA, no mention of pain	Alt		-0.77 (-0.81 to -0.72)	0.46 (0.44 to 0.48)
USA Claims data – 2000	Alt		-2.26 (-2.64 to -1.88)	0.10 (0.072 to 0.15)
USA Claims data – 2010–2016	Alt		-1.60 (-2.43 to -0.77)	0.20 (0.088 to 0.46)

**Adjustment factor is the transformed Beta coefficient in normal space, and can be interpreted as the factor by which the alternative case definition is adjusted to reflect what it would have been if measured as the reference.*

For OA hand, we allowed for alternatives to two dimensions of case definition: affected joint and diagnostic criteria. These alternative case definitions concerned studies reporting on the presence of OA in any single joint type (e.g. distal interphalangeal), present in the first carpometacarpal joint of the thumb specifically, present in multiple joint types, or diagnosed as generalized hand OA. Adjustments were also considered for studies that used X-rays, studies in which a physician diagnosed OA without X-rays, studies that used reported pain, and studies that used self-report. We added two additional covariates for claims data in the USA from the year 2000 and from 2010 onward. The mean and standard error for the coefficients were calculated using the MR-BRT crosswalk adjustment method. Data concerning the presence of OA in the thumb base and through self-report were not included in the final model, as we were unable to find matches to inform a reliable crosswalk. Betas and exponentiated values (which can be interpreted as an odds ratio) for these two covariates are shown in the table below:

Table 3: MR-BRT Crosswalk Adjustment Factors for OA Hand

Data input	Reference or alternative case definition	Gamma	Beta Coefficient, Log (95% CI)	Adjustment factor*
Radiography with pain in a single joint type	Ref	0.36	---	---
OA in a single joint type	Alt		0.32 (0.29 to 0.34)	1.37 (1.34 to 1.40)

OA in multiple joint types	Alt		0.32 (0.30 to 0.34)	1.38 (1.35 to 1.41)
Generalized hand OA	Alt		-0.74 (-0.80 to -0.68)	0.48 (0.45 to 0.51)
Radiography only	Alt		1.09 (1.03 to 1.15)	2.97 (2.79 to 3.16)
Physician diagnosis only	Alt		0.58 (0.51 to 0.65)	1.78 (1.66 to 1.92)
Pain only	Alt		0.055 (0.0077 to 0.10)	1.06 (1.01 to 1.11)
Radiography with pain	Alt		0.31 (0.23 to 0.39)	1.36 (1.26 to 1.48)
Physician diagnosis with pain	Alt		0.28 (0.20 to 0.35)	1.32 (1.22 to 1.42)
USA Claims data – 2000	Alt		-0.48 (-0.49 to -0.47)	0.62 (0.61 to 0.62)
USA Claims data – 2010–2016	Alt		-2.74 (-2.81 to -2.66)	0.065 (0.60 to 0.70)

**Adjustment factor is the transformed Beta coefficient in normal space, and can be interpreted as the factor by which the alternative case definition is adjusted to reflect what it would have been if measured as the reference.*

Modeling strategy

For OA hip and OA knee, prior settings in the DisMod model included setting remission to 0, and it was assumed that there was no incidence or prevalence of OA before the age of 30 years. We assumed that excess mortality is zero. While there are some data on excess mortality risk, the values of hazard ratios or standardised mortality ratios are close to one, with some studies reporting mean estimates less than one.

We made few substantive changes in the modeling strategy from GBD 2017. For OA hip, the coefficient of variation was increased from 0.4 at the Global, Super Region, and Region levels, to 0.8 to allow the model to better follow the data. For OA knee, bounds were set on remission between 0 and 0.05 to account for knee replacement. We included Mean BMI and the SEV scalar for osteoarthritis as country covariates on prevalence. The OA SEV scalar combines the exposure measures for risks estimated to impinge on OA in GBD: increased BMI.

Table 4. Covariates. Summary of covariates used in the OA hip and OA knee DisMod-MR meta-regression models

Covariate	Beta, log (95% Uncertainty Interval), OA Hip	Exponentiated beta (95% Uncertainty Interval), OA Hip	Beta, log (95% Uncertainty Interval), OA Knee	Exponentiated beta (95% Uncertainty Interval), OA Knee
Mean BMI	0.98 (0.94 to 1.00	2.66 (2.56 to 2.72)	0.69 (0.51 to 0.87)	1.99 (1.66 to 2.39)

Log-transformed age-standardized SEV scalar: OA	1.95 (1.25 to 2.00)	7.05 (3.51 to 7.38)	0.78 (0.75 to 0.83)	2.17 (2.12 to 2.29)

For the new OA hand and OA other models, settings in DisMod included setting remission to 0, and assuming no incidence or prevalence of OA before the age of 30 years. In addition, we included the SEV scalar for OA as a country covariate on prevalence for OA other in order to provide a basis for some geographic variation in a model that only has input data in the USA. This covariate was not used in the OA hand model because we did not have reason to believe that there is a reliable relationship between increased BMI and OA in hand joints.

Table 5. Covariates. Summary of covariates used in the OA other DisMod-MR meta-regression model

Covariate	Beta, log (95% Uncertainty Interval)	Exponentiated beta (95% Uncertainty Interval)
Log-transformed age-standardized SEV scalar: OA	1.23 (1.20 to 1.25)	3.42 (3.31 to 3.49)

Severity and Disability

The basis of the GBD disability weight survey assessments are lay descriptions of sequelae highlighting major functional consequences and symptoms. The lay descriptions and disability weights for OA severity levels are shown below.

Table 6. Severity distribution, details on the severity levels for OA in GBD 2019 and the associated disability weight (DW) with that severity.

Severity level	Lay description	DW (95% CI)
Asymptomatic		0
Mild	This person has pain in the leg, which causes some difficulty running, walking long distances, and getting up and down.	0.023 (0.013–0.037)
Moderate	This person has moderate pain in the leg, which makes the person limp, and causes some difficulty walking,	0.079 (0.054–0.110)

	standing, lifting and carrying heavy things, getting up and down, and sleeping.	
Severe	This person has severe pain in the leg, which makes the person limp and causes a lot of difficulty walking, standing, lifting and carrying heavy things, getting up and down, and sleeping.	0.165 (0.112–0.232)

In past GBD rounds, to determine the proportion of people with OA within each of the severity levels, four studies representing the High-income, South Asia, and Southeast Asia, East Asia, and Oceania super regions provided information on the severity of OA. In GBD 2017, data from the USA Osteoarthritis Initiative study were included as well. The OA Initiative is a large cohort study that follows individuals with OA of the knee recruited from four centers around the USA. In all five studies, severity was classified based on the Western Ontario and McMaster Universities Arthritis Index (WOMAC) with scores 0-5 taken as mild, 6-13 as moderate, and 14 and higher as severe. Estimates were pooled across studies using a random effects meta-analysis model. The pooled percentages were mild 47.0% (42.2–51.9), moderate 35.9% (31.3–40.7), and severe 17.1% (12.9–21.6) pooled between patient and physician ratings in a study from Bangladesh, which we apply to low- and middle-income countries. The pooled proportions from three high-income countries were mild 74.3% (64.8–82.7), moderate 24.3% (16.4–33.1), and severe 1.1% (0.6–1.7). After streaming out 1,000 draws assuming a binomial distribution, percentages were scaled to sum to 1 at each draw. For the sake of consistency, the same severity distribution and disability weights were applied to OA hand and OA other, to be reconsidered in the subsequent modeling round.

Low Back Pain

Flowchart

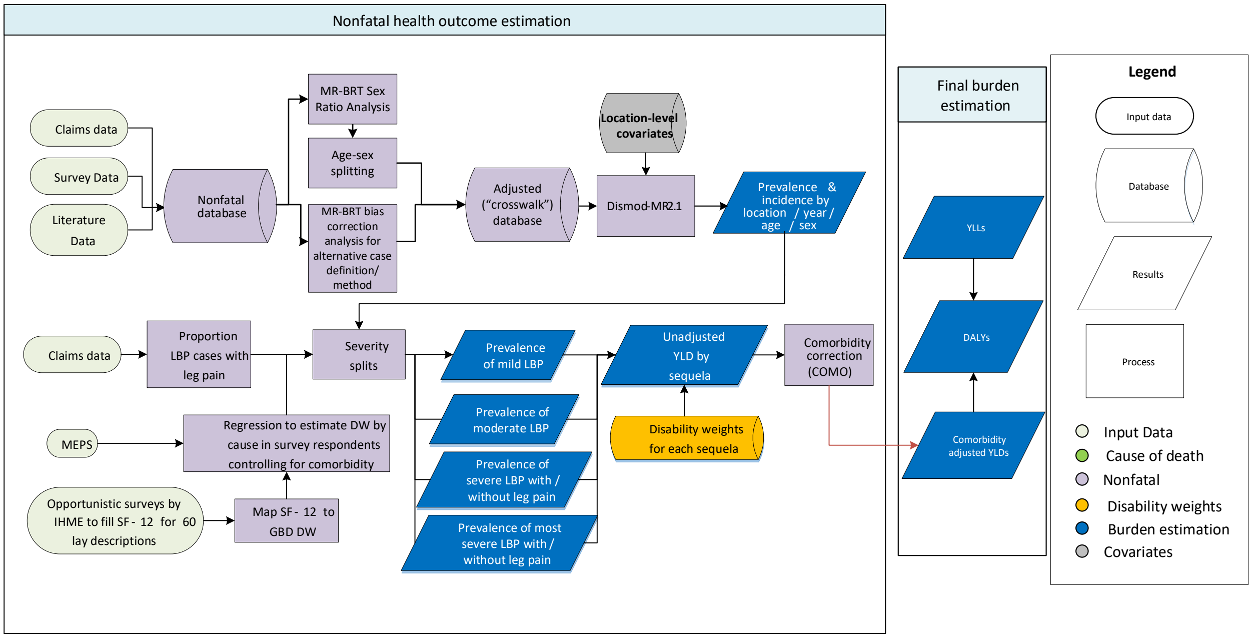

Input Data and Methodological Summary for LBP

Case definition

Low back pain (LBP) is defined as low back pain (with or without pain referred into one or both lower limbs) that lasts for at least one day. The "low back" is defined as the area on the posterior aspect of the body from the lower margin of the twelfth ribs to the lower gluteal folds.

ICD-10 codes for LBP are M54.3, M54.4 and M54.5. The ICD-9 code is 724.

Input data

The last systematic review was conducted from October 2016-October 2017. We searched PUBMED, Ovid Medline, EMBase, and CINAHL electronic databases. There were no age, sex, or language restrictions. The terms "back pain," "lumbar pain," "back ache," "backache," and "lumbago" were used individually and combined with each of the following: "prevalence," "incidence," "cross-sectional," and "epidemiology."

Exclusion criteria were:

1. Sub-populations clearly not representative of the national population
2. Not a population-based study
3. Low sample size (less than 150)
4. Review rather than original studies

Figure 2: PRISMA diagram of low back pain systematic review from 2016–2017

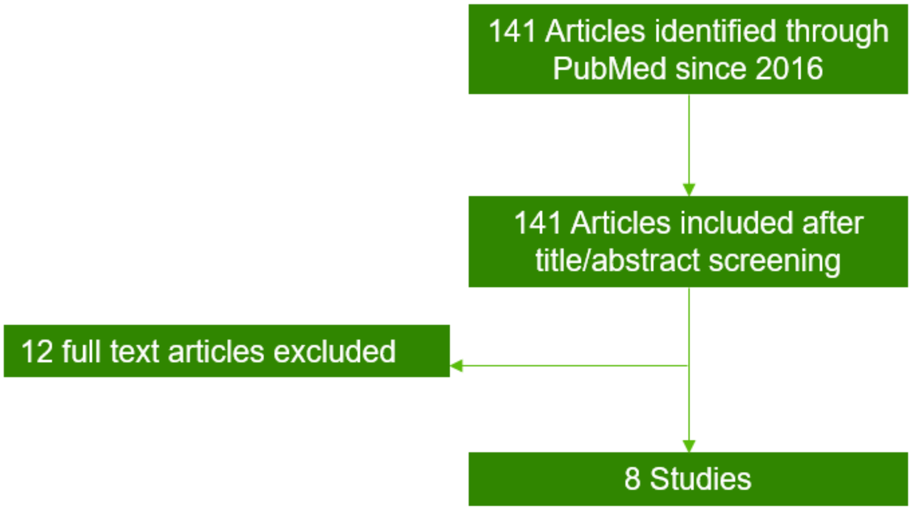

Additional information was derived from unit record data of surveys in the GHDx, GBD's repository of population health data including the World Health surveys and national health surveys. Opportunistically, additional studies encountered during data review were added for GBD 2019. In addition, data from USA claims data for 2000, 2010–2012, and 2014-2016 by state were included.

Table 1: Data Inputs for LBP

Measure	Total sources	Countries with data
All measures	463	103
Prevalence	446	102
Incidence	4	3
Remission	3	2
Proportion	15	1

Age and sex splitting

Reported estimates of prevalence were split by age and sex where possible. First, if studies reported prevalence for broad age groups by sex (eg, prevalence in 15- to 65-year-old males and females separately), and also by specific age groups for both sexes combined (eg, prevalence in 15- to 30-year-olds, then in 31- to 65-year-olds, for males and females combined), age-specific estimates were split by sex using the reported sex ratio and bounds of uncertainty. Second, prevalence data for both sexes that could not be split using a within-study ratio were split using a sex ratio derived from a meta-analysis of existing sex-specific data using MR-BRT. The female to male ratio was 1.18 (1.18 to 1.18). Finally, after the application of bias adjustments, where studies reported estimates across age groups spanning 25 years or more, these were split into five-year age groups using the prevalence age pattern estimated by DisMod-MR 2.1 in GDB 2017.

Data adjustment

We corrected for bias among studies that defined low back back with too broad anatomical region, episode duration of greater than three months, recall periods of one week to one month, recall periods between two months and one year, or as activity-limiting LBP, as well as studies conducted among schoolchildren. We added three additional covariates for claims data in the USA from the year 2000 and from 2010 onward and for Taiwan claims data. These adjustment factors were estimated as the logit difference between the prevalence of alternate case definition data and that of the reference definition for comparable age, sex, year, and location calculated using the MR-BRT network crosswalk adjustment method. Unadjusted low back pain prevalence data is often already close to one, especially for older age groups, and a logit difference strategy ensures that any prevalence data requiring adjustment to a higher value do not exceed one. Claims data from Taiwan were not included in the final model, as we were unable to find matches to inform a reliable crosswalk. Betas and exponentiated values for these covariates are shown in the table below:

Table 2: MR-BRT Crosswalk Adjustment Factors for LBP

Data input	Reference or alternative case definition	Gamma	Beta Coefficient, Logit (95% CI)	Adjustment factor
Point prevalence	Ref	0.86	---	---
Anatomical region too broad	Alt		0.099 (0.080 to 0.12)	0.52 (0.52 to 0.53)
Episode duration >= 3 months	Alt		-0.19 (-1.03 to -0.97)	0.27 (0.26 to 0.28)
Recall periods of 1 week to 1 month	Alt		0.31 (0.28 to 0.34)	0.58 (0.57 to 0.58)
Recall periods between 2 months and one year	Alt		0.80 (0.76 to 0.84)	0.69 (0.68 to 0.70)
Activity-limiting LBP	Alt		-1.53 (-1.55 to -1.51)	0.18 (0.17 to 0.18)
Studies among school children	Alt		0.00 (-0.05 to 0.05)	0.5 (0.49 to 0.51)
USA claims data – 2000	Alt		-1.31 (1.66 to -0.97)	0.21 (0.16 to 0.27)
USA claims data – 2010–2012, 2014-2016	Alt		-0.54 (-0.57 to -0.50)	0.37 (0.36 to 0.38)

After adjusting data for case definition, we outliered data with a median absolute deviation of 1.5 or more above the mean. This was done in a systematic way to cull data that were implausibly high.

Modeling strategy

Prior settings in the DisMod model included setting excess mortality to 0, and it was assumed that there was no incidence or prevalence of low back pain before the age of 5 years. We made no substantive changes in the modeling strategy from GBD 2017. We included the SEV scalar for low back pain as a

country covariate. This combines the exposure measures for risks estimated to impinge on LBP in GBD: occupational ergonomic exposure and increased BMI. We set bounds of 0.75 to 1.25 as the SEV is constructed in a way that if our risk estimates are accurate the value should be 1.

Table 3. Covariates. Summary of covariates used in the LBP DisMod-MR meta-regression model

Covariate	Type	Parameter	Exponentiated beta (95% Uncertainty Interval)
Log-transformed age-standardized SEV scalar: Back pain	Country-level	Prevalence	2.12 (2.12 – 2.13)

Severity and Disability

The basis of the GBD disability weight survey assessments are lay descriptions of sequelae highlighting major functional consequences and symptoms. The lay descriptions and disability weights for LBP severity levels are shown below.

Table 4. Severity distribution, details on the severity levels for LBP in GBD 2019 and the associated disability weight (DW) with that severity.

Severity level	Lay description	DW (95% CI)
Low back pain, mild	This person has mild back pain, which causes some difficulty dressing, standing, and lifting things.	0.020 (0.011–0.035)
Low back pain, moderate	This person has moderate back pain, which causes difficulty dressing, sitting, standing, walking, and lifting things.	0.054 (0.035–0.079)
Low back pain, severe without leg pain	This person has severe back pain, which causes difficulty dressing, sitting, standing, walking, and lifting things. The person sleeps poorly and feels worried.	0.272 (0.182–0.373)
Low back pain, severe with leg pain	This person has severe back and leg pain, which causes difficulty dressing, sitting, standing, walking, and lifting things. The person sleeps poorly and feels worried.	0.325 0.219–0.446)
Low back pain, most severe without leg pain	This person has constant back pain, which causes difficulty dressing, sitting, standing, walking, and lifting things. The person sleeps poorly, is worried, and has lost some enjoyment in life.	0.372 (0.250–0.506)
Low back pain, most severe with leg pain	This person has constant back and leg pain, which causes difficulty dressing, sitting, standing, walking, and lifting things. The person sleeps poorly, is worried, and has lost some enjoyment in life.	0.384 (0.256–0.518)

The severity distributions are derived from an analysis of the Medical Expenditure Panel Surveys (MEPS) in the USA. MEPS is an overlapping continuous panel survey of the United States non-institutionalised population whose primary purpose is to collect information on the use and cost of health care. Panels are two years long and are conducted in five rounds, which are conducted every five to six months. A new panel begins annually, while the last panel is in its second year. Each panel typically contains about

30,000 to 35,000 individual respondents. (http://www.meps.ahrq.gov/survey_comp/hc_data_collection.jsp)

MEPS was initiated in 1996 but only began collecting health status data in the form of SF-12 responses in 2000. For GBD 2016 we used data from 2000–2014. Respondents self-administer the SF-12 twice per panel, at rounds 2 and 4, typically about a year apart. Only adults 18 years and older completed the SF-12. MEPS also usually collects information on diagnoses based on self-report of reasons for encounters with health services. In addition, diagnoses are derived through additional questions on “problems that bother you” or conditions that led to “disability days,” ie, days out of role due to illness. Professional coders translate the verbatim text into three-digit ICD-9 codes. The main reason for LBP being measured in MEPS relates to health care contact. From MEPS, the severity distribution for LBP without leg pain and with leg pain were derived as shown in the below table.

Table 5. Severity distribution, details on the distribution of severity splits for LBP in GBD 2019 with and without leg pain

Severity level	Distribution without leg pain	Distribution with leg pain
Low back pain, mild	0.41 (0.31–0.53)	0.27 (0.19–0.37)
Low back pain, moderate	0.35 (0.25–0.44)	0.36 (0.28–0.43)
Low back pain, severe	0.10 (0.08–0.12)	0.14 (0.10–0.16)
Low back pain, most severe	0.14 (0.09–0.20)	0.23 (0.15–0.32)

We used USA claims data (2012) to derive the proportion of cases with low back pain who report leg pain. The proportions were different by age group as shown in Figure 1. The proportion in each severity level in each age group is calculated by multiplying the proportion in the severity level and the proportion with or without leg pain.

Figure 2: Proportion of LBP with leg pain

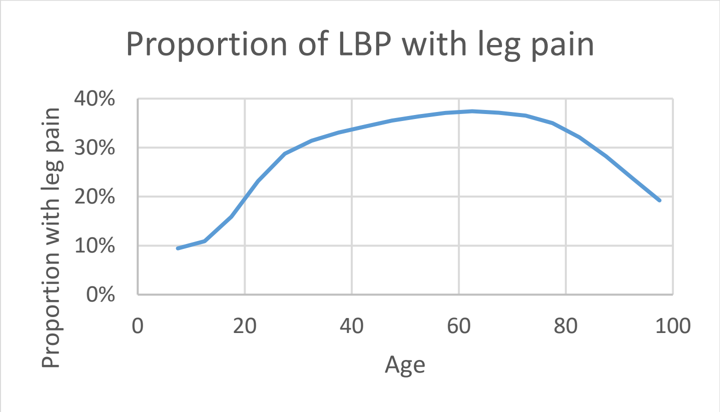

Table 6. Proportion of LBP with leg pain

Age (years)	Proportion with leg pain
5–9	9.4 (9.1–9.8) %
10–14	10.9 (10.7–11.1) %

15–19	15.9 (15.8–16.1) %
20–24	23.2 (23.0–23.4) %
25–29	28.8 (28.6–28.9) %
30–34	31.4 (31.3–31.6) %
35–39	33.1 (32.9–33.2) %
40–44	34.3 (34.2–34.4) %
45–49	35.5 (35.4–35.6) %
50–54	36.4 (36.3–36.5) %
55–59	37.1 (37.0–37.2) %
60–64	37.4 (37.3–37.5) %
65–69	37.1 (36.9–37.3) %
70–74	36.5 (36.4–36.7) %
75–79	35.0 (34.8–35.2) %
80–84	32.1 (31.9–32.4) %
85–89	28.3 (28.0–28.5) %
90–94	23.7 (23.2–24.2) %
95–100	19.2 (18.2–20.2) %

Neck Pain (NP)

Flowchart

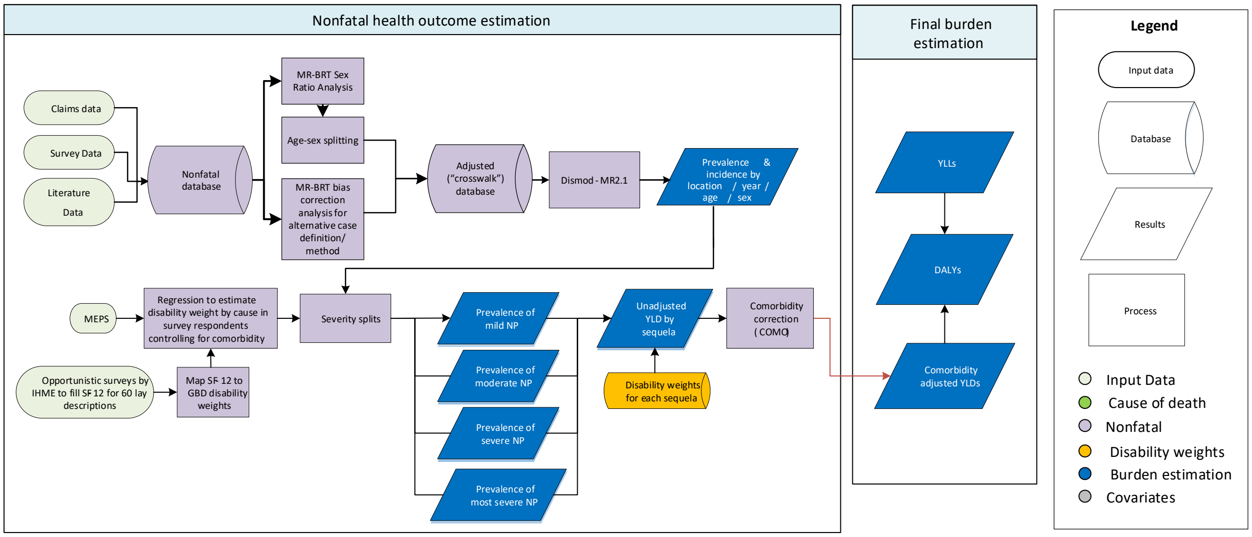

Input Data and Methodological Summary for Neck Pain

Case definition

Neck pain (NP) was defined as: neck pain (+/- pain referred into the upper limb(s)) that lasts for at least one day.

ICD-10 code for neck pain is M54.2. The ICD-9 code is 723.1.

Input data

Ovid MEDLINE, EMBASE, CINAHL, CAB abstracts, WHOLIS, and SIGLE databases were searched for GBD 2010 and PUBMED was searched through October 2017 for GBD 2017. There were no age, sex, or language restrictions. The terms neck pain, neck ache, neckache, and cervical pain individually and combined with each of the following terms: prevalen*, inciden*, cross-sectional, cross sectional, epidemiol*, survey, population-based, population based, population study, population sample.

Exclusion criteria were:

1. Sub-populations clearly not representative of the national population
2. Not a population-based study
3. Studies on a specific type of neck pain (eg, following neck fracture)
4. Low sample size (less than 150)
5. Review rather than original studies

Figure 1: PRISMA diagram of neck pain systematic review from 2016–2017

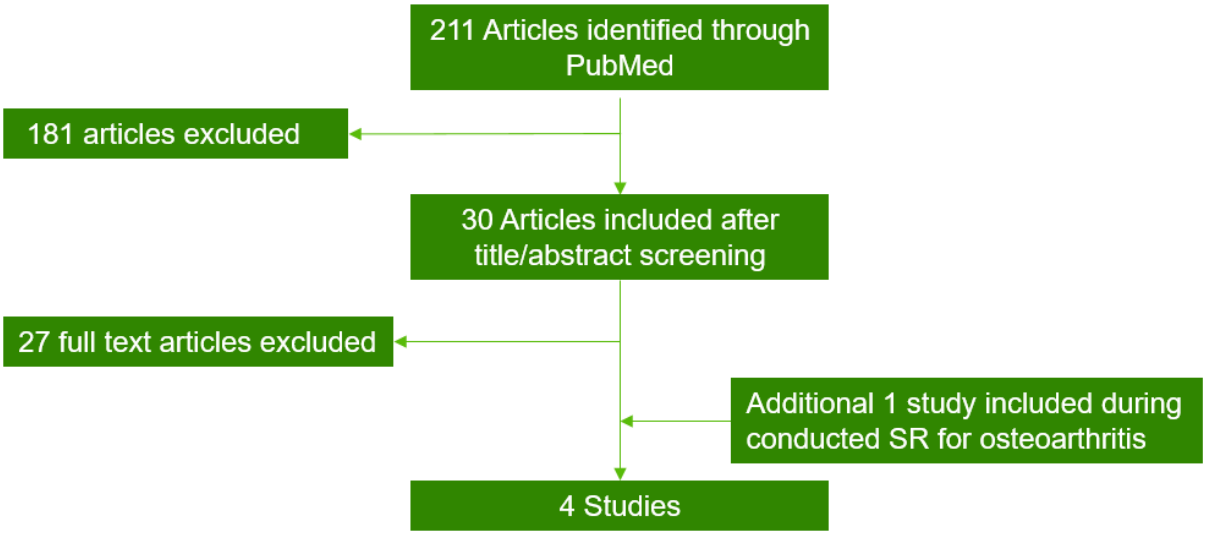

Additional information was derived from unit record data of surveys in the GHDx, GBD's repository of population health data including National Health and Nutrition Examination Survey (NHANES) and National Health Interview Survey (NHIS) in the USA. Opportunistically, additional studies encountered during data review were added for GBD 2019. In addition, data from USA claims data for 2000 and 2010–2015 by state and Taiwan claims data from 2016 were included.

Table 1: Data inputs for neck pain

Measure	Total sources	Countries with data
All measures	92	26
Prevalence	77	26
Remission	1	1
Proportion	15	1

Age and sex splitting

Reported estimates of prevalence were split by age and sex where possible. First, if studies reported prevalence for broad age groups by sex (eg, prevalence in 15- to 65-year-old males and females separately), and also by specific age groups for both sexes combined (eg, prevalence in 15- to 30-year-olds, then in 31- to 65-year-olds, for males and females combined), age-specific estimates were split by sex using the reported sex ratio and bounds of uncertainty. Second, prevalence data for both sexes that could not be split using a within-study ratio were split using a sex ratio derived from a meta-analysis of existing sex-specific data using MR-BRT. The female to male ratio was 1.31 (1.30 to 1.32). Finally, after the application of bias adjustments, where studies reported estimates across age groups spanning 25 years or more, these were split into five-year age groups using the prevalence age pattern estimated by DisMod-MR 2.1 in GBD 2017.

Data adjustment

We used MR-BRT to calculate adjustment factors to correct for biases introduced by alternative case definitions. These alternative case definitions were studies that reported a too broad anatomical region, episode duration of greater than three months, recall periods of one week to one month, recall periods between two months and one year, activity-limiting neck pain, and studies conducted among schoolchildren. We added three additional covariates for claims data in the USA from the year 2000 and from 2010 onward and for Taiwan claims data. The mean and standard error for the coefficients were calculated using the MR-BRT network crosswalk adjustment method. The covariate for claims data from Taiwan was not included in the final adjustments, as we were unable to find matches to inform a reliable crosswalk. Betas and exponentiated values (which can be interpreted as an odds ratio) for these two covariates are shown in the table below:

Table 2: MR-BRT crosswalk adjustment factors for neck pain

Data input	Reference or alternative case definition	Gamma	Beta Coefficient, Log (95% CI)	Adjustment factor*
Point prevalence	Ref	0.30	---	---
Anatomical region too broad	Alt		0.89 (0.66 to 1.12)	2.43 (1.93 to 3.07)
Episode duration >= 3 months	Alt		-0.69 (-0.85 to -0.53)	0.50 (0.43 to 0.59)
Recall periods of 1 week to 1 month	Alt		0.94 (0.51 to 1.38)	2.56 (1.65 to 3.96)
Recall periods between 2 months and one year	Alt		1.23 (0.80 to 1.68)	3.46 (2.24 to 5.36)
Studies among schoolchildren	Alt		0.13 (-0.61 to 0.87)	1.14 (0.54 to 2.39)
Activity-limiting neck pain	Alt		-1.23 (-1.23 to -1.18)	0.30 (0.29 to 0.31)
USA Claims data – 2000	Alt		-1.58 (-2.08 to -1.08)	0.21 (0.13 to 0.34)
USA Claims data – 2010–2016	Alt		-0.65 (-1.09 to -0.21)	0.52 (0.23 to 0.81)

**Adjustment factor is the transformed Beta coefficient in normal space, and can be interpreted as the factor by which the alternative case definition is adjusted to reflect what it would have been if measured as the reference.*

After adjusting data for case definition, we outliered data with a median absolute deviation of 2 or more above or below the mean. This was done in a systematic way to cull data that were implausibly high or low.

Modeling strategy

Prior settings in the DisMod model included setting excess mortality to 0, and it was assumed that there was no incidence or prevalence of neck pain before the age of 5 years. We made no substantive changes in the modeling strategy from GBD 2017, with the exception of increasing the coefficient of variation

from 0.4 to 0.8 for the priors being passed down the geographical hierarchy to allow the model to better follow the data.

Severity and Disability

The basis of the GBD disability weight survey assessments are lay descriptions of health states highlighting major functional consequences and symptoms. The lay descriptions and disability weights for neck pain severity levels are shown below.

Table 3. Severity distribution, details on the severity levels for NP in GBD 2019 and the associated disability weight (DW) with that severity.

Severity level	Lay description	DW (95% CI)	Proportions
Neck pain, mild	This person has neck pain, and has difficulty turning the head and lifting things	0.052 (0.036–0.074)	0.67 (0.57–0.75)
Neck pain, moderate	This person has constant neck pain, and has difficulty turning the head, holding arms up, and lifting things	0.112 (0.079–0.162)	0.12 (0.08–0.19)
Neck pain, severe	This person has severe neck pain, and difficulty turning the head and lifting things. The person gets headaches and arm pain, sleeps poorly, and feels tired and worried	0.226 (0.147–0.323)	0.06 (0.05–0.07)
Neck pain, most severe	This person has constant neck pain and arm pain, and difficulty turning the head, holding arms up, and lifting things. The person gets headaches, sleeps poorly, and feels tired and worried	0.300 0.199–0.434)	0.15 (0.11–0.20)

The severity distributions are derived from an analysis of the Medical Expenditure Panel Surveys (MEPS) in the USA. MEPS is an overlapping continuous panel survey of the United States non-institutionalised population whose primary purpose is to collect information on the use and cost of health care. Panels are two years long and are conducted in five rounds, which are conducted every five to six months. A new panel begins annually, while the last panel is in its second year. Each panel typically contains about 30,000 to 35,000 individual respondents (http://www.meps.ahrq.gov/survey_comp/hc_data_collection.jsp).

MEPS was initiated in 1996 but only began collecting health status data in the form of SF-12 responses in 2000. For GBD 2019 we used data from 2000–2014. Respondents self-administer the SF-12 twice per panel, at rounds two and four, typically about a year apart. Only adults 18 years and older completed the SF-12. MEPS also usually collects information on diagnoses based on self-report of reasons for encounters with health services. In addition, diagnoses are derived through additional questions on "problems that bother you" or conditions that led to "disability days," ie, days out of role due to illness. Professional coders translate the verbatim text into three-digit ICD-9 codes. The main reason for neck pain being measured in MEPS relates to health care contact.

In order to derive a crosswalk of SF-12 values into a scale comparable with that used by the GBD disability weights, small studies on convenience samples were conducted asking respondents to fill in SF-12 to reflect 62 lay descriptions of diverse severity that were used to derive the GBD disability weights. From these responses a relationship between SF-12 summary score and the GBD DWs was

derived. With regression methods, average disability weights were calculated for each of 156 conditions for which there were corresponding diagnoses in MEPS, while controlling for any co-morbid other condition by adding dummy variables for each condition. As our case definition is for point prevalence of neck pain, we ignored the proportion of MEPS respondents with a neck pain diagnosis for whom in our regression we found no disability attributable to neck pain. For the remaining cases we binned the amount of DW attributed to neck pain across the four health states assuming thresholds at the midpoints between DW values.

Gout

Flowchart

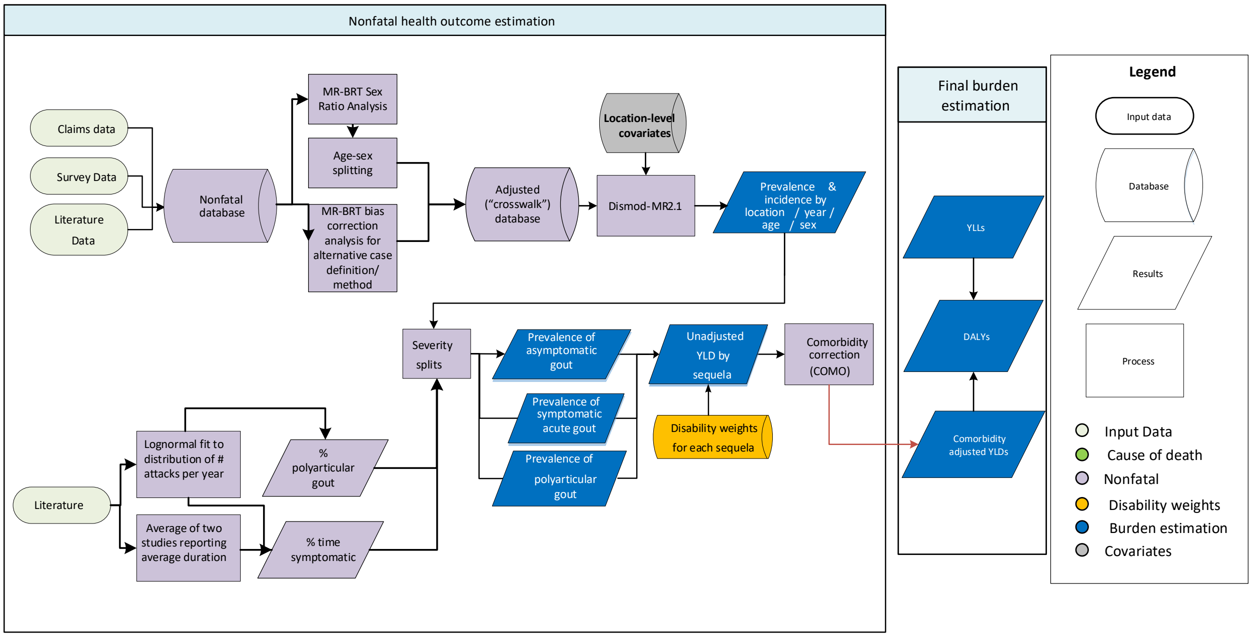

Input Data and Methodological Summary for Gout

Case definition

Gout is a rheumatic disease that is characterised by deposition of monosodium urate (MSU) crystals in the synovial fluid of joints and in other tissues, causing inflammation. The crystal formation is caused by elevated urate levels in extracellular fluids. GBD uses the case definition of primary gout given by the American College of Rheumatology, generally referred to as ARA 1977 survey criteria requiring the presence of MSU crystals in joint fluid or the presence of a tophus proven to contain MSU crystals and at least six of 12 gout symptoms or findings (>1 attack of acute arthritis, development of maximal inflammation within a day, attack of monoarticular arthritis, observation of joint erythema, pain or swelling in the first MTP joint, unilateral attack involving the first MTP joint, unilateral attack involving tarsal joint, suspected tophus, hyperuricemia, asymmetrical swelling within a joint on X-ray and negative culture of joint fluid for microorganisms during attack of joint inflammation) to make a diagnosis.

The ICD-10 code for gout is M10 and the ICD9 code is 274.

Input data

The last systematic review was conducted in GBD 2013 for studies published between 1980 to 2009 using the following search terms on MEDLINE, EMBASE, CINAHL, CAB Abstracts, WHO Library (WHOLIS),

and OpenSIGLE. For prevalence and incidence, the following search terms were used: (gout* OR hyperuricemia) AND (prevalen* OR inciden* OR cross-sectional OR cross sectional OR epidemiol* OR survey OR population-based OR population based OR population study OR population sample OR cohort OR follow-up OR follow up OR longitudinal OR regist*) AND (list of names of all GBD countries).

Exclusion criteria were:

- Sub-populations clearly not representative of the national population
- Not a population-based study
- Low sample size (less than 150)
- Review rather than original studies

For GBD 2019, 14 additional studies shared through the collaborator network were added. In addition, data from USA claims data for 2000 and 2010–2014 by state and Taiwan claims data from 2016 were included.

Table 1: Data inputs for gout

Measure	Total sources	Countries with data
All measures	130	36
Prevalence	113	34
Incidence	15	6
Relative risk	3	2
Standardized mortality ratio	1	1
Proportion	7	3

Age and sex splitting

Reported estimates of prevalence were split by age and sex where possible. First, if studies reported prevalence for broad age groups by sex (eg, prevalence in 15- to 65-year-old males and females separately), and also by specific age groups for both sexes combined (eg, prevalence in 15- to 30-year-olds, then in 31- to 65-year-olds, for males and females combined), age-specific estimates were split by sex using the reported sex ratio and bounds of uncertainty. Second, prevalence data for both sexes that could not be split using a within-study ratio were split using a sex ratio derived from a meta-analysis of existing sex-specific data using MR-BRT. The female to male ratio was 0.33 (0.33 to 0.34). Finally, after the application of bias adjustments, where studies reported estimates across age groups spanning 25 years or more, these were split into five-year age groups using the prevalence age pattern estimated by DisMod-MR 2.1 in GBD 2017.

Data adjustment

We used study covariates for studies relying on self-reported diagnoses and those identifying sources through a diagnostic code in administrative data, which include gout ICD codes as well as read codes used in the UK health system. We used MR-BRT to adjust alternative case definition and claims data in the USA from the year 2000 and from 2010 onward and for Taiwan claims data to the reference case definition. Matched data was based off of age, sex, year, and location. The mean and standard error for

the coefficients were calculated using the MR-BRT crosswalk adjustment method. Betas and exponentiated values (which can be interpreted as an odds ratio) for these covariates are shown in the table below:

Table 2: MR-BRT crosswalk adjustment factors for gout

Data input	Reference or alternative case definition	Gamma	Beta Coefficient, Log (95% CI)	Adjustment factor*
Physician-diagnosed gout	Ref	0.55	---	---
Self-reported gout	Alt		0.33 (0.050 to 0.60)	1.39 (1.05 to 1.83)
Gout identified with administrative data	Alt		0.29 (0.29 to 0.30)	1.34 (1.34 to 1.35)
USA claims data – 2000	Alt		-1.88 (-2.84 to -0.92)	0.15 (0.058 to 0.40)
USA claims data – 2010–2016	Alt		-1.55 (-2.00 to -1.09)	0.22 (0.13 to 0.34)
Taiwan claims data – 2016	Alt		0.30 (0.27 to 0.33)	1.35 (1.31 to 1.40)

**Adjustment factor is the transformed Beta coefficient in normal space, and can be interpreted as the factor by which the alternative case definition is adjusted to reflect what it would have been if measured as the reference.*

Modeling strategy

Prior settings included assuming the excess mortality rate and remission of gout did not exceed 0.01 and 0.2, respectively, and that there was no incidence or prevalence of gout before the age of 15 years. We have made no substantive changes in the modeling strategy from GBD 2017, with the exception of increasing the coefficient of variation from 0.4 at the Global, Super Region, and Region levels to 0.8 to allow the model to better follow the data. We included the summary exposure variable (SEV) scalar for gout which summarises exposure to risks estimated in GBD to impinge on gout, ie, low glomerular filtration rate, as a country covariate. We set bounds of 0.75 to 1.25 as the SEV is constructed in a way that if our risk estimates are accurate the value should be 1.

Table 3. Covariates. Summary of covariates used in the gout DisMod-MR meta-regression model

Covariate	Type	Parameter	Exponentiated beta (95% Uncertainty Interval)
Log-transformed age-standardized SEV scalar: Gout	Country-level	Prevalence	3.47 (3.43 to 3.49)

Severity and Disability

The basis of the GBD disability weight (DW) survey assessments are lay descriptions of sequelae highlighting major functional consequences and symptoms. The lay descriptions and disability weights for gout severity levels are shown below.

Table 4. Severity distribution, details on the severity levels for gout in GBD 2019 and the associated disability weight (DW) with that severity.

Severity level	Lay description	DW (95% CI)
Gout, acute	This person has severe pain and swelling in the leg, making it very difficult to get up and down, stand, walk, lift, and carry heavy things. The person has trouble sleeping because of the pain.	0.295 (0.196–0.409)
Polyarticular gout (same as for severe RA)	This person has severe, constant pain and deformity in most joints, causing difficulty moving around, getting up and down, eating, dressing, lifting, carrying, and using the hands. The person often feels sadness, anxiety, and extreme fatigue.	0.581 (0.403–0.739)
Asymptomatic gout	This person has a diagnosis of gout without pain or functional difficulties	0

To calculate the severity distribution of gout, we used three studies on the distribution of the number of gout attacks per year and fitted a lognormal curve using a least squared differences method.[1,2,3] In the absence of data on the proportion of gout cases who have chronic polyarticular gout, we assumed the proportion is equal to those who would have 52 attacks a year (ie, weekly) or more as implied by the lognormal curve.

The average number of attacks was estimated from the lognormal fit: 5.66 (5.14–6.18). From two studies we derived an average duration of attacks of 6.1 (5.4–6.8) days by simple averaging. The resulting proportion of time symptomatic for acute gout was taken as the multiplication of these two estimates divided by the number of days in a year: 9.4% (8.0–10.9%).

Figure 1: Distribution of cases by frequency

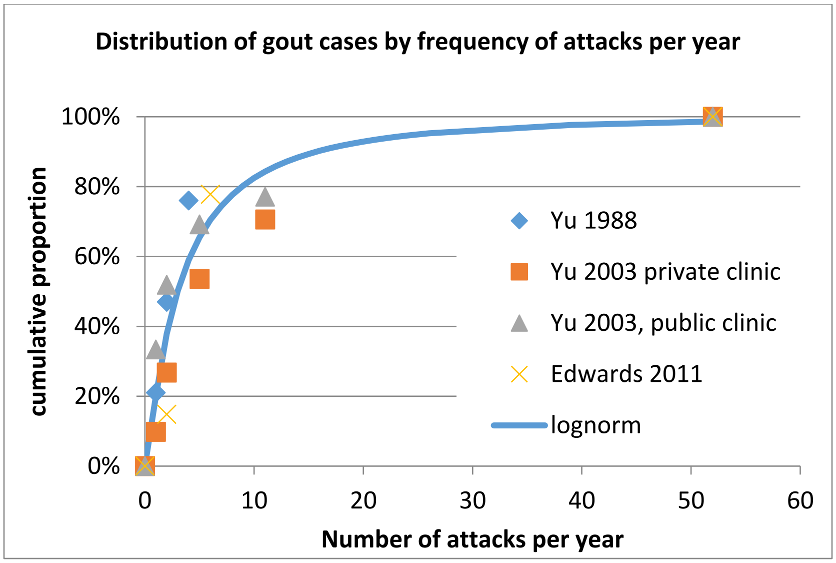

References

1. Edwards NL, Sundy JS, Forsythe A, Blume S, Pan F, Becker MA. Work productivity loss due to flares in patients with chronic gout refractory to conventional therapy. Journal of Medical Economics. 2011; 14(1).
2. Yu, KH, Luo SF, et al. Younger age of onset of gout in Taiwan. Rheumatology. 2003; 42(1): p. 166-170.
3. Yu TF, et al. Diversity of clinical features in gouty arthritis. Seminars in Arthritis and Rheumatism. 1984; 13(4): p. 360-368.

Other Musculoskeletal Disorders (Other MSK)

Flowchart

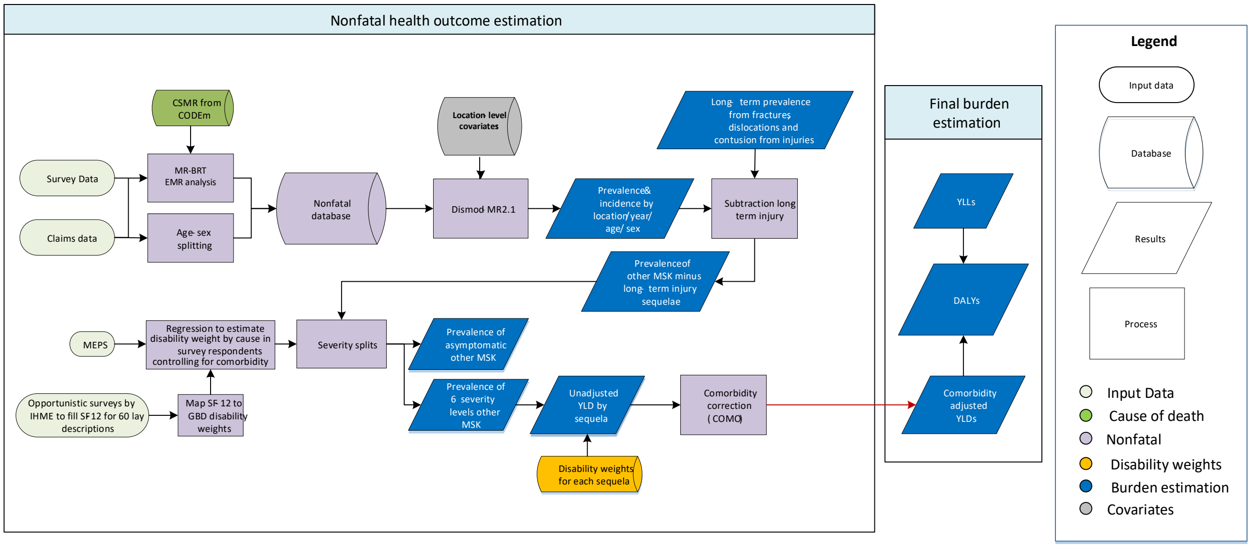

Input Data and Methodological Summary for Other MSK

Case definition

Other musculoskeletal disorders is a heterogeneous rest category comprising a wide range of disorders of muscles, bones, and ligaments that are not included in the five GBD defined musculoskeletal diseases rheumatoid arthritis, osteoarthritis, low back and neck pain, and gout, and are not captured as long-term sequelae of injuries.

The table below provides detail of the ICD-10 and ICD-9 codes included in this category.

ICD-10 codes	ICD-9 codes
L93—Lupus erythematosus	710.0
M00-M02—Infectious arthropathies	711
M08, M11-M13—Inflammatory polyarthropathies	712–713
M20-M25—Other joint disorders	716–719
M30-M35—Systemic connective tissue disorders	710.1-710.9
M40-M43—Deforming dorsopathies	737
M45-M46—Spondylopathies	720–721
M60 -M63—Disorders of muscles	725
M65-M68—Disorders of synovium and tendon	726–728
M70- M73, M75-M79—Other soft tissue disorders	729
M80-M85—Disorders of bone density and structure	733.0-2
M86—Osteomyelitis	730.1-730.3, 730.7-9
M87-M90—Other osteopathies	731, 733.3-9
M91-M94—Chondropathies	732

M95-M99—Other disorders of the MSK system and connective tissue	734–736, 738–739

Input data

The above ICD codes were used to extract other MSK prevalence from USA claims data for 2000 and 2010–2016 by state. The systematic review concentrated on finding health surveys that measured an overall amount of musculoskeletal disorders and complaints and reported information to distinguish a rest category that was not OA, RA, gout, or low back or neck pain. These data sources are based on self-reported musculoskeletal conditions or symptoms and not using the listed ICD codes.

Table 1. Data inputs

Measure	Total sources	Countries with data
All measures	68	23
Prevalence	65	23
Proportion	15	1

Age and sex splitting

Reported estimates of prevalence were split by age and sex where possible. First, if studies reported prevalence for broad age groups by sex (eg, prevalence in 15- to 65-year-old males and females separately), and also by specific age groups but for both sexes combined (eg, prevalence in 15- to 30-year-olds, then in 31- to 65-year-olds, for males and females combined), age-specific estimates were split by sex using the reported sex ratio and bounds of uncertainty. Second, prevalence data for both sexes that could not be split using a within-study ratio were split using a sex ratio derived from a meta-analysis of existing sex-specific data using MR-BRT. The female to male ratio was 1.37 (1.37 to 1.38). Finally, where studies reported estimates across age groups spanning 25 years or more, these were split into five-year age groups using the prevalence age pattern estimated by DisMod-MR 2.1 in GBD 2017.

Data adjustment

In previous rounds, we used two study covariates to adjust claims data from the USA by state from the year 2000 and from 2010 onward. For GBD 2019, we did not carry out bias adjustments for claims data because claims sources are more likely to capture all of the ICD codes included in the other MSK category and reflect the assumed mutual exclusivity of component disorders than study and survey data. In future rounds of the GBD, we intend to begin the process of modeling certain component disorders independently in order to more accurately reflect their prevalence and reduce variability of input data for the remaining disorders in the other MSK model.

Modeling strategy

Prior settings in the DisMod model included the assumption of no incidence or prevalence of other MSK before the age of 10 years. In the absence of any meaningful data on incidence and remission for such a heterogeneous category of disorders, we made a rather arbitrary decision of remission of 0.5–1, ie, an average duration of 1-2 years. We also included the Socio-demographic Index country covariate with bounds set at -1 and 1. These settings were retained for GBD 2019.

In previous rounds, priors on excess mortality rate (EMR) were estimated in DisMod by matching prevalence data points with their corresponding CSMR values within the same age, sex, year, location (by dividing CSMR by prevalence. However, for many causes, DisMod estimated a rather unrealistic pattern of EMR compared to an expected pattern of decreasing EMR with greater access to quality health care. Such unexpected patterns often signal inconsistencies between CSMR estimates and the measures of prevalence and/or incidence. In effort to provide greater guidance to DisMod on the expected pattern of EMR, EMR data generated in the previous round were modeled using the MR-BRT approach by age and sex with a prior on healthcare access and quality index (HAQi) having a negative coefficient. Results from MR-BRT were then predicted for each location year, sex and for ages 0, 10, 20 ….100. We included HAQi as a country-level covariate to inform EMR with a mean and standard deviation produced from MR-BRT.

Despite its inconsistencies between CSMR and prevalence prior to the inclusion of the modeled EMR data, the final other MSK model both excludes the predicted data for the EMR parameter and has last year's DisMod EMR calculation disabled. This is because the input data for the EMR MR-BRT analysis represented a narrow range of relatively high HAQi locations, which resulted in far greater predicted EMR in data sparse, lower HAQi locations than in prior rounds, suppressing prevalence to implausibly low levels. Data for cause-specific mortality rate were also excluded from the model (arguing that the pattern of mortality comes from auto-immune diseases which constitute only a small fraction of the non-fatal manifestations captured in this residual category), a 15-year time window was set, and bounds of 0 to 0 were set on EMR, while retaining the HAQi country covariate on the parameter.

Table 2. Covariates. Summary of covariates used in the other MSK DisMod-MR meta-regression model

Covariate	Type	Parameter	Exponentiated beta (95% Uncertainty Interval)
Health-care access and quality index	Country-level	Excess mortality rate	0.95 (0.94 to 0.96)
Socio-demographic Index	Country-level	Prevalence	2.70 (2.67 to 2.72)

Severity and Disability

The basis of the GBD disability weight survey assessments are lay descriptions of health states highlighting major functional consequences and symptoms. The lay descriptions and disability weights for other MSK severity levels are shown below. They include the three levels of health states that are used for osteoarthritis and rheumatoid arthritis, each.

Table 3. Severity distribution, details on the severity levels for other MSK in GBD 2019 and the associated disability weight (DW) with that severity.

Severity level	Lay description	DW (95% CI)	Proportions
Asymptomatic			0.28 (0.27–0.29)

Musculoskeletal problems, lower limbs, mild	This person has pain in the leg, which causes some difficulty running, walking long distances, and getting up and down.	0.023 (0.013–0.040)	0.22 (0.15–0.30)
Musculoskeletal problems, upper limbs, mild	This person has mild pain and stiffness in the arms and hands. The person has some difficulty lifting, carrying, and holding things.	0.028 (0.017–0.046)	0.20 (0.15–0.29)
Musculoskeletal problems, upper limbs, moderate	This person has moderate pain and stiffness in the arms and hands, which causes difficulty lifting, carrying, and holding things, and trouble sleeping because of the pain.	0.115 (0.079–0.163)	0.10 (0.06–0.15)
Musculoskeletal problems, lower limbs, severe	This person has severe pain in the leg, which makes the person limp and causes a lot of difficulty walking, standing, lifting and carrying heavy things, getting up and down, and sleeping.	0.163 (0.109–0.224)	0.06 (0.04–0.07)
Musculoskeletal problems, generalised, moderate	This person has pain and deformity in most joints, causing difficulty moving around, getting up and down, and using the hands for lifting and carrying. The person often feels fatigue.	0.312 (0.201–0.438)	0.07 (0.06–0.08)
Musculoskeletal problems, generalised, severe	This person has severe, constant pain and deformity in most joints, causing difficulty moving around, getting up and down, eating, dressing, lifting, carrying, and using the hands. The person often feels sadness, anxiety, and extreme fatigue.	0.572 (0.370–0.758)	0.07 (0.07–0.08)

The severity distributions are derived from an analysis of the Medical Expenditure Panel Surveys (MEPS) in the USA. MEPS is an overlapping continuous panel survey of the United States non-institutionalised population whose primary purpose is to collect information on the use and cost of health care. Panels are two years long and are conducted in five rounds, which are conducted every five to six months. A new panel begins annually, while the last panel is in its second year (http://www.meps.ahrq.gov/survey_comp/hc_data_collection.jsp). Each panel typically contains about 30,000 to 35,000 individual respondents.

MEPS was initiated in 1996, but only began collecting health status data in the form of 12-Item Short Form Survey (SF-12) responses in 2000. For GBD 2016 we used data from 2000–2014. Respondents self-administer the SF-12 twice per panel, at rounds two and four, typically about a year apart. Only adults 18 years and older completed the SF-12. MEPS also usually collects information on diagnoses based on self-report of reasons for encounters with health services. In addition, diagnoses are derived through

additional questions on “problems that bother you” or conditions that led to “disability days,” ie, days out of role due to illness. Professional coders translate the verbatim text into three-digit ICD-9 codes. The main reason for other MSK being measured in MEPS relates to health care contact.

In order to derive a crosswalk of SF-12 values into a scale comparable with that used by the GBD disability weights, small studies on convenience samples were conducted asking respondents to fill in SF-12 to reflect 62 lay descriptions of diverse severity that were used to derive the GBD disability weights. From these responses a relationship between SF-12 summary score and the GBD DWs was derived. With regression methods, average disability weights were calculated for each of 156 conditions for which there were corresponding diagnoses in MEPS, while controlling for any comorbid other condition by adding dummy variables for each condition. We binned the amount of DW attributed to other MSK across the seven health states assuming thresholds at the midpoints between DW values.

Congenital birth defects

Overview and Cause List

This write-up covers the following causes: congenital heart defects, neural tube defects, cleft lip and cleft palate, congenital anomalies of the urogenital system, congenital anomalies of the gastrointestinal tract, musculoskeletal congenital anomalies, congenital chromosomal birth defects (Down Syndrome, Turner Syndrome, Klinefelter Syndrome, and other chromosomal abnormalities, genetic syndromes and micro-deletions).

We have estimated the prevalence and associated disability of the following categories of congenital birth defects (those in bold are GBD causes):

1. **Neural tube defects**
    a. Anencephaly
    b. Encephalocele
    c. Spina bifida
2. **Congenital heart defects**
    a. Single ventricle and single ventricle pathway defects
    b. Complex congenital heart defects excluding single ventricle and single ventricle pathway defects
    c. Malformations of great vessels, congenital valvular heart disease and patent ductus arteriosis
    d. Ventricular septal defect and atrial septal defect
    e. Other congenital cardiovascular anomalies
3. **Orofacial clefts: Cleft lip and cleft palate**
4. **Total chromosomal congenital birth defects**
    a. Down Syndrome
    b. Turner Syndrome
    c. Klinefelter Syndrome
    d. Other chromosomal abnormalities, genetic syndromes, and micro-deletions
        i. Edwards Syndrome and Patau Syndrome
        ii. Other chromosomal abnormalities, genetic syndromes, and micro-deletions
5. **Congenital anomalies of the urogenital system**
    a. Congenital urinary anomalies
    b. Congenital genital anomalies
6. **Congenital anomalies of the digestive system**
    a. Congenital diaphragmatic hernia
    b. Congenital malformations of the abdominal wall
    c. Congenital atresia and/or stenosis of the gastrointestinal tract
    d. Other congenital malformations of the gastrointestinal tract
7. **Musculoskeletal congenital anomalies**
    a. Polydactyly and syndactyly
    b. Limb reduction defects
    c. Other musculoskeletal congenital anomalies
8. **Other congenital anomalies: all birth defects (excluding minor anomalies) not contained in the other categories.**

This appendix will first describe the input data sources and aspects of the modelling strategy that are common to all sub-types of congenital anomalies. We will then provide a description of the case definitions, ICD-10 codes, and health states associated with each of the component congenital causes, as well as the specific modelling strategies employed in each congenital cause, including the model settings, study-level and country-level covariates, and other modelling decisions made.

Flowchart

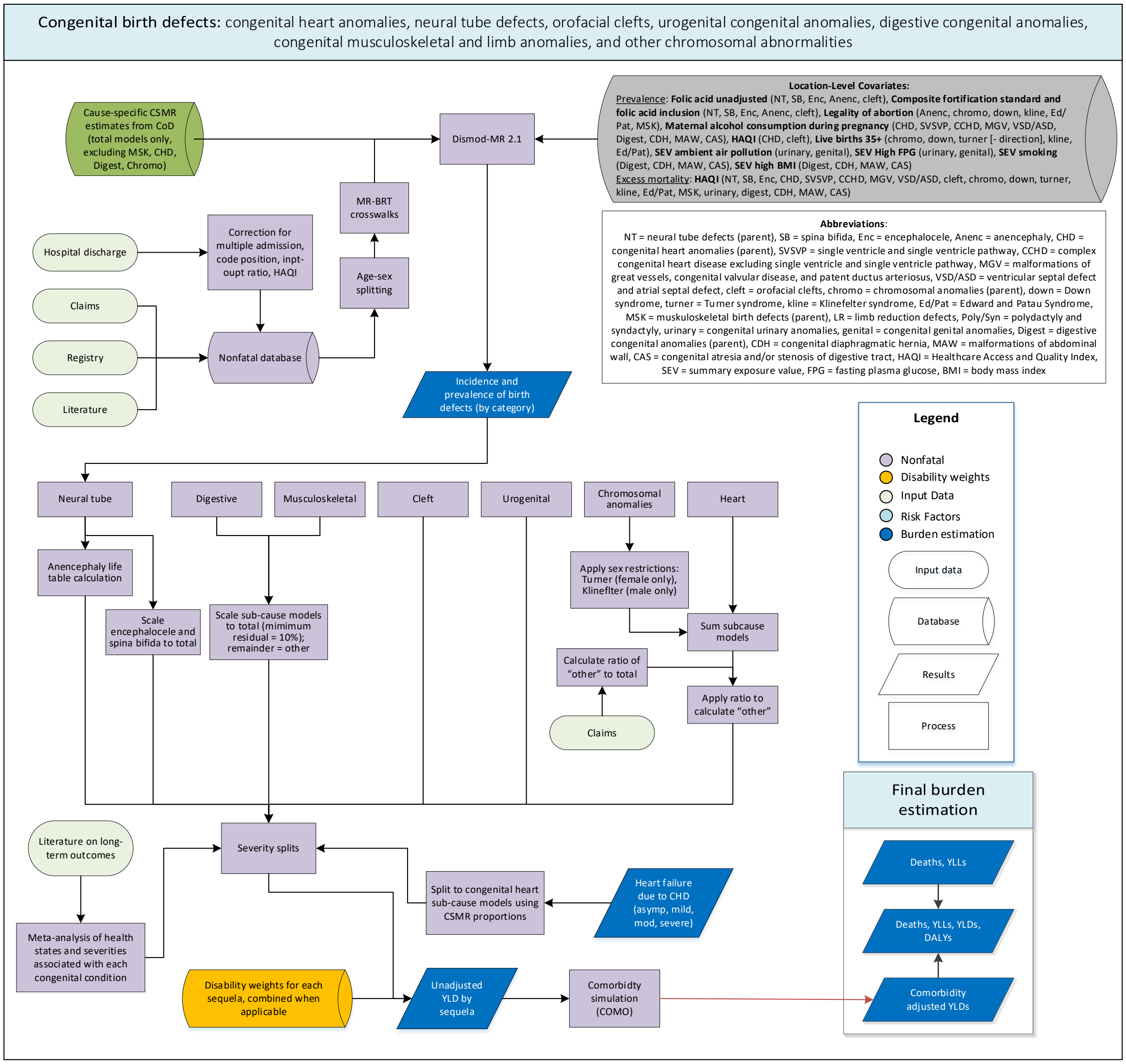

Case Definition

The GBD case definition of congenital anomalies includes any condition present at birth that is a result of abnormalities of embryonic development, excluding those that are directly the result of infections or substance abuse (e.g. fetal alcohol syndrome, congenital syphilis) modeled elsewhere in GBD and excludes minor anomalies as they are defined by EUROCAT.

Input Data

Several types of data sources are used in the estimation of congenital anomalies: literature prevalence, with-condition mortality and excess mortality data, birth prevalence and neonatal with-condition mortality data from a number of international birth defects registries and surveillance systems, inpatient hospital and Marketscan claims data prepared internally by the GBD research team, and cause-specific mortality estimates produced by the causes of death analysis.

First, We extracted data from a number of international birth defects registries. The International Clearinghouse for Birth Defects Surveillance and Research (ICBDSR) reports birth prevalence from a number of international member registries. The World Atlas Report also published birth prevalence estimates from these international registries prior to the publication of ICBDSR reports. The European Surveillance of Congenital Anomalies (EUROCAT) reports the birth prevalence of anomalies for a variety of locations in Western Europe as reported by participating member registries. China's Maternal and Child Health Surveillance survey (MCHS) reports birth prevalence and early neonatal mortality data for all subnational locations of China. The National Birth Defects Prevention Network (NBDPN) reports birth prevalence estimates as compiled by a number of subnational registries within the United States. The Birth Defects Registry of India (BDRI) reports congenital anomalies from participating hospitals within India.

Second, we used ionpatient hospital and claims data (from USA, Taiwan, and Singapore) for all congenital anomalies causes and sub-cause models. These data were prepared centrally by the clinical informatics research team and is described in detail in the Clinical Informatics section of this appendix. Four rounds of data bias correction were employed in the processing of clinical data. This included 1) adjustment for readmission, 2) correction of primary diagnoses to all diagnoses, 3) adjustment for inpatient-to-outpatient ratio, and 4) adjustment based on Healthcare Access and Quality Index (HAQI). Of note, in GBD 2017 we used congenital birth defects data only using the first two corrections, but changed in GBD 2019 to use clinical data that had all four corrections applied. This change was factilitated by improvements in analysis of corrections by the clinical informatics team and was a change made across GBD. Of note, we also changed the mapping of club foot and hip dysplasia in GBD 2019. Previously they were mapped to "limb reduction defects," but in preparatioin for disaggregated models (which is planned for the next time they are estimated in GBD), they are now included only in the total for musculoskeletal birth defects.

Third, we included data from a systematic review of the available literature for all types of congenital birth defects that was completed in GBD 2015 by constructing search strings designed to capture information on the prevalence, associated mortality and long-term health outcomes associated with each sub-category of congenital anomalies. All results were screened – first abstracts, then full-text screenings – to ensure the availability of required information and the representativeness of the reported population, and the exclusion of duplicate data also reported as part of the birth registry data inputs.

Table 1: Data inputs for modeling prevalence of congenital causes

Cause	Total Sources	Countries with Data
Congenital birth defects (all measures)	2065	104
Prevalence	1875	97
With condition mortality rate	160	41
Proportion	52	27
Other	7	5
Neural tube defects (all measures)	1677	88
Prevalence	1663	88
With condition mortality rate	10	6
Proportion	8	3
Congenital heart anomalies (all measures)	1717	93
Prevalence	1623	88
With condition mortality rate	98	28
Other	7	5
Orofacial clefts (all measures)	1619	87
Prevalence	1616	87
With condition mortality rate	5	2
Down syndrome (all measures)	1661	75
Prevalence	1626	73
With condition mortality rate	23	17
Proportion	21	21
Turner syndrome (all measures)	777	46
Prevalence	773	46
With condition mortality rate	2	2
Proportion	3	1
Klinefelter syndrome (all measures)	769	43
Prevalence	766	43
Proportion	3	1
Other chromosomal abnormalities (all measures)	1327	67
Prevalence	1304	65
Proportion	23	22
Congenital musculoskeletal and limb anomalies (all measures)	1639	87
Prevalence	1635	87
With condition mortality rate	2	1
Proportion	2	1
Urogenital congenital anomalies (all measures)	1709	93
Prevalence	1697	92
With condition mortality rate	7	4
Proportion	7	5
Digestive congenital anomalies (all measures)	1758	80
Prevalence	1716	76
With condition mortality rate	45	16
Proportion	7	6

Data processing

Age-sex splitting

Any data that was not sex-specific or did not fit entirely within GBD age-groups were age- and sex-split to fit these groups prior to modeling using empirical age- and sex-patterns derived from previous DisMod-MR 2.1 models of the same condition. This is a change from GBD 2017 when age- and sex-

splitting of data was not completed prior to modeling and had a substantial effect on the magnitude of estimates in those causes for which cause-specific mortality rate (CSMR) data was used in modeling. This is described further below.

Crosswalks in MR-BRT

A number of the input data sources used for the estimation of congenital birth defects are known to have biases leading to under-reporting or over-reporting relative to the true prevalence of congenital anomalies among live births and all subsequent age groups. We used Meta Regression – Bayesian, Regularised Trimmed (MR-BRT) to develop statistical models that were used to adjust non-reference data. The alternate definitions that were crosswalked are described below. The specifics of each MR-BRT crosswalk are shown in the corresponding cause-specific sections.

Live/Stillbirths: Where necessary, we used a crosswalk to adjust for the inclusion of stillbirths in the reported birth prevalence estimates in literature and registry data sources, as stillbirths are not included in our case definition of prevalence among live births. Each of these crosswalks used a spline on log-transformed neonatal mortality rate.

Exclusion of chromosomal conditions: Some sources report birth defects on in isolation (i.e. excluding any persons who have a coexisting genetic or chromosomal disorder). Our reference definition is the inclusion of chromosomal diagnoses. No splines were used in these crosswalks.

Registry to total: For a subset of congenital causes, particularly the congenital heart defects, we noted substantial differences in the lists of case definitions being reported to the various congenital registries. Across all types of congenital heart defects, the National Birth Defects Prevention Network (NBDPN) had the most complete list of reported case definitions – i.e. the highest case ascertainment – and was considered the gold standard among all birth registry data sources. We used registry-specific crosswalks to adjust all other birth defects registries to match the case ascertainment seen in the NBDPN. No splines were used in these crosswalks.

Determining outliers and data thresholds

Underreporting of congenital birth defects is common and can vary by source, location, year, sex, and age. In order to have an empirical, systematic approach to outliering of data, we adapted the non-zero floor approach used by the GBD cause-specific mortatlity analysis. After all age-sex splitting and crosswalking was complete, the first step was to calculate median absolute deviation (MAD) for the age group of birth, where registry and literature data were combined with all clinical data for the early neonatal age group (0 to 6 days). The thresholds chosen were -0.5 MAD and +3 MAD with any data outside of these bounds being identified as outliers. This was determined based on the right skewed distribution observed in most of the congenital data and the expert prior that underreporting is far more prevalent than overreporting – and therefore the bias is asymmetric. In any case where the lower MAD bound was negative, we used a threshold of 0.

For most models, we calculated the MADs using only the EUROCAT data, which we found to be the most reliable source for prevalence of congenital disorders. Exceptions were neural tube defects (all data sources), Urinary birth defects (EUROCAT and USA claims data), musculoskeletal defects (only USA claims data), and chromosomal anomalies, which differed by condition given the high volume of zeroes in the data. For Down Syndrome, we used all data. For Edward Syndrome and Patau Syndrome, we used all non-zero EUROCAT data. For Turner and Klinefelter syndrome, we used EUROCAT data and logged mean absolute deviation and exponentiated this to determine bounds for these data.

To evaluate data for older age groups, we employed two approaches. First, we outliered data from any location-year-source that was outliered for the first stage MAD algorithm. Second, using all clinical and

literature data, we developed a model with fixed effects by age to estimated implied MAD bounds for each non-zero age group and again applied the same thresholds of -0.5 MAD and +3 MAD.

Modelling Strategy

Overview

All available input data was utilized in a series DisMod-MR 2.1 models in order to estimate the prevalence of each category of congenital anomalies across the full life course for each location/age/sex combination. Incidence was set to 0 for all congenital models, as congenital conditions occur at the time of birth and by GBD case definition, congenital cases do not occur after birth. Remission was allowed only in the models of a select subset of causes for which surgical intervention or spontaneous remission can completely eliminate the disability due to that congenital condition. Cause-specific priors and slope priors were used to guide biologically plausible DisMod-MR 2.1 estimates of excess mortality and remission where applicable.

For most of the congenital birth defects causes, we ran DisMod-MR 2.1 models of all defects combined (termed "parent" models). This allowed us to use data on all anomalies within each cause as well as to leverage cause-specific mortality rate (CSMR) results from the GBD cause of death (COD) analysis. When CSMR data is used as an input, DisMod-MR 2.1 pairs each CSMR datum with a mathing prevalence data point by age, sex, location, and year. After matching, CSMR is divided by prevalence to calculate an implied excess-mortality rate (EMR) datum. All EMR data is then used in driving the model. Of note, EMR data is not calculated when prevalence data is of broader than GBD age groups or is for both sexes combined.

We used CSMR as input to all of the models except congenital heart disease, chromosomal anomalies, digestive anomalies, musculoskeletal birth defects, and urogenital congenital anomalies. For congenital heart defects, the reason is that excess mortalilty would be underestimated in older ages if CSMR results are used because despite continuing higher rates of mortality through adolescence and adulthood, many of these deaths are not coded as being due to congenital heart disease. Similarly, musculoskeletal and gastrointestinal anomalies estimates for CSMR in older children, adolescents, and adults are much lower than would be suggested by cohort and cross-sectional studies of survival as few of these deaths are coded as being due to the congenital birth defect present. Finally, for urogenital congenital anomalies, in addition to our modeling urinary and genital anomalies separately, the mechanism of death in older ages will typically be via development of chronic kidney disease and these deaths are classified in GBD as being due to chronic kidney disease due to other conditions. Details are in each cause-specific section below.

Location-level Covariates

Location-level covariates were used in each of the congenital DisMod-MR 2.1 models based on published information about the risk factors for these birth defects. Folic acid availability was used as a covariate on prevalence for all neural tube defects models and a subset of the congenital musculoskeletal anomalies models. A folic acid fortification covariate was used in the neural tube defects and cleft models, which was modelled based on data from the Global Fortification Data Exchange. The legality of abortion was used as a covariate on prevalence for conditions in which prenatal diagnosis is commonly available and the prognosis is severe enough to cause high rate of termination of pregnancy following prenatal diagnosis: these include all chromosomal conditions and a subset of the congenital heart defects. Maternal consumption of alcohol during pregnancy, as a proportion of all pregnancies, was used as a covariate on prevalence for all congenital heart defects. The proportion of live births by mothers age 35+ was used as a covariate on all chromosomal models. Across many of the congenital models, the Health Access and Quality Index (HAQI) covariate was used to guide

the global pattern of with-condition mortality and excess mortality, as was the natural log of the lag-distributed income per capita (LN-LDI). For most of the severe congenital conditions, the mortality associated with the condition is highly dependent on access to adequate surgical interventions and other medical care during the first hours, weeks, and years of life.

Post-model processing

For those causes with a parent model (neural tube defects, We then squeezed the sum of the specific sub-cause prevalence estimates to these total prevalence estimates in order to ensure internal consistency of our cause-level and sub-cause estimates. The prevalence of other heart, musculoskeletal, and gastrointestinal anomalies was derived by reducing the total envelope model for each cause by its sub-causes to derive the difference that was attributable to other anomalies in that category.

Assigning health states and sequelae for long-term outcomes

To determine the distribution of health outcomes associated with the congenital causes, we performed a review of available literature on the long-term health outcomes of survivors in cohorts born with each type of congenital malformation. For conditions requiring surgical intervention shortly after birth to ensure survival, the health states included in the disability weight calculations correspond to the post-surgery outcomes reported in cohorts of individuals born with these life-threatening congenital conditions. Where data was available from multiple cohorts, we pooled these cohorts together to calculate the proportion of individuals with each health state. Where data on the joint distribution of the long-term health outcomes was not available, we assumed independence of each long-term health outcome. Combined disability weights were calculated for all necessary combinations of existing disability weights.

Neural tube defects

Neural tube defects (parent)

In order to ensure internal consistency of the estimates of each sub-type of neural tube defects, we developed a model of the total prevalence of neural tube defects and used these location, year, sex and age-specific prevalence estimates to scale the estimates of anencephaly, encephalocele and spina bifida prevalence. This modelling strategy allowed us to incorporate the cause-specific mortality estimates from the GBD Cause of Death analysis and also allowed us to use literature data where the prevalence and mortality estimates were reported for the total of all neural tube defects only.

Crosswalks

The MR-BRT crosswalk results are shown below.

Table 1: MR-BRT crosswalk betas for alternate definitions (reference = livebirths including those with chromosomal anomalies)

Crosswalk	Beta	Standard Error
Excluding chromosomal diagnoses adjustment	-0.038	0.028
Adjustment for registry specific case definitions (World Atlas)	-0.156	0.005

Figure 1: MR-BRT crosswalk of alternate definition (livebirths and stillbirths included) with spline on log-transformed neonatal mortality rate.

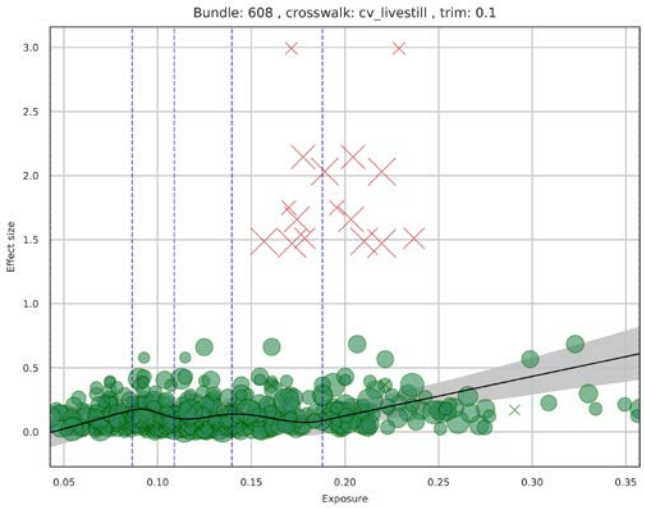

Modelling strategy

The DisMod-MR 2.1 model of total neural tube defects used cause-specific mortality (CSMR) estimates from the GBD cause of death analysis for neural tube defects. This model had a minimum excess mortality of 0.5 for the first week of age and a minimum excess mortality of 0.0003 for ages 1-100 as the risk of death due to neural tube defects is greatest shortly after birth. The model also used an increased smoothness (maximum xi=3) on excess mortality rate in order to allow high excess mortality in the early neonatal age group. Random effects on prevalence were limited to 0-0.75 in order to limit geographic variation in the estimated birth prevalence, and all min cv settings were 0.8.

Table 2. Location-level covariate effects

Covariate Name	Measure	Beta value	Exponentiated value
Folic acid unadjusted (ug)	Prevalence	-0.00121 (-0.00147 - -0.00096)	1.00 (1.00 - 1.00)
Composite fortification standard and folic acid inclusion	Prevalence	-0.00207 (-0.00498 - -0.00018)	1.00 (1.00 - 1.00)
HAQI	EMR	-0.04511 (-0.04574 - -0.04441)	0.96 (0.96 - 0.96)

Anencephaly

Case definition and associated health states

Anencephaly is the absence of a major portion of the brain, skull, and scalp. Anencephaly corresponds to the ICD-10 codes Q00.0 and Q00.2. All infants with anencephaly are assigned the health state of severe motor and cognitive impairment.

Crosswalks

The MR-BRT crosswalk results are shown below.

Table 1: MR-BRT crosswalk betas for alternate definitions (reference = livebirths including those with chromosomal anomalies)

Crosswalk	Beta	Standard Error
Excluding chromosomal diagnoses adjustment	-0.038	0.097

Figure 1: MR-BRT crosswalk of alternate definition (livebirths and stillbirths included) with spline on log-transformed neonatal mortality rate.

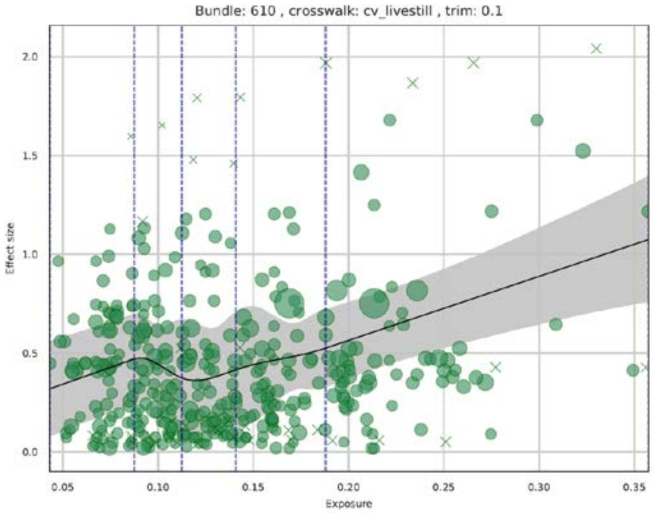

Modelling strategy

The life expectancy for infants born with anencephaly is on the order of hours or days; none of these infants survive past the neonatal age period. Because of the extremely high excess mortality associated with this condition and the short age range over which the prevalence varies, we used a custom modelling process to estimate the prevalence of anencephaly. We first used DisMod-MR 2.1 to model the prevalence of anencephaly at birth for every location, year, age and sex combination. We then used literature data on outcomes largest available cohort of infants born with anencephaly

[1] [2], using the precise time of death information from this cohort to create a life table that applied the high excess mortality rates to all cases of anencephaly at birth.

We applied these mortality rates to both sex and all locations, generating the time lived by infants with anencephaly during the early and late neonatal age groups by location, year and sex. We then used GBD 2019 mortality estimates to calculate the time lived by all infants during the early and late neonatal age groups by location, year and sex, and used these two values to calculate the prevalence of anencephaly in the early and late neonatal age groups; after one month of age, all available literature indicates that no infants born with anencephaly are still alive.

The DisMod-MR 2.1 model for the birth prevalence of anencephaly has random effects on prevalence limited to +- 0.5. As this model was designed to estimate only the prevalence at birth, incidence, remission and excess mortality were set to zero for all ages, and the only age mesh points were 0 and 100 years of age.

Table 2. Location-level covariate effects

Covariate Name	Measure	Beta value	Exponentiated value
Legality of Abortion	Prevalence	-0.00317 (-0.00561 - -0.00078)	1.00 (0.99 - 1.00)
Folic acid unadjusted (ug)	Prevalence	-0.00040 (-0.00103 - -0.00001)	1.00 (1.00 - 1.00)
Composite fortification standard and folic acid inclusion	Prevalence	-0.28278 (-0.40404 - -0.16779)	0.75 (0.67 - 0.85)

Encephalocele

Case definition and associated health states

Encephalocele is characterized by sac-like protrusions of the brain and meninges through openings in the skull. Encephalocele corresponds to the ICD-10 codes Q01.2, Q01.8, and Q01.9. Our case definitions of spina bifida and encephalocele do not consider surgical intervention for either condition as remission.

Cases of spina bifida and encephalocele are split into every combination of mild, moderate, and severe motor impairment, all severities of intellectual disability, and urinary incontinence. These proportions were calculated using a pooled analysis of available literature on the long-term outcomes in cohorts of individuals born with each sub-type of neural tube defects. The distribution of health states associated with encephalocele[3 4 5] was derived separately from the distribution of health states associated with spina bifida[6 7], although these two categories of neural tube defects are associated with the same list of long-term outcome sequela.

Crosswalks

The MR-BRT crosswalk results are shown below.

Table 1: MR-BRT crosswalk betas for alternate definitions (reference = livebirths including those with chromosomal anomalies)

Crosswalk	Beta	Standard Error
Excluding chromosomal diagnoses adjustment	-0.068	0.074

Figure 1: MR-BRT crosswalk of alternate definition (livebirths and stillbirths included) with spline on log-transformed neonatal mortality rate.

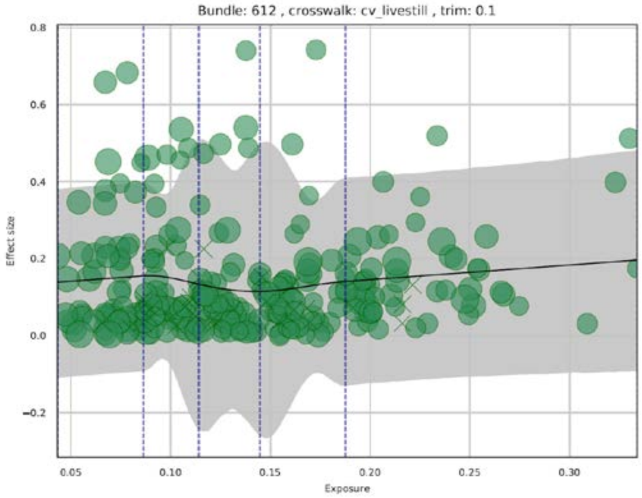

Modelling strategy

The DisMod-MR 2.1 model for encephalocele had a minimum excess mortality prior of 0.2 for the first week of age and a minimum excess mortality prior of 0.0003 for ages 1-54. Excess mortality was set to 0 thereafter, as we believe that those with encephalocele would no longer be dying of this condition past age 55. The model also used an increased smoothness on excess mortality rate (maximum xi=3). Random effects on prevalence were limited to +- 0.5 as we expect limited geographic variation in the birth prevalence of encephalocele.

Table 2. Location-level covariate effects

Covariate Name	Measure	Beta value	Exponentiated value
Legality of Abortion	Prevalence	-0.00441 (-0.00582 - -0.00291)	1.00 (0.99 - 1.00)
Folic acid unadjusted (ug)	Prevalence	-0.00116 (-0.00164 - -0.00068)	1.00 (1.00 - 1.00)

Composite fortification standard and folic acid inclusion	Prevalence	-0.29416 (-0.37040 - -0.22119)	0.75 (0.69 - 0.80)
HAQI	EMR	-0.15055 (-0.29326 - -0.00733)	0.86 (0.75 - 0.99)

Spina bifida

Case definition and associated health states

Spina bifida is when part of the spinal cord and/or meninges are uncovered by skin. Spina bifida occulta, a much less severe form of spina bifida, in which the defect in vertebral column remains covered by skin, is excluded from the GBD case definition of spina bifida. Spina bifida corresponds to the ICD-10 codes Q05.0, Q05.4, Q05.6, Q05.7, Q05.8, and Q05.9. Our case definitions of spina bifida and encephalocele do not consider surgical intervention for either condition as remission.

Cases of spina bifida and encephalocele are split into every combination of mild, moderate, and severe motor impairment, all severities of intellectual disability, and urinary incontinence. These proportions were calculated using a pooled analysis of available literature on the long-term outcomes in cohorts of individuals born with each sub-type of neural tube defects. The distribution of health states associated with encephalocele[8 9 10] was derived separately from the distribution of health states associated with spina bifida[11 12], although these two categories of neural tube defects are associated with the same list of long-term outcome sequela.

Crosswalks

The MR-BRT crosswalk results are shown below.

Table 1: MR-BRT crosswalk betas for alternate definitions (reference = livebirths including those with chromosomal anomalies)

Crosswalk	Beta	Standard Error
Excluding chromosomal diagnoses adjustment	-0.036	0.034

Figure 1: MR-BRT crosswalk of alternate definition (livebirths and stillbirths included) with spline on log-transformed neonatal mortality rate.

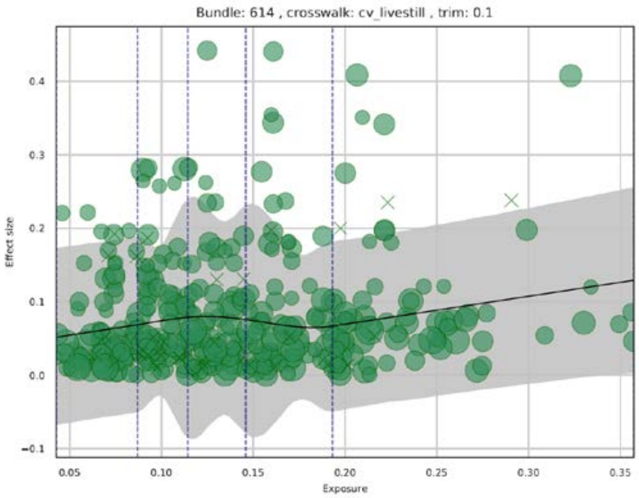

Modelling strategy

The DisMod-MR 2.1 model for spina bifida had a minimum excess mortality of 0.2 for the first week of age, and a minimum of 0.0002 for ages 1+, and a maximum smoothness on excess mortality rate of xi=3. Random effects on prevalence were also limited to +- 0.5.

Table 2. Location-level covariate effects

Covariate Name	Measure	Beta value	Exponentiated value
Legality of Abortion	Prevalence	-0.00789 (-0.00919 - -0.00662)	0.99 (0.99 - 0.99)
Folic acid unadjusted (ug)	Prevalence	-0.00181 (-0.00225 - -0.00132)	1.00 (1.00 - 1.00)
Composite fortification standard and folic acid inclusion	Prevalence	-0.07511 (-0.14490 - -0.01463)	0.93 (0.87 - 0.99)
HAQI	EMR	-0.15033 (-0.29258 - -0.00801)	0.86 (0.75 - 0.99)
HAQI	WCMR	-0.01992 (-0.02211 - -0.01769)	0.98 (0.98 - 0.98)

Post-model processing

Prevalence of spina bifida and encephalocele were summed and scaled to match the total for neural tube defects parent model by location, age group, sex, and year. Age-specific anencephaly prevalence was calculated separately as described above.

Congenital heart anomalies

Summary and associated health states

There are many distinct types of congenital heart anomalies with a range of anatomical patterns, severities, and requirements for medical treatment. For the purpose of estimating nonfatal outcomes, in GBD 2017 congenital heart anomalies were split into five-sub categories based on both the anatomical characteristics and the treatment requirements of each condition.

1. Single ventricle and single ventricle pathway defects
2. Complex congenital heart defects excluding single ventricle and single ventricle pathway defects
3. Malformations of great vessels, congenital valvular heart disease and patent ductus arteriosis
4. Ventricular septal defect and atrial septal defect
5. Other congenital cardiovascular anomalies

We also began development of a model of total congenital heart anomalies, but this was not used in scaling the subcauses for GBD 2019. Instead, we used claims data to calculate a ratio of other-to-total and this was applied to the sum of the other four subcauses for each location, age group, sex, and year.

Every case of congenital heart defects was associated with a health state of congenital heart disease, except for a proportion of ventricular and atrial septal defects which are considered asymptomatic. All congenital heart defects cases were split into a proportion without intellectual disability and a proportion with every severity from borderline to profound intellectual disability. The proportion of congenital heart anomalies cases experiencing each severity of intellectual disability were calculated using available literature sources on the prevalence and severity of intellectual disability in congenital heart defect populations[13] [14] [15] . The proportion of VSD/ASD cases attributed to the asymptomatic category was derived from literature sources on the long-term outcomes of patients diagnosed with septal defects at birth[16] [17] [18]. GBD estimates of congenital heart failure were assigned to the congenital heart defect categories according to the proportion of total congenital heart cause-specific mortality assigned to each category of congenital heart defects.

Total congenital heart anomalies

Crosswalks

The MR-BRT crosswalk results are shown below.

Table 1: MR-BRT crosswalk betas for alternate definitions (reference = livebirths including those with chromosomal anomalies)

Crosswalk	Beta	Standard Error
Excluding chromosomal diagnoses adjustment	-0.096	0.006

Figure 1: MR-BRT crosswalk of alternate definition (livebirths and stilbirths included) with spline on log-transformed neonatal mortality rate.

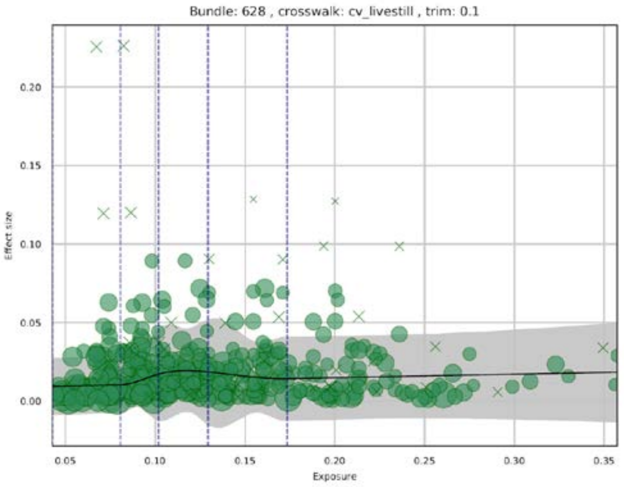

Modelling strategy

In the DisMod-MR 2.1 model of total congenital heart anomalies, random effects on prevalence were limited to +- 0.5 in order to limit geographic variation in the estimates of birth prevalence. The minimum excess mortality rate for the neonatal age range was set to 5.0. The smoothness on excess mortality rate was increased to Xi=5.0 in order to allow high excess mortality in the neonatal age groups and lower excess mortality rates in older ages.

Table 2. Location-level covariate effects

Covariate Name	Measure	Beta value	Exponentiated value
Maternal alcohol consumption during pregnancy (proportion)	Prevalence	0.17046 (0.01367 - 0.37530)	1.19 (1.01 - 1.46)
Healthcare access and quality index	Prevalence	0.00087 (0.00007 - 0.00202)	1.00 (1.00 - 1.00)
Healthcare access and quality index	EMR	-0.15320 (-0.29760 - -0.00718)	0.86 (0.74 - 0.99)

Single ventricle and single ventricle pathway defects

Case definition

Single ventricle and single ventricle pathway defects include tricuspid atresia, hypoplastic left heart syndrome, mitral valve atresia, single left ventricle, double outlet right ventricle, and pulmonary atresia; the corresponding ICD-10 codes are Q20.1, Q20.2, Q20.4, Q22.4, Q22.6 and Q23.4. Each of the single ventricle and single ventricle pathway conditions requires surgical intervention shortly after birth to ensure infant survival.

Crosswalks

The MR-BRT crosswalk results are shown below.

Table 1: MR-BRT crosswalk betas for alternate definitions (reference = livebirths including those with chromosomal anomalies)

Crosswalk	Beta	Standard Error
Excluding chromosomal diagnoses adjustment	-0.066	0.023
Adjustment for registry specific case definitions (World Atlas)	-0.752	0.035
Adjustment for registry specific case definitions (ICBDMS)	-0.751	0.036
Adjustment for registry specific case definitions (Congenital Malformations Worldwide)	-0.754	0.036

Figure 1: MR-BRT crosswalk of alternate definition (livebirths and stillbirths included) with spline on log-transformed neonatal mortality rate.

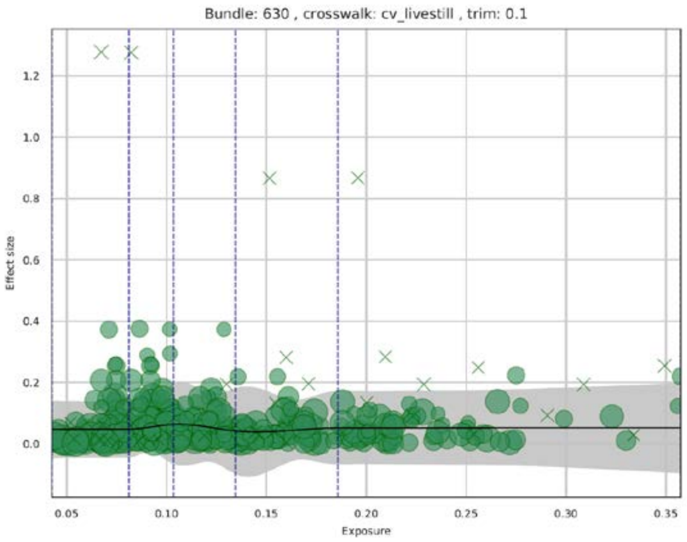

Modelling strategy

In the DisMod-MR 2.1 model of single ventricle and single ventricle pathway heart defects, random effects on prevalence were limited to +- 0.5 in order to limit the estimated geographic variation in birth prevalence. A minimum excess mortality rate of 8 was set for the early neonatal period in order to capture the high mortality risk, based on expert priors and a review available literature on the mortality risk among infants born with single ventricle and single ventricle pathway heart defects. The smoothness on excess mortality rate was set to 5.0 in order to fit steep changes in the excess mortality rate during the first weeks of life, as the risk of death due to these congenital heart anomalies is greatest shortly after birth and diminishes over the life course.

Table 2. Location-level covariate effects

Covariate Name	Measure	Beta value	Exponentiated value
Maternal alcohol consumption during pregnancy (proportion)	Prevalence	0.23369 (0.02821 - 0.45690)	1.26 (1.03 - 1.58)
HAQI	EMR	-0.04909 (-0.09541 - -0.00156)	0.95 (0.91 - 1.00)

Complex congenital heart defects excluding single ventricle and single ventricle pathway defects

Case definition

Complex congenital heart defects excluding single ventricle and single ventricle pathway defects include common arterial trunk, common truncus, discordant ventriculoaterial connection, transposition of great vessels, atrioventricular septal defect, endocardial cushion defect, Tetralogy of fallot, aortopulmonary septal defect, pulmonary valve atresia, congenital stenosis of aortic valve, and total anomalous

pulmonary venous connection. This category of severe congenital heart defects includes ICD-10 codes Q20.0; Q20.3; Q21.2; Q21.3; Q21.4; Q22.0; Q23.0 and Q26.2.

Crosswalks

The MR-BRT crosswalk results are shown below.

Table 1: MR-BRT crosswalk betas for alternate definitions (reference = livebirths including those with chromosomal anomalies)

Crosswalk	Beta	Standard Error
Excluding chromosomal diagnoses adjustment	-0.223	0.014
Adjustment for registry specific case definitions (World Atlas)	-0.626	0.015
Adjustment for registry specific case definitions (ICBDMS)	-0.625	0.016

Figure 1: MR-BRT crosswalk of alternate definition (livebirths and stillbirths included) with spline on log-transformed neonatal mortality rate.

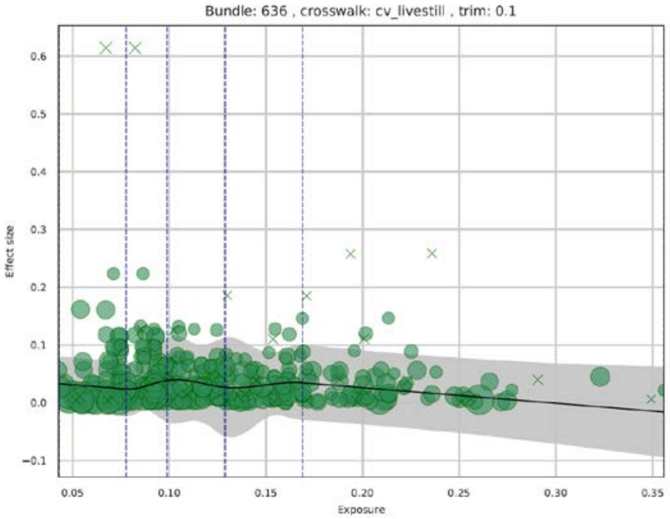

Modelling strategy

In the DisMod-MR 2.1 model of congenital heart defects excluding single ventricle and single ventricle pathway defects, random effects on prevalence were limited to +- 0.5. A minimum excess mortality rate of 1.0 for the early neonatal period was enforced in order to capture the high risk of mortality associated with these conditions, and a decreasing slope prior on excess mortality rate was applied for all ages. The smoothness on excess mortality rate was set to Xi = 3.0 in order to allow the model to fit steep changes in the mortality rate of these conditions in the neonatal age period.

Table 2. Location-level covariate effects

Covariate Name	Measure	Beta value	Exponentiated value
Maternal alcohol consumption during pregnancy (proportion)	Prevalence	0.18871 (0.01810 - 0.43850)	1.21 (1.02 - 1.55)
HAQI	EMR	-0.05045 (-0.09804 - -0.00408)	0.95 (0.91 - 1.00)

Malformations of great vessels, congenital valvular heart disease and patent ductus arteriosis

Case definition

Malformations of great vessels, congenital valvular heart disease and patent ductus arteriosis. The malformations of vessels and valves in this sub-cause category include Ebstein's anomaly, congenital

pulmonary valve stenosis, pulmonary valve insufficiency, other malformations of the pulmonary valve, malformations of the tricuspid valve, tricuspid atresia or stenosis, insufficiency of the aortic valve, mitral stenosis or insufficiency, and other malformations of aortic and mitral valves. Patent ductus arteriosis cases are only included among infants of >37 weeks gestational age, as premature infants often have minor patent ductus arteriosis that closes shortly after birth. The ICD-10 codes corresponding to the critical malformations of great vessels category include Q22.1, Q22.2, Q22.3, Q22.5, Q22.8, Q22.9, Q23.1, Q23.2, Q23.3, Q23.8, Q23, Q25.1, Q25.2, Q25.3, Q25.4, Q25.5, and Q25.0. The majority of these conditions require medical attention shortly within the first few weeks of life.

Crosswalks

The MR-BRT crosswalk results are shown below.

Table 1: MR-BRT crosswalk betas for alternate definitions (reference = livebirths including those with chromosomal anomalies)

Crosswalk	Beta	Standard Error
Excluding chromosomal diagnoses adjustment	-0.094	0.01
Adjustment for registry specific case definitions (World Atlas)	-1.079	0.021
Adjustment for registry specific case definitions (ICBDMS)	-1.08	0.021

Figure 1: MR-BRT crosswalk of alternate definition (livebirths and stillbirths included) with spline on log-transformed neonatal mortality rate.

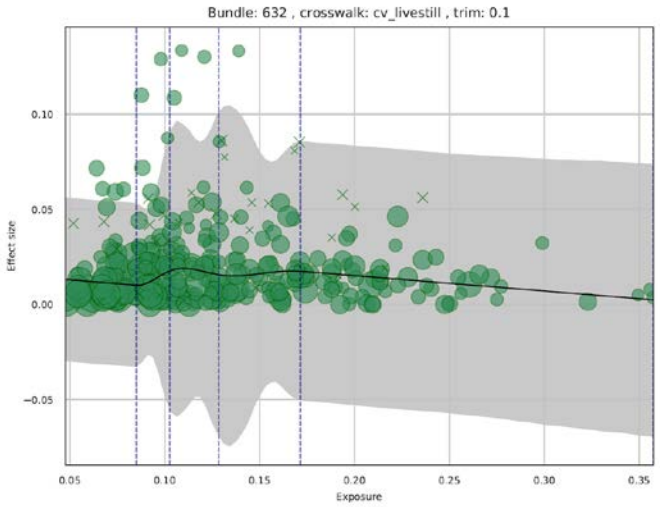

Modelling strategy

In the DisMod-MR 2.1 model of critical malformations of great vessels, congenital valvular heart disease and patent ductus arteriosis, random effects on prevalence were limited to +- 0.5. A minimum excess mortality rate of 1.0 was set for the early neonatal period in order to capture the high mortality risk associated with these conditions. The smoothness on excess mortality was increased to Xi = 3.0 in order to fit steep changes in the mortality associated with these conditions during and after the neonatal period, as the risk of death due to congenital heart anomalies is highest shortly after birth.

Table 2. Location-level covariate effects

Covariate Name	Measure	Beta value	Exponentiated value
Maternal alcohol consumption during pregnancy (proportion)	Prevalence	0.23645 (0.03553 - 0.45853)	1.27 (1.04 - 1.58)
HAQI	EMR	-0.04919 (-0.09692 - 0.00000)	0.95 (0.91 - 1.00)

Ventricular septal defects and atrial septal defects

Case definition

Ventricular septal defects and atrial septal defects, includes holes in the walls separating the chambers of the heart. Many of these septal defects close spontaneously, while other require surgical care. The ICD-10 codes corresponding to ventricular septal defect and atrial septal defect are Q21.0 and Q21.1, respectively.

Crosswalks

The MR-BRT crosswalk results are shown below.

Table 1: MR-BRT crosswalk betas for alternate definitions (reference = livebirths including those with chromosomal anomalies)

Crosswalk	Beta	Standard Error
Excluding chromosomal diagnoses adjustment	-0.082	0.006

Figure 1: MR-BRT crosswalk of alternate definition (livebirths and stillbirths included) with spline on log-transformed neonatal mortality rate.

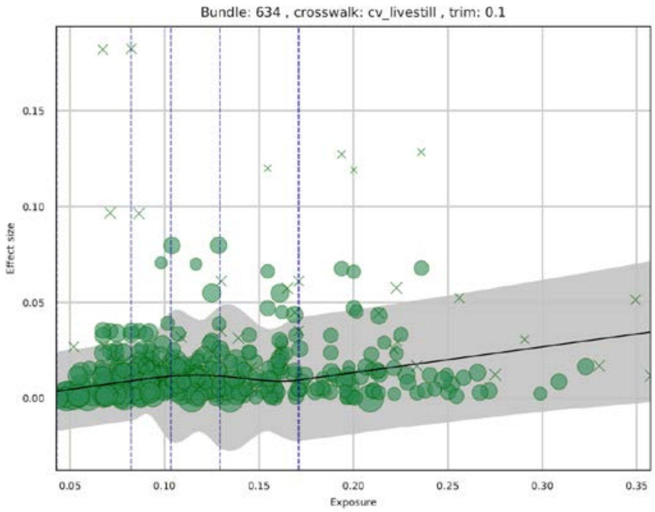

Modelling strategy

In the DisMod-MR 2.1 model of ventricular septal defects and atrial septal defects (VSD/ASD), remission was set to zero for all ages. Cases of septal defects that spontaneously close over time were considered as part of the asymptomatic proportion of VSD/ASD rather than remitted cases. Random effects on prevalence were limited to +- 0.3 in order to limit the random geographic variation in the estimated birth prevalence. No minimum excess mortality rate was set in this model, as VSD/ASD cases are not associated with excess mortality rates as high as the other subtypes of congenital heart defects. The smoothness on excess mortality rate was set to Xi=3.0, and a decreasing slope prior was set on remission for all ages, with remission set to 0 past age 10.

Table 2. Location-level covariate effects

Covariate Name	Measure	Beta value	Exponentiated value
Maternal alcohol consumption during pregnancy (proportion)	Prevalence	0.06761 (0.00336 - 0.17970)	1.07 (1.00 - 1.2)
HAQI	EMR	-0.14973 (-0.29700 - -0.00485)	0.86 (0.74 - 1.0)

Other congenital cardiovascular birth defects

Case definition

The fifth and final sub-cause category of congenital heart defects is other congenital cardiovascular anomalies, which correspond to ICD-10 codes Q27, Q27.1, Q27.2, Q27.3, Q27.30, Q27.31, Q27.32, Q27.33, Q27.34, Q27.39, Q27.4, Q27.8, Q27.9, Q28, Q28.0, Q28.1, Q28.2, Q28.3, Q28.8 and Q28.9.

Modeling strategy

Other congenital cardiovascular anomalies are modeled by applying the ratio of other congenital heart anomalies to total congenital heart anomalies as it is reflected in Marketscan data (a trusted data source), to the sum of the sub-causes of congenital cardiovascular anomalies. The result is prevalence of other congenital cardiovascular anomalies by age/year/sex/location. Specifically, we use claims data to calculate the proportion of cases that are due to the other causes. To do that, we sum the cases for the specified congenital subcauses and the other category subcauses. We divide the number of other subcause cases by the total number of cases to obtain the proportion. In order to have a valid proportion, we only use datapoints for which we have the combination of age, sex, location and year for all subcauses. We then calculate the prevalence of other: p_other = (p_sum_subcauses / 1-prop_other) - p_sub_subcauses.

Orofacial clefts

Case definition and associated health states

Orofacial clefts include isolated cleft lip, isolated cleft palate, and combined cleft lip and cleft palate. Cleft lip is an opening in the upper lip that may extend into the nose, and with cleft palate, the roof of the mouth contains an opening into the nose. Both conditions are the result of the tissues of the face not joining properly during development. The GBD case definition of orofacial clefts includes isolated cleft palate, which corresponds to ICD-10 codes Q35.2, Q35.3, Q35.5, Q35.6, Q35.7, Q35.8, and Q35.9, and cleft palate with or without cleft lip, which corresponds to ICD-10 codes Q36.0, Q36.1, Q36.9, Q37.1, Q37.5, Q37.8, and Q37.9. Craniofacial clefts that do not include the oropharynx are excluded.

These conditions can be successfully treated by surgery, which is typically done during the first few months or years of life but may occasionally be completed later in life. The sequelae associated with orofacial clefts are disfigurement level 1, disfigurement level 2, and disfigurement level 2 with speech problems. Additionally, a proportion of the population with orofacial clefts is considered to be asymptomatic. In the absence of data, we assumed the proportion of each is equal.

Crosswalks

The MR-BRT crosswalk results are shown below.

Table 1: MR-BRT crosswalk betas for alternate definitions (reference = livebirths including those with chromosomal anomalies)

Crosswalk	Beta	Standard Error
Excluding chromosomal diagnoses adjustment	-0.055	0.012

Figure 1: MR-BRT crosswalk of alternate definition (livebirths and stillbirths included) with spline on log-transformed neonatal mortality rate.

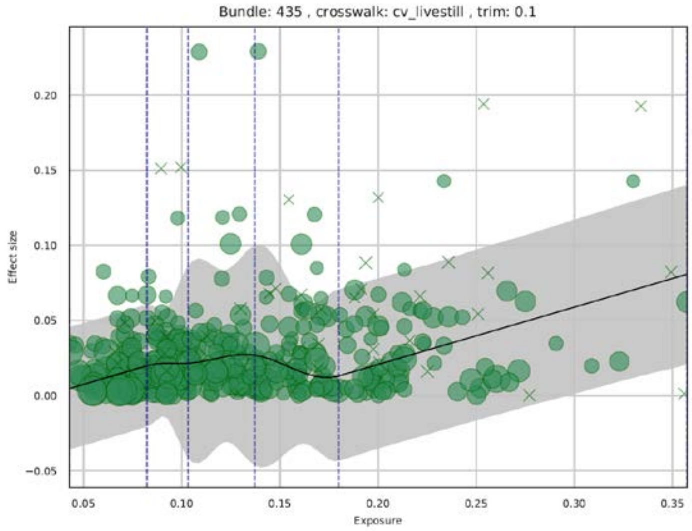

Modelling strategy

The DisMod-MR 2.1 model of orofacial clefts had random effects on prevalence limited to +- 0.8, as we expected limited variation in birth prevalence of orofacial clefts. The model settings allow increased smoothness on both excess mortality rate and remission (maximum Xi = 5.0) in order to fit steep changes in the rates mortality and remission during the first few years of life.

Incidence was set to zero for all ages. Remission was set to zero for the first three months of life, as cleft lip and/or palate are rarely corrected in the first few months of life. A maximum remission of 0.8 was set for ages three months to two years, the age range in which cleft repair is most commonly performed, allowing up to 75% of cleft cases to be repaired between three months and 2 years of age. Remission was bounded from 0 to 0.07 for ages 2 to 5 years, 0 to 0.004 for ages 5 to 20 years, then bounded from 0 to 0.002 for ages 20 to 50 years, and set at 0 for ages 50 years +. These limits on remission reflect our priors that up to 20% of remaining cleft cases are repaired between 2 and 5 years of age, another 5% may be repaired between 5 and 20 years of age, and a maximum 5% of remaining cases are surgically repaired between ages 20 and 50 years.

Priors on excess mortality rate were set at a maximum of 2.5 for the early neonatal period, 0.1 for ages 5-10, and 0.000001 for ages 10+. These limits on excess mortality reflect our priors that up to 5% of individuals with orofacial clefts die in the first week of life, up to 5% die in the following three weeks, up to 20% die in the next 11 months, another maximum of 20% before 5 years of ages, and a maximum of 5% of the remaining individuals die between ages 5 and 10 years.

Table 2. Location-level covariate effects

Covariate Name	Measure	Beta value	Exponentiated value
HAQI	Prevalence	-0.00021 (-0.00041 - -0.00003)	1.00 (1.00 - 1.00)
Folic acid unadjusted (ug)	Prevalence	-0.00039 (-0.00061 - -0.00016)	1.00 (1.00 - 1.00)
Composite fortification standard and folic acid inclusion	Prevalence	-0.02857 (-0.05797 - -0.00365)	0.97 (0.94 - 1.00)
LN-LDI (I$ per capita)	EMR	-0.74958 (-0.75000 - -0.74860)	0.47 (0.47 - 0.47)

Chromosomal Anomalies

In addition to Down syndrome, Turner syndrome, and Klinefelter syndrome, hundreds of different types of chromosomal abnormalities and other genetic syndromes have been identified, described, and categorized. Commonalties between genetic syndromes include the predisposition of affected persons to have dysmorphic body features, congenital heart disease, endocrine problems, and neurodevelopmental abnormalities that can lead to intellectual disability. Many of those with chromosomal abnormalities can be readily recognized or suspected by such features. While each has hallmark physical features and diagnostic criteria, most also require sophisticated laboratory facilities to confirm diagnosis, therefore, especially in lower resource settings, a large number of cases are diagnosed as having "unspecified chromosomal abnormalities" – an ICD code that corresponds to the GBD cause of "other chromosomal abnormalities." Additionally, most congenital birth defects registries have only limited scope as they only track a subset of genetic syndromes.

Total chromosomal anomalies

Crosswalks

The MR-BRT crosswalk results are shown below.

Figure 1: MR-BRT crosswalk of alternate definition (livebirths and stillbirths included) with spline on log-transformed neonatal mortality rate.

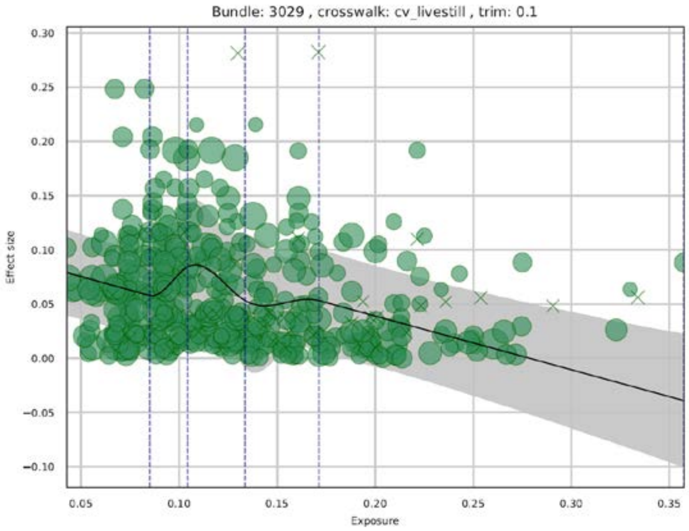

Modelling strategy

In order to maximize the data basis for estimating chromosomal abnormalities and genetic syndromes, we therefore completed an analysis of all chromosomal abnormalities together, leveraging cause-specific mortality results from the GBD COD analysis (for Down syndrome plus "other chromosomal abnormalities"), all prevalence data from registries, and clinical administrative data (hospital and claims). This model estimates total chromosomal abnormalities in DisMod-MR 2.1 and served as the basis for scaling the remaining specific causes (Down, Klinefelter, Turner, Edward/Patau) and estimating the remainder.

Table 2. Location-level covariate effects

Covariate Name	Measure	Beta value	Exponentiated value
Legality of Abortion	Prevalence	-0.00280 (-0.00471 - -0.00157)	1.00 (1.00 - 1.00)
Live Births 35+ (proportion)	Prevalence	0.06443 (0.04042 - 0.08748)	1.07 (1.04 - 1.09)
HAQI	EMR	-0.00012 (-0.00024 - -0.00002)	1.00 (1.00 - 1.00)

Down Syndrome

Case definition and associated health states

Down syndrome, also known as Trisomy 21, is the presence of a third copy of chromosome 21, typically caused by nondisjunction during the production of gametes. Down syndrome is associated with several specific physical characteristics, including decreased muscle tone, flat facial features, an upward slant to the eyes, abnormally shaped ears, a single deep crease across the center of the palm, folded skin on the inner corners of the eyes, and ability to extend joints beyond the usual, among others. The GBD case definition of Down syndrome includes ICD-10 codes Q90.0, Q90.1, Q90.2, and Q90.9.

Individuals with Down syndrome may have several combinations of sequelae: those included in the GBD sequelae list are intellectual disability, congenital heart disease, and dementia. The joint distribution of intellectual disability, congenital heart disease, and dementia associated with cases of Down Syndrome was derived from a review of literature on long-term outcomes in cohorts of Down Syndrome individuals. To calculate the severity distribution of intellectual disability due to Down Syndrome, we used literature values for the IQ distribution of individuals with Down Syndrome[19] and calculated the area under the curve. We obtained age-specific proportions of individuals with Down Syndrome and dementia, and thus global age patterns were modelled to calculate the proportion of the population with each combination of sequelae for each of the following age ranges: 0-44 years, 45-49 years, 50-54 years, 55-69 years, 70-79 years, and 80+ years.

Crosswalks

The MR-BRT crosswalk results are shown below.

Figure 1: MR-BRT crosswalk of alternate definition (livebirths and stillbirths included) with spline on log-transformed neonatal mortality rate.

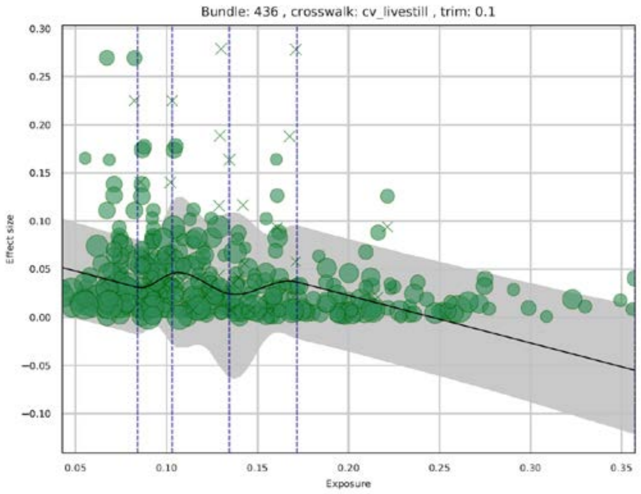

Modelling strategy

The DisMod-MR 2.1 model of Down Syndrome excluded all data with a prevalence of zero as outliers, as we expect that these low values are indicative of under-reporting in the data sources. The DisMod-MR 2.1 model used cause-specific mortality rate data from the corresponding Down Syndrome model in the GBD Cause of Death analysis, and converted these data to excess mortality rate estimates where matching prevalence data is available. Random effects on prevalence and excess mortality rate were limited to +- 0.1 in order to limit the geographic variation in birth prevalence allowed in the model. The maximum smoothness on excess mortality rate was increased to x= 3.0 in order to fit the observed steep decline in the mortality risk associated with Down Syndrome after the neonatal age range.

Of note, the use of cause-specific mortality data in the nonfatal model of Down Syndrome is a substantial change in the modelling strategy as compared to the previous iterations of the GBD, and results in much better-informed excess mortality estimates driving the Down Syndrome prevalence estimates across the life course.

Table 2. Location-level covariate effects

Covariate Name	Measure	Beta value	Exponentiated value
Legality of Abortion	Prevalence	-0.00391 (-0.00477 - -0.00309)	1.00 (1.00 - 1.00)
Live Births 35+ (proportion)	Prevalence	0.00749 (0.00033 - 0.02228)	1.01 (1.00 - 1.02)
HAQI	EMR	-0.06569 (-0.06676 - -0.06465)	0.94 (0.94 - 0.94)

Turner Syndrome

Case definitions and associated health states

Turner syndrome, also known as 45 XO, is a condition in which a female is partly or completely missing an X chromosome. Turner syndrome can lead to a variety of medical and developmental problems, including short height, failure to commence puberty, infertility, heart defects, learning disabilities, and difficulty with social adjustment. The GBD case definition of Turner syndrome includes ICD-10 codes Q96.0, Q96.3, and Q96.9. The sequelae associated with Turner syndrome are congenital heart disease, infertility, and the combination of both congenital heart disease and infertility; additionally, a subset of individuals with Turner syndrome are asymptomatic. The distribution of these sequelae was determined by a review of existing literature on the long-term health consequences of Turner Syndrome.

Crosswalks

The MR-BRT crosswalk results are shown below.

Figure 1: MR-BRT crosswalk of alternate definition (livebirths and stillbirths included) with spline on log-transformed neonatal mortality rate.

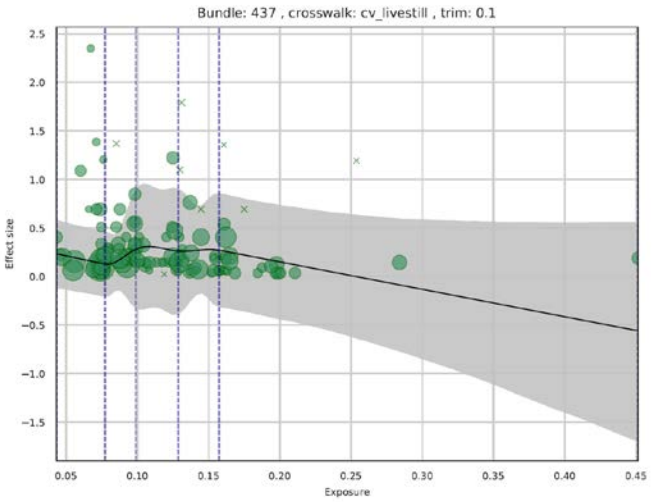

Modelling strategy

One of the known limitations to the use of birth prevalence data on Turner Syndrome is that individuals with Turner Syndrome are commonly diagnosed later in life rather than prenatally or at birth. Thus, we implemented a correction factor to account for under-diagnosis in all birth registry data sources, using available literature on the trends in age pattern of Turner Syndrome diagnosis over time[20] ; although improvements in diagnoses have occurred over time, only between 15% and 30% of all diagnosed Turner Syndrome cases are diagnosed before one year of age. Additionally, the reported denominators

from all birth registries – the number of live births in each registry catchment area – were adjusted to include only female births using the GBD fertility estimates of the age, year, and location-specific proportion of total live births that are female. Furthermore, all prevalence data with values of zero were excluded as outliers, as these low values indicate severe under-reporting in the input data. These modelling strategy changes address known causes of under-reporting of Turner Syndrome in the previous iterations of the GBD and led to higher estimates than reported previously.

The DisMod-MR 2.1 model of Turner Syndrome had an excess mortality rate capped at 0.1 (slightly higher than the highest available literature estimate of excess mortality rate). The model did not have a slope prior set on excess mortality rate as the risk of mortality associated with Turner Syndrome is not specific to the neonatal ages. This model also allows an increased maximum smoothness on excess mortality rate (maximum xi=3.0) and random effects on prevalence limited to +- 0.5 in order to limit random geographic variation in the estimated birth prevalence of Turner Syndrome.

Table 2. Location-level covariate effects

Covariate Name	Measure	Beta value	Exponentiated value
Live Births 35+ (proportion)	Prevalence	-0.11201 (-0.24720 - -0.00707)	0.89 (0.78 - 0.99)
Healthcare access and quality index	EMR	-0.12889 (-0.24833 - -0.00427)	0.88 (0.78 - 1.00)

Klinefelter Syndrome

Case definitions and associated health states

Klinefelter syndrome, also known as 47 XXY, is a condition in which a male is born with an extra X chromosome in all or some of his cells. We also include other genotypes with supranumary X chromosomes, e.g. XXXY, XXXXY, etc. The primary feature of Klinefelter syndrome is sterility, but it can cause a variety of other conditions, including weaker muscles, increased height, poor coordination abilities, smaller genitals, breast growth, and reduced sexual drive as a result of lower testosterone levels. The GBD case definition of Klinefelter syndrome includes ICD-10 codes Q98.0, Q98.5, and Q99.8. The sequelae associated with Klinefelter syndrome are borderline intellectual disability, mild intellectual disability, primary infertility, the combination of borderline intellectual disability and infertility, and the combination of mild intellectual disability and infertility. In addition, a subset of individuals with Klinefelter syndrome are asymptomatic. The distribution of these sequelae was determined by a review of existing literature on the long-term health consequences of Turner Syndrome.

Crosswalks

The MR-BRT crosswalk results are shown below.

Figure 1: MR-BRT crosswalk of alternate definition (livebirths and stillbirths included) with spline on log-transformed neonatal mortality rate.

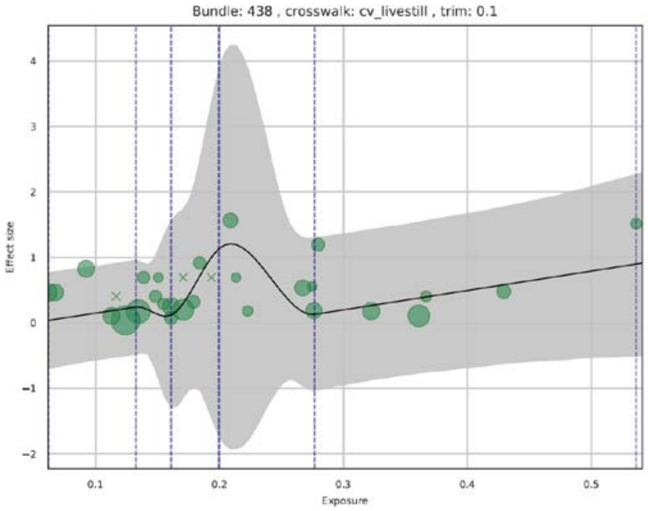

Modelling strategy

As discussed above for Turner Syndrome, one limitation to the use of birth registry data for the estimation of Klinefelter Syndrome is that many individuals with Klinefelter Syndrome are not diagnosed prenatally or at birth. To correct this systematic under-reporting in the birth registry data, we applied a correction factor to all birth registry input data using available literature on the age pattern of Klinefelter Syndrome diagnosis[21]. We also adjusted the both-sex live birth denominators provided in registry data using location, age, and year-specific proportions of all live births that were male. Furthermore, all prevalence data with values of zero were excluded as outliers, as these low values indicate severe under-reporting in the input data. These modelling strategy changes address known causes of under-reporting in the previous iterations of the GBD and resulted in higher estimates of Klinefelter Syndrome than were reported previously.

The DisMod-MR 2.1 model of Klinefelter Syndrome had an excess mortality rate maximum limit of 0.015, allowing the model to fit estimates of excess mortality up to slightly higher than the highest reported literature values. The model did not have a slope prior set on excess mortality and allowed an increased smoothness on excess mortality rate.

Table 2. Location-level covariate effects

Covariate Name	Measure	Beta value	Exponentiated value
Legality of Abortion	Prevalence	-0.00035 (-0.00319 - 0.00000)	1.00 (1.00 - 1.00)
Live Births 35+ (proportion)	Prevalence	0.25507 (0.16529 - 0.29870)	1.29 (1.18 - 1.35)
Healthcare access and quality index	EMR	-0.15166 (-0.29863 - -0.01062)	0.86 (0.74 - 0.99)

Edward and Patau Syndromes

Case definitions and associated health states

Edwards Syndrome, also known as Trisomy 18, is the condition in which infants are born with a third copy of chromosome 18. Patau syndrome, also known as Trisomy 13, is the condition in which infants are born with a third copy of chromosome 13. The GBD estimates the combined prevalence of these two conditions in a single model as they present similarly and are associated with similar rates of excess mortality. Infants with Edwards syndrome typically have low birthweights and a range of associated conditions including a small head and jaw, limb abnormalities, and severe intellectual disability. Infants

with Patau syndrome have a range of associated defects including musculoskeletal anomalies, developmental abnormalities of the nervous system such as microcephaly, congenital heart defects and severe intellectual disability. The ICD-10 code for Edwards syndrome is Q91.3 and the ICD-10 code for Patau syndrome is Q91.7. In the GBD 2017, all cases of Edwards and Patau syndrome are assigned the sequela of severe motor and cognitive impairment, and a proportion of these cases are also associated with congenital heart disease. The proportion of cases with associated congenital heart disease was 0.775, derived by pooling estimates from available literature on the health states associated with the two trisomies[22 23]. This continues to be the case for GBD 2017.

Crosswalks

The MR-BRT crosswalk results are shown below.

Figure 1: MR-BRT crosswalk of alternate definition (livebirths and stillbirths included) with spline on log-transformed neonatal mortality rate.

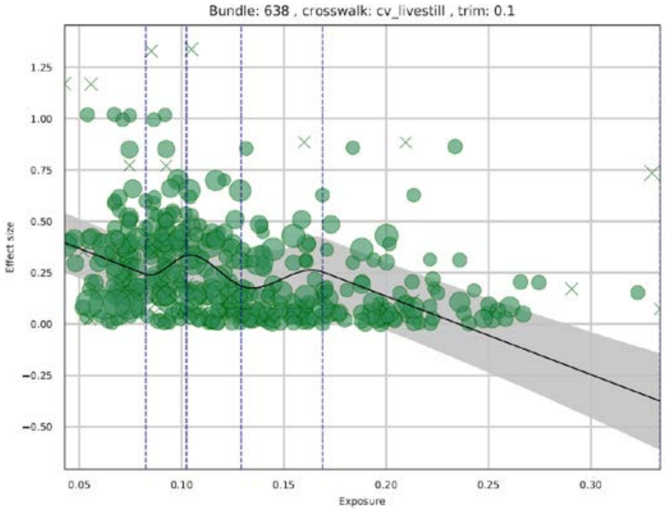

Modelling strategy

In the DisMod-MR 2.1 model of Edwards Syndrome and Patau Syndrome, random effects on prevalence were limited to +- 0.5, reflecting the expectation of limited geographic variation in the birth prevalence of Edwards Syndrome and Patau Syndrome. A decreasing slope prior was set on excess mortality rate for ages 0-1, and an increasing slope prior was set on excess mortality rate for all ages 1+, as individuals with these trisomies generally die within the first few years of life. The model allowed a maximum smoothness of Xi = 3.0 in order to fit high excess mortality in the early age groups.

Table 2. Location-level covariate effects

Covariate Name	Measure	Beta value	Exponentiated value
Legality of Abortion	Prevalence	-0.00127 (-0.00278 - -0.00010)	1.00 (1.00 - 1.00)
Live Births 35+ (proportion)	Prevalence	0.03116 (0.00103 - 0.08521)	1.03 (1.00 - 1.09)
Healthcare access and quality index	EMR	-0.12126 (-0.24418 - -0.00098)	0.89 (0.78 - 1.00)

All input data with birth prevalence values of zero were excluded as outliers, as these values represent under-reporting and low case ascertainment in the input data rather than a true lack of these chromosomal conditions in the corresponding locations.

Other chromosomal abnormalities, genetic syndromes, and microdeletions

Case definitions and associated health states

Unbalanced chromosomal rearrangements are genetic anomalies that typically occur due to meiotic nondisjunction, when homologous chromosomes do not separate normally in nuclear division during gamete formation. The GBD case definition of other chromosomal rearrangements includes 47,XXX (Triple X syndrome), other meiotic nondisjunction events, other female sex chromosome abnormalities, and other unspecified chromosomal abnormalities. The GBD case definition corresponds to the ICD-10 codes Q92.0, Q97.0, Q97.8, and Q99.9. Excluded from this definition are the chromosomal abnormalities of Down syndrome, Turner syndrome, Klinefelter syndrome, Edward syndrome and Patau syndrome, which are each modelled separately. The sequelae associated with other chromosomal rearrangements include intellectual disability, intellectual disability with dementia, intellectual disability with congenital heart disease and dementia, and intellectual disability with congenital heart disease. Additionally, a proportion of the individuals with unbalanced chromosomal rearrangements are asymptomatic. In the absence of available literature on the long-term health outcomes among individuals with other chromosomal conditions, the severity distributions associated with Down Syndrome were used for the sequela associated with other chromosomal anomalies.

Post-model processing

Other chromosomal anomalies were calculated based on reducing the model of total chromosomal anomalies by each of the chromosomal sub-causes, and the remaining prevalence was attributed to other chromosomal anomalies. Specifically, we use claims data to calculate the proportion of cases that are due to the other causes. To do that, we sum the cases for the specific subcauses and the other subcauses cases. We divide the number of other subcause cases by the total number of cases to obtain the proportion. In order to have a valid proportion, we only use datapoints for which we have the combination of age, sex, location and year for all subcauses. We then calculate the prevalence of other: p_other = (p_sum_subcauses / 1-prop_other) - p_sub_subcauses.

Musculoskeletal congenital anomalies

The GBD definition of musculoskeletal congenital anomalies includes any anomalies of the muscles or skeletal system present at birth that are not caused by a defined chromosomal syndrome. Within the range of congenital musculoskeletal anomalies, we explicitly model three sub-categories: polydactyly and syndactyly, limb reduction defects, and all other congenital musculoskeletal anomalies.

Total musculoskeletal birth defects

Crosswalks

The MR-BRT crosswalk results are shown below.

Table 1: MR-BRT crosswalk betas for alternate definitions (reference = livebirths including those with chromosomal anomalies)

Crosswalk	Beta	Standard Error
Excluding chromosomal diagnoses adjustment	-0.053	0.007

Figure 1: MR-BRT crosswalk of alternate definition (livebirths and stilbirths included) with spline on log-transformed neonatal mortality rate.

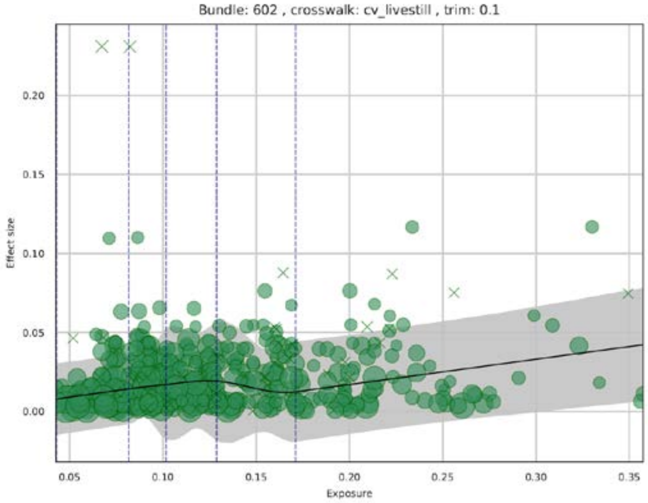

Modelling strategy

The DisMod-MR 2.1 model of total musculoskeletal anomalies used cause-specific mortality estimates from the corresponding model in the GBD Causes of Death analysis, and converted these data to excess mortality estimates where corresponding prevalence data were available. Random effects on prevalence were limited to +- 1.0 in order to limit geographic variation in the birth prevalence of congenital musculoskeletal anomalies. Smoothness on excess mortality rate was increased to Xi= 3.0 to allow the model to fit a steep decrease in excess mortality rate after the earliest age groups.

Table 2. Location-level covariate effects

Covariate Name	Measure	Beta value	Exponentiated value
Legality of Abortion	Prevalence	-0.00043 (-0.00115 - -0.00002)	1.00 (1.00 - 1.00)
Healthcare access and quality index	EMR	-0.02803 (-0.02950 - -0.02692)	0.97 (0.97 - 0.97)

Limb reduction defects

Case definitions and associated health states

Limb reduction defects are the condition where a part or all of the arm or limb of a fetus fails to form during development, so that the limb is either reduced from its normal size or missing entirely. The GBD case definition of limb reduction defects corresponds with ICD-10 codes Q71 (all three-digit codes under Q71), Q72 (all three-digit codes), Q73.0, Q73.1 and Q73.8. Of note, club foot and hip dysplasia are no longer included in this category for GBD 2019.

All cases of limb reduction defects are associated with level 2 disfigurement. A proportion of limb reduction defect cases are associated with no motor impairment, mild motor impairment with and without pain, and moderate motor impairment with and without pain. The distribution of health states associated with congenital limb reduction was derived from an analysis of available literature on the long-term outcomes among individuals with congenital limb reductions[24 25] .

Crosswalks

The MR-BRT crosswalk results are shown below.

Table 1: MR-BRT crosswalk betas for alternate definitions (reference = livebirths including those with chromosomal anomalies)

Crosswalk	Beta	Standard Error
Excluding chromosomal diagnoses adjustment	-0.042	0.034

Figure 1: MR-BRT crosswalk of alternate definition (livebirths and stilbirths included) with spline on log-transformed neonatal mortality rate.

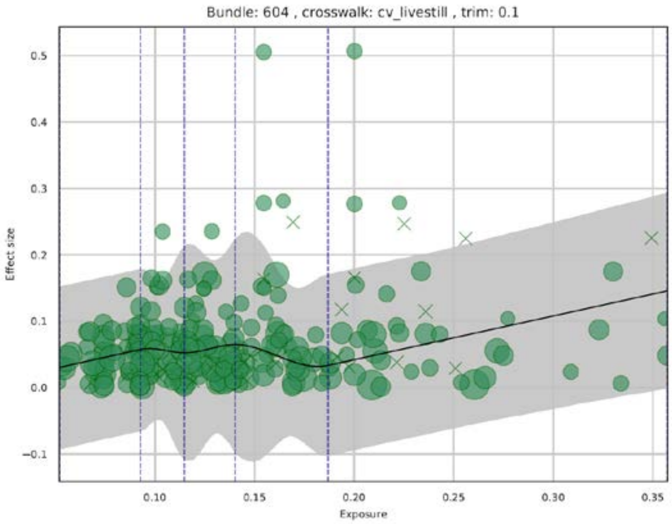

Modelling strategy

In the DisMod-MR 2.1 model of limb reduction defects, random effects on prevalence were limited to +-0.75 in order to limit geographic variation in the estimated birth prevalence. The excess mortality rate was set to a maximum of 0.02 for all ages to reflect the relatively low mortality risk of congenital limb anomalies, and remission was not allowed.

Table 2. Location-level covariate effects

Covariate Name	Measure	Beta value	Exponentiated value
Legality of Abortion	Prevalence	-0.00719 (-0.00911 - -0.00538)	0.99 (0.99 - 0.99)
Healthcare access and quality index	EMR	-0.15313 (-0.29684 - -0.01002)	0.86 (0.74 - 0.99)

Polydactyly and syndactyly

Case definitions and associated health states

Polydactyly is the condition of being born with at least one extra digit on either the hand or the foot, while syndactyly is absence of at least one digit. Our case definition of polydactyly corresponds to ICD-10 code Q69, and syndactyly corresponds to Q70. The sequela associated with all cases of polydactyly and syndactyly is level 1 disfigurement.

All cases of polydactyly and syndactyly are assigned the health state of level 1 disfigurement. Remission is allowed in the model of polydactyly and syndactyly, as individuals born with these conditions may have them surgically corrected and are then no longer considered within our case definition.

Crosswalks

The MR-BRT crosswalk results are shown below.

Table 1: MR-BRT crosswalk betas for alternate definitions (reference = livebirths including those with chromosomal anomalies)

Crosswalk	Beta	Standard Error
Excluding chromosomal diagnoses adjustment	-0.05	0.011
Adjustment for registry specific case definitions (ICBDMS)	-0.379	0.016

Figure 1: MR-BRT crosswalk of alternate definition (livebirths and stilbirths included) with spline on log-transformed neonatal mortality rate.

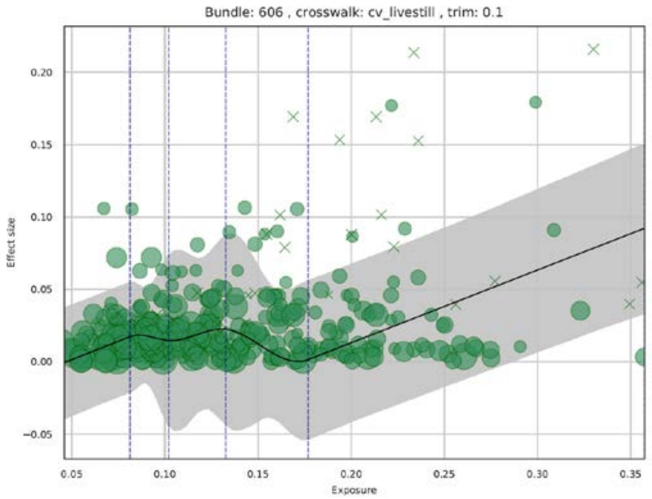

Modelling strategy

The DisMod-MR 2.1 model of polydactly and syndactyly limited random effects on prevalence to +- 0.75, as we expected limited geographic variation in the birth prevalence estimates. Excess mortality priors were set to 0 for ages 0-54 and had a max of 0.1 for ages 55 onwards, as it is not expected that someone will die of these conditions at an early age. The remission rate was bounded from 0 to 0.02 for the first three months of life, as surgical correction of polydactyly or syndactyly rarely occurs in the first few months of life. Remission was bounded between 0 and 0.5 for ages 2 to 5 years, the ages during which surgical correction is most likely to occur, then set to a maximum of 0.02 after 5 years of age. The smoothness on remission was set to Xi= 1.5 in order to facilitate steep changes in remission rates during the first few years of life.

Table 2. Location-level covariate effects

Covariate Name	Measure	Beta value	Exponentiated value
LDI (I$ per capita)	Remission	1.00663 (0.51007 - 1.5)	2.74 (1.67 - 4.48)

Other congenital musculoskeletal defects

Case definitions and associated health states

The other congenital musculoskeletal anomalies included within the total estimate of congenital musculoskeletal anomalies includes clubfoot, skeletal dysplasias, congenital deformities of the spine, congenital dysplasia of the hip, and other congenital musculoskeletal anomalies. This “other” category corresponds to ICD-10 codes Q65, Q65.0, Q65.00, Q65.01, Q65.02, Q65.1; Q65.2; Q65.8; Q65.81; Q65.82; Q65.89; Q65.9; Q66; Q66.0; Q66.1; Q68; Q68.1; Q68.2; Q68.6; Q68.8; Q74; Q74.1; Q74.2; Q74.3; Q74.9; Q75; Q75.0; Q75.5; Q75.9; Q79.8; Q79.9, Q76; Q76.1; Q76.2; Q76.3; Q76.4; Q76.41; Q76.411; Q76.412; Q76.413; Q76.414; Q76.415; Q76.419; Q76.42; Q76.425; Q76.426; Q76.427; Q76.428; Q76.429; Q76.49; Q76.8; Q76.9, Q77; Q77.0; Q77.1; Q77.2; Q77.3; Q77.4; Q77.5; Q77.6; Q77.7; Q77.8; Q77.9; Q78; Q78.0; Q78.1; Q78.2; Q78.3; Q78.4; Q78.5; Q78.6; Q78.8, and Q78.9.

In the absence of comprehensive literature on the long-term outcomes associated with the category of other congenital musculoskeletal anomalies, prevalence estimates of other congenital musculoskeletal anomalies were assigned health states using the proportions derived for limb reduction defects.

Post-model processing

Other congenital musculoskeletal anomalies are modeled by applying the ratio of other congenital digestive anomalies to total congenital digestive anomalies as it is reflected in Marketscan data (a trusted data source), to the sum of the sub-causes of congenital musculoskeletal anomalies. The result is prevalence of other congenital musculoskeletal anomalies by age/year/sex/location. Specifically, we use claims data to calculate the proportion of cases that are due to the other causes. To do that, we sum the cases for the specific subcauses and the other subcause cases. We divide the number of other subcause cases by total number of cases to obtain the proportion. In order to have a valid proportion, we only use datapoints for which we have the combination of age, sex, location and year for all subcauses. We then calculate the prevalence of other: p_other = (p_sum_subcauses / 1-prop_other) - p_sub_subcauses.

Urogenital congenital anomalies

The GBD case definition of urogenital congenital anomalies include anomalies of the genitals and the urinary system that are present at birth. While some types of urogenital congenital anomalies encompass both the urinary and genital systems, we have assigned each congenital condition as a malformation of either the urinary or the genital system in a mutually-exclusive fashion and model anomalies of the urinary and genital systems separately.

Congenital urogenital anomalies were modelled as two distinct categories, with distinct model specifications: urinary congenital anomalies and genital congenital anomalies.

Congenital urinary anomalies

Case definitions and associated health states

Urinary anomalies include congenital malformation of the collecting system, ureter, bladder, and kidney, as well as bladder exstrophy and epispadias. The ICD-10 codes included in the category of urinary anomalies are Q64.0, Q64.1, Q60-Q61 and Q62-Q63.

The total prevalence of congenital urinary anomalies was split into proportions with and without each of the following health states: urinary incontinence, impotence, recurrent urinary tract infections and other recurring abdominal issues, and atypical genitalia (corresponding to disfigurement, level 1 in the GBD Disability Weights Study). The distribution of these long-term outcomes was derived from a review of available literature on the long-term outcomes experienced cohorts of individuals born with a range of congenital urogenital anomalies[26 27 28 29 30 31].

Crosswalks

The MR-BRT crosswalk results are shown below.

Table 1: MR-BRT crosswalk betas for alternate definitions (reference = livebirths including those with chromosomal anomalies)

Crosswalk	Beta	Standard Error
Excluding chromosomal diagnoses adjustment	-0.032	0.008

Figure 1: MR-BRT crosswalk of alternate definition (livebirths and stillbirths included) with spline on log-transformed neonatal mortality rate.

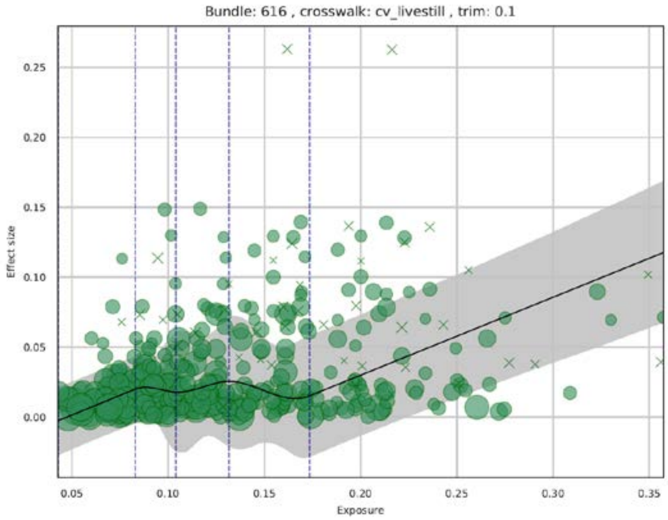

Modelling strategy

In the DisMod-MR 2.1 model of congenital urinary anomalies, random effects on prevalence were limited to +- 0.5 and random effects on with-condition mortality were limited to +- 1.0. The maximum excess mortality rate was set to 0.1 all ages and remission was set to zero. The smoothness on excess mortality rate was set to Xi = 3 in order to fit changes in the excess mortality rate during the neonatal period. Cause-Specific Mortality Rate (CSMR) was also pulled in from our Cause of Death model of congenital urogenital anomalies. As we assume no death due to congenital genital anomalies, this model represents deaths associated with exclusively congenital urinary anomalies.

Table 2. Location-level covariate effects

Covariate Name	Measure	Beta value	Exponentiated value
Age-standardized SEV for Ambient particulate matter	Prevalence	0.03757 (0.00157 - 0.09349)	1.04 (1.00 - 1.10)
Age-standardized SEV for High fasting plasma glucose	Prevalence	2.89926 (2.72300 - 2.99700)	18.16 (15.23 - 20.03)
Healthcare access and quality index	EMR	-0.01352 (-0.01554 - -0.01107)	0.99 (0.98 - 0.99)

Congenital genital anomalies

Case definitions and associated health states

Genital anomalies include hypospadias, ambiguous or indeterminate sex, other congenital abnormalities of the male genitalia, and a variety of female genital malformations. ICD-10 codes Q50-Q52, Q54, Q56, and Q55 (excluding Q55.20-Q55.21) are included in the case definition of congenital genital anomalies. Undescended testicles are excluded from the case definition of genital anomalies, as this is not considered a severe condition.

Cases of congenital genital anomalies was split into proportions with and without primary infertility, impotence, recurrent urinary tract infections and other recurring abdominal issues, and atypical genitalia. Estimates were produced for the prevalence of every possible combination of those long-term sequela, assuming independence between the outcomes. The distribution of these long-term outcomes was derived from a review of available literature on the long-term outcomes experienced cohorts of individuals born with a range of congenital urogenital anomalies[32] [33]

[34] [35] [36] [37].

Crosswalks

The MR-BRT crosswalk results are shown below.

Table 1: MR-BRT crosswalk betas for alternate definitions (reference = livebirths including those with chromosomal anomalies)

Crosswalk	Beta	Standard Error
Excluding chromosomal diagnoses adjustment	-0.019	0.011

Figure 1: MR-BRT crosswalk of alternate definition (livebirths and stilbirths included) with spline on log-transformed neonatal mortality rate.

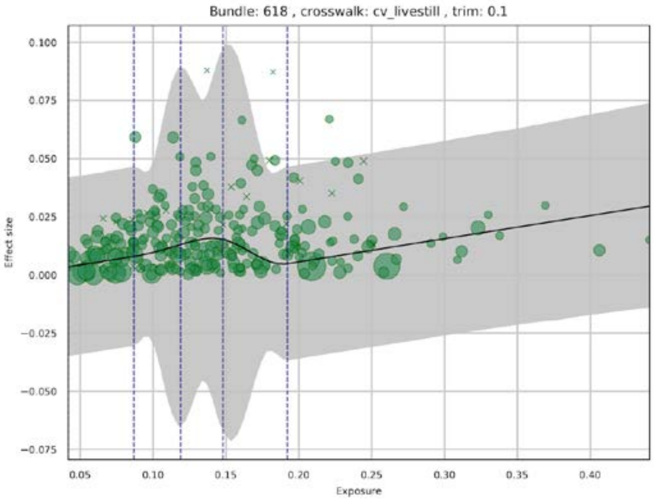

Modelling strategy

In the DisMod-MR 2.1 model of congenital genital anomalies, random effects on prevalence were limited to +- 0.75 in order to limit random geographic variation in the estimates of birth prevalence. Excess mortality was set to 0 for all ages, as we do not believe that individuals are dying due to genital anomalies; this is consistent with our Cause of Death analysis, in which the only causes reflected in our urogenital mortality estimates are congenital urinary conditions.

Table 2. Location-level covariate effects

Covariate Name	Measure	Beta value	Exponentiated value
Age-standardized SEV for Ambient particulates	Prevalence	0.21110 (0.07897 - 0.34302)	1.24 (1.08 - 1.41)
Age-standardized SEV for High FPG	Prevalence	0.22404 (0.00678 - 0.68022)	1.25 (1.01 - 1.97)

Congenital anomalies of the digestive system

Case definitions

Congenital anomalies of the digestive system include any anomalies of the gastrointestinal tract present at birth as the result of abnormal embryonic development. As with the other congenital causes, this variety of digestive system abnormalities is split into four sub-cause categories.

Total digestive congenital anomalies

In order to ensure internal consistency in the estimates of each sub-type of congenital digestive anomalies, we generated a model to estimate the total prevalence and associated mortality due to all

congenital digestive anomalies, then fit the estimates of each sub-type of congenital digestive anomalies proportionally to the envelope of this total model. The prevalence estimates of other congenital digestive anomalies were derived by reducing the total envelope model for each cause by its sub-causes to derive the difference that was attributable to other anomalies in that category. This modelling strategy allowed us to utilize the GBD Cause of Death estimates as input to the total congenital digestive anomalies estimates and also allowed us to incorporate literature data that reported only the total prevalence of all digestive anomalies.

Crosswalks

The MR-BRT crosswalk results are shown below.

Table 1: MR-BRT crosswalk betas for alternate definitions (reference = livebirths including those with chromosomal anomalies)

Crosswalk	Beta	Standard Error
Excluding chromosomal diagnoses adjustment	-0.078	0.011

Figure 1: MR-BRT crosswalk of alternate definition (livebirths and stilbirths included) with spline on log-transformed neonatal mortality rate.

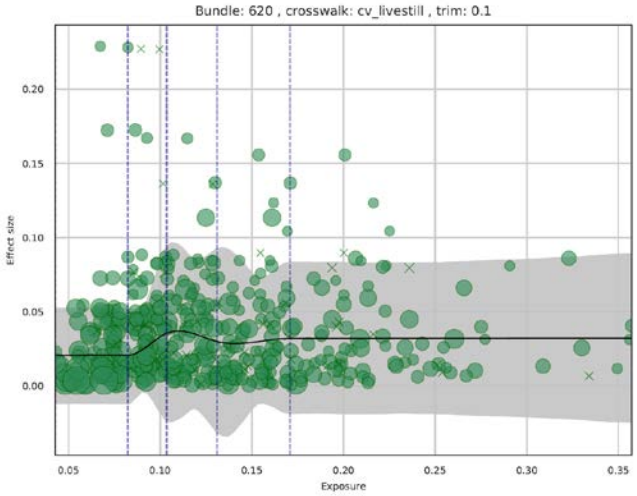

Modelling strategy

The DisMod-MR 2.1 model of total congenital digestive anomalies used cause-specific mortality estimates from the corresponding GBD Cause of Death model of congenital digestive anomalies, and these data were converted to excess mortality estimates where corresponding cause-specific mortality estimates were available. The model had random effects on prevalence limited to +- 0.5 and random effects on excess mortality limited to +/- 0.1. The model also had a slope prior on remission to decrease with age and have an overall all-ages maximum of 1.0. The smoothness on excess mortality rate was increased to Xi = 3.0 in order to fit steep changes in excess mortality rate during the neonatal age period.

Table 2. Location-level covariate effects

Covariate Name	Measure	Beta value	Exponentiated value
Age-standardized SEV for Smoking	Prevalence	0.55810 (0.11556 - 0.96020)	1.75 (1.12 - 2.61)
Age-standardized SEV for High BMI	Prevalence	0.98315 (0.94268 - 0.99960)	2.67 (2.57 - 2.72)
Liters of alcohol consumed per capita	Prevalence	0.06762 (0.06074 - 0.07397)	1.07 (1.06 - 1.08)
Healthcare access and quality index	EMR	-0.07132 (-0.07691 - -0.06839)	0.93 (0.93 - 0.93)

Congenital diaphragmatic hernia

Case definitions and associated health states

Congenital diaphragmatic hernia, a life-threatening malformation of the diaphragm that allows the abdominal organs to push into the chest cavity and obstructs proper formation of the lungs, is modelled separately from all other congenital malformations of the digestive system. Congenital diaphragmatic hernia corresponds to ICD-10 code Q79.0.

The health outcomes associated with congenital diaphragmatic hernia include every combination of disfigurement, chronic abdominal pain, mild chronic respiratory problems and breathlessness, mild intellectual disability, and a proportion of patients who are asymptomatic. The distribution of these long-term health outcomes was derived from a pooled analysis of available literature on the long-term outcomes in surviving patients born with congenital diaphragmatic hernias[38] [39] [40].

Crosswalks

The MR-BRT crosswalk results are shown below.

Table 1: MR-BRT crosswalk betas for alternate definitions (reference = livebirths including those with chromosomal anomalies)

Crosswalk	Beta	Standard Error
Excluding chromosomal diagnoses adjustment	-0.063	0.035

Figure 1: MR-BRT crosswalk of alternate definition (livebirths and stillbirths included) with spline on log-transformed neonatal mortality rate.

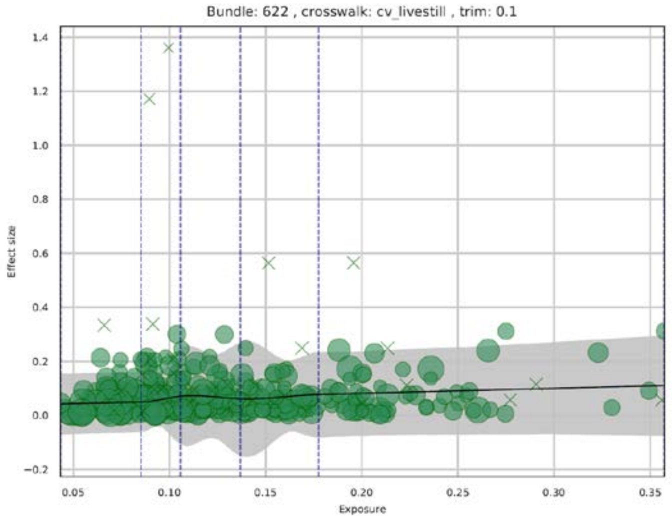

Modelling strategy

In the DisMod-MR 2.1 model of congenital diaphragmatic hernia, random effects on prevalence were set to +- 0.5. The minimum excess mortality for the early neonatal age period was set to 10.0, and to 0.05 for the late neonatal period. A decreasing slope prior on excess mortality rate was set for all ages, as the risk of mortality due to congenital diaphragmatic hernia is highest shortly after birth and diminishes over the life course following surgical correction of the condition. Smoothness on excess mortality rate was increased to Xi= 3.0 in order to fit steep changes in excess mortality rate during the first weeks of life.

Table 2. Location-level covariate effects

Covariate Name	Measure	Beta value	Exponentiated value
Age-standardized SEV for Smoking	Prevalence	0.35808 (0.03364 - 0.78414)	1.43 (1.03 - 2.19)
Age-standardized SEV for High BMI	Prevalence	0.51283 (0.12876 - 0.89302)	1.67 (1.14 - 2.44)

Liters of alcohol consumed per capita	Prevalence	0.00115 (0.00004 - 0.00306)	1.00 (1.00 - 1.00)
Healthcare access and quality index	EMR	-0.05119 (-0.09925 - -0.00208)	0.95 (0.91 - 1.00)

Congenital malformations of the abdominal wall

Case definitions and associated health states

All congenital malformations of the abdominal wall are modelled together as a distinct sub-category. The primary diagnoses in this category are gastroschisis, omphalocele, and prune belly syndrome, corresponding to ICD-10 codes Q79.3, Q79.2, and Q79.4, respectively.

The health outcomes associated with congenital malformations of the abdominal wall include every combination of constipation, chronic abdominal pain, and disfigurement and concern about scars. The distribution of these outcomes was calculated from a pooled analysis of literature sources on the long-term outcomes among surviving individuals born with congenital malformations of the abdominal wall[41] [42].

Crosswalks

The MR-BRT crosswalk results are shown below.

Table 1: MR-BRT crosswalk betas for alternate definitions (reference = livebirths including those with chromosomal anomalies)

Crosswalk	Beta	Standard Error
Excluding chromosomal diagnoses adjustment	-0.069	0.025
Adjustment for registry specific case definitions (NBDPN)	-0.106	0.01

Figure 1: MR-BRT crosswalk of alternate definition (livebirths and stillbirths included) with spline on log-transformed neonatal mortality rate.

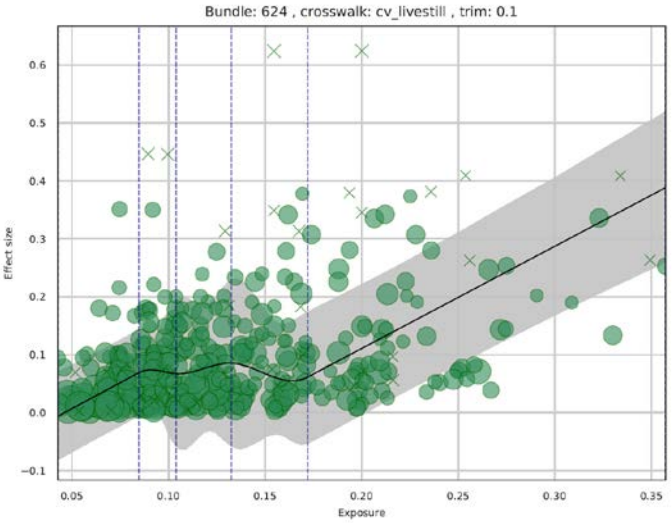

Modelling strategy

The DisMod-MR 2.1 model of congenital malformations of the abdominal wall had random effects on prevalence limited to +- 0.5. The minimum excess mortality rate was set to 0.5 with a maximum excess mortality rate of 10.0, for the early neonatal period. For ages 0.5-100, excess mortality max was set to 0.05. A decreasing slope prior on remission was set for all ages, and the smoothness on excess mortality rate was set to Xi = 3.0, allowing the model to fit a steep decrease in the excess mortality rate after the neonatal age period.

Table 2. Location-level covariate effects

Covariate Name	Measure	Beta value	Exponentiated value
Age-standardized SEV for Smoking	Prevalence	0.37432 (0.03030 - 0.84264)	1.45 (1.03 - 2.32)
Age-standardized SEV for High BMI	Prevalence	0.78693 (0.48530 - 0.98702)	2.20 (1.62 - 2.68)
Liters of alcohol consumed per capita	Prevalence	0.00505 (0.00040 - 0.01204)	1.01 (1.00 - 1.01)
Healthcare access and quality index	EMR	-0.04974 (-0.09745 - -0.00232)	0.95 (0.91 - 1.00)

Congenital atresia and/or stenosis of the digestive tract

Case definitions and associated health states

All variations of atresia and/or stenosis of the digestive tract are modelled together as the third distinct sub-category of digestive congenital anomalies. This includes biliary atresia, esophageal atresia and/or stenosis with and without tracheoesophageal fistula, and atresia and stenosis of the small intestine, large intestine, rectum and anus. The ICD-10 codes included in the atresia and stenosis sub-cause category are Q42.0; Q42.1; Q42.2; Q42.3; Q42.4; Q42.8; Q42.9, Q42.8; Q42.9, Q42.0; Q42.1; Q42.2; Q42.3; Q42.4; Q41 (Q41.0; Q41.1; Q41.2; Q41.8; Q41.9;), Q44.2, Q39.0; Q39.1 and Q39.2.

The outcomes associated with congenital atresia and/or stenosis of the abdominal tract include every combination of dysphagia, acid reflux, chronic abdominal pain and/or nausea, and chronic respiratory problems; the distribution of these long-term outcomes was also derived from available long-term follow-up studies[43 44].

Crosswalks

The MR-BRT crosswalk results are shown below.

Table 1: MR-BRT crosswalk betas for alternate definitions (reference = livebirths including those with chromosomal anomalies)

Crosswalk	Beta	Standard Error
Excluding chromosomal diagnoses adjustment	-0.093	0.016
Adjustment for registry specific case definitions (Cong Malf Wordwide)	-0.304	0.011

Figure 1: MR-BRT crosswalk of alternate definition (livebirths and stilbirths included) with spline on log-transformed neonatal mortality rate.

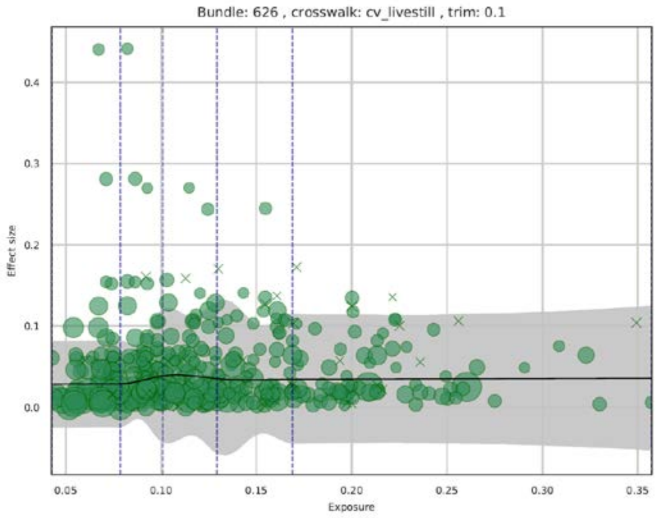

Modelling strategy

In the DisMod-MR 2.1 model of congenital atresia and/or stenosis of the digestive tract, random effects on prevalence were set to +- 0.5 and random effects on with-condition mortality were set to +- 1.0. A

decreasing slope prior on remission was set for all ages, as remission is most likely just after birth. The smoothness on excess mortality rate was increased to Xi = 3.0 in order to fit steep changes in excess mortality rate during the first weeks of life, with value priors set to 2-15 for the early neonatal period, and 0 for ages 70-100.

Table 2. Location-level covariate effects

Covariate Name	Measure	Beta value	Exponentiated value
Age-standardized SEV for Smoking	Prevalence	0.05632 (0.00109 - 0.18860)	1.06 (1.00 - 1.21)
Age-standardized SEV for High BMI	Prevalence	0.96408 (0.88796 - 0.99940)	2.62 (2.43 - 2.72)
Liters of alcohol consumed per capita	Prevalence	0.00095 (0.00002 - 0.00341)	1.00 (1.00 - 1.00)
Healthcare access and quality index	EMR	-0.05106 (-0.09753 - -0.00367)	0.95 (0.91 - 1.00)

Other congenital digestive anomalies

Case definitions and associated health states

Other congenital malformations and diseases of the digestive system includes ICD-10 codes Q38 (Q38.0; Q38.3; Q38.4; Q38.6; Q38.7; Q38.8); Q39(Q39.3; Q39.4; Q39.5; Q39.6; Q39.8; Q39.9); Q40(Q40.0; Q40.1; Q40.2; Q40.3; Q40.8; Q40.9); Q43(Q43.1; Q43.2; Q43.3; Q43.4; Q43.5; Q43.6; Q43.7; Q43.8; Q43.9); Q44(Q44.0; Q44.1; Q44.3; Q44.4; Q44.5; Q44.6; Q44.7); Q45(Q45.0; Q45.1; Q45.2; Q45.3; Q45.8; Q45.9); Q79.1, and Q79.5(Q79.51; Q79.59). Inguinal hernias present at birth are excluded from the case definition of gastrointestinal congenital anomalies and are modelled separately as part of the estimation of inguinal hernias.

The distribution of health outcomes associated with other congenital anomalies of the gastrointestinal tract was considered to be the same as the health outcomes associated with atresia and/or stenosis of the abdominal tract.

Post-model processing

Other congenital digestive anomalies are calculated by summing all of the sub-causes of congenital digestive anomalies and subtracting this sum from the total congenital digestive model (by age/sex/year/location). This residual is the prevalence of other congenital digestive anomalies. If this residual is less than 10% of the total congenital digestive anomalies model, the other sub-causes are squeezed down and other congenital digestive anomalies becomes 10% of the total congenital digestive anomalies model.

Other congenital anomalies

In addition, of the specific types of congenital anomalies outlined in the preceding pages, there are a number of other types of defects that may be present at birth. These other congenital defects include anomalies of the ears, eyes, face and neck, respiratory malformation and diseases, skin disorders, phakomatoses and other neurological disorders that are not included in the case definition of neural tube defects. Estimates of the YLDs attributable to these other congenital anomalies are derived from a YLL:YLD ratio. This ratio was calculated for all congential birth defects combined, but excluding congenital heart defects, as the location-age-sex-year-specific ratio of YLLs from the cause of death (COD) estimates to YLDs from the nonfatal analyses described above. This ratio was then applied to the YLLs estimates for other congenital anomalies to derive estimated YLDs for other congenital anomalies.

References

[1] Jaquier M. Anencephaly Online Survey. Anencephaly.info [Internet]. 2006.

[2] Jaquier M, Klein A, Boltshauser E. Spontaneous pregnancy outcome after prenatal diagnosis of anencephaly. *BJOG*. 2006; 113(8): 951-3.

[3] Lanton AP. The Characteristics of Patients with Encephaloceles. *Z Kinderchir*. 1990; 45(Suppl 1): 18-9.

[4] Da Silva SL, Jeelani Y, Dang H, Krieger MD, McComb JG. Risk factors for hydrocephalus and neurological deficit in children born with an encephalocele. *J Neurosurg Pediatr*. 2015; 15(4): 392–8.

[5] Lo BWY, Kulkarni AV, Rutka JT, Jea A, Drake JM, Lamberti-Pasculli M, Dirks PB, Thabane L. Clinical predictors of developmental outcome in patients with cephaloceles. *J Neurosurg Pediatr*. 2008; 2(4): 254–7.

[6] Moeini Naghani I, Hashemi Zonouz T, Shahjouei S, Homayoun AA, Nejat F, El Khashab M. Congenital cardiac anomalies in myelomeningocele patients. *Acta Med Acad*. 2014; 43(2): 160–4.

[7] Oakeshott P, Hunt GM, Poulton A, Reid F. Open spina bifida: birth findings predict long-term outcome. *Arch Dis Child*. 2012; 97(5): 474–6.

[8] Lanton AP. The Characteristics of Patients with Encephaloceles. *Z Kinderchir*. 1990; 45(Suppl 1): 18-9.

[9] Da Silva SL, Jeelani Y, Dang H, Krieger MD, McComb JG. Risk factors for hydrocephalus and neurological deficit in children born with an encephalocele. *J Neurosurg Pediatr*. 2015; 15(4): 392–8.

[10] Lo BWY, Kulkarni AV, Rutka JT, Jea A, Drake JM, Lamberti-Pasculli M, Dirks PB, Thabane L. Clinical predictors of developmental outcome in patients with cephaloceles. *J Neurosurg Pediatr*. 2008; 2(4): 254–7.

[11] Moeini Naghani I, Hashemi Zonouz T, Shahjouei S, Homayoun AA, Nejat F, El Khashab M. Congenital cardiac anomalies in myelomeningocele patients. *Acta Med Acad*. 2014; 43(2): 160–4.

[12] Oakeshott P, Hunt GM, Poulton A, Reid F. Open spina bifida: birth findings predict long-term outcome. *Arch Dis Child*. 2012; 97(5): 474–6.

[13] Riehle-Colarusso T, Autry A, Razzaghi H, Boyle CA, Mahle WT, Van Naarden Braun K, Correa A. Congenital Heart Defects and Receipt of Special Education Services. *Pediatrics*. 2015; 136(3): 496–504.

[14] Menting ME, Cuypers JAAE, Opić P, Utens EMWJ, Witsenburg M, van den Bosch AE, van Domburg RT, Meijboom FJ, Boersma E, Bogers AJJC, Roos-Hesselink JW. The unnatural history of the ventricular septal defect: outcome up to 40 years after surgical closure. *J Am Coll Cardiol*. 2015; 65(18): 1941–51.

[15] Gaynor JW, Stopp C, Wypij D, Andropoulos DB, Atallah J, Atz AM, Beca J, Donofrio MT, Duncan K, Ghanayem NS, Goldberg CS, Hövels-Gürich H, Ichida F, Jacobs JP, Justo R, Latal B, Li JS, Mahle WT, McQuillen PS, Menon SC, Pemberton VL, Pike NA, Pizarro C, Shekerdemian LS, Synnes A, Williams I, Bellinger DC, Newburger JW, International Cardiac Collaborative on Neurodevelopment (ICCON) Investigators. Neurodevelopmental outcomes after cardiac surgery in infancy. *Pediatrics*. 2015; 135(5): 816–25.

[16] Wren C, O'Sullivan JJ. Survival with congenital heart disease and need for follow up in adult life. *Heart*. 2001; 85(4): 438–43.

[17] Gabriel HM, Heger M, Innerhofer P, Zehetgruber M, Mundigler G, Wimmer M, Maurer G, Baumgartner H. Long-term outcome of patients with ventricular septal defect considered not to require surgical closure during childhood. *J Am Coll Cardiol*. 2002; 39(6): 1066–71.

[18] Neumayer U, Stone S, Somerville J. Small ventricular septal defects in adults. *Eur Heart J*. 1998; 19(10): 1573–82.

[19] Epstein CJ. Down syndrome (trisomy 21). In: Scriver CR, Beaudet AL, Sly WS, Valle D, eds. The Metabolic Basis of Inherited Disease. 7th ed. New York, United States: McGraw Hill Inc., 1995.

[20] Massa G, Verlinde F, De Schepper J, Thomas M, Bourguignon JP, Craen M, de Zegher F, Francois I, Du Caju M, Maes M, Heinrichs C, in collaboration with the Belgian Study Group for Paediatric Endocrinology. Trends in age at diagnosis of Turner syndrome. *Arch Dis Child*. 2005; 90(3): 267-8.

[21] Bojesen A, Juul S, Gravholt CH. Prenatal and Postnatal Prevalence of Klinefelter Syndrome: A National Registry Study. *J Clin Endocrinol Metab*. 2003; 88(2): 622-6.

[22] Petry P, Polli JB, Mattos VF, Rosa RCM, Zen PRG, Graziadio C, Paskulin GA, Rosa RFM. Clinical features and prognosis of a sample of patients with trisomy 13 (Patau syndrome) from Brazil. *Am J Med Genet A*. 2013; 161A(6): 1278–83.

[23] Polli JB, Groff D de P, Petry P, Mattos VF, Rosa RCM, Zen PRG, Graziadio C, Paskulin GA, Rosa RFM. Trisomy 13 (Patau syndrome) and congenital heart defects. *Am J Med Genet A*. 2014; 164A(1): 272–5.

[24] Johansen H, Østlie K, Andersen LØ, Rand-Hendriksen S. Adults with congenital limb deficiency in Norway: demographic and clinical features, pain and the use of health care and welfare services. A cross-sectional study. *Disabil Rehabil*. 2015; 37(22): 2076–82.

[25] Johansen H, Dammann B, Oinæs Andersen L, Andresen I-L. Children with congenital limb deficiency in Norway: issues related to school life and health-related quality of life. A cross-sectional study. *Disabil Rehabil*. 2016; 38(18): 1803–10.

[26] Ching CB, Wood HM, Ross JH, Gao T, Angermeier KW. The Cleveland Clinic experience with adult hypospadias patients undergoing repair: their presentation and a new classification system. *BJU Int*. 2011; 107(7): 1142–6.

[27] Davies MC, Liao L-M, Wilcox DT, Woodhouse CRJ, Creighton SM. Anorectal malformations: what happens in adulthood?. *BJU Int*. 2010; 106(3): 398–404.

[28] Rintala RJ. Congenital cloaca: Long-term follow-up results with emphasis on outcomes beyond childhood. *Semin Pediatr Surg*. 2016; 25(2): 112–6.

[29] Sircili MHP, e Silva FA de Q, Costa EMF, Brito VN, Arnhold IJP, Dénes FT, Inacio M, de Mendonca BB. Long-term surgical outcome of masculinizing genitoplasty in large cohort of patients with disorders of sex development. *J Urol*. 2010; 184(3): 1122–7.

[30] van der Zwan YG, Callens N, van Kuppenveld J, Kwak K, Drop SLS, Kortmann B, Dessens AB, Wolffenbuttel KP, Dutch Study Group on DSD. Long-term outcomes in males with disorders of sex development. *J Urol*. 2013; 190(3): 1038–42.

[31] Warne SA, Wilcox DT, Creighton S, Ransley PG. Long-term gynecological outcome of patients with persistent cloaca. *J Urol*. 2003; 170(4 Pt 2): 1493–6.

[32] Ching CB, Wood HM, Ross JH, Gao T, Angermeier KW. The Cleveland Clinic experience with adult hypospadias patients undergoing repair: their presentation and a new classification system. *BJU Int*. 2011; 107(7): 1142–6.

[33] Davies MC, Liao L-M, Wilcox DT, Woodhouse CRJ, Creighton SM. Anorectal malformations: what happens in adulthood?. *BJU Int*. 2010; 106(3): 398–404.

[34] Rintala RJ. Congenital cloaca: Long-term follow-up results with emphasis on outcomes beyond childhood. *Semin Pediatr Surg*. 2016; 25(2): 112–6.

[35] Sircili MHP, e Silva FA de Q, Costa EMF, Brito VN, Arnhold IJP, Dénes FT, Inacio M, de Mendonca BB. Long-term surgical outcome of masculinizing genitoplasty in large cohort of patients with disorders of sex development. *J Urol*. 2010; 184(3): 1122–7.

[36] van der Zwan YG, Callens N, van Kuppenveld J, Kwak K, Drop SLS, Kortmann B, Dessens AB, Wolffenbuttel KP, Dutch Study Group on DSD. Long-term outcomes in males with disorders of sex development. *J Urol*. 2013; 190(3): 1038–42.

[37] Warne SA, Wilcox DT, Creighton S, Ransley PG. Long-term gynecological outcome of patients with persistent cloaca. *J Urol*. 2003; 170(4 Pt 2): 1493–6.

[38] Crankson SJ, Al Jadaan SA, Namshan MA, Al-Rabeeah AA, Oda O. The immediate and long-term outcomes of newborns with congenital diaphragmatic hernia. *Pediatr Surg Int*. 2006; 22(4): 335-40.

[39] Öst E, Joelsson MÖ, Burgos CM, Frenckner B. Self-assessed physical health among children with congenital diaphragmatic hernia. *Pediatr Surg Int*. 2016; 32(5): 493–503.

[40] Rocha GM, Bianchi RF, Severo M, Rodrigues MM, Baptista MJ, Correia-Pinto J, Guimarães HA. Congenital Diaphragmatic Hernia. The Post-neonatal period. Part II. *Eur J Pediatr Surg*. 2008; 18(5): 307–12.

[41] van Eijck FC, Wijnen RMH, van Goor H. The incidence and morbidity of adhesions after treatment of neonates with gastroschisis and omphalocele: a 30-year review. *J Pediatr Surg*. 2008; 43(3): 479–83.

[42] Harris EL, Minutillo C, Hart S, Warner TM, Ravikumara M, Nathan EA, Dickinson JE. The long term physical consequences of gastroschisis. *J Pediatr Surg*. 2014; 49(10): 1466–70.

[43] Dingemann C, Meyer A, Kircher G, Boemers TM, Vaske B, Till H, Ure BM. Long-term health-related quality of life after complex and/or complicated esophageal atresia in adults and children registered in a German patient support group. *J Pediatr Surg*. 2014; 49(4): 631–8.

[44] Lilja HE, Wester T. Outcome in neonates with esophageal atresia treated over the last 20 years. *Pediatr Surg Int*. 2008; 24(5): 531–6.

Urinary Tract Infection

Flowchart

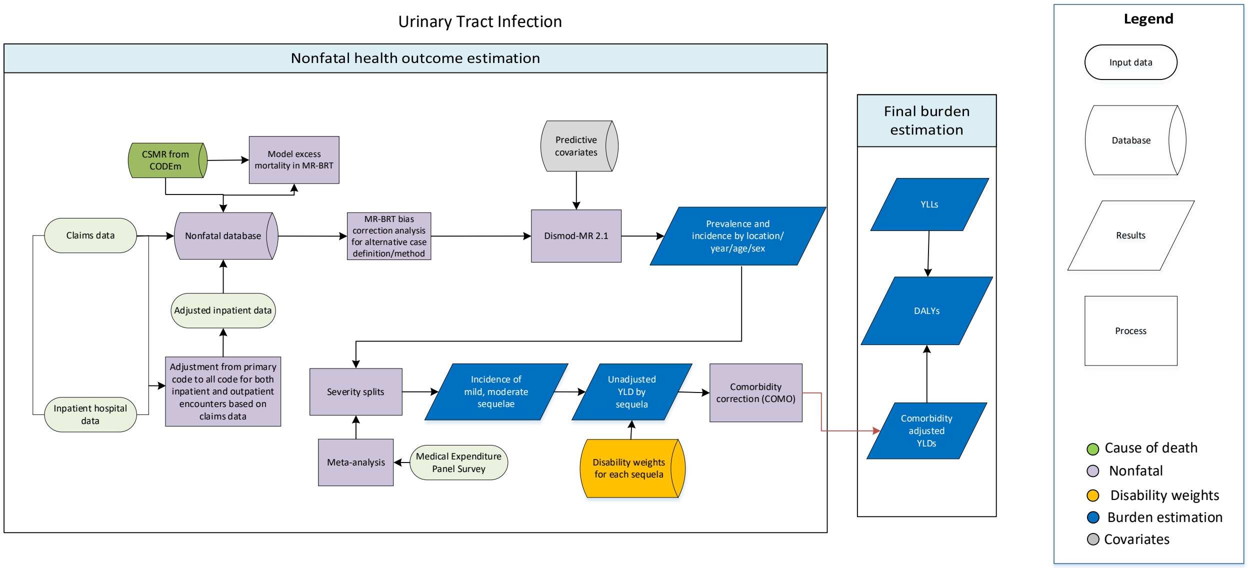

Input Data and Methodological Summary for Urinary Tract Infection

Case definition

Urinary tract infection (UTI) is defined as a kidney infection that can lead to systemic symptoms such as fever and weakness and can cause discomfort and difficulty with daily activities. ICD codes include N10, N10.0, N10.9, N11, N11.0, N11.1, N11.8, N11.9, N12, N12.0, N12.9, N13.6, N15, N15.1, N15.8, N15.9, N16, N16.0-N16.5, N16.8, N30, N30.0-N30.3, N30.8-N30.9, N34, N34.0-N34-3, and N39.0.

Input data and data processing

Input data

Like GBD 2017, the UTI model included data from hospital discharges and claims. No formal literature review has been conducted.

In GBD 2019, we newly added Poland claims data and additional years of data from USA claims (years 2015-2016) and hospital discharges in Mexico, India, New Zealand, Sweden, Georgia, and Ecuador. Notably, we included hospital data from Botswana; southern sub-Saharan Africa previously did not have data.

Table 1. Data Inputs for urinary tract infection morbidity modelling by parameter.

Measure	Total sources	Countries with data
All measures	311	45
Incidence	296	45
Proportion	15	1

Data processing

Similar to GBD 2017, claims data link multiple inpatient and outpatient claims to a single individual; incident cases were extracted if an individual had at least one inpatient or outpatient encounter with an appropriate ICD code as any diagnosis, and correction factors were derived to apply to other data sources. Data from hospital discharges from all other locations were adjusted using correction factors from claims, converting encounters to estimates of cases, correcting for most locations providing only primary diagnostic codes, and estimating outpatient cases from inpatient cases.

The USA claims data from the year 2000 and from the years 2010–2016 were each adjusted to data from hospital discharges outside DisMod using MR-BRT analysis to adjust for selection bias due to commercial insurance.

The process of adjusting for non-reference data using MR-BRT with the logit-transformation method is described below:

1. Identify data points with overlapping year, age, sex, and location between claims (alternative case definition) and hospital discharges (reference case definition)
2. Logit transform overlapping data points of alternative and reference case definitions
3. Convert overlapping data points into a difference in logit space using the following equation: $logit(altnerative) - logit(reference)$
4. Use the delta method to compute standard errors of overlapping data points in logit space, then calculate standard error of logit difference using the following equation: $\sqrt{(variance\ of\ alternative) + (variance\ of\ reference)}$
5. Using MR-BRT, conduct a random effects meta-regression to obtain the pooled logit difference of alternative to reference
6. Apply the pooled logit difference to all data points of alternative case definitions using the following equation:
$$new_{estimate} = inverse.logit((logit(alternative)) - (pooled\ logit\ difference))$$
7. Calculate new standard errors using the delta method, accounting for gamma (between-study heterogeneity)

The table below shows bias correction factors estimated using MR-BRT.

Table 2. MR-BRT Crosswalk Adjustment Factors for Urinary Tract Infection

Data input	Reference or alternative data collection	Gamma	Beta Coefficient, logit difference (95% CI)	Adjustment factor*
Hospital + non-USA claims	Ref	0.36	---	---
USA claims from year 2000	Alt		-0.40 (-1.40, 0.59)	0.40 (0.20, 0.64)
USA claims from year 2010-2016	Alt		-0.18 (-1.03, 0.68)	0.46 (0.26, 0.66)

**Adjustment factor is the inverse-logit transformed Beta coefficient; <0.5 represents that alternative is adjusted upward; >0.5 represents that alternative is adjusted downward*

Data points with an age-standardised incidence rate greater than two median absolute deviations from the median of the age-standardised incidence rate for all inpatient and non-USA claims data were marked as outliers and excluded from analysis. Data points in Taiwan and Indonesia, particularly in older age groups, were also marked as outliers because they were implausibly high when compared to the regional, super-regional, and global rates.

Severity split & disability weight

The basis of the GBD disability weight survey assessments is lay descriptions of sequelae highlighting major functional consequences and symptoms. UTI is split into mild and moderate severity. Mild severity is associated with a disability weight that correlates with low fever and mild discomfort, but no difficulty with daily activities. Moderate discomfort is associated with a disability weight that correlates with systemic symptoms of fever, aches, weakness, and some difficulty with daily activities. The lay descriptions and disability weights for UTI are shown below.

Table 3. Severity Distribution, details on the severity levels for urinary tract infection in GBD 2019 and the associated disability weight (DW) with that severity.

Severity level	Lay description	DW (95% CI)
Mild	Has a low fever and mild discomfort, but no difficulty with daily activities.	0.006 (0.002, 0.012)
Moderate	Has a fever and aches, and feels weak, which causes some difficulty with daily activities.	0.051 (0.032, 0.074)

The severity distribution of UTI was derived from analysis of the Medical Expenditure Panel Surveys (MEPS). MEPS is an overlapping panel survey of the non-institutionalised USA population that collects data on respondents' health service interactions. Panels are initiated every year. Each panel is two years long and consists of five rounds. In 2000, MEPS began using 12-Item Short Form Surveys (SF-12) to collect data on functional health status. The SF-12 survey is administered twice per panel (about once per year).

In order to translate SF-12 scores into GBD disability weights, 62 lay descriptions for conditions representing the full range of disability weight values (from most mild to most severe) were selected. A convenience sample of respondents was then asked to complete an SF-12 form for an individual with the health state described in the lay descriptions of these conditions. Composite mental and physical SF-12 score was regressed on GBD disability weight to derive the relationship between disability weight and SF-12 score. Individual respondent scores were then regressed on reported conditions to obtain a comorbidity-corrected condition-specific disability weight. The distribution of these condition-specific weights was used to derive the proportion of individuals with the conditions that fall within each GBD severity category.

Severity	Distribution
Mild UTI	0.362 (0.258, 0.478)
Moderate UTI	0.638 (0.522, 0.742)

Modeling strategy

Similar to GBD 2017, we ran a DisMod-MR 2.1 model to produce estimates by age, sex, year, and country. Prior settings in DisMod included cure after one week (remission set to 52) between ages 0 and 100 and an upper bound of 0.002 for excess mortality rate (EMR) between ages 0 and 15. We used the function in DisMod-MR 2.1 to pull in cause-specific mortality rate (CSMR) data from our CODEm and CODcorrect analyses. The minimum coefficient of variation at the regional, super-regional, and global-level was changed from 0.4 to 0.8 in GBD 2019 to improve model fit against input data.

In previous rounds, priors on excess mortality rate (EMR) were estimated in DisMod by matching prevalence data points with their corresponding CSMR values within the same age, sex, year, and location (by dividing CSMR by prevalence). For short duration conditions (remission >1), the corresponding prevalence was derived by running an initial model and then applying the same CSMR/prevalence method. However, for many causes, DisMod estimated a rather unrealistic pattern of EMR compared to an expected pattern of decreasing EMR with greater access to quality health care. Such unexpected patterns often signal inconsistencies between CSMR estimates and the measures of prevalence and/or incidence. In effort to provide greater guidance to DisMod on the expected pattern of EMR, EMR data generated in the previous round were modeled using the MR-BRT approach by age and sex with a prior on healthcare access and quality index (HAQi) having a negative coefficient. Results from MR-BRT were then predicted for each location, year, sex, and for ages 0, 10, 20….100. We included HAQi as a predictive covariate to inform EMR with a mean and standard deviation produced from MR-BRT.

The Beta and exponentiated value of the HAQi covariate (which can be interpreted as an odds ratio) are shown in the table below.

Table 4. Covariates. Summary of covariates used in the urinary tract infection DisMod-MR meta-regression model

Covariate	Type	Parameter	Exponentiated beta (95% Uncertainty Interval)
Healthcare access and quality index	Country-level	Excess mortality rate	0.996 (0.994, 0.997)

Acute urolithiasis

Flowchart

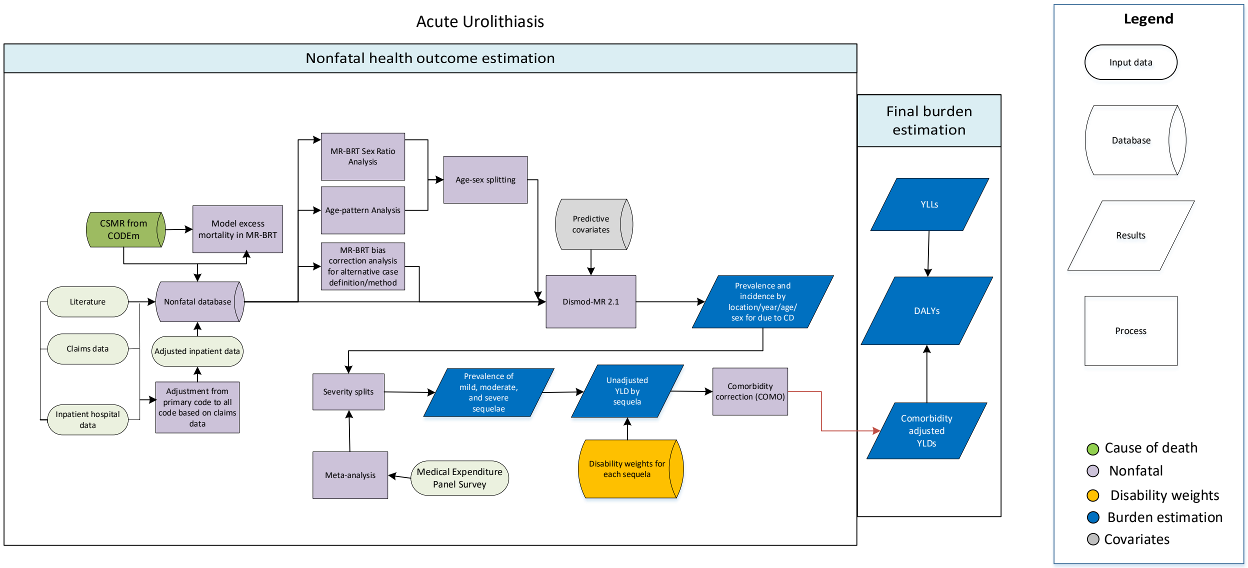

Input Data and Methodological Summary for Acute Urolithiasis

Case definition

Acute urolithiasis (AU) is an acute and usually symptomatic episode of urolithiasis, defined as stone formation located anywhere along the genitourinary tract. Associated ICD codes include N20, N20.0, N20.1, N20.2, N20.9, N21, N21.1, N21.8, N21.9, N22, N22.0, N22.8, N23, and N23.0.

Input data and data processing

Input data

A systematic literature review was first conducted in 2010 and, again, in 2013 and 2016. These data, however, were too scant and provided too little geographic coverage for robust model, thus the model also included data from hospital discharges and claims extracted as incidence.

In addition to claims and hospital discharge data used in GBD 2017, in GBD 2019, we newly added Poland and Russia claims data and additional years of data from USA claims (years 2015-2016) and hospital discharges in Mexico, India, New Zealand, Sweden, Georgia, and Ecuador. Notably, we included hospital data from Botswana; southern sub-Saharan Africa previously did not have data.

Table 1. Data Inputs for acute urolithiasis morbidity modelling by parameter.

Measure	Total sources	Countries with data
All measures	321	47
Incidence	306	47

Proportion	15	1

Data processing

Claims data link multiple inpatient and outpatient encounters to a single individual. In GBD 2017, individuals were extracted as incident cases if they had one or more inpatient encounters with an appropriate ICD code as any diagnosis; repeat encounters within 28 days were assumed to be readmissions for the same episode of illness. Data from hospital discharges with appropriate ICD codes as primary diagnostic code were, then, adjusted using correction factors derived from inpatient claims data, estimating the number of individuals represented by all encounters and adjusting the number of individuals with AU as primary diagnostic code to the number expected if information on all diagnoses had been provided.

In GBD 2019, we improved data processing methods to capture cases that were diagnosed and/or treated in an outpatient setting. Specifically, incident cases were extracted from claims data if an individual had at least one inpatient or outpatient encounter with an appropriate ICD code as any diagnosis; repeat encounters within 28 days, regardless of setting, were assumed to represent care for the same episode. Data from hospital discharges were adjusted using correction factors from claims, converting encounters to estimates of cases, correcting for most locations providing only primary diagnostic codes, and estimating outpatient cases from inpatient cases.

In addition to the improved case ascertainment of AU, the methods for bias adjustment were updated in GBD 2019 to allow a more direct comparison between different case definitions and/or study designs. In the past GBD cycles, we used data from published studies that employed rigorous case definitions for AU as our reference standard, and adjusted clinical administrative data toward this reference standard by marking administrative data with binary covariates, and estimating a fixed effect for this covariate in our DisMod meta-regression modeling process. This amounts to adjusting data using an ecological comparison, and vulnerable to compositional bias; if data from different location-years were collected using different methods or case definitions, true spatiotemporal differences in epidemiology can be erroneously adjusted, and differences truly due to differences in methods can be erroneously estimated as differences in underlying epidemiology. In GBD 2019, we avoided this risk by making pre-modeling bias adjustments and dropping data types that could not be rigorously adjusted. This was done by conducting a meta-regression of the relationship between data points matched with regard to year, age, sex, and location, but differing with regard to one or more study design characteristic. Data from studies that identified cases of AU based on urinalysis and/or imaging findings were scarce, and we were not able to find overlapping data points from administrative data sources to estimate adjustment factors. As a result, these data were excluded and a new case definition was adopted: diagnosis of AU of any etiology as indicated by ICD code in a clinical encounter.

The USA claims data from the year 2000 and from the years 2010–2016 shared a case definition with data from hospital discharges, but were adjusted outside DisMod using MR-BRT to compensate for selection bias due to commercial insurance status.

The process of adjusting for non-reference data using MR-BRT with the logit-transformation method is described below:

1. Identify data points with overlapping year, age, sex, and location between claims (alternative case definition) and hospital discharges (reference case definition)
2. Logit transform overlapping data points of alternative and reference case definitions
3. Convert overlapping data points into a difference in logit space using the following equation: $logit(altnerative) - logit(reference)$
4. Use the delta method to compute standard errors of overlapping data points in logit space, then calculate standard error of logit difference using the following equation: $\sqrt{(variance\ of\ alternative) + (variance\ of\ reference)}$
5. Using MR-BRT, conduct a random effects meta-regression to obtain the pooled logit difference of alternative to reference
6. Apply the pooled logit difference to all data points of alternative case definitions using the following equation:
$$new_{estimate} = inverse.logit((logit(alternative)) - (pooled\ logit\ difference))$$
7. Calculate new standard errors using the delta method, accounting for gamma (between-study heterogeneity)

The table below shows these bias correction factors. Beta coefficients and adjustment factors incorporate study heterogeneity (gamma).

Table 2. MR-BRT Crosswalk Adjustment Factors for Acute Urolithiasis

Data input	Reference or alternative case definition	Gamma	Beta Coefficient, Log (95% CI)	Adjustment factor*
Hospital + non-USA claims	Ref	0	---	---
USA claims from year 2000	Alt		-0.73 (-0.86, -0.60)	0.33 (0.30, 0.35)
USA claims from year 2010-2016	Alt		0.12 (0.09, 0.15)	0.53 (0.52, 0.54)

**Adjustment factor is the inverse-logit transformed Beta coefficient; <0.5 represents that alternative is adjusted upward; >0.5 represents alternative is adjusted downward*

Data points with an age-standardised incidence rate greater than two median absolute deviations from the median of the age-standardised incidence rate for all inpatient and non-USA claims data were marked as outliers and excluded from analysis. Data from Nepal, Iran, Qatar, Turkey, and Russia were also marked as outliers because they were implausibly low when compared to regional, super-regional, and global rates.

Severity split & disability weight

The basis of the GBD disability weight survey assessments is lay descriptions of sequelae highlighting major functional consequences and symptoms. Urolithiasis is split into mild, moderate, and severe categories. The lay descriptions and disability weights for urolithiasis are shown below.

Table 3. Severity Distribution, details on the severity levels for acute urolithiasis in GBD 2019 and the associated disability weight (DW) with that severity.

Severity level	Lay description	DW (95% CI)
Mild	Has some pain in the belly that causes nausea but does not interfere with daily activities.	0.011 (0.005, 0.021)
Moderate	Has pain in the belly and feels nauseous. The person has difficulties with daily activities.	0.114 (0.078, 0.159)
Severe	Has severe pain in the belly and feels nauseous. The person is anxious and unable to carry out daily activities.	0.324 (0.220, 0.442)

The severity distribution of urolithiasis was derived from analysis of the Medical Expenditure Panel Surveys (MEPS). MEPS is an overlapping panel survey of the non-institutionalised USA population that collects data on respondents' health service interactions. Panels are initiated every year. Each panel is two years long and consists of five rounds. In 2000, MEPS began using 12-Item Short Form Surveys (SF-12) to collect data on functional health status. The SF-12 survey is administered twice per panel (about once per year).

In order to translate SF-12 scores into GBD disability weights, 62 lay descriptions for conditions representing the full range of disability weight values (from most mild to most severe) were selected. A convenience sample of respondents was then asked to complete an SF-12 form for an individual with the health state described in the lay descriptions of these conditions. Composite mental and physical SF-12 score was regressed on GBD disability weight to derive the relationship between disability weight and SF-12 score. Individual respondent scores were then regressed on reported conditions to obtain a comorbidity-corrected condition-specific disability weight. The distribution of these condition-specific weights was used to derive the proportion of individuals with the conditions that fall within each GBD severity category.

Severity	Distribution
Mild acute urolithiasis	0.642 (0.536, 0.734)
Moderate acute urolithiasis	0.217 (0.149, 0.296)
Severe acute urolithiasis	0.141 (0.108, 0.178)

Modeling strategy

Similar to GBD 2017, we ran a DisMod-MR 2.1 model to produce estimates by age, sex, year, and country. Prior settings in the DisMod model included setting remission of two weeks. We used the function in DisMod-MR 2.1 to pull in cause-specific mortality rate (CSMR) data from our CODEm and CODcorrect analyses. The minimum coefficient of variation at the regional, super-regional, and global-level was changed from 0.4 to 0.8 in GBD 2019 to improve model fit against input data.

In previous rounds, priors on excess mortality rate (EMR) were estimated in DisMod by matching prevalence data points with their corresponding CSMR values within the same age, sex, year, and location (by dividing CSMR by prevalence). For short duration conditions (remission >1), the corresponding prevalence was derived by running an initial model and then applying the same

CSMR/prevalence method. However, for many causes, DisMod estimated a rather unrealistic pattern of EMR compared to an expected pattern of decreasing EMR with greater access to quality health care. Such unexpected patterns often signal inconsistencies between CSMR estimates and the measures of prevalence and/or incidence. In effort to provide greater guidance to DisMod on the expected pattern of EMR, EMR data generated in the previous round were modeled using the MR-BRT approach by age and sex with a prior on healthcare access and quality index (HAQi) having a negative coefficient. Results from MR-BRT were then predicted for each location, year, sex, and for ages 0, 10, 20….100. We included HAQi as a country-level covariate to inform EMR with a mean and standard deviation produced from MR-BRT. However, even without this setting, DisMod would tend to estimate a coefficient that was consistent with the outputs from the MR-BRT analysis.

The Beta and exponentiated values of this predictive covariate (which can be interpreted as an odds ratio) are shown in the table below.

Table 4. Covariates. Summary of covariates used in the acute urolithiasis DisMod-MR meta-regression model

Covariate	Type	Parameter	Exponentiated beta (95% Uncertainty Interval)
Healthcare access and quality index	Country-level	Excess mortality rate	0.97 (0.97, 0.98)

Benign Prostatic Hyperplasia

Flowchart

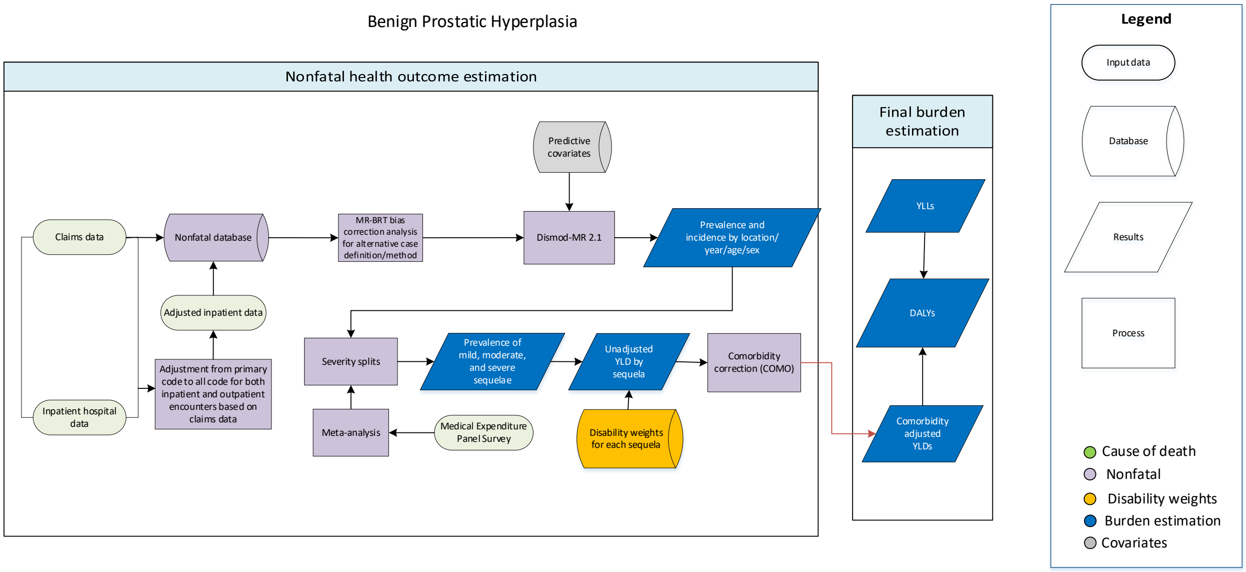

Input Data and Methodological Summary for Benign Prostatic Hyperplasia

Case definition

Benign prostatic hyperplasia (BPH) is defined as a benign proliferation of prostatic tissue, often leading to symptoms such as urinary retention, bladder outlet obstruction, or urinary tract infection. The ICD codes for BPH include N40, N40.0, N40.1, N40.2, N40.3, and N40.9.

Input data and data processing

Input data

Like GBD 2017, the model included data from hospital discharges and claims. No formal literature review has been conducted.

In GBD 2019, we newly added Poland claims data and additional years of data from USA claims (years 2015-2016) and hospital discharges in Mexico, India, New Zealand, Sweden, Georgia, and Ecuador. Notably, we included hospital data from Botswana; southern sub-Saharan Africa previously did not have data.

Table 1. Data Inputs for benign prostatic hyperplasia morbidity modelling by parameter.

Measure	Total sources	Countries with data
All measures	312	46
Prevalence	297	46
Proportion	15	1

Data processing

In GBD 2019, claims data linked multiple inpatient and outpatient claims to a single individual; prevalent cases were extracted if an individual had at least one inpatient or two outpatient encounters with an appropriate ICD code as any diagnosis within a one-year duration. Data from hospital discharges were adjusted using correction factors from claims, converting encounters to estimates of cases, correcting for most locations providing only primary diagnostic codes, and estimating outpatient cases from inpatient cases.

The USA claims data from the year 2000 and from the years 2010–2016 were each adjusted to data from hospital discharges outside DisMod using MR-BRT analysis to adjust for selection bias due to commercial insurance.

The process of adjusting for non-reference data using MR-BRT with the logit-transformation method is described below:

1. Identify data points with overlapping year, age, sex, and location between claims (alternative case definition) and hospital discharges (reference case definition)
2. Logit transform overlapping data points of alternative and reference case definitions
3. Convert overlapping data points into a difference in logit space using the following equation: $logit(altnerative) - logit(reference)$
4. Use the delta method to compute standard errors of overlapping data points in logit space, then calculate standard error of logit difference using the following equation: $\sqrt{(variance\ of\ alternative) + (variance\ of\ reference)}$
5. Using MR-BRT, conduct a random effects meta-regression to obtain the pooled logit difference of alternative to reference
6. Apply the pooled logit difference to all data points of alternative case definitions using the following equation:
$$new_{estimate} = inverse.logit((logit(alternative)) - (pooled\ logit\ difference))$$
7. Calculate new standard errors using the delta method, accounting for gamma (between-study heterogeneity)

The table below shows bias correction factors estimated using MR-BRT.

Table 2. MR-BRT Crosswalk Adjustment Factors for Benign Prostatic Hyperplasia

Data input	Reference or alternative data collection	Gamma	Beta Coefficient, Logit (95% CI)	Adjustment factor*
Hospital + non-USA claims	Ref	0.000025	---	---
USA claims from year 2000	Alt		-0.87 (-0.94, -0.79)	0.29 (0.28, 0.31)
USA claims from year 2010-2016	Alt		-0.28 (-0.36, -0.21)	0.43 (0.41, 0.45)

**Adjustment factor is the inverse-logit transformed Beta coefficient; <0.5 represents that alternative is adjusted upward; >0.5 represents that alternative is adjusted downward*

Data points with an age-standardised prevalence rate greater than two median absolute deviations from the median of the age-standardised prevalence rate for all inpatient and non-USA claims data were marked as outliers and excluded from analysis.

Severity split & disability weight

The basis of the GBD disability weight survey assessments are lay descriptions of sequelae highlighting major functional consequences and symptoms of a given cause. BPH is split into symptomatic and asymptomatic types. There is no disability weight (DW) assigned to asymptomatic cases of BPH. The DW associated with symptomatic BPH, such as urinary frequency, that is sometimes associated with pain – as seen in the table below, which offers further information.

Table 3. Severity Distribution, details on the severity levels for benign prostatic hyperplasia in GBD 2019 and the associated disability weight (DW) with that severity.

Severity level	Lay description	DW (95% CI)
Asymptomatic	N/A	0
Symptomatic	Feels the urge to urinate frequently, but when passing urine it comes out slowly and sometimes is painful.	0.067 (0.043–0.097)

In GBD 2019, the severity distribution of BPH was derived from MR-BRT analysis of the International Prostate Symptom Score (I-PSS) reported in four population-based studies in Japan, USA, France, and Scotland[1]. I-PSS is a widely-used validated questionnaire that is developed to assess severity of lower urinary tract symptoms (LUTS) related to BPH. The questionnaire consists of seven questions on incomplete emptying, frequency, intermittency, urgency, weak stream, straining, and nocturia, and one question on quality of life. Four studies recruited a representative sample of men between ages 40-79 in Japan, USA, and Scotland, ages 50-84 in France, and I-PSS was either self-administered in the presence of a research assistant or through face-to-face interviews. The cumulative distribution of the I-PSS in each country was used to derive the mean proportion of individuals with LUTS.

Severity	Distribution
Asymptomatic BPH	0.673 (0.655, 0.692)
Symptomatic BPH	0.327 (0.245, 0.436)

Modeling strategy

Similar to GBD 2017, we ran a DisMod-MR 2.1 model to produce estimates by age, sex, year, and country. Prior settings in the DisMod model included incidence and remission prior to age 40 years to 0. We set an upper bound on remission after age 40 to 0.1, corresponding to a maximum duration of 10 years. We also assumed that there was no excess mortality related to BPH. The minimum coefficient of variation at the regional, super-regional, and global-level was changed from 0.4 to 0.8 in GBD 2019 to improve model fit against input data.

[1] Sagnier P-P, Girman CJ, Garraway M, Kumamoto Y, Lieber MM, Richard F, et al. International Comparison of the Community Prevalence of Symptoms of Prostatism in Four Countries. EUR. 1996;29:15–20.

In GBD 2019, we included the age-standardized prevalence of diabetes covariate as a predictive covariate to inform prevalence, which was a better predictor than the mean BMI covariate that was used in GBD2017. The Beta and exponentiated values of this covariate (which can be interpreted as an odds ratio) are shown in the table below.

Table 4. Covariates. Summary of covariates used in the benign prostatic hyperplasia DisMod-MR meta-regression model

Covariate	Type	Parameter	Exponentiated beta (95% Uncertainty Interval)
Age-standardized prevalence of diabetes	Country-level	Prevalence	19.50 (18.47, 20.07)

Other urinary diseases

In addition to the urinary diseases described above, there are other types of urinary diseases with a range of severities and associated sequelae. Because these urinary diseases are diverse in their underlying causes and risk factors as well as in their associated health outcomes, modelling them together in a DisMod-MR model would not produce reliable estimates of prevalence. Instead, we calculated the YLDs caused by other urinary disorders directly using a YLD/YLL ratio as a 'place holder'.

We calculated the ratio of YLDs to YLLs across the specified urinary diseases for which non-fatal outcomes were modelled, using YLL estimates from the GBD 2019 cause of death (CoD) analysis. We then multiplied this YLD/YLL ratio by the YLL estimates for other urinary diseases.

Gynaecologic conditions

For GBD 2019, we model gynaecological conditions including uterine fibroids, polycystic ovarian syndrome, endometriosis, genital prolapse, premenstrual syndrome, and other gyneacological diseases that are estimated separately as menstrual disorders and non-menstrual disorders (breast disorders, ovarian cysts, inflammatory and non-inflammatory diseases of the cervix, vagina and vulva). ICD 10 codes for each cause included in the non-fatal estimation are listed in the table below.

ICD 10 codes used in the non-fatal estimation for gynaecological diseases.

Cause	ICD 10 code
Uterine fibroids	D25-D26.9, D28.2
Polycystic ovarian syndrome	E28.2
Endometriosis	N80-N80.9
Genital prolapse	N81-N81.9
Premenstrual syndrome	N94.3
Menstrual disorders	N91-N95.9
Other gynaecological disorders	B37.3-B37.49, N61 - N64.9, N72, N75 – N77.8, N83 – N86, N88 – N90.9

The total number of data sources used for the non-fatal estimation is provided in the following table.

Data inputs for gyneacological diseases morbidity modelling by parameter.

Measures	Total sources	Countries with data
All measures	805	138
Prevalence	742	136
Incidence	30	6
Proportion	34	16

Uterine Fibroids

Flowchart

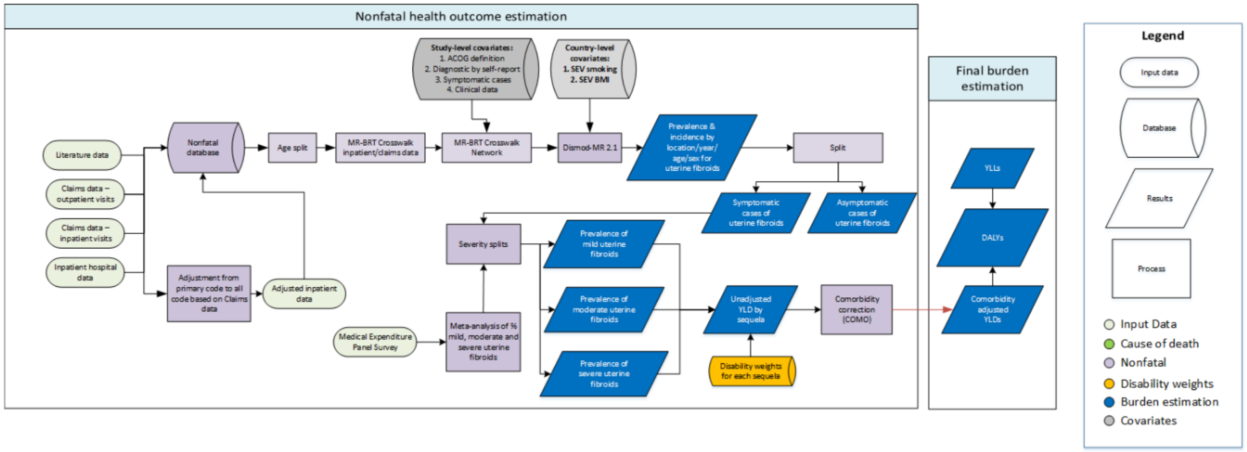

Case definition

Uterine fibroids, also called uterine myomas or leiomyomas, are non-cancerous tumors that develop from the muscle tissue of the *uterus*. Symptoms of uterine fibroids include abdominal/pelvic pain, painful intercourse, infertility and hemorrhages that can lead to anemia. Signs of fibroids can be detected during a routine pelvic exam, but the diagnosis should be confirmed by ultrasonography, hysterectomy, hysterosalpingography, sonohysterography, laparoscopy or imaging tests such as magnetic resonance imaging and compute tomography scans. For GBD 2019, we use the definition proposed by the American College of Obstetricians and Gynecologists (ACOG) as the reference definition/diagnostic method, that is, cases of uterine fibroids diagnosed by pelvic exam follow by or with ultrasonography pelvic, hysteroscopy, hysterosalpingography (X-ray test), sonohysterography or laparoscopy[1]. However, we also incorporate studies that include diagnosis by pelvic exam only and self-report.

Input data

For GBD 2019, we did not conduct a new systematic review for uterine fibroids. Instead, with the express goal of standardizing to ACOG definitions and revising data processing to happen prior to modeling, we focused on re-extracting all sources used in GBD 2017.

The last systematic review was done in GBD 2010, when Ovid MEDLINE, EMBASE, CINAHL, CAB abstracts, WHOLIS, and ISGLE database were searched. An updated PubMed search is planned during the next GBD update of gynaecological disorders. The search strings used in the initial search were as follows:

> **PUBMED**: ("Leiomyoma"[Mesh] OR fibroid OR fibroids OR leiomyoma OR leiomyomas OR leimyoma OR leimyomas OR leyomyoma OR leyomyomas OR fibromyoma OR fibromyomas OR fibroma OR fibromas OR myoma OR myomas) AND ("Genitalia, Female"[Mesh] OR "Gynecology"[Mesh] OR "Uterus"[Mesh] OR genital OR genitals OR genitalia OR gynecology OR gynaecology OR gynecologic OR gynecological OR gynaecologic OR gynaecological OR uterine OR uterus OR hysterectomy) AND ("Prevalence"[Mesh] OR prevalence OR prevalences)
>
> **EMBASE**: ('uterus myoma'/exp OR fibroid OR fibroids OR leiomyoma OR leiomyomas OR leimyoma OR leimyomas OR leyomyoma OR leyomyomas OR fibromyoma OR fibromyomas OR fibroma OR fibromas OR myoma OR myomas) AND ('uterus'/exp OR 'gynecology'/exp OR 'female genital system'/exp OR genital OR genitals OR genitalia OR gynecology OR gynaecology OR gynecologic OR gynecological OR gynaecologic OR gynaecological OR uterine OR uterus OR hysterectomy) AND (prevalence/exp OR prevalence OR prevalences)

Exclusion criteria for the initial systematic review were reviews, studies that did not provide primary data on epidemiological parameters (eg, commentary), and clearly non-representative studies (eg, only high-risk pregnant women).

In addition to literature data, claims data from the USA (MarketScan), Philippines, Taiwan and Poland were included, along with hospital administrative data that were corrected using a scalar that adjusts for inpatient and outpatient care, converting from inpatient primary admissions to inpatient all diagnoses of individuals based on claims data. The total number of data sources used for the non-fatal estimation is provided in the following table.

Data inputs for uterine fibroids morbidity modelling by parameter.

Measures	Total sources	Countries with data
All measures	321	49
Prevalence	305	49
Incidence	2	1
Proportion	15	1

Data processing

The first step of data processing was age splitting. For any datum that did not entirely fit within a GBD age group, the observation was split to be multiple age-specific and sex-specific data points based on the age and sex pattern predicted by GBD 2017 DisMod-MR 2.1 models. It is our intention to update this age-sex splitting with each cycle of GBD.

With changes to the hospital and claims administrative data-processing algorithms implemented since GBD 2017, most notably the addition of a requirement that two outpatient visits coded to a cause are required for a person to count as “a case” of a given disease, the inpatient-to-outpatient corrected administrative data became much more variable. This is hypothesized to be due to differences in care-seeking and health-care provision patterns for women with uterine fibroids, including differences between countries in whether women who have procedures for fibroids are categorized as inpatients or outpatients. We therefore used only inpatient hospital and claims data.

As mentioned before, diagnosis of uterine fibroids by pelvic exam follow by or with ultrasonography pelvic, hysteroscopy, hysterosalpingography (X-ray test), sonohysterography or laparoscopy were set as the reference category. Since we identify that the available data for uterine fibroids may be biased due to the fact that the majority of cases included in the studies are symptomatic or self-reported cases, and only a few cases of asymptomatic fibroids are detected in the general population, we consider clinical data (inpatient hospital and claims only), self-report and symptomatic cases as alternative definitions.

In accordance with GBD 2019 principles for data processing, to make data comparable, we began by evaluating the number of observations of each alternate definition that matched with a corresponding observation from the reference definition. Due to data scarcity, we only found “between study” matches. That means, we matched observations of different studies by age group, location (at the region level), and whether the midpoint of the study was within 5 years of the midpoint of the reference definition observations. All observations that matched were paired with one another and the ratio of the mean values of each, was calculated in logit space. The standard error of the ratio was calculated using the delta method. To perform the crosswalks, we used Meta-Regression - Bayesian, Regularized, Trimmed (MR-BRT), a meta-analytic tool developed for GBD 2019. There were two MR-BRT models informing nonfatal estimation of uterine fibroids. The first model was used to adjust only clinical data by using claims data as the reference definition and inpatient hospital data as the alternative definition. In this model we trimmed 10% of the data and added a quadratic spline on age, assuming non-linear tails. Our final model results for this crosswalk process are illustrated in the next Figure and Table.

Uterine fibroids MR-BRT crosswalk adjustments factors by age for hospital (alternate) to claims (reference) data.

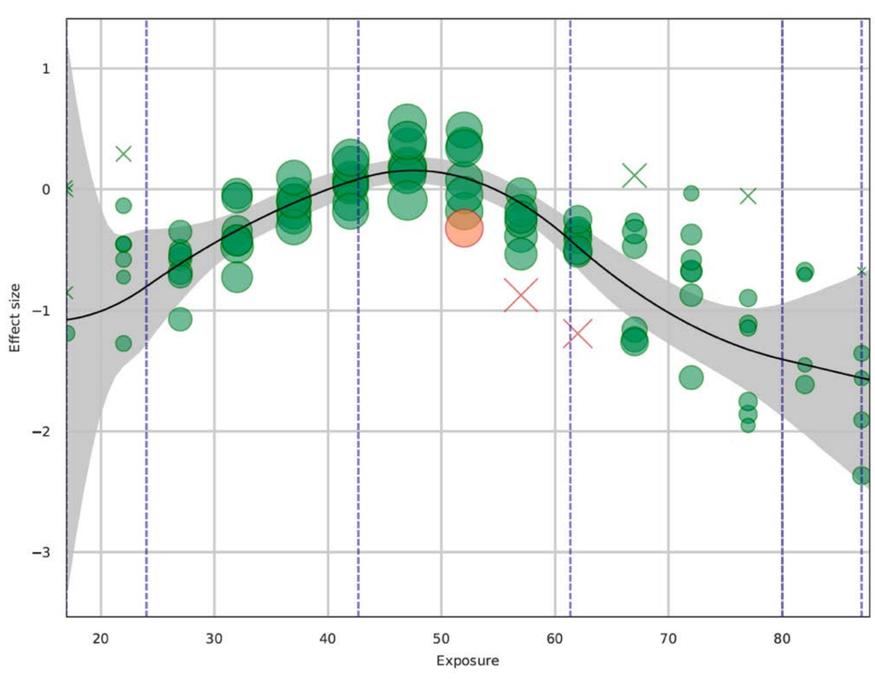

**Exposure on the x-axis is GBD age group and effect size is the logit-transformed ratio of inpatient to claims data.*

MR-BRT Crosswalk Adjustment Factors for uterine fibroids to standardize between different clinical administrative data types.

Data input	Reference or alternative case definition	Gamma	Beta Coefficient, Logit (95% CI)	Adjustment factor*
Claims data	Reference	0	---	---
Inpatient data	Alt		0.041<br>(-0.075 to 0.16)	0.51<br>(0.048 to 0.054)

**Adjustment factor is the transformed Beta coefficient in normal space and can be interpreted as the factor by which the alternative case definition is adjusted to reflect what it would have been if measured as the reference.*

Once the clinical data was adjusted, we performed a network MR-BRT considering the ACOG definition as the reference and clinical data (inpatient hospital and claims only), self-report and symptomatic cases only as alternative definitions. The adjustment factors for each of the included covariates in the models are summarized in the following table.

MR-BRT Crosswalk Adjustment Factors for uterine fibroids network model to standardize to ACOG definition.

Data input	Reference or alternative case definition	Gamma	Beta Coefficient, Logit (95% CI)	Adjustment factor*
ACOG definition	Reference	1.13	---	---
Self-report	Alt		-2.991 (-3.41 to -2.54)	0.049 (0.031 to 0.07)
Symptomatic cases	Alt		-3.558 (-5.22 to -1.83)	0.028 (0.005 to 0.138)
Clinical data	Alt		-1.824 (-2.18 to -1.47)	0.014 (0.102 to 0.0187)

**Adjustment factor is the transformed Beta coefficient in normal space and can be interpreted as the factor by which the alternative case definition is adjusted to reflect what it would have been if measured as the reference.*

Modelling strategy

After standardization to the ACOG definition, we modelled incidence, prevalence, remission, and excess-mortality due to total fibroids in DisMod-MR 2.1. This is a change from GBD 2017 when only symptomatic fibroids were modeled, using clinical data that included *only* inpatient encounters. The assumption at that time was that all inpatient admissions represented fibroids that were symptomatic enough to warrant medical care. Total fibroids in GBD 2017 were then recalculated based on a single study that reported 50% of the total cases of uterine fibroids to symptomatic.[2] For GBD 2019, with changes in the data processing, most notably the addition of a new covariate in the crosswalk that quantifies the bias introduced when estimating the prevalence/incidence of the disease in only symptomatic cases, allow us to model total fibroids cases directly. We then, split total cases of uterine fibroids into symptomatic and asymptomatic fibroids using the beta coefficient of the symptomatic cases obtained from the crosswalk as mentioned below.

As in previous GBD iterations, incidence was set to zero prior to 10 years of age and after 49, and we assumed no excess mortality from uterine fibroids. To allow the model to estimate more accurate uncertainty intervals and to better follow the data, we increased the minimum coefficient of variation from 0.3 to 0.8. In addition, we narrowed the location random effects to +/- 0.5.

Given the lack of established population-level risk factors for uterine fibroids we evaluated the association between the prevalence of uterine fibroids and potential risk factors. Potential risks factors were selected a priori based on a non-systematic literature review and included the summary exposure value (SEV) for smoking, body mass index, systolic blood pressure, physical activity, alcohol consumption, the age-standardized death rate (lnASDR) of sexually transmitted infections (STIs) from GBD 2017 COD analyses, prevalence of pelvic inflammatory disease from GBD 2017 nonfatal analyses, prevalence of contraception, and total fertility rate. From this list we selected two covariates, shown in the following table, as location-level covariates in the Dismod model.

Location-level covariates. Summary of covariates used in the uterine fibroids DisMod-MR meta-regression model.

Covariate name	Type	Measure	Beta value	Exponentiated value
Age-standardized SEV for High body-mass index	Country covariate	Prevalence	-0.406 (-1.102 to 0.421)	0.666 (0.332–1.523)
Age-standardized SEV for smoking	Country covariate	Prevalence	-1.415 (-1.593 to -1.252)	0.243 (0.203–0.286)

Severity splits and disability weights

We split total cases of uterine fibroids in symptomatic and asymptomatic cases of fibroids using the beta coefficient obtained in the crosswalk during data processing. The coefficient suggests that most of uterine fibroids cases (97%) are asymptomatic. This proportion seem to be consistent with other studies that suggest that the majority of women with uterine fibroids do not experience symptoms[3,4], but is a notably significant departure from the proportion identified for GBD 2017. The remaining symptomatic cases were all assumed to have severe symptoms such as abdominal discomfort, severe hemorrhage and consequently, anaemia due to fibroids.

The age-specific anemia prevalence for symptomatic cases uterine fibroids was analyzed as part of overall anemia causal attribution for GBD 2019. The details of the anemia analysis are described separately in the "Anemia Impairment" section. Briefly, after estimating total anemia, a series of counterfactual distributions are generated based on the age- and sex-specific prevalence of each anaemia-causing condition and the quantitative effect that the condition has on haemglobin concentration in the blood, a so-called "haemoglobin shift," that was derived by meta-analyzing cohort studies, observational studies, or trials comparing the haemotologic status of those with as compared to without the disease. Due to limited data on haemologbin shift, all were assumed to be invariant over age, sex, location, and year. It should be noted that anaemia alone is not ascribed to fibroids, but only in conjunction with mild

abdominal pain with the assumption that more severe, symptomatic cases would be more likely to cause anaemia. Disability weights for each sequela are listed below for reference.

Severity distribution, details on the severity levels for uterine fibroids in GBD 2019 and the associated disability weight (DW) with that severity.

Severity	Lay description	DW (95% CI)
Asymptomatic		--
Abdominopelvic problem, mild	Has some pain in the belly that causes nausea but does not interfere with daily activities.	0.011 (0.005–0.021)
Anaemia, mild	Feels slightly tired and weak at times, but this does not interfere with normal daily activities.	0.004 (0.001–0.008)
Anaemia, moderate	Feels moderate fatigue, weakness, and shortness of breath after exercise, making daily activities more difficult.	0.052 (0.034–0.076)
Anaemia, severe	Feels very weak, tired and short of breath, and has problems with activities that require physical effort or deep concentration.	0.149 (0.101–0.21)

Limitations

The main limitation of this analysis is data sparsity, particularly at the population level and the lack of information on the severity distribution of the disease and the proportion of symptomatic and asymptomatic cases that develop anemia. In order to improve our estimates, a systematic review for this cause is planned for next GBD cycle.

Endometriosis

Flowchart

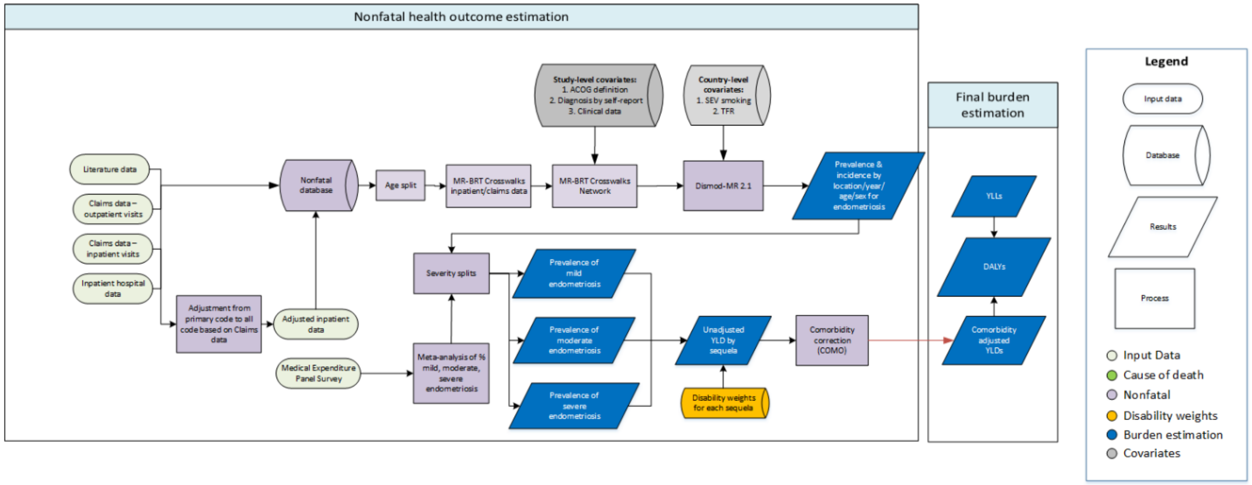

Case definition

Endometriosis is a gynaecological condition defined as growth of tissue that lies inside the uterus (endometrium), outside the uterus. Common symptoms include infertility and chronic abdominal/pelvic pain, especially before and during a menstrual period and during sexual intercourse. For GBD 2019, we define endometriosis cases according to the ACOG guidelines as cases diagnosed by pelvic exam confirmed by laparoscopy or pathology[11]. In previous rounds we only considered diagnosis accompanied by pathological confirmation as the reference.

Input data

For GBD 2019, with the express goal of standardizing all reference definitions for gynaecological diseases to ACOG definitions, we re-extract all sources of endometriosis data used in previous rounds and planned an updated systematic review for the next GBD cycle.

The re-extracted data include all data obtained from the initial review conducted for GBD 2010. The review consisted of a PubMed search and a systematic review of endometriosis throughout the world. Ovid MEDLINE, EMBASE, CINAHL, CAB abstracts, WHOLIS, and ISGLE database were searched. The search strings for PubMed and EMBASE were as follows:

PUBMED: ("Endometriosis"[Mesh] OR Endometriosis OR Endometrioses OR Endometrioma OR Endometriomas OR Adenomyosis) AND (“Incidence”[Mesh] OR Incidence OR Incidences OR “Prevalence”[Mesh] OR Prevalence OR Prevalences)

EMBASE: (‘endometriosis’/exp OR endometriosis OR endometrioses OR endometrioma OR endometriomas OR adenomyosis) AND (‘incidence’/exp OR incidence OR incidences OR ‘prevalence'/exp OR prevalence OR prevalences)

Exclusion criteria for the initial systematic review were reviews, studies that did not provide primary data on epidemiological parameters (eg, commentary), and clearly non-representative studies (eg, only high-risk pregnant women).

As mentioned before, diagnosis confirmed via laparoscopy or histologic pathology from literature studies was set as the reference definition. Self-report cases and clinical administrative data (claims and hospital data) were considered alternative definitions. Hospital administrative data were corrected using a scalar that adjusts for inpatient and outpatient care, converting from inpatient primary admissions to inpatient and outpatient all diagnoses for individuals based on claims data from the USA (MarketScan), Philippines, Taiwan and Poland.

Data inputs for endometriosis morbidity modelling by parameter.

Measures	Total sources	Countries with data
All measures	309	47
Prevalence	302	46
Incidence	8	6

Data processing

To allow for comparisons between different data sources and case definitions, we first split the data by age. We do not split the data by sex because endometriosis only occurs in women. The age splitting algorithm divides up any datum that did not entirely fit within a GBD age group to be multiple by age-specific data points based on the age and sex pattern predicted by GBD 2017 DisMod-MR 2.1 models. This algorithm will be updated in each GBD cycle.

Once the data was age split, we counted the number of observations of each alternate definition that matched with a corresponding observation from the reference definition. We matched observations by age group and location (at the region level), and when the midpoint of the study was within 5 years of the midpoint of the reference definition observation. All matched observations were paired with one another and the ratio of the mean values of each pair, were calculated in logit space. The standard error of the ratio was calculated using the delta method. Then we used the logit transformed mean and standard error as inputs to run a MR-BRT model with a cubic spline on age and 4 knots, trimming 10% of the data and assuming linear tails to crosswalk only the clinical data

considering claims as the reference definition and inpatient hospital data as the alternative definition. Our final model results for this crosswalk process are illustrated in the next figure and table.

Endometriosis MR-BRT crosswalk adjustments factors by age for hospital (alternate) to claims (reference) data.

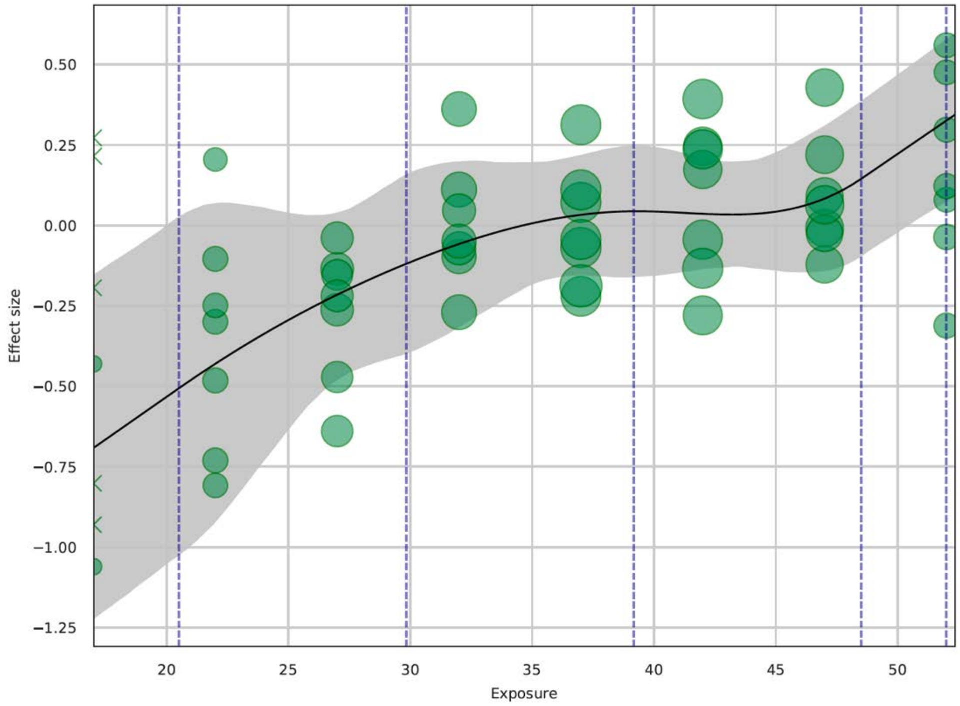

**Exposure on the x-axis is GBD age group and effect size is the logit-transformed ratio of inpatient to claims data.*

MR-BRT Crosswalk Adjustment Factors for endometriosis to standardized between different clinical administrative data types.

Data input	Reference or alternative case definition	Gamma	Beta Coefficient, Logit (95% CI)	Adjustment factor*
Claims data	Reference	0	---	---
Inpatient data	Alt		0.004<br>(-0.20 to 0.21)	0.50<br>(0.45 to 0.55)

**Adjustment factor is the transformed Beta coefficient in normal space and can be interpreted as the factor by which the alternative case definition is adjusted to reflect what it would have been if measured as the reference.*

After the first crosswalk, we performed a network MR-BRT analysis to adjust the data sources that use alternative definitions (clinical data and self-report endometriosis cases) considering the ACOG definition as the reference. The adjustment factors for each of the covariates included in the model are summarized in the following table.

MR-BRT Crosswalk Adjustment Factors for endometriosis to standardized to ACOG definition.

Data input	Reference or alternative case definition	Gamma	Beta Coefficient, Logit (95% CI)	Adjustment factor*
ACOG definition	Reference	1.13	---	---
Self-report	Alt		0.15<br>(0.13 to 0.17)	0.54<br>(0.53 to 0.55)
Clinical data	Alt		-0.22<br>(-0.23 to -0.21)	0.44<br>(0.43 to 0.45)

**Adjustment factor is the transformed Beta coefficient in normal space and can be interpreted as the factor by which the alternative case definition is adjusted to reflect what it would have been if measured as the reference.*

Modelling strategy

We used DisMod-MR 2.1, a Bayesian meta-regression epidemiological tool, to generate incidence, prevalence and remission estimates for endometriosis by age, sex, year, and location.

As in previous GBD iterations, incidence was assumed to be zero except between the ages of 15 years and 50 years. This is because a woman must enter puberty before she can get endometriosis, and the condition remits spontaneously after the onset of menopause. Remission was bounded to be a maximum of 0.2 before the age of 50 years and was set to be equal to 0.2 (1/remission = duration = 5 years). We also bound the excess-mortality rate among the prevalent cases to a maximum of 3 deaths per 10,000 person-years and used the Healthcare Access and Quality Index (HAQI) as the lone location-level covariate on this parameter.

Because in previous GBD rounds no covariates were used to inform the prevalence estimates of endometriosis, we evaluate the relationship between the prevalence of uterine fibroids and associated factors. Associated factors were selected based on a non-systematic literature review and included the summary exposure value (SEV) for smoking, body mass index, physical activity and alcohol consumption, the age-standardized death rate (lnASDR) of sexually transmitted infections (STIs), the prevalence of pelvic inflammatory diseases, prevalence of contraception and total fertility rate (TFR). From the pull of covariates that were tested, we

included TFR and the risk-weight prevalence of smoking as covariates in the final model. Their corresponding beta coefficients and exponentiated values are shown in the following.

Covariates. Summary of covariates used in the endometriosis DisMod-MR meta-regression model.

Covariate Name	Type	Measure	Beta value	Exponentiated value
Total Fertility Rate	Country covariate	Prevalence	0.18 (0.14 - 0.22)	1.20 (1.15 - 1.25)
Age-standardized SEV for Smoking	Country covariate	Prevalence	0.29 (0.22 - 0.37)	1.34 (1.25 - 1.44)
Healthcare access and quality index	Country covariate	Excess mortality rate	-0.00998 (-0.01951 -0.0008)	0.99 (0.98 - 1.00)

Severity splits & disability weights

The basis of the GBD disability weight (DW) survey assessment are lay descriptions of sequelae highlighting major functional consequences and symptoms. The GBD 2010 systematic literature review identified three studies that were combined to inform the severity distribution of those with endometriosis. Only one study reported on the proportion of endometriosis cases with chronic abdominal pain,[12] and another was found to contain data on the distribution of pain severity.[13] Data from each study were combined to calculate a pooled proportion of 69.4% (95% CI 66.5–72.4%) of women with endometriosis who have abdominal pain and, of those who suffer pain, 8.2% (7.3–9.1%) with mild pain; 75.1% (73.6–76.5%) with moderate pain; and 16.8% (15.5–18.0%) with severe pain. No information was available on the proportion of time spent with pain. From the Australian Longitudinal Women's Health Study (ALWHS) we were able to derive an estimate of the proportion of women who have endometriosis and long-term infertility.[14] The excess risk of being permanently infertile with endometriosis (relative to no endometriosis) was calculated as the difference in risk of being infertile with and without endometriosis. This excess risk was 6.2% (95% CI: 4.3–8.3%). Disability weights for each sequela are listed below for reference.

Health states used in estimating YLDs due to endometriosis.

Severity	Lay description	DW (95% CI)
Abdominopelvic problem, mild	Has some pain in the belly that causes nausea but does not interfere with daily activities.	0.011 (0.005–0.021)

Abdominopelvic problem, moderate	Has pain in the belly and feels nauseous. The person has difficulties with daily activities.	0.114 (0.078–0.159)
Abdominopelvic problem, severe	Has severe pain in the belly and feels nauseous. The person is anxious and unable to carry out daily activities.	0.324 (0.219–0.442)
Infertility, primary	Wants to have a child and has a fertile partner, but the couple cannot conceive.	0.008 (0.003–0.015)
Infertility, secondary	Has at least one child and wants to have more children. The person has a fertile partner, but the couple cannot conceive.	0.005 (0.002–0.011)

Limitations

The main limitation of this analysis is data sparsity, particularly at the population level and the lack of information on the severity distribution. In order to improve our estimates, a systematic review for this cause is planned for next GBD cycle.

Polycystic ovarian syndrome (PCOS)

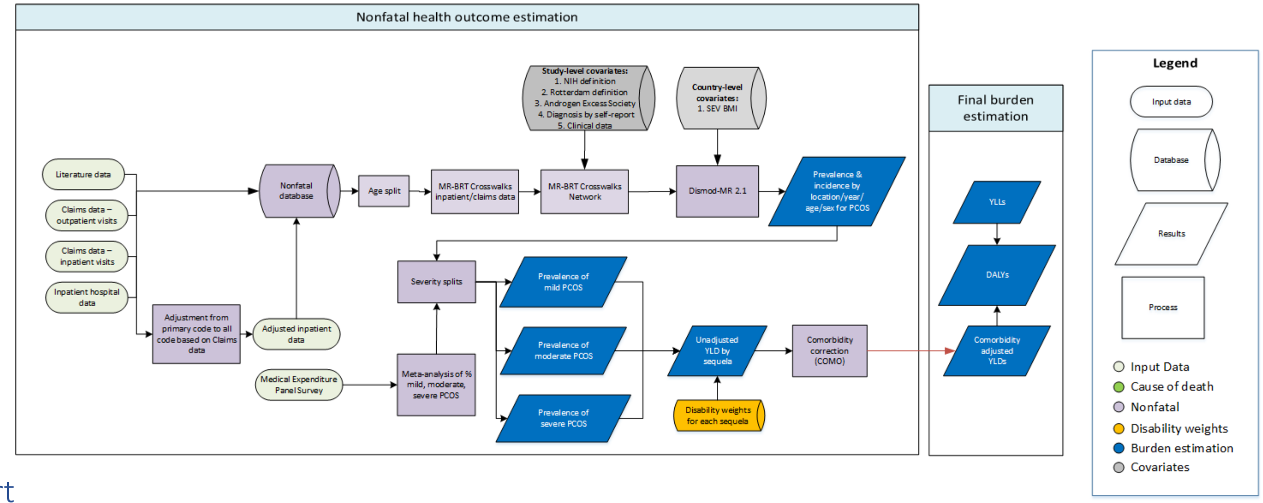

Flowchart

Case definition

Polycystic ovarian syndrome (PCOS) is an endocrinopathy characterized by hyperandrogenism, ovulatory dysfunction, and polycystic ovaries. Women with PCOS often have enlarged ovaries that contain pockets of fluid. Symptoms include infrequent menstruation, excess hair growth, acne, and obesity5.

There is no universally accepted definition of PCOS6. Expert generated diagnostic criteria include the National Institutes of Health (NIH) diagnostic7, the Rotterdam criteria8, and the Androgen Excess Society (AES) definition9. All diagnostic approaches require the presence of more than one sign or symptom and recommend that secondary causes (such as congenital adrenal hyperplasia, hyperprolactinemia, and androgen-secreting neoplasms) should first be excluded.

In GBD 2019, we standardize the reference definition of all gynaecological diseases, including PCOS, to the ACOG definitions. However, according to the ACOG, PCOS diagnosis can generally be accomplished using any of the three diagnostic approaches mentioned prior (NIH, Rotterdam or AES)5. As the Rotterdam and AES definitions have been criticized for including more mild phenotypes10, which can lead to significantly high prevalence estimates, as in GBD 2017, we used the NIH definition, which noted

the disorder as having 1) hyperandrogenism and/or hyperandrogenemia, 2) oligoovulation, and 3) exclusion of known disorders, as our reference definition.

Input data

For GBD 2019, we did not conduct a new systematic review for PCOS. Instead, we reviewed the data used in GBD 2017 and re-extracted the information needed for data processing to happen prior to modeling. Specifically, in addition to the diagnostic criteria, we identified if the cases of PCOS included in the studies were diagnosed by a physician or self-reported.

Data used in this round were first extracted for GBD 2010 purposes. At that time, a systematic review of PCOS throughout the world was conducted. Ovid MEDLINE, EMBASE, CINAHL, CAB abstracts, WHOLIS, and ISGLE and PUBMED database were searched. Search strings were as follows:

> **PUBMED:** (“Polycystic Ovary Syndrome”[Mesh] OR “Polycystic Ovary Syndrome” OR “Sclerocystic Ovary Syndrome” OR “Sclerocystic Ovarian Degeneration” OR “Stein-Leventhal Syndrome” OR “Stein Leventhal Syndrome” OR “Sclerocystic Ovaries” OR “Sclerocystic Ovary”) AND (“Incidence”[Mesh] OR Incidence OR Incidences OR “Prevalence”[Mesh] OR Prevalence OR Prevalences)
> **EMBASE:** (“ovary polycystic disease”/exp OR “cystic ovary” OR “micropolycystic ovary” OR “multiple follicle cyst” OR “ovary polycystic syndrome” OR “ovary, micropolycystic” OR “ovary, polycystic” OR “polycystic ovarian disease” OR “polycystic ovary” OR “polycystic ovary disease” OR “polycystic ovary syndrome”) AND (‘incidence’/exp OR incidence OR incidences OR 'prevalence'/exp OR prevalence OR prevalences)

We excluded reviews and studies that did not provide primary data on epidemiological parameters (eg, commentary) and clearly non-representative studies (eg, only high-risk pregnant women).
In addition to literature, claims data from the USA (MarketScan), Philippines, Taiwan and Poland were included, along with hospital administrative data that were corrected using a scalar that adjusts for inpatient and outpatient care, converting from inpatient primary admissions to inpatient and outpatient all diagnoses for individuals based on claims data. The amount of data included in our model increased significantly since GBD 2016 due to the addition of clinical administrative data.

Data inputs for polycystic ovarian syndrome morbidity modelling by parameter.

Measures	Total sources	Countries with data
All measures	252	30
Prevalence	252	30

Data processing

Prior to modelling, we performed age splitting to ensure all data fit into specific GBD standard age groups. Briefly, the age-sex splitting algorithm uses population weights that are determined by dividing the result predicted by GBD 2017 Dismod-MR 2.1 models for a specific sex and age group by the result for the aggregate age-sex specified in a given input data point. Age-sex specific values were then calculated by multiplying the aggregate input data point by these age specific weights.

Because prevalence and incidence of PCOS among reproductive-aged women varies according to the diagnostic criteria, we use the NIH case definition as the reference definition and adjusted the data from alternative definitions using two MR-BRT models. Acceptable alternate definitions included the Rotterdam definition, AES definition, self-report and clinical data. We started by evaluating the number of observations of each alternate definition that matched with a corresponding observation from the reference definition. Due to data scarcity, we only found "between study" matches (observations of different studies matched by age group and location and when the midpoint of the study was within 5 years of the midpoint of the reference definition observation). To perform the crosswalk, all observations that matched were paired with one another and the ratio of the mean values of each, was calculated in logit space. The standard error of the ratio was calculated using the delta method. The first MR-BRT model was used to adjust/crosswalk clinical data only, considering claims data as the reference definition and inpatient hospital data as the alternative definition. In this model we added a linear spline on age, assumed linear tails, and trimmed 10% of the data. Our final model results for this crosswalk process are illustrated in the following figure and table.

MR-BRT Crosswalk adjustments factors by age for hospital and claims data.

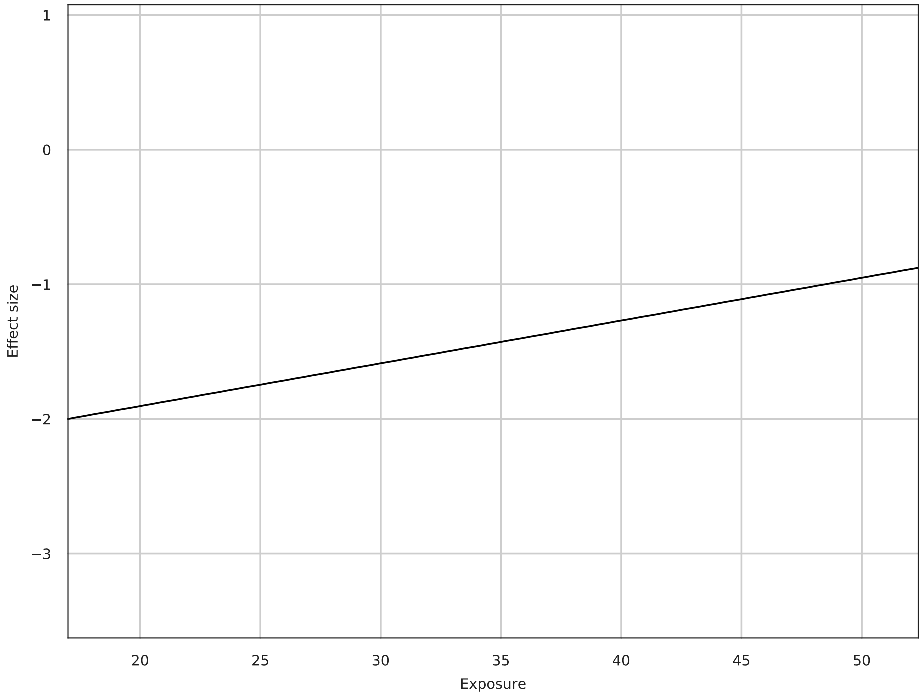

**Exposure on the x-axis is GBD age group and effect size is the logit-transformed ratio of inpatient to claims data.*

MR-BRT Crosswalk adjustment factors for polycystic ovarian syndrome to standardize between different clinical administrative data types

Data input	Reference or alternative case definition	Gamma	Beta Coefficient, Logit (95% CI)	Adjustment factor*
Claims data	Reference	0	---	---
Inpatient data	Alt		-1.52 (-2.05 to -0.95)	0.18 (0.11 to 0.28)

**Adjustment factor is the transformed Beta coefficient in normal space and can be interpreted as the factor by which the alternative case definition is adjusted to reflect what it would have been if measured as the reference.*

For the second MR-BRT model we used a network analysis to crosswalk the different diagnostic criteria including the NIH definition as the reference and the Rotterdam diagnostic criteria, the AES definition, and self-report cases, along with clinical data as alternative definitions. The adjustment factors for each of the included covariates in the models are summarized in the following table.

MR-BRT Crosswalk Adjustment Factors for polycystic ovarian syndrome to standardized between different diagnostic criteria

Data input	Reference or alternative case definition	Gamma	Beta Coefficient, Logit (95% CI)	Adjustment factor*
NIH definition	Reference	0.43	---	---
Rotterdam definition	Alt		0.22 (0.12 to 0.32)	0.45 (0.47 to 0.58)
AES definition	Alt		-0.006 (-0.10 to 0.09)	0.50 (0.47 to 0.52)
Self-report cases	Alt		-0.60 (-0.69 to -0.52)	0.35 (0.33 to 0.37)
Clinical data	Alt		-3.88 (-5.48 to -2.33)	0.02 (0.004 to 0.09)

**Adjustment factor is the transformed Beta coefficient in normal space and can be interpreted as the factor by which the alternative case definition is adjusted to reflect what it would have been if measured as the reference.*

Modelling strategy

We modelled prevalence, incidence and remission of PCOS using DisMod-MR 2.1. Incidence was set to zero prior to 10 years of age and after 55 years of age to reflect that women are only susceptible between menarche and menopause. Remission until age 54 was bounded to have a maximum value of 1 per 10 person-years. After age 55, no priors for remission were set. Unlike GBD 2017, for GBD 2019, PCOS was no longer consider a cause of death, therefore, excess mortality rate was set to 0 and cause-specific mortality rate (CSMR) from the GBD 2017 cause-specific mortality analysis was no longer used in the non-fatal estimation process. To allow the model to estimate more accurate uncertainty intervals and to better follow the data, we increased the minimum coefficient of variation from 0.3 to 0.8 and increased the degree of smoothing over age setting the parameter xi to have a maximum value of 3. In addition, a decreasing slope prior for incidence starting at age 16 was used to help the model to match the highest incidence observed in the data among younger ages (13-20 years). The addition of clinical data allows us to decrease the time span of data used to fit for a particular year to five years. In previous rounds, the time window used to fit the estimates was set to 20 years.

Because the etiology of PCOS remains uncertain, we evaluated the relationship between the prevalence of PCOS and potentially associated factors. Potentially associated factors were selected based on a non-systematic literature review and included the summary exposure value (SEV) for smoking, body mass index, physical activity, alcohol consumption, the age-standardized death rate (lnASDR) of sexually transmitted infections (STIs) from GBD 2017 COD analyses, prevalence of pelvic inflammatory disease from GBD 2017 nonfatal analyses, prevalence of contraception, total fertility rate and the Socio-demographic index (SDI). Most of the covariates from this list were not associated with the prevalence of PMS, therefore and because obesity play an important role in the etiology of the syndrome, we include the relative risk-weight prevalence of high body mass index as a location-level covariate to help drive the magnitude of prevalence estimates in areas of sparse or absent data (the coefficients are shown in the following table).

Covariates. Summary of covariates used in the polycystic ovarian syndrome DisMod-MR meta-regression model.

Covariate Name	Type	Measure	Beta value	Exponentiated value
Age-standardized SEV for body-mass index	Country covariate	Prevalence	0.74<br>(0.57 - 0.89)	2.1<br>(1.76 - 2.44)

Severity splits

The basis of the GBD disability weight (DW) survey assessment are lay descriptions of sequelae highlighting major functional consequences and symptoms. Unfortunately, no health states specific to PCOS were included in the GBD disability weights survey. The main sequelae of PCOS are infertility and hyperandrogenism/hirsutism, the latter of which was approximated with the health state of “disfigurement, level 1.” The NIH definition, which we designated as the reference case definition, consider that all cases of PCOS have hyperandrogenism and hirsutism, and therefore we assumed that 100% of PCOS cases would experience this sequela. Disability weights for each sequela are listed below for reference.

Health states used in estimating YLDs due to polycystic ovarian syndrome.

Severity	Lay description	DW (95% CI)
Disfigurement, level 1	Has a slight, visible physical deformity that others notice, which causes some worry and discomfort.	0.011<br>(0.005–0.021)
Infertility, primary	Wants to have a child and has a fertile partner, but the couple cannot conceive.	0.008<br>(0.003–0.015)
Infertility, secondary	Has at least one child and wants to have more children. The person has a fertile partner, but the couple cannot conceive.	0.005<br>(0.002–0.011)

Limitations

The main limitation of this analysis is data sparsity, particularly at the population level and the lack of information on the severity distribution. In order to improve our estimates, a systematic review for this cause is planned for next GBD cycle.

Genital prolapse

Flowchart

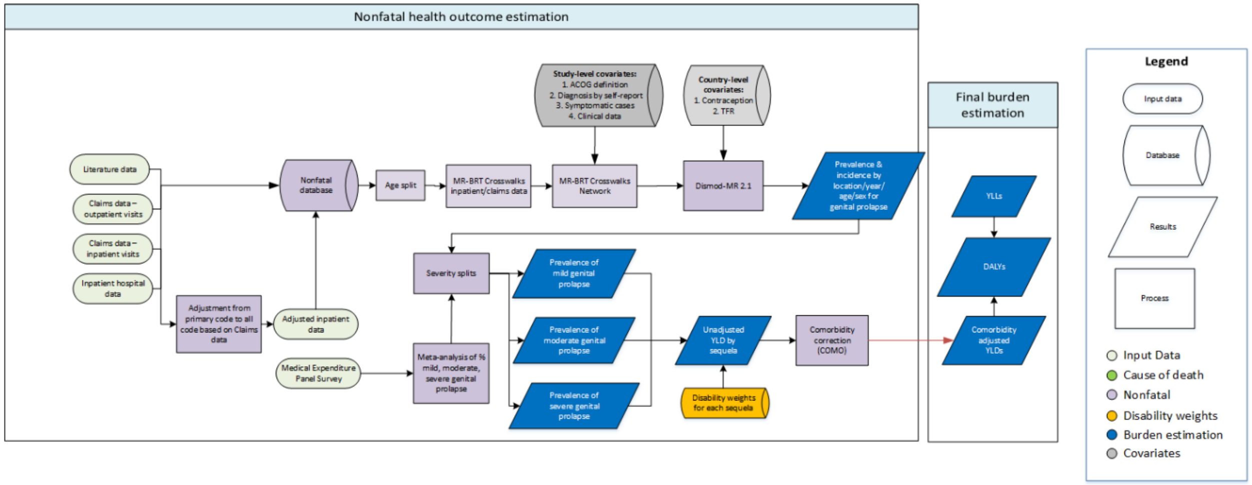

Case definition

Genital prolapse, also called pelvic organ prolapse, is the clinically relevant descent of one or more of the female pelvic structures, including the uterus, bladder, rectum, small or large bowel, or vagina. Risk of prolapse increases with age and can be exacerbated by vaginal childbirth or physical strain. ICD-10 codes associated with genital prolapse include: N81. In an effort to standardize the case definitions of all gyneacological diseases, in GBD 2019, we used the ACOG definition of genital prolapse as the reference definition[15]. The ACOG definition states that mild descent of the pelvic organs should not be considered pathologic unless women experience symptoms such as pressure with or without a bulge, sexual dysfunction or if it is disrupting normal lower urinary tract or bowel function[15].

Input data

Data sources used to inform the genital prolapse non-fatal estimates include literature data (mainly from population-level and community prevalence surveys), claims data and hospital administrative data. The last comprehensive literature review was completed in GBD 2010, where we identified data on prevalence of genital prolapse using the following search strings:

PUBMED: (("genital prolapse" OR "genital prolapses" OR "vaginal prolapse" OR "vaginal prolapses" OR "uterine prolapse" OR "uterine prolapses" OR "uterovaginal prolapse" OR "uterus prolapse" OR "pelvic organ prolapse" OR "urogenital prolapse" OR "vaginal vault prolapse" OR cystocele OR cystoceles OR “Vaginal enterocele” OR “urethrocele” OR “urethroceles”) AND (prevalence OR prevalences OR epidemiology OR incidence OR incidences)) OR ((“Uterine prolapse”[MeSH] OR “Pelvic organ prolapse”[MeSH] OR “cystocele”[MeSH]) AND (“Prevalence”[MeSH] OR “Epidemiology”[MeSH])

EMBASE: (("genital prolapse" OR "genital prolapses" OR "vaginal prolapse" OR "vaginal prolapses" OR "uterine prolapse" OR "uterine prolapses" OR "uterovaginal prolapse" OR "uterus prolapse" OR "pelvic organ prolapse" OR "urogenital prolapse" OR "vaginal vault prolapse" OR cystocele OR cystoceles OR “Vaginal enterocele” OR “urethrocele” OR “urethroceles”) AND (‘incidence’/exp OR incidence OR incidences OR ‘prevalence'/exp OR prevalence OR prevalences)) OR ((‘Uterus prolapse’/exp, ‘Pelvic organ prolapse’/exp, ‘Cystocele’/exp, ‘Enterocele’/exp) AND (‘incidence’/exp OR incidence OR incidences OR ‘prevalence'/exp OR prevalence OR prevalences))

We excluded studies that did not provide primary data on epidemiological parameters (eg, reviews, commentary) and clearly non-representative studies. The extraction and processing of hospital and claims data is described separately. The following table shows the total number of data sources consider in the non-fatal estimation process.

Data inputs for genital prolapse morbidity modelling by parameter.

Measures	Total sources	Countries with data
All measures	309	50
Prevalence	309	50

Data processing

In GBD 2019, the first step to process the data was age-sex splitting. For any datum that did not entirely fit within a GBD age group, the observation was split to be multiple age-specific and sex-specific data points based on the age and sex pattern predicted by GBD 2017 DisMod-MR 2.1 models.

As the prevalence estimates on self-reported symptoms were markedly lower than the prevalence identified by medical examination, we used MR-BRT models to crosswalk the data collected from non-reference definitions including symptomatic cases, self-reported cases and clinical data to the reference definition (cases of genital prolapse diagnosed by medical examination). First, we cross-walked only the clinical data considering claims data as the reference definition and inpatient hospital data as the alternative definition. The settings and results of this model are shown in the following figure and table.

MR-BRT Crosswalk adjustments factors by age for hospital (alternate) and claims (reference) data.

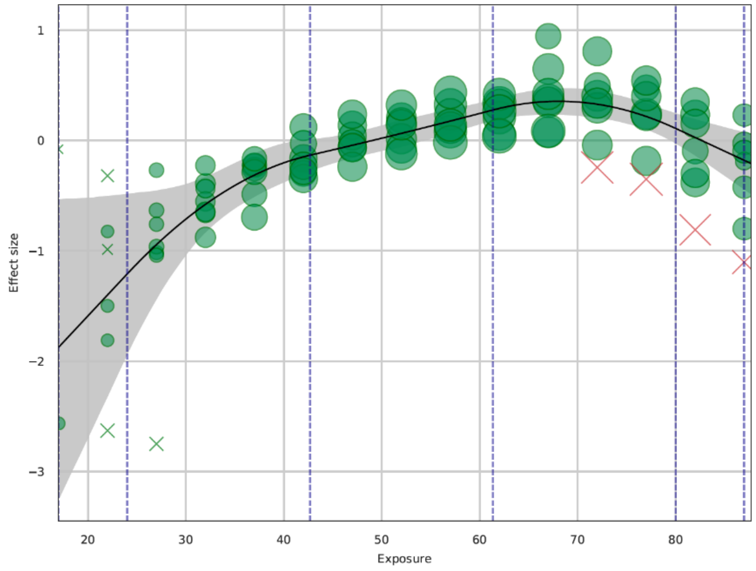

**Exposure on the x-axis is GBD age group and effect size is the logit-transformed ratio of inpatient to claims data. MR-BRT model ran with a quadratic spline on age, linear tails and trimming 10% of the data.*

MR-BRT Crosswalk Adjustment Factors for genital prolapse to standardize between different clinical administrative data types.

Data input	Reference or alternative case definition	Gamma	Beta Coefficient, Logit (95% CI)	Adjustment factor*
Claims data	Reference	0	---	---
Inpatient data	Alt		0.17<br>(0.07 to 0.27)	0.54<br>(0.52 to 0.58)

Clinical data was then included as an alternative definition along with symptomatic and self-reported cases in a network MRBRT model, where the reference definition was cases diagnosed by medical examination. The adjustment factors for each of the covariates included in the model are summarized in the following table.

MR-BRT Crosswalk Adjustment Factors for genital prolapse network model to standardize to ACOG definition.

Data input	Reference or alternative case definition	Gamma	Beta Coefficient, Logit (95% CI)	Adjustment factor*
ACOG definition	Reference	0.51	---	---
Self-report	Alt		-3.48 (-4.55 to -2.43)	0.03 (0.01 to 0.08)
Symptomatic cases	Alt		-2.24 (-3.33 to -1.13)	0.10 (0.03 to 0.24)
Clinical data	Alt		-5.58 (-5.77 to -5.38)	0.004 (0.003 to 0.005)

Modelling strategy

We used DisMod-MR 2.1 to estimate the prevalence, incidence and remission of genital prolapse. As in previous GBD iterations, incidence was set to zero prior to 15 years of age. This is because it is highly unlikely a woman would experience genital prolapse before entering her reproductive years. In an attempt to allow the model to estimate more accurate uncertainty intervals and better follow the data, we increased the minimum coefficient of variation from 0.3 to 0.8 and decrease the time span of data used to fit for a particular year from 20 to 5 years. To ensure that the age pattern of the estimates was consistent with the age pattern observed in the literature and because it is highly unlikely that young women would experience genital prolapse, we outlier all data that reported prevalence values higher than 5% for women under 25 years.

We also conduct a non-systematic literature review to find the main predictors of genital prolapse that could inform Dismod-MR 2.1 estimates. We tested the association between the prevalence of genital prolapse and the summary exposure value (SEV) for smoking, body mass index and physical activity, the prevalence of contraception and total fertility rate (TFR).

In the final model, we used log-transformed total fertility rate and the prevalence of contraception as country covariates as multiparity is a recognized risk factor for prolapse and no significant statistical association was found between the prevalence of prolapse and the rest of the aforementioned covariates. The following table illustrates covariates, measures, parameters, beta, and exponentiated beta values of the final model which was selected based on a combination of qualitative and quantitative goodness of fit to input data, plausibility of geographic and temporal trends and consistency of age pattern.

Location-level covariates. Summary of covariates used in the genital prolapse DisMod-MR meta-regression model.

Covariate Name	Type	Measure	Beta value	Exponentiated value

Contraception (Modern) Prevalence (proportion by age)	Country covariate	Prevalence	1.35 (0.74 - 1.86)	3.85 (2.09 - 6.40)
Total Fertility Rate	Country covariate	Prevalence	0.77 (0.69 - 0.84)	2.15 (2.00 - 2.31)

Severity splits

The basis of the GBD disability weight (DW) survey assessment are lay descriptions of sequelae highlighting major functional consequences and symptoms. To determine the proportion of people within each domain of disability, several studies from the systematic review were identified to contain information on the proportion of women with symptoms. These data were pooled and applied to prevalence estimates. Two studies included information on the proportion of women with prolapse who experience a bulging sensation (pooled proportion = 11.7% [95% CI 6.8–19.4%]),[16,17] three that reported on the proportion with stress incontinence (pooled proportion = 52.8% [40.1–65.1%]),[18–20] and one that reported on the frequency (measured as proportion of the year) of incontinence symptoms (pooled proportion = 7.9% [4.6–13.6%]).[21] Percentages were combined to calculate the proportion of women who fall into both stress incontinence and bulging sensation categories. The lay descriptions and disability weights for genital prolapse are shown below.

Severity distribution, details on the severity levels for genital prolapse in GBD 2019 and the associated disability weight (DW) with that severity.

Severity	Lay description	DW (95% CI)
Stress incontinence	loses small amounts of urine without meaning to when coughing, sneezing, laughing or during physical exercise	0.02 (0.011–0.035)
Abdominopelvic problem, mild	has some pain in the belly that causes nausea but does not interfere with daily activities	0.011 (0.005–0.021)

Limitations

The main limitation of this analysis is data sparsity, particularly at the population level and the lack of information on the severity distribution. In order to improve our estimates, a systematic review for this cause is planned for next GBD cycle.

Premenstrual syndrome (PMS)

Flowchart

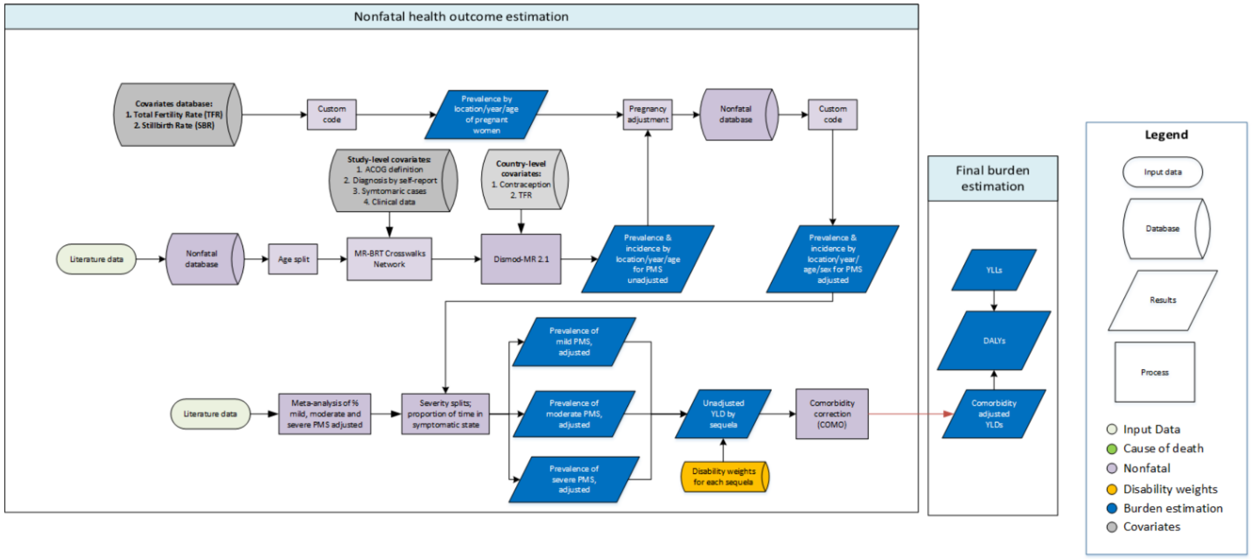

Case definition

Premenstrual syndrome (PMS) refers to psychological and physical symptoms that occur during the luteal phase of the menstrual cycle. Symptoms are extremely varied in nature and severity, but include tenderness, bloating, irritability, fatigue, abdominal pain, and altered mental states. Symptoms cease when a woman is pregnant and once she reaches menopause. The ICD-10 code is N94.3. Lacking a definitive and universally accepted diagnostic criteria for PMS, in GBD 2019, we used the diagnostic criteria proposed by the American College of Obstetricians and Gynaecologists (ACOG), as the reference definition. The ACOG definition of PMS requires at least one emotional or physical symptoms to be experienced by women during the five days before menses and remit within 4 days of onset of menses, with no recurrence at least until day 13 of the cycle, in each of three prior menstrual cycles. Additionally, identifiable dysfunction in social or economic performance and prospective confirmation for two cycles are required.

Input data

With the explicit purpose of systematize the reference definitions of all gynaecological diseases included in GBD to the diagnostic criteria proposed by the ACOG, we re-extract all literature data used in previous rounds. A new systematic literature review in planned for the next GBD cycle.

The last comprehensive literature review was completed in GBD 2010, where we identified data on prevalence of PMS using the following search strings:

PUBMED: "Premenstrual Syndrome"[Mesh] OR (premenstrual AND syndrome) OR (premenstrual AND syndrome) OR (premenstrual AND tension) OR (premenstrual AND tensions) OR (premenstrual AND stress) OR “premenstrual dysphoric disorder” OR “premenstrual dysphoric disorders” OR (menstrual AND distress) AND (("Incidence"[Mesh] OR incidence OR incidences OR onset OR occurrence) OR ("Prevalence"[Mesh] OR prevalence OR prevalences)

EMBASE: 'premenstrual syndrome'/exp OR 'premenstrual dysphoric disorder'/exp OR (premenstrual AND syndrome) OR (premenstrual AND syndromes) OR (premenstrual AND tension) OR (premenstrual AND tensions) OR (premenstrual AND stress) OR “premenstrual dysphoric disorder” OR “premenstrual dysphoric disorders” OR (menstrual AND distress) AND (('incidence'/exp OR incidence OR incidences OR onset OR occurrence) OR (prevalence/exp OR prevalence OR prevalences)

Exclusion criteria for the initial systematic review were studies that did not provide primary data on epidemiological parameters (eg, commentary) and reviews. Inpatient hospital data were not incorporated, as we believed that the likelihood that women with PMS would seek care in the medical system would be far more variable than the true epidemiologic variation.

Data inputs for premenstrual syndrome morbidity modelling by parameter.

Measures	Total sources	Countries with data
All measures	46	23
Prevalence	46	23

Data processing

We performed age-splitting to ensure all data fit into GBD standard age groups. In other words, for any datum that did not entirely fit within a GBD age group, the observation was split to be multiple age-specific and sex-specific data points based on the age and sex pattern predicted by GBD 2017 DisMod-MR 2.1 models.

Because case definitions for PMS vary widely, including varying rosters of symptoms over various time periods, we use as our reference definition the ACOG criteria, which states that the patient reports at least one of each of the following affective and somatic symptoms during the five days before their menses and appear in three consecutive cycles: depression, angry outbursts, irritability, anxiety, confusion, social withdrawal; breast tenderness, abdominal bloating, headache, or swelling of extremities. Alternative PMS definitions included the WHO/ICD-10 definition of having at least one premenstrual symptom during period of assessment, the Premenstrual Symptoms Screening Tool (PSST) definition, cases of premenstrual syndrome describe as “moderate or severe cases”, studies that report point prevalence values of PMS and other definitions of PMS that are not frequently used.

To crosswalk the alternative definitions, we first evaluate the number of observations of each alternate definition that matched with a corresponding observation from the reference definition (direct matches). Due to data scarcity, we only find “between study” matches. That means, we matched observations of different studies by age group, location (at the region level), and when the midpoint of the study was within 20 years of the midpoint of the reference definition observation. Using the same logic, we find all the matches among all possible combinations of alternative definitions. All observations that matched were paired with one another and the ratio of the mean values of each, was calculated in logit space. The standard error of the ratio was calculated using the delta method. To perform the crosswalk, we used Meta-Regression - Bayesian, Regularized, Trimmed (MR-BRT) network model trimming 10% of the data. The adjustment factors for each of the included covariates in the models are summarized in the following table.

MR-BRT Crosswalk Adjustment Factors for premenstrual syndrome to standardize to ACOG definition.

Data input	Reference or alternative case definition	Gamma	Beta Coefficient, Logit(95% CI)	Adjustment factor*
ACOG definition	Reference	1.03	---	---
WHO/ICD 10 definition	Alt		2.08 (1.99 to 2.17)	0.89 (0.87 to 0.90)
Premenstrual syndrome screening tool	Alt		-1.47 (-1.32 to -1.17)	0.19 (0.21 to 0.24)
Other definitions	Alt		-0.42 (-0.33 to -0.05)	0.39 (0.41 to 0.49)
Moderate and severe cases only	Alt		-0.38 (-0.45 to -0.30)	0.41 (0.39 to 0.42)
Period prevalence studies	Alt		-0.60 (-0.67 to -0.52)	0.35 (0.33 to 0.37)

**Adjustment factor is the transformed Beta coefficient in normal space and can be interpreted as the factor by which the alternative case definition is adjusted to reflect what it would have been if measured as the reference.*

Modelling strategy

After the data adjustments, we used Dismod MR-2.1 to estimate the prevalence, incidence and remission of PMS. As in previous GBD iterations, incidence was set to zero prior to 15 years of age and after 49 years for GBD 2019. This is because a woman must enter puberty before she is by definition only susceptible between menarche and menopause. We assumed no excess mortality from PMS and further assumed that the duration of the condition is between 3.3 and 5 years (remission rate = 0.2–0.3 per person-year).

In an attempt to allow the model to estimate more accurate uncertainty intervals and better follow the data, we increased the minimum coefficient of variation from 0.3 to 0.8 and decrease the time span of data used to fit for a particular year from 20 to 5 years.

Given the lack of established population-level risk factors for PMS, we evaluate the association between the prevalence of PMS and potential risk factors. Potential risks factors were selected a priori based on a non-systematic literature review and included the summary exposure value (SEV) for smoking, body mass index, sodium intake, alcohol consumption and physical activity.

The final model included risk-weighted prevalence of BMI and was selected based on a combination of qualitative and quantitative goodness of fit to input data, plausibility of geographic and temporal trends and consistency of age pattern. The following table shows the coefficients for the covariates used at the location level in the PMS model.

Location-level covariates. Summary of covariates used in the DisMod-MR meta-regression model for premenstrual syndrome.

Covariate Name	Type	Measure	Beta value	Exponentiated value
Age-standardized SEV for High body-mass index	Country covariate	Prevalence	-0.23302 (-0.55643 - 0.07201)	0.79 (0.57 - 1.07)

Severity splits and disability weights

Studies on the prevalence of PMS consistently excluded women who were not regularly menstruating. To address this bias, in previous rounds, a post-DisMod MR-2.1 adjustment called “pregnancy adjustment” was made by dividing DisMod estimates of PMS by the prevalence of pregnancy which in turn was estimated through another Dismod model using the UNPOP fertility estimates as input data. For GBD 2019, instead of using the UNPOP fertility data, we estimate the prevalence of pregnancy using the Age-Specific-Fertility-Rate (ASFR) and the Stillbirth Ratio (SBR) obtained from the GBD 2019 covariates dataset. The equation used to compute the prevalence of pregnancy was as follow:

Prevalence of pregnancy = (ASFR + (SBR ASFR)) * 46/52*

Where ASFR is the age-specific fertility rate, SBR is the stillbirth ratio (stillbirths per livebirth) and 46/52 is the proportion of the year spent pregnant (40 weeks) and postpartum (6 weeks). It is our intention to update this adjustment with each GBD cycle using contemporary demographics estimates.

The basis of the GBD disability weight (DW) survey assessment are lay descriptions of sequelae highlighting major functional consequences and symptoms. Unfortunately, no specific disability weights for PMS were estimated during the GBD Disability Weight Measurement Survey. Instead, we identified two health states – abdominopelvic problem (mild) and major depression (mild) – as the closest approximations of the symptoms associated with PMS. To determine the proportion of people within each of these severity levels, five studies were consulted. Three of the prevalence studies in the systematic review provided information on the proportion of PMS cases who feel depressed.[22–24] The pooled proportion was 74.2% (95% CI 69.6–78.3%). Two other studies addressed the proportion of women with PMS who experience abdominal pain.[25,26] The pooled proportion was 41.1% (31.7–51.3%). The lay descriptions and disability weights for premenstrual syndrome are shown below.

Severity distribution, details on the severity levels for premenstrual syndrome in GBD 2019 and the associated disability weight (DW) with that severity.

Severity	Lay description	DW (95% CI)
Major depressive disorder, mild episode	feels persistent sadness and has lost interest in usual activities. The person sometimes sleeps badly, feels tired, or has trouble concentrating but still manages to function in daily life with extra effort.	0.145 (0.099–0.209)
Abdominopelvic problem, mild	has some pain in the belly that causes nausea but does not interfere with daily activities.	0.011 (0.005–0.021)

Limitations

The primary limitations of our estimation are the data availability and the lack of both, information on the severity distribution and evidence of predictors of these conditions. In order to improve our estimates, a systematic review for this cause is planned for next GBD cycle.

Other gynaecological conditions – non menstrual disorders

Flowchart

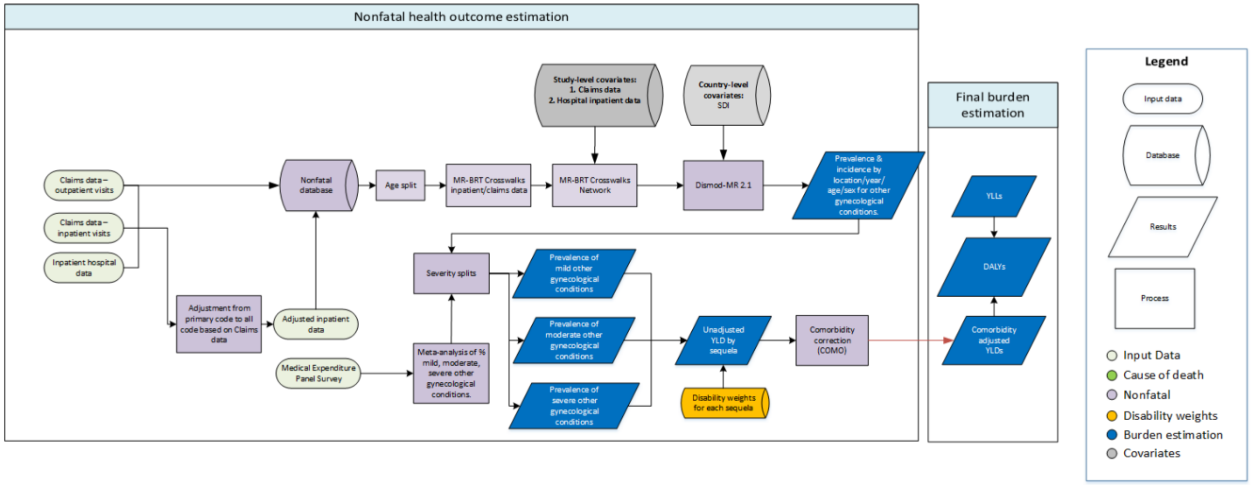

Case definition

Other gynaecological conditions encompasses all disorders that are not menstruation- or bleeding-related that do not fall under the heading of any of the other gynaecological causes. Specifically, other gynaecological disorders include breast disorders; inflammatory disease of cervix uteri; diseases of Bartholin's gland; other inflammation of vagina and vulva; vulvovaginal ulceration and inflammation in diseases classified elsewhere; non-inflammatory disorders of ovary, fallopian tube and broad ligament; other noninflammatory disorders of the uterus, cervix, vagina vulva and perineum; and menopausal and other perimenopausal disorders.

The number of total data sources used in the non-fatal estimation process are shown in the table below.

Data inputs for other gynecological diseases (including other menstrual and non-menstrual related disorders) morbidity modelling by parameter.

Measures	Total sources	Countries with data
All measures	312	46
Prevalence	297	46
Proportion	15	1

Input data

No literature data were used to inform models of other gynaecological conditions. A systematic literature review is planned for the next GBD cycle. We used claims data as the reference category and inpatient hospital data as the alterative definition. Claims data from the USA (MarketScan), Philippines, Taiwan and Poland were included, along with hospital administrative data that were corrected using a scalar that adjusts for inpatient and outpatient care, converting from inpatient primary admissions to inpatient and outpatient all diagnoses individuals based on claims data.

With changes to the hospital and claims administrative data-processing algorithms implemented since GBD 2017, most notably the addition of a requirement that two outpatient visits coded to a cause are required for a person to count as "a case" of a given disease, the inpatient-to-outpatient corrected administrative data became much more variable. This is hypothesized to be due to differences in care-seeking and health-care provision patterns for women with other gynaecological diseases, including differences between countries in whether women who have procedures related to gynaecological diseases are categorized as inpatients or outpatients. We therefore used only inpatient hospital and claims data, considering claims data as the reference definition. The total number of data sources used in the non-fatal estimation process are shown in the following table.

Data processing

A detailed explanation of the clinical data processing is described elsewhere in the appendix. In accordance with GBD 2019 principles for data processing, to make hospital inpatient data and claims data comparable, we began by evaluating the number of observations from hospital inpatient data (alternate definition) that matched with a corresponding observation from claims data (reference definition). We matched the observations by age group, location, and when the midpoint of the study was within 5 years of the midpoint of the reference definition observation. All observations that matched were paired with one another and the ratio of the mean values of each, was calculated in logit space. The standard error of the ratio was calculated using the delta method. To perform the crosswalk, we used a Meta-Regression - Bayesian, Regularized, Trimmed (MR-BRT). In this model we trimmed 10% of the data and added a cubic spline on age, assuming linear tails. Our final model results for this crosswalk process are illustrated in the following figure and table.

MR-BRT Crosswalk adjustments factors by age for hospital (alternate) and claims (reference) data.

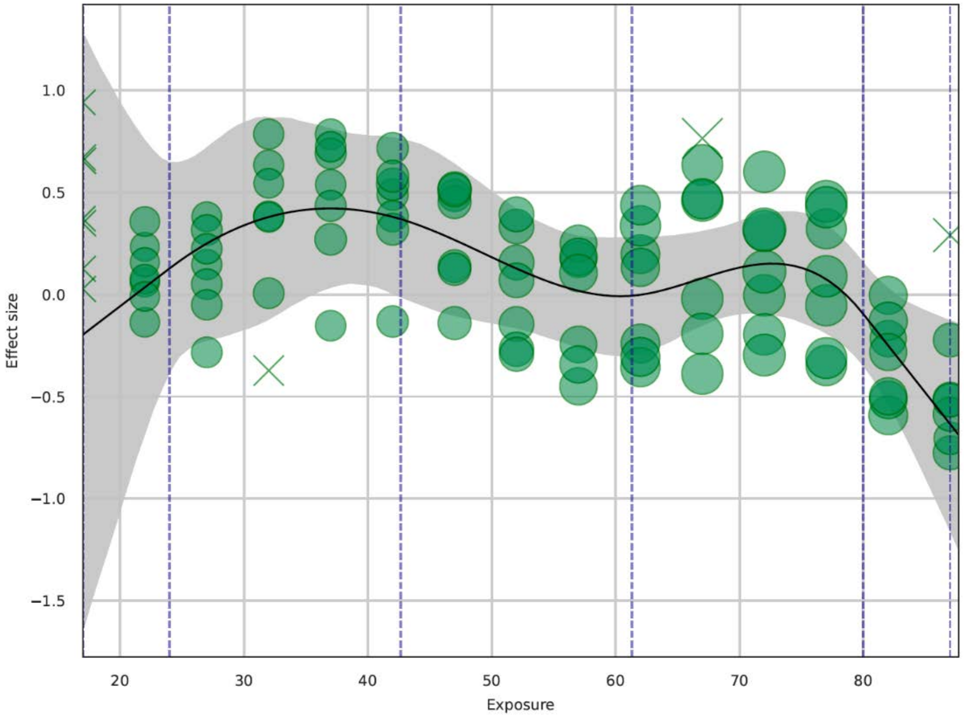

**Exposure on the x-axis is GBD age group and effect size is the logit-transformed ratio of inpatient to claims data.*

MR-BRT Crosswalk Adjustment Factors for other gynaecological diseases to standardize between different clinical administrative data types

Data input	Reference or alternative case definition	Gamma	Beta Coefficient, Logit (95% CI)	Adjustment factor*
Claims data	Reference	0	---	---
Inpatient hospital data	Alt		0.12 (-0.16 to 0.41)	0.53 (0.46 to 0.60)

**Adjustment factor is the transformed Beta coefficient in normal space and can be interpreted as the factor by which the alternative case definition is adjusted to reflect what it would have been if measured as the reference.*

Modelling strategy

We used DisMod-MR 2.1 to estimate the burden of other gynaecological diseases. Incidence was set to zero prior to 15 years of age and we assumed no excess mortality from other gynaecological conditions over the same age range. To allow the model to estimate more accurate uncertainty intervals and to better follow the data, we increased the minimum coefficient of variation from 0.3 to 0.8 and decrease the time span of data used to fit for a particular year from 20 to 5 years.

Given the lack of established population-level risk factors for other gynaecological diseases, we evaluate the association between the prevalence of these conditions and potential risk factors including the summary exposure value (SEV) for smoking, body mass index, sodium intake, alcohol consumption and physical activity, along with sociodemographic index, total fertility rate, use of contraception, the prevalence of pelvic inflammatory diseases and the age-standardized rate of sexually transmitted infections. However, none of the prior mentioned variables, except SDI, were associated with the prevalence of these conditions. The final model included SDI as the only predictor and was selected based on a combination of qualitative and quantitative goodness of fit to input data, plausibility of geographic and temporal trends and consistency of age pattern.

Location-level covariates. Summary of covariates used in the DisMod-MR meta-regression model for other gynaecological diseases.

Covariate Name	Type	Measure	Beta value	Exponentiated value
Socio-demographic index	Country covariate	Prevalence	-0.97<br>(-0.99 - 0.92)	0.38<br>(0.37 – 0.041)

Severity splits & disability weights

The basis of the GBD disability weight (DW) survey assessment are lay descriptions of sequelae highlighting major functional consequences and symptoms. To determine the proportion of women with other gynaecological conditions who fall into each severity level of abdominopelvic problem, data from the Medical Expenditure Panel Survey (MEPS) were used as described elsewhere in the appendix. The lay descriptions and disability weights for other gynaecological conditions are shown in the following table.

Severity distribution, details on the severity levels for other gynaecological diseases in GBD 2019 and the associated disability weight (DW) with that severity.

Severity	Lay description	DW (95% CI)
Abdominopelvic problem, mild	Has some pain in the belly that causes nausea but does not interfere with daily activities.	0.011 (0.005–0.021)
Abdominopelvic problem, moderate	Has pain in the belly and feels nauseous. The person has difficulties with daily activities.	0.114 (0.078–0.159)
Abdominopelvic problem, severe	Has severe pain in the belly and feels nauseous. The person is anxious and unable to carry out daily activities.	0.324 (0.219–0.442)

Limitations

The primary limitations of our estimation are the data availability and the lack of both, information on the severity distribution and evidence of predictors of these conditions. In order to improve our estimates, a systematic review for this cause is planned for next GBD cycle.

Menstrual disorders

Flowchart

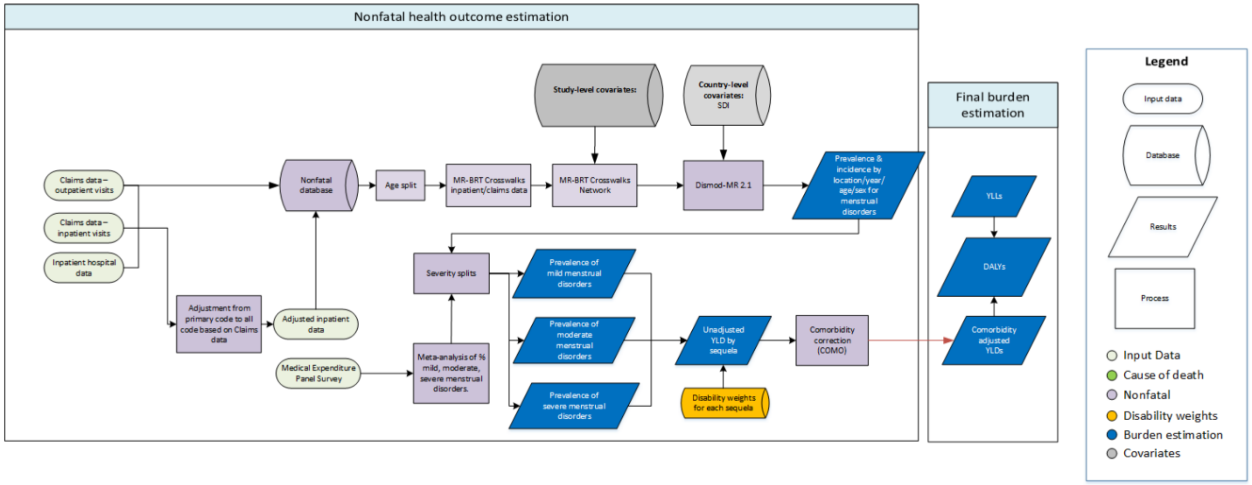

Case definition

Menstrual disorders encompass all disorders that are menstruation- or bleeding-related that do not fall under the heading of any of the other gynaecological causes. Specifically, menstrual disorders include absent, scanty and rare menstruation, pain and other conditions associated with female genital organs and menstrual cycle as define by the ICD.

Input data

This cause was first added to the GBD list for GBD 2017. No literature data are used to inform models of menstrual disorders. A systematic literature review is planned for the next GBD cycle. We used claims data as the reference category. Claims data from the USA (MarketScan), Philippines, Taiwan and Poland were included, along with hospital administrative data that were corrected using a scalar that adjusts for inpatient and outpatient care, converting from inpatient primary admissions to inpatient and outpatient all diagnoses individuals based on MarketScan data.

Data processing

A detailed explanation of the clinical data processing is described elsewhere in the appendix. In accordance with GBD 2019 principles for data processing, to make hospital inpatient data and claims data comparables, we began by evaluating the number of

observations from hospital inpatient data (alternate definition) that matched with a corresponding observation from claims data (reference definition). We matched the observations by age group, location, and when the midpoint of the study was within 5 years of the midpoint of the reference definition observation. All observations that matched were paired with one another and the ratio of the mean values of each, was calculated in logit space. The standard error of the ratio was calculated using the delta method. To perform the crosswalk, we used a Meta-Regression - Bayesian, Regularized, Trimmed (MR-BRT). In this model we trimmed 10% of the data and added a cubic spline on age, assuming linear tails. Our final model results for this crosswalk process are illustrated in the following figure and table.

MR-BRT Crosswalk adjustments factors by age for hospital (alternate) and claims (reference) data.

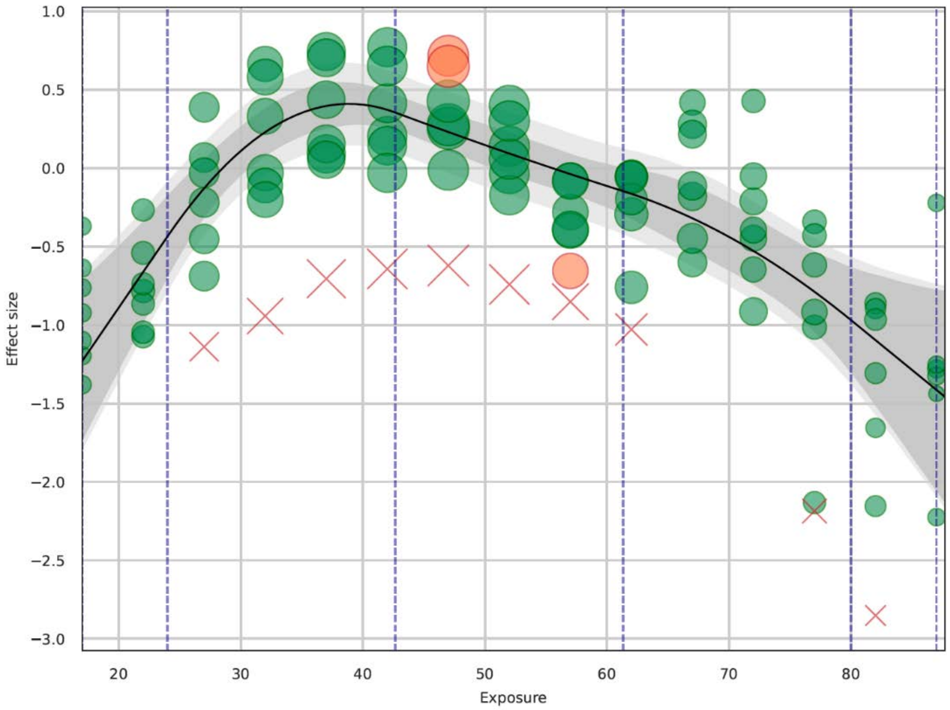

**Exposure on the x-axis is GBD age group and effect size is the logit-transformed ratio of inpatient to claims data.*

MR-BRT Crosswalk Adjustment Factors for menstrual disorders to standardize between different clinical administrative data types

Data input	Reference or alternative case definition	Gamma	Beta Coefficient, Logit (95% CI)	Adjustment factor*
Claims data	Reference	0	---	---
Inpatient hospital data	Alt		-3.05 (-4.16 to -1.96)	0.45 (0.02 to 0.13)

Modelling strategy

We used DisMod-MR 2.1 to estimate the burden of menstrual disorders. Incidence was set to zero prior to 10 years of age and after 55 years. We assume no excess mortality from menstrual disorders. To allow the model to estimate more accurate uncertainty intervals and to better follow the data, we increased the minimum coefficient of variation from 0.3 to 0.8 and decrease the time span of data used to fit for a particular year from 20 to 5 years.

Given the lack of established population-level risk factors for menstrual disorders, we evaluate the association between the prevalence of these conditions and potential risk factors including the summary exposure value (SEV) for smoking, body mass index, sodium intake, alcohol consumption and physical activity, along with sociodemographic index, total fertility rate, use of contraception, the prevalence of pelvic inflammatory diseases and the age-standardized rate of sexually transmitted infections. However, none of the prior mentioned variables, except SDI, were associated with the prevalence of these conditions. From the list of covariates, we included the prevalence of PID and the summary exposure value for body mass index as prevalence predictors in the final model, which was selected based on a combination of qualitative and quantitative goodness of fit to input data, plausibility of geographic and temporal trends and consistency of age pattern.

Location-level covariates. Summary of covariates used in the DisMod-MR meta-regression model for menstrual disorders.

Covariate Name	Type	Measure	Beta value	Exponentiated value
Pelvic inflammatory disease age-standardized prevalence	Country covariate	Prevalence	0.53 (0.078 - 0.95)	1.69 (1.08 – 2.58)
Age-standardized SEV for High body-mass index	Country covariate	Prevalence	-0.91 (-1 — -0.69)	0.40 (0.37 — 0.50)

Severity splits & disability weights

Anaemia causal attribution analysis used prevalence of menstrual disorders and information on the quantitative effect of menstrual disorders on haemoglobin levels to estimate the proportion of overall anaemia by severity that is due to menstrual disorders. The

details of the anemia analysis are described separately in the “Anemia Impairment” section. Briefly, after estimating total anemia, a series of counterfactual distributions are generated based on the age- and sex-specific prevalence of each anaemia-causing condition and the quantitative effect that the condition has on haemglobin concentration in the blood, a so-called “haemoglobin shift,” that was derived by meta-analyzing cohort studies, observational studies, or trials comparing the haemotologic status of those with as compared to without the disease. Due to limited data on haemologbin shift, all were assumed to be invariant over age, sex, location, and year.

Severity distribution, details on the severity levels for menstrual disorders in GBD 2019 and the associated disability weight (DW) with that severity.

Severity	Lay description	DW (95% CI)
Anaemia, mild	feels slightly tired and weak at times, but this does not interfere with normal daily activities.	0.004 (0.001–0.008)
Anaemia, moderate	feels moderate fatigue, weakness, and shortness of breath after exercise, making daily activities more difficult.	0.052 (0.034–0.076)
Anaemia, severe	feels very weak, tired and short of breath, and has problems with activities that require physical effort or deep concentration.	0.149 (0.101–0.21)

Limitations

The main limitation of this analysis is data sparsity, particularly at the population level and the lack of information on the severity distribution. In order to improve our estimates, a systematic review for this cause is planned for next GBD cycle.

References

1 Uterine Fibroids - ACOG. https://www.acog.org/Patients/FAQs/Uterine-Fibroids?IsMobileSet=false (accessed Oct 18, 2019).

2 Divakar H. Asymptomatic uterine fibroids. *Best Pract Res Clin Obstet Gynaecol* 2008; **22**: 643–54.

3 Okolo S. Incidence, aetiology and epidemiology of uterine fibroids. *Best Pract Res Clin Obstet Gynaecol* 2008; **22**: 571–88.

4 Schwartz SM, Marshall LM, Baird DD. Epidemiologic contributions to understanding the etiology of uterine leiomyomata. *Environ Health Perspect* 2000; **108 Suppl 5**: 821–7.

5 Polycystic Ovary Syndrome: ACOG Practice Bulletin, Number 194. *Obstet Gynecol* 2018; **131**: e157.

6 Franks S. Controversy in clinical endocrinology: diagnosis of polycystic ovarian syndrome: in defense of the Rotterdam criteria. *J Clin Endocrinol Metab* 2006; **91**: 786–9.

7 Rotterdam ESHRE/ASRM-Sponsored PCOS Consensus Workshop Group. Revised 2003 consensus on diagnostic criteria and long-term health risks related to polycystic ovary syndrome. *Fertil Steril* 2004; **81**: 19–25.

8 Azziz R, Carmina E, Dewailly D, *et al.* The Androgen Excess and PCOS Society criteria for the polycystic ovary syndrome: the complete task force report. *Fertil Steril* 2009; **91**: 456–88.

9 Endometriosis - ACOG. https://www.acog.org/Patients/FAQs/Endometriosis?IsMobileSet=false (accessed Oct 21, 2019).

10 Sinaii N, Plumb K, Cotton L, *et al.* Differences in characteristics among 1,000 women with endometriosis based on extent of disease. *Fertil Steril* 2008; **89**: 538–45.

11 Sinaii N, Cleary SD, Ballweg ML, Nieman LK, Stratton P. High rates of autoimmune and endocrine disorders, fibromyalgia, chronic fatigue syndrome and atopic diseases among women with endometriosis: a survey analysis. *Hum Reprod Oxf Engl* 2002; **17**: 2715–24.

12 Loxton D, Dobson A, Byles J, Tooth L. Australian Longitudinal Study on Women's Health (ALSWH). http://www.alswh.org.au/.

13 Committee on Practice Bulletins-Gynecology, American Urogynecologic Society. Practice Bulletin No. 185: Pelvic Organ Prolapse. *Obstet Gynecol* 2017; **130**: e234–50.

14 Slieker-ten Hove MCP, Pool-Goudzwaard AL, Eijkemans MJC, Steegers-Theunissen RPM, Burger CW, Vierhout ME. Symptomatic pelvic organ prolapse and possible risk factors in a general population. *Am J Obstet Gynecol* 2009; **200**: 184.e1-7.

15 Scherf C, Morison L, Fiander A, Ekpo G, Walraven G. Epidemiology of pelvic organ prolapse in rural Gambia, West Africa. *BJOG Int J Obstet Gynaecol* 2002; **109**: 431–6.

16 Lawrence JM, Lukacz ES, Nager CW, Hsu J-WY, Luber KM. Prevalence and co-occurrence of pelvic floor disorders in community-dwelling women. *Obstet Gynecol* 2008; **111**: 678–85.

17 Gomman HM, Nossier SA, Fotohi EM, Kholeif AE. Prevalence and factors associated with genital prolapse: a hospital-based study in Alexandria (Part I). *J Egypt Public Health Assoc* 2001; **76**: 313–35.

18 Chuenchompoonut V, Bunyavejchevin S, Wisawasukmongchol W, Taechakraichana N. Prevalence of genital prolapse in Thai menopausal women (using new standardization classification). *J Med Assoc Thail Chotmaihet Thangphaet* 2005; **88**: 1–4.

19 Townsend MK, Danforth KN, Lifford KL, *et al.* Incidence and remission of urinary incontinence in middle-aged women. *Am J Obstet Gynecol* 2007; **197**: 167.e1-5.

20 Nisar N, Zehra N, Haider G, Munir AA, Sohoo NA. Frequency, intensity and impact of premenstrual syndrome in medical students. *J Coll Physicians Surg--Pak JCPSP* 2008; **18**: 481–4.

21 Tabassum S, Afridi B, Aman Z, Tabassum W, Durrani R. Premenstrual syndrome: frequency and severity in young college girls. *JPMA J Pak Med Assoc* 2005; **55**: 546–9.

22 Steiner M, Macdougall M, Brown E. The premenstrual symptoms screening tool (PSST) for clinicians. *Arch Womens Ment Health* 2003; **6**: 203–9.

23 Choi D, Lee D-Y, Lehert P, Lee IS, Kim SH, Dennerstein L. The impact of premenstrual symptoms on activities of daily life in Korean women. *J Psychosom Obstet Gynaecol* 2010; **31**: 10–5.

24 Deuster PA, Adera T, South-Paul J. Biological, social, and behavioral factors associated with premenstrual syndrome. *Arch Fam Med* 1999; **8**: 122–8.

Haemoglobinopathies and haemolytic anaemias

This document describes the nonfatal disease burden modeling process for GBD 2019 for each of sickle cell disorders, thalassaemias, glucose-6-phosphate dehydrogenase (G6PD) deficiency, sickle cell trait, thalassaemia trait, hemizygous G6PD deficiency, and other haemoglobinopathies and haemolytic anaemias.

Flowchart

Hemoglobinopathies and hemolytic anemias: Sickle cell disorders, Thalassemias, Glucose-6-phosphate dehydrogenase (G6PD) deficiency, Sickle cell trait, Thalassemia trait, Hemizygous G6PD deficiency, Other hemoglobinopathies and hemolytic anemias

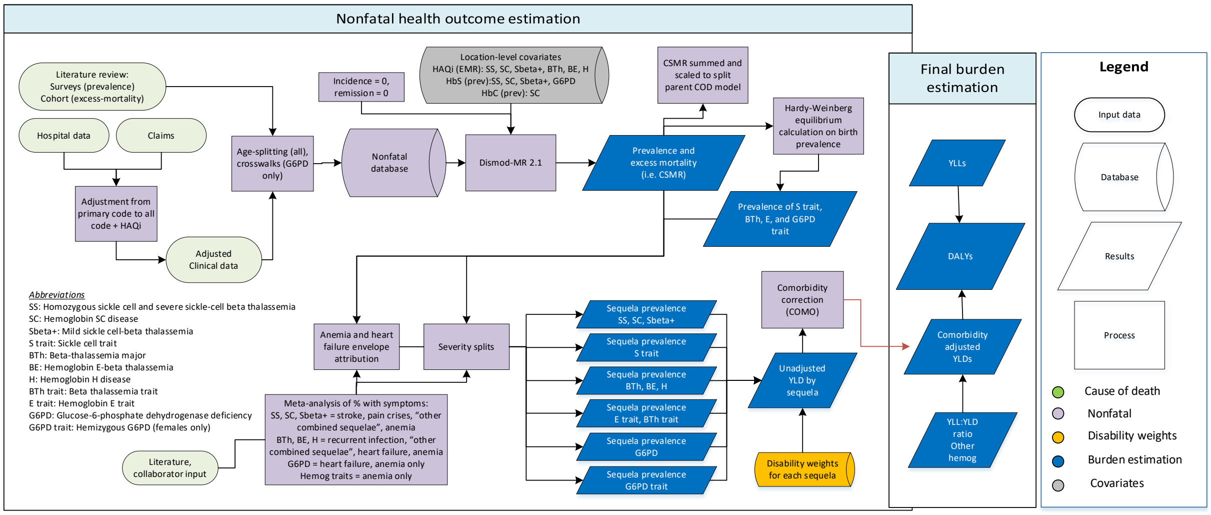

Case definition and overview

Haemoglobinopathies and haemolytic anaemias span four GBD causes: thalassaemias, sickle cell disorders, glucose-6-phosphate dehydrogenase (G6PD) deficiency, and other haemoglobinopathies and haemolytic anaemias. Case definitions for each of the types of thalassemias and sickle cell were based on genotype. G6PD deficiency is an X-linked recessive genetic disease and our reference definition was based on quantitative decline in G6PD activity during reagent (i.e. chemical) testing; genotype or other testing was an acceptable alternate definition and adjusted as described below. Sickle cell trait, thalassemia trait, and hemizygous G6PD deficiency were all similarly defined by genotype. They were estimated from the component disease models' estimates of birth prevalence assuming Hardy-Weinberg equilibrium. YLDs due to other haemoglobinopathies and haemolytic anaemias were estimated assuming the YLD-to-YLL ratio for each age, sex, location, and year was similar to that of the aggregate of sickle cell, thalassaemias, and G6PD deficiency. The primary conditions in this group are aplastic anemias.

Several unique combinations of genetic mutations lead to distinct phenotypes with different natural history, which has led us to estimate several distinct subtypes of thalassaemias and sickle cell disorders. The three thalassaemia models included 1) beta-thalassaemia major, 2) hemoglobin E/beta-thalassaemia, and 3) hemoglobin H disease (genotype = - - / - alpha). Sickle cell models included 1) homozygous sickle cell and severe sickle cell/beta-thalassaemia where the latter genotype had either a severe version of the sickle gene (assumed to always be the case if unspecified and west of the Arabian peninsula) or a nonsense (as opposed to reduced activity) mutation at the other beta haemoglobin gene

locus, 2) haemoglobin SC disease, and 3) “mild” sickle cell-beta thalassaemia. Glucose-6-phosphate dehydrogenase (G6PD) deficiency was estimated in a single model.

Input data

Three sources of data were used for DisMod-MR 2.1 models: literature (generally from community prevalence surveys, birth screening, and cohort studies), claims data, and ICD-9 & ICD-10 hospital discharge data that was adjusted for ICD code position, readmission, inpatient-to-outpatient ratio, and location-specific Healthcare Access and Quality Index (HAQi). We added data from select geographies identified by GBD collaborators for GBD 2019. Of note, there were no hospital data available for haemoglobin E/beta-thalassaemia, haemoglobin H disease, or G6PD deficiency. Our last comprehensive literature review was completed in GBD 2017, where we identified data on prevalence, excess mortality rate, or with-condition mortality rate. Age-specific survival probabilities from cohort studies were converted to corresponding with-condition mortality rates.

A systematic literature reviews was last completed for GBD 2016 using the following search strings in PubMed:

> (G6PD[Title/Abstract] OR G6PD deficiency[Title/Abstract] OR glucose-6 phosphate dehydrogenase[Title/Abstract] OR glucose-6-phosphate dehydrogenase deficiency[Title/Abstract] AND (survival[Title/Abstract] OR mortality[Title/Abstract] OR prevalence[Title/Abstract] OR incidence[Title/Abstract]) AND (2013/01/01[PDat] : 2016/12/31[PDat])) AND "humans"[MeSH Terms]
>
> (sickle cell[Title/Abstract] AND (mortality[Title/Abstract] OR survival[Title/Abstract] OR prevalence[Title/Abstract] OR incidence[Title/Abstract]) AND (2013/04/01[PDat] : 2016/12/31[PDat])) AND "humans"[MeSH Terms]
>
> (thalassemias [Title/Abstract] AND (prevalence[Title/Abstract] OR incidence[Title/Abstract] OR survival[Title/Abstract] OR mortality[Title/Abstract]) AND (2013/01/01[PDat] : 2016/12/31[PDat])) AND "humans"[MeSH Terms]

Inclusion criteria were community or facility-based surveys of prevalence of condition where either genetic testing was completed or The search was completed on July 5, 2016 and supplemented similar searches that were completed for GBD 2010 and GBD 2013. The G6PD deficiency search yielded 120 results of which 57 were selected for full text review and 32 were extracted. The sickle cell search yielded 488 results of which 49 were selected for full text review and 22 were extracted. The thalassemias search yielded 27 results, ten had full text review, and four extracted.

We extracted prevalence data from population-level and community surveys as well as with-condition mortality and excess-mortality data from cohort studies. Age-specific survival proportions were converted to with-condition mortality rates as needed. We also included data from hospital and claims data for a subset of haemoglobinopathy models, including beta-thalassaemia major, haemoglobin E/beta-thalassaemia, homozygous sickle cell and severe sickle cell/beta-thalassaemia, haemoglobin SC disease, and mild sickle cell/beta-thalassaemia.

Processing of clinical administrative data (i.e. hospital and claims) were based on ICD-9 and ICD-10 codes as listed in the table below. The extraction and processing of hospital and claims data is described separately.

Table 1. Data inputs for modeling prevalence of haemoglobinopathies and haemolytic anemias

Condition	Total Sources	Countries with Data
Haemoglobinopathies and haemolytic anaemias (all measures)	856	143
Prevalence	847	143
Other	9	6
Thalassaemias (all measures)	335	93
Prevalence	335	93
Sickle cell disorders (all measures)	484	115
Prevalence	484	115
G6PD deficiency (all measures)	338	93
Prevalence	329	93
Other	9	6

Table 2. International classification of diseases codes for haemoglobinopathies and haemolytic anaemias in GBD 2019 cause of death analysis

Condition	ICD-10 code	ICD-9 code
Thalassaemias	D56	282.4
Sickle cell disorders	D57	282.5-282.6
G6PD deficiency	D55	282.2-282.3
Other haemoglobinopathies and haemolytic anaemias	D58-D64.8	282.0-282.1, 282.7-285.8

Data processing

Data processing strategies changed for GBD 2019 across all diseases, injuries and risk factors to crosswalk non-reference data prior to modeling. Previously, we had used so-called study-level covariates to identify non-reference data and DisMod-MR 2.1 derived adjustment factors.

The first step of the process was age-sex splitting. For any datum that did not entirely fit within a GBD age group or was for both sexes combined, the observation was split to be multiple age-specific and sex-specific data points based on the age and sex pattern predicted by GBD 2017 DisMod-MR 2.1 models. It is our intention to update this age-sex splitting with each cycle of GBD. For thalassemias and sickle cell disorders, this was the only processing completed.

For G6PD deficiency, we crosswalked all data to the reference definition of chemical test. In accordance with GBD 2019 principles for data processing, we began by evaluating the number of observations of each alternate definition that matched with a corresponding observation from the reference definition. A match was considered "within" study if it was from the same data source and also an exact match for age, sex, location, and year. A match was considered "between" study if it was from the same GBD location, GBD age group, sex, and the midpoint of the study was within 5 years of the midpoint of the reference definition observation. Because the prevalence of G6PD deficiency itself can vary between studies, and the difference between reagent and chemical testing is expected to be a largely constant phenomenon, we restricted the crosswalk only to be based on within study matches. There were no matches for diagnostics that were not based on either genetic or reagent testing. All of these data were therefore dropped from the model. The total number of data points and matches is shown in the table below.

Table 3. Data points and matches between alternate and reference definitions

	Reference (cv_dx_chemical)	Alternate #1 (cv_dx_genetic)	Alternate #2 (cv_dx_other)
Number of data points	6370	2578	9
Within-study matches to reference	--	397	0

The ratio of prevalence from alternate:reference was calculated, log-transformed, the standard error of the ratio calculated using the delta method, and all were analysed using MR-BRT (Meta-Regression - Bayesian, Regularized, Trimmed) a meta-regression tool developed for GBD 2019. We tested the relationships as a function of sex, age, and the variability as a function of location (grouped into super-regions). Only sex remained a significant predictor so was the only additional factor included in the final crosswalk model. We trimmed 10% of the data from the MR-BRT model. Our covariate betas for each of the included covariates in the model are summarized in the table below.

Table 4. MR-BRT Crosswalk Adjustment Factors for haemoglobinopathies and haemolytic anaemias

Data input	Reference or alternative case definition	Gamma	Beta Coefficient, Log (95% CI)	Adjustment factor*
Chemical Test	Ref	0.06	---	---
Genetic Test	Alt		0.291 (-0.175 to 0.755)	1.33 (0.84 to 2.13)
Sex	Alt		-0.027	

**Adjustment factor is the transformed Beta coefficient in normal space, and can be interpreted as the factor by which the alternative case definition is adjusted to reflect what it would have been if measured as the reference.*

Modelling strategy

The only substantive changes, in addition to data processing described above, was removal of the Lysenko 1 (holoendemic) covariate and addition of covariates for the prevalence of Haemoglobin S (HbS) and Haemoglobin C (HbC) to the sickle cell and G6PD deficiency models. HbS and HbC rasters were summarized into GBD geographies from Malaria Atlas Project publications on them and assumed to be invariant over time and age. We estimated the non-fatal burden of haemoglobinopathies in four parts.

1. DisMod-MR 2.1 modeling of disease

First, we used the datasets described above to estimate prevalence for each age-sex-location-year in the GBD 2019 location hierarchy using DisMod-MR 2.1. Natural-log transformed lag-distributed income per capita (LN-LDI) was used as a covariate on excess mortality for most models. HbS and HbC were used for each of the subtypes of sickle cell disorders and also for G6PD deficiency, where the effect size and predictive power was expectedly much smaller. Healthcare Access and Quality Index (HAQI) was also used as a covariate for excess mortality rate in the homozygous sickle cell and severe sickle cell/beta-

thalassaemia model. A full table of all of the location-level covariates and their effect sizes are shown below.

In consultation with GBD researchers and collaborators, final models were selected on a combination of qualitative and quantitative goodness of fit to input data, plausibility of geographic and temporal trends, consistency of age pattern, and, when available, comparison with other published studies on haemoglobinopathy epidemiology. Directionality, magnitude, and plausibility of study-level and country-level covariates was also considered in the process of model development. Of note, due to the nature of statistical modelling, final results do not always cover the values reported in input data.

Table 5. Covariate, parameter, beta, and exponentiated beta values for each model

Model	Covariate	Parameter	Beta	Exponentiated beta
Beta-thalassaemia major	LN-LDI	EMR	-0.3 (-0.59 to -0.016)	0.74 (0.55 to 0.98)
Haemoglobin E/beta-thlassaemia	LN-LDI	EMR	0.0091 (-0.26 to 0.27)	1.01 (0.77 to 1.31)
Haemoglobin H disease	--	--	--	--
Homozygous sickle cell and severe sickle cell/beta-thal	(HbS)^2	Prev	49.94 (49.78 to 50.00)	4.86e21 (4.16e21 to 5.18e21)
Homozygous sickle cell and severe sickle cell/beta-thal	LN-LDI	EMR	-0.5 (-0.98 to -0.027)	0.6 (0.38 to 0.97)
Homozygous sickle cell and severe sickle cell/beta-thal	HAQI	EMR	-1.01 (-1.95 to -0.053)	0.36 (0.14 to 0.95)
Haemoglobin SC disease	HbS	Prev	9.99 (9.98 to 10.00)	2.19e4 (2.17e4 to 2.20e4)
Haemoglobin SC disease	HbC	Prev	19.99 (19.97 to 20.00)	4.79e8 (4.71e8 to 4.85e8)
Haemoglobin SC disease	LN-LDI	EMR	-0.026 (-0.05 to -9.7e-4)	0.97 (0.95 to 1.00)
Mild sickle cell/beta-thalassaemia	HbS	Prev	19.99 (19.97 — 20.00)	4.79e8 (4.71e8 to 4.85e8)
Mild sickle cell/beta-thalassaemia	LN-LDI	EMR	-0.15 (-0.29 — 0)	0.86 (0.75 — 1.00)
G6PD deficiency	|Latitude|	Prev	-0.0034 (-0.0048 — -0.0019)	1.00 (1.00 — 1.00)
G6PD deficiency	HbC	Prev	0.074 (0.0019 — 0.21)	1.08 (1.00 — 1.24)
G6PD deficiency	HbS	Prev	0.089 (0.0020 — 0.29)	1.09 (1.00 — 1.33)

Abbreviations: LDI = Lag-distributed income per capita, EMR = Excess mortality rate, HAQI = Healthcare Access and Quality Index; LN = Natural log-transformed; HbS = Haemoglobin S trait prevalence; HbC = Haemoglobin C trait prevalence

1. Hardy-Weinberg equilibrium to estimate carrier prevalence

Second, we calculated prevalence of haemoglobinopathy traits (sickle cell trait, haemoglobin E trait, haemoglobin beta trait, hemizygous G6PD) by back-calculating from birth prevalence estimates from corresponding DisMod-MR 2.1 models, assuming Hardy-Weinberg equilibrium, and no excess mortality. Because G6PD deficiency is an X-linked disease, hemizygous G6PD can only occur in females

2. Severity distributions and sequelae of disease

With the exception of anaemia, only homozygous individuals were considered to experience disability. Estimated sequelae of thalassaemias included anaemia (described separately), heart failure (described separately), and periodic severe infection. Another series of common, but not universal, sequelae also occur in those with thalassaemias, including splenomegaly, skeletal deformity, delayed growth/puberty,

diabetes, hypothyroidism, and leg ulcers. Given sparse data on the occurrence of these sequelae, they were approximated with a health state named "other combined sequelae of thalassaemia," for which we used the disability weight corresponding to a health state of "generic uncomplicated disease, anxiety about diagnosis and daily medication" which, of note, was also used to approximate the disability for those with cancer in remission. For sickle cell disorders, we similarly estimated YLDs for anaemia (described separately), stroke, and pain crises separately and approximated the myriad additional complications of sickle cell disease with the health state "other combined sequelae of sickle cell disease." The only sequelae estimated for G6PD deficiency were anaemia (described separately) and heart failure (described separately). Notably, however, G6PD deficiency is considered to be asymptomatic for a vast majority of those with the condition, with only a very small subset of around 1 in 1,000,000 having chronic haemolysis (Class I disease) and approximately 1% having periodic haemolytic episodes (Class II disease) with exposure to environmental, pharmaceutical, or food products. Females heterozygous for G6PD deficiency exhibit chimerism, as one X chromosome becomes dominant in each of the red blood cells, so we estimated half as many heterozygous females will be symptomatic as homozygous females. The table below has all the disabling health states that were included in calculation of YLDs for haemoglobinopathies and haemolytic anaemias.

3. Anaemia causal attribution

The age- and sex-specific anemia prevalence for each of the haemoglobinopathies, as well as the estimates of anemia due to carrier/ trait state, were analysed as part of overall anemia causal attribution for GBD 2019. The details of the anemia analysis are described separately in the "Anemia Impairment" section. Briefly, after estimating total anemia, a series of counterfactual distributions are generated based on the prevalence of each anaemia-causing condition and the quantitative effect that the condition has on haemglobin concentration in the blood, a so-called "haemoglobin shift," that was derived by meta-analyzing cohort studies, observational studies, or trials comparing the haemotologic status of those with as compared to without the disease. Due to limited data on haemologbin shift, all were assumed to be invariant over age, sex, location, and year.

4. YLL:YLD ratio for other haemoglobinopathies and haemolytic anaemias

Third, and finally, we found the ratio of YLD to YLL ratio for all haemoglobinopathies and then applied it to YLLs estimated for other haemoglobinopathies and haemolytic anaemias in our cause-specific mortality analysis. Quantitative crosswalk results for each model are shown below.

Table 6. Health states for haemoglobinopathies and haemolytic anaemias

Severity level	Lay description	DW (95% CI)	Cause
Mild anaemia	feels slightly tired and weak at times, but this does not interfere with normal daily activities.	0.004 (0.001 - 0.008)	All
Moderate anaemia	feels moderate fatigue, weakness, and shortness of breath after exercise, making daily activities more difficult.	0.052 (0.034 - 0.076)	All
Severe anaemia	feels very weak, tired and short of breath, and has problems with activities that require physical effort or deep concentration.	0.149 (0.101 - 0.209)	All
Severe abdominopelvic problem (proxy for vaso-occlusive crisis)	has severe pain in the belly and feels nauseous. The person is anxious and unable to carry out daily activities.	0.324 (0.22 - 0.442)	Sickle cell disorders
Stroke, long-term consequences, moderate plus cognition problems	has some difficulty in moving around, in using the hands for lifting and holding things, dressing and grooming, and in speaking. The person is often forgetful and confused.	0.316 (0.206 - 0.437)	Sickle cell disorders
Combined sequelae of disease (approximation of all other sequelae)	has a chronic disease that requires medication every day and causes some worry but minimal interference with daily activities.	0.049 (0.031 - 0.072)	Sickle cell disorders, Thalassaemias
Medically managed heart failure	--		Thalassaemias
Mild heart failure	is short of breath and easily tires with moderate physical activity, such as walking uphill or more than a quarter-mile on level ground. The person feels comfortable at rest or during activities requiring less effort.	0.041 (0.026 - 0.062)	Thalassaemias
Moderate heart failure	is short of breath and easily tires with minimal physical activity, such as walking only a short distance. The person feels comfortable at rest but avoids moderate activity.	0.072 (0.047 - 0.103)	Thalassaemias
Severe heart failure	is short of breath and feels tired when at rest. The person avoids any physical activity, for fear of worsening the breathing problems.	0.179 (0.122 - 0.251)	Thalassaemias
Severe infection	has a high fever and pain, and feels very weak, which causes great difficulty with daily activities.	0.133 (0.088 - 0.19)	Thalassaemias

Endocrine, Metabolic, Blood, and Immune Disorders

Flowchart

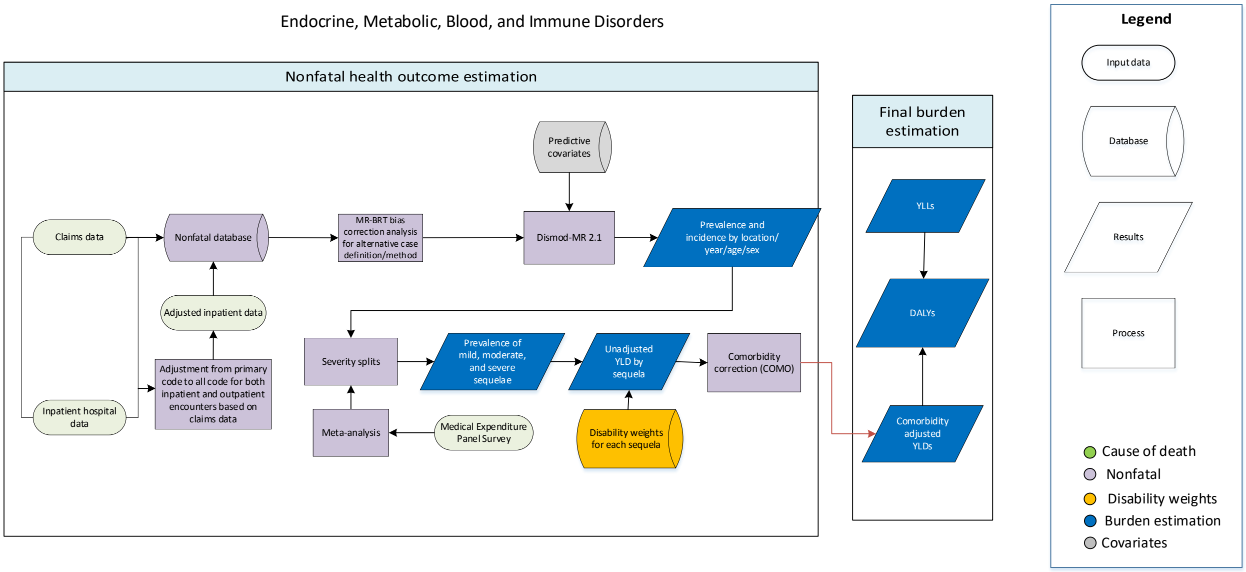

Input Data and Methodological Summary for Endocrine, Metabolic, Blood and Immune Disorders

Case definition

Endocrine, metabolic, blood, and immune disorders (EMBID) is a residual cause consisting of conditions that do not map to other causes within the diabetes, urogenital, blood, and endocrine disease hierarchy. This residual group consists mainly of thyroid disorders, rare metabolic and immune disorders, and blood disorders not resulting in anaemia. From the ICD chapter on endocrine, metabolic, and immune disorders (the E chapter) GBD's definition of EMBID excludes the codes for nutritional deficiencies, diabetes and anaemia which are modelled as separate causes; as well as those for obesity and hypercholesterolemia which are modelled as risk factors, not diseases.

ICD 10 codes for EMBID include: D64.4, D64.8, D68-D68.6, D68.8-D68.9, D69-D69.4, D69.6, D69.8, D70-D70.4, D70.8-D70.9, D72-D72.1, D72.8-D72.9, D73- D73.5, D73.8-D73.9, D74.0, D74.8-D74.9, D75-D75.2, D75.8-D75.9, D76-D76.3, D80-D80.9, D81-D81.9, D82-D82.4, D82.8-D82.9, D83-D83.2, D83.8-D83.9, D84-D84.1, D84.8-D84.9, D86.8, D89-D89.2, D89.8-D89.9, E03-E03.1, E03.3-E03.5, E03.8-E03.9, E04-E04.2, E04.8-E04.9, E05-E05.5, E05.8-E05.9, E06-E06.3, E06.5, E06.9, E07-E07.1, E07.8-E07.9, E16.1-E16.4, E16.8-E16.9, E20-E20.1, E20.8-E20.9, E21-E21.5, E22-E22.2, E22.8-E22.9, E23.0, E23.2-E23.3, E23.6-E23.7, E24-E24.1, E24.3, E24.9, E25.0, E25.8-E25.9, E26-E26.1, E26.8-E26.9, E27-E27.2, E27.4-E27.5, E27.8-E27.9, E28-E28.1, E28.3, E28.8-E28.9, E29-E29.1, E29.8-E29.9, E30-E30.1, E30.8-E30.9, E31-E31.2, E31.8-E31.9, E32-E32.1, E32.8-E32.9, E34-E34.5, E34.8-E34.9, E67-E67.3, E67.8, E70-E70.5, E70.8-

E70.9, E71-E71.5, E72-E72.5, E72.8-E72.9, E73-E73.1, E73.8-E73.9, E74-E74.4, E74.8-E74.9, E75-E75.6, E76-E76.3, E76.8-E76.9, E77-E77.1, E77.8-E77.9, E79-E79.2, E79.8-E79.9, E80-E80.7, E83-E83.9, E84-E84.9, E85-E85.9, E88-E88.9.

Input data and data processing

Input data

Like GBD 2017, the model included data from hospital discharges and claims. In GBD 2019, we newly added Poland claims data and additional years of data from USA claims (years 2015-2016) and hospital discharges in Mexico, India, New Zealand, Sweden, Georgia, and Ecuador. Notably, we included hospital data from Botswana; southern sub-Saharan Africa previously did not have data.

Table 1. Data Inputs for endocrine, metabolic, blood, and immune disorders morbidity modelling by parameter.

Measure	Total sources	Countries with data
All measures	321	47
Prevalence	306	47
Proportion	15	1

Data processing

Hospital discharge data provide observations about encounters, generally with only the primary diagnostic code for the encounter. Claims data, on the other hand, link claims for all inpatient and outpatient encounters for a single individual, and provide primary and secondary diagnoses for all encounters.

In GBD 2017, an individual was extracted from claims data as a prevalent case if that individual had one or more inpatient encounters with an appropriate ICD code as any diagnosis within one year. Data from hospital discharges with appropriate ICD codes as primary diagnostic code were, then, adjusted using correction factors derived from inpatient claims data, estimating the number of individuals represented by each encounter and adjusting the number of individuals with EMBID as primary diagnostic code to the number expected if information on all diagnoses had been provided.

In GBD 2019, claims data linked multiple inpatient and outpatient claims to a single individual; prevalent cases were extracted if an individual had at least one inpatient or two outpatient encounters with an appropriate ICD code as any diagnosis within one year. Data from hospital discharges were adjusted using correction factors from claims, converting encounters to estimates of cases, correcting for most locations providing only primary diagnostic codes, and estimating outpatient cases from inpatient cases.

The USA claims data from the year 2000 and from the years 2010–2016 were each adjusted to data from hospital discharges outside DisMod using MR-BRT analysis to adjust for selection bias due to commercial insurance.

The process of adjusting for non-reference data using MR-BRT with the logit-transformation method is described below:

1. Identify data points with overlapping year, age, sex, and location between claims (alternative case definition) and hospital discharges (reference case definition)
2. Logit transform overlapping data points of alternative and reference case definitions
3. Convert overlapping data points into a difference in logit space using the following equation: $logit(altnerative) - logit(reference)$
4. Use the delta method to compute standard errors of overlapping data points in logit space, then calculate standard error of logit difference using the following equation: $\sqrt{(variance\ of\ alternative) + (variance\ of\ reference)}$
5. Using MR-BRT, conduct a random effects meta-regression to obtain the pooled logit difference of alternative to reference
6. Apply the pooled logit difference to all data points of alternative case definitions using the following equation:
$$new_{estimate} = inverse.logit((logit(alternative)) - (pooled\ logit\ difference))$$
7. Calculate new standard errors using the delta method, accounting for gamma (between-study heterogeneity)

The table below shows bias correction factors of estimated using MR-BRT.

Table 2. MR-BRT Crosswalk Adjustment Factors for Endocrine, Metabolic, Blood, and Immune Disorders

Data input	Reference or alternative data collection	Gamma	Beta Coefficient, Logit-difference (95% CI)	Adjustment factor*
Hospital + non-USA claims	Ref	0.35	---	---
USA claims from year 2000	Alt		-1.26<br>(-2.23, -0.29)	0.22<br>(0.10, 0.43)
USA claims from year 2010-2016	Alt		0.13<br>(-0.83, 1.09)	0.53<br>(0.30, 0.75)

**Adjustment factor is the inverse-logit transformed Beta coefficient; <0.5 represents that alternative is adjusted upward; >0.5 represents that alternative is adjusted downward*

Data points with an age-standardised prevalence rate greater than 1.5 median absolute deviations from the median of the age-standardised prevalence rate for all inpatient and non-USA claims data were marked as outliers and excluded from analysis. Data in Japan, Sweden, Norway, and countries in central Latin America below age 20 were also marked as outliers because their estimates were implausibly high when compared to regional, super-regional, and global rates.

Severity split & disability weight

The basis of the GBD disability weight survey assessments is lay descriptions of sequelae highlighting major functional consequences and symptoms. EMBID is split into asymptomatic, mild, moderate, and severe categories. The lay descriptions and disability weights for EMBID are shown below.

Table 3. Severity Distribution, details on the severity levels for endocrine, metabolic, blood, and immune disorders in GBD 2019 and the associated disability weight (DW) with that severity.

Severity level	Lay description	DW (95% CI)
Asymptomatic	--	--
Mild	Has low energy and feels cold.	0.019 (0.01–0.032)
Moderate	Feels nervous, has palpitations, sweats a lot, and has difficulty sleeping.	0.145 (0.096–0.202)
Severe	Easily bruises and sometimes bleeds from the gums and nose; feels weak and has some difficulty with daily activities.	0.159 (0.106–0.226)

The severity distribution of EMBID was derived from analysis of the Medical Expenditure Panel Surveys (MEPS). MEPS is an overlapping panel survey of the non-institutionalised USA population that collects data on respondents' health service interactions. Panels are initiated every year. Each panel is two years long and consists of five rounds. In 2000, MEPS began using 12-Item Short Form Surveys (SF-12) to collect data on functional health status. The SF-12 survey is administered twice per panel (about once per year).

In order to translate SF-12 scores into GBD disability weights, 62 lay descriptions for conditions representing the full range of disability weight values (from most mild to most severe) were selected. A convenience sample of respondents was then asked to complete an SF-12 form for an individual with the health state described in the lay descriptions of these conditions. Composite mental and physical SF-12 score was regressed on GBD disability weight to derive the relationship between disability weight and SF-12 score. Individual respondent scores were then regressed on reported conditions to obtain a comorbidity-corrected condition-specific disability weight. The distribution of these condition-specific weights was used derive the proportion of individuals with the conditions that fall within each GBD severity category.

Severity	Distribution
Asymptomatic endocrine, metabolic, blood, and immune disorders	0.410 (0.398, 0.423)
Mild endocrine, metabolic, blood, and immune disorders	0.387 (0.328, 0.430)
Moderate endocrine, metabolic, blood, and immune disorders	0.061 (0.042, 0.060)
Severe endocrine, metabolic, blood, and immune disorders	0.142 (0.115, 0.173)

Modeling strategy

Similar to GBD 2017, we ran a DisMod-MR 2.1 model to produce estimates by age, sex, year, and country. Prior settings in the DisMod model included setting maximum remission of four years. It was assumed that no one was born with EMBID. We excluded causes-specific mortality rate (CSMR) data from analysis in GBD 2019 because of implausibly high CSMR estimates in ages below 5 years that was causing overestimation of prevalence in younger age groups. The minimum coefficient of variation at

the regional, super-regional, and global-level was changed from 0.4 to 0.8 in GBD 2019 to improve model fit against input data.

We included Lagged Distributed Income (LDI) as a predictive covariate to inform excess mortality, with a lower bound of -0.5 and an upper bound of -0.1. The Beta and exponentiated values of this covariate (which can be interpreted as an odds ratio) are shown in the table below.

Table 4. Covariates. Summary of covariates used in the endocrine, metabolic, blood, and immune disorders DisMod-MR meta-regression model

Covariate	Type	Parameter	Exponentiated beta (95% Uncertainty Interval)
LDI (I$ per capita)	Country-level	Excess mortality rate	0.74 (0.61, 0.90)

Oral disorders

This document describes the nonfatal disease burden modeling process for GBD 2019 for each of edentulism, caries of deciduous teeth, caries of permanent teeth, chronic periodontal disease, and other oral disorders.

Input data

Data seeking and systematic literature reviews were completed for all oral disorders together given the overlap in data types and data sources that inform the models. An initial literature review was done by the Expert Group for GBD 2010 in PubMed, Embase, LILACS, and SciELO, including published articles as well as the results of national and subnational reports. An updated systematic review was last completed on February 11, 2018 for GBD 2017 in Pubmed and Embase. The search strings used are below:

> **PubMed:** ((((Deciduous caries[Title/Abstract]) OR (milk caries[Title/Abstract]) OR (baby caries[Title/Abstract]) OR (caries[Title/Abstract]) OR (dental health[Title/Abstract]) OR (oral health[Title/Abstract])) OR ((Permanent caries[Title/Abstract]) OR (caries prevalence[Title/Abstract]) OR (dental health[Title/Abstract]) OR (oral health[Title/Abstract])) OR ((Peridontal disease[Title/Abstract]) OR (periodontitis[Title/Abstract]) OR (periodontal[Title/Abstract])) OR ((Edentulism[Title/Abstract]) OR (edentulous[Title/Abstract]) OR (endentulousness[Title/Abstract]) OR (severe tooth loss[Title/Abstract]) OR (total tooth loss[Title/Abstract]) OR (complete tooth loss[Title/Abstract])))) AND ((prevalence[Title/Abstract]) OR (incidence[Title/Abstract])) AND (2013/06/01[PDat] : 2016/12/31[PDat]))

> **Embase:** 'deciduous caries':ab,ti OR 'milk caries':ab,ti OR 'baby caries':ab,ti OR caries:ab,ti OR 'permanent caries':ab,ti OR 'caries prevalence':ab,ti OR 'dental health':ab,ti OR 'oral health':ab,ti OR 'peridontal disease':ab,ti OR periodontitis:ab,ti OR periodontal:ab,ti OR edentulism:ab,ti OR edentulous:ab,ti OR endentulousness:ab,ti OR 'severe tooth loss':ab,ti OR 'total tooth loss':ab,ti OR 'complete tooth loss':ab,ti AND (prevalence:ab,ti OR incidence :ab,ti) AND [2008-2016]/py AND [humans]/lim AND [embase]/lim NOT [medline]/lim

For GBD 2019, we completed an updated systematic review of the Latin American and Caribbean Health Sciences Literature (LILACS) and the Scientific Electronic Library Online (SciELO), focusing first on the most recent period from 2014 to 2018 were subject to full text screening. The search used used for LILACS and SciELO was the same:

> **LILACS/ SciELO:** "(deciduous caries OR milk caries OR baby caries OR caries OR dental health OR oral health OR permanent caries OR caries prevalence OR periodontal disease OR periodontitis OR periodontal OR edentulism OR edentulous OR edentulousness OR complete tooth loss OR tooth loss OR toothloss OR number of teeth OR dentate OR edentate) AND (prevalence OR incidence OR survey OR epidemiology)".

A total of 1696 citations were identified after deduplication, 147 were selected for full text review, and 77 new sources extracted from the following countries: Argentina (1), Brazil (47), Chile (5), Colombia (5), Cuba (5), Ecuador (1), El Salvador (1), Honduras (1), Mexico (5), Peru (5) and Venezuela (1).

We eliminated many data points to avoid repetition in the dataset, while striving to maintain as much data detail as possible. Redundancy tended to arise in three data descriptors: age, gender and urbanicity. Our order of preference for maintaining detail was age, followed by gender, then urbanicity. Additionally, many of the studies presented dmft or DMFT scores, which represent lifetime prevalence

and were often described as “caries experience”. For the purposes of measuring the burden of disability from dental caries, we considered only data on current prevalence to be relevant, and thus converted lifetime prevalence data to current prevalence and incidence where possible. The complete dataset contents for each model are shown in tables for each cause in the corresponding sections below.

Table 1. Total number of sources and countries with data for Oral Disorders, by measure

	Total sources	Countries with data
All measures	945	130
Prevalence	907	130
Incidence	81	40
Proportion	15	1
Other	22	13

Edentulism

Flowchart

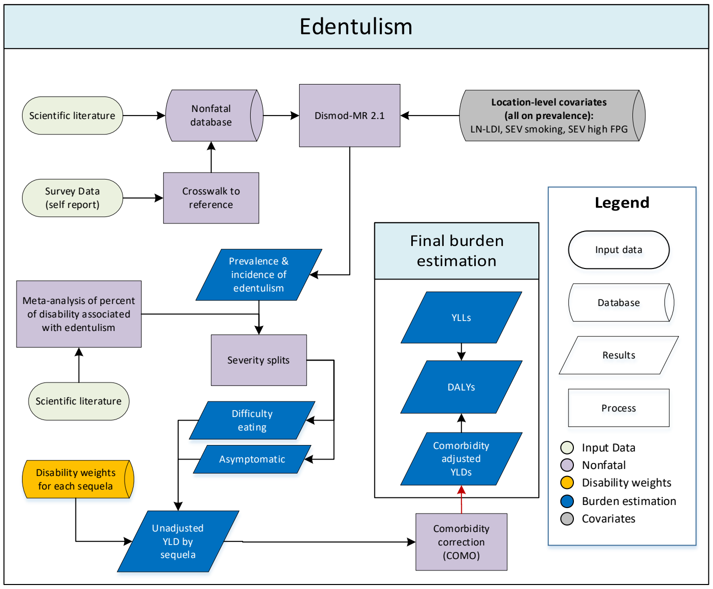

Case definition

The case definition of edentulism includes any individual with zero remaining permanent teeth; toothlessness of infancy is not included. The assessment of this disease includes quantification of the prevalence of the disease as well as estimation of the major sequelae: asymptomatic toothlessness and symptomatic toothlessness leading to “great difficulty in eating meat, fruits, and vegetables.” A small body of evidence has begun to emerge that implicates edentulousness as predisposing individuals to increased risk for ischaemic cardiovascular events including myocardial infarction and stroke. These data are sparse but have been included in models estimating the excess mortality of those with complete tooth loss. Given that the association is believed to be ecological rather than causal, however, edentulism has not been estimated as an underlying cause of death and it is not included in the risk factor analysis for cardiovascular diseases.

Input data and data processing

Details of the systematic literature reviews are above. In addition to published studies, we also utilized self-report data on toothlessness from World Health Survey (WHS) for 47 countries as well as a number of national oral health surveys identified through the Global Health Data Exchange.

Table 1: Total number of sources and countries with data for edentulism, by measure

	Total sources	Countries with data
All measures	254	91
Prevalence	253	91
Incidence	1	1

Age and sex splitting

The first step of data processing was age splitting. For any datum that did not entirely fit within a GBD sex or age group, the observation was split to be multiple age-specific and sex-specific data points based on the age and sex pattern predicted by previous DisMod-MR 2.1 models. It is our intention to update this age-sex splitting with each cycle of GBD.

Crosswalks in MR-BRT

We then crosswalked self-reported (i.e. WHS) data on toothlessness to the reference definition of oral examination. In accordance with GBD 2019 principles for data processing, to make data comparable, we began by evaluating the number of observations of each alternate definition that matched with a corresponding observation from the reference definition. There were no "within" study matches identified so the MR-BRT analysis was based on 60 "between" study matches of alternative and reference definitions where a match was defined as data from different sources from the same GBD location, age group, and midpoint of the study period within 5 years of one another. The ratio of alternative to reference was calculated and logit-transformed. Standard error of the ratio was calculated using the delta method. Sex was included as a fixed effect and Socio-demographic Index (SDI) as a spline. The data matches, adjustment factors, and final input dataset are shown in the tables below.

Table 2: Data points and matches between alternate and reference definitions

	Reference	Alternate #1 (cv_whs)
Number of data points	10028	2874
Within-study matches to reference	--	0
Between-study matches to reference	--	60

Table 3: MR-BRT Crosswalk Adjustment Factors for edentulism, 15% trim

Data input	Reference or alternative case definition	Gamma	Beta Coefficient, Logit (95% CI)	Adjustment factor*
Clinical exam	Reference	0.019	---	---
WHS (self-report)	Alt		-0.126<br>(-0.416 to 0.154)	0.882<br>(0.66 - 1.166)

Modelling strategy

Estimates for the prevalence of edentulism were calculated for each location/year/sex/age using DisMod-MR 2.1. As would be expected for an irreversible condition, remission was fixed at zero for all ages. Mortality and relative risk were both fixed at zero before age 30, as any excess cardiovascular events resulting from severe tooth loss would not be expected at younger ages. We also assigned incidence and prevalence to be zero during childhood. Incidence was allowed to rise beginning at age 15, which was chosen based on the age at which the permanent dentition is expected to have fully formed in all individuals. The random effect limits for all locations were bounded at +/- 1.

As mentioned above, the criteria for diagnosis of edentulism are straightforward, and bias in the dataset was considered negligible. Thus, no study-level covariates were used in modelling the prevalence of edentulism. We included two location-level covariates in the model: 1) Log-transformed lag-distributed income (LDI) with a minimum beta value of 0.02 and 2) Log-transformed age-standardised summary exposure value (SEV) scalar of cardiovascular disease (CVD) in recognition of the common risk factors between CVD and tooth loss.

Table 4: Covariate, parameter, beta, and exponentiated beta values for edentulism

Covariate	Param	Beta	Exponentiated beta
LN-LDI	Prev	-0.16 (-0.17 — -0.16)	0.85 (0.84 — 0.85)
SEV Smoking (age- and sex-specific)	Prev	1.47 (1.34 — 1.60)	4.36 (3.83 — 4.96)
SEV fasting plasma glucose (age- and sex-specific)	Prev	0.25 (0.058 — 0.46)	1.28 (1.06 — 1.58)

Models were vetted based on the plausibility of the results, the extent to which estimates fit the data, and the plausibility of the range of estimates across location hierarchies.

Severity distributions and disability weights

The disability weight used for symptomatic toothlessness leading to “great difficulty in eating meats, fruits, and vegetables” is 0.067 (0.045–0.095) as determined by the GBD disability survey. We considered all those with severe tooth loss and no access to dentures to experience this disability. However, the proportion of those with edentulism and severe tooth loss who have dentures has not been studied extensively.

In order to estimate the proportion of edentulous individuals with no access to dentures, we completed a supplemental literature review of dentures prevalence for GBD 2010. Only six systematic surveys of dentures prevalence were identified, all in high- and middle-income countries. All were completed since 2000. After extracting the data from the studies, we performed linear regressions of denture presence and denture absence against health system access (HSA), a standardised covariate of treatment availability used in many disease estimation models. From the results of the regression, the prevalence of no dentures was calculated for all super-regions. We then completed a population-weighted average of all countries in the super-region based on 2003 populations, the average year of the dentures studies. Uncertainties for the prevalence of dentures were calculated by finding the standard deviation and standard error of the calculated prevalence values.

The estimated prevalence of dentures in each location was used to calculate the proportion of individuals with asymptomatic edentulism and severe tooth loss (ie, those who have access to dentures)

and difficulty eating due to edentulism and severe tooth loss (ie, those without access to dentures). This latter sequela was included as a cause of years lost due to disability (YLDs).

Caries of permanent teeth and caries of deciduous teeth

Separate estimates of caries of deciduous teeth and caries of permanent teeth

The natural histories of deciduous and permanent caries share many similarities, but they also share some important differences. Age patterns of decay in permanent and deciduous dentition are distinct, and duration of a carious lesion in deciduous teeth also tends to be shorter than an untreated episode of permanent caries. Sugar consumption and feeding with formula are both associated with development of deciduous caries, while sugar consumption is associated with the development of caries of permanent teeth. Finally, it is unclear whether the gender patterns and regional differences are the same for both deciduous and permanent caries. For all of these reasons, we elected to model deciduous caries and permanent caries as separate entities and then add the estimates together for an overall estimation of the global burden of dental caries. This is the modelling approach which has been taken in each iteration since GBD 2010.

Flowchart

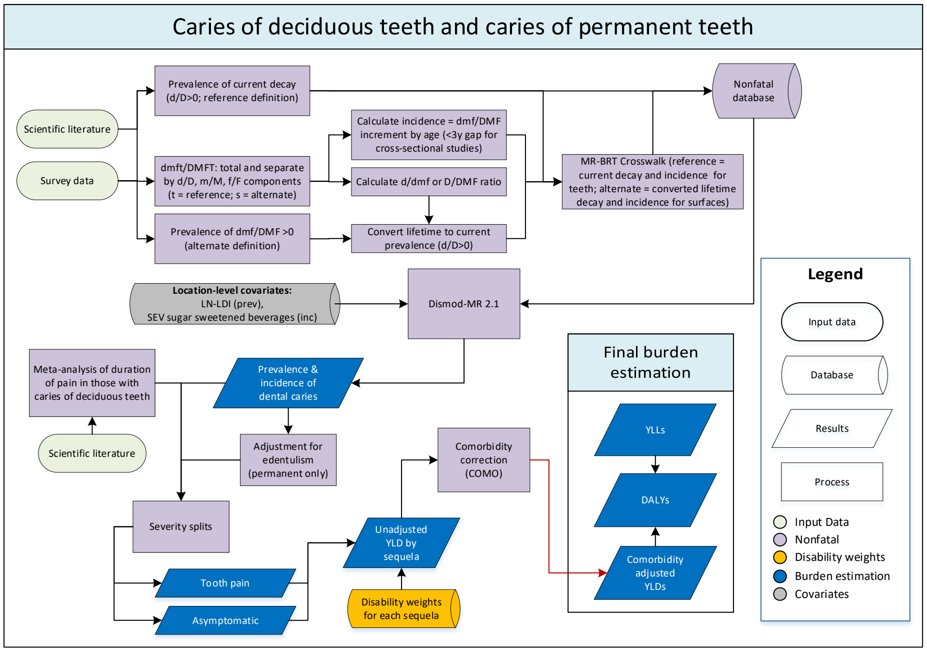

Case definition

The case definition for dental caries is "teeth with unmistakable coronal cavity at dentin level, root cavity in cementum that feel soft or leathery to probing, temporary or permanent restorations, or missing teeth extracted due to a caries lesion." Excluded definitions crowns with isolated cosmetic defects, stained enamel pits or fissures without visible cavitation or softening, fluorosis, and abrasion lesions. This definition corresponds to an ICD-9 code of 521.0 and an ICD-10 code of K02.3 – K02.9. Most caries are subclinical in the sense that they do not cause symptoms a majority of the time. Once a

carious lesion develops, it will occasionally recede without intervention, but often it worsens with time and eventually requires either filling or extraction.

Public health dentists commonly measure dental caries using the dmft/DMFT index, which is an incremental measure of the proportion of unhealthy teeth and is also a measure of an individual's lifetime prevalence of caries. Lowercase letters (dmft) are used for deciduous dentition and uppercase letters (DMFT) for permanent dentition. D is for decayed, M for missing, F for filled, and T for teeth. The maximum dmft score is 20 and the maximum DMFT score is 32. Furthermore, some dentists prefer to measure dental caries in terms of tooth surfaces, rather than number of teeth, and report their results using an analogous dmfs/DMFS index. The maximum dmfs score is 88, and the maximum DMFS score is 128 or 148 depending on whether the third molars are counted.

The DMFT index is easy to measure and inter-rater reliability is high. However, the primary shortcoming of the DMFT is that it does not discriminate well between current and past caries. Strategies we employed to maximally utilise dmf/DMF data for estimating the prevalence of burden due to permanent caries are described below.

Input data and data processing

The approach for systematic literature review is described above. The reference definition for this model was presence of one or more teeth with current decay (for prevalence) whereas each additional carious tooth was counted as a separate incident event.

Table 1: Total number of sources and countries with data for caries of deciduous teeth, by measure

	Total sources	Countries with data
All measures	419	87
Prevalence	384	86
Incidence	75	38
Other	22	13

Table 2: Total number of sources and countries with data for caries of permanent teeth, by measure

	Total sources	Countries with data
All measures	306	91
Prevalence	306	91
Incidence	6	5

Converting lifetime to current prevalence

Many of the studies presented dmft or DMFT scores, which represent lifetime prevalence and were often described as "caries experience." For the purposes of measuring the burden of disability from dental caries, we converted lifetime prevalence data to current prevalence for individuals aged 20 years and less. We did this by multiplying the observed lifetime prevalence by the ratio of d/D to dmf/DMF. When d/dmf or D/DMF information was available from the same study, this ratio was applied. When not available from the same study, the pooled ratio from the closest matching GBD geography was used for the multiplication (country, region, super-region, global).

Calculation of incidence from dmft/DMFT increment

Whereas in the deciduous dentition, a vast majority of the dmf index is accounted for by caries, tooth loss is a major contributor to the DMF index for the permanent dentition. Caries of permanent teeth may not necessarily be the primary driver of this tooth loss, as other factors such as periodontal disease and trauma may contribute significantly. Thus, we performed the conversions of incremental dmf/DMF scores to incidence values for permanent caries only in individuals ages 20 years or less and for all ages in the case of deciduous caries. For longitudinal studies, the difference between the dmf/DMF score in the initial versus subsequent examination was taken to be equivalent to the number of incident caries over that time period. This assumes a negligible proportion of dmf/DMF increment is due to trauma in children. For cross-sectional studies examining children of different ages, we only calculated incidence when the gap in age was three years or fewer given the propensity for strong cohort effects in caries epidemiology.

Age and sex splitting

For any datum that did not entirely fit within a GBD sex or age group, the observation was split to be multiple age-specific and sex-specific data points based on the age and sex pattern predicted by previous DisMod-MR 2.1 models. It is our intention to update with each cycle of GBD.

Crosswalks in MR-BRT

We then crosswalked alternative to reference definitions. In accordance with GBD 2019 principles for data processing, to make data comparable, we began by evaluating the number of observations of each alternate definition that matched with a corresponding observation from the reference definition. The total number of within and between study matches for deciduous caries and permanent caries are shown in the tables below.

Table 3: Data points and matches between alternate and reference definitions for caries of deciduous teeth

	Prev: Reference (d>0)	Prev: Alternate (cv_d_conversion)	Inc: Reference (dmft increment)	Inc: Alternate (cv_dmf_units_surfaces)
Number of data points	3783	1451	1496	174
Within-study matches	--	2157	--	6

Table 4: Data points and matches between alternate and reference definitions for caries of permanent teeth

	Prev: Reference (d>0)	Prev: Alternate (cv_d_conversion)	Inc: Reference (dmft increment)	Inc: Alternate (cv_dmf_units_surfaces)
Number of data points	3282	1347	1650	228
Within-study matches	--	1648	--	2

Owing to the significant heterogeneity in data on caries incidence and prevalence, we limited the comparisons to only "within" study matches where a match was defined as both methods of ascertainment being performed in the identical study population. The ratio of alternative to reference was calculated and logit-transformed. Standard error of the ratio was calculated using the delta method. Sex was included as a fixed effect and, for prevalence only, midpoint of age as a spline. The adjustment factors and spline plots for the crosswalks are shown below.

Table 5: MR-BRT Crosswalk Adjustment Factors for caries of deciduous teeth, 5% trim for prevalence, no trim for incidence

Parameter	Data input	Reference or alternative case definition	Gamma	Beta Coefficient, Logit (95% CI)	Adjustment factor*
Prevalence	Current decay	Reference	0.46	---	---
Prevalence	Converted lifetime decay	Alt		-0.15 (-0.29 - 0)	0.861 (0.748 - 1)
Incidence	dmft increment	Reference	1.11	---	---
Incidence	Increment based on surfaces	Alt		0.01 (-0.16 - 0.18)	1.01 (0.852 - 1.197)

Table 6: MR-BRT Crosswalk Adjustment Factors for caries of permanent teeth, 20% trim for prevalence, no trim for incidence

Parameter	Data input	Reference or alternative case definition	Gamma	Beta Coefficient, Logit (95% CI)	Adjustment factor*
Prevalence	Current decay	Reference	0.46	---	---
Prevalence	Converted lifetime decay	Alt		-0.84 (-2.14 - 0.48)	0.432 (0.118 - 1.616)
Incidence	DMFT increment	Reference	0.01	---	---
Incidence	Increment based on surfaces	Alt		0.03 (-2.27 - 2.21)	1.03 (0.103 - 9.116)

Figure 1: Spline plot showing crosswalk value by age group for alternate case definition of converted lifetime decay for (a) caries of deciduous teeth and (b) caries of permanent teeth

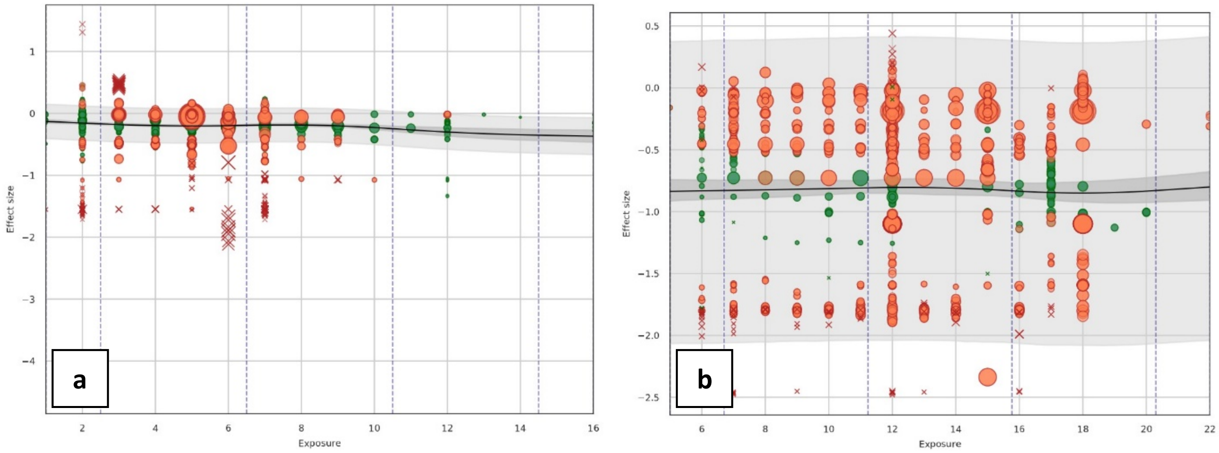

Modelling strategy

DisMod model development

Serious health consequences of caries were also assumed to be uncommon and death very rare. We therefore assigned excess mortality to be zero from age 0 to 100. For both types of caries, most of the model settings were similar. The primary difference between the two models was in value priors. We

assumed zero incident caries in infants under 1 year old and similarly zero incident deciduous caries from age 11 onward. For permanent caries, we assumed zero incident cases in children under 5 years old. Location-level covariates were assigned separately on prevalence and incidence. Sugar availability in food from the GBD diet analysis was used as a covariate on incidence with a positive beta, while prevalence was assigned log-transformed LDI with a negative beta to reflect the association with access to dental care.

Table 6: Covariate, parameter, beta, and exponentiated beta values for dental caries

Cause	Covariate	Param	Beta	Exponentiated beta
Deciduous	LN-LDI	Prev	-0.12 (-0.13 — -0.11)	0.88 (0.87 — 0.89)
Deciduous	SEV High sweetened beverages (age- and sex-specific)	Inc	0.65 (0.079 — 1.31)	1.91 (1.08 — 3.72)
Permanent	LN-LDI	Prev	-0.17 (-0.21 — -0.13)	0.85 (0.81 — 0.88)
Permanent	SEV High sweetened beverages (age- and sex-specific)	Inc	0.80 (0.056 — 1.71)	2.23 (1.06 — 5.52)

Although studies were screened carefully during data extraction to ensure that they specified whether they were measuring permanent or deciduous caries, some data points were marked as outliers during modelling due to their high prevalence values in young ages, as it was deemed likely that some of these studies were reporting deciduous in addition to permanent caries. As with deciduous caries, models for permanent caries were vetted based on the plausibility of the results, the extent to which estimates fit the data, and the plausibility of the range of estimates across location hierarchies.

Correction for edentulism

One systematic source of bias in the literature was the exclusion of edentate individuals from the study populations, which leads to systematic overestimation of caries prevalence when modelled over the entire population. To account for this bias, we used our GBD estimates of edentulism prevalence to adjust YLD estimates for caries of permanent teeth. Final DisMod-MR 2.1 estimates of edentulism prevalence were paired with the corresponding results for caries of permanent teeth by age group, sex, location, and year to adjust for the proportion of the population that was excluded from the denominator of permanent caries models. No adjustment was made to the estimates of caries of deciduous teeth.

Severity distributions and disability weights

As described above, the GBD definition of disability associated with symptomatic dental caries is “this person has a toothache, which causes some difficulty eating.” The disability weight associated with this condition is 0.01 (0.005–0.019), as derived from the GBD disability weights study.

Not all those with dental caries experience this disability all the time. We considered only those with active dentinal decay to experience symptomatic tooth pain. Those with deciduous caries who had undergone exfoliation or had their cavities filled were considered to have no disability. Likewise, those with permanent caries who had received fillings, had their cavities extracted, or lost a carious tooth altogether were considered to have no disability. Thus, two additional pieces of information are required to complete the calculation of years of life lived with disability (YLDs): proportion with symptoms and duration of disability.

To determine which segment of the population has ongoing tooth pain and the proportion of time spent with tooth pain, we considered several different options. First, we examined the data on dental caries symptoms and disability from the Medical Expenditure Panel Survey (MEPS) conducted by the USA Department of Health and Human Services in 2000–2009. MEPS data were widely used in GBD 2010 analyses. Respondents to the survey are asked about all medical conditions. Conditions for which provider care was sought are reported by the respondents at every round, and respondents also report problems for which they did not see a provider if the symptoms were "bothering" them. Conditions can be added to the condition roster if 1) they are reported as a reason for a medical event, 2) the condition was reported as the reason for one or more disability days, or 3) the condition was "bothering" the person during the reference period. Conditions are then recorded as verbatim text and coded to ICD-9CM 3rd digit codes by professional medical coders. These ICD9 codes were mapped to GBD causes, including dental caries. From the MEPS, symptomatic caries in the previous year were reported by 48.4% (95% CI 44.3–52.9) of the respondents. This number is in agreement with our DisMod-MR 2.1 estimates of 1-2 years duration in North America, high-income for permanent caries if we consider people to only have symptoms at the end of a course of caries. The two primary shortcomings of using this approach are 1) it does not provide enough detail to differentiate between the experiences of those with deciduous versus permanent caries, and 2) it indicates the proportion of those with caries who were symptomatic during the previous year, but it does not provide information on the amount of time during that year spent with symptoms (ie, one day versus 12 months). The approach described below addresses both issues.

To determine duration, we adapted the method employed by the Australian Burden of Disease (AusBoD) Study in 1996. For total duration, we used the posterior estimates of duration from final DisMod-MR 2.1 models. For those with symptoms, we split this total duration into two distinct phases of caries disability. The "initial" phase is characterised by *periodic* pain that we assigned to occur an average of one hour per day. The "terminal" phase is a period of *constant* symptoms at the end of an episode. The length of the terminal phase was determined by literature review as described by the AusBoD group. For deciduous caries we used a study by Mason and colleagues of children in the UK presenting to a casualty ward with tooth pain [1]. The length of time each child had been experiencing tooth pain was recorded. Based on the distribution of time courses, a log-normal distribution was plotted that approximated the average duration of *constant* symptoms at 27.6 days leading up to seeking care. For permanent caries, a similar study of the tooth pain experience of adults in New Zealand who presented to hospital dental departments and an emergency clinic [2] resulted in an estimated 55.2 days spent in the terminal phase of caries. For those with severe disease, the length of time spent in the terminal phase was subtracted from the total duration to determine the amount of time spent in the initial phase. For those with mild disease, we considered the entire duration to be spent in the initial phase. These calculations were last completed as part of the GBD 2013 analysis.

To determine proportion with symptoms, we completed a supplemental literature review of tooth pain and caries. We identified a total of 21 studies with data about the prevalence of pain. The studies were grouped according to the type of dentition studied (deciduous or permanent) and the location of the study group (high-income or low- and middle-income countries). We extracted data on the proportion in each group that described symptoms of pain related to their caries as well as a subset who described their symptoms as being severe. The proportions in each group were weighted according to sample size

to give estimates of the relative sizes of three groups: asymptomatic, mild, and severe. The results of this meta-analysis are illustrated in the table below.

We considered asymptomatic individuals to experience no disability. Those with mild disease spent the entire duration in the initial phase of disease (one hour of pain per day). Those with severe disease spent a majority of the duration in the initial phase followed by a period of time in the terminal phase (constant pain). YLDs were calculated by multiplying the prevalence, duration, proportion, and disability weight for each age, country, sex, and year.

Table 7: Duration and distribution of severity for tooth pain due to caries of deciduous and permanent teeth

	# of studies	% symptomatic of total	% severe among symptomatic	% mild of total	% severe of total	% asymptomatic of total
Deciduous caries						
Data-rich	5	0.35	0.257	0.26	0.09	0.65
All others	4	0.555	0.438	0.312	0.243	0.445
Permanent caries						
Data-rich	6	0.602	0.315	0.412	0.189	0.398
All others	6	0.954	0.548	0.432	0.521	0.046
Duration of phases						
Initial phase				1 hour per day		
Terminal phase (deciduous caries)				27.6 days		
Terminal phase (permanent caries)				55.2 days		

Chronic Periodontal Disease

Flowchart

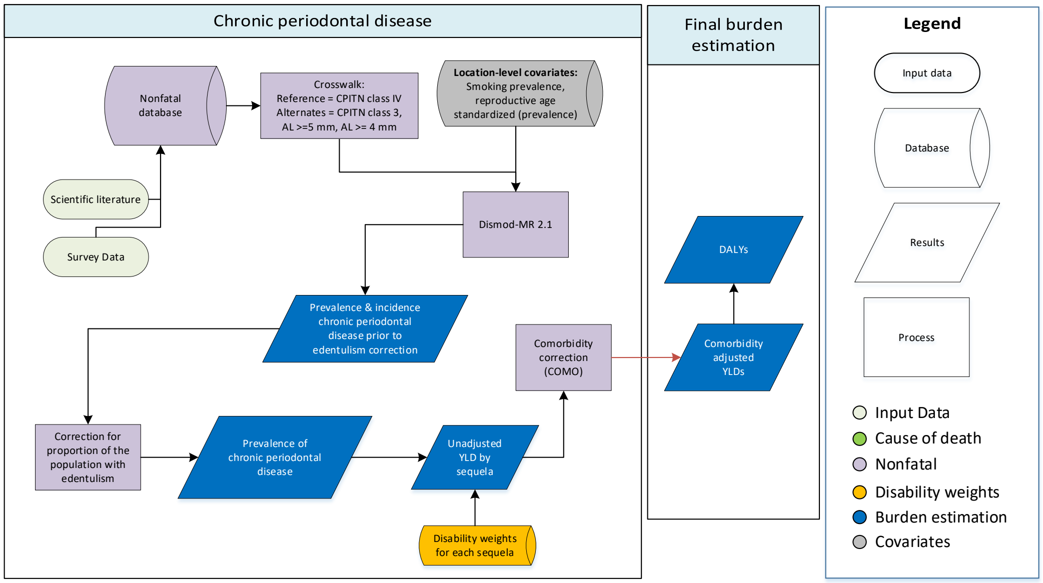

Case definition

Chronic periodontal disease is caused by chronic bacterial infection around the teeth. Symptoms of gingivitis, the mildest form of the disease, include swelling, redness, and propensity of the gums to bleed when perturbed. If the infection is not treated appropriately, it will eventually spread below the

gum line, leading to a chronic inflammatory state of the periodontal tissues. Over time, there will be loss of gingival tissue and alveolar bone destruction. Teeth will become loose and may need to be extracted.

The GBD definition of disability associated with symptomatic severe periodontal disease is "bad breath, a bad taste in the mouth, and gums that bleed a little from time to time, but which does not interfere with daily activities." The ICD-10 codes for periodontal disease are K05.0 – K05.6, and the ICD-9 codes are 523.0 – 523.9.

Defining periodontal disease in a meaningful, reproducible manner has been an ongoing challenge for public health dentists. Attachment loss (AL) and pocket depth (PD) have emerged as the most common metrics of periodontal health measurement. Attachment loss (AL) is measured as the difference between the distance from the gingival margin to the bottom of the pocket and the distance from the cemento-enamel junction to the bottom of the pocket.

The Community Periodontal Index is a classification system that was developed by WHO as a standardised method of periodontal health measurement. CPI classification is based on the examination of all teeth present in the mouth for absence or presence of gingival bleeding and absence or presence of periodontal pockets. A standard-sized probe is used, with depth markings from 0.5 to 11.5 mm. The probe is inserted into the sulcus between a tooth and the gingiva until it meets resistance. The surrounding area is then explored with the probe to determine the maximum depth of the pocket. Multiple areas around each tooth are probed. Pocket scores range from 0 to 2 in order of increasing severity. When the CPI method was employed, we considered those with Class 2 only (pocket of 6mm or more). Additionally, loss of attachment may be collected for specific index teeth by dividing the mouth in sextants. The two molars in each posterior sextant are paired for recording and, if one is missing, there is no replacement. If no index tooth is present in a sextant qualifying for examination, all the teeth that are present in that sextant are examined and the highest score is recorded as the score for the sextant. We excluded studies in which the study population was reported as the number of sextants rather than the number of individuals surveyed. CPI is a modification of CPITN that does not include the assessment of periodontal treatment needs. Also, Class 2 of CPI is equivalent Class 4 of CPITN.

In 2007, a new CDC proposal for gold standard diagnosis of severe, chronic periodontitis was published [1]. This standard specified that a stricter definition of the condition should be implemented. This more exclusive definition of chronic periodontal disease includes $\geq$ 2 interproximal sites with AL $\geq$ 6 mm ***AND*** $\geq$ 1 interproximal site with PD $\geq$ 5 mm. This definition has not been adopted by GBD.

A small body of evidence has begun to emerge that implicates chronic periodontal disease as predisposing individuals to increased risk for ischaemic cardiovascular events including myocardial infarction and stroke. These data are sparse but have been included in models estimating the excess mortality of those with chronic periodontal disease. Given that the association is believed to be ecological rather than causal, however, periodontal disease has not been estimated as an underlying cause of death and it is not included in the risk factor analysis for cardiovascular diseases.

Input data

Details of the systematic review are provided above. We implemented a hierarchical preference for case definitions. We included the following definitions of severe periodontal disease commonly found in the literature:

1. Community Periodontal Index of Treatment Needs (CPITN) – Class 4 only
2. Community Periodontal Index (CPI) – Class 2 only
3. Clinical Attachment Loss (AL) > 6mm
4. Clinical Attachment Loss (AL) > 5mm
5. Clinical Attachment Loss (AL) > 4mm
6. Gingival Pocket Depth (PD) > 5mm

If more than one type of data was included in a study, our first preference was for CPITN = 4, followed by AL >6 mm, and PD >5. All were considered equivalently as reference definitions with no additional crosswalking performed. For those sources that did not provide data on any of the components of CPITN class 4, but did provide data on CPITN class 3, AL >5mm, or AL >4mm, we utilized these data after crosswalking in MR-BRT as described below.

Table 1: Total number of sources and countries with data for chronic periodontal disease, by measure

	Total sources	Countries with data
All measures	116	53
Prevalence	116	53

Age and sex splitting

For any datum that did not entirely fit within a GBD sex or age group, the observation was split to be multiple age-specific and sex-specific data points based on the age and sex pattern predicted by previous DisMod-MR 2.1 models. It is our intention to update with each cycle of GBD.

Crosswalks in MR-BRT

We then crosswalked alternative to reference definitions. In accordance with GBD 2019 principles for data processing, to make data comparable, we began by evaluating the number of observations of each alternate definition that matched with a corresponding observation from the reference definition. All alternative definitions were mutually-exclusive with one another so three separate crosswalks were performed using only within study matches, defined as both methods of ascertainment being performed in the identical study population. The ratio of alternative to reference was calculated and logit-transformed. Standard error of the ratio was calculated using the delta method. Sex was included as a fixed effect and, for prevalence only, midpoint of age as a spline. The total number of matches, the adjustment factors, and the spline plots for periodontal disease crosswalks are shown below.

Table2: Data points and matches between alternate and reference definitions for periodontal disease

	Prev: Reference CPITN 4, AL > 6mm, PD > 5mm	Prev: Alternate (CPITN 3)	Prev: Alternate (AL > 4Mmm)	Prev: Alternate (AL > 5mm)
Data points	3964	592	268	643
Within-study match	--	2290	1399	1434

Table 3: MR-BRT Crosswalk Adjustment Factors for periodontal disease, 10% trim for prevalence

Data input	Reference or alternative case definition	Gamma	Beta Coefficient, Logit (95% CI)	Adjustment factor*
CPITN 4	Reference	0.26	---	---
CPITN 3	Alt		0.13 (-1.34 – 1.62)	1.14 (0.26 – 5.07)
CPITN 4	Reference	0.26	---	---

AL >4 mm	Alt		1.03 (0.01 – 2.08)	2.79 (1.01 – 7.99)
CPITN 4	Reference	0.39	---	---
AL >5 mm	Alt		0.43 (-0.74 – 1.69)	1.53 (0.47 – 5.40)

Figure 1: Spline plot showing crosswalk value by age group for periodontal disease for alternate case definitions of (a) CPITN 3, (b) AL >4 mm, and (c) AL >5mm

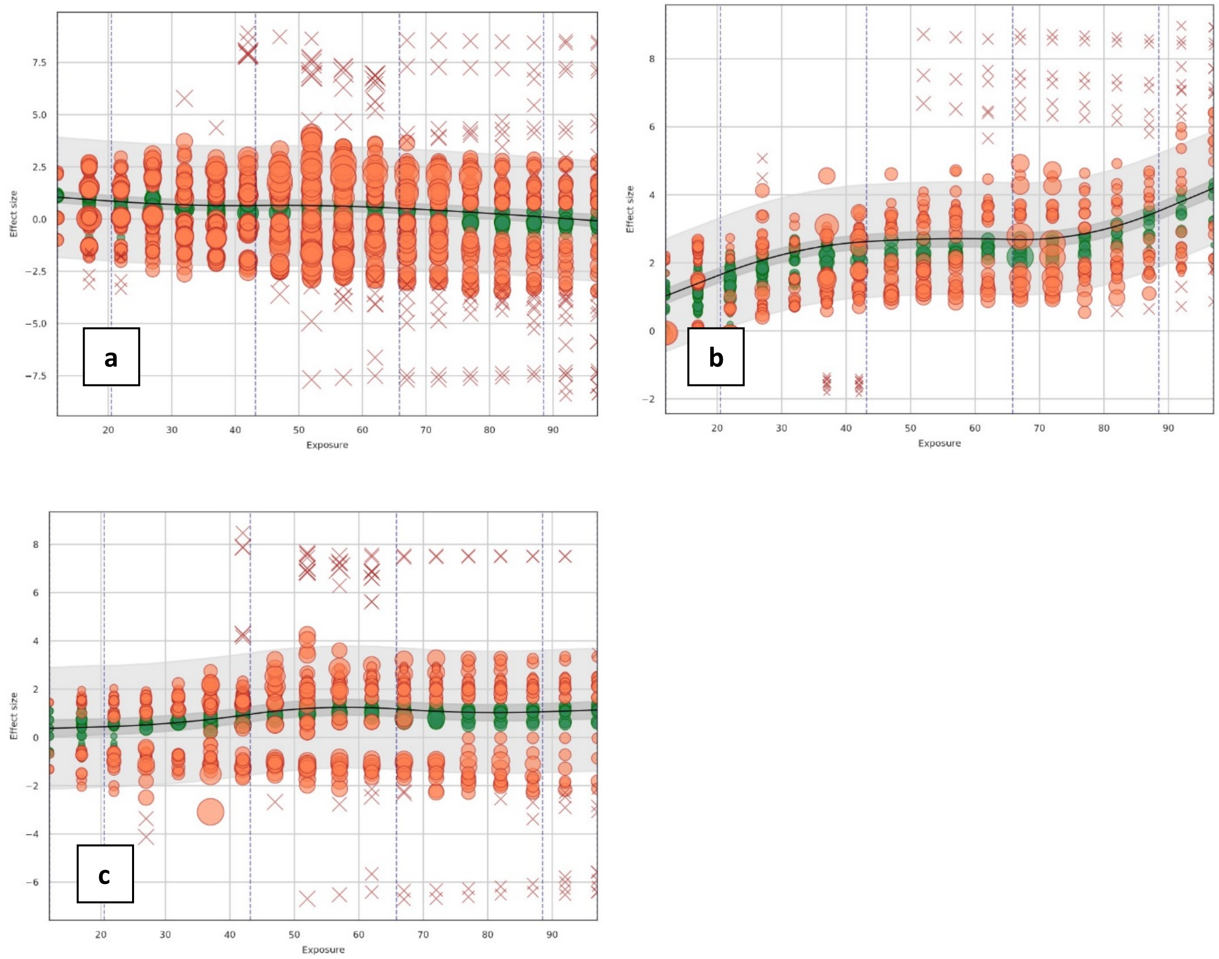

Modelling strategy

First, estimates for the prevalence of chronic periodontal disease were generated for each location/year/sex/age using DisMod-MR 2.1. Mortality was fixed to zero, and relative risk was fixed to 1.0 before age 30, as any excess cardiovascular events that occur in those with severe tooth loss would not be expected at young ages. Incidence and prevalence were assigned to be zero until age 8, as periodontal disease is largely considered to be a disease of adulthood. Incidence was allowed to rise

beginning at age 9, based on the youngest age at which there was a non-zero point estimate for prevalence in the dataset. Additional bounds were assigned for incidence, remission, and excess mortality to improve plausibility in the DisMod estimates. Remission was bounded 0 to 0.05, excess mortality rate from 0 to 0.0001, and incidence from 0 to 0.05. We considered these bounds to reasonably reflect the natural history of the disease. Three location level covariates were used as shown in the table below.

Table 4: Covariate, parameter, beta, and exponentiated beta values for chronic periodontal diseases

Covariate	Param	Beta	Exponentiated beta
LN-LDI	Prev	0.13 (0.093 — 0.16)	1.13 (1.10 — 1.17)
SEV Smoking (age- and sex-specific)	Prev	0.16 (0.0080 — 0.41)	1.17 (1.01 — 1.51)
SEV fasting plasma glucose (age- and sex-specific)	Prev	0.24 (0.012 — 0.61)	1.28 (1.01 — 1.84)

Models were vetted based on the plausibility of the results, the extent to which estimates fit the data, and the plausibility of the range of estimates across location hierarchies.

Correction for edentulism

One systematic source of bias in the literature was the exclusion of edentate individuals from the study populations, which leads to systematic overestimation of periodontal disease prevalence when modelled over the entire population. To account for this bias, we used our GBD estimates of edentulism prevalence to adjust YLD estimates for chronic periodontal disease. Final DisMod-MR 2.1 estimates of edentulism prevalence were paired with the corresponding results for caries of permanent teeth by age group, sex, location, and year to adjust for the proportion of the population that was excluded from the denominator of permanent caries models. No adjustment was made to the estimates of caries of deciduous teeth.

Severity distributions and disability weights

We considered all estimated prevalent cases of chronic periodontal disease to experience the disability described by “bad breath, a bad taste in the mouth, and gums that bleed a little from time to time, but this does not interfere with daily activities.” The GBD disability survey differentiated between those who experience pain and those who do not, but the calculated disability weight was the same for both forms of the condition, 0.007 (0.003–0.014).

Other Oral Disorders

Other oral disorders encompass a wide variety of dental, tongue, and jaw disorders and malformations, including all oral disorders that are not included in the case definitions of permanent or deciduous dental caries, periodontal disease, or edentulism and severe tooth loss. All data on the prevalence of other oral disorders were obtained from the United States Medical Expenditure Panel Surveys (MEPS), a nationally representative survey conducted yearly from 1996 to 2011 by the US Agency for Healthcare Research and Quality. These data were modelled in DisMod-MR 2.1 using a prevalence-only model. Disability weights and severity distribution for these causes were also derived from MEPS.

Table 2: Total number of sources and countries with data for other oral disorders, by measure

	Total sources	Countries with data
All measures	19	1
Prevalence	16	1
Proportion	15	1

References

1. C Mason, SR Porter, G Madland, J Parry. Early management of dental pain in children and adolescents. *J Dent.* Jan 1997; 25(1): 31-4.
2. RA Whyman, ET Treasure, KM Ayers. Dental disease levels and reasons for emergency clinic attendance in patients seeking relief of pain in Auckland. *NZ Dent J.* Dec 1996; 92(410): 114-7
3. RC Page and PI Eke. Case Definitions for Use in Population-Based Surveillance of Periodontitis. *J Periodont*. Jul 2007. 78(7 Suppl): 1387-99
4. Petersen PE, Baez RJ. Oral health surveys: basic methods. Geneva: World Health Organization; 2013.

Injuries

Flowchart

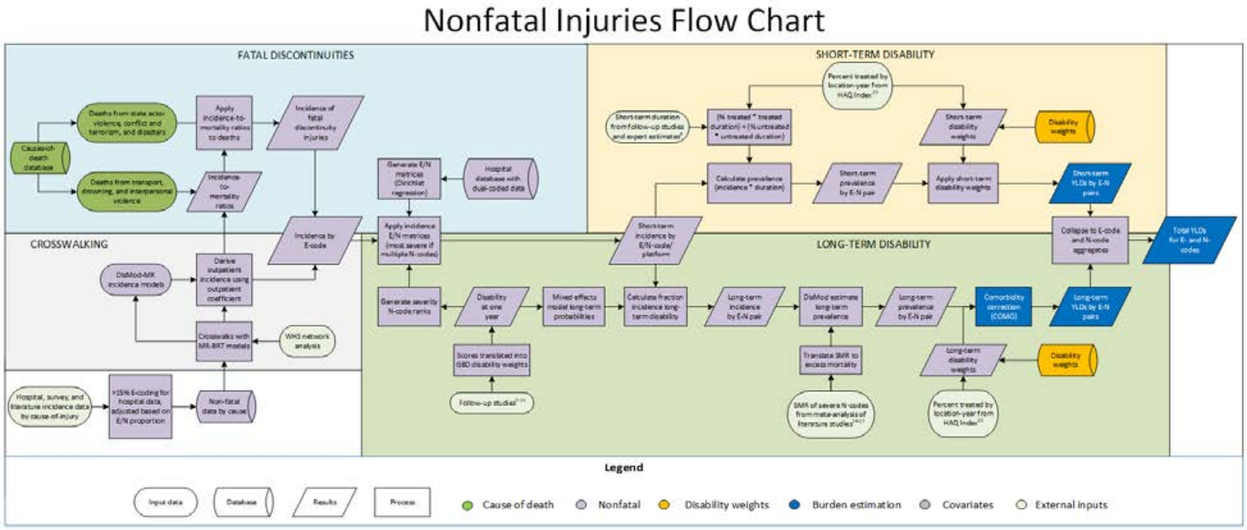

Case definition

For GBD 2019, the Injuries estimation process for non-fatal health outcomes encompasses a range of 30 causes, including transport injuries, falls, drowning, self-harm, interpersonal violence, and animal contact. Injury incidence is defined using ICD-9 codes E000-E999 and ICD-10 chapters V to Y. For non-fatal estimation, Chapters S and T in ICD-10 and codes 800-999 in ICD9 are used to estimate morbidity. Each of these 30 causes of injury can result in a variety of physical injury sequelae (e.g., traumatic brain injury), which we call the "nature of injury." Although the initial DisMod models are at the "cause of injury" level (e.g., drowning), each cause of injury is distributed into cause-nature pairs to capture the actual disability that develops. We report incidence, prevalence, and YLDs due to injuries at the cause-nature pair level.

We make additional distinctions between inpatient and outpatient injuries and between short-term and long-term injuries. Inpatient injuries are defined as injuries that led to overnight hospitalisation, whereas outpatient injuries are defined as ones treated in outpatient settings or emergency care. We define short-term injuries as injuries lasting less than one year and long-term injuries as those lasting longer than one year, at which point we assume lifelong disability.

Input data

Model inputs

To estimate morbidity from injuries, we used data from hospital records, emergency department records, insurance claims, and surveys to produce years lost to disability (YLDs) by country, year, sex, age, external cause-of-injury, and nature-of-injury category. Many countries report hospital data using a mix of cause-of-injury and nature-of-injury codes. In order to retain as much of the data as possible, we included all datasets that had at least 15% of cases coded to the cause of injury. In GBD 2015, we chose 45% as the threshold but have since lowered the threshold to 15%. We made this distinction after assessing the proportions of major injury causes (road injury and falls) in each of the data sources. We concluded that there were no obvious differences between country data with 15%–45% coverage of external cause codes and those with more than 45% coverage. Below the 15% threshold, the cause of nature coding

became more disproportionate when compared to sources with higher cause of nature coding. We assessed the raw hospital data to make sure that there was no disproportionate coding to certain causes in the 15%–45% cause-of-injury coding range. We increased the cause-specific injury cases from these datasets proportionately to sum to the total number of injury cases.

Conflict, war and executions, and police conflict data were obtained from the Uppsala Conflict Data Program [2], the International Institute for Strategic Studies [3], the Armed Conflict Location and Event Dataset [4], the Social Conflict Analysis Database [5], and vital registration systems. Disaster data were obtained from the International Disaster Database from the Center for Research on the Epidemiology of Disasters [6].

Data searches

GBD 2019 utilized the same data as GBD 2017 [1] with some updates to existing data and additions of new data. For GBD 2019, hospital and emergency department records were supplemented with more recent and available site-years, including adding subnational detail in select countries. A hospital utilisation envelope that gave reliable denominators for hospital data allowed for the use of more data sources. We applied correction factors to account for repeat hospital visits within a three-month time window (derived from US claims data) to the incidence estimates to avoid double-counting multiple health service contacts for the same injury. For GBD 2019, we also incorporated a correction for access to health care facilities to account for inidividuals who sustain an injury but do not have access to a hospital or health care facility. This correction is based on the health care access and quality index (HAQi) [29].

Additionally, prior to estimation, we reviewed existing usage in GBD 2017 of other types of data that could be incorporated into nonfatal estimates of injuries. In GBD 2017, we added injury claims data from the Accident Compensation Corporation in New Zealand into the transport, self-harm, and animal contact incidence models [1]. These claims data span ten years (2008-2017) and provide detailed information on age and ethnicity (Maori/non-Maori). We also added national survey data from China, Ghana, India, Mexico, Russian Federation, and South Africa from the World Health Organization's Study on Global AGEing and Adult Health were included in the estimation of injuries due to road accidents and falls. Injury cases from the Vietnam National Injury Survey (VNIS) were also added for GBD 2019. We also added literature studies from India and South Africa based on inputs from the GBD collaborator network.

Infrequently, data points were marked as outliers. Reasons for this were that the data point did not follow the age or time pattern as expected and/or if the incidence rate of people sustaining an injury from a certain cause of injury was not plausible. Table 1 contains information about data coverage for each cause of injury, not including fatal discontinuities: state actor violence, exposure to forces of nature, and conflict and terrorism.

Table 1. Data inputs for injuries incidence modelling

Cause	Total sources	Countries with data
Road injuries	284	75
Pedestrian road injuries	169	23
Cyclist road injuries	178	23
Motorcyclist road injuries	173	23
Motor vehicle road injuries	179	23
Other road injuries	168	19

Other transport injuries	182	20
Falls (EMR)	220	38
Drowning (EMR)	37	11
Fire, heat, and hot substances	212	34
Poisonings	208	34
Poisoning by carbon monoxide (EMR)	154	19
Poisoning by other means	161	20
Exposure to mechanical forces	182	23
Unintentional firearm injuries	178	19
Other exposure to mechanical forces	181	22
Adverse effects of medical treatment	294	44
Animal contact	214	31
Venomous animal contact	180	21
Non-venomous animal contact	180	21
Pulmonary aspiration and foreign body in airway	185	21
Foreign body in eyes	202	21
Foreign body in other body part	203	24
Environmental heat and cold exposure	191	24
Other unintentional injuries	160	21
Self-harm (EMR)	230	38
Self-harm by firearm (EMR)	175	27
Self-harm by other specified means	162	21
Interpersonal violence	212	33
Physical violence by firearm (EMR)	30	6
Physical violence by sharp object	187	25
Physical violence by other means	181	22

Modelling strategy

As in previous GBD iterations, two categories of injury severity were separately modelled: injuries warranting inpatient care and injuries warranting other health care. Injuries warranting inpatient care refer to injury cases of sufficient severity to require inpatient care, if there are no restrictions in access to health care. Injuries warranting other health care refer to injury cases of sufficient severity to require health care attention but not hospitalisation. This category includes emergency department visits. In order to best measure the burden of injuries, the GBD 2019 estimates excluded trivial injuries by restricting morbidity analysis to cases warranting some form of health care in a system with full access to health care. We intended to include cases with injuries that did not receive care in areas with restricted access to health care, but that would have warranted some type of health care in a system with full access to health care. In some surveys, after asking about recall of injuries in the past month or year, respondents were further probed on whether they sought care and why they did not. This allowed us to include cases who cited financial or geographical barriers as reasons for not seeking care.

Cause-of-injury incidence

The list of unique (i.e., not counting aggregate categories like road injuries or interpersonal violence) cause-of-injury categories did not change from the 30 unique causes in GBD 2017 [1]. We treat executions and police conflict ("state actor violence") as a typical cause of injury rather than as a fatal

discontinuity; however, the cause is modelled using the fatal discontinuity estimation strategy using incidence-to-mortality ratios because we do not have incidence data for state actor violence.

The majority of incidence data exist at the external cause-of-injury level. Incidence for cause-of-injury categories was modelled using DisMod-MR 2.1. Multiple datasets from hospital and emergency/outpatient departments, insurance claims, and surveys were fed into these incidence models. We separately estimated two categories of injury severity: inpatient and outpatient injuries.

Excess mortality modeling

In previous rounds, priors on excess mortality rate (EMR) were estimated in DisMod by matching prevalence data points with their corresponding CSMR values within the same age, sex, year, location (by dividing CSMR by prevalence). For short duration conditions like injuries (remission > 1), the corresponding prevalence was derived by running an initial model and then applying the same CSMR/prevalence method. However, for many causes, DisMod estimated a rather unrealistic pattern of EMR compared to an expected pattern of decreasing EMR with greater access to quality health care. Such unexpected patterns often signal inconsistencies between CSMR estimates and the measures of prevalence and/or incidence. This was especially the case for the injuries that we implemented an EMR modeling framework, which included drowning, falls, poisoning by carbon monoxide, assault by firearm, self-harm, and self-harm by firearm.

In effort to provide greater guidance to DisMod on the expected pattern of EMR, EMR data generated in the previous round were modeled using the MR-BRT approach by age and sex with a prior on healthcare access and quality index (HAQi) [29] having a negative coefficient. Results from MR-BRT were then predicted for each location year, sex and for ages 0, 10, 20 … 100. We included HAQi as a country-level covariate to inform EMR with a mean and standard deviation produced from MR-BRT. However, even without this setting DisMod would tend to estimate a coefficient that was consistent with the MR-BRT analysis. For the six injuries using EMR inputs modeled from MR-BRT, we set the trimming parameter to trim 0.1% of the datapoints, added a cubic-spline on age with knots set by data density, and a fixed effect on sex.

Adjusting data

For GBD 2017, we used two covariates in each DisMod-MR 2.1 model as a multiplier from inpatient to outpatient incidence, namely covariates "outpatient" and "in- and outpatient" [1]. For GBD 2019, the adjustment of data via study-level covariates was performed out of DisMod using adjustment coefficients derived from a network analysis on World Health Survey data on road injuries spanning over 50 countries. First, ST-GPR was used to estimate the proportion of people who were able to receive care for their injuries using the ratio of inviduals who received in- or outpatient care to individuals who were injured overall. These proportions allowed us to adjust data to the definition "injuries that received inpatient or outpatient care." Then, MR-BRT was used to crosswalk "received care" incidence and outpatient incidence both to inpatient incidence, using inpatient versus outpatient incidence comparisons from the United States National Hospital Ambulatory Medical Care Survey. This process is summarized in Figure 1, and an example of a MR-BRT output can be seen Figure 2. Country-level covariates are shown in Table 2.

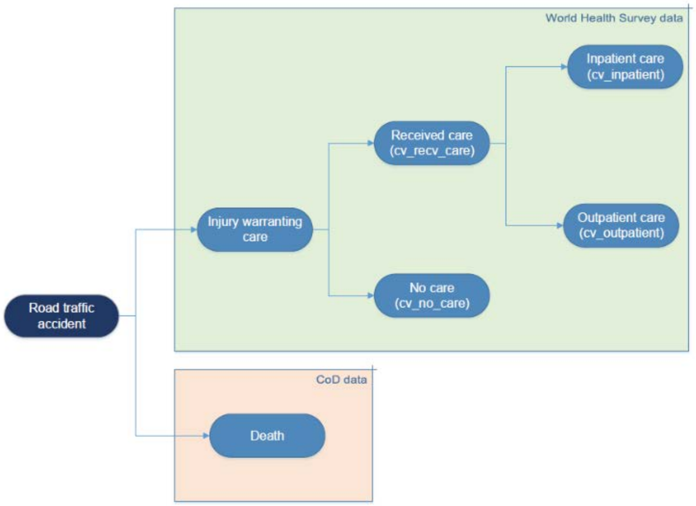

Figure 1. Overview of data adjustment process using road injuries data from World Health Survey data

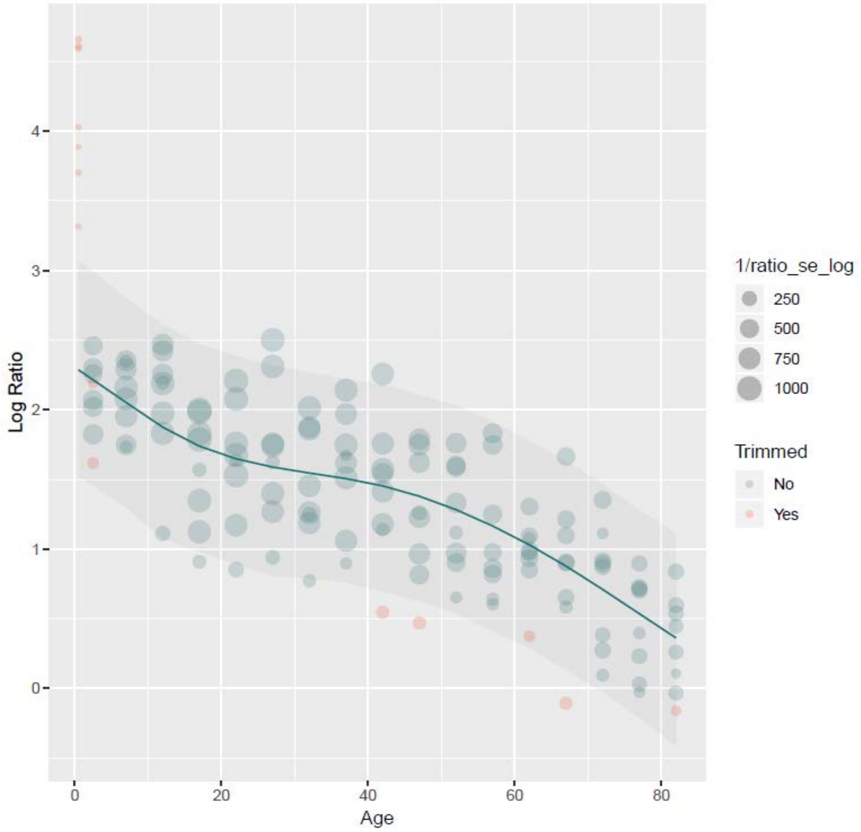

Figure 2. MR-BRT model for road injuries by age. The y-axis shows the log of the ratio of outpatient cases to inpatient cases for each age along the x-axis. This shows how outpatient or ED visits without admission are more probable per inpatient admission in younger ages, while in the oldest ages, it is less likely for a road injury case to be seen only as an outpatient relative to each observed inpatient admission. The red data points show data that were trimmed by MR-BRT. See Figures 5–15 for additional MR-BRT plots.

Table 2. Country-level covariates for DisMod-MR 2.1 incidence models for injuries

Model	Covariate	Exponentiated Value
Road injuries	Log-transformed age-standardized SEV scalar: Road Inj	3.40 (3.29 — 3.48)
	Vehicles - 2+4 wheels (per capita)	1.04 (1.03 — 1.06)
Pedestrian road injuries by road vehicle	Log-transformed age-standardized SEV scalar: Pedest	2.58 (2.14 — 3.18)
Cyclist road injuries	Log-transformed age-standardized SEV scalar: Cyclist	2.43 (2.14 — 2.82)
	Vehicles - 2+4 wheels (per capita)	1.00 (1.00 — 1.01)
Motorcyclist road injuries	Log-transformed age-standardized SEV scalar: Mot Cyc	2.14 (2.12 — 2.20)
	Vehicles - 2 wheels (per capita)	1.54 (1.49 — 1.59)
Motor vehicle road injuries	Log-transformed age-standardized SEV scalar: Mot Veh	2.27 (2.12 — 2.57)
	Vehicles - 4 wheels (per capita)	1.21 (1.18 — 1.23)
Other road injuries	Log-transformed age-standardized SEV scalar: Oth Road	2.17 (2.12 — 2.26)
Other transport injuries	Log-transformed age-standardized SEV scalar: Oth Trans	3.41 (3.26 — 3.49)
Falls (EMR)	Log-transformed age-standardized SEV scalar: Falls	3.48 (3.47 — 3.49)
Drowning (EMR)	Log-transformed age-standardized SEV scalar: Drown	2.77 (2.24 — 3.42)
	Coastal Population within 10km (proportion)	1.02 (1.00 — 1.08)
Fire, heat, and hot substances	Log-transformed age-standardized SEV scalar: Fire	3.39 (3.24 — 3.49)
	Indoor Air Pollution (All Cooking Fuels)	1.05 (0.97 — 1.18)
Poisonings	Log-transformed age-standardized SEV scalar: Poison	3.32 (3.04 — 3.48)
Poisoning by carbon monoxide (EMR)	Log-transformed SEV scalar: Poison	2.35 (2.13 — 2.79)
Poisoning by other means	Log-transformed SEV scalar: Poison	3.07 (2.56 — 3.46)
Exposure to mechanical forces	Log-transformed age-standardized SEV scalar: Mech	3.45 (3.39 — 3.49)
Unintentional firearm injuries	Log-transformed age-standardized SEV scalar: Mech Gun	2.27 (2.13 — 2.52)
Other exposure to mechanical forces	Log-transformed age-standardized SEV scalar: Oth Mech	3.45 (3.38 — 3.49)
Adverse effects of medical treatment	Socio-demographic Index	1.64 (1.63 — 1.65)
Animal contact	Log-transformed age-standardized SEV scalar: Animal	3.45 (3.40 — 3.49)
	LDI (I$ per capita)	0.74 (0.74 — 0.74)

Venomous animal contact	Log-transformed age-standardized SEV scalar: Venom	2.14 (2.12 — 2.19)
Non-venomous animal contact	Log-transformed age-standardized SEV scalar: Non Ven	3.47 (3.43 — 3.49)
Pulmonary aspiration and foreign body in airway	Log-transformed age-standardized SEV scalar: F Body Asp	2.83 (2.25 — 3.44)
Foreign body in eyes	—	—
Foreign body in other body part	Log-transformed SEV scalar: Oth F Body	2.29 (2.12 — 2.69)
Environmental heat and cold exposure	Population-weighted mean temperature	1.17 (1.12 — 1.21)
	90th percentile climatic temperature in the given country-year.	1.54 (1.44 — 1.64)
Other unintentional injuries	Log-transformed age-standardized SEV scalar: Oth Unint	3.29 (2.92 — 3.48)
Self-harm (EMR)	Log-transformed age-standardized SEV scalar: Self Harm	2.15 (2.12 — 2.21)
Self-harm by firearm (EMR)	Log-transformed age-standardized SEV scalar: Self Other	3.36 (3.27 — 3.45)
Self-harm by other specified means	Log-transformed age-standardized SEV scalar: Self Harm	3.43 (3.34 — 3.49)
Interpersonal violence	Log-transformed age-standardized SEV scalar: Violence	2.13 (2.12 — 2.16)
Assault by firearm (EMR)	Log-transformed age-standardized SEV scalar: Viol Gun	2.20 (2.12 — 2.36)
Assault by sharp object	Log-transformed age-standardized SEV scalar: Viol Knife	2.12 (2.12 — 2.14)
Assault by other means	Log-transformed age-standardized SEV scalar: Oth Viol	2.91 (2.74 — 3.10)

Fatal discontinuities

Due to the sporadic nature of the incidence of injuries and a lack of time trend that results from fatal discontinuities, DisMod-MR 2.1 was not used to model incidence due to fatal discontinuities, including state actor violence, exposure to forces of nature (i.e., natural disaster), and conflict and terrorism. Instead, incidence-to-mortality ratios were averaged over super-region, year, and sex to limit the variability in the ratios applied to fatal discontinuities. For disaster incidence, the incidence-to-mortality ratio was calculated as an average of road injuries and drowning if there was a water-related natural disaster in that specific country-year noted in the International Disaster Database [6]. For conflict and terrorism, the incidence-to-mortality ratio was calculated as an average of the road injuries and interpersonal violence causes. We treated executions and police conflict as similar to the fatal discontinuities in that we imputed the incidence using the incidence-to-mortality ratio of interpersonal violence. These incidence-to-mortality ratios were applied to mortality estimates from shock events from the Cause of Death database and shocks modelling process to calculate fatal discontinuity injuries incidence.

Follow-up studies

Similar to GBD 2017, we used follow-up data obtained from a pooled dataset of six follow-up studies from China, the Netherlands, and the US (see Table 3) [1]. These studies followed patients for at least one year

after the injury. We also used the Medical Expenditure Panel Survey (MEPS) [7]. MEPS is a large-scale overlapping continuous panel survey of the US non-institutionalized population that collects information on use and cost of health care and SF-12 responses. SF-12 responses are elicited twice over the two-year period that any individual is part of the study. Thus, MEPS offered the benefit of including health state measures of non-injured and destined to be injured and the benefit of having pre-injury and post-injury SF-12 responses. We pooled all available MEPS data over a 19-year span.

The follow-up studies used different patient reported outcome measures to assess health status, namely the SF-36, Version 1 SF-12, and the EQ-5D. To enable comparison across the six datasets, it was necessary to analyse the data in a standardised patient-reported outcome measure. First, we mapped all patient-reported outcome measures to Version 2 SF-12 (SF-12v2). Second, we normalised the health status measurements by mapping the SF-12 scores to a corresponding disability weight based on several opportunistic surveys asking respondents to score SF-12 based on the lay descriptions for a selection of 60 GBD health states. We ran a regression of logit-transformed disability weight on nature-of-injury category and age group and never-injured status. The pooled dataset informed both the nature-of-injury category hierarchy and the long-term probability of injuries, discussed below.

Table 3. Details of injury follow-up surveys used in GBD 2019

Dataset	Year	Type of data collected	Type of patients	Setting	Sample size* and response
Guangdong follow up survey, China[9]	2006–2007	Follow up survey among sample of ISS patients	Patients (15+ years) who were hospitalized that had been injured by road traffic injury, fall, blunt or penetrating trauma	Based on three national injury surveillance hospitals in Zhuhai, Guangdong Province in China	998 (response 87%)
LIS follow up survey, Netherlands[10]	2001–2002	Follow-up survey among stratified sample of ISS patients (oversampling less common, severe injuries)	Patients (15+ years) who visited the Emergency Department of a hospital and were discharged to the home environment and patients who were admitted to hospital	Based on 17 public hospitals in the Netherlands	8,564 (response 37%)
LIS follow-up survey, Netherlands[11]	2007–2008	Follow-up survey among stratified sample of ISS patients (oversampling less common, severe injuries)	Patients (15+ years) who visited the Emergency Department of a hospital and were discharged to the home environment and patients who were admitted to hospital	Based on 15 public hospitals in the Netherlands	8,057 (response 36%)
NSCOT – National study on Costs and	2001–2002	A prospective cohort study was conducted among a sample of	Patients treated for a moderate to severe injury (as defined by at least one injury	Based on 69 hospitals in 12 states in the US	5,191 (response 61%)

Outcomes of Trauma, USA[12]		adult trauma patients treated at Level I trauma centers and non-trauma center hospitals	of an Abbreviated Injury Scale (AIS) score of 3 or greater		
SCTBIFR – South Carolina Traumatic Brain injury Follow-up Registry, USA[13]	1999–2002	A prospective cohort study was conducted among injured in-patients with a traumatic brain injury-related injury	Patients (15+ years) who were admitted to hospitals and met the CDC case definition of TBI – trauma to the head associated with altered consciousness, amnesia, neurological abnormalities, skull fracture, intracranial lesion, or death	Discharged from all nonfederal in-state acute care hospitals	7,613 (response 28%)
Burns outcome study, Netherlands[14]	2003–2006	A multicenter prospective cohort was conducted among adult (severe) burn patients	Injury patients who sustained severe burns	Three public hospitals with specialized burn units.	311 (response 78%)

*number of patients that met the inclusion criteria; response rate = percentage of patients who responded to the follow-up survey (in case of multiple follow-up times the response rate of the first follow-up moment is reported).

Nature-of-injury category hierarchy

Multiple injuries can occur in one individual. For GBD 2019, a nature-of-injuries severity hierarchy was developed to establish a one-to-one relationship between cause-of-injury and nature-of-injury category. This means that in the case of multiple injuries the nature-of-injury category that was likely to be responsible for the largest burden was selected. To construct the hierarchy, we used data from the pooled dataset of follow-up studies [9–14]. The output of the regression of logit-transformed disability weight on nature-of-injury category and individual characteristics of the follow-up studies were used to calculate the mean long-term disability attributable to each nature-of-injury category. The ranking of nature-of-injury categories by their long-term disability weights formed the basis of our severity hierarchy. Hierarchies were developed separately, for injuries warranting inpatient care and injuries warranting other health care.

Table 4. Nature-of-injury hierarchies: combination of empirical hierarchies estimated from pooled follow-up studies and expert adjustments, for inpatient and outpatient injuries

Rank	Inpatient Hierarchy	Outpatient Hierarchy
1	Spinal cord lesion below neck level	Fracture of pelvis
2	Amputation of lower limbs, bilateral	Fracture of patella, tibia or fibula, or ankle
3	Amputation of upper limbs, bilateral	Fracture of hip
4	Spinal cord lesion at neck level	Fracture of skull
5	Fracture of hip	Amputation of thumb
6	Fracture of femur, other than femoral neck	Fracture of vertebral column
7	Amputation of upper limb, unilateral	Multiple fractures, dislocations, crashes, wounds, sprains, and strains
8	Amputation of lower limb, unilateral	Internal hemorrhage in abdomen and pelvis

9	Multiple fractures, dislocations, crashes, wounds, sprains, and strains	Fracture of femur, other than femoral neck
10	Effect of different environmental factors	Dislocation of hip
11	Fracture of patella, tibia or fibula, or ankle	Amputation of toe/toes
12	Moderate-Severe traumatic brain injury	Fracture of hand (wrist and other distal part of hand)
13	Fracture of foot bones except ankle	Amputation of fingers (excluding thumb)
14	Internal hemorrhage in abdomen and pelvis	Burns, <20% of total burned surface area without lower airway burns
15	Crush injury	Dislocation of knee
16	Minor traumatic brain injury	Contusion in any part of the body
17	Fracture of pelvis	Minor traumatic brain injury
18	Nerve injury	Foreign body in respiratory system
19	Severe chest injury	Severe chest injury
20	Dislocation of hip	Drowning and nonfatal submersion
21	Burns, >= 20% total burned surface area or >= 10% burned surface are if head/neck or hands/wrist involved w/o lower airway burns	Asphyxiation
22	Lower airway burns	Poisoning requiring urgent care
23	Fracture of skull	Effect of different environmental factors
24	Amputation of thumb	Foreign body in GI and urogenital system
25	Fracture of hand (wrist and other distal part of hand)	Fracture of sternum and/or fracture of one or more ribs
26	Fracture of vertebral column	Nerve injury
27	Contusion in any part of the body	Fracture of face bones
28	Open wound(s)	Dislocation of shoulder
29	Amputation of toe/toes	Injury to eyes
30	Dislocation of knee	Fracture of clavicle, scapula, or humerus
31	Amputation of fingers (excluding thumb)	Fracture of radius and/or ulna
32	Drowning and nonfatal submersion	Fracture of foot bones except ankle
33	Asphyxiation	Foreign body in ear
34	Burns, <20% total burned surface area without lower airway burns	Muscle and tendon injuries, including sprains and strains lesser dislocations
35	Muscle and tendon injuries, including sprains and strains lesser dislocations	Superficial injury of any part of the body
36	Fracture of face bones	Open wound(s)
37	Foreign body in respiratory system	Complications following therapeutic procedures
38	Poisoning requiring urgent care	
39	Foreign body in GI and urogenital system	
40	Fracture of sternum and/or fracture of one or more ribs	
41	Dislocation of shoulder	
42	Injury to eyes	
43	Fracture of clavicle, scapula, or humerus	
44	Fracture of radius and/or ulna	
45	Foreign body in ear	
46	Superficial injury of any part of the body	
47	Complications following therapeutic procedures	

Cause-nature matrices
Because injury disability is linked more to the nature of injury than to the cause of injury, matrices were generated to map the proportion of each cause-of-injury category that results in a particular nature-of-injury category. These matrices are based on a collection of dual-coded (i.e., both cause-of-injury and nature-of-injury coded) hospital and emergency department datasets [28]. The data for this step came from inpatient, outpatient, and emergency room discharge data from Argentina, Bulgaria, China, Colombia, Cyprus, Czech Republic, Denmark, Egypt, Estonia, Hungary, Iceland, Iran, Italy, Latvia, Malta, Mauritius, Mexico, Mozambique, Netherlands, Norway, Portugal, Slovenia, Spain, Sweden, Macedonia, Uganda, United States, and Zambia. We applied our nature-of-injury severity hierarchy above to assert that every observation had one cause of injury and one nature of injury.

Dirichlet models were used to estimate all of the nature-of-injury category proportions for one cause of injury simultaneously. These models allow for consistent borrowing of information across age, sex, inpatient/outpatient, and high/low-income countries and assert that the nature-of-injury proportions within a cause-of-injury category must add up to 1. One cause-nature matrix was created for each combination of injury warranting hospital admission versus injury warranting other health care, high/low-income countries, male/female, and age category. Applying these matrices to our cause-of-injury incidence from DisMod-MR, we produced cases of injury warranting hospital admission and incidence of injury warranting other health care by cause and nature of injury.

Probability of permanent health loss
Disability due to injury was assumed to affect all cases in the short term with a proportion having long-term (permanent) outcomes. The probability of long-term outcomes was needed to estimate the incidence and subsequently the prevalence of cases with permanent health loss. In our conceptual model, individuals who suffer a non-fatal injury will, in the long-term, return to either full or partial health. If one-year post-injury patients return to a health status with more disability than their pre-injury health status, injury patients are assumed to have permanent disability from their injury. The difference between the pre-injury health states and health status one year after injury is assumed to be their permanent level of injury-related disability. We assessed the probability of developing permanent health loss using the pooled dataset of follow-up studies [9–14] and the MEPS [7] that were also used to generate the nature-of-injury hierarchy. To assess the probability of permanent health loss, we estimated the effects using a logit-linear mixed effects regression:

$$\begin{aligned} Logit(DW)_{im} = {} & \alpha + \beta(injuries_{im}) + \beta(never\ injured_i) + \beta(never\ injured_i * age_i) \\ & + \beta(fracture\ of\ pelvis_i) + \beta(fracture\ of\ pelvis_i * age_i) + \beta(poisoning_i * age_i) \\ & + \beta(moderate\ to\ severe\ TBI_i * age_i) + RE_c + RE_i \end{aligned}$$

where we included dummies for all the nature-of-injury categories $(injuries_{im})$, with the reference category being no injury (from MEPS dataset). We also included a dummy for never injured prior to the current injury, age, interactions between age and never injured status, and interactions with three long-term nature-of-injury categories that were found to significantly vary with age: pelvis fractures, poisonings, and moderate/severe traumatic brain injuries. In notation, subscript m refers to patient-reported outcome measure, i refers to individual, and c refers to country. Random effects (RE) were included to control for variation between countries and individuals.

After predicting overall disability at one-year follow-up, we estimated a counterfactual by setting all observations to “no injury,” the reference group for $\beta(injuries_{im})$ in our model. The disability attributable to the nature of injury at one year was assumed to be the difference between our

counterfactual of no injury and predicted disability with injury. The probability of treated long-term outcomes was estimated via the ratio of this attributable disability relative to the long-term disability weight for that injury.

$$Probability\ of\ long - term\ disability = \frac{with\ injury\ disability_{im} - counterfactual\ disability_{im}}{DW_m}$$

We developed estimates of the probability of permanent health loss by nature-of-injury category, injury severity level (injuries warranting inpatient admission and injuries warranting other health care), and age. These probabilities are shown in Figure 3 for three selected age groups (25-30, 50-55, 75-80) and selected nature-of-injury categories by inpatient and outpatient. Moderate-severe TBI and spinal cord lesions only have inpatient injury long-term probabilities, and nerve injury, open wounds, and severe chest injury have long-term probabilities of zero for outpatient cases.

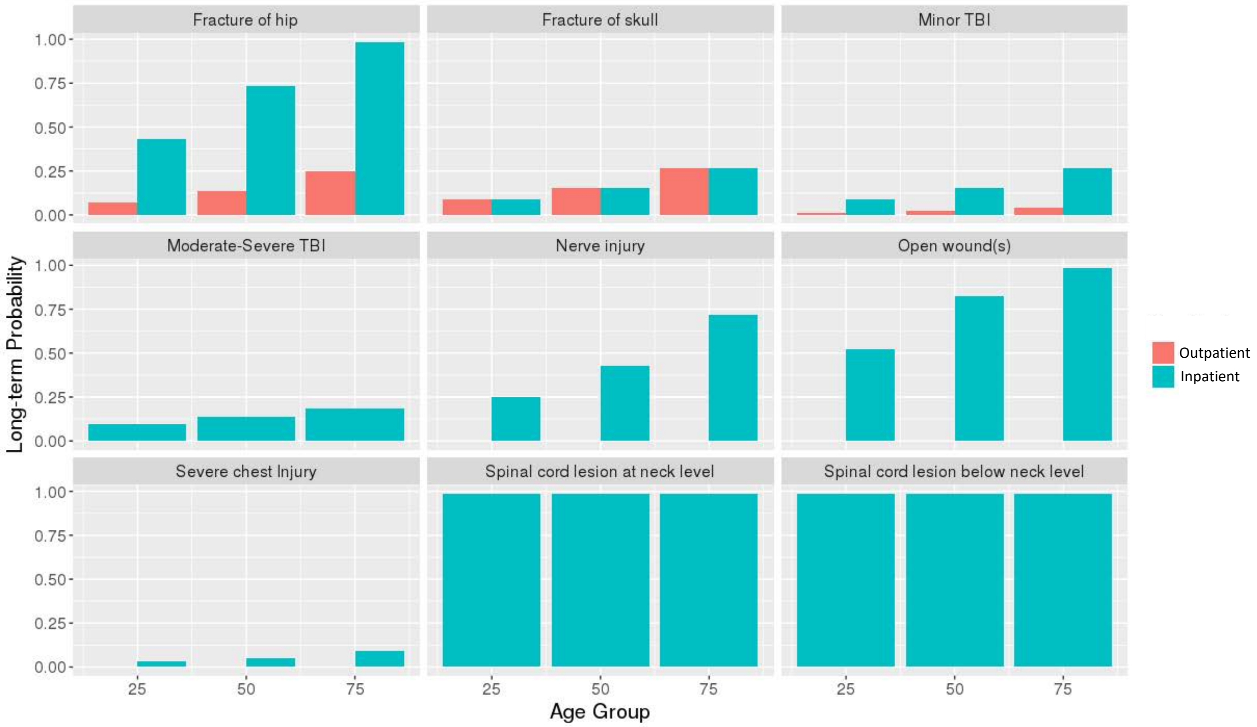

Figure 3. Long-term probabilities derived from the MEPS data for selected nature of injuries and age groups

<u>Disability associated with treated and untreated cases</u>

For many nature-of-injury categories, GBD 2019 has a separate disability weight for treated and for untreated cases. To estimate the percent treated for injuries in a given location-year, we used the Healthcare Access and Quality (HAQ) Index [29] with the same strategy described for the probability of permanent health loss. We chose a reasonable cutoff for the HAQ Index (75 on a scale of 0 – 100) as the threshold at and above which 100% of injuries were treated. This value captured most OECD countries for all years back to 1980. We then scaled all remaining location-years between 10% and 100% treated based on their HAQ Index value and used that as the percent treated in a given location-year. This was done at

the draw level to propagate uncertainty. We made the decision to ignore any long-term disability from injuries with implausibly high estimates of long-term disability.

Duration of short-term health loss

To determine the duration for treated cases of short-term injury, we analysed patient responses from two Dutch Injury Surveillance System follow-up studies conducted from 2001–2003 and 2007–2009 [8]. These studies collected data at 2.5, 5, 9, and 24 months post-injury to determine whether injury patients were still experiencing problems due to their injury. If not, the patients were asked how many days they had experienced problems. The injury patients that still reported having problems one year after the injury were assumed to be captured in our analysis of permanent disability. The duration for treated cases of short-term injury was estimated for injuries warranting inpatient admission and injuries warranting other health care separately. The estimates were supplemented by expert-driven estimates of short-term duration for nature-of-injury categories that did not appear in the Dutch dataset and untreated injuries.

Calculation of prevalence from incidence data – short-term injury

For short-term injury outcomes, which were assumed to be less than one year in duration, the prevalence for each cause-of-injury/nature-of-injury/severity-level grouping was approximated by the incidence for that grouping multiplied by the associated nature-of-injury/severity-level-specific duration.

Calculation of prevalence from incidence data – permanent health loss

For permanent health loss, we assumed no remission and thus integrated incidence over time to arrive at prevalence estimates. We used DisMod ODE (i.e., the “engine” of DisMod-MR 2.1) to carry out this integration for each combination of cause of injury and nature of injury by country, year, and sex. For this step we used random effects meta-analysis to pool data on standardised mortality ratios derived from literature reviews for spinal cord injury, burns covering more than 20% of the body, moderate to severe traumatic brain injury, hip fracture, and multiple significant injuries [14–27]. Here we include examples of these meta-analyses: hip fractures and traumatic brain injuries.

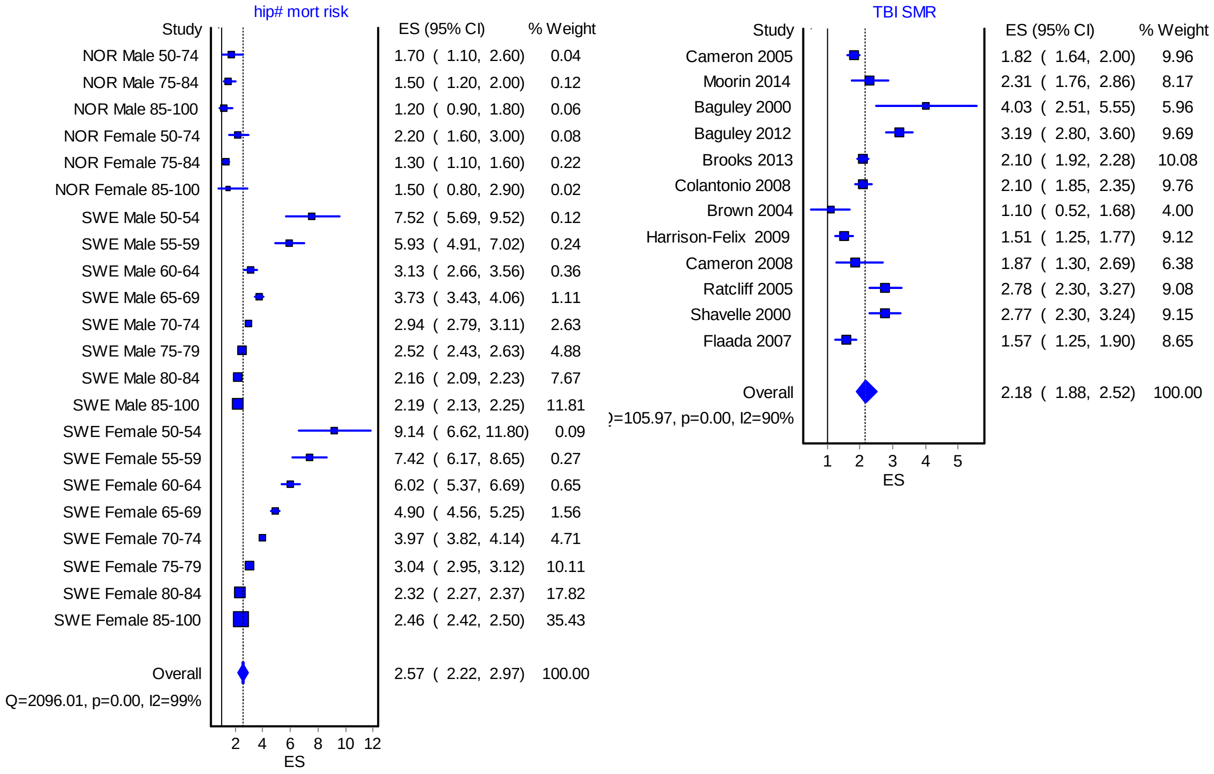

Figure 4. Meta-analyses of standardised mortality ratios derived from literature reviews: hip fractures and traumatic brain injury

For all other nature-of-injury categories, we assumed no long-term excess mortality. For the incidence estimates derived from fatal discontinuities – "exposure to forces of nature" and "conflict and terrorism" – we did not use DisMod as discontinuities by definition violate the assumption of a steady state in DisMod to estimate prevalence from incidence. For these two cause-of-injury categories, we coded the differential equations from DisMod ODE that determine the relationship between incidence, remission, mortality risk, and prevalence into Python and streamed out the prevalence from the incidence in the years of war or disaster by integrating over one year at a time.

MR-BRT models (continued)

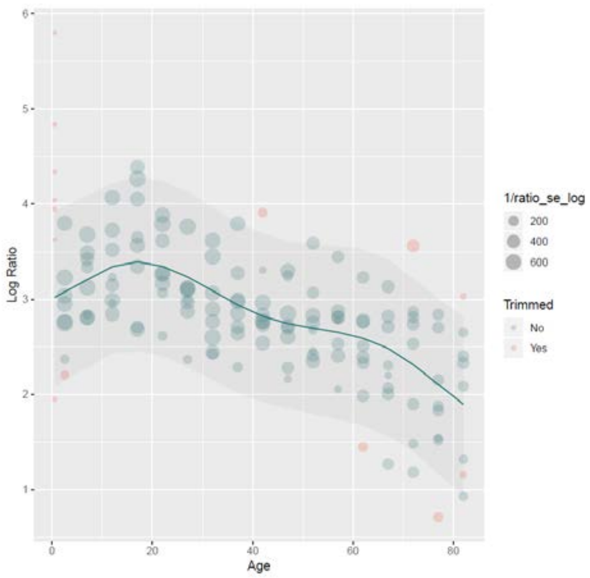

Figure 5. MR-BRT model for animal contact

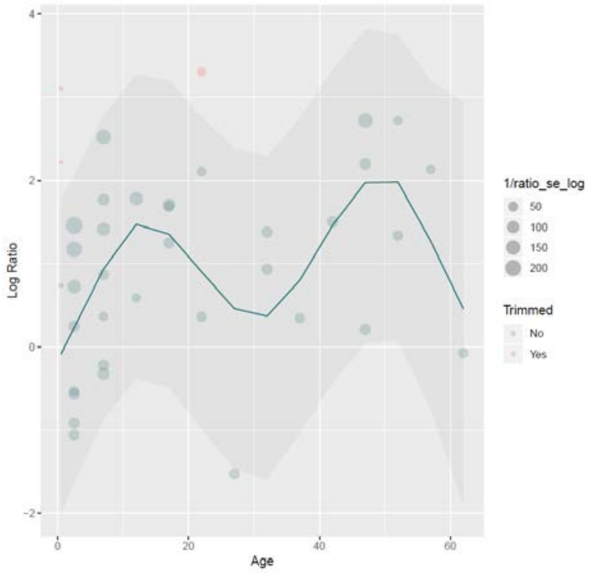

Figure 6. MR-BRT model for drowning

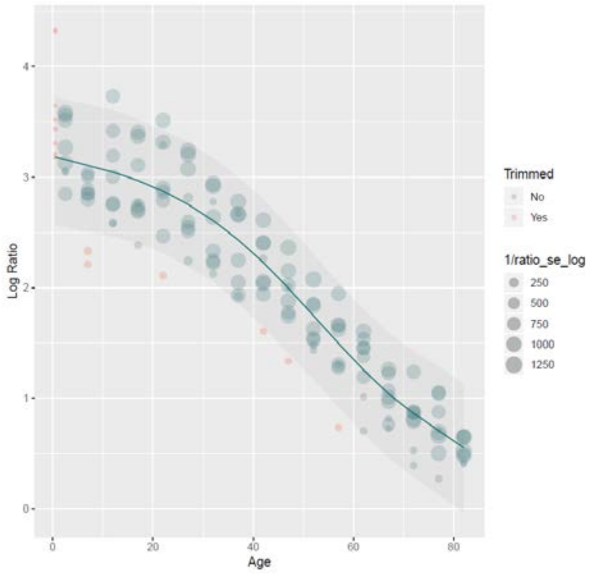

Figure 7. MR-BRT model for falls

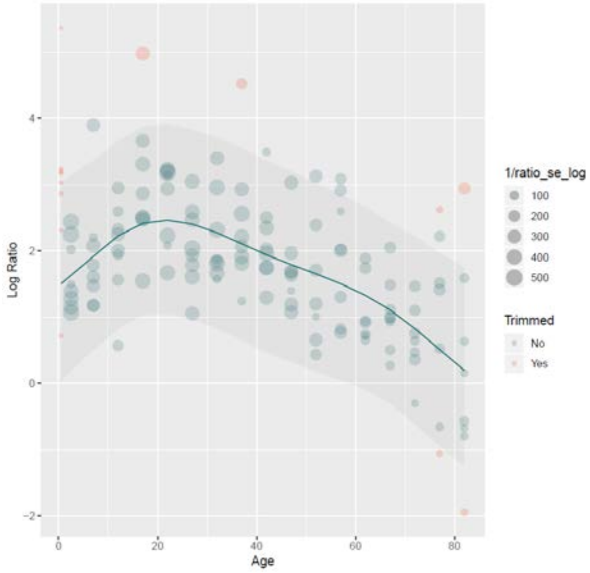

Figure 8. MR-BRT model for fire, heat, and hot substances

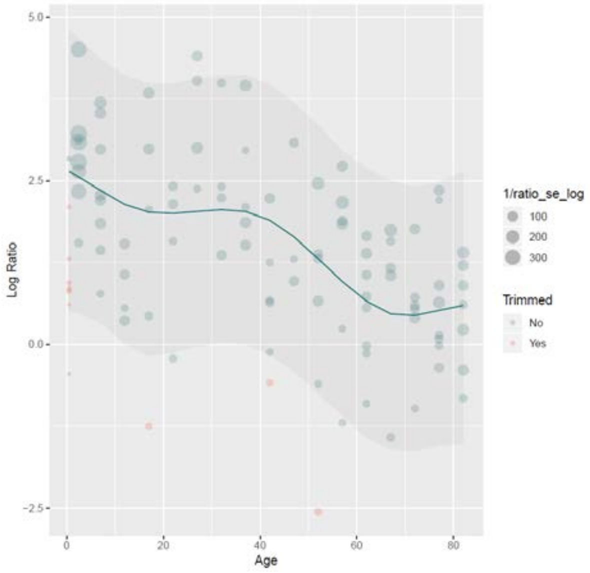

Figure 9. MR-BRT model for pulmonary aspiration and foreign body in airway

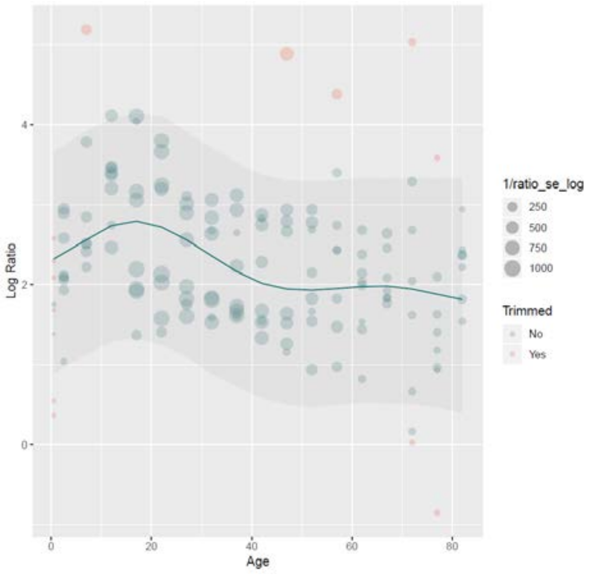

Figure 10. MR-BRT model for interpersonal violence

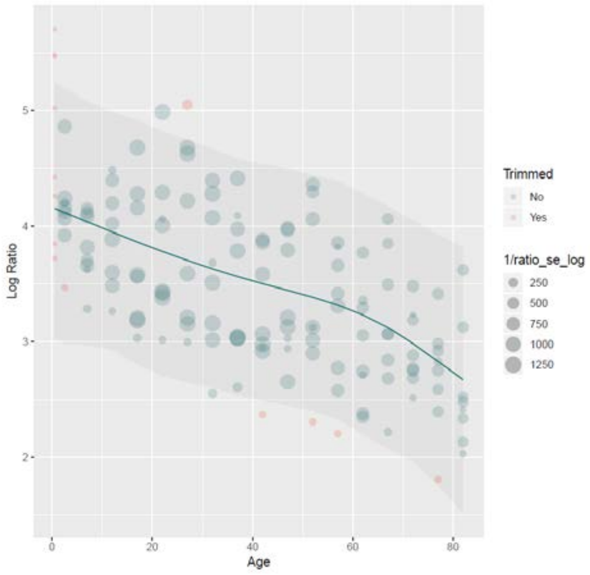

Figure 11. MR-BRT model for exposure to mechanical forces

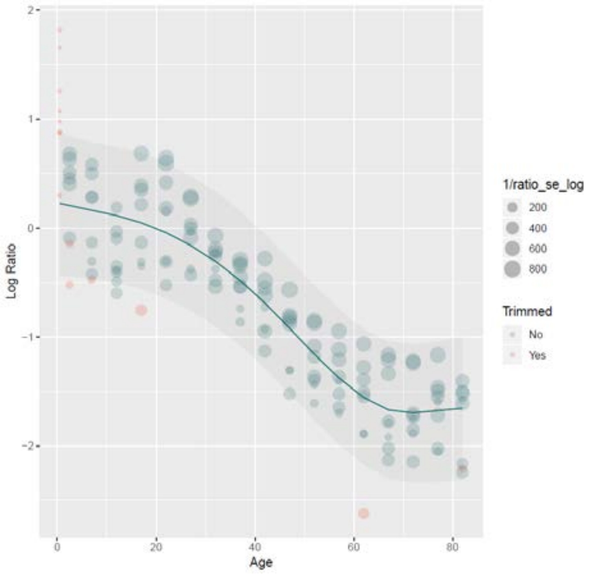

Figure 12. MR-BRT model for adverse effects of medical treatment

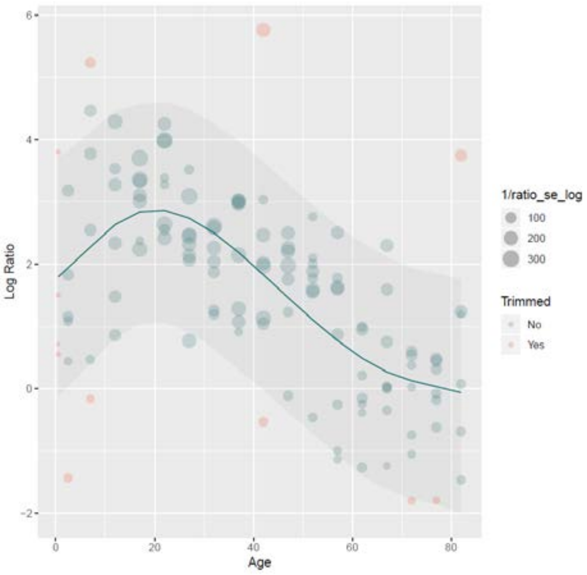

Figure 13. MR-BRT model for exposure to forces of nature

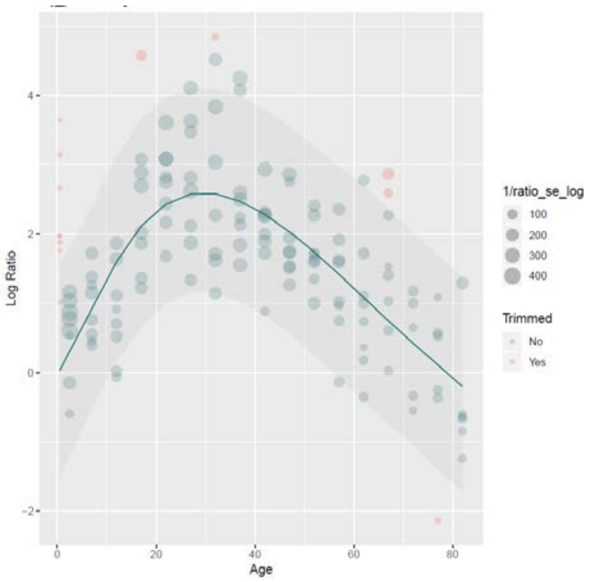

Figure 14. MR-BRT model for poisonings

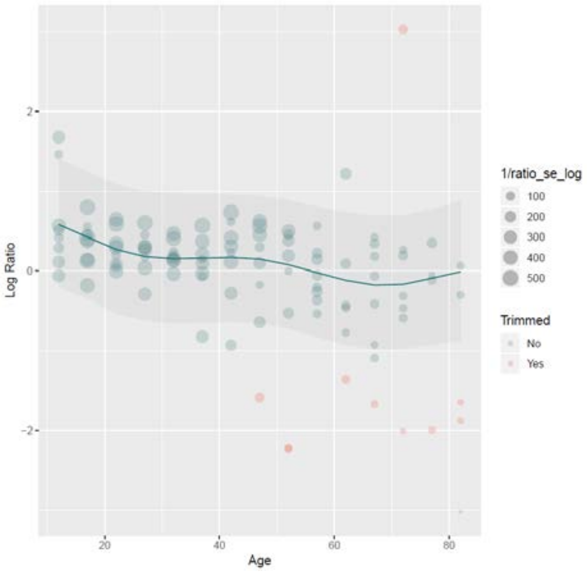

Figure 15. MR-BRT model for self-harm

References

1. James, Spencer L., Degu Abate, Kalkidan Hassen Abate, Solomon M. Abay, Cristiana Abbafati, Nooshin Abbasi, Hedayat Abbastabar, et al. "Global, Regional, and National Incidence, Prevalence, and Years Lived with Disability for 354 Diseases and Injuries for 195 Countries and Territories,

1990–2017: A Systematic Analysis for the Global Burden of Disease Study 2017." The Lancet 392, no. 10159 (November 10, 2018): 1789–1858.

2. Department of Peace and Conflict Research, Uppsala University. UCDP Nonstate Conflict Dataset. Uppsala, Sweden: Department of Peace and Conflict Research, Uppsala University.

3. International Institute for Strategic Studies. International Institute for Strategic Studies Armed Conflict Database. London, United Kingdom: International Institute for Strategic Studies.

4. Climate Change and African Political Stability Project (CCAPS). Armed Conflict Location and Event Dataset, Realtime - Robert S. Strauss Center as referenced in Raleigh, Clionadh, Andrew Linke, Havard Hegre and Joakim Karlsen. 2010. Introducing ACLED-Armed Conflict Location and Event Data. Journal of Peace Research 47(5), 1-10.

5. Salehyan I, Hendrix CS, Hamner J, Case C, Linebarger C, Stull E, Williams J, Robert S. Strauss Center for International Security and Law. Social Conflict in Africa: A New Database. Int Interact. 2012; 38(4): 503-511.

6. Centre for Research on the Epidemiology of Disasters (CRED). EM-DAT: The OFDA/CRED International Disaster Database. Brussels, Belgium: Catholic University of Leuven.

7. Agency for Healthcare Research and Quality. United States Medical Expenditure Panel Survey. Rockville, United States: Agency for Healthcare Research and Quality.

8. Polinder S, van Beeck EF, Essink-Bot ML, Toet H, Looman CW, Mulder S, Meerding WJ. Functional outcome at 2.5, 5, 9, and 24 months after injury in the Netherlands. J Trauma. 2007; 62(1): 133-41.

9. Chinese Center for Disease Control and Prevention (CCDC). China Zhuhai Study 2006-2007 - China CDC.

10. Consumer Safety Institute (Netherlands). Netherlands Injury Surveillance System 2002.

11. Consumer Safety Institute (Netherlands). Netherlands Injury Surveillance System 2008.

12. Mackenzie EJ, Rivara FP, Jurkovich GJ, et al. The National Study on Costs and Outcomes of Trauma. J Trauma 2007; 63: S54-67; discussion S81-86.

13. CDC, Medical University of South Carolina, South Carolina Department of Disabilities and Special Needs, South Carolina Department of Health and Environmental Control. South Carolina Traumatic Brain Injury Follow-up Registry 1999-2013. USA.

14. van Loey NE, van Beeck EF, Faber BW, van de Schoot R, Bremer M. Health-Related Quality of Life After Burns: A Prospective Multicentre Cohort Study With 18 Months Follow-Up. J Trauma. 2011; 72(2): 513-520.

15. Strauss D, Shavelle R, DeVivo MJ, Day S. An analytic method for longitudinal mortality studies. J Insur Med 2000; 32: 217–25.

16. Shavelle R, Strauss D. Comparative mortality of adults with traumatic brain injury in California, 1988–97. J Insur Med 2000; 32: 163–6.

17. Baguley IJ, Nott MT, Howle AA, et al. Late mortality after severe traumatic brain injury in New South Wales: a multicentre study. Med J Aust 2012; 196: 40–5.

18. Middleton JW, Dayton A, Walsh J, Rutkowski SB, Leong G, Duong S. Life expectancy after spinal cord injury: a 50-year study. Spinal Cord 2012; 50: 803–11.

19. Middleton JW, Dayton A, Walsh J, Rutkowski SB, Leong G, Duong S. Life expectancy after spinal cord injury: a 50-year study. Spinal Cord 2012; 50: 803–11.

20. Brooks JC, Strauss DJ, Shavelle RM, Paculdo DR, Hammond FM, Harrison-Felix CL. Long-term disability and survival in traumatic brain injury: results from the National Institute on Disability and Rehabilitation Research Model Systems. Arch Phys Med Rehabil 2013; 94: 2203–9.

21. Baguley I, Slewa-Younan S, Lazarus R, Green A. Long-term mortality trends in patients with traumatic brain injury. Brain Inj 2000; 14: 505–12.

22. Ratcliff G, Colantonio A, Escobar M, Chase S, Vernich L. Long-term survival following traumatic brain injury. Disabil Rehabil 2005; 27: 305–14.

23. Frankel HL, Coll JR, Charlifue SW, et al. Long-term survival in spinal cord injury: a fifty year investigation. Spinal Cord 1998; 36: 266–74.

24. Harrison-Felix CL, Whiteneck GG, Jha A, DeVivo MJ, Hammond FM, Hart DM. Mortality over four decades after traumatic brain injury rehabilitation: a retrospective cohort study. Arch Phys Med Rehabil 2009; 90: 1506–13.

25. Moorin R, Miller TR, Hendrie D. Population-based incidence and 5-year survival for hospital-admitted traumatic brain and spinal cord injury, Western Australia, 2003-2008. J Neurol 2014; 261: 1726–34.

26. Colantonio A, Escobar MD, Chipman M, et al. Predictors of postacute mortality following traumatic brain injury in a seriously injured population. J Trauma 2008; 64: 876–82.

27. Flaada JT, Leibson CL, Mandrekar JN, et al. Relative risk of mortality after traumatic brain injury: a population-based study of the role of age and injury severity. J Neurotrauma 2007; 24: 435–45.

28. GBD 2016 Traumatic Brain Injury and Spinal Cord Injury Collaborators. Global, regional, and national burden of traumatic brain injury and spinal cord injury, 1990–2016: a systematic analysis for the Global Burden of Disease Study 2016. The Lancet Neurology. 26 Nov 2018.

29. GBD 2017 SDG Collaborators. Measuring progress from 1990 to 2017 and projecting attainment to 2030 of the health-related Sustainable Development Goals for 195 countries and territories: a systematic analysis for the Global Burden of Disease Study 2017. The Lancet. 8 Nov 2018; 392:2091–138.

Sexual Violence

Flowchart

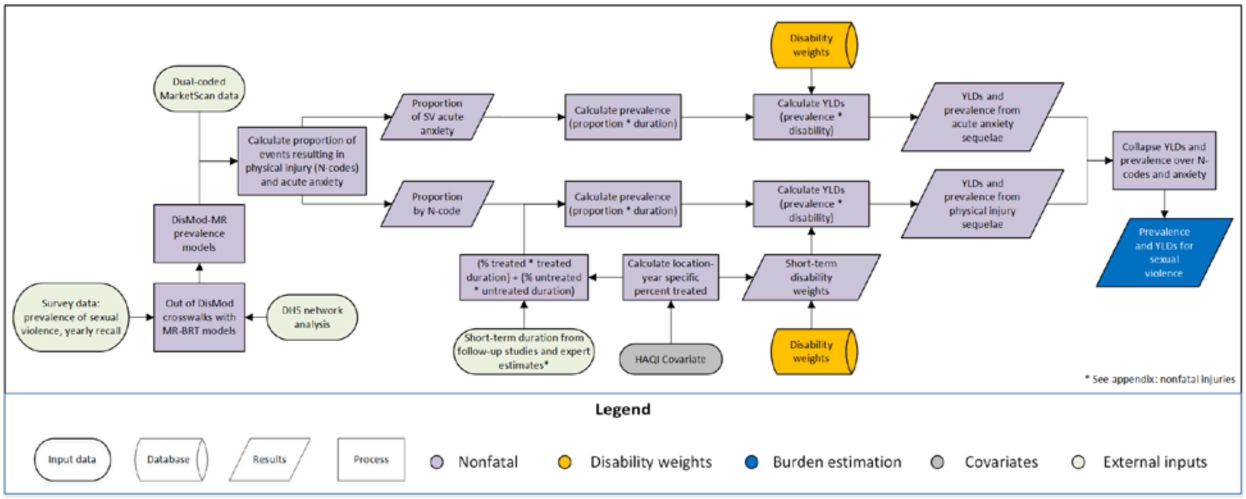

Case definition

For the sexual violence cause, we estimate the yearly prevalence of sexual violence, i.e., the proportion of the population that experienced at least one event of sexual violence in the last year. We define sexual violence as any sexual assault, including both penetrative sexual violence (rape) and non-penetrative sexual violence (other forms of unwanted sexual touching).

Input data

Model inputs

The majority of the data for sexual violence comes from various health and demographic surveys. We include many Demographic and Health Surveys (DHS) and Reproductive Health Surveys (RHS). Other survey series include the US Behavioral Risk Factor Surveillance Survey (BRFSS) and the British Crime Surveys. For GBD 2019, two Philippines Demographic and Health Surveys and two Nigeria Demographic and Health Surveys were re-extracted with greater subnational detail.

The China Health and Family Life Survey from 1999-2000 asks about lifetime prevalence of sexual assault; however, we were able to extract yearly prevalence by pairing a respondent's current age with the reported age of when the sexual assault occurred. Table 1 contains information about our input data for the sexual violence modelling process. Table 2 provides more information about data coverage in the seven Global Burden of Disease super-regions.

Table 1 Data inputs for sexual violence morbidity modelling by parameter

Measure	Total sources	Countries with data
**Prevalence**	119	71

Many other non-survey data sources exist for sexual violence. We explored the use of the United Nations Office on Drugs and Crime (UNODC) Statistics [1] that covers a wide range of geographies from 2003 to 2014. However, these estimates are based only on police reports, and their incidence is about 20 times

lower than the incidence seen in the same location-years from survey data. Although we could include a covariate in our models to adjust for this underreporting, we deemed the source unusable because of the magnitude of the difference between the police reports and survey data. Survey data typically range between 1% and 10% of individuals experiencing sexual violence in the last year. Figure 1 shows the incidence estimates from the UNODC data, where most of the estimates are below about 0.05%. The geographic pattern is the opposite of what we see in survey data, with higher-income countries having higher estimates in the UNODC data. Additionally, the reports were not age-sex-specific, and the definition for what constitutes sexual violence varies across countries.

Figure 1 United Nations Office of Drugs and Crime Statistics: estimates of sexual violence (incidence per person), color by Global Burden of Disease super-regions

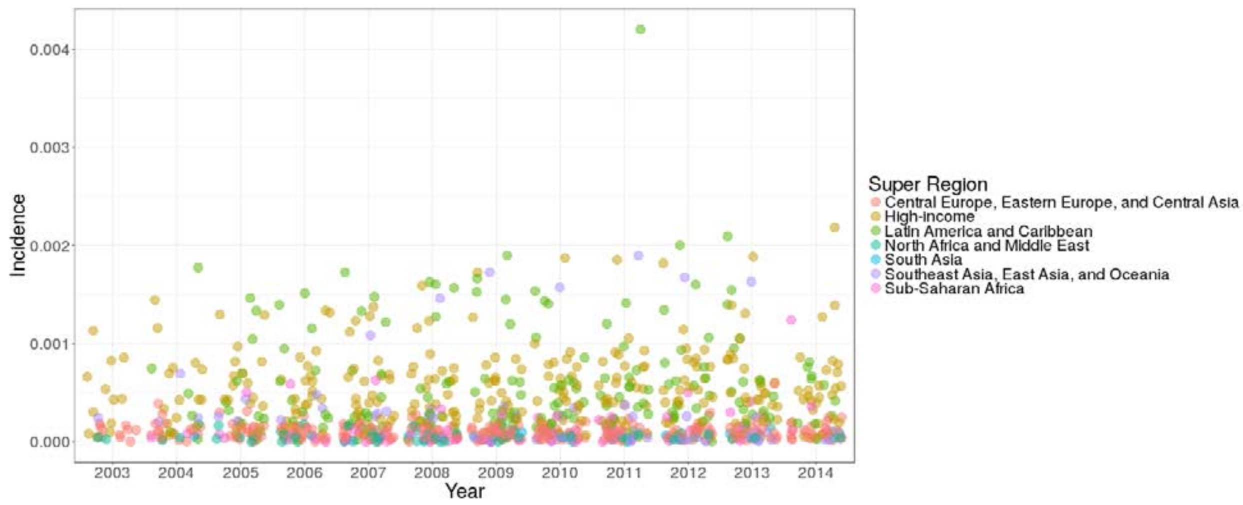

We also chose not to include the Centers for Disease Control non-fatal injury reports of sexual violence. Although this data source includes age- and sex-specific estimates for sexual violence in the United States, only sexual violence cases which resulted in physical injury are reported. These estimates are also systematically lower than the survey data, to the degree at which any adjustment with covariates would be unreliable. Lastly, we excluded a source from the United States Federal Bureau of Investigation: The Uniform Crime Reporting (UCR) program. The FBI estimates are produced at the state level for the United States and are meant to be comparable across states. However, police report data for sexual violence are systematically lower, similar in magnitude to the UNODC data, so we chose to exclude it.

Data searches

To find large data sources for sexual violence, we searched through the Global Health Data Exchange (GHDx) to identify survey series with relevant questions and reviewed surveys that were being used for intimate partner violence (IPV) already. We identified 107 sources with relevant data that were being used for IPV and 33 additional surveys with sexual violence questions. We excluded sources that only asked about lifetime prevalence of sexual violence because our case definition is specific to the past year. We extracted data on the perpetrator of sexual violence where possible (partner versus non-partner).

Additionally, we completed a systematic review of literature sources. Sources were non-representative if they only sampled high-risk populations (war-afflicted, sex workers, intravenous drug users, etc.), sexually abused individuals, or women suffering intimate partner violence; these sources were excluded. We also

excluded studies that only asked about sexual violence in the context of alcohol. After full-text screening, only five literature sources were used since they included yearly recall prevalence.

Modelling strategy

Prevalence of sexual violence

To produce estimates of the yearly prevalence of sexual violence, we used DisMod-MR 2.1. To preserve variation between male- and female-specific estimates, we have separate models for men and women. We make various assumptions within DisMod-MR 2.1, including no excess mortality due to sexual violence and no incidence between 0–2 and 98–100 years of age.

Adjusting data

Because of the different ways that questions about sexual violence in the last year can be asked, we include multiple study-level covariates (for coefficient estimates, see Table 3). We bounded the covariates at logical values to minimize the effect of collinearity between the covariates, i.e., we expect studies that ask about penetrative sexual violence only to have lower estimates of sexual violence overall, so that covariate has an upper bound of 1. Using these study-level covariates, we can extract data that do not meet our case definition and adjust the data accordingly. We performed a network analysis on Demographic Health Survey data to obtain within-study covariate comparisons and used coefficients output by MR-BRT to make necessary adjustments.

Table 2 Study-level covariates for DisMod-MR 2.1 yearly recall prevalence models for sexual violence

Covariate	C
Physically forced sexual violence only	U
Ever-partnered people only	N
Ever-married people only	N
Ever had sex	N
Penetrative sexual violence only	U
Only includes partner sexual violence	U

Years lived with disability (YLDs) due to sexual violence

To calculate the years lived with disability (YLDs) due to having experienced sexual violence in the past year, we utilised claims data from the United States from the years 2000, 2010, and 2012 to assess sexual violence sequelae. We searched through the claims database for the following ICD9 diagnosis codes: 995.53 (child sexual abuse), 995.83 (adult sexual abuse), and E960.1 (rape). We considered sequelae relating to both physical injuries and mental health consequences, in the short-term.

In this process of calculating of years lived with disability due to sexual violence, we currently measure only the short-term physical and psychological effects of sexual violence. In future GBD iterations, we plan to include sexual violence as a risk factor including both sexual violence in the last year and lifetime exposure to sexual violence (independent from, and in interaction with, intimate partner violence) in order to capture the long-term mental health consequences of sexual violence.

Physical injury

For the physical injury sequelae, we looked for any nature-of-injury ICD9 code on the same date of contact with medical service providers for a sexual violence ICD9 code (above) and categorized the

nature-of-injury codes as we do for the general injuries nonfatal modelling process (see appendix: nonfatal injuries). We calculate the proportion of individuals with any sexual violence code that result in each of the physical injuries categories. This strategy is similar to the strategy that we use for the cause-nature of injury matrices in the general injuries modelling process, but we have an additional category for no physical injury result as the majority of sexual violence incidents do not result in physical injury in the claims database. Additionally, because we only have one data source, we do not model these proportions with Dirichlet regression like we do for the injuries cause-nature of injury matrices but just compute them directly from the claims data. To estimate the physical injuries component of YLDs, we multiply the DisMod estimates of yearly prevalence of sexual violence by these proportions and then multiply by each physical injuries' respective short-term duration and disability weight that we use in the general injuries process (see appendix: nonfatal injuries).

Acute anxiety and/or reaction to stress

For the mental and psychological sequelae of sexual violence, we searched an individual being coded to any of the following ICD9 codes at any point *after* a sexual violence incident was noted. The codes are meant to reflect conditions relating to an "acute anxiety and/or reaction to stress" condition following a traumatic incident, displayed in Table 4.

Table 3 ICD9 codes included in the "acute anxiety and/or reaction to stress" condition as a sequela for sexual violence

ICD9 Code	Condition Description
**308**	Acute reaction to stress
**308**	Predominant disturbance of emotions
**308.1**	Predominant disturbance of consciousness
**308.2**	Predominant psychomotor disturbance
**308.3**	Other acute reactions to stress
**308.4**	Mixed disorders as reaction to stress
**308.9**	Unspecified acute reaction to stress
**309**	Adjustment reaction
**309**	Adjustment disorder with depressed mood
**309.1**	Prolonged depressive reaction
**309.2**	Adjustment reaction with predominant disturbance of other emotions
**309.21**	Separation anxiety disorder
**309.22**	Emancipation disorder of adolescence and early adult life
**309.23**	Specific academic or work inhibition
**309.24**	Adjustment disorder with anxiety
**309.28**	Adjustment disorder with mixed anxiety and depressed mood
**309.29**	Other adjustment reactions with predominant disturbance of other emotions
**309.3**	Adjustment disorder with disturbance of conduct
**309.4**	Adjustment disorder with mixed disturbance of emotions and conduct
**309.8**	Other specified adjustment reactions
**309.81**	Posttraumatic stress disorder
**309.82**	Adjustment reaction with physical symptoms

**309.83**	Adjustment reaction with withdrawal
**309.89**	Other specified adjustment reactions
**309.9**	Unspecified adjustment reaction

It is possible that the appearance of one of these ICD9 codes is entirely unrelated to the sexual violence incident. Additionally, the appearance of one of these codes could be related instead to underlying depression and anxiety. To control for these confounding factors, we also searched for these ICD9 codes among individuals that were not victims of sexual violence in the past year. We used Poisson regression with robust standard errors to model the relative risk of the "acute anxiety and/or reaction to stress" comparing individuals with and without sexual violence within the year, controlling for underlying diagnoses of depression and anxiety:

$$\log(\lambda) = \beta_0 + \beta_1(sexual\ violence) + \beta_2(depression) + \beta_3(anxiety) + \beta_4(female) + \beta_5(age)$$

where λ is the risk of "acute anxiety and/or reaction to stress," and e^{β_1} is the relative risk of "acute anxiety and/or reaction to stress" comparing those experiencing at least one sexual violence incident to those with no sexual violence incidence, holding underlying depression, anxiety, sex, and age constant. We can approximate the risk of "acute anxiety and/or reaction to stress" for each age and sex experiencing sexual violence by:

$$\lambda_{age,sex} = e^{\beta_1} * (e^{\beta_0} * e^{sex*\beta_4+age*\beta_5}) - (e^{\beta_0} * e^{sex*\beta_4+age*\beta_5})$$

The claims data had n = 70,6707,63 observations (n = 8,331 sexual violence cases). Using the equation above, the transformed coefficients and transformed robust standard errors (transformations were performed with the Delta method) are shown in Table 5.

Table 4 Estimates of the risk of "acute anxiety and/or reaction to stress" ($\lambda_{age,sex}$) among people experiencing sexual violence over a year time-period, specific to age and sex

Age	Male		Female	
	Estimate	*Standard error*	*Estimate*	*Standard error*
**0-4**	0.0967	0.0023	0.1205	0.0028
**5-9**	0.0933	0.0021	0.1162	0.0027
**10-14**	0.0899	0.0021	0.1120	0.0026
**15-19**	0.0867	0.0020	0.1080	0.0025
**20-24**	0.0836	0.0020	0.1042	0.0024
**25-29**	0.0806	0.0019	0.1004	0.0024
**30-34**	0.0777	0.0018	0.0968	0.0023
**35-39**	0.0749	0.0018	0.0934	0.0022
**40-44**	0.0722	0.0017	0.0900	0.0021
**45-49**	0.0697	0.0016	0.0868	0.0020
**50-54**	0.0672	0.0016	0.0837	0.0020
**55-59**	0.0648	0.0015	0.0807	0.0019
**60-64**	0.0624	0.0015	0.0778	0.0018

**65-69**	0.0602	0.0014	0.0750	0.0018
**70-74**	0.0581	0.0014	0.0723	0.0017
**75-79**	0.0560	0.0013	0.0697	0.0016
**80-84**	0.0540	0.0013	0.0672	0.0016
**85-89**	0.0520	0.0012	0.0648	0.0015
**90-94**	0.0502	0.0012	0.0625	0.0015
**95-99**	0.0484	0.0011	0.0603	0.0014

We multiplied the prevalence of yearly sexual violence by $\lambda_{age,sex}$ to get the prevalence of "acute anxiety and/or reaction to stress" due exclusively to sexual violence. To estimate YLDs for this sexual violence sequela, we used the average of the disability weights for mild depression and anxiety. For simplicity, we assume a duration of one year; thus, the YLDs for the mental and psychological stress component of sexual violence is the product of the residual probability of "acute anxiety and/or reaction to stress" and the disability weight.

Due to data limitations, we are currently unable to capture the long-term disability from sexual violence. However, in future GBD iterations, we plan to address this issue.

References

1. United Nations Office on Drugs and Crime (UNODC). United Nations Office on Drugs and Crime Global Study on Homicide. Vienna, Austria: United Nations Office on Drugs and Crime (UNODC).

Anaemia Impairment (Envelope and causal attribution)

Flowchart

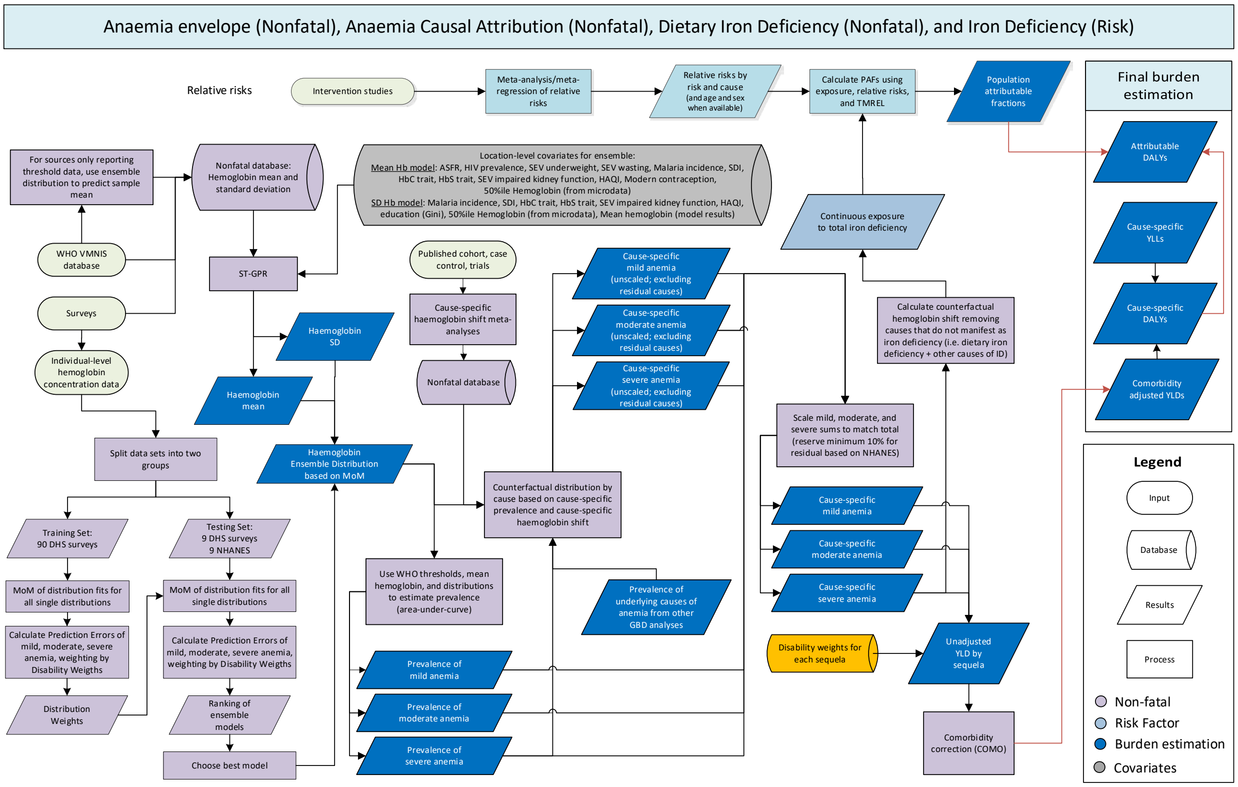

Anaemia envelope: Input data and methodological summary

Case definition

Anaemia is defined as decreased blood concentration of haemoglobin, irrespective of underlying cause, red blood cell morphology, or red blood cell function. Thresholds for defining individuals as being anaemic, as well as thresholds for anaemia severity, are based on WHO thresholds for haemoglobin in g/L.[1] In GBD 2019, we again used a different threshold for the neonatal period than for the rest of the age group <5 years. There are not any international guidelines on appropriate thresholds for diagnosing anaemia in neonates. The thresholds chosen were therefore a blend of those recommended by the WHO for 6 to 59 months and the higher haemoglobin levels typically seen in newborns.

Table 1: Definitions of mild, moderate, and severe anaemia based on blood haemoglobin concentration

Sex	Age	Mild	Moderate	Severe
Both	<28 days	130 - 149 g/L	90 - 129 g/L	< 90 g/L
Both	1 month - 4 years	100 - 109 g/L	70 - 99 g/L	< 70 g/L
Both	5 - 14 years	110 - 114 g/L	80 - 109 g/L	< 80 g/L
Male	15+ years	110 – 129 g/L	80 - 109 g/L	< 80 g/L
Female, non-pregnant	15+ years	110 - 119 g/L	80 - 109 g/L	< 80 g/L
Female, pregnant	15+ years	100 – 109 g/L	70 - 99 g/L	< 70 g/L

Input data

Estimating total anaemia (called "the envelope") utilises data from a variety of sources. Inclusion criteria include quantitative measurement of haemoglobin in either a population-based sample or group judged to adequately represent the sex, age groups, and location of the study.

Population-based surveys including the Demographic and Health Survey (DHS), Multiple Indicator Cluster Survey (MICS) series, national micronutrient surveys, and other national and subnational nutrition surveys comprised the bulk of input data. We supplemented with pertinent sources collated in the WHO Vitamin and Mineral Nutrition Information System (VMNIS) available at http://www.who.int/vmnis/database/anaemia/countries/en/.

All sources with individual-level data were extracted into GBD age groups by sex in seven different formats: mean haemoglobin concentration ([Hb]), standard deviation (SD) of [Hb], severe anaemia prevalence, moderate anaemia prevalence, mod+sev anaemia prevalence, mild anaemia prevalence, and total anaemia prevalence. Pregnancy status was invoked to determine the anaemia prevalence category of an individual, but no adjustment was made to the observed haemoglobin value. Corresponding information on mean and SD [Hb] was extracted from VMNIS and literature sources whenever available. Sources without mean and SD [Hb] were excluded from modeling, but their prevalence data were extracted for purposes of comparing predictive accuracy of model results.

Table 2: Input Data – Anaemia envelope

Measure	Total sources	Countries with data
All measures	703	153
Prevalence	20	37
Continuous	683	153

Data processing

Method of blood sampling and method of testing

Most surveys used a HemoCue test, adjusted for altitude, and excluded those with terminal or acute medical conditions. Published scientific literature studies and those from higher income locations typically measured haemoglobin with a Coulter counter. Both of these methods operate by reacting haemoglobin with a specific reagent (Drabkin's solution) and measuring absorbance wavelengths and were treated as equivalent for this analysis. We also did not make any formal distinction on the data processing side between studies that drew whole blood from participants and those that completed capillary venous sampling. Further investigation is needed to determine if formal data adjustment for HemoCue and capillary venous sampling is needed or if their additional variability, which leads to higher uncertainty in input data sources (and intrinsically lower influence on the model) is sufficient.

Altitude adjustment and smoking adjustment

Haemoglobin concentration increases with increasing elevation, a physiologic response to lower ambient oxygen levels that aims to maintain oxygen delivery throughout the body. Under 1000 meters, there appears to be little effect on [Hb], but previous studies have suggested an exponentially-increasing effect of elevation as illustrated by the following, which is the WHO recommended formula for haemoglobin adjustment:[1]

Figure 1: Haemoglobin adjustment for altitude. Equation: Hb = –0.32 × (altitude in meters × .0033) + 0.22 × (altitude in meters × .0033)^2

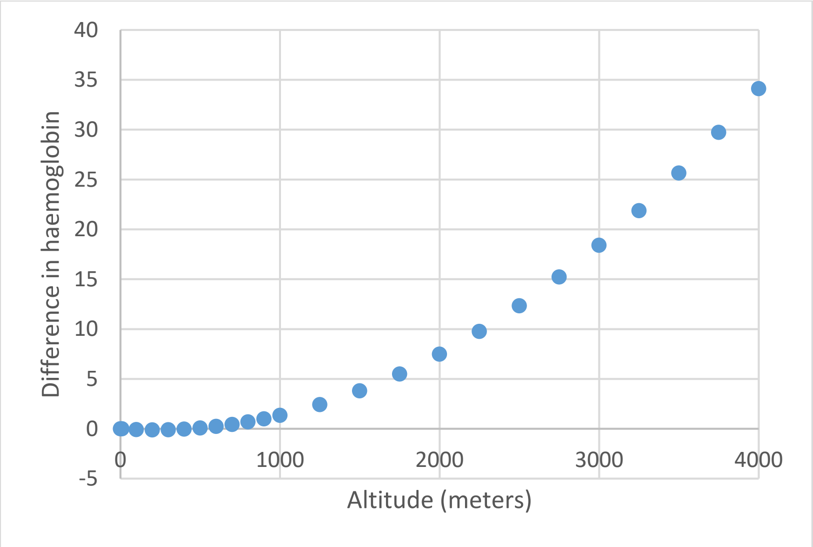

All survey- reported altitude-adjusted haemoglobin data were used directly without further adjustment. Individual level data that did not present altitude-adjusted haemoglobin values, but did present altitude were adjusted using the equation above. Testing alternative approaches for altitude adjustment is an area for further investigation. No adjustment was made for smoking in the GBD analysis.

Age-sex splitting and crosswalking data from pregnant

We divided up any datum that did not entirely fit within a GBD age-sex group to be based on the age and sex pattern observed age- and sex-specific data. This algorithm will be updated in each GBD cycle.

Our approach to [Hb] data from pregnant women depended on the source. Population-based surveys that sampled pregnant women were processed as described above – no adjustment to [Hb] and anaemia prevalence assignment using pregnancy-specific thresholds. Our assumption in these cases was that pregnancy rates in the survey population were representative of the adult female population of the survey. In contrast, studies that *only* included pregnant women were crosswalked to the general population. In GBD 2017, this was completed by mixed effect linear regression between pregnant as compared to general population. For GBD 2019, in concert with other GBD analyses, we changed to using Meta-Regression - Bayesian, Regularized, Trimmed (MR-BRT), a meta-analytic tool developed for GBD 2019. This was completed by first matching observations of pregnant and non-pregnant women by age group and location within studies. The ratios of all matched pairs were log-transformed and standard errors of ratios calculated using the delta method. We then meta-anlaysed the ratios in MR-BRT, trimming 10% of the data. Although age was tested as a potential predictor in the model, we did not observe a significant age dependence of the ratio of [Hb] in pregnant women compared to the general population. The crosswalk effects are illustrated below.

Table 3: MR-BRT crosswalk values

Data input	Reference or alternative case definition	Gamma	Beta Coefficient, Log (95% CI)	Adjustment factor*
Non-Pregnant Women (Hb)	Ref	0.033	---	---
Pregnant Women (Hb)	Alt		-0.08 (-0.15 – -0.02)	0.92 (0.86 – 0.98)

**Adjustment factor is the transformed Beta coefficient in normal space, and can be interpreted as the factor by which the alternative case definition is adjusted to reflect what it would have been if measured as the reference. The adjusted value is calculated as the alternative case definition value divided by this adjustment factor.*

Modelling strategy

Estimation of overall anaemia prevalence occurred in four steps: 1) ST-GPR models of mean and SD of haemoglobin concentration, 2) Calculation of ensemble weights, 3) Generation of ensemble distributions, and 4) Finding the area under the curve to calculate anaemia prevalence.

ST-GPR models of mean and SD of haemoglobin concentration

We completed two ST-GPR models – one for mean haemoglobin and one for standard deviation of haemoglobin. ST-GPR is run in three steps. First, a mixed effects regression with fixed effects on location-level covariates and nested random effects produces a stage 1 prediction. This prediction is revised in the spatiotemporal smoothing step based on data that is differentially weighted based on proximity in time and space. The third step uses Gaussian process regression to smooth residuals between stage 2 predictions and data. The primary change in GBD 2019 for these models was to invoke an ensemble Stage 1 prediction. This included the following covariates for the model of mean haemoglobin, all of which were age- and sex-specific unless otherwise specified: Age-specific Fertility Rate, HIV Prevalence, SEV for Child underweight, SEV for Child wasting, Malaria Incidence, Haemoglobin C (sickle type C) trait (all ages), Haemoglobin S (sickle type S) trait (all ages), Sociodemographic Index, SEV for Impaired kidney function, Healthcare Access and Quality index, Modern contraception prevalence, and 50th percentile of haemoglobin (pooled across all microdata sources). For the SD model, the following covariates were used: Malaria Incidence, Haemoglobin C (sickle type C) trait, Haemoglobin S (sickle type S) trait, Sociodemographic Index, SEV for Impaired kidney function, Healthcare Access and Quality index, Education Relative Inequality (Gini), 50th percentile of haemoglobin (pooled across all microdata sources), and mean haemoglobin (results from mean [Hb] ST-GPR model). The only other change for GBD 2019 was reduction of age-smoothing parameters in ST-GPR that was leading models to generate implausible estimates for very young ages and adult males in locations where only a subset of age groups had data.

Ensemble distribution modeling

We modelled the full distribution of haemoglobin for each population (location/age/year/sex), from which we applied the WHO thresholds to calculate prevalence of each severity of anaemia. In GBD 2015, a Weibull distribution was fit using shape and scale parameters estimated from mean haemoglobin. For GBD 2019, as with GBD 2016 and GBD 2017, we combine multiple two-parameter distributions to create a more precise and unbiased ensemble distribution.

Generation of ensemble weights

First, we created a training and testing set of individual-level haemoglobin measurements. The training set consisted of 90 DHS surveys, providing 290 group-specific samples of microdata from children <5, males 15-45, pregnant females 15-45, and non-pregnant females 15-45 (not all groups were sampled in

each DHS). A set of two-parameter distributions (gamma, mirror gamma, Weibull, mirror lognormal, and mirror gumbel) were fit to the sample's haemoglobin mean and variance. These distributions were combined using weights optimised by a loss function of severity-specific prediction error weighted by the ratio of the severity's disability weight (DW) to mild anaemia DW. Weights were constrained to be positive and sum to 1, so that the resultant ensemble distribution is a proper probability density function. All permutations of the five distributions were tested (ie, we optimised weights for both a mix of all five distributions as well as a gamma-Weibull two-way combination).

The loss function is

$$\sum_{i=1}^{n_i}\sum_{j=1}^{n_j}\sum_{k=1}^{n_k} r_j|p_{ijk} - \hat{p}_{ijk}|$$

Where

$$\hat{p}_{ijk} = \sum_{z=1}^{n_z} w_z \int_{t1_{jk}}^{t2_{jk}} PDF_{ijz}$$

n_i is a list of surveys (in either the training or testing set)

n_j is the list of groups: children <5, males 15-45, pregnant females 15-45, non-pregnant females 15-45, males >45, and females >45

n_k is the list of severities (mild, moderate, severe)

n_z is the list of distributions (gamma, mirror gamma, Weibull, mirror lognormal, and mirror gumbel)

r is the ratio of the severity j disability weight to that of mild anaemia

r_k = 13 for moderate and r_k = 40 for severe

PDF is a probability density function fit to the sample mean and variance

t1 and t2 are the lower and upper bounds to the WHO anaemia definition for the group

w is the set of distribution weights (each constrained to be positive) such that

$\sum_{z=1}^{n_z} w_z = 1$ and all $w_z > 0$

Therefore $\sum_{z=1}^{n_z} w_z * PDF_z$ describes the ensemble probability density function that can be integrated to calculate prevalence for any severity

The testing set consisted of nine NHANES and nine DHS surveys not included in the training data. Inclusion of NHANES as half the testing set ensured out of sample predictive validity by challenging the global weights, as it provided the ensemble distribution with high-income data (DHS is from LMIC countries) and data from adults >45 (DHS did not take blood tests from the elderly). We selected the combination of distributions (including all individual component distributions) that minimised the loss function.

Fitting ensemble distibutions with method of moments

With a set of component distributions and global weights, we then modelled the distribution of haemoglobin in each location/year/age/sex by fitting each component distribution using modelled mean and standard deviation, then weighting to create the ensemble distribution $\sum_{z=1}^{n_z} w_z * PDF_z$. We integrated area under the curve for each group-specific WHO threshold to calculate prevalence of anaemia by severity.

Because anaemia thresholds depend on pregnancy, we separately modelled the distribution of pregnant and non-pregnant females. The method for fitting the ensemble distribution to pregnant women was identical to that of non-pregnant but used the mean and variance from the two DisMod models adjusted by the estimated beta on the pregnancy status fixed effect. The prevalence of anaemia in pregnant and non-pregnant women were weighted by the pregnancy rate and combined to estimate population prevalence of anaemia. The pregnancy rate for each age is estimated as

$$pregnancy = \ (ASFR + SB) * 46/52$$

Where *ASFR* is the location- and age-specific fertility rate and *SB* is the location-specific stillbirth rate .

Finding the area under the curve to calculate anaemia prevalence

Using the WHO anaemia thresholds shown in Table 1, we calculated the prevalence of mild, moderate, and severe anaemia for each age group, sex, location, and year.

Anaemia causal attribution: Input data and methodological summary

Causes and Inputs

Anaemia can arise as a result of many different diseases. Each round of GBD, as evidence is identified and synthesised, additional GBD causes are added to the GBD Anaemia Causal Attribution analysis. Additional changes for specific causes are incorporated to either improve efficiency or reflect changes to modeling approaches for underlying causes. For GBD 2019, the only cause changes were for chronic kidney disease (CKD). End-stage renal disease was added as a cause of anaemia. We also revised CKD inputs to three – one each for for Stage III, IV, and V CKD – instead of fifteen aetiology-stage combinations.

For each cause included in GBD Anaemia Causal Attribution, two inputs were required. The first required input is cause-level prevalence generated from other cause-specific GBD estimates. For estimation details of each cause, see the corresponding section of this methods appendix corresponding to that cause. The second required input is a cause-specific haemoglobin shift. These shifts were derived from published cohort studies, case control studies, or treatment trials (depending on the cause). Data on haemoglobin concentration for diseased versus non-diseased persons in each of the studies were meta-analysed. A majority of the haemolgobin shifts have not changed since GBD 2010 and are described in detail elsewhere.[2] Based on pooling the results of nine iron fortification trials, the hemoglobin shift for iron deficiency was estimate to be 4.01 g/L. There were a few causes shown in Table 3 below that were granted exceptions to the above requirements of needing cause-level prevalence and haemoglobin shift. Exceptions were made because these cause groups – mostly "other" causes in the GBD cause hierarchy – are known or suspected to lead to anaemia. Anaemia burden for each of these causes was assigned from the residual anaemia envelope in a manner analogous to fixed proportion redistribution used in the GBD cause-specific mortality analysis. Our goal is to systematically add all causes of anaemia as specific inputs to GBD Anaemia Causal Attribution and eliminate the need for residual attribution.

Table 4: Causes included in GBD Anaemia Causal Attribution

**Causes with prevalence and haemoglobin shift inputs**
P. falciparum parasitaemia without clinical malaria
P. vivax parasitaemia without clinical malaria
Clinical malaria
Schistosomiasis
Hookworm disease
Other neglected tropical diseases
Maternal haemorrhage
Vitamin A deficiency (under 15 years only)
Other infectious diseases
Peptic ulcer disease
Gastritis
Stage III chronic kidney disease
Stage IV chronic kidney disease
Stage V chronic kidney disease
End stage renal disease
Uterine fibroids
Menstrual disorders
Other haemoglobinopathies and haemolytic anaemias
Other endocrine, nutrition, blood, and immune disorders
G6PD deficiency
Hemizygous G6PD deficiency
Beta-thalassaemia major
Beta-thalassaemia trait
Haemoglobin E trait
Haemoglobin E/beta-thalassaemia
Haemoglobin H disease
Homozygous sickle cell and severe sickle cell/beta-thalassaemia parent
Haemoglobin SC disease
Mild sickle cell/beta-thalassaemia
Sickle cell trait
HIV
Cirrhosis and other chronic liver diseases, decompensated
Ulcerative colitis
Crohn's disease
**Estimated via Fixed Proportion Redistribution Methods* in GBD Anaemia Causal Attribution**
Dietary iron-deficiency
Other infectious diseases
Other neglected tropical diseases
Other endocrine, nutrition, blood, and immune disorders
Other haemoglobinopathies and haemolytic anaemias

* A minimum of 10% of all anaemia was assigned to residual categories based on analysis of NHANES-III data from the United States

Modelling Strategy

Our approach for anaemia causal attribution changed substantially for GBD 2019. Since GBD 2013, we have been using Bayesian contingency table (BCT) modeling, an approach that combined cause-level prevalence and cause-specific haemoglobin shifts with a set of expert priors on the relative severity of different causes of anaemia and their comparative dispersion between mild, moderate, and severe anaemia. In addition to being a computationally expensive algorithm that struggles to scale with increasing geographic and causal detail of annual GBD estimation, BCT modeling was unable to take advantage of the information content of ensemble haemoglobin distribution results.

Starting in GBD 2019, we used the previously modelled full distribution of haemoglobin for each population (location, age, year, and sex), along with WHO thresholds and aetiology prevalence, to calculate the counterfactual prevalence of each anaemia severity for all aetiology-severity pairs, shifting population mean haemoglobin by each aetiology-specific haemoglobin shift times prevalence, but retaining the original estimate of SD. For each anaemia severity, it should hold true that the sum of the difference between the counterfactual and observed prevalence across all contributing causes is equal to the total anaemia prevalence at the same severity.

For all causes with specific population-specific prevalence estimates, we enforced a similar condition where the sum of mild, moderate, and severe anaemia would not exceed the total prevalence within each population. We thus scaled the results to ensure the sum across anaemia severity matched total aetiology prevalence for each cause. As noted in Table 3, we assigned a minimum of 10% of all anaemia to be assigned to residual causes based on review of findings from National Health and Nutrition Examination Survey (NHANES) in the United States.[3,4] The residual envelope was distributed among the remaining five aetiologies.

It is important to take note of the difference between “dietary iron deficiency” as a GBD cause and “iron deficiency” as a GBD risk. Many GBD causes lead to anaemia that clinically manifests as iron deficiency (or microcytosis), but where inadequate intake is not the underlying problem. Examples include neglected tropical diseases such as hookworm, malaria, and schistosomiasis, gastrointestinal disorders, cirrhosis, maternal haemorrhage, menstrual disorders, uterine fibroids, and Vitamin A deficiency. The name “dietary iron deficiency” is intended to differentiate, therefore, between inadequate intake and haemorrhagic or disorders of iron metabolism. Additionally, because we have yet to include 100% of anaemia causes, estimates should be interpreted to also include some acute and chronic haemorrhagic states for which supplementation may be helpful, but poor nutritional intake is not the only underlying problem. Examples include malabsorption syndromes, other micronutrient deficiencies besides Vitamin A deficiency, and injuries with associated acute blood loss anaemia. “Iron deficiency” exposure as estimated for the GBD risk factors analysis, in contrast, includes a combined assessment of the magnitude of haemotologic insult from all causes that manifest as iron deficiency. As mentioned above, our goal is to systematically add all causes of anaemia as specific inputs to GBD Anaemia Causal Attribution, including inadequate iron intake, and eliminate the need for residual attribution.

The anaemia causal attribution process produces estimates for mild, moderate, and severe anaemia due to HIV. Using these estimates, we calculated proportions of HIV with mild, moderate, severe, and no anaemia for each demographic group. GBD produces estimates for seven HIV sub-causes: early HIV, symptomatic HIV, AIDS with antiretroviral treatment, AIDS without antiretroviral treatment, drug--sensitive HIV/AID--tuberculosis, multidrug-resistant HIV/AIDS--tuberculosis, and extensively drug-

resistant HIV/AIDS--tuberculosis. We assumed the anaemia severity proportions were equivalent across the seven sub-causes and estimated the anaemia severity levels for each by multiplying the HIV sub-causes by the anaemia proportions.

References

1 WHO | Haemoglobin concentrations for the diagnosis of anaemia and assessment of severity. Geneva: World Health Organization, 2011 http://www.who.int/vmnis/indicators/haemoglobin. pdf.

2 Kassebaum NJ, Jasrasaria R, Naghavi M, *et al.* A systematic analysis of global anaemia burden from 1990 to 2010. *Blood* 2014; **123**: 615–24.

3 Centers for Disease Control and Prevention (CDC). Iron deficiency--United States, 1999-2000. *MMWR Morb Mortal Wkly Rep* 2002; **51**: 897–9.

4 Looker AC, Dallman PR, Carroll MD, Gunter EW, Johnson CL. PRevalence of iron deficiency in the united states. *JAMA* 1997; **277**: 973–6.

Epilepsy impairment envelope

Flowchart

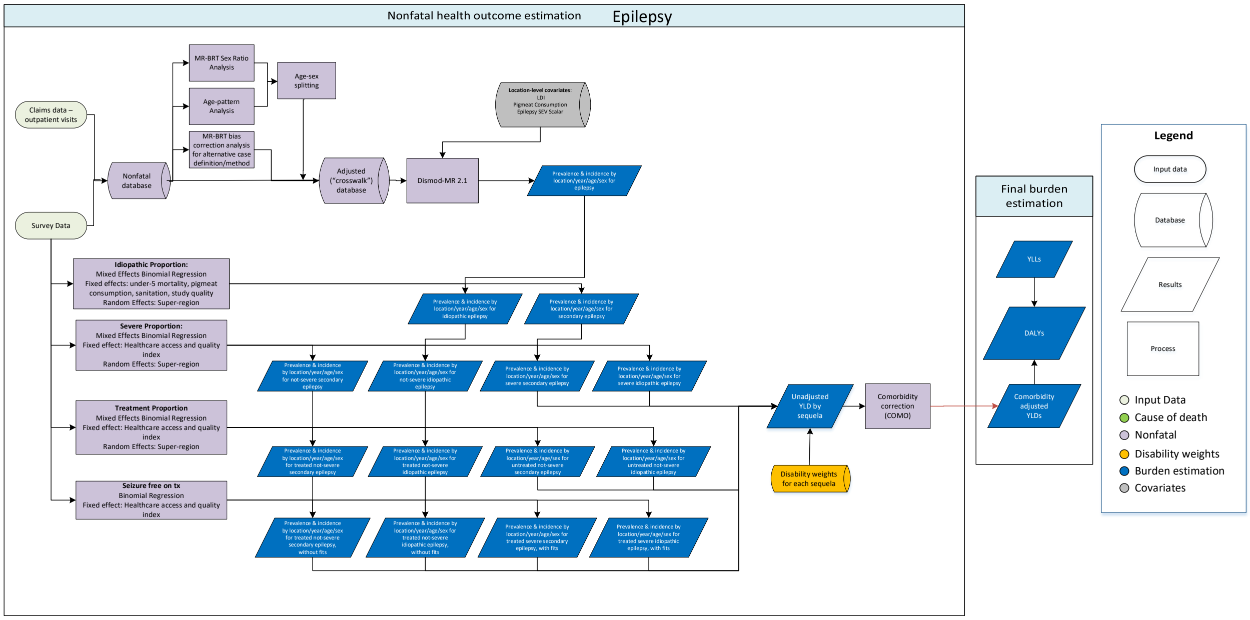

Case definition

Since GBD 2013, we have used the following definitions from the "Guidelines for Epidemiologic Studies on Epilepsy": 1) Epilepsy: a condition characterised by recurrent (two or more) epileptic seizures, unprovoked by any immediate identified cause, and 2) "Active" epilepsy: a prevalent case of active epilepsy is defined as a person with epilepsy who has had at least one epileptic seizure in the previous five years, regardless of antiepileptic drug (AED) treatment. We also use the following ICD-10 codes for epilepsy: G40 (Neuro, epilepsy, total) and G41 (Neuro, epilepsy, status epilepticus). We define severe epilepsy as having seizures one or more times per month.

Input data and processing

Data inputs

The primary data inputs for the epilepsy modeling strategy were measurements of prevalence, incidence, remission rate, excess mortality rate, relative risk of mortality, standardised mortality ratio, or with-condition mortality rate for all epilepsy, regardless of cause, severity or treatment status.

For GBD 2016, we conducted a systematic review covering 10/1/2014 to 10/7/2016 using the following search string:

("2014/10/01"[PDAT] : "2016"[PDAT]) AND ("epilepsy"[MeSH Terms] OR "epilepsy, partial, motor"[MeSH Terms] OR "epilepsy, benign neonatal"[MeSH Terms] OR "epilepsy, reflex"[MeSH Terms] OR "myoclonic epilepsy, juvenile"[MeSH Terms] OR "epilepsy, frontal lobe"[MeSH Terms] OR "epilepsy, complex partial"[MeSH Terms] OR "epilepsy, post-traumatic"[MeSH Terms] OR "epilepsy, temporal lobe"[MeSH

Terms] OR "epilepsy, absence"[MeSH Terms] OR "epilepsy, tonic-clonic"[MeSH Terms] OR "epilepsies, myoclonic"[MeSH Terms] OR "epilepsies, partial"[MeSH Terms] OR epilepsy[Title/Abstract]) AND (incidence[Title/Abstract] OR prevalence[Title/Abstract]) NOT(animals[MeSH] NOT humans[MeSH]).

We included representative, population-based surveys that reported on prevalence, incidence, remission rate, excess mortality rate, relative risk of mortality, standardised mortality ratio, or with-condition mortality rate. We excluded studies with no clearly defined sample (eg, among clinic attenders or patient organisation members with non-specific or non-representative catchment area).

Like GBD 2017, the model included data from hospital discharges and claims. In GBD 2019, we newly added Poland claims data and additional years of data from USA claims (years 2015-2016) and hospital discharges in Mexico, India, New Zealand, Sweden, Georgia, and Ecuador. Notably, we included hospital data from Botswana; southern sub-Saharan Africa previously did not have data.

Additional data inputs include data on the proportion of epilepsy that is primary or idiopathic, the proportion of epilepsy that is severe (one or more fits per month), the proportion of epilepsy that is untreated (the treatment gap), and the proportion of treated epilepsy that is treated without fits (no fits reported in the preceding year). For the proportion of epilepsy that is idiopathic, we have 89 unique sources covering 18 of 21 world regions. For the proportion of epilepsy that is severe, we have 29 unique sources covering 12 unique regions. For the proportion of treated epilepsy that has no fits we have ten unique sources covering six regions. Finally, for the proportion of epilepsy that is treated we have 68 unique studies covering 16 unique regions.

The number of sources used for all epilepsy, and for idiopathic epilepsy specifically, are listed below:

Epilepsy impairment:

Measure	Total sources	Countries with data
All measures	781	106
Prevalence	360	88
Incidence	410	59
Remission	6	6
Excess mortality rate	19	14
Standardized mortality ratio	5	3
With-condition mortality rate	2	2
Proportion	155	55

Idiopathic epilepsy:

Measure	Total sources	Countries with data
All measures	781	106
Prevalence	360	88
Incidence	410	59
Remission	6	6
Excess mortality rate	19	14

Standardized mortality ratio	5	3
With-condition mortality rate	2	2
Proportion	155	55

Data processing

For GBD 2019, we started with the final age split dataset used in GBD 2017 - raw data with large age ranges were split into 5 year age bins using the age pattern generated from a Dismod model with input data of only less than 25 years age range. Standard GBD sex splitting methods were used for studies with only "both" sex data points. We modeled the ratio of female/male prevalence in MR-BRT and calculated male prevalence:

$$prev_{male} = prev_{both} \ * \ \frac{pop_{both}}{(pop_{male} + ratio * pop_{female})}$$

And then calculated female prevalence:

$$prev_{female} = ratio \ * \ prev_{male}$$

For epilepsy, the modeled female/male ratio demonstrated a higher prevalence in males, and was used to proportionally split "both" sex data points into male and female data points (as seen in the figure below).

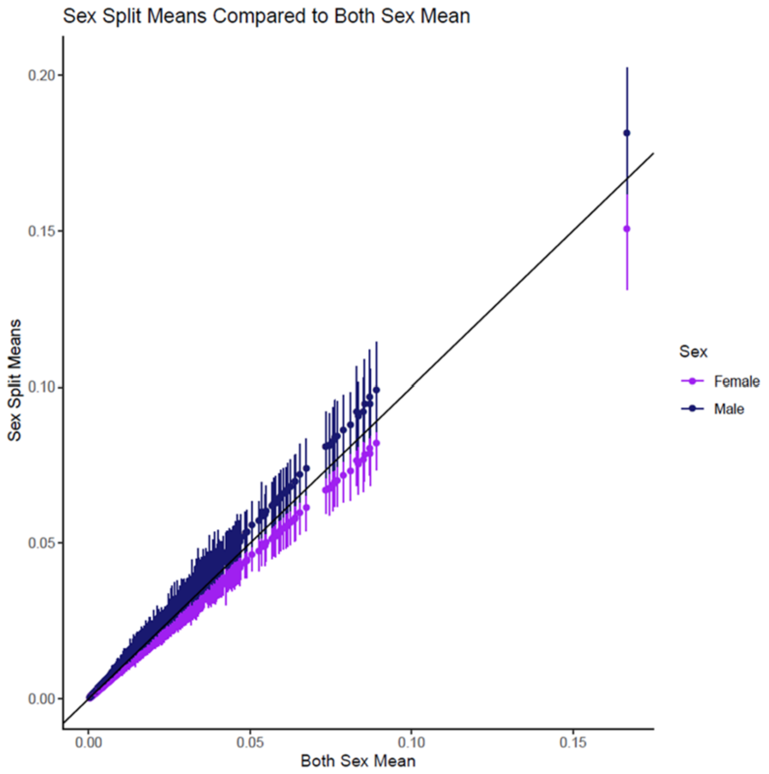

For GBD 2019, adjustment factors for all study-level covariates were determined using matched data (by year, age, sex, location) for reference and alternative case definitions in a log ratio network meta-regression. Studies that asked for lifetime recall, and U.S. Marketscan claims data were crosswalked to the reference definition for epilepsy.

The table below shows adjustment factors estimated using MR-BRT.

MR-BRT Crosswalk Adjustment Factors for Epilepsy Impairment Envelope

Data input	Reference or alternative case definition	Gamma	Beta Coefficient, Log (95% CI)	Adjustment factor*
	Ref	N/A	N/A	N/A
Marketscan	Alt	0.26	-0.86 (-1.40 to -0.33)	0.42
Marketscan 2000	Alt	0.37	-1.15 (-1.90 to -0.40)	0.32
Recall lifetime	Alt	0.25	0.20 (-0.29 to 0.70)	1.22

Adjustment factor is the transformed Beta coefficient in normal space, and can be interpreted as the factor by which the alternative case definition is adjusted to reflect what it would have been if measured as the reference. Note that all of these crosswalks were run separately as opposed to in a network analysis, but all were adjusted to the same reference definition.

Modelling strategy

We modelled the prevalence of epilepsy in two steps: first, we created an epilepsy impairment envelope. Second, we split the envelope into primary (or idiopathic) and secondary epilepsies. Each of these were subdivided into "severe" (on average one or more fits per month) and "non-severe." Non-severe cases were subdivided into "treated" and "un-treated." Finally, "treated" cases were divided into "treated cases with fits" (between one and 11 fits on average in the preceding year) and "treated cases without fits" (no fits reported in the preceding year).

In the first step, we used DisMod-MR 2.1 for the epilepsy impairment envelope to model a consistent fit between incidence, prevalence, remission, and standardised mortality ratio data.

We also included the SEV epilepsy scalar, which summarises the epilepsy risk exposure level for each country, as a predictive covariate on prevalence. We included cause-specific mortality rate (CSMR) results from the epilepsy mortality model as input data to the DisMod model. Where age-specific prevalence data were available, we calculated excess mortality rate (EMR) from prevalence and CSMR. We included the log of the lag-distributed income (LDI) as a covariate on EMR to account for lower mortality in developed countries. We included Bayesian priors on remission to account for the scarcity of remission data. We set bounds on remission from 0 to 0.25 from age 0-60 and 0 to 0.05 from age 61-100. The table below indicates the covariates used in the estimation process, as well as parameters, betas, and exponentiated betas.

Covariates. Summary of covariates used in the epilepsy impairment envelope DisMod-MR meta-regression model

Covariate	Type	Parameter	Exponentiated beta (95% Uncertainty Interval)
Log-transformed age-standardized SEV scalar: Idiopathic epilepsy	Prevalence	0.76	2.14 (2.12-2.18)
LDI ($ per capita)	Excess mortality rate	-0.55	0.58 (0.38-0.87)

In the second step, we used mixed-effects generalised linear models (binomial family) run in GBD 2017 to predict the proportion of idiopathic epilepsy, the proportion of severe epilepsy, the proportion of treated epilepsy and the proportion of epilepsy that is treated without fits.

Because not all of the data on the proportion of idiopathic epilepsy use optimal case finding methods (using CT scans or MRIs in addition to EEGs in order to diagnose secondary epilepsy), we first run an initial linear regression model with a covariate on study quality. We then use the beta from this model to crosswalk studies with non-optimal case finding methods to those with adequate methods. The adjusted data are then used in the regression for the proportion of epilepsy that is idiopathic, with a fixed effect on SDI as well as a random effect on super-region.

We used similar models to predict the proportion of severe epilepsy and treatment gap based on the reported proportions extracted from the systematic review. To predict the proportion of severe epilepsy and the treatment gap, we used mixed-effects models with a fixed effect on the log of HAQ Index and a random effect on super-region.

For the regression to determine the proportion of treated epilepsy cases that have not had a fit in the last year, there is a much smaller dataset, and therefore we cannot use a random effect in the model. Therefore, we use generalised linear model (binomial family) to generate predictions for the proportion of treated epilepsy that is seizure-free with a fixed effect on the log of HAQ Index.

We tested a fixed effect on epilepsy cause-specific mortality, under-5 mortality rate, sanitation, and pig meat consumption as well as random effects on region and country in different models, but they did not improve the models. We generated 1,000 draws of country-specific estimates for each year between 1980 and 2017 for each of the models. The table below shows the betas from these regressions.

Regression	covariate	beta	SE
Idiopathic	Study quality	0.75	0.59
Idiopathic	SDI	1.39	1.12
Severe	HAQ Index	-1.23	1.05
Treatment gap	HAQ Index	-3.54	1.37
Treated without fits	HAQ Index	2.49	1.87

Severity splits & disability weights

The table below illustrates the severity levels, descriptions, and disability weights associated with epilepsy. These are calculated using regressions from literature (ie, frequency of seizures).

Severity level	Lay description	Disability weights (95% CI)
severe (seizures >= once per month)	This person has sudden seizures one or more times each month, with violent muscle contractions and stiffness, loss of consciousness, and loss of urine or bowel control. Between seizures the person has memory loss and difficulty concentrating.	0.552 (0.375–0.71)
less severe (seizures < once per month)	This person has sudden seizures two to five times a year, with violent muscle contractions and stiffness, loss of consciousness, and loss of urine or bowel control.	0.263 (0.173–0.367)
Treated without fits	This person has a chronic disease that requires medication every day and causes some worry but minimal interference with daily activities.	0.049 (0.031–0.072)

Guillain-Barré syndrome (GBS) impairment

Flowchart

Guillain–Barré syndrome (GBS)

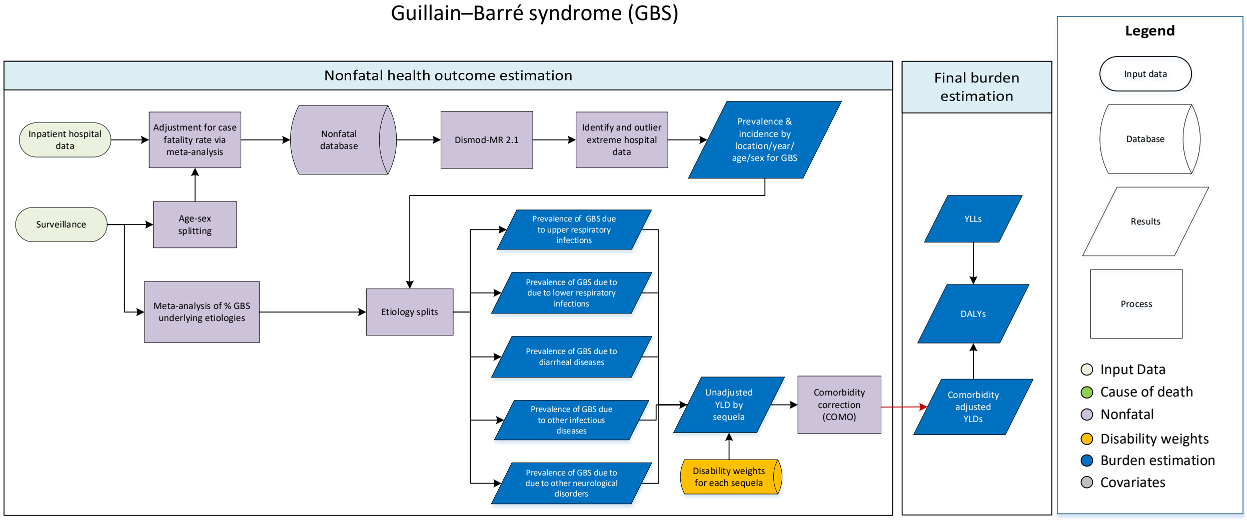

Case definition

Guillain-Barré syndrome is a rare condition that usually occurs as a complication of respiratory or gastrointestinal infection. It is considered an immune-mediated nerve dysfunction with rapid onset of weakness in the feet and legs, and sometimes the arms, which then progresses toward the trunk. In the acute phase, about a quarter of cases required mechanical ventilation for survival. The majority of cases fully recover within months to a year. The following ICD codes are used G61.0 (GBS) and 357.0 (Acute infective polyneuritis). Literature studies are accepted if there is a doctor diagnosed GBS, or other record of GBS.

Input data

Morbidity model inputs

An updated systematic review was done for GBD 2017 from January 2008 to September 2017 using the search string ((((((("guillain barre syndrome"[MeSH Terms] OR ("guillain"[Title/Abstract] AND ("barre"[Title/Abstract] OR "barre"[Title/Abstract]) AND Title/Abstract[All Fields] AND "syndrome"[Title/Abstract])) OR "guillain-barre syndrome"[Title/Abstract]) OR "guillain-barre syndrome"[Title/Abstract]) OR "polyradiculoneuropathy"[Title/Abstract]) OR "Guillain-Barre syndrome"[Title/Abstract]) AND ("prevalence"[Title/Abstract] OR "incidence"[Title/Abstract] OR "epidemiology"[Title/Abstract] OR "remission"[Title/Abstract]))) AND ("2008/01/01"[Date - Publication] : "2017/09/26"[Date - Publication]). This search yielded 436 hits with 25 sources marked for extraction. A flowchart documenting this review is displayed below.

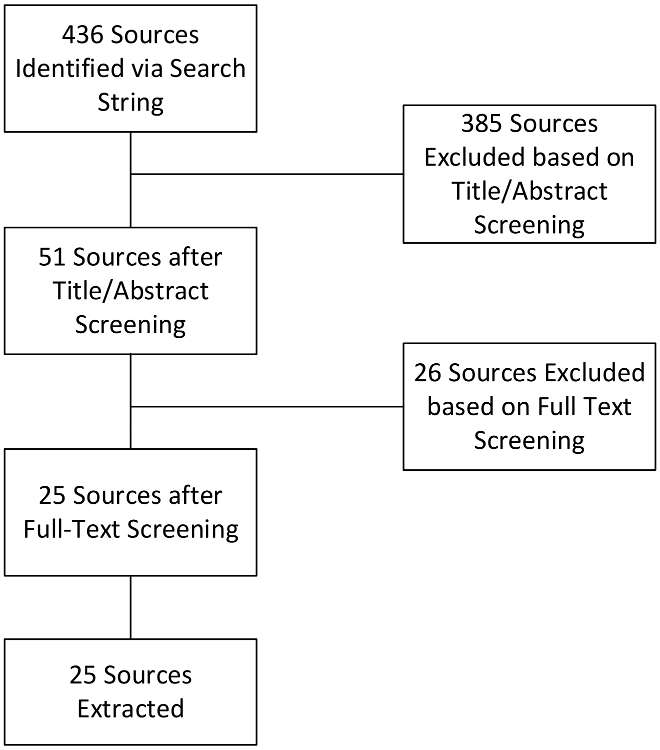

An additional informal search was undertaken for more information on remission and duration of GBS. We extracted remission data from four studies.

Inpatient hospital and claims incidence data were extracted using the ICD codes listed above. Only primary diagnoses were considered. This year we added additional years of claims data from the USA (2015, 2016), and for the first time added claims data from Poland (2015, 2016, 2017).

Aetiology data inputs

Information on aetiology splits come from a systematic review and meta-analysis of the literature completed for GBD 2010. This review searched for articles providing information on the proportion of Guillain-Barré cases with any described aetiological cause, the proportion of Guillain-Barré cases attributed to influenza, the proportion of Guillain-Barré cases attributed to upper respiratory infections, the proportion of Guillain-Barré cases attributed to diarrhoeal diseases and the proportion of Guillain-Barré cases attributed to other infections. This review yielded 35 articles; a breakdown of how many articles inform each proportion contributing to the split is provided below:

Split	Number of sources
All specified aetiologies	31
Influenza	3
Upper respiratory infections	26
Diarrhoeal diseases	25
Other infectious diseases	14

Total source counts for GBS used in GBD 2019 modeling are listed in the table below:

Measure	Total sources	Countries with data
All measures	330	46
Incidence	325	44
Remission	3	3
Case fatality rate	10	8
Proportion	35	19

Data processing

Data extracted from published surveys, disease registries, surveillance studies and medical facilities were sometimes reported for both sexes or broadly defined age-groups in aggregate. In these cases, data were sex split and/or age split. Standard GBD sex splitting methods were used for studies with only “both” sex data points. We modeled the ratio of female/male prevalence in MR-BRT and calculated male prevalence:

$$prev_{male} = prev_{both} * \frac{pop_{both}}{(pop_{male} + ratio * pop_{female})}$$

And then calculated female prevalence:

$$prev_{female} = ratio * prev_{male}$$

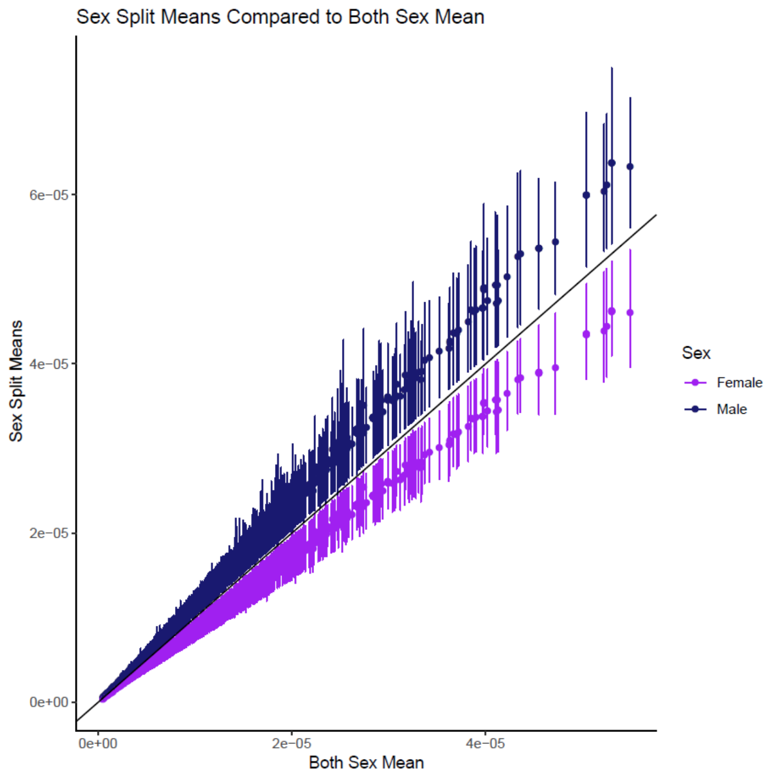

For GBS, the modeled female/male ratio demonstrated a higher prevalence in males, and was used to proportionally split "both" sex data points into male and female data points (as seen in the figure above).

For GBD 2019, raw data with large age ranges were split into 5-year age bins using regional age patterns generated from a Dismod model with only input data with less than a 25-year age range. Finally, we systematically outliered all hospital data-series (entire age span of data) where the age standardized incidence is more than two median absolute deviations away from the median age-standardized incidence across location-years.

Modelling strategy

The first step of our modeling strategy was to correct inputs for survival rate. A random effects meta-analysis calculated a 95% case fatality rate (95% CI 93–98%). A forest plot showing the results of this meta-analysis is displayed below. As mortality mainly occurs during the acute phase of the disease (usually within four weeks of onset), the pooled survival rate was used to get the incidence of the people surviving after the acute phase of the GBS.

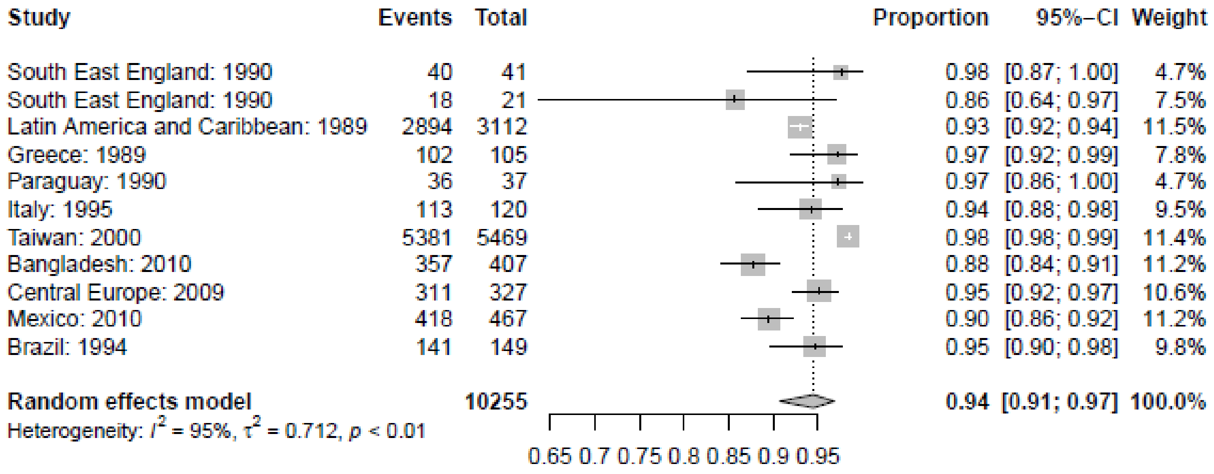

Dismod-MR 2.1 was used to estimate prevalence of Guillain-Barré syndrome for every location, year, age, and sex. We then split the overall prevalence of the impairment by underlying aetiology (upper respiratory infections, influenza, diarrhoeal diseases, other infections, and other neurological causes). We used random effects meta-analysis to pool these proportions. We squeeze the proportions for influenza, diarrhoeal diseases, upper respiratory infections, and other infectious diseases to add to the proportion for all identified infectious underlying diseases. We assigned the complement to one of the proportion with any underlying infectious disease to a rest category of "idiopathic Guillain-Barre syndrome" that is classified under neurological disorders.

First the envelope for Guillain-Barré cases due to all specified aetiologies is established by doing a meta-analysis on the proportions reported in the studies included. Then, the proportions for each of the other splits are squeezed to fit the envelope created in the all specified meta-analysis. Finally, the difference between all specified and 100% is attributed to other neurological disorders. The final results of these aetiology splits are shown below:

Aetiology	Mean	Lower	Upper
Other neurological disorders	0.382	0.331	0.669
Influenza	0.119	0.071	0.192
Upper respiratory infections	0.319	0.27	0.372
Diarrhoeal diseases	0.109	0.086	0.135
Other infectious diseases	0.071	0.054	0.093

Disability weights

The health state for paraplegia was used for all Guillain-Barré cases. It is described as "paralysed from the waist down, cannot feel or move the legs, and has difficulties with urine and bowel control. The person uses a wheelchair to move around". The disability weight is 0.296 (0.198–0.414).

Hearing impairment

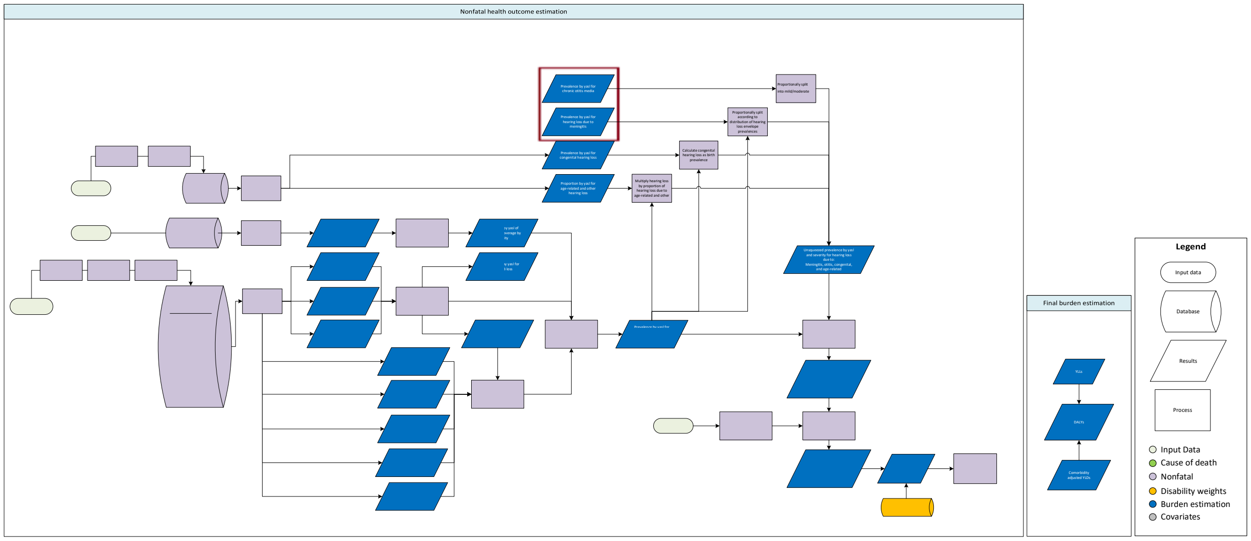

Case definition

Hearing impairment is an estimation of the prevalence of hearing loss at a range of severities, as measured by the softest sound that an individual can hear in their better ear, taken as the average across frequencies from 500 to 4000 Hertz.

Hearing Impairment is modelled for every year, age, sex, and location (y-a-s-l) in the following severity categories:

Table 1: Severity thresholds of hearing loss

Severity thresholds of interest for hearing loss	
**Severity**	**Threshold (in decibels)**
None	0–19
Mild	20–34
Moderate	35–49
Moderately severe	50–64
Severe	65–79
Profound	80–94
Complete	95+

We modelled the following causes of hearing loss: congenital, meningitis, otitis, and age-related and other. Congenital hearing loss is defined as hearing loss present at birth. Age-related and other hearing loss includes causes not identified as meningitis, otitis, or congenital. This includes presbycusis, the gradual loss of hearing with age, caused by breakdown of neurons in the inner ear. For all causes, we estimate hearing loss with and without tinnitus, the perception of noise or ringing in the ears.

Unadjusted estimates of the prevalence of hearing loss due to meningitis and chronic otitis media are produced separately as part of each underlying cause's modeling process, as described in their respective sections. Along with the congenital and age-related etiologies, these unadjusted estimates are incorporated into the overall hearing loss model, as detailed below.

Input data and processing

Studies on hearing loss typically report the prevalence of hearing loss by severity, in categories that are mutually exclusive and exhaustive. The severity grouping that an individual is put into depends on the softest decibel level that they can hear a sound. However, these severity groupings are not standardized across literature. For example, one study may report the prevalence of mild, moderate, and severe hearing loss across the range of decibels. Another study may simply report the prevalence of the study population with no hearing loss, and those that have hearing loss, regardless of range. In order to standardize severity groupings, we established 7 mutually exclusive and exhaustive categories that the GBD would use to model and report the severity of hearing loss. These are referred to as "severity specific envelopes". The range of decibel values applicable to each severity category can be seen in table 1.

For the estimation of severity-specific envelopes, we used prevalence measurements and individual-level data extracted from published surveys identified in a series of systematic reviews, or from sources provided by the GBD collaborator network.

Data sources up to 2008 were identified by a published systematic review (http://www.ncbi.nlm.nih.gov/pubmed/19444763). For GBD 2013, we conducted a systematic review covering 2008–2013 with the following search terms:

> (hearing impairment[Title/Abstract] OR deafness[Title/Abstract] OR hearing loss[Title/Abstract]) AND (prevalence[Title/Abstract]) AND ("2008"[PDAT] : "3000"[PDAT]) AND (cross sectional OR survey)

For GBD 2016, we conducted an additional systematic review using the following search terms:

(hearing impairment[Title/Abstract] OR deafness[Title/Abstract] OR hearing loss[Title/Abstract] OR audiometry[Title/Abstract]) AND (prevalence[Title/Abstract]) AND ("2008/11/26"[PDAT]: "3000"[PDAT]) AND (cross sectional OR survey)

This was conducted on November 30, 2016 and returned 239 results, of which 17 were accepted.

In addition to the search-string hits above, we identified household surveys that measured hearing loss - the United States National Health and Examination Surveys (NHANES) and the Health Survey for England (HSE) – and extracted prevalence measurements from individual-level data.

Self-reported hearing loss data were excluded. This includes censuses in the Integrated Public Use Microdata Series (IPUMS), the WHO Studies on Global Ageing and Adult Health (SAGE), and the WHO Multi-Country Survey Study on Health and Responsiveness (MCSS). Self-reported use of hearing aids (such as in MCSS, SAGE, and NHANES), however, was used to estimate hearing aid coverage.

We focused on improving methods of processing existing data in GBD 2019. An updated systematic review will be performed in a future round.

Table 2: Data inputs

Cause/ Impairment Name	Measure	Total Sources	Countries with data
Hearing Loss	All measures	208	77
	Prevalence	204	77
	Proportion	11	3
Age-related and other hearing loss	All measures	58	34
	Proportion	58	34

Where studies reported hearing loss spanning multiple thresholds (eg, 80+, rather than 80-94 and 95+) or severity categories that did not align with GBD thresholds, we crosswalked data with the MR-BRT methodology to the appropriate GBD severity categories. A description of the MR-BRT methodology can be found in its respective section.

To create adjustment factors between alternate and reference threshold categories, we used microdata extracted from NHANES surveys. This data reported the exact decibel at which each person experienced hearing loss. We estimated the prevalence of each alternate and reference severity category by aggregating microdata into groups specific to age and sex. The prevalent population for each alternate or reference category was comprised of every individual that fell within the range of decibels for a given severity. Adjustment factors were estimated as the logit difference between the prevalence of an alternate category and the prevalence of its corresponding reference category. A table of each adjustment factor can be found below.

Table 3: **MR-BRT crosswalk adjustment factors**

Reference Category (dB)	Alternate Category (dB)	Gamma	Beta Coefficient, Logit (95% CI)
0-19	0-24	0	0.60 (0.54 to 0.67)
	0-25	0	0.70 (0.64 to 0.77)
	0-29	0.23	1.13 (0.68 to 1.59)
	0-30	0.21	1.24 (0.83 to 1.68)
	0-39	0.91	1.67 (-0.04 to 3.58)
	0-40	0.96	1.71 (-0.05 to 3.53)
20-34	0-24	2.50	3.40 (-1.46 to 8.28)
	0-25	2.45	3.49 (-1.53 to 8.29)
	0-29	2.30	3.82 (-0.85 to 8.29)
	0-30	2.27	3.89 (-0.24 to 8.42)
	0-39	1.95	4.48 (0.61 to 8.55)
	0-40	1.91	4.50 (0.86 to 8.14)
	20-39	0.13	0.27 (0.02 to 0.52)
	20-40	0.15	0.29 (0.003 to 0.59)
	20-200	0.41	0.52 (-0.35 to 1.32)
	21-39	0.20	0.12 (-0.29 to 0.52)

	25-39	0.35	-0.39 (-1.04 to 0.34)
	26-40	0.43	-0.50 (-1.36 to 0.28)
	26-99	0.84	-0.03 (-1.65 to 1.73)
	26-200	0.84	-0.03 (-1.74 to 1.54)
	30-40	0.56	-1.06 (-2.24 to 0.007)
	30-200	0.96	-0.37 (-2.12 to 1.43)

35-49	0-39	2.45	5.18 (0.16 to 10.08)
	0-40	2.42	5.24 (0.41 to 10.17)
	20-39	0.71	1.45 (0.04 to 2.85)
	20-40	0.69	1.49 (0.10 to 2.88)
	21-39	0.66	1.31 (0.02 to 2.67)
	25-39	0.54	0.76 (-0.27 to 1.93)
	26-40	0.51	0.67 (-0.30 to 1.75)
	30-40	0.47	0.09 (-0.89 to 1.05)
	31-50	0.52	0.10 (0.29 to 0.74)
	40-64	0.37	-0.10 (-0.85 to 0.61)
	40-69	0.40	-0.04 (-0.82 to 0.811)
	41-55	0.32	-0.45 (-1.06 to 0.23)
	41-60	0.35	-0.29 (-0.99 to 0.37)
	41-70	0.44	-0.12 (-1.06 to 0.76)
50-64	40-64	0.27	1.13 (0.58 to 1.68)
	40-69	0.29	1.22 (0.64 to 1.80)
	41-55	0.4	0.72 (-0.09 to 1.53)
	41-60	0.31	0.92 (0.30 to 1.55)
	41-70	0.32	1.13 (0.49 to 1.77)
	51-70	0.18	0.06 (-0.31 to 0.42)
	55-69	0.29	-0.42 (-1.00 to 0.15)
	56-70	0.33	-0.43 (-1.10 to 0.24)
65-79	40-69	0.77	2.44 (0.92 to 3.99)
	51-70	0.67	1.35 (0.01 to 2.68)
	55-69	0.69	0.86 (-0.53 to 2.24)
	56-70	0.66	0.84 (-0.47 to 2.16)
	61-80	0.19	0.35 (-0.04 to 0.72)
	61-99	0.14	0.46 (0.17 to 0.75)
	65-84	0.02	0.03 (-0.01 to 0.08)
	70-89	0.21	-0.20 (-0.63 to 0.22)
	70-94	0.21	-0.20 (-0.62 to 0.24)
	70-95	0.21	-0.20 (-0.63 to 0.23)
	71-90	0.3	-0.26 (-0.86 to 0.34)
	71-99	0.3	-0.16 (-0.75 to 0.44)
	71-200	0.31	-0.19 (-0.81 to 0.42)
80-94	61-99	1.01	1.58 (-0.42 to 3.58)
	65-84	0.91	0.92 (-0.89 to 2.73)
	70-89	0.81	0.54 (-1.06 to 2.14)

	70-94	0.73	0.44 (-1.01 to 1.88)
	70-95	0.73	0.44 (-1.00 to 1.89)
	71-90	0.61	0.25 (-0.96 to 1.45)
	71-99	0.61	0.37 (-0.83 to 1.58)
	71-200	0.66	0.41 (-0.88 to 1.71)
	80-200	0	0.00 (-0.04 to 0.04)
	81-99	0	$-3.92e^{-16}$ (-0.04 to 0.03)
	81-200	0	0.00 (-0.04 to 0.04)
	85-200	0	$-4.37e^{-24}$ (-0.04 to 0.04)
	90-99	0	0.00 (-0.03 to 0.03)
	90-200	0	0.00 (-0.03 to 0.03)
35-200	20-200	0.15	1.79 (1.48 to 2.10)
	26-200	0.14	1.02 (0.73 to 1.31)
	26-99	0.14	1.02 (0.73 to 1.31)
	30-200	0.07	0.55 (0.40 to 0.70)
	31-200	0.05	0.43 (0.33 to 0.54)
	31-99	0.04	0.44 (0.34 to 0.54)
	40-200	0.04	-0.49 (-0.58 to -0.39)
	40-99	0.05	-0.48 (-0.59 to -0.38)
	41-200	0.09	-0.59 (-0.78 to -0.39)
	41-99	0.10	-0.58 (-0.78 to -0.39)
95-2000	61-99	0.80	2.42 (0.84 to 4.03)
	71-99	0.90	0.65 (-1.14 to 2.43)
	71-200	0.88	0.60 (-1.13 to 2.33)
	80-200	0.22	0.08 (-0.34 to 0.52)
	81-99	0.21	0.08 (-0.35 to 0.50)
	81-200	0.18	0.05 (-0.30 to 0.41)
	85-200	0	0.00 (-0.04 to 0.04)
	90-99	0	0.00 (-0.02 to 0.02)
	90-200	0	0.00 (-0.02 to 0.02)
	91-99	0	0.00 (-0.03 to 0.02)
	91-200	0	0.00 (-0.02 to 0.02)
	95-99	0	0.00 (-0.02 to 0.02)
	96-99	0	0.00 (-0.02 to 0.02)

Modelling strategy

We modelled the prevalence of hearing loss over five steps. First, we ran three DisMod-MR 2.1 models to estimate the total prevalence of the following levels of hearing by y-a-s-l: normal hearing (0–19dB), mild hearing loss (20–34dB), and moderate hearing loss and above (35+ dB). For normal hearing loss (0-19 dB), Dismod-MR 2.1 had trouble fitting prevalence values close to 100% in very young ages. Initial models attempted to follow lower prevalence data points in teen and middle-aged populations, and resulting, estimates of the prevalence of normal hearing in infants were implausible in the face of the data. As a

solution, we modeled all data adjusted to the normal hearing loss category as 1-prevalence, to accommodate for the fact that Dismod interacts better with data points at lower values. We then took the complement of the fitted model at the draw level to obtain normal hearing prevalence estimates. Next, we rescaled the prevalence estimates from the three models (0-19, 20-34, 35+) to sum to 1 for every year, age, sex, and location. We estimated prevalence of normal hearing for the purpose of correctly scaling the other two models only, and hence it did not form part of further analysis.

These three models used Socio-demographic index as a covariate. SDI was also used as a covariate in GBD 2017. The estimated betas are shown in the table below.

Table 4: Covariates

Model	Covariate name	Measure	Beta value	Exponentiated value
Hearing loss impairment at 0-19 dB	Socio-demographic Index	Prevalence	0.013 (0.00067 to 0.033)	1.01 (1.00 to 1.03)
Hearing loss impairment at 35+ dB	Socio-demographic Index	Prevalence	-1.59 (-1.87 to -1.27)	0.20 (0.15 – 0.28)
Hearing loss impairment at 95+ dB	Socio-demographic Index	Prevalence	-1.22 (-1.84 to -0.56)	0.30 (0.16 to 0.57)

Second, we ran five additional DisMod-MR 2.1 models for each severity level of hearing loss above mild: moderate (35–49dB), moderately severe (50–64dB), severe (65–79dB), profound (80–94dB), and complete (95+). We then rescaled the prevalence estimates from these models to fit within the prevalence estimated for 35+dB in the first step. By the end of the second step, we had estimated prevalence of six severity levels of hearing loss, including mild (20–34dB).

Third, we ran two additional Dismod models. The first is a model to estimate the proportion of the hearing impaired that use a hearing aid, deemed "hearing aid coverage". The second estimates the proportion of hearing loss across all severities that is attributable to age-related and other factors.

Fourth, we adjusted the prevalence of each of the six hearing loss severity levels estimated in steps one and two to account for hearing aid use. To do this, we made the assumption that the use of a hearing aid reduces the severity of impairment by one category.

The model used to estimate hearing aid coverage represents *all* severity categories. To estimate the proportion of hearing aid coverage for *each* severity category, we used data obtained from the Nord-Trondelag study and NHANES surveys. These two sources provided detailed information on hearing aid coverage among the impaired by age, sex, and most importantly, severity. We ran a logistic regression on age with binary indicators for severity levels and sex. Outputs of this regression were the proportion of individuals at every severity of hearing impairment that used a hearing aid. We assumed that 0% of people in the completely deaf category (95+) used a hearing aid. We then took estimates of hearing aid coverage that were produced in step 3, and scaled the estimate by dividing the value produced in each location by the value produced for Norway. This was to correct for any bias created by using adjustment factors calculated mostly with data from Norway. From there, we multiplied the scaled value of hearing aid coverage for each location by each of the 6 proportions of severity-specific coverage. This gave us the proportion of individuals in each severity category that use a hearing aid. Lastly, we shifted the identified

fraction of people in each severity category that used a hearing aid to the category directly below. This provided the adjusted prevalence of six severity levels of all-cause hearing loss.

Fifth, we estimated the prevalence of hearing loss due to multiple causes: otitis media, congenital, meningitis, and age-related and other causes not classified elsewhere. In GBD 2017, we estimated the prevalence of hearing loss for each subtype of meningitis (pneumococcal, *H influenzae* type B meningitis, meningococcal, and other bacterial), but in GBD 2019, we estimated the prevalence of hearing loss for meningitis as a whole. See the meningitis cause write-up for further details. For congenital hearing loss, we assumed that all hearing loss occurring at the time of birth are of congenital nature. We also assumed that all hearing loss due to otitis media is at the mild or moderate level. Up to the age of 20, we implemented proportional squeezes to scale cause-specific hearing loss prevalence to the total prevalence of each severity level. Above age 20, we subtracted the prevalence of congenital hearing loss, meningitis, and otitis from the total and called any remainder age-related and other hearing loss. Limitations in the model and underlying data for age-related and other hearing loss required such a step. Since we ensured that congenital prevalence was constant in each age group for every location, year, and sex combination after conducting the proportional squeeze, the sum of the prevalence of all hearing loss aetiologies sometimes exceeded the total prevalence of some severity levels.

Finally, we estimated the percent of people experiencing tinnitus. We determined the proportion of people suffering from tinnitus using data from NHANES years that asked about the frequency each survey respondent heard ringing, roaring, and/or buzzing (1999, 2001, 2003, and 2011–2012). We labeled anyone with mild hearing loss and ringing, roaring, or buzzing “at least once a month” as a mild hearing loss with tinnitus case. Anyone with moderate hearing through to severe hearing loss and ringing, roaring, or buzzing “at least once a day” was labelled as a moderate hearing loss with tinnitus case. Anyone with complete hearing loss who responded that they “almost always” had ringing or buzzing was labelled as a complete hearing loss with tinnitus case. Using the data from NHANES, we calculated confidence intervals assuming a binomial distribution. We assumed the same distribution of tinnitus across all aetiologies of hearing loss. This is the same strategy used in previous GBD cycles.

Table 5: Health states and disability weights

Health state name	Health state description	Disability weight
Hearing loss, mild	has great difficulty hearing and understanding another person talking in a noisy place (for example, on an urban street).	0.01 (0.004–0.019)
Hearing loss, mild, with ringing	has great difficulty hearing and understanding another person talking in a noisy place (for example, on an urban street), and sometimes has annoying ringing in the ears.	0.021 (0.012–0.036)
Hearing loss, moderate	is unable to hear and understand another person talking in a noisy place (for example, on an urban street), and has difficulty hearing another person talking even in a quiet place or on the phone.	0.027 (0.015–0.042)
Hearing loss, moderate, with ringing	is unable to hear and understand another person talking in a noisy place (for example, on an urban street), and has difficulty hearing another person talking even in a quiet place or on the phone, and has annoying ringing in the ears for more than 5 minutes at a time, almost every day.	0.074 (0.048–0.107)
Hearing loss, moderately severe	(custom DW from hearing loss impairment envelope)	0.092 (0.064–0.129)
Hearing loss, moderately severe, with ringing	(custom DW from hearing loss impairment envelope)	0.167 (0.114–0.231)
Hearing loss, severe	is unable to hear and understand another person talking, even in a quiet place, and unable to take part in a phone conversation. Difficulties with communicating and relating to others cause emotional impact at times (for example worry or depression).	0.158 (0.104–0.227)
Hearing loss, severe, with ringing	is unable to hear and understand another person talking, even in a quiet place, and unable to take part in a phone conversation, and has annoying ringing in the ears for more than 5	0.261 (0.174–0.361)

	minutes at a time, almost every day. Difficulties with communicating and relating to others cause emotional impact at times (for example worry or depression).	
Hearing loss, profound	is unable to hear and understand another person talking, even in a quiet place, is unable to take part in a phone conversation, and has great difficulty hearing anything in any other situation. Difficulties with communicating and relating to others often cause worry, depression, and loneliness.	0.204 (0.134–0.288)
Hearing loss, profound, with ringing	is unable to hear and understand another person talking, even in a quiet place, is unable to take part in a phone conversation, has great difficulty hearing anything in any other situation, and has annoying ringing in the ears for more than 5 minutes at a time, several times a day. Difficulties with communicating and relating to others often cause worry, depression, or loneliness.	0.277 (0.182–0.388)
Hearing loss, complete	cannot hear at all in any situation, including even the loudest sounds, and cannot communicate verbally or use a phone. Difficulties with communicating and relating to others often cause worry, depression or loneliness.	0.215 (0.143–0.307)
Hearing loss, complete, with ringing	cannot hear at all in any situation, including even the loudest sounds, and cannot communicate verbally or use a phone, and has very annoying ringing in the ears for more than half of the day. Difficulties with communicating and relating to others often cause worry, depression or loneliness.	0.316 (0.211–0.436)

Heart failure impairment

Flowcharts

Overall modelling strategy

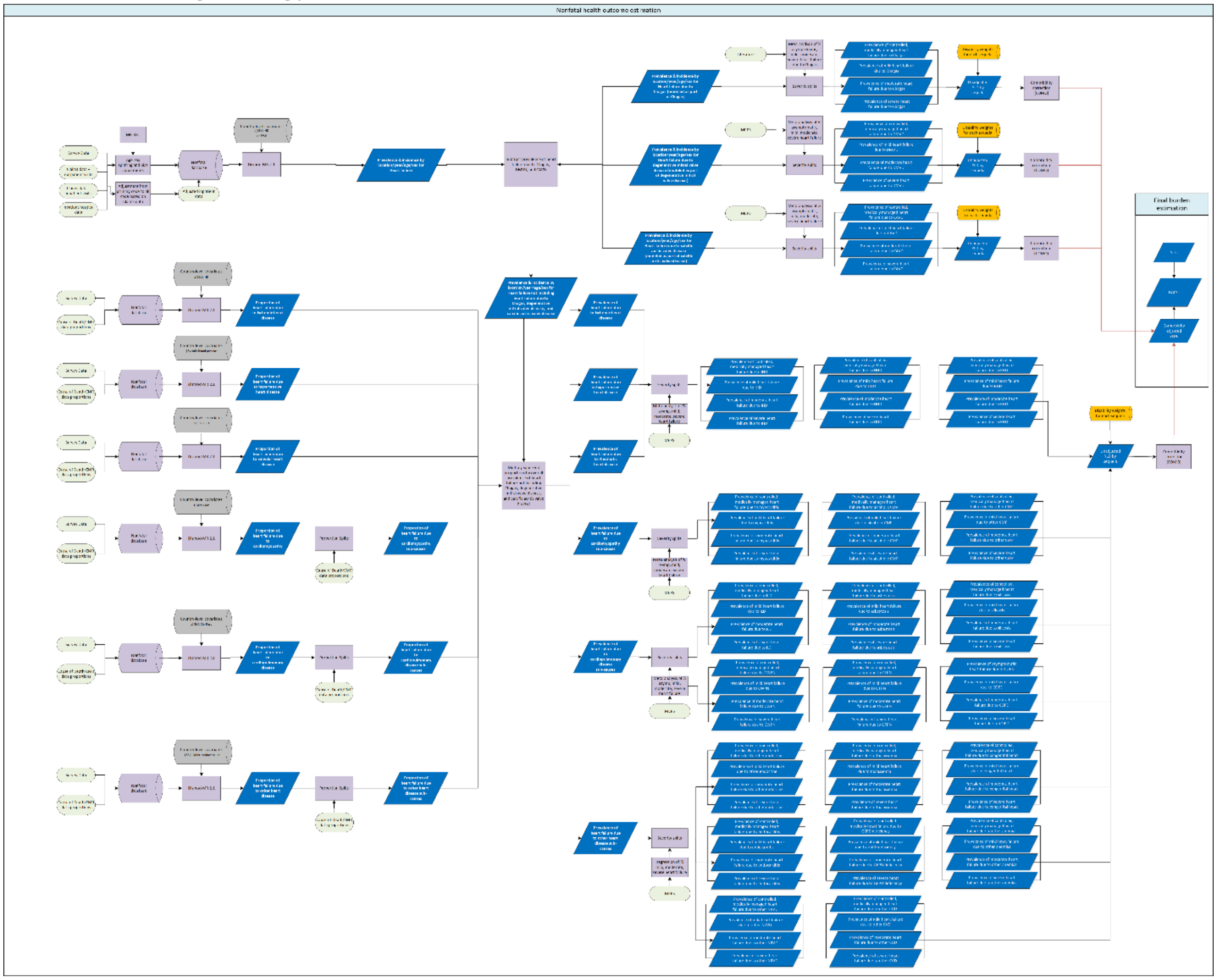

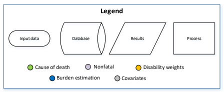

Abbreviations

DMVD: Degenerative mitral valve disease; CAVD: Calcific aortic valve disease; IHD: Ischaemic heart disease; CMP: Cardiomyopathy and myocarditis; HHD: Hypertensive heart disease; ILD: Interstitial lung disease; CWPN: Coal workers pneumoconiosis; OTPN: Other pneumoconiosis; COPD: Chronic obstructive pulmonary disease; RHD: Rheumatic heart disease; CVD: Cardiovascular disease; NRVD: Non-rheumatic valve disease

Proportion splits and correction factor estimation

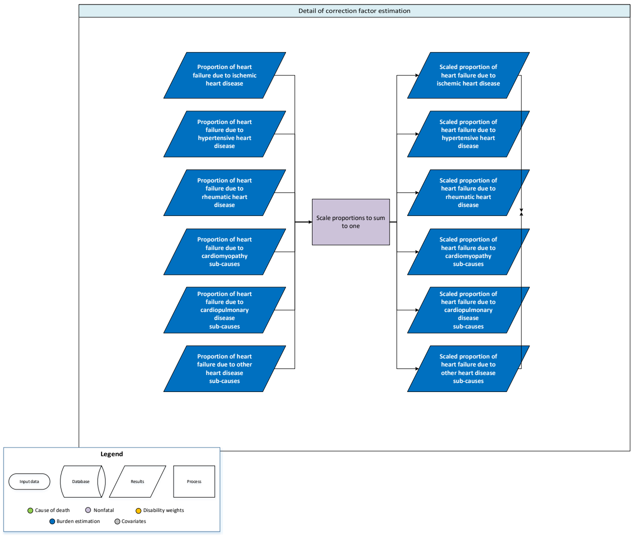

Case definition

Heart failure was diagnosed clinically using structured criteria such as the Framingham or European Society of Cardiology criteria. Previous iterations of GBD modelled symptomatic (i.e. NYHA Class II and above) episodes of HF only. Beginning in GBD 2016, we used ACC/AHA Stage C and above to capture both persons who are currently symptomatic and those who have been diagnosed with heart failure but are currently asymptomatic.

Framingham Criteria (1): Must fulfill two major criteria or one major and two minor criteria.
Major criteria: Paroxysmal nocturnal dyspnoea, neck vein distention, rales, radiographic cardiomegaly, acute pulmonary oedema, S3 gallop, increased central venous pressure (>16 cm H_2O at right atrium), hepatojugular reflux; weight loss >4.5 kg in 5 days in response to treatment
Minor criteria: bilateral ankle oedema, nocturnal cough, dyspnoea on ordinary exertion, hepatomegaly, pleural effusion, decrease in vital capacity by one-third from maximum recorded, tachycardia (heart rate>120 beats/min).

European Society of Cardiology (2): Typical signs (elevated jugular venous pressure, pulmonary crackles and peripheral oedema) and symptoms (eg, breathlessness, ankle swelling, and fatigue) caused by a structural and/or functional cardiac abnormality, resulting in a reduced cardiac output and/or elevated intracardiac pressures at rest or during stress.

Input data

A systematic review was performed GBD 2016, and updated with an unstructured review of the data in 2019. In 2016, the search terms used were: "heart failure"[TIAB] AND (epidemiology[MeSH Terms] OR prevalence[TIAB] OR incidence[TIAB] OR mortality[TIAB]) AND ("1990/01/01"[PDAT] : "2016/09/02"[PDAT]) NOT “animal model” NOT rat NOT mice NOT diabetes[TIAB] NOT “renal transplant”[TIAB]. The dates of the search were 01/01/1990 through 09/02/2016. 37,891 initial hits were returned, and 57 sources were added. An unstructured review yielded an additional 30 sources, of which

six were extracted. In 2019, a review of 8 systematic review articles yielded 519 sources to review, of which 14 were extracted.

The final dataset also included inpatient hospital data and claims data from the US and Taiwan. Inpatient hospital data were corrected for readmission, primary diagnosis to any diagnosis ratios, and inpatient to outpatient utilisation ratios using adjustment factors calculated from individual-level claims data. This methodology is detailed elsewhere in the appendix. Inpatient data were excluded if the facilities were not representative of the national population.

Additionally, we used the following data sources to estimate the proportion of heart failure attributable to each aetiology: Vital Registry data from Mexico, Brazil, Taiwan, Colombia, and the US; Inpatient admissions from Friuli Venezia, Italy; and Linked Vital Registry data from Friuli Venezia, Italy.

For GBD 2019, we used the modeling software Meta-Regression, Baysian Regularized Trimming (MR-BRT) to correct for biases in data types, replacing the in-DisMod crosswalks used in GBD 2017 and earlier. We used a network meta-analysis to adjust MarketScan data from 2010-2016 and MarketScan data from 2000, which used a different sampling methodology than other years, to literature and inpatient data. Table 2 shows MR-BRT crosswalk adjustment factors.

MR-BRT was used to split both-sex data points into sex-specific estimates. This methodology is detailed elsewhere in the appendix. We also split data points where the age range was greater than 25 years. Age splitting was based on the global sex-specific age pattern from a Dismod model that only used input data from scientific literature with less than a 25-year age range.

Table 2: MR-BRT Crosswalk Adjustment Factors for Heart Failure prevalence

$$Estimated\ Reference\ Def = invlogit(logit(Alternative\ Def) - Beta_{Alternative\ Def} - Beta_{Sex} * Sex - Beta_{Age_{scaled}} * Age\ Scaled)$$

Data input	Reference or alternative case definition	Gamma	Beta Coefficient, Logit (95% CI)
Inpatient or Literature data	Reference	0.02	---
MarketScan, 2000	Alternate		-0.59 (-0.51, -0.67)
MarketScan, 2010-2016	Alternate		-0.53 (-0.45, -0.61)
Age, scaled			-0.01 (-0.06, 0.03)
Male sex			-0.03 (-0.08, 0.02)

Table 3. Severity distribution, details on the severity levels for Heart Failure in GBD 2019 and the associated disability weight (DW) with that severity.

Severity level	Lay description	DW (95% CI)
Controlled, medically managed	Has been diagnosed with clinical heart failure, a chronic disease that requires medication every day and causes some worry but minimal interference with daily activities.	0.049 (0.031-0.072)

Mild	Is short of breath and easily tires with moderate physical activity, such as walking uphill or more than a quarter-mile on level ground. The person feels comfortable at rest or during activities requiring less effort.	0.041<br>(0.026–0.062)
Moderate	Is short of breath and easily tires with minimal physical activity, such as walking only a short distance. The person feels comfortable at rest but avoids moderate activity.	0.072<br>(0.047–0.103)
Severe	Is short of breath and feels tired when at rest. The person avoids any physical activity, for fear of worsening the breathing problems.	0.179<br>(0.122–0.251)

Source counts

**Measure**	**Total sources**	**Countries with data**
Prevalence	192	38
Incidence	31	14
Standardized mortality ratio	2	2
With-condition mortality rate	56	22
Proportion	68	51

Modelling strategy

To estimate the burden of heart failure due to each of 23 underlying causes, we first estimated the overall prevalence of heart failure and then the proportion of heart failure that could be attributed to each cause. The latter process includes an initial assessment of the fraction of heart failure cases attributable to each of six high-level parent cause groupings, followed by further division into the detailed causes within each of these groupings. The selection for aetiological causes was based on a review of the literature and expert opinion regarding diseases that lead to congestive heart failure.

Prevalence estimation

Overall prevalence of AHA/ACC stage C or D heart failure was estimated in DisMod-MR 2.1 using literature data, hospital data, and claims data. We set a prior of no remission and capped excess mortality at 1. All data adjustments were done outside of DisMod, described above.

Estimates for the prevalence of heart failure due to Chagas, degenerative mitral valve disease, and calcific aortic valve disease were generated separately as part of the modelling strategy for those causes. We subtracted the prevalence of heart failure due to these causes from the overall heart failure estimates to give an adjusted prevalence of heart failure due to all other aetiologies.

Aetiological fraction estimation

To estimate the proportion of heart failure attributable to each cause, we used Equation 1 to calculate the prevalence of heart failure due to each aetiology, which was then scaled into a proportion.

Equation 1:

$$Prevalence_{HF\ due\ to\ aetiology} = \frac{Cause\ Specific\ Mortality\ Rate_{HF\ due\ to\ aetiology}}{Excess\ Mortality\ Rate_{HF\ due\ to\ aetiology}}$$

First, we calculated the Cause Specific Mortality Rate (CSMR) for heart failure due to each aetiology. We used age-, sex-, and location-specific CSMR (post CoDCorrect) for each aetiology, multiplied by the fraction of deaths that also involved heart failure (Equation 2). This fraction was a modeled quantity, informed by person-level vital registry (VR) data from the United States, Mexico, Brazil, Taiwan, and Colombia, data sources which contained the underlying cause of death as well as all codes in the causal chain. From these sources, we calculated the fraction of underlying deaths from each aetiology in which heart failure was coded in the causal chain. These data were modeled in MR-BRT to generate age- and sex-specific estimates of this proportion. For Hypertensive Heart Disease, Alcoholic Cardiomyopathy, and Other Cardiomyopathy, we set the proportion to be 1, as all deaths due to these causes involve heart failure.

Equation 2:

$$CSMR_{HF\ due\ to\ aetiology} = CSMR_{aetiology} * Proportion\ deaths\ with\ HF_{aetiology}$$

Next, we estimated the Excess Mortality Rate (EMR) for heart failure due to each aetiology. We used uniquely identified person-level hospital discharge data for the entire Italian region of Friuli Venezia Giulia, linked to all death records from the region. Inpatient data contained all primary and non-primary diagnoses associated with the visit, and mortality data contained the underlying cause of death as well as all codes in the causal chain. We identified patients with heart failure due to each aetiology as individuals with hospital coded heart failure concurrent or after a hospital code of the aetiology. Excess Mortality Rate for heart failure due to each aetiology was calculated by subtracting the background mortality rate from the mortality rate of persons with heart failure due to that aetiology. We modelled this quantity in MR-BRT to generate age- and sex-specific estimates of this value. Due to small number of deaths in younger ages, we assumed equal EMR across aetiologies for ages under 45.

We calculated the prevalence of Heart Failure due to each aetiology using Equation 1. These were scaled to sum to one, generating the estimated proportions of Heart Failure due to each aetiology.

These proportions, along with literature data, were used to inform DisMod-MR 2.1 models for the six broadest and mutually exclusive and collectively exhaustive cause groupings: ischaemic heart disease, hypertensive heart disease, cardiomyopathy and myocarditis, rheumatic heart disease, cardiopulmonary disease, and other cardiovascular and circulatory diseases. An exception to this approach was made for sub-Saharan Africa, where we excluded the proportion estimates generated from death data, relying instead on published literature to determine the proportions of heart failure aetiologies. This decision was based on expert opinion that local patterns differed significantly from what would have been determined from death data. The THESUS-HF study, a large-scale, prospective, echocardiographic study of heart failure aetiologies in multiple African countries, provided these proportions (3).

The results of these six proportion models were scaled to sum to one.

For heart failure due to cardiopulmonary disease, heart failure due to cardiomyopathy and myocarditis, and heart failure due to other causes, we calculated the proportion for each sub-cause according to the proportion of that cause within each larger aggregate group.

These estimates were then split into asymptomatic, mild, moderate, and severe heart failure based on an analysis of MEPS data, with the exception of Chagas disease. For that aetiology, we based the severity splits on a meta-analysis of NYHA class among persons diagnosed with heart failure due to Chagas disease in areas where Chagas is endemic.

Models were evaluated based on expert opinion, comparison of results with other rounds of GBD, and model fit.

Limitations

Our estimation of the aetiological causes of heart failure makes several assumptions and has several limitations. First, we assume that each case of heart failure only has one cause. Second, we rely on individually linked inpatient and mortality records from a small region of Italy to calculate aetiology-specific EMR. Third, we rely on multiple cause of death VR data from five countries to inform use the proportion of deaths that contain heart failure in all countries. This approach allows us to produce estimates for all locations and can be updated to include more detailed health record and claims data from additional locations as they become available.

Overall heart failure impairment envelope

Study covariate	Parameter	Beta	Exponentiated beta
Log-transformed age-standardised SEV scalar: Ischemic Heart Disease	Prevalence	0.75 (0.75–0.77)	2.38 (2.21–2.53)
Healthcare access and quality index	Excess mortality rate	-1.05 (-2.00 – -0.12)	0.35 (0.14–0.88)

Six main sub-cause proportion envelopes

Sub-cause	Covariate	Parameter	Beta	Exponentiated beta
Heart failure due to cardiomyopathy impairment envelope	Log-transformed age-standardised SEV scalar: CMP	Proportion	0.75 (0.75–0.75)	2.12 (2.12–2.12)
Heart failure due to cardiopulmonary disease impairment envelope	Log-transformed age-standardised SEV scalar: COPD	Proportion	0.76 (0.75–0.77)	2.13 (2.12–2.15)

Heart failure due to hypertensive heart disease impairment envelope	Systolic blood pressure (mmHg)	Proportion	8.6E-5 (2.7E-6 to 2.9E-4)	1.00 (1.00–1.00)
Heart failure due to ischaemic heart disease impairment envelope	Log-transformed age-standardised SEV scalar: IHD	Proportion	0.75 (0.75–0.75)	2.12 (2.12–2.13)
Heart failure due to other causes impairment envelope	Log-transformed SEV scalar: Oth Cardio	Proportion	0.75 (0.75–0.76)	2.12 (2.12–2.13)
Heart failure due to valvular heart disease impairment envelope	Log-transformed age-standardised SEV scalar: CVD	Proportion	0.75 (0.75–0.76)	2.12 (2.12–2.13)

References

1) http://www.framinghamheartstudy.org/share/protocols/soe0_03s_protocol.pdf
2) 2016 ESC Guidelines for the diagnosis and treatment of acute and chronic heart failure: The Task Force for the diagnosis and treatment of acute and chronic heart failure of the European Society of Cardiology (ESC) Developed with the special contribution of the Heart Failure Association (HFA) of the ESC. Eur Heart J 2016; 37 (27): 2129-2200.
3) Damasceno A, Mayosi BM, Sani M, Ogah OS, Mondo C, Ojji D, Dzudie A, Kouam CK, Suliman A, Schrueder N, Yonga G, Ba SA, Maru F, Alemayehu B, Edwards C, Davison BA, Cotter G, Sliwa K. The Causes, Treatment, and Outcome of Acute Heart Failure in 1006 Africans From 9 Countries. Results of the Sub-Saharan Africa Survey of Heart Failure. Arch Intern Med. 2012;172(18):1386-1394.

Infertility (impairment)

Flowchart

Infertility

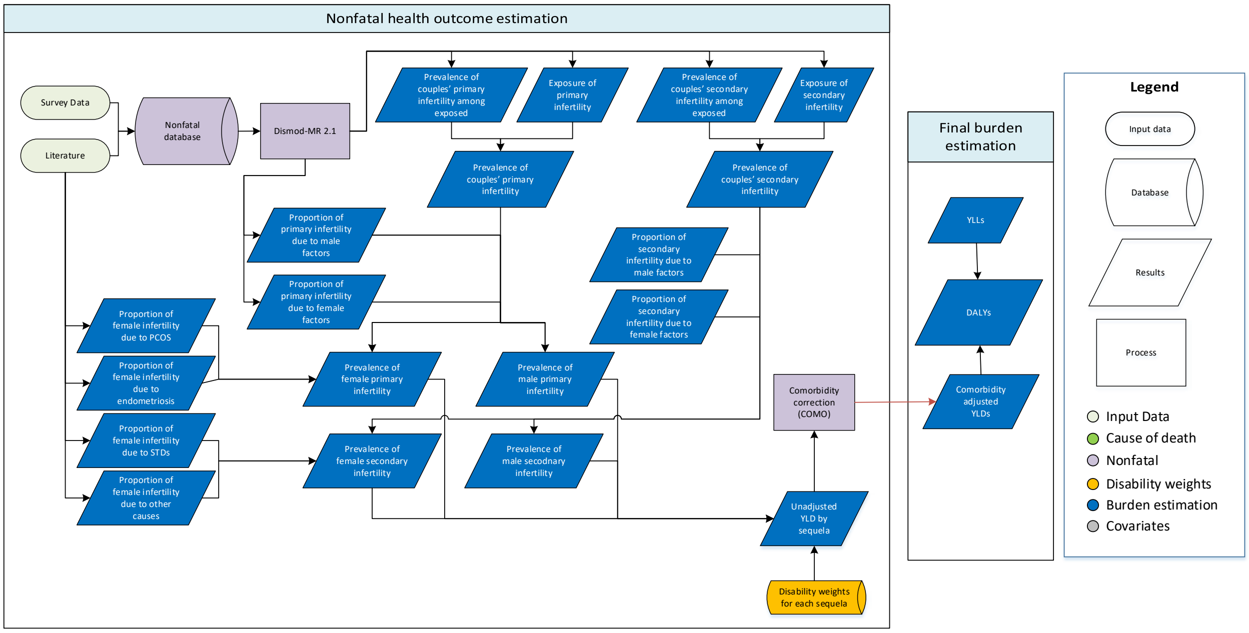

Input Data and Methodological Summary for infertility impairment

Case definition

For GBD 2019, the following case definitions were used for infertility:

1. Primary infertility is defined as a couple who have not had a livebirth, who wish a child, and have been in a union for more than five years without using contraceptives.

2. Secondary infertility is defined in a couple who wish a child and have been in a union for more than five years without using contraceptives since the last livebirth.

Estimation is completed in three steps. First, we estimate total *primary* (unable to have any child) and *secondary* (unable to have an additional child) infertility in *couples*. This is accomplished by first quantifying the rate of infertility among survey respondents who are *married* (the subset to whom such questions are directed) and then quantifying how the married population relates to the overall population. Second, we model which proportion of primary and secondary infertility is due to female and male factors, respectively, to estimate four "envelopes" of infertility: male primary infertility, male secondary infertility, female primary infertility, and female secondary infertility. Third, we execute a "causal attribution" process to assign cases of each envelope to likely underlying causes and assign the remainder to idiopathic infertility (ie, unknown causes).

Input data

Our primary data sources are population surveys. The datasets were last updated for GBD 2015. Data extraction included data for women in five-year age groups between 15 and 49 from population-based surveys including the Demographic and Health Surveys (DHS), World Fertility Surveys (WFS),

Reproductive Health Surveys (RHS), Family and Fertility Survey (FFS), and others (EUR, NSF, PCD, PFM). Such surveys only ask fertility-related questions to married women. Even though only women are interviewed, we treated the responses as a proxy for the infertility of couples in unions because the questions are not structured in a way that it is possible to determine which partner is the cause of the couples' inability to conceive a child. The desire to have a child is the crucial determinant of whether a couple is labeled as infertile (ie, if no child is wanted, infertility is not present).

The combination of variables in surveys that were used to construct each of the four datasets (primary "impairment" and "exposure" and secondary "impairment" and "exposure") are illustrated in the table below. As described below, overall primary and secondary infertility are estimated by multiplying prevalence among those with the "impairment" of infertility (married women who desire a[nother] child) by the prevalence of the "exposure" (being married for 5+ years, not using contraception for 5+ years).

Table 1: Data extraction definitions used in estimation of infertility

Model name	Infertility type	Numerator	Denominator
**Primary (impairment)**	Exposure to primary infertility among married women	Married 5+ years; no contraception for 5+ years prior to survey; no previous births; desires a child.	Married 5+ years
**Primary (exposure)**	Prevalence of exposure	Married 5+ years; no contraception for 5+ plus years prior to survey	All women
**Secondary (impairment)**	Exposure to secondary infertility among married women	Married 5+ years; no contraception for 5+ years prior to survey; last birth 5+ years ago; desires a child.	Married 5+ years; 1+ children
**Secondary (exposure)**	Prevalence of exposure to secondary infertility	married 5+ years; no contraception for 5+ years prior to survey; 1+ children	All women

For GBD 2019, we started extracting data from more recent surveys (2016 and 2017 DHS), but we were not able to incorporate these data in our final model. These data sources will be added in the next GBD cycle.

The second set of four datasets informed estimates of which component of primary and secondary infertility were due to female and male factors, respectively. To obtain data on the sex and cause breakdown for infertility, we systematically searched the literature in GBD 2010 using the following search string:

Causes[Title/abstract] AND infertility[Title] NOT mouse NOT murine NOT rat NOT rodent

We received 626 hits from PubMed and excluded studies according to the following exclusion criteria:

1. studies not representative of the national population;
2. studies that provide no raw data,
3. studies that provide only estimates;
4. studies performed before 1970;
5. case studies or studies with sample size less than 50;
6. studies that provide no data on the sex of the partner responsible for infertility among couples.

The majority of excluded studies were excluded because of the latter criterion. In total, 15 studies were included in our analysis for the sex breakdown among infertile couples.

The total number of data sources included in the analysis are shown in table 2.

Table 2. Data inputs used to estimate infertility.

Measures	Total sources	Countries with data
All measures	350	116
Prevalence	332	114
Proportion	18	15

Data processing

The first step of data processing was age-sex splitting. For any datum that did not entirely fit within a GBD age group or was for both sexes combined, the observation was split to be multiple age-specific and sex-specific data points based on the age and sex pattern predicted by GBD 2017 DisMod-MR 2.1 models. It is our intention to update this age-sex splitting with each cycle of GBD.

Due to the lack of variability among the data sources used to estimate the infertility envelope (all data sources were surveys) we did not perform any crosswalk prior modelling. However, it is our intention to incorporate different data sources and the corresponding crosswalk approach in the infertility pipeline in the next GBD cycle.

Modelling strategy

For GBD 2019, we have made no substantive changes in the modeling strategy from GBD 2017. Infertility among couples was reported as due to one of the following causes: male factor, female factor, both, or unknown. Couples with infertility due to both partners were allocated to both male factor and female factor, and couples with infertility of unknown cause were allocated to male and female factors based on the proportion observed in other couples in the study.

We estimated the proportion of couples' infertility due to male factors and female factors separately in DisMod-MR 2.1. The quantity modelled was the proportion of couples' infertility due to each sex for each of primary and secondary infertility.

We also estimated the prevalence of primary and secondary infertility by sex and cause in three steps: 1) estimation of couples' infertility [four DisMod-MR 2.1 models], 2) estimation of infertility by sex [four DisMod-MR 2.1 models], and 3) causal attribution of infertility. We assumed zero infertility prior to age 15 or after age 50 years as fertility is not expected to be desired outside these age ranges in women; an assumption that was therefore carried over to men as well. All DisMod-MR 2.1 models were run as single parameter models. For all infertility models we tested the prevalence of pelvic inflammatory diseases, the risk-weighted prevalence of smoking, obesity and alcohol use, as measured by the summary exposure value (SEV) for smoking, body mass index and alcohol consumption (%) and the age-standardized death rate (lnASDR) of sexually transmitted infections (STIs) as country-level covariates, but any covariate was statistically significant, therefore, no study or country covariates were used in the final models. As we did not use any study level covariate, no crosswalks were performed.

Estimation of couples' infertility

To estimate the prevalence of primary and secondary infertility among couples, we first run four DisMod-MR 2.1 models to estimate the four parameters detailed above, prevalence of primary infertility (1), prevalence of primary infertility exposure (2), prevalence of secondary infertility (3), and prevalence

of secondary infertility exposure (4). For prevalence of infertility (models 1 and 3), we tried using the covariates mentioned before, but all covariates were not statistically significant, so we did not use them in the final model. We did not use any study- or country-level covariates for these models. Next, we estimated primary and secondary couples' infertility from DisMod-MR 2.1 models by multiplying the estimates for prevalence of infertility among exposed women by the prevalence of exposure to infertility to obtain prevalence of infertility among all women and all men.

Estimation of infertility by sex

After running the four models estimating overall infertility, described above, we ran four DisMod-MR 2.1 models to estimate the proportion of primary and secondary infertility by sex, proportion of primary female infertility, proportion of secondary female infertility, proportion of primary male infertility, and proportion of secondary male infertility. We model sex-specific infertility as a proportion. Because infertility in some couples is attributable to both partners rather than just one, the sum of the proportions due to each partner is greater than one when both partners are infertile. When the sum of the proportions is lower than one, we scale it to be equal to one through custom code. Again, we tried using the covariates previously mentioned, but they were not statistically significant, so we did not use it in the final model. We did not use any study- or country- level covariates for these models. As we did not use any study level covariate, no crosswalks were performed. We multiplied our prevalence of primary and secondary infertility derived in step 1 by the proportion due to male and female factors to estimate primary and secondary infertility by sex.

Causal attribution

There are seven identified causes of female infertility in the GBD 2019 cause list: pelvic inflammatory disease (PID) due to chlamydia, PID due to gonorrhea, PID due to other sexually transmitted diseases, maternal sepsis, polycystic ovarian syndrome, endometriosis, and Turner syndrome. For each of these diseases, we determined the prevalence of infertility by a literature review of the probability of becoming infertile due to that disease. For STIs, we applied a proportion with infertility derived from Westrom and colleagues[1] to incident cases of PID and used DisMod-MR 2.1 to calculate corresponding prevalence for each subsequent age group through the fertile years, assuming zero remission or excess mortality. For the others, we added all the disease-specific estimates of prevalence and assigned the remaining proportion to categories of "female primary infertility due to other causes" and "female secondary infertility due to other causes." We assumed all infertility from Turner syndrome is primary infertility and all infertility following maternal sepsis is secondary infertility. The only recognized cause of male infertility in the GBD 2019 cause list is Klinefelter syndrome. We assigned all other male infertility to "male infertility due to other causes."

Sequelae and disability weights

Every person with infertility was assumed to experience the health state as determined from the GBD disability weights survey. The lay descriptions of primary and secondary infertility are listed below.

Table 3: Health states used in estimation of YLDs due to infertility

Health state name	Health state description	Disability weight
Infertility, primary	This person wants to have a child and has a fertile partner, but the couple cannot conceive.	0.008 (0.003–0.015)
Infertility, secondary	This person has at least one child and wants to have more children. The person has a fertile partner, but the couple cannot conceive.	0.005 (0.002–0.011)

Limitations

The primary limitations of our estimation is data availability and the lack of evidence of predictors of these conditions.

References

1 Weström L, Joesoef R, Reynolds G, Hagdu A, Thompson SE. Pelvic inflammatory disease and fertility. A cohort study of 1,844 women with laparoscopically verified disease and 657 control women with normal laparoscopic results. *Sex Transm Dis* 1992; **19**: 185–92.

Developmental intellectual disability

Flowchart

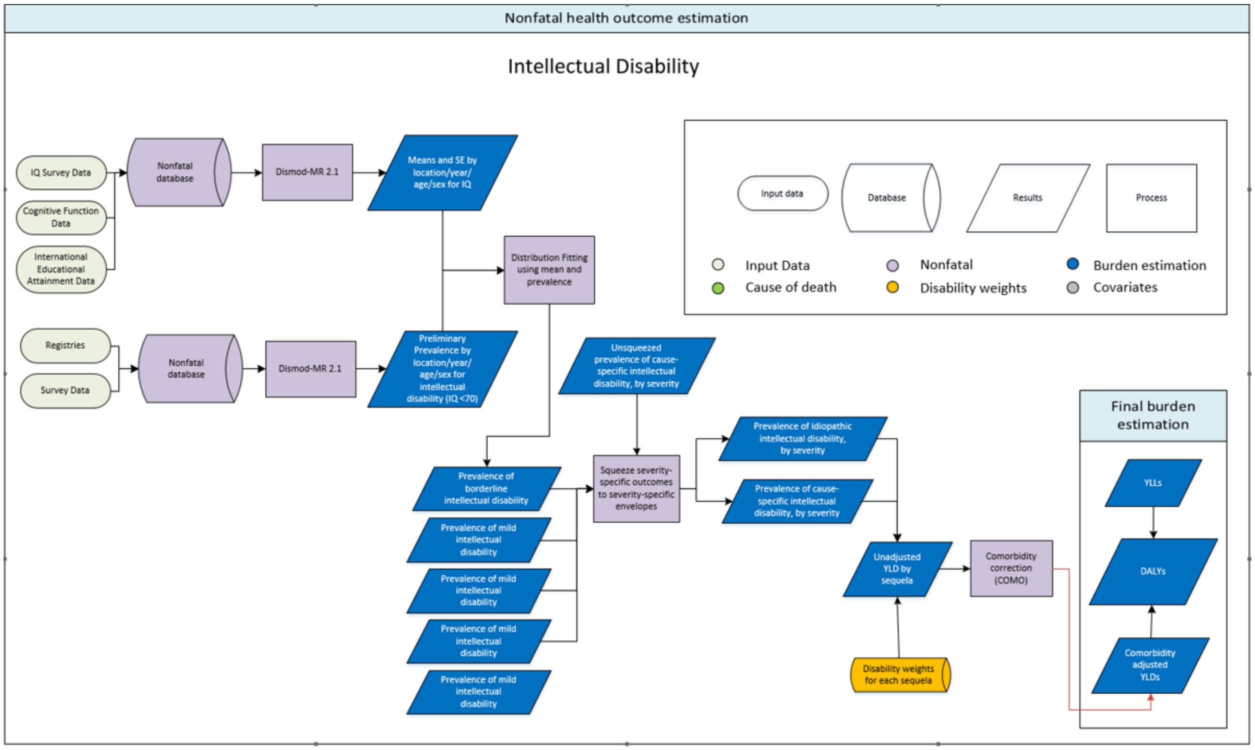

Case definition

Developmental intellectual disability (ID) is a condition of below-average intelligence or mental ability. Consistent with the American Association on Intellectual and Developmental Disabilities, we define developmental intellectual disability as a condition originating before age 18 (as such, it does not include impairment due to stroke, Alzheimer's disease, or other conditions that affect older populations). We model the severities shown in Table 1, as measured by score on intelligence quotient (IQ) tests, which are standardised to have a mean of 100.

Table 1. ID severity definitions

Severity of intellectual disability	IQ score
Profound	0 to 19
Severe	20 to 34
Moderate	35 to 49
Mild	50 to 69
Borderline	70 to 85

Input data

Model inputs

The prevalence of intellectual disability (IQ score <70) came from a systematic review of publications since January 1, 1990, using the following search string: *(((intellectual disability[MeSH Terms]) AND prevalence[Title/Abstract]) AND ('1990'[Date - Publication] : '3000'[Date - Publication]))*. We included studies that estimate the general population prevalence of intellectual disability. We excluded studies that did not use a case definition based on intelligence quotient (IQ) and studies that investigated non-representative groups, such as hospital patients or people of a specific ethnicity. This systematic review was last updated for GBD 2016. Table 2 shows a summary of the input data used.

Table 2. Input data

Measure	Total sources	Countries with data
All measures	58	31
Prevalence	58	31

Data processing

In GBD 2019, we used MR-BRT to split our both-sex data points into sex-specific data. Table 3 has the model coefficient used in sex-splitting.

Table 3. MR-BRT coefficient values (raw and exponentiated)

Sex-split coefficient (95% CI)	Exponentiated sex-split coefficient (95% CI)
-0.10 (-0.14 to -0.07)	0.90 (0.87 to 0.93)

Because we code males as "1" and females as "2", this coefficient means that the observed prevalence of ID is slightly higher in males than in females (i.e., prevalence in females is 0.90 times prevalence in males). To split our both-sex data, we first used the coefficient to get a population-weighted adjustment factor. We then multiplied that adjustment factor by the both-sex data points to get expected prevalence in males, and finally multiplied the coefficient by the expected male prevalence to get expected prevalence in females. In our final modelling dataset, we exclusively used the sex-specific and sex-split data (i.e., no both-sex data were included in the model).

Severity splits – disability weights

Table 4. Intellectual disability severity disability weights

Health state	Description	Disability weight
Borderline intellectual functioning	This person is slow in learning at school. As an adult, the person has some difficulty doing complex or unfamiliar tasks but otherwise functions independently.	0.011 (0.005–0.02)
Intellectual disability/mental retardation, mild	This person has low intelligence and is slow in learning at school. As an adult, the person can live independently, but often needs help to raise children and can only work at simple supervised jobs.	0.043 (0.026–0.064)

Intellectual disability/mental retardation, moderate	This person has low intelligence, and is slow in learning to speak and to do even simple tasks. As an adult, the person requires a lot of support to live independently and raise children. The person can only work at the simplest supervised jobs.	0.1 (0.066–0.142)
Intellectual disability/mental retardation, severe	This person has very low intelligence and cannot speak more than a few words, needs constant supervision and help with most daily activities, and can do only the simplest tasks.	0.16 (0.107–0.226)
Intellectual disability/mental retardation, profound	This person has very low intelligence, has almost no language, and does not understand even the most basic requests or instructions. The person requires constant supervision and help for all activities.	0.2 (0.133–0.283)

Modelling strategy

We modelled the prevalence of ID, both aetiology-specific IDs and idiopathic ID, over multiple steps.

First, we ran a DisMod-MR 2.1 model to estimate the total prevalence of intellectual disability of level IQ <70. We included lagged distributed income and child underweight summary exposure value (SEV) in the model as predictive covariates. Table 5 shows raw and exponentiated model coefficients for the covariates used in the estimation process for the DisMod model. Exponentiated coefficients can be interpreted as odds ratios.

Table 5. Model coefficient values (raw and exponentiated)

Covariate	Parameter	Coefficient (95% CI)	Exponentiated coefficient (95% CI)
Lagged distributed income (LDI) per capita	Prevalence	-0.37 (-0.46 to -0.28)	0.69 (0.63 to 0.76)
Age- and sex-specific SEV for child underweight	Prevalence	1.49 (0.19 to 2.77)	4.42 (1.20 to 15.99)
Sex	Prevalence	0.18 (0.12 to 0.24)	1.19 (1.13 to 1.27)

Second, we split the total prevalence of idiopathic into four severity levels: mild (IQ 50-69), moderate (IQ 35-49), severe (IQ 20-34), and profound (IQ below 20). We pooled a subset of studies that distinguished intellectual disability by these severity levels. We used cumulative severity levels (i.e., IQ <50, IQ <35, and IQ <20) to maximise the number of sources. We estimated these cumulative severities' proportion of the <70 envelope via random effects meta-analyses stratified by two levels of income status (high-income versus low- and middle-income). These proportions were used to estimate discrete severities from the overall intellectual disability (IQ <70) prevalence. We estimated the final severity level, borderline disability (IQ 70-84), via another random-effects meta-analysis of the ratio of IQ 70-84 to IQ <70. The uncertainty of the pooled fractions and ratios were propagated throughout our calculations using 1,000 draws from a normal distribution with mean and standard error estimated by the meta-analysis. The results of the meta-analysis are shown in Table 6.

Table 6. Proportion of intellectual disability cases by severity

Severity	Mean	Standard error
None	0.161	0.034
Borderline	0.161	0.034
Mild	0.375	0.037
Moderate	0.190	0.031
Severe	0.090	0.177
Profound	0.024	0.134

Third, we estimated prevalence of each aetiology-specific intellectual disability using models of the following parent causes. Since we model only developmental intellectual disability, causes that affect older populations such as stroke and Alzheimer's disease are not included in the causal attribution process.

Parent causes included in causal attribution:

- Neonatal preterm birth complications (<28w, 28-32w, 32-36w)
- Neonatal encephalopathy due to birth asphyxia and trauma
- Congenital birth defects (diaphragmatic hernia, cardiovascular anomalies)
- Haemolytic disease and other neonatal jaundice
- Meningitis (pneumococcal, *H influenzae* type B, meningococcal, other bacterial)
- Encephalitis
- Malaria
- Neonatal tetanus
- Neonatal sepsis and other neonatal infections
- Iodine deficiency
- African trypanosomiasis
- Down syndrome
- Klinefelter syndrome
- Chromosomal abnormalities (unbalanced rearrangements, Down syndrome, Edwards syndrome, Patau syndrome, other chromosomal abnormalities)
- Neural tube defects (eg, spina bifida, encephalocele)
- Hypertensive disorders of pregnancy (eclampsia, preeclampsia)
- Autism spectrum disorders (ASD)
- Fetal alcohol syndrome

For autism spectrum disorders (ASD), we identified six studies reporting severity of intellectual disability. We conducted a meta-analysis to produce a severity distribution which we applied to the prevalence of autism to produce severity-specific ID due to autism.[1-6]

[1] Croen LA, Grether JK, Hoogstrate J, Selvin S. The Changing Prevalence of Autism in California. *J Autism Dev Disord*. 2002; 32(3): 207-15.

[2] Fombonne E, du Mazaubrun C. Prevalence of infantile autism in four French regions. *Soc Psychiatry Psychiatr Epidemiol*. 1992; 27(4): 203-10.

We calculated the prevalence of idiopathic ID by subtracting all severity- and aetiology-specific ID from the severity-specific envelope assuming the residuals to represent idiopathic disability. If the residual was less than 5% of the severity-specific envelope, the prevalence of all aetiology-specific ID was proportionally squeezed to fit within 95% of the envelope, leaving 5% for idiopathic ID.

As we estimated the prevalence of individual aetiology-specific ID by models from the respective parent causes, the squeezing may have resulted in a distorted balance of prevalence estimates within their parent causes. With the aim to maintain consistencies of prevalence within each of the parent causes, we added the difference between the original and the squeezed prevalence estimates to the "motor impairment" sequela if the squeezed sequela represented "motor and cognitive impairment." For autism, we obtained the fraction of cases that result in ID from literature (0.29; 95% CI 0.27–0.30) and applied this fraction to the subtraction and squeezing processes. We assumed that all ID cases due to iodine deficiency (cretinism) would result in either severe or profound disability, and that Klinefelter syndrome cases that result in ID would have either borderline or mild severity. Lastly, in GBD 2013, all aetiology-specific models were squeezed into the overall (IQ <70) envelope, while in all subsequent rounds (including GBD 2019), we squeezed each model into its discrete severity envelope.

[3] Ritvo ER, Freeman BJ, Pingree C, Mason-Brothers A, Jorde L, Jenson WR, McMahon WM, Petersen PB, Mo A, Ritvo A. The UCLA-University of Utah epidemiologic survey of autism: prevalence. *Am J Psychiatry*. 1989; 146(2): 194-9.
[4] Yeargin-Allsopp M, Rice C, Karapurkar T, Doernberg N, Boyle C, Murphy C. Prevalence of autism in a US metropolitan area. *JAMA*. 2003; 289(1): 49-55.
[5] Baird G, Simonoff E, Pickles A, Chandler S, Loucas T, Meldrum D, Charman T. Prevalence of disorders of the autism spectrum in a population cohort of children in South Thames: the Special Needs and Autism Project (SNAP). *Lancet*. 2006; 368(9531): 210-5.
[6] Bertrand J, Mars A, Boyle C, Bove F, Yeargin-Allsopp M, Decoufle P. Prevalence of Autism in a United States Population: The Brick Township, New Jersey, Investigation. *Pediatrics*. 2001; 108(5): 1155-61.

Pelvic inflammatory disease (PID)

Flowchart

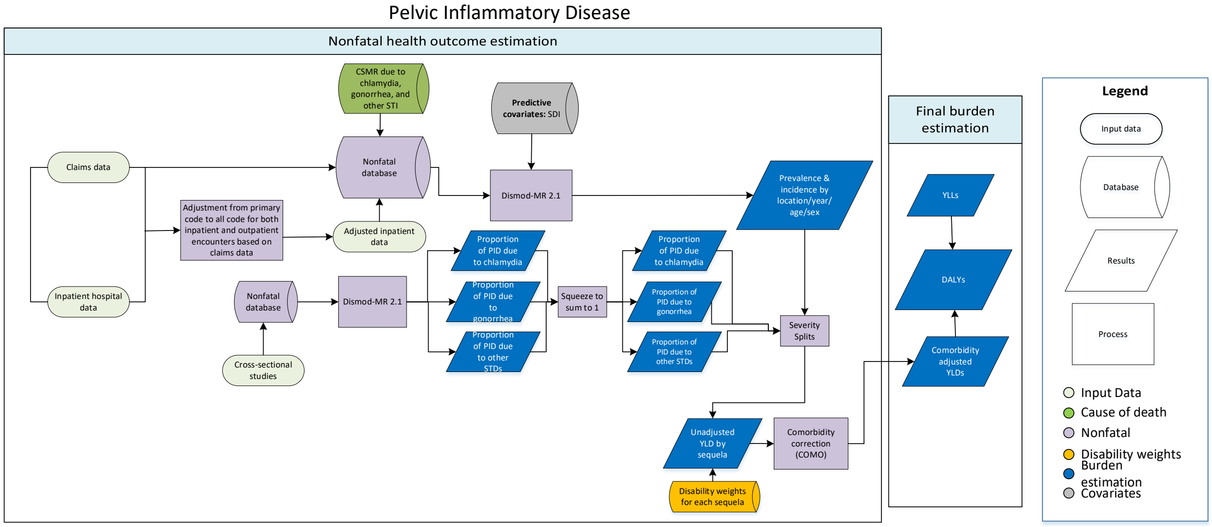

Case definition

Pelvic inflammatory disease (PID) is an infection of the upper portion of the female reproductive tract, and can be caused by multiple sexually transmitted and non-sexually transmitted infections. It causes pain, and can irreversibly damage the uterus, fallopian tubes, or other parts of the female reproductive tract, leading to infertility. In rare instances, it can lead to sepsis and death.

Input data

Model inputs

A systematic review was completed for GBD 2013 on October 28, 2013, using the following search terms:

- (("pelvic inflammatory disease"[Title/Abstract] OR "salpingitis"[Title/Abstract]) AND (“1994”[Date – Publication] : “2013”[Date – Publication]))

In GBD 2013, only data extracted from published studies identified in our systematic review were included. Starting in GBD 2015, data from hospital discharges and claims were exclusively used in the pelvic inflammatory disease envelope model. A subset of the studies from the systematic review reported the underlying etiology of PID, allowing us to estimate the proportion of PID due to chlamydia, gonorrhea, and other sexually transmitted diseases.

Table 1: Data inputs for Pelvic Inflammatory Disease morbidity modelling by parameter

Measure	Total Sources	Countries with data
All measures	1467	195
Prevalence	4	3
Incidence	299	45

Cause-specific mortality rate	1164	195

For the envelope model, In GBD 2015-2017, PID hospital data was corrected to account for secondary diagnoses in an individual. Sources of clinical data often only report primary diagnoses, however individuals with PID may be admitted to the hospital for severe abdominal pain and later diagnosed with the appropriate disease. Because many cases of PID are potentially missed, data on PID incidence was scaled up to include secondary diagnoses. The output represents all cases of PID diagnosed in an inpatient setting. To account for outpatient cases of PID, which would be less severe, a correction factor converting inpatient diagnosis to inpatient and outpatient diagnoses was applied using a ratio of Marketscan data to HCUP data (Healthcare Cost & Utilization Project).

In contrast, in GBD 2019, we adopted a GBD-wide policy of including all diagnosed cases of every disease estimated. This policy was made because hospital discharge data provides observations about encounters, generally with only the primary diagnostic code for the encounter. Claims data, on the other hand, link claims for all inpatient and outpatient encounters for a single individual, and provide primary and secondary diagnoses for all encounters.

This allowed all of the GBD to improve data processing methods to capture cases that were diagnosed and/or treated in an outpatient setting. Specifically, incident cases were extracted from claims data if an individual had at least one inpatient or outpatient encounter with an appropriate ICD code as any diagnosis within one year. Data from hospital discharges were, then, adjusted using correction factors from claims, converting encounters to estimates of cases, accounting for most locations providing only primary diagnostic codes, and estimating outpatient cases from inpatient cases.

Claims data from the United States were adjusted to inpatient hospital data using MR-BRT, prior to analysis in Dismod. A priori, we believed that claims data reflected a certain level of selection bias due to commercial insurance, while inpatient hospital data was more reflective of the general population.

A crosswalk adjusting Marketscan data to inpatient hospital data was made, with differing adjustment factors by age to account for the age pattern seen in the relationship between the incidence of PID reported through Marketscan, and incidence reported from hospital discharges. The result is an estimate of total PID incidence. Adjustment factors were modelled in MR-BRT as a meta-regression of log-transformed ratios between claims data sources and inpatient data sources. Ratios were formed between sources matched by age and location.

Table 2: MR-BRT Crosswalk Adjustment Factors for Pelvic Inflammatory Disease

Data input	Reference or alternative case definition	Gamma	Beta Coefficient, Log (95% CI)	Adjustment Factor*
Inpatient Hospital	Ref	0.08	---	---
Claims (10-14yrs)	Alt		-0.43(-0.65 to -0.21)	0.65(0.52,0.81)
Claims (15-19yrs)	Alt		-0.41(-0.62 to -0.22)	0.66(0.53,0.80)
Claims (20-24yrs)	Alt		-0.39(-0.58 to -0.21)	0.67(0.55,0.81)
Claims (25-29yrs)	Alt		-0.39(-0.57 to -0.20)	0.67(0.56,0.81)

Claims (30-34yrs)	Alt		-0.38(-0.57 to -0.20)	0.68(0.56,0.81)
Claims (35-39yrs)	Alt		-0.34(-0.52 to -0.16)	0.71(0.59,0.85)
Claims (40-44yrs)	Alt		-0.23 (-0.41 to -0.04)	0.79(0.66,0.96)
Claims (45-49yrs)	Alt		-0.01(-0.19 to 0.17)	0.99(0.82,1.18)
Claims (50-54yrs)	Alt		0.29(0.10 to 0.47)	1.33(1.10,1.59)
Claims (55-59yrs)	Alt		0.65(0.45 to 0.84)	1.91(1.56,2.31)
Claims (60-64yrs)	Alt		0.99(0.79 to 1.20)	2.69(2.20,3.32)
Claims (65-69yrs)	Alt		1.26(1.05 to 1.47)	3.52(2.85,4.34)
Claims (70-74yrs)	Alt		1.41(1.21 to 1.63)	4.09(3.35,5.10)
Claims (75-79yrs)	Alt		1.44(1.21 to 1.67)	4.22(3.35,5.31)
Claims (80-84yrs)	Alt		1.39(1.09 to 1.69)	4.01(2.97,5.41)
Claims (85-89yrs)	Alt		1.31(0.95 to 1.68)	3.70(2.58,5.36)
Claims (90-94yrs)	Alt		1.26(0.85 to 1.67)	3.52(2.33,5.31)
Claims (95-99yrs)	Alt		1.23(0.81 to 1.66)	3.42(2.24,5.25)

***Adjustment factor is the transformed Beta coefficient in normal space, and can be interpreted as the factor by which the alternative case definition is adjusted to reflect what it would have been if measured as the reference.**

Table 3: Severity Distribution

Severity Level	Healthstate	Lay Description	Disability weight
Moderate	Abdominopelvic problem, moderate	This person has pain in the belly and feels nauseated. The person has difficulties with daily activities.	0.324 (0.219–0.442)
Severe	Abdominopelvic problem, severe	This person has severe pain in the belly and feels nauseated. The person is anxious and unable to carry out daily activities.	0.114 (0.078–0.159)

Modelling strategy

First, we estimated the total incidence and prevalence of pelvic inflammatory disease using Dismod-MR 2.1. We used a Bayesian prior on remission (13–17), and also set the incidence of PID to 0 for ages 0 to 10 years.

In previous rounds, a prior was set on excess mortality rate from 0 to 0.02. However, for many causes, DisMod estimated a rather unrealistic pattern of EMR compared to an expected pattern of decreasing EMR with greater access to quality health care. Such unexpected patterns often signal inconsistencies between CSMR estimates and the measures of prevalence and/or incidence. In an effort to provide greater guidance to DisMod on the expected pattern of EMR, we aggregated CSMR from each etiology of PID (chlamydia, gonorrhea, and other STIs), and then ran a model to generate EMR data. This EMR model used the MR-BRT approach, with a prior on healthcare access and quality index (HAQi) set to have a negative coefficient; we estimated separate coefficients by age-group. Results from MR-BRT were then predicted for each location, year, sex and for ages 0, 10, 20 ….100. These estimated EMR inputs were then included in our DisMod model, and HAQi also included as a covariate to inform EMR with a mean and standard deviation produced from MR-BRT.

Data points with an age-standardised prevalence greater than one median absolute deviation from the median of the age-standardised prevalence for all inpatient and non-USA claims data were marked as outliers and excluded from analysis.

Table 4: Covariates for pelvic inflammatory disease envelope

Covariate	Parameter	Exponentiated beta (95% Uncertainty Interval)
Health Access & Quality Index	Incidence Hazard	0.97 (0.97-0.97)

Second, we ran three separate DisMod models for the proportion of PID due to the following three causes: chlamydia, gonorrhea, and other STDs. As outlined above, this data came from a systematic review conducted for GBD 2013. A systematic review to inform current estimates of PID etiologies is slated for a future round. No custom adjustment factors are done for these models, however, they are used as inputs to the infertility estimation process. Details can be found in the section allotted for infertility.

Blindness and vision loss

Flowcharts

Vision loss

Nonfatal health outcome estimation

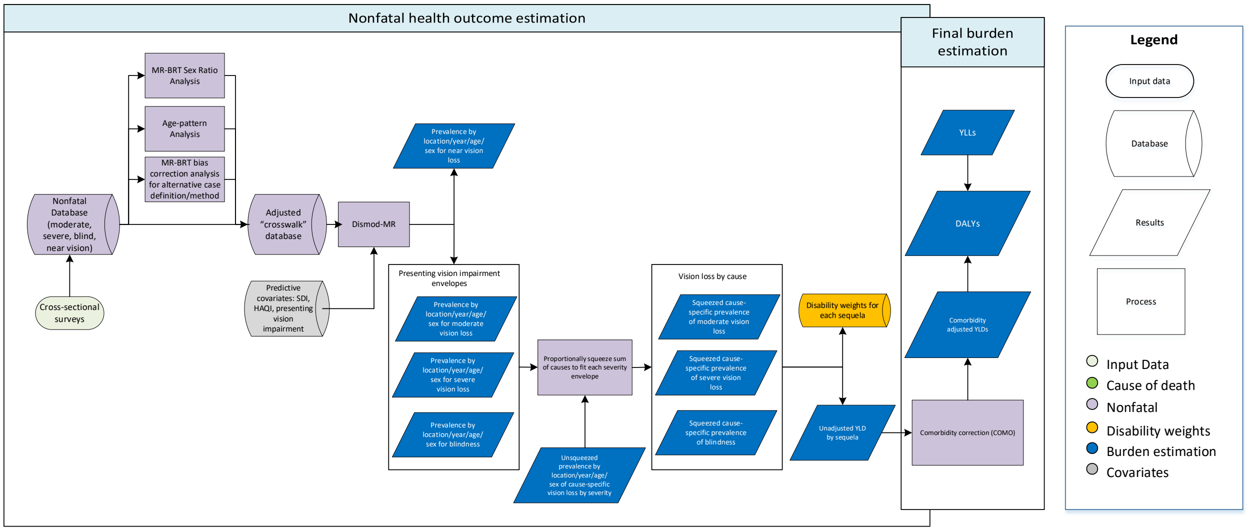

Cause-specific vision loss: Cataract, Glaucoma, Macular Degeneration, Other Vision Loss

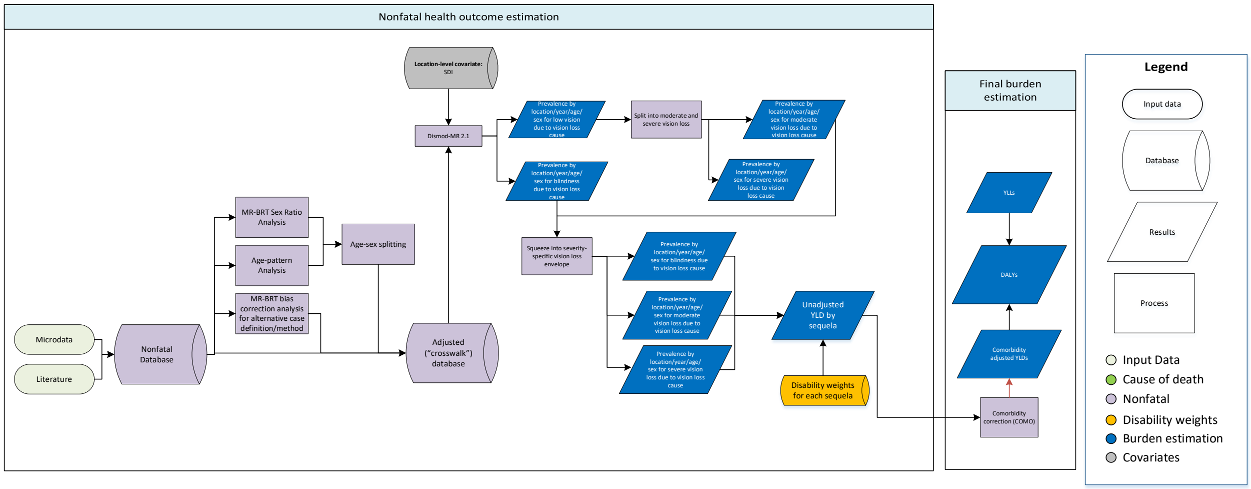

Cause-Specific Vision Loss: Diabetic Retinopathy

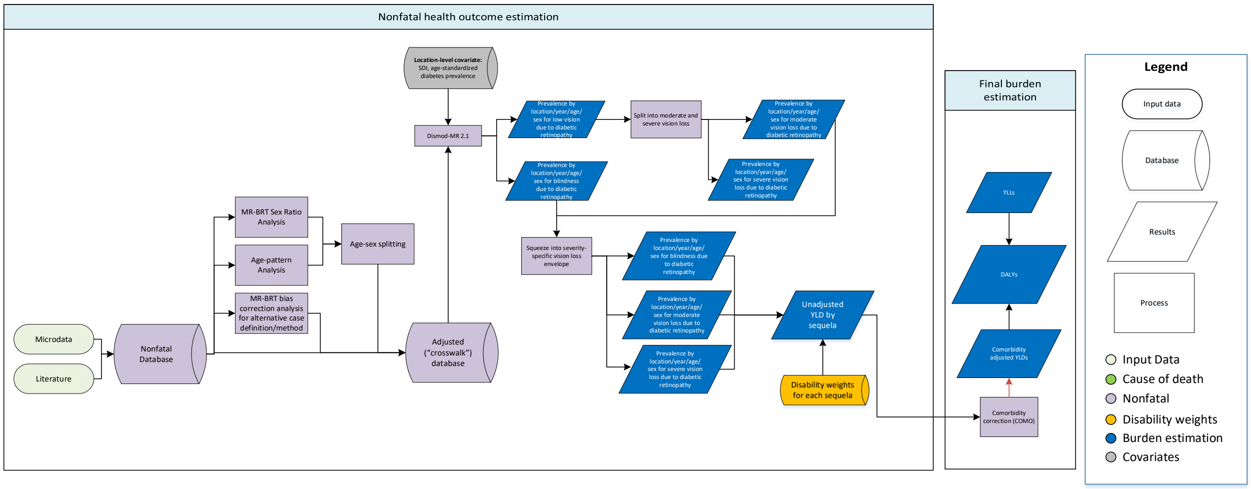

Cause-Specific Vision Loss: Trachoma

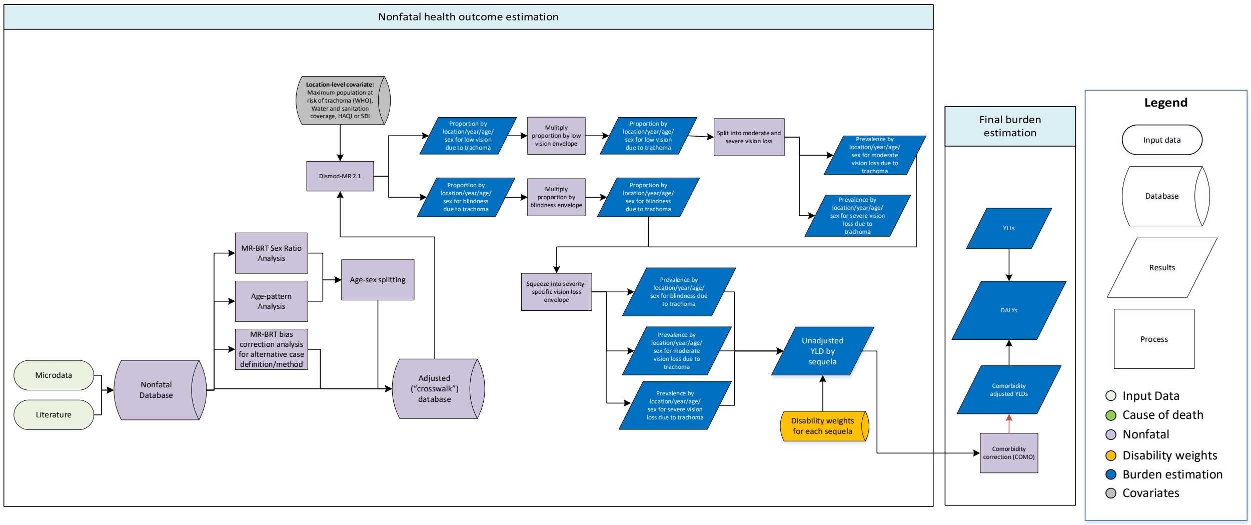

Case definition

We model vision loss with visual acuity <6/18 according to the Snellen chart as our reference case definition. The following levels of severity are modeled:

Condition	Case definition
Blindness	Visual acuity of <3/60 or <10% visual field around central fixation

Severe vision loss	≥3/60 and <6/60
Moderate vision loss	≥6/60 and <6/18
Near vision loss	Near visual acuity of <6/12 distance equivalent

Near vision loss describes the progressive inability to focus on near objects as individuals age (presbyopia). This impairs the ability to read. The majority of presbyopia can be corrected by the use of reading glasses, contact lenses, or refractive surgery.

We model vision loss due to the following causes: uncorrected refractive error, cataract, glaucoma, macular degeneration, diabetic retinopathy, trachoma, vitamin A deficiency, retinopathy of prematurity, meningitis, encephalitis, onchocerciasis, and a residual category of other vision loss. Vision loss due to vitamin A deficiency, retinopathy of prematurity, meningitis, encephalitis, and onchocerciasis are modelled as part of their underlying cause as described in their respective sections.

Refractive error is blurry vision due to the lens's inability to focus. The blurriness caused by refractive error can be addressed through the use of contact lenses, glasses, or refractive surgery. Cataract is clouding of the lens of the eye due to protein buildup that impairs vision. Glaucoma is a condition with increased intraocular pressure which can lead to damage of the optic nerve. Macular degeneration is a deterioration of the macula, leading to central vision loss. Diabetic retinopathy is damage to the retina caused by damaged blood vessels that can leak blood into the retina and cause scarring of the retina. Trachoma results from a conjunctival bacterial infection (*Chlamydia trachomatis*) that produces inflammation and scarring which leads to an inversion of the eyelids and eyelashes scratching the cornea, which, eventually after decades, leads to scarring of the cornea and vision loss or blindness.

Input data

Model inputs

Data on overall vision loss come from surveys measuring visual acuity in representative population-based studies, either from publications in peer-reviewed and grey literature or surveys for which we had the unit record data. Data were excluded if no test was used of visual acuity that can be converted to the Snellen scale, and if a study did not assess "presenting" or "best-corrected" vision. Presenting vision is the visual acuity as measured with the glasses used by an individual. Best corrected vision is with the best possible correction for refractive error, regardless of the strength of glasses used by an individual. A subset of these studies that reported vision loss by cause were used to estimate the prevalence of vision loss due to cataract, glaucoma, macular degeneration, diabetic retinopathy, and other causes.

For GBD 2015, we conducted a systematic review for new sources since GBD 2013 (covering 1/1/2013 – 5/20/2015), using the following search string:

((((glaucoma[Title/Abstract] OR cataract[Title/Abstract] OR macular[Title/Abstract] OR 'refractive error'[Title/Abstract] OR presbyopia[Title/Abstract]) OR (('blindness'[MeSH Terms] OR 'blindness'[All

Fields]) OR 'vision, low'[MeSH Terms])) AND ('2013'[PDAT] : '3000'[PDAT])) AND 'humans'[MeSH Terms]) AND (prevalence[Title/Abstract] OR incidence[Title/Abstract] OR epidemiology[Title/Abstract])

This yielded 1,169 results, of which we extracted 20 sources. Furthermore, we extracted from the following nationally representative surveys measuring visual acuity: the WHO Studies on Global Ageing and Adult Health (SAGE) and the United States National Health and Examination Surveys (NHANES).

For GBD 2016 and GBD 2017, we did a comprehensive extraction of the Rapid Assessment of Avoidable Blindness (RAAB) repository (http://raabdata.info/), a database of vision loss studies in developing settings across the world. There are 266 site-years of data, the majority of which have publicly available reports or publications of the data. A standardized methodology was used by all sources in the repository. This allowed us to use all 185 available reports, 70 of which were newly included for GBD 2017. In addition, we extracted two state-level national surveys from India.

For GBD 2019, we added literature sources from a systematic review conducted by collaborators in the Vision Loss Expert Group (VLEG) where all screened abstracts were sent to regional expert groups to assess data quality for inclusion. Many members of VLEG are also GBD collaborators and for GBD2019 estimates VLEG and GBD estimates are the same. This systematic review was conducted using the search engines MEDLINE, Embase, WHOLIS, SciELO, Open Grey and other grey literature searches commissioned by VLEG from York Health Economics Consortium, UK, an organization that has supported the VLEG by independently conducting these searches in the past. These searches covered the time period of 1980-2018. In total, since 2010 VLEG has provided data extracted from 137 studies, of which 67 came from the most recent systematic review update (2014-2018). In GBD 2019, data from 95 of these literature sources that matched GBD inclusion criteria were newly added to vision models. The Vision Loss Expert Group also provided additional data provided by principle investigators for existing studies, 51 new RAAB surveys, and 5-year disaggregated data for 151 RAAB surveys (previously only data for combined ages 50-99 were available), which better informed the age pattern for vision loss in this year's estimates.

In 2017, near-vision acuity included data from the following nationally representative studies measuring self-reported near vision loss: the Surveys of Health, Ageing, and Retirement in Europe (SHARE); the Multi-Country Survey Study on Health and Responsiveness (MCSS); and the World Health Surveys (WHS). In 2019, we transitioned to measured-only data, and added 11 new sources. The reason for this change in approach was that we could not find a plausible adjustment between measured and self-reported data in SAGE and NHANES surveys, which provide both measured and self-report data on vision loss. A crosswalk using NHANES data demonstrated an over-estimation in self-report data compared to measured data, while a crosswalk using SAGE data demonstrated the opposite.

Several adjustments were made to data extracted from the original data sources.

1) Where studies only reported "both" sex data, a meta-regression in MR-BRT was used to split these data points into sex-specific data points.
2) Where studies reported visual acuity spanning multiple thresholds (e.g., <6/60, rather than separate severe and blind estimates), we applied a logit-difference adjustment meta-regression, using data from studies reporting vision loss by both severity levels.
3) Some studies reported best-corrected vision loss, but not presenting vision loss. We crosswalked these data points using a logit difference meta-regression. This gave us predicted presenting vision loss data points for studies not explicitly reporting presenting vision loss.

4) Where data points spanned more than 25 years of age, we age-split using an algorithm that applies the age-pattern of the super-region (from a DisMod-MR model) to split the data to five-year age groups.

Whereas other vision loss aetiologies are modelled based on prevalence data, vision loss due to trachoma is modelled as a proportion of the overall best-corrected vision loss envelope, a strategy that was chosen based on the nature of available data.

The total source count used in GBD 2019 modeling is listed in the table below:

Total vision loss for each severity

Measure	Total sources
All measures	481
Prevalence	481

Vision loss for the modeled causes of vision loss

Measure	Total sources
All measures	387
Prevalence	369
Proportion	25

Modelling strategy

We modelled the prevalence of vision loss in two steps. In the first step, we estimated the total prevalence estimates of presenting vision loss: moderate vision loss, severe vision loss, blindness, and near vision loss (presbyopia). We directly derived prevalence of near vision loss from this step, whereas the remaining three models that reflect different severity levels of distance vision loss continued to the next step.

1) Estimate severity-specific vision loss (the "envelopes")

First, we ran five DisMod-MR 2.1 models to estimate the total prevalence estimates of presenting vision loss: moderate vision loss, severe vision loss, blindness, near vision loss, and presenting vision loss (moderate + severe + blindness). The presenting vision loss model was used as a covariate in the severity-specific models to improve consistency across severities.

Betas and exponentiated values, which can be interpreted as an odds ratio, are shown in the tables below for each adjustment for alternative case definitions. The best-corrected adjustment factor indicates whether the test measured visual acuity with the level of correction the patient presents with or the ophthalmologist provides additional correction via pinhole or lens correction. Rapid-assessment corrects for potential biases in cause-specific vision loss from studies using expedited visual acuity measurement. The severity covariate splits mixed severity data (moderate/severe, severe/blindness) into severity-specific data. Gamma captures the between study heterogeneity, and the adjustment factor is the inverse-logit transformed beta coefficient where <0.5 represents that the alternative case definition is adjusted upward and >0.5 represents that the alternative case definition is adjusted downward.

MR-BRT Crosswalk Adjustment Factors for Moderate Vision Loss Envelope

Data input	Reference or alternative case definition	Gamma	Beta Coefficient, Logit (95% CI)	Adjustment factor*
Presenting visual acuity, does not use rapid methodology	Ref	0.59	---	---
Best-corrected visual acuity	Alt		-1.11 (-2.27 – 0.06)	0.25
Uses rapid methodology	Alt		-0.06 (-1.23 – 1.11)	0.48

MR-BRT Crosswalk Adjustment Factors for Severe Vision Loss Envelope

Data input	Reference or alternative case definition	Gamma	Beta Coefficient, Logit (95% CI)	Adjustment factor*
Presenting visual acuity, does not use rapid methodology	Ref	0.69	---	---
Best-corrected visual acuity	Alt		-0.94 (-2.30 – 0.42)	0.28
Uses rapid methodology	Alt		0.11 (-1.25 – 1.48)	0.53

MR-BRT Crosswalk Adjustment Factors for Blindness Envelope

Data input	Reference or alternative case definition	Gamma	Beta Coefficient, Logit (95% CI)	Adjustment factor*
Presenting visual acuity, does not use rapid methodology	Ref	0.02	---	---
Best-corrected visual acuity	Alt		-0.15 (-0.19 – -0.15)	0.28
Uses rapid methodology	Alt		0.07 (-0.03 – 0.34)	0.53

MR-BRT Crosswalk Adjustment Factors for Cause-Specific Low Vision Models

Data input	Reference or alternative case definition	Gamma	Beta Coefficient, Logit (95% CI)	Adjustment factor*
Does not use rapid methodology	Ref	0.70	---	---
Uses rapid methodology	Alt		0.12 (-0.03 – 0.34)	0.53

MR-BRT Crosswalk Adjustment Factors for Cause-Specific Blindness Models

Data input	Reference or alternative case definition	Gamma	Beta Coefficient, Logit (95% CI)	Adjustment factor*
Does not use rapid methodology	Ref		---	---
Uses rapid methodology	Alt		0.06 (-0.03 – 0.15)	0.51

MR-BRT Crosswalk Adjustment for Mixed Severity Vision Loss Data

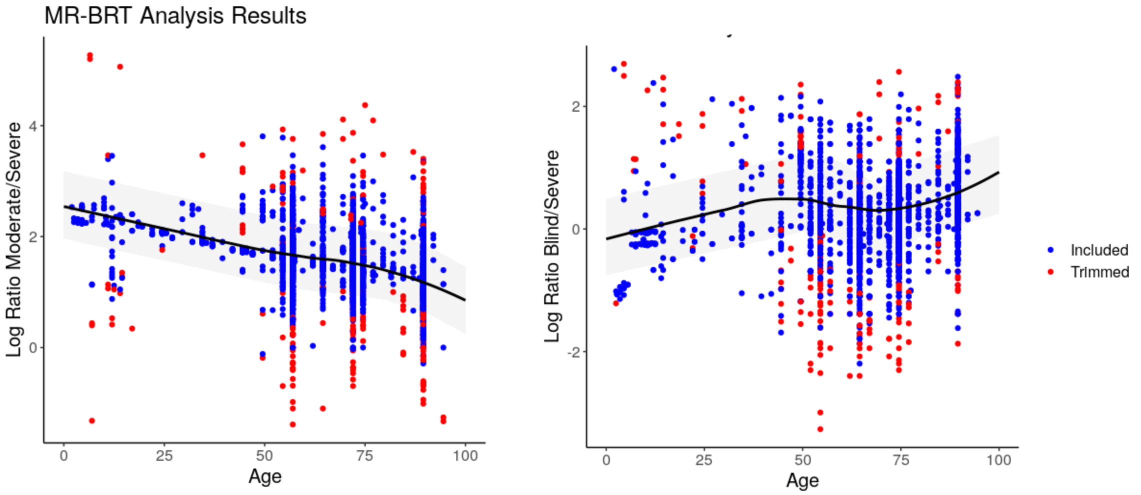

Mixed severity data (either mixed moderate and severe vision loss, or mixed severe vision loss and blindness) was split into severity-specific vision loss using a meta-regression in MR-BRT with a cubic spline on age. The above plots show the underlying data input in each regression, and the model fit over age. These plots demonstrate that the ratio of moderate to severe vision loss decreases with age, and the ratio of blindness to severe vision loss increases slightly with age.

Socio-demographic Index (SDI) and healthcare access and quality index (HAQI) were used as location covariates as a proxy measure of access to eye care such as cataract surgery. All predictors are listed below for each vision model. The exponentiated beta can be interpreted as an odds ratio. For example, in row 1 below, an exponentiated beta of 0.44 for socio-demographic index means that for every 1 unit change in socio-demographic index (measured on a scale from 0 to 1), moderate vision loss is lower by a factor of 0.44.

Summary of predictive covariates used in vision DisMod-MR meta-regression models

Cause	Covariate	Type	Parameter	Exponentiated beta (95% Uncertainty Interval)
Moderate vision loss envelope	Socio-demographic index	Prevalence	-0.83	0.44 (0.37 – 0.53)
Severe vision loss envelope	Socio-demographic index	Prevalence	-1.3	0.27 (0.22 – 0.35)

Blindness loss envelope	Socio-demographic index	Prevalence	-1.51	0.22 (0.18 – 0.28)
Blindness loss envelope	Healthcare access and quality index	Prevalence	-0.01	0.99 (0.99 – 0.99)
Blindness loss envelope	Presenting vision loss	Prevalence	1.20	3.31 (3.01 – 3.61)
Moderate vision loss due to uncorrected refractive error	Socio-demographic index	Prevalence	-1.46	0.23 (0.22 – 0.25)
Severe vision loss due to uncorrected refractive error	Socio-demographic index	Prevalence	-1.94	0.14 (0.14 – 0.16)
Blindness due to uncorrected refractive error	Socio-demographic index	Prevalence	-1.98	0.14 (0.14 – 0.14)
Vision loss due to other vision loss	Socio-demographic index	Prevalence	-1.00	0.37 (0.37-0.37)
Blindness due to other vision loss	Socio-demographic index	Prevalence	-1.00	0.37 (0.37-0.37)
Vision loss due to macular degeneration	Socio-demographic index	Prevalence	-0.94	0.39 (0.37 – 0.45)
Blindness due to macular degeneration	Socio-demographic index	Prevalence	-0.91	0.40 (0.37 – 0.48)
Vision loss due to glaucoma	Socio-demographic index	Prevalence	-0.99	0.37 (0.37 – 0.38)
Blindness due to glaucoma	Socio-demographic index	Prevalence	-1.97	0.14 (0.14 – 0.15)
Vision loss due to cataract	Socio-demographic index	Prevalence	-0.66	0.52 (0.40 – 0.66)
Blindness due to cataract	Socio-demographic index	Prevalence	-2.96	0.052 (0.05 – 0.05)
Vision loss due to diabetes mellitus	Socio-demographic index	Prevalence	-1.7	0.18 (0.14 – 0.29)
Vision loss due to diabetes mellitus	Diabetes age-standard prevalence (proportion)	Prevalence	0.72	2.05 (1.56 – 2.70)
Blindness due to diabetes mellitus	Socio-demographic index	Prevalence	-1.77	0.17 (0.14 – 0.24)
Blindness due to diabetes mellitus	Diabetes age- standard prevalence (proportion)	Prevalence	3.95	52.12 (48.23 – 54.49)
Vision loss due to trachoma	Socio-demographic index	Proportion	-5.99	0.003 (0.003 – 0.003)
Blindness due to trachoma	Healthcare access and quality index	Proportion	-1.98	0.14 (0.11 – 0.17)
Blindness due to trachoma	Max trachoma population at risk	Proportion	-0.66	0.51 (0.30 – 0.82)
Blindness due to trachoma	Improved water source (proportion access)	Proportion	-2.19	0.11 (0.07 – 0.18)

2) Estimate cause-specific vision loss

In the second step, we estimated the prevalence of vision loss due to multiple causes: refractive error, cataract, glaucoma, macular degeneration, diabetic retinopathy, retinopathy due to prematurity, trachoma, vitamin A deficiency, onchocerciasis, meningitis, and other causes not classified elsewhere. The vision loss due to retinopathy of prematurity, vitamin A deficiency, onchocerciasis, meningitis, tetanus, and neonatal conditions was modeled as part of these underlying causes. Vision loss due to trachoma was

modelled as a proportion of the envelope, with separate proportion models for (sever and moderate) vision loss and blindness. For each of cataract, glaucoma, macular degeneration, diabetic retinopathy, and other vision loss, we ran two DisMod-MR 2.1 models: one for the combined category of moderate and severe vision loss due to the cause, and one for blindness due to the cause. Moderate and severe vision loss were modelled together because input data were mostly available for the aggregate. Refractive error was modelled in three models, one for each severity. We used the following age restrictions:

Cause	Minimum age
Cataracts	20
Glaucoma	45
Macular degeneration	45
Diabetic retinopathy	20
Trachoma	15
Other vision loss	0

We estimated the proportions of low vision and blindness due to trachoma using Dismod-MR 2.1 models. Our model included fixed effects on the maximum population at risk for trachoma (proportion) reported by WHO, the proportion of the population with access to sanitation, and HAQI. Finally, we applied geographic and age restrictions to ensure that we estimate zero proportions in non-endemic locations and among those younger than 15 year of age (as scarring of the cornea due to trachoma takes decades to develop). The prevalence of trachoma at each severity level was calculated by multiplying the proportion of vision loss due to trachoma by the corresponding corrected vision loss envelope. For lack of data by level of severity of vision loss this assumes a similar distribution as for all causes of vision loss combined.

We split the moderate plus severe vision loss estimates for each cause into moderate and severe using the ratio of presenting moderate and severe vision loss envelopes. As exceptions, onchocerciasis and retinopathy of prematurity were modelled for moderate and severe vision loss as part of the estimation process of these causes.

We scaled the cause-specific vision loss prevalence to the total prevalence of the vision loss envelopes for each of the three severity levels. The final result is prevalence of vision loss due to each cause by severity.

Health states and disability weights

Health state name	Health state description	Disability weight
Distance vision, severe loss	This person has severe vision loss, which causes difficulty in daily activities, some emotional impact (for example, worry), and some difficulty going outside the home without assistance.	0.184 (0.125–0.259)
Distance vision, moderate loss	This person has vision problems that make it difficult to recognize faces or objects across a room.	0.031 (0.019–0.049)

Distance vision blindness	This person is completely blind, which causes great difficulty in some daily activities, worry and anxiety, and great difficulty going outside the home without assistance.	0.187 (0.124–0.26)
Near Vision Loss	This person has difficulty seeing things that are nearer than 3 feet if uncorrected by reading glasses, but has no difficulty with seeing things at a distance.	0.011 (0.005–0.02)

The following changes have been implemented for GBD 2019:

- We incorporated 151 age-disaggregated RAAB surveys, of which 51 RAAB surveys were newly added this year
- We added new data from 84 literature studies for distance vision and 11 literature studies for near vision loss
- Evaluated alternative case definitions (best-corrected data, studies using Rapid Assessment of Avoidable Blindness methodology, mixed severity data) using new logit difference meta-regression method to determine adjustment factors
- Used new MR-BRT methods to assess sex differences in prevalence for each vision loss cause and the vision loss envelopes, and apply this to “both” sex data points
- Transitioned to only using measured data for near vision loss estimates, and accepted case definition of near vision loss of 6/12 or worse.

Fistula (Impairment)

Flowchart

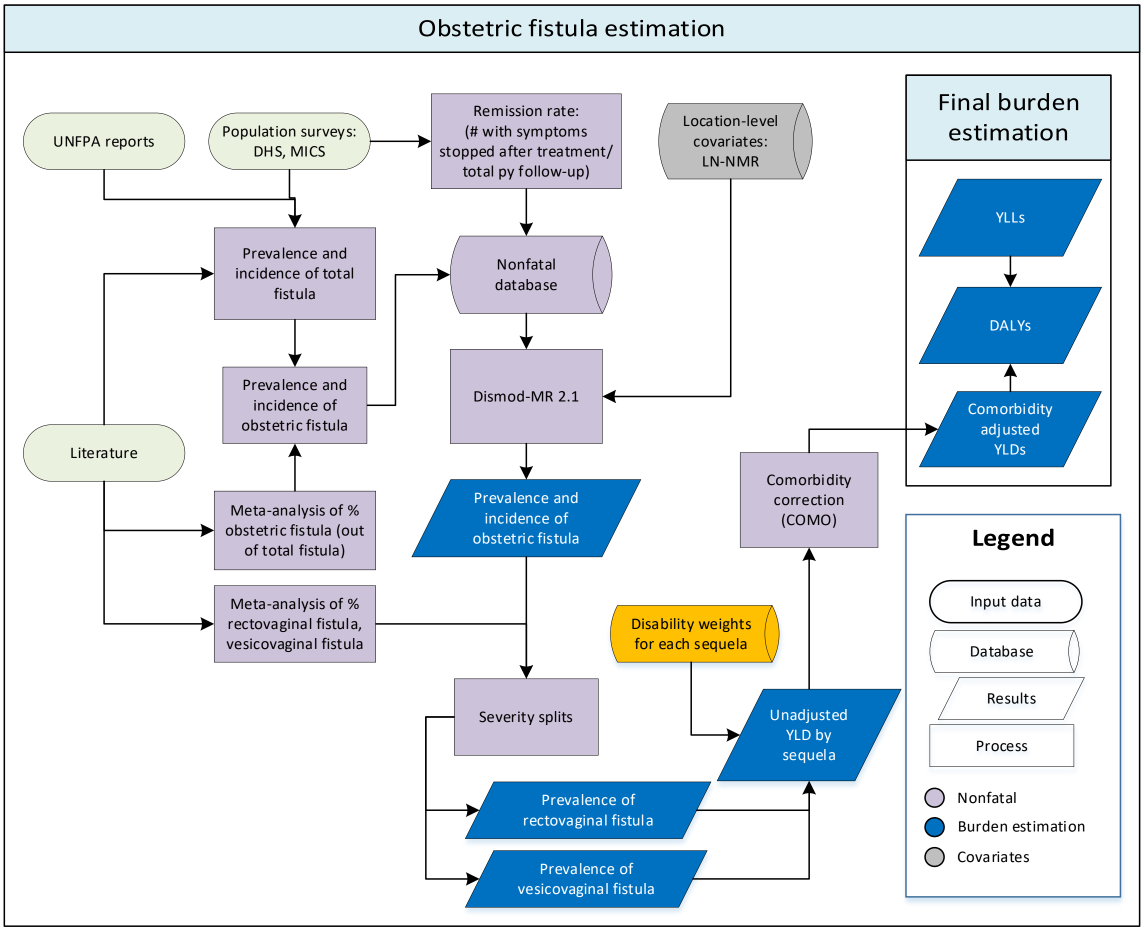

Case Definition

This is estimated as a component of maternal obstructed labour. Obstetric fistula is a severe long-term complication of prolonged obstructed labour in which a fistula (hole) develops between the birth canal and the bladder and/or rectum.

Input data

A systematic review was last conducted for GBD 2015 at which time no additional studies were identified. The PubMed search terms for this search, which were a repeat of those used in GBD 2010 and GBD 2013 were: (('obstetric fistula'[All Fields] OR 'vesicovaginal fistula'[All Fields]) OR 'rectovaginal fistula'[All Fields]) AND ('2013'[PDAT] : '2015'[PDAT]) AND 'humans'[MeSH Terms].

The exclusion criteria were studies that did not provide primary data on epidemiological parameters, eg, commentaries, case series, and reviews. The table below shows the number of literature studies included in GBD 2019, as well as the number of countries or subnational units and GBD world regions represented. In addition to using data from published studies, we also included data from UNFPA

reports and nationally representative Demographic and Health Surveys and Multiple Indicator Cluster Surveys.

The table below shows the number of total sources used in the estimation of obstetric fistula:

Cause/Impairment Name	Measure	Total sources*	Countries with data*
Maternal obstructed labor and uterine rupture	All measures	295	64
Maternal obstructed labor and uterine rupture	Prevalence	33	26
Maternal obstructed labor and uterine rupture	Incidence	249	46
Maternal obstructed labor and uterine rupture	Other	14	6

*These counts include the data sources used in estimating obstructed labour acute event, as well as obstetric fistula. The count of prevalence sources is exclusive to fistula, where the other measures are combined with obstructed labour.

Starting in GBD 2019, we began age splitting all input data where the age range was wider than a single GBD age group using weights derived from our best GBD 2019 Decomposition 1 model results. Weights were determined by dividing the result for a specific age by the result for the aggregate age specified in a given input data point. Age specific values were then calculated by multiplying the aggregate input data point by these age specific weights.

Modelling Strategy

For GBD 2019, obstetric fistula was modelled using DisMod-MR 2.1. We used neonatal mortality rate as a country-level covariate. We assume obstetric fistula is restricted to sub-Saharan Africa, South Asia, Yemen, Afghanistan, and Sudan. Remission was calculated, using the cure data from 11 Demographic and Health surveys, by dividing the number of cured obstetric fistula cases by total person-years of follow-up of all cases (cured, uncured, and untreated). The person-year of follow-up for uncured or untreated fistula cases was calculated as the time interval (in years) between the last birth and the date of interview. For cured cases, we assumed that the person-year of follow up was half the time interval (in years) between the last birth and the date of interview.

Betas and exponentiated values (which can be interpreted as an odds ratio) are shown in the table below:

Covariate Name	Type	Measure	Beta value	Exponentiated value
Neonatal Death Rate Modeled 2 (per 1000)	Country covariate	Prevalence	1.88662 (1.70300 - 1.99500)	6.60 (5.49 - 7.35)
Neonatal Death Rate Modeled 2 (per 1000)	Country covariate	Incidence	0.99474 (0.53650 - 1.44603)	2.70 (1.71 - 4.25)
Neonatal Death Rate Modeled 2 (per 1000)	Country covariate	Remission	-0.49721 (-0.97197 - -0.01657)	0.61 (0.38 - 0.98)

The following severity distributions were assigned based on a meta-analysis of published studies[1-4] and Pakistan Demographic and Health survey (2006–2007): vesicovaginal fistula (90.8%, 95% CI: 85.0 to 95.4%); rectovaginal fistula (9.2%, 95% CI: 4.6 to 15.0%). The lay descriptions and disability weights for severity levels derived from the GBD disability weights study are shown below.

Table 1: Health states for fistula impairment severity distribution

Severity level	Lay description	DW (95% CI)

vesicovaginal fistula	has an abnormal opening between the bladder and the vagina, which makes her unable to control urinating. The woman is anxious and depressed.	0.342 (0.227–0.478)
rectovaginal fistula	has an abnormal opening between her vagina and rectum causing flatulence and feces to escape through the vagina. The person gets infections in her vagina, and has pain when urinating.	0.501 (0.339–0.657)

References

1. Danso KA, Martey J, Wall LL, Elkins TE. The epidemiology of genitourinary fistulae in Kumasi, Ghana, 1977–1992. International Urogynecology Journal. 1996;7(3):117-120.

2. Wall LL, Karshima JA, Kirschner C, Arrowsmith SD. The obstetric vesicovaginal fistula: characteristics of 899 patients from Jos, Nigeria. Am. J. Obstet. Gynecol. 2004;190(4):1011-1016.

3. Das R, Sengupta S. Vesico-vaginal fistula of obstetric origin. J. Obstet. Gynaecol. India. 1969;19:383-389.

4. Mahfouz NP. Urinary and Faecal Fistulae. BJOG. 1938;45(3):405-424.

Section 5: Estimation process for DALYs[3]

Section 5.1: Computing DALYs

To estimate DALYs for GBD 2019, we started by estimating cause-specific mortality and non-fatal health loss. For each year for which YLDs have been estimated, we computed DALYs by adding YLLs and YLDs for each age-sex-location. Uncertainty in YLLs was assumed to be independent of uncertainty in YLDs. We calculated 1000 draws for DALYs by summing the first draw of the 1000 draws for YLLs and YLDs and then repeating for each subsequent draw. 95% UIs were computed by using the 25th and 975th ordered draw of the DALY uncertainty distribution. We calculated DALYs as the sum of YLLs and YLDs for each cause, location, age group, sex, and year. For more information, please refer to the following figure A.

Figure A. DALY burden estimation for GBD 2019

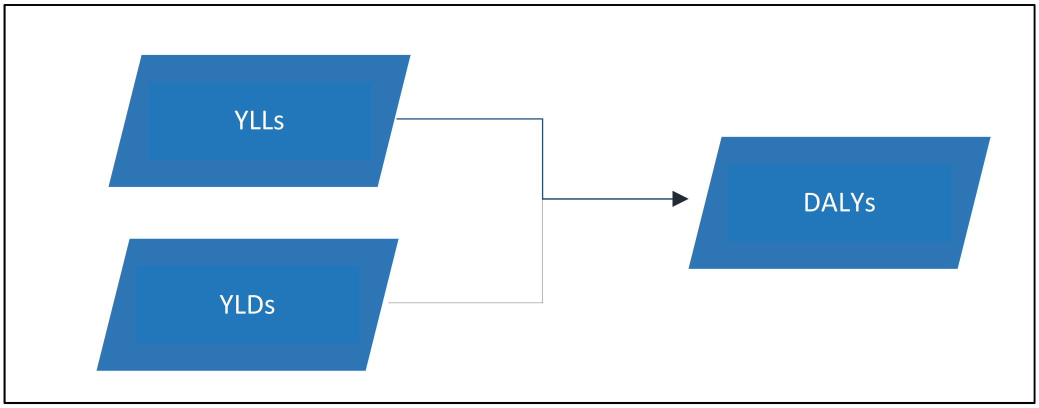

Section 6: SDI analysis[3]

Section 6.1: SDI definition

The Socio-demographic Index (SDI) is a composite indicator of background social and economic conditions that influence health outcomes in each location. In short, it is the geometric mean of 0 to 1 indices of total fertility rate (TFR) for those younger than 25 years old (TFU25), mean education for those 15 years old and older (EDU15+), and lag-distributed income (LDI) per capita. For GBD 2019, after calculating SDI, values were multiplied by 100 for a scale of 0 to 100.

Section 6.2: Development of revised SDI indicator

SDI was originally constructed for GBD 2015 by using the Human Development Index (HDI) methodology, wherein a 0 to 1 index value was determined for each of the original three covariate inputs (TFR in ages 15 to 49 years, EDU15+, and LDI per capita) by using the observed minima and maxima over the estimation period to set the scales.[68]

In response to feedback from collaborators and the evolution of the GBD, we have refined the indicator with each GBD cycle. Beginning in GBD 2017, along with our expanded estimation of age-specific fertility, we replaced TFR with TFU25 as one of the three component indices. The TFU25 provides a better measure of women's status in society because it focuses on ages at which childbearing disrupts the pursuit of education and entrance into the workforce. In addition, we observed that in highly developed countries, the TFU25 has tended to decline consistently over time despite rebounds in TFR driven by increasing

fertility at older ages. The concordance correlation coefficient between SDI based on the GBD 2016 method and the updated method for GBD 2017 was 0.981.

During GBD 2016, we moved from using relative index scales to using absolute scales to enhance the stability of SDI interpretation over time because we noticed that the measure was highly sensitive to the addition of subnational units that tended to stretch the empirical minima and maxima.[21] We selected the minima and maxima of the scales by examining the relationships each of the inputs had with life expectancy at birth and under-5 mortality and by identifying points of limiting returns at both high and low values if they occurred before theoretical limits (eg, a TFU25 of 0) were reached.

Thus, for each covariate input, an index score of 0 represents the minimum level of each covariate input past which selected health outcomes can get no worse, and an index score of 1 represents the maximum level of each covariate input past which selected health outcomes cease to improve. As a composite, a location with an SDI of 0 would have a theoretical minimum level of sociodemographic development relevant to these health outcomes, and a location with an SDI of 1 (before multiplying by 100 for reporting) would have a theoretical maximum level of sociodemographic development relevant to these health outcomes.

We computed the index scores underlying SDI as follows:

$$I_{Cly} = \max\left(\frac{C_{ly} - C_{low}}{C_{high} - C_{low}}, 0.005\right)$$

Where:

> I_{cly} is the index for covariate *C*, location *l*, and year *y* and is equal to the difference between the value of that covariate in that location-year and the lower bound of the covariate divided by the difference between the upper and lower bounds for that covariate

If the values of input covariates fell outside the upper or lower bounds, they were mapped to the respective upper or lower bounds. We also note that the index value for TFU25 was computed as $1 - I_{TFU25ly}$ because lower TFU25s correspond to higher levels of development and thus higher index scores. For GBD 2019, we expanded the computation of SDI to 1062 national and subnational locations spanning the time period 1950–2019.

The composite SDI is the geometric mean of these three indices for a given location-year. The cut-off values used to determine quintiles for analysis were then computed by using country-level estimates of SDI for the year 2019, excluding countries with populations less than 1 million.

For GBD 2019, final SDI values were multiplied by 100 for reporting, in order to improve understanding of and broader engagement with the values. As such, GBD 2019 SDI is calculated as it was in 2017, but multiplied by 100 at the end (see example calculation below). Final reporting values are on a 0 to 100 scale.

Example calculation

We present the equation used to calculate SDI for a hypothetical country in the year 2010:

$$TFU25 = 1.09;\ Mean\ educ\ yrs\ pc = 8.23;\ lnLDI = 9.60$$

$$I_{TFU25} = 1 - \frac{1.09 - 0}{3 - 0} = 0.637$$

$$I_{Educ} = \frac{8.23 - 0}{17 - 0} = 0.484$$

$$I_{lnLDI} = \frac{9.60 - 5.52}{11.00 - 5.52} = 0.744$$

$$SDI = \sqrt[3]{I_{TFU25} * I_{Educ} * I_{lnLDI}} = \sqrt[3]{.637 * .484 * .744} = 0.611$$

$$I_{lnLDI} = \frac{9.58 - 5.52}{11.00 - 5.52} = 0.741$$

$$SDI = \sqrt[3]{I_{TFR} * I_{Educ} * I_{lnLDI}} = \sqrt[3]{.855 * .543 * .741} = 0.701$$

GBD 2019 reporting $SDI = 0.701 * 100 = 70.1$

Section 7: References

References are for the main appendix text. References for cause-specific write-ups are found at the end of each of those sections.

1 Roth GA, Abate D, Abate KH, *et al.* Global, regional, and national age-sex-specific mortality for 282 causes of death in 195 countries and territories, 1980–2017: a systematic analysis for the Global Burden of Disease Study 2017. *The Lancet* 2018; **392**: 1736–88.

2 James SL, Abate D, Abate KH, *et al.* Global, regional, and national incidence, prevalence, and years lived with disability for 354 diseases and injuries for 195 countries and territories, 1990–2017: a systematic analysis for the Global Burden of Disease Study 2017. *The Lancet* 2018; **392**: 1789–858.

3 Kyu HH, Abate D, Abate KH, *et al.* Global, regional, and national disability-adjusted life-years (DALYs) for 359 diseases and injuries and healthy life expectancy (HALE) for 195 countries and territories, 1990–2017: a systematic analysis for the Global Burden of Disease Study 2017. *Lancet* 2018; **392**: 1859–922.

4 Stanaway JD, Afshin A, Gakidou E, *et al.* Global, regional, and national comparative risk assessment of 84 behavioural, environmental and occupational, and metabolic risks or clusters of risks for 195 countries and territories, 1990–2017: a systematic analysis for the Global Burden of Disease Study 2017. *The Lancet* 2018; **392**: 1923–94.

5 Stevens GA, Alkema L, Black RE, *et al.* Guidelines for Accurate and Transparent Health Estimates Reporting: the GATHER statement. *The Lancet* 2016; **388**: e19–23.

6 Sankoh O, Byass P. The INDEPTH Network: filling vital gaps in global epidemiology. *Int J Epidemiol* 2012; **41**: 579–88.

7 Lozano R, Freeman MK, James SL, *et al.* Performance of InterVA for assigning causes of death to verbal autopsies: multisite validation study using clinical diagnostic gold standards. *Popul Health Metr* 2011; **9**: 50.

8 GBD 2019 Demographics Collaborators. Global, regional, and national age-sex-specific fertility, mortality, and population estimates, 1950–2019: a comprehensive demographic analysis for the Global Burden of Disease Study 2019. *The Lancet* In Press.

9 Office of the Registrar General and Census Commissioner. India Medical Certification of Cause of Death Reports 1990-2010. India, 2014.

10 Office of the Registrar General and Census Commissione. India Sample Registration System Statistical Report 2017. India, 2018.

11 Todd S, Barr S, Passmore AP. Cause of death in Alzheimer’s disease: a cohort study. *QJM Mon J Assoc Physicians* 2013; **106**: 747–53.

12 Brunnström HR, Englund EM. Cause of death in patients with dementia disorders. *Eur J Neurol* 2009; **16**: 488–92.

13 Keene J, Hope T, Fairburn CG, Jacoby R. Death and dementia. *Int J Geriatr Psychiatry* 2001; **16**: 969–74.

14 Thomas BM, Starr JM, Whalley LJ. Death certification in treated cases of presenile Alzheimer’s disease and vascular dementia in Scotland. *Age Ageing* 1997; **26**: 401–6.

15 Naghavi M, Makela S, Foreman K, O’Brien J, Pourmalek F, Lozano R. Algorithms for enhancing public health utility of national causes-of-death data. *Popul Health Metr* 2010; **8**: 9.

16 Barker B, Degenhardt L, National Drug and Alcohol Research Centre (Australia). Accidental drug-induced deaths in Australia 1997-2001. Sydney, Australia: National Drug and Alcohol Research Centre, University of New South Wales, 2003.

17 Roxburgh A, Burns L. Accidental drug-induced deaths due to opioids in Australia, 2011. Sydney, Australia: National Drug and Alcohol Research Centre, University of New South Wales, 2015.

18 Roxburgh A, Burns L. Cocaine and methamphetamine related drug-induced deaths in Australia, 2011. Sydney, Australia: National Drug and Alcohol Research Centre, University of New South Wales, 2015.

19 Naghavi M, Wang H, Lozano R, *et al.* Global, regional, and national age-sex specific all-cause and cause-specific mortality for 240 causes of death, 1990–2013: a systematic analysis for the Global Burden of Disease Study 2013. *The Lancet* 2015; **385**: 117–71.

20 Lozano R, Naghavi M, Foreman K, *et al.* Global and regional mortality from 235 causes of death for 20 age groups in 1990 and 2010: a systematic analysis for the Global Burden of Disease Study 2010. *The Lancet* 2012; **380**: 2095–128.

21 Wang H, Abajobir AA, Abate KH, *et al.* Global, regional, and national under-5 mortality, adult mortality, age-specific mortality, and life expectancy, 1970–2016: a systematic analysis for the Global Burden of Disease Study 2016. *The Lancet* 2017; **390**: 1084–150.

22 Calvert C, Ronsmans C. The contribution of HIV to pregnancy-related mortality: a systematic review and meta-analysis. *Aids* 2013; **27**: 1631–9.

23 Figueroa-Damián R. Pregnancy outcome in women infected with the human immunodeficiency virus. *Salud Publica Mex* 1999; **41**: 362–7.

24 Ryder RW, Nsuami M, Nsa W, *et al.* Mortality in HIV-1-seropositive women, their spouses and their newly born children during 36 months of follow-up in Kinshasa, Zaïre. *AIDS Lond Engl* 1994; **8**: 667–72.

25 Zvandasara P, Saungweme G, Mlambo JT, Moyo J. Post Caesarean section infective morbidity in HIV-positive women at a tertiary training hospital in Zimbabwe. *Cent Afr J Med* 2007; **53**: 43–7.

26 Chilongozi D, Wang L, Brown L, *et al.* Morbidity and mortality among a cohort of human immunodeficiency virus type 1-infected and uninfected pregnant women and their infants from Malawi, Zambia, and Tanzania. *Pediatr Infect Dis J* 2008; **27**: 808–14.

27 Leroy V, Ladner J, Nyiraziraje M, *et al.* Effect of HIV-1 infection on pregnancy outcome in women in Kigali, Rwanda, 1992-1994. Pregnancy and HIV Study Group. *AIDS* 1998; **12**: 643–50.

28 Kourtis A, Bansil P, McPheeters M, Meikle S, Posner S, Jamieson D. Hospitalizations of pregnant HIV-infected women in the USA prior to and during the era of HAART, 1994–2003. *AIDS* 2006; **20**: 1823–31.

29 Ticconi C, Mapfumo M, Dorrucci M, *et al.* Effect of maternal HIV and malaria infection on pregnancy and perinatal outcome in Zimbabwe. *Jaids J Acquir Immune Defic Syndr* 2003; **34**: 289–94.

30 Brown T, Peerapatanapokin W. The Asian Epidemic Model: a process model for exploring HIV policy and programme alternatives in Asia. *Sex Transm Infect* 2004; **80**: i19–24.

31 DerSimonian R, Laird N. Meta-analysis in clinical trials. *Control Clin Trials* 1986; **7**: 177–88.

32 Matthews LT, Kaida A, Kanters S, *et al.* HIV-infected women on antiretroviral treatment have increased mortality during pregnant and postpartum periods. *AIDS Lond Engl* 2013; **27 Suppl 1**: S105-112.

33 Westreich D, Maskew M, Evans D, Firnhaber C, Majuba P, Sanne I. Incident pregnancy and time to death or AIDS among HIV-positive women receiving antiretroviral therapy. *PloS One* 2013; **8**: e58117.

34 Stover J. AIM: A computer program for making HIV/AIDS projections and examining the social and economic impact of AIDS. Glastonbury, CT: Futures Group, 2005.

35 Lozano R, Lopez AD, Atkinson C, Naghavi M, Flaxman AD, Murray CJ. Performance of physician-certified verbal autopsies: multisite validation study using clinical diagnostic gold standards. *Popul Health Metr* 2011; **9**: 32.

36 Foreman KJ, Lozano R, Lopez AD, Murray CJ. Modeling causes of death: an integrated approach using CODEm. *Popul Health Metr* 2012; **10**: 1.

37 Bell RM, Koren Y. Lessons from the Netflix Prize Challenge. *SIGKDD Explor Newsl* 2007; **9**: 75–79.

38 Bell RM, Koren Y, Volinsky C. All Together Now: A Perspective on the Netflix Prize. *CHANCE* 2010; **23**: 24–9.

39 Flaxman AD, Vos T, Murray CJL, Kiyono P, editors. An integrative metaregression framework for descriptive epidemiology, 1 edition. Seattle: University of Washington Press, 2015.

40 Murray CJL, Callender CSKH, Kulikoff XR, *et al.* Population and fertility by age and sex for 195 countries and territories, 1950–2017: a systematic analysis for the Global Burden of Disease Study 2017. *Lancet* 2018; **392**: 1995–2051.

41 GBD 2019 Risk Factors Collaborators. Global burden of 87 risk factors in 204 countries and territories, 1990–2019: a systematic analysis for the Global Burden of Disease Study 2019. *The Lancet*; **In press**.

42 Vasudevan S, Ramos F, Nettleton E, Durrant-Whyte H, Blair A. Gaussian Process modeling of large scale terrain. In: 2009 IEEE International Conference on Robotics and Automation. 2009: 1047–53.

43 Rasmussen CE, Williams CKI. Gaussian Processes for Machine Learning. Cambridge, Mass: The MIT Press, 2005.

44 Murray CJ, Lopez AD. Global mortality, disability, and the contribution of risk factors: Global Burden of Disease Study. *The Lancet* 1997; **349**: 1436–42.

45 Ng M, Fleming T, Robinson M, *et al.* Global, regional, and national prevalence of overweight and obesity in children and adults during 1980–2013: a systematic analysis for the Global Burden of Disease Study 2013. *Lancet* 2014; **384**: 766–81.

46 Ng M, Freeman MK, Fleming TD, *et al.* Smoking Prevalence and Cigarette Consumption in 187 Countries, 1980-2012. *JAMA* 2014; **311**: 183–92.

47 Zheng P, Aravkin AY, Barber R, Sorensen RJD, Murray CJL. Trimmed Constrained Mixed Effects Models: Formulations and Algorithms. *ArXiv190910700 Math Stat* 2019; published online Sept 23. http://arxiv.org/abs/1909.10700 (accessed Nov 15, 2019).

48 Aravkin A, Davis D. Trimmed statistical estimation via variance reduction. *Math Oper Res* 2019; published online July 5. https://pubsonline.informs.org/doi/10.1287/moor.2019.0992 (accessed Nov 15, 2019).

49 Rousseeuw P. Multivariate estimation with high breakdown point. 1985. DOI:10.1007/978-94-009-5438-0_20.

50 Wächter A, Biegler LT. On the implementation of an interior-point filter line-search algorithm for large-scale nonlinear programming. *Math Program* 2006; **106**: 25–57.

51 Boor C de. A Practical Guide to Splines. New York: Springer-Verlag, 1978 https://www.springer.com/gp/book/9780387953663 (accessed Nov 15, 2019).

52 Friedman JH. Multivariate Adaptive Regression Splines. *Ann Stat* 1991; **19**: 1–67.

53 Pya N, Wood SN. Shape constrained additive models. *Stat Comput* 2015; **25**: 543–59.

54 Efron B, Tibshirani RJ. An Introduction to the Bootstrap, 1 edition. New York: Chapman and Hall/CRC, 1993.

55 Burstein R, Fleming T, Haagsma J, Salomon JA, Vos T, Murray CJL. Estimating distributions of health state severity for the global burden of disease study. *Popul Health Metr* 2015; **13**: 31.

56 Medical Expenditure Panel Survey Home. https://meps.ahrq.gov/mepsweb/ (accessed Nov 15, 2019).

57 NIAAA Publications. http://pubs.niaaa.nih.gov/publications/arh29-2/74-78.htm (accessed Nov 15, 2019).

58 4326.0 - Mental Health and Wellbeing: Profile of Adults, Australia, 1997. 1998; published online March 12. http://www.abs.gov.au/ausstats/abs@.nsf/ProductsbyReleaseDate/D5A0AC778746378FCA2574EA00122887?OpenDocument (accessed Nov 15, 2019).

59 Salomon JA, Haagsma JA, Davis A, *et al.* Disability weights for the Global Burden of Disease 2013 study. *Lancet Glob Health* 2015; **3**: e712–23.

60 Stouthard MEA, Essink-Bot ML, Bonsel GJ, *et al.* Disability weights for diseases in the Netherlands. AmsterdamInst. Sociale Geneeskunde, 1997 https://dare.uva.nl/search?identifier=e7cbed27-7fab-4104-9b44-1657515747c2 (accessed Nov 15, 2019).

61 Campeau L. The Canadian Cardiovascular Society grading of angina pectoris revisited 30 years later. *Can J Cardiol* 2002; **18**: 371–9.

62 Dolgin M, Committee NYHAC. Nomenclature and criteria for diagnosis of diseases of the heart and great vessels, 9th ed. Boston, MA: Little Brown & Co, 1994 https://trove.nla.gov.au/version/13288061 (accessed Nov 15, 2019).

63 Nord E. Disability weights in the Global Burden of Disease 2010: Unclear meaning and overstatement of international agreement. *Health Policy* 2013; **111**: 99–104.

64 Taylor HR, Jonas JB, Keeffe J, *et al.* Disability weights for vision disorders in Global Burden of Disease study. *The Lancet* 2013; **381**: 23.

65 Voigt K, King NB. Disability weights in the global burden of disease 2010 study: two steps forward, one step back? *Bull World Health Organ* 2014; **92**: 226–8.

66 Kretzschmar M, Mangen M-JJ, Pinheiro P, *et al.* New Methodology for Estimating the Burden of Infectious Diseases in Europe. *PLOS Med* 2012; **9**: e1001205.

67 Vos T, Flaxman AD, Naghavi M, *et al.* Years lived with disability (YLDs) for 1160 sequelae of 289 diseases and injuries 1990–2010: a systematic analysis for the Global Burden of Disease Study 2010. *The Lancet* 2012; **380**: 2163–96.

68 Wang H, Naghavi M, Allen C, *et al.* Global, regional, and national life expectancy, all-cause mortality, and cause-specific mortality for 249 causes of death, 1980–2015: a systematic analysis for the Global Burden of Disease Study 2015. *The Lancet* 2016; **388**: 1459–544.

Figure S1. Analytical flowchart for the development of the GBD 2019 cause of death database (A) and different strategies used to model different causes (B) and ultimately combine them into a consistent set of cause-specific deaths for each location, age, sex, and year.

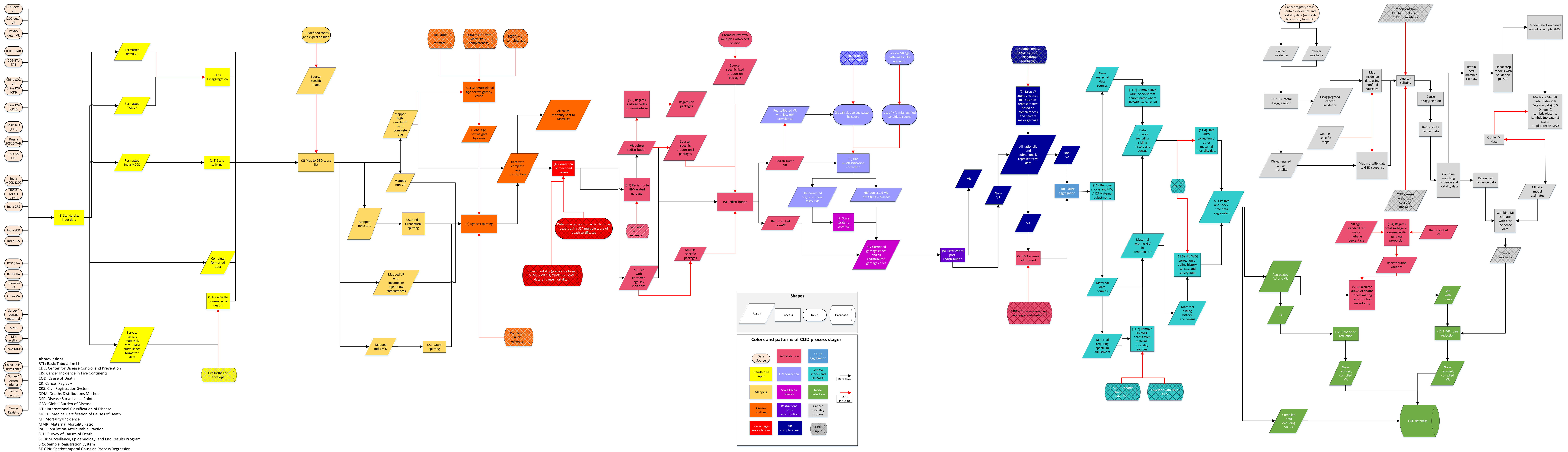

Abbreviations:
BTL: Basic Tabulation List
CDC: Center for Disease Control and Prevention
CI5: Cancer Incidence in Five Continents
COD: Cause of Death
CR: Cancer Registry
CRS: Civil Registration System
DDM: Deaths Distributions Method
DSP: Disease Surveillance Points
GBD: Global Burden of Disease
ICD: International Classification of Disease
MCCD: Medical Certification of Causes of Death
MI: Mortality/Incidence
MMR: Maternal Mortality Ratio
PAF: Population-Attributable Fraction
SCD: Survey of Causes of Death
SEER: Surveillance, Epidemiology, and End Results Program
SRS: Sample Registration System
ST-GPR: Spatiotemporal Gaussian Process Regression
TAB: Tabulation List
VA: Verbal Autopsy
VR: Vital Registration

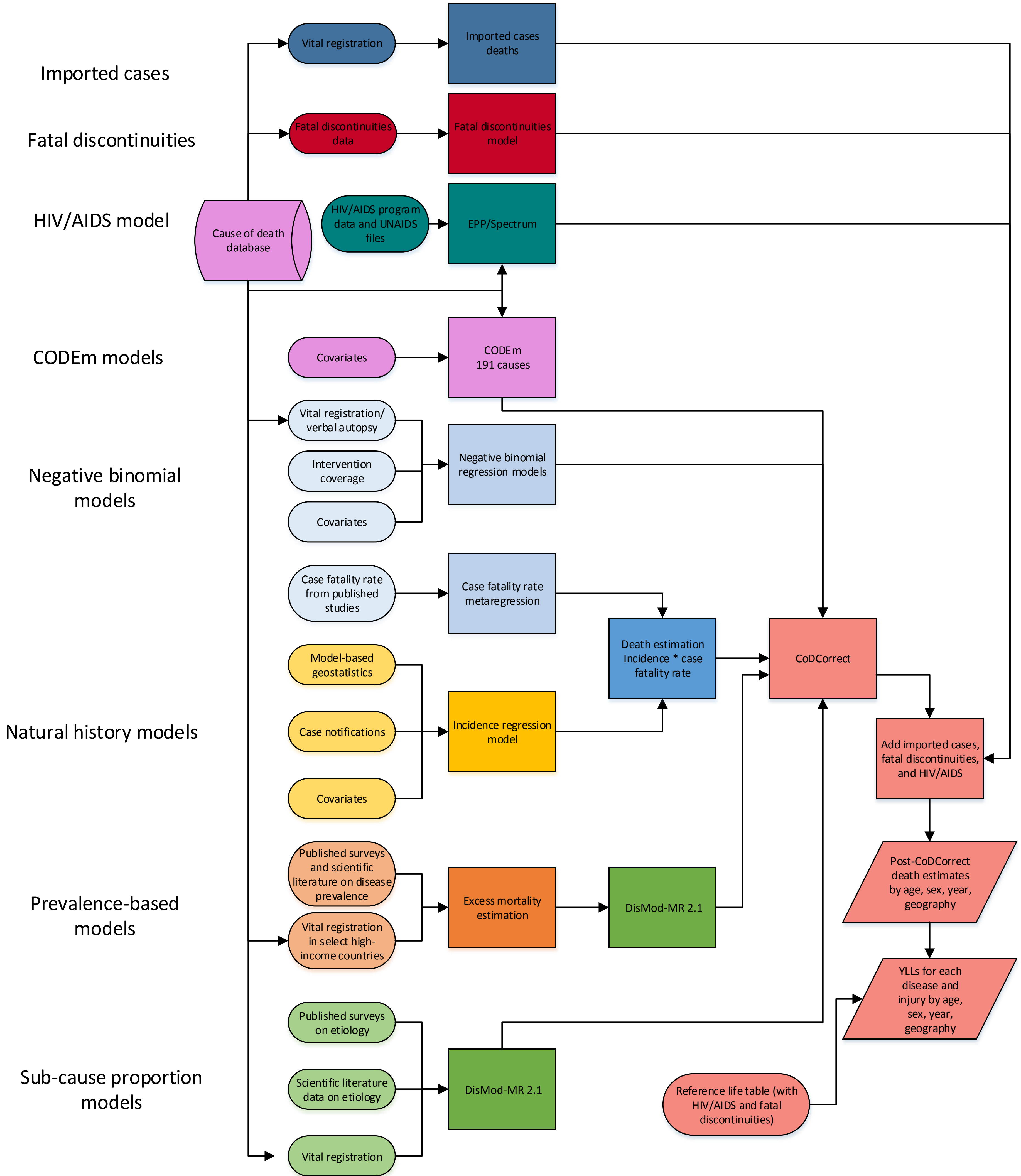

Figure S2. GBD 2019 causes of death estimation flowchart by modelling group

Abbreviations:
CODEm: Cause of death ensemble model
GBD: Global Burden of Disease
YLL: years of life lost

Figure S3. Vital Registration and Verbal Autopsy data availability by country and territory, 1980−2018

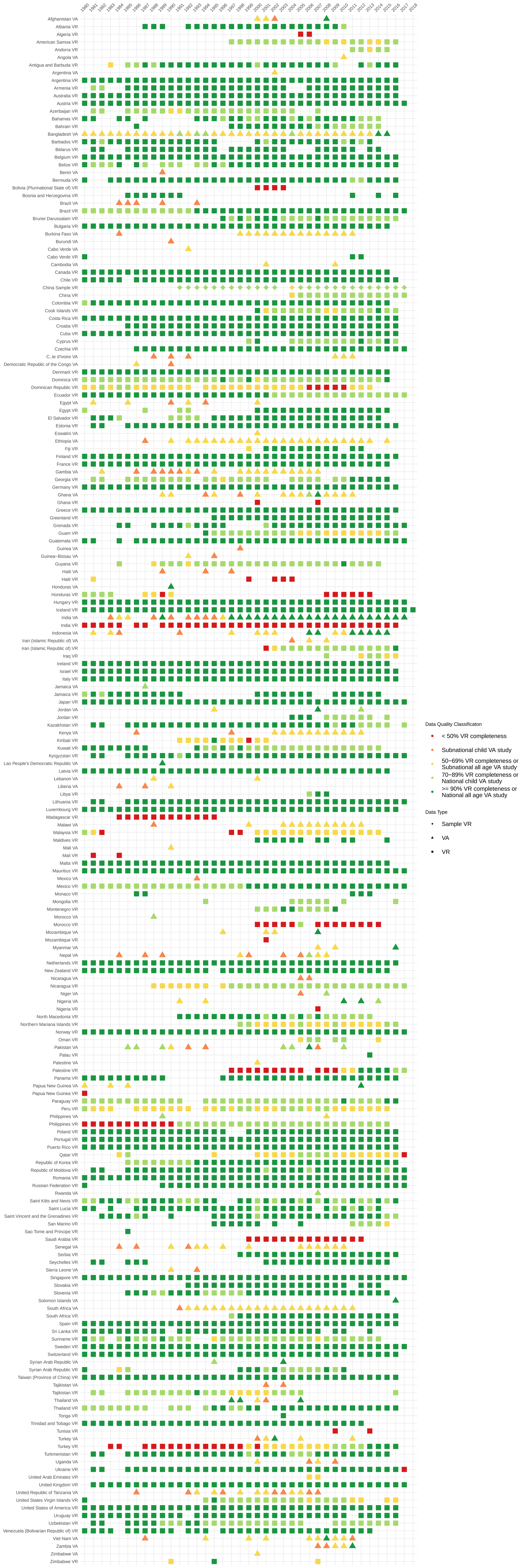

Figure S4: Percentage of vital registration deaths assigned to major garbage codes for all ages and sexes by country and territory, 1980-2018

Year

Country	'80	'81	'82	'83	'84	'85	'86	'87	'88	'89	'90	'91	'92	'93	'94	'95	'96	'97	'98	'99	'00	'01	'02	'03	'04	'05	'06	'07	'08	'09	'10	'11	'12	'13	'14	'15	'16	'17	'18
Albania								37	35	34			36	32	35	30	29	25	27	25	26	28	27	27	26	36	34	25	25	32	23								
Algeria																										51	49												
American Samoa																		26	25	20	17	16	19	15	24	16	30	24	14	20	17	23	21	22	18	15	16		
Andorra																																24	20	18	20	18			
Antigua and Barbuda				29		37	40	38	34	30	25	24	25	24	25	28	20	26	22	21	19	22	21	17	19	28	28	18	22	19			29	27	21	24	26		
Argentina	24	24	27	28	29	30	29	29	29	29	29	30	30	31	30	30	30	32	31	32	32	32	32	31	31	31	31	32	32	32	32	32	31	31	29	26	27		
Armenia		12	13			13	12	13	33	14	13	13	14	17	19	16	15	14	13	13	13	13	14	15			9	11	9	10	10	10	12	12	11	10	11		
Australia	7	7	7	7	7	7	6	7	6	7	7	6	6	7	6	8	6	7	8	8	8	7	8	9	9	9	8	9	9	9	9	9	10	9	9	9	10		
Austria	9	9	9	15	8	7	7	7	7	7	7	7	8	8	8	8	8	8	8	9	9	8	6	5	6	6	7	7	7	8	9	9	10	9	9	11	11	11	
Azerbaijan		13	13			12	11	12	12	14	13	12	11	11	17	14	15	14	14	15	15	8	8	8	10			45											
Bahrain							25											32	42	41	38	39	41	38	41	39	39	38	40	43	47	44	41	38	34				
Barbados	24	25	24	24	25	25	21	22	23	22	22	23	20	22	22	20					23	22	21	25	28	27	18	18	19	19	18	18	21	20					
Belarus		14	14			13	12	12	13	14	20	22	22	22	22	22		22	20	19	20	20	17	18				18	19	17	16	19		18	16				
Belgium	21	20	20	20	21	22	20	19	21	21	20	21	18	16	16	15	14	14	14	15	14	14	14	15	14	14	14	15	14	15	17	17	18	17	17	18	18		
Belize	33	26	36	39	32		29	25		32	39	37		35	38	33	37	30	25	25	30	24	24	26	24	22	20	21	16	13	13	12	13	13	13	13	15		
Bermuda	10			20	7	10	20	10	10	9	11	10	10	12	16	20	5	5	7	9	8	12	7	8	12	12	13	11	10	19	14	14	10	9	7	10	10		
Bolivia																					74	72	70	67															
Bosnia and Herzegovina						40	39	36	35	35	34	35																				34			25		33		
Brazil	38	38	37	38	39	37	37	36	36	35	34	34	34	34	33	31	30	29	30	29	28	28	27	26	25	23	20	20	19	19	19	18	18	17	17	18	18	17	
Brunei																	29	20	15	19	19	18	18	18	19	17	17	19	19	24	22	16	14	15	16	17	18		
Bulgaria	21	20	20	20	19	19	19	21	21	20	19	19	19	19	20	21	23	25	26	26	27	27	26	27	28	25	27	26	26	30	31	34	28	30	29	30			
Canada	11	9	11	11	10	12	9	10	10	10	11	10	10	10	10	11	11	13	11	10	10	10	10	10	10	10	10	10	10	10	10	10	10	10	10	11			
Cape Verde	49																															30	30						
Chile	27	26	26	25	26		24	25	24	24	21	21	20	20	17	16	16	15	15	15	12	11	10	9	9	9	9	10	10	10	10	10	10	10	10	10	10		
China												23	21	18	14	16	16	16	15	14	16	26	25		6	5	9	8	14	13	13	12	11	10	8	8	8	7	
Colombia	29	30	27	25	24	24	24	23	22	22	22	22	20	18	18	18	18	17	13	12	11	11	10	11	11	11	11	11	11	11	11	12	11	10	9	9			
Cook Islands																					40	27	36	30	37	38	30	42	33	24	23	30	25	25	26	21	21		
Costa Rica	24	25	21	19	18	18	17	17	17	17	21	19	17	17	18	17	17	11	10	9	8	8	9	7	8	8	10	9	10	10	9	9	9	10	9	8	10		
Croatia						18	18	18	17	15	14	15	16	18	18	18	18	19	17	15	14	14	15	14	12	12	12	12	11	10	8	8	8	7	7	7	6		
Cuba	14	14	14	14	14	14	13	14	13	13	13	13	14	15	15	15	15	10	10	10	11	10	9	9	9	9	8	8	8	8	8	8	8	8	8	8	8		
Cyprus																				62	65				28	23	22	18	19	20	19	15	16	13	14	14	14		
Czech Republic							9	10	10	10	10	10	11	11	10	14	16	18	16	15	13	14	16	15	16	15	15	13	15	15	15	14	14	12	12	13	14	15	
Denmark	17	16	17	17	17	18	18	19	19	20	21	22	23	22	14	14	17	14	13	12	12	13	15	15	14	13	13	17	16	18	17	17	15	16	16	16			
Dominica	33	26	31	29	29	39	36	37	31	36	33	38	35	34	43	37	40	38	36	31	32	34	35	34	30	13	11	18	18	18	17	14	20	16	28	21			
Dominican Republic	50	47	44	40	37	35	34	34	34	35	34	35	34		33	33	32	30	28	28	26	25	24	23	23	21	19	20	18	15	17	19	22	19					
Ecuador	37	35	34	34	34	33	32	32	31	31	31	29	29	29	34	34	30	34	34	34	34	33	35	33	31	29	27	26	28	25	25	25	24	22	20	17	15	16	
Egypt	68							64				58	58								60	61	59	59	60	59	59	60	60	60	60	56	54	58	53	56			
El Salvador		43	46	49	48						39	37	38	37		35	32	36	36	32	31	32	32	33	31	31	31	33	33	33	34	33	32	36	37				
Estonia		9	9			9	9	9	9	12	16	16	19	19	6	7	7	7	7	7	8	8	9	9	9	9	8	11	7	8	8	8	8	9	9	9	11		
Fiji																				47		57	47	47	46	45	44	43	41	40		35	34						
Finland	17	19	18	18	18	19	19	8	9	10	10	10	9	9	9	9	5	5	4	5	4	5	5	5	5	5	6	6	6	6	6	6	6	6	7	6	6		
France	24	23	23	23	22	21	21	21	20	20	19	19	19	18	18	18	19	19	18	18	18	18	18	19	17	18	17	18	18	18	19	19	20	21	20	20			
Georgia		11	12			11	12	12	9	8	8	9	10		10	10	12	15	14	14	13	12			23	35	40	39		46	69	53	48	48	38	40			
Germany	20	20	20	20	19	19	20	19	19	17	17	15	14	14	14	14	14	14	14	14	15	15	15	15	14	15	14	15	15	15	15	14	14	15	15	15	15		
Ghana																					34							36											
Greece	19	19	18	17	16	16	19	24	24	24	23	23	22	22	21	20	21	21	22	22	21	20	21	20	20	20	19	18	20	18	19	19	19	18	17	17	16		
Greenland																16	16	15	15	14	13	13	15	14	17	12	20	15	16	13	16	22	24	21	36	41			
Grenada					26	42			35	41	38	41	38	35	35	35	30					25	20	26	23	20	24	24	26	18	15	13	14	13	19	17	14	15	
Guam															7	9	11	10	7	8	10	9	10	9	7	9	8	7	7	8	11	9	11	8	11	10	11		
Guatemala	33	36			31		34	32	33	34	35	35	37	38	34	32	33	31	31	30	33	33	30	30	30	30	30	30	28	25	24	23	22	22	22	24	26	26	
Guyana					36				23	25	24	27	24	24	23	22	24	23	26	22	17	20	19	16	17	15	17	18	18	20	19	19	21	20	20				
Haiti		69																		60			46	47	52														
Honduras	55	53	64	76				38	36	43	38																		13	13	13	13	10	11					
Hungary	9	9	10	10	10	11	11	10	10	10	10	10	10	10	9	9	9	8	9	9	8	8	8	8	7	7	7	6	6	6	6	6	6	6	6	6	7	7	
Iceland	15	10	7	8	8	7	9	9	7	7	8	6	6	4	6	7	5	6	7	6	8	7	6	8	6	8	10	8	7	9	9	10	9	11	9	11	10	11	14
India	31	36	40	39	35		29	37		43	41	42	42	40	41	41	41	42	41	42	42	41	40	38	39	41	41	36	43	45	44	38	38	52	43	45	63		
Iran																						42	38	34	26	22	19	18	18	17	18	17	16	18	17	19	14		
Iraq																													46				38	40	40	40	39		
Ireland	10	9	9	9	9	8	8	8	8	8	8	8	8	7	8	8	8	8	8	9	8	9	9	9	8	9	11	7	7	8	8	8	7	8	7	7			
Israel	21	21	20	19	21	19	18	18	19	17	19	19	21	17	17	17	16	20	17	19	18	19	18	18	19	19	20	19	19	19	18	19	20	20	20	21	22		
Italy	10	11	11	11	11	12	12	11	11	10	11	10	10	10	10	11	11	11	11	11	11	11	11	10	10	10	10	10	10	10	10	10	11	11	11	12	11		
Jamaica	34	35	30	28	28	28	27	26	24	26	30	28									21	26	30	22	16	20	13			12	14	11	12	13	12				
Japan	17	17	17	17	18	18	18	18	18	19	19	19	19	19	17	11	12	12	12	12	12	12	12	12	12	13	13	13	14	14	15	14	15	16	16	16	17	17	
Jordan																									22	21	19		19	18	18	20	21	21		21			
Kazakhstan		19	18			18	16	16	16	15	15	12	13	12	12	13	12	13	13	17	15	15	15	15	16	16	17	19	18	22	28	33	34	18	18	17		20	
Kiribati												52	48	46	51	44	54	43	35	46	45	53																	
Kuwait	26	25	31	29	27	24	27	22						23	27	31	24	28	22	29	25	21	24	22	20	18	20	19	21	18	16	16	18	22	18				
Kyrgyzstan		19	17			17	15	15	14	15	18	20	20	22	24	24	23	21	20	19	11	9	8	8	8	8	6	7	7	6	6	6	6	6	6	7	7	7	
Latvia	10	10	10	9	9	9	9	9	9	9	11	15	17	16	18	19	10	9	9	8	9	10	11	11	13	12	13	11	12	11	10	9	8	7	7	6			
Libya																											87	91	89										
Lithuania		10	9			8	8	8	8	8	7	13	13	11	8	7	6	5	7	7	8	7	7	7	7	7	7	8	7	7	7	8	8	7	7	8	8	8	
Luxembourg	16	16	15	13	13	13	12	12	15	14	14	18	13	13	13	12	15	12	17	17	14	15	17	18	16	17	19	18	17	19	17	19	15	15	13	14	13		
Madagascar					54	51	53	50	54	53	49	52	51	52	51	50																							
Malaysia	41	40	26															33	33		37	39	37	39	39	38	38	36	35	27	27	26	26	26	25				
Maldives																					66	59	56	59	60	80		53	51		46	38				47			
Mali		71			74																																		
Malta	18	20	17	18	17	19	25	25	20	12	12	11	10	10	11	9	9	10	10	12	10	9	8	12	8	8	9	8	8	6	6	8	11	7	7	7			
Mauritius	39	34	28	28	27	26	26	24	22	23	25	23	25	28	26	27	30	30	23	23	21	18	18	18	18	16	17	15	16	16	16	15	16	15	13	14	14	15	
Mexico	30	28	26	25	24	23	22	22	19	19	18	18	17	17	16	15	15	14	14	13	13	13	13	12	12	12	12	13	13	12	13	12	12	12	12	12	11	11	
Moldova		10	10			9	9	9	10	11	20	21	23	24	23	19	13	10	10	9	7	5	5	4	3	4	3	4	3	3	3	3	3	3	3	4	4	4	
Monaco							23	24																								30	24	20					
Mongolia															25										76	76	76	74	74		8						7		
Montenegro																					31	35	34	33	32	24	30	25	28	31									
Morocco																					57	57	60	60	57	58		58	58	56	57	60	53	51	51				
Mozambique																						33																	

Country	Year																																						
	'80	'81	'82	'83	'84	'85	'86	'87	'88	'89	'90	'91	'92	'93	'94	'95	'96	'97	'98	'99	'00	'01	'02	'03	'04	'05	'06	'07	'08	'09	'10	'11	'12	'13	'14	'15	'16	'17	'18
Netherlands	12	11	13	14	13	13	13	12	12	13	14	14	14	15	14	15	14	14	15	15	16	16	16	16	15	15	15	14	14	14	14	14	14	15	15	14	15		
New Zealand	5	5	5	5	5	5	5	5	5	5	5	5	5	5	4		4	4	4	3	4	4	3	4	3	4	4	4	4	4	4	4	4	4	4	4			
Nicaragua									26	27	27	25	25	26	26		23	19	17	16	17	14	13	13	13	13	13	14	14	12	11	10	10	10	10	12	9	10	
Nigeria																												96											
North Macedonia												21	21	21	19	20	20	19	18	18	17	17	19	17	28	29	21	21	21	20	21	23	22	23					
Northern Mariana Islands																			22	34	26	18	30	19	22	28	15	13	26	21	23	28	21	14	13	11	11		
Norway	10	10	9	9	9	9	9	9	8	9	9	10	9	10	10	10	9	9	10	11	11	12	12	13	13	14	14	15	15	15	15	15	16	16	16	15	14	14	
Oman																										17	17	18		46	59				56				
Palau																																		49					
Palestine																		43	39	41	40	42	46	44	34	34		34	35	31	31	30	31	31	27	32	31	28	
Panama	26	26	25	26	25	25	23	22	25	24							26	25	18	17	15	15	13	15	16	14	17	14	14	14	16	17	16	17	15	16	18		
Papua New Guinea	29																																						
Paraguay	37	37	38	39	40	39	35	36	36	33	33	31			26	27	27	34	32	34	33	31	33	33	29	28	25	26	26	25	23	21	22	21	21	19	18		
Peru	27	26	27	28			47	50	46	47	49	50	45		48	40	38	41	38	35	31	33	32	29	29	61	64	23	24	27	25	25	25	24	23	19			
Philippines	99	99	99	99	99	99	99	99	99	99	99	82	81	83	83	83	83	84	83	52	52	52	47	51	47	47	16	16	16	16	16	16	18	17	17	16			
Poland	37	36	37	36	36	36	36	36	36	36	36	37	36	36	36	35	35			26	25	24	24	25	24	24	24	25	25	26	26	24	25	25	26	26	26		
Portugal	24	23	23	23	22	22	23	23	24	23	23	23	22	23	23	22	23	24	24	24	25	21	18	18	18	25	26	22	21	21	21	19	20	19	14	14	14		
Puerto Rico	24	22	25	24	24	24	23	23	23	23	23	21	21	22	16	17	17	16	16	17	17	16	17	17	16	17	17	17	17	17	17	16	16	16	18	17	16		
Qatar					81	79										27					100	36	100	100	41	34	33	34	40	30	36	37	41	37	41	36	30	29	
Romania	23	23	23	23	23	23	22	22	22	21	21	22	22	17	16	16	17	16	15	14	14	13	13	13	13	13	13	12	13	13	13	13	14	14	15	15	15	15	
Russia	8									8	10	11	12	13	14	14	14	13	13	13	13	13	13	13	13	12	11	11	11	11	12	12	12	13	15	15	15	15	
Saint Kitts and Nevis	26	29	30	28	31	28	32	31	33	37	41	37	28	32	27	18			19	31	28	39	22	29	34	21	15	13	15	16	11	16	10	11	11	9	8		
Saint Lucia	43	38		31			36	32	33	37	30	28	27	26	26	27	30	27	28	32	23	18	23	22	20	29	26		27	15	18	16	14	18	13				
Saint Vincent and the Grenadines			36	30	46	35	31	40			36					19	23	18	14	16	16	16	14	17	16	15	12	13	20	28	20	17	20	15	12	17	29		
San Marino																24	31	24	29	29	25		23			23						16	21	22	20	18			
Saudi Arabia																				45	46	45	48	49	48	45	46	49	51	51	52	53	55						
Serbia																			22	23	23	23	23	22	24	19	18	18	18	18	18	20	19	20	21	22	21		
Seychelles		28	30			35	34	28														23	22	23	22	22	24	23	21	25	23	21	22	21	22	39			
Singapore	13	11	9	9	10	8	13	8	8	10	8	6	6	6	6	5	5	4	5	5	6	5	5	5	5	5	5	6	6	6	7	6	4	4	3	4	4	4	
Slovakia													17	17	16	18	17	18	18	16	15	15	15	15	15	17	27	22	11	11	11		8	8	9				
Slovenia						7	9	10	9	12	11	7	6	7	7	8	8	9	8	10	11	11	12	12	11	13	13	12	10	10	10	8	10	12	11	12			
South Africa																		37	36	32	32	31	31	31	31	31	32	32	32	32	32	32	32	31	31	31	32		
South Korea						38	37	39	37	34	32	32	28	25	25	27	28	27	27	26	22	19	16	17	18	19	19	17	18	18	17	17	17	17	16	16	17		
Spain	22	22	22	22	21	21	20	19	19	18	18	17	16	16	15	14	14	14	13	14	14	14	14	14	14	14	14	15	15	14	13	14	13	12	12	13	12		
Sri Lanka	56	56	61	57	58	57	56	57	56	57		58	58	58	59	56	57	48	48	47	42	41	41	39	37	40	37	32		36	35			35					
Suriname	48	43	37		35	33	32	30	33	33	30	32	31			32	33	33	31	31	27	25	23	22	24	25	20	21	23	20	21	22	23	23	24				
Sweden	12	12	13	12	13	13	13	9	10	10	10	10	10	10	11	11	11	10	11	11	11	11	11	11	11	11	12	13	13	13	16	15	14	13	13	15	14	13	
Switzerland	25	24	25	24	24	24	24	24	24	25	25	26	26	26	26	14	13	13	13	13	13	13	14	14	14	13	13	12	13	12	13	13	13	13	13	14	14		
Syria	71				77	78													54	47	47	49	48	42	34	41	38	32	34	26	42								
São Tomé and Príncipe						40																																	
Taiwan (province of China)	19	20	20	20	21	21	20	20	20	21	22	22	22	23	22	20	18	18	18	16	16	16	16	15	15	15	15	15	18	17	17	17	15	15	14	15	15		
Tajikistan		16	18			24	23	24	21	20	21	21	27	33	32	31	35	37	40	36	38	35	31	32	34	34											31		
Thailand	72	72	72	72	71	71	72	70			66	65	65		65	66	65	66	66	63	62		56	52	55	55	55	54	54	54	52	47	47	42	40	37	35		
The Bahamas	26	26			21	24		18						24	18	18	25	16	16	12	13	12	24	14	15	16	15	15	14	13	14	12	15	14	15				
Tonga																								47															
Trinidad and Tobago	21	19	18	17	18	18	16	15	16	15	16	15	15	14	14	15	14	14	14	11	10	11	10	10	10	11	11	12	13	12	12	10	10						
Tunisia																														26				35					
Turkey				63	64			60	59	59	60	59	59	57	59	59	59	59	56	59	55	54	54	55	62	59	60	59	60	21	20	21	22	15	16	16	17		
Turkmenistan		12	12			12	12	16	13	16	18	14	15	14	20	20	22	26	29	15	12	10	12	10	11	13	15	16	17	16	17	19	19	21	22	27			
UK		8	7	7	6	6	6	7	6	7	7	6	7	7	7	8	8	8	9	9	9	9	8	9	9	9	8	9	9	9	9	9	8	9	8	9	9	9	
USA	10	10	10	11	11	11	12	12	12	10	10	10	10	10	10	10	11	11	11	11	11	12	12	12	12	12	12	12	13	13	13	13	13	13	13	14	14		
Ukraine		12	12			11	12	14	15	17	21	21	22	20	18	18	19	17	17	16	16	16	17	18	18	12	12	12	11	10	10	9	9	20	9	20	22	6	
United Arab Emirates																											48	46											
Uruguay	23	23	22	23	24	23	23	23	23	22	22	20		23	22	23	22	18	20	21	19	19	20	20	20	19	20	21	21	21	22		23	22	21	20	20		
Uzbekistan		14	14			13	14	14	14	13	13	14	14	15	16	16	18	19	18	18	17	18	22	23	13	12				10	10	11	11	12	14	14	14		
Venezuela	31	30	30	21		32	32	28	27	28	18		18	18	17		13	13	13	12	10	9	9	10	9	9	9	9	9	10	9	9	9	8					
Virgin Islands	19														10	11	12	10	13	10	12	11	12	12	9	14	12	11	12	11	15	15	16			19	11		
Zimbabwe											36					41												25											

Color Scale: 0 | 10 | 20 | 30 | 40 | 50 | 60 | 70 | 80 | 90 | 100

Cells are colour coded by percent of data redistributed in a given country-year from garbage coding to a likely underlying cause of death. Blank white cells indicate lack of VR for that year. High percentages of vaguely-coded deaths highlight the importance of improvements in accurate reporting of mortality data to avoid redistribution of deaths to broad level causes. Countries without VR for 1980-2018 are not listed. VR=vital registration.

Figure S5A. Classification of national time series of vital registration and verbal autopsy data 1980−2018

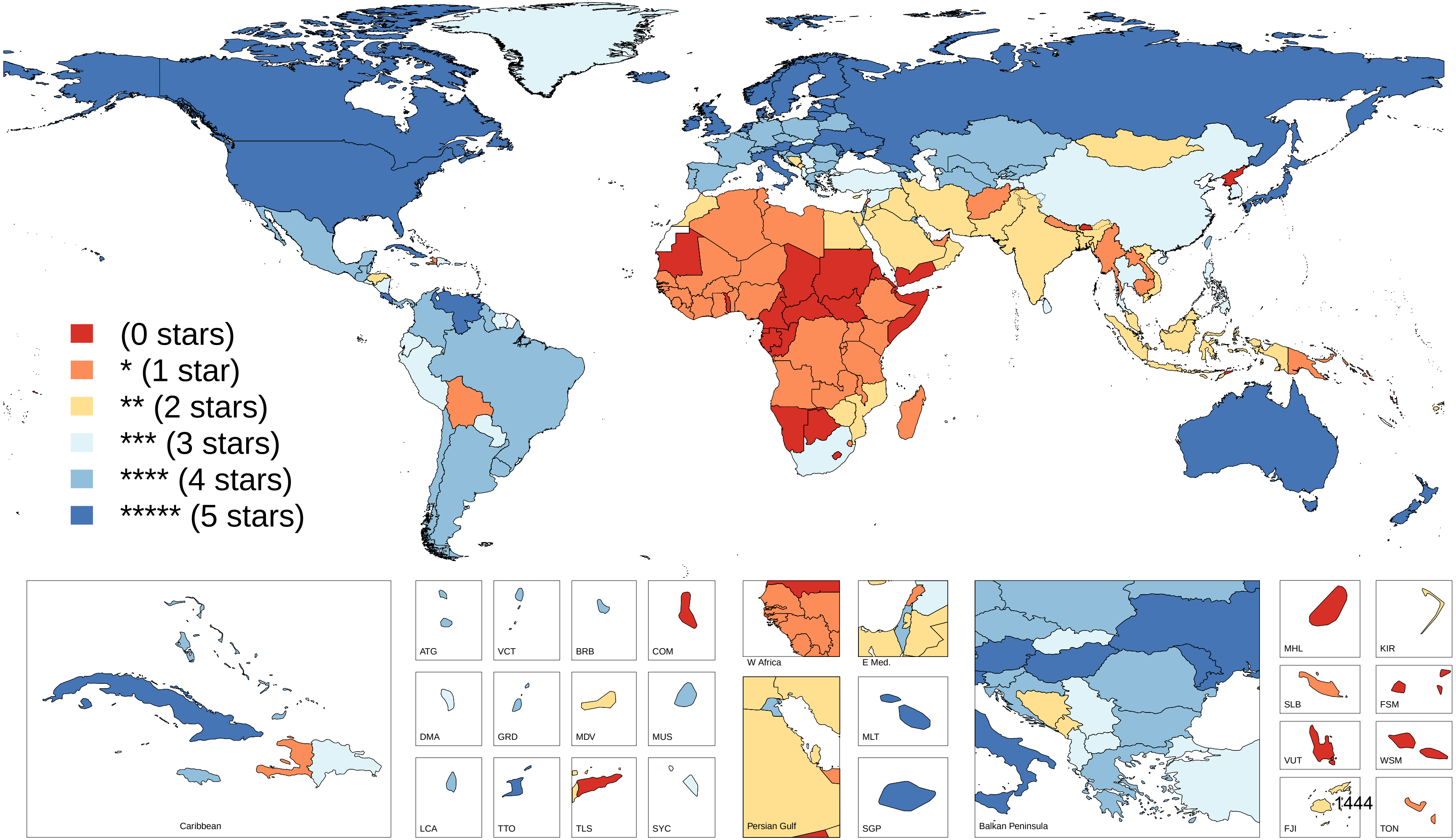

Figure S5B. Classification of national time series of vital registration and verbal autopsy data 2010−2018

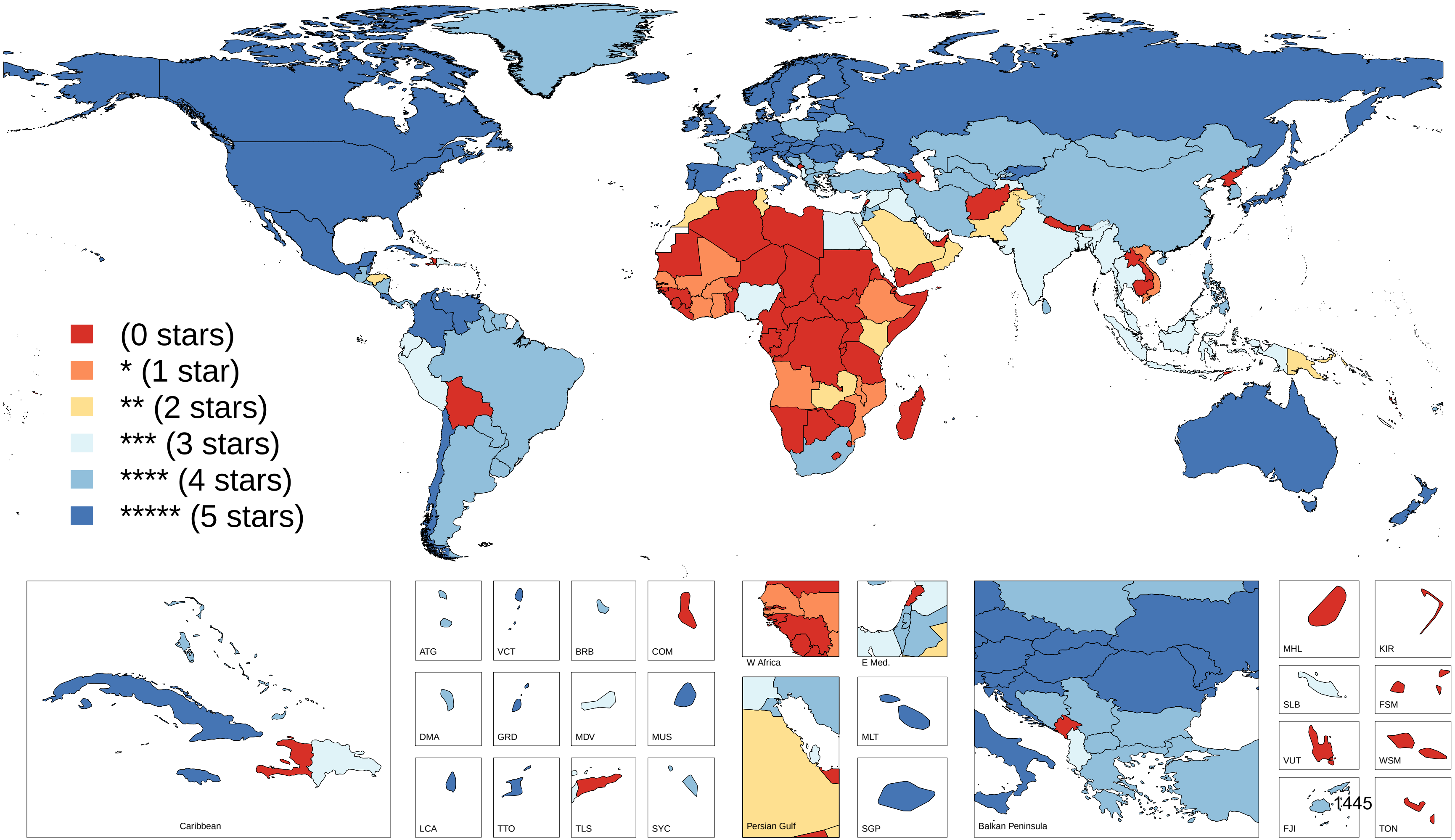

Figure S6. Out-of-sample model performance for CODEm models for GBD 2019 and age-standardised cause-specific mortality rate by level 2 causes

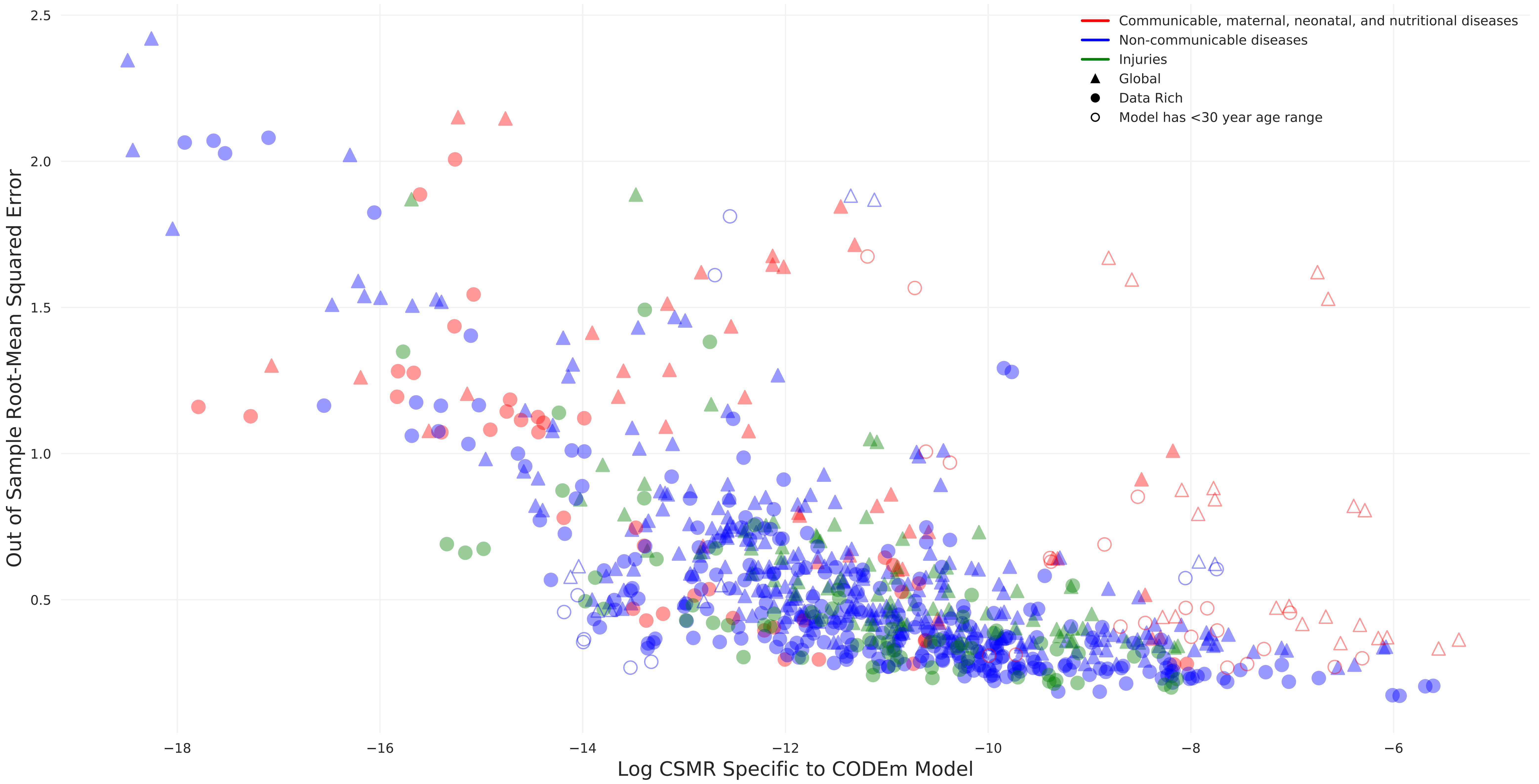

Table S1. GATHER checklist of information that should be included in reports of global health estimates, with description of compliance and location of information for "Global burden of 369 diseases and injuries in 204 countries and territories, 1990–2019: a systematic analysis for the Global Burden of Disease Study 2019"

#	GATHER checklist item	Description of compliance	Reference
**Objectives and funding**			
1	Define the indicators, populations, and time periods for which estimates were made.	Narrative provided in paper and methods appendix describing indicators, definitions, and populations	Main text (Methods—Overview, Geographic units and time periods) and methods appendix
2	List the funding sources for the work.	Funding sources listed in paper	Summary (Funding)
**Data Inputs**			
For all data inputs from multiple sources that are synthesized as part of the study:			
3	Describe how the data were identified and how the data were accessed.	Narrative description of data seeking methods provided	Main text (Methods) and methods appendix
4	Specify the inclusion and exclusion criteria. Identify all ad-hoc exclusions.	Narrative about inclusion and exclusion criteria by data type provided	Main text (Methods) and methods appendix
5	Provide information on all included data sources and their main characteristics. For each data source used, report reference information or contact name/institution, population represented, data collection method, year(s) of data collection, sex and age range, diagnostic criteria or measurement method, and sample size, as relevant.	An interactive, online data source tool that provides metadata for data sources by component, geography, cause, risk, or impairment has been developed	Online data citation tools
6	Identify and describe any categories of input data that have potentially important biases (e.g., based on characteristics listed in item 5).	Summary of known biases by cause included in methods appendix	Methods appendix
For data inputs that contribute to the analysis but were not synthesized as part of the study:			
7	Describe and give sources for any other data inputs.	Included in online data source tool, http://ghdx.healthdata.org/gbd-2019	Online data citation tools
For all data inputs:			
8	Provide all data inputs in a file format from which data can be efficiently extracted (e.g., a spreadsheet as opposed to a PDF), including all relevant meta-data listed in item 5. For any data inputs that cannot be shared due to ethical or legal	Downloads of input data available through online tools, including data visualization tools	Online data visualization tools, data query tools, and

	reasons, such as third-party ownership, provide a contact name or the name of the institution that retains the right to the data.	and data query tools, http://ghdx.healthdata.org/gbd-2019; input data not available in tools will be made available upon request	the Global Health Data Exchange, http://ghdx.healthdata.org
**Data analysis**			
9	Provide a conceptual overview of the data analysis method. A diagram may be helpful.	Flow diagrams of the overall methodological processes, as well as cause-specific modelling processes, have been provided	Main text (Methods) and methods appendix
10	Provide a detailed description of all steps of the analysis, including mathematical formulae. This description should cover, as relevant, data cleaning, data pre-processing, data adjustments and weighting of data sources, and mathematical or statistical model(s).	Flow diagrams and corresponding methodological write-ups for each cause, as well as the demographics and causes of death databases and modelling processes, have been provided	Main text (Methods) and methods appendix
11	Describe how candidate models were evaluated and how the final model(s) were selected.	Provided in the methodological write-ups	Methods appendix
12	Provide the results of an evaluation of model performance, if done, as well as the results of any relevant sensitivity analysis.	Provided in the methodological write-ups	Methods appendix
13	Describe methods for calculating uncertainty of the estimates. State which sources of uncertainty were, and were not, accounted for in the uncertainty analysis.	Provided in the methodological write-ups	Methods appendix
14	State how analytic or statistical source code used to generate estimates can be accessed.	Access statement provided	Code is provided in an online repository
**Results and Discussion**			
15	Provide published estimates in a file format from which data can be efficiently extracted.	Results are available through online data visualization tools, the Global Health Data Exchange, and the online data query tool (http://ghdx.healthdata .org/gbd-2019)	Main text, methods appendix, and online data tools (data visualization tools, data query tools, and the Global Health Data Exchange, http://ghdx.healthdata.org/gbd-2019)
16	Report a quantitative measure of the uncertainty of the estimates (e.g. uncertainty intervals).	Uncertainty intervals are provided with all results	Main text, methods appendix, and online data tools (data

			visualization tools, data query tools, and the Global Health Data Exchange, http://ghdx.healthdata.org/gbd-2019)
17	Interpret results in light of existing evidence. If updating a previous set of estimates, describe the reasons for changes in estimates.	Discussion of methodological changes between GBD rounds provided in the narrative of the Article and methods appendix	Main text (Methods and Discussion) and methods appendix
18	Discuss limitations of the estimates. Include a discussion of any modelling assumptions or data limitations that affect interpretation of the estimates.	Discussion of limitations provided in the narrative of the main paper, as well as in the methodological write-ups in the methods appendix	Main text (Limitations) and methods appendix

Table S2. GBD cause hierarchy with levels

Cause	level
All causes	0
Communicable, maternal, neonatal, and nutritional diseases	1
HIV/AIDS and sexually transmitted infections	2
HIV/AIDS	3
HIV/AIDS–drug-susceptible tuberculosis	4
HIV/AIDS–multidrug-resistant TB without extensive drug resistance	4
HIV/AIDS–extensively drug-resistant tuberculosis	4
HIV/AIDS resulting in other diseases	4
Sexually transmitted infections excluding HIV	3
Syphilis	4
Chlamydial infection	4
Gonococcal infection	4
Trichomoniasis	4
Genital herpes	4
Other sexually transmitted infections	4
Respiratory infections and tuberculosis	2
Tuberculosis	3
Latent tuberculosis infection	4
Drug-susceptible tuberculosis	4
Multidrug resistant TB without extensive drug resistance	4
Extensively drug-resistant tuberculosis	4
Lower respiratory infections	3
Upper respiratory infections	3
Otitis media	3
Enteric infections	2
Diarrhoeal diseases	3
Typhoid and paratyphoid	3
Typhoid fever	4
Paratyphoid fever	4
Invasive non-typhoidal Salmonella (iNTS)	3
Other intestinal infectious diseases	3
Neglected tropical diseases and malaria	2
Malaria	3
Chagas disease	3
Leishmaniasis	3
Visceral leishmaniasis	4
Cutaneous and mucocutaneous leishmaniasis	4
African trypanosomiasis	3
Schistosomiasis	3
Cysticercosis	3
Cystic echinococcosis	3
Lymphatic filariasis	3
Onchocerciasis	3

Trachoma	3
Dengue	3
Yellow fever	3
Rabies	3
Intestinal nematode infections	3
Ascariasis	4
Trichuriasis	4
Hookworm disease	4
Food-borne trematodiases	3
Leprosy	3
Ebola virus disease	3
Zika virus disease	3
Guinea worm disease	3
Other neglected tropical diseases	3
Other infectious diseases	2
Meningitis	3
Encephalitis	3
Diphtheria	3
Whooping cough	3
Tetanus	3
Measles	3
Varicella and herpes zoster	3
Acute hepatitis	3
Acute hepatitis A	4
Acute hepatitis B	4
Acute hepatitis C	4
Acute hepatitis E	4
Other unspecified infectious diseases	3
Maternal and neonatal disorders	2
Maternal disorders	3
Maternal haemorrhage	4
Maternal sepsis and other maternal infections	4
Maternal hypertensive disorders	4
Maternal obstructed labor and uterine rupture	4
Maternal abortion and miscarriage	4
Ectopic pregnancy	4
Indirect maternal deaths	4
Late maternal deaths	4
Maternal deaths aggravated by HIV/AIDS	4
Other maternal disorders	4
Neonatal disorders	3
Neonatal preterm birth	4
Neonatal encephalopathy due to birth asphyxia and trauma	4
Neonatal sepsis and other neonatal infections	4
Haemolytic disease and other neonatal jaundice	4

Other neonatal disorders	4
Nutritional deficiencies	2
Protein-energy malnutrition	3
Iodine deficiency	3
Vitamin A deficiency	3
Dietary iron deficiency	3
Other nutritional deficiencies	3
Non-communicable diseases	1
Neoplasms	2
Lip and oral cavity cancer	3
Nasopharynx cancer	3
Other pharynx cancer	3
Oesophageal cancer	3
Stomach cancer	3
Colon and rectum cancer	3
Liver cancer	3
Liver cancer due to hepatitis B	4
Liver cancer due to hepatitis C	4
Liver cancer due to alcohol use	4
Liver cancer due to NASH	4
Liver cancer due to other causes	4
Gallbladder and biliary tract cancer	3
Pancreatic cancer	3
Larynx cancer	3
Tracheal, bronchus, and lung cancer	3
Malignant skin melanoma	3
Non-melanoma skin cancer	3
Non-melanoma skin cancer (squamous-cell carcinoma)	4
Non-melanoma skin cancer (basal-cell carcinoma)	4
Breast cancer	3
Cervical cancer	3
Uterine cancer	3
Ovarian cancer	3
Prostate cancer	3
Testicular cancer	3
Kidney cancer	3
Bladder cancer	3
Brain and central nervous system cancer	3
Thyroid cancer	3
Mesothelioma	3
Hodgkin lymphoma	3
Non-Hodgkin lymphoma	3
Multiple myeloma	3
Leukaemia	3
Acute lymphoid leukeamia	4

Chronic lymphoid leukaemia	4
Acute myeloid leukaemia	4
Chronic myeloid leukaemia	4
Other leukaemia	4
Other malignant neoplasms	3
Other neoplasms	3
Myelodyspastic, myeloprliferative, and other haematopoietic neoplasms	4
Benign and in situ intestinal neoplasms	4
Benign and in situ cervical and uterine neoplasms	4
Other benign and in situ neoplasms	4
Cardiovascular diseases	2
Rheumatic heart disease	3
Ischaemic heart disease	3
Stroke	3
Ischaemic stroke	4
Intracerebral haemorrhage	4
Subarachnoid haemorrhage	4
Hypertensive heart disease	3
Non-rheumatic valvular heart disease	3
Non-rheumatic calcific aortic valvular heart disease	4
Non-rheumatic degenerative mitral valvular heart disease	4
Other non-rheumatic valvular heart diseases	4
Cardiomyopathy and myocarditis	3
Myocarditis	4
Alcoholic cardiomyopathy	4
Other cardiomyopathy	4
Atrial fibrillation and flutter	3
Aortic aneurysm	3
Peripheral artery disease	3
Endocarditis	3
Other cardiovascular and circulatory diseases	3
Chronic respiratory diseases	2
Chronic obstructive pulmonary disease	3
Pneumoconiosis	3
Silicosis	4
Asbestosis	4
Coal workers pneumoconiosis	4
Other pneumoconiosis	4
Asthma	3
Interstitial lung disease and pulmonary sarcoidosis	3
Other chronic respiratory diseases	3
Digestive diseases	2
Cirrhosis and other chronic liver diseases	3
Cirrhosis and other chronic liver diseases due to hepatitis B	4
Cirrhosis and other chronic liver diseases due to hepatitis C	4

Cirrhosis and other chronic liver diseases due to alcohol use	4
Cirrhosis and other chronic liver diseases due to NAFLD	4
Cirrhosis and other chronic liver diseases due to other causes	4
Upper digestive system diseases	3
Peptic ulcer disease	4
Gastritis and duodenitis	4
Gastro-oesophageal reflux disease	4
Appendicitis	3
Paralytic ileus and intestinal obstruction	3
Inguinal, femoral, and abdominal hernia	3
Inflammatory bowel disease	3
Vascular intestinal disorders	3
Gallbladder and biliary diseases	3
Pancreatitis	3
Other digestive diseases	3
Neurological disorders	2
Alzheimer's disease and other dementias	3
Parkinson's disease	3
Idiopathic epilepsy	3
Multiple sclerosis	3
Motor neuron disease	3
Headache disorders	3
Migraine	4
Tension-type headache	4
Other neurological disorders	3
Mental disorders	2
Schizophrenia	3
Depressive disorders	3
Major depressive disorder	4
Dysthymia	4
Bipolar disorder	3
Anxiety disorders	3
Eating disorders	3
Anorexia nervosa	4
Bulimia nervosa	4
Autism spectrum disorders	3
Attention-deficit/hyperactivity disorder	3
Conduct disorder	3
Idiopathic developmental intellectual disability	3
Other mental disorders	3
Substance use disorders	2
Alcohol use disorders	3
Drug use disorders	3
Opioid use disorders	4
Cocaine use disorders	4

Amphetamine use disorders	4
Cannabis use disorders	4
Other drug use disorders	4
Diabetes and kidney diseases	2
Diabetes mellitus	3
Diabetes mellitus type 1	4
Diabetes mellitus type 2	4
Chronic kidney disease	3
Chronic kidney disease due to diabetes mellitus type 1	4
Chronic kidney disease due to diabetes mellitus type 2	4
Chronic kidney disease due to hypertension	4
Chronic kidney disease due to glomerulonephritis	4
Chronic kidney disease due to other and unspecified causes	4
Acute glomerulonephritis	3
Skin and subcutaneous diseases	2
Dermatitis	3
Atopic dermatitis	4
Contact dermatitis	4
Seborrhoeic dermatitis	4
Psoriasis	3
Bacterial skin diseases	3
Cellulitis	4
Pyoderma	4
Scabies	3
Fungal skin diseases	3
Viral skin diseases	3
Acne vulgaris	3
Alopecia areata	3
Pruritus	3
Urticaria	3
Decubitus ulcer	3
Other skin and subcutaneous diseases	3
Sense organ diseases	2
Blindness and vision loss	3
Glaucoma	4
Cataract	4
Age-related macular degeneration	4
Refraction disorders	4
Near vision loss	4
Other vision loss	4
Age-related and other hearing loss	3
Other sense organ diseases	3
Musculoskeletal disorders	2
Rheumatoid arthritis	3
Osteoarthritis	3

Osteoarthritis hip	4
Osteoarthritis knee	4
Osteoarthritis hand	4
Osteoarthritis other	4
Low back pain	3
Neck pain	3
Gout	3
Other musculoskeletal disorders	3
Other non-communicable diseases	2
Congenital birth defects	3
Neural tube defects	4
Congenital heart anomalies	4
Orofacial clefts	4
Down syndrome	4
Turner syndrome	4
Klinefelter syndrome	4
Other chromosomal abnormalities	4
Congenital musculoskeletal and limb anomalies	4
Urogenital congenital anomalies	4
Digestive congenital anomalies	4
Other congenital birth defects	4
Urinary diseases and male infertility	3
Urinary tract infection and interstitial nephritis	4
Urolithiasis	4
Benign prostatic hyperplasia	4
Male infertility	4
Other urinary diseases	4
Gynaecological diseases	3
Uterine fibroids	4
Polycystic ovarian syndrome	4
Female infertility	4
Endometriosis	4
Genital prolapse	4
Premenstrual syndrome	4
Other gynaecological diseases	4
Haemoglobinopathies and haemolytic anaemias	3
Thalassaemias	4
Thalassaemias trait	4
Sickle cell disorders	4
Sickle cell trait	4
G6PD deficiency	4
G6PD trait	4
Other haemoglobinopathies and haemolytic anaemias	4
Endocrine, metabolic, blood, and immune disorders	3
Oral disorders	3

Caries of deciduous teeth	4
Caries of permanent teeth	4
Periodontal diseases	4
Edentulism and severe tooth loss	4
Other oral disorders	4
Sudden infant death syndrome	3
Injuries	1
Transport injuries	2
Road injuries	3
Pedestrian road injuries	4
Cyclist road injuries	4
Motorcyclist road injuries	4
Motor vehicle road injuries	4
Other road injuries	4
Other transport injuries	3
Unintentional injuries	2
Falls	3
Drowning	3
Fire, heat, and hot substances	3
Poisonings	3
Poisoning by carbon monoxide	4
Poisoning by other means	4
Exposure to mechanical forces	3
Unintentional firearm injuries	4
Other exposure to mechanical forces	4
Adverse effects of medical treatment	3
Animal contact	3
Venomous animal contact	4
Non-venomous animal contact	4
Foreign body	3
Pulmonary aspiration and foreign body in airway	4
Foreign body in eyes	4
Foreign body in other body part	4
Environmental heat and cold exposure	3
Exposure to forces of nature	3
Other unintentional injuries	3
Self-harm and interpersonal violence	2
Self-harm	3
Self-harm by firearm	4
Self-harm by other specified means	4
Interpersonal violence	3
Physical violence by firearm	4
Physical violence by sharp object	4
Sexual violence	4
Physical violence by other means	4

Conflict and terrorism	3
Police conflict and executions	3

Table S3. GBD location hierarchy with levels	
Geography	level
Global	0
Low SDI	1
Low-middle SDI	1
Middle SDI	1
High-middle SDI	1
High SDI	1
Central Europe, eastern Europe, and central Asia	1
Central Asia	2
Armenia	3
Azerbaijan	3
Georgia	3
Kazakhstan	3
Kyrgyzstan	3
Mongolia	3
Tajikistan	3
Turkmenistan	3
Uzbekistan	3
Central Europe	2
Albania	3
Bosnia and Herzegovina	3
Bulgaria	3
Croatia	3
Czech Republic	3
Hungary	3
Montenegro	3
North Macedonia	3
Poland	3
Romania	3
Serbia	3
Slovakia	3
Slovenia	3
Eastern Europe	2
Belarus	3
Estonia	3
Latvia	3
Lithuania	3
Moldova	3
Russia	3
Ukraine	3
High income	1
Australasia	2
Australia	3
New Zealand	3

High-income Asia Pacific	2
Brunei	3
Japan	3
Aichi	4
Akita	4
Aomori	4
Chiba	4
Ehime	4
Fukui	4
Fukuoka	4
Fukushima	4
Gifu	4
Gunma	4
Hiroshima	4
Hokkaidō	4
Hyōgo	4
Ibaraki	4
Ishikawa	4
Iwate	4
Kagawa	4
Kagoshima	4
Kanagawa	4
Kōchi	4
Kumamoto	4
Kyōto	4
Mie	4
Miyagi	4
Miyazaki	4
Nagano	4
Nagasaki	4
Nara	4
Niigata	4
Ōita	4
Okayama	4
Okinawa	4
Ōsaka	4
Saga	4
Saitama	4
Shiga	4
Shimane	4
Shizuoka	4
Tochigi	4
Tokushima	4
Tōkyō	4
Tottori	4

Toyama	4
Wakayama	4
Yamagata	4
Yamaguchi	4
Yamanashi	4
South Korea	3
Singapore	3
High-income North America	2
Canada	3
Greenland	3
USA	3
Alabama	4
Alaska	4
Arizona	4
Arkansas	4
California	4
Colorado	4
Connecticut	4
Delaware	4
Washington, DC	4
Florida	4
Georgia	4
Hawaii	4
Idaho	4
Illinois	4
Indiana	4
Iowa	4
Kansas	4
Kentucky	4
Louisiana	4
Maine	4
Maryland	4
Massachusetts	4
Michigan	4
Minnesota	4
Mississippi	4
Missouri	4
Montana	4
Nebraska	4
Nevada	4
New Hampshire	4
New Jersey	4
New Mexico	4
New York	4
North Carolina	4

North Dakota	4
Ohio	4
Oklahoma	4
Oregon	4
Pennsylvania	4
Rhode Island	4
South Carolina	4
South Dakota	4
Tennessee	4
Texas	4
Utah	4
Vermont	4
Virginia	4
Washington	4
West Virginia	4
Wisconsin	4
Wyoming	4
Southern Latin America	2
Argentina	3
Chile	3
Uruguay	3
Western Europe	2
Andorra	3
Austria	3
Belgium	3
Cyprus	3
Denmark	3
Finland	3
France	3
Germany	3
Greece	3
Iceland	3
Ireland	3
Israel	3
Italy	3
Luxembourg	3
Malta	3
Monaco	3
Netherlands	3
Norway	3
Portugal	3
San Marino	3
Spain	3
Sweden	3
Stockholm	4

Sweden except Stockholm	4
Switzerland	3
UK	3
England	4
East Midlands	5
Derby	6
Derbyshire	6
Leicester	6
Leicestershire	6
Lincolnshire	6
Northamptonshire	6
Nottingham	6
Nottinghamshire	6
Rutland	6
East of England	5
Bedford	6
Cambridgeshire	6
Central Bedfordshire	6
Essex	6
Hertfordshire	6
Luton	6
Norfolk	6
Peterborough	6
Southend-on-Sea	6
Suffolk	6
Thurrock	6
Greater London	5
Barking and Dagenham	6
Barnet	6
Bexley	6
Brent	6
Bromley	6
Camden	6
Croydon	6
Ealing	6
Enfield	6
Greenwich	6
Hackney	6
Hammersmith and Fulham	6
Haringey	6
Harrow	6
Havering	6
Hillingdon	6
Hounslow	6
Islington	6

Kensington and Chelsea	6
Kingston upon Thames	6
Lambeth	6
Lewisham	6
Merton	6
Newham	6
Redbridge	6
Richmond upon Thames	6
Southwark	6
Sutton	6
Tower Hamlets	6
Waltham Forest	6
Wandsworth	6
Westminster	6
North East England	5
County Durham	6
Darlington	6
Gateshead	6
Hartlepool	6
Middlesbrough	6
Newcastle upon Tyne	6
North Tyneside	6
Northumberland	6
Redcar and Cleveland	6
South Tyneside	6
Stockton-on-Tees	6
Sunderland	6
North West England	5
Blackburn with Darwen	6
Blackpool	6
Bolton	6
Bury	6
Cheshire East	6
Cheshire West and Chester	6
Cumbria	6
Halton	6
Knowsley	6
Lancashire	6
Liverpool	6
Manchester	6
Oldham	6
Rochdale	6
Salford	6
Sefton	6
St Helens	6

Stockport	6
Tameside	6
Trafford	6
Warrington	6
Wigan	6
Wirral	6
South East England	5
Bracknell Forest	6
Brighton and Hove	6
Buckinghamshire	6
East Sussex	6
Hampshire	6
Isle of Wight	6
Kent	6
Medway	6
Milton Keynes	6
Oxfordshire	6
Portsmouth	6
Reading	6
Slough	6
Southampton	6
Surrey	6
West Berkshire	6
West Sussex	6
Windsor and Maidenhead	6
Wokingham	6
South West England	5
Bath and North East Somerset	6
Bournemouth	6
Bristol, City of	6
Cornwall	6
Devon	6
Dorset	6
Gloucestershire	6
North Somerset	6
Plymouth	6
Poole	6
Somerset	6
South Gloucestershire	6
Swindon	6
Torbay	6
Wiltshire	6
West Midlands	5
Birmingham	6
Coventry	6

Dudley	6
Herefordshire, County of	6
Sandwell	6
Shropshire	6
Solihull	6
Staffordshire	6
Stoke-on-Trent	6
Telford and Wrekin	6
Walsall	6
Warwickshire	6
Wolverhampton	6
Worcestershire	6
Yorkshire and the Humber	5
Barnsley	6
Bradford	6
Calderdale	6
Doncaster	6
East Riding of Yorkshire	6
Kingston upon Hull, City of	6
Kirklees	6
Leeds	6
North East Lincolnshire	6
North Lincolnshire	6
North Yorkshire	6
Rotherham	6
Sheffield	6
Wakefield	6
York	6
Northern Ireland	4
Scotland	4
Wales	4
Latin America and Caribbean	1
Andean Latin America	2
Bolivia	3
Ecuador	3
Peru	3
Caribbean	2
Antigua and Barbuda	3
The Bahamas	3
Barbados	3
Belize	3
Bermuda	3
Cuba	3
Dominica	3
Dominican Republic	3

Grenada	3
Guyana	3
Haiti	3
Jamaica	3
Puerto Rico	3
Saint Kitts and Nevis	3
Saint Lucia	3
Saint Vincent and the Grenadines	3
Suriname	3
Trinidad and Tobago	3
Virgin Islands	3
Central Latin America	2
Colombia	3
Costa Rica	3
El Salvador	3
Guatemala	3
Honduras	3
Mexico	3
Aguascalientes	4
Baja California	4
Baja California Sur	4
Campeche	4
Chiapas	4
Chihuahua	4
Coahuila	4
Colima	4
Durango	4
Guanajuato	4
Guerrero	4
Hidalgo	4
Jalisco	4
México	4
Mexico City	4
Michoacán de Ocampo	4
Morelos	4
Nayarit	4
Nuevo León	4
Oaxaca	4
Puebla	4
Querétaro	4
Quintana Roo	4
San Luis Potosí	4
Sinaloa	4
Sonora	4
Tabasco	4

Tamaulipas	4
Tlaxcala	4
Veracruz de Ignacio de la Llave	4
Yucatán	4
Zacatecas	4
Nicaragua	3
Panama	3
Venezuela	3
Tropical Latin America	2
Brazil	3
Acre	4
Alagoas	4
Amapá	4
Amazonas	4
Bahia	4
Ceará	4
Distrito Federal	4
Espírito Santo	4
Goiás	4
Maranhão	4
Mato Grosso	4
Mato Grosso do Sul	4
Minas Gerais	4
Pará	4
Paraíba	4
Paraná	4
Pernambuco	4
Piauí	4
Rio de Janeiro	4
Rio Grande do Norte	4
Rio Grande do Sul	4
Rondônia	4
Roraima	4
Santa Catarina	4
São Paulo	4
Sergipe	4
Tocantins	4
Paraguay	3
North Africa and Middle East	1
North Africa and Middle East	2
Afghanistan	3
Algeria	3
Bahrain	3
Egypt	3
Iran	3

Iraq	3
Jordan	3
Kuwait	3
Lebanon	3
Libya	3
Morocco	3
Oman	3
Palestine	3
Qatar	3
Saudi Arabia	3
Sudan	3
Syria	3
Tunisia	3
Turkey	3
United Arab Emirates	3
Yemen	3
South Asia	1
South Asia	2
Bangladesh	3
Bhutan	3
India	3
Andhra Pradesh	4
Arunachal Pradesh	4
Assam	4
Bihar C	4
hhattisgarh	4
Delhi	4
Goa	4
Gujarat	4
Haryana	4
Himachal Pradesh	4
Jammu & Kashmir and Ladakh	4
Jharkhand	4
Karnataka	4
Kerala	4
Madhya Pradesh	4
Maharashtra	4
Manipur	4
Meghalaya	4
Mizoram	4
Nagaland	4
Odisha	4
Punjab	4
Rajasthan	4
Sikkim	4

Tamil Nadu	4
Telangana	4
Tripura	4
Other Union Territories	4
Uttar Pradesh	4
Uttarakhand	4
West Bengal	4
Nepal	3
Pakistan	3
Southeast Asia, east Asia, and Oceania	1
East Asia	2
China	3
North Korea	3
Taiwan (province of China)	3
Oceania	2
American Samoa	3
Cook Islands	3
Fiji	3
Guam	3
Kiribati	3
Marshall Islands	3
Federated States of Micronesia	3
Nauru	3
Niue	3
Northern Mariana Islands	3
Palau	3
Papua New Guinea	3
Samoa	3
Solomon Islands	3
Tokelau	3
Tonga	3
Tuvalu	3
Vanuatu	3
Southeast Asia	2
Cambodia	3
Indonesia	3
Aceh	4
Bali	4
Bangka-Belitung Islands	4
Banten	4
Bengkulu	4
Gorontalo	4
Jakarta	4
Jambi	4
West Java	4

Central Java	4
East Java	4
West Kalimantan	4
South Kalimantan	4
Central Kalimantan	4
East Kalimantan	4
North Kalimantan	4
Riau Islands	4
Lampung	4
Maluku	4
North Maluku	4
West Nusa Tenggara	4
East Nusa Tenggara	4
Papua	4
West Papua	4
Riau	4
West Sulawesi	4
South Sulawesi	4
Central Sulawesi	4
Southeast Sulawesi	4
North Sulawesi	4
West Sumatra	4
South Sumatra	4
North Sumatra	4
Yogyakarta	4
Laos	3
Malaysia	3
Maldives	3
Mauritius	3
Myanmar	3
Philippines	3
Seychelles	3
Sri Lanka	3
Thailand	3
Timor-Leste	3
Vietnam	3
Sub-Saharan Africa	1
Central sub-Saharan Africa	2
Angola	3
Central African Republic	3
Congo (Brazzaville)	3
DR Congo	3
Equatorial Guinea	3
Gabon	3
Eastern sub-Saharan Africa	2

Burundi	3
Comoros	3
Djibouti	3
Eritrea	3
Ethiopia	3
Kenya	3
Baringo	4
Bomet	4
Bungoma	4
Busia	4
Elgeyo Marakwet	4
Embu	4
Garissa	4
Homa Bay	4
Isiolo	4
Kajiado	4
Kakamega	4
Kericho	4
Kiambu	4
Kilifi	4
Kirinyaga	4
Kisii	4
Kisumu	4
Kitui	4
Kwale	4
Laikipia	4
Lamu	4
Machakos	4
Makueni	4
Mandera	4
Marsabit	4
Meru	4
Migori	4
Mombasa	4
Murang'a	4
Nairobi	4
Nakuru	4
Nandi	4
Narok	4
Nyamira	4
Nyandarua	4
Nyeri	4
Samburu	4
Siaya	4
Taita Taveta	4

Tana River	4
Tharaka Nithi	4
Trans Nzoia	4
Turkana	4
Uasin Gishu	4
Vihiga	4
Wajir	4
West Pokot	4
Madagascar	3
Malawi	3
Mozambique	3
Rwanda	3
Somalia	3
South Sudan	3
Uganda	3
Tanzania	3
Zambia	3
Southern sub-Saharan Africa	2
Botswana	3
eSwatini	3
Lesotho	3
Namibia	3
South Africa	3
Zimbabwe	3
Western sub-Saharan Africa	2
Benin	3
Burkina Faso	3
Cape Verde	3
Cameroon	3
Chad	3
Côte d'Ivoire	3
The Gambia	3
Ghana	3
Guinea	3
Guinea-Bissau	3
Liberia	3
Mali	3
Mauritania	3
Niger	3
Nigeria	3
São Tomé and PrÍncipe	3
Senegal	3
Sierra Leone	3
Togo	3

Table S4. Total number of site years by cause and source type for 2019

Cause	Level	Vital Registration	Vital Registration - Sample	Verbal Autopsy	Surveillance	Survey/Census	Sibling History	Cancer Registry	Police Records
All causes	0	24022	825	3072	3514	1534	5675	5385	1625
Communicable, maternal, neonatal, and nutritional diseases	1	24022	825	2815	3514	1467	5675		
HIV/AIDS and sexually transmitted infections	2	23097	825	534	411				
HIV/AIDS	3	23095	825	513	411				
HIV/AIDS–drug-susceptible tuberculosis	4	12067	448		11				
HIV/AIDS resulting in other diseases	4	19349	448		58				
Sexually transmitted infections excluding HIV	3	22556	825	366	239				
Syphilis	4	22067	825		239				
Chlamydial infection	4	20358	825						
Gonococcal infection	4	21798	825						
Genital herpes	4	20217	448						
Other sexually transmitted infections	4	21518	825						
Respiratory infections and tuberculosis	2	23109	825	1792	681				
Tuberculosis	3	23109	825	1728	680				
Drug-susceptible tuberculosis	4	12626	448		679				
Multidrug-resistant tuberculosis without extensive drug resistance	4	12063	448						
Lower respiratory infections	3	23109	825	1766	681				
Upper respiratory infections	3	20371	825						
Otitis media	3	20807	825						
Enteric infections	2	23109	825	1759	575				
Diarrhoeal diseases	3	23109	825	1756	575				
Typhoid and paratyphoid	3	23093	825	393					
Typhoid fever	4	20242	448						
Paratyphoid fever	4	20240	448						
Invasive non-typhoidal Salmonella (iNTS)	3	19972	825						
Other intestinal infectious diseases	3	20856	825	142					
Neglected tropical diseases and malaria	2	23113	825	1537	310				
Malaria	3	23002	825	1453	1				
Chagas disease	3	8764	350						
Leishmaniasis	3	21728	825	186					
Visceral leishmaniasis	4	21689	825	186					
African trypanosomiasis	3	20110	448						
Schistosomiasis	3	20852	448						
Cysticercosis	3	20843	825						
Cystic echinococcosis	3	20992	825						
Dengue	3	22594	825	328	1				
Yellow fever	3	20313	448	123					
Rabies	3	22764	825	937	278				
Intestinal nematode infections	3	21522	825	128					
Ascariasis	4	20111	825						
Ebola virus disease	3	301	56		30				
Zika virus disease	3	12686	825						
Other neglected tropical diseases	3	20378	825						
Other infectious diseases	2	23109	825	1690	912				
Meningitis	3	22568	825	1489	611				
Encephalitis	3	22276	825	469					
Diphtheria	3	22570	825						
Whooping cough	3	22576	825	589					
Tetanus	3	22577	825	1336	396				
Measles	3	22570	825	1207	585				
Varicella and herpes zoster	3	21438	825	439					
Acute hepatitis	3	22574	825	1245					
Acute hepatitis A	4	19351	448						
Acute hepatitis B	4	19353	448						
Acute hepatitis C	4	12063	448						
Acute hepatitis E	4	19344	448						
Other unspecified infectious diseases	3	23105	825	1150	890				
Maternal and neonatal disorders	2	24015	825	2623	3135	1467	5675		
Maternal disorders	3	24006	825	2165	2453	1467	5675		
Maternal haemorrhage	4	21517	825	1121	948	9			
Maternal sepsis and other maternal infections	4	21516	825	826	556	7			
Maternal hypertensive disorders	4	21526	825	981	909	9			
Maternal obstructed labor and uterine rupture	4	21519	825	852	667	8			
Maternal abortion and miscarriage	4	21538	825	353	441	7			
Ectopic pregnancy	4	20981	825	115	629				
Indirect maternal deaths	4	21519	825	909	1036	9			
Late maternal deaths	4	12939	825		152				
Maternal deaths aggravated by HIV/AIDS	4	24006	825	2165	2453	1467	5675		
Other maternal disorders	4	21519	825	321	888	4			
Neonatal disorders	3	22561	823	1658	682				
Neonatal preterm birth	4	20911	823	571	681				
Neonatal encephalopathy due to birth asphyxia and trauma	4	20912	823	554	681				
Neonatal sepsis and other neonatal infections	4	20891	823	168	634				
Haemolytic disease and other neonatal jaundice	4	20788	823	356					
Other neonatal disorders	4	20897	823	175	664				
Nutritional deficiencies	2	22568	825	1443	578				
Protein-energy malnutrition	3	21479	825	1367					
Dietary iron deficiency	3			741					
Other nutritional deficiencies	3	20906	825						
Non-communicable diseases	1	23119	825	1754	756			5385	64
Neoplasms	2	22553	825	1510	723			5385	
Lip and oral cavity cancer	3	21734	825	514				4498	
Nasopharynx cancer	3	21732	825					5247	
Other pharynx cancer	3	21732	825	186				5169	
Oesophageal cancer	3	22020	825	514				5288	
Stomach cancer	3	22024	825	187				5312	
Colon and rectum cancer	3	22024	825	516				5349	
Liver cancer	3	22021	825	539				5320	
Liver cancer due to hepatitis B	4	19344	448						
Liver cancer due to hepatitis C	4	19344	448						
Liver cancer due to alcohol use	4	19344	448						
Liver cancer due to NASH	4	19344	448	329					
Hepatoblastoma	4	20998	824	421				258	
Liver cancer due to other causes (internal)	4	19344	448						
Gallbladder and biliary tract cancer	3	21198	825					5230	
Pancreatic cancer	3	21735	825					5295	
Larynx cancer	3	22020	825	183				5292	
Tracheal, bronchus, and lung cancer	3	22553	825	516				5318	
Malignant skin melanoma	3	21734	825					5266	
Non-melanoma skin cancer	3	21732	825						
Non-melanoma skin cancer (squamous-cell carcinoma)	4	21199	825						
Soft tissue and other extraosseous sarcomas	3	21041	825	538				4732	
Malignant neoplasm of bone and articular cartilage	3	22010	825	532				5281	
Breast cancer	3	22553	825	515	1			5333	
Cervical cancer	3	22024	825	182				4971	
Uterine cancer	3	22021	825	183				4950	
Ovarian cancer	3	21732	825					5007	
Prostate cancer	3	22021	825					4965	
Testicular cancer	3	21041	825	301				4959	
Kidney cancer	3	21732	825					5182	
Bladder cancer	3	21734	825					5146	
Brain and central nervous system cancer	3	21735	825	535				5311	
Eye cancer	3	21183	825	529				770	
Retinoblastoma	4	20988	825	441				386	
Other eye cancers	4	21041	825	528				745	
Neuroblastoma and other peripheral nervous cell tumors	3	21041	825	529				4070	
Thyroid cancer	3	21733	825					5328	
Mesothelioma	3	12939	448					3362	
Hodgkin lymphoma	3	21733	825					5295	
Non-Hodgkin lymphoma	3	22021	825	539				5335	
Burkitt lymphoma	4	21041	825	536				695	
Other non-Hodgkin lymphoma	4	21041	825	539				5105	
Multiple myeloma	3	21104	825					5227	
Leukaemia	3	22552	825	187	650			5319	
Acute lymphoid leukaemia	4	18979	448					2329	
Chronic lymphoid leukaemia	4	18671	434					1062	
Acute myeloid leukaemia	4	18982	448					3122	
Chronic myeloid leukaemia	4	18799	448					2291	
Other leukaemia	4	19328	448					3096	
Other malignant neoplasms (internal)	3	22023	825	488	72			5365	
Other neoplasms	3	19522	448						
Myelodysplastic, myeloproliferative, and other haematopoietic neoplasms	4	19341	448						
Cardiovascular diseases	2	23119	825	1560	2				
Rheumatic heart disease	3	22026	825	418					
Ischaemic heart disease	3	22038	825	1460					
Stroke	3	22567	825	1299	1				
Ischaemic stroke	4	20229	825						
Intracerebral haemorrhage	4	20222	825						
Subarachnoid haemorrhage	4	20222	825						
Hypertensive heart disease	3	21162	825						
Non-rheumatic valvular heart disease	3	20377	825						
Non-rheumatic calcific aortic valvular heart disease	4	20217	448						
Non-rheumatic degenerative mitral valvular heart disease	4	20217	448						
Other non-rheumatic valvular heart diseases	4	20217	448						
Cardiomyopathy and myocarditis	3	21161	825						
Myocarditis	4	20377	448						
Alcoholic cardiomyopathy	4	20377	448						
Other cardiomyopathy	4	20382	448						
Pulmonary arterial hypertension	3	20655	825	301					
Atrial fibrillation and flutter	3	19486	448						
Aortic aneurysm	3	20372	448						
Peripheral artery disease	3	19483	448						
Endocarditis	3	20360	825						
Other cardiovascular and circulatory diseases (internal)	3	21172	825	416					
Chronic respiratory diseases	2	22568	825	1515	549				
Chronic obstructive pulmonary disease	3	20371	825						
Pneumoconiosis	3	20365	448						
Silicosis	4	20216	448						
Asbestosis	4	20216	448						
Coal workers pneumoconiosis	4	20216	448						
Other pneumoconiosis	4	20216	448						
Asthma	3	20364	448						
Interstitial lung disease and pulmonary sarcoidosis	3	20361	448						
Other chronic respiratory diseases	3	20371	825						
Digestive diseases	2	22555	825	1361	602				
Cirrhosis and other chronic liver diseases	3	22555	825	1235					
Cirrhosis and other chronic liver diseases due to hepatitis B	4	7281							
Cirrhosis and other chronic liver diseases due to hepatitis C	4	7281							
Cirrhosis and other chronic liver diseases due to alcohol use	4	7281							
Cirrhosis and other chronic liver diseases due to NAFLD	4	7281							
Cirrhosis and other chronic liver diseases due to other causes	4	7281							
Upper digestive system diseases	3	22554	825	408					
Peptic ulcer disease	4	20644	448						
Gastritis and duodenitis	4	20629	448						
Appendicitis	3	22022	825	187					
Paralytic ileus and intestinal obstruction	3	21733	825	419					
Inguinal, femoral, and abdominal hernia	3	21524	448	746					
Inflammatory bowel disease	3	20368	448						
Vascular intestinal disorders	3	20364	448						
Gallbladder and biliary diseases	3	21820	825						
Pancreatitis	3	21165	448						
Other digestive diseases	3	21693	825						
Neurological disorders	2	22021	825	998					
Alzheimer's disease and other dementias	3	21733	825	181					
Parkinson's disease	3	20997	825						

Table S4. Total number of site years by cause and source type for 2019

Cause	Level	Vital Registration	Vital Registration - Sample	Verbal Autopsy	Surveillance	Survey/Census	Sibling History	Cancer Registry	Police Records
Idiopathic epilepsy	3	21822	825	996					
Multiple sclerosis	3	20993	774						
Motor neuron disease	3	20111	825						
Other neurological disorders	3	20369	825						
Mental disorders	2	20358	825						
Eating disorders	3	18844	392						
Anorexia nervosa	4	17580	392						
Bulimia nervosa	4	14940	84						
Substance use disorders	2	21974	825	393					64
Alcohol use disorders	3	21974	825	381					
Drug use disorders	3	21971	825	378					64
Opioid use disorders	4	20220	448						
Cocaine use disorders	4	20220	448						
Amphetamine use disorders	4	20220	448						
Other drug use disorders	4	20220	448						
Diabetes and kidney diseases	2	22555	825	1432					
Diabetes mellitus	3	22555	825	1224					
Diabetes mellitus type 1	4	20830	448						
Diabetes mellitus type 2	4	20830	448						
Chronic kidney disease	3	22555	825	1359					
Chronic kidney disease due to diabetes mellitus type 1	4	12063	448						
Chronic kidney disease due to diabetes mellitus type 2	4	12063	448						
Chronic kidney disease due to hypertension	4	19346	448						
Chronic kidney disease due to glomerulonephritis	4	19346	448						
Chronic kidney disease due to other and unspecified causes	4	19346	448						
Acute glomerulonephritis	3	20557	825						
Skin and subcutaneous diseases	2	22012	825	426					
Bacterial skin diseases	3	22012	825	407					
Cellulitis	4	19341	448						
Pyoderma	4	19348	448						
Decubitus ulcer	3	20398	825	305					
Other skin and subcutaneous diseases	3	19483	825						
Musculoskeletal disorders	2	22018	825	402					
Rheumatoid arthritis	3	20219	825						
Other musculoskeletal disorders	3	20372	825						
Other non-communicable diseases	2	22578	825	1439	683				
Congenital birth defects	3	22578	825	1390	683				
Neural tube defects	4	21192	825	194	590				
Congenital heart anomalies	4	21192	825	448	680				
Orofacial clefts	4	19876	448						
Down syndrome	4	20224	825		593				
Other chromosomal abnormalities	4	20217	448						
Congenital musculoskeletal and limb anomalies	4	20217	448						
Urogenital congenital anomalies	4	20220	448		500				
Digestive congenital anomalies	4	20222	448						
Other congenital birth defects	4	20222	825	377					
Urinary diseases and male infertility	3	22020	825	403					
Urinary tract infection and interstitial nephritis	4	21391	825	206					
Urolithiasis	4	20363	825	199					
Other urinary diseases	4	19487	825						
Gynaecological diseases	3	21852	825	698					
Uterine fibroids	4	19346	448	151					
Endometriosis	4	19306	448						
Genital prolapse	4	19340	448						
Other gynaecological diseases	4	19346	448						
Haemoglobinopathies and haemolytic anaemias	3	22017	825	1034					
Thalassaemias	4	20636	825	192					
Sickle cell disorders	4	20371	825	387					
G6PD deficiency	4	14832	392						
Other haemoglobinopathies and haemolytic anaemias	4	20368	825						
Endocrine, metabolic, blood, and immune disorders	3	21753	825	754					
Sudden infant death syndrome	3	17250	28						
Injuries	1	22555	825	1846	681	294			1625
Transport injuries	2	22555	825	1664	667	264			68
Road injuries	3	21453	825	188		12			68
Pedestrian road injuries	4	19488	448	188					63
Cyclist road injuries	4	19488	448	188					38
Motorcyclist road injuries	4	19488	448	187		2			43
Motor vehicle road injuries	4	19488	448	188		8			54
Other road injuries	4	19488	448	188					35
Other transport injuries	3	20276	448	187					2
Unintentional injuries	2	22553	825	1755	678	12			223
Falls	3	22024	825	1425	654	4			
Drowning	3	22552	825	1572	647	2			223
Fire, heat, and hot substances	3	22023	825	1577		1			191
Poisonings	3	22023	825	716	582				188
Poisoning by carbon monoxide	4	21240	825	428					188
Poisoning by other means	4	19288	825						
Exposure to mechanical forces	3	21734	825	640	669	8			
Unintentional firearm injuries	4	20023	825	620		1			
Other exposure to mechanical forces	4	19488	825	636	669	4			
Adverse effects of medical treatment	3	21735	825	187					
Animal contact	3	21465	825	1395		1			
Venomous animal contact	4	19760	825	713					
Non-venomous animal contact	4	19488	825						
Foreign body	3	21198	825	642					
Pulmonary aspiration and foreign body in airway	4	19488	825	177					
Foreign body in other body part	4	19483	825						
Environmental heat and cold exposure	3	22007	825	615					
Exposure to forces of nature	3	21978	825	618		5			
Other unintentional injuries	3	21735	825	646		1			
Self-harm and interpersonal violence	2	22555	825	1739	1	12			1334
Self-harm	3	22026	825	1603		3			
Self-harm by firearm	4	19288	448						
Self-harm by other specified means	4	19288	448						
Interpersonal violence	3	22555	825	1545		7			1334
Physical violence by firearm	4	19488	448	626		3			
Physical violence by sharp object	4	19488	448	630					
Physical violence by other means	4	19488	448	631					
Conflict and terrorism	3	21810	825	622		3			
Police conflict and executions	3	21269	825	155					

Table S5: List of International Classification of Diseases (ICD) codes mapped to the Global Burden of Disease cause list for causes of death		
**Cause**	**ICD10**	**ICD9**
Communicable, maternal, neonatal, and nutritional diseases	A00-A00.9, A01.0-A14, A15-A28.9, A32-A39.9, A48.1-A48.2, A48.4-A48.5, A50-A58, A60-A60.9, A63-A63.8, A65-A65.0, A68-A70, A74, A74.8-A75.9, A77-A96.9, A98-A98.8, B00-B06.9, B10-B10.8, B15-B16.2, B17.0, B17.2, B19.1, B20-B27.9, B29.4, B33-B33.1, B33.3-B33.8, B47-B48.8, B50-B54.0, B55.0, B56-B57.5, B60-B60.8, B63, B65-B67.9, B69-B72.0, B74.3-B75, B77-B77.9, B83-B83.8, B90-B91, B94.1, B95-B95.5, B97.4-B97.6, C58-C58.0, D50.1-D50.8, D51-D52.0, D52.8-D53.9, D70.3, D89.3, E00-E02, E40-E46.9, E51-E61.9, E63-E64.0, E64.2-E64.9, F02.1, F02.4, F07.1, G00.0-G00.8, G03-G03.8, G04-G05.8, G14-G14.6, G21.3, H70-H70.9, I00, I02, I02.9, I98.0-I98.1, J00-J02.8, J03-J03.8, J04-J04.2, J05-J05.1, J06.0-J06.8, J09-J15.8, J16-J16.9, J20-J21.9, J36-J36.0, J91.0, K52.1, K67.0-K67.8, K75.3, K76.3, K77.0, K93.0-K93.1, M03.1, M12.1, M49.0-M49.1, M73.0-M73.1, M89.6, N74.1, N96, N98-N98.9, O00-O07.9, O09-O16.9, O20-O26.9, O28-O36.9, O40-O48.1, O60-O77.9, O80-O92.7, O96-O98.6, O98.8-P04.2, P04.5-P05.9, P07-P15.9, P19-P22.9, P23.0-P23.4, P24-P29.9, P35-P37.2, P37.5-P39.9, P50-P61.9, P70-P70.1, P70.3-P72.9, P74-P78.9, P80-P81.9, P83-P84, P90-P94.9, P96, P96.3-P96.4, P96.8, R19.7, U04-U04.9, U06-U06.9, U82-U89, Z16-Z16.3	001-001.9, 002.0-029, 032-034.9, 036-036.3, 036.5-037.9, 040, 040.1-041.0, 042-066.9, 070.0-070.2, 071-075.9, 078.3-078.7, 079-079.7, 080-083.9, 084.0-084.5, 084.7-084.9, 085.0, 086-088, 088.8-088.9, 090-101.6, 104-104.9, 120-124.9, 125.4-125.9, 127-127.1, 128-129.0, 136-136.2, 137-139.0, 181-181.9, 244.2, 260-263.9, 265-269.9, 281.0-281.9, 320.0-320.8, 321-323.9, 381-383.9, 390-390.9, 392, 392.9, 425.6, 460-464.4, 464.8-464.9, 465.0-465.8, 466-469, 470.0, 475-475.9, 476.9, 480-482.8, 483.0-483.9, 484.0-484.7, 487-489, 630-636.9, 638-638.9, 640-679.1, 716.0, 730.4-730.6, 760-760.6, 760.8-768, 768.2-770, 770.1-775.0, 775.4-779.3, 779.6-779.8, V09-V09.9
HIV/AIDS and sexually transmitted infections	A50-A58, A60-A60.9, A63-A63.8, B20-B24.9, B63, F02.4, I98.0, K67.0-K67.2, M03.1, M73.0-M73.1	042-044.9, 054.1, 090-099.9
HIV/AIDS	B20-B24.9, F02.4	042-044.9
HIV/AIDS–drug-susceptible tuberculosis	B20.0	
HIV/AIDS–multidrug-resistant tuberculosis without extensive drug resistance		
HIV/AIDS–extensively drug-resistant tuberculosis		
HIV/AIDS resulting in other diseases	B20, B20.1-B24.9, F02.4	042-044.9
Sexually transmitted infections excluding HIV	A50-A58, A60-A60.9, A63-A63.8, B63, I98.0, K67.0-K67.2, M03.1, M73.0-M73.1	054.1, 090-099.9
Syphilis	A50-A53.9, I98.0, K67.2, M03.1, M73.1	090-097.9
Chlamydial infection	A55-A56.8, K67.0	
Gonococcal infection	A54-A54.9, K67.1, M73.0	098-098.9
Other sexually transmitted infections	A57-A58, A63-A63.8, B63	099-099.9
Respiratory infections and tuberculosis	A10-A14, A15-A19.9, A48.1, A70, B90-B90.9, B97.4-B97.6, H70-H70.9, J00-J02.8, J03-J03.8, J04-J04.2, J05-J05.1, J06.0-J06.8, J09-J15.8, J16-J16.9, J20-J21.9, J36-J36.0, J91.0, K67.3, K93.0, M49.0, N74.1, P23.0-P23.4, P37.0, U04-U04.9, U84.3	010-019.9, 034.0, 079.6, 137-137.9, 138.0-138.9, 381-383.9, 460-464.4, 464.8-464.9, 465.0-465.8, 466-469, 470.0, 475-475.9, 476.9, 480-482.8, 483.0-483.9, 484.1-484.2, 484.6-484.7, 487-489, 730.4-730.6
Tuberculosis	A10-A14, A15-A19.9, B90-B90.9, K67.3, K93.0, M49.0, N74.1, P37.0, U84.3	010-019.9, 137-137.9, 138.0-138.9, 730.4-730.6
Drug-susceptible tuberculosis	A10-A14, A15-A19.9, B90-B90.9, K67.3, K93.0, M49.0, N74.1, P37.0	010-019.9, 137-137.9, 138.0-138.9, 730.4-730.6
Multidrug-resistant tuberculosis without extensive drug resistance	U84.3	
Extensively drug-resistant tuberculosis		
Lower respiratory infections	A48.1, A70, B97.4-B97.6, J09-J15.8, J16-J16.9, J20-J21.9, J91.0, P23.0-P23.4, U04-U04.9	079.6, 466-469, 470.0, 480-482.8, 483.0-483.9, 484.1-484.2, 484.6-484.7, 487-489
Upper respiratory infections	J00-J02.8, J03-J03.8, J04-J04.2, J05-J05.1, J06.0-J06.8, J36-J36.0	034.0, 460-464.4, 464.8-464.9, 465.0-465.8, 475-475.9, 476.9
Otitis media	H70-H70.9	381-383.9
Enteric infections	A00-A00.9, A01.0-A09.9, A80-A80.9, K52.1, R19.7	001-001.9, 002.0-009.9, 045-045.9, 138
Diarrheal diseases	A00-A00.9, A02-A02.0, A02.8-A07, A07.2-A07.4, A08-A09.9, K52.1, R19.7	001-001.9, 003.8-006.9, 007.4-007.8, 008.2-009.9
Typhoid and paratyphoid	A01.0-A01.4	002.0-002.9
Typhoid fever	A01.0	002.0
Paratyphoid fever	A01.1-A01.4	002.1-002.9
Invasive non-typhoidal Salmonella (iNTS)	A02.1-A02.2	003-003.7
Other intestinal infectious diseases	A07.0-A07.1, A07.8-A07.9, A80-A80.9	007-007.3, 007.9-008.1, 045-045.9, 138
Neglected tropical diseases and malaria	A68-A68.9, A69.2-A69.9, A75-A75.9, A77-A79.9, A82-A82.9, A90-A96.9, A98-A98.8, B33.0-B33.1, B50-B54.0, B55.0, B56-B57.5, B60-B60.8, B65-B67.9, B69-B72.0, B74.3-B75, B77-B77.9, B83-B83.8, K93.1, P37.1, U06-U06.9	060-061.8, 065-066.9, 071-071.9, 080-083.9, 084.0-084.5, 084.7-084.9, 085.0, 086-088, 088.8-088.9, 120-124.9, 125.4-125.9, 127-127.1, 128-129.0, 425.6
Malaria	B50-B54.0	084.0-084.5, 084.7-084.9
Leprosy	A30-A30.9	030-030.9
Chagas disease	B57-B57.5, K93.1	086-086.2, 086.9, 425.6
Leishmaniasis	B55.0	085.0
Visceral leishmaniasis	B55.0	085.0
African trypanosomiasis	B56-B56.9	086.3-086.5
Schistosomiasis	B65-B65.9	120-120.9
Cysticercosis	B69-B69.9	123.1
Cystic echinococcosis	B67-B67.4, B67.8-B67.9	122-122.4, 122.8-122.9
Dengue	A90-A91.9	061-061.8
Yellow fever	A95-A95.9	060-060.9
Rabies	A82-A82.9	071-071.9
Intestinal nematode infections	B77-B77.9	127.0
Ascariasis	B77-B77.9	127.0
Ebola virus disease	A98.4	
Zika virus disease	U06-U06.9	
Other neglected tropical diseases	A68-A68.9, A69.2-A69.9, A75-A75.9, A77-A79.9, A92-A94.0, A96-A96.9, A98-A98.3, A98.5-A98.8, B33.0-B33.1, B60-B60.8, B67.5-B67.7, B70-B71.9, B74.3-B75, B83-B83.8, P37.1	065-066.9, 080-083.9, 087-088, 088.8-088.9, 122.5-122.7, 123-123.0, 123.2-124.9, 125.4-125.6, 125.9, 127, 127.1, 128-129.0

Table S5: List of International Classification of Diseases (ICD) codes mapped to the Global Burden of Disease cause list for causes of death		
**Cause**	**ICD10**	**ICD9**
Other infectious diseases	A20-A28.9, A32-A39.9, A48.2, A48.4-A48.5, A65-A65.0, A69-A69.1, A74, A74.8-A74.9, A81-A81.9, A83-A89.9, B00-B06.9, B10-B10.8, B15-B16.2, B17.0, B17.2, B19.1, B25-B27.9, B29.4, B33, B33.3-B33.8, B47-B48.8, B91, B94.1, B95-B95.5, D70.3, D89.3, F02.1, F07.1, G00.0-G00.8, G03-G03.8, G04-G05.8, G14-G14.6, G21.3, I00, I02, I02.9, I98.1, K67.8, K75.3, K76.3, K77.0, M49.1, M89.6, P35-P35.9, P37, P37.2, P37.5-P37.9, U82-U84, U85-U89, Z16-Z16.3	020-029, 032-034, 034.1-034.9, 036-036.3, 036.5-037.9, 040, 040.1-041.0, 046-054.0, 054.2-059.9, 062-064.9, 070.0-070.2, 072-075.9, 078.3-078.7, 079-079.5, 079.7, 100-101.6, 104-104.9, 136-136.2, 139-139.0, 320.0-320.8, 321-323.9, 390-390.9, 392, 392.9, 484.0, 484.3-484.5, 771.0-771.3, V09-V09.9
Meningitis	A39-A39.9, A87-A87.9, G00.0-G00.8, G03-G03.8	036-036.3, 036.5-036.9, 047-049.9, 320.0-320.8, 321-322.9
Encephalitis	A83-A86.4, B94.1, F07.1, G04-G05.8, G21.3	062-064.9, 139.0, 323, 323.4-323.9
Diphtheria	A36-A36.9	032-032.9
Whooping cough	A37-A37.9	033-033.9, 484.3
Tetanus	A33-A35.0	037-037.9, 771.3
Measles	B05-B05.9	055-055.9, 484.0
Varicella and herpes zoster	B01-B02.9, P35.8	052-053.9
Acute hepatitis	B15-B16.2, B17.0, B17.2, B19.1, P35.3	070.0-070.2
Acute hepatitis A	B15-B15.9	070.0-070.1
Acute hepatitis B	B16-B16.2, B17.0, B19.1, P35.3	070.2
Acute hepatitis C		
Acute hepatitis E	B17.2	
Other unspecified infectious diseases	A20-A28.9, A32-A32.9, A38-A38.9, A48.2, A48.4-A48.5, A65-A65.0, A69-A69.1, A74, A74.8-A74.9, A81-A81.9, A88-A89.9, B00-B00.9, B03-B04, B06-B06.9, B10-B10.8, B25-B27.9, B29.4, B33, B33.3-B33.8, B47-B48.8, B91, B95-B95.5, D70.3, D89.3, F02.1, G14-G14.6, I00, I02, I02.9, I98.1, K67.8, K75.3, K76.3, K77.0, M49.1, M89.6, P35-P35.2, P35.9, P37, P37.2, P37.5-P37.9, U82-U84, U85-U89, Z16-Z16.3	020-029, 034, 034.1-034.9, 040, 040.1-041.0, 046-046.9, 050-051.9, 054-054.0, 054.2-054.9, 056-059.9, 072-075.9, 078.3-078.7, 079-079.5, 079.7, 100-101.6, 104-104.9, 136-136.2, 139, 323.0-323.3, 390-390.9, 392, 392.9, 484.4-484.5, 771.0-771.2, V09-V09.9
Maternal and neonatal disorders	C58-C58.0, N96, N98-N98.9, O00-O07.9, O09-O16.9, O20-O26.9, O28-O36.9, O40-O48.1, O60-O77.9, O80-O92.7, O96-O98.6, O98.8-P04.2, P04.5-P05.9, P07-P15.9, P19-P22.9, P24-P29.9, P36-P36.9, P38-P39.9, P50-P61.9, P70-P70.1, P70.3-P72.9, P74-P78.9, P80-P81.9, P83-P84, P90-P94.9, P96, P96.3-P96.4, P96.8	181-181.9, 630-636.9, 638-638.9, 640-679.1, 760-760.6, 760.8-768, 768.2-770, 770.1-771, 771.4-775.0, 775.4-779.3, 779.6-779.8
Maternal disorders	C58-C58.0, N96, N98-N98.9, O00-O07.9, O09-O16.9, O20-O26.9, O28-O36.9, O40-O48.1, O60-O77.9, O80-O92.7, O96-O98.6, O98.8-O99.9	181-181.9, 630-636.9, 638-638.9, 640-679.1
Maternal haemorrhage	O20-O20.9, O43.2, O44-O46.9, O62-O62.9, O67-O67.9, O70, O72-O72.3	640-641.9, 661-661.9, 665, 666-666.9
Maternal sepsis and other maternal infections	O23-O23.9, O85-O86.8, O91-O91.2	659.3, 670-670.9
Maternal hypertensive disorders	O10-O16.9	642-642.9
Maternal obstructed labor and uterine rupture	O32-O33.9, O64-O66.9, O71-O71.9	652-653.9, 660-660.9, 665.0-665.3
Maternal abortion and miscarriage	N96, O01-O07.9	630-632.9, 634-636.9, 638-638.9, 646.3
Ectopic pregnancy	O00-O00.9	633-633.9
Indirect maternal deaths	O24-O25.3, O98-O98.6, O98.8-O99.9	646-646.2, 646.4-649.9
Late maternal deaths	O96-O97.9	
Maternal deaths aggravated by HIV/AIDS		
Other maternal disorders	C58-C58.0, N98-N98.9, O09-O09.9, O21-O22.9, O26-O26.9, O28-O31.8, O34-O36.9, O40-O43.1, O43.8-O43.9, O47-O48.1, O60-O61.9, O63-O63.9, O68-O69.9, O70.0-O70.9, O73-O77.9, O80-O84.9, O87-O90.9, O92-O92.7	181-181.9, 643-645.2, 650-651.9, 654-659.2, 659.4-659.9, 662-664.9, 665.4-665.9, 667-669.9, 671-679.1
Neonatal disorders	P00-P04.2, P04.5-P05.9, P07-P15.9, P19-P22.9, P24-P29.9, P36-P36.9, P38-P39.9, P50-P61.9, P70-P70.1, P70.3-P72.9, P74-P78.9, P80-P81.9, P83-P84, P90-P94.9, P96, P96.3-P96.4, P96.8	760-760.6, 760.8-768, 768.2-770, 770.1-771, 771.4-775.0, 775.4-779.3, 779.6-779.8
Neonatal preterm birth	P01.0-P01.1, P07-P07.3, P22-P22.9, P25-P28.9, P61.2, P77-P77.9	761.0-761.1, 765-765.9, 769-769.9, 770.2-770.9, 776.6, 777.5-777.6
Neonatal encephalopathy due to birth asphyxia and trauma	P01.7, P02-P03.9, P10-P15.9, P20-P21.9, P24-P24.9, P90-P91.9	761.7-763.9, 767-768, 768.2-768.9, 770.1, 772.1-772.9, 779.0-779.2
Neonatal sepsis and other neonatal infections	P36-P36.9, P38-P39.9	771.4-771.9
Hemolytic disease and other neonatal jaundice	P55-P59.9	773-774.9
Other neonatal disorders	P00-P01, P01.2-P01.6, P01.8-P01.9, P04-P04.2, P04.5-P05.9, P08-P09, P19-P19.9, P29-P29.9, P50-P54.9, P60-P61.1, P61.3-P61.9, P70-P70.1, P70.3-P72.9, P74-P76.9, P78-P78.9, P80-P81.9, P83-P84, P92-P94.9, P96, P96.3-P96.4, P96.8	760-760.6, 760.8-761, 761.2-761.6, 764-764.9, 766-766.9, 770, 771, 772-772.0, 775-775.0, 775.4-776.5, 776.7-777.4, 777.7-779, 779.3, 779.6-779.8
Nutritional deficiencies	D50.1-D50.8, D51-D52.0, D52.8-D53.9, E00-E02, E40-E46.9, E51-E61.9, E63-E64.0, E64.2-E64.9, M12.1	244.2, 260-263.9, 265-269.9, 281.0-281.9, 716.0
Protein-energy malnutrition	E40-E46.9, E64.0	260-263.9
Other nutritional deficiencies	D51-D52.0, D52.8-D53.9, E00-E02, E51-E61.9, E63-E64, E64.2-E64.9, M12.1	244.2, 265-269.9, 281.0-281.9, 716.0

Table S5: List of International Classification of Diseases (ICD) codes mapped to the Global Burden of Disease cause list for causes of death		
**Cause**	**ICD10**	**ICD9**
Non-communicable diseases	A46-A46.0, A66-A67.9, B18-B18.9, B33.2, B86, C00-C13.9, C15-C22.8, C23-C25.9, C30-C34.9, C37-C38.8, C40-C41.9, C43-C45.9, C47-C54.9, C56-C57.8, C60-C63.8, C64-C67.9, C68.0-C68.8, C69.0-C69.8, C70-C73.9, C75-C75.8, C81-C86.6, C88-C91.0, C91.2-C91.3, C91.6, C92-C92.6, C93-C93.1, C93.3, C93.8, C94-C96.9, D00.1-D00.2, D01.0-D01.3, D02.0-D02.3, D03-D06.9, D07.0-D07.2, D07.4-D07.5, D09.0, D09.2-D09.3, D09.8, D10.0-D10.7, D11-D12.9, D13.0-D13.7, D14.0-D14.3, D15-D16.9, D22-D27.9, D28.0-D28.7, D29.0-D29.8, D30.0-D30.8, D31-D36, D36.1-D36.7, D37.1-D37.5, D38.0-D38.5, D39.1-D39.2, D39.8, D40.0-D40.8, D41.0-D41.8, D42-D43.9, D44.0-D44.8, D45-D47.9, D48.0-D48.6, D49.2-D49.4, D49.6, D52.1, D55-D58.9, D59.0-D59.3, D59.5-D59.6, D60-D61.9, D63.1, D64.0, D66-D67, D68.0-D69.8, D70-D70.2, D70.4-D75.8, D76-D78.8, D86-D86.9, D89-D89.2, E03-E07.1, E09-E11.9, E15.0, E16.0-E16.9, E20-E34, E34.1-E34.8, E36-E36.8, E65-E68, E70-E85.2, E88-E89.9, F00-F02.0, F02.2-F02.3, F02.8-F03.9, F10-F16.9, F18-F18.9, F24, F50.0-F50.5, G10-G13.8, G20-G20.9, G21.0-G21.1, G23-G26.0, G30-G31.9, G35-G37.9, G40-G41.9, G45-G46.8, G47.3, G61-G61.9, G62.1, G70-G73.7, G90-G90.9, G93.7, G95-G95.9, G97-G97.9, H05.0-H05.1, I01-I01.9, I02.0, I05-I09.9, I11-I13.9, I20-I25.9, I27.0-I27.2, I28-I28.9, I30-I31.1, I31.8-I37.8, I38-I41.9, I42.1-I42.8, I43-I43.9, I47-I48.9, I51.0-I51.4, I60-I63.9, I65-I66.9, I67.0-I67.3, I67.5-I67.7, I68.0-I68.2, I69.0-I69.3, I70.2-I70.8, I71-I73.9, I77-I89.9, I95.2-I95.3, I97-I98, I98.2, I98.9, J30-J35.9, J37-J39.9, J41-J46.9, J60-J63.8, J65-J68.9, J70-J70.9, J82, J84-J84.9, J91, J91.8-J92.9, J95-J95.9, K20-K20.9, K22-K22.6, K22.8-K29.9, K31-K31.8, K35-K38.9, K40-K46.9, K50-K52.0, K52.2-K52.9, K55-K62.9, K63.5, K64-K64.9, K66.8, K67, K68, K70-K70.3, K71.7, K73-K75, K75.1-K75.2, K75.4-K76.2, K76.4-K77, K77.8, K80-K83.9, K85-K86.9, K90-K91.9, K92.8, K93.8-K95.8, L00-L05.9, L08-L08.9, L10-L14.0, L51-L51.9, L88-L89.9, L93-L93.2, L97-L98.4, M00-M03.0, M03.2-M03.6, M05-M09.8, M30-M36.8, M40-M43.1, M65-M65.0, M71.0-M71.1, M72.5-M72.6, M80-M82.8, M86.3-M86.4, M87-M87.1, M88-M89.0, M89.5, M89.7-M89.9, N00-N08.8, N10-N12.9, N13.6, N14-N16.8, N18-N18.9, N20-N23.0, N25-N28.1, N29-N30.3, N30.8-N32.0, N32.3-N32.4, N34-N34.3, N36-N36.9, N39-N39.2, N41-N41.9, N44-N44.0, N45-N45.9, N49-N49.9, N60-N60.9, N65-N65.1, N72-N72.0, N75-N77.8, N80-N81.9, N83-N83.9, N84.0-N84.1, N87-N87.9, N99-N99.9, P04.3-P04.4, P70.2, P96.0-P96.2, P96.5, Q00-Q07.9, Q10.4-Q18.9, Q20-Q28.9, Q30-Q36, Q37-Q45.9, Q50-Q87.8, Q89-Q89.8, Q90-Q93.9, Q95-Q99.8, R50.2, R78.0-R78.5, R95-R95.9, X45-X45.9, X65-X65.9, Y15-Y15.9	035-035.9, 036.4, 102-103.9, 133-133.6, 135-135.9, 140-148.9, 150-155.1, 155.3-158.9, 160-164.9, 170-175.9, 180-180.9, 182-183.8, 184.0-184.4, 184.8, 185-186.9, 187.1-187.8, 188-188.9, 189.0-189.8, 190-190.8, 191-193.9, 194.1-194.8, 200-204.0, 204.2, 205-205.3, 206-206.1, 207-208.9, 209.0-209.1, 209.4-209.5, 210.0-210.9, 211.0-211.8, 212.0-212.8, 213-213.9, 217-220.9, 221.0-221.8, 222.0-222.8, 223.0-223.8, 224-228.9, 229.0, 229.8, 230.1-230.8, 231.0-231.2, 232-232.9, 233.0-233.2, 233.4-233.5, 233.7, 234.0-234.8, 235.0, 235.4, 235.6-235.8, 236.0-236.2, 236.4-236.5, 236.7, 237-237.3, 237.5-237.9, 238.0-238.9, 239.2-239.4, 239.6, 240-243.9, 244.0-244.1, 244.3-244.8, 245-246.9, 251-259.1, 259.3-259.9, 270-273.9, 275-276, 277-277.2, 277.4-277.9, 278.0-278.8, 282-284.9, 286-286.5, 286.7-289.0, 289.4-289.7, 290-292.9, 294.1-294.9, 303-303.9, 304.0-304.8, 305.0, 305.2-305.8, 307.1, 327.2-327.8, 330-331.2, 331.5-332.0, 333-337.9, 340-341.9, 345-345.9, 349, 349.2-349.8, 353.8-353.9, 356-356.9, 357.0-357.1, 357.3-357.7, 358-359.9, 376.0-376.1, 391-391.9, 392.0, 393-398.9, 402-404.9, 410-414.9, 416.0-416.1, 417-417.9, 420-423, 423.1-423.9, 424.0-424.3, 424.8, 425.0-425.3, 425.5, 425.7-425.8, 427.0-427.3, 427.6-427.8, 429.0, 430-435.9, 437.0-437.2, 437.4-437.8, 440.2, 440.4, 441-443.9, 446-457, 457.1-457.9, 459, 459.1-459.3, 470, 470.9-474.9, 476-476.1, 477-479, 491-493.9, 495-504.9, 506-506.9, 508-509, 515, 516-517.8, 518.6-518.7, 518.9, 519.0-519.4, 530-530.0, 530.2-530.6, 531-536.1, 536.4, 537-537.6, 537.8, 538-543.9, 550-553.6, 555-558.9, 560-560.3, 560.8-560.9, 562-562.1, 564-564.7, 565-566.9, 569.0-569.7, 571-571.9, 572.2-573.0, 573.4-577.9, 579-583.9, 585-585.9, 588-590.9, 592-593.8, 594-599.6, 599.8, 601-602.9, 604-604.9, 608.2, 610-610.9, 617-618.9, 620-620.9, 621.4-621.9, 622.1-622.7, 629-629.8, 680-689, 694-695.5, 707-707.9, 710-711.9, 714-714.3, 714.8-714.9, 730.1, 732-732.9, 733.0-733.1, 740-749.0, 749.2-758.9, 759.0-759.8, 760.7, 775.1-775.3, 779.4-779.5, 788.0, 790.3, 798-798.0, E850, E860
Neoplasms	C00-C13.9, C15-C22.8, C23-C25.9, C30-C34.9, C37-C38.8, C40-C41.9, C43-C45.9, C47-C54.9, C56-C57.8, C60-C63.8, C64-C67.9, C68.0-C68.8, C69.0-C69.8, C70-C73.9, C75-C75.8, C81-C86.6, C88-C91.0, C91.2-C91.3, C91.6, C92-C92.6, C93-C93.1, C93.3, C93.8, C94-C96.9, D00.1-D00.2, D01.0-D01.3, D02.0-D02.3, D03-D06.9, D07.0-D07.2, D07.4-D07.5, D09.0, D09.2-D09.3, D09.8, D10.0-D10.7, D11-D12.9, D13.0-D13.7, D14.0-D14.3, D15-D16.9, D22-D24.9, D26.0-D27.9, D28.0-D28.1, D28.7, D29.0-D29.8, D30.0-D30.8, D31-D36, D36.1-D36.7, D37.1-D37.5, D38.0-D38.5, D39.1-D39.2, D39.8, D40.0-D40.8, D41.0-D41.8, D42-D43.9, D44.0-D44.8, D45-D47.9, D48.0-D48.6, D49.2-D49.4, D49.6, K62.0-K62.1, K63.5, N60-N60.9, N84.0-N84.1, N87-N87.9	140-148.9, 150-155.1, 155.3-158.9, 160-164.9, 170-175.9, 180-180.9, 182-183.8, 184.0-184.4, 184.8, 185-186.9, 187.1-187.8, 188-188.9, 189.0-189.8, 190-190.8, 191-193.9, 194.1-194.8, 200-204.0, 204.2, 205-205.3, 206-206.1, 207-208.9, 209.0-209.1, 209.4-209.5, 210.0-210.9, 211.0-211.8, 212.0-212.8, 213-213.9, 217-217.8, 219.0, 220-220.9, 221.0-221.8, 222.0-222.8, 223.0-223.8, 224-228.9, 229.0, 229.8, 230.1-230.8, 231.0-231.2, 232-232.9, 233.0-233.2, 233.4-233.5, 233.7, 234.0-234.8, 235.0, 235.4, 235.6-235.8, 236.1-236.2, 236.4-236.5, 236.7, 237-237.3, 237.5-237.9, 238.0-238.9, 239.2-239.4, 239.6, 569.0, 610-610.9, 622.1-622.2, 622.7
Lip and oral cavity cancer	C00-C08.9, D10.0-D10.5, D11-D11.9	140-145.9, 210.0-210.6, 235.0
Nasopharynx cancer	C11-C11.9, D10.6	147-147.9, 210.7-210.9
Other pharynx cancer	C09-C10.9, C12-C13.9, D10.7	146-146.9, 148-148.9
Oesophageal cancer	C15-C15.9, D00.1, D13.0	150-150.9, 211.0, 230.1
Stomach cancer	C16-C16.9, D00.2, D13.1, D37.1	151-151.9, 211.1, 230.2
Colon and rectum cancer	C18-C21.9, D01.0-D01.3, D12-D12.9, D37.3-D37.5	153-154.9, 209.1, 209.5, 211.3-211.4, 230.3-230.6, 569.0
Liver cancer	C22-C22.8, D13.4	155-155.1, 155.3-155.9, 211.5
Liver cancer due to hepatitis B		
Liver cancer due to hepatitis C		
Liver cancer due to alcohol use		
Liver cancer due to NASH		
Hepatoblastoma	C22.2	
Liver cancer due to other causes (internal)		
Gallbladder and biliary tract cancer	C23-C24.9, D13.5	156-156.9
Pancreatic cancer	C25-C25.9, D13.6-D13.7	157-157.9, 211.6-211.7
Larynx cancer	C32-C32.9, D02.0, D14.1, D38.0	161-161.9, 212.1, 231.0, 235.6
Tracheal, bronchus, and lung cancer	C33-C34.9, D02.1-D02.3, D14.2-D14.3, D38.1	162-162.9, 212.2-212.3, 231.1-231.2, 235.7
Malignant skin melanoma	C43-C43.9, D03-D03.9, D22-D23.9, D48.5	172-172.9
Non-melanoma skin cancer	C44-C44.9, D04-D04.9, D49.2	173-173.9, 222.4, 232-232.9, 238.2
Non-melanoma skin cancer (squamous-cell carcinoma)	C44-C44.9, D04-D04.9, D49.2	173-173.9, 222.4, 232-232.9, 238.2
Soft tissue and other extraosseous sarcomas	C49-C49.9	171-171.9
Malignant neoplasm of bone and articular cartilage	C40-C41.9	170-170.9
Breast cancer	C50-C50.9, D05-D05.9, D24-D24.9, D48.6, D49.3	174-175.9, 217-217.8, 233.0, 238.3, 239.3, 610-610.9
Cervical cancer	C53-C53.9, D06-D06.9, D26.0	180-180.9, 219.0, 233.1, 622.1-622.2, 622.7
Uterine cancer	C54-C54.9, D07.0-D07.2, D26.1-D26.9	182-182.9, 233.2
Ovarian cancer	C56-C56.9, D27-D27.9, D39.1	183-183.0, 220-220.9, 236.2
Prostate cancer	C61-C61.9, D07.5, D29.1, D40.0	185-185.9, 222.2, 236.5
Testicular cancer	C62-C62.9, D29.2-D29.8, D40.1-D40.8	186-186.9, 222.0, 222.3, 236.4
Kidney cancer	C64-C65.9, D30.0-D30.1, D41.0-D41.1	189.0-189.1, 189.5-189.6, 223.0-223.1
Bladder cancer	C67-C67.9, D09.0, D30.3, D41.4-D41.8, D49.4	188-188.9, 223.3, 233.7, 236.7, 239.4
Brain and central nervous system cancer	C70-C72.9	191-192.9
Eye cancer	C69.0-C69.8	190-190.8
Retinoblastoma	C69.2	190.5
Other eye cancers	C69.0-C69.1, C69.3-C69.8	190-190.4, 190.6-190.8

Table S5: List of International Classification of Diseases (ICD) codes mapped to the Global Burden of Disease cause list for causes of death		
**Cause**	**ICD10**	**ICD9**
Neuroblastoma and other peripheral nervous cell tumors	C47-C47.9	
Thyroid cancer	C73-C73.9, D09.3, D09.8, D34-D34.9, D44.0	193-193.9, 226-226.9
Mesothelioma	C45-C45.9	
Hodgkin lymphoma	C81-C81.9	201-201.9
Non-Hodgkin lymphoma	C82-C86.6, C96-C96.9	200-200.9, 202-202.9
Burkitt lymphoma	C83.7-C83.8	200.2
Other non-Hodgkin lymphoma	C82-C83.6, C83.9-C86.6, C96-C96.9	200-200.1, 200.3-200.9, 202-202.9
Multiple myeloma	C88-C90.9	203-203.9
Leukaemia	C91-C91.0, C91.2-C91.3, C91.6, C92-C92.6, C93-C93.1, C93.3, C93.8, C94-C95.9	204-204.0, 204.2, 205-205.3, 206-206.1, 207-208.9
Acute lymphoid leukaemia	C91.0, C91.2-C91.3, C91.6	204.0, 204.2
Chronic lymphoid leukaemia		
Acute myeloid leukaemia	C92.0, C92.3-C92.6, C93.0, C94.0, C94.2, C94.4-C94.5	205.0, 205.2-205.3, 206.0, 207.0, 207.2-207.8
Chronic myeloid leukaemia	C92.1-C92.2	205.1
Other leukaemia	C93.1, C93.3, C93.8, C94.1, C94.3, C94.6-C95.9	206.1, 207.1, 207.9-208.9
Other malignant neoplasms (internal)	C17-C17.9, C30-C31.9, C37-C38.8, C48-C48.9, C4A, C51-C52.9, C57-C57.8, C60-C60.9, C63-C63.8, C66-C66.9, C68.0-C68.8, C75-C75.8, D07.4, D09.2, D13.2-D13.3, D14.0, D15-D16.9, D28.0-D28.1, D28.7, D29.0, D30.2, D30.4-D30.8, D31-D31.9, D35-D35.2, D35.5-D36, D36.1-D36.7, D37.2, D38.2-D38.5, D39.2, D39.8, D41.2-D41.3, D44.1-D44.8, D48.0-D48.4	152-152.9, 158-158.9, 160-160.9, 163-164.9, 183.2-183.8, 184.0-184.4, 184.8, 187.1-187.8, 189.2-189.4, 189.8, 194.1-194.8, 209.0, 209.4, 211.2, 211.8, 212.0, 212.4-212.8, 213-213.9, 221.0-221.8, 222.1, 222.8, 223.2, 223.8, 224-224.9, 227-228.9, 229.0, 229.8, 230.7-230.8, 233.4-233.5, 234.0-234.8, 235.4, 235.8, 236.1, 238.0-238.1, 239.2
Other neoplasms	D32-D33.9, D35.3-D35.4, D42-D43.9, D45-D47.9, D49.6, K62.0-K62.1, K63.5, N60-N60.9, N84.0-N84.1, N87-N87.9	225-225.9, 237-237.3, 237.5-237.9, 238.4-238.9, 239.6
Myelodysplastic, myeloproliferative, and other haematopoietic neoplasms	D45-D47.9	238.4-238.9
Cardiovascular diseases	B33.2, G45-G46.8, I01-I01.9, I02.0, I05-I09.9, I11-I11.9, I20-I25.9, I27.0, I27.2, I28-I28.9, I30-I31.1, I31.8-I37.8, I38-I41.9, I42.1-I42.8, I43-I43.9, I47-I48.9, I51.0-I51.4, I60-I63.9, I65-I66.9, I67.0-I67.3, I67.5-I67.6, I68.0-I68.2, I69.0-I69.3, I70.2-I70.8, I71-I73.9, I77-I83.9, I86-I89.0, I89.9, I98, K75.1	036.4, 391-391.9, 392.0, 393-398.9, 402-402.9, 410-414.9, 416.0, 417-417.9, 420-423, 423.1-423.9, 424.0-424.3, 424.8, 425.0-425.3, 425.5, 425.7-425.8, 427.0-427.3, 427.6-427.8, 429.0, 430-435.9, 437.0-437.2, 437.5-437.8, 440.2, 440.4, 441-443.9, 447-454.9, 456, 456.3-457, 457.1, 457.8-457.9, 459, 459.1-459.3
Rheumatic heart disease	I01-I01.9, I02.0, I05-I09.9	391-391.9, 392.0, 393-398.9
Ischaemic heart disease	I20-I25.9	410-414.9
Stroke	G45-G46.8, I60-I63.9, I65-I66.9, I67.0-I67.3, I67.5-I67.6, I68.1-I68.2, I69.0-I69.3	430-435.9, 437.0-437.2, 437.5-437.8
Ischaemic stroke	G45-G46.8, I63-I63.9, I65-I66.9, I67.2-I67.3, I67.5-I67.6, I69.3	433-435.9, 437.0-437.1, 437.5-437.8
Intracerebral haemorrhage	I61-I62, I62.1-I62.9, I68.1-I68.2, I69.1-I69.2	431-432.9, 437.2
Subarachnoid hemorrhage	I60-I60.9, I62.0, I67.0-I67.1, I69.0	430-430.9
Hypertensive heart disease	I11-I11.9	402-402.9
Non-rheumatic valvular heart disease	I34-I37.8	424.0-424.3, 424.8
Non-rheumatic calcific aortic valvular heart disease	I35-I35.9	424.1
Non-rheumatic degenerative mitral valvular heart disease	I34-I34.9	424.0
Other non-rheumatic valvular heart diseases	I36-I37.8	424.2-424.3, 424.8
Cardiomyopathy and myocarditis	B33.2, I40-I41.9, I42.1-I42.8, I43-I43.9, I51.4	422-422.9, 425.0-425.3, 425.5, 425.7-425.8, 429.0
Myocarditis	B33.2, I40-I41.9, I51.4	422-422.9
Alcoholic cardiomyopathy	I42.6	425.5
Other cardiomyopathy	I42.1-I42.5, I42.7-I42.8, I43-I43.9	425.0-425.3, 425.7-425.8, 429.0
Pulmonary arterial hypertension	I27.0, I27.2	416.0
Atrial fibrillation and flutter	I48-I48.9	427.3
Aortic aneurysm	I71-I71.9	441-441.9
Peripheral artery disease	I70.2-I70.8, I73-I73.9	440.2, 440.4, 443.0-443.9
Endocarditis	I33-I33.9, I38-I39.9	421-421.9
Other cardiovascular and circulatory diseases (internal)	I28-I28.9, I30-I31.1, I31.8-I32.8, I47-I47.9, I51.0-I51.3, I68.0, I72-I72.9, I77-I83.9, I86-I89.0, I89.9, I98, K75.1	036.4, 417-417.9, 420-420.9, 423, 423.1-423.9, 427.0-427.2, 427.6-427.8, 442-443, 447-454.9, 456, 456.3-457, 457.1, 457.8-457.9, 459, 459.1-459.3
Chronic respiratory diseases	D86-D86.2, D86.9, G47.3, J30-J35.9, J37-J39.9, J41-J46.9, J60-J63.8, J65-J68.9, J70, J70.8-J70.9, J82, J84-J84.9, J91, J91.8-J92.9	135-135.9, 327.2-327.8, 470, 470.9-474.9, 476-476.1, 477-479, 491-493.9, 495-504.9, 506-506.9, 508-509, 515, 516-517.8, 518.6, 518.9, 519.1-519.4
Chronic obstructive pulmonary disease	J41-J44.9	491-492.9, 496-499
Pneumoconiosis	J60-J63.8, J65-J65.0, J92.0	500-504.9
Silicosis	J62-J62.9	502-502.9, 503.0, 503.9
Asbestosis	J61-J61.0, J92.0	501
Coal workers pneumoconiosis	J60-J60.0	500-500.9, 501.0-501.9
Other pneumoconiosis	J63-J63.8, J65-J65.0	503, 503.1, 504-504.9
Asthma	J45-J46.9	493-493.9
Interstitial lung disease and pulmonary sarcoidosis	D86-D86.2, D86.9, J84-J84.9	135-135.9, 515, 516-516.9
Other chronic respiratory diseases	G47.3, J30-J35.9, J37-J39.9, J66-J68.9, J70, J70.8-J70.9, J82, J91, J91.8-J92, J92.9	327.2-327.8, 470, 470.9-474.9, 476-476.1, 477-479, 495-495.9, 506-506.9, 508-509, 517-517.8, 518.6, 518.9, 519.1-519.4
Digestive diseases	B18-B18.9, I84-I85.9, I98.2, K20-K20.9, K22-K22.6, K22.8-K29.9, K31-K31.8, K35-K38.9, K40-K42.9, K44-K46.9, K50-K52, K52.2-K52.9, K55-K62, K62.2-K62.6, K62.8-K62.9, K64-K64.9, K66.8, K67, K68, K70-K70.3, K71.7, K73-K75, K75.2, K75.4-K76.2, K76.4-K77, K77.8, K80-K83.9, K85-K86.9, K90-K90.9, K92.8, K93.8, M09.1	455-455.9, 456.0-456.2, 530-530.0, 530.2-530.6, 531-536.1, 537-537.6, 537.8, 538, 540-543.9, 550-551.1, 551.3-552.1, 552.3-553.6, 555-558.9, 560-560.3, 560.8-560.9, 562-562.1, 564-564.1, 564.5-564.7, 565-566.9, 569.1-569.5, 569.7, 571-571.9, 572.2-573.0, 573.4-577.9, 579-579.2, 579.4-579.9
Cirrhosis and other chronic liver diseases	B18-B18.9, I85-I85.9, I98.2, K70-K70.3, K71.7, K73-K75, K75.2, K75.4-K76.2, K76.4-K76.9, K77.8	456.0-456.2, 571-571.9, 572.2-573.0, 573.4-573.9
Cirrhosis and other chronic liver diseases due to hepatitis B		
Cirrhosis and other chronic liver diseases due to hepatitis C		
Cirrhosis and other chronic liver diseases due to alcohol use		

Table S5: List of International Classification of Diseases (ICD) codes mapped to the Global Burden of Disease cause list for causes of death		
**Cause**	**ICD10**	**ICD9**
Cirrhosis and other chronic liver diseases due to NAFLD		
Cirrhosis and other chronic liver diseases due to other causes		
Upper digestive system diseases	K25-K29.9	531-535.9
Peptic ulcer disease	K25-K28.9	531-534.9
Gastritis and duodenitis	K29-K29.9	535-535.9
Appendicitis	K35-K37.9, K38.3-K38.9	540-542.9
Paralytic ileus and intestinal obstruction	K56-K56.9	560-560.3, 560.8-560.9
Inguinal, femoral, and abdominal hernia	K40-K42.9, K44-K46.9	550-551.1, 551.3-552.1, 552.3-553.0, 553.6
Inflammatory bowel disease	K50-K52, K52.8-K52.9, M09.1	555-556.9, 558-558.9, 569.5
Vascular intestinal disorders	K55-K55.9	557-557.9
Gallbladder and biliary diseases	K80-K83.9	574-576.9
Pancreatitis	K85-K86.9	577-577.9, 579.4
Other digestive diseases	I84-I84.9, K20-K20.9, K22-K22.6, K22.8-K24, K31-K31.8, K38-K38.2, K52.2-K52.3, K57-K62, K62.2-K62.6, K62.8-K62.9, K64-K64.9, K66.8, K67, K68, K77, K90-K90.9, K92.8, K93.8	455-455.9, 530-530.0, 530.2-530.6, 536-536.1, 537-537.6, 537.8, 538, 543-543.9, 553.1-553.3, 562-562.1, 564-564.1, 564.5-564.7, 565-566.9, 569.1-569.4, 569.7, 579-579.2, 579.8-579.9
Neurological disorders	F00-F02.0, F02.2-F02.3, F02.8-F03.9, G10-G13.8, G20-G20.9, G23-G24, G24.1-G25.0, G25.2-G25.3, G25.5, G25.8-G26.0, G30-G31.1, G31.8-G31.9, G35-G37.9, G40-G41.9, G61-G61.9, G70-G71.1, G71.3-G72, G72.2-G73.7, G90-G90.9, G95-G95.9, M33-M33.9	290-290.9, 294.1-294.9, 330-331.2, 331.5-332.0, 333-337.9, 340-341.9, 345-345.9, 349, 349.2-349.8, 353.8-353.9, 356-356.9, 357.0-357.1, 357.3-357.4, 357.7, 358-359.9, 775.2
Alzheimer's disease and other dementias	F00-F02.0, F02.8-F03.9, G30-G31.1, G31.8-G31.9	290-290.9, 294.1-294.9, 331-331.2
Parkinson's disease	F02.3, G20-G20.9	332-332.0
Idiopathic epilepsy	G40-G41.9	345-345.9
Multiple sclerosis	G35-G35.9	340-340.9
Motor neuron disease	G12.2-G12.9	335-335.2, 335.8-335.9
Other neurological disorders	F02.2, G10-G12.1, G13-G13.8, G23-G24, G24.1-G25.0, G25.2-G25.3, G25.5, G25.8-G26.0, G36-G37.9, G61-G61.9, G70-G71.1, G71.3-G72, G72.2-G73.7, G90-G90.9, G95-G95.9, M33-M33.9	330-330.9, 331.5-331.9, 333-334.9, 335.3, 336-337.9, 341-341.9, 349, 349.2-349.8, 353.8-353.9, 356-356.9, 357.0-357.1, 357.3-357.4, 357.7, 358-359.9, 775.2
Mental disorders	F24, F50.0-F50.5	307.1
Eating disorders	F50.0-F50.5	307.1
Anorexia nervosa	F50.0-F50.1	307.1
Bulimia nervosa	F50.2-F50.5	
Substance use disorders	E24.4, F10-F16.9, F18-F18.9, G31.2, G62.1, G72.1, P04.3-P04.4, P96.1, Q86.0, R78.0-R78.5, X45-X45.9, X65-X65.9, Y15-Y15.9	291-292.9, 303-303.9, 304.0-304.8, 305.0, 305.2-305.8, 357.5, 760.7, 790.3, E850, E860
Alcohol use disorders	E24.4, F10-F10.9, G31.2, G62.1, G72.1, P04.3, Q86.0, R78.0, X45-X45.9, X65-X65.9, Y15-Y15.9	291-291.9, 303-303.9, 305.0, 357.5, 790.3, E860
Drug use disorders	F11-F16.9, F18-F18.9, P04.4, P96.1, R78.1-R78.5	292-292.9, 304.0-304.8, 305.2-305.8, 760.7, E850
Opioid use disorders	F11-F11.9, P96.1, R78.1	304.0, 305.5
Cocaine use disorders	F14-F14.9, R78.2	304.2, 305.6
Amphetamine use disorders	F15-F15.9	304.4, 305.7
Other drug use disorders	F13-F13.9, F16-F16.9, F18-F18.9, P04.4, R78.3-R78.5	292-292.9, 304.1, 304.5-304.8, 305.3-305.4, 305.8, 760.7
Diabetes and kidney diseases	D63.1, E10-E11.9, I12-I13.9, N00-N08.8, N15.0, N18-N18.9, P70.2, Q61-Q62.8	403-404.9, 580-583.9, 585-585.9, 589-589.9, 753-753.3, 775.1
Diabetes mellitus	E10-E10.1, E10.3-E11.1, E11.3-E11.9, P70.2	775.1
Diabetes mellitus type 1	E10-E10.1, E10.3-E10.9, P70.2	775.1
Diabetes mellitus type 2	E11-E11.1, E11.3-E11.9	
Chronic kidney disease	D63.1, E10.2, E11.2, I12-I13.9, N02-N08.8, N15.0, N18-N18.9, Q61-Q62.8	403-404.9, 581-583.9, 585-585.9, 589-589.9, 753-753.3
Chronic kidney disease due to diabetes mellitus type 1	E10.2	
Chronic kidney disease due to diabetes mellitus type 2	E11.2	
Chronic kidney disease due to hypertension	I12-I13.9	403-404.9
Chronic kidney disease due to glomerulonephritis	N03-N06.9	581-583.9
Chronic kidney disease due to other and unspecified causes	N02-N02.9, N07-N08.8, N15.0, Q61-Q62.8	589-589.9, 753-753.3
Acute glomerulonephritis	N00-N01.9	580-580.9
Skin and subcutaneous diseases	A46-A46.0, A66-A67.9, B86, D86.3, I89.1-I89.8, L00-L05.9, L08-L08.9, L10-L14.0, L51-L51.9, L88-L89.9, L97-L98.4, M72.5-M72.6	035-035.9, 102-103.9, 133-133.6, 457.2-457.3, 680-689, 694-695.3, 707-707.9
Bacterial skin diseases	A46-A46.0, A66-A67.9, I89.1-I89.8, L00-L05.9, L08-L08.9, L88, L97-L98.4, M72.5-M72.6	035-035.9, 102-103.9, 457.2-457.3, 680-689
Cellulitis	L03-L03.9, M72.5-M72.6	681-682.9
Pyoderma	A46-A46.0, A66-A67.9, I89.1-I89.8, L00-L02.9, L04-L05.9, L08-L08.9, L88, L97-L98.4	035-035.9, 102-103.9, 457.2-457.3, 680-680.9, 683-689
Decubitus ulcer	L89-L89.9	707-707.9
Other skin and subcutaneous diseases	D86.3, L10-L14.0, L51-L51.9	694-695.3
Musculoskeletal disorders	I27.1, I67.7, L93-L93.2, M00-M03.0, M03.2-M03.6, M05-M09.0, M09.2-M09.8, M30-M32.9, M34-M36.8, M40-M43.1, M65-M65.0, M71.0-M71.1, M80-M82.8, M86.3-M86.4, M87-M87.0, M88-M89.0, M89.5, M89.7-M89.9	416.1, 437.4, 446-446.9, 695.4-695.5, 710-711.9, 714-714.3, 714.8-714.9, 730.1, 732-732.9, 733.0-733.1
Rheumatoid arthritis	M05-M06.9, M08.0-M08.8	714-714.3, 714.8-714.9
Other musculoskeletal disorders	I27.1, I67.7, L93-L93.2, M00-M03.0, M03.2-M03.6, M07-M08, M08.9-M09.0, M09.2-M09.8, M30-M32.9, M34-M36.8, M40-M43.1, M65-M65.0, M71.0-M71.1, M80-M82.8, M86.3-M86.4, M87-M87.0, M88-M89.0, M89.5, M89.7-M89.9	416.1, 437.4, 446-446.9, 695.4-695.5, 710-711.9, 730.1, 732-732.9, 733.0-733.1

Table S5: List of International Classification of Diseases (ICD) codes mapped to the Global Burden of Disease cause list for causes of death		
Cause	ICD10	ICD9
Other non-communicable diseases	D25-D26, D28.2, D52.1, D55-D58.9, D59.0-D59.3, D59.5-D59.6, D60-D61.9, D64.0, D66-D67, D68.0-D69.8, D70-D70.2, D70.4-D75.8, D76-D78.8, D86.8, D89-D89.2, E03-E07.1, E09-E09.9, E15.0, E16.0-E16.9, E20-E24.3, E24.8-E34, E34.1-E34.8, E36-E36.8, E65-E68, E70-E85.2, E88-E89.9, G21.0-G21.1, G24.0, G25.1, G25.4, G25.6-G25.7, G71.2, G72.0, G93.7, G97-G97.9, I95.2-I95.3, I97-I97.9, I98.9, J70.0-J70.5, J95-J95.9, K43-K43.9, K52.0, K62.7, K91-K91.9, K94-K95.8, M87.1, N10-N12.9, N13.6, N14-N15, N15.1-N16.8, N20-N23.0, N25-N28.1, N29-N30.3, N30.8-N32.0, N32.3-N32.4, N34-N34.3, N36-N36.9, N39-N39.2, N41-N41.9, N44-N44.0, N45-N45.9, N49-N49.9, N65-N65.1, N72-N72.0, N75-N77.8, N80-N81.9, N83-N83.9, N99-N99.9, P96.0, P96.2, P96.5, Q00-Q07.9, Q10.4-Q18.9, Q20-Q28.9, Q30-Q36, Q37-Q45.9, Q50-Q60.6, Q63-Q86, Q86.1-Q87.8, Q89-Q89.8, Q90-Q93.9, Q95-Q99.8, R50.2, R95-R95.9	218-219, 219.1-219.9, 236.0, 240-243.9, 244.0-244.1, 244.3-244.8, 245-246.9, 251-259.1, 259.3-259.9, 270-273.9, 275-276, 277-277.2, 277.4-277.9, 278.0-278.8, 282-284.9, 286-286.5, 286.7-289.0, 289.4-289.7, 357.6, 518.7, 519.0, 536.4, 539-539.9, 551.2, 552.2, 564.2-564.4, 569.6, 579.3, 588-588.9, 590-590.9, 592-593.8, 594-599.6, 599.8, 601-602.9, 604-604.9, 608.2, 617-618.9, 620-620.9, 621.4-621.9, 622.3-622.6, 629-629.8, 740-749.0, 749.2-752.9, 753.4-758.9, 759.0-759.8, 775.3, 779.4-779.5, 788.0, 798-798.0
Congenital birth defects	G71.2, P96.0, Q00-Q07.9, Q10.4-Q18.9, Q20-Q28.9, Q30-Q36, Q37-Q45.9, Q50-Q60.6, Q63-Q86, Q86.1-Q87.8, Q89-Q89.8, Q90-Q93.9, Q95-Q99.8	740-749.0, 749.2-752.9, 753.4-758.9, 759.0-759.8
Neural tube defects	Q00-Q01.9, Q05-Q05.9	740-741.9, 742.0
Congenital heart anomalies	Q20-Q28.9	745-747.9
Orofacial clefts	Q35-Q36, Q37-Q37.9	749-749.0, 749.2-749.9
Down syndrome	Q90-Q90.9	758.0
Other chromosomal abnormalities	Q87-Q87.8, Q91-Q93.9, Q95-Q95.9, Q97-Q97.9, Q99-Q99.8	758, 758.1-758.6, 758.8-758.9
Congenital musculoskeletal and limb anomalies	Q65-Q79, Q79.6-Q79.9	742.5, 754-756.5, 756.8-756.9
Urogenital congenital anomalies	P96.0, Q50-Q60.6, Q63-Q64.9	752-752.9, 753.4-753.9
Digestive congenital anomalies	Q38-Q45.9, Q79.0-Q79.5	750-751.9, 756.6-756.7
Other congenital birth defects	G71.2, Q02-Q04.9, Q06-Q07.9, Q10.4-Q18.9, Q30-Q34.9, Q80-Q86, Q86.1-Q86.8, Q89-Q89.8	742, 742.1-742.4, 742.8-744.9, 748-748.9, 757-757.9, 759.0-759.8
Urinary diseases and male infertility	N10-N12.9, N13.6, N15, N15.1-N16.8, N20-N23.0, N25-N28.1, N29-N30.3, N30.8-N32.0, N32.3-N32.4, N34-N34.3, N36-N36.9, N39-N39.2, N41-N41.9, N44-N44.0, N45-N45.9, N49-N49.9	588-588.9, 590-590.9, 592-593.8, 594-598.1, 598.8-599.6, 599.8, 601-602.9, 604-604.9, 608.2, 788.0
Urinary tract infection and interstitial nephritis	N10-N12.9, N13.6, N15, N15.1-N16.8, N30-N30.3, N30.8-N30.9, N34-N34.3, N39.0-N39.2	590-590.9, 595-595.9, 597-597.9, 599.0
Urolithiasis	N20-N23.0	592-592.9, 594-594.9, 788.0
Other urinary diseases	N25-N28.1, N29-N29.8, N31-N32.0, N32.3-N32.4, N36-N36.9, N39, N41-N41.9, N44-N44.0, N45-N45.9, N49-N49.9	588-588.9, 593-593.8, 596-596.9, 598-598.1, 598.8-599, 599.1-599.6, 599.8, 601-602.9, 604-604.9, 608.2
Gynaecological diseases	D25-D26, D28.2, E28.2, N72-N72.0, N75-N77.8, N80-N81.9, N83-N83.9	218-219, 219.1-219.9, 236.0, 256.4, 617-618.9, 620-620.9, 621.4-621.9, 622.3-622.6, 629-629.8
Uterine fibroids	D25-D26, D28.2	218-219, 219.1-219.9, 236.0
Endometriosis	N80-N80.9	617-617.9
Genital prolapse	N81-N81.9	618-618.9
Other gynaecological diseases	N72-N72.0, N75-N77.8, N83-N83.9	620-620.9, 621.4-621.9, 622.3-622.6, 629-629.8
Haemoglobinopathies and haemolytic anaemias	D55-D58.9, D59.1, D59.3, D59.5, D60-D61.9, D64.0	282-284.9
Thalassaemias	D56-D56.9	282.4-282.5
Sickle cell disorders	D57-D57.8	282.6
G6PD deficiency	D55-D55.2	282.2-282.3
Other haemoglobinopathies and haemolytic anaemias	D55.3-D55.9, D58-D58.9, D59.1, D59.3, D59.5, D60-D61.9, D64.0	282-282.1, 282.7-284.9
Endocrine, metabolic, blood, and immune disorders	D52.1, D59.0, D59.2, D59.6, D66-D67, D68.0-D69.8, D70-D70.2, D70.4-D75.8, D76-D78.8, D86.8, D89-D89.2, E03-E07.1, E09-E09.9, E15.0, E16.0-E16.9, E20-E24.3, E24.8-E28.1, E28.3-E34, E34.1-E34.8, E36-E36.8, E65-E68, E70-E85.2, E88-E89.9, G21.0-G21.1, G24.0, G25.1, G25.4, G25.6-G25.7, G72.0, G93.7, G97-G97.9, I95.2-I95.3, I97-I97.9, I98.9, J70.0-J70.5, J95-J95.9, K43-K43.9, K52.0, K62.7, K91-K91.9, K94-K95.8, M87.1, N14-N14.4, N65-N65.1, N99-N99.9, P96.2, P96.5, R50.2	240-243.9, 244.0-244.1, 244.3-244.8, 245-246.9, 251-256.3, 256.8-259.1, 259.3-259.9, 270-273.9, 275-276, 277-277.2, 277.4-277.9, 278.0-278.8, 286-286.5, 286.7-289.0, 289.4-289.7, 357.6, 518.7, 519.0, 536.4, 539-539.9, 551.2, 552.2, 564.2-564.4, 569.6, 579.3, 598.2, 775.3, 779.4-779.5
Sudden infant death syndrome	R95-R95.9	798-798.0
Injuries	L55-L55.9, L56.3, L56.8-L56.9, L58-L58.9, N30.4, U00-U03, V00-V86.9, V87.2-V87.3, V88.2-V88.3, V90-V98.8, W00-W46.2, W49-W62.9, W64-W70.9, W73-W75.9, W77-W81.9, W83-W94.9, W97.9, W99-X06.9, X08-X39.9, X47-X48.9, X50-X54.9, X57-X58.9, X60-X64.9, X66-X83.9, X85-Y08.9, Y35-Y84.9, Y87.0-Y87.1, Y88-Y88.3, Y89.0-Y89.1	349.0-349.1, 457.0, E800-E807, E830-E838, E840-E849, E856-E857, E861-E865, E867-E869, E870-E876, E878-E879, E880-E886, E888-E928, E930-E979, E990-E999
Transport injuries	V00-V86.9, V87.2-V87.3, V88.2-V88.3, V90-V98.8	E800-E807, E830-E838, E840-E849
Road injuries	V01-V04.9, V06-V80.9, V82-V82.9, V87.2-V87.3	
Pedestrian road injuries	V01-V04.9, V06-V09.9	
Cyclist road injuries	V10-V19.9	
Motorcyclist road injuries	V20-V29.9	
Motor vehicle road injuries	V30-V79.9, V87.2-V87.3	
Other road injuries	V80-V80.9, V82-V82.9	
Other transport injuries	V00-V00.8, V05-V05.9, V81-V81.9, V83-V86.9, V88.2-V88.3, V90-V98.8	E800-E807, E830-E838, E840-E849
Unintentional injuries	L55-L55.9, L56.3, L56.8-L56.9, L58-L58.9, N30.4, W00-W46.2, W49-W62.9, W64-W70.9, W73-W75.9, W77-W81.9, W83-W94.9, W97.9, W99-X06.9, X08-X39.9, X47-X48.9, X50-X54.9, X57-X58.9, Y40-Y84.9, Y88-Y88.3	349.0-349.1, 457.0, E856-E857, E861-E865, E867-E869, E870-E876, E878-E879, E880-E886, E888-E928, E930-E949
Falls	W00-W19.9	E880-E886, E888
Drowning	W65-W70.9, W73-W74.9	E910
Fire, heat, and hot substances	X00-X06.9, X08-X19.9	E890-E899, E924
Poisonings	X47-X48.9	E856-E857, E861-E865, E867-E869
Poisoning by carbon monoxide	X47-X47.9	E862, E868-E869
Poisoning by other means	X48-X48.9	E856-E857, E861, E863-E865, E867
Exposure to mechanical forces	W20-W38.9, W40-W43.9, W45.0-W45.2, W46-W46.2, W49-W52	E916-E922
Unintentional firearm injuries	W32-W34.9	E922

Table S5: List of International Classification of Diseases (ICD) codes mapped to the Global Burden of Disease cause list for causes of death

Cause	ICD10	ICD9
Other exposure to mechanical forces	W20-W31.9, W35-W38.9, W40-W43.9, W45.0-W45.2, W46-W46.2, W49-W52	E916-E921
Adverse effects of medical treatment	N30.4, Y40-Y84.9, Y88-Y88.3	349.0-349.1, 457.0, E870-E876, E878-E879, E930-E949
Animal contact	W52.0-W62.9, W64-W64.9, X20-X29.9	E905-E906
Venomous animal contact	X20-X29.9	E905
Non-venomous animal contact	W52.0-W62.9, W64-W64.9	E906
Foreign body	W44-W45, W45.3-W45.9, W75-W75.9, W78-W80.9, W83-W84.9	E911-E915
Pulmonary aspiration and foreign body in airway	W75-W75.9, W78-W80.9, W83-W84.9	E911-E913
Foreign body in other body part	W44-W45, W45.3-W45.9	E914-E915
Environmental heat and cold exposure	L55-L55.9, L56.3, L56.8-L56.9, L58-L58.9, W88-W94.9, W97.9, W99-W99.9, X30-X32.9, X39-X39.9	E900-E902, E926
Exposure to forces of nature	X33-X38.9	E907-E909
Still Born	P95-P95.9	768.0-768.1
Other unintentional injuries	W39-W39.9, W77-W77.9, W81-W81.9, W85-W87.9, X50-X54.9, X57-X58.9	E903-E904, E923, E925, E927-E928
Self-harm and interpersonal violence	U00-U03, X60-X64.9, X66-X83.9, X85-Y08.9, Y35-Y38.9, Y87.0-Y87.1, Y89.0-Y89.1	E950-E979, E990-E999
Self-harm	X60-X64.9, X66-X83.9, Y87.0	E950-E959
Self-harm by firearm	X72-X74.9	E955
Self-harm by other specified means	X60-X64.9, X66-X71.9, X75-X83.9, Y87.0	E950-E954, E956-E959
Interpersonal violence	X85-Y08.9, Y87.1	E960-E969
Physical violence by firearm	X93-X95.9	E965
Physical violence by sharp object	X99-X99.9	E966
Physical violence by other means	X85-X92.9, X96-X98.9, Y00-Y04.9, Y06-Y08.9, Y87.1	E961-E964, E967-E969
Conflict and terrorism	U00-U03, Y36-Y38.9, Y89.1	E979, E990-E999
Police conflict and executions	Y35-Y35.9, Y89.0	E970-E978
Garbage Code (GBD Level 1)	A40-A41.9, A48.0, A48.3, A49.0-A49.1, A59-A59.9, A71-A71.9, A74.0, B07-B07.9, B30-B30.9, B35-B36.9, B85-B85.4, B87-B88.9, B94.0, D50-D50.0, D50.9, D62-D63.0, D63.8-D64, D64.1-D65.9, D68, D69.9, E15, E16, E50-E50.9, E64.1, E85.3-E87.6, E87.8-E87.9, F06.2-F06.4, F07.2, F09-F09.9, F19-F23.9, F25-F49, F51-F99.0, G06-G08.0, G32-G32.8, G43-G44.2, G44.4-G44.8, G47-G47.2, G47.4-G47.9, G50-G60.9, G62-G62.0, G62.2-G65.2, G80-G83.9, G89-G89.4, G91-G91.2, G91.4-G93, G93.1-G93.2, G93.4-G93.6, G94.0-G94.8, G99-H05, H05.2-H69.9, H71-H99, I26-I26.9, I31.2-I31.4, I46-I46.9, I50.0-I50.4, I76, I95-I95.1, I95.8-I95.9, J69-J69.9, J80-J80.9, J81.0, J85-J85.3, J86-J86.9, J93-J93.1, J93.8-J93.9, J94.2, J96-J96.9, J98.1-J98.3, K00-K19, K30, K65-K66.1, K66.9, K68.1-K68.9, K71-K71.6, K71.8-K72.9, K75.0, L20-L30.9, L40-L50.9, L52-L54.8, L56-L56.2, L56.4-L56.5, L57-L57.9, L59-L68.9, L70-L76.8, L80-L87.9, L90-L92.9, L94-L96, L98.5-L99.8, M04, M10-M12.0, M12.2-M29, M37-M39, M43.2-M49, M49.2-M64, M65.1-M71, M71.2-M72.4, M72.8-M73, M73.8-M79.9, M83-M86.2, M86.5-M86.9, M87.2-M87.9, M89.1-M89.4, M90-M99.9, N17-N17.9, N19-N19.9, N32.1-N32.2, N32.8-N33.8, N35-N35.9, N37-N37.8, N39.3-N39.8, N42-N43.4, N44.1-N44.8, N46-N48.9, N50-N53.9, N61-N64.9, N82-N82.9, N91-N91.5, N95, N95.1-N95.9, N97-N97.9, R02-R02.9, R03.1, R07.0, R08-R09, R09.3, R11-R12.0, R14-R19.6, R19.8-R23, R23.1-R30.9, R32-R50.1, R50.8-R57.9, R58.0-R72.9, R74-R78, R78.6-R94.8, R96-R99.9, U05, U07-U81, U89.9-U99, X40-X44.9, X46-X46.9, X49-X49.9, Y10-Y14.9, Y16-Y19.9, Z00-Z15.8, Z17-unsp.	038-038.9, 040.0, 041.1, 076-078.2, 110-111.9, 125-125.3, 126-126.9, 127.2-127.9, 131-132.9, 133.8-134.9, 136.6, 139.1, 139.9, 247-248, 264-264.9, 274-274.9, 276.0-276.5, 276.7-276.9, 277.3, 280-281, 285-285.9, 286.6, 289.1-289.3, 293, 294-294.0, 295-302.9, 305, 305.9-307.0, 307.2-307.4, 307.6-319.9, 324-327.1, 328-329, 338-339.1, 339.3-339.8, 342-344.9, 346-346.9, 350-353.6, 354-355.9, 360-362, 362.1-376, 376.2-380.9, 384-389.9, 415-415.9, 423.0, 424, 424.4-424.5, 424.9, 427.5, 427.9-428.9, 437.3, 458-458.9, 459.0, 507-507.9, 510-510.9, 512-513.9, 518.1-518.2, 520-529.9, 536.3, 536.8-536.9, 537.7, 537.9, 564.8-564.9, 567-568.9, 570-570.9, 572-572.1, 573.1-573.3, 584-584.9, 586-587.9, 603-603.9, 605-608.1, 608.3-609, 611-612.1, 615-616.9, 619-619.9, 621-621.3, 622-622.0, 622.8-623.6, 623.8-624.5, 624.8-628.9, 629.9, 690-693.9, 695.8-706.9, 708-709.9, 712-713.8, 715-716, 716.2-721.6, 721.8-730.0, 730.2-730.3, 730.7-731.9, 733, 733.2-734.2, 737-738, 738.2-739.9, 780-782.4, 782.6-784.6, 784.9, 785.4-786, 786.6, 786.8, 787, 787.3-788, 788.3-789, 789.1-789.2, 789.5, 790-790.1, 790.4-796.1, 796.3-797.9, 798.1-799, 799.2-799.9, 999.0-999.9, E851-E855, E858, E866, E980-E982, V01-V08, V10-uns
Garbage Code (GBD Level 2)	A14.9, A29-A30.9, A45-A45.9, A47-A48, A48.8-A49, A49.3-A49.9, A61-A62, A72-A73, A76, A97, B08-B09, B11-B14, B28-B29, B31-B32.4, B34-B34.9, B61-B62, B68-B68.9, B73-B74.2, B76-B76.9, B78-B81.8, B84, B92-B94, B94.8-B94.9, B95.6-B97.3, B97.7-B99.9, D59, D59.4, D59.8-D59.9, F17-F17.9, G44.3, G91.3, G93.0, G93.3, I10-I10.9, I15-I15.9, I27, I27.8-I27.9, I50, I50.8-I50.9, I67.4, I70-I70.1, I70.9, I74-I75.8, J81, J81.1, J90-J90.0, J94-J94.1, J94.8-J94.9, K92.0-K92.2, N70-N71.9, N73-N74.0, N74.2-N74.8, R03-R03.0, R04-R06.9, R09.0-R09.2, R09.8-R10.9, R13-R13.9, R23.0, R58, S00-T98.3, W47-W48, W63, W71-W72, W76-W76.9, W82, W95-W97, W98, X07, X55-X56, X59-X59.9, Y20-Y34.9, Y86-Y87, Y87.2, Y89, Y89.9-Y99.9	000-000.9, 030-030.9, 041.2-041.9, 067-069, 078.8-078.9, 079.8-079.9, 089-089.9, 105-109.9, 119, 136.8-136.9, 139.8, 304, 304.9, 305.1, 339.2, 401-401.9, 405-405.9, 416, 416.2-416.9, 440-440.1, 440.3, 440.8-440.9, 444-445.8, 490-490.9, 494-494.9, 511-511.9, 514-514.9, 515.0-515.9, 518-518.0, 518.3-518.5, 518.8, 536.2, 578-578.9, 599.7, 613-614.9, 714.4, 716.1, 721.7, 735-736.9, 738.0-738.1, 784.7-784.8, 786.3, 787.0-787.2, 789.0, 789.3-789.4, 789.6-789.9, 796.2, 799.0-799.1, 800-999, E000-E80, E83, E839, E85, E859, E87, E877, E88, E887, E929, E983-E985, E988-E989

Table S5: List of International Classification of Diseases (ICD) codes mapped to the Global Burden of Disease cause list for causes of death		
**Cause**	**ICD10**	**ICD9**
Garbage Code (GBD Level 3)	A01, A31-A31.9, A42-A44.9, A49.2, A64-A64.0, A99-A99.0, B17, B17.1, B17.8-B17.9, B19-B19.0, B19.2-B19.9, B37-B46.9, B49-B49.9, B55, B55.1-B55.9, B58-B59.9, B89, B94.2, C14-C14.9, C22.9, C26-C29, C35-C36, C39-C39.9, C42, C46-C46.9, C55-C55.9, C57.9, C59, C63.9, C68, C68.9, C74-C74.9, C75.9-C80.9, C87, C97-D00.0, D01, D01.4-D02, D02.4-D02.9, D07, D07.3, D07.6-D09, D09.1, D09.7, D09.9-D10, D10.9, D13, D13.9-D14, D14.4, D17-D21.9, D28, D28.9-D29, D29.9-D30, D30.9, D36.0, D36.9-D37.0, D37.6-D38, D38.6-D39.0, D39.7, D39.9-D40, D40.9-D41, D41.9, D44, D44.9, D48, D48.7-D49.1, D49.5, D49.7-D49.9, D54, D75.9, D79-D85, D87-D88, D89.8-D99, E07.8-E08.9, E17-E19, E34.0, E34.9-E35.8, E37-E39, E47-E49., E62, E69, E87.7, E90-E998, F04-F06.1, F06.5-F07.0, F07.8-F08, F50, F50.8-F50.9, G09-G09.9, G15-G19, G21, G21.2, G21.4-G22.0, G27-G29, G33-G34, G38-G39., G42, G48-G49, G66-G69, G74-G79, G84-G88, G93.8-G94, G96-G96.9, G98-G98.9, I00.0, I03-I04., I14-I14., I16-I19, I29-I29.9, I44-I45.9, I49-I49.9, I51, I51.6-I59, I90-I94, I96-I96.9, I98.4-I98.8, I99-ID5.9, J02.9, J03.9, J04.3, J06, J06.9, J40-J40.9, J47-J59, J71-J79, J81.9, J83, J85.9, J87-J89, J90.9, J93.6, J97-J98.0, J98.4-J99.8, K21-K21.9, K22.7, K31.9-K34, K39, K47-K49, K53-K54, K63-K63.4, K63.8-K63.9, K69, K70.4-K70.9, K78-K79, K84, K87-K89, K92, K92.9-K93, K96-K99, L06-L07, L09, L15-L19, L31-L39, L69, L77-L79, N09, N13-N13.5, N13.7-N13.9, N24, N28.8-N28.9, N38, N39.9-N40.9, N54-N59, N66-N69, N78-N79, N84, N84.2-N86, N88-N90.9, N92-N94.9, N95.0, O08-O08.9, O17-O19, O27, O37-O39, O49-O59, O78-O79, O93-O95.9, P06, P16-P18, P30-P34.2, P40-P49, P62-P69, P73, P79, P82, P85-P89, P96.9-P99.9, Q08-Q10.3, Q19, Q29-Q29., Q36.0-Q36.9, Q46-Q49, Q88, Q89.9, Q94, Q99.9-R01.2, R07, R07.1-R07.9, R31-R31.9	002, 031-031.9, 039-039.9, 070, 070.4-070.9, 085, 085.1-085.9, 088.0-088.7, 112-118.9, 130-130.9, 136.3-136.5, 149-149.9, 155.2, 159-159.9, 165-169, 176-179.9, 183.9-184, 184.5, 184.9, 187, 187.9, 189, 189.9, 190.9, 195-199.9, 209, 209.2-209.3, 209.6-210, 211, 211.9-212, 212.9, 214-216.9, 221, 221.9-222, 222.9-223, 223.9, 229, 229.1, 229.9-230.0, 230.9-231, 231.8-231.9, 233, 233.3, 233.6, 233.9-234, 234.9-235, 235.1-235.3, 235.5, 235.9-236, 236.3, 236.6, 236.9, 237.4, 239-239.1, 239.5, 239.7-239.9, 249-249.9, 259.2, 276.6, 278, 279-279.9, 293.0-293.9, 331.3-331.4, 332.1-332.9, 347-348.9, 349.9, 357, 357.8-357.9, 399-400.0, 406-409.4, 418-419.9, 426-427, 427.4, 429, 429.2-429.9, 459.5-459.9, 464.5, 465, 465.9, 505-505.9, 519, 519.8-519.9, 530.1, 530.7-530.9, 544-549, 553.8-553.9, 559-559.0, 560.4-560.7, 561, 562.2-563, 569, 569.8-569.9, 591-591.9, 593.9, 599.9-600.9, 623.7, 624.6, 637-637.9, 639-639.9, 749.1, 759, 759.9, 779.9, 782.5, 785-785.3, 786.0-786.2, 786.4-786.5, 786.7, 786.9, 788.1-788.2, E986-E987
Garbage Code (GBD Level 4)	B16.9, B64, B82-B82.9, B83.9, C69, C69.9, C91.1, C91.4-C91.5, C91.7-C91.9, C92.7-C92.9, C93.2, C93.5-C93.7, C93.9, E12-E14.9, G00, G00.9-G02.8, G03.9, I37.9, I42-I42.0, I42.9, I51.5, I64-I64.9, I67, I67.8-I68, I68.8-I69, I69.4-I69.9, J07-J08, J15.9, J17-J19.6, J22-J29, J64-J64.9, P23, P23.5-P23.9, P37.3-P37.4, R73-R73.9, V87-V87.1, V87.4-V88.1, V88.4-V89.9, V99-V99.0, X84-X84.9, Y09-Y09.9, Y85-Y85.9	070.3, 084, 084.6, 194-194.0, 194.9, 204.1, 204.5-204.9, 205.8-205.9, 206.2-206.9, 238, 244, 244.9, 250-250.9, 289.8-289.9, 307.5, 320, 320.9, 357.2, 362.0, 425, 425.4, 425.9, 429.1, 436-437, 437.9-439.6, 482.9-483, 484, 484.8-486.9, 770.0, 790.2, E808-E829

Table S6. Restrictions on age and sex by cause for GBD 2019

Cause	Minimum Age	Maximum Age	Sex Restrictions
HIV/AIDS and sexually transmitted infections			
HIV/AIDS	28 days		
HIV/AIDS–drug-susceptible tuberculosis	28 days		
HIV/AIDS–multidrug-resistant TB without extensive drug resistance	28 days		
HIV/AIDS–extensively drug-resistant tuberculosis	28 days		
HIV/AIDS resulting in other diseases	28 days		
Sexually transmitted infections excluding HIV			
Syphilis			
Chlamydial infection	10		
Gonococcal infection	10		
Other sexually transmitted infections	10		
Respiratory infections and tuberculosis	7 days		
Tuberculosis	28 days		
Drug-susceptible tuberculosis	28 days		
Multidrug-resistant tuberculosis without extensive drug resistance	28 days		
Extensively drug-resistant tuberculosis	28 days		
Lower respiratory infections			
Upper respiratory infections			
Otitis media			
Enteric infections			
Diarrhoeal diseases			
Typhoid and paratyphoid	28 days		
Typhoid fever	28 days		
Paratyphoid fever	28 days		
iNTS	7 days		
Other intestinal infectious diseases	28 days		
Neglected tropical diseases and malaria			
Malaria			
Chagas disease	28 days		
Leishmaniasis	28 days		

Visceral leishmaniasis	28 days		
African trypanosomiasis	1		
Schistosomiasis	28 days		
Cysticercosis	1		
Cystic echinococcosis	1		
Dengue	7 days		
Yellow fever	7 days		
Rabies	28 days		
Intestinal nematode infections	28 days		
Ascariasis	28 days		
Ebola virus disease			
Zika virus disease			
Other neglected tropical diseases			
Other infectious diseases			
Meningitis			
Encephalitis			
Diphtheria	28 days	55	
Whooping cough	28 days	55	
Tetanus			
Measles	28 days	55	
Varicella and herpes zoster			
Acute hepatitis	28 days		
Acute hepatitis A	28 days		
Acute hepatitis B	28 days		
Acute hepatitis C	28 days		
Acute hepatitis E	28 days		
Other unspecified infectious diseases			
Maternal and neonatal disorders		50	
Maternal disorders	10	50	Females Only
Maternal haemorrhage	10	50	Females Only
Maternal sepsis and other pregnancy related infections	10	50	Females Only
Maternal hypertensive disorders	10	50	Females Only

Maternal obstructed labour and uterine rupture	10	50	Females Only
Maternal abortive outcome	10	50	Females Only
Ectopic pregnancy	10	50	Females Only
Indirect maternal deaths	10	50	Females Only
Late maternal deaths	10	50	Females Only
Maternal deaths aggravated by HIV/AIDS	10	50	Females Only
Other maternal disorders	10	50	Females Only
Neonatal disorders		1	
Neonatal preterm birth		1	
Neonatal encephalopathy due to birth asphyxia and trauma		1	
Neonatal sepsis and other neonatal infections		1	
Hemolytic disease and other neonatal jaundice		1	
Other neonatal disorders		1	
Nutritional deficiencies	28 days		
Protein-energy malnutrition	28 days		
Other nutritional deficiencies	28 days		
Neoplasms			
Lip and oral cavity cancer	5		
Nasopharynx cancer	5		
Other pharynx cancer	20		
Oesophageal cancer	20		
Stomach cancer	15		
Colon and rectum cancer	5		
Liver cancer			
Liver cancer due to hepatitis B	10		
Liver cancer due to hepatitis C	10		
Liver cancer due to alcohol use	15		
Liver cancer due to NASH	15		
Liver cancer due to other causes			
Gallbladder and biliary tract cancer	20		
Pancreatic cancer	15		
Larynx cancer	20		

Tracheal, bronchus, and lung cancer	10		
Malignant skin melanoma			
Non-melanoma skin cancer	20		
Non-melanoma skin cancer (squamous-cell carcinoma)	20		
Breast cancer	15		
Cervical cancer	15		Females Only
Uterine cancer	20		Females Only
Ovarian cancer	5		Females Only
Prostate cancer	20		Males Only
Testicular cancer			Males Only
Kidney cancer			
Bladder cancer	15		
Brain and nervous system cancer			
Thyroid cancer	5		
Mesothelioma	20		
Hodgkin lymphoma	1		
Non-Hodgkin's lymphoma	1		
Multiple myeloma	20		
Leukaemia			
Acute lymphoid leukaemia			
Chronic lymphoid leukaemia	20		
Acute myeloid leukaemia			
Chronic myeloid leukaemia			
Other leukaemia			
Other malignant neoplasms			
Other neoplasms			
Myelodysplastic, myeloproliferative, and other haematopoietic neoplasms			
Cardiovascular diseases			
Rheumatic heart disease	1		
Ischaemic heart disease	15		
Stroke			
Ischaemic stroke			

Intracerebral hemorrhage			
Subarachnoid hemorrhage			
Hypertensive heart disease	15		
Non-rheumatic valvular heart disease	15		
Non-rheumatic calcific aortic valvular heart disease	15		
Non-rheumatic degenerative mitral valvular heart disease	15		
Other non-rheumatic valvular heart diseases	15		
Cardiomyopathy and myocarditis			
Myocarditis			
Alcoholic cardiomyopathy	15		
Other cardiomyopathy			
Atrial fibrillation and flutter	30		
Aortic aneurysm	15		
Peripheral vascular disease	40		
Endocarditis			
Other cardiovascular and circulatory diseases			
Chronic respiratory diseases			
Chronic obstructive pulmonary disease	28 days		
Pneumoconiosis	15		
Silicosis	15		
Asbestosis	15		
Coal workers pneumoconiosis	15		
Other pneumoconiosis	15		
Asthma	1		
Interstitial lung disease and pulmonary sarcoidosis	1		
Other chronic respiratory diseases			
Digestive diseases			
Cirrhosis and other chronic liver diseases	1		
Cirrhosis and other chronic liver diseases due to hepatitis B	1		
Cirrhosis and other chronic liver diseases due to hepatitis C	1		
Cirrhosis and other chronic liver diseases due to alcohol use	15		
Cirrhosis and other chronic liver diseases due to NAFLD	15		

Cirrhosis and other chronic liver diseases due to other causes	1		
Upper digestive system diseases	1		
Peptic ulcer disease	1		
Gastritis and duodenitis	1		
Appendicitis	1		
Paralytic ileus and intestinal obstruction			
Inguinal, femoral, and abdominal hernia			
Inflammatory bowel disease	1		
Vascular intestinal disorders	1		
Gallbladder and biliary diseases	1		
Pancreatitis	1		
Other digestive diseases	1		
Neurological disorders	28 days		
Alzheimer's disease and other dementias	40		
Parkinson's disease	20		
Idiopathic epilepsy	28 days		
Multiple sclerosis	5		
Motor neuron disease			
Other neurological disorders	28 days		
Mental disorders			
Eating disorders	5	45	
Anorexia nervosa	5	45	
Bulimia nervosa	5	45	
Substance use disorders			
Alcohol use disorders	10		
Drug use disorders			
Opioid use disorders			
Cocaine use disorders	10		
Amphetamine use disorders	10		
Other drug use disorders	10		
Diabetes and kidney diseases			
Diabetes mellitus			

Diabetes mellitus type 1			
Diabetes mellitus type 2	15		
Chronic kidney disease	28 days		
Chronic kidney disease due to diabetes mellitus type 1	28 days		
Chronic kidney disease due to diabetes mellitus type 2	15		
Chronic kidney disease due to hypertension	15		
Chronic kidney disease due to glomerulonephritis	28 days		
Chronic kidney disease due to other and unspecified causes	28 days		
Acute glomerulonephritis	28 days		
Skin and subcutaneous diseases	28 days		
Bacterial skin diseases			
Cellulitis			
Pyoderma			
Decubitus ulcer	1		
Other skin and subcutaneous diseases			
Musculoskeletal disorders	5		
Rheumatoid arthritis	5		
Other musculoskeletal disorders	5		
Other non-communicable diseases			
Congenital anomalies		65	
Neural tube defects		65	
Congenital heart anomalies		65	
Orofacial clefts		1	
Down syndrome		65	
Other chromosomal abnormalities		65	
Congenital musculoskeletal and limb anomalies		65	
Urogenital congenital anomalies		65	
Digestive congenital anomalies		65	
Other congenital anomalies		65	
Urinary diseases and male infertility			
Urinary tract infection and interstitial nephritis			
Urolithiasis	1		

Other urinary diseases			
Gynaecological diseases	10		Females Only
Uterine fibroids	10		Females Only
Endometriosis	10	50	Females Only
Genital prolapse	10		Females Only
Other gynaecological diseases	10		Females Only
Haemoglobinopathies and haemolytic anaemias			
Thalassaemias			
Sickle cell disorders			
G6PD deficiency			
Other haemoglobinopathies and haemolytic anaemias			
Endocrine, metabolic, blood, and immune disorders			
Sudden infant death syndrome	7 days	364 days	
Transport injuries			
Road injuries			
Pedestrian road injuries			
Cyclist road injuries	1		
Motorcyclist road injuries			
Motor vehicle road injuries			
Other road injuries			
Other transport injuries			
Unintentional injuries			
Falls			
Drowning			
Fire, heat, and hot substances			
Poisonings			
Poisoning by carbon monoxide			
Poisoning by other means			
Exposure to mechanical forces			
Unintentional firearm injuries			
Other exposure to mechanical forces			
Adverse effects of medical treatment			

Animal contact			
Venomous animal contact			
Non-venomous animal contact			
Foreign body			
Pulmonary aspiration and foreign body in airway			
Foreign body in other body part			
Environmental heat and cold exposure			
Exposure to forces of nature			
Other unintentional injuries			
Self-harm and interpersonal violence			
Self-harm	10		
Self-harm by firearm	10		
Self-harm by other specified means	10		
Interpersonal violence			
Physical violence by firearm			
Physical violence by sharp object			
Physical violence by other means			
Conflict and terrorism			
Police conflict and executions			

Table s7. Data quality rating from 0 to 5 stars, maximum percent well certified per 5-year interval and percent well certified across time series for 204 countries, 1980-2019.

Country	Data Quality Rating	1980-1984	1985-1989	1990-1994	1995-1999	2000-2004	2005-2009	2010-2019	1980-2019
Afghanistan	1	0.0	0.0	0.0	0.0	3.4	28.7	0.0	4.6
Albania	3	0.0	65.9	67.8	74.8	73.8	72.5	54.6	58.5
Algeria	1	0.0	0.0	0.0	0.0	0.0	15.8	0.0	2.3
American Samoa	3	0.0	0.0	0.0	65.9	72.6	69.7	66.2	39.2
Andorra	1	0.0	0.0	0.0	0.0	0.0	0.0	65.3	9.3
Angola	1	0.0	0.0	0.0	0.0	0.0	0.0	2.8	0.4
Antigua and Barbuda	4	48.9	69.6	76.0	79.3	81.3	79.6	78.6	73.3
Argentina	4	76.1	71.5	70.6	69.9	69.1	69.1	73.7	71.4
Armenia	5	75.6	84.5	86.2	86.1	86.4	90.9	89.6	85.6
Australia	5	93.3	93.7	93.6	93.5	92.5	91.8	91.1	92.8
Austria	5	92.3	93.2	92.7	92.3	94.8	93.6	91.1	92.8
Azerbaijan	3	69.7	69.1	68.0	65.4	72.1	42.7	0.0	55.3
Bahrain	3	0.0	75.2	0.0	62.9	61.8	58.8	54.9	44.8
Bangladesh	2	2.2	3.4	30.2	3.9	19.1	6.3	53.3	16.9
Barbados	4	76.1	78.2	80.1	80.1	77.2	78.0	76.8	78.1
Belarus	4	85.0	87.9	80.0	80.7	82.6	83.4	83.7	83.3
Belgium	4	80.3	80.8	84.3	86.3	86.2	86.4	82.9	83.9
Belize	4	61.5	66.7	57.9	74.9	75.9	87.2	87.6	73.1
Benin	1	0.0	2.1	0.0	0.0	0.0	0.0	0.0	0.3
Bermuda	5	92.5	90.9	90.4	95.0	93.0	88.8	90.4	91.6
Bhutan	0	0.0	0.0	0.0	0.0	0.0	0.0	0.0	0.0
Venezuela	5	78.9	72.9	83.2	88.5	90.7	91.1	91.3	85.2
Bosnia and Herzegovina	2	0.0	65.1	66.5	0.0	0.0	0.0	75.4	29.6
Botswana	0	0.0	0.0	0.0	0.0	0.0	0.0	0.0	0.0
Brazil	4	51.3	55.5	62.0	66.2	70.9	77.8	82.3	66.6
Brunei	3	0.0	0.0	0.0	78.9	76.4	73.9	73.8	43.3
Bulgaria	4	81.1	81.4	81.3	78.6	70.1	70.2	68.1	75.8
Burkina Faso	1	3.7	0.0	0.0	4.6	6.3	2.4	0.3	2.5
Burundi	1	0.0	0.0	2.9	0.0	0.0	0.0	0.0	0.4
Cambodia	1	0.0	0.0	0.0	0.0	0.9	4.2	0.0	0.7
Cameroon	0	0.0	0.0	0.0	0.0	0.0	0.0	0.0	0.0
Canada	5	90.5	90.7	89.9	89.6	90.0	90.5	90.2	90.2
Central African Republic	0	0.0	0.0	0.0	0.0	0.0	0.0	0.0	0.0
Chad	0	0.0	0.0	0.0	0.0	0.0	0.0	0.0	0.0
Chile	4	75.3	75.9	82.0	85.1	91.2	91.5	90.4	84.5
China	3	0.0	1.0	70.0	73.7	66.8	72.1	71.7	50.8
Colombia	4	69.2	72.0	76.3	88.3	89.7	88.5	90.8	82.1
The Bahamas	4	74.3	82.3	80.1	84.1	83.4	82.3	82.5	81.3
Comoros	0	0.0	0.0	0.0	0.0	0.0	0.0	0.0	0.0
Congo (Brazzaville)	0	0.0	0.0	0.0	0.0	0.0	0.0	0.0	0.0
Cook Islands	2	0.0	0.0	0.0	0.0	60.0	53.9	68.5	26.1
Costa Rica	5	81.7	83.2	83.3	91.4	92.7	91.6	91.6	87.9
Croatia	4	0.0	84.9	85.9	84.9	88.2	89.9	93.7	75.4
Cuba	5	85.9	87.2	86.8	89.9	91.3	92.2	92.3	89.4
Cyprus	2	0.0	0.0	0.0	32.2	60.7	69.7	78.4	34.4
Czech Republic	4	0.0	91.2	90.5	85.9	86.5	86.6	87.9	75.5
North Korea	0	0.0	0.0	0.0	0.0	0.0	0.0	0.0	0.0
DR Congo	1	0.0	0.9	3.6	0.0	0.0	0.0	0.0	0.6
Denmark	5	83.6	82.3	85.7	87.3	87.8	87.0	85.1	85.6
Djibouti	0	0.0	0.0	0.0	0.0	0.0	0.0	0.0	0.0
Dominica	3	60.1	55.6	55.9	63.3	58.9	72.3	74.2	62.9
Dominican Republic	3	46.3	46.7	39.3	42.9	45.9	44.6	45.6	44.5
Ecuador	3	65.9	65.3	66.5	63.4	60.8	55.2	63.4	62.9
Egypt	2	25.0	30.8	35.4	0.0	39.1	38.4	46.6	30.8
El Salvador	3	57.4	0.0	55.8	62.7	66.8	65.7	65.4	53.4
Equatorial Guinea	0	0.0	0.0	0.0	0.0	0.0	0.0	0.0	0.0
Eritrea	0	0.0	0.0	0.0	0.0	0.0	0.0	0.0	0.0
Estonia	5	90.9	91.2	93.1	93.2	92.3	92.8	91.9	92.2
Ethiopia	1	0.0	3.0	3.2	0.9	3.3	4.0	4.0	2.6
Federated States of Micronesia	0	0.0	0.0	0.0	0.0	0.0	0.0	0.0	0.0
Fiji	2	0.0	0.0	0.0	32.9	53.5	59.7	66.0	30.3
Finland	5	82.7	91.6	91.3	96.1	95.3	94.3	94.4	92.2
France	4	78.0	80.3	82.0	81.9	82.3	82.4	80.7	81.1
Gabon	0	0.0	0.0	0.0	0.0	0.0	0.0	0.0	0.0
Georgia	4	78.3	76.5	71.1	67.3	69.6	48.3	58.9	67.1
Germany	4	80.5	83.1	86.1	86.1	85.4	85.7	85.6	84.7
Ghana	1	0.0	0.4	2.1	0.9	9.1	21.4	0.4	4.9
Greece	4	83.6	84.0	78.8	79.6	80.2	82.3	82.7	81.6
Greenland	3	0.0	0.0	0.0	86.3	87.4	88.0	83.6	49.3
Grenada	4	69.0	64.0	65.3	65.3	80.2	82.3	87.5	73.4
Guam	3	0.0	0.0	85.8	80.5	71.6	65.6	63.3	52.4
Guatemala	4	68.8	66.1	66.1	69.7	69.9	75.0	77.7	70.5
Guinea	1	0.0	0.0	0.0	3.4	0.0	0.0	0.0	0.5
Guinea-Bissau	1	0.0	0.0	0.4	2.3	0.0	0.0	0.0	0.4
Guyana	4	49.2	65.9	61.4	68.1	73.5	75.1	74.2	66.8
Haiti	1	15.6	1.9	1.7	11.9	5.4	0.0	0.0	5.2
Honduras	2	35.7	42.8	39.1	0.0	0.0	12.4	13.0	20.4
Hungary	5	91.2	90.4	90.5	91.6	93.3	93.9	94.2	92.1
Iceland	5	91.7	93.3	96.0	95.0	94.3	92.6	91.3	93.5
India	2	2.6	4.6	2.7	4.8	6.3	49.7	46.0	16.7
Indonesia	2	1.7	0.0	1.8	0.6	0.3	53.9	63.4	17.4
Iraq	2	0.0	0.0	0.0	0.0	0.0	39.2	46.2	12.2
Ireland	5	91.4	92.2	92.6	92.0	91.6	92.7	92.9	92.2
Iran	2	0.0	0.0	0.0	0.0	57.5	66.1	80.0	29.1
Israel	4	80.8	82.5	83.5	83.5	82.3	81.0	82.3	82.3
Italy	5	89.6	89.6	90.1	89.2	89.7	90.1	89.8	89.7
Jamaica	4	72.0	74.2	67.0	0.0	83.9	87.2	89.4	67.7
Japan	5	82.9	82.1	83.5	89.1	87.6	87.0	85.5	85.4
Jordan	2	0.0	0.0	0.0	1.7	72.8	79.3	65.2	31.3
Kazakhstan	4	79.8	83.6	87.3	85.5	79.7	76.9	73.6	80.9
Kenya	1	0.0	3.4	0.0	2.4	4.3	5.1	23.4	5.5
eSwatini	1	0.0	0.0	0.0	0.0	0.5	0.0	0.0	0.1
Kiribati	2	0.0	0.0	35.6	55.6	32.2	0.0	0.0	17.6
Kuwait	4	74.8	76.9	71.9	71.7	69.9	67.7	68.5	71.7
Kyrgyzstan	4	75.5	83.8	74.5	78.7	88.3	94.4	94.1	84.2
Laos	1	0.0	3.8	0.0	0.0	0.0	0.0	0.0	0.5
Latvia	5	91.3	91.3	88.8	92.0	91.3	89.2	93.8	91.1
Lebanon	1	0.0	3.6	0.0	0.0	0.3	0.0	0.0	0.6
Lesotho	0	0.0	0.0	0.0	0.0	0.0	0.0	0.0	0.0
Liberia	1	4.1	4.1	4.3	0.0	0.0	0.0	0.0	1.8
Libya	1	0.0	0.0	0.0	0.0	0.0	11.5	0.0	1.6
Lithuania	5	90.6	92.2	92.7	94.8	93.2	92.8	93.5	92.8
Luxembourg	4	86.9	87.5	85.1	85.0	82.8	82.2	83.1	84.7
Madagascar	1	2.3	2.9	2.1	2.2	0.0	0.0	0.0	1.4
Malawi	1	0.0	3.8	0.0	0.7	3.2	3.4	0.6	1.7
Malaysia	2	44.0	0.0	0.0	31.4	36.0	40.5	40.4	27.5
Maldives	2	0.0	0.0	0.0	0.0	44.0	49.1	61.9	22.1
Mali	1	2.3	0.0	0.3	0.0	0.0	0.0	5.6	1.2
Malta	5	83.0	87.9	89.9	90.8	92.3	94.5	93.5	90.2
Marshall Islands	0	0.0	0.0	0.0	0.0	0.0	0.0	0.0	0.0
Mauritania	0	0.0	0.0	0.0	0.0	0.0	0.0	0.0	0.0
Mauritius	4	73.5	77.7	76.8	77.4	82.4	84.4	87.0	79.9
Mexico	4	64.0	71.4	73.0	78.8	80.6	82.4	89.4	77.1
Mongolia	2	0.0	0.0	59.1	0.0	19.5	20.9	79.4	25.6

Table S7. Data quality rating from 0 to 5 stars, maximum percent well certified per 5-year interval and percent well certified across time series for 204 countries, 1980-2019.

Country	Data Quality Rating	1980-1984	1985-1989	1990-1994	1995-1999	2000-2004	2005-2009	2010-2019	1980-2019
Montenegro	2	0.0	0.0	0.0	0.0	62.0	62.6	0.0	17.8
Morocco	2	0.0	24.7	0.0	0.0	10.7	29.6	12.1	11.0
Mozambique	2	0.0	0.0	0.0	0.3	14.9	63.3	3.5	11.7
Myanmar	1	0.0	0.0	0.0	0.0	0.0	2.9	48.9	7.4
Namibia	0	0.0	0.0	0.0	0.0	0.0	0.0	0.0	0.0
Nepal	1	3.3	3.2	0.0	1.2	1.0	16.4	0.0	3.6
Netherlands	5	88.8	88.0	86.5	86.0	84.2	84.5	84.2	86.0
New Zealand	5	95.4	95.0	95.5	96.7	96.9	96.4	96.0	96.0
Nicaragua	3	0.0	49.0	50.4	64.6	66.8	68.6	79.5	54.1
Niger	1	0.0	0.0	0.0	0.0	0.0	42.6	0.0	6.1
Nigeria	1	0.0	0.0	4.3	0.0	0.0	0.0	40.4	6.4
Northern Mariana Islands	2	0.0	0.0	0.0	59.2	54.2	61.2	64.9	34.2
Norway	5	91.0	91.5	91.0	90.8	88.4	86.1	85.7	89.2
Oman	2	0.0	0.0	0.0	0.0	0.0	63.3	32.4	13.7
Pakistan	2	0.0	31.5	2.1	0.0	13.8	23.4	21.6	13.2
Palestine	2	0.0	0.0	0.0	28.5	31.6	33.8	72.8	23.8
Panama	4	74.1	77.1	0.0	83.3	86.7	86.5	84.6	70.3
Papua New Guinea	1	6.3	3.6	0.0	0.0	0.0	0.0	29.5	5.6
Paraguay	3	51.7	54.5	61.5	62.3	63.1	67.2	82.2	63.2
Peru	3	51.6	34.4	34.5	45.8	55.4	56.2	52.1	47.1
Philippines	3	0.4	12.9	15.4	40.4	46.2	74.1	75.4	37.8
Bolivia	1	0.0	0.0	0.0	0.0	12.4	0.0	0.0	1.8
Poland	4	64.3	64.4	64.2	73.5	75.8	75.9	75.9	70.6
Portugal	4	77.6	77.9	77.6	77.6	82.4	79.5	86.0	79.8
Monaco	2	0.0	76.8	0.0	0.0	0.0	0.0	80.3	22.4
Puerto Rico	4	78.1	77.3	84.1	83.8	84.0	83.4	84.5	82.2
Qatar	2	12.2	16.0	0.0	49.0	43.6	50.2	38.9	30.0
Cape Verde	2	48.7	0.0	0.3	0.0	0.0	0.0	70.2	17.0
Côte d'Ivoire	1	0.0	3.7	3.6	0.0	0.0	0.2	0.1	1.1
South Korea	3	0.0	53.0	72.1	74.2	83.9	82.8	84.0	64.3
Moldova	5	89.5	91.0	79.5	86.9	89.9	87.8	90.1	87.8
Nauru	0	0.0	0.0	0.0	0.0	0.0	0.0	0.0	0.0
Niue	0	0.0	0.0	0.0	0.0	0.0	0.0	0.0	0.0
Palau	1	0.0	0.0	0.0	0.0	0.0	0.0	51.0	7.3
San Marino	3	0.0	0.0	0.0	75.7	77.4	77.4	65.8	42.3
The Gambia	1	3.9	3.5	3.4	2.8	2.5	2.2	0.0	2.6
Romania	4	77.3	79.0	83.8	86.1	87.2	87.5	86.8	84.0
Russia	5	92.5	86.6	90.2	87.0	87.4	88.9	88.5	88.7
Rwanda	1	0.0	0.0	0.0	0.0	0.0	28.3	0.0	4.0
Saint Kitts and Nevis	4	65.3	68.4	68.0	78.4	67.3	78.5	84.8	73.0
Saint Lucia	4	67.7	68.0	73.9	73.3	80.4	74.0	86.5	74.9
Saint Vincent and the Grenadines	4	69.7	60.8	59.1	85.7	83.4	84.8	87.2	75.8
Samoa	0	0.0	0.0	0.0	0.0	0.0	0.0	0.0	0.0
São Tomé and Príncipe	1	0.0	60.1	0.0	0.0	0.0	0.0	0.0	8.6
Saudi Arabia	2	0.0	0.0	0.0	19.2	20.7	21.8	20.6	11.8
Senegal	1	2.8	3.4	3.5	3.3	0.0	0.0	0.0	1.9
Serbia	3	0.0	0.0	0.0	77.9	77.7	81.3	81.4	45.5
Seychelles	3	70.3	71.3	0.0	0.0	77.8	79.1	79.4	54.0
Sierra Leone	1	0.0	0.0	4.0	0.0	0.0	0.0	0.0	0.6
Singapore	5	91.1	91.6	90.6	92.5	93.4	92.2	93.8	92.2
Slovakia	3	0.0	0.0	83.8	83.7	85.3	88.5	92.0	61.9
Slovenia	4	0.0	86.2	85.4	81.5	85.0	86.1	87.0	73.0
Vietnam	2	0.0	1.3	0.9	1.2	1.5	62.0	4.8	10.3
Solomon Islands	1	0.0	0.0	0.0	0.0	0.0	0.0	38.0	5.4
Somalia	0	0.0	0.0	0.0	0.0	0.0	0.0	0.0	0.0
South Africa	3	0.0	0.0	1.2	66.0	69.3	69.2	69.1	39.2
South Sudan	0	0.0	0.0	0.0	0.0	0.0	0.0	0.0	0.0
Spain	4	79.4	81.7	85.3	86.7	86.2	86.2	88.0	84.8
Sri Lanka	3	43.9	43.7	41.9	52.7	62.7	67.9	65.3	54.0
Sudan	0	0.0	0.0	0.0	0.0	0.0	0.0	0.0	0.0
Suriname	3	58.1	62.9	57.7	60.1	68.4	67.0	67.1	63.1
Sweden	5	88.4	90.5	90.1	89.7	88.9	88.1	86.0	88.8
Switzerland	4	76.2	76.0	75.3	87.3	86.4	87.4	87.5	82.3
Syria	3	29.5	18.0	0.0	51.0	54.8	64.3	52.5	38.6
Taiwan (province of China)	4	75.7	79.4	78.5	83.5	84.9	85.2	85.6	81.8
Tajikistan	3	67.4	65.8	66.5	51.8	48.3	48.4	50.5	57.0
Thailand	3	24.6	25.0	31.3	62.6	48.3	61.6	64.6	45.4
North Macedonia	3	0.0	0.0	75.9	75.5	77.1	71.9	68.8	52.7
Timor-Leste	0	0.0	0.0	0.0	0.0	0.0	0.0	0.0	0.0
Togo	0	0.0	0.0	0.0	0.0	0.0	0.0	0.0	0.0
Tokelau	0	0.0	0.0	0.0	0.0	0.0	0.0	0.0	0.0
Tonga	1	0.0	0.0	0.0	0.0	52.9	0.0	0.0	7.6
Trinidad and Tobago	5	78.9	83.1	85.9	89.4	90.4	88.8	89.8	86.6
Tunisia	1	0.0	0.0	0.0	0.0	0.0	28.3	24.0	7.5
Turkey	3	12.2	15.8	17.4	20.8	59.2	63.7	79.5	38.4
Turkmenistan	4	86.9	88.0	86.2	79.7	85.9	79.9	82.0	84.1
Tuvalu	0	0.0	0.0	0.0	0.0	0.0	0.0	0.0	0.0
Uganda	1	0.0	0.0	0.0	0.0	0.4	3.3	0.0	0.5
Ukraine	5	87.9	88.5	81.7	83.5	84.1	89.8	91.2	86.7
United Arab Emirates	1	0.0	0.0	0.0	0.0	0.0	35.8	0.0	5.1
UK	5	93.7	93.7	93.6	92.1	91.6	91.5	91.6	92.5
Northern Ireland	5	93.8	94.0	94.5	94.3	92.4	92.7	92.8	93.5
Scotland	5	94.1	94.4	93.9	92.9	93.6	92.9	91.4	93.3
Wales	5	93.3	93.7	93.7	93.1	92.0	91.3	91.7	92.7
England	5	93.8	93.7	93.6	91.9	91.4	91.3	91.6	92.5
Tanzania	1	0.0	3.8	3.3	2.3	6.3	3.1	0.0	2.7
Virgin Islands	3	76.3	0.0	79.3	82.3	71.3	67.9	61.2	62.6
USA	5	90.4	89.6	90.1	89.5	88.6	87.8	87.2	89.0
Uruguay	4	77.6	77.4	79.5	81.2	80.8	80.6	80.4	79.6
Uzbekistan	4	81.8	86.4	77.7	69.9	63.5	69.7	73.0	74.6
Vanuatu	0	0.0	0.0	0.0	0.0	0.0	0.0	0.0	0.0
Yemen	0	0.0	0.0	0.0	0.0	0.0	0.0	0.0	0.0
Zambia	1	0.0	0.0	0.0	0.0	0.0	6.4	15.7	3.1
Zimbabwe	2	0.0	0.0	38.9	58.7	0.4	44.3	0.0	20.3

Table S8. HIV/AIDS-related garbage code redistribution packages		
**Package Name**	**ICD10 codes**	**ICD9 codes**
Actinomycosis	A42-A42.9	039-039.9, 113-113.6
Bartonellosis	A44-A44.9	088.0-088.7
Urogenital Candidiasis	B37.3-B37.4	112.1-112.2
Candidiasis	B37-B37.2, B37.5-B37.9	112-112.0, 112.3-112.9
Coccidioidomycosis	B38-B38.9	114-114.9
Histoplasmosis	B39-B39.9	115-115.9
Blastomycosis	B40-B40.9	116-116.0, 116.2-116.9
Paracoccidioidomycosis	B41-B41.9	116.1
Sporotrichosis and Chromomycosis	B42-B43.9	117.1
Zygomycosis	B46-B46.9	117.3
Aspergillosis	B44-B44.9	117.7
Toxoplasmosis	B58-B58.9	130-130.9
Pneumocystosis	B59-B59.9	136.3-136.5
Cryptococcosis	B45-B45.9	117.5
Nocardiosis	A43-A43.9	117.2
Unspecified mycosis	B49-B49.9	117-117.0, 117.4, 117.6, 117.8-118.9
Cutaneous leishmaniasis	B55, B55.1-B55.9	085.1-085.5
Mycobacterial skin infection	A31.1-A31.2	031.1
Other Mycobacterial infection	A31-A31.0, A31.8-A31.9	031-031.0, 031.2-031.9
Immunodeficiency cell	D81-D82.9	279.2-279.4
Immunodeficiency antibody	D80-D80.9	279.0-279.1
Immunodeficiency other	D83-D84.9, D89.8-D89.9	279, 279.5-279.9
Kaposi's sarcoma	C46-C46.9	176-176.9

Table S9. Underlying indicators for percent well-certified for data source with maximum percent well certified in each 5-year time interval for 204 countries, 1980-2019.

Location	Time Window	Stars	Percent Well-Certified [PWC] (%)	Max PWC Data Year	Max PWC Data Source	Completeness (%)	Percent Major Garbage (%)	Verbal Autopsy Adjustment (None for VR) (%)
Afghanistan	1980-1984	0						
Afghanistan	1985-1989	0						
Afghanistan	1990-1994	0						
Afghanistan	1995-1999	0						
Afghanistan	2000-2004	1	3.4	2001	Afghanistan - Badghis Nutrition and Health Survey 2002		47.1	6.4
Afghanistan	2005-2009	2	28.7	2008	Afghanistan Special Demographic and Health Survey 2010		55.2	64.0
Afghanistan	2010-2019	0						
Albania	1980-1984	0						
Albania	1985-1989	4	65.9	1989	Vital Registration	100.0	34.1	
Albania	1990-1994	4	67.8	1993	Vital Registration	100.0	32.2	
Albania	1995-1999	4	74.8	1997	Vital Registration	100.0	25.2	
Albania	2000-2004	4	73.8	2004	Vital Registration	100.0	26.2	
Albania	2005-2009	4	72.5	2008	Vital Registration	97.0	25.3	
Albania	2010-2019	3	54.6	2010	Vital Registration	70.6	22.7	
Algeria	1980-1984	0						
Algeria	1985-1989	0						
Algeria	1990-1994	0						
Algeria	1995-1999	0						
Algeria	2000-2004	0						
Algeria	2005-2009	2	15.8	2006	Vital Registration	31.3	49.4	
Algeria	2010-2019	0						
American Samoa	1980-1984	0						
American Samoa	1985-1989	0						
American Samoa	1990-1994	0						
American Samoa	1995-1999	4	65.9	1997	Vital Registration	88.8	25.9	
American Samoa	2000-2004	4	72.6	2002	Vital Registration	89.4	18.9	
American Samoa	2005-2009	4	69.7	2009	Vital Registration	87.1	20.0	
American Samoa	2010-2019	4	66.2	2015	Vital Registration	77.9	15.1	
Andorra	1980-1984	0						
Andorra	1985-1989	0						
Andorra	1990-1994	0						
Andorra	1995-1999	0						
Andorra	2000-2004	0						
Andorra	2005-2009	0						
Andorra	2010-2019	4	65.3	2012	Vital Registration	81.3	19.6	
Angola	1980-1984	0						
Angola	1985-1989	0						
Angola	1990-1994	0						
Angola	1995-1999	0						
Angola	2000-2004	0						
Angola	2005-2009	0						
Angola	2010-2019	1	2.8	2010	Angola - Dande Health and Demographic Surveillance System		56.9	6.4
Antigua and Barbuda	1980-1984	3	48.9	1983	Vital Registration	68.6	28.8	
Antigua and Barbuda	1985-1989	4	69.6	1989	Vital Registration	99.0	29.7	
Antigua and Barbuda	1990-1994	4	76.0	1993	Vital Registration	100.0	24.0	
Antigua and Barbuda	1995-1999	4	79.3	1999	Vital Registration	100.0	20.7	
Antigua and Barbuda	2000-2004	4	81.3	2004	Vital Registration	100.0	18.7	
Antigua and Barbuda	2005-2009	4	79.6	2007	Vital Registration	96.7	17.7	
Antigua and Barbuda	2010-2019	4	78.6	2014	Vital Registration	100.0	21.4	
Argentina	1980-1984	4	76.1	1981	Vital Registration	100.0	23.9	
Argentina	1985-1989	4	71.5	1988	Vital Registration	100.0	28.5	
Argentina	1990-1994	4	70.6	1990	Vital Registration	100.0	29.4	
Argentina	1995-1999	4	69.9	1996	Vital Registration	100.0	30.1	
Argentina	2000-2004	4	69.1	2004	Vital Registration	100.0	30.9	
Argentina	2005-2009	4	69.1	2005	Vital Registration	100.0	30.9	
Argentina	2010-2019	4	73.7	2015	Vital Registration	100.0	26.3	
Armenia	1980-1984	4	75.6	1982	Vital Registration	86.9	13.0	
Armenia	1985-1989	4	84.5	1987	Vital Registration	96.7	12.6	
Armenia	1990-1994	5	86.2	1992	Vital Registration	100.0	13.8	
Armenia	1995-1999	5	86.1	1999	Vital Registration	99.1	13.2	
Armenia	2000-2004	5	86.4	2000	Vital Registration	99.1	12.9	
Armenia	2005-2009	5	90.9	2008	Vital Registration	100.0	9.1	
Armenia	2010-2019	5	89.6	2010	Vital Registration	100.0	10.4	
Australia	1980-1984	5	93.3	1982	Vital Registration	100.0	6.7	
Australia	1985-1989	5	93.7	1986	Vital Registration	100.0	6.3	
Australia	1990-1994	5	93.6	1994	Vital Registration	100.0	6.4	
Australia	1995-1999	5	93.5	1996	Vital Registration	100.0	6.5	
Australia	2000-2004	5	92.5	2001	Vital Registration	100.0	7.5	
Australia	2005-2009	5	91.8	2006	Vital Registration	100.0	8.2	
Australia	2010-2019	5	91.1	2013	Vital Registration	100.0	8.9	
Austria	1980-1984	5	92.3	1984	Vital Registration	100.0	7.7	
Austria	1985-1989	5	93.2	1987	Vital Registration	100.0	6.8	
Austria	1990-1994	5	92.7	1990	Vital Registration	100.0	7.3	
Austria	1995-1999	5	92.3	1996	Vital Registration	100.0	7.7	
Austria	2000-2004	5	94.8	2003	Vital Registration	100.0	5.2	
Austria	2005-2009	5	93.6	2005	Vital Registration	100.0	6.4	
Austria	2010-2019	5	91.1	2010	Vital Registration	100.0	8.9	
Azerbaijan	1980-1984	4	69.7	1981	Vital Registration	79.6	12.5	
Azerbaijan	1985-1989	4	69.1	1985	Vital Registration	78.9	12.4	
Azerbaijan	1990-1994	4	68.0	1993	Vital Registration	76.5	11.1	
Azerbaijan	1995-1999	4	65.4	1999	Vital Registration	76.7	14.7	
Azerbaijan	2000-2004	4	72.1	2003	Vital Registration	78.4	8.0	
Azerbaijan	2005-2009	3	42.7	2007	Vital Registration	77.7	45.0	
Azerbaijan	2010-2019	0						
Bahrain	1980-1984	0						
Bahrain	1985-1989	4	75.2	1986	Vital Registration	100.0	24.8	
Bahrain	1990-1994	0						
Bahrain	1995-1999	3	62.9	1997	Vital Registration	93.0	32.4	
Bahrain	2000-2004	3	61.8	2000	Vital Registration	98.9	37.5	
Bahrain	2005-2009	3	58.8	2005	Vital Registration	95.8	38.6	
Bahrain	2010-2019	3	54.9	2014	Vital Registration	83.2	34.1	
Bangladesh	1980-1984	1	2.2	1983	Bangladesh - Matlab Health and Demographic Surveillance System		66.3	6.4
Bangladesh	1985-1989	1	3.4	1989	Bangladesh - Matlab Health and Demographic Surveillance System		47.5	6.4
Bangladesh	1990-1994	2	30.2	1991	Causes of childhood deaths in Bangladesh: an update		2.0	30.8
Bangladesh	1995-1999	1	3.9	1998	Bangladesh - Matlab Health and Demographic Surveillance System		38.8	6.4
Bangladesh	2000-2004	2	19.1	2004	Bangladesh Demographic and Health Survey 2004		4.8	20.0
Bangladesh	2005-2009	1	6.3	2006	Bangladesh - Matlab Health and Demographic Surveillance System		2.2	6.4
Bangladesh	2010-2019	3	53.3	2015	Verbal Autopsy		1.7	54.3
Barbados	1980-1984	4	76.1	1980	Vital Registration	100.0	23.9	
Barbados	1985-1989	4	78.2	1987	Vital Registration	100.0	21.8	
Barbados	1990-1994	4	80.1	1992	Vital Registration	100.0	19.9	
Barbados	1995-1999	4	80.1	1995	Vital Registration	100.0	19.9	
Barbados	2000-2004	4	77.2	2000	Vital Registration	100.0	22.8	
Barbados	2005-2009	4	78.0	2008	Vital Registration	96.4	19.2	
Barbados	2010-2019	4	76.8	2011	Vital Registration	93.7	18.1	
Belarus	1980-1984	5	85.0	1982	Vital Registration	99.2	14.3	
Belarus	1985-1989	5	87.9	1986	Vital Registration	100.0	12.1	
Belarus	1990-1994	4	80.0	1990	Vital Registration	100.0	20.0	
Belarus	1995-1999	4	80.7	1999	Vital Registration	100.0	19.3	
Belarus	2000-2004	4	82.6	2002	Vital Registration	100.0	17.4	
Belarus	2005-2009	4	83.4	2009	Vital Registration	100.0	16.6	
Belarus	2010-2019	4	83.7	2010	Vital Registration	100.0	16.3	
Belgium	1980-1984	4	80.3	1981	Vital Registration	100.0	19.7	
Belgium	1985-1989	4	80.8	1987	Vital Registration	100.0	19.2	
Belgium	1990-1994	4	84.3	1994	Vital Registration	100.0	15.7	
Belgium	1995-1999	5	86.3	1998	Vital Registration	100.0	13.7	
Belgium	2000-2004	5	86.2	2001	Vital Registration	100.0	13.8	
Belgium	2005-2009	5	86.4	2008	Vital Registration	100.0	13.6	
Belgium	2010-2019	4	82.9	2011	Vital Registration	99.8	16.9	
Belize	1980-1984	3	61.5	1984	Vital Registration	90.5	32.1	
Belize	1985-1989	4	66.7	1987	Vital Registration	88.5	24.7	
Belize	1990-1994	3	57.9	1993	Vital Registration	89.5	35.3	
Belize	1995-1999	4	74.9	1998	Vital Registration	100.0	25.1	
Belize	2000-2004	4	75.9	2001	Vital Registration	100.0	24.1	
Belize	2005-2009	5	87.2	2009	Vital Registration	100.0	12.8	
Belize	2010-2019	5	87.6	2011	Vital Registration	100.0	12.4	
Benin	1980-1984	0						
Benin	1985-1989	1	2.1	1989	Incidence de décès de 0 à 1 an dans une cohorte de 802 enfants en milieu rural au sud du Bénin		6.5	2.3
Benin	1990-1994	0						
Benin	1995-1999	0						
Benin	2000-2004	0						
Benin	2005-2009	0						
Benin	2010-2019	0						
Bermuda	1980-1984	5	92.5	1984	Vital Registration	100.0	7.5	
Bermuda	1985-1989	5	90.9	1989	Vital Registration	100.0	9.1	
Bermuda	1990-1994	5	90.4	1992	Vital Registration	100.0	9.6	
Bermuda	1995-1999	5	95.0	1996	Vital Registration	100.0	5.0	
Bermuda	2000-2004	5	93.0	2002	Vital Registration	99.9	6.8	
Bermuda	2005-2009	5	88.8	2007	Vital Registration	100.0	11.2	
Bermuda	2010-2019	5	90.4	2014	Vital Registration	97.0	6.9	
Bhutan	1980-1984	0						
Bhutan	1985-1989	0						
Bhutan	1990-1994	0						
Bhutan	1995-1999	0						
Bhutan	2000-2004	0						
Bhutan	2005-2009	0						
Bhutan	2010-2019	0						
Bolivia	1980-1984	0						
Bolivia	1985-1989	0						
Bolivia	1990-1994	0						
Bolivia	1995-1999	0						
Bolivia	2000-2004	2	12.4	2003	Vital Registration	38.1	67.3	
Bolivia	2005-2009	0						
Bolivia	2010-2019	0						
Bosnia and Herzegovina	1980-1984	0						
Bosnia and Herzegovina	1985-1989	4	65.1	1988	Vital Registration	100.0	34.9	
Bosnia and Herzegovina	1990-1994	4	66.5	1990	Vital Registration	100.0	33.5	
Bosnia and Herzegovina	1995-1999	0						
Bosnia and Herzegovina	2000-2004	0						
Bosnia and Herzegovina	2005-2009	0						
Bosnia and Herzegovina	2010-2019	4	75.4	2014	Vital Registration	100.0	24.6	
Botswana	1980-1984	0						
Botswana	1985-1989	0						
Botswana	1990-1994	0						
Botswana	1995-1999	0						
Botswana	2000-2004	0						
Botswana	2005-2009	0						
Botswana	2010-2019	0						
Brazil	1980-1984	3	51.3	1984	Vital Registration	83.6	38.6	
Brazil	1985-1989	3	55.5	1989	Vital Registration	85.2	34.8	
Brazil	1990-1994	3	62.0	1994	Vital Registration	92.1	32.7	
Brazil	1995-1999	4	66.2	1999	Vital Registration	93.6	29.3	
Brazil	2000-2004	4	70.9	2004	Vital Registration	94.5	25.0	
Brazil	2005-2009	4	77.8	2009	Vital Registration	96.1	19.0	
Brazil	2010-2019	4	82.3	2014	Vital Registration	99.3	17.2	
Brunei	1980-1984	0						
Brunei	1985-1989	0						
Brunei	1990-1994	0						
Brunei	1995-1999	4	78.9	1998	Vital Registration	93.1	15.3	
Brunei	2000-2004	4	76.4	2002	Vital Registration	92.8	17.7	
Brunei	2005-2009	4	73.9	2007	Vital Registration	91.2	19.0	
Brunei	2010-2019	4	73.8	2016	Vital Registration	89.6	17.6	
Bulgaria	1980-1984	4	81.1	1984	Vital Registration	100.0	18.9	
Bulgaria	1985-1989	4	81.4	1986	Vital Registration	100.0	18.6	

Table S9. Underlying indicators for percent well-certified for data source with maximum percent well certified in each 5-year time interval for 204 countries, 1980-2019.

Location	Time Window	Stars	Percent Well-Certified [PWC] (%)	Max PWC Data Year	Max PWC Data Source	Completeness (%)	Percent Major Garbage (%)	Verbal Autopsy Adjustment (None for VR) (%)
Bulgaria	1990-1994	4	81.3	1993	Vital Registration	100.0	18.7	
Bulgaria	1995-1999	4	78.6	1995	Vital Registration	99.9	21.3	
Bulgaria	2000-2004	4	70.1	2002	Vital Registration	94.9	26.2	
Bulgaria	2005-2009	4	70.2	2005	Vital Registration	93.8	25.1	
Bulgaria	2010-2019	4	68.1	2012	Vital Registration	94.1	27.6	
Burkina Faso	1980-1984	1	3.7	1984	The burden of malaria mortality among African children in the year 2000		11.1	4.1
Burkina Faso	1985-1989	0						
Burkina Faso	1990-1994	0						
Burkina Faso	1995-1999	1	4.6	1998	Measuring the local burden of disease. A study of years of life lost in sub-Saharan Africa		27.6	6.4
Burkina Faso	2000-2004	1	6.3	2000	Burkina Faso - Nouna Health and Demographic Surveillance System		0.9	6.4
Burkina Faso	2005-2009	1	2.4	2009	An improved method for physician-certified verbal autopsy reduces the rate of discrepancy: experiences in the Nouna Health and Demographic Surveillance Site (NHDSS), Burkina Faso		62.5	6.4
Burkina Faso	2010-2019	1	0.3	2010	Africa, Asia, Oceania - INDEPTH Network Cause-Specific Mortality - Release 2014		95.6	6.4
Burundi	1980-1984	0						
Burundi	1985-1989	0						
Burundi	1990-1994	1	2.9	1990	Mortality and morbidity at young ages in a stable hyperendemic malaria region, community Nyanza-Lac, Imbo South, Burundi		5.0	3.1
Burundi	1995-1999	0						
Burundi	2000-2004	0						
Burundi	2005-2009	0						
Burundi	2010-2019	0						
Cambodia	1980-1984	0						
Cambodia	1985-1989	0						
Cambodia	1990-1994	0						
Cambodia	1995-1999	0						
Cambodia	2000-2004	1	0.9	2001	Community-based surveillance: a pilot study from rural Cambodia		86.6	6.4
Cambodia	2005-2009	1	4.2	2009	Mortality in Cambodia An 18-Month Prospective Community-based Surveillance of All-age Deaths Using Verbal Autopsies		34.7	6.4
Cambodia	2010-2019	0						
Cameroon	1980-1984	0						
Cameroon	1985-1989	0						
Cameroon	1990-1994	0						
Cameroon	1995-1999	0						
Cameroon	2000-2004	0						
Cameroon	2005-2009	0						
Cameroon	2010-2019	0						
Canada	1980-1984	5	90.5	1981	Vital Registration	100.0	9.5	
Canada	1985-1989	5	90.7	1986	Vital Registration	100.0	9.3	
Canada	1990-1994	5	89.9	1992	Vital Registration	100.0	10.1	
Canada	1995-1999	5	89.6	1999	Vital Registration	100.0	10.4	
Canada	2000-2004	5	90.0	2001	Vital Registration	100.0	10.0	
Canada	2005-2009	5	90.5	2009	Vital Registration	100.0	9.5	
Canada	2010-2019	5	90.2	2013	Vital Registration	100.0	9.8	
Cape Verde	1980-1984	3	48.7	1980	Vital Registration	96.2	49.3	
Cape Verde	1985-1989	0						
Cape Verde	1990-1994	1	0.3	1992	Deaths among women of reproductive age in Cape Verde: causes and avoidability		6.4	0.3
Cape Verde	1995-1999	0						
Cape Verde	2000-2004	0						
Cape Verde	2005-2009	0						
Cape Verde	2010-2019	4	70.2	2012	Vital Registration	100.0	29.8	
Central African Republic	1980-1984	0						
Central African Republic	1985-1989	0						
Central African Republic	1990-1994	0						
Central African Republic	1995-1999	0						
Central African Republic	2000-2004	0						
Central African Republic	2005-2009	0						
Central African Republic	2010-2019	0						
Chad	1980-1984	0						
Chad	1985-1989	0						
Chad	1990-1994	0						
Chad	1995-1999	0						
Chad	2000-2004	0						
Chad	2005-2009	0						
Chad	2010-2019	0						
Chile	1980-1984	4	75.3	1983	Vital Registration	100.0	24.7	
Chile	1985-1989	4	75.9	1988	Vital Registration	100.0	24.1	
Chile	1990-1994	4	82.0	1994	Vital Registration	99.1	17.3	
Chile	1995-1999	5	85.1	1999	Vital Registration	100.0	14.9	
Chile	2000-2004	5	91.2	2003	Vital Registration	100.0	8.8	
Chile	2005-2009	5	91.5	2006	Vital Registration	100.0	8.5	
Chile	2010-2019	5	90.4	2012	Vital Registration	100.0	9.6	
China	1980-1984	0						
China	1985-1989	1	1.0	1986	Infant mortality among various nationalities in the middle part of Guizhou, China		1.6	1.0
China	1990-1994	4	70.0	1994	Vital Registration - Sample	81.5	14.1	
China	1995-1999	4	73.7	1998	Vital Registration - Sample	86.4	14.7	
China	2000-2004	4	66.8	2000	Vital Registration - Sample	80.0	16.5	
China	2005-2009	4	72.1	2009	Vital Registration - Sample	76.9	6.3	
China	2010-2019	4	71.7	2010	Vital Registration - Sample	76.6	6.3	
Colombia	1980-1984	4	69.2	1983	Vital Registration	92.4	25.0	
Colombia	1985-1989	4	72.0	1987	Vital Registration	93.5	23.0	
Colombia	1990-1994	4	76.3	1993	Vital Registration	93.5	18.4	
Colombia	1995-1999	5	88.3	1999	Vital Registration	100.0	11.7	
Colombia	2000-2004	5	89.7	2002	Vital Registration	100.0	10.3	
Colombia	2005-2009	5	88.5	2008	Vital Registration	99.2	10.8	
Colombia	2010-2019	5	90.8	2015	Vital Registration	100.0	9.2	
Comoros	1980-1984	0						
Comoros	1985-1989	0						
Comoros	1990-1994	0						
Comoros	1995-1999	0						
Comoros	2000-2004	0						
Comoros	2005-2009	0						
Comoros	2010-2019	0						
Congo (Brazzaville)	1980-1984	0						
Congo (Brazzaville)	1985-1989	0						
Congo (Brazzaville)	1990-1994	0						
Congo (Brazzaville)	1995-1999	0						
Congo (Brazzaville)	2000-2004	0						
Congo (Brazzaville)	2005-2009	0						
Congo (Brazzaville)	2010-2019	0						
Cook Islands	1980-1984	0						
Cook Islands	1985-1989	0						
Cook Islands	1990-1994	0						
Cook Islands	1995-1999	0						
Cook Islands	2000-2004	3	60.0	2000	Vital Registration	100.0	40.0	
Cook Islands	2005-2009	3	53.9	2006	Vital Registration	76.6	29.6	
Cook Islands	2010-2019	4	68.5	2014	Vital Registration	92.3	25.8	
Costa Rica	1980-1984	4	81.7	1984	Vital Registration	100.0	18.3	
Costa Rica	1985-1989	4	83.2	1988	Vital Registration	100.0	16.8	
Costa Rica	1990-1994	4	83.3	1993	Vital Registration	100.0	16.7	
Costa Rica	1995-1999	5	91.4	1999	Vital Registration	100.0	8.6	
Costa Rica	2000-2004	5	92.7	2003	Vital Registration	100.0	7.3	
Costa Rica	2005-2009	5	91.6	2005	Vital Registration	100.0	8.4	
Costa Rica	2010-2019	5	91.6	2015	Vital Registration	100.0	8.4	
Croatia	1980-1984	0						
Croatia	1985-1989	4	84.9	1989	Vital Registration	100.0	15.1	
Croatia	1990-1994	5	85.9	1990	Vital Registration	100.0	14.1	
Croatia	1995-1999	4	84.9	1999	Vital Registration	100.0	15.1	
Croatia	2000-2004	5	88.2	2004	Vital Registration	100.0	11.8	
Croatia	2005-2009	5	89.9	2009	Vital Registration	100.0	10.1	
Croatia	2010-2019	5	93.7	2016	Vital Registration	100.0	6.3	
Cuba	1980-1984	5	85.9	1984	Vital Registration	99.6	13.7	
Cuba	1985-1989	5	87.2	1988	Vital Registration	100.0	12.8	
Cuba	1990-1994	5	86.8	1990	Vital Registration	100.0	13.2	
Cuba	1995-1999	5	89.9	1997	Vital Registration	100.0	10.1	
Cuba	2000-2004	5	91.3	2004	Vital Registration	100.0	8.7	
Cuba	2005-2009	5	92.2	2009	Vital Registration	100.0	7.8	
Cuba	2010-2019	5	92.3	2012	Vital Registration	100.0	7.7	
Cyprus	1980-1984	0						
Cyprus	1985-1989	0						
Cyprus	1990-1994	0						
Cyprus	1995-1999	2	32.2	1999	Vital Registration	85.7	62.4	
Cyprus	2000-2004	3	60.7	2004	Vital Registration	84.7	28.4	
Cyprus	2005-2009	4	69.7	2007	Vital Registration	85.0	17.9	
Cyprus	2010-2019	4	78.4	2015	Vital Registration	90.7	13.6	
Czech Republic	1980-1984	0						
Czech Republic	1985-1989	5	91.2	1986	Vital Registration	100.0	8.8	
Czech Republic	1990-1994	5	90.5	1990	Vital Registration	100.0	9.5	
Czech Republic	1995-1999	5	85.9	1995	Vital Registration	100.0	14.1	
Czech Republic	2000-2004	5	86.5	2000	Vital Registration	99.8	13.3	
Czech Republic	2005-2009	5	86.6	2007	Vital Registration	100.0	13.4	
Czech Republic	2010-2019	5	87.9	2013	Vital Registration	100.0	12.1	
Côte d'Ivoire	1980-1984	0						
Côte d'Ivoire	1985-1989	1	3.7	1988	Effet de l'observance des d'approvisionnement en eau et de la therapie par voie orale sur les diarrhees chez les enfants de moins de 5 de la Cote d'Ivoire		8.6	4.0
Côte d'Ivoire	1990-1994	1	3.6	1990	Effet de l'observance des d'approvisionnement en eau et de la therapie par voie orale sur les diarrhees chez les enfants de moins de 5 de la Cote d'Ivoire		9.0	3.9
Côte d'Ivoire	1995-1999	0						
Côte d'Ivoire	2000-2004	0						
Côte d'Ivoire	2005-2009	1	0.2	2009	Africa, Asia, Oceania - INDEPTH Network Cause-Specific Mortality - Release 2014		97.2	6.4
Côte d'Ivoire	2010-2019	1	0.1	2010	Africa, Asia, Oceania - INDEPTH Network Cause-Specific Mortality - Release 2014		98.2	6.4
DR Congo	1980-1984	0						
DR Congo	1985-1989	1	0.9	1986	Etude de la mortalité globale et de la mortalité liée au paludisme dans le Kivu montagneux, Zaïre		86.2	6.4
DR Congo	1990-1994	1	3.6	1990	Influence of nutritional status on child mortality in rural Zaire		2.5	3.7
DR Congo	1995-1999	0						
DR Congo	2000-2004	0						
DR Congo	2005-2009	0						
DR Congo	2010-2019	0						
Denmark	1980-1984	4	83.6	1981	Vital Registration	100.0	16.4	
Denmark	1985-1989	4	82.3	1985	Vital Registration	100.0	17.7	
Denmark	1990-1994	5	85.7	1994	Vital Registration	100.0	14.3	
Denmark	1995-1999	5	87.3	1999	Vital Registration	99.0	11.8	
Denmark	2000-2004	5	87.8	2000	Vital Registration	100.0	12.2	
Denmark	2005-2009	5	87.0	2005	Vital Registration	100.0	13.0	
Denmark	2010-2019	5	85.1	2012	Vital Registration	100.0	14.9	
Djibouti	1980-1984	0						
Djibouti	1985-1989	0						
Djibouti	1990-1994	0						
Djibouti	1995-1999	0						
Djibouti	2000-2004	0						
Djibouti	2005-2009	0						
Djibouti	2010-2019	0						
Dominica	1980-1984	3	60.1	1984	Vital Registration	84.5	28.9	
Dominica	1985-1989	3	55.6	1986	Vital Registration	87.4	36.4	
Dominica	1990-1994	3	55.9	1992	Vital Registration	86.6	35.4	
Dominica	1995-1999	3	63.3	1999	Vital Registration	91.4	30.8	
Dominica	2000-2004	3	58.9	2004	Vital Registration	83.8	29.7	
Dominica	2005-2009	4	72.3	2006	Vital Registration	81.2	11.0	
Dominica	2010-2019	4	74.2	2011	Vital Registration	86.4	14.1	
Dominican Republic	1980-1984	3	46.3	1984	Vital Registration	73.5	37.0	
Dominican Republic	1985-1989	3	46.7	1985	Vital Registration	72.4	35.4	

Table S9. Underlying indicators for percent well-certified for data source with maximum percent well certified in each 5-year time interval for 204 countries, 1980-2019.

Location	Time Window	Stars	Percent Well-Certified [PWC] (%)	Max PWC Data Year	Max PWC Data Source	Completeness (%)	Percent Major Garbage (%)	Verbal Autopsy Adjustment (None for VR) (%)
Dominican Republic	1990-1994	3	39.3	1990	Vital Registration	59.5	34.0	
Dominican Republic	1995-1999	3	42.9	1999	Vital Registration	59.3	27.7	
Dominican Republic	2000-2004	3	45.9	2004	Vital Registration	59.8	23.3	
Dominican Republic	2005-2009	3	44.6	2005	Vital Registration	56.8	21.4	
Dominican Republic	2010-2019	3	45.6	2012	Vital Registration	58.6	22.2	
Ecuador	1980-1984	4	65.9	1983	Vital Registration	99.9	34.0	
Ecuador	1985-1989	4	65.3	1989	Vital Registration	95.0	31.3	
Ecuador	1990-1994	4	66.5	1991	Vital Registration	94.4	29.5	
Ecuador	1995-1999	3	63.4	1996	Vital Registration	90.4	29.9	
Ecuador	2000-2004	3	60.8	2001	Vital Registration	91.0	33.2	
Ecuador	2005-2009	3	55.2	2006	Vital Registration	75.8	27.2	
Ecuador	2010-2019	3	63.4	2016	Vital Registration	74.2	14.6	
Egypt	1980-1984	2	25.0	1980	Vital Registration	77.6	67.8	
Egypt	1985-1989	2	30.8	1987	Vital Registration	85.2	63.9	
Egypt	1990-1994	3	35.4	1992	Vital Registration	83.5	57.6	
Egypt	1995-1999	0						
Egypt	2000-2004	3	39.1	2003	Vital Registration	95.1	58.9	
Egypt	2005-2009	3	38.4	2005	Vital Registration	93.5	58.9	
Egypt	2010-2019	3	46.6	2014	Vital Registration	100.0	53.4	
El Salvador	1980-1984	3	57.4	1981	Vital Registration	100.0	42.6	
El Salvador	1985-1989	0						
El Salvador	1990-1994	3	55.8	1993	Vital Registration	88.8	37.2	
El Salvador	1995-1999	3	62.7	1996	Vital Registration	91.8	31.7	
El Salvador	2000-2004	4	66.8	2001	Vital Registration	97.8	31.8	
El Salvador	2005-2009	4	65.7	2005	Vital Registration	94.7	30.6	
El Salvador	2010-2019	4	65.4	2012	Vital Registration	96.8	32.4	
Equatorial Guinea	1980-1984	0						
Equatorial Guinea	1985-1989	0						
Equatorial Guinea	1990-1994	0						
Equatorial Guinea	1995-1999	0						
Equatorial Guinea	2000-2004	0						
Equatorial Guinea	2005-2009	0						
Equatorial Guinea	2010-2019	0						
Eritrea	1980-1984	0						
Eritrea	1985-1989	0						
Eritrea	1990-1994	0						
Eritrea	1995-1999	0						
Eritrea	2000-2004	0						
Eritrea	2005-2009	0						
Eritrea	2010-2019	0						
Estonia	1980-1984	5	90.9	1982	Vital Registration	100.0	9.1	
Estonia	1985-1989	5	91.2	1986	Vital Registration	100.0	8.8	
Estonia	1990-1994	5	93.1	1994	Vital Registration	99.3	6.3	
Estonia	1995-1999	5	93.2	1995	Vital Registration	100.0	6.8	
Estonia	2000-2004	5	92.3	2000	Vital Registration	100.0	7.7	
Estonia	2005-2009	5	92.8	2008	Vital Registration	100.0	7.2	
Estonia	2010-2019	5	91.9	2010	Vital Registration	100.0	8.1	
Ethiopia	1980-1984	0						
Ethiopia	1985-1989	1	3.0	1987	The Butajira rural health project in Ethiopia: mortality pattern of the under fives		7.7	3.3
Ethiopia	1990-1994	1	3.2	1992	Patterns of childhood mortality in three districts of north Gondar Administrative Zone. A community based study using the verbal autopsy method		2.1	3.3
Ethiopia	1995-1999	1	0.9	1997	The use of simplified verbal autopsy in identifying causes of adult death in a predominantly rural population in Ethiopia		7.1	1.0
Ethiopia	2000-2004	1	3.3	2004	Determinants of under-five mortality in Gilgel Gibe Field Research Center, Southwest Ethiopia		0.9	3.3
Ethiopia	2005-2009	1	4.0	2009	Ethiopia Demographic Surveillance Verbal Autopsy Data 2009-2013		37.0	6.4
Ethiopia	2010-2019	1	4.0	2010	Ethiopia Demographic Surveillance Verbal Autopsy Data 2009-2013		37.5	6.4
Federated States of Micronesia	1980-1984	0						
Federated States of Micronesia	1985-1989	0						
Federated States of Micronesia	1990-1994	0						
Federated States of Micronesia	1995-1999	0						
Federated States of Micronesia	2000-2004	0						
Federated States of Micronesia	2005-2009	0						
Federated States of Micronesia	2010-2019	0						
Fiji	1980-1984	0						
Fiji	1985-1989	0						
Fiji	1990-1994	0						
Fiji	1995-1999	2	32.9	1999	Vital Registration	61.8	46.7	
Fiji	2000-2004	3	53.5	2003	Vital Registration	100.0	46.5	
Fiji	2005-2009	3	59.7	2009	Vital Registration	100.0	40.3	
Fiji	2010-2019	4	66.0	2012	Vital Registration	100.0	34.0	
Finland	1980-1984	4	82.7	1980	Vital Registration	100.0	17.3	
Finland	1985-1989	5	91.6	1987	Vital Registration	100.0	8.4	
Finland	1990-1994	5	91.3	1992	Vital Registration	99.9	8.6	
Finland	1995-1999	5	96.1	1998	Vital Registration	100.0	3.9	
Finland	2000-2004	5	95.3	2000	Vital Registration	99.7	4.4	
Finland	2005-2009	5	94.3	2006	Vital Registration	100.0	5.7	
Finland	2010-2019	5	94.4	2016	Vital Registration	100.0	5.6	
France	1980-1984	4	78.0	1984	Vital Registration	100.0	22.0	
France	1985-1989	4	80.3	1989	Vital Registration	100.0	19.7	
France	1990-1994	4	82.0	1993	Vital Registration	100.0	18.0	
France	1995-1999	4	81.9	1999	Vital Registration	100.0	18.1	
France	2000-2004	4	82.3	2000	Vital Registration	100.0	17.7	
France	2005-2009	4	82.4	2005	Vital Registration	100.0	17.6	
France	2010-2019	4	80.7	2010	Vital Registration	100.0	19.3	
Gabon	1980-1984	0						
Gabon	1985-1989	0						
Gabon	1990-1994	0						
Gabon	1995-1999	0						
Gabon	2000-2004	0						
Gabon	2005-2009	0						
Gabon	2010-2019	0						
Georgia	1980-1984	4	78.3	1981	Vital Registration	87.5	10.5	
Georgia	1985-1989	4	76.5	1985	Vital Registration	86.4	11.4	
Georgia	1990-1994	4	71.1	1990	Vital Registration	77.2	7.9	
Georgia	1995-1999	4	67.3	1999	Vital Registration	78.5	14.2	
Georgia	2000-2004	4	69.6	2000	Vital Registration	79.8	12.8	
Georgia	2005-2009	3	48.3	2005	Vital Registration	74.1	34.9	
Georgia	2010-2019	3	58.9	2014	Vital Registration	94.3	37.6	
Germany	1980-1984	4	80.5	1984	Vital Registration	100.0	19.5	
Germany	1985-1989	4	83.1	1989	Vital Registration	100.0	16.9	
Germany	1990-1994	5	86.1	1994	Vital Registration	100.0	13.9	
Germany	1995-1999	5	86.1	1995	Vital Registration	100.0	13.9	
Germany	2000-2004	5	85.4	2002	Vital Registration	100.0	14.6	
Germany	2005-2009	5	85.7	2006	Vital Registration	100.0	14.3	
Germany	2010-2019	5	85.6	2011	Vital Registration	99.8	14.2	
Ghana	1980-1984	0						
Ghana	1985-1989	1	0.4	1989	Maternal mortality among the Kassena-Nankana of northern Ghana		10.4	0.5
Ghana	1990-1994	1	2.1	1990	Vitamin A supplementation in northern Ghana: effects on clinic attendances, hospital admissions, and child mortality		2.0	2.2
Ghana	1995-1999	1	0.9	1998	Trend and causes of neonatal mortality in the Kassena-Nankana district of northern Ghana, 1995-2002		0.3	0.9
Ghana	2000-2004	1	9.1	2000	Vital Registration	13.7	33.9	
Ghana	2005-2009	2	21.4	2006	Ghana Child Verbal Autopsy Study 2008		0.8	21.6
Ghana	2010-2019	1	0.4	2011	Africa, Asia, Oceania - INDEPTH Network Cause-Specific Mortality - Release 2014		94.5	6.4
Greece	1980-1984	4	83.6	1984	Vital Registration	100.0	16.4	
Greece	1985-1989	4	84.0	1985	Vital Registration	100.0	16.0	
Greece	1990-1994	4	78.8	1994	Vital Registration	100.0	21.2	
Greece	1995-1999	4	79.6	1995	Vital Registration	100.0	20.4	
Greece	2000-2004	4	80.2	2004	Vital Registration	100.0	19.8	
Greece	2005-2009	4	82.3	2009	Vital Registration	100.0	17.7	
Greece	2010-2019	4	82.7	2014	Vital Registration	100.0	17.3	
Greenland	1980-1984	0						
Greenland	1985-1989	0						
Greenland	1990-1994	0						
Greenland	1995-1999	5	86.3	1999	Vital Registration	100.0	13.7	
Greenland	2000-2004	5	87.4	2001	Vital Registration	100.0	12.6	
Greenland	2005-2009	5	88.0	2005	Vital Registration	100.0	12.0	
Greenland	2010-2019	4	83.6	2010	Vital Registration	100.0	16.4	
Grenada	1980-1984	4	69.0	1984	Vital Registration	93.2	26.0	
Grenada	1985-1989	3	64.0	1988	Vital Registration	99.2	35.4	
Grenada	1990-1994	4	65.3	1994	Vital Registration	100.0	34.7	
Grenada	1995-1999	4	65.3	1996	Vital Registration	93.4	30.1	
Grenada	2000-2004	4	80.2	2002	Vital Registration	100.0	19.8	
Grenada	2005-2009	4	82.3	2009	Vital Registration	100.0	17.7	
Grenada	2010-2019	5	87.5	2011	Vital Registration	100.0	12.5	
Guam	1980-1984	0						
Guam	1985-1989	0						
Guam	1990-1994	5	85.8	1994	Vital Registration	92.5	7.3	
Guam	1995-1999	4	80.5	1999	Vital Registration	87.4	7.9	
Guam	2000-2004	4	71.6	2000	Vital Registration	79.9	10.4	
Guam	2005-2009	4	65.6	2007	Vital Registration	70.5	6.9	
Guam	2010-2019	3	63.3	2016	Vital Registration	71.5	11.4	
Guatemala	1980-1984	4	68.8	1984	Vital Registration	99.2	30.6	
Guatemala	1985-1989	4	66.1	1987	Vital Registration	97.5	32.1	
Guatemala	1990-1994	4	66.1	1994	Vital Registration	100.0	33.9	
Guatemala	1995-1999	4	69.7	1999	Vital Registration	100.0	30.3	
Guatemala	2000-2004	4	69.9	2002	Vital Registration	100.0	30.1	
Guatemala	2005-2009	4	75.0	2009	Vital Registration	99.8	24.9	
Guatemala	2010-2019	4	77.7	2013	Vital Registration	100.0	22.3	
Guinea	1980-1984	0						
Guinea	1985-1989	0						
Guinea	1990-1994	0						
Guinea	1995-1999	1	3.4	1998	Guinea - Mandiana Mortality Study 1998-1999		0.7	3.4
Guinea	2000-2004	0						
Guinea	2005-2009	0						
Guinea	2010-2019	0						
Guinea-Bissau	1980-1984	0						
Guinea-Bissau	1985-1989	0						
Guinea-Bissau	1990-1994	1	0.4	1992	Maternal mortality in Guinea-Bissau: the use of verbal autopsy in a multi-ethnic population		11.1	0.4
Guinea-Bissau	1995-1999	1	2.3	1995	BCG vaccination scar associated with better childhood survival in Guinea-Bissau		3.0	2.4
Guinea-Bissau	2000-2004	0						
Guinea-Bissau	2005-2009	0						
Guinea-Bissau	2010-2019	0						
Guyana	1980-1984	3	49.2	1984	Vital Registration	77.2	36.3	
Guyana	1985-1989	4	65.9	1989	Vital Registration	88.0	25.0	
Guyana	1990-1994	3	61.4	1990	Vital Registration	81.2	24.4	
Guyana	1995-1999	4	68.1	1995	Vital Registration	87.7	22.4	
Guyana	2000-2004	4	73.5	2004	Vital Registration	88.6	17.0	
Guyana	2005-2009	4	75.1	2005	Vital Registration	88.8	15.5	
Guyana	2010-2019	4	74.2	2010	Vital Registration	91.2	18.6	
Haiti	1980-1984	2	15.6	1981	Vital Registration	50.2	68.8	
Haiti	1985-1989	1	1.9	1989	The utility of verbal autopsies for identifying HIV-1-related deaths in Haitian children		3.4	1.9
Haiti	1990-1994	1	1.7	1994	Survey on Infant mortality in Mirebalais, Haiti		4.6	1.8
Haiti	1995-1999	2	11.9	1999	Vital Registration	29.7	60.1	
Haiti	2000-2004	1	5.4	2003	Vital Registration	10.3	47.5	
Haiti	2005-2009	0						
Haiti	2010-2019	0						
Honduras	1980-1984	3	35.7	1981	Vital Registration	76.7	53.4	
Honduras	1985-1989	3	42.8	1988	Vital Registration	67.1	36.3	
Honduras	1990-1994	3	39.1	1990	Vital Registration	63.0	37.9	
Honduras	1995-1999	0						
Honduras	2000-2004	0						
Honduras	2005-2009	2	12.4	2008	Vital Registration	14.3	13.5	

Table S9. Underlying indicators for percent well-certified for data source with maximum percent well certified in each 5-year time interval for 204 countries, 1980-2019.

Location	Time Window	Stars	Percent Well-Certified [PWC] (%)	Max PWC Data Year	Max PWC Data Source	Completeness (%)	Percent Major Garbage (%)	Verbal Autopsy Adjustment (None for VR) (%)
Honduras	2010-2019	2	13.0	2013	Vital Registration	14.7	11.4	
Hungary	1980-1984	5	91.2	1981	Vital Registration	100.0	8.8	
Hungary	1985-1989	5	90.4	1988	Vital Registration	100.0	9.6	
Hungary	1990-1994	5	90.5	1994	Vital Registration	100.0	9.5	
Hungary	1995-1999	5	91.6	1997	Vital Registration	100.0	8.4	
Hungary	2000-2004	5	93.3	2004	Vital Registration	100.0	6.7	
Hungary	2005-2009	5	93.9	2008	Vital Registration	100.0	6.1	
Hungary	2010-2019	5	94.2	2011	Vital Registration	100.0	5.8	
Iceland	1980-1984	5	91.7	1983	Vital Registration	100.0	8.3	
Iceland	1985-1989	5	93.3	1988	Vital Registration	100.0	6.7	
Iceland	1990-1994	5	96.0	1993	Vital Registration	100.0	4.0	
Iceland	1995-1999	5	95.0	1996	Vital Registration	100.0	5.0	
Iceland	2000-2004	5	94.3	2004	Vital Registration	100.0	5.7	
Iceland	2005-2009	5	92.6	2008	Vital Registration	100.0	7.4	
Iceland	2010-2019	5	91.3	2010	Vital Registration	100.0	8.7	
India	1980-1984	1	2.6	1983	Morbidity and mortality in diarrhea in rural Haryana Indian		1.9	2.6
India	1985-1989	1	4.6	1989	Maternal mortality in seven districts of Uttar Pradesh - an ICMR task force study		10.8	5.2
India	1990-1994	1	2.7	1994	Vital Registration	4.5	40.7	
India	1995-1999	1	4.8	1999	India SRS Maternal Mortality: Trends, Causes and Risk Factors 1997-2003		11.7	5.4
India	2000-2004	1	6.3	2003	India Study on Causes of Death by Verbal Autopsy 2003		1.8	6.4
India	2005-2009	3	49.7	2005	Verbal Autopsy		22.3	64.0
India	2010-2019	3	46.0	2012	Verbal Autopsy		28.2	64.0
Indonesia	1980-1984	1	1.7	1984	The pattern of the causes of death in children in rural swampy area of South Sumatra. Indonesia		0.9	1.7
Indonesia	1985-1989	0						
Indonesia	1990-1994	1	1.8	1991	Care-seeking for fatal illnesses in young children in Indramayu, West Java. Indonesia		3.8	1.8
Indonesia	1995-1999	1	0.6	1997	Age- and cause-specific childhood mortality in Lombok, Indonesia, as a factor for determining the appropriateness of introducing Haemophilus influenzae type b and pneumococcal vaccines		1.8	0.6
Indonesia	2000-2004	1	0.3	2001	Determining the Cause of Death: Mortality Surveillance Using Verbal Autopsy in Indonesia		95.9	6.4
Indonesia	2005-2009	3	53.9	2006	Indonesia Basic Health Research 2007-2008		2.1	55.1
Indonesia	2010-2019	3	63.4	2013	Indonesia Sample Registration System - Deaths 2013		0.9	64.0
Iran	1980-1984	0						
Iran	1985-1989	0						
Iran	1990-1994	0						
Iran	1995-1999	0						
Iran	2000-2004	3	57.5	2004	Vital Registration	78.0	26.2	
Iran	2005-2009	4	66.1	2007	Vital Registration	81.0	18.4	
Iran	2010-2019	4	80.0	2016	Vital Registration	92.7	13.8	
Iraq	1980-1984	0						
Iraq	1985-1989	0						
Iraq	1990-1994	0						
Iraq	1995-1999	0						
Iraq	2000-2004	0						
Iraq	2005-2009	3	39.2	2008	Vital Registration	71.9	45.5	
Iraq	2010-2019	3	46.2	2014	Vital Registration	76.4	39.5	
Ireland	1980-1984	5	91.4	1984	Vital Registration	100.0	8.6	
Ireland	1985-1989	5	92.2	1988	Vital Registration	100.0	7.8	
Ireland	1990-1994	5	92.6	1993	Vital Registration	100.0	7.4	
Ireland	1995-1999	5	92.0	1997	Vital Registration	100.0	8.0	
Ireland	2000-2004	5	91.6	2000	Vital Registration	100.0	8.4	
Ireland	2005-2009	5	92.7	2007	Vital Registration	100.0	7.3	
Ireland	2010-2019	5	92.9	2015	Vital Registration	100.0	7.1	
Israel	1980-1984	4	80.8	1983	Vital Registration	100.0	19.2	
Israel	1985-1989	4	82.5	1989	Vital Registration	100.0	17.5	
Israel	1990-1994	4	83.5	1994	Vital Registration	100.0	16.5	
Israel	1995-1999	4	83.5	1996	Vital Registration	99.7	16.2	
Israel	2000-2004	4	82.3	2003	Vital Registration	100.0	17.7	
Israel	2005-2009	4	81.0	2009	Vital Registration	99.6	18.6	
Israel	2010-2019	4	82.3	2010	Vital Registration	100.0	17.7	
Italy	1980-1984	5	89.6	1980	Vital Registration	100.0	10.4	
Italy	1985-1989	5	89.6	1989	Vital Registration	100.0	10.4	
Italy	1990-1994	5	90.1	1994	Vital Registration	100.0	9.9	
Italy	1995-1999	5	89.2	1995	Vital Registration	100.0	10.8	
Italy	2000-2004	5	89.7	2003	Vital Registration	100.0	10.3	
Italy	2005-2009	5	90.1	2008	Vital Registration	100.0	9.9	
Italy	2010-2019	5	89.8	2010	Vital Registration	100.0	10.2	
Jamaica	1980-1984	4	72.0	1983	Vital Registration	100.0	28.0	
Jamaica	1985-1989	4	74.2	1987	Vital Registration	99.8	25.7	
Jamaica	1990-1994	4	67.0	1990	Vital Registration	95.8	30.1	
Jamaica	1995-1999	0						
Jamaica	2000-2004	4	83.9	2004	Vital Registration	100.0	16.1	
Jamaica	2005-2009	5	87.2	2006	Vital Registration	99.8	12.6	
Jamaica	2010-2019	5	89.4	2011	Vital Registration	100.0	10.6	
Japan	1980-1984	4	82.9	1982	Vital Registration	100.0	17.1	
Japan	1985-1989	4	82.1	1985	Vital Registration	100.0	17.9	
Japan	1990-1994	4	83.5	1994	Vital Registration	100.0	16.5	
Japan	1995-1999	5	89.1	1995	Vital Registration	100.0	10.9	
Japan	2000-2004	5	87.6	2001	Vital Registration	99.4	11.9	
Japan	2005-2009	5	87.0	2005	Vital Registration	100.0	13.0	
Japan	2010-2019	5	85.5	2011	Vital Registration	100.0	14.5	
Jordan	1980-1984	0						
Jordan	1985-1989	0						
Jordan	1990-1994	0						
Jordan	1995-1999	1	1.7	1995	Mortality and causes of death in Jordan 1995-96: assessment by verbal autopsy		73.9	6.4
Jordan	2000-2004	4	72.8	2004	Vital Registration	93.8	22.4	
Jordan	2005-2009	4	79.3	2006	Vital Registration	97.6	18.8	
Jordan	2010-2019	4	65.2	2011	Vital Registration	81.3	19.7	
Kazakhstan	1980-1984	4	79.8	1982	Vital Registration	96.7	17.6	
Kazakhstan	1985-1989	4	83.6	1988	Vital Registration	100.0	16.3	
Kazakhstan	1990-1994	5	87.3	1993	Vital Registration	99.0	11.8	
Kazakhstan	1995-1999	5	85.5	1995	Vital Registration	97.8	12.6	
Kazakhstan	2000-2004	4	79.7	2000	Vital Registration	93.8	15.0	
Kazakhstan	2005-2009	4	76.9	2005	Vital Registration	91.6	16.1	
Kazakhstan	2010-2019	4	73.6	2015	Vital Registration	88.3	16.7	
Kenya	1980-1984	0						
Kenya	1985-1989	1	3.4	1986	Mortality patterns in a rural Kenyan community		1.5	3.4
Kenya	1990-1994	0						
Kenya	1995-1999	1	2.4	1997	The burden of malaria mortality among African children in the year 2000		9.8	2.7
Kenya	2000-2004	1	4.3	2003	Kenya - Nairobi Urban Health and Demographic Surveillance System		30.6	6.2
Kenya	2005-2009	1	5.1	2006	Kenya - Nairobi Urban Health and Demographic Surveillance System		20.7	6.4
Kenya	2010-2019	2	23.4	2018	Child Health and Mortality Prevention Surveillance (CHAMPS) Network Program		1.3	23.7
Kiribati	1980-1984	0						
Kiribati	1985-1989	0						
Kiribati	1990-1994	3	35.6	1993	Vital Registration	65.5	45.7	
Kiribati	1995-1999	3	55.6	1995	Vital Registration	100.0	44.4	
Kiribati	2000-2004	2	32.2	2000	Vital Registration	58.8	45.2	
Kiribati	2005-2009	0						
Kiribati	2010-2019	0						
Kuwait	1980-1984	4	74.8	1981	Vital Registration	100.0	25.2	
Kuwait	1985-1989	4	76.9	1987	Vital Registration	98.2	21.6	
Kuwait	1990-1994	4	71.9	1993	Vital Registration	93.9	23.4	
Kuwait	1995-1999	4	71.7	1998	Vital Registration	91.5	21.6	
Kuwait	2000-2004	4	69.9	2001	Vital Registration	88.6	21.1	
Kuwait	2005-2009	4	67.7	2009	Vital Registration	82.3	17.7	
Kuwait	2010-2019	4	68.5	2012	Vital Registration	83.1	17.6	
Kyrgyzstan	1980-1984	4	75.5	1981	Vital Registration	93.0	18.9	
Kyrgyzstan	1985-1989	4	83.8	1988	Vital Registration	97.1	13.7	
Kyrgyzstan	1990-1994	4	74.5	1990	Vital Registration	91.0	18.1	
Kyrgyzstan	1995-1999	4	78.7	1998	Vital Registration	98.4	20.0	
Kyrgyzstan	2000-2004	5	88.3	2002	Vital Registration	95.9	7.9	
Kyrgyzstan	2005-2009	5	94.4	2009	Vital Registration	100.0	5.6	
Kyrgyzstan	2010-2019	5	94.1	2014	Vital Registration	100.0	5.9	
Laos	1980-1984	0						
Laos	1985-1989	1	3.8	1989	The Lao People's Democratic Republic: maternal mortality and female mortality: determining causes of deaths		9.8	4.2
Laos	1990-1994	0						
Laos	1995-1999	0						
Laos	2000-2004	0						
Laos	2005-2009	0						
Laos	2010-2019	0						
Latvia	1980-1984	5	91.3	1983	Vital Registration	100.0	8.7	
Latvia	1985-1989	5	91.3	1985	Vital Registration	100.0	8.7	
Latvia	1990-1994	5	88.8	1990	Vital Registration	100.0	11.2	
Latvia	1995-1999	5	92.0	1999	Vital Registration	100.0	8.0	
Latvia	2000-2004	5	91.3	2000	Vital Registration	100.0	8.7	
Latvia	2005-2009	5	89.2	2007	Vital Registration	100.0	10.8	
Latvia	2010-2019	5	93.8	2015	Vital Registration	100.0	6.2	
Lebanon	1980-1984	0						
Lebanon	1985-1989	1	3.6	1988	Non-communicable disease mortality rates using the verbal autopsy in a cohort of middle aged and older populations in Beirut during wartime. 1983-93		12.8	4.1
Lebanon	1990-1994	0						
Lebanon	1995-1999	0						
Lebanon	2000-2004	1	0.3	2000	Facility-based audit of maternal mortality in Lebanon: A feasibility study		12.5	0.4
Lebanon	2005-2009	0						
Lebanon	2010-2019	0						
Lesotho	1980-1984	0						
Lesotho	1985-1989	0						
Lesotho	1990-1994	0						
Lesotho	1995-1999	0						
Lesotho	2000-2004	0						
Lesotho	2005-2009	0						
Lesotho	2010-2019	0						
Liberia	1980-1984	1	4.1	1984	Infant and child mortality in two counties of Liberia: results of a survey in 1988 and trends since 1984		6.4	4.4
Liberia	1985-1989	1	4.1	1987	Infant and child mortality in two counties of Liberia: results of a survey in 1988 and trends since 1984		6.7	4.4
Liberia	1990-1994	1	4.3	1990	Application of the verbal autopsy during a clinical trial		10.1	4.8
Liberia	1995-1999	0						
Liberia	2000-2004	0						
Liberia	2005-2009	0						
Liberia	2010-2019	0						
Libya	1980-1984	0						
Libya	1985-1989	0						
Libya	1990-1994	0						
Libya	1995-1999	0						
Libya	2000-2004	0						
Libya	2005-2009	2	11.5	2006	Vital Registration	88.0	86.9	
Libya	2010-2019	0						
Lithuania	1980-1984	5	90.6	1982	Vital Registration	99.6	9.1	
Lithuania	1985-1989	5	92.2	1988	Vital Registration	100.0	7.8	
Lithuania	1990-1994	5	92.7	1990	Vital Registration	100.0	7.3	
Lithuania	1995-1999	5	94.8	1997	Vital Registration	100.0	5.2	
Lithuania	2000-2004	5	93.2	2003	Vital Registration	100.0	6.8	
Lithuania	2005-2009	5	92.8	2006	Vital Registration	100.0	7.2	
Lithuania	2010-2019	5	93.5	2013	Vital Registration	100.0	6.5	
Luxembourg	1980-1984	5	86.9	1984	Vital Registration	100.0	13.1	
Luxembourg	1985-1989	5	87.5	1987	Vital Registration	100.0	12.5	
Luxembourg	1990-1994	5	85.1	1992	Vital Registration	97.5	12.7	
Luxembourg	1995-1999	5	85.0	1997	Vital Registration	96.7	12.1	
Luxembourg	2000-2004	4	82.8	2000	Vital Registration	96.7	14.4	
Luxembourg	2005-2009	4	82.2	2007	Vital Registration	100.0	17.8	
Luxembourg	2010-2019	4	83.1	2012	Vital Registration	97.2	14.6	

Table S9. Underlying indicators for percent well-certified for data source with maximum percent well certified in each 5-year time interval for 204 countries, 1980-2019.

Location	Time Window	Stars	Percent Well-Certified [PWC] (%)	Max PWC Data Year	Max PWC Data Source	Completeness (%)	Percent Major Garbage (%)	Verbal Autopsy Adjustment (None for VR) (%)
Madagascar	1980-1984	1	2.3	1984	Vital Registration	4.9	53.9	
Madagascar	1985-1989	1	2.9	1986	Vital Registration	6.0	52.8	
Madagascar	1990-1994	1	2.1	1990	Vital Registration	4.2	49.3	
Madagascar	1995-1999	1	2.2	1995	Vital Registration	4.4	49.5	
Madagascar	2000-2004	0						
Madagascar	2005-2009	0						
Madagascar	2010-2019	0						
Malawi	1980-1984	0						
Malawi	1985-1989	1	3.8	1988	Infant and second-year mortality in rural Malawi: causes and descriptive epidemiology		5.1	4.0
Malawi	1990-1994	0						
Malawi	1995-1999	1	0.7	1999	Estimation of AIDS adult mortality by verbal autopsy in rural Malawi		3.0	0.7
Malawi	2000-2004	1	3.2	2004	Declining child mortality in northern Malawi despite high rates of infection with HIV		3.7	3.3
Malawi	2005-2009	1	3.4	2008	Rates and causes of death in Chiradzulu District, Malawi, 2008: a key informant study		46.7	6.4
Malawi	2010-2019	1	0.6	2010	Measuring causes of adult mortality in rural northern Malawi over a decade of change		81.2	3.3
Malaysia	1980-1984	3	44.0	1980	Vital Registration	74.3	40.9	
Malaysia	1985-1989	0						
Malaysia	1990-1994	0						
Malaysia	1995-1999	2	31.4	1997	Vital Registration	46.6	32.6	
Malaysia	2000-2004	3	36.0	2004	Vital Registration	59.2	39.2	
Malaysia	2005-2009	3	40.5	2008	Vital Registration	62.8	35.4	
Malaysia	2010-2019	3	40.4	2011	Vital Registration	54.7	26.1	
Maldives	1980-1984	0						
Maldives	1985-1989	0						
Maldives	1990-1994	0						
Maldives	1995-1999	0						
Maldives	2000-2004	3	44.0	2002	Vital Registration	100.0	56.0	
Maldives	2005-2009	3	49.1	2008	Vital Registration	100.0	50.9	
Maldives	2010-2019	3	61.9	2011	Vital Registration	100.0	38.1	
Mali	1980-1984	1	2.3	1981	Vital Registration	8.0	70.6	
Mali	1985-1989	0						
Mali	1990-1994	1	0.3	1990	Assessment of maternal mortality and late maternal mortality among a cohort of pregnant women in Bamako, Mali		10.5	0.3
Mali	1995-1999	0						
Mali	2000-2004	0						
Mali	2005-2009	0						
Mali	2010-2019	1	5.6	2017	Child Health and Mortality Prevention Surveillance (CHAMPS) Network Program		2.1	5.7
Malta	1980-1984	4	83.0	1984	Vital Registration	100.0	17.0	
Malta	1985-1989	5	87.9	1989	Vital Registration	100.0	12.1	
Malta	1990-1994	5	89.9	1992	Vital Registration	100.0	10.1	
Malta	1995-1999	5	90.8	1996	Vital Registration	100.0	9.2	
Malta	2000-2004	5	92.3	2004	Vital Registration	100.0	7.7	
Malta	2005-2009	5	94.5	2009	Vital Registration	100.0	5.5	
Malta	2010-2019	5	93.5	2015	Vital Registration	100.0	6.5	
Marshall Islands	1980-1984	0						
Marshall Islands	1985-1989	0						
Marshall Islands	1990-1994	0						
Marshall Islands	1995-1999	0						
Marshall Islands	2000-2004	0						
Marshall Islands	2005-2009	0						
Marshall Islands	2010-2019	0						
Mauritania	1980-1984	0						
Mauritania	1985-1989	0						
Mauritania	1990-1994	0						
Mauritania	1995-1999	0						
Mauritania	2000-2004	0						
Mauritania	2005-2009	0						
Mauritania	2010-2019	0						
Mauritius	1980-1984	4	73.5	1984	Vital Registration	100.0	26.5	
Mauritius	1985-1989	4	77.7	1988	Vital Registration	100.0	22.3	
Mauritius	1990-1994	4	76.8	1991	Vital Registration	100.0	23.2	
Mauritius	1995-1999	4	77.4	1998	Vital Registration	100.0	22.6	
Mauritius	2000-2004	4	82.4	2002	Vital Registration	100.0	17.6	
Mauritius	2005-2009	4	84.4	2009	Vital Registration	100.0	15.6	
Mauritius	2010-2019	5	87.0	2014	Vital Registration	99.8	12.8	
Mexico	1980-1984	3	64.0	1984	Vital Registration	84.7	24.4	
Mexico	1985-1989	4	71.4	1989	Vital Registration	87.7	18.5	
Mexico	1990-1994	4	73.0	1994	Vital Registration	86.8	16.0	
Mexico	1995-1999	4	78.8	1999	Vital Registration	90.6	13.1	
Mexico	2000-2004	4	80.6	2004	Vital Registration	91.6	12.0	
Mexico	2005-2009	4	82.4	2009	Vital Registration	94.2	12.5	
Mexico	2010-2019	5	89.4	2017	Vital Registration	100.0	10.6	
Moldova	1980-1984	5	89.5	1981	Vital Registration	100.0	10.5	
Moldova	1985-1989	5	91.0	1985	Vital Registration	100.0	9.0	
Moldova	1990-1994	4	79.5	1990	Vital Registration	99.4	20.1	
Moldova	1995-1999	5	86.9	1996	Vital Registration	100.0	13.1	
Moldova	2000-2004	5	89.9	2003	Vital Registration	93.7	4.1	
Moldova	2005-2009	5	87.8	2009	Vital Registration	91.0	3.5	
Moldova	2010-2019	5	90.1	2012	Vital Registration	93.0	3.0	
Monaco	1980-1984	0						
Monaco	1985-1989	4	76.8	1986	Vital Registration	100.0	23.2	
Monaco	1990-1994	0						
Monaco	1995-1999	0						
Monaco	2000-2004	0						
Monaco	2005-2009	0						
Monaco	2010-2019	4	80.3	2013	Vital Registration	100.0	19.7	
Mongolia	1980-1984	0						
Mongolia	1985-1989	0						
Mongolia	1990-1994	3	59.1	1994	Vital Registration	78.5	24.6	
Mongolia	1995-1999	0						
Mongolia	2000-2004	2	19.5	2004	Vital Registration	80.7	75.9	
Mongolia	2005-2009	2	20.9	2007	Vital Registration	81.4	74.3	
Mongolia	2010-2019	4	79.4	2010	Vital Registration	86.1	7.8	
Montenegro	1980-1984	0						
Montenegro	1985-1989	0						
Montenegro	1990-1994	0						
Montenegro	1995-1999	0						
Montenegro	2000-2004	3	62.0	2004	Vital Registration	91.2	31.9	
Montenegro	2005-2009	3	62.6	2009	Vital Registration	90.2	30.6	
Montenegro	2010-2019	0						
Morocco	1980-1984	0						
Morocco	1985-1989	2	24.7	1988	Morocco National Survey on Causes and Circumstances of Infant and Child Deaths 1988-1989		2.9	25.5
Morocco	1990-1994	0						
Morocco	1995-1999	0						
Morocco	2000-2004	2	10.7	2004	Vital Registration	24.9	57.0	
Morocco	2005-2009	2	29.6	2005	Vital Registration	70.0	57.7	
Morocco	2010-2019	2	12.1	2014	Vital Registration	24.6	50.7	
Mozambique	1980-1984	0						
Mozambique	1985-1989	0						
Mozambique	1990-1994	0						
Mozambique	1995-1999	1	0.3	1996	Quality of registration of maternal deaths in Mozambique: a community-based study in rural and urban areas		11.2	0.3
Mozambique	2000-2004	2	14.9	2001	Vital Registration	22.3	33.2	
Mozambique	2005-2009	3	63.3	2007	Mozambique National Survey on the Causes of Death 2007-2008		1.0	64.0
Mozambique	2010-2019	1	3.5	2017	Child Health and Mortality Prevention Surveillance (CHAMPS) Network Program		0.4	3.5
Myanmar	1980-1984	0						
Myanmar	1985-1989	0						
Myanmar	1990-1994	0						
Myanmar	1995-1999	0						
Myanmar	2000-2004	0						
Myanmar	2005-2009	1	2.9	2007	Cause of Death Verification Study in Myanmar		54.5	6.3
Myanmar	2010-2019	3	48.9	2016	Myanmar National Mortality Survey 2016		23.6	64.0
Namibia	1980-1984	0						
Namibia	1985-1989	0						
Namibia	1990-1994	0						
Namibia	1995-1999	0						
Namibia	2000-2004	0						
Namibia	2005-2009	0						
Namibia	2010-2019	0						
Nauru	1980-1984	0						
Nauru	1985-1989	0						
Nauru	1990-1994	0						
Nauru	1995-1999	0						
Nauru	2000-2004	0						
Nauru	2005-2009	0						
Nauru	2010-2019	0						
Nepal	1980-1984	1	3.3	1984	Impact of a pilot acute respiratory infection (ARI) control programme in a rural community of the hill region of Nepal		3.4	3.4
Nepal	1985-1989	1	3.2	1987	Reduction in total under-five mortality in western Nepal through community-based antimicrobial treatment of pneumonia		2.0	3.2
Nepal	1990-1994	0						
Nepal	1995-1999	1	1.2	1999	Evaluation of neonatal verbal autopsy using physician review versus algorithm-based cause-of-death assignment in rural Nepal		2.2	1.2
Nepal	2000-2004	1	1.0	2003	Effect of daily zinc supplementation on child mortality in southern Nepal: a community-based, cluster randomised, placebo-controlled trial		2.2	1.0
Nepal	2005-2009	2	16.4	2006	Nepal Demographic and Health Survey 2006		4.9	17.2
Nepal	2010-2019	0						
Netherlands	1980-1984	5	88.8	1981	Vital Registration	100.0	11.2	
Netherlands	1985-1989	5	88.0	1987	Vital Registration	100.0	12.0	
Netherlands	1990-1994	5	86.5	1991	Vital Registration	100.0	13.5	
Netherlands	1995-1999	5	86.0	1996	Vital Registration	100.0	14.0	
Netherlands	2000-2004	4	84.2	2000	Vital Registration	100.0	15.8	
Netherlands	2005-2009	4	84.5	2005	Vital Registration	99.2	14.8	
Netherlands	2010-2019	4	84.2	2012	Vital Registration	97.6	13.7	
New Zealand	1980-1984	5	95.4	1984	Vital Registration	100.0	4.6	
New Zealand	1985-1989	5	95.0	1988	Vital Registration	100.0	5.0	
New Zealand	1990-1994	5	95.5	1994	Vital Registration	100.0	4.5	
New Zealand	1995-1999	5	96.7	1999	Vital Registration	100.0	3.3	
New Zealand	2000-2004	5	96.9	2004	Vital Registration	100.0	3.1	
New Zealand	2005-2009	5	96.4	2006	Vital Registration	100.0	3.6	
New Zealand	2010-2019	5	96.0	2012	Vital Registration	100.0	4.0	
Nicaragua	1980-1984	0						
Nicaragua	1985-1989	3	49.0	1988	Vital Registration	66.6	26.5	
Nicaragua	1990-1994	3	50.4	1990	Vital Registration	68.7	26.6	
Nicaragua	1995-1999	3	64.6	1998	Vital Registration	77.6	16.8	
Nicaragua	2000-2004	4	66.8	2003	Vital Registration	76.5	12.7	
Nicaragua	2005-2009	4	68.6	2008	Vital Registration	79.5	13.6	
Nicaragua	2010-2019	4	79.5	2016	Vital Registration	87.4	9.1	
Niger	1980-1984	0						
Niger	1985-1989	0						
Niger	1990-1994	0						
Niger	1995-1999	0						
Niger	2000-2004	0						
Niger	2005-2009	3	42.6	2008	Direct estimates of national neonatal and child cause-specific mortality proportions in Niger by expert algorithm and physician-coded analysis of verbal autopsy interviews		1.0	43.0
Niger	2010-2019	0						
Nigeria	1980-1984	0						
Nigeria	1985-1989	0						
Nigeria	1990-1994	1	4.3	1991	Community-based surveillance of paediatric deaths in Cross River State, Nigeria		2.3	4.4
Nigeria	1995-1999	0						
Nigeria	2000-2004	0						

Table S9. Underlying indicators for percent well-certified for data source with maximum percent well certified in each 5-year time interval for 204 countries, 1980-2019.

Location	Time Window	Stars	Percent Well-Certified [PWC] (%)	Max PWC Data Year	Max PWC Data Source	Completeness (%)	Percent Major Garbage (%)	Verbal Autopsy Adjustment (None for VR) (%)
Nigeria	2005-2009	1	0.0	2007	Vital Registration	1.3	96.4	
Nigeria	2010-2019	3	40.4	2014	Direct estimates of cause-specific mortality fractions and rates of under-five deaths in the northern and southern regions of Nigeria by verbal autopsy interview		0.8	40.7
Niue	1980-1984	0						
Niue	1985-1989	0						
Niue	1990-1994	0						
Niue	1995-1999	0						
Niue	2000-2004	0						
Niue	2005-2009	0						
Niue	2010-2019	0						
North Korea	1980-1984	0						
North Korea	1985-1989	0						
North Korea	1990-1994	0						
North Korea	1995-1999	0						
North Korea	2000-2004	0						
North Korea	2005-2009	0						
North Korea	2010-2019	0						
North Macedonia	1980-1984	0						
North Macedonia	1985-1989	0						
North Macedonia	1990-1994	4	75.9	1992	Vital Registration	95.9	20.8	
North Macedonia	1995-1999	4	75.5	1998	Vital Registration	91.6	17.6	
North Macedonia	2000-2004	4	77.1	2000	Vital Registration	92.7	16.8	
North Macedonia	2005-2009	4	71.9	2007	Vital Registration	91.2	21.2	
North Macedonia	2010-2019	4	68.8	2010	Vital Registration	86.9	20.8	
Northern Mariana Islands	1980-1984	0						
Northern Mariana Islands	1985-1989	0						
Northern Mariana Islands	1990-1994	0						
Northern Mariana Islands	1995-1999	3	59.2	1998	Vital Registration	75.7	21.8	
Northern Mariana Islands	2000-2004	3	54.2	2004	Vital Registration	69.5	22.1	
Northern Mariana Islands	2005-2009	3	61.2	2009	Vital Registration	77.4	20.9	
Northern Mariana Islands	2010-2019	3	64.9	2016	Vital Registration	73.0	11.2	
Norway	1980-1984	5	91.0	1984	Vital Registration	100.0	9.0	
Norway	1985-1989	5	91.5	1988	Vital Registration	100.0	8.5	
Norway	1990-1994	5	91.0	1990	Vital Registration	100.0	9.0	
Norway	1995-1999	5	90.8	1997	Vital Registration	100.0	9.2	
Norway	2000-2004	5	88.4	2000	Vital Registration	99.4	11.0	
Norway	2005-2009	5	86.1	2005	Vital Registration	100.0	13.9	
Norway	2010-2019	5	85.7	2016	Vital Registration	100.0	14.3	
Oman	1980-1984	0						
Oman	1985-1989	0						
Oman	1990-1994	0						
Oman	1995-1999	0						
Oman	2000-2004	0						
Oman	2005-2009	3	63.3	2007	Vital Registration	77.3	18.2	
Oman	2010-2019	2	32.4	2010	Vital Registration	79.2	59.1	
Pakistan	1980-1984	0						
Pakistan	1985-1989	2	31.5	1986	Acute respiratory infections in children: a case management intervention in Abbottabad District. Pakistan		2.0	32.2
Pakistan	1990-1994	1	2.1	1990	Time to focus child survival programmes on the newborn: assessment of levels and causes of infant mortality in rural Pakistan		2.6	2.1
Pakistan	1995-1999	0						
Pakistan	2000-2004	2	13.8	2003	Impact of a community-based perinatal and newborn preventive care package on perinatal and neonatal mortality in a remote mountainous district in Northern Pakistan		0.0	13.8
Pakistan	2005-2009	2	23.4	2006	Pakistan Demographic and Health Survey 2006-2007		3.9	24.3
Pakistan	2010-2019	2	21.6	2010	Cause of Death in under 5 Children in a Demographic Surveillance Site in Pakistan		3.1	22.3
Palau	1980-1984	0						
Palau	1985-1989	0						
Palau	1990-1994	0						
Palau	1995-1999	0						
Palau	2000-2004	0						
Palau	2005-2009	0						
Palau	2010-2019	3	51.0	2013	Vital Registration	100.0	49.0	
Palestine	1980-1984	0						
Palestine	1985-1989	0						
Palestine	1990-1994	0						
Palestine	1995-1999	2	28.5	1998	Vital Registration	46.4	38.5	
Palestine	2000-2004	2	31.6	2004	Vital Registration	47.9	34.0	
Palestine	2005-2009	2	33.8	2009	Vital Registration	49.1	31.0	
Palestine	2010-2019	4	72.8	2014	Vital Registration	100.0	27.2	
Panama	1980-1984	4	74.1	1983	Vital Registration	99.6	25.6	
Panama	1985-1989	4	77.1	1987	Vital Registration	99.4	22.4	
Panama	1990-1994	0						
Panama	1995-1999	4	83.3	1999	Vital Registration	100.0	16.7	
Panama	2000-2004	5	86.7	2002	Vital Registration	100.0	13.3	
Panama	2005-2009	5	86.5	2007	Vital Registration	100.0	13.5	
Panama	2010-2019	4	84.6	2014	Vital Registration	100.0	15.4	
Papua New Guinea	1980-1984	1	6.3	1980	Vital Registration	8.9	29.5	
Papua New Guinea	1985-1989	1	3.6	1985	Mortality rates and the utilization of health services during terminal illness in the Asaro Valley, Eastern Highlands Province, Papua New Guinea		43.4	6.4
Papua New Guinea	1990-1994	0						
Papua New Guinea	1995-1999	0						
Papua New Guinea	2000-2004	0						
Papua New Guinea	2005-2009	0						
Papua New Guinea	2010-2019	2	29.5	2012	Verbal Autopsy		39.3	48.6
Paraguay	1980-1984	3	51.7	1984	Vital Registration	86.7	40.4	
Paraguay	1985-1989	3	54.5	1989	Vital Registration	81.8	33.3	
Paraguay	1990-1994	3	61.5	1994	Vital Registration	82.8	25.8	
Paraguay	1995-1999	3	62.3	1996	Vital Registration	85.3	27.0	
Paraguay	2000-2004	3	63.1	2004	Vital Registration	88.5	28.7	
Paraguay	2005-2009	4	67.2	2009	Vital Registration	89.4	24.8	
Paraguay	2010-2019	4	82.2	2016	Vital Registration	100.0	17.8	
Peru	1980-1984	3	51.6	1980	Vital Registration	70.7	27.0	
Peru	1985-1989	2	34.4	1989	Vital Registration	64.4	46.5	
Peru	1990-1994	2	34.5	1992	Vital Registration	62.3	44.7	
Peru	1995-1999	3	45.8	1999	Vital Registration	70.8	35.3	
Peru	2000-2004	3	55.4	2004	Vital Registration	78.4	29.4	
Peru	2005-2009	3	56.2	2007	Vital Registration	73.3	23.3	
Peru	2010-2019	3	52.1	2015	Vital Registration	64.4	19.1	
Philippines	1980-1984	1	0.4	1981	Vital Registration	41.1	99.1	
Philippines	1985-1989	2	12.9	1989	Effect of not breastfeeding on the risk of diarrheal and respiratory mortality in children under 2 years of age in Metro Cebu, The Philippines		6.2	13.7
Philippines	1990-1994	2	15.4	1992	Vital Registration	82.7	81.4	
Philippines	1995-1999	3	40.4	1999	Vital Registration	84.3	52.1	
Philippines	2000-2004	3	46.2	2002	Vital Registration	87.0	46.9	
Philippines	2005-2009	4	74.1	2009	Vital Registration	88.0	15.8	
Philippines	2010-2019	4	75.4	2015	Vital Registration	89.3	15.6	
Poland	1980-1984	3	64.3	1984	Vital Registration	100.0	35.7	
Poland	1985-1989	3	64.4	1986	Vital Registration	100.0	35.6	
Poland	1990-1994	3	64.2	1994	Vital Registration	100.0	35.8	
Poland	1995-1999	4	73.5	1999	Vital Registration	100.0	26.5	
Poland	2000-2004	4	75.8	2002	Vital Registration	100.0	24.2	
Poland	2005-2009	4	75.9	2006	Vital Registration	100.0	24.1	
Poland	2010-2019	4	75.9	2011	Vital Registration	100.0	24.1	
Portugal	1980-1984	4	77.6	1984	Vital Registration	100.0	22.4	
Portugal	1985-1989	4	77.9	1985	Vital Registration	100.0	22.1	
Portugal	1990-1994	4	77.6	1992	Vital Registration	100.0	22.4	
Portugal	1995-1999	4	77.6	1995	Vital Registration	100.0	22.4	
Portugal	2000-2004	4	82.4	2002	Vital Registration	100.0	17.6	
Portugal	2005-2009	4	79.5	2009	Vital Registration	100.0	20.5	
Portugal	2010-2019	5	86.0	2014	Vital Registration	100.0	14.0	
Puerto Rico	1980-1984	4	78.1	1981	Vital Registration	100.0	21.9	
Puerto Rico	1985-1989	4	77.3	1989	Vital Registration	100.0	22.7	
Puerto Rico	1990-1994	4	84.1	1994	Vital Registration	100.0	15.9	
Puerto Rico	1995-1999	4	83.8	1997	Vital Registration	100.0	16.2	
Puerto Rico	2000-2004	4	84.0	2004	Vital Registration	100.0	16.0	
Puerto Rico	2005-2009	4	83.4	2008	Vital Registration	100.0	16.6	
Puerto Rico	2010-2019	4	84.5	2012	Vital Registration	100.0	15.5	
Qatar	1980-1984	2	12.2	1984	Vital Registration	64.5	81.1	
Qatar	1985-1989	2	16.0	1985	Vital Registration	75.8	78.8	
Qatar	1990-1994	0						
Qatar	1995-1999	3	49.0	1995	Vital Registration	67.5	27.4	
Qatar	2000-2004	3	43.6	2001	Vital Registration	68.5	36.4	
Qatar	2005-2009	3	50.2	2006	Vital Registration	75.2	33.3	
Qatar	2010-2019	3	38.9	2010	Vital Registration	60.8	36.0	
Romania	1980-1984	4	77.3	1980	Vital Registration	100.0	22.7	
Romania	1985-1989	4	79.0	1989	Vital Registration	100.0	21.0	
Romania	1990-1994	4	83.8	1994	Vital Registration	100.0	16.2	
Romania	1995-1999	5	86.1	1999	Vital Registration	100.0	13.9	
Romania	2000-2004	5	87.2	2004	Vital Registration	100.0	12.8	
Romania	2005-2009	5	87.5	2007	Vital Registration	100.0	12.5	
Romania	2010-2019	5	86.8	2010	Vital Registration	100.0	13.2	
Russia	1980-1984	5	92.5	1980	Vital Registration	100.0	7.5	
Russia	1985-1989	5	86.6	1989	Vital Registration	94.4	8.2	
Russia	1990-1994	5	90.2	1990	Vital Registration	100.0	9.8	
Russia	1995-1999	5	87.0	1999	Vital Registration	100.0	13.0	
Russia	2000-2004	5	87.4	2004	Vital Registration	100.0	12.6	
Russia	2005-2009	5	88.9	2009	Vital Registration	100.0	11.1	
Russia	2010-2019	5	88.5	2010	Vital Registration	100.0	11.5	
Rwanda	1980-1984	0						
Rwanda	1985-1989	0						
Rwanda	1990-1994	0						
Rwanda	1995-1999	0						
Rwanda	2000-2004	0						
Rwanda	2005-2009	2	28.3	2007	Rwanda Child Verbal Autopsy Study 2008		1.2	28.6
Rwanda	2010-2019	0						
Saint Kitts and Nevis	1980-1984	4	65.3	1983	Vital Registration	90.6	28.0	
Saint Kitts and Nevis	1985-1989	4	68.4	1987	Vital Registration	99.0	31.0	
Saint Kitts and Nevis	1990-1994	4	68.0	1994	Vital Registration	93.3	27.1	
Saint Kitts and Nevis	1995-1999	4	78.4	1998	Vital Registration	97.0	19.2	
Saint Kitts and Nevis	2000-2004	4	67.3	2002	Vital Registration	86.3	22.0	
Saint Kitts and Nevis	2005-2009	4	78.5	2008	Vital Registration	92.1	14.7	
Saint Kitts and Nevis	2010-2019	4	84.8	2014	Vital Registration	95.1	10.8	
Saint Lucia	1980-1984	4	67.7	1983	Vital Registration	97.8	30.8	
Saint Lucia	1985-1989	4	68.0	1987	Vital Registration	100.0	32.0	
Saint Lucia	1990-1994	4	73.9	1994	Vital Registration	100.0	26.1	
Saint Lucia	1995-1999	4	73.3	1997	Vital Registration	100.0	26.7	
Saint Lucia	2000-2004	4	80.4	2004	Vital Registration	100.0	19.6	
Saint Lucia	2005-2009	4	74.0	2009	Vital Registration	87.5	15.4	
Saint Lucia	2010-2019	5	86.5	2014	Vital Registration	100.0	13.5	
Saint Vincent and the Grenadines	1980-1984	4	69.7	1983	Vital Registration	100.0	30.3	
Saint Vincent and the Grenadines	1985-1989	3	60.8	1986	Vital Registration	88.5	31.3	
Saint Vincent and the Grenadines	1990-1994	3	59.1	1990	Vital Registration	92.8	36.3	
Saint Vincent and the Grenadines	1995-1999	5	85.7	1998	Vital Registration	100.0	14.3	
Saint Vincent and the Grenadines	2000-2004	4	83.4	2002	Vital Registration	97.3	14.3	
Saint Vincent and the Grenadines	2005-2009	4	84.8	2005	Vital Registration	100.0	15.2	
Saint Vincent and the Grenadines	2010-2019	5	87.2	2014	Vital Registration	99.2	12.0	
Samoa	1980-1984	0						
Samoa	1985-1989	0						
Samoa	1990-1994	0						
Samoa	1995-1999	0						
Samoa	2000-2004	0						
Samoa	2005-2009	0						
Samoa	2010-2019	0						
San Marino	1980-1984	0						
San Marino	1985-1989	0						

Table S9. Underlying indicators for percent well-certified for data source with maximum percent well certified in each 5-year time interval for 204 countries, 1980-2019.								
Location	Time Window	Stars	Percent Well-Certified [PWC] (%)	Max PWC Data Year	Max PWC Data Source	Completeness (%)	Percent Major Garbage (%)	Verbal Autopsy Adjustment (None for VR) (%)
San Marino	1990-1994	0						
San Marino	1995-1999	4	75.7	1995	Vital Registration	100.0	24.3	
San Marino	2000-2004	4	77.4	2002	Vital Registration	100.0	22.6	
San Marino	2005-2009	4	77.4	2005	Vital Registration	100.0	22.6	
San Marino	2010-2019	4	65.8	2012	Vital Registration	82.8	20.6	
Saudi Arabia	1980-1984	0						
Saudi Arabia	1985-1989	0						
Saudi Arabia	1990-1994	0						
Saudi Arabia	1995-1999	2	19.2	1999	Vital Registration	34.9	45.0	
Saudi Arabia	2000-2004	2	20.7	2004	Vital Registration	40.1	48.4	
Saudi Arabia	2005-2009	2	21.8	2005	Vital Registration	40.0	45.4	
Saudi Arabia	2010-2019	2	20.6	2010	Vital Registration	43.1	52.1	
Senegal	1980-1984	1	2.8	1984	Senegal - Risk of Death Associated with Different Nutritional States in Children of Preschool age: Study Conducted in Niakhar (Senegal) 1983-1986		1.9	2.8
Senegal	1985-1989	1	3.4	1986	International differences in clinical patterns of diarrhoeal deaths: a comparison of children from Brazil, Senegal, Bangladesh, and India		5.5	3.6
Senegal	1990-1994	1	3.5	1990	Childhood mortality and probable causes of death using verbal autopsy in Niakhar, Senegal, 1989-2000		4.2	3.6
Senegal	1995-1999	1	3.3	1996	Childhood mortality and probable causes of death using verbal autopsy in Niakhar, Senegal, 1989-2000		2.8	3.4
Senegal	2000-2004	0						
Senegal	2005-2009	1	0.0	2008	Africa, Asia, Oceania - INDEPTH Network Cause-Specific Mortality - Release 2014		99.7	6.4
Senegal	2010-2019	1	0.0	2010	Africa, Asia, Oceania - INDEPTH Network Cause-Specific Mortality - Release 2014		100.0	6.4
Serbia	1980-1984	0						
Serbia	1985-1989	0						
Serbia	1990-1994	0						
Serbia	1995-1999	4	77.9	1998	Vital Registration	100.0	22.1	
Serbia	2000-2004	4	77.7	2003	Vital Registration	100.0	22.3	
Serbia	2005-2009	4	81.3	2005	Vital Registration	100.0	18.7	
Serbia	2010-2019	4	81.4	2010	Vital Registration	98.7	17.5	
Seychelles	1980-1984	4	70.3	1981	Vital Registration	98.3	28.4	
Seychelles	1985-1989	4	71.3	1987	Vital Registration	99.7	28.5	
Seychelles	1990-1994	0						
Seychelles	1995-1999	0						
Seychelles	2000-2004	4	77.8	2004	Vital Registration	100.0	22.2	
Seychelles	2005-2009	4	79.1	2008	Vital Registration	100.0	20.9	
Seychelles	2010-2019	4	79.4	2011	Vital Registration	100.0	20.6	
Sierra Leone	1980-1984	0						
Sierra Leone	1985-1989	0						
Sierra Leone	1990-1994	1	4.0	1990	Malaria in a rural area of Sierra Leone. I. Initial results		1.3	4.1
Sierra Leone	1995-1999	0						
Sierra Leone	2000-2004	0						
Sierra Leone	2005-2009	0						
Sierra Leone	2010-2019	0						
Singapore	1980-1984	5	91.1	1983	Vital Registration	100.0	8.9	
Singapore	1985-1989	5	91.6	1985	Vital Registration	99.7	8.1	
Singapore	1990-1994	5	90.6	1992	Vital Registration	96.8	6.4	
Singapore	1995-1999	5	92.5	1998	Vital Registration	97.6	5.2	
Singapore	2000-2004	5	93.4	2003	Vital Registration	98.2	4.9	
Singapore	2005-2009	5	92.2	2007	Vital Registration	97.9	5.8	
Singapore	2010-2019	5	93.8	2014	Vital Registration	97.0	3.2	
Slovakia	1980-1984	0						
Slovakia	1985-1989	0						
Slovakia	1990-1994	4	83.8	1994	Vital Registration	100.0	16.2	
Slovakia	1995-1999	4	83.7	1999	Vital Registration	100.0	16.3	
Slovakia	2000-2004	5	85.3	2000	Vital Registration	100.0	14.7	
Slovakia	2005-2009	5	88.5	2008	Vital Registration	99.3	10.8	
Slovakia	2010-2019	5	92.0	2012	Vital Registration	100.0	8.0	
Slovenia	1980-1984	0						
Slovenia	1985-1989	5	86.2	1985	Vital Registration	93.2	7.5	
Slovenia	1990-1994	5	85.4	1992	Vital Registration	91.1	6.2	
Slovenia	1995-1999	4	81.5	1998	Vital Registration	89.1	8.5	
Slovenia	2000-2004	5	85.0	2003	Vital Registration	96.1	11.5	
Slovenia	2005-2009	5	86.1	2009	Vital Registration	96.1	10.4	
Slovenia	2010-2019	5	87.0	2011	Vital Registration	94.8	8.3	
Solomon Islands	1980-1984	0						
Solomon Islands	1985-1989	0						
Solomon Islands	1990-1994	0						
Solomon Islands	1995-1999	0						
Solomon Islands	2000-2004	0						
Solomon Islands	2005-2009	0						
Solomon Islands	2010-2019	3	38.0	2016	Verbal Autopsy		30.7	54.8
Somalia	1980-1984	0						
Somalia	1985-1989	0						
Somalia	1990-1994	0						
Somalia	1995-1999	0						
Somalia	2000-2004	0						
Somalia	2005-2009	0						
Somalia	2010-2019	0						
South Africa	1980-1984	0						
South Africa	1985-1989	0						
South Africa	1990-1994	1	1.2	1991	'A bothersome death' -- narrative accounts of infant mortality in Cape Town, South Africa		2.3	1.2
South Africa	1995-1999	4	66.0	1999	Vital Registration	97.7	32.5	
South Africa	2000-2004	4	69.3	2004	Vital Registration	100.0	30.7	
South Africa	2005-2009	4	69.2	2005	Vital Registration	100.0	30.8	
South Africa	2010-2019	4	69.1	2014	Vital Registration	100.0	30.9	
South Korea	1980-1984	0						
South Korea	1985-1989	3	53.0	1989	Vital Registration	80.2	34.0	
South Korea	1990-1994	4	72.1	1994	Vital Registration	96.6	25.3	
South Korea	1995-1999	4	74.2	1999	Vital Registration	100.0	25.8	
South Korea	2000-2004	4	83.9	2002	Vital Registration	100.0	16.1	
South Korea	2005-2009	4	82.8	2007	Vital Registration	100.0	17.2	
South Korea	2010-2019	4	84.0	2015	Vital Registration	100.0	16.0	
South Sudan	1980-1984	0						
South Sudan	1985-1989	0						
South Sudan	1990-1994	0						
South Sudan	1995-1999	0						
South Sudan	2000-2004	0						
South Sudan	2005-2009	0						
South Sudan	2010-2019	0						
Spain	1980-1984	4	79.4	1984	Vital Registration	100.0	20.6	
Spain	1985-1989	4	81.7	1989	Vital Registration	100.0	18.3	
Spain	1990-1994	5	85.3	1994	Vital Registration	100.0	14.7	
Spain	1995-1999	5	86.7	1998	Vital Registration	100.0	13.3	
Spain	2000-2004	5	86.2	2004	Vital Registration	99.9	13.7	
Spain	2005-2009	5	86.2	2009	Vital Registration	100.0	13.8	
Spain	2010-2019	5	88.0	2014	Vital Registration	100.0	12.0	
Sri Lanka	1980-1984	3	43.9	1980	Vital Registration	100.0	56.1	
Sri Lanka	1985-1989	3	43.7	1988	Vital Registration	99.0	55.9	
Sri Lanka	1990-1994	3	41.9	1993	Vital Registration	100.0	58.1	
Sri Lanka	1995-1999	3	52.7	1999	Vital Registration	100.0	47.3	
Sri Lanka	2000-2004	3	62.7	2004	Vital Registration	100.0	37.3	
Sri Lanka	2005-2009	4	67.9	2007	Vital Registration	100.0	32.1	
Sri Lanka	2010-2019	4	65.3	2010	Vital Registration	100.0	34.7	
Sudan	1980-1984	0						
Sudan	1985-1989	0						
Sudan	1990-1994	0						
Sudan	1995-1999	0						
Sudan	2000-2004	0						
Sudan	2005-2009	0						
Sudan	2010-2019	0						
Suriname	1980-1984	3	58.1	1984	Vital Registration	90.0	35.4	
Suriname	1985-1989	3	62.9	1989	Vital Registration	94.1	33.1	
Suriname	1990-1994	3	57.7	1990	Vital Registration	82.1	29.8	
Suriname	1995-1999	3	60.1	1999	Vital Registration	86.9	30.8	
Suriname	2000-2004	4	68.4	2002	Vital Registration	89.4	23.4	
Suriname	2005-2009	4	67.0	2008	Vital Registration	87.2	23.1	
Suriname	2010-2019	4	67.1	2010	Vital Registration	85.0	21.0	
Sweden	1980-1984	5	88.4	1980	Vital Registration	100.0	11.6	
Sweden	1985-1989	5	90.5	1987	Vital Registration	99.9	9.4	
Sweden	1990-1994	5	90.1	1990	Vital Registration	100.0	9.9	
Sweden	1995-1999	5	89.7	1997	Vital Registration	100.0	10.3	
Sweden	2000-2004	5	88.9	2003	Vital Registration	100.0	11.1	
Sweden	2005-2009	5	88.1	2005	Vital Registration	99.3	11.3	
Sweden	2010-2019	5	86.0	2012	Vital Registration	100.0	14.0	
Switzerland	1980-1984	4	76.2	1983	Vital Registration	100.0	23.8	
Switzerland	1985-1989	4	76.0	1986	Vital Registration	100.0	24.0	
Switzerland	1990-1994	4	75.3	1990	Vital Registration	100.0	24.7	
Switzerland	1995-1999	5	87.3	1997	Vital Registration	100.0	12.7	
Switzerland	2000-2004	5	86.4	2000	Vital Registration	99.9	13.5	
Switzerland	2005-2009	5	87.4	2009	Vital Registration	99.9	12.5	
Switzerland	2010-2019	5	87.5	2012	Vital Registration	100.0	12.5	
Syria	1980-1984	2	29.5	1980	Vital Registration	100.0	70.5	
Syria	1985-1989	2	18.0	1985	Vital Registration	81.1	77.8	
Syria	1990-1994	0						
Syria	1995-1999	3	51.0	1999	Vital Registration	96.0	46.9	
Syria	2000-2004	3	54.8	2004	Vital Registration	82.9	33.9	
Syria	2005-2009	3	64.3	2009	Vital Registration	86.7	25.8	
Syria	2010-2019	3	52.5	2010	Vital Registration	90.3	41.8	
São Tomé and PrÌncipe	1980-1984	0						
São Tomé and PrÌncipe	1985-1989	3	60.1	1985	Vital Registration	100.0	39.9	
São Tomé and PrÌncipe	1990-1994	0						
São Tomé and PrÌncipe	1995-1999	0						
São Tomé and PrÌncipe	2000-2004	0						
São Tomé and PrÌncipe	2005-2009	0						
São Tomé and PrÌncipe	2010-2019	0						
Taiwan (province of China)	1980-1984	4	75.7	1983	Vital Registration	94.9	20.3	
Taiwan (province of China)	1985-1989	4	79.4	1988	Vital Registration	99.9	20.5	
Taiwan (province of China)	1990-1994	4	78.5	1994	Vital Registration	100.0	21.5	
Taiwan (province of China)	1995-1999	4	83.5	1999	Vital Registration	100.0	16.5	
Taiwan (province of China)	2000-2004	4	84.9	2004	Vital Registration	100.0	15.1	
Taiwan (province of China)	2005-2009	5	85.2	2005	Vital Registration	100.0	14.8	
Taiwan (province of China)	2010-2019	5	85.6	2014	Vital Registration	100.0	14.4	
Tajikistan	1980-1984	4	67.4	1981	Vital Registration	80.7	16.5	
Tajikistan	1985-1989	4	65.8	1988	Vital Registration	83.7	21.4	
Tajikistan	1990-1994	4	66.5	1993	Vital Registration	100.0	33.5	
Tajikistan	1995-1999	3	51.8	1995	Vital Registration	75.5	31.3	
Tajikistan	2000-2004	3	48.3	2002	Vital Registration	70.4	31.5	
Tajikistan	2005-2009	3	48.4	2005	Vital Registration	73.7	34.4	
Tajikistan	2010-2019	3	50.5	2016	Vital Registration	73.4	31.2	
Tanzania	1980-1984	0						
Tanzania	1985-1989	1	3.8	1986	Risk factors for deaths in children under 5 years old in Bagamoyo district, Tanzania		2.9	3.9
Tanzania	1990-1994	1	3.3	1993	Community based studies on childhood mortality in a malaria holoendemic area on the Tanzanian coast		7.3	3.6
Tanzania	1995-1999	1	2.3	1995	The Policy Implications of Tanzania's Mortality Burden		64.2	6.4
Tanzania	2000-2004	1	6.3	2000	Tanzania - Rufiji Health and Demographic Surveillance System		0.9	6.4
Tanzania	2005-2009	1	3.1	2005	The contribution of reduction in malaria as a cause of rapid decline of under-five mortality: evidence from the Rufiji Health and Demographic Surveillance System (HDSS) in rural Tanzania		0.9	3.1
Tanzania	2010-2019	0						
Thailand	1980-1984	2	24.6	1983	Vital Registration	87.4	71.8	
Thailand	1985-1989	2	25.0	1987	Vital Registration	83.3	70.0	
Thailand	1990-1994	2	31.3	1994	Vital Registration	88.7	64.7	
Thailand	1995-1999	3	62.6	1998	Thailand Burden of Disease and Injuries 1998-1999		2.1	64.0
Thailand	2000-2004	3	48.3	2003	Vital Registration	100.0	51.7	
Thailand	2005-2009	3	61.6	2005	Thailand Verbal Autopsy Study 2005		3.7	64.0

Table S9. Underlying indicators for percent well-certified for data source with maximum percent well certified in each 5-year time interval for 204 countries, 1980-2019.

Location	Time Window	Stars	Percent Well-Certified [PWC] (%)	Max PWC Data Year	Max PWC Data Source	Completeness (%)	Percent Major Garbage (%)	Verbal Autopsy Adjustment (None for VR) (%)
Thailand	2010-2019	3	64.6	2016	Vital Registration	100.0	35.4	
The Bahamas	1980-1984	4	74.3	1980	Vital Registration	100.0	25.7	
The Bahamas	1985-1989	4	82.3	1987	Vital Registration	100.0	17.7	
The Bahamas	1990-1994	4	80.1	1994	Vital Registration	97.5	17.8	
The Bahamas	1995-1999	4	84.1	1998	Vital Registration	100.0	15.9	
The Bahamas	2000-2004	4	83.4	2001	Vital Registration	95.1	12.4	
The Bahamas	2005-2009	4	82.3	2009	Vital Registration	94.7	13.1	
The Bahamas	2010-2019	4	82.5	2011	Vital Registration	93.7	12.0	
The Gambia	1980-1984	1	3.9	1982	Deaths in infancy and early childhood in a well-vaccinated, rural, West African population		1.9	4.0
The Gambia	1985-1989	1	3.5	1989	Changes in the pattern of infant and childhood mortality in upper river division. The Gambia. from 1989 to 1993		4.5	3.6
The Gambia	1990-1994	1	3.4	1990	Changes in the pattern of infant and childhood mortality in upper river division. The Gambia. from 1989 to 1993		5.6	3.6
The Gambia	1995-1999	1	2.8	1999	Reaching millennium development goal 4 - the Gambia		8.3	3.0
The Gambia	2000-2004	1	2.5	2002	Reaching millennium development goal 4 - the Gambia		8.9	2.7
The Gambia	2005-2009	1	2.2	2006	Preventive measures in infancy to reduce under-five mortality: a case-control study in The Gambia		7.1	2.4
The Gambia	2010-2019	0						
Timor-Leste	1980-1984	0						
Timor-Leste	1985-1989	0						
Timor-Leste	1990-1994	0						
Timor-Leste	1995-1999	0						
Timor-Leste	2000-2004	0						
Timor-Leste	2005-2009	0						
Timor-Leste	2010-2019	0						
Togo	1980-1984	0						
Togo	1985-1989	0						
Togo	1990-1994	0						
Togo	1995-1999	0						
Togo	2000-2004	0						
Togo	2005-2009	0						
Togo	2010-2019	0						
Tokelau	1980-1984	0						
Tokelau	1985-1989	0						
Tokelau	1990-1994	0						
Tokelau	1995-1999	0						
Tokelau	2000-2004	0						
Tokelau	2005-2009	0						
Tokelau	2010-2019	0						
Tonga	1980-1984	0						
Tonga	1985-1989	0						
Tonga	1990-1994	0						
Tonga	1995-1999	0						
Tonga	2000-2004	3	52.9	2003	Vital Registration	100.0	47.1	
Tonga	2005-2009	0						
Tonga	2010-2019	0						
Trinidad and Tobago	1980-1984	4	78.9	1983	Vital Registration	95.1	17.0	
Trinidad and Tobago	1985-1989	4	83.1	1987	Vital Registration	98.3	15.5	
Trinidad and Tobago	1990-1994	5	85.9	1994	Vital Registration	100.0	14.1	
Trinidad and Tobago	1995-1999	5	89.4	1999	Vital Registration	100.0	10.6	
Trinidad and Tobago	2000-2004	5	90.4	2002	Vital Registration	100.0	9.6	
Trinidad and Tobago	2005-2009	5	88.8	2005	Vital Registration	100.0	11.2	
Trinidad and Tobago	2010-2019	5	89.8	2012	Vital Registration	100.0	10.2	
Tunisia	1980-1984	0						
Tunisia	1985-1989	0						
Tunisia	1990-1994	0						
Tunisia	1995-1999	0						
Tunisia	2000-2004	0						
Tunisia	2005-2009	2	28.3	2009	Vital Registration	38.0	25.7	
Tunisia	2010-2019	2	24.0	2013	Vital Registration	36.9	35.1	
Turkey	1980-1984	2	12.2	1983	Vital Registration	33.1	63.0	
Turkey	1985-1989	2	15.8	1989	Vital Registration	38.6	59.0	
Turkey	1990-1994	2	17.4	1993	Vital Registration	40.7	57.3	
Turkey	1995-1999	2	20.8	1999	Vital Registration	50.4	58.7	
Turkey	2000-2004	3	59.2	2002	Turkey Verbal Autopsy Survey 2003		7.5	64.0
Turkey	2005-2009	3	63.7	2009	Vital Registration	80.5	20.9	
Turkey	2010-2019	4	79.5	2016	Vital Registration	95.4	16.7	
Turkmenistan	1980-1984	5	86.9	1981	Vital Registration	98.2	11.5	
Turkmenistan	1985-1989	5	88.0	1986	Vital Registration	100.0	12.0	
Turkmenistan	1990-1994	5	86.2	1993	Vital Registration	100.0	13.8	
Turkmenistan	1995-1999	4	79.7	1995	Vital Registration	100.0	20.3	
Turkmenistan	2000-2004	5	85.9	2001	Vital Registration	95.4	10.0	
Turkmenistan	2005-2009	4	79.9	2009	Vital Registration	95.5	16.3	
Turkmenistan	2010-2019	4	82.0	2010	Vital Registration	98.5	16.8	
Tuvalu	1980-1984	0						
Tuvalu	1985-1989	0						
Tuvalu	1990-1994	0						
Tuvalu	1995-1999	0						
Tuvalu	2000-2004	0						
Tuvalu	2005-2009	0						
Tuvalu	2010-2019	0						
UK	1980-1984	5	93.7	1984	Vital Registration	100.0	6.3	
UK	1985-1989	5	93.7	1985	Vital Registration	100.0	6.3	
UK	1990-1994	5	93.6	1991	Vital Registration	100.0	6.4	
UK	1995-1999	5	92.1	1995	Vital Registration	100.0	7.9	
UK	2000-2004	5	91.6	2002	Vital Registration	100.0	8.4	
UK	2005-2009	5	91.5	2006	Vital Registration	100.0	8.5	
UK	2010-2019	5	91.6	2012	Vital Registration	100.0	8.4	
USA	1980-1984	5	90.4	1980	Vital Registration	100.0	9.6	
USA	1985-1989	5	89.6	1989	Vital Registration	100.0	10.4	
USA	1990-1994	5	90.1	1990	Vital Registration	100.0	9.9	
USA	1995-1999	5	89.5	1995	Vital Registration	100.0	10.5	
USA	2000-2004	5	88.6	2000	Vital Registration	100.0	11.4	
USA	2005-2009	5	87.8	2005	Vital Registration	100.0	12.2	
USA	2010-2019	5	87.2	2010	Vital Registration	99.9	12.8	
Uganda	1980-1984	0						
Uganda	1985-1989	0						
Uganda	1990-1994	0						
Uganda	1995-1999	0						
Uganda	2000-2004	1	0.4	2000	Effect of HIV infection on pregnancy-related mortality in sub-Saharan Africa: secondary analyses of pooled community-based data from the network for Analysing Longitudinal Population-based HIV/AIDS data on Africa (ALPHA)		12.7	0.4
Uganda	2005-2009	1	3.3	2006	Uganda Child Verbal Autopsy Study 2007		1.3	3.4
Uganda	2010-2019	0						
Ukraine	1980-1984	5	87.9	1982	Vital Registration	100.0	12.1	
Ukraine	1985-1989	5	88.5	1985	Vital Registration	100.0	11.5	
Ukraine	1990-1994	4	81.7	1994	Vital Registration	100.0	18.3	
Ukraine	1995-1999	4	83.5	1999	Vital Registration	100.0	16.5	
Ukraine	2000-2004	4	84.1	2000	Vital Registration	100.0	15.9	
Ukraine	2005-2009	5	89.8	2009	Vital Registration	100.0	10.2	
Ukraine	2010-2019	5	91.2	2012	Vital Registration	100.0	8.8	
United Arab Emirates	1980-1984	0						
United Arab Emirates	1985-1989	0						
United Arab Emirates	1990-1994	0						
United Arab Emirates	1995-1999	0						
United Arab Emirates	2000-2004	0						
United Arab Emirates	2005-2009	3	35.8	2007	Vital Registration	66.6	46.2	
United Arab Emirates	2010-2019	0						
Uruguay	1980-1984	4	77.6	1982	Vital Registration	100.0	22.4	
Uruguay	1985-1989	4	77.4	1989	Vital Registration	99.4	22.1	
Uruguay	1990-1994	4	79.5	1991	Vital Registration	100.0	20.5	
Uruguay	1995-1999	4	81.2	1997	Vital Registration	99.4	18.4	
Uruguay	2000-2004	4	80.8	2001	Vital Registration	100.0	19.2	
Uruguay	2005-2009	4	80.6	2005	Vital Registration	100.0	19.4	
Uruguay	2010-2019	4	80.4	2015	Vital Registration	100.0	19.6	
Uzbekistan	1980-1984	4	81.8	1982	Vital Registration	94.6	13.6	
Uzbekistan	1985-1989	5	86.4	1986	Vital Registration	100.0	13.6	
Uzbekistan	1990-1994	4	77.7	1992	Vital Registration	90.8	14.4	
Uzbekistan	1995-1999	4	69.9	1995	Vital Registration	83.2	16.0	
Uzbekistan	2000-2004	3	63.5	2004	Vital Registration	72.6	12.6	
Uzbekistan	2005-2009	4	69.7	2009	Vital Registration	77.1	9.6	
Uzbekistan	2010-2019	4	73.0	2011	Vital Registration	81.7	10.6	
Vanuatu	1980-1984	0						
Vanuatu	1985-1989	0						
Vanuatu	1990-1994	0						
Vanuatu	1995-1999	0						
Vanuatu	2000-2004	0						
Vanuatu	2005-2009	0						
Vanuatu	2010-2019	0						
Venezuela	1980-1984	4	78.9	1983	Vital Registration	100.0	21.1	
Venezuela	1985-1989	4	72.9	1988	Vital Registration	99.3	26.5	
Venezuela	1990-1994	4	83.2	1994	Vital Registration	100.0	16.8	
Venezuela	1995-1999	5	88.5	1999	Vital Registration	100.0	11.5	
Venezuela	2000-2004	5	90.7	2001	Vital Registration	100.0	9.3	
Venezuela	2005-2009	5	91.1	2007	Vital Registration	100.0	8.9	
Venezuela	2010-2019	5	91.3	2013	Vital Registration	99.8	8.5	
Vietnam	1980-1984	0						
Vietnam	1985-1989	1	1.3	1987	Are there social inequities in child morbidity and mortality in rural Vietnam		5.2	1.4
Vietnam	1990-1994	1	0.9	1994	Maternal mortality in Vietnam in 1994-95		10.1	1.0
Vietnam	1995-1999	1	1.2	1999	Applying verbal autopsy to determine cause of death in rural Vietnam		80.9	6.4
Vietnam	2000-2004	1	1.5	2001	Socio-economic status inequality and major causes of death in adults: a 5-year follow-up study in rural Vietnam		73.1	5.6
Vietnam	2005-2009	3	62.0	2008	Mortality measures from sample-based surveillance: evidence of the epidemiological transition in Viet Nam, Unpublished data		3.1	64.0
Vietnam	2010-2019	1	4.8	2010	The causes of deaths in Chililab between 2008-2010 based on verbal autopsy method		24.3	6.4
Virgin Islands	1980-1984	4	76.3	1980	Vital Registration	93.9	18.7	
Virgin Islands	1985-1989	0						
Virgin Islands	1990-1994	4	79.3	1994	Vital Registration	87.7	9.6	
Virgin Islands	1995-1999	4	82.3	1995	Vital Registration	92.5	10.9	
Virgin Islands	2000-2004	4	71.3	2000	Vital Registration	81.2	12.1	
Virgin Islands	2005-2009	4	67.9	2007	Vital Registration	76.2	10.9	
Virgin Islands	2010-2019	3	61.2	2010	Vital Registration	71.6	14.5	
Yemen	1980-1984	0						
Yemen	1985-1989	0						
Yemen	1990-1994	0						
Yemen	1995-1999	0						
Yemen	2000-2004	0						
Yemen	2005-2009	0						
Yemen	2010-2019	0						
Zambia	1980-1984	0						
Zambia	1985-1989	0						
Zambia	1990-1994	0						
Zambia	1995-1999	0						
Zambia	2000-2004	0						
Zambia	2005-2009	1	6.4	2009	Zambia Sample Vital Registration with Verbal Autopsy (SAVVY) Data 2010		0.7	6.4
Zambia	2010-2019	2	15.7	2011	Adult Mortality in Sub-saharan Africa, Zambia: Where Do Adults Die?		11.7	17.8
Zimbabwe	1980-1984	0						
Zimbabwe	1985-1989	0						
Zimbabwe	1990-1994	3	38.9	1990	Vital Registration	60.3	35.5	
Zimbabwe	1995-1999	3	58.7	1995	Vital Registration	100.0	41.3	
Zimbabwe	2000-2004	1	0.4	2000	Effect of HIV infection on pregnancy-related mortality in sub-Saharan Africa: secondary analyses of pooled community-based data from the network for Analysing Longitudinal Population-based HIV/AIDS data on Africa (ALPHA)		12.8	0.5
Zimbabwe	2005-2009	3	44.3	2007	Vital Registration	59.4	25.3	

Table S9. Underlying indicators for percent well-certified for data source with maximum percent well certified in each 5-year time interval for 204 countries, 1980-2019.

Location	Time Window	Stars	Percent Well-Certified [PWC] (%)	Max PWC Data Year	Max PWC Data Source	Completeness (%)	Percent Major Garbage (%)	Verbal Autopsy Adjustment (None for VR) (%)
Zimbabwe	2010-2019	0						
eSwatini	1980-1984	0						
eSwatini	1985-1989	0						
eSwatini	1990-1994	0						
eSwatini	1995-1999	0						
eSwatini	2000-2004	1	0.5	2000	Effect of HIV infection on pregnancy-related mortality in sub-Saharan Africa: secondary analyses of pooled community-based data from the network for Analysing Longitudinal Population-based HIV/AIDS data on Africa (ALPHA)		12.6	0.6
eSwatini	2005-2009	0						
eSwatini	2010-2019	0						

Table S10. CodCorrect cause hierarchy with levels	
Cause Name	CodCorrect Level
All causes	0
Sexually transmitted infections excluding HIV	1
Syphilis	2
Chlamydial infection	2
Gonococcal infection	2
Other sexually transmitted infections	2
Tuberculosis	1
Drug-susceptible tuberculosis	2
Multidrug-resistant tuberculosis without extensive drug resistance	2
Extensively drug-resistant tuberculosis	2
Lower respiratory infections	1
Upper respiratory infections	1
Otitis media	1
Diarrhoeal diseases	1
Typhoid fever	1
Paratyphoid fever	1
Invasive non-typhoidal Salmonella (iNTS)	1
Other intestinal infectious diseases	1
Malaria	1
Chagas disease	1
Visceral leishmaniasis	1
African trypanosomiasis	1
Schistosomiasis	1
Cysticercosis	1
Cystic echinococcosis	1
Dengue	1
Yellow fever	1
Rabies	1
Ascariasis	1
Zika virus disease	1
Other neglected tropical diseases	1
Meningitis	1
Encephalitis	1
Diphtheria	1
Whooping cough	1
Tetanus	1
Measles	1
Varicella and herpes zoster	1
Acute hepatitis	1
Acute hepatitis A	2
Acute hepatitis B	2
Acute hepatitis C	2
Acute hepatitis E	2
Other unspecified infectious diseases	1
Maternal disorders	1
Maternal haemorrhage	2

Maternal sepsis and other maternal infections	2
Maternal hypertensive disorders	2
Maternal obstructed labor and uterine rupture	2
Maternal abortion and miscarriage	2
Ectopic pregnancy	2
Indirect maternal deaths	2
Late maternal deaths	2
Maternal deaths aggravated by HIV/AIDS	2
Other maternal disorders	2
Neonatal disorders	1
Neonatal preterm birth	2
Neonatal encephalopathy due to birth asphyxia and trauma	2
Neonatal sepsis and other neonatal infections	2
Hemolytic disease and other neonatal jaundice	2
Other neonatal disorders	2
Nutritional deficiencies	1
Protein-energy malnutrition	2
Other nutritional deficiencies	2
Lip and oral cavity cancer	1
Nasopharynx cancer	1
Other pharynx cancer	1
Oesophageal cancer	1
Stomach cancer	1
Colon and rectum cancer	1
Liver cancer due to hepatitis B	1
Liver cancer due to hepatitis C	1
Liver cancer due to alcohol use	1
Liver cancer due to NASH	1
Hepatoblastoma	1
Liver cancer due to other causes (internal)	1
Gallbladder and biliary tract cancer	1
Pancreatic cancer	1
Larynx cancer	1
Tracheal, bronchus, and lung cancer	1
Malignant skin melanoma	1
Non-melanoma skin cancer (squamous-cell carcinoma)	1
Soft tissue and other extraosseous sarcomas	1
Malignant neoplasm of bone and articular cartilage	1
Breast cancer	1
Cervical cancer	1
Uterine cancer	1
Ovarian cancer	1
Prostate cancer	1
Testicular cancer	1
Kidney cancer	1
Bladder cancer	1
Brain and central nervous system cancer	1
Eye cancer	1

Retinoblastoma	2
Other eye cancers	2
Neuroblastoma and other peripheral nervous cell tumors	1
Thyroid cancer	1
Mesothelioma	1
Hodgkin lymphoma	1
Non-Hodgkin lymphoma	1
Burkitt lymphoma	2
Other non-Hodgkin lymphoma	2
Multiple myeloma	1
Leukaemia	1
Acute lymphoid leukeamia	2
Chronic lymphoid leukaemia	2
Acute myeloid leukaemia	2
Chronic myeloid leukaemia	2
Other leukaemia	2
Other malignant neoplasms (internal)	1
Myelodysplastic, myeloproliferative, and other haematopoietic neoplasms	1
Cardiovascular diseases	1
Rheumatic heart disease	2
Ischaemic heart disease	2
Stroke	2
Ischaemic stroke	3
Intracerebral haemorrhage	3
Subarachnoid haemorrhage	3
Hypertensive heart disease	2
Non-rheumatic valvular heart disease	2
Non-rheumatic calcific aortic valvular heart disease	3
Non-rheumatic degenerative mitral valvular heart disease	3
Other non-rheumatic valvular heart diseases	3
Cardiomyopathy and myocarditis	2
Myocarditis	3
Alcoholic cardiomyopathy	3
Other cardiomyopathy	3
Pulmonary Arterial Hypertension	2
Atrial fibrillation and flutter	2
Aortic aneurysm	2
Peripheral artery disease	2
Endocarditis	2
Other cardiovascular and circulatory diseases (internal)	2
Chronic respiratory diseases	1
Chronic obstructive pulmonary disease	2
Pneumoconiosis	2
Silicosis	3
Asbestosis	3
Coal workers pneumoconiosis	3
Other pneumoconiosis	3
Asthma	2

Interstitial lung disease and pulmonary sarcoidosis	2
Other chronic respiratory diseases	2
Digestive diseases	1
Cirrhosis and other chronic liver diseases	2
Cirrhosis and other chronic liver diseases due to hepatitis B	3
Cirrhosis and other chronic liver diseases due to hepatitis C	3
Cirrhosis and other chronic liver diseases due to alcohol use	3
Cirrhosis and other chronic liver diseases due to NAFLD	3
Cirrhosis and other chronic liver diseases due to other causes	3
Upper digestive system diseases	2
Peptic ulcer disease	3
Gastritis and duodenitis	3
Appendicitis	2
Paralytic ileus and intestinal obstruction	2
Inguinal, femoral, and abdominal hernia	2
Inflammatory bowel disease	2
Vascular intestinal disorders	2
Gallbladder and biliary diseases	2
Pancreatitis	2
Other digestive diseases	2
Alzheimer's disease and other dementias	1
Parkinson's disease	1
Idiopathic epilepsy	1
Multiple sclerosis	1
Motor neuron disease	1
Other neurological disorders	1
Eating disorders	1
Anorexia nervosa	2
Bulimia nervosa	2
Alcohol use disorders	1
Drug use disorders	1
Opioid use disorders	2
Cocaine use disorders	2
Amphetamine use disorders	2
Other drug use disorders	2
Diabetes mellitus	1
Diabetes mellitus type 1	2
Diabetes mellitus type 2	2
Chronic kidney disease	1
Chronic kidney disease due to diabetes mellitus type 1	2
Chronic kidney disease due to diabetes mellitus type 2	2
Chronic kidney disease due to hypertension	2
Chronic kidney disease due to glomerulonephritis	2
Chronic kidney disease due to other and unspecified causes	2
Acute glomerulonephritis	1
Skin and subcutaneous diseases	1
Bacterial skin diseases	2
Cellulitis	3

Pyoderma	3
Decubitus ulcer	2
Other skin and subcutaneous diseases	2
Musculoskeletal disorders	1
Rheumatoid arthritis	2
Other musculoskeletal disorders	2
Congenital birth defects	1
Neural tube defects	2
Congenital heart anomalies	2
Orofacial clefts	2
Down syndrome	2
Other chromosomal abnormalities	2
Congenital musculoskeletal and limb anomalies	2
Urogenital congenital anomalies	2
Digestive congenital anomalies	2
Other congenital birth defects	2
Urinary diseases and male infertility	1
Urinary tract infections and interstitial nephritis	2
Urolithiasis	2
Other urinary diseases	2
Gynaecological diseases	1
Uterine fibroids	2
Endometriosis	2
Genital prolapse	2
Other gynaecological diseases	2
Haemoglobinopathies and haemolytic anaemias	1
Thalassemias	2
Sickle cell disorders	2
G6PD deficiency	2
Other haemoglobinopathies and haemolytic anaemias	2
Endocrine, metabolic, blood, and immune disorders	1
Sudden infant death syndrome	1
Transport injuries	1
Road injuries	2
Pedestrian road injuries	3
Cyclist road injuries	3
Motorcyclist road injuries	3
Motor vehicle road injuries	3
Other road injuries	3
Other transport injuries	2
Falls	1
Drowning	1
Fire, heat, and hot substances	1
Poisonings	1
Poisoning by carbon monoxide	2
Poisoning by other means	2
Exposure to mechanical forces	1
Unintentional firearm injuries	2

Other exposure to mechanical forces	2
Adverse effects of medical treatment	1
Animal contact	1
Venomous animal contact	2
Non-venomous animal contact	2
Foreign body	1
Pulmonary aspiration and foreign body in airway	2
Foreign body in other body part	2
Environmental heat and cold exposure	1
Other unintentional injuries	1
Self-harm	1
Self-harm by firearm	2
Self-harm by other specified means	2
Interpersonal violence	1
Physical violence by firearm	2
Physical violence by sharp object	2
Physical violence by other means	2
Police conflict and executions	1

Table S11. Modelling strategy for individual cause of death models in GBD 2019		
**Cause Name**	**Level**	**Model type**
Communicable, maternal, neonatal, and nutritional diseases	Aggregate	
HIV/AIDS and sexually transmitted infections	Aggregate	
HIV/AIDS	3	EPP-ASM, Spectrum
HIV-AIDS–drug-susceptible tuberculosis	4	Data proportion
HIV/AIDS–multidrug-resistant tuberculosis without extensive drug resistance	4	Data proportion
HIV/AIDS–extensively drug-resistant tuberculosis	4	Data proportion
HIV/AIDS resulting in other diseases	4	Data proportion
Sexually transmitted infections excluding HIV	3	CODEm; natural history model (congenital syphilis)
Syphilis	4	Data proportion (age/sex-specific VR); natural history model (congenital syphilis)
Chlamydial infection	4	Data proportion (age/sex-specific VR)
Gonococcal infection	4	Data proportion (age/sex-specific VR)
Other sexually transmitted diseases	4	Data proportion (age/sex-specific VR)
Respiratory infections and tuberculosis	Aggregate	
Tuberculosis	3	CODEm
Drug-susceptible tuberculosis	4	Spatio-temporal Gaussian process regression proportion
Multidrug-resistant tuberculosis without extensive drug resistance	4	Spatio-temporal Gaussian process regression proportion
Extensively drug-resistant tuberculosis	4	Spatio-temporal Gaussian process regression proportion
Lower respiratory infections	3	CODEm
Upper respiratory infections	3	CODEm
Otitis media	3	CODEm
Enteric infections	Aggregate	
Diarrhoeal diseases	3	CODEm; Fatal Discontinuity
Typhoid and paratyphoid	Aggregate	
Typhoid fever	4	CODEm (data rich countries); natural history model (non-data rich countries)
Paratyphoid fever	4	CODEm (data rich countries); natural history model (non-data rich countries)
Invasive non-typhoidal Salmonella (iNTS)	3	CODEm (data rich countries); natural history model (non-data rich countries)
Other intestinal infectious diseases	3	Negative binomial regression
Neglected tropical diseases and malaria	Aggregate	
Malaria	3	CODEm (P. falciparum outside of Africa); natural history model (P. falciparum within Africa); negative binomial regression (P. vivax), Fatal Discontinuity
Chagas disease	3	CODEm

Table S11. Modeling strategy for individual cause of death models in GBD 2019		
**Cause Name**	**Level**	**Model type**
Leishmaniasis	Aggregate	
Visceral leishmaniasis	4	Natural history model
African trypanosomiasis	3	Natural history model
Schistosomiasis	3	Negative binomial regression
Cysticercosis	3	Negative binomial regression
Cystic echinococcosis	3	Negative binomial regression
Dengue	3	CODEm, Fatal Discontinuity
Yellow fever	3	Natural history model
Rabies	3	CODEm
Intestinal nematode infections	Aggregate	
Ascariasis	4	Negative binomial regression
Ebola virus disease	3	Fatal Discontinuity
Zika virus disease	3	Natural history model
Other neglected tropical diseases	3	CODEm
Other infectious diseases	Aggregate	
Meningitis	3	CODEm, Fatal Discontinuity
Encephalitis	3	CODEm
Diphtheria	3	CODEm (data rich countries); negative binomial regression (non-data rich countries)
Whooping cough	3	CODEm (data rich countries); natural history model (non-data rich countries)
Tetanus	3	CODEm
Measles	3	CODEm (data rich countries); natural history model (non-data rich countries), Fatal Discontinuity
Varicella and herpes zoster	3	CODEm (data rich countries); negative binomial regression (non-data rich countries)
Acute hepatitis	3	CODEm
Acute hepatitis A	4	CODEm
Acute hepatitis B	4	CODEm
Acute hepatitis C	4	CODEm
Acute hepatitis E	4	CODEm
Other unspecified infectious diseases	3	CODEm, Fatal Discontinuity
Maternal and neonatal disorders	Aggregate	
Maternal disorders	3	CODEm
Maternal haemorrhage	4	Spatio-temporal Gaussian process regression
Maternal sepsis and other maternal infections	4	Spatio-temporal Gaussian process regression
Maternal hypertensive disorders	4	Spatio-temporal Gaussian process regression
Maternal obstructed labor and uterine rupture	4	Spatio-temporal Gaussian process regression
Maternal abortion and miscarriage	4	Spatio-temporal Gaussian process regression
Ectopic pregnancy	4	Spatio-temporal Gaussian process regression proportion

Table S11. Modeling strategy for individual cause of death models in GBD 2019		
**Cause Name**	**Level**	**Model type**
Indirect maternal deaths	4	Spatio-temporal Gaussian process regression
Late maternal deaths	4	DisMod MR-2.1 proportion model
Maternal deaths aggravated by HIV/AIDS	4	Spatio-temporal Gaussian process regression
Other maternal disorders	4	Spatio-temporal Gaussian process regression
Neonatal disorders	3	CODEm
Neonatal preterm birth	4	CODEm
Neonatal encephalopathy due to birth asphyxia and trauma	4	CODEm
Neonatal sepsis and other neonatal infections	4	CODEm
Haemolytic disease and other neonatal jaundice	4	CODEm
Other neonatal disorders	4	CODEm
Nutritional deficiencies	2	CODEm
Protein-energy malnutrition	3	CODEm; Fatal Discontinuity
Other nutritional deficiencies	3	CODEm
Non-communicable diseases	Aggregate	
Neoplasms	Aggregate	
Lip and oral cavity cancer	3	CODEm
Nasopharynx cancer	3	CODEm
Other pharynx cancer	3	CODEm
Oesophageal cancer	3	CODEm
Stomach cancer	3	CODEm
Colon and rectum cancer	3	CODEm
Liver cancer	3	CODEm
Liver cancer due to hepatitis B	4	DisMod MR-2.1 proportion model
Liver cancer due to hepatitis C	4	DisMod MR-2.1 proportion model
Liver cancer due to alcohol use	4	DisMod MR-2.1 proportion model
Liver cancer due to NASH	4	DisMod MR-2.1 proportion model
Liver cancer due to other causes	4	DisMod MR-2.1 proportion model
Gallbladder and biliary tract cancer	3	CODEm
Pancreatic cancer	3	CODEm
Larynx cancer	3	CODEm
Tracheal, bronchus, and lung cancer	3	CODEm
Malignant skin melanoma	3	CODEm
Non-melanoma skin cancer	Aggregate	
Non-melanoma skin cancer (squamous-cell carcinoma)	4	CODEm
Breast cancer	3	CODEm
Cervical cancer	3	CODEm
Uterine cancer	3	CODEm
Ovarian cancer	3	CODEm
Prostate cancer	3	CODEm
Testicular cancer	3	CODEm
Kidney cancer	3	CODEm

Table S11. Modeling strategy for individual cause of death models in GBD 2019		
**Cause Name**	**Level**	**Model type**
Bladder cancer	3	CODEm
Brain and nervous system cancer	3	CODEm
Thyroid cancer	3	CODEm
Mesothelioma	3	CODEm
Hodgkin lymphoma	3	CODEm
Non-Hodgkin lymphoma	3	CODEm
Multiple myeloma	3	CODEm
Leukaemia	3	CODEm
Acute lymphoid leukaemia	4	CODEm
Chronic lymphoid leukaemia	4	CODEm
Acute myeloid leukaemia	4	CODEm
Chronic myeloid leukaemia	4	CODEm
Other leukaemia	4	CODEm
Other malignant neoplasms	3	CODEm
Other neoplasms	Aggregate	
Myelodysplastic, myeloproliferative, and other haematopoietic neoplasms	4	CODEm
Other benign and in situ neoplasms	4	CODEm
Cardiovascular diseases	2	CODEm
Rheumatic heart disease	3	CODEm
Ischaemic heart disease	3	CODEm
Stroke	3	CODEm
Ischemic stroke	4	CODEm
Intracerebral haemorrhage	4	CODEm
Subarachnoid haemorrhage	4	CODEm
Hypertensive heart disease	3	CODEm
Non-rheumatic valvular heart disease	3	CODEm
Non-rheumatic calcific aortic valvular heart disease	4	CODEm
Non-rheumatic degenerative mitral valvular heart disease	4	CODEm
Other non-rheumatic valvular heart diseases	4	CODEm
Cardiomyopathy and myocarditis	3	CODEm
Myocarditis	4	CODEm
Alcoholic cardiomyopathy	4	CODEm
Other cardiomyopathy	4	CODEm
Atrial fibrillation and flutter	3	CODEm
Aortic aneurysm	3	CODEm
Peripheral artery disease	3	CODEm
Endocarditis	3	CODEm
Other cardiovascular and circulatory diseases	3	CODEm
Chronic respiratory diseases	2	CODEm
Chronic obstructive pulmonary disease	3	CODEm
Pneumoconiosis	3	CODEm
Silicosis	4	CODEm
Asbestosis	4	CODEm

Table S11. Modeling strategy for individual cause of death models in GBD 2019		
**Cause Name**	**Level**	**Model type**
Coal workers pneumoconiosis	4	CODEm
Other pneumoconiosis	4	CODEm
Asthma	3	CODEm
Interstitial lung disease and pulmonary sarcoidosis	3	CODEm
Other chronic respiratory diseases	3	CODEm
Digestive diseases	2	CODEm
Cirrhosis and other chronic liver diseases	3	CODEm
Cirrhosis and other chronic liver diseases due to hepatitis B	4	DisMod MR-2.1 proportion model
Cirrhosis and other chronic liver diseases due to hepatitis C	4	DisMod MR-2.1 proportion model
Cirrhosis and other chronic liver diseases due to alcohol use	4	DisMod MR-2.1 proportion model
Cirrhosis due to NASH	4	DisMod MR-2.1 proportion model
Cirrhosis and other chronic liver diseases due to other causes	4	DisMod MR-2.1 proportion model
Upper digestive system diseases	3	CODEm
Peptic ulcer disease	4	CODEm
Gastritis and duodenitis	4	CODEm
Appendicitis	3	CODEm
Paralytic ileus and intestinal obstruction	3	CODEm
Inguinal, femoral, and abdominal hernia	3	CODEm
Inflammatory bowel disease	3	CODEm
Vascular intestinal disorders	3	CODEm
Gallbladder and biliary diseases	3	CODEm
Pancreatitis	3	CODEm
Other digestive diseases	3	CODEm
Neurological disorders	Aggregate	
Alzheimer's disease and other dementias	3	DisMod MR-2.1; custom excess mortality analysis
Parkinson's disease	3	CODEm
Idiopathic epilepsy	3	CODEm
Multiple sclerosis	3	CODEm
Motor neuron disease	3	CODEm
Other neurological disorders	3	CODEm
Mental disorders	Aggregate	
Eating disorders	3	CODEm
Anorexia nervosa	4	CODEm
Bulimia nervosa	4	CODEm
Substance use disorders	Aggregate	
Alcohol use disorders	3	CODEm
Drug use disorders	3	CODEm
Opioid use disorders	4	CODEm
Cocaine use disorders	4	CODEm
Amphetamine use disorders	4	CODEm
Other drug use disorders	4	CODEm

Table S11. Modeling strategy for individual cause of death models in GBD 2019		
**Cause Name**	**Level**	**Model type**
Diabetes and kidney diseases	Aggregate	
Diabetes mellitus	3	CODEm
Diabetes mellitus type 1	4	CODEm
Diabetes mellitus type 2	4	CODEm
Chronic kidney disease	3	CODEm
Chronic kidney disease due to diabetes mellitus type 1	4	DisMod MR-2.1 proportion model
Chronic kidney disease due to diabetes mellitus type 2	4	DisMod MR-2.1 proportion model
Chronic kidney disease due to hypertension	4	DisMod MR-2.1 proportion model
Chronic kidney disease due to glomerulonephritis	4	DisMod MR-2.1 proportion model
Chronic kidney disease due to other causes	4	DisMod MR-2.1 proportion model
Acute glomerulonephritis	3	CODEm
Skin and subcutaneous diseases	2	CODEm
Bacterial skin diseases	3	CODEm
Cellulitis	4	CODEm
Pyoderma	4	CODEm
Decubitus ulcer	3	CODEm
Other skin and subcutaneous diseases	3	CODEm
Musculoskeletal disorders	2	CODEm
Rheumatoid arthritis	3	CODEm
Other musculoskeletal disorders	3	CODEm
Other non-communicable diseases	Aggregate	
Congenital birth defects	3	CODEm
Neural tube defects	4	CODEm
Congenital heart anomalies	4	CODEm
Orofacial clefts	4	CODEm
Down syndrome	4	CODEm
Other chromosomal abnormalities	4	CODEm
Congenital musculoskeletal and limb anomalies	4	CODEm
Urogenital congenital anomalies	4	CODEm
Digestive congenital anomalies	4	CODEm
Other congenital birth defects	4	CODEm
Urinary diseases and male infertility	3	CODEm
Urinary tract infection and interstitial nephritis	4	CODEm
Urolithiasis	4	CODEm
Other urinary diseases	4	CODEm
Gynaecological diseases	3	CODEm
Uterine fibroids	4	CODEm
Endometriosis	4	CODEm
Genital prolapse	4	CODEm
Other gynaecological diseases	4	CODEm
Haemoglobinopathies and haemolytic anaemias	3	CODEm

Table S11. Modeling strategy for individual cause of death models in GBD 2019		
**Cause Name**	**Level**	**Model type**
Thalassaemias	4	DisMod MR-2.1 cause-specific mortality model
Sickle cell disorders	4	DisMod MR-2.1 cause-specific mortality model
G6PD deficiency	4	DisMod MR-2.1 cause-specific mortality model
Other haemoglobinopathies and haemolytic aneamias	4	Data proportion
Endocrine, metabolic, blood, and immune disorders	3	CODEm
Sudden infant death syndrome	3	CODEm
Injuries	Aggregate	
Transport injuries	2	CODEm
Road injuries	3	CODEm
Pedestrian road injuries	4	CODEm
Cyclist road injuries	4	CODEm
Motorcyclist road injuries	4	CODEm
Motor vehicle road injuries	4	CODEm
Other road injuries	4	CODEm
Other transport injuries	3	CODEm; Fatal Discontinuity
Unintentional injuries	Aggregate	
Falls	3	CODEm
Drowning	3	CODEm
Fire, heat, and hot substances	3	CODEm; Fatal Discontinuity
Poisonings	3	CODEm
Poisoning by carbon monoxide	4	CODEm
Poisoning by other means	4	CODEm, Fatal Discontinuity
Exposure to mechanical forces	Aggregate	
Unintentional firearm injuries	4	CODEm
Other exposure to mechanical forces	4	CODEm; Fatal Discontinuity
Adverse effects of medical treatment	3	CODEm
Animal contact	3	CODEm
Venomous animal contact	4	CODEm
Non-venomous animal contact	4	CODEm, Fatal Discontinuity
Foreign body	Aggregate	
Pulmonary aspiration and foreign body in airway	4	CODEm
Foreign body in other body part	4	CODEm
Environmental heat and cold exposure	3	CODEm; Fatal Discontinuity
Exposure to forces of nature	3	Fatal Discontinuity
Other unintentional injuries	3	CODEm
Self-harm and interpersonal violence	Aggregate	
Self-harm	3	CODEm
Self-harm by firearm	4	CODEm
Self-harm by other specified means	4	CODEm
Interpersonal violence	3	CODEm
Physical violence by firearm	4	CODEm, Fatal Discontinuity
Physical violence by sharp object	4	CODEm, Fatal Discontinuity

Table S11. Modeling strategy for individual cause of death models in GBD 2019		
**Cause Name**	**Level**	**Model type**
Physical violence by other means	4	CODEm, Fatal Discontinuity
Conflict and terrorism	3	Fatal Discontinuity
Police conflict and executions	3	CODEm; Fatal Discontinuity

Table S12. Percent change before and after CoDCorrect by cause for all ages, both sexes, global, 2019		
**Cause name**	**CoDCorrect level**	**Percent change**
All causes	0	4.67<br>-1.56 to 8.31
Communicable, maternal, neonatal, and nutritional disorders	1	13.65<br>6.36 to 19.47
Tuberculosis	3	9.82<br>5.82 to 14.26
Diarrhoeal diseases	3	4.07<br>-3.72 to 12.52
Typhoid fever	4	0.51<br>-11.12 to 14.1
Paratyphoid fever	4	-0.95<br>-13.08 to 13.68
Other intestinal infectious diseases	3	12.97<br>1.02 to 24.66
Lower respiratory infections	3	5.61<br>-0.78 to 10.22
Upper respiratory infections	3	7.13<br>-1.5 to 15.78
Otitis media	3	2.14<br>-4.63 to 9.64
Meningitis	3	11.79<br>2.71 to 19.57
Encephalitis	3	5.21<br>-0.2 to 10.7
Diphtheria	3	22.88<br>1.53 to 42.43
Whooping cough	3	1.49<br>-10.61 to 16.0
Tetanus	3	0.95<br>-8.42 to 7.18
Measles	3	13.51<br>-2.28 to 38.83
Varicella and herpes zoster	3	7.61<br>-0.46 to 14.62
Neglected tropical diseases and malaria	2	-0.36<br>-19.13 to 19.13
Malaria	3	-1.84<br>-20.69 to 20.58
Chagas disease	3	0.7<br>-5.49 to 3.98
Leishmaniasis	3	-1.21<br>-10.01 to 11.28
Visceral leishmaniasis	4	-1.48<br>-10.26 to 10.82
African trypanosomiasis	3	8.78<br>-5.05 to 20.98
Schistosomiasis	3	12.1<br>4.7 to 18.67
Cysticercosis	3	7.83<br>2.0 to 13.32
Cystic echinococcosis	3	9.38<br>4.43 to 14.31
Dengue	3	1.75<br>-4.48 to 17.91
Yellow fever	3	12.02<br>-0.46 to 23.17
Rabies	3	9.1<br>1.01 to 18.0
Intestinal nematode infections	3	18.56<br>1.78 to 33.23

Table S12. Percent change before and after CoDCorrect by cause for all ages, both sexes, global, 2019		
**Cause name**	**CoDCorrect level**	**Percent change**
Ascariasis	4	18.56<br>1.78 to 33.23
Other neglected tropical diseases	3	12.73<br>0.04 to 24.84
Maternal disorders	3	2.27<br>-4.29 to 7.53
Maternal haemorrhage	4	3.42<br>-3.58 to 8.68
Maternal sepsis and other pregnancy related infections	4	-1.56<br>-8.59 to 3.84
Maternal hypertensive disorders	4	4.98<br>-0.73 to 9.96
Maternal obstructed labour and uterine rupture	4	4.02<br>-3.63 to 10.68
Ectopic pregnancy	4	0.58<br>-6.99 to 6.51
Indirect maternal deaths	4	6.62<br>0.27 to 11.73
Late maternal deaths	4	-0.68<br>-8.96 to 5.93
Other maternal disorders	4	2.63<br>-4.25 to 7.81
Neonatal disorders	3	-1.13<br>-5.47 to 5.6
Neonatal preterm birth	4	3.35<br>-1.84 to 8.99
Neonatal encephalopathy due to birth asphyxia and trauma	4	6.4<br>0.45 to 13.38
Neonatal sepsis and other neonatal infections	4	-0.11<br>-6.49 to 7.01
Hemolytic disease and other neonatal jaundice	4	4.88<br>-1.73 to 13.02
Other neonatal disorders	4	-1.91<br>-8.84 to 4.69
Nutritional deficiencies	2	8.29<br>0.44 to 14.95
Protein-energy malnutrition	3	18.81<br>-5.37 to 35.73
Other nutritional deficiencies	3	22.8<br>5.78 to 45.34
Sexually transmitted infections excluding HIV	3	1.29<br>-4.71 to 10.83
Syphilis	4	0.89<br>-5.33 to 11.1
Chlamydial infection	4	6.69<br>-0.09 to 11.82
Gonococcal infection	4	6.73<br>0.17 to 11.67
Other sexually transmitted infections	4	7.13<br>1.64 to 11.38
Acute hepatitis	3	6.61<br>0.48 to 12.95
Acute hepatitis A	4	487.55<br>267.82 to 1063.85
Acute hepatitis B	4	260.62<br>162.64 to 486.37
Acute hepatitis C	4	572.41<br>368.51 to 884.63
Acute hepatitis E	4	466.32<br>286.72 to 743.67

Table S12. Percent change before and after CoDCorrect by cause for all ages, both sexes, global, 2019		
**Cause name**	**CoDCorrect level**	**Percent change**
Other unspecified infectious diseases	3	6.25<br>1.96 to 10.29
Non-communicable diseases	1	2.5<br>-4.77 to 6.21
Neoplasms	2	1.74<br>-3.24 to 4.88
Oesophageal cancer	3	1.66<br>-2.51 to 4.87
Stomach cancer	3	1.88<br>-3.22 to 5.18
Liver cancer	3	1.19<br>-3.38 to 4.41
Liver cancer due to hepatitis B	4	1.4<br>-1.53 to 4.55
Liver cancer due to hepatitis C	4	0.67<br>-6.42 to 4.83
Liver cancer due to alcohol use	4	1.33<br>-2.55 to 3.91
Larynx cancer	3	4.03<br>0.75 to 7.15
Tracheal, bronchus, and lung cancer	3	0.72<br>-3.95 to 3.6
Breast cancer	3	2.12<br>-3.22 to 5.63
Cervical cancer	3	3.36<br>-0.98 to 7.2
Uterine cancer	3	1.41<br>-4.77 to 4.97
Prostate cancer	3	2.13<br>-4.09 to 5.99
Colon and rectum cancer	3	1.4<br>-4.72 to 4.84
Lip and oral cavity cancer	3	4.93<br>0.68 to 8.65
Nasopharynx cancer	3	3.53<br>0.64 to 7.06
Other pharynx cancer	3	5.54<br>2.32 to 9.49
Gallbladder and biliary tract cancer	3	1.96<br>-5.22 to 6.36
Pancreatic cancer	3	0.85<br>-5.11 to 4.06
Malignant skin melanoma	3	0.86<br>-4.06 to 3.72
Non-melanoma skin cancer	3	1.56<br>-6.01 to 5.78
Ovarian cancer	3	1.46<br>-3.95 to 5.04
Testicular cancer	3	5.8<br>2.71 to 10.87
Kidney cancer	3	0.89<br>-4.18 to 3.85
Bladder cancer	3	1.56<br>-5.03 to 5.33
Brain and nervous system cancer	3	1.41<br>-1.74 to 4.93
Thyroid cancer	3	3.33<br>-2.39 to 7.08
Mesothelioma	3	0.99<br>-4.19 to 3.99

Table S12. Percent change before and after CoDCorrect by cause for all ages, both sexes, global, 2019		
**Cause name**	**CoDCorrect level**	**Percent change**
Hodgkin lymphoma	3	5.82<br>1.76 to 9.73
Non-Hodgkin's lymphoma	3	2.06<br>-3.12 to 5.64
Multiple myeloma	3	1.23<br>-4.67 to 4.6
Leukaemia	3	2.29<br>-2.23 to 5.78
Other neoplasms	3	0.76<br>-8.08 to 5.67
Cardiovascular diseases	2	2.66<br>-4.27 to 6.44
Rheumatic heart disease	3	0.83<br>-5.18 to 7.2
Ischaemic heart disease	3	2.88<br>-4.56 to 7.18
Stroke	3	4.0<br>-3.58 to 9.07
Ischaemic stroke	4	0.78<br>-8.6 to 6.88
Intracerebral hemorrhage	4	1.02<br>-5.17 to 7.82
Subarachnoid hemorrhage	4	1.56<br>-4.35 to 7.15
Hypertensive heart disease	3	4.3<br>-4.54 to 9.79
Cardiomyopathy and myocarditis	3	3.53<br>-3.42 to 10.07
Atrial fibrillation and flutter	3	2.93<br>-9.66 to 10.2
Aortic aneurysm	3	2.27<br>-5.81 to 7.43
Peripheral vascular disease	3	1.93<br>-9.12 to 8.87
Endocarditis	3	1.79<br>-6.32 to 7.7
Non-rheumatic valvular heart disease	3	1.7<br>-11.2 to 9.68
Chronic respiratory diseases	2	3.96<br>-3.46 to 8.65
Chronic obstructive pulmonary disease	3	29.72<br>13.89 to 40.87
Pneumoconiosis	3	13.53<br>4.3 to 25.27
Silicosis	4	17.65<br>-9.93 to 84.09
Asbestosis	4	14.55<br>-6.97 to 98.75
Coal workers pneumoconiosis	4	25.76<br>7.17 to 94.91
Other pneumoconiosis	4	57.03<br>21.43 to 147.84
Asthma	3	62.77<br>36.61 to 80.39
Interstitial lung disease and pulmonary sarcoidosis	3	39.63<br>20.32 to 69.5
Other chronic respiratory diseases	3	27.44<br>14.37 to 40.48
Cirrhosis and other chronic liver diseases	3	10.81<br>4.99 to 17.24

Table S12. Percent change before and after CoDCorrect by cause for all ages, both sexes, global, 2019		
Cause name	CoDCorrect level	Percent change
Cirrhosis and other chronic liver diseases due to hepatitis B	4	10.75<br>3.66 to 18.56
Cirrhosis and other chronic liver diseases due to hepatitis C	4	9.31<br>3.45 to 15.8
Cirrhosis and other chronic liver diseases due to alcohol use	4	7.68<br>2.55 to 13.06
Cirrhosis and other chronic liver diseases due to other causes	4	12.26<br>6.13 to 19.71
Digestive diseases	2	4.23<br>-0.72 to 7.83
Peptic ulcer disease	4	4.81<br>-5.13 to 19.07
Gastritis and duodenitis	4	2.19<br>-7.23 to 14.53
Appendicitis	3	21.62<br>12.91 to 31.04
Paralytic ileus and intestinal obstruction	3	19.07<br>10.83 to 27.4
Inguinal, femoral, and abdominal hernia	3	19.64<br>10.36 to 27.62
Inflammatory bowel disease	3	6.96<br>-2.23 to 13.9
Vascular intestinal disorders	3	7.1<br>-2.86 to 15.66
Gallbladder and biliary diseases	3	10.69<br>0.15 to 17.9
Pancreatitis	3	10.9<br>5.33 to 17.16
Other digestive diseases	3	9.62<br>-0.69 to 17.94
Neurological disorders	2	-1.27<br>-12.02 to 7.78
Alzheimer's disease and other dementias	3	-2.74<br>-12.72 to 9.52
Parkinson's disease	3	2.58<br>-6.48 to 7.42
Idiopathic epilepsy	3	6.02<br>1.36 to 11.08
Multiple sclerosis	3	1.79<br>-1.64 to 4.81
Motor neuron disease	3	-0.1<br>-4.74 to 2.64
Other neurological disorders	3	2.17<br>-2.18 to 5.65
Mental disorders	2	-1.11<br>-2.57 to 1.09
Alcohol use disorders	3	3.22<br>1.08 to 5.75
Drug use disorders	3	-4.37<br>-7.13 to -2.28
Opioid use disorders	4	6.08<br>-1.04 to 14.71
Cocaine use disorders	4	1.47<br>-5.09 to 12.31
Amphetamine use disorders	4	5.37<br>-0.64 to 14.8
Other drug use disorders	4	22.1<br>16.16 to 27.33
Eating disorders	3	-1.11<br>-2.57 to 1.09

Table S12. Percent change before and after CoDCorrect by cause for all ages, both sexes, global, 2019		
**Cause name**	**CoDCorrect level**	**Percent change**
Anorexia nervosa	4	-4.49<br>-13.63 to 12.09
Bulimia nervosa	4	-54.32<br>-66.98 to -29.65
Diabetes mellitus	3	3.8<br>-2.05 to 7.82
Acute glomerulonephritis	3	1.49<br>-3.21 to 4.96
Chronic kidney disease	3	3.5<br>-3.39 to 7.48
Chronic kidney disease due to hypertension	4	3.07<br>-5.55 to 7.74
Chronic kidney disease due to glomerulonephritis	4	4.94<br>0.08 to 8.42
Chronic kidney disease due to other and unspecified causes	4	3.83<br>-3.17 to 7.99
Urinary diseases and male infertility	3	4.09<br>-4.61 to 8.82
Urinary tract infections and interstitial nephritis	4	27.45<br>10.77 to 64.33
Urolithiasis	4	23.14<br>9.42 to 52.75
Other urinary diseases	4	47.02<br>24.23 to 108.24
Gynaecological diseases	3	6.24<br>-0.51 to 11.66
Uterine fibroids	4	209.79<br>74.66 to 458.84
Endometriosis	4	40.89<br>-14.43 to 135.05
Genital prolapse	4	299.1<br>122.0 to 601.85
Other gynaecological diseases	4	51.53<br>-10.9 to 120.61
Haemoglobinopathies and haemolytic anaemias	3	7.18<br>-2.03 to 15.6
Thalassaemias	4	9.11<br>2.02 to 16.87
Sickle cell disorders	4	7.91<br>-9.24 to 24.0
G6PD deficiency	4	8.77<br>4.6 to 13.23
Other haemoglobinopathies and haemolytic anaemias	4	4.7<br>-3.35 to 9.28
Endocrine, metabolic, blood, and immune disorders	3	1.17<br>-4.9 to 4.96
Musculoskeletal disorders	2	3.16<br>-3.95 to 7.37
Rheumatoid arthritis	3	33.6<br>7.48 to 56.02
Other musculoskeletal disorders	3	8.31<br>-12.38 to 29.3
Other non-communicable diseases	2	3.1<br>-1.54 to 7.66
Congenital anomalies	3	2.21<br>-3.3 to 8.15
Neural tube defects	4	-25.02<br>-45.37 to -0.88
Congenital heart anomalies	4	-13.09<br>-21.51 to -2.67

Table S12. Percent change before and after CoDCorrect by cause for all ages, both sexes, global, 2019		
Cause name	CoDCorrect level	Percent change
Orofacial clefts	4	-12.82<br>-24.71 to 3.49
Down syndrome	4	-7.94<br>-19.19 to 6.8
Other chromosomal abnormalities	4	-10.48<br>-19.84 to 0.81
Congenital musculoskeletal and limb anomalies	4	-9.6<br>-22.24 to 5.48
Urogenital congenital anomalies	4	-12.29<br>-23.78 to 1.66
Digestive congenital anomalies	4	-9.29<br>-21.24 to 6.21
Other congenital anomalies	4	-13.45<br>-25.83 to -0.99
Skin and subcutaneous diseases	2	3.4<br>-4.73 to 8.26
Cellulitis	4	14.85<br>-27.08 to 71.51
Pyoderma	4	22.82<br>-9.36 to 67.13
Decubitus ulcer	3	-2.97<br>-36.62 to 29.91
Other skin and subcutaneous diseases	3	9.5<br>-17.27 to 49.25
Sudden infant death syndrome	3	7.98<br>-4.51 to 17.19
Injuries	1	6.71<br>3.2 to 11.19
Transport injuries	2	4.83<br>2.03 to 9.7
Road injuries	3	-2.18<br>-12.97 to 2.22
Pedestrian road injuries	4	-0.67<br>-11.78 to 11.16
Cyclist road injuries	4	1.05<br>-13.21 to 12.7
Motorcyclist road injuries	4	2.07<br>-12.98 to 11.25
Motor vehicle road injuries	4	1.26<br>-8.56 to 14.02
Other road injuries	4	1.3<br>-8.46 to 13.61
Other transport injuries	3	1.99<br>-9.41 to 7.04
Unintentional injuries	2	5.44<br>0.18 to 9.79
Falls	3	4.31<br>-3.39 to 8.98
Drowning	3	3.16<br>-1.53 to 8.85
Fire, heat, and hot substances	3	6.92<br>2.38 to 11.2
Poisonings	3	5.92<br>2.0 to 10.33
Poisoning by carbon monoxide	4	17.39<br>8.26 to 52.62
Poisoning by other means	4	31.94<br>17.48 to 75.29
Exposure to mechanical forces	3	6.25<br>2.62 to 12.26

Table S12. Percent change before and after CoDCorrect by cause for all ages, both sexes, global, 2019		
**Cause name**	**CoDCorrect level**	**Percent change**
Unintentional firearm injuries	4	17.7<br>-3.15 to 38.52
Other exposure to mechanical forces	4	13.47<br>-1.41 to 35.65
Adverse effects of medical treatment	3	6.92<br>1.8 to 11.11
Animal contact	3	8.38<br>2.66 to 14.23
Venomous animal contact	4	47.76<br>-4.87 to 75.47
Non-venomous animal contact	4	4.5<br>-26.37 to 50.85
Foreign body	3	3.18<br>-2.53 to 7.44
Pulmonary aspiration and foreign body in airway	4	2.05<br>-6.37 to 19.02
Foreign body in other body part	4	4.73<br>-11.81 to 24.11
Other unintentional injuries	3	6.61<br>3.41 to 12.25
Self-harm and interpersonal violence	2	10.62<br>7.39 to 14.78
Self-harm	3	4.44<br>1.39 to 8.39
Self-harm by firearm	4	1.57<br>-4.97 to 14.6
Self-harm by other specified means	4	3.94<br>-6.94 to 13.64
Interpersonal violence	3	5.38<br>2.31 to 9.14
Physical violence by firearm	4	9.35<br>5.51 to 14.61
Physical violence by sharp object	4	16.45<br>5.99 to 30.28
Physical violence by other means	4	26.76<br>18.01 to 36.26
Maternal deaths aggravated by HIV/AIDS	4	-1.84<br>-11.24 to 5.58
Environmental heat and cold exposure	3	9.76<br>5.56 to 13.38
Acute lymphoid leukaemia	4	19.83<br>13.0 to 36.88
Chronic lymphoid leukaemia	4	-17.23<br>-24.08 to -8.72
Acute myeloid leukaemia	4	11.06<br>4.42 to 25.8
Chronic myeloid leukaemia	4	19.24<br>9.22 to 30.99
Non-melanoma skin cancer (squamous-cell carcinoma)	4	1.56<br>-6.01 to 5.78
Police conflict and executions	3	56.83<br>46.81 to 70.26
Drug-susceptible tuberculosis	4	8.9<br>4.93 to 13.31
Zika virus disease	3	1.92<br>-0.36 to 4.27
Alcoholic cardiomyopathy	4	14.85<br>-5.14 to 35.53
Myocarditis	4	3.47<br>-11.26 to 27.59

Table S12. Percent change before and after CoDCorrect by cause for all ages, both sexes, global, 2019		
**Cause name**	**CoDCorrect level**	**Percent change**
Other leukaemia	4	16.12<br>7.78 to 30.62
Other cardiomyopathy	4	1.99<br>-9.79 to 19.44
Multidrug-resistant tuberculosis without extensive drug resistance	4	9.71<br>5.46 to 14.45
Extensively drug-resistant tuberculosis	4	7.32<br>4.09 to 11.16
HIV/AIDS and sexually transmitted infections	2	972.59<br>478.7 to 2517.85
Respiratory infections and tuberculosis	2	6.92<br>1.45 to 11.15
Enteric infections	2	4.2<br>-3.73 to 12.56
Typhoid and paratyphoid	3	0.25<br>-11.45 to 13.89
iNTS	3	14.58<br>-4.28 to 30.2
Other infectious diseases	2	7.71<br>0.04 to 15.47
Maternal and neonatal disorders	2	-0.82<br>-4.76 to 5.1
Myelodysplastic, myeloproliferative, and other haematopoietic neoplasms	4	0.76<br>-8.08 to 5.67
Non-rheumatic calcific aortic valvular heart disease	4	5.95<br>-12.08 to 35.29
Non-rheumatic degenerative mitral valvular heart disease	4	15.93<br>0.18 to 49.48
Other non-rheumatic valvular heart diseases	4	29.34<br>10.54 to 45.98
Cirrhosis and other chronic liver diseases due to NAFLD	4	10.0<br>3.83 to 15.28
Substance use disorders	2	-0.21<br>-2.39 to 1.93
Diabetes and kidney diseases	2	3.65<br>-2.7 to 7.5
Diabetes mellitus type 1	4	14.54<br>5.08 to 33.31
Diabetes mellitus type 2	4	10.01<br>0.26 to 23.24
Bacterial skin diseases	3	1.52<br>-25.62 to 27.08
Upper digestive system diseases	3	11.39<br>4.25 to 19.28
Maternal abortive outcome	4	-4.21<br>-11.66 to 1.84
Liver cancer due to NASH	4	1.81<br>-4.0 to 5.81
Chronic kidney disease due to diabetes mellitus type 1	4	4.09<br>1.84 to 7.58
Chronic kidney disease due to diabetes mellitus type 2	4	3.11<br>-4.13 to 7.12
Liver cancer due to other causes	4	1.14<br>-2.74 to 4.33
Other malignant neoplasms	3	3.71<br>-0.73 to 7.64
Other cardiovascular and circulatory diseases	3	4.27<br>-2.69 to 9.96

Table S13. GBD 2019 sequelae, health states, health state lay descriptions, and disability weights			
**Health state name**		**Health state lay description**	**Disability Weight**
HIV/AIDS - Drug-susceptible Tuberculosis without anemia	Tuberculosis, HIV infected	has a persistent cough and fever, shortness of breath, night sweats, weakness and fatigue and severe weight loss.	0.408 (0.274-0.549)
HIV/AIDS - Drug-susceptible Tuberculosis with mild anemia	Tuberculosis, HIV infected and anemia, mild	(combined DW)	0.411 (0.278-0.551)
HIV/AIDS - Drug-susceptible Tuberculosis with moderate anemia	Tuberculosis, HIV infected and anemia, moderate	(combined DW)	0.439 (0.307-0.577)
HIV/AIDS - Drug-susceptible Tuberculosis with severe anemia	Tuberculosis, HIV infected and anemia, severe	(combined DW)	0.495 (0.353-0.64)
HIV/AIDS - Multidrug-resistant Tuberculosis without extensive drug resistance without anemia	Tuberculosis, HIV infected	has a persistent cough and fever, shortness of breath, night sweats, weakness and fatigue and severe weight loss.	0.408 (0.274-0.549)
HIV/AIDS - Multidrug-resistant Tuberculosis without extensive drug resistance with mild anemia	Tuberculosis, HIV infected and anemia, mild	(combined DW)	0.411 (0.278-0.551)
HIV/AIDS - Multidrug-resistant Tuberculosis without extensive drug resistance with moderate anemia	Tuberculosis, HIV infected and anemia, moderate	(combined DW)	0.439 (0.307-0.577)
HIV/AIDS - Multidrug-resistant Tuberculosis without extensive drug resistance with severe anemia	Tuberculosis, HIV infected and anemia, severe	(combined DW)	0.495 (0.353-0.64)
HIV/AIDS - Extensively drug-resistant Tuberculosis without anemia	Tuberculosis, HIV infected	has a persistent cough and fever, shortness of breath, night sweats, weakness and fatigue and severe weight loss.	0.408 (0.274-0.549)
HIV/AIDS - Extensively drug-resistant Tuberculosis with mild anemia	Tuberculosis, HIV infected and anemia, mild	(combined DW)	0.411 (0.278-0.551)
HIV/AIDS - Extensively drug-resistant Tuberculosis with moderate anemia	Tuberculosis, HIV infected and anemia, moderate	(combined DW)	0.439 (0.307-0.577)
HIV/AIDS - Extensively drug-resistant Tuberculosis with severe anemia	Tuberculosis, HIV infected and anemia, severe	(combined DW)	0.495 (0.353-0.64)
Symptomatic HIV without anemia	HIV cases, symptomatic, pre-AIDS	has weight loss, fatigue, and frequent infections.	0.274 (0.184-0.377)
AIDS without anemia	AIDS cases, not receiving ARV treatment	has severe weight loss, weakness, fatigue, cough and fever, and frequent infections, skin rashes and diarrhea.	0.582 (0.406-0.743)
Early HIV without anemia	Generic uncomplicated disease: anxiety about diagnosis	has a disease diagnosis that causes some worry but minimal interference with daily activities.	0.012 (0.006-0.023)
Early HIV with mild anemia	Anemia, mild; Generic uncomplicated disease anxiety		0.016 (0.008-0.031)
Early HIV with moderate anemia	Anemia, moderate; Generic uncomplicated disease anxiety		0.063 (0.04-0.095)
Early HIV with severe anemia	Anemia, severe; Generic uncomplicated disease anxiety		0.159 (0.109-0.22)
Symptomatic HIV with mild anemia	HIV cases, symptomatic, pre-AIDS and anemia, mild	(combined DW)	0.277 (0.189-0.379)
Symptomatic HIV with moderate anemia	HIV cases, symptomatic, pre-AIDS and anemia, moderate	(combined DW)	0.312 (0.217-0.418)
Symptomatic HIV with severe anemia	HIV cases, symptomatic, pre-AIDS and anemia, severe	(combined DW)	0.381 (0.269-0.505)
AIDS with mild anemia	AIDS cases, not receiving ARV treatment and anemia, mild	(combined DW)	0.583 (0.409-0.743)
AIDS with moderate anemia	AIDS cases, not receiving ARV treatment and anemia, moderate	(combined DW)	0.603 (0.43-0.758)
AIDS with severe anemia	AIDS cases, not receiving ARV treatment and anemia, severe	(combined DW)	0.642 (0.47-0.792)
HIV/AIDS with antiretroviral treatment without anemia	HIV/AIDS cases, receiving ARV treatment	has occasional fevers and infections. The person takes daily medication that sometimes causes diarrhea.	0.078 (0.052-0.111)
HIV/AIDS with antiretroviral treatment with mild anemia	HIV/AIDS cases, receiving ARV treatment and anemia, mild	(combined DW)	0.081 (0.054-0.116)
HIV/AIDS with antiretroviral treatment with moderate anemia	HIV/AIDS cases, receiving ARV treatment and anemia, moderate	(combined DW)	0.125 (0.085-0.176)
HIV/AIDS with antiretroviral treatment with severe anemia	HIV/AIDS cases, receiving ARV treatment and anemia, severe	(combined DW)	0.215 (0.148-0.295)
Mild early syphilis infection	Infectious disease, acute episode, mild	has a low fever and mild discomfort , but no difficulty with daily activities.	0.006 (0.002-0.012)
Asymptomatic early syphilis infection	Asymptomatic		0 (0-0)
Cardiovascular complications due to adult tertiary syphilis	Infectious disease, acute episode, moderate	has a fever and aches, and feels weak, which causes some difficulty with daily activities.	0.051 (0.032-0.074)
Severe disfigurement due to adult tertiary syphilis	Disfigurement, level 3	has an obvious physical deformity that makes others uncomfortable, which causes the person to avoid social contact, feel worried, sleep poorly, and think about suicide.	0.405 (0.275-0.546)
Neurological problems due to adult tertiary syphilis	Motor plus cognitive impairments, moderate	has some difficulty in moving around, holding objects, dressing and sitting upright, but can walk without help. The person has low intelligence and is slow in learning to speak and to do simple tasks.	0.203 (0.134-0.29)
Asymptomatic adult tertiary syphilis	Asymptomatic		0 (0-0)
Neurological problems and cardiovascular complications due to adult tertiary syphilis	Moderate motor plus cognitive impairments and moderate infectious disease, acute episode	(combined DW)	0.243 (0.168-0.333)
Severe disfigurement and cardiovascular complications due to adult tertiary syphilis	Level 3 disfigurement and moderate infectious disease, acute episode	(combined DW)	0.435 (0.306-0.571)
Severe disfigurement and neurological problems due to adult tertiary syphilis	Level 3 disfigurement and moderate motor plus cognitive impairments	(combined DW)	0.523 (0.378-0.669)
Severe disfigurement, neurological problems, and cardiovascular complications due to adult tertiary syphilis	Level 3 disfigurement, moderate motor plus cognitive impairments, and moderate infectious disease, acute episode	(combined DW)	0.547 (0.402-0.691)
Mild chlamydial infection	Infectious disease, acute episode, mild	has a low fever and mild discomfort , but no difficulty with daily activities.	0.006 (0.002-0.012)
Asymptomatic chlamydial infection	Asymptomatic		0 (0-0)
Epididymo-orchitis due to chlamydial infection	Epididymo-orchitis	has swelling and tenderness in the testicles and pain during urination.	0.128 (0.086-0.18)
Primary infertility due to chlamydial infection	Infertility, primary	wants to have a child and has a fertile partner, but the couple cannot conceive.	0.008 (0.003-0.015)
Secondary infertility due to chlamydial infection	Infertility, secondary	has at least one child, and wants to have more children. The person has a fertile partner, but the couple cannot conceive.	0.005 (0.002-0.011)
Moderate pelvic inflammatory diseases due to chlamydial infection	Abdominopelvic problem, moderate	has pain in the belly and feels nauseous. The person has difficulties with daily activities.	0.114 (0.078-0.159)
Severe pelvic inflammatory diseases due to chlamydial infection	Abdominopelvic problem, severe	has severe pain in the belly and feels nauseous. The person is anxious and unable to carry out daily activities.	0.324 (0.22-0.442)
Epididymo-orchitis due to gonococcal infection	Epididymo-orchitis	has swelling and tenderness in the testicles and pain during urination.	0.128 (0.086-0.18)
Primary infertility due to gonococcal infection	Infertility, primary	wants to have a child and has a fertile partner, but the couple cannot conceive.	0.008 (0.003-0.015)
Secondary infertility due to gonococcal infection	Infertility, secondary	has at least one child, and wants to have more children. The person has a fertile partner, but the couple cannot conceive.	0.005 (0.002-0.011)
Moderate pelvic inflammatory diseases due to gonococcal infection	Abdominopelvic problem, moderate	has pain in the belly and feels nauseous. The person has difficulties with daily activities.	0.114 (0.078-0.159)
Severe pelvic inflammatory diseases due to gonococcal infection	Abdominopelvic problem, severe	has severe pain in the belly and feels nauseous. The person is anxious and unable to carry out daily activities.	0.324 (0.22-0.442)
Mild gonococcal infection	Infectious disease, acute episode, mild	has a low fever and mild discomfort , but no difficulty with daily activities.	0.006 (0.002-0.012)
Asymptomatic gonococcal infection	Asymptomatic		0 (0-0)
Acute trichomoniasis infection	Infectious disease, acute episode, mild	has a low fever and mild discomfort , but no difficulty with daily activities.	0.006 (0.002-0.012)
Asymptomatic trichomoniasis infection	Asymptomatic		0 (0-0)
Symptomatic genital herpes	Infectious disease, acute episode, mild	has a low fever and mild discomfort , but no difficulty with daily activities.	0.006 (0.002-0.012)
Moderate infection due to initial genital herpes episode	Infectious disease, acute episode, moderate	has a fever and aches, and feels weak, which causes some difficulty with daily activities.	0.051 (0.032-0.074)
Asymptomatic genital herpes	Asymptomatic		0 (0-0)
Primary infertility due to other sexually transmitted diseases	Infertility, primary	wants to have a child and has a fertile partner, but the couple cannot conceive.	0.008 (0.003-0.015)
Secondary infertility due to other sexually transmitted diseases	Infertility, secondary	has at least one child, and wants to have more children. The person has a fertile partner, but the couple cannot conceive.	0.005 (0.002-0.011)

Moderate pelvic inflammatory diseases due to other sexually transmitted diseases	Abdominopelvic problem, moderate	has pain in the belly and feels nauseous. The person has difficulties with daily activities.	0.114 (0.078-0.159)
Severe pelvic inflammatory diseases due to other sexually transmitted diseases	Abdominopelvic problem, severe	has severe pain in the belly and feels nauseous. The person is anxious and unable to carry out daily activities.	0.324 (0.22-0.442)
Other sexually transmitted diseases residual	Post-COMO calculation for residuals (YLL/YLD ratio, other methods)		--
Latent tuberculosis infection	Asymptomatic		0 (0-0)
Drug-susceptible tuberculosis	Tuberculosis, not HIV infected	has a persistent cough and fever, is short of breath, feels weak, and has lost a lot of weight.	0.333 (0.224-0.454)
Multidrug-resistant tuberculosis without extensive drug resistance	Tuberculosis, not HIV infected	has a persistent cough and fever, is short of breath, feels weak, and has lost a lot of weight.	0.333 (0.224-0.454)
Extensively drug-resistant tuberculosis	Tuberculosis, not HIV infected	has a persistent cough and fever, is short of breath, feels weak, and has lost a lot of weight.	0.333 (0.224-0.454)
Guillain-Barré syndrome due to lower respiratory infections	Spinal cord lesion below neck level (treated)	is paralyzed from the waist down, cannot feel or move the legs and has difficulties with urine and bowel control. The person uses a wheelchair to move around.	0.296 (0.198-0.414)
Moderate lower respiratory infections	Infectious disease, acute episode, moderate	has a fever and aches, and feels weak, which causes some difficulty with daily activities.	0.051 (0.032-0.074)
Severe lower respiratory infections	Infectious disease, acute episode, severe	has a high fever and pain, and feels very weak, which causes great difficulty with daily activities.	0.133 (0.088-0.19)
Guillain-Barré syndrome due to upper respiratory infections	Spinal cord lesion below neck level (treated)	is paralyzed from the waist down, cannot feel or move the legs and has difficulties with urine and bowel control. The person uses a wheelchair to move around.	0.296 (0.198-0.414)
Mild upper respiratory infections	Infectious disease, acute episode, mild	has a low fever and mild discomfort , but no difficulty with daily activities.	0.006 (0.002-0.012)
Moderate upper respiratory infections	Infectious disease, acute episode, moderate	has a fever and aches, and feels weak, which causes some difficulty with daily activities.	0.051 (0.032-0.074)
Acute otitis media	Ear pain	has an ear-ache that causes some difficulty with daily activities.	0.013 (0.007-0.024)
Severe infectious complications due to chronic otitis media	Ear pain	has an ear-ache that causes some difficulty with daily activities.	0.013 (0.007-0.024)
Mild hearing loss due to chronic otitis media	Hearing loss, mild	has great difficulty hearing and understanding another person talking in a noisy place (for example, on an urban street).	0.01 (0.004-0.019)
Moderate hearing loss due to chronic otitis media	Hearing loss, moderate	is unable to hear and understand another person talking in a noisy place (for example, on an urban street), and has difficulty hearing another person talking even in a quiet place or on the phone.	0.027 (0.015-0.042)
Mild hearing loss with ringing due to chronic otitis media	Hearing loss, mild, with ringing	has great difficulty hearing and understanding another person talking in a noisy place (for example, on an urban street), and sometimes has annoying ringing in the ears.	0.021 (0.012-0.036)
Moderate hearing loss with ringing due to chronic otitis media	Hearing loss, moderate, with ringing	is unable to hear and understand another person talking in a noisy place (for example, on an urban street), and has difficulty hearing another person talking even in a quiet place or on the phone, and has annoying ringing in the ears for more than 5 minutes at a time, almost everyday.	0.074 (0.049-0.107)
Vertigo with mild hearing loss due to chronic otitis media	Vertigo with mild hearing loss		0.122 (0.079-0.17)
Vertigo with mild hearing loss and ringing due to chronic otitis media	Vertigo with mild hearing loss and ringing		0.132 (0.086-0.184)
Vertigo with moderate hearing loss due to chronic otitis media	Vertigo with moderate hearing loss		0.137 (0.089-0.189)
Vertigo with moderate hearing loss and ringing due to chronic otitis media	Vertigo with moderate hearing loss and ringing		0.179 (0.12-0.247)
Guillain-Barré syndrome due to diarrheal diseases	Spinal cord lesion below neck level (treated)	is paralyzed from the waist down, cannot feel or move the legs and has difficulties with urine and bowel control. The person uses a wheelchair to move around.	0.296 (0.198-0.414)
Mild diarrheal diseases	Diarrhea, mild	has diarrhea three or more times a day with occasional discomfort in the belly.	0.074 (0.049-0.104)
Moderate diarrheal diseases	Diarrhea, moderate	has diarrhea three or more times a day, with painful cramps in the belly and feeling thirsty	0.188 (0.125-0.264)
Severe diarrheal diseases	Diarrhea, severe	has diarrhea three or more times a day with severe belly cramps. The person is very thirsty and feels nauseous and tired.	0.247 (0.164-0.348)
Gastrointestinal bleeding due to typhoid	Gastric bleeding	vomits blood and feels nauseous.	0.325 (0.209-0.462)
Intestinal perforation due to typhoid	Abdominopelvic problem, severe	has severe pain in the belly and feels nauseous. The person is anxious and unable to carry out daily activities.	0.324 (0.22-0.442)
Acute typhoid infection	Infectious disease, acute episode, moderate	has a fever and aches, and feels weak, which causes some difficulty with daily activities.	0.051 (0.032-0.074)
Severe typhoid fever	Infectious disease, acute episode, severe	has a high fever and pain, and feels very weak, which causes great difficulty with daily activities.	0.133 (0.088-0.19)
Intestinal perforation due to paratyphoid	Abdominopelvic problem, moderate	has pain in the belly and feels nauseous. The person has difficulties with daily activities.	0.114 (0.078-0.159)
Acute paratyphoid infection	Infectious disease, acute episode, mild	has a low fever and mild discomfort , but no difficulty with daily activities.	0.006 (0.002-0.012)
Moderate paratyphoid fever	Infectious disease, acute episode, moderate	has a fever and aches, and feels weak, which causes some difficulty with daily activities.	0.051 (0.032-0.074)
Severe paratyphoid fever	Infectious disease, acute episode, severe	has a high fever and pain, and feels very weak, which causes great difficulty with daily activities.	0.133 (0.088-0.19)
Severe acute iNTS	Infectious disease, acute episode, severe	has a high fever and pain, and feels very weak, which causes great difficulty with daily activities.	0.133 (0.088-0.19)
Other intestinal infectious diseases	Post-COMO calculation for residuals (YLL/YLD ratio, other methods)		--
Moderate motor impairment due to malaria	Motor impairment, moderate	has some difficulty in moving around, and difficulty in lifting and holding objects, dressing and sitting upright, but is able to walk without help.	0.061 (0.04-0.089)
Severe motor impairment due to malaria	Motor impairment, severe	is unable to move around without help, and is not able to lift or hold objects, get dressed or sit upright.	0.402 (0.268-0.545)
Moderate motor impairment with blindness due to malaria	Moderate motor impairment with blindness	(combined DW)	0.236 (0.165-0.323)
Moderate motor impairment with epilepsy due to malaria	Moderate motor impairment with epilepsy	(combined DW)	--
Moderate motor impairment with epilepsy due to malaria	Moderate motor impairment with epilepsy	(combined DW)	--
Moderate motor impairment with epilepsy due to malaria	Moderate motor impairment with epilepsy	(combined DW)	--
Moderate motor impairment with blindness and epilepsy due to malaria	Moderate motor impairment with blindness and epilepsy	(combined DW)	--
Moderate motor impairment with blindness and epilepsy due to malaria	Moderate motor impairment with blindness and epilepsy	(combined DW)	--
Moderate motor impairment with blindness and epilepsy due to malaria	Moderate motor impairment with blindness and epilepsy	(combined DW)	--
Moderate motor impairment with blindness and epilepsy due to malaria	Moderate motor impairment with blindness and epilepsy	(combined DW)	--
Moderate motor plus cognitive impairment with blindness due to malaria	Moderate motor plus cognitive impairment with blindness	(combined DW)	0.351 (0.245-0.467)
Moderate motor plus cognitive impairment with blindness due to malaria	Moderate motor plus cognitive impairment with blindness	(combined DW)	0.351 (0.245-0.467)
Moderate motor plus cognitive impairment with epilepsy due to malaria	Moderate motor plus cognitive impairment with epilepsy	(combined DW)	--
Moderate motor plus cognitive impairment with epilepsy due to malaria	Moderate motor plus cognitive impairment with epilepsy	(combined DW)	--
Moderate motor plus cognitive impairment with epilepsy due to malaria	Moderate motor plus cognitive impairment with epilepsy	(combined DW)	--
Moderate motor plus cognitive impairment with epilepsy due to malaria	Moderate motor plus cognitive impairment with epilepsy	(combined DW)	--
Moderate motor plus cognitive impairment with blindness and epilepsy due to malaria	Moderate motor plus cognitive impairment with blindness and epilepsy	(combined DW)	--
Moderate motor plus cognitive impairment with blindness and epilepsy due to malaria	Moderate motor plus cognitive impairment with blindness and epilepsy	(combined DW)	--
Moderate motor plus cognitive impairment with blindness and epilepsy due to malaria	Moderate motor plus cognitive impairment with blindness and epilepsy	(combined DW)	--
Moderate motor plus cognitive impairment with blindness and epilepsy due to malaria	Moderate motor plus cognitive impairment with blindness and epilepsy	(combined DW)	--
Moderate motor plus cognitive impairment with blindness and epilepsy due to malaria	Moderate motor plus cognitive impairment with blindness and epilepsy	(combined DW)	--
Severe motor impairment with blindness due to malaria	Severe motor impairment with blindness	(combined DW)	0.512 (0.365-0.658)
Severe motor impairment with epilepsy due to malaria	Severe motor impairment with epilepsy	(combined DW)	--
Severe motor impairment with epilepsy due to malaria	Severe motor impairment with epilepsy	(combined DW)	--
Severe motor impairment with epilepsy due to malaria	Severe motor impairment with epilepsy	(combined DW)	--
Severe motor impairment with blindness and epilepsy due to malaria	Severe motor impairment with blindness and epilepsy	(combined DW)	--
Severe motor impairment with blindness and epilepsy due to malaria	Severe motor impairment with blindness and epilepsy	(combined DW)	--
Severe motor impairment with blindness and epilepsy due to malaria	Severe motor impairment with blindness and epilepsy	(combined DW)	--
Severe motor impairment with blindness and epilepsy due to malaria	Severe motor impairment with blindness and epilepsy	(combined DW)	--
Severe motor plus cognitive impairment with blindness due to malaria	Severe motor plus cognitive impairment with blindness	(combined DW)	0.625 (0.454-0.778)

Severe motor plus cognitive impairment with blindness due to malaria	Severe motor plus cognitive impairment with blindness	(combined DW)	0.625 (0.454-0.778)
Severe motor plus cognitive impairment with epilepsy due to malaria	Severe motor plus cognitive impairment with epilepsy	(combined DW)	--
Severe motor plus cognitive impairment with epilepsy due to malaria	Severe motor plus cognitive impairment with epilepsy	(combined DW)	--
Severe motor plus cognitive impairment with epilepsy due to malaria	Severe motor plus cognitive impairment with epilepsy	(combined DW)	--
Severe motor plus cognitive impairment with epilepsy due to malaria	Severe motor plus cognitive impairment with epilepsy	(combined DW)	--
Severe motor plus cognitive impairment with blindness and epilepsy due to malaria	Severe motor plus cognitive impairment with blindness and epilepsy	(combined DW)	--
Severe motor plus cognitive impairment with blindness and epilepsy due to malaria	Severe motor plus cognitive impairment with blindness and epilepsy	(combined DW)	--
Severe motor plus cognitive impairment with blindness and epilepsy due to malaria	Severe motor plus cognitive impairment with blindness and epilepsy	(combined DW)	--
Severe motor plus cognitive impairment with blindness and epilepsy due to malaria	Severe motor plus cognitive impairment with blindness and epilepsy	(combined DW)	--
Severe motor plus cognitive impairment with blindness and epilepsy due to malaria	Severe motor plus cognitive impairment with blindness and epilepsy	(combined DW)	--
Mild malaria	Infectious disease, acute episode, mild	has a low fever and mild discomfort , but no difficulty with daily activities.	0.006 (0.002-0.012)
Moderate malaria	Infectious disease, acute episode, moderate	has a fever and aches, and feels weak, which causes some difficulty with daily activities.	0.051 (0.032-0.074)
Severe malaria	Infectious disease, acute episode, severe	has a high fever and pain, and feels very weak, which causes great difficulty with daily activities.	0.133 (0.088-0.19)
Severe malaria with mild anemia	Infectious disease, acute episode, severe, with mild anemia	(combined DW)	0.137 (0.091-0.192)
Severe malaria with moderate anemia	Infectious disease, acute episode, severe, with moderate anemia	(combined DW)	0.178 (0.122-0.247)
Severe malaria with severe anemia	Infectious disease, acute episode, severe, with severe anemia	(combined DW)	0.262 (0.184-0.359)
Mild malaria with mild anemia	Infectious disease, acute episode, mild, with mild anemia	(combined DW)	0.009 (0.004-0.02)
Mild malaria with moderate anemia	Infectious disease, acute episode, mild, with moderate anemia	(combined DW)	0.057 (0.037-0.085)
Mild malaria with severe anemia	Infectious disease, acute episode, mild, with severe anemia	(combined DW)	0.154 (0.105-0.214)
Moderate malaria with mild anemia	Infectious disease, acute episode, moderate, with mild anemia	(combined DW)	0.054 (0.034-0.079)
Moderate malaria with moderate anemia	Infectious disease, acute episode, moderate, with moderate anemia	(combined DW)	0.099 (0.065-0.142)
Moderate malaria with severe anemia	Infectious disease, acute episode, moderate, with severe anemia	(combined DW)	0.192 (0.133-0.263)
Mild anemia due to malaria parasitemia (PfPR)	Anemia, mild	feels slightly tired and weak at times, but this does not interfere with normal daily activities.	0.004 (0.001-0.008)
Mild anemia due to malaria vivax (PvPR)	Anemia, mild	feels slightly tired and weak at times, but this does not interfere with normal daily activities.	0.004 (0.001-0.008)
Moderate anemia due to malaria parasitemia (PfPR)	Anemia, moderate	feels moderate fatigue, weakness, and shortness of breath after exercise, making daily activities more difficult.	0.052 (0.034-0.076)
Moderate anemia due to malaria vivax (PvPR)	Anemia, moderate	feels moderate fatigue, weakness, and shortness of breath after exercise, making daily activities more difficult.	0.052 (0.034-0.076)
Severe anemia due to malaria parasitemia (PfPR)	Anemia, severe	feels very weak, tired and short of breath, and has problems with activities that require physical effort or deep concentration.	0.149 (0.101-0.209)
Severe anemia due to malaria vivax (PvPR)	Anemia, severe	feels very weak, tired and short of breath, and has problems with activities that require physical effort or deep concentration.	0.149 (0.101-0.209)
Asymptomatic malaria parasitemia (PfPR)	Asymptomatic		0 (0-0)
Asymptomatic malaria vivax (PvPR)	Asymptomatic		0 (0-0)
Acute Chagas disease	Infectious disease, acute episode, moderate	has a fever and aches, and feels weak, which causes some difficulty with daily activities.	0.051 (0.032-0.074)
Asymptomatic Chagas disease	Asymptomatic		0 (0-0)
Atrial fibrillation and flutter due to Chagas disease	Cardiac conduction disorders and cardiac dysrhythmias	has periods of rapid and irregular heartbeats and occasional fainting.	0.224 (0.151-0.312)
Mild heart failure due to Chagas disease	Heart failure, mild	is short of breath and easily tires with moderate physical activity, such as walking uphill or more than a quarter-mile on level ground. The person feels comfortable at rest or during activities requiring less effort.	0.041 (0.026-0.062)
Moderate heart failure due to Chagas disease	Heart failure, moderate	is short of breath and easily tires with minimal physical activity, such as walking only a short distance. The person feels comfortable at rest but avoids moderate activity.	0.072 (0.047-0.103)
Severe heart failure due to Chagas disease	Heart failure, severe	is short of breath and feels tired when at rest. The person avoids any physical activity, for fear of worsening the breathing problems.	0.179 (0.122-0.251)
Mild chronic digestive disease due to Chagas disease	Abdominopelvic problem, mild	has some pain in the belly that causes nausea but does not interfere with daily activities.	0.011 (0.005-0.021)
Moderate chronic digestive disease due to Chagas disease	Abdominopelvic problem, moderate	has pain in the belly and feels nauseous. The person has difficulties with daily activities.	0.114 (0.078-0.159)
Controlled, medically managed heart failure due to Chagas disease	Generic uncomplicated disease: worry and daily medication	has a chronic disease that requires medication every day and causes some worry but minimal interference with daily activities.	0.049 (0.031-0.072)
Moderate visceral leishmaniasis	Infectious disease, acute episode, moderate	has a fever and aches, and feels weak, which causes some difficulty with daily activities.	0.051 (0.032-0.074)
Severe visceral leishmaniasis	Infectious disease, acute episode, severe	has a high fever and pain, and feels very weak, which causes great difficulty with daily activities.	0.133 (0.088-0.19)
Cutaneous and mucocutaneous leishmaniasis	Disfigurement, level 2	has a visible physical deformity that causes others to stare and comment. As a result, the person is worried and has trouble sleeping and concentrating.	0.067 (0.044-0.096)
Skin disfigurement due to Trypanosoma brucei gambiense	Disfigurement, level 1 with itch/pain	has a slight, visible physical deformity that is sometimes sore or itchy. Others notice the deformity, which causes some worry and discomfort.	0.027 (0.015-0.042)
Sleeping sickness due to Trypanosoma brucei gambiense	Motor plus cognitive impairments, severe	cannot move around without help, and cannot lift or hold objects, get dressed or sit upright. The person also has very low intelligence, speaks few words, and needs constant supervision and help with all daily activities.	0.542 (0.374-0.702)
Sleeping sickness due to Trypanosoma brucei rhodesiense	Motor plus cognitive impairments, severe	cannot move around without help, and cannot lift or hold objects, get dressed or sit upright. The person also has very low intelligence, speaks few words, and needs constant supervision and help with all daily activities.	0.542 (0.374-0.702)
Skin disfigurement due to Trypanosoma brucei rhodesiense	Disfigurement, level 1 with itch/pain	has a slight, visible physical deformity that is sometimes sore or itchy. Others notice the deformity, which causes some worry and discomfort.	0.027 (0.015-0.042)
Mild schistosomiasis	Infectious disease, acute episode, mild	has a low fever and mild discomfort , but no difficulty with daily activities.	0.006 (0.002-0.012)
Mild anemia due to schistosomiasis	Anemia, mild	feels slightly tired and weak at times, but this does not interfere with normal daily activities.	0.004 (0.001-0.008)
Moderate anemia due to schistosomiasis	Anemia, moderate	feels moderate fatigue, weakness, and shortness of breath after exercise, making daily activities more difficult.	0.052 (0.034-0.076)
Severe anemia due to schistosomiasis	Anemia, severe	feels very weak, tired and short of breath, and has problems with activities that require physical effort or deep concentration.	0.149 (0.101-0.209)
Mild diarrhea due to schistosomiasis	Diarrhea, mild	has diarrhea three or more times a day with occasional discomfort in the belly.	0.074 (0.049-0.104)
Hematemesis due to schistosomiasis	Gastric bleeding	vomits blood and feels nauseous.	0.325 (0.209-0.462)
Hepatomegaly due to schistosomiasis	Abdominopelvic problem, mild	has some pain in the belly that causes nausea but does not interfere with daily activities.	0.011 (0.005-0.021)
Dysuria due to schistosomiasis	Abdominopelvic problem, mild	has some pain in the belly that causes nausea but does not interfere with daily activities.	0.011 (0.005-0.021)
Bladder pathology due to schistosomiasis	Abdominopelvic problem, mild	has some pain in the belly that causes nausea but does not interfere with daily activities.	0.011 (0.005-0.021)
Hydronephrosis due to schistosomiasis	Abdominopelvic problem, mild	has some pain in the belly that causes nausea but does not interfere with daily activities.	0.011 (0.005-0.021)
Ascites due to schistosomiasis	Abdominopelvic problem, moderate	has pain in the belly and feels nauseous. The person has difficulties with daily activities.	0.114 (0.078-0.159)
Neurocysticercosis with epilepsy	Epilepsy	(combined DW)	--
Neurocysticercosis with epilepsy	Epilepsy	(combined DW)	--
Neurocysticercosis with epilepsy	Epilepsy	(combined DW)	--
Chronic respiratory disease due to cystic echinococcosis	COPD and other chronic respiratory problems, mild	has cough and shortness of breath after heavy physical activity, but is able to walk long distances and climb stairs.	0.019 (0.011-0.033)
Abdominal problems due to cystic echinococcosis	Abdominopelvic problem, moderate	has pain in the belly and feels nauseous. The person has difficulties with daily activities.	0.114 (0.078-0.159)
Epilepsy due to echinococcosis	Epilepsy	(combined DW)	--
Epilepsy due to echinococcosis	Epilepsy	(combined DW)	--
Epilepsy due to echinococcosis	Epilepsy	(combined DW)	--

Prevalence of detectable microfiliaria due to lymphatic filariasis	Asymptomatic		0 (0-0)
Acute adenolymphangitis due to lymphatic filariasis	Infectious disease, acute episode, moderate	has a fever and aches, and feels weak, which causes some difficulty with daily activities.	0.051 (0.032-0.074)
Hydrocele due to lymphatic filariasis	Epididymo-orchitis	has swelling and tenderness in the testicles and pain during urination.	0.128 (0.086-0.18)
Lymphedema due to lymphatic filariasis	Lymphatic filariasis, symptomatic	has swollen legs with hard and thick skin, which causes difficulty in moving around.	0.109 (0.073-0.154)
Asymptomatic onchocerciasis	Asymptomatic		0 (0-0)
Mild skin disease without itch due to onchocerciasis	Disfigurement, level 1	has a slight, visible physical deformity that others notice, which causes some worry and discomfort.	0.011 (0.005-0.021)
Severe skin disease without itch due to onchocerciasis	Disfigurement, level 3	has an obvious physical deformity that makes others uncomfortable, which causes the person to avoid social contact, feel worried, sleep poorly, and think about suicide.	0.405 (0.275-0.546)
Mild skin disease due to onchocerciasis	Disfigurement, level 1 with itch/pain	has a slight, visible physical deformity that is sometimes sore or itchy. Others notice the deformity, which causes some worry and discomfort.	0.027 (0.015-0.042)
Moderate skin disease due to onchocerciasis	Disfigurement, level 2, with itch/pain	has a visible physical deformity that is sore and itchy. Other people stare and comment, which causes the person to worry. The person has trouble sleeping and concentrating.	0.188 (0.125-0.267)
Severe skin disease due to onchocerciasis	Disfigurement, level 2, with itch/pain	has a visible physical deformity that is sore and itchy. Other people stare and comment, which causes the person to worry. The person has trouble sleeping and concentrating.	0.188 (0.125-0.267)
Moderate vision impairment due to onchocerciasis	Distance vision, moderate impairment	has vision problems that make it difficult to recognize faces or objects across a room.	0.031 (0.019-0.049)
Severe vision impairment due to onchocerciasis	Distance vision, severe impairment	has severe vision loss, which causes difficulty in daily activities, some emotional impact (for example worry), and some difficulty going outside the home without assistance.	0.184 (0.125-0.258)
Blindness due to onchocerciasis	Distance vision blindness	is completely blind, which causes great difficulty in some daily activities, worry and anxiety, and great difficulty going outside the home without assistance.	0.187 (0.124-0.26)
Moderate vision impairment due to trachoma	Distance vision, moderate impairment	has vision problems that make it difficult to recognize faces or objects across a room.	0.031 (0.019-0.049)
Severe vision impairment due to trachoma	Distance vision, severe impairment	has severe vision loss, which causes difficulty in daily activities, some emotional impact (for example worry), and some difficulty going outside the home without assistance.	0.184 (0.125-0.258)
Blindness due to trachoma	Distance vision blindness	is completely blind, which causes great difficulty in some daily activities, worry and anxiety, and great difficulty going outside the home without assistance.	0.187 (0.124-0.26)
Post-dengue chronic fatigue syndrome	Infectious disease, post-acute consequences (fatigue, emotional lability, insomnia)	is always tired and easily upset. The person feels pain all over the body and is depressed.	0.219 (0.148-0.308)
Moderate dengue	Infectious disease, acute episode, moderate	has a fever and aches, and feels weak, which causes some difficulty with daily activities.	0.051 (0.032-0.074)
Severe dengue	Infectious disease, acute episode, severe	has a high fever and pain, and feels very weak, which causes great difficulty with daily activities.	0.133 (0.088-0.19)
Asymptomatic yellow fever	Asymptomatic		0 (0-0)
Moderate yellow fever	Infectious disease, acute episode, moderate	has a fever and aches, and feels weak, which causes some difficulty with daily activities.	0.051 (0.032-0.074)
Severe yellow fever	Infectious disease, acute episode, severe	has a high fever and pain, and feels very weak, which causes great difficulty with daily activities.	0.133 (0.088-0.19)
Rabies	Infectious disease, acute episode, severe	has a high fever and pain, and feels very weak, which causes great difficulty with daily activities.	0.133 (0.088-0.19)
Asymptomatic ascariasis	Asymptomatic		0 (0-0)
Heavy infestation of ascariasis	Intestinal nematode infections, symptomatic	has cramping pain and a bloated feeling in the belly.	0.027 (0.015-0.043)
Mild abdominopelvic problems due to ascariasis	Abdominopelvic problem, mild	has some pain in the belly that causes nausea but does not interfere with daily activities.	0.011 (0.005-0.021)
Severe wasting due to ascariasis	Severe wasting	is extremely skinny and has no energy.	0.128 (0.082-0.183)
Asymptomatic trichuriasis	Asymptomatic		0 (0-0)
Heavy infestation of trichuriasis	Intestinal nematode infections, symptomatic	has cramping pain and a bloated feeling in the belly.	0.027 (0.015-0.043)
Mild abdominopelvic problems due to trichuriasis	Abdominopelvic problem, mild	has some pain in the belly that causes nausea but does not interfere with daily activities.	0.011 (0.005-0.021)
Severe wasting due to trichuriasis	Severe wasting	is extremely skinny and has no energy.	0.128 (0.082-0.183)
Asymptomatic hookworm disease	Asymptomatic		0 (0-0)
Mild anemia due to hookworm disease	Anemia, mild	feels slightly tired and weak at times, but this does not interfere with normal daily activities.	0.004 (0.001-0.008)
Moderate anemia due to hookworm disease	Anemia, moderate	feels moderate fatigue, weakness, and shortness of breath after exercise, making daily activities more difficult.	0.052 (0.034-0.076)
Severe anemia due to hookworm disease	Anemia, severe	feels very weak, tired and short of breath, and has problems with activities that require physical effort or deep concentration.	0.149 (0.101-0.209)
Heavy infestation of hookworm	Intestinal nematode infections, symptomatic	has cramping pain and a bloated feeling in the belly.	0.027 (0.015-0.043)
Mild abdominopelvic problems due to hookworm disease	Abdominopelvic problem, mild	has some pain in the belly that causes nausea but does not interfere with daily activities.	0.011 (0.005-0.021)
Severe wasting due to hookworm disease	Severe wasting	is extremely skinny and has no energy.	0.128 (0.082-0.183)
Asymptomatic clonorchiasis	Asymptomatic		0 (0-0)
Asymptomatic fascioliasis	Asymptomatic		0 (0-0)
Asymptomatic intestinal fluke infection	Asymptomatic		0 (0-0)
Asymptomatic opisthorchiasis	Asymptomatic		0 (0-0)
Asymptomatic paragonimiasis	Asymptomatic		0 (0-0)
Mild paragonimiasis due to food-borne trematodiases	COPD and other chronic respiratory problems, mild	has cough and shortness of breath after heavy physical activity, but is able to walk long distances and climb stairs.	0.019 (0.011-0.033)
Moderate paragonimiasis due to food-borne trematodiases	COPD and other chronic respiratory problems, moderate	has cough, wheezing and shortness of breath, even after light physical activity. The person feels tired and can walk only short distances or climb only a few stairs.	0.225 (0.153-0.31)
Severe paragonimiasis due to food-borne trematodiases	COPD and other chronic respiratory problems, severe	has cough, wheezing and shortness of breath all the time. The person has great difficulty walking even short distances or climbing any stairs, feels tired when at rest, and is anxious.	0.408 (0.273-0.556)
Heavy clonorchiasis due to food-borne trematodiases	Abdominopelvic problem, moderate	has pain in the belly and feels nauseous. The person has difficulties with daily activities.	0.114 (0.078-0.159)
Heavy fascioliasis due to food-borne trematodiases	Abdominopelvic problem, moderate	has pain in the belly and feels nauseous. The person has difficulties with daily activities.	0.114 (0.078-0.159)
Heavy intestinal fluke infection due to food-borne trematodiases	Abdominopelvic problem, moderate	has pain in the belly and feels nauseous. The person has difficulties with daily activities.	0.114 (0.078-0.159)
Heavy opisthorchiasis due to food-borne trematodiases	Abdominopelvic problem, moderate	has pain in the belly and feels nauseous. The person has difficulties with daily activities.	0.114 (0.078-0.159)
Cerebral paragonimiasis	Epilepsy	(combined DW)	--
Cerebral paragonimiasis	Epilepsy	(combined DW)	--
Cerebral paragonimiasis	Epilepsy	(combined DW)	--
Disfigurement level 1 due to leprosy	Disfigurement, level 1	has a slight, visible physical deformity that others notice, which causes some worry and discomfort.	0.011 (0.005-0.021)
Disfigurement level 2 due to leprosy	Disfigurement, level 2	has a visible physical deformity that causes others to stare and comment. As a result, the person is worried and has trouble sleeping and concentrating.	0.067 (0.044-0.096)
Ebola cases	Infectious disease, acute episode, severe	has a high fever and pain, and feels very weak, which causes great difficulty with daily activities.	0.133 (0.088-0.19)
Post-Ebola chronic fatigue syndrome	Infectious disease, post-acute consequences (fatigue, emotional lability, insomnia)	is always tired and easily upset. The person feels pain all over the body and is depressed.	0.219 (0.148-0.308)
Guillain Barré syndrome due to Zika infection	Spinal cord lesion below neck level (treated)	is paralyzed from the waist down, cannot feel or move the legs and has difficulties with urine and bowel control. The person uses a wheelchair to move around.	0.296 (0.198-0.414)

Congenital Zika syndrome	Severe motor plus cognitive impairment with epilepsy	(combined DW)	--
Congenital Zika syndrome	Severe motor plus cognitive impairment with epilepsy	(combined DW)	--
Acute Zika infection	Infectious disease, acute episode, moderate	has a fever and aches, and feels weak, which causes some difficulty with daily activities.	0.051 (0.032-0.074)
Asymptomatic Zika infection	Asymptomatic		0 (0-0)
Moderate pain and limited mobility due to guinea worm	Musculoskeletal problems, lower limbs, moderate	has moderate pain in the leg, which makes the person limp, and causes some difficulty walking, standing, lifting and carrying heavy things, getting up and down and sleeping.	0.079 (0.054-0.11)
Mild pain due to Guinea worm emergence	Disfigurement, level 1 with itch/pain	has a slight, visible physical deformity that is sometimes sore or itchy. Others notice the deformity, which causes some worry and discomfort.	0.027 (0.015-0.042)
Acute infection due to other neglected tropical diseases	Post-COMO calculation for residuals (YLL/YLD ratio, other methods)		--
Mild anemia due to other neglected tropical diseases	Anemia, mild	feels slightly tired and weak at times, but this does not interfere with normal daily activities.	0.004 (0.001-0.008)
Moderate anemia due to other neglected tropical diseases	Anemia, moderate	feels moderate fatigue, weakness, and shortness of breath after exercise, making daily activities more difficult.	0.052 (0.034-0.076)
Severe anemia due to other neglected tropical diseases	Anemia, severe	feels very weak, tired and short of breath, and has problems with activities that require physical effort or deep concentration.	0.149 (0.101-0.209)
Acute meningitis	Infectious disease, acute episode, severe	has a high fever and pain, and feels very weak, which causes great difficulty with daily activities.	0.133 (0.088-0.19)
Acute viral meningitis	Infectious disease, acute episode, severe	has a high fever and pain, and feels very weak, which causes great difficulty with daily activities.	0.133 (0.088-0.19)
Mild behavioral problems due to meningitis	Attention deficit hyperactivity disorder	is hyperactive and has difficulty concentrating, remembering things, and completing tasks.	0.045 (0.028-0.066)
Borderline intellectual disability due to meningitis	Borderline intellectual functioning	is slow in learning at school. As an adult, the person has some difficulty doing complex or unfamiliar tasks but otherwise functions independently.	0.011 (0.005-0.02)
Mild intellectual disability due to meningitis	Intellectual disability / mental retardation, mild	has low intelligence and is slow in learning at school. As an adult, the person can live independently, but often needs help to raise children and can only work at simple supervised jobs.	0.043 (0.026-0.064)
Mild hearing loss due to meningitis	Hearing loss, mild	has great difficulty hearing and understanding another person talking in a noisy place (for example, on an urban street).	0.01 (0.004-0.019)
Moderate hearing loss due to meningitis	Hearing loss, moderate	is unable to hear and understand another person talking in a noisy place (for example, on an urban street), and has difficulty hearing another person talking even in a quiet place or on the phone.	0.027 (0.015-0.042)
Severe hearing loss due to meningitis	Hearing loss, severe	is unable to hear and understand another person talking, even in a quiet place, and unable to take part in a phone conversation. Difficulties with communicating and relating to others cause emotional impact at times (for example worry or depression).	0.158 (0.105-0.227)
Profound hearing loss due to meningitis	Hearing loss, profound	is unable to hear and understand another person talking, even in a quiet place, is unable to take part in a phone conversation, and has great difficulty hearing anything in any other situation. Difficulties with communicating and relating to othersoften cause worry, depression, and loneliness.	0.204 (0.134-0.288)
Complete hearing loss due to meningitis	Hearing loss, complete	cannot hear at all in any situation, including even the loudest sounds, and cannot communicate verbally or use a phone. Difficulties with communicating and relating to others often cause worry, depression or loneliness.	0.215 (0.144-0.307)
Mild hearing loss with ringing due to meningitis	Hearing loss, mild, with ringing	has great difficulty hearing and understanding another person talking in a noisy place (for example, on an urban street), and sometimes has annoying ringing in the ears.	0.021 (0.012-0.036)
Moderate hearing loss with ringing due to meningitis	Hearing loss, moderate, with ringing	is unable to hear and understand another person talking in a noisy place (for example, on an urban street), and has difficulty hearing another person talking even in a quiet place or on the phone, and has annoying ringing in the ears for more than 5 minutes at a time, almost everyday.	0.074 (0.049-0.107)
Severe hearing loss with ringing due to meningitis	Hearing loss, severe, with ringing	is unable to hear and understand another person talking, even in a quiet place, and unable to take part in a phone conversation, and has annoying ringing in the ears for more than 5 minutes at a time, almost everyday. Difficulties with communicating and relating to others cause emotional impact at times (for example worry or depression).	0.261 (0.175-0.36)
Profound hearing loss with ringing due to meningitis	Hearing loss, profound, with ringing	is unable to hear and understand another person talking, even in a quiet place, is unable to take part in a phone conversation, has great difficulty hearing anything in any other situation, and has annoying ringing in the ears for more than 5 minutes at a time, several times a day. Difficulties with communicating and relating to others often cause worry, depression, or loneliness.	0.277 (0.182-0.387)
Complete hearing loss with ringing due to meningitis	Hearing loss, complete, with ringing	cannot hear at all in any situation, including even the loudest sounds, and cannot communicate verbally or use a phone, and has very annoying ringing in the ears for more than half of the day. Difficulties with communicating and relating to others often cause worry, depression or loneliness.	0.316 (0.212-0.435)
Moderate vision impairment due to meningitis	Distance vision, moderate impairment	has vision problems that make it difficult to recognize faces or objects across a room.	0.031 (0.019-0.049)
Severe vision impairment due to meningitis	Distance vision, severe impairment	has severe vision loss, which causes difficulty in daily activities, some emotional impact (for example worry), and some difficulty going outside the home without assistance.	0.184 (0.125-0.258)
Blindness due to meningitis	Distance vision blindness	is completely blind, which causes great difficulty in some daily activities, worry and anxiety, and great difficulty going outside the home without assistance.	0.187 (0.124-0.26)
Mild motor impairment due to long term due to meningitis	Motor impairment, mild	has some difficulty in moving around but is able to walk without help.	0.01 (0.005-0.019)
Moderate motor impairment due to meningitis	Motor impairment, moderate	has some difficulty in moving around, and difficulty in lifting and holding objects, dressing and sitting upright, but is able to walk without help.	0.061 (0.04-0.089)
Severe motor impairment due to meningitis	Motor impairment, severe	is unable to move around without help, and is not able to lift or hold objects, get dressed or sit upright.	0.402 (0.268-0.545)
Mild motor plus cognitive impairments due to meningitis	Motor plus cognitive impairments, mild	has some difficulty in moving around but is able to walk without help. The person is slow in learning at school. As an adult, the person has some difficulty doing complex or unfamiliar tasks but otherwise functions independently.	0.031 (0.018-0.05)
Moderate motor plus cognitive impairments due to meningitis	Motor plus cognitive impairments, moderate	has some difficulty in moving around, holding objects, dressing and sitting upright, but can walk without help. The person has low intelligence and is slow in learning to speak and to do simple tasks.	0.203 (0.134-0.29)
Severe motor plus cognitive impairments due to meningitis	Motor plus cognitive impairments, severe	cannot move around without help, and cannot lift or hold objects, get dressed or sit upright. The person also has very low intelligence, speaks few words, and needs constant supervision and help with all daily activities.	0.542 (0.374-0.702)
Moderately severe hearing loss due to meningitis	Hearing loss, moderately severe	(custom DW from hearing loss impairment envelope)	0.092 (0.064-0.129)
Moderately severe hearing loss with ringing due to meningitis	Hearing loss, moderately severe, with ringing	(custom DW from hearing loss impairment envelope)	0.167 (0.115-0.231)
Monocular distance vision loss due to meningitis	Distance vision, monocular	is blind in one eye and has difficulty judging distances	0.017 (0.009-0.029)
Epilepsy due to meningitis	Epilepsy	(combined DW)	--
Epilepsy due to meningitis	Epilepsy	(combined DW)	--
Epilepsy due to meningitis	Epilepsy	(combined DW)	--
Acute encephalitis	Infectious disease, acute episode, severe	has a high fever and pain, and feels very weak, which causes great difficulty with daily activities.	0.133 (0.088-0.19)
Mild behavioral problems due to encephalitis	Attention deficit hyperactivity disorder	is hyperactive and has difficulty concentrating, remembering things, and completing tasks.	0.045 (0.028-0.066)
Borderline intellectual disability due to encephalitis	Borderline intellectual functioning	is slow in learning at school. As an adult, the person has some difficulty doing complex or unfamiliar tasks but otherwise functions independently.	0.011 (0.005-0.02)
Mild intellectual disability due to encephalitis	Intellectual disability / mental retardation, mild	has low intelligence and is slow in learning at school. As an adult, the person can live independently, but often needs help to raise children and can only work at simple supervised jobs.	0.043 (0.026-0.064)
Moderate vision impairment due to encephalitis	Distance vision, moderate impairment	has vision problems that make it difficult to recognize faces or objects across a room.	0.031 (0.019-0.049)
Severe vision impairment due to encephalitis	Distance vision, severe impairment	has severe vision loss, which causes difficulty in daily activities, some emotional impact (for example worry), and some difficulty going outside the home without assistance.	0.184 (0.125-0.258)
Blindness due to encephalitis	Distance vision blindness	is completely blind, which causes great difficulty in some daily activities, worry and anxiety, and great difficulty going outside the home without assistance.	0.187 (0.124-0.26)
Mild motor impairment due to long term due to encephalitis	Motor impairment, mild	has some difficulty in moving around but is able to walk without help.	0.01 (0.005-0.019)
Moderate motor impairment due to encephalitis	Motor impairment, moderate	has some difficulty in moving around, and difficulty in lifting and holding objects, dressing and sitting upright, but is able to walk without help.	0.061 (0.04-0.089)

Severe motor impairment due to encephalitis	Motor impairment, severe	is unable to move around without help, and is not able to lift or hold objects, get dressed or sit upright.	0.402 (0.268-0.545)
Mild motor plus cognitive impairments due to encephalitis	Motor plus cognitive impairments, mild	has some difficulty in moving around but is able to walk without help. The person is slow in learning at school. As an adult, the person has some difficulty doing complex or unfamiliar tasks but otherwise functions independently.	0.031 (0.018-0.05)
Moderate motor plus cognitive impairments due to encephalitis	Motor plus cognitive impairments, moderate	has some difficulty in moving around, holding objects, dressing and sitting upright, but can walk without help. The person has low intelligence and is slow in learning to speak and to do simple tasks.	0.203 (0.134-0.29)
Severe motor plus cognitive impairments due to encephalitis	Motor plus cognitive impairments, severe	cannot move around without help, and cannot lift or hold objects, get dressed or sit upright. The person also has very low intelligence, speaks few words, and needs constant supervision and help with all daily activities.	0.542 (0.374-0.702)
Monocular distance vision loss due to encephalitis	Distance vision, monocular	is blind in one eye and has difficulty judging distances	0.017 (0.009-0.029)
Epilepsy due to encephalitis	Epilepsy	(combined DW)	--
Epilepsy due to encephalitis	Epilepsy	(combined DW)	--
Epilepsy due to encephalitis	Epilepsy	(combined DW)	--
Moderate diphtheria	Infectious disease, acute episode, moderate	has a fever and aches, and feels weak, which causes some difficulty with daily activities.	0.051 (0.032-0.074)
Severe diphtheria	Infectious disease, acute episode, severe	has a high fever and pain, and feels very weak, which causes great difficulty with daily activities.	0.133 (0.088-0.19)
Whooping cough	Infectious disease, acute episode, moderate	has a fever and aches, and feels weak, which causes some difficulty with daily activities.	0.051 (0.032-0.074)
Severe tetanus	Infectious disease, acute episode, severe	has a high fever and pain, and feels very weak, which causes great difficulty with daily activities.	0.133 (0.088-0.19)
Mild motor impairment due to neonatal tetanus	Motor impairment, mild	has some difficulty in moving around but is able to walk without help.	0.01 (0.005-0.019)
Moderate motor impairment due to neonatal tetanus	Motor impairment, moderate	has some difficulty in moving around, and difficulty in lifting and holding objects, dressing and sitting upright, but is able to walk without help.	0.061 (0.04-0.089)
Severe motor impairment due to neonatal tetanus	Motor impairment, severe	is unable to move around without help, and is not able to lift or hold objects, get dressed or sit upright.	0.402 (0.268-0.545)
Mild motor plus cognitive impairments due to neonatal tetanus	Motor plus cognitive impairments, mild	has some difficulty in moving around but is able to walk without help. The person is slow in learning at school. As an adult, the person has some difficulty doing complex or unfamiliar tasks but otherwise functions independently.	0.031 (0.018-0.05)
Moderate motor impairment with blindness due to neonatal tetanus	Moderate motor impairment with blindness	(combined DW)	0.236 (0.165-0.323)
Moderate motor impairment with epilepsy due to neonatal tetanus	Moderate motor impairment with epilepsy	(combined DW)	--
Moderate motor impairment with epilepsy due to neonatal tetanus	Moderate motor impairment with epilepsy	(combined DW)	--
Moderate motor impairment with epilepsy due to neonatal tetanus	Moderate motor impairment with epilepsy	(combined DW)	--
Moderate motor impairment with blindness and epilepsy due to neonatal tetanus	Moderate motor impairment with blindness and epilepsy	(combined DW)	--
Moderate motor impairment with blindness and epilepsy due to neonatal tetanus	Moderate motor impairment with blindness and epilepsy	(combined DW)	--
Moderate motor impairment with blindness and epilepsy due to neonatal tetanus	Moderate motor impairment with blindness and epilepsy	(combined DW)	--
Moderate motor impairment with blindness and epilepsy due to neonatal tetanus	Moderate motor impairment with blindness and epilepsy	(combined DW)	--
Moderate motor plus cognitive impairment with blindness due to neonatal tetanus	Moderate motor plus cognitive impairment with blindness	(combined DW)	0.351 (0.245-0.467)
Moderate motor plus cognitive impairment with blindness due to neonatal tetanus	Moderate motor plus cognitive impairment with blindness	(combined DW)	0.351 (0.245-0.467)
Moderate motor plus cognitive impairment with epilepsy due to neonatal tetanus	Moderate motor plus cognitive impairment with epilepsy	(combined DW)	--
Moderate motor plus cognitive impairment with epilepsy due to neonatal tetanus	Moderate motor plus cognitive impairment with epilepsy	(combined DW)	--
Moderate motor plus cognitive impairment with epilepsy due to neonatal tetanus	Moderate motor plus cognitive impairment with epilepsy	(combined DW)	--
Moderate motor plus cognitive impairment with epilepsy due to neonatal tetanus	Moderate motor plus cognitive impairment with epilepsy	(combined DW)	--
Moderate motor plus cognitive impairment with blindness and epilepsy due to neonatal tetanus	Moderate motor plus cognitive impairment with blindness and epilepsy	(combined DW)	--
Moderate motor plus cognitive impairment with blindness and epilepsy due to neonatal tetanus	Moderate motor plus cognitive impairment with blindness and epilepsy	(combined DW)	--
Moderate motor plus cognitive impairment with blindness and epilepsy due to neonatal tetanus	Moderate motor plus cognitive impairment with blindness and epilepsy	(combined DW)	--
Moderate motor plus cognitive impairment with blindness and epilepsy due to neonatal tetanus	Moderate motor plus cognitive impairment with blindness and epilepsy	(combined DW)	--
Moderate motor plus cognitive impairment with blindness and epilepsy due to neonatal tetanus	Moderate motor plus cognitive impairment with blindness and epilepsy	(combined DW)	--
Severe motor impairment with blindness due to neonatal tetanus	Severe motor impairment with blindness	(combined DW)	0.512 (0.365-0.658)
Severe motor impairment with epilepsy due to neonatal tetanus	Severe motor impairment with epilepsy	(combined DW)	--
Severe motor impairment with epilepsy due to neonatal tetanus	Severe motor impairment with epilepsy	(combined DW)	--
Severe motor impairment with epilepsy due to neonatal tetanus	Severe motor impairment with epilepsy	(combined DW)	--
Severe motor impairment with blindness and epilepsy due to neonatal tetanus	Severe motor impairment with blindness and epilepsy	(combined DW)	--
Severe motor impairment with blindness and epilepsy due to neonatal tetanus	Severe motor impairment with blindness and epilepsy	(combined DW)	--
Severe motor impairment with blindness and epilepsy due to neonatal tetanus	Severe motor impairment with blindness and epilepsy	(combined DW)	--
Severe motor impairment with blindness and epilepsy due to neonatal tetanus	Severe motor impairment with blindness and epilepsy	(combined DW)	--
Severe motor plus cognitive impairment with blindness due to neonatal tetanus	Severe motor plus cognitive impairment with blindness	(combined DW)	0.625 (0.454-0.778)
Severe motor plus cognitive impairment with blindness due to neonatal tetanus	Severe motor plus cognitive impairment with blindness	(combined DW)	0.625 (0.454-0.778)
Severe motor plus cognitive impairment with epilepsy due to neonatal tetanus	Severe motor plus cognitive impairment with epilepsy	(combined DW)	--
Severe motor plus cognitive impairment with epilepsy due to neonatal tetanus	Severe motor plus cognitive impairment with epilepsy	(combined DW)	--
Severe motor plus cognitive impairment with epilepsy due to neonatal tetanus	Severe motor plus cognitive impairment with epilepsy	(combined DW)	--
Severe motor plus cognitive impairment with epilepsy due to neonatal tetanus	Severe motor plus cognitive impairment with epilepsy	(combined DW)	--
Severe motor plus cognitive impairment with blindness and epilepsy due to neonatal tetanus	Severe motor plus cognitive impairment with blindness and epilepsy	(combined DW)	--
Severe motor plus cognitive impairment with blindness and epilepsy due to neonatal tetanus	Severe motor plus cognitive impairment with blindness and epilepsy	(combined DW)	--
Severe motor plus cognitive impairment with blindness and epilepsy due to neonatal tetanus	Severe motor plus cognitive impairment with blindness and epilepsy	(combined DW)	--
Severe motor plus cognitive impairment with blindness and epilepsy due to neonatal tetanus	Severe motor plus cognitive impairment with blindness and epilepsy	(combined DW)	--
Severe motor plus cognitive impairment with blindness and epilepsy due to neonatal tetanus	Severe motor plus cognitive impairment with blindness and epilepsy	(combined DW)	--
Moderate measles	Infectious disease, acute episode, moderate	has a fever and aches, and feels weak, which causes some difficulty with daily activities.	0.051 (0.032-0.074)
Severe measles	Infectious disease, acute episode, severe	has a high fever and pain, and feels very weak, which causes great difficulty with daily activities.	0.133 (0.088-0.19)
Chickenpox	Infectious disease, acute episode, mild	has a low fever and mild discomfort , but no difficulty with daily activities.	0.006 (0.002-0.012)
Herpes zoster	Herpes zoster	has a blistering skin rash that causes pain, with some burning and itching.	0.058 (0.035-0.09)
Moderate acute hepatitis A	Infectious disease, acute episode, moderate	has a fever and aches, and feels weak, which causes some difficulty with daily activities.	0.051 (0.032-0.074)
Severe acute hepatitis A	Infectious disease, acute episode, severe	has a high fever and pain, and feels very weak, which causes great difficulty with daily activities.	0.133 (0.088-0.19)
Asymptomatic acute hepatitis A	Asymptomatic		0 (0-0)
Moderate acute hepatitis B	Infectious disease, acute episode, moderate	has a fever and aches, and feels weak, which causes some difficulty with daily activities.	0.051 (0.032-0.074)
Severe acute hepatitis B	Infectious disease, acute episode, severe	has a high fever and pain, and feels very weak, which causes great difficulty with daily activities.	0.133 (0.088-0.19)
Asymptomatic acute hepatitis B	Asymptomatic		0 (0-0)
Moderate acute hepatitis C	Infectious disease, acute episode, moderate	has a fever and aches, and feels weak, which causes some difficulty with daily activities.	0.051 (0.032-0.074)
Severe acute hepatitis C	Infectious disease, acute episode, severe	has a high fever and pain, and feels very weak, which causes great difficulty with daily activities.	0.133 (0.088-0.19)
Asymptomatic acute hepatitis C	Asymptomatic		0 (0-0)
Moderate acute hepatitis E	Infectious disease, acute episode, moderate	has a fever and aches, and feels weak, which causes some difficulty with daily activities.	0.051 (0.032-0.074)
Severe acute hepatitis E	Infectious disease, acute episode, severe	has a high fever and pain, and feels very weak, which causes great difficulty with daily activities.	0.133 (0.088-0.19)
Asymptomatic acute hepatitis E	Asymptomatic		0 (0-0)

Guillain-Barré syndrome due to other infectious diseases	Spinal cord lesion below neck level (treated)	is paralyzed from the waist down, cannot feel or move the legs and has difficulties with urine and bowel control. The person uses a wheelchair to move around.	0.296 (0.198-0.414)
Other infectious diseases	Post-COMO calculation for residuals (YLL/YLD ratio, other methods)		--
Mild anemia due to other infectious diseases	Anemia, mild	feels slightly tired and weak at times, but this does not interfere with normal daily activities.	0.004 (0.001-0.008)
Moderate anemia due to other infectious diseases	Anemia, moderate	feels moderate fatigue, weakness, and shortness of breath after exercise, making daily activities more difficult.	0.052 (0.034-0.076)
Severe anemia due to other infectious diseases	Anemia, severe	feels very weak, tired and short of breath, and has problems with activities that require physical effort or deep concentration.	0.149 (0.101-0.209)
Mild anemia due to maternal hemorrhage	Anemia, mild	feels slightly tired and weak at times, but this does not interfere with normal daily activities.	0.004 (0.001-0.008)
Moderate anemia due to maternal hemorrhage	Anemia, moderate	feels moderate fatigue, weakness, and shortness of breath after exercise, making daily activities more difficult.	0.052 (0.034-0.076)
Severe anemia due to maternal hemorrhage	Anemia, severe	feels very weak, tired and short of breath, and has problems with activities that require physical effort or deep concentration.	0.149 (0.101-0.209)
Maternal hemorrhage (< 1L blood lost)	Abdominopelvic problem, moderate	has pain in the belly and feels nauseous. The person has difficulties with daily activities.	0.114 (0.078-0.159)
Maternal hemorrhage (> 1L blood lost)	Abdominopelvic problem, severe	has severe pain in the belly and feels nauseous. The person is anxious and unable to carry out daily activities.	0.324 (0.22-0.442)
Infertility due to puerperal sepsis	Infertility, secondary	has at least one child, and wants to have more children. The person has a fertile partner, but the couple cannot conceive.	0.005 (0.002-0.011)
Other maternal infections	Infectious disease, acute episode, moderate	has a fever and aches, and feels weak, which causes some difficulty with daily activities.	0.051 (0.032-0.074)
Puerperal sepsis	Infectious disease, acute episode, severe	has a high fever and pain, and feels very weak, which causes great difficulty with daily activities.	0.133 (0.088-0.19)
Long term sequelae of severe pre-eclampsia	Tension-type headaches, mild motor plus cognitive impairment	(combined DW)	0.067 (0.041-0.103)
Long term sequelae of eclampsia	Tension-type headaches, mild motor plus cognitive impairment	(combined DW)	0.067 (0.041-0.103)
Other hypertensive disorders of pregnancy	Generic uncomplicated disease: worry and daily medication	has a chronic disease that requires medication every day and causes some worry but minimal interference with daily activities.	0.049 (0.031-0.072)
Severe pre-eclampsia	Moderate abdominal pain, tension-type headaches, mild motor plus cognitive impairment	(combined DW)	0.174 (0.12-0.239)
Eclampsia	Moderate abdominal pain and severe epilepsy	(combined DW)	0.602 (0.427-0.753)
Obstructed labor, acute event	Abdominopelvic problem, severe	has severe pain in the belly and feels nauseous. The person is anxious and unable to carry out daily activities.	0.324 (0.22-0.442)
Rectovaginal fistula	Rectovaginal fistula	has an abnormal opening between her vagina and rectum causing flatulence and feces to escape through the vagina. The person gets infections in her vagina, and has pain when urinating.	0.501 (0.339-0.657)
Vesicovaginal fistula	Vesicovaginal fistula	has an abnormal opening between the bladder and the vagina, which makes her unable to control urinating. The woman is anxious and depressed.	0.342 (0.227-0.478)
Maternal abortive outcome	Abdominopelvic problem, moderate	has pain in the belly and feels nauseous. The person has difficulties with daily activities.	0.114 (0.078-0.159)
Ectopic Pregnancy	Abdominopelvic problem, moderate	has pain in the belly and feels nauseous. The person has difficulties with daily activities.	0.114 (0.078-0.159)
Other maternal disorders	Post-COMO calculation for residuals (YLL/YLD ratio, other methods)		--
Mild motor impairment due to neonatal preterm birth complications <28wks	Motor impairment, mild	has some difficulty in moving around but is able to walk without help.	0.01 (0.005-0.019)
Mild motor impairment due to neonatal preterm birth complications 28-32wks	Motor impairment, mild	has some difficulty in moving around but is able to walk without help.	0.01 (0.005-0.019)
Mild motor impairment due to neonatal preterm birth complications 32-36wks	Motor impairment, mild	has some difficulty in moving around but is able to walk without help.	0.01 (0.005-0.019)
Moderate motor impairment due to neonatal preterm birth complications 28-32wks	Motor impairment, moderate	has some difficulty in moving around, and difficulty in lifting and holding objects, dressing and sitting upright, but is able to walk without help.	0.061 (0.04-0.089)
Moderate motor impairment due to neonatal preterm birth complications <28wks	Motor impairment, moderate	has some difficulty in moving around, and difficulty in lifting and holding objects, dressing and sitting upright, but is able to walk without help.	0.061 (0.04-0.089)
Moderate motor impairment due to neonatal preterm birth complications 32-36wks	Motor impairment, moderate	has some difficulty in moving around, and difficulty in lifting and holding objects, dressing and sitting upright, but is able to walk without help.	0.061 (0.04-0.089)
Severe motor impairment due to neonatal preterm birth complications <28wks	Motor impairment, severe	is unable to move around without help, and is not able to lift or hold objects, get dressed or sit upright.	0.402 (0.268-0.545)
Severe motor impairment due to neonatal preterm birth complications 32-36wks	Motor impairment, severe	is unable to move around without help, and is not able to lift or hold objects, get dressed or sit upright.	0.402 (0.268-0.545)
Severe motor impairment due to neonatal preterm birth complications 28-32wks	Motor impairment, severe	is unable to move around without help, and is not able to lift or hold objects, get dressed or sit upright.	0.402 (0.268-0.545)
Mild motor plus cognitive impairments due to neonatal preterm birth complications <28wks	Motor plus cognitive impairments, mild	has some difficulty in moving around but is able to walk without help. The person is slow in learning at school. As an adult, the person has some difficulty doing complex or unfamiliar tasks but otherwise functions independently.	0.031 (0.018-0.05)
Mild motor plus cognitive impairments due to neonatal preterm birth complications 28-32wks	Motor plus cognitive impairments, mild	has some difficulty in moving around but is able to walk without help. The person is slow in learning at school. As an adult, the person has some difficulty doing complex or unfamiliar tasks but otherwise functions independently.	0.031 (0.018-0.05)
Mild motor plus cognitive impairments due to neonatal preterm birth complications 32-36wks	Motor plus cognitive impairments, mild	has some difficulty in moving around but is able to walk without help. The person is slow in learning at school. As an adult, the person has some difficulty doing complex or unfamiliar tasks but otherwise functions independently.	0.031 (0.018-0.05)
Moderate motor impairment with blindness due to neonatal preterm birth complications <28wks	Moderate motor impairment with blindness	(combined DW)	0.236 (0.165-0.323)
Moderate motor impairment with blindness due to neonatal preterm birth complications 28-32wks	Moderate motor impairment with blindness	(combined DW)	0.236 (0.165-0.323)
Moderate motor impairment with blindness due to neonatal preterm birth complications 32-36wks	Moderate motor impairment with blindness	(combined DW)	0.236 (0.165-0.323)
Moderate motor impairment with epilepsy due to neonatal preterm birth complications 32-36wks	Moderate motor impairment with epilepsy	(combined DW)	--
Moderate motor impairment with epilepsy due to neonatal preterm birth complications 32-36wks	Moderate motor impairment with epilepsy	(combined DW)	--
Moderate motor impairment with epilepsy due to neonatal preterm birth complications 32-36wks	Moderate motor impairment with epilepsy	(combined DW)	--
Moderate motor impairment with epilepsy due to neonatal preterm birth complications 28-32wks	Moderate motor impairment with epilepsy	(combined DW)	--
Moderate motor impairment with epilepsy due to neonatal preterm birth complications 28-32wks	Moderate motor impairment with epilepsy	(combined DW)	--
Moderate motor impairment with epilepsy due to neonatal preterm birth complications 28-32wks	Moderate motor impairment with epilepsy	(combined DW)	--
Moderate motor impairment with epilepsy due to neonatal preterm birth complications <28wks	Moderate motor impairment with epilepsy	(combined DW)	--
Moderate motor impairment with epilepsy due to neonatal preterm birth complications <28wks	Moderate motor impairment with epilepsy	(combined DW)	--
Moderate motor impairment with epilepsy due to neonatal preterm birth complications <28wks	Moderate motor impairment with epilepsy	(combined DW)	--
Moderate motor impairment with blindness and epilepsy due to neonatal preterm birth complications 32-36wks	Moderate motor impairment with blindness and epilepsy	(combined DW)	--
Moderate motor impairment with blindness and epilepsy due to neonatal preterm birth complications 32-36wks	Moderate motor impairment with blindness and epilepsy	(combined DW)	--
Moderate motor impairment with blindness and epilepsy due to neonatal preterm birth complications 32-36wks	Moderate motor impairment with blindness and epilepsy	(combined DW)	--
Moderate motor impairment with blindness and epilepsy due to neonatal preterm birth complications 32-36wks	Moderate motor impairment with blindness and epilepsy	(combined DW)	--
Moderate motor impairment with blindness and epilepsy due to neonatal preterm birth complications 28-32wks	Moderate motor impairment with blindness and epilepsy	(combined DW)	--
Moderate motor impairment with blindness and epilepsy due to neonatal preterm birth complications 28-32wks	Moderate motor impairment with blindness and epilepsy	(combined DW)	--
Moderate motor impairment with blindness and epilepsy due to neonatal preterm birth complications 28-32wks	Moderate motor impairment with blindness and epilepsy	(combined DW)	--
Moderate motor impairment with blindness and epilepsy due to neonatal preterm birth complications 28-32wks	Moderate motor impairment with blindness and epilepsy	(combined DW)	--
Moderate motor impairment with blindness and epilepsy due to neonatal preterm birth complications <28wks	Moderate motor impairment with blindness and epilepsy	(combined DW)	--
Moderate motor impairment with blindness and epilepsy due to neonatal preterm birth complications <28wks	Moderate motor impairment with blindness and epilepsy	(combined DW)	--

Moderate motor impairment with blindness and epilepsy due to neonatal preterm birth complications <28wks	Moderate motor impairment with blindness and epilepsy	(combined DW)	--
Moderate motor impairment with blindness and epilepsy due to neonatal preterm birth complications <28wks	Moderate motor impairment with blindness and epilepsy	(combined DW)	--
Moderate motor plus cognitive impairment with blindness due to neonatal preterm birth complications 28-32wks	Moderate motor plus cognitive impairment with blindness	(combined DW)	0.351 (0.245-0.467)
Moderate motor plus cognitive impairment with blindness due to neonatal preterm birth complications 28-32wks	Moderate motor plus cognitive impairment with blindness	(combined DW)	0.351 (0.245-0.467)
Moderate motor plus cognitive impairment with blindness due to neonatal preterm birth complications <28wks	Moderate motor plus cognitive impairment with blindness	(combined DW)	0.351 (0.245-0.467)
Moderate motor plus cognitive impairment with blindness due to neonatal preterm birth complications <28wks	Moderate motor plus cognitive impairment with blindness	(combined DW)	0.351 (0.245-0.467)
Moderate motor plus cognitive impairment with blindness due to neonatal preterm birth complications 32-36wks	Moderate motor plus cognitive impairment with blindness	(combined DW)	0.351 (0.245-0.467)
Moderate motor plus cognitive impairment with blindness due to neonatal preterm birth complications 32-36wks	Moderate motor plus cognitive impairment with blindness	(combined DW)	0.351 (0.245-0.467)
Moderate motor plus cognitive impairment with epilepsy due to neonatal preterm birth complications 32-36wks	Moderate motor plus cognitive impairment with epilepsy	(combined DW)	--
Moderate motor plus cognitive impairment with epilepsy due to neonatal preterm birth complications 32-36wks	Moderate motor plus cognitive impairment with epilepsy	(combined DW)	--
Moderate motor plus cognitive impairment with epilepsy due to neonatal preterm birth complications 32-36wks	Moderate motor plus cognitive impairment with epilepsy	(combined DW)	--
Moderate motor plus cognitive impairment with epilepsy due to neonatal preterm birth complications 32-36wks	Moderate motor plus cognitive impairment with epilepsy	(combined DW)	--
Moderate motor plus cognitive impairment with epilepsy due to neonatal preterm birth complications <28wks	Moderate motor plus cognitive impairment with epilepsy	(combined DW)	--
Moderate motor plus cognitive impairment with epilepsy due to neonatal preterm birth complications <28wks	Moderate motor plus cognitive impairment with epilepsy	(combined DW)	--
Moderate motor plus cognitive impairment with epilepsy due to neonatal preterm birth complications <28wks	Moderate motor plus cognitive impairment with epilepsy	(combined DW)	--
Moderate motor plus cognitive impairment with epilepsy due to neonatal preterm birth complications <28wks	Moderate motor plus cognitive impairment with epilepsy	(combined DW)	--
Moderate motor plus cognitive impairment with epilepsy due to neonatal preterm birth complications 28-32wks	Moderate motor plus cognitive impairment with epilepsy	(combined DW)	--
Moderate motor plus cognitive impairment with epilepsy due to neonatal preterm birth complications 28-32wks	Moderate motor plus cognitive impairment with epilepsy	(combined DW)	--
Moderate motor plus cognitive impairment with epilepsy due to neonatal preterm birth complications 28-32wks	Moderate motor plus cognitive impairment with epilepsy	(combined DW)	--
Moderate motor plus cognitive impairment with epilepsy due to neonatal preterm birth complications 28-32wks	Moderate motor plus cognitive impairment with epilepsy	(combined DW)	--
Moderate motor plus cognitive impairment with blindness and epilepsy due to neonatal preterm birth complications 32-36wks	Moderate motor plus cognitive impairment with blindness and epilepsy	(combined DW)	--
Moderate motor plus cognitive impairment with blindness and epilepsy due to neonatal preterm birth complications 32-36wks	Moderate motor plus cognitive impairment with blindness and epilepsy	(combined DW)	--
Moderate motor plus cognitive impairment with blindness and epilepsy due to neonatal preterm birth complications 32-36wks	Moderate motor plus cognitive impairment with blindness and epilepsy	(combined DW)	--
Moderate motor plus cognitive impairment with blindness and epilepsy due to neonatal preterm birth complications 32-36wks	Moderate motor plus cognitive impairment with blindness and epilepsy	(combined DW)	--
Moderate motor plus cognitive impairment with blindness and epilepsy due to neonatal preterm birth complications 32-36wks	Moderate motor plus cognitive impairment with blindness and epilepsy	(combined DW)	--
Moderate motor plus cognitive impairment with blindness and epilepsy due to neonatal preterm birth complications <28wks	Moderate motor plus cognitive impairment with blindness and epilepsy	(combined DW)	--
Moderate motor plus cognitive impairment with blindness and epilepsy due to neonatal preterm birth complications <28wks	Moderate motor plus cognitive impairment with blindness and epilepsy	(combined DW)	--
Moderate motor plus cognitive impairment with blindness and epilepsy due to neonatal preterm birth complications <28wks	Moderate motor plus cognitive impairment with blindness and epilepsy	(combined DW)	--
Moderate motor plus cognitive impairment with blindness and epilepsy due to neonatal preterm birth complications <28wks	Moderate motor plus cognitive impairment with blindness and epilepsy	(combined DW)	--
Moderate motor plus cognitive impairment with blindness and epilepsy due to neonatal preterm birth complications <28wks	Moderate motor plus cognitive impairment with blindness and epilepsy	(combined DW)	--
Moderate motor plus cognitive impairment with blindness and epilepsy due to neonatal preterm birth complications 28-32wks	Moderate motor plus cognitive impairment with blindness and epilepsy	(combined DW)	--
Moderate motor plus cognitive impairment with blindness and epilepsy due to neonatal preterm birth complications 28-32wks	Moderate motor plus cognitive impairment with blindness and epilepsy	(combined DW)	--
Moderate motor plus cognitive impairment with blindness and epilepsy due to neonatal preterm birth complications 28-32wks	Moderate motor plus cognitive impairment with blindness and epilepsy	(combined DW)	--
Moderate motor plus cognitive impairment with blindness and epilepsy due to neonatal preterm birth complications 28-32wks	Moderate motor plus cognitive impairment with blindness and epilepsy	(combined DW)	--
Moderate motor plus cognitive impairment with blindness and epilepsy due to neonatal preterm birth complications 28-32wks	Moderate motor plus cognitive impairment with blindness and epilepsy	(combined DW)	--
Severe motor impairment with blindness due to neonatal preterm birth complications 28-32wks	Severe motor impairment with blindness	(combined DW)	0.512 (0.365-0.658)
Severe motor impairment with blindness due to neonatal preterm birth complications <28wks	Severe motor impairment with blindness	(combined DW)	0.512 (0.365-0.658)
Severe motor impairment with blindness due to neonatal preterm birth complications 32-36wks	Severe motor impairment with blindness	(combined DW)	0.512 (0.365-0.658)
Severe motor impairment with epilepsy due to neonatal preterm birth complications 32-36wks	Severe motor impairment with epilepsy	(combined DW)	--
Severe motor impairment with epilepsy due to neonatal preterm birth complications 32-36wks	Severe motor impairment with epilepsy	(combined DW)	--
Severe motor impairment with epilepsy due to neonatal preterm birth complications 32-36wks	Severe motor impairment with epilepsy	(combined DW)	--
Severe motor impairment with epilepsy due to neonatal preterm birth complications <28wks	Severe motor impairment with epilepsy	(combined DW)	--
Severe motor impairment with epilepsy due to neonatal preterm birth complications <28wks	Severe motor impairment with epilepsy	(combined DW)	--
Severe motor impairment with epilepsy due to neonatal preterm birth complications <28wks	Severe motor impairment with epilepsy	(combined DW)	--
Severe motor impairment with epilepsy due to neonatal preterm birth complications 28-32wks	Severe motor impairment with epilepsy	(combined DW)	--
Severe motor impairment with epilepsy due to neonatal preterm birth complications 28-32wks	Severe motor impairment with epilepsy	(combined DW)	--
Severe motor impairment with epilepsy due to neonatal preterm birth complications 28-32wks	Severe motor impairment with epilepsy	(combined DW)	--
Severe motor impairment with blindness and epilepsy due to neonatal preterm birth complications <28wks	Severe motor impairment with blindness and epilepsy	(combined DW)	--
Severe motor impairment with blindness and epilepsy due to neonatal preterm birth complications <28wks	Severe motor impairment with blindness and epilepsy	(combined DW)	--
Severe motor impairment with blindness and epilepsy due to neonatal preterm birth complications <28wks	Severe motor impairment with blindness and epilepsy	(combined DW)	--
Severe motor impairment with blindness and epilepsy due to neonatal preterm birth complications <28wks	Severe motor impairment with blindness and epilepsy	(combined DW)	--
Severe motor impairment with blindness and epilepsy due to neonatal preterm birth complications 28-32wks	Severe motor impairment with blindness and epilepsy	(combined DW)	--
Severe motor impairment with blindness and epilepsy due to neonatal preterm birth complications 28-32wks	Severe motor impairment with blindness and epilepsy	(combined DW)	--
Severe motor impairment with blindness and epilepsy due to neonatal preterm birth complications 28-32wks	Severe motor impairment with blindness and epilepsy	(combined DW)	--
Severe motor impairment with blindness and epilepsy due to neonatal preterm birth complications 28-32wks	Severe motor impairment with blindness and epilepsy	(combined DW)	--
Severe motor impairment with blindness and epilepsy due to neonatal preterm birth complications 32-36wks	Severe motor impairment with blindness and epilepsy	(combined DW)	--
Severe motor impairment with blindness and epilepsy due to neonatal preterm birth complications 32-36wks	Severe motor impairment with blindness and epilepsy	(combined DW)	--
Severe motor impairment with blindness and epilepsy due to neonatal preterm birth complications 32-36wks	Severe motor impairment with blindness and epilepsy	(combined DW)	--
Severe motor impairment with blindness and epilepsy due to neonatal preterm birth complications 32-36wks	Severe motor impairment with blindness and epilepsy	(combined DW)	--
Severe motor plus cognitive impairment with blindness due to neonatal preterm birth complications 28-32wks	Severe motor plus cognitive impairment with blindness	(combined DW)	0.625 (0.454-0.778)
Severe motor plus cognitive impairment with blindness due to neonatal preterm birth complications 28-32wks	Severe motor plus cognitive impairment with blindness	(combined DW)	0.625 (0.454-0.778)
Severe motor plus cognitive impairment with blindness due to neonatal preterm birth complications 32-36wks	Severe motor plus cognitive impairment with blindness	(combined DW)	0.625 (0.454-0.778)

Severe motor plus cognitive impairment with blindness due to neonatal preterm birth complications 32-36wks	Severe motor plus cognitive impairment with blindness	(combined DW)	0.625 (0.454-0.778)
Severe motor plus cognitive impairment with blindness due to neonatal preterm birth complications <28wks	Severe motor plus cognitive impairment with blindness	(combined DW)	0.625 (0.454-0.778)
Severe motor plus cognitive impairment with blindness due to neonatal preterm birth complications <28wks	Severe motor plus cognitive impairment with blindness	(combined DW)	0.625 (0.454-0.778)
Severe motor plus cognitive impairment with epilepsy due to neonatal preterm birth complications <28wks	Severe motor plus cognitive impairment with epilepsy	(combined DW)	--
Severe motor plus cognitive impairment with epilepsy due to neonatal preterm birth complications <28wks	Severe motor plus cognitive impairment with epilepsy	(combined DW)	--
Severe motor plus cognitive impairment with epilepsy due to neonatal preterm birth complications <28wks	Severe motor plus cognitive impairment with epilepsy	(combined DW)	--
Severe motor plus cognitive impairment with epilepsy due to neonatal preterm birth complications <28wks	Severe motor plus cognitive impairment with epilepsy	(combined DW)	--
Severe motor plus cognitive impairment with epilepsy due to neonatal preterm birth complications 32-36wks	Severe motor plus cognitive impairment with epilepsy	(combined DW)	--
Severe motor plus cognitive impairment with epilepsy due to neonatal preterm birth complications 32-36wks	Severe motor plus cognitive impairment with epilepsy	(combined DW)	--
Severe motor plus cognitive impairment with epilepsy due to neonatal preterm birth complications 32-36wks	Severe motor plus cognitive impairment with epilepsy	(combined DW)	--
Severe motor plus cognitive impairment with epilepsy due to neonatal preterm birth complications 32-36wks	Severe motor plus cognitive impairment with epilepsy	(combined DW)	--
Severe motor plus cognitive impairment with epilepsy due to neonatal preterm birth complications 28-32wks	Severe motor plus cognitive impairment with epilepsy	(combined DW)	--
Severe motor plus cognitive impairment with epilepsy due to neonatal preterm birth complications 28-32wks	Severe motor plus cognitive impairment with epilepsy	(combined DW)	--
Severe motor plus cognitive impairment with epilepsy due to neonatal preterm birth complications 28-32wks	Severe motor plus cognitive impairment with epilepsy	(combined DW)	--
Severe motor plus cognitive impairment with epilepsy due to neonatal preterm birth complications 28-32wks	Severe motor plus cognitive impairment with epilepsy	(combined DW)	--
Severe motor plus cognitive impairment with blindness and epilepsy due to neonatal preterm birth complications 28-32wks	Severe motor plus cognitive impairment with blindness and epilepsy	(combined DW)	--
Severe motor plus cognitive impairment with blindness and epilepsy due to neonatal preterm birth complications 28-32wks	Severe motor plus cognitive impairment with blindness and epilepsy	(combined DW)	--
Severe motor plus cognitive impairment with blindness and epilepsy due to neonatal preterm birth complications 28-32wks	Severe motor plus cognitive impairment with blindness and epilepsy	(combined DW)	--
Severe motor plus cognitive impairment with blindness and epilepsy due to neonatal preterm birth complications 28-32wks	Severe motor plus cognitive impairment with blindness and epilepsy	(combined DW)	--
Severe motor plus cognitive impairment with blindness and epilepsy due to neonatal preterm birth complications 28-32wks	Severe motor plus cognitive impairment with blindness and epilepsy	(combined DW)	--
Severe motor plus cognitive impairment with blindness and epilepsy due to neonatal preterm birth complications <28wks	Severe motor plus cognitive impairment with blindness and epilepsy	(combined DW)	--
Severe motor plus cognitive impairment with blindness and epilepsy due to neonatal preterm birth complications <28wks	Severe motor plus cognitive impairment with blindness and epilepsy	(combined DW)	--
Severe motor plus cognitive impairment with blindness and epilepsy due to neonatal preterm birth complications <28wks	Severe motor plus cognitive impairment with blindness and epilepsy	(combined DW)	--
Severe motor plus cognitive impairment with blindness and epilepsy due to neonatal preterm birth complications <28wks	Severe motor plus cognitive impairment with blindness and epilepsy	(combined DW)	--
Severe motor plus cognitive impairment with blindness and epilepsy due to neonatal preterm birth complications <28wks	Severe motor plus cognitive impairment with blindness and epilepsy	(combined DW)	--
Severe motor plus cognitive impairment with blindness and epilepsy due to neonatal preterm birth complications 32-36wks	Severe motor plus cognitive impairment with blindness and epilepsy	(combined DW)	--
Severe motor plus cognitive impairment with blindness and epilepsy due to neonatal preterm birth complications 32-36wks	Severe motor plus cognitive impairment with blindness and epilepsy	(combined DW)	--
Severe motor plus cognitive impairment with blindness and epilepsy due to neonatal preterm birth complications 32-36wks	Severe motor plus cognitive impairment with blindness and epilepsy	(combined DW)	--
Severe motor plus cognitive impairment with blindness and epilepsy due to neonatal preterm birth complications 32-36wks	Severe motor plus cognitive impairment with blindness and epilepsy	(combined DW)	--
Severe motor plus cognitive impairment with blindness and epilepsy due to neonatal preterm birth complications 32-36wks	Severe motor plus cognitive impairment with blindness and epilepsy	(combined DW)	--
Mild vision impairment due to retinopathy of prematurity	Distance vision, mild impairment	has some difficulty with distance vision, for example reading signs, but no other problems with eyesight.	0.003 (0.001-0.007)
Moderate vision impairment due to retinopathy of prematurity	Distance vision, moderate impairment	has vision problems that make it difficult to recognize faces or objects across a room.	0.031 (0.019-0.049)
Severe vision impairment due to retinopathy of prematurity	Distance vision, severe impairment	has severe vision loss, which causes difficulty in daily activities, some emotional impact (for example worry), and some difficulty going outside the home without assistance.	0.184 (0.125-0.258)
Blindness due to retinopathy of prematurity	Distance vision blindness	is completely blind, which causes great difficulty in some daily activities, worry and anxiety, and great difficulty going outside the home without assistance.	0.187 (0.124-0.26)
Asymptomatic retinopathy of prematurity	Asymptomatic		0 (0-0)
Asymptomatic neonatal preterm birth <28 weeks	Asymptomatic		0 (0-0)
Asymptomatic neonatal preterm birth 28-<32 wks	Asymptomatic		0 (0-0)
Asymptomatic neonatal preterm birth 32-<37wks	Asymptomatic		0 (0-0)
Mild motor impairment due to neonatal encephalopathy due to birth asphyxia and trauma	Motor impairment, mild	has some difficulty in moving around but is able to walk without help.	0.01 (0.005-0.019)
Moderate motor impairment due to neonatal encephalopathy due to birth asphyxia and trauma	Motor impairment, moderate	has some difficulty in moving around, and difficulty in lifting and holding objects, dressing and sitting upright, but is able to walk without help.	0.061 (0.04-0.089)
Severe motor impairment due to neonatal encephalopathy due to birth asphyxia and trauma	Motor impairment, severe	is unable to move around without help, and is not able to lift or hold objects, get dressed or sit upright.	0.402 (0.268-0.545)
Mild motor plus cognitive impairments due to neonatal encephalopathy due to birth asphyxia and trauma	Motor plus cognitive impairments, mild	has some difficulty in moving around but is able to walk without help. The person is slow in learning at school. As an adult, the person has some difficulty doing complex or unfamiliar tasks but otherwise functions independently.	0.031 (0.018-0.05)
Moderate motor impairment with blindness due to neonatal encephalopathy due to birth asphyxia and trauma	Moderate motor impairment with blindness	(combined DW)	0.236 (0.165-0.323)
Moderate motor impairment with epilepsy due to neonatal encephalopathy due to birth asphyxia and trauma	Moderate motor impairment with epilepsy	(combined DW)	--
Moderate motor impairment with epilepsy due to neonatal encephalopathy due to birth asphyxia and trauma	Moderate motor impairment with epilepsy	(combined DW)	--
Moderate motor impairment with epilepsy due to neonatal encephalopathy due to birth asphyxia and trauma	Moderate motor impairment with epilepsy	(combined DW)	--
Moderate motor impairment with blindness and epilepsy due to neonatal encephalopathy due to birth asphyxia and trauma	Moderate motor impairment with blindness and epilepsy	(combined DW)	--
Moderate motor impairment with blindness and epilepsy due to neonatal encephalopathy due to birth asphyxia and trauma	Moderate motor impairment with blindness and epilepsy	(combined DW)	--
Moderate motor impairment with blindness and epilepsy due to neonatal encephalopathy due to birth asphyxia and trauma	Moderate motor impairment with blindness and epilepsy	(combined DW)	--
Moderate motor impairment with blindness and epilepsy due to neonatal encephalopathy due to birth asphyxia and trauma	Moderate motor impairment with blindness and epilepsy	(combined DW)	--
Moderate motor plus cognitive impairment with blindness due to neonatal encephalopathy due to birth asphyxia and trauma	Moderate motor plus cognitive impairment with blindness	(combined DW)	0.351 (0.245-0.467)
Moderate motor plus cognitive impairment with blindness due to neonatal encephalopathy due to birth asphyxia and trauma	Moderate motor plus cognitive impairment with blindness	(combined DW)	0.351 (0.245-0.467)
Moderate motor plus cognitive impairment with epilepsy due to neonatal encephalopathy due to birth asphyxia and trauma	Moderate motor plus cognitive impairment with epilepsy	(combined DW)	--
Moderate motor plus cognitive impairment with epilepsy due to neonatal encephalopathy due to birth asphyxia and trauma	Moderate motor plus cognitive impairment with epilepsy	(combined DW)	--
Moderate motor plus cognitive impairment with epilepsy due to neonatal encephalopathy due to birth asphyxia and trauma	Moderate motor plus cognitive impairment with epilepsy	(combined DW)	--
Moderate motor plus cognitive impairment with epilepsy due to neonatal encephalopathy due to birth asphyxia and trauma	Moderate motor plus cognitive impairment with epilepsy	(combined DW)	--
Moderate motor plus cognitive impairment with blindness and epilepsy due to neonatal encephalopathy due to birth asphyxia and trauma	Moderate motor plus cognitive impairment with blindness and epilepsy	(combined DW)	--
Moderate motor plus cognitive impairment with blindness and epilepsy due to neonatal encephalopathy due to birth asphyxia and trauma	Moderate motor plus cognitive impairment with blindness and epilepsy	(combined DW)	--
Moderate motor plus cognitive impairment with blindness and epilepsy due to neonatal encephalopathy due to birth asphyxia and trauma	Moderate motor plus cognitive impairment with blindness and epilepsy	(combined DW)	--
Moderate motor plus cognitive impairment with blindness and epilepsy due to neonatal encephalopathy due to birth asphyxia and trauma	Moderate motor plus cognitive impairment with blindness and epilepsy	(combined DW)	--

Moderate motor plus cognitive impairment with blindness and epilepsy due to neonatal encephalopathy due to birth asphyxia and trauma	Moderate motor plus cognitive impairment with blindness and epilepsy	(combined DW)	--
Severe motor impairment with blindness due to neonatal encephalopathy due to birth asphyxia and trauma	Severe motor impairment with blindness	(combined DW)	0.512 (0.365-0.658)
Severe motor impairment with epilepsy due to neonatal encephalopathy due to birth asphyxia and trauma	Severe motor impairment with epilepsy	(combined DW)	--
Severe motor impairment with epilepsy due to neonatal encephalopathy due to birth asphyxia and trauma	Severe motor impairment with epilepsy	(combined DW)	--
Severe motor impairment with epilepsy due to neonatal encephalopathy due to birth asphyxia and trauma	Severe motor impairment with epilepsy	(combined DW)	--
Severe motor impairment with blindness and epilepsy due to neonatal encephalopathy due to birth asphyxia and trauma	Severe motor impairment with blindness and epilepsy	(combined DW)	--
Severe motor impairment with blindness and epilepsy due to neonatal encephalopathy due to birth asphyxia and trauma	Severe motor impairment with blindness and epilepsy	(combined DW)	--
Severe motor impairment with blindness and epilepsy due to neonatal encephalopathy due to birth asphyxia and trauma	Severe motor impairment with blindness and epilepsy	(combined DW)	--
Severe motor impairment with blindness and epilepsy due to neonatal encephalopathy due to birth asphyxia and trauma	Severe motor impairment with blindness and epilepsy	(combined DW)	--
Severe motor plus cognitive impairment with blindness due to neonatal encephalopathy due to birth asphyxia and trauma	Severe motor plus cognitive impairment with blindness	(combined DW)	0.625 (0.454-0.778)
Severe motor plus cognitive impairment with blindness due to neonatal encephalopathy due to birth asphyxia and trauma	Severe motor plus cognitive impairment with blindness	(combined DW)	0.625 (0.454-0.778)
Severe motor plus cognitive impairment with epilepsy due to neonatal encephalopathy due to birth asphyxia and trauma	Severe motor plus cognitive impairment with epilepsy	(combined DW)	--
Severe motor plus cognitive impairment with epilepsy due to neonatal encephalopathy due to birth asphyxia and trauma	Severe motor plus cognitive impairment with epilepsy	(combined DW)	--
Severe motor plus cognitive impairment with epilepsy due to neonatal encephalopathy due to birth asphyxia and trauma	Severe motor plus cognitive impairment with epilepsy	(combined DW)	--
Severe motor plus cognitive impairment with epilepsy due to neonatal encephalopathy due to birth asphyxia and trauma	Severe motor plus cognitive impairment with epilepsy	(combined DW)	--
Severe motor plus cognitive impairment with blindness and epilepsy due to neonatal encephalopathy due to birth asphyxia and trauma	Severe motor plus cognitive impairment with blindness and epilepsy	(combined DW)	--
Severe motor plus cognitive impairment with blindness and epilepsy due to neonatal encephalopathy due to birth asphyxia and trauma	Severe motor plus cognitive impairment with blindness and epilepsy	(combined DW)	--
Severe motor plus cognitive impairment with blindness and epilepsy due to neonatal encephalopathy due to birth asphyxia and trauma	Severe motor plus cognitive impairment with blindness and epilepsy	(combined DW)	--
Severe motor plus cognitive impairment with blindness and epilepsy due to neonatal encephalopathy due to birth asphyxia and trauma	Severe motor plus cognitive impairment with blindness and epilepsy	(combined DW)	--
Severe motor plus cognitive impairment with blindness and epilepsy due to neonatal encephalopathy due to birth asphyxia and trauma	Severe motor plus cognitive impairment with blindness and epilepsy	(combined DW)	--
Asymptomatic neonatal encephalopathy due to birth asphyxia and trauma	Asymptomatic		0 (0-0)
Mild motor impairment due to neonatal sepsis and other neonatal infections	Motor impairment, mild	has some difficulty in moving around but is able to walk without help.	0.01 (0.005-0.019)
Moderate motor impairment due to neonatal sepsis and other neonatal infections	Motor impairment, moderate	has some difficulty in moving around, and difficulty in lifting and holding objects, dressing and sitting upright, but is able to walk without help.	0.061 (0.04-0.089)
Severe motor impairment due to neonatal sepsis and other neonatal infections	Motor impairment, severe	is unable to move around without help, and is not able to lift or hold objects, get dressed or sit upright.	0.402 (0.268-0.545)
Mild motor plus cognitive impairments due to neonatal sepsis and other neonatal infections	Motor plus cognitive impairments, mild	has some difficulty in moving around but is able to walk without help. The person is slow in learning at school. As an adult, the person has some difficulty doing complex or unfamiliar tasks but otherwise functions independently.	0.031 (0.018-0.05)
Moderate motor impairment with blindness due to neonatal sepsis and other neonatal infections	Moderate motor impairment with blindness	(combined DW)	0.236 (0.165-0.323)
Moderate motor impairment with blindness due to neonatal sepsis and other neonatal infections	Moderate motor impairment with blindness	(combined DW)	0.236 (0.165-0.323)
Moderate motor impairment with epilepsy due to neonatal sepsis and other neonatal infections	Moderate motor impairment with epilepsy	(combined DW)	--
Moderate motor impairment with epilepsy due to neonatal sepsis and other neonatal infections	Moderate motor impairment with epilepsy	(combined DW)	--
Moderate motor impairment with epilepsy due to neonatal sepsis and other neonatal infections	Moderate motor impairment with epilepsy	(combined DW)	--
Moderate motor impairment with epilepsy due to neonatal sepsis and other neonatal infections	Moderate motor impairment with epilepsy	(combined DW)	--
Moderate motor impairment with blindness and epilepsy due to neonatal sepsis and other neonatal infections	Moderate motor impairment with blindness and epilepsy	(combined DW)	--
Moderate motor impairment with blindness and epilepsy due to neonatal sepsis and other neonatal infections	Moderate motor impairment with blindness and epilepsy	(combined DW)	--
Moderate motor impairment with blindness and epilepsy due to neonatal sepsis and other neonatal infections	Moderate motor impairment with blindness and epilepsy	(combined DW)	--
Moderate motor impairment with blindness and epilepsy due to neonatal sepsis and other neonatal infections	Moderate motor impairment with blindness and epilepsy	(combined DW)	--
Moderate motor impairment with blindness and epilepsy due to neonatal sepsis and other neonatal infections	Moderate motor impairment with blindness and epilepsy	(combined DW)	--
Moderate motor plus cognitive impairment with blindness due to neonatal sepsis and other neonatal infections	Moderate motor plus cognitive impairment with blindness	(combined DW)	0.351 (0.245-0.467)
Moderate motor plus cognitive impairment with blindness due to neonatal sepsis and other neonatal infections	Moderate motor plus cognitive impairment with blindness	(combined DW)	0.351 (0.245-0.467)
Moderate motor plus cognitive impairment with epilepsy due to neonatal sepsis and other neonatal infections	Moderate motor plus cognitive impairment with epilepsy	(combined DW)	--
Moderate motor plus cognitive impairment with epilepsy due to neonatal sepsis and other neonatal infections	Moderate motor plus cognitive impairment with epilepsy	(combined DW)	--
Moderate motor plus cognitive impairment with epilepsy due to neonatal sepsis and other neonatal infections	Moderate motor plus cognitive impairment with epilepsy	(combined DW)	--
Moderate motor plus cognitive impairment with epilepsy due to neonatal sepsis and other neonatal infections	Moderate motor plus cognitive impairment with epilepsy	(combined DW)	--
Moderate motor plus cognitive impairment with blindness and epilepsy due to neonatal sepsis and other neonatal infections	Moderate motor plus cognitive impairment with blindness and epilepsy	(combined DW)	--
Moderate motor plus cognitive impairment with blindness and epilepsy due to neonatal sepsis and other neonatal infections	Moderate motor plus cognitive impairment with blindness and epilepsy	(combined DW)	--
Moderate motor plus cognitive impairment with blindness and epilepsy due to neonatal sepsis and other neonatal infections	Moderate motor plus cognitive impairment with blindness and epilepsy	(combined DW)	--
Moderate motor plus cognitive impairment with blindness and epilepsy due to neonatal sepsis and other neonatal infections	Moderate motor plus cognitive impairment with blindness and epilepsy	(combined DW)	--
Moderate motor plus cognitive impairment with blindness and epilepsy due to neonatal sepsis and other neonatal infections	Moderate motor plus cognitive impairment with blindness and epilepsy	(combined DW)	--
Severe motor impairment with blindness due to neonatal sepsis and other neonatal infections	Severe motor impairment with blindness	(combined DW)	0.512 (0.365-0.658)
Severe motor impairment with blindness due to neonatal sepsis and other neonatal infections	Severe motor impairment with blindness	(combined DW)	0.512 (0.365-0.658)
Severe motor impairment with epilepsy due to neonatal sepsis and other neonatal infections	Severe motor impairment with epilepsy	(combined DW)	--
Severe motor impairment with epilepsy due to neonatal sepsis and other neonatal infections	Severe motor impairment with epilepsy	(combined DW)	--
Severe motor impairment with epilepsy due to neonatal sepsis and other neonatal infections	Severe motor impairment with epilepsy	(combined DW)	--
Severe motor impairment with epilepsy due to neonatal sepsis and other neonatal infections	Severe motor impairment with epilepsy	(combined DW)	--
Severe motor impairment with blindness and epilepsy due to neonatal sepsis and other neonatal infections	Severe motor impairment with blindness and epilepsy	(combined DW)	--
Severe motor impairment with blindness and epilepsy due to neonatal sepsis and other neonatal infections	Severe motor impairment with blindness and epilepsy	(combined DW)	--
Severe motor impairment with blindness and epilepsy due to neonatal sepsis and other neonatal infections	Severe motor impairment with blindness and epilepsy	(combined DW)	--
Severe motor impairment with blindness and epilepsy due to neonatal sepsis and other neonatal infections	Severe motor impairment with blindness and epilepsy	(combined DW)	--
Severe motor impairment with blindness and epilepsy due to neonatal sepsis and other neonatal infections	Severe motor impairment with blindness and epilepsy	(combined DW)	--
Severe motor plus cognitive impairment with blindness due to neonatal sepsis and other neonatal infections	Severe motor plus cognitive impairment with blindness	(combined DW)	0.625 (0.454-0.778)
Severe motor plus cognitive impairment with blindness due to neonatal sepsis and other neonatal infections	Severe motor plus cognitive impairment with blindness	(combined DW)	0.625 (0.454-0.778)
Severe motor plus cognitive impairment with epilepsy due to neonatal sepsis and other neonatal infections	Severe motor plus cognitive impairment with epilepsy	(combined DW)	--
Severe motor plus cognitive impairment with epilepsy due to neonatal sepsis and other neonatal infections	Severe motor plus cognitive impairment with epilepsy	(combined DW)	--

Severe motor plus cognitive impairment with epilepsy due to neonatal sepsis and other neonatal infections	Severe motor plus cognitive impairment with epilepsy	(combined DW)	--
Severe motor plus cognitive impairment with epilepsy due to neonatal sepsis and other neonatal infections	Severe motor plus cognitive impairment with epilepsy	(combined DW)	--
Severe motor plus cognitive impairment with blindness and epilepsy due to neonatal sepsis and other neonatal infections	Severe motor plus cognitive impairment with blindness and epilepsy	(combined DW)	--
Severe motor plus cognitive impairment with blindness and epilepsy due to neonatal sepsis and other neonatal infections	Severe motor plus cognitive impairment with blindness and epilepsy	(combined DW)	--
Severe motor plus cognitive impairment with blindness and epilepsy due to neonatal sepsis and other neonatal infections	Severe motor plus cognitive impairment with blindness and epilepsy	(combined DW)	--
Severe motor plus cognitive impairment with blindness and epilepsy due to neonatal sepsis and other neonatal infections	Severe motor plus cognitive impairment with blindness and epilepsy	(combined DW)	--
Severe motor plus cognitive impairment with blindness and epilepsy due to neonatal sepsis and other neonatal infections	Severe motor plus cognitive impairment with blindness and epilepsy	(combined DW)	--
Asymptomatic neonatal sepsis and other neonatal infections	Asymptomatic		0 (0-0)
Extreme hyperbilirubinemia due to hemolytic disease and other neonatal jaundice, without kernicterus	Abdominopelvic problem, severe	has severe pain in the belly and feels nauseous. The person is anxious and unable to carry out daily activities.	0.324 (0.22-0.442)
Moderate motor impairment due to hemolytic disease and other neonatal jaundice	Motor impairment, moderate	has some difficulty in moving around, and difficulty in lifting and holding objects, dressing and sitting upright, but is able to walk without help.	0.061 (0.04-0.089)
Severe motor impairment severe due to hemolytic disease and other neonatal jaundice	Motor impairment, severe	is unable to move around without help, and is not able to lift or hold objects, get dressed or sit upright.	0.402 (0.268-0.545)
Moderate motor impairment with blindness due to hemolytic disease and other neonatal jaundice	Moderate motor impairment with blindness	(combined DW)	0.236 (0.165-0.323)
Moderate motor impairment with epilepsy due to hemolytic disease and other neonatal jaundice	Moderate motor impairment with epilepsy	(combined DW)	--
Moderate motor impairment with epilepsy due to hemolytic disease and other neonatal jaundice	Moderate motor impairment with epilepsy	(combined DW)	--
Moderate motor impairment with epilepsy due to hemolytic disease and other neonatal jaundice	Moderate motor impairment with epilepsy	(combined DW)	--
Moderate motor impairment with blindness and epilepsy due to hemolytic disease and other neonatal jaundice	Moderate motor impairment with blindness and epilepsy	(combined DW)	--
Moderate motor impairment with blindness and epilepsy due to hemolytic disease and other neonatal jaundice	Moderate motor impairment with blindness and epilepsy	(combined DW)	--
Moderate motor impairment with blindness and epilepsy due to hemolytic disease and other neonatal jaundice	Moderate motor impairment with blindness and epilepsy	(combined DW)	--
Moderate motor impairment with blindness and epilepsy due to hemolytic disease and other neonatal jaundice	Moderate motor impairment with blindness and epilepsy	(combined DW)	--
Moderate motor plus cognitive impairment with blindness due to hemolytic disease and other neonatal jaundice	Moderate motor plus cognitive impairment with blindness	(combined DW)	0.351 (0.245-0.467)
Moderate motor plus cognitive impairment with blindness due to hemolytic disease and other neonatal jaundice	Moderate motor plus cognitive impairment with blindness	(combined DW)	0.351 (0.245-0.467)
Moderate motor plus cognitive impairment with epilepsy due to hemolytic disease and other neonatal jaundice	Moderate motor plus cognitive impairment with epilepsy	(combined DW)	--
Moderate motor plus cognitive impairment with epilepsy due to hemolytic disease and other neonatal jaundice	Moderate motor plus cognitive impairment with epilepsy	(combined DW)	--
Moderate motor plus cognitive impairment with epilepsy due to hemolytic disease and other neonatal jaundice	Moderate motor plus cognitive impairment with epilepsy	(combined DW)	--
Moderate motor plus cognitive impairment with epilepsy due to hemolytic disease and other neonatal jaundice	Moderate motor plus cognitive impairment with epilepsy	(combined DW)	--
Moderate motor plus cognitive impairment with blindness and epilepsy due to hemolytic disease and other neonatal jaundice	Moderate motor plus cognitive impairment with blindness and epilepsy	(combined DW)	--
Moderate motor plus cognitive impairment with blindness and epilepsy due to hemolytic disease and other neonatal jaundice	Moderate motor plus cognitive impairment with blindness and epilepsy	(combined DW)	--
Moderate motor plus cognitive impairment with blindness and epilepsy due to hemolytic disease and other neonatal jaundice	Moderate motor plus cognitive impairment with blindness and epilepsy	(combined DW)	--
Moderate motor plus cognitive impairment with blindness and epilepsy due to hemolytic disease and other neonatal jaundice	Moderate motor plus cognitive impairment with blindness and epilepsy	(combined DW)	--
Moderate motor plus cognitive impairment with blindness and epilepsy due to hemolytic disease and other neonatal jaundice	Moderate motor plus cognitive impairment with blindness and epilepsy	(combined DW)	--
Severe motor impairment with blindness due to hemolytic disease and other neonatal jaundice	Severe motor impairment with blindness	(combined DW)	0.512 (0.365-0.658)
Severe motor impairment with epilepsy due to hemolytic disease and other neonatal jaundice	Severe motor impairment with epilepsy	(combined DW)	--
Severe motor impairment with epilepsy due to hemolytic disease and other neonatal jaundice	Severe motor impairment with epilepsy	(combined DW)	--
Severe motor impairment with epilepsy due to hemolytic disease and other neonatal jaundice	Severe motor impairment with epilepsy	(combined DW)	--
Severe motor impairment with blindness and epilepsy due to hemolytic disease and other neonatal jaundice	Severe motor impairment with blindness and epilepsy	(combined DW)	--
Severe motor impairment with blindness and epilepsy due to hemolytic disease and other neonatal jaundice	Severe motor impairment with blindness and epilepsy	(combined DW)	--
Severe motor impairment with blindness and epilepsy due to hemolytic disease and other neonatal jaundice	Severe motor impairment with blindness and epilepsy	(combined DW)	--
Severe motor impairment with blindness and epilepsy due to hemolytic disease and other neonatal jaundice	Severe motor impairment with blindness and epilepsy	(combined DW)	--
Severe motor plus cognitive impairment with blindness due to hemolytic disease and other neonatal jaundice	Severe motor plus cognitive impairment with blindness	(combined DW)	0.625 (0.454-0.778)
Severe motor plus cognitive impairment with blindness due to hemolytic disease and other neonatal jaundice	Severe motor plus cognitive impairment with blindness	(combined DW)	0.625 (0.454-0.778)
Severe motor plus cognitive impairment with epilepsy due to hemolytic disease and other neonatal jaundice	Severe motor plus cognitive impairment with epilepsy	(combined DW)	--
Severe motor plus cognitive impairment with epilepsy due to hemolytic disease and other neonatal jaundice	Severe motor plus cognitive impairment with epilepsy	(combined DW)	--
Severe motor plus cognitive impairment with epilepsy due to hemolytic disease and other neonatal jaundice	Severe motor plus cognitive impairment with epilepsy	(combined DW)	--
Severe motor plus cognitive impairment with epilepsy due to hemolytic disease and other neonatal jaundice	Severe motor plus cognitive impairment with epilepsy	(combined DW)	--
Severe motor plus cognitive impairment with blindness and epilepsy due to hemolytic disease and other neonatal jaundice	Severe motor plus cognitive impairment with blindness and epilepsy	(combined DW)	--
Severe motor plus cognitive impairment with blindness and epilepsy due to hemolytic disease and other neonatal jaundice	Severe motor plus cognitive impairment with blindness and epilepsy	(combined DW)	--
Severe motor plus cognitive impairment with blindness and epilepsy due to hemolytic disease and other neonatal jaundice	Severe motor plus cognitive impairment with blindness and epilepsy	(combined DW)	--
Severe motor plus cognitive impairment with blindness and epilepsy due to hemolytic disease and other neonatal jaundice	Severe motor plus cognitive impairment with blindness and epilepsy	(combined DW)	--
Severe motor plus cognitive impairment with blindness and epilepsy due to hemolytic disease and other neonatal jaundice	Severe motor plus cognitive impairment with blindness and epilepsy	(combined DW)	--
Other neonatal disorders	Post-COMO calculation for residuals (YLL/YLD ratio, other methods)		--
Moderate wasting with edema	Kwashiorkor	is very tired and irritable and has diarrhea.	0.051 (0.031-0.079)
Severe wasting without edema	Severe wasting	is extremely skinny and has no energy.	0.128 (0.082-0.183)
Moderate wasting without edema	Asymptomatic		0 (0-0)
Severe wasting with edema	Kwashiorkor and severe wasting	(combined DW)	0.172 (0.115-0.238)
Visible goiter without symptoms	Disfigurement, level 1	has a slight, visible physical deformity that others notice, which causes some worry and discomfort.	0.011 (0.005-0.021)
Visible goiter with profound intellectual disability due to iodine deficiency	Intellectual disability / mental retardation, profound and Iodine-deficiency goiter	(combined DW)	0.358 (0.252-0.475)
Visible goiter with severe intellectual disability due to iodine deficiency	Intellectual disability / mental retardation, severe and Iodine-deficiency goiter	(combined DW)	0.326 (0.233-0.438)
Asymptomatic vitamin A deficiency	Asymptomatic		0 (0-0)
Moderate vision impairment loss due to vitamin A deficiency	Distance vision, moderate impairment	has vision problems that make it difficult to recognize faces or objects across a room.	0.031 (0.019-0.049)
Severe vision impairment loss due to vitamin A deficiency	Distance vision, severe impairment	has severe vision loss, which causes difficulty in daily activities, some emotional impact (for example worry), and some difficulty going outside the home without assistance.	0.184 (0.125-0.258)
Blindness due to vitamin A deficiency	Distance vision blindness	is completely blind, which causes great difficulty in some daily activities, worry and anxiety, and great difficulty going outside the home without assistance.	0.187 (0.124-0.26)

Vitamin A deficiency with mild anemia	Anemia, mild	feels slightly tired and weak at times, but this does not interfere with normal daily activities.	0.004 (0.001-0.008)
Vitamin A deficiency with moderate anemia	Anemia, moderate	feels moderate fatigue, weakness, and shortness of breath after exercise, making daily activities more difficult.	0.052 (0.034-0.076)
Vitamin A deficiency with severe anemia	Anemia, severe	feels very weak, tired and short of breath, and has problems with activities that require physical effort or deep concentration.	0.149 (0.101-0.209)
Mild iron-deficiency anemia	Anemia, mild	feels slightly tired and weak at times, but this does not interfere with normal daily activities.	0.004 (0.001-0.008)
Moderate iron-deficiency anemia	Anemia, moderate	feels moderate fatigue, weakness, and shortness of breath after exercise, making daily activities more difficult.	0.052 (0.034-0.076)
Severe iron-deficiency anemia	Anemia, severe	feels very weak, tired and short of breath, and has problems with activities that require physical effort or deep concentration.	0.149 (0.101-0.209)
Other nutritional deficiencies	Post-COMO calculation for residuals (YLL/YLD ratio, other methods)		--
Diagnosis and primary therapy phase of mouth cancer	Cancer, diagnosis and primary therapy	has pain, nausea, fatigue, weight loss and high anxiety.	0.288 (0.193-0.399)
Metastatic phase of mouth cancer	Cancer, metastatic	has severe pain, extreme fatigue, weight loss and high anxiety.	0.451 (0.307-0.6)
Terminal phase of mouth cancer	Terminal phase, with medication (for cancers, end-stage kidney/liver disease)	has lost a lot of weight and regularly uses strong medication to avoid constant pain. The person has no appetite, feels nauseous, and needs to spend most of the day in bed.	0.54 (0.377-0.687)
Controlled phase of mouth cancer	Generic uncomplicated disease: worry and daily medication	has a chronic disease that requires medication every day and causes some worry but minimal interference with daily activities.	0.049 (0.031-0.072)
Diagnosis and primary therapy phase of nasopharynx cancer	Cancer, diagnosis and primary therapy	has pain, nausea, fatigue, weight loss and high anxiety.	0.288 (0.193-0.399)
Metastatic phase of nasopharynx cancer	Cancer, metastatic	has severe pain, extreme fatigue, weight loss and high anxiety.	0.451 (0.307-0.6)
Terminal phase of nasopharynx cancer	Terminal phase, with medication (for cancers, end-stage kidney/liver disease)	has lost a lot of weight and regularly uses strong medication to avoid constant pain. The person has no appetite, feels nauseous, and needs to spend most of the day in bed.	0.54 (0.377-0.687)
Controlled phase of nasopharynx cancer	Generic uncomplicated disease: worry and daily medication	has a chronic disease that requires medication every day and causes some worry but minimal interference with daily activities.	0.049 (0.031-0.072)
Diagnosis and primary therapy phase of other pharynx cancer	Cancer, diagnosis and primary therapy	has pain, nausea, fatigue, weight loss and high anxiety.	0.288 (0.193-0.399)
Metastatic phase of other pharynx cancer	Cancer, metastatic	has severe pain, extreme fatigue, weight loss and high anxiety.	0.451 (0.307-0.6)
Terminal phase of other pharynx cancer	Terminal phase, with medication (for cancers, end-stage kidney/liver disease)	has lost a lot of weight and regularly uses strong medication to avoid constant pain. The person has no appetite, feels nauseous, and needs to spend most of the day in bed.	0.54 (0.377-0.687)
Controlled phase of other pharynx cancer	Generic uncomplicated disease: worry and daily medication	has a chronic disease that requires medication every day and causes some worry but minimal interference with daily activities.	0.049 (0.031-0.072)
Diagnosis and primary therapy phase of esophageal cancer	Cancer, diagnosis and primary therapy	has pain, nausea, fatigue, weight loss and high anxiety.	0.288 (0.193-0.399)
Metastatic phase of esophageal cancer	Cancer, metastatic	has severe pain, extreme fatigue, weight loss and high anxiety.	0.451 (0.307-0.6)
Terminal phase of esophageal cancer	Terminal phase, with medication (for cancers, end-stage kidney/liver disease)	has lost a lot of weight and regularly uses strong medication to avoid constant pain. The person has no appetite, feels nauseous, and needs to spend most of the day in bed.	0.54 (0.377-0.687)
Controlled phase of esophageal cancer	Generic uncomplicated disease: worry and daily medication	has a chronic disease that requires medication every day and causes some worry but minimal interference with daily activities.	0.049 (0.031-0.072)
Diagnosis and primary therapy phase of stomach cancer	Cancer, diagnosis and primary therapy	has pain, nausea, fatigue, weight loss and high anxiety.	0.288 (0.193-0.399)
Metastatic phase of stomach cancer	Cancer, metastatic	has severe pain, extreme fatigue, weight loss and high anxiety.	0.451 (0.307-0.6)
Terminal phase of stomach cancer	Terminal phase, with medication (for cancers, end-stage kidney/liver disease)	has lost a lot of weight and regularly uses strong medication to avoid constant pain. The person has no appetite, feels nauseous, and needs to spend most of the day in bed.	0.54 (0.377-0.687)
Controlled phase of stomach cancer	Generic uncomplicated disease: worry and daily medication	has a chronic disease that requires medication every day and causes some worry but minimal interference with daily activities.	0.049 (0.031-0.072)
Diagnosis and primary therapy phase of colon and rectum cancers	Cancer, diagnosis and primary therapy	has pain, nausea, fatigue, weight loss and high anxiety.	0.288 (0.193-0.399)
Metastatic phase of colon and rectum cancers	Cancer, metastatic	has severe pain, extreme fatigue, weight loss and high anxiety.	0.451 (0.307-0.6)
Stoma from colon and rectum cancers, beyond 10 years	Stoma	has a pouch attached to an opening in the belly to collect and empty stools.	0.095 (0.063-0.131)
Terminal phase of colon and rectum cancers	Terminal phase, with medication (for cancers, end-stage kidney/liver disease)	has lost a lot of weight and regularly uses strong medication to avoid constant pain. The person has no appetite, feels nauseous, and needs to spend most of the day in bed.	0.54 (0.377-0.687)
Controlled phase of colon and rectum cancers, without stoma	Generic uncomplicated disease: worry and daily medication	has a chronic disease that requires medication every day and causes some worry but minimal interference with daily activities.	0.049 (0.031-0.072)
Controlled phase of colon and rectum cancers, with stoma	Stoma and generic medication	(combined DW)	0.139 (0.094-0.192)
Diagnosis and primary therapy phase of liver cancer due to hepatitis B	Cancer, diagnosis and primary therapy	has pain, nausea, fatigue, weight loss and high anxiety.	0.288 (0.193-0.399)
Metastatic phase of liver cancer due to hepatitis B	Cancer, metastatic	has severe pain, extreme fatigue, weight loss and high anxiety.	0.451 (0.307-0.6)
Terminal phase of liver cancer due to hepatitis B	Terminal phase, with medication (for cancers, end-stage kidney/liver disease)	has lost a lot of weight and regularly uses strong medication to avoid constant pain. The person has no appetite, feels nauseous, and needs to spend most of the day in bed.	0.54 (0.377-0.687)
Controlled phase of liver cancer due to hepatitis B	Generic uncomplicated disease: worry and daily medication	has a chronic disease that requires medication every day and causes some worry but minimal interference with daily activities.	0.049 (0.031-0.072)
Diagnosis and primary therapy phase of liver cancer due to hepatitis C	Cancer, diagnosis and primary therapy	has pain, nausea, fatigue, weight loss and high anxiety.	0.288 (0.193-0.399)
Metastatic phase of liver cancer due to hepatitis C	Cancer, metastatic	has severe pain, extreme fatigue, weight loss and high anxiety.	0.451 (0.307-0.6)
Terminal phase of liver cancer due to hepatitis C	Terminal phase, with medication (for cancers, end-stage kidney/liver disease)	has lost a lot of weight and regularly uses strong medication to avoid constant pain. The person has no appetite, feels nauseous, and needs to spend most of the day in bed.	0.54 (0.377-0.687)
Controlled phase of liver cancer due to hepatitis C	Generic uncomplicated disease: worry and daily medication	has a chronic disease that requires medication every day and causes some worry but minimal interference with daily activities.	0.049 (0.031-0.072)
Diagnosis and primary therapy phase of liver cancer due to alcohol use	Cancer, diagnosis and primary therapy	has pain, nausea, fatigue, weight loss and high anxiety.	0.288 (0.193-0.399)
Metastatic phase of liver cancer due to alcohol use	Cancer, metastatic	has severe pain, extreme fatigue, weight loss and high anxiety.	0.451 (0.307-0.6)
Terminal phase of liver cancer due to alcohol use	Terminal phase, with medication (for cancers, end-stage kidney/liver disease)	has lost a lot of weight and regularly uses strong medication to avoid constant pain. The person has no appetite, feels nauseous, and needs to spend most of the day in bed.	0.54 (0.377-0.687)
Controlled phase of liver cancer due to alcohol use	Generic uncomplicated disease: worry and daily medication	has a chronic disease that requires medication every day and causes some worry but minimal interference with daily activities.	0.049 (0.031-0.072)
Diagnosis and primary therapy phase of liver cancer due to NASH	Cancer, diagnosis and primary therapy	has pain, nausea, fatigue, weight loss and high anxiety.	0.288 (0.193-0.399)
Metastatic phase of liver cancer due to NASH	Cancer, metastatic	has severe pain, extreme fatigue, weight loss and high anxiety.	0.451 (0.307-0.6)
Terminal phase of liver cancer due to NASH	Terminal phase, without medication (for cancers, end-stage kidney/liver disease)	has lost a lot of weight and has constant pain. The person has no appetite, feels nauseous, and needs to spend most of the day in bed.	0.569 (0.389-0.727)
Controlled phase of liver cancer due to NASH	Generic uncomplicated disease: worry and daily medication	has a chronic disease that requires medication every day and causes some worry but minimal interference with daily activities.	0.049 (0.031-0.072)
Diagnosis and primary therapy phase of hepatoblastoma	Cancer, diagnosis and primary therapy	has pain, nausea, fatigue, weight loss and high anxiety.	0.288 (0.193-0.399)
Metastatic phase of hepatoblastoma	Cancer, metastatic	has severe pain, extreme fatigue, weight loss and high anxiety.	0.451 (0.307-0.6)
Terminal phase of hepatoblastoma	Terminal phase, with medication (for cancers, end-stage kidney/liver disease)	has lost a lot of weight and regularly uses strong medication to avoid constant pain. The person has no appetite, feels nauseous, and needs to spend most of the day in bed.	0.54 (0.377-0.687)
Controlled phase of hepatoblastoma	Generic uncomplicated disease: worry and daily medication	has a chronic disease that requires medication every day and causes some worry but minimal interference with daily activities.	0.049 (0.031-0.072)
Diagnosis and primary therapy phase of liver cancer due to other causes	Cancer, diagnosis and primary therapy	has pain, nausea, fatigue, weight loss and high anxiety.	0.288 (0.193-0.399)
Metastatic phase of liver cancer due to other causes	Cancer, metastatic	has severe pain, extreme fatigue, weight loss and high anxiety.	0.451 (0.307-0.6)
Terminal phase of liver cancer due to other causes	Terminal phase, with medication (for cancers, end-stage kidney/liver disease)	has lost a lot of weight and regularly uses strong medication to avoid constant pain. The person has no appetite, feels nauseous, and needs to spend most of the day in bed.	0.54 (0.377-0.687)
Controlled phase of liver cancer due to other causes	Generic uncomplicated disease: worry and daily medication	has a chronic disease that requires medication every day and causes some worry but minimal interference with daily activities.	0.049 (0.031-0.072)

Diagnosis and primary therapy phase of gallbladder and biliary tract cancer	Cancer, diagnosis and primary therapy	has pain, nausea, fatigue, weight loss and high anxiety.	0.288 (0.193-0.399)
Metastatic phase of gallbladder and biliary tract cancer	Cancer, metastatic	has severe pain, extreme fatigue, weight loss and high anxiety.	0.451 (0.307-0.6)
Terminal phase of gallbladder and biliary tract cancer	Terminal phase, with medication (for cancers, end-stage kidney/liver disease)	has lost a lot of weight and regularly uses strong medication to avoid constant pain. The person has no appetite, feels nauseous, and needs to spend most of the day in bed.	0.54 (0.377-0.687)
Controlled phase of gallbladder and biliary tract cancer	Generic uncomplicated disease: worry and daily medication	has a chronic disease that requires medication every day and causes some worry but minimal interference with daily activities.	0.049 (0.031-0.072)
Diagnosis and primary therapy phase of pancreatic cancer	Cancer, diagnosis and primary therapy	has pain, nausea, fatigue, weight loss and high anxiety.	0.288 (0.193-0.399)
Metastatic phase of pancreatic cancer	Cancer, metastatic	has severe pain, extreme fatigue, weight loss and high anxiety.	0.451 (0.307-0.6)
Terminal phase of pancreatic cancer	Terminal phase, with medication (for cancers, end-stage kidney/liver disease)	has lost a lot of weight and regularly uses strong medication to avoid constant pain. The person has no appetite, feels nauseous, and needs to spend most of the day in bed.	0.54 (0.377-0.687)
Controlled phase of pancreatic cancer	Generic uncomplicated disease: worry and daily medication	has a chronic disease that requires medication every day and causes some worry but minimal interference with daily activities.	0.049 (0.031-0.072)
Diagnosis and primary therapy phase of larynx cancer	Cancer, diagnosis and primary therapy	has pain, nausea, fatigue, weight loss and high anxiety.	0.288 (0.193-0.399)
Metastatic phase of larynx cancer	Cancer, metastatic	has severe pain, extreme fatigue, weight loss and high anxiety.	0.451 (0.307-0.6)
Terminal phase of larynx cancer	Terminal phase, with medication (for cancers, end-stage kidney/liver disease)	has lost a lot of weight and regularly uses strong medication to avoid constant pain. The person has no appetite, feels nauseous, and needs to spend most of the day in bed.	0.54 (0.377-0.687)
Laryngectomy from larynx cancer, beyond 10 years	Speech problems	has difficulty speaking, and others find it difficult to understand.	0.051 (0.032-0.078)
Controlled phase of larynx cancer, without laryngectomy	Generic uncomplicated disease: worry and daily medication	has a chronic disease that requires medication every day and causes some worry but minimal interference with daily activities.	0.049 (0.031-0.072)
Controlled phase of larynx cancer, with laryngectomy	Speech problems and generic medication	(combined DW)	0.098 (0.063-0.145)
Diagnosis and primary therapy phase of lung, bronchus, and trachea cancer	Cancer, diagnosis and primary therapy	has pain, nausea, fatigue, weight loss and high anxiety.	0.288 (0.193-0.399)
Metastatic phase of lung, bronchus, and trachea cancer	Cancer, metastatic	has severe pain, extreme fatigue, weight loss and high anxiety.	0.451 (0.307-0.6)
Terminal phase of lung, bronchus, and trachea cancer	Terminal phase, with medication (for cancers, end-stage kidney/liver disease)	has lost a lot of weight and regularly uses strong medication to avoid constant pain. The person has no appetite, feels nauseous, and needs to spend most of the day in bed.	0.54 (0.377-0.687)
Controlled phase of lung, bronchus, and trachea cancer	Generic uncomplicated disease: worry and daily medication	has a chronic disease that requires medication every day and causes some worry but minimal interference with daily activities.	0.049 (0.031-0.072)
Diagnosis and primary therapy phase of malignant skin melanoma	Cancer, diagnosis and primary therapy	has pain, nausea, fatigue, weight loss and high anxiety.	0.288 (0.193-0.399)
Metastatic phase of malignant skin melanoma	Cancer, metastatic	has severe pain, extreme fatigue, weight loss and high anxiety.	0.451 (0.307-0.6)
Terminal phase of malignant skin melanoma	Terminal phase, with medication (for cancers, end-stage kidney/liver disease)	has lost a lot of weight and regularly uses strong medication to avoid constant pain. The person has no appetite, feels nauseous, and needs to spend most of the day in bed.	0.54 (0.377-0.687)
Controlled phase of malignant skin melanoma	Generic uncomplicated disease: worry and daily medication	has a chronic disease that requires medication every day and causes some worry but minimal interference with daily activities.	0.049 (0.031-0.072)
Mild disfigurement due to squamous cell carcinoma	Disfigurement, level 1	has a slight, visible physical deformity that others notice, which causes some worry and discomfort.	0.011 (0.005-0.021)
Moderate disfigurement due to squamous cell carcinoma	Disfigurement, level 2	has a visible physical deformity that causes others to stare and comment. As a result, the person is worried and has trouble sleeping and concentrating.	0.067 (0.044-0.096)
Severe disfigurement due to squamous cell carcinoma	Disfigurement, level 3, with itch/pain	has an obvious physical deformity that is very painful and itchy. The physical deformity makes others uncomfortable, which causes the person to avoid social contact, feel worried, sleep poorly, and think about suicide.	0.576 (0.401-0.731)
Disfigurement due to basal cell carcinoma	Disfigurement, level 1	has a slight, visible physical deformity that others notice, which causes some worry and discomfort.	0.011 (0.005-0.021)
Basal cell carcinoma without disfigurement	Asymptomatic		0 (0-0)
Diagnosis and primary therapy phase of soft tissue and other extraosseous sarcomas	Cancer, diagnosis and primary therapy	has pain, nausea, fatigue, weight loss and high anxiety.	0.288 (0.193-0.399)
Metastatic phase of soft tissue and other extraosseous sarcomas	Cancer, metastatic	has severe pain, extreme fatigue, weight loss and high anxiety.	0.451 (0.307-0.6)
Terminal phase of soft tissue and other extraosseous sarcomas	Terminal phase, with medication (for cancers, end-stage kidney/liver disease)	has lost a lot of weight and regularly uses strong medication to avoid constant pain. The person has no appetite, feels nauseous, and needs to spend most of the day in bed.	0.54 (0.377-0.687)
Controlled phase of soft tissue and other extraosseous sarcomas	Generic uncomplicated disease: worry and daily medication	has a chronic disease that requires medication every day and causes some worry but minimal interference with daily activities.	0.049 (0.031-0.072)
Diagnosis and primary therapy phase of malignant bone tumors	Cancer, diagnosis and primary therapy	has pain, nausea, fatigue, weight loss and high anxiety.	0.288 (0.193-0.399)
Metastatic phase of malignant bone tumors	Cancer, metastatic	has severe pain, extreme fatigue, weight loss and high anxiety.	0.451 (0.307-0.6)
Terminal phase of malignant bone tumors	Terminal phase, with medication (for cancers, end-stage kidney/liver disease)	has lost a lot of weight and regularly uses strong medication to avoid constant pain. The person has no appetite, feels nauseous, and needs to spend most of the day in bed.	0.54 (0.377-0.687)
Controlled phase of malignant bone tumors	Generic uncomplicated disease: worry and daily medication	has a chronic disease that requires medication every day and causes some worry but minimal interference with daily activities.	0.049 (0.031-0.072)
Diagnosis and primary therapy phase of breast cancer	Cancer, diagnosis and primary therapy	has pain, nausea, fatigue, weight loss and high anxiety.	0.288 (0.193-0.399)
Metastatic phase of breast cancer	Cancer, metastatic	has severe pain, extreme fatigue, weight loss and high anxiety.	0.451 (0.307-0.6)
Mastectomy from breast cancer, beyond 10 years	Mastectomy	had one of her breasts removed and sometimes has pain or swelling in the arms.	0.036 (0.02-0.057)
Terminal phase of breast cancer	Terminal phase, with medication (for cancers, end-stage kidney/liver disease)	has lost a lot of weight and regularly uses strong medication to avoid constant pain. The person has no appetite, feels nauseous, and needs to spend most of the day in bed.	0.54 (0.377-0.687)
Controlled phase of breast cancer, without mastectomy	Generic uncomplicated disease: worry and daily medication	has a chronic disease that requires medication every day and causes some worry but minimal interference with daily activities.	0.049 (0.031-0.072)
Controlled phase of breast cancer, with mastectomy	Mastectomy and generic medication	(combined DW)	0.083 (0.052-0.124)
Diagnosis and primary therapy phase of cervical cancer	Cancer, diagnosis and primary therapy	has pain, nausea, fatigue, weight loss and high anxiety.	0.288 (0.193-0.399)
Metastatic phase of cervical cancer	Cancer, metastatic	has severe pain, extreme fatigue, weight loss and high anxiety.	0.451 (0.307-0.6)
Terminal phase of cervical cancer	Terminal phase, with medication (for cancers, end-stage kidney/liver disease)	has lost a lot of weight and regularly uses strong medication to avoid constant pain. The person has no appetite, feels nauseous, and needs to spend most of the day in bed.	0.54 (0.377-0.687)
Controlled phase of cervical cancer	Generic uncomplicated disease: worry and daily medication	has a chronic disease that requires medication every day and causes some worry but minimal interference with daily activities.	0.049 (0.031-0.072)
Diagnosis and primary therapy phase of uterine cancer	Cancer, diagnosis and primary therapy	has pain, nausea, fatigue, weight loss and high anxiety.	0.288 (0.193-0.399)
Metastatic phase of uterine cancer	Cancer, metastatic	has severe pain, extreme fatigue, weight loss and high anxiety.	0.451 (0.307-0.6)
Terminal phase of uterine cancer	Terminal phase, with medication (for cancers, end-stage kidney/liver disease)	has lost a lot of weight and regularly uses strong medication to avoid constant pain. The person has no appetite, feels nauseous, and needs to spend most of the day in bed.	0.54 (0.377-0.687)
Controlled phase of uterine cancer	Generic uncomplicated disease: worry and daily medication	has a chronic disease that requires medication every day and causes some worry but minimal interference with daily activities.	0.049 (0.031-0.072)
Diagnosis and primary therapy phase of ovarian cancer	Cancer, diagnosis and primary therapy	has pain, nausea, fatigue, weight loss and high anxiety.	0.288 (0.193-0.399)
Metastatic phase of ovarian cancer	Cancer, metastatic	has severe pain, extreme fatigue, weight loss and high anxiety.	0.451 (0.307-0.6)
Terminal phase of ovarian cancer	Terminal phase, with medication (for cancers, end-stage kidney/liver disease)	has lost a lot of weight and regularly uses strong medication to avoid constant pain. The person has no appetite, feels nauseous, and needs to spend most of the day in bed.	0.54 (0.377-0.687)
Controlled phase of ovarian cancer	Generic uncomplicated disease: worry and daily medication	has a chronic disease that requires medication every day and causes some worry but minimal interference with daily activities.	0.049 (0.031-0.072)
Diagnosis and primary therapy phase of prostate cancer	Cancer, diagnosis and primary therapy	has pain, nausea, fatigue, weight loss and high anxiety.	0.288 (0.193-0.399)
Metastatic phase of prostate cancer	Cancer, metastatic	has severe pain, extreme fatigue, weight loss and high anxiety.	0.451 (0.307-0.6)

Terminal phase of prostate cancer	Terminal phase, with medication (for cancers, end-stage kidney/liver disease)	has lost a lot of weight and regularly uses strong medication to avoid constant pain. The person has no appetite, feels nauseous, and needs to spend most of the day in bed.	0.54 (0.377-0.687)
Controlled phase of prostate cancer, without impotence or incontinence	Generic uncomplicated disease: worry and daily medication	has a chronic disease that requires medication every day and causes some worry but minimal interference with daily activities.	0.049 (0.031-0.072)
Controlled phase of prostate cancer, with impotence	Impotence and generic medication	(combined DW)	0.065 (0.04-0.1)
Controlled phase of prostate cancer, with incontinence	Incontinence and generic medication	(combined DW)	0.181 (0.124-0.248)
Incontinence from prostate cancer, beyond 10 years	Urinary incontinence	cannot control urinating.	0.139 (0.094-0.198)
Impotence from prostate cancer, beyond 10 years	Impotence	has difficulty in obtaining or maintaining an erection.	0.017 (0.009-0.03)
Diagnosis and primary therapy phase of testicular cancer	Cancer, diagnosis and primary therapy	has pain, nausea, fatigue, weight loss and high anxiety.	0.288 (0.193-0.399)
Metastatic phase of testicular cancer	Cancer, metastatic	has severe pain, extreme fatigue, weight loss and high anxiety.	0.451 (0.307-0.6)
Terminal phase of testicular cancer	Terminal phase, with medication (for cancers, end-stage kidney/liver disease)	has lost a lot of weight and regularly uses strong medication to avoid constant pain. The person has no appetite, feels nauseous, and needs to spend most of the day in bed.	0.54 (0.377-0.687)
Controlled phase of testicular cancer	Generic uncomplicated disease: worry and daily medication	has a chronic disease that requires medication every day and causes some worry but minimal interference with daily activities.	0.049 (0.031-0.072)
Diagnosis and primary therapy phase of kidney cancer	Cancer, diagnosis and primary therapy	has pain, nausea, fatigue, weight loss and high anxiety.	0.288 (0.193-0.399)
Metastatic phase of kidney cancer	Cancer, metastatic	has severe pain, extreme fatigue, weight loss and high anxiety.	0.451 (0.307-0.6)
Terminal phase of kidney cancer	Terminal phase, with medication (for cancers, end-stage kidney/liver disease)	has lost a lot of weight and regularly uses strong medication to avoid constant pain. The person has no appetite, feels nauseous, and needs to spend most of the day in bed.	0.54 (0.377-0.687)
Controlled phase of kidney cancer	Generic uncomplicated disease: worry and daily medication	has a chronic disease that requires medication every day and causes some worry but minimal interference with daily activities.	0.049 (0.031-0.072)
Diagnosis and primary therapy phase of bladder cancer	Cancer, diagnosis and primary therapy	has pain, nausea, fatigue, weight loss and high anxiety.	0.288 (0.193-0.399)
Metastatic phase of bladder cancer	Cancer, metastatic	has severe pain, extreme fatigue, weight loss and high anxiety.	0.451 (0.307-0.6)
Terminal phase of bladder cancer	Terminal phase, with medication (for cancers, end-stage kidney/liver disease)	has lost a lot of weight and regularly uses strong medication to avoid constant pain. The person has no appetite, feels nauseous, and needs to spend most of the day in bed.	0.54 (0.377-0.687)
Incontinence from bladder cancer, beyond 10 years	Urinary incontinence	cannot control urinating.	0.139 (0.094-0.198)
Controlled phase of bladder cancer, without incontinence	Generic uncomplicated disease: worry and daily medication	has a chronic disease that requires medication every day and causes some worry but minimal interference with daily activities.	0.049 (0.031-0.072)
Controlled phase of bladder cancer, with incontinence	Incontinence and generic medication	(combined DW)	0.181 (0.124-0.248)
Diagnosis and primary therapy phase of brain and central nervous system cancers	Cancer, diagnosis and primary therapy	has pain, nausea, fatigue, weight loss and high anxiety.	0.288 (0.193-0.399)
Metastatic phase of brain and central nervous system cancers	Cancer, metastatic	has severe pain, extreme fatigue, weight loss and high anxiety.	0.451 (0.307-0.6)
Terminal phase of brain and central nervous system cancers	Terminal phase, with medication (for cancers, end-stage kidney/liver disease)	has lost a lot of weight and regularly uses strong medication to avoid constant pain. The person has no appetite, feels nauseous, and needs to spend most of the day in bed.	0.54 (0.377-0.687)
Controlled phase of brain and central nervous system cancers	Generic uncomplicated disease: worry and daily medication	has a chronic disease that requires medication every day and causes some worry but minimal interference with daily activities.	0.049 (0.031-0.072)
Diagnosis and primary therapy phase of retinoblastoma	Cancer, diagnosis and primary therapy	has pain, nausea, fatigue, weight loss and high anxiety.	0.288 (0.193-0.399)
Metastatic phase of retinoblastoma	Cancer, metastatic	has severe pain, extreme fatigue, weight loss and high anxiety.	0.451 (0.307-0.6)
Terminal phase of retinoblastoma	Terminal phase, with medication (for cancers, end-stage kidney/liver disease)	has lost a lot of weight and regularly uses strong medication to avoid constant pain. The person has no appetite, feels nauseous, and needs to spend most of the day in bed.	0.54 (0.377-0.687)
Controlled phase of retinoblastoma	Generic uncomplicated disease: worry and daily medication	has a chronic disease that requires medication every day and causes some worry but minimal interference with daily activities.	0.049 (0.031-0.072)
Diagnosis and primary therapy phase of other eye cancers	Cancer, diagnosis and primary therapy	has pain, nausea, fatigue, weight loss and high anxiety.	0.288 (0.193-0.399)
Metastatic phase of other eye cancers	Cancer, metastatic	has severe pain, extreme fatigue, weight loss and high anxiety.	0.451 (0.307-0.6)
Terminal phase of other eye cancers	Terminal phase, with medication (for cancers, end-stage kidney/liver disease)	has lost a lot of weight and regularly uses strong medication to avoid constant pain. The person has no appetite, feels nauseous, and needs to spend most of the day in bed.	0.54 (0.377-0.687)
Controlled phase of other eye cancers	Generic uncomplicated disease: worry and daily medication	has a chronic disease that requires medication every day and causes some worry but minimal interference with daily activities.	0.049 (0.031-0.072)
Diagnosis and primary therapy phase of neuroblastoma and other peripheral nervous cell tumors	Cancer, diagnosis and primary therapy	has pain, nausea, fatigue, weight loss and high anxiety.	0.288 (0.193-0.399)
Metastatic phase of neuroblastoma and other peripheral nervous cell tumors	Cancer, metastatic	has severe pain, extreme fatigue, weight loss and high anxiety.	0.451 (0.307-0.6)
Terminal phase of neuroblastoma and other peripheral nervous cell tumors	Terminal phase, with medication (for cancers, end-stage kidney/liver disease)	has lost a lot of weight and regularly uses strong medication to avoid constant pain. The person has no appetite, feels nauseous, and needs to spend most of the day in bed.	0.54 (0.377-0.687)
Controlled phase of neuroblastoma and other peripheral nervous cell tumors	Generic uncomplicated disease: worry and daily medication	has a chronic disease that requires medication every day and causes some worry but minimal interference with daily activities.	0.049 (0.031-0.072)
Diagnosis and primary therapy phase of thyroid cancer	Cancer, diagnosis and primary therapy	has pain, nausea, fatigue, weight loss and high anxiety.	0.288 (0.193-0.399)
Metastatic phase of thyroid cancer	Cancer, metastatic	has severe pain, extreme fatigue, weight loss and high anxiety.	0.451 (0.307-0.6)
Terminal phase of thyroid cancer	Terminal phase, with medication (for cancers, end-stage kidney/liver disease)	has lost a lot of weight and regularly uses strong medication to avoid constant pain. The person has no appetite, feels nauseous, and needs to spend most of the day in bed.	0.54 (0.377-0.687)
Controlled phase of thyroid cancer	Generic uncomplicated disease: worry and daily medication	has a chronic disease that requires medication every day and causes some worry but minimal interference with daily activities.	0.049 (0.031-0.072)
Diagnosis and primary therapy phase of mesothelioma	Cancer, diagnosis and primary therapy	has pain, nausea, fatigue, weight loss and high anxiety.	0.288 (0.193-0.399)
Metastatic phase of mesothelioma	Cancer, metastatic	has severe pain, extreme fatigue, weight loss and high anxiety.	0.451 (0.307-0.6)
Terminal phase of mesothelioma	Terminal phase, with medication (for cancers, end-stage kidney/liver disease)	has lost a lot of weight and regularly uses strong medication to avoid constant pain. The person has no appetite, feels nauseous, and needs to spend most of the day in bed.	0.54 (0.377-0.687)
Controlled phase of mesothelioma	Generic uncomplicated disease: worry and daily medication	has a chronic disease that requires medication every day and causes some worry but minimal interference with daily activities.	0.049 (0.031-0.072)
Diagnosis and primary therapy phase of Hodgkin disease	Cancer, diagnosis and primary therapy	has pain, nausea, fatigue, weight loss and high anxiety.	0.288 (0.193-0.399)
Metastatic phase of Hodgkin disease	Cancer, metastatic	has severe pain, extreme fatigue, weight loss and high anxiety.	0.451 (0.307-0.6)
Terminal phase of Hodgkin disease	Terminal phase, with medication (for cancers, end-stage kidney/liver disease)	has lost a lot of weight and regularly uses strong medication to avoid constant pain. The person has no appetite, feels nauseous, and needs to spend most of the day in bed.	0.54 (0.377-0.687)
Controlled phase of Hodgkin disease	Generic uncomplicated disease: worry and daily medication	has a chronic disease that requires medication every day and causes some worry but minimal interference with daily activities.	0.049 (0.031-0.072)
Diagnosis and primary therapy phase of Burkitt lymphoma	Cancer, diagnosis and primary therapy	has pain, nausea, fatigue, weight loss and high anxiety.	0.288 (0.193-0.399)
Metastatic phase of Burkitt lymphoma	Cancer, metastatic	has severe pain, extreme fatigue, weight loss and high anxiety.	0.451 (0.307-0.6)
Terminal phase of Burkitt lymphoma	Terminal phase, with medication (for cancers, end-stage kidney/liver disease)	has lost a lot of weight and regularly uses strong medication to avoid constant pain. The person has no appetite, feels nauseous, and needs to spend most of the day in bed.	0.54 (0.377-0.687)
Controlled phase of Burkitt lymphoma	Generic uncomplicated disease: worry and daily medication	has a chronic disease that requires medication every day and causes some worry but minimal interference with daily activities.	0.049 (0.031-0.072)
Diagnosis and primary therapy phase of other non-Hodgkin lymphoma	Cancer, diagnosis and primary therapy	has pain, nausea, fatigue, weight loss and high anxiety.	0.288 (0.193-0.399)
Metastatic phase of other non-Hodgkin lymphoma	Cancer, metastatic	has severe pain, extreme fatigue, weight loss and high anxiety.	0.451 (0.307-0.6)
Terminal phase of other non-Hodgkin lymphoma	Terminal phase, with medication (for cancers, end-stage kidney/liver disease)	has lost a lot of weight and regularly uses strong medication to avoid constant pain. The person has no appetite, feels nauseous, and needs to spend most of the day in bed.	0.54 (0.377-0.687)

Controlled phase of other non-Hodgkin lymphoma	Generic uncomplicated disease: worry and daily medication	has a chronic disease that requires medication every day and causes some worry but minimal interference with daily activities.	0.049 (0.031-0.072)
Diagnosis and primary therapy phase of multiple myeloma	Cancer, diagnosis and primary therapy	has pain, nausea, fatigue, weight loss and high anxiety.	0.288 (0.193-0.399)
Metastatic phase of multiple myeloma	Cancer, metastatic	has severe pain, extreme fatigue, weight loss and high anxiety.	0.451 (0.307-0.6)
Terminal phase of multiple myeloma	Terminal phase, with medication (for cancers, end-stage kidney/liver disease)	has lost a lot of weight and regularly uses strong medication to avoid constant pain. The person has no appetite, feels nauseous, and needs to spend most of the day in bed.	0.54 (0.377-0.687)
Controlled phase of multiple myeloma	Generic uncomplicated disease: worry and daily medication	has a chronic disease that requires medication every day and causes some worry but minimal interference with daily activities.	0.049 (0.031-0.072)
Diagnosis and primary therapy phase of acute lymphoid leukemia	Cancer, diagnosis and primary therapy	has pain, nausea, fatigue, weight loss and high anxiety.	0.288 (0.193-0.399)
Metastatic phase of acute lymphoid leukemia	Cancer, metastatic	has severe pain, extreme fatigue, weight loss and high anxiety.	0.451 (0.307-0.6)
Terminal phase of acute lymphoid leukemia	Terminal phase, with medication (for cancers, end-stage kidney/liver disease)	has lost a lot of weight and regularly uses strong medication to avoid constant pain. The person has no appetite, feels nauseous, and needs to spend most of the day in bed.	0.54 (0.377-0.687)
Controlled phase of acute lymphoid leukemia	Generic uncomplicated disease: worry and daily medication	has a chronic disease that requires medication every day and causes some worry but minimal interference with daily activities.	0.049 (0.031-0.072)
Diagnosis and primary therapy phase of chronic lymphoid leukemia	Cancer, diagnosis and primary therapy	has pain, nausea, fatigue, weight loss and high anxiety.	0.288 (0.193-0.399)
Metastatic phase of chronic lymphoid leukemia	Cancer, metastatic	has severe pain, extreme fatigue, weight loss and high anxiety.	0.451 (0.307-0.6)
Terminal phase of chronic lymphoid leukemia	Terminal phase, with medication (for cancers, end-stage kidney/liver disease)	has lost a lot of weight and regularly uses strong medication to avoid constant pain. The person has no appetite, feels nauseous, and needs to spend most of the day in bed.	0.54 (0.377-0.687)
Controlled phase of chronic lymphoid leukemia	Generic uncomplicated disease: worry and daily medication	has a chronic disease that requires medication every day and causes some worry but minimal interference with daily activities.	0.049 (0.031-0.072)
Diagnosis and primary therapy phase of acute myeloid leukemia	Cancer, diagnosis and primary therapy	has pain, nausea, fatigue, weight loss and high anxiety.	0.288 (0.193-0.399)
Metastatic phase of acute myeloid leukemia	Cancer, metastatic	has severe pain, extreme fatigue, weight loss and high anxiety.	0.451 (0.307-0.6)
Terminal phase of acute myeloid leukemia	Terminal phase, with medication (for cancers, end-stage kidney/liver disease)	has lost a lot of weight and regularly uses strong medication to avoid constant pain. The person has no appetite, feels nauseous, and needs to spend most of the day in bed.	0.54 (0.377-0.687)
Controlled phase of acute myeloid leukemia	Generic uncomplicated disease: worry and daily medication	has a chronic disease that requires medication every day and causes some worry but minimal interference with daily activities.	0.049 (0.031-0.072)
Diagnosis and primary therapy phase of chronic myeloid leukemia	Cancer, diagnosis and primary therapy	has pain, nausea, fatigue, weight loss and high anxiety.	0.288 (0.193-0.399)
Metastatic phase of chronic myeloid leukemia	Cancer, metastatic	has severe pain, extreme fatigue, weight loss and high anxiety.	0.451 (0.307-0.6)
Terminal phase of chronic myeloid leukemia	Terminal phase, with medication (for cancers, end-stage kidney/liver disease)	has lost a lot of weight and regularly uses strong medication to avoid constant pain. The person has no appetite, feels nauseous, and needs to spend most of the day in bed.	0.54 (0.377-0.687)
Controlled phase of chronic myeloid leukemia	Generic uncomplicated disease: worry and daily medication	has a chronic disease that requires medication every day and causes some worry but minimal interference with daily activities.	0.049 (0.031-0.072)
Diagnosis and primary therapy phase of other leukemia	Cancer, diagnosis and primary therapy	has pain, nausea, fatigue, weight loss and high anxiety.	0.288 (0.193-0.399)
Metastatic phase of other leukemia	Cancer, metastatic	has severe pain, extreme fatigue, weight loss and high anxiety.	0.451 (0.307-0.6)
Terminal phase of other leukemia	Terminal phase, with medication (for cancers, end-stage kidney/liver disease)	has lost a lot of weight and regularly uses strong medication to avoid constant pain. The person has no appetite, feels nauseous, and needs to spend most of the day in bed.	0.54 (0.377-0.687)
Controlled phase of other leukemia	Generic uncomplicated disease: worry and daily medication	has a chronic disease that requires medication every day and causes some worry but minimal interference with daily activities.	0.049 (0.031-0.072)
Diagnosis and primary therapy phase of other malignant neoplasms	Cancer, diagnosis and primary therapy	has pain, nausea, fatigue, weight loss and high anxiety.	0.288 (0.193-0.399)
Metastatic phase of other malignant neoplasms	Cancer, metastatic	has severe pain, extreme fatigue, weight loss and high anxiety.	0.451 (0.307-0.6)
Terminal phase of other malignant neoplasms	Terminal phase, with medication (for cancers, end-stage kidney/liver disease)	has lost a lot of weight and regularly uses strong medication to avoid constant pain. The person has no appetite, feels nauseous, and needs to spend most of the day in bed.	0.54 (0.377-0.687)
Controlled phase of other malignant neoplasms	Generic uncomplicated disease: worry and daily medication	has a chronic disease that requires medication every day and causes some worry but minimal interference with daily activities.	0.049 (0.031-0.072)
Myelodysplastic, myeloproliferative, and other hematopoietic neoplasms	Generic uncomplicated disease: worry and daily medication	has a chronic disease that requires medication every day and causes some worry but minimal interference with daily activities.	0.049 (0.031-0.072)
Benign and in situ intestinal neoplasms	Asymptomatic		0 (0-0)
Benign and in situ cervical and uterine neoplasms	Asymptomatic		0 (0-0)
Other benign and in situ neoplasms	Asymptomatic		0 (0-0)
Rheumatic heart disease, without heart failure	Generic uncomplicated disease: worry and daily medication	has a chronic disease that requires medication every day and causes some worry but minimal interference with daily activities.	0.049 (0.031-0.072)
Mild heart failure due to rheumatic heart disease	Heart failure, mild	is short of breath and easily tires with moderate physical activity, such as walking uphill or more than a quarter-mile on level ground. The person feels comfortable at rest or during activities requiring less effort.	0.041 (0.026-0.062)
Moderate heart failure due to rheumatic heart disease	Heart failure, moderate	is short of breath and easily tires with minimal physical activity, such as walking only a short distance. The person feels comfortable at rest but avoids moderate activity.	0.072 (0.047-0.103)
Severe heart failure due to rheumatic heart disease	Heart failure, severe	is short of breath and feels tired when at rest. The person avoids any physical activity, for fear of worsening the breathing problems.	0.179 (0.122-0.251)
Controlled, medically managed heart failure due to rheumatic heart disease	Generic uncomplicated disease: worry and daily medication	has a chronic disease that requires medication every day and causes some worry but minimal interference with daily activities.	0.049 (0.031-0.072)
Asymptomatic ischemic heart disease following myocardial infarction	Asymptomatic		0 (0-0)
Mild angina due to ischemic heart disease	Angina pectoris, mild	has chest pain that occurs with strenuous physical activity, such as running or lifting heavy objects. After a brief rest, the pain goes away.	0.033 (0.02-0.052)
Moderate angina due to ischemic heart disease	Angina pectoris, moderate	has chest pain that occurs with moderate physical activity, such as walking uphill or more than half a kilometer (around a quarter-mile) on level ground. After a brief rest, the pain goes away.	0.08 (0.052-0.113)
Severe angina due to ischemic heart disease	Angina pectoris, severe	has chest pain that occurs with minimal physical activity, such as walking only a short distance. After a brief rest, the pain goes away. The person avoids most physical activities because of the pain.	0.167 (0.11-0.24)
Asymptomatic angina due to ischemic heart disease	Asymptomatic		0 (0-0)
Mild heart failure due to ischemic heart disease	Heart failure, mild	is short of breath and easily tires with moderate physical activity, such as walking uphill or more than a quarter-mile on level ground. The person feels comfortable at rest or during activities requiring less effort.	0.041 (0.026-0.062)
Moderate heart failure due to ischemic heart disease	Heart failure, moderate	is short of breath and easily tires with minimal physical activity, such as walking only a short distance. The person feels comfortable at rest but avoids moderate activity.	0.072 (0.047-0.103)
Severe heart failure due to ischemic heart disease	Heart failure, severe	is short of breath and feels tired when at rest. The person avoids any physical activity, for fear of worsening the breathing problems.	0.179 (0.122-0.251)
Controlled, medically managed heart failure due ischemic heart disease	Generic uncomplicated disease: worry and daily medication	has a chronic disease that requires medication every day and causes some worry but minimal interference with daily activities.	0.049 (0.031-0.072)
Acute myocardial infarction first 2 days	Acute myocardial infarction, days 1-2	has severe chest pain that becomes worse with any physical activity,. The person feels nauseous, short of breath, and very anxious.	0.432 (0.288-0.579)
Acute myocardial infarction 3 to 28 days	Acute myocardial infarction, days 3-28	gets short of breath after heavy physical activity, and tires easily, but has no problems when at rest. The person has to take medication every day and has some anxiety.	0.074 (0.049-0.105)
Acute ischemic stroke severity level 1	Stroke, long-term consequences, mild	has some difficulty in moving around and some weakness in one hand, but is able to walk without help.	0.019 (0.01-0.032)
Acute ischemic stroke severity level 2	Stroke, long-term consequences, moderate	has some difficulty in moving around, and in using the hands for lifting and holding things, dressing and grooming.	0.07 (0.046-0.099)
Acute ischemic stroke severity level 3	Stroke, long-term consequences, moderate plus cognition problems	has some difficulty in moving around, in using the hands for lifting and holding things, dressing and grooming, and in speaking. The person is often forgetful and confused.	0.316 (0.206-0.437)
Acute ischemic stroke severity level 4	Stroke, long-term consequences, severe	is confined to bed or a wheelchair, has difficulty speaking and depends on others for feeding, toileting and dressing.	0.552 (0.377-0.707)
Acute ischemic stroke severity level 5	Stroke, long-term consequences, severe plus cognition problems	is confined to bed or a wheelchair, depends on others for feeding, toileting and dressing, and has difficulty speaking, thinking clearly and remembering things.	0.588 (0.411-0.744)

Chronic ischemic stroke severity level 1	Stroke, long-term consequences, mild	has some difficulty in moving around and some weakness in one hand, but is able to walk without help.	0.019 (0.01-0.032)
Chronic ischemic stroke severity level 2	Stroke, long-term consequences, moderate	has some difficulty in moving around, and in using the hands for lifting and holding things, dressing and grooming.	0.07 (0.046-0.099)
Chronic ischemic stroke severity level 3	Stroke, long-term consequences, moderate plus cognition problems	has some difficulty in moving around, in using the hands for lifting and holding things, dressing and grooming, and in speaking. The person is often forgetful and confused.	0.316 (0.206-0.437)
Chronic ischemic stroke severity level 4	Stroke, long-term consequences, severe	is confined to bed or a wheelchair, has difficulty speaking and depends on others for feeding, toileting and dressing.	0.552 (0.377-0.707)
Chronic ischemic stroke severity level 5	Stroke, long-term consequences, severe plus cognition problems	is confined to bed or a wheelchair, depends on others for feeding, toileting and dressing, and has difficulty speaking, thinking clearly and remembering things.	0.588 (0.411-0.744)
Asymptomatic chronic ischemic stroke	Asymptomatic		0 (0-0)
Acute intracerebral hemorrhage severity level 1	Stroke, long-term consequences, mild	has some difficulty in moving around and some weakness in one hand, but is able to walk without help.	0.019 (0.01-0.032)
Acute intracerebral hemorrhage severity level 2	Stroke, long-term consequences, moderate	has some difficulty in moving around, and in using the hands for lifting and holding things, dressing and grooming.	0.07 (0.046-0.099)
Acute intracerebral hemorrhage severity level 3	Stroke, long-term consequences, moderate plus cognition problems	has some difficulty in moving around, in using the hands for lifting and holding things, dressing and grooming, and in speaking. The person is often forgetful and confused.	0.316 (0.206-0.437)
Acute intracerebral hemorrhage severity level 4	Stroke, long-term consequences, severe	is confined to bed or a wheelchair, has difficulty speaking and depends on others for feeding, toileting and dressing.	0.552 (0.377-0.707)
Acute intracerebral hemorrhage severity level 5	Stroke, long-term consequences, severe plus cognition problems	is confined to bed or a wheelchair, depends on others for feeding, toileting and dressing, and has difficulty speaking, thinking clearly and remembering things.	0.588 (0.411-0.744)
Chronic intracerebral hemorrhage severity level 1	Stroke, long-term consequences, mild	has some difficulty in moving around and some weakness in one hand, but is able to walk without help.	0.019 (0.01-0.032)
Chronic intracerebral hemorrhage severity level 2	Stroke, long-term consequences, moderate	has some difficulty in moving around, and in using the hands for lifting and holding things, dressing and grooming.	0.07 (0.046-0.099)
Chronic intracerebral hemorrhage severity level 3	Stroke, long-term consequences, moderate plus cognition problems	has some difficulty in moving around, in using the hands for lifting and holding things, dressing and grooming, and in speaking. The person is often forgetful and confused.	0.316 (0.206-0.437)
Chronic intracerebral hemorrhage severity level 4	Stroke, long-term consequences, severe	is confined to bed or a wheelchair, has difficulty speaking and depends on others for feeding, toileting and dressing.	0.552 (0.377-0.707)
Chronic intracerebral hemorrhage severity level 5	Stroke, long-term consequences, severe plus cognition problems	is confined to bed or a wheelchair, depends on others for feeding, toileting and dressing, and has difficulty speaking, thinking clearly and remembering things.	0.588 (0.411-0.744)
Asymptomatic chronic intracerebral hemorrhage	Asymptomatic		0 (0-0)
Acute subarachnoid hemorrhage severity level 1	Stroke, long-term consequences, mild	has some difficulty in moving around and some weakness in one hand, but is able to walk without help.	0.019 (0.01-0.032)
Acute subarachnoid hemorrhage severity level 2	Stroke, long-term consequences, moderate	has some difficulty in moving around, and in using the hands for lifting and holding things, dressing and grooming.	0.07 (0.046-0.099)
Acute subarachnoid hemorrhage severity level 3	Stroke, long-term consequences, moderate plus cognition problems	has some difficulty in moving around, in using the hands for lifting and holding things, dressing and grooming, and in speaking. The person is often forgetful and confused.	0.316 (0.206-0.437)
Acute subarachnoid hemorrhage severity level 4	Stroke, long-term consequences, severe	is confined to bed or a wheelchair, has difficulty speaking and depends on others for feeding, toileting and dressing.	0.552 (0.377-0.707)
Acute subarachnoid hemorrhage severity level 5	Stroke, long-term consequences, severe plus cognition problems	is confined to bed or a wheelchair, depends on others for feeding, toileting and dressing, and has difficulty speaking, thinking clearly and remembering things.	0.588 (0.411-0.744)
Chronic subarachnoid hemorrhage severity level 1	Stroke, long-term consequences, mild	has some difficulty in moving around and some weakness in one hand, but is able to walk without help.	0.019 (0.01-0.032)
Chronic subarachnoid hemorrhage severity level 2	Stroke, long-term consequences, moderate	has some difficulty in moving around, and in using the hands for lifting and holding things, dressing and grooming.	0.07 (0.046-0.099)
Chronic subarachnoid hemorrhage severity level 3	Stroke, long-term consequences, moderate plus cognition problems	has some difficulty in moving around, in using the hands for lifting and holding things, dressing and grooming, and in speaking. The person is often forgetful and confused.	0.316 (0.206-0.437)
Chronic subarachnoid hemorrhage severity level 4	Stroke, long-term consequences, severe	is confined to bed or a wheelchair, has difficulty speaking and depends on others for feeding, toileting and dressing.	0.552 (0.377-0.707)
Chronic subarachnoid hemorrhage severity level 5	Stroke, long-term consequences, severe plus cognition problems	is confined to bed or a wheelchair, depends on others for feeding, toileting and dressing, and has difficulty speaking, thinking clearly and remembering things.	0.588 (0.411-0.744)
Asymptomatic chronic subarachnoid hemorrhage	Asymptomatic		0 (0-0)
Mild heart failure due to hypertensive heart disease	Heart failure, mild	is short of breath and easily tires with moderate physical activity, such as walking uphill or more than a quarter-mile on level ground. The person feels comfortable at rest or during activities requiring less effort.	0.041 (0.026-0.062)
Moderate heart failure due to hypertensive heart disease	Heart failure, moderate	is short of breath and easily tires with minimal physical activity, such as walking only a short distance. The person feels comfortable at rest but avoids moderate activity.	0.072 (0.047-0.103)
Severe heart failure due to hypertensive heart disease	Heart failure, severe	is short of breath and feels tired when at rest. The person avoids any physical activity, for fear of worsening the breathing problems.	0.179 (0.122-0.251)
Controlled, medically managed heart failure due to hypertensive heart disease	Generic uncomplicated disease: worry and daily medication	has a chronic disease that requires medication every day and causes some worry but minimal interference with daily activities.	0.049 (0.031-0.072)
Mild heart failure due to calcific aortic valve disease	Heart failure, mild	is short of breath and easily tires with moderate physical activity, such as walking uphill or more than a quarter-mile on level ground. The person feels comfortable at rest or during activities requiring less effort.	0.041 (0.026-0.062)
Moderate heart failure due to calcific aortic valve disease	Heart failure, moderate	is short of breath and easily tires with minimal physical activity, such as walking only a short distance. The person feels comfortable at rest but avoids moderate activity.	0.072 (0.047-0.103)
Severe heart failure due to calcific aortic valve disease	Heart failure, severe	is short of breath and feels tired when at rest. The person avoids any physical activity, for fear of worsening the breathing problems.	0.179 (0.122-0.251)
Controlled, medically managed heart failure due to calcific aortic valve disease	Generic uncomplicated disease: worry and daily medication	has a chronic disease that requires medication every day and causes some worry but minimal interference with daily activities.	0.049 (0.031-0.072)
Calcific aortic valve disease after valve intervention	Generic uncomplicated disease: worry and daily medication	has a chronic disease that requires medication every day and causes some worry but minimal interference with daily activities.	0.049 (0.031-0.072)
Asymptomatic calcific aortic valve disease	Asymptomatic		0 (0-0)
Mild heart failure due to degenerative mitral valve disease	Heart failure, mild	is short of breath and easily tires with moderate physical activity, such as walking uphill or more than a quarter-mile on level ground. The person feels comfortable at rest or during activities requiring less effort.	0.041 (0.026-0.062)
Moderate heart failure due to degenerative mitral valve disease	Heart failure, moderate	is short of breath and easily tires with minimal physical activity, such as walking only a short distance. The person feels comfortable at rest but avoids moderate activity.	0.072 (0.047-0.103)
Severe heart failure due to degenerative mitral valve disease	Heart failure, severe	is short of breath and feels tired when at rest. The person avoids any physical activity, for fear of worsening the breathing problems.	0.179 (0.122-0.251)
Controlled, medically managed heart failure due to degenerative mitral valve disease	Generic uncomplicated disease: worry and daily medication	has a chronic disease that requires medication every day and causes some worry but minimal interference with daily activities.	0.049 (0.031-0.072)
Degenerative mitral valve disease after valve intervention	Generic uncomplicated disease: worry and daily medication	has a chronic disease that requires medication every day and causes some worry but minimal interference with daily activities.	0.049 (0.031-0.072)
Asymptomatic degenerative mitral valve disease	Asymptomatic		0 (0-0)
Mild heart failure due to other non-rheumatic valve disease	Heart failure, mild	is short of breath and easily tires with moderate physical activity, such as walking uphill or more than a quarter-mile on level ground. The person feels comfortable at rest or during activities requiring less effort.	0.041 (0.026-0.062)
Moderate heart failure due to other non-rheumatic valve disease	Heart failure, moderate	is short of breath and easily tires with minimal physical activity, such as walking only a short distance. The person feels comfortable at rest but avoids moderate activity.	0.072 (0.047-0.103)
Severe heart failure due to other non-rheumatic valve disease	Heart failure, severe	is short of breath and feels tired when at rest. The person avoids any physical activity, for fear of worsening the breathing problems.	0.179 (0.122-0.251)
Controlled, medically managed heart failure due to other non-rheumatic valve disease	Generic uncomplicated disease: worry and daily medication	has a chronic disease that requires medication every day and causes some worry but minimal interference with daily activities.	0.049 (0.031-0.072)
Acute myocarditis	Infectious disease, acute episode, moderate	has a fever and aches, and feels weak, which causes some difficulty with daily activities.	0.051 (0.032-0.074)
Mild heart failure due to myocarditis	Heart failure, mild	is short of breath and easily tires with moderate physical activity, such as walking uphill or more than a quarter-mile on level ground. The person feels comfortable at rest or during activities requiring less effort.	0.041 (0.026-0.062)
Moderate heart failure due to myocarditis	Heart failure, moderate	is short of breath and easily tires with minimal physical activity, such as walking only a short distance. The person feels comfortable at rest but avoids moderate activity.	0.072 (0.047-0.103)
Severe heart failure due to myocarditis	Heart failure, severe	is short of breath and feels tired when at rest. The person avoids any physical activity, for fear of worsening the breathing problems.	0.179 (0.122-0.251)

Controlled, medically managed heart failure due to myocarditis	Generic uncomplicated disease: worry and daily medication	has a chronic disease that requires medication every day and causes some worry but minimal interference with daily activities.	0.049 (0.031-0.072)
Mild heart failure due to alcoholic cardiomyopathy	Heart failure, mild	is short of breath and easily tires with moderate physical activity, such as walking uphill or more than a quarter-mile on level ground. The person feels comfortable at rest or during activities requiring less effort.	0.041 (0.026-0.062)
Moderate heart failure due to alcoholic cardiomyopathy	Heart failure, moderate	is short of breath and easily tires with minimal physical activity, such as walking only a short distance. The person feels comfortable at rest but avoids moderate activity.	0.072 (0.047-0.103)
Severe heart failure due to alcoholic cardiomyopathy	Heart failure, severe	is short of breath and feels tired when at rest. The person avoids any physical activity, for fear of worsening the breathing problems.	0.179 (0.122-0.251)
Controlled, medically managed heart failure due to alcoholic cardiomyopathy	Generic uncomplicated disease: worry and daily medication	has a chronic disease that requires medication every day and causes some worry but minimal interference with daily activities.	0.049 (0.031-0.072)
Mild heart failure due to other cardiomyopathy	Heart failure, mild	is short of breath and easily tires with moderate physical activity, such as walking uphill or more than a quarter-mile on level ground. The person feels comfortable at rest or during activities requiring less effort.	0.041 (0.026-0.062)
Moderate heart failure due to other cardiomyopathy	Heart failure, moderate	is short of breath and easily tires with minimal physical activity, such as walking only a short distance. The person feels comfortable at rest but avoids moderate activity.	0.072 (0.047-0.103)
Severe heart failure due to other cardiomyopathy	Heart failure, severe	is short of breath and feels tired when at rest. The person avoids any physical activity, for fear of worsening the breathing problems.	0.179 (0.122-0.251)
Controlled, medically managed heart failure due to other cardiomyopathy	Generic uncomplicated disease: worry and daily medication	has a chronic disease that requires medication every day and causes some worry but minimal interference with daily activities.	0.049 (0.031-0.072)
Mild heart failure due to Pulmonary Arterial Hypertension	Heart failure, mild	is short of breath and easily tires with moderate physical activity, such as walking uphill or more than a quarter-mile on level ground. The person feels comfortable at rest or during activities requiring less effort.	0.041 (0.026-0.062)
Moderate heart failure due to Pulmonary Arterial Hypertension	Heart failure, moderate	is short of breath and easily tires with minimal physical activity, such as walking only a short distance. The person feels comfortable at rest but avoids moderate activity.	0.072 (0.047-0.103)
Severe heart failure due to Pulmonary Arterial Hypertension	Heart failure, severe	is short of breath and feels tired when at rest. The person avoids any physical activity, for fear of worsening the breathing problems.	0.179 (0.122-0.251)
Controlled, medically managed heart failure due to Pulmonary Arterial Hypertension	Generic uncomplicated disease: worry and daily medication	has a chronic disease that requires medication every day and causes some worry but minimal interference with daily activities.	0.049 (0.031-0.072)
Symptomatic atrial fibrillation and flutter	Cardiac conduction disorders and cardiac dysrhythmias	has periods of rapid and irregular heartbeats and occasional fainting.	0.224 (0.151-0.312)
Asymptomatic atrial fibrillation and flutter	Asymptomatic		0 (0-0)
Symptomatic claudication due to peripheral arterial disease	Claudication	has cramping pains in the legs after walking a medium distance. The pain goes away after a short rest.	0.014 (0.007-0.025)
Asymptomatic peripheral arterial disease	Asymptomatic		0 (0-0)
Moderate endocarditis	Infectious disease, acute episode, moderate	has a fever and aches, and feels weak, which causes some difficulty with daily activities.	0.051 (0.032-0.074)
Severe endocarditis	Infectious disease, acute episode, severe	has a high fever and pain, and feels very weak, which causes great difficulty with daily activities.	0.133 (0.088-0.19)
Mild heart failure due to endocarditis	Heart failure, mild	is short of breath and easily tires with moderate physical activity, such as walking uphill or more than a quarter-mile on level ground. The person feels comfortable at rest or during activities requiring less effort.	0.041 (0.026-0.062)
Moderate heart failure due to endocarditis	Heart failure, moderate	is short of breath and easily tires with minimal physical activity, such as walking only a short distance. The person feels comfortable at rest but avoids moderate activity.	0.072 (0.047-0.103)
Severe heart failure due to endocarditis	Heart failure, severe	is short of breath and feels tired when at rest. The person avoids any physical activity, for fear of worsening the breathing problems.	0.179 (0.122-0.251)
Controlled, medically managed heart failure due to endocarditis	Generic uncomplicated disease: worry and daily medication	has a chronic disease that requires medication every day and causes some worry but minimal interference with daily activities.	0.049 (0.031-0.072)
Mild heart failure due to other cardiovascular diseases	Heart failure, mild	is short of breath and easily tires with moderate physical activity, such as walking uphill or more than a quarter-mile on level ground. The person feels comfortable at rest or during activities requiring less effort.	0.041 (0.026-0.062)
Moderate heart failure due to other cardiovascular diseases	Heart failure, moderate	is short of breath and easily tires with minimal physical activity, such as walking only a short distance. The person feels comfortable at rest but avoids moderate activity.	0.072 (0.047-0.103)
Severe heart failure due to other cardiovascular diseases	Heart failure, severe	is short of breath and feels tired when at rest. The person avoids any physical activity, for fear of worsening the breathing problems.	0.179 (0.122-0.251)
Controlled, medically managed heart failure due to other cardiovascular disease	Generic uncomplicated disease: worry and daily medication	has a chronic disease that requires medication every day and causes some worry but minimal interference with daily activities.	0.049 (0.031-0.072)
Mild other cardiovascular diseases	Heart failure, mild	is short of breath and easily tires with moderate physical activity, such as walking uphill or more than a quarter-mile on level ground. The person feels comfortable at rest or during activities requiring less effort.	0.041 (0.026-0.062)
Moderate other cardiovascular diseases	Heart failure, moderate	is short of breath and easily tires with minimal physical activity, such as walking only a short distance. The person feels comfortable at rest but avoids moderate activity.	0.072 (0.047-0.103)
Severe other cardiovascular diseases	Heart failure, severe	is short of breath and feels tired when at rest. The person avoids any physical activity, for fear of worsening the breathing problems.	0.179 (0.122-0.251)
Asymptomatic other cardiovascular diseases	Asymptomatic		0 (0-0)
Mild heart failure due to severe chronic obstructive pulmonary disease	Severe COPD and other chronic respiratory, with mild heart failure		0.432 (0.3-0.577)
Moderate heart failure due to severe chronic obstructive pulmonary disease	Severe COPD and other chronic respiratory, with moderate heart failure		0.45 (0.315-0.597)
Severe heart failure due to severe chronic obstructive pulmonary disease	Severe COPD and other chronic respiratory, with severe heart failure		0.512 (0.365-0.666)
Controlled, medically managed heart failure due to severe chronic obstructive pulmonary disease	COPD and other chronic respiratory problems, severe and generic uncomplicated disease: worry and daily medication	(combined DW)	0.437 (0.301-0.581)
Mild chronic obstructive pulmonary disease	COPD and other chronic respiratory problems, mild	has cough and shortness of breath after heavy physical activity, but is able to walk long distances and climb stairs.	0.019 (0.011-0.033)
Moderate chronic obstructive pulmonary disease	COPD and other chronic respiratory problems, moderate	has cough, wheezing and shortness of breath, even after light physical activity. The person feels tired and can walk only short distances or climb only a few stairs.	0.225 (0.153-0.31)
Severe chronic obstructive pulmonary disease without heart failure	COPD and other chronic respiratory problems, severe	has cough, wheezing and shortness of breath all the time. The person has great difficulty walking even short distances or climbing any stairs, feels tired when at rest, and is anxious.	0.408 (0.273-0.556)
Asymptomatic chronic obstructive pulmonary disease	Asymptomatic		0 (0-0)
Mild heart failure due to severe silicosis	Severe COPD and other chronic respiratory, with mild heart failure		0.432 (0.3-0.577)
Moderate heart failure due to severe silicosis	Severe COPD and other chronic respiratory, with moderate heart failure		0.45 (0.315-0.597)
Severe heart failure due to severe silicosis	Severe COPD and other chronic respiratory, with severe heart failure		0.512 (0.365-0.666)
Controlled, medically managed heart failure due to severe silicosis	COPD and other chronic respiratory problems, severe and generic uncomplicated disease: worry and daily medication	(combined DW)	0.437 (0.301-0.581)
Mild silicosis	COPD and other chronic respiratory problems, mild	has cough and shortness of breath after heavy physical activity, but is able to walk long distances and climb stairs.	0.019 (0.011-0.033)
Moderate silicosis	COPD and other chronic respiratory problems, moderate	has cough, wheezing and shortness of breath, even after light physical activity. The person feels tired and can walk only short distances or climb only a few stairs.	0.225 (0.153-0.31)
Severe silicosis without heart failure	COPD and other chronic respiratory problems, severe	has cough, wheezing and shortness of breath all the time. The person has great difficulty walking even short distances or climbing any stairs, feels tired when at rest, and is anxious.	0.408 (0.273-0.556)
Asymptomatic silicosis	Asymptomatic		0 (0-0)
Mild asbestosis	COPD and other chronic respiratory problems, mild	has cough and shortness of breath after heavy physical activity, but is able to walk long distances and climb stairs.	0.019 (0.011-0.033)
Moderate asbestosis	COPD and other chronic respiratory problems, moderate	has cough, wheezing and shortness of breath, even after light physical activity. The person feels tired and can walk only short distances or climb only a few stairs.	0.225 (0.153-0.31)
Severe asbestosis without heart failure	COPD and other chronic respiratory problems, severe	has cough, wheezing and shortness of breath all the time. The person has great difficulty walking even short distances or climbing any stairs, feels tired when at rest, and is anxious.	0.408 (0.273-0.556)
Asymptomatic asbestosis	Asymptomatic		0 (0-0)
Mild heart failure due to severe asbestosis	Severe COPD and other chronic respiratory, with mild heart failure		0.432 (0.3-0.577)
Moderate heart failure due to severe asbestosis	Severe COPD and other chronic respiratory, with moderate heart failure		0.45 (0.315-0.597)

Severe heart failure due to severe asbestosis	Severe COPD and other chronic respiratory, with severe heart failure		0.512 (0.365-0.666)
Controlled, medically managed heart failure due to severe asbestosis	COPD and other chronic respiratory problems, severe and generic uncomplicated disease: worry and daily medication	(combined DW)	0.437 (0.301-0.581)
Mild coal workers pneumoconiosis	COPD and other chronic respiratory problems, mild	has cough and shortness of breath after heavy physical activity, but is able to walk long distances and climb stairs.	0.019 (0.011-0.033)
Moderate coal workers pneumoconiosis	COPD and other chronic respiratory problems, moderate	has cough, wheezing and shortness of breath, even after light physical activity. The person feels tired and can walk only short distances or climb only a few stairs.	0.225 (0.153-0.31)
Severe coal workers pneumoconiosis without heart failure	COPD and other chronic respiratory problems, severe	has cough, wheezing and shortness of breath all the time. The person has great difficulty walking even short distances or climbing any stairs, feels tired when at rest, and is anxious.	0.408 (0.273-0.556)
Asymptomatic coal workers pneumoconiosis	Asymptomatic		0 (0-0)
Mild heart failure due to severe coal workers pneumoconiosis	Severe COPD and other chronic respiratory, with mild heart failure		0.432 (0.3-0.577)
Moderate heart failure due to severe coal workers pneumoconiosis	Severe COPD and other chronic respiratory, with moderate heart failure		0.45 (0.315-0.597)
Severe heart failure due to severe coal workers pneumoconiosis	Severe COPD and other chronic respiratory, with severe heart failure		0.512 (0.365-0.666)
Controlled, medically managed heart failure due to severe coal workers pneumoconiosis	COPD and other chronic respiratory problems, severe and generic uncomplicated disease: worry and daily medication	(combined DW)	0.437 (0.301-0.581)
Mild heart failure due to severe other pneumoconiosis	Severe COPD and other chronic respiratory, with mild heart failure		0.432 (0.3-0.577)
Moderate heart failure due to severe other pneumoconiosis	Severe COPD and other chronic respiratory, with moderate heart failure		0.45 (0.315-0.597)
Severe heart failure due to severe other pneumoconiosis	Severe COPD and other chronic respiratory, with severe heart failure		0.512 (0.365-0.666)
Controlled, medically managed heart failure due to severe other pneumoconiosis	COPD and other chronic respiratory problems, severe and generic uncomplicated disease: worry and daily medication	(combined DW)	0.437 (0.301-0.581)
Mild other pneumoconiosis	COPD and other chronic respiratory problems, mild	has cough and shortness of breath after heavy physical activity, but is able to walk long distances and climb stairs.	0.019 (0.011-0.033)
Moderate other pneumoconiosis	COPD and other chronic respiratory problems, moderate	has cough, wheezing and shortness of breath, even after light physical activity. The person feels tired and can walk only short distances or climb only a few stairs.	0.225 (0.153-0.31)
Severe other pneumoconiosis without heart failure	COPD and other chronic respiratory problems, severe	has cough, wheezing and shortness of breath all the time. The person has great difficulty walking even short distances or climbing any stairs, feels tired when at rest, and is anxious.	0.408 (0.273-0.556)
Asymptomatic other pneumoconiosis	Asymptomatic		0 (0-0)
Asymptomatic asthma	Asymptomatic		0 (0-0)
Controlled asthma	Asthma, controlled	has wheezing and cough once a month, which does not cause difficulty with daily activities.	0.015 (0.007-0.026)
Partially controlled asthma	Asthma, partially controlled	has wheezing and cough once a week, which causes some difficulty with daily activities.	0.036 (0.022-0.055)
Uncontrolled asthma	Asthma, uncontrolled	has wheezing, cough and shortness of breath more than twice a week, which causes difficulty with daily activities and sometimes wakes the person at night.	0.133 (0.086-0.192)
Mild heart failure due to severe interstitial lung disease and pulmonary sarcoidosis	Severe COPD and other chronic respiratory, with mild heart failure		0.432 (0.3-0.577)
Moderate heart failure due to severe interstitial lung disease and pulmonary sarcoidosis	Severe COPD and other chronic respiratory, with moderate heart failure		0.45 (0.315-0.597)
Severe heart failure due to severe interstitial lung disease and pulmonary sarcoidosis	Severe COPD and other chronic respiratory, with severe heart failure		0.512 (0.365-0.666)
Controlled, medically managed heart failure due to severe interstitial lung disease and pulmonary sarcoidosis	COPD and other chronic respiratory problems, severe and generic uncomplicated disease: worry and daily medication	(combined DW)	0.437 (0.301-0.581)
Mild interstitial lung disease and pulmonary sarcoidosis	COPD and other chronic respiratory problems, mild	has cough and shortness of breath after heavy physical activity, but is able to walk long distances and climb stairs.	0.019 (0.011-0.033)
Moderate interstitial lung disease and pulmonary sarcoidosis	COPD and other chronic respiratory problems, moderate	has cough, wheezing and shortness of breath, even after light physical activity. The person feels tired and can walk only short distances or climb only a few stairs.	0.225 (0.153-0.31)
Severe interstitial lung disease and pulmonary sarcoidosis without heart failure	COPD and other chronic respiratory problems, severe	has cough, wheezing and shortness of breath all the time. The person has great difficulty walking even short distances or climbing any stairs, feels tired when at rest, and is anxious.	0.408 (0.273-0.556)
Asymptomatic interstitial lung disease and pulmonary sarcoidosis	Asymptomatic		0 (0-0)
Other chronic respiratory diseases	Post-COMO calculation for residuals (YLL/YLD ratio, other methods)		--
Cirrhosis and other chronic liver diseases due to hepatitis B, decompensated, without anemia	Decompensated cirrhosis of the liver	has a swollen belly and swollen legs. The person feels weakness, fatigue and loss of appetite.	0.178 (0.123-0.25)
Cirrhosis and other chronic liver diseases due to hepatitis B, decompensated, with mild anemia	Decompensated cirrhosis of the liver and mild anemia	(combined DW)	0.181 (0.126-0.252)
Cirrhosis and other chronic liver diseases due to hepatitis B, decompensated, with moderate anemia	Decompensated cirrhosis of the liver and moderate anemia	(combined DW)	0.22 (0.156-0.298)
Cirrhosis and other chronic liver diseases due to hepatitis B, decompensated, with severe anemia	Decompensated cirrhosis of the liver and severe anemia	(combined DW)	0.3 (0.212-0.404)
Cirrhosis and other chronic liver diseases due to hepatitis B, compensated	Asymptomatic		0 (0-0)
Chronic hepatitis B without cirrhosis	Asymptomatic		0 (0-0)
Cirrhosis and other chronic liver diseases due to hepatitis C, decompensated, with no anemia	Decompensated cirrhosis of the liver	has a swollen belly and swollen legs. The person feels weakness, fatigue and loss of appetite.	0.178 (0.123-0.25)
Cirrhosis and other chronic liver diseases due to hepatitis C, decompensated, with mild anemia	Decompensated cirrhosis of the liver and mild anemia	(combined DW)	0.181 (0.126-0.252)
Cirrhosis and other chronic liver diseases due to hepatitis C, decompensated, with moderate anemia	Decompensated cirrhosis of the liver and moderate anemia	(combined DW)	0.22 (0.156-0.298)
Cirrhosis and other chronic liver diseases due to hepatitis C, decompensated, with severe anemia	Decompensated cirrhosis of the liver and severe anemia	(combined DW)	0.3 (0.212-0.404)
Cirrhosis and other chronic liver diseases due to hepatitis C, compensated	Asymptomatic		0 (0-0)
Chronic hepatitis C without cirrhosis	Asymptomatic		0 (0-0)
Cirrhosis and other chronic liver diseases due to alcohol, decompensated, without anemia	Decompensated cirrhosis of the liver	has a swollen belly and swollen legs. The person feels weakness, fatigue and loss of appetite.	0.178 (0.123-0.25)
Cirrhosis and other chronic liver diseases due to alcohol, decompensated, with mild anemia	Decompensated cirrhosis of the liver and mild anemia	(combined DW)	0.181 (0.126-0.252)
Cirrhosis and other chronic liver diseases due to alcohol, decompensated, with moderate anemia	Decompensated cirrhosis of the liver and moderate anemia	(combined DW)	0.22 (0.156-0.298)
Cirrhosis and other chronic liver diseases due to alcohol, decompensated, with severe anemia	Decompensated cirrhosis of the liver and severe anemia	(combined DW)	0.3 (0.212-0.404)
Cirrhosis and other chronic liver diseases due to alcohol, compensated	Asymptomatic		0 (0-0)
Non-alcoholic fatty liver disease (NAFLD) / Non-alcoholic steatohepatitis (NASH)	Asymptomatic		0 (0-0)
Cirrhosis and other chronic liver diseases due to NASH, decompensated, with no anemia	Decompensated cirrhosis of the liver	has a swollen belly and swollen legs. The person feels weakness, fatigue and loss of appetite.	0.178 (0.123-0.25)
Cirrhosis and other chronic liver diseases due to NASH, decompensated, with mild anemia	Decompensated cirrhosis of the liver and mild anemia	(combined DW)	0.181 (0.126-0.252)
Cirrhosis and other chronic liver diseases due to NASH, decompensated, with moderate anemia	Decompensated cirrhosis of the liver and moderate anemia	(combined DW)	0.22 (0.156-0.298)
Cirrhosis and other chronic liver diseases due to NASH, decompensated, with severe anemia	Decompensated cirrhosis of the liver and severe anemia	(combined DW)	0.3 (0.212-0.404)
Cirrhosis and other chronic liver diseases due to NASH, compensated	Asymptomatic		0 (0-0)
Cirrhosis and other chronic liver diseases due to other, decompensated, with no anemia	Decompensated cirrhosis of the liver	has a swollen belly and swollen legs. The person feels weakness, fatigue and loss of appetite.	0.178 (0.123-0.25)
Cirrhosis and other chronic liver diseases due to other, decompensated, with mild anemia	Decompensated cirrhosis of the liver and mild anemia	(combined DW)	0.181 (0.126-0.252)
Cirrhosis and other chronic liver diseases due to other, decompensated, with moderate anemia	Decompensated cirrhosis of the liver and moderate anemia	(combined DW)	0.22 (0.156-0.298)
Cirrhosis and other chronic liver diseases due to other, decompensated, with severe anemia	Decompensated cirrhosis of the liver and severe anemia	(combined DW)	0.3 (0.212-0.404)
Cirrhosis and other chronic liver diseases due to other cause, compensated	Asymptomatic		0 (0-0)

Severe, acute, uncomplicated PUD with no anemia	Abdominopelvic problem, severe	has severe pain in the belly and feels nauseous. The person is anxious and unable to carry out daily activities.	0.324 (0.22-0.442)
Severe, acute, uncomplicated PUD with mild anemia	Abdominopelvic problem, severe and mild anemia	(combined DW)	0.327 (0.224-0.443)
Severe, acute, uncomplicated PUD with moderate anemia	Abdominopelvic problem, severe and moderate anemia	(combined DW)	0.359 (0.254-0.476)
Severe, acute, uncomplicated PUD with severe anemia	Abdominopelvic problem, severe and severe anemia	(combined DW)	0.423 (0.302-0.556)
Complicated PUD with no anemia	Gastric bleeding	vomits blood and feels nauseous.	0.325 (0.209-0.462)
Mildly symptomatic PUD with no anemia	Abdominopelvic problem, mild	has some pain in the belly that causes nausea but does not interfere with daily activities.	0.011 (0.005-0.021)
Moderately symptomatic PUD with no anemia	Abdominopelvic problem, moderate	has pain in the belly and feels nauseous. The person has difficulties with daily activities.	0.114 (0.078-0.159)
Asymptomatic PUD with mild anemia	Anemia, mild	feels slightly tired and weak at times, but this does not interfere with normal daily activities.	0.004 (0.001-0.008)
Asymptomatic PUD with moderate anemia	Anemia, moderate	feels moderate fatigue, weakness, and shortness of breath after exercise, making daily activities more difficult.	0.052 (0.034-0.076)
Asymptomatic PUD with severe anemia	Anemia, severe	feels very weak, tired and short of breath, and has problems with activities that require physical effort or deep concentration.	0.149 (0.101-0.209)
Asymptomatic PUD with no anemia	Asymptomatic		0 (0-0)
Mildly symptomatic PUD with mild anemia	Mild abdominal pain with mild anemia	(combined DW)	0.015 (0.007-0.029)
Mildly symptomatic PUD with moderate anemia	Mild abdominal pain with moderate anemia	(combined DW)	0.062 (0.04-0.093)
Mildly symptomatic PUD with severe anemia	Mild abdominal pain with severe anemia	(combined DW)	0.158 (0.109-0.219)
Moderately symptomatic PUD with severe anemia	Mild abdominal pain with severe anemia	(combined DW)	0.158 (0.109-0.219)
Moderately symptomatic PUD with mild anemia	Moderate abdominal pain with mild anemia	(combined DW)	0.118 (0.081-0.163)
Moderately symptomatic PUD with moderate anemia	Moderate abdominal pain with moderate anemia	(combined DW)	0.16 (0.109-0.22)
Complicated PUD with mild anemia	Gastric bleeding and anemia, mild	(combined DW)	0.327 (0.213-0.463)
Complicated PUD with moderate anemia	Gastric bleeding and anemia, moderate	(combined DW)	0.359 (0.242-0.497)
Complicated PUD with severe anemia	Gastric bleeding and anemia, severe	(combined DW)	0.424 (0.293-0.57)
Severe, acute, uncomplicated gastritis/duodenitis with no anemia	Abdominopelvic problem, severe	has severe pain in the belly and feels nauseous. The person is anxious and unable to carry out daily activities.	0.324 (0.22-0.442)
Severe, acute, uncomplicated gastritis/duodenitis with mild anemia	Abdominopelvic problem, severe and mild anemia	(combined DW)	0.327 (0.224-0.443)
Severe, acute, uncomplicated gastritis/duodenitis with moderate anemia	Abdominopelvic problem, severe and moderate anemia	(combined DW)	0.359 (0.254-0.476)
Severe, acute, uncomplicated gastritis/duodenitis with severe anemia	Abdominopelvic problem, severe and severe anemia	(combined DW)	0.423 (0.302-0.556)
Complicated gastritis/duodenitis with no anemia	Gastric bleeding	vomits blood and feels nauseous.	0.325 (0.209-0.462)
Mildly symptomatic gastritis/duodenitis with no anemia	Abdominopelvic problem, mild	has some pain in the belly that causes nausea but does not interfere with daily activities.	0.011 (0.005-0.021)
Moderately symptomatic gastritis/duodenitis with no anemia	Abdominopelvic problem, moderate	has pain in the belly and feels nauseous. The person has difficulties with daily activities.	0.114 (0.078-0.159)
Asymptomatic gastritis/duodenitis with mild anemia	Anemia, mild	feels slightly tired and weak at times, but this does not interfere with normal daily activities.	0.004 (0.001-0.008)
Asymptomatic gastritis/duodenitis with moderate anemia	Anemia, moderate	feels moderate fatigue, weakness, and shortness of breath after exercise, making daily activities more difficult.	0.052 (0.034-0.076)
Asymptomatic gastritis/duodenitis with severe anemia	Anemia, severe	feels very weak, tired and short of breath, and has problems with activities that require physical effort or deep concentration.	0.149 (0.101-0.209)
Asymptomatic gastritis/duodenitis with no anemia	Asymptomatic		0 (0-0)
Mildy symptomatic gastritis/duodenitis with mild anemia	Mild abdominal pain with mild anemia	(combined DW)	0.015 (0.007-0.029)
Mildly symptomatic gastritis/duodenitis with moderate anemia	Mild abdominal pain with moderate anemia	(combined DW)	0.062 (0.04-0.093)
Mildy symptomatic gastritis/duodenitis with severe anemia	Mild abdominal pain with severe anemia	(combined DW)	0.158 (0.109-0.219)
Moderately symptomatic gastritis/duodenitis with mild anemia	Moderate abdominal pain with mild anemia	(combined DW)	0.118 (0.081-0.163)
Moderately symptomatic gastritis/duodenitis with moderate anemia	Moderate abdominal pain with moderate anemia	(combined DW)	0.16 (0.109-0.22)
Moderately symptomatic gastritis/duodenitis with severe anemia	Moderate abdominal pain with severe anemia	(combined DW)	0.246 (0.171-0.334)
Complicated gastritis/duodenitis with mild anemia	Gastric bleeding and anemia, mild	(combined DW)	0.327 (0.213-0.463)
Complicated gastritis/duodenitis with moderate anemia	Gastric bleeding and anemia, moderate	(combined DW)	0.359 (0.242-0.497)
Complicated gastritis/duodenitis with severe anemia	Gastric bleeding and anemia, severe	(combined DW)	0.424 (0.293-0.57)
Mild to moderate GERD, symptomatic days	Abdominopelvic problem, mild	has some pain in the belly that causes nausea but does not interfere with daily activities.	0.011 (0.005-0.021)
Mild to moderate GERD, asymptomatic days	Asymptomatic		0 (0-0)
Severe GERD, asymptomatic days	Asymptomatic		0 (0-0)
Severe GERD, symptomatic days	Often has a burning sensation in the back of the chest after eating	Standard	0.026 (0.015-0.042)
Appendicitis	Abdominopelvic problem, severe	has severe pain in the belly and feels nauseous. The person is anxious and unable to carry out daily activities.	0.324 (0.22-0.442)
Paralytic ileus and intestinal obstruction	Abdominopelvic problem, severe	has severe pain in the belly and feels nauseous. The person is anxious and unable to carry out daily activities.	0.324 (0.22-0.442)
Mild symptomatic inguinal, femoral and abdominal hernia	Abdominopelvic problem, mild	has some pain in the belly that causes nausea but does not interfere with daily activities.	0.011 (0.005-0.021)
Moderate symptomatic inguinal, femoral and abdominal hernia	Abdominopelvic problem, moderate	has pain in the belly and feels nauseous. The person has difficulties with daily activities.	0.114 (0.078-0.159)
Severe symptomatic inguinal, femoral and abdominal hernia	Abdominopelvic problem, severe	has severe pain in the belly and feels nauseous. The person is anxious and unable to carry out daily activities.	0.324 (0.22-0.442)
Asymptomatic symptomatic inguinal, femoral and abdominal hernia	Asymptomatic		0 (0-0)
Ulcerative colitis with mild anemia	Crohn disease or ulcerative colitis	has cramping abdominal pain, has diarrhea several times a day, and feels very tired for two months every year. When the person does not have symptoms, there is anxiety about them returning.	0.231 (0.156-0.32)
Ulcerative colitis with moderate anemia	Crohn disease or ulcerative colitis	has cramping abdominal pain, has diarrhea several times a day, and feels very tired for two months every year. When the person does not have symptoms, there is anxiety about them returning.	0.231 (0.156-0.32)
Ulcerative colitis with severe anemia	Crohn disease or ulcerative colitis	has cramping abdominal pain, has diarrhea several times a day, and feels very tired for two months every year. When the person does not have symptoms, there is anxiety about them returning.	0.231 (0.156-0.32)
Ulcerative colitis without anemia	Crohn disease or ulcerative colitis	has cramping abdominal pain, has diarrhea several times a day, and feels very tired for two months every year. When the person does not have symptoms, there is anxiety about them returning.	0.231 (0.156-0.32)
Asymptomatic ulcerative colitis	Asymptomatic		0 (0-0)
Crohn's disease with mild anemia	Crohn disease or ulcerative colitis	has cramping abdominal pain, has diarrhea several times a day, and feels very tired for two months every year. When the person does not have symptoms, there is anxiety about them returning.	0.231 (0.156-0.32)
Crohn's disease with moderate anemia	Crohn disease or ulcerative colitis	has cramping abdominal pain, has diarrhea several times a day, and feels very tired for two months every year. When the person does not have symptoms, there is anxiety about them returning.	0.231 (0.156-0.32)
Crohn's disease with severe anemia	Crohn disease or ulcerative colitis	has cramping abdominal pain, has diarrhea several times a day, and feels very tired for two months every year. When the person does not have symptoms, there is anxiety about them returning.	0.231 (0.156-0.32)

Crohn's disease without anemia	Crohn disease or ulcerative colitis	has cramping abdominal pain, has diarrhea several times a day, and feels very tired for two months every year. When the person does not have symptoms, there is anxiety about them returning.	0.231 (0.156-0.32)
Asymptomatic Crohn's disease	Asymptomatic		0 (0-0)
Vascular intestinal disorders	Abdominopelvic problem, severe	has severe pain in the belly and feels nauseous. The person is anxious and unable to carry out daily activities.	0.324 (0.22-0.442)
Mild symptomatic episodes gallbladder and biliary diseases	Abdominopelvic problem, mild	has some pain in the belly that causes nausea but does not interfere with daily activities.	0.011 (0.005-0.021)
Moderate symptomatic episodes gallbladder and biliary diseases	Abdominopelvic problem, moderate	has pain in the belly and feels nauseous. The person has difficulties with daily activities.	0.114 (0.078-0.159)
Severe symptomatic episodes gallbladder and biliary diseases	Abdominopelvic problem, severe	has severe pain in the belly and feels nauseous. The person is anxious and unable to carry out daily activities.	0.324 (0.22-0.442)
Asymptomatic gallbladder and biliary diseases	Asymptomatic		0 (0-0)
Acute pancreatitis	Infectious disease, acute episode, severe and abdominopelvic problem, severe	(combined DW)	0.413 (0.296-0.541)
Mild chronic pancreatitis	Abdominopelvic problem, mild	has some pain in the belly that causes nausea but does not interfere with daily activities.	0.011 (0.005-0.021)
Moderate chronic pancreatitis	Abdominopelvic problem, moderate	has pain in the belly and feels nauseous. The person has difficulties with daily activities.	0.114 (0.078-0.159)
Severe chronic pancreatitis	Abdominopelvic problem, severe	has severe pain in the belly and feels nauseous. The person is anxious and unable to carry out daily activities.	0.324 (0.22-0.442)
Asymptomatic chronic pancreatitis	Asymptomatic		0 (0-0)
Other digestive diseases	Post-COMO calculation for residuals (YLL/YLD ratio, other methods)		--
Mild Alzheimer's disease and other dementias	Dementia, mild	has some trouble remembering recent events, and finds it hard to concentrate and make decisions and plans.	0.069 (0.046-0.099)
Moderate Alzheimer's disease and other dementias	Dementia, moderate	has memory problems and confusion, feels disoriented, at times hears voices that are not real, and needs help with some daily activities.	0.377 (0.252-0.508)
Severe Alzheimer's disease and other dementias	Dementia, severe	has complete memory loss; no longer recognizes close family members; and requires help with all daily activities.	0.449 (0.304-0.595)
Mild Parkinson's disease	Parkinson disease, mild	has mild tremors and moves a little slowly, but is able to walk and do daily activities without assistance.	0.01 (0.005-0.019)
Moderate Parkinson's disease	Parkinson disease, moderate	has moderate tremors and moves slowly, which causes some difficulty in walking and daily activities. The person has some trouble swallowing, talking, sleeping, and remembering things.	0.267 (0.181-0.372)
Severe Parkinson's disease	Parkinson disease, severe	has severe tremors and moves very slowly, which causes great difficulty in walking and daily activities. The person falls easily and has a lot of difficulty talking, swallowing, sleeping, and remembering things.	0.575 (0.396-0.73)
Idiopathic, seizure-free, treated epilepsy	Generic uncomplicated disease: worry and daily medication	has a chronic disease that requires medication every day and causes some worry but minimal interference with daily activities.	0.049 (0.031-0.072)
Idiopathic, severe epilepsy	Epilepsy, seizures >= once a month	has sudden seizures one or more times each month, with violent muscle contractions and stiffness, loss of consciousness, and loss of urine or bowel control. Between seizures the person has memory loss and difficulty concentrating.	0.552 (0.375-0.71)
Idiopathic, less severe epilepsy	Epilepsy, seizures 1-11 per year	has sudden seizures two to five times a year, with violent muscle contractions and stiffness, loss of consciousness, and loss of urine or bowel control.	0.263 (0.173-0.367)
Mild multiple sclerosis	Multiple sclerosis, mild	has mild loss of feeling in one hand, is a little unsteady while walking, has slight loss of vision in one eye, and often needs to urinate urgently.	0.183 (0.124-0.253)
Moderate multiple sclerosis	Multiple sclerosis, moderate	needs help walking, has difficulty with writing and arm coordination, has loss of vision in one eye and cannot control urinating.	0.463 (0.313-0.613)
Severe multiple sclerosis	Multiple sclerosis, severe	has slurred speech and difficulty swallowing. The person has weak arms and hands, very limited and stiff leg movement, has loss of vision in both eyes and cannot control urinating.	0.719 (0.534-0.858)
Asymptomatic multiple sclerosis	Asymptomatic		0 (0-0)
Mild respiratory problems due to motor neuron disease	COPD and other chronic respiratory problems, mild	has cough and shortness of breath after heavy physical activity, but is able to walk long distances and climb stairs.	0.019 (0.011-0.033)
Moderate respiratory problems due to motor neuron disease	COPD and other chronic respiratory problems, moderate	has cough, wheezing and shortness of breath, even after light physical activity. The person feels tired and can walk only short distances or climb only a few stairs.	0.225 (0.153-0.31)
Severe respiratory problems due to motor neuron disease	COPD and other chronic respiratory problems, severe	has cough, wheezing and shortness of breath all the time. The person has great difficulty walking even short distances or climbing any stairs, feels tired when at rest, and is anxious.	0.408 (0.273-0.556)
Diagnosis of motor neuron disease	Generic uncomplicated disease: anxiety about diagnosis	has a disease diagnosis that causes some worry but minimal interference with daily activities.	0.012 (0.006-0.023)
Speech problems due to motor neuron disease	Speech problems	has difficulty speaking, and others find it difficult to understand.	0.051 (0.032-0.078)
Mild motor impairment due to motor neuron disease	Motor impairment, mild	has some difficulty in moving around but is able to walk without help.	0.01 (0.005-0.019)
Moderate motor impairment due to motor neuron disease	Motor impairment, moderate	has some difficulty in moving around, and difficulty in lifting and holding objects, dressing and sitting upright, but is able to walk without help.	0.061 (0.04-0.089)
Severe motor impairment due to motor neuron disease	Motor impairment, severe	is unable to move around without help, and is not able to lift or hold objects, get dressed or sit upright.	0.402 (0.268-0.545)
Mild motor impairment, mild respiratory problems, and speech problems due to motor neuron disease	Mild motor impairment with mild respiratory problems and speech problems	(combined dw)	0.079 (0.049-0.123)
Mild motor impairment, moderate respiratory problems, and speech problems due to motor neuron disease	Mild motor impairment with moderate respiratory problems and speech problems	(combined dw)	0.272 (0.191-0.369)
Mild motor impairment, severe respiratory problems and speech problems due to motor neuron disease	Mild motor impairment with severe respiratory problems and speech problems	(combined DW)	0.444 (0.311-0.585)
Moderate motor impairment, mild respiratory problems, and speech problems due to motor neuron disease	Moderate motor impairment with mild respiratory problems and speech problems	(combined DW)	0.126 (0.081-0.183)
Moderate motor impairment, moderate respiratory problems, and speech problems due to motor neuron disease	Moderate motor impairment with moderate respiratory problems and speech problems	(combined DW)	0.309 (0.221-0.414)
Moderate motor impairment, severe respiratory problems, and speech problems due to motor neuron disease	Moderate motor impairment with severe respiratory problems and speech problems	(combined DW)	0.472 (0.339-0.611)
Severe motor impairment, mild respiratory problems, and speech problems due to motor neuron disease	Severe motor impairment with mild respiratory problems and speech problems	(combined DW)	0.443 (0.316-0.58)
Severe motor impairment, moderate respiratory problems, and speech problems due to motor neuron disease	Severe motor impairment with moderate respiratory problems and speech problems	(combined DW)	0.557 (0.412-0.705)
Severe motor impairment, severe respiratory problems, and speech problems due to motor neuron disease	Severe motor impairment with severe respiratory problems and speech problems	(combined DW)	0.659 (0.495-0.809)
Mild motor impairment and mild respiratory problems due to motor neuron disease	Mild motor impairment and mild respiratory problems	(combined DW)	0.079 (0.05-0.117)
Mild motor impairment and moderate respiratory problems due to motor neuron disease	Mild motor impairment and moderate respiratory problems	(combined DW)	0.272 (0.19-0.371)
Mild motor impairment and severe respiratory problems due to motor neuron disease	Mild motor impairment and severe respiratory problems	(combined DW)	0.443 (0.311-0.587)
Moderate motor impairment and mild respiratory problems due to motor neuron disease	Moderate motor impairment and mild respiratory problems	(combined DW)	0.029 (0.015-0.051)
Moderate motor impairment and moderate respiratory problems due to motor neuron disease	Moderate motor impairment and moderate respiratory problems	(combined DW)	0.233 (0.16-0.322)
Moderate motor impairment and severe respiratory problems due to motor neuron disease	Moderate motor impairment and severe respiratory problems	(combined DW)	0.414 (0.281-0.559)
Severe motor impairment and mild respiratory problems due to motor neuron disease	Severe motor impairment and mild respiratory problems	(combined DW)	0.413 (0.286-0.553)
Severe motor impairment and moderate respiratory problems due to motor neuron disease	Severe motor impairment and moderate respiratory problems	(combined DW)	0.534 (0.382-0.685)
Severe motor impairment and severe respiratory problems due to motor neuron disease	Severe motor impairment and severe respiratory problems	(combined DW)	0.641 (0.47-0.796)
Mild motor impairment and speech problems due to motor neuron disease	Mild motor impairment and speech problems	(combined DW)	0.061 (0.038-0.094)
Moderate motor impairment and speech problems due to motor neuron disease	Moderate motor impairment and speech problems	(combined DW)	0.109 (0.071-0.158)
Severe motor impairment and speech problems due to motor neuron disease	Severe motor impairment and speech problems	(combined DW)	0.432 (0.306-0.572)
Mild respiratory problems and speech problems due to motor neuron disease	Mild respiratory and speech problems	(combined DW)	0.069 (0.043-0.106)
Moderate respiratory problems and speech problems due to motor neuron disease	Moderate respiratory and speech problems	(combined DW)	0.265 (0.184-0.36)
Severe respiratory problems and speech problems due to motor neuron disease	Severe respiratory and speech problems	(combined DW)	0.438 (0.304-0.581)

Symptomatic medication overuse headache due to migraine	Headache, medication overuse	has daily headaches, felt as dull pain and often lasting all day, with poor sleep, nausea and fatigue. The person takes medicine for the headaches, which provides little relief but is needed to avoid having worse symptoms.	0.223 (0.146-0.313)
Asymptomatic medication overuse headache due to migraine	Asymptomatic		0 (0-0)
Symptomatic probable migraine	Headache, migraine	has severe, throbbing head pain and nausea that cause great difficulty in daily activities and sometimes confine the person to bed. Moving around, light, and noise make it worse.	0.441 (0.294-0.588)
Symptomatic definite migraine	Headache, migraine	has severe, throbbing head pain and nausea that cause great difficulty in daily activities and sometimes confine the person to bed. Moving around, light, and noise make it worse.	0.441 (0.294-0.588)
Asymptomatic probable migraine	Asymptomatic		0 (0-0)
Asymptomatic definite migraine	Asymptomatic		0 (0-0)
Symptomatic medication overuse headache due to tension-type headache	Headache, medication overuse	has daily headaches, felt as dull pain and often lasting all day, with poor sleep, nausea and fatigue. The person takes medicine for the headaches, which provides little relief but is needed to avoid having worse symptoms.	0.223 (0.146-0.313)
Asymptomatic medication overuse headache due to tension-type headache	Asymptomatic		0 (0-0)
Symptomatic probable tension-type headache	Headache, tension-type	has a moderate headache that also affects the neck, which causes difficulty in daily activities.	0.037 (0.022-0.057)
Symptomatic definite tension-type headache	Headache, tension-type	has a moderate headache that also affects the neck, which causes difficulty in daily activities.	0.037 (0.022-0.057)
Asymptomatic probable tension-type headache	Asymptomatic		0 (0-0)
Asymptomatic definite tension-type headache	Asymptomatic		0 (0-0)
Guillain-BarrÃ© syndrome due to other neurological disorders	Spinal cord lesion below neck level (treated)	is paralyzed from the waist down, cannot feel or move the legs and has difficulties with urine and bowel control. The person uses a wheelchair to move around.	0.296 (0.198-0.414)
Other neurological disorders	Post-COMO calculation for residuals (YLL/YLD ratio, other methods)		--
Schizophrenia acute state	Schizophrenia, acute state	hears and sees things that are not real and is afraid, confused, and sometimes violent. The person has great difficulty with communication and daily activities, and sometimes wants to harm or kill himself (or herself).	0.778 (0.606-0.9)
Schizophrenia residual state	Schizophrenia, residual state	hears and sees things that are not real and has trouble communicating. The person can be forgetful, has difficulty with daily activities, and thinks about hurting himself (or herself).	0.588 (0.411-0.754)
Mild major depressive disorder	Major depressive disorder, mild episode	feels persistent sadness and has lost interest in usual activities. The person sometimes sleeps badly, feels tired, or has trouble concentrating but still manages to function in daily life with extra effort.	0.145 (0.099-0.209)
Moderate major depressive disorder	Major depressive disorder, moderate episode	has constant sadness and has lost interest in usual activities. The person has some difficulty in daily life, sleeps badly, has trouble concentrating, and sometimes thinks about harming himself (or herself).	0.396 (0.267-0.531)
Severe major depressive disorder	Major depressive disorder, severe episode	has overwhelming, constant sadness and cannot function in daily life. The person sometimes loses touch with reality and wants to harm or kill himself (or herself).	0.658 (0.477-0.807)
Major depressive disorder, currently without symptoms	Asymptomatic		0 (0-0)
Symptomatic dysthymia	Major depressive disorder, mild episode	feels persistent sadness and has lost interest in usual activities. The person sometimes sleeps badly, feels tired, or has trouble concentrating but still manages to function in daily life with extra effort.	0.145 (0.099-0.209)
Dysthymia, currently without symptoms	Asymptomatic		0 (0-0)
Bipolar disorder depressive state	Major depressive disorder, moderate episode	has constant sadness and has lost interest in usual activities. The person has some difficulty in daily life, sleeps badly, has trouble concentrating, and sometimes thinks about harming himself (or herself).	0.396 (0.267-0.531)
Bipolar disorder manic state	Bipolar disorder, manic episode	is hyperactive, hears and believes things that are not real, and engages in impulsive and aggressive behavior that endanger the person and others.	0.492 (0.341-0.646)
Bipolar disorder residual state	Bipolar disorder, residual state	has mild mood swings, irritability and some difficulty with daily activities.	0.032 (0.018-0.051)
Mild anxiety disorders	Anxiety disorders, mild	feels mildly anxious and worried, which makes it slightly difficult to concentrate, remember things, and sleep. The person tires easily but is able to perform daily activities.	0.03 (0.018-0.046)
Moderate anxiety disorders	Anxiety disorders, moderate	feels anxious and worried, which makes it difficult to concentrate, remember things, and sleep. The person tires easily and finds it difficult to perform daily activities.	0.133 (0.091-0.186)
Severe anxiety disorders	Anxiety disorders, severe	constantly feels very anxious and worried, which makes it difficult to concentrate, remember things and sleep. The person has lost pleasure in life and thinks about suicide.	0.523 (0.362-0.677)
Anxiety disorders, currently without symptoms	Asymptomatic		0 (0-0)
Anorexia nervosa	Anorexia nervosa	feels an overwhelming need to starve and exercises excessively to lose weight. The person is very thin, weak and anxious.	0.224 (0.15-0.312)
Bulimia nervosa	Bulimia nervosa	has uncontrolled overeating followed by guilt, starving, and vomiting to lose weight.	0.223 (0.149-0.311)
Autism spectrum disorders without intellectual disability	Autism spectrum disorder without intellectual disability	(combined DW)	0.13 (0.087-0.184)
Autism spectrum disorders with borderline intellectual disability	Autism spectrum disorder with borderline intellectual disability	(combined DW)	0.139 (0.095-0.194)
Autism spectrum disorders with mild intellectual disability	Autism spectrum disorder with mild intellectual disability	(combined DW)	0.166 (0.116-0.226)
Autism spectrum disorders with moderate intellectual disability	Autism spectrum disorder with moderate intellectual disability	(combined DW)	0.216 (0.152-0.293)
Autism spectrum disorders with severe intellectual disability	Autism spectrum disorder with severe intellectual disability	(combined DW)	0.268 (0.188-0.367)
Autism spectrum disorders with profound intellectual disability	Autism spectrum disorder with profound intellectual disability	(combined DW)	0.303 (0.209-0.405)
Symptomatic attention-deficit/hyperactivity disorder	Attention deficit hyperactivity disorder	is hyperactive and has difficulty concentrating, remembering things, and completing tasks.	0.045 (0.028-0.066)
Attention-deficit/hyperactivity disorder, currently without symptoms	Asymptomatic		0 (0-0)
Symptomatic conduct disorder	Conduct disorder	has frequent behavior problems, which are sometimes violent. The person often has difficulty interacting with other people and feels irritable.	0.241 (0.159-0.341)
Conduct disorder, currently without symptoms	Asymptomatic		0 (0-0)
Borderline idiopathic developmental intellectual disability	Borderline intellectual functioning	is slow in learning at school. As an adult, the person has some difficulty doing complex or unfamiliar tasks but otherwise functions independently.	0.011 (0.005-0.02)
Mild idiopathic developmental intellectual disability	Intellectual disability / mental retardation, mild	has low intelligence and is slow in learning at school. As an adult, the person can live independently, but often needs help to raise children and can only work at simple supervised jobs.	0.043 (0.026-0.064)
Moderate idiopathic developmental intellectual disability	Intellectual disability / mental retardation, moderate	has low intelligence, and is slow in learning to speak and to do even simple tasks. As an adult, the person requires a lot of support to live independently and raise children. The person can only work at the simplest supervised jobs.	0.1 (0.066-0.142)
Severe idiopathic developmental intellectual disability	Intellectual disability / mental retardation, severe	has very low intelligence and cannot speak more than a few words, needs constant supervision and help with most daily activities, and can do only the simplest tasks.	0.16 (0.107-0.226)
Profound idiopathic developmental intellectual disability	Intellectual disability / mental retardation, profound	has very low intelligence, has almost no language, and does not understand even the most basic requests or instructions. The person requires constant supervision and help for all activities.	0.2 (0.133-0.283)
Mild other mental disorders	Anxiety disorders, mild	feels mildly anxious and worried, which makes it slightly difficult to concentrate, remember things, and sleep. The person tires easily but is able to perform daily activities.	0.03 (0.018-0.046)
Moderate other mental disorders	Anxiety disorders, moderate	feels anxious and worried, which makes it difficult to concentrate, remember things, and sleep. The person tires easily and finds it difficult to perform daily activities.	0.133 (0.091-0.186)
Severe other mental disorders	Anxiety disorders, severe	constantly feels very anxious and worried, which makes it difficult to concentrate, remember things and sleep. The person has lost pleasure in life and thinks about suicide.	0.523 (0.362-0.677)
Other mental disorders, currently without symptoms	Asymptomatic		0 (0-0)
Mild alcohol dependence	Alcohol use disorder, mild	drinks a lot of alcohol and sometimes has difficulty controlling the urge to drink. While intoxicated, the person has difficulty performing daily activities.	0.235 (0.16-0.327)

Moderate alcohol dependence	Alcohol use disorder, moderate	drinks a lot, gets drunk almost every week and has great difficulty controlling the urge to drink. Drinking and recovering cause great difficulty in daily activities, sleep loss, and fatigue.	0.373 (0.248-0.508)
Severe alcohol dependence	Alcohol use disorder, severe	gets drunk almost every day and is unable to control the urge to drink. Drinking and recovering replace most daily activities. The person has difficulty thinking, remembering and communicating, and feels constant pain and fatigue.	0.57 (0.396-0.732)
Very mild alcohol dependence	Alcohol use disorder, very mild	drinks alcohol daily and has difficulty controlling the urge to drink. When sober, the person functions normally.	0.123 (0.082-0.177)
Asymptomatic alcohol dependence	Asymptomatic		0 (0-0)
Mild fetal alcohol syndrome	Fetal alcohol syndrome, mild	is a little slow in developing physically and mentally, which causes some difficulty in learning but no other difficulties in daily activities.	0.016 (0.008-0.03)
Moderate fetal alcohol syndrome	Fetal alcohol syndrome, moderate	is slow in developing physically and mentally, which causes some difficulty in daily activities.	0.056 (0.035-0.083)
Severe fetal alcohol syndrome	Fetal alcohol syndrome, severe	is very slow in developing physically and mentally, which causes great difficulty in daily activities.	0.179 (0.119-0.257)
Asymptomatic fetal alcohol syndrome	Asymptomatic		0 (0-0)
Severe opioid dependence	Heroin and other opioid dependence	uses heroin daily and has difficulty controlling the habit. When the effects wear off, the person feels severe nausea, agitation, vomiting and fever. The person has a lot of difficulty in daily activities.	0.697 (0.51-0.843)
Mild opioid dependence	Heroin and other opioid dependence, mild	uses heroin (or methadone)Ã‚Â daily and has difficulty controlling the habit. When not using, the person functions normally.	0.335 (0.221-0.473)
Asymptomatic opioid dependence	Asymptomatic		0 (0-0)
Severe cocaine dependence	Cocaine dependence	uses cocaine and has difficulty controlling the habit. The person sometimes has mood swings, anxiety, paranoia, hallucinations and sleep problems, and has some difficulty in daily activities.	0.479 (0.324-0.634)
Mild cocaine dependence	Cocaine dependence, mild	uses cocaine at least once a week and has some difficulty controlling the habit. When not using, the person functions normally.	0.116 (0.074-0.165)
Asymptomatic cocaine dependence	Asymptomatic		0 (0-0)
Severe amphetamine dependence	Amphetamine dependence	uses stimulants (drugs) and has difficulty controlling the habit. The person sometimes has depression, hallucinations and mood swings, and has difficulty in daily activities.	0.486 (0.329-0.637)
Mild amphetamine dependence	Amphetamine dependence, mild	uses stimulants (drugs) at least once a week and has some difficulty controlling the habit. When not using, the person functions normally.	0.079 (0.051-0.114)
Asymptomatic amphetamine dependence	Asymptomatic		0 (0-0)
Severe cannabis dependence	Cannabis dependence	uses marijuana daily and has difficulty controlling the habit. The person sometimes has mood swings, anxiety and hallucinations, and has some difficulty in daily activities.	0.266 (0.178-0.364)
Mild cannabis dependence	Cannabis dependence, mild	uses marijuana at least once a week and has some difficulty controlling the habit. When not using, the person functions normally.	0.039 (0.024-0.06)
Asymptomatic cannabis dependence	Asymptomatic		0 (0-0)
Other drug use disorders	Cocaine dependence, mild	uses cocaine at least once a week and has some difficulty controlling the habit. When not using, the person functions normally.	0.116 (0.074-0.165)
Uncomplicated diabetes mellitus type 1	Generic uncomplicated disease: worry and daily medication	has a chronic disease that requires medication every day and causes some worry but minimal interference with daily activities.	0.049 (0.031-0.072)
Moderate vision impairment due to diabetes mellitus type 1 retinopathy	Distance vision, moderate impairment	has vision problems that make it difficult to recognize faces or objects across a room.	0.031 (0.019-0.049)
Severe vision impairment due to diabetes mellitus type 1 retinopathy	Distance vision, severe impairment	has severe vision loss, which causes difficulty in daily activities, some emotional impact (for example worry), and some difficulty going outside the home without assistance.	0.184 (0.125-0.258)
Blindness due to diabetes mellitus type 1 retinopathy	Distance vision blindness	is completely blind, which causes great difficulty in some daily activities, worry and anxiety, and great difficulty going outside the home without assistance.	0.187 (0.124-0.26)
Diabetic neuropathy due to diabetes mellitus type 1, without diabetic foot or amputation	Diabetic neuropathy	has pain, tingling and numbness in the arms, legs, hands and feet. The person sometimes gets cramps and muscle weakness.	0.133 (0.089-0.187)
Diabetic foot due to neuropathy due to diabetes mellitus type 1	Diabetic neuropathy with diabetic foot		0.15 (0.103-0.208)
Diabetic neuropathy and amputation with treatment due to diabetes mellitus type 1	Diabetic neuropathy with treated amputation		0.167 (0.114-0.229)
Diabetic neuropathy and amputation without treatment due to diabetes mellitus type 1	Diabetic neuropathy with untreated amputation		0.282 (0.198-0.379)
Uncomplicated diabetes mellitus type 2	Generic uncomplicated disease: worry and daily medication	has a chronic disease that requires medication every day and causes some worry but minimal interference with daily activities.	0.049 (0.031-0.072)
Moderate vision impairment due to diabetes mellitus type 2 retinopathy	Distance vision, moderate impairment	has vision problems that make it difficult to recognize faces or objects across a room.	0.031 (0.019-0.049)
Severe vision impairment due to diabetes mellitus type 2 retinopathy	Distance vision, severe impairment	has severe vision loss, which causes difficulty in daily activities, some emotional impact (for example worry), and some difficulty going outside the home without assistance.	0.184 (0.125-0.258)
Blindness due to diabetes mellitus type 2 retinopathy	Distance vision blindness	is completely blind, which causes great difficulty in some daily activities, worry and anxiety, and great difficulty going outside the home without assistance.	0.187 (0.124-0.26)
Diabetic neuropathy due to diabetes mellitus type 2, without diabetic foot or amputation	Diabetic neuropathy	has pain, tingling and numbness in the arms, legs, hands and feet. The person sometimes gets cramps and muscle weakness.	0.133 (0.089-0.187)
Diabetic foot due to neuropathy due to diabetes mellitus type 2	Diabetic neuropathy with diabetic foot		0.15 (0.103-0.208)
Diabetic neuropathy and amputation with treatment due to diabetes mellitus type 2	Diabetic neuropathy with treated amputation		0.167 (0.114-0.229)
Diabetic neuropathy and amputation without treatment due to diabetes mellitus type 2	Diabetic neuropathy with untreated amputation		0.282 (0.198-0.379)
Stage 1-2 chronic kidney disease with preserved GFR due to type 1 diabetes mellitus	Asymptomatic		0 (0-0)
End-stage renal disease after transplant due to type 1 diabetes mellitus	End-stage renal disease, with kidney transplant	sometimes feels tired and down, and has some difficulty with daily activities.	0.024 (0.014-0.039)
End-stage renal disease on dialysis without anemia due to type 1 diabetes mellitus	End-stage renal disease, on dialysis	is tired and has itching, cramps, headache, joint pains and shortness of breath. The person needs intensive medical care every other day lasting about half a day.	0.571 (0.398-0.725)
End-stage renal disease on dialysis and mild anemia due to type 1 diabetes mellitus	End-stage renal disease, on dialysis and mild anemia	(combined DW)	0.573 (0.403-0.726)
End-stage renal disease on dialysis and moderate anemia due to type 1 diabetes mellitus	End-stage renal disease, on dialysis and moderate anemia	(combined DW)	0.593 (0.424-0.742)
End-stage renal disease on dialysis and severe anemia due to type 1 diabetes mellitus	End-stage renal disease, on dialysis and severe anemia	(combined DW)	0.633 (0.462-0.781)
Stage 3 chronic kidney disease and mild anemia due to type 1 diabetes mellitus	Anemia, mild	feels slightly tired and weak at times, but this does not interfere with normal daily activities.	0.004 (0.001-0.008)
Stage 3 chronic kidney disease and moderate anemia due to type 1 diabetes mellitus	Anemia, moderate	feels moderate fatigue, weakness, and shortness of breath after exercise, making daily activities more difficult.	0.052 (0.034-0.076)
Stage 3 chronic kidney disease and severe anemia due to type 1 diabetes mellitus	Anemia, severe	feels very weak, tired and short of breath, and has problems with activities that require physical effort or deep concentration.	0.149 (0.101-0.209)
Stage 3 chronic kidney disease without anemia due to type 1 diabetes mellitus	Asymptomatic		0 (0-0)
Stage 4 chronic kidney disease untreated without anemia due to type 1 diabetes mellitus	Chronic kidney disease (stage IV)	tires easily, has nausea, reduced appetite and difficulty sleeping.	0.104 (0.07-0.147)
Stage 4 chronic kidney disease untreated and mild anemia due to type 1 diabetes mellitus	Mild anemia with Stage IV CKD		0.108 (0.072-0.151)
Stage 4 chronic kidney disease untreated and moderate anemia due to type 1 diabetes mellitus	Moderate anemia with Stage IV CKD		0.15 (0.103-0.207)
Stage 4 chronic kidney disease untreated and severe anemia due to type 1 diabetes mellitus	Severe anemia with Stage IV CKD		0.237 (0.165-0.324)
Stage 5 chronic kidney disease untreated without anemia due to type 1 diabetes mellitus	Terminal phase, without medication (for cancers, end-stage kidney/liver disease)	has lost a lot of weight and has constant pain. The person has no appetite, feels nauseous, and needs to spend most of the day in bed.	0.569 (0.389-0.727)
Stage 5 chronic kidney disease untreated and mild anemia due to type 1 diabetes mellitus	Mild anemia and terminal phase, without medication (for cancers, end-stage kidney/liver disease)	(combined DW)	0.57 (0.391-0.727)
Stage 5 chronic kidney disease untreated and moderate anemia due to type 1 diabetes mellitus	Moderate anemia and terminal phase, without medication (for cancers, end-stage kidney/liver disease)	(combined DW)	0.591 (0.414-0.743)
Stage 5 chronic kidney disease untreated and severe anemia due to type 1 diabetes mellitus	Severe anemia and terminal phase, without medication (for cancers, end-stage kidney/liver disease)	(combined DW)	0.631 (0.456-0.782)
Stage 1-2 chronic kidney disease with preserved GFR due to type 2 diabetes mellitus	Asymptomatic		0 (0-0)

End-stage renal disease after transplant due to type 2 diabetes mellitus	End-stage renal disease, with kidney transplant	sometimes feels tired and down, and has some difficulty with daily activities.	0.024 (0.014-0.039)
End-stage renal disease on dialysis without anemia due to type 2 diabetes mellitus	End-stage renal disease, on dialysis	is tired and has itching, cramps, headache, joint pains and shortness of breath. The person needs intensive medical care every other day lasting about half a day.	0.571 (0.398-0.725)
End-stage renal disease on dialysis and mild anemia due to type 2 diabetes mellitus	End-stage renal disease, on dialysis and mild anemia	(combined DW)	0.573 (0.403-0.726)
End-stage renal disease on dialysis and moderate anemia due to type 2 diabetes mellitus	End-stage renal disease, on dialysis and moderate anemia	(combined DW)	0.593 (0.424-0.742)
End-stage renal disease on dialysis and severe anemia due to type 2 diabetes mellitus	End-stage renal disease, on dialysis and severe anemia	(combined DW)	0.633 (0.462-0.781)
Stage 3 chronic kidney disease and mild anemia due to type 2 diabetes mellitus	Anemia, mild	feels slightly tired and weak at times, but this does not interfere with normal daily activities.	0.004 (0.001-0.008)
Stage 3 chronic kidney disease and moderate anemia due to type 2 diabetes mellitus	Anemia, moderate	feels moderate fatigue, weakness, and shortness of breath after exercise, making daily activities more difficult.	0.052 (0.034-0.076)
Stage 3 chronic kidney disease and severe anemia due to type 2 diabetes mellitus	Anemia, severe	feels very weak, tired and short of breath, and has problems with activities that require physical effort or deep concentration.	0.149 (0.101-0.209)
Stage 3 chronic kidney disease without anemia due to type 2 diabetes mellitus	Asymptomatic		0 (0-0)
Stage 4 chronic kidney disease untreated without anemia due to type 2 diabetes mellitus	Chronic kidney disease (stage IV)	tires easily, has nausea, reduced appetite and difficulty sleeping.	0.104 (0.07-0.147)
Stage 4 chronic kidney disease untreated and mild anemia due to type 2 diabetes mellitus	Mild anemia with Stage IV CKD		0.108 (0.072-0.151)
Stage 4 chronic kidney disease untreated and moderate anemia due to type 2 diabetes mellitus	Moderate anemia with Stage IV CKD		0.15 (0.103-0.207)
Stage 4 chronic kidney disease untreated and severe anemia due to type 2 diabetes mellitus	Severe anemia with Stage IV CKD		0.237 (0.165-0.324)
Stage 5 chronic kidney disease untreated without anemia due to type 2 diabetes mellitus	Terminal phase, without medication (for cancers, end-stage kidney/liver disease)	has lost a lot of weight and has constant pain. The person has no appetite, feels nauseous, and needs to spend most of the day in bed.	0.569 (0.389-0.727)
Stage 5 chronic kidney disease untreated and mild anemia due to type 2 diabetes mellitus	Mild anemia and terminal phase, without medication (for cancers, end-stage kidney/liver disease)	(combined DW)	0.57 (0.391-0.727)
Stage 5 chronic kidney disease untreated and moderate anemia due to type 2 diabetes mellitus	Moderate anemia and terminal phase, without medication (for cancers, end-stage kidney/liver disease)	(combined DW)	0.591 (0.414-0.743)
Stage 5 chronic kidney disease untreated and severe anemia due to type 2 diabetes mellitus	Severe anemia and terminal phase, without medication (for cancers, end-stage kidney/liver disease)	(combined DW)	0.631 (0.456-0.782)
Stage 1-2 chronic kidney disease with preserved GFR due to hypertension	Asymptomatic		0 (0-0)
End-stage renal disease after transplant due to hypertension	End-stage renal disease, with kidney transplant	sometimes feels tired and down, and has some difficulty with daily activities.	0.024 (0.014-0.039)
End-stage renal disease on dialysis without anemia due to hypertension	End-stage renal disease, on dialysis	is tired and has itching, cramps, headache, joint pains and shortness of breath. The person needs intensive medical care every other day lasting about half a day.	0.571 (0.398-0.725)
End-stage renal disease on dialysis and mild anemia due to hypertension	End-stage renal disease, on dialysis and mild anemia	(combined DW)	0.573 (0.403-0.726)
End-stage renal disease on dialysis and moderate anemia due to hypertension	End-stage renal disease, on dialysis and moderate anemia	(combined DW)	0.593 (0.424-0.742)
End-stage renal disease on dialysis and severe anemia due to hypertension	End-stage renal disease, on dialysis and severe anemia	(combined DW)	0.633 (0.462-0.781)
Stage 3 chronic kidney disease and mild anemia due to hypertension	Anemia, mild	feels slightly tired and weak at times, but this does not interfere with normal daily activities.	0.004 (0.001-0.008)
Stage 3 chronic kidney disease and moderate anemia due to hypertension	Anemia, moderate	feels moderate fatigue, weakness, and shortness of breath after exercise, making daily activities more difficult.	0.052 (0.034-0.076)
Stage 3 chronic kidney disease and severe anemia due to hypertension	Anemia, severe	feels very weak, tired and short of breath, and has problems with activities that require physical effort or deep concentration.	0.149 (0.101-0.209)
Stage 3 chronic kidney disease without anemia due to hypertension	Asymptomatic		0 (0-0)
Stage 4 chronic kidney disease untreated without anemia due to hypertension	Chronic kidney disease (stage IV)	tires easily, has nausea, reduced appetite and difficulty sleeping.	0.104 (0.07-0.147)
Stage 4 chronic kidney disease untreated and mild anemia due to hypertension	Mild anemia with Stage IV CKD		0.108 (0.072-0.151)
Stage 4 chronic kidney disease untreated and moderate anemia due to hypertension	Moderate anemia with Stage IV CKD		0.15 (0.103-0.207)
Stage 4 chronic kidney disease untreated and severe anemia due to hypertension	Severe anemia with Stage IV CKD		0.237 (0.165-0.324)
Stage 5 chronic kidney disease untreated without anemia due to hypertension	Terminal phase, without medication (for cancers, end-stage kidney/liver disease)	has lost a lot of weight and has constant pain. The person has no appetite, feels nauseous, and needs to spend most of the day in bed.	0.569 (0.389-0.727)
Stage 5 chronic kidney disease untreated and mild anemia due to hypertension	Mild anemia and terminal phase, without medication (for cancers, end-stage kidney/liver disease)	(combined DW)	0.57 (0.391-0.727)
Stage 5 chronic kidney disease untreated and moderate anemia due to hypertension	Moderate anemia and terminal phase, without medication (for cancers, end-stage kidney/liver disease)	(combined DW)	0.591 (0.414-0.743)
Stage 5 chronic kidney disease untreated and severe anemia due to hypertension	Severe anemia and terminal phase, without medication (for cancers, end-stage kidney/liver disease)	(combined DW)	0.631 (0.456-0.782)
Stage 1-2 chronic kidney disease with preserved GFR due to glomerulonephritis	Asymptomatic		0 (0-0)
End-stage renal disease after transplant due to glomerulonephritis	End-stage renal disease, with kidney transplant	sometimes feels tired and down, and has some difficulty with daily activities.	0.024 (0.014-0.039)
End-stage renal disease on dialysis without anemia due to glomerulonephritis	End-stage renal disease, on dialysis	is tired and has itching, cramps, headache, joint pains and shortness of breath. The person needs intensive medical care every other day lasting about half a day.	0.571 (0.398-0.725)
End-stage renal disease on dialysis and mild anemia due to glomerulonephritis	End-stage renal disease, on dialysis and mild anemia	(combined DW)	0.573 (0.403-0.726)
End-stage renal disease on dialysis and moderate anemia due to glomerulonephritis	End-stage renal disease, on dialysis and moderate anemia	(combined DW)	0.593 (0.424-0.742)
End-stage renal disease on dialysis and severe anemia due to glomerulonephritis	End-stage renal disease, on dialysis and severe anemia	(combined DW)	0.633 (0.462-0.781)
Stage 3 chronic kidney disease and mild anemia due to glomerulonephritis	Anemia, mild	feels slightly tired and weak at times, but this does not interfere with normal daily activities.	0.004 (0.001-0.008)
Stage 3 chronic kidney disease and moderate anemia due to glomerulonephritis	Anemia, moderate	feels moderate fatigue, weakness, and shortness of breath after exercise, making daily activities more difficult.	0.052 (0.034-0.076)
Stage 3 chronic kidney disease and severe anemia due to glomerulonephritis	Anemia, severe	feels very weak, tired and short of breath, and has problems with activities that require physical effort or deep concentration.	0.149 (0.101-0.209)
Stage 3 chronic kidney disease without anemia due to glomerulonephritis	Asymptomatic		0 (0-0)
Stage 4 chronic kidney disease untreated without anemia due to glomerulonephritis	Chronic kidney disease (stage IV)	tires easily, has nausea, reduced appetite and difficulty sleeping.	0.104 (0.07-0.147)
Stage 4 chronic kidney disease untreated and mild anemia due to glomerulonephritis	Mild anemia with Stage IV CKD		0.108 (0.072-0.151)
Stage 4 chronic kidney disease untreated and moderate anemia due to glomerulonephritis	Moderate anemia with Stage IV CKD		0.15 (0.103-0.207)
Stage 4 chronic kidney disease untreated and severe anemia due to glomerulonephritis	Severe anemia with Stage IV CKD		0.237 (0.165-0.324)
Stage 5 chronic kidney disease untreated without anemia due to glomerulonephritis	Terminal phase, without medication (for cancers, end-stage kidney/liver disease)	has lost a lot of weight and has constant pain. The person has no appetite, feels nauseous, and needs to spend most of the day in bed.	0.569 (0.389-0.727)
Stage 5 chronic kidney disease untreated and mild anemia due to glomerulonephritis	Mild anemia and terminal phase, without medication (for cancers, end-stage kidney/liver disease)	(combined DW)	0.57 (0.391-0.727)
Stage 5 chronic kidney disease untreated and moderate anemia due to glomerulonephritis	Moderate anemia and terminal phase, without medication (for cancers, end-stage kidney/liver disease)	(combined DW)	0.591 (0.414-0.743)
Stage 5 chronic kidney disease untreated and severe anemia due to glomerulonephritis	Severe anemia and terminal phase, without medication (for cancers, end-stage kidney/liver disease)	(combined DW)	0.631 (0.456-0.782)
Stage 1-2 chronic kidney disease with preserved GFR due to other causes	Asymptomatic		0 (0-0)
End-stage renal disease after transplant due to other and unspecified causes	End-stage renal disease, with kidney transplant	sometimes feels tired and down, and has some difficulty with daily activities.	0.024 (0.014-0.039)
End-stage renal disease on dialysis without anemia due to other and unspecified causes	End-stage renal disease, on dialysis	is tired and has itching, cramps, headache, joint pains and shortness of breath. The person needs intensive medical care every other day lasting about half a day.	0.571 (0.398-0.725)
End-stage renal disease on dialysis and mild anemia due to other and unspecified causes	End-stage renal disease, on dialysis and mild anemia	(combined DW)	0.573 (0.403-0.726)
End-stage renal disease on dialysis and moderate anemia due to other and unspecified causes	End-stage renal disease, on dialysis and moderate anemia	(combined DW)	0.593 (0.424-0.742)
End-stage renal disease on dialysis and severe anemia due to other and unspecified causes	End-stage renal disease, on dialysis and severe anemia	(combined DW)	0.633 (0.462-0.781)
Stage 3 chronic kidney disease and mild anemia due to other causes	Anemia, mild	feels slightly tired and weak at times, but this does not interfere with normal daily activities.	0.004 (0.001-0.008)

Stage 3 chronic kidney disease and moderate anemia due to other causes	Anemia, moderate	feels moderate fatigue, weakness, and shortness of breath after exercise, making daily activities more difficult.	0.052 (0.034-0.076)
Stage 3 chronic kidney disease and severe anemia due to other causes	Anemia, severe	feels very weak, tired and short of breath, and has problems with activities that require physical effort or deep concentration.	0.149 (0.101-0.209)
Stage 3 chronic kidney disease without anemia due to other causes	Asymptomatic		0 (0-0)
Stage 4 chronic kidney disease untreated without anemia due to other causes	Chronic kidney disease (stage IV)	tires easily, has nausea, reduced appetite and difficulty sleeping.	0.104 (0.07-0.147)
Stage 4 chronic kidney disease untreated and mild anemia due to other causes	Mild anemia with Stage IV CKD		0.108 (0.072-0.151)
Stage 4 chronic kidney disease untreated and moderate anemia due to other causes	Moderate anemia with Stage IV CKD		0.15 (0.103-0.207)
Stage 4 chronic kidney disease untreated and severe anemia due to other causes	Severe anemia with Stage IV CKD		0.237 (0.165-0.324)
Stage 5 chronic kidney disease untreated without anemia due to other causes	Terminal phase, without medication (for cancers, end-stage kidney/liver disease)	has lost a lot of weight and has constant pain. The person has no appetite, feels nauseous, and needs to spend most of the day in bed.	0.569 (0.389-0.727)
Stage 5 chronic kidney disease untreated and mild anemia due to other causes	Mild anemia and terminal phase, without medication (for cancers, end-stage kidney/liver disease)	(combined DW)	0.57 (0.391-0.727)
Stage 5 chronic kidney disease untreated and moderate anemia due to other causes	Moderate anemia and terminal phase, without medication (for cancers, end-stage kidney/liver disease)	(combined DW)	0.591 (0.414-0.743)
Stage 5 chronic kidney disease untreated and severe anemia due to other causes	Severe anemia and terminal phase, without medication (for cancers, end-stage kidney/liver disease)	(combined DW)	0.631 (0.456-0.782)
Acute glomerulonephritis	Infectious disease, acute episode, moderate	has a fever and aches, and feels weak, which causes some difficulty with daily activities.	0.051 (0.032-0.074)
Mild atopic dermatitis	Disfigurement, level 1 with itch/pain	has a slight, visible physical deformity that is sometimes sore or itchy. Others notice the deformity, which causes some worry and discomfort.	0.027 (0.015-0.042)
Moderate atopic dermatitis	Disfigurement, level 2, with itch/pain	has a visible physical deformity that is sore and itchy. Other people stare and comment, which causes the person to worry. The person has trouble sleeping and concentrating.	0.188 (0.125-0.267)
Severe atopic dermatitis	Disfigurement, level 3, with itch/pain	has an obvious physical deformity that is very painful and itchy. The physical deformity makes others uncomfortable, which causes the person to avoid social contact, feel worried, sleep poorly, and think about suicide.	0.576 (0.401-0.731)
Mild contact dermatitis	Disfigurement, level 1 with itch/pain	has a slight, visible physical deformity that is sometimes sore or itchy. Others notice the deformity, which causes some worry and discomfort.	0.027 (0.015-0.042)
Moderate contact dermatitis	Disfigurement, level 2, with itch/pain	has a visible physical deformity that is sore and itchy. Other people stare and comment, which causes the person to worry. The person has trouble sleeping and concentrating.	0.188 (0.125-0.267)
Asymptomatic contact dermatitis	Asymptomatic		0 (0-0)
Symptomatic seborrhoeic dermatitis	Disfigurement, level 1 with itch/pain	has a slight, visible physical deformity that is sometimes sore or itchy. Others notice the deformity, which causes some worry and discomfort.	0.027 (0.015-0.042)
Asymptomatic seborrhoeic dermatitis	Asymptomatic		0 (0-0)
Mild psoriasis	Disfigurement, level 1 with itch/pain	has a slight, visible physical deformity that is sometimes sore or itchy. Others notice the deformity, which causes some worry and discomfort.	0.027 (0.015-0.042)
Moderate psoriasis	Disfigurement, level 2, with itch/pain	has a visible physical deformity that is sore and itchy. Other people stare and comment, which causes the person to worry. The person has trouble sleeping and concentrating.	0.188 (0.125-0.267)
Severe psoriasis	Disfigurement, level 3, with itch/pain	has an obvious physical deformity that is very painful and itchy. The physical deformity makes others uncomfortable, which causes the person to avoid social contact, feel worried, sleep poorly, and think about suicide.	0.576 (0.401-0.731)
Mild cellulitis	Infectious disease, acute episode, mild	has a low fever and mild discomfort , but no difficulty with daily activities.	0.006 (0.002-0.012)
Moderate cellulitis	Infectious disease, acute episode, moderate	has a fever and aches, and feels weak, which causes some difficulty with daily activities.	0.051 (0.032-0.074)
Severe cellulitis	Infectious disease, acute episode, severe	has a high fever and pain, and feels very weak, which causes great difficulty with daily activities.	0.133 (0.088-0.19)
Impetigo	Infectious disease, acute episode, mild	has a low fever and mild discomfort , but no difficulty with daily activities.	0.006 (0.002-0.012)
Abscess and other bacterial skin diseases	Infectious disease, acute episode, mild	has a low fever and mild discomfort , but no difficulty with daily activities.	0.006 (0.002-0.012)
Scabies	Disfigurement, level 1 with itch/pain	has a slight, visible physical deformity that is sometimes sore or itchy. Others notice the deformity, which causes some worry and discomfort.	0.027 (0.015-0.042)
Tinea capitis	Infectious disease, acute episode, mild	has a low fever and mild discomfort , but no difficulty with daily activities.	0.006 (0.002-0.012)
Other fungal skin diseases	Infectious disease, acute episode, mild	has a low fever and mild discomfort , but no difficulty with daily activities.	0.006 (0.002-0.012)
Mild viral warts	Infectious disease, acute episode, mild	has a low fever and mild discomfort , but no difficulty with daily activities.	0.006 (0.002-0.012)
Severe viral warts	Disfigurement, level 2	has a visible physical deformity that causes others to stare and comment. As a result, the person is worried and has trouble sleeping and concentrating.	0.067 (0.044-0.096)
Mild molluscum contagiosum	Infectious disease, acute episode, mild	has a low fever and mild discomfort , but no difficulty with daily activities.	0.006 (0.002-0.012)
Severe molluscum contagiosum	Disfigurement, level 2	has a visible physical deformity that causes others to stare and comment. As a result, the person is worried and has trouble sleeping and concentrating.	0.067 (0.044-0.096)
Mild acne vulgaris	Disfigurement, level 1	has a slight, visible physical deformity that others notice, which causes some worry and discomfort.	0.011 (0.005-0.021)
Non-disabling symptomatic acne	Disfigurement, level 1	has a slight, visible physical deformity that others notice, which causes some worry and discomfort.	0.011 (0.005-0.021)
Moderate acne vulgaris	Disfigurement, level 2	has a visible physical deformity that causes others to stare and comment. As a result, the person is worried and has trouble sleeping and concentrating.	0.067 (0.044-0.096)
Severe acne vulgaris	Disfigurement, level 3	has an obvious physical deformity that makes others uncomfortable, which causes the person to avoid social contact, feel worried, sleep poorly, and think about suicide.	0.405 (0.275-0.546)
Mild alopecia areata	Disfigurement, level 1	has a slight, visible physical deformity that others notice, which causes some worry and discomfort.	0.011 (0.005-0.021)
Severe alopecia areata	Disfigurement, level 2	has a visible physical deformity that causes others to stare and comment. As a result, the person is worried and has trouble sleeping and concentrating.	0.067 (0.044-0.096)
Pruritus	Disfigurement, level 1	has a slight, visible physical deformity that others notice, which causes some worry and discomfort.	0.011 (0.005-0.021)
Mild urticaria	Disfigurement, level 1 with itch/pain	has a slight, visible physical deformity that is sometimes sore or itchy. Others notice the deformity, which causes some worry and discomfort.	0.027 (0.015-0.042)
Severe urticaria	Disfigurement, level 2, with itch/pain	has a visible physical deformity that is sore and itchy. Other people stare and comment, which causes the person to worry. The person has trouble sleeping and concentrating.	0.188 (0.125-0.267)
Mild decubitus ulcer	Disfigurement, level 1 with itch/pain	has a slight, visible physical deformity that is sometimes sore or itchy. Others notice the deformity, which causes some worry and discomfort.	0.027 (0.015-0.042)
Moderate decubitus ulcer	Disfigurement, level 2, with itch/pain	has a visible physical deformity that is sore and itchy. Other people stare and comment, which causes the person to worry. The person has trouble sleeping and concentrating.	0.188 (0.125-0.267)
Severe decubitus ulcer	Disfigurement, level 3, with itch/pain	has an obvious physical deformity that is very painful and itchy. The physical deformity makes others uncomfortable, which causes the person to avoid social contact, feel worried, sleep poorly, and think about suicide.	0.576 (0.401-0.731)
Symptomatic other skin and subcutaneous diseases	Disfigurement, level 1	has a slight, visible physical deformity that others notice, which causes some worry and discomfort.	0.011 (0.005-0.021)
Asymptomatic other skin and subcutaneous diseases	Asymptomatic		0 (0-0)
Moderate vision impairment due to glaucoma	Distance vision, moderate impairment	has vision problems that make it difficult to recognize faces or objects across a room.	0.031 (0.019-0.049)
Severe vision impairment due to glaucoma	Distance vision, severe impairment	has severe vision loss, which causes difficulty in daily activities, some emotional impact (for example worry), and some difficulty going outside the home without assistance.	0.184 (0.125-0.258)
Blindness due to glaucoma	Distance vision blindness	is completely blind, which causes great difficulty in some daily activities, worry and anxiety, and great difficulty going outside the home without assistance.	0.187 (0.124-0.26)
Moderate vision impairment due to cataract	Distance vision, moderate impairment	has vision problems that make it difficult to recognize faces or objects across a room.	0.031 (0.019-0.049)
Severe vision impairment due to cataract	Distance vision, severe impairment	has severe vision loss, which causes difficulty in daily activities, some emotional impact (for example worry), and some difficulty going outside the home without assistance.	0.184 (0.125-0.258)
Blindness due to cataract	Distance vision blindness	is completely blind, which causes great difficulty in some daily activities, worry and anxiety, and great difficulty going outside the home without assistance.	0.187 (0.124-0.26)

Moderate vision impairment due to macular degeneration	Distance vision, moderate impairment	has vision problems that make it difficult to recognize faces or objects across a room.	0.031 (0.019-0.049)
Severe vision impairment due to macular degeneration	Distance vision, severe impairment	has severe vision loss, which causes difficulty in daily activities, some emotional impact (for example worry), and some difficulty going outside the home without assistance.	0.184 (0.125-0.258)
Blindness due to macular degeneration	Distance vision blindness	is completely blind, which causes great difficulty in some daily activities, worry and anxiety, and great difficulty going outside the home without assistance.	0.187 (0.124-0.26)
Moderate vision impairment due to uncorrected refractive error	Distance vision, moderate impairment	has vision problems that make it difficult to recognize faces or objects across a room.	0.031 (0.019-0.049)
Severe vision impairment due to uncorrected refractive error	Distance vision, severe impairment	has severe vision loss, which causes difficulty in daily activities, some emotional impact (for example worry), and some difficulty going outside the home without assistance.	0.184 (0.125-0.258)
Blindness due to uncorrected refractive error	Distance vision blindness	is completely blind, which causes great difficulty in some daily activities, worry and anxiety, and great difficulty going outside the home without assistance.	0.187 (0.124-0.26)
Near vision loss	Presbyopia	has difficulty seeing things that are nearer than 3 feet, but has no difficulty with seeing things at a distance.	0.011 (0.005-0.02)
Moderate vision impairment due to other vision loss	Distance vision, moderate impairment	has vision problems that make it difficult to recognize faces or objects across a room.	0.031 (0.019-0.049)
Severe vision impairment due to other vision loss	Distance vision, severe impairment	has severe vision loss, which causes difficulty in daily activities, some emotional impact (for example worry), and some difficulty going outside the home without assistance.	0.184 (0.125-0.258)
Blindness due to other vision loss	Distance vision blindness	is completely blind, which causes great difficulty in some daily activities, worry and anxiety, and great difficulty going outside the home without assistance.	0.187 (0.124-0.26)
Mild hearing loss due to age-related and other hearing loss	Hearing loss, mild	has great difficulty hearing and understanding another person talking in a noisy place (for example, on an urban street).	0.01 (0.004-0.019)
Moderate hearing loss due to age-related and other hearing loss	Hearing loss, moderate	is unable to hear and understand another person talking in a noisy place (for example, on an urban street), and has difficulty hearing another person talking even in a quiet place or on the phone.	0.027 (0.015-0.042)
Severe hearing loss due to age-related and other hearing loss	Hearing loss, severe	is unable to hear and understand another person talking, even in a quiet place, and unable to take part in a phone conversation. Difficulties with communicating and relating to others cause emotional impact at times (for example worry or depression).	0.158 (0.105-0.227)
Profound hearing loss due to age-related and other hearing loss	Hearing loss, profound	is unable to hear and understand another person talking, even in a quiet place, is unable to take part in a phone conversation, and has great difficulty hearing anything in any other situation. Difficulties with communicating and relating to othersoften cause worry, depression, and loneliness.	0.204 (0.134-0.288)
Complete hearing loss due to age-related and other hearing loss	Hearing loss, complete	cannot hear at all in any situation, including even the loudest sounds, and cannot communicate verbally or use a phone. Difficulties with communicating and relating to others often cause worry, depression or loneliness.	0.215 (0.144-0.307)
Mild hearing loss with ringing due to age-related and other hearing loss	Hearing loss, mild, with ringing	has great difficulty hearing and understanding another person talking in a noisy place (for example, on an urban street), and sometimes has annoying ringing in the ears.	0.021 (0.012-0.036)
Moderate hearing loss with ringing due to age-related and other hearing loss	Hearing loss, moderate, with ringing	is unable to hear and understand another person talking in a noisy place (for example, on an urban street), and has difficulty hearing another person talking even in a quiet place or on the phone, and has annoying ringing in the ears for more than 5 minutes at a time, almost everyday.	0.074 (0.049-0.107)
Severe hearing loss with ringing due to age-related and other hearing loss	Hearing loss, severe, with ringing	is unable to hear and understand another person talking, even in a quiet place, and unable to take part in a phone conversation, and has annoying ringing in the ears for more than 5 minutes at a time, almost everyday. Difficulties with communicating and relating to others cause emotional impact at times (for example worry or depression).	0.261 (0.175-0.36)
Profound hearing loss with ringing due to age-related and other hearing loss	Hearing loss, profound, with ringing	is unable to hear and understand another person talking, even in a quiet place, is unable to take part in a phone conversation, has great difficulty hearing anything in any other situation, and has annoying ringing in the ears for more than 5 minutes at a time, several times a day. Difficulties with communicating and relating to others often cause worry, depression, or loneliness.	0.277 (0.182-0.387)
Complete hearing loss with ringing due to age-related and other hearing loss	Hearing loss, complete, with ringing	cannot hear at all in any situation, including even the loudest sounds, and cannot communicate verbally or use a phone, and has very annoying ringing in the ears for more than half of the day. Difficulties with communicating and relating to others often cause worry, depression or loneliness.	0.316 (0.212-0.435)
Moderately severe hearing loss due to age-related and other hearing loss	Hearing loss, moderately severe	(custom DW from hearing loss impairment envelope)	0.092 (0.064-0.129)
Moderately severe hearing loss with ringing due to age-related and other hearing loss	Hearing loss, moderately severe, with ringing	(custom DW from hearing loss impairment envelope)	0.167 (0.115-0.231)
Mild chronic other sense organ diseases	Disfigurement, level 1	has a slight, visible physical deformity that others notice, which causes some worry and discomfort.	0.011 (0.005-0.021)
Asymptomatic chronic other sense organ diseases	Asymptomatic		0 (0-0)
Moderate chronic other sense organ diseases	Vertigo		0.113 (0.074-0.158)
Mild acute other sense organ diseases	Infectious disease, acute episode, mild	has a low fever and mild discomfort , but no difficulty with daily activities.	0.006 (0.002-0.012)
Moderate acute other sense organ diseases	Infectious disease, acute episode, moderate	has a fever and aches, and feels weak, which causes some difficulty with daily activities.	0.051 (0.032-0.074)
Asymptomatic acute other sense organ diseases	Asymptomatic		0 (0-0)
Mild rheumatoid arthritis	Musculoskeletal problems, upper limbs, moderate	has moderate pain and stiffness in the arms and hands, which causes difficulty lifting, carrying, and holding things, and trouble sleeping because of the pain.	0.117 (0.08-0.163)
Moderate rheumatoid arthritis	Musculoskeletal problems, generalized, moderate	has pain and deformity in most joints, causing difficulty moving around, getting up and down, and using the hands for lifting and carrying. The person often feels fatigue.	0.317 (0.216-0.44)
Severe rheumatoid arthritis	Musculoskeletal problems, generalized, severe	has severe, constant pain and deformity in most joints, causing difficulty moving around, getting up and down, eating, dressing, lifting, carrying and using the hands. The person often feels sadness, anxiety and extreme fatigue.	0.581 (0.403-0.739)
Asymptomatic rheumatoid arthritis	Asymptomatic		0 (0-0)
Mild osteoarthritis of the hip	Musculoskeletal problems, lower limbs, mild	has pain in the leg, which causes some difficulty running, walking long distances, and getting up and down.	0.023 (0.013-0.037)
Moderate osteoarthritis of the hip	Musculoskeletal problems, lower limbs, moderate	has moderate pain in the leg, which makes the person limp, and causes some difficulty walking, standing, lifting and carrying heavy things, getting up and down and sleeping.	0.079 (0.054-0.11)
Severe osteoarthritis of the hip	Musculoskeletal problems, lower limbs, severe	has severe pain in the leg, which makes the person limp and causes a lot of difficulty walking, standing, lifting and carrying heavy things, getting up and down, and sleeping.	0.165 (0.112-0.232)
Asymptomatic osteoarthritis of the hip	Asymptomatic		0 (0-0)
Mild osteoarthritis of the knee	Musculoskeletal problems, lower limbs, mild	has pain in the leg, which causes some difficulty running, walking long distances, and getting up and down.	0.023 (0.013-0.037)
Moderate osteoarthritis of the knee	Musculoskeletal problems, lower limbs, moderate	has moderate pain in the leg, which makes the person limp, and causes some difficulty walking, standing, lifting and carrying heavy things, getting up and down and sleeping.	0.079 (0.054-0.11)
Severe osteoarthritis of the knee	Musculoskeletal problems, lower limbs, severe	has severe pain in the leg, which makes the person limp and causes a lot of difficulty walking, standing, lifting and carrying heavy things, getting up and down, and sleeping.	0.165 (0.112-0.232)
Asymptomatic osteoarthritis of the knee	Asymptomatic		0 (0-0)
Mild osteoarthritis of the hand and foot	Musculoskeletal problems, lower limbs, mild	has pain in the leg, which causes some difficulty running, walking long distances, and getting up and down.	0.023 (0.013-0.037)
Moderate osteoarthritis of the hand and foot	Musculoskeletal problems, lower limbs, moderate	has moderate pain in the leg, which makes the person limp, and causes some difficulty walking, standing, lifting and carrying heavy things, getting up and down and sleeping.	0.079 (0.054-0.11)
Severe osteoarthritis of the hand and foot	Musculoskeletal problems, lower limbs, severe	has severe pain in the leg, which makes the person limp and causes a lot of difficulty walking, standing, lifting and carrying heavy things, getting up and down, and sleeping.	0.165 (0.112-0.232)
Asymptomatic osteoarthritis of the hand and foot	Asymptomatic		0 (0-0)
Mild osteoarthritis other	Musculoskeletal problems, lower limbs, mild	has pain in the leg, which causes some difficulty running, walking long distances, and getting up and down.	0.023 (0.013-0.037)

Moderate osteoarthritis other	Musculoskeletal problems, lower limbs, moderate	has moderate pain in the leg, which makes the person limp, and causes some difficulty walking, standing, lifting and carrying heavy things, getting up and down and sleeping.	0.079 (0.054-0.11)
Severe osteoarthritis other	Musculoskeletal problems, lower limbs, severe	has severe pain in the leg, which makes the person limp and causes a lot of difficulty walking, standing, lifting and carrying heavy things, getting up and down, and sleeping.	0.165 (0.112-0.232)
Asymptomatic osteoarthritis other	Asymptomatic		0 (0-0)
Severe low back pain with leg pain	Back pain, severe, with leg pain	has severe back and leg pain, which causes difficulty dressing, sitting, standing, walking, and lifting things. The person sleeps poorly and feels worried.	0.325 (0.219-0.446)
Most severe low back pain with leg pain	Back pain, most severe, with leg pain	has constant back and leg pain, which causes difficulty dressing, sitting, standing, walking, and lifting things. The person sleeps poorly, is worried, and has lost some enjoyment in life.	0.384 (0.256-0.518)
Mild low back pain with leg pain	Mild low back pain with leg pain	(combined DW)	0.02 (0.011-0.035)
Moderate low back pain with leg pain	Moderate low back pain with leg pain	(combined DW)	0.054 (0.035-0.079)
Severe low back pain without leg pain	Back pain, severe, without leg pain	has severe back pain, which causes difficulty dressing, sitting, standing, walking, and lifting things. The person sleeps poorly and feels worried.	0.272 (0.182-0.373)
Most severe low back pain without leg pain	Back pain, most severe, without leg pain	has constant back pain, which causes difficulty dressing, sitting, standing, walking, and lifting things. The person sleeps poorly, is worried, and has lost some enjoyment in life.	0.372 (0.25-0.506)
Mild low back pain without leg pain	Low back pain, mild	has mild back pain, which causes some difficulty dressing, standing, and lifting things.	0.02 (0.011-0.035)
Moderate low back pain without leg pain	Low back pain, moderate	has moderate back pain, which causes difficulty dressing, sitting, standing, walking, and lifting things.	0.054 (0.035-0.079)
Mild neck pain	Neck pain, mild	has neck pain, and has difficulty turning the head and lifting things.	0.053 (0.034-0.078)
Severe neck pain	Neck pain, severe	has severe neck pain, and difficulty turning the head and lifting things. The person gets headaches and arm pain, sleeps poorly, and feels tired and worried.	0.229 (0.153-0.317)
Moderate neck pain	Neck pain, moderate	has constant neck pain, and has difficulty turning the head, holding arms up, and lifting things	0.114 (0.075-0.162)
Most severe neck pain	Neck pain, most severe	has constant neck pain and arm pain, and difficulty turning the head, holding arms up, and lifting things. The person gets headaches, sleeps poorly, and feels tired and worried.	0.304 (0.202-0.415)
Polyarticular gout	Musculoskeletal problems, generalized, severe	has severe, constant pain and deformity in most joints, causing difficulty moving around, getting up and down, eating, dressing, lifting, carrying and using the hands. The person often feels sadness, anxiety and extreme fatigue.	0.581 (0.403-0.739)
Symptomatic episodes of gout	Gout, acute	has severe pain and swelling in the leg, making it very difficult to get up and down, stand, walk, lift, and carry heavy things. The person has trouble sleeping because of the pain.	0.295 (0.196-0.409)
Asymptomatic gout	Asymptomatic		0 (0-0)
Other musculoskeletal disorders severity level 1	Musculoskeletal problems, lower limbs, mild	has pain in the leg, which causes some difficulty running, walking long distances, and getting up and down.	0.023 (0.013-0.037)
Other musculoskeletal disorders severity level 4	Musculoskeletal problems, lower limbs, severe	has severe pain in the leg, which makes the person limp and causes a lot of difficulty walking, standing, lifting and carrying heavy things, getting up and down, and sleeping.	0.165 (0.112-0.232)
Other musculoskeletal disorders severity level 2	Musculoskeletal problems, upper limbs, mild	has mild pain and stiffness in the arms and hands. The person has some difficulty lifting, carrying and holding things.	0.028 (0.017-0.045)
Other musculoskeletal disorders severity level 3	Musculoskeletal problems, upper limbs, moderate	has moderate pain and stiffness in the arms and hands, which causes difficulty lifting, carrying, and holding things, and trouble sleeping because of the pain.	0.117 (0.08-0.163)
Other musculoskeletal disorders severity level 5	Musculoskeletal problems, generalized, moderate	has pain and deformity in most joints, causing difficulty moving around, getting up and down, and using the hands for lifting and carrying. The person often feels fatigue.	0.317 (0.216-0.44)
Other musculoskeletal disorders severity level 6	Musculoskeletal problems, generalized, severe	has severe, constant pain and deformity in most joints, causing difficulty moving around, getting up and down, eating, dressing, lifting, carrying and using the hands. The person often feels sadness, anxiety and extreme fatigue.	0.581 (0.403-0.739)
Asymptomatic other musculoskeletal disorders	Asymptomatic		0 (0-0)
Severe motor and cognitive impairment due to anencephaly	Motor plus cognitive impairments, severe	cannot move around without help, and cannot lift or hold objects, get dressed or sit upright. The person also has very low intelligence, speaks few words, and needs constant supervision and help with all daily activities.	0.542 (0.374-0.702)
Incontinence due to encephalocele	Urinary incontinence	cannot control urinating.	0.139 (0.094-0.198)
Borderline intellectual disability due to encephalocele	Borderline intellectual functioning	is slow in learning at school. As an adult, the person has some difficulty doing complex or unfamiliar tasks but otherwise functions independently.	0.011 (0.005-0.02)
Mild intellectual disability due to encephalocele	Intellectual disability / mental retardation, mild	has low intelligence and is slow in learning at school. As an adult, the person can live independently, but often needs help to raise children and can only work at simple supervised jobs.	0.043 (0.026-0.064)
Moderate intellectual disability due to encephalocele	Intellectual disability / mental retardation, moderate	has low intelligence, and is slow in learning to speak and to do even simple tasks. As an adult, the person requires a lot of support to live independently and raise children. The person can only work at the simplest supervised jobs.	0.1 (0.066-0.142)
Severe intellectual disability due to encephalocele	Intellectual disability / mental retardation, severe	has very low intelligence and cannot speak more than a few words, needs constant supervision and help with most daily activities, and can do only the simplest tasks.	0.16 (0.107-0.226)
Profound intellectual disability due to encephalocele	Intellectual disability / mental retardation, profound	has very low intelligence, has almost no language, and does not understand even the most basic requests or instructions. The person requires constant supervision and help for all activities.	0.2 (0.133-0.283)
Mild motor impairment due to encephalocele	Motor impairment, mild	has some difficulty in moving around but is able to walk without help.	0.01 (0.005-0.019)
Moderate motor impairment due to encephalocele	Motor impairment, moderate	has some difficulty in moving around, and difficulty in lifting and holding objects, dressing and sitting upright, but is able to walk without help.	0.061 (0.04-0.089)
Severe motor impairment due to encephalocele	Motor impairment, severe	is unable to move around without help, and is not able to lift or hold objects, get dressed or sit upright.	0.402 (0.268-0.545)
Mild motor impairment and mild intellectual disability due to encephalocele	Motor plus cognitive impairments, mild	has some difficulty in moving around but is able to walk without help. The person is slow in learning at school. As an adult, the person has some difficulty doing complex or unfamiliar tasks but otherwise functions independently.	0.031 (0.018-0.05)
Moderate motor impairment and moderate intellectual disability due to encephalocele	Motor plus cognitive impairments, moderate	has some difficulty in moving around, holding objects, dressing and sitting upright, but can walk without help. The person has low intelligence and is slow in learning to speak and to do simple tasks.	0.203 (0.134-0.29)
Severe motor impairment and incontinence due to encephalocele	Severe motor impairment with incontinence	(combined DW)	0.483 (0.346-0.629)
Mild motor impairment and profound intellectual disability due to encephalocele	Severe motor impairment with incontinence	(combined DW)	0.483 (0.346-0.629)
Moderate motor impairment and profound intellectual disability due to encephalocele	Severe motor impairment with incontinence	(combined DW)	0.483 (0.346-0.629)
Severe motor impairment and profound intellectual disability due to encephalocele	Severe motor impairment with incontinence	(combined DW)	0.483 (0.346-0.629)
Mild motor impairment, profound intellectual disability and incontinence due to encephalocele	Severe motor impairment with incontinence	(combined DW)	0.483 (0.346-0.629)
Asymptomatic encephalocele following treatment	Asymptomatic		0 (0-0)
Severe motor impairment, mild intellectual disability and incontinence due to encephalocele	Severe motor impairment with mild intellectual disability and incontinence	(combined DW)	0.505 (0.367-0.647)
Severe motor impairment, moderate intellectual disability and incontinence due to encephalocele	Severe motor impairment with moderate intellectual disability and incontinence	(combined DW)	0.534 (0.391-0.675)
Severe motor impairment, severe intellectual disability and incontinence due to encephalocele	Severe motor impairment with severe intellectual disability and incontinence	(combined DW)	0.564 (0.418-0.71)
Severe motor impairment, profound intellectual disability and incontinence due to encephalocele	Severe motor impairment with severe intellectual disability and incontinence	(combined DW)	0.564 (0.418-0.71)
Severe motor impairment and mild intellectual disability due to encephalocele	Severe motor impairment with mild intellectual disability	(combined DW)	0.427 (0.3-0.567)
Severe motor impairment and moderate intellectual disability due to encephalocele	Severe motor impairment with moderate intellectual disability	(combined DW)	0.461 (0.324-0.603)
Severe motor impairment and severe intellectual disability due to encephalocele	Severe motor impairment with severe intellectual disability	(combined DW)	0.496 (0.355-0.641)
Borderline intellectual disability and incontinence due to encephalocele	Borderline intellectual functioning and urinary incontinence	(combined DW)	0.148 (0.101-0.206)

Mild intellectual disability and incontinence due to encephalocele	Mild intellectual disability and urinary incontinence	(combined DW)	0.176<br>(0.12-0.242)
Moderate intellectual disability and incontinence due to encephalocele	Moderate intellectual disability and urinary incontinence	(combined DW)	0.225<br>(0.156-0.304)
Severe intellectual disability and incontinence due to encephalocele	Severe intellectual disability and urinary incontinence	(combined DW)	0.276<br>(0.194-0.376)
Profound intellectual disability and incontinence due to encephalocele	Profound intellectual disability and urinary incontinence	(combined DW)	0.311<br>(0.217-0.418)
Mild motor impairment and borderline intellectual disability due to encephalocele	Mild motor impairment and borderline intellectual functioning	(combined DW)	0.021<br>(0.01-0.039)
Moderate motor impairment and borderline intellectual disability due to encephalocele	Moderate motor impairment and borderline intellectual functioning	(combined DW)	0.071<br>(0.045-0.106)
Severe motor impairment and borderline intellectual disability due to encephalocele	Severe motor impairment and borderline intellectual functioning	(combined DW)	0.408<br>(0.279-0.55)
Moderate motor impairment and mild intellectual disability due to encephalocele	Moderate motor impairment and mild intellectual functioning	(combined DW)	0.101<br>(0.066-0.146)
Mild motor impairment and moderate intellectual disability due to encephalocele	Mild motor impairment and moderate intellectual functioning	(combined DW)	0.109<br>(0.073-0.154)
Mild motor impairment and severe intellectual disability due to encephalocele	Mild motor impairment and severe intellectual functioning	(combined DW)	0.169<br>(0.113-0.237)
Moderate motor impairment and severe intellectual disability due to encephalocele	Moderate motor impairment and severe intellectual functioning	(combined DW)	0.211<br>(0.145-0.293)
Mild motor impairment, borderline intellectual disability and incontinence due to encephalocele	Mild motor impairment, borderline intellectual functioning, and urinary incontinence	(combined DW)	0.157<br>(0.108-0.218)
Moderate motor impairment, borderline intellectual disability and incontinence due to encephalocele	Moderate motor impairment, borderline intellectual functioning, and urinary incontinence	(combined DW)	0.2<br>(0.139-0.273)
Severe motor impairment, borderline intellectual disability and incontinence due to encephalocele	Severe motor impairment, borderline intellectual functioning, and urinary incontinence	(combined DW)	0.489<br>(0.353-0.632)
Mild motor impairment, mild intellectual disability and incontinence due to encephalocele	Mild motor impairment, mild intellectual functioning, and urinary incontinence	(combined DW)	0.184<br>(0.128-0.253)
Moderate motor impairment, mild intellectual disability and incontinence due to encephalocele	Moderate motor impairment, mild intellectual functioning, and urinary incontinence	(combined DW)	0.272<br>(0.191-0.364)
Mild motor impairment, moderate intellectual disability and incontinence due to encephalocele	Mild motor impairment, moderate intellectual functioning, and urinary incontinence	(combined DW)	0.233<br>(0.161-0.314)
Moderate motor impairment, moderate intellectual disability and incontinence due to encephalocele	Moderate motor impairment, moderate intellectual functioning, and urinary incontinence	(combined DW)	0.272<br>(0.191-0.364)
Mild motor impairment, severe intellectual disability and incontinence due to encephalocele	Mild motor impairment, servere intellectual functioning, and urinary incontinence	(combined DW)	0.284<br>(0.201-0.385)
Moderate motor impairment, severe intellectual disability and incontinence due to encephalocele	Moderate motor impairment, servere intellectual functioning, and urinary incontinence	(combined DW)	0.32<br>(0.228-0.429)
Mild motor impairment and incontinence due to encephalocele	Mild motor impairment and urinary incontinence	(combined DW)	0.148<br>(0.1-0.207)
Moderate motor impairment and incontinence due to encephalocele	Moderate motor impairment and urinary incontinence	(combined DW)	0.191<br>(0.132-0.263)
Moderate motor impairment, profound intellectual disability and incontinence due to encephalocele	Mild motor impairment, profound intellectual functioning, and urinary incontinence	(combined DW)	0.318<br>(0.224-0.426)
Mild motor impairment due to spina bifida	Motor impairment, mild	has some difficulty in moving around but is able to walk without help.	0.01<br>(0.005-0.019)
Moderate motor impairment due to spina bifida	Motor impairment, moderate	has some difficulty in moving around, and difficulty in lifting and holding objects, dressing and sitting upright, but is able to walk without help.	0.061<br>(0.04-0.089)
Severe motor impairment due to spina bifida	Motor impairment, severe	is unable to move around without help, and is not able to lift or hold objects, get dressed or sit upright.	0.402<br>(0.268-0.545)
Mild motor impairment and mild intellectual disability due to spina bifida	Motor plus cognitive impairments, mild	has some difficulty in moving around but is able to walk without help. The person is slow in learning at school. As an adult, the person has some difficulty doing complex or unfamiliar tasks but otherwise functions independently.	0.031<br>(0.018-0.05)
Moderate motor impairment and moderate intellectual disability due to spina bifida	Motor plus cognitive impairments, moderate	has some difficulty in moving around, holding objects, dressing and sitting upright, but can walk without help. The person has low intelligence and is slow in learning to speak and to do simple tasks.	0.203<br>(0.134-0.29)
Severe motor impairment and incontinence due to spina bifida	Severe motor impairment with incontinence	(combined DW)	0.483<br>(0.346-0.629)
Severe motor impairment, mild intellectual disability and incontinence due to spina bifida	Severe motor impairment with mild intellectual disability and incontinence	(combined DW)	0.505<br>(0.367-0.647)
Severe motor impairment, moderate intellectual disability and incontinence due to spina bifida	Severe motor impairment with moderate intellectual disability and incontinence	(combined DW)	0.534<br>(0.391-0.675)
Severe motor impairment, severe intellectual disability and incontinence due to spina bifida	Severe motor impairment with severe intellectual disability and incontinence	(combined DW)	0.564<br>(0.418-0.71)
Severe motor impairment, profound intellectual disability and incontinence due to spina bifida	Severe motor impairment with profound intellectual disability and incontinence	(combined DW)	0.584<br>(0.435-0.73)
Severe motor impairment and mild intellectual disability due to spina bifida	Severe motor impairment with mild intellectual disability	(combined DW)	0.427<br>(0.3-0.567)
Severe motor impairment and moderate intellectual disability due to spina bifida	Severe motor impairment with moderate intellectual disability	(combined DW)	0.461<br>(0.324-0.603)
Severe motor impairment and severe intellectual disability due to spina bifida	Severe motor impairment with severe intellectual disability	(combined DW)	0.496<br>(0.355-0.641)
Severe motor impairment and profound intellectual disability due to spina bifida	Severe motor impairment with profound intellectual disability	(combined DW)	0.519<br>(0.37-0.668)
Mild motor impairment and borderline intellectual disability due to spina bifida	Mild motor impairment and borderline intellectual functioning	(combined DW)	0.021<br>(0.01-0.039)
Moderate motor impairment and borderline intellectual disability due to spina bifida	Moderate motor impairment and borderline intellectual functioning	(combined DW)	0.071<br>(0.045-0.106)
Severe motor impairment and borderline intellectual disability due to spina bifida	Severe motor impairment and borderline intellectual functioning	(combined DW)	0.408<br>(0.279-0.55)
Moderate motor impairment and mild intellectual disability due to spina bifida	Moderate motor impairment and mild intellectual functioning	(combined DW)	0.101<br>(0.066-0.146)
Mild motor impairment and moderate intellectual disability due to spina bifida	Mild motor impairment and moderate intellectual functioning	(combined DW)	0.109<br>(0.073-0.154)
Mild motor impairment and severe intellectual disability due to spina bifida	Mild motor impairment and severe intellectual functioning	(combined DW)	0.169<br>(0.113-0.237)
Moderate motor impairment and severe intellectual disability due to spina bifida	Moderate motor impairment and severe intellectual functioning	(combined DW)	0.211<br>(0.145-0.293)
Mild motor impairment, borderline intellectual disability and incontinence due to spina bifida	Mild motor impairment, borderline intellectual functioning, and urinary incontinence	(combined DW)	0.157<br>(0.108-0.218)
Moderate motor impairment, borderline intellectual disability and incontinence due to spina bifida	Moderate motor impairment, borderline intellectual functioning, and urinary incontinence	(combined DW)	0.2<br>(0.139-0.273)
Severe motor impairment, borderline intellectual disability and incontinence due to spina bifida	Severe motor impairment, borderline intellectual functioning, and urinary incontinence	(combined DW)	0.489<br>(0.353-0.632)
Mild motor impairment, mild intellectual disability and incontinence due to spina bifida	Mild motor impairment, mild intellectual functioning, and urinary incontinence	(combined DW)	0.184<br>(0.128-0.253)
Moderate motor impairment, mild intellectual disability and incontinence due to spina bifida	Moderate motor impairment, mild intellectual functioning, and urinary incontinence	(combined DW)	0.272<br>(0.191-0.364)
Mild motor impairment, moderate intellectual disability and incontinence due to spina bifida	Mild motor impairment, moderate intellectual functioning, and urinary incontinence	(combined DW)	0.233<br>(0.161-0.314)
Moderate motor impairment, moderate intellectual disability and incontinence due to spina bifida	Moderate motor impairment, moderate intellectual functioning, and urinary incontinence	(combined DW)	0.272<br>(0.191-0.364)
Mild motor impairment, severe intellectual disability and incontinence due to spina bifida	Mild motor impairment, servere intellectual functioning, and urinary incontinence	(combined DW)	0.284<br>(0.201-0.385)
Moderate motor impairment, severe intellectual disability and incontinence due to spina bifida	Moderate motor impairment, servere intellectual functioning, and urinary incontinence	(combined DW)	0.32<br>(0.228-0.429)
Mild motor impairment and incontinence due to spina bifida	Mild motor impairment and urinary incontinence	(combined DW)	0.148<br>(0.1-0.207)
Moderate motor impairment and incontinence due to spina bifida	Moderate motor impairment and urinary incontinence	(combined DW)	0.191<br>(0.132-0.263)
Mild motor impairment, profound intellectual disability and incontinence due to spina bifida	Mild motor impairment, profound intellectual functioning, and urinary incontinence	(combined DW)	0.318<br>(0.224-0.426)
Mild motor impairment and profound intellectual disability due to spina bifida	Mild motor impairment with profound intellectual disability	(combined DW)	0.208<br>(0.142-0.289)
Moderate motor impairment and profound intellectual disability due to spina bifida	Moderate motor impairment with profound intellectual disability	(combined DW)	0.249<br>(0.174-0.338)
Moderate motor impairment, profound intellectual disability and incontinence due to spina bifida	Moderate motor impairment with profound intellectual disability and incontinence	(combined DW)	0.352<br>(0.254-0.465)
Congenital heart disease without heart failure or intellectual disability due to critical malformations of great vessels, congenital valvular heart disease and patent ductus arteriosus	Congenital heart disease	(custom DW from MEPS)	0.061<br>(0.024-0.107)

Congenital heart disease and mild heart failure without intellectual disability due to critical malformations of great vessels, congenital valvular heart disease and patent ductus arteriosus	Congenital heart disease and mild heart failure	(combined DW)	0.041<br>(0.026-0.062)
Congenital heart disease and moderate heart failure without intellectual disability due to critical malformations of great vessels, congenital valvular heart disease and patent ductus arteriosus	Congenital heart disease and moderate heart failure	(combined DW)	0.072<br>(0.047-0.103)
Congenital heart disease and severe heart failure without intellectual disability due to critical malformations of great vessels, congenital valvular heart disease and patent ductus arteriosus	Congenital heart disease and severe heart failure	(combined DW)	0.179<br>(0.122-0.251)
Congenital heart disease, boderline intellectual disability and mild heart failure due to critical malformations of great vessels, congenital valvular heart disease and patent ductus arteriosus	Congenital heart disease, borderline intellectual functioning, and mild heart failure	(combined DW)	0.052<br>(0.032-0.081)
Congenital heart disease, boderline intellectual disability and mild heart failure due to critical malformations of great vessels, congenital valvular heart disease and patent ductus arteriosus	Congenital heart disease, borderline intellectual functioning, and mild heart failure	(combined DW)	0.052<br>(0.032-0.081)
Congenital heart disease, borderline intellectual disability and moderate heart failure due to critical malformations of great vessels, congenital valvular heart disease and patent ductus arteriosus	Congenital heart disease, borderline intellectual functioning, and moderate heart failure	(combined DW)	0.082<br>(0.053-0.12)
Congenital heart disease, borderline intellectual disability and moderate heart failure due to critical malformations of great vessels, congenital valvular heart disease and patent ductus arteriosus	Congenital heart disease, borderline intellectual functioning, and moderate heart failure	(combined DW)	0.082<br>(0.053-0.12)
Congenital heart disease, borderline intellectual disability and severe heart failure due to critical malformations of great vessels, congenital valvular heart disease and patent ductus arteriosus	Congenital heart disease,borderline intellectual functioning, and severe heart failure	(combined DW)	0.188<br>(0.13-0.258)
Congenital heart disease, borderline intellectual disability and severe heart failure due to critical malformations of great vessels, congenital valvular heart disease and patent ductus arteriosus	Congenital heart disease,borderline intellectual functioning, and severe heart failure	(combined DW)	0.188<br>(0.13-0.258)
Congenital heart disease, mild intellectual disability and mild heart failure due to critical malformations of great vessels, congenital valvular heart disease and patent ductus arteriosus	Congenital heart disease, mild intellectual disability, and mild heart failure	(combined DW)	0.082<br>(0.052-0.121)
Congenital heart disease, mild intellectual disability and mild heart failure due to critical malformations of great vessels, congenital valvular heart disease and patent ductus arteriosus	Congenital heart disease, mild intellectual disability, and mild heart failure	(combined DW)	0.082<br>(0.052-0.121)
Congenital heart disease, mild intellectual disability and moderate heart failure due to critical malformations of great vessels, congenital valvular heart disease and patent ductus arteriosus	Congenital heart disease, mild intellectual disability, and moderate heart failure	(combined DW)	0.111<br>(0.072-0.161)
Congenital heart disease, mild intellectual disability and moderate heart failure due to critical malformations of great vessels, congenital valvular heart disease and patent ductus arteriosus	Congenital heart disease, mild intellectual disability, and moderate heart failure	(combined DW)	0.111<br>(0.072-0.161)
Congenital heart disease, mild intellectual disability and severe heart failure due to critical malformations of great vessels, congenital valvular heart disease and patent ductus arteriosus	Congenital heart disease, mild intellectual disability, and servere heart failure	(combined DW)	0.214<br>(0.148-0.291)
Congenital heart disease, mild intellectual disability and severe heart failure due to critical malformations of great vessels, congenital valvular heart disease and patent ductus arteriosus	Congenital heart disease, mild intellectual disability, and servere heart failure	(combined DW)	0.214<br>(0.148-0.291)
Congenital heart disease, moderate intellectual disability and mild heart failure due to critical malformations of great vessels, congenital valvular heart disease and patent ductus arteriosus	Congenital heart disease, moderate intellectual disability, and mild heart failure	(combined DW)	0.137<br>(0.093-0.191)
Congenital heart disease, moderate intellectual disability and mild heart failure due to critical malformations of great vessels, congenital valvular heart disease and patent ductus arteriosus	Congenital heart disease, moderate intellectual disability, and mild heart failure	(combined DW)	0.137<br>(0.093-0.191)
Congenital heart disease, moderate intellectual disability and moderate heart failure due to critical malformations of great vessels, congenital valvular heart disease and patent ductus arteriosus	Congenital heart disease, moderate intellectual disability, and moderate heart failure	(combined DW)	0.164<br>(0.112-0.225)
Congenital heart disease, moderate intellectual disability and moderate heart failure due to critical malformations of great vessels, congenital valvular heart disease and patent ductus arteriosus	Congenital heart disease, moderate intellectual disability, and moderate heart failure	(combined DW)	0.164<br>(0.112-0.225)
Congenital heart disease, moderate intellectual disability and severe heart failure due to critical malformations of great vessels, congenital valvular heart disease and patent ductus arteriosus	Congenital heart disease, moderate intellectual disability, and severe heart failure	(combined DW)	0.261<br>(0.182-0.352)
Congenital heart disease, moderate intellectual disability and severe heart failure due to critical malformations of great vessels, congenital valvular heart disease and patent ductus arteriosus	Congenital heart disease, moderate intellectual disability, and severe heart failure	(combined DW)	0.261<br>(0.182-0.352)
Congenital heart disease, severe intellectual disability and mild heart failure due to critical malformations of great vessels, congenital valvular heart disease and patent ductus arteriosus	Congenital heart disease, severe intellectual disability, and mild heart failure	(combined DW)	0.195<br>(0.134-0.269)
Congenital heart disease, severe intellectual disability and mild heart failure due to critical malformations of great vessels, congenital valvular heart disease and patent ductus arteriosus	Congenital heart disease, severe intellectual disability, and mild heart failure	(combined DW)	0.195<br>(0.134-0.269)
Congenital heart disease, severe intellectual disability and moderate heart failure due to critical malformations of great vessels, congenital valvular heart disease and patent ductus arteriosus	Congenital heart disease, severe intellectual disability, and moderate heart failure	(combined DW)	0.22<br>(0.151-0.302)
Congenital heart disease, severe intellectual disability and moderate heart failure due to critical malformations of great vessels, congenital valvular heart disease and patent ductus arteriosus	Congenital heart disease, severe intellectual disability, and moderate heart failure	(combined DW)	0.22<br>(0.151-0.302)
Congenital heart disease, severe intellectual disability and severe heart failure due to critical malformations of great vessels, congenital valvular heart disease and patent ductus arteriosus	Congenital heart disease, severe intellectual disability, and severe heart failure	(combined DW)	0.31<br>(0.22-0.419)
Congenital heart disease, severe intellectual disability and severe heart failure due to critical malformations of great vessels, congenital valvular heart disease and patent ductus arteriosus	Congenital heart disease, severe intellectual disability, and severe heart failure	(combined DW)	0.31<br>(0.22-0.419)
Congenital heart disease, profound intellectual disability and mild heart failure due to critical malformations of great vessels, congenital valvular heart disease and patent ductus arteriosus	Congenital heart disease, profound intellectual disability, and mild heart failure	(combined DW)	0.233<br>(0.162-0.317)
Congenital heart disease, profound intellectual disability and mild heart failure due to critical malformations of great vessels, congenital valvular heart disease and patent ductus arteriosus	Congenital heart disease, profound intellectual disability, and mild heart failure	(combined DW)	0.233<br>(0.162-0.317)
Congenital heart disease, profound intellectual disability and moderate heart failure due to critical malformations of great vessels, congenital valvular heart disease and patent ductus arteriosus	Congenital heart disease, profound intellectual disability, and moderate heart failure	(combined DW)	0.257<br>(0.181-0.351)
Congenital heart disease, profound intellectual disability and moderate heart failure due to critical malformations of great vessels, congenital valvular heart disease and patent ductus arteriosus	Congenital heart disease, profound intellectual disability, and moderate heart failure	(combined DW)	0.257<br>(0.181-0.351)
Congenital heart disease, profound intellectual disability and severe heart failure due to critical malformations of great vessels, congenital valvular heart disease and patent ductus arteriosus	Congenital heart disease, profound intellectual disability, and severe heart failure	(combined DW)	0.342<br>(0.239-0.457)
Congenital heart disease, profound intellectual disability and severe heart failure due to critical malformations of great vessels, congenital valvular heart disease and patent ductus arteriosus	Congenital heart disease, profound intellectual disability, and severe heart failure	(combined DW)	0.342<br>(0.239-0.457)
Congenital heart disease and borderline intellectual disability without heart failure due to critical malformations of great vessels, congenital valvular heart disease and patent ductus arteriosus	Congenital heart disease and borderline intellectual functioning	(combined DW)	0.011<br>(0.005-0.02)
Congenital heart disease and mild intellectual disability without heart failure due to critical malformations of great vessels, congenital valvular heart disease and patent ductus arteriosus	Congenital heart disease and Intellectual disability / mental retardation, mild	(combined DW)	0.043<br>(0.026-0.064)
Congenital heart disease and moderate intellectual disability without heart failure due to critical malformations of great vessels, congenital valvular heart disease and patent ductus arteriosus	Congenital heart disease and Intellectual disability / mental retardation, moderate	(combined DW)	0.1<br>(0.066-0.142)
Congenital heart disease and severe intellectual disability without heart failure due to critical malformations of great vessels, congenital valvular heart disease and patent ductus arteriosus	Congenital heart disease and Intellectual disability / mental retardation, severe	(combined DW)	0.16<br>(0.107-0.226)
Congenital heart disease and profound intellectual disability without heart failure due to critical malformations of great vessels, congenital valvular heart disease and patent ductus arteriosus	Congenital heart disease and Intellectual disability / mental retardation, profound	(combined DW)	0.2<br>(0.133-0.283)
Congenital heart disease and controlled, medically managed heart failure without intellectual disability due to critical malformations of great vessels, congenital valvular heart disease and patent ductus arteriosus	Congenital heart disease and generic uncomplicated disease: worry and daily medication	(combined DW)	0.049<br>(0.031-0.072)
Congenital heart disease, boderline intellectual disability and controlled, medically managed heart failure due to critical malformations of great vessels, congenital valvular heart disease and patent ductus arteriosus	Congenital heart disease and borderline intellectual functioning and generic uncomplicated disease: worry and daily medication	(combined DW)	0.059<br>(0.037-0.09)
Congenital heart disease, boderline intellectual disability and controlled, medically managed heart failure due to critical malformations of great vessels, congenital valvular heart disease and patent ductus arteriosus	Congenital heart disease and borderline intellectual functioning and generic uncomplicated disease: worry and daily medication	(combined DW)	0.059<br>(0.037-0.09)

Congenital heart disease, mild intellectual disability and controlled, medically managed heart failure due to critical malformations of great vessels, congenital valvular heart disease and patent ductus arteriosus	Congenital heart disease and Intellectual disability / mental retardation, mild and generic uncomplicated disease: worry and daily medication	(combined DW)	0.089<br>(0.056-0.133)
Congenital heart disease, mild intellectual disability and controlled, medically managed heart failure due to critical malformations of great vessels, congenital valvular heart disease and patent ductus arteriosus	Congenital heart disease and Intellectual disability / mental retardation, mild and generic uncomplicated disease: worry and daily medication	(combined DW)	0.089<br>(0.056-0.133)
Congenital heart disease, moderate intellectual disability and controlled, medically managed heart failure due to critical malformations of great vessels, congenital valvular heart disease and patent ductus arteriosus	Congenital heart disease and Intellectual disability / mental retardation, moderate and generic uncomplicated disease: worry and daily medication	(combined DW)	0.144<br>(0.098-0.199)
Congenital heart disease, moderate intellectual disability and controlled, medically managed heart failure due to critical malformations of great vessels, congenital valvular heart disease and patent ductus arteriosus	Congenital heart disease and Intellectual disability / mental retardation, moderate and generic uncomplicated disease: worry and daily medication	(combined DW)	0.144<br>(0.098-0.199)
Congenital heart disease, profound intellectual disability and controlled, medically managed heart failure due to critical malformations of great vessels, congenital valvular heart disease and patent ductus arteriosus	Congenital heart disease and Intellectual disability / mental retardation, severe and generic uncomplicated disease: worry and daily medication	(combined DW)	0.201<br>(0.138-0.276)
Congenital heart disease, profound intellectual disability and controlled, medically managed heart failure due to critical malformations of great vessels, congenital valvular heart disease and patent ductus arteriosus	Congenital heart disease and Intellectual disability / mental retardation, severe and generic uncomplicated disease: worry and daily medication	(combined DW)	0.201<br>(0.138-0.276)
Congenital heart disease, severe intellectual disability and controlled, medically managed heart failure due to critical malformations of great vessels, congenital valvular heart disease and patent ductus arteriosus	Congenital heart disease and Intellectual disability / mental retardation, profound and generic uncomplicated disease: worry and daily medication	(combined DW)	0.239<br>(0.167-0.325)
Congenital heart disease, severe intellectual disability and controlled, medically managed heart failure due to critical malformations of great vessels, congenital valvular heart disease and patent ductus arteriosus	Congenital heart disease and Intellectual disability / mental retardation, profound and generic uncomplicated disease: worry and daily medication	(combined DW)	0.239<br>(0.167-0.325)
Congenital heart disease without intellectual disability or heart failure due to other congenital cardiovascular anomalies	Congenital heart disease	(custom DW from MEPS)	0.061<br>(0.024-0.107)
Congenital heart disease and borderline intellectual disability without heart failure due to other congenital cardiovascular anomalies	Congenital heart disease and borderline intellectual functioning	(combined DW)	0.011<br>(0.005-0.02)
Congenital heart disease and mild intellectual disability without heart failure due to other congenital cardiovascular anomalies	Congenital heart disease and Intellectual disability / mental retardation, mild	(combined DW)	0.043<br>(0.026-0.064)
Congenital heart disease and moderate intellectual disability without heart failure due to other congenital cardiovascular anomalies	Congenital heart disease and Intellectual disability / mental retardation, moderate	(combined DW)	0.1<br>(0.066-0.142)
Congenital heart disease and severe intellectual disability without heart failure due to other congenital cardiovascular anomalies	Congenital heart disease and Intellectual disability / mental retardation, severe	(combined DW)	0.16<br>(0.107-0.226)
Congenital heart disease and profound intellectual disability without heart failure due to other congenital cardiovascular anomalies	Congenital heart disease and Intellectual disability / mental retardation, profound	(combined DW)	0.2<br>(0.133-0.283)
Congenital heart disease without intellectual disability or heart failure due to severe congenital heart anomalies excluding single ventricle heart defects	Congenital heart disease	(custom DW from MEPS)	0.061<br>(0.024-0.107)
Congenital heart disease and mild heart failure without intellectual disability due to severe congenital heart anomalies excluding single ventricle heart defects	Congenital heart disease and mild heart failure	(combined DW)	0.041<br>(0.026-0.062)
Congenital heart disease and moderate heart failure without intellectual disability due to severe congenital heart anomalies excluding single ventricle heart defects	Congenital heart disease and moderate heart failure	(combined DW)	0.072<br>(0.047-0.103)
Congenital heart disease and severe heart failure without intellectual disability due to severe congenital heart anomalies excluding single ventricle heart defects	Congenital heart disease and severe heart failure	(combined DW)	0.179<br>(0.122-0.251)
Congenital heart disease, boderline intellectual disability and mild heart failure due to severe congenital heart anomalies excluding single ventricle heart defects	Congenital heart disease, borderline intellectual functioning, and mild heart failure	(combined DW)	0.052<br>(0.032-0.081)
Congenital heart disease, boderline intellectual disability and mild heart failure due to severe congenital heart anomalies excluding single ventricle heart defects	Congenital heart disease, borderline intellectual functioning, and mild heart failure	(combined DW)	0.052<br>(0.032-0.081)
Congenital heart disease, borderline intellectual disability and moderate heart failure due to severe congenital heart anomalies excluding single ventricle heart defects	Congenital heart disease, borderline intellectual functioning, and moderate heart failure	(combined DW)	0.082<br>(0.053-0.12)
Congenital heart disease, borderline intellectual disability and moderate heart failure due to severe congenital heart anomalies excluding single ventricle heart defects	Congenital heart disease, borderline intellectual functioning, and moderate heart failure	(combined DW)	0.082<br>(0.053-0.12)
Congenital heart disease, borderline intellectual disability and severe heart failure due to severe congenital heart anomalies excluding single ventricle heart defects	Congenital heart disease,borderline intellectual functioning, and severe heart failure	(combined DW)	0.188<br>(0.13-0.258)
Congenital heart disease, borderline intellectual disability and severe heart failure due to severe congenital heart anomalies excluding single ventricle heart defects	Congenital heart disease,borderline intellectual functioning, and severe heart failure	(combined DW)	0.188<br>(0.13-0.258)
Congenital heart disease, mild intellectual disability and mild heart failure due to severe congenital heart anomalies excluding single ventricle heart defects	Congenital heart disease, mild intellectual disability, and mild heart failure	(combined DW)	0.082<br>(0.052-0.121)
Congenital heart disease, mild intellectual disability and mild heart failure due to severe congenital heart anomalies excluding single ventricle heart defects	Congenital heart disease, mild intellectual disability, and mild heart failure	(combined DW)	0.082<br>(0.052-0.121)
Congenital heart disease, mild intellectual disability and moderate heart failure due to severe congenital heart anomalies excluding single ventricle heart defects	Congenital heart disease, mild intellectual disability, and moderate heart failure	(combined DW)	0.111<br>(0.072-0.161)
Congenital heart disease, mild intellectual disability and moderate heart failure due to severe congenital heart anomalies excluding single ventricle heart defects	Congenital heart disease, mild intellectual disability, and moderate heart failure	(combined DW)	0.111<br>(0.072-0.161)
Congenital heart disease, mild intellectual disability and severe heart failure due to severe congenital heart anomalies excluding single ventricle heart defects	Congenital heart disease, mild intellectual disability, and servere heart failure	(combined DW)	0.214<br>(0.148-0.291)
Congenital heart disease, mild intellectual disability and severe heart failure due to severe congenital heart anomalies excluding single ventricle heart defects	Congenital heart disease, mild intellectual disability, and servere heart failure	(combined DW)	0.214<br>(0.148-0.291)
Congenital heart disease, moderate intellectual disability and mild heart failure due to severe congenital heart anomalies excluding single ventricle heart defects	Congenital heart disease, moderate intellectual disability, and mild heart failure	(combined DW)	0.137<br>(0.093-0.191)
Congenital heart disease, moderate intellectual disability and mild heart failure due to severe congenital heart anomalies excluding single ventricle heart defects	Congenital heart disease, moderate intellectual disability, and mild heart failure	(combined DW)	0.137<br>(0.093-0.191)
Congenital heart disease, moderate intellectual disability and moderate heart failure due to severe congenital heart anomalies excluding single ventricle heart defects	Congenital heart disease, moderate intellectual disability, and moderate heart failure	(combined DW)	0.164<br>(0.112-0.225)
Congenital heart disease, moderate intellectual disability and moderate heart failure due to severe congenital heart anomalies excluding single ventricle heart defects	Congenital heart disease, moderate intellectual disability, and moderate heart failure	(combined DW)	0.164<br>(0.112-0.225)
Congenital heart disease, moderate intellectual disability and severe heart failure due to severe congenital heart anomalies excluding single ventricle heart defects	Congenital heart disease, moderate intellectual disability, and severe heart failure	(combined DW)	0.261<br>(0.182-0.352)
Congenital heart disease, moderate intellectual disability and severe heart failure due to severe congenital heart anomalies excluding single ventricle heart defects	Congenital heart disease, moderate intellectual disability, and severe heart failure	(combined DW)	0.261<br>(0.182-0.352)
Congenital heart disease, severe intellectual disability and mild heart failure due to severe congenital heart anomalies excluding single ventricle heart defects	Congenital heart disease, severe intellectual disability, and mild heart failure	(combined DW)	0.195<br>(0.134-0.269)
Congenital heart disease, severe intellectual disability and mild heart failure due to severe congenital heart anomalies excluding single ventricle heart defects	Congenital heart disease, severe intellectual disability, and mild heart failure	(combined DW)	0.195<br>(0.134-0.269)
Congenital heart disease, severe intellectual disability and moderate heart failure due to severe congenital heart anomalies excluding single ventricle heart defects	Congenital heart disease, severe intellectual disability, and moderate heart failure	(combined DW)	0.22<br>(0.151-0.302)
Congenital heart disease, severe intellectual disability and moderate heart failure due to severe congenital heart anomalies excluding single ventricle heart defects	Congenital heart disease, severe intellectual disability, and moderate heart failure	(combined DW)	0.22<br>(0.151-0.302)
Congenital heart disease, severe intellectual disability and severe heart failure due to severe congenital heart anomalies excluding single ventricle heart defects	Congenital heart disease, severe intellectual disability, and severe heart failure	(combined DW)	0.31<br>(0.22-0.419)
Congenital heart disease, severe intellectual disability and severe heart failure due to severe congenital heart anomalies excluding single ventricle heart defects	Congenital heart disease, severe intellectual disability, and severe heart failure	(combined DW)	0.31<br>(0.22-0.419)
Congenital heart disease, profound intellectual disability and mild heart failure due to severe congenital heart anomalies excluding single ventricle heart defects	Congenital heart disease, profound intellectual disability, and mild heart failure	(combined DW)	0.233<br>(0.162-0.317)
Congenital heart disease, profound intellectual disability and mild heart failure due to severe congenital heart anomalies excluding single ventricle heart defects	Congenital heart disease, profound intellectual disability, and mild heart failure	(combined DW)	0.233<br>(0.162-0.317)
Congenital heart disease, profound intellectual disability and moderate heart failure due to severe congenital heart anomalies excluding single ventricle heart defects	Congenital heart disease, profound intellectual disability, and moderate heart failure	(combined DW)	0.257<br>(0.181-0.351)
Congenital heart disease, profound intellectual disability and moderate heart failure due to severe congenital heart anomalies excluding single ventricle heart defects	Congenital heart disease, profound intellectual disability, and moderate heart failure	(combined DW)	0.257<br>(0.181-0.351)
Congenital heart disease, profound intellectual disability and severe heart failure due to severe congenital heart anomalies excluding single ventricle heart defects	Congenital heart disease, profound intellectual disability, and severe heart failure	(combined DW)	0.342<br>(0.239-0.457)
Congenital heart disease, profound intellectual disability and severe heart failure due to severe congenital heart anomalies excluding single ventricle heart defects	Congenital heart disease, profound intellectual disability, and severe heart failure	(combined DW)	0.342<br>(0.239-0.457)
Congenital heart disease and borderline intellectual disability without heart failure due to severe congenital heart anomalies excluding single ventricle heart defects	Congenital heart disease and borderline intellectual functioning	(combined DW)	0.011<br>(0.005-0.02)
Congenital heart disease and mild intellectual disability without heart failure due to severe congenital heart anomalies excluding single ventricle heart defects	Congenital heart disease and Intellectual disability / mental retardation, mild	(combined DW)	0.043<br>(0.026-0.064)
Congenital heart disease and moderate intellectual disability without heart failure due to severe congenital heart anomalies excluding single ventricle heart defects	Congenital heart disease and Intellectual disability / mental retardation, moderate	(combined DW)	0.1<br>(0.066-0.142)
Congenital heart disease and severe intellectual disability without heart failure due to severe congenital heart anomalies excluding single ventricle heart defects	Congenital heart disease and Intellectual disability / mental retardation, severe	(combined DW)	0.16<br>(0.107-0.226)
Congenital heart disease and profound intellectual disability without heart failure due to severe congenital heart anomalies excluding single ventricle heart defects	Congenital heart disease and Intellectual disability / mental retardation, profound	(combined DW)	0.2<br>(0.133-0.283)
Congenital heart disease and controlled, medically managed heart failure without intellectual disability due to severe congenital heart anomalies excluding single ventricle heart defects	Congenital heart disease and generic uncomplicated disease: worry and daily medication	(combined DW)	0.049<br>(0.031-0.072)
Congenital heart disease, boderline intellectual disability and controlled, medically managed heart failure due to severe congenital heart anomalies excluding single ventricle heart defects	Congenital heart disease and borderline intellectual functioning and generic uncomplicated disease: worry and daily medication	(combined DW)	0.059<br>(0.037-0.09)
Congenital heart disease, boderline intellectual disability and controlled, medically managed heart failure due to severe congenital heart anomalies excluding single ventricle heart defects	Congenital heart disease and borderline intellectual functioning and generic uncomplicated disease: worry and daily medication	(combined DW)	0.059<br>(0.037-0.09)

Congenital heart disease, mild intellectual disability and controlled, medically managed heart failure due to severe congenital heart anomalies excluding single ventricle heart defects	Congenital heart disease and Intellectual disability / mental retardation, mild and generic uncomplicated disease: worry and daily medication	(combined DW)	0.089 (0.056-0.133)
Congenital heart disease, mild intellectual disability and controlled, medically managed heart failure due to severe congenital heart anomalies excluding single ventricle heart defects	Congenital heart disease and Intellectual disability / mental retardation, mild and generic uncomplicated disease: worry and daily medication	(combined DW)	0.089 (0.056-0.133)
Congenital heart disease, moderate intellectual disability and controlled, medically managed heart failure due to severe congenital heart anomalies excluding single ventricle heart defects	Congenital heart disease and Intellectual disability / mental retardation, moderate and generic uncomplicated disease: worry and daily medication	(combined DW)	0.144 (0.098-0.199)
Congenital heart disease, moderate intellectual disability and controlled, medically managed heart failure due to severe congenital heart anomalies excluding single ventricle heart defects	Congenital heart disease and Intellectual disability / mental retardation, moderate and generic uncomplicated disease: worry and daily medication	(combined DW)	0.144 (0.098-0.199)
Congenital heart disease, profound intellectual disability and controlled, medically managed heart failure due to severe congenital heart anomalies excluding single ventricle heart defects	Congenital heart disease and Intellectual disability / mental retardation, severe and generic uncomplicated disease: worry and daily medication	(combined DW)	0.201 (0.138-0.276)
Congenital heart disease, profound intellectual disability and controlled, medically managed heart failure due to severe congenital heart anomalies excluding single ventricle heart defects	Congenital heart disease and Intellectual disability / mental retardation, severe and generic uncomplicated disease: worry and daily medication	(combined DW)	0.201 (0.138-0.276)
Congenital heart disease, severe intellectual disability and controlled, medically managed heart failure due to severe congenital heart anomalies excluding single ventricle heart defects	Congenital heart disease and Intellectual disability / mental retardation, profound and generic uncomplicated disease: worry and daily medication	(combined DW)	0.239 (0.167-0.325)
Congenital heart disease, severe intellectual disability and controlled, medically managed heart failure due to severe congenital heart anomalies excluding single ventricle heart defects	Congenital heart disease and Intellectual disability / mental retardation, profound and generic uncomplicated disease: worry and daily medication	(combined DW)	0.239 (0.167-0.325)
Congenital heart disease without intellectual disability or heart failure due to single ventricle and single ventricle pathway heart defects	Congenital heart disease	(custom DW from MEPS)	0.061 (0.024-0.107)
Congenital heart disease and mild heart failure without intellectual disability due to single ventricle and single ventricle pathway heart defects	Congenital heart disease and mild heart failure	(combined DW)	0.041 (0.026-0.062)
Congenital heart disease and moderate heart failure without intellectual disability due to single ventricle and single ventricle pathway heart defects	Congenital heart disease and moderate heart failure	(combined DW)	0.072 (0.047-0.103)
Congenital heart disease and severe heart failure without intellectual disability due to single ventricle and single ventricle pathway heart defects	Congenital heart disease and severe heart failure	(combined DW)	0.179 (0.122-0.251)
Congenital heart disease, boderline intellectual disability and mild heart failure due to single ventricle and single ventricle pathway heart defects	Congenital heart disease, borderline intellectual functioning, and mild heart failure	(combined DW)	0.052 (0.032-0.081)
Congenital heart disease, boderline intellectual disability and mild heart failure due to single ventricle and single ventricle pathway heart defects	Congenital heart disease, borderline intellectual functioning, and mild heart failure	(combined DW)	0.052 (0.032-0.081)
Congenital heart disease, borderline intellectual disability and moderate heart failure due to single ventricle and single ventricle pathway heart defects	Congenital heart disease, borderline intellectual functioning, and moderate heart failure	(combined DW)	0.082 (0.053-0.12)
Congenital heart disease, borderline intellectual disability and moderate heart failure due to single ventricle and single ventricle pathway heart defects	Congenital heart disease, borderline intellectual functioning, and moderate heart failure	(combined DW)	0.082 (0.053-0.12)
Congenital heart disease, borderline intellectual disability and severe heart failure due to single ventricle and single ventricle pathway heart defects	Congenital heart disease,borderline intellectual functioning, and severe heart failure	(combined DW)	0.188 (0.13-0.258)
Congenital heart disease, borderline intellectual disability and severe heart failure due to single ventricle and single ventricle pathway heart defects	Congenital heart disease,borderline intellectual functioning, and severe heart failure	(combined DW)	0.188 (0.13-0.258)
Congenital heart disease, mild intellectual disability and mild heart failure due to single ventricle and single ventricle pathway heart defects	Congenital heart disease, mild intellectual disability, and mild heart failure	(combined DW)	0.082 (0.052-0.121)
Congenital heart disease, mild intellectual disability and mild heart failure due to single ventricle and single ventricle pathway heart defects	Congenital heart disease, mild intellectual disability, and mild heart failure	(combined DW)	0.082 (0.052-0.121)
Congenital heart disease, mild intellectual disability and moderate heart failure due to single ventricle and single ventricle pathway heart defects	Congenital heart disease, mild intellectual disability, and moderate heart failure	(combined DW)	0.111 (0.072-0.161)
Congenital heart disease, mild intellectual disability and moderate heart failure due to single ventricle and single ventricle pathway heart defects	Congenital heart disease, mild intellectual disability, and moderate heart failure	(combined DW)	0.111 (0.072-0.161)
Congenital heart disease, mild intellectual disability and severe heart failure due to single ventricle and single ventricle pathway heart defects	Congenital heart disease, mild intellectual disability, and servere heart failure	(combined DW)	0.214 (0.148-0.291)
Congenital heart disease, mild intellectual disability and severe heart failure due to single ventricle and single ventricle pathway heart defects	Congenital heart disease, mild intellectual disability, and servere heart failure	(combined DW)	0.214 (0.148-0.291)
Congenital heart disease, moderate intellectual disability and mild heart failure due to single ventricle and single ventricle pathway heart defects	Congenital heart disease, moderate intellectual disability, and mild heart failure	(combined DW)	0.137 (0.093-0.191)
Congenital heart disease, moderate intellectual disability and mild heart failure due to single ventricle and single ventricle pathway heart defects	Congenital heart disease, moderate intellectual disability, and mild heart failure	(combined DW)	0.137 (0.093-0.191)
Congenital heart disease, moderate intellectual disability and moderate heart failure due to single ventricle and single ventricle pathway heart defects	Congenital heart disease, moderate intellectual disability, and moderate heart failure	(combined DW)	0.164 (0.112-0.225)
Congenital heart disease, moderate intellectual disability and moderate heart failure due to single ventricle and single ventricle pathway heart defects	Congenital heart disease, moderate intellectual disability, and moderate heart failure	(combined DW)	0.164 (0.112-0.225)
Congenital heart disease, moderate intellectual disability and severe heart failure due to single ventricle and single ventricle pathway heart defects	Congenital heart disease, moderate intellectual disability, and severe heart failure	(combined DW)	0.261 (0.182-0.352)
Congenital heart disease, moderate intellectual disability and severe heart failure due to single ventricle and single ventricle pathway heart defects	Congenital heart disease, moderate intellectual disability, and severe heart failure	(combined DW)	0.261 (0.182-0.352)
Congenital heart disease, severe intellectual disability and mild heart failure due to single ventricle and single ventricle pathway heart defects	Congenital heart disease, severe intellectual disability, and mild heart failure	(combined DW)	0.195 (0.134-0.269)
Congenital heart disease, severe intellectual disability and mild heart failure due to single ventricle and single ventricle pathway heart defects	Congenital heart disease, severe intellectual disability, and mild heart failure	(combined DW)	0.195 (0.134-0.269)
Congenital heart disease, severe intellectual disability and moderate heart failure due to single ventricle and single ventricle pathway heart defects	Congenital heart disease, severe intellectual disability, and moderate heart failure	(combined DW)	0.22 (0.151-0.302)
Congenital heart disease, severe intellectual disability and moderate heart failure due to single ventricle and single ventricle pathway heart defects	Congenital heart disease, severe intellectual disability, and moderate heart failure	(combined DW)	0.22 (0.151-0.302)
Congenital heart disease, severe intellectual disability and severe heart failure due to single ventricle and single ventricle pathway heart defects	Congenital heart disease, severe intellectual disability, and severe heart failure	(combined DW)	0.31 (0.22-0.419)
Congenital heart disease, severe intellectual disability and severe heart failure due to single ventricle and single ventricle pathway heart defects	Congenital heart disease, severe intellectual disability, and severe heart failure	(combined DW)	0.31 (0.22-0.419)
Congenital heart disease, profound intellectual disability and mild heart failure due to single ventricle and single ventricle pathway heart defects	Congenital heart disease, profound intellectual disability, and mild heart failure	(combined DW)	0.233 (0.162-0.317)
Congenital heart disease, profound intellectual disability and mild heart failure due to single ventricle and single ventricle pathway heart defects	Congenital heart disease, profound intellectual disability, and mild heart failure	(combined DW)	0.233 (0.162-0.317)
Congenital heart disease, profound intellectual disability and moderate heart failure due to single ventricle and single ventricle pathway heart defects	Congenital heart disease, profound intellectual disability, and moderate heart failure	(combined DW)	0.257 (0.181-0.351)
Congenital heart disease, profound intellectual disability and moderate heart failure due to single ventricle and single ventricle pathway heart defects	Congenital heart disease, profound intellectual disability, and moderate heart failure	(combined DW)	0.257 (0.181-0.351)
Congenital heart disease, profound intellectual disability and severe heart failure due to single ventricle and single ventricle pathway heart defects	Congenital heart disease, profound intellectual disability, and severe heart failure	(combined DW)	0.342 (0.239-0.457)
Congenital heart disease, profound intellectual disability and severe heart failure due to single ventricle and single ventricle pathway heart defects	Congenital heart disease, profound intellectual disability, and severe heart failure	(combined DW)	0.342 (0.239-0.457)
Congenital heart disease and borderline intellectual disability without heart failure due to single ventricle and single ventricle pathway heart defects	Congenital heart disease and borderline intellectual functioning	(combined DW)	0.011 (0.005-0.02)
Congenital heart disease and mild intellectual disability without heart failure due to single ventricle and single ventricle pathway heart defects	Congenital heart disease and Intellectual disability / mental retardation, mild	(combined DW)	0.043 (0.026-0.064)
Congenital heart disease and moderate intellectual disability without heart failure due to single ventricle and single ventricle pathway heart defects	Congenital heart disease and Intellectual disability / mental retardation, moderate	(combined DW)	0.1 (0.066-0.142)
Congenital heart disease and severe intellectual disability without heart failure due to single ventricle and single ventricle pathway heart defects	Congenital heart disease and Intellectual disability / mental retardation, severe	(combined DW)	0.16 (0.107-0.226)
Congenital heart disease and profound intellectual disability without heart failure due to single ventricle and single ventricle pathway heart defects	Congenital heart disease and Intellectual disability / mental retardation, profound	(combined DW)	0.2 (0.133-0.283)
Congenital heart disease and controlled, medically managed heart failure without intellectual disability due to single ventricle and single ventricle pathway heart defects	Congenital heart disease and generic uncomplicated disease: worry and daily medication	(combined DW)	0.049 (0.031-0.072)
Congenital heart disease, boderline intellectual disability and controlled, medically managed heart failure due to single ventricle and single ventricle pathway heart defects	Congenital heart disease and borderline intellectual functioning and generic uncomplicated disease: worry and daily medication	(combined DW)	0.059 (0.037-0.09)
Congenital heart disease, boderline intellectual disability and controlled, medically managed heart failure due to single ventricle and single ventricle pathway heart defects	Congenital heart disease and borderline intellectual functioning and generic uncomplicated disease: worry and daily medication	(combined DW)	0.059 (0.037-0.09)
Congenital heart disease, mild intellectual disability and controlled, medically managed heart failure due to single ventricle and single ventricle pathway heart defects	Congenital heart disease and Intellectual disability / mental retardation, mild and generic uncomplicated disease: worry and daily medication	(combined DW)	0.089 (0.056-0.133)
Congenital heart disease, mild intellectual disability and controlled, medically managed heart failure due to single ventricle and single ventricle pathway heart defects	Congenital heart disease and Intellectual disability / mental retardation, mild and generic uncomplicated disease: worry and daily medication	(combined DW)	0.089 (0.056-0.133)
Congenital heart disease, moderate intellectual disability and controlled, medically managed heart failure due to single ventricle and single ventricle pathway heart defects	Congenital heart disease and Intellectual disability / mental retardation, moderate and generic uncomplicated disease: worry and daily medication	(combined DW)	0.144 (0.098-0.199)
Congenital heart disease, moderate intellectual disability and controlled, medically managed heart failure due to single ventricle and single ventricle pathway heart defects	Congenital heart disease and Intellectual disability / mental retardation, moderate and generic uncomplicated disease: worry and daily medication	(combined DW)	0.144 (0.098-0.199)
Congenital heart disease, profound intellectual disability and controlled, medically managed heart failure due to single ventricle and single ventricle pathway heart defects	Congenital heart disease and Intellectual disability / mental retardation, severe and generic uncomplicated disease: worry and daily medication	(combined DW)	0.201 (0.138-0.276)
Congenital heart disease, profound intellectual disability and controlled, medically managed heart failure due to single ventricle and single ventricle pathway heart defects	Congenital heart disease and Intellectual disability / mental retardation, severe and generic uncomplicated disease: worry and daily medication	(combined DW)	0.201 (0.138-0.276)
Congenital heart disease, severe intellectual disability and controlled, medically managed heart failure due to single ventricle and single ventricle pathway heart defects	Congenital heart disease and Intellectual disability / mental retardation, profound and generic uncomplicated disease: worry and daily medication	(combined DW)	0.239 (0.167-0.325)
Congenital heart disease, severe intellectual disability and controlled, medically managed heart failure due to single ventricle and single ventricle pathway heart defects	Congenital heart disease and Intellectual disability / mental retardation, profound and generic uncomplicated disease: worry and daily medication	(combined DW)	0.239 (0.167-0.325)

Congenital heart disease without heart failure or intellectual disability due to ventricular septal defect and atrial septal defect	Congenital heart disease	(custom DW from MEPS)	0.061 (0.024-0.107)
Asymptomatic ventricular septal defect and atrial septal defect	Asymptomatic		0 (0-0)
Congenital heart disease and mild heart failure without intellectual disability due to ventricular septal defect and atrial septal defect	Congenital heart disease and mild heart failure	(combined DW)	0.041 (0.026-0.062)
Congenital heart disease and moderate heart failure without intellectual disability due to ventricular septal defect and atrial septal defect	Congenital heart disease and moderate heart failure	(combined DW)	0.072 (0.047-0.103)
Congenital heart disease and severe heart without intellectual disability due to ventricular septal defect and atrial septal defect	Congenital heart disease and severe heart failure	(combined DW)	0.179 (0.122-0.251)
Congenital heart disease, boderline intellectual disability and mild heart failure due to ventricular septal defect and atrial septal defect	Congenital heart disease, borderline intellectual functioning, and mild heart failure	(combined DW)	0.052 (0.032-0.081)
Congenital heart disease, boderline intellectual disability and mild heart failure due to ventricular septal defect and atrial septal defect	Congenital heart disease, borderline intellectual functioning, and mild heart failure	(combined DW)	0.052 (0.032-0.081)
Congenital heart disease, borderline intellectual disability and moderate heart failure due to ventricular septal defect and atrial septal defect	Congenital heart disease, borderline intellectual functioning, and moderate heart failure	(combined DW)	0.082 (0.053-0.12)
Congenital heart disease, borderline intellectual disability and moderate heart failure due to ventricular septal defect and atrial septal defect	Congenital heart disease, borderline intellectual functioning, and moderate heart failure	(combined DW)	0.082 (0.053-0.12)
Congenital heart disease, borderline intellectual disability and severe heart failure due to ventricular septal defect and atrial septal defect	Congenital heart disease,borderline intellectual functioning, and severe heart failure	(combined DW)	0.188 (0.13-0.258)
Congenital heart disease, borderline intellectual disability and severe heart failure due to ventricular septal defect and atrial septal defect	Congenital heart disease,borderline intellectual functioning, and severe heart failure	(combined DW)	0.188 (0.13-0.258)
Congenital heart disease, mild intellectual disability and mild heart failure due to ventricular septal defect and atrial septal defect	Congenital heart disease, mild intellectual disability, and mild heart failure	(combined DW)	0.082 (0.052-0.121)
Congenital heart disease, mild intellectual disability and mild heart failure due to ventricular septal defect and atrial septal defect	Congenital heart disease, mild intellectual disability, and mild heart failure	(combined DW)	0.082 (0.052-0.121)
Congenital heart disease, mild intellectual disability and moderate heart failure due to ventricular septal defect and atrial septal defect	Congenital heart disease, mild intellectual disability, and moderate heart failure	(combined DW)	0.111 (0.072-0.161)
Congenital heart disease, mild intellectual disability and moderate heart failure due to ventricular septal defect and atrial septal defect	Congenital heart disease, mild intellectual disability, and moderate heart failure	(combined DW)	0.111 (0.072-0.161)
Congenital heart disease, mild intellectual disability and severe heart failure due to ventricular septal defect and atrial septal defect	Congenital heart disease, mild intellectual disability, and servere heart failure	(combined DW)	0.214 (0.148-0.291)
Congenital heart disease, mild intellectual disability and severe heart failure due to ventricular septal defect and atrial septal defect	Congenital heart disease, mild intellectual disability, and servere heart failure	(combined DW)	0.214 (0.148-0.291)
Congenital heart disease, moderate intellectual disability and mild heart failure due to ventricular septal defect and atrial septal defect	Congenital heart disease, moderate intellectual disability, and mild heart failure	(combined DW)	0.137 (0.093-0.191)
Congenital heart disease, moderate intellectual disability and mild heart failure due to ventricular septal defect and atrial septal defect	Congenital heart disease, moderate intellectual disability, and mild heart failure	(combined DW)	0.137 (0.093-0.191)
Congenital heart disease, moderate intellectual disability and moderate heart failure due to ventricular septal defect and atrial septal defect	Congenital heart disease, moderate intellectual disability, and moderate heart failure	(combined DW)	0.164 (0.112-0.225)
Congenital heart disease, moderate intellectual disability and moderate heart failure due to ventricular septal defect and atrial septal defect	Congenital heart disease, moderate intellectual disability, and moderate heart failure	(combined DW)	0.164 (0.112-0.225)
Congenital heart disease, moderate intellectual disability and severe heart failure due to ventricular septal defect and atrial septal defect	Congenital heart disease, moderate intellectual disability, and severe heart failure	(combined DW)	0.261 (0.182-0.352)
Congenital heart disease, moderate intellectual disability and severe heart failure due to ventricular septal defect and atrial septal defect	Congenital heart disease, moderate intellectual disability, and severe heart failure	(combined DW)	0.261 (0.182-0.352)
Congenital heart disease, severe intellectual disability and mild heart failure due to ventricular septal defect and atrial septal defect	Congenital heart disease, severe intellectual disability, and mild heart failure	(combined DW)	0.195 (0.134-0.269)
Congenital heart disease, severe intellectual disability and mild heart failure due to ventricular septal defect and atrial septal defect	Congenital heart disease, severe intellectual disability, and mild heart failure	(combined DW)	0.195 (0.134-0.269)
Congenital heart disease, severe intellectual disability and moderate heart failure due to ventricular septal defect and atrial septal defect	Congenital heart disease, severe intellectual disability, and moderate heart failure	(combined DW)	0.22 (0.151-0.302)
Congenital heart disease, severe intellectual disability and moderate heart failure due to ventricular septal defect and atrial septal defect	Congenital heart disease, severe intellectual disability, and moderate heart failure	(combined DW)	0.22 (0.151-0.302)
Congenital heart disease, severe intellectual disability and severe heart failure due to ventricular septal defect and atrial septal defect	Congenital heart disease, severe intellectual disability, and severe heart failure	(combined DW)	0.31 (0.22-0.419)
Congenital heart disease, severe intellectual disability and severe heart failure due to ventricular septal defect and atrial septal defect	Congenital heart disease, severe intellectual disability, and severe heart failure	(combined DW)	0.31 (0.22-0.419)
Congenital heart disease, profound intellectual disability and mild heart failure due to ventricular septal defect and atrial septal defect	Congenital heart disease, profound intellectual disability, and mild heart failure	(combined DW)	0.233 (0.162-0.317)
Congenital heart disease, profound intellectual disability and mild heart failure due to ventricular septal defect and atrial septal defect	Congenital heart disease, profound intellectual disability, and mild heart failure	(combined DW)	0.233 (0.162-0.317)
Congenital heart disease, profound intellectual disability and moderate heart failure due to ventricular septal defect and atrial septal defect	Congenital heart disease, profound intellectual disability, and moderate heart failure	(combined DW)	0.257 (0.181-0.351)
Congenital heart disease, profound intellectual disability and moderate heart failure due to ventricular septal defect and atrial septal defect	Congenital heart disease, profound intellectual disability, and moderate heart failure	(combined DW)	0.257 (0.181-0.351)
Congenital heart disease, profound intellectual disability and severe heart failure due to ventricular septal defect and atrial septal defect	Congenital heart disease, profound intellectual disability, and severe heart failure	(combined DW)	0.342 (0.239-0.457)
Congenital heart disease, profound intellectual disability and severe heart failure due to ventricular septal defect and atrial septal defect	Congenital heart disease, profound intellectual disability, and severe heart failure	(combined DW)	0.342 (0.239-0.457)
Congenital heart disease and borderline intellectual disability without heart failure due to ventricular septal defect and atrial septal defect	Congenital heart disease and borderline intellectual functioning	(combined DW)	0.011 (0.005-0.02)
Congenital heart disease and mild intellectual disability without heart failure due to ventricular septal defect and atrial septal defect	Congenital heart disease and Intellectual disability / mental retardation, mild	(combined DW)	0.043 (0.026-0.064)
Congenital heart disease and moderate intellectual disability without heart failure due to ventricular septal defect and atrial septal defect	Congenital heart disease and Intellectual disability / mental retardation, moderate	(combined DW)	0.1 (0.066-0.142)
Congenital heart disease and severe intellectual disability without heart failure due to ventricular septal defect and atrial septal defect	Congenital heart disease and Intellectual disability / mental retardation, severe	(combined DW)	0.16 (0.107-0.226)
Congenital heart disease and profound intellectual disability without heart failure due to ventricular septal defect and atrial septal defect	Congenital heart disease and Intellectual disability / mental retardation, profound	(combined DW)	0.2 (0.133-0.283)
Congenital heart disease and controlled, medically managed heart failure without intellectual disability due to ventricular septal defect and atrial septal defect	Congenital heart disease and generic uncomplicated disease: worry and daily medication	(combined DW)	0.049 (0.031-0.072)
Congenital heart disease, boderline intellectual disability and controlled, medically managed heart failure due to ventricular septal defect and atrial septal defect	Congenital heart disease and borderline intellectual functioning and generic uncomplicated disease: worry and daily medication	(combined DW)	0.059 (0.037-0.09)
Congenital heart disease, boderline intellectual disability and controlled, medically managed heart failure due to ventricular septal defect and atrial septal defect	Congenital heart disease and borderline intellectual functioning and generic uncomplicated disease: worry and daily medication	(combined DW)	0.059 (0.037-0.09)
Congenital heart disease, mild intellectual disability and controlled, medically managed heart failure due to ventricular septal defect and atrial septal defect	Congenital heart disease and Intellectual disability / mental retardation, mild and generic uncomplicated disease: worry and daily medication	(combined DW)	0.089 (0.056-0.133)
Congenital heart disease, mild intellectual disability and controlled, medically managed heart failure due to ventricular septal defect and atrial septal defect	Congenital heart disease and Intellectual disability / mental retardation, mild and generic uncomplicated disease: worry and daily medication	(combined DW)	0.089 (0.056-0.133)
Congenital heart disease, moderate intellectual disability and controlled, medically managed heart failure due to ventricular septal defect and atrial septal defect	Congenital heart disease and Intellectual disability / mental retardation, moderate and generic uncomplicated disease: worry and daily medication	(combined DW)	0.144 (0.098-0.199)
Congenital heart disease, moderate intellectual disability and controlled, medically managed heart failure due to ventricular septal defect and atrial septal defect	Congenital heart disease and Intellectual disability / mental retardation, moderate and generic uncomplicated disease: worry and daily medication	(combined DW)	0.144 (0.098-0.199)
Congenital heart disease, profound intellectual disability and controlled, medically managed heart failure due to ventricular septal defect and atrial septal defect	Congenital heart disease and Intellectual disability / mental retardation, severe and generic uncomplicated disease: worry and daily medication	(combined DW)	0.201 (0.138-0.276)
Congenital heart disease, profound intellectual disability and controlled, medically managed heart failure due to ventricular septal defect and atrial septal defect	Congenital heart disease and Intellectual disability / mental retardation, severe and generic uncomplicated disease: worry and daily medication	(combined DW)	0.201 (0.138-0.276)
Congenital heart disease, severe intellectual disability and controlled, medically managed heart failure due to ventricular septal defect and atrial septal defect	Congenital heart disease and Intellectual disability / mental retardation, profound and generic uncomplicated disease: worry and daily medication	(combined DW)	0.239 (0.167-0.325)
Congenital heart disease, severe intellectual disability and controlled, medically managed heart failure due to ventricular septal defect and atrial septal defect	Congenital heart disease and Intellectual disability / mental retardation, profound and generic uncomplicated disease: worry and daily medication	(combined DW)	0.239 (0.167-0.325)
Disfigurement level 1 due to orofacial clefts	Disfigurement, level 1	has a slight, visible physical deformity that others notice, which causes some worry and discomfort.	0.011 (0.005-0.021)
Disfigurement level 2 due to orofacial clefts	Disfigurement, level 2	has a visible physical deformity that causes others to stare and comment. As a result, the person is worried and has trouble sleeping and concentrating.	0.067 (0.044-0.096)
Disfigurement level 2 and speech problems due to orofacial clefts	Speech problems with disfigurement level 2	(combined DW)	0.115 (0.076-0.164)
Mild dementia due to Down syndrome	Dementia, mild	has some trouble remembering recent events, and finds it hard to concentrate and make decisions and plans.	0.069 (0.046-0.099)
Moderate dementia due to Down syndrome	Dementia, moderate	has memory problems and confusion, feels disoriented, at times hears voices that are not real, and needs help with some daily activities.	0.377 (0.252-0.508)
Severe dementia due to Down syndrome	Dementia, severe	has complete memory loss; no longer recognizes close family members; and requires help with all daily activities.	0.449 (0.304-0.595)
Borderline intellectual disability due to Down syndrome	Borderline intellectual functioning	is slow in learning at school. As an adult, the person has some difficulty doing complex or unfamiliar tasks but otherwise functions independently.	0.011 (0.005-0.02)
Mild intellectual disability due to Down syndrome	Intellectual disability / mental retardation, mild	has low intelligence and is slow in learning at school. As an adult, the person can live independently, but often needs help to raise children and can only work at simple supervised jobs.	0.043 (0.026-0.064)
Moderate intellectual disability due to Down syndrome	Intellectual disability / mental retardation, moderate	has low intelligence, and is slow in learning to speak and to do even simple tasks. As an adult, the person requires a lot of support to live independently and raise children. The person can only work at the simplest supervised jobs.	0.1 (0.066-0.142)

Severe intellectual disability due to Down syndrome	Intellectual disability / mental retardation, severe	has very low intelligence and cannot speak more than a few words, needs constant supervision and help with most daily activities, and can do only the simplest tasks.	0.16 (0.107-0.226)
Profound intellectual disability due to Down syndrome	Intellectual disability / mental retardation, profound	has very low intelligence, has almost no language, and does not understand even the most basic requests or instructions. The person requires constant supervision and help for all activities.	0.2 (0.133-0.283)
Isolated congenital heart disease due to Down syndrome	Congenital heart disease	(custom DW from MEPS)	0.061 (0.024-0.107)
Asymptomatic Down syndrome	Asymptomatic		0 (0-0)
Borderline intellectual disability with congenital heart disease due to Down syndrome	Borderline intellectual disability with congenital heart disease	(combined DW)	0.011 (0.005-0.02)
Mild intellectual disability with congenital heart disease due to Down syndrome	Mild intellectual disability with congenital heart disease	(combined DW)	0.043 (0.026-0.064)
Moderate intellectual disability with congenital heart disease due to Down syndrome	Moderate intellectual disability with congenital heart disease	(combined DW)	0.1 (0.066-0.142)
Severe intellectual disability with congenital heart disease due to Down syndrome	Severe intellectual disability with congenital heart disease	(combined DW)	0.16 (0.107-0.226)
Profound intellectual disability with congenital heart disease due to Down syndrome	Profound intellectual disability with congenital heart disease	(combined DW)	0.2 (0.133-0.283)
Congenital heart disease and mild dementia due to Down syndrome	Congenital heart disease and mild dementia	(combined DW)	0.069 (0.046-0.099)
Congenital heart disease and moderate dementia due to Down syndrome	Congenital heart disease and moderate dementia	(combined DW)	0.377 (0.252-0.508)
Congenital heart disease and severe dementia due to Down syndrome	Congenital heart disease and severe dementia	(combined DW)	0.449 (0.304-0.595)
Borderline intellectual disability, mild dementia, and congenital heart disease due to Down syndrome	Congenital heart disease, mild dementia, borderline intellectual disability	(combined DW)	0.079 (0.051-0.115)
Mild intellectual disability, mild dementia, and congenital heart disease due to Down syndrome	Congenital heart disease, mild dementia, mild intellectual disability	(combined DW)	0.109 (0.071-0.159)
Moderate intellectual disability, mild dementia, and congenital heart disease due to Down syndrome	Congenital heart disease, mild dementia, moderate intellectual disability	(combined DW)	0.162 (0.11-0.222)
Severe intellectual disability, mild dementia, and congenital heart disease due to Down syndrome	Congenital heart disease, mild dementia, severe intellectual disability	(combined DW)	0.218 (0.149-0.299)
Profound intellectual disability, mild dementia, and congenital heart disease due to Down syndrome	Congenital heart disease, mild dementia, profound intellectual disability	(combined DW)	0.255 (0.178-0.346)
Borderline intellectual disability, moderate dementia, and congenital heart disease due to Down syndrome	Congenital heart disease, moderate dementia, borderline intellectual disability	(combined DW)	0.384 (0.262-0.517)
Mild intellectual disability, moderate dementia, and congenital heart disease due to Down syndrome	Congenital heart disease, moderate dementia, mild intellectual disability	(combined DW)	0.403 (0.281-0.536)
Moderate intellectual disability, moderate dementia, and congenital heart disease due to Down syndrome	Congenital heart disease, moderate dementia, moderate intellectual disability	(combined DW)	0.438 (0.311-0.576)
Severe intellectual disability, moderate dementia, and congenital heart disease due to Down syndrome	Congenital heart disease, moderate dementia, severe intellectual disability	(combined DW)	0.475 (0.34-0.614)
Profound intellectual disability, moderate dementia, and congenital heart disease due to Down syndrome	Congenital heart disease, moderate dementia, profound intellectual disability	(combined DW)	0.499 (0.358-0.645)
Borderline intellectual disability, severe dementia, and congenital heart disease due to Down syndrome	Congenital heart disease, severe dementia, borderline intellectual disability	(combined DW)	0.455 (0.316-0.597)
Mild intellectual disability, severe dementia, and congenital heart disease due to Down syndrome	Congenital heart disease, severe dementia, mild intellectual disability	(combined DW)	0.472 (0.332-0.615)
Moderate intellectual disability, severe dementia, and congenital heart disease due to Down syndrome	Congenital heart disease, severe dementia, moderate intellectual disability	(combined DW)	0.503 (0.355-0.646)
Severe intellectual disability, severe dementia, and congenital heart disease due to Down syndrome	Congenital heart disease, severe dementia, severe intellectual disability	(combined DW)	0.535 (0.384-0.681)
Profound intellectual disability, severe dementia, and congenital heart disease due to Down syndrome	Congenital heart disease, severe dementia, profound intellectual disability	(combined DW)	0.557 (0.401-0.703)
Borderline intellectual disability and mild dementia due to Down syndrome	Mild dementia, borderline intellectual disability	(combined DW)	0.079 (0.051-0.115)
Mild intellectual disability and mild dementia due to Down syndrome	Mild dementia, mild intellectual disability	(combined DW)	0.109 (0.071-0.159)
Moderate intellectual disability and mild dementia due to Down syndrome	Mild dementia, moderate intellectual disability	(combined DW)	0.162 (0.11-0.222)
Severe intellectual disability and mild dementia due to Down syndrome	Mild dementia, severe intellectual disability	(combined DW)	0.218 (0.149-0.299)
Profound intellectual disability and mild dementia due to Down syndrome	Mild dementia, profound intellectual disability	(combined DW)	0.255 (0.178-0.346)
Borderline intellectual disability and moderate dementia due to Down syndrome	Moderate dementia, borderline intellectual disability	(combined DW)	0.384 (0.262-0.517)
Mild intellectual disability and moderate dementia due to Down syndrome	Moderate dementia, mild intellectual disability	(combined DW)	0.403 (0.281-0.536)
Moderate intellectual disability and moderate dementia due to Down syndrome	Moderate dementia, moderate intellectual disability	(combined DW)	0.438 (0.311-0.576)
Severe intellectual disability and moderate dementia due to Down syndrome	Moderate dementia, severe intellectual disability	(combined DW)	0.475 (0.34-0.614)
Profound intellectual disability and moderate dementia due to Down syndrome	Moderate dementia, profound intellectual disability	(combined DW)	0.499 (0.358-0.645)
Borderline intellectual disability and severe dementia due to Down syndrome	Severe dementia, borderline intellectual disability	(combined DW)	0.455 (0.316-0.597)
Mild intellectual disability and severe dementia due to Down syndrome	Severe dementia, mild intellectual disability	(combined DW)	0.472 (0.332-0.615)
Moderate intellectual disability and severe dementia due to Down syndrome	Severe dementia, moderate intellectual disability	(combined DW)	0.503 (0.355-0.646)
Severe intellectual disability and severe dementia due to Down syndrome	Severe dementia, severe intellectual disability	(combined DW)	0.535 (0.384-0.681)
Profound intellectual disability and severe dementia due to Down syndrome	Severe dementia, profound intellectual disability	(combined DW)	0.557 (0.401-0.703)
Primary infertility due to Turner syndrome	Infertility, primary	wants to have a child and has a fertile partner, but the couple cannot conceive.	0.008 (0.003-0.015)
Congenital heart disease due to Turner syndrome	Congenital heart disease	(custom DW from MEPS)	0.061 (0.024-0.107)
Congenital heart disease with infertility due to Turner syndrome	Congenital heart disease with primary infertility	(combined DW)	0.068 (0.031-0.114)
Asymptomatic Turner syndrome	Asymptomatic		0 (0-0)
Primary infertility due to Klinefelter syndrome	Infertility, primary	wants to have a child and has a fertile partner, but the couple cannot conceive.	0.008 (0.003-0.015)
Borderline intellectual disability due to Klinefelter syndrome	Borderline intellectual functioning	is slow in learning at school. As an adult, the person has some difficulty doing complex or unfamiliar tasks but otherwise functions independently.	0.011 (0.005-0.02)
Mild intellectual disability due to Klinefelter syndrome	Intellectual disability / mental retardation, mild	has low intelligence and is slow in learning at school. As an adult, the person can live independently, but often needs help to raise children and can only work at simple supervised jobs.	0.043 (0.026-0.064)
Mild intellectual disability with infertility due to Klinefelter syndrome	Mild intellectual disability with primary infertility	(combined DW)	0.05 (0.03-0.078)
Mild intellectual disability with infertility due to Klinefelter syndrome	Mild intellectual disability with primary infertility	(combined DW)	0.05 (0.03-0.078)
Borderline intellectual disability with infertility due to Klinefelter syndrome	Borderline intellectual disability with primary infertility	(combined DW)	0.018 (0.009-0.034)
Borderline intellectual disability with infertility due to Klinefelter syndrome	Borderline intellectual disability with primary infertility	(combined DW)	0.018 (0.009-0.034)
Asymptomatic Klinefelter syndrome	Asymptomatic		0 (0-0)
Mild dementia due to other chromosomal abnormalities	Dementia, mild	has some trouble remembering recent events, and finds it hard to concentrate and make decisions and plans.	0.069 (0.046-0.099)
Moderate dementia due to other chromosomal abnormalities	Dementia, moderate	has memory problems and confusion, feels disoriented, at times hears voices that are not real, and needs help with some daily activities.	0.377 (0.252-0.508)
Severe dementia due to other chromosomal abnormalities	Dementia, severe	has complete memory loss; no longer recognizes close family members; and requires help with all daily activities.	0.449 (0.304-0.595)
Borderline intellectual disability due to other chromosomal abnormalities	Borderline intellectual functioning	is slow in learning at school. As an adult, the person has some difficulty doing complex or unfamiliar tasks but otherwise functions independently.	0.011 (0.005-0.02)
Mild intellectual disability due to other chromosomal abnormalities	Intellectual disability / mental retardation, mild	has low intelligence and is slow in learning at school. As an adult, the person can live independently, but often needs help to raise children and can only work at simple supervised jobs.	0.043 (0.026-0.064)

Moderate intellectual disability due to other chromosomal abnormalities	Intellectual disability / mental retardation, moderate	has low intelligence, and is slow in learning to speak and to do even simple tasks. As an adult, the person requires a lot of support to live independently and raise children. The person can only work at the simplest supervised jobs.	0.1 (0.066-0.142)
Severe intellectual disability due to other chromosomal abnormalities	Intellectual disability / mental retardation, severe	has very low intelligence and cannot speak more than a few words, needs constant supervision and help with most daily activities, and can do only the simplest tasks.	0.16 (0.107-0.226)
Profound intellectual disability due to other chromosomal abnormalities	Intellectual disability / mental retardation, profound	has very low intelligence, has almost no language, and does not understand even the most basic requests or instructions. The person requires constant supervision and help for all activities.	0.2 (0.133-0.283)
Severe motor and cognitive impairment due to Edward Syndrome or Patau Syndrome	Motor plus cognitive impairments, severe	cannot move around without help, and cannot lift or hold objects, get dressed or sit upright. The person also has very low intelligence, speaks few words, and needs constant supervision and help with all daily activities.	0.542 (0.374-0.702)
Isolated congenital heart disease due to other chromosomal abnormalities	Congenital heart disease	(custom DW from MEPS)	0.061 (0.024-0.107)
Asymptomatic other chromosomal abnormalities	Asymptomatic		0 (0-0)
Borderline intellectual disability with congenital heart disease due to other chromosomal abnormalities	Borderline intellectual disability with congenital heart disease	(combined DW)	0.011 (0.005-0.02)
Mild intellectual disability with congenital heart disease due to other chromosomal abnormalities	Mild intellectual disability with congenital heart disease	(combined DW)	0.043 (0.026-0.064)
Moderate intellectual disability with congenital heart disease due to other chromosomal abnormalities	Moderate intellectual disability with congenital heart disease	(combined DW)	0.1 (0.066-0.142)
Severe intellectual disability with congenital heart disease due to other chromosomal abnormalities	Severe intellectual disability with congenital heart disease	(combined DW)	0.16 (0.107-0.226)
Profound intellectual disability with congenital heart disease due to other chromosomal abnormalities	Profound intellectual disability with congenital heart disease	(combined DW)	0.2 (0.133-0.283)
Congenital heart disease and mild dementia due to other chromosomal abnormalities	Congenital heart disease and mild dementia	(combined DW)	0.069 (0.046-0.099)
Congenital heart disease and moderate dementia due to other chromosomal abnormalities	Congenital heart disease and moderate dementia	(combined DW)	0.377 (0.252-0.508)
Congenital heart disease and severe dementia due to other chromosomal abnormalities	Congenital heart disease and severe dementia	(combined DW)	0.449 (0.304-0.595)
Borderline intellectual disability, mild dementia, and congenital heart disease due to other chromosomal abnormalities	Congenital heart disease, mild dementia, borderline intellectual disability	(combined DW)	0.079 (0.051-0.115)
Mild intellectual disability, mild dementia, and congenital heart disease due to other chromosomal abnormalities	Congenital heart disease, mild dementia, mild intellectual disability	(combined DW)	0.109 (0.071-0.159)
Moderate intellectual disability, mild dementia, and congenital heart disease due to other chromosomal abnormalities	Congenital heart disease, mild dementia, moderate intellectual disability	(combined DW)	0.162 (0.11-0.222)
Severe intellectual disability, mild dementia, and congenital heart disease due to other chromosomal abnormalities	Congenital heart disease, mild dementia, severe intellectual disability	(combined DW)	0.218 (0.149-0.299)
Profound intellectual disability, mild dementia, and congenital heart disease due to other chromosomal abnormalities	Congenital heart disease, mild dementia, profound intellectual disability	(combined DW)	0.255 (0.178-0.346)
Borderline intellectual disability, moderate dementia, and congenital heart disease due to other chromosomal abnormalities	Congenital heart disease, moderate dementia, borderline intellectual disability	(combined DW)	0.384 (0.262-0.517)
Mild intellectual disability, moderate dementia, and congenital heart disease due to other chromosomal abnormalities	Congenital heart disease, moderate dementia, mild intellectual disability	(combined DW)	0.403 (0.281-0.536)
Moderate intellectual disability, moderate dementia, and congenital heart disease due to other chromosomal abnormalities	Congenital heart disease, moderate dementia, moderate intellectual disability	(combined DW)	0.438 (0.311-0.576)
Severe intellectual disability, moderate dementia, and congenital heart disease due to other chromosomal abnormalities	Congenital heart disease, moderate dementia, severe intellectual disability	(combined DW)	0.475 (0.34-0.614)
Profound intellectual disability, moderate dementia, and congenital heart disease due to other chromosomal abnormalities	Congenital heart disease, moderate dementia, profound intellectual disability	(combined DW)	0.499 (0.358-0.645)
Borderline intellectual disability, severe dementia, and congenital heart disease due to other chromosomal abnormalities	Congenital heart disease, severe dementia, borderline intellectual disability	(combined DW)	0.455 (0.316-0.597)
Mild intellectual disability, severe dementia, and congenital heart disease due to other chromosomal abnormalities	Congenital heart disease, severe dementia, mild intellectual disability	(combined DW)	0.472 (0.332-0.615)
Moderate intellectual disability, severe dementia, and congenital heart disease due to other chromosomal abnormalities	Congenital heart disease, severe dementia, moderate intellectual disability	(combined DW)	0.503 (0.355-0.646)
Severe intellectual disability, severe dementia, and congenital heart disease due to other chromosomal abnormalities	Congenital heart disease, severe dementia, severe intellectual disability	(combined DW)	0.535 (0.384-0.681)
Profound intellectual disability, severe dementia, and congenital heart disease due to other chromosomal abnormalities	Congenital heart disease, severe dementia, profound intellectual disability	(combined DW)	0.557 (0.401-0.703)
Borderline intellectual disability and mild dementia due to other chromosomal abnormalities	Mild dementia, borderline intellectual disability	(combined DW)	0.079 (0.051-0.115)
Mild intellectual disability and mild dementia due to other chromosomal abnormalities	Mild dementia, mild intellectual disability	(combined DW)	0.109 (0.071-0.159)
Moderate intellectual disability and mild dementia due to other chromosomal abnormalities	Mild dementia, moderate intellectual disability	(combined DW)	0.162 (0.11-0.222)
Severe intellectual disability and mild dementia due to other chromosomal abnormalities	Mild dementia, severe intellectual disability	(combined DW)	0.218 (0.149-0.299)
Profound intellectual disability and mild dementia due to other chromosomal abnormalities	Mild dementia, profound intellectual disability	(combined DW)	0.255 (0.178-0.346)
Borderline intellectual disability and moderate dementia due to other chromosomal abnormalities	Moderate dementia, borderline intellectual disability	(combined DW)	0.384 (0.262-0.517)
Mild intellectual disability and moderate dementia due to other chromosomal abnormalities	Moderate dementia, mild intellectual disability	(combined DW)	0.403 (0.281-0.536)
Moderate intellectual disability and moderate dementia due to other chromosomal abnormalities	Moderate dementia, moderate intellectual disability	(combined DW)	0.438 (0.311-0.576)
Severe intellectual disability and moderate dementia due to other chromosomal abnormalities	Moderate dementia, severe intellectual disability	(combined DW)	0.475 (0.34-0.614)
Profound intellectual disability and moderate dementia due to other chromosomal abnormalities	Moderate dementia, profound intellectual disability	(combined DW)	0.499 (0.358-0.645)
Borderline intellectual disability and severe dementia due to other chromosomal abnormalities	Severe dementia, borderline intellectual disability	(combined DW)	0.455 (0.316-0.597)
Mild intellectual disability and severe dementia due to other chromosomal abnormalities	Severe dementia, mild intellectual disability	(combined DW)	0.472 (0.332-0.615)
Moderate intellectual disability and severe dementia due to other chromosomal abnormalities	Severe dementia, moderate intellectual disability	(combined DW)	0.503 (0.355-0.646)
Severe intellectual disability and severe dementia due to other chromosomal abnormalities	Severe dementia, severe intellectual disability	(combined DW)	0.535 (0.384-0.681)
Profound intellectual disability and severe dementia due to other chromosomal abnormalities	Severe dementia, profound intellectual disability	(combined DW)	0.557 (0.401-0.703)
Severe motor and cognitive impairment with congenital heart disease due to Edward Syndrome or Patau Syndrome	Severe motor plus cognitive impairments and congenital heart disease	(combined DW)	0.542 (0.374-0.702)
Disfigurement level 1 due to polydactyly and syndactyly	Disfigurement, level 1	has a slight, visible physical deformity that others notice, which causes some worry and discomfort.	0.011 (0.005-0.021)
Disfigurement level 2 due to congenital limb deficiency	Disfigurement, level 2	has a visible physical deformity that causes others to stare and comment. As a result, the person is worried and has trouble sleeping and concentrating.	0.067 (0.044-0.096)
Disfigurement level 2 with pain due to congenital limb deficiency	Disfigurement, level 2, with itch/pain	has a visible physical deformity that is sore and itchy. Other people stare and comment, which causes the person to worry. The person has trouble sleeping and concentrating.	0.188 (0.125-0.267)
Disfigurement level 2 and mild motor impairment due to congenital limb deficiency	Level 2 disfigurement with mild motor impairment	(combined DW)	0.076 (0.051-0.112)
Disfigurement level 2 and moderate motor impairment due to congenital limb deficiency	Level 2 disfigurement with moderate motor impairment	(combined DW)	0.124 (0.083-0.175)
Disfigurement level 2 with pain and mild motor impairment due to congenital limb deficiency	Level 2 disfigurement with itch/pain and mild motor impairment	(combined DW)	0.196 (0.132-0.275)
Disfigurement level 2 with pain and moderate motor impairment due to congenital limb deficiency	Level 2 disfigurement with itch/pain and moderate motor impairment	(combined DW)	0.237 (0.163-0.324)
Disfigurement level 2 due to other congenital musculoskeletal anomalies	Disfigurement, level 2	has a visible physical deformity that causes others to stare and comment. As a result, the person is worried and has trouble sleeping and concentrating.	0.067 (0.044-0.096)
Disfigurement level 2 with pain due to other congenital musculoskeletal anomalies	Disfigurement, level 2, with itch/pain	has a visible physical deformity that is sore and itchy. Other people stare and comment, which causes the person to worry. The person has trouble sleeping and concentrating.	0.188 (0.125-0.267)
Disfigurement level 2 and mild motor impairment due to other congenital musculoskeletal anomalies	Level 2 disfigurement with mild motor impairment	(combined DW)	0.076 (0.051-0.112)
Disfigurement level 2 and moderate motor impairment due to other congenital musculoskeletal anomalies	Level 2 disfigurement with moderate motor impairment	(combined DW)	0.124 (0.083-0.175)
Disfigurement level 2 with pain and mild motor impairment due to other congenital musculoskeletal anomalies	Level 2 disfigurement with itch/pain and mild motor impairment	(combined DW)	0.196 (0.132-0.275)
Disfigurement level 2 with pain and moderate motor impairment due to other congenital musculoskeletal anomalies	Level 2 disfigurement with itch/pain and moderate motor impairment	(combined DW)	0.237 (0.163-0.324)
Incontinence due to congenital anomalies of the urinary tract	Urinary incontinence	cannot control urinating.	0.139 (0.094-0.198)

Impotence due to congenital genital anomalies	Impotence	has difficulty in obtaining or maintaining an erection.	0.017 (0.009-0.03)
Impotence due to congenital anomalies of the urinary tract	Impotence	has difficulty in obtaining or maintaining an erection.	0.017 (0.009-0.03)
Primary infertility due to congenital genital anomalies	Infertility, primary	wants to have a child and has a fertile partner, but the couple cannot conceive.	0.008 (0.003-0.015)
Recurrent urinary tract infections or other abdominal issues due to congenital genital anomalies	Abdominopelvic problem, mild	has some pain in the belly that causes nausea but does not interfere with daily activities.	0.011 (0.005-0.021)
Recurrent urinary tract infections or other abdominal issues due to congenital anomalies of the urinary tract	Abdominopelvic problem, mild	has some pain in the belly that causes nausea but does not interfere with daily activities.	0.011 (0.005-0.021)
Atypical genitalia due to congenital genital anomalies	Disfigurement, level 1	has a slight, visible physical deformity that others notice, which causes some worry and discomfort.	0.011 (0.005-0.021)
Atypical genitalia due to congenital anomalies of the urinary tract	Disfigurement, level 1	has a slight, visible physical deformity that others notice, which causes some worry and discomfort.	0.011 (0.005-0.021)
Asymptomatic congenital genital anomalies	Asymptomatic		0 (0-0)
Asymptomatic congenital anomalies of the urinary tract	Asymptomatic		0 (0-0)
Atypical genitalia and primary infertility due to congenital genital anomalies	Disfigurement level 1 and primary infertility	(combined DW)	0.018 (0.009-0.035)
Primary infertility and recurrent urinary tract infections or other abdominal issues due to congenital genital anomalies	Mild abdominal pain and primary infertility	(combined DW)	0.018 (0.009-0.036)
Atypical genitalia, infertility, impotence, and recurrent urinary tract infections or other abdominal issues and impotence due to congenital genital anomalies	Mild abdominopelvic problem, primary infertility, impotence, and level 1 disfigurement	(combined DW)	0.046 (0.023-0.083)
Infertility, impotence, and recurrent urinary tract infections or other abdominal issues and impotence due to congenital genital anomalies	Mild abdominopelvic problem, primary infertility, and impotence	(combined DW)	0.035 (0.018-0.064)
Impotence and recurrent urinary tract infections or other abdominal issues due to congenital genital anomalies	Mild abdominopelvic problem and impotence	(combined DW)	0.028 (0.014-0.05)
Impotence and recurrent urinary tract infections or other abdominal issues due to congenital anomalies of the urinary tract	Mild abdominopelvic problem and impotence	(combined DW)	0.028 (0.014-0.05)
Atypical genitalia, recurrent urinary tract infections or other abdominal issues and infertility due to congenital genital anomalies	Mild abdominopelvic problem, level 1 disfigurement and primary infertility	(combined DW)	0.029 (0.014-0.055)
Atypical genitalia, infertility and impotence due to congenital genital anomalies	Mild abdominopelvic problem, level 1 disfigurement and primary infertility	(combined DW)	0.029 (0.014-0.055)
Atypical genitalia and impotence due to congenital genital anomalies	Level 1 disfigurement and impotence	(combined DW)	0.028 (0.014-0.05)
Atypical genitalia and impotence due to congenital anomalies of the urinary tract	Level 1 disfigurement and impotence	(combined DW)	0.028 (0.014-0.05)
Atypical genitalia, recurrent urinary tract infections or other abdominal issues and impotence due to congenital genital anomalies	Mild abdominopelvic problem, level 1 disfigurement and impotence	(combined DW)	0.039 (0.02-0.07)
Atypical genitalia, recurrent urinary tract infections or other abdominal issues and impotence due to congenital anomalies of the urinary tract	Mild abdominopelvic problem, level 1 disfigurement and impotence	(combined DW)	0.039 (0.02-0.07)
Infertility and impotence due to congenital genital anomalies	Primary infertility and impotence	(combined DW)	0.025 (0.012-0.045)
Atypical genital and recurrent urinary tract infections and other abdominal issues due to congenital genital anomalies	Mild abdominopelvic problem and level 1 disfigurement	(combined DW)	0.022 (0.011-0.041)
Atypical genital and recurrent urinary tract infections and other abdominal issues due to congenital anomalies of the urinary tract	Mild abdominopelvic problem and level 1 disfigurement	(combined DW)	0.022 (0.011-0.041)
Atypical genitalia and incontinence due to congenital anomalies of the urinary tract	Mild abdominopelvic problem and level 1 disfigurement	(combined DW)	0.022 (0.011-0.041)
Incontinence and recurrent urinary tract infections or other abdominal issues due to congenital anomalies of the urinary tract	Level 1 disfigurement and urinary incontinence	(combined DW)	0.149 (0.101-0.206)
Incontinence and impotence due to congenital anomalies of the urinary tract	urinary incontinence and impotence	(combined DW)	0.154 (0.105-0.214)
Atypical genitalia, recurrent urinary tract infections or other abdominal issues and incontinence due to congenital anomalies of the urinary tract	Mild abdominopelvic problem, urinary incontinence, and level 1 disfigurement	(combined DW)	0.158 (0.108-0.218)
Atypical genitalia, incontinence and impotence due to congenital anomalies of the urinary tract	Mild abdominopelvic problem, urinary incontinence, and level 1 disfigurement	(combined DW)	0.158 (0.108-0.218)
Incontinence, impotence, and recurrent urinary tract infections or other abdominal issues and impotence due to congenital anomalies of the urinary tract	Mild abdominopelvic problem, urinary incontinence, and impotence	(combined DW)	0.163 (0.112-0.225)
Atypical genitalia, incontinence, impotence, and recurrent urinary tract infections or other abdominal issues and impotence due to congenital anomalies of the urinary tract	Mild abdominopelvic problem, urinary incontinence, impotence, and level 1 disfigurement	(combined DW)	0.172 (0.118-0.239)
Mild chronic respiratory problems and breathlessness due to congenital diaphragmatic hernia	COPD and other chronic respiratory problems, mild	has cough and shortness of breath after heavy physical activity, but is able to walk long distances and climb stairs.	0.019 (0.011-0.033)
Developmental delay or mild intellectual disability due to congenital diaphragmatic hernia	Intellectual disability / mental retardation, mild	has low intelligence and is slow in learning at school. As an adult, the person can live independently, but often needs help to raise children and can only work at simple supervised jobs.	0.043 (0.026-0.064)
Chronic abdominal pain due to congenital diaphragmatic hernia	Abdominopelvic problem, mild	has some pain in the belly that causes nausea but does not interfere with daily activities.	0.011 (0.005-0.021)
Disfigurement due to congenital diaphragmatic hernia	Disfigurement, level 1	has a slight, visible physical deformity that others notice, which causes some worry and discomfort.	0.011 (0.005-0.021)
Asymptomatic congenital diaphragmatic hernia	Asymptomatic		0 (0-0)
Chronic abdominal pain and disfigurement due to congenital diaphragmatic hernia	Level 1 disfigurement with itch/pain and mild abdominopelvic problem	(combined DW)	0.037 (0.02-0.062)
Chronic abdominal pain and mild chronic respiratory problems due to congenital diaphragmatic hernia	Mild abdominopelvic problem and mild COPD and other chronic respiratory problems	(combined DW)	0.03 (0.016-0.053)
Chronic abdominal pain and developmental delay due to congenital diaphragmatic hernia	Mild abdominopelvic problem and mild intellectual disability	(combined DW)	0.053 (0.032-0.083)
Disfigurement and mild chronic respiratory problems due to congenital diaphragmatic hernia	Level 1 disfigurement with itch/pain and mild COPD and other chronic respiratory problems	(combined DW)	0.045 (0.026-0.073)
Disfigurement and developmental delay due to congenital diaphragmatic hernia	Level 1 disfigurement with itch/pain and mild intellectual disability	(combined DW)	0.068 (0.041-0.102)
Mild chronic respiratory problems and developmental delay due to congenital diaphragmatic hernia	Mild intellectual disability and mild COPD and other chronic respiratory problem	(combined DW)	0.061 (0.037-0.093)
Chronic abdominal pain, disfigurement and chronic respiratory problems due to congenital diaphragmatic hernia	Mild abdominopelvic problem, mild COPD and other chronic respiratory problems, and level 1 disfigurement with itch/pain	(combined DW)	0.056 (0.031-0.092)
Chronic abdominal pain, chronic respiratory problems and developmental delay due to congenital diaphragmatic hernia	Mild abdominopelvic problem, mild COPD and other chronic respiratory problems, and level 1 disfigurement with itch/pain	(combined DW)	0.056 (0.031-0.092)
Chronic abdominal pain, disfigurement and developmental delay due to congenital diaphragmatic hernia	Mild abdominopelvic problem, mild intellectual disability, and level 1 disfigurement with itch/pain	(combined DW)	0.078 (0.046-0.12)
Disfigurement, chronic respiratory problems and developmental delay due to congenital diaphragmatic hernia	Mild COPD and other chronic respiratory problems, mild intellectual disability, and level 1 disfigurement with itch/pain	(combined DW)	0.086 (0.052-0.131)
Chronic abdominal pain, disfigurement, developmental delay and chronic respiratory problems due to congenital diaphragmatic hernia	Mild abdominopelvic problem, mild COPD and other chronic respiratory problems, mild intellectual disability, and level 1 disfigurement with itch/pain	(combined DW)	0.096 (0.057-0.148)
Chronic respiratory problems including difficulty breaking and recurrent upper respiratory infections due to atresia and/or stenosis of the digestive tract	COPD and other chronic respiratory problems, mild	has cough and shortness of breath after heavy physical activity, but is able to walk long distances and climb stairs.	0.019 (0.011-0.033)
Dysphagia or acid reflux due to congenital atresia and/or stenosis of the digestive tract	Abdominopelvic problem, mild	has some pain in the belly that causes nausea but does not interfere with daily activities.	0.011 (0.005-0.021)
Chronic abdominal pain due to congenital atresia and/or stenosis of the digestive tract	Abdominopelvic problem, moderate	has pain in the belly and feels nauseous. The person has difficulties with daily activities.	0.114 (0.078-0.159)
Asymptomatic congenital atresia and/or stenosis of the digestive tract	Asymptomatic		0 (0-0)
Chronic respiratory problems and dysphagia or acid reflux due to congenital atresia and/or stenosis of the digestive tract	Mild abdominopelvic problem and mild COPD and other chronic respiratory problems	(combined DW)	0.03 (0.016-0.053)
Chronic respiratory problems and abdominal pain due to congenital atresia and/or stenosis of the digestive tract	Mild abdominopelvic problem and mild COPD and other chronic respiratory problems	(combined DW)	0.03 (0.016-0.053)
Dysphagia or acid reflux, chronic abdominal pain and chronic respiratory problems due to congenital atresia and/or stenosis of the digestive tract	Mild abdominopelvic problem, moderate abdominopelvic problem, and mild COPD and other chronic respiratory problems	(combined DW)	0.141 (0.096-0.198)
Dysphagia or acid reflux and chronic abdominal pain due to congenital atresia and/or stenosis of the digestive tract	Mild abdominopelvic problem and moderate abdominopelvic problem	(combined DW)	0.124 (0.085-0.171)
Chronic abdominal pain due to congenital malformations of the abdominal wall	Abdominopelvic problem, mild	has some pain in the belly that causes nausea but does not interfere with daily activities.	0.011 (0.005-0.021)
Constipation due to congenital malformations of the abdominal wall	Abdominopelvic problem, moderate	has pain in the belly and feels nauseous. The person has difficulties with daily activities.	0.114 (0.078-0.159)
Disfigurement from scars following treatment for congenital malformations of the abdominal wall	Disfigurement, level 1	has a slight, visible physical deformity that others notice, which causes some worry and discomfort.	0.011 (0.005-0.021)
Asymptomatic congenital malformations of the abdominal wall after treatment	Asymptomatic		0 (0-0)
Chronic abdominal pain and concern about scars due to congenital malformations of the abdominal wall	Moderate abdominopelvic problem and level 1 disfigurement	(combined DW)	0.124 (0.085-0.172)
Constipation and concern about scars due to congenital malformations of the abdominal wall	Mild abdominopelvic problem and level 1 disfigurement	(combined DW)	0.022 (0.011-0.041)

Constipation, chronic abdominal pain and concern about scars due to congenital malformations of the abdominal wall	Mild abdominopelvic problem, moderate abdominopelvic problem, and level 1 disfigurement	(combined DW)	0.206 (0.143-0.283)
Constipation and chronic abdominal pain due to congenital malformations of the abdominal wall	Mild abdominopelvic problem and moderate abdominopelvic problem	(combined DW)	0.124 (0.085-0.171)
Acid reflux, dyspahgia, and/or constipation due to other congenital malformations of the digestive tract	Abdominopelvic problem, mild	has some pain in the belly that causes nausea but does not interfere with daily activities.	0.011 (0.005-0.021)
Chronic abdominal pain and/or nausea due to other congenital malformations of the digestive tract	Abdominopelvic problem, moderate	has pain in the belly and feels nauseous. The person has difficulties with daily activities.	0.114 (0.078-0.159)
Asymptomatic other congenital malformations of the digestive tract	Asymptomatic		0 (0-0)
Chronic abdominal pain and/or nausea with acid reflux, dyspahgia, and/or constipation due to other congenital malformations of the digestive tract	Mild abdominopelvic problem and moderate abdominopelvic problem	(combined DW)	0.124 (0.085-0.171)
Other congenital birth defects	Post-COMO calculation for residuals (YLL/YLD ratio, other methods)		--
Mild hearing loss due to other congenital anomalies	Hearing loss, mild	has great difficulty hearing and understanding another person talking in a noisy place (for example, on an urban street).	0.01 (0.004-0.019)
Moderate hearing loss due to other congenital anomalies	Hearing loss, moderate	is unable to hear and understand another person talking in a noisy place (for example, on an urban street), and has difficulty hearing another person talking even in a quiet place or on the phone.	0.027 (0.015-0.042)
Severe hearing loss due to other congenital anomalies	Hearing loss, severe	is unable to hear and understand another person talking, even in a quiet place, and unable to take part in a phone conversation. Difficulties with communicating and relating to others cause emotional impact at times (for example worry or depression).	0.158 (0.105-0.227)
Profound hearing loss due to other congenital anomalies	Hearing loss, profound	is unable to hear and understand another person talking, even in a quiet place, is unable to take part in a phone conversation, and has great difficulty hearing anything in any other situation. Difficulties with communicating and relating to othersoften cause worry, depression, and loneliness.	0.204 (0.134-0.288)
Complete hearing loss due to other congenital anomalies	Hearing loss, complete	cannot hear at all in any situation, including even the loudest sounds, and cannot communicate verbally or use a phone. Difficulties with communicating and relating to others often cause worry, depression or loneliness.	0.215 (0.144-0.307)
Mild hearing loss with ringing due to other congenital anomalies	Hearing loss, mild, with ringing	has great difficulty hearing and understanding another person talking in a noisy place (for example, on an urban street), and sometimes has annoying ringing in the ears.	0.021 (0.012-0.036)
Moderate hearing loss with ringing due to other congenital anomalies	Hearing loss, moderate, with ringing	is unable to hear and understand another person talking in a noisy place (for example, on an urban street), and has difficulty hearing another person talking even in a quiet place or on the phone, and has annoying ringing in the ears for more than 5 minutes at a time, almost everyday.	0.074 (0.049-0.107)
Severe hearing loss with ringing due to other congenital anomalies	Hearing loss, severe, with ringing	is unable to hear and understand another person talking, even in a quiet place, and unable to take part in a phone conversation, and has annoying ringing in the ears for more than 5 minutes at a time, almost everyday. Difficulties with communicating and relating to others cause emotional impact at times (for example worry or depression).	0.261 (0.175-0.36)
Profound hearing loss with ringing due to other congenital anomalies	Hearing loss, profound, with ringing	is unable to hear and understand another person talking, even in a quiet place, is unable to take part in a phone conversation, has great difficulty hearing anything in any other situation, and has annoying ringing in the ears for more than 5 minutes at a time, several times a day. Difficulties with communicating and relating to others often cause worry, depression, or loneliness.	0.277 (0.182-0.387)
Complete hearing loss with ringing due to other congenital anomalies	Hearing loss, complete, with ringing	cannot hear at all in any situation, including even the loudest sounds, and cannot communicate verbally or use a phone, and has very annoying ringing in the ears for more than half of the day. Difficulties with communicating and relating to others often cause worry, depression or loneliness.	0.316 (0.212-0.435)
Moderately severe hearing loss due to other congenital anomalies	Hearing loss, moderately severe	(custom DW from hearing loss impairment envelope)	0.092 (0.064-0.129)
Moderately severe hearing loss with ringing due to other congenital anomalies	Hearing loss, moderately severe, with ringing	(custom DW from hearing loss impairment envelope)	0.167 (0.115-0.231)
Mild urinary tract infections	Infectious disease, acute episode, mild	has a low fever and mild discomfort , but no difficulty with daily activities.	0.006 (0.002-0.012)
Moderate urinary tract infections	Infectious disease, acute episode, moderate	has a fever and aches, and feels weak, which causes some difficulty with daily activities.	0.051 (0.032-0.074)
Mild urolithiasis episodes	Abdominopelvic problem, mild	has some pain in the belly that causes nausea but does not interfere with daily activities.	0.011 (0.005-0.021)
Moderate urolithiasis episodes	Abdominopelvic problem, moderate	has pain in the belly and feels nauseous. The person has difficulties with daily activities.	0.114 (0.078-0.159)
Severe urolithiasis episodes	Abdominopelvic problem, severe	has severe pain in the belly and feels nauseous. The person is anxious and unable to carry out daily activities.	0.324 (0.22-0.442)
Symptomatic benign prostatic hyperplasia	Benign prostatic hypertrophy, symptomatic cases	feels the urge to urinate frequently, but when passing urine it comes out slowly and sometimes is painful.	0.067 (0.043-0.097)
Asymptomatic benign prostatic hyperplasia	Asymptomatic		0 (0-0)
Idiopathic primary male infertility	Infertility, primary	wants to have a child and has a fertile partner, but the couple cannot conceive.	0.008 (0.003-0.015)
Idiopathic secondary male infertility	Infertility, secondary	has at least one child, and wants to have more children. The person has a fertile partner, but the couple cannot conceive.	0.005 (0.002-0.011)
Other urinary diseases	Post-COMO calculation for residuals (YLL/YLD ratio, other methods)		--
Mild abdominal pain due to uterine fibroids, without anemia	Abdominopelvic problem, mild	has some pain in the belly that causes nausea but does not interfere with daily activities.	0.011 (0.005-0.021)
Asymptomatic uterine fibroids	Asymptomatic		0 (0-0)
Mild abdominal pain due to uterine fibroids, with mild anemia	Mild abdominal pain with mild anemia	(combined DW)	0.015 (0.007-0.029)
Mild abdominal pain due to uterine fibroids, with moderate anemia	Mild abdominal pain with moderate anemia	(combined DW)	0.062 (0.04-0.093)
Mild abdominal pain due to uterine fibroids, with severe anemia	Mild abdominal pain with severe anemia	(combined DW)	0.158 (0.109-0.219)
Primary infertility due to polycystic ovarian syndrome	Infertility, primary	wants to have a child and has a fertile partner, but the couple cannot conceive.	0.008 (0.003-0.015)
Hirsutism and secondary infertility due to polycystic ovarian syndrome	Infertility, secondary	has at least one child, and wants to have more children. The person has a fertile partner, but the couple cannot conceive.	0.005 (0.002-0.011)
Hirsutism due to polycystic ovarian syndrome	Disfigurement, level 1	has a slight, visible physical deformity that others notice, which causes some worry and discomfort.	0.011 (0.005-0.021)
Asymptomatic polycystic ovarian syndrome	Asymptomatic		0 (0-0)
Hirsutism and primary infertility due to polycystic ovarian syndrome	Disfigurement level 1 and primary infertility	(combined DW)	0.018 (0.009-0.035)
Secondary infertility due to polycystic ovarian syndrome	Disfigurement level 1 and secondary infertility	(combined DW)	0.016 (0.007-0.031)
Idiopathic primary female infertility	Infertility, primary	wants to have a child and has a fertile partner, but the couple cannot conceive.	0.008 (0.003-0.015)
Idiopathic secondary female infertility	Infertility, secondary	has at least one child, and wants to have more children. The person has a fertile partner, but the couple cannot conceive.	0.005 (0.002-0.011)
Primary infertility due to endometriosis	Infertility, primary	wants to have a child and has a fertile partner, but the couple cannot conceive.	0.008 (0.003-0.015)
Secondary infertility due to endometriosis	Infertility, secondary	has at least one child, and wants to have more children. The person has a fertile partner, but the couple cannot conceive.	0.005 (0.002-0.011)
Mild abdominal pain due to endometriosis	Abdominopelvic problem, mild	has some pain in the belly that causes nausea but does not interfere with daily activities.	0.011 (0.005-0.021)
Moderate abdominal pain due to endometriosis	Abdominopelvic problem, moderate	has pain in the belly and feels nauseous. The person has difficulties with daily activities.	0.114 (0.078-0.159)
Severe abdominal pain due to endometriosis	Abdominopelvic problem, severe	has severe pain in the belly and feels nauseous. The person is anxious and unable to carry out daily activities.	0.324 (0.22-0.442)
Asymptomatic endometriosis	Asymptomatic		0 (0-0)
Mild abdominal pain and primary infertility due to endometriosis	Mild abdominal pain and primary infertility	(combined DW)	0.018 (0.009-0.036)
Moderate abdominal pain and primary infertility due to endometriosis	Moderate abdominal pain and primary infertility	(combined DW)	0.121 (0.083-0.168)
Severe abdominal pain and primary infertility due to endometriosis	Severe abdominal pain and primary infertility	(combined DW)	0.329 (0.227-0.445)
Mild abdominal pain and secondary infertility due to endometriosis	Mild abdominal pain and secondary infertility	(combined DW)	0.016 (0.007-0.031)
Moderate abdominal pain and secondary infertility due to endometriosis	Moderate abdominal pain and secondary infertility	(combined DW)	0.119 (0.081-0.164)

Severe abdominal pain and secondary infertility due to endometriosis	Severe abdominal pain and secondary infertility	(combined DW)	0.328 (0.225-0.444)
Abdominal pain due to genital prolapse	Abdominopelvic problem, mild	has some pain in the belly that causes nausea but does not interfere with daily activities.	0.011 (0.005-0.021)
Stress incontinence due to genital prolapse	Stress incontinence	loses small amounts of urine without meaning to when coughing, sneezing, laughing or during physical exercise.	0.02 (0.011-0.035)
Asymptomatic genital prolapse	Asymptomatic		0 (0-0)
Abdominal pain and stress incontinence due to genital prolapse	Mild abdominal pain and stress incontinence	(combined DW)	0.031 (0.016-0.054)
Depression due to premenstrual syndrome	Major depressive disorder, mild episode	feels persistent sadness and has lost interest in usual activities. The person sometimes sleeps badly, feels tired, or has trouble concentrating but still manages to function in daily life with extra effort.	0.145 (0.099-0.209)
Abdominal pain due to premenstrual syndrome	Abdominopelvic problem, mild	has some pain in the belly that causes nausea but does not interfere with daily activities.	0.011 (0.005-0.021)
Asymptomatic premenstrual syndrome	Asymptomatic		0 (0-0)
Abdominal pain and depression due to premenstrual syndrome	Mild abdominal pain and mild depression	(combined DW)	0.155 (0.107-0.22)
Mild other gynecological disorders	Abdominopelvic problem, mild	has some pain in the belly that causes nausea but does not interfere with daily activities.	0.011 (0.005-0.021)
Menstrual disorders without anemia	Asymptomatic		0 (0-0)
Moderate other gynecological disorders	Abdominopelvic problem, moderate	has pain in the belly and feels nauseous. The person has difficulties with daily activities.	0.114 (0.078-0.159)
Severe other gynecological disorders	Abdominopelvic problem, severe	has severe pain in the belly and feels nauseous. The person is anxious and unable to carry out daily activities.	0.324 (0.22-0.442)
Asymptomatic other gynecological disorders	Asymptomatic		0 (0-0)
Mild anemia due to menstrual disorders	Anemia, mild	feels slightly tired and weak at times, but this does not interfere with normal daily activities.	0.004 (0.001-0.008)
Moderate anemia due to menstrual disorders	Anemia, moderate	feels moderate fatigue, weakness, and shortness of breath after exercise, making daily activities more difficult.	0.052 (0.034-0.076)
Severe anemia due to menstrual disorders	Anemia, severe	feels very weak, tired and short of breath, and has problems with activities that require physical effort or deep concentration.	0.149 (0.101-0.209)
Mild heart failure due to thalassemias	Heart failure, mild	is short of breath and easily tires with moderate physical activity, such as walking uphill or more than a quarter-mile on level ground. The person feels comfortable at rest or during activities requiring less effort.	0.041 (0.026-0.062)
Moderate heart failure due to thalassemias	Heart failure, moderate	is short of breath and easily tires with minimal physical activity, such as walking only a short distance. The person feels comfortable at rest but avoids moderate activity.	0.072 (0.047-0.103)
Severe heart failure due to thalassemias	Heart failure, severe	is short of breath and feels tired when at rest. The person avoids any physical activity, for fear of worsening the breathing problems.	0.179 (0.122-0.251)
Controlled, medically managed heart failure due to thalassemias	Generic uncomplicated disease: worry and daily medication	has a chronic disease that requires medication every day and causes some worry but minimal interference with daily activities.	0.049 (0.031-0.072)
Hemoglobin H disease, without anemia	Generic uncomplicated disease: anxiety about diagnosis	has a disease diagnosis that causes some worry but minimal interference with daily activities.	0.012 (0.006-0.023)
Beta-thalassemia major, without anemia	Generic uncomplicated disease: anxiety about diagnosis	has a disease diagnosis that causes some worry but minimal interference with daily activities.	0.012 (0.006-0.023)
Hemoglobin E/Beta-thalassemia, without anemia	Generic uncomplicated disease: anxiety about diagnosis	has a disease diagnosis that causes some worry but minimal interference with daily activities.	0.012 (0.006-0.023)
Beta-thalassemia major, severe infection with severe anemia	Infectious disease, acute episode, severe; Generic uncomplicated disease anxiety; Anemia, severe		0.271 (0.19-0.37)
Hemoglobin E/beta-thalassemia, severe infection with severe anemia	Infectious disease, acute episode, severe; Generic uncomplicated disease anxiety; Anemia, severe		0.271 (0.19-0.37)
Hemoglobin H disease, severe infection with severe anemia	Infectious disease, acute episode, severe; Generic uncomplicated disease anxiety; Anemia, severe		0.271 (0.19-0.37)
Beta-thalassemia major, with mild anemia	Anemia, mild; Generic uncomplicated disease anxiety		0.016 (0.008-0.031)
Hemoglobin E/beta-thalassemia, with mild anemia	Anemia, mild; Generic uncomplicated disease anxiety		0.016 (0.008-0.031)
Hemoglobin H disease, with mild anemia	Anemia, mild; Generic uncomplicated disease anxiety		0.016 (0.008-0.031)
Beta-thalassemia major, with moderate anemia	Anemia, moderate; Generic uncomplicated disease anxiety		0.063 (0.04-0.095)
Hemoglobin E/beta-thalassemia, with moderate anemia	Anemia, moderate; Generic uncomplicated disease anxiety		0.063 (0.04-0.095)
Hemoglobin H disease, with moderate anemia	Anemia, moderate; Generic uncomplicated disease anxiety		0.063 (0.04-0.095)
Beta-thalassemia major, with severe anemia	Anemia, severe; Generic uncomplicated disease anxiety		0.159 (0.109-0.22)
Hemoglobin E/beta-thalassemia, with severe anemia	Anemia, severe; Generic uncomplicated disease anxiety		0.159 (0.109-0.22)
Hemoglobin H disease, with severe anemia	Anemia, severe; Generic uncomplicated disease anxiety		0.159 (0.109-0.22)
Mild anemia due to B-thalassemia trait	Anemia, mild	feels slightly tired and weak at times, but this does not interfere with normal daily activities.	0.004 (0.001-0.008)
Mild anemia due to hemoglobin E trait	Anemia, mild	feels slightly tired and weak at times, but this does not interfere with normal daily activities.	0.004 (0.001-0.008)
Moderate anemia due to B-thalassemia trait	Anemia, moderate	feels moderate fatigue, weakness, and shortness of breath after exercise, making daily activities more difficult.	0.052 (0.034-0.076)
Moderate anemia due to hemoglobin E trait	Anemia, moderate	feels moderate fatigue, weakness, and shortness of breath after exercise, making daily activities more difficult.	0.052 (0.034-0.076)
Severe anemia due to B-thalassemia trait	Anemia, severe	feels very weak, tired and short of breath, and has problems with activities that require physical effort or deep concentration.	0.149 (0.101-0.209)
Severe anemia due to hemoglobin E trait	Anemia, severe	feels very weak, tired and short of breath, and has problems with activities that require physical effort or deep concentration.	0.149 (0.101-0.209)
Asymptomatic B-thalassemia trait	Asymptomatic		0 (0-0)
Asymptomatic hemoglobin E trait	Asymptomatic		0 (0-0)
Homozygous sickle cell and severe sickle cell/beta-thalassemia, without anemia	Generic uncomplicated disease: anxiety about diagnosis	has a disease diagnosis that causes some worry but minimal interference with daily activities.	0.012 (0.006-0.023)
Other combined sequelae of homozygous sickle cell and severe sickle cell/beta-thalassemia	Generic uncomplicated disease: anxiety about diagnosis	has a disease diagnosis that causes some worry but minimal interference with daily activities.	0.012 (0.006-0.023)
Hemoglobin SC disease, without anemia	Generic uncomplicated disease: anxiety about diagnosis	has a disease diagnosis that causes some worry but minimal interference with daily activities.	0.012 (0.006-0.023)
Other combined sequelae of hemoglobin SC disease	Generic uncomplicated disease: anxiety about diagnosis	has a disease diagnosis that causes some worry but minimal interference with daily activities.	0.012 (0.006-0.023)
Mild sickle cell/beta-thalassemia, without anemia	Generic uncomplicated disease: anxiety about diagnosis	has a disease diagnosis that causes some worry but minimal interference with daily activities.	0.012 (0.006-0.023)
Other combined sequelae of mild sickle cell/beta-thalassemia exclusivity adjustment	Generic uncomplicated disease: anxiety about diagnosis	has a disease diagnosis that causes some worry but minimal interference with daily activities.	0.012 (0.006-0.023)
Homozygous sickle cell and severe sickle cell/beta-thalassemia, with vaso-occlusive crisis, without anemia	Generic uncomplicated disease anxiety and severe abdominopelvic problem		0.333 (0.231-0.448)
Hemoglobin SC disease, with vaso-occlusive crisis, without anemia	Generic uncomplicated disease anxiety and severe abdominopelvic problem		0.333 (0.231-0.448)
Mild sickle cell/beta-thalassemia, with vaso-occlusive crisis, without anemia	Generic uncomplicated disease anxiety and severe abdominopelvic problem		0.333 (0.231-0.448)
Homozygous sickle cell and severe sickle cell/beta-thalassemia, with stroke, without anemia	Generic uncomplicated disease anxiety; long-term consequences due to stroke		0.325 (0.219-0.443)
Hemoglobin SC disease, with stroke, without anemia	Generic uncomplicated disease anxiety; long-term consequences due to stroke		0.325 (0.219-0.443)
Mild sickle cell/beta-thalassemia, with stroke, without anemia	Generic uncomplicated disease anxiety; long-term consequences due to stroke		0.325 (0.219-0.443)
Homozygous sickle cell and severe sickle cell/beta-thalassemia, with vaso-occlusive crisis and stroke, without anemia	Generic uncomplicated disease anxiety; long-term consequences due to stroke; severe abdominopelvic problem		0.541 (0.39-0.685)
Hemoglobin SC disease, with vaso-occlusive crisis and stroke, without anemia	Generic uncomplicated disease anxiety; long-term consequences due to stroke; severe abdominopelvic problem		0.541 (0.39-0.685)
Mild sickle cell/beta-thalassemia, with vaso-occlusive crisis and stroke, without anemia	Generic uncomplicated disease anxiety; long-term consequences due to stroke; severe abdominopelvic problem		0.541 (0.39-0.685)
Homozygous sickle cell and severe sickle cell/beta-thalassemia, with vaso-occlusive crisis and severe anemia	Moderate abdominal pain; Anemia, severe; Generic uncomplicated disease anxiety		0.431 (0.309-0.562)

Hemoglobin SC disease, with vaso-occlusive crisis and severe anemia	Moderate abdominal pain; Anemia, severe; Generic uncomplicated disease anxiety		0.431 (0.309-0.562)
Mild sickle cell/beta-thalassemia, with vaso-occlusive crisis and severe anemia	Moderate abdominal pain; Anemia, severe; Generic uncomplicated disease anxiety		0.431 (0.309-0.562)
Homozygous sickle cell and severe sickle cell/beta-thalassemia, with stroke and severe anemia	Generic uncomplicated disease anxiety; Long-term consequences due to stroke; Anemia, severe		0.424 (0.302-0.554)
Hemoglobin SC disease, with stroke and severe anemia	Generic uncomplicated disease anxiety; Long-term consequences due to stroke; Anemia, severe		0.424 (0.302-0.554)
Mild sickle cell/beta-thalassemia, with stroke and severe anemia	Generic uncomplicated disease anxiety; Long-term consequences due to stroke; Anemia, severe		0.424 (0.302-0.554)
Homozygous sickle cell and severe sickle cell/beta-thalassemia, with vaso-occlusive crisis, stroke, and severe anemia	Generic uncomplicated disease anxiety; long-term consequences due to stroke; severe abdominopelvic problem; Anemia, severe		0.607 (0.454-0.747)
Hemoglobin SC disease, with vaso-occlusive crisis, stroke, and severe anemia	Generic uncomplicated disease anxiety; long-term consequences due to stroke; severe abdominopelvic problem; Anemia, severe		0.607 (0.454-0.747)
Mild sickle cell/beta-thalassemia, with vaso-occlusive crisis, stroke, and severe anemia	Generic uncomplicated disease anxiety; long-term consequences due to stroke; severe abdominopelvic problem; Anemia, severe		0.607 (0.454-0.747)
Homozygous sickle cell and severe sickle cell/beta-thalassemia, with mild anemia	Anemia, mild; Generic uncomplicated disease anxiety		0.016 (0.008-0.031)
Hemoglobin SC disease, with mild anemia	Anemia, mild; Generic uncomplicated disease anxiety		0.016 (0.008-0.031)
Mild sickle cell/beta-thalassemia, with mild anemia	Anemia, mild; Generic uncomplicated disease anxiety		0.016 (0.008-0.031)
Homozygous sickle cell and severe sickle cell/beta-thalassemia, with moderate anemia	Anemia, moderate; Generic uncomplicated disease anxiety		0.063 (0.04-0.095)
Hemoglobin SC disease, with moderate anemia	Anemia, moderate; Generic uncomplicated disease anxiety		0.063 (0.04-0.095)
Mild sickle cell/beta-thalassemia, with moderate anemia	Anemia, moderate; Generic uncomplicated disease anxiety		0.063 (0.04-0.095)
Homozygous sickle cell and severe sickle cell/beta-thalassemia, with severe anemia	Anemia, severe; Generic uncomplicated disease anxiety		0.159 (0.109-0.22)
Hemoglobin SC disease, with severe anemia	Anemia, severe; Generic uncomplicated disease anxiety		0.159 (0.109-0.22)
Mild sickle cell/beta-thalassemia, with severe anemia	Anemia, severe; Generic uncomplicated disease anxiety		0.159 (0.109-0.22)
Mild anemia due to sickle cell trait	Anemia, mild	feels slightly tired and weak at times, but this does not interfere with normal daily activities.	0.004 (0.001-0.008)
Moderate anemia due to sickle cell trait	Anemia, moderate	feels moderate fatigue, weakness, and shortness of breath after exercise, making daily activities more difficult.	0.052 (0.034-0.076)
Severe anemia due to sickle cell trait	Anemia, severe	feels very weak, tired and short of breath, and has problems with activities that require physical effort or deep concentration.	0.149 (0.101-0.209)
Asymptomatic sickle cell trait	Asymptomatic		0 (0-0)
Mild heart failure due to G6PD deficiency	Heart failure, mild	is short of breath and easily tires with moderate physical activity, such as walking uphill or more than a quarter-mile on level ground. The person feels comfortable at rest or during activities requiring less effort.	0.041 (0.026-0.062)
Moderate heart failure due to G6PD deficiency	Heart failure, moderate	is short of breath and easily tires with minimal physical activity, such as walking only a short distance. The person feels comfortable at rest but avoids moderate activity.	0.072 (0.047-0.103)
Severe heart failure due to G6PD deficiency	Heart failure, severe	is short of breath and feels tired when at rest. The person avoids any physical activity, for fear of worsening the breathing problems.	0.179 (0.122-0.251)
Mild anemia due to G6PD deficiency	Anemia, mild	feels slightly tired and weak at times, but this does not interfere with normal daily activities.	0.004 (0.001-0.008)
Moderate anemia due to G6PD deficiency	Anemia, moderate	feels moderate fatigue, weakness, and shortness of breath after exercise, making daily activities more difficult.	0.052 (0.034-0.076)
Severe anemia due to G6PD deficiency	Anemia, severe	feels very weak, tired and short of breath, and has problems with activities that require physical effort or deep concentration.	0.149 (0.101-0.209)
Controlled, medically managed heart failure due to G6PD deficiency	Generic uncomplicated disease: worry and daily medication	has a chronic disease that requires medication every day and causes some worry but minimal interference with daily activities.	0.049 (0.031-0.072)
Asymptomatic G6PD deficiency	Asymptomatic		0 (0-0)
Mild anemia due to hemizygous G6PD trait	Anemia, mild	feels slightly tired and weak at times, but this does not interfere with normal daily activities.	0.004 (0.001-0.008)
Moderate anemia due to hemizygous G6PD trait	Anemia, moderate	feels moderate fatigue, weakness, and shortness of breath after exercise, making daily activities more difficult.	0.052 (0.034-0.076)
Severe anemia due to hemizygous G6PD trait	Anemia, severe	feels very weak, tired and short of breath, and has problems with activities that require physical effort or deep concentration.	0.149 (0.101-0.209)
Asymptomatic hemizygous G6PD trait	Asymptomatic		0 (0-0)
Mild heart failure due to other hemoglobinopathies and hemolytic anemias	Heart failure, mild	is short of breath and easily tires with moderate physical activity, such as walking uphill or more than a quarter-mile on level ground. The person feels comfortable at rest or during activities requiring less effort.	0.041 (0.026-0.062)
Moderate heart failure due to other hemoglobinopathies and hemolytic anemias	Heart failure, moderate	is short of breath and easily tires with minimal physical activity, such as walking only a short distance. The person feels comfortable at rest but avoids moderate activity.	0.072 (0.047-0.103)
Severe heart failure due to other hemoglobinopathies and hemolytic anemias	Heart failure, severe	is short of breath and feels tired when at rest. The person avoids any physical activity, for fear of worsening the breathing problems.	0.179 (0.122-0.251)
Mild anemia due to other hemoglobinopathies and hemolytic anemias	Anemia, mild	feels slightly tired and weak at times, but this does not interfere with normal daily activities.	0.004 (0.001-0.008)
Moderate anemia due to other hemoglobinopathies and hemolytic anemias	Anemia, moderate	feels moderate fatigue, weakness, and shortness of breath after exercise, making daily activities more difficult.	0.052 (0.034-0.076)
Severe anemia due to other hemoglobinopathies and hemolytic anemias	Anemia, severe	feels very weak, tired and short of breath, and has problems with activities that require physical effort or deep concentration.	0.149 (0.101-0.209)
Controlled, medically managed heart failure due to other hemoglobinopathies and hemolytic anemias	Generic uncomplicated disease: worry and daily medication	has a chronic disease that requires medication every day and causes some worry but minimal interference with daily activities.	0.049 (0.031-0.072)
Other hemoglobinopathies and hemolytic anemias residual	Post-COMO calculation for residuals (YLL/YLD ratio, other methods)		--
Mild anemia due to endocrine, metabolic, blood, and immune disorders	Anemia, mild	feels slightly tired and weak at times, but this does not interfere with normal daily activities.	0.004 (0.001-0.008)
Moderate anemia due to endocrine, metabolic, blood, and immune disorders	Anemia, moderate	feels moderate fatigue, weakness, and shortness of breath after exercise, making daily activities more difficult.	0.052 (0.034-0.076)
Severe anemia due to endocrine, metabolic, blood, and immune disorders	Anemia, severe	feels very weak, tired and short of breath, and has problems with activities that require physical effort or deep concentration.	0.149 (0.101-0.209)
Asymptomatic endocrine, metabolic, blood, and immune disorders	Asymptomatic		0 (0-0)
Severe endocrine, metabolic, blood, and immune disorders	Thrombocytopenic purpura	easily bruises and sometimes bleeds from the gums and nose; feels weak and has some difficulty with daily activities.	0.159 (0.106-0.226)
Mild endocrine, metabolic, blood, and immune disorders	Hypothyroidism	has low energy and feels cold.	0.019 (0.01-0.032)
Moderate endocrine, metabolic, blood, and immune disorders	Hyperthyroidism	feels nervous, has palpitations, sweats a lot and has difficulty sleeping.	0.145 (0.096-0.202)
Mild heart failure due to endocrine, metabolic, blood, and immune disorders	Heart failure, mild	is short of breath and easily tires with moderate physical activity, such as walking uphill or more than a quarter-mile on level ground. The person feels comfortable at rest or during activities requiring less effort.	0.041 (0.026-0.062)
Moderate heart failure due to endocrine, metabolic, blood, and immune disorders	Heart failure, moderate	is short of breath and easily tires with minimal physical activity, such as walking only a short distance. The person feels comfortable at rest but avoids moderate activity.	0.072 (0.047-0.103)
Severe heart failure due to endocrine, metabolic, blood, and immune disorders	Heart failure, severe	is short of breath and feels tired when at rest. The person avoids any physical activity, for fear of worsening the breathing problems.	0.179 (0.122-0.251)
Controlled, medically managed heart failure due to endocrine, metabolic, blood, and immune disorders	Generic uncomplicated disease: worry and daily medication	has a chronic disease that requires medication every day and causes some worry but minimal interference with daily activities.	0.049 (0.031-0.072)
Pain due to caries of deciduous teeth	Dental caries, symptomatic	has a toothache, which causes some difficulty in eating.	0.01 (0.005-0.019)
Asymptomatic caries of deciduous teeth	Asymptomatic		0 (0-0)
Pain due to caries of permanent teeth	Dental caries, symptomatic	has a toothache, which causes some difficulty in eating.	0.01 (0.005-0.019)
Asymptomatic caries of permanent teeth	Asymptomatic		0 (0-0)
Chronic periodontal diseases	Periodontitis	has minor bleeding of the gums from time to time, with mild discomfort.	0.007 (0.003-0.014)
Difficulty eating due to edentulism and severe tooth loss	Severe tooth loss	has lost more than 20 teeth including front and back, and has great difficulty in eating meat, fruits, and vegetables.	0.067 (0.045-0.095)
Asymptomatic edentulism and severe tooth loss	Asymptomatic		0 (0-0)

Mild other oral disorders	Infectious disease, acute episode, mild	has a low fever and mild discomfort , but no difficulty with daily activities.	0.006 (0.002-0.012)
Severe other oral disorders	Infectious disease, acute episode, moderate	has a fever and aches, and feels weak, which causes some difficulty with daily activities.	0.051 (0.032-0.074)

Table S14: GBD 2019 methods of estimating years lived with disability (YLDs) for 34 residual categories	
**Residual**	**Method**
	**Estimation: YLD to YLL ratio method**
Other intestinal infectious diseases	YLD to YLL ratio of explicitly estimated intestinal infectious diseases by geography, country, sex applied to YLL from other intestinal infectious diseases by geography, country, sex, and age. Causes used for estimation include: Typhoid fever, Paratyphoid fever.
Other neglected tropical diseases	YLD to YLL ratio of explicitly estimated neglected tropical disease causes by geography, country, sex applied to YLL from other neglected tropical diseases by geography, country, sex, and age. Causes used for estimation include: Chagas disease, Visceral leishmaniasis, Dengue, Yellow fever, Rabies.
Other maternal disorders	YLD to YLL ratio of explicitly estimated maternal disorder causes by geography, country, sex applied to YLL from other maternal disorders by geography, country, sex, and age. Causes used for estimation include: Maternal haemorrhage, Maternal sepsis and other maternal infections, Maternal hypertensive disorders, Maternal obstructed labor and uterine rupture, Maternal abortion, miscarriage, and ectopic pregnancy, Indirect maternal deaths, Late maternal deaths, Maternal deaths aggravated by HIV/AIDS.
Other neonatal disorders	YLD to YLL ratio of explicitly estimated neonatal disorders by geography, country, sex applied to YLL from other neonatal disorders by geography, country, sex, and age. Causes used for estimation include: Neonatal preterm birth complications, Neonatal encephalopathy due to birth asphyxia and trauma, Neonatal sepsis and other neonatal infections, Haemolytic disease and other neonatal jaundice.
Other nutritional deficiencies	YLD to YLL ratio of explicitly estimated nutritional deficiencies by geography, country, sex applied to YLL from other nutritional deficiencies by geography, country, sex, and age. Causes used for estimation include Protein-energy malnutrition.
Other sexually transmitted infections	YLD to YLL ratio of gonococcal and chlamydial infection by geography, country, sex applied to YLL from other sexually transmitted diseases by geography, country, sex, and age. Causes used for estimation include: Chlamydial infection, Gonococcal infection.
Other unspecified infectious diseases	YLD to YLL ratio of HIV/AIDS and tuberculosis, diarrhea, lower respiratory and other common infectious diseases, neglected tropical diseases and malaria, sexually transmitted diseases and hepatitis by geography, country, sex applied to YLL from other infectious diseases by geography, country, sex, and age. Causes used for estimation include: Typhoid fever, Paratyphoid fever, Lower respiratory infections, Upper respiratory infections, Varicella and herpes zoster, Malaria, Acute hepatitis A, Hepatitis B, Hepatitis C, Acute hepatitis E, Chlamydial infection, Gonococcal infection, Diphtheria, Whooping cough, Measles, Chagas disease, Visceral leishmaniasis, Dengue, Yellow fever, Maternal sepsis and other maternal infections, Pneumococcal meningitis, H influenzae type B meningitis, Meningococcal meningitis, Other meningitis, Encephalitis, Tetanus, Ascariasis, Neonatal sepsis and other neonatal infections.
Other chronic respiratory diseases	YLD to YLL ratio of COPD, pneumoconiosis and interstitial lung disease and pulmonary sarcoidosis by geography, country, sex applied to YLL from other chronic respiratory diseases by geography, country, sex, and age. Causes used for estimation include: Chronic obstructive pulmonary disease, Silicosis, Asbestosis, Coal workers pneumoconiosis, Other pneumoconiosis, Interstitial lung disease and pulmonary sarcoidosis.
Other digestive disorders	YLD to YLL ratio of explicitly estimated digestive disorders by geography, country, sex applied to YLL from other digestive disorders by geography, country, sex, and age. Causes used for estimation include: Gastritis and duodenitis, Appendicitis, Paralytic ileus and intestinal obstruction, Inguinal, femoral, and abdominal hernia, Inflammatory bowel disease, Vascular intestinal disorders, Gallbladder and biliary diseases, Pancreatitis.
Other neurological disorders	YLD to YLL ratio of Alzheimer and other dementias, Parkinson disease, multiple sclerosis and motor-neuron disease by geography, country, sex applied to YLL from other neurological disorders by geography, country, sex, and age. Causes used for estimation include: Alzheimer disease and other dementias, Parkinson disease, Epilepsy, Multiple sclerosis.
Other urinary diseases	YLD to YLL ratio of explicitly estimated urinary diseases by geography, country, sex applied to YLL from other urinary diseases by geography, country, sex, and age. Causes used for estimation include: Interstitial nephritis and urinary tract infections, Urolithiasis.
Other haemoglobinopathies and haemolytic anaemias	YLD to YLL ratio of explicitly estimated haemoglobinopathies and haemolytic anaemias by geography, country, sex applied to YLL from other haemoglobinopathies and haemolytic anaemias by geography, country, sex and age. Causes used for estimation include: Thalassemias, Sickle cell disorders, G6PD deficiency.
Other congenital anomalies	YLD to YLL ratio of explicitly estimated congenital anomalies by geography, country, sex applied to YLL from other congenital anomalies by geography, country, sex, and age. Causes used for estimation include: Neural tube defects, Congenital heart anomalies.

Table S14: GBD 2019 methods of estimating years lived with disability (YLDs) for 34 residual categories	
Residual	Method
	Estimation: based on epidemiological data
Pelvic inflammatory disease due to other causes	DisMod-MR 2.1 model of the proportion of pelvic inflammatory disease due to other causes, constrained to 100% with proportions of pelvic inflammatory disease due to gonococcal and chlamydial infection and applied to the DisMod-MR 2.1 model for all pelvic inflammatory disease.
Other malignant neoplasms	Similar to all other cancers: mortality to incidence ratio method applied to cancer registry data for other neoplasms.
Liver cancer due to other causes	Data on proportion of liver cancer due to other causes modelled in DisMod-MR 2.1, forced to sum to 100% at 1,000 draw level for each geography, year, age, and sex with the proportions for liver cancer due to hepatitis B, hepatitis C and alcohol, and applied to total liver cancer estimates from cancer analyses using mortality to incidence ratios.
Other cardiovascular diseases	Ratio of prevalence of ICD-9 coded other cardiovascular diseases in MEPS and 2005 USA outpatient data to prevalence of heart failure due to other cardiovascular diseases (estimated as part of the heart failure envelope), and applied to prevalence of heart failure due to other CVD estimates for all other locations and years.
Cirrhosis and other chronic liver diseases due to other causes	Data on proportion of cirrhosis and other chronic liver diseases due to other causes modelled in DisMod-MR 2.1, forced to sum to 100% at 1,000 draw level for each geography-year-age-sex with the proportions for cirrhosis and other chronic liver diseases due to hepatitis B, hepatitis C and alcohol, and applied to total cirrhosis and other chronic liver diseases estimates from DisMod-MR 2.1 analysis.
Other pneumoconiosis	DisMod-MR 2.1 model based on hospital admission and claims data.
Other drug use disorders	NESARC prevalence of drug dependence other than cannabis, opioids, amphetamines and cocaine multiplied by ratio of YLD to prevalence for cocaine and amphetamine by geography, year, age, and sex.
Other mental disorders	Other mental disorders: Prevalence of personality disorders not comorbid with GBD mental disorder categories and severity distribution from NESARC and the Australian National Survey of Mental Health and Wellbeing 1997.
Other drug use disorders	Other drug use disorders: Prevalence of drug use disorders not comorbid with GBD drug use disorder categories from NESARC and the Australian National Survey of Mental Health and Wellbeing 1997.
Chronic kidney disease due to other causes	Data on proportion of chronic kidney disease due to other causes from renal registries modelled in DisMod-MR 2.1, forced to sum to 100% at 1,000 draw level for each geography-year-age-sex with the proportions of chronic kidney disease due to diabetes, hypertension and glomerulonephritis, and applied to total chronic kidney disease estimates from DisMod-MR 2.1 analyses.
Other gynaecological disorders	Dismod MR 2.1 using US claims data.
Other musculoskeletal disorders	DisMod-MR 2.1 model of survey and US claims data on prevalence of all musculoskeletal symptoms and diseases minus rheumatoid arthritis, osteoarthritis, gout, low back pain, and neck pain. Long-term sequelae of fractures, dislocations and contusions due to injuries are subtracted out of other musculoskeletal disorders to avoid double counting.
Other skin	Dismod-MR 2.1 model using outpatient and US claims data.
Age-related and other hearing loss	Survey data on the proportion of hearing loss due to age-related and other hearing loss modelled in dismod MR 2.1 and forced to sum to total hearing loss by geography, year, age, and sex.
Other vision loss	Survey data on vision loss due to other causes modelled in DisMod-MR 2.1 and forced to sum to total vision loss by geography, year, age, and sex.

Table S14: GBD 2019 methods of estimating years lived with disability (YLDs) for 34 residual categories	
**Residual**	**Method**
Other sense organ disorders	DisMod-MR 2.1 model using outpatient and US claims data.
Other oral disorders	DisMod-MR 2.1 model using US Medical Expenditure Panel Surveys (MEPS) data.
Other road injuries	DisMod-MR 2.1 model using data from surveys, hospital admission and outpatient/emergency department visits.
Other transport injuries	DisMod-MR 2.1 model using data from surveys, hospital admission and outpatient/emergency department visits.
Poisoning by other means	DisMod-MR 2.1 model using data from surveys, hospital admission and outpatient/emergency department visits.
Other exposure to mechanical forces	DisMod-MR 2.1 model using data from surveys, hospital admission and outpatient/emergency department visits.
Foreign body in other part	DisMod-MR 2.1 model using data from surveys, hospital admission and outpatient/emergency department visits.
Other unintentional injuries	DisMod-MR 2.1 model using data from surveys, hospital admission and outpatient/emergency department visits.
Self-harm by other specified means	DisMod-MR 2.1 model using data from surveys, hospital admission and outpatient/emergency department visits.
Physical violence by other means	DisMod-MR 2.1 model using data from surveys, hospital admission and outpatient/emergency department visits.

Table S15: List of GBD 2019 non-fatal causes with prevalence at birth
**Causes**
HIV/AIDS resulting in other diseases
Chagas disease
Zika virus disease
Brain and nervous system cancer
Acute myeloid leukaemia
Other malignant cancers
Alcohol use disorders
Autism spectrum disorders
Acute Hepatitis B
Acute Hepatitis E
Neonatal preterm birth complications
Neonatal encephalopathy due to birth asphyxia and trauma
Neonatal sepsis and other neonatal infections
Haemolytic disease and other neonatal jaundice
Iodine deficiency
Neural tube defects
Congenital heart anomalies
Orofacial clefts
Down syndrome
Turner syndrome
Klinefelter syndrome
Other chromosomal abnormalities
Congenital musculoskeletal and limb anomalies
Urogenital congenital anomalies
Digestive congenital anomalies
Thalassaemias
Thalassaemias trait
Sickle cell disorders
Sickle cell trait
G6PD deficiency
G6PD trait
Protein-energy malnutrition

Table S16. CODEm covariates used, level of covariate, and expected direction of covariate by cause, sex, and age
Covariates that CODEm did not select during the covariate selection process have no draw counts listed.

Cause	Sex	Age Start	Age End	Model Type	Direction	Level	Covariate Name	Number of Draws
Sexually transmitted infections excluding HIV	Female	10-14 years	95+ years	Data Rich	1	1	Syphilis prevalence (proportion)	--
Sexually transmitted infections excluding HIV	Female	10-14 years	95+ years	Data Rich	-1	2	Education (years per capita)	57
Sexually transmitted infections excluding HIV	Female	10-14 years	95+ years	Data Rich	-1	2	Healthcare access and quality index	112
Sexually transmitted infections excluding HIV	Female	10-14 years	95+ years	Data Rich	-1	2	Legality of Abortion	618
Sexually transmitted infections excluding HIV	Female	10-14 years	95+ years	Data Rich	-1	2	Maternal care and immunization	--
Sexually transmitted infections excluding HIV	Female	10-14 years	95+ years	Data Rich	1	2	Age-Specific Fertility Rate	--
Sexually transmitted infections excluding HIV	Female	10-14 years	95+ years	Data Rich	1	2	Total Fertility Rate	--
Sexually transmitted infections excluding HIV	Female	10-14 years	95+ years	Data Rich	-1	3	Antenatal Care (4 visits) Coverage (proportion)	138
Sexually transmitted infections excluding HIV	Female	10-14 years	95+ years	Data Rich	-1	3	Antenatal Care (1 visit) Coverage (proportion)	531
Sexually transmitted infections excluding HIV	Female	10-14 years	95+ years	Data Rich	-1	3	LDI (I$ per capita)	--
Sexually transmitted infections excluding HIV	Female	10-14 years	95+ years	Global	1	1	Syphilis prevalence (proportion)	--
Sexually transmitted infections excluding HIV	Female	10-14 years	95+ years	Global	-1	2	Education (years per capita)	59
Sexually transmitted infections excluding HIV	Female	10-14 years	95+ years	Global	-1	2	Healthcare access and quality index	284
Sexually transmitted infections excluding HIV	Female	10-14 years	95+ years	Global	-1	2	Legality of Abortion	343
Sexually transmitted infections excluding HIV	Female	10-14 years	95+ years	Global	-1	2	Maternal care and immunization	--
Sexually transmitted infections excluding HIV	Female	10-14 years	95+ years	Global	1	2	Age-Specific Fertility Rate	--
Sexually transmitted infections excluding HIV	Female	10-14 years	95+ years	Global	1	2	Total Fertility Rate	--
Sexually transmitted infections excluding HIV	Female	10-14 years	95+ years	Global	-1	3	Antenatal Care (1 visit) Coverage (proportion)	336
Sexually transmitted infections excluding HIV	Female	10-14 years	95+ years	Global	-1	3	Antenatal Care (4 visits) Coverage (proportion)	359
Sexually transmitted infections excluding HIV	Female	10-14 years	95+ years	Global	-1	3	LDI (I$ per capita)	--
Sexually transmitted infections excluding HIV	Male	10-14 years	95+ years	Data Rich	1	1	Syphilis prevalence (proportion)	--
Sexually transmitted infections excluding HIV	Male	10-14 years	95+ years	Data Rich	-1	2	Healthcare access and quality index	404
Sexually transmitted infections excluding HIV	Male	10-14 years	95+ years	Data Rich	-1	2	Education (years per capita)	449
Sexually transmitted infections excluding HIV	Male	10-14 years	95+ years	Data Rich	-1	2	Legality of Abortion	643
Sexually transmitted infections excluding HIV	Male	10-14 years	95+ years	Data Rich	-1	2	Maternal care and immunization	--
Sexually transmitted infections excluding HIV	Male	10-14 years	95+ years	Data Rich	1	2	Age-Specific Fertility Rate	--
Sexually transmitted infections excluding HIV	Male	10-14 years	95+ years	Data Rich	1	2	Total Fertility Rate	--
Sexually transmitted infections excluding HIV	Male	10-14 years	95+ years	Data Rich	-1	3	Antenatal Care (1 visit) Coverage (proportion)	175
Sexually transmitted infections excluding HIV	Male	10-14 years	95+ years	Data Rich	-1	3	Antenatal Care (4 visits) Coverage (proportion)	221
Sexually transmitted infections excluding HIV	Male	10-14 years	95+ years	Data Rich	-1	3	LDI (I$ per capita)	--
Sexually transmitted infections excluding HIV	Male	10-14 years	95+ years	Global	1	1	Syphilis prevalence (proportion)	--
Sexually transmitted infections excluding HIV	Male	10-14 years	95+ years	Global	-1	2	Education (years per capita)	385
Sexually transmitted infections excluding HIV	Male	10-14 years	95+ years	Global	-1	2	Healthcare access and quality index	385
Sexually transmitted infections excluding HIV	Male	10-14 years	95+ years	Global	-1	2	Legality of Abortion	385
Sexually transmitted infections excluding HIV	Male	10-14 years	95+ years	Global	-1	2	Maternal care and immunization	--
Sexually transmitted infections excluding HIV	Male	10-14 years	95+ years	Global	1	2	Age-Specific Fertility Rate	--
Sexually transmitted infections excluding HIV	Male	10-14 years	95+ years	Global	1	2	Total Fertility Rate	--
Sexually transmitted infections excluding HIV	Male	10-14 years	95+ years	Global	-1	3	Antenatal Care (4 visits) Coverage (proportion)	99
Sexually transmitted infections excluding HIV	Male	10-14 years	95+ years	Global	-1	3	Antenatal Care (1 visit) Coverage (proportion)	335
Sexually transmitted infections excluding HIV	Male	10-14 years	95+ years	Global	-1	3	LDI (I$ per capita)	--

Table S16. CODEm covariates used, level of covariate, and expected direction of covariate by cause, sex, and age
Covariates that CODEm did not select during the covariate selection process have no draw counts listed.

Cause	Sex	Age Start	Age End	Model Type	Direction	Level	Covariate Name	Number of Draws
Lower respiratory infections	Female	0-6 days	1-4 years	Data Rich	-1	1	Antibiotics for LRI	94
Lower respiratory infections	Female	0-6 days	1-4 years	Data Rich	-1	1	PCV3 Coverage (proportion)	401
Lower respiratory infections	Female	0-6 days	1-4 years	Data Rich	-1	1	Hib3 Vaccine Coverage (proportion)	506
Lower respiratory infections	Female	0-6 days	1-4 years	Data Rich	1	1	Indoor Air Pollution (All Cooking Fuels)	208
Lower respiratory infections	Female	0-6 days	1-4 years	Data Rich	1	1	Short gestation SEV (all ages, by sex)	328
Lower respiratory infections	Female	0-6 days	1-4 years	Data Rich	1	1	Low birth weight SEV (all ages, by sex)	384
Lower respiratory infections	Female	0-6 days	1-4 years	Data Rich	1	1	Age- and sex-specific SEV for Child stunting	--
Lower respiratory infections	Female	0-6 days	1-4 years	Data Rich	1	1	Age- and sex-specific SEV for Child underweight	--
Lower respiratory infections	Female	0-6 days	1-4 years	Data Rich	1	1	Age- and sex-specific SEV for Child wasting	--
Lower respiratory infections	Female	0-6 days	1-4 years	Data Rich	1	1	Log-transformed SEV scalar: LRI	--
Lower respiratory infections	Female	0-6 days	1-4 years	Data Rich	-1	2	Healthcare access and quality index	126
Lower respiratory infections	Female	0-6 days	1-4 years	Data Rich	-1	2	DTP3 Coverage (proportion)	407
Lower respiratory infections	Female	0-6 days	1-4 years	Data Rich	1	2	Zinc deficiency	0
Lower respiratory infections	Female	0-6 days	1-4 years	Data Rich	1	2	Vitamin A Deficiency Prevalence (age-standardized)	145
Lower respiratory infections	Female	0-6 days	1-4 years	Data Rich	1	2	Secondhand smoke	330
Lower respiratory infections	Female	0-6 days	1-4 years	Data Rich	1	2	Outdoor Air Pollution (PM2.5)	382
Lower respiratory infections	Female	0-6 days	1-4 years	Data Rich	1	2	Discontinued breastfeeding SEV	--
Lower respiratory infections	Female	0-6 days	1-4 years	Data Rich	-1	3	Maternal Education (years per capita)	0
Lower respiratory infections	Female	0-6 days	1-4 years	Data Rich	-1	3	Socio-demographic Index	90
Lower respiratory infections	Female	0-6 days	1-4 years	Data Rich	1	3	Population Density (over 1000 ppl/sqkm, proportion)	38
Lower respiratory infections	Female	0-6 days	1-4 years	Data Rich	1	3	No access to handwashing facility	404
Lower respiratory infections	Female	0-6 days	1-4 years	Data Rich	1	3	Age- and sex-specific SEV for Unsafe sanitation	596
Lower respiratory infections	Female	0-6 days	1-4 years	Global	-1	1	Hib3 Vaccine Coverage (proportion)	259
Lower respiratory infections	Female	0-6 days	1-4 years	Global	-1	1	Antibiotics for LRI	389
Lower respiratory infections	Female	0-6 days	1-4 years	Global	-1	1	PCV3 Coverage (proportion)	428
Lower respiratory infections	Female	0-6 days	1-4 years	Global	1	1	Short gestation SEV (all ages, by sex)	143
Lower respiratory infections	Female	0-6 days	1-4 years	Global	1	1	Indoor Air Pollution (All Cooking Fuels)	290
Lower respiratory infections	Female	0-6 days	1-4 years	Global	1	1	Low birth weight SEV (all ages, by sex)	382
Lower respiratory infections	Female	0-6 days	1-4 years	Global	1	1	Age- and sex-specific SEV for Child stunting	--
Lower respiratory infections	Female	0-6 days	1-4 years	Global	1	1	Age- and sex-specific SEV for Child underweight	--
Lower respiratory infections	Female	0-6 days	1-4 years	Global	1	1	Age- and sex-specific SEV for Child wasting	--
Lower respiratory infections	Female	0-6 days	1-4 years	Global	1	1	Log-transformed SEV scalar: LRI	--
Lower respiratory infections	Female	0-6 days	1-4 years	Global	-1	2	Healthcare access and quality index	4
Lower respiratory infections	Female	0-6 days	1-4 years	Global	-1	2	DTP3 Coverage (proportion)	69
Lower respiratory infections	Female	0-6 days	1-4 years	Global	1	2	Vitamin A Deficiency Prevalence (age-standardized)	3
Lower respiratory infections	Female	0-6 days	1-4 years	Global	1	2	Secondhand smoke	34
Lower respiratory infections	Female	0-6 days	1-4 years	Global	1	2	Zinc deficiency	77
Lower respiratory infections	Female	0-6 days	1-4 years	Global	1	2	Discontinued breastfeeding SEV	--
Lower respiratory infections	Female	0-6 days	1-4 years	Global	1	2	Outdoor Air Pollution (PM2.5)	--
Lower respiratory infections	Female	0-6 days	1-4 years	Global	-1	3	Socio-demographic Index	375
Lower respiratory infections	Female	0-6 days	1-4 years	Global	-1	3	Maternal Education (years per capita)	385
Lower respiratory infections	Female	0-6 days	1-4 years	Global	1	3	No access to handwashing facility	381
Lower respiratory infections	Female	0-6 days	1-4 years	Global	1	3	Age- and sex-specific SEV for Unsafe sanitation	553
Lower respiratory infections	Female	0-6 days	1-4 years	Global	1	3	Population Density (over 1000 ppl/sqkm, proportion)	--
Lower respiratory infections	Female	5-9 years	95+ years	Data Rich	1	1	Secondhand smoke	3
Lower respiratory infections	Female	5-9 years	95+ years	Data Rich	1	1	Outdoor Air Pollution (PM2.5)	19
Lower respiratory infections	Female	5-9 years	95+ years	Data Rich	1	1	Smoking Prevalence	562
Lower respiratory infections	Female	5-9 years	95+ years	Data Rich	1	1	Indoor Air Pollution (All Cooking Fuels)	828
Lower respiratory infections	Female	5-9 years	95+ years	Data Rich	1	1	Log-transformed SEV scalar: LRI	--

Table S16. CODEm covariates used, level of covariate, and expected direction of covariate by cause, sex, and age
Covariates that CODEm did not select during the covariate selection process have no draw counts listed.

Cause	Sex	Age Start	Age End	Model Type	Direction	Level	Covariate Name	Number of Draws
Lower respiratory infections	Female	5-9 years	95+ years	Data Rich	-1	2	Mean BMI	1
Lower respiratory infections	Female	5-9 years	95+ years	Data Rich	-1	2	DTP3 Coverage (proportion)	51
Lower respiratory infections	Female	5-9 years	95+ years	Data Rich	-1	2	Healthcare access and quality index	52
Lower respiratory infections	Female	5-9 years	95+ years	Data Rich	-1	2	PCV3 Coverage (proportion)	--
Lower respiratory infections	Female	5-9 years	95+ years	Data Rich	1	2	No access to handwashing facility	346
Lower respiratory infections	Female	5-9 years	95+ years	Data Rich	-1	3	Socio-demographic Index	28
Lower respiratory infections	Female	5-9 years	95+ years	Data Rich	-1	3	Education (years per capita)	35
Lower respiratory infections	Female	5-9 years	95+ years	Data Rich	-1	3	LDI (I$ per capita)	--
Lower respiratory infections	Female	5-9 years	95+ years	Data Rich	1	3	Age- and sex-specific SEV for Unsafe sanitation	45
Lower respiratory infections	Female	5-9 years	95+ years	Data Rich	1	3	Alcohol (liters per capita)	520
Lower respiratory infections	Female	5-9 years	95+ years	Global	1	1	Secondhand smoke	3
Lower respiratory infections	Female	5-9 years	95+ years	Global	1	1	Indoor Air Pollution (All Cooking Fuels)	7
Lower respiratory infections	Female	5-9 years	95+ years	Global	1	1	Smoking Prevalence	879
Lower respiratory infections	Female	5-9 years	95+ years	Global	1	1	Log-transformed SEV scalar: LRI	--
Lower respiratory infections	Female	5-9 years	95+ years	Global	1	1	Outdoor Air Pollution (PM2.5)	--
Lower respiratory infections	Female	5-9 years	95+ years	Global	-1	2	DTP3 Coverage (proportion)	0
Lower respiratory infections	Female	5-9 years	95+ years	Global	-1	2	PCV3 Coverage (proportion)	72
Lower respiratory infections	Female	5-9 years	95+ years	Global	-1	2	Mean BMI	191
Lower respiratory infections	Female	5-9 years	95+ years	Global	-1	2	Healthcare access and quality index	296
Lower respiratory infections	Female	5-9 years	95+ years	Global	1	2	No access to handwashing facility	254
Lower respiratory infections	Female	5-9 years	95+ years	Global	-1	3	Education (years per capita)	0
Lower respiratory infections	Female	5-9 years	95+ years	Global	-1	3	Socio-demographic Index	100
Lower respiratory infections	Female	5-9 years	95+ years	Global	-1	3	LDI (I$ per capita)	--
Lower respiratory infections	Female	5-9 years	95+ years	Global	1	3	Age- and sex-specific SEV for Unsafe sanitation	287
Lower respiratory infections	Female	5-9 years	95+ years	Global	1	3	Alcohol (liters per capita)	655
Lower respiratory infections	Male	0-6 days	1-4 years	Data Rich	-1	1	Antibiotics for LRI	119
Lower respiratory infections	Male	0-6 days	1-4 years	Data Rich	-1	1	PCV3 Coverage (proportion)	268
Lower respiratory infections	Male	0-6 days	1-4 years	Data Rich	-1	1	Hib3 Vaccine Coverage (proportion)	421
Lower respiratory infections	Male	0-6 days	1-4 years	Data Rich	1	1	Indoor Air Pollution (All Cooking Fuels)	0
Lower respiratory infections	Male	0-6 days	1-4 years	Data Rich	1	1	Low birth weight SEV (all ages, by sex)	179
Lower respiratory infections	Male	0-6 days	1-4 years	Data Rich	1	1	Short gestation SEV (all ages, by sex)	270
Lower respiratory infections	Male	0-6 days	1-4 years	Data Rich	1	1	Age- and sex-specific SEV for Child stunting	--
Lower respiratory infections	Male	0-6 days	1-4 years	Data Rich	1	1	Age- and sex-specific SEV for Child underweight	--
Lower respiratory infections	Male	0-6 days	1-4 years	Data Rich	1	1	Age- and sex-specific SEV for Child wasting	--
Lower respiratory infections	Male	0-6 days	1-4 years	Data Rich	1	1	Log-transformed SEV scalar: LRI	--
Lower respiratory infections	Male	0-6 days	1-4 years	Data Rich	-1	2	DTP3 Coverage (proportion)	254
Lower respiratory infections	Male	0-6 days	1-4 years	Data Rich	-1	2	Healthcare access and quality index	338
Lower respiratory infections	Male	0-6 days	1-4 years	Data Rich	1	2	Zinc deficiency	0
Lower respiratory infections	Male	0-6 days	1-4 years	Data Rich	1	2	Vitamin A Deficiency Prevalence (age-standardized)	3
Lower respiratory infections	Male	0-6 days	1-4 years	Data Rich	1	2	Discontinued breastfeeding SEV	366
Lower respiratory infections	Male	0-6 days	1-4 years	Data Rich	1	2	Secondhand smoke	563
Lower respiratory infections	Male	0-6 days	1-4 years	Data Rich	1	2	Outdoor Air Pollution (PM2.5)	638
Lower respiratory infections	Male	0-6 days	1-4 years	Data Rich	-1	3	Maternal Education (years per capita)	66
Lower respiratory infections	Male	0-6 days	1-4 years	Data Rich	-1	3	Socio-demographic Index	232
Lower respiratory infections	Male	0-6 days	1-4 years	Data Rich	1	3	Population Density (over 1000 ppl/sqkm, proportion)	558
Lower respiratory infections	Male	0-6 days	1-4 years	Data Rich	1	3	No access to handwashing facility	679
Lower respiratory infections	Male	0-6 days	1-4 years	Data Rich	1	3	Age- and sex-specific SEV for Unsafe sanitation	771
Lower respiratory infections	Male	0-6 days	1-4 years	Global	-1	1	Hib3 Vaccine Coverage (proportion)	269
Lower respiratory infections	Male	0-6 days	1-4 years	Global	-1	1	Antibiotics for LRI	408
Lower respiratory infections	Male	0-6 days	1-4 years	Global	-1	1	PCV3 Coverage (proportion)	754
Lower respiratory infections	Male	0-6 days	1-4 years	Global	1	1	Indoor Air Pollution (All Cooking Fuels)	159
Lower respiratory infections	Male	0-6 days	1-4 years	Global	1	1	Short gestation SEV (all ages, by sex)	185

**Table S16. CODEm covariates used, level of covariate, and expected direction of covariate by cause, sex, and age** *Covariates that CODEm did not select during the covariate selection process have no draw counts listed.*								
Cause	Sex	Age Start	Age End	Model Type	Direction	Level	Covariate Name	Number of Draws
Lower respiratory infections	Male	0-6 days	1-4 years	Global	1	1	Low birth weight SEV (all ages, by sex)	423
Lower respiratory infections	Male	0-6 days	1-4 years	Global	1	1	Age- and sex-specific SEV for Child stunting	--
Lower respiratory infections	Male	0-6 days	1-4 years	Global	1	1	Age- and sex-specific SEV for Child underweight	--
Lower respiratory infections	Male	0-6 days	1-4 years	Global	1	1	Age- and sex-specific SEV for Child wasting	--
Lower respiratory infections	Male	0-6 days	1-4 years	Global	1	1	Log-transformed SEV scalar: LRI	--
Lower respiratory infections	Male	0-6 days	1-4 years	Global	-1	2	Healthcare access and quality index	0
Lower respiratory infections	Male	0-6 days	1-4 years	Global	-1	2	DTP3 Coverage (proportion)	3
Lower respiratory infections	Male	0-6 days	1-4 years	Global	1	2	Outdoor Air Pollution (PM2.5)	0
Lower respiratory infections	Male	0-6 days	1-4 years	Global	1	2	Secondhand smoke	0
Lower respiratory infections	Male	0-6 days	1-4 years	Global	1	2	Vitamin A Deficiency Prevalence (age-standardized)	0
Lower respiratory infections	Male	0-6 days	1-4 years	Global	1	2	Zinc deficiency	3
Lower respiratory infections	Male	0-6 days	1-4 years	Global	1	2	Discontinued breastfeeding SEV	--
Lower respiratory infections	Male	0-6 days	1-4 years	Global	-1	3	Socio-demographic Index	310
Lower respiratory infections	Male	0-6 days	1-4 years	Global	-1	3	Maternal Education (years per capita)	353
Lower respiratory infections	Male	0-6 days	1-4 years	Global	1	3	No access to handwashing facility	403
Lower respiratory infections	Male	0-6 days	1-4 years	Global	1	3	Age- and sex-specific SEV for Unsafe sanitation	812
Lower respiratory infections	Male	0-6 days	1-4 years	Global	1	3	Population Density (over 1000 ppl/sqkm, proportion)	--
Lower respiratory infections	Male	5-9 years	95+ years	Data Rich	1	1	Outdoor Air Pollution (PM2.5)	0
Lower respiratory infections	Male	5-9 years	95+ years	Data Rich	1	1	Smoking Prevalence	326
Lower respiratory infections	Male	5-9 years	95+ years	Data Rich	1	1	Indoor Air Pollution (All Cooking Fuels)	699
Lower respiratory infections	Male	5-9 years	95+ years	Data Rich	1	1	Secondhand smoke	730
Lower respiratory infections	Male	5-9 years	95+ years	Data Rich	1	1	Log-transformed SEV scalar: LRI	--
Lower respiratory infections	Male	5-9 years	95+ years	Data Rich	-1	2	Healthcare access and quality index	16
Lower respiratory infections	Male	5-9 years	95+ years	Data Rich	-1	2	DTP3 Coverage (proportion)	56
Lower respiratory infections	Male	5-9 years	95+ years	Data Rich	-1	2	PCV3 Coverage (proportion)	125
Lower respiratory infections	Male	5-9 years	95+ years	Data Rich	-1	2	Mean BMI	141
Lower respiratory infections	Male	5-9 years	95+ years	Data Rich	1	2	No access to handwashing facility	1
Lower respiratory infections	Male	5-9 years	95+ years	Data Rich	-1	3	Socio-demographic Index	35
Lower respiratory infections	Male	5-9 years	95+ years	Data Rich	-1	3	Education (years per capita)	95
Lower respiratory infections	Male	5-9 years	95+ years	Data Rich	-1	3	LDI (I$ per capita)	--
Lower respiratory infections	Male	5-9 years	95+ years	Data Rich	1	3	Age- and sex-specific SEV for Unsafe sanitation	1
Lower respiratory infections	Male	5-9 years	95+ years	Data Rich	1	3	Alcohol (liters per capita)	491
Lower respiratory infections	Male	5-9 years	95+ years	Global	1	1	Smoking Prevalence	5
Lower respiratory infections	Male	5-9 years	95+ years	Global	1	1	Secondhand smoke	577
Lower respiratory infections	Male	5-9 years	95+ years	Global	1	1	Indoor Air Pollution (All Cooking Fuels)	647
Lower respiratory infections	Male	5-9 years	95+ years	Global	1	1	Log-transformed SEV scalar: LRI	--
Lower respiratory infections	Male	5-9 years	95+ years	Global	1	1	Outdoor Air Pollution (PM2.5)	--
Lower respiratory infections	Male	5-9 years	95+ years	Global	-1	2	DTP3 Coverage (proportion)	3
Lower respiratory infections	Male	5-9 years	95+ years	Global	-1	2	Mean BMI	8
Lower respiratory infections	Male	5-9 years	95+ years	Global	-1	2	Healthcare access and quality index	21
Lower respiratory infections	Male	5-9 years	95+ years	Global	-1	2	PCV3 Coverage (proportion)	331
Lower respiratory infections	Male	5-9 years	95+ years	Global	1	2	No access to handwashing facility	0
Lower respiratory infections	Male	5-9 years	95+ years	Global	-1	3	Socio-demographic Index	1
Lower respiratory infections	Male	5-9 years	95+ years	Global	-1	3	Education (years per capita)	2
Lower respiratory infections	Male	5-9 years	95+ years	Global	-1	3	LDI (I$ per capita)	--
Lower respiratory infections	Male	5-9 years	95+ years	Global	1	3	Age- and sex-specific SEV for Unsafe sanitation	46
Lower respiratory infections	Male	5-9 years	95+ years	Global	1	3	Alcohol (liters per capita)	624
Upper respiratory infections	Female	0-6 days	95+ years	Data Rich	1	1	Smoking Prevalence	1000
Upper respiratory infections	Female	0-6 days	95+ years	Data Rich	-1	2	Healthcare access and quality index	--
Upper respiratory infections	Female	0-6 days	95+ years	Data Rich	1	2	Indoor Air Pollution (All Cooking Fuels)	488
Upper respiratory infections	Female	0-6 days	95+ years	Data Rich	1	2	Outdoor Air Pollution (PM2.5)	488
Upper respiratory infections	Female	0-6 days	95+ years	Data Rich	-1	3	Socio-demographic Index	145
Upper respiratory infections	Female	0-6 days	95+ years	Data Rich	-1	3	Education (years per capita)	--
Upper respiratory infections	Female	0-6 days	95+ years	Data Rich	-1	3	LDI (I$ per capita)	--

Table S16. CODEm covariates used, level of covariate, and expected direction of covariate by cause, sex, and age
Covariates that CODEm did not select during the covariate selection process have no draw counts listed.

Cause	Sex	Age Start	Age End	Model Type	Direction	Level	Covariate Name	Number of Draws
Upper respiratory infections	Female	0-6 days	95+ years	Global	1	1	Smoking Prevalence	1000
Upper respiratory infections	Female	0-6 days	95+ years	Global	-1	2	Healthcare access and quality index	--
Upper respiratory infections	Female	0-6 days	95+ years	Global	1	2	Indoor Air Pollution (All Cooking Fuels)	356
Upper respiratory infections	Female	0-6 days	95+ years	Global	1	2	Outdoor Air Pollution (PM2.5)	--
Upper respiratory infections	Female	0-6 days	95+ years	Global	-1	3	Socio-demographic Index	160
Upper respiratory infections	Female	0-6 days	95+ years	Global	-1	3	Education (years per capita)	--
Upper respiratory infections	Female	0-6 days	95+ years	Global	-1	3	LDI (I$ per capita)	--
Upper respiratory infections	Male	0-6 days	95+ years	Data Rich	1	1	Smoking Prevalence	1000
Upper respiratory infections	Male	0-6 days	95+ years	Data Rich	-1	2	Healthcare access and quality index	324
Upper respiratory infections	Male	0-6 days	95+ years	Data Rich	1	2	Indoor Air Pollution (All Cooking Fuels)	351
Upper respiratory infections	Male	0-6 days	95+ years	Data Rich	1	2	Outdoor Air Pollution (PM2.5)	--
Upper respiratory infections	Male	0-6 days	95+ years	Data Rich	-1	3	Socio-demographic Index	151
Upper respiratory infections	Male	0-6 days	95+ years	Data Rich	-1	3	Education (years per capita)	751
Upper respiratory infections	Male	0-6 days	95+ years	Data Rich	-1	3	LDI (I$ per capita)	--
Upper respiratory infections	Male	0-6 days	95+ years	Global	1	1	Smoking Prevalence	1000
Upper respiratory infections	Male	0-6 days	95+ years	Global	-1	2	Healthcare access and quality index	336
Upper respiratory infections	Male	0-6 days	95+ years	Global	1	2	Indoor Air Pollution (All Cooking Fuels)	336
Upper respiratory infections	Male	0-6 days	95+ years	Global	1	2	Outdoor Air Pollution (PM2.5)	--
Upper respiratory infections	Male	0-6 days	95+ years	Global	-1	3	Socio-demographic Index	208
Upper respiratory infections	Male	0-6 days	95+ years	Global	-1	3	Education (years per capita)	328
Upper respiratory infections	Male	0-6 days	95+ years	Global	-1	3	LDI (I$ per capita)	--
Otitis media	Female	0-6 days	95+ years	Data Rich	1	1	Smoking Prevalence	786
Otitis media	Female	0-6 days	95+ years	Data Rich	1	1	Log-transformed SEV scalar: Otitis	--
Otitis media	Female	0-6 days	95+ years	Data Rich	-1	2	Healthcare access and quality index	940
Otitis media	Female	0-6 days	95+ years	Data Rich	1	2	Indoor Air Pollution (All Cooking Fuels)	17
Otitis media	Female	0-6 days	95+ years	Data Rich	1	2	Outdoor Air Pollution (PM2.5)	209
Otitis media	Female	0-6 days	95+ years	Data Rich	-1	3	Socio-demographic Index	40
Otitis media	Female	0-6 days	95+ years	Data Rich	-1	3	Education (years per capita)	595
Otitis media	Female	0-6 days	95+ years	Data Rich	-1	3	LDI (I$ per capita)	--
Otitis media	Female	0-6 days	95+ years	Global	1	1	Smoking Prevalence	889
Otitis media	Female	0-6 days	95+ years	Global	1	1	Log-transformed SEV scalar: Otitis	--
Otitis media	Female	0-6 days	95+ years	Global	-1	2	Healthcare access and quality index	641
Otitis media	Female	0-6 days	95+ years	Global	1	2	Indoor Air Pollution (All Cooking Fuels)	175
Otitis media	Female	0-6 days	95+ years	Global	1	2	Outdoor Air Pollution (PM2.5)	361
Otitis media	Female	0-6 days	95+ years	Global	-1	3	Socio-demographic Index	278
Otitis media	Female	0-6 days	95+ years	Global	-1	3	Education (years per capita)	655
Otitis media	Female	0-6 days	95+ years	Global	-1	3	LDI (I$ per capita)	--
Otitis media	Male	0-6 days	95+ years	Data Rich	1	1	Smoking Prevalence	874
Otitis media	Male	0-6 days	95+ years	Data Rich	1	1	Log-transformed SEV scalar: Otitis	--
Otitis media	Male	0-6 days	95+ years	Data Rich	-1	2	Healthcare access and quality index	913
Otitis media	Male	0-6 days	95+ years	Data Rich	1	2	Indoor Air Pollution (All Cooking Fuels)	46
Otitis media	Male	0-6 days	95+ years	Data Rich	1	2	Outdoor Air Pollution (PM2.5)	812
Otitis media	Male	0-6 days	95+ years	Data Rich	-1	3	Socio-demographic Index	66
Otitis media	Male	0-6 days	95+ years	Data Rich	-1	3	Education (years per capita)	512
Otitis media	Male	0-6 days	95+ years	Data Rich	-1	3	LDI (I$ per capita)	--
Otitis media	Male	0-6 days	95+ years	Global	1	1	Smoking Prevalence	673
Otitis media	Male	0-6 days	95+ years	Global	1	1	Log-transformed SEV scalar: Otitis	--
Otitis media	Male	0-6 days	95+ years	Global	-1	2	Healthcare access and quality index	736
Otitis media	Male	0-6 days	95+ years	Global	1	2	Indoor Air Pollution (All Cooking Fuels)	116
Otitis media	Male	0-6 days	95+ years	Global	1	2	Outdoor Air Pollution (PM2.5)	669
Otitis media	Male	0-6 days	95+ years	Global	-1	3	Socio-demographic Index	170
Otitis media	Male	0-6 days	95+ years	Global	-1	3	Education (years per capita)	467
Otitis media	Male	0-6 days	95+ years	Global	-1	3	LDI (I$ per capita)	--
Diarrhoeal diseases	Female	0-6 days	1-4 years	Data Rich	-1	1	Improved Water Source (proportion with access)	0
Diarrhoeal diseases	Female	0-6 days	1-4 years	Data Rich	-1	1	Rotavirus coverage (proportion)	0
Diarrhoeal diseases	Female	0-6 days	1-4 years	Data Rich	-1	1	ORS (oral rehydration)	550

Table S16. CODEm covariates used, level of covariate, and expected direction of covariate by cause, sex, and age *Covariates that CODEm did not select during the covariate selection process have no draw counts listed.*								
**Cause**	**Sex**	**Age Start**	**Age End**	**Model Type**	**Direction**	**Level**	**Covariate Name**	**Number of Draws**
Diarrhoeal diseases	Female	0-6 days	1-4 years	Data Rich	-1	1	Sanitation (proportion with access)	553
Diarrhoeal diseases	Female	0-6 days	1-4 years	Data Rich	1	1	Short gestation SEV (all ages, by sex)	130
Diarrhoeal diseases	Female	0-6 days	1-4 years	Data Rich	1	1	Age- and sex-specific SEV for Unsafe sanitation	190
Diarrhoeal diseases	Female	0-6 days	1-4 years	Data Rich	1	1	Low birth weight SEV (all ages, by sex)	615
Diarrhoeal diseases	Female	0-6 days	1-4 years	Data Rich	1	1	Age- and sex-specific SEV for Unsafe water	810
Diarrhoeal diseases	Female	0-6 days	1-4 years	Data Rich	1	1	Age- and sex-specific SEV for Child stunting	--
Diarrhoeal diseases	Female	0-6 days	1-4 years	Data Rich	1	1	Age- and sex-specific SEV for Child underweight	--
Diarrhoeal diseases	Female	0-6 days	1-4 years	Data Rich	1	1	Age- and sex-specific SEV for Child wasting	--
Diarrhoeal diseases	Female	0-6 days	1-4 years	Data Rich	1	1	Log-transformed SEV scalar: Diarrhea	--
Diarrhoeal diseases	Female	0-6 days	1-4 years	Data Rich	-1	2	Healthcare access and quality index	0
Diarrhoeal diseases	Female	0-6 days	1-4 years	Data Rich	-1	2	Zinc treatment for diarrhea	74
Diarrhoeal diseases	Female	0-6 days	1-4 years	Data Rich	1	2	Vitamin A Deficiency Prevalence (age-standardized)	13
Diarrhoeal diseases	Female	0-6 days	1-4 years	Data Rich	1	2	Discontinued breastfeeding SEV	17
Diarrhoeal diseases	Female	0-6 days	1-4 years	Data Rich	1	2	Zinc deficiency	416
Diarrhoeal diseases	Female	0-6 days	1-4 years	Data Rich	-1	3	Maternal Education (years per capita)	0
Diarrhoeal diseases	Female	0-6 days	1-4 years	Data Rich	-1	3	Socio-demographic Index	0
Diarrhoeal diseases	Female	0-6 days	1-4 years	Data Rich	-1	3	LDI (I$ per capita)	--
Diarrhoeal diseases	Female	0-6 days	1-4 years	Data Rich	1	3	No access to handwashing facility	661
Diarrhoeal diseases	Female	0-6 days	1-4 years	Global	-1	1	Improved Water Source (proportion with access)	0
Diarrhoeal diseases	Female	0-6 days	1-4 years	Global	-1	1	Rotavirus coverage (proportion)	12
Diarrhoeal diseases	Female	0-6 days	1-4 years	Global	-1	1	ORS (oral rehydration)	541
Diarrhoeal diseases	Female	0-6 days	1-4 years	Global	-1	1	Sanitation (proportion with access)	708
Diarrhoeal diseases	Female	0-6 days	1-4 years	Global	1	1	Low birth weight SEV (all ages, by sex)	154
Diarrhoeal diseases	Female	0-6 days	1-4 years	Global	1	1	Short gestation SEV (all ages, by sex)	262
Diarrhoeal diseases	Female	0-6 days	1-4 years	Global	1	1	Age- and sex-specific SEV for Unsafe water	441
Diarrhoeal diseases	Female	0-6 days	1-4 years	Global	1	1	Age- and sex-specific SEV for Unsafe sanitation	514
Diarrhoeal diseases	Female	0-6 days	1-4 years	Global	1	1	Age- and sex-specific SEV for Child stunting	--
Diarrhoeal diseases	Female	0-6 days	1-4 years	Global	1	1	Age- and sex-specific SEV for Child underweight	--
Diarrhoeal diseases	Female	0-6 days	1-4 years	Global	1	1	Age- and sex-specific SEV for Child wasting	--
Diarrhoeal diseases	Female	0-6 days	1-4 years	Global	1	1	Log-transformed SEV scalar: Diarrhea	--
Diarrhoeal diseases	Female	0-6 days	1-4 years	Global	-1	2	Healthcare access and quality index	0
Diarrhoeal diseases	Female	0-6 days	1-4 years	Global	-1	2	Zinc treatment for diarrhea	--
Diarrhoeal diseases	Female	0-6 days	1-4 years	Global	1	2	Zinc deficiency	44
Diarrhoeal diseases	Female	0-6 days	1-4 years	Global	1	2	Vitamin A Deficiency Prevalence (age-standardized)	131
Diarrhoeal diseases	Female	0-6 days	1-4 years	Global	1	2	Discontinued breastfeeding SEV	659
Diarrhoeal diseases	Female	0-6 days	1-4 years	Global	-1	3	Maternal Education (years per capita)	0
Diarrhoeal diseases	Female	0-6 days	1-4 years	Global	-1	3	Socio-demographic Index	0
Diarrhoeal diseases	Female	0-6 days	1-4 years	Global	-1	3	LDI (I$ per capita)	--
Diarrhoeal diseases	Female	0-6 days	1-4 years	Global	1	3	No access to handwashing facility	421
Diarrhoeal diseases	Female	5-9 years	95+ years	Data Rich	-1	1	Improved Water Source (proportion with access)	0
Diarrhoeal diseases	Female	5-9 years	95+ years	Data Rich	-1	1	Sanitation (proportion with access)	558
Diarrhoeal diseases	Female	5-9 years	95+ years	Data Rich	1	1	Age- and sex-specific SEV for Unsafe sanitation	369
Diarrhoeal diseases	Female	5-9 years	95+ years	Data Rich	1	1	Age- and sex-specific SEV for Unsafe water	525
Diarrhoeal diseases	Female	5-9 years	95+ years	Data Rich	1	1	Log-transformed SEV scalar: Diarrhea	--
Diarrhoeal diseases	Female	5-9 years	95+ years	Data Rich	-1	2	Healthcare access and quality index	--
Diarrhoeal diseases	Female	5-9 years	95+ years	Data Rich	-1	2	Rotavirus coverage (proportion)	--
Diarrhoeal diseases	Female	5-9 years	95+ years	Data Rich	-1	3	Education (years per capita)	--
Diarrhoeal diseases	Female	5-9 years	95+ years	Data Rich	-1	3	LDI (I$ per capita)	--

Table S16. CODEm covariates used, level of covariate, and expected direction of covariate by cause, sex, and age *Covariates that CODEm did not select during the covariate selection process have no draw counts listed.*								
**Cause**	**Sex**	**Age Start**	**Age End**	**Model Type**	**Direction**	**Level**	**Covariate Name**	**Number of Draws**
Diarrhoeal diseases	Female	5-9 years	95+ years	Data Rich	-1	3	Mean BMI	--
Diarrhoeal diseases	Female	5-9 years	95+ years	Data Rich	-1	3	Socio-demographic Index	--
Diarrhoeal diseases	Female	5-9 years	95+ years	Data Rich	0	3	Population Density (over 1000 ppl/sqkm, proportion)	446
Diarrhoeal diseases	Female	5-9 years	95+ years	Global	-1	1	Sanitation (proportion with access)	508
Diarrhoeal diseases	Female	5-9 years	95+ years	Global	-1	1	Improved Water Source (proportion with access)	--
Diarrhoeal diseases	Female	5-9 years	95+ years	Global	1	1	Age- and sex-specific SEV for Unsafe sanitation	455
Diarrhoeal diseases	Female	5-9 years	95+ years	Global	1	1	Age- and sex-specific SEV for Unsafe water	602
Diarrhoeal diseases	Female	5-9 years	95+ years	Global	1	1	Log-transformed SEV scalar: Diarrhea	--
Diarrhoeal diseases	Female	5-9 years	95+ years	Global	-1	2	Healthcare access and quality index	--
Diarrhoeal diseases	Female	5-9 years	95+ years	Global	-1	2	Rotavirus coverage (proportion)	--
Diarrhoeal diseases	Female	5-9 years	95+ years	Global	-1	3	Education (years per capita)	--
Diarrhoeal diseases	Female	5-9 years	95+ years	Global	-1	3	LDI (I$ per capita)	--
Diarrhoeal diseases	Female	5-9 years	95+ years	Global	-1	3	Mean BMI	--
Diarrhoeal diseases	Female	5-9 years	95+ years	Global	-1	3	Socio-demographic Index	--
Diarrhoeal diseases	Female	5-9 years	95+ years	Global	0	3	Population Density (over 1000 ppl/sqkm, proportion)	348
Diarrhoeal diseases	Male	0-6 days	1-4 years	Data Rich	-1	1	Improved Water Source (proportion with access)	0
Diarrhoeal diseases	Male	0-6 days	1-4 years	Data Rich	-1	1	Rotavirus coverage (proportion)	0
Diarrhoeal diseases	Male	0-6 days	1-4 years	Data Rich	-1	1	ORS (oral rehydration)	301
Diarrhoeal diseases	Male	0-6 days	1-4 years	Data Rich	-1	1	Sanitation (proportion with access)	558
Diarrhoeal diseases	Male	0-6 days	1-4 years	Data Rich	1	1	Short gestation SEV (all ages, by sex)	0
Diarrhoeal diseases	Male	0-6 days	1-4 years	Data Rich	1	1	Low birth weight SEV (all ages, by sex)	1
Diarrhoeal diseases	Male	0-6 days	1-4 years	Data Rich	1	1	Age- and sex-specific SEV for Unsafe sanitation	260
Diarrhoeal diseases	Male	0-6 days	1-4 years	Data Rich	1	1	Age- and sex-specific SEV for Unsafe water	878
Diarrhoeal diseases	Male	0-6 days	1-4 years	Data Rich	1	1	Age- and sex-specific SEV for Child stunting	--
Diarrhoeal diseases	Male	0-6 days	1-4 years	Data Rich	1	1	Age- and sex-specific SEV for Child underweight	--
Diarrhoeal diseases	Male	0-6 days	1-4 years	Data Rich	1	1	Age- and sex-specific SEV for Child wasting	--
Diarrhoeal diseases	Male	0-6 days	1-4 years	Data Rich	1	1	Log-transformed SEV scalar: Diarrhea	--
Diarrhoeal diseases	Male	0-6 days	1-4 years	Data Rich	-1	2	Healthcare access and quality index	0
Diarrhoeal diseases	Male	0-6 days	1-4 years	Data Rich	-1	2	Zinc treatment for diarrhea	35
Diarrhoeal diseases	Male	0-6 days	1-4 years	Data Rich	1	2	Zinc deficiency	34
Diarrhoeal diseases	Male	0-6 days	1-4 years	Data Rich	1	2	Vitamin A Deficiency Prevalence (age-standardized)	67
Diarrhoeal diseases	Male	0-6 days	1-4 years	Data Rich	1	2	Discontinued breastfeeding SEV	76
Diarrhoeal diseases	Male	0-6 days	1-4 years	Data Rich	-1	3	Maternal Education (years per capita)	0
Diarrhoeal diseases	Male	0-6 days	1-4 years	Data Rich	-1	3	Socio-demographic Index	0
Diarrhoeal diseases	Male	0-6 days	1-4 years	Data Rich	-1	3	LDI (I$ per capita)	--
Diarrhoeal diseases	Male	0-6 days	1-4 years	Data Rich	1	3	No access to handwashing facility	378
Diarrhoeal diseases	Male	0-6 days	1-4 years	Global	-1	1	Improved Water Source (proportion with access)	0
Diarrhoeal diseases	Male	0-6 days	1-4 years	Global	-1	1	Rotavirus coverage (proportion)	56
Diarrhoeal diseases	Male	0-6 days	1-4 years	Global	-1	1	ORS (oral rehydration)	562
Diarrhoeal diseases	Male	0-6 days	1-4 years	Global	-1	1	Sanitation (proportion with access)	841
Diarrhoeal diseases	Male	0-6 days	1-4 years	Global	1	1	Age- and sex-specific SEV for Unsafe sanitation	153
Diarrhoeal diseases	Male	0-6 days	1-4 years	Global	1	1	Short gestation SEV (all ages, by sex)	207
Diarrhoeal diseases	Male	0-6 days	1-4 years	Global	1	1	Low birth weight SEV (all ages, by sex)	425
Diarrhoeal diseases	Male	0-6 days	1-4 years	Global	1	1	Age- and sex-specific SEV for Unsafe water	776
Diarrhoeal diseases	Male	0-6 days	1-4 years	Global	1	1	Age- and sex-specific SEV for Child stunting	--
Diarrhoeal diseases	Male	0-6 days	1-4 years	Global	1	1	Age- and sex-specific SEV for Child underweight	--
Diarrhoeal diseases	Male	0-6 days	1-4 years	Global	1	1	Age- and sex-specific SEV for Child wasting	--

Table S16. CODEm covariates used, level of covariate, and expected direction of covariate by cause, sex, and age<br>*Covariates that CODEm did not select during the covariate selection process have no draw counts listed.*								
**Cause**	**Sex**	**Age Start**	**Age End**	**Model Type**	**Direction**	**Level**	**Covariate Name**	**Number of Draws**
Diarrhoeal diseases	Male	0-6 days	1-4 years	Global	1	1	Log-transformed SEV scalar: Diarrhea	--
Diarrhoeal diseases	Male	0-6 days	1-4 years	Global	-1	2	Healthcare access and quality index	33
Diarrhoeal diseases	Male	0-6 days	1-4 years	Global	-1	2	Zinc treatment for diarrhea	--
Diarrhoeal diseases	Male	0-6 days	1-4 years	Global	1	2	Zinc deficiency	1
Diarrhoeal diseases	Male	0-6 days	1-4 years	Global	1	2	Vitamin A Deficiency Prevalence (age-standardized)	123
Diarrhoeal diseases	Male	0-6 days	1-4 years	Global	1	2	Discontinued breastfeeding SEV	363
Diarrhoeal diseases	Male	0-6 days	1-4 years	Global	-1	3	Maternal Education (years per capita)	77
Diarrhoeal diseases	Male	0-6 days	1-4 years	Global	-1	3	Socio-demographic Index	92
Diarrhoeal diseases	Male	0-6 days	1-4 years	Global	-1	3	LDI (I$ per capita)	--
Diarrhoeal diseases	Male	0-6 days	1-4 years	Global	1	3	No access to handwashing facility	149
Diarrhoeal diseases	Male	5-9 years	95+ years	Data Rich	-1	1	Sanitation (proportion with access)	395
Diarrhoeal diseases	Male	5-9 years	95+ years	Data Rich	-1	1	Improved Water Source (proportion with access)	--
Diarrhoeal diseases	Male	5-9 years	95+ years	Data Rich	1	1	Age- and sex-specific SEV for Unsafe water	142
Diarrhoeal diseases	Male	5-9 years	95+ years	Data Rich	1	1	Age- and sex-specific SEV for Unsafe sanitation	562
Diarrhoeal diseases	Male	5-9 years	95+ years	Data Rich	1	1	Log-transformed SEV scalar: Diarrhea	--
Diarrhoeal diseases	Male	5-9 years	95+ years	Data Rich	-1	2	Healthcare access and quality index	--
Diarrhoeal diseases	Male	5-9 years	95+ years	Data Rich	-1	2	Rotavirus coverage (proportion)	--
Diarrhoeal diseases	Male	5-9 years	95+ years	Data Rich	-1	3	Education (years per capita)	--
Diarrhoeal diseases	Male	5-9 years	95+ years	Data Rich	-1	3	LDI (I$ per capita)	--
Diarrhoeal diseases	Male	5-9 years	95+ years	Data Rich	-1	3	Mean BMI	--
Diarrhoeal diseases	Male	5-9 years	95+ years	Data Rich	-1	3	Socio-demographic Index	--
Diarrhoeal diseases	Male	5-9 years	95+ years	Data Rich	0	3	Population Density (over 1000 ppl/sqkm, proportion)	443
Diarrhoeal diseases	Male	5-9 years	95+ years	Global	-1	1	Improved Water Source (proportion with access)	0
Diarrhoeal diseases	Male	5-9 years	95+ years	Global	-1	1	Sanitation (proportion with access)	466
Diarrhoeal diseases	Male	5-9 years	95+ years	Global	1	1	Age- and sex-specific SEV for Unsafe water	544
Diarrhoeal diseases	Male	5-9 years	95+ years	Global	1	1	Age- and sex-specific SEV for Unsafe sanitation	564
Diarrhoeal diseases	Male	5-9 years	95+ years	Global	1	1	Log-transformed SEV scalar: Diarrhea	--
Diarrhoeal diseases	Male	5-9 years	95+ years	Global	-1	2	Healthcare access and quality index	0
Diarrhoeal diseases	Male	5-9 years	95+ years	Global	-1	2	Rotavirus coverage (proportion)	--
Diarrhoeal diseases	Male	5-9 years	95+ years	Global	-1	3	Education (years per capita)	--
Diarrhoeal diseases	Male	5-9 years	95+ years	Global	-1	3	LDI (I$ per capita)	--
Diarrhoeal diseases	Male	5-9 years	95+ years	Global	-1	3	Mean BMI	--
Diarrhoeal diseases	Male	5-9 years	95+ years	Global	-1	3	Socio-demographic Index	--
Diarrhoeal diseases	Male	5-9 years	95+ years	Global	0	3	Population Density (over 1000 ppl/sqkm, proportion)	471
Dengue	Female	28-364 days	95+ years	Data Rich	1	1	Population weighted probability of dengue transmission	720
Dengue	Female	28-364 days	95+ years	Data Rich	1	1	Population Density (over 1000 ppl/sqkm, proportion)	--
Dengue	Female	28-364 days	95+ years	Data Rich	0	2	Health System Access (unitless)	740
Dengue	Female	28-364 days	95+ years	Data Rich	1	2	Elevation Under 100m (proportion)	259
Dengue	Female	28-364 days	95+ years	Data Rich	1	2	Latitude Under 15 (proportion)	528
Dengue	Female	28-364 days	95+ years	Data Rich	1	2	Dengue outbreaks (binary)	787
Dengue	Female	28-364 days	95+ years	Data Rich	1	2	Rainfall Quintile 4 (proportion)	--
Dengue	Female	28-364 days	95+ years	Data Rich	1	2	Rainfall Quintile 5 (proportion)	--
Dengue	Female	28-364 days	95+ years	Data Rich	0	3	Education (years per capita)	299
Dengue	Female	28-364 days	95+ years	Data Rich	0	3	LDI (I$ per capita)	--
Dengue	Female	28-364 days	95+ years	Global	1	1	Population weighted probability of dengue transmission	1000
Dengue	Female	28-364 days	95+ years	Global	1	1	Population Density (over 1000 ppl/sqkm, proportion)	--
Dengue	Female	28-364 days	95+ years	Global	0	2	Health System Access (unitless)	647

Table S16. CODEm covariates used, level of covariate, and expected direction of covariate by cause, sex, and age<br>*Covariates that CODEm did not select during the covariate selection process have no draw counts listed.*								
**Cause**	**Sex**	**Age Start**	**Age End**	**Model Type**	**Direction**	**Level**	**Covariate Name**	**Number of Draws**
Dengue	Female	28-364 days	95+ years	Global	1	2	Elevation Under 100m (proportion)	129
Dengue	Female	28-364 days	95+ years	Global	1	2	Dengue outbreaks (binary)	725
Dengue	Female	28-364 days	95+ years	Global	1	2	Latitude Under 15 (proportion)	725
Dengue	Female	28-364 days	95+ years	Global	1	2	Rainfall Quintile 4 (proportion)	--
Dengue	Female	28-364 days	95+ years	Global	1	2	Rainfall Quintile 5 (proportion)	--
Dengue	Female	28-364 days	95+ years	Global	0	3	Education (years per capita)	--
Dengue	Female	28-364 days	95+ years	Global	0	3	LDI (I$ per capita)	--
Dengue	Male	28-364 days	95+ years	Data Rich	1	1	Population weighted probability of dengue transmission	668
Dengue	Male	28-364 days	95+ years	Data Rich	1	1	Population Density (over 1000 ppl/sqkm, proportion)	--
Dengue	Male	28-364 days	95+ years	Data Rich	0	2	Health System Access (unitless)	672
Dengue	Male	28-364 days	95+ years	Data Rich	1	2	Elevation Under 100m (proportion)	244
Dengue	Male	28-364 days	95+ years	Data Rich	1	2	Rainfall Quintile 5 (proportion)	244
Dengue	Male	28-364 days	95+ years	Data Rich	1	2	Dengue outbreaks (binary)	428
Dengue	Male	28-364 days	95+ years	Data Rich	1	2	Latitude Under 15 (proportion)	672
Dengue	Male	28-364 days	95+ years	Data Rich	1	2	Rainfall Quintile 4 (proportion)	--
Dengue	Male	28-364 days	95+ years	Data Rich	0	3	Education (years per capita)	600
Dengue	Male	28-364 days	95+ years	Data Rich	0	3	LDI (I$ per capita)	--
Dengue	Male	28-364 days	95+ years	Global	1	1	Population Density (over 1000 ppl/sqkm, proportion)	101
Dengue	Male	28-364 days	95+ years	Global	1	1	Population weighted probability of dengue transmission	899
Dengue	Male	28-364 days	95+ years	Global	0	2	Health System Access (unitless)	483
Dengue	Male	28-364 days	95+ years	Global	1	2	Elevation Under 100m (proportion)	87
Dengue	Male	28-364 days	95+ years	Global	1	2	Rainfall Quintile 4 (proportion)	570
Dengue	Male	28-364 days	95+ years	Global	1	2	Dengue outbreaks (binary)	594
Dengue	Male	28-364 days	95+ years	Global	1	2	Latitude Under 15 (proportion)	612
Dengue	Male	28-364 days	95+ years	Global	1	2	Rainfall Quintile 5 (proportion)	--
Dengue	Male	28-364 days	95+ years	Global	0	3	Education (years per capita)	381
Dengue	Male	28-364 days	95+ years	Global	0	3	LDI (I$ per capita)	--
Rabies	Female	28-364 days	95+ years	Data Rich	-1	1	Antenatal Care (4 visits) Coverage (proportion)	206
Rabies	Female	28-364 days	95+ years	Data Rich	-1	1	In-Facility Delivery (proportion)	384
Rabies	Female	28-364 days	95+ years	Data Rich	-1	1	Health System Access (unitless)	700
Rabies	Female	28-364 days	95+ years	Data Rich	-1	2	Healthcare access and quality index	82
Rabies	Female	28-364 days	95+ years	Data Rich	-1	2	Skilled Birth Attendance (proportion)	952
Rabies	Female	28-364 days	95+ years	Data Rich	-1	2	Maternal care and immunization	--
Rabies	Female	28-364 days	95+ years	Data Rich	-1	3	Socio-demographic Index	44
Rabies	Female	28-364 days	95+ years	Data Rich	0	3	Population Density (500-1000 ppl/sqkm, proportion)	--
Rabies	Female	28-364 days	95+ years	Data Rich	0	3	Population Density (under 150 ppl/sqkm, proportion)	--
Rabies	Female	28-364 days	95+ years	Global	-1	1	Antenatal Care (4 visits) Coverage (proportion)	352
Rabies	Female	28-364 days	95+ years	Global	-1	1	In-Facility Delivery (proportion)	919
Rabies	Female	28-364 days	95+ years	Global	-1	2	Skilled Birth Attendance (proportion)	246
Rabies	Female	28-364 days	95+ years	Global	-1	2	Health System Access (unitless)	342

Table S16. CODEm covariates used, level of covariate, and expected direction of covariate by cause, sex, and age
Covariates that CODEm did not select during the covariate selection process have no draw counts listed.

Cause	Sex	Age Start	Age End	Model Type	Direction	Level	Covariate Name	Number of Draws
Rabies	Female	28-364 days	95+ years	Global	1	2	Healthcare access and quality index	--
Rabies	Female	28-364 days	95+ years	Global	-1	3	Socio-demographic Index	329
Rabies	Female	28-364 days	95+ years	Global	0	3	Population Density (500-1000 ppl/sqkm, proportion)	--
Rabies	Female	28-364 days	95+ years	Global	0	3	Population Density (under 150 ppl/sqkm, proportion)	--
Rabies	Male	28-364 days	95+ years	Data Rich	-1	1	Health System Access (unitless)	288
Rabies	Male	28-364 days	95+ years	Data Rich	-1	1	Antenatal Care (4 visits) Coverage (proportion)	444
Rabies	Male	28-364 days	95+ years	Data Rich	-1	1	In-Facility Delivery (proportion)	793
Rabies	Male	28-364 days	95+ years	Data Rich	-1	2	Healthcare access and quality index	353
Rabies	Male	28-364 days	95+ years	Data Rich	-1	2	Skilled Birth Attendance (proportion)	959
Rabies	Male	28-364 days	95+ years	Data Rich	-1	2	Maternal care and immunization	--
Rabies	Male	28-364 days	95+ years	Data Rich	-1	3	Socio-demographic Index	57
Rabies	Male	28-364 days	95+ years	Data Rich	0	3	Population Density (500-1000 ppl/sqkm, proportion)	--
Rabies	Male	28-364 days	95+ years	Data Rich	0	3	Population Density (under 150 ppl/sqkm, proportion)	--
Rabies	Male	28-364 days	95+ years	Global	-1	1	In-Facility Delivery (proportion)	697
Rabies	Male	28-364 days	95+ years	Global	-1	1	Antenatal Care (4 visits) Coverage (proportion)	747
Rabies	Male	28-364 days	95+ years	Global	-1	2	Skilled Birth Attendance (proportion)	38
Rabies	Male	28-364 days	95+ years	Global	-1	2	Health System Access (unitless)	130
Rabies	Male	28-364 days	95+ years	Global	1	2	Healthcare access and quality index	231
Rabies	Male	28-364 days	95+ years	Global	-1	3	Socio-demographic Index	735
Rabies	Male	28-364 days	95+ years	Global	0	3	Population Density (500-1000 ppl/sqkm, proportion)	--
Rabies	Male	28-364 days	95+ years	Global	0	3	Population Density (under 150 ppl/sqkm, proportion)	--
Other neglected tropical diseases	Female	0-6 days	95+ years	Data Rich	-1	1	Healthcare access and quality index	521
Other neglected tropical diseases	Female	0-6 days	95+ years	Data Rich	1	1	Latitude Under 15 (proportion)	728
Other neglected tropical diseases	Female	0-6 days	95+ years	Data Rich	-1	2	Sanitation (proportion with access)	155
Other neglected tropical diseases	Female	0-6 days	95+ years	Data Rich	1	2	Rainfall Quintile 5 (proportion)	377
Other neglected tropical diseases	Female	0-6 days	95+ years	Data Rich	-1	3	Socio-demographic Index	173
Other neglected tropical diseases	Female	0-6 days	95+ years	Data Rich	-1	3	Education (years per capita)	--
Other neglected tropical diseases	Female	0-6 days	95+ years	Data Rich	-1	3	LDI (I$ per capita)	--
Other neglected tropical diseases	Female	0-6 days	95+ years	Global	-1	1	Healthcare access and quality index	674
Other neglected tropical diseases	Female	0-6 days	95+ years	Global	1	1	Latitude Under 15 (proportion)	252
Other neglected tropical diseases	Female	0-6 days	95+ years	Global	-1	2	Sanitation (proportion with access)	237
Other neglected tropical diseases	Female	0-6 days	95+ years	Global	1	2	Rainfall Quintile 5 (proportion)	563
Other neglected tropical diseases	Female	0-6 days	95+ years	Global	-1	3	Education (years per capita)	--
Other neglected tropical diseases	Female	0-6 days	95+ years	Global	-1	3	LDI (I$ per capita)	--
Other neglected tropical diseases	Female	0-6 days	95+ years	Global	-1	3	Socio-demographic Index	--
Other neglected tropical diseases	Male	0-6 days	95+ years	Data Rich	-1	1	Healthcare access and quality index	487
Other neglected tropical diseases	Male	0-6 days	95+ years	Data Rich	1	1	Latitude Under 15 (proportion)	646
Other neglected tropical diseases	Male	0-6 days	95+ years	Data Rich	-1	2	Sanitation (proportion with access)	475
Other neglected tropical diseases	Male	0-6 days	95+ years	Data Rich	1	2	Rainfall Quintile 5 (proportion)	475
Other neglected tropical diseases	Male	0-6 days	95+ years	Data Rich	-1	3	Education (years per capita)	--
Other neglected tropical diseases	Male	0-6 days	95+ years	Data Rich	-1	3	LDI (I$ per capita)	--
Other neglected tropical diseases	Male	0-6 days	95+ years	Data Rich	-1	3	Socio-demographic Index	--
Other neglected tropical diseases	Male	0-6 days	95+ years	Global	-1	1	Healthcare access and quality index	537
Other neglected tropical diseases	Male	0-6 days	95+ years	Global	1	1	Latitude Under 15 (proportion)	629
Other neglected tropical diseases	Male	0-6 days	95+ years	Global	-1	2	Sanitation (proportion with access)	406
Other neglected tropical diseases	Male	0-6 days	95+ years	Global	1	2	Rainfall Quintile 5 (proportion)	100
Other neglected tropical diseases	Male	0-6 days	95+ years	Global	-1	3	Education (years per capita)	--
Other neglected tropical diseases	Male	0-6 days	95+ years	Global	-1	3	LDI (I$ per capita)	--
Other neglected tropical diseases	Male	0-6 days	95+ years	Global	-1	3	Socio-demographic Index	--

Table S16. CODEm covariates used, level of covariate, and expected direction of covariate by cause, sex, and age<br>*Covariates that CODEm did not select during the covariate selection process have no draw counts listed.*								
**Cause**	**Sex**	**Age Start**	**Age End**	**Model Type**	**Direction**	**Level**	**Covariate Name**	**Number of Draws**
Meningitis	Female	0-6 days	1-4 years	Data Rich	-1	1	Hib3 Vaccine Coverage (proportion)	1000
Meningitis	Female	0-6 days	1-4 years	Data Rich	-1	1	Proportion of total population covered by menafrivac initiative (meningitis meningococcal type A vaccine)	--
Meningitis	Female	0-6 days	1-4 years	Data Rich	1	1	meningitis belt (proportion)	--
Meningitis	Female	0-6 days	1-4 years	Data Rich	-1	2	Healthcare access and quality index	233
Meningitis	Female	0-6 days	1-4 years	Data Rich	-1	2	Improved Water Source (proportion with access)	--
Meningitis	Female	0-6 days	1-4 years	Data Rich	-1	2	Maternal care and immunization	--
Meningitis	Female	0-6 days	1-4 years	Data Rich	1	2	Underweight (proportion <2SD weight for age, <5 years)	502
Meningitis	Female	0-6 days	1-4 years	Data Rich	-1	3	DTP3 Coverage (proportion)	65
Meningitis	Female	0-6 days	1-4 years	Data Rich	-1	3	Sanitation (proportion with access)	192
Meningitis	Female	0-6 days	1-4 years	Data Rich	-1	3	Maternal Education (years per capita)	229
Meningitis	Female	0-6 days	1-4 years	Data Rich	-1	3	Socio-demographic Index	274
Meningitis	Female	0-6 days	1-4 years	Data Rich	-1	3	LDI (I$ per capita)	--
Meningitis	Female	0-6 days	1-4 years	Global	-1	1	Proportion of total population covered by menafrivac initiative (meningitis meningococcal type A vaccine)	3
Meningitis	Female	0-6 days	1-4 years	Global	-1	1	Hib3 Vaccine Coverage (proportion)	819
Meningitis	Female	0-6 days	1-4 years	Global	1	1	meningitis belt (proportion)	421
Meningitis	Female	0-6 days	1-4 years	Global	-1	2	Healthcare access and quality index	252
Meningitis	Female	0-6 days	1-4 years	Global	-1	2	Improved Water Source (proportion with access)	--
Meningitis	Female	0-6 days	1-4 years	Global	-1	2	Maternal care and immunization	--
Meningitis	Female	0-6 days	1-4 years	Global	1	2	Underweight (proportion <2SD weight for age, <5 years)	193
Meningitis	Female	0-6 days	1-4 years	Global	-1	3	Maternal Education (years per capita)	65
Meningitis	Female	0-6 days	1-4 years	Global	-1	3	Socio-demographic Index	73
Meningitis	Female	0-6 days	1-4 years	Global	-1	3	Sanitation (proportion with access)	254
Meningitis	Female	0-6 days	1-4 years	Global	-1	3	DTP3 Coverage (proportion)	283
Meningitis	Female	0-6 days	1-4 years	Global	-1	3	LDI (I$ per capita)	--
Meningitis	Female	5-9 years	95+ years	Data Rich	-1	1	Hib3 Vaccine Coverage (proportion)	1000
Meningitis	Female	5-9 years	95+ years	Data Rich	-1	1	Proportion of total population covered by menafrivac initiative (meningitis meningococcal type A vaccine)	--
Meningitis	Female	5-9 years	95+ years	Data Rich	1	1	meningitis belt (proportion)	--
Meningitis	Female	5-9 years	95+ years	Data Rich	-1	2	Healthcare access and quality index	40
Meningitis	Female	5-9 years	95+ years	Data Rich	-1	2	Improved Water Source (proportion with access)	--
Meningitis	Female	5-9 years	95+ years	Data Rich	-1	2	Maternal care and immunization	--
Meningitis	Female	5-9 years	95+ years	Data Rich	1	2	Underweight (proportion <2SD weight for age, <5 years)	542
Meningitis	Female	5-9 years	95+ years	Data Rich	-1	3	DTP3 Coverage (proportion)	13
Meningitis	Female	5-9 years	95+ years	Data Rich	-1	3	Maternal Education (years per capita)	176
Meningitis	Female	5-9 years	95+ years	Data Rich	-1	3	Sanitation (proportion with access)	408
Meningitis	Female	5-9 years	95+ years	Data Rich	-1	3	Socio-demographic Index	414
Meningitis	Female	5-9 years	95+ years	Data Rich	-1	3	LDI (I$ per capita)	--
Meningitis	Female	5-9 years	95+ years	Global	-1	1	Hib3 Vaccine Coverage (proportion)	992
Meningitis	Female	5-9 years	95+ years	Global	-1	1	Proportion of total population covered by menafrivac initiative (meningitis meningococcal type A vaccine)	--
Meningitis	Female	5-9 years	95+ years	Global	1	1	meningitis belt (proportion)	8
Meningitis	Female	5-9 years	95+ years	Global	-1	2	Healthcare access and quality index	90
Meningitis	Female	5-9 years	95+ years	Global	-1	2	Improved Water Source (proportion with access)	--
Meningitis	Female	5-9 years	95+ years	Global	-1	2	Maternal care and immunization	--
Meningitis	Female	5-9 years	95+ years	Global	1	2	Underweight (proportion <2SD weight for age, <5 years)	525
Meningitis	Female	5-9 years	95+ years	Global	-1	3	DTP3 Coverage (proportion)	26

Table S16. CODEm covariates used, level of covariate, and expected direction of covariate by cause, sex, and age *Covariates that CODEm did not select during the covariate selection process have no draw counts listed.*								
**Cause**	**Sex**	**Age Start**	**Age End**	**Model Type**	**Direction**	**Level**	**Covariate Name**	**Number of Draws**
Meningitis	Female	5-9 years	95+ years	Global	-1	3	Maternal Education (years per capita)	70
Meningitis	Female	5-9 years	95+ years	Global	-1	3	Socio-demographic Index	302
Meningitis	Female	5-9 years	95+ years	Global	-1	3	Sanitation (proportion with access)	507
Meningitis	Female	5-9 years	95+ years	Global	-1	3	LDI (I$ per capita)	--
Meningitis	Male	0-6 days	1-4 years	Data Rich	-1	1	Hib3 Vaccine Coverage (proportion)	1000
Meningitis	Male	0-6 days	1-4 years	Data Rich	-1	1	Proportion of total population covered by menafrivac initiative (meningitis meningococcal type A vaccine)	--
Meningitis	Male	0-6 days	1-4 years	Data Rich	1	1	meningitis belt (proportion)	--
Meningitis	Male	0-6 days	1-4 years	Data Rich	-1	2	Healthcare access and quality index	305
Meningitis	Male	0-6 days	1-4 years	Data Rich	-1	2	Improved Water Source (proportion with access)	--
Meningitis	Male	0-6 days	1-4 years	Data Rich	-1	2	Maternal care and immunization	--
Meningitis	Male	0-6 days	1-4 years	Data Rich	1	2	Underweight (proportion <2SD weight for age, <5 years)	531
Meningitis	Male	0-6 days	1-4 years	Data Rich	-1	3	DTP3 Coverage (proportion)	108
Meningitis	Male	0-6 days	1-4 years	Data Rich	-1	3	Sanitation (proportion with access)	279
Meningitis	Male	0-6 days	1-4 years	Data Rich	-1	3	Maternal Education (years per capita)	306
Meningitis	Male	0-6 days	1-4 years	Data Rich	-1	3	Socio-demographic Index	375
Meningitis	Male	0-6 days	1-4 years	Data Rich	-1	3	LDI (I$ per capita)	--
Meningitis	Male	0-6 days	1-4 years	Global	-1	1	Hib3 Vaccine Coverage (proportion)	635
Meningitis	Male	0-6 days	1-4 years	Global	-1	1	Proportion of total population covered by menafrivac initiative (meningitis meningococcal type A vaccine)	--
Meningitis	Male	0-6 days	1-4 years	Global	1	1	meningitis belt (proportion)	606
Meningitis	Male	0-6 days	1-4 years	Global	-1	2	Healthcare access and quality index	372
Meningitis	Male	0-6 days	1-4 years	Global	-1	2	Improved Water Source (proportion with access)	--
Meningitis	Male	0-6 days	1-4 years	Global	-1	2	Maternal care and immunization	--
Meningitis	Male	0-6 days	1-4 years	Global	1	2	Underweight (proportion <2SD weight for age, <5 years)	145
Meningitis	Male	0-6 days	1-4 years	Global	-1	3	DTP3 Coverage (proportion)	80
Meningitis	Male	0-6 days	1-4 years	Global	-1	3	Maternal Education (years per capita)	113
Meningitis	Male	0-6 days	1-4 years	Global	-1	3	Socio-demographic Index	126
Meningitis	Male	0-6 days	1-4 years	Global	-1	3	Sanitation (proportion with access)	525
Meningitis	Male	0-6 days	1-4 years	Global	-1	3	LDI (I$ per capita)	--
Meningitis	Male	5-9 years	95+ years	Data Rich	-1	1	Hib3 Vaccine Coverage (proportion)	1000
Meningitis	Male	5-9 years	95+ years	Data Rich	-1	1	Proportion of total population covered by menafrivac initiative (meningitis meningococcal type A vaccine)	--
Meningitis	Male	5-9 years	95+ years	Data Rich	1	1	meningitis belt (proportion)	--
Meningitis	Male	5-9 years	95+ years	Data Rich	-1	2	Healthcare access and quality index	73
Meningitis	Male	5-9 years	95+ years	Data Rich	-1	2	Improved Water Source (proportion with access)	--
Meningitis	Male	5-9 years	95+ years	Data Rich	-1	2	Maternal care and immunization	--
Meningitis	Male	5-9 years	95+ years	Data Rich	1	2	Underweight (proportion <2SD weight for age, <5 years)	153
Meningitis	Male	5-9 years	95+ years	Data Rich	-1	3	Maternal Education (years per capita)	202
Meningitis	Male	5-9 years	95+ years	Data Rich	-1	3	Socio-demographic Index	241
Meningitis	Male	5-9 years	95+ years	Data Rich	-1	3	Sanitation (proportion with access)	358
Meningitis	Male	5-9 years	95+ years	Data Rich	-1	3	DTP3 Coverage (proportion)	--
Meningitis	Male	5-9 years	95+ years	Data Rich	-1	3	LDI (I$ per capita)	--
Meningitis	Male	5-9 years	95+ years	Global	-1	1	Hib3 Vaccine Coverage (proportion)	--
Meningitis	Male	5-9 years	95+ years	Global	-1	1	Proportion of total population covered by menafrivac initiative (meningitis meningococcal type A vaccine)	--
Meningitis	Male	5-9 years	95+ years	Global	1	1	meningitis belt (proportion)	24
Meningitis	Male	5-9 years	95+ years	Global	-1	2	Healthcare access and quality index	420
Meningitis	Male	5-9 years	95+ years	Global	-1	2	Improved Water Source (proportion with access)	--

Table S16. CODEm covariates used, level of covariate, and expected direction of covariate by cause, sex, and age<br>*Covariates that CODEm did not select during the covariate selection process have no draw counts listed.*								
Cause	Sex	Age Start	Age End	Model Type	Direction	Level	Covariate Name	Number of Draws
Meningitis	Male	5-9 years	95+ years	Global	-1	2	Maternal care and immunization	--
Meningitis	Male	5-9 years	95+ years	Global	1	2	Underweight (proportion <2SD weight for age, <5 years)	325
Meningitis	Male	5-9 years	95+ years	Global	-1	3	DTP3 Coverage (proportion)	182
Meningitis	Male	5-9 years	95+ years	Global	-1	3	Socio-demographic Index	285
Meningitis	Male	5-9 years	95+ years	Global	-1	3	Sanitation (proportion with access)	381
Meningitis	Male	5-9 years	95+ years	Global	-1	3	LDI (I$ per capita)	--
Meningitis	Male	5-9 years	95+ years	Global	-1	3	Maternal Education (years per capita)	--
Encephalitis	Female	0-6 days	95+ years	Data Rich	1	1	Japanese encelphalitis endemic area (binary)	611
Encephalitis	Female	0-6 days	95+ years	Data Rich	1	1	Underweight (proportion <2SD weight for age, <5 years)	698
Encephalitis	Female	0-6 days	95+ years	Data Rich	-1	2	Healthcare access and quality index	--
Encephalitis	Female	0-6 days	95+ years	Data Rich	-1	2	LDI (I$ per capita)	--
Encephalitis	Female	0-6 days	95+ years	Data Rich	-1	2	Maternal care and immunization	--
Encephalitis	Female	0-6 days	95+ years	Data Rich	-1	3	Improved Water Source (proportion with access)	69
Encephalitis	Female	0-6 days	95+ years	Data Rich	-1	3	Sanitation (proportion with access)	658
Encephalitis	Female	0-6 days	95+ years	Data Rich	-1	3	DTP3 Coverage (proportion)	--
Encephalitis	Female	0-6 days	95+ years	Data Rich	-1	3	In-Facility Delivery (proportion)	--
Encephalitis	Female	0-6 days	95+ years	Data Rich	-1	3	Maternal Education (years per capita)	--
Encephalitis	Female	0-6 days	95+ years	Data Rich	-1	3	Socio-demographic Index	--
Encephalitis	Female	0-6 days	95+ years	Global	1	1	Japanese encelphalitis endemic area (binary)	800
Encephalitis	Female	0-6 days	95+ years	Global	1	1	Underweight (proportion <2SD weight for age, <5 years)	--
Encephalitis	Female	0-6 days	95+ years	Global	-1	2	Healthcare access and quality index	322
Encephalitis	Female	0-6 days	95+ years	Global	-1	2	LDI (I$ per capita)	--
Encephalitis	Female	0-6 days	95+ years	Global	-1	2	Maternal care and immunization	--
Encephalitis	Female	0-6 days	95+ years	Global	-1	3	Socio-demographic Index	19
Encephalitis	Female	0-6 days	95+ years	Global	-1	3	DTP3 Coverage (proportion)	--
Encephalitis	Female	0-6 days	95+ years	Global	-1	3	Improved Water Source (proportion with access)	--
Encephalitis	Female	0-6 days	95+ years	Global	-1	3	In-Facility Delivery (proportion)	--
Encephalitis	Female	0-6 days	95+ years	Global	-1	3	Maternal Education (years per capita)	--
Encephalitis	Female	0-6 days	95+ years	Global	-1	3	Sanitation (proportion with access)	--
Encephalitis	Male	0-6 days	95+ years	Data Rich	1	1	Underweight (proportion <2SD weight for age, <5 years)	546
Encephalitis	Male	0-6 days	95+ years	Data Rich	1	1	Japanese encelphalitis endemic area (binary)	695
Encephalitis	Male	0-6 days	95+ years	Data Rich	-1	2	Healthcare access and quality index	51
Encephalitis	Male	0-6 days	95+ years	Data Rich	-1	2	LDI (I$ per capita)	--
Encephalitis	Male	0-6 days	95+ years	Data Rich	-1	2	Maternal care and immunization	--
Encephalitis	Male	0-6 days	95+ years	Data Rich	-1	3	Improved Water Source (proportion with access)	38
Encephalitis	Male	0-6 days	95+ years	Data Rich	-1	3	Sanitation (proportion with access)	418
Encephalitis	Male	0-6 days	95+ years	Data Rich	-1	3	DTP3 Coverage (proportion)	--
Encephalitis	Male	0-6 days	95+ years	Data Rich	-1	3	In-Facility Delivery (proportion)	--
Encephalitis	Male	0-6 days	95+ years	Data Rich	-1	3	Maternal Education (years per capita)	--
Encephalitis	Male	0-6 days	95+ years	Data Rich	-1	3	Socio-demographic Index	--
Encephalitis	Male	0-6 days	95+ years	Global	1	1	Japanese encelphalitis endemic area (binary)	548
Encephalitis	Male	0-6 days	95+ years	Global	1	1	Underweight (proportion <2SD weight for age, <5 years)	--
Encephalitis	Male	0-6 days	95+ years	Global	-1	2	Healthcare access and quality index	276
Encephalitis	Male	0-6 days	95+ years	Global	-1	2	LDI (I$ per capita)	--
Encephalitis	Male	0-6 days	95+ years	Global	-1	2	Maternal care and immunization	--
Encephalitis	Male	0-6 days	95+ years	Global	-1	3	Socio-demographic Index	33
Encephalitis	Male	0-6 days	95+ years	Global	-1	3	DTP3 Coverage (proportion)	--
Encephalitis	Male	0-6 days	95+ years	Global	-1	3	Improved Water Source (proportion with access)	--
Encephalitis	Male	0-6 days	95+ years	Global	-1	3	In-Facility Delivery (proportion)	--
Encephalitis	Male	0-6 days	95+ years	Global	-1	3	Maternal Education (years per capita)	--
Encephalitis	Male	0-6 days	95+ years	Global	-1	3	Sanitation (proportion with access)	--

Table S16. CODEm covariates used, level of covariate, and expected direction of covariate by cause, sex, and age<br>*Covariates that CODEm did not select during the covariate selection process have no draw counts listed.*								
**Cause**	**Sex**	**Age Start**	**Age End**	**Model Type**	**Direction**	**Level**	**Covariate Name**	**Number of Draws**
Tetanus	Female	0-6 days	28-364 days	Data Rich	-1	1	DTP3 Coverage (proportion)	13
Tetanus	Female	0-6 days	28-364 days	Data Rich	-1	1	Tetanus Toxoid Coverage Smooth (proportion)	1000
Tetanus	Female	0-6 days	28-364 days	Data Rich	-1	2	In-Facility Delivery (proportion)	115
Tetanus	Female	0-6 days	28-364 days	Data Rich	-1	2	Skilled Birth Attendance (proportion)	133
Tetanus	Female	0-6 days	28-364 days	Data Rich	-1	2	Healthcare access and quality index	768
Tetanus	Female	0-6 days	28-364 days	Data Rich	-1	2	Maternal care and immunization	--
Tetanus	Female	0-6 days	28-364 days	Data Rich	-1	3	Education (years per capita)	238
Tetanus	Female	0-6 days	28-364 days	Data Rich	-1	3	Socio-demographic Index	479
Tetanus	Female	0-6 days	28-364 days	Data Rich	-1	3	LDI (I$ per capita)	--
Tetanus	Female	0-6 days	28-364 days	Global	-1	1	DTP3 Coverage (proportion)	613
Tetanus	Female	0-6 days	28-364 days	Global	-1	1	Tetanus Toxoid Coverage Smooth (proportion)	670
Tetanus	Female	0-6 days	28-364 days	Global	-1	2	In-Facility Delivery (proportion)	339
Tetanus	Female	0-6 days	28-364 days	Global	-1	2	Healthcare access and quality index	496
Tetanus	Female	0-6 days	28-364 days	Global	-1	2	Skilled Birth Attendance (proportion)	629
Tetanus	Female	0-6 days	28-364 days	Global	-1	2	Maternal care and immunization	--
Tetanus	Female	0-6 days	28-364 days	Global	-1	3	Socio-demographic Index	353
Tetanus	Female	0-6 days	28-364 days	Global	-1	3	Education (years per capita)	454
Tetanus	Female	0-6 days	28-364 days	Global	-1	3	LDI (I$ per capita)	--
Tetanus	Female	1-4 years	95+ years	Data Rich	-1	1	DTP3 Coverage (proportion)	1000
Tetanus	Female	1-4 years	95+ years	Data Rich	-1	2	Healthcare access and quality index	828
Tetanus	Female	1-4 years	95+ years	Data Rich	-1	2	Maternal care and immunization	--
Tetanus	Female	1-4 years	95+ years	Data Rich	-1	3	Socio-demographic Index	537
Tetanus	Female	1-4 years	95+ years	Data Rich	-1	3	Education (years per capita)	625
Tetanus	Female	1-4 years	95+ years	Data Rich	-1	3	Sanitation (proportion with access)	745
Tetanus	Female	1-4 years	95+ years	Data Rich	-1	3	LDI (I$ per capita)	--
Tetanus	Female	1-4 years	95+ years	Global	-1	1	DTP3 Coverage (proportion)	1000
Tetanus	Female	1-4 years	95+ years	Global	-1	2	Healthcare access and quality index	540
Tetanus	Female	1-4 years	95+ years	Global	-1	2	Maternal care and immunization	--
Tetanus	Female	1-4 years	95+ years	Global	-1	3	Socio-demographic Index	402
Tetanus	Female	1-4 years	95+ years	Global	-1	3	Education (years per capita)	496
Tetanus	Female	1-4 years	95+ years	Global	-1	3	Sanitation (proportion with access)	569
Tetanus	Female	1-4 years	95+ years	Global	-1	3	LDI (I$ per capita)	--
Tetanus	Male	0-6 days	28-364 days	Data Rich	-1	1	DTP3 Coverage (proportion)	428
Tetanus	Male	0-6 days	28-364 days	Data Rich	-1	1	Tetanus Toxoid Coverage Smooth (proportion)	819
Tetanus	Male	0-6 days	28-364 days	Data Rich	-1	2	Skilled Birth Attendance (proportion)	124
Tetanus	Male	0-6 days	28-364 days	Data Rich	-1	2	In-Facility Delivery (proportion)	199
Tetanus	Male	0-6 days	28-364 days	Data Rich	-1	2	Healthcare access and quality index	216
Tetanus	Male	0-6 days	28-364 days	Data Rich	-1	2	Maternal care and immunization	--
Tetanus	Male	0-6 days	28-364 days	Data Rich	-1	3	Socio-demographic Index	550
Tetanus	Male	0-6 days	28-364 days	Data Rich	-1	3	Education (years per capita)	728
Tetanus	Male	0-6 days	28-364 days	Data Rich	-1	3	LDI (I$ per capita)	--
Tetanus	Male	0-6 days	28-364 days	Global	-1	1	Tetanus Toxoid Coverage Smooth (proportion)	615
Tetanus	Male	0-6 days	28-364 days	Global	-1	1	DTP3 Coverage (proportion)	688
Tetanus	Male	0-6 days	28-364 days	Global	-1	2	Skilled Birth Attendance (proportion)	714
Tetanus	Male	0-6 days	28-364 days	Global	-1	2	In-Facility Delivery (proportion)	744

Table S16. CODEm covariates used, level of covariate, and expected direction of covariate by cause, sex, and age
Covariates that CODEm did not select during the covariate selection process have no draw counts listed.

Cause	Sex	Age Start	Age End	Model Type	Direction	Level	Covariate Name	Number of Draws
Tetanus	Male	0-6 days	28-364 days	Global	-1	2	Healthcare access and quality index	766
Tetanus	Male	0-6 days	28-364 days	Global	-1	2	Maternal care and immunization	--
Tetanus	Male	0-6 days	28-364 days	Global	-1	3	Education (years per capita)	238
Tetanus	Male	0-6 days	28-364 days	Global	-1	3	Socio-demographic Index	327
Tetanus	Male	0-6 days	28-364 days	Global	-1	3	LDI (I$ per capita)	--
Tetanus	Male	1-4 years	95+ years	Data Rich	-1	1	DTP3 Coverage (proportion)	1000
Tetanus	Male	1-4 years	95+ years	Data Rich	-1	2	Healthcare access and quality index	--
Tetanus	Male	1-4 years	95+ years	Data Rich	-1	2	Maternal care and immunization	--
Tetanus	Male	1-4 years	95+ years	Data Rich	-1	3	Socio-demographic Index	548
Tetanus	Male	1-4 years	95+ years	Data Rich	-1	3	Education (years per capita)	--
Tetanus	Male	1-4 years	95+ years	Data Rich	-1	3	LDI (I$ per capita)	--
Tetanus	Male	1-4 years	95+ years	Data Rich	-1	3	Sanitation (proportion with access)	--
Tetanus	Male	1-4 years	95+ years	Global	-1	1	DTP3 Coverage (proportion)	665
Tetanus	Male	1-4 years	95+ years	Global	-1	2	Healthcare access and quality index	576
Tetanus	Male	1-4 years	95+ years	Global	-1	2	Maternal care and immunization	--
Tetanus	Male	1-4 years	95+ years	Global	-1	3	Socio-demographic Index	660
Tetanus	Male	1-4 years	95+ years	Global	-1	3	Education (years per capita)	786
Tetanus	Male	1-4 years	95+ years	Global	-1	3	Sanitation (proportion with access)	786
Tetanus	Male	1-4 years	95+ years	Global	-1	3	LDI (I$ per capita)	--
Acute hepatitis	Female	28-364 days	95+ years	Data Rich	1	1	Seroprevalence of anti-HAV (IgG)	474
Acute hepatitis	Female	28-364 days	95+ years	Data Rich	1	1	Hepatitis B (HBsAg) Seroprevalence	770
Acute hepatitis	Female	28-364 days	95+ years	Data Rich	1	1	Hepatitis C (IgG) Seroprevalence	--
Acute hepatitis	Female	28-364 days	95+ years	Data Rich	1	1	Log-transformed SEV scalar: Hep	--
Acute hepatitis	Female	28-364 days	95+ years	Data Rich	1	1	Seroprevalence of anti-HEV (IgG)	--
Acute hepatitis	Female	28-364 days	95+ years	Data Rich	-1	2	Socio-demographic Index	170
Acute hepatitis	Female	28-364 days	95+ years	Data Rich	-1	2	Healthcare access and quality index	194
Acute hepatitis	Female	28-364 days	95+ years	Data Rich	-1	2	Hepatitis B vaccine coverage (proportion), aged through time	282
Acute hepatitis	Female	28-364 days	95+ years	Data Rich	1	2	Age- and sex-specific SEV for Unsafe sanitation	122
Acute hepatitis	Female	28-364 days	95+ years	Data Rich	1	2	Age- and sex-specific SEV for Unsafe water	304
Acute hepatitis	Female	28-364 days	95+ years	Data Rich	-1	3	Education (years per capita)	24
Acute hepatitis	Female	28-364 days	95+ years	Data Rich	-1	3	LDI (I$ per capita)	--
Acute hepatitis	Female	28-364 days	95+ years	Global	1	1	Hepatitis B (HBsAg) Seroprevalence	369
Acute hepatitis	Female	28-364 days	95+ years	Global	1	1	Seroprevalence of anti-HAV (IgG)	725
Acute hepatitis	Female	28-364 days	95+ years	Global	1	1	Hepatitis C (IgG) Seroprevalence	--
Acute hepatitis	Female	28-364 days	95+ years	Global	1	1	Log-transformed SEV scalar: Hep	--
Acute hepatitis	Female	28-364 days	95+ years	Global	1	1	Seroprevalence of anti-HEV (IgG)	--
Acute hepatitis	Female	28-364 days	95+ years	Global	-1	2	Healthcare access and quality index	221
Acute hepatitis	Female	28-364 days	95+ years	Global	-1	2	Hepatitis B vaccine coverage (proportion), aged through time	313
Acute hepatitis	Female	28-364 days	95+ years	Global	-1	2	Socio-demographic Index	323
Acute hepatitis	Female	28-364 days	95+ years	Global	1	2	Age- and sex-specific SEV for Unsafe sanitation	396
Acute hepatitis	Female	28-364 days	95+ years	Global	1	2	Age- and sex-specific SEV for Unsafe water	621
Acute hepatitis	Female	28-364 days	95+ years	Global	-1	3	Education (years per capita)	295
Acute hepatitis	Female	28-364 days	95+ years	Global	-1	3	LDI (I$ per capita)	--
Acute hepatitis	Male	28-364 days	95+ years	Data Rich	1	1	Seroprevalence of anti-HAV (IgG)	172
Acute hepatitis	Male	28-364 days	95+ years	Data Rich	1	1	Hepatitis B (HBsAg) Seroprevalence	865

Table S16. CODEm covariates used, level of covariate, and expected direction of covariate by cause, sex, and age<br>*Covariates that CODEm did not select during the covariate selection process have no draw counts listed.*								
**Cause**	**Sex**	**Age Start**	**Age End**	**Model Type**	**Direction**	**Level**	**Covariate Name**	**Number of Draws**
Acute hepatitis	Male	28-364 days	95+ years	Data Rich	1	1	Hepatitis C (IgG) Seroprevalence	--
Acute hepatitis	Male	28-364 days	95+ years	Data Rich	1	1	Seroprevalence of anti-HEV (IgG)	--
Acute hepatitis	Male	28-364 days	95+ years	Data Rich	-1	2	Healthcare access and quality index	20
Acute hepatitis	Male	28-364 days	95+ years	Data Rich	-1	2	Hepatitis B vaccine coverage (proportion), aged through time	453
Acute hepatitis	Male	28-364 days	95+ years	Data Rich	1	2	Age- and sex-specific SEV for Unsafe sanitation	231
Acute hepatitis	Male	28-364 days	95+ years	Data Rich	1	2	Age- and sex-specific SEV for Unsafe water	233
Acute hepatitis	Male	28-364 days	95+ years	Data Rich	1	2	Log-transformed SEV scalar: Hep	--
Acute hepatitis	Male	28-364 days	95+ years	Data Rich	-1	3	Education (years per capita)	43
Acute hepatitis	Male	28-364 days	95+ years	Data Rich	-1	3	Socio-demographic Index	133
Acute hepatitis	Male	28-364 days	95+ years	Data Rich	-1	3	LDI (I$ per capita)	--
Acute hepatitis	Male	28-364 days	95+ years	Global	1	1	Hepatitis B (HBsAg) Seroprevalence	523
Acute hepatitis	Male	28-364 days	95+ years	Global	1	1	Seroprevalence of anti-HAV (IgG)	603
Acute hepatitis	Male	28-364 days	95+ years	Global	1	1	Hepatitis C (IgG) Seroprevalence	--
Acute hepatitis	Male	28-364 days	95+ years	Global	1	1	Log-transformed SEV scalar: Hep	--
Acute hepatitis	Male	28-364 days	95+ years	Global	1	1	Seroprevalence of anti-HEV (IgG)	--
Acute hepatitis	Male	28-364 days	95+ years	Global	-1	2	Socio-demographic Index	31
Acute hepatitis	Male	28-364 days	95+ years	Global	-1	2	Healthcare access and quality index	238
Acute hepatitis	Male	28-364 days	95+ years	Global	-1	2	Hepatitis B vaccine coverage (proportion), aged through time	453
Acute hepatitis	Male	28-364 days	95+ years	Global	1	2	Age- and sex-specific SEV for Unsafe sanitation	227
Acute hepatitis	Male	28-364 days	95+ years	Global	1	2	Age- and sex-specific SEV for Unsafe water	299
Acute hepatitis	Male	28-364 days	95+ years	Global	-1	3	Education (years per capita)	36
Acute hepatitis	Male	28-364 days	95+ years	Global	-1	3	LDI (I$ per capita)	--
Other unspecified infectious diseases	Female	0-6 days	95+ years	Data Rich	-1	1	DTP3 Coverage (proportion)	525
Other unspecified infectious diseases	Female	0-6 days	95+ years	Data Rich	-1	1	Health System Access (unitless)	--
Other unspecified infectious diseases	Female	0-6 days	95+ years	Data Rich	-1	1	Measles Vaccine Coverage (proportion)	--
Other unspecified infectious diseases	Female	0-6 days	95+ years	Data Rich	-1	2	Antenatal Care (1 visit) Coverage (proportion)	--
Other unspecified infectious diseases	Female	0-6 days	95+ years	Data Rich	-1	2	Healthcare access and quality index	--
Other unspecified infectious diseases	Female	0-6 days	95+ years	Data Rich	-1	2	Improved Water Source (proportion with access)	--
Other unspecified infectious diseases	Female	0-6 days	95+ years	Data Rich	-1	2	Latitude Over 45 (proportion)	--
Other unspecified infectious diseases	Female	0-6 days	95+ years	Data Rich	-1	2	Maternal care and immunization	--
Other unspecified infectious diseases	Female	0-6 days	95+ years	Data Rich	-1	2	Rainfall Quintile 1 (proportion)	--
Other unspecified infectious diseases	Female	0-6 days	95+ years	Data Rich	-1	2	Sanitation (proportion with access)	--
Other unspecified infectious diseases	Female	0-6 days	95+ years	Data Rich	0	2	Rainfall Quintile 2 (proportion)	236
Other unspecified infectious diseases	Female	0-6 days	95+ years	Data Rich	0	2	Rainfall Quintile 4 (proportion)	236
Other unspecified infectious diseases	Female	0-6 days	95+ years	Data Rich	0	2	Latitude 30 to 45 (proportion)	246
Other unspecified infectious diseases	Female	0-6 days	95+ years	Data Rich	0	2	Latitude 15 to 30 (proportion)	452
Other unspecified infectious diseases	Female	0-6 days	95+ years	Data Rich	0	2	Rainfall Quintile 3 (proportion)	620
Other unspecified infectious diseases	Female	0-6 days	95+ years	Data Rich	1	2	Latitude Under 15 (proportion)	431
Other unspecified infectious diseases	Female	0-6 days	95+ years	Data Rich	1	2	Rainfall Quintile 5 (proportion)	--
Other unspecified infectious diseases	Female	0-6 days	95+ years	Data Rich	1	2	Underweight (proportion <2SD weight for age, <5 years)	--
Other unspecified infectious diseases	Female	0-6 days	95+ years	Data Rich	-1	3	Education (years per capita)	--
Other unspecified infectious diseases	Female	0-6 days	95+ years	Data Rich	-1	3	LDI (I$ per capita)	--
Other unspecified infectious diseases	Female	0-6 days	95+ years	Data Rich	-1	3	Socio-demographic Index	--
Other unspecified infectious diseases	Female	0-6 days	95+ years	Global	-1	1	Antenatal Care (1 visit) Coverage (proportion)	--
Other unspecified infectious diseases	Female	0-6 days	95+ years	Global	1	1	Underweight (proportion <2SD weight for age, <5 years)	1000
Other unspecified infectious diseases	Female	0-6 days	95+ years	Global	-1	2	Sanitation (proportion with access)	459

Table S16. CODEm covariates used, level of covariate, and expected direction of covariate by cause, sex, and age<br>*Covariates that CODEm did not select during the covariate selection process have no draw counts listed.*								
**Cause**	**Sex**	**Age Start**	**Age End**	**Model Type**	**Direction**	**Level**	**Covariate Name**	**Number of Draws**
Other unspecified infectious diseases	Female	0-6 days	95+ years	Global	-1	2	Latitude Over 45 (proportion)	662
Other unspecified infectious diseases	Female	0-6 days	95+ years	Global	-1	2	Healthcare access and quality index	--
Other unspecified infectious diseases	Female	0-6 days	95+ years	Global	-1	2	Improved Water Source (proportion with access)	--
Other unspecified infectious diseases	Female	0-6 days	95+ years	Global	-1	3	DTP3 Coverage (proportion)	--
Other unspecified infectious diseases	Female	0-6 days	95+ years	Global	-1	3	Socio-demographic Index	--
Other unspecified infectious diseases	Female	0-6 days	95+ years	Global	0	3	Latitude 30 to 45 (proportion)	557
Other unspecified infectious diseases	Female	0-6 days	95+ years	Global	0	3	Latitude 15 to 30 (proportion)	582
Other unspecified infectious diseases	Female	0-6 days	95+ years	Global	1	3	Latitude Under 15 (proportion)	573
Other unspecified infectious diseases	Male	0-6 days	95+ years	Data Rich	-1	1	Measles Vaccine Coverage (proportion)	892
Other unspecified infectious diseases	Male	0-6 days	95+ years	Data Rich	-1	1	DTP3 Coverage (proportion)	--
Other unspecified infectious diseases	Male	0-6 days	95+ years	Data Rich	-1	1	Health System Access (unitless)	--
Other unspecified infectious diseases	Male	0-6 days	95+ years	Data Rich	-1	2	Antenatal Care (1 visit) Coverage (proportion)	--
Other unspecified infectious diseases	Male	0-6 days	95+ years	Data Rich	-1	2	Improved Water Source (proportion with access)	--
Other unspecified infectious diseases	Male	0-6 days	95+ years	Data Rich	-1	2	Latitude Over 45 (proportion)	--
Other unspecified infectious diseases	Male	0-6 days	95+ years	Data Rich	-1	2	Rainfall Quintile 1 (proportion)	--
Other unspecified infectious diseases	Male	0-6 days	95+ years	Data Rich	-1	2	Sanitation (proportion with access)	--
Other unspecified infectious diseases	Male	0-6 days	95+ years	Data Rich	0	2	Latitude 15 to 30 (proportion)	108
Other unspecified infectious diseases	Male	0-6 days	95+ years	Data Rich	0	2	Latitude 30 to 45 (proportion)	108
Other unspecified infectious diseases	Male	0-6 days	95+ years	Data Rich	0	2	Rainfall Quintile 4 (proportion)	162
Other unspecified infectious diseases	Male	0-6 days	95+ years	Data Rich	0	2	Rainfall Quintile 3 (proportion)	434
Other unspecified infectious diseases	Male	0-6 days	95+ years	Data Rich	0	2	Rainfall Quintile 2 (proportion)	492
Other unspecified infectious diseases	Male	0-6 days	95+ years	Data Rich	1	2	Rainfall Quintile 5 (proportion)	374
Other unspecified infectious diseases	Male	0-6 days	95+ years	Data Rich	1	2	Latitude Under 15 (proportion)	--
Other unspecified infectious diseases	Male	0-6 days	95+ years	Data Rich	1	2	Underweight (proportion <2SD weight for age, <5 years)	--
Other unspecified infectious diseases	Male	0-6 days	95+ years	Data Rich	-1	3	Education (years per capita)	--
Other unspecified infectious diseases	Male	0-6 days	95+ years	Data Rich	-1	3	LDI (I$ per capita)	--
Other unspecified infectious diseases	Male	0-6 days	95+ years	Data Rich	-1	3	Socio-demographic Index	--
Other unspecified infectious diseases	Male	0-6 days	95+ years	Global	-1	1	Antenatal Care (1 visit) Coverage (proportion)	381
Other unspecified infectious diseases	Male	0-6 days	95+ years	Global	1	1	Underweight (proportion <2SD weight for age, <5 years)	884
Other unspecified infectious diseases	Male	0-6 days	95+ years	Global	-1	2	Improved Water Source (proportion with access)	358
Other unspecified infectious diseases	Male	0-6 days	95+ years	Global	-1	2	Sanitation (proportion with access)	358
Other unspecified infectious diseases	Male	0-6 days	95+ years	Global	-1	2	Latitude Over 45 (proportion)	532
Other unspecified infectious diseases	Male	0-6 days	95+ years	Global	-1	3	DTP3 Coverage (proportion)	--
Other unspecified infectious diseases	Male	0-6 days	95+ years	Global	-1	3	Socio-demographic Index	--
Other unspecified infectious diseases	Male	0-6 days	95+ years	Global	0	3	Latitude 30 to 45 (proportion)	545
Other unspecified infectious diseases	Male	0-6 days	95+ years	Global	0	3	Latitude 15 to 30 (proportion)	576
Other unspecified infectious diseases	Male	0-6 days	95+ years	Global	1	3	Latitude Under 15 (proportion)	165
Neonatal disorders	Female	0-6 days	1-4 years	Data Rich	1	1	Indoor Air Pollution (All Cooking Fuels)	177
Neonatal disorders	Female	0-6 days	1-4 years	Data Rich	1	1	Smoking Prevalence (Reproductive Age Standardized)	743
Neonatal disorders	Female	0-6 days	1-4 years	Data Rich	-1	2	In-Facility Delivery (proportion)	61
Neonatal disorders	Female	0-6 days	1-4 years	Data Rich	-1	2	Antenatal Care (4 visits) Coverage (proportion)	103
Neonatal disorders	Female	0-6 days	1-4 years	Data Rich	-1	2	Skilled Birth Attendance (proportion)	150
Neonatal disorders	Female	0-6 days	1-4 years	Data Rich	-1	2	Healthcare access and quality index	253
Neonatal disorders	Female	0-6 days	1-4 years	Data Rich	-1	2	Maternal care and immunization	--
Neonatal disorders	Female	0-6 days	1-4 years	Data Rich	1	2	Live Births 35+ (proportion)	187
Neonatal disorders	Female	0-6 days	1-4 years	Data Rich	1	2	Age-standardized SEV for Child underweight	--
Neonatal disorders	Female	0-6 days	1-4 years	Data Rich	-1	3	Socio-demographic Index	80
Neonatal disorders	Female	0-6 days	1-4 years	Data Rich	-1	3	Education (years per capita)	126
Neonatal disorders	Female	0-6 days	1-4 years	Data Rich	-1	3	LDI (I$ per capita)	--
Neonatal disorders	Female	0-6 days	1-4 years	Data Rich	1	3	Total Fertility Rate	--
Neonatal disorders	Female	0-6 days	1-4 years	Global	1	1	Indoor Air Pollution (All Cooking Fuels)	169
Neonatal disorders	Female	0-6 days	1-4 years	Global	1	1	Smoking Prevalence (Reproductive Age Standardized)	581

Table S16. CODEm covariates used, level of covariate, and expected direction of covariate by cause, sex, and age<br>*Covariates that CODEm did not select during the covariate selection process have no draw counts listed.*								
**Cause**	**Sex**	**Age Start**	**Age End**	**Model Type**	**Direction**	**Level**	**Covariate Name**	**Number of Draws**
Neonatal disorders	Female	0-6 days	1-4 years	Global	-1	2	Antenatal Care (4 visits) Coverage (proportion)	48
Neonatal disorders	Female	0-6 days	1-4 years	Global	-1	2	In-Facility Delivery (proportion)	89
Neonatal disorders	Female	0-6 days	1-4 years	Global	-1	2	Skilled Birth Attendance (proportion)	89
Neonatal disorders	Female	0-6 days	1-4 years	Global	-1	2	Healthcare access and quality index	356
Neonatal disorders	Female	0-6 days	1-4 years	Global	-1	2	Maternal care and immunization	--
Neonatal disorders	Female	0-6 days	1-4 years	Global	1	2	Live Births 35+ (proportion)	323
Neonatal disorders	Female	0-6 days	1-4 years	Global	1	2	Age-standardized SEV for Child underweight	--
Neonatal disorders	Female	0-6 days	1-4 years	Global	-1	3	Education (years per capita)	125
Neonatal disorders	Female	0-6 days	1-4 years	Global	-1	3	Socio-demographic Index	150
Neonatal disorders	Female	0-6 days	1-4 years	Global	-1	3	LDI (I$ per capita)	--
Neonatal disorders	Female	0-6 days	1-4 years	Global	1	3	Total Fertility Rate	--
Neonatal disorders	Male	0-6 days	1-4 years	Data Rich	1	1	Smoking Prevalence (Reproductive Age Standardized)	316
Neonatal disorders	Male	0-6 days	1-4 years	Data Rich	1	1	Indoor Air Pollution (All Cooking Fuels)	498
Neonatal disorders	Male	0-6 days	1-4 years	Data Rich	-1	2	Antenatal Care (4 visits) Coverage (proportion)	8
Neonatal disorders	Male	0-6 days	1-4 years	Data Rich	-1	2	Skilled Birth Attendance (proportion)	166
Neonatal disorders	Male	0-6 days	1-4 years	Data Rich	-1	2	In-Facility Delivery (proportion)	240
Neonatal disorders	Male	0-6 days	1-4 years	Data Rich	-1	2	Healthcare access and quality index	--
Neonatal disorders	Male	0-6 days	1-4 years	Data Rich	-1	2	Maternal care and immunization	--
Neonatal disorders	Male	0-6 days	1-4 years	Data Rich	1	2	Live Births 35+ (proportion)	250
Neonatal disorders	Male	0-6 days	1-4 years	Data Rich	1	2	Age-standardized SEV for Child underweight	--
Neonatal disorders	Male	0-6 days	1-4 years	Data Rich	-1	3	Socio-demographic Index	61
Neonatal disorders	Male	0-6 days	1-4 years	Data Rich	-1	3	Education (years per capita)	70
Neonatal disorders	Male	0-6 days	1-4 years	Data Rich	-1	3	LDI (I$ per capita)	--
Neonatal disorders	Male	0-6 days	1-4 years	Data Rich	1	3	Total Fertility Rate	--
Neonatal disorders	Male	0-6 days	1-4 years	Global	1	1	Indoor Air Pollution (All Cooking Fuels)	387
Neonatal disorders	Male	0-6 days	1-4 years	Global	1	1	Smoking Prevalence (Reproductive Age Standardized)	394
Neonatal disorders	Male	0-6 days	1-4 years	Global	-1	2	Antenatal Care (4 visits) Coverage (proportion)	57
Neonatal disorders	Male	0-6 days	1-4 years	Global	-1	2	In-Facility Delivery (proportion)	92
Neonatal disorders	Male	0-6 days	1-4 years	Global	-1	2	Skilled Birth Attendance (proportion)	210
Neonatal disorders	Male	0-6 days	1-4 years	Global	-1	2	Healthcare access and quality index	--
Neonatal disorders	Male	0-6 days	1-4 years	Global	-1	2	Maternal care and immunization	--
Neonatal disorders	Male	0-6 days	1-4 years	Global	1	2	Live Births 35+ (proportion)	323
Neonatal disorders	Male	0-6 days	1-4 years	Global	1	2	Age-standardized SEV for Child underweight	--
Neonatal disorders	Male	0-6 days	1-4 years	Global	-1	3	Socio-demographic Index	85
Neonatal disorders	Male	0-6 days	1-4 years	Global	-1	3	Education (years per capita)	156
Neonatal disorders	Male	0-6 days	1-4 years	Global	-1	3	LDI (I$ per capita)	--
Neonatal disorders	Male	0-6 days	1-4 years	Global	1	3	Total Fertility Rate	--
Neonatal preterm birth	Female	0-6 days	1-4 years	Data Rich	-1	1	Maternal care and immunization	--
Neonatal preterm birth	Female	0-6 days	1-4 years	Data Rich	1	1	Indoor Air Pollution (All Cooking Fuels)	12
Neonatal preterm birth	Female	0-6 days	1-4 years	Data Rich	1	1	Smoking Prevalence (Reproductive Age Standardized)	396
Neonatal preterm birth	Female	0-6 days	1-4 years	Data Rich	-1	2	In-Facility Delivery (proportion)	1
Neonatal preterm birth	Female	0-6 days	1-4 years	Data Rich	-1	2	Skilled Birth Attendance (proportion)	53
Neonatal preterm birth	Female	0-6 days	1-4 years	Data Rich	-1	2	Antenatal Care (4 visits) Coverage (proportion)	106
Neonatal preterm birth	Female	0-6 days	1-4 years	Data Rich	-1	2	Healthcare access and quality index	483
Neonatal preterm birth	Female	0-6 days	1-4 years	Data Rich	1	2	Live Births 35+ (proportion)	191
Neonatal preterm birth	Female	0-6 days	1-4 years	Data Rich	1	2	Age-standardized SEV for Child underweight	--
Neonatal preterm birth	Female	0-6 days	1-4 years	Data Rich	-1	3	Education (years per capita)	27
Neonatal preterm birth	Female	0-6 days	1-4 years	Data Rich	-1	3	Socio-demographic Index	197
Neonatal preterm birth	Female	0-6 days	1-4 years	Data Rich	-1	3	LDI (I$ per capita)	--
Neonatal preterm birth	Female	0-6 days	1-4 years	Data Rich	1	3	Total Fertility Rate	--
Neonatal preterm birth	Female	0-6 days	1-4 years	Global	-1	1	Maternal care and immunization	--

Table S16. CODEm covariates used, level of covariate, and expected direction of covariate by cause, sex, and age
Covariates that CODEm did not select during the covariate selection process have no draw counts listed.

Cause	Sex	Age Start	Age End	Model Type	Direction	Level	Covariate Name	Number of Draws
Neonatal preterm birth	Female	0-6 days	1-4 years	Global	1	1	Indoor Air Pollution (All Cooking Fuels)	79
Neonatal preterm birth	Female	0-6 days	1-4 years	Global	1	1	Smoking Prevalence (Reproductive Age Standardized)	199
Neonatal preterm birth	Female	0-6 days	1-4 years	Global	-1	2	Antenatal Care (4 visits) Coverage (proportion)	18
Neonatal preterm birth	Female	0-6 days	1-4 years	Global	-1	2	In-Facility Delivery (proportion)	106
Neonatal preterm birth	Female	0-6 days	1-4 years	Global	-1	2	Skilled Birth Attendance (proportion)	114
Neonatal preterm birth	Female	0-6 days	1-4 years	Global	-1	2	Healthcare access and quality index	412
Neonatal preterm birth	Female	0-6 days	1-4 years	Global	1	2	Live Births 35+ (proportion)	324
Neonatal preterm birth	Female	0-6 days	1-4 years	Global	1	2	Age-standardized SEV for Child underweight	--
Neonatal preterm birth	Female	0-6 days	1-4 years	Global	-1	3	Education (years per capita)	53
Neonatal preterm birth	Female	0-6 days	1-4 years	Global	-1	3	Socio-demographic Index	131
Neonatal preterm birth	Female	0-6 days	1-4 years	Global	-1	3	LDI (I$ per capita)	--
Neonatal preterm birth	Female	0-6 days	1-4 years	Global	1	3	Total Fertility Rate	--
Neonatal preterm birth	Male	0-6 days	1-4 years	Data Rich	-1	1	Maternal care and immunization	--
Neonatal preterm birth	Male	0-6 days	1-4 years	Data Rich	1	1	Indoor Air Pollution (All Cooking Fuels)	19
Neonatal preterm birth	Male	0-6 days	1-4 years	Data Rich	1	1	Smoking Prevalence (Reproductive Age Standardized)	32
Neonatal preterm birth	Male	0-6 days	1-4 years	Data Rich	-1	2	Antenatal Care (4 visits) Coverage (proportion)	42
Neonatal preterm birth	Male	0-6 days	1-4 years	Data Rich	-1	2	Healthcare access and quality index	122
Neonatal preterm birth	Male	0-6 days	1-4 years	Data Rich	-1	2	In-Facility Delivery (proportion)	122
Neonatal preterm birth	Male	0-6 days	1-4 years	Data Rich	-1	2	Skilled Birth Attendance (proportion)	187
Neonatal preterm birth	Male	0-6 days	1-4 years	Data Rich	1	2	Live Births 35+ (proportion)	193
Neonatal preterm birth	Male	0-6 days	1-4 years	Data Rich	1	2	Age-standardized SEV for Child underweight	--
Neonatal preterm birth	Male	0-6 days	1-4 years	Data Rich	-1	3	Education (years per capita)	44
Neonatal preterm birth	Male	0-6 days	1-4 years	Data Rich	-1	3	Socio-demographic Index	172
Neonatal preterm birth	Male	0-6 days	1-4 years	Data Rich	-1	3	LDI (I$ per capita)	--
Neonatal preterm birth	Male	0-6 days	1-4 years	Data Rich	1	3	Total Fertility Rate	--
Neonatal preterm birth	Male	0-6 days	1-4 years	Global	-1	1	Maternal care and immunization	--
Neonatal preterm birth	Male	0-6 days	1-4 years	Global	1	1	Smoking Prevalence (Reproductive Age Standardized)	33
Neonatal preterm birth	Male	0-6 days	1-4 years	Global	1	1	Indoor Air Pollution (All Cooking Fuels)	66
Neonatal preterm birth	Male	0-6 days	1-4 years	Global	-1	2	Antenatal Care (4 visits) Coverage (proportion)	45
Neonatal preterm birth	Male	0-6 days	1-4 years	Global	-1	2	In-Facility Delivery (proportion)	58
Neonatal preterm birth	Male	0-6 days	1-4 years	Global	-1	2	Healthcare access and quality index	121
Neonatal preterm birth	Male	0-6 days	1-4 years	Global	-1	2	Skilled Birth Attendance (proportion)	160
Neonatal preterm birth	Male	0-6 days	1-4 years	Global	1	2	Live Births 35+ (proportion)	324
Neonatal preterm birth	Male	0-6 days	1-4 years	Global	1	2	Age-standardized SEV for Child underweight	--
Neonatal preterm birth	Male	0-6 days	1-4 years	Global	-1	3	Education (years per capita)	67
Neonatal preterm birth	Male	0-6 days	1-4 years	Global	-1	3	Socio-demographic Index	102
Neonatal preterm birth	Male	0-6 days	1-4 years	Global	-1	3	LDI (I$ per capita)	--
Neonatal preterm birth	Male	0-6 days	1-4 years	Global	1	3	Total Fertility Rate	--
Neonatal encephalopathy due to birth asphyxia and trauma	Female	0-6 days	1-4 years	Data Rich	1	1	Indoor Air Pollution (All Cooking Fuels)	326
Neonatal encephalopathy due to birth asphyxia and trauma	Female	0-6 days	1-4 years	Data Rich	1	1	Smoking Prevalence (Reproductive Age Standardized)	854
Neonatal encephalopathy due to birth asphyxia and trauma	Female	0-6 days	1-4 years	Data Rich	-1	2	Antenatal Care (4 visits) Coverage (proportion)	37
Neonatal encephalopathy due to birth asphyxia and trauma	Female	0-6 days	1-4 years	Data Rich	-1	2	In-Facility Delivery (proportion)	78
Neonatal encephalopathy due to birth asphyxia and trauma	Female	0-6 days	1-4 years	Data Rich	-1	2	Healthcare access and quality index	239
Neonatal encephalopathy due to birth asphyxia and trauma	Female	0-6 days	1-4 years	Data Rich	-1	2	Skilled Birth Attendance (proportion)	256
Neonatal encephalopathy due to birth asphyxia and trauma	Female	0-6 days	1-4 years	Data Rich	-1	2	Maternal care and immunization	--
Neonatal encephalopathy due to birth asphyxia and trauma	Female	0-6 days	1-4 years	Data Rich	1	2	Age-standardized SEV for Child underweight	--
Neonatal encephalopathy due to birth asphyxia and trauma	Female	0-6 days	1-4 years	Data Rich	1	2	Live Births 35+ (proportion)	--
Neonatal encephalopathy due to birth asphyxia and trauma	Female	0-6 days	1-4 years	Data Rich	-1	3	Socio-demographic Index	284
Neonatal encephalopathy due to birth asphyxia and trauma	Female	0-6 days	1-4 years	Data Rich	-1	3	Education (years per capita)	--
Neonatal encephalopathy due to birth asphyxia and trauma	Female	0-6 days	1-4 years	Data Rich	-1	3	LDI (I$ per capita)	--

Table S16. CODEm covariates used, level of covariate, and expected direction of covariate by cause, sex, and age
Covariates that CODEm did not select during the covariate selection process have no draw counts listed.

Cause	Sex	Age Start	Age End	Model Type	Direction	Level	Covariate Name	Number of Draws
Neonatal encephalopathy due to birth asphyxia and trauma	Female	0-6 days	1-4 years	Data Rich	1	3	Total Fertility Rate	--
Neonatal encephalopathy due to birth asphyxia and trauma	Female	0-6 days	1-4 years	Global	1	1	Smoking Prevalence (Reproductive Age Standardized)	384
Neonatal encephalopathy due to birth asphyxia and trauma	Female	0-6 days	1-4 years	Global	1	1	Indoor Air Pollution (All Cooking Fuels)	412
Neonatal encephalopathy due to birth asphyxia and trauma	Female	0-6 days	1-4 years	Global	-1	2	Skilled Birth Attendance (proportion)	106
Neonatal encephalopathy due to birth asphyxia and trauma	Female	0-6 days	1-4 years	Global	-1	2	Antenatal Care (4 visits) Coverage (proportion)	112
Neonatal encephalopathy due to birth asphyxia and trauma	Female	0-6 days	1-4 years	Global	-1	2	In-Facility Delivery (proportion)	160
Neonatal encephalopathy due to birth asphyxia and trauma	Female	0-6 days	1-4 years	Global	-1	2	Healthcare access and quality index	--
Neonatal encephalopathy due to birth asphyxia and trauma	Female	0-6 days	1-4 years	Global	-1	2	Maternal care and immunization	--
Neonatal encephalopathy due to birth asphyxia and trauma	Female	0-6 days	1-4 years	Global	1	2	Live Births 35+ (proportion)	324
Neonatal encephalopathy due to birth asphyxia and trauma	Female	0-6 days	1-4 years	Global	1	2	Age-standardized SEV for Child underweight	--
Neonatal encephalopathy due to birth asphyxia and trauma	Female	0-6 days	1-4 years	Global	-1	3	Socio-demographic Index	115
Neonatal encephalopathy due to birth asphyxia and trauma	Female	0-6 days	1-4 years	Global	-1	3	Education (years per capita)	--
Neonatal encephalopathy due to birth asphyxia and trauma	Female	0-6 days	1-4 years	Global	-1	3	LDI (I$ per capita)	--
Neonatal encephalopathy due to birth asphyxia and trauma	Female	0-6 days	1-4 years	Global	1	3	Total Fertility Rate	--
Neonatal encephalopathy due to birth asphyxia and trauma	Male	0-6 days	1-4 years	Data Rich	1	1	Indoor Air Pollution (All Cooking Fuels)	364
Neonatal encephalopathy due to birth asphyxia and trauma	Male	0-6 days	1-4 years	Data Rich	1	1	Smoking Prevalence (Reproductive Age Standardized)	550
Neonatal encephalopathy due to birth asphyxia and trauma	Male	0-6 days	1-4 years	Data Rich	-1	2	Antenatal Care (4 visits) Coverage (proportion)	75
Neonatal encephalopathy due to birth asphyxia and trauma	Male	0-6 days	1-4 years	Data Rich	-1	2	Skilled Birth Attendance (proportion)	103
Neonatal encephalopathy due to birth asphyxia and trauma	Male	0-6 days	1-4 years	Data Rich	-1	2	In-Facility Delivery (proportion)	152
Neonatal encephalopathy due to birth asphyxia and trauma	Male	0-6 days	1-4 years	Data Rich	-1	2	Healthcare access and quality index	--
Neonatal encephalopathy due to birth asphyxia and trauma	Male	0-6 days	1-4 years	Data Rich	-1	2	Maternal care and immunization	--
Neonatal encephalopathy due to birth asphyxia and trauma	Male	0-6 days	1-4 years	Data Rich	1	2	Age-standardized SEV for Child underweight	--
Neonatal encephalopathy due to birth asphyxia and trauma	Male	0-6 days	1-4 years	Data Rich	1	2	Live Births 35+ (proportion)	--
Neonatal encephalopathy due to birth asphyxia and trauma	Male	0-6 days	1-4 years	Data Rich	-1	3	Socio-demographic Index	140
Neonatal encephalopathy due to birth asphyxia and trauma	Male	0-6 days	1-4 years	Data Rich	-1	3	Education (years per capita)	207
Neonatal encephalopathy due to birth asphyxia and trauma	Male	0-6 days	1-4 years	Data Rich	-1	3	LDI (I$ per capita)	--
Neonatal encephalopathy due to birth asphyxia and trauma	Male	0-6 days	1-4 years	Data Rich	1	3	Total Fertility Rate	--
Neonatal encephalopathy due to birth asphyxia and trauma	Male	0-6 days	1-4 years	Global	1	1	Indoor Air Pollution (All Cooking Fuels)	226
Neonatal encephalopathy due to birth asphyxia and trauma	Male	0-6 days	1-4 years	Global	1	1	Smoking Prevalence (Reproductive Age Standardized)	558
Neonatal encephalopathy due to birth asphyxia and trauma	Male	0-6 days	1-4 years	Global	-1	2	Antenatal Care (4 visits) Coverage (proportion)	12
Neonatal encephalopathy due to birth asphyxia and trauma	Male	0-6 days	1-4 years	Global	-1	2	Skilled Birth Attendance (proportion)	74
Neonatal encephalopathy due to birth asphyxia and trauma	Male	0-6 days	1-4 years	Global	-1	2	In-Facility Delivery (proportion)	172
Neonatal encephalopathy due to birth asphyxia and trauma	Male	0-6 days	1-4 years	Global	-1	2	Healthcare access and quality index	213
Neonatal encephalopathy due to birth asphyxia and trauma	Male	0-6 days	1-4 years	Global	-1	2	Maternal care and immunization	--
Neonatal encephalopathy due to birth asphyxia and trauma	Male	0-6 days	1-4 years	Global	1	2	Age-standardized SEV for Child underweight	--
Neonatal encephalopathy due to birth asphyxia and trauma	Male	0-6 days	1-4 years	Global	1	2	Live Births 35+ (proportion)	--
Neonatal encephalopathy due to birth asphyxia and trauma	Male	0-6 days	1-4 years	Global	-1	3	Socio-demographic Index	164
Neonatal encephalopathy due to birth asphyxia and trauma	Male	0-6 days	1-4 years	Global	-1	3	Education (years per capita)	--
Neonatal encephalopathy due to birth asphyxia and trauma	Male	0-6 days	1-4 years	Global	-1	3	LDI (I$ per capita)	--
Neonatal encephalopathy due to birth asphyxia and trauma	Male	0-6 days	1-4 years	Global	1	3	Total Fertility Rate	--
Neonatal sepsis and other neonatal infections	Female	0-6 days	1-4 years	Data Rich	1	1	Smoking Prevalence (Reproductive Age Standardized)	953
Neonatal sepsis and other neonatal infections	Female	0-6 days	1-4 years	Data Rich	1	1	Indoor Air Pollution (All Cooking Fuels)	--
Neonatal sepsis and other neonatal infections	Female	0-6 days	1-4 years	Data Rich	-1	2	Healthcare access and quality index	127
Neonatal sepsis and other neonatal infections	Female	0-6 days	1-4 years	Data Rich	-1	2	Antenatal Care (4 visits) Coverage (proportion)	187
Neonatal sepsis and other neonatal infections	Female	0-6 days	1-4 years	Data Rich	-1	2	In-Facility Delivery (proportion)	--
Neonatal sepsis and other neonatal infections	Female	0-6 days	1-4 years	Data Rich	-1	2	Maternal care and immunization	--
Neonatal sepsis and other neonatal infections	Female	0-6 days	1-4 years	Data Rich	-1	2	Skilled Birth Attendance (proportion)	--
Neonatal sepsis and other neonatal infections	Female	0-6 days	1-4 years	Data Rich	1	2	Live Births 35+ (proportion)	47
Neonatal sepsis and other neonatal infections	Female	0-6 days	1-4 years	Data Rich	1	2	Age-standardized SEV for Child underweight	--
Neonatal sepsis and other neonatal infections	Female	0-6 days	1-4 years	Data Rich	-1	3	Education (years per capita)	111

Table S16. CODEm covariates used, level of covariate, and expected direction of covariate by cause, sex, and age
Covariates that CODEm did not select during the covariate selection process have no draw counts listed.

Cause	Sex	Age Start	Age End	Model Type	Direction	Level	Covariate Name	Number of Draws
Neonatal sepsis and other neonatal infections	Female	0-6 days	1-4 years	Data Rich	-1	3	Socio-demographic Index	204
Neonatal sepsis and other neonatal infections	Female	0-6 days	1-4 years	Data Rich	-1	3	LDI (I$ per capita)	--
Neonatal sepsis and other neonatal infections	Female	0-6 days	1-4 years	Data Rich	1	3	Total Fertility Rate	--
Neonatal sepsis and other neonatal infections	Female	0-6 days	1-4 years	Global	1	1	Smoking Prevalence (Reproductive Age Standardized)	920
Neonatal sepsis and other neonatal infections	Female	0-6 days	1-4 years	Global	1	1	Indoor Air Pollution (All Cooking Fuels)	--
Neonatal sepsis and other neonatal infections	Female	0-6 days	1-4 years	Global	-1	2	Antenatal Care (4 visits) Coverage (proportion)	38
Neonatal sepsis and other neonatal infections	Female	0-6 days	1-4 years	Global	-1	2	Healthcare access and quality index	269
Neonatal sepsis and other neonatal infections	Female	0-6 days	1-4 years	Global	-1	2	In-Facility Delivery (proportion)	--
Neonatal sepsis and other neonatal infections	Female	0-6 days	1-4 years	Global	-1	2	Maternal care and immunization	--
Neonatal sepsis and other neonatal infections	Female	0-6 days	1-4 years	Global	-1	2	Skilled Birth Attendance (proportion)	--
Neonatal sepsis and other neonatal infections	Female	0-6 days	1-4 years	Global	1	2	Live Births 35+ (proportion)	80
Neonatal sepsis and other neonatal infections	Female	0-6 days	1-4 years	Global	1	2	Age-standardized SEV for Child underweight	--
Neonatal sepsis and other neonatal infections	Female	0-6 days	1-4 years	Global	-1	3	Education (years per capita)	129
Neonatal sepsis and other neonatal infections	Female	0-6 days	1-4 years	Global	-1	3	Socio-demographic Index	129
Neonatal sepsis and other neonatal infections	Female	0-6 days	1-4 years	Global	-1	3	LDI (I$ per capita)	--
Neonatal sepsis and other neonatal infections	Female	0-6 days	1-4 years	Global	1	3	Total Fertility Rate	--
Neonatal sepsis and other neonatal infections	Male	0-6 days	1-4 years	Data Rich	1	1	Indoor Air Pollution (All Cooking Fuels)	89
Neonatal sepsis and other neonatal infections	Male	0-6 days	1-4 years	Data Rich	1	1	Smoking Prevalence (Reproductive Age Standardized)	765
Neonatal sepsis and other neonatal infections	Male	0-6 days	1-4 years	Data Rich	1	1	Age-standardized SEV for Child underweight	--
Neonatal sepsis and other neonatal infections	Male	0-6 days	1-4 years	Data Rich	-1	2	Skilled Birth Attendance (proportion)	24
Neonatal sepsis and other neonatal infections	Male	0-6 days	1-4 years	Data Rich	-1	2	Antenatal Care (4 visits) Coverage (proportion)	107
Neonatal sepsis and other neonatal infections	Male	0-6 days	1-4 years	Data Rich	-1	2	Healthcare access and quality index	324
Neonatal sepsis and other neonatal infections	Male	0-6 days	1-4 years	Data Rich	-1	2	In-Facility Delivery (proportion)	--
Neonatal sepsis and other neonatal infections	Male	0-6 days	1-4 years	Data Rich	-1	2	Maternal care and immunization	--
Neonatal sepsis and other neonatal infections	Male	0-6 days	1-4 years	Data Rich	1	2	Live Births 35+ (proportion)	33
Neonatal sepsis and other neonatal infections	Male	0-6 days	1-4 years	Data Rich	-1	3	Socio-demographic Index	118
Neonatal sepsis and other neonatal infections	Male	0-6 days	1-4 years	Data Rich	-1	3	Education (years per capita)	202
Neonatal sepsis and other neonatal infections	Male	0-6 days	1-4 years	Data Rich	-1	3	LDI (I$ per capita)	--
Neonatal sepsis and other neonatal infections	Male	0-6 days	1-4 years	Data Rich	1	3	Total Fertility Rate	--
Neonatal sepsis and other neonatal infections	Male	0-6 days	1-4 years	Global	1	1	Indoor Air Pollution (All Cooking Fuels)	62
Neonatal sepsis and other neonatal infections	Male	0-6 days	1-4 years	Global	1	1	Smoking Prevalence (Reproductive Age Standardized)	772
Neonatal sepsis and other neonatal infections	Male	0-6 days	1-4 years	Global	-1	2	Skilled Birth Attendance (proportion)	26
Neonatal sepsis and other neonatal infections	Male	0-6 days	1-4 years	Global	-1	2	In-Facility Delivery (proportion)	32
Neonatal sepsis and other neonatal infections	Male	0-6 days	1-4 years	Global	-1	2	Antenatal Care (4 visits) Coverage (proportion)	39
Neonatal sepsis and other neonatal infections	Male	0-6 days	1-4 years	Global	-1	2	Healthcare access and quality index	440
Neonatal sepsis and other neonatal infections	Male	0-6 days	1-4 years	Global	-1	2	Maternal care and immunization	--
Neonatal sepsis and other neonatal infections	Male	0-6 days	1-4 years	Global	1	2	Live Births 35+ (proportion)	190
Neonatal sepsis and other neonatal infections	Male	0-6 days	1-4 years	Global	1	2	Age-standardized SEV for Child underweight	--
Neonatal sepsis and other neonatal infections	Male	0-6 days	1-4 years	Global	-1	3	Socio-demographic Index	249
Neonatal sepsis and other neonatal infections	Male	0-6 days	1-4 years	Global	-1	3	Education (years per capita)	281
Neonatal sepsis and other neonatal infections	Male	0-6 days	1-4 years	Global	-1	3	LDI (I$ per capita)	--
Neonatal sepsis and other neonatal infections	Male	0-6 days	1-4 years	Global	1	3	Total Fertility Rate	--
Hemolytic disease and other neonatal jaundice	Female	0-6 days	1-4 years	Data Rich	1	1	Indoor Air Pollution (All Cooking Fuels)	332
Hemolytic disease and other neonatal jaundice	Female	0-6 days	1-4 years	Data Rich	1	1	Smoking Prevalence (Reproductive Age Standardized)	737
Hemolytic disease and other neonatal jaundice	Female	0-6 days	1-4 years	Data Rich	-1	2	Antenatal Care (4 visits) Coverage (proportion)	84
Hemolytic disease and other neonatal jaundice	Female	0-6 days	1-4 years	Data Rich	-1	2	Healthcare access and quality index	141
Hemolytic disease and other neonatal jaundice	Female	0-6 days	1-4 years	Data Rich	-1	2	Skilled Birth Attendance (proportion)	174
Hemolytic disease and other neonatal jaundice	Female	0-6 days	1-4 years	Data Rich	-1	2	In-Facility Delivery (proportion)	446
Hemolytic disease and other neonatal jaundice	Female	0-6 days	1-4 years	Data Rich	-1	2	Maternal care and immunization	--
Hemolytic disease and other neonatal jaundice	Female	0-6 days	1-4 years	Data Rich	1	2	Age-standardized SEV for Child underweight	--

Table S16. CODEm covariates used, level of covariate, and expected direction of covariate by cause, sex, and age<br>*Covariates that CODEm did not select during the covariate selection process have no draw counts listed.*								
**Cause**	**Sex**	**Age Start**	**Age End**	**Model Type**	**Direction**	**Level**	**Covariate Name**	**Number of Draws**
Hemolytic disease and other neonatal jaundice	Female	0-6 days	1-4 years	Data Rich	1	2	Live Births 35+ (proportion)	--
Hemolytic disease and other neonatal jaundice	Female	0-6 days	1-4 years	Data Rich	-1	3	Education (years per capita)	187
Hemolytic disease and other neonatal jaundice	Female	0-6 days	1-4 years	Data Rich	-1	3	Socio-demographic Index	338
Hemolytic disease and other neonatal jaundice	Female	0-6 days	1-4 years	Data Rich	-1	3	LDI (I$ per capita)	--
Hemolytic disease and other neonatal jaundice	Female	0-6 days	1-4 years	Data Rich	1	3	Total Fertility Rate	--
Hemolytic disease and other neonatal jaundice	Female	0-6 days	1-4 years	Global	1	1	Indoor Air Pollution (All Cooking Fuels)	269
Hemolytic disease and other neonatal jaundice	Female	0-6 days	1-4 years	Global	1	1	Smoking Prevalence (Reproductive Age Standardized)	911
Hemolytic disease and other neonatal jaundice	Female	0-6 days	1-4 years	Global	1	1	Age-standardized SEV for Child underweight	--
Hemolytic disease and other neonatal jaundice	Female	0-6 days	1-4 years	Global	-1	2	Antenatal Care (4 visits) Coverage (proportion)	39
Hemolytic disease and other neonatal jaundice	Female	0-6 days	1-4 years	Global	-1	2	Skilled Birth Attendance (proportion)	68
Hemolytic disease and other neonatal jaundice	Female	0-6 days	1-4 years	Global	-1	2	Healthcare access and quality index	115
Hemolytic disease and other neonatal jaundice	Female	0-6 days	1-4 years	Global	-1	2	In-Facility Delivery (proportion)	741
Hemolytic disease and other neonatal jaundice	Female	0-6 days	1-4 years	Global	-1	2	Maternal care and immunization	--
Hemolytic disease and other neonatal jaundice	Female	0-6 days	1-4 years	Global	1	2	Live Births 35+ (proportion)	--
Hemolytic disease and other neonatal jaundice	Female	0-6 days	1-4 years	Global	-1	3	Education (years per capita)	5
Hemolytic disease and other neonatal jaundice	Female	0-6 days	1-4 years	Global	-1	3	Socio-demographic Index	279
Hemolytic disease and other neonatal jaundice	Female	0-6 days	1-4 years	Global	-1	3	LDI (I$ per capita)	--
Hemolytic disease and other neonatal jaundice	Female	0-6 days	1-4 years	Global	1	3	Total Fertility Rate	--
Hemolytic disease and other neonatal jaundice	Male	0-6 days	1-4 years	Data Rich	1	1	Indoor Air Pollution (All Cooking Fuels)	354
Hemolytic disease and other neonatal jaundice	Male	0-6 days	1-4 years	Data Rich	1	1	Smoking Prevalence (Reproductive Age Standardized)	854
Hemolytic disease and other neonatal jaundice	Male	0-6 days	1-4 years	Data Rich	-1	2	Skilled Birth Attendance (proportion)	5
Hemolytic disease and other neonatal jaundice	Male	0-6 days	1-4 years	Data Rich	-1	2	Antenatal Care (4 visits) Coverage (proportion)	89
Hemolytic disease and other neonatal jaundice	Male	0-6 days	1-4 years	Data Rich	-1	2	In-Facility Delivery (proportion)	156
Hemolytic disease and other neonatal jaundice	Male	0-6 days	1-4 years	Data Rich	-1	2	Healthcare access and quality index	242
Hemolytic disease and other neonatal jaundice	Male	0-6 days	1-4 years	Data Rich	-1	2	Maternal care and immunization	--
Hemolytic disease and other neonatal jaundice	Male	0-6 days	1-4 years	Data Rich	1	2	Age-standardized SEV for Child underweight	--
Hemolytic disease and other neonatal jaundice	Male	0-6 days	1-4 years	Data Rich	1	2	Live Births 35+ (proportion)	--
Hemolytic disease and other neonatal jaundice	Male	0-6 days	1-4 years	Data Rich	-1	3	Education (years per capita)	218
Hemolytic disease and other neonatal jaundice	Male	0-6 days	1-4 years	Data Rich	-1	3	Socio-demographic Index	350
Hemolytic disease and other neonatal jaundice	Male	0-6 days	1-4 years	Data Rich	-1	3	LDI (I$ per capita)	--
Hemolytic disease and other neonatal jaundice	Male	0-6 days	1-4 years	Data Rich	1	3	Total Fertility Rate	--
Hemolytic disease and other neonatal jaundice	Male	0-6 days	1-4 years	Global	1	1	Indoor Air Pollution (All Cooking Fuels)	419
Hemolytic disease and other neonatal jaundice	Male	0-6 days	1-4 years	Global	1	1	Smoking Prevalence (Reproductive Age Standardized)	791
Hemolytic disease and other neonatal jaundice	Male	0-6 days	1-4 years	Global	-1	2	In-Facility Delivery (proportion)	82
Hemolytic disease and other neonatal jaundice	Male	0-6 days	1-4 years	Global	-1	2	Healthcare access and quality index	121
Hemolytic disease and other neonatal jaundice	Male	0-6 days	1-4 years	Global	-1	2	Skilled Birth Attendance (proportion)	143
Hemolytic disease and other neonatal jaundice	Male	0-6 days	1-4 years	Global	-1	2	Antenatal Care (4 visits) Coverage (proportion)	241
Hemolytic disease and other neonatal jaundice	Male	0-6 days	1-4 years	Global	-1	2	Maternal care and immunization	--
Hemolytic disease and other neonatal jaundice	Male	0-6 days	1-4 years	Global	1	2	Age-standardized SEV for Child underweight	--
Hemolytic disease and other neonatal jaundice	Male	0-6 days	1-4 years	Global	1	2	Live Births 35+ (proportion)	--
Hemolytic disease and other neonatal jaundice	Male	0-6 days	1-4 years	Global	-1	3	Socio-demographic Index	142
Hemolytic disease and other neonatal jaundice	Male	0-6 days	1-4 years	Global	-1	3	Education (years per capita)	194
Hemolytic disease and other neonatal jaundice	Male	0-6 days	1-4 years	Global	-1	3	LDI (I$ per capita)	--
Hemolytic disease and other neonatal jaundice	Male	0-6 days	1-4 years	Global	1	3	Total Fertility Rate	--
Other neonatal disorders	Female	0-6 days	1-4 years	Data Rich	1	1	Indoor Air Pollution (All Cooking Fuels)	484
Other neonatal disorders	Female	0-6 days	1-4 years	Data Rich	1	1	Smoking Prevalence (Reproductive Age Standardized)	679
Other neonatal disorders	Female	0-6 days	1-4 years	Data Rich	-1	2	Skilled Birth Attendance (proportion)	49
Other neonatal disorders	Female	0-6 days	1-4 years	Data Rich	-1	2	In-Facility Delivery (proportion)	50
Other neonatal disorders	Female	0-6 days	1-4 years	Data Rich	-1	2	Antenatal Care (4 visits) Coverage (proportion)	268
Other neonatal disorders	Female	0-6 days	1-4 years	Data Rich	-1	2	Healthcare access and quality index	550
Other neonatal disorders	Female	0-6 days	1-4 years	Data Rich	-1	2	Maternal care and immunization	--

Table S16. CODEm covariates used, level of covariate, and expected direction of covariate by cause, sex, and age
Covariates that CODEm did not select during the covariate selection process have no draw counts listed.

Cause	Sex	Age Start	Age End	Model Type	Direction	Level	Covariate Name	Number of Draws
Other neonatal disorders	Female	0-6 days	1-4 years	Data Rich	1	2	Live Births 35+ (proportion)	186
Other neonatal disorders	Female	0-6 days	1-4 years	Data Rich	1	2	Age-standardized SEV for Child underweight	--
Other neonatal disorders	Female	0-6 days	1-4 years	Data Rich	-1	3	Socio-demographic Index	67
Other neonatal disorders	Female	0-6 days	1-4 years	Data Rich	-1	3	Education (years per capita)	164
Other neonatal disorders	Female	0-6 days	1-4 years	Data Rich	-1	3	LDI (I$ per capita)	--
Other neonatal disorders	Female	0-6 days	1-4 years	Data Rich	1	3	Total Fertility Rate	--
Other neonatal disorders	Female	0-6 days	1-4 years	Global	1	1	Indoor Air Pollution (All Cooking Fuels)	320
Other neonatal disorders	Female	0-6 days	1-4 years	Global	1	1	Smoking Prevalence (Reproductive Age Standardized)	490
Other neonatal disorders	Female	0-6 days	1-4 years	Global	-1	2	Skilled Birth Attendance (proportion)	165
Other neonatal disorders	Female	0-6 days	1-4 years	Global	-1	2	In-Facility Delivery (proportion)	376
Other neonatal disorders	Female	0-6 days	1-4 years	Global	-1	2	Antenatal Care (4 visits) Coverage (proportion)	464
Other neonatal disorders	Female	0-6 days	1-4 years	Global	-1	2	Healthcare access and quality index	--
Other neonatal disorders	Female	0-6 days	1-4 years	Global	-1	2	Maternal care and immunization	--
Other neonatal disorders	Female	0-6 days	1-4 years	Global	1	2	Live Births 35+ (proportion)	190
Other neonatal disorders	Female	0-6 days	1-4 years	Global	1	2	Age-standardized SEV for Child underweight	--
Other neonatal disorders	Female	0-6 days	1-4 years	Global	-1	3	Education (years per capita)	74
Other neonatal disorders	Female	0-6 days	1-4 years	Global	-1	3	Socio-demographic Index	79
Other neonatal disorders	Female	0-6 days	1-4 years	Global	-1	3	LDI (I$ per capita)	--
Other neonatal disorders	Female	0-6 days	1-4 years	Global	1	3	Total Fertility Rate	--
Other neonatal disorders	Male	0-6 days	1-4 years	Data Rich	1	1	Indoor Air Pollution (All Cooking Fuels)	451
Other neonatal disorders	Male	0-6 days	1-4 years	Data Rich	1	1	Smoking Prevalence (Reproductive Age Standardized)	719
Other neonatal disorders	Male	0-6 days	1-4 years	Data Rich	-1	2	Antenatal Care (4 visits) Coverage (proportion)	51
Other neonatal disorders	Male	0-6 days	1-4 years	Data Rich	-1	2	In-Facility Delivery (proportion)	321
Other neonatal disorders	Male	0-6 days	1-4 years	Data Rich	-1	2	Skilled Birth Attendance (proportion)	359
Other neonatal disorders	Male	0-6 days	1-4 years	Data Rich	-1	2	Healthcare access and quality index	--
Other neonatal disorders	Male	0-6 days	1-4 years	Data Rich	-1	2	Maternal care and immunization	--
Other neonatal disorders	Male	0-6 days	1-4 years	Data Rich	1	2	Live Births 35+ (proportion)	188
Other neonatal disorders	Male	0-6 days	1-4 years	Data Rich	1	2	Age-standardized SEV for Child underweight	--
Other neonatal disorders	Male	0-6 days	1-4 years	Data Rich	-1	3	Education (years per capita)	--
Other neonatal disorders	Male	0-6 days	1-4 years	Data Rich	-1	3	LDI (I$ per capita)	--
Other neonatal disorders	Male	0-6 days	1-4 years	Data Rich	-1	3	Socio-demographic Index	--
Other neonatal disorders	Male	0-6 days	1-4 years	Data Rich	1	3	Total Fertility Rate	--
Other neonatal disorders	Male	0-6 days	1-4 years	Global	1	1	Smoking Prevalence (Reproductive Age Standardized)	234
Other neonatal disorders	Male	0-6 days	1-4 years	Global	1	1	Indoor Air Pollution (All Cooking Fuels)	807
Other neonatal disorders	Male	0-6 days	1-4 years	Global	-1	2	Antenatal Care (4 visits) Coverage (proportion)	173
Other neonatal disorders	Male	0-6 days	1-4 years	Global	-1	2	Skilled Birth Attendance (proportion)	228
Other neonatal disorders	Male	0-6 days	1-4 years	Global	-1	2	In-Facility Delivery (proportion)	230
Other neonatal disorders	Male	0-6 days	1-4 years	Global	-1	2	Healthcare access and quality index	--
Other neonatal disorders	Male	0-6 days	1-4 years	Global	-1	2	Maternal care and immunization	--
Other neonatal disorders	Male	0-6 days	1-4 years	Global	1	2	Live Births 35+ (proportion)	193
Other neonatal disorders	Male	0-6 days	1-4 years	Global	1	2	Age-standardized SEV for Child underweight	--
Other neonatal disorders	Male	0-6 days	1-4 years	Global	-1	3	Education (years per capita)	--
Other neonatal disorders	Male	0-6 days	1-4 years	Global	-1	3	LDI (I$ per capita)	--
Other neonatal disorders	Male	0-6 days	1-4 years	Global	-1	3	Socio-demographic Index	--
Other neonatal disorders	Male	0-6 days	1-4 years	Global	1	3	Total Fertility Rate	--
Nutritional deficiencies	Female	28-364 days	95+ years	Data Rich	-1	1	Proportion of households using iodized salt (adjusted)	0
Nutritional deficiencies	Female	28-364 days	95+ years	Data Rich	-1	1	energy unadjusted(kcal)	0
Nutritional deficiencies	Female	28-364 days	95+ years	Data Rich	1	1	Age-Standardize Prevalence of Severe Anemia	0
Nutritional deficiencies	Female	28-364 days	95+ years	Data Rich	1	1	Underweight, age and sex specific	358
Nutritional deficiencies	Female	28-364 days	95+ years	Data Rich	1	1	Age-standardized SEV for Child underweight	--

Table S16. CODEm covariates used, level of covariate, and expected direction of covariate by cause, sex, and age<br>*Covariates that CODEm did not select during the covariate selection process have no draw counts listed.*								
**Cause**	**Sex**	**Age Start**	**Age End**	**Model Type**	**Direction**	**Level**	**Covariate Name**	**Number of Draws**
Nutritional deficiencies	Female	28-364 days	95+ years	Data Rich	1	1	Age-standardized SEV for Child wasting	--
Nutritional deficiencies	Female	28-364 days	95+ years	Data Rich	-1	2	Healthcare access and quality index	0
Nutritional deficiencies	Female	28-364 days	95+ years	Data Rich	-1	2	Maternal care and immunization	--
Nutritional deficiencies	Female	28-364 days	95+ years	Data Rich	0	2	Rainfall Quintile 2 (proportion)	49
Nutritional deficiencies	Female	28-364 days	95+ years	Data Rich	0	2	Rainfall Quintile 1 (proportion)	74
Nutritional deficiencies	Female	28-364 days	95+ years	Data Rich	1	2	Age- and sex-specific SEV for Unsafe water	17
Nutritional deficiencies	Female	28-364 days	95+ years	Data Rich	1	2	Age- and sex-specific SEV for Unsafe sanitation	27
Nutritional deficiencies	Female	28-364 days	95+ years	Data Rich	1	2	Age- and sex-specific SEV for Alcohol use	--
Nutritional deficiencies	Female	28-364 days	95+ years	Data Rich	1	2	Log-transformed SEV scalar: Diarrhea	--
Nutritional deficiencies	Female	28-364 days	95+ years	Data Rich	1	2	Mortality Rate Due to War Shocks (per 1 person)	--
Nutritional deficiencies	Female	28-364 days	95+ years	Data Rich	-1	3	Maternal Education (years per capita)	0
Nutritional deficiencies	Female	28-364 days	95+ years	Data Rich	-1	3	Education (years per capita)	167
Nutritional deficiencies	Female	28-364 days	95+ years	Data Rich	-1	3	Socio-demographic Index	269
Nutritional deficiencies	Female	28-364 days	95+ years	Data Rich	-1	3	LDI (I$ per capita)	--
Nutritional deficiencies	Female	28-364 days	95+ years	Global	-1	1	energy unadjusted(kcal)	73
Nutritional deficiencies	Female	28-364 days	95+ years	Global	-1	1	Proportion of households using iodized salt (adjusted)	511
Nutritional deficiencies	Female	28-364 days	95+ years	Global	1	1	Age-Standardize Prevalence of Severe Anemia	60
Nutritional deficiencies	Female	28-364 days	95+ years	Global	1	1	Underweight, age and sex specific	949
Nutritional deficiencies	Female	28-364 days	95+ years	Global	1	1	Age-standardized SEV for Child underweight	--
Nutritional deficiencies	Female	28-364 days	95+ years	Global	1	1	Age-standardized SEV for Child wasting	--
Nutritional deficiencies	Female	28-364 days	95+ years	Global	-1	2	Healthcare access and quality index	7
Nutritional deficiencies	Female	28-364 days	95+ years	Global	-1	2	Maternal care and immunization	--
Nutritional deficiencies	Female	28-364 days	95+ years	Global	0	2	Rainfall Quintile 1 (proportion)	70
Nutritional deficiencies	Female	28-364 days	95+ years	Global	0	2	Rainfall Quintile 2 (proportion)	399
Nutritional deficiencies	Female	28-364 days	95+ years	Global	1	2	Age- and sex-specific SEV for Unsafe sanitation	388
Nutritional deficiencies	Female	28-364 days	95+ years	Global	1	2	Age- and sex-specific SEV for Unsafe water	388
Nutritional deficiencies	Female	28-364 days	95+ years	Global	1	2	Age- and sex-specific SEV for Alcohol use	--
Nutritional deficiencies	Female	28-364 days	95+ years	Global	1	2	Log-transformed SEV scalar: Diarrhea	--
Nutritional deficiencies	Female	28-364 days	95+ years	Global	1	2	Mortality Rate Due to War Shocks (per 1 person)	--
Nutritional deficiencies	Female	28-364 days	95+ years	Global	-1	3	Socio-demographic Index	2
Nutritional deficiencies	Female	28-364 days	95+ years	Global	-1	3	Education (years per capita)	158
Nutritional deficiencies	Female	28-364 days	95+ years	Global	-1	3	LDI (I$ per capita)	--
Nutritional deficiencies	Male	28-364 days	95+ years	Data Rich	-1	1	Proportion of households using iodized salt (adjusted)	3
Nutritional deficiencies	Male	28-364 days	95+ years	Data Rich	-1	1	energy unadjusted(kcal)	138
Nutritional deficiencies	Male	28-364 days	95+ years	Data Rich	1	1	Age-Standardize Prevalence of Severe Anemia	350
Nutritional deficiencies	Male	28-364 days	95+ years	Data Rich	1	1	Underweight, age and sex specific	920
Nutritional deficiencies	Male	28-364 days	95+ years	Data Rich	1	1	Age-standardized SEV for Child underweight	--
Nutritional deficiencies	Male	28-364 days	95+ years	Data Rich	1	1	Age-standardized SEV for Child wasting	--
Nutritional deficiencies	Male	28-364 days	95+ years	Data Rich	-1	2	Healthcare access and quality index	0
Nutritional deficiencies	Male	28-364 days	95+ years	Data Rich	-1	2	Sanitation (proportion with access)	0

Table S16. CODEm covariates used, level of covariate, and expected direction of covariate by cause, sex, and age
Covariates that CODEm did not select during the covariate selection process have no draw counts listed.

Cause	Sex	Age Start	Age End	Model Type	Direction	Level	Covariate Name	Number of Draws
Nutritional deficiencies	Male	28-364 days	95+ years	Data Rich	-1	2	Improved Water Source (proportion with access)	12
Nutritional deficiencies	Male	28-364 days	95+ years	Data Rich	-1	2	Maternal care and immunization	--
Nutritional deficiencies	Male	28-364 days	95+ years	Data Rich	0	2	Rainfall Quintile 2 (proportion)	34
Nutritional deficiencies	Male	28-364 days	95+ years	Data Rich	0	2	Rainfall Quintile 1 (proportion)	285
Nutritional deficiencies	Male	28-364 days	95+ years	Data Rich	1	2	Age- and sex-specific SEV for Alcohol use	--
Nutritional deficiencies	Male	28-364 days	95+ years	Data Rich	1	2	Mortality Rate Due to War Shocks (per 1 person)	--
Nutritional deficiencies	Male	28-364 days	95+ years	Data Rich	-1	3	Education (years per capita)	0
Nutritional deficiencies	Male	28-364 days	95+ years	Data Rich	-1	3	Socio-demographic Index	4
Nutritional deficiencies	Male	28-364 days	95+ years	Data Rich	-1	3	Maternal Education (years per capita)	27
Nutritional deficiencies	Male	28-364 days	95+ years	Data Rich	-1	3	LDI (I$ per capita)	--
Nutritional deficiencies	Male	28-364 days	95+ years	Global	-1	1	Proportion of households using iodized salt (adjusted)	0
Nutritional deficiencies	Male	28-364 days	95+ years	Global	-1	1	energy unadjusted(kcal)	16
Nutritional deficiencies	Male	28-364 days	95+ years	Global	1	1	Age-Standardize Prevalence of Severe Anemia	212
Nutritional deficiencies	Male	28-364 days	95+ years	Global	1	1	Underweight, age and sex specific	467
Nutritional deficiencies	Male	28-364 days	95+ years	Global	1	1	Age-standardized SEV for Child underweight	--
Nutritional deficiencies	Male	28-364 days	95+ years	Global	1	1	Age-standardized SEV for Child wasting	--
Nutritional deficiencies	Male	28-364 days	95+ years	Global	-1	2	Healthcare access and quality index	174
Nutritional deficiencies	Male	28-364 days	95+ years	Global	-1	2	Maternal care and immunization	--
Nutritional deficiencies	Male	28-364 days	95+ years	Global	0	2	Rainfall Quintile 1 (proportion)	177
Nutritional deficiencies	Male	28-364 days	95+ years	Global	0	2	Rainfall Quintile 2 (proportion)	215
Nutritional deficiencies	Male	28-364 days	95+ years	Global	1	2	Age- and sex-specific SEV for Unsafe water	101
Nutritional deficiencies	Male	28-364 days	95+ years	Global	1	2	Age- and sex-specific SEV for Unsafe sanitation	189
Nutritional deficiencies	Male	28-364 days	95+ years	Global	1	2	Age- and sex-specific SEV for Alcohol use	--
Nutritional deficiencies	Male	28-364 days	95+ years	Global	1	2	Log-transformed SEV scalar: Diarrhea	--
Nutritional deficiencies	Male	28-364 days	95+ years	Global	1	2	Mortality Rate Due to War Shocks (per 1 person)	--
Nutritional deficiencies	Male	28-364 days	95+ years	Global	-1	3	Education (years per capita)	3
Nutritional deficiencies	Male	28-364 days	95+ years	Global	-1	3	Socio-demographic Index	114
Nutritional deficiencies	Male	28-364 days	95+ years	Global	-1	3	Maternal Education (years per capita)	147
Nutritional deficiencies	Male	28-364 days	95+ years	Global	-1	3	LDI (I$ per capita)	--
Protein-energy malnutrition	Female	28-364 days	1-4 years	Data Rich	-1	1	energy unadjusted(kcal)	--
Protein-energy malnutrition	Female	28-364 days	1-4 years	Data Rich	1	1	Age-Standardize Prevalence of Severe Anemia	342
Protein-energy malnutrition	Female	28-364 days	1-4 years	Data Rich	1	1	Underweight, age and sex specific	502
Protein-energy malnutrition	Female	28-364 days	1-4 years	Data Rich	1	1	Age- and sex-specific SEV for Child wasting	--
Protein-energy malnutrition	Female	28-364 days	1-4 years	Data Rich	1	1	Malnutrition Shock mortality rate	--
Protein-energy malnutrition	Female	28-364 days	1-4 years	Data Rich	-1	2	Healthcare access and quality index	115
Protein-energy malnutrition	Female	28-364 days	1-4 years	Data Rich	-1	2	Maternal care and immunization	--
Protein-energy malnutrition	Female	28-364 days	1-4 years	Data Rich	0	2	Rainfall Quintile 1 (proportion)	0
Protein-energy malnutrition	Female	28-364 days	1-4 years	Data Rich	0	2	Rainfall Quintile 2 (proportion)	365
Protein-energy malnutrition	Female	28-364 days	1-4 years	Data Rich	1	2	Age- and sex-specific SEV for Unsafe sanitation	115
Protein-energy malnutrition	Female	28-364 days	1-4 years	Data Rich	1	2	Age- and sex-specific SEV for Unsafe water	115

Table S16. CODEm covariates used, level of covariate, and expected direction of covariate by cause, sex, and age<br>*Covariates that CODEm did not select during the covariate selection process have no draw counts listed.*								
**Cause**	**Sex**	**Age Start**	**Age End**	**Model Type**	**Direction**	**Level**	**Covariate Name**	**Number of Draws**
Protein-energy malnutrition	Female	28-364 days	1-4 years	Data Rich	1	2	Age- and sex-specific SEV for Alcohol use	--
Protein-energy malnutrition	Female	28-364 days	1-4 years	Data Rich	1	2	Log-transformed SEV scalar: Diarrhea	--
Protein-energy malnutrition	Female	28-364 days	1-4 years	Data Rich	1	2	Mortality Rate Due to War Shocks (per 1 person)	--
Protein-energy malnutrition	Female	28-364 days	1-4 years	Data Rich	-1	3	Antenatal Care (4 visits) Coverage (proportion)	76
Protein-energy malnutrition	Female	28-364 days	1-4 years	Data Rich	-1	3	Socio-demographic Index	179
Protein-energy malnutrition	Female	28-364 days	1-4 years	Data Rich	-1	3	Education (years per capita)	212
Protein-energy malnutrition	Female	28-364 days	1-4 years	Data Rich	-1	3	LDI (I$ per capita)	--
Protein-energy malnutrition	Female	28-364 days	1-4 years	Global	-1	1	energy unadjusted(kcal)	--
Protein-energy malnutrition	Female	28-364 days	1-4 years	Global	1	1	Age-Standardize Prevalence of Severe Anemia	320
Protein-energy malnutrition	Female	28-364 days	1-4 years	Global	1	1	Underweight, age and sex specific	576
Protein-energy malnutrition	Female	28-364 days	1-4 years	Global	1	1	Age- and sex-specific SEV for Child wasting	--
Protein-energy malnutrition	Female	28-364 days	1-4 years	Global	1	1	Malnutrition Shock mortality rate	--
Protein-energy malnutrition	Female	28-364 days	1-4 years	Global	-1	2	Healthcare access and quality index	0
Protein-energy malnutrition	Female	28-364 days	1-4 years	Global	-1	2	Maternal care and immunization	--
Protein-energy malnutrition	Female	28-364 days	1-4 years	Global	0	2	Rainfall Quintile 1 (proportion)	178
Protein-energy malnutrition	Female	28-364 days	1-4 years	Global	0	2	Rainfall Quintile 2 (proportion)	179
Protein-energy malnutrition	Female	28-364 days	1-4 years	Global	1	2	Age- and sex-specific SEV for Unsafe water	34
Protein-energy malnutrition	Female	28-364 days	1-4 years	Global	1	2	Age- and sex-specific SEV for Unsafe sanitation	125
Protein-energy malnutrition	Female	28-364 days	1-4 years	Global	1	2	Age- and sex-specific SEV for Alcohol use	--
Protein-energy malnutrition	Female	28-364 days	1-4 years	Global	1	2	Log-transformed SEV scalar: Diarrhea	--
Protein-energy malnutrition	Female	28-364 days	1-4 years	Global	1	2	Mortality Rate Due to War Shocks (per 1 person)	--
Protein-energy malnutrition	Female	28-364 days	1-4 years	Global	-1	3	Education (years per capita)	0
Protein-energy malnutrition	Female	28-364 days	1-4 years	Global	-1	3	Socio-demographic Index	0
Protein-energy malnutrition	Female	28-364 days	1-4 years	Global	-1	3	Antenatal Care (4 visits) Coverage (proportion)	504
Protein-energy malnutrition	Female	28-364 days	1-4 years	Global	-1	3	LDI (I$ per capita)	--
Protein-energy malnutrition	Female	5-9 years	95+ years	Data Rich	-1	1	energy unadjusted(kcal)	--
Protein-energy malnutrition	Female	5-9 years	95+ years	Data Rich	1	1	Age-Standardize Prevalence of Severe Anemia	--
Protein-energy malnutrition	Female	5-9 years	95+ years	Data Rich	1	1	Age-standardized SEV for Child wasting	--
Protein-energy malnutrition	Female	5-9 years	95+ years	Data Rich	1	1	Malnutrition Shock mortality rate	--
Protein-energy malnutrition	Female	5-9 years	95+ years	Data Rich	1	1	Underweight, age and sex specific	--
Protein-energy malnutrition	Female	5-9 years	95+ years	Data Rich	-1	2	Healthcare access and quality index	--
Protein-energy malnutrition	Female	5-9 years	95+ years	Data Rich	-1	2	Maternal care and immunization	--
Protein-energy malnutrition	Female	5-9 years	95+ years	Data Rich	0	2	Rainfall Quintile 2 (proportion)	1000
Protein-energy malnutrition	Female	5-9 years	95+ years	Data Rich	0	2	Rainfall Quintile 1 (proportion)	--
Protein-energy malnutrition	Female	5-9 years	95+ years	Data Rich	1	2	Age- and sex-specific SEV for Alcohol use	--
Protein-energy malnutrition	Female	5-9 years	95+ years	Data Rich	1	2	Age- and sex-specific SEV for Unsafe sanitation	--
Protein-energy malnutrition	Female	5-9 years	95+ years	Data Rich	1	2	Age- and sex-specific SEV for Unsafe water	--
Protein-energy malnutrition	Female	5-9 years	95+ years	Data Rich	1	2	Log-transformed SEV scalar: Diarrhea	--
Protein-energy malnutrition	Female	5-9 years	95+ years	Data Rich	1	2	Mortality Rate Due to War Shocks (per 1 person)	--
Protein-energy malnutrition	Female	5-9 years	95+ years	Data Rich	-1	3	Antenatal Care (4 visits) Coverage (proportion)	--
Protein-energy malnutrition	Female	5-9 years	95+ years	Data Rich	-1	3	Education (years per capita)	--
Protein-energy malnutrition	Female	5-9 years	95+ years	Data Rich	-1	3	LDI (I$ per capita)	--
Protein-energy malnutrition	Female	5-9 years	95+ years	Data Rich	-1	3	Socio-demographic Index	--
Protein-energy malnutrition	Female	5-9 years	95+ years	Global	-1	1	energy unadjusted(kcal)	--

Table S16. CODEm covariates used, level of covariate, and expected direction of covariate by cause, sex, and age
Covariates that CODEm did not select during the covariate selection process have no draw counts listed.

Cause	Sex	Age Start	Age End	Model Type	Direction	Level	Covariate Name	Number of Draws
Protein-energy malnutrition	Female	5-9 years	95+ years	Global	1	1	Age-Standardize Prevalence of Severe Anemia	197
Protein-energy malnutrition	Female	5-9 years	95+ years	Global	1	1	Underweight, age and sex specific	783
Protein-energy malnutrition	Female	5-9 years	95+ years	Global	1	1	Age-standardized SEV for Child wasting	--
Protein-energy malnutrition	Female	5-9 years	95+ years	Global	1	1	Malnutrition Shock mortality rate	--
Protein-energy malnutrition	Female	5-9 years	95+ years	Global	-1	2	Healthcare access and quality index	--
Protein-energy malnutrition	Female	5-9 years	95+ years	Global	-1	2	Maternal care and immunization	--
Protein-energy malnutrition	Female	5-9 years	95+ years	Global	0	2	Rainfall Quintile 1 (proportion)	255
Protein-energy malnutrition	Female	5-9 years	95+ years	Global	0	2	Rainfall Quintile 2 (proportion)	597
Protein-energy malnutrition	Female	5-9 years	95+ years	Global	1	2	Age- and sex-specific SEV for Unsafe sanitation	84
Protein-energy malnutrition	Female	5-9 years	95+ years	Global	1	2	Age- and sex-specific SEV for Unsafe water	279
Protein-energy malnutrition	Female	5-9 years	95+ years	Global	1	2	Age- and sex-specific SEV for Alcohol use	--
Protein-energy malnutrition	Female	5-9 years	95+ years	Global	1	2	Log-transformed SEV scalar: Diarrhea	--
Protein-energy malnutrition	Female	5-9 years	95+ years	Global	1	2	Mortality Rate Due to War Shocks (per 1 person)	--
Protein-energy malnutrition	Female	5-9 years	95+ years	Global	-1	3	Socio-demographic Index	2
Protein-energy malnutrition	Female	5-9 years	95+ years	Global	-1	3	Education (years per capita)	5
Protein-energy malnutrition	Female	5-9 years	95+ years	Global	-1	3	Antenatal Care (4 visits) Coverage (proportion)	426
Protein-energy malnutrition	Female	5-9 years	95+ years	Global	-1	3	LDI (I$ per capita)	--
Protein-energy malnutrition	Male	28-364 days	1-4 years	Data Rich	-1	1	energy unadjusted(kcal)	--
Protein-energy malnutrition	Male	28-364 days	1-4 years	Data Rich	1	1	Underweight, age and sex specific	337
Protein-energy malnutrition	Male	28-364 days	1-4 years	Data Rich	1	1	Age-Standardize Prevalence of Severe Anemia	388
Protein-energy malnutrition	Male	28-364 days	1-4 years	Data Rich	1	1	Age- and sex-specific SEV for Child wasting	--
Protein-energy malnutrition	Male	28-364 days	1-4 years	Data Rich	1	1	Malnutrition Shock mortality rate	--
Protein-energy malnutrition	Male	28-364 days	1-4 years	Data Rich	-1	2	Healthcare access and quality index	9
Protein-energy malnutrition	Male	28-364 days	1-4 years	Data Rich	-1	2	Maternal care and immunization	--
Protein-energy malnutrition	Male	28-364 days	1-4 years	Data Rich	0	2	Rainfall Quintile 1 (proportion)	294
Protein-energy malnutrition	Male	28-364 days	1-4 years	Data Rich	0	2	Rainfall Quintile 2 (proportion)	359
Protein-energy malnutrition	Male	28-364 days	1-4 years	Data Rich	1	2	Age- and sex-specific SEV for Unsafe water	11
Protein-energy malnutrition	Male	28-364 days	1-4 years	Data Rich	1	2	Age- and sex-specific SEV for Unsafe sanitation	52
Protein-energy malnutrition	Male	28-364 days	1-4 years	Data Rich	1	2	Age- and sex-specific SEV for Alcohol use	--
Protein-energy malnutrition	Male	28-364 days	1-4 years	Data Rich	1	2	Log-transformed SEV scalar: Diarrhea	--
Protein-energy malnutrition	Male	28-364 days	1-4 years	Data Rich	1	2	Mortality Rate Due to War Shocks (per 1 person)	--
Protein-energy malnutrition	Male	28-364 days	1-4 years	Data Rich	-1	3	Education (years per capita)	17
Protein-energy malnutrition	Male	28-364 days	1-4 years	Data Rich	-1	3	Socio-demographic Index	18
Protein-energy malnutrition	Male	28-364 days	1-4 years	Data Rich	-1	3	LDI (I$ per capita)	--
Protein-energy malnutrition	Male	28-364 days	1-4 years	Global	-1	1	energy unadjusted(kcal)	--
Protein-energy malnutrition	Male	28-364 days	1-4 years	Global	1	1	Age-Standardize Prevalence of Severe Anemia	190
Protein-energy malnutrition	Male	28-364 days	1-4 years	Global	1	1	Underweight, age and sex specific	759
Protein-energy malnutrition	Male	28-364 days	1-4 years	Global	1	1	Age- and sex-specific SEV for Child wasting	--
Protein-energy malnutrition	Male	28-364 days	1-4 years	Global	1	1	Malnutrition Shock mortality rate	--
Protein-energy malnutrition	Male	28-364 days	1-4 years	Global	-1	2	Healthcare access and quality index	0
Protein-energy malnutrition	Male	28-364 days	1-4 years	Global	-1	2	Maternal care and immunization	--
Protein-energy malnutrition	Male	28-364 days	1-4 years	Global	0	2	Rainfall Quintile 1 (proportion)	755
Protein-energy malnutrition	Male	28-364 days	1-4 years	Global	0	2	Rainfall Quintile 2 (proportion)	803

Table S16. CODEm covariates used, level of covariate, and expected direction of covariate by cause, sex, and age
Covariates that CODEm did not select during the covariate selection process have no draw counts listed.

Cause	Sex	Age Start	Age End	Model Type	Direction	Level	Covariate Name	Number of Draws
Protein-energy malnutrition	Male	28-364 days	1-4 years	Global	1	2	Age- and sex-specific SEV for Unsafe water	15
Protein-energy malnutrition	Male	28-364 days	1-4 years	Global	1	2	Age- and sex-specific SEV for Unsafe sanitation	716
Protein-energy malnutrition	Male	28-364 days	1-4 years	Global	1	2	Age- and sex-specific SEV for Alcohol use	--
Protein-energy malnutrition	Male	28-364 days	1-4 years	Global	1	2	Log-transformed SEV scalar: Diarrhea	--
Protein-energy malnutrition	Male	28-364 days	1-4 years	Global	1	2	Mortality Rate Due to War Shocks (per 1 person)	--
Protein-energy malnutrition	Male	28-364 days	1-4 years	Global	-1	3	Education (years per capita)	0
Protein-energy malnutrition	Male	28-364 days	1-4 years	Global	-1	3	Socio-demographic Index	0
Protein-energy malnutrition	Male	28-364 days	1-4 years	Global	-1	3	LDI (I$ per capita)	--
Protein-energy malnutrition	Male	5-9 years	95+ years	Data Rich	-1	1	energy unadjusted(kcal)	--
Protein-energy malnutrition	Male	5-9 years	95+ years	Data Rich	1	1	Age-Standardize Prevalence of Severe Anemia	254
Protein-energy malnutrition	Male	5-9 years	95+ years	Data Rich	1	1	Underweight, age and sex specific	254
Protein-energy malnutrition	Male	5-9 years	95+ years	Data Rich	1	1	Malnutrition Shock mortality rate	--
Protein-energy malnutrition	Male	5-9 years	95+ years	Data Rich	-1	2	Healthcare access and quality index	--
Protein-energy malnutrition	Male	5-9 years	95+ years	Data Rich	-1	2	Maternal care and immunization	--
Protein-energy malnutrition	Male	5-9 years	95+ years	Data Rich	0	2	Rainfall Quintile 2 (proportion)	294
Protein-energy malnutrition	Male	5-9 years	95+ years	Data Rich	0	2	Rainfall Quintile 1 (proportion)	--
Protein-energy malnutrition	Male	5-9 years	95+ years	Data Rich	1	2	Age- and sex-specific SEV for Unsafe sanitation	185
Protein-energy malnutrition	Male	5-9 years	95+ years	Data Rich	1	2	Age- and sex-specific SEV for Alcohol use	--
Protein-energy malnutrition	Male	5-9 years	95+ years	Data Rich	1	2	Age- and sex-specific SEV for Unsafe water	--
Protein-energy malnutrition	Male	5-9 years	95+ years	Data Rich	1	2	Log-transformed SEV scalar: Diarrhea	--
Protein-energy malnutrition	Male	5-9 years	95+ years	Data Rich	1	2	Mortality Rate Due to War Shocks (per 1 person)	--
Protein-energy malnutrition	Male	5-9 years	95+ years	Data Rich	-1	3	Education (years per capita)	--
Protein-energy malnutrition	Male	5-9 years	95+ years	Data Rich	-1	3	LDI (I$ per capita)	--
Protein-energy malnutrition	Male	5-9 years	95+ years	Data Rich	-1	3	Socio-demographic Index	--
Protein-energy malnutrition	Male	5-9 years	95+ years	Global	-1	1	energy unadjusted(kcal)	--
Protein-energy malnutrition	Male	5-9 years	95+ years	Global	1	1	Age-Standardize Prevalence of Severe Anemia	25
Protein-energy malnutrition	Male	5-9 years	95+ years	Global	1	1	Underweight, age and sex specific	535
Protein-energy malnutrition	Male	5-9 years	95+ years	Global	1	1	Malnutrition Shock mortality rate	--
Protein-energy malnutrition	Male	5-9 years	95+ years	Global	-1	2	Healthcare access and quality index	--
Protein-energy malnutrition	Male	5-9 years	95+ years	Global	-1	2	Maternal care and immunization	--
Protein-energy malnutrition	Male	5-9 years	95+ years	Global	0	2	Rainfall Quintile 2 (proportion)	5
Protein-energy malnutrition	Male	5-9 years	95+ years	Global	0	2	Rainfall Quintile 1 (proportion)	13
Protein-energy malnutrition	Male	5-9 years	95+ years	Global	1	2	Age- and sex-specific SEV for Unsafe sanitation	56
Protein-energy malnutrition	Male	5-9 years	95+ years	Global	1	2	Age- and sex-specific SEV for Unsafe water	410
Protein-energy malnutrition	Male	5-9 years	95+ years	Global	1	2	Age- and sex-specific SEV for Alcohol use	--
Protein-energy malnutrition	Male	5-9 years	95+ years	Global	1	2	Log-transformed SEV scalar: Diarrhea	--
Protein-energy malnutrition	Male	5-9 years	95+ years	Global	1	2	Mortality Rate Due to War Shocks (per 1 person)	--
Protein-energy malnutrition	Male	5-9 years	95+ years	Global	-1	3	Education (years per capita)	10
Protein-energy malnutrition	Male	5-9 years	95+ years	Global	-1	3	Socio-demographic Index	94
Protein-energy malnutrition	Male	5-9 years	95+ years	Global	-1	3	Maternal Education (years per capita)	155
Protein-energy malnutrition	Male	5-9 years	95+ years	Global	-1	3	LDI (I$ per capita)	--
Other nutritional deficiencies	Female	28-364 days	95+ years	Data Rich	1	1	Age-Standardize Prevalence of Severe Anemia	191
Other nutritional deficiencies	Female	28-364 days	95+ years	Data Rich	1	1	Underweight, age and sex specific	879
Other nutritional deficiencies	Female	28-364 days	95+ years	Data Rich	1	1	Age-standardized SEV for Child underweight	--
Other nutritional deficiencies	Female	28-364 days	95+ years	Data Rich	1	1	Malnutrition Shock mortality rate	--
Other nutritional deficiencies	Female	28-364 days	95+ years	Data Rich	-1	2	energy unadjusted(kcal)	22
Other nutritional deficiencies	Female	28-364 days	95+ years	Data Rich	-1	2	Healthcare access and quality index	459

Table S16. CODEm covariates used, level of covariate, and expected direction of covariate by cause, sex, and age
Covariates that CODEm did not select during the covariate selection process have no draw counts listed.

Cause	Sex	Age Start	Age End	Model Type	Direction	Level	Covariate Name	Number of Draws
Other nutritional deficiencies	Female	28-364 days	95+ years	Data Rich	-1	2	Maternal care and immunization	--
Other nutritional deficiencies	Female	28-364 days	95+ years	Data Rich	0	2	Rainfall Quintile 2 (proportion)	92
Other nutritional deficiencies	Female	28-364 days	95+ years	Data Rich	0	2	Rainfall Quintile 1 (proportion)	496
Other nutritional deficiencies	Female	28-364 days	95+ years	Data Rich	1	2	Age- and sex-specific SEV for Unsafe sanitation	13
Other nutritional deficiencies	Female	28-364 days	95+ years	Data Rich	1	2	Age- and sex-specific SEV for Unsafe water	28
Other nutritional deficiencies	Female	28-364 days	95+ years	Data Rich	1	2	Age- and sex-specific SEV for Alcohol use	--
Other nutritional deficiencies	Female	28-364 days	95+ years	Data Rich	1	2	Log-transformed SEV scalar: Diarrhea	--
Other nutritional deficiencies	Female	28-364 days	95+ years	Data Rich	1	2	Mortality Rate Due to War Shocks (per 1 person)	--
Other nutritional deficiencies	Female	28-364 days	95+ years	Data Rich	-1	3	Socio-demographic Index	453
Other nutritional deficiencies	Female	28-364 days	95+ years	Data Rich	-1	3	Education (years per capita)	522
Other nutritional deficiencies	Female	28-364 days	95+ years	Data Rich	-1	3	LDI (I$ per capita)	--
Other nutritional deficiencies	Female	28-364 days	95+ years	Global	1	1	Age-Standardize Prevalence of Severe Anemia	323
Other nutritional deficiencies	Female	28-364 days	95+ years	Global	1	1	Underweight, age and sex specific	744
Other nutritional deficiencies	Female	28-364 days	95+ years	Global	1	1	Age-standardized SEV for Child underweight	--
Other nutritional deficiencies	Female	28-364 days	95+ years	Global	1	1	Malnutrition Shock mortality rate	--
Other nutritional deficiencies	Female	28-364 days	95+ years	Global	-1	2	energy unadjusted(kcal)	32
Other nutritional deficiencies	Female	28-364 days	95+ years	Global	-1	2	Healthcare access and quality index	535
Other nutritional deficiencies	Female	28-364 days	95+ years	Global	-1	2	Maternal care and immunization	--
Other nutritional deficiencies	Female	28-364 days	95+ years	Global	0	2	Rainfall Quintile 1 (proportion)	86
Other nutritional deficiencies	Female	28-364 days	95+ years	Global	0	2	Rainfall Quintile 2 (proportion)	582
Other nutritional deficiencies	Female	28-364 days	95+ years	Global	1	2	Age- and sex-specific SEV for Unsafe sanitation	4
Other nutritional deficiencies	Female	28-364 days	95+ years	Global	1	2	Age- and sex-specific SEV for Unsafe water	40
Other nutritional deficiencies	Female	28-364 days	95+ years	Global	1	2	Age- and sex-specific SEV for Alcohol use	--
Other nutritional deficiencies	Female	28-364 days	95+ years	Global	1	2	Log-transformed SEV scalar: Diarrhea	--
Other nutritional deficiencies	Female	28-364 days	95+ years	Global	1	2	Mortality Rate Due to War Shocks (per 1 person)	--
Other nutritional deficiencies	Female	28-364 days	95+ years	Global	-1	3	Socio-demographic Index	170
Other nutritional deficiencies	Female	28-364 days	95+ years	Global	-1	3	Education (years per capita)	471
Other nutritional deficiencies	Female	28-364 days	95+ years	Global	-1	3	LDI (I$ per capita)	--
Other nutritional deficiencies	Male	28-364 days	95+ years	Data Rich	1	1	Age-Standardize Prevalence of Severe Anemia	191
Other nutritional deficiencies	Male	28-364 days	95+ years	Data Rich	1	1	Underweight, age and sex specific	899
Other nutritional deficiencies	Male	28-364 days	95+ years	Data Rich	1	1	Age-standardized SEV for Child underweight	--
Other nutritional deficiencies	Male	28-364 days	95+ years	Data Rich	1	1	Malnutrition Shock mortality rate	--
Other nutritional deficiencies	Male	28-364 days	95+ years	Data Rich	-1	2	Healthcare access and quality index	7
Other nutritional deficiencies	Male	28-364 days	95+ years	Data Rich	-1	2	energy unadjusted(kcal)	299
Other nutritional deficiencies	Male	28-364 days	95+ years	Data Rich	-1	2	Maternal care and immunization	--
Other nutritional deficiencies	Male	28-364 days	95+ years	Data Rich	0	2	Rainfall Quintile 2 (proportion)	411
Other nutritional deficiencies	Male	28-364 days	95+ years	Data Rich	0	2	Rainfall Quintile 1 (proportion)	500
Other nutritional deficiencies	Male	28-364 days	95+ years	Data Rich	1	2	Age- and sex-specific SEV for Unsafe sanitation	22
Other nutritional deficiencies	Male	28-364 days	95+ years	Data Rich	1	2	Age- and sex-specific SEV for Unsafe water	324
Other nutritional deficiencies	Male	28-364 days	95+ years	Data Rich	1	2	Age- and sex-specific SEV for Alcohol use	--

Table S16. CODEm covariates used, level of covariate, and expected direction of covariate by cause, sex, and age<br>*Covariates that CODEm did not select during the covariate selection process have no draw counts listed.*								
**Cause**	**Sex**	**Age Start**	**Age End**	**Model Type**	**Direction**	**Level**	**Covariate Name**	**Number of Draws**
Other nutritional deficiencies	Male	28-364 days	95+ years	Data Rich	1	2	Log-transformed SEV scalar: Diarrhea	--
Other nutritional deficiencies	Male	28-364 days	95+ years	Data Rich	1	2	Mortality Rate Due to War Shocks (per 1 person)	--
Other nutritional deficiencies	Male	28-364 days	95+ years	Data Rich	-1	3	Socio-demographic Index	249
Other nutritional deficiencies	Male	28-364 days	95+ years	Data Rich	-1	3	Education (years per capita)	--
Other nutritional deficiencies	Male	28-364 days	95+ years	Data Rich	-1	3	LDI (I$ per capita)	--
Other nutritional deficiencies	Male	28-364 days	95+ years	Global	1	1	Age-Standardize Prevalence of Severe Anemia	412
Other nutritional deficiencies	Male	28-364 days	95+ years	Global	1	1	Underweight, age and sex specific	949
Other nutritional deficiencies	Male	28-364 days	95+ years	Global	1	1	Age-standardized SEV for Child underweight	--
Other nutritional deficiencies	Male	28-364 days	95+ years	Global	1	1	Malnutrition Shock mortality rate	--
Other nutritional deficiencies	Male	28-364 days	95+ years	Global	-1	2	Healthcare access and quality index	15
Other nutritional deficiencies	Male	28-364 days	95+ years	Global	-1	2	energy unadjusted(kcal)	170
Other nutritional deficiencies	Male	28-364 days	95+ years	Global	-1	2	Maternal care and immunization	--
Other nutritional deficiencies	Male	28-364 days	95+ years	Global	0	2	Rainfall Quintile 1 (proportion)	120
Other nutritional deficiencies	Male	28-364 days	95+ years	Global	0	2	Rainfall Quintile 2 (proportion)	383
Other nutritional deficiencies	Male	28-364 days	95+ years	Global	1	2	Age- and sex-specific SEV for Unsafe sanitation	1
Other nutritional deficiencies	Male	28-364 days	95+ years	Global	1	2	Age- and sex-specific SEV for Unsafe water	24
Other nutritional deficiencies	Male	28-364 days	95+ years	Global	1	2	Age- and sex-specific SEV for Alcohol use	--
Other nutritional deficiencies	Male	28-364 days	95+ years	Global	1	2	Log-transformed SEV scalar: Diarrhea	--
Other nutritional deficiencies	Male	28-364 days	95+ years	Global	1	2	Mortality Rate Due to War Shocks (per 1 person)	--
Other nutritional deficiencies	Male	28-364 days	95+ years	Global	-1	3	Education (years per capita)	202
Other nutritional deficiencies	Male	28-364 days	95+ years	Global	-1	3	LDI (I$ per capita)	--
Other nutritional deficiencies	Male	28-364 days	95+ years	Global	-1	3	Socio-demographic Index	--
Lip and oral cavity cancer	Female	15-19 years	95+ years	Data Rich	-1	1	vegetables adjusted(g)	434
Lip and oral cavity cancer	Female	15-19 years	95+ years	Data Rich	1	1	Cumulative Cigarettes (10 Years)	0
Lip and oral cavity cancer	Female	15-19 years	95+ years	Data Rich	1	1	Cumulative Cigarettes (5 Years)	0
Lip and oral cavity cancer	Female	15-19 years	95+ years	Data Rich	1	1	Cumulative Cigarettes (15 Years)	16
Lip and oral cavity cancer	Female	15-19 years	95+ years	Data Rich	1	1	Smoking Prevalence	35
Lip and oral cavity cancer	Female	15-19 years	95+ years	Data Rich	1	1	Alcohol (liters per capita)	201
Lip and oral cavity cancer	Female	15-19 years	95+ years	Data Rich	1	1	Cumulative Cigarettes (20 Years)	278
Lip and oral cavity cancer	Female	15-19 years	95+ years	Data Rich	1	1	Log-transformed SEV scalar: Lip Oral C	--
Lip and oral cavity cancer	Female	15-19 years	95+ years	Data Rich	1	1	Tobacco (cigarettes per capita)	--
Lip and oral cavity cancer	Female	15-19 years	95+ years	Data Rich	-1	2	Healthcare access and quality index	22
Lip and oral cavity cancer	Female	15-19 years	95+ years	Data Rich	-1	2	Health System Access 2 (unitless)	366
Lip and oral cavity cancer	Female	15-19 years	95+ years	Data Rich	-1	2	fruits adjusted(g)	--
Lip and oral cavity cancer	Female	15-19 years	95+ years	Data Rich	1	2	red meats adjusted(g)	479
Lip and oral cavity cancer	Female	15-19 years	95+ years	Data Rich	-1	3	Education (years per capita)	--
Lip and oral cavity cancer	Female	15-19 years	95+ years	Data Rich	0	3	Socio-demographic Index	810
Lip and oral cavity cancer	Female	15-19 years	95+ years	Data Rich	0	3	LDI (I$ per capita)	--
Lip and oral cavity cancer	Female	15-19 years	95+ years	Global	-1	1	vegetables adjusted(g)	1000
Lip and oral cavity cancer	Female	15-19 years	95+ years	Global	1	1	Cumulative Cigarettes (5 Years)	0

Table S16. CODEm covariates used, level of covariate, and expected direction of covariate by cause, sex, and age
Covariates that CODEm did not select during the covariate selection process have no draw counts listed.

Cause	Sex	Age Start	Age End	Model Type	Direction	Level	Covariate Name	Number of Draws
Lip and oral cavity cancer	Female	15-19 years	95+ years	Global	1	1	Tobacco (cigarettes per capita)	0
Lip and oral cavity cancer	Female	15-19 years	95+ years	Global	1	1	Cumulative Cigarettes (15 Years)	26
Lip and oral cavity cancer	Female	15-19 years	95+ years	Global	1	1	Cumulative Cigarettes (10 Years)	102
Lip and oral cavity cancer	Female	15-19 years	95+ years	Global	1	1	Cumulative Cigarettes (20 Years)	304
Lip and oral cavity cancer	Female	15-19 years	95+ years	Global	1	1	Smoking Prevalence	566
Lip and oral cavity cancer	Female	15-19 years	95+ years	Global	1	1	Alcohol (liters per capita)	849
Lip and oral cavity cancer	Female	15-19 years	95+ years	Global	1	1	Log-transformed SEV scalar: Lip Oral C	--
Lip and oral cavity cancer	Female	15-19 years	95+ years	Global	-1	2	fruits adjusted(g)	0
Lip and oral cavity cancer	Female	15-19 years	95+ years	Global	-1	2	Healthcare access and quality index	298
Lip and oral cavity cancer	Female	15-19 years	95+ years	Global	1	2	red meats adjusted(g)	99
Lip and oral cavity cancer	Female	15-19 years	95+ years	Global	-1	3	Education (years per capita)	0
Lip and oral cavity cancer	Female	15-19 years	95+ years	Global	0	3	Socio-demographic Index	684
Lip and oral cavity cancer	Female	15-19 years	95+ years	Global	0	3	LDI (I$ per capita)	--
Lip and oral cavity cancer	Male	15-19 years	95+ years	Data Rich	-1	1	vegetables adjusted(g)	60
Lip and oral cavity cancer	Male	15-19 years	95+ years	Data Rich	1	1	Tobacco (cigarettes per capita)	0
Lip and oral cavity cancer	Male	15-19 years	95+ years	Data Rich	1	1	Cumulative Cigarettes (10 Years)	6
Lip and oral cavity cancer	Male	15-19 years	95+ years	Data Rich	1	1	Cumulative Cigarettes (5 Years)	12
Lip and oral cavity cancer	Male	15-19 years	95+ years	Data Rich	1	1	Smoking Prevalence	13
Lip and oral cavity cancer	Male	15-19 years	95+ years	Data Rich	1	1	Cumulative Cigarettes (15 Years)	16
Lip and oral cavity cancer	Male	15-19 years	95+ years	Data Rich	1	1	Cumulative Cigarettes (20 Years)	30
Lip and oral cavity cancer	Male	15-19 years	95+ years	Data Rich	1	1	Alcohol (liters per capita)	458
Lip and oral cavity cancer	Male	15-19 years	95+ years	Data Rich	1	1	Log-transformed SEV scalar: Lip Oral C	--
Lip and oral cavity cancer	Male	15-19 years	95+ years	Data Rich	-1	2	Healthcare access and quality index	0
Lip and oral cavity cancer	Male	15-19 years	95+ years	Data Rich	-1	2	fruits adjusted(g)	--
Lip and oral cavity cancer	Male	15-19 years	95+ years	Data Rich	1	2	red meats adjusted(g)	604
Lip and oral cavity cancer	Male	15-19 years	95+ years	Data Rich	-1	3	Education (years per capita)	7
Lip and oral cavity cancer	Male	15-19 years	95+ years	Data Rich	0	3	Socio-demographic Index	623
Lip and oral cavity cancer	Male	15-19 years	95+ years	Data Rich	0	3	LDI (I$ per capita)	--
Lip and oral cavity cancer	Male	15-19 years	95+ years	Global	-1	1	vegetables adjusted(g)	293
Lip and oral cavity cancer	Male	15-19 years	95+ years	Global	1	1	Cumulative Cigarettes (5 Years)	0
Lip and oral cavity cancer	Male	15-19 years	95+ years	Global	1	1	Cumulative Cigarettes (10 Years)	1
Lip and oral cavity cancer	Male	15-19 years	95+ years	Global	1	1	Smoking Prevalence	4
Lip and oral cavity cancer	Male	15-19 years	95+ years	Global	1	1	Cumulative Cigarettes (15 Years)	41
Lip and oral cavity cancer	Male	15-19 years	95+ years	Global	1	1	Cumulative Cigarettes (20 Years)	233
Lip and oral cavity cancer	Male	15-19 years	95+ years	Global	1	1	Tobacco (cigarettes per capita)	254
Lip and oral cavity cancer	Male	15-19 years	95+ years	Global	1	1	Alcohol (liters per capita)	568
Lip and oral cavity cancer	Male	15-19 years	95+ years	Global	1	1	Log-transformed SEV scalar: Lip Oral C	--
Lip and oral cavity cancer	Male	15-19 years	95+ years	Global	-1	2	Healthcare access and quality index	329
Lip and oral cavity cancer	Male	15-19 years	95+ years	Global	-1	2	fruits adjusted(g)	--
Lip and oral cavity cancer	Male	15-19 years	95+ years	Global	1	2	red meats adjusted(g)	659

**Table S16. CODEm covariates used, level of covariate, and expected direction of covariate by cause, sex, and age** *Covariates that CODEm did not select during the covariate selection process have no draw counts listed.*								
**Cause**	**Sex**	**Age Start**	**Age End**	**Model Type**	**Direction**	**Level**	**Covariate Name**	**Number of Draws**
Lip and oral cavity cancer	Male	15-19 years	95+ years	Global	-1	3	Education (years per capita)	0
Lip and oral cavity cancer	Male	15-19 years	95+ years	Global	0	3	Socio-demographic Index	572
Lip and oral cavity cancer	Male	15-19 years	95+ years	Global	0	3	LDI (I$ per capita)	--
Nasopharynx cancer	Female	5-9 years	95+ years	Data Rich	1	1	Tobacco (cigarettes per capita)	0
Nasopharynx cancer	Female	5-9 years	95+ years	Data Rich	1	1	Cumulative Cigarettes (10 Years)	47
Nasopharynx cancer	Female	5-9 years	95+ years	Data Rich	1	1	Cumulative Cigarettes (15 Years)	163
Nasopharynx cancer	Female	5-9 years	95+ years	Data Rich	1	1	Smoking Prevalence	286
Nasopharynx cancer	Female	5-9 years	95+ years	Data Rich	1	1	Cumulative Cigarettes (5 Years)	295
Nasopharynx cancer	Female	5-9 years	95+ years	Data Rich	1	1	Cumulative Cigarettes (20 Years)	738
Nasopharynx cancer	Female	5-9 years	95+ years	Data Rich	1	1	Alcohol (liters per capita)	--
Nasopharynx cancer	Female	5-9 years	95+ years	Data Rich	1	1	Log-transformed SEV scalar: Nasoph C	--
Nasopharynx cancer	Female	5-9 years	95+ years	Data Rich	-1	2	Healthcare access and quality index	0
Nasopharynx cancer	Female	5-9 years	95+ years	Data Rich	-1	2	fruits adjusted(g)	0
Nasopharynx cancer	Female	5-9 years	95+ years	Data Rich	-1	2	vegetables adjusted(g)	0
Nasopharynx cancer	Female	5-9 years	95+ years	Data Rich	1	2	Population Density (over 1000 ppl/sqkm, proportion)	1
Nasopharynx cancer	Female	5-9 years	95+ years	Data Rich	1	2	Population Density (under 150 ppl/sqkm, proportion)	--
Nasopharynx cancer	Female	5-9 years	95+ years	Data Rich	-1	3	Education (years per capita)	0
Nasopharynx cancer	Female	5-9 years	95+ years	Data Rich	0	3	Socio-demographic Index	608
Nasopharynx cancer	Female	5-9 years	95+ years	Data Rich	0	3	LDI (I$ per capita)	--
Nasopharynx cancer	Female	5-9 years	95+ years	Global	1	1	Tobacco (cigarettes per capita)	39
Nasopharynx cancer	Female	5-9 years	95+ years	Global	1	1	Smoking Prevalence	57
Nasopharynx cancer	Female	5-9 years	95+ years	Global	1	1	Cumulative Cigarettes (10 Years)	200
Nasopharynx cancer	Female	5-9 years	95+ years	Global	1	1	Cumulative Cigarettes (20 Years)	212
Nasopharynx cancer	Female	5-9 years	95+ years	Global	1	1	Cumulative Cigarettes (5 Years)	237
Nasopharynx cancer	Female	5-9 years	95+ years	Global	1	1	Cumulative Cigarettes (15 Years)	311
Nasopharynx cancer	Female	5-9 years	95+ years	Global	1	1	Alcohol (liters per capita)	--
Nasopharynx cancer	Female	5-9 years	95+ years	Global	1	1	Log-transformed SEV scalar: Nasoph C	--
Nasopharynx cancer	Female	5-9 years	95+ years	Global	-1	2	fruits adjusted(g)	0
Nasopharynx cancer	Female	5-9 years	95+ years	Global	-1	2	Healthcare access and quality index	40
Nasopharynx cancer	Female	5-9 years	95+ years	Global	-1	2	vegetables adjusted(g)	449
Nasopharynx cancer	Female	5-9 years	95+ years	Global	1	2	Population Density (over 1000 ppl/sqkm, proportion)	496
Nasopharynx cancer	Female	5-9 years	95+ years	Global	1	2	Population Density (under 150 ppl/sqkm, proportion)	--
Nasopharynx cancer	Female	5-9 years	95+ years	Global	-1	3	Education (years per capita)	0
Nasopharynx cancer	Female	5-9 years	95+ years	Global	0	3	Socio-demographic Index	223
Nasopharynx cancer	Female	5-9 years	95+ years	Global	0	3	LDI (I$ per capita)	--
Nasopharynx cancer	Male	5-9 years	95+ years	Data Rich	1	1	Cumulative Cigarettes (15 Years)	4
Nasopharynx cancer	Male	5-9 years	95+ years	Data Rich	1	1	Smoking Prevalence	10
Nasopharynx cancer	Male	5-9 years	95+ years	Data Rich	1	1	Cumulative Cigarettes (5 Years)	12
Nasopharynx cancer	Male	5-9 years	95+ years	Data Rich	1	1	Cumulative Cigarettes (10 Years)	81
Nasopharynx cancer	Male	5-9 years	95+ years	Data Rich	1	1	Tobacco (cigarettes per capita)	110
Nasopharynx cancer	Male	5-9 years	95+ years	Data Rich	1	1	Cumulative Cigarettes (20 Years)	332
Nasopharynx cancer	Male	5-9 years	95+ years	Data Rich	1	1	Alcohol (liters per capita)	--
Nasopharynx cancer	Male	5-9 years	95+ years	Data Rich	1	1	Log-transformed SEV scalar: Nasoph C	--
Nasopharynx cancer	Male	5-9 years	95+ years	Data Rich	-1	2	Healthcare access and quality index	27
Nasopharynx cancer	Male	5-9 years	95+ years	Data Rich	-1	2	fruits adjusted(g)	193
Nasopharynx cancer	Male	5-9 years	95+ years	Data Rich	-1	2	vegetables adjusted(g)	257
Nasopharynx cancer	Male	5-9 years	95+ years	Data Rich	1	2	Population Density (over 1000 ppl/sqkm, proportion)	532
Nasopharynx cancer	Male	5-9 years	95+ years	Data Rich	1	2	Population Density (under 150 ppl/sqkm, proportion)	--
Nasopharynx cancer	Male	5-9 years	95+ years	Data Rich	-1	3	Education (years per capita)	27
Nasopharynx cancer	Male	5-9 years	95+ years	Data Rich	0	3	Socio-demographic Index	46
Nasopharynx cancer	Male	5-9 years	95+ years	Data Rich	0	3	LDI (I$ per capita)	--
Nasopharynx cancer	Male	5-9 years	95+ years	Global	1	1	Cumulative Cigarettes (5 Years)	0
Nasopharynx cancer	Male	5-9 years	95+ years	Global	1	1	Tobacco (cigarettes per capita)	5
Nasopharynx cancer	Male	5-9 years	95+ years	Global	1	1	Cumulative Cigarettes (20 Years)	84

Table S16. CODEm covariates used, level of covariate, and expected direction of covariate by cause, sex, and age
Covariates that CODEm did not select during the covariate selection process have no draw counts listed.

Cause	Sex	Age Start	Age End	Model Type	Direction	Level	Covariate Name	Number of Draws
Nasopharynx cancer	Male	5-9 years	95+ years	Global	1	1	Cumulative Cigarettes (10 Years)	87
Nasopharynx cancer	Male	5-9 years	95+ years	Global	1	1	Cumulative Cigarettes (15 Years)	189
Nasopharynx cancer	Male	5-9 years	95+ years	Global	1	1	Smoking Prevalence	241
Nasopharynx cancer	Male	5-9 years	95+ years	Global	1	1	Alcohol (liters per capita)	650
Nasopharynx cancer	Male	5-9 years	95+ years	Global	1	1	Log-transformed SEV scalar: Nasoph C	--
Nasopharynx cancer	Male	5-9 years	95+ years	Global	-1	2	fruits adjusted(g)	50
Nasopharynx cancer	Male	5-9 years	95+ years	Global	-1	2	Healthcare access and quality index	360
Nasopharynx cancer	Male	5-9 years	95+ years	Global	-1	2	vegetables adjusted(g)	602
Nasopharynx cancer	Male	5-9 years	95+ years	Global	1	2	Population Density (over 1000 ppl/sqkm, proportion)	311
Nasopharynx cancer	Male	5-9 years	95+ years	Global	1	2	Population Density (under 150 ppl/sqkm, proportion)	--
Nasopharynx cancer	Male	5-9 years	95+ years	Global	-1	3	Education (years per capita)	129
Nasopharynx cancer	Male	5-9 years	95+ years	Global	0	3	Socio-demographic Index	278
Nasopharynx cancer	Male	5-9 years	95+ years	Global	0	3	LDI (I$ per capita)	--
Other pharynx cancer	Female	15-19 years	95+ years	Data Rich	1	1	Alcohol (liters per capita)	225
Other pharynx cancer	Female	15-19 years	95+ years	Data Rich	1	1	Log-transformed SEV scalar: Oth Phar C	--
Other pharynx cancer	Female	15-19 years	95+ years	Data Rich	1	1	Smoking Prevalence	--
Other pharynx cancer	Female	15-19 years	95+ years	Data Rich	-1	2	Healthcare access and quality index	189
Other pharynx cancer	Female	15-19 years	95+ years	Data Rich	-1	2	vegetables adjusted(g)	236
Other pharynx cancer	Female	15-19 years	95+ years	Data Rich	-1	2	fruits adjusted(g)	--
Other pharynx cancer	Female	15-19 years	95+ years	Data Rich	1	2	Population Density (over 1000 ppl/sqkm, proportion)	309
Other pharynx cancer	Female	15-19 years	95+ years	Data Rich	1	2	Cumulative Cigarettes (5 Years)	--
Other pharynx cancer	Female	15-19 years	95+ years	Data Rich	1	2	Population Density (under 150 ppl/sqkm, proportion)	--
Other pharynx cancer	Female	15-19 years	95+ years	Data Rich	-1	3	Education (years per capita)	97
Other pharynx cancer	Female	15-19 years	95+ years	Data Rich	0	3	Socio-demographic Index	271
Other pharynx cancer	Female	15-19 years	95+ years	Data Rich	0	3	LDI (I$ per capita)	--
Other pharynx cancer	Female	15-19 years	95+ years	Global	1	1	Smoking Prevalence	170
Other pharynx cancer	Female	15-19 years	95+ years	Global	1	1	Alcohol (liters per capita)	729
Other pharynx cancer	Female	15-19 years	95+ years	Global	1	1	Log-transformed SEV scalar: Oth Phar C	--
Other pharynx cancer	Female	15-19 years	95+ years	Global	-1	2	fruits adjusted(g)	17
Other pharynx cancer	Female	15-19 years	95+ years	Global	-1	2	vegetables adjusted(g)	125
Other pharynx cancer	Female	15-19 years	95+ years	Global	-1	2	Healthcare access and quality index	205
Other pharynx cancer	Female	15-19 years	95+ years	Global	1	2	Cumulative Cigarettes (5 Years)	144
Other pharynx cancer	Female	15-19 years	95+ years	Global	1	2	Population Density (over 1000 ppl/sqkm, proportion)	636
Other pharynx cancer	Female	15-19 years	95+ years	Global	1	2	Population Density (under 150 ppl/sqkm, proportion)	--
Other pharynx cancer	Female	15-19 years	95+ years	Global	-1	3	Education (years per capita)	61
Other pharynx cancer	Female	15-19 years	95+ years	Global	0	3	Socio-demographic Index	315
Other pharynx cancer	Female	15-19 years	95+ years	Global	0	3	LDI (I$ per capita)	--
Other pharynx cancer	Male	15-19 years	95+ years	Data Rich	1	1	Alcohol (liters per capita)	799
Other pharynx cancer	Male	15-19 years	95+ years	Data Rich	1	1	Log-transformed SEV scalar: Oth Phar C	--
Other pharynx cancer	Male	15-19 years	95+ years	Data Rich	1	1	Smoking Prevalence	--
Other pharynx cancer	Male	15-19 years	95+ years	Data Rich	-1	2	Healthcare access and quality index	--
Other pharynx cancer	Male	15-19 years	95+ years	Data Rich	-1	2	fruits adjusted(g)	--
Other pharynx cancer	Male	15-19 years	95+ years	Data Rich	-1	2	vegetables adjusted(g)	--

Table S16. CODEm covariates used, level of covariate, and expected direction of covariate by cause, sex, and age<br>*Covariates that CODEm did not select during the covariate selection process have no draw counts listed.*								
**Cause**	**Sex**	**Age Start**	**Age End**	**Model Type**	**Direction**	**Level**	**Covariate Name**	**Number of Draws**
Other pharynx cancer	Male	15-19 years	95+ years	Data Rich	1	2	Population Density (over 1000 ppl/sqkm, proportion)	423
Other pharynx cancer	Male	15-19 years	95+ years	Data Rich	1	2	Cumulative Cigarettes (5 Years)	--
Other pharynx cancer	Male	15-19 years	95+ years	Data Rich	1	2	Population Density (under 150 ppl/sqkm, proportion)	--
Other pharynx cancer	Male	15-19 years	95+ years	Data Rich	-1	3	Education (years per capita)	--
Other pharynx cancer	Male	15-19 years	95+ years	Data Rich	0	3	Socio-demographic Index	406
Other pharynx cancer	Male	15-19 years	95+ years	Data Rich	0	3	LDI (I$ per capita)	--
Other pharynx cancer	Male	15-19 years	95+ years	Global	1	1	Alcohol (liters per capita)	784
Other pharynx cancer	Male	15-19 years	95+ years	Global	1	1	Log-transformed SEV scalar: Oth Phar C	--
Other pharynx cancer	Male	15-19 years	95+ years	Global	1	1	Smoking Prevalence	--
Other pharynx cancer	Male	15-19 years	95+ years	Global	-1	2	Healthcare access and quality index	--
Other pharynx cancer	Male	15-19 years	95+ years	Global	-1	2	fruits adjusted(g)	--
Other pharynx cancer	Male	15-19 years	95+ years	Global	-1	2	vegetables adjusted(g)	--
Other pharynx cancer	Male	15-19 years	95+ years	Global	1	2	Population Density (over 1000 ppl/sqkm, proportion)	276
Other pharynx cancer	Male	15-19 years	95+ years	Global	1	2	Cumulative Cigarettes (5 Years)	--
Other pharynx cancer	Male	15-19 years	95+ years	Global	1	2	Population Density (under 150 ppl/sqkm, proportion)	--
Other pharynx cancer	Male	15-19 years	95+ years	Global	-1	3	Education (years per capita)	--
Other pharynx cancer	Male	15-19 years	95+ years	Global	0	3	Socio-demographic Index	198
Other pharynx cancer	Male	15-19 years	95+ years	Global	0	3	LDI (I$ per capita)	--
Oesophageal cancer	Female	15-19 years	95+ years	Data Rich	-1	1	fruits adjusted(g)	--
Oesophageal cancer	Female	15-19 years	95+ years	Data Rich	1	1	Smoking Prevalence	0
Oesophageal cancer	Female	15-19 years	95+ years	Data Rich	1	1	Tobacco (cigarettes per capita)	0
Oesophageal cancer	Female	15-19 years	95+ years	Data Rich	1	1	Mean BMI	62
Oesophageal cancer	Female	15-19 years	95+ years	Data Rich	1	1	Alcohol (liters per capita)	833
Oesophageal cancer	Female	15-19 years	95+ years	Data Rich	1	1	Log-transformed SEV scalar: Esophag C	--
Oesophageal cancer	Female	15-19 years	95+ years	Data Rich	1	1	Log-transformed age-standardized SEV scalar: Esophag C	--
Oesophageal cancer	Female	15-19 years	95+ years	Data Rich	-1	2	Healthcare access and quality index	0
Oesophageal cancer	Female	15-19 years	95+ years	Data Rich	-1	2	Improved Water Source (proportion with access)	0
Oesophageal cancer	Female	15-19 years	95+ years	Data Rich	-1	2	vegetables adjusted(g)	7
Oesophageal cancer	Female	15-19 years	95+ years	Data Rich	-1	2	Sanitation (proportion with access)	379
Oesophageal cancer	Female	15-19 years	95+ years	Data Rich	1	2	Indoor Air Pollution (All Cooking Fuels)	77
Oesophageal cancer	Female	15-19 years	95+ years	Data Rich	-1	3	Education (years per capita)	0
Oesophageal cancer	Female	15-19 years	95+ years	Data Rich	0	3	Socio-demographic Index	357
Oesophageal cancer	Female	15-19 years	95+ years	Data Rich	0	3	LDI (I$ per capita)	--
Oesophageal cancer	Female	15-19 years	95+ years	Global	-1	1	fruits adjusted(g)	313
Oesophageal cancer	Female	15-19 years	95+ years	Global	1	1	Smoking Prevalence	43
Oesophageal cancer	Female	15-19 years	95+ years	Global	1	1	Tobacco (cigarettes per capita)	53
Oesophageal cancer	Female	15-19 years	95+ years	Global	1	1	Alcohol (liters per capita)	538
Oesophageal cancer	Female	15-19 years	95+ years	Global	1	1	Mean BMI	685
Oesophageal cancer	Female	15-19 years	95+ years	Global	1	1	Log-transformed SEV scalar: Esophag C	--
Oesophageal cancer	Female	15-19 years	95+ years	Global	1	1	Log-transformed age-standardized SEV scalar: Esophag C	--

Table S16. CODEm covariates used, level of covariate, and expected direction of covariate by cause, sex, and age
Covariates that CODEm did not select during the covariate selection process have no draw counts listed.

Cause	Sex	Age Start	Age End	Model Type	Direction	Level	Covariate Name	Number of Draws
Oesophageal cancer	Female	15-19 years	95+ years	Global	-1	2	Improved Water Source (proportion with access)	0
Oesophageal cancer	Female	15-19 years	95+ years	Global	-1	2	Healthcare access and quality index	57
Oesophageal cancer	Female	15-19 years	95+ years	Global	-1	2	vegetables adjusted(g)	207
Oesophageal cancer	Female	15-19 years	95+ years	Global	-1	2	Sanitation (proportion with access)	442
Oesophageal cancer	Female	15-19 years	95+ years	Global	1	2	Indoor Air Pollution (All Cooking Fuels)	431
Oesophageal cancer	Female	15-19 years	95+ years	Global	-1	3	Education (years per capita)	170
Oesophageal cancer	Female	15-19 years	95+ years	Global	0	3	Socio-demographic Index	488
Oesophageal cancer	Female	15-19 years	95+ years	Global	0	3	LDI (I$ per capita)	--
Oesophageal cancer	Male	15-19 years	95+ years	Data Rich	-1	1	fruits adjusted(g)	--
Oesophageal cancer	Male	15-19 years	95+ years	Data Rich	1	1	Tobacco (cigarettes per capita)	3
Oesophageal cancer	Male	15-19 years	95+ years	Data Rich	1	1	Smoking Prevalence	7
Oesophageal cancer	Male	15-19 years	95+ years	Data Rich	1	1	Alcohol (liters per capita)	147
Oesophageal cancer	Male	15-19 years	95+ years	Data Rich	1	1	Mean BMI	981
Oesophageal cancer	Male	15-19 years	95+ years	Data Rich	1	1	Log-transformed SEV scalar: Esophag C	--
Oesophageal cancer	Male	15-19 years	95+ years	Data Rich	1	1	Log-transformed age-standardized SEV scalar: Esophag C	--
Oesophageal cancer	Male	15-19 years	95+ years	Data Rich	-1	2	Healthcare access and quality index	1
Oesophageal cancer	Male	15-19 years	95+ years	Data Rich	-1	2	vegetables adjusted(g)	288
Oesophageal cancer	Male	15-19 years	95+ years	Data Rich	-1	2	Sanitation (proportion with access)	460
Oesophageal cancer	Male	15-19 years	95+ years	Data Rich	-1	2	Improved Water Source (proportion with access)	--
Oesophageal cancer	Male	15-19 years	95+ years	Data Rich	1	2	Indoor Air Pollution (All Cooking Fuels)	305
Oesophageal cancer	Male	15-19 years	95+ years	Data Rich	-1	3	Education (years per capita)	0
Oesophageal cancer	Male	15-19 years	95+ years	Data Rich	0	3	Socio-demographic Index	547
Oesophageal cancer	Male	15-19 years	95+ years	Data Rich	0	3	LDI (I$ per capita)	--
Oesophageal cancer	Male	15-19 years	95+ years	Global	-1	1	fruits adjusted(g)	259
Oesophageal cancer	Male	15-19 years	95+ years	Global	1	1	Smoking Prevalence	0
Oesophageal cancer	Male	15-19 years	95+ years	Global	1	1	Tobacco (cigarettes per capita)	0
Oesophageal cancer	Male	15-19 years	95+ years	Global	1	1	Alcohol (liters per capita)	196
Oesophageal cancer	Male	15-19 years	95+ years	Global	1	1	Mean BMI	999
Oesophageal cancer	Male	15-19 years	95+ years	Global	1	1	Log-transformed SEV scalar: Esophag C	--
Oesophageal cancer	Male	15-19 years	95+ years	Global	1	1	Log-transformed age-standardized SEV scalar: Esophag C	--
Oesophageal cancer	Male	15-19 years	95+ years	Global	-1	2	Healthcare access and quality index	0
Oesophageal cancer	Male	15-19 years	95+ years	Global	-1	2	Improved Water Source (proportion with access)	0
Oesophageal cancer	Male	15-19 years	95+ years	Global	-1	2	vegetables adjusted(g)	344
Oesophageal cancer	Male	15-19 years	95+ years	Global	-1	2	Sanitation (proportion with access)	447
Oesophageal cancer	Male	15-19 years	95+ years	Global	1	2	Indoor Air Pollution (All Cooking Fuels)	379
Oesophageal cancer	Male	15-19 years	95+ years	Global	-1	3	Education (years per capita)	0
Oesophageal cancer	Male	15-19 years	95+ years	Global	0	3	Socio-demographic Index	583
Oesophageal cancer	Male	15-19 years	95+ years	Global	0	3	LDI (I$ per capita)	--
Stomach cancer	Female	15-19 years	95+ years	Data Rich	1	1	Smoking Prevalence	0
Stomach cancer	Female	15-19 years	95+ years	Data Rich	1	1	Age- and sex-specific SEV for Unsafe water	6

Table S16. CODEm covariates used, level of covariate, and expected direction of covariate by cause, sex, and age
Covariates that CODEm did not select during the covariate selection process have no draw counts listed.

Cause	Sex	Age Start	Age End	Model Type	Direction	Level	Covariate Name	Number of Draws
Stomach cancer	Female	15-19 years	95+ years	Data Rich	1	1	Cumulative Cigarettes (5 Years)	15
Stomach cancer	Female	15-19 years	95+ years	Data Rich	1	1	Cumulative Cigarettes (15 Years)	54
Stomach cancer	Female	15-19 years	95+ years	Data Rich	1	1	Age- and sex-specific SEV for Unsafe sanitation	178
Stomach cancer	Female	15-19 years	95+ years	Data Rich	1	1	Cumulative Cigarettes (10 Years)	215
Stomach cancer	Female	15-19 years	95+ years	Data Rich	1	1	Tobacco (cigarettes per capita)	367
Stomach cancer	Female	15-19 years	95+ years	Data Rich	1	1	Cumulative Cigarettes (20 Years)	368
Stomach cancer	Female	15-19 years	95+ years	Data Rich	1	1	Diet high in sodium	--
Stomach cancer	Female	15-19 years	95+ years	Data Rich	1	1	Log-transformed SEV scalar: Stomach C	--
Stomach cancer	Female	15-19 years	95+ years	Data Rich	-1	2	fruits adjusted(g)	20
Stomach cancer	Female	15-19 years	95+ years	Data Rich	-1	2	vegetables adjusted(g)	23
Stomach cancer	Female	15-19 years	95+ years	Data Rich	-1	2	Healthcare access and quality index	27
Stomach cancer	Female	15-19 years	95+ years	Data Rich	-1	2	Sanitation (proportion with access)	152
Stomach cancer	Female	15-19 years	95+ years	Data Rich	-1	2	Improved Water Source (proportion with access)	153
Stomach cancer	Female	15-19 years	95+ years	Data Rich	1	2	Mean BMI	--
Stomach cancer	Female	15-19 years	95+ years	Data Rich	-1	3	Education (years per capita)	699
Stomach cancer	Female	15-19 years	95+ years	Data Rich	0	3	Socio-demographic Index	551
Stomach cancer	Female	15-19 years	95+ years	Data Rich	0	3	LDI (I$ per capita)	--
Stomach cancer	Female	15-19 years	95+ years	Global	1	1	Cumulative Cigarettes (5 Years)	0
Stomach cancer	Female	15-19 years	95+ years	Global	1	1	Smoking Prevalence	0
Stomach cancer	Female	15-19 years	95+ years	Global	1	1	Cumulative Cigarettes (10 Years)	2
Stomach cancer	Female	15-19 years	95+ years	Global	1	1	Age- and sex-specific SEV for Unsafe sanitation	69
Stomach cancer	Female	15-19 years	95+ years	Global	1	1	Age- and sex-specific SEV for Unsafe water	85
Stomach cancer	Female	15-19 years	95+ years	Global	1	1	Cumulative Cigarettes (15 Years)	281
Stomach cancer	Female	15-19 years	95+ years	Global	1	1	Cumulative Cigarettes (20 Years)	367
Stomach cancer	Female	15-19 years	95+ years	Global	1	1	Diet high in sodium	769
Stomach cancer	Female	15-19 years	95+ years	Global	1	1	Tobacco (cigarettes per capita)	946
Stomach cancer	Female	15-19 years	95+ years	Global	1	1	Log-transformed SEV scalar: Stomach C	--
Stomach cancer	Female	15-19 years	95+ years	Global	-1	2	fruits adjusted(g)	1
Stomach cancer	Female	15-19 years	95+ years	Global	-1	2	Sanitation (proportion with access)	12
Stomach cancer	Female	15-19 years	95+ years	Global	-1	2	Improved Water Source (proportion with access)	19
Stomach cancer	Female	15-19 years	95+ years	Global	-1	2	Healthcare access and quality index	28
Stomach cancer	Female	15-19 years	95+ years	Global	-1	2	vegetables adjusted(g)	129
Stomach cancer	Female	15-19 years	95+ years	Global	1	2	Mean BMI	--
Stomach cancer	Female	15-19 years	95+ years	Global	-1	3	Education (years per capita)	38
Stomach cancer	Female	15-19 years	95+ years	Global	0	3	Socio-demographic Index	234
Stomach cancer	Female	15-19 years	95+ years	Global	0	3	LDI (I$ per capita)	--
Stomach cancer	Male	15-19 years	95+ years	Data Rich	1	1	Age- and sex-specific SEV for Unsafe water	0
Stomach cancer	Male	15-19 years	95+ years	Data Rich	1	1	Cumulative Cigarettes (20 Years)	22
Stomach cancer	Male	15-19 years	95+ years	Data Rich	1	1	Age- and sex-specific SEV for Unsafe sanitation	33
Stomach cancer	Male	15-19 years	95+ years	Data Rich	1	1	Cumulative Cigarettes (15 Years)	99

Table S16. CODEm covariates used, level of covariate, and expected direction of covariate by cause, sex, and age
Covariates that CODEm did not select during the covariate selection process have no draw counts listed.

Cause	Sex	Age Start	Age End	Model Type	Direction	Level	Covariate Name	Number of Draws
Stomach cancer	Male	15-19 years	95+ years	Data Rich	1	1	Cumulative Cigarettes (10 Years)	105
Stomach cancer	Male	15-19 years	95+ years	Data Rich	1	1	Tobacco (cigarettes per capita)	185
Stomach cancer	Male	15-19 years	95+ years	Data Rich	1	1	Smoking Prevalence	297
Stomach cancer	Male	15-19 years	95+ years	Data Rich	1	1	Cumulative Cigarettes (5 Years)	452
Stomach cancer	Male	15-19 years	95+ years	Data Rich	1	1	Diet high in sodium	--
Stomach cancer	Male	15-19 years	95+ years	Data Rich	1	1	Log-transformed SEV scalar: Stomach C	--
Stomach cancer	Male	15-19 years	95+ years	Data Rich	-1	2	Healthcare access and quality index	14
Stomach cancer	Male	15-19 years	95+ years	Data Rich	-1	2	fruits adjusted(g)	14
Stomach cancer	Male	15-19 years	95+ years	Data Rich	-1	2	Sanitation (proportion with access)	138
Stomach cancer	Male	15-19 years	95+ years	Data Rich	-1	2	vegetables adjusted(g)	183
Stomach cancer	Male	15-19 years	95+ years	Data Rich	-1	2	Improved Water Source (proportion with access)	302
Stomach cancer	Male	15-19 years	95+ years	Data Rich	1	2	Mean BMI	--
Stomach cancer	Male	15-19 years	95+ years	Data Rich	-1	3	Education (years per capita)	751
Stomach cancer	Male	15-19 years	95+ years	Data Rich	0	3	Socio-demographic Index	868
Stomach cancer	Male	15-19 years	95+ years	Data Rich	0	3	LDI (I$ per capita)	--
Stomach cancer	Male	15-19 years	95+ years	Global	1	1	Cumulative Cigarettes (5 Years)	87
Stomach cancer	Male	15-19 years	95+ years	Global	1	1	Diet high in sodium	180
Stomach cancer	Male	15-19 years	95+ years	Global	1	1	Smoking Prevalence	210
Stomach cancer	Male	15-19 years	95+ years	Global	1	1	Age- and sex-specific SEV for Unsafe sanitation	217
Stomach cancer	Male	15-19 years	95+ years	Global	1	1	Age- and sex-specific SEV for Unsafe water	231
Stomach cancer	Male	15-19 years	95+ years	Global	1	1	Cumulative Cigarettes (10 Years)	235
Stomach cancer	Male	15-19 years	95+ years	Global	1	1	Cumulative Cigarettes (15 Years)	268
Stomach cancer	Male	15-19 years	95+ years	Global	1	1	Cumulative Cigarettes (20 Years)	385
Stomach cancer	Male	15-19 years	95+ years	Global	1	1	Tobacco (cigarettes per capita)	568
Stomach cancer	Male	15-19 years	95+ years	Global	1	1	Log-transformed SEV scalar: Stomach C	--
Stomach cancer	Male	15-19 years	95+ years	Global	-1	2	vegetables adjusted(g)	39
Stomach cancer	Male	15-19 years	95+ years	Global	-1	2	Sanitation (proportion with access)	80
Stomach cancer	Male	15-19 years	95+ years	Global	-1	2	fruits adjusted(g)	83
Stomach cancer	Male	15-19 years	95+ years	Global	-1	2	Healthcare access and quality index	221
Stomach cancer	Male	15-19 years	95+ years	Global	-1	2	Improved Water Source (proportion with access)	223
Stomach cancer	Male	15-19 years	95+ years	Global	1	2	Mean BMI	--
Stomach cancer	Male	15-19 years	95+ years	Global	-1	3	Education (years per capita)	73
Stomach cancer	Male	15-19 years	95+ years	Global	0	3	Socio-demographic Index	108
Stomach cancer	Male	15-19 years	95+ years	Global	0	3	LDI (I$ per capita)	--
Colon and rectum cancer	Female	15-19 years	95+ years	Data Rich	-1	1	Total Physical Activity (MET-min/week), Age-standardized	7
Colon and rectum cancer	Female	15-19 years	95+ years	Data Rich	-1	1	Total Physical Activity (MET-min/week), Age-specific	174
Colon and rectum cancer	Female	15-19 years	95+ years	Data Rich	1	1	Tobacco (cigarettes per capita)	0
Colon and rectum cancer	Female	15-19 years	95+ years	Data Rich	1	1	Smoking Prevalence	79
Colon and rectum cancer	Female	15-19 years	95+ years	Data Rich	1	1	Alcohol (liters per capita)	137
Colon and rectum cancer	Female	15-19 years	95+ years	Data Rich	1	1	Mean BMI	739

Table S16. CODEm covariates used, level of covariate, and expected direction of covariate by cause, sex, and age<br>*Covariates that CODEm did not select during the covariate selection process have no draw counts listed.*								
**Cause**	**Sex**	**Age Start**	**Age End**	**Model Type**	**Direction**	**Level**	**Covariate Name**	**Number of Draws**
Colon and rectum cancer	Female	15-19 years	95+ years	Data Rich	1	1	red meats adjusted(g)	818
Colon and rectum cancer	Female	15-19 years	95+ years	Data Rich	1	1	Log-transformed SEV scalar: Colorect C	--
Colon and rectum cancer	Female	15-19 years	95+ years	Data Rich	-1	2	Healthcare access and quality index	0
Colon and rectum cancer	Female	15-19 years	95+ years	Data Rich	-1	2	fruits adjusted(g)	0
Colon and rectum cancer	Female	15-19 years	95+ years	Data Rich	-1	2	nuts seeds adjusted(g)	150
Colon and rectum cancer	Female	15-19 years	95+ years	Data Rich	-1	2	pufa adjusted(percent)	257
Colon and rectum cancer	Female	15-19 years	95+ years	Data Rich	-1	2	vegetables adjusted(g)	545
Colon and rectum cancer	Female	15-19 years	95+ years	Data Rich	-1	2	milk adjusted(g)	594
Colon and rectum cancer	Female	15-19 years	95+ years	Data Rich	-1	2	fiber adjusted(g)	815
Colon and rectum cancer	Female	15-19 years	95+ years	Data Rich	-1	2	calcium adjusted(g)	--
Colon and rectum cancer	Female	15-19 years	95+ years	Data Rich	1	2	Cumulative Cigarettes (10 Years)	21
Colon and rectum cancer	Female	15-19 years	95+ years	Data Rich	1	2	Diabetes Age-Specific Prevalence (proportion)	32
Colon and rectum cancer	Female	15-19 years	95+ years	Data Rich	1	2	Cumulative Cigarettes (5 Years)	156
Colon and rectum cancer	Female	15-19 years	95+ years	Data Rich	1	2	Cumulative Cigarettes (15 Years)	266
Colon and rectum cancer	Female	15-19 years	95+ years	Data Rich	1	2	Cumulative Cigarettes (20 Years)	410
Colon and rectum cancer	Female	15-19 years	95+ years	Data Rich	1	2	Diabetes Fasting Plasma Glucose (mmol/L), age-standardized 25+	592
Colon and rectum cancer	Female	15-19 years	95+ years	Data Rich	-1	3	Education (years per capita)	67
Colon and rectum cancer	Female	15-19 years	95+ years	Data Rich	0	3	Socio-demographic Index	0
Colon and rectum cancer	Female	15-19 years	95+ years	Data Rich	0	3	LDI (I$ per capita)	--
Colon and rectum cancer	Female	15-19 years	95+ years	Global	-1	1	Total Physical Activity (MET-min/week), Age-specific	163
Colon and rectum cancer	Female	15-19 years	95+ years	Global	-1	1	Total Physical Activity (MET-min/week), Age-standardized	370
Colon and rectum cancer	Female	15-19 years	95+ years	Global	1	1	Mean BMI	0
Colon and rectum cancer	Female	15-19 years	95+ years	Global	1	1	Tobacco (cigarettes per capita)	78
Colon and rectum cancer	Female	15-19 years	95+ years	Global	1	1	Smoking Prevalence	84
Colon and rectum cancer	Female	15-19 years	95+ years	Global	1	1	Alcohol (liters per capita)	242
Colon and rectum cancer	Female	15-19 years	95+ years	Global	1	1	red meats adjusted(g)	408
Colon and rectum cancer	Female	15-19 years	95+ years	Global	1	1	Log-transformed SEV scalar: Colorect C	--
Colon and rectum cancer	Female	15-19 years	95+ years	Global	-1	2	milk adjusted(g)	0
Colon and rectum cancer	Female	15-19 years	95+ years	Global	-1	2	fruits adjusted(g)	4
Colon and rectum cancer	Female	15-19 years	95+ years	Global	-1	2	vegetables adjusted(g)	17
Colon and rectum cancer	Female	15-19 years	95+ years	Global	-1	2	calcium adjusted(g)	22
Colon and rectum cancer	Female	15-19 years	95+ years	Global	-1	2	pufa adjusted(percent)	23
Colon and rectum cancer	Female	15-19 years	95+ years	Global	-1	2	Healthcare access and quality index	46
Colon and rectum cancer	Female	15-19 years	95+ years	Global	-1	2	nuts seeds adjusted(g)	612
Colon and rectum cancer	Female	15-19 years	95+ years	Global	-1	2	fiber adjusted(g)	961
Colon and rectum cancer	Female	15-19 years	95+ years	Global	1	2	Cumulative Cigarettes (5 Years)	1
Colon and rectum cancer	Female	15-19 years	95+ years	Global	1	2	Cumulative Cigarettes (15 Years)	215
Colon and rectum cancer	Female	15-19 years	95+ years	Global	1	2	Cumulative Cigarettes (20 Years)	216
Colon and rectum cancer	Female	15-19 years	95+ years	Global	1	2	Cumulative Cigarettes (10 Years)	244
Colon and rectum cancer	Female	15-19 years	95+ years	Global	1	2	Diabetes Fasting Plasma Glucose (mmol/L), age-standardized 25+	291

Table S16. CODEm covariates used, level of covariate, and expected direction of covariate by cause, sex, and age<br>*Covariates that CODEm did not select during the covariate selection process have no draw counts listed.*								
**Cause**	**Sex**	**Age Start**	**Age End**	**Model Type**	**Direction**	**Level**	**Covariate Name**	**Number of Draws**
Colon and rectum cancer	Female	15-19 years	95+ years	Global	1	2	Diabetes Age-Specific Prevalence (proportion)	654
Colon and rectum cancer	Female	15-19 years	95+ years	Global	-1	3	Education (years per capita)	262
Colon and rectum cancer	Female	15-19 years	95+ years	Global	0	3	Socio-demographic Index	498
Colon and rectum cancer	Female	15-19 years	95+ years	Global	0	3	LDI (I$ per capita)	--
Colon and rectum cancer	Male	15-19 years	95+ years	Data Rich	-1	1	Total Physical Activity (MET-min/week), Age-specific	42
Colon and rectum cancer	Male	15-19 years	95+ years	Data Rich	-1	1	Total Physical Activity (MET-min/week), Age-standardized	199
Colon and rectum cancer	Male	15-19 years	95+ years	Data Rich	1	1	Alcohol (liters per capita)	0
Colon and rectum cancer	Male	15-19 years	95+ years	Data Rich	1	1	Tobacco (cigarettes per capita)	0
Colon and rectum cancer	Male	15-19 years	95+ years	Data Rich	1	1	Smoking Prevalence	79
Colon and rectum cancer	Male	15-19 years	95+ years	Data Rich	1	1	Mean BMI	397
Colon and rectum cancer	Male	15-19 years	95+ years	Data Rich	1	1	red meats adjusted(g)	401
Colon and rectum cancer	Male	15-19 years	95+ years	Data Rich	1	1	Log-transformed SEV scalar: Colorect C	--
Colon and rectum cancer	Male	15-19 years	95+ years	Data Rich	-1	2	Healthcare access and quality index	0
Colon and rectum cancer	Male	15-19 years	95+ years	Data Rich	-1	2	fruits adjusted(g)	4
Colon and rectum cancer	Male	15-19 years	95+ years	Data Rich	-1	2	pufa adjusted(percent)	96
Colon and rectum cancer	Male	15-19 years	95+ years	Data Rich	-1	2	milk adjusted(g)	138
Colon and rectum cancer	Male	15-19 years	95+ years	Data Rich	-1	2	vegetables adjusted(g)	246
Colon and rectum cancer	Male	15-19 years	95+ years	Data Rich	-1	2	nuts seeds adjusted(g)	270
Colon and rectum cancer	Male	15-19 years	95+ years	Data Rich	-1	2	fiber adjusted(g)	436
Colon and rectum cancer	Male	15-19 years	95+ years	Data Rich	-1	2	calcium adjusted(g)	--
Colon and rectum cancer	Male	15-19 years	95+ years	Data Rich	1	2	Cumulative Cigarettes (5 Years)	0
Colon and rectum cancer	Male	15-19 years	95+ years	Data Rich	1	2	Cumulative Cigarettes (15 Years)	4
Colon and rectum cancer	Male	15-19 years	95+ years	Data Rich	1	2	Cumulative Cigarettes (10 Years)	13
Colon and rectum cancer	Male	15-19 years	95+ years	Data Rich	1	2	Diabetes Age-Specific Prevalence (proportion)	24
Colon and rectum cancer	Male	15-19 years	95+ years	Data Rich	1	2	Cumulative Cigarettes (20 Years)	317
Colon and rectum cancer	Male	15-19 years	95+ years	Data Rich	1	2	Diabetes Fasting Plasma Glucose (mmol/L), age-standardized 25+	544
Colon and rectum cancer	Male	15-19 years	95+ years	Data Rich	-1	3	Education (years per capita)	0
Colon and rectum cancer	Male	15-19 years	95+ years	Data Rich	0	3	Socio-demographic Index	515
Colon and rectum cancer	Male	15-19 years	95+ years	Data Rich	0	3	LDI (I$ per capita)	--
Colon and rectum cancer	Male	15-19 years	95+ years	Global	-1	1	Total Physical Activity (MET-min/week), Age-standardized	84
Colon and rectum cancer	Male	15-19 years	95+ years	Global	-1	1	Total Physical Activity (MET-min/week), Age-specific	107
Colon and rectum cancer	Male	15-19 years	95+ years	Global	1	1	Alcohol (liters per capita)	0
Colon and rectum cancer	Male	15-19 years	95+ years	Global	1	1	Smoking Prevalence	57
Colon and rectum cancer	Male	15-19 years	95+ years	Global	1	1	Tobacco (cigarettes per capita)	77
Colon and rectum cancer	Male	15-19 years	95+ years	Global	1	1	red meats adjusted(g)	504
Colon and rectum cancer	Male	15-19 years	95+ years	Global	1	1	Mean BMI	709
Colon and rectum cancer	Male	15-19 years	95+ years	Global	1	1	Log-transformed SEV scalar: Colorect C	--
Colon and rectum cancer	Male	15-19 years	95+ years	Global	-1	2	Healthcare access and quality index	0
Colon and rectum cancer	Male	15-19 years	95+ years	Global	-1	2	fruits adjusted(g)	5
Colon and rectum cancer	Male	15-19 years	95+ years	Global	-1	2	nuts seeds adjusted(g)	157

Table S16. CODEm covariates used, level of covariate, and expected direction of covariate by cause, sex, and age<br>*Covariates that CODEm did not select during the covariate selection process have no draw counts listed.*								
**Cause**	**Sex**	**Age Start**	**Age End**	**Model Type**	**Direction**	**Level**	**Covariate Name**	**Number of Draws**
Colon and rectum cancer	Male	15-19 years	95+ years	Global	-1	2	pufa adjusted(percent)	296
Colon and rectum cancer	Male	15-19 years	95+ years	Global	-1	2	milk adjusted(g)	364
Colon and rectum cancer	Male	15-19 years	95+ years	Global	-1	2	vegetables adjusted(g)	381
Colon and rectum cancer	Male	15-19 years	95+ years	Global	-1	2	fiber adjusted(g)	623
Colon and rectum cancer	Male	15-19 years	95+ years	Global	-1	2	calcium adjusted(g)	--
Colon and rectum cancer	Male	15-19 years	95+ years	Global	1	2	Cumulative Cigarettes (5 Years)	0
Colon and rectum cancer	Male	15-19 years	95+ years	Global	1	2	Diabetes Age-Specific Prevalence (proportion)	175
Colon and rectum cancer	Male	15-19 years	95+ years	Global	1	2	Cumulative Cigarettes (10 Years)	190
Colon and rectum cancer	Male	15-19 years	95+ years	Global	1	2	Cumulative Cigarettes (20 Years)	255
Colon and rectum cancer	Male	15-19 years	95+ years	Global	1	2	Cumulative Cigarettes (15 Years)	294
Colon and rectum cancer	Male	15-19 years	95+ years	Global	1	2	Diabetes Fasting Plasma Glucose (mmol/L), age-standardized 25+	878
Colon and rectum cancer	Male	15-19 years	95+ years	Global	-1	3	Education (years per capita)	0
Colon and rectum cancer	Male	15-19 years	95+ years	Global	0	3	Socio-demographic Index	468
Colon and rectum cancer	Male	15-19 years	95+ years	Global	0	3	LDI (I$ per capita)	--
Liver cancer	Female	5-9 years	95+ years	Data Rich	1	1	Hepatitis B (HBsAg) Seroprevalence	10
Liver cancer	Female	5-9 years	95+ years	Data Rich	1	1	Alcohol (liters per capita)	196
Liver cancer	Female	5-9 years	95+ years	Data Rich	1	1	HIV age-standardized prevalence	--
Liver cancer	Female	5-9 years	95+ years	Data Rich	1	1	Hepatitis C (IgG) Seroprevalence	--
Liver cancer	Female	5-9 years	95+ years	Data Rich	1	1	Log-transformed SEV scalar: Liver C	--
Liver cancer	Female	5-9 years	95+ years	Data Rich	-1	2	Hepatitis B 3-dose coverage (proportion)	303
Liver cancer	Female	5-9 years	95+ years	Data Rich	-1	2	Hepatitis B 3-dose coverage (proportion), lagged 10 years	304
Liver cancer	Female	5-9 years	95+ years	Data Rich	-1	2	Hepatitis B vaccine coverage (proportion), aged through time	462
Liver cancer	Female	5-9 years	95+ years	Data Rich	-1	2	Healthcare access and quality index	--
Liver cancer	Female	5-9 years	95+ years	Data Rich	-1	2	Hepatitis B 3-dose coverage (proportion), lagged 5 years	--
Liver cancer	Female	5-9 years	95+ years	Data Rich	1	2	Diabetes Fasting Plasma Glucose (mmol/L), age-standardized 25+	94
Liver cancer	Female	5-9 years	95+ years	Data Rich	1	2	red meats adjusted(g)	252
Liver cancer	Female	5-9 years	95+ years	Data Rich	1	2	Intravenous drug use (age-standardized proportion)	333
Liver cancer	Female	5-9 years	95+ years	Data Rich	1	2	Diabetes Age-Standardized Prevalence (proportion)	502
Liver cancer	Female	5-9 years	95+ years	Data Rich	1	2	Mean BMI	671
Liver cancer	Female	5-9 years	95+ years	Data Rich	1	2	Cumulative Cigarettes (10 Years)	--
Liver cancer	Female	5-9 years	95+ years	Data Rich	1	2	Cumulative Cigarettes (15 Years)	--
Liver cancer	Female	5-9 years	95+ years	Data Rich	1	2	Cumulative Cigarettes (20 Years)	--
Liver cancer	Female	5-9 years	95+ years	Data Rich	1	2	Cumulative Cigarettes (5 Years)	--
Liver cancer	Female	5-9 years	95+ years	Data Rich	1	2	Tobacco (cigarettes per capita)	--
Liver cancer	Female	5-9 years	95+ years	Data Rich	-1	3	Education (years per capita)	--
Liver cancer	Female	5-9 years	95+ years	Data Rich	0	3	Socio-demographic Index	61
Liver cancer	Female	5-9 years	95+ years	Data Rich	0	3	LDI (I$ per capita)	--
Liver cancer	Female	5-9 years	95+ years	Global	1	1	Alcohol (liters per capita)	--
Liver cancer	Female	5-9 years	95+ years	Global	1	1	HIV age-standardized prevalence	--
Liver cancer	Female	5-9 years	95+ years	Global	1	1	Hepatitis B (HBsAg) Seroprevalence	--
Liver cancer	Female	5-9 years	95+ years	Global	1	1	Hepatitis C (IgG) Seroprevalence	--
Liver cancer	Female	5-9 years	95+ years	Global	1	1	Log-transformed SEV scalar: Liver C	--
Liver cancer	Female	5-9 years	95+ years	Global	-1	2	Healthcare access and quality index	--
Liver cancer	Female	5-9 years	95+ years	Global	-1	2	Hepatitis B 3-dose coverage (proportion)	--
Liver cancer	Female	5-9 years	95+ years	Global	-1	2	Hepatitis B 3-dose coverage (proportion), lagged 10 years	--
Liver cancer	Female	5-9 years	95+ years	Global	-1	2	Hepatitis B 3-dose coverage (proportion), lagged 5 years	--
Liver cancer	Female	5-9 years	95+ years	Global	-1	2	Hepatitis B vaccine coverage (proportion), aged through time	--

Table S16. CODEm covariates used, level of covariate, and expected direction of covariate by cause, sex, and age<br>*Covariates that CODEm did not select during the covariate selection process have no draw counts listed.*								
**Cause**	**Sex**	**Age Start**	**Age End**	**Model Type**	**Direction**	**Level**	**Covariate Name**	**Number of Draws**
Liver cancer	Female	5-9 years	95+ years	Global	1	2	Cumulative Cigarettes (10 Years)	--
Liver cancer	Female	5-9 years	95+ years	Global	1	2	Cumulative Cigarettes (15 Years)	--
Liver cancer	Female	5-9 years	95+ years	Global	1	2	Cumulative Cigarettes (20 Years)	--
Liver cancer	Female	5-9 years	95+ years	Global	1	2	Cumulative Cigarettes (5 Years)	--
Liver cancer	Female	5-9 years	95+ years	Global	1	2	Diabetes Age-Standardized Prevalence (proportion)	--
Liver cancer	Female	5-9 years	95+ years	Global	1	2	Diabetes Fasting Plasma Glucose (mmol/L), age-standardized 25+	--
Liver cancer	Female	5-9 years	95+ years	Global	1	2	Intravenous drug use (age-standardized proportion)	--
Liver cancer	Female	5-9 years	95+ years	Global	1	2	Mean BMI	--
Liver cancer	Female	5-9 years	95+ years	Global	1	2	Tobacco (cigarettes per capita)	--
Liver cancer	Female	5-9 years	95+ years	Global	1	2	red meats adjusted(g)	--
Liver cancer	Female	5-9 years	95+ years	Global	-1	3	Education (years per capita)	--
Liver cancer	Female	5-9 years	95+ years	Global	0	3	LDI (I$ per capita)	--
Liver cancer	Female	5-9 years	95+ years	Global	0	3	Socio-demographic Index	--
Liver cancer	Male	5-9 years	95+ years	Data Rich	1	1	Alcohol (liters per capita)	579
Liver cancer	Male	5-9 years	95+ years	Data Rich	1	1	HIV age-standardized prevalence	--
Liver cancer	Male	5-9 years	95+ years	Data Rich	1	1	Hepatitis B (HBsAg) Seroprevalence	--
Liver cancer	Male	5-9 years	95+ years	Data Rich	1	1	Hepatitis C (IgG) Seroprevalence	--
Liver cancer	Male	5-9 years	95+ years	Data Rich	1	1	Log-transformed SEV scalar: Liver C	--
Liver cancer	Male	5-9 years	95+ years	Data Rich	-1	2	Hepatitis B vaccine coverage (proportion), aged through time	235
Liver cancer	Male	5-9 years	95+ years	Data Rich	-1	2	Hepatitis B 3-dose coverage (proportion), lagged 5 years	344
Liver cancer	Male	5-9 years	95+ years	Data Rich	-1	2	Hepatitis B 3-dose coverage (proportion), lagged 10 years	354
Liver cancer	Male	5-9 years	95+ years	Data Rich	-1	2	Hepatitis B 3-dose coverage (proportion)	413
Liver cancer	Male	5-9 years	95+ years	Data Rich	-1	2	Healthcare access and quality index	--
Liver cancer	Male	5-9 years	95+ years	Data Rich	1	2	Diabetes Fasting Plasma Glucose (mmol/L), age-standardized 25+	2
Liver cancer	Male	5-9 years	95+ years	Data Rich	1	2	red meats adjusted(g)	2
Liver cancer	Male	5-9 years	95+ years	Data Rich	1	2	Intravenous drug use (age-standardized proportion)	173
Liver cancer	Male	5-9 years	95+ years	Data Rich	1	2	Diabetes Age-Standardized Prevalence (proportion)	293
Liver cancer	Male	5-9 years	95+ years	Data Rich	1	2	Mean BMI	979
Liver cancer	Male	5-9 years	95+ years	Data Rich	1	2	Cumulative Cigarettes (10 Years)	--
Liver cancer	Male	5-9 years	95+ years	Data Rich	1	2	Cumulative Cigarettes (15 Years)	--
Liver cancer	Male	5-9 years	95+ years	Data Rich	1	2	Cumulative Cigarettes (20 Years)	--
Liver cancer	Male	5-9 years	95+ years	Data Rich	1	2	Cumulative Cigarettes (5 Years)	--
Liver cancer	Male	5-9 years	95+ years	Data Rich	1	2	Tobacco (cigarettes per capita)	--
Liver cancer	Male	5-9 years	95+ years	Data Rich	-1	3	Education (years per capita)	73
Liver cancer	Male	5-9 years	95+ years	Data Rich	0	3	Socio-demographic Index	252
Liver cancer	Male	5-9 years	95+ years	Data Rich	0	3	LDI (I$ per capita)	--
Liver cancer	Male	5-9 years	95+ years	Global	1	1	Alcohol (liters per capita)	--
Liver cancer	Male	5-9 years	95+ years	Global	1	1	HIV age-standardized prevalence	--
Liver cancer	Male	5-9 years	95+ years	Global	1	1	Hepatitis B (HBsAg) Seroprevalence	--
Liver cancer	Male	5-9 years	95+ years	Global	1	1	Hepatitis C (IgG) Seroprevalence	--
Liver cancer	Male	5-9 years	95+ years	Global	1	1	Log-transformed SEV scalar: Liver C	--
Liver cancer	Male	5-9 years	95+ years	Global	-1	2	Healthcare access and quality index	--
Liver cancer	Male	5-9 years	95+ years	Global	-1	2	Hepatitis B 3-dose coverage (proportion)	--
Liver cancer	Male	5-9 years	95+ years	Global	-1	2	Hepatitis B 3-dose coverage (proportion), lagged 10 years	--
Liver cancer	Male	5-9 years	95+ years	Global	-1	2	Hepatitis B 3-dose coverage (proportion), lagged 5 years	--
Liver cancer	Male	5-9 years	95+ years	Global	-1	2	Hepatitis B vaccine coverage (proportion), aged through time	--
Liver cancer	Male	5-9 years	95+ years	Global	1	2	Cumulative Cigarettes (10 Years)	--
Liver cancer	Male	5-9 years	95+ years	Global	1	2	Cumulative Cigarettes (15 Years)	--
Liver cancer	Male	5-9 years	95+ years	Global	1	2	Cumulative Cigarettes (20 Years)	--
Liver cancer	Male	5-9 years	95+ years	Global	1	2	Cumulative Cigarettes (5 Years)	--
Liver cancer	Male	5-9 years	95+ years	Global	1	2	Diabetes Age-Standardized Prevalence (proportion)	--

Table S16. CODEm covariates used, level of covariate, and expected direction of covariate by cause, sex, and age<br>*Covariates that CODEm did not select during the covariate selection process have no draw counts listed.*								
**Cause**	**Sex**	**Age Start**	**Age End**	**Model Type**	**Direction**	**Level**	**Covariate Name**	**Number of Draws**
Liver cancer	Male	5-9 years	95+ years	Global	1	2	Diabetes Fasting Plasma Glucose (mmol/L), age-standardized 25+	--
Liver cancer	Male	5-9 years	95+ years	Global	1	2	Intravenous drug use (age-standardized proportion)	--
Liver cancer	Male	5-9 years	95+ years	Global	1	2	Mean BMI	--
Liver cancer	Male	5-9 years	95+ years	Global	1	2	Tobacco (cigarettes per capita)	--
Liver cancer	Male	5-9 years	95+ years	Global	1	2	red meats adjusted(g)	--
Liver cancer	Male	5-9 years	95+ years	Global	-1	3	Education (years per capita)	--
Liver cancer	Male	5-9 years	95+ years	Global	0	3	LDI (I$ per capita)	--
Liver cancer	Male	5-9 years	95+ years	Global	0	3	Socio-demographic Index	--
Gallbladder and biliary tract cancer	Female	15-19 years	95+ years	Data Rich	1	1	Log-transformed SEV scalar: Gallblad C	--
Gallbladder and biliary tract cancer	Female	15-19 years	95+ years	Data Rich	1	1	Mean BMI	--
Gallbladder and biliary tract cancer	Female	15-19 years	95+ years	Data Rich	-1	2	fruits adjusted(g)	25
Gallbladder and biliary tract cancer	Female	15-19 years	95+ years	Data Rich	-1	2	Healthcare access and quality index	79
Gallbladder and biliary tract cancer	Female	15-19 years	95+ years	Data Rich	-1	2	vegetables adjusted(g)	125
Gallbladder and biliary tract cancer	Female	15-19 years	95+ years	Data Rich	1	2	Smoking Prevalence	354
Gallbladder and biliary tract cancer	Female	15-19 years	95+ years	Data Rich	1	2	Cumulative Cigarettes (10 Years)	502
Gallbladder and biliary tract cancer	Female	15-19 years	95+ years	Data Rich	1	2	Alcohol (liters per capita)	--
Gallbladder and biliary tract cancer	Female	15-19 years	95+ years	Data Rich	1	2	Cumulative Cigarettes (5 Years)	--
Gallbladder and biliary tract cancer	Female	15-19 years	95+ years	Data Rich	1	2	Diabetes Age-Standardized Prevalence (proportion)	--
Gallbladder and biliary tract cancer	Female	15-19 years	95+ years	Data Rich	1	2	Tobacco (cigarettes per capita)	--
Gallbladder and biliary tract cancer	Female	15-19 years	95+ years	Data Rich	-1	3	Education (years per capita)	113
Gallbladder and biliary tract cancer	Female	15-19 years	95+ years	Data Rich	0	3	Socio-demographic Index	77
Gallbladder and biliary tract cancer	Female	15-19 years	95+ years	Data Rich	0	3	LDI (I$ per capita)	--
Gallbladder and biliary tract cancer	Female	15-19 years	95+ years	Global	1	1	Log-transformed SEV scalar: Gallblad C	--
Gallbladder and biliary tract cancer	Female	15-19 years	95+ years	Global	1	1	Mean BMI	--
Gallbladder and biliary tract cancer	Female	15-19 years	95+ years	Global	-1	2	Healthcare access and quality index	532
Gallbladder and biliary tract cancer	Female	15-19 years	95+ years	Global	-1	2	fruits adjusted(g)	--
Gallbladder and biliary tract cancer	Female	15-19 years	95+ years	Global	-1	2	vegetables adjusted(g)	--
Gallbladder and biliary tract cancer	Female	15-19 years	95+ years	Global	1	2	Cumulative Cigarettes (10 Years)	262
Gallbladder and biliary tract cancer	Female	15-19 years	95+ years	Global	1	2	Smoking Prevalence	412
Gallbladder and biliary tract cancer	Female	15-19 years	95+ years	Global	1	2	Cumulative Cigarettes (5 Years)	420
Gallbladder and biliary tract cancer	Female	15-19 years	95+ years	Global	1	2	Alcohol (liters per capita)	682
Gallbladder and biliary tract cancer	Female	15-19 years	95+ years	Global	1	2	Diabetes Age-Standardized Prevalence (proportion)	--
Gallbladder and biliary tract cancer	Female	15-19 years	95+ years	Global	1	2	Tobacco (cigarettes per capita)	--
Gallbladder and biliary tract cancer	Female	15-19 years	95+ years	Global	-1	3	Education (years per capita)	282
Gallbladder and biliary tract cancer	Female	15-19 years	95+ years	Global	0	3	Socio-demographic Index	343
Gallbladder and biliary tract cancer	Female	15-19 years	95+ years	Global	0	3	LDI (I$ per capita)	--
Gallbladder and biliary tract cancer	Male	15-19 years	95+ years	Data Rich	1	1	Log-transformed SEV scalar: Gallblad C	--
Gallbladder and biliary tract cancer	Male	15-19 years	95+ years	Data Rich	1	1	Mean BMI	--
Gallbladder and biliary tract cancer	Male	15-19 years	95+ years	Data Rich	-1	2	fruits adjusted(g)	97
Gallbladder and biliary tract cancer	Male	15-19 years	95+ years	Data Rich	-1	2	vegetables adjusted(g)	497
Gallbladder and biliary tract cancer	Male	15-19 years	95+ years	Data Rich	-1	2	Healthcare access and quality index	--
Gallbladder and biliary tract cancer	Male	15-19 years	95+ years	Data Rich	1	2	Cumulative Cigarettes (5 Years)	155

Table S16. CODEm covariates used, level of covariate, and expected direction of covariate by cause, sex, and age
Covariates that CODEm did not select during the covariate selection process have no draw counts listed.

Cause	Sex	Age Start	Age End	Model Type	Direction	Level	Covariate Name	Number of Draws
Gallbladder and biliary tract cancer	Male	15-19 years	95+ years	Data Rich	1	2	Alcohol (liters per capita)	198
Gallbladder and biliary tract cancer	Male	15-19 years	95+ years	Data Rich	1	2	Cumulative Cigarettes (10 Years)	241
Gallbladder and biliary tract cancer	Male	15-19 years	95+ years	Data Rich	1	2	Smoking Prevalence	396
Gallbladder and biliary tract cancer	Male	15-19 years	95+ years	Data Rich	1	2	Tobacco (cigarettes per capita)	396
Gallbladder and biliary tract cancer	Male	15-19 years	95+ years	Data Rich	1	2	Diabetes Age-Standardized Prevalence (proportion)	--
Gallbladder and biliary tract cancer	Male	15-19 years	95+ years	Data Rich	-1	3	Education (years per capita)	324
Gallbladder and biliary tract cancer	Male	15-19 years	95+ years	Data Rich	0	3	Socio-demographic Index	207
Gallbladder and biliary tract cancer	Male	15-19 years	95+ years	Data Rich	0	3	LDI (I$ per capita)	--
Gallbladder and biliary tract cancer	Male	15-19 years	95+ years	Global	1	1	Log-transformed SEV scalar: Gallblad C	--
Gallbladder and biliary tract cancer	Male	15-19 years	95+ years	Global	1	1	Mean BMI	--
Gallbladder and biliary tract cancer	Male	15-19 years	95+ years	Global	-1	2	vegetables adjusted(g)	100
Gallbladder and biliary tract cancer	Male	15-19 years	95+ years	Global	-1	2	Healthcare access and quality index	--
Gallbladder and biliary tract cancer	Male	15-19 years	95+ years	Global	-1	2	fruits adjusted(g)	--
Gallbladder and biliary tract cancer	Male	15-19 years	95+ years	Global	1	2	Alcohol (liters per capita)	245
Gallbladder and biliary tract cancer	Male	15-19 years	95+ years	Global	1	2	Cumulative Cigarettes (10 Years)	562
Gallbladder and biliary tract cancer	Male	15-19 years	95+ years	Global	1	2	Cumulative Cigarettes (5 Years)	--
Gallbladder and biliary tract cancer	Male	15-19 years	95+ years	Global	1	2	Diabetes Age-Standardized Prevalence (proportion)	--
Gallbladder and biliary tract cancer	Male	15-19 years	95+ years	Global	1	2	Smoking Prevalence	--
Gallbladder and biliary tract cancer	Male	15-19 years	95+ years	Global	1	2	Tobacco (cigarettes per capita)	--
Gallbladder and biliary tract cancer	Male	15-19 years	95+ years	Global	-1	3	Education (years per capita)	293
Gallbladder and biliary tract cancer	Male	15-19 years	95+ years	Global	0	3	Socio-demographic Index	173
Gallbladder and biliary tract cancer	Male	15-19 years	95+ years	Global	0	3	LDI (I$ per capita)	--
Pancreatic cancer	Female	15-19 years	95+ years	Data Rich	1	1	Alcohol (liters per capita)	0
Pancreatic cancer	Female	15-19 years	95+ years	Data Rich	1	1	Tobacco (cigarettes per capita)	5
Pancreatic cancer	Female	15-19 years	95+ years	Data Rich	1	1	Cumulative Cigarettes (5 Years)	105
Pancreatic cancer	Female	15-19 years	95+ years	Data Rich	1	1	Cumulative Cigarettes (10 Years)	177
Pancreatic cancer	Female	15-19 years	95+ years	Data Rich	1	1	Cumulative Cigarettes (15 Years)	230
Pancreatic cancer	Female	15-19 years	95+ years	Data Rich	1	1	Cumulative Cigarettes (20 Years)	279
Pancreatic cancer	Female	15-19 years	95+ years	Data Rich	1	1	Smoking Prevalence	446
Pancreatic cancer	Female	15-19 years	95+ years	Data Rich	1	1	Mean BMI	810
Pancreatic cancer	Female	15-19 years	95+ years	Data Rich	1	1	Log-transformed SEV scalar: Pancreas C	--
Pancreatic cancer	Female	15-19 years	95+ years	Data Rich	-1	2	Healthcare access and quality index	--
Pancreatic cancer	Female	15-19 years	95+ years	Data Rich	-1	2	fruits adjusted(g)	--
Pancreatic cancer	Female	15-19 years	95+ years	Data Rich	-1	2	vegetables adjusted(g)	--
Pancreatic cancer	Female	15-19 years	95+ years	Data Rich	1	2	red meats adjusted(g)	0
Pancreatic cancer	Female	15-19 years	95+ years	Data Rich	1	2	Diabetes Fasting Plasma Glucose (mmol/L), age-standardized 25+	75
Pancreatic cancer	Female	15-19 years	95+ years	Data Rich	1	2	energy unadjusted(kcal)	186
Pancreatic cancer	Female	15-19 years	95+ years	Data Rich	1	2	Diabetes Age-Standardized Prevalence (proportion)	192
Pancreatic cancer	Female	15-19 years	95+ years	Data Rich	-1	3	Education (years per capita)	--
Pancreatic cancer	Female	15-19 years	95+ years	Data Rich	0	3	Socio-demographic Index	980

Table S16. CODEm covariates used, level of covariate, and expected direction of covariate by cause, sex, and age
Covariates that CODEm did not select during the covariate selection process have no draw counts listed.

Cause	Sex	Age Start	Age End	Model Type	Direction	Level	Covariate Name	Number of Draws
Pancreatic cancer	Female	15-19 years	95+ years	Data Rich	0	3	LDI (I$ per capita)	--
Pancreatic cancer	Female	15-19 years	95+ years	Global	1	1	Alcohol (liters per capita)	0
Pancreatic cancer	Female	15-19 years	95+ years	Global	1	1	Cumulative Cigarettes (15 Years)	131
Pancreatic cancer	Female	15-19 years	95+ years	Global	1	1	Cumulative Cigarettes (5 Years)	133
Pancreatic cancer	Female	15-19 years	95+ years	Global	1	1	Cumulative Cigarettes (20 Years)	157
Pancreatic cancer	Female	15-19 years	95+ years	Global	1	1	Cumulative Cigarettes (10 Years)	179
Pancreatic cancer	Female	15-19 years	95+ years	Global	1	1	Tobacco (cigarettes per capita)	502
Pancreatic cancer	Female	15-19 years	95+ years	Global	1	1	Smoking Prevalence	542
Pancreatic cancer	Female	15-19 years	95+ years	Global	1	1	Mean BMI	999
Pancreatic cancer	Female	15-19 years	95+ years	Global	1	1	Log-transformed SEV scalar: Pancreas C	--
Pancreatic cancer	Female	15-19 years	95+ years	Global	-1	2	Healthcare access and quality index	--
Pancreatic cancer	Female	15-19 years	95+ years	Global	-1	2	fruits adjusted(g)	--
Pancreatic cancer	Female	15-19 years	95+ years	Global	-1	2	vegetables adjusted(g)	--
Pancreatic cancer	Female	15-19 years	95+ years	Global	1	2	energy unadjusted(kcal)	0
Pancreatic cancer	Female	15-19 years	95+ years	Global	1	2	Diabetes Age-Standardized Prevalence (proportion)	1
Pancreatic cancer	Female	15-19 years	95+ years	Global	1	2	Diabetes Fasting Plasma Glucose (mmol/L), age-standardized 25+	1
Pancreatic cancer	Female	15-19 years	95+ years	Global	1	2	red meats adjusted(g)	138
Pancreatic cancer	Female	15-19 years	95+ years	Global	-1	3	Education (years per capita)	--
Pancreatic cancer	Female	15-19 years	95+ years	Global	0	3	Socio-demographic Index	999
Pancreatic cancer	Female	15-19 years	95+ years	Global	0	3	LDI (I$ per capita)	--
Pancreatic cancer	Male	15-19 years	95+ years	Data Rich	1	1	Cumulative Cigarettes (5 Years)	12
Pancreatic cancer	Male	15-19 years	95+ years	Data Rich	1	1	Tobacco (cigarettes per capita)	46
Pancreatic cancer	Male	15-19 years	95+ years	Data Rich	1	1	Cumulative Cigarettes (10 Years)	93
Pancreatic cancer	Male	15-19 years	95+ years	Data Rich	1	1	Smoking Prevalence	165
Pancreatic cancer	Male	15-19 years	95+ years	Data Rich	1	1	Cumulative Cigarettes (20 Years)	205
Pancreatic cancer	Male	15-19 years	95+ years	Data Rich	1	1	Cumulative Cigarettes (15 Years)	702
Pancreatic cancer	Male	15-19 years	95+ years	Data Rich	1	1	Mean BMI	923
Pancreatic cancer	Male	15-19 years	95+ years	Data Rich	1	1	Alcohol (liters per capita)	--
Pancreatic cancer	Male	15-19 years	95+ years	Data Rich	1	1	Log-transformed SEV scalar: Pancreas C	--
Pancreatic cancer	Male	15-19 years	95+ years	Data Rich	-1	2	Healthcare access and quality index	--
Pancreatic cancer	Male	15-19 years	95+ years	Data Rich	-1	2	fruits adjusted(g)	--
Pancreatic cancer	Male	15-19 years	95+ years	Data Rich	-1	2	vegetables adjusted(g)	--
Pancreatic cancer	Male	15-19 years	95+ years	Data Rich	1	2	Diabetes Age-Standardized Prevalence (proportion)	46
Pancreatic cancer	Male	15-19 years	95+ years	Data Rich	1	2	energy unadjusted(kcal)	66
Pancreatic cancer	Male	15-19 years	95+ years	Data Rich	1	2	red meats adjusted(g)	84
Pancreatic cancer	Male	15-19 years	95+ years	Data Rich	1	2	Diabetes Fasting Plasma Glucose (mmol/L), age-standardized 25+	436
Pancreatic cancer	Male	15-19 years	95+ years	Data Rich	-1	3	Education (years per capita)	--
Pancreatic cancer	Male	15-19 years	95+ years	Data Rich	0	3	Socio-demographic Index	588
Pancreatic cancer	Male	15-19 years	95+ years	Data Rich	0	3	LDI (I$ per capita)	--
Pancreatic cancer	Male	15-19 years	95+ years	Global	1	1	Alcohol (liters per capita)	0

Table S16. CODEm covariates used, level of covariate, and expected direction of covariate by cause, sex, and age
Covariates that CODEm did not select during the covariate selection process have no draw counts listed.

Cause	Sex	Age Start	Age End	Model Type	Direction	Level	Covariate Name	Number of Draws
Pancreatic cancer	Male	15-19 years	95+ years	Global	1	1	Tobacco (cigarettes per capita)	38
Pancreatic cancer	Male	15-19 years	95+ years	Global	1	1	Cumulative Cigarettes (20 Years)	82
Pancreatic cancer	Male	15-19 years	95+ years	Global	1	1	Cumulative Cigarettes (10 Years)	85
Pancreatic cancer	Male	15-19 years	95+ years	Global	1	1	Smoking Prevalence	214
Pancreatic cancer	Male	15-19 years	95+ years	Global	1	1	Cumulative Cigarettes (15 Years)	327
Pancreatic cancer	Male	15-19 years	95+ years	Global	1	1	Cumulative Cigarettes (5 Years)	532
Pancreatic cancer	Male	15-19 years	95+ years	Global	1	1	Mean BMI	935
Pancreatic cancer	Male	15-19 years	95+ years	Global	1	1	Log-transformed SEV scalar: Pancreas C	--
Pancreatic cancer	Male	15-19 years	95+ years	Global	-1	2	Healthcare access and quality index	--
Pancreatic cancer	Male	15-19 years	95+ years	Global	-1	2	fruits adjusted(g)	--
Pancreatic cancer	Male	15-19 years	95+ years	Global	-1	2	vegetables adjusted(g)	--
Pancreatic cancer	Male	15-19 years	95+ years	Global	1	2	energy unadjusted(kcal)	67
Pancreatic cancer	Male	15-19 years	95+ years	Global	1	2	Diabetes Fasting Plasma Glucose (mmol/L), age-standardized 25+	122
Pancreatic cancer	Male	15-19 years	95+ years	Global	1	2	Diabetes Age-Standardized Prevalence (proportion)	254
Pancreatic cancer	Male	15-19 years	95+ years	Global	1	2	red meats adjusted(g)	337
Pancreatic cancer	Male	15-19 years	95+ years	Global	-1	3	Education (years per capita)	--
Pancreatic cancer	Male	15-19 years	95+ years	Global	0	3	Socio-demographic Index	679
Pancreatic cancer	Male	15-19 years	95+ years	Global	0	3	LDI (I$ per capita)	--
Larynx cancer	Female	15-19 years	95+ years	Data Rich	1	1	Alcohol (liters per capita)	180
Larynx cancer	Female	15-19 years	95+ years	Data Rich	1	1	Log-transformed SEV scalar: Larynx C	--
Larynx cancer	Female	15-19 years	95+ years	Data Rich	-1	2	Healthcare access and quality index	0
Larynx cancer	Female	15-19 years	95+ years	Data Rich	-1	2	fruits adjusted(g)	0
Larynx cancer	Female	15-19 years	95+ years	Data Rich	-1	2	vegetables adjusted(g)	0
Larynx cancer	Female	15-19 years	95+ years	Data Rich	1	2	Tobacco (cigarettes per capita)	0
Larynx cancer	Female	15-19 years	95+ years	Data Rich	1	2	Smoking Prevalence	10
Larynx cancer	Female	15-19 years	95+ years	Data Rich	1	2	Cumulative Cigarettes (20 Years)	120
Larynx cancer	Female	15-19 years	95+ years	Data Rich	1	2	Cumulative Cigarettes (15 Years)	123
Larynx cancer	Female	15-19 years	95+ years	Data Rich	1	2	Cumulative Cigarettes (10 Years)	161
Larynx cancer	Female	15-19 years	95+ years	Data Rich	1	2	Cumulative Cigarettes (5 Years)	212
Larynx cancer	Female	15-19 years	95+ years	Data Rich	1	2	Population Density (over 1000 ppl/sqkm, proportion)	529
Larynx cancer	Female	15-19 years	95+ years	Data Rich	1	2	Asbestos consumption (metric tons per year per capita)	--
Larynx cancer	Female	15-19 years	95+ years	Data Rich	1	2	Population Density (under 150 ppl/sqkm, proportion)	--
Larynx cancer	Female	15-19 years	95+ years	Data Rich	-1	3	Education (years per capita)	8
Larynx cancer	Female	15-19 years	95+ years	Data Rich	0	3	Socio-demographic Index	375
Larynx cancer	Female	15-19 years	95+ years	Data Rich	0	3	LDI (I$ per capita)	--
Larynx cancer	Female	15-19 years	95+ years	Global	1	1	Alcohol (liters per capita)	253
Larynx cancer	Female	15-19 years	95+ years	Global	1	1	Log-transformed SEV scalar: Larynx C	--
Larynx cancer	Female	15-19 years	95+ years	Global	-1	2	vegetables adjusted(g)	0
Larynx cancer	Female	15-19 years	95+ years	Global	-1	2	fruits adjusted(g)	138
Larynx cancer	Female	15-19 years	95+ years	Global	-1	2	Healthcare access and quality index	372

Table S16. CODEm covariates used, level of covariate, and expected direction of covariate by cause, sex, and age<br>*Covariates that CODEm did not select during the covariate selection process have no draw counts listed.*								
**Cause**	**Sex**	**Age Start**	**Age End**	**Model Type**	**Direction**	**Level**	**Covariate Name**	**Number of Draws**
Larynx cancer	Female	15-19 years	95+ years	Global	1	2	Tobacco (cigarettes per capita)	94
Larynx cancer	Female	15-19 years	95+ years	Global	1	2	Cumulative Cigarettes (20 Years)	152
Larynx cancer	Female	15-19 years	95+ years	Global	1	2	Cumulative Cigarettes (15 Years)	181
Larynx cancer	Female	15-19 years	95+ years	Global	1	2	Cumulative Cigarettes (10 Years)	233
Larynx cancer	Female	15-19 years	95+ years	Global	1	2	Cumulative Cigarettes (5 Years)	285
Larynx cancer	Female	15-19 years	95+ years	Global	1	2	Smoking Prevalence	413
Larynx cancer	Female	15-19 years	95+ years	Global	1	2	Population Density (over 1000 ppl/sqkm, proportion)	618
Larynx cancer	Female	15-19 years	95+ years	Global	1	2	Asbestos consumption (metric tons per year per capita)	--
Larynx cancer	Female	15-19 years	95+ years	Global	1	2	Population Density (under 150 ppl/sqkm, proportion)	--
Larynx cancer	Female	15-19 years	95+ years	Global	-1	3	Education (years per capita)	165
Larynx cancer	Female	15-19 years	95+ years	Global	0	3	Socio-demographic Index	39
Larynx cancer	Female	15-19 years	95+ years	Global	0	3	LDI (I$ per capita)	--
Larynx cancer	Male	15-19 years	95+ years	Data Rich	1	1	Alcohol (liters per capita)	982
Larynx cancer	Male	15-19 years	95+ years	Data Rich	1	1	Log-transformed SEV scalar: Larynx C	--
Larynx cancer	Male	15-19 years	95+ years	Data Rich	-1	2	fruits adjusted(g)	0
Larynx cancer	Male	15-19 years	95+ years	Data Rich	-1	2	Healthcare access and quality index	57
Larynx cancer	Male	15-19 years	95+ years	Data Rich	-1	2	vegetables adjusted(g)	732
Larynx cancer	Male	15-19 years	95+ years	Data Rich	1	2	Tobacco (cigarettes per capita)	97
Larynx cancer	Male	15-19 years	95+ years	Data Rich	1	2	Cumulative Cigarettes (10 Years)	143
Larynx cancer	Male	15-19 years	95+ years	Data Rich	1	2	Smoking Prevalence	347
Larynx cancer	Male	15-19 years	95+ years	Data Rich	1	2	Cumulative Cigarettes (15 Years)	400
Larynx cancer	Male	15-19 years	95+ years	Data Rich	1	2	Cumulative Cigarettes (5 Years)	467
Larynx cancer	Male	15-19 years	95+ years	Data Rich	1	2	Cumulative Cigarettes (20 Years)	468
Larynx cancer	Male	15-19 years	95+ years	Data Rich	1	2	Population Density (over 1000 ppl/sqkm, proportion)	789
Larynx cancer	Male	15-19 years	95+ years	Data Rich	1	2	Asbestos consumption (metric tons per year per capita)	--
Larynx cancer	Male	15-19 years	95+ years	Data Rich	1	2	Population Density (under 150 ppl/sqkm, proportion)	--
Larynx cancer	Male	15-19 years	95+ years	Data Rich	-1	3	Education (years per capita)	22
Larynx cancer	Male	15-19 years	95+ years	Data Rich	0	3	Socio-demographic Index	32
Larynx cancer	Male	15-19 years	95+ years	Data Rich	0	3	LDI (I$ per capita)	--
Larynx cancer	Male	15-19 years	95+ years	Global	1	1	Alcohol (liters per capita)	587
Larynx cancer	Male	15-19 years	95+ years	Global	1	1	Log-transformed SEV scalar: Larynx C	--
Larynx cancer	Male	15-19 years	95+ years	Global	-1	2	fruits adjusted(g)	0
Larynx cancer	Male	15-19 years	95+ years	Global	-1	2	Healthcare access and quality index	168
Larynx cancer	Male	15-19 years	95+ years	Global	-1	2	vegetables adjusted(g)	390
Larynx cancer	Male	15-19 years	95+ years	Global	1	2	Cumulative Cigarettes (20 Years)	79
Larynx cancer	Male	15-19 years	95+ years	Global	1	2	Cumulative Cigarettes (10 Years)	377
Larynx cancer	Male	15-19 years	95+ years	Global	1	2	Smoking Prevalence	466
Larynx cancer	Male	15-19 years	95+ years	Global	1	2	Population Density (over 1000 ppl/sqkm, proportion)	471
Larynx cancer	Male	15-19 years	95+ years	Global	1	2	Cumulative Cigarettes (5 Years)	484
Larynx cancer	Male	15-19 years	95+ years	Global	1	2	Tobacco (cigarettes per capita)	693

Table S16. CODEm covariates used, level of covariate, and expected direction of covariate by cause, sex, and age<br>*Covariates that CODEm did not select during the covariate selection process have no draw counts listed.*								
**Cause**	**Sex**	**Age Start**	**Age End**	**Model Type**	**Direction**	**Level**	**Covariate Name**	**Number of Draws**
Larynx cancer	Male	15-19 years	95+ years	Global	1	2	Cumulative Cigarettes (15 Years)	727
Larynx cancer	Male	15-19 years	95+ years	Global	1	2	Asbestos consumption (metric tons per year per capita)	--
Larynx cancer	Male	15-19 years	95+ years	Global	1	2	Population Density (under 150 ppl/sqkm, proportion)	--
Larynx cancer	Male	15-19 years	95+ years	Global	-1	3	Education (years per capita)	155
Larynx cancer	Male	15-19 years	95+ years	Global	0	3	Socio-demographic Index	245
Larynx cancer	Male	15-19 years	95+ years	Global	0	3	LDI (I$ per capita)	--
Tracheal, bronchus, and lung cancer	Female	15-19 years	95+ years	Data Rich	1	1	Cumulative Cigarettes (10 Years)	15
Tracheal, bronchus, and lung cancer	Female	15-19 years	95+ years	Data Rich	1	1	Cumulative Cigarettes (15 Years)	89
Tracheal, bronchus, and lung cancer	Female	15-19 years	95+ years	Data Rich	1	1	Cumulative Cigarettes (20 Years)	120
Tracheal, bronchus, and lung cancer	Female	15-19 years	95+ years	Data Rich	1	1	Smoking Prevalence	167
Tracheal, bronchus, and lung cancer	Female	15-19 years	95+ years	Data Rich	1	1	Cumulative Cigarettes (5 Years)	179
Tracheal, bronchus, and lung cancer	Female	15-19 years	95+ years	Data Rich	1	1	Asbestos consumption (metric tons per year per capita)	429
Tracheal, bronchus, and lung cancer	Female	15-19 years	95+ years	Data Rich	1	1	Log-transformed SEV scalar: Lung C	--
Tracheal, bronchus, and lung cancer	Female	15-19 years	95+ years	Data Rich	1	1	Log-transformed age-standardized SEV scalar: Lung C	--
Tracheal, bronchus, and lung cancer	Female	15-19 years	95+ years	Data Rich	1	1	Secondhand smoke	--
Tracheal, bronchus, and lung cancer	Female	15-19 years	95+ years	Data Rich	1	1	Tobacco (cigarettes per capita)	--
Tracheal, bronchus, and lung cancer	Female	15-19 years	95+ years	Data Rich	-1	2	Healthcare access and quality index	--
Tracheal, bronchus, and lung cancer	Female	15-19 years	95+ years	Data Rich	1	2	Outdoor Air Pollution (PM2.5)	0
Tracheal, bronchus, and lung cancer	Female	15-19 years	95+ years	Data Rich	1	2	Diabetes Fasting Plasma Glucose (mmol/L), age-standardized 25+	605
Tracheal, bronchus, and lung cancer	Female	15-19 years	95+ years	Data Rich	1	2	Indoor Air Pollution (All Cooking Fuels)	--
Tracheal, bronchus, and lung cancer	Female	15-19 years	95+ years	Data Rich	1	2	Residential radon	--
Tracheal, bronchus, and lung cancer	Female	15-19 years	95+ years	Data Rich	-1	3	Education (years per capita)	--
Tracheal, bronchus, and lung cancer	Female	15-19 years	95+ years	Data Rich	0	3	Socio-demographic Index	571
Tracheal, bronchus, and lung cancer	Female	15-19 years	95+ years	Data Rich	0	3	LDI (I$ per capita)	--
Tracheal, bronchus, and lung cancer	Female	15-19 years	95+ years	Global	1	1	Asbestos consumption (metric tons per year per capita)	0
Tracheal, bronchus, and lung cancer	Female	15-19 years	95+ years	Global	1	1	Secondhand smoke	0
Tracheal, bronchus, and lung cancer	Female	15-19 years	95+ years	Global	1	1	Cumulative Cigarettes (10 Years)	27
Tracheal, bronchus, and lung cancer	Female	15-19 years	95+ years	Global	1	1	Cumulative Cigarettes (15 Years)	27
Tracheal, bronchus, and lung cancer	Female	15-19 years	95+ years	Global	1	1	Cumulative Cigarettes (20 Years)	71
Tracheal, bronchus, and lung cancer	Female	15-19 years	95+ years	Global	1	1	Cumulative Cigarettes (5 Years)	163
Tracheal, bronchus, and lung cancer	Female	15-19 years	95+ years	Global	1	1	Smoking Prevalence	404
Tracheal, bronchus, and lung cancer	Female	15-19 years	95+ years	Global	1	1	Log-transformed SEV scalar: Lung C	--
Tracheal, bronchus, and lung cancer	Female	15-19 years	95+ years	Global	1	1	Log-transformed age-standardized SEV scalar: Lung C	--
Tracheal, bronchus, and lung cancer	Female	15-19 years	95+ years	Global	1	1	Tobacco (cigarettes per capita)	--
Tracheal, bronchus, and lung cancer	Female	15-19 years	95+ years	Global	-1	2	Healthcare access and quality index	--
Tracheal, bronchus, and lung cancer	Female	15-19 years	95+ years	Global	1	2	Outdoor Air Pollution (PM2.5)	138
Tracheal, bronchus, and lung cancer	Female	15-19 years	95+ years	Global	1	2	Diabetes Fasting Plasma Glucose (mmol/L), age-standardized 25+	472
Tracheal, bronchus, and lung cancer	Female	15-19 years	95+ years	Global	1	2	Indoor Air Pollution (All Cooking Fuels)	--
Tracheal, bronchus, and lung cancer	Female	15-19 years	95+ years	Global	1	2	Residential radon	--
Tracheal, bronchus, and lung cancer	Female	15-19 years	95+ years	Global	-1	3	Education (years per capita)	--

Table S16. CODEm covariates used, level of covariate, and expected direction of covariate by cause, sex, and age
Covariates that CODEm did not select during the covariate selection process have no draw counts listed.

Cause	Sex	Age Start	Age End	Model Type	Direction	Level	Covariate Name	Number of Draws
Tracheal, bronchus, and lung cancer	Female	15-19 years	95+ years	Global	0	3	Socio-demographic Index	999
Tracheal, bronchus, and lung cancer	Female	15-19 years	95+ years	Global	0	3	LDI (I$ per capita)	--
Tracheal, bronchus, and lung cancer	Male	15-19 years	95+ years	Data Rich	1	1	Smoking Prevalence	0
Tracheal, bronchus, and lung cancer	Male	15-19 years	95+ years	Data Rich	1	1	Tobacco (cigarettes per capita)	0
Tracheal, bronchus, and lung cancer	Male	15-19 years	95+ years	Data Rich	1	1	Cumulative Cigarettes (5 Years)	27
Tracheal, bronchus, and lung cancer	Male	15-19 years	95+ years	Data Rich	1	1	Secondhand smoke	69
Tracheal, bronchus, and lung cancer	Male	15-19 years	95+ years	Data Rich	1	1	Cumulative Cigarettes (10 Years)	118
Tracheal, bronchus, and lung cancer	Male	15-19 years	95+ years	Data Rich	1	1	Cumulative Cigarettes (20 Years)	224
Tracheal, bronchus, and lung cancer	Male	15-19 years	95+ years	Data Rich	1	1	Cumulative Cigarettes (15 Years)	494
Tracheal, bronchus, and lung cancer	Male	15-19 years	95+ years	Data Rich	1	1	Asbestos consumption (metric tons per year per capita)	690
Tracheal, bronchus, and lung cancer	Male	15-19 years	95+ years	Data Rich	1	1	Log-transformed SEV scalar: Lung C	--
Tracheal, bronchus, and lung cancer	Male	15-19 years	95+ years	Data Rich	1	1	Log-transformed age-standardized SEV scalar: Lung C	--
Tracheal, bronchus, and lung cancer	Male	15-19 years	95+ years	Data Rich	-1	2	Healthcare access and quality index	0
Tracheal, bronchus, and lung cancer	Male	15-19 years	95+ years	Data Rich	1	2	Outdoor Air Pollution (PM2.5)	46
Tracheal, bronchus, and lung cancer	Male	15-19 years	95+ years	Data Rich	1	2	Diabetes Fasting Plasma Glucose (mmol/L), age-standardized 25+	599
Tracheal, bronchus, and lung cancer	Male	15-19 years	95+ years	Data Rich	1	2	Indoor Air Pollution (All Cooking Fuels)	--
Tracheal, bronchus, and lung cancer	Male	15-19 years	95+ years	Data Rich	1	2	Residential radon	--
Tracheal, bronchus, and lung cancer	Male	15-19 years	95+ years	Data Rich	-1	3	Education (years per capita)	547
Tracheal, bronchus, and lung cancer	Male	15-19 years	95+ years	Data Rich	0	3	Socio-demographic Index	673
Tracheal, bronchus, and lung cancer	Male	15-19 years	95+ years	Data Rich	0	3	LDI (I$ per capita)	--
Tracheal, bronchus, and lung cancer	Male	15-19 years	95+ years	Global	1	1	Tobacco (cigarettes per capita)	63
Tracheal, bronchus, and lung cancer	Male	15-19 years	95+ years	Global	1	1	Cumulative Cigarettes (15 Years)	68
Tracheal, bronchus, and lung cancer	Male	15-19 years	95+ years	Global	1	1	Smoking Prevalence	68
Tracheal, bronchus, and lung cancer	Male	15-19 years	95+ years	Global	1	1	Cumulative Cigarettes (10 Years)	231
Tracheal, bronchus, and lung cancer	Male	15-19 years	95+ years	Global	1	1	Cumulative Cigarettes (5 Years)	239
Tracheal, bronchus, and lung cancer	Male	15-19 years	95+ years	Global	1	1	Cumulative Cigarettes (20 Years)	268
Tracheal, bronchus, and lung cancer	Male	15-19 years	95+ years	Global	1	1	Asbestos consumption (metric tons per year per capita)	361
Tracheal, bronchus, and lung cancer	Male	15-19 years	95+ years	Global	1	1	Secondhand smoke	365
Tracheal, bronchus, and lung cancer	Male	15-19 years	95+ years	Global	1	1	Log-transformed SEV scalar: Lung C	--
Tracheal, bronchus, and lung cancer	Male	15-19 years	95+ years	Global	1	1	Log-transformed age-standardized SEV scalar: Lung C	--
Tracheal, bronchus, and lung cancer	Male	15-19 years	95+ years	Global	-1	2	Healthcare access and quality index	0
Tracheal, bronchus, and lung cancer	Male	15-19 years	95+ years	Global	1	2	Diabetes Fasting Plasma Glucose (mmol/L), age-standardized 25+	324
Tracheal, bronchus, and lung cancer	Male	15-19 years	95+ years	Global	1	2	Outdoor Air Pollution (PM2.5)	353
Tracheal, bronchus, and lung cancer	Male	15-19 years	95+ years	Global	1	2	Indoor Air Pollution (All Cooking Fuels)	--
Tracheal, bronchus, and lung cancer	Male	15-19 years	95+ years	Global	1	2	Residential radon	--
Tracheal, bronchus, and lung cancer	Male	15-19 years	95+ years	Global	-1	3	Education (years per capita)	9
Tracheal, bronchus, and lung cancer	Male	15-19 years	95+ years	Global	0	3	Socio-demographic Index	538
Tracheal, bronchus, and lung cancer	Male	15-19 years	95+ years	Global	0	3	LDI (I$ per capita)	--
Malignant skin melanoma	Female	15-19 years	95+ years	Data Rich	1	1	Alcohol (liters per capita)	--
Malignant skin melanoma	Female	15-19 years	95+ years	Data Rich	-1	2	Latitude 30 to 45 (proportion)	310

Table S16. CODEm covariates used, level of covariate, and expected direction of covariate by cause, sex, and age
Covariates that CODEm did not select during the covariate selection process have no draw counts listed.

Cause	Sex	Age Start	Age End	Model Type	Direction	Level	Covariate Name	Number of Draws
Malignant skin melanoma	Female	15-19 years	95+ years	Data Rich	-1	2	Healthcare access and quality index	--
Malignant skin melanoma	Female	15-19 years	95+ years	Data Rich	-1	2	Latitude Over 45 (proportion)	--
Malignant skin melanoma	Female	15-19 years	95+ years	Data Rich	-1	2	fruits adjusted(g)	--
Malignant skin melanoma	Female	15-19 years	95+ years	Data Rich	-1	2	vegetables adjusted(g)	--
Malignant skin melanoma	Female	15-19 years	95+ years	Data Rich	0	2	Latitude Under 15 (proportion)	367
Malignant skin melanoma	Female	15-19 years	95+ years	Data Rich	0	2	Latitude 15 to 30 (proportion)	--
Malignant skin melanoma	Female	15-19 years	95+ years	Data Rich	-1	3	Education (years per capita)	--
Malignant skin melanoma	Female	15-19 years	95+ years	Data Rich	0	3	Socio-demographic Index	566
Malignant skin melanoma	Female	15-19 years	95+ years	Data Rich	0	3	LDI (I$ per capita)	--
Malignant skin melanoma	Female	15-19 years	95+ years	Global	1	1	Alcohol (liters per capita)	181
Malignant skin melanoma	Female	15-19 years	95+ years	Global	-1	2	vegetables adjusted(g)	433
Malignant skin melanoma	Female	15-19 years	95+ years	Global	-1	2	Healthcare access and quality index	--
Malignant skin melanoma	Female	15-19 years	95+ years	Global	-1	2	Latitude 30 to 45 (proportion)	--
Malignant skin melanoma	Female	15-19 years	95+ years	Global	-1	2	Latitude Over 45 (proportion)	--
Malignant skin melanoma	Female	15-19 years	95+ years	Global	-1	2	fruits adjusted(g)	--
Malignant skin melanoma	Female	15-19 years	95+ years	Global	0	2	Latitude 15 to 30 (proportion)	472
Malignant skin melanoma	Female	15-19 years	95+ years	Global	0	2	Latitude Under 15 (proportion)	--
Malignant skin melanoma	Female	15-19 years	95+ years	Global	-1	3	Education (years per capita)	--
Malignant skin melanoma	Female	15-19 years	95+ years	Global	0	3	Socio-demographic Index	331
Malignant skin melanoma	Female	15-19 years	95+ years	Global	0	3	LDI (I$ per capita)	--
Malignant skin melanoma	Male	15-19 years	95+ years	Data Rich	1	1	Alcohol (liters per capita)	224
Malignant skin melanoma	Male	15-19 years	95+ years	Data Rich	-1	2	Healthcare access and quality index	--
Malignant skin melanoma	Male	15-19 years	95+ years	Data Rich	-1	2	Latitude 30 to 45 (proportion)	--
Malignant skin melanoma	Male	15-19 years	95+ years	Data Rich	-1	2	Latitude Over 45 (proportion)	--
Malignant skin melanoma	Male	15-19 years	95+ years	Data Rich	-1	2	fruits adjusted(g)	--
Malignant skin melanoma	Male	15-19 years	95+ years	Data Rich	-1	2	vegetables adjusted(g)	--
Malignant skin melanoma	Male	15-19 years	95+ years	Data Rich	0	2	Latitude Under 15 (proportion)	520
Malignant skin melanoma	Male	15-19 years	95+ years	Data Rich	0	2	Latitude 15 to 30 (proportion)	--
Malignant skin melanoma	Male	15-19 years	95+ years	Data Rich	-1	3	Education (years per capita)	--
Malignant skin melanoma	Male	15-19 years	95+ years	Data Rich	0	3	Socio-demographic Index	362
Malignant skin melanoma	Male	15-19 years	95+ years	Data Rich	0	3	LDI (I$ per capita)	--
Malignant skin melanoma	Male	15-19 years	95+ years	Global	1	1	Alcohol (liters per capita)	1000
Malignant skin melanoma	Male	15-19 years	95+ years	Global	-1	2	Healthcare access and quality index	--
Malignant skin melanoma	Male	15-19 years	95+ years	Global	-1	2	Latitude 30 to 45 (proportion)	--
Malignant skin melanoma	Male	15-19 years	95+ years	Global	-1	2	Latitude Over 45 (proportion)	--
Malignant skin melanoma	Male	15-19 years	95+ years	Global	-1	2	fruits adjusted(g)	--
Malignant skin melanoma	Male	15-19 years	95+ years	Global	-1	2	vegetables adjusted(g)	--
Malignant skin melanoma	Male	15-19 years	95+ years	Global	0	2	Latitude Under 15 (proportion)	398
Malignant skin melanoma	Male	15-19 years	95+ years	Global	0	2	Latitude 15 to 30 (proportion)	--
Malignant skin melanoma	Male	15-19 years	95+ years	Global	-1	3	Education (years per capita)	--

Table S16. CODEm covariates used, level of covariate, and expected direction of covariate by cause, sex, and age
Covariates that CODEm did not select during the covariate selection process have no draw counts listed.

Cause	Sex	Age Start	Age End	Model Type	Direction	Level	Covariate Name	Number of Draws
Malignant skin melanoma	Male	15-19 years	95+ years	Global	0	3	LDI (I$ per capita)	--
Malignant skin melanoma	Male	15-19 years	95+ years	Global	0	3	Socio-demographic Index	--
Non-melanoma skin cancer	Female	15-19 years	95+ years	Data Rich	1	1	Cumulative Cigarettes (15 Years)	99
Non-melanoma skin cancer	Female	15-19 years	95+ years	Data Rich	1	1	Cumulative Cigarettes (10 Years)	182
Non-melanoma skin cancer	Female	15-19 years	95+ years	Data Rich	1	1	Smoking Prevalence	353
Non-melanoma skin cancer	Female	15-19 years	95+ years	Data Rich	1	1	Cumulative Cigarettes (5 Years)	519
Non-melanoma skin cancer	Female	15-19 years	95+ years	Data Rich	-1	2	Healthcare access and quality index	355
Non-melanoma skin cancer	Female	15-19 years	95+ years	Data Rich	0	2	Average latitude	215
Non-melanoma skin cancer	Female	15-19 years	95+ years	Data Rich	-1	3	Education (years per capita)	262
Non-melanoma skin cancer	Female	15-19 years	95+ years	Data Rich	0	3	Socio-demographic Index	957
Non-melanoma skin cancer	Female	15-19 years	95+ years	Data Rich	0	3	LDI (I$ per capita)	--
Non-melanoma skin cancer	Female	15-19 years	95+ years	Global	1	1	Smoking Prevalence	572
Non-melanoma skin cancer	Female	15-19 years	95+ years	Global	1	1	Cumulative Cigarettes (10 Years)	--
Non-melanoma skin cancer	Female	15-19 years	95+ years	Global	1	1	Cumulative Cigarettes (15 Years)	--
Non-melanoma skin cancer	Female	15-19 years	95+ years	Global	1	1	Cumulative Cigarettes (5 Years)	--
Non-melanoma skin cancer	Female	15-19 years	95+ years	Global	-1	2	Healthcare access and quality index	282
Non-melanoma skin cancer	Female	15-19 years	95+ years	Global	0	2	Average latitude	192
Non-melanoma skin cancer	Female	15-19 years	95+ years	Global	-1	3	Education (years per capita)	107
Non-melanoma skin cancer	Female	15-19 years	95+ years	Global	0	3	Socio-demographic Index	465
Non-melanoma skin cancer	Female	15-19 years	95+ years	Global	0	3	LDI (I$ per capita)	--
Non-melanoma skin cancer	Male	15-19 years	95+ years	Data Rich	1	1	Cumulative Cigarettes (5 Years)	73
Non-melanoma skin cancer	Male	15-19 years	95+ years	Data Rich	1	1	Cumulative Cigarettes (10 Years)	130
Non-melanoma skin cancer	Male	15-19 years	95+ years	Data Rich	1	1	Smoking Prevalence	153
Non-melanoma skin cancer	Male	15-19 years	95+ years	Data Rich	1	1	Cumulative Cigarettes (15 Years)	211
Non-melanoma skin cancer	Male	15-19 years	95+ years	Data Rich	-1	2	Healthcare access and quality index	392
Non-melanoma skin cancer	Male	15-19 years	95+ years	Data Rich	0	2	Average latitude	778
Non-melanoma skin cancer	Male	15-19 years	95+ years	Data Rich	-1	3	Education (years per capita)	2
Non-melanoma skin cancer	Male	15-19 years	95+ years	Data Rich	0	3	Socio-demographic Index	646
Non-melanoma skin cancer	Male	15-19 years	95+ years	Data Rich	0	3	LDI (I$ per capita)	--
Non-melanoma skin cancer	Male	15-19 years	95+ years	Global	1	1	Cumulative Cigarettes (5 Years)	70
Non-melanoma skin cancer	Male	15-19 years	95+ years	Global	1	1	Cumulative Cigarettes (10 Years)	151
Non-melanoma skin cancer	Male	15-19 years	95+ years	Global	1	1	Cumulative Cigarettes (15 Years)	237
Non-melanoma skin cancer	Male	15-19 years	95+ years	Global	1	1	Smoking Prevalence	473
Non-melanoma skin cancer	Male	15-19 years	95+ years	Global	-1	2	Healthcare access and quality index	250
Non-melanoma skin cancer	Male	15-19 years	95+ years	Global	0	2	Average latitude	562
Non-melanoma skin cancer	Male	15-19 years	95+ years	Global	-1	3	Education (years per capita)	4
Non-melanoma skin cancer	Male	15-19 years	95+ years	Global	0	3	Socio-demographic Index	605
Non-melanoma skin cancer	Male	15-19 years	95+ years	Global	0	3	LDI (I$ per capita)	--
Non-melanoma skin cancer (squamous-cell carcinoma)	Female	28-364 days	95+ years	Data Rich	1	1	Cumulative Cigarettes (5 Years)	195
Non-melanoma skin cancer (squamous-cell carcinoma)	Female	28-364 days	95+ years	Data Rich	1	1	Cumulative Cigarettes (10 Years)	217

Table S16. CODEm covariates used, level of covariate, and expected direction of covariate by cause, sex, and age<br>*Covariates that CODEm did not select during the covariate selection process have no draw counts listed.*								
**Cause**	**Sex**	**Age Start**	**Age End**	**Model Type**	**Direction**	**Level**	**Covariate Name**	**Number of Draws**
Non-melanoma skin cancer (squamous-cell carcinoma)	Female	28-364 days	95+ years	Data Rich	1	1	Cumulative Cigarettes (15 Years)	260
Non-melanoma skin cancer (squamous-cell carcinoma)	Female	28-364 days	95+ years	Data Rich	1	1	Smoking Prevalence	264
Non-melanoma skin cancer (squamous-cell carcinoma)	Female	28-364 days	95+ years	Data Rich	-1	2	Healthcare access and quality index	516
Non-melanoma skin cancer (squamous-cell carcinoma)	Female	28-364 days	95+ years	Data Rich	0	2	Average latitude	307
Non-melanoma skin cancer (squamous-cell carcinoma)	Female	28-364 days	95+ years	Data Rich	-1	3	Education (years per capita)	30
Non-melanoma skin cancer (squamous-cell carcinoma)	Female	28-364 days	95+ years	Data Rich	0	3	Socio-demographic Index	501
Non-melanoma skin cancer (squamous-cell carcinoma)	Female	28-364 days	95+ years	Data Rich	0	3	LDI (I$ per capita)	621
Non-melanoma skin cancer (squamous-cell carcinoma)	Female	28-364 days	95+ years	Global	1	1	Smoking Prevalence	31
Non-melanoma skin cancer (squamous-cell carcinoma)	Female	28-364 days	95+ years	Global	1	1	Cumulative Cigarettes (10 Years)	150
Non-melanoma skin cancer (squamous-cell carcinoma)	Female	28-364 days	95+ years	Global	1	1	Cumulative Cigarettes (15 Years)	192
Non-melanoma skin cancer (squamous-cell carcinoma)	Female	28-364 days	95+ years	Global	1	1	Cumulative Cigarettes (5 Years)	198
Non-melanoma skin cancer (squamous-cell carcinoma)	Female	28-364 days	95+ years	Global	-1	2	Healthcare access and quality index	80
Non-melanoma skin cancer (squamous-cell carcinoma)	Female	28-364 days	95+ years	Global	0	2	Average latitude	669
Non-melanoma skin cancer (squamous-cell carcinoma)	Female	28-364 days	95+ years	Global	-1	3	Education (years per capita)	45
Non-melanoma skin cancer (squamous-cell carcinoma)	Female	28-364 days	95+ years	Global	0	3	Socio-demographic Index	375
Non-melanoma skin cancer (squamous-cell carcinoma)	Female	28-364 days	95+ years	Global	0	3	LDI (I$ per capita)	501
Non-melanoma skin cancer (squamous-cell carcinoma)	Male	28-364 days	95+ years	Data Rich	1	1	Cumulative Cigarettes (5 Years)	68
Non-melanoma skin cancer (squamous-cell carcinoma)	Male	28-364 days	95+ years	Data Rich	1	1	Cumulative Cigarettes (10 Years)	116
Non-melanoma skin cancer (squamous-cell carcinoma)	Male	28-364 days	95+ years	Data Rich	1	1	Smoking Prevalence	211
Non-melanoma skin cancer (squamous-cell carcinoma)	Male	28-364 days	95+ years	Data Rich	1	1	Cumulative Cigarettes (15 Years)	269
Non-melanoma skin cancer (squamous-cell carcinoma)	Male	28-364 days	95+ years	Data Rich	-1	2	Healthcare access and quality index	275
Non-melanoma skin cancer (squamous-cell carcinoma)	Male	28-364 days	95+ years	Data Rich	0	2	Average latitude	668
Non-melanoma skin cancer (squamous-cell carcinoma)	Male	28-364 days	95+ years	Data Rich	-1	3	Education (years per capita)	16
Non-melanoma skin cancer (squamous-cell carcinoma)	Male	28-364 days	95+ years	Data Rich	0	3	LDI (I$ per capita)	412
Non-melanoma skin cancer (squamous-cell carcinoma)	Male	28-364 days	95+ years	Data Rich	0	3	Socio-demographic Index	649
Non-melanoma skin cancer (squamous-cell carcinoma)	Male	28-364 days	95+ years	Global	1	1	Cumulative Cigarettes (10 Years)	141
Non-melanoma skin cancer (squamous-cell carcinoma)	Male	28-364 days	95+ years	Global	1	1	Cumulative Cigarettes (5 Years)	153
Non-melanoma skin cancer (squamous-cell carcinoma)	Male	28-364 days	95+ years	Global	1	1	Cumulative Cigarettes (15 Years)	473
Non-melanoma skin cancer (squamous-cell carcinoma)	Male	28-364 days	95+ years	Global	1	1	Smoking Prevalence	724
Non-melanoma skin cancer (squamous-cell carcinoma)	Male	28-364 days	95+ years	Global	-1	2	Healthcare access and quality index	292
Non-melanoma skin cancer (squamous-cell carcinoma)	Male	28-364 days	95+ years	Global	0	2	Average latitude	16
Non-melanoma skin cancer (squamous-cell carcinoma)	Male	28-364 days	95+ years	Global	-1	3	Education (years per capita)	15
Non-melanoma skin cancer (squamous-cell carcinoma)	Male	28-364 days	95+ years	Global	0	3	Socio-demographic Index	308
Non-melanoma skin cancer (squamous-cell carcinoma)	Male	28-364 days	95+ years	Global	0	3	LDI (I$ per capita)	395
Breast cancer	Female	15-19 years	95+ years	Data Rich	1	1	Mean BMI	388
Breast cancer	Female	15-19 years	95+ years	Data Rich	1	1	Alcohol (liters per capita)	669
Breast cancer	Female	15-19 years	95+ years	Data Rich	1	1	Log-transformed SEV scalar: Breast C	--
Breast cancer	Female	15-19 years	95+ years	Data Rich	-1	2	Healthcare access and quality index	0
Breast cancer	Female	15-19 years	95+ years	Data Rich	-1	2	vegetables adjusted(g)	0
Breast cancer	Female	15-19 years	95+ years	Data Rich	-1	2	Total Fertility Rate	626

Table S16. CODEm covariates used, level of covariate, and expected direction of covariate by cause, sex, and age
Covariates that CODEm did not select during the covariate selection process have no draw counts listed.

Cause	Sex	Age Start	Age End	Model Type	Direction	Level	Covariate Name	Number of Draws
Breast cancer	Female	15-19 years	95+ years	Data Rich	-1	2	Age-Specific Fertility Rate	811
Breast cancer	Female	15-19 years	95+ years	Data Rich	-1	2	fruits adjusted(g)	--
Breast cancer	Female	15-19 years	95+ years	Data Rich	1	2	Secondhand smoke	0
Breast cancer	Female	15-19 years	95+ years	Data Rich	1	2	Smoking Prevalence	0
Breast cancer	Female	15-19 years	95+ years	Data Rich	1	2	Cumulative Cigarettes (10 Years)	2
Breast cancer	Female	15-19 years	95+ years	Data Rich	1	2	Cumulative Cigarettes (5 Years)	40
Breast cancer	Female	15-19 years	95+ years	Data Rich	1	2	Cumulative Cigarettes (15 Years)	140
Breast cancer	Female	15-19 years	95+ years	Data Rich	1	2	Cumulative Cigarettes (20 Years)	350
Breast cancer	Female	15-19 years	95+ years	Data Rich	1	2	Diabetes Fasting Plasma Glucose (mmol/L), age-standardized 25+	814
Breast cancer	Female	15-19 years	95+ years	Data Rich	-1	3	Education (years per capita)	0
Breast cancer	Female	15-19 years	95+ years	Data Rich	0	3	Socio-demographic Index	0
Breast cancer	Female	15-19 years	95+ years	Data Rich	0	3	LDI (I$ per capita)	--
Breast cancer	Female	15-19 years	95+ years	Global	1	1	Mean BMI	289
Breast cancer	Female	15-19 years	95+ years	Global	1	1	Alcohol (liters per capita)	694
Breast cancer	Female	15-19 years	95+ years	Global	1	1	Log-transformed SEV scalar: Breast C	--
Breast cancer	Female	15-19 years	95+ years	Global	-1	2	Healthcare access and quality index	203
Breast cancer	Female	15-19 years	95+ years	Global	-1	2	Age-Specific Fertility Rate	382
Breast cancer	Female	15-19 years	95+ years	Global	-1	2	vegetables adjusted(g)	423
Breast cancer	Female	15-19 years	95+ years	Global	-1	2	Total Fertility Rate	718
Breast cancer	Female	15-19 years	95+ years	Global	-1	2	fruits adjusted(g)	--
Breast cancer	Female	15-19 years	95+ years	Global	1	2	Cumulative Cigarettes (5 Years)	155
Breast cancer	Female	15-19 years	95+ years	Global	1	2	Cumulative Cigarettes (20 Years)	202
Breast cancer	Female	15-19 years	95+ years	Global	1	2	Cumulative Cigarettes (10 Years)	219
Breast cancer	Female	15-19 years	95+ years	Global	1	2	Cumulative Cigarettes (15 Years)	253
Breast cancer	Female	15-19 years	95+ years	Global	1	2	Diabetes Fasting Plasma Glucose (mmol/L), age-standardized 25+	329
Breast cancer	Female	15-19 years	95+ years	Global	1	2	Smoking Prevalence	488
Breast cancer	Female	15-19 years	95+ years	Global	1	2	Secondhand smoke	--
Breast cancer	Female	15-19 years	95+ years	Global	-1	3	Education (years per capita)	8
Breast cancer	Female	15-19 years	95+ years	Global	0	3	Socio-demographic Index	269
Breast cancer	Female	15-19 years	95+ years	Global	0	3	LDI (I$ per capita)	--
Breast cancer	Male	15-19 years	95+ years	Data Rich	1	1	Mean BMI	784
Breast cancer	Male	15-19 years	95+ years	Data Rich	1	1	Alcohol (liters per capita)	--
Breast cancer	Male	15-19 years	95+ years	Data Rich	1	1	Log-transformed SEV scalar: Breast C	--
Breast cancer	Male	15-19 years	95+ years	Data Rich	-1	2	Healthcare access and quality index	21
Breast cancer	Male	15-19 years	95+ years	Data Rich	-1	2	fruits adjusted(g)	64
Breast cancer	Male	15-19 years	95+ years	Data Rich	-1	2	vegetables adjusted(g)	--
Breast cancer	Male	15-19 years	95+ years	Data Rich	1	2	Cumulative Cigarettes (15 Years)	10
Breast cancer	Male	15-19 years	95+ years	Data Rich	1	2	Cumulative Cigarettes (10 Years)	12
Breast cancer	Male	15-19 years	95+ years	Data Rich	1	2	Secondhand smoke	17
Breast cancer	Male	15-19 years	95+ years	Data Rich	1	2	Cumulative Cigarettes (5 Years)	24

Table S16. CODEm covariates used, level of covariate, and expected direction of covariate by cause, sex, and age<br>*Covariates that CODEm did not select during the covariate selection process have no draw counts listed.*								
**Cause**	**Sex**	**Age Start**	**Age End**	**Model Type**	**Direction**	**Level**	**Covariate Name**	**Number of Draws**
Breast cancer	Male	15-19 years	95+ years	Data Rich	1	2	Smoking Prevalence	34
Breast cancer	Male	15-19 years	95+ years	Data Rich	1	2	Diabetes Fasting Plasma Glucose (mmol/L), age-standardized 25+	356
Breast cancer	Male	15-19 years	95+ years	Data Rich	1	2	Cumulative Cigarettes (20 Years)	--
Breast cancer	Male	15-19 years	95+ years	Data Rich	-1	3	Education (years per capita)	9
Breast cancer	Male	15-19 years	95+ years	Data Rich	0	3	Socio-demographic Index	94
Breast cancer	Male	15-19 years	95+ years	Data Rich	0	3	LDI (I$ per capita)	--
Breast cancer	Male	15-19 years	95+ years	Global	1	1	Mean BMI	541
Breast cancer	Male	15-19 years	95+ years	Global	1	1	Alcohol (liters per capita)	--
Breast cancer	Male	15-19 years	95+ years	Global	1	1	Log-transformed SEV scalar: Breast C	--
Breast cancer	Male	15-19 years	95+ years	Global	-1	2	Healthcare access and quality index	248
Breast cancer	Male	15-19 years	95+ years	Global	-1	2	fruits adjusted(g)	--
Breast cancer	Male	15-19 years	95+ years	Global	-1	2	vegetables adjusted(g)	--
Breast cancer	Male	15-19 years	95+ years	Global	1	2	Cumulative Cigarettes (5 Years)	10
Breast cancer	Male	15-19 years	95+ years	Global	1	2	Cumulative Cigarettes (10 Years)	25
Breast cancer	Male	15-19 years	95+ years	Global	1	2	Cumulative Cigarettes (15 Years)	96
Breast cancer	Male	15-19 years	95+ years	Global	1	2	Smoking Prevalence	112
Breast cancer	Male	15-19 years	95+ years	Global	1	2	Cumulative Cigarettes (20 Years)	177
Breast cancer	Male	15-19 years	95+ years	Global	1	2	Secondhand smoke	221
Breast cancer	Male	15-19 years	95+ years	Global	1	2	Diabetes Fasting Plasma Glucose (mmol/L), age-standardized 25+	603
Breast cancer	Male	15-19 years	95+ years	Global	-1	3	Education (years per capita)	153
Breast cancer	Male	15-19 years	95+ years	Global	0	3	Socio-demographic Index	159
Breast cancer	Male	15-19 years	95+ years	Global	0	3	LDI (I$ per capita)	--
Cervical cancer	Female	15-19 years	95+ years	Data Rich	1	1	Cumulative Cigarettes (5 Years)	348
Cervical cancer	Female	15-19 years	95+ years	Data Rich	1	1	Cumulative Cigarettes (10 Years)	633
Cervical cancer	Female	15-19 years	95+ years	Data Rich	1	1	Cumulative Cigarettes (15 Years)	--
Cervical cancer	Female	15-19 years	95+ years	Data Rich	1	1	HIV age-standardized prevalence	--
Cervical cancer	Female	15-19 years	95+ years	Data Rich	-1	2	fruits adjusted(g)	139
Cervical cancer	Female	15-19 years	95+ years	Data Rich	-1	2	vegetables adjusted(g)	216
Cervical cancer	Female	15-19 years	95+ years	Data Rich	-1	2	Healthcare access and quality index	465
Cervical cancer	Female	15-19 years	95+ years	Data Rich	1	2	Age-Specific Fertility Rate	4
Cervical cancer	Female	15-19 years	95+ years	Data Rich	1	2	Total Fertility Rate	243
Cervical cancer	Female	15-19 years	95+ years	Data Rich	1	2	Smoking Prevalence	300
Cervical cancer	Female	15-19 years	95+ years	Data Rich	-1	3	Education (years per capita)	474
Cervical cancer	Female	15-19 years	95+ years	Data Rich	0	3	Socio-demographic Index	295
Cervical cancer	Female	15-19 years	95+ years	Data Rich	0	3	LDI (I$ per capita)	--
Cervical cancer	Female	15-19 years	95+ years	Global	1	1	Cumulative Cigarettes (5 Years)	487
Cervical cancer	Female	15-19 years	95+ years	Global	1	1	Cumulative Cigarettes (15 Years)	513
Cervical cancer	Female	15-19 years	95+ years	Global	1	1	Cumulative Cigarettes (10 Years)	--
Cervical cancer	Female	15-19 years	95+ years	Global	1	1	HIV age-standardized prevalence	--
Cervical cancer	Female	15-19 years	95+ years	Global	-1	2	vegetables adjusted(g)	9

Table S16. CODEm covariates used, level of covariate, and expected direction of covariate by cause, sex, and age
Covariates that CODEm did not select during the covariate selection process have no draw counts listed.

Cause	Sex	Age Start	Age End	Model Type	Direction	Level	Covariate Name	Number of Draws
Cervical cancer	Female	15-19 years	95+ years	Global	-1	2	fruits adjusted(g)	15
Cervical cancer	Female	15-19 years	95+ years	Global	-1	2	Healthcare access and quality index	295
Cervical cancer	Female	15-19 years	95+ years	Global	1	2	Age-Specific Fertility Rate	15
Cervical cancer	Female	15-19 years	95+ years	Global	1	2	Total Fertility Rate	369
Cervical cancer	Female	15-19 years	95+ years	Global	1	2	Smoking Prevalence	--
Cervical cancer	Female	15-19 years	95+ years	Global	-1	3	Education (years per capita)	362
Cervical cancer	Female	15-19 years	95+ years	Global	0	3	Socio-demographic Index	302
Cervical cancer	Female	15-19 years	95+ years	Global	0	3	LDI (I$ per capita)	--
Uterine cancer	Female	15-19 years	95+ years	Data Rich	1	1	Mean BMI	369
Uterine cancer	Female	15-19 years	95+ years	Data Rich	1	1	Log-transformed SEV scalar: Uterus C	--
Uterine cancer	Female	15-19 years	95+ years	Data Rich	-1	2	fruits adjusted(g)	35
Uterine cancer	Female	15-19 years	95+ years	Data Rich	-1	2	Healthcare access and quality index	43
Uterine cancer	Female	15-19 years	95+ years	Data Rich	-1	2	vegetables adjusted(g)	655
Uterine cancer	Female	15-19 years	95+ years	Data Rich	0	2	Total Fertility Rate	674
Uterine cancer	Female	15-19 years	95+ years	Data Rich	1	2	Cumulative Cigarettes (10 Years)	2
Uterine cancer	Female	15-19 years	95+ years	Data Rich	1	2	Smoking Prevalence	2
Uterine cancer	Female	15-19 years	95+ years	Data Rich	1	2	Cumulative Cigarettes (5 Years)	5
Uterine cancer	Female	15-19 years	95+ years	Data Rich	1	2	Tobacco (cigarettes per capita)	52
Uterine cancer	Female	15-19 years	95+ years	Data Rich	1	2	Diabetes Age-Standardized Prevalence (proportion)	--
Uterine cancer	Female	15-19 years	95+ years	Data Rich	-1	3	Education (years per capita)	1
Uterine cancer	Female	15-19 years	95+ years	Data Rich	0	3	Socio-demographic Index	20
Uterine cancer	Female	15-19 years	95+ years	Data Rich	0	3	LDI (I$ per capita)	--
Uterine cancer	Female	15-19 years	95+ years	Global	1	1	Mean BMI	368
Uterine cancer	Female	15-19 years	95+ years	Global	1	1	Log-transformed SEV scalar: Uterus C	--
Uterine cancer	Female	15-19 years	95+ years	Global	-1	2	fruits adjusted(g)	265
Uterine cancer	Female	15-19 years	95+ years	Global	-1	2	Healthcare access and quality index	267
Uterine cancer	Female	15-19 years	95+ years	Global	-1	2	vegetables adjusted(g)	269
Uterine cancer	Female	15-19 years	95+ years	Global	0	2	Total Fertility Rate	340
Uterine cancer	Female	15-19 years	95+ years	Global	1	2	Smoking Prevalence	14
Uterine cancer	Female	15-19 years	95+ years	Global	1	2	Cumulative Cigarettes (10 Years)	17
Uterine cancer	Female	15-19 years	95+ years	Global	1	2	Cumulative Cigarettes (5 Years)	28
Uterine cancer	Female	15-19 years	95+ years	Global	1	2	Tobacco (cigarettes per capita)	294
Uterine cancer	Female	15-19 years	95+ years	Global	1	2	Diabetes Age-Standardized Prevalence (proportion)	--
Uterine cancer	Female	15-19 years	95+ years	Global	-1	3	Education (years per capita)	33
Uterine cancer	Female	15-19 years	95+ years	Global	0	3	Socio-demographic Index	144
Uterine cancer	Female	15-19 years	95+ years	Global	0	3	LDI (I$ per capita)	--
Ovarian cancer	Female	15-19 years	95+ years	Data Rich	-1	1	Contraception (Modern) Prevalence (proportion)	--
Ovarian cancer	Female	15-19 years	95+ years	Data Rich	1	1	Tobacco (cigarettes per capita)	0
Ovarian cancer	Female	15-19 years	95+ years	Data Rich	1	1	Cumulative Cigarettes (20 Years)	1
Ovarian cancer	Female	15-19 years	95+ years	Data Rich	1	1	Cumulative Cigarettes (15 Years)	8

**Table S16. CODEm covariates used, level of covariate, and expected direction of covariate by cause, sex, and age** *Covariates that CODEm did not select during the covariate selection process have no draw counts listed.*								
**Cause**	**Sex**	**Age Start**	**Age End**	**Model Type**	**Direction**	**Level**	**Covariate Name**	**Number of Draws**
Ovarian cancer	Female	15-19 years	95+ years	Data Rich	1	1	Cumulative Cigarettes (10 Years)	90
Ovarian cancer	Female	15-19 years	95+ years	Data Rich	1	1	Cumulative Cigarettes (5 Years)	230
Ovarian cancer	Female	15-19 years	95+ years	Data Rich	1	1	Alcohol (liters per capita)	--
Ovarian cancer	Female	15-19 years	95+ years	Data Rich	1	1	Log-transformed SEV scalar: Ovary C	--
Ovarian cancer	Female	15-19 years	95+ years	Data Rich	-1	2	fruits adjusted(g)	95
Ovarian cancer	Female	15-19 years	95+ years	Data Rich	-1	2	vegetables adjusted(g)	123
Ovarian cancer	Female	15-19 years	95+ years	Data Rich	-1	2	Healthcare access and quality index	--
Ovarian cancer	Female	15-19 years	95+ years	Data Rich	0	2	Total Fertility Rate	285
Ovarian cancer	Female	15-19 years	95+ years	Data Rich	1	2	energy unadjusted(kcal)	0
Ovarian cancer	Female	15-19 years	95+ years	Data Rich	1	2	Diabetes Fasting Plasma Glucose (mmol/L), age-standardized 25+	95
Ovarian cancer	Female	15-19 years	95+ years	Data Rich	1	2	Asbestos consumption (metric tons per year per capita)	329
Ovarian cancer	Female	15-19 years	95+ years	Data Rich	1	2	Diabetes Age-Standardized Prevalence (proportion)	329
Ovarian cancer	Female	15-19 years	95+ years	Data Rich	1	2	Smoking Prevalence	329
Ovarian cancer	Female	15-19 years	95+ years	Data Rich	1	2	Mean BMI	371
Ovarian cancer	Female	15-19 years	95+ years	Data Rich	-1	3	Education (years per capita)	56
Ovarian cancer	Female	15-19 years	95+ years	Data Rich	0	3	Socio-demographic Index	429
Ovarian cancer	Female	15-19 years	95+ years	Data Rich	0	3	LDI (I$ per capita)	--
Ovarian cancer	Female	15-19 years	95+ years	Global	-1	1	Contraception (Modern) Prevalence (proportion)	--
Ovarian cancer	Female	15-19 years	95+ years	Global	1	1	Tobacco (cigarettes per capita)	6
Ovarian cancer	Female	15-19 years	95+ years	Global	1	1	Cumulative Cigarettes (5 Years)	52
Ovarian cancer	Female	15-19 years	95+ years	Global	1	1	Cumulative Cigarettes (10 Years)	66
Ovarian cancer	Female	15-19 years	95+ years	Global	1	1	Cumulative Cigarettes (15 Years)	223
Ovarian cancer	Female	15-19 years	95+ years	Global	1	1	Cumulative Cigarettes (20 Years)	325
Ovarian cancer	Female	15-19 years	95+ years	Global	1	1	Alcohol (liters per capita)	--
Ovarian cancer	Female	15-19 years	95+ years	Global	1	1	Log-transformed SEV scalar: Ovary C	--
Ovarian cancer	Female	15-19 years	95+ years	Global	-1	2	Healthcare access and quality index	--
Ovarian cancer	Female	15-19 years	95+ years	Global	-1	2	fruits adjusted(g)	--
Ovarian cancer	Female	15-19 years	95+ years	Global	-1	2	vegetables adjusted(g)	--
Ovarian cancer	Female	15-19 years	95+ years	Global	0	2	Total Fertility Rate	216
Ovarian cancer	Female	15-19 years	95+ years	Global	1	2	energy unadjusted(kcal)	12
Ovarian cancer	Female	15-19 years	95+ years	Global	1	2	Smoking Prevalence	42
Ovarian cancer	Female	15-19 years	95+ years	Global	1	2	Diabetes Fasting Plasma Glucose (mmol/L), age-standardized 25+	290
Ovarian cancer	Female	15-19 years	95+ years	Global	1	2	Diabetes Age-Standardized Prevalence (proportion)	300
Ovarian cancer	Female	15-19 years	95+ years	Global	1	2	Mean BMI	323
Ovarian cancer	Female	15-19 years	95+ years	Global	1	2	Asbestos consumption (metric tons per year per capita)	400
Ovarian cancer	Female	15-19 years	95+ years	Global	-1	3	Education (years per capita)	77
Ovarian cancer	Female	15-19 years	95+ years	Global	0	3	Socio-demographic Index	576
Ovarian cancer	Female	15-19 years	95+ years	Global	0	3	LDI (I$ per capita)	--
Prostate cancer	Male	15-19 years	95+ years	Data Rich	1	1	Log-transformed SEV scalar: Prostate C	--
Prostate cancer	Male	15-19 years	95+ years	Data Rich	-1	2	Healthcare access and quality index	--

**Table S16. CODEm covariates used, level of covariate, and expected direction of covariate by cause, sex, and age** *Covariates that CODEm did not select during the covariate selection process have no draw counts listed.*								
**Cause**	**Sex**	**Age Start**	**Age End**	**Model Type**	**Direction**	**Level**	**Covariate Name**	**Number of Draws**
Prostate cancer	Male	15-19 years	95+ years	Data Rich	1	2	Smoking Prevalence	--
Prostate cancer	Male	15-19 years	95+ years	Data Rich	-1	3	Education (years per capita)	--
Prostate cancer	Male	15-19 years	95+ years	Data Rich	0	3	Socio-demographic Index	692
Prostate cancer	Male	15-19 years	95+ years	Data Rich	0	3	LDI (I$ per capita)	--
Prostate cancer	Male	15-19 years	95+ years	Global	1	1	Log-transformed SEV scalar: Prostate C	--
Prostate cancer	Male	15-19 years	95+ years	Global	-1	2	Healthcare access and quality index	352
Prostate cancer	Male	15-19 years	95+ years	Global	1	2	Smoking Prevalence	218
Prostate cancer	Male	15-19 years	95+ years	Global	-1	3	Education (years per capita)	--
Prostate cancer	Male	15-19 years	95+ years	Global	0	3	Socio-demographic Index	504
Prostate cancer	Male	15-19 years	95+ years	Global	0	3	LDI (I$ per capita)	--
Testicular cancer	Male	15-19 years	95+ years	Data Rich	-1	2	Healthcare access and quality index	0
Testicular cancer	Male	15-19 years	95+ years	Data Rich	-1	2	vegetables adjusted(g)	440
Testicular cancer	Male	15-19 years	95+ years	Data Rich	-1	2	fruits adjusted(g)	--
Testicular cancer	Male	15-19 years	95+ years	Data Rich	1	2	Cumulative Cigarettes (5 Years)	4
Testicular cancer	Male	15-19 years	95+ years	Data Rich	1	2	Cumulative Cigarettes (15 Years)	11
Testicular cancer	Male	15-19 years	95+ years	Data Rich	1	2	Cumulative Cigarettes (20 Years)	18
Testicular cancer	Male	15-19 years	95+ years	Data Rich	1	2	Cumulative Cigarettes (10 Years)	40
Testicular cancer	Male	15-19 years	95+ years	Data Rich	1	2	Smoking Prevalence	99
Testicular cancer	Male	15-19 years	95+ years	Data Rich	1	2	Tobacco (cigarettes per capita)	893
Testicular cancer	Male	15-19 years	95+ years	Data Rich	-1	3	Education (years per capita)	280
Testicular cancer	Male	15-19 years	95+ years	Data Rich	0	3	Socio-demographic Index	695
Testicular cancer	Male	15-19 years	95+ years	Data Rich	0	3	LDI (I$ per capita)	--
Testicular cancer	Male	15-19 years	95+ years	Global	-1	2	Healthcare access and quality index	66
Testicular cancer	Male	15-19 years	95+ years	Global	-1	2	vegetables adjusted(g)	375
Testicular cancer	Male	15-19 years	95+ years	Global	-1	2	fruits adjusted(g)	--
Testicular cancer	Male	15-19 years	95+ years	Global	1	2	Cumulative Cigarettes (15 Years)	71
Testicular cancer	Male	15-19 years	95+ years	Global	1	2	Cumulative Cigarettes (5 Years)	152
Testicular cancer	Male	15-19 years	95+ years	Global	1	2	Cumulative Cigarettes (20 Years)	201
Testicular cancer	Male	15-19 years	95+ years	Global	1	2	Cumulative Cigarettes (10 Years)	217
Testicular cancer	Male	15-19 years	95+ years	Global	1	2	Tobacco (cigarettes per capita)	316
Testicular cancer	Male	15-19 years	95+ years	Global	1	2	Smoking Prevalence	322
Testicular cancer	Male	15-19 years	95+ years	Global	-1	3	Education (years per capita)	60
Testicular cancer	Male	15-19 years	95+ years	Global	0	3	Socio-demographic Index	221
Testicular cancer	Male	15-19 years	95+ years	Global	0	3	LDI (I$ per capita)	--
Kidney cancer	Female	0-6 days	95+ years	Data Rich	1	1	Mean BMI	433
Kidney cancer	Female	0-6 days	95+ years	Data Rich	1	1	Cumulative Cigarettes (10 Years)	--
Kidney cancer	Female	0-6 days	95+ years	Data Rich	1	1	Cumulative Cigarettes (15 Years)	--
Kidney cancer	Female	0-6 days	95+ years	Data Rich	1	1	Cumulative Cigarettes (5 Years)	--
Kidney cancer	Female	0-6 days	95+ years	Data Rich	1	1	Log-transformed SEV scalar: Kidney C	--
Kidney cancer	Female	0-6 days	95+ years	Data Rich	-1	2	Healthcare access and quality index	266
Kidney cancer	Female	0-6 days	95+ years	Data Rich	1	2	Alcohol (liters per capita)	313
Kidney cancer	Female	0-6 days	95+ years	Data Rich	1	2	Diabetes Age-Standardized Prevalence (proportion)	--

Table S16. CODEm covariates used, level of covariate, and expected direction of covariate by cause, sex, and age
Covariates that CODEm did not select during the covariate selection process have no draw counts listed.

Cause	Sex	Age Start	Age End	Model Type	Direction	Level	Covariate Name	Number of Draws
Kidney cancer	Female	0-6 days	95+ years	Data Rich	1	2	Smoking Prevalence	--
Kidney cancer	Female	0-6 days	95+ years	Data Rich	1	2	Systolic Blood Pressure (mmHg)	--
Kidney cancer	Female	0-6 days	95+ years	Data Rich	-1	3	Education (years per capita)	--
Kidney cancer	Female	0-6 days	95+ years	Data Rich	0	3	Socio-demographic Index	374
Kidney cancer	Female	0-6 days	95+ years	Data Rich	0	3	LDI (I$ per capita)	--
Kidney cancer	Female	0-6 days	95+ years	Global	1	1	Mean BMI	395
Kidney cancer	Female	0-6 days	95+ years	Global	1	1	Cumulative Cigarettes (10 Years)	--
Kidney cancer	Female	0-6 days	95+ years	Global	1	1	Cumulative Cigarettes (15 Years)	--
Kidney cancer	Female	0-6 days	95+ years	Global	1	1	Cumulative Cigarettes (5 Years)	--
Kidney cancer	Female	0-6 days	95+ years	Global	1	1	Log-transformed SEV scalar: Kidney C	--
Kidney cancer	Female	0-6 days	95+ years	Global	-1	2	Healthcare access and quality index	--
Kidney cancer	Female	0-6 days	95+ years	Global	1	2	Diabetes Age-Standardized Prevalence (proportion)	80
Kidney cancer	Female	0-6 days	95+ years	Global	1	2	Alcohol (liters per capita)	263
Kidney cancer	Female	0-6 days	95+ years	Global	1	2	Smoking Prevalence	--
Kidney cancer	Female	0-6 days	95+ years	Global	1	2	Systolic Blood Pressure (mmHg)	--
Kidney cancer	Female	0-6 days	95+ years	Global	-1	3	Education (years per capita)	--
Kidney cancer	Female	0-6 days	95+ years	Global	0	3	Socio-demographic Index	404
Kidney cancer	Female	0-6 days	95+ years	Global	0	3	LDI (I$ per capita)	--
Kidney cancer	Male	0-6 days	95+ years	Data Rich	1	1	Mean BMI	607
Kidney cancer	Male	0-6 days	95+ years	Data Rich	1	1	Cumulative Cigarettes (10 Years)	--
Kidney cancer	Male	0-6 days	95+ years	Data Rich	1	1	Cumulative Cigarettes (15 Years)	--
Kidney cancer	Male	0-6 days	95+ years	Data Rich	1	1	Cumulative Cigarettes (5 Years)	--
Kidney cancer	Male	0-6 days	95+ years	Data Rich	1	1	Log-transformed SEV scalar: Kidney C	--
Kidney cancer	Male	0-6 days	95+ years	Data Rich	-1	2	Healthcare access and quality index	--
Kidney cancer	Male	0-6 days	95+ years	Data Rich	1	2	Systolic Blood Pressure (mmHg)	22
Kidney cancer	Male	0-6 days	95+ years	Data Rich	1	2	Diabetes Age-Standardized Prevalence (proportion)	97
Kidney cancer	Male	0-6 days	95+ years	Data Rich	1	2	Alcohol (liters per capita)	--
Kidney cancer	Male	0-6 days	95+ years	Data Rich	1	2	Smoking Prevalence	--
Kidney cancer	Male	0-6 days	95+ years	Data Rich	-1	3	Education (years per capita)	--
Kidney cancer	Male	0-6 days	95+ years	Data Rich	0	3	Socio-demographic Index	720
Kidney cancer	Male	0-6 days	95+ years	Data Rich	0	3	LDI (I$ per capita)	--
Kidney cancer	Male	0-6 days	95+ years	Global	1	1	Mean BMI	619
Kidney cancer	Male	0-6 days	95+ years	Global	1	1	Cumulative Cigarettes (10 Years)	--
Kidney cancer	Male	0-6 days	95+ years	Global	1	1	Cumulative Cigarettes (15 Years)	--
Kidney cancer	Male	0-6 days	95+ years	Global	1	1	Cumulative Cigarettes (5 Years)	--
Kidney cancer	Male	0-6 days	95+ years	Global	1	1	Log-transformed SEV scalar: Kidney C	--
Kidney cancer	Male	0-6 days	95+ years	Global	-1	2	Healthcare access and quality index	--
Kidney cancer	Male	0-6 days	95+ years	Global	1	2	Diabetes Age-Standardized Prevalence (proportion)	57
Kidney cancer	Male	0-6 days	95+ years	Global	1	2	Systolic Blood Pressure (mmHg)	271
Kidney cancer	Male	0-6 days	95+ years	Global	1	2	Alcohol (liters per capita)	--
Kidney cancer	Male	0-6 days	95+ years	Global	1	2	Smoking Prevalence	--
Kidney cancer	Male	0-6 days	95+ years	Global	-1	3	Education (years per capita)	--
Kidney cancer	Male	0-6 days	95+ years	Global	0	3	Socio-demographic Index	481
Kidney cancer	Male	0-6 days	95+ years	Global	0	3	LDI (I$ per capita)	--
Bladder cancer	Female	15-19 years	95+ years	Data Rich	1	1	Cumulative Cigarettes (10 Years)	15
Bladder cancer	Female	15-19 years	95+ years	Data Rich	1	1	Cumulative Cigarettes (5 Years)	47
Bladder cancer	Female	15-19 years	95+ years	Data Rich	1	1	Schistosomiasis Prevalence (proportion)	71
Bladder cancer	Female	15-19 years	95+ years	Data Rich	1	1	Cumulative Cigarettes (15 Years)	87
Bladder cancer	Female	15-19 years	95+ years	Data Rich	1	1	Smoking Prevalence	189
Bladder cancer	Female	15-19 years	95+ years	Data Rich	1	1	Log-transformed SEV scalar: Bladder C	--
Bladder cancer	Female	15-19 years	95+ years	Data Rich	-1	2	fruits adjusted(g)	0
Bladder cancer	Female	15-19 years	95+ years	Data Rich	-1	2	Healthcare access and quality index	249
Bladder cancer	Female	15-19 years	95+ years	Data Rich	-1	2	vegetables adjusted(g)	249

Table S16. CODEm covariates used, level of covariate, and expected direction of covariate by cause, sex, and age
Covariates that CODEm did not select during the covariate selection process have no draw counts listed.

Cause	Sex	Age Start	Age End	Model Type	Direction	Level	Covariate Name	Number of Draws
Bladder cancer	Female	15-19 years	95+ years	Data Rich	1	2	Diabetes Fasting Plasma Glucose (mmol/L), age-standardized 25+	269
Bladder cancer	Female	15-19 years	95+ years	Data Rich	1	2	Alcohol (liters per capita)	509
Bladder cancer	Female	15-19 years	95+ years	Data Rich	-1	3	Education (years per capita)	180
Bladder cancer	Female	15-19 years	95+ years	Data Rich	0	3	LDI (I$ per capita)	341
Bladder cancer	Female	15-19 years	95+ years	Data Rich	0	3	Socio-demographic Index	678
Bladder cancer	Female	15-19 years	95+ years	Global	1	1	Cumulative Cigarettes (10 Years)	72
Bladder cancer	Female	15-19 years	95+ years	Global	1	1	Cumulative Cigarettes (5 Years)	100
Bladder cancer	Female	15-19 years	95+ years	Global	1	1	Cumulative Cigarettes (15 Years)	102
Bladder cancer	Female	15-19 years	95+ years	Global	1	1	Smoking Prevalence	235
Bladder cancer	Female	15-19 years	95+ years	Global	1	1	Schistosomiasis Prevalence (proportion)	251
Bladder cancer	Female	15-19 years	95+ years	Global	1	1	Log-transformed SEV scalar: Bladder C	--
Bladder cancer	Female	15-19 years	95+ years	Global	-1	2	fruits adjusted(g)	31
Bladder cancer	Female	15-19 years	95+ years	Global	-1	2	Healthcare access and quality index	387
Bladder cancer	Female	15-19 years	95+ years	Global	-1	2	vegetables adjusted(g)	413
Bladder cancer	Female	15-19 years	95+ years	Global	1	2	Diabetes Fasting Plasma Glucose (mmol/L), age-standardized 25+	301
Bladder cancer	Female	15-19 years	95+ years	Global	1	2	Alcohol (liters per capita)	526
Bladder cancer	Female	15-19 years	95+ years	Global	-1	3	Education (years per capita)	63
Bladder cancer	Female	15-19 years	95+ years	Global	0	3	LDI (I$ per capita)	533
Bladder cancer	Female	15-19 years	95+ years	Global	0	3	Socio-demographic Index	761
Bladder cancer	Male	15-19 years	95+ years	Data Rich	1	1	Cumulative Cigarettes (5 Years)	8
Bladder cancer	Male	15-19 years	95+ years	Data Rich	1	1	Smoking Prevalence	78
Bladder cancer	Male	15-19 years	95+ years	Data Rich	1	1	Cumulative Cigarettes (10 Years)	198
Bladder cancer	Male	15-19 years	95+ years	Data Rich	1	1	Cumulative Cigarettes (15 Years)	361
Bladder cancer	Male	15-19 years	95+ years	Data Rich	1	1	Schistosomiasis Prevalence (proportion)	722
Bladder cancer	Male	15-19 years	95+ years	Data Rich	1	1	Log-transformed SEV scalar: Bladder C	--
Bladder cancer	Male	15-19 years	95+ years	Data Rich	-1	2	fruits adjusted(g)	0
Bladder cancer	Male	15-19 years	95+ years	Data Rich	-1	2	Healthcare access and quality index	571
Bladder cancer	Male	15-19 years	95+ years	Data Rich	-1	2	vegetables adjusted(g)	571
Bladder cancer	Male	15-19 years	95+ years	Data Rich	1	2	Alcohol (liters per capita)	499
Bladder cancer	Male	15-19 years	95+ years	Data Rich	1	2	Diabetes Fasting Plasma Glucose (mmol/L), age-standardized 25+	571
Bladder cancer	Male	15-19 years	95+ years	Data Rich	-1	3	Education (years per capita)	0
Bladder cancer	Male	15-19 years	95+ years	Data Rich	0	3	Socio-demographic Index	282
Bladder cancer	Male	15-19 years	95+ years	Data Rich	0	3	LDI (I$ per capita)	315
Bladder cancer	Male	15-19 years	95+ years	Global	1	1	Schistosomiasis Prevalence (proportion)	0
Bladder cancer	Male	15-19 years	95+ years	Global	1	1	Cumulative Cigarettes (15 Years)	117
Bladder cancer	Male	15-19 years	95+ years	Global	1	1	Cumulative Cigarettes (10 Years)	175
Bladder cancer	Male	15-19 years	95+ years	Global	1	1	Cumulative Cigarettes (5 Years)	219
Bladder cancer	Male	15-19 years	95+ years	Global	1	1	Smoking Prevalence	639
Bladder cancer	Male	15-19 years	95+ years	Global	1	1	Log-transformed SEV scalar: Bladder C	--
Bladder cancer	Male	15-19 years	95+ years	Global	-1	2	fruits adjusted(g)	138

Table S16. CODEm covariates used, level of covariate, and expected direction of covariate by cause, sex, and age<br>*Covariates that CODEm did not select during the covariate selection process have no draw counts listed.*								
**Cause**	**Sex**	**Age Start**	**Age End**	**Model Type**	**Direction**	**Level**	**Covariate Name**	**Number of Draws**
Bladder cancer	Male	15-19 years	95+ years	Global	-1	2	vegetables adjusted(g)	546
Bladder cancer	Male	15-19 years	95+ years	Global	-1	2	Healthcare access and quality index	658
Bladder cancer	Male	15-19 years	95+ years	Global	1	2	Alcohol (liters per capita)	923
Bladder cancer	Male	15-19 years	95+ years	Global	1	2	Diabetes Fasting Plasma Glucose (mmol/L), age-standardized 25+	995
Bladder cancer	Male	15-19 years	95+ years	Global	-1	3	Education (years per capita)	0
Bladder cancer	Male	15-19 years	95+ years	Global	0	3	LDI (I$ per capita)	819
Bladder cancer	Male	15-19 years	95+ years	Global	0	3	Socio-demographic Index	982
Brain and nervous system cancer	Female	0-6 days	95+ years	Data Rich	1	1	Alcohol (liters per capita)	1000
Brain and nervous system cancer	Female	0-6 days	95+ years	Data Rich	1	1	Cumulative Cigarettes (10 Years)	--
Brain and nervous system cancer	Female	0-6 days	95+ years	Data Rich	1	1	Cumulative Cigarettes (15 Years)	--
Brain and nervous system cancer	Female	0-6 days	95+ years	Data Rich	1	1	Smoking Prevalence	--
Brain and nervous system cancer	Female	0-6 days	95+ years	Data Rich	-1	2	Healthcare access and quality index	--
Brain and nervous system cancer	Female	0-6 days	95+ years	Data Rich	-1	2	fruits adjusted(g)	--
Brain and nervous system cancer	Female	0-6 days	95+ years	Data Rich	-1	2	vegetables adjusted(g)	--
Brain and nervous system cancer	Female	0-6 days	95+ years	Data Rich	1	2	Cholesterol (total, mean per capita)	471
Brain and nervous system cancer	Female	0-6 days	95+ years	Data Rich	1	2	red meats adjusted(g)	471
Brain and nervous system cancer	Female	0-6 days	95+ years	Data Rich	1	2	Systolic Blood Pressure (mmHg)	--
Brain and nervous system cancer	Female	0-6 days	95+ years	Data Rich	-1	3	Education (years per capita)	--
Brain and nervous system cancer	Female	0-6 days	95+ years	Data Rich	0	3	LDI (I$ per capita)	624
Brain and nervous system cancer	Female	0-6 days	95+ years	Data Rich	0	3	Socio-demographic Index	--
Brain and nervous system cancer	Female	0-6 days	95+ years	Global	1	1	Alcohol (liters per capita)	602
Brain and nervous system cancer	Female	0-6 days	95+ years	Global	1	1	Cumulative Cigarettes (10 Years)	--
Brain and nervous system cancer	Female	0-6 days	95+ years	Global	1	1	Cumulative Cigarettes (15 Years)	--
Brain and nervous system cancer	Female	0-6 days	95+ years	Global	1	1	Smoking Prevalence	--
Brain and nervous system cancer	Female	0-6 days	95+ years	Global	-1	2	Healthcare access and quality index	--
Brain and nervous system cancer	Female	0-6 days	95+ years	Global	-1	2	fruits adjusted(g)	--
Brain and nervous system cancer	Female	0-6 days	95+ years	Global	-1	2	vegetables adjusted(g)	--
Brain and nervous system cancer	Female	0-6 days	95+ years	Global	1	2	red meats adjusted(g)	328
Brain and nervous system cancer	Female	0-6 days	95+ years	Global	1	2	Cholesterol (total, mean per capita)	--
Brain and nervous system cancer	Female	0-6 days	95+ years	Global	1	2	Systolic Blood Pressure (mmHg)	--
Brain and nervous system cancer	Female	0-6 days	95+ years	Global	-1	3	Education (years per capita)	--
Brain and nervous system cancer	Female	0-6 days	95+ years	Global	0	3	LDI (I$ per capita)	398
Brain and nervous system cancer	Female	0-6 days	95+ years	Global	0	3	Socio-demographic Index	398
Brain and nervous system cancer	Male	0-6 days	95+ years	Data Rich	1	1	Alcohol (liters per capita)	--
Brain and nervous system cancer	Male	0-6 days	95+ years	Data Rich	1	1	Cumulative Cigarettes (10 Years)	--
Brain and nervous system cancer	Male	0-6 days	95+ years	Data Rich	1	1	Cumulative Cigarettes (15 Years)	--
Brain and nervous system cancer	Male	0-6 days	95+ years	Data Rich	1	1	Smoking Prevalence	--
Brain and nervous system cancer	Male	0-6 days	95+ years	Data Rich	-1	2	Healthcare access and quality index	--
Brain and nervous system cancer	Male	0-6 days	95+ years	Data Rich	-1	2	fruits adjusted(g)	--
Brain and nervous system cancer	Male	0-6 days	95+ years	Data Rich	-1	2	vegetables adjusted(g)	--
Brain and nervous system cancer	Male	0-6 days	95+ years	Data Rich	1	2	Cholesterol (total, mean per capita)	326
Brain and nervous system cancer	Male	0-6 days	95+ years	Data Rich	1	2	red meats adjusted(g)	434
Brain and nervous system cancer	Male	0-6 days	95+ years	Data Rich	1	2	Systolic Blood Pressure (mmHg)	--
Brain and nervous system cancer	Male	0-6 days	95+ years	Data Rich	-1	3	Education (years per capita)	--
Brain and nervous system cancer	Male	0-6 days	95+ years	Data Rich	0	3	Socio-demographic Index	566
Brain and nervous system cancer	Male	0-6 days	95+ years	Data Rich	0	3	LDI (I$ per capita)	742
Brain and nervous system cancer	Male	0-6 days	95+ years	Global	1	1	Alcohol (liters per capita)	454
Brain and nervous system cancer	Male	0-6 days	95+ years	Global	1	1	Cumulative Cigarettes (10 Years)	--
Brain and nervous system cancer	Male	0-6 days	95+ years	Global	1	1	Cumulative Cigarettes (15 Years)	--
Brain and nervous system cancer	Male	0-6 days	95+ years	Global	1	1	Smoking Prevalence	--
Brain and nervous system cancer	Male	0-6 days	95+ years	Global	-1	2	Healthcare access and quality index	--
Brain and nervous system cancer	Male	0-6 days	95+ years	Global	-1	2	fruits adjusted(g)	--
Brain and nervous system cancer	Male	0-6 days	95+ years	Global	-1	2	vegetables adjusted(g)	--
Brain and nervous system cancer	Male	0-6 days	95+ years	Global	1	2	red meats adjusted(g)	546
Brain and nervous system cancer	Male	0-6 days	95+ years	Global	1	2	Cholesterol (total, mean per capita)	--
Brain and nervous system cancer	Male	0-6 days	95+ years	Global	1	2	Systolic Blood Pressure (mmHg)	--
Brain and nervous system cancer	Male	0-6 days	95+ years	Global	-1	3	Education (years per capita)	--

Table S16. CODEm covariates used, level of covariate, and expected direction of covariate by cause, sex, and age<br>*Covariates that CODEm did not select during the covariate selection process have no draw counts listed.*								
**Cause**	**Sex**	**Age Start**	**Age End**	**Model Type**	**Direction**	**Level**	**Covariate Name**	**Number of Draws**
Brain and nervous system cancer	Male	0-6 days	95+ years	Global	0	3	Socio-demographic Index	546
Brain and nervous system cancer	Male	0-6 days	95+ years	Global	0	3	LDI (I$ per capita)	--
Thyroid cancer	Female	10-14 years	95+ years	Data Rich	1	1	Alcohol (liters per capita)	665
Thyroid cancer	Female	10-14 years	95+ years	Data Rich	1	1	Log-transformed SEV scalar: Thyroid C	--
Thyroid cancer	Female	10-14 years	95+ years	Data Rich	-1	2	Sanitation (proportion with access)	5
Thyroid cancer	Female	10-14 years	95+ years	Data Rich	-1	2	Healthcare access and quality index	55
Thyroid cancer	Female	10-14 years	95+ years	Data Rich	-1	2	Improved Water Source (proportion with access)	55
Thyroid cancer	Female	10-14 years	95+ years	Data Rich	-1	2	fruits adjusted(g)	55
Thyroid cancer	Female	10-14 years	95+ years	Data Rich	-1	2	vegetables adjusted(g)	--
Thyroid cancer	Female	10-14 years	95+ years	Data Rich	1	2	Smoking Prevalence	27
Thyroid cancer	Female	10-14 years	95+ years	Data Rich	1	2	Tobacco (cigarettes per capita)	33
Thyroid cancer	Female	10-14 years	95+ years	Data Rich	1	2	red meats adjusted(g)	68
Thyroid cancer	Female	10-14 years	95+ years	Data Rich	1	2	Mean BMI	192
Thyroid cancer	Female	10-14 years	95+ years	Data Rich	-1	3	Education (years per capita)	31
Thyroid cancer	Female	10-14 years	95+ years	Data Rich	0	3	Socio-demographic Index	279
Thyroid cancer	Female	10-14 years	95+ years	Data Rich	0	3	LDI (I$ per capita)	--
Thyroid cancer	Female	10-14 years	95+ years	Global	1	1	Alcohol (liters per capita)	796
Thyroid cancer	Female	10-14 years	95+ years	Global	1	1	Log-transformed SEV scalar: Thyroid C	--
Thyroid cancer	Female	10-14 years	95+ years	Global	-1	2	Sanitation (proportion with access)	72
Thyroid cancer	Female	10-14 years	95+ years	Global	-1	2	Healthcare access and quality index	134
Thyroid cancer	Female	10-14 years	95+ years	Global	-1	2	Improved Water Source (proportion with access)	134
Thyroid cancer	Female	10-14 years	95+ years	Global	-1	2	vegetables adjusted(g)	167
Thyroid cancer	Female	10-14 years	95+ years	Global	-1	2	fruits adjusted(g)	173
Thyroid cancer	Female	10-14 years	95+ years	Global	1	2	Tobacco (cigarettes per capita)	70
Thyroid cancer	Female	10-14 years	95+ years	Global	1	2	Smoking Prevalence	136
Thyroid cancer	Female	10-14 years	95+ years	Global	1	2	Mean BMI	234
Thyroid cancer	Female	10-14 years	95+ years	Global	1	2	red meats adjusted(g)	234
Thyroid cancer	Female	10-14 years	95+ years	Global	-1	3	Education (years per capita)	64
Thyroid cancer	Female	10-14 years	95+ years	Global	0	3	Socio-demographic Index	125
Thyroid cancer	Female	10-14 years	95+ years	Global	0	3	LDI (I$ per capita)	--
Thyroid cancer	Male	10-14 years	95+ years	Data Rich	1	1	Alcohol (liters per capita)	369
Thyroid cancer	Male	10-14 years	95+ years	Data Rich	1	1	Log-transformed SEV scalar: Thyroid C	--
Thyroid cancer	Male	10-14 years	95+ years	Data Rich	-1	2	Sanitation (proportion with access)	19
Thyroid cancer	Male	10-14 years	95+ years	Data Rich	-1	2	Improved Water Source (proportion with access)	43
Thyroid cancer	Male	10-14 years	95+ years	Data Rich	-1	2	Healthcare access and quality index	97
Thyroid cancer	Male	10-14 years	95+ years	Data Rich	-1	2	fruits adjusted(g)	384
Thyroid cancer	Male	10-14 years	95+ years	Data Rich	-1	2	vegetables adjusted(g)	421
Thyroid cancer	Male	10-14 years	95+ years	Data Rich	1	2	Tobacco (cigarettes per capita)	25
Thyroid cancer	Male	10-14 years	95+ years	Data Rich	1	2	Smoking Prevalence	43
Thyroid cancer	Male	10-14 years	95+ years	Data Rich	1	2	Mean BMI	663
Thyroid cancer	Male	10-14 years	95+ years	Data Rich	1	2	red meats adjusted(g)	--

Table S16. CODEm covariates used, level of covariate, and expected direction of covariate by cause, sex, and age<br>*Covariates that CODEm did not select during the covariate selection process have no draw counts listed.*								
**Cause**	**Sex**	**Age Start**	**Age End**	**Model Type**	**Direction**	**Level**	**Covariate Name**	**Number of Draws**
Thyroid cancer	Male	10-14 years	95+ years	Data Rich	-1	3	Education (years per capita)	25
Thyroid cancer	Male	10-14 years	95+ years	Data Rich	0	3	Socio-demographic Index	149
Thyroid cancer	Male	10-14 years	95+ years	Data Rich	0	3	LDI (I$ per capita)	--
Thyroid cancer	Male	10-14 years	95+ years	Global	1	1	Alcohol (liters per capita)	483
Thyroid cancer	Male	10-14 years	95+ years	Global	1	1	Log-transformed SEV scalar: Thyroid C	--
Thyroid cancer	Male	10-14 years	95+ years	Global	2	1	Smoking Prevalence	--
Thyroid cancer	Male	10-14 years	95+ years	Global	-1	2	Improved Water Source (proportion with access)	17
Thyroid cancer	Male	10-14 years	95+ years	Global	-1	2	Sanitation (proportion with access)	19
Thyroid cancer	Male	10-14 years	95+ years	Global	-1	2	vegetables adjusted(g)	53
Thyroid cancer	Male	10-14 years	95+ years	Global	-1	2	Healthcare access and quality index	226
Thyroid cancer	Male	10-14 years	95+ years	Global	-1	2	fruits adjusted(g)	232
Thyroid cancer	Male	10-14 years	95+ years	Global	1	2	Tobacco (cigarettes per capita)	68
Thyroid cancer	Male	10-14 years	95+ years	Global	1	2	red meats adjusted(g)	116
Thyroid cancer	Male	10-14 years	95+ years	Global	1	2	Mean BMI	--
Thyroid cancer	Male	10-14 years	95+ years	Global	-1	3	Education (years per capita)	125
Thyroid cancer	Male	10-14 years	95+ years	Global	0	3	Socio-demographic Index	297
Thyroid cancer	Male	10-14 years	95+ years	Global	0	3	LDI (I$ per capita)	--
Mesothelioma	Female	15-19 years	95+ years	Data Rich	1	1	Asbestos consumption (metric tons per year per capita)	0
Mesothelioma	Female	15-19 years	95+ years	Data Rich	1	1	Cumulative Cigarettes (5 Years)	39
Mesothelioma	Female	15-19 years	95+ years	Data Rich	1	1	Smoking Prevalence	130
Mesothelioma	Female	15-19 years	95+ years	Data Rich	1	1	Asbestos production (binary)	--
Mesothelioma	Female	15-19 years	95+ years	Data Rich	1	1	Indoor Air Pollution (All Cooking Fuels)	--
Mesothelioma	Female	15-19 years	95+ years	Data Rich	1	1	Log-transformed SEV scalar: Mesothel	--
Mesothelioma	Female	15-19 years	95+ years	Data Rich	1	1	Log-transformed age-standardized SEV scalar: Mesothel	--
Mesothelioma	Female	15-19 years	95+ years	Data Rich	-1	2	Healthcare access and quality index	--
Mesothelioma	Female	15-19 years	95+ years	Data Rich	1	2	Gold production (kg) per capita	0
Mesothelioma	Female	15-19 years	95+ years	Data Rich	1	2	Gold production (binary)	498
Mesothelioma	Female	15-19 years	95+ years	Data Rich	1	2	Population Density (over 1000 ppl/sqkm, proportion)	498
Mesothelioma	Female	15-19 years	95+ years	Data Rich	1	2	Asbestos production (kg) per capita	--
Mesothelioma	Female	15-19 years	95+ years	Data Rich	-1	3	Education (years per capita)	--
Mesothelioma	Female	15-19 years	95+ years	Data Rich	0	3	Socio-demographic Index	648
Mesothelioma	Female	15-19 years	95+ years	Data Rich	0	3	LDI (I$ per capita)	--
Mesothelioma	Female	15-19 years	95+ years	Global	1	1	Asbestos consumption (metric tons per year per capita)	5
Mesothelioma	Female	15-19 years	95+ years	Global	1	1	Smoking Prevalence	86
Mesothelioma	Female	15-19 years	95+ years	Global	1	1	Cumulative Cigarettes (5 Years)	211
Mesothelioma	Female	15-19 years	95+ years	Global	1	1	Asbestos production (binary)	--
Mesothelioma	Female	15-19 years	95+ years	Global	1	1	Indoor Air Pollution (All Cooking Fuels)	--
Mesothelioma	Female	15-19 years	95+ years	Global	1	1	Log-transformed SEV scalar: Mesothel	--
Mesothelioma	Female	15-19 years	95+ years	Global	1	1	Log-transformed age-standardized SEV scalar: Mesothel	--
Mesothelioma	Female	15-19 years	95+ years	Global	-1	2	Healthcare access and quality index	--

Table S16. CODEm covariates used, level of covariate, and expected direction of covariate by cause, sex, and age
Covariates that CODEm did not select during the covariate selection process have no draw counts listed.

Cause	Sex	Age Start	Age End	Model Type	Direction	Level	Covariate Name	Number of Draws
Mesothelioma	Female	15-19 years	95+ years	Global	1	2	Gold production (binary)	113
Mesothelioma	Female	15-19 years	95+ years	Global	1	2	Gold production (kg) per capita	131
Mesothelioma	Female	15-19 years	95+ years	Global	1	2	Population Density (over 1000 ppl/sqkm, proportion)	463
Mesothelioma	Female	15-19 years	95+ years	Global	1	2	Asbestos production (kg) per capita	--
Mesothelioma	Female	15-19 years	95+ years	Global	-1	3	Education (years per capita)	--
Mesothelioma	Female	15-19 years	95+ years	Global	0	3	Socio-demographic Index	332
Mesothelioma	Female	15-19 years	95+ years	Global	0	3	LDI (I$ per capita)	--
Mesothelioma	Male	15-19 years	95+ years	Data Rich	1	1	Asbestos consumption (metric tons per year per capita)	0
Mesothelioma	Male	15-19 years	95+ years	Data Rich	1	1	Smoking Prevalence	0
Mesothelioma	Male	15-19 years	95+ years	Data Rich	1	1	Cumulative Cigarettes (5 Years)	375
Mesothelioma	Male	15-19 years	95+ years	Data Rich	1	1	Asbestos production (binary)	--
Mesothelioma	Male	15-19 years	95+ years	Data Rich	1	1	Indoor Air Pollution (All Cooking Fuels)	--
Mesothelioma	Male	15-19 years	95+ years	Data Rich	1	1	Log-transformed SEV scalar: Mesothel	--
Mesothelioma	Male	15-19 years	95+ years	Data Rich	1	1	Log-transformed age-standardized SEV scalar: Mesothel	--
Mesothelioma	Male	15-19 years	95+ years	Data Rich	-1	2	Healthcare access and quality index	--
Mesothelioma	Male	15-19 years	95+ years	Data Rich	1	2	Population Density (over 1000 ppl/sqkm, proportion)	6
Mesothelioma	Male	15-19 years	95+ years	Data Rich	1	2	Gold production (binary)	246
Mesothelioma	Male	15-19 years	95+ years	Data Rich	1	2	Asbestos production (kg) per capita	--
Mesothelioma	Male	15-19 years	95+ years	Data Rich	1	2	Gold production (kg) per capita	--
Mesothelioma	Male	15-19 years	95+ years	Data Rich	-1	3	Education (years per capita)	--
Mesothelioma	Male	15-19 years	95+ years	Data Rich	0	3	Socio-demographic Index	694
Mesothelioma	Male	15-19 years	95+ years	Data Rich	0	3	LDI (I$ per capita)	--
Mesothelioma	Male	15-19 years	95+ years	Global	1	1	Asbestos consumption (metric tons per year per capita)	0
Mesothelioma	Male	15-19 years	95+ years	Global	1	1	Smoking Prevalence	11
Mesothelioma	Male	15-19 years	95+ years	Global	1	1	Indoor Air Pollution (All Cooking Fuels)	167
Mesothelioma	Male	15-19 years	95+ years	Global	1	1	Cumulative Cigarettes (5 Years)	443
Mesothelioma	Male	15-19 years	95+ years	Global	1	1	Asbestos production (binary)	--
Mesothelioma	Male	15-19 years	95+ years	Global	1	1	Log-transformed SEV scalar: Mesothel	--
Mesothelioma	Male	15-19 years	95+ years	Global	1	1	Log-transformed age-standardized SEV scalar: Mesothel	--
Mesothelioma	Male	15-19 years	95+ years	Global	-1	2	Healthcare access and quality index	--
Mesothelioma	Male	15-19 years	95+ years	Global	1	2	Gold production (binary)	0
Mesothelioma	Male	15-19 years	95+ years	Global	1	2	Gold production (kg) per capita	0
Mesothelioma	Male	15-19 years	95+ years	Global	1	2	Population Density (over 1000 ppl/sqkm, proportion)	84
Mesothelioma	Male	15-19 years	95+ years	Global	1	2	Asbestos production (kg) per capita	--
Mesothelioma	Male	15-19 years	95+ years	Global	-1	3	Education (years per capita)	--
Mesothelioma	Male	15-19 years	95+ years	Global	0	3	Socio-demographic Index	630
Mesothelioma	Male	15-19 years	95+ years	Global	0	3	LDI (I$ per capita)	--
Hodgkin lymphoma	Female	0-6 days	95+ years	Data Rich	-1	2	Healthcare access and quality index	1000
Hodgkin lymphoma	Female	0-6 days	95+ years	Data Rich	-1	3	Education (years per capita)	308
Hodgkin lymphoma	Female	0-6 days	95+ years	Data Rich	0	3	Socio-demographic Index	308
Hodgkin lymphoma	Female	0-6 days	95+ years	Data Rich	0	3	LDI (I$ per capita)	--
Hodgkin lymphoma	Female	0-6 days	95+ years	Global	-1	2	Healthcare access and quality index	1000

Table S16. CODEm covariates used, level of covariate, and expected direction of covariate by cause, sex, and age<br>*Covariates that CODEm did not select during the covariate selection process have no draw counts listed.*								
**Cause**	**Sex**	**Age Start**	**Age End**	**Model Type**	**Direction**	**Level**	**Covariate Name**	**Number of Draws**
Hodgkin lymphoma	Female	0-6 days	95+ years	Global	-1	3	Education (years per capita)	328
Hodgkin lymphoma	Female	0-6 days	95+ years	Global	0	3	Socio-demographic Index	328
Hodgkin lymphoma	Female	0-6 days	95+ years	Global	0	3	LDI (I$ per capita)	--
Hodgkin lymphoma	Male	0-6 days	95+ years	Data Rich	-1	2	Healthcare access and quality index	--
Hodgkin lymphoma	Male	0-6 days	95+ years	Data Rich	-1	3	Education (years per capita)	381
Hodgkin lymphoma	Male	0-6 days	95+ years	Data Rich	0	3	Socio-demographic Index	1000
Hodgkin lymphoma	Male	0-6 days	95+ years	Data Rich	0	3	LDI (I$ per capita)	--
Hodgkin lymphoma	Male	0-6 days	95+ years	Global	-1	2	Healthcare access and quality index	1000
Hodgkin lymphoma	Male	0-6 days	95+ years	Global	-1	3	Education (years per capita)	--
Hodgkin lymphoma	Male	0-6 days	95+ years	Global	0	3	Socio-demographic Index	452
Hodgkin lymphoma	Male	0-6 days	95+ years	Global	0	3	LDI (I$ per capita)	--
Non-Hodgkin's lymphoma	Female	0-6 days	95+ years	Data Rich	-1	2	Healthcare access and quality index	128
Non-Hodgkin's lymphoma	Female	0-6 days	95+ years	Data Rich	1	2	Cumulative Cigarettes (15 Years)	3
Non-Hodgkin's lymphoma	Female	0-6 days	95+ years	Data Rich	1	2	Cumulative Cigarettes (10 Years)	22
Non-Hodgkin's lymphoma	Female	0-6 days	95+ years	Data Rich	1	2	Smoking Prevalence	24
Non-Hodgkin's lymphoma	Female	0-6 days	95+ years	Data Rich	1	2	Cumulative Cigarettes (5 Years)	44
Non-Hodgkin's lymphoma	Female	0-6 days	95+ years	Data Rich	1	2	Cumulative Cigarettes (20 Years)	117
Non-Hodgkin's lymphoma	Female	0-6 days	95+ years	Data Rich	1	2	Mean BMI	407
Non-Hodgkin's lymphoma	Female	0-6 days	95+ years	Data Rich	1	2	Alcohol (liters per capita)	902
Non-Hodgkin's lymphoma	Female	0-6 days	95+ years	Data Rich	0	3	Total Fertility Rate	107
Non-Hodgkin's lymphoma	Female	0-6 days	95+ years	Data Rich	0	3	Socio-demographic Index	366
Non-Hodgkin's lymphoma	Female	0-6 days	95+ years	Data Rich	0	3	LDI (I$ per capita)	--
Non-Hodgkin's lymphoma	Female	0-6 days	95+ years	Global	-1	2	Healthcare access and quality index	247
Non-Hodgkin's lymphoma	Female	0-6 days	95+ years	Global	1	2	Cumulative Cigarettes (5 Years)	38
Non-Hodgkin's lymphoma	Female	0-6 days	95+ years	Global	1	2	Cumulative Cigarettes (10 Years)	86
Non-Hodgkin's lymphoma	Female	0-6 days	95+ years	Global	1	2	Smoking Prevalence	103
Non-Hodgkin's lymphoma	Female	0-6 days	95+ years	Global	1	2	Cumulative Cigarettes (20 Years)	161
Non-Hodgkin's lymphoma	Female	0-6 days	95+ years	Global	1	2	Cumulative Cigarettes (15 Years)	283
Non-Hodgkin's lymphoma	Female	0-6 days	95+ years	Global	1	2	Mean BMI	532
Non-Hodgkin's lymphoma	Female	0-6 days	95+ years	Global	1	2	Alcohol (liters per capita)	749
Non-Hodgkin's lymphoma	Female	0-6 days	95+ years	Global	0	3	Socio-demographic Index	361
Non-Hodgkin's lymphoma	Female	0-6 days	95+ years	Global	0	3	Total Fertility Rate	458
Non-Hodgkin's lymphoma	Female	0-6 days	95+ years	Global	0	3	LDI (I$ per capita)	--
Non-Hodgkin's lymphoma	Male	0-6 days	95+ years	Data Rich	-1	2	Healthcare access and quality index	233
Non-Hodgkin's lymphoma	Male	0-6 days	95+ years	Data Rich	1	2	Cumulative Cigarettes (10 Years)	13
Non-Hodgkin's lymphoma	Male	0-6 days	95+ years	Data Rich	1	2	Cumulative Cigarettes (20 Years)	17
Non-Hodgkin's lymphoma	Male	0-6 days	95+ years	Data Rich	1	2	Cumulative Cigarettes (15 Years)	23
Non-Hodgkin's lymphoma	Male	0-6 days	95+ years	Data Rich	1	2	Cumulative Cigarettes (5 Years)	169
Non-Hodgkin's lymphoma	Male	0-6 days	95+ years	Data Rich	1	2	Mean BMI	249
Non-Hodgkin's lymphoma	Male	0-6 days	95+ years	Data Rich	1	2	Alcohol (liters per capita)	568
Non-Hodgkin's lymphoma	Male	0-6 days	95+ years	Data Rich	1	2	Smoking Prevalence	--
Non-Hodgkin's lymphoma	Male	0-6 days	95+ years	Data Rich	0	3	Socio-demographic Index	344
Non-Hodgkin's lymphoma	Male	0-6 days	95+ years	Data Rich	0	3	LDI (I$ per capita)	--
Non-Hodgkin's lymphoma	Male	0-6 days	95+ years	Global	-1	2	Healthcare access and quality index	364
Non-Hodgkin's lymphoma	Male	0-6 days	95+ years	Global	1	2	Cumulative Cigarettes (10 Years)	50
Non-Hodgkin's lymphoma	Male	0-6 days	95+ years	Global	1	2	Cumulative Cigarettes (5 Years)	60
Non-Hodgkin's lymphoma	Male	0-6 days	95+ years	Global	1	2	Cumulative Cigarettes (20 Years)	73
Non-Hodgkin's lymphoma	Male	0-6 days	95+ years	Global	1	2	Cumulative Cigarettes (15 Years)	183
Non-Hodgkin's lymphoma	Male	0-6 days	95+ years	Global	1	2	Mean BMI	324
Non-Hodgkin's lymphoma	Male	0-6 days	95+ years	Global	1	2	Alcohol (liters per capita)	349
Non-Hodgkin's lymphoma	Male	0-6 days	95+ years	Global	1	2	Smoking Prevalence	--
Non-Hodgkin's lymphoma	Male	0-6 days	95+ years	Global	0	3	Socio-demographic Index	622
Non-Hodgkin's lymphoma	Male	0-6 days	95+ years	Global	0	3	LDI (I$ per capita)	--
Multiple myeloma	Female	15-19 years	95+ years	Data Rich	1	1	Smoking Prevalence	287
Multiple myeloma	Female	15-19 years	95+ years	Data Rich	1	1	Alcohol (liters per capita)	713
Multiple myeloma	Female	15-19 years	95+ years	Data Rich	1	1	Tobacco (cigarettes per capita)	--
Multiple myeloma	Female	15-19 years	95+ years	Data Rich	-1	2	Healthcare access and quality index	--
Multiple myeloma	Female	15-19 years	95+ years	Data Rich	-1	2	Improved Water Source (proportion with access)	--

Table S16. CODEm covariates used, level of covariate, and expected direction of covariate by cause, sex, and age
Covariates that CODEm did not select during the covariate selection process have no draw counts listed.

Cause	Sex	Age Start	Age End	Model Type	Direction	Level	Covariate Name	Number of Draws
Multiple myeloma	Female	15-19 years	95+ years	Data Rich	-1	2	Sanitation (proportion with access)	--
Multiple myeloma	Female	15-19 years	95+ years	Data Rich	-1	2	fruits adjusted(g)	--
Multiple myeloma	Female	15-19 years	95+ years	Data Rich	-1	2	vegetables adjusted(g)	--
Multiple myeloma	Female	15-19 years	95+ years	Data Rich	1	2	red meats adjusted(g)	191
Multiple myeloma	Female	15-19 years	95+ years	Data Rich	1	2	Mean BMI	379
Multiple myeloma	Female	15-19 years	95+ years	Data Rich	-1	3	Education (years per capita)	--
Multiple myeloma	Female	15-19 years	95+ years	Data Rich	0	3	Socio-demographic Index	370
Multiple myeloma	Female	15-19 years	95+ years	Data Rich	0	3	LDI (I$ per capita)	--
Multiple myeloma	Female	15-19 years	95+ years	Global	1	1	Smoking Prevalence	643
Multiple myeloma	Female	15-19 years	95+ years	Global	1	1	Alcohol (liters per capita)	796
Multiple myeloma	Female	15-19 years	95+ years	Global	1	1	Tobacco (cigarettes per capita)	--
Multiple myeloma	Female	15-19 years	95+ years	Global	-1	2	Healthcare access and quality index	--
Multiple myeloma	Female	15-19 years	95+ years	Global	-1	2	Improved Water Source (proportion with access)	--
Multiple myeloma	Female	15-19 years	95+ years	Global	-1	2	Sanitation (proportion with access)	--
Multiple myeloma	Female	15-19 years	95+ years	Global	-1	2	fruits adjusted(g)	--
Multiple myeloma	Female	15-19 years	95+ years	Global	-1	2	vegetables adjusted(g)	--
Multiple myeloma	Female	15-19 years	95+ years	Global	1	2	red meats adjusted(g)	430
Multiple myeloma	Female	15-19 years	95+ years	Global	1	2	Mean BMI	648
Multiple myeloma	Female	15-19 years	95+ years	Global	-1	3	Education (years per capita)	--
Multiple myeloma	Female	15-19 years	95+ years	Global	0	3	Socio-demographic Index	327
Multiple myeloma	Female	15-19 years	95+ years	Global	0	3	LDI (I$ per capita)	--
Multiple myeloma	Male	15-19 years	95+ years	Data Rich	1	1	Alcohol (liters per capita)	677
Multiple myeloma	Male	15-19 years	95+ years	Data Rich	1	1	Smoking Prevalence	--
Multiple myeloma	Male	15-19 years	95+ years	Data Rich	1	1	Tobacco (cigarettes per capita)	--
Multiple myeloma	Male	15-19 years	95+ years	Data Rich	-1	2	Healthcare access and quality index	--
Multiple myeloma	Male	15-19 years	95+ years	Data Rich	-1	2	Improved Water Source (proportion with access)	--
Multiple myeloma	Male	15-19 years	95+ years	Data Rich	-1	2	Sanitation (proportion with access)	--
Multiple myeloma	Male	15-19 years	95+ years	Data Rich	-1	2	fruits adjusted(g)	--
Multiple myeloma	Male	15-19 years	95+ years	Data Rich	-1	2	vegetables adjusted(g)	--
Multiple myeloma	Male	15-19 years	95+ years	Data Rich	1	2	red meats adjusted(g)	707
Multiple myeloma	Male	15-19 years	95+ years	Data Rich	1	2	Mean BMI	--
Multiple myeloma	Male	15-19 years	95+ years	Data Rich	-1	3	Education (years per capita)	--
Multiple myeloma	Male	15-19 years	95+ years	Data Rich	0	3	Socio-demographic Index	751
Multiple myeloma	Male	15-19 years	95+ years	Data Rich	0	3	LDI (I$ per capita)	--
Multiple myeloma	Male	15-19 years	95+ years	Global	1	1	Alcohol (liters per capita)	1000
Multiple myeloma	Male	15-19 years	95+ years	Global	1	1	Smoking Prevalence	--
Multiple myeloma	Male	15-19 years	95+ years	Global	1	1	Tobacco (cigarettes per capita)	--
Multiple myeloma	Male	15-19 years	95+ years	Global	-1	2	Healthcare access and quality index	--
Multiple myeloma	Male	15-19 years	95+ years	Global	-1	2	Improved Water Source (proportion with access)	--
Multiple myeloma	Male	15-19 years	95+ years	Global	-1	2	Sanitation (proportion with access)	--

Table S16. CODEm covariates used, level of covariate, and expected direction of covariate by cause, sex, and age
Covariates that CODEm did not select during the covariate selection process have no draw counts listed.

Cause	Sex	Age Start	Age End	Model Type	Direction	Level	Covariate Name	Number of Draws
Multiple myeloma	Male	15-19 years	95+ years	Global	-1	2	fruits adjusted(g)	--
Multiple myeloma	Male	15-19 years	95+ years	Global	-1	2	vegetables adjusted(g)	--
Multiple myeloma	Male	15-19 years	95+ years	Global	1	2	Mean BMI	535
Multiple myeloma	Male	15-19 years	95+ years	Global	1	2	red meats adjusted(g)	535
Multiple myeloma	Male	15-19 years	95+ years	Global	-1	3	Education (years per capita)	--
Multiple myeloma	Male	15-19 years	95+ years	Global	0	3	Socio-demographic Index	463
Multiple myeloma	Male	15-19 years	95+ years	Global	0	3	LDI (I$ per capita)	--
Leukaemia	Female	0-6 days	95+ years	Data Rich	1	1	Log-transformed SEV scalar: leukaemia	--
Leukaemia	Female	0-6 days	95+ years	Data Rich	1	1	Log-transformed age-standardized SEV scalar: leukaemia	--
Leukaemia	Female	0-6 days	95+ years	Data Rich	-1	2	Healthcare access and quality index	351
Leukaemia	Female	0-6 days	95+ years	Data Rich	1	2	Tobacco (cigarettes per capita)	51
Leukaemia	Female	0-6 days	95+ years	Data Rich	1	2	Smoking Prevalence	184
Leukaemia	Female	0-6 days	95+ years	Data Rich	1	2	Cumulative Cigarettes (15 Years)	197
Leukaemia	Female	0-6 days	95+ years	Data Rich	1	2	Alcohol (liters per capita)	759
Leukaemia	Female	0-6 days	95+ years	Data Rich	1	2	Cumulative Cigarettes (10 Years)	--
Leukaemia	Female	0-6 days	95+ years	Data Rich	1	2	Cumulative Cigarettes (20 Years)	--
Leukaemia	Female	0-6 days	95+ years	Data Rich	1	2	Cumulative Cigarettes (5 Years)	--
Leukaemia	Female	0-6 days	95+ years	Data Rich	1	2	Mean BMI	--
Leukaemia	Female	0-6 days	95+ years	Data Rich	-1	3	Education (years per capita)	256
Leukaemia	Female	0-6 days	95+ years	Data Rich	0	3	Socio-demographic Index	191
Leukaemia	Female	0-6 days	95+ years	Data Rich	0	3	LDI (I$ per capita)	--
Leukaemia	Female	0-6 days	95+ years	Global	1	1	Log-transformed SEV scalar: leukaemia	--
Leukaemia	Female	0-6 days	95+ years	Global	1	1	Log-transformed age-standardized SEV scalar: leukaemia	--
Leukaemia	Female	0-6 days	95+ years	Global	-1	2	Healthcare access and quality index	525
Leukaemia	Female	0-6 days	95+ years	Global	1	2	Cumulative Cigarettes (15 Years)	144
Leukaemia	Female	0-6 days	95+ years	Global	1	2	Cumulative Cigarettes (20 Years)	381
Leukaemia	Female	0-6 days	95+ years	Global	1	2	Alcohol (liters per capita)	--
Leukaemia	Female	0-6 days	95+ years	Global	1	2	Cumulative Cigarettes (10 Years)	--
Leukaemia	Female	0-6 days	95+ years	Global	1	2	Cumulative Cigarettes (5 Years)	--
Leukaemia	Female	0-6 days	95+ years	Global	1	2	Mean BMI	--
Leukaemia	Female	0-6 days	95+ years	Global	1	2	Smoking Prevalence	--
Leukaemia	Female	0-6 days	95+ years	Global	1	2	Tobacco (cigarettes per capita)	--
Leukaemia	Female	0-6 days	95+ years	Global	-1	3	Education (years per capita)	328
Leukaemia	Female	0-6 days	95+ years	Global	0	3	Socio-demographic Index	220
Leukaemia	Female	0-6 days	95+ years	Global	0	3	LDI (I$ per capita)	--
Leukaemia	Male	0-6 days	95+ years	Data Rich	1	1	Log-transformed SEV scalar: leukaemia	--
Leukaemia	Male	0-6 days	95+ years	Data Rich	1	1	Log-transformed age-standardized SEV scalar: leukaemia	--
Leukaemia	Male	0-6 days	95+ years	Data Rich	-1	2	Healthcare access and quality index	329
Leukaemia	Male	0-6 days	95+ years	Data Rich	1	2	Smoking Prevalence	0
Leukaemia	Male	0-6 days	95+ years	Data Rich	1	2	Cumulative Cigarettes (10 Years)	55
Leukaemia	Male	0-6 days	95+ years	Data Rich	1	2	Cumulative Cigarettes (5 Years)	99
Leukaemia	Male	0-6 days	95+ years	Data Rich	1	2	Cumulative Cigarettes (20 Years)	103
Leukaemia	Male	0-6 days	95+ years	Data Rich	1	2	Cumulative Cigarettes (15 Years)	105
Leukaemia	Male	0-6 days	95+ years	Data Rich	1	2	Tobacco (cigarettes per capita)	296
Leukaemia	Male	0-6 days	95+ years	Data Rich	1	2	Mean BMI	429
Leukaemia	Male	0-6 days	95+ years	Data Rich	1	2	Alcohol (liters per capita)	--
Leukaemia	Male	0-6 days	95+ years	Data Rich	-1	3	Education (years per capita)	465
Leukaemia	Male	0-6 days	95+ years	Data Rich	0	3	Socio-demographic Index	636
Leukaemia	Male	0-6 days	95+ years	Data Rich	0	3	LDI (I$ per capita)	--
Leukaemia	Male	0-6 days	95+ years	Global	1	1	Log-transformed SEV scalar: leukaemia	--
Leukaemia	Male	0-6 days	95+ years	Global	1	1	Log-transformed age-standardized SEV scalar: leukaemia	--
Leukaemia	Male	0-6 days	95+ years	Global	-1	2	Healthcare access and quality index	416
Leukaemia	Male	0-6 days	95+ years	Global	1	2	Cumulative Cigarettes (5 Years)	198

Table S16. CODEm covariates used, level of covariate, and expected direction of covariate by cause, sex, and age
Covariates that CODEm did not select during the covariate selection process have no draw counts listed.

Cause	Sex	Age Start	Age End	Model Type	Direction	Level	Covariate Name	Number of Draws
Leukaemia	Male	0-6 days	95+ years	Global	1	2	Smoking Prevalence	230
Leukaemia	Male	0-6 days	95+ years	Global	1	2	Cumulative Cigarettes (10 Years)	256
Leukaemia	Male	0-6 days	95+ years	Global	1	2	Tobacco (cigarettes per capita)	324
Leukaemia	Male	0-6 days	95+ years	Global	1	2	Cumulative Cigarettes (20 Years)	514
Leukaemia	Male	0-6 days	95+ years	Global	1	2	Alcohol (liters per capita)	--
Leukaemia	Male	0-6 days	95+ years	Global	1	2	Cumulative Cigarettes (15 Years)	--
Leukaemia	Male	0-6 days	95+ years	Global	1	2	Mean BMI	--
Leukaemia	Male	0-6 days	95+ years	Global	-1	3	Education (years per capita)	32
Leukaemia	Male	0-6 days	95+ years	Global	0	3	Socio-demographic Index	457
Leukaemia	Male	0-6 days	95+ years	Global	0	3	LDI (I$ per capita)	--
Acute lymphoid leukaemia	Female	0-6 days	95+ years	Data Rich	1	1	Log-transformed SEV scalar: leukaemia	--
Acute lymphoid leukaemia	Female	0-6 days	95+ years	Data Rich	1	1	Log-transformed age-standardized SEV scalar: leukaemia	--
Acute lymphoid leukaemia	Female	0-6 days	95+ years	Data Rich	-1	2	Healthcare access and quality index	18
Acute lymphoid leukaemia	Female	0-6 days	95+ years	Data Rich	1	2	Alcohol (liters per capita)	18
Acute lymphoid leukaemia	Female	0-6 days	95+ years	Data Rich	1	2	Cumulative Cigarettes (20 Years)	22
Acute lymphoid leukaemia	Female	0-6 days	95+ years	Data Rich	1	2	Mean BMI	199
Acute lymphoid leukaemia	Female	0-6 days	95+ years	Data Rich	1	2	Tobacco (cigarettes per capita)	383
Acute lymphoid leukaemia	Female	0-6 days	95+ years	Data Rich	1	2	Cumulative Cigarettes (10 Years)	--
Acute lymphoid leukaemia	Female	0-6 days	95+ years	Data Rich	1	2	Cumulative Cigarettes (15 Years)	--
Acute lymphoid leukaemia	Female	0-6 days	95+ years	Data Rich	1	2	Cumulative Cigarettes (5 Years)	--
Acute lymphoid leukaemia	Female	0-6 days	95+ years	Data Rich	1	2	Smoking Prevalence	--
Acute lymphoid leukaemia	Female	0-6 days	95+ years	Data Rich	-1	3	Education (years per capita)	295
Acute lymphoid leukaemia	Female	0-6 days	95+ years	Data Rich	0	3	Socio-demographic Index	612
Acute lymphoid leukaemia	Female	0-6 days	95+ years	Data Rich	0	3	LDI (I$ per capita)	646
Acute lymphoid leukaemia	Female	0-6 days	95+ years	Global	1	1	Log-transformed SEV scalar: leukaemia	--
Acute lymphoid leukaemia	Female	0-6 days	95+ years	Global	1	1	Log-transformed age-standardized SEV scalar: leukaemia	--
Acute lymphoid leukaemia	Female	0-6 days	95+ years	Global	-1	2	Healthcare access and quality index	--
Acute lymphoid leukaemia	Female	0-6 days	95+ years	Global	1	2	Alcohol (liters per capita)	34
Acute lymphoid leukaemia	Female	0-6 days	95+ years	Global	1	2	Cumulative Cigarettes (20 Years)	109
Acute lymphoid leukaemia	Female	0-6 days	95+ years	Global	1	2	Mean BMI	229
Acute lymphoid leukaemia	Female	0-6 days	95+ years	Global	1	2	Tobacco (cigarettes per capita)	265
Acute lymphoid leukaemia	Female	0-6 days	95+ years	Global	1	2	Cumulative Cigarettes (10 Years)	--
Acute lymphoid leukaemia	Female	0-6 days	95+ years	Global	1	2	Cumulative Cigarettes (15 Years)	--
Acute lymphoid leukaemia	Female	0-6 days	95+ years	Global	1	2	Cumulative Cigarettes (5 Years)	--
Acute lymphoid leukaemia	Female	0-6 days	95+ years	Global	1	2	Smoking Prevalence	--
Acute lymphoid leukaemia	Female	0-6 days	95+ years	Global	-1	3	Education (years per capita)	463
Acute lymphoid leukaemia	Female	0-6 days	95+ years	Global	0	3	Socio-demographic Index	341
Acute lymphoid leukaemia	Female	0-6 days	95+ years	Global	0	3	LDI (I$ per capita)	462
Acute lymphoid leukaemia	Male	0-6 days	95+ years	Data Rich	1	1	Log-transformed SEV scalar: leukaemia	--
Acute lymphoid leukaemia	Male	0-6 days	95+ years	Data Rich	1	1	Log-transformed age-standardized SEV scalar: leukaemia	--
Acute lymphoid leukaemia	Male	0-6 days	95+ years	Data Rich	-1	2	Healthcare access and quality index	3
Acute lymphoid leukaemia	Male	0-6 days	95+ years	Data Rich	1	2	Tobacco (cigarettes per capita)	141
Acute lymphoid leukaemia	Male	0-6 days	95+ years	Data Rich	1	2	Mean BMI	508
Acute lymphoid leukaemia	Male	0-6 days	95+ years	Data Rich	1	2	Alcohol (liters per capita)	--
Acute lymphoid leukaemia	Male	0-6 days	95+ years	Data Rich	1	2	Cumulative Cigarettes (10 Years)	--
Acute lymphoid leukaemia	Male	0-6 days	95+ years	Data Rich	1	2	Cumulative Cigarettes (15 Years)	--
Acute lymphoid leukaemia	Male	0-6 days	95+ years	Data Rich	1	2	Cumulative Cigarettes (20 Years)	--
Acute lymphoid leukaemia	Male	0-6 days	95+ years	Data Rich	1	2	Cumulative Cigarettes (5 Years)	--
Acute lymphoid leukaemia	Male	0-6 days	95+ years	Data Rich	1	2	Smoking Prevalence	--
Acute lymphoid leukaemia	Male	0-6 days	95+ years	Data Rich	-1	3	Education (years per capita)	171
Acute lymphoid leukaemia	Male	0-6 days	95+ years	Data Rich	0	3	Socio-demographic Index	539
Acute lymphoid leukaemia	Male	0-6 days	95+ years	Data Rich	0	3	LDI (I$ per capita)	579
Acute lymphoid leukaemia	Male	0-6 days	95+ years	Global	1	1	Log-transformed SEV scalar: leukaemia	--
Acute lymphoid leukaemia	Male	0-6 days	95+ years	Global	1	1	Log-transformed age-standardized SEV scalar: leukaemia	--
Acute lymphoid leukaemia	Male	0-6 days	95+ years	Global	-1	2	Healthcare access and quality index	--
Acute lymphoid leukaemia	Male	0-6 days	95+ years	Global	1	2	Cumulative Cigarettes (20 Years)	12

Table S16. CODEm covariates used, level of covariate, and expected direction of covariate by cause, sex, and age
Covariates that CODEm did not select during the covariate selection process have no draw counts listed.

Cause	Sex	Age Start	Age End	Model Type	Direction	Level	Covariate Name	Number of Draws
Acute lymphoid leukaemia	Male	0-6 days	95+ years	Global	1	2	Smoking Prevalence	17
Acute lymphoid leukaemia	Male	0-6 days	95+ years	Global	1	2	Tobacco (cigarettes per capita)	131
Acute lymphoid leukaemia	Male	0-6 days	95+ years	Global	1	2	Mean BMI	289
Acute lymphoid leukaemia	Male	0-6 days	95+ years	Global	1	2	Alcohol (liters per capita)	--
Acute lymphoid leukaemia	Male	0-6 days	95+ years	Global	1	2	Cumulative Cigarettes (10 Years)	--
Acute lymphoid leukaemia	Male	0-6 days	95+ years	Global	1	2	Cumulative Cigarettes (15 Years)	--
Acute lymphoid leukaemia	Male	0-6 days	95+ years	Global	1	2	Cumulative Cigarettes (5 Years)	--
Acute lymphoid leukaemia	Male	0-6 days	95+ years	Global	-1	3	Education (years per capita)	103
Acute lymphoid leukaemia	Male	0-6 days	95+ years	Global	0	3	Socio-demographic Index	235
Acute lymphoid leukaemia	Male	0-6 days	95+ years	Global	0	3	LDI (I$ per capita)	536
Chronic lymphoid leukaemia	Female	15-19 years	95+ years	Data Rich	1	1	Log-transformed SEV scalar: leukaemia	--
Chronic lymphoid leukaemia	Female	15-19 years	95+ years	Data Rich	1	1	Log-transformed age-standardized SEV scalar: leukaemia	--
Chronic lymphoid leukaemia	Female	15-19 years	95+ years	Data Rich	-1	2	Healthcare access and quality index	102
Chronic lymphoid leukaemia	Female	15-19 years	95+ years	Data Rich	1	2	Cumulative Cigarettes (20 Years)	40
Chronic lymphoid leukaemia	Female	15-19 years	95+ years	Data Rich	1	2	Cumulative Cigarettes (5 Years)	53
Chronic lymphoid leukaemia	Female	15-19 years	95+ years	Data Rich	1	2	Cumulative Cigarettes (10 Years)	109
Chronic lymphoid leukaemia	Female	15-19 years	95+ years	Data Rich	1	2	Tobacco (cigarettes per capita)	180
Chronic lymphoid leukaemia	Female	15-19 years	95+ years	Data Rich	1	2	Smoking Prevalence	322
Chronic lymphoid leukaemia	Female	15-19 years	95+ years	Data Rich	1	2	Mean BMI	429
Chronic lymphoid leukaemia	Female	15-19 years	95+ years	Data Rich	1	2	Alcohol (liters per capita)	568
Chronic lymphoid leukaemia	Female	15-19 years	95+ years	Data Rich	1	2	Cumulative Cigarettes (15 Years)	594
Chronic lymphoid leukaemia	Female	15-19 years	95+ years	Data Rich	-1	3	Education (years per capita)	212
Chronic lymphoid leukaemia	Female	15-19 years	95+ years	Data Rich	0	3	Socio-demographic Index	46
Chronic lymphoid leukaemia	Female	15-19 years	95+ years	Data Rich	0	3	LDI (I$ per capita)	347
Chronic lymphoid leukaemia	Female	15-19 years	95+ years	Global	1	1	Healthcare access and quality index	814
Chronic lymphoid leukaemia	Female	15-19 years	95+ years	Global	1	1	Log-transformed SEV scalar: leukaemia	--
Chronic lymphoid leukaemia	Female	15-19 years	95+ years	Global	1	1	Log-transformed age-standardized SEV scalar: leukaemia	--
Chronic lymphoid leukaemia	Female	15-19 years	95+ years	Global	1	2	Mean BMI	0
Chronic lymphoid leukaemia	Female	15-19 years	95+ years	Global	1	2	Cumulative Cigarettes (5 Years)	111
Chronic lymphoid leukaemia	Female	15-19 years	95+ years	Global	1	2	Cumulative Cigarettes (15 Years)	206
Chronic lymphoid leukaemia	Female	15-19 years	95+ years	Global	1	2	Tobacco (cigarettes per capita)	239
Chronic lymphoid leukaemia	Female	15-19 years	95+ years	Global	1	2	Smoking Prevalence	338
Chronic lymphoid leukaemia	Female	15-19 years	95+ years	Global	1	2	Cumulative Cigarettes (10 Years)	552
Chronic lymphoid leukaemia	Female	15-19 years	95+ years	Global	1	2	Cumulative Cigarettes (20 Years)	600
Chronic lymphoid leukaemia	Female	15-19 years	95+ years	Global	1	2	Alcohol (liters per capita)	676
Chronic lymphoid leukaemia	Female	15-19 years	95+ years	Global	-1	3	Education (years per capita)	25
Chronic lymphoid leukaemia	Female	15-19 years	95+ years	Global	0	3	LDI (I$ per capita)	223
Chronic lymphoid leukaemia	Female	15-19 years	95+ years	Global	0	3	Socio-demographic Index	229
Chronic lymphoid leukaemia	Male	15-19 years	95+ years	Data Rich	1	1	Log-transformed SEV scalar: leukaemia	--
Chronic lymphoid leukaemia	Male	15-19 years	95+ years	Data Rich	1	1	Log-transformed age-standardized SEV scalar: leukaemia	--
Chronic lymphoid leukaemia	Male	15-19 years	95+ years	Data Rich	-1	2	Healthcare access and quality index	226
Chronic lymphoid leukaemia	Male	15-19 years	95+ years	Data Rich	1	2	Tobacco (cigarettes per capita)	16
Chronic lymphoid leukaemia	Male	15-19 years	95+ years	Data Rich	1	2	Cumulative Cigarettes (10 Years)	42

Table S16. CODEm covariates used, level of covariate, and expected direction of covariate by cause, sex, and age<br>*Covariates that CODEm did not select during the covariate selection process have no draw counts listed.*								
**Cause**	**Sex**	**Age Start**	**Age End**	**Model Type**	**Direction**	**Level**	**Covariate Name**	**Number of Draws**
Chronic lymphoid leukaemia	Male	15-19 years	95+ years	Data Rich	1	2	Cumulative Cigarettes (20 Years)	75
Chronic lymphoid leukaemia	Male	15-19 years	95+ years	Data Rich	1	2	Cumulative Cigarettes (15 Years)	107
Chronic lymphoid leukaemia	Male	15-19 years	95+ years	Data Rich	1	2	Mean BMI	578
Chronic lymphoid leukaemia	Male	15-19 years	95+ years	Data Rich	1	2	Alcohol (liters per capita)	--
Chronic lymphoid leukaemia	Male	15-19 years	95+ years	Data Rich	1	2	Cumulative Cigarettes (5 Years)	--
Chronic lymphoid leukaemia	Male	15-19 years	95+ years	Data Rich	1	2	Smoking Prevalence	--
Chronic lymphoid leukaemia	Male	15-19 years	95+ years	Data Rich	-1	3	Education (years per capita)	12
Chronic lymphoid leukaemia	Male	15-19 years	95+ years	Data Rich	0	3	LDI (I$ per capita)	253
Chronic lymphoid leukaemia	Male	15-19 years	95+ years	Data Rich	0	3	Socio-demographic Index	373
Chronic lymphoid leukaemia	Male	15-19 years	95+ years	Global	1	1	Log-transformed SEV scalar: leukaemia	--
Chronic lymphoid leukaemia	Male	15-19 years	95+ years	Global	1	1	Log-transformed age-standardized SEV scalar: leukaemia	--
Chronic lymphoid leukaemia	Male	15-19 years	95+ years	Global	-1	2	Healthcare access and quality index	250
Chronic lymphoid leukaemia	Male	15-19 years	95+ years	Global	1	2	Tobacco (cigarettes per capita)	22
Chronic lymphoid leukaemia	Male	15-19 years	95+ years	Global	1	2	Cumulative Cigarettes (15 Years)	27
Chronic lymphoid leukaemia	Male	15-19 years	95+ years	Global	1	2	Cumulative Cigarettes (10 Years)	137
Chronic lymphoid leukaemia	Male	15-19 years	95+ years	Global	1	2	Cumulative Cigarettes (20 Years)	137
Chronic lymphoid leukaemia	Male	15-19 years	95+ years	Global	1	2	Cumulative Cigarettes (5 Years)	146
Chronic lymphoid leukaemia	Male	15-19 years	95+ years	Global	1	2	Mean BMI	287
Chronic lymphoid leukaemia	Male	15-19 years	95+ years	Global	1	2	Alcohol (liters per capita)	--
Chronic lymphoid leukaemia	Male	15-19 years	95+ years	Global	1	2	Smoking Prevalence	--
Chronic lymphoid leukaemia	Male	15-19 years	95+ years	Global	-1	3	Education (years per capita)	55
Chronic lymphoid leukaemia	Male	15-19 years	95+ years	Global	0	3	Socio-demographic Index	345
Chronic lymphoid leukaemia	Male	15-19 years	95+ years	Global	0	3	LDI (I$ per capita)	469
Acute myeloid leukaemia	Female	0-6 days	95+ years	Data Rich	1	1	Log-transformed SEV scalar: leukaemia	--
Acute myeloid leukaemia	Female	0-6 days	95+ years	Data Rich	1	1	Log-transformed age-standardized SEV scalar: leukaemia	--
Acute myeloid leukaemia	Female	0-6 days	95+ years	Data Rich	-1	2	Healthcare access and quality index	--
Acute myeloid leukaemia	Female	0-6 days	95+ years	Data Rich	1	2	Tobacco (cigarettes per capita)	147
Acute myeloid leukaemia	Female	0-6 days	95+ years	Data Rich	1	2	Alcohol (liters per capita)	356
Acute myeloid leukaemia	Female	0-6 days	95+ years	Data Rich	1	2	Cumulative Cigarettes (10 Years)	--
Acute myeloid leukaemia	Female	0-6 days	95+ years	Data Rich	1	2	Cumulative Cigarettes (15 Years)	--
Acute myeloid leukaemia	Female	0-6 days	95+ years	Data Rich	1	2	Cumulative Cigarettes (20 Years)	--
Acute myeloid leukaemia	Female	0-6 days	95+ years	Data Rich	1	2	Cumulative Cigarettes (5 Years)	--
Acute myeloid leukaemia	Female	0-6 days	95+ years	Data Rich	1	2	Mean BMI	--
Acute myeloid leukaemia	Female	0-6 days	95+ years	Data Rich	1	2	Smoking Prevalence	--
Acute myeloid leukaemia	Female	0-6 days	95+ years	Data Rich	-1	3	Education (years per capita)	--
Acute myeloid leukaemia	Female	0-6 days	95+ years	Data Rich	0	3	LDI (I$ per capita)	--
Acute myeloid leukaemia	Female	0-6 days	95+ years	Data Rich	0	3	Socio-demographic Index	--
Acute myeloid leukaemia	Female	0-6 days	95+ years	Global	1	1	Log-transformed SEV scalar: leukaemia	--
Acute myeloid leukaemia	Female	0-6 days	95+ years	Global	1	1	Log-transformed age-standardized SEV scalar: leukaemia	--
Acute myeloid leukaemia	Female	0-6 days	95+ years	Global	-1	2	Healthcare access and quality index	--
Acute myeloid leukaemia	Female	0-6 days	95+ years	Global	1	2	Tobacco (cigarettes per capita)	42
Acute myeloid leukaemia	Female	0-6 days	95+ years	Global	1	2	Cumulative Cigarettes (10 Years)	46
Acute myeloid leukaemia	Female	0-6 days	95+ years	Global	1	2	Cumulative Cigarettes (5 Years)	49
Acute myeloid leukaemia	Female	0-6 days	95+ years	Global	1	2	Alcohol (liters per capita)	485
Acute myeloid leukaemia	Female	0-6 days	95+ years	Global	1	2	Cumulative Cigarettes (15 Years)	--
Acute myeloid leukaemia	Female	0-6 days	95+ years	Global	1	2	Cumulative Cigarettes (20 Years)	--
Acute myeloid leukaemia	Female	0-6 days	95+ years	Global	1	2	Mean BMI	--

Table S16. CODEm covariates used, level of covariate, and expected direction of covariate by cause, sex, and age<br>*Covariates that CODEm did not select during the covariate selection process have no draw counts listed.*								
**Cause**	**Sex**	**Age Start**	**Age End**	**Model Type**	**Direction**	**Level**	**Covariate Name**	**Number of Draws**
Acute myeloid leukaemia	Female	0-6 days	95+ years	Global	1	2	Smoking Prevalence	--
Acute myeloid leukaemia	Female	0-6 days	95+ years	Global	-1	3	Education (years per capita)	--
Acute myeloid leukaemia	Female	0-6 days	95+ years	Global	0	3	Socio-demographic Index	192
Acute myeloid leukaemia	Female	0-6 days	95+ years	Global	0	3	LDI (I$ per capita)	--
Acute myeloid leukaemia	Male	0-6 days	95+ years	Data Rich	1	1	Log-transformed SEV scalar: leukaemia	--
Acute myeloid leukaemia	Male	0-6 days	95+ years	Data Rich	1	1	Log-transformed age-standardized SEV scalar: leukaemia	--
Acute myeloid leukaemia	Male	0-6 days	95+ years	Data Rich	-1	2	Healthcare access and quality index	--
Acute myeloid leukaemia	Male	0-6 days	95+ years	Data Rich	1	2	Tobacco (cigarettes per capita)	105
Acute myeloid leukaemia	Male	0-6 days	95+ years	Data Rich	1	2	Alcohol (liters per capita)	271
Acute myeloid leukaemia	Male	0-6 days	95+ years	Data Rich	1	2	Cumulative Cigarettes (5 Years)	271
Acute myeloid leukaemia	Male	0-6 days	95+ years	Data Rich	1	2	Mean BMI	705
Acute myeloid leukaemia	Male	0-6 days	95+ years	Data Rich	1	2	Cumulative Cigarettes (10 Years)	--
Acute myeloid leukaemia	Male	0-6 days	95+ years	Data Rich	1	2	Cumulative Cigarettes (15 Years)	--
Acute myeloid leukaemia	Male	0-6 days	95+ years	Data Rich	1	2	Cumulative Cigarettes (20 Years)	--
Acute myeloid leukaemia	Male	0-6 days	95+ years	Data Rich	1	2	Smoking Prevalence	--
Acute myeloid leukaemia	Male	0-6 days	95+ years	Data Rich	-1	3	Education (years per capita)	12
Acute myeloid leukaemia	Male	0-6 days	95+ years	Data Rich	0	3	Socio-demographic Index	281
Acute myeloid leukaemia	Male	0-6 days	95+ years	Data Rich	0	3	LDI (I$ per capita)	--
Acute myeloid leukaemia	Male	0-6 days	95+ years	Global	1	1	Log-transformed SEV scalar: leukaemia	--
Acute myeloid leukaemia	Male	0-6 days	95+ years	Global	1	1	Log-transformed age-standardized SEV scalar: leukaemia	--
Acute myeloid leukaemia	Male	0-6 days	95+ years	Global	-1	2	Healthcare access and quality index	--
Acute myeloid leukaemia	Male	0-6 days	95+ years	Global	1	2	Cumulative Cigarettes (5 Years)	184
Acute myeloid leukaemia	Male	0-6 days	95+ years	Global	1	2	Mean BMI	189
Acute myeloid leukaemia	Male	0-6 days	95+ years	Global	1	2	Tobacco (cigarettes per capita)	259
Acute myeloid leukaemia	Male	0-6 days	95+ years	Global	1	2	Alcohol (liters per capita)	288
Acute myeloid leukaemia	Male	0-6 days	95+ years	Global	1	2	Cumulative Cigarettes (10 Years)	--
Acute myeloid leukaemia	Male	0-6 days	95+ years	Global	1	2	Cumulative Cigarettes (15 Years)	--
Acute myeloid leukaemia	Male	0-6 days	95+ years	Global	1	2	Cumulative Cigarettes (20 Years)	--
Acute myeloid leukaemia	Male	0-6 days	95+ years	Global	1	2	Smoking Prevalence	--
Acute myeloid leukaemia	Male	0-6 days	95+ years	Global	-1	3	Education (years per capita)	--
Acute myeloid leukaemia	Male	0-6 days	95+ years	Global	0	3	Socio-demographic Index	307
Acute myeloid leukaemia	Male	0-6 days	95+ years	Global	0	3	LDI (I$ per capita)	--
Chronic myeloid leukaemia	Female	28-364 days	95+ years	Data Rich	1	1	Log-transformed SEV scalar: leukaemia	--
Chronic myeloid leukaemia	Female	28-364 days	95+ years	Data Rich	1	1	Log-transformed age-standardized SEV scalar: leukaemia	--
Chronic myeloid leukaemia	Female	28-364 days	95+ years	Data Rich	-1	2	Healthcare access and quality index	702
Chronic myeloid leukaemia	Female	28-364 days	95+ years	Data Rich	1	2	Cumulative Cigarettes (5 Years)	50
Chronic myeloid leukaemia	Female	28-364 days	95+ years	Data Rich	1	2	Cumulative Cigarettes (10 Years)	118
Chronic myeloid leukaemia	Female	28-364 days	95+ years	Data Rich	1	2	Cumulative Cigarettes (20 Years)	239
Chronic myeloid leukaemia	Female	28-364 days	95+ years	Data Rich	1	2	Cumulative Cigarettes (15 Years)	317
Chronic myeloid leukaemia	Female	28-364 days	95+ years	Data Rich	1	2	Smoking Prevalence	327
Chronic myeloid leukaemia	Female	28-364 days	95+ years	Data Rich	1	2	Alcohol (liters per capita)	432
Chronic myeloid leukaemia	Female	28-364 days	95+ years	Data Rich	1	2	Tobacco (cigarettes per capita)	507
Chronic myeloid leukaemia	Female	28-364 days	95+ years	Data Rich	1	2	Mean BMI	--
Chronic myeloid leukaemia	Female	28-364 days	95+ years	Data Rich	-1	3	Education (years per capita)	418
Chronic myeloid leukaemia	Female	28-364 days	95+ years	Data Rich	0	3	Socio-demographic Index	673
Chronic myeloid leukaemia	Female	28-364 days	95+ years	Data Rich	0	3	LDI (I$ per capita)	--
Chronic myeloid leukaemia	Female	28-364 days	95+ years	Global	1	1	Log-transformed SEV scalar: leukaemia	--
Chronic myeloid leukaemia	Female	28-364 days	95+ years	Global	1	1	Log-transformed age-standardized SEV scalar: leukaemia	--
Chronic myeloid leukaemia	Female	28-364 days	95+ years	Global	-1	2	Healthcare access and quality index	939

**Table S16. CODEm covariates used, level of covariate, and expected direction of covariate by cause, sex, and age** *Covariates that CODEm did not select during the covariate selection process have no draw counts listed.*								
**Cause**	**Sex**	**Age Start**	**Age End**	**Model Type**	**Direction**	**Level**	**Covariate Name**	**Number of Draws**
Chronic myeloid leukaemia	Female	28-364 days	95+ years	Global	1	2	Cumulative Cigarettes (5 Years)	109
Chronic myeloid leukaemia	Female	28-364 days	95+ years	Global	1	2	Cumulative Cigarettes (15 Years)	148
Chronic myeloid leukaemia	Female	28-364 days	95+ years	Global	1	2	Cumulative Cigarettes (20 Years)	265
Chronic myeloid leukaemia	Female	28-364 days	95+ years	Global	1	2	Cumulative Cigarettes (10 Years)	417
Chronic myeloid leukaemia	Female	28-364 days	95+ years	Global	1	2	Smoking Prevalence	501
Chronic myeloid leukaemia	Female	28-364 days	95+ years	Global	1	2	Tobacco (cigarettes per capita)	669
Chronic myeloid leukaemia	Female	28-364 days	95+ years	Global	1	2	Alcohol (liters per capita)	778
Chronic myeloid leukaemia	Female	28-364 days	95+ years	Global	1	2	Mean BMI	--
Chronic myeloid leukaemia	Female	28-364 days	95+ years	Global	-1	3	Education (years per capita)	131
Chronic myeloid leukaemia	Female	28-364 days	95+ years	Global	0	3	Socio-demographic Index	217
Chronic myeloid leukaemia	Female	28-364 days	95+ years	Global	0	3	LDI (I$ per capita)	--
Chronic myeloid leukaemia	Male	28-364 days	95+ years	Data Rich	1	1	Log-transformed SEV scalar: leukaemia	--
Chronic myeloid leukaemia	Male	28-364 days	95+ years	Data Rich	1	1	Log-transformed age-standardized SEV scalar: leukaemia	--
Chronic myeloid leukaemia	Male	28-364 days	95+ years	Data Rich	-1	2	Healthcare access and quality index	274
Chronic myeloid leukaemia	Male	28-364 days	95+ years	Data Rich	1	2	Cumulative Cigarettes (20 Years)	37
Chronic myeloid leukaemia	Male	28-364 days	95+ years	Data Rich	1	2	Cumulative Cigarettes (15 Years)	47
Chronic myeloid leukaemia	Male	28-364 days	95+ years	Data Rich	1	2	Tobacco (cigarettes per capita)	54
Chronic myeloid leukaemia	Male	28-364 days	95+ years	Data Rich	1	2	Cumulative Cigarettes (10 Years)	79
Chronic myeloid leukaemia	Male	28-364 days	95+ years	Data Rich	1	2	Cumulative Cigarettes (5 Years)	111
Chronic myeloid leukaemia	Male	28-364 days	95+ years	Data Rich	1	2	Alcohol (liters per capita)	321
Chronic myeloid leukaemia	Male	28-364 days	95+ years	Data Rich	1	2	Smoking Prevalence	496
Chronic myeloid leukaemia	Male	28-364 days	95+ years	Data Rich	1	2	Mean BMI	--
Chronic myeloid leukaemia	Male	28-364 days	95+ years	Data Rich	-1	3	Education (years per capita)	274
Chronic myeloid leukaemia	Male	28-364 days	95+ years	Data Rich	0	3	Socio-demographic Index	286
Chronic myeloid leukaemia	Male	28-364 days	95+ years	Data Rich	0	3	LDI (I$ per capita)	--
Chronic myeloid leukaemia	Male	28-364 days	95+ years	Global	1	1	Log-transformed SEV scalar: leukaemia	--
Chronic myeloid leukaemia	Male	28-364 days	95+ years	Global	1	1	Log-transformed age-standardized SEV scalar: leukaemia	--
Chronic myeloid leukaemia	Male	28-364 days	95+ years	Global	-1	2	Healthcare access and quality index	224
Chronic myeloid leukaemia	Male	28-364 days	95+ years	Global	1	2	Alcohol (liters per capita)	55
Chronic myeloid leukaemia	Male	28-364 days	95+ years	Global	1	2	Cumulative Cigarettes (5 Years)	83
Chronic myeloid leukaemia	Male	28-364 days	95+ years	Global	1	2	Cumulative Cigarettes (10 Years)	187
Chronic myeloid leukaemia	Male	28-364 days	95+ years	Global	1	2	Cumulative Cigarettes (15 Years)	293
Chronic myeloid leukaemia	Male	28-364 days	95+ years	Global	1	2	Smoking Prevalence	320
Chronic myeloid leukaemia	Male	28-364 days	95+ years	Global	1	2	Cumulative Cigarettes (20 Years)	397
Chronic myeloid leukaemia	Male	28-364 days	95+ years	Global	1	2	Tobacco (cigarettes per capita)	790
Chronic myeloid leukaemia	Male	28-364 days	95+ years	Global	1	2	Mean BMI	--
Chronic myeloid leukaemia	Male	28-364 days	95+ years	Global	-1	3	Education (years per capita)	374
Chronic myeloid leukaemia	Male	28-364 days	95+ years	Global	0	3	Socio-demographic Index	302
Chronic myeloid leukaemia	Male	28-364 days	95+ years	Global	0	3	LDI (I$ per capita)	--
Other leukaemia	Female	0-6 days	95+ years	Data Rich	1	1	Log-transformed SEV scalar: leukaemia	--

Table S16. CODEm covariates used, level of covariate, and expected direction of covariate by cause, sex, and age<br>*Covariates that CODEm did not select during the covariate selection process have no draw counts listed.*								
**Cause**	**Sex**	**Age Start**	**Age End**	**Model Type**	**Direction**	**Level**	**Covariate Name**	**Number of Draws**
Other leukaemia	Female	0-6 days	95+ years	Data Rich	1	1	Log-transformed age-standardized SEV scalar: leukaemia	--
Other leukaemia	Female	0-6 days	95+ years	Data Rich	-1	2	Healthcare access and quality index	380
Other leukaemia	Female	0-6 days	95+ years	Data Rich	1	2	Cumulative Cigarettes (15 Years)	206
Other leukaemia	Female	0-6 days	95+ years	Data Rich	1	2	Cumulative Cigarettes (20 Years)	245
Other leukaemia	Female	0-6 days	95+ years	Data Rich	1	2	Smoking Prevalence	267
Other leukaemia	Female	0-6 days	95+ years	Data Rich	1	2	Cumulative Cigarettes (5 Years)	279
Other leukaemia	Female	0-6 days	95+ years	Data Rich	1	2	Cumulative Cigarettes (10 Years)	459
Other leukaemia	Female	0-6 days	95+ years	Data Rich	1	2	Alcohol (liters per capita)	--
Other leukaemia	Female	0-6 days	95+ years	Data Rich	1	2	Mean BMI	--
Other leukaemia	Female	0-6 days	95+ years	Data Rich	1	2	Tobacco (cigarettes per capita)	--
Other leukaemia	Female	0-6 days	95+ years	Data Rich	-1	3	Education (years per capita)	18
Other leukaemia	Female	0-6 days	95+ years	Data Rich	0	3	LDI (I$ per capita)	327
Other leukaemia	Female	0-6 days	95+ years	Data Rich	0	3	Socio-demographic Index	357
Other leukaemia	Female	0-6 days	95+ years	Global	1	1	Log-transformed SEV scalar: leukaemia	--
Other leukaemia	Female	0-6 days	95+ years	Global	1	1	Log-transformed age-standardized SEV scalar: leukaemia	--
Other leukaemia	Female	0-6 days	95+ years	Global	-1	2	Healthcare access and quality index	--
Other leukaemia	Female	0-6 days	95+ years	Global	1	2	Smoking Prevalence	51
Other leukaemia	Female	0-6 days	95+ years	Global	1	2	Cumulative Cigarettes (10 Years)	97
Other leukaemia	Female	0-6 days	95+ years	Global	1	2	Cumulative Cigarettes (20 Years)	151
Other leukaemia	Female	0-6 days	95+ years	Global	1	2	Cumulative Cigarettes (15 Years)	174
Other leukaemia	Female	0-6 days	95+ years	Global	1	2	Cumulative Cigarettes (5 Years)	458
Other leukaemia	Female	0-6 days	95+ years	Global	1	2	Alcohol (liters per capita)	--
Other leukaemia	Female	0-6 days	95+ years	Global	1	2	Mean BMI	--
Other leukaemia	Female	0-6 days	95+ years	Global	1	2	Tobacco (cigarettes per capita)	--
Other leukaemia	Female	0-6 days	95+ years	Global	-1	3	Education (years per capita)	249
Other leukaemia	Female	0-6 days	95+ years	Global	0	3	LDI (I$ per capita)	401
Other leukaemia	Female	0-6 days	95+ years	Global	0	3	Socio-demographic Index	513
Other leukaemia	Male	0-6 days	95+ years	Data Rich	1	1	Log-transformed SEV scalar: leukaemia	--
Other leukaemia	Male	0-6 days	95+ years	Data Rich	1	1	Log-transformed age-standardized SEV scalar: leukaemia	--
Other leukaemia	Male	0-6 days	95+ years	Data Rich	-1	2	Healthcare access and quality index	70
Other leukaemia	Male	0-6 days	95+ years	Data Rich	1	2	Cumulative Cigarettes (10 Years)	38
Other leukaemia	Male	0-6 days	95+ years	Data Rich	1	2	Smoking Prevalence	39
Other leukaemia	Male	0-6 days	95+ years	Data Rich	1	2	Tobacco (cigarettes per capita)	103
Other leukaemia	Male	0-6 days	95+ years	Data Rich	1	2	Cumulative Cigarettes (20 Years)	125
Other leukaemia	Male	0-6 days	95+ years	Data Rich	1	2	Cumulative Cigarettes (15 Years)	182
Other leukaemia	Male	0-6 days	95+ years	Data Rich	1	2	Cumulative Cigarettes (5 Years)	493
Other leukaemia	Male	0-6 days	95+ years	Data Rich	1	2	Alcohol (liters per capita)	--
Other leukaemia	Male	0-6 days	95+ years	Data Rich	1	2	Mean BMI	--
Other leukaemia	Male	0-6 days	95+ years	Data Rich	-1	3	Education (years per capita)	96
Other leukaemia	Male	0-6 days	95+ years	Data Rich	0	3	Socio-demographic Index	382
Other leukaemia	Male	0-6 days	95+ years	Data Rich	0	3	LDI (I$ per capita)	439
Other leukaemia	Male	0-6 days	95+ years	Global	1	1	Log-transformed SEV scalar: leukaemia	--
Other leukaemia	Male	0-6 days	95+ years	Global	1	1	Log-transformed age-standardized SEV scalar: leukaemia	--
Other leukaemia	Male	0-6 days	95+ years	Global	-1	2	Healthcare access and quality index	122
Other leukaemia	Male	0-6 days	95+ years	Global	1	2	Mean BMI	38
Other leukaemia	Male	0-6 days	95+ years	Global	1	2	Cumulative Cigarettes (20 Years)	107
Other leukaemia	Male	0-6 days	95+ years	Global	1	2	Tobacco (cigarettes per capita)	122
Other leukaemia	Male	0-6 days	95+ years	Global	1	2	Cumulative Cigarettes (15 Years)	181
Other leukaemia	Male	0-6 days	95+ years	Global	1	2	Cumulative Cigarettes (10 Years)	361
Other leukaemia	Male	0-6 days	95+ years	Global	1	2	Cumulative Cigarettes (5 Years)	452
Other leukaemia	Male	0-6 days	95+ years	Global	1	2	Alcohol (liters per capita)	510
Other leukaemia	Male	0-6 days	95+ years	Global	1	2	Smoking Prevalence	--
Other leukaemia	Male	0-6 days	95+ years	Global	-1	3	Education (years per capita)	82
Other leukaemia	Male	0-6 days	95+ years	Global	0	3	Socio-demographic Index	82
Other leukaemia	Male	0-6 days	95+ years	Global	0	3	LDI (I$ per capita)	498
Other malignant cancers	Female	0-6 days	95+ years	Data Rich	1	1	Tobacco (cigarettes per capita)	764
Other malignant cancers	Female	0-6 days	95+ years	Data Rich	1	1	Smoking Prevalence	--

Table S16. CODEm covariates used, level of covariate, and expected direction of covariate by cause, sex, and age<br>*Covariates that CODEm did not select during the covariate selection process have no draw counts listed.*								
**Cause**	**Sex**	**Age Start**	**Age End**	**Model Type**	**Direction**	**Level**	**Covariate Name**	**Number of Draws**
Other malignant cancers	Female	0-6 days	95+ years	Data Rich	-1	2	vegetables adjusted(g)	111
Other malignant cancers	Female	0-6 days	95+ years	Data Rich	-1	2	Healthcare access and quality index	352
Other malignant cancers	Female	0-6 days	95+ years	Data Rich	-1	2	fruits adjusted(g)	--
Other malignant cancers	Female	0-6 days	95+ years	Data Rich	-1	2	nuts seeds adjusted(g)	--
Other malignant cancers	Female	0-6 days	95+ years	Data Rich	-1	2	pufa adjusted(percent)	--
Other malignant cancers	Female	0-6 days	95+ years	Data Rich	-1	3	Education (years per capita)	--
Other malignant cancers	Female	0-6 days	95+ years	Data Rich	0	3	LDI (I$ per capita)	328
Other malignant cancers	Female	0-6 days	95+ years	Data Rich	0	3	Socio-demographic Index	529
Other malignant cancers	Female	0-6 days	95+ years	Global	1	1	Tobacco (cigarettes per capita)	669
Other malignant cancers	Female	0-6 days	95+ years	Global	1	1	Smoking Prevalence	--
Other malignant cancers	Female	0-6 days	95+ years	Global	-1	2	fruits adjusted(g)	51
Other malignant cancers	Female	0-6 days	95+ years	Global	-1	2	nuts seeds adjusted(g)	78
Other malignant cancers	Female	0-6 days	95+ years	Global	-1	2	Healthcare access and quality index	233
Other malignant cancers	Female	0-6 days	95+ years	Global	-1	2	pufa adjusted(percent)	233
Other malignant cancers	Female	0-6 days	95+ years	Global	-1	2	vegetables adjusted(g)	233
Other malignant cancers	Female	0-6 days	95+ years	Global	-1	3	Education (years per capita)	100
Other malignant cancers	Female	0-6 days	95+ years	Global	0	3	Socio-demographic Index	580
Other malignant cancers	Female	0-6 days	95+ years	Global	0	3	LDI (I$ per capita)	680
Other malignant cancers	Male	0-6 days	95+ years	Data Rich	1	1	Smoking Prevalence	392
Other malignant cancers	Male	0-6 days	95+ years	Data Rich	1	1	Tobacco (cigarettes per capita)	394
Other malignant cancers	Male	0-6 days	95+ years	Data Rich	-1	2	vegetables adjusted(g)	57
Other malignant cancers	Male	0-6 days	95+ years	Data Rich	-1	2	nuts seeds adjusted(g)	251
Other malignant cancers	Male	0-6 days	95+ years	Data Rich	-1	2	pufa adjusted(percent)	251
Other malignant cancers	Male	0-6 days	95+ years	Data Rich	-1	2	Healthcare access and quality index	--
Other malignant cancers	Male	0-6 days	95+ years	Data Rich	-1	2	fruits adjusted(g)	--
Other malignant cancers	Male	0-6 days	95+ years	Data Rich	-1	3	Education (years per capita)	388
Other malignant cancers	Male	0-6 days	95+ years	Data Rich	0	3	LDI (I$ per capita)	341
Other malignant cancers	Male	0-6 days	95+ years	Data Rich	0	3	Socio-demographic Index	580
Other malignant cancers	Male	0-6 days	95+ years	Global	1	1	Tobacco (cigarettes per capita)	745
Other malignant cancers	Male	0-6 days	95+ years	Global	1	1	Smoking Prevalence	--
Other malignant cancers	Male	0-6 days	95+ years	Global	-1	2	fruits adjusted(g)	105
Other malignant cancers	Male	0-6 days	95+ years	Global	-1	2	Healthcare access and quality index	333
Other malignant cancers	Male	0-6 days	95+ years	Global	-1	2	pufa adjusted(percent)	438
Other malignant cancers	Male	0-6 days	95+ years	Global	-1	2	nuts seeds adjusted(g)	--
Other malignant cancers	Male	0-6 days	95+ years	Global	-1	2	vegetables adjusted(g)	--
Other malignant cancers	Male	0-6 days	95+ years	Global	-1	3	Education (years per capita)	374
Other malignant cancers	Male	0-6 days	95+ years	Global	0	3	Socio-demographic Index	437
Other malignant cancers	Male	0-6 days	95+ years	Global	0	3	LDI (I$ per capita)	629
Other neoplasms	Female	0-6 days	95+ years	Data Rich	-1	2	Healthcare access and quality index	--
Other neoplasms	Female	0-6 days	95+ years	Data Rich	-1	3	Education (years per capita)	--
Other neoplasms	Female	0-6 days	95+ years	Data Rich	0	3	LDI (I$ per capita)	1000
Other neoplasms	Female	0-6 days	95+ years	Data Rich	0	3	Socio-demographic Index	1000
Other neoplasms	Female	0-6 days	95+ years	Global	-1	2	Healthcare access and quality index	--
Other neoplasms	Female	0-6 days	95+ years	Global	-1	3	Education (years per capita)	--
Other neoplasms	Female	0-6 days	95+ years	Global	0	3	Socio-demographic Index	546
Other neoplasms	Female	0-6 days	95+ years	Global	0	3	LDI (I$ per capita)	1000
Other neoplasms	Male	0-6 days	95+ years	Data Rich	-1	2	Healthcare access and quality index	--
Other neoplasms	Male	0-6 days	95+ years	Data Rich	-1	3	Education (years per capita)	--
Other neoplasms	Male	0-6 days	95+ years	Data Rich	0	3	Socio-demographic Index	591
Other neoplasms	Male	0-6 days	95+ years	Data Rich	0	3	LDI (I$ per capita)	1000
Other neoplasms	Male	0-6 days	95+ years	Global	-1	2	Healthcare access and quality index	--
Other neoplasms	Male	0-6 days	95+ years	Global	-1	3	Education (years per capita)	--
Other neoplasms	Male	0-6 days	95+ years	Global	0	3	LDI (I$ per capita)	1000
Other neoplasms	Male	0-6 days	95+ years	Global	0	3	Socio-demographic Index	1000
Myelodysplastic, myeloproliferative, and other hematopoietic neoplasms	Female	0-6 days	95+ years	Data Rich	1	1	Log-transformed SEV scalar: leukaemia	--
Myelodysplastic, myeloproliferative, and other hematopoietic neoplasms	Female	0-6 days	95+ years	Data Rich	1	1	Log-transformed age-standardized SEV scalar: leukaemia	--
Myelodysplastic, myeloproliferative, and other hematopoietic neoplasms	Female	0-6 days	95+ years	Data Rich	-1	2	Healthcare access and quality index	--
Myelodysplastic, myeloproliferative, and other hematopoietic neoplasms	Female	0-6 days	95+ years	Data Rich	1	2	Alcohol (liters per capita)	74

Table S16. CODEm covariates used, level of covariate, and expected direction of covariate by cause, sex, and age
Covariates that CODEm did not select during the covariate selection process have no draw counts listed.

Cause	Sex	Age Start	Age End	Model Type	Direction	Level	Covariate Name	Number of Draws
Myelodysplastic, myeloproliferative, and other hematopoietic neoplasms	Female	0-6 days	95+ years	Data Rich	1	2	Cumulative Cigarettes (10 Years)	186
Myelodysplastic, myeloproliferative, and other hematopoietic neoplasms	Female	0-6 days	95+ years	Data Rich	1	2	Cumulative Cigarettes (5 Years)	231
Myelodysplastic, myeloproliferative, and other hematopoietic neoplasms	Female	0-6 days	95+ years	Data Rich	1	2	Cumulative Cigarettes (15 Years)	--
Myelodysplastic, myeloproliferative, and other hematopoietic neoplasms	Female	0-6 days	95+ years	Data Rich	1	2	Cumulative Cigarettes (20 Years)	--
Myelodysplastic, myeloproliferative, and other hematopoietic neoplasms	Female	0-6 days	95+ years	Data Rich	1	2	Smoking Prevalence	--
Myelodysplastic, myeloproliferative, and other hematopoietic neoplasms	Female	0-6 days	95+ years	Data Rich	1	2	Tobacco (cigarettes per capita)	--
Myelodysplastic, myeloproliferative, and other hematopoietic neoplasms	Female	0-6 days	95+ years	Data Rich	-1	3	Education (years per capita)	--
Myelodysplastic, myeloproliferative, and other hematopoietic neoplasms	Female	0-6 days	95+ years	Data Rich	0	3	Socio-demographic Index	449
Myelodysplastic, myeloproliferative, and other hematopoietic neoplasms	Female	0-6 days	95+ years	Data Rich	0	3	LDI (I$ per capita)	--
Myelodysplastic, myeloproliferative, and other hematopoietic neoplasms	Female	0-6 days	95+ years	Global	1	1	Log-transformed SEV scalar: leukaemia	--
Myelodysplastic, myeloproliferative, and other hematopoietic neoplasms	Female	0-6 days	95+ years	Global	1	1	Log-transformed age-standardized SEV scalar: leukaemia	--
Myelodysplastic, myeloproliferative, and other hematopoietic neoplasms	Female	0-6 days	95+ years	Global	-1	2	Healthcare access and quality index	--
Myelodysplastic, myeloproliferative, and other hematopoietic neoplasms	Female	0-6 days	95+ years	Global	1	2	Cumulative Cigarettes (5 Years)	3
Myelodysplastic, myeloproliferative, and other hematopoietic neoplasms	Female	0-6 days	95+ years	Global	1	2	Cumulative Cigarettes (10 Years)	19
Myelodysplastic, myeloproliferative, and other hematopoietic neoplasms	Female	0-6 days	95+ years	Global	1	2	Cumulative Cigarettes (15 Years)	33
Myelodysplastic, myeloproliferative, and other hematopoietic neoplasms	Female	0-6 days	95+ years	Global	1	2	Cumulative Cigarettes (20 Years)	92
Myelodysplastic, myeloproliferative, and other hematopoietic neoplasms	Female	0-6 days	95+ years	Global	1	2	Smoking Prevalence	139
Myelodysplastic, myeloproliferative, and other hematopoietic neoplasms	Female	0-6 days	95+ years	Global	1	2	Alcohol (liters per capita)	195
Myelodysplastic, myeloproliferative, and other hematopoietic neoplasms	Female	0-6 days	95+ years	Global	1	2	Tobacco (cigarettes per capita)	--
Myelodysplastic, myeloproliferative, and other hematopoietic neoplasms	Female	0-6 days	95+ years	Global	-1	3	Education (years per capita)	--
Myelodysplastic, myeloproliferative, and other hematopoietic neoplasms	Female	0-6 days	95+ years	Global	0	3	Socio-demographic Index	186
Myelodysplastic, myeloproliferative, and other hematopoietic neoplasms	Female	0-6 days	95+ years	Global	0	3	LDI (I$ per capita)	--
Myelodysplastic, myeloproliferative, and other hematopoietic neoplasms	Male	0-6 days	95+ years	Data Rich	1	1	Log-transformed SEV scalar: leukaemia	--
Myelodysplastic, myeloproliferative, and other hematopoietic neoplasms	Male	0-6 days	95+ years	Data Rich	1	1	Log-transformed age-standardized SEV scalar: leukaemia	--
Myelodysplastic, myeloproliferative, and other hematopoietic neoplasms	Male	0-6 days	95+ years	Data Rich	-1	2	Healthcare access and quality index	--
Myelodysplastic, myeloproliferative, and other hematopoietic neoplasms	Male	0-6 days	95+ years	Data Rich	1	2	Cumulative Cigarettes (20 Years)	88
Myelodysplastic, myeloproliferative, and other hematopoietic neoplasms	Male	0-6 days	95+ years	Data Rich	1	2	Cumulative Cigarettes (15 Years)	107
Myelodysplastic, myeloproliferative, and other hematopoietic neoplasms	Male	0-6 days	95+ years	Data Rich	1	2	Cumulative Cigarettes (10 Years)	128
Myelodysplastic, myeloproliferative, and other hematopoietic neoplasms	Male	0-6 days	95+ years	Data Rich	1	2	Alcohol (liters per capita)	--
Myelodysplastic, myeloproliferative, and other hematopoietic neoplasms	Male	0-6 days	95+ years	Data Rich	1	2	Cumulative Cigarettes (5 Years)	--
Myelodysplastic, myeloproliferative, and other hematopoietic neoplasms	Male	0-6 days	95+ years	Data Rich	1	2	Smoking Prevalence	--
Myelodysplastic, myeloproliferative, and other hematopoietic neoplasms	Male	0-6 days	95+ years	Data Rich	1	2	Tobacco (cigarettes per capita)	--
Myelodysplastic, myeloproliferative, and other hematopoietic neoplasms	Male	0-6 days	95+ years	Data Rich	-1	3	Education (years per capita)	--
Myelodysplastic, myeloproliferative, and other hematopoietic neoplasms	Male	0-6 days	95+ years	Data Rich	0	3	Socio-demographic Index	228
Myelodysplastic, myeloproliferative, and other hematopoietic neoplasms	Male	0-6 days	95+ years	Data Rich	0	3	LDI (I$ per capita)	--
Myelodysplastic, myeloproliferative, and other hematopoietic neoplasms	Male	0-6 days	95+ years	Global	1	1	Log-transformed SEV scalar: leukaemia	--
Myelodysplastic, myeloproliferative, and other hematopoietic neoplasms	Male	0-6 days	95+ years	Global	1	1	Log-transformed age-standardized SEV scalar: leukaemia	--
Myelodysplastic, myeloproliferative, and other hematopoietic neoplasms	Male	0-6 days	95+ years	Global	-1	2	Healthcare access and quality index	--
Myelodysplastic, myeloproliferative, and other hematopoietic neoplasms	Male	0-6 days	95+ years	Global	1	2	Alcohol (liters per capita)	--
Myelodysplastic, myeloproliferative, and other hematopoietic neoplasms	Male	0-6 days	95+ years	Global	1	2	Cumulative Cigarettes (10 Years)	--

Table S16. CODEm covariates used, level of covariate, and expected direction of covariate by cause, sex, and age
Covariates that CODEm did not select during the covariate selection process have no draw counts listed.

Cause	Sex	Age Start	Age End	Model Type	Direction	Level	Covariate Name	Number of Draws
Myelodysplastic, myeloproliferative, and other hematopoietic neoplasms	Male	0-6 days	95+ years	Global	1	2	Cumulative Cigarettes (15 Years)	--
Myelodysplastic, myeloproliferative, and other hematopoietic neoplasms	Male	0-6 days	95+ years	Global	1	2	Cumulative Cigarettes (20 Years)	--
Myelodysplastic, myeloproliferative, and other hematopoietic neoplasms	Male	0-6 days	95+ years	Global	1	2	Cumulative Cigarettes (5 Years)	--
Myelodysplastic, myeloproliferative, and other hematopoietic neoplasms	Male	0-6 days	95+ years	Global	1	2	Smoking Prevalence	--
Myelodysplastic, myeloproliferative, and other hematopoietic neoplasms	Male	0-6 days	95+ years	Global	1	2	Tobacco (cigarettes per capita)	--
Myelodysplastic, myeloproliferative, and other hematopoietic neoplasms	Male	0-6 days	95+ years	Global	-1	3	Education (years per capita)	--
Myelodysplastic, myeloproliferative, and other hematopoietic neoplasms	Male	0-6 days	95+ years	Global	0	3	LDI (I$ per capita)	--
Myelodysplastic, myeloproliferative, and other hematopoietic neoplasms	Male	0-6 days	95+ years	Global	0	3	Socio-demographic Index	--
Other benign and in situ neoplasms	Female	0-6 days	95+ years	Data Rich	-1	2	Healthcare access and quality index	--
Other benign and in situ neoplasms	Female	0-6 days	95+ years	Data Rich	-1	3	Education (years per capita)	--
Other benign and in situ neoplasms	Female	0-6 days	95+ years	Data Rich	0	3	LDI (I$ per capita)	1000
Other benign and in situ neoplasms	Female	0-6 days	95+ years	Data Rich	0	3	Socio-demographic Index	1000
Other benign and in situ neoplasms	Female	0-6 days	95+ years	Global	-1	2	Healthcare access and quality index	546
Other benign and in situ neoplasms	Female	0-6 days	95+ years	Global	-1	3	Education (years per capita)	--
Other benign and in situ neoplasms	Female	0-6 days	95+ years	Global	0	3	LDI (I$ per capita)	454
Other benign and in situ neoplasms	Female	0-6 days	95+ years	Global	0	3	Socio-demographic Index	454
Other benign and in situ neoplasms	Male	0-6 days	95+ years	Data Rich	-1	2	Healthcare access and quality index	--
Other benign and in situ neoplasms	Male	0-6 days	95+ years	Data Rich	-1	3	Education (years per capita)	--
Other benign and in situ neoplasms	Male	0-6 days	95+ years	Data Rich	0	3	Socio-demographic Index	546
Other benign and in situ neoplasms	Male	0-6 days	95+ years	Data Rich	0	3	LDI (I$ per capita)	1000
Other benign and in situ neoplasms	Male	0-6 days	95+ years	Global	-1	2	Healthcare access and quality index	--
Other benign and in situ neoplasms	Male	0-6 days	95+ years	Global	-1	3	Education (years per capita)	--
Other benign and in situ neoplasms	Male	0-6 days	95+ years	Global	0	3	LDI (I$ per capita)	1000
Other benign and in situ neoplasms	Male	0-6 days	95+ years	Global	0	3	Socio-demographic Index	1000
Cardiovascular diseases	Female	0-6 days	95+ years	Data Rich	1	1	Cholesterol (total, mean per capita)	195
Cardiovascular diseases	Female	0-6 days	95+ years	Data Rich	1	1	Systolic Blood Pressure (mmHg)	681
Cardiovascular diseases	Female	0-6 days	95+ years	Data Rich	1	1	Smoking Prevalence	997
Cardiovascular diseases	Female	0-6 days	95+ years	Data Rich	1	1	Log-transformed SEV scalar: CVD	--
Cardiovascular diseases	Female	0-6 days	95+ years	Data Rich	-1	2	Healthcare access and quality index	0
Cardiovascular diseases	Female	0-6 days	95+ years	Data Rich	-1	2	Elevation Over 1500m (proportion)	256
Cardiovascular diseases	Female	0-6 days	95+ years	Data Rich	1	2	Indoor Air Pollution (All Cooking Fuels)	26
Cardiovascular diseases	Female	0-6 days	95+ years	Data Rich	1	2	Outdoor Air Pollution (PM2.5)	283
Cardiovascular diseases	Female	0-6 days	95+ years	Data Rich	1	2	Diabetes Fasting Plasma Glucose (mmol/L), age-standardized 25+	--
Cardiovascular diseases	Female	0-6 days	95+ years	Data Rich	1	2	Mean BMI	--
Cardiovascular diseases	Female	0-6 days	95+ years	Data Rich	-1	3	pulses legumes adjusted(g)	0
Cardiovascular diseases	Female	0-6 days	95+ years	Data Rich	-1	3	LDI (I$ per capita)	71
Cardiovascular diseases	Female	0-6 days	95+ years	Data Rich	-1	3	nuts seeds adjusted(g)	144
Cardiovascular diseases	Female	0-6 days	95+ years	Data Rich	-1	3	fruits adjusted(g)	172
Cardiovascular diseases	Female	0-6 days	95+ years	Data Rich	-1	3	pufa adjusted(percent)	211
Cardiovascular diseases	Female	0-6 days	95+ years	Data Rich	-1	3	omega 3 adjusted(g)	--
Cardiovascular diseases	Female	0-6 days	95+ years	Data Rich	-1	3	vegetables adjusted(g)	--
Cardiovascular diseases	Female	0-6 days	95+ years	Data Rich	0	3	Socio-demographic Index	0
Cardiovascular diseases	Female	0-6 days	95+ years	Data Rich	0	3	Alcohol (liters per capita)	168
Cardiovascular diseases	Female	0-6 days	95+ years	Data Rich	1	3	Diet high in trans fatty acids	15
Cardiovascular diseases	Female	0-6 days	95+ years	Global	1	1	Systolic Blood Pressure (mmHg)	45
Cardiovascular diseases	Female	0-6 days	95+ years	Global	1	1	Cholesterol (total, mean per capita)	139
Cardiovascular diseases	Female	0-6 days	95+ years	Global	1	1	Smoking Prevalence	652
Cardiovascular diseases	Female	0-6 days	95+ years	Global	1	1	Log-transformed SEV scalar: CVD	--
Cardiovascular diseases	Female	0-6 days	95+ years	Global	-1	2	Elevation Over 1500m (proportion)	0
Cardiovascular diseases	Female	0-6 days	95+ years	Global	-1	2	Healthcare access and quality index	798
Cardiovascular diseases	Female	0-6 days	95+ years	Global	1	2	Outdoor Air Pollution (PM2.5)	25
Cardiovascular diseases	Female	0-6 days	95+ years	Global	1	2	Indoor Air Pollution (All Cooking Fuels)	47
Cardiovascular diseases	Female	0-6 days	95+ years	Global	1	2	Diabetes Fasting Plasma Glucose (mmol/L), age-standardized 25+	--

Table S16. CODEm covariates used, level of covariate, and expected direction of covariate by cause, sex, and age<br>*Covariates that CODEm did not select during the covariate selection process have no draw counts listed.*								
**Cause**	**Sex**	**Age Start**	**Age End**	**Model Type**	**Direction**	**Level**	**Covariate Name**	**Number of Draws**
Cardiovascular diseases	Female	0-6 days	95+ years	Global	1	2	Mean BMI	--
Cardiovascular diseases	Female	0-6 days	95+ years	Global	-1	3	fruits adjusted(g)	0
Cardiovascular diseases	Female	0-6 days	95+ years	Global	-1	3	pulses legumes adjusted(g)	0
Cardiovascular diseases	Female	0-6 days	95+ years	Global	-1	3	nuts seeds adjusted(g)	140
Cardiovascular diseases	Female	0-6 days	95+ years	Global	-1	3	LDI (I$ per capita)	288
Cardiovascular diseases	Female	0-6 days	95+ years	Global	-1	3	pufa adjusted(percent)	360
Cardiovascular diseases	Female	0-6 days	95+ years	Global	-1	3	omega 3 adjusted(g)	--
Cardiovascular diseases	Female	0-6 days	95+ years	Global	-1	3	vegetables adjusted(g)	--
Cardiovascular diseases	Female	0-6 days	95+ years	Global	0	3	Socio-demographic Index	326
Cardiovascular diseases	Female	0-6 days	95+ years	Global	0	3	Alcohol (liters per capita)	476
Cardiovascular diseases	Female	0-6 days	95+ years	Global	1	3	Diet high in trans fatty acids	515
Cardiovascular diseases	Male	0-6 days	95+ years	Data Rich	1	1	Cholesterol (total, mean per capita)	265
Cardiovascular diseases	Male	0-6 days	95+ years	Data Rich	1	1	Systolic Blood Pressure (mmHg)	265
Cardiovascular diseases	Male	0-6 days	95+ years	Data Rich	1	1	Smoking Prevalence	1000
Cardiovascular diseases	Male	0-6 days	95+ years	Data Rich	1	1	Log-transformed SEV scalar: CVD	--
Cardiovascular diseases	Male	0-6 days	95+ years	Data Rich	-1	2	Healthcare access and quality index	116
Cardiovascular diseases	Male	0-6 days	95+ years	Data Rich	-1	2	Elevation Over 1500m (proportion)	121
Cardiovascular diseases	Male	0-6 days	95+ years	Data Rich	1	2	Indoor Air Pollution (All Cooking Fuels)	84
Cardiovascular diseases	Male	0-6 days	95+ years	Data Rich	1	2	Outdoor Air Pollution (PM2.5)	121
Cardiovascular diseases	Male	0-6 days	95+ years	Data Rich	1	2	Diabetes Fasting Plasma Glucose (mmol/L), age-standardized 25+	--
Cardiovascular diseases	Male	0-6 days	95+ years	Data Rich	1	2	Mean BMI	--
Cardiovascular diseases	Male	0-6 days	95+ years	Data Rich	-1	3	fruits adjusted(g)	0
Cardiovascular diseases	Male	0-6 days	95+ years	Data Rich	-1	3	LDI (I$ per capita)	39
Cardiovascular diseases	Male	0-6 days	95+ years	Data Rich	-1	3	nuts seeds adjusted(g)	113
Cardiovascular diseases	Male	0-6 days	95+ years	Data Rich	-1	3	pufa adjusted(percent)	454
Cardiovascular diseases	Male	0-6 days	95+ years	Data Rich	-1	3	pulses legumes adjusted(g)	516
Cardiovascular diseases	Male	0-6 days	95+ years	Data Rich	-1	3	omega 3 adjusted(g)	--
Cardiovascular diseases	Male	0-6 days	95+ years	Data Rich	-1	3	vegetables adjusted(g)	--
Cardiovascular diseases	Male	0-6 days	95+ years	Data Rich	0	3	Socio-demographic Index	45
Cardiovascular diseases	Male	0-6 days	95+ years	Data Rich	0	3	Alcohol (liters per capita)	511
Cardiovascular diseases	Male	0-6 days	95+ years	Data Rich	1	3	Diet high in trans fatty acids	0
Cardiovascular diseases	Male	0-6 days	95+ years	Global	1	1	Systolic Blood Pressure (mmHg)	19
Cardiovascular diseases	Male	0-6 days	95+ years	Global	1	1	Cholesterol (total, mean per capita)	626
Cardiovascular diseases	Male	0-6 days	95+ years	Global	1	1	Smoking Prevalence	979
Cardiovascular diseases	Male	0-6 days	95+ years	Global	1	1	Log-transformed SEV scalar: CVD	--
Cardiovascular diseases	Male	0-6 days	95+ years	Global	-1	2	Elevation Over 1500m (proportion)	285
Cardiovascular diseases	Male	0-6 days	95+ years	Global	-1	2	Healthcare access and quality index	411
Cardiovascular diseases	Male	0-6 days	95+ years	Global	1	2	Indoor Air Pollution (All Cooking Fuels)	264
Cardiovascular diseases	Male	0-6 days	95+ years	Global	1	2	Diabetes Fasting Plasma Glucose (mmol/L), age-standardized 25+	285
Cardiovascular diseases	Male	0-6 days	95+ years	Global	1	2	Outdoor Air Pollution (PM2.5)	285
Cardiovascular diseases	Male	0-6 days	95+ years	Global	1	2	Mean BMI	--
Cardiovascular diseases	Male	0-6 days	95+ years	Global	-1	3	fruits adjusted(g)	0
Cardiovascular diseases	Male	0-6 days	95+ years	Global	-1	3	LDI (I$ per capita)	216
Cardiovascular diseases	Male	0-6 days	95+ years	Global	-1	3	pufa adjusted(percent)	408
Cardiovascular diseases	Male	0-6 days	95+ years	Global	-1	3	nuts seeds adjusted(g)	494
Cardiovascular diseases	Male	0-6 days	95+ years	Global	-1	3	pulses legumes adjusted(g)	950
Cardiovascular diseases	Male	0-6 days	95+ years	Global	-1	3	omega 3 adjusted(g)	--
Cardiovascular diseases	Male	0-6 days	95+ years	Global	-1	3	vegetables adjusted(g)	--
Cardiovascular diseases	Male	0-6 days	95+ years	Global	0	3	Socio-demographic Index	351
Cardiovascular diseases	Male	0-6 days	95+ years	Global	0	3	Alcohol (liters per capita)	923
Cardiovascular diseases	Male	0-6 days	95+ years	Global	1	3	Diet high in trans fatty acids	0
Rheumatic heart disease	Female	1-4 years	95+ years	Data Rich	-1	1	Improved Water Source (proportion with access)	165
Rheumatic heart disease	Female	1-4 years	95+ years	Data Rich	-1	1	Sanitation (proportion with access)	606
Rheumatic heart disease	Female	1-4 years	95+ years	Data Rich	1	1	Underweight (proportion <2SD weight for age, <5 years)	281
Rheumatic heart disease	Female	1-4 years	95+ years	Data Rich	1	1	Log-transformed SEV scalar: RHD	--
Rheumatic heart disease	Female	1-4 years	95+ years	Data Rich	-1	2	Healthcare access and quality index	199

Table S16. CODEm covariates used, level of covariate, and expected direction of covariate by cause, sex, and age
Covariates that CODEm did not select during the covariate selection process have no draw counts listed.

Cause	Sex	Age Start	Age End	Model Type	Direction	Level	Covariate Name	Number of Draws
Rheumatic heart disease	Female	1-4 years	95+ years	Data Rich	-1	3	Socio-demographic Index	424
Rheumatic heart disease	Female	1-4 years	95+ years	Data Rich	-1	3	Education (years per capita)	782
Rheumatic heart disease	Female	1-4 years	95+ years	Data Rich	-1	3	LDI (I$ per capita)	--
Rheumatic heart disease	Female	1-4 years	95+ years	Global	-1	1	Improved Water Source (proportion with access)	162
Rheumatic heart disease	Female	1-4 years	95+ years	Global	-1	1	Sanitation (proportion with access)	395
Rheumatic heart disease	Female	1-4 years	95+ years	Global	1	1	Underweight (proportion <2SD weight for age, <5 years)	221
Rheumatic heart disease	Female	1-4 years	95+ years	Global	1	1	Log-transformed SEV scalar: RHD	--
Rheumatic heart disease	Female	1-4 years	95+ years	Global	-1	2	Healthcare access and quality index	282
Rheumatic heart disease	Female	1-4 years	95+ years	Global	-1	3	Socio-demographic Index	333
Rheumatic heart disease	Female	1-4 years	95+ years	Global	-1	3	Education (years per capita)	756
Rheumatic heart disease	Female	1-4 years	95+ years	Global	-1	3	LDI (I$ per capita)	--
Rheumatic heart disease	Male	1-4 years	95+ years	Data Rich	-1	1	Improved Water Source (proportion with access)	39
Rheumatic heart disease	Male	1-4 years	95+ years	Data Rich	-1	1	Sanitation (proportion with access)	772
Rheumatic heart disease	Male	1-4 years	95+ years	Data Rich	1	1	Underweight (proportion <2SD weight for age, <5 years)	478
Rheumatic heart disease	Male	1-4 years	95+ years	Data Rich	1	1	Log-transformed SEV scalar: RHD	--
Rheumatic heart disease	Male	1-4 years	95+ years	Data Rich	-1	2	Healthcare access and quality index	555
Rheumatic heart disease	Male	1-4 years	95+ years	Data Rich	-1	3	Education (years per capita)	336
Rheumatic heart disease	Male	1-4 years	95+ years	Data Rich	-1	3	Socio-demographic Index	363
Rheumatic heart disease	Male	1-4 years	95+ years	Data Rich	-1	3	LDI (I$ per capita)	--
Rheumatic heart disease	Male	1-4 years	95+ years	Global	-1	1	Improved Water Source (proportion with access)	23
Rheumatic heart disease	Male	1-4 years	95+ years	Global	-1	1	Sanitation (proportion with access)	546
Rheumatic heart disease	Male	1-4 years	95+ years	Global	1	1	Underweight (proportion <2SD weight for age, <5 years)	377
Rheumatic heart disease	Male	1-4 years	95+ years	Global	1	1	Log-transformed SEV scalar: RHD	--
Rheumatic heart disease	Male	1-4 years	95+ years	Global	-1	2	Healthcare access and quality index	681
Rheumatic heart disease	Male	1-4 years	95+ years	Global	-1	3	Socio-demographic Index	136
Rheumatic heart disease	Male	1-4 years	95+ years	Global	-1	3	Education (years per capita)	281
Rheumatic heart disease	Male	1-4 years	95+ years	Global	-1	3	LDI (I$ per capita)	--
Ischaemic heart disease	Female	15-19 years	95+ years	Data Rich	1	1	Cholesterol (total, mean per capita)	628
Ischaemic heart disease	Female	15-19 years	95+ years	Data Rich	1	1	Systolic Blood Pressure (mmHg)	723
Ischaemic heart disease	Female	15-19 years	95+ years	Data Rich	1	1	Smoking Prevalence	972
Ischaemic heart disease	Female	15-19 years	95+ years	Data Rich	1	1	Log-transformed SEV scalar: IHD	--
Ischaemic heart disease	Female	15-19 years	95+ years	Data Rich	-1	2	Healthcare access and quality index	460
Ischaemic heart disease	Female	15-19 years	95+ years	Data Rich	-1	2	Elevation Over 1500m (proportion)	624
Ischaemic heart disease	Female	15-19 years	95+ years	Data Rich	1	2	Indoor Air Pollution (All Cooking Fuels)	374
Ischaemic heart disease	Female	15-19 years	95+ years	Data Rich	1	2	Outdoor Air Pollution (PM2.5)	648
Ischaemic heart disease	Female	15-19 years	95+ years	Data Rich	1	2	Diabetes Fasting Plasma Glucose (mmol/L), age-standardized 25+	--
Ischaemic heart disease	Female	15-19 years	95+ years	Data Rich	1	2	Mean BMI	--
Ischaemic heart disease	Female	15-19 years	95+ years	Data Rich	-1	3	nuts seeds adjusted(g)	12
Ischaemic heart disease	Female	15-19 years	95+ years	Data Rich	-1	3	fruits adjusted(g)	13
Ischaemic heart disease	Female	15-19 years	95+ years	Data Rich	-1	3	pufa adjusted(percent)	14
Ischaemic heart disease	Female	15-19 years	95+ years	Data Rich	-1	3	pulses legumes adjusted(g)	527
Ischaemic heart disease	Female	15-19 years	95+ years	Data Rich	-1	3	LDI (I$ per capita)	--
Ischaemic heart disease	Female	15-19 years	95+ years	Data Rich	-1	3	omega 3 adjusted(g)	--
Ischaemic heart disease	Female	15-19 years	95+ years	Data Rich	-1	3	vegetables adjusted(g)	--
Ischaemic heart disease	Female	15-19 years	95+ years	Data Rich	0	3	Socio-demographic Index	370
Ischaemic heart disease	Female	15-19 years	95+ years	Data Rich	0	3	Alcohol (liters per capita)	711
Ischaemic heart disease	Female	15-19 years	95+ years	Data Rich	1	3	Diet high in trans fatty acids	371

Table S16. CODEm covariates used, level of covariate, and expected direction of covariate by cause, sex, and age
Covariates that CODEm did not select during the covariate selection process have no draw counts listed.

Cause	Sex	Age Start	Age End	Model Type	Direction	Level	Covariate Name	Number of Draws
Ischaemic heart disease	Female	15-19 years	95+ years	Global	1	1	Cholesterol (total, mean per capita)	551
Ischaemic heart disease	Female	15-19 years	95+ years	Global	1	1	Smoking Prevalence	735
Ischaemic heart disease	Female	15-19 years	95+ years	Global	1	1	Systolic Blood Pressure (mmHg)	924
Ischaemic heart disease	Female	15-19 years	95+ years	Global	1	1	Log-transformed SEV scalar: IHD	--
Ischaemic heart disease	Female	15-19 years	95+ years	Global	-1	2	Healthcare access and quality index	71
Ischaemic heart disease	Female	15-19 years	95+ years	Global	-1	2	Elevation Over 1500m (proportion)	424
Ischaemic heart disease	Female	15-19 years	95+ years	Global	1	2	Indoor Air Pollution (All Cooking Fuels)	0
Ischaemic heart disease	Female	15-19 years	95+ years	Global	1	2	Outdoor Air Pollution (PM2.5)	210
Ischaemic heart disease	Female	15-19 years	95+ years	Global	1	2	Diabetes Fasting Plasma Glucose (mmol/L), age-standardized 25+	281
Ischaemic heart disease	Female	15-19 years	95+ years	Global	1	2	Mean BMI	--
Ischaemic heart disease	Female	15-19 years	95+ years	Global	-1	3	fruits adjusted(g)	0
Ischaemic heart disease	Female	15-19 years	95+ years	Global	-1	3	nuts seeds adjusted(g)	24
Ischaemic heart disease	Female	15-19 years	95+ years	Global	-1	3	pufa adjusted(percent)	32
Ischaemic heart disease	Female	15-19 years	95+ years	Global	-1	3	pulses legumes adjusted(g)	422
Ischaemic heart disease	Female	15-19 years	95+ years	Global	-1	3	LDI (I$ per capita)	--
Ischaemic heart disease	Female	15-19 years	95+ years	Global	-1	3	omega 3 adjusted(g)	--
Ischaemic heart disease	Female	15-19 years	95+ years	Global	-1	3	vegetables adjusted(g)	--
Ischaemic heart disease	Female	15-19 years	95+ years	Global	0	3	Alcohol (liters per capita)	182
Ischaemic heart disease	Female	15-19 years	95+ years	Global	0	3	Socio-demographic Index	275
Ischaemic heart disease	Female	15-19 years	95+ years	Global	1	3	Diet high in trans fatty acids	168
Ischaemic heart disease	Male	15-19 years	95+ years	Data Rich	1	1	Systolic Blood Pressure (mmHg)	81
Ischaemic heart disease	Male	15-19 years	95+ years	Data Rich	1	1	Cholesterol (total, mean per capita)	611
Ischaemic heart disease	Male	15-19 years	95+ years	Data Rich	1	1	Smoking Prevalence	1000
Ischaemic heart disease	Male	15-19 years	95+ years	Data Rich	1	1	Log-transformed SEV scalar: IHD	--
Ischaemic heart disease	Male	15-19 years	95+ years	Data Rich	-1	2	Elevation Over 1500m (proportion)	728
Ischaemic heart disease	Male	15-19 years	95+ years	Data Rich	-1	2	Healthcare access and quality index	757
Ischaemic heart disease	Male	15-19 years	95+ years	Data Rich	1	2	Indoor Air Pollution (All Cooking Fuels)	541
Ischaemic heart disease	Male	15-19 years	95+ years	Data Rich	1	2	Outdoor Air Pollution (PM2.5)	730
Ischaemic heart disease	Male	15-19 years	95+ years	Data Rich	1	2	Diabetes Fasting Plasma Glucose (mmol/L), age-standardized 25+	--
Ischaemic heart disease	Male	15-19 years	95+ years	Data Rich	1	2	Mean BMI	--
Ischaemic heart disease	Male	15-19 years	95+ years	Data Rich	-1	3	fruits adjusted(g)	1
Ischaemic heart disease	Male	15-19 years	95+ years	Data Rich	-1	3	pufa adjusted(percent)	180
Ischaemic heart disease	Male	15-19 years	95+ years	Data Rich	-1	3	nuts seeds adjusted(g)	620
Ischaemic heart disease	Male	15-19 years	95+ years	Data Rich	-1	3	pulses legumes adjusted(g)	896
Ischaemic heart disease	Male	15-19 years	95+ years	Data Rich	-1	3	LDI (I$ per capita)	--
Ischaemic heart disease	Male	15-19 years	95+ years	Data Rich	-1	3	omega 3 adjusted(g)	--
Ischaemic heart disease	Male	15-19 years	95+ years	Data Rich	-1	3	vegetables adjusted(g)	--
Ischaemic heart disease	Male	15-19 years	95+ years	Data Rich	0	3	Socio-demographic Index	234
Ischaemic heart disease	Male	15-19 years	95+ years	Data Rich	0	3	Alcohol (liters per capita)	843
Ischaemic heart disease	Male	15-19 years	95+ years	Data Rich	1	3	Diet high in trans fatty acids	783

Table S16. CODEm covariates used, level of covariate, and expected direction of covariate by cause, sex, and age
Covariates that CODEm did not select during the covariate selection process have no draw counts listed.

Cause	Sex	Age Start	Age End	Model Type	Direction	Level	Covariate Name	Number of Draws
Ischaemic heart disease	Male	15-19 years	95+ years	Global	1	1	Cholesterol (total, mean per capita)	851
Ischaemic heart disease	Male	15-19 years	95+ years	Global	1	1	Smoking Prevalence	939
Ischaemic heart disease	Male	15-19 years	95+ years	Global	1	1	Systolic Blood Pressure (mmHg)	966
Ischaemic heart disease	Male	15-19 years	95+ years	Global	1	1	Log-transformed SEV scalar: IHD	--
Ischaemic heart disease	Male	15-19 years	95+ years	Global	-1	2	Elevation Over 1500m (proportion)	667
Ischaemic heart disease	Male	15-19 years	95+ years	Global	-1	2	Healthcare access and quality index	674
Ischaemic heart disease	Male	15-19 years	95+ years	Global	1	2	Indoor Air Pollution (All Cooking Fuels)	233
Ischaemic heart disease	Male	15-19 years	95+ years	Global	1	2	Outdoor Air Pollution (PM2.5)	671
Ischaemic heart disease	Male	15-19 years	95+ years	Global	1	2	Diabetes Fasting Plasma Glucose (mmol/L), age-standardized 25+	786
Ischaemic heart disease	Male	15-19 years	95+ years	Global	1	2	Mean BMI	--
Ischaemic heart disease	Male	15-19 years	95+ years	Global	-1	3	fruits adjusted(g)	0
Ischaemic heart disease	Male	15-19 years	95+ years	Global	-1	3	nuts seeds adjusted(g)	298
Ischaemic heart disease	Male	15-19 years	95+ years	Global	-1	3	pufa adjusted(percent)	427
Ischaemic heart disease	Male	15-19 years	95+ years	Global	-1	3	pulses legumes adjusted(g)	553
Ischaemic heart disease	Male	15-19 years	95+ years	Global	-1	3	LDI (I$ per capita)	--
Ischaemic heart disease	Male	15-19 years	95+ years	Global	-1	3	omega 3 adjusted(g)	--
Ischaemic heart disease	Male	15-19 years	95+ years	Global	-1	3	vegetables adjusted(g)	--
Ischaemic heart disease	Male	15-19 years	95+ years	Global	0	3	Socio-demographic Index	65
Ischaemic heart disease	Male	15-19 years	95+ years	Global	0	3	Alcohol (liters per capita)	552
Ischaemic heart disease	Male	15-19 years	95+ years	Global	1	3	Diet high in trans fatty acids	33
Stroke	Female	0-6 days	95+ years	Data Rich	1	1	Cholesterol (total, mean per capita)	0
Stroke	Female	0-6 days	95+ years	Data Rich	1	1	Smoking Prevalence	910
Stroke	Female	0-6 days	95+ years	Data Rich	1	1	Systolic Blood Pressure (mmHg)	1000
Stroke	Female	0-6 days	95+ years	Data Rich	1	1	Log-transformed SEV scalar: Stroke	--
Stroke	Female	0-6 days	95+ years	Data Rich	-1	2	Elevation Over 1500m (proportion)	0
Stroke	Female	0-6 days	95+ years	Data Rich	-1	2	fruits adjusted(g)	386
Stroke	Female	0-6 days	95+ years	Data Rich	-1	2	Healthcare access and quality index	416
Stroke	Female	0-6 days	95+ years	Data Rich	-1	2	vegetables adjusted(g)	--
Stroke	Female	0-6 days	95+ years	Data Rich	1	2	Outdoor Air Pollution (PM2.5)	0
Stroke	Female	0-6 days	95+ years	Data Rich	1	2	Indoor Air Pollution (All Cooking Fuels)	416
Stroke	Female	0-6 days	95+ years	Data Rich	1	2	Diabetes Fasting Plasma Glucose (mmol/L), age-standardized 25+	--
Stroke	Female	0-6 days	95+ years	Data Rich	1	2	Mean BMI	--
Stroke	Female	0-6 days	95+ years	Data Rich	-1	3	pufa adjusted(percent)	61
Stroke	Female	0-6 days	95+ years	Data Rich	-1	3	pulses legumes adjusted(g)	160
Stroke	Female	0-6 days	95+ years	Data Rich	-1	3	nuts seeds adjusted(g)	316
Stroke	Female	0-6 days	95+ years	Data Rich	-1	3	LDI (I$ per capita)	--
Stroke	Female	0-6 days	95+ years	Data Rich	-1	3	omega 3 adjusted(g)	--
Stroke	Female	0-6 days	95+ years	Data Rich	0	3	Socio-demographic Index	488
Stroke	Female	0-6 days	95+ years	Data Rich	0	3	Alcohol (liters per capita)	849
Stroke	Female	0-6 days	95+ years	Data Rich	1	3	Diet high in trans fatty acids	285
Stroke	Female	0-6 days	95+ years	Global	1	1	Cholesterol (total, mean per capita)	28
Stroke	Female	0-6 days	95+ years	Global	1	1	Smoking Prevalence	738
Stroke	Female	0-6 days	95+ years	Global	1	1	Systolic Blood Pressure (mmHg)	940
Stroke	Female	0-6 days	95+ years	Global	1	1	Log-transformed SEV scalar: Stroke	--
Stroke	Female	0-6 days	95+ years	Global	-1	2	fruits adjusted(g)	245
Stroke	Female	0-6 days	95+ years	Global	-1	2	Healthcare access and quality index	817
Stroke	Female	0-6 days	95+ years	Global	-1	2	Elevation Over 1500m (proportion)	--
Stroke	Female	0-6 days	95+ years	Global	-1	2	vegetables adjusted(g)	--

Table S16. CODEm covariates used, level of covariate, and expected direction of covariate by cause, sex, and age
Covariates that CODEm did not select during the covariate selection process have no draw counts listed.

Cause	Sex	Age Start	Age End	Model Type	Direction	Level	Covariate Name	Number of Draws
Stroke	Female	0-6 days	95+ years	Global	1	2	Outdoor Air Pollution (PM2.5)	51
Stroke	Female	0-6 days	95+ years	Global	1	2	Indoor Air Pollution (All Cooking Fuels)	208
Stroke	Female	0-6 days	95+ years	Global	1	2	Diabetes Fasting Plasma Glucose (mmol/L), age-standardized 25+	--
Stroke	Female	0-6 days	95+ years	Global	1	2	Mean BMI	--
Stroke	Female	0-6 days	95+ years	Global	-1	3	pulses legumes adjusted(g)	180
Stroke	Female	0-6 days	95+ years	Global	-1	3	nuts seeds adjusted(g)	208
Stroke	Female	0-6 days	95+ years	Global	-1	3	pufa adjusted(percent)	317
Stroke	Female	0-6 days	95+ years	Global	-1	3	LDI (I$ per capita)	--
Stroke	Female	0-6 days	95+ years	Global	-1	3	omega 3 adjusted(g)	--
Stroke	Female	0-6 days	95+ years	Global	0	3	Socio-demographic Index	233
Stroke	Female	0-6 days	95+ years	Global	0	3	Alcohol (liters per capita)	432
Stroke	Female	0-6 days	95+ years	Global	1	3	Diet high in trans fatty acids	18
Stroke	Male	0-6 days	95+ years	Data Rich	1	1	Cholesterol (total, mean per capita)	18
Stroke	Male	0-6 days	95+ years	Data Rich	1	1	Systolic Blood Pressure (mmHg)	234
Stroke	Male	0-6 days	95+ years	Data Rich	1	1	Smoking Prevalence	999
Stroke	Male	0-6 days	95+ years	Data Rich	1	1	Log-transformed SEV scalar: Stroke	--
Stroke	Male	0-6 days	95+ years	Data Rich	-1	2	fruits adjusted(g)	148
Stroke	Male	0-6 days	95+ years	Data Rich	-1	2	Elevation Over 1500m (proportion)	170
Stroke	Male	0-6 days	95+ years	Data Rich	-1	2	Healthcare access and quality index	170
Stroke	Male	0-6 days	95+ years	Data Rich	-1	2	vegetables adjusted(g)	--
Stroke	Male	0-6 days	95+ years	Data Rich	1	2	Outdoor Air Pollution (PM2.5)	0
Stroke	Male	0-6 days	95+ years	Data Rich	1	2	Indoor Air Pollution (All Cooking Fuels)	33
Stroke	Male	0-6 days	95+ years	Data Rich	1	2	Diabetes Fasting Plasma Glucose (mmol/L), age-standardized 25+	--
Stroke	Male	0-6 days	95+ years	Data Rich	1	2	Mean BMI	--
Stroke	Male	0-6 days	95+ years	Data Rich	-1	3	nuts seeds adjusted(g)	157
Stroke	Male	0-6 days	95+ years	Data Rich	-1	3	pufa adjusted(percent)	310
Stroke	Male	0-6 days	95+ years	Data Rich	-1	3	pulses legumes adjusted(g)	612
Stroke	Male	0-6 days	95+ years	Data Rich	-1	3	LDI (I$ per capita)	--
Stroke	Male	0-6 days	95+ years	Data Rich	-1	3	omega 3 adjusted(g)	--
Stroke	Male	0-6 days	95+ years	Data Rich	0	3	Socio-demographic Index	304
Stroke	Male	0-6 days	95+ years	Data Rich	0	3	Alcohol (liters per capita)	915
Stroke	Male	0-6 days	95+ years	Data Rich	1	3	Diet high in trans fatty acids	255
Stroke	Male	0-6 days	95+ years	Global	1	1	Cholesterol (total, mean per capita)	107
Stroke	Male	0-6 days	95+ years	Global	1	1	Smoking Prevalence	467
Stroke	Male	0-6 days	95+ years	Global	1	1	Systolic Blood Pressure (mmHg)	565
Stroke	Male	0-6 days	95+ years	Global	1	1	Log-transformed SEV scalar: Stroke	--
Stroke	Male	0-6 days	95+ years	Global	-1	2	fruits adjusted(g)	102
Stroke	Male	0-6 days	95+ years	Global	-1	2	Elevation Over 1500m (proportion)	258
Stroke	Male	0-6 days	95+ years	Global	-1	2	Healthcare access and quality index	761
Stroke	Male	0-6 days	95+ years	Global	-1	2	vegetables adjusted(g)	--
Stroke	Male	0-6 days	95+ years	Global	1	2	Indoor Air Pollution (All Cooking Fuels)	3
Stroke	Male	0-6 days	95+ years	Global	1	2	Outdoor Air Pollution (PM2.5)	261
Stroke	Male	0-6 days	95+ years	Global	1	2	Diabetes Fasting Plasma Glucose (mmol/L), age-standardized 25+	--
Stroke	Male	0-6 days	95+ years	Global	1	2	Mean BMI	--
Stroke	Male	0-6 days	95+ years	Global	-1	3	pufa adjusted(percent)	179
Stroke	Male	0-6 days	95+ years	Global	-1	3	nuts seeds adjusted(g)	324
Stroke	Male	0-6 days	95+ years	Global	-1	3	pulses legumes adjusted(g)	483
Stroke	Male	0-6 days	95+ years	Global	-1	3	LDI (I$ per capita)	--
Stroke	Male	0-6 days	95+ years	Global	-1	3	omega 3 adjusted(g)	--
Stroke	Male	0-6 days	95+ years	Global	0	3	Alcohol (liters per capita)	261
Stroke	Male	0-6 days	95+ years	Global	0	3	Socio-demographic Index	371
Stroke	Male	0-6 days	95+ years	Global	1	3	Diet high in trans fatty acids	136
Ischaemic stroke	Female	0-6 days	95+ years	Data Rich	1	1	Cholesterol (total, mean per capita)	41
Ischaemic stroke	Female	0-6 days	95+ years	Data Rich	1	1	Smoking Prevalence	239
Ischaemic stroke	Female	0-6 days	95+ years	Data Rich	1	1	Systolic Blood Pressure (mmHg)	736
Ischaemic stroke	Female	0-6 days	95+ years	Data Rich	1	1	Log-transformed SEV scalar: Isch Stroke	--

Table S16. CODEm covariates used, level of covariate, and expected direction of covariate by cause, sex, and age
Covariates that CODEm did not select during the covariate selection process have no draw counts listed.

Cause	Sex	Age Start	Age End	Model Type	Direction	Level	Covariate Name	Number of Draws
Ischaemic stroke	Female	0-6 days	95+ years	Data Rich	-1	2	Elevation Over 1500m (proportion)	577
Ischaemic stroke	Female	0-6 days	95+ years	Data Rich	-1	2	Healthcare access and quality index	577
Ischaemic stroke	Female	0-6 days	95+ years	Data Rich	1	2	Outdoor Air Pollution (PM2.5)	0
Ischaemic stroke	Female	0-6 days	95+ years	Data Rich	1	2	Indoor Air Pollution (All Cooking Fuels)	604
Ischaemic stroke	Female	0-6 days	95+ years	Data Rich	1	2	Diabetes Fasting Plasma Glucose (mmol/L), age-standardized 25+	--
Ischaemic stroke	Female	0-6 days	95+ years	Data Rich	1	2	Mean BMI	--
Ischaemic stroke	Female	0-6 days	95+ years	Data Rich	-1	3	pulses legumes adjusted(g)	0
Ischaemic stroke	Female	0-6 days	95+ years	Data Rich	-1	3	pufa adjusted(percent)	219
Ischaemic stroke	Female	0-6 days	95+ years	Data Rich	-1	3	nuts seeds adjusted(g)	343
Ischaemic stroke	Female	0-6 days	95+ years	Data Rich	-1	3	LDI (I$ per capita)	--
Ischaemic stroke	Female	0-6 days	95+ years	Data Rich	-1	3	fruits adjusted(g)	--
Ischaemic stroke	Female	0-6 days	95+ years	Data Rich	-1	3	omega 3 adjusted(g)	--
Ischaemic stroke	Female	0-6 days	95+ years	Data Rich	-1	3	vegetables adjusted(g)	--
Ischaemic stroke	Female	0-6 days	95+ years	Data Rich	0	3	Alcohol (liters per capita)	92
Ischaemic stroke	Female	0-6 days	95+ years	Data Rich	0	3	Socio-demographic Index	443
Ischaemic stroke	Female	0-6 days	95+ years	Data Rich	1	3	Diet high in trans fatty acids	424
Ischaemic stroke	Female	0-6 days	95+ years	Global	1	1	Systolic Blood Pressure (mmHg)	184
Ischaemic stroke	Female	0-6 days	95+ years	Global	1	1	Cholesterol (total, mean per capita)	527
Ischaemic stroke	Female	0-6 days	95+ years	Global	1	1	Smoking Prevalence	553
Ischaemic stroke	Female	0-6 days	95+ years	Global	1	1	Log-transformed SEV scalar: Isch Stroke	--
Ischaemic stroke	Female	0-6 days	95+ years	Global	-1	2	Elevation Over 1500m (proportion)	728
Ischaemic stroke	Female	0-6 days	95+ years	Global	-1	2	Healthcare access and quality index	815
Ischaemic stroke	Female	0-6 days	95+ years	Global	1	2	Indoor Air Pollution (All Cooking Fuels)	138
Ischaemic stroke	Female	0-6 days	95+ years	Global	1	2	Outdoor Air Pollution (PM2.5)	457
Ischaemic stroke	Female	0-6 days	95+ years	Global	1	2	Diabetes Fasting Plasma Glucose (mmol/L), age-standardized 25+	--
Ischaemic stroke	Female	0-6 days	95+ years	Global	1	2	Mean BMI	--
Ischaemic stroke	Female	0-6 days	95+ years	Global	-1	3	nuts seeds adjusted(g)	324
Ischaemic stroke	Female	0-6 days	95+ years	Global	-1	3	pufa adjusted(percent)	483
Ischaemic stroke	Female	0-6 days	95+ years	Global	-1	3	LDI (I$ per capita)	--
Ischaemic stroke	Female	0-6 days	95+ years	Global	-1	3	fruits adjusted(g)	--
Ischaemic stroke	Female	0-6 days	95+ years	Global	-1	3	omega 3 adjusted(g)	--
Ischaemic stroke	Female	0-6 days	95+ years	Global	-1	3	pulses legumes adjusted(g)	--
Ischaemic stroke	Female	0-6 days	95+ years	Global	-1	3	vegetables adjusted(g)	--
Ischaemic stroke	Female	0-6 days	95+ years	Global	0	3	Socio-demographic Index	500
Ischaemic stroke	Female	0-6 days	95+ years	Global	0	3	Alcohol (liters per capita)	650
Ischaemic stroke	Female	0-6 days	95+ years	Global	1	3	Diet high in trans fatty acids	509
Ischaemic stroke	Male	0-6 days	95+ years	Data Rich	1	1	Cholesterol (total, mean per capita)	64
Ischaemic stroke	Male	0-6 days	95+ years	Data Rich	1	1	Systolic Blood Pressure (mmHg)	918
Ischaemic stroke	Male	0-6 days	95+ years	Data Rich	1	1	Smoking Prevalence	1000
Ischaemic stroke	Male	0-6 days	95+ years	Data Rich	1	1	Log-transformed SEV scalar: Isch Stroke	--
Ischaemic stroke	Male	0-6 days	95+ years	Data Rich	-1	2	Elevation Over 1500m (proportion)	454
Ischaemic stroke	Male	0-6 days	95+ years	Data Rich	-1	2	Healthcare access and quality index	454
Ischaemic stroke	Male	0-6 days	95+ years	Data Rich	1	2	Outdoor Air Pollution (PM2.5)	0
Ischaemic stroke	Male	0-6 days	95+ years	Data Rich	1	2	Indoor Air Pollution (All Cooking Fuels)	454
Ischaemic stroke	Male	0-6 days	95+ years	Data Rich	1	2	Diabetes Fasting Plasma Glucose (mmol/L), age-standardized 25+	--
Ischaemic stroke	Male	0-6 days	95+ years	Data Rich	1	2	Mean BMI	--
Ischaemic stroke	Male	0-6 days	95+ years	Data Rich	-1	3	pulses legumes adjusted(g)	32
Ischaemic stroke	Male	0-6 days	95+ years	Data Rich	-1	3	nuts seeds adjusted(g)	219
Ischaemic stroke	Male	0-6 days	95+ years	Data Rich	-1	3	pufa adjusted(percent)	661
Ischaemic stroke	Male	0-6 days	95+ years	Data Rich	-1	3	LDI (I$ per capita)	--
Ischaemic stroke	Male	0-6 days	95+ years	Data Rich	-1	3	fruits adjusted(g)	--
Ischaemic stroke	Male	0-6 days	95+ years	Data Rich	-1	3	omega 3 adjusted(g)	--
Ischaemic stroke	Male	0-6 days	95+ years	Data Rich	-1	3	vegetables adjusted(g)	--
Ischaemic stroke	Male	0-6 days	95+ years	Data Rich	0	3	Alcohol (liters per capita)	764
Ischaemic stroke	Male	0-6 days	95+ years	Data Rich	0	3	Socio-demographic Index	825

Table S16. CODEm covariates used, level of covariate, and expected direction of covariate by cause, sex, and age
Covariates that CODEm did not select during the covariate selection process have no draw counts listed.

Cause	Sex	Age Start	Age End	Model Type	Direction	Level	Covariate Name	Number of Draws
Ischaemic stroke	Male	0-6 days	95+ years	Data Rich	1	3	Diet high in trans fatty acids	50
Ischaemic stroke	Male	0-6 days	95+ years	Global	1	1	Cholesterol (total, mean per capita)	384
Ischaemic stroke	Male	0-6 days	95+ years	Global	1	1	Systolic Blood Pressure (mmHg)	548
Ischaemic stroke	Male	0-6 days	95+ years	Global	1	1	Smoking Prevalence	952
Ischaemic stroke	Male	0-6 days	95+ years	Global	1	1	Log-transformed SEV scalar: Isch Stroke	--
Ischaemic stroke	Male	0-6 days	95+ years	Global	-1	2	Elevation Over 1500m (proportion)	408
Ischaemic stroke	Male	0-6 days	95+ years	Global	-1	2	Healthcare access and quality index	489
Ischaemic stroke	Male	0-6 days	95+ years	Global	1	2	Indoor Air Pollution (All Cooking Fuels)	250
Ischaemic stroke	Male	0-6 days	95+ years	Global	1	2	Outdoor Air Pollution (PM2.5)	557
Ischaemic stroke	Male	0-6 days	95+ years	Global	1	2	Diabetes Fasting Plasma Glucose (mmol/L), age-standardized 25+	574
Ischaemic stroke	Male	0-6 days	95+ years	Global	1	2	Mean BMI	--
Ischaemic stroke	Male	0-6 days	95+ years	Global	-1	3	pufa adjusted(percent)	90
Ischaemic stroke	Male	0-6 days	95+ years	Global	-1	3	nuts seeds adjusted(g)	274
Ischaemic stroke	Male	0-6 days	95+ years	Global	-1	3	LDI (I$ per capita)	--
Ischaemic stroke	Male	0-6 days	95+ years	Global	-1	3	fruits adjusted(g)	--
Ischaemic stroke	Male	0-6 days	95+ years	Global	-1	3	omega 3 adjusted(g)	--
Ischaemic stroke	Male	0-6 days	95+ years	Global	-1	3	pulses legumes adjusted(g)	--
Ischaemic stroke	Male	0-6 days	95+ years	Global	-1	3	vegetables adjusted(g)	--
Ischaemic stroke	Male	0-6 days	95+ years	Global	0	3	Socio-demographic Index	299
Ischaemic stroke	Male	0-6 days	95+ years	Global	0	3	Alcohol (liters per capita)	643
Ischaemic stroke	Male	0-6 days	95+ years	Global	1	3	Diet high in trans fatty acids	226
Intracerebral hemorrhage	Female	0-6 days	95+ years	Data Rich	0	1	Cholesterol (total, mean per capita)	821
Intracerebral hemorrhage	Female	0-6 days	95+ years	Data Rich	1	1	Smoking Prevalence	993
Intracerebral hemorrhage	Female	0-6 days	95+ years	Data Rich	1	1	Systolic Blood Pressure (mmHg)	1000
Intracerebral hemorrhage	Female	0-6 days	95+ years	Data Rich	1	1	Log-transformed SEV scalar: Intrahem Stroke	--
Intracerebral hemorrhage	Female	0-6 days	95+ years	Data Rich	-1	2	Elevation Over 1500m (proportion)	255
Intracerebral hemorrhage	Female	0-6 days	95+ years	Data Rich	-1	2	Healthcare access and quality index	402
Intracerebral hemorrhage	Female	0-6 days	95+ years	Data Rich	1	2	Outdoor Air Pollution (PM2.5)	441
Intracerebral hemorrhage	Female	0-6 days	95+ years	Data Rich	1	2	Indoor Air Pollution (All Cooking Fuels)	445
Intracerebral hemorrhage	Female	0-6 days	95+ years	Data Rich	1	2	Diabetes Fasting Plasma Glucose (mmol/L), age-standardized 25+	--
Intracerebral hemorrhage	Female	0-6 days	95+ years	Data Rich	1	2	Mean BMI	--
Intracerebral hemorrhage	Female	0-6 days	95+ years	Data Rich	-1	3	pulses legumes adjusted(g)	160
Intracerebral hemorrhage	Female	0-6 days	95+ years	Data Rich	-1	3	fruits adjusted(g)	299
Intracerebral hemorrhage	Female	0-6 days	95+ years	Data Rich	-1	3	nuts seeds adjusted(g)	422
Intracerebral hemorrhage	Female	0-6 days	95+ years	Data Rich	-1	3	LDI (I$ per capita)	--
Intracerebral hemorrhage	Female	0-6 days	95+ years	Data Rich	-1	3	omega 3 adjusted(g)	--
Intracerebral hemorrhage	Female	0-6 days	95+ years	Data Rich	-1	3	pufa adjusted(percent)	--
Intracerebral hemorrhage	Female	0-6 days	95+ years	Data Rich	-1	3	vegetables adjusted(g)	--
Intracerebral hemorrhage	Female	0-6 days	95+ years	Data Rich	0	3	Socio-demographic Index	145
Intracerebral hemorrhage	Female	0-6 days	95+ years	Data Rich	0	3	Alcohol (liters per capita)	755
Intracerebral hemorrhage	Female	0-6 days	95+ years	Data Rich	1	3	Diet high in trans fatty acids	0
Intracerebral hemorrhage	Female	0-6 days	95+ years	Global	0	1	Cholesterol (total, mean per capita)	960
Intracerebral hemorrhage	Female	0-6 days	95+ years	Global	1	1	Smoking Prevalence	930
Intracerebral hemorrhage	Female	0-6 days	95+ years	Global	1	1	Systolic Blood Pressure (mmHg)	996
Intracerebral hemorrhage	Female	0-6 days	95+ years	Global	1	1	Log-transformed SEV scalar: Intrahem Stroke	--
Intracerebral hemorrhage	Female	0-6 days	95+ years	Global	-1	2	Healthcare access and quality index	427
Intracerebral hemorrhage	Female	0-6 days	95+ years	Global	-1	2	Elevation Over 1500m (proportion)	--
Intracerebral hemorrhage	Female	0-6 days	95+ years	Global	1	2	Indoor Air Pollution (All Cooking Fuels)	36
Intracerebral hemorrhage	Female	0-6 days	95+ years	Global	1	2	Outdoor Air Pollution (PM2.5)	40
Intracerebral hemorrhage	Female	0-6 days	95+ years	Global	1	2	Diabetes Fasting Plasma Glucose (mmol/L), age-standardized 25+	--
Intracerebral hemorrhage	Female	0-6 days	95+ years	Global	1	2	Mean BMI	--
Intracerebral hemorrhage	Female	0-6 days	95+ years	Global	-1	3	fruits adjusted(g)	10
Intracerebral hemorrhage	Female	0-6 days	95+ years	Global	-1	3	nuts seeds adjusted(g)	364
Intracerebral hemorrhage	Female	0-6 days	95+ years	Global	-1	3	pulses legumes adjusted(g)	698

Table S16. CODEm covariates used, level of covariate, and expected direction of covariate by cause, sex, and age<br>*Covariates that CODEm did not select during the covariate selection process have no draw counts listed.*								
**Cause**	**Sex**	**Age Start**	**Age End**	**Model Type**	**Direction**	**Level**	**Covariate Name**	**Number of Draws**
Intracerebral hemorrhage	Female	0-6 days	95+ years	Global	-1	3	LDI (I$ per capita)	--
Intracerebral hemorrhage	Female	0-6 days	95+ years	Global	-1	3	omega 3 adjusted(g)	--
Intracerebral hemorrhage	Female	0-6 days	95+ years	Global	-1	3	pufa adjusted(percent)	--
Intracerebral hemorrhage	Female	0-6 days	95+ years	Global	-1	3	vegetables adjusted(g)	--
Intracerebral hemorrhage	Female	0-6 days	95+ years	Global	0	3	Alcohol (liters per capita)	230
Intracerebral hemorrhage	Female	0-6 days	95+ years	Global	0	3	Socio-demographic Index	481
Intracerebral hemorrhage	Female	0-6 days	95+ years	Global	1	3	Diet high in trans fatty acids	0
Intracerebral hemorrhage	Male	0-6 days	95+ years	Data Rich	0	1	Cholesterol (total, mean per capita)	811
Intracerebral hemorrhage	Male	0-6 days	95+ years	Data Rich	1	1	Systolic Blood Pressure (mmHg)	815
Intracerebral hemorrhage	Male	0-6 days	95+ years	Data Rich	1	1	Smoking Prevalence	1000
Intracerebral hemorrhage	Male	0-6 days	95+ years	Data Rich	1	1	Log-transformed SEV scalar: Intrahem Stroke	--
Intracerebral hemorrhage	Male	0-6 days	95+ years	Data Rich	-1	2	Healthcare access and quality index	805
Intracerebral hemorrhage	Male	0-6 days	95+ years	Data Rich	-1	2	Elevation Over 1500m (proportion)	833
Intracerebral hemorrhage	Male	0-6 days	95+ years	Data Rich	1	2	Indoor Air Pollution (All Cooking Fuels)	222
Intracerebral hemorrhage	Male	0-6 days	95+ years	Data Rich	1	2	Outdoor Air Pollution (PM2.5)	930
Intracerebral hemorrhage	Male	0-6 days	95+ years	Data Rich	1	2	Diabetes Fasting Plasma Glucose (mmol/L), age-standardized 25+	--
Intracerebral hemorrhage	Male	0-6 days	95+ years	Data Rich	1	2	Mean BMI	--
Intracerebral hemorrhage	Male	0-6 days	95+ years	Data Rich	-1	3	fruits adjusted(g)	46
Intracerebral hemorrhage	Male	0-6 days	95+ years	Data Rich	-1	3	nuts seeds adjusted(g)	89
Intracerebral hemorrhage	Male	0-6 days	95+ years	Data Rich	-1	3	pulses legumes adjusted(g)	681
Intracerebral hemorrhage	Male	0-6 days	95+ years	Data Rich	-1	3	LDI (I$ per capita)	--
Intracerebral hemorrhage	Male	0-6 days	95+ years	Data Rich	-1	3	omega 3 adjusted(g)	--
Intracerebral hemorrhage	Male	0-6 days	95+ years	Data Rich	-1	3	pufa adjusted(percent)	--
Intracerebral hemorrhage	Male	0-6 days	95+ years	Data Rich	-1	3	vegetables adjusted(g)	--
Intracerebral hemorrhage	Male	0-6 days	95+ years	Data Rich	0	3	Alcohol (liters per capita)	300
Intracerebral hemorrhage	Male	0-6 days	95+ years	Data Rich	0	3	Socio-demographic Index	585
Intracerebral hemorrhage	Male	0-6 days	95+ years	Data Rich	1	3	Diet high in trans fatty acids	0
Intracerebral hemorrhage	Male	0-6 days	95+ years	Global	0	1	Cholesterol (total, mean per capita)	509
Intracerebral hemorrhage	Male	0-6 days	95+ years	Global	1	1	Systolic Blood Pressure (mmHg)	435
Intracerebral hemorrhage	Male	0-6 days	95+ years	Global	1	1	Smoking Prevalence	998
Intracerebral hemorrhage	Male	0-6 days	95+ years	Global	1	1	Log-transformed SEV scalar: Intrahem Stroke	--
Intracerebral hemorrhage	Male	0-6 days	95+ years	Global	-1	2	Healthcare access and quality index	458
Intracerebral hemorrhage	Male	0-6 days	95+ years	Global	-1	2	Elevation Over 1500m (proportion)	--
Intracerebral hemorrhage	Male	0-6 days	95+ years	Global	1	2	Indoor Air Pollution (All Cooking Fuels)	478
Intracerebral hemorrhage	Male	0-6 days	95+ years	Global	1	2	Outdoor Air Pollution (PM2.5)	864
Intracerebral hemorrhage	Male	0-6 days	95+ years	Global	1	2	Diabetes Fasting Plasma Glucose (mmol/L), age-standardized 25+	--
Intracerebral hemorrhage	Male	0-6 days	95+ years	Global	1	2	Mean BMI	--
Intracerebral hemorrhage	Male	0-6 days	95+ years	Global	-1	3	fruits adjusted(g)	0
Intracerebral hemorrhage	Male	0-6 days	95+ years	Global	-1	3	nuts seeds adjusted(g)	247
Intracerebral hemorrhage	Male	0-6 days	95+ years	Global	-1	3	pulses legumes adjusted(g)	855
Intracerebral hemorrhage	Male	0-6 days	95+ years	Global	-1	3	LDI (I$ per capita)	--
Intracerebral hemorrhage	Male	0-6 days	95+ years	Global	-1	3	omega 3 adjusted(g)	--
Intracerebral hemorrhage	Male	0-6 days	95+ years	Global	-1	3	pufa adjusted(percent)	--
Intracerebral hemorrhage	Male	0-6 days	95+ years	Global	-1	3	vegetables adjusted(g)	--
Intracerebral hemorrhage	Male	0-6 days	95+ years	Global	0	3	Socio-demographic Index	492
Intracerebral hemorrhage	Male	0-6 days	95+ years	Global	0	3	Alcohol (liters per capita)	713
Intracerebral hemorrhage	Male	0-6 days	95+ years	Global	1	3	Diet high in trans fatty acids	242
Subarachnoid hemorrhage	Female	0-6 days	95+ years	Data Rich	1	1	Systolic Blood Pressure (mmHg)	468
Subarachnoid hemorrhage	Female	0-6 days	95+ years	Data Rich	1	1	Smoking Prevalence	866
Subarachnoid hemorrhage	Female	0-6 days	95+ years	Data Rich	-1	2	Healthcare access and quality index	308
Subarachnoid hemorrhage	Female	0-6 days	95+ years	Data Rich	-1	3	LDI (I$ per capita)	--
Subarachnoid hemorrhage	Female	0-6 days	95+ years	Data Rich	0	3	Socio-demographic Index	178
Subarachnoid hemorrhage	Female	0-6 days	95+ years	Data Rich	0	3	Alcohol (liters per capita)	237
Subarachnoid hemorrhage	Female	0-6 days	95+ years	Global	1	1	Systolic Blood Pressure (mmHg)	564
Subarachnoid hemorrhage	Female	0-6 days	95+ years	Global	1	1	Smoking Prevalence	936
Subarachnoid hemorrhage	Female	0-6 days	95+ years	Global	-1	2	Healthcare access and quality index	647

Table S16. CODEm covariates used, level of covariate, and expected direction of covariate by cause, sex, and age
Covariates that CODEm did not select during the covariate selection process have no draw counts listed.

Cause	Sex	Age Start	Age End	Model Type	Direction	Level	Covariate Name	Number of Draws
Subarachnoid hemorrhage	Female	0-6 days	95+ years	Global	-1	3	LDI (I$ per capita)	--
Subarachnoid hemorrhage	Female	0-6 days	95+ years	Global	0	3	Alcohol (liters per capita)	351
Subarachnoid hemorrhage	Female	0-6 days	95+ years	Global	0	3	Socio-demographic Index	446
Subarachnoid hemorrhage	Male	0-6 days	95+ years	Data Rich	1	1	Smoking Prevalence	330
Subarachnoid hemorrhage	Male	0-6 days	95+ years	Data Rich	1	1	Systolic Blood Pressure (mmHg)	1000
Subarachnoid hemorrhage	Male	0-6 days	95+ years	Data Rich	-1	2	Healthcare access and quality index	401
Subarachnoid hemorrhage	Male	0-6 days	95+ years	Data Rich	-1	3	LDI (I$ per capita)	--
Subarachnoid hemorrhage	Male	0-6 days	95+ years	Data Rich	0	3	Socio-demographic Index	476
Subarachnoid hemorrhage	Male	0-6 days	95+ years	Data Rich	0	3	Alcohol (liters per capita)	554
Subarachnoid hemorrhage	Male	0-6 days	95+ years	Global	1	1	Systolic Blood Pressure (mmHg)	66
Subarachnoid hemorrhage	Male	0-6 days	95+ years	Global	1	1	Smoking Prevalence	245
Subarachnoid hemorrhage	Male	0-6 days	95+ years	Global	-1	2	Healthcare access and quality index	592
Subarachnoid hemorrhage	Male	0-6 days	95+ years	Global	-1	3	LDI (I$ per capita)	--
Subarachnoid hemorrhage	Male	0-6 days	95+ years	Global	0	3	Alcohol (liters per capita)	228
Subarachnoid hemorrhage	Male	0-6 days	95+ years	Global	0	3	Socio-demographic Index	371
Hypertensive heart disease	Female	15-19 years	95+ years	Data Rich	1	1	Systolic Blood Pressure (mmHg)	615
Hypertensive heart disease	Female	15-19 years	95+ years	Data Rich	-1	2	Healthcare access and quality index	214
Hypertensive heart disease	Female	15-19 years	95+ years	Data Rich	1	2	Smoking Prevalence	141
Hypertensive heart disease	Female	15-19 years	95+ years	Data Rich	1	2	Cholesterol (total, mean per capita)	--
Hypertensive heart disease	Female	15-19 years	95+ years	Data Rich	1	2	Mean BMI	--
Hypertensive heart disease	Female	15-19 years	95+ years	Data Rich	-1	3	LDI (I$ per capita)	--
Hypertensive heart disease	Female	15-19 years	95+ years	Data Rich	-1	3	fruits adjusted(g)	--
Hypertensive heart disease	Female	15-19 years	95+ years	Data Rich	-1	3	nuts seeds adjusted(g)	--
Hypertensive heart disease	Female	15-19 years	95+ years	Data Rich	-1	3	omega 3 adjusted(g)	--
Hypertensive heart disease	Female	15-19 years	95+ years	Data Rich	-1	3	pufa adjusted(percent)	--
Hypertensive heart disease	Female	15-19 years	95+ years	Data Rich	-1	3	pulses legumes adjusted(g)	--
Hypertensive heart disease	Female	15-19 years	95+ years	Data Rich	-1	3	vegetables adjusted(g)	--
Hypertensive heart disease	Female	15-19 years	95+ years	Data Rich	0	3	Socio-demographic Index	433
Hypertensive heart disease	Female	15-19 years	95+ years	Data Rich	0	3	Alcohol (liters per capita)	448
Hypertensive heart disease	Female	15-19 years	95+ years	Data Rich	1	3	Diet high in trans fatty acids	--
Hypertensive heart disease	Female	15-19 years	95+ years	Global	1	1	Systolic Blood Pressure (mmHg)	480
Hypertensive heart disease	Female	15-19 years	95+ years	Global	-1	2	Healthcare access and quality index	389
Hypertensive heart disease	Female	15-19 years	95+ years	Global	1	2	Smoking Prevalence	277
Hypertensive heart disease	Female	15-19 years	95+ years	Global	1	2	Cholesterol (total, mean per capita)	--
Hypertensive heart disease	Female	15-19 years	95+ years	Global	1	2	Mean BMI	--
Hypertensive heart disease	Female	15-19 years	95+ years	Global	-1	3	nuts seeds adjusted(g)	14
Hypertensive heart disease	Female	15-19 years	95+ years	Global	-1	3	fruits adjusted(g)	47
Hypertensive heart disease	Female	15-19 years	95+ years	Global	-1	3	LDI (I$ per capita)	--
Hypertensive heart disease	Female	15-19 years	95+ years	Global	-1	3	omega 3 adjusted(g)	--
Hypertensive heart disease	Female	15-19 years	95+ years	Global	-1	3	pufa adjusted(percent)	--
Hypertensive heart disease	Female	15-19 years	95+ years	Global	-1	3	pulses legumes adjusted(g)	--
Hypertensive heart disease	Female	15-19 years	95+ years	Global	-1	3	vegetables adjusted(g)	--
Hypertensive heart disease	Female	15-19 years	95+ years	Global	0	3	Alcohol (liters per capita)	425
Hypertensive heart disease	Female	15-19 years	95+ years	Global	0	3	Socio-demographic Index	560
Hypertensive heart disease	Female	15-19 years	95+ years	Global	1	3	Diet high in trans fatty acids	--

Table S16. CODEm covariates used, level of covariate, and expected direction of covariate by cause, sex, and age<br>*Covariates that CODEm did not select during the covariate selection process have no draw counts listed.*								
**Cause**	**Sex**	**Age Start**	**Age End**	**Model Type**	**Direction**	**Level**	**Covariate Name**	**Number of Draws**
Hypertensive heart disease	Male	15-19 years	95+ years	Data Rich	1	1	Systolic Blood Pressure (mmHg)	--
Hypertensive heart disease	Male	15-19 years	95+ years	Data Rich	-1	2	Healthcare access and quality index	285
Hypertensive heart disease	Male	15-19 years	95+ years	Data Rich	1	2	Smoking Prevalence	647
Hypertensive heart disease	Male	15-19 years	95+ years	Data Rich	1	2	Cholesterol (total, mean per capita)	--
Hypertensive heart disease	Male	15-19 years	95+ years	Data Rich	1	2	Mean BMI	--
Hypertensive heart disease	Male	15-19 years	95+ years	Data Rich	-1	3	pulses legumes adjusted(g)	145
Hypertensive heart disease	Male	15-19 years	95+ years	Data Rich	-1	3	LDI (I$ per capita)	--
Hypertensive heart disease	Male	15-19 years	95+ years	Data Rich	-1	3	fruits adjusted(g)	--
Hypertensive heart disease	Male	15-19 years	95+ years	Data Rich	-1	3	nuts seeds adjusted(g)	--
Hypertensive heart disease	Male	15-19 years	95+ years	Data Rich	-1	3	omega 3 adjusted(g)	--
Hypertensive heart disease	Male	15-19 years	95+ years	Data Rich	-1	3	pufa adjusted(percent)	--
Hypertensive heart disease	Male	15-19 years	95+ years	Data Rich	-1	3	vegetables adjusted(g)	--
Hypertensive heart disease	Male	15-19 years	95+ years	Data Rich	0	3	Alcohol (liters per capita)	248
Hypertensive heart disease	Male	15-19 years	95+ years	Data Rich	0	3	Socio-demographic Index	477
Hypertensive heart disease	Male	15-19 years	95+ years	Data Rich	1	3	Diet high in trans fatty acids	--
Hypertensive heart disease	Male	15-19 years	95+ years	Global	1	1	Systolic Blood Pressure (mmHg)	110
Hypertensive heart disease	Male	15-19 years	95+ years	Global	-1	2	Healthcare access and quality index	557
Hypertensive heart disease	Male	15-19 years	95+ years	Global	1	2	Mean BMI	430
Hypertensive heart disease	Male	15-19 years	95+ years	Global	1	2	Smoking Prevalence	547
Hypertensive heart disease	Male	15-19 years	95+ years	Global	1	2	Cholesterol (total, mean per capita)	--
Hypertensive heart disease	Male	15-19 years	95+ years	Global	-1	3	fruits adjusted(g)	31
Hypertensive heart disease	Male	15-19 years	95+ years	Global	-1	3	LDI (I$ per capita)	--
Hypertensive heart disease	Male	15-19 years	95+ years	Global	-1	3	nuts seeds adjusted(g)	--
Hypertensive heart disease	Male	15-19 years	95+ years	Global	-1	3	omega 3 adjusted(g)	--
Hypertensive heart disease	Male	15-19 years	95+ years	Global	-1	3	pufa adjusted(percent)	--
Hypertensive heart disease	Male	15-19 years	95+ years	Global	-1	3	pulses legumes adjusted(g)	--
Hypertensive heart disease	Male	15-19 years	95+ years	Global	-1	3	vegetables adjusted(g)	--
Hypertensive heart disease	Male	15-19 years	95+ years	Global	0	3	Socio-demographic Index	251
Hypertensive heart disease	Male	15-19 years	95+ years	Global	0	3	Alcohol (liters per capita)	429
Hypertensive heart disease	Male	15-19 years	95+ years	Global	1	3	Diet high in trans fatty acids	--
Non-rheumatic valvular heart disease	Female	15-19 years	95+ years	Data Rich	1	1	Systolic Blood Pressure (mmHg)	370
Non-rheumatic valvular heart disease	Female	15-19 years	95+ years	Data Rich	1	1	Smoking Prevalence	416
Non-rheumatic valvular heart disease	Female	15-19 years	95+ years	Data Rich	-1	2	Healthcare access and quality index	--
Non-rheumatic valvular heart disease	Female	15-19 years	95+ years	Data Rich	1	2	Mean BMI	276
Non-rheumatic valvular heart disease	Female	15-19 years	95+ years	Data Rich	1	2	Cholesterol (total, mean per capita)	--
Non-rheumatic valvular heart disease	Female	15-19 years	95+ years	Data Rich	-1	3	LDI (I$ per capita)	--
Non-rheumatic valvular heart disease	Female	15-19 years	95+ years	Data Rich	0	3	Socio-demographic Index	420
Non-rheumatic valvular heart disease	Female	15-19 years	95+ years	Data Rich	0	3	Alcohol (liters per capita)	431
Non-rheumatic valvular heart disease	Female	15-19 years	95+ years	Global	1	1	Systolic Blood Pressure (mmHg)	350
Non-rheumatic valvular heart disease	Female	15-19 years	95+ years	Global	1	1	Smoking Prevalence	414

Table S16. CODEm covariates used, level of covariate, and expected direction of covariate by cause, sex, and age
Covariates that CODEm did not select during the covariate selection process have no draw counts listed.

Cause	Sex	Age Start	Age End	Model Type	Direction	Level	Covariate Name	Number of Draws
Non-rheumatic valvular heart disease	Female	15-19 years	95+ years	Global	-1	2	Healthcare access and quality index	183
Non-rheumatic valvular heart disease	Female	15-19 years	95+ years	Global	1	2	Mean BMI	525
Non-rheumatic valvular heart disease	Female	15-19 years	95+ years	Global	1	2	Cholesterol (total, mean per capita)	--
Non-rheumatic valvular heart disease	Female	15-19 years	95+ years	Global	-1	3	LDI (I$ per capita)	--
Non-rheumatic valvular heart disease	Female	15-19 years	95+ years	Global	0	3	Socio-demographic Index	513
Non-rheumatic valvular heart disease	Female	15-19 years	95+ years	Global	0	3	Alcohol (liters per capita)	673
Non-rheumatic valvular heart disease	Male	15-19 years	95+ years	Data Rich	1	1	Systolic Blood Pressure (mmHg)	149
Non-rheumatic valvular heart disease	Male	15-19 years	95+ years	Data Rich	1	1	Smoking Prevalence	272
Non-rheumatic valvular heart disease	Male	15-19 years	95+ years	Data Rich	-1	2	Healthcare access and quality index	77
Non-rheumatic valvular heart disease	Male	15-19 years	95+ years	Data Rich	1	2	Mean BMI	599
Non-rheumatic valvular heart disease	Male	15-19 years	95+ years	Data Rich	1	2	Cholesterol (total, mean per capita)	--
Non-rheumatic valvular heart disease	Male	15-19 years	95+ years	Data Rich	-1	3	LDI (I$ per capita)	--
Non-rheumatic valvular heart disease	Male	15-19 years	95+ years	Data Rich	0	3	Alcohol (liters per capita)	280
Non-rheumatic valvular heart disease	Male	15-19 years	95+ years	Data Rich	0	3	Socio-demographic Index	401
Non-rheumatic valvular heart disease	Male	15-19 years	95+ years	Global	1	1	Smoking Prevalence	248
Non-rheumatic valvular heart disease	Male	15-19 years	95+ years	Global	1	1	Systolic Blood Pressure (mmHg)	395
Non-rheumatic valvular heart disease	Male	15-19 years	95+ years	Global	-1	2	Healthcare access and quality index	268
Non-rheumatic valvular heart disease	Male	15-19 years	95+ years	Global	1	2	Mean BMI	299
Non-rheumatic valvular heart disease	Male	15-19 years	95+ years	Global	1	2	Cholesterol (total, mean per capita)	--
Non-rheumatic valvular heart disease	Male	15-19 years	95+ years	Global	-1	3	LDI (I$ per capita)	--
Non-rheumatic valvular heart disease	Male	15-19 years	95+ years	Global	0	3	Alcohol (liters per capita)	402
Non-rheumatic valvular heart disease	Male	15-19 years	95+ years	Global	0	3	Socio-demographic Index	576
Non-rheumatic calcific aortic valve disease	Female	15-19 years	95+ years	Data Rich	1	1	Smoking Prevalence	277
Non-rheumatic calcific aortic valve disease	Female	15-19 years	95+ years	Data Rich	1	1	Mean BMI	1000
Non-rheumatic calcific aortic valve disease	Female	15-19 years	95+ years	Data Rich	1	1	Systolic Blood Pressure (mmHg)	--
Non-rheumatic calcific aortic valve disease	Female	15-19 years	95+ years	Data Rich	-1	2	Healthcare access and quality index	--
Non-rheumatic calcific aortic valve disease	Female	15-19 years	95+ years	Data Rich	1	2	Cholesterol (total, mean per capita)	--
Non-rheumatic calcific aortic valve disease	Female	15-19 years	95+ years	Data Rich	1	2	Diabetes Fasting Plasma Glucose (mmol/L), age-standardized 25+	--
Non-rheumatic calcific aortic valve disease	Female	15-19 years	95+ years	Data Rich	-1	3	LDI (I$ per capita)	--
Non-rheumatic calcific aortic valve disease	Female	15-19 years	95+ years	Data Rich	0	3	Socio-demographic Index	463
Non-rheumatic calcific aortic valve disease	Female	15-19 years	95+ years	Data Rich	0	3	Alcohol (liters per capita)	596
Non-rheumatic calcific aortic valve disease	Female	15-19 years	95+ years	Global	1	1	Mean BMI	513
Non-rheumatic calcific aortic valve disease	Female	15-19 years	95+ years	Global	1	1	Smoking Prevalence	513
Non-rheumatic calcific aortic valve disease	Female	15-19 years	95+ years	Global	1	1	Systolic Blood Pressure (mmHg)	--
Non-rheumatic calcific aortic valve disease	Female	15-19 years	95+ years	Global	-1	2	Healthcare access and quality index	--
Non-rheumatic calcific aortic valve disease	Female	15-19 years	95+ years	Global	1	2	Diabetes Fasting Plasma Glucose (mmol/L), age-standardized 25+	334
Non-rheumatic calcific aortic valve disease	Female	15-19 years	95+ years	Global	1	2	Cholesterol (total, mean per capita)	--
Non-rheumatic calcific aortic valve disease	Female	15-19 years	95+ years	Global	-1	3	LDI (I$ per capita)	--
Non-rheumatic calcific aortic valve disease	Female	15-19 years	95+ years	Global	0	3	Socio-demographic Index	282
Non-rheumatic calcific aortic valve disease	Female	15-19 years	95+ years	Global	0	3	Alcohol (liters per capita)	628

Table S16. CODEm covariates used, level of covariate, and expected direction of covariate by cause, sex, and age
Covariates that CODEm did not select during the covariate selection process have no draw counts listed.

Cause	Sex	Age Start	Age End	Model Type	Direction	Level	Covariate Name	Number of Draws
Non-rheumatic calcific aortic valve disease	Male	15-19 years	95+ years	Data Rich	1	1	Smoking Prevalence	278
Non-rheumatic calcific aortic valve disease	Male	15-19 years	95+ years	Data Rich	1	1	Mean BMI	1000
Non-rheumatic calcific aortic valve disease	Male	15-19 years	95+ years	Data Rich	1	1	Systolic Blood Pressure (mmHg)	--
Non-rheumatic calcific aortic valve disease	Male	15-19 years	95+ years	Data Rich	-1	2	Healthcare access and quality index	--
Non-rheumatic calcific aortic valve disease	Male	15-19 years	95+ years	Data Rich	1	2	Cholesterol (total, mean per capita)	--
Non-rheumatic calcific aortic valve disease	Male	15-19 years	95+ years	Data Rich	1	2	Diabetes Fasting Plasma Glucose (mmol/L), age-standardized 25+	--
Non-rheumatic calcific aortic valve disease	Male	15-19 years	95+ years	Data Rich	-1	3	LDI (I$ per capita)	--
Non-rheumatic calcific aortic valve disease	Male	15-19 years	95+ years	Data Rich	0	3	Socio-demographic Index	317
Non-rheumatic calcific aortic valve disease	Male	15-19 years	95+ years	Data Rich	0	3	Alcohol (liters per capita)	572
Non-rheumatic calcific aortic valve disease	Male	15-19 years	95+ years	Global	1	1	Smoking Prevalence	278
Non-rheumatic calcific aortic valve disease	Male	15-19 years	95+ years	Global	1	1	Mean BMI	1000
Non-rheumatic calcific aortic valve disease	Male	15-19 years	95+ years	Global	1	1	Systolic Blood Pressure (mmHg)	--
Non-rheumatic calcific aortic valve disease	Male	15-19 years	95+ years	Global	-1	2	Healthcare access and quality index	--
Non-rheumatic calcific aortic valve disease	Male	15-19 years	95+ years	Global	1	2	Cholesterol (total, mean per capita)	--
Non-rheumatic calcific aortic valve disease	Male	15-19 years	95+ years	Global	1	2	Diabetes Fasting Plasma Glucose (mmol/L), age-standardized 25+	--
Non-rheumatic calcific aortic valve disease	Male	15-19 years	95+ years	Global	-1	3	LDI (I$ per capita)	--
Non-rheumatic calcific aortic valve disease	Male	15-19 years	95+ years	Global	0	3	Alcohol (liters per capita)	572
Non-rheumatic calcific aortic valve disease	Male	15-19 years	95+ years	Global	0	3	Socio-demographic Index	572
Non-rheumatic degenerative mitral valve disease	Female	15-19 years	95+ years	Data Rich	-1	1	Healthcare access and quality index	557
Non-rheumatic degenerative mitral valve disease	Female	15-19 years	95+ years	Data Rich	0	1	Socio-demographic Index	619
Non-rheumatic degenerative mitral valve disease	Female	15-19 years	95+ years	Data Rich	0	1	LDI (I$ per capita)	--
Non-rheumatic degenerative mitral valve disease	Female	15-19 years	95+ years	Global	-1	1	Healthcare access and quality index	445
Non-rheumatic degenerative mitral valve disease	Female	15-19 years	95+ years	Global	0	1	Socio-demographic Index	533
Non-rheumatic degenerative mitral valve disease	Female	15-19 years	95+ years	Global	0	1	LDI (I$ per capita)	--
Non-rheumatic degenerative mitral valve disease	Male	15-19 years	95+ years	Data Rich	-1	1	Healthcare access and quality index	600
Non-rheumatic degenerative mitral valve disease	Male	15-19 years	95+ years	Data Rich	0	1	Socio-demographic Index	410
Non-rheumatic degenerative mitral valve disease	Male	15-19 years	95+ years	Data Rich	0	1	LDI (I$ per capita)	--
Non-rheumatic degenerative mitral valve disease	Male	15-19 years	95+ years	Global	-1	1	Healthcare access and quality index	443
Non-rheumatic degenerative mitral valve disease	Male	15-19 years	95+ years	Global	0	1	Socio-demographic Index	621
Non-rheumatic degenerative mitral valve disease	Male	15-19 years	95+ years	Global	0	1	LDI (I$ per capita)	--
Other non-rheumatic valve diseases	Female	15-19 years	95+ years	Data Rich	1	1	Log-transformed SEV scalar: CVD	--
Other non-rheumatic valve diseases	Female	15-19 years	95+ years	Global	1	1	Log-transformed SEV scalar: CVD	--
Other non-rheumatic valve diseases	Male	15-19 years	95+ years	Data Rich	1	1	Log-transformed SEV scalar: CVD	--
Other non-rheumatic valve diseases	Male	15-19 years	95+ years	Global	1	1	Log-transformed SEV scalar: CVD	--
Cardiomyopathy and myocarditis	Female	0-6 days	95+ years	Data Rich	1	1	Smoking Prevalence	193
Cardiomyopathy and myocarditis	Female	0-6 days	95+ years	Data Rich	1	1	Systolic Blood Pressure (mmHg)	449
Cardiomyopathy and myocarditis	Female	0-6 days	95+ years	Data Rich	1	1	Log-transformed SEV scalar: CMP	--
Cardiomyopathy and myocarditis	Female	0-6 days	95+ years	Data Rich	-1	2	Healthcare access and quality index	314
Cardiomyopathy and myocarditis	Female	0-6 days	95+ years	Data Rich	1	2	Mean BMI	422
Cardiomyopathy and myocarditis	Female	0-6 days	95+ years	Data Rich	0	3	Socio-demographic Index	438
Cardiomyopathy and myocarditis	Female	0-6 days	95+ years	Data Rich	0	3	Alcohol (liters per capita)	467
Cardiomyopathy and myocarditis	Female	0-6 days	95+ years	Data Rich	0	3	LDI (I$ per capita)	--
Cardiomyopathy and myocarditis	Female	0-6 days	95+ years	Global	0	1	Smoking Prevalence	755

Table S16. CODEm covariates used, level of covariate, and expected direction of covariate by cause, sex, and age
Covariates that CODEm did not select during the covariate selection process have no draw counts listed.

Cause	Sex	Age Start	Age End	Model Type	Direction	Level	Covariate Name	Number of Draws
Cardiomyopathy and myocarditis	Female	0-6 days	95+ years	Global	0	1	Systolic Blood Pressure (mmHg)	783
Cardiomyopathy and myocarditis	Female	0-6 days	95+ years	Global	0	1	Log-transformed SEV scalar: CMP	--
Cardiomyopathy and myocarditis	Female	0-6 days	95+ years	Global	-1	2	Healthcare access and quality index	49
Cardiomyopathy and myocarditis	Female	0-6 days	95+ years	Global	1	2	Mean BMI	554
Cardiomyopathy and myocarditis	Female	0-6 days	95+ years	Global	0	3	Socio-demographic Index	78
Cardiomyopathy and myocarditis	Female	0-6 days	95+ years	Global	0	3	Alcohol (liters per capita)	253
Cardiomyopathy and myocarditis	Female	0-6 days	95+ years	Global	0	3	LDI (I$ per capita)	--
Cardiomyopathy and myocarditis	Male	0-6 days	95+ years	Data Rich	1	1	Smoking Prevalence	208
Cardiomyopathy and myocarditis	Male	0-6 days	95+ years	Data Rich	1	1	Systolic Blood Pressure (mmHg)	298
Cardiomyopathy and myocarditis	Male	0-6 days	95+ years	Data Rich	1	1	Log-transformed SEV scalar: CMP	--
Cardiomyopathy and myocarditis	Male	0-6 days	95+ years	Data Rich	-1	2	Healthcare access and quality index	--
Cardiomyopathy and myocarditis	Male	0-6 days	95+ years	Data Rich	1	2	Mean BMI	600
Cardiomyopathy and myocarditis	Male	0-6 days	95+ years	Data Rich	0	3	Socio-demographic Index	527
Cardiomyopathy and myocarditis	Male	0-6 days	95+ years	Data Rich	0	3	Alcohol (liters per capita)	705
Cardiomyopathy and myocarditis	Male	0-6 days	95+ years	Data Rich	0	3	LDI (I$ per capita)	--
Cardiomyopathy and myocarditis	Male	0-6 days	95+ years	Global	1	1	Smoking Prevalence	187
Cardiomyopathy and myocarditis	Male	0-6 days	95+ years	Global	1	1	Systolic Blood Pressure (mmHg)	424
Cardiomyopathy and myocarditis	Male	0-6 days	95+ years	Global	1	1	Log-transformed SEV scalar: CMP	--
Cardiomyopathy and myocarditis	Male	0-6 days	95+ years	Global	-1	2	Healthcare access and quality index	273
Cardiomyopathy and myocarditis	Male	0-6 days	95+ years	Global	1	2	Mean BMI	265
Cardiomyopathy and myocarditis	Male	0-6 days	95+ years	Global	0	3	Alcohol (liters per capita)	487
Cardiomyopathy and myocarditis	Male	0-6 days	95+ years	Global	0	3	Socio-demographic Index	636
Cardiomyopathy and myocarditis	Male	0-6 days	95+ years	Global	0	3	LDI (I$ per capita)	--
Myocarditis	Female	0-6 days	95+ years	Data Rich	1	1	Log-transformed SEV scalar: CMP	--
Myocarditis	Female	0-6 days	95+ years	Data Rich	1	1	Systolic Blood Pressure (mmHg)	--
Myocarditis	Female	0-6 days	95+ years	Data Rich	-1	2	Healthcare access and quality index	489
Myocarditis	Female	0-6 days	95+ years	Data Rich	-1	3	LDI (I$ per capita)	--
Myocarditis	Female	0-6 days	95+ years	Data Rich	0	3	Socio-demographic Index	778
Myocarditis	Female	0-6 days	95+ years	Global	1	1	Log-transformed SEV scalar: CMP	--
Myocarditis	Female	0-6 days	95+ years	Global	1	1	Systolic Blood Pressure (mmHg)	--
Myocarditis	Female	0-6 days	95+ years	Global	-1	2	Healthcare access and quality index	424
Myocarditis	Female	0-6 days	95+ years	Global	-1	3	LDI (I$ per capita)	--
Myocarditis	Female	0-6 days	95+ years	Global	0	3	Socio-demographic Index	674
Myocarditis	Male	0-6 days	95+ years	Data Rich	1	1	Log-transformed SEV scalar: CMP	--
Myocarditis	Male	0-6 days	95+ years	Data Rich	1	1	Systolic Blood Pressure (mmHg)	--
Myocarditis	Male	0-6 days	95+ years	Data Rich	-1	2	Healthcare access and quality index	--
Myocarditis	Male	0-6 days	95+ years	Data Rich	-1	3	LDI (I$ per capita)	--
Myocarditis	Male	0-6 days	95+ years	Data Rich	0	3	Socio-demographic Index	1000
Myocarditis	Male	0-6 days	95+ years	Global	1	1	Log-transformed SEV scalar: CMP	--
Myocarditis	Male	0-6 days	95+ years	Global	1	1	Systolic Blood Pressure (mmHg)	--
Myocarditis	Male	0-6 days	95+ years	Global	-1	2	Healthcare access and quality index	489
Myocarditis	Male	0-6 days	95+ years	Global	-1	3	LDI (I$ per capita)	--
Myocarditis	Male	0-6 days	95+ years	Global	0	3	Socio-demographic Index	778
Alcoholic cardiomyopathy	Female	15-19 years	95+ years	Data Rich	1	1	Smoking Prevalence	182
Alcoholic cardiomyopathy	Female	15-19 years	95+ years	Data Rich	1	1	Alcohol (liters per capita)	899
Alcoholic cardiomyopathy	Female	15-19 years	95+ years	Data Rich	1	1	Log-transformed SEV scalar: CMP	--
Alcoholic cardiomyopathy	Female	15-19 years	95+ years	Data Rich	-1	2	Healthcare access and quality index	193
Alcoholic cardiomyopathy	Female	15-19 years	95+ years	Data Rich	-1	3	LDI (I$ per capita)	--
Alcoholic cardiomyopathy	Female	15-19 years	95+ years	Data Rich	0	3	Socio-demographic Index	168
Alcoholic cardiomyopathy	Female	15-19 years	95+ years	Global	1	1	Smoking Prevalence	453
Alcoholic cardiomyopathy	Female	15-19 years	95+ years	Global	1	1	Alcohol (liters per capita)	605
Alcoholic cardiomyopathy	Female	15-19 years	95+ years	Global	1	1	Log-transformed SEV scalar: CMP	--
Alcoholic cardiomyopathy	Female	15-19 years	95+ years	Global	-1	2	Healthcare access and quality index	169
Alcoholic cardiomyopathy	Female	15-19 years	95+ years	Global	-1	3	LDI (I$ per capita)	--

Table S16. CODEm covariates used, level of covariate, and expected direction of covariate by cause, sex, and age<br>*Covariates that CODEm did not select during the covariate selection process have no draw counts listed.*								
**Cause**	**Sex**	**Age Start**	**Age End**	**Model Type**	**Direction**	**Level**	**Covariate Name**	**Number of Draws**
Alcoholic cardiomyopathy	Female	15-19 years	95+ years	Global	0	3	Socio-demographic Index	204
Alcoholic cardiomyopathy	Male	15-19 years	95+ years	Data Rich	1	1	Smoking Prevalence	84
Alcoholic cardiomyopathy	Male	15-19 years	95+ years	Data Rich	1	1	Alcohol (liters per capita)	648
Alcoholic cardiomyopathy	Male	15-19 years	95+ years	Data Rich	1	1	Log-transformed SEV scalar: CMP	--
Alcoholic cardiomyopathy	Male	15-19 years	95+ years	Data Rich	-1	2	Healthcare access and quality index	--
Alcoholic cardiomyopathy	Male	15-19 years	95+ years	Data Rich	-1	3	LDI (I$ per capita)	--
Alcoholic cardiomyopathy	Male	15-19 years	95+ years	Data Rich	0	3	Socio-demographic Index	714
Alcoholic cardiomyopathy	Male	15-19 years	95+ years	Global	1	1	Smoking Prevalence	186
Alcoholic cardiomyopathy	Male	15-19 years	95+ years	Global	1	1	Alcohol (liters per capita)	378
Alcoholic cardiomyopathy	Male	15-19 years	95+ years	Global	1	1	Log-transformed SEV scalar: CMP	--
Alcoholic cardiomyopathy	Male	15-19 years	95+ years	Global	-1	2	Healthcare access and quality index	50
Alcoholic cardiomyopathy	Male	15-19 years	95+ years	Global	-1	3	LDI (I$ per capita)	--
Alcoholic cardiomyopathy	Male	15-19 years	95+ years	Global	0	3	Socio-demographic Index	623
Other cardiomyopathy	Female	0-6 days	95+ years	Data Rich	1	1	Smoking Prevalence	316
Other cardiomyopathy	Female	0-6 days	95+ years	Data Rich	1	1	Systolic Blood Pressure (mmHg)	334
Other cardiomyopathy	Female	0-6 days	95+ years	Data Rich	1	1	Log-transformed SEV scalar: CMP	--
Other cardiomyopathy	Female	0-6 days	95+ years	Data Rich	-1	2	Healthcare access and quality index	--
Other cardiomyopathy	Female	0-6 days	95+ years	Data Rich	1	2	Mean BMI	426
Other cardiomyopathy	Female	0-6 days	95+ years	Data Rich	0	3	Socio-demographic Index	634
Other cardiomyopathy	Female	0-6 days	95+ years	Data Rich	0	3	LDI (I$ per capita)	--
Other cardiomyopathy	Female	0-6 days	95+ years	Global	1	1	Smoking Prevalence	718
Other cardiomyopathy	Female	0-6 days	95+ years	Global	1	1	Systolic Blood Pressure (mmHg)	718
Other cardiomyopathy	Female	0-6 days	95+ years	Global	1	1	Log-transformed SEV scalar: CMP	--
Other cardiomyopathy	Female	0-6 days	95+ years	Global	-1	2	Healthcare access and quality index	--
Other cardiomyopathy	Female	0-6 days	95+ years	Global	1	2	Mean BMI	364
Other cardiomyopathy	Female	0-6 days	95+ years	Global	0	3	Socio-demographic Index	585
Other cardiomyopathy	Female	0-6 days	95+ years	Global	0	3	LDI (I$ per capita)	--
Other cardiomyopathy	Male	0-6 days	95+ years	Data Rich	1	1	Systolic Blood Pressure (mmHg)	364
Other cardiomyopathy	Male	0-6 days	95+ years	Data Rich	1	1	Log-transformed SEV scalar: CMP	--
Other cardiomyopathy	Male	0-6 days	95+ years	Data Rich	1	1	Smoking Prevalence	--
Other cardiomyopathy	Male	0-6 days	95+ years	Data Rich	-1	2	Healthcare access and quality index	--
Other cardiomyopathy	Male	0-6 days	95+ years	Data Rich	1	2	Mean BMI	692
Other cardiomyopathy	Male	0-6 days	95+ years	Data Rich	0	3	Socio-demographic Index	463
Other cardiomyopathy	Male	0-6 days	95+ years	Data Rich	0	3	LDI (I$ per capita)	--
Other cardiomyopathy	Male	0-6 days	95+ years	Global	1	1	Systolic Blood Pressure (mmHg)	364
Other cardiomyopathy	Male	0-6 days	95+ years	Global	1	1	Log-transformed SEV scalar: CMP	--
Other cardiomyopathy	Male	0-6 days	95+ years	Global	1	1	Smoking Prevalence	--
Other cardiomyopathy	Male	0-6 days	95+ years	Global	-1	2	Healthcare access and quality index	--
Other cardiomyopathy	Male	0-6 days	95+ years	Global	1	2	Mean BMI	523
Other cardiomyopathy	Male	0-6 days	95+ years	Global	0	3	Socio-demographic Index	607
Other cardiomyopathy	Male	0-6 days	95+ years	Global	0	3	LDI (I$ per capita)	--
Atrial fibrillation and flutter	Female	30-34 years	95+ years	Data Rich	1	1	Smoking Prevalence	633
Atrial fibrillation and flutter	Female	30-34 years	95+ years	Data Rich	1	1	Log-transformed SEV scalar: A Fib	--
Atrial fibrillation and flutter	Female	30-34 years	95+ years	Data Rich	1	1	Systolic Blood Pressure (mmHg)	--
Atrial fibrillation and flutter	Female	30-34 years	95+ years	Data Rich	-1	2	Healthcare access and quality index	--
Atrial fibrillation and flutter	Female	30-34 years	95+ years	Data Rich	1	2	Mean BMI	334
Atrial fibrillation and flutter	Female	30-34 years	95+ years	Data Rich	1	2	Diabetes Fasting Plasma Glucose (mmol/L), age-standardized 25+	361
Atrial fibrillation and flutter	Female	30-34 years	95+ years	Data Rich	1	2	Cholesterol (total, mean per capita)	--
Atrial fibrillation and flutter	Female	30-34 years	95+ years	Data Rich	-1	3	vegetables adjusted(g)	7

Table S16. CODEm covariates used, level of covariate, and expected direction of covariate by cause, sex, and age
Covariates that CODEm did not select during the covariate selection process have no draw counts listed.

Cause	Sex	Age Start	Age End	Model Type	Direction	Level	Covariate Name	Number of Draws
Atrial fibrillation and flutter	Female	30-34 years	95+ years	Data Rich	-1	3	nuts seeds adjusted(g)	46
Atrial fibrillation and flutter	Female	30-34 years	95+ years	Data Rich	-1	3	pulses legumes adjusted(g)	194
Atrial fibrillation and flutter	Female	30-34 years	95+ years	Data Rich	-1	3	LDI (I$ per capita)	--
Atrial fibrillation and flutter	Female	30-34 years	95+ years	Data Rich	-1	3	fruits adjusted(g)	--
Atrial fibrillation and flutter	Female	30-34 years	95+ years	Data Rich	-1	3	omega 3 adjusted(g)	--
Atrial fibrillation and flutter	Female	30-34 years	95+ years	Data Rich	-1	3	pufa adjusted(percent)	--
Atrial fibrillation and flutter	Female	30-34 years	95+ years	Data Rich	0	3	Alcohol (liters per capita)	279
Atrial fibrillation and flutter	Female	30-34 years	95+ years	Data Rich	0	3	Socio-demographic Index	410
Atrial fibrillation and flutter	Female	30-34 years	95+ years	Data Rich	1	3	Diet high in trans fatty acids	96
Atrial fibrillation and flutter	Female	30-34 years	95+ years	Global	1	1	Smoking Prevalence	485
Atrial fibrillation and flutter	Female	30-34 years	95+ years	Global	1	1	Log-transformed SEV scalar: A Fib	--
Atrial fibrillation and flutter	Female	30-34 years	95+ years	Global	1	1	Systolic Blood Pressure (mmHg)	--
Atrial fibrillation and flutter	Female	30-34 years	95+ years	Global	-1	2	Healthcare access and quality index	378
Atrial fibrillation and flutter	Female	30-34 years	95+ years	Global	1	2	Mean BMI	250
Atrial fibrillation and flutter	Female	30-34 years	95+ years	Global	1	2	Diabetes Fasting Plasma Glucose (mmol/L), age-standardized 25+	378
Atrial fibrillation and flutter	Female	30-34 years	95+ years	Global	1	2	Cholesterol (total, mean per capita)	--
Atrial fibrillation and flutter	Female	30-34 years	95+ years	Global	-1	3	vegetables adjusted(g)	100
Atrial fibrillation and flutter	Female	30-34 years	95+ years	Global	-1	3	pulses legumes adjusted(g)	189
Atrial fibrillation and flutter	Female	30-34 years	95+ years	Global	-1	3	LDI (I$ per capita)	--
Atrial fibrillation and flutter	Female	30-34 years	95+ years	Global	-1	3	fruits adjusted(g)	--
Atrial fibrillation and flutter	Female	30-34 years	95+ years	Global	-1	3	nuts seeds adjusted(g)	--
Atrial fibrillation and flutter	Female	30-34 years	95+ years	Global	-1	3	omega 3 adjusted(g)	--
Atrial fibrillation and flutter	Female	30-34 years	95+ years	Global	-1	3	pufa adjusted(percent)	--
Atrial fibrillation and flutter	Female	30-34 years	95+ years	Global	0	3	Alcohol (liters per capita)	316
Atrial fibrillation and flutter	Female	30-34 years	95+ years	Global	0	3	Socio-demographic Index	487
Atrial fibrillation and flutter	Female	30-34 years	95+ years	Global	1	3	Diet high in trans fatty acids	67
Atrial fibrillation and flutter	Male	30-34 years	95+ years	Data Rich	1	1	Log-transformed SEV scalar: A Fib	--
Atrial fibrillation and flutter	Male	30-34 years	95+ years	Data Rich	1	1	Smoking Prevalence	--
Atrial fibrillation and flutter	Male	30-34 years	95+ years	Data Rich	1	1	Systolic Blood Pressure (mmHg)	--
Atrial fibrillation and flutter	Male	30-34 years	95+ years	Data Rich	-1	2	Healthcare access and quality index	--
Atrial fibrillation and flutter	Male	30-34 years	95+ years	Data Rich	1	2	Diabetes Fasting Plasma Glucose (mmol/L), age-standardized 25+	244
Atrial fibrillation and flutter	Male	30-34 years	95+ years	Data Rich	1	2	Mean BMI	428
Atrial fibrillation and flutter	Male	30-34 years	95+ years	Data Rich	1	2	Cholesterol (total, mean per capita)	--
Atrial fibrillation and flutter	Male	30-34 years	95+ years	Data Rich	-1	3	pulses legumes adjusted(g)	192
Atrial fibrillation and flutter	Male	30-34 years	95+ years	Data Rich	-1	3	LDI (I$ per capita)	--
Atrial fibrillation and flutter	Male	30-34 years	95+ years	Data Rich	-1	3	fruits adjusted(g)	--
Atrial fibrillation and flutter	Male	30-34 years	95+ years	Data Rich	-1	3	nuts seeds adjusted(g)	--
Atrial fibrillation and flutter	Male	30-34 years	95+ years	Data Rich	-1	3	omega 3 adjusted(g)	--
Atrial fibrillation and flutter	Male	30-34 years	95+ years	Data Rich	-1	3	pufa adjusted(percent)	--
Atrial fibrillation and flutter	Male	30-34 years	95+ years	Data Rich	-1	3	vegetables adjusted(g)	--

Table S16. CODEm covariates used, level of covariate, and expected direction of covariate by cause, sex, and age
Covariates that CODEm did not select during the covariate selection process have no draw counts listed.

Cause	Sex	Age Start	Age End	Model Type	Direction	Level	Covariate Name	Number of Draws
Atrial fibrillation and flutter	Male	30-34 years	95+ years	Data Rich	0	3	Socio-demographic Index	160
Atrial fibrillation and flutter	Male	30-34 years	95+ years	Data Rich	0	3	Alcohol (liters per capita)	680
Atrial fibrillation and flutter	Male	30-34 years	95+ years	Data Rich	1	3	Diet high in trans fatty acids	444
Atrial fibrillation and flutter	Male	30-34 years	95+ years	Global	1	1	Log-transformed SEV scalar: A Fib	--
Atrial fibrillation and flutter	Male	30-34 years	95+ years	Global	1	1	Smoking Prevalence	--
Atrial fibrillation and flutter	Male	30-34 years	95+ years	Global	1	1	Systolic Blood Pressure (mmHg)	--
Atrial fibrillation and flutter	Male	30-34 years	95+ years	Global	-1	2	Healthcare access and quality index	--
Atrial fibrillation and flutter	Male	30-34 years	95+ years	Global	1	2	Diabetes Fasting Plasma Glucose (mmol/L), age-standardized 25+	428
Atrial fibrillation and flutter	Male	30-34 years	95+ years	Global	1	2	Mean BMI	757
Atrial fibrillation and flutter	Male	30-34 years	95+ years	Global	1	2	Cholesterol (total, mean per capita)	--
Atrial fibrillation and flutter	Male	30-34 years	95+ years	Global	-1	3	pulses legumes adjusted(g)	36
Atrial fibrillation and flutter	Male	30-34 years	95+ years	Global	-1	3	LDI (I$ per capita)	--
Atrial fibrillation and flutter	Male	30-34 years	95+ years	Global	-1	3	fruits adjusted(g)	--
Atrial fibrillation and flutter	Male	30-34 years	95+ years	Global	-1	3	nuts seeds adjusted(g)	--
Atrial fibrillation and flutter	Male	30-34 years	95+ years	Global	-1	3	omega 3 adjusted(g)	--
Atrial fibrillation and flutter	Male	30-34 years	95+ years	Global	-1	3	pufa adjusted(percent)	--
Atrial fibrillation and flutter	Male	30-34 years	95+ years	Global	-1	3	vegetables adjusted(g)	--
Atrial fibrillation and flutter	Male	30-34 years	95+ years	Global	0	3	Socio-demographic Index	177
Atrial fibrillation and flutter	Male	30-34 years	95+ years	Global	0	3	Alcohol (liters per capita)	691
Atrial fibrillation and flutter	Male	30-34 years	95+ years	Global	1	3	Diet high in trans fatty acids	36
Aortic aneurysm	Female	15-19 years	95+ years	Data Rich	1	1	Systolic Blood Pressure (mmHg)	65
Aortic aneurysm	Female	15-19 years	95+ years	Data Rich	1	1	Cumulative Cigarettes (10 Years)	272
Aortic aneurysm	Female	15-19 years	95+ years	Data Rich	1	1	Cholesterol (total, mean per capita)	602
Aortic aneurysm	Female	15-19 years	95+ years	Data Rich	1	1	Log-transformed SEV scalar: Aort An	--
Aortic aneurysm	Female	15-19 years	95+ years	Data Rich	-1	2	Healthcare access and quality index	--
Aortic aneurysm	Female	15-19 years	95+ years	Data Rich	1	2	Mean BMI	187
Aortic aneurysm	Female	15-19 years	95+ years	Data Rich	-1	3	nuts seeds adjusted(g)	1
Aortic aneurysm	Female	15-19 years	95+ years	Data Rich	-1	3	vegetables adjusted(g)	1
Aortic aneurysm	Female	15-19 years	95+ years	Data Rich	-1	3	pulses legumes adjusted(g)	66
Aortic aneurysm	Female	15-19 years	95+ years	Data Rich	-1	3	LDI (I$ per capita)	--
Aortic aneurysm	Female	15-19 years	95+ years	Data Rich	-1	3	fruits adjusted(g)	--
Aortic aneurysm	Female	15-19 years	95+ years	Data Rich	-1	3	omega 3 adjusted(g)	--
Aortic aneurysm	Female	15-19 years	95+ years	Data Rich	-1	3	pufa adjusted(percent)	--
Aortic aneurysm	Female	15-19 years	95+ years	Data Rich	0	3	Socio-demographic Index	685
Aortic aneurysm	Female	15-19 years	95+ years	Data Rich	0	3	Alcohol (liters per capita)	871
Aortic aneurysm	Female	15-19 years	95+ years	Global	1	1	Systolic Blood Pressure (mmHg)	113
Aortic aneurysm	Female	15-19 years	95+ years	Global	1	1	Cumulative Cigarettes (10 Years)	198
Aortic aneurysm	Female	15-19 years	95+ years	Global	1	1	Cholesterol (total, mean per capita)	795
Aortic aneurysm	Female	15-19 years	95+ years	Global	1	1	Log-transformed SEV scalar: Aort An	--
Aortic aneurysm	Female	15-19 years	95+ years	Global	-1	2	Healthcare access and quality index	39

Table S16. CODEm covariates used, level of covariate, and expected direction of covariate by cause, sex, and age<br>*Covariates that CODEm did not select during the covariate selection process have no draw counts listed.*								
**Cause**	**Sex**	**Age Start**	**Age End**	**Model Type**	**Direction**	**Level**	**Covariate Name**	**Number of Draws**
Aortic aneurysm	Female	15-19 years	95+ years	Global	1	2	Mean BMI	253
Aortic aneurysm	Female	15-19 years	95+ years	Global	-1	3	nuts seeds adjusted(g)	33
Aortic aneurysm	Female	15-19 years	95+ years	Global	-1	3	pulses legumes adjusted(g)	96
Aortic aneurysm	Female	15-19 years	95+ years	Global	-1	3	vegetables adjusted(g)	337
Aortic aneurysm	Female	15-19 years	95+ years	Global	-1	3	LDI (I$ per capita)	--
Aortic aneurysm	Female	15-19 years	95+ years	Global	-1	3	fruits adjusted(g)	--
Aortic aneurysm	Female	15-19 years	95+ years	Global	-1	3	omega 3 adjusted(g)	--
Aortic aneurysm	Female	15-19 years	95+ years	Global	-1	3	pufa adjusted(percent)	--
Aortic aneurysm	Female	15-19 years	95+ years	Global	0	3	Socio-demographic Index	558
Aortic aneurysm	Female	15-19 years	95+ years	Global	0	3	Alcohol (liters per capita)	795
Aortic aneurysm	Male	15-19 years	95+ years	Data Rich	1	1	Systolic Blood Pressure (mmHg)	112
Aortic aneurysm	Male	15-19 years	95+ years	Data Rich	1	1	Cumulative Cigarettes (10 Years)	121
Aortic aneurysm	Male	15-19 years	95+ years	Data Rich	1	1	Cholesterol (total, mean per capita)	1000
Aortic aneurysm	Male	15-19 years	95+ years	Data Rich	1	1	Log-transformed SEV scalar: Aort An	--
Aortic aneurysm	Male	15-19 years	95+ years	Data Rich	-1	2	Healthcare access and quality index	122
Aortic aneurysm	Male	15-19 years	95+ years	Data Rich	1	2	Mean BMI	253
Aortic aneurysm	Male	15-19 years	95+ years	Data Rich	-1	3	nuts seeds adjusted(g)	76
Aortic aneurysm	Male	15-19 years	95+ years	Data Rich	-1	3	vegetables adjusted(g)	89
Aortic aneurysm	Male	15-19 years	95+ years	Data Rich	-1	3	LDI (I$ per capita)	--
Aortic aneurysm	Male	15-19 years	95+ years	Data Rich	-1	3	fruits adjusted(g)	--
Aortic aneurysm	Male	15-19 years	95+ years	Data Rich	-1	3	omega 3 adjusted(g)	--
Aortic aneurysm	Male	15-19 years	95+ years	Data Rich	-1	3	pufa adjusted(percent)	--
Aortic aneurysm	Male	15-19 years	95+ years	Data Rich	-1	3	pulses legumes adjusted(g)	--
Aortic aneurysm	Male	15-19 years	95+ years	Data Rich	0	3	Socio-demographic Index	504
Aortic aneurysm	Male	15-19 years	95+ years	Data Rich	0	3	Alcohol (liters per capita)	586
Aortic aneurysm	Male	15-19 years	95+ years	Global	1	1	Cumulative Cigarettes (10 Years)	8
Aortic aneurysm	Male	15-19 years	95+ years	Global	1	1	Systolic Blood Pressure (mmHg)	158
Aortic aneurysm	Male	15-19 years	95+ years	Global	1	1	Cholesterol (total, mean per capita)	978
Aortic aneurysm	Male	15-19 years	95+ years	Global	1	1	Log-transformed SEV scalar: Aort An	--
Aortic aneurysm	Male	15-19 years	95+ years	Global	-1	2	Healthcare access and quality index	99
Aortic aneurysm	Male	15-19 years	95+ years	Global	1	2	Mean BMI	374
Aortic aneurysm	Male	15-19 years	95+ years	Global	-1	3	nuts seeds adjusted(g)	127
Aortic aneurysm	Male	15-19 years	95+ years	Global	-1	3	vegetables adjusted(g)	248
Aortic aneurysm	Male	15-19 years	95+ years	Global	-1	3	LDI (I$ per capita)	--
Aortic aneurysm	Male	15-19 years	95+ years	Global	-1	3	fruits adjusted(g)	--
Aortic aneurysm	Male	15-19 years	95+ years	Global	-1	3	omega 3 adjusted(g)	--
Aortic aneurysm	Male	15-19 years	95+ years	Global	-1	3	pufa adjusted(percent)	--
Aortic aneurysm	Male	15-19 years	95+ years	Global	-1	3	pulses legumes adjusted(g)	--
Aortic aneurysm	Male	15-19 years	95+ years	Global	0	3	Socio-demographic Index	517
Aortic aneurysm	Male	15-19 years	95+ years	Global	0	3	Alcohol (liters per capita)	718

Table S16. CODEm covariates used, level of covariate, and expected direction of covariate by cause, sex, and age
Covariates that CODEm did not select during the covariate selection process have no draw counts listed.

Cause	Sex	Age Start	Age End	Model Type	Direction	Level	Covariate Name	Number of Draws
Peripheral vascular disease	Female	40-44 years	95+ years	Data Rich	1	1	Cholesterol (total, mean per capita)	--
Peripheral vascular disease	Female	40-44 years	95+ years	Data Rich	1	1	Log-transformed SEV scalar: PAD	--
Peripheral vascular disease	Female	40-44 years	95+ years	Data Rich	1	1	Smoking Prevalence	--
Peripheral vascular disease	Female	40-44 years	95+ years	Data Rich	1	1	Systolic Blood Pressure (mmHg)	--
Peripheral vascular disease	Female	40-44 years	95+ years	Data Rich	-1	2	Healthcare access and quality index	--
Peripheral vascular disease	Female	40-44 years	95+ years	Data Rich	1	2	Diabetes Fasting Plasma Glucose (mmol/L), age-standardized 25+	149
Peripheral vascular disease	Female	40-44 years	95+ years	Data Rich	1	2	Mean BMI	578
Peripheral vascular disease	Female	40-44 years	95+ years	Data Rich	-1	3	pulses legumes adjusted(g)	16
Peripheral vascular disease	Female	40-44 years	95+ years	Data Rich	-1	3	LDI (I$ per capita)	--
Peripheral vascular disease	Female	40-44 years	95+ years	Data Rich	-1	3	fruits adjusted(g)	--
Peripheral vascular disease	Female	40-44 years	95+ years	Data Rich	-1	3	nuts seeds adjusted(g)	--
Peripheral vascular disease	Female	40-44 years	95+ years	Data Rich	-1	3	omega 3 adjusted(g)	--
Peripheral vascular disease	Female	40-44 years	95+ years	Data Rich	-1	3	pufa adjusted(percent)	--
Peripheral vascular disease	Female	40-44 years	95+ years	Data Rich	-1	3	vegetables adjusted(g)	--
Peripheral vascular disease	Female	40-44 years	95+ years	Data Rich	0	3	Alcohol (liters per capita)	527
Peripheral vascular disease	Female	40-44 years	95+ years	Data Rich	0	3	Socio-demographic Index	666
Peripheral vascular disease	Female	40-44 years	95+ years	Global	1	1	Cholesterol (total, mean per capita)	--
Peripheral vascular disease	Female	40-44 years	95+ years	Global	1	1	Log-transformed SEV scalar: PAD	--
Peripheral vascular disease	Female	40-44 years	95+ years	Global	1	1	Smoking Prevalence	--
Peripheral vascular disease	Female	40-44 years	95+ years	Global	1	1	Systolic Blood Pressure (mmHg)	--
Peripheral vascular disease	Female	40-44 years	95+ years	Global	-1	2	Healthcare access and quality index	--
Peripheral vascular disease	Female	40-44 years	95+ years	Global	1	2	Diabetes Fasting Plasma Glucose (mmol/L), age-standardized 25+	142
Peripheral vascular disease	Female	40-44 years	95+ years	Global	1	2	Mean BMI	591
Peripheral vascular disease	Female	40-44 years	95+ years	Global	-1	3	pulses legumes adjusted(g)	12
Peripheral vascular disease	Female	40-44 years	95+ years	Global	-1	3	LDI (I$ per capita)	--
Peripheral vascular disease	Female	40-44 years	95+ years	Global	-1	3	fruits adjusted(g)	--
Peripheral vascular disease	Female	40-44 years	95+ years	Global	-1	3	nuts seeds adjusted(g)	--
Peripheral vascular disease	Female	40-44 years	95+ years	Global	-1	3	omega 3 adjusted(g)	--
Peripheral vascular disease	Female	40-44 years	95+ years	Global	-1	3	pufa adjusted(percent)	--
Peripheral vascular disease	Female	40-44 years	95+ years	Global	-1	3	vegetables adjusted(g)	--
Peripheral vascular disease	Female	40-44 years	95+ years	Global	0	3	Socio-demographic Index	664
Peripheral vascular disease	Female	40-44 years	95+ years	Global	0	3	Alcohol (liters per capita)	754
Peripheral vascular disease	Male	40-44 years	95+ years	Data Rich	1	1	Cholesterol (total, mean per capita)	--
Peripheral vascular disease	Male	40-44 years	95+ years	Data Rich	1	1	Log-transformed SEV scalar: PAD	--
Peripheral vascular disease	Male	40-44 years	95+ years	Data Rich	1	1	Smoking Prevalence	--
Peripheral vascular disease	Male	40-44 years	95+ years	Data Rich	1	1	Systolic Blood Pressure (mmHg)	--
Peripheral vascular disease	Male	40-44 years	95+ years	Data Rich	-1	2	Healthcare access and quality index	--
Peripheral vascular disease	Male	40-44 years	95+ years	Data Rich	1	2	Mean BMI	738
Peripheral vascular disease	Male	40-44 years	95+ years	Data Rich	1	2	Diabetes Fasting Plasma Glucose (mmol/L), age-standardized 25+	--
Peripheral vascular disease	Male	40-44 years	95+ years	Data Rich	-1	3	pulses legumes adjusted(g)	34

Table S16. CODEm covariates used, level of covariate, and expected direction of covariate by cause, sex, and age
Covariates that CODEm did not select during the covariate selection process have no draw counts listed.

Cause	Sex	Age Start	Age End	Model Type	Direction	Level	Covariate Name	Number of Draws
Peripheral vascular disease	Male	40-44 years	95+ years	Data Rich	-1	3	LDI (I$ per capita)	--
Peripheral vascular disease	Male	40-44 years	95+ years	Data Rich	-1	3	fruits adjusted(g)	--
Peripheral vascular disease	Male	40-44 years	95+ years	Data Rich	-1	3	nuts seeds adjusted(g)	--
Peripheral vascular disease	Male	40-44 years	95+ years	Data Rich	-1	3	omega 3 adjusted(g)	--
Peripheral vascular disease	Male	40-44 years	95+ years	Data Rich	-1	3	pufa adjusted(percent)	--
Peripheral vascular disease	Male	40-44 years	95+ years	Data Rich	-1	3	vegetables adjusted(g)	--
Peripheral vascular disease	Male	40-44 years	95+ years	Data Rich	0	3	Alcohol (liters per capita)	610
Peripheral vascular disease	Male	40-44 years	95+ years	Data Rich	0	3	Socio-demographic Index	648
Peripheral vascular disease	Male	40-44 years	95+ years	Global	1	1	Cholesterol (total, mean per capita)	--
Peripheral vascular disease	Male	40-44 years	95+ years	Global	1	1	Log-transformed SEV scalar: PAD	--
Peripheral vascular disease	Male	40-44 years	95+ years	Global	1	1	Smoking Prevalence	--
Peripheral vascular disease	Male	40-44 years	95+ years	Global	1	1	Systolic Blood Pressure (mmHg)	--
Peripheral vascular disease	Male	40-44 years	95+ years	Global	-1	2	Healthcare access and quality index	--
Peripheral vascular disease	Male	40-44 years	95+ years	Global	1	2	Mean BMI	511
Peripheral vascular disease	Male	40-44 years	95+ years	Global	1	2	Diabetes Fasting Plasma Glucose (mmol/L), age-standardized 25+	--
Peripheral vascular disease	Male	40-44 years	95+ years	Global	-1	3	LDI (I$ per capita)	--
Peripheral vascular disease	Male	40-44 years	95+ years	Global	-1	3	fruits adjusted(g)	--
Peripheral vascular disease	Male	40-44 years	95+ years	Global	-1	3	nuts seeds adjusted(g)	--
Peripheral vascular disease	Male	40-44 years	95+ years	Global	-1	3	omega 3 adjusted(g)	--
Peripheral vascular disease	Male	40-44 years	95+ years	Global	-1	3	pufa adjusted(percent)	--
Peripheral vascular disease	Male	40-44 years	95+ years	Global	-1	3	pulses legumes adjusted(g)	--
Peripheral vascular disease	Male	40-44 years	95+ years	Global	-1	3	vegetables adjusted(g)	--
Peripheral vascular disease	Male	40-44 years	95+ years	Global	0	3	Alcohol (liters per capita)	815
Peripheral vascular disease	Male	40-44 years	95+ years	Global	0	3	Socio-demographic Index	815
Endocarditis	Female	0-6 days	95+ years	Data Rich	-1	1	Improved Water Source (proportion with access)	602
Endocarditis	Female	0-6 days	95+ years	Data Rich	-1	1	Healthcare access and quality index	--
Endocarditis	Female	0-6 days	95+ years	Data Rich	-1	1	Sanitation (proportion with access)	--
Endocarditis	Female	0-6 days	95+ years	Data Rich	1	1	Log-transformed SEV scalar: Endocar	--
Endocarditis	Female	0-6 days	95+ years	Data Rich	-1	3	LDI (I$ per capita)	--
Endocarditis	Female	0-6 days	95+ years	Data Rich	0	3	Socio-demographic Index	726
Endocarditis	Female	0-6 days	95+ years	Global	0	1	Healthcare access and quality index	599
Endocarditis	Female	0-6 days	95+ years	Global	0	1	Improved Water Source (proportion with access)	648
Endocarditis	Female	0-6 days	95+ years	Global	0	1	Sanitation (proportion with access)	677
Endocarditis	Female	0-6 days	95+ years	Global	0	1	Log-transformed SEV scalar: Endocar	--
Endocarditis	Female	0-6 days	95+ years	Global	0	3	Socio-demographic Index	469
Endocarditis	Female	0-6 days	95+ years	Global	0	3	LDI (I$ per capita)	--
Endocarditis	Male	0-6 days	95+ years	Data Rich	-1	1	Healthcare access and quality index	--
Endocarditis	Male	0-6 days	95+ years	Data Rich	0	1	Sanitation (proportion with access)	689
Endocarditis	Male	0-6 days	95+ years	Data Rich	0	1	Improved Water Source (proportion with access)	763
Endocarditis	Male	0-6 days	95+ years	Data Rich	0	1	Log-transformed SEV scalar: Endocar	--
Endocarditis	Male	0-6 days	95+ years	Data Rich	-1	3	LDI (I$ per capita)	--
Endocarditis	Male	0-6 days	95+ years	Data Rich	0	3	Socio-demographic Index	700
Endocarditis	Male	0-6 days	95+ years	Global	0	1	Healthcare access and quality index	417
Endocarditis	Male	0-6 days	95+ years	Global	0	1	Improved Water Source (proportion with access)	545
Endocarditis	Male	0-6 days	95+ years	Global	0	1	Sanitation (proportion with access)	639

Table S16. CODEm covariates used, level of covariate, and expected direction of covariate by cause, sex, and age
Covariates that CODEm did not select during the covariate selection process have no draw counts listed.

Cause	Sex	Age Start	Age End	Model Type	Direction	Level	Covariate Name	Number of Draws
Endocarditis	Male	0-6 days	95+ years	Global	0	1	Log-transformed SEV scalar: Endocar	--
Endocarditis	Male	0-6 days	95+ years	Global	0	3	Socio-demographic Index	659
Endocarditis	Male	0-6 days	95+ years	Global	0	3	LDI (I$ per capita)	--
Other cardiovascular and circulatory diseases	Female	0-6 days	95+ years	Data Rich	1	1	Cholesterol (total, mean per capita)	177
Other cardiovascular and circulatory diseases	Female	0-6 days	95+ years	Data Rich	1	1	Systolic Blood Pressure (mmHg)	337
Other cardiovascular and circulatory diseases	Female	0-6 days	95+ years	Data Rich	1	1	Smoking Prevalence	359
Other cardiovascular and circulatory diseases	Female	0-6 days	95+ years	Data Rich	1	1	Log-transformed SEV scalar: Oth Cardio	--
Other cardiovascular and circulatory diseases	Female	0-6 days	95+ years	Data Rich	-1	2	Healthcare access and quality index	381
Other cardiovascular and circulatory diseases	Female	0-6 days	95+ years	Data Rich	-1	2	Elevation Over 1500m (proportion)	--
Other cardiovascular and circulatory diseases	Female	0-6 days	95+ years	Data Rich	1	2	Indoor Air Pollution (All Cooking Fuels)	126
Other cardiovascular and circulatory diseases	Female	0-6 days	95+ years	Data Rich	1	2	Diabetes Fasting Plasma Glucose (mmol/L), age-standardized 25+	301
Other cardiovascular and circulatory diseases	Female	0-6 days	95+ years	Data Rich	1	2	Outdoor Air Pollution (PM2.5)	507
Other cardiovascular and circulatory diseases	Female	0-6 days	95+ years	Data Rich	1	2	Mean BMI	--
Other cardiovascular and circulatory diseases	Female	0-6 days	95+ years	Data Rich	-1	3	nuts seeds adjusted(g)	10
Other cardiovascular and circulatory diseases	Female	0-6 days	95+ years	Data Rich	-1	3	vegetables adjusted(g)	16
Other cardiovascular and circulatory diseases	Female	0-6 days	95+ years	Data Rich	-1	3	LDI (I$ per capita)	--
Other cardiovascular and circulatory diseases	Female	0-6 days	95+ years	Data Rich	-1	3	fruits adjusted(g)	--
Other cardiovascular and circulatory diseases	Female	0-6 days	95+ years	Data Rich	-1	3	omega 3 adjusted(g)	--
Other cardiovascular and circulatory diseases	Female	0-6 days	95+ years	Data Rich	-1	3	pufa adjusted(percent)	--
Other cardiovascular and circulatory diseases	Female	0-6 days	95+ years	Data Rich	-1	3	pulses legumes adjusted(g)	--
Other cardiovascular and circulatory diseases	Female	0-6 days	95+ years	Data Rich	0	3	Socio-demographic Index	626
Other cardiovascular and circulatory diseases	Female	0-6 days	95+ years	Data Rich	0	3	Alcohol (liters per capita)	739
Other cardiovascular and circulatory diseases	Female	0-6 days	95+ years	Global	1	1	Smoking Prevalence	327
Other cardiovascular and circulatory diseases	Female	0-6 days	95+ years	Global	1	1	Systolic Blood Pressure (mmHg)	649
Other cardiovascular and circulatory diseases	Female	0-6 days	95+ years	Global	1	1	Cholesterol (total, mean per capita)	--
Other cardiovascular and circulatory diseases	Female	0-6 days	95+ years	Global	1	1	Log-transformed SEV scalar: Oth Cardio	--
Other cardiovascular and circulatory diseases	Female	0-6 days	95+ years	Global	-1	2	Healthcare access and quality index	359
Other cardiovascular and circulatory diseases	Female	0-6 days	95+ years	Global	-1	2	Elevation Over 1500m (proportion)	--
Other cardiovascular and circulatory diseases	Female	0-6 days	95+ years	Global	1	2	Indoor Air Pollution (All Cooking Fuels)	167
Other cardiovascular and circulatory diseases	Female	0-6 days	95+ years	Global	1	2	Diabetes Fasting Plasma Glucose (mmol/L), age-standardized 25+	--
Other cardiovascular and circulatory diseases	Female	0-6 days	95+ years	Global	1	2	Mean BMI	--
Other cardiovascular and circulatory diseases	Female	0-6 days	95+ years	Global	1	2	Outdoor Air Pollution (PM2.5)	--
Other cardiovascular and circulatory diseases	Female	0-6 days	95+ years	Global	-1	3	nuts seeds adjusted(g)	3
Other cardiovascular and circulatory diseases	Female	0-6 days	95+ years	Global	-1	3	vegetables adjusted(g)	23
Other cardiovascular and circulatory diseases	Female	0-6 days	95+ years	Global	-1	3	LDI (I$ per capita)	--
Other cardiovascular and circulatory diseases	Female	0-6 days	95+ years	Global	-1	3	fruits adjusted(g)	--
Other cardiovascular and circulatory diseases	Female	0-6 days	95+ years	Global	-1	3	omega 3 adjusted(g)	--
Other cardiovascular and circulatory diseases	Female	0-6 days	95+ years	Global	-1	3	pufa adjusted(percent)	--
Other cardiovascular and circulatory diseases	Female	0-6 days	95+ years	Global	-1	3	pulses legumes adjusted(g)	--
Other cardiovascular and circulatory diseases	Female	0-6 days	95+ years	Global	0	3	Socio-demographic Index	708
Other cardiovascular and circulatory diseases	Female	0-6 days	95+ years	Global	0	3	Alcohol (liters per capita)	719
Other cardiovascular and circulatory diseases	Male	0-6 days	95+ years	Data Rich	1	1	Smoking Prevalence	245
Other cardiovascular and circulatory diseases	Male	0-6 days	95+ years	Data Rich	1	1	Systolic Blood Pressure (mmHg)	429
Other cardiovascular and circulatory diseases	Male	0-6 days	95+ years	Data Rich	1	1	Cholesterol (total, mean per capita)	536
Other cardiovascular and circulatory diseases	Male	0-6 days	95+ years	Data Rich	1	1	Log-transformed SEV scalar: Oth Cardio	--
Other cardiovascular and circulatory diseases	Male	0-6 days	95+ years	Data Rich	-1	2	Healthcare access and quality index	176
Other cardiovascular and circulatory diseases	Male	0-6 days	95+ years	Data Rich	-1	2	Elevation Over 1500m (proportion)	--
Other cardiovascular and circulatory diseases	Male	0-6 days	95+ years	Data Rich	1	2	Indoor Air Pollution (All Cooking Fuels)	206
Other cardiovascular and circulatory diseases	Male	0-6 days	95+ years	Data Rich	1	2	Diabetes Fasting Plasma Glucose (mmol/L), age-standardized 25+	253
Other cardiovascular and circulatory diseases	Male	0-6 days	95+ years	Data Rich	1	2	Mean BMI	253
Other cardiovascular and circulatory diseases	Male	0-6 days	95+ years	Data Rich	1	2	Outdoor Air Pollution (PM2.5)	329
Other cardiovascular and circulatory diseases	Male	0-6 days	95+ years	Data Rich	-1	3	vegetables adjusted(g)	2
Other cardiovascular and circulatory diseases	Male	0-6 days	95+ years	Data Rich	-1	3	nuts seeds adjusted(g)	6
Other cardiovascular and circulatory diseases	Male	0-6 days	95+ years	Data Rich	-1	3	LDI (I$ per capita)	--

Table S16. CODEm covariates used, level of covariate, and expected direction of covariate by cause, sex, and age<br>*Covariates that CODEm did not select during the covariate selection process have no draw counts listed.*								
**Cause**	**Sex**	**Age Start**	**Age End**	**Model Type**	**Direction**	**Level**	**Covariate Name**	**Number of Draws**
Other cardiovascular and circulatory diseases	Male	0-6 days	95+ years	Data Rich	-1	3	fruits adjusted(g)	--
Other cardiovascular and circulatory diseases	Male	0-6 days	95+ years	Data Rich	-1	3	omega 3 adjusted(g)	--
Other cardiovascular and circulatory diseases	Male	0-6 days	95+ years	Data Rich	-1	3	pufa adjusted(percent)	--
Other cardiovascular and circulatory diseases	Male	0-6 days	95+ years	Data Rich	-1	3	pulses legumes adjusted(g)	--
Other cardiovascular and circulatory diseases	Male	0-6 days	95+ years	Data Rich	0	3	Socio-demographic Index	301
Other cardiovascular and circulatory diseases	Male	0-6 days	95+ years	Data Rich	0	3	Alcohol (liters per capita)	725
Other cardiovascular and circulatory diseases	Male	0-6 days	95+ years	Global	1	1	Smoking Prevalence	81
Other cardiovascular and circulatory diseases	Male	0-6 days	95+ years	Global	1	1	Cholesterol (total, mean per capita)	122
Other cardiovascular and circulatory diseases	Male	0-6 days	95+ years	Global	1	1	Systolic Blood Pressure (mmHg)	150
Other cardiovascular and circulatory diseases	Male	0-6 days	95+ years	Global	1	1	Log-transformed SEV scalar: Oth Cardio	--
Other cardiovascular and circulatory diseases	Male	0-6 days	95+ years	Global	-1	2	Healthcare access and quality index	171
Other cardiovascular and circulatory diseases	Male	0-6 days	95+ years	Global	-1	2	Elevation Over 1500m (proportion)	--
Other cardiovascular and circulatory diseases	Male	0-6 days	95+ years	Global	1	2	Indoor Air Pollution (All Cooking Fuels)	121
Other cardiovascular and circulatory diseases	Male	0-6 days	95+ years	Global	1	2	Diabetes Fasting Plasma Glucose (mmol/L), age-standardized 25+	486
Other cardiovascular and circulatory diseases	Male	0-6 days	95+ years	Global	1	2	Mean BMI	486
Other cardiovascular and circulatory diseases	Male	0-6 days	95+ years	Global	1	2	Outdoor Air Pollution (PM2.5)	671
Other cardiovascular and circulatory diseases	Male	0-6 days	95+ years	Global	-1	3	nuts seeds adjusted(g)	0
Other cardiovascular and circulatory diseases	Male	0-6 days	95+ years	Global	-1	3	vegetables adjusted(g)	0
Other cardiovascular and circulatory diseases	Male	0-6 days	95+ years	Global	-1	3	LDI (I$ per capita)	--
Other cardiovascular and circulatory diseases	Male	0-6 days	95+ years	Global	-1	3	fruits adjusted(g)	--
Other cardiovascular and circulatory diseases	Male	0-6 days	95+ years	Global	-1	3	omega 3 adjusted(g)	--
Other cardiovascular and circulatory diseases	Male	0-6 days	95+ years	Global	-1	3	pufa adjusted(percent)	--
Other cardiovascular and circulatory diseases	Male	0-6 days	95+ years	Global	-1	3	pulses legumes adjusted(g)	--
Other cardiovascular and circulatory diseases	Male	0-6 days	95+ years	Global	0	3	Socio-demographic Index	394
Other cardiovascular and circulatory diseases	Male	0-6 days	95+ years	Global	0	3	Alcohol (liters per capita)	647
Chronic respiratory diseases	Female	1-4 years	95+ years	Data Rich	1	1	Cumulative Cigarettes (10 Years)	26
Chronic respiratory diseases	Female	1-4 years	95+ years	Data Rich	1	1	Cumulative Cigarettes (5 Years)	48
Chronic respiratory diseases	Female	1-4 years	95+ years	Data Rich	1	1	Healthcare access and quality index	--
Chronic respiratory diseases	Female	1-4 years	95+ years	Data Rich	1	1	Log-transformed SEV scalar: Chr Resp	--
Chronic respiratory diseases	Female	1-4 years	95+ years	Data Rich	1	2	Smoking Prevalence	3
Chronic respiratory diseases	Female	1-4 years	95+ years	Data Rich	1	2	Indoor Air Pollution (All Cooking Fuels)	120
Chronic respiratory diseases	Female	1-4 years	95+ years	Data Rich	1	2	Outdoor Air Pollution (PM2.5)	242
Chronic respiratory diseases	Female	1-4 years	95+ years	Data Rich	1	2	Elevation Over 1500m (proportion)	365
Chronic respiratory diseases	Female	1-4 years	95+ years	Data Rich	-1	3	Education (years per capita)	142
Chronic respiratory diseases	Female	1-4 years	95+ years	Data Rich	-1	3	LDI (I$ per capita)	--
Chronic respiratory diseases	Female	1-4 years	95+ years	Data Rich	0	3	Socio-demographic Index	246
Chronic respiratory diseases	Female	1-4 years	95+ years	Data Rich	1	3	Population Density (over 1000 ppl/sqkm, proportion)	0
Chronic respiratory diseases	Female	1-4 years	95+ years	Data Rich	1	3	Elevation 500 to 1500m (proportion)	434
Chronic respiratory diseases	Female	1-4 years	95+ years	Global	1	1	Cumulative Cigarettes (10 Years)	22
Chronic respiratory diseases	Female	1-4 years	95+ years	Global	1	1	Cumulative Cigarettes (5 Years)	23
Chronic respiratory diseases	Female	1-4 years	95+ years	Global	1	1	Healthcare access and quality index	--
Chronic respiratory diseases	Female	1-4 years	95+ years	Global	1	1	Log-transformed SEV scalar: Chr Resp	--
Chronic respiratory diseases	Female	1-4 years	95+ years	Global	1	2	Smoking Prevalence	11
Chronic respiratory diseases	Female	1-4 years	95+ years	Global	1	2	Elevation Over 1500m (proportion)	622
Chronic respiratory diseases	Female	1-4 years	95+ years	Global	1	2	Indoor Air Pollution (All Cooking Fuels)	622
Chronic respiratory diseases	Female	1-4 years	95+ years	Global	1	2	Outdoor Air Pollution (PM2.5)	--
Chronic respiratory diseases	Female	1-4 years	95+ years	Global	-1	3	Education (years per capita)	400
Chronic respiratory diseases	Female	1-4 years	95+ years	Global	-1	3	LDI (I$ per capita)	--
Chronic respiratory diseases	Female	1-4 years	95+ years	Global	0	3	Socio-demographic Index	153
Chronic respiratory diseases	Female	1-4 years	95+ years	Global	1	3	Elevation 500 to 1500m (proportion)	684
Chronic respiratory diseases	Female	1-4 years	95+ years	Global	1	3	Population Density (over 1000 ppl/sqkm, proportion)	--
Chronic respiratory diseases	Male	1-4 years	95+ years	Data Rich	1	1	Cumulative Cigarettes (10 Years)	337
Chronic respiratory diseases	Male	1-4 years	95+ years	Data Rich	1	1	Cumulative Cigarettes (5 Years)	526

**Table S16. CODEm covariates used, level of covariate, and expected direction of covariate by cause, sex, and age** *Covariates that CODEm did not select during the covariate selection process have no draw counts listed.*								
**Cause**	**Sex**	**Age Start**	**Age End**	**Model Type**	**Direction**	**Level**	**Covariate Name**	**Number of Draws**
Chronic respiratory diseases	Male	1-4 years	95+ years	Data Rich	1	1	Log-transformed SEV scalar: Chr Resp	--
Chronic respiratory diseases	Male	1-4 years	95+ years	Data Rich	-1	2	Healthcare access and quality index	2
Chronic respiratory diseases	Male	1-4 years	95+ years	Data Rich	1	2	Indoor Air Pollution (All Cooking Fuels)	0
Chronic respiratory diseases	Male	1-4 years	95+ years	Data Rich	1	2	Smoking Prevalence	775
Chronic respiratory diseases	Male	1-4 years	95+ years	Data Rich	1	2	Elevation Over 1500m (proportion)	777
Chronic respiratory diseases	Male	1-4 years	95+ years	Data Rich	1	2	Outdoor Air Pollution (PM2.5)	777
Chronic respiratory diseases	Male	1-4 years	95+ years	Data Rich	-1	3	Education (years per capita)	140
Chronic respiratory diseases	Male	1-4 years	95+ years	Data Rich	-1	3	LDI (I$ per capita)	--
Chronic respiratory diseases	Male	1-4 years	95+ years	Data Rich	0	3	Socio-demographic Index	116
Chronic respiratory diseases	Male	1-4 years	95+ years	Data Rich	1	3	Population Density (over 1000 ppl/sqkm, proportion)	158
Chronic respiratory diseases	Male	1-4 years	95+ years	Data Rich	1	3	Elevation 500 to 1500m (proportion)	484
Chronic respiratory diseases	Male	1-4 years	95+ years	Global	1	1	Cumulative Cigarettes (10 Years)	75
Chronic respiratory diseases	Male	1-4 years	95+ years	Global	1	1	Indoor Air Pollution (All Cooking Fuels)	165
Chronic respiratory diseases	Male	1-4 years	95+ years	Global	1	1	Cumulative Cigarettes (5 Years)	234
Chronic respiratory diseases	Male	1-4 years	95+ years	Global	1	1	Smoking Prevalence	296
Chronic respiratory diseases	Male	1-4 years	95+ years	Global	1	1	Log-transformed SEV scalar: Chr Resp	--
Chronic respiratory diseases	Male	1-4 years	95+ years	Global	-1	2	Healthcare access and quality index	253
Chronic respiratory diseases	Male	1-4 years	95+ years	Global	1	2	Outdoor Air Pollution (PM2.5)	0
Chronic respiratory diseases	Male	1-4 years	95+ years	Global	1	2	Elevation Over 1500m (proportion)	548
Chronic respiratory diseases	Male	1-4 years	95+ years	Global	-1	3	Education (years per capita)	467
Chronic respiratory diseases	Male	1-4 years	95+ years	Global	-1	3	LDI (I$ per capita)	--
Chronic respiratory diseases	Male	1-4 years	95+ years	Global	0	3	Socio-demographic Index	262
Chronic respiratory diseases	Male	1-4 years	95+ years	Global	1	3	Population Density (over 1000 ppl/sqkm, proportion)	202
Chronic respiratory diseases	Male	1-4 years	95+ years	Global	1	3	Elevation 500 to 1500m (proportion)	846
Chronic obstructive pulmonary disease	Female	1-4 years	95+ years	Data Rich	1	1	Outdoor Air Pollution (PM2.5)	446
Chronic obstructive pulmonary disease	Female	1-4 years	95+ years	Data Rich	1	1	Elevation Over 1500m (proportion)	678
Chronic obstructive pulmonary disease	Female	1-4 years	95+ years	Data Rich	1	1	Healthcare access and quality index	706
Chronic obstructive pulmonary disease	Female	1-4 years	95+ years	Data Rich	1	1	Cumulative Cigarettes (10 Years)	--
Chronic obstructive pulmonary disease	Female	1-4 years	95+ years	Data Rich	1	1	Cumulative Cigarettes (5 Years)	--
Chronic obstructive pulmonary disease	Female	1-4 years	95+ years	Data Rich	1	1	Log-transformed SEV scalar: COPD	--
Chronic obstructive pulmonary disease	Female	1-4 years	95+ years	Data Rich	1	2	Indoor Air Pollution (All Cooking Fuels)	1
Chronic obstructive pulmonary disease	Female	1-4 years	95+ years	Data Rich	1	2	Smoking Prevalence	3
Chronic obstructive pulmonary disease	Female	1-4 years	95+ years	Data Rich	-1	3	Education (years per capita)	2
Chronic obstructive pulmonary disease	Female	1-4 years	95+ years	Data Rich	-1	3	LDI (I$ per capita)	--
Chronic obstructive pulmonary disease	Female	1-4 years	95+ years	Data Rich	0	3	Socio-demographic Index	782
Chronic obstructive pulmonary disease	Female	1-4 years	95+ years	Global	1	1	Healthcare access and quality index	588
Chronic obstructive pulmonary disease	Female	1-4 years	95+ years	Global	1	1	Elevation Over 1500m (proportion)	803
Chronic obstructive pulmonary disease	Female	1-4 years	95+ years	Global	1	1	Cumulative Cigarettes (10 Years)	--
Chronic obstructive pulmonary disease	Female	1-4 years	95+ years	Global	1	1	Cumulative Cigarettes (5 Years)	--
Chronic obstructive pulmonary disease	Female	1-4 years	95+ years	Global	1	1	Log-transformed SEV scalar: COPD	--
Chronic obstructive pulmonary disease	Female	1-4 years	95+ years	Global	1	1	Outdoor Air Pollution (PM2.5)	--
Chronic obstructive pulmonary disease	Female	1-4 years	95+ years	Global	1	2	Smoking Prevalence	3
Chronic obstructive pulmonary disease	Female	1-4 years	95+ years	Global	1	2	Indoor Air Pollution (All Cooking Fuels)	206
Chronic obstructive pulmonary disease	Female	1-4 years	95+ years	Global	-1	3	Education (years per capita)	35
Chronic obstructive pulmonary disease	Female	1-4 years	95+ years	Global	-1	3	LDI (I$ per capita)	--
Chronic obstructive pulmonary disease	Female	1-4 years	95+ years	Global	0	3	Socio-demographic Index	140
Chronic obstructive pulmonary disease	Male	1-4 years	95+ years	Data Rich	1	1	Cumulative Cigarettes (10 Years)	0
Chronic obstructive pulmonary disease	Male	1-4 years	95+ years	Data Rich	1	1	Cumulative Cigarettes (20 Years)	0
Chronic obstructive pulmonary disease	Male	1-4 years	95+ years	Data Rich	1	1	Cumulative Cigarettes (5 Years)	0
Chronic obstructive pulmonary disease	Male	1-4 years	95+ years	Data Rich	1	1	Elevation Over 1500m (proportion)	485
Chronic obstructive pulmonary disease	Male	1-4 years	95+ years	Data Rich	1	1	Outdoor Air Pollution (PM2.5)	551
Chronic obstructive pulmonary disease	Male	1-4 years	95+ years	Data Rich	1	1	Log-transformed SEV scalar: COPD	--
Chronic obstructive pulmonary disease	Male	1-4 years	95+ years	Data Rich	-1	2	Healthcare access and quality index	0

Table S16. CODEm covariates used, level of covariate, and expected direction of covariate by cause, sex, and age
Covariates that CODEm did not select during the covariate selection process have no draw counts listed.

Cause	Sex	Age Start	Age End	Model Type	Direction	Level	Covariate Name	Number of Draws
Chronic obstructive pulmonary disease	Male	1-4 years	95+ years	Data Rich	1	2	Indoor Air Pollution (All Cooking Fuels)	0
Chronic obstructive pulmonary disease	Male	1-4 years	95+ years	Data Rich	1	2	Smoking Prevalence	488
Chronic obstructive pulmonary disease	Male	1-4 years	95+ years	Data Rich	-1	3	Education (years per capita)	813
Chronic obstructive pulmonary disease	Male	1-4 years	95+ years	Data Rich	-1	3	LDI (I$ per capita)	--
Chronic obstructive pulmonary disease	Male	1-4 years	95+ years	Data Rich	0	3	Socio-demographic Index	940
Chronic obstructive pulmonary disease	Male	1-4 years	95+ years	Global	1	1	Cumulative Cigarettes (20 Years)	0
Chronic obstructive pulmonary disease	Male	1-4 years	95+ years	Global	1	1	Cumulative Cigarettes (10 Years)	4
Chronic obstructive pulmonary disease	Male	1-4 years	95+ years	Global	1	1	Cumulative Cigarettes (5 Years)	7
Chronic obstructive pulmonary disease	Male	1-4 years	95+ years	Global	1	1	Outdoor Air Pollution (PM2.5)	124
Chronic obstructive pulmonary disease	Male	1-4 years	95+ years	Global	1	1	Elevation Over 1500m (proportion)	939
Chronic obstructive pulmonary disease	Male	1-4 years	95+ years	Global	1	1	Log-transformed SEV scalar: COPD	--
Chronic obstructive pulmonary disease	Male	1-4 years	95+ years	Global	-1	2	Healthcare access and quality index	477
Chronic obstructive pulmonary disease	Male	1-4 years	95+ years	Global	1	2	Indoor Air Pollution (All Cooking Fuels)	71
Chronic obstructive pulmonary disease	Male	1-4 years	95+ years	Global	1	2	Smoking Prevalence	380
Chronic obstructive pulmonary disease	Male	1-4 years	95+ years	Global	-1	3	Education (years per capita)	437
Chronic obstructive pulmonary disease	Male	1-4 years	95+ years	Global	-1	3	LDI (I$ per capita)	--
Chronic obstructive pulmonary disease	Male	1-4 years	95+ years	Global	0	3	Socio-demographic Index	664
Pneumoconiosis	Female	15-19 years	95+ years	Data Rich	1	1	Asbestos consumption (metric tons per year per capita)	260
Pneumoconiosis	Female	15-19 years	95+ years	Data Rich	1	1	Coal Production (per capita)	--
Pneumoconiosis	Female	15-19 years	95+ years	Data Rich	1	1	Gold production (kg) per capita	--
Pneumoconiosis	Female	15-19 years	95+ years	Data Rich	-1	2	Healthcare access and quality index	43
Pneumoconiosis	Female	15-19 years	95+ years	Data Rich	1	2	Cumulative Cigarettes (5 Years)	38
Pneumoconiosis	Female	15-19 years	95+ years	Data Rich	1	2	Indoor Air Pollution (All Cooking Fuels)	38
Pneumoconiosis	Female	15-19 years	95+ years	Data Rich	1	2	Smoking Prevalence	404
Pneumoconiosis	Female	15-19 years	95+ years	Data Rich	-1	3	Education (years per capita)	106
Pneumoconiosis	Female	15-19 years	95+ years	Data Rich	-1	3	Socio-demographic Index	430
Pneumoconiosis	Female	15-19 years	95+ years	Data Rich	-1	3	LDI (I$ per capita)	--
Pneumoconiosis	Female	15-19 years	95+ years	Global	1	1	Asbestos consumption (metric tons per year per capita)	1000
Pneumoconiosis	Female	15-19 years	95+ years	Global	1	1	Coal Production (per capita)	--
Pneumoconiosis	Female	15-19 years	95+ years	Global	1	1	Gold production (kg) per capita	--
Pneumoconiosis	Female	15-19 years	95+ years	Global	-1	2	Healthcare access and quality index	190
Pneumoconiosis	Female	15-19 years	95+ years	Global	1	2	Cumulative Cigarettes (5 Years)	24
Pneumoconiosis	Female	15-19 years	95+ years	Global	1	2	Indoor Air Pollution (All Cooking Fuels)	24
Pneumoconiosis	Female	15-19 years	95+ years	Global	1	2	Smoking Prevalence	193
Pneumoconiosis	Female	15-19 years	95+ years	Global	-1	3	Socio-demographic Index	144
Pneumoconiosis	Female	15-19 years	95+ years	Global	-1	3	Education (years per capita)	266
Pneumoconiosis	Female	15-19 years	95+ years	Global	-1	3	LDI (I$ per capita)	--
Pneumoconiosis	Male	15-19 years	95+ years	Data Rich	1	1	Asbestos consumption (metric tons per year per capita)	365
Pneumoconiosis	Male	15-19 years	95+ years	Data Rich	1	1	Coal Production (per capita)	805
Pneumoconiosis	Male	15-19 years	95+ years	Data Rich	1	1	Gold production (kg) per capita	--
Pneumoconiosis	Male	15-19 years	95+ years	Data Rich	-1	2	Healthcare access and quality index	34
Pneumoconiosis	Male	15-19 years	95+ years	Data Rich	1	2	Indoor Air Pollution (All Cooking Fuels)	0
Pneumoconiosis	Male	15-19 years	95+ years	Data Rich	1	2	Smoking Prevalence	154
Pneumoconiosis	Male	15-19 years	95+ years	Data Rich	1	2	Cumulative Cigarettes (5 Years)	167
Pneumoconiosis	Male	15-19 years	95+ years	Data Rich	-1	3	Socio-demographic Index	29

Table S16. CODEm covariates used, level of covariate, and expected direction of covariate by cause, sex, and age<br>*Covariates that CODEm did not select during the covariate selection process have no draw counts listed.*								
**Cause**	**Sex**	**Age Start**	**Age End**	**Model Type**	**Direction**	**Level**	**Covariate Name**	**Number of Draws**
Pneumoconiosis	Male	15-19 years	95+ years	Data Rich	-1	3	Education (years per capita)	323
Pneumoconiosis	Male	15-19 years	95+ years	Data Rich	-1	3	LDI (I$ per capita)	--
Pneumoconiosis	Male	15-19 years	95+ years	Global	1	1	Coal Production (per capita)	567
Pneumoconiosis	Male	15-19 years	95+ years	Global	1	1	Asbestos consumption (metric tons per year per capita)	750
Pneumoconiosis	Male	15-19 years	95+ years	Global	1	1	Gold production (kg) per capita	--
Pneumoconiosis	Male	15-19 years	95+ years	Global	-1	2	Healthcare access and quality index	160
Pneumoconiosis	Male	15-19 years	95+ years	Global	1	2	Indoor Air Pollution (All Cooking Fuels)	2
Pneumoconiosis	Male	15-19 years	95+ years	Global	1	2	Smoking Prevalence	126
Pneumoconiosis	Male	15-19 years	95+ years	Global	1	2	Cumulative Cigarettes (5 Years)	154
Pneumoconiosis	Male	15-19 years	95+ years	Global	-1	3	Education (years per capita)	299
Pneumoconiosis	Male	15-19 years	95+ years	Global	-1	3	Socio-demographic Index	413
Pneumoconiosis	Male	15-19 years	95+ years	Global	-1	3	LDI (I$ per capita)	--
Silicosis	Female	15-19 years	95+ years	Data Rich	1	1	Gold production (kg) per capita	--
Silicosis	Female	15-19 years	95+ years	Data Rich	-1	2	Healthcare access and quality index	568
Silicosis	Female	15-19 years	95+ years	Data Rich	1	2	Cumulative Cigarettes (5 Years)	--
Silicosis	Female	15-19 years	95+ years	Data Rich	1	2	Indoor Air Pollution (All Cooking Fuels)	--
Silicosis	Female	15-19 years	95+ years	Data Rich	1	2	Smoking Prevalence	--
Silicosis	Female	15-19 years	95+ years	Data Rich	-1	3	Socio-demographic Index	568
Silicosis	Female	15-19 years	95+ years	Data Rich	-1	3	Education (years per capita)	--
Silicosis	Female	15-19 years	95+ years	Data Rich	-1	3	LDI (I$ per capita)	--
Silicosis	Female	15-19 years	95+ years	Global	1	1	Gold production (kg) per capita	--
Silicosis	Female	15-19 years	95+ years	Global	-1	2	Healthcare access and quality index	546
Silicosis	Female	15-19 years	95+ years	Global	1	2	Cumulative Cigarettes (5 Years)	--
Silicosis	Female	15-19 years	95+ years	Global	1	2	Indoor Air Pollution (All Cooking Fuels)	--
Silicosis	Female	15-19 years	95+ years	Global	1	2	Smoking Prevalence	--
Silicosis	Female	15-19 years	95+ years	Global	-1	3	Socio-demographic Index	546
Silicosis	Female	15-19 years	95+ years	Global	-1	3	Education (years per capita)	--
Silicosis	Female	15-19 years	95+ years	Global	-1	3	LDI (I$ per capita)	--
Silicosis	Male	15-19 years	95+ years	Data Rich	1	1	Gold production (kg) per capita	823
Silicosis	Male	15-19 years	95+ years	Data Rich	-1	2	Healthcare access and quality index	110
Silicosis	Male	15-19 years	95+ years	Data Rich	1	2	Indoor Air Pollution (All Cooking Fuels)	10
Silicosis	Male	15-19 years	95+ years	Data Rich	1	2	Cumulative Cigarettes (5 Years)	70
Silicosis	Male	15-19 years	95+ years	Data Rich	1	2	Smoking Prevalence	504
Silicosis	Male	15-19 years	95+ years	Data Rich	-1	3	Education (years per capita)	277
Silicosis	Male	15-19 years	95+ years	Data Rich	-1	3	Socio-demographic Index	384
Silicosis	Male	15-19 years	95+ years	Data Rich	-1	3	LDI (I$ per capita)	--
Silicosis	Male	15-19 years	95+ years	Global	1	1	Gold production (kg) per capita	1000
Silicosis	Male	15-19 years	95+ years	Global	-1	2	Healthcare access and quality index	321
Silicosis	Male	15-19 years	95+ years	Global	1	2	Indoor Air Pollution (All Cooking Fuels)	121
Silicosis	Male	15-19 years	95+ years	Global	1	2	Cumulative Cigarettes (5 Years)	215

Table S16. CODEm covariates used, level of covariate, and expected direction of covariate by cause, sex, and age
Covariates that CODEm did not select during the covariate selection process have no draw counts listed.

Cause	Sex	Age Start	Age End	Model Type	Direction	Level	Covariate Name	Number of Draws
Silicosis	Male	15-19 years	95+ years	Global	1	2	Smoking Prevalence	--
Silicosis	Male	15-19 years	95+ years	Global	-1	3	Socio-demographic Index	192
Silicosis	Male	15-19 years	95+ years	Global	-1	3	Education (years per capita)	225
Silicosis	Male	15-19 years	95+ years	Global	-1	3	LDI (I$ per capita)	--
Asbestosis	Female	15-19 years	95+ years	Data Rich	1	1	Asbestos consumption (metric tons per year per capita)	--
Asbestosis	Female	15-19 years	95+ years	Data Rich	-1	2	Healthcare access and quality index	--
Asbestosis	Female	15-19 years	95+ years	Data Rich	1	2	Elevation Over 1500m (proportion)	744
Asbestosis	Female	15-19 years	95+ years	Data Rich	1	2	Cumulative Cigarettes (10 Years)	--
Asbestosis	Female	15-19 years	95+ years	Data Rich	1	2	Cumulative Cigarettes (5 Years)	--
Asbestosis	Female	15-19 years	95+ years	Data Rich	1	2	Elevation 500 to 1500m (proportion)	--
Asbestosis	Female	15-19 years	95+ years	Data Rich	1	2	Indoor Air Pollution (All Cooking Fuels)	--
Asbestosis	Female	15-19 years	95+ years	Data Rich	1	2	Smoking Prevalence	--
Asbestosis	Female	15-19 years	95+ years	Data Rich	-1	3	Education (years per capita)	--
Asbestosis	Female	15-19 years	95+ years	Data Rich	-1	3	LDI (I$ per capita)	--
Asbestosis	Female	15-19 years	95+ years	Data Rich	-1	3	Socio-demographic Index	--
Asbestosis	Female	15-19 years	95+ years	Global	1	1	Asbestos consumption (metric tons per year per capita)	--
Asbestosis	Female	15-19 years	95+ years	Global	-1	2	Healthcare access and quality index	546
Asbestosis	Female	15-19 years	95+ years	Global	1	2	Elevation Over 1500m (proportion)	546
Asbestosis	Female	15-19 years	95+ years	Global	1	2	Cumulative Cigarettes (10 Years)	--
Asbestosis	Female	15-19 years	95+ years	Global	1	2	Cumulative Cigarettes (5 Years)	--
Asbestosis	Female	15-19 years	95+ years	Global	1	2	Elevation 500 to 1500m (proportion)	--
Asbestosis	Female	15-19 years	95+ years	Global	1	2	Indoor Air Pollution (All Cooking Fuels)	--
Asbestosis	Female	15-19 years	95+ years	Global	1	2	Smoking Prevalence	--
Asbestosis	Female	15-19 years	95+ years	Global	-1	3	Education (years per capita)	--
Asbestosis	Female	15-19 years	95+ years	Global	-1	3	LDI (I$ per capita)	--
Asbestosis	Female	15-19 years	95+ years	Global	-1	3	Socio-demographic Index	--
Asbestosis	Male	15-19 years	95+ years	Data Rich	1	1	Asbestos consumption (metric tons per year per capita)	--
Asbestosis	Male	15-19 years	95+ years	Data Rich	1	1	Smoking Prevalence	--
Asbestosis	Male	15-19 years	95+ years	Data Rich	-1	2	Healthcare access and quality index	--
Asbestosis	Male	15-19 years	95+ years	Data Rich	1	2	Elevation 500 to 1500m (proportion)	516
Asbestosis	Male	15-19 years	95+ years	Data Rich	1	2	Elevation Over 1500m (proportion)	516
Asbestosis	Male	15-19 years	95+ years	Data Rich	1	2	Cumulative Cigarettes (5 Years)	--
Asbestosis	Male	15-19 years	95+ years	Data Rich	1	2	Indoor Air Pollution (All Cooking Fuels)	--
Asbestosis	Male	15-19 years	95+ years	Data Rich	-1	3	Education (years per capita)	--
Asbestosis	Male	15-19 years	95+ years	Data Rich	-1	3	LDI (I$ per capita)	--
Asbestosis	Male	15-19 years	95+ years	Data Rich	0	3	Socio-demographic Index	742
Asbestosis	Male	15-19 years	95+ years	Global	1	1	Asbestos consumption (metric tons per year per capita)	--
Asbestosis	Male	15-19 years	95+ years	Global	-1	2	Healthcare access and quality index	--
Asbestosis	Male	15-19 years	95+ years	Global	1	2	Elevation 500 to 1500m (proportion)	1000
Asbestosis	Male	15-19 years	95+ years	Global	1	2	Elevation Over 1500m (proportion)	1000

Table S16. CODEm covariates used, level of covariate, and expected direction of covariate by cause, sex, and age
Covariates that CODEm did not select during the covariate selection process have no draw counts listed.

Cause	Sex	Age Start	Age End	Model Type	Direction	Level	Covariate Name	Number of Draws
Asbestosis	Male	15-19 years	95+ years	Global	1	2	Cumulative Cigarettes (5 Years)	--
Asbestosis	Male	15-19 years	95+ years	Global	1	2	Indoor Air Pollution (All Cooking Fuels)	--
Asbestosis	Male	15-19 years	95+ years	Global	1	2	Smoking Prevalence	--
Asbestosis	Male	15-19 years	95+ years	Global	-1	3	Education (years per capita)	--
Asbestosis	Male	15-19 years	95+ years	Global	-1	3	LDI (I$ per capita)	--
Asbestosis	Male	15-19 years	95+ years	Global	-1	3	Socio-demographic Index	--
Coal workers pneumoconiosis	Female	15-19 years	95+ years	Data Rich	1	1	Coal Production (per capita)	--
Coal workers pneumoconiosis	Female	15-19 years	95+ years	Data Rich	-1	2	Healthcare access and quality index	325
Coal workers pneumoconiosis	Female	15-19 years	95+ years	Data Rich	1	2	Smoking Prevalence	325
Coal workers pneumoconiosis	Female	15-19 years	95+ years	Data Rich	1	2	Cumulative Cigarettes (5 Years)	--
Coal workers pneumoconiosis	Female	15-19 years	95+ years	Data Rich	1	2	Indoor Air Pollution (All Cooking Fuels)	--
Coal workers pneumoconiosis	Female	15-19 years	95+ years	Data Rich	-1	3	Education (years per capita)	--
Coal workers pneumoconiosis	Female	15-19 years	95+ years	Data Rich	-1	3	LDI (I$ per capita)	--
Coal workers pneumoconiosis	Female	15-19 years	95+ years	Data Rich	-1	3	Socio-demographic Index	--
Coal workers pneumoconiosis	Female	15-19 years	95+ years	Global	1	1	Coal Production (per capita)	--
Coal workers pneumoconiosis	Female	15-19 years	95+ years	Global	-1	2	Healthcare access and quality index	105
Coal workers pneumoconiosis	Female	15-19 years	95+ years	Global	1	2	Smoking Prevalence	354
Coal workers pneumoconiosis	Female	15-19 years	95+ years	Global	1	2	Cumulative Cigarettes (5 Years)	--
Coal workers pneumoconiosis	Female	15-19 years	95+ years	Global	1	2	Indoor Air Pollution (All Cooking Fuels)	--
Coal workers pneumoconiosis	Female	15-19 years	95+ years	Global	-1	3	Socio-demographic Index	281
Coal workers pneumoconiosis	Female	15-19 years	95+ years	Global	-1	3	Education (years per capita)	--
Coal workers pneumoconiosis	Female	15-19 years	95+ years	Global	-1	3	LDI (I$ per capita)	--
Coal workers pneumoconiosis	Male	15-19 years	95+ years	Data Rich	1	1	Coal Production (per capita)	1000
Coal workers pneumoconiosis	Male	15-19 years	95+ years	Data Rich	-1	2	Healthcare access and quality index	327
Coal workers pneumoconiosis	Male	15-19 years	95+ years	Data Rich	1	2	Indoor Air Pollution (All Cooking Fuels)	68
Coal workers pneumoconiosis	Male	15-19 years	95+ years	Data Rich	1	2	Cumulative Cigarettes (5 Years)	250
Coal workers pneumoconiosis	Male	15-19 years	95+ years	Data Rich	1	2	Smoking Prevalence	274
Coal workers pneumoconiosis	Male	15-19 years	95+ years	Data Rich	-1	3	Socio-demographic Index	195
Coal workers pneumoconiosis	Male	15-19 years	95+ years	Data Rich	-1	3	Education (years per capita)	464
Coal workers pneumoconiosis	Male	15-19 years	95+ years	Data Rich	-1	3	LDI (I$ per capita)	--
Coal workers pneumoconiosis	Male	15-19 years	95+ years	Global	1	1	Coal Production (per capita)	1000
Coal workers pneumoconiosis	Male	15-19 years	95+ years	Global	-1	2	Healthcare access and quality index	461
Coal workers pneumoconiosis	Male	15-19 years	95+ years	Global	1	2	Indoor Air Pollution (All Cooking Fuels)	64
Coal workers pneumoconiosis	Male	15-19 years	95+ years	Global	1	2	Cumulative Cigarettes (5 Years)	334
Coal workers pneumoconiosis	Male	15-19 years	95+ years	Global	1	2	Smoking Prevalence	339
Coal workers pneumoconiosis	Male	15-19 years	95+ years	Global	-1	3	Socio-demographic Index	191
Coal workers pneumoconiosis	Male	15-19 years	95+ years	Global	-1	3	Education (years per capita)	232
Coal workers pneumoconiosis	Male	15-19 years	95+ years	Global	-1	3	LDI (I$ per capita)	--
Other pneumoconiosis	Female	15-19 years	95+ years	Data Rich	-1	2	Healthcare access and quality index	89
Other pneumoconiosis	Female	15-19 years	95+ years	Data Rich	1	2	Indoor Air Pollution (All Cooking Fuels)	38

Table S16. CODEm covariates used, level of covariate, and expected direction of covariate by cause, sex, and age<br>*Covariates that CODEm did not select during the covariate selection process have no draw counts listed.*								
Cause	Sex	Age Start	Age End	Model Type	Direction	Level	Covariate Name	Number of Draws
Other pneumoconiosis	Female	15-19 years	95+ years	Data Rich	1	2	Smoking Prevalence	408
Other pneumoconiosis	Female	15-19 years	95+ years	Data Rich	1	2	Cumulative Cigarettes (5 Years)	575
Other pneumoconiosis	Female	15-19 years	95+ years	Data Rich	-1	3	Socio-demographic Index	137
Other pneumoconiosis	Female	15-19 years	95+ years	Data Rich	-1	3	Education (years per capita)	--
Other pneumoconiosis	Female	15-19 years	95+ years	Data Rich	-1	3	LDI (I$ per capita)	--
Other pneumoconiosis	Female	15-19 years	95+ years	Global	-1	2	Healthcare access and quality index	38
Other pneumoconiosis	Female	15-19 years	95+ years	Global	1	2	Indoor Air Pollution (All Cooking Fuels)	63
Other pneumoconiosis	Female	15-19 years	95+ years	Global	1	2	Cumulative Cigarettes (5 Years)	431
Other pneumoconiosis	Female	15-19 years	95+ years	Global	1	2	Smoking Prevalence	625
Other pneumoconiosis	Female	15-19 years	95+ years	Global	-1	3	Education (years per capita)	22
Other pneumoconiosis	Female	15-19 years	95+ years	Global	-1	3	Socio-demographic Index	236
Other pneumoconiosis	Female	15-19 years	95+ years	Global	-1	3	LDI (I$ per capita)	--
Other pneumoconiosis	Male	15-19 years	95+ years	Data Rich	-1	2	Healthcare access and quality index	508
Other pneumoconiosis	Male	15-19 years	95+ years	Data Rich	1	2	Indoor Air Pollution (All Cooking Fuels)	169
Other pneumoconiosis	Male	15-19 years	95+ years	Data Rich	1	2	Cumulative Cigarettes (5 Years)	323
Other pneumoconiosis	Male	15-19 years	95+ years	Data Rich	1	2	Smoking Prevalence	323
Other pneumoconiosis	Male	15-19 years	95+ years	Data Rich	-1	3	Education (years per capita)	141
Other pneumoconiosis	Male	15-19 years	95+ years	Data Rich	-1	3	LDI (I$ per capita)	--
Other pneumoconiosis	Male	15-19 years	95+ years	Data Rich	-1	3	Socio-demographic Index	--
Other pneumoconiosis	Male	15-19 years	95+ years	Global	-1	2	Healthcare access and quality index	265
Other pneumoconiosis	Male	15-19 years	95+ years	Global	1	2	Indoor Air Pollution (All Cooking Fuels)	216
Other pneumoconiosis	Male	15-19 years	95+ years	Global	1	2	Cumulative Cigarettes (5 Years)	435
Other pneumoconiosis	Male	15-19 years	95+ years	Global	1	2	Smoking Prevalence	719
Other pneumoconiosis	Male	15-19 years	95+ years	Global	-1	3	Socio-demographic Index	52
Other pneumoconiosis	Male	15-19 years	95+ years	Global	-1	3	Education (years per capita)	201
Other pneumoconiosis	Male	15-19 years	95+ years	Global	-1	3	LDI (I$ per capita)	--
Asthma	Female	1-4 years	95+ years	Data Rich	-1	1	Healthcare access and quality index	566
Asthma	Female	1-4 years	95+ years	Data Rich	1	1	Cumulative Cigarettes (10 Years)	108
Asthma	Female	1-4 years	95+ years	Data Rich	1	1	Cumulative Cigarettes (5 Years)	199
Asthma	Female	1-4 years	95+ years	Data Rich	1	1	Log-transformed SEV scalar: Asthma	--
Asthma	Female	1-4 years	95+ years	Data Rich	1	2	Smoking Prevalence	5
Asthma	Female	1-4 years	95+ years	Data Rich	1	2	Indoor Air Pollution (All Cooking Fuels)	29
Asthma	Female	1-4 years	95+ years	Data Rich	1	2	Outdoor Air Pollution (PM2.5)	537
Asthma	Female	1-4 years	95+ years	Data Rich	-1	3	Socio-demographic Index	2
Asthma	Female	1-4 years	95+ years	Data Rich	-1	3	Education (years per capita)	180
Asthma	Female	1-4 years	95+ years	Data Rich	-1	3	LDI (I$ per capita)	--
Asthma	Female	1-4 years	95+ years	Global	-1	1	Healthcare access and quality index	593
Asthma	Female	1-4 years	95+ years	Global	1	1	Cumulative Cigarettes (5 Years)	145
Asthma	Female	1-4 years	95+ years	Global	1	1	Cumulative Cigarettes (10 Years)	160
Asthma	Female	1-4 years	95+ years	Global	1	1	Log-transformed SEV scalar: Asthma	--
Asthma	Female	1-4 years	95+ years	Global	1	2	Smoking Prevalence	2
Asthma	Female	1-4 years	95+ years	Global	1	2	Indoor Air Pollution (All Cooking Fuels)	91
Asthma	Female	1-4 years	95+ years	Global	1	2	Outdoor Air Pollution (PM2.5)	300
Asthma	Female	1-4 years	95+ years	Global	-1	3	Socio-demographic Index	12
Asthma	Female	1-4 years	95+ years	Global	-1	3	Education (years per capita)	273

Table S16. CODEm covariates used, level of covariate, and expected direction of covariate by cause, sex, and age
Covariates that CODEm did not select during the covariate selection process have no draw counts listed.

Cause	Sex	Age Start	Age End	Model Type	Direction	Level	Covariate Name	Number of Draws
Asthma	Female	1-4 years	95+ years	Global	-1	3	LDI (I$ per capita)	--
Asthma	Male	1-4 years	95+ years	Data Rich	-1	1	Healthcare access and quality index	159
Asthma	Male	1-4 years	95+ years	Data Rich	1	1	Cumulative Cigarettes (5 Years)	143
Asthma	Male	1-4 years	95+ years	Data Rich	1	1	Cumulative Cigarettes (10 Years)	283
Asthma	Male	1-4 years	95+ years	Data Rich	1	1	Log-transformed SEV scalar: Asthma	--
Asthma	Male	1-4 years	95+ years	Data Rich	1	2	Outdoor Air Pollution (PM2.5)	9
Asthma	Male	1-4 years	95+ years	Data Rich	1	2	Smoking Prevalence	27
Asthma	Male	1-4 years	95+ years	Data Rich	1	2	Indoor Air Pollution (All Cooking Fuels)	110
Asthma	Male	1-4 years	95+ years	Data Rich	-1	3	Socio-demographic Index	432
Asthma	Male	1-4 years	95+ years	Data Rich	-1	3	Education (years per capita)	571
Asthma	Male	1-4 years	95+ years	Data Rich	-1	3	LDI (I$ per capita)	--
Asthma	Male	1-4 years	95+ years	Global	-1	1	Healthcare access and quality index	788
Asthma	Male	1-4 years	95+ years	Global	1	1	Cumulative Cigarettes (5 Years)	268
Asthma	Male	1-4 years	95+ years	Global	1	1	Cumulative Cigarettes (10 Years)	427
Asthma	Male	1-4 years	95+ years	Global	1	1	Log-transformed SEV scalar: Asthma	--
Asthma	Male	1-4 years	95+ years	Global	1	2	Smoking Prevalence	63
Asthma	Male	1-4 years	95+ years	Global	1	2	Indoor Air Pollution (All Cooking Fuels)	70
Asthma	Male	1-4 years	95+ years	Global	1	2	Outdoor Air Pollution (PM2.5)	198
Asthma	Male	1-4 years	95+ years	Global	-1	3	Socio-demographic Index	76
Asthma	Male	1-4 years	95+ years	Global	-1	3	Education (years per capita)	549
Asthma	Male	1-4 years	95+ years	Global	-1	3	LDI (I$ per capita)	--
Interstitial lung disease and pulmonary sarcoidosis	Female	1-4 years	95+ years	Data Rich	1	1	Log-transformed SEV scalar: ILD	414
Interstitial lung disease and pulmonary sarcoidosis	Female	1-4 years	95+ years	Data Rich	1	1	Cumulative Cigarettes (10 Years)	--
Interstitial lung disease and pulmonary sarcoidosis	Female	1-4 years	95+ years	Data Rich	1	1	Cumulative Cigarettes (5 Years)	--
Interstitial lung disease and pulmonary sarcoidosis	Female	1-4 years	95+ years	Data Rich	1	1	Smoking Prevalence	--
Interstitial lung disease and pulmonary sarcoidosis	Female	1-4 years	95+ years	Data Rich	-1	2	Healthcare access and quality index	--
Interstitial lung disease and pulmonary sarcoidosis	Female	1-4 years	95+ years	Data Rich	1	2	Outdoor Air Pollution (PM2.5)	586
Interstitial lung disease and pulmonary sarcoidosis	Female	1-4 years	95+ years	Data Rich	1	2	Indoor Air Pollution (All Cooking Fuels)	--
Interstitial lung disease and pulmonary sarcoidosis	Female	1-4 years	95+ years	Data Rich	-1	3	Education (years per capita)	--
Interstitial lung disease and pulmonary sarcoidosis	Female	1-4 years	95+ years	Data Rich	-1	3	LDI (I$ per capita)	--
Interstitial lung disease and pulmonary sarcoidosis	Female	1-4 years	95+ years	Data Rich	0	3	Socio-demographic Index	618
Interstitial lung disease and pulmonary sarcoidosis	Female	1-4 years	95+ years	Global	1	1	Outdoor Air Pollution (PM2.5)	681
Interstitial lung disease and pulmonary sarcoidosis	Female	1-4 years	95+ years	Global	1	1	Log-transformed SEV scalar: ILD	739
Interstitial lung disease and pulmonary sarcoidosis	Female	1-4 years	95+ years	Global	1	1	Cumulative Cigarettes (10 Years)	--
Interstitial lung disease and pulmonary sarcoidosis	Female	1-4 years	95+ years	Global	1	1	Cumulative Cigarettes (5 Years)	--
Interstitial lung disease and pulmonary sarcoidosis	Female	1-4 years	95+ years	Global	1	1	Indoor Air Pollution (All Cooking Fuels)	--
Interstitial lung disease and pulmonary sarcoidosis	Female	1-4 years	95+ years	Global	1	1	Smoking Prevalence	--
Interstitial lung disease and pulmonary sarcoidosis	Female	1-4 years	95+ years	Global	-1	2	Healthcare access and quality index	--
Interstitial lung disease and pulmonary sarcoidosis	Female	1-4 years	95+ years	Global	-1	3	Education (years per capita)	--
Interstitial lung disease and pulmonary sarcoidosis	Female	1-4 years	95+ years	Global	-1	3	LDI (I$ per capita)	--
Interstitial lung disease and pulmonary sarcoidosis	Female	1-4 years	95+ years	Global	0	3	Socio-demographic Index	693
Interstitial lung disease and pulmonary sarcoidosis	Male	1-4 years	95+ years	Data Rich	1	1	Cumulative Cigarettes (10 Years)	--
Interstitial lung disease and pulmonary sarcoidosis	Male	1-4 years	95+ years	Data Rich	1	1	Cumulative Cigarettes (5 Years)	--
Interstitial lung disease and pulmonary sarcoidosis	Male	1-4 years	95+ years	Data Rich	1	1	Log-transformed SEV scalar: ILD	--
Interstitial lung disease and pulmonary sarcoidosis	Male	1-4 years	95+ years	Data Rich	1	1	Smoking Prevalence	--
Interstitial lung disease and pulmonary sarcoidosis	Male	1-4 years	95+ years	Data Rich	-1	2	Healthcare access and quality index	--
Interstitial lung disease and pulmonary sarcoidosis	Male	1-4 years	95+ years	Data Rich	1	2	Outdoor Air Pollution (PM2.5)	1000
Interstitial lung disease and pulmonary sarcoidosis	Male	1-4 years	95+ years	Data Rich	1	2	Indoor Air Pollution (All Cooking Fuels)	--
Interstitial lung disease and pulmonary sarcoidosis	Male	1-4 years	95+ years	Data Rich	-1	3	Education (years per capita)	--
Interstitial lung disease and pulmonary sarcoidosis	Male	1-4 years	95+ years	Data Rich	-1	3	LDI (I$ per capita)	--
Interstitial lung disease and pulmonary sarcoidosis	Male	1-4 years	95+ years	Data Rich	0	3	Socio-demographic Index	1000
Interstitial lung disease and pulmonary sarcoidosis	Male	1-4 years	95+ years	Global	1	1	Cumulative Cigarettes (10 Years)	--
Interstitial lung disease and pulmonary sarcoidosis	Male	1-4 years	95+ years	Global	1	1	Cumulative Cigarettes (5 Years)	--
Interstitial lung disease and pulmonary sarcoidosis	Male	1-4 years	95+ years	Global	1	1	Log-transformed SEV scalar: ILD	--
Interstitial lung disease and pulmonary sarcoidosis	Male	1-4 years	95+ years	Global	1	1	Smoking Prevalence	--
Interstitial lung disease and pulmonary sarcoidosis	Male	1-4 years	95+ years	Global	-1	2	Healthcare access and quality index	--
Interstitial lung disease and pulmonary sarcoidosis	Male	1-4 years	95+ years	Global	1	2	Outdoor Air Pollution (PM2.5)	422

Table S16. CODEm covariates used, level of covariate, and expected direction of covariate by cause, sex, and age<br>*Covariates that CODEm did not select during the covariate selection process have no draw counts listed.*								
**Cause**	**Sex**	**Age Start**	**Age End**	**Model Type**	**Direction**	**Level**	**Covariate Name**	**Number of Draws**
Interstitial lung disease and pulmonary sarcoidosis	Male	1-4 years	95+ years	Global	1	2	Indoor Air Pollution (All Cooking Fuels)	--
Interstitial lung disease and pulmonary sarcoidosis	Male	1-4 years	95+ years	Global	-1	3	Education (years per capita)	--
Interstitial lung disease and pulmonary sarcoidosis	Male	1-4 years	95+ years	Global	-1	3	LDI (I$ per capita)	--
Interstitial lung disease and pulmonary sarcoidosis	Male	1-4 years	95+ years	Global	0	3	Socio-demographic Index	815
Other chronic respiratory diseases	Female	1-4 years	95+ years	Data Rich	1	1	Indoor Air Pollution (All Cooking Fuels)	242
Other chronic respiratory diseases	Female	1-4 years	95+ years	Data Rich	1	1	Outdoor Air Pollution (PM2.5)	536
Other chronic respiratory diseases	Female	1-4 years	95+ years	Data Rich	1	1	Cumulative Cigarettes (5 Years)	--
Other chronic respiratory diseases	Female	1-4 years	95+ years	Data Rich	1	1	Log-transformed SEV scalar: Oth Resp	--
Other chronic respiratory diseases	Female	1-4 years	95+ years	Data Rich	1	1	Smoking Prevalence	--
Other chronic respiratory diseases	Female	1-4 years	95+ years	Data Rich	-1	2	Healthcare access and quality index	58
Other chronic respiratory diseases	Female	1-4 years	95+ years	Data Rich	1	2	Elevation Over 1500m (proportion)	12
Other chronic respiratory diseases	Female	1-4 years	95+ years	Data Rich	1	2	Elevation 500 to 1500m (proportion)	306
Other chronic respiratory diseases	Female	1-4 years	95+ years	Data Rich	1	2	Population Density (over 1000 ppl/sqkm, proportion)	--
Other chronic respiratory diseases	Female	1-4 years	95+ years	Data Rich	-1	3	Education (years per capita)	17
Other chronic respiratory diseases	Female	1-4 years	95+ years	Data Rich	-1	3	Socio-demographic Index	44
Other chronic respiratory diseases	Female	1-4 years	95+ years	Data Rich	-1	3	LDI (I$ per capita)	--
Other chronic respiratory diseases	Female	1-4 years	95+ years	Global	1	1	Indoor Air Pollution (All Cooking Fuels)	134
Other chronic respiratory diseases	Female	1-4 years	95+ years	Global	1	1	Cumulative Cigarettes (5 Years)	--
Other chronic respiratory diseases	Female	1-4 years	95+ years	Global	1	1	Log-transformed SEV scalar: Oth Resp	--
Other chronic respiratory diseases	Female	1-4 years	95+ years	Global	1	1	Outdoor Air Pollution (PM2.5)	--
Other chronic respiratory diseases	Female	1-4 years	95+ years	Global	1	1	Smoking Prevalence	--
Other chronic respiratory diseases	Female	1-4 years	95+ years	Global	-1	2	Healthcare access and quality index	215
Other chronic respiratory diseases	Female	1-4 years	95+ years	Global	1	2	Elevation 500 to 1500m (proportion)	337
Other chronic respiratory diseases	Female	1-4 years	95+ years	Global	1	2	Elevation Over 1500m (proportion)	405
Other chronic respiratory diseases	Female	1-4 years	95+ years	Global	1	2	Population Density (over 1000 ppl/sqkm, proportion)	--
Other chronic respiratory diseases	Female	1-4 years	95+ years	Global	-1	3	Socio-demographic Index	70
Other chronic respiratory diseases	Female	1-4 years	95+ years	Global	-1	3	Education (years per capita)	300
Other chronic respiratory diseases	Female	1-4 years	95+ years	Global	-1	3	LDI (I$ per capita)	--
Other chronic respiratory diseases	Male	1-4 years	95+ years	Data Rich	1	1	Smoking Prevalence	19
Other chronic respiratory diseases	Male	1-4 years	95+ years	Data Rich	1	1	Indoor Air Pollution (All Cooking Fuels)	230
Other chronic respiratory diseases	Male	1-4 years	95+ years	Data Rich	1	1	Outdoor Air Pollution (PM2.5)	253
Other chronic respiratory diseases	Male	1-4 years	95+ years	Data Rich	1	1	Cumulative Cigarettes (5 Years)	401
Other chronic respiratory diseases	Male	1-4 years	95+ years	Data Rich	1	1	Log-transformed SEV scalar: Oth Resp	--
Other chronic respiratory diseases	Male	1-4 years	95+ years	Data Rich	-1	2	Healthcare access and quality index	0
Other chronic respiratory diseases	Male	1-4 years	95+ years	Data Rich	1	2	Elevation Over 1500m (proportion)	60
Other chronic respiratory diseases	Male	1-4 years	95+ years	Data Rich	1	2	Elevation 500 to 1500m (proportion)	--
Other chronic respiratory diseases	Male	1-4 years	95+ years	Data Rich	1	2	Population Density (over 1000 ppl/sqkm, proportion)	--
Other chronic respiratory diseases	Male	1-4 years	95+ years	Data Rich	-1	3	Education (years per capita)	0
Other chronic respiratory diseases	Male	1-4 years	95+ years	Data Rich	-1	3	Socio-demographic Index	0
Other chronic respiratory diseases	Male	1-4 years	95+ years	Data Rich	-1	3	LDI (I$ per capita)	--
Other chronic respiratory diseases	Male	1-4 years	95+ years	Global	1	1	Cumulative Cigarettes (5 Years)	132
Other chronic respiratory diseases	Male	1-4 years	95+ years	Global	1	1	Smoking Prevalence	139
Other chronic respiratory diseases	Male	1-4 years	95+ years	Global	1	1	Indoor Air Pollution (All Cooking Fuels)	256
Other chronic respiratory diseases	Male	1-4 years	95+ years	Global	1	1	Log-transformed SEV scalar: Oth Resp	--
Other chronic respiratory diseases	Male	1-4 years	95+ years	Global	1	1	Outdoor Air Pollution (PM2.5)	--
Other chronic respiratory diseases	Male	1-4 years	95+ years	Global	-1	2	Healthcare access and quality index	182
Other chronic respiratory diseases	Male	1-4 years	95+ years	Global	1	2	Elevation Over 1500m (proportion)	390
Other chronic respiratory diseases	Male	1-4 years	95+ years	Global	1	2	Elevation 500 to 1500m (proportion)	410
Other chronic respiratory diseases	Male	1-4 years	95+ years	Global	1	2	Population Density (over 1000 ppl/sqkm, proportion)	--
Other chronic respiratory diseases	Male	1-4 years	95+ years	Global	-1	3	Socio-demographic Index	69

Table S16. CODEm covariates used, level of covariate, and expected direction of covariate by cause, sex, and age
Covariates that CODEm did not select during the covariate selection process have no draw counts listed.

Cause	Sex	Age Start	Age End	Model Type	Direction	Level	Covariate Name	Number of Draws
Other chronic respiratory diseases	Male	1-4 years	95+ years	Global	-1	3	Education (years per capita)	168
Other chronic respiratory diseases	Male	1-4 years	95+ years	Global	-1	3	LDI (I$ per capita)	--
Digestive diseases	Female	0-6 days	95+ years	Data Rich	-1	1	Sanitation (proportion with access)	417
Digestive diseases	Female	0-6 days	95+ years	Data Rich	1	1	Cumulative Cigarettes (5 Years)	596
Digestive diseases	Female	0-6 days	95+ years	Data Rich	1	1	Alcohol (liters per capita)	693
Digestive diseases	Female	0-6 days	95+ years	Data Rich	-1	2	fruits adjusted(g)	7
Digestive diseases	Female	0-6 days	95+ years	Data Rich	-1	2	Healthcare access and quality index	--
Digestive diseases	Female	0-6 days	95+ years	Data Rich	1	2	red meats adjusted(g)	--
Digestive diseases	Female	0-6 days	95+ years	Data Rich	-1	3	LDI (I$ per capita)	156
Digestive diseases	Female	0-6 days	95+ years	Data Rich	-1	3	Socio-demographic Index	317
Digestive diseases	Female	0-6 days	95+ years	Data Rich	-1	3	Education (years per capita)	472
Digestive diseases	Female	0-6 days	95+ years	Global	-1	1	Sanitation (proportion with access)	477
Digestive diseases	Female	0-6 days	95+ years	Global	1	1	Cumulative Cigarettes (5 Years)	150
Digestive diseases	Female	0-6 days	95+ years	Global	1	1	Alcohol (liters per capita)	814
Digestive diseases	Female	0-6 days	95+ years	Global	-1	2	fruits adjusted(g)	71
Digestive diseases	Female	0-6 days	95+ years	Global	-1	2	Healthcare access and quality index	211
Digestive diseases	Female	0-6 days	95+ years	Global	1	2	red meats adjusted(g)	--
Digestive diseases	Female	0-6 days	95+ years	Global	-1	3	LDI (I$ per capita)	137
Digestive diseases	Female	0-6 days	95+ years	Global	-1	3	Socio-demographic Index	168
Digestive diseases	Female	0-6 days	95+ years	Global	-1	3	Education (years per capita)	210
Digestive diseases	Male	0-6 days	95+ years	Data Rich	-1	1	Sanitation (proportion with access)	141
Digestive diseases	Male	0-6 days	95+ years	Data Rich	1	1	Cumulative Cigarettes (5 Years)	278
Digestive diseases	Male	0-6 days	95+ years	Data Rich	1	1	Alcohol (liters per capita)	905
Digestive diseases	Male	0-6 days	95+ years	Data Rich	-1	2	fruits adjusted(g)	85
Digestive diseases	Male	0-6 days	95+ years	Data Rich	-1	2	Healthcare access and quality index	183
Digestive diseases	Male	0-6 days	95+ years	Data Rich	1	2	red meats adjusted(g)	--
Digestive diseases	Male	0-6 days	95+ years	Data Rich	-1	3	LDI (I$ per capita)	27
Digestive diseases	Male	0-6 days	95+ years	Data Rich	-1	3	Education (years per capita)	142
Digestive diseases	Male	0-6 days	95+ years	Data Rich	-1	3	Socio-demographic Index	186
Digestive diseases	Male	0-6 days	95+ years	Global	-1	1	Sanitation (proportion with access)	414
Digestive diseases	Male	0-6 days	95+ years	Global	1	1	Cumulative Cigarettes (5 Years)	111
Digestive diseases	Male	0-6 days	95+ years	Global	1	1	Alcohol (liters per capita)	924
Digestive diseases	Male	0-6 days	95+ years	Global	-1	2	Healthcare access and quality index	116
Digestive diseases	Male	0-6 days	95+ years	Global	-1	2	fruits adjusted(g)	160
Digestive diseases	Male	0-6 days	95+ years	Global	1	2	red meats adjusted(g)	--
Digestive diseases	Male	0-6 days	95+ years	Global	-1	3	LDI (I$ per capita)	129
Digestive diseases	Male	0-6 days	95+ years	Global	-1	3	Socio-demographic Index	216
Digestive diseases	Male	0-6 days	95+ years	Global	-1	3	Education (years per capita)	314
Cirrhosis and other chronic liver diseases	Female	1-4 years	95+ years	Data Rich	-1	1	Hepatitis B 3-dose coverage (proportion)	--
Cirrhosis and other chronic liver diseases	Female	1-4 years	95+ years	Data Rich	1	1	Alcohol (liters per capita)	881
Cirrhosis and other chronic liver diseases	Female	1-4 years	95+ years	Data Rich	1	1	Schistosomiasis Prevalence (proportion)	931
Cirrhosis and other chronic liver diseases	Female	1-4 years	95+ years	Data Rich	1	1	Hepatitis B (HBsAg) Seroprevalence	--
Cirrhosis and other chronic liver diseases	Female	1-4 years	95+ years	Data Rich	1	1	Hepatitis C (IgG) Seroprevalence	--
Cirrhosis and other chronic liver diseases	Female	1-4 years	95+ years	Data Rich	-1	2	Healthcare access and quality index	187
Cirrhosis and other chronic liver diseases	Female	1-4 years	95+ years	Data Rich	1	2	Diabetes Age-Standardized Prevalence (proportion)	397
Cirrhosis and other chronic liver diseases	Female	1-4 years	95+ years	Data Rich	1	2	Mean BMI	--
Cirrhosis and other chronic liver diseases	Female	1-4 years	95+ years	Data Rich	-1	3	Health System Access 2 (unitless)	79
Cirrhosis and other chronic liver diseases	Female	1-4 years	95+ years	Data Rich	-1	3	Education (years per capita)	--
Cirrhosis and other chronic liver diseases	Female	1-4 years	95+ years	Data Rich	-1	3	LDI (I$ per capita)	--
Cirrhosis and other chronic liver diseases	Female	1-4 years	95+ years	Data Rich	0	3	Socio-demographic Index	363
Cirrhosis and other chronic liver diseases	Female	1-4 years	95+ years	Global	-1	1	Hepatitis B 3-dose coverage (proportion)	33
Cirrhosis and other chronic liver diseases	Female	1-4 years	95+ years	Global	1	1	Hepatitis B (HBsAg) Seroprevalence	22
Cirrhosis and other chronic liver diseases	Female	1-4 years	95+ years	Global	1	1	Schistosomiasis Prevalence (proportion)	247
Cirrhosis and other chronic liver diseases	Female	1-4 years	95+ years	Global	1	1	Alcohol (liters per capita)	937
Cirrhosis and other chronic liver diseases	Female	1-4 years	95+ years	Global	1	1	Hepatitis C (IgG) Seroprevalence	--
Cirrhosis and other chronic liver diseases	Female	1-4 years	95+ years	Global	-1	2	Healthcare access and quality index	54
Cirrhosis and other chronic liver diseases	Female	1-4 years	95+ years	Global	1	2	Mean BMI	365

Table S16. CODEm covariates used, level of covariate, and expected direction of covariate by cause, sex, and age
Covariates that CODEm did not select during the covariate selection process have no draw counts listed.

Cause	Sex	Age Start	Age End	Model Type	Direction	Level	Covariate Name	Number of Draws
Cirrhosis and other chronic liver diseases	Female	1-4 years	95+ years	Global	1	2	Diabetes Age-Standardized Prevalence (proportion)	--
Cirrhosis and other chronic liver diseases	Female	1-4 years	95+ years	Global	-1	3	Education (years per capita)	36
Cirrhosis and other chronic liver diseases	Female	1-4 years	95+ years	Global	-1	3	Health System Access 2 (unitless)	67
Cirrhosis and other chronic liver diseases	Female	1-4 years	95+ years	Global	-1	3	LDI (I$ per capita)	--
Cirrhosis and other chronic liver diseases	Female	1-4 years	95+ years	Global	0	3	Socio-demographic Index	579
Cirrhosis and other chronic liver diseases	Male	1-4 years	95+ years	Data Rich	-1	1	Hepatitis B 3-dose coverage (proportion)	42
Cirrhosis and other chronic liver diseases	Male	1-4 years	95+ years	Data Rich	1	1	Alcohol (liters per capita)	640
Cirrhosis and other chronic liver diseases	Male	1-4 years	95+ years	Data Rich	1	1	Schistosomiasis Prevalence (proportion)	683
Cirrhosis and other chronic liver diseases	Male	1-4 years	95+ years	Data Rich	1	1	Hepatitis B (HBsAg) Seroprevalence	--
Cirrhosis and other chronic liver diseases	Male	1-4 years	95+ years	Data Rich	1	1	Hepatitis C (IgG) Seroprevalence	--
Cirrhosis and other chronic liver diseases	Male	1-4 years	95+ years	Data Rich	-1	2	Healthcare access and quality index	50
Cirrhosis and other chronic liver diseases	Male	1-4 years	95+ years	Data Rich	1	2	Mean BMI	272
Cirrhosis and other chronic liver diseases	Male	1-4 years	95+ years	Data Rich	1	2	Diabetes Age-Standardized Prevalence (proportion)	--
Cirrhosis and other chronic liver diseases	Male	1-4 years	95+ years	Data Rich	-1	3	Health System Access 2 (unitless)	105
Cirrhosis and other chronic liver diseases	Male	1-4 years	95+ years	Data Rich	-1	3	Education (years per capita)	--
Cirrhosis and other chronic liver diseases	Male	1-4 years	95+ years	Data Rich	-1	3	LDI (I$ per capita)	--
Cirrhosis and other chronic liver diseases	Male	1-4 years	95+ years	Data Rich	0	3	Socio-demographic Index	739
Cirrhosis and other chronic liver diseases	Male	1-4 years	95+ years	Global	-1	1	Hepatitis B 3-dose coverage (proportion)	65
Cirrhosis and other chronic liver diseases	Male	1-4 years	95+ years	Global	1	1	Hepatitis B (HBsAg) Seroprevalence	63
Cirrhosis and other chronic liver diseases	Male	1-4 years	95+ years	Global	1	1	Schistosomiasis Prevalence (proportion)	415
Cirrhosis and other chronic liver diseases	Male	1-4 years	95+ years	Global	1	1	Alcohol (liters per capita)	837
Cirrhosis and other chronic liver diseases	Male	1-4 years	95+ years	Global	1	1	Hepatitis C (IgG) Seroprevalence	--
Cirrhosis and other chronic liver diseases	Male	1-4 years	95+ years	Global	-1	2	Healthcare access and quality index	60
Cirrhosis and other chronic liver diseases	Male	1-4 years	95+ years	Global	1	2	Mean BMI	535
Cirrhosis and other chronic liver diseases	Male	1-4 years	95+ years	Global	1	2	Diabetes Age-Standardized Prevalence (proportion)	--
Cirrhosis and other chronic liver diseases	Male	1-4 years	95+ years	Global	-1	3	Education (years per capita)	14
Cirrhosis and other chronic liver diseases	Male	1-4 years	95+ years	Global	-1	3	Health System Access 2 (unitless)	148
Cirrhosis and other chronic liver diseases	Male	1-4 years	95+ years	Global	-1	3	LDI (I$ per capita)	--
Cirrhosis and other chronic liver diseases	Male	1-4 years	95+ years	Global	0	3	Socio-demographic Index	517
Upper digestive system diseases	Female	1-4 years	95+ years	Data Rich	1	1	Cumulative Cigarettes (5 Years)	82
Upper digestive system diseases	Female	1-4 years	95+ years	Data Rich	1	1	Cumulative Cigarettes (10 Years)	288
Upper digestive system diseases	Female	1-4 years	95+ years	Data Rich	1	1	Smoking Prevalence	409
Upper digestive system diseases	Female	1-4 years	95+ years	Data Rich	1	1	Alcohol (liters per capita)	426
Upper digestive system diseases	Female	1-4 years	95+ years	Data Rich	-1	2	Sanitation (proportion with access)	18
Upper digestive system diseases	Female	1-4 years	95+ years	Data Rich	-1	2	Healthcare access and quality index	981
Upper digestive system diseases	Female	1-4 years	95+ years	Data Rich	0	2	vegetables adjusted(g)	999
Upper digestive system diseases	Female	1-4 years	95+ years	Data Rich	1	2	Age- and sex-specific SEV for Unsafe water	807
Upper digestive system diseases	Female	1-4 years	95+ years	Data Rich	-1	3	Maternal Education (years per capita)	0
Upper digestive system diseases	Female	1-4 years	95+ years	Data Rich	-1	3	Socio-demographic Index	528
Upper digestive system diseases	Female	1-4 years	95+ years	Data Rich	-1	3	LDI (I$ per capita)	--
Upper digestive system diseases	Female	1-4 years	95+ years	Global	1	1	Smoking Prevalence	216
Upper digestive system diseases	Female	1-4 years	95+ years	Global	1	1	Cumulative Cigarettes (5 Years)	299
Upper digestive system diseases	Female	1-4 years	95+ years	Global	1	1	Cumulative Cigarettes (10 Years)	394
Upper digestive system diseases	Female	1-4 years	95+ years	Global	1	1	Alcohol (liters per capita)	640
Upper digestive system diseases	Female	1-4 years	95+ years	Global	-1	2	Sanitation (proportion with access)	69
Upper digestive system diseases	Female	1-4 years	95+ years	Global	-1	2	Healthcare access and quality index	654
Upper digestive system diseases	Female	1-4 years	95+ years	Global	0	2	vegetables adjusted(g)	699
Upper digestive system diseases	Female	1-4 years	95+ years	Global	1	2	Age- and sex-specific SEV for Unsafe water	313
Upper digestive system diseases	Female	1-4 years	95+ years	Global	-1	3	Socio-demographic Index	286
Upper digestive system diseases	Female	1-4 years	95+ years	Global	-1	3	Maternal Education (years per capita)	344
Upper digestive system diseases	Female	1-4 years	95+ years	Global	-1	3	LDI (I$ per capita)	--
Upper digestive system diseases	Male	1-4 years	95+ years	Data Rich	1	1	Alcohol (liters per capita)	1
Upper digestive system diseases	Male	1-4 years	95+ years	Data Rich	1	1	Cumulative Cigarettes (10 Years)	362
Upper digestive system diseases	Male	1-4 years	95+ years	Data Rich	1	1	Smoking Prevalence	605
Upper digestive system diseases	Male	1-4 years	95+ years	Data Rich	1	1	Cumulative Cigarettes (5 Years)	637

Table S16. CODEm covariates used, level of covariate, and expected direction of covariate by cause, sex, and age<br>*Covariates that CODEm did not select during the covariate selection process have no draw counts listed.*								
**Cause**	**Sex**	**Age Start**	**Age End**	**Model Type**	**Direction**	**Level**	**Covariate Name**	**Number of Draws**
Upper digestive system diseases	Male	1-4 years	95+ years	Data Rich	-1	2	Healthcare access and quality index	2
Upper digestive system diseases	Male	1-4 years	95+ years	Data Rich	-1	2	Sanitation (proportion with access)	690
Upper digestive system diseases	Male	1-4 years	95+ years	Data Rich	0	2	vegetables adjusted(g)	591
Upper digestive system diseases	Male	1-4 years	95+ years	Data Rich	1	2	Age- and sex-specific SEV for Unsafe water	448
Upper digestive system diseases	Male	1-4 years	95+ years	Data Rich	-1	3	Maternal Education (years per capita)	153
Upper digestive system diseases	Male	1-4 years	95+ years	Data Rich	-1	3	Socio-demographic Index	337
Upper digestive system diseases	Male	1-4 years	95+ years	Data Rich	-1	3	LDI (I$ per capita)	--
Upper digestive system diseases	Male	1-4 years	95+ years	Global	1	1	Cumulative Cigarettes (5 Years)	242
Upper digestive system diseases	Male	1-4 years	95+ years	Global	1	1	Smoking Prevalence	286
Upper digestive system diseases	Male	1-4 years	95+ years	Global	1	1	Cumulative Cigarettes (10 Years)	406
Upper digestive system diseases	Male	1-4 years	95+ years	Global	1	1	Alcohol (liters per capita)	753
Upper digestive system diseases	Male	1-4 years	95+ years	Global	-1	2	Sanitation (proportion with access)	783
Upper digestive system diseases	Male	1-4 years	95+ years	Global	-1	2	Healthcare access and quality index	852
Upper digestive system diseases	Male	1-4 years	95+ years	Global	0	2	vegetables adjusted(g)	793
Upper digestive system diseases	Male	1-4 years	95+ years	Global	1	2	Age- and sex-specific SEV for Unsafe water	100
Upper digestive system diseases	Male	1-4 years	95+ years	Global	-1	3	Maternal Education (years per capita)	85
Upper digestive system diseases	Male	1-4 years	95+ years	Global	-1	3	Socio-demographic Index	124
Upper digestive system diseases	Male	1-4 years	95+ years	Global	-1	3	LDI (I$ per capita)	--
Peptic ulcer disease	Female	1-4 years	95+ years	Data Rich	1	1	Cumulative Cigarettes (10 Years)	288
Peptic ulcer disease	Female	1-4 years	95+ years	Data Rich	1	1	Cumulative Cigarettes (5 Years)	296
Peptic ulcer disease	Female	1-4 years	95+ years	Data Rich	1	1	Alcohol (liters per capita)	478
Peptic ulcer disease	Female	1-4 years	95+ years	Data Rich	1	1	Smoking Prevalence	741
Peptic ulcer disease	Female	1-4 years	95+ years	Data Rich	-1	2	Sanitation (proportion with access)	0
Peptic ulcer disease	Female	1-4 years	95+ years	Data Rich	-1	2	Healthcare access and quality index	961
Peptic ulcer disease	Female	1-4 years	95+ years	Data Rich	0	2	vegetables adjusted(g)	956
Peptic ulcer disease	Female	1-4 years	95+ years	Data Rich	1	2	Age- and sex-specific SEV for Unsafe water	965
Peptic ulcer disease	Female	1-4 years	95+ years	Data Rich	-1	3	Maternal Education (years per capita)	48
Peptic ulcer disease	Female	1-4 years	95+ years	Data Rich	-1	3	Socio-demographic Index	448
Peptic ulcer disease	Female	1-4 years	95+ years	Data Rich	-1	3	LDI (I$ per capita)	--
Peptic ulcer disease	Female	1-4 years	95+ years	Global	1	1	Cumulative Cigarettes (5 Years)	244
Peptic ulcer disease	Female	1-4 years	95+ years	Global	1	1	Cumulative Cigarettes (10 Years)	381
Peptic ulcer disease	Female	1-4 years	95+ years	Global	1	1	Smoking Prevalence	684
Peptic ulcer disease	Female	1-4 years	95+ years	Global	1	1	Alcohol (liters per capita)	966
Peptic ulcer disease	Female	1-4 years	95+ years	Global	-1	2	Sanitation (proportion with access)	15
Peptic ulcer disease	Female	1-4 years	95+ years	Global	-1	2	Healthcare access and quality index	930
Peptic ulcer disease	Female	1-4 years	95+ years	Global	0	2	vegetables adjusted(g)	933
Peptic ulcer disease	Female	1-4 years	95+ years	Global	1	2	Age- and sex-specific SEV for Unsafe water	930
Peptic ulcer disease	Female	1-4 years	95+ years	Global	-1	3	Maternal Education (years per capita)	36
Peptic ulcer disease	Female	1-4 years	95+ years	Global	-1	3	Socio-demographic Index	368
Peptic ulcer disease	Female	1-4 years	95+ years	Global	-1	3	LDI (I$ per capita)	--
Peptic ulcer disease	Male	1-4 years	95+ years	Data Rich	1	1	Cumulative Cigarettes (10 Years)	101
Peptic ulcer disease	Male	1-4 years	95+ years	Data Rich	1	1	Smoking Prevalence	505
Peptic ulcer disease	Male	1-4 years	95+ years	Data Rich	1	1	Cumulative Cigarettes (5 Years)	580
Peptic ulcer disease	Male	1-4 years	95+ years	Data Rich	1	1	Alcohol (liters per capita)	775
Peptic ulcer disease	Male	1-4 years	95+ years	Data Rich	-1	2	Healthcare access and quality index	955
Peptic ulcer disease	Male	1-4 years	95+ years	Data Rich	-1	2	Sanitation (proportion with access)	979
Peptic ulcer disease	Male	1-4 years	95+ years	Data Rich	0	2	vegetables adjusted(g)	923
Peptic ulcer disease	Male	1-4 years	95+ years	Data Rich	1	2	Age- and sex-specific SEV for Unsafe water	944
Peptic ulcer disease	Male	1-4 years	95+ years	Data Rich	-1	3	Maternal Education (years per capita)	75
Peptic ulcer disease	Male	1-4 years	95+ years	Data Rich	-1	3	Socio-demographic Index	129
Peptic ulcer disease	Male	1-4 years	95+ years	Data Rich	-1	3	LDI (I$ per capita)	--
Peptic ulcer disease	Male	1-4 years	95+ years	Global	1	1	Cumulative Cigarettes (5 Years)	346
Peptic ulcer disease	Male	1-4 years	95+ years	Global	1	1	Cumulative Cigarettes (10 Years)	409
Peptic ulcer disease	Male	1-4 years	95+ years	Global	1	1	Smoking Prevalence	465
Peptic ulcer disease	Male	1-4 years	95+ years	Global	1	1	Alcohol (liters per capita)	719

Table S16. CODEm covariates used, level of covariate, and expected direction of covariate by cause, sex, and age
Covariates that CODEm did not select during the covariate selection process have no draw counts listed.

Cause	Sex	Age Start	Age End	Model Type	Direction	Level	Covariate Name	Number of Draws
Peptic ulcer disease	Male	1-4 years	95+ years	Global	-1	2	Sanitation (proportion with access)	789
Peptic ulcer disease	Male	1-4 years	95+ years	Global	-1	2	Healthcare access and quality index	852
Peptic ulcer disease	Male	1-4 years	95+ years	Global	0	2	vegetables adjusted(g)	855
Peptic ulcer disease	Male	1-4 years	95+ years	Global	1	2	Age- and sex-specific SEV for Unsafe water	525
Peptic ulcer disease	Male	1-4 years	95+ years	Global	-1	3	Maternal Education (years per capita)	125
Peptic ulcer disease	Male	1-4 years	95+ years	Global	-1	3	Socio-demographic Index	135
Peptic ulcer disease	Male	1-4 years	95+ years	Global	-1	3	LDI (I$ per capita)	--
Gastritis and duodenitis	Female	1-4 years	95+ years	Data Rich	1	1	Cumulative Cigarettes (5 Years)	5
Gastritis and duodenitis	Female	1-4 years	95+ years	Data Rich	1	1	Cumulative Cigarettes (10 Years)	161
Gastritis and duodenitis	Female	1-4 years	95+ years	Data Rich	1	1	Alcohol (liters per capita)	907
Gastritis and duodenitis	Female	1-4 years	95+ years	Data Rich	1	1	Smoking Prevalence	--
Gastritis and duodenitis	Female	1-4 years	95+ years	Data Rich	-1	2	Sanitation (proportion with access)	142
Gastritis and duodenitis	Female	1-4 years	95+ years	Data Rich	-1	2	Healthcare access and quality index	185
Gastritis and duodenitis	Female	1-4 years	95+ years	Data Rich	0	2	vegetables adjusted(g)	81
Gastritis and duodenitis	Female	1-4 years	95+ years	Data Rich	1	2	Age- and sex-specific SEV for Unsafe water	162
Gastritis and duodenitis	Female	1-4 years	95+ years	Data Rich	-1	3	Education (years per capita)	15
Gastritis and duodenitis	Female	1-4 years	95+ years	Data Rich	-1	3	Socio-demographic Index	62
Gastritis and duodenitis	Female	1-4 years	95+ years	Data Rich	-1	3	LDI (I$ per capita)	--
Gastritis and duodenitis	Female	1-4 years	95+ years	Global	1	1	Cumulative Cigarettes (10 Years)	100
Gastritis and duodenitis	Female	1-4 years	95+ years	Global	1	1	Alcohol (liters per capita)	--
Gastritis and duodenitis	Female	1-4 years	95+ years	Global	1	1	Cumulative Cigarettes (5 Years)	--
Gastritis and duodenitis	Female	1-4 years	95+ years	Global	1	1	Smoking Prevalence	--
Gastritis and duodenitis	Female	1-4 years	95+ years	Global	-1	2	Healthcare access and quality index	151
Gastritis and duodenitis	Female	1-4 years	95+ years	Global	-1	2	Sanitation (proportion with access)	463
Gastritis and duodenitis	Female	1-4 years	95+ years	Global	0	2	vegetables adjusted(g)	614
Gastritis and duodenitis	Female	1-4 years	95+ years	Global	1	2	Age- and sex-specific SEV for Unsafe water	--
Gastritis and duodenitis	Female	1-4 years	95+ years	Global	-1	3	Socio-demographic Index	120
Gastritis and duodenitis	Female	1-4 years	95+ years	Global	-1	3	Education (years per capita)	--
Gastritis and duodenitis	Female	1-4 years	95+ years	Global	-1	3	LDI (I$ per capita)	--
Gastritis and duodenitis	Male	1-4 years	95+ years	Data Rich	1	1	Cumulative Cigarettes (5 Years)	28
Gastritis and duodenitis	Male	1-4 years	95+ years	Data Rich	1	1	Alcohol (liters per capita)	435
Gastritis and duodenitis	Male	1-4 years	95+ years	Data Rich	1	1	Smoking Prevalence	596
Gastritis and duodenitis	Male	1-4 years	95+ years	Data Rich	1	1	Cumulative Cigarettes (10 Years)	671
Gastritis and duodenitis	Male	1-4 years	95+ years	Data Rich	-1	2	Healthcare access and quality index	9
Gastritis and duodenitis	Male	1-4 years	95+ years	Data Rich	-1	2	Sanitation (proportion with access)	164
Gastritis and duodenitis	Male	1-4 years	95+ years	Data Rich	0	2	vegetables adjusted(g)	249
Gastritis and duodenitis	Male	1-4 years	95+ years	Data Rich	1	2	Age- and sex-specific SEV for Unsafe water	16
Gastritis and duodenitis	Male	1-4 years	95+ years	Data Rich	-1	3	Education (years per capita)	5
Gastritis and duodenitis	Male	1-4 years	95+ years	Data Rich	-1	3	Socio-demographic Index	14
Gastritis and duodenitis	Male	1-4 years	95+ years	Data Rich	-1	3	LDI (I$ per capita)	--
Gastritis and duodenitis	Male	1-4 years	95+ years	Global	1	1	Cumulative Cigarettes (5 Years)	24
Gastritis and duodenitis	Male	1-4 years	95+ years	Global	1	1	Alcohol (liters per capita)	241
Gastritis and duodenitis	Male	1-4 years	95+ years	Global	1	1	Cumulative Cigarettes (10 Years)	465
Gastritis and duodenitis	Male	1-4 years	95+ years	Global	1	1	Smoking Prevalence	508
Gastritis and duodenitis	Male	1-4 years	95+ years	Global	-1	2	Healthcare access and quality index	83
Gastritis and duodenitis	Male	1-4 years	95+ years	Global	-1	2	Sanitation (proportion with access)	656
Gastritis and duodenitis	Male	1-4 years	95+ years	Global	0	2	vegetables adjusted(g)	484
Gastritis and duodenitis	Male	1-4 years	95+ years	Global	1	2	Age- and sex-specific SEV for Unsafe water	3
Gastritis and duodenitis	Male	1-4 years	95+ years	Global	-1	3	Socio-demographic Index	8
Gastritis and duodenitis	Male	1-4 years	95+ years	Global	-1	3	Education (years per capita)	12
Gastritis and duodenitis	Male	1-4 years	95+ years	Global	-1	3	LDI (I$ per capita)	--
Appendicitis	Female	1-4 years	95+ years	Data Rich	-1	2	vegetables adjusted(g)	265
Appendicitis	Female	1-4 years	95+ years	Data Rich	-1	2	fruits adjusted(g)	425
Appendicitis	Female	1-4 years	95+ years	Data Rich	-1	2	Healthcare access and quality index	595
Appendicitis	Female	1-4 years	95+ years	Data Rich	-1	3	Education (years per capita)	12
Appendicitis	Female	1-4 years	95+ years	Data Rich	-1	3	Socio-demographic Index	48
Appendicitis	Female	1-4 years	95+ years	Data Rich	-1	3	LDI (I$ per capita)	--

**Table S16. CODEm covariates used, level of covariate, and expected direction of covariate by cause, sex, and age** *Covariates that CODEm did not select during the covariate selection process have no draw counts listed.*								
**Cause**	**Sex**	**Age Start**	**Age End**	**Model Type**	**Direction**	**Level**	**Covariate Name**	**Number of Draws**
Appendicitis	Female	1-4 years	95+ years	Data Rich	-1	3	Maternal care and immunization	--
Appendicitis	Female	1-4 years	95+ years	Global	-1	2	fruits adjusted(g)	225
Appendicitis	Female	1-4 years	95+ years	Global	-1	2	vegetables adjusted(g)	433
Appendicitis	Female	1-4 years	95+ years	Global	-1	2	Healthcare access and quality index	653
Appendicitis	Female	1-4 years	95+ years	Global	-1	3	Education (years per capita)	94
Appendicitis	Female	1-4 years	95+ years	Global	-1	3	Socio-demographic Index	310
Appendicitis	Female	1-4 years	95+ years	Global	-1	3	LDI (I$ per capita)	--
Appendicitis	Female	1-4 years	95+ years	Global	-1	3	Maternal care and immunization	--
Appendicitis	Male	1-4 years	95+ years	Data Rich	-1	2	fruits adjusted(g)	283
Appendicitis	Male	1-4 years	95+ years	Data Rich	-1	2	vegetables adjusted(g)	557
Appendicitis	Male	1-4 years	95+ years	Data Rich	-1	2	Healthcare access and quality index	654
Appendicitis	Male	1-4 years	95+ years	Data Rich	-1	3	Education (years per capita)	12
Appendicitis	Male	1-4 years	95+ years	Data Rich	-1	3	Socio-demographic Index	502
Appendicitis	Male	1-4 years	95+ years	Data Rich	-1	3	LDI (I$ per capita)	--
Appendicitis	Male	1-4 years	95+ years	Data Rich	-1	3	Maternal care and immunization	--
Appendicitis	Male	1-4 years	95+ years	Global	-1	2	vegetables adjusted(g)	217
Appendicitis	Male	1-4 years	95+ years	Global	-1	2	fruits adjusted(g)	394
Appendicitis	Male	1-4 years	95+ years	Global	-1	2	Healthcare access and quality index	728
Appendicitis	Male	1-4 years	95+ years	Global	-1	3	Education (years per capita)	58
Appendicitis	Male	1-4 years	95+ years	Global	-1	3	Socio-demographic Index	257
Appendicitis	Male	1-4 years	95+ years	Global	-1	3	LDI (I$ per capita)	--
Appendicitis	Male	1-4 years	95+ years	Global	-1	3	Maternal care and immunization	--
Paralytic ileus and intestinal obstruction	Female	0-6 days	95+ years	Data Rich	-1	2	Healthcare access and quality index	1000
Paralytic ileus and intestinal obstruction	Female	0-6 days	95+ years	Data Rich	-1	2	vegetables adjusted(g)	1000
Paralytic ileus and intestinal obstruction	Female	0-6 days	95+ years	Data Rich	-1	2	fruits adjusted(g)	--
Paralytic ileus and intestinal obstruction	Female	0-6 days	95+ years	Data Rich	-1	3	Education (years per capita)	--
Paralytic ileus and intestinal obstruction	Female	0-6 days	95+ years	Data Rich	-1	3	LDI (I$ per capita)	--
Paralytic ileus and intestinal obstruction	Female	0-6 days	95+ years	Data Rich	-1	3	Maternal care and immunization	--
Paralytic ileus and intestinal obstruction	Female	0-6 days	95+ years	Data Rich	-1	3	Socio-demographic Index	--
Paralytic ileus and intestinal obstruction	Female	0-6 days	95+ years	Global	-1	2	Healthcare access and quality index	463
Paralytic ileus and intestinal obstruction	Female	0-6 days	95+ years	Global	-1	2	fruits adjusted(g)	537
Paralytic ileus and intestinal obstruction	Female	0-6 days	95+ years	Global	-1	2	vegetables adjusted(g)	559
Paralytic ileus and intestinal obstruction	Female	0-6 days	95+ years	Global	-1	3	Socio-demographic Index	133
Paralytic ileus and intestinal obstruction	Female	0-6 days	95+ years	Global	-1	3	Education (years per capita)	166
Paralytic ileus and intestinal obstruction	Female	0-6 days	95+ years	Global	-1	3	LDI (I$ per capita)	--
Paralytic ileus and intestinal obstruction	Female	0-6 days	95+ years	Global	-1	3	Maternal care and immunization	--
Paralytic ileus and intestinal obstruction	Male	0-6 days	95+ years	Data Rich	-1	2	Healthcare access and quality index	326
Paralytic ileus and intestinal obstruction	Male	0-6 days	95+ years	Data Rich	-1	2	vegetables adjusted(g)	674
Paralytic ileus and intestinal obstruction	Male	0-6 days	95+ years	Data Rich	-1	2	fruits adjusted(g)	--
Paralytic ileus and intestinal obstruction	Male	0-6 days	95+ years	Data Rich	-1	3	Education (years per capita)	267
Paralytic ileus and intestinal obstruction	Male	0-6 days	95+ years	Data Rich	-1	3	LDI (I$ per capita)	--
Paralytic ileus and intestinal obstruction	Male	0-6 days	95+ years	Data Rich	-1	3	Maternal care and immunization	--
Paralytic ileus and intestinal obstruction	Male	0-6 days	95+ years	Data Rich	-1	3	Socio-demographic Index	--
Paralytic ileus and intestinal obstruction	Male	0-6 days	95+ years	Global	-1	2	vegetables adjusted(g)	514
Paralytic ileus and intestinal obstruction	Male	0-6 days	95+ years	Global	-1	2	Healthcare access and quality index	706
Paralytic ileus and intestinal obstruction	Male	0-6 days	95+ years	Global	-1	2	fruits adjusted(g)	796
Paralytic ileus and intestinal obstruction	Male	0-6 days	95+ years	Global	-1	3	Socio-demographic Index	161
Paralytic ileus and intestinal obstruction	Male	0-6 days	95+ years	Global	-1	3	Education (years per capita)	--
Paralytic ileus and intestinal obstruction	Male	0-6 days	95+ years	Global	-1	3	LDI (I$ per capita)	--
Paralytic ileus and intestinal obstruction	Male	0-6 days	95+ years	Global	-1	3	Maternal care and immunization	--
Inguinal, femoral, and abdominal hernia	Female	1-4 years	95+ years	Data Rich	-1	1	Mean BMI	751
Inguinal, femoral, and abdominal hernia	Female	1-4 years	95+ years	Data Rich	1	1	Smoking Prevalence	243
Inguinal, femoral, and abdominal hernia	Female	1-4 years	95+ years	Data Rich	1	1	Cumulative Cigarettes (10 Years)	249
Inguinal, femoral, and abdominal hernia	Female	1-4 years	95+ years	Data Rich	1	1	Cumulative Cigarettes (5 Years)	--
Inguinal, femoral, and abdominal hernia	Female	1-4 years	95+ years	Data Rich	-1	2	Healthcare access and quality index	--
Inguinal, femoral, and abdominal hernia	Female	1-4 years	95+ years	Data Rich	-1	3	Education (years per capita)	--
Inguinal, femoral, and abdominal hernia	Female	1-4 years	95+ years	Data Rich	-1	3	LDI (I$ per capita)	--
Inguinal, femoral, and abdominal hernia	Female	1-4 years	95+ years	Data Rich	0	3	Socio-demographic Index	--
Inguinal, femoral, and abdominal hernia	Female	1-4 years	95+ years	Global	-1	1	Mean BMI	816
Inguinal, femoral, and abdominal hernia	Female	1-4 years	95+ years	Global	1	1	Cumulative Cigarettes (10 Years)	409

Table S16. CODEm covariates used, level of covariate, and expected direction of covariate by cause, sex, and age
Covariates that CODEm did not select during the covariate selection process have no draw counts listed.

Cause	Sex	Age Start	Age End	Model Type	Direction	Level	Covariate Name	Number of Draws
Inguinal, femoral, and abdominal hernia	Female	1-4 years	95+ years	Global	1	1	Cumulative Cigarettes (5 Years)	485
Inguinal, femoral, and abdominal hernia	Female	1-4 years	95+ years	Global	1	1	Smoking Prevalence	--
Inguinal, femoral, and abdominal hernia	Female	1-4 years	95+ years	Global	-1	2	Healthcare access and quality index	--
Inguinal, femoral, and abdominal hernia	Female	1-4 years	95+ years	Global	-1	3	Education (years per capita)	330
Inguinal, femoral, and abdominal hernia	Female	1-4 years	95+ years	Global	-1	3	LDI (I$ per capita)	--
Inguinal, femoral, and abdominal hernia	Female	1-4 years	95+ years	Global	0	3	Socio-demographic Index	227
Inguinal, femoral, and abdominal hernia	Male	1-4 years	95+ years	Data Rich	-1	1	Mean BMI	185
Inguinal, femoral, and abdominal hernia	Male	1-4 years	95+ years	Data Rich	1	1	Smoking Prevalence	87
Inguinal, femoral, and abdominal hernia	Male	1-4 years	95+ years	Data Rich	1	1	Cumulative Cigarettes (10 Years)	175
Inguinal, femoral, and abdominal hernia	Male	1-4 years	95+ years	Data Rich	1	1	Cumulative Cigarettes (5 Years)	681
Inguinal, femoral, and abdominal hernia	Male	1-4 years	95+ years	Data Rich	-1	2	Healthcare access and quality index	430
Inguinal, femoral, and abdominal hernia	Male	1-4 years	95+ years	Data Rich	-1	3	Education (years per capita)	496
Inguinal, femoral, and abdominal hernia	Male	1-4 years	95+ years	Data Rich	-1	3	LDI (I$ per capita)	--
Inguinal, femoral, and abdominal hernia	Male	1-4 years	95+ years	Data Rich	0	3	Socio-demographic Index	340
Inguinal, femoral, and abdominal hernia	Male	1-4 years	95+ years	Global	-1	1	Mean BMI	635
Inguinal, femoral, and abdominal hernia	Male	1-4 years	95+ years	Global	1	1	Cumulative Cigarettes (5 Years)	73
Inguinal, femoral, and abdominal hernia	Male	1-4 years	95+ years	Global	1	1	Cumulative Cigarettes (10 Years)	380
Inguinal, femoral, and abdominal hernia	Male	1-4 years	95+ years	Global	1	1	Smoking Prevalence	560
Inguinal, femoral, and abdominal hernia	Male	1-4 years	95+ years	Global	-1	2	Healthcare access and quality index	67
Inguinal, femoral, and abdominal hernia	Male	1-4 years	95+ years	Global	-1	3	Education (years per capita)	191
Inguinal, femoral, and abdominal hernia	Male	1-4 years	95+ years	Global	-1	3	LDI (I$ per capita)	--
Inguinal, femoral, and abdominal hernia	Male	1-4 years	95+ years	Global	0	3	Socio-demographic Index	361
Inflammatory bowel disease	Female	1-4 years	95+ years	Data Rich	-1	1	fruits adjusted(g)	--
Inflammatory bowel disease	Female	1-4 years	95+ years	Data Rich	-1	1	vegetables adjusted(g)	--
Inflammatory bowel disease	Female	1-4 years	95+ years	Data Rich	1	1	saturated fats adjusted(percent)	455
Inflammatory bowel disease	Female	1-4 years	95+ years	Data Rich	1	1	red meats adjusted(g)	--
Inflammatory bowel disease	Female	1-4 years	95+ years	Data Rich	-1	2	Healthcare access and quality index	411
Inflammatory bowel disease	Female	1-4 years	95+ years	Data Rich	-1	2	Latitude 15 to 30 (proportion)	--
Inflammatory bowel disease	Female	1-4 years	95+ years	Data Rich	1	2	Latitude Over 45 (proportion)	623
Inflammatory bowel disease	Female	1-4 years	95+ years	Data Rich	1	2	Latitude 30 to 45 (proportion)	--
Inflammatory bowel disease	Female	1-4 years	95+ years	Data Rich	-1	3	Education (years per capita)	--
Inflammatory bowel disease	Female	1-4 years	95+ years	Data Rich	0	3	Socio-demographic Index	430
Inflammatory bowel disease	Female	1-4 years	95+ years	Data Rich	0	3	LDI (I$ per capita)	--
Inflammatory bowel disease	Female	1-4 years	95+ years	Global	-1	1	vegetables adjusted(g)	369
Inflammatory bowel disease	Female	1-4 years	95+ years	Global	-1	1	fruits adjusted(g)	428
Inflammatory bowel disease	Female	1-4 years	95+ years	Global	1	1	saturated fats adjusted(percent)	338
Inflammatory bowel disease	Female	1-4 years	95+ years	Global	1	1	red meats adjusted(g)	--
Inflammatory bowel disease	Female	1-4 years	95+ years	Global	-1	2	Healthcare access and quality index	382
Inflammatory bowel disease	Female	1-4 years	95+ years	Global	-1	2	Latitude 15 to 30 (proportion)	--
Inflammatory bowel disease	Female	1-4 years	95+ years	Global	1	2	Latitude Over 45 (proportion)	501
Inflammatory bowel disease	Female	1-4 years	95+ years	Global	1	2	Latitude 30 to 45 (proportion)	--
Inflammatory bowel disease	Female	1-4 years	95+ years	Global	-1	3	Education (years per capita)	72
Inflammatory bowel disease	Female	1-4 years	95+ years	Global	0	3	Socio-demographic Index	281
Inflammatory bowel disease	Female	1-4 years	95+ years	Global	0	3	LDI (I$ per capita)	--
Inflammatory bowel disease	Male	1-4 years	95+ years	Data Rich	-1	1	fruits adjusted(g)	751
Inflammatory bowel disease	Male	1-4 years	95+ years	Data Rich	-1	1	vegetables adjusted(g)	--
Inflammatory bowel disease	Male	1-4 years	95+ years	Data Rich	1	1	saturated fats adjusted(percent)	249
Inflammatory bowel disease	Male	1-4 years	95+ years	Data Rich	1	1	red meats adjusted(g)	--
Inflammatory bowel disease	Male	1-4 years	95+ years	Data Rich	-1	2	Healthcare access and quality index	--
Inflammatory bowel disease	Male	1-4 years	95+ years	Data Rich	-1	2	Latitude 15 to 30 (proportion)	--
Inflammatory bowel disease	Male	1-4 years	95+ years	Data Rich	1	2	Latitude Over 45 (proportion)	485
Inflammatory bowel disease	Male	1-4 years	95+ years	Data Rich	1	2	Latitude 30 to 45 (proportion)	--
Inflammatory bowel disease	Male	1-4 years	95+ years	Data Rich	-1	3	Education (years per capita)	453
Inflammatory bowel disease	Male	1-4 years	95+ years	Data Rich	0	3	Socio-demographic Index	453
Inflammatory bowel disease	Male	1-4 years	95+ years	Data Rich	0	3	LDI (I$ per capita)	--
Inflammatory bowel disease	Male	1-4 years	95+ years	Global	-1	1	fruits adjusted(g)	337
Inflammatory bowel disease	Male	1-4 years	95+ years	Global	-1	1	vegetables adjusted(g)	432
Inflammatory bowel disease	Male	1-4 years	95+ years	Global	1	1	saturated fats adjusted(percent)	342
Inflammatory bowel disease	Male	1-4 years	95+ years	Global	1	1	red meats adjusted(g)	--
Inflammatory bowel disease	Male	1-4 years	95+ years	Global	-1	2	Healthcare access and quality index	387

Table S16. CODEm covariates used, level of covariate, and expected direction of covariate by cause, sex, and age<br>*Covariates that CODEm did not select during the covariate selection process have no draw counts listed.*								
**Cause**	**Sex**	**Age Start**	**Age End**	**Model Type**	**Direction**	**Level**	**Covariate Name**	**Number of Draws**
Inflammatory bowel disease	Male	1-4 years	95+ years	Global	-1	2	Latitude 15 to 30 (proportion)	--
Inflammatory bowel disease	Male	1-4 years	95+ years	Global	1	2	Latitude Over 45 (proportion)	629
Inflammatory bowel disease	Male	1-4 years	95+ years	Global	1	2	Latitude 30 to 45 (proportion)	--
Inflammatory bowel disease	Male	1-4 years	95+ years	Global	-1	3	Education (years per capita)	66
Inflammatory bowel disease	Male	1-4 years	95+ years	Global	0	3	Socio-demographic Index	305
Inflammatory bowel disease	Male	1-4 years	95+ years	Global	0	3	LDI (I$ per capita)	--
Vascular intestinal disorders	Female	1-4 years	95+ years	Data Rich	1	1	saturated fats adjusted(percent)	87
Vascular intestinal disorders	Female	1-4 years	95+ years	Data Rich	1	1	Diabetes Fasting Plasma Glucose (mmol/L), age-standardized 25+	496
Vascular intestinal disorders	Female	1-4 years	95+ years	Data Rich	1	1	Diabetes Age-Standardized Prevalence (proportion)	504
Vascular intestinal disorders	Female	1-4 years	95+ years	Data Rich	1	1	Cholesterol (total, mean per capita)	--
Vascular intestinal disorders	Female	1-4 years	95+ years	Data Rich	1	1	Systolic Blood Pressure (mmHg)	--
Vascular intestinal disorders	Female	1-4 years	95+ years	Data Rich	-1	2	Healthcare access and quality index	--
Vascular intestinal disorders	Female	1-4 years	95+ years	Data Rich	-1	2	fruits adjusted(g)	--
Vascular intestinal disorders	Female	1-4 years	95+ years	Data Rich	-1	2	vegetables adjusted(g)	--
Vascular intestinal disorders	Female	1-4 years	95+ years	Data Rich	1	2	Alcohol (liters per capita)	--
Vascular intestinal disorders	Female	1-4 years	95+ years	Data Rich	-1	3	Education (years per capita)	--
Vascular intestinal disorders	Female	1-4 years	95+ years	Data Rich	-1	3	LDI (I$ per capita)	--
Vascular intestinal disorders	Female	1-4 years	95+ years	Data Rich	0	3	Socio-demographic Index	262
Vascular intestinal disorders	Female	1-4 years	95+ years	Data Rich	1	3	Latitude Over 45 (proportion)	535
Vascular intestinal disorders	Female	1-4 years	95+ years	Global	1	1	saturated fats adjusted(percent)	330
Vascular intestinal disorders	Female	1-4 years	95+ years	Global	1	1	Diabetes Age-Standardized Prevalence (proportion)	417
Vascular intestinal disorders	Female	1-4 years	95+ years	Global	1	1	Diabetes Fasting Plasma Glucose (mmol/L), age-standardized 25+	493
Vascular intestinal disorders	Female	1-4 years	95+ years	Global	1	1	Cholesterol (total, mean per capita)	--
Vascular intestinal disorders	Female	1-4 years	95+ years	Global	1	1	Systolic Blood Pressure (mmHg)	--
Vascular intestinal disorders	Female	1-4 years	95+ years	Global	-1	2	Healthcare access and quality index	--
Vascular intestinal disorders	Female	1-4 years	95+ years	Global	-1	2	fruits adjusted(g)	--
Vascular intestinal disorders	Female	1-4 years	95+ years	Global	-1	2	vegetables adjusted(g)	--
Vascular intestinal disorders	Female	1-4 years	95+ years	Global	1	2	Alcohol (liters per capita)	--
Vascular intestinal disorders	Female	1-4 years	95+ years	Global	-1	3	Education (years per capita)	--
Vascular intestinal disorders	Female	1-4 years	95+ years	Global	-1	3	LDI (I$ per capita)	--
Vascular intestinal disorders	Female	1-4 years	95+ years	Global	0	3	Socio-demographic Index	102
Vascular intestinal disorders	Female	1-4 years	95+ years	Global	1	3	Latitude Over 45 (proportion)	493
Vascular intestinal disorders	Male	1-4 years	95+ years	Data Rich	1	1	saturated fats adjusted(percent)	98
Vascular intestinal disorders	Male	1-4 years	95+ years	Data Rich	1	1	Diabetes Fasting Plasma Glucose (mmol/L), age-standardized 25+	167
Vascular intestinal disorders	Male	1-4 years	95+ years	Data Rich	1	1	Diabetes Age-Standardized Prevalence (proportion)	572
Vascular intestinal disorders	Male	1-4 years	95+ years	Data Rich	1	1	Cholesterol (total, mean per capita)	--
Vascular intestinal disorders	Male	1-4 years	95+ years	Data Rich	1	1	Systolic Blood Pressure (mmHg)	--
Vascular intestinal disorders	Male	1-4 years	95+ years	Data Rich	-1	2	Healthcare access and quality index	--
Vascular intestinal disorders	Male	1-4 years	95+ years	Data Rich	-1	2	fruits adjusted(g)	--
Vascular intestinal disorders	Male	1-4 years	95+ years	Data Rich	-1	2	vegetables adjusted(g)	--
Vascular intestinal disorders	Male	1-4 years	95+ years	Data Rich	1	2	Alcohol (liters per capita)	523
Vascular intestinal disorders	Male	1-4 years	95+ years	Data Rich	-1	3	Education (years per capita)	--
Vascular intestinal disorders	Male	1-4 years	95+ years	Data Rich	-1	3	LDI (I$ per capita)	--
Vascular intestinal disorders	Male	1-4 years	95+ years	Data Rich	0	3	Socio-demographic Index	180
Vascular intestinal disorders	Male	1-4 years	95+ years	Data Rich	1	3	Latitude Over 45 (proportion)	531
Vascular intestinal disorders	Male	1-4 years	95+ years	Global	1	1	saturated fats adjusted(percent)	331
Vascular intestinal disorders	Male	1-4 years	95+ years	Global	1	1	Diabetes Fasting Plasma Glucose (mmol/L), age-standardized 25+	498
Vascular intestinal disorders	Male	1-4 years	95+ years	Global	1	1	Diabetes Age-Standardized Prevalence (proportion)	645
Vascular intestinal disorders	Male	1-4 years	95+ years	Global	1	1	Cholesterol (total, mean per capita)	--
Vascular intestinal disorders	Male	1-4 years	95+ years	Global	1	1	Systolic Blood Pressure (mmHg)	--
Vascular intestinal disorders	Male	1-4 years	95+ years	Global	-1	2	vegetables adjusted(g)	24
Vascular intestinal disorders	Male	1-4 years	95+ years	Global	-1	2	Healthcare access and quality index	29
Vascular intestinal disorders	Male	1-4 years	95+ years	Global	-1	2	fruits adjusted(g)	--
Vascular intestinal disorders	Male	1-4 years	95+ years	Global	1	2	Alcohol (liters per capita)	622
Vascular intestinal disorders	Male	1-4 years	95+ years	Global	-1	3	Education (years per capita)	--
Vascular intestinal disorders	Male	1-4 years	95+ years	Global	-1	3	LDI (I$ per capita)	--

Table S16. CODEm covariates used, level of covariate, and expected direction of covariate by cause, sex, and age
Covariates that CODEm did not select during the covariate selection process have no draw counts listed.

Cause	Sex	Age Start	Age End	Model Type	Direction	Level	Covariate Name	Number of Draws
Vascular intestinal disorders	Male	1-4 years	95+ years	Global	0	3	Socio-demographic Index	40
Vascular intestinal disorders	Male	1-4 years	95+ years	Global	1	3	Latitude Over 45 (proportion)	536
Gallbladder and biliary diseases	Female	1-4 years	95+ years	Data Rich	1	1	saturated fats adjusted(percent)	408
Gallbladder and biliary diseases	Female	1-4 years	95+ years	Data Rich	1	1	Mean BMI	473
Gallbladder and biliary diseases	Female	1-4 years	95+ years	Data Rich	-1	2	Healthcare access and quality index	365
Gallbladder and biliary diseases	Female	1-4 years	95+ years	Data Rich	1	2	red meats adjusted(g)	365
Gallbladder and biliary diseases	Female	1-4 years	95+ years	Data Rich	1	2	Alcohol (liters per capita)	--
Gallbladder and biliary diseases	Female	1-4 years	95+ years	Data Rich	1	2	Population Over 65 (proportion)	--
Gallbladder and biliary diseases	Female	1-4 years	95+ years	Data Rich	0	3	Socio-demographic Index	263
Gallbladder and biliary diseases	Female	1-4 years	95+ years	Data Rich	0	3	Education (years per capita)	304
Gallbladder and biliary diseases	Female	1-4 years	95+ years	Data Rich	0	3	LDI (I$ per capita)	--
Gallbladder and biliary diseases	Female	1-4 years	95+ years	Global	1	1	Mean BMI	276
Gallbladder and biliary diseases	Female	1-4 years	95+ years	Global	1	1	saturated fats adjusted(percent)	813
Gallbladder and biliary diseases	Female	1-4 years	95+ years	Global	-1	2	Healthcare access and quality index	650
Gallbladder and biliary diseases	Female	1-4 years	95+ years	Global	1	2	red meats adjusted(g)	83
Gallbladder and biliary diseases	Female	1-4 years	95+ years	Global	1	2	Alcohol (liters per capita)	--
Gallbladder and biliary diseases	Female	1-4 years	95+ years	Global	1	2	Population Over 65 (proportion)	--
Gallbladder and biliary diseases	Female	1-4 years	95+ years	Global	0	3	Socio-demographic Index	530
Gallbladder and biliary diseases	Female	1-4 years	95+ years	Global	0	3	Education (years per capita)	649
Gallbladder and biliary diseases	Female	1-4 years	95+ years	Global	0	3	LDI (I$ per capita)	--
Gallbladder and biliary diseases	Male	1-4 years	95+ years	Data Rich	1	1	Mean BMI	404
Gallbladder and biliary diseases	Male	1-4 years	95+ years	Data Rich	1	1	saturated fats adjusted(percent)	966
Gallbladder and biliary diseases	Male	1-4 years	95+ years	Data Rich	-1	2	Healthcare access and quality index	3
Gallbladder and biliary diseases	Male	1-4 years	95+ years	Data Rich	1	2	Alcohol (liters per capita)	3
Gallbladder and biliary diseases	Male	1-4 years	95+ years	Data Rich	1	2	red meats adjusted(g)	25
Gallbladder and biliary diseases	Male	1-4 years	95+ years	Data Rich	1	2	Population Over 65 (proportion)	492
Gallbladder and biliary diseases	Male	1-4 years	95+ years	Data Rich	0	3	Education (years per capita)	108
Gallbladder and biliary diseases	Male	1-4 years	95+ years	Data Rich	0	3	Socio-demographic Index	391
Gallbladder and biliary diseases	Male	1-4 years	95+ years	Data Rich	0	3	LDI (I$ per capita)	--
Gallbladder and biliary diseases	Male	1-4 years	95+ years	Global	1	1	Mean BMI	417
Gallbladder and biliary diseases	Male	1-4 years	95+ years	Global	1	1	saturated fats adjusted(percent)	713
Gallbladder and biliary diseases	Male	1-4 years	95+ years	Global	-1	2	Healthcare access and quality index	200
Gallbladder and biliary diseases	Male	1-4 years	95+ years	Global	-1	2	Maternal care and immunization	--
Gallbladder and biliary diseases	Male	1-4 years	95+ years	Global	1	2	red meats adjusted(g)	104
Gallbladder and biliary diseases	Male	1-4 years	95+ years	Global	1	2	Alcohol (liters per capita)	248
Gallbladder and biliary diseases	Male	1-4 years	95+ years	Global	1	2	Population Over 65 (proportion)	267
Gallbladder and biliary diseases	Male	1-4 years	95+ years	Global	0	3	Education (years per capita)	200
Gallbladder and biliary diseases	Male	1-4 years	95+ years	Global	0	3	Socio-demographic Index	233
Gallbladder and biliary diseases	Male	1-4 years	95+ years	Global	0	3	LDI (I$ per capita)	--
Pancreatitis	Female	1-4 years	95+ years	Data Rich	1	1	Alcohol (liters per capita)	--
Pancreatitis	Female	1-4 years	95+ years	Data Rich	1	1	Log-transformed SEV scalar: Pancreatit	--
Pancreatitis	Female	1-4 years	95+ years	Data Rich	-1	2	Healthcare access and quality index	428
Pancreatitis	Female	1-4 years	95+ years	Data Rich	1	2	Mean BMI	--
Pancreatitis	Female	1-4 years	95+ years	Data Rich	-1	3	Education (years per capita)	134
Pancreatitis	Female	1-4 years	95+ years	Data Rich	0	3	Socio-demographic Index	358
Pancreatitis	Female	1-4 years	95+ years	Data Rich	0	3	LDI (I$ per capita)	--
Pancreatitis	Female	1-4 years	95+ years	Global	1	1	Alcohol (liters per capita)	--
Pancreatitis	Female	1-4 years	95+ years	Global	1	1	Log-transformed SEV scalar: Pancreatit	--
Pancreatitis	Female	1-4 years	95+ years	Global	-1	2	Healthcare access and quality index	424
Pancreatitis	Female	1-4 years	95+ years	Global	1	2	Mean BMI	--
Pancreatitis	Female	1-4 years	95+ years	Global	-1	3	Education (years per capita)	133
Pancreatitis	Female	1-4 years	95+ years	Global	0	3	Socio-demographic Index	392
Pancreatitis	Female	1-4 years	95+ years	Global	0	3	LDI (I$ per capita)	--
Pancreatitis	Male	1-4 years	95+ years	Data Rich	1	1	Alcohol (liters per capita)	547
Pancreatitis	Male	1-4 years	95+ years	Data Rich	1	1	Log-transformed SEV scalar: Pancreatit	--
Pancreatitis	Male	1-4 years	95+ years	Data Rich	-1	2	Healthcare access and quality index	74
Pancreatitis	Male	1-4 years	95+ years	Data Rich	1	2	Mean BMI	706
Pancreatitis	Male	1-4 years	95+ years	Data Rich	-1	3	Education (years per capita)	5
Pancreatitis	Male	1-4 years	95+ years	Data Rich	0	3	Socio-demographic Index	626

Table S16. CODEm covariates used, level of covariate, and expected direction of covariate by cause, sex, and age *Covariates that CODEm did not select during the covariate selection process have no draw counts listed.*								
**Cause**	**Sex**	**Age Start**	**Age End**	**Model Type**	**Direction**	**Level**	**Covariate Name**	**Number of Draws**
Pancreatitis	Male	1-4 years	95+ years	Data Rich	0	3	LDI (I$ per capita)	--
Pancreatitis	Male	1-4 years	95+ years	Global	1	1	Alcohol (liters per capita)	114
Pancreatitis	Male	1-4 years	95+ years	Global	1	1	Log-transformed SEV scalar: Pancreatit	--
Pancreatitis	Male	1-4 years	95+ years	Global	-1	2	Healthcare access and quality index	207
Pancreatitis	Male	1-4 years	95+ years	Global	-1	2	Maternal care and immunization	--
Pancreatitis	Male	1-4 years	95+ years	Global	1	2	Mean BMI	513
Pancreatitis	Male	1-4 years	95+ years	Global	-1	3	Education (years per capita)	52
Pancreatitis	Male	1-4 years	95+ years	Global	0	3	Socio-demographic Index	456
Pancreatitis	Male	1-4 years	95+ years	Global	0	3	LDI (I$ per capita)	--
Other digestive diseases	Female	1-4 years	95+ years	Data Rich	1	1	Cumulative Cigarettes (5 Years)	19
Other digestive diseases	Female	1-4 years	95+ years	Data Rich	1	1	Cumulative Cigarettes (10 Years)	28
Other digestive diseases	Female	1-4 years	95+ years	Data Rich	1	1	Alcohol (liters per capita)	451
Other digestive diseases	Female	1-4 years	95+ years	Data Rich	1	1	Smoking Prevalence	906
Other digestive diseases	Female	1-4 years	95+ years	Data Rich	-1	2	Healthcare access and quality index	--
Other digestive diseases	Female	1-4 years	95+ years	Data Rich	-1	2	Improved Water Source (proportion with access)	--
Other digestive diseases	Female	1-4 years	95+ years	Data Rich	-1	2	Sanitation (proportion with access)	--
Other digestive diseases	Female	1-4 years	95+ years	Data Rich	-1	2	fruits adjusted(g)	--
Other digestive diseases	Female	1-4 years	95+ years	Data Rich	0	2	vegetables adjusted(g)	256
Other digestive diseases	Female	1-4 years	95+ years	Data Rich	1	2	Diabetes Age-Standardized Prevalence (proportion)	98
Other digestive diseases	Female	1-4 years	95+ years	Data Rich	1	2	Mean BMI	420
Other digestive diseases	Female	1-4 years	95+ years	Data Rich	1	2	red meats adjusted(g)	--
Other digestive diseases	Female	1-4 years	95+ years	Data Rich	1	2	saturated fats adjusted(percent)	--
Other digestive diseases	Female	1-4 years	95+ years	Data Rich	-1	3	Health System Access 2 (unitless)	192
Other digestive diseases	Female	1-4 years	95+ years	Data Rich	-1	3	Education (years per capita)	--
Other digestive diseases	Female	1-4 years	95+ years	Data Rich	-1	3	LDI (I$ per capita)	--
Other digestive diseases	Female	1-4 years	95+ years	Data Rich	0	3	Socio-demographic Index	547
Other digestive diseases	Female	1-4 years	95+ years	Global	1	1	Alcohol (liters per capita)	659
Other digestive diseases	Female	1-4 years	95+ years	Global	1	1	Smoking Prevalence	767
Other digestive diseases	Female	1-4 years	95+ years	Global	1	1	Cumulative Cigarettes (10 Years)	--
Other digestive diseases	Female	1-4 years	95+ years	Global	1	1	Cumulative Cigarettes (5 Years)	--
Other digestive diseases	Female	1-4 years	95+ years	Global	-1	2	Improved Water Source (proportion with access)	7
Other digestive diseases	Female	1-4 years	95+ years	Global	-1	2	Sanitation (proportion with access)	12
Other digestive diseases	Female	1-4 years	95+ years	Global	-1	2	fruits adjusted(g)	12
Other digestive diseases	Female	1-4 years	95+ years	Global	-1	2	Healthcare access and quality index	15
Other digestive diseases	Female	1-4 years	95+ years	Global	0	2	vegetables adjusted(g)	596
Other digestive diseases	Female	1-4 years	95+ years	Global	1	2	Mean BMI	720
Other digestive diseases	Female	1-4 years	95+ years	Global	1	2	Diabetes Age-Standardized Prevalence (proportion)	--
Other digestive diseases	Female	1-4 years	95+ years	Global	1	2	red meats adjusted(g)	--
Other digestive diseases	Female	1-4 years	95+ years	Global	1	2	saturated fats adjusted(percent)	--
Other digestive diseases	Female	1-4 years	95+ years	Global	-1	3	Education (years per capita)	10
Other digestive diseases	Female	1-4 years	95+ years	Global	-1	3	Health System Access 2 (unitless)	416
Other digestive diseases	Female	1-4 years	95+ years	Global	-1	3	LDI (I$ per capita)	--
Other digestive diseases	Female	1-4 years	95+ years	Global	0	3	Socio-demographic Index	610
Other digestive diseases	Male	1-4 years	95+ years	Data Rich	1	1	Cumulative Cigarettes (5 Years)	40
Other digestive diseases	Male	1-4 years	95+ years	Data Rich	1	1	Smoking Prevalence	288
Other digestive diseases	Male	1-4 years	95+ years	Data Rich	1	1	Alcohol (liters per capita)	924
Other digestive diseases	Male	1-4 years	95+ years	Data Rich	1	1	Cumulative Cigarettes (10 Years)	--
Other digestive diseases	Male	1-4 years	95+ years	Data Rich	-1	2	Healthcare access and quality index	--
Other digestive diseases	Male	1-4 years	95+ years	Data Rich	-1	2	Improved Water Source (proportion with access)	--
Other digestive diseases	Male	1-4 years	95+ years	Data Rich	-1	2	Sanitation (proportion with access)	--
Other digestive diseases	Male	1-4 years	95+ years	Data Rich	-1	2	fruits adjusted(g)	--
Other digestive diseases	Male	1-4 years	95+ years	Data Rich	0	2	vegetables adjusted(g)	263
Other digestive diseases	Male	1-4 years	95+ years	Data Rich	1	2	Diabetes Age-Standardized Prevalence (proportion)	409
Other digestive diseases	Male	1-4 years	95+ years	Data Rich	1	2	Mean BMI	592
Other digestive diseases	Male	1-4 years	95+ years	Data Rich	1	2	red meats adjusted(g)	--
Other digestive diseases	Male	1-4 years	95+ years	Data Rich	1	2	saturated fats adjusted(percent)	--
Other digestive diseases	Male	1-4 years	95+ years	Data Rich	-1	3	Health System Access 2 (unitless)	303

Table S16. CODEm covariates used, level of covariate, and expected direction of covariate by cause, sex, and age<br>*Covariates that CODEm did not select during the covariate selection process have no draw counts listed.*								
**Cause**	**Sex**	**Age Start**	**Age End**	**Model Type**	**Direction**	**Level**	**Covariate Name**	**Number of Draws**
Other digestive diseases	Male	1-4 years	95+ years	Data Rich	-1	3	Education (years per capita)	--
Other digestive diseases	Male	1-4 years	95+ years	Data Rich	-1	3	LDI (I$ per capita)	--
Other digestive diseases	Male	1-4 years	95+ years	Data Rich	0	3	Socio-demographic Index	437
Other digestive diseases	Male	1-4 years	95+ years	Global	1	1	Smoking Prevalence	50
Other digestive diseases	Male	1-4 years	95+ years	Global	1	1	Cumulative Cigarettes (5 Years)	96
Other digestive diseases	Male	1-4 years	95+ years	Global	1	1	Cumulative Cigarettes (10 Years)	175
Other digestive diseases	Male	1-4 years	95+ years	Global	1	1	Alcohol (liters per capita)	854
Other digestive diseases	Male	1-4 years	95+ years	Global	-1	2	Healthcare access and quality index	67
Other digestive diseases	Male	1-4 years	95+ years	Global	-1	2	Improved Water Source (proportion with access)	82
Other digestive diseases	Male	1-4 years	95+ years	Global	-1	2	Sanitation (proportion with access)	88
Other digestive diseases	Male	1-4 years	95+ years	Global	-1	2	fruits adjusted(g)	93
Other digestive diseases	Male	1-4 years	95+ years	Global	0	2	vegetables adjusted(g)	470
Other digestive diseases	Male	1-4 years	95+ years	Global	1	2	Mean BMI	326
Other digestive diseases	Male	1-4 years	95+ years	Global	1	2	Diabetes Age-Standardized Prevalence (proportion)	--
Other digestive diseases	Male	1-4 years	95+ years	Global	1	2	red meats adjusted(g)	--
Other digestive diseases	Male	1-4 years	95+ years	Global	1	2	saturated fats adjusted(percent)	--
Other digestive diseases	Male	1-4 years	95+ years	Global	-1	3	Education (years per capita)	47
Other digestive diseases	Male	1-4 years	95+ years	Global	-1	3	Health System Access 2 (unitless)	233
Other digestive diseases	Male	1-4 years	95+ years	Global	-1	3	LDI (I$ per capita)	--
Other digestive diseases	Male	1-4 years	95+ years	Global	0	3	Socio-demographic Index	407
Alzheimer's disease and other dementias	Female	40-44 years	95+ years	Data Rich	-1	1	Education (years per capita)	125
Alzheimer's disease and other dementias	Female	40-44 years	95+ years	Data Rich	1	1	Systolic Blood Pressure (mmHg)	0
Alzheimer's disease and other dementias	Female	40-44 years	95+ years	Data Rich	1	1	Diabetes Age-Specific Prevalence (proportion)	214
Alzheimer's disease and other dementias	Female	40-44 years	95+ years	Data Rich	1	1	Smoking Prevalence	673
Alzheimer's disease and other dementias	Female	40-44 years	95+ years	Data Rich	1	1	Mean BMI	685
Alzheimer's disease and other dementias	Female	40-44 years	95+ years	Data Rich	1	1	Cholesterol (total, mean per capita)	--
Alzheimer's disease and other dementias	Female	40-44 years	95+ years	Data Rich	-1	2	Total Physical Activity (MET-min/week), Age-specific	178
Alzheimer's disease and other dementias	Female	40-44 years	95+ years	Data Rich	-1	2	Healthcare access and quality index	378
Alzheimer's disease and other dementias	Female	40-44 years	95+ years	Data Rich	1	2	red meats adjusted(g)	138
Alzheimer's disease and other dementias	Female	40-44 years	95+ years	Data Rich	1	2	Latitude Over 45 (proportion)	222
Alzheimer's disease and other dementias	Female	40-44 years	95+ years	Data Rich	1	2	Outdoor Air Pollution (PM2.5)	--
Alzheimer's disease and other dementias	Female	40-44 years	95+ years	Data Rich	-1	3	Sanitation (proportion with access)	54
Alzheimer's disease and other dementias	Female	40-44 years	95+ years	Data Rich	-1	3	Improved Water Source (proportion with access)	243
Alzheimer's disease and other dementias	Female	40-44 years	95+ years	Data Rich	0	3	LDI (I$ per capita)	--
Alzheimer's disease and other dementias	Female	40-44 years	95+ years	Global	-1	1	Education (years per capita)	314
Alzheimer's disease and other dementias	Female	40-44 years	95+ years	Global	1	1	Systolic Blood Pressure (mmHg)	7
Alzheimer's disease and other dementias	Female	40-44 years	95+ years	Global	1	1	Diabetes Age-Specific Prevalence (proportion)	104
Alzheimer's disease and other dementias	Female	40-44 years	95+ years	Global	1	1	Mean BMI	487
Alzheimer's disease and other dementias	Female	40-44 years	95+ years	Global	1	1	Smoking Prevalence	578
Alzheimer's disease and other dementias	Female	40-44 years	95+ years	Global	1	1	Cholesterol (total, mean per capita)	--
Alzheimer's disease and other dementias	Female	40-44 years	95+ years	Global	-1	2	Healthcare access and quality index	141
Alzheimer's disease and other dementias	Female	40-44 years	95+ years	Global	-1	2	Total Physical Activity (MET-min/week), Age-specific	294
Alzheimer's disease and other dementias	Female	40-44 years	95+ years	Global	1	2	red meats adjusted(g)	138
Alzheimer's disease and other dementias	Female	40-44 years	95+ years	Global	1	2	Latitude Over 45 (proportion)	246
Alzheimer's disease and other dementias	Female	40-44 years	95+ years	Global	1	2	Outdoor Air Pollution (PM2.5)	--
Alzheimer's disease and other dementias	Female	40-44 years	95+ years	Global	-1	3	Sanitation (proportion with access)	123

Table S16. CODEm covariates used, level of covariate, and expected direction of covariate by cause, sex, and age<br>*Covariates that CODEm did not select during the covariate selection process have no draw counts listed.*								
**Cause**	**Sex**	**Age Start**	**Age End**	**Model Type**	**Direction**	**Level**	**Covariate Name**	**Number of Draws**
Alzheimer's disease and other dementias	Female	40-44 years	95+ years	Global	-1	3	Improved Water Source (proportion with access)	221
Alzheimer's disease and other dementias	Female	40-44 years	95+ years	Global	0	3	LDI (I$ per capita)	--
Alzheimer's disease and other dementias	Male	40-44 years	95+ years	Data Rich	-1	1	Education (years per capita)	87
Alzheimer's disease and other dementias	Male	40-44 years	95+ years	Data Rich	1	1	Diabetes Age-Specific Prevalence (proportion)	23
Alzheimer's disease and other dementias	Male	40-44 years	95+ years	Data Rich	1	1	Smoking Prevalence	278
Alzheimer's disease and other dementias	Male	40-44 years	95+ years	Data Rich	1	1	Mean BMI	687
Alzheimer's disease and other dementias	Male	40-44 years	95+ years	Data Rich	1	1	Cholesterol (total, mean per capita)	--
Alzheimer's disease and other dementias	Male	40-44 years	95+ years	Data Rich	1	1	Systolic Blood Pressure (mmHg)	--
Alzheimer's disease and other dementias	Male	40-44 years	95+ years	Data Rich	-1	2	Healthcare access and quality index	0
Alzheimer's disease and other dementias	Male	40-44 years	95+ years	Data Rich	-1	2	Total Physical Activity (MET-min/week), Age-specific	--
Alzheimer's disease and other dementias	Male	40-44 years	95+ years	Data Rich	1	2	red meats adjusted(g)	115
Alzheimer's disease and other dementias	Male	40-44 years	95+ years	Data Rich	1	2	Latitude Over 45 (proportion)	296
Alzheimer's disease and other dementias	Male	40-44 years	95+ years	Data Rich	1	2	Outdoor Air Pollution (PM2.5)	--
Alzheimer's disease and other dementias	Male	40-44 years	95+ years	Data Rich	-1	3	Sanitation (proportion with access)	138
Alzheimer's disease and other dementias	Male	40-44 years	95+ years	Data Rich	-1	3	Improved Water Source (proportion with access)	194
Alzheimer's disease and other dementias	Male	40-44 years	95+ years	Data Rich	0	3	LDI (I$ per capita)	--
Alzheimer's disease and other dementias	Male	40-44 years	95+ years	Global	-1	1	Education (years per capita)	273
Alzheimer's disease and other dementias	Male	40-44 years	95+ years	Global	1	1	Smoking Prevalence	54
Alzheimer's disease and other dementias	Male	40-44 years	95+ years	Global	1	1	Diabetes Age-Specific Prevalence (proportion)	524
Alzheimer's disease and other dementias	Male	40-44 years	95+ years	Global	1	1	Mean BMI	547
Alzheimer's disease and other dementias	Male	40-44 years	95+ years	Global	1	1	Cholesterol (total, mean per capita)	--
Alzheimer's disease and other dementias	Male	40-44 years	95+ years	Global	1	1	Systolic Blood Pressure (mmHg)	--
Alzheimer's disease and other dementias	Male	40-44 years	95+ years	Global	-1	2	Healthcare access and quality index	5
Alzheimer's disease and other dementias	Male	40-44 years	95+ years	Global	-1	2	Total Physical Activity (MET-min/week), Age-specific	--
Alzheimer's disease and other dementias	Male	40-44 years	95+ years	Global	1	2	red meats adjusted(g)	72
Alzheimer's disease and other dementias	Male	40-44 years	95+ years	Global	1	2	Latitude Over 45 (proportion)	670
Alzheimer's disease and other dementias	Male	40-44 years	95+ years	Global	1	2	Outdoor Air Pollution (PM2.5)	--
Alzheimer's disease and other dementias	Male	40-44 years	95+ years	Global	-1	3	Sanitation (proportion with access)	87
Alzheimer's disease and other dementias	Male	40-44 years	95+ years	Global	-1	3	Improved Water Source (proportion with access)	98
Alzheimer's disease and other dementias	Male	40-44 years	95+ years	Global	0	3	LDI (I$ per capita)	--
Parkinson's disease	Female	20-24 years	95+ years	Data Rich	-1	1	Cumulative Cigarettes (10 Years)	704
Parkinson's disease	Female	20-24 years	95+ years	Data Rich	-1	2	fruits adjusted(g)	17
Parkinson's disease	Female	20-24 years	95+ years	Data Rich	-1	2	Healthcare access and quality index	--
Parkinson's disease	Female	20-24 years	95+ years	Data Rich	0	2	Improved Water Source (proportion with access)	410
Parkinson's disease	Female	20-24 years	95+ years	Data Rich	0	2	Sanitation (proportion with access)	419
Parkinson's disease	Female	20-24 years	95+ years	Data Rich	1	2	Absolute value of average latitude	--
Parkinson's disease	Female	20-24 years	95+ years	Data Rich	1	2	Cholesterol (total, mean per capita)	--
Parkinson's disease	Female	20-24 years	95+ years	Data Rich	-1	3	Education (years per capita)	83
Parkinson's disease	Female	20-24 years	95+ years	Data Rich	0	3	LDI (I$ per capita)	--
Parkinson's disease	Female	20-24 years	95+ years	Data Rich	1	3	Socio-demographic Index	259

Table S16. CODEm covariates used, level of covariate, and expected direction of covariate by cause, sex, and age
Covariates that CODEm did not select during the covariate selection process have no draw counts listed.

Cause	Sex	Age Start	Age End	Model Type	Direction	Level	Covariate Name	Number of Draws
Parkinson's disease	Female	20-24 years	95+ years	Global	-1	1	Cumulative Cigarettes (10 Years)	680
Parkinson's disease	Female	20-24 years	95+ years	Global	-1	2	Healthcare access and quality index	188
Parkinson's disease	Female	20-24 years	95+ years	Global	-1	2	fruits adjusted(g)	--
Parkinson's disease	Female	20-24 years	95+ years	Global	0	2	Improved Water Source (proportion with access)	553
Parkinson's disease	Female	20-24 years	95+ years	Global	0	2	Sanitation (proportion with access)	553
Parkinson's disease	Female	20-24 years	95+ years	Global	1	2	Cholesterol (total, mean per capita)	77
Parkinson's disease	Female	20-24 years	95+ years	Global	1	2	Absolute value of average latitude	265
Parkinson's disease	Female	20-24 years	95+ years	Global	-1	3	Education (years per capita)	66
Parkinson's disease	Female	20-24 years	95+ years	Global	0	3	LDI (I$ per capita)	--
Parkinson's disease	Female	20-24 years	95+ years	Global	1	3	Socio-demographic Index	406
Parkinson's disease	Male	20-24 years	95+ years	Data Rich	-1	1	Cumulative Cigarettes (10 Years)	300
Parkinson's disease	Male	20-24 years	95+ years	Data Rich	-1	2	fruits adjusted(g)	119
Parkinson's disease	Male	20-24 years	95+ years	Data Rich	-1	2	Healthcare access and quality index	--
Parkinson's disease	Male	20-24 years	95+ years	Data Rich	0	2	Improved Water Source (proportion with access)	599
Parkinson's disease	Male	20-24 years	95+ years	Data Rich	0	2	Sanitation (proportion with access)	--
Parkinson's disease	Male	20-24 years	95+ years	Data Rich	1	2	Absolute value of average latitude	119
Parkinson's disease	Male	20-24 years	95+ years	Data Rich	1	2	Cholesterol (total, mean per capita)	--
Parkinson's disease	Male	20-24 years	95+ years	Data Rich	-1	3	Education (years per capita)	--
Parkinson's disease	Male	20-24 years	95+ years	Data Rich	0	3	LDI (I$ per capita)	--
Parkinson's disease	Male	20-24 years	95+ years	Data Rich	1	3	Socio-demographic Index	496
Parkinson's disease	Male	20-24 years	95+ years	Global	-1	1	Cumulative Cigarettes (10 Years)	1000
Parkinson's disease	Male	20-24 years	95+ years	Global	-1	2	Healthcare access and quality index	130
Parkinson's disease	Male	20-24 years	95+ years	Global	-1	2	fruits adjusted(g)	130
Parkinson's disease	Male	20-24 years	95+ years	Global	0	2	Sanitation (proportion with access)	312
Parkinson's disease	Male	20-24 years	95+ years	Global	0	2	Improved Water Source (proportion with access)	372
Parkinson's disease	Male	20-24 years	95+ years	Global	1	2	Absolute value of average latitude	130
Parkinson's disease	Male	20-24 years	95+ years	Global	1	2	Cholesterol (total, mean per capita)	--
Parkinson's disease	Male	20-24 years	95+ years	Global	-1	3	Education (years per capita)	--
Parkinson's disease	Male	20-24 years	95+ years	Global	0	3	LDI (I$ per capita)	--
Parkinson's disease	Male	20-24 years	95+ years	Global	1	3	Socio-demographic Index	398
Idiopathic epilepsy	Female	28-364 days	95+ years	Data Rich	1	1	Pig Meat (kg per capita)	630
Idiopathic epilepsy	Female	28-364 days	95+ years	Data Rich	1	1	Pigs (per capita)	687
Idiopathic epilepsy	Female	28-364 days	95+ years	Data Rich	1	1	Log-transformed SEV scalar: Idiopathic epilepsy	--
Idiopathic epilepsy	Female	28-364 days	95+ years	Data Rich	1	1	Systolic Blood Pressure (mmHg)	--
Idiopathic epilepsy	Female	28-364 days	95+ years	Data Rich	-1	2	Healthcare access and quality index	28
Idiopathic epilepsy	Female	28-364 days	95+ years	Data Rich	1	2	Mean BMI	582
Idiopathic epilepsy	Female	28-364 days	95+ years	Data Rich	1	2	Cholesterol (total, mean per capita)	--
Idiopathic epilepsy	Female	28-364 days	95+ years	Data Rich	-1	3	Education (years per capita)	12
Idiopathic epilepsy	Female	28-364 days	95+ years	Data Rich	-1	3	Socio-demographic Index	16
Idiopathic epilepsy	Female	28-364 days	95+ years	Data Rich	-1	3	LDI (I$ per capita)	--

Table S16. CODEm covariates used, level of covariate, and expected direction of covariate by cause, sex, and age
Covariates that CODEm did not select during the covariate selection process have no draw counts listed.

Cause	Sex	Age Start	Age End	Model Type	Direction	Level	Covariate Name	Number of Draws
Idiopathic epilepsy	Female	28-364 days	95+ years	Data Rich	1	3	Cumulative Cigarettes (10 Years)	--
Idiopathic epilepsy	Female	28-364 days	95+ years	Data Rich	1	3	Cumulative Cigarettes (5 Years)	--
Idiopathic epilepsy	Female	28-364 days	95+ years	Global	1	1	Pig Meat (kg per capita)	493
Idiopathic epilepsy	Female	28-364 days	95+ years	Global	1	1	Pigs (per capita)	667
Idiopathic epilepsy	Female	28-364 days	95+ years	Global	1	1	Log-transformed SEV scalar: Idiopathic epilepsy	--
Idiopathic epilepsy	Female	28-364 days	95+ years	Global	1	1	Systolic Blood Pressure (mmHg)	--
Idiopathic epilepsy	Female	28-364 days	95+ years	Global	-1	2	Healthcare access and quality index	52
Idiopathic epilepsy	Female	28-364 days	95+ years	Global	1	2	Mean BMI	429
Idiopathic epilepsy	Female	28-364 days	95+ years	Global	1	2	Cholesterol (total, mean per capita)	--
Idiopathic epilepsy	Female	28-364 days	95+ years	Global	-1	3	Education (years per capita)	39
Idiopathic epilepsy	Female	28-364 days	95+ years	Global	-1	3	Socio-demographic Index	45
Idiopathic epilepsy	Female	28-364 days	95+ years	Global	-1	3	LDI (I$ per capita)	--
Idiopathic epilepsy	Female	28-364 days	95+ years	Global	1	3	Cumulative Cigarettes (10 Years)	--
Idiopathic epilepsy	Female	28-364 days	95+ years	Global	1	3	Cumulative Cigarettes (5 Years)	--
Idiopathic epilepsy	Male	28-364 days	95+ years	Data Rich	1	1	Pig Meat (kg per capita)	580
Idiopathic epilepsy	Male	28-364 days	95+ years	Data Rich	1	1	Pigs (per capita)	662
Idiopathic epilepsy	Male	28-364 days	95+ years	Data Rich	1	1	Log-transformed SEV scalar: Idiopathic epilepsy	--
Idiopathic epilepsy	Male	28-364 days	95+ years	Data Rich	1	1	Systolic Blood Pressure (mmHg)	--
Idiopathic epilepsy	Male	28-364 days	95+ years	Data Rich	-1	2	Healthcare access and quality index	13
Idiopathic epilepsy	Male	28-364 days	95+ years	Data Rich	1	2	Mean BMI	522
Idiopathic epilepsy	Male	28-364 days	95+ years	Data Rich	1	2	Cholesterol (total, mean per capita)	--
Idiopathic epilepsy	Male	28-364 days	95+ years	Data Rich	-1	3	Education (years per capita)	14
Idiopathic epilepsy	Male	28-364 days	95+ years	Data Rich	-1	3	Socio-demographic Index	14
Idiopathic epilepsy	Male	28-364 days	95+ years	Data Rich	-1	3	LDI (I$ per capita)	--
Idiopathic epilepsy	Male	28-364 days	95+ years	Data Rich	1	3	Cumulative Cigarettes (10 Years)	26
Idiopathic epilepsy	Male	28-364 days	95+ years	Data Rich	1	3	Cumulative Cigarettes (5 Years)	33
Idiopathic epilepsy	Male	28-364 days	95+ years	Global	1	1	Pig Meat (kg per capita)	468
Idiopathic epilepsy	Male	28-364 days	95+ years	Global	1	1	Pigs (per capita)	712
Idiopathic epilepsy	Male	28-364 days	95+ years	Global	1	1	Log-transformed SEV scalar: Idiopathic epilepsy	--
Idiopathic epilepsy	Male	28-364 days	95+ years	Global	1	1	Systolic Blood Pressure (mmHg)	--
Idiopathic epilepsy	Male	28-364 days	95+ years	Global	-1	2	Healthcare access and quality index	54
Idiopathic epilepsy	Male	28-364 days	95+ years	Global	1	2	Mean BMI	394
Idiopathic epilepsy	Male	28-364 days	95+ years	Global	1	2	Cholesterol (total, mean per capita)	--
Idiopathic epilepsy	Male	28-364 days	95+ years	Global	-1	3	Socio-demographic Index	43
Idiopathic epilepsy	Male	28-364 days	95+ years	Global	-1	3	Education (years per capita)	89
Idiopathic epilepsy	Male	28-364 days	95+ years	Global	-1	3	LDI (I$ per capita)	--
Idiopathic epilepsy	Male	28-364 days	95+ years	Global	1	3	Cumulative Cigarettes (5 Years)	33
Idiopathic epilepsy	Male	28-364 days	95+ years	Global	1	3	Cumulative Cigarettes (10 Years)	--
Multiple sclerosis	Female	20-24 years	95+ years	Data Rich	1	1	Absolute value of average latitude	1000
Multiple sclerosis	Female	20-24 years	95+ years	Data Rich	-1	2	Healthcare access and quality index	--

Table S16. CODEm covariates used, level of covariate, and expected direction of covariate by cause, sex, and age<br>*Covariates that CODEm did not select during the covariate selection process have no draw counts listed.*								
**Cause**	**Sex**	**Age Start**	**Age End**	**Model Type**	**Direction**	**Level**	**Covariate Name**	**Number of Draws**
Multiple sclerosis	Female	20-24 years	95+ years	Data Rich	1	2	Cholesterol (total, mean per capita)	--
Multiple sclerosis	Female	20-24 years	95+ years	Data Rich	-1	3	Education (years per capita)	--
Multiple sclerosis	Female	20-24 years	95+ years	Data Rich	-1	3	LDI (I$ per capita)	--
Multiple sclerosis	Female	20-24 years	95+ years	Data Rich	1	3	Socio-demographic Index	618
Multiple sclerosis	Female	20-24 years	95+ years	Data Rich	1	3	Cumulative Cigarettes (10 Years)	--
Multiple sclerosis	Female	20-24 years	95+ years	Data Rich	1	3	Cumulative Cigarettes (5 Years)	--
Multiple sclerosis	Female	20-24 years	95+ years	Data Rich	1	3	Smoking Prevalence	--
Multiple sclerosis	Female	20-24 years	95+ years	Global	1	1	Absolute value of average latitude	1000
Multiple sclerosis	Female	20-24 years	95+ years	Global	-1	2	Healthcare access and quality index	--
Multiple sclerosis	Female	20-24 years	95+ years	Global	1	2	Cholesterol (total, mean per capita)	--
Multiple sclerosis	Female	20-24 years	95+ years	Global	-1	3	Education (years per capita)	--
Multiple sclerosis	Female	20-24 years	95+ years	Global	-1	3	LDI (I$ per capita)	--
Multiple sclerosis	Female	20-24 years	95+ years	Global	1	3	Socio-demographic Index	569
Multiple sclerosis	Female	20-24 years	95+ years	Global	1	3	Cumulative Cigarettes (10 Years)	--
Multiple sclerosis	Female	20-24 years	95+ years	Global	1	3	Cumulative Cigarettes (5 Years)	--
Multiple sclerosis	Female	20-24 years	95+ years	Global	1	3	Smoking Prevalence	--
Multiple sclerosis	Male	20-24 years	95+ years	Data Rich	1	1	Absolute value of average latitude	1000
Multiple sclerosis	Male	20-24 years	95+ years	Data Rich	-1	2	Healthcare access and quality index	--
Multiple sclerosis	Male	20-24 years	95+ years	Data Rich	1	2	Cholesterol (total, mean per capita)	--
Multiple sclerosis	Male	20-24 years	95+ years	Data Rich	-1	3	Education (years per capita)	--
Multiple sclerosis	Male	20-24 years	95+ years	Data Rich	-1	3	LDI (I$ per capita)	--
Multiple sclerosis	Male	20-24 years	95+ years	Data Rich	1	3	Socio-demographic Index	324
Multiple sclerosis	Male	20-24 years	95+ years	Data Rich	1	3	Cumulative Cigarettes (10 Years)	--
Multiple sclerosis	Male	20-24 years	95+ years	Data Rich	1	3	Cumulative Cigarettes (5 Years)	--
Multiple sclerosis	Male	20-24 years	95+ years	Data Rich	1	3	Smoking Prevalence	--
Multiple sclerosis	Male	20-24 years	95+ years	Global	1	1	Absolute value of average latitude	1000
Multiple sclerosis	Male	20-24 years	95+ years	Global	-1	2	Healthcare access and quality index	--
Multiple sclerosis	Male	20-24 years	95+ years	Global	1	2	Cholesterol (total, mean per capita)	--
Multiple sclerosis	Male	20-24 years	95+ years	Global	-1	3	Education (years per capita)	--
Multiple sclerosis	Male	20-24 years	95+ years	Global	-1	3	LDI (I$ per capita)	--
Multiple sclerosis	Male	20-24 years	95+ years	Global	1	3	Socio-demographic Index	569
Multiple sclerosis	Male	20-24 years	95+ years	Global	1	3	Cumulative Cigarettes (10 Years)	--
Multiple sclerosis	Male	20-24 years	95+ years	Global	1	3	Cumulative Cigarettes (5 Years)	--
Multiple sclerosis	Male	20-24 years	95+ years	Global	1	3	Smoking Prevalence	--
Motor neuron disease	Female	0-6 days	95+ years	Data Rich	-1	1	Mean BMI	--
Motor neuron disease	Female	0-6 days	95+ years	Data Rich	0	1	fruits adjusted(g)	758
Motor neuron disease	Female	0-6 days	95+ years	Data Rich	0	1	Cholesterol (total, mean per capita)	761
Motor neuron disease	Female	0-6 days	95+ years	Data Rich	1	1	Diabetes Fasting Plasma Glucose (mmol/L), age-standardized 25+	286
Motor neuron disease	Female	0-6 days	95+ years	Data Rich	1	1	Absolute value of average latitude	312
Motor neuron disease	Female	0-6 days	95+ years	Data Rich	1	1	Socio-demographic Index	659
Motor neuron disease	Female	0-6 days	95+ years	Data Rich	-1	2	Healthcare access and quality index	--
Motor neuron disease	Female	0-6 days	95+ years	Data Rich	0	2	Population-weighted mean temperature	270

Table S16. CODEm covariates used, level of covariate, and expected direction of covariate by cause, sex, and age
Covariates that CODEm did not select during the covariate selection process have no draw counts listed.

Cause	Sex	Age Start	Age End	Model Type	Direction	Level	Covariate Name	Number of Draws
Motor neuron disease	Female	0-6 days	95+ years	Data Rich	0	2	Improved Water Source (proportion with access)	--
Motor neuron disease	Female	0-6 days	95+ years	Data Rich	0	2	Sanitation (proportion with access)	--
Motor neuron disease	Female	0-6 days	95+ years	Data Rich	0	3	Education (years per capita)	--
Motor neuron disease	Female	0-6 days	95+ years	Data Rich	0	3	LDI (I$ per capita)	--
Motor neuron disease	Female	0-6 days	95+ years	Global	0	1	fruits adjusted(g)	543
Motor neuron disease	Female	0-6 days	95+ years	Global	0	1	Cholesterol (total, mean per capita)	750
Motor neuron disease	Female	0-6 days	95+ years	Global	1	1	Absolute value of average latitude	240
Motor neuron disease	Female	0-6 days	95+ years	Global	1	1	Diabetes Fasting Plasma Glucose (mmol/L), age-standardized 25+	465
Motor neuron disease	Female	0-6 days	95+ years	Global	1	1	Socio-demographic Index	811
Motor neuron disease	Female	0-6 days	95+ years	Global	-1	2	Healthcare access and quality index	--
Motor neuron disease	Female	0-6 days	95+ years	Global	-1	2	Mean BMI	--
Motor neuron disease	Female	0-6 days	95+ years	Global	0	2	Improved Water Source (proportion with access)	40
Motor neuron disease	Female	0-6 days	95+ years	Global	0	2	Population-weighted mean temperature	50
Motor neuron disease	Female	0-6 days	95+ years	Global	0	2	Sanitation (proportion with access)	74
Motor neuron disease	Female	0-6 days	95+ years	Global	0	3	Education (years per capita)	194
Motor neuron disease	Female	0-6 days	95+ years	Global	0	3	LDI (I$ per capita)	--
Motor neuron disease	Male	0-6 days	95+ years	Data Rich	-1	1	Mean BMI	--
Motor neuron disease	Male	0-6 days	95+ years	Data Rich	0	1	Cholesterol (total, mean per capita)	134
Motor neuron disease	Male	0-6 days	95+ years	Data Rich	0	1	fruits adjusted(g)	743
Motor neuron disease	Male	0-6 days	95+ years	Data Rich	1	1	Diabetes Fasting Plasma Glucose (mmol/L), age-standardized 25+	165
Motor neuron disease	Male	0-6 days	95+ years	Data Rich	1	1	Absolute value of average latitude	473
Motor neuron disease	Male	0-6 days	95+ years	Data Rich	1	1	Socio-demographic Index	889
Motor neuron disease	Male	0-6 days	95+ years	Data Rich	-1	2	Healthcare access and quality index	--
Motor neuron disease	Male	0-6 days	95+ years	Data Rich	0	2	Sanitation (proportion with access)	4
Motor neuron disease	Male	0-6 days	95+ years	Data Rich	0	2	Improved Water Source (proportion with access)	20
Motor neuron disease	Male	0-6 days	95+ years	Data Rich	0	2	Population-weighted mean temperature	--
Motor neuron disease	Male	0-6 days	95+ years	Data Rich	0	3	Education (years per capita)	260
Motor neuron disease	Male	0-6 days	95+ years	Data Rich	0	3	LDI (I$ per capita)	--
Motor neuron disease	Male	0-6 days	95+ years	Global	-1	1	Mean BMI	--
Motor neuron disease	Male	0-6 days	95+ years	Global	0	1	Cholesterol (total, mean per capita)	420
Motor neuron disease	Male	0-6 days	95+ years	Global	0	1	fruits adjusted(g)	762
Motor neuron disease	Male	0-6 days	95+ years	Global	1	1	Diabetes Fasting Plasma Glucose (mmol/L), age-standardized 25+	37
Motor neuron disease	Male	0-6 days	95+ years	Global	1	1	Absolute value of average latitude	413
Motor neuron disease	Male	0-6 days	95+ years	Global	1	1	Socio-demographic Index	705
Motor neuron disease	Male	0-6 days	95+ years	Global	-1	2	Healthcare access and quality index	--
Motor neuron disease	Male	0-6 days	95+ years	Global	0	2	Sanitation (proportion with access)	164
Motor neuron disease	Male	0-6 days	95+ years	Global	0	2	Improved Water Source (proportion with access)	--
Motor neuron disease	Male	0-6 days	95+ years	Global	0	2	Population-weighted mean temperature	--
Motor neuron disease	Male	0-6 days	95+ years	Global	0	3	Education (years per capita)	--
Motor neuron disease	Male	0-6 days	95+ years	Global	0	3	LDI (I$ per capita)	--
Other neurological disorders	Female	28-364 days	95+ years	Data Rich	1	1	Pig Meat (kg per capita)	154
Other neurological disorders	Female	28-364 days	95+ years	Data Rich	1	1	Cholesterol (total, mean per capita)	395
Other neurological disorders	Female	28-364 days	95+ years	Data Rich	1	1	red meats adjusted(g)	398
Other neurological disorders	Female	28-364 days	95+ years	Data Rich	1	1	Mean BMI	960
Other neurological disorders	Female	28-364 days	95+ years	Data Rich	1	1	Systolic Blood Pressure (mmHg)	--
Other neurological disorders	Female	28-364 days	95+ years	Data Rich	1	1	Underweight (proportion <2SD weight for age, <5 years)	--
Other neurological disorders	Female	28-364 days	95+ years	Data Rich	-1	2	fruits adjusted(g)	119
Other neurological disorders	Female	28-364 days	95+ years	Data Rich	-1	2	Healthcare access and quality index	--
Other neurological disorders	Female	28-364 days	95+ years	Data Rich	1	2	Alcohol (liters per capita)	--
Other neurological disorders	Female	28-364 days	95+ years	Data Rich	1	2	Population Density (over 1000 ppl/sqkm, proportion)	--

Table S16. CODEm covariates used, level of covariate, and expected direction of covariate by cause, sex, and age<br>*Covariates that CODEm did not select during the covariate selection process have no draw counts listed.*								
**Cause**	**Sex**	**Age Start**	**Age End**	**Model Type**	**Direction**	**Level**	**Covariate Name**	**Number of Draws**
Other neurological disorders	Female	28-364 days	95+ years	Data Rich	-1	3	Education (years per capita)	--
Other neurological disorders	Female	28-364 days	95+ years	Data Rich	-1	3	LDI (I$ per capita)	--
Other neurological disorders	Female	28-364 days	95+ years	Data Rich	0	3	Socio-demographic Index	179
Other neurological disorders	Female	28-364 days	95+ years	Data Rich	1	3	Smoking Prevalence	207
Other neurological disorders	Female	28-364 days	95+ years	Data Rich	1	3	Cumulative Cigarettes (10 Years)	--
Other neurological disorders	Female	28-364 days	95+ years	Data Rich	1	3	Cumulative Cigarettes (5 Years)	--
Other neurological disorders	Female	28-364 days	95+ years	Global	1	1	red meats adjusted(g)	176
Other neurological disorders	Female	28-364 days	95+ years	Global	1	1	Pig Meat (kg per capita)	222
Other neurological disorders	Female	28-364 days	95+ years	Global	1	1	Cholesterol (total, mean per capita)	544
Other neurological disorders	Female	28-364 days	95+ years	Global	1	1	Mean BMI	963
Other neurological disorders	Female	28-364 days	95+ years	Global	1	1	Systolic Blood Pressure (mmHg)	--
Other neurological disorders	Female	28-364 days	95+ years	Global	1	1	Underweight (proportion <2SD weight for age, <5 years)	--
Other neurological disorders	Female	28-364 days	95+ years	Global	-1	2	fruits adjusted(g)	62
Other neurological disorders	Female	28-364 days	95+ years	Global	-1	2	Healthcare access and quality index	--
Other neurological disorders	Female	28-364 days	95+ years	Global	1	2	Population Density (over 1000 ppl/sqkm, proportion)	10
Other neurological disorders	Female	28-364 days	95+ years	Global	1	2	Alcohol (liters per capita)	--
Other neurological disorders	Female	28-364 days	95+ years	Global	-1	3	Education (years per capita)	--
Other neurological disorders	Female	28-364 days	95+ years	Global	-1	3	LDI (I$ per capita)	--
Other neurological disorders	Female	28-364 days	95+ years	Global	0	3	Socio-demographic Index	436
Other neurological disorders	Female	28-364 days	95+ years	Global	1	3	Smoking Prevalence	101
Other neurological disorders	Female	28-364 days	95+ years	Global	1	3	Cumulative Cigarettes (10 Years)	--
Other neurological disorders	Female	28-364 days	95+ years	Global	1	3	Cumulative Cigarettes (5 Years)	--
Other neurological disorders	Male	28-364 days	95+ years	Data Rich	1	1	Pig Meat (kg per capita)	7
Other neurological disorders	Male	28-364 days	95+ years	Data Rich	1	1	Cholesterol (total, mean per capita)	87
Other neurological disorders	Male	28-364 days	95+ years	Data Rich	1	1	Systolic Blood Pressure (mmHg)	468
Other neurological disorders	Male	28-364 days	95+ years	Data Rich	1	1	Mean BMI	715
Other neurological disorders	Male	28-364 days	95+ years	Data Rich	1	1	Underweight (proportion <2SD weight for age, <5 years)	--
Other neurological disorders	Male	28-364 days	95+ years	Data Rich	1	1	red meats adjusted(g)	--
Other neurological disorders	Male	28-364 days	95+ years	Data Rich	-1	2	Healthcare access and quality index	21
Other neurological disorders	Male	28-364 days	95+ years	Data Rich	-1	2	fruits adjusted(g)	21
Other neurological disorders	Male	28-364 days	95+ years	Data Rich	1	2	Alcohol (liters per capita)	293
Other neurological disorders	Male	28-364 days	95+ years	Data Rich	1	2	Population Density (over 1000 ppl/sqkm, proportion)	--
Other neurological disorders	Male	28-364 days	95+ years	Data Rich	-1	3	Education (years per capita)	--
Other neurological disorders	Male	28-364 days	95+ years	Data Rich	-1	3	LDI (I$ per capita)	--
Other neurological disorders	Male	28-364 days	95+ years	Data Rich	0	3	Socio-demographic Index	534
Other neurological disorders	Male	28-364 days	95+ years	Data Rich	1	3	Cumulative Cigarettes (10 Years)	--
Other neurological disorders	Male	28-364 days	95+ years	Data Rich	1	3	Cumulative Cigarettes (5 Years)	--
Other neurological disorders	Male	28-364 days	95+ years	Data Rich	1	3	Smoking Prevalence	--
Other neurological disorders	Male	28-364 days	95+ years	Global	1	1	Pig Meat (kg per capita)	102
Other neurological disorders	Male	28-364 days	95+ years	Global	1	1	Cholesterol (total, mean per capita)	245

Table S16. CODEm covariates used, level of covariate, and expected direction of covariate by cause, sex, and age<br>*Covariates that CODEm did not select during the covariate selection process have no draw counts listed.*								
**Cause**	**Sex**	**Age Start**	**Age End**	**Model Type**	**Direction**	**Level**	**Covariate Name**	**Number of Draws**
Other neurological disorders	Male	28-364 days	95+ years	Global	1	1	Systolic Blood Pressure (mmHg)	260
Other neurological disorders	Male	28-364 days	95+ years	Global	1	1	Mean BMI	935
Other neurological disorders	Male	28-364 days	95+ years	Global	1	1	Underweight (proportion <2SD weight for age, <5 years)	--
Other neurological disorders	Male	28-364 days	95+ years	Global	1	1	red meats adjusted(g)	--
Other neurological disorders	Male	28-364 days	95+ years	Global	-1	2	Healthcare access and quality index	140
Other neurological disorders	Male	28-364 days	95+ years	Global	-1	2	fruits adjusted(g)	140
Other neurological disorders	Male	28-364 days	95+ years	Global	1	2	Population Density (over 1000 ppl/sqkm, proportion)	9
Other neurological disorders	Male	28-364 days	95+ years	Global	1	2	Alcohol (liters per capita)	140
Other neurological disorders	Male	28-364 days	95+ years	Global	-1	3	Education (years per capita)	--
Other neurological disorders	Male	28-364 days	95+ years	Global	-1	3	LDI (I$ per capita)	--
Other neurological disorders	Male	28-364 days	95+ years	Global	0	3	Socio-demographic Index	296
Other neurological disorders	Male	28-364 days	95+ years	Global	1	3	Cumulative Cigarettes (10 Years)	--
Other neurological disorders	Male	28-364 days	95+ years	Global	1	3	Cumulative Cigarettes (5 Years)	--
Other neurological disorders	Male	28-364 days	95+ years	Global	1	3	Smoking Prevalence	--
Eating disorders	Female	5-9 years	45-49 years	Data Rich	-1	1	Underweight (proportion <2SD weight for age, <5 years)	37
Eating disorders	Female	5-9 years	45-49 years	Data Rich	1	1	Maternal Education (years per capita)	568
Eating disorders	Female	5-9 years	45-49 years	Data Rich	1	1	Education (years per capita)	--
Eating disorders	Female	5-9 years	45-49 years	Data Rich	1	1	LDI (I$ per capita)	--
Eating disorders	Female	5-9 years	45-49 years	Data Rich	1	1	Sanitation (proportion with access)	--
Eating disorders	Female	5-9 years	45-49 years	Data Rich	-1	2	Healthcare access and quality index	138
Eating disorders	Female	5-9 years	45-49 years	Data Rich	1	3	Socio-demographic Index	161
Eating disorders	Female	5-9 years	45-49 years	Global	-1	1	Underweight (proportion <2SD weight for age, <5 years)	105
Eating disorders	Female	5-9 years	45-49 years	Global	1	1	Maternal Education (years per capita)	477
Eating disorders	Female	5-9 years	45-49 years	Global	1	1	Education (years per capita)	--
Eating disorders	Female	5-9 years	45-49 years	Global	1	1	LDI (I$ per capita)	--
Eating disorders	Female	5-9 years	45-49 years	Global	1	1	Sanitation (proportion with access)	--
Eating disorders	Female	5-9 years	45-49 years	Global	-1	2	Healthcare access and quality index	119
Eating disorders	Female	5-9 years	45-49 years	Global	1	3	Socio-demographic Index	211
Eating disorders	Male	5-9 years	45-49 years	Data Rich	-1	1	Underweight (proportion <2SD weight for age, <5 years)	--
Eating disorders	Male	5-9 years	45-49 years	Data Rich	1	1	Maternal Education (years per capita)	510
Eating disorders	Male	5-9 years	45-49 years	Data Rich	1	1	Education (years per capita)	--
Eating disorders	Male	5-9 years	45-49 years	Data Rich	1	1	LDI (I$ per capita)	--
Eating disorders	Male	5-9 years	45-49 years	Data Rich	1	1	Sanitation (proportion with access)	--
Eating disorders	Male	5-9 years	45-49 years	Data Rich	-1	2	Healthcare access and quality index	718
Eating disorders	Male	5-9 years	45-49 years	Data Rich	1	3	Socio-demographic Index	351
Eating disorders	Male	5-9 years	45-49 years	Global	-1	1	Underweight (proportion <2SD weight for age, <5 years)	15
Eating disorders	Male	5-9 years	45-49 years	Global	1	1	Maternal Education (years per capita)	542
Eating disorders	Male	5-9 years	45-49 years	Global	1	1	Education (years per capita)	--
Eating disorders	Male	5-9 years	45-49 years	Global	1	1	LDI (I$ per capita)	--
Eating disorders	Male	5-9 years	45-49 years	Global	1	1	Sanitation (proportion with access)	--

Table S16. CODEm covariates used, level of covariate, and expected direction of covariate by cause, sex, and age
Covariates that CODEm did not select during the covariate selection process have no draw counts listed.

Cause	Sex	Age Start	Age End	Model Type	Direction	Level	Covariate Name	Number of Draws
Eating disorders	Male	5-9 years	45-49 years	Global	-1	2	Healthcare access and quality index	162
Eating disorders	Male	5-9 years	45-49 years	Global	1	3	Socio-demographic Index	451
Anorexia nervosa	Female	5-9 years	45-49 years	Data Rich	-1	1	Underweight (proportion <2SD weight for age, <5 years)	27
Anorexia nervosa	Female	5-9 years	45-49 years	Data Rich	1	1	Maternal Education (years per capita)	751
Anorexia nervosa	Female	5-9 years	45-49 years	Data Rich	1	1	Education (years per capita)	--
Anorexia nervosa	Female	5-9 years	45-49 years	Data Rich	1	1	LDI (I$ per capita)	--
Anorexia nervosa	Female	5-9 years	45-49 years	Data Rich	1	1	Sanitation (proportion with access)	--
Anorexia nervosa	Female	5-9 years	45-49 years	Data Rich	-1	2	Healthcare access and quality index	386
Anorexia nervosa	Female	5-9 years	45-49 years	Data Rich	1	3	Socio-demographic Index	47
Anorexia nervosa	Female	5-9 years	45-49 years	Global	-1	1	Underweight (proportion <2SD weight for age, <5 years)	92
Anorexia nervosa	Female	5-9 years	45-49 years	Global	1	1	Maternal Education (years per capita)	565
Anorexia nervosa	Female	5-9 years	45-49 years	Global	1	1	Education (years per capita)	--
Anorexia nervosa	Female	5-9 years	45-49 years	Global	1	1	LDI (I$ per capita)	--
Anorexia nervosa	Female	5-9 years	45-49 years	Global	1	1	Sanitation (proportion with access)	--
Anorexia nervosa	Female	5-9 years	45-49 years	Global	-1	2	Healthcare access and quality index	185
Anorexia nervosa	Female	5-9 years	45-49 years	Global	1	3	Socio-demographic Index	174
Anorexia nervosa	Male	5-9 years	45-49 years	Data Rich	-1	1	Underweight (proportion <2SD weight for age, <5 years)	--
Anorexia nervosa	Male	5-9 years	45-49 years	Data Rich	1	1	Maternal Education (years per capita)	45
Anorexia nervosa	Male	5-9 years	45-49 years	Data Rich	1	1	Education (years per capita)	--
Anorexia nervosa	Male	5-9 years	45-49 years	Data Rich	1	1	LDI (I$ per capita)	--
Anorexia nervosa	Male	5-9 years	45-49 years	Data Rich	1	1	Sanitation (proportion with access)	--
Anorexia nervosa	Male	5-9 years	45-49 years	Data Rich	-1	2	Healthcare access and quality index	3
Anorexia nervosa	Male	5-9 years	45-49 years	Data Rich	1	3	Socio-demographic Index	71
Anorexia nervosa	Male	5-9 years	45-49 years	Global	-1	1	Underweight (proportion <2SD weight for age, <5 years)	47
Anorexia nervosa	Male	5-9 years	45-49 years	Global	1	1	Maternal Education (years per capita)	133
Anorexia nervosa	Male	5-9 years	45-49 years	Global	1	1	Education (years per capita)	--
Anorexia nervosa	Male	5-9 years	45-49 years	Global	1	1	LDI (I$ per capita)	--
Anorexia nervosa	Male	5-9 years	45-49 years	Global	1	1	Sanitation (proportion with access)	--
Anorexia nervosa	Male	5-9 years	45-49 years	Global	-1	2	Healthcare access and quality index	7
Anorexia nervosa	Male	5-9 years	45-49 years	Global	1	3	Socio-demographic Index	69
Bulimia nervosa	Female	5-9 years	45-49 years	Data Rich	-1	1	Underweight (proportion <2SD weight for age, <5 years)	432
Bulimia nervosa	Female	5-9 years	45-49 years	Data Rich	1	1	Maternal Education (years per capita)	1000
Bulimia nervosa	Female	5-9 years	45-49 years	Data Rich	1	1	Education (years per capita)	--
Bulimia nervosa	Female	5-9 years	45-49 years	Data Rich	1	1	LDI (I$ per capita)	--
Bulimia nervosa	Female	5-9 years	45-49 years	Data Rich	1	1	Sanitation (proportion with access)	--
Bulimia nervosa	Female	5-9 years	45-49 years	Data Rich	-1	2	Healthcare access and quality index	--
Bulimia nervosa	Female	5-9 years	45-49 years	Data Rich	1	3	Socio-demographic Index	--
Bulimia nervosa	Female	5-9 years	45-49 years	Global	-1	1	Underweight (proportion <2SD weight for age, <5 years)	231
Bulimia nervosa	Female	5-9 years	45-49 years	Global	1	1	Maternal Education (years per capita)	331
Bulimia nervosa	Female	5-9 years	45-49 years	Global	1	1	Education (years per capita)	--

Table S16. CODEm covariates used, level of covariate, and expected direction of covariate by cause, sex, and age
Covariates that CODEm did not select during the covariate selection process have no draw counts listed.

Cause	Sex	Age Start	Age End	Model Type	Direction	Level	Covariate Name	Number of Draws
Bulimia nervosa	Female	5-9 years	45-49 years	Global	1	1	LDI (I$ per capita)	--
Bulimia nervosa	Female	5-9 years	45-49 years	Global	1	1	Sanitation (proportion with access)	--
Bulimia nervosa	Female	5-9 years	45-49 years	Global	-1	2	Healthcare access and quality index	--
Bulimia nervosa	Female	5-9 years	45-49 years	Global	1	3	Socio-demographic Index	--
Bulimia nervosa	Male	5-9 years	45-49 years	Data Rich	-1	1	Underweight (proportion <2SD weight for age, <5 years)	795
Bulimia nervosa	Male	5-9 years	45-49 years	Data Rich	1	1	Maternal Education (years per capita)	144
Bulimia nervosa	Male	5-9 years	45-49 years	Data Rich	1	1	Education (years per capita)	--
Bulimia nervosa	Male	5-9 years	45-49 years	Data Rich	1	1	LDI (I$ per capita)	--
Bulimia nervosa	Male	5-9 years	45-49 years	Data Rich	1	1	Sanitation (proportion with access)	--
Bulimia nervosa	Male	5-9 years	45-49 years	Data Rich	-1	2	Healthcare access and quality index	--
Bulimia nervosa	Male	5-9 years	45-49 years	Data Rich	1	3	Socio-demographic Index	256
Bulimia nervosa	Male	5-9 years	45-49 years	Global	-1	1	Underweight (proportion <2SD weight for age, <5 years)	155
Bulimia nervosa	Male	5-9 years	45-49 years	Global	1	1	Maternal Education (years per capita)	311
Bulimia nervosa	Male	5-9 years	45-49 years	Global	1	1	Education (years per capita)	--
Bulimia nervosa	Male	5-9 years	45-49 years	Global	1	1	LDI (I$ per capita)	--
Bulimia nervosa	Male	5-9 years	45-49 years	Global	1	1	Sanitation (proportion with access)	--
Bulimia nervosa	Male	5-9 years	45-49 years	Global	-1	2	Healthcare access and quality index	73
Bulimia nervosa	Male	5-9 years	45-49 years	Global	1	3	Socio-demographic Index	266
Alcohol use disorders	Female	15-19 years	95+ years	Data Rich	1	1	Alcohol (liters per capita)	38
Alcohol use disorders	Female	15-19 years	95+ years	Data Rich	1	1	Alcohol binge drinker proportion, age-standardized	962
Alcohol use disorders	Female	15-19 years	95+ years	Data Rich	-1	2	Health System Access 2 (unitless)	--
Alcohol use disorders	Female	15-19 years	95+ years	Data Rich	-1	2	Healthcare access and quality index	--
Alcohol use disorders	Female	15-19 years	95+ years	Data Rich	-1	2	Religion (binary, >50% Muslim)	--
Alcohol use disorders	Female	15-19 years	95+ years	Data Rich	0	2	Smoking Prevalence	157
Alcohol use disorders	Female	15-19 years	95+ years	Data Rich	0	2	Cumulative Cigarettes (10 Years)	481
Alcohol use disorders	Female	15-19 years	95+ years	Data Rich	-1	3	Education (years per capita)	--
Alcohol use disorders	Female	15-19 years	95+ years	Data Rich	-1	3	LDI (I$ per capita)	--
Alcohol use disorders	Female	15-19 years	95+ years	Data Rich	0	3	Socio-demographic Index	691
Alcohol use disorders	Female	15-19 years	95+ years	Global	1	1	Alcohol (liters per capita)	290
Alcohol use disorders	Female	15-19 years	95+ years	Global	1	1	Alcohol binge drinker proportion, age-standardized	710
Alcohol use disorders	Female	15-19 years	95+ years	Global	-1	2	Healthcare access and quality index	--
Alcohol use disorders	Female	15-19 years	95+ years	Global	-1	2	Religion (binary, >50% Muslim)	--
Alcohol use disorders	Female	15-19 years	95+ years	Global	0	2	Smoking Prevalence	378
Alcohol use disorders	Female	15-19 years	95+ years	Global	0	2	Cumulative Cigarettes (10 Years)	670
Alcohol use disorders	Female	15-19 years	95+ years	Global	-1	3	Education (years per capita)	--
Alcohol use disorders	Female	15-19 years	95+ years	Global	-1	3	LDI (I$ per capita)	--
Alcohol use disorders	Female	15-19 years	95+ years	Global	0	3	Socio-demographic Index	521
Alcohol use disorders	Male	15-19 years	95+ years	Data Rich	1	1	Alcohol (liters per capita)	647
Alcohol use disorders	Male	15-19 years	95+ years	Data Rich	1	1	Alcohol binge drinker proportion, age-standardized	754
Alcohol use disorders	Male	15-19 years	95+ years	Data Rich	-1	2	Health System Access 2 (unitless)	--

Table S16. CODEm covariates used, level of covariate, and expected direction of covariate by cause, sex, and age<br>*Covariates that CODEm did not select during the covariate selection process have no draw counts listed.*								
**Cause**	**Sex**	**Age Start**	**Age End**	**Model Type**	**Direction**	**Level**	**Covariate Name**	**Number of Draws**
Alcohol use disorders	Male	15-19 years	95+ years	Data Rich	-1	2	Healthcare access and quality index	--
Alcohol use disorders	Male	15-19 years	95+ years	Data Rich	-1	2	Religion (binary, >50% Muslim)	--
Alcohol use disorders	Male	15-19 years	95+ years	Data Rich	0	2	Cumulative Cigarettes (10 Years)	309
Alcohol use disorders	Male	15-19 years	95+ years	Data Rich	0	2	Smoking Prevalence	374
Alcohol use disorders	Male	15-19 years	95+ years	Data Rich	-1	3	Education (years per capita)	--
Alcohol use disorders	Male	15-19 years	95+ years	Data Rich	-1	3	LDI (I$ per capita)	--
Alcohol use disorders	Male	15-19 years	95+ years	Data Rich	0	3	Socio-demographic Index	780
Alcohol use disorders	Male	15-19 years	95+ years	Global	1	1	Alcohol binge drinker proportion, age-standardized	624
Alcohol use disorders	Male	15-19 years	95+ years	Global	1	1	Alcohol (liters per capita)	662
Alcohol use disorders	Male	15-19 years	95+ years	Global	-1	2	Religion (binary, >50% Muslim)	409
Alcohol use disorders	Male	15-19 years	95+ years	Global	-1	2	Healthcare access and quality index	--
Alcohol use disorders	Male	15-19 years	95+ years	Global	0	2	Cumulative Cigarettes (10 Years)	723
Alcohol use disorders	Male	15-19 years	95+ years	Global	0	2	Smoking Prevalence	723
Alcohol use disorders	Male	15-19 years	95+ years	Global	-1	3	Education (years per capita)	--
Alcohol use disorders	Male	15-19 years	95+ years	Global	-1	3	LDI (I$ per capita)	--
Alcohol use disorders	Male	15-19 years	95+ years	Global	0	3	Socio-demographic Index	583
Drug use disorders	Female	15-19 years	95+ years	Data Rich	1	1	Intravenous drug use (age-standardized proportion)	6
Drug use disorders	Female	15-19 years	95+ years	Data Rich	1	1	Intravenous drug use (proportion by age)	160
Drug use disorders	Female	15-19 years	95+ years	Data Rich	1	1	Opioids per million population per day (10 year lag)	1000
Drug use disorders	Female	15-19 years	95+ years	Data Rich	0	2	Healthcare access and quality index	962
Drug use disorders	Female	15-19 years	95+ years	Data Rich	1	2	Opium Cultivation (binary)	0
Drug use disorders	Female	15-19 years	95+ years	Data Rich	1	2	Cumulative Cigarettes (10 Years)	--
Drug use disorders	Female	15-19 years	95+ years	Data Rich	1	2	Cumulative Cigarettes (5 Years)	--
Drug use disorders	Female	15-19 years	95+ years	Data Rich	1	2	Smoking Prevalence	--
Drug use disorders	Female	15-19 years	95+ years	Data Rich	0	3	Education (years per capita)	1000
Drug use disorders	Female	15-19 years	95+ years	Data Rich	0	3	Socio-demographic Index	1000
Drug use disorders	Female	15-19 years	95+ years	Data Rich	0	3	LDI (I$ per capita)	--
Drug use disorders	Female	15-19 years	95+ years	Global	1	1	Intravenous drug use (age-standardized proportion)	220
Drug use disorders	Female	15-19 years	95+ years	Global	1	1	Intravenous drug use (proportion by age)	634
Drug use disorders	Female	15-19 years	95+ years	Global	1	1	Opioids per million population per day (10 year lag)	988
Drug use disorders	Female	15-19 years	95+ years	Global	0	2	Healthcare access and quality index	332
Drug use disorders	Female	15-19 years	95+ years	Global	1	2	Opium Cultivation (binary)	0
Drug use disorders	Female	15-19 years	95+ years	Global	1	2	Cumulative Cigarettes (10 Years)	--
Drug use disorders	Female	15-19 years	95+ years	Global	1	2	Cumulative Cigarettes (5 Years)	--
Drug use disorders	Female	15-19 years	95+ years	Global	1	2	Smoking Prevalence	--
Drug use disorders	Female	15-19 years	95+ years	Global	0	3	Socio-demographic Index	950
Drug use disorders	Female	15-19 years	95+ years	Global	0	3	Education (years per capita)	993
Drug use disorders	Female	15-19 years	95+ years	Global	0	3	LDI (I$ per capita)	--
Drug use disorders	Male	15-19 years	95+ years	Data Rich	1	1	Intravenous drug use (proportion by age)	199
Drug use disorders	Male	15-19 years	95+ years	Data Rich	1	1	Intravenous drug use (age-standardized proportion)	839

Table S16. CODEm covariates used, level of covariate, and expected direction of covariate by cause, sex, and age
Covariates that CODEm did not select during the covariate selection process have no draw counts listed.

Cause	Sex	Age Start	Age End	Model Type	Direction	Level	Covariate Name	Number of Draws
Drug use disorders	Male	15-19 years	95+ years	Data Rich	1	1	Opioids per million population per day (10 year lag)	999
Drug use disorders	Male	15-19 years	95+ years	Data Rich	0	2	Healthcare access and quality index	994
Drug use disorders	Male	15-19 years	95+ years	Data Rich	1	2	Opium Cultivation (binary)	0
Drug use disorders	Male	15-19 years	95+ years	Data Rich	1	2	Cumulative Cigarettes (10 Years)	--
Drug use disorders	Male	15-19 years	95+ years	Data Rich	1	2	Cumulative Cigarettes (5 Years)	--
Drug use disorders	Male	15-19 years	95+ years	Data Rich	1	2	Smoking Prevalence	--
Drug use disorders	Male	15-19 years	95+ years	Data Rich	0	3	Socio-demographic Index	968
Drug use disorders	Male	15-19 years	95+ years	Data Rich	0	3	Education (years per capita)	1000
Drug use disorders	Male	15-19 years	95+ years	Data Rich	0	3	LDI (I$ per capita)	--
Drug use disorders	Male	15-19 years	95+ years	Global	1	1	Intravenous drug use (proportion by age)	613
Drug use disorders	Male	15-19 years	95+ years	Global	1	1	Intravenous drug use (age-standardized proportion)	872
Drug use disorders	Male	15-19 years	95+ years	Global	1	1	Opioids per million population per day (10 year lag)	997
Drug use disorders	Male	15-19 years	95+ years	Global	0	2	Healthcare access and quality index	346
Drug use disorders	Male	15-19 years	95+ years	Global	1	2	Cumulative Cigarettes (10 Years)	--
Drug use disorders	Male	15-19 years	95+ years	Global	1	2	Cumulative Cigarettes (5 Years)	--
Drug use disorders	Male	15-19 years	95+ years	Global	1	2	Opium Cultivation (binary)	--
Drug use disorders	Male	15-19 years	95+ years	Global	1	2	Smoking Prevalence	--
Drug use disorders	Male	15-19 years	95+ years	Global	0	3	Socio-demographic Index	928
Drug use disorders	Male	15-19 years	95+ years	Global	0	3	Education (years per capita)	971
Drug use disorders	Male	15-19 years	95+ years	Global	0	3	LDI (I$ per capita)	--
Opioid use disorders	Female	15-19 years	95+ years	Data Rich	1	1	Opioids per million population per day (10 year lag)	190
Opioid use disorders	Female	15-19 years	95+ years	Data Rich	1	1	Opioids per million population per day	281
Opioid use disorders	Female	15-19 years	95+ years	Data Rich	1	1	Opioids per million population per day (5 year lag)	529
Opioid use disorders	Female	15-19 years	95+ years	Data Rich	0	2	Healthcare access and quality index	32
Opioid use disorders	Female	15-19 years	95+ years	Data Rich	1	2	Opium Cultivation (binary)	38
Opioid use disorders	Female	15-19 years	95+ years	Data Rich	1	2	Cumulative Cigarettes (10 Years)	--
Opioid use disorders	Female	15-19 years	95+ years	Data Rich	1	2	Cumulative Cigarettes (5 Years)	--
Opioid use disorders	Female	15-19 years	95+ years	Data Rich	1	2	Smoking Prevalence	--
Opioid use disorders	Female	15-19 years	95+ years	Data Rich	0	3	Socio-demographic Index	875
Opioid use disorders	Female	15-19 years	95+ years	Data Rich	0	3	Education (years per capita)	999
Opioid use disorders	Female	15-19 years	95+ years	Data Rich	0	3	LDI (I$ per capita)	--
Opioid use disorders	Female	15-19 years	95+ years	Global	1	1	Opioids per million population per day (10 year lag)	153
Opioid use disorders	Female	15-19 years	95+ years	Global	1	1	Opioids per million population per day (5 year lag)	332
Opioid use disorders	Female	15-19 years	95+ years	Global	1	1	Opioids per million population per day	515
Opioid use disorders	Female	15-19 years	95+ years	Global	0	2	Healthcare access and quality index	471
Opioid use disorders	Female	15-19 years	95+ years	Global	1	2	Opium Cultivation (binary)	25
Opioid use disorders	Female	15-19 years	95+ years	Global	1	2	Cumulative Cigarettes (10 Years)	--
Opioid use disorders	Female	15-19 years	95+ years	Global	1	2	Cumulative Cigarettes (5 Years)	--
Opioid use disorders	Female	15-19 years	95+ years	Global	1	2	Smoking Prevalence	--
Opioid use disorders	Female	15-19 years	95+ years	Global	0	3	Socio-demographic Index	700

Table S16. CODEm covariates used, level of covariate, and expected direction of covariate by cause, sex, and age
Covariates that CODEm did not select during the covariate selection process have no draw counts listed.

Cause	Sex	Age Start	Age End	Model Type	Direction	Level	Covariate Name	Number of Draws
Opioid use disorders	Female	15-19 years	95+ years	Global	0	3	Education (years per capita)	857
Opioid use disorders	Female	15-19 years	95+ years	Global	0	3	LDI (I$ per capita)	--
Opioid use disorders	Male	15-19 years	95+ years	Data Rich	1	1	Intravenous drug use (age-standardized proportion)	7
Opioid use disorders	Male	15-19 years	95+ years	Data Rich	1	1	Intravenous drug use (proportion by age)	833
Opioid use disorders	Male	15-19 years	95+ years	Data Rich	1	1	Opioids per million population per day (10 year lag)	1000
Opioid use disorders	Male	15-19 years	95+ years	Data Rich	0	2	Healthcare access and quality index	802
Opioid use disorders	Male	15-19 years	95+ years	Data Rich	1	2	Cumulative Cigarettes (10 Years)	--
Opioid use disorders	Male	15-19 years	95+ years	Data Rich	1	2	Cumulative Cigarettes (5 Years)	--
Opioid use disorders	Male	15-19 years	95+ years	Data Rich	1	2	Opium Cultivation (binary)	--
Opioid use disorders	Male	15-19 years	95+ years	Data Rich	1	2	Smoking Prevalence	--
Opioid use disorders	Male	15-19 years	95+ years	Data Rich	0	3	Socio-demographic Index	967
Opioid use disorders	Male	15-19 years	95+ years	Data Rich	0	3	Education (years per capita)	994
Opioid use disorders	Male	15-19 years	95+ years	Data Rich	0	3	LDI (I$ per capita)	--
Opioid use disorders	Male	15-19 years	95+ years	Global	1	1	Intravenous drug use (age-standardized proportion)	466
Opioid use disorders	Male	15-19 years	95+ years	Global	1	1	Intravenous drug use (proportion by age)	543
Opioid use disorders	Male	15-19 years	95+ years	Global	1	1	Opioids per million population per day (10 year lag)	997
Opioid use disorders	Male	15-19 years	95+ years	Global	0	2	Healthcare access and quality index	88
Opioid use disorders	Male	15-19 years	95+ years	Global	1	2	Opium Cultivation (binary)	0
Opioid use disorders	Male	15-19 years	95+ years	Global	1	2	Cumulative Cigarettes (10 Years)	--
Opioid use disorders	Male	15-19 years	95+ years	Global	1	2	Cumulative Cigarettes (5 Years)	--
Opioid use disorders	Male	15-19 years	95+ years	Global	1	2	Smoking Prevalence	--
Opioid use disorders	Male	15-19 years	95+ years	Global	0	3	Socio-demographic Index	571
Opioid use disorders	Male	15-19 years	95+ years	Global	0	3	Education (years per capita)	839
Opioid use disorders	Male	15-19 years	95+ years	Global	0	3	LDI (I$ per capita)	--
Cocaine use disorders	Female	15-19 years	95+ years	Data Rich	1	1	Alcohol (liters per capita)	1000
Cocaine use disorders	Female	15-19 years	95+ years	Data Rich	1	1	Cumulative Cigarettes (10 Years)	--
Cocaine use disorders	Female	15-19 years	95+ years	Data Rich	1	1	Cumulative Cigarettes (5 Years)	--
Cocaine use disorders	Female	15-19 years	95+ years	Data Rich	1	1	Smoking Prevalence	--
Cocaine use disorders	Female	15-19 years	95+ years	Data Rich	-1	2	Healthcare access and quality index	--
Cocaine use disorders	Female	15-19 years	95+ years	Data Rich	0	3	Education (years per capita)	926
Cocaine use disorders	Female	15-19 years	95+ years	Data Rich	0	3	LDI (I$ per capita)	--
Cocaine use disorders	Female	15-19 years	95+ years	Data Rich	1	3	Socio-demographic Index	--
Cocaine use disorders	Female	15-19 years	95+ years	Global	1	1	Alcohol (liters per capita)	1000
Cocaine use disorders	Female	15-19 years	95+ years	Global	1	1	Cumulative Cigarettes (10 Years)	--
Cocaine use disorders	Female	15-19 years	95+ years	Global	1	1	Cumulative Cigarettes (5 Years)	--
Cocaine use disorders	Female	15-19 years	95+ years	Global	1	1	Smoking Prevalence	--
Cocaine use disorders	Female	15-19 years	95+ years	Global	-1	2	Healthcare access and quality index	--
Cocaine use disorders	Female	15-19 years	95+ years	Global	0	3	Education (years per capita)	724
Cocaine use disorders	Female	15-19 years	95+ years	Global	0	3	LDI (I$ per capita)	--
Cocaine use disorders	Female	15-19 years	95+ years	Global	1	3	Socio-demographic Index	--

Table S16. CODEm covariates used, level of covariate, and expected direction of covariate by cause, sex, and age
Covariates that CODEm did not select during the covariate selection process have no draw counts listed.

Cause	Sex	Age Start	Age End	Model Type	Direction	Level	Covariate Name	Number of Draws
Cocaine use disorders	Male	15-19 years	95+ years	Data Rich	1	1	Alcohol (liters per capita)	193
Cocaine use disorders	Male	15-19 years	95+ years	Data Rich	1	1	Cumulative Cigarettes (10 Years)	--
Cocaine use disorders	Male	15-19 years	95+ years	Data Rich	1	1	Cumulative Cigarettes (5 Years)	--
Cocaine use disorders	Male	15-19 years	95+ years	Data Rich	1	1	Smoking Prevalence	--
Cocaine use disorders	Male	15-19 years	95+ years	Data Rich	-1	2	Healthcare access and quality index	--
Cocaine use disorders	Male	15-19 years	95+ years	Data Rich	0	3	Education (years per capita)	968
Cocaine use disorders	Male	15-19 years	95+ years	Data Rich	0	3	LDI (I$ per capita)	--
Cocaine use disorders	Male	15-19 years	95+ years	Data Rich	1	3	Socio-demographic Index	807
Cocaine use disorders	Male	15-19 years	95+ years	Global	1	1	Alcohol (liters per capita)	1000
Cocaine use disorders	Male	15-19 years	95+ years	Global	1	1	Cumulative Cigarettes (10 Years)	--
Cocaine use disorders	Male	15-19 years	95+ years	Global	1	1	Cumulative Cigarettes (5 Years)	--
Cocaine use disorders	Male	15-19 years	95+ years	Global	1	1	Smoking Prevalence	--
Cocaine use disorders	Male	15-19 years	95+ years	Global	-1	2	Healthcare access and quality index	--
Cocaine use disorders	Male	15-19 years	95+ years	Global	0	3	Education (years per capita)	724
Cocaine use disorders	Male	15-19 years	95+ years	Global	0	3	LDI (I$ per capita)	--
Cocaine use disorders	Male	15-19 years	95+ years	Global	1	3	Socio-demographic Index	449
Amphetamine use disorders	Female	15-19 years	95+ years	Data Rich	1	1	Alcohol (liters per capita)	166
Amphetamine use disorders	Female	15-19 years	95+ years	Data Rich	1	1	Cumulative Cigarettes (10 Years)	--
Amphetamine use disorders	Female	15-19 years	95+ years	Data Rich	1	1	Cumulative Cigarettes (5 Years)	--
Amphetamine use disorders	Female	15-19 years	95+ years	Data Rich	1	1	Smoking Prevalence	--
Amphetamine use disorders	Female	15-19 years	95+ years	Data Rich	-1	2	Healthcare access and quality index	--
Amphetamine use disorders	Female	15-19 years	95+ years	Data Rich	0	3	Education (years per capita)	834
Amphetamine use disorders	Female	15-19 years	95+ years	Data Rich	0	3	LDI (I$ per capita)	--
Amphetamine use disorders	Female	15-19 years	95+ years	Data Rich	1	3	Socio-demographic Index	834
Amphetamine use disorders	Female	15-19 years	95+ years	Global	1	1	Alcohol (liters per capita)	382
Amphetamine use disorders	Female	15-19 years	95+ years	Global	1	1	Cumulative Cigarettes (10 Years)	--
Amphetamine use disorders	Female	15-19 years	95+ years	Global	1	1	Cumulative Cigarettes (5 Years)	--
Amphetamine use disorders	Female	15-19 years	95+ years	Global	1	1	Smoking Prevalence	--
Amphetamine use disorders	Female	15-19 years	95+ years	Global	-1	2	Healthcare access and quality index	--
Amphetamine use disorders	Female	15-19 years	95+ years	Global	0	3	Education (years per capita)	618
Amphetamine use disorders	Female	15-19 years	95+ years	Global	0	3	LDI (I$ per capita)	--
Amphetamine use disorders	Female	15-19 years	95+ years	Global	1	3	Socio-demographic Index	618
Amphetamine use disorders	Male	15-19 years	95+ years	Data Rich	1	1	Alcohol (liters per capita)	1000
Amphetamine use disorders	Male	15-19 years	95+ years	Data Rich	1	1	Cumulative Cigarettes (10 Years)	--
Amphetamine use disorders	Male	15-19 years	95+ years	Data Rich	1	1	Cumulative Cigarettes (5 Years)	--
Amphetamine use disorders	Male	15-19 years	95+ years	Data Rich	1	1	Smoking Prevalence	--
Amphetamine use disorders	Male	15-19 years	95+ years	Data Rich	-1	2	Healthcare access and quality index	--
Amphetamine use disorders	Male	15-19 years	95+ years	Data Rich	0	3	Education (years per capita)	--
Amphetamine use disorders	Male	15-19 years	95+ years	Data Rich	0	3	LDI (I$ per capita)	--
Amphetamine use disorders	Male	15-19 years	95+ years	Data Rich	1	3	Socio-demographic Index	--

Table S16. CODEm covariates used, level of covariate, and expected direction of covariate by cause, sex, and age
Covariates that CODEm did not select during the covariate selection process have no draw counts listed.

Cause	Sex	Age Start	Age End	Model Type	Direction	Level	Covariate Name	Number of Draws
Amphetamine use disorders	Male	15-19 years	95+ years	Global	1	1	Alcohol (liters per capita)	1000
Amphetamine use disorders	Male	15-19 years	95+ years	Global	1	1	Cumulative Cigarettes (10 Years)	--
Amphetamine use disorders	Male	15-19 years	95+ years	Global	1	1	Cumulative Cigarettes (5 Years)	--
Amphetamine use disorders	Male	15-19 years	95+ years	Global	1	1	Smoking Prevalence	--
Amphetamine use disorders	Male	15-19 years	95+ years	Global	-1	2	Healthcare access and quality index	--
Amphetamine use disorders	Male	15-19 years	95+ years	Global	0	3	Education (years per capita)	500
Amphetamine use disorders	Male	15-19 years	95+ years	Global	0	3	LDI (I$ per capita)	--
Amphetamine use disorders	Male	15-19 years	95+ years	Global	1	3	Socio-demographic Index	--
Other drug use disorders	Female	15-19 years	95+ years	Data Rich	1	1	Alcohol (liters per capita)	1000
Other drug use disorders	Female	15-19 years	95+ years	Data Rich	1	1	Cumulative Cigarettes (10 Years)	--
Other drug use disorders	Female	15-19 years	95+ years	Data Rich	1	1	Cumulative Cigarettes (5 Years)	--
Other drug use disorders	Female	15-19 years	95+ years	Data Rich	1	1	Smoking Prevalence	--
Other drug use disorders	Female	15-19 years	95+ years	Data Rich	-1	2	Healthcare access and quality index	--
Other drug use disorders	Female	15-19 years	95+ years	Data Rich	0	3	Education (years per capita)	807
Other drug use disorders	Female	15-19 years	95+ years	Data Rich	0	3	LDI (I$ per capita)	--
Other drug use disorders	Female	15-19 years	95+ years	Data Rich	0	3	Socio-demographic Index	--
Other drug use disorders	Female	15-19 years	95+ years	Global	1	1	Alcohol (liters per capita)	1000
Other drug use disorders	Female	15-19 years	95+ years	Global	1	1	Cumulative Cigarettes (10 Years)	--
Other drug use disorders	Female	15-19 years	95+ years	Global	1	1	Cumulative Cigarettes (5 Years)	--
Other drug use disorders	Female	15-19 years	95+ years	Global	1	1	Smoking Prevalence	--
Other drug use disorders	Female	15-19 years	95+ years	Global	-1	2	Healthcare access and quality index	--
Other drug use disorders	Female	15-19 years	95+ years	Global	0	3	Education (years per capita)	724
Other drug use disorders	Female	15-19 years	95+ years	Global	0	3	LDI (I$ per capita)	--
Other drug use disorders	Female	15-19 years	95+ years	Global	0	3	Socio-demographic Index	--
Other drug use disorders	Male	15-19 years	95+ years	Data Rich	1	1	Alcohol (liters per capita)	1000
Other drug use disorders	Male	15-19 years	95+ years	Data Rich	1	1	Cumulative Cigarettes (10 Years)	--
Other drug use disorders	Male	15-19 years	95+ years	Data Rich	1	1	Cumulative Cigarettes (5 Years)	--
Other drug use disorders	Male	15-19 years	95+ years	Data Rich	1	1	Smoking Prevalence	--
Other drug use disorders	Male	15-19 years	95+ years	Data Rich	-1	2	Healthcare access and quality index	--
Other drug use disorders	Male	15-19 years	95+ years	Data Rich	0	3	Socio-demographic Index	802
Other drug use disorders	Male	15-19 years	95+ years	Data Rich	0	3	Education (years per capita)	962
Other drug use disorders	Male	15-19 years	95+ years	Data Rich	0	3	LDI (I$ per capita)	--
Other drug use disorders	Male	15-19 years	95+ years	Global	1	1	Alcohol (liters per capita)	1000
Other drug use disorders	Male	15-19 years	95+ years	Global	1	1	Cumulative Cigarettes (10 Years)	--
Other drug use disorders	Male	15-19 years	95+ years	Global	1	1	Cumulative Cigarettes (5 Years)	--
Other drug use disorders	Male	15-19 years	95+ years	Global	1	1	Smoking Prevalence	--
Other drug use disorders	Male	15-19 years	95+ years	Global	-1	2	Healthcare access and quality index	--
Other drug use disorders	Male	15-19 years	95+ years	Global	0	3	Education (years per capita)	724
Other drug use disorders	Male	15-19 years	95+ years	Global	0	3	LDI (I$ per capita)	--
Other drug use disorders	Male	15-19 years	95+ years	Global	0	3	Socio-demographic Index	--

**Table S16. CODEm covariates used, level of covariate, and expected direction of covariate by cause, sex, and age**<br>***Covariates that CODEm did not select during the covariate selection process have no draw counts listed.***								
**Cause**	**Sex**	**Age Start**	**Age End**	**Model Type**	**Direction**	**Level**	**Covariate Name**	**Number of Draws**
Diabetes mellitus	Female	0-6 days	10-14 years	Data Rich	-1	1	Healthcare access and quality index	199
Diabetes mellitus	Female	0-6 days	10-14 years	Data Rich	-1	2	Mean birth weight	186
Diabetes mellitus	Female	0-6 days	10-14 years	Data Rich	-1	2	Age-standardized SEV for Child stunting	--
Diabetes mellitus	Female	0-6 days	10-14 years	Data Rich	-1	2	Age-standardized SEV for Child underweight	--
Diabetes mellitus	Female	0-6 days	10-14 years	Data Rich	1	2	Age-Specific Fertility Rate	945
Diabetes mellitus	Female	0-6 days	10-14 years	Data Rich	1	2	Absolute value of average latitude	--
Diabetes mellitus	Female	0-6 days	10-14 years	Data Rich	1	2	Live Births 35+ (proportion)	--
Diabetes mellitus	Female	0-6 days	10-14 years	Data Rich	1	2	Live Births 40+ (proportion)	--
Diabetes mellitus	Female	0-6 days	10-14 years	Data Rich	-1	3	Education (years per capita)	198
Diabetes mellitus	Female	0-6 days	10-14 years	Data Rich	-1	3	Socio-demographic Index	252
Diabetes mellitus	Female	0-6 days	10-14 years	Global	-1	1	Healthcare access and quality index	632
Diabetes mellitus	Female	0-6 days	10-14 years	Global	-1	2	Age-standardized SEV for Child stunting	--
Diabetes mellitus	Female	0-6 days	10-14 years	Global	-1	2	Age-standardized SEV for Child underweight	--
Diabetes mellitus	Female	0-6 days	10-14 years	Global	-1	2	Mean birth weight	--
Diabetes mellitus	Female	0-6 days	10-14 years	Global	1	2	Age-Specific Fertility Rate	733
Diabetes mellitus	Female	0-6 days	10-14 years	Global	1	2	Absolute value of average latitude	--
Diabetes mellitus	Female	0-6 days	10-14 years	Global	1	2	Live Births 35+ (proportion)	--
Diabetes mellitus	Female	0-6 days	10-14 years	Global	1	2	Live Births 40+ (proportion)	--
Diabetes mellitus	Female	0-6 days	10-14 years	Global	-1	3	Education (years per capita)	354
Diabetes mellitus	Female	0-6 days	10-14 years	Global	-1	3	Socio-demographic Index	354
Diabetes mellitus	Female	15-19 years	95+ years	Data Rich	1	1	Mean BMI	483
Diabetes mellitus	Female	15-19 years	95+ years	Data Rich	1	1	Diabetes Age-Standardized Prevalence (proportion)	517
Diabetes mellitus	Female	15-19 years	95+ years	Data Rich	1	1	Diabetes Fasting Plasma Glucose (mmol/L), age-standardized 25+	530
Diabetes mellitus	Female	15-19 years	95+ years	Data Rich	1	1	Prevalence of obesity	592
Diabetes mellitus	Female	15-19 years	95+ years	Data Rich	0	2	vegetables adjusted(g)	390
Diabetes mellitus	Female	15-19 years	95+ years	Data Rich	0	2	Cholesterol (total, mean per capita)	606
Diabetes mellitus	Female	15-19 years	95+ years	Data Rich	0	2	Systolic Blood Pressure (mmHg)	803
Diabetes mellitus	Female	15-19 years	95+ years	Data Rich	0	2	fruits adjusted(g)	--
Diabetes mellitus	Female	15-19 years	95+ years	Data Rich	1	2	sugar adjusted(g)	504
Diabetes mellitus	Female	15-19 years	95+ years	Data Rich	0	3	Education (years per capita)	284
Diabetes mellitus	Female	15-19 years	95+ years	Data Rich	0	3	Healthcare access and quality index	313
Diabetes mellitus	Female	15-19 years	95+ years	Data Rich	0	3	LDI (I$ per capita)	--
Diabetes mellitus	Female	15-19 years	95+ years	Global	1	1	Mean BMI	99
Diabetes mellitus	Female	15-19 years	95+ years	Global	1	1	Diabetes Fasting Plasma Glucose (mmol/L), age-standardized 25+	442
Diabetes mellitus	Female	15-19 years	95+ years	Global	1	1	Prevalence of obesity	620
Diabetes mellitus	Female	15-19 years	95+ years	Global	1	1	Diabetes Age-Standardized Prevalence (proportion)	985
Diabetes mellitus	Female	15-19 years	95+ years	Global	0	2	vegetables adjusted(g)	245
Diabetes mellitus	Female	15-19 years	95+ years	Global	0	2	Systolic Blood Pressure (mmHg)	915
Diabetes mellitus	Female	15-19 years	95+ years	Global	0	2	Cholesterol (total, mean per capita)	928
Diabetes mellitus	Female	15-19 years	95+ years	Global	0	2	fruits adjusted(g)	--

Table S16. CODEm covariates used, level of covariate, and expected direction of covariate by cause, sex, and age<br>*Covariates that CODEm did not select during the covariate selection process have no draw counts listed.*								
**Cause**	**Sex**	**Age Start**	**Age End**	**Model Type**	**Direction**	**Level**	**Covariate Name**	**Number of Draws**
Diabetes mellitus	Female	15-19 years	95+ years	Global	1	2	sugar adjusted(g)	781
Diabetes mellitus	Female	15-19 years	95+ years	Global	0	3	Education (years per capita)	504
Diabetes mellitus	Female	15-19 years	95+ years	Global	0	3	Healthcare access and quality index	670
Diabetes mellitus	Female	15-19 years	95+ years	Global	0	3	LDI (I$ per capita)	--
Diabetes mellitus	Male	0-6 days	10-14 years	Data Rich	-1	1	Healthcare access and quality index	793
Diabetes mellitus	Male	0-6 days	10-14 years	Data Rich	-1	2	Mean birth weight	718
Diabetes mellitus	Male	0-6 days	10-14 years	Data Rich	-1	2	Age-standardized SEV for Child stunting	--
Diabetes mellitus	Male	0-6 days	10-14 years	Data Rich	-1	2	Age-standardized SEV for Child underweight	--
Diabetes mellitus	Male	0-6 days	10-14 years	Data Rich	1	2	Live Births 35+ (proportion)	207
Diabetes mellitus	Male	0-6 days	10-14 years	Data Rich	1	2	Absolute value of average latitude	--
Diabetes mellitus	Male	0-6 days	10-14 years	Data Rich	1	2	Age-Specific Fertility Rate	--
Diabetes mellitus	Male	0-6 days	10-14 years	Data Rich	1	2	Live Births 40+ (proportion)	--
Diabetes mellitus	Male	0-6 days	10-14 years	Data Rich	-1	3	Education (years per capita)	265
Diabetes mellitus	Male	0-6 days	10-14 years	Data Rich	-1	3	Socio-demographic Index	347
Diabetes mellitus	Male	0-6 days	10-14 years	Global	-1	1	Healthcare access and quality index	718
Diabetes mellitus	Male	0-6 days	10-14 years	Global	-1	2	Mean birth weight	666
Diabetes mellitus	Male	0-6 days	10-14 years	Global	-1	2	Age-standardized SEV for Child stunting	--
Diabetes mellitus	Male	0-6 days	10-14 years	Global	-1	2	Age-standardized SEV for Child underweight	--
Diabetes mellitus	Male	0-6 days	10-14 years	Global	1	2	Live Births 35+ (proportion)	282
Diabetes mellitus	Male	0-6 days	10-14 years	Global	1	2	Absolute value of average latitude	--
Diabetes mellitus	Male	0-6 days	10-14 years	Global	1	2	Age-Specific Fertility Rate	--
Diabetes mellitus	Male	0-6 days	10-14 years	Global	1	2	Live Births 40+ (proportion)	--
Diabetes mellitus	Male	0-6 days	10-14 years	Global	-1	3	Education (years per capita)	424
Diabetes mellitus	Male	0-6 days	10-14 years	Global	-1	3	Socio-demographic Index	424
Diabetes mellitus	Male	15-19 years	95+ years	Data Rich	1	1	Prevalence of obesity	76
Diabetes mellitus	Male	15-19 years	95+ years	Data Rich	1	1	Diabetes Age-Standardized Prevalence (proportion)	297
Diabetes mellitus	Male	15-19 years	95+ years	Data Rich	1	1	Diabetes Fasting Plasma Glucose (mmol/L), age-standardized 25+	417
Diabetes mellitus	Male	15-19 years	95+ years	Data Rich	1	1	Mean BMI	879
Diabetes mellitus	Male	15-19 years	95+ years	Data Rich	0	2	vegetables adjusted(g)	755
Diabetes mellitus	Male	15-19 years	95+ years	Data Rich	0	2	Cholesterol (total, mean per capita)	775
Diabetes mellitus	Male	15-19 years	95+ years	Data Rich	0	2	Systolic Blood Pressure (mmHg)	931
Diabetes mellitus	Male	15-19 years	95+ years	Data Rich	0	2	fruits adjusted(g)	--
Diabetes mellitus	Male	15-19 years	95+ years	Data Rich	1	2	sugar adjusted(g)	0
Diabetes mellitus	Male	15-19 years	95+ years	Data Rich	0	3	Education (years per capita)	387
Diabetes mellitus	Male	15-19 years	95+ years	Data Rich	0	3	Healthcare access and quality index	410
Diabetes mellitus	Male	15-19 years	95+ years	Data Rich	0	3	LDI (I$ per capita)	--
Diabetes mellitus	Male	15-19 years	95+ years	Global	1	1	Prevalence of obesity	208
Diabetes mellitus	Male	15-19 years	95+ years	Global	1	1	Mean BMI	660
Diabetes mellitus	Male	15-19 years	95+ years	Global	1	1	Diabetes Fasting Plasma Glucose (mmol/L), age-standardized 25+	670
Diabetes mellitus	Male	15-19 years	95+ years	Global	1	1	Diabetes Age-Standardized Prevalence (proportion)	1000

Table S16. CODEm covariates used, level of covariate, and expected direction of covariate by cause, sex, and age<br>*Covariates that CODEm did not select during the covariate selection process have no draw counts listed.*								
**Cause**	**Sex**	**Age Start**	**Age End**	**Model Type**	**Direction**	**Level**	**Covariate Name**	**Number of Draws**
Diabetes mellitus	Male	15-19 years	95+ years	Global	0	2	Systolic Blood Pressure (mmHg)	295
Diabetes mellitus	Male	15-19 years	95+ years	Global	0	2	vegetables adjusted(g)	586
Diabetes mellitus	Male	15-19 years	95+ years	Global	0	2	Cholesterol (total, mean per capita)	615
Diabetes mellitus	Male	15-19 years	95+ years	Global	0	2	fruits adjusted(g)	--
Diabetes mellitus	Male	15-19 years	95+ years	Global	1	2	sugar adjusted(g)	--
Diabetes mellitus	Male	15-19 years	95+ years	Global	0	3	Education (years per capita)	529
Diabetes mellitus	Male	15-19 years	95+ years	Global	0	3	Healthcare access and quality index	593
Diabetes mellitus	Male	15-19 years	95+ years	Global	0	3	LDI (I$ per capita)	--
Diabetes mellitus type 1	Female	0-6 days	95+ years	Data Rich	-1	1	Healthcare access and quality index	1000
Diabetes mellitus type 1	Female	0-6 days	95+ years	Data Rich	-1	2	Age-standardized SEV for Child stunting	--
Diabetes mellitus type 1	Female	0-6 days	95+ years	Data Rich	-1	2	Age-standardized SEV for Child underweight	--
Diabetes mellitus type 1	Female	0-6 days	95+ years	Data Rich	-1	2	Mean birth weight	--
Diabetes mellitus type 1	Female	0-6 days	95+ years	Data Rich	1	2	Absolute value of average latitude	158
Diabetes mellitus type 1	Female	0-6 days	95+ years	Data Rich	1	2	Age-Specific Fertility Rate	--
Diabetes mellitus type 1	Female	0-6 days	95+ years	Data Rich	1	2	Live Births 35+ (proportion)	--
Diabetes mellitus type 1	Female	0-6 days	95+ years	Data Rich	1	2	Live Births 40+ (proportion)	--
Diabetes mellitus type 1	Female	0-6 days	95+ years	Data Rich	-1	3	Socio-demographic Index	145
Diabetes mellitus type 1	Female	0-6 days	95+ years	Data Rich	-1	3	Education (years per capita)	491
Diabetes mellitus type 1	Female	0-6 days	95+ years	Global	-1	1	Healthcare access and quality index	1000
Diabetes mellitus type 1	Female	0-6 days	95+ years	Global	-1	2	Age-standardized SEV for Child stunting	--
Diabetes mellitus type 1	Female	0-6 days	95+ years	Global	-1	2	Age-standardized SEV for Child underweight	--
Diabetes mellitus type 1	Female	0-6 days	95+ years	Global	-1	2	Mean birth weight	--
Diabetes mellitus type 1	Female	0-6 days	95+ years	Global	1	2	Absolute value of average latitude	269
Diabetes mellitus type 1	Female	0-6 days	95+ years	Global	1	2	Age-Specific Fertility Rate	--
Diabetes mellitus type 1	Female	0-6 days	95+ years	Global	1	2	Live Births 35+ (proportion)	--
Diabetes mellitus type 1	Female	0-6 days	95+ years	Global	1	2	Live Births 40+ (proportion)	--
Diabetes mellitus type 1	Female	0-6 days	95+ years	Global	-1	3	Education (years per capita)	450
Diabetes mellitus type 1	Female	0-6 days	95+ years	Global	-1	3	Socio-demographic Index	--
Diabetes mellitus type 1	Male	0-6 days	95+ years	Data Rich	-1	1	Healthcare access and quality index	1000
Diabetes mellitus type 1	Male	0-6 days	95+ years	Data Rich	-1	2	Mean birth weight	177
Diabetes mellitus type 1	Male	0-6 days	95+ years	Data Rich	-1	2	Age-standardized SEV for Child stunting	--
Diabetes mellitus type 1	Male	0-6 days	95+ years	Data Rich	-1	2	Age-standardized SEV for Child underweight	--
Diabetes mellitus type 1	Male	0-6 days	95+ years	Data Rich	1	2	Absolute value of average latitude	195
Diabetes mellitus type 1	Male	0-6 days	95+ years	Data Rich	1	2	Age-Specific Fertility Rate	--
Diabetes mellitus type 1	Male	0-6 days	95+ years	Data Rich	1	2	Live Births 35+ (proportion)	--
Diabetes mellitus type 1	Male	0-6 days	95+ years	Data Rich	1	2	Live Births 40+ (proportion)	--
Diabetes mellitus type 1	Male	0-6 days	95+ years	Data Rich	-1	3	Socio-demographic Index	72
Diabetes mellitus type 1	Male	0-6 days	95+ years	Data Rich	-1	3	Education (years per capita)	492
Diabetes mellitus type 1	Male	0-6 days	95+ years	Global	-1	1	Healthcare access and quality index	1000
Diabetes mellitus type 1	Male	0-6 days	95+ years	Global	-1	2	Mean birth weight	440
Diabetes mellitus type 1	Male	0-6 days	95+ years	Global	-1	2	Age-standardized SEV for Child stunting	--
Diabetes mellitus type 1	Male	0-6 days	95+ years	Global	-1	2	Age-standardized SEV for Child underweight	--
Diabetes mellitus type 1	Male	0-6 days	95+ years	Global	1	2	Absolute value of average latitude	119
Diabetes mellitus type 1	Male	0-6 days	95+ years	Global	1	2	Age-Specific Fertility Rate	--
Diabetes mellitus type 1	Male	0-6 days	95+ years	Global	1	2	Live Births 35+ (proportion)	--
Diabetes mellitus type 1	Male	0-6 days	95+ years	Global	1	2	Live Births 40+ (proportion)	--
Diabetes mellitus type 1	Male	0-6 days	95+ years	Global	-1	3	Education (years per capita)	414
Diabetes mellitus type 1	Male	0-6 days	95+ years	Global	-1	3	Socio-demographic Index	--
Diabetes mellitus type 2	Female	15-19 years	95+ years	Data Rich	1	1	Prevalence of obesity	487
Diabetes mellitus type 2	Female	15-19 years	95+ years	Data Rich	1	1	Mean BMI	489
Diabetes mellitus type 2	Female	15-19 years	95+ years	Data Rich	1	1	Diabetes Fasting Plasma Glucose (mmol/L), age-standardized 25+	742

Table S16. CODEm covariates used, level of covariate, and expected direction of covariate by cause, sex, and age
Covariates that CODEm did not select during the covariate selection process have no draw counts listed.

Cause	Sex	Age Start	Age End	Model Type	Direction	Level	Covariate Name	Number of Draws
Diabetes mellitus type 2	Female	15-19 years	95+ years	Data Rich	1	1	Diabetes Age-Standardized Prevalence (proportion)	942
Diabetes mellitus type 2	Female	15-19 years	95+ years	Data Rich	0	2	fruits adjusted(g)	654
Diabetes mellitus type 2	Female	15-19 years	95+ years	Data Rich	0	2	Cholesterol (total, mean per capita)	869
Diabetes mellitus type 2	Female	15-19 years	95+ years	Data Rich	0	2	Systolic Blood Pressure (mmHg)	900
Diabetes mellitus type 2	Female	15-19 years	95+ years	Data Rich	0	2	vegetables adjusted(g)	951
Diabetes mellitus type 2	Female	15-19 years	95+ years	Data Rich	0	2	Age- and sex-specific SEV for Alcohol use	--
Diabetes mellitus type 2	Female	15-19 years	95+ years	Data Rich	1	2	sugar adjusted(g)	1
Diabetes mellitus type 2	Female	15-19 years	95+ years	Data Rich	0	3	Healthcare access and quality index	333
Diabetes mellitus type 2	Female	15-19 years	95+ years	Data Rich	0	3	Education (years per capita)	334
Diabetes mellitus type 2	Female	15-19 years	95+ years	Data Rich	0	3	LDI (I$ per capita)	--
Diabetes mellitus type 2	Female	15-19 years	95+ years	Global	1	1	Prevalence of obesity	241
Diabetes mellitus type 2	Female	15-19 years	95+ years	Global	1	1	Mean BMI	537
Diabetes mellitus type 2	Female	15-19 years	95+ years	Global	1	1	Diabetes Fasting Plasma Glucose (mmol/L), age-standardized 25+	556
Diabetes mellitus type 2	Female	15-19 years	95+ years	Global	1	1	Diabetes Age-Standardized Prevalence (proportion)	837
Diabetes mellitus type 2	Female	15-19 years	95+ years	Global	0	2	Cholesterol (total, mean per capita)	219
Diabetes mellitus type 2	Female	15-19 years	95+ years	Global	0	2	vegetables adjusted(g)	253
Diabetes mellitus type 2	Female	15-19 years	95+ years	Global	0	2	Systolic Blood Pressure (mmHg)	256
Diabetes mellitus type 2	Female	15-19 years	95+ years	Global	0	2	fruits adjusted(g)	412
Diabetes mellitus type 2	Female	15-19 years	95+ years	Global	0	2	Age- and sex-specific SEV for Alcohol use	--
Diabetes mellitus type 2	Female	15-19 years	95+ years	Global	1	2	sugar adjusted(g)	252
Diabetes mellitus type 2	Female	15-19 years	95+ years	Global	0	3	Education (years per capita)	379
Diabetes mellitus type 2	Female	15-19 years	95+ years	Global	0	3	Healthcare access and quality index	443
Diabetes mellitus type 2	Female	15-19 years	95+ years	Global	0	3	LDI (I$ per capita)	--
Diabetes mellitus type 2	Male	15-19 years	95+ years	Data Rich	1	1	Diabetes Age-Standardized Prevalence (proportion)	83
Diabetes mellitus type 2	Male	15-19 years	95+ years	Data Rich	1	1	Prevalence of obesity	88
Diabetes mellitus type 2	Male	15-19 years	95+ years	Data Rich	1	1	Diabetes Fasting Plasma Glucose (mmol/L), age-standardized 25+	102
Diabetes mellitus type 2	Male	15-19 years	95+ years	Data Rich	1	1	Mean BMI	912
Diabetes mellitus type 2	Male	15-19 years	95+ years	Data Rich	0	2	Cholesterol (total, mean per capita)	703
Diabetes mellitus type 2	Male	15-19 years	95+ years	Data Rich	0	2	fruits adjusted(g)	720
Diabetes mellitus type 2	Male	15-19 years	95+ years	Data Rich	0	2	vegetables adjusted(g)	879
Diabetes mellitus type 2	Male	15-19 years	95+ years	Data Rich	0	2	Systolic Blood Pressure (mmHg)	984
Diabetes mellitus type 2	Male	15-19 years	95+ years	Data Rich	0	2	Age- and sex-specific SEV for Alcohol use	--
Diabetes mellitus type 2	Male	15-19 years	95+ years	Data Rich	1	2	sugar adjusted(g)	0
Diabetes mellitus type 2	Male	15-19 years	95+ years	Data Rich	0	3	Education (years per capita)	163
Diabetes mellitus type 2	Male	15-19 years	95+ years	Data Rich	0	3	Healthcare access and quality index	501
Diabetes mellitus type 2	Male	15-19 years	95+ years	Data Rich	0	3	LDI (I$ per capita)	--
Diabetes mellitus type 2	Male	15-19 years	95+ years	Global	1	1	Diabetes Fasting Plasma Glucose (mmol/L), age-standardized 25+	184
Diabetes mellitus type 2	Male	15-19 years	95+ years	Global	1	1	Prevalence of obesity	270
Diabetes mellitus type 2	Male	15-19 years	95+ years	Global	1	1	Mean BMI	418
Diabetes mellitus type 2	Male	15-19 years	95+ years	Global	1	1	Diabetes Age-Standardized Prevalence (proportion)	1000

Table S16. CODEm covariates used, level of covariate, and expected direction of covariate by cause, sex, and age
Covariates that CODEm did not select during the covariate selection process have no draw counts listed.

Cause	Sex	Age Start	Age End	Model Type	Direction	Level	Covariate Name	Number of Draws
Diabetes mellitus type 2	Male	15-19 years	95+ years	Global	0	2	Cholesterol (total, mean per capita)	361
Diabetes mellitus type 2	Male	15-19 years	95+ years	Global	0	2	Systolic Blood Pressure (mmHg)	542
Diabetes mellitus type 2	Male	15-19 years	95+ years	Global	0	2	fruits adjusted(g)	590
Diabetes mellitus type 2	Male	15-19 years	95+ years	Global	0	2	vegetables adjusted(g)	590
Diabetes mellitus type 2	Male	15-19 years	95+ years	Global	0	2	Age- and sex-specific SEV for Alcohol use	--
Diabetes mellitus type 2	Male	15-19 years	95+ years	Global	1	2	sugar adjusted(g)	48
Diabetes mellitus type 2	Male	15-19 years	95+ years	Global	0	3	Healthcare access and quality index	471
Diabetes mellitus type 2	Male	15-19 years	95+ years	Global	0	3	Education (years per capita)	507
Diabetes mellitus type 2	Male	15-19 years	95+ years	Global	0	3	LDI (I$ per capita)	--
Chronic kidney disease	Female	28-364 days	95+ years	Data Rich	-1	1	Healthcare access and quality index	9
Chronic kidney disease	Female	28-364 days	95+ years	Data Rich	1	1	Systolic Blood Pressure (mmHg)	1
Chronic kidney disease	Female	28-364 days	95+ years	Data Rich	1	1	Mean BMI	41
Chronic kidney disease	Female	28-364 days	95+ years	Data Rich	1	1	Diabetes Age-Standardized Prevalence (proportion)	248
Chronic kidney disease	Female	28-364 days	95+ years	Data Rich	1	1	Diabetes Fasting Plasma Glucose (mmol/L), age-standardized 25+	921
Chronic kidney disease	Female	28-364 days	95+ years	Data Rich	0	2	red meats adjusted(g)	138
Chronic kidney disease	Female	28-364 days	95+ years	Data Rich	1	2	Cholesterol (total, mean per capita)	0
Chronic kidney disease	Female	28-364 days	95+ years	Data Rich	1	2	energy unadjusted(kcal)	518
Chronic kidney disease	Female	28-364 days	95+ years	Data Rich	-1	3	Education (years per capita)	116
Chronic kidney disease	Female	28-364 days	95+ years	Data Rich	-1	3	LDI (I$ per capita)	--
Chronic kidney disease	Female	28-364 days	95+ years	Data Rich	0	3	Socio-demographic Index	163
Chronic kidney disease	Female	28-364 days	95+ years	Global	-1	1	Healthcare access and quality index	388
Chronic kidney disease	Female	28-364 days	95+ years	Global	1	1	Systolic Blood Pressure (mmHg)	19
Chronic kidney disease	Female	28-364 days	95+ years	Global	1	1	Diabetes Age-Standardized Prevalence (proportion)	59
Chronic kidney disease	Female	28-364 days	95+ years	Global	1	1	Diabetes Fasting Plasma Glucose (mmol/L), age-standardized 25+	954
Chronic kidney disease	Female	28-364 days	95+ years	Global	1	1	Mean BMI	1000
Chronic kidney disease	Female	28-364 days	95+ years	Global	0	2	red meats adjusted(g)	444
Chronic kidney disease	Female	28-364 days	95+ years	Global	1	2	Cholesterol (total, mean per capita)	0
Chronic kidney disease	Female	28-364 days	95+ years	Global	1	2	energy unadjusted(kcal)	0
Chronic kidney disease	Female	28-364 days	95+ years	Global	-1	3	Education (years per capita)	647
Chronic kidney disease	Female	28-364 days	95+ years	Global	-1	3	LDI (I$ per capita)	--
Chronic kidney disease	Female	28-364 days	95+ years	Global	0	3	Socio-demographic Index	489
Chronic kidney disease	Male	28-364 days	95+ years	Data Rich	-1	1	Healthcare access and quality index	48
Chronic kidney disease	Male	28-364 days	95+ years	Data Rich	1	1	Systolic Blood Pressure (mmHg)	1
Chronic kidney disease	Male	28-364 days	95+ years	Data Rich	1	1	Mean BMI	130
Chronic kidney disease	Male	28-364 days	95+ years	Data Rich	1	1	Diabetes Fasting Plasma Glucose (mmol/L), age-standardized 25+	427
Chronic kidney disease	Male	28-364 days	95+ years	Data Rich	1	1	Diabetes Age-Standardized Prevalence (proportion)	790
Chronic kidney disease	Male	28-364 days	95+ years	Data Rich	0	2	red meats adjusted(g)	273
Chronic kidney disease	Male	28-364 days	95+ years	Data Rich	1	2	Cholesterol (total, mean per capita)	3
Chronic kidney disease	Male	28-364 days	95+ years	Data Rich	1	2	energy unadjusted(kcal)	105
Chronic kidney disease	Male	28-364 days	95+ years	Data Rich	-1	3	Education (years per capita)	68

Table S16. CODEm covariates used, level of covariate, and expected direction of covariate by cause, sex, and age
Covariates that CODEm did not select during the covariate selection process have no draw counts listed.

Cause	Sex	Age Start	Age End	Model Type	Direction	Level	Covariate Name	Number of Draws
Chronic kidney disease	Male	28-364 days	95+ years	Data Rich	-1	3	LDI (I$ per capita)	--
Chronic kidney disease	Male	28-364 days	95+ years	Data Rich	0	3	Socio-demographic Index	114
Chronic kidney disease	Male	28-364 days	95+ years	Global	-1	1	Healthcare access and quality index	363
Chronic kidney disease	Male	28-364 days	95+ years	Global	1	1	Systolic Blood Pressure (mmHg)	0
Chronic kidney disease	Male	28-364 days	95+ years	Global	1	1	Diabetes Fasting Plasma Glucose (mmol/L), age-standardized 25+	452
Chronic kidney disease	Male	28-364 days	95+ years	Global	1	1	Mean BMI	828
Chronic kidney disease	Male	28-364 days	95+ years	Global	1	1	Diabetes Age-Standardized Prevalence (proportion)	860
Chronic kidney disease	Male	28-364 days	95+ years	Global	0	2	red meats adjusted(g)	319
Chronic kidney disease	Male	28-364 days	95+ years	Global	1	2	Cholesterol (total, mean per capita)	0
Chronic kidney disease	Male	28-364 days	95+ years	Global	1	2	energy unadjusted(kcal)	107
Chronic kidney disease	Male	28-364 days	95+ years	Global	-1	3	Education (years per capita)	215
Chronic kidney disease	Male	28-364 days	95+ years	Global	-1	3	LDI (I$ per capita)	--
Chronic kidney disease	Male	28-364 days	95+ years	Global	0	3	Socio-demographic Index	257
Acute glomerulonephritis	Female	28-364 days	95+ years	Data Rich	-1	2	Sanitation (proportion with access)	601
Acute glomerulonephritis	Female	28-364 days	95+ years	Data Rich	-1	2	Improved Water Source (proportion with access)	760
Acute glomerulonephritis	Female	28-364 days	95+ years	Data Rich	-1	2	Healthcare access and quality index	935
Acute glomerulonephritis	Female	28-364 days	95+ years	Data Rich	1	2	Systolic Blood Pressure (mmHg)	0
Acute glomerulonephritis	Female	28-364 days	95+ years	Data Rich	1	2	Diabetes Age-Standardized Prevalence (proportion)	--
Acute glomerulonephritis	Female	28-364 days	95+ years	Data Rich	-1	3	Education (years per capita)	16
Acute glomerulonephritis	Female	28-364 days	95+ years	Data Rich	-1	3	Socio-demographic Index	43
Acute glomerulonephritis	Female	28-364 days	95+ years	Data Rich	-1	3	LDI (I$ per capita)	--
Acute glomerulonephritis	Female	28-364 days	95+ years	Global	-1	2	Healthcare access and quality index	492
Acute glomerulonephritis	Female	28-364 days	95+ years	Global	-1	2	Sanitation (proportion with access)	558
Acute glomerulonephritis	Female	28-364 days	95+ years	Global	-1	2	Improved Water Source (proportion with access)	728
Acute glomerulonephritis	Female	28-364 days	95+ years	Global	1	2	Systolic Blood Pressure (mmHg)	15
Acute glomerulonephritis	Female	28-364 days	95+ years	Global	1	2	Diabetes Age-Standardized Prevalence (proportion)	--
Acute glomerulonephritis	Female	28-364 days	95+ years	Global	-1	3	Education (years per capita)	207
Acute glomerulonephritis	Female	28-364 days	95+ years	Global	-1	3	Socio-demographic Index	219
Acute glomerulonephritis	Female	28-364 days	95+ years	Global	-1	3	LDI (I$ per capita)	--
Acute glomerulonephritis	Male	28-364 days	95+ years	Data Rich	-1	2	Sanitation (proportion with access)	491
Acute glomerulonephritis	Male	28-364 days	95+ years	Data Rich	-1	2	Improved Water Source (proportion with access)	592
Acute glomerulonephritis	Male	28-364 days	95+ years	Data Rich	-1	2	Healthcare access and quality index	941
Acute glomerulonephritis	Male	28-364 days	95+ years	Data Rich	1	2	Systolic Blood Pressure (mmHg)	0
Acute glomerulonephritis	Male	28-364 days	95+ years	Data Rich	1	2	Diabetes Age-Standardized Prevalence (proportion)	242
Acute glomerulonephritis	Male	28-364 days	95+ years	Data Rich	-1	3	Socio-demographic Index	1
Acute glomerulonephritis	Male	28-364 days	95+ years	Data Rich	-1	3	Education (years per capita)	58
Acute glomerulonephritis	Male	28-364 days	95+ years	Data Rich	-1	3	LDI (I$ per capita)	--
Acute glomerulonephritis	Male	28-364 days	95+ years	Global	-1	2	Sanitation (proportion with access)	520
Acute glomerulonephritis	Male	28-364 days	95+ years	Global	-1	2	Improved Water Source (proportion with access)	538
Acute glomerulonephritis	Male	28-364 days	95+ years	Global	-1	2	Healthcare access and quality index	744

Table S16. CODEm covariates used, level of covariate, and expected direction of covariate by cause, sex, and age
Covariates that CODEm did not select during the covariate selection process have no draw counts listed.

Cause	Sex	Age Start	Age End	Model Type	Direction	Level	Covariate Name	Number of Draws
Acute glomerulonephritis	Male	28-364 days	95+ years	Global	1	2	Systolic Blood Pressure (mmHg)	154
Acute glomerulonephritis	Male	28-364 days	95+ years	Global	1	2	Diabetes Age-Standardized Prevalence (proportion)	--
Acute glomerulonephritis	Male	28-364 days	95+ years	Global	-1	3	Education (years per capita)	44
Acute glomerulonephritis	Male	28-364 days	95+ years	Global	-1	3	Socio-demographic Index	142
Acute glomerulonephritis	Male	28-364 days	95+ years	Global	-1	3	LDI (I$ per capita)	--
Skin and subcutaneous diseases	Female	28-364 days	95+ years	Data Rich	-1	1	Improved Water Source (proportion with access)	--
Skin and subcutaneous diseases	Female	28-364 days	95+ years	Data Rich	1	1	Age- and sex-specific SEV for Unsafe sanitation	--
Skin and subcutaneous diseases	Female	28-364 days	95+ years	Data Rich	-1	2	Healthcare access and quality index	--
Skin and subcutaneous diseases	Female	28-364 days	95+ years	Data Rich	1	2	Alcohol (liters per capita)	1000
Skin and subcutaneous diseases	Female	28-364 days	95+ years	Data Rich	1	2	Cumulative Cigarettes (10 Years)	--
Skin and subcutaneous diseases	Female	28-364 days	95+ years	Data Rich	1	2	Cumulative Cigarettes (5 Years)	--
Skin and subcutaneous diseases	Female	28-364 days	95+ years	Data Rich	1	2	Smoking Prevalence	--
Skin and subcutaneous diseases	Female	28-364 days	95+ years	Data Rich	-1	3	Education (years per capita)	--
Skin and subcutaneous diseases	Female	28-364 days	95+ years	Data Rich	-1	3	LDI (I$ per capita)	--
Skin and subcutaneous diseases	Female	28-364 days	95+ years	Data Rich	0	3	Socio-demographic Index	568
Skin and subcutaneous diseases	Female	28-364 days	95+ years	Global	-1	1	Improved Water Source (proportion with access)	--
Skin and subcutaneous diseases	Female	28-364 days	95+ years	Global	1	1	Age- and sex-specific SEV for Unsafe sanitation	--
Skin and subcutaneous diseases	Female	28-364 days	95+ years	Global	-1	2	Healthcare access and quality index	--
Skin and subcutaneous diseases	Female	28-364 days	95+ years	Global	1	2	Alcohol (liters per capita)	--
Skin and subcutaneous diseases	Female	28-364 days	95+ years	Global	1	2	Cumulative Cigarettes (10 Years)	--
Skin and subcutaneous diseases	Female	28-364 days	95+ years	Global	1	2	Cumulative Cigarettes (5 Years)	--
Skin and subcutaneous diseases	Female	28-364 days	95+ years	Global	1	2	Smoking Prevalence	--
Skin and subcutaneous diseases	Female	28-364 days	95+ years	Global	-1	3	Education (years per capita)	--
Skin and subcutaneous diseases	Female	28-364 days	95+ years	Global	-1	3	LDI (I$ per capita)	--
Skin and subcutaneous diseases	Female	28-364 days	95+ years	Global	0	3	Socio-demographic Index	1000
Skin and subcutaneous diseases	Male	28-364 days	95+ years	Data Rich	-1	1	Improved Water Source (proportion with access)	--
Skin and subcutaneous diseases	Male	28-364 days	95+ years	Data Rich	1	1	Age- and sex-specific SEV for Unsafe sanitation	--
Skin and subcutaneous diseases	Male	28-364 days	95+ years	Data Rich	-1	2	Healthcare access and quality index	--
Skin and subcutaneous diseases	Male	28-364 days	95+ years	Data Rich	1	2	Alcohol (liters per capita)	1000
Skin and subcutaneous diseases	Male	28-364 days	95+ years	Data Rich	1	2	Cumulative Cigarettes (10 Years)	--
Skin and subcutaneous diseases	Male	28-364 days	95+ years	Data Rich	1	2	Cumulative Cigarettes (5 Years)	--
Skin and subcutaneous diseases	Male	28-364 days	95+ years	Data Rich	1	2	Smoking Prevalence	--
Skin and subcutaneous diseases	Male	28-364 days	95+ years	Data Rich	-1	3	Education (years per capita)	--
Skin and subcutaneous diseases	Male	28-364 days	95+ years	Data Rich	-1	3	LDI (I$ per capita)	--
Skin and subcutaneous diseases	Male	28-364 days	95+ years	Data Rich	0	3	Socio-demographic Index	568
Skin and subcutaneous diseases	Male	28-364 days	95+ years	Global	-1	1	Improved Water Source (proportion with access)	--
Skin and subcutaneous diseases	Male	28-364 days	95+ years	Global	1	1	Age- and sex-specific SEV for Unsafe sanitation	674
Skin and subcutaneous diseases	Male	28-364 days	95+ years	Global	-1	2	Healthcare access and quality index	--
Skin and subcutaneous diseases	Male	28-364 days	95+ years	Global	1	2	Alcohol (liters per capita)	452
Skin and subcutaneous diseases	Male	28-364 days	95+ years	Global	1	2	Cumulative Cigarettes (10 Years)	--

Table S16. CODEm covariates used, level of covariate, and expected direction of covariate by cause, sex, and age<br>*Covariates that CODEm did not select during the covariate selection process have no draw counts listed.*								
**Cause**	**Sex**	**Age Start**	**Age End**	**Model Type**	**Direction**	**Level**	**Covariate Name**	**Number of Draws**
Skin and subcutaneous diseases	Male	28-364 days	95+ years	Global	1	2	Cumulative Cigarettes (5 Years)	--
Skin and subcutaneous diseases	Male	28-364 days	95+ years	Global	1	2	Smoking Prevalence	--
Skin and subcutaneous diseases	Male	28-364 days	95+ years	Global	-1	3	Education (years per capita)	--
Skin and subcutaneous diseases	Male	28-364 days	95+ years	Global	-1	3	LDI (I$ per capita)	--
Skin and subcutaneous diseases	Male	28-364 days	95+ years	Global	0	3	Socio-demographic Index	593
Bacterial skin diseases	Female	0-6 days	95+ years	Data Rich	-1	1	Improved Water Source (proportion with access)	--
Bacterial skin diseases	Female	0-6 days	95+ years	Data Rich	1	1	Age- and sex-specific SEV for Unsafe sanitation	--
Bacterial skin diseases	Female	0-6 days	95+ years	Data Rich	-1	2	Healthcare access and quality index	--
Bacterial skin diseases	Female	0-6 days	95+ years	Data Rich	1	2	Alcohol (liters per capita)	1000
Bacterial skin diseases	Female	0-6 days	95+ years	Data Rich	1	2	Cumulative Cigarettes (10 Years)	--
Bacterial skin diseases	Female	0-6 days	95+ years	Data Rich	1	2	Cumulative Cigarettes (5 Years)	--
Bacterial skin diseases	Female	0-6 days	95+ years	Data Rich	1	2	Smoking Prevalence	--
Bacterial skin diseases	Female	0-6 days	95+ years	Data Rich	-1	3	Education (years per capita)	--
Bacterial skin diseases	Female	0-6 days	95+ years	Data Rich	-1	3	LDI (I$ per capita)	--
Bacterial skin diseases	Female	0-6 days	95+ years	Data Rich	0	3	Socio-demographic Index	--
Bacterial skin diseases	Female	0-6 days	95+ years	Global	-1	1	Improved Water Source (proportion with access)	--
Bacterial skin diseases	Female	0-6 days	95+ years	Global	1	1	Age- and sex-specific SEV for Unsafe sanitation	726
Bacterial skin diseases	Female	0-6 days	95+ years	Global	-1	2	Healthcare access and quality index	--
Bacterial skin diseases	Female	0-6 days	95+ years	Global	1	2	Alcohol (liters per capita)	274
Bacterial skin diseases	Female	0-6 days	95+ years	Global	1	2	Cumulative Cigarettes (10 Years)	--
Bacterial skin diseases	Female	0-6 days	95+ years	Global	1	2	Cumulative Cigarettes (5 Years)	--
Bacterial skin diseases	Female	0-6 days	95+ years	Global	1	2	Smoking Prevalence	--
Bacterial skin diseases	Female	0-6 days	95+ years	Global	-1	3	Education (years per capita)	--
Bacterial skin diseases	Female	0-6 days	95+ years	Global	-1	3	LDI (I$ per capita)	--
Bacterial skin diseases	Female	0-6 days	95+ years	Global	0	3	Socio-demographic Index	398
Bacterial skin diseases	Male	0-6 days	95+ years	Data Rich	-1	1	Improved Water Source (proportion with access)	--
Bacterial skin diseases	Male	0-6 days	95+ years	Data Rich	1	1	Age- and sex-specific SEV for Unsafe sanitation	--
Bacterial skin diseases	Male	0-6 days	95+ years	Data Rich	-1	2	Healthcare access and quality index	--
Bacterial skin diseases	Male	0-6 days	95+ years	Data Rich	1	2	Alcohol (liters per capita)	691
Bacterial skin diseases	Male	0-6 days	95+ years	Data Rich	1	2	Cumulative Cigarettes (10 Years)	--
Bacterial skin diseases	Male	0-6 days	95+ years	Data Rich	1	2	Cumulative Cigarettes (5 Years)	--
Bacterial skin diseases	Male	0-6 days	95+ years	Data Rich	1	2	Smoking Prevalence	--
Bacterial skin diseases	Male	0-6 days	95+ years	Data Rich	-1	3	Education (years per capita)	--
Bacterial skin diseases	Male	0-6 days	95+ years	Data Rich	-1	3	LDI (I$ per capita)	--
Bacterial skin diseases	Male	0-6 days	95+ years	Data Rich	0	3	Socio-demographic Index	593
Bacterial skin diseases	Male	0-6 days	95+ years	Global	-1	1	Improved Water Source (proportion with access)	--
Bacterial skin diseases	Male	0-6 days	95+ years	Global	1	1	Age- and sex-specific SEV for Unsafe sanitation	1000
Bacterial skin diseases	Male	0-6 days	95+ years	Global	-1	2	Healthcare access and quality index	--
Bacterial skin diseases	Male	0-6 days	95+ years	Global	1	2	Alcohol (liters per capita)	491
Bacterial skin diseases	Male	0-6 days	95+ years	Global	1	2	Cumulative Cigarettes (10 Years)	--
Bacterial skin diseases	Male	0-6 days	95+ years	Global	1	2	Cumulative Cigarettes (5 Years)	--
Bacterial skin diseases	Male	0-6 days	95+ years	Global	1	2	Smoking Prevalence	--
Bacterial skin diseases	Male	0-6 days	95+ years	Global	-1	3	Education (years per capita)	--
Bacterial skin diseases	Male	0-6 days	95+ years	Global	-1	3	LDI (I$ per capita)	--
Bacterial skin diseases	Male	0-6 days	95+ years	Global	0	3	Socio-demographic Index	--
Cellulitis	Female	28-364 days	95+ years	Data Rich	-1	2	Healthcare access and quality index	--
Cellulitis	Female	28-364 days	95+ years	Data Rich	0	3	Education (years per capita)	--
Cellulitis	Female	28-364 days	95+ years	Data Rich	0	3	LDI (I$ per capita)	--
Cellulitis	Female	28-364 days	95+ years	Global	-1	2	Healthcare access and quality index	--
Cellulitis	Female	28-364 days	95+ years	Global	0	3	Education (years per capita)	546
Cellulitis	Female	28-364 days	95+ years	Global	0	3	LDI (I$ per capita)	--

**Table S16. CODEm covariates used, level of covariate, and expected direction of covariate by cause, sex, and age** *Covariates that CODEm did not select during the covariate selection process have no draw counts listed.*								
**Cause**	**Sex**	**Age Start**	**Age End**	**Model Type**	**Direction**	**Level**	**Covariate Name**	**Number of Draws**
Cellulitis	Male	28-364 days	95+ years	Data Rich	-1	2	Healthcare access and quality index	--
Cellulitis	Male	28-364 days	95+ years	Data Rich	0	3	Education (years per capita)	546
Cellulitis	Male	28-364 days	95+ years	Data Rich	0	3	LDI (I$ per capita)	--
Cellulitis	Male	28-364 days	95+ years	Global	-1	2	Healthcare access and quality index	--
Cellulitis	Male	28-364 days	95+ years	Global	0	3	Education (years per capita)	454
Cellulitis	Male	28-364 days	95+ years	Global	0	3	LDI (I$ per capita)	--
Pyoderma	Female	0-6 days	95+ years	Data Rich	-1	1	Improved Water Source (proportion with access)	--
Pyoderma	Female	0-6 days	95+ years	Data Rich	1	1	Age- and sex-specific SEV for Unsafe sanitation	1000
Pyoderma	Female	0-6 days	95+ years	Data Rich	-1	2	Healthcare access and quality index	--
Pyoderma	Female	0-6 days	95+ years	Data Rich	1	2	Alcohol (liters per capita)	--
Pyoderma	Female	0-6 days	95+ years	Data Rich	1	2	Cumulative Cigarettes (10 Years)	--
Pyoderma	Female	0-6 days	95+ years	Data Rich	1	2	Cumulative Cigarettes (5 Years)	--
Pyoderma	Female	0-6 days	95+ years	Data Rich	1	2	Smoking Prevalence	--
Pyoderma	Female	0-6 days	95+ years	Data Rich	-1	3	Education (years per capita)	--
Pyoderma	Female	0-6 days	95+ years	Data Rich	-1	3	LDI (I$ per capita)	--
Pyoderma	Female	0-6 days	95+ years	Data Rich	0	3	Socio-demographic Index	546
Pyoderma	Female	0-6 days	95+ years	Global	-1	1	Improved Water Source (proportion with access)	--
Pyoderma	Female	0-6 days	95+ years	Global	1	1	Age- and sex-specific SEV for Unsafe sanitation	1000
Pyoderma	Female	0-6 days	95+ years	Global	-1	2	Healthcare access and quality index	--
Pyoderma	Female	0-6 days	95+ years	Global	1	2	Alcohol (liters per capita)	--
Pyoderma	Female	0-6 days	95+ years	Global	1	2	Cumulative Cigarettes (10 Years)	--
Pyoderma	Female	0-6 days	95+ years	Global	1	2	Cumulative Cigarettes (5 Years)	--
Pyoderma	Female	0-6 days	95+ years	Global	1	2	Smoking Prevalence	--
Pyoderma	Female	0-6 days	95+ years	Global	-1	3	Education (years per capita)	--
Pyoderma	Female	0-6 days	95+ years	Global	-1	3	LDI (I$ per capita)	--
Pyoderma	Female	0-6 days	95+ years	Global	0	3	Socio-demographic Index	546
Pyoderma	Male	0-6 days	95+ years	Data Rich	-1	1	Improved Water Source (proportion with access)	--
Pyoderma	Male	0-6 days	95+ years	Data Rich	1	1	Age- and sex-specific SEV for Unsafe sanitation	--
Pyoderma	Male	0-6 days	95+ years	Data Rich	-1	2	Healthcare access and quality index	--
Pyoderma	Male	0-6 days	95+ years	Data Rich	1	2	Alcohol (liters per capita)	--
Pyoderma	Male	0-6 days	95+ years	Data Rich	1	2	Cumulative Cigarettes (10 Years)	--
Pyoderma	Male	0-6 days	95+ years	Data Rich	1	2	Cumulative Cigarettes (5 Years)	--
Pyoderma	Male	0-6 days	95+ years	Data Rich	1	2	Smoking Prevalence	--
Pyoderma	Male	0-6 days	95+ years	Data Rich	-1	3	Education (years per capita)	--
Pyoderma	Male	0-6 days	95+ years	Data Rich	-1	3	LDI (I$ per capita)	--
Pyoderma	Male	0-6 days	95+ years	Data Rich	0	3	Socio-demographic Index	1000
Pyoderma	Male	0-6 days	95+ years	Global	-1	1	Improved Water Source (proportion with access)	--
Pyoderma	Male	0-6 days	95+ years	Global	1	1	Age- and sex-specific SEV for Unsafe sanitation	1000
Pyoderma	Male	0-6 days	95+ years	Global	-1	2	Healthcare access and quality index	--
Pyoderma	Male	0-6 days	95+ years	Global	1	2	Alcohol (liters per capita)	--
Pyoderma	Male	0-6 days	95+ years	Global	1	2	Cumulative Cigarettes (10 Years)	--
Pyoderma	Male	0-6 days	95+ years	Global	1	2	Cumulative Cigarettes (5 Years)	--
Pyoderma	Male	0-6 days	95+ years	Global	1	2	Smoking Prevalence	--
Pyoderma	Male	0-6 days	95+ years	Global	-1	3	Education (years per capita)	--
Pyoderma	Male	0-6 days	95+ years	Global	-1	3	LDI (I$ per capita)	--
Pyoderma	Male	0-6 days	95+ years	Global	0	3	Socio-demographic Index	--
Decubitus ulcer	Female	1-4 years	95+ years	Data Rich	-1	1	Improved Water Source (proportion with access)	--
Decubitus ulcer	Female	1-4 years	95+ years	Data Rich	1	1	Diabetes Fasting Plasma Glucose (mmol/L), age-standardized 25+	--
Decubitus ulcer	Female	1-4 years	95+ years	Data Rich	1	1	Prevalence of obesity	--
Decubitus ulcer	Female	1-4 years	95+ years	Data Rich	-1	2	Healthcare access and quality index	--
Decubitus ulcer	Female	1-4 years	95+ years	Data Rich	1	2	Smoking Prevalence	105
Decubitus ulcer	Female	1-4 years	95+ years	Data Rich	1	2	Cumulative Cigarettes (10 Years)	128

Table S16. CODEm covariates used, level of covariate, and expected direction of covariate by cause, sex, and age<br>*Covariates that CODEm did not select during the covariate selection process have no draw counts listed.*								
**Cause**	**Sex**	**Age Start**	**Age End**	**Model Type**	**Direction**	**Level**	**Covariate Name**	**Number of Draws**
Decubitus ulcer	Female	1-4 years	95+ years	Data Rich	1	2	Alcohol (liters per capita)	498
Decubitus ulcer	Female	1-4 years	95+ years	Data Rich	1	2	Cumulative Cigarettes (5 Years)	584
Decubitus ulcer	Female	1-4 years	95+ years	Data Rich	-1	3	Health System Access 2 (unitless)	113
Decubitus ulcer	Female	1-4 years	95+ years	Data Rich	-1	3	Education (years per capita)	334
Decubitus ulcer	Female	1-4 years	95+ years	Data Rich	-1	3	LDI (I$ per capita)	--
Decubitus ulcer	Female	1-4 years	95+ years	Data Rich	0	3	Socio-demographic Index	189
Decubitus ulcer	Female	1-4 years	95+ years	Data Rich	1	3	Age- and sex-specific SEV for Unsafe sanitation	42
Decubitus ulcer	Female	1-4 years	95+ years	Global	-1	1	Improved Water Source (proportion with access)	194
Decubitus ulcer	Female	1-4 years	95+ years	Global	1	1	Diabetes Fasting Plasma Glucose (mmol/L), age-standardized 25+	194
Decubitus ulcer	Female	1-4 years	95+ years	Global	1	1	Prevalence of obesity	--
Decubitus ulcer	Female	1-4 years	95+ years	Global	-1	2	Healthcare access and quality index	--
Decubitus ulcer	Female	1-4 years	95+ years	Global	1	2	Cumulative Cigarettes (5 Years)	170
Decubitus ulcer	Female	1-4 years	95+ years	Global	1	2	Cumulative Cigarettes (10 Years)	557
Decubitus ulcer	Female	1-4 years	95+ years	Global	1	2	Alcohol (liters per capita)	727
Decubitus ulcer	Female	1-4 years	95+ years	Global	1	2	Smoking Prevalence	--
Decubitus ulcer	Female	1-4 years	95+ years	Global	-1	3	Education (years per capita)	279
Decubitus ulcer	Female	1-4 years	95+ years	Global	-1	3	Health System Access 2 (unitless)	361
Decubitus ulcer	Female	1-4 years	95+ years	Global	-1	3	LDI (I$ per capita)	--
Decubitus ulcer	Female	1-4 years	95+ years	Global	0	3	Socio-demographic Index	375
Decubitus ulcer	Female	1-4 years	95+ years	Global	1	3	Age- and sex-specific SEV for Unsafe sanitation	104
Decubitus ulcer	Male	1-4 years	95+ years	Data Rich	-1	1	Improved Water Source (proportion with access)	572
Decubitus ulcer	Male	1-4 years	95+ years	Data Rich	1	1	Diabetes Fasting Plasma Glucose (mmol/L), age-standardized 25+	572
Decubitus ulcer	Male	1-4 years	95+ years	Data Rich	1	1	Prevalence of obesity	--
Decubitus ulcer	Male	1-4 years	95+ years	Data Rich	-1	2	Healthcare access and quality index	--
Decubitus ulcer	Male	1-4 years	95+ years	Data Rich	1	2	Cumulative Cigarettes (5 Years)	428
Decubitus ulcer	Male	1-4 years	95+ years	Data Rich	1	2	Alcohol (liters per capita)	--
Decubitus ulcer	Male	1-4 years	95+ years	Data Rich	1	2	Cumulative Cigarettes (10 Years)	--
Decubitus ulcer	Male	1-4 years	95+ years	Data Rich	1	2	Smoking Prevalence	--
Decubitus ulcer	Male	1-4 years	95+ years	Data Rich	-1	3	Health System Access 2 (unitless)	428
Decubitus ulcer	Male	1-4 years	95+ years	Data Rich	-1	3	Education (years per capita)	--
Decubitus ulcer	Male	1-4 years	95+ years	Data Rich	-1	3	LDI (I$ per capita)	--
Decubitus ulcer	Male	1-4 years	95+ years	Data Rich	0	3	Socio-demographic Index	--
Decubitus ulcer	Male	1-4 years	95+ years	Data Rich	1	3	Age- and sex-specific SEV for Unsafe sanitation	325
Decubitus ulcer	Male	1-4 years	95+ years	Global	-1	1	Improved Water Source (proportion with access)	249
Decubitus ulcer	Male	1-4 years	95+ years	Global	1	1	Diabetes Fasting Plasma Glucose (mmol/L), age-standardized 25+	970
Decubitus ulcer	Male	1-4 years	95+ years	Global	1	1	Prevalence of obesity	--
Decubitus ulcer	Male	1-4 years	95+ years	Global	-1	2	Healthcare access and quality index	563
Decubitus ulcer	Male	1-4 years	95+ years	Global	1	2	Cumulative Cigarettes (10 Years)	121
Decubitus ulcer	Male	1-4 years	95+ years	Global	1	2	Smoking Prevalence	176
Decubitus ulcer	Male	1-4 years	95+ years	Global	1	2	Alcohol (liters per capita)	383
Decubitus ulcer	Male	1-4 years	95+ years	Global	1	2	Cumulative Cigarettes (5 Years)	529
Decubitus ulcer	Male	1-4 years	95+ years	Global	-1	3	Health System Access 2 (unitless)	32
Decubitus ulcer	Male	1-4 years	95+ years	Global	-1	3	Education (years per capita)	300
Decubitus ulcer	Male	1-4 years	95+ years	Global	-1	3	LDI (I$ per capita)	--
Decubitus ulcer	Male	1-4 years	95+ years	Global	0	3	Socio-demographic Index	177
Decubitus ulcer	Male	1-4 years	95+ years	Global	1	3	Age- and sex-specific SEV for Unsafe sanitation	132
Other skin and subcutaneous diseases	Female	28-364 days	95+ years	Data Rich	-1	1	Improved Water Source (proportion with access)	--
Other skin and subcutaneous diseases	Female	28-364 days	95+ years	Data Rich	1	1	Age- and sex-specific SEV for Unsafe sanitation	1000
Other skin and subcutaneous diseases	Female	28-364 days	95+ years	Data Rich	1	1	Age-standardized SEV for Child underweight	--
Other skin and subcutaneous diseases	Female	28-364 days	95+ years	Data Rich	-1	2	Healthcare access and quality index	--
Other skin and subcutaneous diseases	Female	28-364 days	95+ years	Data Rich	1	2	Alcohol (liters per capita)	--
Other skin and subcutaneous diseases	Female	28-364 days	95+ years	Data Rich	1	2	Cumulative Cigarettes (10 Years)	--

Table S16. CODEm covariates used, level of covariate, and expected direction of covariate by cause, sex, and age
Covariates that CODEm did not select during the covariate selection process have no draw counts listed.

Cause	Sex	Age Start	Age End	Model Type	Direction	Level	Covariate Name	Number of Draws
Other skin and subcutaneous diseases	Female	28-364 days	95+ years	Data Rich	1	2	Cumulative Cigarettes (5 Years)	--
Other skin and subcutaneous diseases	Female	28-364 days	95+ years	Data Rich	1	2	Smoking Prevalence	--
Other skin and subcutaneous diseases	Female	28-364 days	95+ years	Data Rich	-1	3	Education (years per capita)	--
Other skin and subcutaneous diseases	Female	28-364 days	95+ years	Data Rich	-1	3	Health System Access 2 (unitless)	--
Other skin and subcutaneous diseases	Female	28-364 days	95+ years	Data Rich	-1	3	LDI (I$ per capita)	--
Other skin and subcutaneous diseases	Female	28-364 days	95+ years	Data Rich	0	3	Socio-demographic Index	--
Other skin and subcutaneous diseases	Female	28-364 days	95+ years	Global	-1	1	Improved Water Source (proportion with access)	1000
Other skin and subcutaneous diseases	Female	28-364 days	95+ years	Global	1	1	Age- and sex-specific SEV for Unsafe sanitation	--
Other skin and subcutaneous diseases	Female	28-364 days	95+ years	Global	1	1	Age-standardized SEV for Child underweight	--
Other skin and subcutaneous diseases	Female	28-364 days	95+ years	Global	-1	2	Healthcare access and quality index	--
Other skin and subcutaneous diseases	Female	28-364 days	95+ years	Global	1	2	Cumulative Cigarettes (5 Years)	407
Other skin and subcutaneous diseases	Female	28-364 days	95+ years	Global	1	2	Cumulative Cigarettes (10 Years)	414
Other skin and subcutaneous diseases	Female	28-364 days	95+ years	Global	1	2	Alcohol (liters per capita)	--
Other skin and subcutaneous diseases	Female	28-364 days	95+ years	Global	1	2	Smoking Prevalence	--
Other skin and subcutaneous diseases	Female	28-364 days	95+ years	Global	-1	3	Health System Access 2 (unitless)	275
Other skin and subcutaneous diseases	Female	28-364 days	95+ years	Global	-1	3	Education (years per capita)	--
Other skin and subcutaneous diseases	Female	28-364 days	95+ years	Global	-1	3	LDI (I$ per capita)	--
Other skin and subcutaneous diseases	Female	28-364 days	95+ years	Global	0	3	Socio-demographic Index	--
Other skin and subcutaneous diseases	Male	28-364 days	95+ years	Data Rich	-1	1	Improved Water Source (proportion with access)	1000
Other skin and subcutaneous diseases	Male	28-364 days	95+ years	Data Rich	1	1	Age- and sex-specific SEV for Unsafe sanitation	--
Other skin and subcutaneous diseases	Male	28-364 days	95+ years	Data Rich	1	1	Age-standardized SEV for Child underweight	--
Other skin and subcutaneous diseases	Male	28-364 days	95+ years	Data Rich	-1	2	Healthcare access and quality index	--
Other skin and subcutaneous diseases	Male	28-364 days	95+ years	Data Rich	1	2	Cumulative Cigarettes (10 Years)	275
Other skin and subcutaneous diseases	Male	28-364 days	95+ years	Data Rich	1	2	Alcohol (liters per capita)	--
Other skin and subcutaneous diseases	Male	28-364 days	95+ years	Data Rich	1	2	Cumulative Cigarettes (5 Years)	--
Other skin and subcutaneous diseases	Male	28-364 days	95+ years	Data Rich	1	2	Smoking Prevalence	--
Other skin and subcutaneous diseases	Male	28-364 days	95+ years	Data Rich	-1	3	Education (years per capita)	171
Other skin and subcutaneous diseases	Male	28-364 days	95+ years	Data Rich	-1	3	Health System Access 2 (unitless)	--
Other skin and subcutaneous diseases	Male	28-364 days	95+ years	Data Rich	-1	3	LDI (I$ per capita)	--
Other skin and subcutaneous diseases	Male	28-364 days	95+ years	Data Rich	0	3	Socio-demographic Index	361
Other skin and subcutaneous diseases	Male	28-364 days	95+ years	Global	-1	1	Improved Water Source (proportion with access)	570
Other skin and subcutaneous diseases	Male	28-364 days	95+ years	Global	1	1	Age- and sex-specific SEV for Unsafe sanitation	430
Other skin and subcutaneous diseases	Male	28-364 days	95+ years	Global	1	1	Age-standardized SEV for Child underweight	--
Other skin and subcutaneous diseases	Male	28-364 days	95+ years	Global	-1	2	Healthcare access and quality index	82
Other skin and subcutaneous diseases	Male	28-364 days	95+ years	Global	1	2	Cumulative Cigarettes (5 Years)	256
Other skin and subcutaneous diseases	Male	28-364 days	95+ years	Global	1	2	Smoking Prevalence	337
Other skin and subcutaneous diseases	Male	28-364 days	95+ years	Global	1	2	Alcohol (liters per capita)	--
Other skin and subcutaneous diseases	Male	28-364 days	95+ years	Global	1	2	Cumulative Cigarettes (10 Years)	--
Other skin and subcutaneous diseases	Male	28-364 days	95+ years	Global	-1	3	Education (years per capita)	28
Other skin and subcutaneous diseases	Male	28-364 days	95+ years	Global	-1	3	Health System Access 2 (unitless)	226

Table S16. CODEm covariates used, level of covariate, and expected direction of covariate by cause, sex, and age
Covariates that CODEm did not select during the covariate selection process have no draw counts listed.

Cause	Sex	Age Start	Age End	Model Type	Direction	Level	Covariate Name	Number of Draws
Other skin and subcutaneous diseases	Male	28-364 days	95+ years	Global	-1	3	LDI (I$ per capita)	--
Other skin and subcutaneous diseases	Male	28-364 days	95+ years	Global	0	3	Socio-demographic Index	46
Musculoskeletal disorders	Female	5-9 years	95+ years	Data Rich	1	1	Mean BMI	--
Musculoskeletal disorders	Female	5-9 years	95+ years	Data Rich	-1	2	Healthcare access and quality index	--
Musculoskeletal disorders	Female	5-9 years	95+ years	Data Rich	0	2	Education (years per capita)	--
Musculoskeletal disorders	Female	5-9 years	95+ years	Data Rich	0	2	LDI (I$ per capita)	--
Musculoskeletal disorders	Female	5-9 years	95+ years	Data Rich	0	2	vegetables adjusted(g)	--
Musculoskeletal disorders	Female	5-9 years	95+ years	Data Rich	1	2	Age-standardized bone mineral density among population age 60+ years	--
Musculoskeletal disorders	Female	5-9 years	95+ years	Data Rich	1	2	Alcohol (liters per capita)	--
Musculoskeletal disorders	Female	5-9 years	95+ years	Data Rich	1	2	Cholesterol (total, mean per capita)	--
Musculoskeletal disorders	Female	5-9 years	95+ years	Data Rich	1	2	Cumulative Cigarettes (10 Years)	--
Musculoskeletal disorders	Female	5-9 years	95+ years	Data Rich	1	2	Cumulative Cigarettes (5 Years)	--
Musculoskeletal disorders	Female	5-9 years	95+ years	Data Rich	1	2	Low bone mineral density	--
Musculoskeletal disorders	Female	5-9 years	95+ years	Data Rich	1	2	Smoking Prevalence	--
Musculoskeletal disorders	Female	5-9 years	95+ years	Data Rich	0	3	Socio-demographic Index	--
Musculoskeletal disorders	Female	5-9 years	95+ years	Global	1	1	Mean BMI	282
Musculoskeletal disorders	Female	5-9 years	95+ years	Global	-1	2	Healthcare access and quality index	--
Musculoskeletal disorders	Female	5-9 years	95+ years	Global	0	2	vegetables adjusted(g)	344
Musculoskeletal disorders	Female	5-9 years	95+ years	Global	0	2	Education (years per capita)	362
Musculoskeletal disorders	Female	5-9 years	95+ years	Global	0	2	LDI (I$ per capita)	--
Musculoskeletal disorders	Female	5-9 years	95+ years	Global	1	2	Smoking Prevalence	52
Musculoskeletal disorders	Female	5-9 years	95+ years	Global	1	2	Cumulative Cigarettes (5 Years)	66
Musculoskeletal disorders	Female	5-9 years	95+ years	Global	1	2	Cumulative Cigarettes (10 Years)	79
Musculoskeletal disorders	Female	5-9 years	95+ years	Global	1	2	Alcohol (liters per capita)	95
Musculoskeletal disorders	Female	5-9 years	95+ years	Global	1	2	Low bone mineral density	623
Musculoskeletal disorders	Female	5-9 years	95+ years	Global	1	2	Age-standardized bone mineral density among population age 60+ years	--
Musculoskeletal disorders	Female	5-9 years	95+ years	Global	1	2	Cholesterol (total, mean per capita)	--
Musculoskeletal disorders	Female	5-9 years	95+ years	Global	0	3	Socio-demographic Index	246
Musculoskeletal disorders	Male	5-9 years	95+ years	Data Rich	1	1	Mean BMI	678
Musculoskeletal disorders	Male	5-9 years	95+ years	Data Rich	-1	2	Healthcare access and quality index	285
Musculoskeletal disorders	Male	5-9 years	95+ years	Data Rich	0	2	Education (years per capita)	545
Musculoskeletal disorders	Male	5-9 years	95+ years	Data Rich	0	2	vegetables adjusted(g)	593
Musculoskeletal disorders	Male	5-9 years	95+ years	Data Rich	0	2	LDI (I$ per capita)	--
Musculoskeletal disorders	Male	5-9 years	95+ years	Data Rich	1	2	Cumulative Cigarettes (10 Years)	9
Musculoskeletal disorders	Male	5-9 years	95+ years	Data Rich	1	2	Smoking Prevalence	9
Musculoskeletal disorders	Male	5-9 years	95+ years	Data Rich	1	2	Cumulative Cigarettes (5 Years)	12
Musculoskeletal disorders	Male	5-9 years	95+ years	Data Rich	1	2	Low bone mineral density	359
Musculoskeletal disorders	Male	5-9 years	95+ years	Data Rich	1	2	Alcohol (liters per capita)	613
Musculoskeletal disorders	Male	5-9 years	95+ years	Data Rich	1	2	Age-standardized bone mineral density among population age 60+ years	--
Musculoskeletal disorders	Male	5-9 years	95+ years	Data Rich	1	2	Cholesterol (total, mean per capita)	--
Musculoskeletal disorders	Male	5-9 years	95+ years	Data Rich	0	3	Socio-demographic Index	487
Musculoskeletal disorders	Male	5-9 years	95+ years	Global	1	1	Mean BMI	605
Musculoskeletal disorders	Male	5-9 years	95+ years	Global	-1	2	Healthcare access and quality index	144
Musculoskeletal disorders	Male	5-9 years	95+ years	Global	0	2	vegetables adjusted(g)	309
Musculoskeletal disorders	Male	5-9 years	95+ years	Global	0	2	Education (years per capita)	625
Musculoskeletal disorders	Male	5-9 years	95+ years	Global	0	2	LDI (I$ per capita)	--
Musculoskeletal disorders	Male	5-9 years	95+ years	Global	1	2	Smoking Prevalence	66
Musculoskeletal disorders	Male	5-9 years	95+ years	Global	1	2	Cumulative Cigarettes (10 Years)	99
Musculoskeletal disorders	Male	5-9 years	95+ years	Global	1	2	Alcohol (liters per capita)	309
Musculoskeletal disorders	Male	5-9 years	95+ years	Global	1	2	Cholesterol (total, mean per capita)	--
Musculoskeletal disorders	Male	5-9 years	95+ years	Global	1	2	Cumulative Cigarettes (5 Years)	--
Musculoskeletal disorders	Male	5-9 years	95+ years	Global	0	3	Socio-demographic Index	491
Rheumatoid arthritis	Female	5-9 years	95+ years	Data Rich	-1	1	Healthcare access and quality index	329
Rheumatoid arthritis	Female	5-9 years	95+ years	Data Rich	1	1	Smoking Prevalence	118
Rheumatoid arthritis	Female	5-9 years	95+ years	Data Rich	1	1	Alcohol (liters per capita)	137

Table S16. CODEm covariates used, level of covariate, and expected direction of covariate by cause, sex, and age
Covariates that CODEm did not select during the covariate selection process have no draw counts listed.

Cause	Sex	Age Start	Age End	Model Type	Direction	Level	Covariate Name	Number of Draws
Rheumatoid arthritis	Female	5-9 years	95+ years	Data Rich	1	1	Cumulative Cigarettes (10 Years)	149
Rheumatoid arthritis	Female	5-9 years	95+ years	Data Rich	1	1	Cumulative Cigarettes (5 Years)	839
Rheumatoid arthritis	Female	5-9 years	95+ years	Data Rich	1	2	Cholesterol (total, mean per capita)	12
Rheumatoid arthritis	Female	5-9 years	95+ years	Data Rich	1	2	Mean BMI	603
Rheumatoid arthritis	Female	5-9 years	95+ years	Data Rich	-1	3	Education (years per capita)	47
Rheumatoid arthritis	Female	5-9 years	95+ years	Data Rich	-1	3	LDI (I$ per capita)	--
Rheumatoid arthritis	Female	5-9 years	95+ years	Data Rich	0	3	milk adjusted(g)	61
Rheumatoid arthritis	Female	5-9 years	95+ years	Data Rich	0	3	Socio-demographic Index	361
Rheumatoid arthritis	Female	5-9 years	95+ years	Global	-1	1	Healthcare access and quality index	309
Rheumatoid arthritis	Female	5-9 years	95+ years	Global	1	1	Smoking Prevalence	36
Rheumatoid arthritis	Female	5-9 years	95+ years	Global	1	1	Cumulative Cigarettes (5 Years)	258
Rheumatoid arthritis	Female	5-9 years	95+ years	Global	1	1	Cumulative Cigarettes (10 Years)	563
Rheumatoid arthritis	Female	5-9 years	95+ years	Global	1	1	Alcohol (liters per capita)	--
Rheumatoid arthritis	Female	5-9 years	95+ years	Global	1	2	Cholesterol (total, mean per capita)	11
Rheumatoid arthritis	Female	5-9 years	95+ years	Global	1	2	Mean BMI	51
Rheumatoid arthritis	Female	5-9 years	95+ years	Global	-1	3	Education (years per capita)	13
Rheumatoid arthritis	Female	5-9 years	95+ years	Global	-1	3	LDI (I$ per capita)	--
Rheumatoid arthritis	Female	5-9 years	95+ years	Global	0	3	Socio-demographic Index	36
Rheumatoid arthritis	Female	5-9 years	95+ years	Global	0	3	milk adjusted(g)	205
Rheumatoid arthritis	Male	5-9 years	95+ years	Data Rich	-1	1	Healthcare access and quality index	260
Rheumatoid arthritis	Male	5-9 years	95+ years	Data Rich	1	1	Smoking Prevalence	36
Rheumatoid arthritis	Male	5-9 years	95+ years	Data Rich	1	1	Cumulative Cigarettes (10 Years)	149
Rheumatoid arthritis	Male	5-9 years	95+ years	Data Rich	1	1	Cumulative Cigarettes (5 Years)	704
Rheumatoid arthritis	Male	5-9 years	95+ years	Data Rich	1	1	Alcohol (liters per capita)	--
Rheumatoid arthritis	Male	5-9 years	95+ years	Data Rich	1	2	Mean BMI	642
Rheumatoid arthritis	Male	5-9 years	95+ years	Data Rich	1	2	Cholesterol (total, mean per capita)	--
Rheumatoid arthritis	Male	5-9 years	95+ years	Data Rich	-1	3	Education (years per capita)	--
Rheumatoid arthritis	Male	5-9 years	95+ years	Data Rich	-1	3	LDI (I$ per capita)	--
Rheumatoid arthritis	Male	5-9 years	95+ years	Data Rich	0	3	milk adjusted(g)	311
Rheumatoid arthritis	Male	5-9 years	95+ years	Data Rich	0	3	Socio-demographic Index	516
Rheumatoid arthritis	Male	5-9 years	95+ years	Global	-1	1	Healthcare access and quality index	444
Rheumatoid arthritis	Male	5-9 years	95+ years	Global	1	1	Smoking Prevalence	50
Rheumatoid arthritis	Male	5-9 years	95+ years	Global	1	1	Cumulative Cigarettes (5 Years)	379
Rheumatoid arthritis	Male	5-9 years	95+ years	Global	1	1	Cumulative Cigarettes (10 Years)	415
Rheumatoid arthritis	Male	5-9 years	95+ years	Global	1	1	Alcohol (liters per capita)	--
Rheumatoid arthritis	Male	5-9 years	95+ years	Global	1	2	Cholesterol (total, mean per capita)	--
Rheumatoid arthritis	Male	5-9 years	95+ years	Global	1	2	Mean BMI	--
Rheumatoid arthritis	Male	5-9 years	95+ years	Global	-1	3	Education (years per capita)	224
Rheumatoid arthritis	Male	5-9 years	95+ years	Global	-1	3	LDI (I$ per capita)	--
Rheumatoid arthritis	Male	5-9 years	95+ years	Global	0	3	Socio-demographic Index	13
Rheumatoid arthritis	Male	5-9 years	95+ years	Global	0	3	milk adjusted(g)	134
Other musculoskeletal disorders	Female	5-9 years	95+ years	Data Rich	1	1	Mean BMI	508
Other musculoskeletal disorders	Female	5-9 years	95+ years	Data Rich	-1	2	Healthcare access and quality index	--
Other musculoskeletal disorders	Female	5-9 years	95+ years	Data Rich	0	2	vegetables adjusted(g)	352
Other musculoskeletal disorders	Female	5-9 years	95+ years	Data Rich	0	2	Education (years per capita)	683
Other musculoskeletal disorders	Female	5-9 years	95+ years	Data Rich	0	2	LDI (I$ per capita)	--
Other musculoskeletal disorders	Female	5-9 years	95+ years	Data Rich	1	2	Alcohol (liters per capita)	95
Other musculoskeletal disorders	Female	5-9 years	95+ years	Data Rich	1	2	Cumulative Cigarettes (5 Years)	179
Other musculoskeletal disorders	Female	5-9 years	95+ years	Data Rich	1	2	Cumulative Cigarettes (10 Years)	239
Other musculoskeletal disorders	Female	5-9 years	95+ years	Data Rich	1	2	Cholesterol (total, mean per capita)	--
Other musculoskeletal disorders	Female	5-9 years	95+ years	Data Rich	1	2	Smoking Prevalence	--
Other musculoskeletal disorders	Female	5-9 years	95+ years	Data Rich	0	3	Socio-demographic Index	341
Other musculoskeletal disorders	Female	5-9 years	95+ years	Global	1	1	Mean BMI	359
Other musculoskeletal disorders	Female	5-9 years	95+ years	Global	-1	2	Healthcare access and quality index	--
Other musculoskeletal disorders	Female	5-9 years	95+ years	Global	0	2	vegetables adjusted(g)	253
Other musculoskeletal disorders	Female	5-9 years	95+ years	Global	0	2	Education (years per capita)	572
Other musculoskeletal disorders	Female	5-9 years	95+ years	Global	0	2	LDI (I$ per capita)	--
Other musculoskeletal disorders	Female	5-9 years	95+ years	Global	1	2	Alcohol (liters per capita)	47
Other musculoskeletal disorders	Female	5-9 years	95+ years	Global	1	2	Cumulative Cigarettes (5 Years)	85
Other musculoskeletal disorders	Female	5-9 years	95+ years	Global	1	2	Cumulative Cigarettes (10 Years)	102

**Table S16. CODEm covariates used, level of covariate, and expected direction of covariate by cause, sex, and age** *Covariates that CODEm did not select during the covariate selection process have no draw counts listed.*								
**Cause**	**Sex**	**Age Start**	**Age End**	**Model Type**	**Direction**	**Level**	**Covariate Name**	**Number of Draws**
Other musculoskeletal disorders	Female	5-9 years	95+ years	Global	1	2	Cholesterol (total, mean per capita)	--
Other musculoskeletal disorders	Female	5-9 years	95+ years	Global	1	2	Smoking Prevalence	--
Other musculoskeletal disorders	Female	5-9 years	95+ years	Global	0	3	Socio-demographic Index	470
Other musculoskeletal disorders	Male	5-9 years	95+ years	Data Rich	1	1	Mean BMI	141
Other musculoskeletal disorders	Male	5-9 years	95+ years	Data Rich	-1	2	Healthcare access and quality index	--
Other musculoskeletal disorders	Male	5-9 years	95+ years	Data Rich	0	2	vegetables adjusted(g)	378
Other musculoskeletal disorders	Male	5-9 years	95+ years	Data Rich	0	2	Education (years per capita)	496
Other musculoskeletal disorders	Male	5-9 years	95+ years	Data Rich	0	2	LDI (I$ per capita)	--
Other musculoskeletal disorders	Male	5-9 years	95+ years	Data Rich	1	2	Alcohol (liters per capita)	580
Other musculoskeletal disorders	Male	5-9 years	95+ years	Data Rich	1	2	Cholesterol (total, mean per capita)	--
Other musculoskeletal disorders	Male	5-9 years	95+ years	Data Rich	1	2	Cumulative Cigarettes (10 Years)	--
Other musculoskeletal disorders	Male	5-9 years	95+ years	Data Rich	1	2	Cumulative Cigarettes (5 Years)	--
Other musculoskeletal disorders	Male	5-9 years	95+ years	Data Rich	1	2	Smoking Prevalence	--
Other musculoskeletal disorders	Male	5-9 years	95+ years	Data Rich	0	3	Socio-demographic Index	618
Other musculoskeletal disorders	Male	5-9 years	95+ years	Global	1	1	Mean BMI	--
Other musculoskeletal disorders	Male	5-9 years	95+ years	Global	-1	2	Healthcare access and quality index	--
Other musculoskeletal disorders	Male	5-9 years	95+ years	Global	0	2	Education (years per capita)	264
Other musculoskeletal disorders	Male	5-9 years	95+ years	Global	0	2	vegetables adjusted(g)	381
Other musculoskeletal disorders	Male	5-9 years	95+ years	Global	0	2	LDI (I$ per capita)	--
Other musculoskeletal disorders	Male	5-9 years	95+ years	Global	1	2	Alcohol (liters per capita)	264
Other musculoskeletal disorders	Male	5-9 years	95+ years	Global	1	2	Cholesterol (total, mean per capita)	--
Other musculoskeletal disorders	Male	5-9 years	95+ years	Global	1	2	Cumulative Cigarettes (10 Years)	--
Other musculoskeletal disorders	Male	5-9 years	95+ years	Global	1	2	Cumulative Cigarettes (5 Years)	--
Other musculoskeletal disorders	Male	5-9 years	95+ years	Global	1	2	Smoking Prevalence	--
Other musculoskeletal disorders	Male	5-9 years	95+ years	Global	0	3	Socio-demographic Index	707
Congenital anomalies	Female	0-6 days	65-69 years	Data Rich	-1	1	Composite fortification standard and folic acid inclusion	86
Congenital anomalies	Female	0-6 days	65-69 years	Data Rich	-1	1	Folic acid unadjusted (ug)	743
Congenital anomalies	Female	0-6 days	65-69 years	Data Rich	-1	1	In-Facility Delivery (proportion)	--
Congenital anomalies	Female	0-6 days	65-69 years	Data Rich	1	1	Live Births 35+ (proportion)	460
Congenital anomalies	Female	0-6 days	65-69 years	Data Rich	1	1	Birth prevalence of congenital chromosomal anomalies	584
Congenital anomalies	Female	0-6 days	65-69 years	Data Rich	1	1	Birth prevalence of CHD	--
Congenital anomalies	Female	0-6 days	65-69 years	Data Rich	1	1	Maternal alcohol consumption during pregnancy (proportion)	--
Congenital anomalies	Female	0-6 days	65-69 years	Data Rich	-1	2	Antenatal Care (4 visits) Coverage (proportion)	68
Congenital anomalies	Female	0-6 days	65-69 years	Data Rich	-1	2	Healthcare access and quality index	--
Congenital anomalies	Female	0-6 days	65-69 years	Data Rich	-1	2	Legality of Abortion	--
Congenital anomalies	Female	0-6 days	65-69 years	Data Rich	0	2	Antenatal Care (1 visit) Coverage (proportion)	495
Congenital anomalies	Female	0-6 days	65-69 years	Data Rich	1	2	Smoking Prevalence (Reproductive Age Standardized)	10
Congenital anomalies	Female	0-6 days	65-69 years	Data Rich	-1	3	Socio-demographic Index	11
Congenital anomalies	Female	0-6 days	65-69 years	Data Rich	-1	3	Maternal Education (years per capita)	23
Congenital anomalies	Female	0-6 days	65-69 years	Data Rich	1	3	Outdoor Air Pollution (PM2.5)	21
Congenital anomalies	Female	0-6 days	65-69 years	Data Rich	1	3	Alcohol (liters per capita)	92
Congenital anomalies	Female	0-6 days	65-69 years	Data Rich	1	3	fruits unadjusted(g)	151
Congenital anomalies	Female	0-6 days	65-69 years	Data Rich	1	3	vegetables unadjusted(g)	319
Congenital anomalies	Female	0-6 days	65-69 years	Data Rich	1	3	Indoor Air Pollution (All Cooking Fuels)	--
Congenital anomalies	Female	0-6 days	65-69 years	Global	-1	1	Composite fortification standard and folic acid inclusion	0
Congenital anomalies	Female	0-6 days	65-69 years	Global	-1	1	In-Facility Delivery (proportion)	0
Congenital anomalies	Female	0-6 days	65-69 years	Global	-1	1	Folic acid unadjusted (ug)	855
Congenital anomalies	Female	0-6 days	65-69 years	Global	1	1	Birth prevalence of CHD	0

Table S16. CODEm covariates used, level of covariate, and expected direction of covariate by cause, sex, and age
Covariates that CODEm did not select during the covariate selection process have no draw counts listed.

Cause	Sex	Age Start	Age End	Model Type	Direction	Level	Covariate Name	Number of Draws
Congenital anomalies	Female	0-6 days	65-69 years	Global	1	1	Birth prevalence of congenital chromosomal anomalies	748
Congenital anomalies	Female	0-6 days	65-69 years	Global	1	1	Live Births 35+ (proportion)	758
Congenital anomalies	Female	0-6 days	65-69 years	Global	1	1	Maternal alcohol consumption during pregnancy (proportion)	--
Congenital anomalies	Female	0-6 days	65-69 years	Global	-1	2	Antenatal Care (4 visits) Coverage (proportion)	0
Congenital anomalies	Female	0-6 days	65-69 years	Global	-1	2	Healthcare access and quality index	0
Congenital anomalies	Female	0-6 days	65-69 years	Global	-1	2	Legality of Abortion	--
Congenital anomalies	Female	0-6 days	65-69 years	Global	0	2	Antenatal Care (1 visit) Coverage (proportion)	977
Congenital anomalies	Female	0-6 days	65-69 years	Global	1	2	Smoking Prevalence (Reproductive Age Standardized)	0
Congenital anomalies	Female	0-6 days	65-69 years	Global	-1	3	Maternal Education (years per capita)	0
Congenital anomalies	Female	0-6 days	65-69 years	Global	-1	3	Socio-demographic Index	0
Congenital anomalies	Female	0-6 days	65-69 years	Global	1	3	Alcohol (liters per capita)	0
Congenital anomalies	Female	0-6 days	65-69 years	Global	1	3	Indoor Air Pollution (All Cooking Fuels)	0
Congenital anomalies	Female	0-6 days	65-69 years	Global	1	3	Outdoor Air Pollution (PM2.5)	73
Congenital anomalies	Female	0-6 days	65-69 years	Global	1	3	vegetables unadjusted(g)	185
Congenital anomalies	Female	0-6 days	65-69 years	Global	1	3	fruits unadjusted(g)	268
Congenital anomalies	Male	0-6 days	65-69 years	Data Rich	-1	1	Composite fortification standard and folic acid inclusion	93
Congenital anomalies	Male	0-6 days	65-69 years	Data Rich	-1	1	Folic acid unadjusted (ug)	827
Congenital anomalies	Male	0-6 days	65-69 years	Data Rich	-1	1	In-Facility Delivery (proportion)	--
Congenital anomalies	Male	0-6 days	65-69 years	Data Rich	1	1	Birth prevalence of CHD	25
Congenital anomalies	Male	0-6 days	65-69 years	Data Rich	1	1	Birth prevalence of congenital chromosomal anomalies	402
Congenital anomalies	Male	0-6 days	65-69 years	Data Rich	1	1	Live Births 35+ (proportion)	644
Congenital anomalies	Male	0-6 days	65-69 years	Data Rich	1	1	Maternal alcohol consumption during pregnancy (proportion)	--
Congenital anomalies	Male	0-6 days	65-69 years	Data Rich	-1	2	Healthcare access and quality index	21
Congenital anomalies	Male	0-6 days	65-69 years	Data Rich	-1	2	Antenatal Care (4 visits) Coverage (proportion)	53
Congenital anomalies	Male	0-6 days	65-69 years	Data Rich	-1	2	Legality of Abortion	--
Congenital anomalies	Male	0-6 days	65-69 years	Data Rich	0	2	Antenatal Care (1 visit) Coverage (proportion)	624
Congenital anomalies	Male	0-6 days	65-69 years	Data Rich	1	2	Smoking Prevalence (Reproductive Age Standardized)	5
Congenital anomalies	Male	0-6 days	65-69 years	Data Rich	-1	3	Socio-demographic Index	23
Congenital anomalies	Male	0-6 days	65-69 years	Data Rich	-1	3	Maternal Education (years per capita)	25
Congenital anomalies	Male	0-6 days	65-69 years	Data Rich	1	3	Outdoor Air Pollution (PM2.5)	44
Congenital anomalies	Male	0-6 days	65-69 years	Data Rich	1	3	vegetables unadjusted(g)	236
Congenital anomalies	Male	0-6 days	65-69 years	Data Rich	1	3	fruits unadjusted(g)	286
Congenital anomalies	Male	0-6 days	65-69 years	Data Rich	1	3	Alcohol (liters per capita)	--
Congenital anomalies	Male	0-6 days	65-69 years	Data Rich	1	3	Indoor Air Pollution (All Cooking Fuels)	--
Congenital anomalies	Male	0-6 days	65-69 years	Global	-1	1	Composite fortification standard and folic acid inclusion	0
Congenital anomalies	Male	0-6 days	65-69 years	Global	-1	1	Folic acid unadjusted (ug)	524
Congenital anomalies	Male	0-6 days	65-69 years	Global	-1	1	In-Facility Delivery (proportion)	--
Congenital anomalies	Male	0-6 days	65-69 years	Global	1	1	Birth prevalence of CHD	0
Congenital anomalies	Male	0-6 days	65-69 years	Global	1	1	Birth prevalence of congenital chromosomal anomalies	491
Congenital anomalies	Male	0-6 days	65-69 years	Global	1	1	Live Births 35+ (proportion)	911

Table S16. CODEm covariates used, level of covariate, and expected direction of covariate by cause, sex, and age
Covariates that CODEm did not select during the covariate selection process have no draw counts listed.

Cause	Sex	Age Start	Age End	Model Type	Direction	Level	Covariate Name	Number of Draws
Congenital anomalies	Male	0-6 days	65-69 years	Global	1	1	Maternal alcohol consumption during pregnancy (proportion)	--
Congenital anomalies	Male	0-6 days	65-69 years	Global	-1	2	Antenatal Care (4 visits) Coverage (proportion)	0
Congenital anomalies	Male	0-6 days	65-69 years	Global	-1	2	Healthcare access and quality index	0
Congenital anomalies	Male	0-6 days	65-69 years	Global	-1	2	Legality of Abortion	--
Congenital anomalies	Male	0-6 days	65-69 years	Global	0	2	Antenatal Care (1 visit) Coverage (proportion)	894
Congenital anomalies	Male	0-6 days	65-69 years	Global	1	2	Smoking Prevalence (Reproductive Age Standardized)	0
Congenital anomalies	Male	0-6 days	65-69 years	Global	-1	3	Maternal Education (years per capita)	0
Congenital anomalies	Male	0-6 days	65-69 years	Global	-1	3	Socio-demographic Index	0
Congenital anomalies	Male	0-6 days	65-69 years	Global	1	3	Indoor Air Pollution (All Cooking Fuels)	0
Congenital anomalies	Male	0-6 days	65-69 years	Global	1	3	vegetables unadjusted(g)	251
Congenital anomalies	Male	0-6 days	65-69 years	Global	1	3	Outdoor Air Pollution (PM2.5)	317
Congenital anomalies	Male	0-6 days	65-69 years	Global	1	3	fruits unadjusted(g)	398
Congenital anomalies	Male	0-6 days	65-69 years	Global	1	3	Alcohol (liters per capita)	--
Neural tube defects	Female	0-6 days	65-69 years	Data Rich	-1	1	In-Facility Delivery (proportion)	29
Neural tube defects	Female	0-6 days	65-69 years	Data Rich	-1	1	Socio-demographic Index	186
Neural tube defects	Female	0-6 days	65-69 years	Data Rich	-1	1	Composite fortification standard and folic acid inclusion	573
Neural tube defects	Female	0-6 days	65-69 years	Data Rich	-1	1	Folic acid unadjusted (ug)	848
Neural tube defects	Female	0-6 days	65-69 years	Data Rich	-1	2	Antenatal Care (1 visit) Coverage (proportion)	10
Neural tube defects	Female	0-6 days	65-69 years	Data Rich	-1	2	Healthcare access and quality index	15
Neural tube defects	Female	0-6 days	65-69 years	Data Rich	-1	2	Antenatal Care (4 visits) Coverage (proportion)	18
Neural tube defects	Female	0-6 days	65-69 years	Data Rich	-1	2	vegetables unadjusted(g)	366
Neural tube defects	Female	0-6 days	65-69 years	Data Rich	-1	2	Legality of Abortion	389
Neural tube defects	Female	0-6 days	65-69 years	Data Rich	1	2	Smoking Prevalence (Reproductive Age Standardized)	215
Neural tube defects	Female	0-6 days	65-69 years	Data Rich	-1	3	Maternal Education (years per capita)	80
Neural tube defects	Female	0-6 days	65-69 years	Data Rich	0	3	fruits unadjusted(g)	263
Neural tube defects	Female	0-6 days	65-69 years	Data Rich	1	3	Indoor Air Pollution (All Cooking Fuels)	0
Neural tube defects	Female	0-6 days	65-69 years	Data Rich	1	3	Diabetes Age-Standardized Prevalence (proportion)	77
Neural tube defects	Female	0-6 days	65-69 years	Data Rich	1	3	Maternal alcohol consumption during pregnancy (proportion)	--
Neural tube defects	Female	0-6 days	65-69 years	Global	-1	1	Socio-demographic Index	250
Neural tube defects	Female	0-6 days	65-69 years	Global	-1	1	In-Facility Delivery (proportion)	287
Neural tube defects	Female	0-6 days	65-69 years	Global	-1	1	Folic acid unadjusted (ug)	479
Neural tube defects	Female	0-6 days	65-69 years	Global	-1	1	Composite fortification standard and folic acid inclusion	712
Neural tube defects	Female	0-6 days	65-69 years	Global	-1	2	Antenatal Care (1 visit) Coverage (proportion)	0
Neural tube defects	Female	0-6 days	65-69 years	Global	-1	2	Antenatal Care (4 visits) Coverage (proportion)	0
Neural tube defects	Female	0-6 days	65-69 years	Global	-1	2	Healthcare access and quality index	13
Neural tube defects	Female	0-6 days	65-69 years	Global	-1	2	Legality of Abortion	275
Neural tube defects	Female	0-6 days	65-69 years	Global	-1	2	vegetables unadjusted(g)	324
Neural tube defects	Female	0-6 days	65-69 years	Global	1	2	Smoking Prevalence (Reproductive Age Standardized)	764
Neural tube defects	Female	0-6 days	65-69 years	Global	-1	3	Maternal Education (years per capita)	12
Neural tube defects	Female	0-6 days	65-69 years	Global	0	3	fruits unadjusted(g)	115

Table S16. CODEm covariates used, level of covariate, and expected direction of covariate by cause, sex, and age
Covariates that CODEm did not select during the covariate selection process have no draw counts listed.

Cause	Sex	Age Start	Age End	Model Type	Direction	Level	Covariate Name	Number of Draws
Neural tube defects	Female	0-6 days	65-69 years	Global	1	3	Indoor Air Pollution (All Cooking Fuels)	11
Neural tube defects	Female	0-6 days	65-69 years	Global	1	3	Diabetes Age-Standardized Prevalence (proportion)	138
Neural tube defects	Female	0-6 days	65-69 years	Global	1	3	Maternal alcohol consumption during pregnancy (proportion)	--
Neural tube defects	Male	0-6 days	65-69 years	Data Rich	-1	1	In-Facility Delivery (proportion)	0
Neural tube defects	Male	0-6 days	65-69 years	Data Rich	-1	1	Socio-demographic Index	193
Neural tube defects	Male	0-6 days	65-69 years	Data Rich	-1	1	Composite fortification standard and folic acid inclusion	587
Neural tube defects	Male	0-6 days	65-69 years	Data Rich	-1	1	Folic acid unadjusted (ug)	687
Neural tube defects	Male	0-6 days	65-69 years	Data Rich	-1	2	Antenatal Care (1 visit) Coverage (proportion)	0
Neural tube defects	Male	0-6 days	65-69 years	Data Rich	-1	2	Healthcare access and quality index	23
Neural tube defects	Male	0-6 days	65-69 years	Data Rich	-1	2	Antenatal Care (4 visits) Coverage (proportion)	53
Neural tube defects	Male	0-6 days	65-69 years	Data Rich	-1	2	vegetables unadjusted(g)	65
Neural tube defects	Male	0-6 days	65-69 years	Data Rich	-1	2	Legality of Abortion	161
Neural tube defects	Male	0-6 days	65-69 years	Data Rich	1	2	Smoking Prevalence (Reproductive Age Standardized)	373
Neural tube defects	Male	0-6 days	65-69 years	Data Rich	-1	3	Maternal Education (years per capita)	165
Neural tube defects	Male	0-6 days	65-69 years	Data Rich	0	3	fruits unadjusted(g)	18
Neural tube defects	Male	0-6 days	65-69 years	Data Rich	1	3	Indoor Air Pollution (All Cooking Fuels)	0
Neural tube defects	Male	0-6 days	65-69 years	Data Rich	1	3	Maternal alcohol consumption during pregnancy (proportion)	41
Neural tube defects	Male	0-6 days	65-69 years	Data Rich	1	3	Diabetes Age-Standardized Prevalence (proportion)	82
Neural tube defects	Male	0-6 days	65-69 years	Global	-1	1	Folic acid unadjusted (ug)	18
Neural tube defects	Male	0-6 days	65-69 years	Global	-1	1	Composite fortification standard and folic acid inclusion	397
Neural tube defects	Male	0-6 days	65-69 years	Global	-1	1	In-Facility Delivery (proportion)	647
Neural tube defects	Male	0-6 days	65-69 years	Global	-1	1	Socio-demographic Index	797
Neural tube defects	Male	0-6 days	65-69 years	Global	-1	2	Antenatal Care (1 visit) Coverage (proportion)	4
Neural tube defects	Male	0-6 days	65-69 years	Global	-1	2	vegetables unadjusted(g)	23
Neural tube defects	Male	0-6 days	65-69 years	Global	-1	2	Antenatal Care (4 visits) Coverage (proportion)	43
Neural tube defects	Male	0-6 days	65-69 years	Global	-1	2	Healthcare access and quality index	157
Neural tube defects	Male	0-6 days	65-69 years	Global	-1	2	Legality of Abortion	187
Neural tube defects	Male	0-6 days	65-69 years	Global	1	2	Smoking Prevalence (Reproductive Age Standardized)	337
Neural tube defects	Male	0-6 days	65-69 years	Global	-1	3	Maternal Education (years per capita)	204
Neural tube defects	Male	0-6 days	65-69 years	Global	0	3	fruits unadjusted(g)	49
Neural tube defects	Male	0-6 days	65-69 years	Global	1	3	Diabetes Age-Standardized Prevalence (proportion)	10
Neural tube defects	Male	0-6 days	65-69 years	Global	1	3	Indoor Air Pollution (All Cooking Fuels)	491
Neural tube defects	Male	0-6 days	65-69 years	Global	1	3	Maternal alcohol consumption during pregnancy (proportion)	--
Congenital heart anomalies	Female	0-6 days	65-69 years	Data Rich	1	1	Birth prevalence of CHD	--
Congenital heart anomalies	Female	0-6 days	65-69 years	Data Rich	1	1	Maternal alcohol consumption during pregnancy (proportion)	--
Congenital heart anomalies	Female	0-6 days	65-69 years	Data Rich	-1	2	Healthcare access and quality index	235
Congenital heart anomalies	Female	0-6 days	65-69 years	Data Rich	-1	2	Socio-demographic Index	235
Congenital heart anomalies	Female	0-6 days	65-69 years	Data Rich	-1	2	Antenatal Care (1 visit) Coverage (proportion)	--
Congenital heart anomalies	Female	0-6 days	65-69 years	Data Rich	-1	2	In-Facility Delivery (proportion)	--
Congenital heart anomalies	Female	0-6 days	65-69 years	Data Rich	-1	2	Legality of Abortion	--

Table S16. CODEm covariates used, level of covariate, and expected direction of covariate by cause, sex, and age
Covariates that CODEm did not select during the covariate selection process have no draw counts listed.

Cause	Sex	Age Start	Age End	Model Type	Direction	Level	Covariate Name	Number of Draws
Congenital heart anomalies	Female	0-6 days	65-69 years	Data Rich	1	2	Smoking Prevalence (Reproductive Age Standardized)	367
Congenital heart anomalies	Female	0-6 days	65-69 years	Data Rich	1	2	Diabetes Age-Standardized Prevalence (proportion)	--
Congenital heart anomalies	Female	0-6 days	65-69 years	Data Rich	-1	3	Antenatal Care (4 visits) Coverage (proportion)	76
Congenital heart anomalies	Female	0-6 days	65-69 years	Data Rich	-1	3	Maternal Education (years per capita)	101
Congenital heart anomalies	Female	0-6 days	65-69 years	Data Rich	-1	3	Skilled Birth Attendance (proportion)	--
Congenital heart anomalies	Female	0-6 days	65-69 years	Data Rich	1	3	Alcohol (liters per capita)	516
Congenital heart anomalies	Female	0-6 days	65-69 years	Data Rich	1	3	Live Births 35+ (proportion)	--
Congenital heart anomalies	Female	0-6 days	65-69 years	Global	1	1	Birth prevalence of CHD	--
Congenital heart anomalies	Female	0-6 days	65-69 years	Global	1	1	Maternal alcohol consumption during pregnancy (proportion)	--
Congenital heart anomalies	Female	0-6 days	65-69 years	Global	-1	2	Antenatal Care (1 visit) Coverage (proportion)	25
Congenital heart anomalies	Female	0-6 days	65-69 years	Global	-1	2	In-Facility Delivery (proportion)	38
Congenital heart anomalies	Female	0-6 days	65-69 years	Global	-1	2	Healthcare access and quality index	168
Congenital heart anomalies	Female	0-6 days	65-69 years	Global	-1	2	Socio-demographic Index	168
Congenital heart anomalies	Female	0-6 days	65-69 years	Global	-1	2	Legality of Abortion	--
Congenital heart anomalies	Female	0-6 days	65-69 years	Global	1	2	Diabetes Age-Standardized Prevalence (proportion)	435
Congenital heart anomalies	Female	0-6 days	65-69 years	Global	1	2	Smoking Prevalence (Reproductive Age Standardized)	603
Congenital heart anomalies	Female	0-6 days	65-69 years	Global	-1	3	Antenatal Care (4 visits) Coverage (proportion)	32
Congenital heart anomalies	Female	0-6 days	65-69 years	Global	-1	3	Skilled Birth Attendance (proportion)	33
Congenital heart anomalies	Female	0-6 days	65-69 years	Global	-1	3	Maternal Education (years per capita)	101
Congenital heart anomalies	Female	0-6 days	65-69 years	Global	1	3	Alcohol (liters per capita)	494
Congenital heart anomalies	Female	0-6 days	65-69 years	Global	1	3	Live Births 35+ (proportion)	--
Congenital heart anomalies	Male	0-6 days	65-69 years	Data Rich	1	1	Birth prevalence of CHD	--
Congenital heart anomalies	Male	0-6 days	65-69 years	Data Rich	1	1	Maternal alcohol consumption during pregnancy (proportion)	--
Congenital heart anomalies	Male	0-6 days	65-69 years	Data Rich	-1	2	Healthcare access and quality index	329
Congenital heart anomalies	Male	0-6 days	65-69 years	Data Rich	-1	2	Socio-demographic Index	372
Congenital heart anomalies	Male	0-6 days	65-69 years	Data Rich	-1	2	Antenatal Care (1 visit) Coverage (proportion)	--
Congenital heart anomalies	Male	0-6 days	65-69 years	Data Rich	-1	2	In-Facility Delivery (proportion)	--
Congenital heart anomalies	Male	0-6 days	65-69 years	Data Rich	-1	2	Legality of Abortion	--
Congenital heart anomalies	Male	0-6 days	65-69 years	Data Rich	1	2	Smoking Prevalence (Reproductive Age Standardized)	782
Congenital heart anomalies	Male	0-6 days	65-69 years	Data Rich	1	2	Diabetes Age-Standardized Prevalence (proportion)	--
Congenital heart anomalies	Male	0-6 days	65-69 years	Data Rich	-1	3	Antenatal Care (4 visits) Coverage (proportion)	60
Congenital heart anomalies	Male	0-6 days	65-69 years	Data Rich	-1	3	Maternal Education (years per capita)	333
Congenital heart anomalies	Male	0-6 days	65-69 years	Data Rich	-1	3	Skilled Birth Attendance (proportion)	--
Congenital heart anomalies	Male	0-6 days	65-69 years	Data Rich	1	3	Alcohol (liters per capita)	--
Congenital heart anomalies	Male	0-6 days	65-69 years	Data Rich	1	3	Live Births 35+ (proportion)	--
Congenital heart anomalies	Male	0-6 days	65-69 years	Global	1	1	Birth prevalence of CHD	--
Congenital heart anomalies	Male	0-6 days	65-69 years	Global	1	1	Maternal alcohol consumption during pregnancy (proportion)	--
Congenital heart anomalies	Male	0-6 days	65-69 years	Global	-1	2	Healthcare access and quality index	237
Congenital heart anomalies	Male	0-6 days	65-69 years	Global	-1	2	Socio-demographic Index	237
Congenital heart anomalies	Male	0-6 days	65-69 years	Global	-1	2	Antenatal Care (1 visit) Coverage (proportion)	--

Table S16. CODEm covariates used, level of covariate, and expected direction of covariate by cause, sex, and age<br>*Covariates that CODEm did not select during the covariate selection process have no draw counts listed.*								
**Cause**	**Sex**	**Age Start**	**Age End**	**Model Type**	**Direction**	**Level**	**Covariate Name**	**Number of Draws**
Congenital heart anomalies	Male	0-6 days	65-69 years	Global	-1	2	In-Facility Delivery (proportion)	--
Congenital heart anomalies	Male	0-6 days	65-69 years	Global	-1	2	Legality of Abortion	--
Congenital heart anomalies	Male	0-6 days	65-69 years	Global	1	2	Diabetes Age-Standardized Prevalence (proportion)	402
Congenital heart anomalies	Male	0-6 days	65-69 years	Global	1	2	Smoking Prevalence (Reproductive Age Standardized)	639
Congenital heart anomalies	Male	0-6 days	65-69 years	Global	-1	3	Antenatal Care (4 visits) Coverage (proportion)	164
Congenital heart anomalies	Male	0-6 days	65-69 years	Global	-1	3	Maternal Education (years per capita)	197
Congenital heart anomalies	Male	0-6 days	65-69 years	Global	-1	3	Skilled Birth Attendance (proportion)	--
Congenital heart anomalies	Male	0-6 days	65-69 years	Global	1	3	Alcohol (liters per capita)	--
Congenital heart anomalies	Male	0-6 days	65-69 years	Global	1	3	Live Births 35+ (proportion)	--
Orofacial clefts	Female	0-6 days	1-4 years	Data Rich	-1	1	Socio-demographic Index	574
Orofacial clefts	Female	0-6 days	1-4 years	Data Rich	-1	1	Composite fortification standard and folic acid inclusion	766
Orofacial clefts	Female	0-6 days	1-4 years	Data Rich	1	1	Indoor Air Pollution (All Cooking Fuels)	15
Orofacial clefts	Female	0-6 days	1-4 years	Data Rich	-1	2	Skilled Birth Attendance (proportion)	0
Orofacial clefts	Female	0-6 days	1-4 years	Data Rich	-1	2	Healthcare access and quality index	597
Orofacial clefts	Female	0-6 days	1-4 years	Data Rich	-1	2	Legality of Abortion	--
Orofacial clefts	Female	0-6 days	1-4 years	Data Rich	1	2	Smoking Prevalence (Reproductive Age Standardized)	619
Orofacial clefts	Female	0-6 days	1-4 years	Data Rich	1	2	Diabetes Age-Standardized Prevalence (proportion)	--
Orofacial clefts	Female	0-6 days	1-4 years	Data Rich	1	2	Maternal alcohol consumption during pregnancy (proportion)	--
Orofacial clefts	Female	0-6 days	1-4 years	Data Rich	-1	3	Antenatal Care (4 visits) Coverage (proportion)	10
Orofacial clefts	Female	0-6 days	1-4 years	Data Rich	-1	3	Maternal Education (years per capita)	450
Orofacial clefts	Female	0-6 days	1-4 years	Data Rich	-1	3	Antenatal Care (1 visit) Coverage (proportion)	--
Orofacial clefts	Female	0-6 days	1-4 years	Data Rich	0	3	vegetables unadjusted(g)	386
Orofacial clefts	Female	0-6 days	1-4 years	Data Rich	0	3	fruits unadjusted(g)	583
Orofacial clefts	Female	0-6 days	1-4 years	Data Rich	1	3	Alcohol (liters per capita)	--
Orofacial clefts	Female	0-6 days	1-4 years	Global	-1	1	Socio-demographic Index	533
Orofacial clefts	Female	0-6 days	1-4 years	Global	-1	1	Composite fortification standard and folic acid inclusion	818
Orofacial clefts	Female	0-6 days	1-4 years	Global	-1	2	Skilled Birth Attendance (proportion)	2
Orofacial clefts	Female	0-6 days	1-4 years	Global	-1	2	Healthcare access and quality index	569
Orofacial clefts	Female	0-6 days	1-4 years	Global	-1	2	Legality of Abortion	--
Orofacial clefts	Female	0-6 days	1-4 years	Global	1	2	Smoking Prevalence (Reproductive Age Standardized)	569
Orofacial clefts	Female	0-6 days	1-4 years	Global	1	2	Diabetes Age-Standardized Prevalence (proportion)	--
Orofacial clefts	Female	0-6 days	1-4 years	Global	1	2	Maternal alcohol consumption during pregnancy (proportion)	--
Orofacial clefts	Female	0-6 days	1-4 years	Global	-1	3	Antenatal Care (4 visits) Coverage (proportion)	2
Orofacial clefts	Female	0-6 days	1-4 years	Global	-1	3	Maternal Education (years per capita)	498
Orofacial clefts	Female	0-6 days	1-4 years	Global	-1	3	Antenatal Care (1 visit) Coverage (proportion)	--
Orofacial clefts	Female	0-6 days	1-4 years	Global	0	3	fruits unadjusted(g)	543
Orofacial clefts	Female	0-6 days	1-4 years	Global	0	3	vegetables unadjusted(g)	663
Orofacial clefts	Female	0-6 days	1-4 years	Global	1	3	Indoor Air Pollution (All Cooking Fuels)	2
Orofacial clefts	Female	0-6 days	1-4 years	Global	1	3	Alcohol (liters per capita)	--
Orofacial clefts	Male	0-6 days	1-4 years	Data Rich	-1	1	Composite fortification standard and folic acid inclusion	313
Orofacial clefts	Male	0-6 days	1-4 years	Data Rich	-1	1	Socio-demographic Index	535
Orofacial clefts	Male	0-6 days	1-4 years	Data Rich	-1	1	Folic acid unadjusted (ug)	950
Orofacial clefts	Male	0-6 days	1-4 years	Data Rich	-1	2	Legality of Abortion	0
Orofacial clefts	Male	0-6 days	1-4 years	Data Rich	-1	2	Skilled Birth Attendance (proportion)	0
Orofacial clefts	Male	0-6 days	1-4 years	Data Rich	-1	2	Healthcare access and quality index	79

Table S16. CODEm covariates used, level of covariate, and expected direction of covariate by cause, sex, and age
Covariates that CODEm did not select during the covariate selection process have no draw counts listed.

Cause	Sex	Age Start	Age End	Model Type	Direction	Level	Covariate Name	Number of Draws
Orofacial clefts	Male	0-6 days	1-4 years	Data Rich	1	2	Diabetes Age-Standardized Prevalence (proportion)	0
Orofacial clefts	Male	0-6 days	1-4 years	Data Rich	1	2	Smoking Prevalence (Reproductive Age Standardized)	215
Orofacial clefts	Male	0-6 days	1-4 years	Data Rich	1	2	Maternal alcohol consumption during pregnancy (proportion)	--
Orofacial clefts	Male	0-6 days	1-4 years	Data Rich	-1	3	Antenatal Care (1 visit) Coverage (proportion)	0
Orofacial clefts	Male	0-6 days	1-4 years	Data Rich	-1	3	Antenatal Care (4 visits) Coverage (proportion)	2
Orofacial clefts	Male	0-6 days	1-4 years	Data Rich	-1	3	Maternal Education (years per capita)	375
Orofacial clefts	Male	0-6 days	1-4 years	Data Rich	0	3	fruits unadjusted(g)	391
Orofacial clefts	Male	0-6 days	1-4 years	Data Rich	0	3	vegetables unadjusted(g)	627
Orofacial clefts	Male	0-6 days	1-4 years	Data Rich	1	3	Indoor Air Pollution (All Cooking Fuels)	2
Orofacial clefts	Male	0-6 days	1-4 years	Data Rich	1	3	Alcohol (liters per capita)	--
Orofacial clefts	Male	0-6 days	1-4 years	Global	-1	1	Composite fortification standard and folic acid inclusion	495
Orofacial clefts	Male	0-6 days	1-4 years	Global	-1	1	Socio-demographic Index	521
Orofacial clefts	Male	0-6 days	1-4 years	Global	-1	1	Folic acid unadjusted (ug)	604
Orofacial clefts	Male	0-6 days	1-4 years	Global	-1	2	Skilled Birth Attendance (proportion)	0
Orofacial clefts	Male	0-6 days	1-4 years	Global	-1	2	Healthcare access and quality index	511
Orofacial clefts	Male	0-6 days	1-4 years	Global	-1	2	Legality of Abortion	--
Orofacial clefts	Male	0-6 days	1-4 years	Global	1	2	Diabetes Age-Standardized Prevalence (proportion)	223
Orofacial clefts	Male	0-6 days	1-4 years	Global	1	2	Smoking Prevalence (Reproductive Age Standardized)	556
Orofacial clefts	Male	0-6 days	1-4 years	Global	1	2	Maternal alcohol consumption during pregnancy (proportion)	--
Orofacial clefts	Male	0-6 days	1-4 years	Global	-1	3	Antenatal Care (1 visit) Coverage (proportion)	0
Orofacial clefts	Male	0-6 days	1-4 years	Global	-1	3	Antenatal Care (4 visits) Coverage (proportion)	4
Orofacial clefts	Male	0-6 days	1-4 years	Global	-1	3	Maternal Education (years per capita)	64
Orofacial clefts	Male	0-6 days	1-4 years	Global	0	3	fruits unadjusted(g)	631
Orofacial clefts	Male	0-6 days	1-4 years	Global	0	3	vegetables unadjusted(g)	757
Orofacial clefts	Male	0-6 days	1-4 years	Global	1	3	Indoor Air Pollution (All Cooking Fuels)	0
Orofacial clefts	Male	0-6 days	1-4 years	Global	1	3	Alcohol (liters per capita)	--
Down syndrome	Female	0-6 days	65-69 years	Data Rich	-1	1	Legality of Abortion	--
Down syndrome	Female	0-6 days	65-69 years	Data Rich	1	1	Live Births 40+ (proportion)	269
Down syndrome	Female	0-6 days	65-69 years	Data Rich	1	1	Live Births 35+ (proportion)	752
Down syndrome	Female	0-6 days	65-69 years	Data Rich	1	1	Birth prevalence of congenital chromosomal anomalies	902
Down syndrome	Female	0-6 days	65-69 years	Data Rich	-1	2	Healthcare access and quality index	--
Down syndrome	Female	0-6 days	65-69 years	Data Rich	-1	2	In-Facility Delivery (proportion)	--
Down syndrome	Female	0-6 days	65-69 years	Data Rich	-1	2	Socio-demographic Index	--
Down syndrome	Female	0-6 days	65-69 years	Data Rich	-1	3	vegetables unadjusted(g)	249
Down syndrome	Female	0-6 days	65-69 years	Data Rich	-1	3	Antenatal Care (1 visit) Coverage (proportion)	--
Down syndrome	Female	0-6 days	65-69 years	Data Rich	-1	3	Antenatal Care (4 visits) Coverage (proportion)	--
Down syndrome	Female	0-6 days	65-69 years	Data Rich	-1	3	Maternal Education (years per capita)	--
Down syndrome	Female	0-6 days	65-69 years	Data Rich	1	3	Indoor Air Pollution (All Cooking Fuels)	--
Down syndrome	Female	0-6 days	65-69 years	Data Rich	1	3	Maternal alcohol consumption during pregnancy (proportion)	--
Down syndrome	Female	0-6 days	65-69 years	Data Rich	1	3	Smoking Prevalence (Reproductive Age Standardized)	--
Down syndrome	Female	0-6 days	65-69 years	Global	-1	1	Legality of Abortion	--
Down syndrome	Female	0-6 days	65-69 years	Global	1	1	Live Births 40+ (proportion)	332
Down syndrome	Female	0-6 days	65-69 years	Global	1	1	Live Births 35+ (proportion)	758

Table S16. CODEm covariates used, level of covariate, and expected direction of covariate by cause, sex, and age
Covariates that CODEm did not select during the covariate selection process have no draw counts listed.

Cause	Sex	Age Start	Age End	Model Type	Direction	Level	Covariate Name	Number of Draws
Down syndrome	Female	0-6 days	65-69 years	Global	1	1	Birth prevalence of congenital chromosomal anomalies	778
Down syndrome	Female	0-6 days	65-69 years	Global	-1	2	Healthcare access and quality index	--
Down syndrome	Female	0-6 days	65-69 years	Global	-1	2	In-Facility Delivery (proportion)	--
Down syndrome	Female	0-6 days	65-69 years	Global	-1	2	Socio-demographic Index	--
Down syndrome	Female	0-6 days	65-69 years	Global	-1	3	vegetables unadjusted(g)	227
Down syndrome	Female	0-6 days	65-69 years	Global	-1	3	Antenatal Care (1 visit) Coverage (proportion)	--
Down syndrome	Female	0-6 days	65-69 years	Global	-1	3	Antenatal Care (4 visits) Coverage (proportion)	--
Down syndrome	Female	0-6 days	65-69 years	Global	-1	3	Maternal Education (years per capita)	--
Down syndrome	Female	0-6 days	65-69 years	Global	1	3	Indoor Air Pollution (All Cooking Fuels)	--
Down syndrome	Female	0-6 days	65-69 years	Global	1	3	Maternal alcohol consumption during pregnancy (proportion)	--
Down syndrome	Female	0-6 days	65-69 years	Global	1	3	Smoking Prevalence (Reproductive Age Standardized)	--
Down syndrome	Male	0-6 days	65-69 years	Data Rich	-1	1	Legality of Abortion	--
Down syndrome	Male	0-6 days	65-69 years	Data Rich	1	1	Live Births 40+ (proportion)	432
Down syndrome	Male	0-6 days	65-69 years	Data Rich	1	1	Birth prevalence of congenital chromosomal anomalies	756
Down syndrome	Male	0-6 days	65-69 years	Data Rich	1	1	Live Births 35+ (proportion)	793
Down syndrome	Male	0-6 days	65-69 years	Data Rich	-1	2	Healthcare access and quality index	--
Down syndrome	Male	0-6 days	65-69 years	Data Rich	-1	2	In-Facility Delivery (proportion)	--
Down syndrome	Male	0-6 days	65-69 years	Data Rich	-1	2	Socio-demographic Index	--
Down syndrome	Male	0-6 days	65-69 years	Data Rich	-1	3	vegetables unadjusted(g)	292
Down syndrome	Male	0-6 days	65-69 years	Data Rich	-1	3	Antenatal Care (1 visit) Coverage (proportion)	--
Down syndrome	Male	0-6 days	65-69 years	Data Rich	-1	3	Antenatal Care (4 visits) Coverage (proportion)	--
Down syndrome	Male	0-6 days	65-69 years	Data Rich	-1	3	Maternal Education (years per capita)	--
Down syndrome	Male	0-6 days	65-69 years	Data Rich	1	3	Indoor Air Pollution (All Cooking Fuels)	--
Down syndrome	Male	0-6 days	65-69 years	Data Rich	1	3	Maternal alcohol consumption during pregnancy (proportion)	--
Down syndrome	Male	0-6 days	65-69 years	Data Rich	1	3	Smoking Prevalence (Reproductive Age Standardized)	--
Down syndrome	Male	0-6 days	65-69 years	Global	-1	1	Legality of Abortion	--
Down syndrome	Male	0-6 days	65-69 years	Global	1	1	Live Births 40+ (proportion)	216
Down syndrome	Male	0-6 days	65-69 years	Global	1	1	Birth prevalence of congenital chromosomal anomalies	574
Down syndrome	Male	0-6 days	65-69 years	Global	1	1	Live Births 35+ (proportion)	863
Down syndrome	Male	0-6 days	65-69 years	Global	-1	2	Healthcare access and quality index	--
Down syndrome	Male	0-6 days	65-69 years	Global	-1	2	In-Facility Delivery (proportion)	--
Down syndrome	Male	0-6 days	65-69 years	Global	-1	2	Socio-demographic Index	--
Down syndrome	Male	0-6 days	65-69 years	Global	-1	3	vegetables unadjusted(g)	270
Down syndrome	Male	0-6 days	65-69 years	Global	-1	3	Antenatal Care (1 visit) Coverage (proportion)	--
Down syndrome	Male	0-6 days	65-69 years	Global	-1	3	Antenatal Care (4 visits) Coverage (proportion)	--
Down syndrome	Male	0-6 days	65-69 years	Global	-1	3	Maternal Education (years per capita)	--
Down syndrome	Male	0-6 days	65-69 years	Global	1	3	Indoor Air Pollution (All Cooking Fuels)	--
Down syndrome	Male	0-6 days	65-69 years	Global	1	3	Maternal alcohol consumption during pregnancy (proportion)	--
Down syndrome	Male	0-6 days	65-69 years	Global	1	3	Smoking Prevalence (Reproductive Age Standardized)	--
Other chromosomal abnormalities	Female	0-6 days	65-69 years	Data Rich	-1	1	Legality of Abortion	--

Table S16. CODEm covariates used, level of covariate, and expected direction of covariate by cause, sex, and age
Covariates that CODEm did not select during the covariate selection process have no draw counts listed.

Cause	Sex	Age Start	Age End	Model Type	Direction	Level	Covariate Name	Number of Draws
Other chromosomal abnormalities	Female	0-6 days	65-69 years	Data Rich	1	1	Live Births 35+ (proportion)	1000
Other chromosomal abnormalities	Female	0-6 days	65-69 years	Data Rich	1	1	Live Births 40+ (proportion)	--
Other chromosomal abnormalities	Female	0-6 days	65-69 years	Data Rich	-1	2	Antenatal Care (1 visit) Coverage (proportion)	--
Other chromosomal abnormalities	Female	0-6 days	65-69 years	Data Rich	-1	2	Antenatal Care (4 visits) Coverage (proportion)	--
Other chromosomal abnormalities	Female	0-6 days	65-69 years	Data Rich	-1	2	Healthcare access and quality index	--
Other chromosomal abnormalities	Female	0-6 days	65-69 years	Data Rich	-1	2	In-Facility Delivery (proportion)	--
Other chromosomal abnormalities	Female	0-6 days	65-69 years	Data Rich	-1	2	LDI (I$ per capita)	--
Other chromosomal abnormalities	Female	0-6 days	65-69 years	Data Rich	1	2	Maternal alcohol consumption during pregnancy (proportion)	--
Other chromosomal abnormalities	Female	0-6 days	65-69 years	Data Rich	-1	3	Maternal Education (years per capita)	--
Other chromosomal abnormalities	Female	0-6 days	65-69 years	Data Rich	-1	3	Skilled Birth Attendance (proportion)	--
Other chromosomal abnormalities	Female	0-6 days	65-69 years	Data Rich	0	3	Socio-demographic Index	--
Other chromosomal abnormalities	Female	0-6 days	65-69 years	Data Rich	1	3	Alcohol (liters per capita)	481
Other chromosomal abnormalities	Female	0-6 days	65-69 years	Data Rich	1	3	Indoor Air Pollution (All Cooking Fuels)	--
Other chromosomal abnormalities	Female	0-6 days	65-69 years	Data Rich	1	3	Smoking Prevalence (Reproductive Age Standardized)	--
Other chromosomal abnormalities	Female	0-6 days	65-69 years	Global	-1	1	Legality of Abortion	--
Other chromosomal abnormalities	Female	0-6 days	65-69 years	Global	1	1	Live Births 35+ (proportion)	1000
Other chromosomal abnormalities	Female	0-6 days	65-69 years	Global	1	1	Live Births 40+ (proportion)	--
Other chromosomal abnormalities	Female	0-6 days	65-69 years	Global	-1	2	Antenatal Care (1 visit) Coverage (proportion)	--
Other chromosomal abnormalities	Female	0-6 days	65-69 years	Global	-1	2	Antenatal Care (4 visits) Coverage (proportion)	--
Other chromosomal abnormalities	Female	0-6 days	65-69 years	Global	-1	2	Healthcare access and quality index	--
Other chromosomal abnormalities	Female	0-6 days	65-69 years	Global	-1	2	In-Facility Delivery (proportion)	--
Other chromosomal abnormalities	Female	0-6 days	65-69 years	Global	-1	2	LDI (I$ per capita)	--
Other chromosomal abnormalities	Female	0-6 days	65-69 years	Global	1	2	Maternal alcohol consumption during pregnancy (proportion)	--
Other chromosomal abnormalities	Female	0-6 days	65-69 years	Global	-1	3	Maternal Education (years per capita)	--
Other chromosomal abnormalities	Female	0-6 days	65-69 years	Global	-1	3	Skilled Birth Attendance (proportion)	--
Other chromosomal abnormalities	Female	0-6 days	65-69 years	Global	0	3	Socio-demographic Index	326
Other chromosomal abnormalities	Female	0-6 days	65-69 years	Global	1	3	Alcohol (liters per capita)	593
Other chromosomal abnormalities	Female	0-6 days	65-69 years	Global	1	3	Indoor Air Pollution (All Cooking Fuels)	--
Other chromosomal abnormalities	Female	0-6 days	65-69 years	Global	1	3	Smoking Prevalence (Reproductive Age Standardized)	--
Other chromosomal abnormalities	Male	0-6 days	65-69 years	Data Rich	-1	1	Legality of Abortion	707
Other chromosomal abnormalities	Male	0-6 days	65-69 years	Data Rich	1	1	Live Births 35+ (proportion)	293
Other chromosomal abnormalities	Male	0-6 days	65-69 years	Data Rich	1	1	Live Births 40+ (proportion)	--
Other chromosomal abnormalities	Male	0-6 days	65-69 years	Data Rich	-1	2	Antenatal Care (1 visit) Coverage (proportion)	--
Other chromosomal abnormalities	Male	0-6 days	65-69 years	Data Rich	-1	2	Antenatal Care (4 visits) Coverage (proportion)	--
Other chromosomal abnormalities	Male	0-6 days	65-69 years	Data Rich	-1	2	Healthcare access and quality index	--
Other chromosomal abnormalities	Male	0-6 days	65-69 years	Data Rich	-1	2	In-Facility Delivery (proportion)	--
Other chromosomal abnormalities	Male	0-6 days	65-69 years	Data Rich	-1	2	LDI (I$ per capita)	--
Other chromosomal abnormalities	Male	0-6 days	65-69 years	Data Rich	1	2	Maternal alcohol consumption during pregnancy (proportion)	--
Other chromosomal abnormalities	Male	0-6 days	65-69 years	Data Rich	-1	3	Maternal Education (years per capita)	--
Other chromosomal abnormalities	Male	0-6 days	65-69 years	Data Rich	-1	3	Skilled Birth Attendance (proportion)	--

Table S16. CODEm covariates used, level of covariate, and expected direction of covariate by cause, sex, and age
Covariates that CODEm did not select during the covariate selection process have no draw counts listed.

Cause	Sex	Age Start	Age End	Model Type	Direction	Level	Covariate Name	Number of Draws
Other chromosomal abnormalities	Male	0-6 days	65-69 years	Data Rich	0	3	Socio-demographic Index	486
Other chromosomal abnormalities	Male	0-6 days	65-69 years	Data Rich	1	3	Alcohol (liters per capita)	--
Other chromosomal abnormalities	Male	0-6 days	65-69 years	Data Rich	1	3	Indoor Air Pollution (All Cooking Fuels)	--
Other chromosomal abnormalities	Male	0-6 days	65-69 years	Data Rich	1	3	Smoking Prevalence (Reproductive Age Standardized)	--
Other chromosomal abnormalities	Male	0-6 days	65-69 years	Global	-1	1	Legality of Abortion	328
Other chromosomal abnormalities	Male	0-6 days	65-69 years	Global	1	1	Live Births 35+ (proportion)	672
Other chromosomal abnormalities	Male	0-6 days	65-69 years	Global	1	1	Live Births 40+ (proportion)	--
Other chromosomal abnormalities	Male	0-6 days	65-69 years	Global	-1	2	Antenatal Care (1 visit) Coverage (proportion)	--
Other chromosomal abnormalities	Male	0-6 days	65-69 years	Global	-1	2	Antenatal Care (4 visits) Coverage (proportion)	--
Other chromosomal abnormalities	Male	0-6 days	65-69 years	Global	-1	2	Healthcare access and quality index	--
Other chromosomal abnormalities	Male	0-6 days	65-69 years	Global	-1	2	In-Facility Delivery (proportion)	--
Other chromosomal abnormalities	Male	0-6 days	65-69 years	Global	-1	2	LDI (I$ per capita)	--
Other chromosomal abnormalities	Male	0-6 days	65-69 years	Global	1	2	Maternal alcohol consumption during pregnancy (proportion)	--
Other chromosomal abnormalities	Male	0-6 days	65-69 years	Global	-1	3	Maternal Education (years per capita)	--
Other chromosomal abnormalities	Male	0-6 days	65-69 years	Global	-1	3	Skilled Birth Attendance (proportion)	--
Other chromosomal abnormalities	Male	0-6 days	65-69 years	Global	0	3	Socio-demographic Index	636
Other chromosomal abnormalities	Male	0-6 days	65-69 years	Global	1	3	Alcohol (liters per capita)	--
Other chromosomal abnormalities	Male	0-6 days	65-69 years	Global	1	3	Indoor Air Pollution (All Cooking Fuels)	--
Other chromosomal abnormalities	Male	0-6 days	65-69 years	Global	1	3	Smoking Prevalence (Reproductive Age Standardized)	--
Congenital musculoskeletal and limb anomalies	Female	0-6 days	65-69 years	Data Rich	-1	1	Legality of Abortion	674
Congenital musculoskeletal and limb anomalies	Female	0-6 days	65-69 years	Data Rich	1	1	Maternal alcohol consumption during pregnancy (proportion)	--
Congenital musculoskeletal and limb anomalies	Female	0-6 days	65-69 years	Data Rich	-1	2	Healthcare access and quality index	--
Congenital musculoskeletal and limb anomalies	Female	0-6 days	65-69 years	Data Rich	-1	2	In-Facility Delivery (proportion)	--
Congenital musculoskeletal and limb anomalies	Female	0-6 days	65-69 years	Data Rich	-1	2	Socio-demographic Index	--
Congenital musculoskeletal and limb anomalies	Female	0-6 days	65-69 years	Data Rich	1	2	Diabetes Age-Standardized Prevalence (proportion)	185
Congenital musculoskeletal and limb anomalies	Female	0-6 days	65-69 years	Data Rich	1	2	Indoor Air Pollution (All Cooking Fuels)	185
Congenital musculoskeletal and limb anomalies	Female	0-6 days	65-69 years	Data Rich	1	2	Smoking Prevalence (Reproductive Age Standardized)	--
Congenital musculoskeletal and limb anomalies	Female	0-6 days	65-69 years	Data Rich	-1	3	Antenatal Care (1 visit) Coverage (proportion)	--
Congenital musculoskeletal and limb anomalies	Female	0-6 days	65-69 years	Data Rich	-1	3	Antenatal Care (4 visits) Coverage (proportion)	--
Congenital musculoskeletal and limb anomalies	Female	0-6 days	65-69 years	Data Rich	-1	3	LDI (I$ per capita)	--
Congenital musculoskeletal and limb anomalies	Female	0-6 days	65-69 years	Data Rich	-1	3	Maternal Education (years per capita)	--
Congenital musculoskeletal and limb anomalies	Female	0-6 days	65-69 years	Data Rich	0	3	fruits unadjusted(g)	326
Congenital musculoskeletal and limb anomalies	Female	0-6 days	65-69 years	Data Rich	0	3	vegetables unadjusted(g)	--
Congenital musculoskeletal and limb anomalies	Female	0-6 days	65-69 years	Data Rich	1	3	Alcohol (liters per capita)	326
Congenital musculoskeletal and limb anomalies	Female	0-6 days	65-69 years	Global	-1	1	Legality of Abortion	254
Congenital musculoskeletal and limb anomalies	Female	0-6 days	65-69 years	Global	1	1	Maternal alcohol consumption during pregnancy (proportion)	--
Congenital musculoskeletal and limb anomalies	Female	0-6 days	65-69 years	Global	-1	2	Healthcare access and quality index	--
Congenital musculoskeletal and limb anomalies	Female	0-6 days	65-69 years	Global	-1	2	In-Facility Delivery (proportion)	--
Congenital musculoskeletal and limb anomalies	Female	0-6 days	65-69 years	Global	-1	2	Socio-demographic Index	--
Congenital musculoskeletal and limb anomalies	Female	0-6 days	65-69 years	Global	1	2	Diabetes Age-Standardized Prevalence (proportion)	541

Table S16. CODEm covariates used, level of covariate, and expected direction of covariate by cause, sex, and age
Covariates that CODEm did not select during the covariate selection process have no draw counts listed.

Cause	Sex	Age Start	Age End	Model Type	Direction	Level	Covariate Name	Number of Draws
Congenital musculoskeletal and limb anomalies	Female	0-6 days	65-69 years	Global	1	2	Indoor Air Pollution (All Cooking Fuels)	--
Congenital musculoskeletal and limb anomalies	Female	0-6 days	65-69 years	Global	1	2	Smoking Prevalence (Reproductive Age Standardized)	--
Congenital musculoskeletal and limb anomalies	Female	0-6 days	65-69 years	Global	-1	3	Antenatal Care (1 visit) Coverage (proportion)	--
Congenital musculoskeletal and limb anomalies	Female	0-6 days	65-69 years	Global	-1	3	Antenatal Care (4 visits) Coverage (proportion)	--
Congenital musculoskeletal and limb anomalies	Female	0-6 days	65-69 years	Global	-1	3	LDI (I$ per capita)	--
Congenital musculoskeletal and limb anomalies	Female	0-6 days	65-69 years	Global	-1	3	Maternal Education (years per capita)	--
Congenital musculoskeletal and limb anomalies	Female	0-6 days	65-69 years	Global	0	3	vegetables unadjusted(g)	197
Congenital musculoskeletal and limb anomalies	Female	0-6 days	65-69 years	Global	0	3	fruits unadjusted(g)	336
Congenital musculoskeletal and limb anomalies	Female	0-6 days	65-69 years	Global	1	3	Alcohol (liters per capita)	239
Congenital musculoskeletal and limb anomalies	Male	0-6 days	65-69 years	Data Rich	-1	1	Legality of Abortion	807
Congenital musculoskeletal and limb anomalies	Male	0-6 days	65-69 years	Data Rich	1	1	Maternal alcohol consumption during pregnancy (proportion)	--
Congenital musculoskeletal and limb anomalies	Male	0-6 days	65-69 years	Data Rich	-1	2	Healthcare access and quality index	44
Congenital musculoskeletal and limb anomalies	Male	0-6 days	65-69 years	Data Rich	-1	2	Socio-demographic Index	264
Congenital musculoskeletal and limb anomalies	Male	0-6 days	65-69 years	Data Rich	-1	2	In-Facility Delivery (proportion)	--
Congenital musculoskeletal and limb anomalies	Male	0-6 days	65-69 years	Data Rich	1	2	Indoor Air Pollution (All Cooking Fuels)	35
Congenital musculoskeletal and limb anomalies	Male	0-6 days	65-69 years	Data Rich	1	2	Smoking Prevalence (Reproductive Age Standardized)	79
Congenital musculoskeletal and limb anomalies	Male	0-6 days	65-69 years	Data Rich	1	2	Diabetes Age-Standardized Prevalence (proportion)	--
Congenital musculoskeletal and limb anomalies	Male	0-6 days	65-69 years	Data Rich	-1	3	Antenatal Care (1 visit) Coverage (proportion)	16
Congenital musculoskeletal and limb anomalies	Male	0-6 days	65-69 years	Data Rich	-1	3	Antenatal Care (4 visits) Coverage (proportion)	52
Congenital musculoskeletal and limb anomalies	Male	0-6 days	65-69 years	Data Rich	-1	3	Maternal Education (years per capita)	329
Congenital musculoskeletal and limb anomalies	Male	0-6 days	65-69 years	Data Rich	-1	3	LDI (I$ per capita)	--
Congenital musculoskeletal and limb anomalies	Male	0-6 days	65-69 years	Data Rich	0	3	fruits unadjusted(g)	240
Congenital musculoskeletal and limb anomalies	Male	0-6 days	65-69 years	Data Rich	0	3	vegetables unadjusted(g)	399
Congenital musculoskeletal and limb anomalies	Male	0-6 days	65-69 years	Data Rich	1	3	Alcohol (liters per capita)	--
Congenital musculoskeletal and limb anomalies	Male	0-6 days	65-69 years	Global	-1	1	Legality of Abortion	809
Congenital musculoskeletal and limb anomalies	Male	0-6 days	65-69 years	Global	1	1	Maternal alcohol consumption during pregnancy (proportion)	--
Congenital musculoskeletal and limb anomalies	Male	0-6 days	65-69 years	Global	-1	2	In-Facility Delivery (proportion)	58
Congenital musculoskeletal and limb anomalies	Male	0-6 days	65-69 years	Global	-1	2	Socio-demographic Index	143
Congenital musculoskeletal and limb anomalies	Male	0-6 days	65-69 years	Global	-1	2	Healthcare access and quality index	268
Congenital musculoskeletal and limb anomalies	Male	0-6 days	65-69 years	Global	1	2	Indoor Air Pollution (All Cooking Fuels)	140
Congenital musculoskeletal and limb anomalies	Male	0-6 days	65-69 years	Global	1	2	Diabetes Age-Standardized Prevalence (proportion)	191
Congenital musculoskeletal and limb anomalies	Male	0-6 days	65-69 years	Global	1	2	Smoking Prevalence (Reproductive Age Standardized)	350
Congenital musculoskeletal and limb anomalies	Male	0-6 days	65-69 years	Global	-1	3	Antenatal Care (4 visits) Coverage (proportion)	11
Congenital musculoskeletal and limb anomalies	Male	0-6 days	65-69 years	Global	-1	3	Antenatal Care (1 visit) Coverage (proportion)	84
Congenital musculoskeletal and limb anomalies	Male	0-6 days	65-69 years	Global	-1	3	Maternal Education (years per capita)	125
Congenital musculoskeletal and limb anomalies	Male	0-6 days	65-69 years	Global	-1	3	LDI (I$ per capita)	--
Congenital musculoskeletal and limb anomalies	Male	0-6 days	65-69 years	Global	0	3	fruits unadjusted(g)	86
Congenital musculoskeletal and limb anomalies	Male	0-6 days	65-69 years	Global	0	3	vegetables unadjusted(g)	482
Congenital musculoskeletal and limb anomalies	Male	0-6 days	65-69 years	Global	1	3	Alcohol (liters per capita)	314
Urogenital congenital anomalies	Female	0-6 days	65-69 years	Data Rich	1	1	Maternal alcohol consumption during pregnancy (proportion)	--

Table S16. CODEm covariates used, level of covariate, and expected direction of covariate by cause, sex, and age
Covariates that CODEm did not select during the covariate selection process have no draw counts listed.

Cause	Sex	Age Start	Age End	Model Type	Direction	Level	Covariate Name	Number of Draws
Urogenital congenital anomalies	Female	0-6 days	65-69 years	Data Rich	1	1	Smoking Prevalence (Reproductive Age Standardized)	--
Urogenital congenital anomalies	Female	0-6 days	65-69 years	Data Rich	-1	2	Healthcare access and quality index	153
Urogenital congenital anomalies	Female	0-6 days	65-69 years	Data Rich	-1	2	Socio-demographic Index	480
Urogenital congenital anomalies	Female	0-6 days	65-69 years	Data Rich	-1	2	In-Facility Delivery (proportion)	--
Urogenital congenital anomalies	Female	0-6 days	65-69 years	Data Rich	1	2	Diabetes Age-Standardized Prevalence (proportion)	807
Urogenital congenital anomalies	Female	0-6 days	65-69 years	Data Rich	1	2	Indoor Air Pollution (All Cooking Fuels)	--
Urogenital congenital anomalies	Female	0-6 days	65-69 years	Data Rich	1	2	Outdoor Air Pollution (PM2.5)	--
Urogenital congenital anomalies	Female	0-6 days	65-69 years	Data Rich	-1	3	Maternal Education (years per capita)	193
Urogenital congenital anomalies	Female	0-6 days	65-69 years	Data Rich	-1	3	Antenatal Care (4 visits) Coverage (proportion)	211
Urogenital congenital anomalies	Female	0-6 days	65-69 years	Data Rich	-1	3	Antenatal Care (1 visit) Coverage (proportion)	--
Urogenital congenital anomalies	Female	0-6 days	65-69 years	Data Rich	-1	3	LDI (I$ per capita)	--
Urogenital congenital anomalies	Female	0-6 days	65-69 years	Data Rich	1	3	Alcohol (liters per capita)	--
Urogenital congenital anomalies	Female	0-6 days	65-69 years	Global	1	1	Maternal alcohol consumption during pregnancy (proportion)	--
Urogenital congenital anomalies	Female	0-6 days	65-69 years	Global	1	1	Smoking Prevalence (Reproductive Age Standardized)	--
Urogenital congenital anomalies	Female	0-6 days	65-69 years	Global	-1	2	In-Facility Delivery (proportion)	171
Urogenital congenital anomalies	Female	0-6 days	65-69 years	Global	-1	2	Healthcare access and quality index	180
Urogenital congenital anomalies	Female	0-6 days	65-69 years	Global	-1	2	Socio-demographic Index	364
Urogenital congenital anomalies	Female	0-6 days	65-69 years	Global	1	2	Indoor Air Pollution (All Cooking Fuels)	535
Urogenital congenital anomalies	Female	0-6 days	65-69 years	Global	1	2	Diabetes Age-Standardized Prevalence (proportion)	940
Urogenital congenital anomalies	Female	0-6 days	65-69 years	Global	1	2	Outdoor Air Pollution (PM2.5)	--
Urogenital congenital anomalies	Female	0-6 days	65-69 years	Global	-1	3	Antenatal Care (4 visits) Coverage (proportion)	172
Urogenital congenital anomalies	Female	0-6 days	65-69 years	Global	-1	3	Maternal Education (years per capita)	195
Urogenital congenital anomalies	Female	0-6 days	65-69 years	Global	-1	3	Antenatal Care (1 visit) Coverage (proportion)	--
Urogenital congenital anomalies	Female	0-6 days	65-69 years	Global	-1	3	LDI (I$ per capita)	--
Urogenital congenital anomalies	Female	0-6 days	65-69 years	Global	1	3	Alcohol (liters per capita)	--
Urogenital congenital anomalies	Male	0-6 days	65-69 years	Data Rich	1	1	Maternal alcohol consumption during pregnancy (proportion)	--
Urogenital congenital anomalies	Male	0-6 days	65-69 years	Data Rich	1	1	Smoking Prevalence (Reproductive Age Standardized)	--
Urogenital congenital anomalies	Male	0-6 days	65-69 years	Data Rich	-1	2	Healthcare access and quality index	546
Urogenital congenital anomalies	Male	0-6 days	65-69 years	Data Rich	-1	2	In-Facility Delivery (proportion)	--
Urogenital congenital anomalies	Male	0-6 days	65-69 years	Data Rich	-1	2	Socio-demographic Index	--
Urogenital congenital anomalies	Male	0-6 days	65-69 years	Data Rich	1	2	Outdoor Air Pollution (PM2.5)	546
Urogenital congenital anomalies	Male	0-6 days	65-69 years	Data Rich	1	2	Diabetes Age-Standardized Prevalence (proportion)	1000
Urogenital congenital anomalies	Male	0-6 days	65-69 years	Data Rich	1	2	Indoor Air Pollution (All Cooking Fuels)	--
Urogenital congenital anomalies	Male	0-6 days	65-69 years	Data Rich	-1	3	Antenatal Care (1 visit) Coverage (proportion)	--
Urogenital congenital anomalies	Male	0-6 days	65-69 years	Data Rich	-1	3	Antenatal Care (4 visits) Coverage (proportion)	--
Urogenital congenital anomalies	Male	0-6 days	65-69 years	Data Rich	-1	3	LDI (I$ per capita)	--
Urogenital congenital anomalies	Male	0-6 days	65-69 years	Data Rich	-1	3	Maternal Education (years per capita)	--
Urogenital congenital anomalies	Male	0-6 days	65-69 years	Data Rich	1	3	Alcohol (liters per capita)	--
Urogenital congenital anomalies	Male	0-6 days	65-69 years	Global	1	1	Maternal alcohol consumption during pregnancy (proportion)	--
Urogenital congenital anomalies	Male	0-6 days	65-69 years	Global	1	1	Smoking Prevalence (Reproductive Age Standardized)	--

Table S16. CODEm covariates used, level of covariate, and expected direction of covariate by cause, sex, and age<br>*Covariates that CODEm did not select during the covariate selection process have no draw counts listed.*								
**Cause**	**Sex**	**Age Start**	**Age End**	**Model Type**	**Direction**	**Level**	**Covariate Name**	**Number of Draws**
Urogenital congenital anomalies	Male	0-6 days	65-69 years	Global	-1	2	Socio-demographic Index	131
Urogenital congenital anomalies	Male	0-6 days	65-69 years	Global	-1	2	Healthcare access and quality index	160
Urogenital congenital anomalies	Male	0-6 days	65-69 years	Global	-1	2	In-Facility Delivery (proportion)	385
Urogenital congenital anomalies	Male	0-6 days	65-69 years	Global	1	2	Outdoor Air Pollution (PM2.5)	214
Urogenital congenital anomalies	Male	0-6 days	65-69 years	Global	1	2	Indoor Air Pollution (All Cooking Fuels)	414
Urogenital congenital anomalies	Male	0-6 days	65-69 years	Global	1	2	Diabetes Age-Standardized Prevalence (proportion)	759
Urogenital congenital anomalies	Male	0-6 days	65-69 years	Global	-1	3	Maternal Education (years per capita)	49
Urogenital congenital anomalies	Male	0-6 days	65-69 years	Global	-1	3	Antenatal Care (4 visits) Coverage (proportion)	160
Urogenital congenital anomalies	Male	0-6 days	65-69 years	Global	-1	3	Antenatal Care (1 visit) Coverage (proportion)	--
Urogenital congenital anomalies	Male	0-6 days	65-69 years	Global	-1	3	LDI (I$ per capita)	--
Urogenital congenital anomalies	Male	0-6 days	65-69 years	Global	1	3	Alcohol (liters per capita)	--
Digestive congenital anomalies	Female	0-6 days	65-69 years	Data Rich	1	1	Smoking Prevalence (Reproductive Age Standardized)	532
Digestive congenital anomalies	Female	0-6 days	65-69 years	Data Rich	1	1	Maternal alcohol consumption during pregnancy (proportion)	--
Digestive congenital anomalies	Female	0-6 days	65-69 years	Data Rich	-1	2	Socio-demographic Index	114
Digestive congenital anomalies	Female	0-6 days	65-69 years	Data Rich	-1	2	Healthcare access and quality index	340
Digestive congenital anomalies	Female	0-6 days	65-69 years	Data Rich	-1	2	In-Facility Delivery (proportion)	--
Digestive congenital anomalies	Female	0-6 days	65-69 years	Data Rich	1	2	Diabetes Age-Standardized Prevalence (proportion)	187
Digestive congenital anomalies	Female	0-6 days	65-69 years	Data Rich	1	2	Prevalence of obesity (age-standardized)	768
Digestive congenital anomalies	Female	0-6 days	65-69 years	Data Rich	1	2	Indoor Air Pollution (All Cooking Fuels)	--
Digestive congenital anomalies	Female	0-6 days	65-69 years	Data Rich	-1	3	Maternal Education (years per capita)	20
Digestive congenital anomalies	Female	0-6 days	65-69 years	Data Rich	-1	3	Antenatal Care (1 visit) Coverage (proportion)	--
Digestive congenital anomalies	Female	0-6 days	65-69 years	Data Rich	-1	3	Antenatal Care (4 visits) Coverage (proportion)	--
Digestive congenital anomalies	Female	0-6 days	65-69 years	Data Rich	-1	3	LDI (I$ per capita)	--
Digestive congenital anomalies	Female	0-6 days	65-69 years	Data Rich	-1	3	Maternal care and immunization	--
Digestive congenital anomalies	Female	0-6 days	65-69 years	Data Rich	0	3	vegetables unadjusted(g)	285
Digestive congenital anomalies	Female	0-6 days	65-69 years	Data Rich	0	3	fruits unadjusted(g)	522
Digestive congenital anomalies	Female	0-6 days	65-69 years	Data Rich	1	3	Alcohol (liters per capita)	356
Digestive congenital anomalies	Female	0-6 days	65-69 years	Global	1	1	Smoking Prevalence (Reproductive Age Standardized)	282
Digestive congenital anomalies	Female	0-6 days	65-69 years	Global	1	1	Maternal alcohol consumption during pregnancy (proportion)	--
Digestive congenital anomalies	Female	0-6 days	65-69 years	Global	-1	2	Healthcare access and quality index	141
Digestive congenital anomalies	Female	0-6 days	65-69 years	Global	-1	2	In-Facility Delivery (proportion)	--
Digestive congenital anomalies	Female	0-6 days	65-69 years	Global	-1	2	Socio-demographic Index	--
Digestive congenital anomalies	Female	0-6 days	65-69 years	Global	1	2	Prevalence of obesity (age-standardized)	348
Digestive congenital anomalies	Female	0-6 days	65-69 years	Global	1	2	Diabetes Age-Standardized Prevalence (proportion)	397
Digestive congenital anomalies	Female	0-6 days	65-69 years	Global	1	2	Indoor Air Pollution (All Cooking Fuels)	--
Digestive congenital anomalies	Female	0-6 days	65-69 years	Global	-1	3	Antenatal Care (1 visit) Coverage (proportion)	--
Digestive congenital anomalies	Female	0-6 days	65-69 years	Global	-1	3	Antenatal Care (4 visits) Coverage (proportion)	--
Digestive congenital anomalies	Female	0-6 days	65-69 years	Global	-1	3	LDI (I$ per capita)	--
Digestive congenital anomalies	Female	0-6 days	65-69 years	Global	-1	3	Maternal Education (years per capita)	--
Digestive congenital anomalies	Female	0-6 days	65-69 years	Global	-1	3	Maternal care and immunization	--

Table S16. CODEm covariates used, level of covariate, and expected direction of covariate by cause, sex, and age
Covariates that CODEm did not select during the covariate selection process have no draw counts listed.

Cause	Sex	Age Start	Age End	Model Type	Direction	Level	Covariate Name	Number of Draws
Digestive congenital anomalies	Female	0-6 days	65-69 years	Global	0	3	fruits unadjusted(g)	414
Digestive congenital anomalies	Female	0-6 days	65-69 years	Global	0	3	vegetables unadjusted(g)	459
Digestive congenital anomalies	Female	0-6 days	65-69 years	Global	1	3	Alcohol (liters per capita)	472
Digestive congenital anomalies	Male	0-6 days	65-69 years	Data Rich	1	1	Smoking Prevalence (Reproductive Age Standardized)	333
Digestive congenital anomalies	Male	0-6 days	65-69 years	Data Rich	1	1	Maternal alcohol consumption during pregnancy (proportion)	--
Digestive congenital anomalies	Male	0-6 days	65-69 years	Data Rich	-1	2	Healthcare access and quality index	96
Digestive congenital anomalies	Male	0-6 days	65-69 years	Data Rich	-1	2	Socio-demographic Index	121
Digestive congenital anomalies	Male	0-6 days	65-69 years	Data Rich	-1	2	In-Facility Delivery (proportion)	--
Digestive congenital anomalies	Male	0-6 days	65-69 years	Data Rich	1	2	Diabetes Age-Standardized Prevalence (proportion)	307
Digestive congenital anomalies	Male	0-6 days	65-69 years	Data Rich	1	2	Prevalence of obesity (age-standardized)	347
Digestive congenital anomalies	Male	0-6 days	65-69 years	Data Rich	1	2	Indoor Air Pollution (All Cooking Fuels)	--
Digestive congenital anomalies	Male	0-6 days	65-69 years	Data Rich	-1	3	Maternal Education (years per capita)	24
Digestive congenital anomalies	Male	0-6 days	65-69 years	Data Rich	-1	3	Antenatal Care (1 visit) Coverage (proportion)	--
Digestive congenital anomalies	Male	0-6 days	65-69 years	Data Rich	-1	3	Antenatal Care (4 visits) Coverage (proportion)	--
Digestive congenital anomalies	Male	0-6 days	65-69 years	Data Rich	-1	3	LDI (I$ per capita)	--
Digestive congenital anomalies	Male	0-6 days	65-69 years	Data Rich	-1	3	Maternal care and immunization	--
Digestive congenital anomalies	Male	0-6 days	65-69 years	Data Rich	0	3	vegetables unadjusted(g)	406
Digestive congenital anomalies	Male	0-6 days	65-69 years	Data Rich	0	3	fruits unadjusted(g)	467
Digestive congenital anomalies	Male	0-6 days	65-69 years	Data Rich	1	3	Alcohol (liters per capita)	342
Digestive congenital anomalies	Male	0-6 days	65-69 years	Global	1	1	Maternal alcohol consumption during pregnancy (proportion)	260
Digestive congenital anomalies	Male	0-6 days	65-69 years	Global	1	1	Smoking Prevalence (Reproductive Age Standardized)	--
Digestive congenital anomalies	Male	0-6 days	65-69 years	Global	-1	2	Socio-demographic Index	88
Digestive congenital anomalies	Male	0-6 days	65-69 years	Global	-1	2	Healthcare access and quality index	118
Digestive congenital anomalies	Male	0-6 days	65-69 years	Global	-1	2	In-Facility Delivery (proportion)	--
Digestive congenital anomalies	Male	0-6 days	65-69 years	Global	1	2	Prevalence of obesity (age-standardized)	359
Digestive congenital anomalies	Male	0-6 days	65-69 years	Global	1	2	Diabetes Age-Standardized Prevalence (proportion)	487
Digestive congenital anomalies	Male	0-6 days	65-69 years	Global	1	2	Indoor Air Pollution (All Cooking Fuels)	--
Digestive congenital anomalies	Male	0-6 days	65-69 years	Global	-1	3	Maternal Education (years per capita)	39
Digestive congenital anomalies	Male	0-6 days	65-69 years	Global	-1	3	Antenatal Care (1 visit) Coverage (proportion)	--
Digestive congenital anomalies	Male	0-6 days	65-69 years	Global	-1	3	Antenatal Care (4 visits) Coverage (proportion)	--
Digestive congenital anomalies	Male	0-6 days	65-69 years	Global	-1	3	LDI (I$ per capita)	--
Digestive congenital anomalies	Male	0-6 days	65-69 years	Global	-1	3	Maternal care and immunization	--
Digestive congenital anomalies	Male	0-6 days	65-69 years	Global	0	3	vegetables unadjusted(g)	137
Digestive congenital anomalies	Male	0-6 days	65-69 years	Global	0	3	fruits unadjusted(g)	178
Digestive congenital anomalies	Male	0-6 days	65-69 years	Global	1	3	Alcohol (liters per capita)	461
Other congenital anomalies	Female	0-6 days	65-69 years	Data Rich	1	1	Live Births 35+ (proportion)	282
Other congenital anomalies	Female	0-6 days	65-69 years	Data Rich	1	1	Maternal alcohol consumption during pregnancy (proportion)	--
Other congenital anomalies	Female	0-6 days	65-69 years	Data Rich	-1	2	Healthcare access and quality index	354
Other congenital anomalies	Female	0-6 days	65-69 years	Data Rich	-1	2	In-Facility Delivery (proportion)	--
Other congenital anomalies	Female	0-6 days	65-69 years	Data Rich	-1	2	Legality of Abortion	--

Table S16. CODEm covariates used, level of covariate, and expected direction of covariate by cause, sex, and age
Covariates that CODEm did not select during the covariate selection process have no draw counts listed.

Cause	Sex	Age Start	Age End	Model Type	Direction	Level	Covariate Name	Number of Draws
Other congenital anomalies	Female	0-6 days	65-69 years	Data Rich	1	2	Smoking Prevalence (Reproductive Age Standardized)	354
Other congenital anomalies	Female	0-6 days	65-69 years	Data Rich	1	2	Indoor Air Pollution (All Cooking Fuels)	--
Other congenital anomalies	Female	0-6 days	65-69 years	Data Rich	-1	3	Maternal Education (years per capita)	153
Other congenital anomalies	Female	0-6 days	65-69 years	Data Rich	-1	3	Socio-demographic Index	211
Other congenital anomalies	Female	0-6 days	65-69 years	Data Rich	-1	3	Antenatal Care (1 visit) Coverage (proportion)	--
Other congenital anomalies	Female	0-6 days	65-69 years	Data Rich	-1	3	Antenatal Care (4 visits) Coverage (proportion)	--
Other congenital anomalies	Female	0-6 days	65-69 years	Data Rich	-1	3	LDI (I$ per capita)	--
Other congenital anomalies	Female	0-6 days	65-69 years	Data Rich	1	3	Diabetes Age-Standardized Prevalence (proportion)	415
Other congenital anomalies	Female	0-6 days	65-69 years	Data Rich	1	3	Alcohol (liters per capita)	--
Other congenital anomalies	Female	0-6 days	65-69 years	Global	1	1	Live Births 35+ (proportion)	405
Other congenital anomalies	Female	0-6 days	65-69 years	Global	1	1	Maternal alcohol consumption during pregnancy (proportion)	--
Other congenital anomalies	Female	0-6 days	65-69 years	Global	-1	2	Healthcare access and quality index	236
Other congenital anomalies	Female	0-6 days	65-69 years	Global	-1	2	In-Facility Delivery (proportion)	--
Other congenital anomalies	Female	0-6 days	65-69 years	Global	-1	2	Legality of Abortion	--
Other congenital anomalies	Female	0-6 days	65-69 years	Global	1	2	Smoking Prevalence (Reproductive Age Standardized)	236
Other congenital anomalies	Female	0-6 days	65-69 years	Global	1	2	Indoor Air Pollution (All Cooking Fuels)	--
Other congenital anomalies	Female	0-6 days	65-69 years	Global	-1	3	Antenatal Care (4 visits) Coverage (proportion)	133
Other congenital anomalies	Female	0-6 days	65-69 years	Global	-1	3	Socio-demographic Index	148
Other congenital anomalies	Female	0-6 days	65-69 years	Global	-1	3	Maternal Education (years per capita)	161
Other congenital anomalies	Female	0-6 days	65-69 years	Global	-1	3	Antenatal Care (1 visit) Coverage (proportion)	--
Other congenital anomalies	Female	0-6 days	65-69 years	Global	-1	3	LDI (I$ per capita)	--
Other congenital anomalies	Female	0-6 days	65-69 years	Global	1	3	Diabetes Age-Standardized Prevalence (proportion)	674
Other congenital anomalies	Female	0-6 days	65-69 years	Global	1	3	Alcohol (liters per capita)	--
Other congenital anomalies	Male	0-6 days	65-69 years	Data Rich	1	1	Live Births 35+ (proportion)	428
Other congenital anomalies	Male	0-6 days	65-69 years	Data Rich	1	1	Maternal alcohol consumption during pregnancy (proportion)	--
Other congenital anomalies	Male	0-6 days	65-69 years	Data Rich	-1	2	Healthcare access and quality index	196
Other congenital anomalies	Male	0-6 days	65-69 years	Data Rich	-1	2	In-Facility Delivery (proportion)	--
Other congenital anomalies	Male	0-6 days	65-69 years	Data Rich	-1	2	Legality of Abortion	--
Other congenital anomalies	Male	0-6 days	65-69 years	Data Rich	1	2	Smoking Prevalence (Reproductive Age Standardized)	196
Other congenital anomalies	Male	0-6 days	65-69 years	Data Rich	1	2	Indoor Air Pollution (All Cooking Fuels)	--
Other congenital anomalies	Male	0-6 days	65-69 years	Data Rich	-1	3	Socio-demographic Index	227
Other congenital anomalies	Male	0-6 days	65-69 years	Data Rich	-1	3	Maternal Education (years per capita)	256
Other congenital anomalies	Male	0-6 days	65-69 years	Data Rich	-1	3	Antenatal Care (1 visit) Coverage (proportion)	--
Other congenital anomalies	Male	0-6 days	65-69 years	Data Rich	-1	3	Antenatal Care (4 visits) Coverage (proportion)	--
Other congenital anomalies	Male	0-6 days	65-69 years	Data Rich	-1	3	LDI (I$ per capita)	--
Other congenital anomalies	Male	0-6 days	65-69 years	Data Rich	1	3	Diabetes Age-Standardized Prevalence (proportion)	719
Other congenital anomalies	Male	0-6 days	65-69 years	Data Rich	1	3	Alcohol (liters per capita)	--
Other congenital anomalies	Male	0-6 days	65-69 years	Global	1	1	Live Births 35+ (proportion)	405
Other congenital anomalies	Male	0-6 days	65-69 years	Global	1	1	Maternal alcohol consumption during pregnancy (proportion)	--
Other congenital anomalies	Male	0-6 days	65-69 years	Global	-1	2	Healthcare access and quality index	274

Table S16. CODEm covariates used, level of covariate, and expected direction of covariate by cause, sex, and age
Covariates that CODEm did not select during the covariate selection process have no draw counts listed.

Cause	Sex	Age Start	Age End	Model Type	Direction	Level	Covariate Name	Number of Draws
Other congenital anomalies	Male	0-6 days	65-69 years	Global	-1	2	In-Facility Delivery (proportion)	--
Other congenital anomalies	Male	0-6 days	65-69 years	Global	-1	2	Legality of Abortion	--
Other congenital anomalies	Male	0-6 days	65-69 years	Global	1	2	Smoking Prevalence (Reproductive Age Standardized)	274
Other congenital anomalies	Male	0-6 days	65-69 years	Global	1	2	Indoor Air Pollution (All Cooking Fuels)	--
Other congenital anomalies	Male	0-6 days	65-69 years	Global	-1	3	Antenatal Care (4 visits) Coverage (proportion)	65
Other congenital anomalies	Male	0-6 days	65-69 years	Global	-1	3	Maternal Education (years per capita)	161
Other congenital anomalies	Male	0-6 days	65-69 years	Global	-1	3	Socio-demographic Index	173
Other congenital anomalies	Male	0-6 days	65-69 years	Global	-1	3	Antenatal Care (1 visit) Coverage (proportion)	--
Other congenital anomalies	Male	0-6 days	65-69 years	Global	-1	3	LDI (I$ per capita)	--
Other congenital anomalies	Male	0-6 days	65-69 years	Global	1	3	Diabetes Age-Standardized Prevalence (proportion)	581
Other congenital anomalies	Male	0-6 days	65-69 years	Global	1	3	Alcohol (liters per capita)	--
Urinary diseases and male infertility	Female	0-6 days	95+ years	Data Rich	-1	2	Healthcare access and quality index	0
Urinary diseases and male infertility	Female	0-6 days	95+ years	Data Rich	0	2	Latitude Under 15 (proportion)	340
Urinary diseases and male infertility	Female	0-6 days	95+ years	Data Rich	0	2	Latitude 30 to 45 (proportion)	447
Urinary diseases and male infertility	Female	0-6 days	95+ years	Data Rich	0	2	Latitude Over 45 (proportion)	497
Urinary diseases and male infertility	Female	0-6 days	95+ years	Data Rich	0	2	Latitude 15 to 30 (proportion)	502
Urinary diseases and male infertility	Female	0-6 days	95+ years	Data Rich	1	2	Mean BMI	971
Urinary diseases and male infertility	Female	0-6 days	95+ years	Data Rich	-1	3	Education (years per capita)	--
Urinary diseases and male infertility	Female	0-6 days	95+ years	Data Rich	-1	3	LDI (I$ per capita)	--
Urinary diseases and male infertility	Female	0-6 days	95+ years	Data Rich	0	3	Socio-demographic Index	180
Urinary diseases and male infertility	Female	0-6 days	95+ years	Global	-1	2	Healthcare access and quality index	0
Urinary diseases and male infertility	Female	0-6 days	95+ years	Global	0	2	Latitude 15 to 30 (proportion)	305
Urinary diseases and male infertility	Female	0-6 days	95+ years	Global	0	2	Latitude Over 45 (proportion)	332
Urinary diseases and male infertility	Female	0-6 days	95+ years	Global	0	2	Latitude Under 15 (proportion)	508
Urinary diseases and male infertility	Female	0-6 days	95+ years	Global	0	2	Latitude 30 to 45 (proportion)	557
Urinary diseases and male infertility	Female	0-6 days	95+ years	Global	1	2	Mean BMI	996
Urinary diseases and male infertility	Female	0-6 days	95+ years	Global	-1	3	Education (years per capita)	0
Urinary diseases and male infertility	Female	0-6 days	95+ years	Global	-1	3	LDI (I$ per capita)	--
Urinary diseases and male infertility	Female	0-6 days	95+ years	Global	0	3	Socio-demographic Index	884
Urinary diseases and male infertility	Male	0-6 days	95+ years	Data Rich	-1	2	Healthcare access and quality index	0
Urinary diseases and male infertility	Male	0-6 days	95+ years	Data Rich	0	2	Latitude Under 15 (proportion)	258
Urinary diseases and male infertility	Male	0-6 days	95+ years	Data Rich	0	2	Latitude 15 to 30 (proportion)	330
Urinary diseases and male infertility	Male	0-6 days	95+ years	Data Rich	0	2	Latitude Over 45 (proportion)	344
Urinary diseases and male infertility	Male	0-6 days	95+ years	Data Rich	0	2	Latitude 30 to 45 (proportion)	618
Urinary diseases and male infertility	Male	0-6 days	95+ years	Data Rich	1	2	Mean BMI	952
Urinary diseases and male infertility	Male	0-6 days	95+ years	Data Rich	-1	3	Education (years per capita)	0
Urinary diseases and male infertility	Male	0-6 days	95+ years	Data Rich	-1	3	LDI (I$ per capita)	--
Urinary diseases and male infertility	Male	0-6 days	95+ years	Data Rich	0	3	Socio-demographic Index	86
Urinary diseases and male infertility	Male	0-6 days	95+ years	Global	-1	2	Healthcare access and quality index	507
Urinary diseases and male infertility	Male	0-6 days	95+ years	Global	0	2	Latitude 30 to 45 (proportion)	545
Urinary diseases and male infertility	Male	0-6 days	95+ years	Global	0	2	Latitude Over 45 (proportion)	549
Urinary diseases and male infertility	Male	0-6 days	95+ years	Global	0	2	Latitude Under 15 (proportion)	665
Urinary diseases and male infertility	Male	0-6 days	95+ years	Global	0	2	Latitude 15 to 30 (proportion)	673
Urinary diseases and male infertility	Male	0-6 days	95+ years	Global	1	2	Mean BMI	953
Urinary diseases and male infertility	Male	0-6 days	95+ years	Global	-1	3	Education (years per capita)	0
Urinary diseases and male infertility	Male	0-6 days	95+ years	Global	-1	3	LDI (I$ per capita)	--
Urinary diseases and male infertility	Male	0-6 days	95+ years	Global	0	3	Socio-demographic Index	548
Urinary tract infection and interstitial nephritis	Female	0-6 days	95+ years	Data Rich	1	1	Sanitation (proportion with access)	1000
Urinary tract infection and interstitial nephritis	Female	0-6 days	95+ years	Data Rich	-1	2	Education (years per capita)	--
Urinary tract infection and interstitial nephritis	Female	0-6 days	95+ years	Data Rich	-1	2	Healthcare access and quality index	--
Urinary tract infection and interstitial nephritis	Female	0-6 days	95+ years	Data Rich	-1	2	LDI (I$ per capita)	--
Urinary tract infection and interstitial nephritis	Female	0-6 days	95+ years	Data Rich	0	3	Socio-demographic Index	--
Urinary tract infection and interstitial nephritis	Female	0-6 days	95+ years	Global	1	1	Sanitation (proportion with access)	326
Urinary tract infection and interstitial nephritis	Female	0-6 days	95+ years	Global	-1	2	Healthcare access and quality index	489
Urinary tract infection and interstitial nephritis	Female	0-6 days	95+ years	Global	-1	2	Education (years per capita)	--

Table S16. CODEm covariates used, level of covariate, and expected direction of covariate by cause, sex, and age
Covariates that CODEm did not select during the covariate selection process have no draw counts listed.

Cause	Sex	Age Start	Age End	Model Type	Direction	Level	Covariate Name	Number of Draws
Urinary tract infection and interstitial nephritis	Female	0-6 days	95+ years	Global	-1	2	LDI (I$ per capita)	--
Urinary tract infection and interstitial nephritis	Female	0-6 days	95+ years	Global	0	3	Socio-demographic Index	452
Urinary tract infection and interstitial nephritis	Male	0-6 days	95+ years	Data Rich	1	1	Sanitation (proportion with access)	1000
Urinary tract infection and interstitial nephritis	Male	0-6 days	95+ years	Data Rich	-1	2	Education (years per capita)	--
Urinary tract infection and interstitial nephritis	Male	0-6 days	95+ years	Data Rich	-1	2	Healthcare access and quality index	--
Urinary tract infection and interstitial nephritis	Male	0-6 days	95+ years	Data Rich	-1	2	LDI (I$ per capita)	--
Urinary tract infection and interstitial nephritis	Male	0-6 days	95+ years	Data Rich	-1	2	Maternal care and immunization	--
Urinary tract infection and interstitial nephritis	Male	0-6 days	95+ years	Data Rich	0	3	Socio-demographic Index	--
Urinary tract infection and interstitial nephritis	Male	0-6 days	95+ years	Global	1	1	Sanitation (proportion with access)	326
Urinary tract infection and interstitial nephritis	Male	0-6 days	95+ years	Global	-1	2	Healthcare access and quality index	489
Urinary tract infection and interstitial nephritis	Male	0-6 days	95+ years	Global	-1	2	Education (years per capita)	--
Urinary tract infection and interstitial nephritis	Male	0-6 days	95+ years	Global	-1	2	LDI (I$ per capita)	--
Urinary tract infection and interstitial nephritis	Male	0-6 days	95+ years	Global	0	3	Socio-demographic Index	452
Urolithiasis	Female	5-9 years	95+ years	Data Rich	1	1	Healthcare access and quality index	620
Urolithiasis	Female	5-9 years	95+ years	Data Rich	1	1	90th percentile climatic temperature in the given country-year.	683
Urolithiasis	Female	5-9 years	95+ years	Data Rich	-1	2	fruits adjusted(g)	369
Urolithiasis	Female	5-9 years	95+ years	Data Rich	-1	2	vegetables adjusted(g)	822
Urolithiasis	Female	5-9 years	95+ years	Data Rich	1	2	red meats adjusted(g)	903
Urolithiasis	Female	5-9 years	95+ years	Data Rich	-1	3	Education (years per capita)	5
Urolithiasis	Female	5-9 years	95+ years	Data Rich	-1	3	LDI (I$ per capita)	--
Urolithiasis	Female	5-9 years	95+ years	Data Rich	0	3	Socio-demographic Index	319
Urolithiasis	Female	5-9 years	95+ years	Global	1	1	Healthcare access and quality index	333
Urolithiasis	Female	5-9 years	95+ years	Global	1	1	90th percentile climatic temperature in the given country-year.	864
Urolithiasis	Female	5-9 years	95+ years	Global	-1	2	fruits adjusted(g)	466
Urolithiasis	Female	5-9 years	95+ years	Global	-1	2	vegetables adjusted(g)	466
Urolithiasis	Female	5-9 years	95+ years	Global	1	2	red meats adjusted(g)	466
Urolithiasis	Female	5-9 years	95+ years	Global	-1	3	Education (years per capita)	88
Urolithiasis	Female	5-9 years	95+ years	Global	-1	3	LDI (I$ per capita)	--
Urolithiasis	Female	5-9 years	95+ years	Global	0	3	Socio-demographic Index	267
Urolithiasis	Male	5-9 years	95+ years	Data Rich	-1	1	Healthcare access and quality index	267
Urolithiasis	Male	5-9 years	95+ years	Data Rich	1	1	90th percentile climatic temperature in the given country-year.	381
Urolithiasis	Male	5-9 years	95+ years	Data Rich	1	1	red meats adjusted(g)	935
Urolithiasis	Male	5-9 years	95+ years	Data Rich	-1	2	fruits adjusted(g)	18
Urolithiasis	Male	5-9 years	95+ years	Data Rich	-1	2	vegetables adjusted(g)	398
Urolithiasis	Male	5-9 years	95+ years	Data Rich	-1	3	Education (years per capita)	0
Urolithiasis	Male	5-9 years	95+ years	Data Rich	-1	3	LDI (I$ per capita)	--
Urolithiasis	Male	5-9 years	95+ years	Data Rich	0	3	Socio-demographic Index	397
Urolithiasis	Male	5-9 years	95+ years	Global	1	1	90th percentile climatic temperature in the given country-year.	1000
Urolithiasis	Male	5-9 years	95+ years	Global	-1	2	vegetables adjusted(g)	189
Urolithiasis	Male	5-9 years	95+ years	Global	-1	2	Healthcare access and quality index	414
Urolithiasis	Male	5-9 years	95+ years	Global	-1	2	fruits adjusted(g)	414
Urolithiasis	Male	5-9 years	95+ years	Global	1	2	red meats adjusted(g)	225
Urolithiasis	Male	5-9 years	95+ years	Global	-1	3	Education (years per capita)	222
Urolithiasis	Male	5-9 years	95+ years	Global	-1	3	LDI (I$ per capita)	--
Urolithiasis	Male	5-9 years	95+ years	Global	0	3	Socio-demographic Index	283
Other urinary diseases	Female	0-6 days	95+ years	Data Rich	1	1	Education (years per capita)	--
Other urinary diseases	Female	0-6 days	95+ years	Data Rich	1	1	LDI (I$ per capita)	--
Other urinary diseases	Female	0-6 days	95+ years	Data Rich	1	1	Mean BMI	--
Other urinary diseases	Female	0-6 days	95+ years	Data Rich	-1	2	Healthcare access and quality index	653
Other urinary diseases	Female	0-6 days	95+ years	Data Rich	0	3	Socio-demographic Index	614
Other urinary diseases	Female	0-6 days	95+ years	Global	1	1	Education (years per capita)	--
Other urinary diseases	Female	0-6 days	95+ years	Global	1	1	LDI (I$ per capita)	--
Other urinary diseases	Female	0-6 days	95+ years	Global	1	1	Mean BMI	--
Other urinary diseases	Female	0-6 days	95+ years	Global	-1	2	Healthcare access and quality index	807
Other urinary diseases	Female	0-6 days	95+ years	Global	0	3	Socio-demographic Index	557

Table S16. CODEm covariates used, level of covariate, and expected direction of covariate by cause, sex, and age
Covariates that CODEm did not select during the covariate selection process have no draw counts listed.

Cause	Sex	Age Start	Age End	Model Type	Direction	Level	Covariate Name	Number of Draws
Other urinary diseases	Male	0-6 days	95+ years	Data Rich	1	1	Mean BMI	--
Other urinary diseases	Male	0-6 days	95+ years	Data Rich	-1	2	LDI (I$ per capita)	89
Other urinary diseases	Male	0-6 days	95+ years	Data Rich	-1	2	Education (years per capita)	344
Other urinary diseases	Male	0-6 days	95+ years	Data Rich	-1	2	Healthcare access and quality index	453
Other urinary diseases	Male	0-6 days	95+ years	Data Rich	0	3	Socio-demographic Index	662
Other urinary diseases	Male	0-6 days	95+ years	Global	1	1	Mean BMI	--
Other urinary diseases	Male	0-6 days	95+ years	Global	-1	2	LDI (I$ per capita)	251
Other urinary diseases	Male	0-6 days	95+ years	Global	-1	2	Education (years per capita)	598
Other urinary diseases	Male	0-6 days	95+ years	Global	-1	2	Healthcare access and quality index	635
Other urinary diseases	Male	0-6 days	95+ years	Global	0	3	Socio-demographic Index	435
Gynecological diseases	Female	15-19 years	95+ years	Data Rich	0	1	Smoking Prevalence	486
Gynecological diseases	Female	15-19 years	95+ years	Data Rich	-1	2	Skilled Birth Attendance (proportion)	407
Gynecological diseases	Female	15-19 years	95+ years	Data Rich	-1	2	Healthcare access and quality index	514
Gynecological diseases	Female	15-19 years	95+ years	Data Rich	-1	2	Maternal care and immunization	--
Gynecological diseases	Female	15-19 years	95+ years	Data Rich	1	2	Total Fertility Rate	407
Gynecological diseases	Female	15-19 years	95+ years	Data Rich	1	2	Live Births 35+ (proportion)	--
Gynecological diseases	Female	15-19 years	95+ years	Data Rich	-1	3	Socio-demographic Index	274
Gynecological diseases	Female	15-19 years	95+ years	Data Rich	-1	3	Education (years per capita)	418
Gynecological diseases	Female	15-19 years	95+ years	Data Rich	-1	3	LDI (I$ per capita)	--
Gynecological diseases	Female	15-19 years	95+ years	Global	0	1	Smoking Prevalence	1000
Gynecological diseases	Female	15-19 years	95+ years	Global	-1	2	Skilled Birth Attendance (proportion)	145
Gynecological diseases	Female	15-19 years	95+ years	Global	-1	2	Healthcare access and quality index	307
Gynecological diseases	Female	15-19 years	95+ years	Global	-1	2	Maternal care and immunization	--
Gynecological diseases	Female	15-19 years	95+ years	Global	1	2	Total Fertility Rate	201
Gynecological diseases	Female	15-19 years	95+ years	Global	1	2	Live Births 35+ (proportion)	--
Gynecological diseases	Female	15-19 years	95+ years	Global	-1	3	Socio-demographic Index	171
Gynecological diseases	Female	15-19 years	95+ years	Global	-1	3	Education (years per capita)	379
Gynecological diseases	Female	15-19 years	95+ years	Global	-1	3	LDI (I$ per capita)	--
Uterine fibroids	Female	15-19 years	95+ years	Data Rich	0	1	Smoking Prevalence	--
Uterine fibroids	Female	15-19 years	95+ years	Data Rich	-1	2	Skilled Birth Attendance (proportion)	300
Uterine fibroids	Female	15-19 years	95+ years	Data Rich	-1	2	Healthcare access and quality index	--
Uterine fibroids	Female	15-19 years	95+ years	Data Rich	-1	2	Maternal care and immunization	--
Uterine fibroids	Female	15-19 years	95+ years	Data Rich	1	2	Live Births 35+ (proportion)	--
Uterine fibroids	Female	15-19 years	95+ years	Data Rich	1	2	Total Fertility Rate	--
Uterine fibroids	Female	15-19 years	95+ years	Data Rich	-1	3	Socio-demographic Index	221
Uterine fibroids	Female	15-19 years	95+ years	Data Rich	-1	3	Education (years per capita)	380
Uterine fibroids	Female	15-19 years	95+ years	Data Rich	-1	3	LDI (I$ per capita)	--
Uterine fibroids	Female	15-19 years	95+ years	Global	0	1	Smoking Prevalence	1000
Uterine fibroids	Female	15-19 years	95+ years	Global	-1	2	Skilled Birth Attendance (proportion)	168
Uterine fibroids	Female	15-19 years	95+ years	Global	-1	2	Healthcare access and quality index	430
Uterine fibroids	Female	15-19 years	95+ years	Global	-1	2	Maternal care and immunization	--
Uterine fibroids	Female	15-19 years	95+ years	Global	1	2	Total Fertility Rate	36
Uterine fibroids	Female	15-19 years	95+ years	Global	1	2	Live Births 35+ (proportion)	--

Table S16. CODEm covariates used, level of covariate, and expected direction of covariate by cause, sex, and age
Covariates that CODEm did not select during the covariate selection process have no draw counts listed.

Cause	Sex	Age Start	Age End	Model Type	Direction	Level	Covariate Name	Number of Draws
Uterine fibroids	Female	15-19 years	95+ years	Global	-1	3	LDI (I$ per capita)	31
Uterine fibroids	Female	15-19 years	95+ years	Global	-1	3	Socio-demographic Index	137
Uterine fibroids	Female	15-19 years	95+ years	Global	-1	3	Education (years per capita)	428
Polycystic ovarian syndrome	Female	15-19 years	50-54 years	Data Rich	0	1	Smoking Prevalence	454
Polycystic ovarian syndrome	Female	15-19 years	50-54 years	Data Rich	-1	2	Healthcare access and quality index	--
Polycystic ovarian syndrome	Female	15-19 years	50-54 years	Data Rich	-1	2	Maternal care and immunization	--
Polycystic ovarian syndrome	Female	15-19 years	50-54 years	Data Rich	-1	2	Skilled Birth Attendance (proportion)	--
Polycystic ovarian syndrome	Female	15-19 years	50-54 years	Data Rich	1	2	Live Births 35+ (proportion)	--
Polycystic ovarian syndrome	Female	15-19 years	50-54 years	Data Rich	1	2	Total Fertility Rate	--
Polycystic ovarian syndrome	Female	15-19 years	50-54 years	Data Rich	-1	3	LDI (I$ per capita)	546
Polycystic ovarian syndrome	Female	15-19 years	50-54 years	Data Rich	-1	3	Education (years per capita)	--
Polycystic ovarian syndrome	Female	15-19 years	50-54 years	Data Rich	-1	3	Socio-demographic Index	--
Polycystic ovarian syndrome	Female	15-19 years	50-54 years	Global	0	1	Smoking Prevalence	1000
Polycystic ovarian syndrome	Female	15-19 years	50-54 years	Global	-1	2	Healthcare access and quality index	--
Polycystic ovarian syndrome	Female	15-19 years	50-54 years	Global	-1	2	Maternal care and immunization	--
Polycystic ovarian syndrome	Female	15-19 years	50-54 years	Global	-1	2	Skilled Birth Attendance (proportion)	--
Polycystic ovarian syndrome	Female	15-19 years	50-54 years	Global	1	2	Live Births 35+ (proportion)	--
Polycystic ovarian syndrome	Female	15-19 years	50-54 years	Global	1	2	Total Fertility Rate	--
Polycystic ovarian syndrome	Female	15-19 years	50-54 years	Global	-1	3	Education (years per capita)	--
Polycystic ovarian syndrome	Female	15-19 years	50-54 years	Global	-1	3	LDI (I$ per capita)	--
Polycystic ovarian syndrome	Female	15-19 years	50-54 years	Global	-1	3	Socio-demographic Index	--
Endometriosis	Female	15-19 years	50-54 years	Data Rich	0	1	Smoking Prevalence	1000
Endometriosis	Female	15-19 years	50-54 years	Data Rich	-1	2	Skilled Birth Attendance (proportion)	76
Endometriosis	Female	15-19 years	50-54 years	Data Rich	-1	2	Healthcare access and quality index	559
Endometriosis	Female	15-19 years	50-54 years	Data Rich	-1	2	Maternal care and immunization	--
Endometriosis	Female	15-19 years	50-54 years	Data Rich	1	2	Total Fertility Rate	76
Endometriosis	Female	15-19 years	50-54 years	Data Rich	1	2	Live Births 35+ (proportion)	--
Endometriosis	Female	15-19 years	50-54 years	Data Rich	-1	3	Education (years per capita)	193
Endometriosis	Female	15-19 years	50-54 years	Data Rich	-1	3	Socio-demographic Index	312
Endometriosis	Female	15-19 years	50-54 years	Data Rich	-1	3	LDI (I$ per capita)	564
Endometriosis	Female	15-19 years	50-54 years	Global	0	1	Smoking Prevalence	1000
Endometriosis	Female	15-19 years	50-54 years	Global	-1	2	Skilled Birth Attendance (proportion)	210
Endometriosis	Female	15-19 years	50-54 years	Global	-1	2	Healthcare access and quality index	302
Endometriosis	Female	15-19 years	50-54 years	Global	-1	2	Maternal care and immunization	--
Endometriosis	Female	15-19 years	50-54 years	Global	1	2	Total Fertility Rate	192
Endometriosis	Female	15-19 years	50-54 years	Global	1	2	Live Births 35+ (proportion)	--
Endometriosis	Female	15-19 years	50-54 years	Global	-1	3	Education (years per capita)	122
Endometriosis	Female	15-19 years	50-54 years	Global	-1	3	Socio-demographic Index	218
Endometriosis	Female	15-19 years	50-54 years	Global	-1	3	LDI (I$ per capita)	311
Genital prolapse	Female	15-19 years	95+ years	Data Rich	0	1	Smoking Prevalence	1000

Table S16. CODEm covariates used, level of covariate, and expected direction of covariate by cause, sex, and age
Covariates that CODEm did not select during the covariate selection process have no draw counts listed.

Cause	Sex	Age Start	Age End	Model Type	Direction	Level	Covariate Name	Number of Draws
Genital prolapse	Female	15-19 years	95+ years	Data Rich	-1	2	Healthcare access and quality index	397
Genital prolapse	Female	15-19 years	95+ years	Data Rich	-1	2	Skilled Birth Attendance (proportion)	405
Genital prolapse	Female	15-19 years	95+ years	Data Rich	-1	2	Maternal care and immunization	--
Genital prolapse	Female	15-19 years	95+ years	Data Rich	1	2	Total Fertility Rate	35
Genital prolapse	Female	15-19 years	95+ years	Data Rich	1	2	Live Births 35+ (proportion)	--
Genital prolapse	Female	15-19 years	95+ years	Data Rich	-1	3	LDI (I$ per capita)	198
Genital prolapse	Female	15-19 years	95+ years	Data Rich	-1	3	Socio-demographic Index	384
Genital prolapse	Female	15-19 years	95+ years	Data Rich	-1	3	Education (years per capita)	713
Genital prolapse	Female	15-19 years	95+ years	Global	0	1	Smoking Prevalence	1000
Genital prolapse	Female	15-19 years	95+ years	Global	-1	2	Skilled Birth Attendance (proportion)	382
Genital prolapse	Female	15-19 years	95+ years	Global	-1	2	Healthcare access and quality index	438
Genital prolapse	Female	15-19 years	95+ years	Global	-1	2	Maternal care and immunization	--
Genital prolapse	Female	15-19 years	95+ years	Global	1	2	Total Fertility Rate	326
Genital prolapse	Female	15-19 years	95+ years	Global	1	2	Live Births 35+ (proportion)	--
Genital prolapse	Female	15-19 years	95+ years	Global	-1	3	Socio-demographic Index	168
Genital prolapse	Female	15-19 years	95+ years	Global	-1	3	Education (years per capita)	304
Genital prolapse	Female	15-19 years	95+ years	Global	-1	3	LDI (I$ per capita)	400
Other gynecological diseases	Female	15-19 years	95+ years	Data Rich	0	1	Smoking Prevalence	1000
Other gynecological diseases	Female	15-19 years	95+ years	Data Rich	-1	2	Healthcare access and quality index	347
Other gynecological diseases	Female	15-19 years	95+ years	Data Rich	-1	2	Skilled Birth Attendance (proportion)	375
Other gynecological diseases	Female	15-19 years	95+ years	Data Rich	-1	2	Maternal care and immunization	--
Other gynecological diseases	Female	15-19 years	95+ years	Data Rich	1	2	Live Births 35+ (proportion)	262
Other gynecological diseases	Female	15-19 years	95+ years	Data Rich	1	2	Total Fertility Rate	375
Other gynecological diseases	Female	15-19 years	95+ years	Data Rich	-1	3	LDI (I$ per capita)	78
Other gynecological diseases	Female	15-19 years	95+ years	Data Rich	-1	3	Education (years per capita)	151
Other gynecological diseases	Female	15-19 years	95+ years	Data Rich	-1	3	Socio-demographic Index	193
Other gynecological diseases	Female	15-19 years	95+ years	Global	0	1	Smoking Prevalence	1000
Other gynecological diseases	Female	15-19 years	95+ years	Global	-1	2	Healthcare access and quality index	86
Other gynecological diseases	Female	15-19 years	95+ years	Global	-1	2	Skilled Birth Attendance (proportion)	339
Other gynecological diseases	Female	15-19 years	95+ years	Global	-1	2	Maternal care and immunization	--
Other gynecological diseases	Female	15-19 years	95+ years	Global	1	2	Live Births 35+ (proportion)	339
Other gynecological diseases	Female	15-19 years	95+ years	Global	1	2	Total Fertility Rate	457
Other gynecological diseases	Female	15-19 years	95+ years	Global	-1	3	LDI (I$ per capita)	151
Other gynecological diseases	Female	15-19 years	95+ years	Global	-1	3	Education (years per capita)	188
Other gynecological diseases	Female	15-19 years	95+ years	Global	-1	3	Socio-demographic Index	204
Hemoglobinopathies and hemolytic anaemias	Female	0-6 days	95+ years	Data Rich	-1	2	Healthcare access and quality index	--
Hemoglobinopathies and hemolytic anaemias	Female	0-6 days	95+ years	Data Rich	-1	2	Maternal care and immunization	--
Hemoglobinopathies and hemolytic anaemias	Female	0-6 days	95+ years	Data Rich	-1	3	Education (years per capita)	1000
Hemoglobinopathies and hemolytic anaemias	Female	0-6 days	95+ years	Data Rich	-1	3	Latitude Over 45 (proportion)	1000
Hemoglobinopathies and hemolytic anaemias	Female	0-6 days	95+ years	Data Rich	-1	3	Socio-demographic Index	1000
Hemoglobinopathies and hemolytic anaemias	Female	0-6 days	95+ years	Data Rich	-1	3	LDI (I$ per capita)	--
Hemoglobinopathies and hemolytic anaemias	Female	0-6 days	95+ years	Data Rich	0	3	Latitude 30 to 45 (proportion)	1000
Hemoglobinopathies and hemolytic anaemias	Female	0-6 days	95+ years	Data Rich	1	3	Latitude 15 to 30 (proportion)	--

Table S16. CODEm covariates used, level of covariate, and expected direction of covariate by cause, sex, and age
Covariates that CODEm did not select during the covariate selection process have no draw counts listed.

Cause	Sex	Age Start	Age End	Model Type	Direction	Level	Covariate Name	Number of Draws
Hemoglobinopathies and hemolytic anaemias	Female	0-6 days	95+ years	Data Rich	1	3	Latitude Under 15 (proportion)	--
Hemoglobinopathies and hemolytic anaemias	Female	0-6 days	95+ years	Data Rich	1	3	Malaria Lysenko PFPR 1 (Holoendemic)	--
Hemoglobinopathies and hemolytic anaemias	Female	0-6 days	95+ years	Global	1	1	Hemoglobinopathies Prevalence x Excess Mortality	119
Hemoglobinopathies and hemolytic anaemias	Female	0-6 days	95+ years	Global	1	1	Hemoglobinopathies Prevalence x Excess Mortality (excluding G6PD deficiency)	201
Hemoglobinopathies and hemolytic anaemias	Female	0-6 days	95+ years	Global	-1	2	Healthcare access and quality index	257
Hemoglobinopathies and hemolytic anaemias	Female	0-6 days	95+ years	Global	-1	2	Maternal care and immunization	--
Hemoglobinopathies and hemolytic anaemias	Female	0-6 days	95+ years	Global	-1	3	Socio-demographic Index	221
Hemoglobinopathies and hemolytic anaemias	Female	0-6 days	95+ years	Global	-1	3	Education (years per capita)	305
Hemoglobinopathies and hemolytic anaemias	Female	0-6 days	95+ years	Global	-1	3	Latitude Over 45 (proportion)	374
Hemoglobinopathies and hemolytic anaemias	Female	0-6 days	95+ years	Global	-1	3	LDI (I$ per capita)	--
Hemoglobinopathies and hemolytic anaemias	Female	0-6 days	95+ years	Global	0	3	Latitude 30 to 45 (proportion)	633
Hemoglobinopathies and hemolytic anaemias	Female	0-6 days	95+ years	Global	1	3	Latitude 15 to 30 (proportion)	173
Hemoglobinopathies and hemolytic anaemias	Female	0-6 days	95+ years	Global	1	3	Malaria Lysenko PFPR 1 (Holoendemic)	247
Hemoglobinopathies and hemolytic anaemias	Female	0-6 days	95+ years	Global	1	3	Latitude Under 15 (proportion)	--
Hemoglobinopathies and hemolytic anaemias	Male	0-6 days	95+ years	Data Rich	-1	2	Healthcare access and quality index	613
Hemoglobinopathies and hemolytic anaemias	Male	0-6 days	95+ years	Data Rich	-1	2	Maternal care and immunization	--
Hemoglobinopathies and hemolytic anaemias	Male	0-6 days	95+ years	Data Rich	-1	3	Socio-demographic Index	50
Hemoglobinopathies and hemolytic anaemias	Male	0-6 days	95+ years	Data Rich	-1	3	Education (years per capita)	60
Hemoglobinopathies and hemolytic anaemias	Male	0-6 days	95+ years	Data Rich	-1	3	Latitude Over 45 (proportion)	445
Hemoglobinopathies and hemolytic anaemias	Male	0-6 days	95+ years	Data Rich	-1	3	LDI (I$ per capita)	--
Hemoglobinopathies and hemolytic anaemias	Male	0-6 days	95+ years	Data Rich	0	3	Latitude 30 to 45 (proportion)	477
Hemoglobinopathies and hemolytic anaemias	Male	0-6 days	95+ years	Data Rich	1	3	Latitude Under 15 (proportion)	202
Hemoglobinopathies and hemolytic anaemias	Male	0-6 days	95+ years	Data Rich	1	3	Latitude 15 to 30 (proportion)	312
Hemoglobinopathies and hemolytic anaemias	Male	0-6 days	95+ years	Data Rich	1	3	Malaria Lysenko PFPR 1 (Holoendemic)	--
Hemoglobinopathies and hemolytic anaemias	Male	0-6 days	95+ years	Global	1	1	Hemoglobinopathies Prevalence x Excess Mortality (excluding G6PD deficiency)	159
Hemoglobinopathies and hemolytic anaemias	Male	0-6 days	95+ years	Global	1	1	Hemoglobinopathies Prevalence x Excess Mortality	522
Hemoglobinopathies and hemolytic anaemias	Male	0-6 days	95+ years	Global	-1	2	Healthcare access and quality index	386
Hemoglobinopathies and hemolytic anaemias	Male	0-6 days	95+ years	Global	-1	2	Maternal care and immunization	--
Hemoglobinopathies and hemolytic anaemias	Male	0-6 days	95+ years	Global	-1	3	Socio-demographic Index	184
Hemoglobinopathies and hemolytic anaemias	Male	0-6 days	95+ years	Global	-1	3	Education (years per capita)	271
Hemoglobinopathies and hemolytic anaemias	Male	0-6 days	95+ years	Global	-1	3	Latitude Over 45 (proportion)	692
Hemoglobinopathies and hemolytic anaemias	Male	0-6 days	95+ years	Global	-1	3	LDI (I$ per capita)	--
Hemoglobinopathies and hemolytic anaemias	Male	0-6 days	95+ years	Global	0	3	Latitude 30 to 45 (proportion)	585
Hemoglobinopathies and hemolytic anaemias	Male	0-6 days	95+ years	Global	1	3	Malaria Lysenko PFPR 1 (Holoendemic)	462
Hemoglobinopathies and hemolytic anaemias	Male	0-6 days	95+ years	Global	1	3	Latitude 15 to 30 (proportion)	654
Hemoglobinopathies and hemolytic anaemias	Male	0-6 days	95+ years	Global	1	3	Latitude Under 15 (proportion)	--
Endocrine, metabolic, blood, and immune disorders	Female	0-6 days	95+ years	Data Rich	1	1	Mean BMI	--
Endocrine, metabolic, blood, and immune disorders	Female	0-6 days	95+ years	Data Rich	-1	2	Healthcare access and quality index	699
Endocrine, metabolic, blood, and immune disorders	Female	0-6 days	95+ years	Data Rich	1	2	Alcohol (liters per capita)	--
Endocrine, metabolic, blood, and immune disorders	Female	0-6 days	95+ years	Data Rich	1	2	Cholesterol (total, mean per capita)	--
Endocrine, metabolic, blood, and immune disorders	Female	0-6 days	95+ years	Data Rich	-1	3	Education (years per capita)	--
Endocrine, metabolic, blood, and immune disorders	Female	0-6 days	95+ years	Data Rich	-1	3	LDI (I$ per capita)	--
Endocrine, metabolic, blood, and immune disorders	Female	0-6 days	95+ years	Data Rich	0	3	Socio-demographic Index	572
Endocrine, metabolic, blood, and immune disorders	Female	0-6 days	95+ years	Global	1	1	Mean BMI	--
Endocrine, metabolic, blood, and immune disorders	Female	0-6 days	95+ years	Global	-1	2	Healthcare access and quality index	317
Endocrine, metabolic, blood, and immune disorders	Female	0-6 days	95+ years	Global	1	2	Alcohol (liters per capita)	--
Endocrine, metabolic, blood, and immune disorders	Female	0-6 days	95+ years	Global	1	2	Cholesterol (total, mean per capita)	--
Endocrine, metabolic, blood, and immune disorders	Female	0-6 days	95+ years	Global	-1	3	Education (years per capita)	271
Endocrine, metabolic, blood, and immune disorders	Female	0-6 days	95+ years	Global	-1	3	LDI (I$ per capita)	--
Endocrine, metabolic, blood, and immune disorders	Female	0-6 days	95+ years	Global	0	3	Socio-demographic Index	479
Endocrine, metabolic, blood, and immune disorders	Male	0-6 days	95+ years	Data Rich	1	1	Mean BMI	--
Endocrine, metabolic, blood, and immune disorders	Male	0-6 days	95+ years	Data Rich	-1	2	Healthcare access and quality index	137
Endocrine, metabolic, blood, and immune disorders	Male	0-6 days	95+ years	Data Rich	1	2	Cholesterol (total, mean per capita)	137
Endocrine, metabolic, blood, and immune disorders	Male	0-6 days	95+ years	Data Rich	1	2	Alcohol (liters per capita)	--
Endocrine, metabolic, blood, and immune disorders	Male	0-6 days	95+ years	Data Rich	-1	3	Education (years per capita)	78

Table S16. CODEm covariates used, level of covariate, and expected direction of covariate by cause, sex, and age
Covariates that CODEm did not select during the covariate selection process have no draw counts listed.

Cause	Sex	Age Start	Age End	Model Type	Direction	Level	Covariate Name	Number of Draws
Endocrine, metabolic, blood, and immune disorders	Male	0-6 days	95+ years	Data Rich	-1	3	LDI (I$ per capita)	--
Endocrine, metabolic, blood, and immune disorders	Male	0-6 days	95+ years	Data Rich	0	3	Socio-demographic Index	348
Endocrine, metabolic, blood, and immune disorders	Male	0-6 days	95+ years	Global	1	1	Mean BMI	--
Endocrine, metabolic, blood, and immune disorders	Male	0-6 days	95+ years	Global	-1	2	Healthcare access and quality index	317
Endocrine, metabolic, blood, and immune disorders	Male	0-6 days	95+ years	Global	1	2	Alcohol (liters per capita)	--
Endocrine, metabolic, blood, and immune disorders	Male	0-6 days	95+ years	Global	1	2	Cholesterol (total, mean per capita)	--
Endocrine, metabolic, blood, and immune disorders	Male	0-6 days	95+ years	Global	-1	3	Education (years per capita)	286
Endocrine, metabolic, blood, and immune disorders	Male	0-6 days	95+ years	Global	-1	3	LDI (I$ per capita)	--
Endocrine, metabolic, blood, and immune disorders	Male	0-6 days	95+ years	Global	0	3	Socio-demographic Index	531
Sudden infant death syndrome	Female	7-27 days	28-364 days	Data Rich	-1	1	In-Facility Delivery (proportion)	251
Sudden infant death syndrome	Female	7-27 days	28-364 days	Data Rich	1	1	Tobacco (cigarettes per capita)	900
Sudden infant death syndrome	Female	7-27 days	28-364 days	Data Rich	-1	2	Skilled Birth Attendance (proportion)	120
Sudden infant death syndrome	Female	7-27 days	28-364 days	Data Rich	1	2	Underweight (proportion <2SD weight for age, <5 years)	--
Sudden infant death syndrome	Female	7-27 days	28-364 days	Data Rich	-1	3	Education (years per capita)	208
Sudden infant death syndrome	Female	7-27 days	28-364 days	Data Rich	0	3	Socio-demographic Index	208
Sudden infant death syndrome	Female	7-27 days	28-364 days	Data Rich	0	3	LDI (I$ per capita)	--
Sudden infant death syndrome	Female	7-27 days	28-364 days	Data Rich	1	3	Total Fertility Rate	--
Sudden infant death syndrome	Female	7-27 days	28-364 days	Global	-1	1	In-Facility Delivery (proportion)	412
Sudden infant death syndrome	Female	7-27 days	28-364 days	Global	1	1	Tobacco (cigarettes per capita)	689
Sudden infant death syndrome	Female	7-27 days	28-364 days	Global	-1	2	Skilled Birth Attendance (proportion)	240
Sudden infant death syndrome	Female	7-27 days	28-364 days	Global	1	2	Underweight (proportion <2SD weight for age, <5 years)	--
Sudden infant death syndrome	Female	7-27 days	28-364 days	Global	-1	3	Education (years per capita)	146
Sudden infant death syndrome	Female	7-27 days	28-364 days	Global	0	3	Socio-demographic Index	146
Sudden infant death syndrome	Female	7-27 days	28-364 days	Global	0	3	LDI (I$ per capita)	--
Sudden infant death syndrome	Female	7-27 days	28-364 days	Global	1	3	Total Fertility Rate	--
Sudden infant death syndrome	Male	7-27 days	28-364 days	Data Rich	-1	1	In-Facility Delivery (proportion)	40
Sudden infant death syndrome	Male	7-27 days	28-364 days	Data Rich	1	1	Tobacco (cigarettes per capita)	960
Sudden infant death syndrome	Male	7-27 days	28-364 days	Data Rich	-1	2	Healthcare access and quality index	368
Sudden infant death syndrome	Male	7-27 days	28-364 days	Data Rich	-1	2	Maternal care and immunization	--
Sudden infant death syndrome	Male	7-27 days	28-364 days	Data Rich	-1	2	Skilled Birth Attendance (proportion)	--
Sudden infant death syndrome	Male	7-27 days	28-364 days	Data Rich	1	2	Underweight (proportion <2SD weight for age, <5 years)	--
Sudden infant death syndrome	Male	7-27 days	28-364 days	Data Rich	-1	3	Education (years per capita)	433
Sudden infant death syndrome	Male	7-27 days	28-364 days	Data Rich	0	3	Socio-demographic Index	242
Sudden infant death syndrome	Male	7-27 days	28-364 days	Data Rich	0	3	LDI (I$ per capita)	--
Sudden infant death syndrome	Male	7-27 days	28-364 days	Data Rich	1	3	Total Fertility Rate	--
Sudden infant death syndrome	Male	7-27 days	28-364 days	Global	-1	1	In-Facility Delivery (proportion)	120
Sudden infant death syndrome	Male	7-27 days	28-364 days	Global	1	1	Tobacco (cigarettes per capita)	880
Sudden infant death syndrome	Male	7-27 days	28-364 days	Global	-1	2	Healthcare access and quality index	352
Sudden infant death syndrome	Male	7-27 days	28-364 days	Global	-1	2	Maternal care and immunization	--
Sudden infant death syndrome	Male	7-27 days	28-364 days	Global	-1	2	Skilled Birth Attendance (proportion)	--
Sudden infant death syndrome	Male	7-27 days	28-364 days	Global	1	2	Underweight (proportion <2SD weight for age, <5 years)	--
Sudden infant death syndrome	Male	7-27 days	28-364 days	Global	-1	3	Education (years per capita)	212
Sudden infant death syndrome	Male	7-27 days	28-364 days	Global	0	3	Socio-demographic Index	116

Table S16. CODEm covariates used, level of covariate, and expected direction of covariate by cause, sex, and age *Covariates that CODEm did not select during the covariate selection process have no draw counts listed.*								
**Cause**	**Sex**	**Age Start**	**Age End**	**Model Type**	**Direction**	**Level**	**Covariate Name**	**Number of Draws**
Sudden infant death syndrome	Male	7-27 days	28-364 days	Global	0	3	LDI (I$ per capita)	--
Sudden infant death syndrome	Male	7-27 days	28-364 days	Global	1	3	Total Fertility Rate	--
Transport injuries	Female	0-6 days	95+ years	Data Rich	1	1	Alcohol (liters per capita)	410
Transport injuries	Female	0-6 days	95+ years	Data Rich	1	1	Vehicles - 2 wheels fraction (proportion)	694
Transport injuries	Female	0-6 days	95+ years	Data Rich	1	1	Vehicles - 2+4 wheels (per capita)	--
Transport injuries	Female	0-6 days	95+ years	Data Rich	-1	2	Healthcare access and quality index	462
Transport injuries	Female	0-6 days	95+ years	Data Rich	0	2	Population Density (300-500 ppl/sqkm, proportion)	462
Transport injuries	Female	0-6 days	95+ years	Data Rich	0	2	Population Density (500-1000 ppl/sqkm, proportion)	462
Transport injuries	Female	0-6 days	95+ years	Data Rich	0	2	LDI (I$ per capita)	--
Transport injuries	Female	0-6 days	95+ years	Data Rich	-1	3	Education (years per capita)	149
Transport injuries	Female	0-6 days	95+ years	Data Rich	-1	3	Socio-demographic Index	342
Transport injuries	Female	0-6 days	95+ years	Data Rich	1	3	Rainfall Quintile 5 (proportion)	--
Transport injuries	Female	0-6 days	95+ years	Global	1	1	Alcohol (liters per capita)	671
Transport injuries	Female	0-6 days	95+ years	Global	1	1	Vehicles - 2 wheels fraction (proportion)	812
Transport injuries	Female	0-6 days	95+ years	Global	1	1	Vehicles - 2+4 wheels (per capita)	--
Transport injuries	Female	0-6 days	95+ years	Global	-1	2	Healthcare access and quality index	323
Transport injuries	Female	0-6 days	95+ years	Global	0	2	Population Density (300-500 ppl/sqkm, proportion)	527
Transport injuries	Female	0-6 days	95+ years	Global	0	2	Population Density (500-1000 ppl/sqkm, proportion)	531
Transport injuries	Female	0-6 days	95+ years	Global	0	2	LDI (I$ per capita)	--
Transport injuries	Female	0-6 days	95+ years	Global	-1	3	Socio-demographic Index	294
Transport injuries	Female	0-6 days	95+ years	Global	-1	3	Education (years per capita)	344
Transport injuries	Female	0-6 days	95+ years	Global	1	3	Rainfall Quintile 5 (proportion)	--
Transport injuries	Male	0-6 days	95+ years	Data Rich	1	1	Alcohol (liters per capita)	1000
Transport injuries	Male	0-6 days	95+ years	Data Rich	1	1	Vehicles - 2 wheels fraction (proportion)	--
Transport injuries	Male	0-6 days	95+ years	Data Rich	1	1	Vehicles - 2+4 wheels (per capita)	--
Transport injuries	Male	0-6 days	95+ years	Data Rich	-1	2	Healthcare access and quality index	466
Transport injuries	Male	0-6 days	95+ years	Data Rich	0	2	Population Density (500-1000 ppl/sqkm, proportion)	373
Transport injuries	Male	0-6 days	95+ years	Data Rich	0	2	Population Density (300-500 ppl/sqkm, proportion)	424
Transport injuries	Male	0-6 days	95+ years	Data Rich	0	2	LDI (I$ per capita)	--
Transport injuries	Male	0-6 days	95+ years	Data Rich	-1	3	Education (years per capita)	385
Transport injuries	Male	0-6 days	95+ years	Data Rich	-1	3	Socio-demographic Index	437
Transport injuries	Male	0-6 days	95+ years	Data Rich	1	3	Rainfall Quintile 5 (proportion)	--
Transport injuries	Male	0-6 days	95+ years	Global	1	1	Alcohol (liters per capita)	470
Transport injuries	Male	0-6 days	95+ years	Global	1	1	Vehicles - 2 wheels fraction (proportion)	834
Transport injuries	Male	0-6 days	95+ years	Global	1	1	Vehicles - 2+4 wheels (per capita)	--
Transport injuries	Male	0-6 days	95+ years	Global	-1	2	Healthcare access and quality index	535
Transport injuries	Male	0-6 days	95+ years	Global	0	2	Population Density (300-500 ppl/sqkm, proportion)	352
Transport injuries	Male	0-6 days	95+ years	Global	0	2	Population Density (500-1000 ppl/sqkm, proportion)	481
Transport injuries	Male	0-6 days	95+ years	Global	0	2	LDI (I$ per capita)	--
Transport injuries	Male	0-6 days	95+ years	Global	-1	3	Socio-demographic Index	309
Transport injuries	Male	0-6 days	95+ years	Global	-1	3	Education (years per capita)	338
Transport injuries	Male	0-6 days	95+ years	Global	1	3	Rainfall Quintile 5 (proportion)	--
Road injuries	Female	0-6 days	95+ years	Data Rich	1	1	Alcohol (liters per capita)	3
Road injuries	Female	0-6 days	95+ years	Data Rich	1	1	Vehicles - 2 wheels fraction (proportion)	961
Road injuries	Female	0-6 days	95+ years	Data Rich	1	1	Log-transformed SEV scalar: Road Inj	--
Road injuries	Female	0-6 days	95+ years	Data Rich	1	1	Vehicles - 2 wheels (per capita)	--
Road injuries	Female	0-6 days	95+ years	Data Rich	1	1	Vehicles - 2+4 wheels (per capita)	--
Road injuries	Female	0-6 days	95+ years	Data Rich	1	1	Vehicles - 4 wheels (per capita)	--
Road injuries	Female	0-6 days	95+ years	Data Rich	-1	2	Healthcare access and quality index	240
Road injuries	Female	0-6 days	95+ years	Data Rich	0	2	Population Density (500-1000 ppl/sqkm, proportion)	115
Road injuries	Female	0-6 days	95+ years	Data Rich	0	2	Population Density (300-500 ppl/sqkm, proportion)	240

Table S16. CODEm covariates used, level of covariate, and expected direction of covariate by cause, sex, and age
Covariates that CODEm did not select during the covariate selection process have no draw counts listed.

Cause	Sex	Age Start	Age End	Model Type	Direction	Level	Covariate Name	Number of Draws
Road injuries	Female	0-6 days	95+ years	Data Rich	1	2	Population 15 to 30 (proportion)	240
Road injuries	Female	0-6 days	95+ years	Data Rich	-1	3	Education (years per capita)	0
Road injuries	Female	0-6 days	95+ years	Data Rich	-1	3	Socio-demographic Index	0
Road injuries	Female	0-6 days	95+ years	Data Rich	0	3	LDI (I$ per capita)	--
Road injuries	Female	0-6 days	95+ years	Global	1	1	Alcohol (liters per capita)	338
Road injuries	Female	0-6 days	95+ years	Global	1	1	Vehicles - 2 wheels fraction (proportion)	908
Road injuries	Female	0-6 days	95+ years	Global	1	1	Log-transformed SEV scalar: Road Inj	--
Road injuries	Female	0-6 days	95+ years	Global	1	1	Vehicles - 2 wheels (per capita)	--
Road injuries	Female	0-6 days	95+ years	Global	1	1	Vehicles - 2+4 wheels (per capita)	--
Road injuries	Female	0-6 days	95+ years	Global	1	1	Vehicles - 4 wheels (per capita)	--
Road injuries	Female	0-6 days	95+ years	Global	-1	2	Healthcare access and quality index	500
Road injuries	Female	0-6 days	95+ years	Global	0	2	Population Density (300-500 ppl/sqkm, proportion)	534
Road injuries	Female	0-6 days	95+ years	Global	0	2	Population Density (500-1000 ppl/sqkm, proportion)	534
Road injuries	Female	0-6 days	95+ years	Global	1	2	Population 15 to 30 (proportion)	71
Road injuries	Female	0-6 days	95+ years	Global	-1	3	Socio-demographic Index	184
Road injuries	Female	0-6 days	95+ years	Global	-1	3	Education (years per capita)	242
Road injuries	Female	0-6 days	95+ years	Global	0	3	LDI (I$ per capita)	--
Road injuries	Male	0-6 days	95+ years	Data Rich	1	1	Alcohol (liters per capita)	519
Road injuries	Male	0-6 days	95+ years	Data Rich	1	1	Vehicles - 2 wheels fraction (proportion)	866
Road injuries	Male	0-6 days	95+ years	Data Rich	1	1	Log-transformed SEV scalar: Road Inj	--
Road injuries	Male	0-6 days	95+ years	Data Rich	1	1	Vehicles - 2 wheels (per capita)	--
Road injuries	Male	0-6 days	95+ years	Data Rich	1	1	Vehicles - 2+4 wheels (per capita)	--
Road injuries	Male	0-6 days	95+ years	Data Rich	1	1	Vehicles - 4 wheels (per capita)	--
Road injuries	Male	0-6 days	95+ years	Data Rich	-1	2	Healthcare access and quality index	726
Road injuries	Male	0-6 days	95+ years	Data Rich	0	2	Population Density (500-1000 ppl/sqkm, proportion)	68
Road injuries	Male	0-6 days	95+ years	Data Rich	0	2	Population Density (300-500 ppl/sqkm, proportion)	326
Road injuries	Male	0-6 days	95+ years	Data Rich	1	2	Population 15 to 30 (proportion)	382
Road injuries	Male	0-6 days	95+ years	Data Rich	-1	3	Socio-demographic Index	1
Road injuries	Male	0-6 days	95+ years	Data Rich	-1	3	Education (years per capita)	361
Road injuries	Male	0-6 days	95+ years	Data Rich	0	3	LDI (I$ per capita)	--
Road injuries	Male	0-6 days	95+ years	Global	1	1	Vehicles - 2 wheels fraction (proportion)	398
Road injuries	Male	0-6 days	95+ years	Global	1	1	Alcohol (liters per capita)	963
Road injuries	Male	0-6 days	95+ years	Global	1	1	Log-transformed SEV scalar: Road Inj	--
Road injuries	Male	0-6 days	95+ years	Global	1	1	Vehicles - 2 wheels (per capita)	--
Road injuries	Male	0-6 days	95+ years	Global	1	1	Vehicles - 2+4 wheels (per capita)	--
Road injuries	Male	0-6 days	95+ years	Global	1	1	Vehicles - 4 wheels (per capita)	--
Road injuries	Male	0-6 days	95+ years	Global	-1	2	Healthcare access and quality index	433
Road injuries	Male	0-6 days	95+ years	Global	0	2	Population Density (300-500 ppl/sqkm, proportion)	549
Road injuries	Male	0-6 days	95+ years	Global	0	2	Population Density (500-1000 ppl/sqkm, proportion)	615
Road injuries	Male	0-6 days	95+ years	Global	1	2	Population 15 to 30 (proportion)	397
Road injuries	Male	0-6 days	95+ years	Global	-1	3	Education (years per capita)	328
Road injuries	Male	0-6 days	95+ years	Global	-1	3	Socio-demographic Index	343
Road injuries	Male	0-6 days	95+ years	Global	0	3	LDI (I$ per capita)	--
Pedestrian road injuries	Female	0-6 days	95+ years	Data Rich	1	1	Vehicles - 2 wheels fraction (proportion)	246
Pedestrian road injuries	Female	0-6 days	95+ years	Data Rich	1	1	Alcohol (liters per capita)	298
Pedestrian road injuries	Female	0-6 days	95+ years	Data Rich	1	1	Log-transformed SEV scalar: Pedest	--
Pedestrian road injuries	Female	0-6 days	95+ years	Data Rich	1	1	Vehicles - 2+4 wheels (per capita)	--
Pedestrian road injuries	Female	0-6 days	95+ years	Data Rich	-1	2	Healthcare access and quality index	10
Pedestrian road injuries	Female	0-6 days	95+ years	Data Rich	0	2	Population Density (300-500 ppl/sqkm, proportion)	10
Pedestrian road injuries	Female	0-6 days	95+ years	Data Rich	0	2	Population Density (500-1000 ppl/sqkm, proportion)	10
Pedestrian road injuries	Female	0-6 days	95+ years	Data Rich	0	2	LDI (I$ per capita)	--
Pedestrian road injuries	Female	0-6 days	95+ years	Data Rich	-1	3	Socio-demographic Index	24
Pedestrian road injuries	Female	0-6 days	95+ years	Data Rich	-1	3	Education (years per capita)	976

Table S16. CODEm covariates used, level of covariate, and expected direction of covariate by cause, sex, and age
Covariates that CODEm did not select during the covariate selection process have no draw counts listed.

Cause	Sex	Age Start	Age End	Model Type	Direction	Level	Covariate Name	Number of Draws
Pedestrian road injuries	Female	0-6 days	95+ years	Data Rich	1	3	Rainfall Quintile 5 (proportion)	--
Pedestrian road injuries	Female	0-6 days	95+ years	Global	1	1	Vehicles - 2 wheels fraction (proportion)	479
Pedestrian road injuries	Female	0-6 days	95+ years	Global	1	1	Alcohol (liters per capita)	656
Pedestrian road injuries	Female	0-6 days	95+ years	Global	1	1	Log-transformed SEV scalar: Pedest	--
Pedestrian road injuries	Female	0-6 days	95+ years	Global	1	1	Vehicles - 2+4 wheels (per capita)	--
Pedestrian road injuries	Female	0-6 days	95+ years	Global	-1	2	Healthcare access and quality index	139
Pedestrian road injuries	Female	0-6 days	95+ years	Global	0	2	Population Density (300-500 ppl/sqkm, proportion)	298
Pedestrian road injuries	Female	0-6 days	95+ years	Global	0	2	Population Density (500-1000 ppl/sqkm, proportion)	440
Pedestrian road injuries	Female	0-6 days	95+ years	Global	0	2	LDI (I$ per capita)	--
Pedestrian road injuries	Female	0-6 days	95+ years	Global	-1	3	Socio-demographic Index	120
Pedestrian road injuries	Female	0-6 days	95+ years	Global	-1	3	Education (years per capita)	694
Pedestrian road injuries	Female	0-6 days	95+ years	Global	1	3	Rainfall Quintile 5 (proportion)	3
Pedestrian road injuries	Male	0-6 days	95+ years	Data Rich	1	1	Alcohol (liters per capita)	38
Pedestrian road injuries	Male	0-6 days	95+ years	Data Rich	1	1	Log-transformed SEV scalar: Pedest	--
Pedestrian road injuries	Male	0-6 days	95+ years	Data Rich	1	1	Vehicles - 2 wheels fraction (proportion)	--
Pedestrian road injuries	Male	0-6 days	95+ years	Data Rich	1	1	Vehicles - 2+4 wheels (per capita)	--
Pedestrian road injuries	Male	0-6 days	95+ years	Data Rich	-1	2	Healthcare access and quality index	7
Pedestrian road injuries	Male	0-6 days	95+ years	Data Rich	0	2	Population Density (500-1000 ppl/sqkm, proportion)	0
Pedestrian road injuries	Male	0-6 days	95+ years	Data Rich	0	2	Population Density (300-500 ppl/sqkm, proportion)	808
Pedestrian road injuries	Male	0-6 days	95+ years	Data Rich	0	2	LDI (I$ per capita)	--
Pedestrian road injuries	Male	0-6 days	95+ years	Data Rich	-1	3	Socio-demographic Index	834
Pedestrian road injuries	Male	0-6 days	95+ years	Data Rich	-1	3	Education (years per capita)	999
Pedestrian road injuries	Male	0-6 days	95+ years	Data Rich	1	3	Rainfall Quintile 5 (proportion)	--
Pedestrian road injuries	Male	0-6 days	95+ years	Global	1	1	Vehicles - 2 wheels fraction (proportion)	465
Pedestrian road injuries	Male	0-6 days	95+ years	Global	1	1	Alcohol (liters per capita)	928
Pedestrian road injuries	Male	0-6 days	95+ years	Global	1	1	Log-transformed SEV scalar: Pedest	--
Pedestrian road injuries	Male	0-6 days	95+ years	Global	1	1	Vehicles - 2+4 wheels (per capita)	--
Pedestrian road injuries	Male	0-6 days	95+ years	Global	-1	2	Healthcare access and quality index	578
Pedestrian road injuries	Male	0-6 days	95+ years	Global	0	2	Population Density (500-1000 ppl/sqkm, proportion)	425
Pedestrian road injuries	Male	0-6 days	95+ years	Global	0	2	Population Density (300-500 ppl/sqkm, proportion)	476
Pedestrian road injuries	Male	0-6 days	95+ years	Global	0	2	LDI (I$ per capita)	--
Pedestrian road injuries	Male	0-6 days	95+ years	Global	-1	3	Socio-demographic Index	384
Pedestrian road injuries	Male	0-6 days	95+ years	Global	-1	3	Education (years per capita)	525
Pedestrian road injuries	Male	0-6 days	95+ years	Global	1	3	Rainfall Quintile 5 (proportion)	--
Cyclist road injuries	Female	1-4 years	95+ years	Data Rich	1	1	Vehicles - 2+4 wheels (per capita)	251
Cyclist road injuries	Female	1-4 years	95+ years	Data Rich	1	1	Vehicles - 2 wheels fraction (proportion)	385
Cyclist road injuries	Female	1-4 years	95+ years	Data Rich	1	1	Alcohol (liters per capita)	773
Cyclist road injuries	Female	1-4 years	95+ years	Data Rich	1	1	Log-transformed SEV scalar: Cyclist	--
Cyclist road injuries	Female	1-4 years	95+ years	Data Rich	-1	2	Healthcare access and quality index	0
Cyclist road injuries	Female	1-4 years	95+ years	Data Rich	0	2	Population Density (300-500 ppl/sqkm, proportion)	179
Cyclist road injuries	Female	1-4 years	95+ years	Data Rich	0	2	Population Density (500-1000 ppl/sqkm, proportion)	179
Cyclist road injuries	Female	1-4 years	95+ years	Data Rich	0	2	LDI (I$ per capita)	--
Cyclist road injuries	Female	1-4 years	95+ years	Data Rich	-1	3	Education (years per capita)	0
Cyclist road injuries	Female	1-4 years	95+ years	Data Rich	0	3	Socio-demographic Index	714
Cyclist road injuries	Female	1-4 years	95+ years	Global	1	1	Vehicles - 2+4 wheels (per capita)	267
Cyclist road injuries	Female	1-4 years	95+ years	Global	1	1	Alcohol (liters per capita)	684
Cyclist road injuries	Female	1-4 years	95+ years	Global	1	1	Vehicles - 2 wheels fraction (proportion)	745
Cyclist road injuries	Female	1-4 years	95+ years	Global	1	1	Log-transformed SEV scalar: Cyclist	--
Cyclist road injuries	Female	1-4 years	95+ years	Global	-1	2	Healthcare access and quality index	74
Cyclist road injuries	Female	1-4 years	95+ years	Global	0	2	Population Density (500-1000 ppl/sqkm, proportion)	376
Cyclist road injuries	Female	1-4 years	95+ years	Global	0	2	Population Density (300-500 ppl/sqkm, proportion)	468
Cyclist road injuries	Female	1-4 years	95+ years	Global	0	2	LDI (I$ per capita)	--
Cyclist road injuries	Female	1-4 years	95+ years	Global	-1	3	Education (years per capita)	42

Table S16. CODEm covariates used, level of covariate, and expected direction of covariate by cause, sex, and age
Covariates that CODEm did not select during the covariate selection process have no draw counts listed.

Cause	Sex	Age Start	Age End	Model Type	Direction	Level	Covariate Name	Number of Draws
Cyclist road injuries	Female	1-4 years	95+ years	Global	0	3	Socio-demographic Index	475
Cyclist road injuries	Male	1-4 years	95+ years	Data Rich	1	1	Vehicles - 2+4 wheels (per capita)	0
Cyclist road injuries	Male	1-4 years	95+ years	Data Rich	1	1	Vehicles - 2 wheels fraction (proportion)	191
Cyclist road injuries	Male	1-4 years	95+ years	Data Rich	1	1	Alcohol (liters per capita)	1000
Cyclist road injuries	Male	1-4 years	95+ years	Data Rich	1	1	Log-transformed SEV scalar: Cyclist	--
Cyclist road injuries	Male	1-4 years	95+ years	Data Rich	-1	2	Healthcare access and quality index	0
Cyclist road injuries	Male	1-4 years	95+ years	Data Rich	0	2	Population Density (300-500 ppl/sqkm, proportion)	803
Cyclist road injuries	Male	1-4 years	95+ years	Data Rich	0	2	Population Density (500-1000 ppl/sqkm, proportion)	962
Cyclist road injuries	Male	1-4 years	95+ years	Data Rich	0	2	LDI (I$ per capita)	--
Cyclist road injuries	Male	1-4 years	95+ years	Data Rich	-1	3	Education (years per capita)	0
Cyclist road injuries	Male	1-4 years	95+ years	Data Rich	0	3	Socio-demographic Index	999
Cyclist road injuries	Male	1-4 years	95+ years	Global	1	1	Vehicles - 2+4 wheels (per capita)	246
Cyclist road injuries	Male	1-4 years	95+ years	Global	1	1	Vehicles - 2 wheels fraction (proportion)	575
Cyclist road injuries	Male	1-4 years	95+ years	Global	1	1	Alcohol (liters per capita)	682
Cyclist road injuries	Male	1-4 years	95+ years	Global	1	1	Log-transformed SEV scalar: Cyclist	--
Cyclist road injuries	Male	1-4 years	95+ years	Global	-1	2	Healthcare access and quality index	110
Cyclist road injuries	Male	1-4 years	95+ years	Global	0	2	Population Density (500-1000 ppl/sqkm, proportion)	524
Cyclist road injuries	Male	1-4 years	95+ years	Global	0	2	Population Density (300-500 ppl/sqkm, proportion)	559
Cyclist road injuries	Male	1-4 years	95+ years	Global	0	2	LDI (I$ per capita)	--
Cyclist road injuries	Male	1-4 years	95+ years	Global	-1	3	Education (years per capita)	32
Cyclist road injuries	Male	1-4 years	95+ years	Global	0	3	Socio-demographic Index	438
Motorcyclist road injuries	Female	0-6 days	95+ years	Data Rich	1	1	Alcohol (liters per capita)	1000
Motorcyclist road injuries	Female	0-6 days	95+ years	Data Rich	1	1	Log-transformed SEV scalar: Mot Cyc	--
Motorcyclist road injuries	Female	0-6 days	95+ years	Data Rich	1	1	Vehicles - 2 wheels (per capita)	--
Motorcyclist road injuries	Female	0-6 days	95+ years	Data Rich	-1	2	Healthcare access and quality index	8
Motorcyclist road injuries	Female	0-6 days	95+ years	Data Rich	0	2	Population Density (300-500 ppl/sqkm, proportion)	344
Motorcyclist road injuries	Female	0-6 days	95+ years	Data Rich	0	2	Population Density (500-1000 ppl/sqkm, proportion)	851
Motorcyclist road injuries	Female	0-6 days	95+ years	Data Rich	0	2	LDI (I$ per capita)	--
Motorcyclist road injuries	Female	0-6 days	95+ years	Data Rich	-1	3	Education (years per capita)	998
Motorcyclist road injuries	Female	0-6 days	95+ years	Data Rich	0	3	Socio-demographic Index	17
Motorcyclist road injuries	Female	0-6 days	95+ years	Data Rich	1	3	Rainfall Quintile 5 (proportion)	--
Motorcyclist road injuries	Female	0-6 days	95+ years	Global	1	1	Alcohol (liters per capita)	426
Motorcyclist road injuries	Female	0-6 days	95+ years	Global	1	1	Log-transformed SEV scalar: Mot Cyc	--
Motorcyclist road injuries	Female	0-6 days	95+ years	Global	1	1	Vehicles - 2 wheels (per capita)	--
Motorcyclist road injuries	Female	0-6 days	95+ years	Global	-1	2	Healthcare access and quality index	486
Motorcyclist road injuries	Female	0-6 days	95+ years	Global	0	2	Population Density (500-1000 ppl/sqkm, proportion)	288
Motorcyclist road injuries	Female	0-6 days	95+ years	Global	0	2	Population Density (300-500 ppl/sqkm, proportion)	450
Motorcyclist road injuries	Female	0-6 days	95+ years	Global	0	2	LDI (I$ per capita)	--
Motorcyclist road injuries	Female	0-6 days	95+ years	Global	-1	3	Education (years per capita)	596
Motorcyclist road injuries	Female	0-6 days	95+ years	Global	0	3	Socio-demographic Index	297
Motorcyclist road injuries	Female	0-6 days	95+ years	Global	1	3	Rainfall Quintile 5 (proportion)	--
Motorcyclist road injuries	Male	0-6 days	95+ years	Data Rich	1	1	Alcohol (liters per capita)	1000
Motorcyclist road injuries	Male	0-6 days	95+ years	Data Rich	1	1	Log-transformed SEV scalar: Mot Cyc	--
Motorcyclist road injuries	Male	0-6 days	95+ years	Data Rich	1	1	Vehicles - 2 wheels (per capita)	--
Motorcyclist road injuries	Male	0-6 days	95+ years	Data Rich	-1	2	Healthcare access and quality index	1
Motorcyclist road injuries	Male	0-6 days	95+ years	Data Rich	0	2	Population Density (300-500 ppl/sqkm, proportion)	0
Motorcyclist road injuries	Male	0-6 days	95+ years	Data Rich	0	2	Population Density (500-1000 ppl/sqkm, proportion)	161
Motorcyclist road injuries	Male	0-6 days	95+ years	Data Rich	0	2	LDI (I$ per capita)	--
Motorcyclist road injuries	Male	0-6 days	95+ years	Data Rich	-1	3	Education (years per capita)	32
Motorcyclist road injuries	Male	0-6 days	95+ years	Data Rich	0	3	Socio-demographic Index	801
Motorcyclist road injuries	Male	0-6 days	95+ years	Data Rich	1	3	Rainfall Quintile 5 (proportion)	--
Motorcyclist road injuries	Male	0-6 days	95+ years	Global	1	1	Alcohol (liters per capita)	1000

Table S16. CODEm covariates used, level of covariate, and expected direction of covariate by cause, sex, and age<br>*Covariates that CODEm did not select during the covariate selection process have no draw counts listed.*								
**Cause**	**Sex**	**Age Start**	**Age End**	**Model Type**	**Direction**	**Level**	**Covariate Name**	**Number of Draws**
Motorcyclist road injuries	Male	0-6 days	95+ years	Global	1	1	Log-transformed SEV scalar: Mot Cyc	--
Motorcyclist road injuries	Male	0-6 days	95+ years	Global	1	1	Vehicles - 2 wheels (per capita)	--
Motorcyclist road injuries	Male	0-6 days	95+ years	Global	-1	2	Healthcare access and quality index	266
Motorcyclist road injuries	Male	0-6 days	95+ years	Global	0	2	Population Density (300-500 ppl/sqkm, proportion)	615
Motorcyclist road injuries	Male	0-6 days	95+ years	Global	0	2	Population Density (500-1000 ppl/sqkm, proportion)	615
Motorcyclist road injuries	Male	0-6 days	95+ years	Global	0	2	LDI (I$ per capita)	--
Motorcyclist road injuries	Male	0-6 days	95+ years	Global	-1	3	Education (years per capita)	328
Motorcyclist road injuries	Male	0-6 days	95+ years	Global	0	3	Socio-demographic Index	180
Motorcyclist road injuries	Male	0-6 days	95+ years	Global	1	3	Rainfall Quintile 5 (proportion)	--
Motor vehicle road injuries	Female	0-6 days	95+ years	Data Rich	1	1	Alcohol (liters per capita)	1000
Motor vehicle road injuries	Female	0-6 days	95+ years	Data Rich	1	1	Log-transformed SEV scalar: Mot Veh	--
Motor vehicle road injuries	Female	0-6 days	95+ years	Data Rich	1	1	Vehicles - 4 wheels (per capita)	--
Motor vehicle road injuries	Female	0-6 days	95+ years	Data Rich	-1	2	Healthcare access and quality index	57
Motor vehicle road injuries	Female	0-6 days	95+ years	Data Rich	0	2	Population Density (300-500 ppl/sqkm, proportion)	471
Motor vehicle road injuries	Female	0-6 days	95+ years	Data Rich	0	2	Population Density (500-1000 ppl/sqkm, proportion)	471
Motor vehicle road injuries	Female	0-6 days	95+ years	Data Rich	0	3	Education (years per capita)	609
Motor vehicle road injuries	Female	0-6 days	95+ years	Data Rich	0	3	Socio-demographic Index	762
Motor vehicle road injuries	Female	0-6 days	95+ years	Data Rich	0	3	LDI (I$ per capita)	--
Motor vehicle road injuries	Female	0-6 days	95+ years	Data Rich	1	3	Rainfall Quintile 5 (proportion)	--
Motor vehicle road injuries	Female	0-6 days	95+ years	Global	1	1	Alcohol (liters per capita)	1000
Motor vehicle road injuries	Female	0-6 days	95+ years	Global	1	1	Log-transformed SEV scalar: Mot Veh	--
Motor vehicle road injuries	Female	0-6 days	95+ years	Global	1	1	Vehicles - 4 wheels (per capita)	--
Motor vehicle road injuries	Female	0-6 days	95+ years	Global	-1	2	Healthcare access and quality index	199
Motor vehicle road injuries	Female	0-6 days	95+ years	Global	0	2	Population Density (300-500 ppl/sqkm, proportion)	665
Motor vehicle road injuries	Female	0-6 days	95+ years	Global	0	2	Population Density (500-1000 ppl/sqkm, proportion)	665
Motor vehicle road injuries	Female	0-6 days	95+ years	Global	0	3	Socio-demographic Index	399
Motor vehicle road injuries	Female	0-6 days	95+ years	Global	0	3	Education (years per capita)	477
Motor vehicle road injuries	Female	0-6 days	95+ years	Global	0	3	LDI (I$ per capita)	--
Motor vehicle road injuries	Female	0-6 days	95+ years	Global	1	3	Rainfall Quintile 5 (proportion)	--
Motor vehicle road injuries	Male	0-6 days	95+ years	Data Rich	1	1	Alcohol (liters per capita)	766
Motor vehicle road injuries	Male	0-6 days	95+ years	Data Rich	1	1	Log-transformed SEV scalar: Mot Veh	--
Motor vehicle road injuries	Male	0-6 days	95+ years	Data Rich	1	1	Vehicles - 4 wheels (per capita)	--
Motor vehicle road injuries	Male	0-6 days	95+ years	Data Rich	-1	2	Healthcare access and quality index	219
Motor vehicle road injuries	Male	0-6 days	95+ years	Data Rich	0	2	Population Density (300-500 ppl/sqkm, proportion)	--
Motor vehicle road injuries	Male	0-6 days	95+ years	Data Rich	0	2	Population Density (500-1000 ppl/sqkm, proportion)	--
Motor vehicle road injuries	Male	0-6 days	95+ years	Data Rich	0	3	Education (years per capita)	304
Motor vehicle road injuries	Male	0-6 days	95+ years	Data Rich	0	3	Socio-demographic Index	382
Motor vehicle road injuries	Male	0-6 days	95+ years	Data Rich	0	3	LDI (I$ per capita)	--
Motor vehicle road injuries	Male	0-6 days	95+ years	Data Rich	1	3	Rainfall Quintile 5 (proportion)	--
Motor vehicle road injuries	Male	0-6 days	95+ years	Global	1	1	Alcohol (liters per capita)	353
Motor vehicle road injuries	Male	0-6 days	95+ years	Global	1	1	Log-transformed SEV scalar: Mot Veh	--
Motor vehicle road injuries	Male	0-6 days	95+ years	Global	1	1	Vehicles - 4 wheels (per capita)	--
Motor vehicle road injuries	Male	0-6 days	95+ years	Global	-1	2	Healthcare access and quality index	242
Motor vehicle road injuries	Male	0-6 days	95+ years	Global	0	2	Population Density (300-500 ppl/sqkm, proportion)	770
Motor vehicle road injuries	Male	0-6 days	95+ years	Global	0	2	Population Density (500-1000 ppl/sqkm, proportion)	770
Motor vehicle road injuries	Male	0-6 days	95+ years	Global	0	3	Education (years per capita)	432
Motor vehicle road injuries	Male	0-6 days	95+ years	Global	0	3	Socio-demographic Index	571
Motor vehicle road injuries	Male	0-6 days	95+ years	Global	0	3	LDI (I$ per capita)	--
Motor vehicle road injuries	Male	0-6 days	95+ years	Global	1	3	Rainfall Quintile 5 (proportion)	--
Other road injuries	Female	0-6 days	95+ years	Data Rich	1	1	Vehicles - 2 wheels fraction (proportion)	1000
Other road injuries	Female	0-6 days	95+ years	Data Rich	1	1	Alcohol (liters per capita)	--
Other road injuries	Female	0-6 days	95+ years	Data Rich	1	1	Log-transformed SEV scalar: Oth Road	--

Table S16. CODEm covariates used, level of covariate, and expected direction of covariate by cause, sex, and age<br>*Covariates that CODEm did not select during the covariate selection process have no draw counts listed.*								
**Cause**	**Sex**	**Age Start**	**Age End**	**Model Type**	**Direction**	**Level**	**Covariate Name**	**Number of Draws**
Other road injuries	Female	0-6 days	95+ years	Data Rich	1	1	Vehicles - 2+4 wheels (per capita)	--
Other road injuries	Female	0-6 days	95+ years	Data Rich	-1	2	Healthcare access and quality index	1
Other road injuries	Female	0-6 days	95+ years	Data Rich	0	2	LDI (I$ per capita)	--
Other road injuries	Female	0-6 days	95+ years	Data Rich	-1	3	Socio-demographic Index	166
Other road injuries	Female	0-6 days	95+ years	Data Rich	1	3	Rainfall Quintile 5 (proportion)	--
Other road injuries	Female	0-6 days	95+ years	Global	1	1	Vehicles - 2 wheels fraction (proportion)	1000
Other road injuries	Female	0-6 days	95+ years	Global	1	1	Alcohol (liters per capita)	--
Other road injuries	Female	0-6 days	95+ years	Global	1	1	Log-transformed SEV scalar: Oth Road	--
Other road injuries	Female	0-6 days	95+ years	Global	1	1	Vehicles - 2+4 wheels (per capita)	--
Other road injuries	Female	0-6 days	95+ years	Global	-1	2	Healthcare access and quality index	161
Other road injuries	Female	0-6 days	95+ years	Global	0	2	LDI (I$ per capita)	--
Other road injuries	Female	0-6 days	95+ years	Global	-1	3	Socio-demographic Index	193
Other road injuries	Female	0-6 days	95+ years	Global	1	3	Rainfall Quintile 5 (proportion)	--
Other road injuries	Male	0-6 days	95+ years	Data Rich	1	1	Vehicles - 2 wheels fraction (proportion)	6
Other road injuries	Male	0-6 days	95+ years	Data Rich	1	1	Alcohol (liters per capita)	--
Other road injuries	Male	0-6 days	95+ years	Data Rich	1	1	Log-transformed SEV scalar: Oth Road	--
Other road injuries	Male	0-6 days	95+ years	Data Rich	1	1	Vehicles - 2+4 wheels (per capita)	--
Other road injuries	Male	0-6 days	95+ years	Data Rich	-1	2	Healthcare access and quality index	1
Other road injuries	Male	0-6 days	95+ years	Data Rich	0	2	LDI (I$ per capita)	--
Other road injuries	Male	0-6 days	95+ years	Data Rich	-1	3	Socio-demographic Index	967
Other road injuries	Male	0-6 days	95+ years	Data Rich	1	3	Rainfall Quintile 5 (proportion)	--
Other road injuries	Male	0-6 days	95+ years	Global	1	1	Vehicles - 2 wheels fraction (proportion)	294
Other road injuries	Male	0-6 days	95+ years	Global	1	1	Alcohol (liters per capita)	--
Other road injuries	Male	0-6 days	95+ years	Global	1	1	Log-transformed SEV scalar: Oth Road	--
Other road injuries	Male	0-6 days	95+ years	Global	1	1	Vehicles - 2+4 wheels (per capita)	--
Other road injuries	Male	0-6 days	95+ years	Global	-1	2	Healthcare access and quality index	--
Other road injuries	Male	0-6 days	95+ years	Global	0	2	LDI (I$ per capita)	--
Other road injuries	Male	0-6 days	95+ years	Global	-1	3	Socio-demographic Index	674
Other road injuries	Male	0-6 days	95+ years	Global	1	3	Rainfall Quintile 5 (proportion)	--
Other transport injuries	Female	0-6 days	95+ years	Data Rich	1	1	Vehicles - 2 wheels fraction (proportion)	4
Other transport injuries	Female	0-6 days	95+ years	Data Rich	1	1	Alcohol (liters per capita)	702
Other transport injuries	Female	0-6 days	95+ years	Data Rich	1	1	Log-transformed SEV scalar: Oth Trans	--
Other transport injuries	Female	0-6 days	95+ years	Data Rich	1	1	Vehicles - 2+4 wheels (per capita)	--
Other transport injuries	Female	0-6 days	95+ years	Data Rich	-1	2	Healthcare access and quality index	4
Other transport injuries	Female	0-6 days	95+ years	Data Rich	0	2	Population Density (300-500 ppl/sqkm, proportion)	233
Other transport injuries	Female	0-6 days	95+ years	Data Rich	0	2	Population Density (500-1000 ppl/sqkm, proportion)	234
Other transport injuries	Female	0-6 days	95+ years	Data Rich	0	3	Education (years per capita)	36
Other transport injuries	Female	0-6 days	95+ years	Data Rich	0	3	Socio-demographic Index	62
Other transport injuries	Female	0-6 days	95+ years	Data Rich	0	3	LDI (I$ per capita)	--
Other transport injuries	Female	0-6 days	95+ years	Data Rich	1	3	Rainfall Quintile 5 (proportion)	--
Other transport injuries	Female	0-6 days	95+ years	Global	1	1	Vehicles - 2 wheels fraction (proportion)	436
Other transport injuries	Female	0-6 days	95+ years	Global	1	1	Alcohol (liters per capita)	702
Other transport injuries	Female	0-6 days	95+ years	Global	1	1	Log-transformed SEV scalar: Oth Trans	--
Other transport injuries	Female	0-6 days	95+ years	Global	1	1	Vehicles - 2+4 wheels (per capita)	--
Other transport injuries	Female	0-6 days	95+ years	Global	-1	2	Healthcare access and quality index	1
Other transport injuries	Female	0-6 days	95+ years	Global	0	2	Population Density (300-500 ppl/sqkm, proportion)	481
Other transport injuries	Female	0-6 days	95+ years	Global	0	2	Population Density (500-1000 ppl/sqkm, proportion)	481
Other transport injuries	Female	0-6 days	95+ years	Global	0	3	Education (years per capita)	136
Other transport injuries	Female	0-6 days	95+ years	Global	0	3	Socio-demographic Index	150
Other transport injuries	Female	0-6 days	95+ years	Global	0	3	LDI (I$ per capita)	--
Other transport injuries	Female	0-6 days	95+ years	Global	1	3	Rainfall Quintile 5 (proportion)	--
Other transport injuries	Male	0-6 days	95+ years	Data Rich	1	1	Alcohol (liters per capita)	564
Other transport injuries	Male	0-6 days	95+ years	Data Rich	1	1	Vehicles - 2 wheels fraction (proportion)	999

Table S16. CODEm covariates used, level of covariate, and expected direction of covariate by cause, sex, and age<br>*Covariates that CODEm did not select during the covariate selection process have no draw counts listed.*								
**Cause**	**Sex**	**Age Start**	**Age End**	**Model Type**	**Direction**	**Level**	**Covariate Name**	**Number of Draws**
Other transport injuries	Male	0-6 days	95+ years	Data Rich	1	1	Log-transformed SEV scalar: Oth Trans	--
Other transport injuries	Male	0-6 days	95+ years	Data Rich	1	1	Vehicles - 2+4 wheels (per capita)	--
Other transport injuries	Male	0-6 days	95+ years	Data Rich	-1	2	Healthcare access and quality index	11
Other transport injuries	Male	0-6 days	95+ years	Data Rich	0	2	Population Density (300-500 ppl/sqkm, proportion)	414
Other transport injuries	Male	0-6 days	95+ years	Data Rich	0	2	Population Density (500-1000 ppl/sqkm, proportion)	586
Other transport injuries	Male	0-6 days	95+ years	Data Rich	0	3	Socio-demographic Index	70
Other transport injuries	Male	0-6 days	95+ years	Data Rich	0	3	Education (years per capita)	178
Other transport injuries	Male	0-6 days	95+ years	Data Rich	1	3	LDI (I$ per capita)	--
Other transport injuries	Male	0-6 days	95+ years	Data Rich	1	3	Rainfall Quintile 5 (proportion)	--
Other transport injuries	Male	0-6 days	95+ years	Global	1	1	Alcohol (liters per capita)	97
Other transport injuries	Male	0-6 days	95+ years	Global	1	1	Vehicles - 2 wheels fraction (proportion)	938
Other transport injuries	Male	0-6 days	95+ years	Global	1	1	Log-transformed SEV scalar: Oth Trans	--
Other transport injuries	Male	0-6 days	95+ years	Global	1	1	Vehicles - 2+4 wheels (per capita)	--
Other transport injuries	Male	0-6 days	95+ years	Global	-1	2	Healthcare access and quality index	103
Other transport injuries	Male	0-6 days	95+ years	Global	0	2	Population Density (300-500 ppl/sqkm, proportion)	507
Other transport injuries	Male	0-6 days	95+ years	Global	0	2	Population Density (500-1000 ppl/sqkm, proportion)	507
Other transport injuries	Male	0-6 days	95+ years	Global	0	3	Socio-demographic Index	376
Other transport injuries	Male	0-6 days	95+ years	Global	0	3	Education (years per capita)	486
Other transport injuries	Male	0-6 days	95+ years	Global	1	3	LDI (I$ per capita)	--
Other transport injuries	Male	0-6 days	95+ years	Global	1	3	Rainfall Quintile 5 (proportion)	--
Falls	Female	0-6 days	95+ years	Data Rich	1	1	Alcohol (liters per capita)	967
Falls	Female	0-6 days	95+ years	Data Rich	1	1	Log-transformed SEV scalar: Falls	--
Falls	Female	0-6 days	95+ years	Data Rich	-1	2	Healthcare access and quality index	12
Falls	Female	0-6 days	95+ years	Data Rich	-1	2	milk adjusted(g)	432
Falls	Female	0-6 days	95+ years	Data Rich	0	3	Socio-demographic Index	21
Falls	Female	0-6 days	95+ years	Data Rich	0	3	LDI (I$ per capita)	--
Falls	Female	0-6 days	95+ years	Data Rich	1	3	Elevation Over 1500m (proportion)	276
Falls	Female	0-6 days	95+ years	Global	1	1	Alcohol (liters per capita)	836
Falls	Female	0-6 days	95+ years	Global	1	1	Log-transformed SEV scalar: Falls	--
Falls	Female	0-6 days	95+ years	Global	-1	2	milk adjusted(g)	45
Falls	Female	0-6 days	95+ years	Global	-1	2	Healthcare access and quality index	388
Falls	Female	0-6 days	95+ years	Global	0	3	Socio-demographic Index	658
Falls	Female	0-6 days	95+ years	Global	0	3	LDI (I$ per capita)	--
Falls	Female	0-6 days	95+ years	Global	1	3	Elevation Over 1500m (proportion)	494
Falls	Male	0-6 days	95+ years	Data Rich	1	1	Alcohol (liters per capita)	806
Falls	Male	0-6 days	95+ years	Data Rich	1	1	Log-transformed SEV scalar: Falls	--
Falls	Male	0-6 days	95+ years	Data Rich	-1	2	Healthcare access and quality index	48
Falls	Male	0-6 days	95+ years	Data Rich	-1	2	milk adjusted(g)	--
Falls	Male	0-6 days	95+ years	Data Rich	0	3	Socio-demographic Index	727
Falls	Male	0-6 days	95+ years	Data Rich	0	3	LDI (I$ per capita)	--
Falls	Male	0-6 days	95+ years	Data Rich	1	3	Elevation Over 1500m (proportion)	737
Falls	Male	0-6 days	95+ years	Global	1	1	Alcohol (liters per capita)	1000
Falls	Male	0-6 days	95+ years	Global	1	1	Log-transformed SEV scalar: Falls	--
Falls	Male	0-6 days	95+ years	Global	-1	2	Healthcare access and quality index	317
Falls	Male	0-6 days	95+ years	Global	-1	2	milk adjusted(g)	--
Falls	Male	0-6 days	95+ years	Global	0	3	Socio-demographic Index	561
Falls	Male	0-6 days	95+ years	Global	0	3	LDI (I$ per capita)	--
Falls	Male	0-6 days	95+ years	Global	1	3	Elevation Over 1500m (proportion)	561
Drowning	Female	0-6 days	95+ years	Data Rich	-1	1	Landlocked Nation (binary)	--
Drowning	Female	0-6 days	95+ years	Data Rich	-1	1	Rainfall Quintile 1 (proportion)	--
Drowning	Female	0-6 days	95+ years	Data Rich	1	1	Coastal Population within 10km (proportion)	945
Drowning	Female	0-6 days	95+ years	Data Rich	1	1	Alcohol (liters per capita)	1000
Drowning	Female	0-6 days	95+ years	Data Rich	1	1	Log-transformed SEV scalar: Drown	--
Drowning	Female	0-6 days	95+ years	Data Rich	1	1	Rainfall Quintile 5 (proportion)	--

Table S16. CODEm covariates used, level of covariate, and expected direction of covariate by cause, sex, and age
Covariates that CODEm did not select during the covariate selection process have no draw counts listed.

Cause	Sex	Age Start	Age End	Model Type	Direction	Level	Covariate Name	Number of Draws
Drowning	Female	0-6 days	95+ years	Data Rich	1	2	Elevation Under 100m (proportion)	--
Drowning	Female	0-6 days	95+ years	Data Rich	-1	3	Socio-demographic Index	38
Drowning	Female	0-6 days	95+ years	Data Rich	-1	3	Education (years per capita)	641
Drowning	Female	0-6 days	95+ years	Data Rich	0	3	LDI (I$ per capita)	--
Drowning	Female	0-6 days	95+ years	Global	-1	1	Landlocked Nation (binary)	--
Drowning	Female	0-6 days	95+ years	Global	-1	1	Rainfall Quintile 1 (proportion)	--
Drowning	Female	0-6 days	95+ years	Global	1	1	Alcohol (liters per capita)	393
Drowning	Female	0-6 days	95+ years	Global	1	1	Coastal Population within 10km (proportion)	754
Drowning	Female	0-6 days	95+ years	Global	1	1	Rainfall Quintile 5 (proportion)	767
Drowning	Female	0-6 days	95+ years	Global	1	1	Log-transformed SEV scalar: Drown	--
Drowning	Female	0-6 days	95+ years	Global	1	2	Elevation Under 100m (proportion)	22
Drowning	Female	0-6 days	95+ years	Global	-1	3	Socio-demographic Index	355
Drowning	Female	0-6 days	95+ years	Global	-1	3	Education (years per capita)	543
Drowning	Female	0-6 days	95+ years	Global	0	3	LDI (I$ per capita)	--
Drowning	Male	0-6 days	95+ years	Data Rich	-1	1	Landlocked Nation (binary)	--
Drowning	Male	0-6 days	95+ years	Data Rich	-1	1	Rainfall Quintile 1 (proportion)	--
Drowning	Male	0-6 days	95+ years	Data Rich	1	1	Coastal Population within 10km (proportion)	626
Drowning	Male	0-6 days	95+ years	Data Rich	1	1	Rainfall Quintile 5 (proportion)	858
Drowning	Male	0-6 days	95+ years	Data Rich	1	1	Alcohol (liters per capita)	1000
Drowning	Male	0-6 days	95+ years	Data Rich	1	1	Log-transformed SEV scalar: Drown	--
Drowning	Male	0-6 days	95+ years	Data Rich	1	2	Elevation Under 100m (proportion)	336
Drowning	Male	0-6 days	95+ years	Data Rich	-1	3	Education (years per capita)	17
Drowning	Male	0-6 days	95+ years	Data Rich	-1	3	Socio-demographic Index	983
Drowning	Male	0-6 days	95+ years	Data Rich	0	3	LDI (I$ per capita)	--
Drowning	Male	0-6 days	95+ years	Global	-1	1	Rainfall Quintile 1 (proportion)	546
Drowning	Male	0-6 days	95+ years	Global	-1	1	Landlocked Nation (binary)	--
Drowning	Male	0-6 days	95+ years	Global	1	1	Coastal Population within 10km (proportion)	259
Drowning	Male	0-6 days	95+ years	Global	1	1	Rainfall Quintile 5 (proportion)	339
Drowning	Male	0-6 days	95+ years	Global	1	1	Alcohol (liters per capita)	644
Drowning	Male	0-6 days	95+ years	Global	1	1	Log-transformed SEV scalar: Drown	--
Drowning	Male	0-6 days	95+ years	Global	1	2	Elevation Under 100m (proportion)	672
Drowning	Male	0-6 days	95+ years	Global	-1	3	Education (years per capita)	306
Drowning	Male	0-6 days	95+ years	Global	-1	3	Socio-demographic Index	500
Drowning	Male	0-6 days	95+ years	Global	0	3	LDI (I$ per capita)	--
Fire, heat, and hot substances	Female	0-6 days	95+ years	Data Rich	1	1	Log-transformed SEV scalar: Fire	--
Fire, heat, and hot substances	Female	0-6 days	95+ years	Data Rich	-1	2	Healthcare access and quality index	967
Fire, heat, and hot substances	Female	0-6 days	95+ years	Data Rich	0	2	Population Density (over 1000 ppl/sqkm, proportion)	967
Fire, heat, and hot substances	Female	0-6 days	95+ years	Data Rich	1	2	Alcohol (liters per capita)	967
Fire, heat, and hot substances	Female	0-6 days	95+ years	Data Rich	1	2	Indoor Air Pollution (All Cooking Fuels)	967
Fire, heat, and hot substances	Female	0-6 days	95+ years	Data Rich	1	2	Tobacco (cigarettes per capita)	967
Fire, heat, and hot substances	Female	0-6 days	95+ years	Data Rich	-1	3	Socio-demographic Index	33
Fire, heat, and hot substances	Female	0-6 days	95+ years	Data Rich	-1	3	Education (years per capita)	--
Fire, heat, and hot substances	Female	0-6 days	95+ years	Data Rich	0	3	LDI (I$ per capita)	--
Fire, heat, and hot substances	Female	0-6 days	95+ years	Global	1	1	Log-transformed SEV scalar: Fire	--
Fire, heat, and hot substances	Female	0-6 days	95+ years	Global	-1	2	Healthcare access and quality index	740
Fire, heat, and hot substances	Female	0-6 days	95+ years	Global	0	2	Population Density (over 1000 ppl/sqkm, proportion)	740
Fire, heat, and hot substances	Female	0-6 days	95+ years	Global	1	2	Alcohol (liters per capita)	740
Fire, heat, and hot substances	Female	0-6 days	95+ years	Global	1	2	Indoor Air Pollution (All Cooking Fuels)	740
Fire, heat, and hot substances	Female	0-6 days	95+ years	Global	1	2	Tobacco (cigarettes per capita)	740
Fire, heat, and hot substances	Female	0-6 days	95+ years	Global	-1	3	Education (years per capita)	260
Fire, heat, and hot substances	Female	0-6 days	95+ years	Global	-1	3	Socio-demographic Index	260
Fire, heat, and hot substances	Female	0-6 days	95+ years	Global	0	3	LDI (I$ per capita)	--
Fire, heat, and hot substances	Male	0-6 days	95+ years	Data Rich	1	1	Log-transformed SEV scalar: Fire	--
Fire, heat, and hot substances	Male	0-6 days	95+ years	Data Rich	-1	2	Healthcare access and quality index	366
Fire, heat, and hot substances	Male	0-6 days	95+ years	Data Rich	0	2	Population Density (over 1000 ppl/sqkm, proportion)	57
Fire, heat, and hot substances	Male	0-6 days	95+ years	Data Rich	1	2	Indoor Air Pollution (All Cooking Fuels)	3

Table S16. CODEm covariates used, level of covariate, and expected direction of covariate by cause, sex, and age
Covariates that CODEm did not select during the covariate selection process have no draw counts listed.

Cause	Sex	Age Start	Age End	Model Type	Direction	Level	Covariate Name	Number of Draws
Fire, heat, and hot substances	Male	0-6 days	95+ years	Data Rich	1	2	Tobacco (cigarettes per capita)	313
Fire, heat, and hot substances	Male	0-6 days	95+ years	Data Rich	1	2	Alcohol (liters per capita)	368
Fire, heat, and hot substances	Male	0-6 days	95+ years	Data Rich	-1	3	Education (years per capita)	140
Fire, heat, and hot substances	Male	0-6 days	95+ years	Data Rich	-1	3	Socio-demographic Index	509
Fire, heat, and hot substances	Male	0-6 days	95+ years	Data Rich	0	3	LDI (I$ per capita)	--
Fire, heat, and hot substances	Male	0-6 days	95+ years	Global	1	1	Log-transformed SEV scalar: Fire	--
Fire, heat, and hot substances	Male	0-6 days	95+ years	Global	-1	2	Healthcare access and quality index	303
Fire, heat, and hot substances	Male	0-6 days	95+ years	Global	0	2	Population Density (over 1000 ppl/sqkm, proportion)	303
Fire, heat, and hot substances	Male	0-6 days	95+ years	Global	1	2	Alcohol (liters per capita)	303
Fire, heat, and hot substances	Male	0-6 days	95+ years	Global	1	2	Indoor Air Pollution (All Cooking Fuels)	303
Fire, heat, and hot substances	Male	0-6 days	95+ years	Global	1	2	Tobacco (cigarettes per capita)	557
Fire, heat, and hot substances	Male	0-6 days	95+ years	Global	-1	3	Education (years per capita)	150
Fire, heat, and hot substances	Male	0-6 days	95+ years	Global	-1	3	Socio-demographic Index	593
Fire, heat, and hot substances	Male	0-6 days	95+ years	Global	0	3	LDI (I$ per capita)	--
Poisonings	Female	0-6 days	95+ years	Data Rich	1	1	Opium Cultivation (binary)	999
Poisonings	Female	0-6 days	95+ years	Data Rich	1	1	Log-transformed SEV scalar: Poison	--
Poisonings	Female	0-6 days	95+ years	Data Rich	-1	2	Healthcare access and quality index	962
Poisonings	Female	0-6 days	95+ years	Data Rich	0	2	Population Density (under 150 ppl/sqkm, proportion)	961
Poisonings	Female	0-6 days	95+ years	Data Rich	0	2	Population Density (over 1000 ppl/sqkm, proportion)	962
Poisonings	Female	0-6 days	95+ years	Data Rich	-1	3	Socio-demographic Index	32
Poisonings	Female	0-6 days	95+ years	Data Rich	-1	3	Education (years per capita)	160
Poisonings	Female	0-6 days	95+ years	Data Rich	0	3	LDI (I$ per capita)	--
Poisonings	Female	0-6 days	95+ years	Global	1	1	Opium Cultivation (binary)	1000
Poisonings	Female	0-6 days	95+ years	Global	1	1	Log-transformed SEV scalar: Poison	--
Poisonings	Female	0-6 days	95+ years	Global	-1	2	Healthcare access and quality index	218
Poisonings	Female	0-6 days	95+ years	Global	0	2	Population Density (over 1000 ppl/sqkm, proportion)	432
Poisonings	Female	0-6 days	95+ years	Global	0	2	Population Density (under 150 ppl/sqkm, proportion)	--
Poisonings	Female	0-6 days	95+ years	Global	-1	3	Socio-demographic Index	141
Poisonings	Female	0-6 days	95+ years	Global	-1	3	Education (years per capita)	264
Poisonings	Female	0-6 days	95+ years	Global	0	3	LDI (I$ per capita)	--
Poisonings	Male	0-6 days	95+ years	Data Rich	1	1	Log-transformed SEV scalar: Poison	--
Poisonings	Male	0-6 days	95+ years	Data Rich	1	1	Opium Cultivation (binary)	--
Poisonings	Male	0-6 days	95+ years	Data Rich	-1	2	Healthcare access and quality index	864
Poisonings	Male	0-6 days	95+ years	Data Rich	0	2	Population Density (over 1000 ppl/sqkm, proportion)	864
Poisonings	Male	0-6 days	95+ years	Data Rich	0	2	Population Density (under 150 ppl/sqkm, proportion)	864
Poisonings	Male	0-6 days	95+ years	Data Rich	-1	3	Education (years per capita)	25
Poisonings	Male	0-6 days	95+ years	Data Rich	-1	3	Socio-demographic Index	164
Poisonings	Male	0-6 days	95+ years	Data Rich	0	3	LDI (I$ per capita)	--
Poisonings	Male	0-6 days	95+ years	Global	1	1	Opium Cultivation (binary)	412
Poisonings	Male	0-6 days	95+ years	Global	1	1	Log-transformed SEV scalar: Poison	--
Poisonings	Male	0-6 days	95+ years	Global	-1	2	Healthcare access and quality index	352
Poisonings	Male	0-6 days	95+ years	Global	0	2	Population Density (over 1000 ppl/sqkm, proportion)	560
Poisonings	Male	0-6 days	95+ years	Global	0	2	Population Density (under 150 ppl/sqkm, proportion)	560
Poisonings	Male	0-6 days	95+ years	Global	-1	3	Education (years per capita)	236
Poisonings	Male	0-6 days	95+ years	Global	-1	3	Socio-demographic Index	236
Poisonings	Male	0-6 days	95+ years	Global	0	3	LDI (I$ per capita)	--
Poisoning by carbon monoxide	Female	0-6 days	95+ years	Data Rich	-1	3	Education (years per capita)	6
Poisoning by carbon monoxide	Female	0-6 days	95+ years	Data Rich	-1	3	Healthcare access and quality index	31
Poisoning by carbon monoxide	Female	0-6 days	95+ years	Data Rich	0	3	Socio-demographic Index	962
Poisoning by carbon monoxide	Female	0-6 days	95+ years	Data Rich	0	3	LDI (I$ per capita)	--
Poisoning by carbon monoxide	Female	0-6 days	95+ years	Global	-1	3	Education (years per capita)	444
Poisoning by carbon monoxide	Female	0-6 days	95+ years	Global	-1	3	Healthcare access and quality index	530
Poisoning by carbon monoxide	Female	0-6 days	95+ years	Global	0	3	Socio-demographic Index	395
Poisoning by carbon monoxide	Female	0-6 days	95+ years	Global	0	3	LDI (I$ per capita)	--
Poisoning by carbon monoxide	Male	0-6 days	95+ years	Data Rich	-1	3	Healthcare access and quality index	1

Table S16. CODEm covariates used, level of covariate, and expected direction of covariate by cause, sex, and age<br>*Covariates that CODEm did not select during the covariate selection process have no draw counts listed.*								
**Cause**	**Sex**	**Age Start**	**Age End**	**Model Type**	**Direction**	**Level**	**Covariate Name**	**Number of Draws**
Poisoning by carbon monoxide	Male	0-6 days	95+ years	Data Rich	-1	3	Education (years per capita)	961
Poisoning by carbon monoxide	Male	0-6 days	95+ years	Data Rich	0	3	Socio-demographic Index	999
Poisoning by carbon monoxide	Male	0-6 days	95+ years	Data Rich	0	3	LDI (I$ per capita)	--
Poisoning by carbon monoxide	Male	0-6 days	95+ years	Global	-1	3	Healthcare access and quality index	274
Poisoning by carbon monoxide	Male	0-6 days	95+ years	Global	-1	3	Education (years per capita)	768
Poisoning by carbon monoxide	Male	0-6 days	95+ years	Global	0	3	Socio-demographic Index	410
Poisoning by carbon monoxide	Male	0-6 days	95+ years	Global	0	3	LDI (I$ per capita)	--
Poisoning by other means	Female	0-6 days	95+ years	Data Rich	-1	3	Education (years per capita)	161
Poisoning by other means	Female	0-6 days	95+ years	Data Rich	-1	3	Healthcare access and quality index	967
Poisoning by other means	Female	0-6 days	95+ years	Data Rich	0	3	Socio-demographic Index	193
Poisoning by other means	Female	0-6 days	95+ years	Data Rich	0	3	LDI (I$ per capita)	--
Poisoning by other means	Female	0-6 days	95+ years	Global	-1	3	Education (years per capita)	365
Poisoning by other means	Female	0-6 days	95+ years	Global	-1	3	Healthcare access and quality index	399
Poisoning by other means	Female	0-6 days	95+ years	Global	0	3	Socio-demographic Index	444
Poisoning by other means	Female	0-6 days	95+ years	Global	0	3	LDI (I$ per capita)	--
Poisoning by other means	Male	0-6 days	95+ years	Data Rich	-1	3	Education (years per capita)	967
Poisoning by other means	Male	0-6 days	95+ years	Data Rich	-1	3	Healthcare access and quality index	1000
Poisoning by other means	Male	0-6 days	95+ years	Data Rich	0	3	Socio-demographic Index	993
Poisoning by other means	Male	0-6 days	95+ years	Data Rich	0	3	LDI (I$ per capita)	--
Poisoning by other means	Male	0-6 days	95+ years	Global	-1	3	Education (years per capita)	501
Poisoning by other means	Male	0-6 days	95+ years	Global	-1	3	Healthcare access and quality index	536
Poisoning by other means	Male	0-6 days	95+ years	Global	0	3	Socio-demographic Index	524
Poisoning by other means	Male	0-6 days	95+ years	Global	0	3	LDI (I$ per capita)	--
Exposure to mechanical forces	Female	0-6 days	95+ years	Data Rich	-1	2	Healthcare access and quality index	0
Exposure to mechanical forces	Female	0-6 days	95+ years	Data Rich	0	2	Population Density (under 150 ppl/sqkm, proportion)	839
Exposure to mechanical forces	Female	0-6 days	95+ years	Data Rich	0	2	Population Density (over 1000 ppl/sqkm, proportion)	968
Exposure to mechanical forces	Female	0-6 days	95+ years	Data Rich	1	2	Alcohol (liters per capita)	7
Exposure to mechanical forces	Female	0-6 days	95+ years	Data Rich	-1	3	Education (years per capita)	0
Exposure to mechanical forces	Female	0-6 days	95+ years	Data Rich	-1	3	Socio-demographic Index	0
Exposure to mechanical forces	Female	0-6 days	95+ years	Data Rich	0	3	LDI (I$ per capita)	--
Exposure to mechanical forces	Female	0-6 days	95+ years	Global	-1	2	Healthcare access and quality index	847
Exposure to mechanical forces	Female	0-6 days	95+ years	Global	0	2	Population Density (under 150 ppl/sqkm, proportion)	436
Exposure to mechanical forces	Female	0-6 days	95+ years	Global	0	2	Population Density (over 1000 ppl/sqkm, proportion)	556
Exposure to mechanical forces	Female	0-6 days	95+ years	Global	1	2	Alcohol (liters per capita)	258
Exposure to mechanical forces	Female	0-6 days	95+ years	Global	-1	3	Education (years per capita)	51
Exposure to mechanical forces	Female	0-6 days	95+ years	Global	-1	3	Socio-demographic Index	168
Exposure to mechanical forces	Female	0-6 days	95+ years	Global	0	3	LDI (I$ per capita)	--
Exposure to mechanical forces	Male	0-6 days	95+ years	Data Rich	-1	2	Healthcare access and quality index	1000
Exposure to mechanical forces	Male	0-6 days	95+ years	Data Rich	0	2	Population Density (under 150 ppl/sqkm, proportion)	967
Exposure to mechanical forces	Male	0-6 days	95+ years	Data Rich	0	2	Population Density (over 1000 ppl/sqkm, proportion)	1000
Exposure to mechanical forces	Male	0-6 days	95+ years	Data Rich	1	2	Alcohol (liters per capita)	199
Exposure to mechanical forces	Male	0-6 days	95+ years	Data Rich	-1	3	Education (years per capita)	0
Exposure to mechanical forces	Male	0-6 days	95+ years	Data Rich	-1	3	Socio-demographic Index	161
Exposure to mechanical forces	Male	0-6 days	95+ years	Data Rich	0	3	LDI (I$ per capita)	--
Exposure to mechanical forces	Male	0-6 days	95+ years	Global	-1	2	Healthcare access and quality index	535
Exposure to mechanical forces	Male	0-6 days	95+ years	Global	0	2	Population Density (under 150 ppl/sqkm, proportion)	487
Exposure to mechanical forces	Male	0-6 days	95+ years	Global	0	2	Population Density (over 1000 ppl/sqkm, proportion)	694
Exposure to mechanical forces	Male	0-6 days	95+ years	Global	1	2	Alcohol (liters per capita)	482
Exposure to mechanical forces	Male	0-6 days	95+ years	Global	-1	3	Socio-demographic Index	154
Exposure to mechanical forces	Male	0-6 days	95+ years	Global	-1	3	Education (years per capita)	511
Exposure to mechanical forces	Male	0-6 days	95+ years	Global	0	3	LDI (I$ per capita)	--
Unintentional firearm injuries	Female	0-6 days	95+ years	Data Rich	1	1	Log-transformed SEV scalar: Mech Gun	--
Unintentional firearm injuries	Female	0-6 days	95+ years	Data Rich	-1	2	Healthcare access and quality index	689
Unintentional firearm injuries	Female	0-6 days	95+ years	Data Rich	-1	2	Health System Access (unitless)	--
Unintentional firearm injuries	Female	0-6 days	95+ years	Data Rich	1	2	Alcohol (liters per capita)	--
Unintentional firearm injuries	Female	0-6 days	95+ years	Data Rich	-1	3	Socio-demographic Index	150

Table S16. CODEm covariates used, level of covariate, and expected direction of covariate by cause, sex, and age
Covariates that CODEm did not select during the covariate selection process have no draw counts listed.

Cause	Sex	Age Start	Age End	Model Type	Direction	Level	Covariate Name	Number of Draws
Unintentional firearm injuries	Female	0-6 days	95+ years	Data Rich	-1	3	Education (years per capita)	--
Unintentional firearm injuries	Female	0-6 days	95+ years	Data Rich	0	3	Population Density (over 1000 ppl/sqkm, proportion)	235
Unintentional firearm injuries	Female	0-6 days	95+ years	Data Rich	0	3	Population Density (under 150 ppl/sqkm, proportion)	369
Unintentional firearm injuries	Female	0-6 days	95+ years	Data Rich	0	3	LDI (I$ per capita)	--
Unintentional firearm injuries	Female	0-6 days	95+ years	Global	1	1	Log-transformed SEV scalar: Mech Gun	--
Unintentional firearm injuries	Female	0-6 days	95+ years	Global	-1	2	Healthcare access and quality index	392
Unintentional firearm injuries	Female	0-6 days	95+ years	Global	-1	2	Health System Access (unitless)	--
Unintentional firearm injuries	Female	0-6 days	95+ years	Global	1	2	Alcohol (liters per capita)	--
Unintentional firearm injuries	Female	0-6 days	95+ years	Global	-1	3	Education (years per capita)	133
Unintentional firearm injuries	Female	0-6 days	95+ years	Global	-1	3	Socio-demographic Index	475
Unintentional firearm injuries	Female	0-6 days	95+ years	Global	0	3	Population Density (over 1000 ppl/sqkm, proportion)	513
Unintentional firearm injuries	Female	0-6 days	95+ years	Global	0	3	Population Density (under 150 ppl/sqkm, proportion)	839
Unintentional firearm injuries	Female	0-6 days	95+ years	Global	0	3	LDI (I$ per capita)	--
Unintentional firearm injuries	Male	0-6 days	95+ years	Data Rich	1	1	Log-transformed SEV scalar: Mech Gun	--
Unintentional firearm injuries	Male	0-6 days	95+ years	Data Rich	-1	2	Health System Access (unitless)	725
Unintentional firearm injuries	Male	0-6 days	95+ years	Data Rich	-1	2	Healthcare access and quality index	--
Unintentional firearm injuries	Male	0-6 days	95+ years	Data Rich	1	2	Alcohol (liters per capita)	224
Unintentional firearm injuries	Male	0-6 days	95+ years	Data Rich	-1	3	Socio-demographic Index	48
Unintentional firearm injuries	Male	0-6 days	95+ years	Data Rich	-1	3	Education (years per capita)	188
Unintentional firearm injuries	Male	0-6 days	95+ years	Data Rich	0	3	Population Density (under 150 ppl/sqkm, proportion)	113
Unintentional firearm injuries	Male	0-6 days	95+ years	Data Rich	0	3	Population Density (over 1000 ppl/sqkm, proportion)	551
Unintentional firearm injuries	Male	0-6 days	95+ years	Data Rich	0	3	LDI (I$ per capita)	--
Unintentional firearm injuries	Male	0-6 days	95+ years	Global	1	1	Log-transformed SEV scalar: Mech Gun	--
Unintentional firearm injuries	Male	0-6 days	95+ years	Global	-1	2	Health System Access (unitless)	582
Unintentional firearm injuries	Male	0-6 days	95+ years	Global	-1	2	Healthcare access and quality index	692
Unintentional firearm injuries	Male	0-6 days	95+ years	Global	1	2	Alcohol (liters per capita)	504
Unintentional firearm injuries	Male	0-6 days	95+ years	Global	-1	3	Education (years per capita)	91
Unintentional firearm injuries	Male	0-6 days	95+ years	Global	-1	3	Socio-demographic Index	373
Unintentional firearm injuries	Male	0-6 days	95+ years	Global	0	3	Population Density (under 150 ppl/sqkm, proportion)	383
Unintentional firearm injuries	Male	0-6 days	95+ years	Global	0	3	Population Density (over 1000 ppl/sqkm, proportion)	609
Unintentional firearm injuries	Male	0-6 days	95+ years	Global	0	3	LDI (I$ per capita)	--
Other exposure to mechanical forces	Female	0-6 days	95+ years	Data Rich	1	1	Log-transformed SEV scalar: Oth Mech	--
Other exposure to mechanical forces	Female	0-6 days	95+ years	Data Rich	-1	2	Health System Access (unitless)	132
Other exposure to mechanical forces	Female	0-6 days	95+ years	Data Rich	-1	2	Healthcare access and quality index	132
Other exposure to mechanical forces	Female	0-6 days	95+ years	Data Rich	0	2	Population Density (over 1000 ppl/sqkm, proportion)	397
Other exposure to mechanical forces	Female	0-6 days	95+ years	Data Rich	0	2	Population Density (under 150 ppl/sqkm, proportion)	397
Other exposure to mechanical forces	Female	0-6 days	95+ years	Data Rich	1	2	Alcohol (liters per capita)	265
Other exposure to mechanical forces	Female	0-6 days	95+ years	Data Rich	-1	3	Education (years per capita)	6
Other exposure to mechanical forces	Female	0-6 days	95+ years	Data Rich	-1	3	Socio-demographic Index	14
Other exposure to mechanical forces	Female	0-6 days	95+ years	Data Rich	0	3	LDI (I$ per capita)	--
Other exposure to mechanical forces	Female	0-6 days	95+ years	Global	1	1	Log-transformed SEV scalar: Oth Mech	--
Other exposure to mechanical forces	Female	0-6 days	95+ years	Global	-1	2	Health System Access (unitless)	354
Other exposure to mechanical forces	Female	0-6 days	95+ years	Global	-1	2	Healthcare access and quality index	354
Other exposure to mechanical forces	Female	0-6 days	95+ years	Global	0	2	Population Density (over 1000 ppl/sqkm, proportion)	614
Other exposure to mechanical forces	Female	0-6 days	95+ years	Global	0	2	Population Density (under 150 ppl/sqkm, proportion)	614
Other exposure to mechanical forces	Female	0-6 days	95+ years	Global	1	2	Alcohol (liters per capita)	614
Other exposure to mechanical forces	Female	0-6 days	95+ years	Global	-1	3	Education (years per capita)	104
Other exposure to mechanical forces	Female	0-6 days	95+ years	Global	-1	3	Socio-demographic Index	--
Other exposure to mechanical forces	Female	0-6 days	95+ years	Global	0	3	LDI (I$ per capita)	--
Other exposure to mechanical forces	Male	0-6 days	95+ years	Data Rich	1	1	Log-transformed SEV scalar: Oth Mech	--
Other exposure to mechanical forces	Male	0-6 days	95+ years	Data Rich	-1	2	Healthcare access and quality index	804

Table S16. CODEm covariates used, level of covariate, and expected direction of covariate by cause, sex, and age
Covariates that CODEm did not select during the covariate selection process have no draw counts listed.

Cause	Sex	Age Start	Age End	Model Type	Direction	Level	Covariate Name	Number of Draws
Other exposure to mechanical forces	Male	0-6 days	95+ years	Data Rich	-1	2	Health System Access (unitless)	--
Other exposure to mechanical forces	Male	0-6 days	95+ years	Data Rich	0	2	Population Density (over 1000 ppl/sqkm, proportion)	812
Other exposure to mechanical forces	Male	0-6 days	95+ years	Data Rich	0	2	Population Density (under 150 ppl/sqkm, proportion)	812
Other exposure to mechanical forces	Male	0-6 days	95+ years	Data Rich	1	2	Alcohol (liters per capita)	8
Other exposure to mechanical forces	Male	0-6 days	95+ years	Data Rich	-1	3	Education (years per capita)	251
Other exposure to mechanical forces	Male	0-6 days	95+ years	Data Rich	-1	3	Socio-demographic Index	302
Other exposure to mechanical forces	Male	0-6 days	95+ years	Data Rich	0	3	LDI (I$ per capita)	--
Other exposure to mechanical forces	Male	0-6 days	95+ years	Global	1	1	Log-transformed SEV scalar: Oth Mech	--
Other exposure to mechanical forces	Male	0-6 days	95+ years	Global	-1	2	Health System Access (unitless)	449
Other exposure to mechanical forces	Male	0-6 days	95+ years	Global	-1	2	Healthcare access and quality index	449
Other exposure to mechanical forces	Male	0-6 days	95+ years	Global	0	2	Population Density (over 1000 ppl/sqkm, proportion)	449
Other exposure to mechanical forces	Male	0-6 days	95+ years	Global	0	2	Population Density (under 150 ppl/sqkm, proportion)	449
Other exposure to mechanical forces	Male	0-6 days	95+ years	Global	1	2	Alcohol (liters per capita)	449
Other exposure to mechanical forces	Male	0-6 days	95+ years	Global	-1	3	Education (years per capita)	218
Other exposure to mechanical forces	Male	0-6 days	95+ years	Global	-1	3	Socio-demographic Index	251
Other exposure to mechanical forces	Male	0-6 days	95+ years	Global	0	3	LDI (I$ per capita)	--
Adverse effects of medical treatment	Female	0-6 days	95+ years	Data Rich	0	2	Healthcare access and quality index	--
Adverse effects of medical treatment	Female	0-6 days	95+ years	Data Rich	0	3	Socio-demographic Index	166
Adverse effects of medical treatment	Female	0-6 days	95+ years	Data Rich	0	3	LDI (I$ per capita)	--
Adverse effects of medical treatment	Female	0-6 days	95+ years	Global	0	2	Healthcare access and quality index	602
Adverse effects of medical treatment	Female	0-6 days	95+ years	Global	0	3	Socio-demographic Index	692
Adverse effects of medical treatment	Female	0-6 days	95+ years	Global	0	3	LDI (I$ per capita)	--
Adverse effects of medical treatment	Male	0-6 days	95+ years	Data Rich	0	2	Healthcare access and quality index	--
Adverse effects of medical treatment	Male	0-6 days	95+ years	Data Rich	0	3	LDI (I$ per capita)	--
Adverse effects of medical treatment	Male	0-6 days	95+ years	Data Rich	0	3	Socio-demographic Index	--
Adverse effects of medical treatment	Male	0-6 days	95+ years	Global	0	2	Healthcare access and quality index	602
Adverse effects of medical treatment	Male	0-6 days	95+ years	Global	0	3	Socio-demographic Index	726
Adverse effects of medical treatment	Male	0-6 days	95+ years	Global	0	3	LDI (I$ per capita)	--
Animal contact	Female	0-6 days	95+ years	Data Rich	1	1	Alcohol (liters per capita)	--
Animal contact	Female	0-6 days	95+ years	Data Rich	1	1	Log-transformed SEV scalar: Animal	--
Animal contact	Female	0-6 days	95+ years	Data Rich	-1	2	Healthcare access and quality index	38
Animal contact	Female	0-6 days	95+ years	Data Rich	1	2	Population 15 to 30 (proportion)	38
Animal contact	Female	0-6 days	95+ years	Data Rich	-1	3	Socio-demographic Index	1
Animal contact	Female	0-6 days	95+ years	Data Rich	-1	3	Education (years per capita)	160
Animal contact	Female	0-6 days	95+ years	Data Rich	0	3	Elevation Over 1500m (proportion)	192
Animal contact	Female	0-6 days	95+ years	Data Rich	0	3	Population Density (over 1000 ppl/sqkm, proportion)	801
Animal contact	Female	0-6 days	95+ years	Data Rich	0	3	Population Density (under 150 ppl/sqkm, proportion)	801
Animal contact	Female	0-6 days	95+ years	Data Rich	0	3	Elevation Under 100m (proportion)	--
Animal contact	Female	0-6 days	95+ years	Data Rich	0	3	LDI (I$ per capita)	--
Animal contact	Female	0-6 days	95+ years	Global	1	1	Alcohol (liters per capita)	--
Animal contact	Female	0-6 days	95+ years	Global	1	1	Log-transformed SEV scalar: Animal	--
Animal contact	Female	0-6 days	95+ years	Global	-1	2	Healthcare access and quality index	424
Animal contact	Female	0-6 days	95+ years	Global	1	2	Population 15 to 30 (proportion)	424
Animal contact	Female	0-6 days	95+ years	Global	-1	3	Education (years per capita)	133
Animal contact	Female	0-6 days	95+ years	Global	-1	3	Socio-demographic Index	392
Animal contact	Female	0-6 days	95+ years	Global	0	3	Population Density (over 1000 ppl/sqkm, proportion)	282
Animal contact	Female	0-6 days	95+ years	Global	0	3	Population Density (under 150 ppl/sqkm, proportion)	282
Animal contact	Female	0-6 days	95+ years	Global	0	3	Elevation Over 1500m (proportion)	513
Animal contact	Female	0-6 days	95+ years	Global	0	3	Elevation Under 100m (proportion)	807
Animal contact	Female	0-6 days	95+ years	Global	0	3	LDI (I$ per capita)	--
Animal contact	Male	0-6 days	95+ years	Data Rich	1	1	Alcohol (liters per capita)	969
Animal contact	Male	0-6 days	95+ years	Data Rich	1	1	Log-transformed SEV scalar: Animal	--
Animal contact	Male	0-6 days	95+ years	Data Rich	-1	2	Healthcare access and quality index	0

Table S16. CODEm covariates used, level of covariate, and expected direction of covariate by cause, sex, and age
Covariates that CODEm did not select during the covariate selection process have no draw counts listed.

Cause	Sex	Age Start	Age End	Model Type	Direction	Level	Covariate Name	Number of Draws
Animal contact	Male	0-6 days	95+ years	Data Rich	1	2	Population 15 to 30 (proportion)	0
Animal contact	Male	0-6 days	95+ years	Data Rich	-1	3	Education (years per capita)	0
Animal contact	Male	0-6 days	95+ years	Data Rich	-1	3	Socio-demographic Index	0
Animal contact	Male	0-6 days	95+ years	Data Rich	0	3	Population Density (under 150 ppl/sqkm, proportion)	1
Animal contact	Male	0-6 days	95+ years	Data Rich	0	3	Elevation Under 100m (proportion)	803
Animal contact	Male	0-6 days	95+ years	Data Rich	0	3	Population Density (over 1000 ppl/sqkm, proportion)	803
Animal contact	Male	0-6 days	95+ years	Data Rich	0	3	Elevation Over 1500m (proportion)	804
Animal contact	Male	0-6 days	95+ years	Data Rich	0	3	LDI (I$ per capita)	--
Animal contact	Male	0-6 days	95+ years	Global	1	1	Alcohol (liters per capita)	491
Animal contact	Male	0-6 days	95+ years	Global	1	1	Log-transformed SEV scalar: Animal	--
Animal contact	Male	0-6 days	95+ years	Global	-1	2	Healthcare access and quality index	170
Animal contact	Male	0-6 days	95+ years	Global	1	2	Population 15 to 30 (proportion)	161
Animal contact	Male	0-6 days	95+ years	Global	-1	3	Socio-demographic Index	195
Animal contact	Male	0-6 days	95+ years	Global	-1	3	Education (years per capita)	196
Animal contact	Male	0-6 days	95+ years	Global	0	3	Elevation Over 1500m (proportion)	114
Animal contact	Male	0-6 days	95+ years	Global	0	3	Elevation Under 100m (proportion)	207
Animal contact	Male	0-6 days	95+ years	Global	0	3	Population Density (under 150 ppl/sqkm, proportion)	352
Animal contact	Male	0-6 days	95+ years	Global	0	3	Population Density (over 1000 ppl/sqkm, proportion)	495
Animal contact	Male	0-6 days	95+ years	Global	0	3	LDI (I$ per capita)	--
Venomous animal contact	Female	0-6 days	95+ years	Data Rich	1	1	Alcohol (liters per capita)	--
Venomous animal contact	Female	0-6 days	95+ years	Data Rich	1	1	Log-transformed SEV scalar: Venom	--
Venomous animal contact	Female	0-6 days	95+ years	Data Rich	-1	2	Healthcare access and quality index	--
Venomous animal contact	Female	0-6 days	95+ years	Data Rich	-1	3	Education (years per capita)	181
Venomous animal contact	Female	0-6 days	95+ years	Data Rich	-1	3	Socio-demographic Index	181
Venomous animal contact	Female	0-6 days	95+ years	Data Rich	0	3	Elevation Over 1500m (proportion)	819
Venomous animal contact	Female	0-6 days	95+ years	Data Rich	0	3	Elevation Under 100m (proportion)	819
Venomous animal contact	Female	0-6 days	95+ years	Data Rich	0	3	Population Density (under 150 ppl/sqkm, proportion)	1000
Venomous animal contact	Female	0-6 days	95+ years	Data Rich	0	3	LDI (I$ per capita)	--
Venomous animal contact	Female	0-6 days	95+ years	Data Rich	0	3	Population Density (over 1000 ppl/sqkm, proportion)	--
Venomous animal contact	Female	0-6 days	95+ years	Global	1	1	Alcohol (liters per capita)	--
Venomous animal contact	Female	0-6 days	95+ years	Global	1	1	Log-transformed SEV scalar: Venom	--
Venomous animal contact	Female	0-6 days	95+ years	Global	-1	2	Healthcare access and quality index	--
Venomous animal contact	Female	0-6 days	95+ years	Global	-1	3	Education (years per capita)	546
Venomous animal contact	Female	0-6 days	95+ years	Global	-1	3	Socio-demographic Index	546
Venomous animal contact	Female	0-6 days	95+ years	Global	0	3	Elevation Under 100m (proportion)	454
Venomous animal contact	Female	0-6 days	95+ years	Global	0	3	Population Density (over 1000 ppl/sqkm, proportion)	454
Venomous animal contact	Female	0-6 days	95+ years	Global	0	3	Elevation Over 1500m (proportion)	--
Venomous animal contact	Female	0-6 days	95+ years	Global	0	3	LDI (I$ per capita)	--
Venomous animal contact	Female	0-6 days	95+ years	Global	0	3	Population Density (under 150 ppl/sqkm, proportion)	--
Venomous animal contact	Male	0-6 days	95+ years	Data Rich	1	1	Alcohol (liters per capita)	738
Venomous animal contact	Male	0-6 days	95+ years	Data Rich	1	1	Log-transformed SEV scalar: Venom	--
Venomous animal contact	Male	0-6 days	95+ years	Data Rich	-1	2	Healthcare access and quality index	241
Venomous animal contact	Male	0-6 days	95+ years	Data Rich	-1	3	Socio-demographic Index	21
Venomous animal contact	Male	0-6 days	95+ years	Data Rich	-1	3	Education (years per capita)	86
Venomous animal contact	Male	0-6 days	95+ years	Data Rich	0	3	Elevation Over 1500m (proportion)	16
Venomous animal contact	Male	0-6 days	95+ years	Data Rich	0	3	Elevation Under 100m (proportion)	111
Venomous animal contact	Male	0-6 days	95+ years	Data Rich	0	3	Population Density (under 150 ppl/sqkm, proportion)	399
Venomous animal contact	Male	0-6 days	95+ years	Data Rich	0	3	Population Density (over 1000 ppl/sqkm, proportion)	466
Venomous animal contact	Male	0-6 days	95+ years	Data Rich	0	3	LDI (I$ per capita)	--
Venomous animal contact	Male	0-6 days	95+ years	Global	1	1	Alcohol (liters per capita)	661
Venomous animal contact	Male	0-6 days	95+ years	Global	1	1	Log-transformed SEV scalar: Venom	--
Venomous animal contact	Male	0-6 days	95+ years	Global	-1	2	Healthcare access and quality index	566

Table S16. CODEm covariates used, level of covariate, and expected direction of covariate by cause, sex, and age
Covariates that CODEm did not select during the covariate selection process have no draw counts listed.

Cause	Sex	Age Start	Age End	Model Type	Direction	Level	Covariate Name	Number of Draws
Venomous animal contact	Male	0-6 days	95+ years	Global	-1	3	Education (years per capita)	125
Venomous animal contact	Male	0-6 days	95+ years	Global	-1	3	Socio-demographic Index	225
Venomous animal contact	Male	0-6 days	95+ years	Global	0	3	Population Density (under 150 ppl/sqkm, proportion)	575
Venomous animal contact	Male	0-6 days	95+ years	Global	0	3	Elevation Over 1500m (proportion)	579
Venomous animal contact	Male	0-6 days	95+ years	Global	0	3	Population Density (over 1000 ppl/sqkm, proportion)	755
Venomous animal contact	Male	0-6 days	95+ years	Global	0	3	Elevation Under 100m (proportion)	780
Venomous animal contact	Male	0-6 days	95+ years	Global	0	3	LDI (I$ per capita)	--
Non-venomous animal contact	Female	0-6 days	95+ years	Data Rich	1	1	Alcohol (liters per capita)	211
Non-venomous animal contact	Female	0-6 days	95+ years	Data Rich	1	1	Log-transformed SEV scalar: Non Ven	--
Non-venomous animal contact	Female	0-6 days	95+ years	Data Rich	-1	2	Healthcare access and quality index	--
Non-venomous animal contact	Female	0-6 days	95+ years	Data Rich	-1	3	Education (years per capita)	--
Non-venomous animal contact	Female	0-6 days	95+ years	Data Rich	-1	3	Socio-demographic Index	--
Non-venomous animal contact	Female	0-6 days	95+ years	Data Rich	0	3	Population Density (under 150 ppl/sqkm, proportion)	39
Non-venomous animal contact	Female	0-6 days	95+ years	Data Rich	0	3	Elevation Over 1500m (proportion)	828
Non-venomous animal contact	Female	0-6 days	95+ years	Data Rich	0	3	Elevation Under 100m (proportion)	828
Non-venomous animal contact	Female	0-6 days	95+ years	Data Rich	0	3	Population Density (over 1000 ppl/sqkm, proportion)	828
Non-venomous animal contact	Female	0-6 days	95+ years	Data Rich	0	3	LDI (I$ per capita)	--
Non-venomous animal contact	Female	0-6 days	95+ years	Global	1	1	Alcohol (liters per capita)	1000
Non-venomous animal contact	Female	0-6 days	95+ years	Global	1	1	Log-transformed SEV scalar: Non Ven	--
Non-venomous animal contact	Female	0-6 days	95+ years	Global	-1	2	Healthcare access and quality index	--
Non-venomous animal contact	Female	0-6 days	95+ years	Global	-1	3	Education (years per capita)	161
Non-venomous animal contact	Female	0-6 days	95+ years	Global	-1	3	Socio-demographic Index	193
Non-venomous animal contact	Female	0-6 days	95+ years	Global	0	3	Elevation Over 1500m (proportion)	282
Non-venomous animal contact	Female	0-6 days	95+ years	Global	0	3	Population Density (over 1000 ppl/sqkm, proportion)	282
Non-venomous animal contact	Female	0-6 days	95+ years	Global	0	3	Elevation Under 100m (proportion)	636
Non-venomous animal contact	Female	0-6 days	95+ years	Global	0	3	Population Density (under 150 ppl/sqkm, proportion)	636
Non-venomous animal contact	Female	0-6 days	95+ years	Global	0	3	LDI (I$ per capita)	--
Non-venomous animal contact	Male	0-6 days	95+ years	Data Rich	1	1	Alcohol (liters per capita)	468
Non-venomous animal contact	Male	0-6 days	95+ years	Data Rich	1	1	Log-transformed SEV scalar: Non Ven	--
Non-venomous animal contact	Male	0-6 days	95+ years	Data Rich	-1	2	Healthcare access and quality index	277
Non-venomous animal contact	Male	0-6 days	95+ years	Data Rich	-1	3	Socio-demographic Index	255
Non-venomous animal contact	Male	0-6 days	95+ years	Data Rich	-1	3	Education (years per capita)	--
Non-venomous animal contact	Male	0-6 days	95+ years	Data Rich	0	3	Elevation Over 1500m (proportion)	374
Non-venomous animal contact	Male	0-6 days	95+ years	Data Rich	0	3	Elevation Under 100m (proportion)	643
Non-venomous animal contact	Male	0-6 days	95+ years	Data Rich	0	3	Population Density (over 1000 ppl/sqkm, proportion)	643
Non-venomous animal contact	Male	0-6 days	95+ years	Data Rich	0	3	Population Density (under 150 ppl/sqkm, proportion)	643
Non-venomous animal contact	Male	0-6 days	95+ years	Data Rich	0	3	LDI (I$ per capita)	--
Non-venomous animal contact	Male	0-6 days	95+ years	Global	1	1	Log-transformed SEV scalar: Non Ven	--
Non-venomous animal contact	Male	0-6 days	95+ years	Global	-1	3	Education (years per capita)	454
Non-venomous animal contact	Male	0-6 days	95+ years	Global	-1	3	Healthcare access and quality index	454
Non-venomous animal contact	Male	0-6 days	95+ years	Global	-1	3	Socio-demographic Index	454
Non-venomous animal contact	Male	0-6 days	95+ years	Global	0	3	LDI (I$ per capita)	--
Non-venomous animal contact	Male	0-6 days	95+ years	Global	1	3	Alcohol (liters per capita)	546
Foreign body	Female	0-6 days	95+ years	Data Rich	1	1	LDI (I$ per capita)	999
Foreign body	Female	0-6 days	95+ years	Data Rich	1	1	Education (years per capita)	1000
Foreign body	Female	0-6 days	95+ years	Data Rich	1	1	Indoor Air Pollution (All Cooking Fuels)	1000
Foreign body	Female	0-6 days	95+ years	Data Rich	1	1	Population Over 65 (proportion)	1000
Foreign body	Female	0-6 days	95+ years	Data Rich	1	1	Population Density (over 1000 ppl/sqkm, proportion)	--
Foreign body	Female	0-6 days	95+ years	Data Rich	-1	2	Healthcare access and quality index	165
Foreign body	Female	0-6 days	95+ years	Data Rich	0	3	Socio-demographic Index	963
Foreign body	Female	0-6 days	95+ years	Global	1	1	Indoor Air Pollution (All Cooking Fuels)	55

Table S16. CODEm covariates used, level of covariate, and expected direction of covariate by cause, sex, and age
Covariates that CODEm did not select during the covariate selection process have no draw counts listed.

Cause	Sex	Age Start	Age End	Model Type	Direction	Level	Covariate Name	Number of Draws
Foreign body	Female	0-6 days	95+ years	Global	1	1	Population Over 65 (proportion)	611
Foreign body	Female	0-6 days	95+ years	Global	1	1	LDI (I$ per capita)	614
Foreign body	Female	0-6 days	95+ years	Global	1	1	Education (years per capita)	748
Foreign body	Female	0-6 days	95+ years	Global	1	1	Population Density (over 1000 ppl/sqkm, proportion)	--
Foreign body	Female	0-6 days	95+ years	Global	-1	2	Healthcare access and quality index	34
Foreign body	Female	0-6 days	95+ years	Global	0	3	Socio-demographic Index	539
Foreign body	Male	0-6 days	95+ years	Data Rich	1	1	Education (years per capita)	395
Foreign body	Male	0-6 days	95+ years	Data Rich	1	1	Indoor Air Pollution (All Cooking Fuels)	992
Foreign body	Male	0-6 days	95+ years	Data Rich	1	1	LDI (I$ per capita)	1000
Foreign body	Male	0-6 days	95+ years	Data Rich	1	1	Population Over 65 (proportion)	1000
Foreign body	Male	0-6 days	95+ years	Data Rich	1	1	Population Density (over 1000 ppl/sqkm, proportion)	--
Foreign body	Male	0-6 days	95+ years	Data Rich	-1	2	Healthcare access and quality index	61
Foreign body	Male	0-6 days	95+ years	Data Rich	0	3	Socio-demographic Index	884
Foreign body	Male	0-6 days	95+ years	Global	1	1	Population Density (over 1000 ppl/sqkm, proportion)	21
Foreign body	Male	0-6 days	95+ years	Global	1	1	Indoor Air Pollution (All Cooking Fuels)	467
Foreign body	Male	0-6 days	95+ years	Global	1	1	Population Over 65 (proportion)	614
Foreign body	Male	0-6 days	95+ years	Global	1	1	LDI (I$ per capita)	648
Foreign body	Male	0-6 days	95+ years	Global	1	1	Education (years per capita)	835
Foreign body	Male	0-6 days	95+ years	Global	-1	2	Healthcare access and quality index	--
Foreign body	Male	0-6 days	95+ years	Global	0	3	Socio-demographic Index	475
Pulmonary aspiration and foreign body in airway	Female	0-6 days	95+ years	Data Rich	1	1	Log-transformed SEV scalar: F Body Asp	--
Pulmonary aspiration and foreign body in airway	Female	0-6 days	95+ years	Data Rich	-1	2	Healthcare access and quality index	32
Pulmonary aspiration and foreign body in airway	Female	0-6 days	95+ years	Data Rich	1	2	Mean BMI	32
Pulmonary aspiration and foreign body in airway	Female	0-6 days	95+ years	Data Rich	1	2	Alcohol (liters per capita)	--
Pulmonary aspiration and foreign body in airway	Female	0-6 days	95+ years	Data Rich	0	3	Socio-demographic Index	770
Pulmonary aspiration and foreign body in airway	Female	0-6 days	95+ years	Data Rich	0	3	LDI (I$ per capita)	--
Pulmonary aspiration and foreign body in airway	Female	0-6 days	95+ years	Global	1	1	Log-transformed SEV scalar: F Body Asp	--
Pulmonary aspiration and foreign body in airway	Female	0-6 days	95+ years	Global	-1	2	Healthcare access and quality index	264
Pulmonary aspiration and foreign body in airway	Female	0-6 days	95+ years	Global	1	2	Alcohol binge drinker proportion, age-standardized	428
Pulmonary aspiration and foreign body in airway	Female	0-6 days	95+ years	Global	1	2	Mean BMI	692
Pulmonary aspiration and foreign body in airway	Female	0-6 days	95+ years	Global	1	2	Alcohol (liters per capita)	--
Pulmonary aspiration and foreign body in airway	Female	0-6 days	95+ years	Global	-1	3	Education (years per capita)	100
Pulmonary aspiration and foreign body in airway	Female	0-6 days	95+ years	Global	0	3	Socio-demographic Index	352
Pulmonary aspiration and foreign body in airway	Female	0-6 days	95+ years	Global	0	3	LDI (I$ per capita)	--
Pulmonary aspiration and foreign body in airway	Male	0-6 days	95+ years	Data Rich	1	1	Log-transformed SEV scalar: F Body Asp	--
Pulmonary aspiration and foreign body in airway	Male	0-6 days	95+ years	Data Rich	-1	2	Healthcare access and quality index	250
Pulmonary aspiration and foreign body in airway	Male	0-6 days	95+ years	Data Rich	1	2	Mean BMI	750
Pulmonary aspiration and foreign body in airway	Male	0-6 days	95+ years	Data Rich	1	2	Alcohol (liters per capita)	1000
Pulmonary aspiration and foreign body in airway	Male	0-6 days	95+ years	Data Rich	0	3	Socio-demographic Index	1000
Pulmonary aspiration and foreign body in airway	Male	0-6 days	95+ years	Data Rich	0	3	LDI (I$ per capita)	--
Pulmonary aspiration and foreign body in airway	Male	0-6 days	95+ years	Global	1	1	Log-transformed SEV scalar: F Body Asp	--
Pulmonary aspiration and foreign body in airway	Male	0-6 days	95+ years	Global	-1	2	Healthcare access and quality index	585
Pulmonary aspiration and foreign body in airway	Male	0-6 days	95+ years	Global	1	2	Alcohol (liters per capita)	282
Pulmonary aspiration and foreign body in airway	Male	0-6 days	95+ years	Global	1	2	Mean BMI	--
Pulmonary aspiration and foreign body in airway	Male	0-6 days	95+ years	Global	0	3	Socio-demographic Index	475
Pulmonary aspiration and foreign body in airway	Male	0-6 days	95+ years	Global	0	3	LDI (I$ per capita)	--
Foreign body in other body part	Female	0-6 days	95+ years	Data Rich	1	1	Alcohol (liters per capita)	889
Foreign body in other body part	Female	0-6 days	95+ years	Data Rich	1	1	Log-transformed SEV scalar: Oth F Body	--
Foreign body in other body part	Female	0-6 days	95+ years	Data Rich	-1	2	Healthcare access and quality index	298
Foreign body in other body part	Female	0-6 days	95+ years	Data Rich	-1	3	Socio-demographic Index	239
Foreign body in other body part	Female	0-6 days	95+ years	Data Rich	-1	3	Education (years per capita)	--
Foreign body in other body part	Female	0-6 days	95+ years	Data Rich	0	3	Population Density (over 1000 ppl/sqkm, proportion)	290
Foreign body in other body part	Female	0-6 days	95+ years	Data Rich	0	3	Elevation Over 1500m (proportion)	350
Foreign body in other body part	Female	0-6 days	95+ years	Data Rich	0	3	Elevation Under 100m (proportion)	418

Table S16. CODEm covariates used, level of covariate, and expected direction of covariate by cause, sex, and age<br>*Covariates that CODEm did not select during the covariate selection process have no draw counts listed.*								
**Cause**	**Sex**	**Age Start**	**Age End**	**Model Type**	**Direction**	**Level**	**Covariate Name**	**Number of Draws**
Foreign body in other body part	Female	0-6 days	95+ years	Data Rich	0	3	Population Density (under 150 ppl/sqkm, proportion)	529
Foreign body in other body part	Female	0-6 days	95+ years	Data Rich	0	3	LDI (I$ per capita)	--
Foreign body in other body part	Female	0-6 days	95+ years	Global	1	1	Alcohol (liters per capita)	1000
Foreign body in other body part	Female	0-6 days	95+ years	Global	1	1	Log-transformed SEV scalar: Oth F Body	--
Foreign body in other body part	Female	0-6 days	95+ years	Global	-1	2	Healthcare access and quality index	293
Foreign body in other body part	Female	0-6 days	95+ years	Global	-1	3	Education (years per capita)	92
Foreign body in other body part	Female	0-6 days	95+ years	Global	-1	3	Socio-demographic Index	111
Foreign body in other body part	Female	0-6 days	95+ years	Global	0	3	Elevation Over 1500m (proportion)	388
Foreign body in other body part	Female	0-6 days	95+ years	Global	0	3	Population Density (under 150 ppl/sqkm, proportion)	388
Foreign body in other body part	Female	0-6 days	95+ years	Global	0	3	Elevation Under 100m (proportion)	591
Foreign body in other body part	Female	0-6 days	95+ years	Global	0	3	Population Density (over 1000 ppl/sqkm, proportion)	591
Foreign body in other body part	Female	0-6 days	95+ years	Global	0	3	LDI (I$ per capita)	--
Foreign body in other body part	Male	0-6 days	95+ years	Data Rich	1	1	Alcohol (liters per capita)	968
Foreign body in other body part	Male	0-6 days	95+ years	Data Rich	1	1	Log-transformed SEV scalar: Oth F Body	--
Foreign body in other body part	Male	0-6 days	95+ years	Data Rich	-1	2	Healthcare access and quality index	153
Foreign body in other body part	Male	0-6 days	95+ years	Data Rich	-1	3	Socio-demographic Index	249
Foreign body in other body part	Male	0-6 days	95+ years	Data Rich	-1	3	Education (years per capita)	--
Foreign body in other body part	Male	0-6 days	95+ years	Data Rich	0	3	Elevation Under 100m (proportion)	46
Foreign body in other body part	Male	0-6 days	95+ years	Data Rich	0	3	Population Density (under 150 ppl/sqkm, proportion)	69
Foreign body in other body part	Male	0-6 days	95+ years	Data Rich	0	3	Population Density (over 1000 ppl/sqkm, proportion)	355
Foreign body in other body part	Male	0-6 days	95+ years	Data Rich	0	3	Elevation Over 1500m (proportion)	409
Foreign body in other body part	Male	0-6 days	95+ years	Data Rich	0	3	LDI (I$ per capita)	--
Foreign body in other body part	Male	0-6 days	95+ years	Global	1	1	Alcohol (liters per capita)	636
Foreign body in other body part	Male	0-6 days	95+ years	Global	1	1	Log-transformed SEV scalar: Oth F Body	--
Foreign body in other body part	Male	0-6 days	95+ years	Global	-1	2	Healthcare access and quality index	264
Foreign body in other body part	Male	0-6 days	95+ years	Global	-1	3	Socio-demographic Index	100
Foreign body in other body part	Male	0-6 days	95+ years	Global	-1	3	Education (years per capita)	--
Foreign body in other body part	Male	0-6 days	95+ years	Global	0	3	Population Density (under 150 ppl/sqkm, proportion)	426
Foreign body in other body part	Male	0-6 days	95+ years	Global	0	3	Elevation Over 1500m (proportion)	437
Foreign body in other body part	Male	0-6 days	95+ years	Global	0	3	Population Density (over 1000 ppl/sqkm, proportion)	499
Foreign body in other body part	Male	0-6 days	95+ years	Global	0	3	Elevation Under 100m (proportion)	581
Foreign body in other body part	Male	0-6 days	95+ years	Global	0	3	LDI (I$ per capita)	--
Environmental heat and cold exposure	Female	0-6 days	95+ years	Data Rich	-1	2	Healthcare access and quality index	1000
Environmental heat and cold exposure	Female	0-6 days	95+ years	Data Rich	-1	3	Education (years per capita)	6
Environmental heat and cold exposure	Female	0-6 days	95+ years	Data Rich	-1	3	Socio-demographic Index	--
Environmental heat and cold exposure	Female	0-6 days	95+ years	Data Rich	0	3	Elevation Over 1500m (proportion)	6
Environmental heat and cold exposure	Female	0-6 days	95+ years	Data Rich	0	3	LDI (I$ per capita)	6
Environmental heat and cold exposure	Female	0-6 days	95+ years	Data Rich	0	3	Population-weighted mean temperature	161
Environmental heat and cold exposure	Female	0-6 days	95+ years	Data Rich	0	3	Sanitation (proportion with access)	161
Environmental heat and cold exposure	Female	0-6 days	95+ years	Data Rich	0	3	90th percentile climatic temperature in the given country-year.	167
Environmental heat and cold exposure	Female	0-6 days	95+ years	Data Rich	0	3	Elevation 500 to 1500m (proportion)	167
Environmental heat and cold exposure	Female	0-6 days	95+ years	Data Rich	0	3	Population Density (150-300 ppl/sqkm, proportion)	167
Environmental heat and cold exposure	Female	0-6 days	95+ years	Data Rich	0	3	Rainfall (Quintiles 4-5)	167
Environmental heat and cold exposure	Female	0-6 days	95+ years	Global	-1	2	Healthcare access and quality index	1000
Environmental heat and cold exposure	Female	0-6 days	95+ years	Global	-1	3	Education (years per capita)	196
Environmental heat and cold exposure	Female	0-6 days	95+ years	Global	-1	3	Socio-demographic Index	231
Environmental heat and cold exposure	Female	0-6 days	95+ years	Global	0	3	Population-weighted mean temperature	107
Environmental heat and cold exposure	Female	0-6 days	95+ years	Global	0	3	LDI (I$ per capita)	200
Environmental heat and cold exposure	Female	0-6 days	95+ years	Global	0	3	Sanitation (proportion with access)	200

Table S16. CODEm covariates used, level of covariate, and expected direction of covariate by cause, sex, and age
Covariates that CODEm did not select during the covariate selection process have no draw counts listed.

Cause	Sex	Age Start	Age End	Model Type	Direction	Level	Covariate Name	Number of Draws
Environmental heat and cold exposure	Female	0-6 days	95+ years	Global	0	3	Elevation Over 1500m (proportion)	299
Environmental heat and cold exposure	Female	0-6 days	95+ years	Global	0	3	Population Density (150-300 ppl/sqkm, proportion)	320
Environmental heat and cold exposure	Female	0-6 days	95+ years	Global	0	3	Elevation 500 to 1500m (proportion)	324
Environmental heat and cold exposure	Female	0-6 days	95+ years	Global	0	3	90th percentile climatic temperature in the given country-year.	--
Environmental heat and cold exposure	Female	0-6 days	95+ years	Global	0	3	Rainfall (Quintiles 4-5)	--
Environmental heat and cold exposure	Male	0-6 days	95+ years	Data Rich	-1	2	Healthcare access and quality index	1000
Environmental heat and cold exposure	Male	0-6 days	95+ years	Data Rich	-1	3	Education (years per capita)	--
Environmental heat and cold exposure	Male	0-6 days	95+ years	Data Rich	-1	3	Socio-demographic Index	--
Environmental heat and cold exposure	Male	0-6 days	95+ years	Data Rich	0	3	Rainfall (Quintiles 4-5)	15
Environmental heat and cold exposure	Male	0-6 days	95+ years	Data Rich	0	3	LDI (I$ per capita)	62
Environmental heat and cold exposure	Male	0-6 days	95+ years	Data Rich	0	3	Population-weighted mean temperature	62
Environmental heat and cold exposure	Male	0-6 days	95+ years	Data Rich	0	3	Elevation 500 to 1500m (proportion)	132
Environmental heat and cold exposure	Male	0-6 days	95+ years	Data Rich	0	3	90th percentile climatic temperature in the given country-year.	253
Environmental heat and cold exposure	Male	0-6 days	95+ years	Data Rich	0	3	Population Density (150-300 ppl/sqkm, proportion)	385
Environmental heat and cold exposure	Male	0-6 days	95+ years	Data Rich	0	3	Elevation Over 1500m (proportion)	--
Environmental heat and cold exposure	Male	0-6 days	95+ years	Data Rich	0	3	Sanitation (proportion with access)	--
Environmental heat and cold exposure	Male	0-6 days	95+ years	Global	-1	2	Healthcare access and quality index	674
Environmental heat and cold exposure	Male	0-6 days	95+ years	Global	-1	3	Education (years per capita)	566
Environmental heat and cold exposure	Male	0-6 days	95+ years	Global	-1	3	Socio-demographic Index	--
Environmental heat and cold exposure	Male	0-6 days	95+ years	Global	0	3	Elevation 500 to 1500m (proportion)	326
Environmental heat and cold exposure	Male	0-6 days	95+ years	Global	0	3	Elevation Over 1500m (proportion)	326
Environmental heat and cold exposure	Male	0-6 days	95+ years	Global	0	3	LDI (I$ per capita)	326
Environmental heat and cold exposure	Male	0-6 days	95+ years	Global	0	3	Population Density (150-300 ppl/sqkm, proportion)	326
Environmental heat and cold exposure	Male	0-6 days	95+ years	Global	0	3	Sanitation (proportion with access)	452
Environmental heat and cold exposure	Male	0-6 days	95+ years	Global	0	3	90th percentile climatic temperature in the given country-year.	538
Environmental heat and cold exposure	Male	0-6 days	95+ years	Global	0	3	Population-weighted mean temperature	538
Environmental heat and cold exposure	Male	0-6 days	95+ years	Global	0	3	Rainfall (Quintiles 4-5)	--
Other unintentional injuries	Female	0-6 days	95+ years	Data Rich	0	1	Vehicles - 4 wheels (per capita)	1000
Other unintentional injuries	Female	0-6 days	95+ years	Data Rich	1	1	Alcohol (liters per capita)	0
Other unintentional injuries	Female	0-6 days	95+ years	Data Rich	1	1	Vehicles - 2 wheels (per capita)	1000
Other unintentional injuries	Female	0-6 days	95+ years	Data Rich	1	1	Log-transformed SEV scalar: Oth Unint	--
Other unintentional injuries	Female	0-6 days	95+ years	Data Rich	-1	2	Healthcare access and quality index	834
Other unintentional injuries	Female	0-6 days	95+ years	Data Rich	-1	3	Education (years per capita)	1000
Other unintentional injuries	Female	0-6 days	95+ years	Data Rich	0	3	Population Density (over 1000 ppl/sqkm, proportion)	166
Other unintentional injuries	Female	0-6 days	95+ years	Data Rich	0	3	Population Density (under 150 ppl/sqkm, proportion)	198
Other unintentional injuries	Female	0-6 days	95+ years	Data Rich	0	3	Socio-demographic Index	962
Other unintentional injuries	Female	0-6 days	95+ years	Data Rich	0	3	Elevation Under 100m (proportion)	999
Other unintentional injuries	Female	0-6 days	95+ years	Data Rich	0	3	Elevation Over 1500m (proportion)	1000
Other unintentional injuries	Female	0-6 days	95+ years	Data Rich	0	3	LDI (I$ per capita)	--
Other unintentional injuries	Female	0-6 days	95+ years	Global	0	1	Vehicles - 4 wheels (per capita)	668
Other unintentional injuries	Female	0-6 days	95+ years	Global	1	1	Alcohol (liters per capita)	509
Other unintentional injuries	Female	0-6 days	95+ years	Global	1	1	Vehicles - 2 wheels (per capita)	551
Other unintentional injuries	Female	0-6 days	95+ years	Global	1	1	Log-transformed SEV scalar: Oth Unint	--
Other unintentional injuries	Female	0-6 days	95+ years	Global	-1	2	Healthcare access and quality index	277
Other unintentional injuries	Female	0-6 days	95+ years	Global	-1	3	Education (years per capita)	717
Other unintentional injuries	Female	0-6 days	95+ years	Global	0	3	Elevation Under 100m (proportion)	463
Other unintentional injuries	Female	0-6 days	95+ years	Global	0	3	Population Density (over 1000 ppl/sqkm, proportion)	680

Table S16. CODEm covariates used, level of covariate, and expected direction of covariate by cause, sex, and age
Covariates that CODEm did not select during the covariate selection process have no draw counts listed.

Cause	Sex	Age Start	Age End	Model Type	Direction	Level	Covariate Name	Number of Draws
Other unintentional injuries	Female	0-6 days	95+ years	Global	0	3	Population Density (under 150 ppl/sqkm, proportion)	700
Other unintentional injuries	Female	0-6 days	95+ years	Global	0	3	Elevation Over 1500m (proportion)	797
Other unintentional injuries	Female	0-6 days	95+ years	Global	0	3	Socio-demographic Index	821
Other unintentional injuries	Female	0-6 days	95+ years	Global	0	3	LDI (I$ per capita)	--
Other unintentional injuries	Male	0-6 days	95+ years	Data Rich	0	1	Vehicles - 4 wheels (per capita)	1000
Other unintentional injuries	Male	0-6 days	95+ years	Data Rich	1	1	Alcohol (liters per capita)	189
Other unintentional injuries	Male	0-6 days	95+ years	Data Rich	1	1	Vehicles - 2 wheels (per capita)	1000
Other unintentional injuries	Male	0-6 days	95+ years	Data Rich	1	1	Log-transformed SEV scalar: Oth Unint	--
Other unintentional injuries	Male	0-6 days	95+ years	Data Rich	-1	2	Healthcare access and quality index	765
Other unintentional injuries	Male	0-6 days	95+ years	Data Rich	-1	3	Education (years per capita)	765
Other unintentional injuries	Male	0-6 days	95+ years	Data Rich	0	3	Population Density (over 1000 ppl/sqkm, proportion)	233
Other unintentional injuries	Male	0-6 days	95+ years	Data Rich	0	3	Elevation Under 100m (proportion)	811
Other unintentional injuries	Male	0-6 days	95+ years	Data Rich	0	3	Elevation Over 1500m (proportion)	989
Other unintentional injuries	Male	0-6 days	95+ years	Data Rich	0	3	Population Density (under 150 ppl/sqkm, proportion)	1000
Other unintentional injuries	Male	0-6 days	95+ years	Data Rich	0	3	Socio-demographic Index	1000
Other unintentional injuries	Male	0-6 days	95+ years	Data Rich	0	3	LDI (I$ per capita)	--
Other unintentional injuries	Male	0-6 days	95+ years	Global	0	1	Vehicles - 4 wheels (per capita)	1000
Other unintentional injuries	Male	0-6 days	95+ years	Global	1	1	Alcohol (liters per capita)	401
Other unintentional injuries	Male	0-6 days	95+ years	Global	1	1	Vehicles - 2 wheels (per capita)	922
Other unintentional injuries	Male	0-6 days	95+ years	Global	1	1	Log-transformed SEV scalar: Oth Unint	--
Other unintentional injuries	Male	0-6 days	95+ years	Global	-1	2	Healthcare access and quality index	156
Other unintentional injuries	Male	0-6 days	95+ years	Global	-1	3	Education (years per capita)	724
Other unintentional injuries	Male	0-6 days	95+ years	Global	0	3	Population Density (over 1000 ppl/sqkm, proportion)	116
Other unintentional injuries	Male	0-6 days	95+ years	Global	0	3	Elevation Under 100m (proportion)	603
Other unintentional injuries	Male	0-6 days	95+ years	Global	0	3	Socio-demographic Index	605
Other unintentional injuries	Male	0-6 days	95+ years	Global	0	3	Population Density (under 150 ppl/sqkm, proportion)	735
Other unintentional injuries	Male	0-6 days	95+ years	Global	0	3	Elevation Over 1500m (proportion)	890
Other unintentional injuries	Male	0-6 days	95+ years	Global	0	3	LDI (I$ per capita)	--
Self-harm	Female	10-14 years	95+ years	Data Rich	1	1	Alcohol (liters per capita)	38
Self-harm	Female	10-14 years	95+ years	Data Rich	1	1	Major depressive disorder	1000
Self-harm	Female	10-14 years	95+ years	Data Rich	1	1	Log-transformed SEV scalar: Self Harm	--
Self-harm	Female	10-14 years	95+ years	Data Rich	-1	2	Healthcare access and quality index	0
Self-harm	Female	10-14 years	95+ years	Data Rich	-1	2	Religion (binary, >50% Muslim)	--
Self-harm	Female	10-14 years	95+ years	Data Rich	0	2	Population Density (300-500 ppl/sqkm, proportion)	0
Self-harm	Female	10-14 years	95+ years	Data Rich	0	2	Population Density (500-1000 ppl/sqkm, proportion)	31
Self-harm	Female	10-14 years	95+ years	Data Rich	0	2	Population Density (under 150 ppl/sqkm, proportion)	31
Self-harm	Female	10-14 years	95+ years	Data Rich	0	2	Population Density (over 1000 ppl/sqkm, proportion)	803
Self-harm	Female	10-14 years	95+ years	Data Rich	0	2	Population Density (150-300 ppl/sqkm, proportion)	804
Self-harm	Female	10-14 years	95+ years	Data Rich	0	3	Education (years per capita)	0
Self-harm	Female	10-14 years	95+ years	Data Rich	0	3	Socio-demographic Index	0
Self-harm	Female	10-14 years	95+ years	Data Rich	0	3	LDI (I$ per capita)	--
Self-harm	Female	10-14 years	95+ years	Global	1	1	Risk of selfharm due to major depressive disorder	0
Self-harm	Female	10-14 years	95+ years	Global	1	1	Non-partner lifetime prevalence of sexual violence (female-only)	371
Self-harm	Female	10-14 years	95+ years	Global	1	1	Alcohol (liters per capita)	404
Self-harm	Female	10-14 years	95+ years	Global	1	1	Major depressive disorder	998
Self-harm	Female	10-14 years	95+ years	Global	1	1	Log-transformed SEV scalar: Self Harm	--

Table S16. CODEm covariates used, level of covariate, and expected direction of covariate by cause, sex, and age
Covariates that CODEm did not select during the covariate selection process have no draw counts listed.

Cause	Sex	Age Start	Age End	Model Type	Direction	Level	Covariate Name	Number of Draws
Self-harm	Female	10-14 years	95+ years	Global	-1	2	Healthcare access and quality index	0
Self-harm	Female	10-14 years	95+ years	Global	-1	2	Religion (binary, >50% Muslim)	417
Self-harm	Female	10-14 years	95+ years	Global	0	2	Population Density (500-1000 ppl/sqkm, proportion)	136
Self-harm	Female	10-14 years	95+ years	Global	0	2	Population Density (300-500 ppl/sqkm, proportion)	291
Self-harm	Female	10-14 years	95+ years	Global	0	2	Population Density (over 1000 ppl/sqkm, proportion)	354
Self-harm	Female	10-14 years	95+ years	Global	0	2	Population Density (under 150 ppl/sqkm, proportion)	369
Self-harm	Female	10-14 years	95+ years	Global	0	2	Population Density (150-300 ppl/sqkm, proportion)	417
Self-harm	Female	10-14 years	95+ years	Global	0	3	Socio-demographic Index	678
Self-harm	Female	10-14 years	95+ years	Global	0	3	Education (years per capita)	754
Self-harm	Female	10-14 years	95+ years	Global	0	3	LDI (I$ per capita)	--
Self-harm	Male	10-14 years	95+ years	Data Rich	1	1	Alcohol (liters per capita)	--
Self-harm	Male	10-14 years	95+ years	Data Rich	-1	2	Religion (binary, >50% Muslim)	15
Self-harm	Male	10-14 years	95+ years	Data Rich	-1	2	Healthcare access and quality index	--
Self-harm	Male	10-14 years	95+ years	Data Rich	0	2	Population Density (500-1000 ppl/sqkm, proportion)	84
Self-harm	Male	10-14 years	95+ years	Data Rich	0	2	Population Density (under 150 ppl/sqkm, proportion)	703
Self-harm	Male	10-14 years	95+ years	Data Rich	0	2	Population Density (300-500 ppl/sqkm, proportion)	718
Self-harm	Male	10-14 years	95+ years	Data Rich	0	2	Population Density (150-300 ppl/sqkm, proportion)	--
Self-harm	Male	10-14 years	95+ years	Data Rich	0	2	Population Density (over 1000 ppl/sqkm, proportion)	--
Self-harm	Male	10-14 years	95+ years	Data Rich	0	3	Education (years per capita)	760
Self-harm	Male	10-14 years	95+ years	Data Rich	0	3	Socio-demographic Index	791
Self-harm	Male	10-14 years	95+ years	Data Rich	0	3	LDI (I$ per capita)	--
Self-harm	Male	10-14 years	95+ years	Global	1	1	Alcohol (liters per capita)	--
Self-harm	Male	10-14 years	95+ years	Global	-1	2	Muslim Religion (proportion of population)	668
Self-harm	Male	10-14 years	95+ years	Global	-1	2	Healthcare access and quality index	--
Self-harm	Male	10-14 years	95+ years	Global	0	2	Population Density (under 150 ppl/sqkm, proportion)	436
Self-harm	Male	10-14 years	95+ years	Global	0	2	Population Density (500-1000 ppl/sqkm, proportion)	512
Self-harm	Male	10-14 years	95+ years	Global	0	2	Population Density (300-500 ppl/sqkm, proportion)	572
Self-harm	Male	10-14 years	95+ years	Global	0	2	Population Density (150-300 ppl/sqkm, proportion)	760
Self-harm	Male	10-14 years	95+ years	Global	0	2	Population Density (over 1000 ppl/sqkm, proportion)	760
Self-harm	Male	10-14 years	95+ years	Global	0	3	Education (years per capita)	677
Self-harm	Male	10-14 years	95+ years	Global	0	3	Socio-demographic Index	722
Self-harm	Male	10-14 years	95+ years	Global	0	3	LDI (I$ per capita)	--
Self-harm by firearm	Female	10-14 years	95+ years	Data Rich	1	1	Major depressive disorder	1000
Self-harm by firearm	Female	10-14 years	95+ years	Data Rich	1	1	Alcohol (liters per capita)	--
Self-harm by firearm	Female	10-14 years	95+ years	Data Rich	1	1	Log-transformed SEV scalar: Self Harm	--
Self-harm by firearm	Female	10-14 years	95+ years	Data Rich	-1	2	Healthcare access and quality index	14
Self-harm by firearm	Female	10-14 years	95+ years	Data Rich	-1	2	Religion (binary, >50% Muslim)	--
Self-harm by firearm	Female	10-14 years	95+ years	Data Rich	0	2	Population Density (over 1000 ppl/sqkm, proportion)	14
Self-harm by firearm	Female	10-14 years	95+ years	Data Rich	0	2	Population Density (under 150 ppl/sqkm, proportion)	319
Self-harm by firearm	Female	10-14 years	95+ years	Data Rich	0	2	Population Density (150-300 ppl/sqkm, proportion)	588

Table S16. CODEm covariates used, level of covariate, and expected direction of covariate by cause, sex, and age
Covariates that CODEm did not select during the covariate selection process have no draw counts listed.

Cause	Sex	Age Start	Age End	Model Type	Direction	Level	Covariate Name	Number of Draws
Self-harm by firearm	Female	10-14 years	95+ years	Data Rich	0	2	Population Density (300-500 ppl/sqkm, proportion)	641
Self-harm by firearm	Female	10-14 years	95+ years	Data Rich	0	2	Population Density (500-1000 ppl/sqkm, proportion)	698
Self-harm by firearm	Female	10-14 years	95+ years	Data Rich	0	3	Education (years per capita)	872
Self-harm by firearm	Female	10-14 years	95+ years	Data Rich	0	3	Socio-demographic Index	938
Self-harm by firearm	Female	10-14 years	95+ years	Data Rich	0	3	LDI (I$ per capita)	--
Self-harm by firearm	Female	10-14 years	95+ years	Global	1	1	Major depressive disorder	1000
Self-harm by firearm	Female	10-14 years	95+ years	Global	1	1	Alcohol (liters per capita)	--
Self-harm by firearm	Female	10-14 years	95+ years	Global	1	1	Log-transformed SEV scalar: Self Harm	--
Self-harm by firearm	Female	10-14 years	95+ years	Global	-1	2	Healthcare access and quality index	200
Self-harm by firearm	Female	10-14 years	95+ years	Global	-1	2	Religion (binary, >50% Muslim)	--
Self-harm by firearm	Female	10-14 years	95+ years	Global	0	2	Population Density (150-300 ppl/sqkm, proportion)	149
Self-harm by firearm	Female	10-14 years	95+ years	Global	0	2	Population Density (300-500 ppl/sqkm, proportion)	687
Self-harm by firearm	Female	10-14 years	95+ years	Global	0	2	Population Density (500-1000 ppl/sqkm, proportion)	700
Self-harm by firearm	Female	10-14 years	95+ years	Global	0	2	Population Density (over 1000 ppl/sqkm, proportion)	711
Self-harm by firearm	Female	10-14 years	95+ years	Global	0	2	Population Density (under 150 ppl/sqkm, proportion)	749
Self-harm by firearm	Female	10-14 years	95+ years	Global	0	3	Socio-demographic Index	390
Self-harm by firearm	Female	10-14 years	95+ years	Global	0	3	Education (years per capita)	517
Self-harm by firearm	Female	10-14 years	95+ years	Global	0	3	LDI (I$ per capita)	--
Self-harm by firearm	Male	10-14 years	95+ years	Data Rich	1	1	Major depressive disorder	1000
Self-harm by firearm	Male	10-14 years	95+ years	Data Rich	1	1	Alcohol (liters per capita)	--
Self-harm by firearm	Male	10-14 years	95+ years	Data Rich	1	1	Log-transformed SEV scalar: Self Harm	--
Self-harm by firearm	Male	10-14 years	95+ years	Data Rich	-1	2	Healthcare access and quality index	--
Self-harm by firearm	Male	10-14 years	95+ years	Data Rich	-1	2	Religion (binary, >50% Muslim)	--
Self-harm by firearm	Male	10-14 years	95+ years	Data Rich	0	2	Population Density (under 150 ppl/sqkm, proportion)	47
Self-harm by firearm	Male	10-14 years	95+ years	Data Rich	0	2	Population Density (300-500 ppl/sqkm, proportion)	181
Self-harm by firearm	Male	10-14 years	95+ years	Data Rich	0	2	Population Density (150-300 ppl/sqkm, proportion)	211
Self-harm by firearm	Male	10-14 years	95+ years	Data Rich	0	2	Population Density (over 1000 ppl/sqkm, proportion)	218
Self-harm by firearm	Male	10-14 years	95+ years	Data Rich	0	2	Population Density (500-1000 ppl/sqkm, proportion)	219
Self-harm by firearm	Male	10-14 years	95+ years	Data Rich	0	3	Socio-demographic Index	210
Self-harm by firearm	Male	10-14 years	95+ years	Data Rich	0	3	Education (years per capita)	991
Self-harm by firearm	Male	10-14 years	95+ years	Data Rich	0	3	LDI (I$ per capita)	--
Self-harm by firearm	Male	10-14 years	95+ years	Global	1	1	Alcohol (liters per capita)	--
Self-harm by firearm	Male	10-14 years	95+ years	Global	1	1	Log-transformed SEV scalar: Self Harm	--
Self-harm by firearm	Male	10-14 years	95+ years	Global	1	1	Major depressive disorder	--
Self-harm by firearm	Male	10-14 years	95+ years	Global	-1	2	Healthcare access and quality index	96
Self-harm by firearm	Male	10-14 years	95+ years	Global	-1	2	Religion (binary, >50% Muslim)	--
Self-harm by firearm	Male	10-14 years	95+ years	Global	0	2	Population Density (over 1000 ppl/sqkm, proportion)	85
Self-harm by firearm	Male	10-14 years	95+ years	Global	0	2	Population Density (500-1000 ppl/sqkm, proportion)	98
Self-harm by firearm	Male	10-14 years	95+ years	Global	0	2	Population Density (150-300 ppl/sqkm, proportion)	546
Self-harm by firearm	Male	10-14 years	95+ years	Global	0	2	Population Density (300-500 ppl/sqkm, proportion)	--

**Table S16. CODEm covariates used, level of covariate, and expected direction of covariate by cause, sex, and age** *Covariates that CODEm did not select during the covariate selection process have no draw counts listed.*								
**Cause**	**Sex**	**Age Start**	**Age End**	**Model Type**	**Direction**	**Level**	**Covariate Name**	**Number of Draws**
Self-harm by firearm	Male	10-14 years	95+ years	Global	0	2	Population Density (under 150 ppl/sqkm, proportion)	--
Self-harm by firearm	Male	10-14 years	95+ years	Global	0	3	Education (years per capita)	177
Self-harm by firearm	Male	10-14 years	95+ years	Global	0	3	Socio-demographic Index	295
Self-harm by firearm	Male	10-14 years	95+ years	Global	0	3	LDI (I$ per capita)	--
Self-harm by other specified means	Female	10-14 years	95+ years	Data Rich	1	1	Alcohol (liters per capita)	0
Self-harm by other specified means	Female	10-14 years	95+ years	Data Rich	1	1	Major depressive disorder	1000
Self-harm by other specified means	Female	10-14 years	95+ years	Data Rich	1	1	Log-transformed SEV scalar: Self Harm	--
Self-harm by other specified means	Female	10-14 years	95+ years	Data Rich	-1	2	Healthcare access and quality index	0
Self-harm by other specified means	Female	10-14 years	95+ years	Data Rich	-1	2	Religion (binary, >50% Muslim)	--
Self-harm by other specified means	Female	10-14 years	95+ years	Data Rich	0	2	Population Density (over 1000 ppl/sqkm, proportion)	32
Self-harm by other specified means	Female	10-14 years	95+ years	Data Rich	0	2	Population Density (150-300 ppl/sqkm, proportion)	198
Self-harm by other specified means	Female	10-14 years	95+ years	Data Rich	0	2	Population Density (300-500 ppl/sqkm, proportion)	839
Self-harm by other specified means	Female	10-14 years	95+ years	Data Rich	0	2	Population Density (500-1000 ppl/sqkm, proportion)	967
Self-harm by other specified means	Female	10-14 years	95+ years	Data Rich	0	2	Population Density (under 150 ppl/sqkm, proportion)	993
Self-harm by other specified means	Female	10-14 years	95+ years	Data Rich	0	3	Education (years per capita)	0
Self-harm by other specified means	Female	10-14 years	95+ years	Data Rich	0	3	Socio-demographic Index	0
Self-harm by other specified means	Female	10-14 years	95+ years	Data Rich	0	3	LDI (I$ per capita)	--
Self-harm by other specified means	Female	10-14 years	95+ years	Global	1	1	Alcohol (liters per capita)	200
Self-harm by other specified means	Female	10-14 years	95+ years	Global	1	1	Major depressive disorder	910
Self-harm by other specified means	Female	10-14 years	95+ years	Global	1	1	Log-transformed SEV scalar: Self Harm	--
Self-harm by other specified means	Female	10-14 years	95+ years	Global	-1	2	Healthcare access and quality index	7
Self-harm by other specified means	Female	10-14 years	95+ years	Global	-1	2	Religion (binary, >50% Muslim)	673
Self-harm by other specified means	Female	10-14 years	95+ years	Global	0	2	Population Density (300-500 ppl/sqkm, proportion)	41
Self-harm by other specified means	Female	10-14 years	95+ years	Global	0	2	Population Density (150-300 ppl/sqkm, proportion)	438
Self-harm by other specified means	Female	10-14 years	95+ years	Global	0	2	Population Density (500-1000 ppl/sqkm, proportion)	444
Self-harm by other specified means	Female	10-14 years	95+ years	Global	0	2	Population Density (under 150 ppl/sqkm, proportion)	462
Self-harm by other specified means	Female	10-14 years	95+ years	Global	0	2	Population Density (over 1000 ppl/sqkm, proportion)	523
Self-harm by other specified means	Female	10-14 years	95+ years	Global	0	3	Education (years per capita)	268
Self-harm by other specified means	Female	10-14 years	95+ years	Global	0	3	Socio-demographic Index	537
Self-harm by other specified means	Female	10-14 years	95+ years	Global	0	3	LDI (I$ per capita)	--
Self-harm by other specified means	Male	10-14 years	95+ years	Data Rich	1	1	Major depressive disorder	736
Self-harm by other specified means	Male	10-14 years	95+ years	Data Rich	1	1	Alcohol (liters per capita)	--
Self-harm by other specified means	Male	10-14 years	95+ years	Data Rich	1	1	Log-transformed SEV scalar: Self Harm	--
Self-harm by other specified means	Male	10-14 years	95+ years	Data Rich	-1	2	Religion (binary, >50% Muslim)	66
Self-harm by other specified means	Male	10-14 years	95+ years	Data Rich	-1	2	Healthcare access and quality index	--
Self-harm by other specified means	Male	10-14 years	95+ years	Data Rich	0	2	Population Density (over 1000 ppl/sqkm, proportion)	198
Self-harm by other specified means	Male	10-14 years	95+ years	Data Rich	0	2	Population Density (300-500 ppl/sqkm, proportion)	330
Self-harm by other specified means	Male	10-14 years	95+ years	Data Rich	0	2	Population Density (150-300 ppl/sqkm, proportion)	396
Self-harm by other specified means	Male	10-14 years	95+ years	Data Rich	0	2	Population Density (500-1000 ppl/sqkm, proportion)	462
Self-harm by other specified means	Male	10-14 years	95+ years	Data Rich	0	2	Population Density (under 150 ppl/sqkm, proportion)	--

Table S16. CODEm covariates used, level of covariate, and expected direction of covariate by cause, sex, and age
Covariates that CODEm did not select during the covariate selection process have no draw counts listed.

Cause	Sex	Age Start	Age End	Model Type	Direction	Level	Covariate Name	Number of Draws
Self-harm by other specified means	Male	10-14 years	95+ years	Data Rich	0	3	Socio-demographic Index	66
Self-harm by other specified means	Male	10-14 years	95+ years	Data Rich	0	3	Education (years per capita)	264
Self-harm by other specified means	Male	10-14 years	95+ years	Data Rich	0	3	LDI (I$ per capita)	--
Self-harm by other specified means	Male	10-14 years	95+ years	Global	1	1	Major depressive disorder	839
Self-harm by other specified means	Male	10-14 years	95+ years	Global	1	1	Alcohol (liters per capita)	--
Self-harm by other specified means	Male	10-14 years	95+ years	Global	1	1	Log-transformed SEV scalar: Self Harm	--
Self-harm by other specified means	Male	10-14 years	95+ years	Global	-1	2	Religion (binary, >50% Muslim)	75
Self-harm by other specified means	Male	10-14 years	95+ years	Global	-1	2	Healthcare access and quality index	--
Self-harm by other specified means	Male	10-14 years	95+ years	Global	0	2	Population Density (300-500 ppl/sqkm, proportion)	101
Self-harm by other specified means	Male	10-14 years	95+ years	Global	0	2	Population Density (150-300 ppl/sqkm, proportion)	176
Self-harm by other specified means	Male	10-14 years	95+ years	Global	0	2	Population Density (over 1000 ppl/sqkm, proportion)	318
Self-harm by other specified means	Male	10-14 years	95+ years	Global	0	2	Population Density (under 150 ppl/sqkm, proportion)	419
Self-harm by other specified means	Male	10-14 years	95+ years	Global	0	2	Population Density (500-1000 ppl/sqkm, proportion)	--
Self-harm by other specified means	Male	10-14 years	95+ years	Global	0	3	Education (years per capita)	427
Self-harm by other specified means	Male	10-14 years	95+ years	Global	0	3	LDI (I$ per capita)	--
Self-harm by other specified means	Male	10-14 years	95+ years	Global	0	3	Socio-demographic Index	--
Interpersonal violence	Female	0-6 days	95+ years	Data Rich	1	1	Alcohol (liters per capita)	--
Interpersonal violence	Female	0-6 days	95+ years	Data Rich	1	1	Log-transformed SEV scalar: Violence	--
Interpersonal violence	Female	0-6 days	95+ years	Data Rich	-1	2	Healthcare access and quality index	483
Interpersonal violence	Female	0-6 days	95+ years	Data Rich	1	2	Opium Cultivation (binary)	10
Interpersonal violence	Female	0-6 days	95+ years	Data Rich	1	2	Population Density (over 1000 ppl/sqkm, proportion)	--
Interpersonal violence	Female	0-6 days	95+ years	Data Rich	0	3	Socio-demographic Index	148
Interpersonal violence	Female	0-6 days	95+ years	Data Rich	0	3	Education (years per capita)	156
Interpersonal violence	Female	0-6 days	95+ years	Data Rich	0	3	LDI (I$ per capita)	--
Interpersonal violence	Female	0-6 days	95+ years	Global	1	1	Alcohol (liters per capita)	--
Interpersonal violence	Female	0-6 days	95+ years	Global	1	1	Log-transformed SEV scalar: Violence	--
Interpersonal violence	Female	0-6 days	95+ years	Global	-1	2	Healthcare access and quality index	203
Interpersonal violence	Female	0-6 days	95+ years	Global	1	2	Population Density (over 1000 ppl/sqkm, proportion)	331
Interpersonal violence	Female	0-6 days	95+ years	Global	1	2	Opium Cultivation (binary)	--
Interpersonal violence	Female	0-6 days	95+ years	Global	0	3	Education (years per capita)	76
Interpersonal violence	Female	0-6 days	95+ years	Global	0	3	Socio-demographic Index	312
Interpersonal violence	Female	0-6 days	95+ years	Global	0	3	LDI (I$ per capita)	--
Interpersonal violence	Male	0-6 days	95+ years	Data Rich	1	1	Alcohol (liters per capita)	161
Interpersonal violence	Male	0-6 days	95+ years	Data Rich	1	1	Log-transformed SEV scalar: Violence	--
Interpersonal violence	Male	0-6 days	95+ years	Data Rich	-1	2	Healthcare access and quality index	6
Interpersonal violence	Male	0-6 days	95+ years	Data Rich	1	2	Population Density (over 1000 ppl/sqkm, proportion)	0
Interpersonal violence	Male	0-6 days	95+ years	Data Rich	1	2	Opium Cultivation (binary)	801
Interpersonal violence	Male	0-6 days	95+ years	Data Rich	0	3	Education (years per capita)	38
Interpersonal violence	Male	0-6 days	95+ years	Data Rich	0	3	Socio-demographic Index	961
Interpersonal violence	Male	0-6 days	95+ years	Data Rich	0	3	LDI (I$ per capita)	--
Interpersonal violence	Male	0-6 days	95+ years	Global	1	1	Alcohol (liters per capita)	--
Interpersonal violence	Male	0-6 days	95+ years	Global	1	1	Log-transformed SEV scalar: Violence	--
Interpersonal violence	Male	0-6 days	95+ years	Global	-1	2	Healthcare access and quality index	150
Interpersonal violence	Male	0-6 days	95+ years	Global	1	2	Population Density (over 1000 ppl/sqkm, proportion)	266
Interpersonal violence	Male	0-6 days	95+ years	Global	1	2	Opium Cultivation (binary)	486
Interpersonal violence	Male	0-6 days	95+ years	Global	0	3	Education (years per capita)	49
Interpersonal violence	Male	0-6 days	95+ years	Global	0	3	Socio-demographic Index	222
Interpersonal violence	Male	0-6 days	95+ years	Global	0	3	LDI (I$ per capita)	--

Table S16. CODEm covariates used, level of covariate, and expected direction of covariate by cause, sex, and age
Covariates that CODEm did not select during the covariate selection process have no draw counts listed.

Cause	Sex	Age Start	Age End	Model Type	Direction	Level	Covariate Name	Number of Draws
Physical violence by firearm	Female	0-6 days	95+ years	Data Rich	1	1	Alcohol (liters per capita)	--
Physical violence by firearm	Female	0-6 days	95+ years	Data Rich	1	1	Log-transformed SEV scalar: Viol Gun	--
Physical violence by firearm	Female	0-6 days	95+ years	Data Rich	-1	2	Healthcare access and quality index	38
Physical violence by firearm	Female	0-6 days	95+ years	Data Rich	1	2	Opium Cultivation (binary)	1
Physical violence by firearm	Female	0-6 days	95+ years	Data Rich	1	2	Population Density (over 1000 ppl/sqkm, proportion)	--
Physical violence by firearm	Female	0-6 days	95+ years	Data Rich	0	3	Socio-demographic Index	160
Physical violence by firearm	Female	0-6 days	95+ years	Data Rich	0	3	Education (years per capita)	--
Physical violence by firearm	Female	0-6 days	95+ years	Data Rich	0	3	LDI (I$ per capita)	--
Physical violence by firearm	Female	0-6 days	95+ years	Global	1	1	Alcohol (liters per capita)	--
Physical violence by firearm	Female	0-6 days	95+ years	Global	1	1	Log-transformed SEV scalar: Viol Gun	--
Physical violence by firearm	Female	0-6 days	95+ years	Global	-1	2	Healthcare access and quality index	416
Physical violence by firearm	Female	0-6 days	95+ years	Global	1	2	Opium Cultivation (binary)	416
Physical violence by firearm	Female	0-6 days	95+ years	Global	1	2	Population Density (over 1000 ppl/sqkm, proportion)	--
Physical violence by firearm	Female	0-6 days	95+ years	Global	0	3	Education (years per capita)	391
Physical violence by firearm	Female	0-6 days	95+ years	Global	0	3	Socio-demographic Index	391
Physical violence by firearm	Female	0-6 days	95+ years	Global	0	3	LDI (I$ per capita)	--
Physical violence by firearm	Male	0-6 days	95+ years	Data Rich	1	1	Alcohol (liters per capita)	765
Physical violence by firearm	Male	0-6 days	95+ years	Data Rich	1	1	Log-transformed SEV scalar: Viol Gun	--
Physical violence by firearm	Male	0-6 days	95+ years	Data Rich	-1	2	Healthcare access and quality index	--
Physical violence by firearm	Male	0-6 days	95+ years	Data Rich	1	2	Population Density (over 1000 ppl/sqkm, proportion)	47
Physical violence by firearm	Male	0-6 days	95+ years	Data Rich	1	2	Opium Cultivation (binary)	--
Physical violence by firearm	Male	0-6 days	95+ years	Data Rich	0	3	Socio-demographic Index	349
Physical violence by firearm	Male	0-6 days	95+ years	Data Rich	0	3	Education (years per capita)	--
Physical violence by firearm	Male	0-6 days	95+ years	Data Rich	0	3	LDI (I$ per capita)	--
Physical violence by firearm	Male	0-6 days	95+ years	Global	1	1	Alcohol (liters per capita)	593
Physical violence by firearm	Male	0-6 days	95+ years	Global	1	1	Log-transformed SEV scalar: Viol Gun	--
Physical violence by firearm	Male	0-6 days	95+ years	Global	-1	2	Healthcare access and quality index	57
Physical violence by firearm	Male	0-6 days	95+ years	Global	1	2	Opium Cultivation (binary)	228
Physical violence by firearm	Male	0-6 days	95+ years	Global	1	2	Population Density (over 1000 ppl/sqkm, proportion)	324
Physical violence by firearm	Male	0-6 days	95+ years	Global	0	3	Education (years per capita)	259
Physical violence by firearm	Male	0-6 days	95+ years	Global	0	3	Socio-demographic Index	267
Physical violence by firearm	Male	0-6 days	95+ years	Global	0	3	LDI (I$ per capita)	--
Physical violence by sharp object	Female	0-6 days	95+ years	Data Rich	1	1	Alcohol (liters per capita)	7
Physical violence by sharp object	Female	0-6 days	95+ years	Data Rich	1	1	Log-transformed SEV scalar: Viol Knife	--
Physical violence by sharp object	Female	0-6 days	95+ years	Data Rich	-1	2	Healthcare access and quality index	32
Physical violence by sharp object	Female	0-6 days	95+ years	Data Rich	1	2	Opium Cultivation (binary)	0
Physical violence by sharp object	Female	0-6 days	95+ years	Data Rich	1	2	Population Density (over 1000 ppl/sqkm, proportion)	32
Physical violence by sharp object	Female	0-6 days	95+ years	Data Rich	0	3	Socio-demographic Index	803
Physical violence by sharp object	Female	0-6 days	95+ years	Data Rich	0	3	Education (years per capita)	809
Physical violence by sharp object	Female	0-6 days	95+ years	Data Rich	0	3	LDI (I$ per capita)	--
Physical violence by sharp object	Female	0-6 days	95+ years	Global	1	1	Alcohol (liters per capita)	1000
Physical violence by sharp object	Female	0-6 days	95+ years	Global	1	1	Log-transformed SEV scalar: Viol Knife	--
Physical violence by sharp object	Female	0-6 days	95+ years	Global	-1	2	Healthcare access and quality index	269
Physical violence by sharp object	Female	0-6 days	95+ years	Global	1	2	Opium Cultivation (binary)	126
Physical violence by sharp object	Female	0-6 days	95+ years	Global	1	2	Population Density (over 1000 ppl/sqkm, proportion)	479
Physical violence by sharp object	Female	0-6 days	95+ years	Global	0	3	Socio-demographic Index	156
Physical violence by sharp object	Female	0-6 days	95+ years	Global	0	3	Education (years per capita)	239
Physical violence by sharp object	Female	0-6 days	95+ years	Global	0	3	LDI (I$ per capita)	--
Physical violence by sharp object	Male	0-6 days	95+ years	Data Rich	1	1	Alcohol (liters per capita)	288
Physical violence by sharp object	Male	0-6 days	95+ years	Data Rich	1	1	Log-transformed SEV scalar: Viol Knife	--
Physical violence by sharp object	Male	0-6 days	95+ years	Data Rich	-1	2	Healthcare access and quality index	172
Physical violence by sharp object	Male	0-6 days	95+ years	Data Rich	1	2	Opium Cultivation (binary)	0
Physical violence by sharp object	Male	0-6 days	95+ years	Data Rich	1	2	Population Density (over 1000 ppl/sqkm, proportion)	172

Table S16. CODEm covariates used, level of covariate, and expected direction of covariate by cause, sex, and age
Covariates that CODEm did not select during the covariate selection process have no draw counts listed.

Cause	Sex	Age Start	Age End	Model Type	Direction	Level	Covariate Name	Number of Draws
Physical violence by sharp object	Male	0-6 days	95+ years	Data Rich	0	3	Education (years per capita)	22
Physical violence by sharp object	Male	0-6 days	95+ years	Data Rich	0	3	Socio-demographic Index	529
Physical violence by sharp object	Male	0-6 days	95+ years	Data Rich	0	3	LDI (I$ per capita)	--
Physical violence by sharp object	Male	0-6 days	95+ years	Global	1	1	Alcohol (liters per capita)	399
Physical violence by sharp object	Male	0-6 days	95+ years	Global	1	1	Log-transformed SEV scalar: Viol Knife	--
Physical violence by sharp object	Male	0-6 days	95+ years	Global	-1	2	Healthcare access and quality index	666
Physical violence by sharp object	Male	0-6 days	95+ years	Global	1	2	Opium Cultivation (binary)	41
Physical violence by sharp object	Male	0-6 days	95+ years	Global	1	2	Population Density (over 1000 ppl/sqkm, proportion)	666
Physical violence by sharp object	Male	0-6 days	95+ years	Global	0	3	Education (years per capita)	291
Physical violence by sharp object	Male	0-6 days	95+ years	Global	0	3	Socio-demographic Index	411
Physical violence by sharp object	Male	0-6 days	95+ years	Global	0	3	LDI (I$ per capita)	--
Physical violence by other means	Female	0-6 days	95+ years	Data Rich	1	1	Alcohol (liters per capita)	905
Physical violence by other means	Female	0-6 days	95+ years	Data Rich	1	1	Log-transformed SEV scalar: Oth Viol	--
Physical violence by other means	Female	0-6 days	95+ years	Data Rich	-1	2	Healthcare access and quality index	298
Physical violence by other means	Female	0-6 days	95+ years	Data Rich	1	2	Population Density (over 1000 ppl/sqkm, proportion)	63
Physical violence by other means	Female	0-6 days	95+ years	Data Rich	1	2	Opium Cultivation (binary)	--
Physical violence by other means	Female	0-6 days	95+ years	Data Rich	0	3	Socio-demographic Index	150
Physical violence by other means	Female	0-6 days	95+ years	Data Rich	0	3	Education (years per capita)	396
Physical violence by other means	Female	0-6 days	95+ years	Data Rich	0	3	LDI (I$ per capita)	--
Physical violence by other means	Female	0-6 days	95+ years	Global	1	1	Alcohol (liters per capita)	614
Physical violence by other means	Female	0-6 days	95+ years	Global	1	1	Log-transformed SEV scalar: Oth Viol	--
Physical violence by other means	Female	0-6 days	95+ years	Global	-1	2	Healthcare access and quality index	311
Physical violence by other means	Female	0-6 days	95+ years	Global	1	2	Population Density (over 1000 ppl/sqkm, proportion)	468
Physical violence by other means	Female	0-6 days	95+ years	Global	1	2	Opium Cultivation (binary)	--
Physical violence by other means	Female	0-6 days	95+ years	Global	0	3	Socio-demographic Index	476
Physical violence by other means	Female	0-6 days	95+ years	Global	0	3	Education (years per capita)	528
Physical violence by other means	Female	0-6 days	95+ years	Global	0	3	LDI (I$ per capita)	--
Physical violence by other means	Male	0-6 days	95+ years	Data Rich	1	1	Alcohol (liters per capita)	144
Physical violence by other means	Male	0-6 days	95+ years	Data Rich	1	1	Log-transformed SEV scalar: Oth Viol	--
Physical violence by other means	Male	0-6 days	95+ years	Data Rich	-1	2	Healthcare access and quality index	--
Physical violence by other means	Male	0-6 days	95+ years	Data Rich	1	2	Population Density (over 1000 ppl/sqkm, proportion)	207
Physical violence by other means	Male	0-6 days	95+ years	Data Rich	1	2	Opium Cultivation (binary)	381
Physical violence by other means	Male	0-6 days	95+ years	Data Rich	0	3	Socio-demographic Index	457
Physical violence by other means	Male	0-6 days	95+ years	Data Rich	0	3	Education (years per capita)	504
Physical violence by other means	Male	0-6 days	95+ years	Data Rich	0	3	LDI (I$ per capita)	--
Physical violence by other means	Male	0-6 days	95+ years	Global	1	1	Alcohol (liters per capita)	--
Physical violence by other means	Male	0-6 days	95+ years	Global	1	1	Log-transformed SEV scalar: Oth Viol	--
Physical violence by other means	Male	0-6 days	95+ years	Global	-1	2	Healthcare access and quality index	770
Physical violence by other means	Male	0-6 days	95+ years	Global	1	2	Population Density (over 1000 ppl/sqkm, proportion)	345
Physical violence by other means	Male	0-6 days	95+ years	Global	1	2	Opium Cultivation (binary)	606
Physical violence by other means	Male	0-6 days	95+ years	Global	0	3	Socio-demographic Index	111
Physical violence by other means	Male	0-6 days	95+ years	Global	0	3	Education (years per capita)	488
Physical violence by other means	Male	0-6 days	95+ years	Global	0	3	LDI (I$ per capita)	--
Police conflict and executions	Female	28-364 days	95+ years	Data Rich	-1	2	Healthcare access and quality index	312
Police conflict and executions	Female	28-364 days	95+ years	Data Rich	0	2	Socio-demographic Index	1000
Police conflict and executions	Female	28-364 days	95+ years	Data Rich	1	2	Alcohol (liters per capita)	--
Police conflict and executions	Female	28-364 days	95+ years	Data Rich	1	2	Population Density (over 1000 ppl/sqkm, proportion)	--
Police conflict and executions	Female	28-364 days	95+ years	Data Rich	0	3	LDI (I$ per capita)	587
Police conflict and executions	Female	28-364 days	95+ years	Data Rich	1	3	Education (years per capita)	80
Police conflict and executions	Female	28-364 days	95+ years	Global	-1	2	Healthcare access and quality index	502
Police conflict and executions	Female	28-364 days	95+ years	Global	0	2	Socio-demographic Index	772

Table S16. CODEm covariates used, level of covariate, and expected direction of covariate by cause, sex, and age
Covariates that CODEm did not select during the covariate selection process have no draw counts listed.

Cause	Sex	Age Start	Age End	Model Type	Direction	Level	Covariate Name	Number of Draws
Police conflict and executions	Female	28-364 days	95+ years	Global	1	2	Alcohol (liters per capita)	--
Police conflict and executions	Female	28-364 days	95+ years	Global	1	2	Population Density (over 1000 ppl/sqkm, proportion)	--
Police conflict and executions	Female	28-364 days	95+ years	Global	0	3	LDI (I$ per capita)	135
Police conflict and executions	Female	28-364 days	95+ years	Global	1	3	Education (years per capita)	586
Police conflict and executions	Male	28-364 days	95+ years	Data Rich	-1	2	Healthcare access and quality index	994
Police conflict and executions	Male	28-364 days	95+ years	Data Rich	0	2	Socio-demographic Index	994
Police conflict and executions	Male	28-364 days	95+ years	Data Rich	1	2	Population Density (over 1000 ppl/sqkm, proportion)	839
Police conflict and executions	Male	28-364 days	95+ years	Data Rich	1	2	Alcohol (liters per capita)	--
Police conflict and executions	Male	28-364 days	95+ years	Data Rich	0	3	LDI (I$ per capita)	1
Police conflict and executions	Male	28-364 days	95+ years	Data Rich	1	3	Education (years per capita)	33
Police conflict and executions	Male	28-364 days	95+ years	Global	-1	2	Healthcare access and quality index	420
Police conflict and executions	Male	28-364 days	95+ years	Global	0	2	Socio-demographic Index	833
Police conflict and executions	Male	28-364 days	95+ years	Global	1	2	Population Density (over 1000 ppl/sqkm, proportion)	489
Police conflict and executions	Male	28-364 days	95+ years	Global	1	2	Alcohol (liters per capita)	--
Police conflict and executions	Male	28-364 days	95+ years	Global	0	3	LDI (I$ per capita)	498
Police conflict and executions	Male	28-364 days	95+ years	Global	1	3	Education (years per capita)	381

Table S17. CODEm predictive validity results by cause, model type, sex, and age										
Cause	Sex	Age Start	Age End	RMSE In-Sample	RMSE Out-of-Sample	Trend In-Sample	Trend Out-of-Sample	Coverage In-Sample	Coverage Out-of-Sample
Tuberculosis [Data Rich]	Female	28-364 days	95+ years	0.206562	0.280965	0.154252	0.168709	0.999793	0.999576
Tuberculosis [Global]	Female	28-364 days	95+ years	0.253134	0.516713	0.171939	0.17648	0.999406	0.989562
Diarrhoeal diseases [Data Rich]	Female	5-9 years	95+ years	0.267962	0.555842	0.168908	0.20347	0.993262	0.992955
Diarrhoeal diseases [Global]	Female	5-9 years	95+ years	0.946461	1.00979	0.218124	0.218459	0.972715	0.961151
Diarrhoeal diseases [Global]	Female	0-6 days	1-4 years	0.338654	0.82101	0.260989	0.261756	0.99913	0.974752
Diarrhoeal diseases [Data Rich]	Female	0-6 days	1-4 years	0.263718	0.472222	0.172617	0.18172	0.999503	0.998797
Diarrhoeal diseases [Data Rich]	Male	5-9 years	95+ years	0.275552	0.52717	0.17736	0.217199	0.996544	0.995955
Diarrhoeal diseases [Global]	Male	5-9 years	95+ years	0.532718	0.912065	0.213655	0.225917	0.997123	0.973163
Diarrhoeal diseases [Global]	Male	0-6 days	1-4 years	0.343406	0.806498	0.256788	0.263058	0.998923	0.974338
Diarrhoeal diseases [Data Rich]	Male	0-6 days	1-4 years	0.276211	0.470756	0.182019	0.205576	0.999451	0.998518
Lower respiratory infections [Global]	Male	5-9 years	95+ years	0.233835	0.372007	0.157264	0.168835	0.999732	0.986302
Lower respiratory infections [Data Rich]	Male	5-9 years	95+ years	0.195994	0.280541	0.141916	0.183488	0.999871	0.999298
Lower respiratory infections [Global]	Male	0-6 days	1-4 years	0.246261	0.371696	0.181242	0.182526	0.999454	0.992937
Lower respiratory infections [Data Rich]	Male	0-6 days	1-4 years	0.198117	0.280088	0.147597	0.163785	0.999639	0.998955
Lower respiratory infections [Data Rich]	Female	0-6 days	1-4 years	0.189501	0.267831	0.141415	0.156921	0.999605	0.999166
Lower respiratory infections [Data Rich]	Female	5-9 years	95+ years	0.194538	0.278575	0.141984	0.183507	0.99979	0.999231
Lower respiratory infections [Global]	Female	0-6 days	1-4 years	0.237926	0.369056	0.177361	0.179462	0.999437	0.993271
Lower respiratory infections [Global]	Female	5-9 years	95+ years	0.233915	0.366942	0.156885	0.166662	0.999732	0.987765
Upper respiratory infections [Data Rich]	Female	0-6 days	95+ years	0.409515	0.74643	0.311372	0.31454	0.99589	0.991964
Upper respiratory infections [Global]	Female	0-6 days	95+ years	0.740944	1.19316	0.372364	0.371693	0.993088	0.965899
Upper respiratory infections [Data Rich]	Male	0-6 days	95+ years	0.410695	0.684708	0.314005	0.329996	0.995162	0.990103
Upper respiratory infections [Global]	Male	0-6 days	95+ years	0.678248	1.07699	0.359962	0.358833	0.992637	0.968986
Otitis media [Global]	Male	0-6 days	95+ years	1.09489	2.14666	0.907887	0.916465	0.962487	0.88326
Otitis media [Data Rich]	Male	0-6 days	95+ years	0.996688	2.00658	0.838285	0.852156	0.955808	0.898367
Otitis media [Data Rich]	Female	0-6 days	95+ years	0.958193	1.88642	0.795705	0.79886	0.962783	0.916748
Otitis media [Global]	Female	0-6 days	95+ years	1.1735	2.15092	0.865078	0.901068	0.970286	0.893655
Meningitis [Data Rich]	Female	0-6 days	1-4 years	0.197611	0.309363	0.144891	0.163292	0.999927	0.999834
Meningitis [Global]	Female	0-6 days	1-4 years	0.301179	0.441192	0.228687	0.222753	0.999731	0.995981
Meningitis [Data Rich]	Female	5-9 years	95+ years	0.207363	0.295814	0.161228	0.182587	0.999911	0.999847
Meningitis [Global]	Female	5-9 years	95+ years	0.28112	0.433759	0.190208	0.191913	0.999385	0.994034
Meningitis [Data Rich]	Male	0-6 days	1-4 years	0.20637	0.311984	0.152886	0.164701	0.999899	0.999726
Meningitis [Global]	Male	0-6 days	1-4 years	0.276673	0.443547	0.218119	0.214207	0.999701	0.995984
Meningitis [Global]	Male	5-9 years	95+ years	0.268533	0.422466	0.17972	0.183575	0.999597	0.994471
Meningitis [Data Rich]	Male	5-9 years	95+ years	0.198518	0.295727	0.152303	0.165657	0.999918	0.999861
Encephalitis [Global]	Male	0-6 days	95+ years	0.344791	0.602549	0.21016	0.217604	0.999585	0.991647
Encephalitis [Data Rich]	Male	0-6 days	95+ years	0.253958	0.395214	0.183748	0.219688	0.999956	0.999836
Encephalitis [Global]	Female	0-6 days	95+ years	0.35522	0.605301	0.215156	0.222333	0.999718	0.993507
Encephalitis [Data Rich]	Female	0-6 days	95+ years	0.322353	0.437497	0.198812	0.231857	0.999925	0.999776
Tetanus [Data Rich]	Female	0-6 days	28-364 days	0.700186	1.00679	0.567971	0.630997	0.98284	0.972445
Tetanus [Data Rich]	Female	1-4 years	95+ years	0.827586	1.10572	0.687602	0.721723	0.972116	0.95629
Tetanus [Global]	Female	0-6 days	28-364 days	0.907172	1.62084	0.827049	0.693144	0.80206	0.765017
Tetanus [Global]	Female	1-4 years	95+ years	1.0077	1.84559	0.704603	0.708938	0.664441	0.63174
Tetanus [Data Rich]	Male	0-6 days	28-364 days	0.689845	0.96931	0.57961	0.653809	0.989518	0.981408
Tetanus [Data Rich]	Male	1-4 years	95+ years	0.813941	1.12105	0.625641	0.710585	0.991094	0.984213
Tetanus [Global]	Male	0-6 days	28-364 days	0.928513	1.52978	0.897469	0.832859	0.826393	0.802291
Tetanus [Global]	Male	1-4 years	95+ years	1.01865	1.71446	0.647613	0.66581	0.694878	0.668301
Dengue [Data Rich]	Male	7-27 days	95+ years	0.901355	1.54436	0.723825	0.730733	0.984554	0.931832
Dengue [Global]	Male	7-27 days	95+ years	0.947303	1.67647	0.708956	0.723415	0.985286	0.926821
Dengue [Data Rich]	Female	7-27 days	95+ years	0.799433	1.43549	0.624162	0.655389	0.985552	0.935666
Dengue [Global]	Female	7-27 days	95+ years	0.882841	1.63922	0.64375	0.669486	0.984151	0.927956
Rabies [Data Rich]	Female	28-364 days	95+ years	0.774408	1.28191	0.625035	0.686678	0.985559	0.952313
Rabies [Global]	Female	28-364 days	95+ years	0.90367	1.62042	0.637319	0.662412	0.985695	0.928314
Rabies [Data Rich]	Male	28-364 days	95+ years	0.781858	1.27627	0.619517	0.664019	0.982661	0.946348
Rabies [Global]	Male	28-364 days	95+ years	0.880934	1.64647	0.626647	0.657639	0.983957	0.920952
Other neglected tropical diseases [Global]	Female	0-6 days	95+ years	0.697695	1.28353	0.469389	0.486504	0.993361	0.953246
Other neglected tropical diseases [Data Rich]	Female	0-6 days	95+ years	0.599875	1.18458	0.43117	0.497025	0.994634	0.98997
Other neglected tropical diseases [Global]	Male	0-6 days	95+ years	0.724892	1.2866	0.482361	0.51805	0.992913	0.953579
Other neglected tropical diseases [Data Rich]	Male	0-6 days	95+ years	0.685485	1.12508	0.459144	0.516495	0.994452	0.990454
Neonatal disorders [Global]	Male	0-6 days	1-4 years	0.223407	0.363574	0.134095	0.145121	0.999815	0.998132
Neonatal disorders [Data Rich]	Male	0-6 days	1-4 years	0.178228	0.299778	0.113882	0.1708	0.999898	0.999761
Neonatal disorders [Data Rich]	Female	0-6 days	1-4 years	0.165796	0.26999	0.111568	0.16854	0.999941	0.999768
Neonatal disorders [Global]	Female	0-6 days	1-4 years	0.213316	0.333332	0.13417	0.144784	0.999918	0.998163
Neonatal preterm birth [Data Rich]	Female	0-6 days	1-4 years	0.171858	0.331385	0.114432	0.148523	0.999905	0.999448
Neonatal preterm birth [Global]	Female	0-6 days	1-4 years	0.223466	0.351629	0.152962	0.15945	0.99977	0.997383
Neonatal preterm birth [Data Rich]	Male	0-6 days	1-4 years	0.200303	0.455621	0.126129	0.186376	0.99972	0.999128
Neonatal preterm birth [Global]	Male	0-6 days	1-4 years	0.24689	0.413992	0.163626	0.177629	0.999647	0.995931
Neonatal encephalopathy due to birth asphyxia and trauma [Global]	Female	0-6 days	1-4 years	0.255751	0.417218	0.166116	0.17737	0.999858	0.99716
Neonatal encephalopathy due to birth asphyxia and trauma [Data Rich]	Female	0-6 days	1-4 years	0.194481	0.373202	0.135818	0.188625	0.99989	0.999365
Neonatal encephalopathy due to birth asphyxia and trauma [Global]	Male	0-6 days	1-4 years	0.286983	0.442253	0.169497	0.174627	0.999644	0.99696
Neonatal encephalopathy due to birth asphyxia and trauma [Data Rich]	Male	0-6 days	1-4 years	0.192375	0.394955	0.134988	0.175231	0.999891	0.999503
Neonatal sepsis and other neonatal infections [Data Rich]	Male	0-6 days	1-4 years	0.36362	0.852341	0.233021	0.280139	0.991306	0.990599
Neonatal sepsis and other neonatal infections [Global]	Male	0-6 days	1-4 years	0.432695	0.843775	0.290304	0.301943	0.992343	0.983681
Neonatal sepsis and other neonatal infections [Data Rich]	Female	0-6 days	1-4 years	0.33686	0.689239	0.225129	0.275412	0.991683	0.990302
Neonatal sepsis and other neonatal infections [Global]	Female	0-6 days	1-4 years	0.408698	0.793763	0.279383	0.293966	0.99283	0.985794
Hemolytic disease and other neonatal jaundice [Data Rich]	Female	0-6 days	1-4 years	0.635106	1.67453	0.49628	0.599443	0.878677	0.880441
Hemolytic disease and other neonatal jaundice [Global]	Female	0-6 days	1-4 years	0.674505	1.67005	0.503322	0.545197	0.898713	0.870306
Hemolytic disease and other neonatal jaundice [Global]	Male	0-6 days	1-4 years	0.675515	1.5955	0.4698	0.508309	0.901595	0.886826
Hemolytic disease and other neonatal jaundice [Data Rich]	Male	0-6 days	1-4 years	0.630603	1.56665	0.45587	0.562964	0.886151	0.887837
Other neonatal disorders [Global]	Female	0-6 days	1-4 years	0.280165	0.472823	0.196535	0.212695	0.999399	0.991765
Other neonatal disorders [Data Rich]	Female	0-6 days	1-4 years	0.232659	0.408029	0.170029	0.248479	0.999685	0.998645
Other neonatal disorders [Global]	Male	0-6 days	1-4 years	0.27641	0.478867	0.191903	0.210661	0.99942	0.992001
Other neonatal disorders [Data Rich]	Male	0-6 days	1-4 years	0.237135	0.420685	0.172545	0.260083	0.999534	0.998316
Nutritional deficiencies [Data Rich]	Male	28-364 days	95+ years	0.242917	0.359665	0.171536	0.199307	0.992342	0.992063

Table S17. CODEm predictive validity results by cause, model type, sex, and age									
Cause	Sex	Age Start	Age End	RMSE In-Sample	RMSE Out-of-Sample	Trend In-Sample	Trend Out-of-Sample	Coverage In-Sample	Coverage Out-of-Sample
Nutritional deficiencies [Global]	Male	28-364 days	95+ years	0.294144	0.641735	0.206056	0.213275	0.992977	0.97421
Nutritional deficiencies [Data Rich]	Female	28-364 days	95+ years	0.235436	0.362519	0.161846	0.192605	0.993092	0.992776
Nutritional deficiencies [Global]	Female	28-364 days	95+ years	0.29154	0.641457	0.199112	0.207264	0.993451	0.974251
Protein-energy malnutrition [Data Rich]	Female	5-9 years	95+ years	0.361979	0.618673	0.218598	0.252527	0.953666	0.955963
Protein-energy malnutrition [Global]	Female	28-364 days	1-4 years	0.39543	0.882098	0.269134	0.280973	0.958516	0.940979
Protein-energy malnutrition [Global]	Female	5-9 years	95+ years	0.375193	0.860551	0.239434	0.248188	0.95895	0.932307
Protein-energy malnutrition [Data Rich]	Female	28-364 days	1-4 years	0.324789	0.630198	0.229798	0.263789	0.950792	0.950284
Protein-energy malnutrition [Data Rich]	Male	5-9 years	95+ years	0.36509	0.644086	0.223318	0.263929	0.963884	0.965399
Protein-energy malnutrition [Global]	Male	28-364 days	1-4 years	0.395207	0.875889	0.280503	0.29861	0.960976	0.940415
Protein-energy malnutrition [Global]	Male	5-9 years	95+ years	0.376228	0.821023	0.248686	0.258939	0.968036	0.938583
Protein-energy malnutrition [Data Rich]	Male	28-364 days	1-4 years	0.34113	0.642772	0.247344	0.261942	0.952864	0.952935
Other nutritional deficiencies [Global]	Male	28-364 days	95+ years	0.357495	0.788037	0.252884	0.248967	0.938229	0.928448
Other nutritional deficiencies [Data Rich]	Female	28-364 days	95+ years	0.285916	0.536286	0.204603	0.218949	0.945009	0.944556
Other nutritional deficiencies [Global]	Female	28-364 days	95+ years	0.36151	0.797588	0.249762	0.246126	0.950318	0.939809
Other nutritional deficiencies [Data Rich]	Male	28-364 days	95+ years	0.292935	0.514494	0.212683	0.222198	0.929017	0.928212
Sexually transmitted infections excluding HIV [Global]	Male	10-14 years	95+ years	0.877725	1.4136	0.604581	0.595492	0.883863	0.87141
Sexually transmitted infections excluding HIV [Data Rich]	Male	10-14 years	95+ years	0.624781	0.780362	0.499349	0.440304	0.981393	0.970433
Sexually transmitted infections excluding HIV [Global]	Female	10-14 years	95+ years	0.460052	0.684514	0.365756	0.348792	0.98868	0.980303
Sexually transmitted infections excluding HIV [Data Rich]	Female	10-14 years	95+ years	0.306893	0.428865	0.237292	0.273327	0.99307	0.992605
Acute hepatitis [Data Rich]	Female	28-364 days	95+ years	0.322041	0.469235	0.239226	0.272176	0.999312	0.998065
Acute hepatitis [Global]	Female	28-364 days	95+ years	0.39288	0.734184	0.266393	0.280944	0.998885	0.983011
Acute hepatitis [Data Rich]	Male	28-364 days	95+ years	0.312094	0.452053	0.230292	0.265018	0.999373	0.998317
Acute hepatitis [Global]	Male	28-364 days	95+ years	0.370699	0.732325	0.253056	0.265059	0.998852	0.984859
Acute hepatitis A [Global]	Female	28-364 days	95+ years	0.926238	1.51312	0.655099	0.678676	0.989491	0.926366
Acute hepatitis A [Data Rich]	Female	28-364 days	95+ years	0.837062	1.14403	0.636858	0.691001	0.99162	0.980728
Acute hepatitis A [Global]	Male	28-364 days	95+ years	0.807388	1.43534	0.577944	0.606168	0.990762	0.920911
Acute hepatitis A [Data Rich]	Male	28-364 days	95+ years	0.775937	1.11482	0.571393	0.645065	0.992516	0.985164
Acute hepatitis B [Data Rich]	Male	28-364 days	95+ years	0.673442	1.07351	0.378588	0.454143	0.997137	0.99421
Acute hepatitis B [Global]	Male	28-364 days	95+ years	0.575521	1.09202	0.391313	0.419656	0.995758	0.965372
Acute hepatitis B [Data Rich]	Female	28-364 days	95+ years	0.649525	1.08177	0.379986	0.439736	0.996345	0.993134
Acute hepatitis B [Global]	Female	28-364 days	95+ years	0.634082	1.19479	0.409297	0.444515	0.9938	0.960078
Acute hepatitis C [Data Rich]	Female	28-364 days	95+ years	0.740775	1.1944	0.461718	0.499459	0.992104	0.959234
Acute hepatitis C [Global]	Female	28-364 days	95+ years	0.691147	1.20484	0.436413	0.459577	0.991252	0.946145
Acute hepatitis C [Data Rich]	Male	28-364 days	95+ years	0.685294	1.07283	0.408837	0.485843	0.996695	0.984625
Acute hepatitis C [Global]	Male	28-364 days	95+ years	0.610673	1.07761	0.391915	0.430869	0.995941	0.959874
Acute hepatitis E [Global]	Female	28-364 days	95+ years	0.896101	1.30117	0.642954	0.628529	0.98783	0.949998
Acute hepatitis E [Data Rich]	Female	28-364 days	95+ years	0.841882	1.15979	0.634751	0.593008	0.989583	0.96419
Acute hepatitis E [Data Rich]	Male	28-364 days	95+ years	0.816535	1.12773	0.629791	0.574443	0.993702	0.979263
Acute hepatitis E [Global]	Male	28-364 days	95+ years	0.786134	1.26096	0.62045	0.614191	0.991896	0.944839
Other unspecified infectious diseases [Data Rich]	Male	0-6 days	95+ years	0.374021	0.429616	0.184066	0.208151	0.999379	0.999045
Other unspecified infectious diseases [Global]	Male	0-6 days	95+ years	0.408637	0.651826	0.292936	0.291108	0.998577	0.990478
Other unspecified infectious diseases [Data Rich]	Female	0-6 days	95+ years	0.343679	0.40553	0.18431	0.204473	0.999296	0.99902
Other unspecified infectious diseases [Global]	Female	0-6 days	95+ years	0.420851	0.629324	0.299457	0.296333	0.998613	0.993358
Oesophageal cancer [Global]	Female	20-24 years	95+ years	0.262599	0.439	0.209188	0.207226	0.998757	0.984349
Oesophageal cancer [Data Rich]	Female	20-24 years	95+ years	0.216536	0.272917	0.181223	0.22027	0.99904	0.998262
Oesophageal cancer [Global]	Male	20-24 years	95+ years	0.240983	0.396882	0.188189	0.190213	0.998341	0.981691
Oesophageal cancer [Data Rich]	Male	20-24 years	95+ years	0.197786	0.243235	0.162392	0.178707	0.998139	0.996859
Stomach cancer [Global]	Female	15-19 years	95+ years	0.224046	0.327492	0.176793	0.173164	0.997996	0.984052
Stomach cancer [Data Rich]	Female	15-19 years	95+ years	0.172379	0.213413	0.142051	0.160748	0.997414	0.995481
Stomach cancer [Global]	Male	15-19 years	95+ years	0.221437	0.342098	0.175544	0.175098	0.997261	0.97773
Stomach cancer [Data Rich]	Male	15-19 years	95+ years	0.174823	0.216585	0.143368	0.163153	0.996825	0.994387
Liver cancer [Data Rich]	Male	0-6 days	95+ years	0.277096	0.371486	0.229624	0.264623	0.996156	0.993083
Liver cancer [Global]	Male	0-6 days	95+ years	0.327027	0.644127	0.26654	0.263789	0.995576	0.974485
Liver cancer [Data Rich]	Female	0-6 days	95+ years	0.316171	0.408322	0.22516	0.260284	0.99793	0.995804
Liver cancer [Global]	Female	0-6 days	95+ years	0.319711	0.603808	0.25925	0.259977	0.997464	0.983341
Larynx cancer [Global]	Female	20-24 years	95+ years	0.35978	0.515003	0.290513	0.292237	0.994603	0.989751
Larynx cancer [Data Rich]	Female	20-24 years	95+ years	0.293451	0.352203	0.246924	0.275323	0.99328	0.993707
Larynx cancer [Global]	Male	20-24 years	95+ years	0.269215	0.393217	0.215016	0.213305	0.9994	0.990692
Larynx cancer [Data Rich]	Male	20-24 years	95+ years	0.220487	0.264833	0.184581	0.198495	0.999327	0.998869
Tracheal, bronchus, and lung cancer [Data Rich]	Female	10-14 years	95+ years	0.211586	0.278726	0.1735	0.187087	0.997126	0.994813
Tracheal, bronchus, and lung cancer [Global]	Female	10-14 years	95+ years	0.259003	0.380143	0.20628	0.196712	0.998099	0.984358
Tracheal, bronchus, and lung cancer [Data Rich]	Male	10-14 years	95+ years	0.20052	0.252085	0.164035	0.168916	0.995802	0.992387
Tracheal, bronchus, and lung cancer [Global]	Male	10-14 years	95+ years	0.256649	0.350376	0.202454	0.193446	0.997257	0.979902
Breast cancer [Global]	Male	15-19 years	95+ years	0.769967	0.895063	0.67604	0.665045	0.939103	0.925064
Breast cancer [Data Rich]	Female	15-19 years	95+ years	0.190097	0.238463	0.156457	0.180158	0.995296	0.992005
Breast cancer [Global]	Female	15-19 years	95+ years	0.213415	0.292349	0.168595	0.171745	0.995641	0.986556
Breast cancer [Data Rich]	Male	15-19 years	95+ years	0.706904	0.839615	0.646192	0.755323	0.928699	0.92097
Cervical cancer [Global]	Female	15-19 years	95+ years	0.242419	0.360121	0.195079	0.201005	0.999068	0.991885
Cervical cancer [Data Rich]	Female	15-19 years	95+ years	0.205345	0.259868	0.169338	0.203447	0.998678	0.997579
Uterine cancer [Data Rich]	Female	20-24 years	95+ years	0.228228	0.279147	0.190758	0.20872	0.999054	0.998101
Uterine cancer [Global]	Female	20-24 years	95+ years	0.31705	0.413482	0.255661	0.25166	0.9992	0.994406
Prostate cancer [Data Rich]	Male	20-24 years	95+ years	0.1923	0.229505	0.147543	0.160288	0.997635	0.995545
Prostate cancer [Global]	Male	20-24 years	95+ years	0.219589	0.279819	0.155811	0.152698	0.997501	0.991061
Colon and rectum cancer [Data Rich]	Female	5-9 years	95+ years	0.18306	0.231029	0.151894	0.158584	0.99588	0.993197
Colon and rectum cancer [Global]	Female	5-9 years	95+ years	0.206598	0.280277	0.168597	0.167246	0.996496	0.991145
Colon and rectum cancer [Data Rich]	Male	5-9 years	95+ years	0.190946	0.24147	0.157788	0.159771	0.995286	0.992178
Colon and rectum cancer [Global]	Male	5-9 years	95+ years	0.227892	0.304014	0.184744	0.177172	0.996427	0.989429
Lip and oral cavity cancer [Global]	Male	5-9 years	95+ years	0.268411	0.385903	0.22366	0.222025	0.997854	0.993797
Lip and oral cavity cancer [Data Rich]	Male	5-9 years	95+ years	0.254061	0.321112	0.216056	0.205439	0.997306	0.995437
Lip and oral cavity cancer [Data Rich]	Female	5-9 years	95+ years	0.217157	0.297755	0.177057	0.17384	0.998593	0.997568
Lip and oral cavity cancer [Global]	Female	5-9 years	95+ years	0.245299	0.35369	0.191169	0.192107	0.998795	0.996958
Nasopharynx cancer [Global]	Female	5-9 years	95+ years	0.33418	0.503304	0.273791	0.274855	0.994869	0.987136
Nasopharynx cancer [Data Rich]	Female	5-9 years	95+ years	0.301697	0.369424	0.259233	0.289024	0.995143	0.994079

Table S17. CODEm predictive validity results by cause, model type, sex, and age

Cause	Sex	Age Start	Age End	RMSE In-Sample	RMSE Out-of-Sample	Trend In-Sample	Trend Out-of-Sample	Coverage In-Sample	Coverage Out-of-Sample
Nasopharynx cancer [Global]	Male	5-9 years	95+ years	0.303888	0.463655	0.255738	0.252325	0.999663	0.990558
Nasopharynx cancer [Data Rich]	Male	5-9 years	95+ years	0.277888	0.328338	0.243308	0.246639	0.999822	0.999556
Other pharynx cancer [Data Rich]	Male	20-24 years	95+ years	0.244485	0.307088	0.20426	0.213738	0.999408	0.998881
Other pharynx cancer [Global]	Female	20-24 years	95+ years	0.309356	0.441873	0.253513	0.247686	0.999056	0.996806
Other pharynx cancer [Data Rich]	Female	20-24 years	95+ years	0.280274	0.3384	0.238221	0.250264	0.998726	0.998526
Other pharynx cancer [Global]	Male	20-24 years	95+ years	0.277714	0.443553	0.222129	0.217025	0.999432	0.991696
Gallbladder and biliary tract cancer [Global]	Male	20-24 years	95+ years	0.251943	0.357674	0.200561	0.195415	0.999264	0.992854
Gallbladder and biliary tract cancer [Data Rich]	Male	20-24 years	95+ years	0.210762	0.257952	0.170754	0.179093	0.99896	0.997991
Gallbladder and biliary tract cancer [Data Rich]	Female	20-24 years	95+ years	0.213419	0.254766	0.168672	0.176916	0.998906	0.997939
Gallbladder and biliary tract cancer [Global]	Female	20-24 years	95+ years	0.239816	0.352588	0.187002	0.183674	0.998964	0.988385
Pancreatic cancer [Data Rich]	Female	15-19 years	95+ years	0.211322	0.264799	0.179225	0.205886	0.997031	0.995275
Pancreatic cancer [Global]	Female	15-19 years	95+ years	0.229988	0.327964	0.18741	0.191431	0.997558	0.994161
Pancreatic cancer [Data Rich]	Male	15-19 years	95+ years	0.208363	0.263915	0.171769	0.200205	0.99645	0.994616
Pancreatic cancer [Global]	Male	15-19 years	95+ years	0.243467	0.353046	0.195591	0.196526	0.99724	0.992451
Malignant skin melanoma [Data Rich]	Male	0-6 days	95+ years	0.282741	0.405971	0.221329	0.232564	0.998476	0.997567
Malignant skin melanoma [Global]	Female	0-6 days	95+ years	0.323326	0.441126	0.225913	0.220791	0.99899	0.995255
Malignant skin melanoma [Data Rich]	Female	0-6 days	95+ years	0.248595	0.333716	0.193576	0.193995	0.999166	0.998469
Malignant skin melanoma [Global]	Male	0-6 days	95+ years	0.341866	0.461807	0.244628	0.239671	0.99866	0.993613
Non-melanoma skin cancer [Global]	Male	20-24 years	95+ years	0.216512	0.353766	0.150853	0.153729	0.999772	0.996557
Non-melanoma skin cancer [Data Rich]	Male	20-24 years	95+ years	0.183951	0.270625	0.132073	0.153122	0.999798	0.999571
Non-melanoma skin cancer [Global]	Female	20-24 years	95+ years	0.279219	0.448854	0.197015	0.198423	0.997966	0.992238
Non-melanoma skin cancer [Data Rich]	Female	20-24 years	95+ years	0.206143	0.295132	0.152355	0.168725	0.999076	0.998797
Ovarian cancer [Data Rich]	Female	5-9 years	95+ years	0.207516	0.273279	0.168154	0.184435	0.998087	0.996715
Ovarian cancer [Global]	Female	5-9 years	95+ years	0.240434	0.348621	0.180684	0.182016	0.998618	0.994472
Testicular cancer [Data Rich]	Male	0-6 days	95+ years	0.336663	0.454064	0.286962	0.284092	0.999204	0.998398
Testicular cancer [Global]	Male	0-6 days	95+ years	0.451796	0.651728	0.38196	0.382064	0.995026	0.989898
Kidney cancer [Global]	Male	0-6 days	95+ years	0.28997	0.414506	0.23986	0.255253	0.998188	0.99382
Kidney cancer [Data Rich]	Male	0-6 days	95+ years	0.270148	0.374426	0.231028	0.292066	0.997861	0.996453
Kidney cancer [Global]	Female	0-6 days	95+ years	0.305091	0.46851	0.244577	0.267581	0.99812	0.993281
Kidney cancer [Data Rich]	Female	0-6 days	95+ years	0.282258	0.435576	0.234894	0.322233	0.99821	0.996758
Bladder cancer [Data Rich]	Male	15-19 years	95+ years	0.240898	0.285527	0.199469	0.213802	0.998131	0.996401
Bladder cancer [Global]	Male	15-19 years	95+ years	0.274484	0.368138	0.225794	0.224493	0.998225	0.990777
Bladder cancer [Data Rich]	Female	15-19 years	95+ years	0.219065	0.27044	0.179335	0.199428	0.99817	0.997553
Bladder cancer [Global]	Female	15-19 years	95+ years	0.253852	0.361089	0.201613	0.206813	0.998074	0.995339
Brain and nervous system cancer [Global]	Male	0-6 days	95+ years	0.317265	0.455977	0.221404	0.227775	0.998775	0.992953
Brain and nervous system cancer [Data Rich]	Male	0-6 days	95+ years	0.256468	0.339441	0.198232	0.243717	0.998414	0.997352
Brain and nervous system cancer [Global]	Female	0-6 days	95+ years	0.301083	0.44498	0.2191	0.22137	0.99905	0.994921
Brain and nervous system cancer [Data Rich]	Female	0-6 days	95+ years	0.275224	0.35502	0.190857	0.22379	0.99882	0.997871
Thyroid cancer [Data Rich]	Female	5-9 years	95+ years	0.374735	0.424526	0.329318	0.307338	0.994602	0.992091
Thyroid cancer [Global]	Female	5-9 years	95+ years	0.395627	0.504645	0.330969	0.329624	0.995041	0.991576
Thyroid cancer [Data Rich]	Male	5-9 years	95+ years	0.301986	0.364563	0.25634	0.275841	0.999081	0.998499
Thyroid cancer [Global]	Male	5-9 years	95+ years	0.325978	0.451676	0.268093	0.275032	0.999028	0.997434
Mesothelioma [Global]	Female	20-24 years	95+ years	0.440471	0.744811	0.415638	0.499273	0.998004	0.987963
Mesothelioma [Data Rich]	Female	20-24 years	95+ years	0.455723	0.910995	0.454968	0.689974	0.999968	0.991245
Mesothelioma [Global]	Male	20-24 years	95+ years	0.264438	0.437326	0.19753	0.214515	0.999683	0.996791
Mesothelioma [Data Rich]	Male	20-24 years	95+ years	0.200856	0.360612	0.175292	0.234068	0.999987	0.999943
Hodgkin lymphoma [Global]	Male	1-4 years	95+ years	0.403778	0.527964	0.308644	0.308939	0.999444	0.995815
Hodgkin lymphoma [Data Rich]	Male	1-4 years	95+ years	0.338556	0.440404	0.288515	0.347947	0.997264	0.997151
Hodgkin lymphoma [Global]	Female	1-4 years	95+ years	0.460638	0.612558	0.325305	0.337065	0.992827	0.987712
Hodgkin lymphoma [Data Rich]	Female	1-4 years	95+ years	0.425215	0.529984	0.29507	0.366626	0.990635	0.991235
Non-Hodgkin's lymphoma [Data Rich]	Female	1-4 years	95+ years	0.1921	0.275211	0.158491	0.182009	0.99926	0.998202
Non-Hodgkin's lymphoma [Global]	Female	1-4 years	95+ years	0.218556	0.335889	0.168391	0.166175	0.999419	0.996138
Non-Hodgkin's lymphoma [Data Rich]	Male	1-4 years	95+ years	0.190296	0.26584	0.155396	0.176023	0.998955	0.997787
Non-Hodgkin's lymphoma [Global]	Male	1-4 years	95+ years	0.212483	0.326477	0.164107	0.162797	0.99916	0.995587
Multiple myeloma [Data Rich]	Female	20-24 years	95+ years	0.242432	0.287812	0.200849	0.200434	0.999643	0.999242
Multiple myeloma [Global]	Female	20-24 years	95+ years	0.304571	0.388807	0.226423	0.228057	0.999523	0.995996
Multiple myeloma [Data Rich]	Male	20-24 years	95+ years	0.232883	0.275872	0.189341	0.190352	0.999453	0.999132
Multiple myeloma [Global]	Male	20-24 years	95+ years	0.299219	0.383876	0.223369	0.218695	0.999491	0.995722
Leukaemia [Data Rich]	Male	0-6 days	95+ years	0.214077	0.263591	0.186937	0.222432	0.999145	0.99826
Leukaemia [Global]	Male	0-6 days	95+ years	0.271541	0.335096	0.211088	0.213378	0.999578	0.99719
Leukaemia [Data Rich]	Female	0-6 days	95+ years	0.198555	0.244279	0.170536	0.197341	0.999169	0.998474
Leukaemia [Global]	Female	0-6 days	95+ years	0.253005	0.323649	0.211247	0.20947	0.999523	0.997907
Other malignant cancers [Data Rich]	Female	0-6 days	95+ years	0.198916	0.238695	0.166856	0.173074	0.99734	0.994796
Other malignant cancers [Global]	Female	0-6 days	95+ years	0.220775	0.335985	0.175327	0.179505	0.997653	0.993175
Other malignant cancers [Data Rich]	Male	0-6 days	95+ years	0.209241	0.256764	0.172726	0.190063	0.996091	0.993792
Other malignant cancers [Global]	Male	0-6 days	95+ years	0.249786	0.354058	0.189328	0.193065	0.997607	0.991761
Other neoplasms [Data Rich]	Female	0-6 days	95+ years	0.386466	0.696833	0.164675	0.19074	0.999916	0.998958
Other neoplasms [Global]	Female	0-6 days	95+ years	0.363195	0.614383	0.17654	0.18455	0.999779	0.987539
Other neoplasms [Data Rich]	Male	0-6 days	95+ years	0.447516	0.747584	0.181515	0.210552	0.999821	0.999435
Other neoplasms [Global]	Male	0-6 days	95+ years	0.398412	0.674041	0.183668	0.195692	0.999776	0.98492
Cardiovascular diseases [Global]	Male	0-6 days	95+ years	0.212604	0.339243	0.129793	0.135304	0.997889	0.979652
Cardiovascular diseases [Data Rich]	Male	0-6 days	95+ years	0.131374	0.203841	0.0967905	0.118744	0.999829	0.999384
Cardiovascular diseases [Global]	Female	0-6 days	95+ years	0.215366	0.337097	0.129874	0.137819	0.997999	0.981473
Cardiovascular diseases [Data Rich]	Female	0-6 days	95+ years	0.130569	0.205433	0.0976755	0.127386	0.999857	0.999595
Rheumatic heart disease [Data Rich]	Female	1-4 years	95+ years	0.183708	0.299876	0.133141	0.147016	0.999912	0.99949
Rheumatic heart disease [Global]	Female	1-4 years	95+ years	0.22206	0.453451	0.152066	0.155058	0.999637	0.983861
Rheumatic heart disease [Data Rich]	Male	1-4 years	95+ years	0.177883	0.292269	0.127791	0.14432	0.999905	0.999463
Rheumatic heart disease [Global]	Male	1-4 years	95+ years	0.222803	0.458438	0.151269	0.155534	0.999625	0.988458
Ischaemic heart disease [Data Rich]	Female	15-19 years	95+ years	0.129752	0.173343	0.102214	0.119189	0.999687	0.999002
Ischaemic heart disease [Global]	Female	15-19 years	95+ years	0.153186	0.267212	0.114306	0.117402	0.999545	0.979119
Ischaemic heart disease [Data Rich]	Male	15-19 years	95+ years	0.125142	0.171354	0.0962461	0.105212	0.999728	0.99886
Ischaemic heart disease [Global]	Male	15-19 years	95+ years	0.147175	0.278002	0.108871	0.111373	0.999558	0.968286
Stroke [Data Rich]	Male	0-6 days	95+ years	0.1507	0.21953	0.111058	0.143345	0.99992	0.999609

Table S17. CODEm predictive validity results by cause, model type, sex, and age										
Cause	Sex	Age Start	Age End	RMSE In-Sample	RMSE Out-of-Sample	Trend In-Sample	Trend Out-of-Sample	Coverage In-Sample	Coverage Out-of-Sample	
Stroke [Global]	Male	0-6 days	95+ years	0.192683	0.335403	0.138293	0.138574	0.999633	0.99082	
Stroke [Data Rich]	Female	0-6 days	95+ years	0.152405	0.232001	0.110413	0.147889	0.999855	0.999503	
Stroke [Global]	Female	0-6 days	95+ years	0.184475	0.326423	0.132448	0.136416	0.999709	0.990868	
Ischaemic stroke [Data Rich]	Female	0-6 days	95+ years	0.160454	0.27705	0.111638	0.147142	0.99994	0.999854	
Ischaemic stroke [Global]	Female	0-6 days	95+ years	0.188542	0.346608	0.120593	0.12659	0.999734	0.995348	
Ischaemic stroke [Data Rich]	Male	0-6 days	95+ years	0.156217	0.259613	0.111485	0.144504	0.999985	0.99992	
Ischaemic stroke [Global]	Male	0-6 days	95+ years	0.185777	0.327909	0.123588	0.12927	0.999837	0.996042	
Intracerebral hemorrhage [Data Rich]	Female	0-6 days	95+ years	0.153065	0.255446	0.106928	0.127816	0.999868	0.999534	
Intracerebral hemorrhage [Global]	Female	0-6 days	95+ years	0.1865	0.387945	0.135989	0.139266	0.99952	0.989936	
Intracerebral hemorrhage [Data Rich]	Male	0-6 days	95+ years	0.150265	0.238784	0.105639	0.12532	0.999949	0.9996	
Intracerebral hemorrhage [Global]	Male	0-6 days	95+ years	0.194229	0.382618	0.142012	0.14102	0.999453	0.990966	
Subarachnoid hemorrhage [Global]	Male	0-6 days	95+ years	0.241515	0.387143	0.140294	0.140749	0.999575	0.99461	
Subarachnoid hemorrhage [Data Rich]	Male	0-6 days	95+ years	0.199444	0.306771	0.115521	0.127942	0.999951	0.999766	
Subarachnoid hemorrhage [Data Rich]	Female	0-6 days	95+ years	0.167589	0.267196	0.111003	0.123797	0.999811	0.999297	
Subarachnoid hemorrhage [Global]	Female	0-6 days	95+ years	0.202573	0.356349	0.131228	0.132256	0.999464	0.994073	
Hypertensive heart disease [Global]	Female	15-19 years	95+ years	0.229658	0.508923	0.149977	0.153463	0.998692	0.966153	
Hypertensive heart disease [Data Rich]	Female	15-19 years	95+ years	0.204759	0.351845	0.125696	0.151711	0.99946	0.998638	
Hypertensive heart disease [Global]	Male	15-19 years	95+ years	0.247179	0.537665	0.166361	0.168586	0.998822	0.967409	
Hypertensive heart disease [Data Rich]	Male	15-19 years	95+ years	0.214418	0.370156	0.138076	0.165093	0.999727	0.998904	
Cardiomyopathy and myocarditis [Data Rich]	Female	0-6 days	95+ years	0.232786	0.408733	0.145137	0.171305	0.999783	0.999366	
Cardiomyopathy and myocarditis [Global]	Female	0-6 days	95+ years	0.346595	0.608799	0.169077	0.175422	0.999651	0.983548	
Cardiomyopathy and myocarditis [Data Rich]	Male	0-6 days	95+ years	0.244325	0.406318	0.137611	0.161214	0.999719	0.999072	
Cardiomyopathy and myocarditis [Global]	Male	0-6 days	95+ years	0.336695	0.553028	0.15863	0.165125	0.9995	0.982275	
Atrial fibrillation and flutter [Global]	Male	30-34 years	95+ years	0.14133	0.257955	0.0552125	0.0563417	0.998752	0.997355	
Atrial fibrillation and flutter [Data Rich]	Male	30-34 years	95+ years	0.141172	0.186072	0.0488075	0.0517754	0.998846	0.99878	
Atrial fibrillation and flutter [Data Rich]	Female	30-34 years	95+ years	0.138822	0.18542	0.0440076	0.0460562	0.983569	0.983561	
Atrial fibrillation and flutter [Global]	Female	30-34 years	95+ years	0.14744	0.316148	0.0563393	0.0579589	0.985406	0.984763	
Aortic aneurysm [Data Rich]	Female	15-19 years	95+ years	0.179972	0.275389	0.135065	0.141501	0.99969	0.999434	
Aortic aneurysm [Global]	Female	15-19 years	95+ years	0.210515	0.391637	0.149952	0.152942	0.999622	0.990755	
Aortic aneurysm [Data Rich]	Male	15-19 years	95+ years	0.162706	0.26486	0.123749	0.136058	0.999841	0.999604	
Aortic aneurysm [Global]	Male	15-19 years	95+ years	0.183656	0.343422	0.133179	0.137016	0.999825	0.988962	
Peripheral vascular disease [Global]	Female	40-44 years	95+ years	0.499342	1.00545	0.275311	0.300942	0.992189	0.935799	
Peripheral vascular disease [Data Rich]	Female	40-44 years	95+ years	0.850021	1.29231	0.266585	0.38414	0.991551	0.98733	
Peripheral vascular disease [Global]	Male	40-44 years	95+ years	0.434297	0.990786	0.271455	0.302762	0.995112	0.928661	
Peripheral vascular disease [Data Rich]	Male	40-44 years	95+ years	0.944254	1.27924	0.282689	0.391109	0.967228	0.960803	
Endocarditis [Data Rich]	Male	0-6 days	95+ years	0.318494	0.532874	0.142295	0.150771	0.999822	0.999751	
Endocarditis [Global]	Male	0-6 days	95+ years	0.394844	0.647721	0.160024	0.167322	0.999837	0.994046	
Endocarditis [Data Rich]	Female	0-6 days	95+ years	0.332203	0.584285	0.149131	0.152551	0.999838	0.999725	
Endocarditis [Global]	Female	0-6 days	95+ years	0.380107	0.657835	0.16421	0.168534	0.999857	0.992047	
Non-rheumatic valvular heart disease [Data Rich]	Female	15-19 years	95+ years	0.176892	0.297563	0.130031	0.136234	0.999908	0.999654	
Non-rheumatic valvular heart disease [Global]	Female	15-19 years	95+ years	0.213225	0.419784	0.146504	0.149936	0.999729	0.991542	
Non-rheumatic valvular heart disease [Data Rich]	Male	15-19 years	95+ years	0.176565	0.301217	0.1314	0.144603	0.999925	0.999644	
Non-rheumatic valvular heart disease [Global]	Male	15-19 years	95+ years	0.212093	0.387368	0.144937	0.14767	0.999848	0.995092	
Other cardiovascular and circulatory diseases [Data Rich]	Female	0-6 days	95+ years	0.155088	0.235906	0.110678	0.14061	0.999785	0.999466	
Other cardiovascular and circulatory diseases [Global]	Female	0-6 days	95+ years	0.204676	0.363271	0.133584	0.138248	0.998894	0.992744	
Other cardiovascular and circulatory diseases [Global]	Male	0-6 days	95+ years	0.214023	0.387858	0.14012	0.142589	0.999014	0.991734	
Other cardiovascular and circulatory diseases [Data Rich]	Male	0-6 days	95+ years	0.159256	0.242472	0.114931	0.147102	0.999874	0.999718	
Chronic respiratory diseases [Global]	Male	1-4 years	95+ years	0.190914	0.322058	0.144039	0.142706	0.999731	0.982789	
Chronic respiratory diseases [Data Rich]	Female	1-4 years	95+ years	0.16999	0.246363	0.118218	0.131793	0.999941	0.999447	
Chronic respiratory diseases [Global]	Female	1-4 years	95+ years	0.204659	0.38079	0.144364	0.149635	0.999751	0.977439	
Chronic respiratory diseases [Data Rich]	Male	1-4 years	95+ years	0.156895	0.219126	0.121201	0.138644	0.999855	0.999266	
Chronic obstructive pulmonary disease [Global]	Male	1-4 years	95+ years	0.179314	0.342609	0.133154	0.137174	0.99986	0.983274	
Chronic obstructive pulmonary disease [Data Rich]	Male	1-4 years	95+ years	0.157288	0.245909	0.119085	0.13688	0.999892	0.999346	
Chronic obstructive pulmonary disease [Data Rich]	Female	1-4 years	95+ years	0.162207	0.300372	0.117064	0.125568	0.999938	0.999602	
Chronic obstructive pulmonary disease [Global]	Female	1-4 years	95+ years	0.222445	0.414214	0.129946	0.133419	0.999923	0.982292	
Pneumoconiosis [Data Rich]	Female	15-19 years	95+ years	0.41819	0.725925	0.32477	0.402408	0.996943	0.994137	
Pneumoconiosis [Global]	Female	15-19 years	95+ years	0.501946	1.09792	0.376388	0.390646	0.994283	0.946877	
Pneumoconiosis [Data Rich]	Male	15-19 years	95+ years	0.297714	0.477136	0.213029	0.199667	0.998546	0.997156	
Pneumoconiosis [Global]	Male	15-19 years	95+ years	0.381357	0.928255	0.2654	0.263138	0.996432	0.964539	
Silicosis [Global]	Female	15-19 years	95+ years	0.93327	1.5062	0.663847	0.703496	0.989143	0.930261	
Silicosis [Data Rich]	Female	15-19 years	95+ years	0.819731	1.06105	0.627646	0.746427	0.99171	0.984249	
Silicosis [Global]	Male	15-19 years	95+ years	0.619779	1.26792	0.390424	0.375566	0.993347	0.932347	
Silicosis [Data Rich]	Male	15-19 years	95+ years	0.440411	0.708694	0.332482	0.295288	0.994846	0.99045	
Asbestosis [Global]	Female	15-19 years	95+ years	0.900294	1.53303	0.547837	0.57427	0.988049	0.902885	
Asbestosis [Global]	Male	15-19 years	95+ years	0.635125	1.305	0.457086	0.469886	0.989707	0.940432	
Asbestosis [Data Rich]	Female	15-19 years	95+ years	0.887552	1.17514	0.506889	0.53745	0.990368	0.982453	
Asbestosis [Data Rich]	Male	15-19 years	95+ years	0.529414	0.921171	0.375836	0.431486	0.99392	0.989396	
Coal workers pneumoconiosis [Data Rich]	Female	15-19 years	95+ years	0.866332	1.16405	0.613371	0.75798	0.990773	0.980416	
Coal workers pneumoconiosis [Global]	Female	15-19 years	95+ years	0.892844	1.50902	0.651242	0.71079	0.989478	0.928003	
Coal workers pneumoconiosis [Data Rich]	Male	15-19 years	95+ years	0.5278	0.846271	0.405557	0.39432	0.990557	0.984569	
Coal workers pneumoconiosis [Global]	Male	15-19 years	95+ years	0.659432	1.43131	0.47202	0.476981	0.987363	0.924818	
Other pneumoconiosis [Data Rich]	Male	15-19 years	95+ years	0.543322	0.846171	0.420842	0.42554	0.993061	0.986965	
Other pneumoconiosis [Global]	Female	15-19 years	95+ years	0.782798	1.51913	0.493057	0.52644	0.991118	0.910409	
Other pneumoconiosis [Data Rich]	Female	15-19 years	95+ years	0.66134	1.07644	0.435207	0.498874	0.993963	0.989419	
Other pneumoconiosis [Global]	Male	15-19 years	95+ years	0.639306	1.39639	0.480949	0.496232	0.989272	0.916825	
Asthma [Data Rich]	Male	1-4 years	95+ years	0.196744	0.398389	0.142828	0.160173	0.999362	0.999236	
Asthma [Global]	Male	1-4 years	95+ years	0.223725	0.523245	0.156602	0.162203	0.999503	0.981262	
Asthma [Global]	Female	1-4 years	95+ years	0.214571	0.452264	0.149365	0.155743	0.99981	0.989924	
Asthma [Data Rich]	Female	1-4 years	95+ years	0.18646	0.355499	0.134353	0.155318	0.999729	0.999632	
Interstitial lung disease and pulmonary sarcoidosis [Global]	Female	1-4 years	95+ years	0.361754	0.559166	0.161806	0.161562	0.999828	0.992338	
Interstitial lung disease and pulmonary sarcoidosis [Data Rich]	Female	1-4 years	95+ years	0.343458	0.571361	0.149257	0.146609	0.999935	0.999718	
Interstitial lung disease and pulmonary sarcoidosis [Data Rich]	Male	1-4 years	95+ years	0.368608	0.611904	0.151037	0.153149	0.999942	0.99971	
Interstitial lung disease and pulmonary sarcoidosis [Global]	Male	1-4 years	95+ years	0.35179	0.583111	0.161571	0.162397	0.999858	0.991028	

Table S17. CODEm predictive validity results by cause, model type, sex, and age									
**Cause**	**Sex**	**Age Start**	**Age End**	**RMSE In-Sample**	**RMSE Out-of-Sample**	**Trend In-Sample**	**Trend Out-of-Sample**	**Coverage In-Sample**	**Coverage Out-of-Sample**
Other chronic respiratory diseases [Data Rich]	Female	1-4 years	95+ years	0.440027	0.74686	0.254205	0.291167	0.998244	0.99695
Other chronic respiratory diseases [Global]	Female	1-4 years	95+ years	0.534936	0.78245	0.301904	0.314898	0.997921	0.983704
Other chronic respiratory diseases [Data Rich]	Male	1-4 years	95+ years	0.356424	0.708911	0.226925	0.274618	0.999095	0.998008
Other chronic respiratory diseases [Global]	Male	1-4 years	95+ years	0.512463	0.731044	0.2746	0.283581	0.99857	0.986813
Cirrhosis and other chronic liver diseases [Global]	Male	1-4 years	95+ years	0.201648	0.415282	0.131227	0.13331	0.99966	0.979333
Cirrhosis and other chronic liver diseases [Data Rich]	Male	1-4 years	95+ years	0.158053	0.257548	0.115627	0.129832	0.999891	0.999385
Cirrhosis and other chronic liver diseases [Global]	Female	1-4 years	95+ years	0.21436	0.399444	0.138113	0.141487	0.999818	0.985832
Cirrhosis and other chronic liver diseases [Data Rich]	Female	1-4 years	95+ years	0.162632	0.253283	0.122329	0.142266	0.999915	0.999446
Digestive diseases [Data Rich]	Female	0-6 days	95+ years	0.158222	0.231152	0.120107	0.147548	0.999899	0.999327
Digestive diseases [Global]	Female	0-6 days	95+ years	0.200389	0.355594	0.150999	0.151227	0.999736	0.986077
Digestive diseases [Data Rich]	Male	0-6 days	95+ years	0.155716	0.231276	0.116889	0.142888	0.99992	0.999318
Digestive diseases [Global]	Male	0-6 days	95+ years	0.200361	0.367457	0.148648	0.150233	0.99959	0.98209
Peptic ulcer disease [Data Rich]	Female	1-4 years	95+ years	0.164987	0.285529	0.112946	0.135897	0.999991	0.999839
Peptic ulcer disease [Global]	Female	1-4 years	95+ years	0.228268	0.391494	0.142522	0.144142	0.9998	0.99416
Peptic ulcer disease [Data Rich]	Male	1-4 years	95+ years	0.160493	0.262661	0.115222	0.131114	0.99998	0.999799
Peptic ulcer disease [Global]	Male	1-4 years	95+ years	0.225163	0.368685	0.144633	0.145768	0.999711	0.994317
Gastritis and duodenitis [Data Rich]	Male	1-4 years	95+ years	0.28856	0.585478	0.197182	0.219928	0.999356	0.998592
Gastritis and duodenitis [Global]	Male	1-4 years	95+ years	0.402501	0.825899	0.249913	0.25724	0.996488	0.978843
Gastritis and duodenitis [Data Rich]	Female	1-4 years	95+ years	0.281323	0.614256	0.19658	0.215048	0.999323	0.998499
Gastritis and duodenitis [Global]	Female	1-4 years	95+ years	0.401666	0.850832	0.266043	0.274913	0.995208	0.97294
Appendicitis [Data Rich]	Male	1-4 years	95+ years	0.272774	0.367551	0.175748	0.202399	0.999874	0.999652
Appendicitis [Global]	Male	1-4 years	95+ years	0.270593	0.477314	0.192221	0.199391	0.999578	0.995844
Appendicitis [Global]	Female	1-4 years	95+ years	0.298402	0.535344	0.202999	0.208917	0.997732	0.994865
Appendicitis [Data Rich]	Female	1-4 years	95+ years	0.238872	0.355677	0.176377	0.210167	0.999785	0.999435
Paralytic ileus and intestinal obstruction [Data Rich]	Female	0-6 days	95+ years	0.255999	0.341778	0.133508	0.151103	0.999975	0.999871
Paralytic ileus and intestinal obstruction [Global]	Female	0-6 days	95+ years	0.277631	0.434326	0.151835	0.155206	0.999689	0.996969
Paralytic ileus and intestinal obstruction [Data Rich]	Male	0-6 days	95+ years	0.240209	0.341392	0.136948	0.156561	0.999972	0.999846
Paralytic ileus and intestinal obstruction [Global]	Male	0-6 days	95+ years	0.269732	0.423015	0.152853	0.157649	0.999763	0.997697
Inguinal, femoral, and abdominal hernia [Data Rich]	Female	0-6 days	95+ years	0.201257	0.35474	0.124643	0.140592	0.999924	0.99973
Inguinal, femoral, and abdominal hernia [Global]	Female	0-6 days	95+ years	0.382954	0.588246	0.185563	0.187086	0.998005	0.991565
Inguinal, femoral, and abdominal hernia [Global]	Male	0-6 days	95+ years	0.290658	0.531022	0.165751	0.165657	0.998606	0.992247
Inguinal, femoral, and abdominal hernia [Data Rich]	Male	0-6 days	95+ years	0.203615	0.358228	0.124157	0.151167	0.999891	0.999348
Inflammatory bowel disease [Data Rich]	Male	1-4 years	95+ years	0.254141	0.411299	0.156174	0.181028	0.999958	0.999359
Inflammatory bowel disease [Global]	Male	1-4 years	95+ years	0.316115	0.512997	0.193065	0.194561	0.999563	0.995149
Inflammatory bowel disease [Global]	Female	1-4 years	95+ years	0.310389	0.530444	0.193473	0.196982	0.999446	0.993982
Inflammatory bowel disease [Data Rich]	Female	1-4 years	95+ years	0.221065	0.420532	0.153192	0.171482	0.999897	0.998612
Vascular intestinal disorders [Data Rich]	Female	1-4 years	95+ years	0.191273	0.419728	0.133261	0.156704	0.999823	0.999026
Vascular intestinal disorders [Global]	Female	1-4 years	95+ years	0.236496	0.516146	0.14867	0.155412	0.999602	0.989979
Vascular intestinal disorders [Data Rich]	Male	1-4 years	95+ years	0.190233	0.389245	0.125778	0.144911	0.999902	0.999671
Vascular intestinal disorders [Global]	Male	1-4 years	95+ years	0.22585	0.460265	0.141474	0.148841	0.999557	0.99165
Gallbladder and biliary diseases [Data Rich]	Female	1-4 years	95+ years	0.22016	0.319722	0.126926	0.139626	0.999983	0.999887
Gallbladder and biliary diseases [Global]	Female	1-4 years	95+ years	0.284357	0.477952	0.158988	0.166111	0.999457	0.993698
Gallbladder and biliary diseases [Data Rich]	Male	1-4 years	95+ years	0.206656	0.279615	0.12505	0.142425	0.999978	0.999862
Gallbladder and biliary diseases [Global]	Male	1-4 years	95+ years	0.275835	0.44593	0.159522	0.168058	0.999543	0.994672
Pancreatitis [Data Rich]	Male	1-4 years	95+ years	0.183794	0.291412	0.129909	0.143267	0.99998	0.999796
Pancreatitis [Global]	Male	1-4 years	95+ years	0.2803	0.43972	0.150246	0.15317	0.999648	0.994059
Pancreatitis [Global]	Female	1-4 years	95+ years	0.29383	0.439627	0.157849	0.157462	0.9997	0.995311
Pancreatitis [Data Rich]	Female	1-4 years	95+ years	0.203904	0.294052	0.131637	0.138688	0.999968	0.999848
Other digestive diseases [Data Rich]	Female	1-4 years	95+ years	0.225883	0.307904	0.130225	0.147633	0.999908	0.999783
Other digestive diseases [Global]	Female	1-4 years	95+ years	0.258476	0.512571	0.166193	0.171511	0.999563	0.993372
Other digestive diseases [Global]	Male	1-4 years	95+ years	0.25315	0.46687	0.163603	0.170959	0.999565	0.994247
Other digestive diseases [Data Rich]	Male	1-4 years	95+ years	0.255143	0.327306	0.137841	0.152086	0.99985	0.99974
Parkinson's disease [Global]	Male	20-24 years	95+ years	0.171569	0.340454	0.061058	0.0737203	0.99981	0.994358
Parkinson's disease [Data Rich]	Female	20-24 years	95+ years	0.143129	0.262618	0.0581757	0.0987957	0.999844	0.999611
Parkinson's disease [Global]	Female	20-24 years	95+ years	0.202819	0.38544	0.0593999	0.0678716	0.999867	0.993548
Parkinson's disease [Data Rich]	Male	20-24 years	95+ years	0.137244	0.278179	0.0604832	0.112034	0.99975	0.999508
Idiopathic epilepsy [Global]	Female	28-364 days	95+ years	0.241267	0.414302	0.153129	0.160348	0.999708	0.99506
Idiopathic epilepsy [Data Rich]	Female	28-364 days	95+ years	0.200646	0.283581	0.137039	0.174515	0.999995	0.999929
Idiopathic epilepsy [Global]	Male	28-364 days	95+ years	0.230485	0.410444	0.148803	0.157883	0.999693	0.994499
Idiopathic epilepsy [Data Rich]	Male	28-364 days	95+ years	0.182283	0.272792	0.133698	0.168566	0.999979	0.999864
Multiple sclerosis [Global]	Male	5-9 years	95+ years	0.443449	0.759118	0.186593	0.19569	0.994596	0.987507
Multiple sclerosis [Data Rich]	Male	5-9 years	95+ years	0.358873	0.588065	0.151193	0.184106	0.995254	0.995506
Multiple sclerosis [Global]	Female	5-9 years	95+ years	0.374434	0.73511	0.168823	0.178538	0.995873	0.990507
Multiple sclerosis [Data Rich]	Female	5-9 years	95+ years	0.381726	0.681902	0.150984	0.190432	0.996254	0.996377
Motor neuron disease [Global]	Male	0-6 days	95+ years	0.34065	0.705736	0.204874	0.20645	0.997474	0.985702
Motor neuron disease [Data Rich]	Male	0-6 days	95+ years	0.254648	0.524858	0.159359	0.168159	0.999034	0.997774
Motor neuron disease [Global]	Female	0-6 days	95+ years	0.355689	0.712456	0.213156	0.215724	0.976249	0.969903
Motor neuron disease [Data Rich]	Female	0-6 days	95+ years	0.265094	0.611092	0.163935	0.180144	0.998729	0.997523
Other neurological disorders [Global]	Female	28-364 days	95+ years	0.252011	0.408071	0.170079	0.17257	0.999209	0.99648
Other neurological disorders [Global]	Male	28-364 days	95+ years	0.23664	0.397346	0.155584	0.158741	0.999579	0.99639
Other neurological disorders [Data Rich]	Female	28-364 days	95+ years	0.217115	0.371859	0.150202	0.173302	0.999547	0.998944
Other neurological disorders [Data Rich]	Male	28-364 days	95+ years	0.202373	0.352994	0.139636	0.162844	0.999786	0.999199
Alcohol use disorders [Global]	Male	15-19 years	95+ years	0.248569	0.61304	0.154868	0.163101	0.999605	0.96966
Alcohol use disorders [Data Rich]	Male	15-19 years	95+ years	0.202065	0.353967	0.140839	0.17082	0.999226	0.996287
Alcohol use disorders [Data Rich]	Female	15-19 years	95+ years	0.226536	0.36873	0.163597	0.212988	0.99961	0.998356
Alcohol use disorders [Global]	Female	15-19 years	95+ years	0.256948	0.661423	0.18141	0.189411	0.999506	0.97685
Drug use disorders [Data Rich]	Female	15-19 years	95+ years	0.226404	0.339675	0.160038	0.199283	0.999928	0.99979
Drug use disorders [Global]	Female	15-19 years	95+ years	0.264599	0.565506	0.184009	0.200765	0.999753	0.992261
Drug use disorders [Data Rich]	Male	15-19 years	95+ years	0.236271	0.353972	0.158505	0.19548	0.999902	0.999767
Drug use disorders [Global]	Male	15-19 years	95+ years	0.261384	0.576913	0.173748	0.189452	0.99975	0.989708
Opioid use disorders [Data Rich]	Female	15-19 years	95+ years	0.186057	0.456549	0.132543	0.204713	0.999962	0.999626
Opioid use disorders [Global]	Female	15-19 years	95+ years	0.260574	0.596691	0.181748	0.196987	0.999352	0.991017
Opioid use disorders [Data Rich]	Male	15-19 years	95+ years	0.242361	0.478018	0.152908	0.183021	0.999922	0.999829

Table S17. CODEm predictive validity results by cause, model type, sex, and age									
Cause	Sex	Age Start	Age End	RMSE In-Sample	RMSE Out-of-Sample	Trend In-Sample	Trend Out-of-Sample	Coverage In-Sample	Coverage Out-of-Sample
Opioid use disorders [Global]	Male	15-19 years	95+ years	0.259563	0.572114	0.16524	0.18041	0.999753	0.993191
Cocaine use disorders [Data Rich]	Female	15-19 years	95+ years	0.299559	0.63854	0.21168	0.248099	0.997853	0.996335
Cocaine use disorders [Global]	Female	15-19 years	95+ years	0.391245	0.821799	0.267302	0.280228	0.995261	0.978889
Cocaine use disorders [Data Rich]	Male	15-19 years	95+ years	0.286565	0.585871	0.175354	0.208299	0.999229	0.998421
Cocaine use disorders [Global]	Male	15-19 years	95+ years	0.356246	0.770239	0.219514	0.235481	0.997929	0.9856
Amphetamine use disorders [Global]	Male	15-19 years	95+ years	0.35858	0.739732	0.215124	0.225448	0.998164	0.98664
Amphetamine use disorders [Data Rich]	Male	15-19 years	95+ years	0.263658	0.746558	0.164977	0.210133	0.999465	0.996506
Amphetamine use disorders [Data Rich]	Female	15-19 years	95+ years	0.26707	0.88871	0.182591	0.2053	0.998767	0.996239
Amphetamine use disorders [Global]	Female	15-19 years	95+ years	0.363582	0.806592	0.237206	0.247872	0.997427	0.980178
Other drug use disorders [Data Rich]	Female	15-19 years	95+ years	0.188393	0.427684	0.124963	0.184353	0.999794	0.999657
Other drug use disorders [Global]	Female	15-19 years	95+ years	0.227259	0.533317	0.141666	0.155657	0.999623	0.995274
Other drug use disorders [Data Rich]	Male	15-19 years	95+ years	0.210454	0.488731	0.124201	0.171226	0.999959	0.999815
Other drug use disorders [Global]	Male	15-19 years	95+ years	0.247974	0.589678	0.138206	0.157408	0.999859	0.992215
Eating disorders [Data Rich]	Female	5-9 years	45-49 years	0.607657	1.16563	0.498575	0.57109	0.985094	0.973677
Eating disorders [Global]	Female	5-9 years	45-49 years	0.694263	1.53906	0.560702	0.579644	0.983226	0.902194
Eating disorders [Data Rich]	Male	5-9 years	45-49 years	1.11845	2.02733	0.924589	1.17402	0.924486	0.883449
Eating disorders [Global]	Male	5-9 years	45-49 years	1.1758	2.42016	0.962521	1.03502	0.93646	0.784609
Anorexia nervosa [Data Rich]	Male	5-9 years	45-49 years	1.10499	2.07038	0.912384	1.08833	0.912635	0.834675
Anorexia nervosa [Global]	Male	5-9 years	45-49 years	1.16754	2.34579	0.951295	1.01544	0.925012	0.780405
Anorexia nervosa [Data Rich]	Female	5-9 years	45-49 years	0.640919	1.40345	0.528919	0.560703	0.983273	0.937665
Anorexia nervosa [Global]	Female	5-9 years	45-49 years	0.707581	1.59041	0.595071	0.610886	0.981314	0.875789
Bulimia nervosa [Global]	Female	5-9 years	45-49 years	0.955997	2.03854	0.673573	0.653241	0.226086	0.218445
Bulimia nervosa [Data Rich]	Female	5-9 years	45-49 years	0.842432	2.08061	0.630021	0.742582	0.588567	0.549971
Bulimia nervosa [Global]	Male	5-9 years	45-49 years	1.09987	1.76943	0.834781	0.86806	0.35803	0.317302
Bulimia nervosa [Data Rich]	Male	5-9 years	45-49 years	0.99323	2.0642	0.78435	0.861065	0.703345	0.626324
Diabetes mellitus [Global]	Male	15-19 years	95+ years	0.21313	0.363743	0.148044	0.152406	0.999137	0.983893
Diabetes mellitus [Data Rich]	Male	15-19 years	95+ years	0.173304	0.2539	0.127587	0.158844	0.999258	0.997852
Diabetes mellitus [Global]	Male	0-6 days	10-14 years	0.334571	0.495507	0.149654	0.142303	0.99958	0.99631
Diabetes mellitus [Data Rich]	Male	0-6 days	10-14 years	0.201199	0.267777	0.0959537	0.114776	1	1
Diabetes mellitus [Global]	Female	15-19 years	95+ years	0.221594	0.371016	0.145318	0.151113	0.999048	0.983088
Diabetes mellitus [Data Rich]	Female	15-19 years	95+ years	0.175942	0.267173	0.125225	0.150746	0.998713	0.996492
Diabetes mellitus [Data Rich]	Female	0-6 days	10-14 years	0.212445	0.287721	0.0947399	0.122515	1	0.999993
Diabetes mellitus [Global]	Female	0-6 days	10-14 years	0.361787	0.549999	0.159806	0.154645	0.999587	0.994974
Acute glomerulonephritis [Global]	Female	28-364 days	95+ years	0.794331	1.46729	0.587676	0.580506	0.966386	0.918298
Acute glomerulonephritis [Data Rich]	Female	28-364 days	95+ years	0.690702	1.01083	0.544591	0.487876	0.969151	0.960151
Acute glomerulonephritis [Global]	Male	28-364 days	95+ years	0.812912	1.45548	0.581157	0.577485	0.967803	0.923836
Acute glomerulonephritis [Data Rich]	Male	28-364 days	95+ years	0.696861	1.00728	0.538751	0.494628	0.970297	0.961742
Chronic kidney disease [Data Rich]	Male	28-364 days	95+ years	0.170831	0.266238	0.126779	0.149812	0.999896	0.999526
Chronic kidney disease [Data Rich]	Female	28-364 days	95+ years	0.169167	0.273276	0.123632	0.140709	0.999843	0.999367
Chronic kidney disease [Global]	Female	28-364 days	95+ years	0.193716	0.363295	0.134681	0.137729	0.999621	0.987905
Chronic kidney disease [Global]	Male	28-364 days	95+ years	0.192453	0.362695	0.136133	0.141805	0.999692	0.988585
Urinary diseases and male infertility [Data Rich]	Male	0-6 days	95+ years	0.225293	0.323195	0.13182	0.142333	0.999873	0.999447
Urinary diseases and male infertility [Global]	Male	0-6 days	95+ years	0.296913	0.549031	0.176313	0.175737	0.998029	0.986096
Urinary diseases and male infertility [Data Rich]	Female	0-6 days	95+ years	0.193989	0.303199	0.12719	0.135876	0.999823	0.99919
Urinary diseases and male infertility [Global]	Female	0-6 days	95+ years	0.289458	0.559517	0.165223	0.163565	0.999076	0.983366
Urinary tract infections and interstitial nephritis [Data Rich]	Female	0-6 days	95+ years	0.294583	0.454113	0.135202	0.14956	0.999879	0.999308
Urinary tract infections and interstitial nephritis [Global]	Female	0-6 days	95+ years	0.312993	0.55414	0.155721	0.161548	0.99972	0.987033
Urinary tract infections and interstitial nephritis [Global]	Male	0-6 days	95+ years	0.368938	0.595435	0.168249	0.174432	0.999356	0.989454
Urinary tract infections and interstitial nephritis [Data Rich]	Male	0-6 days	95+ years	0.280407	0.45617	0.139919	0.160266	0.999567	0.999222
Urolithiasis [Data Rich]	Female	1-4 years	95+ years	0.280002	0.468409	0.198432	0.211855	0.997234	0.996275
Urolithiasis [Global]	Female	1-4 years	95+ years	0.432147	0.755462	0.26187	0.259496	0.993767	0.980956
Urolithiasis [Global]	Male	1-4 years	95+ years	0.506568	0.865165	0.291792	0.283294	0.990692	0.977963
Urolithiasis [Data Rich]	Male	1-4 years	95+ years	0.299288	0.486487	0.2199	0.219133	0.995928	0.994695
Other urinary diseases [Data Rich]	Male	0-6 days	95+ years	0.311859	0.567115	0.182358	0.201551	0.986491	0.986107
Other urinary diseases [Global]	Male	0-6 days	95+ years	0.472715	0.697533	0.219868	0.230407	0.981413	0.97519
Other urinary diseases [Data Rich]	Female	0-6 days	95+ years	0.406242	0.682842	0.244773	0.221737	0.970231	0.968476
Other urinary diseases [Global]	Female	0-6 days	95+ years	0.591199	0.860265	0.296755	0.302692	0.965101	0.957387
Gynecological diseases [Global]	Female	10-14 years	95+ years	0.470499	0.730102	0.312105	0.304992	0.995856	0.985445
Gynecological diseases [Data Rich]	Female	10-14 years	95+ years	0.330418	0.50367	0.257598	0.286479	0.99744	0.996262
Uterine fibroids [Global]	Female	10-14 years	95+ years	0.59418	1.26405	0.416268	0.404672	0.942289	0.879682
Uterine fibroids [Data Rich]	Female	10-14 years	95+ years	0.502974	0.956617	0.394817	0.418895	0.940327	0.931248
Endometriosis [Global]	Female	10-14 years	50-54 years	1.25278	2.02181	1.03131	1.03268	0.878317	0.830312
Endometriosis [Data Rich]	Female	10-14 years	50-54 years	1.1452	1.82458	0.967154	0.947587	0.866965	0.834971
Genital prolapse [Global]	Female	10-14 years	95+ years	0.834588	1.5277	0.658068	0.656233	0.656159	0.654258
Genital prolapse [Data Rich]	Female	10-14 years	95+ years	0.680114	1.16377	0.551501	0.541821	0.946296	0.932004
Other gynecological diseases [Global]	Female	10-14 years	95+ years	0.525218	0.915327	0.413289	0.415503	0.991277	0.963539
Other gynecological diseases [Data Rich]	Female	10-14 years	95+ years	0.502651	0.772155	0.393329	0.372534	0.993151	0.969757
Hemoglobinopathies and hemolytic anaemias [Data Rich]	Male	0-6 days	95+ years	0.201972	0.31062	0.15552	0.176728	0.99997	0.999913
Hemoglobinopathies and hemolytic anaemias [Global]	Male	0-6 days	95+ years	0.234689	0.438419	0.17616	0.179089	0.999813	0.996404
Hemoglobinopathies and hemolytic anaemias [Data Rich]	Female	0-6 days	95+ years	0.201564	0.302577	0.151245	0.177805	0.999972	0.999883
Hemoglobinopathies and hemolytic anaemias [Global]	Female	0-6 days	95+ years	0.238765	0.445117	0.174538	0.179569	0.999762	0.996089
Endocrine, metabolic, blood, and immune disorders [Data Rich]	Female	0-6 days	95+ years	0.21015	0.315863	0.142498	0.171845	0.999904	0.99962
Endocrine, metabolic, blood, and immune disorders [Global]	Female	0-6 days	95+ years	0.280394	0.467776	0.169405	0.177541	0.999733	0.992096
Endocrine, metabolic, blood, and immune disorders [Data Rich]	Male	0-6 days	95+ years	0.250901	0.357414	0.151689	0.183718	0.999824	0.999517
Endocrine, metabolic, blood, and immune disorders [Global]	Male	0-6 days	95+ years	0.307709	0.466987	0.172964	0.180822	0.99964	0.993635
Musculoskeletal disorders [Data Rich]	Female	5-9 years	95+ years	0.247997	0.3574	0.141479	0.159736	0.999861	0.999367
Musculoskeletal disorders [Global]	Female	5-9 years	95+ years	0.255147	0.443132	0.157736	0.165475	0.999664	0.989678
Musculoskeletal disorders [Data Rich]	Male	5-9 years	95+ years	0.238081	0.345891	0.154493	0.181247	0.999886	0.999673
Musculoskeletal disorders [Global]	Male	5-9 years	95+ years	0.244193	0.427844	0.172573	0.176424	0.999701	0.995348
Rheumatoid arthritis [Global]	Male	5-9 years	95+ years	0.300562	0.586702	0.201967	0.20434	0.99836	0.986897
Rheumatoid arthritis [Data Rich]	Male	5-9 years	95+ years	0.238339	0.406339	0.168648	0.199327	0.999247	0.998663
Rheumatoid arthritis [Data Rich]	Female	5-9 years	95+ years	0.210777	0.413402	0.138463	0.165909	0.999888	0.999586
Rheumatoid arthritis [Global]	Female	5-9 years	95+ years	0.332003	0.54499	0.169872	0.171945	0.999318	0.988752

Table S17. CODEm predictive validity results by cause, model type, sex, and age									
Cause	Sex	Age Start	Age End	RMSE In-Sample	RMSE Out-of-Sample	Trend In-Sample	Trend Out-of-Sample	Coverage In-Sample	Coverage Out-of-Sample
Other musculoskeletal disorders [Data Rich]	Female	5-9 years	95+ years	0.206931	0.495397	0.144438	0.172543	0.999817	0.999445
Other musculoskeletal disorders [Global]	Female	5-9 years	95+ years	0.243896	0.475132	0.161345	0.165792	0.999781	0.991562
Other musculoskeletal disorders [Data Rich]	Male	5-9 years	95+ years	0.213819	0.384873	0.15753	0.179753	0.999929	0.999792
Other musculoskeletal disorders [Global]	Male	5-9 years	95+ years	0.254828	0.445307	0.180468	0.181014	0.999875	0.997591
Congenital anomalies [Data Rich]	Female	0-6 days	65-69 years	0.185234	0.271245	0.125042	0.140953	0.999989	0.999907
Congenital anomalies [Global]	Female	0-6 days	65-69 years	0.197492	0.324243	0.140257	0.144732	0.999949	0.998655
Congenital anomalies [Data Rich]	Male	0-6 days	65-69 years	0.199805	0.287734	0.127024	0.141173	0.999979	0.999825
Congenital anomalies [Global]	Male	0-6 days	65-69 years	0.203241	0.335381	0.141248	0.144162	0.999925	0.997592
Neural tube defects [Global]	Male	0-6 days	65-69 years	0.488627	0.892953	0.310274	0.313411	0.943625	0.94329
Neural tube defects [Data Rich]	Male	0-6 days	65-69 years	0.423819	0.706173	0.289044	0.283808	0.944707	0.939726
Neural tube defects [Data Rich]	Female	0-6 days	65-69 years	0.423495	0.743967	0.282342	0.313835	0.935409	0.928095
Neural tube defects [Global]	Female	0-6 days	65-69 years	0.490117	1.011	0.302365	0.32992	0.933316	0.9371
Congenital heart anomalies [Global]	Female	0-6 days	65-69 years	0.211489	0.347074	0.154141	0.155898	0.999901	0.999064
Congenital heart anomalies [Data Rich]	Female	0-6 days	65-69 years	0.205228	0.288289	0.138049	0.153381	0.999962	0.999886
Congenital heart anomalies [Data Rich]	Male	0-6 days	65-69 years	0.191426	0.294912	0.131737	0.146644	0.999976	0.99991
Congenital heart anomalies [Global]	Male	0-6 days	65-69 years	0.204052	0.348417	0.148609	0.150972	0.999925	0.998685
Orofacial clefts [Data Rich]	Female	0-6 days	1-4 years	1.20934	1.81172	0.995279	1.01108	0.922387	0.896064
Orofacial clefts [Global]	Female	0-6 days	1-4 years	1.22025	1.88245	1.00586	0.990533	0.858482	0.818503
Orofacial clefts [Data Rich]	Male	0-6 days	1-4 years	1.11263	1.61051	0.899226	0.880822	0.925234	0.904273
Orofacial clefts [Global]	Male	0-6 days	1-4 years	1.10879	1.86868	0.907931	0.921708	0.853687	0.81768
Down syndrome [Data Rich]	Female	0-6 days	65-69 years	0.273198	0.678825	0.193314	0.230003	0.999038	0.998712
Down syndrome [Global]	Female	0-6 days	65-69 years	0.334674	0.739715	0.217849	0.219606	0.999241	0.986757
Down syndrome [Global]	Male	0-6 days	65-69 years	0.314526	0.749929	0.215639	0.229473	0.99949	0.981808
Down syndrome [Data Rich]	Male	0-6 days	65-69 years	0.271946	0.679587	0.195372	0.231695	0.999687	0.999358
Other chromosomal abnormalities [Global]	Male	0-6 days	65-69 years	0.398714	0.760967	0.234051	0.247823	0.999168	0.989847
Other chromosomal abnormalities [Data Rich]	Male	0-6 days	65-69 years	0.343217	0.578248	0.209189	0.237079	0.999718	0.999254
Other chromosomal abnormalities [Data Rich]	Female	0-6 days	65-69 years	0.335362	0.53562	0.189159	0.22491	0.999782	0.999442
Other chromosomal abnormalities [Global]	Female	0-6 days	65-69 years	0.373967	0.718724	0.206655	0.219701	0.999445	0.988507
Congenital musculoskeletal and limb anomalies [Data Rich]	Female	0-6 days	65-69 years	0.323359	0.433982	0.252026	0.235937	0.998183	0.996326
Congenital musculoskeletal and limb anomalies [Global]	Female	0-6 days	65-69 years	0.386884	0.658264	0.278564	0.28107	0.997622	0.98875
Congenital musculoskeletal and limb anomalies [Data Rich]	Male	0-6 days	65-69 years	0.371765	0.465438	0.253884	0.242624	0.998274	0.996633
Congenital musculoskeletal and limb anomalies [Global]	Male	0-6 days	65-69 years	0.368935	0.663823	0.27127	0.264885	0.997759	0.989971
Urogenital congenital anomalies [Data Rich]	Female	0-6 days	65-69 years	0.582512	0.631124	0.327396	0.36132	0.909415	0.909621
Urogenital congenital anomalies [Global]	Female	0-6 days	65-69 years	0.484225	0.809536	0.353116	0.379889	0.92112	0.931609
Urogenital congenital anomalies [Global]	Male	0-6 days	65-69 years	0.431287	0.699673	0.291215	0.296358	0.980562	0.978785
Urogenital congenital anomalies [Data Rich]	Male	0-6 days	65-69 years	0.391149	0.476932	0.237936	0.253194	0.981578	0.980231
Digestive congenital anomalies [Data Rich]	Male	0-6 days	65-69 years	0.263856	0.333894	0.1602	0.156885	0.993002	0.992006
Digestive congenital anomalies [Global]	Male	0-6 days	65-69 years	0.299751	0.53759	0.175202	0.180131	0.999508	0.992346
Digestive congenital anomalies [Data Rich]	Female	0-6 days	65-69 years	0.293473	0.375693	0.160283	0.169952	0.989098	0.987918
Digestive congenital anomalies [Global]	Female	0-6 days	65-69 years	0.313488	0.561957	0.17244	0.169256	0.999532	0.991997
Other congenital anomalies [Data Rich]	Female	0-6 days	65-69 years	0.254708	0.322431	0.165252	0.183289	0.999986	0.999941
Other congenital anomalies [Global]	Female	0-6 days	65-69 years	0.255795	0.394686	0.175292	0.179866	0.999876	0.99818
Other congenital anomalies [Global]	Male	0-6 days	65-69 years	0.265279	0.397039	0.178947	0.186101	0.999949	0.998082
Other congenital anomalies [Data Rich]	Male	0-6 days	65-69 years	0.237512	0.323874	0.168772	0.199416	0.999993	0.999946
Skin and subcutaneous diseases [Data Rich]	Female	28-364 days	95+ years	0.26551	0.425777	0.143915	0.169247	0.996163	0.996623
Skin and subcutaneous diseases [Global]	Female	28-364 days	95+ years	0.311971	0.604418	0.180336	0.189792	0.995184	0.985585
Skin and subcutaneous diseases [Data Rich]	Male	28-364 days	95+ years	0.314509	0.466318	0.147893	0.172982	0.997715	0.997888
Skin and subcutaneous diseases [Global]	Male	28-364 days	95+ years	0.321593	0.615463	0.181954	0.190039	0.997397	0.990149
Cellulitis [Global]	Male	0-6 days	95+ years	0.623399	1.08806	0.255736	0.268147	0.997213	0.973429
Cellulitis [Data Rich]	Male	0-6 days	95+ years	0.423335	1.119	0.228591	0.270132	0.997756	0.993906
Cellulitis [Global]	Female	0-6 days	95+ years	0.578589	1.01721	0.245097	0.249677	0.998127	0.969507
Cellulitis [Data Rich]	Female	0-6 days	95+ years	0.366275	0.986221	0.222007	0.249962	0.998742	0.994139
Pyoderma [Global]	Female	0-6 days	95+ years	0.388238	0.850436	0.20805	0.231518	0.999474	0.983259
Pyoderma [Data Rich]	Female	0-6 days	95+ years	0.273169	0.80969	0.182479	0.244284	0.99967	0.99265
Pyoderma [Data Rich]	Male	0-6 days	95+ years	0.294732	0.781775	0.182355	0.252501	0.999762	0.995254
Pyoderma [Global]	Male	0-6 days	95+ years	0.39082	0.814123	0.205397	0.233166	0.999595	0.987616
Decubitus ulcer [Global]	Female	1-4 years	95+ years	0.623324	1.14625	0.271622	0.278301	0.975388	0.950747
Decubitus ulcer [Data Rich]	Female	1-4 years	95+ years	0.329926	0.728105	0.198384	0.212926	0.975246	0.976259
Decubitus ulcer [Global]	Male	1-4 years	95+ years	0.533066	1.03325	0.308952	0.307131	0.975092	0.951109
Decubitus ulcer [Data Rich]	Male	1-4 years	95+ years	0.48381	0.759563	0.220599	0.269892	0.977738	0.977637
Other skin and subcutaneous diseases [Global]	Male	28-364 days	95+ years	0.530437	0.981397	0.379774	0.396112	0.988907	0.964709
Other skin and subcutaneous diseases [Data Rich]	Male	28-364 days	95+ years	0.393522	0.567931	0.307144	0.307694	0.994846	0.991828
Other skin and subcutaneous diseases [Global]	Female	28-364 days	95+ years	0.489996	0.939049	0.34252	0.356743	0.992544	0.972312
Other skin and subcutaneous diseases [Data Rich]	Female	28-364 days	95+ years	0.398379	0.600226	0.279222	0.294581	0.996886	0.9945
Sudden infant death syndrome [Global]	Female	7-27 days	28-364 days	0.34112	0.630679	0.200747	0.206543	0.999311	0.980433
Sudden infant death syndrome [Data Rich]	Female	7-27 days	28-364 days	0.259347	0.574111	0.18414	0.213612	0.999533	0.992591
Sudden infant death syndrome [Global]	Male	7-27 days	28-364 days	0.336379	0.622713	0.189068	0.195957	0.999351	0.979872
Sudden infant death syndrome [Data Rich]	Male	7-27 days	28-364 days	0.247303	0.604693	0.171396	0.209557	0.99944	0.991368
Transport injuries [Global]	Female	0-6 days	95+ years	0.214058	0.359901	0.168225	0.160343	0.999634	0.991933
Transport injuries [Data Rich]	Female	0-6 days	95+ years	0.156442	0.212877	0.117678	0.137265	0.999884	0.999634
Transport injuries [Global]	Male	0-6 days	95+ years	0.217146	0.341897	0.161485	0.155068	0.999591	0.989194
Transport injuries [Data Rich]	Male	0-6 days	95+ years	0.146089	0.199775	0.108364	0.127709	0.999807	0.999221
Road injuries [Global]	Male	0-6 days	95+ years	0.198187	0.336464	0.131309	0.134642	0.99946	0.990948
Road injuries [Data Rich]	Male	0-6 days	95+ years	0.150743	0.209355	0.110869	0.129792	0.99976	0.999257
Road injuries [Global]	Female	0-6 days	95+ years	0.199966	0.357491	0.138784	0.143033	0.999657	0.991953
Road injuries [Data Rich]	Female	0-6 days	95+ years	0.159882	0.220487	0.119362	0.139424	0.999797	0.999499
Pedestrian road injuries [Global]	Female	0-6 days	95+ years	0.230421	0.403209	0.157031	0.159299	0.999442	0.994568
Pedestrian road injuries [Data Rich]	Female	0-6 days	95+ years	0.180508	0.349747	0.131914	0.154024	0.999962	0.999776
Pedestrian road injuries [Global]	Male	0-6 days	95+ years	0.282796	0.451275	0.156837	0.156195	0.999157	0.993069
Pedestrian road injuries [Data Rich]	Male	0-6 days	95+ years	0.17317	0.330608	0.125625	0.144518	0.999876	0.999569
Cyclist road injuries [Data Rich]	Female	1-4 years	95+ years	0.241916	0.480987	0.175486	0.187739	0.999891	0.999522
Cyclist road injuries [Global]	Female	1-4 years	95+ years	0.300804	0.59035	0.200017	0.19735	0.99876	0.991642
Cyclist road injuries [Data Rich]	Male	1-4 years	95+ years	0.211617	0.534084	0.147913	0.180228	0.999905	0.999655

Table S17. CODEm predictive validity results by cause, model type, sex, and age									
Cause	Sex	Age Start	Age End	RMSE In-Sample	RMSE Out-of-Sample	Trend In-Sample	Trend Out-of-Sample	Coverage In-Sample	Coverage Out-of-Sample
Cyclist road injuries [Global]	Male	1-4 years	95+ years	0.270523	0.553351	0.17119	0.176831	0.998829	0.988734
Motorcyclist road injuries [Data Rich]	Male	0-6 days	95+ years	0.203092	0.441889	0.137629	0.169413	0.999842	0.999492
Motorcyclist road injuries [Global]	Male	0-6 days	95+ years	0.276111	0.530317	0.165732	0.173294	0.999172	0.988364
Motorcyclist road injuries [Global]	Female	0-6 days	95+ years	0.360373	0.757909	0.221986	0.239534	0.998998	0.989434
Motorcyclist road injuries [Data Rich]	Female	0-6 days	95+ years	0.272866	0.755627	0.192692	0.24068	0.999639	0.998782
Motor vehicle road injuries [Global]	Female	0-6 days	95+ years	0.253451	0.416466	0.143252	0.149021	0.999737	0.995403
Motor vehicle road injuries [Data Rich]	Female	0-6 days	95+ years	0.16998	0.335293	0.122168	0.141869	0.999938	0.999729
Motor vehicle road injuries [Data Rich]	Male	0-6 days	95+ years	0.160769	0.31748	0.115869	0.138412	0.999928	0.999591
Motor vehicle road injuries [Global]	Male	0-6 days	95+ years	0.243881	0.401531	0.138933	0.144582	0.999568	0.992747
Other road injuries [Global]	Female	0-6 days	95+ years	0.56197	0.961528	0.325455	0.38623	0.990963	0.959611
Other road injuries [Data Rich]	Female	0-6 days	95+ years	0.448998	1.13967	0.296464	0.459198	0.990829	0.966724
Other road injuries [Data Rich]	Male	0-6 days	95+ years	0.520433	1.38232	0.341609	0.566476	0.98183	0.944303
Other road injuries [Global]	Male	0-6 days	95+ years	0.590232	1.16874	0.367749	0.458176	0.977432	0.916622
Other transport injuries [Global]	Male	0-6 days	95+ years	0.266146	0.528431	0.174719	0.180349	0.998594	0.989599
Other transport injuries [Data Rich]	Male	0-6 days	95+ years	0.199862	0.399993	0.146412	0.187068	0.999581	0.999269
Other transport injuries [Data Rich]	Female	0-6 days	95+ years	0.259916	0.412868	0.202384	0.269466	0.999558	0.998947
Other transport injuries [Global]	Female	0-6 days	95+ years	0.321291	0.567386	0.231637	0.251538	0.998729	0.991747
Falls [Data Rich]	Female	0-6 days	95+ years	0.156051	0.22465	0.115018	0.142981	0.999931	0.999833
Falls [Global]	Female	0-6 days	95+ years	0.257036	0.431767	0.174971	0.177066	0.999473	0.992369
Falls [Data Rich]	Male	0-6 days	95+ years	0.154114	0.215355	0.11475	0.132317	0.999946	0.999855
Falls [Global]	Male	0-6 days	95+ years	0.25899	0.376263	0.155476	0.149083	0.999723	0.992408
Drowning [Data Rich]	Female	0-6 days	95+ years	0.188844	0.268832	0.14619	0.176156	0.999911	0.999801
Drowning [Global]	Female	0-6 days	95+ years	0.248734	0.453804	0.193315	0.192433	0.999452	0.992923
Drowning [Data Rich]	Male	0-6 days	95+ years	0.17262	0.234738	0.132227	0.15964	0.999819	0.999437
Drowning [Global]	Male	0-6 days	95+ years	0.238382	0.399327	0.182746	0.175907	0.999466	0.990295
Fire, heat, and hot substances [Global]	Male	0-6 days	95+ years	0.288899	0.418171	0.176852	0.171942	0.999545	0.996308
Fire, heat, and hot substances [Data Rich]	Male	0-6 days	95+ years	0.173545	0.232402	0.133091	0.159792	0.999934	0.999821
Fire, heat, and hot substances [Global]	Female	0-6 days	95+ years	0.354272	0.490069	0.18366	0.181319	0.999406	0.995179
Fire, heat, and hot substances [Data Rich]	Female	0-6 days	95+ years	0.177061	0.242463	0.134734	0.164622	0.999943	0.999878
Poisonings [Data Rich]	Female	0-6 days	95+ years	0.205629	0.301798	0.154039	0.190686	0.999878	0.999724
Poisonings [Global]	Female	0-6 days	95+ years	0.30075	0.574972	0.226617	0.237466	0.99936	0.992092
Poisonings [Data Rich]	Male	0-6 days	95+ years	0.199935	0.303251	0.14231	0.171029	0.999914	0.999779
Poisonings [Global]	Male	0-6 days	95+ years	0.315298	0.61206	0.228028	0.218435	0.999309	0.988793
Poisoning by carbon monoxide [Global]	Female	0-6 days	95+ years	0.453934	0.735433	0.276277	0.281053	0.998079	0.98846
Poisoning by carbon monoxide [Data Rich]	Female	0-6 days	95+ years	0.295548	0.40834	0.227937	0.253742	0.998689	0.997117
Poisoning by carbon monoxide [Global]	Male	0-6 days	95+ years	0.408392	0.720391	0.244484	0.256824	0.998796	0.990085
Poisoning by carbon monoxide [Data Rich]	Male	0-6 days	95+ years	0.258571	0.354936	0.197136	0.224647	0.999413	0.998589
Poisoning by other means [Global]	Female	0-6 days	95+ years	0.335078	0.675863	0.197042	0.208002	0.999639	0.987215
Poisoning by other means [Data Rich]	Female	0-6 days	95+ years	0.232485	0.638927	0.168819	0.210919	0.999698	0.998256
Poisoning by other means [Data Rich]	Male	0-6 days	95+ years	0.257829	0.675807	0.173133	0.201233	0.999806	0.999609
Poisoning by other means [Global]	Male	0-6 days	95+ years	0.315947	0.716124	0.19627	0.20863	0.99962	0.98411
Exposure to mechanical forces [Global]	Male	0-6 days	95+ years	0.356949	0.530351	0.314304	0.303348	0.999339	0.990532
Exposure to mechanical forces [Data Rich]	Male	0-6 days	95+ years	0.169819	0.268274	0.12322	0.155274	0.99975	0.999152
Exposure to mechanical forces [Data Rich]	Female	0-6 days	95+ years	0.185423	0.303729	0.134263	0.167614	0.999932	0.999826
Exposure to mechanical forces [Global]	Female	0-6 days	95+ years	0.360979	0.539613	0.338577	0.312942	0.99951	0.996063
Unintentional firearm injuries [Global]	Female	0-6 days	95+ years	0.368724	0.668486	0.233756	0.239057	0.99622	0.987597
Unintentional firearm injuries [Data Rich]	Female	0-6 days	95+ years	0.238845	0.4686	0.177056	0.202255	0.99657	0.996024
Unintentional firearm injuries [Data Rich]	Male	0-6 days	95+ years	0.267167	0.457065	0.178903	0.188009	0.997959	0.997533
Unintentional firearm injuries [Global]	Male	0-6 days	95+ years	0.403093	0.680238	0.236258	0.234265	0.997326	0.986007
Other exposure to mechanical forces [Data Rich]	Female	0-6 days	95+ years	0.209185	0.421027	0.147727	0.177286	0.999899	0.999845
Other exposure to mechanical forces [Global]	Female	0-6 days	95+ years	0.399804	0.560665	0.361142	0.337643	0.999571	0.997132
Other exposure to mechanical forces [Data Rich]	Male	0-6 days	95+ years	0.167479	0.325609	0.122737	0.147504	0.999868	0.999709
Other exposure to mechanical forces [Global]	Male	0-6 days	95+ years	0.364539	0.485405	0.330419	0.274298	0.999427	0.995951
Adverse effects of medical treatment [Global]	Male	0-6 days	95+ years	0.324712	0.517894	0.161046	0.172493	0.999695	0.994661
Adverse effects of medical treatment [Data Rich]	Male	0-6 days	95+ years	0.211591	0.344473	0.143151	0.178642	0.999871	0.999584
Adverse effects of medical treatment [Data Rich]	Female	0-6 days	95+ years	0.239367	0.36404	0.14507	0.177367	0.999912	0.999674
Adverse effects of medical treatment [Global]	Female	0-6 days	95+ years	0.286872	0.459545	0.156632	0.165677	0.999801	0.995188
Animal contact [Data Rich]	Female	0-6 days	95+ years	0.318335	0.494807	0.251101	0.338075	0.998992	0.998172
Animal contact [Global]	Female	0-6 days	95+ years	0.38602	0.783539	0.274873	0.306107	0.99838	0.982376
Animal contact [Data Rich]	Male	0-6 days	95+ years	0.258121	0.429758	0.193404	0.223663	0.999583	0.999238
Animal contact [Global]	Male	0-6 days	95+ years	0.33357	0.709047	0.225008	0.238333	0.998731	0.98736
Venomous animal contact [Global]	Female	0-6 days	95+ years	0.561634	1.04952	0.434315	0.488131	0.825959	0.786413
Venomous animal contact [Data Rich]	Female	0-6 days	95+ years	0.52173	0.873655	0.420506	0.54523	0.789268	0.788221
Venomous animal contact [Data Rich]	Male	0-6 days	95+ years	0.47259	0.84719	0.398255	0.534195	0.879611	0.881596
Venomous animal contact [Global]	Male	0-6 days	95+ years	0.524331	1.04014	0.416541	0.478512	0.899543	0.860224
Non-venomous animal contact [Data Rich]	Male	0-6 days	95+ years	0.308983	0.575639	0.237933	0.278082	0.997342	0.996688
Non-venomous animal contact [Global]	Male	0-6 days	95+ years	0.353457	0.755122	0.259593	0.273029	0.997205	0.98794
Non-venomous animal contact [Data Rich]	Female	0-6 days	95+ years	0.352435	0.674139	0.282908	0.363772	0.982106	0.982144
Non-venomous animal contact [Global]	Female	0-6 days	95+ years	0.392446	0.792286	0.298698	0.327196	0.985851	0.971692
Foreign body [Global]	Female	0-6 days	95+ years	0.217283	0.416663	0.149616	0.155518	0.999699	0.994635
Foreign body [Data Rich]	Female	0-6 days	95+ years	0.172104	0.275533	0.12581	0.152347	0.999952	0.999839
Foreign body [Global]	Male	0-6 days	95+ years	0.220339	0.416569	0.149944	0.15174	0.999576	0.991569
Foreign body [Data Rich]	Male	0-6 days	95+ years	0.169497	0.26081	0.12527	0.146469	0.999916	0.999626
Pulmonary aspiration and foreign body in airway [Global]	Female	0-6 days	95+ years	0.263506	0.420752	0.148223	0.149809	0.999898	0.996769
Pulmonary aspiration and foreign body in airway [Data Rich]	Female	0-6 days	95+ years	0.190077	0.357004	0.128534	0.147111	0.999989	0.999904
Pulmonary aspiration and foreign body in airway [Data Rich]	Male	0-6 days	95+ years	0.172339	0.336392	0.125357	0.140925	0.999978	0.999848
Pulmonary aspiration and foreign body in airway [Global]	Male	0-6 days	95+ years	0.311043	0.434704	0.147404	0.150074	0.999915	0.994915
Foreign body in other body part [Global]	Male	0-6 days	95+ years	0.469825	0.896892	0.356018	0.383179	0.930649	0.920747
Foreign body in other body part [Data Rich]	Male	0-6 days	95+ years	0.417373	0.690326	0.329949	0.388592	0.924011	0.926802
Foreign body in other body part [Global]	Female	0-6 days	95+ years	0.435247	0.843408	0.319684	0.344451	0.917063	0.897481
Foreign body in other body part [Data Rich]	Female	0-6 days	95+ years	0.371451	0.660901	0.287667	0.336908	0.904573	0.908371
Other unintentional injuries [Data Rich]	Female	0-6 days	95+ years	0.246133	0.413934	0.169375	0.222561	0.99985	0.999524
Other unintentional injuries [Global]	Female	0-6 days	95+ years	0.363735	0.700982	0.216034	0.224115	0.998826	0.983305

Table S17. CODEm predictive validity results by cause, model type, sex, and age									
**Cause**	**Sex**	**Age Start**	**Age End**	**RMSE In-Sample**	**RMSE Out-of-Sample**	**Trend In-Sample**	**Trend Out-of-Sample**	**Coverage In-Sample**	**Coverage Out-of-Sample**
Other unintentional injuries [Data Rich]	Male	0-6 days	95+ years	0.213331	0.354879	0.143459	0.221394	0.999805	0.999318
Other unintentional injuries [Global]	Male	0-6 days	95+ years	0.33444	0.610738	0.186459	0.196234	0.998884	0.9863
Self-harm [Data Rich]	Female	10-14 years	95+ years	0.165406	0.242606	0.125151	0.150981	0.999891	0.999619
Self-harm [Global]	Female	10-14 years	95+ years	0.223342	0.402908	0.160643	0.166195	0.998733	0.986068
Self-harm [Global]	Male	10-14 years	95+ years	0.206921	0.353461	0.140408	0.143617	0.999538	0.986016
Self-harm [Data Rich]	Male	10-14 years	95+ years	0.156753	0.228973	0.118317	0.142756	0.99979	0.999173
Self-harm by firearm [Data Rich]	Male	10-14 years	95+ years	0.187663	0.385863	0.141881	0.165656	0.998112	0.997882
Self-harm by firearm [Global]	Male	10-14 years	95+ years	0.267703	0.576453	0.156854	0.15818	0.998141	0.983009
Self-harm by firearm [Data Rich]	Female	10-14 years	95+ years	0.257458	0.452385	0.199523	0.204178	0.989702	0.989969
Self-harm by firearm [Global]	Female	10-14 years	95+ years	0.310444	0.662749	0.224363	0.243834	0.989367	0.980667
Self-harm by other specified means [Global]	Female	10-14 years	95+ years	0.198693	0.391509	0.139992	0.145319	0.999817	0.989008
Self-harm by other specified means [Data Rich]	Female	10-14 years	95+ years	0.171354	0.35115	0.126892	0.149712	0.999911	0.99957
Self-harm by other specified means [Global]	Male	10-14 years	95+ years	0.179822	0.357085	0.128823	0.134974	0.999774	0.981077
Self-harm by other specified means [Data Rich]	Male	10-14 years	95+ years	0.188653	0.322392	0.122416	0.139397	0.999912	0.999692
Interpersonal violence [Data Rich]	Female	0-6 days	95+ years	0.165825	0.299819	0.152514	0.219707	0.998887	0.997359
Interpersonal violence [Global]	Female	0-6 days	95+ years	0.285729	0.469644	0.220603	0.220326	0.998539	0.988856
Interpersonal violence [Data Rich]	Male	0-6 days	95+ years	0.16221	0.306068	0.139585	0.206067	0.998585	0.996178
Interpersonal violence [Global]	Male	0-6 days	95+ years	0.292197	0.544166	0.211726	0.224819	0.997719	0.981479
Physical violence by firearm [Global]	Male	0-6 days	95+ years	0.338638	0.731346	0.232166	0.236482	0.994403	0.97352
Physical violence by firearm [Data Rich]	Male	0-6 days	95+ years	0.177454	0.548399	0.117896	0.227031	0.995765	0.994523
Physical violence by firearm [Data Rich]	Female	0-6 days	95+ years	0.169416	0.506381	0.115492	0.224667	0.999006	0.998122
Physical violence by firearm [Global]	Female	0-6 days	95+ years	0.357133	0.643855	0.237262	0.225878	0.996861	0.987426
Physical violence by sharp object [Data Rich]	Female	0-6 days	95+ years	0.155736	0.431121	0.107611	0.216911	0.999986	0.999438
Physical violence by sharp object [Global]	Female	0-6 days	95+ years	0.286709	0.520707	0.201216	0.21261	0.999163	0.994159
Physical violence by sharp object [Data Rich]	Male	0-6 days	95+ years	0.17192	0.516148	0.113438	0.204915	0.999981	0.999172
Physical violence by sharp object [Global]	Male	0-6 days	95+ years	0.329542	0.597916	0.200449	0.202967	0.999119	0.990213
Physical violence by other means [Global]	Male	0-6 days	95+ years	0.251126	0.466663	0.159096	0.160575	0.99935	0.993019
Physical violence by other means [Data Rich]	Male	0-6 days	95+ years	0.132481	0.394042	0.0872444	0.164275	0.999973	0.999586
Physical violence by other means [Data Rich]	Female	0-6 days	95+ years	0.132125	0.319908	0.0950185	0.180708	0.999986	0.999846
Physical violence by other means [Global]	Female	0-6 days	95+ years	0.241622	0.415163	0.170075	0.173039	0.999517	0.995206
Environmental heat and cold exposure [Global]	Female	0-6 days	95+ years	0.457416	0.767594	0.239618	0.246869	0.9975	0.985222
Environmental heat and cold exposure [Data Rich]	Female	0-6 days	95+ years	0.263702	0.469594	0.185412	0.229909	0.997331	0.997066
Environmental heat and cold exposure [Global]	Male	0-6 days	95+ years	0.364885	0.620518	0.223264	0.225784	0.999417	0.990837
Environmental heat and cold exposure [Data Rich]	Male	0-6 days	95+ years	0.235748	0.363401	0.171699	0.215468	0.999693	0.999322
Acute lymphoid leukaemia [Data Rich]	Male	0-6 days	95+ years	0.310748	0.421052	0.257037	0.250826	0.999775	0.999201
Acute lymphoid leukaemia [Global]	Female	0-6 days	95+ years	0.387468	0.532984	0.309633	0.305578	0.999247	0.995595
Acute lymphoid leukaemia [Data Rich]	Female	0-6 days	95+ years	0.330709	0.469462	0.291051	0.295814	0.999639	0.99753
Acute lymphoid leukaemia [Global]	Male	0-6 days	95+ years	0.340238	0.481488	0.269561	0.266842	0.999598	0.996258
Chronic lymphoid leukaemia [Data Rich]	Male	20-24 years	95+ years	0.281179	0.549864	0.215486	0.224895	0.993027	0.986267
Chronic lymphoid leukaemia [Global]	Male	20-24 years	95+ years	0.320083	0.610391	0.242085	0.241267	0.992505	0.982641
Chronic lymphoid leukaemia [Data Rich]	Female	20-24 years	95+ years	0.39135	0.586716	0.370043	0.380051	0.988081	0.980056
Chronic lymphoid leukaemia [Global]	Female	20-24 years	95+ years	0.412761	0.641749	0.374616	0.418784	0.989395	0.977703
Acute myeloid leukaemia [Global]	Male	0-6 days	95+ years	0.442375	0.54861	0.372251	0.356678	0.998107	0.996117
Acute myeloid leukaemia [Data Rich]	Male	0-6 days	95+ years	0.41862	0.467416	0.371762	0.271103	0.998507	0.997028
Acute myeloid leukaemia [Global]	Female	0-6 days	95+ years	0.407571	0.523704	0.353383	0.33313	0.998587	0.995357
Acute myeloid leukaemia [Data Rich]	Female	0-6 days	95+ years	0.394568	0.448862	0.352312	0.249893	0.998964	0.997453
Chronic myeloid leukaemia [Global]	Female	0-6 days	95+ years	0.398875	0.739473	0.313448	0.315678	0.987321	0.984064
Chronic myeloid leukaemia [Data Rich]	Female	0-6 days	95+ years	0.350665	0.590275	0.289082	0.278623	0.984769	0.985895
Chronic myeloid leukaemia [Global]	Male	0-6 days	95+ years	0.367785	0.691518	0.303339	0.313485	0.988074	0.985079
Chronic myeloid leukaemia [Data Rich]	Male	0-6 days	95+ years	0.337209	0.602014	0.292333	0.283856	0.985279	0.98508
Non-melanoma skin cancer (squamous-cell carcinoma) [Data Rich]	Female	20-24 years	95+ years	0.213103	0.305569	0.15533	0.168031	0.999125	0.998895
Non-melanoma skin cancer (squamous-cell carcinoma) [Global]	Female	20-24 years	95+ years	0.267942	0.451328	0.188854	0.185703	0.998427	0.992207
Non-melanoma skin cancer (squamous-cell carcinoma) [Data Rich]	Male	20-24 years	95+ years	0.187014	0.271342	0.132362	0.153868	0.999799	0.999579
Non-melanoma skin cancer (squamous-cell carcinoma) [Global]	Male	20-24 years	95+ years	0.215805	0.354613	0.150469	0.152022	0.999798	0.997394
Police conflict and executions [Global]	Male	28-364 days	95+ years	1.02049	1.88613	0.771676	0.789582	0.658613	0.63731
Police conflict and executions [Data Rich]	Male	28-364 days	95+ years	0.943007	1.49198	0.769747	0.773765	0.609659	0.593569
Police conflict and executions [Global]	Female	28-364 days	95+ years	1.28753	1.87029	0.699587	0.742916	0.47453	0.475053
Police conflict and executions [Data Rich]	Female	28-364 days	95+ years	0.807008	1.34854	0.65945	0.738254	0.432443	0.441836
Alcoholic cardiomyopathy [Global]	Female	15-19 years	95+ years	0.427458	0.834555	0.296157	0.297787	0.994826	0.97718
Alcoholic cardiomyopathy [Data Rich]	Female	15-19 years	95+ years	0.407459	0.704454	0.286336	0.202602	0.996617	0.995009
Alcoholic cardiomyopathy [Global]	Male	15-19 years	95+ years	0.377154	0.658182	0.287322	0.273992	0.997438	0.981515
Alcoholic cardiomyopathy [Data Rich]	Male	15-19 years	95+ years	0.34268	0.58175	0.269727	0.164646	0.997666	0.994412
Myocarditis [Data Rich]	Female	0-6 days	95+ years	0.504879	0.618706	0.234237	0.235234	0.999791	0.999228
Myocarditis [Global]	Female	0-6 days	95+ years	0.488177	0.831609	0.251019	0.260179	0.999671	0.984337
Myocarditis [Data Rich]	Male	0-6 days	95+ years	0.454295	0.538226	0.226681	0.228834	0.999783	0.99916
Myocarditis [Global]	Male	0-6 days	95+ years	0.447338	0.743749	0.240321	0.24841	0.999582	0.990285
Other leukaemia [Global]	Male	0-6 days	95+ years	0.309665	0.463243	0.251833	0.245941	0.99957	0.993564
Other leukaemia [Data Rich]	Male	0-6 days	95+ years	0.282494	0.381942	0.241361	0.229578	0.999734	0.999253
Other leukaemia [Data Rich]	Female	0-6 days	95+ years	0.279342	0.386578	0.238006	0.240633	0.999753	0.999095
Other leukaemia [Global]	Female	0-6 days	95+ years	0.337468	0.46428	0.252373	0.245634	0.999625	0.994124
Other cardiomyopathy [Data Rich]	Female	0-6 days	95+ years	0.249505	0.468767	0.147086	0.169643	0.999933	0.999778
Other cardiomyopathy [Global]	Female	0-6 days	95+ years	0.26514	0.62649	0.169672	0.1757	0.999799	0.984607
Other cardiomyopathy [Data Rich]	Male	0-6 days	95+ years	0.299804	0.465719	0.150968	0.16148	0.999867	0.999603
Other cardiomyopathy [Global]	Male	0-6 days	95+ years	0.303166	0.585465	0.169605	0.172274	0.99977	0.987044
Myelodysplastic, myeloproliferative, and other hematopoietic neoplasms [Global]	Male	0-6 days	95+ years	0.393475	0.62733	0.175898	0.180363	0.993561	0.986727
Myelodysplastic, myeloproliferative, and other hematopoietic neoplasms [Data Rich]	Female	0-6 days	95+ years	0.233481	0.602649	0.14587	0.160022	0.996729	0.995889
Myelodysplastic, myeloproliferative, and other hematopoietic neoplasms [Global]	Female	0-6 days	95+ years	0.286466	0.611284	0.185013	0.19146	0.994493	0.98866
Myelodysplastic, myeloproliferative, and other hematopoietic neoplasms [Data Rich]	Male	0-6 days	95+ years	0.30167	0.66623	0.143972	0.152493	0.996302	0.995486
Non-rheumatic calcific aortic valvular heart disease [Global]	Male	15-19 years	95+ years	0.219259	0.434492	0.146809	0.151405	0.999733	0.990228
Non-rheumatic calcific aortic valvular heart disease [Data Rich]	Male	15-19 years	95+ years	0.192372	0.366708	0.133302	0.141795	0.999983	0.999883
Non-rheumatic calcific aortic valvular heart disease [Global]	Female	15-19 years	95+ years	0.233419	0.48052	0.153055	0.156823	0.999131	0.990256
Non-rheumatic calcific aortic valvular heart disease [Data Rich]	Female	15-19 years	95+ years	0.202508	0.36618	0.131717	0.137969	0.99964	0.999567

Table S17. CODEm predictive validity results by cause, model type, sex, and age									
Cause	Sex	Age Start	Age End	RMSE In-Sample	RMSE Out-of-Sample	Trend In-Sample	Trend Out-of-Sample	Coverage In-Sample	Coverage Out-of-Sample
Non-rheumatic degenerative mitral valvular heart disease [Global]	Male	15-19 years	95+ years	0.357049	0.540794	0.214198	0.221077	0.999509	0.994103
Non-rheumatic degenerative mitral valvular heart disease [Data Rich]	Female	15-19 years	95+ years	0.365176	0.539582	0.205161	0.228993	0.999317	0.99819
Non-rheumatic degenerative mitral valvular heart disease [Global]	Female	15-19 years	95+ years	0.386556	0.57383	0.223202	0.230698	0.998652	0.991079
Non-rheumatic degenerative mitral valvular heart disease [Data Rich]	Male	15-19 years	95+ years	0.330883	0.511146	0.195821	0.228605	0.99985	0.999524
Other non-rheumatic valvular heart diseases [Data Rich]	Male	15-19 years	95+ years	0.818543	1.03296	0.493847	0.526873	0.991983	0.986877
Other non-rheumatic valvular heart diseases [Global]	Male	15-19 years	95+ years	0.807673	1.14825	0.505234	0.531731	0.990465	0.965444
Other non-rheumatic valvular heart diseases [Data Rich]	Female	15-19 years	95+ years	0.787884	1.00007	0.461616	0.501656	0.995308	0.992147
Other non-rheumatic valvular heart diseases [Global]	Female	15-19 years	95+ years	0.732013	1.0773	0.460801	0.499458	0.99482	0.971047
Diabetes mellitus type 1 [Global]	Male	0-6 days	95+ years	0.258039	0.425434	0.117026	0.120614	0.999416	0.991828
Diabetes mellitus type 1 [Data Rich]	Male	0-6 days	95+ years	0.211973	0.320813	0.105664	0.116303	0.999385	0.999368
Diabetes mellitus type 1 [Data Rich]	Female	0-6 days	95+ years	0.241148	0.402287	0.103867	0.1156	0.999218	0.999144
Diabetes mellitus type 1 [Global]	Female	0-6 days	95+ years	0.237461	0.459372	0.117734	0.118935	0.999177	0.992557
Diabetes mellitus type 2 [Global]	Female	15-19 years	95+ years	0.21785	0.387509	0.140692	0.142845	0.998898	0.99047
Diabetes mellitus type 2 [Data Rich]	Female	15-19 years	95+ years	0.206536	0.363513	0.121761	0.134536	0.999858	0.998514
Diabetes mellitus type 2 [Data Rich]	Male	15-19 years	95+ years	0.210553	0.324721	0.118312	0.128967	0.999883	0.99907
Diabetes mellitus type 2 [Global]	Male	15-19 years	95+ years	0.209379	0.375507	0.135622	0.137967	0.999385	0.992014
Bacterial skin diseases [Global]	Male	0-6 days	95+ years	0.378286	0.8219	0.202353	0.203804	0.989492	0.972978
Bacterial skin diseases [Data Rich]	Female	0-6 days	95+ years	0.244357	0.59414	0.157693	0.198142	0.999736	0.999391
Bacterial skin diseases [Global]	Female	0-6 days	95+ years	0.359199	0.859112	0.203892	0.213703	0.988477	0.969042
Bacterial skin diseases [Data Rich]	Male	0-6 days	95+ years	0.261327	0.610034	0.148336	0.180457	0.999769	0.999368
Upper digestive system diseases [Global]	Male	1-4 years	95+ years	0.20806	0.376699	0.139412	0.141514	0.999332	0.991884
Upper digestive system diseases [Data Rich]	Male	1-4 years	95+ years	0.160402	0.22216	0.117802	0.135604	0.999939	0.999658
Upper digestive system diseases [Data Rich]	Female	1-4 years	95+ years	0.164839	0.238133	0.117028	0.141948	0.999986	0.999817
Upper digestive system diseases [Global]	Female	1-4 years	95+ years	0.218633	0.386509	0.142072	0.144972	0.999449	0.992746
Pulmonary Arterial Hypertension [Data Rich]	Male	0-6 days	95+ years	0.403058	0.702748	0.224406	0.245136	0.999143	0.99839
Pulmonary Arterial Hypertension [Data Rich]	Female	0-6 days	95+ years	0.430758	0.741586	0.227039	0.231136	0.999071	0.998338
Pulmonary Arterial Hypertension [Global]	Female	0-6 days	95+ years	0.482193	0.872406	0.26086	0.26452	0.998577	0.976619
Pulmonary Arterial Hypertension [Global]	Male	0-6 days	95+ years	0.536452	0.871513	0.265027	0.26939	0.998694	0.978926
Hepatoblastoma [Data Rich]	Male	0-6 days	10-14 years	0.5047	0.515512	0.230663	0.176857	0.997917	0.995525
Hepatoblastoma [Global]	Male	0-6 days	10-14 years	0.407048	0.6147	0.246613	0.236964	0.997367	0.988149
Hepatoblastoma [Global]	Female	0-6 days	10-14 years	0.38034	0.578585	0.245267	0.236488	0.996607	0.991566
Hepatoblastoma [Data Rich]	Female	0-6 days	10-14 years	0.466592	0.457909	0.234648	0.188151	0.997274	0.99569
Burkitt lymphoma [Data Rich]	Female	1-4 years	95+ years	0.333096	0.405636	0.2217	0.165721	0.998457	0.99622
Burkitt lymphoma [Global]	Female	1-4 years	95+ years	0.341783	0.500535	0.244252	0.228822	0.996709	0.993751
Burkitt lymphoma [Data Rich]	Male	1-4 years	95+ years	0.436396	0.539359	0.280039	0.212951	0.99742	0.994168
Burkitt lymphoma [Global]	Male	1-4 years	95+ years	0.454874	0.605781	0.31072	0.297928	0.995415	0.990787
Other non-Hodgkin lymphoma [Data Rich]	Male	1-4 years	95+ years	0.31554	0.370473	0.223201	0.227704	0.998468	0.997258
Other non-Hodgkin lymphoma [Global]	Male	1-4 years	95+ years	0.462158	0.52209	0.340932	0.320114	0.997454	0.987238
Other non-Hodgkin lymphoma [Global]	Female	1-4 years	95+ years	0.440335	0.533262	0.326964	0.324062	0.998392	0.987694
Other non-Hodgkin lymphoma [Data Rich]	Female	1-4 years	95+ years	0.291424	0.345982	0.229882	0.241386	0.998663	0.997484
Eye cancer [Global]	Female	0-6 days	95+ years	0.34235	0.469228	0.238212	0.230413	0.997837	0.994456
Eye cancer [Data Rich]	Female	0-6 days	95+ years	0.297684	0.354061	0.21198	0.193013	0.998879	0.997378
Eye cancer [Global]	Male	0-6 days	95+ years	0.377216	0.490653	0.254858	0.246219	0.997199	0.993821
Eye cancer [Data Rich]	Male	0-6 days	95+ years	0.296902	0.366581	0.218129	0.205273	0.998994	0.997045
Retinoblastoma [Data Rich]	Male	0-6 days	15-19 years	0.301279	0.364823	0.221298	0.228031	0.998057	0.996361
Retinoblastoma [Global]	Female	0-6 days	15-19 years	0.34258	0.463108	0.22999	0.228006	0.997595	0.994302
Retinoblastoma [Data Rich]	Female	0-6 days	15-19 years	0.304516	0.35512	0.219797	0.228061	0.998126	0.99682
Retinoblastoma [Global]	Male	0-6 days	15-19 years	0.364393	0.465386	0.238859	0.233329	0.996922	0.994125
Other eye cancers [Global]	Male	1-4 years	95+ years	0.414725	0.487974	0.27379	0.26204	0.996996	0.993444
Other eye cancers [Data Rich]	Male	1-4 years	95+ years	0.310161	0.350546	0.233455	0.201847	0.998635	0.996671
Other eye cancers [Data Rich]	Female	1-4 years	95+ years	0.297757	0.334036	0.225201	0.207256	0.998909	0.997212
Other eye cancers [Global]	Female	1-4 years	95+ years	0.367887	0.491649	0.254692	0.252611	0.997737	0.994332
Soft tissue and other extraosseous sarcomas [Data Rich]	Male	0-6 days	95+ years	0.411639	0.477938	0.27752	0.25336	0.999761	0.999222
Soft tissue and other extraosseous sarcomas [Global]	Male	0-6 days	95+ years	0.448588	0.613314	0.349539	0.342343	0.997836	0.98907
Soft tissue and other extraosseous sarcomas [Global]	Female	0-6 days	95+ years	0.463558	0.611126	0.338864	0.320885	0.998912	0.991599
Soft tissue and other extraosseous sarcomas [Data Rich]	Female	0-6 days	95+ years	0.396538	0.46168	0.266218	0.256151	0.999765	0.999298
Malignant neoplasm of bone and articular cartilage [Data Rich]	Male	1-4 years	95+ years	0.379173	0.442497	0.29039	0.306139	0.998655	0.997623
Malignant neoplasm of bone and articular cartilage [Global]	Male	1-4 years	95+ years	0.482772	0.647766	0.368325	0.358684	0.997787	0.987218
Malignant neoplasm of bone and articular cartilage [Data Rich]	Female	1-4 years	95+ years	0.358499	0.420605	0.29032	0.300079	0.99913	0.998307
Malignant neoplasm of bone and articular cartilage [Global]	Female	1-4 years	95+ years	0.475759	0.627016	0.359386	0.346431	0.998467	0.988843
Neuroblastoma and other peripheral nervous cell tumors [Data Rich]	Female	0-6 days	95+ years	0.417733	0.498987	0.368879	0.378147	0.998856	0.996795
Neuroblastoma and other peripheral nervous cell tumors [Global]	Male	0-6 days	95+ years	0.478096	0.603992	0.385829	0.369484	0.999182	0.995694
Neuroblastoma and other peripheral nervous cell tumors [Global]	Female	0-6 days	95+ years	0.464457	0.580749	0.368289	0.350985	0.998855	0.994193
Neuroblastoma and other peripheral nervous cell tumors [Data Rich]	Male	0-6 days	95+ years	0.451585	0.53109	0.388807	0.377342	0.998741	0.996324

Table S18. Comparison of GBD 2017 and GBD 2019 covariates and level of covariates used in cause of death modeling								
**Cause**	**Sex**	**Covariate**	**Level 1: GBD 2019**	**Level 2: GBD 2019**	**Level 3: GBD 2019**	**Level 1: GBD 2017**	**Level 2: GBD 2017**	**Level 3: GBD 2017**
Diarrhoeal diseases	Male	Rotavirus coverage (proportion)		X		X		
Diarrhoeal diseases	Male	Rotavirus coverage (proportion)		X			X	
Diarrhoeal diseases	Male	Rotavirus coverage (proportion)		X		X		
Diarrhoeal diseases	Male	Rotavirus coverage (proportion)	X			X		
Diarrhoeal diseases	Female	Rotavirus coverage (proportion)		X			X	
Diarrhoeal diseases	Female	Rotavirus coverage (proportion)		X		X		
Diarrhoeal diseases	Female	Rotavirus coverage (proportion)	X				X	
Diarrhoeal diseases	Female	Rotavirus coverage (proportion)	X			X		
Diarrhoeal diseases	Female	Rotavirus coverage (proportion)		X			X	
Diarrhoeal diseases	Female	Rotavirus coverage (proportion)		X		X		
Diarrhoeal diseases	Female	Rotavirus coverage (proportion)	X				X	
Diarrhoeal diseases	Female	Rotavirus coverage (proportion)	X			X		
Diarrhoeal diseases	Male	Vitamin A Deficiency Prevalence (age-standardized)		X			X	
Diarrhoeal diseases	Female	Vitamin A Deficiency Prevalence (age-standardized)		X			X	
Diarrhoeal diseases	Male	ORS (oral rehydration)	X				X	
Diarrhoeal diseases	Male	ORS (oral rehydration)	X			X		
Diarrhoeal diseases	Female	ORS (oral rehydration)	X				X	
Diarrhoeal diseases	Female	ORS (oral rehydration)	X			X		
Diarrhoeal diseases	Male	No access to handwashing facility			X			X
Diarrhoeal diseases	Female	No access to handwashing facility			X			X
Diarrhoeal diseases	Male	Zinc deficiency		X			X	
Diarrhoeal diseases	Female	Zinc deficiency		X			X	
Diarrhoeal diseases	Male	Zinc treatment for diarrhea		X			X	
Diarrhoeal diseases	Female	Zinc treatment for diarrhea		X			X	
Diarrhoeal diseases	Male	Rotavirus coverage (proportion)	X			X		
Diarrhoeal diseases	Male	Rotavirus coverage (proportion)	X				X	
Diarrhoeal diseases	Male	Rotavirus coverage (proportion)	X				X	
Diarrhoeal diseases	Female	Age- and sex-specific SEV for Unsafe sanitation	X			X		
Diarrhoeal diseases	Male	Education (years per capita)			X			X
Diarrhoeal diseases	Female	Education (years per capita)			X			X
Diarrhoeal diseases	Male	LDI (I$ per capita)			X			X
Diarrhoeal diseases	Female	LDI (I$ per capita)			X			X
Diarrhoeal diseases	Male	Socio-demographic Index			X			X
Diarrhoeal diseases	Female	Socio-demographic Index			X			X
Diarrhoeal diseases	Male	Rotavirus coverage (proportion)		X			X	
Diarrhoeal diseases	Male	Healthcare access and quality index		X				X
Diarrhoeal diseases	Female	Healthcare access and quality index		X			X	
Diarrhoeal diseases	Female	Healthcare access and quality index		X				X
Diarrhoeal diseases	Male	Maternal Education (years per capita)			X			X
Diarrhoeal diseases	Female	Maternal Education (years per capita)			X			X
Diarrhoeal diseases	Male	Healthcare access and quality index		X			X	
Diarrhoeal diseases	Female	Sanitation (proportion with access)	X			X		
Diarrhoeal diseases	Male	Improved Water Source (proportion with access)	X			X		
Diarrhoeal diseases	Female	Improved Water Source (proportion with access)	X			X		
Diarrhoeal diseases	Male	Population Density (over 1000 ppl/sqkm, proportion)			X			X
Diarrhoeal diseases	Female	Population Density (over 1000 ppl/sqkm, proportion)			X			X
Diarrhoeal diseases	Male	Log-transformed SEV scalar: Diarrhea	X			X		
Diarrhoeal diseases	Female	Log-transformed SEV scalar: Diarrhea	X			X		
Diarrhoeal diseases	Male	Age- and sex-specific SEV for Unsafe water	X			X		
Diarrhoeal diseases	Female	Age- and sex-specific SEV for Unsafe water	X			X		
Diarrhoeal diseases	Male	Age- and sex-specific SEV for Unsafe sanitation	X			X		
Diarrhoeal diseases	Male	Sanitation (proportion with access)	X			X		
Lower respiratory infections	Female	Secondhand smoke	X			X		
Lower respiratory infections	Female	Secondhand smoke		X			X	
Lower respiratory infections	Female	Secondhand smoke		X		X		
Lower respiratory infections	Male	Hib3 Vaccine Coverage (proportion)	X			X		
Lower respiratory infections	Male	Hib3 Vaccine Coverage (proportion)	X			X		
Lower respiratory infections	Female	Education (years per capita)			X			X
Lower respiratory infections	Female	Hib3 Vaccine Coverage (proportion)	X			X		
Lower respiratory infections	Male	Antibiotics for LRI	X			X		
Lower respiratory infections	Female	Antibiotics for LRI	X			X		
Lower respiratory infections	Female	Secondhand smoke	X				X	
Lower respiratory infections	Female	Hib3 Vaccine Coverage (proportion)	X			X		
Lower respiratory infections	Male	Secondhand smoke		X		X		
Lower respiratory infections	Male	Education (years per capita)			X			X
Lower respiratory infections	Male	Secondhand smoke	X			X		
Lower respiratory infections	Female	Vitamin A Deficiency Prevalence (age-standardized)		X			X	
Lower respiratory infections	Female	Healthcare access and quality index		X			X	
Lower respiratory infections	Male	No access to handwashing facility		X			X	
Lower respiratory infections	Male	No access to handwashing facility			X		X	
Lower respiratory infections	Female	No access to handwashing facility		X			X	
Lower respiratory infections	Female	No access to handwashing facility			X		X	
Lower respiratory infections	Male	Zinc deficiency		X			X	
Lower respiratory infections	Female	Zinc deficiency		X			X	
Lower respiratory infections	Male	Age- and sex-specific SEV for Child underweight	X			X		
Lower respiratory infections	Female	Age- and sex-specific SEV for Child underweight	X			X		

Table S18. Comparison of GBD 2017 and GBD 2019 covariates and level of covariates used in cause of death modeling								
**Cause**	**Sex**	**Covariate**	**Level 1: GBD 2019**	**Level 2: GBD 2019**	**Level 3: GBD 2019**	**Level 1: GBD 2017**	**Level 2: GBD 2017**	**Level 3: GBD 2017**
Lower respiratory infections	Male	Age- and sex-specific SEV for Child stunting	X			X		
Lower respiratory infections	Male	Age- and sex-specific SEV for Child wasting	X			X		
Lower respiratory infections	Female	Age- and sex-specific SEV for Child wasting	X			X		
Lower respiratory infections	Male	DTP3 Coverage (proportion)		X			X	
Lower respiratory infections	Male	DTP3 Coverage (proportion)		X			X	
Lower respiratory infections	Male	Vitamin A Deficiency Prevalence (age-standardized)		X			X	
Lower respiratory infections	Female	Age- and sex-specific SEV for Unsafe sanitation			X			X
Lower respiratory infections	Male	Age- and sex-specific SEV for Unsafe sanitation			X			X
Lower respiratory infections	Female	Population Density (over 1000 ppl/sqkm, proportion)			X			X
Lower respiratory infections	Male	Secondhand smoke		X			X	
Lower respiratory infections	Male	Outdoor Air Pollution (PM2.5)	X				X	
Lower respiratory infections	Female	Indoor Air Pollution (All Cooking Fuels)	X			X		
Lower respiratory infections	Male	Indoor Air Pollution (All Cooking Fuels)	X			X		
Lower respiratory infections	Male	Outdoor Air Pollution (PM2.5)	X			X		
Lower respiratory infections	Male	Outdoor Air Pollution (PM2.5)		X			X	
Lower respiratory infections	Male	Outdoor Air Pollution (PM2.5)		X		X		
Lower respiratory infections	Female	DTP3 Coverage (proportion)		X			X	
Lower respiratory infections	Female	Outdoor Air Pollution (PM2.5)	X				X	
Lower respiratory infections	Female	Outdoor Air Pollution (PM2.5)		X			X	
Lower respiratory infections	Female	Outdoor Air Pollution (PM2.5)		X		X		
Lower respiratory infections	Male	Maternal Education (years per capita)			X			X
Lower respiratory infections	Female	Maternal Education (years per capita)			X			X
Lower respiratory infections	Male	Smoking Prevalence	X			X		
Lower respiratory infections	Female	Smoking Prevalence	X			X		
Lower respiratory infections	Male	Population Density (over 1000 ppl/sqkm, proportion)			X			X
Lower respiratory infections	Female	Outdoor Air Pollution (PM2.5)	X			X		
Lower respiratory infections	Female	DTP3 Coverage (proportion)		X			X	
Lower respiratory infections	Female	Age- and sex-specific SEV for Child stunting	X			X		
Lower respiratory infections	Male	PCV3 Coverage (proportion)		X			X	
Lower respiratory infections	Female	PCV3 Coverage (proportion)	X			X		
Lower respiratory infections	Female	PCV3 Coverage (proportion)		X			X	
Lower respiratory infections	Female	PCV3 Coverage (proportion)		X		X		
Lower respiratory infections	Female	PCV3 Coverage (proportion)	X				X	
Lower respiratory infections	Female	PCV3 Coverage (proportion)	X			X		
Lower respiratory infections	Female	Log-transformed SEV scalar: LRI	X			X		
Lower respiratory infections	Male	PCV3 Coverage (proportion)		X		X		
Lower respiratory infections	Female	PCV3 Coverage (proportion)	X				X	
Lower respiratory infections	Male	Secondhand smoke	X				X	
Lower respiratory infections	Male	LDI (I$ per capita)			X			X
Lower respiratory infections	Female	LDI (I$ per capita)			X			X
Lower respiratory infections	Male	Log-transformed SEV scalar: LRI	X			X		
Lower respiratory infections	Female	Socio-demographic Index			X			X
Lower respiratory infections	Male	Healthcare access and quality index		X			X	
Lower respiratory infections	Female	PCV3 Coverage (proportion)		X			X	
Lower respiratory infections	Male	PCV3 Coverage (proportion)	X			X		
Lower respiratory infections	Female	PCV3 Coverage (proportion)		X		X		
Lower respiratory infections	Male	PCV3 Coverage (proportion)	X				X	
Lower respiratory infections	Male	PCV3 Coverage (proportion)		X		X		
Lower respiratory infections	Male	PCV3 Coverage (proportion)		X			X	
Lower respiratory infections	Male	PCV3 Coverage (proportion)	X			X		
Lower respiratory infections	Male	Socio-demographic Index			X			X
Lower respiratory infections	Male	PCV3 Coverage (proportion)	X				X	
Upper respiratory infections	Male	Outdoor Air Pollution (PM2.5)		X			X	
Upper respiratory infections	Female	Education (years per capita)			X			X
Upper respiratory infections	Female	LDI (I$ per capita)			X			X
Upper respiratory infections	Male	LDI (I$ per capita)			X			X
Upper respiratory infections	Female	Socio-demographic Index			X			X
Upper respiratory infections	Male	Socio-demographic Index			X			X
Upper respiratory infections	Female	Healthcare access and quality index		X			X	
Upper respiratory infections	Male	Healthcare access and quality index		X			X	
Upper respiratory infections	Female	Indoor Air Pollution (All Cooking Fuels)		X			X	
Upper respiratory infections	Female	Outdoor Air Pollution (PM2.5)		X			X	
Upper respiratory infections	Male	Education (years per capita)			X			X
Upper respiratory infections	Male	Indoor Air Pollution (All Cooking Fuels)		X			X	
Upper respiratory infections	Male	Smoking Prevalence	X			X		
Upper respiratory infections	Female	Smoking Prevalence	X			X		
Otitis media	Male	Log-transformed SEV scalar: Otitis	X			X		
Otitis media	Female	Log-transformed SEV scalar: Otitis	X			X		
Otitis media	Female	Indoor Air Pollution (All Cooking Fuels)		X			X	
Otitis media	Male	Indoor Air Pollution (All Cooking Fuels)		X			X	
Otitis media	Female	Healthcare access and quality index		X			X	
Otitis media	Male	Healthcare access and quality index		X			X	
Otitis media	Female	Socio-demographic Index			X			X
Otitis media	Male	Socio-demographic Index			X			X
Otitis media	Female	LDI (I$ per capita)			X			X
Otitis media	Male	LDI (I$ per capita)			X			X
Otitis media	Female	Education (years per capita)			X			X
Otitis media	Male	Education (years per capita)			X			X

Table S18. Comparison of GBD 2017 and GBD 2019 covariates and level of covariates used in cause of death modeling								
Cause	Sex	Covariate	Level 1: GBD 2019	Level 2: GBD 2019	Level 3: GBD 2019	Level 1: GBD 2017	Level 2: GBD 2017	Level 3: GBD 2017
Otitis media	Female	Smoking Prevalence	X			X		
Otitis media	Male	Outdoor Air Pollution (PM2.5)		X			X	
Otitis media	Female	Outdoor Air Pollution (PM2.5)		X			X	
Otitis media	Male	Smoking Prevalence	X			X		
Meningitis	Female	Healthcare access and quality index		X			X	
Meningitis	Female	Maternal care and immunization		X			X	
Meningitis	Female	Sanitation (proportion with access)			X			X
Meningitis	Male	Sanitation (proportion with access)			X			X
Meningitis	Female	Improved Water Source (proportion with access)		X			X	
Meningitis	Male	Improved Water Source (proportion with access)		X			X	
Meningitis	Female	Hib3 Vaccine Coverage (proportion)	X			X		
Meningitis	Female	Hib3 Vaccine Coverage (proportion)	X			X		
Meningitis	Male	Hib3 Vaccine Coverage (proportion)	X			X		
Meningitis	Male	Hib3 Vaccine Coverage (proportion)	X			X		
Meningitis	Female	meningitis belt (proportion)	X			X		
Meningitis	Male	meningitis belt (proportion)	X			X		
Meningitis	Female	Proportion of total population covered by menafrivac initiative (meningitis meningococcal type A vaccine)	X			X		
Meningitis	Male	Healthcare access and quality index		X			X	
Meningitis	Female	Proportion of total population covered by menafrivac initiative (meningitis meningococcal type A vaccine)	X			X		
Meningitis	Male	Proportion of total population covered by menafrivac initiative (meningitis meningococcal type A vaccine)	X			X		
Meningitis	Male	Proportion of total population covered by menafrivac initiative (meningitis meningococcal type A vaccine)	X			X		
Meningitis	Male	Maternal Education (years per capita)			X			X
Meningitis	Female	Maternal Education (years per capita)			X			X
Meningitis	Male	Maternal care and immunization		X			X	
Meningitis	Female	LDI (I$ per capita)			X			X
Meningitis	Male	LDI (I$ per capita)			X			X
Meningitis	Female	Socio-demographic Index			X			X
Meningitis	Male	Socio-demographic Index			X			X
Encephalitis	Female	Socio-demographic Index			X			X
Encephalitis	Female	Japanese encelphalitis endemic area (binary)	X			X		
Encephalitis	Male	Japanese encelphalitis endemic area (binary)	X			X		
Encephalitis	Female	DTP3 Coverage (proportion)			X			X
Encephalitis	Male	DTP3 Coverage (proportion)			X			X
Encephalitis	Male	Improved Water Source (proportion with access)			X			X
Encephalitis	Female	Sanitation (proportion with access)			X			X
Encephalitis	Female	Improved Water Source (proportion with access)			X			X
Encephalitis	Female	Maternal care and immunization		X			X	
Encephalitis	Male	Socio-demographic Index			X			X
Encephalitis	Female	LDI (I$ per capita)		X			X	
Encephalitis	Male	Sanitation (proportion with access)			X			X
Encephalitis	Female	Healthcare access and quality index		X			X	
Encephalitis	Male	In-Facility Delivery (proportion)			X			X
Encephalitis	Male	LDI (I$ per capita)		X			X	
Encephalitis	Male	Maternal Education (years per capita)			X			X
Encephalitis	Female	Maternal Education (years per capita)			X			X
Encephalitis	Male	Maternal care and immunization		X			X	
Encephalitis	Female	In-Facility Delivery (proportion)			X			X
Encephalitis	Male	Healthcare access and quality index		X			X	
Tetanus	Female	Socio-demographic Index			X			X
Tetanus	Male	Education (years per capita)			X			X
Tetanus	Female	Education (years per capita)			X			X
Tetanus	Male	LDI (I$ per capita)			X			X
Tetanus	Female	LDI (I$ per capita)			X			X
Tetanus	Male	Socio-demographic Index			X			X
Tetanus	Male	Healthcare access and quality index		X			X	
Tetanus	Male	In-Facility Delivery (proportion)		X			X	
Tetanus	Female	Healthcare access and quality index		X			X	
Tetanus	Female	Tetanus Toxoid Coverage Smooth (proportion)	X			X		
Tetanus	Male	Tetanus Toxoid Coverage Smooth (proportion)	X			X		
Tetanus	Female	DTP3 Coverage (proportion)	X			X		
Tetanus	Male	DTP3 Coverage (proportion)	X			X		
Tetanus	Female	DTP3 Coverage (proportion)	X			X		
Tetanus	Female	Sanitation (proportion with access)			X			X
Tetanus	Male	Sanitation (proportion with access)			X			X
Tetanus	Female	Skilled Birth Attendance (proportion)		X			X	
Tetanus	Male	Skilled Birth Attendance (proportion)		X			X	
Tetanus	Female	In-Facility Delivery (proportion)		X			X	
Tetanus	Male	DTP3 Coverage (proportion)	X			X		
Dengue	Male	Education (years per capita)			X			X
Dengue	Female	Education (years per capita)			X			X
Dengue	Male	LDI (I$ per capita)			X			X
Dengue	Female	LDI (I$ per capita)			X			X
Dengue	Male	Population Density (over 1000 ppl/sqkm, proportion)	X			X		

Table S18. Comparison of GBD 2017 and GBD 2019 covariates and level of covariates used in cause of death modeling

Cause	Sex	Covariate	Level 1: GBD 2019	Level 2: GBD 2019	Level 3: GBD 2019	Level 1: GBD 2017	Level 2: GBD 2017	Level 3: GBD 2017
Dengue	Female	Population Density (over 1000 ppl/sqkm, proportion)	X			X		
Dengue	Male	Latitude Under 15 (proportion)		X			X	
Dengue	Female	Latitude Under 15 (proportion)		X			X	
Dengue	Female	Rainfall Quintile 5 (proportion)		X			X	
Dengue	Male	Rainfall Quintile 4 (proportion)		X			X	
Dengue	Female	Rainfall Quintile 4 (proportion)		X			X	
Dengue	Male	Elevation Under 100m (proportion)		X			X	
Dengue	Male	Population weighted probability of dengue transmission	X			X		
Dengue	Female	Population weighted probability of dengue transmission	X			X		
Dengue	Male	Dengue outbreaks (binary)		X			X	
Dengue	Female	Dengue outbreaks (binary)		X			X	
Dengue	Male	Rainfall Quintile 5 (proportion)		X			X	
Dengue	Female	Elevation Under 100m (proportion)		X			X	
Rabies	Male	Population Density (500-1000 ppl/sqkm, proportion)			X			X
Rabies	Female	Population Density (500-1000 ppl/sqkm, proportion)			X			X
Rabies	Male	Antenatal Care (4 visits) Coverage (proportion)	X			X		
Rabies	Female	Population Density (under 150 ppl/sqkm, proportion)			X			X
Rabies	Male	Population Density (under 150 ppl/sqkm, proportion)			X			X
Rabies	Female	Maternal care and immunization		X			X	
Rabies	Male	Maternal care and immunization		X			X	
Rabies	Female	Skilled Birth Attendance (proportion)		X			X	
Rabies	Male	Skilled Birth Attendance (proportion)		X			X	
Rabies	Female	In-Facility Delivery (proportion)	X			X		
Rabies	Male	In-Facility Delivery (proportion)	X			X		
Rabies	Female	Antenatal Care (4 visits) Coverage (proportion)	X			X		
Rabies	Male	Socio-demographic Index			X			X
Rabies	Female	Socio-demographic Index			X			X
Rabies	Male	Healthcare access and quality index		X			X	
Rabies	Female	Healthcare access and quality index		X			X	
Other neglected tropical diseases	Female	Socio-demographic Index			X			X
Other neglected tropical diseases	Male	Healthcare access and quality index	X			X		
Other neglected tropical diseases	Female	Sanitation (proportion with access)		X			X	
Other neglected tropical diseases	Male	Sanitation (proportion with access)		X			X	
Other neglected tropical diseases	Male	Socio-demographic Index			X			X
Other neglected tropical diseases	Female	Healthcare access and quality index	X			X		
Other neglected tropical diseases	Female	LDI (I$ per capita)			X			X
Other neglected tropical diseases	Male	Rainfall Quintile 5 (proportion)		X			X	
Other neglected tropical diseases	Female	Education (years per capita)			X			X
Other neglected tropical diseases	Male	Education (years per capita)			X			X
Other neglected tropical diseases	Female	Rainfall Quintile 5 (proportion)		X			X	
Other neglected tropical diseases	Male	Latitude Under 15 (proportion)	X			X		
Other neglected tropical diseases	Male	LDI (I$ per capita)			X			X
Other neglected tropical diseases	Female	Latitude Under 15 (proportion)	X			X		
Neonatal disorders	Male	Maternal care and immunization		X		X		
Neonatal disorders	Female	Skilled Birth Attendance (proportion)		X				X
Neonatal disorders	Male	Skilled Birth Attendance (proportion)		X				X
Neonatal disorders	Female	Maternal care and immunization		X		X		
Neonatal disorders	Male	Total Fertility Rate			X			X
Neonatal disorders	Female	Total Fertility Rate			X			X
Neonatal disorders	Male	Healthcare access and quality index		X			X	
Neonatal disorders	Female	Live Births 35+ (proportion)		X			X	
Neonatal disorders	Male	Live Births 35+ (proportion)		X			X	
Neonatal disorders	Female	Healthcare access and quality index		X			X	
Neonatal disorders	Male	In-Facility Delivery (proportion)		X				X
Neonatal disorders	Female	Socio-demographic Index			X		X	
Neonatal disorders	Male	Socio-demographic Index			X		X	
Neonatal disorders	Female	In-Facility Delivery (proportion)		X				X
Neonatal disorders	Male	LDI (I$ per capita)			X			X
Neonatal disorders	Male	Antenatal Care (4 visits) Coverage (proportion)		X				X
Neonatal disorders	Female	LDI (I$ per capita)			X			X
Neonatal disorders	Female	Antenatal Care (4 visits) Coverage (proportion)		X				X
Neonatal preterm birth	Female	LDI (I$ per capita)			X			X
Neonatal preterm birth	Female	Maternal care and immunization	X			X		
Neonatal preterm birth	Male	Maternal care and immunization	X			X		
Neonatal preterm birth	Male	Antenatal Care (4 visits) Coverage (proportion)		X				X
Neonatal preterm birth	Female	Skilled Birth Attendance (proportion)		X				X
Neonatal preterm birth	Male	Skilled Birth Attendance (proportion)		X				X
Neonatal preterm birth	Female	Live Births 35+ (proportion)		X			X	
Neonatal preterm birth	Male	Live Births 35+ (proportion)		X			X	
Neonatal preterm birth	Female	In-Facility Delivery (proportion)		X				X
Neonatal preterm birth	Male	In-Facility Delivery (proportion)		X				X
Neonatal preterm birth	Female	Healthcare access and quality index		X			X	
Neonatal preterm birth	Male	Healthcare access and quality index		X			X	
Neonatal preterm birth	Male	Total Fertility Rate			X			X
Neonatal preterm birth	Male	LDI (I$ per capita)			X			X
Neonatal preterm birth	Female	Antenatal Care (4 visits) Coverage (proportion)		X				X

Table S18. Comparison of GBD 2017 and GBD 2019 covariates and level of covariates used in cause of death modeling								
Cause	Sex	Covariate	Level 1: GBD 2019	Level 2: GBD 2019	Level 3: GBD 2019	Level 1: GBD 2017	Level 2: GBD 2017	Level 3: GBD 2017
Neonatal preterm birth	Male	Socio-demographic Index			X		X	
Neonatal preterm birth	Female	Total Fertility Rate			X			X
Neonatal preterm birth	Female	Socio-demographic Index			X		X	
Neonatal encephalopathy due to birth asphyxia and trauma	Male	Socio-demographic Index			X		X	
Neonatal encephalopathy due to birth asphyxia and trauma	Male	Skilled Birth Attendance (proportion)		X				X
Neonatal encephalopathy due to birth asphyxia and trauma	Female	In-Facility Delivery (proportion)		X				X
Neonatal encephalopathy due to birth asphyxia and trauma	Male	In-Facility Delivery (proportion)		X				X
Neonatal encephalopathy due to birth asphyxia and trauma	Female	Antenatal Care (4 visits) Coverage (proportion)		X				X
Neonatal encephalopathy due to birth asphyxia and trauma	Male	Antenatal Care (4 visits) Coverage (proportion)		X				X
Neonatal encephalopathy due to birth asphyxia and trauma	Female	Healthcare access and quality index		X			X	
Neonatal encephalopathy due to birth asphyxia and trauma	Male	Healthcare access and quality index		X			X	
Neonatal encephalopathy due to birth asphyxia and trauma	Female	Live Births 35+ (proportion)		X			X	
Neonatal encephalopathy due to birth asphyxia and trauma	Female	Socio-demographic Index			X		X	
Neonatal encephalopathy due to birth asphyxia and trauma	Female	LDI (I$ per capita)			X			X
Neonatal encephalopathy due to birth asphyxia and trauma	Male	LDI (I$ per capita)			X			X
Neonatal encephalopathy due to birth asphyxia and trauma	Female	Skilled Birth Attendance (proportion)		X				X
Neonatal encephalopathy due to birth asphyxia and trauma	Male	Maternal care and immunization		X		X		
Neonatal encephalopathy due to birth asphyxia and trauma	Female	Maternal care and immunization		X		X		
Neonatal encephalopathy due to birth asphyxia and trauma	Male	Total Fertility Rate			X			X
Neonatal encephalopathy due to birth asphyxia and trauma	Female	Total Fertility Rate			X			X
Neonatal encephalopathy due to birth asphyxia and trauma	Male	Live Births 35+ (proportion)		X			X	
Neonatal sepsis and other neonatal infections	Male	Antenatal Care (4 visits) Coverage (proportion)		X				X
Neonatal sepsis and other neonatal infections	Female	Healthcare access and quality index		X			X	
Neonatal sepsis and other neonatal infections	Male	Total Fertility Rate			X			X
Neonatal sepsis and other neonatal infections	Female	Total Fertility Rate			X			X
Neonatal sepsis and other neonatal infections	Male	Healthcare access and quality index		X			X	
Neonatal sepsis and other neonatal infections	Female	Socio-demographic Index			X		X	
Neonatal sepsis and other neonatal infections	Male	Socio-demographic Index			X		X	
Neonatal sepsis and other neonatal infections	Female	LDI (I$ per capita)			X			X
Neonatal sepsis and other neonatal infections	Male	LDI (I$ per capita)			X			X
Neonatal sepsis and other neonatal infections	Female	Antenatal Care (4 visits) Coverage (proportion)		X				X
Neonatal sepsis and other neonatal infections	Female	Live Births 35+ (proportion)		X			X	
Neonatal sepsis and other neonatal infections	Female	In-Facility Delivery (proportion)		X				X
Neonatal sepsis and other neonatal infections	Male	Live Births 35+ (proportion)		X			X	
Neonatal sepsis and other neonatal infections	Male	Skilled Birth Attendance (proportion)		X				X
Neonatal sepsis and other neonatal infections	Female	Skilled Birth Attendance (proportion)		X				X
Neonatal sepsis and other neonatal infections	Male	Maternal care and immunization		X		X		
Neonatal sepsis and other neonatal infections	Female	Maternal care and immunization		X		X		
Neonatal sepsis and other neonatal infections	Male	In-Facility Delivery (proportion)		X				X
Hemolytic disease and other neonatal jaundice	Female	Total Fertility Rate			X			X
Hemolytic disease and other neonatal jaundice	Male	Total Fertility Rate			X			X
Hemolytic disease and other neonatal jaundice	Female	Maternal care and immunization		X		X		
Hemolytic disease and other neonatal jaundice	Female	Skilled Birth Attendance (proportion)		X				X
Hemolytic disease and other neonatal jaundice	Male	Skilled Birth Attendance (proportion)		X				X
Hemolytic disease and other neonatal jaundice	Female	Live Births 35+ (proportion)		X			X	
Hemolytic disease and other neonatal jaundice	Male	Live Births 35+ (proportion)		X			X	
Hemolytic disease and other neonatal jaundice	Male	Antenatal Care (4 visits) Coverage (proportion)		X				X
Hemolytic disease and other neonatal jaundice	Male	In-Facility Delivery (proportion)		X				X
Hemolytic disease and other neonatal jaundice	Female	Antenatal Care (4 visits) Coverage (proportion)		X				X
Hemolytic disease and other neonatal jaundice	Female	Healthcare access and quality index		X			X	
Hemolytic disease and other neonatal jaundice	Male	Healthcare access and quality index		X			X	
Hemolytic disease and other neonatal jaundice	Female	Socio-demographic Index			X		X	
Hemolytic disease and other neonatal jaundice	Female	LDI (I$ per capita)			X			X
Hemolytic disease and other neonatal jaundice	Male	LDI (I$ per capita)			X			X
Hemolytic disease and other neonatal jaundice	Female	In-Facility Delivery (proportion)		X				X
Hemolytic disease and other neonatal jaundice	Male	Socio-demographic Index			X		X	
Hemolytic disease and other neonatal jaundice	Male	Maternal care and immunization		X		X		
Other neonatal disorders	Female	In-Facility Delivery (proportion)		X				X
Other neonatal disorders	Male	LDI (I$ per capita)			X			X
Other neonatal disorders	Female	LDI (I$ per capita)			X			X
Other neonatal disorders	Male	Socio-demographic Index			X		X	
Other neonatal disorders	Female	Socio-demographic Index			X		X	
Other neonatal disorders	Male	Healthcare access and quality index		X			X	
Other neonatal disorders	Female	Healthcare access and quality index		X			X	
Other neonatal disorders	Male	Total Fertility Rate			X			X
Other neonatal disorders	Male	Antenatal Care (4 visits) Coverage (proportion)		X				X
Other neonatal disorders	Female	Maternal care and immunization		X		X		
Other neonatal disorders	Male	Maternal care and immunization		X		X		
Other neonatal disorders	Female	Skilled Birth Attendance (proportion)		X				X
Other neonatal disorders	Male	Skilled Birth Attendance (proportion)		X				X
Other neonatal disorders	Female	Live Births 35+ (proportion)		X			X	
Other neonatal disorders	Male	Live Births 35+ (proportion)		X			X	
Other neonatal disorders	Female	Antenatal Care (4 visits) Coverage (proportion)		X				X
Other neonatal disorders	Female	Total Fertility Rate			X			X
Other neonatal disorders	Male	In-Facility Delivery (proportion)		X				X
Nutritional deficiencies	Male	Mortality Rate Due to War Shocks (per 1 person)		X			X	
Nutritional deficiencies	Male	Proportion of households using iodized salt (adjusted)	X			X		

Table S18. Comparison of GBD 2017 and GBD 2019 covariates and level of covariates used in cause of death modeling								
Cause	Sex	Covariate	Level 1: GBD 2019	Level 2: GBD 2019	Level 3: GBD 2019	Level 1: GBD 2017	Level 2: GBD 2017	Level 3: GBD 2017
Nutritional deficiencies	Male	Socio-demographic Index			X			X
Nutritional deficiencies	Female	Socio-demographic Index			X			X
Nutritional deficiencies	Male	LDI (I$ per capita)			X			X
Nutritional deficiencies	Female	LDI (I$ per capita)			X			X
Nutritional deficiencies	Male	Education (years per capita)			X			X
Nutritional deficiencies	Male	Education (years per capita)			X			X
Nutritional deficiencies	Female	Education (years per capita)			X			X
Nutritional deficiencies	Female	Mortality Rate Due to War Shocks (per 1 person)		X			X	
Nutritional deficiencies	Female	Proportion of households using iodized salt (adjusted)	X			X		
Nutritional deficiencies	Male	Rainfall Quintile 1 (proportion)		X			X	
Nutritional deficiencies	Male	Healthcare access and quality index		X			X	
Nutritional deficiencies	Female	Rainfall Quintile 1 (proportion)		X			X	
Nutritional deficiencies	Female	Rainfall Quintile 2 (proportion)		X			X	
Nutritional deficiencies	Male	Age- and sex-specific SEV for Unsafe sanitation		X			X	
Nutritional deficiencies	Female	Age- and sex-specific SEV for Unsafe sanitation		X			X	
Nutritional deficiencies	Male	Age- and sex-specific SEV for Unsafe water		X			X	
Nutritional deficiencies	Female	Age- and sex-specific SEV for Unsafe water		X			X	
Nutritional deficiencies	Male	Log-transformed SEV scalar: Diarrhea		X			X	
Nutritional deficiencies	Female	Log-transformed SEV scalar: Diarrhea		X			X	
Nutritional deficiencies	Male	Age- and sex-specific SEV for Alcohol use		X			X	
Nutritional deficiencies	Female	Age- and sex-specific SEV for Alcohol use		X			X	
Nutritional deficiencies	Male	Age-standardized SEV for Child underweight	X			X		
Nutritional deficiencies	Female	Age-standardized SEV for Child underweight	X			X		
Nutritional deficiencies	Male	Rainfall Quintile 2 (proportion)		X			X	
Nutritional deficiencies	Female	energy unadjusted(kcal)	X			X		
Nutritional deficiencies	Female	Healthcare access and quality index		X			X	
Nutritional deficiencies	Male	Maternal Education (years per capita)			X			X
Nutritional deficiencies	Male	energy unadjusted(kcal)	X			X		
Nutritional deficiencies	Male	Age-standardized SEV for Child wasting	X			X		
Nutritional deficiencies	Female	Age-standardized SEV for Child wasting	X			X		
Nutritional deficiencies	Female	Age-Standardize Prevalence of Severe Anemia	X			X		
Nutritional deficiencies	Male	Improved Water Source (proportion with access)		X			X	
Nutritional deficiencies	Male	Age-Standardize Prevalence of Severe Anemia	X			X		
Nutritional deficiencies	Male	Maternal care and immunization		X			X	
Nutritional deficiencies	Female	Maternal care and immunization		X			X	
Nutritional deficiencies	Female	Maternal Education (years per capita)			X			X
Nutritional deficiencies	Male	Maternal Education (years per capita)			X			X
Nutritional deficiencies	Male	Sanitation (proportion with access)		X			X	
Protein-energy malnutrition	Male	Healthcare access and quality index		X			X	
Protein-energy malnutrition	Female	Age-standardized SEV for Child wasting	X			X		
Protein-energy malnutrition	Male	Malnutrition Shock mortality rate	X			X		
Protein-energy malnutrition	Female	Malnutrition Shock mortality rate	X			X		
Protein-energy malnutrition	Female	Healthcare access and quality index		X			X	
Protein-energy malnutrition	Female	Socio-demographic Index			X			X
Protein-energy malnutrition	Female	Education (years per capita)			X			X
Protein-energy malnutrition	Female	LDI (I$ per capita)			X			X
Protein-energy malnutrition	Male	LDI (I$ per capita)			X			X
Protein-energy malnutrition	Female	Education (years per capita)			X			X
Protein-energy malnutrition	Male	energy unadjusted(kcal)	X			X		
Protein-energy malnutrition	Male	Education (years per capita)			X			X
Protein-energy malnutrition	Male	Education (years per capita)			X			X
Protein-energy malnutrition	Male	Socio-demographic Index			X			X
Protein-energy malnutrition	Female	Age-Standardize Prevalence of Severe Anemia	X			X		
Protein-energy malnutrition	Female	Rainfall Quintile 1 (proportion)		X			X	
Protein-energy malnutrition	Female	Mortality Rate Due to War Shocks (per 1 person)		X			X	
Protein-energy malnutrition	Male	Maternal Education (years per capita)			X			X
Protein-energy malnutrition	Female	Antenatal Care (4 visits) Coverage (proportion)			X			X
Protein-energy malnutrition	Male	Maternal care and immunization		X			X	
Protein-energy malnutrition	Female	energy unadjusted(kcal)	X			X		
Protein-energy malnutrition	Male	Age- and sex-specific SEV for Alcohol use		X			X	
Protein-energy malnutrition	Female	Age- and sex-specific SEV for Alcohol use		X			X	
Protein-energy malnutrition	Male	Log-transformed SEV scalar: Diarrhea		X			X	
Protein-energy malnutrition	Female	Log-transformed SEV scalar: Diarrhea		X			X	
Protein-energy malnutrition	Male	Age-Standardize Prevalence of Severe Anemia	X			X		
Protein-energy malnutrition	Male	Age- and sex-specific SEV for Unsafe water		X			X	
Protein-energy malnutrition	Male	Age- and sex-specific SEV for Unsafe sanitation		X			X	
Protein-energy malnutrition	Female	Age- and sex-specific SEV for Unsafe sanitation		X			X	
Protein-energy malnutrition	Male	Age- and sex-specific SEV for Child wasting	X			X		
Protein-energy malnutrition	Female	Age- and sex-specific SEV for Child wasting	X			X		
Protein-energy malnutrition	Male	Rainfall Quintile 2 (proportion)		X			X	
Protein-energy malnutrition	Female	Rainfall Quintile 2 (proportion)		X			X	
Protein-energy malnutrition	Male	Rainfall Quintile 1 (proportion)		X			X	

Table S18. Comparison of GBD 2017 and GBD 2019 covariates and level of covariates used in cause of death modeling

Cause	Sex	Covariate	Level 1: GBD 2019	Level 2: GBD 2019	Level 3: GBD 2019	Level 1: GBD 2017	Level 2: GBD 2017	Level 3: GBD 2017
Protein-energy malnutrition	Male	Mortality Rate Due to War Shocks (per 1 person)		X			X	
Protein-energy malnutrition	Female	Age- and sex-specific SEV for Unsafe water		X			X	
Protein-energy malnutrition	Female	Maternal care and immunization		X			X	
Other nutritional deficiencies	Male	Healthcare access and quality index		X			X	
Other nutritional deficiencies	Male	Log-transformed SEV scalar: Diarrhea		X			X	
Other nutritional deficiencies	Male	Age- and sex-specific SEV for Unsafe sanitation		X			X	
Other nutritional deficiencies	Female	Age- and sex-specific SEV for Unsafe water		X			X	
Other nutritional deficiencies	Male	Age- and sex-specific SEV for Unsafe water		X			X	
Other nutritional deficiencies	Female	Log-transformed SEV scalar: Diarrhea		X			X	
Other nutritional deficiencies	Female	Education (years per capita)			X			X
Other nutritional deficiencies	Female	Age- and sex-specific SEV for Alcohol use		X			X	
Other nutritional deficiencies	Male	Age- and sex-specific SEV for Alcohol use		X			X	
Other nutritional deficiencies	Female	Age- and sex-specific SEV for Unsafe sanitation		X			X	
Other nutritional deficiencies	Female	Age-standardized SEV for Child underweight	X			X		
Other nutritional deficiencies	Female	Maternal care and immunization		X			X	
Other nutritional deficiencies	Male	Maternal care and immunization		X			X	
Other nutritional deficiencies	Female	energy unadjusted(kcal)		X			X	
Other nutritional deficiencies	Male	energy unadjusted(kcal)		X			X	
Other nutritional deficiencies	Female	Healthcare access and quality index		X			X	
Other nutritional deficiencies	Male	LDI (I$ per capita)			X			X
Other nutritional deficiencies	Female	Socio-demographic Index			X			X
Other nutritional deficiencies	Male	Age-standardized SEV for Child underweight	X			X		
Other nutritional deficiencies	Male	Socio-demographic Index			X			X
Other nutritional deficiencies	Male	Rainfall Quintile 2 (proportion)		X			X	
Other nutritional deficiencies	Male	Rainfall Quintile 1 (proportion)		X			X	
Other nutritional deficiencies	Male	Education (years per capita)			X			X
Other nutritional deficiencies	Female	Rainfall Quintile 2 (proportion)		X			X	
Other nutritional deficiencies	Female	Education (years per capita)			X			X
Other nutritional deficiencies	Female	Malnutrition Shock mortality rate	X			X		
Other nutritional deficiencies	Male	Malnutrition Shock mortality rate	X			X		
Other nutritional deficiencies	Male	Education (years per capita)			X			X
Other nutritional deficiencies	Male	Age-Standardize Prevalence of Severe Anemia	X			X		
Other nutritional deficiencies	Female	Mortality Rate Due to War Shocks (per 1 person)		X			X	
Other nutritional deficiencies	Male	Mortality Rate Due to War Shocks (per 1 person)		X			X	
Other nutritional deficiencies	Female	Rainfall Quintile 1 (proportion)		X			X	
Other nutritional deficiencies	Female	Age-Standardize Prevalence of Severe Anemia	X			X		
Other nutritional deficiencies	Female	LDI (I$ per capita)			X			X
Sexually transmitted infections excluding HIV	Male	Maternal care and immunization		X			X	
Sexually transmitted infections excluding HIV	Female	Maternal care and immunization		X			X	
Sexually transmitted infections excluding HIV	Male	Age-Specific Fertility Rate		X			X	
Sexually transmitted infections excluding HIV	Female	Age-Specific Fertility Rate		X			X	
Sexually transmitted infections excluding HIV	Male	Total Fertility Rate		X			X	
Sexually transmitted infections excluding HIV	Female	Total Fertility Rate		X			X	
Sexually transmitted infections excluding HIV	Male	Syphilis prevalence (proportion)	X			X		
Sexually transmitted infections excluding HIV	Female	Syphilis prevalence (proportion)	X			X		
Sexually transmitted infections excluding HIV	Male	Antenatal Care (4 visits) Coverage (proportion)			X			X
Sexually transmitted infections excluding HIV	Female	Antenatal Care (1 visit) Coverage (proportion)			X			X
Sexually transmitted infections excluding HIV	Male	Antenatal Care (1 visit) Coverage (proportion)			X			X
Sexually transmitted infections excluding HIV	Female	Legality of Abortion		X			X	
Sexually transmitted infections excluding HIV	Male	Legality of Abortion		X			X	
Sexually transmitted infections excluding HIV	Female	Healthcare access and quality index		X			X	
Sexually transmitted infections excluding HIV	Male	Healthcare access and quality index		X			X	
Sexually transmitted infections excluding HIV	Female	LDI (I$ per capita)			X			X
Sexually transmitted infections excluding HIV	Male	LDI (I$ per capita)			X			X
Sexually transmitted infections excluding HIV	Female	Antenatal Care (4 visits) Coverage (proportion)			X			X
Sexually transmitted infections excluding HIV	Male	Education (years per capita)		X			X	
Sexually transmitted infections excluding HIV	Female	Education (years per capita)		X			X	
Acute hepatitis	Female	Socio-demographic Index		X			X	
Acute hepatitis	Female	Age- and sex-specific SEV for Unsafe water		X			X	
Acute hepatitis	Male	Age- and sex-specific SEV for Unsafe water		X			X	
Acute hepatitis	Female	Healthcare access and quality index		X			X	
Acute hepatitis	Male	Healthcare access and quality index		X			X	
Acute hepatitis	Male	Log-transformed SEV scalar: Hep		X		X		
Acute hepatitis	Male	Socio-demographic Index		X			X	
Acute hepatitis	Male	Socio-demographic Index			X		X	
Acute hepatitis	Female	LDI (I$ per capita)			X			X
Acute hepatitis	Male	LDI (I$ per capita)			X			X
Acute hepatitis	Female	Education (years per capita)			X			X
Acute hepatitis	Female	Education (years per capita)			X			X
Acute hepatitis	Male	Education (years per capita)			X			X
Acute hepatitis	Male	Log-transformed SEV scalar: Hep	X			X		
Acute hepatitis	Female	Log-transformed SEV scalar: Hep	X			X		

Table S18. Comparison of GBD 2017 and GBD 2019 covariates and level of covariates used in cause of death modeling								
**Cause**	**Sex**	**Covariate**	**Level 1: GBD 2019**	**Level 2: GBD 2019**	**Level 3: GBD 2019**	**Level 1: GBD 2017**	**Level 2: GBD 2017**	**Level 3: GBD 2017**
Acute hepatitis	Male	Hepatitis B vaccine coverage (proportion), aged through time		X			X	
Acute hepatitis	Male	Hepatitis B vaccine coverage (proportion), aged through time		X			X	
Acute hepatitis	Female	Hepatitis B vaccine coverage (proportion), aged through time		X			X	
Acute hepatitis	Female	Hepatitis B vaccine coverage (proportion), aged through time		X			X	
Acute hepatitis	Male	Age- and sex-specific SEV for Unsafe sanitation		X			X	
Acute hepatitis	Female	Age- and sex-specific SEV for Unsafe sanitation		X			X	
Other unspecified infectious diseases	Male	DTP3 Coverage (proportion)			X	X		
Other unspecified infectious diseases	Male	DTP3 Coverage (proportion)	X			X		
Other unspecified infectious diseases	Female	DTP3 Coverage (proportion)			X	X		
Other unspecified infectious diseases	Male	Antenatal Care (1 visit) Coverage (proportion)		X				X
Other unspecified infectious diseases	Female	Improved Water Source (proportion with access)		X			X	
Other unspecified infectious diseases	Male	Improved Water Source (proportion with access)		X			X	
Other unspecified infectious diseases	Female	Sanitation (proportion with access)		X			X	
Other unspecified infectious diseases	Male	Sanitation (proportion with access)		X			X	
Other unspecified infectious diseases	Female	Antenatal Care (1 visit) Coverage (proportion)		X				X
Other unspecified infectious diseases	Female	Antenatal Care (1 visit) Coverage (proportion)	X					X
Other unspecified infectious diseases	Male	Socio-demographic Index			X			X
Other unspecified infectious diseases	Female	Socio-demographic Index			X			X
Other unspecified infectious diseases	Female	Healthcare access and quality index		X			X	
Other unspecified infectious diseases	Female	DTP3 Coverage (proportion)	X			X		
Other unspecified infectious diseases	Male	Antenatal Care (1 visit) Coverage (proportion)	X					X
Oesophageal cancer	Female	Log-transformed age-standardized SEV scalar: Esophag C	X			X		
Oesophageal cancer	Male	Socio-demographic Index			X			X
Oesophageal cancer	Male	Healthcare access and quality index		X			X	
Oesophageal cancer	Female	Socio-demographic Index			X			X
Oesophageal cancer	Female	Indoor Air Pollution (All Cooking Fuels)		X			X	
Oesophageal cancer	Female	Mean BMI	X			X		
Oesophageal cancer	Male	Mean BMI	X			X		
Oesophageal cancer	Female	LDI (I$ per capita)			X			X
Oesophageal cancer	Male	LDI (I$ per capita)			X			X
Oesophageal cancer	Female	Education (years per capita)			X			X
Oesophageal cancer	Male	Education (years per capita)			X			X
Oesophageal cancer	Female	Healthcare access and quality index		X			X	
Oesophageal cancer	Male	Smoking Prevalence	X			X		
Oesophageal cancer	Male	Sanitation (proportion with access)		X				X
Oesophageal cancer	Female	Sanitation (proportion with access)		X				X
Oesophageal cancer	Male	Improved Water Source (proportion with access)		X				X
Oesophageal cancer	Female	Improved Water Source (proportion with access)		X				X
Oesophageal cancer	Female	Tobacco (cigarettes per capita)	X				X	
Oesophageal cancer	Female	Tobacco (cigarettes per capita)	X				X	
Oesophageal cancer	Male	Tobacco (cigarettes per capita)	X				X	
Oesophageal cancer	Male	Tobacco (cigarettes per capita)	X				X	
Oesophageal cancer	Male	Log-transformed age-standardized SEV scalar: Esophag C	X			X		
Oesophageal cancer	Female	Smoking Prevalence	X			X		
Oesophageal cancer	Male	Indoor Air Pollution (All Cooking Fuels)		X			X	
Stomach cancer	Male	Mean BMI		X			X	
Stomach cancer	Female	Age- and sex-specific SEV for Unsafe sanitation	X				X	
Stomach cancer	Male	Age- and sex-specific SEV for Unsafe sanitation	X				X	
Stomach cancer	Female	Age- and sex-specific SEV for Unsafe water	X				X	
Stomach cancer	Male	Age- and sex-specific SEV for Unsafe water	X				X	
Stomach cancer	Female	Improved Water Source (proportion with access)		X			X	
Stomach cancer	Male	Improved Water Source (proportion with access)		X			X	
Stomach cancer	Female	Sanitation (proportion with access)		X			X	
Stomach cancer	Male	Sanitation (proportion with access)		X			X	
Stomach cancer	Female	Healthcare access and quality index		X			X	
Stomach cancer	Male	Healthcare access and quality index		X			X	
Stomach cancer	Female	Socio-demographic Index			X			X
Stomach cancer	Male	Socio-demographic Index			X			X
Stomach cancer	Female	Mean BMI		X			X	
Stomach cancer	Male	Tobacco (cigarettes per capita)	X			X		
Stomach cancer	Male	LDI (I$ per capita)			X			X
Stomach cancer	Male	Tobacco (cigarettes per capita)	X			X		
Stomach cancer	Female	LDI (I$ per capita)			X			X
Stomach cancer	Female	Diet high in sodium	X			X		
Stomach cancer	Male	Diet high in sodium	X			X		
Stomach cancer	Male	Log-transformed SEV scalar: Stomach C	X			X		
Stomach cancer	Female	Log-transformed SEV scalar: Stomach C	X			X		
Stomach cancer	Male	Cumulative Cigarettes (20 Years)	X				X	
Stomach cancer	Female	Tobacco (cigarettes per capita)	X			X		
Stomach cancer	Female	Tobacco (cigarettes per capita)	X			X		
Stomach cancer	Male	Education (years per capita)			X			X

Table S18. Comparison of GBD 2017 and GBD 2019 covariates and level of covariates used in cause of death modeling								
Cause	Sex	Covariate	Level 1: GBD 2019	Level 2: GBD 2019	Level 3: GBD 2019	Level 1: GBD 2017	Level 2: GBD 2017	Level 3: GBD 2017
Stomach cancer	Female	Education (years per capita)			X			X
Stomach cancer	Female	Cumulative Cigarettes (20 Years)	X				X	
Liver cancer	Female	Healthcare access and quality index		X			X	
Liver cancer	Female	Hepatitis B 3-dose coverage (proportion)		X			X	
Liver cancer	Male	Healthcare access and quality index		X			X	
Liver cancer	Male	Socio-demographic Index			X			X
Liver cancer	Male	Education (years per capita)			X			X
Liver cancer	Male	Mean BMI		X			X	
Liver cancer	Female	Mean BMI		X			X	
Liver cancer	Male	LDI (I$ per capita)			X			X
Liver cancer	Female	LDI (I$ per capita)			X			X
Liver cancer	Female	Socio-demographic Index			X			X
Liver cancer	Female	Hepatitis B 3-dose coverage (proportion)		X			X	
Liver cancer	Female	Diabetes Fasting Plasma Glucose (mmol/L), age-standardized 25+		X			X	
Liver cancer	Male	Hepatitis B 3-dose coverage (proportion)		X			X	
Liver cancer	Female	Log-transformed SEV scalar: Liver C	X			X		
Liver cancer	Male	HIV age-standardized prevalence	X			X		
Liver cancer	Male	HIV age-standardized prevalence	X			X		
Liver cancer	Female	Education (years per capita)			X			X
Liver cancer	Female	HIV age-standardized prevalence	X			X		
Liver cancer	Male	Cumulative Cigarettes (20 Years)		X			X	
Liver cancer	Female	Cumulative Cigarettes (20 Years)		X			X	
Liver cancer	Male	Intravenous drug use (age-standardized proportion)		X			X	
Liver cancer	Male	Hepatitis B 3-dose coverage (proportion)		X			X	
Liver cancer	Female	Intravenous drug use (age-standardized proportion)		X			X	
Liver cancer	Female	Tobacco (cigarettes per capita)		X			X	
Liver cancer	Male	Tobacco (cigarettes per capita)		X			X	
Liver cancer	Male	Tobacco (cigarettes per capita)		X			X	
Liver cancer	Male	Hepatitis B vaccine coverage (proportion), aged through time		X			X	
Liver cancer	Male	Hepatitis B vaccine coverage (proportion), aged through time		X			X	
Liver cancer	Female	Hepatitis B vaccine coverage (proportion), aged through time		X			X	
Liver cancer	Female	Hepatitis B vaccine coverage (proportion), aged through time		X			X	
Liver cancer	Male	Diabetes Fasting Plasma Glucose (mmol/L), age-standardized 25+		X			X	
Liver cancer	Female	Tobacco (cigarettes per capita)		X			X	
Liver cancer	Female	HIV age-standardized prevalence	X			X		
Liver cancer	Male	Log-transformed SEV scalar: Liver C	X			X		
Larynx cancer	Female	Population Density (over 1000 ppl/sqkm, proportion)		X			X	
Larynx cancer	Female	Asbestos consumption (metric tons per year per capita)		X			X	
Larynx cancer	Male	Asbestos consumption (metric tons per year per capita)		X			X	
Larynx cancer	Female	Log-transformed SEV scalar: Larynx C	X			X		
Larynx cancer	Male	Log-transformed SEV scalar: Larynx C	X			X		
Larynx cancer	Female	Cumulative Cigarettes (20 Years)		X			X	
Larynx cancer	Male	Cumulative Cigarettes (20 Years)		X			X	
Larynx cancer	Male	LDI (I$ per capita)			X			X
Larynx cancer	Male	Population Density (over 1000 ppl/sqkm, proportion)		X			X	
Larynx cancer	Female	Cumulative Cigarettes (10 Years)		X			X	
Larynx cancer	Male	Cumulative Cigarettes (10 Years)		X			X	
Larynx cancer	Female	Smoking Prevalence		X			X	
Larynx cancer	Male	Smoking Prevalence		X			X	
Larynx cancer	Female	Healthcare access and quality index		X			X	
Larynx cancer	Male	Healthcare access and quality index		X			X	
Larynx cancer	Female	Socio-demographic Index			X			X
Larynx cancer	Male	Socio-demographic Index			X			X
Larynx cancer	Female	LDI (I$ per capita)			X			X
Tracheal, bronchus, and lung cancer	Female	Log-transformed age-standardized SEV scalar: Lung C	X			X		
Tracheal, bronchus, and lung cancer	Male	Residential radon		X			X	
Tracheal, bronchus, and lung cancer	Female	Socio-demographic Index			X			X
Tracheal, bronchus, and lung cancer	Male	Socio-demographic Index			X			X
Tracheal, bronchus, and lung cancer	Female	Residential radon		X			X	
Tracheal, bronchus, and lung cancer	Female	Log-transformed SEV scalar: Lung C	X			X		
Tracheal, bronchus, and lung cancer	Male	Log-transformed age-standardized SEV scalar: Lung C	X			X		
Tracheal, bronchus, and lung cancer	Female	Asbestos consumption (metric tons per year per capita)	X			X		
Tracheal, bronchus, and lung cancer	Female	LDI (I$ per capita)			X			X
Tracheal, bronchus, and lung cancer	Male	LDI (I$ per capita)			X			X
Tracheal, bronchus, and lung cancer	Female	Education (years per capita)			X			X
Tracheal, bronchus, and lung cancer	Male	Education (years per capita)			X			X
Tracheal, bronchus, and lung cancer	Male	Healthcare access and quality index		X			X	
Tracheal, bronchus, and lung cancer	Female	Healthcare access and quality index		X			X	
Tracheal, bronchus, and lung cancer	Male	Diabetes Fasting Plasma Glucose (mmol/L), age-standardized 25+		X			X	
Tracheal, bronchus, and lung cancer	Female	Diabetes Fasting Plasma Glucose (mmol/L), age-standardized 25+		X			X	
Tracheal, bronchus, and lung cancer	Male	Indoor Air Pollution (All Cooking Fuels)		X			X	
Tracheal, bronchus, and lung cancer	Female	Indoor Air Pollution (All Cooking Fuels)		X			X	
Tracheal, bronchus, and lung cancer	Male	Outdoor Air Pollution (PM2.5)		X			X	

Table S18. Comparison of GBD 2017 and GBD 2019 covariates and level of covariates used in cause of death modeling								
Cause	Sex	Covariate	Level 1: GBD 2019	Level 2: GBD 2019	Level 3: GBD 2019	Level 1: GBD 2017	Level 2: GBD 2017	Level 3: GBD 2017
Tracheal, bronchus, and lung cancer	Male	Log-transformed SEV scalar: Lung C	X			X		
Tracheal, bronchus, and lung cancer	Female	Outdoor Air Pollution (PM2.5)		X			X	
Tracheal, bronchus, and lung cancer	Female	Smoking Prevalence	X			X		
Tracheal, bronchus, and lung cancer	Male	Cumulative Cigarettes (10 Years)	X				X	
Tracheal, bronchus, and lung cancer	Female	Cumulative Cigarettes (10 Years)	X				X	
Tracheal, bronchus, and lung cancer	Male	Secondhand smoke	X				X	
Tracheal, bronchus, and lung cancer	Female	Secondhand smoke	X				X	
Tracheal, bronchus, and lung cancer	Male	Cumulative Cigarettes (20 Years)	X				X	
Tracheal, bronchus, and lung cancer	Female	Cumulative Cigarettes (20 Years)	X				X	
Tracheal, bronchus, and lung cancer	Male	Asbestos consumption (metric tons per year per capita)	X			X		
Tracheal, bronchus, and lung cancer	Male	Smoking Prevalence	X			X		
Breast cancer	Female	Healthcare access and quality index		X			X	
Breast cancer	Male	Diabetes Fasting Plasma Glucose (mmol/L), age-standardized 25+		X			X	
Breast cancer	Male	Smoking Prevalence		X			X	
Breast cancer	Male	Healthcare access and quality index		X			X	
Breast cancer	Male	LDI (I$ per capita)			X			X
Breast cancer	Male	Socio-demographic Index			X			X
Breast cancer	Female	Mean BMI	X			X		
Breast cancer	Male	Mean BMI	X			X		
Breast cancer	Female	LDI (I$ per capita)			X			X
Breast cancer	Female	Smoking Prevalence		X			X	
Breast cancer	Female	Socio-demographic Index			X			X
Breast cancer	Male	Cumulative Cigarettes (10 Years)		X			X	
Breast cancer	Female	Diabetes Fasting Plasma Glucose (mmol/L), age-standardized 25+		X			X	
Breast cancer	Female	Age-Specific Fertility Rate		X			X	
Breast cancer	Female	Age-Specific Fertility Rate		X			X	
Breast cancer	Female	Total Fertility Rate		X			X	
Breast cancer	Female	Total Fertility Rate		X			X	
Breast cancer	Male	Cumulative Cigarettes (20 Years)		X			X	
Breast cancer	Female	Cumulative Cigarettes (20 Years)		X			X	
Breast cancer	Male	Log-transformed SEV scalar: Breast C	X			X		
Breast cancer	Female	Log-transformed SEV scalar: Breast C	X			X		
Breast cancer	Female	Cumulative Cigarettes (10 Years)		X			X	
Cervical cancer	Female	Smoking Prevalence		X			X	
Cervical cancer	Female	Age-Specific Fertility Rate		X			X	
Cervical cancer	Female	Age-Specific Fertility Rate		X			X	
Cervical cancer	Female	Cumulative Cigarettes (5 Years)	X			X		
Cervical cancer	Female	HIV age-standardized prevalence	X			X		
Cervical cancer	Female	Total Fertility Rate		X			X	
Cervical cancer	Female	HIV age-standardized prevalence	X			X		
Cervical cancer	Female	Healthcare access and quality index		X			X	
Cervical cancer	Female	Total Fertility Rate		X			X	
Cervical cancer	Female	Socio-demographic Index			X			X
Cervical cancer	Female	LDI (I$ per capita)			X			X
Cervical cancer	Female	Education (years per capita)			X			X
Uterine cancer	Female	Tobacco (cigarettes per capita)		X			X	
Uterine cancer	Female	Healthcare access and quality index		X			X	
Uterine cancer	Female	Tobacco (cigarettes per capita)		X			X	
Uterine cancer	Female	Log-transformed SEV scalar: Uterus C	X			X		
Uterine cancer	Female	Total Fertility Rate		X			X	
Uterine cancer	Female	Cumulative Cigarettes (10 Years)		X			X	
Uterine cancer	Female	Smoking Prevalence		X			X	
Uterine cancer	Female	Socio-demographic Index			X			X
Uterine cancer	Female	Mean BMI	X			X		
Uterine cancer	Female	LDI (I$ per capita)			X			X
Uterine cancer	Female	Education (years per capita)			X			X
Uterine cancer	Female	Diabetes Age-Standardized Prevalence (proportion)		X			X	
Uterine cancer	Female	Cumulative Cigarettes (5 Years)		X			X	
Uterine cancer	Female	Total Fertility Rate		X			X	
Prostate cancer	Male	Education (years per capita)			X			X
Prostate cancer	Male	LDI (I$ per capita)			X			X
Prostate cancer	Male	Socio-demographic Index			X			X
Prostate cancer	Male	Healthcare access and quality index		X			X	
Prostate cancer	Male	Smoking Prevalence		X			X	
Prostate cancer	Male	Log-transformed SEV scalar: Prostate C	X			X		
Colon and rectum cancer	Female	pufa adjusted(percent)		X			X	
Colon and rectum cancer	Male	pufa adjusted(percent)		X			X	
Colon and rectum cancer	Female	Cumulative Cigarettes (5 Years)		X			X	
Colon and rectum cancer	Male	Cumulative Cigarettes (5 Years)		X			X	
Colon and rectum cancer	Female	Tobacco (cigarettes per capita)	X			X		
Colon and rectum cancer	Male	Tobacco (cigarettes per capita)	X			X		
Colon and rectum cancer	Male	Cumulative Cigarettes (20 Years)		X			X	
Colon and rectum cancer	Female	Log-transformed SEV scalar: Colorect C	X			X		
Colon and rectum cancer	Male	Log-transformed SEV scalar: Colorect C	X			X		
Colon and rectum cancer	Female	Total Physical Activity (MET-min/week), Age-specific	X			X		
Colon and rectum cancer	Male	Total Physical Activity (MET-min/week), Age-specific	X			X		
Colon and rectum cancer	Female	Education (years per capita)			X			X
Colon and rectum cancer	Male	Education (years per capita)			X			X
Colon and rectum cancer	Female	LDI (I$ per capita)			X			X
Colon and rectum cancer	Male	LDI (I$ per capita)			X			X

Table S18. Comparison of GBD 2017 and GBD 2019 covariates and level of covariates used in cause of death modeling

Cause	Sex	Covariate	Level 1: GBD 2019	Level 2: GBD 2019	Level 3: GBD 2019	Level 1: GBD 2017	Level 2: GBD 2017	Level 3: GBD 2017
Colon and rectum cancer	Female	Mean BMI	X			X		
Colon and rectum cancer	Male	Mean BMI	X			X		
Colon and rectum cancer	Female	Socio-demographic Index			X			X
Colon and rectum cancer	Male	Socio-demographic Index			X			X
Colon and rectum cancer	Female	Healthcare access and quality index		X				X
Colon and rectum cancer	Male	Healthcare access and quality index		X				X
Colon and rectum cancer	Female	Diabetes Fasting Plasma Glucose (mmol/L), age-standardized 25+		X			X	
Colon and rectum cancer	Male	Diabetes Fasting Plasma Glucose (mmol/L), age-standardized 25+		X			X	
Lip and oral cavity cancer	Male	Education (years per capita)			X			X
Lip and oral cavity cancer	Female	LDI (I$ per capita)			X			X
Lip and oral cavity cancer	Female	Education (years per capita)			X			X
Lip and oral cavity cancer	Female	Socio-demographic Index			X			X
Lip and oral cavity cancer	Male	Socio-demographic Index			X			X
Lip and oral cavity cancer	Male	LDI (I$ per capita)			X			X
Lip and oral cavity cancer	Male	Log-transformed SEV scalar: Lip Oral C	X			X		
Lip and oral cavity cancer	Female	Log-transformed SEV scalar: Lip Oral C	X			X		
Lip and oral cavity cancer	Male	Cumulative Cigarettes (20 Years)	X			X		
Lip and oral cavity cancer	Female	Cumulative Cigarettes (20 Years)	X			X		
Lip and oral cavity cancer	Male	Tobacco (cigarettes per capita)	X			X		
Lip and oral cavity cancer	Female	Tobacco (cigarettes per capita)	X			X		
Lip and oral cavity cancer	Male	Cumulative Cigarettes (10 Years)	X			X		
Lip and oral cavity cancer	Female	Cumulative Cigarettes (10 Years)	X			X		
Lip and oral cavity cancer	Male	Healthcare access and quality index		X			X	
Lip and oral cavity cancer	Female	Healthcare access and quality index		X			X	
Nasopharynx cancer	Female	Education (years per capita)			X			X
Nasopharynx cancer	Male	Socio-demographic Index			X			X
Nasopharynx cancer	Male	Log-transformed SEV scalar: Nasoph C	X			X		
Nasopharynx cancer	Female	Cumulative Cigarettes (20 Years)	X			X		
Nasopharynx cancer	Male	Cumulative Cigarettes (20 Years)	X			X		
Nasopharynx cancer	Female	Tobacco (cigarettes per capita)	X			X		
Nasopharynx cancer	Male	Tobacco (cigarettes per capita)	X			X		
Nasopharynx cancer	Female	Population Density (over 1000 ppl/sqkm, proportion)		X			X	
Nasopharynx cancer	Male	Population Density (over 1000 ppl/sqkm, proportion)		X			X	
Nasopharynx cancer	Female	Cumulative Cigarettes (10 Years)	X			X		
Nasopharynx cancer	Male	Cumulative Cigarettes (10 Years)	X			X		
Nasopharynx cancer	Female	Healthcare access and quality index		X			X	
Nasopharynx cancer	Male	Healthcare access and quality index		X			X	
Nasopharynx cancer	Female	Socio-demographic Index			X			X
Nasopharynx cancer	Female	LDI (I$ per capita)			X			X
Nasopharynx cancer	Female	Log-transformed SEV scalar: Nasoph C	X			X		
Nasopharynx cancer	Male	LDI (I$ per capita)			X			X
Nasopharynx cancer	Male	Education (years per capita)			X			X
Other pharynx cancer	Female	LDI (I$ per capita)			X			X
Other pharynx cancer	Female	Log-transformed SEV scalar: Oth Phar C	X			X		
Other pharynx cancer	Male	Population Density (under 150 ppl/sqkm, proportion)		X			X	
Other pharynx cancer	Female	Socio-demographic Index			X			X
Other pharynx cancer	Male	Cumulative Cigarettes (5 Years)		X			X	
Other pharynx cancer	Female	Cumulative Cigarettes (5 Years)		X			X	
Other pharynx cancer	Male	Population Density (over 1000 ppl/sqkm, proportion)		X			X	
Other pharynx cancer	Female	Population Density (over 1000 ppl/sqkm, proportion)		X			X	
Other pharynx cancer	Female	Smoking Prevalence	X			X		
Other pharynx cancer	Male	Healthcare access and quality index		X			X	
Other pharynx cancer	Female	Healthcare access and quality index		X			X	
Other pharynx cancer	Male	Socio-demographic Index			X			X
Other pharynx cancer	Female	Education (years per capita)			X			X
Other pharynx cancer	Male	Education (years per capita)			X			X
Other pharynx cancer	Male	Smoking Prevalence	X			X		
Other pharynx cancer	Male	Log-transformed SEV scalar: Oth Phar C	X			X		
Other pharynx cancer	Female	Population Density (under 150 ppl/sqkm, proportion)		X			X	
Other pharynx cancer	Male	LDI (I$ per capita)			X			X
Gallbladder and biliary tract cancer	Female	Socio-demographic Index			X			X
Gallbladder and biliary tract cancer	Male	Healthcare access and quality index		X			X	
Gallbladder and biliary tract cancer	Male	Mean BMI	X			X		
Gallbladder and biliary tract cancer	Female	LDI (I$ per capita)			X			X
Gallbladder and biliary tract cancer	Male	LDI (I$ per capita)			X			X
Gallbladder and biliary tract cancer	Female	Education (years per capita)			X			X
Gallbladder and biliary tract cancer	Male	Education (years per capita)			X			X
Gallbladder and biliary tract cancer	Female	Diabetes Age-Standardized Prevalence (proportion)		X			X	
Gallbladder and biliary tract cancer	Male	Diabetes Age-Standardized Prevalence (proportion)		X			X	
Gallbladder and biliary tract cancer	Female	Log-transformed SEV scalar: Gallblad C	X			X		
Gallbladder and biliary tract cancer	Male	Socio-demographic Index			X			X
Gallbladder and biliary tract cancer	Male	Log-transformed SEV scalar: Gallblad C	X			X		
Gallbladder and biliary tract cancer	Male	Tobacco (cigarettes per capita)		X			X	
Gallbladder and biliary tract cancer	Female	Cumulative Cigarettes (5 Years)		X			X	
Gallbladder and biliary tract cancer	Female	Healthcare access and quality index		X			X	
Gallbladder and biliary tract cancer	Male	Smoking Prevalence		X			X	
Gallbladder and biliary tract cancer	Female	Smoking Prevalence		X			X	

Table S18. Comparison of GBD 2017 and GBD 2019 covariates and level of covariates used in cause of death modeling								
Cause	Sex	Covariate	Level 1: GBD 2019	Level 2: GBD 2019	Level 3: GBD 2019	Level 1: GBD 2017	Level 2: GBD 2017	Level 3: GBD 2017
Gallbladder and biliary tract cancer	Male	Cumulative Cigarettes (10 Years)		X			X	
Gallbladder and biliary tract cancer	Female	Cumulative Cigarettes (10 Years)		X			X	
Gallbladder and biliary tract cancer	Male	Cumulative Cigarettes (5 Years)		X			X	
Gallbladder and biliary tract cancer	Female	Tobacco (cigarettes per capita)		X			X	
Gallbladder and biliary tract cancer	Female	Mean BMI	X			X		
Pancreatic cancer	Male	Diabetes Age-Standardized Prevalence (proportion)		X			X	
Pancreatic cancer	Female	Healthcare access and quality index		X			X	
Pancreatic cancer	Male	Healthcare access and quality index		X			X	
Pancreatic cancer	Female	Socio-demographic Index			X			X
Pancreatic cancer	Female	LDI (I$ per capita)			X			X
Pancreatic cancer	Female	Diabetes Fasting Plasma Glucose (mmol/L), age-standardized 25+		X			X	
Pancreatic cancer	Male	LDI (I$ per capita)			X			X
Pancreatic cancer	Female	Education (years per capita)			X			X
Pancreatic cancer	Male	Education (years per capita)			X			X
Pancreatic cancer	Female	Diabetes Age-Standardized Prevalence (proportion)		X			X	
Pancreatic cancer	Female	Mean BMI	X			X		
Pancreatic cancer	Female	Log-transformed SEV scalar: Pancreas C	X			X		
Pancreatic cancer	Male	Log-transformed SEV scalar: Pancreas C	X			X		
Pancreatic cancer	Female	Cumulative Cigarettes (20 Years)	X			X		
Pancreatic cancer	Male	Cumulative Cigarettes (20 Years)	X			X		
Pancreatic cancer	Female	Tobacco (cigarettes per capita)	X			X		
Pancreatic cancer	Male	Tobacco (cigarettes per capita)	X			X		
Pancreatic cancer	Female	Cumulative Cigarettes (10 Years)	X			X		
Pancreatic cancer	Male	Cumulative Cigarettes (10 Years)	X			X		
Pancreatic cancer	Female	energy unadjusted(kcal)		X			X	
Pancreatic cancer	Male	energy unadjusted(kcal)		X			X	
Pancreatic cancer	Male	Mean BMI	X			X		
Pancreatic cancer	Male	Diabetes Fasting Plasma Glucose (mmol/L), age-standardized 25+		X			X	
Pancreatic cancer	Male	Socio-demographic Index			X			X
Malignant skin melanoma	Female	Latitude Under 15 (proportion)		X			X	
Malignant skin melanoma	Female	LDI (I$ per capita)			X			X
Malignant skin melanoma	Male	Education (years per capita)			X			X
Malignant skin melanoma	Male	LDI (I$ per capita)			X			X
Malignant skin melanoma	Female	Socio-demographic Index			X			X
Malignant skin melanoma	Female	Healthcare access and quality index		X			X	
Malignant skin melanoma	Male	Healthcare access and quality index		X			X	
Malignant skin melanoma	Male	Socio-demographic Index			X			X
Malignant skin melanoma	Male	Latitude Under 15 (proportion)		X			X	
Malignant skin melanoma	Male	Latitude 15 to 30 (proportion)		X			X	
Malignant skin melanoma	Female	Latitude 30 to 45 (proportion)		X			X	
Malignant skin melanoma	Male	Latitude 30 to 45 (proportion)		X			X	
Malignant skin melanoma	Female	Latitude Over 45 (proportion)		X			X	
Malignant skin melanoma	Female	Education (years per capita)			X			X
Malignant skin melanoma	Female	Latitude 15 to 30 (proportion)		X			X	
Malignant skin melanoma	Male	Latitude Over 45 (proportion)		X			X	
Non-melanoma skin cancer	Female	Socio-demographic Index			X			X
Non-melanoma skin cancer	Male	Education (years per capita)			X			X
Non-melanoma skin cancer	Female	Education (years per capita)			X			X
Non-melanoma skin cancer	Male	LDI (I$ per capita)			X			X
Non-melanoma skin cancer	Female	LDI (I$ per capita)			X			X
Non-melanoma skin cancer	Male	Socio-demographic Index			X			X
Non-melanoma skin cancer	Male	Healthcare access and quality index		X			X	
Non-melanoma skin cancer	Male	Smoking Prevalence	X			X		
Non-melanoma skin cancer	Female	Smoking Prevalence	X			X		
Non-melanoma skin cancer	Female	Average latitude		X			X	
Non-melanoma skin cancer	Male	Average latitude		X			X	
Non-melanoma skin cancer	Female	Cumulative Cigarettes (15 Years)	X			X		
Non-melanoma skin cancer	Male	Cumulative Cigarettes (15 Years)	X			X		
Non-melanoma skin cancer	Female	Cumulative Cigarettes (5 Years)	X			X		
Non-melanoma skin cancer	Male	Cumulative Cigarettes (5 Years)	X			X		
Non-melanoma skin cancer	Male	Cumulative Cigarettes (10 Years)	X			X		
Non-melanoma skin cancer	Female	Cumulative Cigarettes (10 Years)	X			X		
Non-melanoma skin cancer	Female	Healthcare access and quality index		X			X	
Ovarian cancer	Female	Log-transformed SEV scalar: Ovary C	X			X		
Ovarian cancer	Female	Contraception (Modern) Prevalence (proportion)	X				X	
Ovarian cancer	Female	Total Fertility Rate		X			X	
Ovarian cancer	Female	Cumulative Cigarettes (10 Years)	X				X	
Ovarian cancer	Female	Diabetes Age-Standardized Prevalence (proportion)		X			X	
Ovarian cancer	Female	Education (years per capita)			X			X
Ovarian cancer	Female	LDI (I$ per capita)			X			X
Ovarian cancer	Female	Mean BMI		X			X	
Ovarian cancer	Female	Socio-demographic Index			X			X
Ovarian cancer	Female	Healthcare access and quality index		X			X	
Ovarian cancer	Female	energy unadjusted(kcal)		X			X	
Ovarian cancer	Female	Smoking Prevalence		X			X	
Ovarian cancer	Female	Cumulative Cigarettes (20 Years)	X				X	
Ovarian cancer	Female	Asbestos consumption (metric tons per year per capita)		X			X	
Testicular cancer	Male	Tobacco (cigarettes per capita)		X			X	
Testicular cancer	Male	Healthcare access and quality index		X			X	

Table S18. Comparison of GBD 2017 and GBD 2019 covariates and level of covariates used in cause of death modeling								
Cause	Sex	Covariate	Level 1: GBD 2019	Level 2: GBD 2019	Level 3: GBD 2019	Level 1: GBD 2017	Level 2: GBD 2017	Level 3: GBD 2017
Testicular cancer	Male	Socio-demographic Index			X			X
Testicular cancer	Male	LDI (I$ per capita)			X			X
Testicular cancer	Male	Education (years per capita)			X			X
Testicular cancer	Male	Cumulative Cigarettes (20 Years)		X			X	
Testicular cancer	Male	Cumulative Cigarettes (15 Years)		X			X	
Testicular cancer	Male	Cumulative Cigarettes (5 Years)		X			X	
Testicular cancer	Male	Cumulative Cigarettes (10 Years)		X			X	
Testicular cancer	Male	Smoking Prevalence		X			X	
Kidney cancer	Female	Systolic Blood Pressure (mmHg)		X			X	
Kidney cancer	Male	Healthcare access and quality index		X			X	
Kidney cancer	Female	Log-transformed SEV scalar: Kidney C	X			X		
Kidney cancer	Male	Cumulative Cigarettes (10 Years)	X			X		
Kidney cancer	Female	Cumulative Cigarettes (10 Years)	X			X		
Kidney cancer	Male	Log-transformed SEV scalar: Kidney C	X			X		
Kidney cancer	Female	Healthcare access and quality index		X			X	
Kidney cancer	Male	LDI (I$ per capita)			X			X
Kidney cancer	Male	Diabetes Age-Standardized Prevalence (proportion)		X			X	
Kidney cancer	Female	Diabetes Age-Standardized Prevalence (proportion)		X			X	
Kidney cancer	Male	Education (years per capita)			X			X
Kidney cancer	Female	Education (years per capita)			X			X
Kidney cancer	Female	LDI (I$ per capita)			X			X
Kidney cancer	Male	Systolic Blood Pressure (mmHg)		X			X	
Kidney cancer	Female	Mean BMI	X			X		
Kidney cancer	Male	Socio-demographic Index			X			X
Kidney cancer	Female	Socio-demographic Index			X			X
Kidney cancer	Male	Mean BMI	X			X		
Bladder cancer	Male	Socio-demographic Index			X			X
Bladder cancer	Male	LDI (I$ per capita)			X			X
Bladder cancer	Female	LDI (I$ per capita)			X			X
Bladder cancer	Female	Schistosomiasis Prevalence (proportion)	X			X		
Bladder cancer	Male	Schistosomiasis Prevalence (proportion)	X			X		
Bladder cancer	Female	Socio-demographic Index			X			X
Bladder cancer	Female	Cumulative Cigarettes (10 Years)	X				X	
Bladder cancer	Male	Log-transformed SEV scalar: Bladder C	X			X		
Bladder cancer	Female	Diabetes Fasting Plasma Glucose (mmol/L), age-standardized 25+		X			X	
Bladder cancer	Male	Diabetes Fasting Plasma Glucose (mmol/L), age-standardized 25+		X			X	
Bladder cancer	Female	Smoking Prevalence	X			X		
Bladder cancer	Male	Smoking Prevalence	X			X		
Bladder cancer	Female	Log-transformed SEV scalar: Bladder C	X			X		
Bladder cancer	Male	Cumulative Cigarettes (10 Years)	X				X	
Bladder cancer	Female	Healthcare access and quality index		X			X	
Bladder cancer	Male	Healthcare access and quality index		X			X	
Brain and nervous system cancer	Male	Education (years per capita)			X			X
Brain and nervous system cancer	Female	Education (years per capita)			X			X
Brain and nervous system cancer	Female	Socio-demographic Index			X			X
Brain and nervous system cancer	Male	Smoking Prevalence	X			X		
Brain and nervous system cancer	Female	Smoking Prevalence	X			X		
Brain and nervous system cancer	Male	Systolic Blood Pressure (mmHg)		X			X	
Brain and nervous system cancer	Female	Systolic Blood Pressure (mmHg)		X			X	
Brain and nervous system cancer	Male	Cholesterol (total, mean per capita)		X			X	
Brain and nervous system cancer	Female	Cholesterol (total, mean per capita)		X			X	
Brain and nervous system cancer	Male	Healthcare access and quality index		X			X	
Brain and nervous system cancer	Female	Healthcare access and quality index		X			X	
Brain and nervous system cancer	Male	Socio-demographic Index			X			X
Brain and nervous system cancer	Female	Cumulative Cigarettes (10 Years)	X			X		
Brain and nervous system cancer	Male	Cumulative Cigarettes (10 Years)	X			X		
Brain and nervous system cancer	Female	LDI (I$ per capita)			X			X
Brain and nervous system cancer	Male	LDI (I$ per capita)			X			X
Thyroid cancer	Female	Tobacco (cigarettes per capita)		X			X	
Thyroid cancer	Female	Mean BMI		X			X	
Thyroid cancer	Male	Tobacco (cigarettes per capita)		X			X	
Thyroid cancer	Male	Education (years per capita)			X			X
Thyroid cancer	Female	Education (years per capita)			X			X
Thyroid cancer	Male	LDI (I$ per capita)			X			X
Thyroid cancer	Female	LDI (I$ per capita)			X			X
Thyroid cancer	Female	Log-transformed SEV scalar: Thyroid C	X			X		
Thyroid cancer	Male	Mean BMI		X			X	
Thyroid cancer	Male	Log-transformed SEV scalar: Thyroid C	X			X		
Thyroid cancer	Male	Socio-demographic Index			X			X
Thyroid cancer	Female	Improved Water Source (proportion with access)		X				X
Thyroid cancer	Female	Socio-demographic Index			X			X
Thyroid cancer	Male	Healthcare access and quality index		X			X	
Thyroid cancer	Female	Healthcare access and quality index		X			X	
Thyroid cancer	Male	Sanitation (proportion with access)		X				X
Thyroid cancer	Female	Sanitation (proportion with access)		X				X
Thyroid cancer	Male	Improved Water Source (proportion with access)		X				X
Mesothelioma	Female	Education (years per capita)			X			X
Mesothelioma	Male	Log-transformed age-standardized SEV scalar: Mesothel	X			X		
Mesothelioma	Male	Log-transformed SEV scalar: Mesothel	X			X		

Table S18. Comparison of GBD 2017 and GBD 2019 covariates and level of covariates used in cause of death modeling								
**Cause**	**Sex**	**Covariate**	**Level 1: GBD 2019**	**Level 2: GBD 2019**	**Level 3: GBD 2019**	**Level 1: GBD 2017**	**Level 2: GBD 2017**	**Level 3: GBD 2017**
Mesothelioma	Female	Gold production (binary)		X			X	
Mesothelioma	Female	Asbestos consumption (metric tons per year per capita)	X			X		
Mesothelioma	Male	Asbestos consumption (metric tons per year per capita)	X			X		
Mesothelioma	Female	Cumulative Cigarettes (5 Years)	X				X	
Mesothelioma	Male	Cumulative Cigarettes (5 Years)	X				X	
Mesothelioma	Female	Population Density (over 1000 ppl/sqkm, proportion)		X			X	
Mesothelioma	Male	Population Density (over 1000 ppl/sqkm, proportion)		X			X	
Mesothelioma	Female	Smoking Prevalence	X			X		
Mesothelioma	Male	Smoking Prevalence	X			X		
Mesothelioma	Female	Indoor Air Pollution (All Cooking Fuels)	X				X	
Mesothelioma	Male	Indoor Air Pollution (All Cooking Fuels)	X				X	
Mesothelioma	Female	Healthcare access and quality index		X			X	
Mesothelioma	Male	Healthcare access and quality index		X			X	
Mesothelioma	Female	Socio-demographic Index			X			X
Mesothelioma	Male	Socio-demographic Index			X			X
Mesothelioma	Male	LDI (I$ per capita)			X			X
Mesothelioma	Male	Education (years per capita)			X			X
Mesothelioma	Female	LDI (I$ per capita)			X			X
Mesothelioma	Male	Gold production (binary)		X			X	
Hodgkin lymphoma	Female	LDI (I$ per capita)			X			X
Hodgkin lymphoma	Female	Healthcare access and quality index		X			X	
Hodgkin lymphoma	Male	Education (years per capita)			X			X
Hodgkin lymphoma	Female	Education (years per capita)			X			X
Hodgkin lymphoma	Male	LDI (I$ per capita)			X			X
Hodgkin lymphoma	Male	Healthcare access and quality index		X			X	
Hodgkin lymphoma	Male	Socio-demographic Index			X			X
Hodgkin lymphoma	Female	Socio-demographic Index			X			X
Non-Hodgkin's lymphoma	Male	Healthcare access and quality index		X			X	
Non-Hodgkin's lymphoma	Female	Healthcare access and quality index		X			X	
Non-Hodgkin's lymphoma	Male	Smoking Prevalence		X			X	
Non-Hodgkin's lymphoma	Female	Cumulative Cigarettes (20 Years)		X			X	
Non-Hodgkin's lymphoma	Female	Smoking Prevalence		X			X	
Non-Hodgkin's lymphoma	Male	Cumulative Cigarettes (10 Years)		X			X	
Non-Hodgkin's lymphoma	Female	Cumulative Cigarettes (10 Years)		X			X	
Non-Hodgkin's lymphoma	Male	Cumulative Cigarettes (5 Years)		X			X	
Non-Hodgkin's lymphoma	Female	Socio-demographic Index			X			X
Non-Hodgkin's lymphoma	Male	Socio-demographic Index			X			X
Non-Hodgkin's lymphoma	Female	LDI (I$ per capita)			X			X
Non-Hodgkin's lymphoma	Male	Mean BMI		X			X	
Non-Hodgkin's lymphoma	Male	LDI (I$ per capita)			X			X
Non-Hodgkin's lymphoma	Female	Cumulative Cigarettes (5 Years)		X			X	
Non-Hodgkin's lymphoma	Female	Total Fertility Rate			X			X
Non-Hodgkin's lymphoma	Male	Cumulative Cigarettes (15 Years)		X			X	
Non-Hodgkin's lymphoma	Female	Cumulative Cigarettes (15 Years)		X			X	
Non-Hodgkin's lymphoma	Male	Cumulative Cigarettes (20 Years)		X			X	
Non-Hodgkin's lymphoma	Female	Mean BMI		X			X	
Multiple myeloma	Male	LDI (I$ per capita)			X			X
Multiple myeloma	Female	Tobacco (cigarettes per capita)	X			X		
Multiple myeloma	Female	LDI (I$ per capita)			X			X
Multiple myeloma	Female	Mean BMI		X			X	
Multiple myeloma	Male	Mean BMI		X			X	
Multiple myeloma	Female	Education (years per capita)			X			X
Multiple myeloma	Female	Healthcare access and quality index		X			X	
Multiple myeloma	Male	Tobacco (cigarettes per capita)	X			X		
Multiple myeloma	Female	Improved Water Source (proportion with access)		X			X	
Multiple myeloma	Male	Improved Water Source (proportion with access)		X			X	
Multiple myeloma	Female	Sanitation (proportion with access)		X			X	
Multiple myeloma	Male	Sanitation (proportion with access)		X			X	
Multiple myeloma	Female	Smoking Prevalence	X			X		
Multiple myeloma	Male	Smoking Prevalence	X			X		
Multiple myeloma	Male	Healthcare access and quality index		X			X	
Multiple myeloma	Female	Socio-demographic Index			X			X
Multiple myeloma	Male	Socio-demographic Index			X			X
Multiple myeloma	Male	Education (years per capita)			X			X
Leukaemia	Male	Tobacco (cigarettes per capita)		X			X	
Leukaemia	Female	Cumulative Cigarettes (10 Years)		X			X	
Leukaemia	Male	Cumulative Cigarettes (10 Years)		X			X	
Leukaemia	Male	Log-transformed age-standardized SEV scalar: Leukemia	X			X		
Leukaemia	Male	Healthcare access and quality index		X			X	
Leukaemia	Male	Tobacco (cigarettes per capita)		X			X	
Leukaemia	Female	Socio-demographic Index			X			X
Leukaemia	Male	Socio-demographic Index			X			X
Leukaemia	Female	Healthcare access and quality index		X			X	
Leukaemia	Female	Tobacco (cigarettes per capita)		X			X	
Leukaemia	Female	Education (years per capita)			X			X
Leukaemia	Male	Cumulative Cigarettes (20 Years)		X			X	
Leukaemia	Female	Cumulative Cigarettes (20 Years)		X			X	
Leukaemia	Male	Log-transformed SEV scalar: Leukemia	X			X		
Leukaemia	Male	Education (years per capita)			X			X

Table S18. Comparison of GBD 2017 and GBD 2019 covariates and level of covariates used in cause of death modeling

Cause	Sex	Covariate	Level 1: GBD 2019	Level 2: GBD 2019	Level 3: GBD 2019	Level 1: GBD 2017	Level 2: GBD 2017	Level 3: GBD 2017
Leukaemia	Male	LDI (I$ per capita)			X			X
Leukaemia	Female	LDI (I$ per capita)			X			X
Leukaemia	Female	Log-transformed SEV scalar: Leukemia	X			X		
Leukaemia	Male	Mean BMI		X			X	
Leukaemia	Female	Tobacco (cigarettes per capita)		X			X	
Leukaemia	Female	Mean BMI		X			X	
Leukaemia	Female	Log-transformed age-standardized SEV scalar: Leukemia	X			X		
Other malignant cancers	Female	Socio-demographic Index			X			X
Other malignant cancers	Female	Education (years per capita)			X			X
Other malignant cancers	Male	Education (years per capita)			X			X
Other malignant cancers	Female	LDI (I$ per capita)			X			X
Other malignant cancers	Male	LDI (I$ per capita)			X			X
Other malignant cancers	Male	Socio-demographic Index			X			X
Other malignant cancers	Female	Healthcare access and quality index		X			X	
Other malignant cancers	Female	Smoking Prevalence	X			X		
Other malignant cancers	Male	Healthcare access and quality index		X			X	
Other malignant cancers	Female	pufa adjusted(percent)		X			X	
Other malignant cancers	Male	pufa adjusted(percent)		X			X	
Other malignant cancers	Male	Tobacco (cigarettes per capita)	X			X		
Other malignant cancers	Male	Tobacco (cigarettes per capita)	X			X		
Other malignant cancers	Female	Tobacco (cigarettes per capita)	X			X		
Other malignant cancers	Female	Tobacco (cigarettes per capita)	X			X		
Other malignant cancers	Male	Smoking Prevalence	X			X		
Other neoplasms	Female	LDI (I$ per capita)			X			X
Other neoplasms	Male	LDI (I$ per capita)			X			X
Other neoplasms	Male	Socio-demographic Index			X			X
Other neoplasms	Female	Education (years per capita)			X			X
Other neoplasms	Male	Healthcare access and quality index		X			X	
Other neoplasms	Female	Healthcare access and quality index		X			X	
Other neoplasms	Female	Socio-demographic Index			X			X
Other neoplasms	Male	Education (years per capita)			X			X
Cardiovascular diseases	Female	LDI (I$ per capita)			X			X
Cardiovascular diseases	Male	LDI (I$ per capita)			X			X
Cardiovascular diseases	Male	Systolic Blood Pressure (mmHg)	X			X		
Cardiovascular diseases	Male	Smoking Prevalence	X			X		
Cardiovascular diseases	Female	Smoking Prevalence	X			X		
Cardiovascular diseases	Male	Elevation Over 1500m (proportion)		X			X	
Cardiovascular diseases	Female	Elevation Over 1500m (proportion)		X			X	
Cardiovascular diseases	Female	Outdoor Air Pollution (PM2.5)		X			X	
Cardiovascular diseases	Male	Indoor Air Pollution (All Cooking Fuels)		X			X	
Cardiovascular diseases	Female	Indoor Air Pollution (All Cooking Fuels)		X			X	
Cardiovascular diseases	Female	Diet high in trans fatty acids			X			X
Cardiovascular diseases	Female	Systolic Blood Pressure (mmHg)	X			X		
Cardiovascular diseases	Female	Cholesterol (total, mean per capita)	X			X		
Cardiovascular diseases	Male	Diabetes Fasting Plasma Glucose (mmol/L), age-standardized 25+		X			X	
Cardiovascular diseases	Female	Diabetes Fasting Plasma Glucose (mmol/L), age-standardized 25+		X			X	
Cardiovascular diseases	Male	Healthcare access and quality index		X			X	
Cardiovascular diseases	Female	Healthcare access and quality index		X			X	
Cardiovascular diseases	Male	Mean BMI		X			X	
Cardiovascular diseases	Female	Mean BMI		X			X	
Cardiovascular diseases	Male	Cholesterol (total, mean per capita)	X			X		
Cardiovascular diseases	Male	Diet high in trans fatty acids			X			X
Cardiovascular diseases	Male	Outdoor Air Pollution (PM2.5)		X			X	
Rheumatic heart disease	Male	LDI (I$ per capita)			X			X
Rheumatic heart disease	Female	Log-transformed SEV scalar: RHD	X			X		
Rheumatic heart disease	Male	Log-transformed SEV scalar: RHD	X			X		
Rheumatic heart disease	Female	Improved Water Source (proportion with access)	X			X		
Rheumatic heart disease	Male	Improved Water Source (proportion with access)	X			X		
Rheumatic heart disease	Female	Education (years per capita)			X			X
Rheumatic heart disease	Male	Education (years per capita)			X			X
Rheumatic heart disease	Female	Sanitation (proportion with access)	X			X		
Rheumatic heart disease	Female	LDI (I$ per capita)			X			X
Rheumatic heart disease	Male	Socio-demographic Index			X			X
Rheumatic heart disease	Male	Healthcare access and quality index		X			X	
Rheumatic heart disease	Female	Healthcare access and quality index		X			X	
Rheumatic heart disease	Male	Sanitation (proportion with access)	X			X		
Ischaemic heart disease	Male	Mean BMI		X			X	
Ischaemic heart disease	Male	Diabetes Fasting Plasma Glucose (mmol/L), age-standardized 25+		X			X	
Ischaemic heart disease	Female	Cholesterol (total, mean per capita)	X			X		
Ischaemic heart disease	Male	Cholesterol (total, mean per capita)	X			X		
Ischaemic heart disease	Female	Systolic Blood Pressure (mmHg)	X			X		
Ischaemic heart disease	Male	Systolic Blood Pressure (mmHg)	X			X		
Ischaemic heart disease	Female	Indoor Air Pollution (All Cooking Fuels)		X			X	
Ischaemic heart disease	Male	Indoor Air Pollution (All Cooking Fuels)		X			X	
Ischaemic heart disease	Female	Outdoor Air Pollution (PM2.5)		X			X	
Ischaemic heart disease	Female	Mean BMI		X			X	
Ischaemic heart disease	Male	Healthcare access and quality index		X			X	
Ischaemic heart disease	Male	Outdoor Air Pollution (PM2.5)		X			X	
Ischaemic heart disease	Male	LDI (I$ per capita)			X			X
Ischaemic heart disease	Female	LDI (I$ per capita)			X			X

Table S18. Comparison of GBD 2017 and GBD 2019 covariates and level of covariates used in cause of death modeling								
Cause	Sex	Covariate	Level 1: GBD 2019	Level 2: GBD 2019	Level 3: GBD 2019	Level 1: GBD 2017	Level 2: GBD 2017	Level 3: GBD 2017
Ischaemic heart disease	Female	Healthcare access and quality index		X			X	
Ischaemic heart disease	Male	Log-transformed SEV scalar: IHD	X			X		
Ischaemic heart disease	Female	Log-transformed SEV scalar: IHD	X			X		
Ischaemic heart disease	Female	Diabetes Fasting Plasma Glucose (mmol/L), age-standardized 25+		X			X	
Ischaemic heart disease	Female	Diet high in trans fatty acids			X			X
Ischaemic heart disease	Male	Smoking Prevalence	X			X		
Ischaemic heart disease	Female	Smoking Prevalence	X			X		
Ischaemic heart disease	Male	Elevation Over 1500m (proportion)		X			X	
Ischaemic heart disease	Male	Diet high in trans fatty acids			X			X
Ischaemic heart disease	Female	Elevation Over 1500m (proportion)		X			X	
Stroke	Female	Outdoor Air Pollution (PM2.5)		X			X	
Stroke	Male	Elevation Over 1500m (proportion)		X			X	
Stroke	Female	Elevation Over 1500m (proportion)		X			X	
Stroke	Female	Smoking Prevalence	X			X		
Stroke	Male	Outdoor Air Pollution (PM2.5)		X			X	
Stroke	Male	Diet high in trans fatty acids			X			X
Stroke	Female	Diet high in trans fatty acids			X			X
Stroke	Male	Log-transformed SEV scalar: Stroke	X			X		
Stroke	Female	Log-transformed SEV scalar: Stroke	X			X		
Stroke	Male	Smoking Prevalence	X			X		
Stroke	Female	Indoor Air Pollution (All Cooking Fuels)		X			X	
Stroke	Male	Systolic Blood Pressure (mmHg)	X			X		
Stroke	Female	Systolic Blood Pressure (mmHg)	X			X		
Stroke	Female	Cholesterol (total, mean per capita)	X			X		
Stroke	Male	Cholesterol (total, mean per capita)	X			X		
Stroke	Female	Diabetes Fasting Plasma Glucose (mmol/L), age-standardized 25+		X			X	
Stroke	Female	Healthcare access and quality index		X			X	
Stroke	Male	Healthcare access and quality index		X			X	
Stroke	Female	Mean BMI		X			X	
Stroke	Male	Mean BMI		X			X	
Stroke	Female	LDI (I$ per capita)			X			X
Stroke	Male	LDI (I$ per capita)			X			X
Stroke	Male	Indoor Air Pollution (All Cooking Fuels)		X			X	
Stroke	Male	Diabetes Fasting Plasma Glucose (mmol/L), age-standardized 25+		X			X	
Ischaemic stroke	Female	Smoking Prevalence	X			X		
Ischaemic stroke	Female	Mean BMI		X			X	
Ischaemic stroke	Male	Outdoor Air Pollution (PM2.5)		X			X	
Ischaemic stroke	Female	Outdoor Air Pollution (PM2.5)		X			X	
Ischaemic stroke	Male	Elevation Over 1500m (proportion)		X			X	
Ischaemic stroke	Female	Elevation Over 1500m (proportion)		X			X	
Ischaemic stroke	Male	Smoking Prevalence	X			X		
Ischaemic stroke	Female	Systolic Blood Pressure (mmHg)	X			X		
Ischaemic stroke	Male	Diet high in trans fatty acids			X			X
Ischaemic stroke	Female	Diet high in trans fatty acids			X			X
Ischaemic stroke	Male	Log-transformed SEV scalar: Isch Stroke	X			X		
Ischaemic stroke	Female	Log-transformed SEV scalar: Isch Stroke	X			X		
Ischaemic stroke	Male	Systolic Blood Pressure (mmHg)	X			X		
Ischaemic stroke	Female	Cholesterol (total, mean per capita)	X			X		
Ischaemic stroke	Male	Cholesterol (total, mean per capita)	X			X		
Ischaemic stroke	Female	Diabetes Fasting Plasma Glucose (mmol/L), age-standardized 25+		X			X	
Ischaemic stroke	Female	Indoor Air Pollution (All Cooking Fuels)		X			X	
Ischaemic stroke	Male	Diabetes Fasting Plasma Glucose (mmol/L), age-standardized 25+		X			X	
Ischaemic stroke	Male	Healthcare access and quality index		X			X	
Ischaemic stroke	Male	Mean BMI		X			X	
Ischaemic stroke	Female	LDI (I$ per capita)			X			X
Ischaemic stroke	Male	LDI (I$ per capita)			X			X
Ischaemic stroke	Female	Healthcare access and quality index		X			X	
Ischaemic stroke	Male	Indoor Air Pollution (All Cooking Fuels)		X			X	
Intracerebral hemorrhage	Female	Log-transformed SEV scalar: Intrahem Stroke	X			X		
Intracerebral hemorrhage	Male	Log-transformed SEV scalar: Intrahem Stroke	X			X		
Intracerebral hemorrhage	Female	Diet high in trans fatty acids			X			X
Intracerebral hemorrhage	Male	Diet high in trans fatty acids			X			X
Intracerebral hemorrhage	Female	Smoking Prevalence	X			X		
Intracerebral hemorrhage	Male	Smoking Prevalence	X			X		
Intracerebral hemorrhage	Female	Mean BMI		X			X	
Intracerebral hemorrhage	Male	Mean BMI		X			X	
Intracerebral hemorrhage	Female	LDI (I$ per capita)			X			X
Intracerebral hemorrhage	Male	LDI (I$ per capita)			X			X
Intracerebral hemorrhage	Male	Elevation Over 1500m (proportion)		X			X	
Intracerebral hemorrhage	Female	Cholesterol (total, mean per capita)	X					X
Intracerebral hemorrhage	Female	Elevation Over 1500m (proportion)		X			X	
Intracerebral hemorrhage	Female	Outdoor Air Pollution (PM2.5)		X			X	
Intracerebral hemorrhage	Male	Outdoor Air Pollution (PM2.5)		X			X	
Intracerebral hemorrhage	Female	Indoor Air Pollution (All Cooking Fuels)		X			X	
Intracerebral hemorrhage	Male	Indoor Air Pollution (All Cooking Fuels)		X			X	
Intracerebral hemorrhage	Female	Systolic Blood Pressure (mmHg)	X			X		
Intracerebral hemorrhage	Male	Systolic Blood Pressure (mmHg)	X			X		
Intracerebral hemorrhage	Male	Cholesterol (total, mean per capita)	X					X
Intracerebral hemorrhage	Male	Healthcare access and quality index		X			X	

Table S18. Comparison of GBD 2017 and GBD 2019 covariates and level of covariates used in cause of death modeling

Cause	Sex	Covariate	Level 1: GBD 2019	Level 2: GBD 2019	Level 3: GBD 2019	Level 1: GBD 2017	Level 2: GBD 2017	Level 3: GBD 2017
Intracerebral hemorrhage	Female	Healthcare access and quality index		X			X	
Intracerebral hemorrhage	Male	Diabetes Fasting Plasma Glucose (mmol/L), age-standardized 25+		X			X	
Intracerebral hemorrhage	Female	Diabetes Fasting Plasma Glucose (mmol/L), age-standardized 25+		X			X	
Subarachnoid hemorrhage	Male	LDI (I$ per capita)			X			X
Subarachnoid hemorrhage	Male	Smoking Prevalence	X			X		
Subarachnoid hemorrhage	Female	Systolic Blood Pressure (mmHg)	X			X		
Subarachnoid hemorrhage	Male	Systolic Blood Pressure (mmHg)	X			X		
Subarachnoid hemorrhage	Female	Healthcare access and quality index		X			X	
Subarachnoid hemorrhage	Male	Healthcare access and quality index		X			X	
Subarachnoid hemorrhage	Female	LDI (I$ per capita)			X			X
Subarachnoid hemorrhage	Female	Smoking Prevalence	X			X		
Hypertensive heart disease	Male	Healthcare access and quality index		X			X	
Hypertensive heart disease	Male	LDI (I$ per capita)			X			X
Hypertensive heart disease	Female	LDI (I$ per capita)			X			X
Hypertensive heart disease	Male	Mean BMI		X			X	
Hypertensive heart disease	Female	Mean BMI		X			X	
Hypertensive heart disease	Male	Socio-demographic Index			X			X
Hypertensive heart disease	Female	Socio-demographic Index			X			X
Hypertensive heart disease	Female	Healthcare access and quality index		X			X	
Hypertensive heart disease	Male	Smoking Prevalence		X			X	
Hypertensive heart disease	Female	Cholesterol (total, mean per capita)		X			X	
Hypertensive heart disease	Male	Systolic Blood Pressure (mmHg)	X			X		
Hypertensive heart disease	Female	Systolic Blood Pressure (mmHg)	X			X		
Hypertensive heart disease	Female	Smoking Prevalence		X			X	
Hypertensive heart disease	Male	Diet high in trans fatty acids			X			X
Hypertensive heart disease	Female	Diet high in trans fatty acids			X			X
Hypertensive heart disease	Male	Cholesterol (total, mean per capita)		X			X	
Cardiomyopathy and myocarditis	Male	Mean BMI		X			X	
Cardiomyopathy and myocarditis	Female	Mean BMI		X			X	
Cardiomyopathy and myocarditis	Male	Socio-demographic Index			X			X
Cardiomyopathy and myocarditis	Female	Socio-demographic Index			X			X
Cardiomyopathy and myocarditis	Male	Healthcare access and quality index		X			X	
Cardiomyopathy and myocarditis	Female	Smoking Prevalence	X			X		
Cardiomyopathy and myocarditis	Female	Systolic Blood Pressure (mmHg)	X				X	
Cardiomyopathy and myocarditis	Male	Smoking Prevalence	X			X		
Cardiomyopathy and myocarditis	Male	Log-transformed SEV scalar: CMP	X			X		
Cardiomyopathy and myocarditis	Female	Log-transformed SEV scalar: CMP	X			X		
Cardiomyopathy and myocarditis	Female	LDI (I$ per capita)			X			X
Cardiomyopathy and myocarditis	Female	Healthcare access and quality index		X			X	
Cardiomyopathy and myocarditis	Male	LDI (I$ per capita)			X			X
Cardiomyopathy and myocarditis	Male	Systolic Blood Pressure (mmHg)	X				X	
Atrial fibrillation and flutter	Female	Diabetes Fasting Plasma Glucose (mmol/L), age-standardized 25+		X			X	
Atrial fibrillation and flutter	Female	Diet high in trans fatty acids			X			X
Atrial fibrillation and flutter	Male	Diet high in trans fatty acids			X			X
Atrial fibrillation and flutter	Female	Smoking Prevalence	X			X		
Atrial fibrillation and flutter	Male	Smoking Prevalence	X			X		
Atrial fibrillation and flutter	Female	Systolic Blood Pressure (mmHg)	X			X		
Atrial fibrillation and flutter	Male	Systolic Blood Pressure (mmHg)	X			X		
Atrial fibrillation and flutter	Female	Cholesterol (total, mean per capita)		X			X	
Atrial fibrillation and flutter	Male	Cholesterol (total, mean per capita)		X			X	
Atrial fibrillation and flutter	Male	Log-transformed SEV scalar: A Fib	X			X		
Atrial fibrillation and flutter	Male	Diabetes Fasting Plasma Glucose (mmol/L), age-standardized 25+		X			X	
Atrial fibrillation and flutter	Male	Healthcare access and quality index		X			X	
Atrial fibrillation and flutter	Female	Socio-demographic Index			X			X
Atrial fibrillation and flutter	Male	Socio-demographic Index			X			X
Atrial fibrillation and flutter	Male	LDI (I$ per capita)			X			X
Atrial fibrillation and flutter	Female	LDI (I$ per capita)			X			X
Atrial fibrillation and flutter	Male	Mean BMI		X			X	
Atrial fibrillation and flutter	Female	Mean BMI		X			X	
Atrial fibrillation and flutter	Female	Healthcare access and quality index		X			X	
Atrial fibrillation and flutter	Female	Log-transformed SEV scalar: A Fib	X			X		
Aortic aneurysm	Female	Cumulative Cigarettes (10 Years)	X			X		
Aortic aneurysm	Male	Log-transformed SEV scalar: Aort An	X			X		
Aortic aneurysm	Male	Socio-demographic Index			X			X
Aortic aneurysm	Female	Systolic Blood Pressure (mmHg)	X			X		
Aortic aneurysm	Male	Cumulative Cigarettes (10 Years)	X			X		
Aortic aneurysm	Female	Log-transformed SEV scalar: Aort An	X			X		
Aortic aneurysm	Male	Cholesterol (total, mean per capita)	X			X		
Aortic aneurysm	Female	Healthcare access and quality index		X			X	
Aortic aneurysm	Male	Healthcare access and quality index		X			X	
Aortic aneurysm	Female	Socio-demographic Index			X			X
Aortic aneurysm	Female	Cholesterol (total, mean per capita)	X			X		
Aortic aneurysm	Female	Mean BMI		X			X	
Aortic aneurysm	Male	Mean BMI		X			X	
Aortic aneurysm	Female	LDI (I$ per capita)			X			X
Aortic aneurysm	Male	Systolic Blood Pressure (mmHg)	X			X		
Aortic aneurysm	Male	LDI (I$ per capita)			X			X
Peripheral vascular disease	Female	Cholesterol (total, mean per capita)	X			X		
Peripheral vascular disease	Female	Socio-demographic Index			X			X
Peripheral vascular disease	Male	Socio-demographic Index			X			X
Peripheral vascular disease	Female	Mean BMI		X			X	

Table S18. Comparison of GBD 2017 and GBD 2019 covariates and level of covariates used in cause of death modeling

Cause	Sex	Covariate	Level 1: GBD 2019	Level 2: GBD 2019	Level 3: GBD 2019	Level 1: GBD 2017	Level 2: GBD 2017	Level 3: GBD 2017
Peripheral vascular disease	Female	Log-transformed SEV scalar: PAD	X			X		
Peripheral vascular disease	Male	Log-transformed SEV scalar: PAD	X			X		
Peripheral vascular disease	Female	Smoking Prevalence	X			X		
Peripheral vascular disease	Male	Smoking Prevalence	X			X		
Peripheral vascular disease	Female	Systolic Blood Pressure (mmHg)	X			X		
Peripheral vascular disease	Female	LDI (I$ per capita)			X			X
Peripheral vascular disease	Male	LDI (I$ per capita)			X			X
Peripheral vascular disease	Male	Diabetes Fasting Plasma Glucose (mmol/L), age-standardized 25+		X			X	
Peripheral vascular disease	Male	Systolic Blood Pressure (mmHg)	X			X		
Peripheral vascular disease	Male	Healthcare access and quality index		X			X	
Peripheral vascular disease	Female	Diabetes Fasting Plasma Glucose (mmol/L), age-standardized 25+		X			X	
Peripheral vascular disease	Male	Cholesterol (total, mean per capita)	X			X		
Peripheral vascular disease	Male	Mean BMI		X			X	
Peripheral vascular disease	Female	Healthcare access and quality index		X			X	
Endocarditis	Male	Sanitation (proportion with access)	X			X		
Endocarditis	Male	LDI (I$ per capita)			X			X
Endocarditis	Female	Sanitation (proportion with access)	X			X		
Endocarditis	Male	Improved Water Source (proportion with access)	X			X		
Endocarditis	Female	Improved Water Source (proportion with access)	X			X		
Endocarditis	Female	Healthcare access and quality index	X				X	
Endocarditis	Male	Log-transformed SEV scalar: Endocar	X			X		
Endocarditis	Female	Log-transformed SEV scalar: Endocar	X			X		
Endocarditis	Male	Healthcare access and quality index	X				X	
Endocarditis	Female	Socio-demographic Index			X			X
Endocarditis	Male	Socio-demographic Index			X			X
Endocarditis	Female	LDI (I$ per capita)			X			X
Non-rheumatic valvular heart disease	Female	LDI (I$ per capita)			X			X
Non-rheumatic valvular heart disease	Male	Systolic Blood Pressure (mmHg)	X			X		
Non-rheumatic valvular heart disease	Female	Systolic Blood Pressure (mmHg)	X			X		
Non-rheumatic valvular heart disease	Male	Cholesterol (total, mean per capita)		X			X	
Non-rheumatic valvular heart disease	Female	Cholesterol (total, mean per capita)		X			X	
Non-rheumatic valvular heart disease	Male	Healthcare access and quality index		X			X	
Non-rheumatic valvular heart disease	Female	Healthcare access and quality index		X			X	
Non-rheumatic valvular heart disease	Female	Socio-demographic Index			X			X
Non-rheumatic valvular heart disease	Male	Mean BMI		X			X	
Non-rheumatic valvular heart disease	Female	Mean BMI		X			X	
Non-rheumatic valvular heart disease	Male	LDI (I$ per capita)			X			X
Non-rheumatic valvular heart disease	Male	Socio-demographic Index			X			X
Non-rheumatic valvular heart disease	Female	Smoking Prevalence	X			X		
Non-rheumatic valvular heart disease	Male	Smoking Prevalence	X			X		
Other cardiovascular and circulatory diseases	Male	Log-transformed SEV scalar: Oth Cardio	X			X		
Other cardiovascular and circulatory diseases	Male	LDI (I$ per capita)			X			X
Other cardiovascular and circulatory diseases	Female	LDI (I$ per capita)			X			X
Other cardiovascular and circulatory diseases	Male	Mean BMI		X			X	
Other cardiovascular and circulatory diseases	Female	Mean BMI		X			X	
Other cardiovascular and circulatory diseases	Male	Socio-demographic Index			X			X
Other cardiovascular and circulatory diseases	Female	Socio-demographic Index			X			X
Other cardiovascular and circulatory diseases	Male	Healthcare access and quality index		X			X	
Other cardiovascular and circulatory diseases	Female	Healthcare access and quality index		X			X	
Other cardiovascular and circulatory diseases	Female	Diabetes Fasting Plasma Glucose (mmol/L), age-standardized 25+		X			X	
Other cardiovascular and circulatory diseases	Male	Cholesterol (total, mean per capita)	X			X		
Other cardiovascular and circulatory diseases	Female	Log-transformed SEV scalar: Oth Cardio	X			X		
Other cardiovascular and circulatory diseases	Female	Cholesterol (total, mean per capita)	X			X		
Other cardiovascular and circulatory diseases	Female	Systolic Blood Pressure (mmHg)	X			X		
Other cardiovascular and circulatory diseases	Male	Indoor Air Pollution (All Cooking Fuels)		X			X	
Other cardiovascular and circulatory diseases	Female	Indoor Air Pollution (All Cooking Fuels)		X			X	
Other cardiovascular and circulatory diseases	Male	Outdoor Air Pollution (PM2.5)		X			X	
Other cardiovascular and circulatory diseases	Female	Outdoor Air Pollution (PM2.5)		X			X	
Other cardiovascular and circulatory diseases	Male	Elevation Over 1500m (proportion)		X			X	
Other cardiovascular and circulatory diseases	Female	Elevation Over 1500m (proportion)		X			X	
Other cardiovascular and circulatory diseases	Male	Smoking Prevalence	X			X		
Other cardiovascular and circulatory diseases	Female	Smoking Prevalence	X			X		
Other cardiovascular and circulatory diseases	Male	pufa adjusted(percent)			X			X
Other cardiovascular and circulatory diseases	Female	pufa adjusted(percent)			X			X
Other cardiovascular and circulatory diseases	Male	Systolic Blood Pressure (mmHg)	X			X		
Other cardiovascular and circulatory diseases	Male	Diabetes Fasting Plasma Glucose (mmol/L), age-standardized 25+		X			X	
Chronic respiratory diseases	Male	Outdoor Air Pollution (PM2.5)		X			X	
Chronic respiratory diseases	Male	Smoking Prevalence		X			X	
Chronic respiratory diseases	Female	Population Density (over 1000 ppl/sqkm, proportion)			X			X
Chronic respiratory diseases	Male	Population Density (over 1000 ppl/sqkm, proportion)			X			X
Chronic respiratory diseases	Female	Cumulative Cigarettes (5 Years)	X			X		
Chronic respiratory diseases	Male	Cumulative Cigarettes (5 Years)	X			X		
Chronic respiratory diseases	Female	Elevation 500 to 1500m (proportion)			X			X
Chronic respiratory diseases	Male	Elevation 500 to 1500m (proportion)			X			X
Chronic respiratory diseases	Male	Smoking Prevalence	X			X		
Chronic respiratory diseases	Male	Smoking Prevalence	X				X	
Chronic respiratory diseases	Male	Smoking Prevalence		X		X		
Chronic respiratory diseases	Female	Smoking Prevalence		X			X	
Chronic respiratory diseases	Male	Elevation Over 1500m (proportion)		X			X	

Table S18. Comparison of GBD 2017 and GBD 2019 covariates and level of covariates used in cause of death modeling								
Cause	Sex	Covariate	Level 1: GBD 2019	Level 2: GBD 2019	Level 3: GBD 2019	Level 1: GBD 2017	Level 2: GBD 2017	Level 3: GBD 2017
Chronic respiratory diseases	Female	Elevation Over 1500m (proportion)		X			X	
Chronic respiratory diseases	Female	Outdoor Air Pollution (PM2.5)		X			X	
Chronic respiratory diseases	Male	Indoor Air Pollution (All Cooking Fuels)	X			X		
Chronic respiratory diseases	Male	Indoor Air Pollution (All Cooking Fuels)	X				X	
Chronic respiratory diseases	Male	Indoor Air Pollution (All Cooking Fuels)		X		X		
Chronic respiratory diseases	Male	Indoor Air Pollution (All Cooking Fuels)		X			X	
Chronic respiratory diseases	Female	Indoor Air Pollution (All Cooking Fuels)		X			X	
Chronic respiratory diseases	Male	Healthcare access and quality index		X			X	
Chronic respiratory diseases	Female	Healthcare access and quality index	X			X		
Chronic respiratory diseases	Male	Socio-demographic Index			X			X
Chronic respiratory diseases	Female	Socio-demographic Index			X			X
Chronic respiratory diseases	Male	LDI (I$ per capita)			X			X
Chronic respiratory diseases	Female	LDI (I$ per capita)			X			X
Chronic respiratory diseases	Male	Education (years per capita)			X			X
Chronic respiratory diseases	Female	Education (years per capita)			X			X
Chronic respiratory diseases	Male	Cumulative Cigarettes (10 Years)	X			X		
Chronic respiratory diseases	Female	Cumulative Cigarettes (10 Years)	X			X		
Chronic obstructive pulmonary disease	Female	Cumulative Cigarettes (5 Years)	X			X		
Chronic obstructive pulmonary disease	Male	Cumulative Cigarettes (20 Years)	X			X		
Chronic obstructive pulmonary disease	Female	Education (years per capita)			X			X
Chronic obstructive pulmonary disease	Male	Cumulative Cigarettes (5 Years)	X			X		
Chronic obstructive pulmonary disease	Female	Cumulative Cigarettes (10 Years)	X			X		
Chronic obstructive pulmonary disease	Male	Education (years per capita)			X			X
Chronic obstructive pulmonary disease	Female	Smoking Prevalence		X			X	
Chronic obstructive pulmonary disease	Male	Smoking Prevalence		X			X	
Chronic obstructive pulmonary disease	Female	Elevation Over 1500m (proportion)	X			X		
Chronic obstructive pulmonary disease	Male	Elevation Over 1500m (proportion)	X			X		
Chronic obstructive pulmonary disease	Female	Outdoor Air Pollution (PM2.5)	X			X		
Chronic obstructive pulmonary disease	Male	Outdoor Air Pollution (PM2.5)	X			X		
Chronic obstructive pulmonary disease	Female	Indoor Air Pollution (All Cooking Fuels)		X			X	
Chronic obstructive pulmonary disease	Male	Cumulative Cigarettes (10 Years)	X			X		
Chronic obstructive pulmonary disease	Male	Log-transformed SEV scalar: COPD	X			X		
Chronic obstructive pulmonary disease	Male	Indoor Air Pollution (All Cooking Fuels)		X			X	
Chronic obstructive pulmonary disease	Female	Healthcare access and quality index	X			X		
Chronic obstructive pulmonary disease	Male	Healthcare access and quality index		X			X	
Chronic obstructive pulmonary disease	Female	Socio-demographic Index			X			X
Chronic obstructive pulmonary disease	Male	Socio-demographic Index			X			X
Chronic obstructive pulmonary disease	Female	LDI (I$ per capita)			X			X
Chronic obstructive pulmonary disease	Male	LDI (I$ per capita)			X			X
Chronic obstructive pulmonary disease	Female	Log-transformed SEV scalar: COPD	X			X		
Pneumoconiosis	Female	Cumulative Cigarettes (5 Years)		X			X	
Pneumoconiosis	Male	Cumulative Cigarettes (5 Years)		X			X	
Pneumoconiosis	Female	Smoking Prevalence		X			X	
Pneumoconiosis	Female	Indoor Air Pollution (All Cooking Fuels)		X			X	
Pneumoconiosis	Male	Indoor Air Pollution (All Cooking Fuels)		X			X	
Pneumoconiosis	Female	Healthcare access and quality index		X			X	
Pneumoconiosis	Female	LDI (I$ per capita)			X			X
Pneumoconiosis	Female	Socio-demographic Index			X			X
Pneumoconiosis	Male	Socio-demographic Index			X			X
Pneumoconiosis	Male	LDI (I$ per capita)			X			X
Pneumoconiosis	Female	Education (years per capita)			X			X
Pneumoconiosis	Male	Education (years per capita)			X			X
Pneumoconiosis	Male	Education (years per capita)			X			X
Pneumoconiosis	Male	Asbestos consumption (metric tons per year per capita)	X			X		
Pneumoconiosis	Male	Healthcare access and quality index		X			X	
Pneumoconiosis	Female	Asbestos consumption (metric tons per year per capita)	X			X		
Pneumoconiosis	Male	Smoking Prevalence		X			X	
Silicosis	Female	Smoking Prevalence		X			X	
Silicosis	Female	Education (years per capita)			X			X
Silicosis	Male	LDI (I$ per capita)			X			X
Silicosis	Female	LDI (I$ per capita)			X			X
Silicosis	Female	Socio-demographic Index			X			X
Silicosis	Male	Healthcare access and quality index		X			X	
Silicosis	Male	Socio-demographic Index			X			X
Silicosis	Male	Indoor Air Pollution (All Cooking Fuels)		X			X	
Silicosis	Female	Indoor Air Pollution (All Cooking Fuels)		X			X	
Silicosis	Male	Smoking Prevalence		X			X	
Silicosis	Male	Education (years per capita)			X			X
Silicosis	Female	Cumulative Cigarettes (5 Years)		X			X	
Silicosis	Female	Healthcare access and quality index		X			X	
Silicosis	Male	Cumulative Cigarettes (5 Years)		X			X	
Asbestosis	Female	Socio-demographic Index			X			X
Asbestosis	Male	Education (years per capita)			X			X
Asbestosis	Female	Education (years per capita)			X			X
Asbestosis	Male	LDI (I$ per capita)			X			X
Asbestosis	Female	LDI (I$ per capita)			X			X
Asbestosis	Male	Socio-demographic Index			X			X
Asbestosis	Female	Healthcare access and quality index		X			X	
Asbestosis	Male	Indoor Air Pollution (All Cooking Fuels)		X			X	
Asbestosis	Female	Asbestos consumption (metric tons per year per capita)	X			X		
Asbestosis	Male	Asbestos consumption (metric tons per year per capita)	X			X		

Table S18. Comparison of GBD 2017 and GBD 2019 covariates and level of covariates used in cause of death modeling								
**Cause**	**Sex**	**Covariate**	**Level 1: GBD 2019**	**Level 2: GBD 2019**	**Level 3: GBD 2019**	**Level 1: GBD 2017**	**Level 2: GBD 2017**	**Level 3: GBD 2017**
Asbestosis	Female	Elevation 500 to 1500m (proportion)		X			X	
Asbestosis	Male	Elevation 500 to 1500m (proportion)		X			X	
Asbestosis	Female	Cumulative Cigarettes (5 Years)		X			X	
Asbestosis	Male	Cumulative Cigarettes (5 Years)		X			X	
Asbestosis	Male	Healthcare access and quality index		X			X	
Asbestosis	Female	Cumulative Cigarettes (10 Years)		X			X	
Asbestosis	Male	Smoking Prevalence	X				X	
Asbestosis	Male	Smoking Prevalence	X			X		
Asbestosis	Male	Smoking Prevalence		X			X	
Asbestosis	Male	Smoking Prevalence		X		X		
Asbestosis	Female	Elevation Over 1500m (proportion)		X			X	
Asbestosis	Male	Elevation Over 1500m (proportion)		X			X	
Asbestosis	Female	Indoor Air Pollution (All Cooking Fuels)		X			X	
Asbestosis	Female	Smoking Prevalence		X			X	
Coal workers pneumoconiosis	Female	LDI (I$ per capita)			X			X
Coal workers pneumoconiosis	Male	LDI (I$ per capita)			X			X
Coal workers pneumoconiosis	Female	Socio-demographic Index			X			X
Coal workers pneumoconiosis	Male	Cumulative Cigarettes (5 Years)		X			X	
Coal workers pneumoconiosis	Female	Education (years per capita)			X			X
Coal workers pneumoconiosis	Male	Education (years per capita)			X			X
Coal workers pneumoconiosis	Female	Cumulative Cigarettes (5 Years)		X			X	
Coal workers pneumoconiosis	Male	Healthcare access and quality index		X			X	
Coal workers pneumoconiosis	Male	Socio-demographic Index			X			X
Coal workers pneumoconiosis	Male	Indoor Air Pollution (All Cooking Fuels)		X			X	
Coal workers pneumoconiosis	Female	Indoor Air Pollution (All Cooking Fuels)		X			X	
Coal workers pneumoconiosis	Male	Smoking Prevalence		X			X	
Coal workers pneumoconiosis	Female	Smoking Prevalence		X			X	
Coal workers pneumoconiosis	Female	Healthcare access and quality index		X			X	
Other pneumoconiosis	Male	Indoor Air Pollution (All Cooking Fuels)		X			X	
Other pneumoconiosis	Female	Cumulative Cigarettes (5 Years)		X			X	
Other pneumoconiosis	Male	Cumulative Cigarettes (5 Years)		X			X	
Other pneumoconiosis	Male	Smoking Prevalence		X			X	
Other pneumoconiosis	Female	Indoor Air Pollution (All Cooking Fuels)		X			X	
Other pneumoconiosis	Female	Healthcare access and quality index		X			X	
Other pneumoconiosis	Female	Smoking Prevalence		X			X	
Other pneumoconiosis	Male	Education (years per capita)			X			X
Other pneumoconiosis	Female	Education (years per capita)			X			X
Other pneumoconiosis	Male	LDI (I$ per capita)			X			X
Other pneumoconiosis	Female	LDI (I$ per capita)			X			X
Other pneumoconiosis	Male	Socio-demographic Index			X			X
Other pneumoconiosis	Female	Socio-demographic Index			X			X
Other pneumoconiosis	Male	Healthcare access and quality index		X			X	
Asthma	Female	Cumulative Cigarettes (5 Years)	X			X		
Asthma	Male	Education (years per capita)			X			X
Asthma	Male	Cumulative Cigarettes (5 Years)	X			X		
Asthma	Female	Education (years per capita)			X			X
Asthma	Male	LDI (I$ per capita)			X			X
Asthma	Female	LDI (I$ per capita)			X			X
Asthma	Male	Socio-demographic Index			X			X
Asthma	Male	Log-transformed SEV scalar: Asthma	X			X		
Asthma	Female	Socio-demographic Index			X			X
Asthma	Male	Healthcare access and quality index	X			X		
Asthma	Female	Healthcare access and quality index	X			X		
Asthma	Male	Indoor Air Pollution (All Cooking Fuels)		X			X	
Asthma	Female	Indoor Air Pollution (All Cooking Fuels)		X			X	
Asthma	Male	Outdoor Air Pollution (PM2.5)		X			X	
Asthma	Female	Outdoor Air Pollution (PM2.5)		X			X	
Asthma	Male	Smoking Prevalence		X			X	
Asthma	Female	Smoking Prevalence		X			X	
Asthma	Male	Cumulative Cigarettes (10 Years)	X			X		
Asthma	Female	Cumulative Cigarettes (10 Years)	X			X		
Asthma	Female	Log-transformed SEV scalar: Asthma	X			X		
Interstitial lung disease and pulmonary sarcoidosis	Male	Cumulative Cigarettes (10 Years)	X			X		
Interstitial lung disease and pulmonary sarcoidosis	Female	Cumulative Cigarettes (10 Years)	X			X		
Interstitial lung disease and pulmonary sarcoidosis	Female	Indoor Air Pollution (All Cooking Fuels)		X			X	
Interstitial lung disease and pulmonary sarcoidosis	Female	Cumulative Cigarettes (5 Years)	X			X		
Interstitial lung disease and pulmonary sarcoidosis	Male	Indoor Air Pollution (All Cooking Fuels)		X			X	
Interstitial lung disease and pulmonary sarcoidosis	Female	Healthcare access and quality index		X			X	
Interstitial lung disease and pulmonary sarcoidosis	Male	Healthcare access and quality index		X			X	
Interstitial lung disease and pulmonary sarcoidosis	Female	Socio-demographic Index			X			X
Interstitial lung disease and pulmonary sarcoidosis	Male	Socio-demographic Index			X			X
Interstitial lung disease and pulmonary sarcoidosis	Female	LDI (I$ per capita)			X			X
Interstitial lung disease and pulmonary sarcoidosis	Female	Indoor Air Pollution (All Cooking Fuels)		X		X		
Interstitial lung disease and pulmonary sarcoidosis	Male	LDI (I$ per capita)			X			X
Interstitial lung disease and pulmonary sarcoidosis	Female	Education (years per capita)			X			X
Interstitial lung disease and pulmonary sarcoidosis	Female	Education (years per capita)			X			X
Interstitial lung disease and pulmonary sarcoidosis	Male	Education (years per capita)			X			X
Interstitial lung disease and pulmonary sarcoidosis	Female	Indoor Air Pollution (All Cooking Fuels)	X			X		
Interstitial lung disease and pulmonary sarcoidosis	Female	Indoor Air Pollution (All Cooking Fuels)	X				X	
Interstitial lung disease and pulmonary sarcoidosis	Male	Outdoor Air Pollution (PM2.5)		X			X	
Interstitial lung disease and pulmonary sarcoidosis	Female	Outdoor Air Pollution (PM2.5)		X		X		
Interstitial lung disease and pulmonary sarcoidosis	Female	Outdoor Air Pollution (PM2.5)		X			X	
Interstitial lung disease and pulmonary sarcoidosis	Female	Outdoor Air Pollution (PM2.5)	X			X		

Table S18. Comparison of GBD 2017 and GBD 2019 covariates and level of covariates used in cause of death modeling

Cause	Sex	Covariate	Level 1: GBD 2019	Level 2: GBD 2019	Level 3: GBD 2019	Level 1: GBD 2017	Level 2: GBD 2017	Level 3: GBD 2017
Interstitial lung disease and pulmonary sarcoidosis	Male	Smoking Prevalence	X			X		
Interstitial lung disease and pulmonary sarcoidosis	Female	Smoking Prevalence	X			X		
Interstitial lung disease and pulmonary sarcoidosis	Male	Cumulative Cigarettes (5 Years)	X			X		
Interstitial lung disease and pulmonary sarcoidosis	Female	Outdoor Air Pollution (PM2.5)	X				X	
Other chronic respiratory diseases	Male	Outdoor Air Pollution (PM2.5)	X			X		
Other chronic respiratory diseases	Female	Outdoor Air Pollution (PM2.5)	X			X		
Other chronic respiratory diseases	Female	Elevation Over 1500m (proportion)		X			X	
Other chronic respiratory diseases	Male	Elevation Over 1500m (proportion)		X			X	
Other chronic respiratory diseases	Male	Indoor Air Pollution (All Cooking Fuels)	X			X		
Other chronic respiratory diseases	Female	Indoor Air Pollution (All Cooking Fuels)	X			X		
Other chronic respiratory diseases	Male	Healthcare access and quality index		X			X	
Other chronic respiratory diseases	Female	Healthcare access and quality index		X			X	
Other chronic respiratory diseases	Male	Socio-demographic Index			X			X
Other chronic respiratory diseases	Female	Socio-demographic Index			X			X
Other chronic respiratory diseases	Male	LDI (I$ per capita)			X			X
Other chronic respiratory diseases	Female	Population Density (over 1000 ppl/sqkm, proportion)		X			X	
Other chronic respiratory diseases	Male	Population Density (over 1000 ppl/sqkm, proportion)		X			X	
Other chronic respiratory diseases	Female	Cumulative Cigarettes (5 Years)	X			X		
Other chronic respiratory diseases	Male	Cumulative Cigarettes (5 Years)	X			X		
Other chronic respiratory diseases	Female	Elevation 500 to 1500m (proportion)		X			X	
Other chronic respiratory diseases	Male	Elevation 500 to 1500m (proportion)		X			X	
Other chronic respiratory diseases	Female	LDI (I$ per capita)			X			X
Other chronic respiratory diseases	Male	Education (years per capita)			X			X
Other chronic respiratory diseases	Female	Education (years per capita)			X			X
Other chronic respiratory diseases	Female	Education (years per capita)			X			X
Other chronic respiratory diseases	Female	Smoking Prevalence	X			X		
Other chronic respiratory diseases	Male	Smoking Prevalence	X			X		
Cirrhosis and other chronic liver diseases	Male	Diabetes Age-Standardized Prevalence (proportion)		X			X	
Cirrhosis and other chronic liver diseases	Female	Diabetes Age-Standardized Prevalence (proportion)		X			X	
Cirrhosis and other chronic liver diseases	Male	Education (years per capita)			X			X
Cirrhosis and other chronic liver diseases	Female	Education (years per capita)			X			X
Cirrhosis and other chronic liver diseases	Male	LDI (I$ per capita)			X			X
Cirrhosis and other chronic liver diseases	Female	LDI (I$ per capita)			X			X
Cirrhosis and other chronic liver diseases	Male	Mean BMI		X			X	
Cirrhosis and other chronic liver diseases	Female	Mean BMI		X			X	
Cirrhosis and other chronic liver diseases	Male	Schistosomiasis Prevalence (proportion)	X				X	
Cirrhosis and other chronic liver diseases	Female	Schistosomiasis Prevalence (proportion)	X				X	
Cirrhosis and other chronic liver diseases	Male	Socio-demographic Index			X			X
Cirrhosis and other chronic liver diseases	Female	Socio-demographic Index			X			X
Cirrhosis and other chronic liver diseases	Male	Healthcare access and quality index		X			X	
Cirrhosis and other chronic liver diseases	Female	Healthcare access and quality index		X			X	
Digestive diseases	Female	Education (years per capita)			X			X
Digestive diseases	Male	Education (years per capita)			X			X
Digestive diseases	Male	Sanitation (proportion with access)	X			X		
Digestive diseases	Male	LDI (I$ per capita)			X			X
Digestive diseases	Female	LDI (I$ per capita)			X			X
Digestive diseases	Male	Socio-demographic Index			X			X
Digestive diseases	Female	Socio-demographic Index			X			X
Digestive diseases	Male	Healthcare access and quality index		X			X	
Digestive diseases	Female	Healthcare access and quality index		X			X	
Digestive diseases	Female	Sanitation (proportion with access)	X			X		
Digestive diseases	Male	Cumulative Cigarettes (5 Years)	X			X		
Digestive diseases	Female	Cumulative Cigarettes (5 Years)	X			X		
Peptic ulcer disease	Female	Smoking Prevalence	X			X		
Peptic ulcer disease	Female	LDI (I$ per capita)			X			X
Peptic ulcer disease	Male	Healthcare access and quality index		X			X	
Peptic ulcer disease	Male	LDI (I$ per capita)			X			X
Peptic ulcer disease	Male	Socio-demographic Index			X			X
Peptic ulcer disease	Male	Cumulative Cigarettes (5 Years)	X			X		
Peptic ulcer disease	Female	Cumulative Cigarettes (5 Years)	X			X		
Peptic ulcer disease	Male	Age- and sex-specific SEV for Unsafe water		X		X		
Peptic ulcer disease	Female	Age- and sex-specific SEV for Unsafe water		X		X		
Peptic ulcer disease	Male	Sanitation (proportion with access)		X		X		
Peptic ulcer disease	Female	Healthcare access and quality index		X			X	
Peptic ulcer disease	Male	Cumulative Cigarettes (10 Years)	X			X		
Peptic ulcer disease	Female	Cumulative Cigarettes (10 Years)	X			X		
Peptic ulcer disease	Male	Smoking Prevalence	X			X		
Peptic ulcer disease	Female	Socio-demographic Index			X			X
Peptic ulcer disease	Female	Sanitation (proportion with access)		X		X		
Gastritis and duodenitis	Male	Smoking Prevalence	X				X	
Gastritis and duodenitis	Female	LDI (I$ per capita)			X			X
Gastritis and duodenitis	Male	LDI (I$ per capita)			X			X
Gastritis and duodenitis	Male	Healthcare access and quality index		X			X	
Gastritis and duodenitis	Female	Socio-demographic Index			X			X
Gastritis and duodenitis	Male	Socio-demographic Index			X			X
Gastritis and duodenitis	Male	Education (years per capita)			X			X
Gastritis and duodenitis	Female	Cumulative Cigarettes (5 Years)	X				X	
Gastritis and duodenitis	Male	Cumulative Cigarettes (5 Years)	X				X	
Gastritis and duodenitis	Female	Education (years per capita)			X			X

Table S18. Comparison of GBD 2017 and GBD 2019 covariates and level of covariates used in cause of death modeling

Cause	Sex	Covariate	Level 1: GBD 2019	Level 2: GBD 2019	Level 3: GBD 2019	Level 1: GBD 2017	Level 2: GBD 2017	Level 3: GBD 2017
Gastritis and duodenitis	Male	Age- and sex-specific SEV for Unsafe water		X		X		
Gastritis and duodenitis	Female	Sanitation (proportion with access)		X		X		
Gastritis and duodenitis	Male	Sanitation (proportion with access)		X		X		
Gastritis and duodenitis	Female	Healthcare access and quality index		X			X	
Gastritis and duodenitis	Female	Cumulative Cigarettes (10 Years)	X				X	
Gastritis and duodenitis	Male	Cumulative Cigarettes (10 Years)	X				X	
Gastritis and duodenitis	Female	Smoking Prevalence	X				X	
Gastritis and duodenitis	Female	Age- and sex-specific SEV for Unsafe water		X		X		
Appendicitis	Male	Healthcare access and quality index		X			X	
Appendicitis	Female	Education (years per capita)			X			X
Appendicitis	Male	Education (years per capita)			X			X
Appendicitis	Male	Socio-demographic Index			X			X
Appendicitis	Female	Socio-demographic Index			X			X
Appendicitis	Female	Healthcare access and quality index		X			X	
Appendicitis	Female	LDI (I$ per capita)			X			X
Appendicitis	Male	LDI (I$ per capita)			X			X
Paralytic ileus and intestinal obstruction	Male	Education (years per capita)			X			X
Paralytic ileus and intestinal obstruction	Female	LDI (I$ per capita)			X			X
Paralytic ileus and intestinal obstruction	Male	LDI (I$ per capita)			X			X
Paralytic ileus and intestinal obstruction	Female	Socio-demographic Index			X			X
Paralytic ileus and intestinal obstruction	Male	Socio-demographic Index			X			X
Paralytic ileus and intestinal obstruction	Female	Healthcare access and quality index		X			X	
Paralytic ileus and intestinal obstruction	Female	Education (years per capita)			X			X
Paralytic ileus and intestinal obstruction	Male	Healthcare access and quality index		X			X	
Inguinal, femoral, and abdominal hernia	Female	Education (years per capita)			X			X
Inguinal, femoral, and abdominal hernia	Female	Healthcare access and quality index		X			X	
Inguinal, femoral, and abdominal hernia	Male	Education (years per capita)			X			X
Inguinal, femoral, and abdominal hernia	Female	LDI (I$ per capita)			X			X
Inguinal, femoral, and abdominal hernia	Male	Cumulative Cigarettes (5 Years)	X			X		
Inguinal, femoral, and abdominal hernia	Male	LDI (I$ per capita)			X			X
Inguinal, femoral, and abdominal hernia	Female	Mean BMI	X			X		
Inguinal, femoral, and abdominal hernia	Male	Mean BMI	X			X		
Inguinal, femoral, and abdominal hernia	Female	Socio-demographic Index			X			X
Inguinal, femoral, and abdominal hernia	Male	Socio-demographic Index			X			X
Inguinal, femoral, and abdominal hernia	Female	Cumulative Cigarettes (5 Years)	X			X		
Inguinal, femoral, and abdominal hernia	Male	Healthcare access and quality index		X			X	
Inguinal, femoral, and abdominal hernia	Female	Smoking Prevalence	X			X		
Inguinal, femoral, and abdominal hernia	Male	Smoking Prevalence	X			X		
Inguinal, femoral, and abdominal hernia	Female	Cumulative Cigarettes (10 Years)	X			X		
Inguinal, femoral, and abdominal hernia	Male	Cumulative Cigarettes (10 Years)	X			X		
Inflammatory bowel disease	Male	Latitude 30 to 45 (proportion)		X			X	
Inflammatory bowel disease	Male	Latitude Over 45 (proportion)		X			X	
Inflammatory bowel disease	Female	Latitude Over 45 (proportion)		X			X	
Inflammatory bowel disease	Female	Latitude 30 to 45 (proportion)		X			X	
Inflammatory bowel disease	Female	Healthcare access and quality index		X			X	
Inflammatory bowel disease	Female	LDI (I$ per capita)			X			X
Inflammatory bowel disease	Male	Latitude 15 to 30 (proportion)		X			X	
Inflammatory bowel disease	Female	Latitude 15 to 30 (proportion)		X			X	
Inflammatory bowel disease	Male	Healthcare access and quality index		X			X	
Inflammatory bowel disease	Male	Socio-demographic Index			X			X
Inflammatory bowel disease	Female	Socio-demographic Index			X			X
Inflammatory bowel disease	Male	LDI (I$ per capita)			X			X
Inflammatory bowel disease	Male	Education (years per capita)			X			X
Inflammatory bowel disease	Female	Education (years per capita)			X			X
Vascular intestinal disorders	Female	LDI (I$ per capita)			X			X
Vascular intestinal disorders	Male	LDI (I$ per capita)			X			X
Vascular intestinal disorders	Female	Education (years per capita)			X			X
Vascular intestinal disorders	Female	Healthcare access and quality index		X			X	
Vascular intestinal disorders	Male	Socio-demographic Index			X			X
Vascular intestinal disorders	Female	Systolic Blood Pressure (mmHg)	X			X		
Vascular intestinal disorders	Male	Education (years per capita)			X			X
Vascular intestinal disorders	Female	Socio-demographic Index			X			X
Vascular intestinal disorders	Male	Cholesterol (total, mean per capita)	X			X		
Vascular intestinal disorders	Male	Diabetes Fasting Plasma Glucose (mmol/L), age-standardized 25+	X			X		
Vascular intestinal disorders	Female	Diabetes Fasting Plasma Glucose (mmol/L), age-standardized 25+	X			X		
Vascular intestinal disorders	Female	Cholesterol (total, mean per capita)	X			X		
Vascular intestinal disorders	Male	Systolic Blood Pressure (mmHg)	X			X		
Vascular intestinal disorders	Male	Healthcare access and quality index		X			X	
Gallbladder and biliary diseases	Male	Mean BMI	X			X		
Gallbladder and biliary diseases	Female	Mean BMI	X			X		
Gallbladder and biliary diseases	Female	Socio-demographic Index			X			X
Gallbladder and biliary diseases	Male	LDI (I$ per capita)			X			X
Gallbladder and biliary diseases	Female	Population Over 65 (proportion)		X			X	
Gallbladder and biliary diseases	Male	Education (years per capita)			X			X
Gallbladder and biliary diseases	Male	Socio-demographic Index			X			X
Gallbladder and biliary diseases	Male	Population Over 65 (proportion)		X			X	
Gallbladder and biliary diseases	Female	LDI (I$ per capita)			X			X
Gallbladder and biliary diseases	Female	Healthcare access and quality index		X			X	
Gallbladder and biliary diseases	Female	Education (years per capita)			X			X
Gallbladder and biliary diseases	Male	Healthcare access and quality index		X			X	
Pancreatitis	Male	Education (years per capita)			X			X

Table S18. Comparison of GBD 2017 and GBD 2019 covariates and level of covariates used in cause of death modeling								
Cause	Sex	Covariate	Level 1: GBD 2019	Level 2: GBD 2019	Level 3: GBD 2019	Level 1: GBD 2017	Level 2: GBD 2017	Level 3: GBD 2017
Pancreatitis	Female	LDI (I$ per capita)			X			X
Pancreatitis	Male	Log-transformed SEV scalar: Pancreatit	X			X		
Pancreatitis	Male	Healthcare access and quality index		X			X	
Pancreatitis	Female	Healthcare access and quality index		X			X	
Pancreatitis	Female	Log-transformed SEV scalar: Pancreatit	X			X		
Pancreatitis	Female	Socio-demographic Index			X			X
Pancreatitis	Male	Mean BMI		X			X	
Pancreatitis	Female	Mean BMI		X			X	
Pancreatitis	Male	LDI (I$ per capita)			X			X
Pancreatitis	Female	Education (years per capita)			X			X
Pancreatitis	Male	Socio-demographic Index			X			X
Other digestive diseases	Female	Education (years per capita)			X			X
Other digestive diseases	Male	LDI (I$ per capita)			X			X
Other digestive diseases	Female	LDI (I$ per capita)			X			X
Other digestive diseases	Male	Mean BMI		X			X	
Other digestive diseases	Female	Mean BMI		X			X	
Other digestive diseases	Male	Socio-demographic Index			X			X
Other digestive diseases	Female	Socio-demographic Index			X			X
Other digestive diseases	Female	Cumulative Cigarettes (10 Years)	X			X		
Other digestive diseases	Female	Healthcare access and quality index		X			X	
Other digestive diseases	Male	Smoking Prevalence	X			X		
Other digestive diseases	Female	Smoking Prevalence	X			X		
Other digestive diseases	Male	Cumulative Cigarettes (10 Years)	X			X		
Other digestive diseases	Male	Education (years per capita)			X			X
Other digestive diseases	Male	Sanitation (proportion with access)		X			X	
Other digestive diseases	Female	Sanitation (proportion with access)		X			X	
Other digestive diseases	Male	Improved Water Source (proportion with access)		X			X	
Other digestive diseases	Female	Improved Water Source (proportion with access)		X			X	
Other digestive diseases	Male	Healthcare access and quality index		X			X	
Other digestive diseases	Female	Diabetes Age-Standardized Prevalence (proportion)		X			X	
Other digestive diseases	Female	Cumulative Cigarettes (5 Years)	X			X		
Other digestive diseases	Male	Cumulative Cigarettes (5 Years)	X			X		
Other digestive diseases	Male	Diabetes Age-Standardized Prevalence (proportion)		X			X	
Parkinson's disease	Male	Education (years per capita)			X			X
Parkinson's disease	Male	Absolute value of average latitude		X			X	
Parkinson's disease	Female	Improved Water Source (proportion with access)		X			X	
Parkinson's disease	Female	Absolute value of average latitude		X			X	
Parkinson's disease	Male	Improved Water Source (proportion with access)		X			X	
Parkinson's disease	Female	Sanitation (proportion with access)		X			X	
Parkinson's disease	Male	Sanitation (proportion with access)		X			X	
Parkinson's disease	Female	Cumulative Cigarettes (10 Years)	X			X		
Parkinson's disease	Male	Cumulative Cigarettes (10 Years)	X			X		
Parkinson's disease	Female	Cholesterol (total, mean per capita)		X			X	
Parkinson's disease	Female	Healthcare access and quality index		X			X	
Parkinson's disease	Male	Healthcare access and quality index		X			X	
Parkinson's disease	Female	Socio-demographic Index			X			X
Parkinson's disease	Male	Socio-demographic Index			X			X
Parkinson's disease	Female	LDI (I$ per capita)			X			X
Parkinson's disease	Female	Education (years per capita)			X			X
Parkinson's disease	Female	Education (years per capita)			X			X
Parkinson's disease	Male	Education (years per capita)			X			X
Parkinson's disease	Male	Cholesterol (total, mean per capita)		X			X	
Parkinson's disease	Male	LDI (I$ per capita)			X			X
Idiopathic epilepsy	Female	Systolic Blood Pressure (mmHg)	X			X		
Idiopathic epilepsy	Female	Cumulative Cigarettes (10 Years)			X			X
Idiopathic epilepsy	Male	Log-transformed SEV scalar: Idiopathic epilepsy	X			X		
Idiopathic epilepsy	Female	Log-transformed SEV scalar: Idiopathic epilepsy	X			X		
Idiopathic epilepsy	Male	Pigs (per capita)	X			X		
Idiopathic epilepsy	Female	Pigs (per capita)	X			X		
Idiopathic epilepsy	Male	Cumulative Cigarettes (5 Years)			X			X
Idiopathic epilepsy	Female	Cumulative Cigarettes (5 Years)			X			X
Idiopathic epilepsy	Male	Cumulative Cigarettes (10 Years)			X			X
Idiopathic epilepsy	Male	Systolic Blood Pressure (mmHg)	X			X		
Idiopathic epilepsy	Male	Cholesterol (total, mean per capita)		X			X	
Idiopathic epilepsy	Female	Cholesterol (total, mean per capita)		X			X	
Idiopathic epilepsy	Female	Healthcare access and quality index		X			X	
Idiopathic epilepsy	Male	Socio-demographic Index			X			X
Idiopathic epilepsy	Female	Socio-demographic Index			X			X
Idiopathic epilepsy	Male	Mean BMI		X			X	
Idiopathic epilepsy	Female	Mean BMI		X			X	
Idiopathic epilepsy	Male	LDI (I$ per capita)			X			X
Idiopathic epilepsy	Female	LDI (I$ per capita)			X			X
Idiopathic epilepsy	Male	Education (years per capita)			X			X
Idiopathic epilepsy	Female	Education (years per capita)			X			X
Idiopathic epilepsy	Male	Healthcare access and quality index		X			X	
Multiple sclerosis	Female	LDI (I$ per capita)			X			X
Multiple sclerosis	Male	Socio-demographic Index			X			X
Multiple sclerosis	Male	LDI (I$ per capita)			X			X
Multiple sclerosis	Female	Healthcare access and quality index		X			X	

Table S18. Comparison of GBD 2017 and GBD 2019 covariates and level of covariates used in cause of death modeling

Cause	Sex	Covariate	Level 1: GBD 2019	Level 2: GBD 2019	Level 3: GBD 2019	Level 1: GBD 2017	Level 2: GBD 2017	Level 3: GBD 2017
Multiple sclerosis	Male	Education (years per capita)			X			X
Multiple sclerosis	Female	Socio-demographic Index			X			X
Multiple sclerosis	Female	Education (years per capita)			X			X
Multiple sclerosis	Male	Healthcare access and quality index		X			X	
Multiple sclerosis	Female	Cumulative Cigarettes (5 Years)			X			X
Multiple sclerosis	Female	Cholesterol (total, mean per capita)		X			X	
Multiple sclerosis	Male	Smoking Prevalence			X			X
Multiple sclerosis	Female	Smoking Prevalence			X			X
Multiple sclerosis	Male	Cumulative Cigarettes (10 Years)			X			X
Multiple sclerosis	Female	Cumulative Cigarettes (10 Years)			X			X
Multiple sclerosis	Male	Absolute value of average latitude	X			X		
Multiple sclerosis	Female	Absolute value of average latitude	X			X		
Multiple sclerosis	Male	Cumulative Cigarettes (5 Years)			X			X
Multiple sclerosis	Male	Cholesterol (total, mean per capita)		X			X	
Motor neuron disease	Female	Cholesterol (total, mean per capita)	X			X		
Motor neuron disease	Male	Population-weighted mean temperature		X			X	
Motor neuron disease	Female	Population-weighted mean temperature		X			X	
Motor neuron disease	Male	Absolute value of average latitude	X			X		
Motor neuron disease	Male	Sanitation (proportion with access)		X			X	
Motor neuron disease	Male	Improved Water Source (proportion with access)		X			X	
Motor neuron disease	Female	Improved Water Source (proportion with access)		X			X	
Motor neuron disease	Male	Diabetes Fasting Plasma Glucose (mmol/L), age-standardized 25+	X			X		
Motor neuron disease	Female	Absolute value of average latitude	X			X		
Motor neuron disease	Female	Diabetes Fasting Plasma Glucose (mmol/L), age-standardized 25+	X			X		
Motor neuron disease	Male	Mean BMI	X			X		
Motor neuron disease	Female	Healthcare access and quality index		X			X	
Motor neuron disease	Male	Socio-demographic Index	X			X		
Motor neuron disease	Female	Socio-demographic Index	X			X		
Motor neuron disease	Female	Mean BMI	X			X		
Motor neuron disease	Female	Mean BMI		X		X		
Motor neuron disease	Male	LDI (I$ per capita)			X			X
Motor neuron disease	Female	LDI (I$ per capita)			X			X
Motor neuron disease	Male	Education (years per capita)			X			X
Motor neuron disease	Female	Education (years per capita)			X			X
Motor neuron disease	Male	Healthcare access and quality index		X			X	
Motor neuron disease	Male	Cholesterol (total, mean per capita)	X			X		
Motor neuron disease	Female	Sanitation (proportion with access)		X			X	
Other neurological disorders	Female	LDI (I$ per capita)			X			X
Other neurological disorders	Male	Education (years per capita)			X			X
Other neurological disorders	Female	Education (years per capita)			X			X
Other neurological disorders	Male	LDI (I$ per capita)			X			X
Other neurological disorders	Male	Mean BMI	X			X		
Other neurological disorders	Female	Mean BMI	X			X		
Other neurological disorders	Male	Socio-demographic Index			X			X
Other neurological disorders	Female	Socio-demographic Index			X			X
Other neurological disorders	Female	Healthcare access and quality index		X			X	
Other neurological disorders	Male	Cholesterol (total, mean per capita)	X			X		
Other neurological disorders	Female	Cholesterol (total, mean per capita)	X			X		
Other neurological disorders	Male	Healthcare access and quality index		X			X	
Other neurological disorders	Female	Systolic Blood Pressure (mmHg)	X			X		
Other neurological disorders	Female	Cumulative Cigarettes (5 Years)			X			X
Other neurological disorders	Male	Cumulative Cigarettes (5 Years)			X			X
Other neurological disorders	Male	Systolic Blood Pressure (mmHg)	X			X		
Other neurological disorders	Male	Population Density (over 1000 ppl/sqkm, proportion)		X			X	
Other neurological disorders	Female	Population Density (over 1000 ppl/sqkm, proportion)		X			X	
Other neurological disorders	Male	Cumulative Cigarettes (10 Years)			X			X
Other neurological disorders	Female	Smoking Prevalence			X			X
Other neurological disorders	Male	Smoking Prevalence			X			X
Other neurological disorders	Female	Cumulative Cigarettes (10 Years)			X			X
Alcohol use disorders	Female	Alcohol binge drinker proportion, age-standardized	X			X		
Alcohol use disorders	Male	Smoking Prevalence		X			X	
Alcohol use disorders	Male	Alcohol binge drinker proportion, age-standardized	X			X		
Alcohol use disorders	Female	Cumulative Cigarettes (10 Years)		X			X	
Alcohol use disorders	Male	Cumulative Cigarettes (10 Years)		X			X	
Alcohol use disorders	Female	Smoking Prevalence		X			X	
Alcohol use disorders	Female	Healthcare access and quality index		X			X	
Alcohol use disorders	Male	Education (years per capita)			X			X
Alcohol use disorders	Male	Healthcare access and quality index		X			X	
Alcohol use disorders	Female	Socio-demographic Index			X			X
Alcohol use disorders	Male	Socio-demographic Index			X			X
Alcohol use disorders	Female	LDI (I$ per capita)			X			X
Alcohol use disorders	Male	LDI (I$ per capita)			X			X
Alcohol use disorders	Female	Education (years per capita)			X			X
Drug use disorders	Female	Education (years per capita)			X			X
Drug use disorders	Male	Education (years per capita)			X			X
Drug use disorders	Male	LDI (I$ per capita)			X			X
Drug use disorders	Male	Cumulative Cigarettes (5 Years)		X			X	
Drug use disorders	Male	Socio-demographic Index			X			X

Table S18. Comparison of GBD 2017 and GBD 2019 covariates and level of covariates used in cause of death modeling

Cause	Sex	Covariate	Level 1: GBD 2019	Level 2: GBD 2019	Level 3: GBD 2019	Level 1: GBD 2017	Level 2: GBD 2017	Level 3: GBD 2017
Drug use disorders	Female	Opioids per million population per day (10 year lag)	X			X		
Drug use disorders	Male	Opioids per million population per day (10 year lag)	X			X		
Drug use disorders	Female	Intravenous drug use (age-standardized proportion)	X			X		
Drug use disorders	Male	Intravenous drug use (age-standardized proportion)	X			X		
Drug use disorders	Female	Intravenous drug use (proportion by age)	X			X		
Drug use disorders	Male	Opium Cultivation (binary)		X			X	
Drug use disorders	Female	LDI (I$ per capita)			X			X
Drug use disorders	Female	Cumulative Cigarettes (5 Years)		X			X	
Drug use disorders	Male	Cumulative Cigarettes (10 Years)		X			X	
Drug use disorders	Female	Smoking Prevalence		X			X	
Drug use disorders	Male	Smoking Prevalence		X			X	
Drug use disorders	Female	Healthcare access and quality index		X			X	
Drug use disorders	Male	Healthcare access and quality index		X			X	
Drug use disorders	Female	Socio-demographic Index			X			X
Drug use disorders	Female	Cumulative Cigarettes (10 Years)		X			X	
Drug use disorders	Male	Intravenous drug use (proportion by age)	X			X		
Drug use disorders	Female	Opium Cultivation (binary)		X			X	
Opioid use disorders	Male	Socio-demographic Index			X			X
Opioid use disorders	Male	Healthcare access and quality index		X			X	
Opioid use disorders	Female	Healthcare access and quality index		X			X	
Opioid use disorders	Female	Opioids per million population per day (5 year lag)	X			X		
Opioid use disorders	Female	Socio-demographic Index			X			X
Opioid use disorders	Male	LDI (I$ per capita)			X			X
Opioid use disorders	Female	LDI (I$ per capita)			X			X
Opioid use disorders	Male	Education (years per capita)			X			X
Opioid use disorders	Female	Education (years per capita)			X			X
Opioid use disorders	Female	Cumulative Cigarettes (10 Years)		X			X	
Opioid use disorders	Male	Cumulative Cigarettes (10 Years)		X			X	
Opioid use disorders	Female	Cumulative Cigarettes (5 Years)		X			X	
Opioid use disorders	Male	Cumulative Cigarettes (5 Years)		X			X	
Opioid use disorders	Female	Opium Cultivation (binary)		X			X	
Opioid use disorders	Male	Opium Cultivation (binary)		X			X	
Opioid use disorders	Male	Intravenous drug use (proportion by age)	X			X		
Opioid use disorders	Male	Intravenous drug use (age-standardized proportion)	X			X		
Opioid use disorders	Female	Opioids per million population per day (10 year lag)	X			X		
Opioid use disorders	Male	Opioids per million population per day (10 year lag)	X			X		
Opioid use disorders	Female	Opioids per million population per day	X			X		
Opioid use disorders	Female	Smoking Prevalence		X			X	
Opioid use disorders	Male	Smoking Prevalence		X			X	
Cocaine use disorders	Male	Smoking Prevalence	X			X		
Cocaine use disorders	Male	Cumulative Cigarettes (5 Years)	X			X		
Cocaine use disorders	Female	Cumulative Cigarettes (10 Years)	X			X		
Cocaine use disorders	Male	Cumulative Cigarettes (10 Years)	X			X		
Cocaine use disorders	Female	Smoking Prevalence	X			X		
Cocaine use disorders	Female	Healthcare access and quality index		X			X	
Cocaine use disorders	Male	Education (years per capita)			X			X
Cocaine use disorders	Female	Education (years per capita)			X			X
Cocaine use disorders	Male	LDI (I$ per capita)			X			X
Cocaine use disorders	Female	LDI (I$ per capita)			X			X
Cocaine use disorders	Male	Socio-demographic Index			X			X
Cocaine use disorders	Female	Cumulative Cigarettes (5 Years)	X			X		
Cocaine use disorders	Female	Socio-demographic Index			X			X
Cocaine use disorders	Male	Healthcare access and quality index		X			X	
Amphetamine use disorders	Female	Cumulative Cigarettes (5 Years)	X			X		
Amphetamine use disorders	Male	Cumulative Cigarettes (5 Years)	X			X		
Amphetamine use disorders	Female	Cumulative Cigarettes (10 Years)	X			X		
Amphetamine use disorders	Male	Cumulative Cigarettes (10 Years)	X			X		
Amphetamine use disorders	Female	Smoking Prevalence	X			X		
Amphetamine use disorders	Male	Smoking Prevalence	X			X		
Amphetamine use disorders	Female	Healthcare access and quality index		X			X	
Amphetamine use disorders	Male	Healthcare access and quality index		X			X	
Amphetamine use disorders	Female	Socio-demographic Index			X			X
Amphetamine use disorders	Male	Socio-demographic Index			X			X
Amphetamine use disorders	Female	LDI (I$ per capita)			X			X
Amphetamine use disorders	Male	LDI (I$ per capita)			X			X
Amphetamine use disorders	Female	Education (years per capita)			X			X
Amphetamine use disorders	Male	Education (years per capita)			X			X
Other drug use disorders	Female	Cumulative Cigarettes (5 Years)	X			X		
Other drug use disorders	Male	Cumulative Cigarettes (5 Years)	X			X		
Other drug use disorders	Female	Education (years per capita)			X			X
Other drug use disorders	Male	Healthcare access and quality index		X			X	
Other drug use disorders	Male	Education (years per capita)			X			X
Other drug use disorders	Female	LDI (I$ per capita)			X			X
Other drug use disorders	Male	LDI (I$ per capita)			X			X
Other drug use disorders	Female	Socio-demographic Index			X			X
Other drug use disorders	Male	Cumulative Cigarettes (10 Years)	X			X		
Other drug use disorders	Female	Cumulative Cigarettes (10 Years)	X			X		
Other drug use disorders	Male	Smoking Prevalence	X			X		
Other drug use disorders	Female	Smoking Prevalence	X			X		

Table S18. Comparison of GBD 2017 and GBD 2019 covariates and level of covariates used in cause of death modeling

Cause	Sex	Covariate	Level 1: GBD 2019	Level 2: GBD 2019	Level 3: GBD 2019	Level 1: GBD 2017	Level 2: GBD 2017	Level 3: GBD 2017
Other drug use disorders	Male	Socio-demographic Index			X			X
Other drug use disorders	Female	Healthcare access and quality index		X			X	
Eating disorders	Female	Education (years per capita)	X			X		
Eating disorders	Male	Education (years per capita)	X			X		
Eating disorders	Female	Socio-demographic Index			X			X
Eating disorders	Male	LDI (I$ per capita)	X			X		
Eating disorders	Female	Sanitation (proportion with access)	X			X		
Eating disorders	Male	Sanitation (proportion with access)	X			X		
Eating disorders	Female	Maternal Education (years per capita)	X			X		
Eating disorders	Male	Maternal Education (years per capita)	X			X		
Eating disorders	Female	Healthcare access and quality index		X			X	
Eating disorders	Male	Healthcare access and quality index		X			X	
Eating disorders	Female	LDI (I$ per capita)	X			X		
Eating disorders	Male	Socio-demographic Index			X			X
Anorexia nervosa	Male	Healthcare access and quality index		X			X	
Anorexia nervosa	Female	LDI (I$ per capita)	X			X		
Anorexia nervosa	Female	Sanitation (proportion with access)	X			X		
Anorexia nervosa	Male	Sanitation (proportion with access)	X			X		
Anorexia nervosa	Female	Maternal Education (years per capita)	X			X		
Anorexia nervosa	Male	Maternal Education (years per capita)	X			X		
Anorexia nervosa	Female	Healthcare access and quality index		X			X	
Anorexia nervosa	Female	Socio-demographic Index			X			X
Anorexia nervosa	Male	Socio-demographic Index			X			X
Anorexia nervosa	Male	LDI (I$ per capita)	X			X		
Anorexia nervosa	Female	Education (years per capita)	X			X		
Anorexia nervosa	Male	Education (years per capita)	X			X		
Bulimia nervosa	Female	Maternal Education (years per capita)	X			X		
Bulimia nervosa	Female	LDI (I$ per capita)	X			X		
Bulimia nervosa	Female	Education (years per capita)	X			X		
Bulimia nervosa	Male	Sanitation (proportion with access)	X			X		
Bulimia nervosa	Male	Socio-demographic Index			X			X
Bulimia nervosa	Male	Maternal Education (years per capita)	X			X		
Bulimia nervosa	Male	Education (years per capita)	X			X		
Bulimia nervosa	Male	LDI (I$ per capita)	X			X		
Bulimia nervosa	Female	Sanitation (proportion with access)	X			X		
Bulimia nervosa	Male	Healthcare access and quality index		X			X	
Bulimia nervosa	Female	Healthcare access and quality index		X			X	
Bulimia nervosa	Female	Socio-demographic Index			X			X
Diabetes mellitus	Female	Healthcare access and quality index			X	X		
Diabetes mellitus	Male	Live Births 40+ (proportion)		X			X	
Diabetes mellitus	Female	Live Births 40+ (proportion)		X			X	
Diabetes mellitus	Male	Prevalence of obesity	X			X		
Diabetes mellitus	Female	Prevalence of obesity	X			X		
Diabetes mellitus	Male	Absolute value of average latitude		X			X	
Diabetes mellitus	Female	Absolute value of average latitude		X			X	
Diabetes mellitus	Male	Age-Specific Fertility Rate		X			X	
Diabetes mellitus	Female	Age-Specific Fertility Rate		X			X	
Diabetes mellitus	Male	Mean birth weight		X			X	
Diabetes mellitus	Female	Mean birth weight		X			X	
Diabetes mellitus	Female	LDI (I$ per capita)			X			X
Diabetes mellitus	Male	LDI (I$ per capita)			X			X
Diabetes mellitus	Female	Education (years per capita)			X			X
Diabetes mellitus	Male	Education (years per capita)			X			X
Diabetes mellitus	Female	Diabetes Age-Standardized Prevalence (proportion)	X			X		
Diabetes mellitus	Male	Age-standardized SEV for Child underweight		X			X	
Diabetes mellitus	Female	Age-standardized SEV for Child underweight		X			X	
Diabetes mellitus	Male	Age-standardized SEV for Child stunting		X			X	
Diabetes mellitus	Female	Age-standardized SEV for Child stunting		X			X	
Diabetes mellitus	Male	Healthcare access and quality index	X			X		
Diabetes mellitus	Male	Healthcare access and quality index			X			X
Diabetes mellitus	Male	Healthcare access and quality index			X	X		
Diabetes mellitus	Female	Socio-demographic Index			X			X
Diabetes mellitus	Male	Socio-demographic Index			X			X
Diabetes mellitus	Female	Mean BMI	X			X		
Diabetes mellitus	Female	Healthcare access and quality index	X			X		
Diabetes mellitus	Male	Diabetes Age-Standardized Prevalence (proportion)	X			X		
Diabetes mellitus	Female	Healthcare access and quality index	X					X
Diabetes mellitus	Female	Diabetes Fasting Plasma Glucose (mmol/L), age-standardized 25+	X			X		
Diabetes mellitus	Male	Cholesterol (total, mean per capita)		X			X	
Diabetes mellitus	Female	Cholesterol (total, mean per capita)		X			X	
Diabetes mellitus	Male	Systolic Blood Pressure (mmHg)		X			X	
Diabetes mellitus	Female	Systolic Blood Pressure (mmHg)		X			X	
Diabetes mellitus	Male	Live Births 35+ (proportion)		X			X	
Diabetes mellitus	Female	Live Births 35+ (proportion)		X			X	
Diabetes mellitus	Male	Diabetes Fasting Plasma Glucose (mmol/L), age-standardized 25+	X			X		
Diabetes mellitus	Female	Healthcare access and quality index			X			X
Diabetes mellitus	Male	Mean BMI	X			X		
Diabetes mellitus	Male	Healthcare access and quality index	X					X
Acute glomerulonephritis	Male	Diabetes Age-Standardized Prevalence (proportion)		X			X	

Table S18. Comparison of GBD 2017 and GBD 2019 covariates and level of covariates used in cause of death modeling								
Cause	Sex	Covariate	Level 1: GBD 2019	Level 2: GBD 2019	Level 3: GBD 2019	Level 1: GBD 2017	Level 2: GBD 2017	Level 3: GBD 2017
Acute glomerulonephritis	Male	Improved Water Source (proportion with access)		X			X	
Acute glomerulonephritis	Female	Improved Water Source (proportion with access)		X			X	
Acute glomerulonephritis	Male	Sanitation (proportion with access)		X			X	
Acute glomerulonephritis	Female	Diabetes Age-Standardized Prevalence (proportion)		X			X	
Acute glomerulonephritis	Male	Systolic Blood Pressure (mmHg)		X			X	
Acute glomerulonephritis	Female	Education (years per capita)			X			X
Acute glomerulonephritis	Female	Sanitation (proportion with access)		X			X	
Acute glomerulonephritis	Male	Healthcare access and quality index		X			X	
Acute glomerulonephritis	Female	Healthcare access and quality index		X			X	
Acute glomerulonephritis	Male	Socio-demographic Index			X			X
Acute glomerulonephritis	Female	Socio-demographic Index			X			X
Acute glomerulonephritis	Male	LDI (I$ per capita)			X			X
Acute glomerulonephritis	Female	LDI (I$ per capita)			X			X
Acute glomerulonephritis	Male	Education (years per capita)			X			X
Acute glomerulonephritis	Female	Systolic Blood Pressure (mmHg)		X			X	
Chronic kidney disease	Female	Education (years per capita)			X			X
Chronic kidney disease	Male	Education (years per capita)			X			X
Chronic kidney disease	Male	Education (years per capita)			X			X
Chronic kidney disease	Female	Diabetes Age-Standardized Prevalence (proportion)	X			X		
Chronic kidney disease	Male	Diabetes Age-Standardized Prevalence (proportion)	X			X		
Chronic kidney disease	Female	Diabetes Fasting Plasma Glucose (mmol/L), age-standardized 25+	X			X		
Chronic kidney disease	Female	energy unadjusted(kcal)		X			X	
Chronic kidney disease	Female	Cholesterol (total, mean per capita)		X			X	
Chronic kidney disease	Male	Systolic Blood Pressure (mmHg)	X			X		
Chronic kidney disease	Female	Systolic Blood Pressure (mmHg)	X			X		
Chronic kidney disease	Male	energy unadjusted(kcal)		X			X	
Chronic kidney disease	Female	Education (years per capita)			X			X
Chronic kidney disease	Male	Cholesterol (total, mean per capita)		X			X	
Chronic kidney disease	Male	LDI (I$ per capita)			X			X
Chronic kidney disease	Female	LDI (I$ per capita)			X			X
Chronic kidney disease	Female	LDI (I$ per capita)			X			X
Chronic kidney disease	Male	Mean BMI	X			X		
Chronic kidney disease	Female	Mean BMI	X			X		
Chronic kidney disease	Male	Socio-demographic Index			X			X
Chronic kidney disease	Female	Socio-demographic Index			X			X
Chronic kidney disease	Male	Healthcare access and quality index	X			X		
Chronic kidney disease	Female	Healthcare access and quality index	X			X		
Chronic kidney disease	Male	Diabetes Fasting Plasma Glucose (mmol/L), age-standardized 25+	X			X		
Chronic kidney disease	Male	LDI (I$ per capita)			X			X
Urinary diseases and male infertility	Male	Mean BMI		X			X	
Urinary diseases and male infertility	Female	Healthcare access and quality index		X			X	
Urinary diseases and male infertility	Male	Healthcare access and quality index		X			X	
Urinary diseases and male infertility	Female	Socio-demographic Index			X			X
Urinary diseases and male infertility	Male	Socio-demographic Index			X			X
Urinary diseases and male infertility	Female	Education (years per capita)			X			X
Urinary diseases and male infertility	Female	LDI (I$ per capita)			X			X
Urinary diseases and male infertility	Male	LDI (I$ per capita)			X			X
Urinary diseases and male infertility	Male	Education (years per capita)			X			X
Urinary diseases and male infertility	Female	Mean BMI		X			X	
Urinary tract infections and interstitial nephritis	Male	Healthcare access and quality index		X			X	
Urinary tract infections and interstitial nephritis	Female	Sanitation (proportion with access)	X			X		
Urinary tract infections and interstitial nephritis	Female	Socio-demographic Index			X			X
Urinary tract infections and interstitial nephritis	Female	Healthcare access and quality index		X			X	
Urinary tract infections and interstitial nephritis	Female	LDI (I$ per capita)		X			X	
Urinary tract infections and interstitial nephritis	Male	LDI (I$ per capita)		X			X	
Urinary tract infections and interstitial nephritis	Female	Education (years per capita)		X			X	
Urinary tract infections and interstitial nephritis	Male	Socio-demographic Index			X			X
Urinary tract infections and interstitial nephritis	Male	Sanitation (proportion with access)	X			X		
Urinary tract infections and interstitial nephritis	Male	Education (years per capita)		X			X	
Urolithiasis	Male	Education (years per capita)			X			X
Urolithiasis	Male	90th percentile climatic temperature in the given country-year.	X			X		
Urolithiasis	Female	90th percentile climatic temperature in the given country-year.	X			X		
Urolithiasis	Male	Healthcare access and quality index	X				X	
Urolithiasis	Male	Healthcare access and quality index		X			X	
Urolithiasis	Female	Healthcare access and quality index	X				X	
Urolithiasis	Male	Socio-demographic Index			X			X
Urolithiasis	Female	Socio-demographic Index			X			X
Urolithiasis	Male	LDI (I$ per capita)			X			X
Urolithiasis	Female	LDI (I$ per capita)			X			X
Urolithiasis	Female	Education (years per capita)			X			X
Other urinary diseases	Male	LDI (I$ per capita)		X			X	
Other urinary diseases	Female	LDI (I$ per capita)	X				X	
Other urinary diseases	Male	Mean BMI	X			X		
Other urinary diseases	Female	Mean BMI	X			X		
Other urinary diseases	Female	Healthcare access and quality index		X			X	
Other urinary diseases	Female	Socio-demographic Index			X			X
Other urinary diseases	Male	Healthcare access and quality index		X			X	
Other urinary diseases	Female	Education (years per capita)	X				X	

Table S18. Comparison of GBD 2017 and GBD 2019 covariates and level of covariates used in cause of death modeling

Cause	Sex	Covariate	Level 1: GBD 2019	Level 2: GBD 2019	Level 3: GBD 2019	Level 1: GBD 2017	Level 2: GBD 2017	Level 3: GBD 2017
Other urinary diseases	Male	Socio-demographic Index			X			X
Other urinary diseases	Male	Education (years per capita)		X			X	
Gynecological diseases	Female	LDI (I$ per capita)			X			X
Gynecological diseases	Female	Total Fertility Rate		X			X	
Gynecological diseases	Female	Socio-demographic Index			X			X
Gynecological diseases	Female	Education (years per capita)			X			X
Gynecological diseases	Female	Healthcare access and quality index		X			X	
Gynecological diseases	Female	Live Births 35+ (proportion)		X			X	
Gynecological diseases	Female	Total Fertility Rate		X			X	
Gynecological diseases	Female	Skilled Birth Attendance (proportion)		X			X	
Gynecological diseases	Female	Maternal care and immunization		X			X	
Gynecological diseases	Female	Live Births 35+ (proportion)		X			X	
Uterine fibroids	Female	Total Fertility Rate		X			X	
Uterine fibroids	Female	Maternal care and immunization		X			X	
Uterine fibroids	Female	Skilled Birth Attendance (proportion)		X			X	
Uterine fibroids	Female	Live Births 35+ (proportion)		X			X	
Uterine fibroids	Female	Healthcare access and quality index		X			X	
Uterine fibroids	Female	Socio-demographic Index			X			X
Uterine fibroids	Female	LDI (I$ per capita)			X			X
Uterine fibroids	Female	Education (years per capita)			X			X
Endometriosis	Female	Maternal care and immunization		X			X	
Endometriosis	Female	Skilled Birth Attendance (proportion)		X			X	
Endometriosis	Female	Live Births 35+ (proportion)		X			X	
Endometriosis	Female	Healthcare access and quality index		X			X	
Endometriosis	Female	Socio-demographic Index			X			X
Endometriosis	Female	LDI (I$ per capita)			X			X
Endometriosis	Female	Education (years per capita)			X			X
Endometriosis	Female	Total Fertility Rate		X			X	
Genital prolapse	Female	Education (years per capita)			X			X
Genital prolapse	Female	LDI (I$ per capita)			X			X
Genital prolapse	Female	Socio-demographic Index			X			X
Genital prolapse	Female	Live Births 35+ (proportion)		X			X	
Genital prolapse	Female	Skilled Birth Attendance (proportion)		X			X	
Genital prolapse	Female	Maternal care and immunization		X			X	
Genital prolapse	Female	Total Fertility Rate		X			X	
Genital prolapse	Female	Healthcare access and quality index		X			X	
Other gynecological diseases	Female	Total Fertility Rate		X			X	
Other gynecological diseases	Female	Maternal care and immunization		X			X	
Other gynecological diseases	Female	Skilled Birth Attendance (proportion)		X			X	
Other gynecological diseases	Female	Live Births 35+ (proportion)		X			X	
Other gynecological diseases	Female	Healthcare access and quality index		X			X	
Other gynecological diseases	Female	Socio-demographic Index			X			X
Other gynecological diseases	Female	LDI (I$ per capita)			X			X
Other gynecological diseases	Female	Education (years per capita)			X			X
Hemoglobinopathies and hemolytic anaemias	Male	Latitude Over 45 (proportion)			X			X
Hemoglobinopathies and hemolytic anaemias	Female	Latitude Over 45 (proportion)			X			X
Hemoglobinopathies and hemolytic anaemias	Male	Latitude Under 15 (proportion)			X			X
Hemoglobinopathies and hemolytic anaemias	Female	Latitude Under 15 (proportion)			X			X
Hemoglobinopathies and hemolytic anaemias	Female	Hemoglobinopathies Prevalence x Excess Mortality (excluding G6PD deficiency)	X			X		
Hemoglobinopathies and hemolytic anaemias	Female	Malaria Lysenko PFPR 1 (Holoendemic)			X	X		
Hemoglobinopathies and hemolytic anaemias	Male	Hemoglobinopathies Prevalence x Excess Mortality	X			X		
Hemoglobinopathies and hemolytic anaemias	Female	Hemoglobinopathies Prevalence x Excess Mortality	X			X		
Hemoglobinopathies and hemolytic anaemias	Male	Hemoglobinopathies Prevalence x Excess Mortality (excluding G6PD deficiency)	X			X		
Hemoglobinopathies and hemolytic anaemias	Male	Malaria Lysenko PFPR 1 (Holoendemic)			X	X		
Hemoglobinopathies and hemolytic anaemias	Male	Latitude 30 to 45 (proportion)			X			X
Hemoglobinopathies and hemolytic anaemias	Female	Latitude 30 to 45 (proportion)			X			X
Hemoglobinopathies and hemolytic anaemias	Male	Latitude 15 to 30 (proportion)			X			X
Hemoglobinopathies and hemolytic anaemias	Female	Maternal care and immunization		X			X	
Hemoglobinopathies and hemolytic anaemias	Male	Maternal care and immunization		X			X	
Hemoglobinopathies and hemolytic anaemias	Male	Education (years per capita)			X			X
Hemoglobinopathies and hemolytic anaemias	Female	Education (years per capita)			X			X
Hemoglobinopathies and hemolytic anaemias	Male	LDI (I$ per capita)			X			X
Hemoglobinopathies and hemolytic anaemias	Female	LDI (I$ per capita)			X			X
Hemoglobinopathies and hemolytic anaemias	Male	Socio-demographic Index			X			X
Hemoglobinopathies and hemolytic anaemias	Female	Socio-demographic Index			X			X
Hemoglobinopathies and hemolytic anaemias	Male	Healthcare access and quality index		X			X	
Hemoglobinopathies and hemolytic anaemias	Female	Healthcare access and quality index		X			X	
Hemoglobinopathies and hemolytic anaemias	Female	Latitude 15 to 30 (proportion)			X			X
Endocrine, metabolic, blood, and immune disorders	Female	Cholesterol (total, mean per capita)		X			X	
Endocrine, metabolic, blood, and immune disorders	Female	Healthcare access and quality index		X			X	
Endocrine, metabolic, blood, and immune disorders	Male	Healthcare access and quality index		X			X	
Endocrine, metabolic, blood, and immune disorders	Female	Socio-demographic Index			X			X
Endocrine, metabolic, blood, and immune disorders	Male	Socio-demographic Index			X			X
Endocrine, metabolic, blood, and immune disorders	Female	Mean BMI	X			X		
Endocrine, metabolic, blood, and immune disorders	Male	Mean BMI	X			X		
Endocrine, metabolic, blood, and immune disorders	Female	LDI (I$ per capita)			X			X
Endocrine, metabolic, blood, and immune disorders	Male	LDI (I$ per capita)			X			X
Endocrine, metabolic, blood, and immune disorders	Female	Education (years per capita)			X			X
Endocrine, metabolic, blood, and immune disorders	Male	Education (years per capita)			X			X
Endocrine, metabolic, blood, and immune disorders	Male	Cholesterol (total, mean per capita)		X			X	
Musculoskeletal disorders	Male	LDI (I$ per capita)		X			X	

Table S18. Comparison of GBD 2017 and GBD 2019 covariates and level of covariates used in cause of death modeling								
Cause	Sex	Covariate	Level 1: GBD 2019	Level 2: GBD 2019	Level 3: GBD 2019	Level 1: GBD 2017	Level 2: GBD 2017	Level 3: GBD 2017
Musculoskeletal disorders	Female	LDI (I$ per capita)		X			X	
Musculoskeletal disorders	Male	Low bone mineral density		X			X	
Musculoskeletal disorders	Male	Cholesterol (total, mean per capita)		X			X	
Musculoskeletal disorders	Female	Healthcare access and quality index		X			X	
Musculoskeletal disorders	Male	Healthcare access and quality index		X			X	
Musculoskeletal disorders	Female	Socio-demographic Index			X			X
Musculoskeletal disorders	Male	Socio-demographic Index			X			X
Musculoskeletal disorders	Female	Smoking Prevalence		X			X	
Musculoskeletal disorders	Male	Cumulative Cigarettes (10 Years)		X			X	
Musculoskeletal disorders	Female	Cumulative Cigarettes (10 Years)		X			X	
Musculoskeletal disorders	Male	Cumulative Cigarettes (5 Years)		X			X	
Musculoskeletal disorders	Male	Smoking Prevalence		X			X	
Musculoskeletal disorders	Female	Cumulative Cigarettes (5 Years)		X			X	
Musculoskeletal disorders	Female	Cholesterol (total, mean per capita)		X			X	
Musculoskeletal disorders	Male	LDI (I$ per capita)		X			X	
Musculoskeletal disorders	Female	Education (years per capita)		X			X	
Musculoskeletal disorders	Male	Education (years per capita)		X			X	
Musculoskeletal disorders	Male	Education (years per capita)		X			X	
Musculoskeletal disorders	Male	Mean BMI	X			X		
Musculoskeletal disorders	Female	Mean BMI	X			X		
Musculoskeletal disorders	Female	Low bone mineral density		X			X	
Musculoskeletal disorders	Male	Age-standardized bone mineral density among population age 60+ years		X			X	
Musculoskeletal disorders	Female	Age-standardized bone mineral density among population age 60+ years		X			X	
Rheumatoid arthritis	Female	Education (years per capita)			X			X
Rheumatoid arthritis	Male	LDI (I$ per capita)			X			X
Rheumatoid arthritis	Female	Cholesterol (total, mean per capita)		X			X	
Rheumatoid arthritis	Male	Cholesterol (total, mean per capita)		X			X	
Rheumatoid arthritis	Male	Healthcare access and quality index	X			X		
Rheumatoid arthritis	Female	Socio-demographic Index			X			X
Rheumatoid arthritis	Male	Education (years per capita)			X			X
Rheumatoid arthritis	Male	Socio-demographic Index			X			X
Rheumatoid arthritis	Female	Mean BMI		X			X	
Rheumatoid arthritis	Male	Mean BMI		X			X	
Rheumatoid arthritis	Female	LDI (I$ per capita)			X			X
Rheumatoid arthritis	Male	Smoking Prevalence	X			X		
Rheumatoid arthritis	Female	Smoking Prevalence	X			X		
Rheumatoid arthritis	Female	Healthcare access and quality index	X			X		
Rheumatoid arthritis	Male	Cumulative Cigarettes (10 Years)	X			X		
Rheumatoid arthritis	Female	Cumulative Cigarettes (10 Years)	X			X		
Rheumatoid arthritis	Male	Cumulative Cigarettes (5 Years)	X			X		
Rheumatoid arthritis	Female	Cumulative Cigarettes (5 Years)	X			X		
Other musculoskeletal disorders	Female	Healthcare access and quality index		X			X	
Other musculoskeletal disorders	Male	LDI (I$ per capita)		X			X	
Other musculoskeletal disorders	Female	LDI (I$ per capita)		X			X	
Other musculoskeletal disorders	Female	LDI (I$ per capita)		X			X	
Other musculoskeletal disorders	Male	Mean BMI	X			X		
Other musculoskeletal disorders	Female	Mean BMI	X			X		
Other musculoskeletal disorders	Male	Socio-demographic Index			X			X
Other musculoskeletal disorders	Female	Socio-demographic Index			X			X
Other musculoskeletal disorders	Male	Healthcare access and quality index		X			X	
Other musculoskeletal disorders	Female	Cholesterol (total, mean per capita)		X			X	
Other musculoskeletal disorders	Male	Cholesterol (total, mean per capita)		X			X	
Other musculoskeletal disorders	Female	Education (years per capita)		X			X	
Other musculoskeletal disorders	Male	Smoking Prevalence		X			X	
Other musculoskeletal disorders	Female	Smoking Prevalence		X			X	
Other musculoskeletal disorders	Male	Cumulative Cigarettes (10 Years)		X			X	
Other musculoskeletal disorders	Female	Cumulative Cigarettes (10 Years)		X			X	
Other musculoskeletal disorders	Male	Cumulative Cigarettes (5 Years)		X			X	
Other musculoskeletal disorders	Female	Cumulative Cigarettes (5 Years)		X			X	
Other musculoskeletal disorders	Male	Education (years per capita)		X			X	
Other musculoskeletal disorders	Female	Education (years per capita)		X			X	
Congenital anomalies	Female	Maternal Education (years per capita)			X			X
Congenital anomalies	Male	Folic acid unadjusted (ug)	X			X		
Congenital anomalies	Female	Folic acid unadjusted (ug)	X			X		
Congenital anomalies	Female	Composite fortification standard and folic acid inclusion	X			X		
Congenital anomalies	Female	Maternal alcohol consumption during pregnancy (proportion)	X			X		
Congenital anomalies	Male	Socio-demographic Index			X			X
Congenital anomalies	Female	Socio-demographic Index			X			X
Congenital anomalies	Male	Healthcare access and quality index		X			X	
Congenital anomalies	Female	Healthcare access and quality index		X			X	
Congenital anomalies	Male	Legality of Abortion		X			X	
Congenital anomalies	Female	Legality of Abortion		X			X	
Congenital anomalies	Male	Antenatal Care (1 visit) Coverage (proportion)		X			X	
Congenital anomalies	Female	Antenatal Care (1 visit) Coverage (proportion)		X			X	
Congenital anomalies	Male	Antenatal Care (4 visits) Coverage (proportion)		X			X	
Congenital anomalies	Female	Antenatal Care (4 visits) Coverage (proportion)		X			X	
Congenital anomalies	Male	In-Facility Delivery (proportion)	X			X		
Congenital anomalies	Female	In-Facility Delivery (proportion)	X			X		
Congenital anomalies	Male	Live Births 35+ (proportion)	X			X		

Table S18. Comparison of GBD 2017 and GBD 2019 covariates and level of covariates used in cause of death modeling

Cause	Sex	Covariate	Level 1: GBD 2019	Level 2: GBD 2019	Level 3: GBD 2019	Level 1: GBD 2017	Level 2: GBD 2017	Level 3: GBD 2017
Congenital anomalies	Female	Live Births 35+ (proportion)	X			X		
Congenital anomalies	Male	Outdoor Air Pollution (PM2.5)			X			X
Congenital anomalies	Male	Birth prevalence of CHD	X			X		
Congenital anomalies	Female	Birth prevalence of CHD	X			X		
Congenital anomalies	Male	Birth prevalence of congenital chromosomal anomalies	X			X		
Congenital anomalies	Female	Birth prevalence of congenital chromosomal anomalies	X			X		
Congenital anomalies	Male	Maternal Education (years per capita)			X			X
Congenital anomalies	Male	Composite fortification standard and folic acid inclusion	X			X		
Congenital anomalies	Male	Maternal alcohol consumption during pregnancy (proportion)	X			X		
Neural tube defects	Male	Composite fortification standard and folic acid inclusion	X			X		
Neural tube defects	Female	Maternal alcohol consumption during pregnancy (proportion)			X			X
Neural tube defects	Female	Healthcare access and quality index		X			X	
Neural tube defects	Female	Socio-demographic Index	X			X		
Neural tube defects	Male	Socio-demographic Index	X			X		
Neural tube defects	Male	Healthcare access and quality index		X			X	
Neural tube defects	Female	Legality of Abortion		X			X	
Neural tube defects	Male	Legality of Abortion		X			X	
Neural tube defects	Female	Antenatal Care (1 visit) Coverage (proportion)		X			X	
Neural tube defects	Male	Antenatal Care (1 visit) Coverage (proportion)		X			X	
Neural tube defects	Female	Antenatal Care (4 visits) Coverage (proportion)		X			X	
Neural tube defects	Male	Antenatal Care (4 visits) Coverage (proportion)		X			X	
Neural tube defects	Female	In-Facility Delivery (proportion)	X			X		
Neural tube defects	Male	Maternal Education (years per capita)			X			X
Neural tube defects	Female	Maternal Education (years per capita)			X			X
Neural tube defects	Male	Maternal alcohol consumption during pregnancy (proportion)			X			X
Neural tube defects	Female	Folic acid unadjusted (ug)	X			X		
Neural tube defects	Male	Folic acid unadjusted (ug)	X			X		
Neural tube defects	Female	Composite fortification standard and folic acid inclusion	X			X		
Neural tube defects	Male	In-Facility Delivery (proportion)	X			X		
Congenital heart anomalies	Female	Socio-demographic Index		X			X	
Congenital heart anomalies	Male	Healthcare access and quality index		X			X	
Congenital heart anomalies	Female	Healthcare access and quality index		X			X	
Congenital heart anomalies	Male	Legality of Abortion		X			X	
Congenital heart anomalies	Female	Legality of Abortion		X			X	
Congenital heart anomalies	Male	Antenatal Care (1 visit) Coverage (proportion)		X			X	
Congenital heart anomalies	Male	Skilled Birth Attendance (proportion)			X			X
Congenital heart anomalies	Female	Maternal alcohol consumption during pregnancy (proportion)	X			X		
Congenital heart anomalies	Female	Antenatal Care (1 visit) Coverage (proportion)		X			X	
Congenital heart anomalies	Male	Antenatal Care (4 visits) Coverage (proportion)			X			X
Congenital heart anomalies	Male	Socio-demographic Index		X			X	
Congenital heart anomalies	Male	Maternal alcohol consumption during pregnancy (proportion)	X			X		
Congenital heart anomalies	Female	Maternal Education (years per capita)			X			X
Congenital heart anomalies	Male	Maternal Education (years per capita)			X			X
Congenital heart anomalies	Female	Skilled Birth Attendance (proportion)			X			X
Congenital heart anomalies	Female	In-Facility Delivery (proportion)		X			X	
Congenital heart anomalies	Male	Birth prevalence of CHD	X			X		
Congenital heart anomalies	Female	Live Births 35+ (proportion)			X			X
Congenital heart anomalies	Male	In-Facility Delivery (proportion)		X			X	
Congenital heart anomalies	Female	Birth prevalence of CHD	X			X		
Congenital heart anomalies	Female	Antenatal Care (4 visits) Coverage (proportion)			X			X
Congenital heart anomalies	Male	Live Births 35+ (proportion)			X			X
Orofacial clefts	Male	Maternal Education (years per capita)			X			X
Orofacial clefts	Male	Composite fortification standard and folic acid inclusion	X			X		
Orofacial clefts	Female	Maternal Education (years per capita)			X			X
Orofacial clefts	Female	Smoking Prevalence (Reproductive Age Standardized)		X			X	
Orofacial clefts	Male	Indoor Air Pollution (All Cooking Fuels)			X			X
Orofacial clefts	Female	Indoor Air Pollution (All Cooking Fuels)			X	X		
Orofacial clefts	Female	vegetables unadjusted(g)			X			X
Orofacial clefts	Female	Maternal alcohol consumption during pregnancy (proportion)		X			X	
Orofacial clefts	Male	Maternal alcohol consumption during pregnancy (proportion)		X			X	
Orofacial clefts	Male	Folic acid unadjusted (ug)	X			X		
Orofacial clefts	Female	Composite fortification standard and folic acid inclusion	X			X		
Orofacial clefts	Female	Skilled Birth Attendance (proportion)		X			X	
Orofacial clefts	Male	Skilled Birth Attendance (proportion)		X			X	
Orofacial clefts	Female	Indoor Air Pollution (All Cooking Fuels)	X			X		
Orofacial clefts	Male	Antenatal Care (1 visit) Coverage (proportion)			X			X
Orofacial clefts	Female	Antenatal Care (4 visits) Coverage (proportion)			X			X
Orofacial clefts	Female	fruits unadjusted(g)			X			X

Table S18. Comparison of GBD 2017 and GBD 2019 covariates and level of covariates used in cause of death modeling

Cause	Sex	Covariate	Level 1: GBD 2019	Level 2: GBD 2019	Level 3: GBD 2019	Level 1: GBD 2017	Level 2: GBD 2017	Level 3: GBD 2017
Orofacial clefts	Female	Diabetes Age-Standardized Prevalence (proportion)		X			X	
Orofacial clefts	Male	Antenatal Care (4 visits) Coverage (proportion)			X			X
Orofacial clefts	Male	Socio-demographic Index	X			X		
Orofacial clefts	Female	Healthcare access and quality index		X			X	
Orofacial clefts	Female	Socio-demographic Index	X			X		
Orofacial clefts	Female	Legality of Abortion		X			X	
Orofacial clefts	Male	Legality of Abortion		X			X	
Orofacial clefts	Female	Antenatal Care (1 visit) Coverage (proportion)			X			X
Orofacial clefts	Male	Healthcare access and quality index		X			X	
Down syndrome	Male	Live Births 40+ (proportion)	X			X		
Down syndrome	Male	Birth prevalence of congenital chromosomal anomalies	X			X		
Down syndrome	Female	Maternal alcohol consumption during pregnancy (proportion)			X			X
Down syndrome	Male	Maternal alcohol consumption during pregnancy (proportion)			X			X
Down syndrome	Female	Maternal Education (years per capita)			X			X
Down syndrome	Male	Maternal Education (years per capita)			X			X
Down syndrome	Female	Birth prevalence of congenital chromosomal anomalies	X			X		
Down syndrome	Female	Live Births 40+ (proportion)	X			X		
Down syndrome	Male	Live Births 35+ (proportion)	X			X		
Down syndrome	Female	In-Facility Delivery (proportion)		X			X	
Down syndrome	Female	Legality of Abortion	X			X		
Down syndrome	Male	Legality of Abortion	X			X		
Down syndrome	Male	Healthcare access and quality index		X			X	
Down syndrome	Female	Socio-demographic Index		X			X	
Down syndrome	Male	Socio-demographic Index		X			X	
Down syndrome	Female	Healthcare access and quality index		X			X	
Down syndrome	Male	Antenatal Care (1 visit) Coverage (proportion)			X			X
Down syndrome	Female	Antenatal Care (1 visit) Coverage (proportion)			X			X
Down syndrome	Male	Antenatal Care (4 visits) Coverage (proportion)			X			X
Down syndrome	Female	Antenatal Care (4 visits) Coverage (proportion)			X			X
Down syndrome	Male	In-Facility Delivery (proportion)		X			X	
Down syndrome	Female	Live Births 35+ (proportion)	X			X		
Other chromosomal abnormalities	Female	Antenatal Care (4 visits) Coverage (proportion)		X			X	
Other chromosomal abnormalities	Female	Maternal alcohol consumption during pregnancy (proportion)		X			X	
Other chromosomal abnormalities	Male	Maternal Education (years per capita)			X			X
Other chromosomal abnormalities	Female	Maternal Education (years per capita)			X			X
Other chromosomal abnormalities	Male	In-Facility Delivery (proportion)		X			X	
Other chromosomal abnormalities	Female	In-Facility Delivery (proportion)		X			X	
Other chromosomal abnormalities	Male	Antenatal Care (4 visits) Coverage (proportion)		X			X	
Other chromosomal abnormalities	Male	Antenatal Care (1 visit) Coverage (proportion)		X			X	
Other chromosomal abnormalities	Female	Socio-demographic Index			X			X
Other chromosomal abnormalities	Female	Legality of Abortion	X			X		
Other chromosomal abnormalities	Male	Healthcare access and quality index		X			X	
Other chromosomal abnormalities	Female	Healthcare access and quality index		X			X	
Other chromosomal abnormalities	Male	Socio-demographic Index			X			X
Other chromosomal abnormalities	Male	Maternal alcohol consumption during pregnancy (proportion)		X			X	
Other chromosomal abnormalities	Male	LDI (I$ per capita)		X			X	
Other chromosomal abnormalities	Female	LDI (I$ per capita)		X			X	
Other chromosomal abnormalities	Male	Legality of Abortion	X			X		
Other chromosomal abnormalities	Female	Live Births 35+ (proportion)	X			X		
Other chromosomal abnormalities	Female	Antenatal Care (1 visit) Coverage (proportion)		X			X	
Other chromosomal abnormalities	Female	Live Births 40+ (proportion)	X			X		
Other chromosomal abnormalities	Male	Live Births 40+ (proportion)	X			X		
Other chromosomal abnormalities	Female	Skilled Birth Attendance (proportion)			X			X
Other chromosomal abnormalities	Male	Skilled Birth Attendance (proportion)			X			X
Other chromosomal abnormalities	Male	Live Births 35+ (proportion)	X			X		
Congenital musculoskeletal and limb anomalies	Male	Diabetes Age-Standardized Prevalence (proportion)		X			X	
Congenital musculoskeletal and limb anomalies	Male	LDI (I$ per capita)			X			X
Congenital musculoskeletal and limb anomalies	Female	LDI (I$ per capita)			X			X
Congenital musculoskeletal and limb anomalies	Male	Socio-demographic Index		X			X	
Congenital musculoskeletal and limb anomalies	Female	Socio-demographic Index		X			X	
Congenital musculoskeletal and limb anomalies	Female	Maternal alcohol consumption during pregnancy (proportion)	X			X		
Congenital musculoskeletal and limb anomalies	Male	Maternal alcohol consumption during pregnancy (proportion)	X			X		
Congenital musculoskeletal and limb anomalies	Female	Maternal Education (years per capita)			X			X
Congenital musculoskeletal and limb anomalies	Male	Maternal Education (years per capita)			X			X
Congenital musculoskeletal and limb anomalies	Female	In-Facility Delivery (proportion)		X			X	
Congenital musculoskeletal and limb anomalies	Male	In-Facility Delivery (proportion)		X			X	
Congenital musculoskeletal and limb anomalies	Female	Antenatal Care (4 visits) Coverage (proportion)			X			X
Congenital musculoskeletal and limb anomalies	Male	Antenatal Care (4 visits) Coverage (proportion)			X			X
Congenital musculoskeletal and limb anomalies	Female	Antenatal Care (1 visit) Coverage (proportion)			X			X
Congenital musculoskeletal and limb anomalies	Male	Healthcare access and quality index		X			X	

Table S18. Comparison of GBD 2017 and GBD 2019 covariates and level of covariates used in cause of death modeling

Cause	Sex	Covariate	Level 1: GBD 2019	Level 2: GBD 2019	Level 3: GBD 2019	Level 1: GBD 2017	Level 2: GBD 2017	Level 3: GBD 2017
Congenital musculoskeletal and limb anomalies	Female	Healthcare access and quality index		X			X	
Congenital musculoskeletal and limb anomalies	Male	Legality of Abortion	X			X		
Congenital musculoskeletal and limb anomalies	Female	Legality of Abortion	X			X		
Congenital musculoskeletal and limb anomalies	Male	Antenatal Care (1 visit) Coverage (proportion)			X			X
Urogenital congenital anomalies	Male	In-Facility Delivery (proportion)		X			X	
Urogenital congenital anomalies	Female	In-Facility Delivery (proportion)		X			X	
Urogenital congenital anomalies	Male	Antenatal Care (4 visits) Coverage (proportion)			X			X
Urogenital congenital anomalies	Female	Antenatal Care (4 visits) Coverage (proportion)			X			X
Urogenital congenital anomalies	Female	Maternal alcohol consumption during pregnancy (proportion)	X			X		
Urogenital congenital anomalies	Female	Maternal Education (years per capita)			X			X
Urogenital congenital anomalies	Male	Antenatal Care (1 visit) Coverage (proportion)			X			X
Urogenital congenital anomalies	Female	Antenatal Care (1 visit) Coverage (proportion)			X			X
Urogenital congenital anomalies	Male	Healthcare access and quality index		X			X	
Urogenital congenital anomalies	Female	Healthcare access and quality index		X			X	
Urogenital congenital anomalies	Female	Diabetes Age-Standardized Prevalence (proportion)		X			X	
Urogenital congenital anomalies	Male	Diabetes Age-Standardized Prevalence (proportion)		X			X	
Urogenital congenital anomalies	Female	LDI (I$ per capita)			X			X
Urogenital congenital anomalies	Male	LDI (I$ per capita)			X			X
Urogenital congenital anomalies	Female	Smoking Prevalence (Reproductive Age Standardized)	X			X		
Urogenital congenital anomalies	Female	Socio-demographic Index		X			X	
Urogenital congenital anomalies	Male	Socio-demographic Index		X			X	
Urogenital congenital anomalies	Male	Maternal alcohol consumption during pregnancy (proportion)	X			X		
Urogenital congenital anomalies	Male	Maternal Education (years per capita)			X			X
Digestive congenital anomalies	Male	Healthcare access and quality index		X			X	
Digestive congenital anomalies	Female	Socio-demographic Index		X			X	
Digestive congenital anomalies	Male	Socio-demographic Index		X			X	
Digestive congenital anomalies	Female	LDI (I$ per capita)			X			X
Digestive congenital anomalies	Male	Diabetes Age-Standardized Prevalence (proportion)		X			X	
Digestive congenital anomalies	Male	LDI (I$ per capita)			X			X
Digestive congenital anomalies	Female	Healthcare access and quality index		X			X	
Digestive congenital anomalies	Male	Antenatal Care (1 visit) Coverage (proportion)			X			X
Digestive congenital anomalies	Female	Antenatal Care (4 visits) Coverage (proportion)			X			X
Digestive congenital anomalies	Male	Antenatal Care (4 visits) Coverage (proportion)			X			X
Digestive congenital anomalies	Male	Prevalence of obesity (age-standardized)		X			X	
Digestive congenital anomalies	Female	Maternal care and immunization			X			X
Digestive congenital anomalies	Male	Maternal care and immunization			X			X
Digestive congenital anomalies	Female	Maternal alcohol consumption during pregnancy (proportion)	X			X		
Digestive congenital anomalies	Male	Maternal alcohol consumption during pregnancy (proportion)	X			X		
Digestive congenital anomalies	Female	Maternal Education (years per capita)			X			X
Digestive congenital anomalies	Male	Maternal Education (years per capita)			X			X
Digestive congenital anomalies	Female	In-Facility Delivery (proportion)		X			X	
Digestive congenital anomalies	Male	In-Facility Delivery (proportion)		X			X	
Digestive congenital anomalies	Female	Antenatal Care (1 visit) Coverage (proportion)			X			X
Other congenital anomalies	Male	Antenatal Care (4 visits) Coverage (proportion)			X			X
Other congenital anomalies	Female	In-Facility Delivery (proportion)		X			X	
Other congenital anomalies	Male	In-Facility Delivery (proportion)		X			X	
Other congenital anomalies	Female	Maternal Education (years per capita)			X			X
Other congenital anomalies	Male	Live Births 35+ (proportion)	X			X		
Other congenital anomalies	Female	Maternal alcohol consumption during pregnancy (proportion)	X			X		
Other congenital anomalies	Male	Maternal alcohol consumption during pregnancy (proportion)	X			X		
Other congenital anomalies	Female	Live Births 35+ (proportion)	X			X		
Other congenital anomalies	Male	Maternal Education (years per capita)			X			X
Other congenital anomalies	Female	Antenatal Care (4 visits) Coverage (proportion)			X			X
Other congenital anomalies	Female	Antenatal Care (1 visit) Coverage (proportion)			X			X
Other congenital anomalies	Male	Legality of Abortion		X			X	
Other congenital anomalies	Female	Legality of Abortion		X			X	
Other congenital anomalies	Male	Healthcare access and quality index		X			X	
Other congenital anomalies	Female	Healthcare access and quality index		X			X	
Other congenital anomalies	Male	Socio-demographic Index			X			X
Other congenital anomalies	Female	Socio-demographic Index			X			X
Other congenital anomalies	Male	LDI (I$ per capita)			X			X
Other congenital anomalies	Female	LDI (I$ per capita)			X			X
Other congenital anomalies	Male	Antenatal Care (1 visit) Coverage (proportion)			X			X
Skin and subcutaneous diseases	Male	Cumulative Cigarettes (10 Years)		X			X	
Skin and subcutaneous diseases	Female	Improved Water Source (proportion with access)	X			X		
Skin and subcutaneous diseases	Male	Improved Water Source (proportion with access)	X			X		
Skin and subcutaneous diseases	Male	Cumulative Cigarettes (5 Years)		X			X	
Skin and subcutaneous diseases	Male	Age- and sex-specific SEV for Unsafe sanitation	X			X		

Table S18. Comparison of GBD 2017 and GBD 2019 covariates and level of covariates used in cause of death modeling								
Cause	Sex	Covariate	Level 1: GBD 2019	Level 2: GBD 2019	Level 3: GBD 2019	Level 1: GBD 2017	Level 2: GBD 2017	Level 3: GBD 2017
Skin and subcutaneous diseases	Female	Cumulative Cigarettes (10 Years)		X			X	
Skin and subcutaneous diseases	Female	Age- and sex-specific SEV for Unsafe sanitation	X			X		
Skin and subcutaneous diseases	Male	Smoking Prevalence		X			X	
Skin and subcutaneous diseases	Female	Cumulative Cigarettes (5 Years)		X			X	
Skin and subcutaneous diseases	Male	Healthcare access and quality index		X		X		
Skin and subcutaneous diseases	Female	Healthcare access and quality index		X		X		
Skin and subcutaneous diseases	Male	Socio-demographic Index			X			X
Skin and subcutaneous diseases	Female	Socio-demographic Index			X			X
Skin and subcutaneous diseases	Male	LDI (I$ per capita)			X			X
Skin and subcutaneous diseases	Female	LDI (I$ per capita)			X			X
Skin and subcutaneous diseases	Male	Education (years per capita)			X			X
Skin and subcutaneous diseases	Female	Education (years per capita)			X			X
Skin and subcutaneous diseases	Female	Smoking Prevalence		X			X	
Cellulitis	Female	Education (years per capita)			X			X
Cellulitis	Male	Education (years per capita)			X			X
Cellulitis	Female	LDI (I$ per capita)			X		X	
Cellulitis	Male	Healthcare access and quality index		X		X		
Cellulitis	Female	Healthcare access and quality index		X		X		
Cellulitis	Male	LDI (I$ per capita)			X		X	
Pyoderma	Female	Age- and sex-specific SEV for Unsafe sanitation	X			X		
Pyoderma	Male	Cumulative Cigarettes (5 Years)		X			X	
Pyoderma	Female	Cumulative Cigarettes (5 Years)		X			X	
Pyoderma	Male	Age- and sex-specific SEV for Unsafe sanitation	X			X		
Pyoderma	Male	Improved Water Source (proportion with access)	X			X		
Pyoderma	Female	Improved Water Source (proportion with access)	X			X		
Pyoderma	Male	Education (years per capita)			X			X
Pyoderma	Female	Cumulative Cigarettes (10 Years)		X			X	
Pyoderma	Male	Smoking Prevalence		X			X	
Pyoderma	Female	Smoking Prevalence		X			X	
Pyoderma	Male	Healthcare access and quality index		X		X		
Pyoderma	Female	Healthcare access and quality index		X		X		
Pyoderma	Male	Socio-demographic Index			X			X
Pyoderma	Female	Socio-demographic Index			X			X
Pyoderma	Male	LDI (I$ per capita)			X			X
Pyoderma	Female	LDI (I$ per capita)			X			X
Pyoderma	Male	Cumulative Cigarettes (10 Years)		X			X	
Pyoderma	Female	Education (years per capita)			X			X
Decubitus ulcer	Female	Cumulative Cigarettes (10 Years)		X			X	
Decubitus ulcer	Male	Improved Water Source (proportion with access)	X			X		
Decubitus ulcer	Female	Improved Water Source (proportion with access)	X			X		
Decubitus ulcer	Male	Prevalence of obesity	X			X		
Decubitus ulcer	Male	Cumulative Cigarettes (10 Years)		X			X	
Decubitus ulcer	Male	Age- and sex-specific SEV for Unsafe sanitation			X			X
Decubitus ulcer	Male	Cumulative Cigarettes (5 Years)		X			X	
Decubitus ulcer	Female	Cumulative Cigarettes (5 Years)		X			X	
Decubitus ulcer	Female	Prevalence of obesity	X			X		
Decubitus ulcer	Female	Smoking Prevalence		X			X	
Decubitus ulcer	Female	Age- and sex-specific SEV for Unsafe sanitation			X			X
Decubitus ulcer	Female	Diabetes Fasting Plasma Glucose (mmol/L), age-standardized 25+	X			X		
Decubitus ulcer	Male	Education (years per capita)			X			X
Decubitus ulcer	Female	Education (years per capita)			X			X
Decubitus ulcer	Male	LDI (I$ per capita)			X			X
Decubitus ulcer	Female	LDI (I$ per capita)			X			X
Decubitus ulcer	Male	Smoking Prevalence		X			X	
Decubitus ulcer	Female	Socio-demographic Index			X			X
Decubitus ulcer	Male	Healthcare access and quality index		X			X	
Decubitus ulcer	Female	Healthcare access and quality index		X			X	
Decubitus ulcer	Male	Diabetes Fasting Plasma Glucose (mmol/L), age-standardized 25+	X			X		
Decubitus ulcer	Male	Socio-demographic Index			X			X
Other skin and subcutaneous diseases	Female	LDI (I$ per capita)			X			X
Other skin and subcutaneous diseases	Male	Socio-demographic Index			X			X
Other skin and subcutaneous diseases	Female	Socio-demographic Index			X			X
Other skin and subcutaneous diseases	Male	Healthcare access and quality index		X		X		
Other skin and subcutaneous diseases	Female	Healthcare access and quality index		X		X		
Other skin and subcutaneous diseases	Male	Smoking Prevalence		X			X	
Other skin and subcutaneous diseases	Female	Smoking Prevalence		X			X	
Other skin and subcutaneous diseases	Male	Cumulative Cigarettes (10 Years)		X			X	
Other skin and subcutaneous diseases	Male	Improved Water Source (proportion with access)	X			X		
Other skin and subcutaneous diseases	Female	Improved Water Source (proportion with access)	X			X		
Other skin and subcutaneous diseases	Male	Age-standardized SEV for Child underweight	X			X		
Other skin and subcutaneous diseases	Male	LDI (I$ per capita)			X			X
Other skin and subcutaneous diseases	Male	Age- and sex-specific SEV for Unsafe sanitation	X			X		
Other skin and subcutaneous diseases	Female	Age- and sex-specific SEV for Unsafe sanitation	X			X		
Other skin and subcutaneous diseases	Male	Cumulative Cigarettes (5 Years)		X			X	

Table S18. Comparison of GBD 2017 and GBD 2019 covariates and level of covariates used in cause of death modeling								
Cause	Sex	Covariate	Level 1: GBD 2019	Level 2: GBD 2019	Level 3: GBD 2019	Level 1: GBD 2017	Level 2: GBD 2017	Level 3: GBD 2017
Other skin and subcutaneous diseases	Female	Cumulative Cigarettes (5 Years)		X			X	
Other skin and subcutaneous diseases	Female	Cumulative Cigarettes (10 Years)		X			X	
Other skin and subcutaneous diseases	Female	Age-standardized SEV for Child underweight	X			X		
Other skin and subcutaneous diseases	Female	Education (years per capita)			X			X
Other skin and subcutaneous diseases	Male	Education (years per capita)			X			X
Sudden infant death syndrome	Male	Total Fertility Rate			X			X
Sudden infant death syndrome	Male	Tobacco (cigarettes per capita)	X			X		
Sudden infant death syndrome	Female	Total Fertility Rate			X			X
Sudden infant death syndrome	Male	Maternal care and immunization		X			X	
Sudden infant death syndrome	Female	Skilled Birth Attendance (proportion)		X			X	
Sudden infant death syndrome	Male	Skilled Birth Attendance (proportion)		X			X	
Sudden infant death syndrome	Male	In-Facility Delivery (proportion)	X			X		
Sudden infant death syndrome	Male	Healthcare access and quality index		X			X	
Sudden infant death syndrome	Female	In-Facility Delivery (proportion)	X			X		
Sudden infant death syndrome	Male	Socio-demographic Index			X			X
Sudden infant death syndrome	Female	LDI (I$ per capita)			X			X
Sudden infant death syndrome	Male	LDI (I$ per capita)			X			X
Sudden infant death syndrome	Female	Education (years per capita)			X			X
Sudden infant death syndrome	Female	Tobacco (cigarettes per capita)	X			X		
Sudden infant death syndrome	Male	Education (years per capita)			X			X
Sudden infant death syndrome	Female	Socio-demographic Index			X			X
Transport injuries	Female	Population Density (300-500 ppl/sqkm, proportion)		X			X	
Transport injuries	Female	Vehicles - 2+4 wheels (per capita)	X			X		
Transport injuries	Male	Vehicles - 2+4 wheels (per capita)	X			X		
Transport injuries	Female	Population Density (500-1000 ppl/sqkm, proportion)		X			X	
Transport injuries	Male	Population Density (500-1000 ppl/sqkm, proportion)		X			X	
Transport injuries	Male	Population Density (300-500 ppl/sqkm, proportion)		X			X	
Transport injuries	Male	Healthcare access and quality index		X			X	
Transport injuries	Male	Socio-demographic Index			X		X	
Transport injuries	Female	Rainfall Quintile 5 (proportion)			X			X
Transport injuries	Male	Rainfall Quintile 5 (proportion)			X			X
Transport injuries	Female	Healthcare access and quality index		X			X	
Transport injuries	Male	Vehicles - 2 wheels fraction (proportion)	X			X		
Transport injuries	Female	LDI (I$ per capita)		X			X	
Transport injuries	Female	Socio-demographic Index			X		X	
Transport injuries	Female	Vehicles - 2 wheels fraction (proportion)	X			X		
Transport injuries	Male	LDI (I$ per capita)		X			X	
Transport injuries	Female	Education (years per capita)			X		X	
Transport injuries	Male	Education (years per capita)			X		X	
Road injuries	Male	Education (years per capita)			X		X	
Road injuries	Male	Education (years per capita)			X			X
Road injuries	Female	Education (years per capita)			X		X	
Road injuries	Female	Education (years per capita)			X			X
Road injuries	Male	LDI (I$ per capita)			X		X	
Road injuries	Male	LDI (I$ per capita)			X			X
Road injuries	Female	LDI (I$ per capita)			X			X
Road injuries	Female	LDI (I$ per capita)			X		X	
Road injuries	Male	Healthcare access and quality index		X			X	
Road injuries	Female	Healthcare access and quality index		X			X	
Road injuries	Male	Population 15 to 30 (proportion)		X			X	
Road injuries	Female	Population 15 to 30 (proportion)		X			X	
Road injuries	Female	Log-transformed SEV scalar: Road Inj	X			X		
Road injuries	Male	Log-transformed SEV scalar: Road Inj	X			X		
Road injuries	Male	Socio-demographic Index			X			X
Road injuries	Female	Vehicles - 2+4 wheels (per capita)	X			X		
Road injuries	Male	Vehicles - 2+4 wheels (per capita)	X			X		
Road injuries	Female	Population Density (500-1000 ppl/sqkm, proportion)		X			X	
Road injuries	Male	Population Density (500-1000 ppl/sqkm, proportion)		X			X	
Road injuries	Female	Population Density (300-500 ppl/sqkm, proportion)		X			X	
Road injuries	Male	Population Density (300-500 ppl/sqkm, proportion)		X			X	
Road injuries	Female	Vehicles - 4 wheels (per capita)	X			X		
Road injuries	Male	Vehicles - 4 wheels (per capita)	X			X		
Road injuries	Female	Vehicles - 2 wheels (per capita)	X			X		
Road injuries	Male	Vehicles - 2 wheels (per capita)	X			X		
Road injuries	Female	Socio-demographic Index			X		X	
Road injuries	Female	Socio-demographic Index			X			X
Road injuries	Male	Socio-demographic Index			X		X	
Road injuries	Male	Vehicles - 2 wheels fraction (proportion)	X			X		
Road injuries	Female	Vehicles - 2 wheels fraction (proportion)	X			X		
Pedestrian road injuries	Female	Population Density (300-500 ppl/sqkm, proportion)		X			X	
Pedestrian road injuries	Female	Socio-demographic Index			X		X	
Pedestrian road injuries	Male	Log-transformed SEV scalar: Pedest	X			X		
Pedestrian road injuries	Female	Vehicles - 2 wheels fraction (proportion)	X			X		
Pedestrian road injuries	Male	Vehicles - 2 wheels fraction (proportion)	X			X		
Pedestrian road injuries	Female	Vehicles - 2+4 wheels (per capita)	X			X		
Pedestrian road injuries	Male	Vehicles - 2+4 wheels (per capita)	X			X		
Pedestrian road injuries	Female	Population Density (500-1000 ppl/sqkm, proportion)		X			X	

Table S18. Comparison of GBD 2017 and GBD 2019 covariates and level of covariates used in cause of death modeling								
Cause	Sex	Covariate	Level 1: GBD 2019	Level 2: GBD 2019	Level 3: GBD 2019	Level 1: GBD 2017	Level 2: GBD 2017	Level 3: GBD 2017
Pedestrian road injuries	Male	Population Density (500-1000 ppl/sqkm, proportion)		X			X	
Pedestrian road injuries	Male	Population Density (300-500 ppl/sqkm, proportion)		X			X	
Pedestrian road injuries	Male	Socio-demographic Index			X		X	
Pedestrian road injuries	Male	Education (years per capita)			X		X	
Pedestrian road injuries	Female	Education (years per capita)			X		X	
Pedestrian road injuries	Male	LDI (I$ per capita)		X			X	
Pedestrian road injuries	Female	LDI (I$ per capita)		X			X	
Pedestrian road injuries	Male	Healthcare access and quality index		X			X	
Pedestrian road injuries	Female	Healthcare access and quality index		X			X	
Pedestrian road injuries	Female	Log-transformed SEV scalar: Pedest	X			X		
Pedestrian road injuries	Male	Rainfall Quintile 5 (proportion)			X			X
Pedestrian road injuries	Female	Rainfall Quintile 5 (proportion)			X			X
Cyclist road injuries	Female	Healthcare access and quality index		X			X	
Cyclist road injuries	Male	Education (years per capita)			X		X	
Cyclist road injuries	Male	Education (years per capita)			X		X	
Cyclist road injuries	Female	Education (years per capita)			X		X	
Cyclist road injuries	Male	LDI (I$ per capita)		X			X	
Cyclist road injuries	Female	LDI (I$ per capita)		X			X	
Cyclist road injuries	Male	Healthcare access and quality index		X			X	
Cyclist road injuries	Male	Socio-demographic Index			X		X	
Cyclist road injuries	Female	Socio-demographic Index			X		X	
Cyclist road injuries	Female	Education (years per capita)			X		X	
Cyclist road injuries	Male	Population Density (300-500 ppl/sqkm, proportion)		X			X	
Cyclist road injuries	Male	Population Density (500-1000 ppl/sqkm, proportion)		X			X	
Cyclist road injuries	Female	Population Density (500-1000 ppl/sqkm, proportion)		X			X	
Cyclist road injuries	Male	Vehicles - 2+4 wheels (per capita)	X			X		
Cyclist road injuries	Female	Vehicles - 2+4 wheels (per capita)	X			X		
Cyclist road injuries	Male	Vehicles - 2 wheels fraction (proportion)	X			X		
Cyclist road injuries	Female	Vehicles - 2 wheels fraction (proportion)	X			X		
Cyclist road injuries	Male	Log-transformed SEV scalar: Cyclist	X			X		
Cyclist road injuries	Female	Log-transformed SEV scalar: Cyclist	X			X		
Cyclist road injuries	Female	Population Density (300-500 ppl/sqkm, proportion)		X			X	
Motorcyclist road injuries	Female	Population Density (500-1000 ppl/sqkm, proportion)		X			X	
Motorcyclist road injuries	Male	Population Density (500-1000 ppl/sqkm, proportion)		X			X	
Motorcyclist road injuries	Male	LDI (I$ per capita)		X			X	
Motorcyclist road injuries	Male	Log-transformed SEV scalar: Mot Cyc	X			X		
Motorcyclist road injuries	Male	Population Density (300-500 ppl/sqkm, proportion)		X			X	
Motorcyclist road injuries	Female	Log-transformed SEV scalar: Mot Cyc	X			X		
Motorcyclist road injuries	Female	Population Density (300-500 ppl/sqkm, proportion)		X			X	
Motorcyclist road injuries	Male	Education (years per capita)			X		X	
Motorcyclist road injuries	Female	Socio-demographic Index			X		X	
Motorcyclist road injuries	Male	Rainfall Quintile 5 (proportion)			X			X
Motorcyclist road injuries	Female	Rainfall Quintile 5 (proportion)			X			X
Motorcyclist road injuries	Male	Healthcare access and quality index		X			X	
Motorcyclist road injuries	Female	Healthcare access and quality index		X			X	
Motorcyclist road injuries	Female	LDI (I$ per capita)		X			X	
Motorcyclist road injuries	Female	Education (years per capita)			X		X	
Motorcyclist road injuries	Male	Socio-demographic Index			X		X	
Motor vehicle road injuries	Male	Population Density (300-500 ppl/sqkm, proportion)		X			X	
Motor vehicle road injuries	Female	Population Density (300-500 ppl/sqkm, proportion)		X			X	
Motor vehicle road injuries	Male	Population Density (500-1000 ppl/sqkm, proportion)		X			X	
Motor vehicle road injuries	Male	Log-transformed SEV scalar: Mot Veh	X			X		
Motor vehicle road injuries	Female	Log-transformed SEV scalar: Mot Veh	X			X		
Motor vehicle road injuries	Female	Vehicles - 4 wheels (per capita)	X			X		
Motor vehicle road injuries	Female	Population Density (500-1000 ppl/sqkm, proportion)		X			X	
Motor vehicle road injuries	Male	Vehicles - 4 wheels (per capita)	X			X		
Motor vehicle road injuries	Male	Rainfall Quintile 5 (proportion)			X			X
Motor vehicle road injuries	Male	Socio-demographic Index			X		X	
Motor vehicle road injuries	Female	Socio-demographic Index			X		X	
Motor vehicle road injuries	Male	Education (years per capita)			X		X	
Motor vehicle road injuries	Female	Education (years per capita)			X		X	
Motor vehicle road injuries	Male	LDI (I$ per capita)			X		X	
Motor vehicle road injuries	Male	Education (years per capita)			X		X	
Motor vehicle road injuries	Male	Healthcare access and quality index		X			X	
Motor vehicle road injuries	Female	Healthcare access and quality index		X			X	
Motor vehicle road injuries	Female	Rainfall Quintile 5 (proportion)			X			X
Motor vehicle road injuries	Female	LDI (I$ per capita)			X		X	
Other road injuries	Male	Log-transformed SEV scalar: Oth Road	X			X		
Other road injuries	Female	Log-transformed SEV scalar: Oth Road	X			X		
Other road injuries	Female	Vehicles - 2 wheels fraction (proportion)	X			X		
Other road injuries	Male	Vehicles - 2 wheels fraction (proportion)	X			X		
Other road injuries	Female	Vehicles - 2+4 wheels (per capita)	X			X		
Other road injuries	Male	Vehicles - 2+4 wheels (per capita)	X			X		
Other road injuries	Female	Socio-demographic Index			X			X
Other road injuries	Male	Socio-demographic Index			X		X	

Table S18. Comparison of GBD 2017 and GBD 2019 covariates and level of covariates used in cause of death modeling

Cause	Sex	Covariate	Level 1: GBD 2019	Level 2: GBD 2019	Level 3: GBD 2019	Level 1: GBD 2017	Level 2: GBD 2017	Level 3: GBD 2017
Other road injuries	Male	Socio-demographic Index			X			X
Other road injuries	Female	Rainfall Quintile 5 (proportion)			X			X
Other road injuries	Male	LDI (I$ per capita)		X			X	
Other road injuries	Male	Rainfall Quintile 5 (proportion)			X			X
Other road injuries	Male	Healthcare access and quality index		X			X	
Other road injuries	Female	Healthcare access and quality index		X			X	
Other road injuries	Female	LDI (I$ per capita)		X			X	
Other transport injuries	Male	Education (years per capita)			X		X	
Other transport injuries	Male	Education (years per capita)			X		X	
Other transport injuries	Female	Education (years per capita)			X		X	
Other transport injuries	Male	LDI (I$ per capita)			X		X	
Other transport injuries	Female	LDI (I$ per capita)			X		X	
Other transport injuries	Male	Socio-demographic Index			X		X	
Other transport injuries	Female	Socio-demographic Index			X		X	
Other transport injuries	Male	Healthcare access and quality index		X			X	
Other transport injuries	Female	Healthcare access and quality index		X			X	
Other transport injuries	Male	Rainfall Quintile 5 (proportion)			X			X
Other transport injuries	Male	Population Density (300-500 ppl/sqkm, proportion)		X			X	
Other transport injuries	Female	Rainfall Quintile 5 (proportion)			X			X
Other transport injuries	Male	Log-transformed SEV scalar: Oth Trans	X			X		
Other transport injuries	Female	Log-transformed SEV scalar: Oth Trans	X			X		
Other transport injuries	Male	Population Density (500-1000 ppl/sqkm, proportion)		X			X	
Other transport injuries	Female	Population Density (500-1000 ppl/sqkm, proportion)		X			X	
Other transport injuries	Male	Vehicles - 2+4 wheels (per capita)	X			X		
Other transport injuries	Female	Population Density (300-500 ppl/sqkm, proportion)		X			X	
Other transport injuries	Male	Vehicles - 2 wheels fraction (proportion)	X			X		
Other transport injuries	Female	Vehicles - 2 wheels fraction (proportion)	X			X		
Other transport injuries	Female	Vehicles - 2+4 wheels (per capita)	X			X		
Falls	Female	Healthcare access and quality index		X			X	
Falls	Male	Elevation Over 1500m (proportion)			X			X
Falls	Male	Healthcare access and quality index		X			X	
Falls	Male	Log-transformed SEV scalar: Falls	X			X		
Falls	Female	Log-transformed SEV scalar: Falls	X			X		
Falls	Female	Socio-demographic Index			X			X
Falls	Female	Socio-demographic Index			X			X
Falls	Male	Socio-demographic Index			X			X
Falls	Male	Socio-demographic Index			X			X
Falls	Female	LDI (I$ per capita)			X			X
Falls	Male	LDI (I$ per capita)			X			X
Falls	Female	Elevation Over 1500m (proportion)			X			X
Drowning	Female	Rainfall Quintile 5 (proportion)	X			X		
Drowning	Female	Elevation Under 100m (proportion)		X			X	
Drowning	Female	Landlocked Nation (binary)	X			X		
Drowning	Male	Landlocked Nation (binary)	X			X		
Drowning	Male	Education (years per capita)			X			X
Drowning	Male	Education (years per capita)			X			X
Drowning	Female	Education (years per capita)			X			X
Drowning	Male	Elevation Under 100m (proportion)		X			X	
Drowning	Female	Rainfall Quintile 1 (proportion)	X			X		
Drowning	Male	Rainfall Quintile 1 (proportion)	X			X		
Drowning	Female	Education (years per capita)			X			X
Drowning	Female	Log-transformed SEV scalar: Drown	X			X		
Drowning	Female	Socio-demographic Index			X			X
Drowning	Male	Coastal Population within 10km (proportion)	X			X		
Drowning	Female	Coastal Population within 10km (proportion)	X			X		
Drowning	Male	Rainfall Quintile 5 (proportion)	X			X		
Drowning	Male	Socio-demographic Index			X			X
Drowning	Male	Socio-demographic Index			X			X
Drowning	Female	LDI (I$ per capita)			X			X
Drowning	Male	LDI (I$ per capita)			X			X
Drowning	Male	Log-transformed SEV scalar: Drown	X			X		
Fire, heat, and hot substances	Male	Log-transformed SEV scalar: Fire	X			X		
Fire, heat, and hot substances	Female	Log-transformed SEV scalar: Fire	X			X		
Fire, heat, and hot substances	Male	Education (years per capita)			X			X
Fire, heat, and hot substances	Male	Healthcare access and quality index		X			X	
Fire, heat, and hot substances	Female	LDI (I$ per capita)			X			X
Fire, heat, and hot substances	Male	LDI (I$ per capita)			X			X
Fire, heat, and hot substances	Female	Socio-demographic Index			X			X
Fire, heat, and hot substances	Female	Socio-demographic Index			X			X
Fire, heat, and hot substances	Male	Socio-demographic Index			X			X
Fire, heat, and hot substances	Male	Tobacco (cigarettes per capita)		X			X	
Fire, heat, and hot substances	Male	Socio-demographic Index			X			X
Fire, heat, and hot substances	Female	Healthcare access and quality index		X			X	
Fire, heat, and hot substances	Female	Education (years per capita)			X			X
Fire, heat, and hot substances	Female	Indoor Air Pollution (All Cooking Fuels)		X			X	
Fire, heat, and hot substances	Male	Indoor Air Pollution (All Cooking Fuels)		X			X	
Fire, heat, and hot substances	Female	Population Density (over 1000 ppl/sqkm, proportion)		X			X	
Fire, heat, and hot substances	Male	Population Density (over 1000 ppl/sqkm, proportion)		X			X	
Fire, heat, and hot substances	Female	Tobacco (cigarettes per capita)		X			X	

Table S18. Comparison of GBD 2017 and GBD 2019 covariates and level of covariates used in cause of death modeling

Cause	Sex	Covariate	Level 1: GBD 2019	Level 2: GBD 2019	Level 3: GBD 2019	Level 1: GBD 2017	Level 2: GBD 2017	Level 3: GBD 2017
Poisonings	Female	Population Density (over 1000 ppl/sqkm, proportion)		X			X	
Poisonings	Male	Population Density (over 1000 ppl/sqkm, proportion)		X			X	
Poisonings	Female	Population Density (under 150 ppl/sqkm, proportion)		X			X	
Poisonings	Male	Healthcare access and quality index		X			X	
Poisonings	Female	Socio-demographic Index			X			X
Poisonings	Male	Socio-demographic Index			X			X
Poisonings	Female	Education (years per capita)			X			X
Poisonings	Male	Education (years per capita)			X			X
Poisonings	Female	LDI (I$ per capita)			X			X
Poisonings	Male	LDI (I$ per capita)			X			X
Poisonings	Female	Socio-demographic Index			X			X
Poisonings	Male	Population Density (under 150 ppl/sqkm, proportion)		X			X	
Poisonings	Male	Socio-demographic Index			X			X
Poisonings	Female	Opium Cultivation (binary)	X			X		
Poisonings	Male	Log-transformed SEV scalar: Poison	X			X		
Poisonings	Female	Log-transformed SEV scalar: Poison	X			X		
Poisonings	Male	Opium Cultivation (binary)	X			X		
Poisonings	Female	Healthcare access and quality index		X			X	
Poisoning by carbon monoxide	Male	LDI (I$ per capita)			X			X
Poisoning by carbon monoxide	Female	LDI (I$ per capita)			X			X
Poisoning by carbon monoxide	Male	Education (years per capita)			X			X
Poisoning by carbon monoxide	Female	Education (years per capita)			X			X
Poisoning by carbon monoxide	Female	Socio-demographic Index			X			X
Poisoning by carbon monoxide	Female	Healthcare access and quality index			X			X
Poisoning by carbon monoxide	Male	Socio-demographic Index			X			X
Poisoning by carbon monoxide	Male	Healthcare access and quality index			X			X
Poisoning by other means	Male	Education (years per capita)			X			X
Poisoning by other means	Female	Education (years per capita)			X			X
Poisoning by other means	Male	LDI (I$ per capita)			X			X
Poisoning by other means	Female	LDI (I$ per capita)			X			X
Poisoning by other means	Female	Socio-demographic Index			X			X
Poisoning by other means	Male	Healthcare access and quality index			X			X
Poisoning by other means	Female	Healthcare access and quality index			X			X
Poisoning by other means	Male	Socio-demographic Index			X			X
Exposure to mechanical forces	Male	LDI (I$ per capita)			X			X
Exposure to mechanical forces	Female	Education (years per capita)			X			X
Exposure to mechanical forces	Female	Education (years per capita)			X			X
Exposure to mechanical forces	Male	Education (years per capita)			X			X
Exposure to mechanical forces	Male	Education (years per capita)			X			X
Exposure to mechanical forces	Female	LDI (I$ per capita)			X			X
Exposure to mechanical forces	Male	Socio-demographic Index			X			X
Exposure to mechanical forces	Female	Socio-demographic Index			X			X
Exposure to mechanical forces	Male	Healthcare access and quality index		X			X	
Exposure to mechanical forces	Female	Population Density (over 1000 ppl/sqkm, proportion)		X			X	
Exposure to mechanical forces	Male	Population Density (over 1000 ppl/sqkm, proportion)		X			X	
Exposure to mechanical forces	Female	Population Density (under 150 ppl/sqkm, proportion)		X			X	
Exposure to mechanical forces	Male	Population Density (under 150 ppl/sqkm, proportion)		X			X	
Exposure to mechanical forces	Female	Healthcare access and quality index		X			X	
Unintentional firearm injuries	Female	Population Density (over 1000 ppl/sqkm, proportion)			X			X
Unintentional firearm injuries	Male	Socio-demographic Index			X			X
Unintentional firearm injuries	Female	Healthcare access and quality index		X			X	
Unintentional firearm injuries	Male	Healthcare access and quality index		X			X	
Unintentional firearm injuries	Male	Population Density (over 1000 ppl/sqkm, proportion)			X			X
Unintentional firearm injuries	Female	Education (years per capita)			X			X
Unintentional firearm injuries	Male	Population Density (under 150 ppl/sqkm, proportion)			X			X
Unintentional firearm injuries	Female	Log-transformed SEV scalar: Mech Gun	X			X		
Unintentional firearm injuries	Male	Log-transformed SEV scalar: Mech Gun	X			X		
Unintentional firearm injuries	Female	Socio-demographic Index			X			X
Unintentional firearm injuries	Female	Population Density (under 150 ppl/sqkm, proportion)			X			X
Unintentional firearm injuries	Male	LDI (I$ per capita)			X			X
Unintentional firearm injuries	Female	LDI (I$ per capita)			X			X
Unintentional firearm injuries	Male	Education (years per capita)			X			X
Other exposure to mechanical forces	Female	Log-transformed SEV scalar: Oth Mech	X			X		
Other exposure to mechanical forces	Male	Population Density (under 150 ppl/sqkm, proportion)		X			X	
Other exposure to mechanical forces	Female	Population Density (under 150 ppl/sqkm, proportion)		X			X	
Other exposure to mechanical forces	Male	Population Density (over 1000 ppl/sqkm, proportion)		X			X	
Other exposure to mechanical forces	Female	Population Density (over 1000 ppl/sqkm, proportion)		X			X	
Other exposure to mechanical forces	Male	Healthcare access and quality index		X			X	
Other exposure to mechanical forces	Female	Healthcare access and quality index		X			X	
Other exposure to mechanical forces	Male	Socio-demographic Index			X			X
Other exposure to mechanical forces	Female	Socio-demographic Index			X			X
Other exposure to mechanical forces	Male	LDI (I$ per capita)			X			X
Other exposure to mechanical forces	Female	LDI (I$ per capita)			X			X
Other exposure to mechanical forces	Male	Education (years per capita)			X			X

Table S18. Comparison of GBD 2017 and GBD 2019 covariates and level of covariates used in cause of death modeling								
Cause	Sex	Covariate	Level 1: GBD 2019	Level 2: GBD 2019	Level 3: GBD 2019	Level 1: GBD 2017	Level 2: GBD 2017	Level 3: GBD 2017
Other exposure to mechanical forces	Male	Education (years per capita)			X			X
Other exposure to mechanical forces	Female	Education (years per capita)			X			X
Other exposure to mechanical forces	Male	Log-transformed SEV scalar: Oth Mech	X			X		
Adverse effects of medical treatment	Male	LDI (I$ per capita)			X			X
Adverse effects of medical treatment	Female	LDI (I$ per capita)			X			X
Adverse effects of medical treatment	Male	Socio-demographic Index			X			X
Adverse effects of medical treatment	Female	Socio-demographic Index			X			X
Adverse effects of medical treatment	Male	Healthcare access and quality index		X			X	
Adverse effects of medical treatment	Female	Healthcare access and quality index		X			X	
Animal contact	Female	Elevation Under 100m (proportion)			X			X
Animal contact	Male	Elevation Under 100m (proportion)			X			X
Animal contact	Female	Log-transformed SEV scalar: Animal	X			X		
Animal contact	Male	Log-transformed SEV scalar: Animal	X			X		
Animal contact	Male	Population Density (under 150 ppl/sqkm, proportion)			X			X
Animal contact	Female	Population 15 to 30 (proportion)		X			X	
Animal contact	Male	Socio-demographic Index			X			X
Animal contact	Female	Healthcare access and quality index		X			X	
Animal contact	Male	Healthcare access and quality index		X			X	
Animal contact	Female	Elevation Over 1500m (proportion)			X			X
Animal contact	Male	Elevation Over 1500m (proportion)			X			X
Animal contact	Female	Population Density (over 1000 ppl/sqkm, proportion)			X			X
Animal contact	Male	Population Density (over 1000 ppl/sqkm, proportion)			X			X
Animal contact	Male	Population 15 to 30 (proportion)		X			X	
Animal contact	Female	Socio-demographic Index			X			X
Animal contact	Male	Socio-demographic Index			X			X
Animal contact	Male	LDI (I$ per capita)			X			X
Animal contact	Male	LDI (I$ per capita)			X			X
Animal contact	Female	LDI (I$ per capita)			X			X
Animal contact	Female	LDI (I$ per capita)			X			X
Animal contact	Female	Socio-demographic Index			X			X
Animal contact	Male	Education (years per capita)			X			X
Animal contact	Female	Education (years per capita)			X			X
Animal contact	Female	Education (years per capita)			X			X
Animal contact	Female	Population Density (under 150 ppl/sqkm, proportion)			X			X
Animal contact	Male	Education (years per capita)			X			X
Venomous animal contact	Female	Population Density (over 1000 ppl/sqkm, proportion)			X			X
Venomous animal contact	Male	Population Density (over 1000 ppl/sqkm, proportion)			X			X
Venomous animal contact	Female	Population Density (under 150 ppl/sqkm, proportion)			X			X
Venomous animal contact	Male	Population Density (under 150 ppl/sqkm, proportion)			X			X
Venomous animal contact	Female	Elevation Under 100m (proportion)			X			X
Venomous animal contact	Male	Elevation Under 100m (proportion)			X			X
Venomous animal contact	Female	Log-transformed SEV scalar: Venom	X			X		
Venomous animal contact	Male	Log-transformed SEV scalar: Venom	X			X		
Venomous animal contact	Female	Socio-demographic Index			X			X
Venomous animal contact	Male	Socio-demographic Index			X			X
Venomous animal contact	Male	Socio-demographic Index			X			X
Venomous animal contact	Male	Education (years per capita)			X			X
Venomous animal contact	Female	Healthcare access and quality index		X			X	
Venomous animal contact	Male	Healthcare access and quality index		X			X	
Venomous animal contact	Female	Elevation Over 1500m (proportion)			X			X
Venomous animal contact	Male	Elevation Over 1500m (proportion)			X			X
Venomous animal contact	Female	Socio-demographic Index			X			X
Venomous animal contact	Male	LDI (I$ per capita)			X			X
Venomous animal contact	Female	LDI (I$ per capita)			X			X
Venomous animal contact	Female	Education (years per capita)			X			X
Non-venomous animal contact	Male	Population Density (under 150 ppl/sqkm, proportion)			X	X		
Non-venomous animal contact	Female	Education (years per capita)			X			X
Non-venomous animal contact	Male	Education (years per capita)			X			X
Non-venomous animal contact	Female	LDI (I$ per capita)			X			X
Non-venomous animal contact	Male	LDI (I$ per capita)			X			X
Non-venomous animal contact	Female	Socio-demographic Index			X			X
Non-venomous animal contact	Male	Socio-demographic Index			X			X
Non-venomous animal contact	Female	Healthcare access and quality index		X			X	
Non-venomous animal contact	Male	Healthcare access and quality index			X		X	
Non-venomous animal contact	Male	Healthcare access and quality index			X			X
Non-venomous animal contact	Male	Population Density (under 150 ppl/sqkm, proportion)			X			X
Non-venomous animal contact	Male	Log-transformed SEV scalar: Non Ven	X			X		
Non-venomous animal contact	Female	Elevation Under 100m (proportion)			X			X
Non-venomous animal contact	Male	Elevation Under 100m (proportion)			X			X
Non-venomous animal contact	Female	Population Density (over 1000 ppl/sqkm, proportion)			X			X
Non-venomous animal contact	Male	Elevation Under 100m (proportion)			X	X		
Non-venomous animal contact	Male	Elevation Over 1500m (proportion)			X	X		
Non-venomous animal contact	Female	Elevation Over 1500m (proportion)			X			X
Non-venomous animal contact	Male	Elevation Over 1500m (proportion)			X			X
Non-venomous animal contact	Male	Population Density (over 1000 ppl/sqkm, proportion)			X			X
Non-venomous animal contact	Male	Population Density (over 1000 ppl/sqkm, proportion)			X	X		

Table S18. Comparison of GBD 2017 and GBD 2019 covariates and level of covariates used in cause of death modeling

Cause	Sex	Covariate	Level 1: GBD 2019	Level 2: GBD 2019	Level 3: GBD 2019	Level 1: GBD 2017	Level 2: GBD 2017	Level 3: GBD 2017
Non-venomous animal contact	Female	Log-transformed SEV scalar: Non Ven	X			X		
Non-venomous animal contact	Male	Healthcare access and quality index		X			X	
Non-venomous animal contact	Male	Healthcare access and quality index		X				X
Non-venomous animal contact	Female	Population Density (under 150 ppl/sqkm, proportion)			X			X
Foreign body	Female	Education (years per capita)	X			X		
Foreign body	Male	Education (years per capita)	X			X		
Foreign body	Female	LDI (I$ per capita)	X			X		
Foreign body	Male	Socio-demographic Index			X			X
Foreign body	Female	Socio-demographic Index			X			X
Foreign body	Male	Healthcare access and quality index		X			X	
Foreign body	Female	Healthcare access and quality index		X			X	
Foreign body	Male	Indoor Air Pollution (All Cooking Fuels)	X			X		
Foreign body	Female	Indoor Air Pollution (All Cooking Fuels)	X			X		
Foreign body	Male	LDI (I$ per capita)	X			X		
Foreign body	Female	Population Over 65 (proportion)	X			X		
Foreign body	Male	Population Over 65 (proportion)	X			X		
Pulmonary aspiration and foreign body in airway	Female	Log-transformed SEV scalar: F Body Asp	X			X		
Pulmonary aspiration and foreign body in airway	Male	Log-transformed SEV scalar: F Body Asp	X			X		
Pulmonary aspiration and foreign body in airway	Male	LDI (I$ per capita)			X			X
Pulmonary aspiration and foreign body in airway	Female	LDI (I$ per capita)			X			X
Pulmonary aspiration and foreign body in airway	Female	Education (years per capita)			X	X		
Pulmonary aspiration and foreign body in airway	Female	Education (years per capita)			X			X
Pulmonary aspiration and foreign body in airway	Male	Healthcare access and quality index		X			X	
Pulmonary aspiration and foreign body in airway	Female	Healthcare access and quality index		X			X	
Pulmonary aspiration and foreign body in airway	Male	Socio-demographic Index			X			X
Pulmonary aspiration and foreign body in airway	Female	Socio-demographic Index			X			X
Pulmonary aspiration and foreign body in airway	Male	Mean BMI		X			X	
Pulmonary aspiration and foreign body in airway	Female	Mean BMI		X			X	
Pulmonary aspiration and foreign body in airway	Female	Alcohol binge drinker proportion, age-standardized		X			X	
Pulmonary aspiration and foreign body in airway	Male	LDI (I$ per capita)			X			X
Foreign body in other body part	Female	Log-transformed SEV scalar: Oth F Body	X			X		
Foreign body in other body part	Female	Education (years per capita)			X			X
Foreign body in other body part	Male	LDI (I$ per capita)			X			X
Foreign body in other body part	Female	LDI (I$ per capita)			X			X
Foreign body in other body part	Male	Socio-demographic Index			X			X
Foreign body in other body part	Female	Socio-demographic Index			X			X
Foreign body in other body part	Male	Healthcare access and quality index		X			X	
Foreign body in other body part	Female	Healthcare access and quality index		X			X	
Foreign body in other body part	Male	Log-transformed SEV scalar: Oth F Body	X			X		
Foreign body in other body part	Male	Education (years per capita)			X			X
Foreign body in other body part	Male	LDI (I$ per capita)			X			X
Foreign body in other body part	Male	Education (years per capita)			X			X
Other unintentional injuries	Male	Log-transformed SEV scalar: Oth Unint	X			X		
Other unintentional injuries	Female	Vehicles - 4 wheels (per capita)	X			X		
Other unintentional injuries	Male	Vehicles - 2 wheels (per capita)	X			X		
Other unintentional injuries	Female	Vehicles - 2 wheels (per capita)	X			X		
Other unintentional injuries	Male	Population Density (under 150 ppl/sqkm, proportion)			X			X
Other unintentional injuries	Female	Population Density (under 150 ppl/sqkm, proportion)			X			X
Other unintentional injuries	Male	Population Density (over 1000 ppl/sqkm, proportion)			X			X
Other unintentional injuries	Female	Population Density (over 1000 ppl/sqkm, proportion)			X			X
Other unintentional injuries	Male	Healthcare access and quality index		X			X	
Other unintentional injuries	Female	Healthcare access and quality index		X			X	
Other unintentional injuries	Male	Socio-demographic Index			X			X
Other unintentional injuries	Female	Socio-demographic Index			X			X
Other unintentional injuries	Male	LDI (I$ per capita)			X			X
Other unintentional injuries	Male	LDI (I$ per capita)			X			X
Other unintentional injuries	Female	LDI (I$ per capita)			X			X
Other unintentional injuries	Female	LDI (I$ per capita)			X			X
Other unintentional injuries	Male	Education (years per capita)			X			X
Other unintentional injuries	Male	Education (years per capita)			X			X
Other unintentional injuries	Female	Education (years per capita)			X			X
Other unintentional injuries	Female	Education (years per capita)			X			X
Other unintentional injuries	Male	Vehicles - 4 wheels (per capita)	X			X		
Other unintentional injuries	Female	Log-transformed SEV scalar: Oth Unint	X			X		
Self-harm	Female	Education (years per capita)			X			X
Self-harm	Female	Major depressive disorder	X			X		
Self-harm	Female	Education (years per capita)			X			X
Self-harm	Male	LDI (I$ per capita)			X			X
Self-harm	Female	LDI (I$ per capita)			X			X
Self-harm	Female	LDI (I$ per capita)			X			X
Self-harm	Male	Socio-demographic Index			X			X
Self-harm	Male	Socio-demographic Index			X			X
Self-harm	Female	Socio-demographic Index			X			X
Self-harm	Male	Healthcare access and quality index		X			X	
Self-harm	Female	Healthcare access and quality index		X			X	
Self-harm	Male	Education (years per capita)			X			X
Self-harm	Male	Population Density (over 1000 ppl/sqkm, proportion)		X			X	
Self-harm	Male	Population Density (under 150 ppl/sqkm, proportion)		X			X	

Table S18. Comparison of GBD 2017 and GBD 2019 covariates and level of covariates used in cause of death modeling

Cause	Sex	Covariate	Level 1: GBD 2019	Level 2: GBD 2019	Level 3: GBD 2019	Level 1: GBD 2017	Level 2: GBD 2017	Level 3: GBD 2017
Self-harm	Female	Population Density (under 150 ppl/sqkm, proportion)		X			X	
Self-harm	Male	Population Density (150-300 ppl/sqkm, proportion)		X			X	
Self-harm	Female	Population Density (150-300 ppl/sqkm, proportion)		X			X	
Self-harm	Male	Population Density (300-500 ppl/sqkm, proportion)		X			X	
Self-harm	Female	Population Density (300-500 ppl/sqkm, proportion)		X			X	
Self-harm	Male	Population Density (500-1000 ppl/sqkm, proportion)		X			X	
Self-harm	Female	Population Density (500-1000 ppl/sqkm, proportion)		X			X	
Self-harm	Female	Log-transformed SEV scalar: Self Harm	X			X		
Self-harm	Female	Population Density (over 1000 ppl/sqkm, proportion)		X			X	
Self-harm	Female	Socio-demographic Index			X			X
Self-harm	Male	Muslim Religion (proportion of population)		X		X		
Self-harm	Male	Muslim Religion (proportion of population)		X			X	
Self-harm by firearm	Female	Population Density (500-1000 ppl/sqkm, proportion)		X			X	
Self-harm by firearm	Female	Major depressive disorder	X			X		
Self-harm by firearm	Male	Major depressive disorder	X			X		
Self-harm by firearm	Female	Log-transformed SEV scalar: Self Harm	X			X		
Self-harm by firearm	Male	Log-transformed SEV scalar: Self Harm	X			X		
Self-harm by firearm	Male	Population Density (500-1000 ppl/sqkm, proportion)		X			X	
Self-harm by firearm	Female	Population Density (300-500 ppl/sqkm, proportion)		X			X	
Self-harm by firearm	Male	Population Density (300-500 ppl/sqkm, proportion)		X			X	
Self-harm by firearm	Male	Population Density (150-300 ppl/sqkm, proportion)		X			X	
Self-harm by firearm	Female	Population Density (under 150 ppl/sqkm, proportion)		X			X	
Self-harm by firearm	Female	Population Density (150-300 ppl/sqkm, proportion)		X			X	
Self-harm by firearm	Female	Population Density (over 1000 ppl/sqkm, proportion)		X			X	
Self-harm by firearm	Male	Education (years per capita)			X			X
Self-harm by firearm	Female	Education (years per capita)			X			X
Self-harm by firearm	Male	LDI (I$ per capita)			X			X
Self-harm by firearm	Male	Population Density (under 150 ppl/sqkm, proportion)		X			X	
Self-harm by firearm	Male	Socio-demographic Index			X			X
Self-harm by firearm	Female	LDI (I$ per capita)			X			X
Self-harm by firearm	Female	Socio-demographic Index			X			X
Self-harm by firearm	Male	Healthcare access and quality index		X			X	
Self-harm by firearm	Female	Healthcare access and quality index		X			X	
Self-harm by firearm	Male	Population Density (over 1000 ppl/sqkm, proportion)		X			X	
Self-harm by other specified means	Female	Population Density (over 1000 ppl/sqkm, proportion)		X			X	
Self-harm by other specified means	Female	Population Density (150-300 ppl/sqkm, proportion)		X			X	
Self-harm by other specified means	Female	Population Density (under 150 ppl/sqkm, proportion)		X			X	
Self-harm by other specified means	Male	Population Density (over 1000 ppl/sqkm, proportion)		X			X	
Self-harm by other specified means	Male	Population Density (150-300 ppl/sqkm, proportion)		X			X	
Self-harm by other specified means	Male	Healthcare access and quality index		X			X	
Self-harm by other specified means	Male	LDI (I$ per capita)			X			X
Self-harm by other specified means	Male	Socio-demographic Index			X			X
Self-harm by other specified means	Female	Socio-demographic Index			X			X
Self-harm by other specified means	Female	LDI (I$ per capita)			X			X
Self-harm by other specified means	Male	Education (years per capita)			X			X
Self-harm by other specified means	Female	Education (years per capita)			X			X
Self-harm by other specified means	Female	Population Density (300-500 ppl/sqkm, proportion)		X			X	
Self-harm by other specified means	Female	Healthcare access and quality index		X			X	
Self-harm by other specified means	Male	Population Density (300-500 ppl/sqkm, proportion)		X			X	
Self-harm by other specified means	Male	Population Density (under 150 ppl/sqkm, proportion)		X			X	
Self-harm by other specified means	Male	Population Density (500-1000 ppl/sqkm, proportion)		X			X	
Self-harm by other specified means	Female	Log-transformed SEV scalar: Self Harm	X			X		
Self-harm by other specified means	Male	Log-transformed SEV scalar: Self Harm	X			X		
Self-harm by other specified means	Female	Major depressive disorder	X			X		
Self-harm by other specified means	Male	Major depressive disorder	X			X		
Self-harm by other specified means	Female	Population Density (500-1000 ppl/sqkm, proportion)		X			X	
Interpersonal violence	Female	Opium Cultivation (binary)		X			X	
Interpersonal violence	Male	Opium Cultivation (binary)		X			X	
Interpersonal violence	Female	Socio-demographic Index			X			X
Interpersonal violence	Male	Socio-demographic Index			X			X
Interpersonal violence	Female	Population Density (over 1000 ppl/sqkm, proportion)		X			X	
Interpersonal violence	Male	Healthcare access and quality index		X			X	
Interpersonal violence	Male	Log-transformed SEV scalar: Violence	X			X		
Interpersonal violence	Female	LDI (I$ per capita)			X			X
Interpersonal violence	Male	LDI (I$ per capita)			X			X
Interpersonal violence	Female	Education (years per capita)			X			X
Interpersonal violence	Female	Healthcare access and quality index		X			X	

Table S18. Comparison of GBD 2017 and GBD 2019 covariates and level of covariates used in cause of death modeling								
**Cause**	**Sex**	**Covariate**	**Level 1: GBD 2019**	**Level 2: GBD 2019**	**Level 3: GBD 2019**	**Level 1: GBD 2017**	**Level 2: GBD 2017**	**Level 3: GBD 2017**
Interpersonal violence	Female	Log-transformed SEV scalar: Violence	X			X		
Interpersonal violence	Male	Population Density (over 1000 ppl/sqkm, proportion)		X			X	
Interpersonal violence	Male	Education (years per capita)			X			X
Physical violence by firearm	Female	Socio-demographic Index			X			X
Physical violence by firearm	Female	Log-transformed SEV scalar: Viol Gun	X			X		
Physical violence by firearm	Male	Log-transformed SEV scalar: Viol Gun	X			X		
Physical violence by firearm	Female	Opium Cultivation (binary)		X			X	
Physical violence by firearm	Male	Opium Cultivation (binary)		X			X	
Physical violence by firearm	Female	Population Density (over 1000 ppl/sqkm, proportion)		X			X	
Physical violence by firearm	Male	Socio-demographic Index			X			X
Physical violence by firearm	Female	Healthcare access and quality index		X			X	
Physical violence by firearm	Male	Healthcare access and quality index		X			X	
Physical violence by firearm	Male	Education (years per capita)			X			X
Physical violence by firearm	Female	Education (years per capita)			X			X
Physical violence by firearm	Male	LDI (I$ per capita)			X			X
Physical violence by firearm	Male	Population Density (over 1000 ppl/sqkm, proportion)		X			X	
Physical violence by firearm	Female	LDI (I$ per capita)			X			X
Physical violence by sharp object	Female	Log-transformed SEV scalar: Viol Knife	X			X		
Physical violence by sharp object	Male	Log-transformed SEV scalar: Viol Knife	X			X		
Physical violence by sharp object	Male	Opium Cultivation (binary)		X			X	
Physical violence by sharp object	Female	Population Density (over 1000 ppl/sqkm, proportion)		X			X	
Physical violence by sharp object	Male	Population Density (over 1000 ppl/sqkm, proportion)		X			X	
Physical violence by sharp object	Female	Opium Cultivation (binary)		X			X	
Physical violence by sharp object	Female	Socio-demographic Index			X			X
Physical violence by sharp object	Male	Healthcare access and quality index		X			X	
Physical violence by sharp object	Male	Education (years per capita)			X			X
Physical violence by sharp object	Female	Education (years per capita)			X			X
Physical violence by sharp object	Female	Healthcare access and quality index		X			X	
Physical violence by sharp object	Female	LDI (I$ per capita)			X			X
Physical violence by sharp object	Male	Socio-demographic Index			X			X
Physical violence by sharp object	Male	LDI (I$ per capita)			X			X
Physical violence by other means	Female	Log-transformed SEV scalar: Oth Viol	X			X		
Physical violence by other means	Male	Log-transformed SEV scalar: Oth Viol	X			X		
Physical violence by other means	Female	Opium Cultivation (binary)		X			X	
Physical violence by other means	Male	Opium Cultivation (binary)		X			X	
Physical violence by other means	Female	Population Density (over 1000 ppl/sqkm, proportion)		X			X	
Physical violence by other means	Male	Population Density (over 1000 ppl/sqkm, proportion)		X			X	
Physical violence by other means	Male	Healthcare access and quality index		X			X	
Physical violence by other means	Female	Socio-demographic Index			X			X
Physical violence by other means	Female	LDI (I$ per capita)			X			X
Physical violence by other means	Male	LDI (I$ per capita)			X			X
Physical violence by other means	Female	Education (years per capita)			X			X
Physical violence by other means	Male	Education (years per capita)			X			X
Physical violence by other means	Female	Healthcare access and quality index		X			X	
Physical violence by other means	Male	Socio-demographic Index			X			X
Environmental heat and cold exposure	Male	Population Density (150-300 ppl/sqkm, proportion)			X			X
Environmental heat and cold exposure	Female	Elevation 500 to 1500m (proportion)			X			X
Environmental heat and cold exposure	Female	Elevation Over 1500m (proportion)			X			X
Environmental heat and cold exposure	Male	Population-weighted mean temperature			X			X
Environmental heat and cold exposure	Female	Sanitation (proportion with access)			X			X
Environmental heat and cold exposure	Male	Sanitation (proportion with access)			X			X
Environmental heat and cold exposure	Male	Elevation Over 1500m (proportion)			X			X
Environmental heat and cold exposure	Female	Healthcare access and quality index		X			X	
Environmental heat and cold exposure	Male	Healthcare access and quality index		X			X	
Environmental heat and cold exposure	Female	90th percentile climatic temperature in the given country-year.			X			X
Environmental heat and cold exposure	Male	Elevation 500 to 1500m (proportion)			X			X
Environmental heat and cold exposure	Male	90th percentile climatic temperature in the given country-year.			X			X
Environmental heat and cold exposure	Male	Rainfall (Quintiles 4-5)			X			X
Environmental heat and cold exposure	Female	Population Density (150-300 ppl/sqkm, proportion)			X			X
Environmental heat and cold exposure	Male	Education (years per capita)			X			X
Environmental heat and cold exposure	Female	Education (years per capita)			X			X
Environmental heat and cold exposure	Male	LDI (I$ per capita)			X			X
Environmental heat and cold exposure	Female	Socio-demographic Index			X			X
Environmental heat and cold exposure	Female	LDI (I$ per capita)			X			X
Environmental heat and cold exposure	Male	Socio-demographic Index			X			X
Environmental heat and cold exposure	Female	Rainfall (Quintiles 4-5)			X			X
Environmental heat and cold exposure	Female	Population-weighted mean temperature			X			X
Acute lymphoid leukaemia	Female	Mean BMI		X			X	
Acute lymphoid leukaemia	Male	Mean BMI		X			X	
Acute lymphoid leukaemia	Female	Log-transformed age-standardized SEV scalar: Leukemia	X			X		
Acute lymphoid leukaemia	Male	LDI (I$ per capita)			X			X
Acute lymphoid leukaemia	Female	LDI (I$ per capita)			X			X
Acute lymphoid leukaemia	Male	Education (years per capita)			X			X
Acute lymphoid leukaemia	Female	Education (years per capita)			X			X
Acute lymphoid leukaemia	Male	Log-transformed age-standardized SEV scalar: Leukemia	X			X		
Acute lymphoid leukaemia	Male	Socio-demographic Index			X			X

Table S18. Comparison of GBD 2017 and GBD 2019 covariates and level of covariates used in cause of death modeling

Cause	Sex	Covariate	Level 1: GBD 2019	Level 2: GBD 2019	Level 3: GBD 2019	Level 1: GBD 2017	Level 2: GBD 2017	Level 3: GBD 2017
Acute lymphoid leukaemia	Female	Healthcare access and quality index		X			X	
Acute lymphoid leukaemia	Male	Healthcare access and quality index		X			X	
Acute lymphoid leukaemia	Female	Socio-demographic Index			X			X
Acute lymphoid leukaemia	Male	Cumulative Cigarettes (10 Years)		X			X	
Acute lymphoid leukaemia	Female	Tobacco (cigarettes per capita)		X			X	
Acute lymphoid leukaemia	Male	Tobacco (cigarettes per capita)		X			X	
Acute lymphoid leukaemia	Female	Cumulative Cigarettes (20 Years)		X			X	
Acute lymphoid leukaemia	Male	Cumulative Cigarettes (20 Years)		X			X	
Acute lymphoid leukaemia	Female	Log-transformed SEV scalar: Leukemia	X			X		
Acute lymphoid leukaemia	Male	Log-transformed SEV scalar: Leukemia	X			X		
Acute lymphoid leukaemia	Female	Cumulative Cigarettes (10 Years)		X			X	
Chronic lymphoid leukaemia	Female	Cumulative Cigarettes (15 Years)		X			X	
Chronic lymphoid leukaemia	Male	Cumulative Cigarettes (20 Years)		X			X	
Chronic lymphoid leukaemia	Female	Cumulative Cigarettes (20 Years)		X			X	
Chronic lymphoid leukaemia	Female	Log-transformed age-standardized SEV scalar: Leukemia	X			X		
Chronic lymphoid leukaemia	Female	Log-transformed SEV scalar: Leukemia	X			X		
Chronic lymphoid leukaemia	Male	Log-transformed age-standardized SEV scalar: Leukemia	X			X		
Chronic lymphoid leukaemia	Male	Cumulative Cigarettes (15 Years)		X			X	
Chronic lymphoid leukaemia	Male	Log-transformed SEV scalar: Leukemia	X			X		
Chronic lymphoid leukaemia	Female	Tobacco (cigarettes per capita)		X			X	
Chronic lymphoid leukaemia	Female	Smoking Prevalence		X			X	
Chronic lymphoid leukaemia	Female	Cumulative Cigarettes (5 Years)		X			X	
Chronic lymphoid leukaemia	Male	Education (years per capita)			X			X
Chronic lymphoid leukaemia	Male	Tobacco (cigarettes per capita)		X			X	
Chronic lymphoid leukaemia	Female	Education (years per capita)			X			X
Chronic lymphoid leukaemia	Male	LDI (I$ per capita)			X			X
Chronic lymphoid leukaemia	Female	LDI (I$ per capita)			X			X
Chronic lymphoid leukaemia	Male	Mean BMI		X			X	
Chronic lymphoid leukaemia	Male	Socio-demographic Index			X			X
Chronic lymphoid leukaemia	Female	Socio-demographic Index			X			X
Chronic lymphoid leukaemia	Male	Healthcare access and quality index		X			X	
Chronic lymphoid leukaemia	Female	Mean BMI		X			X	
Chronic lymphoid leukaemia	Female	Healthcare access and quality index		X		X		
Chronic lymphoid leukaemia	Female	Healthcare access and quality index	X				X	
Chronic lymphoid leukaemia	Female	Healthcare access and quality index	X			X		
Chronic lymphoid leukaemia	Male	Smoking Prevalence		X			X	
Chronic lymphoid leukaemia	Male	Cumulative Cigarettes (10 Years)		X			X	
Chronic lymphoid leukaemia	Female	Cumulative Cigarettes (10 Years)		X			X	
Chronic lymphoid leukaemia	Male	Cumulative Cigarettes (5 Years)		X			X	
Chronic lymphoid leukaemia	Female	Healthcare access and quality index		X			X	
Acute myeloid leukaemia	Female	Smoking Prevalence		X			X	
Acute myeloid leukaemia	Male	Log-transformed SEV scalar: Leukemia	X			X		
Acute myeloid leukaemia	Male	Cumulative Cigarettes (20 Years)		X			X	
Acute myeloid leukaemia	Female	Cumulative Cigarettes (20 Years)		X			X	
Acute myeloid leukaemia	Male	Cumulative Cigarettes (10 Years)		X			X	
Acute myeloid leukaemia	Female	Cumulative Cigarettes (10 Years)		X			X	
Acute myeloid leukaemia	Male	Smoking Prevalence		X			X	
Acute myeloid leukaemia	Male	Healthcare access and quality index		X			X	
Acute myeloid leukaemia	Female	Education (years per capita)			X			X
Acute myeloid leukaemia	Male	Socio-demographic Index			X			X
Acute myeloid leukaemia	Female	Socio-demographic Index			X			X
Acute myeloid leukaemia	Male	Mean BMI		X			X	
Acute myeloid leukaemia	Female	Mean BMI		X			X	
Acute myeloid leukaemia	Male	LDI (I$ per capita)			X			X
Acute myeloid leukaemia	Female	LDI (I$ per capita)			X			X
Acute myeloid leukaemia	Male	Education (years per capita)			X			X
Acute myeloid leukaemia	Female	Log-transformed age-standardized SEV scalar: Leukemia	X			X		
Acute myeloid leukaemia	Female	Healthcare access and quality index		X			X	
Acute myeloid leukaemia	Male	Log-transformed age-standardized SEV scalar: Leukemia	X			X		
Acute myeloid leukaemia	Female	Log-transformed SEV scalar: Leukemia	X			X		
Chronic myeloid leukaemia	Male	Education (years per capita)			X			X
Chronic myeloid leukaemia	Female	Smoking Prevalence		X			X	
Chronic myeloid leukaemia	Male	Smoking Prevalence		X			X	
Chronic myeloid leukaemia	Female	Cumulative Cigarettes (10 Years)		X			X	
Chronic myeloid leukaemia	Female	Education (years per capita)			X			X
Chronic myeloid leukaemia	Female	Cumulative Cigarettes (5 Years)		X			X	
Chronic myeloid leukaemia	Male	Cumulative Cigarettes (5 Years)		X			X	
Chronic myeloid leukaemia	Female	Tobacco (cigarettes per capita)		X			X	
Chronic myeloid leukaemia	Male	Healthcare access and quality index		X			X	
Chronic myeloid leukaemia	Male	Tobacco (cigarettes per capita)		X			X	
Chronic myeloid leukaemia	Male	Cumulative Cigarettes (15 Years)		X			X	
Chronic myeloid leukaemia	Female	Cumulative Cigarettes (20 Years)		X			X	
Chronic myeloid leukaemia	Male	Cumulative Cigarettes (20 Years)		X			X	
Chronic myeloid leukaemia	Female	Log-transformed SEV scalar: Leukemia	X			X		
Chronic myeloid leukaemia	Male	Log-transformed SEV scalar: Leukemia	X			X		
Chronic myeloid leukaemia	Female	Log-transformed age-standardized SEV scalar: Leukemia	X			X		
Chronic myeloid leukaemia	Male	Log-transformed age-standardized SEV scalar: Leukemia	X			X		
Chronic myeloid leukaemia	Female	Cumulative Cigarettes (15 Years)		X			X	
Chronic myeloid leukaemia	Female	Healthcare access and quality index		X			X	
Chronic myeloid leukaemia	Male	Cumulative Cigarettes (10 Years)		X			X	

Table S18. Comparison of GBD 2017 and GBD 2019 covariates and level of covariates used in cause of death modeling								
Cause	Sex	Covariate	Level 1: GBD 2019	Level 2: GBD 2019	Level 3: GBD 2019	Level 1: GBD 2017	Level 2: GBD 2017	Level 3: GBD 2017
Chronic myeloid leukaemia	Female	LDI (I$ per capita)			X			X
Chronic myeloid leukaemia	Male	LDI (I$ per capita)			X			X
Chronic myeloid leukaemia	Female	Mean BMI		X			X	
Chronic myeloid leukaemia	Male	Mean BMI		X			X	
Chronic myeloid leukaemia	Female	Socio-demographic Index			X			X
Chronic myeloid leukaemia	Male	Socio-demographic Index			X			X
Non-melanoma skin cancer (squamous-cell carcinoma)	Male	LDI (I$ per capita)			X			X
Non-melanoma skin cancer (squamous-cell carcinoma)	Female	Education (years per capita)			X			X
Non-melanoma skin cancer (squamous-cell carcinoma)	Male	Education (years per capita)			X			X
Non-melanoma skin cancer (squamous-cell carcinoma)	Female	LDI (I$ per capita)			X			X
Non-melanoma skin cancer (squamous-cell carcinoma)	Female	Socio-demographic Index			X			X
Non-melanoma skin cancer (squamous-cell carcinoma)	Male	Socio-demographic Index			X			X
Non-melanoma skin cancer (squamous-cell carcinoma)	Female	Healthcare access and quality index		X			X	
Non-melanoma skin cancer (squamous-cell carcinoma)	Male	Healthcare access and quality index		X			X	
Non-melanoma skin cancer (squamous-cell carcinoma)	Female	Smoking Prevalence	X			X		
Non-melanoma skin cancer (squamous-cell carcinoma)	Male	Smoking Prevalence	X			X		
Non-melanoma skin cancer (squamous-cell carcinoma)	Female	Cumulative Cigarettes (5 Years)	X			X		
Non-melanoma skin cancer (squamous-cell carcinoma)	Male	Cumulative Cigarettes (10 Years)	X			X		
Non-melanoma skin cancer (squamous-cell carcinoma)	Male	Cumulative Cigarettes (5 Years)	X			X		
Non-melanoma skin cancer (squamous-cell carcinoma)	Female	Cumulative Cigarettes (15 Years)	X			X		
Non-melanoma skin cancer (squamous-cell carcinoma)	Male	Cumulative Cigarettes (15 Years)	X			X		
Non-melanoma skin cancer (squamous-cell carcinoma)	Female	Average latitude		X			X	
Non-melanoma skin cancer (squamous-cell carcinoma)	Male	Average latitude		X			X	
Non-melanoma skin cancer (squamous-cell carcinoma)	Female	Cumulative Cigarettes (10 Years)	X			X		
Police conflict and executions	Male	Education (years per capita)			X			X
Police conflict and executions	Male	Education (years per capita)			X			X
Police conflict and executions	Male	Healthcare access and quality index		X			X	
Police conflict and executions	Female	Education (years per capita)			X			X
Police conflict and executions	Female	Healthcare access and quality index		X			X	
Police conflict and executions	Male	LDI (I$ per capita)			X			X
Police conflict and executions	Male	LDI (I$ per capita)			X			X
Police conflict and executions	Female	LDI (I$ per capita)			X			X
Police conflict and executions	Female	LDI (I$ per capita)			X			X
Police conflict and executions	Male	Socio-demographic Index		X			X	
Police conflict and executions	Female	Socio-demographic Index		X			X	
Police conflict and executions	Female	Population Density (over 1000 ppl/sqkm, proportion)		X			X	
Police conflict and executions	Male	Population Density (over 1000 ppl/sqkm, proportion)		X			X	
Police conflict and executions	Female	Education (years per capita)			X			X
Alcoholic cardiomyopathy	Female	Log-transformed SEV scalar: CMP	X			X		
Alcoholic cardiomyopathy	Male	Healthcare access and quality index		X			X	
Alcoholic cardiomyopathy	Female	Healthcare access and quality index		X			X	
Alcoholic cardiomyopathy	Male	Smoking Prevalence	X			X		
Alcoholic cardiomyopathy	Female	Smoking Prevalence	X			X		
Alcoholic cardiomyopathy	Male	Log-transformed SEV scalar: CMP	X			X		
Alcoholic cardiomyopathy	Male	LDI (I$ per capita)			X			X
Alcoholic cardiomyopathy	Female	LDI (I$ per capita)			X			X
Myocarditis	Female	Systolic Blood Pressure (mmHg)	X			X		
Myocarditis	Male	Log-transformed SEV scalar: CMP	X			X		
Myocarditis	Female	LDI (I$ per capita)			X			X
Myocarditis	Male	LDI (I$ per capita)			X			X
Myocarditis	Female	Socio-demographic Index			X			X
Myocarditis	Male	Socio-demographic Index			X			X
Myocarditis	Female	Healthcare access and quality index		X			X	
Myocarditis	Male	Healthcare access and quality index		X			X	
Myocarditis	Male	Systolic Blood Pressure (mmHg)	X			X		
Myocarditis	Female	Log-transformed SEV scalar: CMP	X			X		
Other leukaemia	Female	Log-transformed SEV scalar: Leukemia	X			X		
Other leukaemia	Female	Healthcare access and quality index		X			X	
Other leukaemia	Female	Education (years per capita)			X			X
Other leukaemia	Male	Education (years per capita)			X			X
Other leukaemia	Female	LDI (I$ per capita)			X			X
Other leukaemia	Male	LDI (I$ per capita)			X			X
Other leukaemia	Female	Mean BMI		X			X	
Other leukaemia	Male	Healthcare access and quality index		X			X	
Other leukaemia	Female	Cumulative Cigarettes (10 Years)		X			X	
Other leukaemia	Male	Mean BMI		X			X	
Other leukaemia	Male	Cumulative Cigarettes (10 Years)		X			X	
Other leukaemia	Female	Tobacco (cigarettes per capita)		X			X	
Other leukaemia	Male	Tobacco (cigarettes per capita)		X			X	
Other leukaemia	Female	Socio-demographic Index			X			X
Other leukaemia	Female	Cumulative Cigarettes (20 Years)		X			X	
Other leukaemia	Male	Log-transformed SEV scalar: Leukemia	X			X		
Other leukaemia	Male	Cumulative Cigarettes (20 Years)		X			X	
Other leukaemia	Male	Socio-demographic Index			X			X
Other leukaemia	Female	Log-transformed age-standardized SEV scalar: Leukemia	X			X		
Other leukaemia	Male	Log-transformed age-standardized SEV scalar: Leukemia	X			X		
Other cardiomyopathy	Male	Healthcare access and quality index		X			X	
Other cardiomyopathy	Female	Log-transformed SEV scalar: CMP	X			X		
Other cardiomyopathy	Male	LDI (I$ per capita)			X			X
Other cardiomyopathy	Female	LDI (I$ per capita)			X			X
Other cardiomyopathy	Male	Mean BMI		X			X	

Table S18. Comparison of GBD 2017 and GBD 2019 covariates and level of covariates used in cause of death modeling

Cause	Sex	Covariate	Level 1: GBD 2019	Level 2: GBD 2019	Level 3: GBD 2019	Level 1: GBD 2017	Level 2: GBD 2017	Level 3: GBD 2017
Other cardiomyopathy	Female	Mean BMI		X			X	
Other cardiomyopathy	Male	Socio-demographic Index			X			X
Other cardiomyopathy	Female	Socio-demographic Index			X			X
Other cardiomyopathy	Male	Log-transformed SEV scalar: CMP	X			X		
Other cardiomyopathy	Female	Healthcare access and quality index		X			X	
Other cardiomyopathy	Male	Systolic Blood Pressure (mmHg)	X			X		
Other cardiomyopathy	Female	Systolic Blood Pressure (mmHg)	X			X		
Other cardiomyopathy	Male	Smoking Prevalence	X			X		
Other cardiomyopathy	Female	Smoking Prevalence	X			X		
Myelodysplastic, myeloproliferative, and other hematopoietic neoplasms	Female	Log-transformed age-standardized SEV scalar: Leukemia	X			X		
Myelodysplastic, myeloproliferative, and other hematopoietic neoplasms	Male	Log-transformed age-standardized SEV scalar: Leukemia	X			X		
Myelodysplastic, myeloproliferative, and other hematopoietic neoplasms	Female	Education (years per capita)			X			X
Myelodysplastic, myeloproliferative, and other hematopoietic neoplasms	Male	Education (years per capita)			X			X
Myelodysplastic, myeloproliferative, and other hematopoietic neoplasms	Female	Healthcare access and quality index		X			X	
Myelodysplastic, myeloproliferative, and other hematopoietic neoplasms	Male	LDI (I$ per capita)			X			X
Myelodysplastic, myeloproliferative, and other hematopoietic neoplasms	Female	Tobacco (cigarettes per capita)		X			X	
Myelodysplastic, myeloproliferative, and other hematopoietic neoplasms	Male	Cumulative Cigarettes (5 Years)		X			X	
Myelodysplastic, myeloproliferative, and other hematopoietic neoplasms	Female	Cumulative Cigarettes (5 Years)		X			X	
Myelodysplastic, myeloproliferative, and other hematopoietic neoplasms	Male	Cumulative Cigarettes (10 Years)		X			X	
Myelodysplastic, myeloproliferative, and other hematopoietic neoplasms	Female	Cumulative Cigarettes (10 Years)		X			X	
Myelodysplastic, myeloproliferative, and other hematopoietic neoplasms	Male	Smoking Prevalence		X			X	
Myelodysplastic, myeloproliferative, and other hematopoietic neoplasms	Female	Smoking Prevalence		X			X	
Myelodysplastic, myeloproliferative, and other hematopoietic neoplasms	Male	Healthcare access and quality index		X			X	
Myelodysplastic, myeloproliferative, and other hematopoietic neoplasms	Male	Socio-demographic Index			X			X
Myelodysplastic, myeloproliferative, and other hematopoietic neoplasms	Male	Cumulative Cigarettes (15 Years)		X			X	
Myelodysplastic, myeloproliferative, and other hematopoietic neoplasms	Female	Cumulative Cigarettes (20 Years)		X			X	
Myelodysplastic, myeloproliferative, and other hematopoietic neoplasms	Male	Cumulative Cigarettes (20 Years)		X			X	
Myelodysplastic, myeloproliferative, and other hematopoietic neoplasms	Male	Tobacco (cigarettes per capita)		X			X	
Myelodysplastic, myeloproliferative, and other hematopoietic neoplasms	Female	Log-transformed SEV scalar: Leukemia	X			X		
Myelodysplastic, myeloproliferative, and other hematopoietic neoplasms	Female	Cumulative Cigarettes (15 Years)		X			X	
Myelodysplastic, myeloproliferative, and other hematopoietic neoplasms	Female	Socio-demographic Index			X			X
Myelodysplastic, myeloproliferative, and other hematopoietic neoplasms	Male	Log-transformed SEV scalar: Leukemia	X			X		
Myelodysplastic, myeloproliferative, and other hematopoietic neoplasms	Female	LDI (I$ per capita)			X			X
Non-rheumatic calcific aortic valve disease	Female	Smoking Prevalence	X			X		
Non-rheumatic calcific aortic valve disease	Male	Smoking Prevalence	X			X		
Non-rheumatic calcific aortic valve disease	Female	Systolic Blood Pressure (mmHg)	X			X		
Non-rheumatic calcific aortic valve disease	Female	Cholesterol (total, mean per capita)		X			X	
Non-rheumatic calcific aortic valve disease	Female	Mean BMI	X			X		
Non-rheumatic calcific aortic valve disease	Female	Diabetes Fasting Plasma Glucose (mmol/L), age-standardized 25+		X			X	
Non-rheumatic calcific aortic valve disease	Male	Diabetes Fasting Plasma Glucose (mmol/L), age-standardized 25+		X			X	
Non-rheumatic calcific aortic valve disease	Female	Healthcare access and quality index		X			X	
Non-rheumatic calcific aortic valve disease	Male	Healthcare access and quality index		X			X	
Non-rheumatic calcific aortic valve disease	Male	LDI (I$ per capita)			X			X
Non-rheumatic calcific aortic valve disease	Female	LDI (I$ per capita)			X			X
Non-rheumatic calcific aortic valve disease	Female	Socio-demographic Index			X			X
Non-rheumatic calcific aortic valve disease	Male	Socio-demographic Index			X			X
Non-rheumatic calcific aortic valve disease	Male	Cholesterol (total, mean per capita)		X			X	
Non-rheumatic calcific aortic valve disease	Male	Systolic Blood Pressure (mmHg)	X			X		
Non-rheumatic calcific aortic valve disease	Male	Mean BMI	X			X		
Non-rheumatic degenerative mitral valve disease	Female	Healthcare access and quality index	X			X		
Non-rheumatic degenerative mitral valve disease	Male	Healthcare access and quality index	X			X		
Non-rheumatic degenerative mitral valve disease	Female	Socio-demographic Index	X			X		
Non-rheumatic degenerative mitral valve disease	Male	Socio-demographic Index	X			X		
Non-rheumatic degenerative mitral valve disease	Female	LDI (I$ per capita)	X			X		
Non-rheumatic degenerative mitral valve disease	Male	LDI (I$ per capita)	X			X		
Diabetes mellitus type 1	Female	Absolute value of average latitude		X			X	
Diabetes mellitus type 1	Male	Age-Specific Fertility Rate		X			X	
Diabetes mellitus type 1	Female	Age-Specific Fertility Rate		X			X	
Diabetes mellitus type 1	Male	Mean birth weight		X			X	
Diabetes mellitus type 1	Female	Age-standardized SEV for Child underweight		X			X	
Diabetes mellitus type 1	Male	Age-standardized SEV for Child underweight		X			X	
Diabetes mellitus type 1	Male	Absolute value of average latitude		X			X	
Diabetes mellitus type 1	Male	Age-standardized SEV for Child stunting		X			X	
Diabetes mellitus type 1	Female	Age-standardized SEV for Child stunting		X			X	
Diabetes mellitus type 1	Female	Live Births 40+ (proportion)		X			X	
Diabetes mellitus type 1	Female	Mean birth weight		X			X	
Diabetes mellitus type 1	Female	Live Births 35+ (proportion)		X			X	
Diabetes mellitus type 1	Male	Live Births 35+ (proportion)		X			X	
Diabetes mellitus type 1	Female	Healthcare access and quality index	X			X		
Diabetes mellitus type 1	Male	Healthcare access and quality index	X			X		
Diabetes mellitus type 1	Female	Socio-demographic Index			X			X
Diabetes mellitus type 1	Male	Socio-demographic Index			X			X
Diabetes mellitus type 1	Female	Education (years per capita)			X			X
Diabetes mellitus type 1	Male	Education (years per capita)			X			X
Diabetes mellitus type 1	Male	Live Births 40+ (proportion)		X			X	
Diabetes mellitus type 2	Male	Cholesterol (total, mean per capita)		X			X	
Diabetes mellitus type 2	Female	Systolic Blood Pressure (mmHg)		X			X	
Diabetes mellitus type 2	Male	Systolic Blood Pressure (mmHg)		X			X	
Diabetes mellitus type 2	Male	Age- and sex-specific SEV for Alcohol use		X			X	
Diabetes mellitus type 2	Male	Prevalence of obesity	X			X		

Table S18. Comparison of GBD 2017 and GBD 2019 covariates and level of covariates used in cause of death modeling

Cause	Sex	Covariate	Level 1: GBD 2019	Level 2: GBD 2019	Level 3: GBD 2019	Level 1: GBD 2017	Level 2: GBD 2017	Level 3: GBD 2017
Diabetes mellitus type 2	Female	Age- and sex-specific SEV for Alcohol use		X			X	
Diabetes mellitus type 2	Female	Cholesterol (total, mean per capita)		X			X	
Diabetes mellitus type 2	Female	Prevalence of obesity	X			X		
Diabetes mellitus type 2	Male	Diabetes Fasting Plasma Glucose (mmol/L), age-standardized 25+	X			X		
Diabetes mellitus type 2	Female	Diabetes Age-Standardized Prevalence (proportion)	X			X		
Diabetes mellitus type 2	Male	Healthcare access and quality index			X			X
Diabetes mellitus type 2	Female	Healthcare access and quality index			X			X
Diabetes mellitus type 2	Male	Mean BMI	X			X		
Diabetes mellitus type 2	Male	Diabetes Age-Standardized Prevalence (proportion)	X			X		
Diabetes mellitus type 2	Female	Education (years per capita)			X			X
Diabetes mellitus type 2	Male	Education (years per capita)			X			X
Diabetes mellitus type 2	Female	LDI (I$ per capita)			X			X
Diabetes mellitus type 2	Male	LDI (I$ per capita)			X			X
Diabetes mellitus type 2	Female	Mean BMI	X			X		
Diabetes mellitus type 2	Female	Diabetes Fasting Plasma Glucose (mmol/L), age-standardized 25+	X			X		
Bacterial skin diseases	Female	Cumulative Cigarettes (5 Years)		X			X	
Bacterial skin diseases	Male	LDI (I$ per capita)			X			X
Bacterial skin diseases	Female	Age- and sex-specific SEV for Unsafe sanitation	X			X		
Bacterial skin diseases	Female	LDI (I$ per capita)			X			X
Bacterial skin diseases	Male	Socio-demographic Index			X			X
Bacterial skin diseases	Female	Socio-demographic Index			X			X
Bacterial skin diseases	Male	Healthcare access and quality index		X		X		
Bacterial skin diseases	Female	Healthcare access and quality index		X		X		
Bacterial skin diseases	Male	Smoking Prevalence		X			X	
Bacterial skin diseases	Male	Education (years per capita)			X			X
Bacterial skin diseases	Male	Cumulative Cigarettes (5 Years)		X			X	
Bacterial skin diseases	Female	Smoking Prevalence		X			X	
Bacterial skin diseases	Male	Cumulative Cigarettes (10 Years)		X			X	
Bacterial skin diseases	Female	Cumulative Cigarettes (10 Years)		X			X	
Bacterial skin diseases	Female	Education (years per capita)			X			X
Bacterial skin diseases	Female	Improved Water Source (proportion with access)	X			X		
Bacterial skin diseases	Male	Age- and sex-specific SEV for Unsafe sanitation	X			X		
Bacterial skin diseases	Male	Improved Water Source (proportion with access)	X			X		
Upper digestive system diseases	Male	Cumulative Cigarettes (10 Years)	X			X		
Upper digestive system diseases	Female	Cumulative Cigarettes (10 Years)	X			X		
Upper digestive system diseases	Male	Sanitation (proportion with access)		X		X		
Upper digestive system diseases	Female	Sanitation (proportion with access)		X		X		
Upper digestive system diseases	Male	Age- and sex-specific SEV for Unsafe water		X		X		
Upper digestive system diseases	Female	Age- and sex-specific SEV for Unsafe water		X		X		
Upper digestive system diseases	Male	Cumulative Cigarettes (5 Years)	X			X		
Upper digestive system diseases	Male	LDI (I$ per capita)			X			X
Upper digestive system diseases	Male	Socio-demographic Index			X			X
Upper digestive system diseases	Female	Socio-demographic Index			X			X
Upper digestive system diseases	Male	Healthcare access and quality index		X			X	
Upper digestive system diseases	Female	Healthcare access and quality index		X			X	
Upper digestive system diseases	Male	Smoking Prevalence	X			X		
Upper digestive system diseases	Female	Cumulative Cigarettes (5 Years)	X			X		
Upper digestive system diseases	Female	LDI (I$ per capita)			X			X
Upper digestive system diseases	Female	Smoking Prevalence	X			X		

Table S19. Socio-demographic Index R-squared values with lags up to 10 years

Lag	e(0)	ln(35q15)	ln(20q50)	ln(5q0)
0	0.66759977	0.34235576	0.53994049	0.65329693
1	0.67418777	0.348246629	0.545044199	0.653181113
2	0.67809727	0.35181489	0.54164147	0.65301258
3	0.666798558	0.334170682	0.52351018	0.65281289
4	0.66903423	0.33090096	0.52260514	0.66006772
5	0.665793242	0.325679768	0.514505368	0.662069101
6	0.67200966	0.33021662	0.51787493	0.66650974
7	0.658956804	0.317390154	0.500897055	0.664478616
8	0.64744290	0.29667684	0.47739526	0.66599732
9	0.616907591	0.252479626	0.434787203	0.664699099
10	0.62612861	0.25826863	0.43860339	0.67060422
