# Supplementary material for: Global burden of 369 diseases and injuries in 204 countries and territories, 1990–2019: a systematic analysis for the Global Burden of Disease Study 2019
Source: Lancet. 2020 Oct 17;396(10258):1204–22. doi: 10.1016/S0140-6736(20)30925-9 (PMC7567026; doi:10.1016/S0140-6736(20)30925-9)
Supplement: Supplementary appendix 2 [file mmc2a.pdf]

THE LANCET

Supplementary appendix 2a (Part 1 of 5)

This appendix formed part of the original submission and has been peer reviewed. We post it as supplied by the authors.

Supplement to: GBD 2019 Diseases and Injuries Collaborators. Global burden of 369 diseases and injuries in 204 countries and territories, 1990–2019: a systematic analysis for the Global Burden of Disease Study 2019. *Lancet* 2020; **396:** 1204–22.

Appendix 2A: supplementary results appendix to “Global burden of 369 diseases and injuries in 204 countries and territories, 1990–2019: a systematic analysis for the Global Burden of Disease Study 2019”

This supplement provides additional figures and more detailed results for “Global burden of 369 diseases and injuries in 204 countries and territories, 1990–2019: a systematic analysis for the Global Burden of Disease Study 2019."

List of supplementary results appendix figures and tables:

Appendix 2A

Figure S1: Leading GBD Level 3 causes of age-standardised YLDs rates by location for a) females and b) males, 2019. 1

Figure S2: Leading GBD Level 3 causes of age-standardised prevalence rates by location for a) females and b) males, 2019. 3

Figure S3: Trends of age-standardised YLD rates per 100,000 for the top eight Level 3 causes of non-fatal burden in 2019 by SDI quintile for both sexes, from 1990 to 2019. 5

Figure S4: Death counts due to fatal discontinuities by type, 1980–2019. 6

Figure S5: Leading level 3 causes of all-age DALY counts by location for females in 1990 (A) males in 1990 (B) females in 2019 (C) males in 2019 (D) 10

Figure S6: Leading 20 GBD cause hierarchy Level 3 causes of YLLs by SDI grouping and sex for 1990, 2010, and 2019, with percentage change in number of YLLs, and all-age and age-standardised rates. 14

Figure S7: Global distribution of total YLDs by 23 GBD age groups for Level 2 causes of non-fatal burden in 1990 and 2019 for a) females and b) males. 24

Figure S8: Global leading 30 level 3 causes of DALYs for 1990, 2010, and 2019, with percentage change in number of DALYs, and age-standardised rates, by sex and SDI quintile 26

Table S1: All-age DALYs counts and age standardised DALY rates per 100,000 individuals for all causes and level 1 cause groups by location for 1990, 2010, 2019 , both sexes combined. 36

Appendix 2B

Table S2a: Global DALYS by cause and age group for males with 95% uncertainty intervals, by location for 2019. 4734

Appendix 2C

Table S2b: Global DALYS by cause and age group for females with 95% uncertainty intervals, by location for 2019. 6708

Appendix 2D

Table S2c: Global DALYS by cause and age group for both sexes combined with 95% uncertainty intervals, by location for 2019. 8680

Appendix 2E

Table S3: Global deaths and YLL numbers, age-standardised rates per 100,000, and percentage change between 2010 and 2019 for males for all GBD causes and Levels 1 through 4 of the cause hierarchy 10630

Table S4: Global deaths by cause and age group for females (1990, 2010, 2019) 10635

Table S5: Global deaths by cause and age group for males (1990, 2010, 2019) 10642

Table S6: Global deaths by cause and age group for both sexes combined (1990, 2010, 2019) 10648

Table S7: Global YLLs by cause and age group for females. 10654

Table S8: Global YLLs by cause and age group for males. 10656

Table S9: Global YLLs by cause and age group for both sexes combined 10658

Table S10: Deaths due to fatal discontinuities by country, all ages, for both sexes combined 10660

Table S11: Diarrhoeal diseases and lower respiratory infection aetiologies deaths, mortality rates, and annual percentage change from 1990 to 2019. 10676

Table S12: Deaths by location, cause, and age group for males. 10677

Table S13: Deaths by location, cause, and age group for females. 13007

Table S14: Deaths by location, cause, and age group for both sexes combined. 15354

Table S15: Distribution of percentage change in age-standardised deaths for Level 1 causes by location from 1990-2010 and 2010-2019, both sexes. 17543

Table S16: Global deaths and YLL numbers, age-standardised rates per 100,000, and percentage change between 2010 and 2019 for females for all GBD causes and Levels 1 through 4 of the cause hierarchy. 17561

Table S17: Global prevalence, incidence and YLDs for 2019, percentage change of YLDs counts, and percentage change of YLD rates between 2010 and 2019 by cause and by age 17566

Table S18: Prevalence, incidence, and YLDs for 2019, percentage change of YLD counts, and percentage change of age-standardised YLD rates between 2010 and 2019 by cause and GBD location. 17707

SR Figure 1a−b. Leading GBD 2019 Level 3 Causes of Age−Standardised YLD rates by location for a) Females, 2019

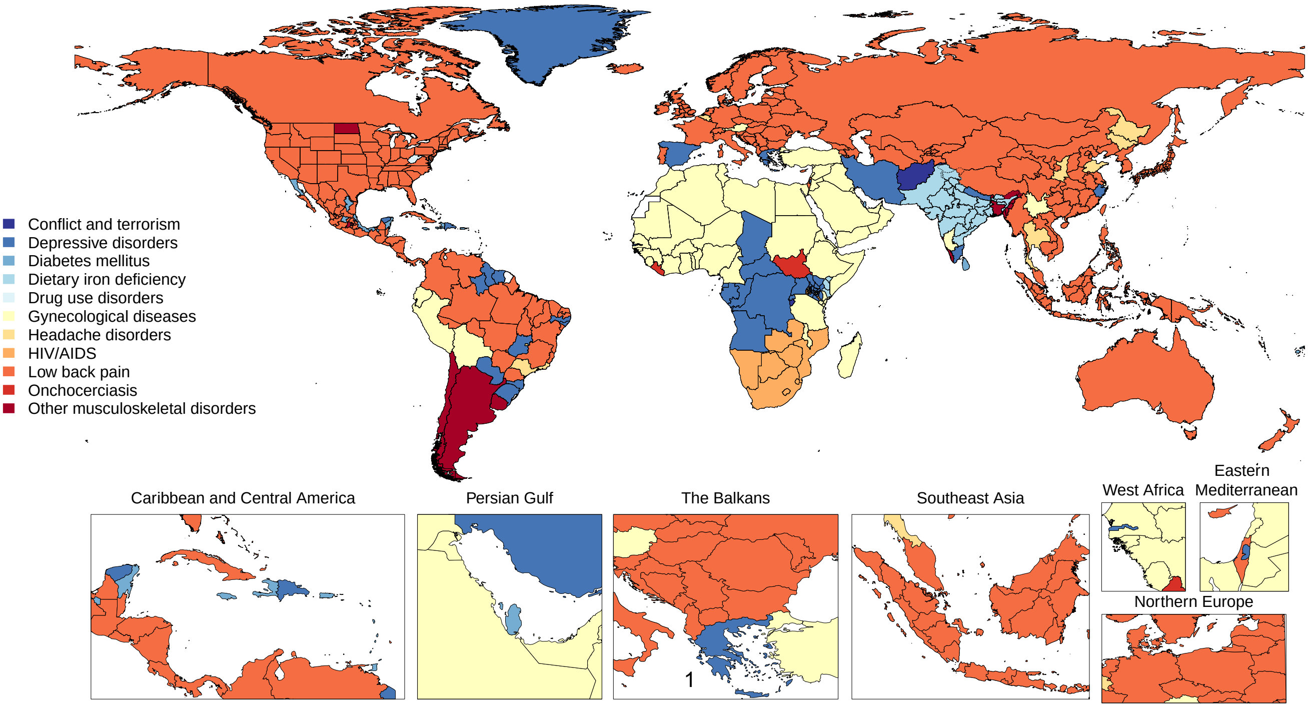

SR Figure 1a−b. Leading GBD 2019 Level 3 Causes of Age−Standardised YLD rates by location for b) Males, 2019

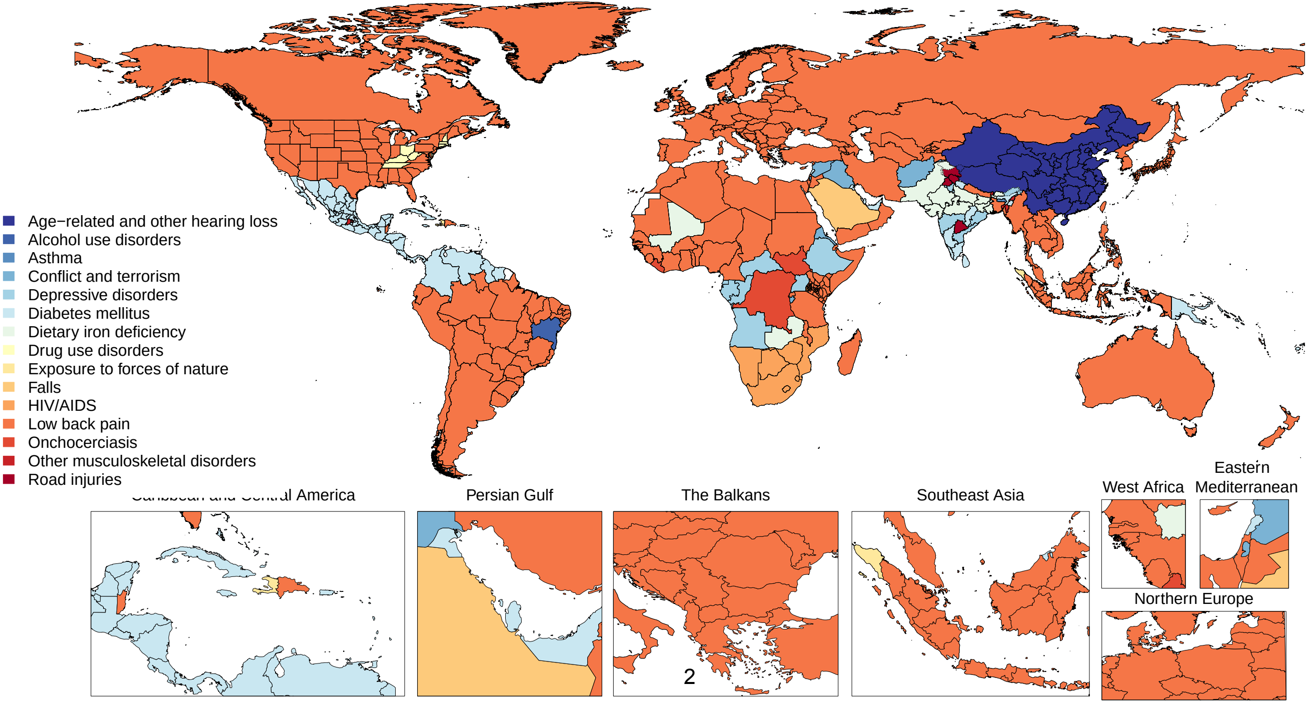

Figure 2a−b. Leading GBD 2019 Level 3 Causes of Age−Standardised Prevalence rates by location for a) Females, 2019

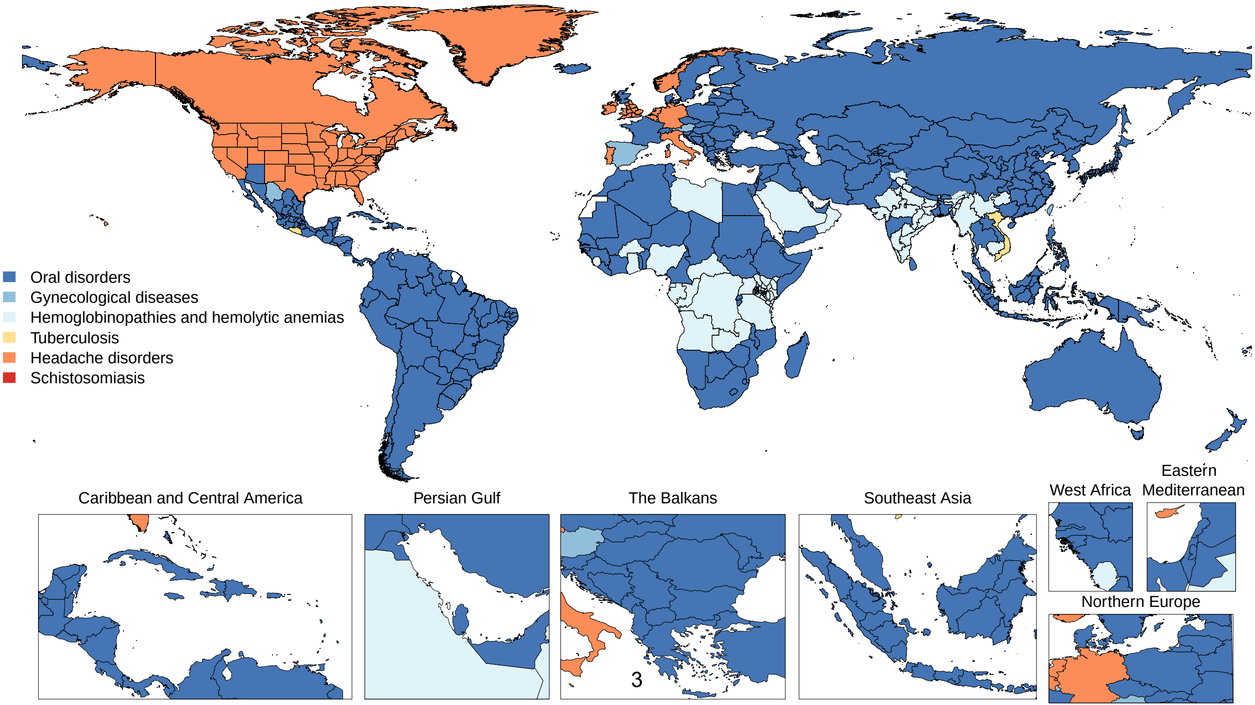

Figure 2a−b. Leading GBD 2019 Level 3 Causes of Age−Standardised Prevalence rates by location for b) Males, 2019

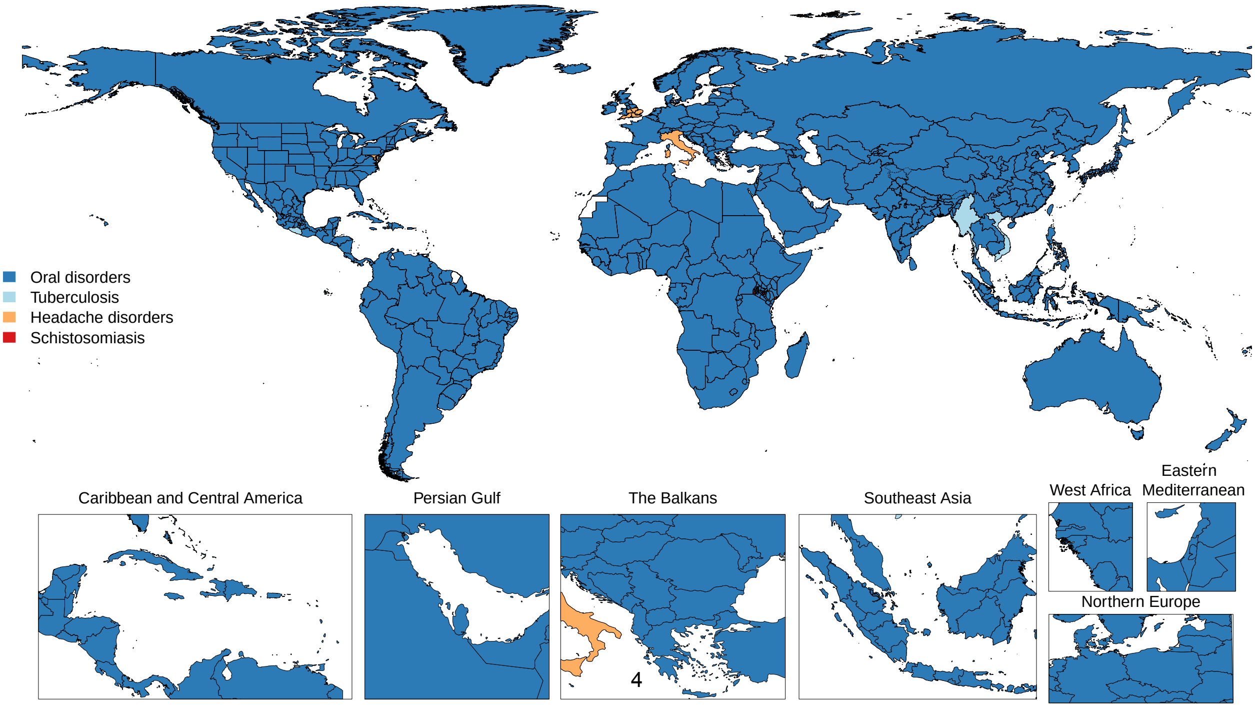

Figure 3. Trends in age−standardized YLD rates per 100,000 for the top eight level 3 causes of non−fatal burden in 2019 by SDI quintile for both sexes, from 1990 to 2019.

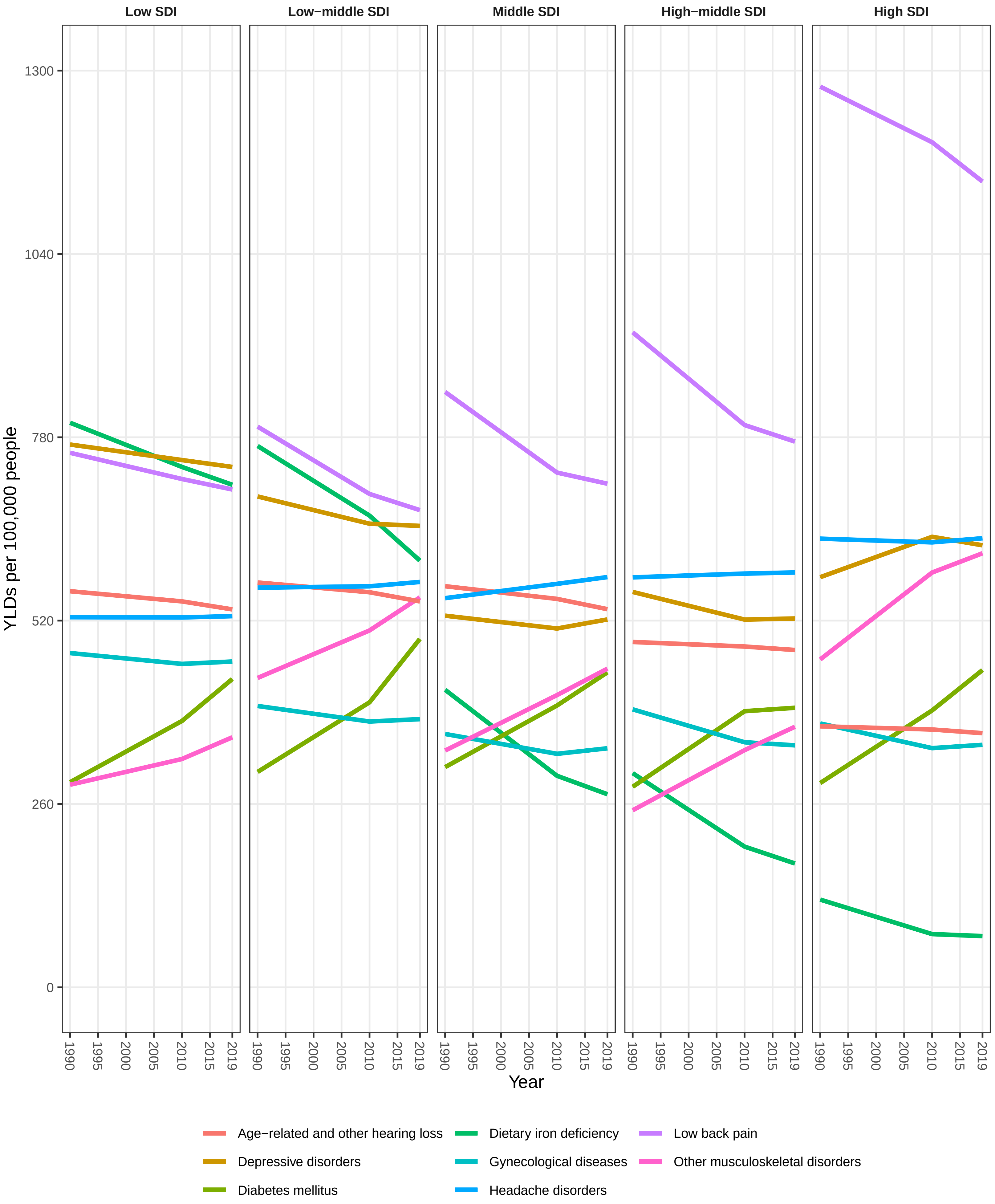

GBD=Global Burden of Disease. YLDs=years lived disability. SDI=Socio−demographic Index. Mean estimates are shown

SR Figure 4a. Death counts due to disasters, 1980–2019
Causes included: Exposure to forces of nature, Fire, heat, and hot substances, Environmental heat and cold exposure, Poisonings, Other transport injuries, and Road injuries

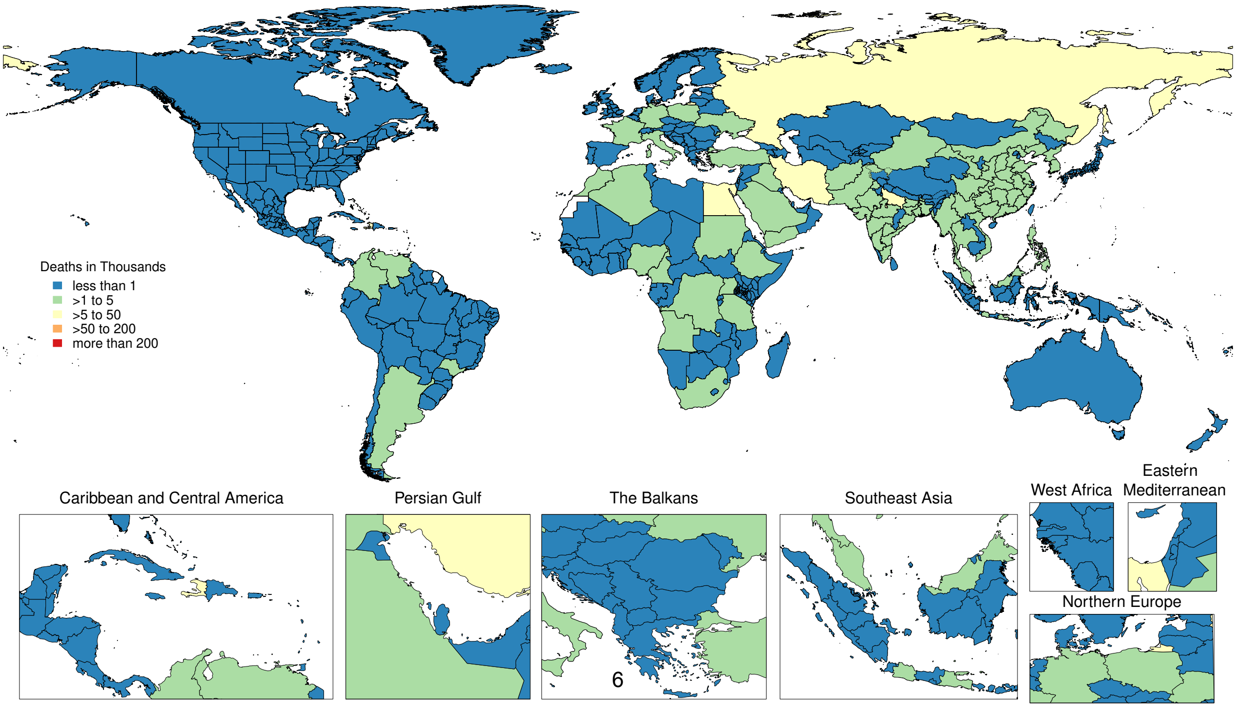

SR Figure 4b. Death counts due to disease outbreak, 1980–2019
Causes included: Malaria, Measles, Meningococcal meningitis, Diarrheal diseases, Dengue Ebola, and Other unspecified infectious diseases

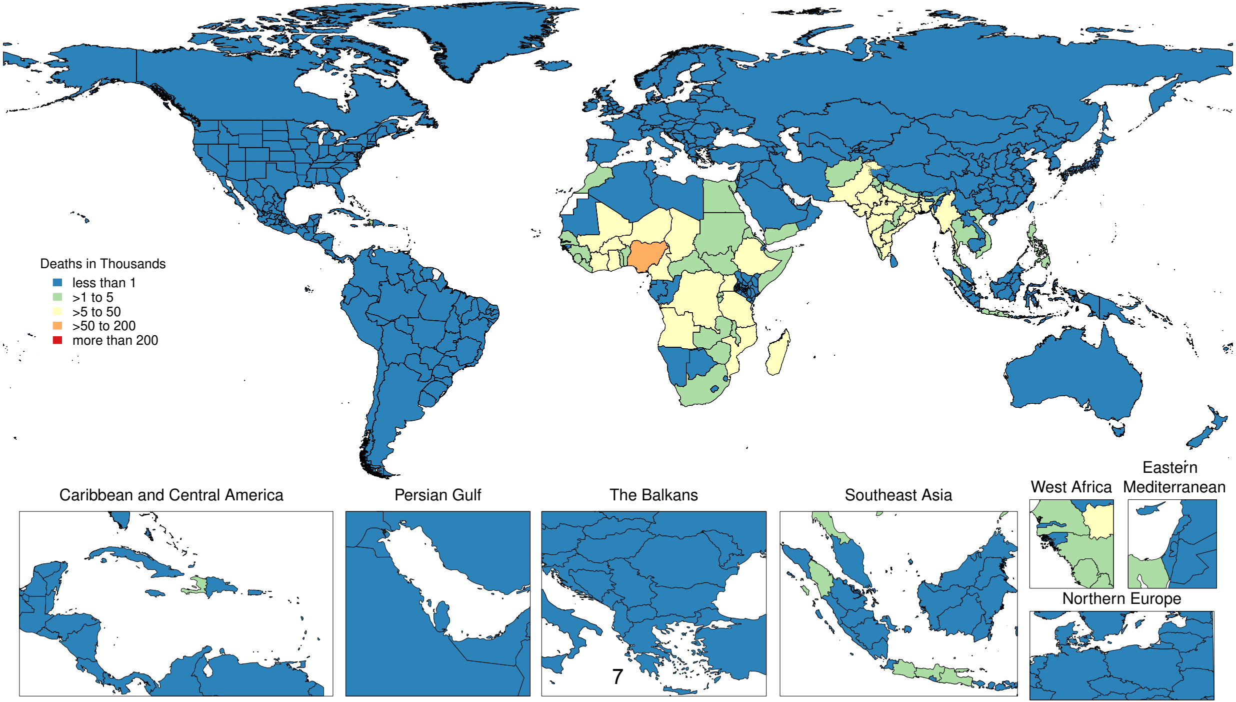

SR Figure 4c. Death counts due to famine, 1980–2019
Cause included: Protein–energy malnutrition

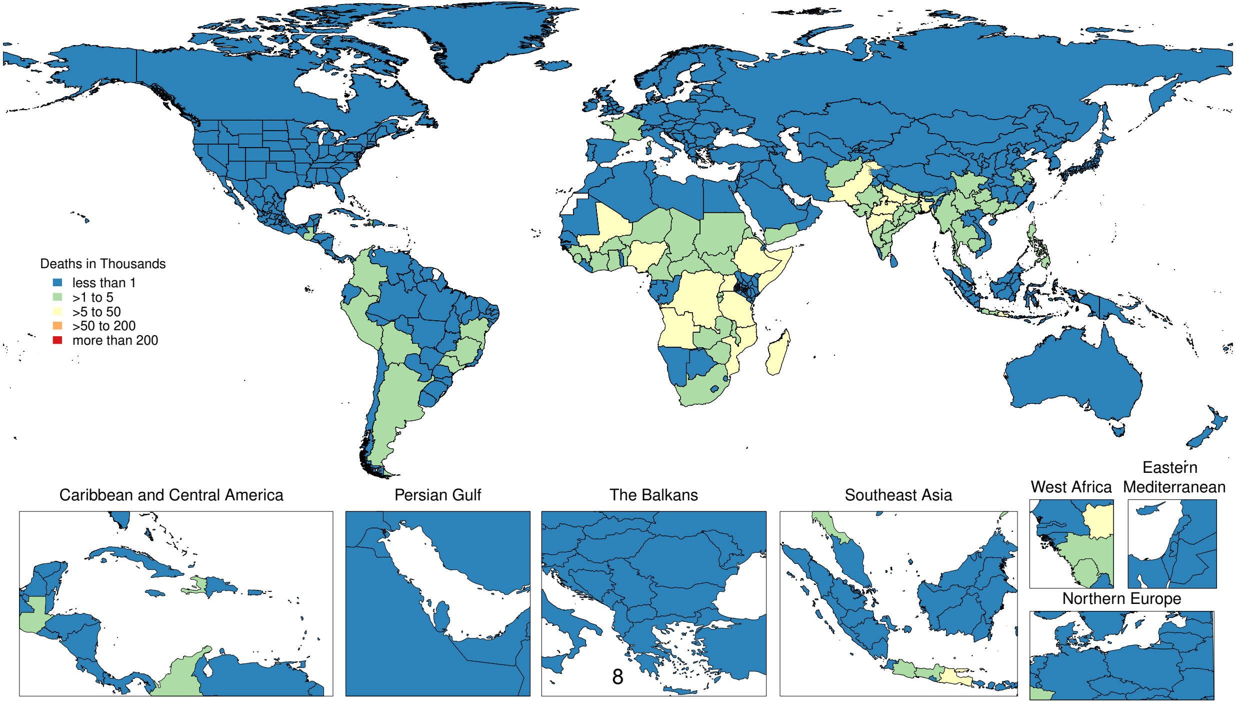

SR Figure 4d. Death counts due to violence, 1980–2019
Causes included: Conflict and terrorism, Interpersonal violence, and Executions and police violence

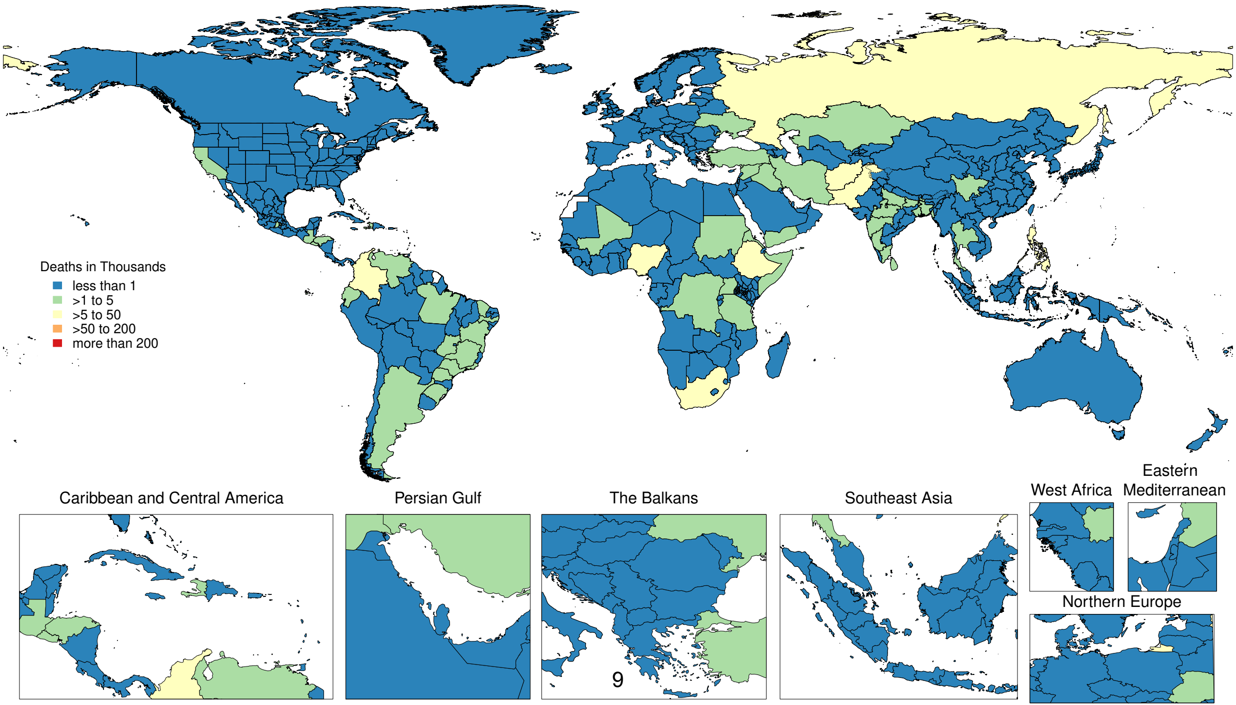

Figure 5: Leading level 3 causes of all−age DALY counts by location for females in 1990 (A)

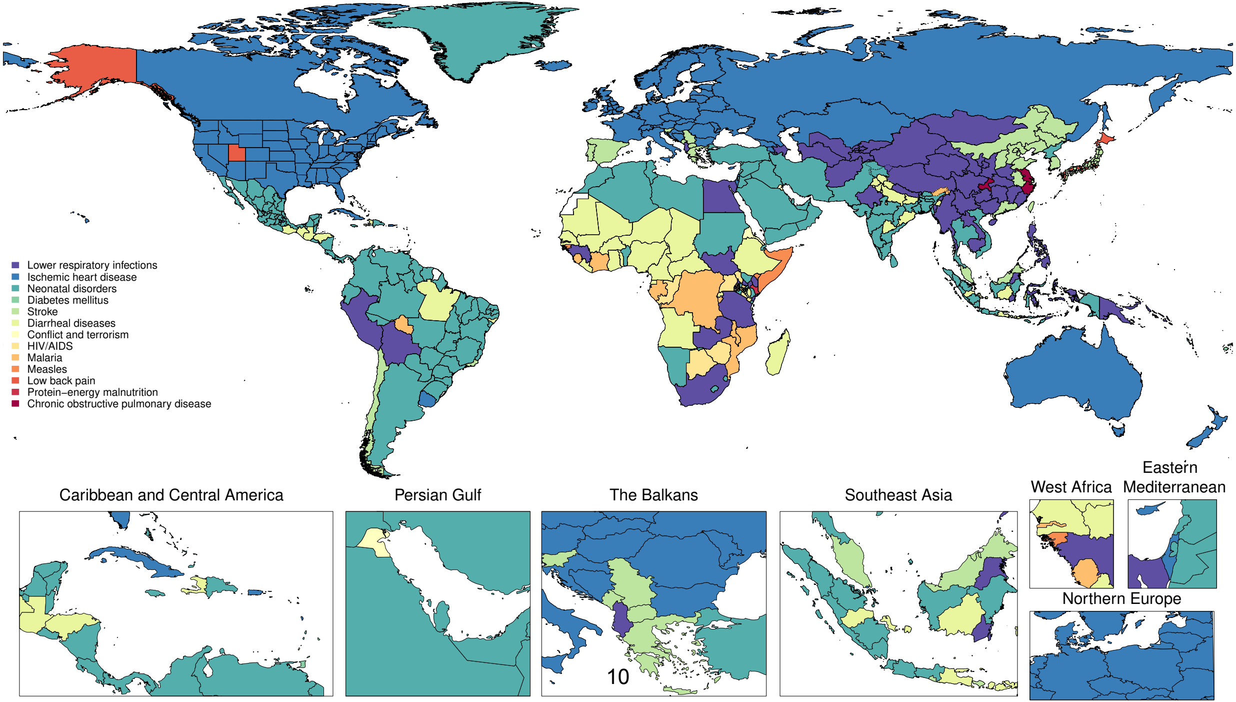

Figure 5: Leading level 3 causes of all–age DALY counts by location for males in 1990 (B)

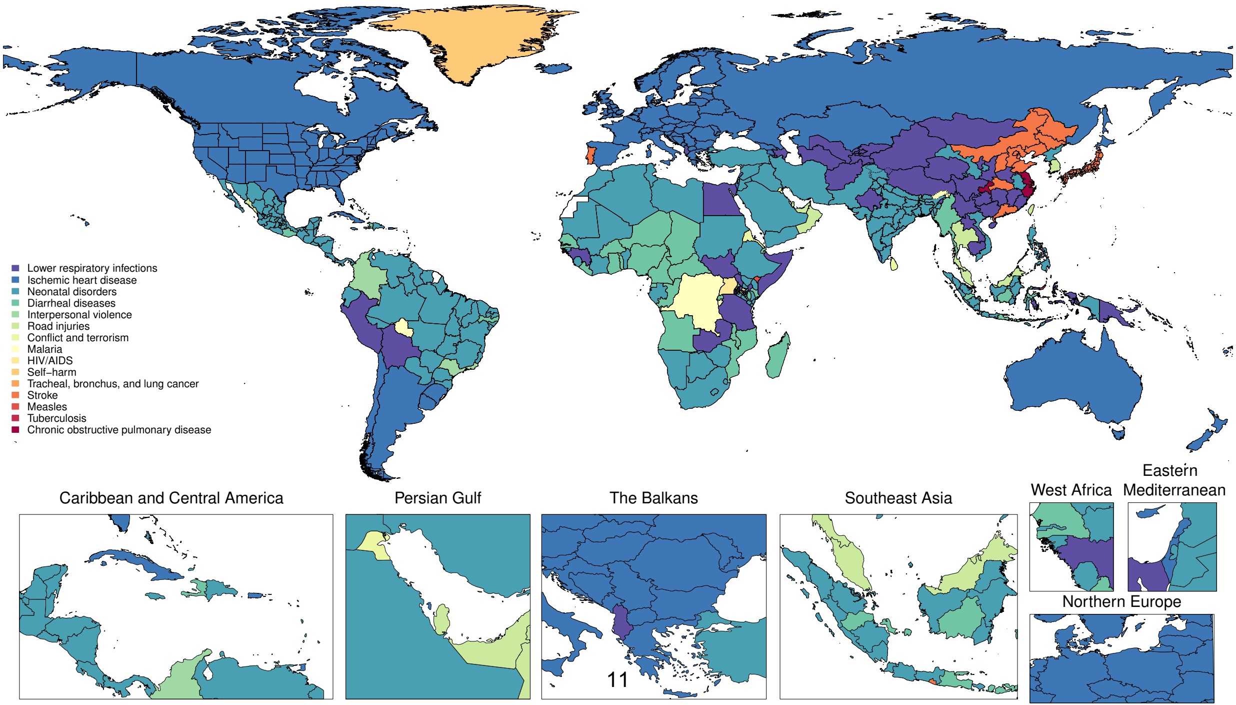

Figure 5: Leading level 3 causes of all–age DALY counts by location for females in 2019 (C)

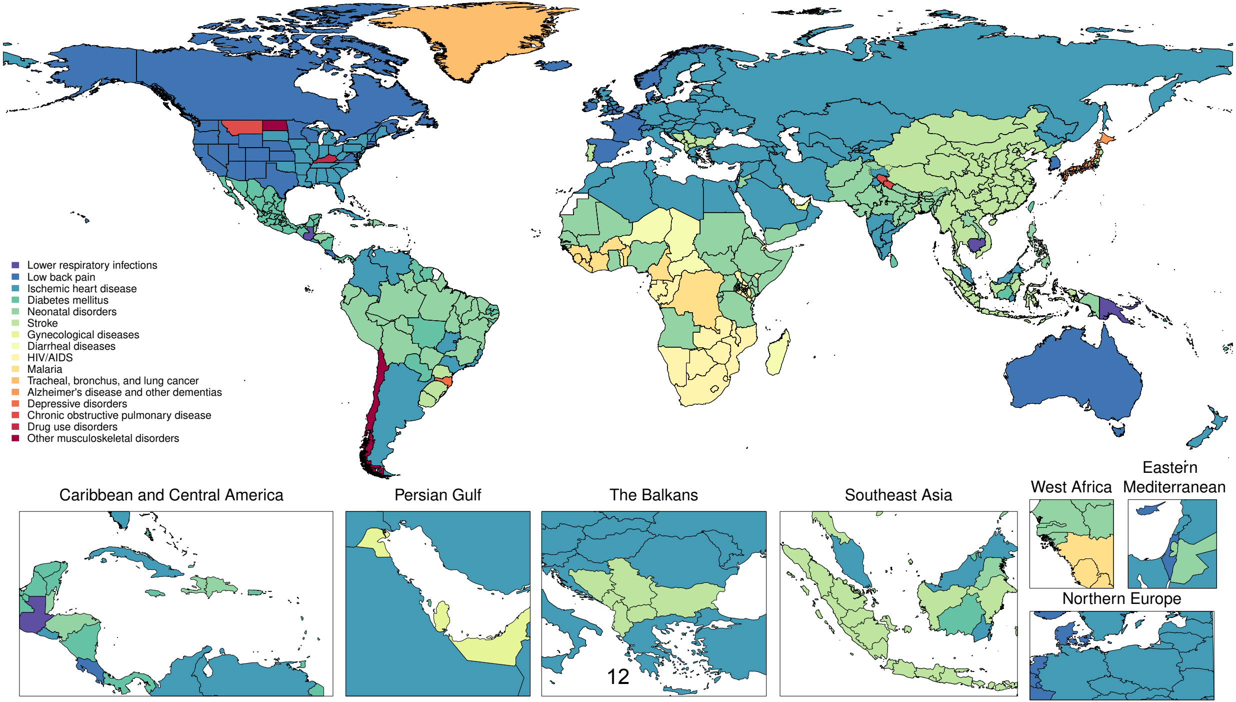

Figure 5: Leading level 3 causes of all–age DALY counts by location for males in 2019 (D)

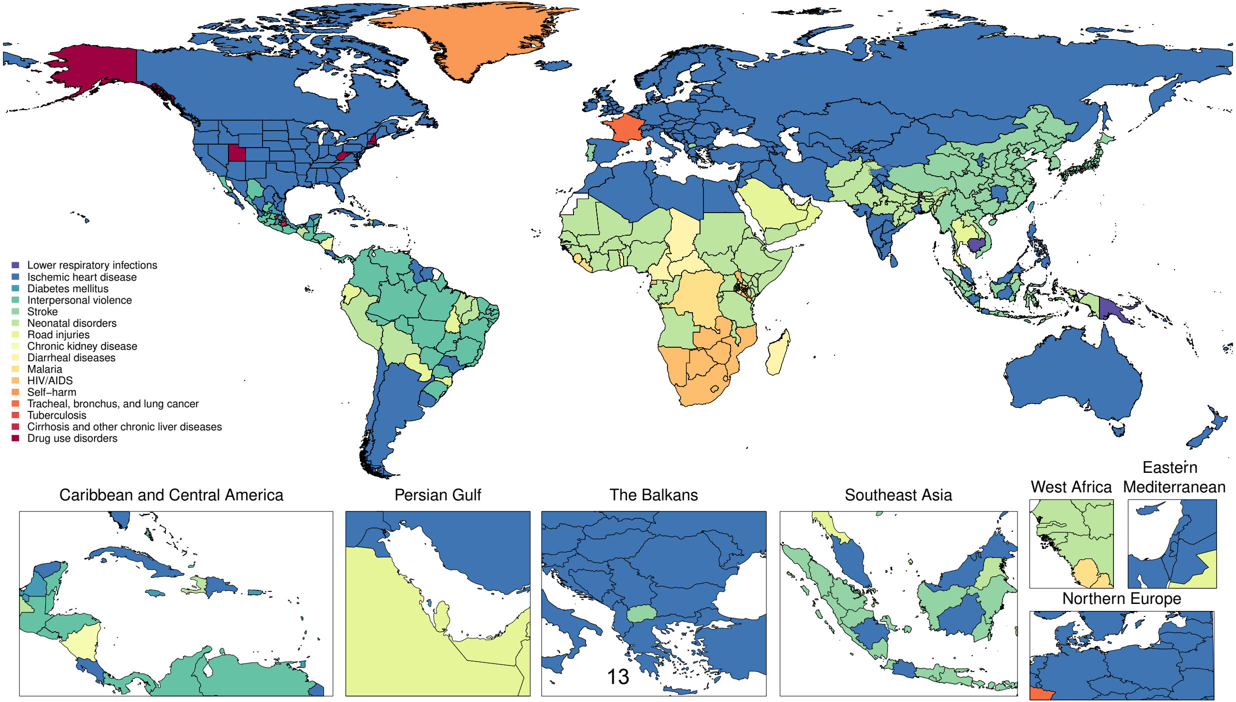

SR Figure 6a: Leading 20 GBD cause hierarchy level 3 causes of Low SDI SDI YLLs for females for 1990, 2010, and 2019

Causes are connected by arrows between time periods. Communicable maternal, neonatal and nutritional disorders are shown in red, non-communicable causes in blue and injuries in green. For the time period 1990 to 2010 and for 2010 to 2019, three measures of change are shown: percent change in the number of YLLs, percent change in the all-age YLL rate and percent change in the age-standardized YLL rate. Numbers in bold highlight statistically significant changes.

Leading causes 1990	Leading causes 2010	Mean % change number of YLLs 1990-2010	Mean % change all-age YLL rate 1990-2010	Mean % change age-standardized YLL rate 1990-2010	Leading causes 2019	Mean % change number of YLLs 2010-2019	Mean % change all-age YLL rate 2010-2019	Mean % change age-standardized YLL rate 2010-2019
1 Diarrhoeal diseases	1 Neonatal disorders	**16.1**	**-32.1**	**-21.2**	1 Neonatal disorders	-9.7	**-28.1**	**-16.9**
2 Neonatal disorders	2 Diarrhoeal diseases	-23.9	**-55.5**	**-52.4**	2 Lower respiratory infections	**-19.4**	**-35.9**	**-24.8**
3 Lower respiratory infections	3 Lower respiratory infections	**-17.8**	**-51.9**	**-43.7**	3 Diarrhoeal diseases	**-25.6**	**-40.8**	**-32.6**
4 Malaria	4 Malaria	20.0	**-29.8**	-22.1	4 Malaria	**-35.9**	**-49.0**	**-42.1**
5 Measles	5 HIV/AIDS	**137.6**	**39.0**	**54.4**	5 Congenital anomalies	-3.5	**-23.3**	-12.4
6 Protein-energy malnutrition	6 Congenital anomalies	16.9	**-31.6**	-23.5	6 HIV/AIDS	**-52.5**	**-62.2**	**-62.1**
7 Tuberculosis	7 Tuberculosis	-16.8	**-51.3**	**-47.4**	7 Tuberculosis	**-20.8**	**-37.0**	**-33.5**
8 Congenital anomalies	8 Maternal disorders	**18.0**	**-31.0**	**-33.7**	8 Maternal disorders	-11.2	**-29.3**	**-31.6**
9 HIV/AIDS	9 Meningitis	-4.8	**-44.3**	**-39.8**	9 Ischaemic heart disease	**28.6**	2.3	-3.8
10 Meningitis	10 Exposure to forces of nature	**43540.9**	**25427.2**	**26754.4**	10 Stroke	**23.4**	-1.8	-7.0
11 Maternal disorders	11 Measles	**-68.2**	**-81.4**	**-80.1**	11 Meningitis	**-26.7**	**-41.7**	**-34.1**
12 Whooping cough	12 Protein-energy malnutrition	**-42.9**	**-66.6**	**-62.8**	12 Protein-energy malnutrition	**-31.8**	**-45.7**	**-37.6**
13 Stroke	13 Stroke	**34.2**	**-21.5**	**-18.5**	13 Whooping cough	-12.1	-30.1	-20.8
14 Tetanus	14 Ischaemic heart disease	**60.6**	-6.1	-5.2	14 Road injuries	-1.0	**-21.3**	**-15.6**
15 Ischaemic heart disease	15 Whooping cough	-12.1	**-48.6**	-44.2	15 Measles	**-50.0**	**-60.2**	**-54.3**
16 Road injuries	16 Road injuries	16.3	**-32.0**	**-24.4**	16 Cirrhosis and other chronic liver diseases	12.5	-10.5	**-12.8**
17 Sexually transmitted infections excluding HIV	17 iNTS	**123.1**	**30.5**	**32.8**	17 iNTS	-15.1	**-32.5**	**-27.4**
18 Conflict and terrorism	18 Cirrhosis and other chronic liver diseases	**34.8**	**-21.1**	**-19.7**	18 Chronic obstructive pulmonary disease	**23.0**	-2.2	-5.6
19 Drowning	19 Sexually transmitted infections excluding HIV	-8.3	**-46.4**	**-38.8**	19 Sexually transmitted infections excluding HIV	4.9	**-16.5**	-4.8
20 Cirrhosis and other chronic liver diseases	20 Chronic obstructive pulmonary disease	**45.7**	-14.8	-10.9	20 Diabetes mellitus	**33.0**	5.8	0.2
23 Chronic obstructive pulmonary disease	23 Diabetes mellitus				101 Exposure to forces of nature			
25 iNTS	28 Drowning							
32 Diabetes mellitus	32 Tetanus							
117 Exposure to forces of nature	44 Conflict and terrorism							

SR Figure 6b: Leading 20 GBD cause hierarchy level 3 causes of Low-middle SDI SDI YLLs for females for 1990, 2010, and 2019

Causes are connected by arrows between time periods. Communicable maternal, neonatal and nutritional disorders are shown in red, non-communicable causes in blue and injuries in green. For the time period 1990 to 2010 and for 2010 to 2019, three measures of change are shown: percent change in the number of YLLs, percent change in the all-age YLL rate and percent change in the age-standardized YLL rate. Numbers in bold highlight statistically significant changes.

Leading causes 1990	Leading causes 2010	Mean % change number of YLLs 1990-2010	Mean % change all-age YLL rate 1990-2010	Mean % change age-standardized YLL rate 1990-2010	Leading causes 2019	Mean % change number of YLLs 2010-2019	Mean % change all-age YLL rate 2010-2019	Mean % change age-standardized YLL rate 2010-2019
1 Neonatal disorders	1 Neonatal disorders	**-24.0**	**-45.7**	**-23.0**	1 Neonatal disorders	**-27.8**	**-36.3**	**-23.9**
2 Diarrhoeal diseases	2 Lower respiratory infections	**-47.8**	**-62.7**	**-50.0**	2 Ischaemic heart disease	**35.2**	**19.4**	1.6
3 Lower respiratory infections	3 Diarrhoeal diseases	**-50.3**	**-64.5**	**-58.2**	3 Lower respiratory infections	**-31.6**	**-39.6**	**-31.6**
4 Tuberculosis	4 HIV/AIDS	**467.8**	**305.7**	**339.5**	4 Stroke	**19.3**	5.3	-10.4
5 Measles	5 Ischaemic heart disease	**55.8**	11.3	**-12.0**	5 Diarrhoeal diseases	**-39.6**	**-46.7**	**-42.6**
6 Protein-energy malnutrition	6 Stroke	**34.8**	-3.7	**-22.4**	6 Chronic obstructive pulmonary disease	**25.8**	11.0	-7.4
7 Congenital anomalies	7 Tuberculosis	**-41.3**	**-58.0**	**-58.8**	7 HIV/AIDS	**-40.3**	**-47.3**	**-47.9**
8 Malaria	8 Congenital anomalies	-23.3	**-45.2**	-25.3	8 Tuberculosis	**-20.9**	**-30.2**	**-32.6**
9 Stroke	9 Malaria	-26.7	**-47.6**	**-37.0**	9 Congenital anomalies	**-21.9**	**-31.1**	-18.9
10 Maternal disorders	10 Chronic obstructive pulmonary disease	**27.8**	-8.7	**-29.4**	10 Malaria	-25.8	**-34.5**	-27.1
11 Ischaemic heart disease	11 Maternal disorders	**-33.7**	**-52.6**	**-55.6**	11 Diabetes mellitus	**37.8**	**21.6**	5.1
12 Meningitis	12 Self-harm	-3.0	**-30.7**	**-33.9**	12 Self-harm	-5.7	**-16.8**	**-17.4**
13 Tetanus	13 Cirrhosis and other chronic liver diseases	**26.3**	-9.7	**-20.4**	13 Cirrhosis and other chronic liver diseases	10.8	-2.2	-10.2
14 Whooping cough	14 Meningitis	**-39.3**	**-56.7**	**-46.8**	14 Maternal disorders	**-21.0**	**-30.3**	**-30.9**
15 Chronic obstructive pulmonary disease	15 Road injuries	0.5	**-28.2**	**-23.9**	15 Breast cancer	**41.2**	**24.7**	10.3
16 Self-harm	16 Diabetes mellitus	**82.9**	**30.7**	8.2	16 Chronic kidney disease	**26.9**	12.0	0.9
17 Drowning	17 Measles	**-77.3**	**-83.8**	**-78.8**	17 Road injuries	-5.4	**-16.5**	**-16.0**
18 Typhoid and paratyphoid	18 Typhoid and paratyphoid	**-27.8**	**-48.4**	**-41.7**	18 Asthma	6.8	-5.7	-16.8
19 Road injuries	19 Chronic kidney disease	**50.9**	7.9	-3.7	19 Hypertensive heart disease	**29.5**	14.3	-4.6
20 Cirrhosis and other chronic liver diseases	20 Breast cancer	**83.1**	**30.8**	7.8	20 Cervical cancer	**22.2**	7.8	-3.8
23 HIV/AIDS	21 Protein-energy malnutrition				21 Typhoid and paratyphoid			
26 Chronic kidney disease	22 Asthma				24 Meningitis			
27 Diabetes mellitus	23 Whooping cough				31 Protein-energy malnutrition			
29 Breast cancer	25 Drowning				53 Measles			
	26 Cervical cancer							
	28 Hypertensive heart disease							
	44 Tetanus							

SR Figure 6c: Leading 20 GBD cause hierarchy level 3 causes of Middle SDI SDI YLLs for females for 1990, 2010, and 2019

Causes are connected by arrows between time periods. Communicable maternal, neonatal and nutritional disorders are shown in red, non-communicable causes in blue and injuries in green. For the time period 1990 to 2010 and for 2010 to 2019, three measures of change are shown: percent change in the number of YLLs, percent change in the all-age YLL rate and percent change in the age-standardized YLL rate. Numbers in bold highlight statistically significant changes.

Leading causes 1990
1 Neonatal disorders
2 Lower respiratory infections
3 Stroke
4 Diarrhoeal diseases
5 Congenital anomalies
6 Ischaemic heart disease
7 Chronic obstructive pulmonary disease
8 Tuberculosis
9 Road injuries
10 Self-harm
11 Measles
12 Drowning
13 Malaria
14 Maternal disorders
15 Cirrhosis and other chronic liver diseases
16 Protein-energy malnutrition
17 Chronic kidney disease
18 Hypertensive heart disease
19 Diabetes mellitus
20 Meningitis
22 Stomach cancer
24 Breast cancer
26 Cervical cancer
27 Tracheal, bronchus, and lung cancer
57 HIV/AIDS

Leading causes 2010	Mean % change number of YLLs 1990-2010	Mean % change all-age YLL rate 1990-2010	Mean % change age-standardized YLL rate 1990-2010
1 Stroke	**20.2**	-6.9	**-31.0**
2 Ischaemic heart disease	**65.4**	**28.0**	-7.7
3 Neonatal disorders	**-41.8**	**-54.9**	**-32.9**
4 HIV/AIDS	**2001.8**	**1526.8**	**1464.0**
5 Lower respiratory infections	**-63.0**	**-71.4**	**-61.7**
6 Chronic obstructive pulmonary disease	-17.6	**-36.2**	**-54.0**
7 Congenital anomalies	**-44.1**	**-56.7**	**-37.5**
8 Road injuries	**-16.9**	**-35.7**	**-33.4**
9 Diarrhoeal diseases	**-63.1**	**-71.5**	**-63.8**
10 Diabetes mellitus	**76.6**	**36.7**	1.5
11 Chronic kidney disease	**53.6**	**18.9**	-2.8
12 Breast cancer	**84.2**	**42.6**	3.5
13 Cirrhosis and other chronic liver diseases	8.2	**-16.3**	**-33.9**
14 Tuberculosis	**-48.9**	**-60.4**	**-63.9**
15 Tracheal, bronchus, and lung cancer	**90.7**	**47.6**	9.0
16 Malaria	-24.4	**-41.5**	-25.3
17 Self-harm	**-38.0**	**-52.0**	**-55.2**
18 Hypertensive heart disease	4.1	**-19.4**	**-43.2**
19 Stomach cancer	8.5	**-16.0**	**-38.0**
20 Cervical cancer	**39.7**	8.2	**-21.1**
21 Maternal disorders			
22 Colon and rectum cancer			
23 Alzheimer's disease and other dementias			
26 Drowning			
30 Meningitis			
36 Protein-energy malnutrition			
41 Measles			

Leading causes 2019	Mean % change number of YLLs 2010-2019	Mean % change all-age YLL rate 2010-2019	Mean % change age-standardized YLL rate 2010-2019
1 Ischaemic heart disease	**22.8**	**12.5**	**-9.4**
2 Stroke	7.3	-1.7	**-20.9**
3 Neonatal disorders	**-35.6**	**-41.1**	**-32.6**
4 Lower respiratory infections	**-27.3**	**-33.5**	**-35.0**
5 Diabetes mellitus	**31.2**	**20.2**	-1.8
6 Chronic obstructive pulmonary disease	1.0	-7.5	**-27.4**
7 HIV/AIDS	**-41.4**	**-46.3**	**-46.5**
8 Chronic kidney disease	**20.4**	**10.3**	-6.2
9 Breast cancer	**25.0**	**14.5**	-0.1
10 Congenital anomalies	**-29.4**	**-35.3**	**-27.5**
11 Road injuries	**-20.2**	**-26.9**	**-28.0**
12 Tracheal, bronchus, and lung cancer	**36.0**	**24.6**	2.3
13 Cirrhosis and other chronic liver diseases	5.5	-3.4	**-16.4**
14 Hypertensive heart disease	**25.0**	14.5	-8.5
15 Diarrhoeal diseases	**-36.9**	**-42.2**	**-43.3**
16 Alzheimer's disease and other dementias	**41.5**	**29.6**	-2.1
17 Cervical cancer	11.7	2.3	-10.7
18 Colon and rectum cancer	**29.7**	**18.8**	-1.3
19 Tuberculosis	**-30.1**	**-36.0**	**-41.0**
20 Stomach cancer	-1.7	-10.0	**-25.4**
21 Self-harm			
22 Malaria			
26 Maternal disorders			

SR Figure 6d: Leading 20 GBD cause hierarchy level 3 causes of High-middle SDI SDI YLLs for females for 1990, 2010, and 2019

Causes are connected by arrows between time periods. Communicable maternal, neonatal and nutritional disorders are shown in red, non-communicable causes in blue and injuries in green. For the time period 1990 to 2010 and for 2010 to 2019, three measures of change are shown: percent change in the number of YLLs, percent change in the all-age YLL rate and percent change in the age-standardized YLL rate. Numbers in bold highlight statistically significant changes.

Leading causes 1990	Leading causes 2010	Mean % change number of YLLs 1990-2010	Mean % change all-age YLL rate 1990-2010	Mean % change age-standardized YLL rate 1990-2010
1 Ischaemic heart disease	1 Ischaemic heart disease	**19.8**	1.8	**-23.5**
2 Stroke	2 Stroke	-4.0	**-18.4**	**-37.8**
3 Neonatal disorders	3 Breast cancer	**28.7**	**9.4**	**-13.3**
4 Lower respiratory infections	4 Neonatal disorders	**-58.3**	**-64.6**	**-46.7**
5 Congenital anomalies	5 Chronic obstructive pulmonary disease	-32.3	**-42.4**	**-56.1**
6 Chronic obstructive pulmonary disease	6 Tracheal, bronchus, and lung cancer	**66.3**	**41.3**	**13.1**
7 Breast cancer	7 Lower respiratory infections	**-61.1**	**-67.0**	**-67.2**
8 Road injuries	8 Road injuries	**-10.1**	**-23.6**	**-25.6**
9 Self-harm	9 Colon and rectum cancer	**34.8**	**14.6**	**-9.0**
10 Stomach cancer	10 Cirrhosis and other chronic liver diseases	**29.1**	**9.7**	**-9.6**
11 Diarrhoeal diseases	11 Alzheimer's disease and other dementias	**78.6**	**51.8**	3.1
12 Tracheal, bronchus, and lung cancer	12 Congenital anomalies	**-56.8**	**-63.3**	**-46.7**
13 Cirrhosis and other chronic liver diseases	13 Diabetes mellitus	**21.6**	3.4	**-17.3**
14 Colon and rectum cancer	14 Stomach cancer	**-15.0**	**-27.7**	**-42.2**
15 Diabetes mellitus	15 Chronic kidney disease	**28.3**	9.0	**-12.1**
16 Chronic kidney disease	16 Self-harm	**-32.8**	**-42.9**	**-47.6**
17 Hypertensive heart disease	17 Hypertensive heart disease	21.5	3.3	**-21.5**
18 Tuberculosis	18 Cardiomyopathy and myocarditis	**49.6**	27.2	0.6
19 Alzheimer's disease and other dementias	19 Cervical cancer	14.9	-2.4	**-21.1**
20 Leukaemia	20 Ovarian cancer	**39.9**	**18.9**	-4.2
22 Cervical cancer	21 Pancreatic cancer			
25 Cardiomyopathy and myocarditis	24 Leukaemia			
29 Ovarian cancer	28 Tuberculosis			
34 Pancreatic cancer	31 Diarrhoeal diseases			

Leading causes 2019	Mean % change number of YLLs 2010-2019	Mean % change all-age YLL rate 2010-2019	Mean % change age-standardized YLL rate 2010-2019
1 Ischaemic heart disease	0.6	-4.4	**-20.5**
2 Stroke	-5.2	-9.8	**-24.4**
3 Breast cancer	8.1	2.7	**-8.8**
4 Tracheal, bronchus, and lung cancer	**22.1**	**16.0**	-0.7
5 Chronic obstructive pulmonary disease	-2.7	-7.5	**-23.8**
6 Alzheimer's disease and other dementias	**29.1**	**22.7**	-4.4
7 Colon and rectum cancer	**12.1**	6.6	-8.3
8 Lower respiratory infections	**-9.0**	**-13.5**	**-30.6**
9 Diabetes mellitus	**16.2**	**10.4**	-6.7
10 Neonatal disorders	**-37.5**	**-40.6**	**-35.5**
11 Cirrhosis and other chronic liver diseases	**-10.0**	**-14.4**	**-23.1**
12 Chronic kidney disease	**13.2**	7.6	-8.3
13 Stomach cancer	-8.4	-12.9	**-24.5**
14 Hypertensive heart disease	**12.7**	7.1	**-12.9**
15 Road injuries	**-26.5**	**-30.1**	**-30.0**
16 Congenital anomalies	**-29.2**	**-32.7**	**-28.3**
17 Cervical cancer	4.6	-0.6	-9.8
18 Pancreatic cancer	**26.4**	**20.2**	2.1
19 Self-harm	**-21.6**	**-25.5**	**-23.8**
20 Ovarian cancer	**13.5**	7.9	-4.7
21 Cardiomyopathy and myocarditis			

SR Figure 6e: Leading 20 GBD cause hierarchy level 3 causes of High SDI SDI YLLs for females for 1990, 2010, and 2019

Causes are connected by arrows between time periods. Communicable maternal, neonatal and nutritional disorders are shown in red, non-communicable causes in blue and injuries in green. For the time period 1990 to 2010 and for 2010 to 2019, three measures of change are shown: percent change in the number of YLLs, percent change in the all-age YLL rate and percent change in the age-standardized YLL rate. Numbers in bold highlight statistically significant changes.

Leading causes 1990
1 Ischaemic heart disease
2 Stroke
3 Breast cancer
4 Tracheal, bronchus, and lung cancer
5 Colon and rectum cancer
6 Road injuries
7 Lower respiratory infections
8 Neonatal disorders
9 Chronic obstructive pulmonary disease
10 Alzheimer's disease and other dementias
11 Congenital anomalies
12 Self-harm
13 Diabetes mellitus
14 Cirrhosis and other chronic liver diseases
15 Stomach cancer
16 Ovarian cancer
17 Chronic kidney disease
18 Pancreatic cancer
19 Leukaemia
20 Cervical cancer
21 Hypertensive heart disease
22 Other malignant neoplasms
24 Falls

Leading causes 2010	Mean % change number of YLLs 1990-2010	Mean % change all-age YLL rate 1990-2010	Mean % change age-standardized YLL rate 1990-2010
1 Ischaemic heart disease	**-34.4**	**-43.1**	**-55.9**
2 Stroke	**-30.7**	**-39.9**	**-53.4**
3 Tracheal, bronchus, and lung cancer	**47.5**	**28.0**	**4.2**
4 Breast cancer	0.8	**-12.5**	**-27.3**
5 Alzheimer's disease and other dementias	**76.2**	**52.9**	4.8
6 Chronic obstructive pulmonary disease	**49.3**	**29.6**	3.2
7 Colon and rectum cancer	**7.4**	**-6.8**	**-23.9**
8 Lower respiratory infections	**-7.7**	**-19.9**	**-40.7**
9 Self-harm	**6.6**	**-7.5**	**-8.3**
10 Chronic kidney disease	**73.6**	**50.6**	**15.7**
11 Cirrhosis and other chronic liver diseases	**7.8**	**-6.4**	**-21.6**
12 Diabetes mellitus	-0.1	**-13.3**	**-29.0**
13 Pancreatic cancer	**55.1**	**34.6**	**10.8**
14 Road injuries	**-40.1**	**-48.0**	**-48.1**
15 Ovarian cancer	**7.5**	-6.7	**-22.4**
16 Neonatal disorders	**-47.2**	**-54.1**	**-42.4**
17 Stomach cancer	**-28.4**	**-37.9**	**-49.9**
18 Congenital anomalies	**-45.0**	**-52.2**	**-43.9**
19 Hypertensive heart disease	**24.4**	8.0	**-18.1**
20 Other malignant neoplasms	**17.3**	1.8	**-11.8**
21 Falls			
22 Leukaemia			
23 Drug use disorders			
24 Cervical cancer			

Leading causes 2019	Mean % change number of YLLs 2010-2019	Mean % change all-age YLL rate 2010-2019	Mean % change age-standardized YLL rate 2010-2019
1 Ischaemic heart disease	**4.9**	-0.7	**-12.6**
2 Stroke	**5.9**	0.3	**-11.9**
3 Tracheal, bronchus, and lung cancer	**11.9**	**5.9**	**-5.6**
4 Breast cancer	**4.1**	-1.4	**-8.7**
5 Alzheimer's disease and other dementias	**22.9**	**16.4**	-2.0
6 Chronic obstructive pulmonary disease	**19.4**	**13.1**	-0.0
7 Colon and rectum cancer	**10.0**	**4.1**	**-6.2**
8 Lower respiratory infections	**13.1**	**7.1**	**-8.1**
9 Chronic kidney disease	**22.6**	**16.1**	3.1
10 Pancreatic cancer	**19.9**	**13.5**	0.8
11 Cirrhosis and other chronic liver diseases	**7.3**	1.6	**-5.4**
12 Diabetes mellitus	**5.7**	0.1	**-9.8**
13 Self-harm	**-9.1**	**-14.0**	**-13.3**
14 Ovarian cancer	**7.3**	1.5	**-7.0**
15 Road injuries	-4.8	**-9.9**	**-9.2**
16 Drug use disorders	**74.6**	**65.3**	**72.2**
17 Hypertensive heart disease	**20.6**	**14.2**	0.5
18 Stomach cancer	**-3.1**	**-8.2**	**-18.4**
19 Falls	**15.7**	**9.6**	**-5.1**
20 Neonatal disorders	**-21.5**	**-25.7**	**-18.0**
21 Other malignant neoplasms			
22 Congenital anomalies			

SR Figure 6f: Leading 20 GBD cause hierarchy level 3 causes of Low SDI SDI YLLs for males for 1990, 2010, and 2019

Causes are connected by arrows between time periods. Communicable maternal, neonatal and nutritional disorders are shown in red, non-communicable causes in blue and injuries in green. For the time period 1990 to 2010 and for 2010 to 2019, three measures of change are shown: percent change in the number of YLLs, percent change in the all-age YLL rate and percent change in the age-standardized YLL rate. Numbers in bold highlight statistically significant changes.

Leading causes 1990
1 Neonatal disorders
2 Diarrhoeal diseases
3 Lower respiratory infections
4 Malaria
5 Measles
6 Tuberculosis
7 Protein-energy malnutrition
8 Congenital anomalies
9 Meningitis
10 HIV/AIDS
11 Ischaemic heart disease
12 Road injuries
13 Stroke
14 Whooping cough
15 Tetanus
16 Conflict and terrorism
17 Cirrhosis and other chronic liver diseases
18 Sexually transmitted infections excluding HIV
19 Leishmaniasis
20 Chronic obstructive pulmonary disease
23 Interpersonal violence
24 Self-harm
25 iNTS
111 Exposure to forces of nature

Leading causes 2010	Mean % change number of YLLs 1990-2010	Mean % change all-age YLL rate 1990-2010	Mean % change age-standardized YLL rate 1990-2010
1 Neonatal disorders	**25.5**	**-26.3**	**-15.0**
2 Diarrhoeal diseases	**-20.2**	**-53.1**	**-46.9**
3 Lower respiratory infections	**-21.7**	**-54.0**	**-44.4**
4 Malaria	25.0	-26.6	-17.0
5 HIV/AIDS	**113.9**	**25.6**	**36.3**
6 Tuberculosis	**-10.7**	**-47.6**	**-44.0**
7 Congenital anomalies	20.0	-29.5	-21.4
8 Exposure to forces of nature	**42013.3**	**24625.9**	**25194.3**
9 Meningitis	2.7	**-39.7**	**-34.4**
10 Ischaemic heart disease	**55.8**	-8.5	-4.1
11 Road injuries	**45.2**	**-14.8**	-9.5
12 Measles	**-66.7**	**-80.4**	**-79.2**
13 Protein-energy malnutrition	**-44.7**	**-67.5**	**-63.6**
14 Stroke	**28.7**	**-24.4**	**-18.4**
15 Cirrhosis and other chronic liver diseases	**43.3**	-15.9	-13.8
16 Whooping cough	-8.2	-46.1	-41.7
17 iNTS	**132.2**	**36.4**	**39.5**
18 Interpersonal violence	**77.7**	4.3	1.2
19 Chronic obstructive pulmonary disease	**33.6**	**-21.6**	**-14.8**
20 Sexually transmitted infections excluding HIV	-8.0	**-46.0**	**-38.5**
21 Self-harm			
28 Tetanus			
31 Conflict and terrorism			
56 Leishmaniasis			

Leading causes 2019	Mean % change number of YLLs 2010-2019	Mean % change all-age YLL rate 2010-2019	Mean % change age-standardized YLL rate 2010-2019
1 Neonatal disorders	-8.7	**-26.8**	**-16.0**
2 Diarrhoeal diseases	**-24.8**	**-39.7**	**-32.4**
3 Lower respiratory infections	**-21.4**	**-37.0**	**-26.4**
4 Malaria	**-34.6**	**-47.6**	**-41.6**
5 Congenital anomalies	-4.3	-23.3	-12.9
6 Tuberculosis	**-12.4**	**-29.8**	**-29.6**
7 Ischaemic heart disease	**23.0**	-1.4	-7.1
8 Road injuries	5.6	**-15.3**	**-14.2**
9 HIV/AIDS	**-42.1**	**-53.6**	**-51.5**
10 Stroke	**17.6**	-5.8	**-10.8**
11 Meningitis	**-24.3**	**-39.3**	**-32.0**
12 Cirrhosis and other chronic liver diseases	8.9	-12.7	**-16.2**
13 Protein-energy malnutrition	**-39.3**	**-51.3**	**-44.4**
14 Interpersonal violence	**19.2**	-4.4	-7.2
15 Chronic obstructive pulmonary disease	**15.2**	-7.7	**-12.4**
16 Whooping cough	-10.7	-28.5	-19.6
17 Measles	**-49.1**	**-59.2**	**-53.3**
18 iNTS	-12.3	**-29.7**	**-26.0**
19 Self-harm	**18.0**	-5.4	-9.2
20 Sexually transmitted infections excluding HIV	6.1	-15.0	-3.3
94 Exposure to forces of nature			

SR Figure 6g: Leading 20 GBD cause hierarchy level 3 causes of Low-middle SDI SDI YLLs for males for 1990, 2010, and 2019

Causes are connected by arrows between time periods. Communicable maternal, neonatal and nutritional disorders are shown in red, non-communicable causes in blue and injuries in green. For the time period 1990 to 2010 and for 2010 to 2019, three measures of change are shown: percent change in the number of YLLs, percent change in the all-age YLL rate and percent change in the age-standardized YLL rate. Numbers in bold highlight statistically significant changes.

Leading causes 1990	Leading causes 2010	Mean % change number of YLLs 1990-2010	Mean % change all-age YLL rate 1990-2010	Mean % change age-standardized YLL rate 1990-2010	Leading causes 2019	Mean % change number of YLLs 2010-2019	Mean % change all-age YLL rate 2010-2019	Mean % change age-standardized YLL rate 2010-2019
1 Neonatal disorders	1 Neonatal disorders	**-20.8**	**-42.4**	**-20.2**	1 Neonatal disorders	**-27.8**	**-35.5**	**-23.9**
2 Lower respiratory infections	2 Lower respiratory infections	**-49.4**	**-63.2**	**-50.0**	2 Ischaemic heart disease	**21.8**	8.8	-4.4
3 Diarrhoeal diseases	3 Ischaemic heart disease	**68.7**	**22.7**	1.3	3 Stroke	**11.4**	-0.5	**-13.1**
4 Tuberculosis	4 Diarrhoeal diseases	**-52.0**	**-65.1**	**-57.6**	4 Lower respiratory infections	**-32.5**	**-39.7**	**-31.8**
5 Measles	5 Stroke	**42.6**	3.7	**-12.2**	5 Tuberculosis	**-16.6**	**-25.5**	**-30.4**
6 Congenital anomalies	6 Tuberculosis	**-25.6**	**-45.9**	**-49.9**	6 Road injuries	-4.2	**-14.4**	**-16.2**
7 Ischaemic heart disease	7 Road injuries	**35.0**	-1.8	-2.3	7 Chronic obstructive pulmonary disease	11.9	0.0	**-14.1**
8 Protein-energy malnutrition	8 Chronic obstructive pulmonary disease	**27.0**	-7.6	**-23.9**	8 Diarrhoeal diseases	**-44.6**	**-50.5**	**-46.3**
9 Stroke	9 HIV/AIDS	**427.8**	**284.0**	**315.0**	9 Cirrhosis and other chronic liver diseases	7.8	-3.7	**-11.8**
10 Malaria	10 Congenital anomalies	-23.7	**-44.5**	-25.8	10 Congenital anomalies	-23.3	**-31.4**	-20.2
11 Road injuries	11 Cirrhosis and other chronic liver diseases	**39.7**	1.6	**-10.1**	11 Self-harm	0.9	-9.9	**-13.5**
12 Chronic obstructive pulmonary disease	12 Malaria	-21.2	**-42.7**	-31.3	12 HIV/AIDS	**-34.9**	**-41.8**	**-42.4**
13 Drowning	13 Self-harm	**30.3**	-5.2	**-11.8**	13 Interpersonal violence	-0.7	**-11.3**	**-13.1**
14 Tetanus	14 Interpersonal violence	**61.4**	**17.4**	**10.2**	14 Malaria	-23.7	**-31.8**	-25.8
15 Cirrhosis and other chronic liver diseases	15 Drowning	**-40.2**	**-56.5**	**-48.7**	15 Chronic kidney disease	**17.2**	4.7	-4.7
16 Meningitis	16 Chronic kidney disease	**61.3**	**17.3**	7.1	16 Diabetes mellitus	**32.9**	**18.8**	4.1
17 Typhoid and paratyphoid	17 Meningitis	**-33.2**	**-51.4**	**-40.9**	17 Falls	6.2	-5.1	-8.4
18 Self-harm	18 Typhoid and paratyphoid	**-31.7**	**-50.3**	**-45.2**	18 Tracheal, bronchus, and lung cancer	**28.2**	**14.6**	-0.4
19 Whooping cough	19 Diabetes mellitus	**96.1**	**42.7**	**20.6**	19 Drowning	**-30.7**	**-38.1**	**-34.2**
20 Interpersonal violence	20 Falls	**22.0**	-11.3	-11.4	20 Typhoid and paratyphoid	**-27.6**	**-35.3**	**-30.0**
22 Falls	21 Measles				22 Meningitis			
23 Chronic kidney disease	22 Tracheal, bronchus, and lung cancer				61 Measles			
28 HIV/AIDS	24 Protein-energy malnutrition							
29 Diabetes mellitus	26 Whooping cough							
	44 Tetanus							

SR Figure 6h: Leading 20 GBD cause hierarchy level 3 causes of Middle SDI SDI YLLs for males for 1990, 2010, and 2019

Causes are connected by arrows between time periods. Communicable maternal, neonatal and nutritional disorders are shown in red, non-communicable causes in blue and injuries in green. For the time period 1990 to 2010 and for 2010 to 2019, three measures of change are shown: percent change in the number of YLLs, percent change in the all-age YLL rate and percent change in the age-standardized YLL rate. Numbers in bold highlight statistically significant changes.

Leading causes 1990	Leading causes 2010	Mean % change number of YLLs 1990-2010	Mean % change all-age YLL rate 1990-2010	Mean % change age-standardized YLL rate 1990-2010	Leading causes 2019	Mean % change number of YLLs 2010-2019	Mean % change all-age YLL rate 2010-2019	Mean % change age-standardized YLL rate 2010-2019
1 Neonatal disorders	1 Ischaemic heart disease	**79.9**	**41.0**	0.9	1 Ischaemic heart disease	**20.5**	**11.3**	-6.8
2 Lower respiratory infections	2 Stroke	**44.8**	**13.5**	**-15.6**	2 Stroke	9.4	1.1	**-16.5**
3 Stroke	3 Neonatal disorders	**-40.1**	**-53.1**	**-31.8**	3 Road injuries	**-16.5**	**-22.9**	**-22.6**
4 Road injuries	4 Road injuries	9.5	**-14.2**	**-15.7**	4 Neonatal disorders	**-36.6**	**-41.4**	**-33.5**
5 Diarrhoeal diseases	5 Lower respiratory infections	**-60.6**	**-69.1**	**-58.8**	5 Chronic obstructive pulmonary disease	2.1	-5.7	**-24.4**
6 Ischaemic heart disease	6 Chronic obstructive pulmonary disease	4.3	-18.3	**-40.8**	6 Tracheal, bronchus, and lung cancer	**25.0**	15.5	-4.7
7 Congenital anomalies	7 HIV/AIDS	**1139.5**	**871.4**	**801.2**	7 Cirrhosis and other chronic liver diseases	7.0	-1.2	**-12.0**
8 Tuberculosis	8 Cirrhosis and other chronic liver diseases	**27.6**	0.0	**-22.7**	8 Lower respiratory infections	**-25.7**	**-31.4**	**-32.1**
9 Chronic obstructive pulmonary disease	9 Tracheal, bronchus, and lung cancer	**94.7**	**52.6**	13.6	9 HIV/AIDS	**-29.1**	**-34.5**	**-35.8**
10 Drowning	10 Congenital anomalies	**-43.8**	**-55.9**	**-37.9**	10 Chronic kidney disease	**21.6**	**12.3**	-2.6
11 Cirrhosis and other chronic liver diseases	11 Tuberculosis	**-32.2**	**-46.8**	**-55.3**	11 Diabetes mellitus	**38.5**	**27.9**	6.8
12 Interpersonal violence	12 Interpersonal violence	0.7	**-21.1**	**-23.6**	12 Interpersonal violence	-1.3	**-8.8**	-5.5
13 Self-harm	13 Stomach cancer	**38.1**	8.3	**-20.4**	13 Stomach cancer	-4.2	-11.5	**-25.9**
14 Measles	14 Diarrhoeal diseases	**-66.2**	**-73.5**	**-66.5**	14 Congenital anomalies	**-30.0**	**-35.3**	**-28.0**
15 Tracheal, bronchus, and lung cancer	15 Chronic kidney disease	**68.8**	**32.3**	7.8	15 Tuberculosis	**-23.8**	**-29.6**	**-35.7**
16 Stomach cancer	16 Self-harm	2.8	-19.5	**-26.6**	16 Self-harm	-2.9	**-10.3**	**-11.7**
17 Malaria	17 Diabetes mellitus	**100.6**	**57.2**	**17.1**	17 Liver cancer	**29.6**	19.7	3.4
18 Liver cancer	18 Drowning	**-50.3**	**-61.1**	**-53.9**	18 Colon and rectum cancer	**31.1**	**21.1**	2.5
19 Meningitis	19 Falls	17.4	-8.0	-13.6	19 Hypertensive heart disease	**31.5**	**21.4**	-0.6
20 Chronic kidney disease	20 Oesophageal cancer	**45.0**	13.7	-17.1	20 Falls	2.7	-5.2	-10.4
22 Falls	21 Malaria				21 Diarrhoeal diseases			
24 Oesophageal cancer	22 Liver cancer				22 Oesophageal cancer			
25 Diabetes mellitus	23 Colon and rectum cancer				23 Drowning			
49 HIV/AIDS	24 Hypertensive heart disease				24 Malaria			
	29 Meningitis							
	50 Measles							

SR Figure 6i: Leading 20 GBD cause hierarchy level 3 causes of High-middle SDI SDI YLLs for males for 1990, 2010, and 2019

Causes are connected by arrows between time periods. Communicable maternal, neonatal and nutritional disorders are shown in red, non-communicable causes in blue and injuries in green. For the time period 1990 to 2010 and for 2010 to 2019, three measures of change are shown: percent change in the number of YLLs, percent change in the all-age YLL rate and percent change in the age-standardized YLL rate. Numbers in bold highlight statistically significant changes.

Leading causes 1990
1 Ischaemic heart disease
2 Stroke
3 Neonatal disorders
4 Road injuries
5 Lower respiratory infections
6 Tracheal, bronchus, and lung cancer
7 Chronic obstructive pulmonary disease
8 Congenital anomalies
9 Self-harm
10 Stomach cancer
11 Cirrhosis and other chronic liver diseases
12 Drowning
13 Interpersonal violence
14 Tuberculosis
15 Diarrhoeal diseases
16 Liver cancer
17 Falls
18 Colon and rectum cancer
19 Alcohol use disorders
20 Cardiomyopathy and myocarditis
21 Chronic kidney disease
22 Oesophageal cancer
24 Diabetes mellitus
41 HIV/AIDS

Leading causes 2010	Mean % change number of YLLs 1990-2010	Mean % change all-age YLL rate 1990-2010	Mean % change age-standardized YLL rate 1990-2010
1 Ischaemic heart disease	**26.7**	**7.4**	**-20.1**
2 Stroke	**15.2**	-2.4	**-27.4**
3 Tracheal, bronchus, and lung cancer	**20.1**	1.8	**-19.7**
4 Road injuries	-7.3	**-21.5**	**-25.3**
5 Cirrhosis and other chronic liver diseases	**34.1**	**13.6**	**-10.2**
6 Self-harm	-0.1	**-15.3**	**-24.1**
7 Chronic obstructive pulmonary disease	**-22.4**	**-34.3**	**-50.4**
8 Neonatal disorders	**-59.5**	**-65.7**	**-49.0**
9 Lower respiratory infections	**-49.2**	**-56.9**	**-56.6**
10 Stomach cancer	1.1	**-14.4**	**-33.7**
11 Cardiomyopathy and myocarditis	**106.6**	**75.1**	31.6
12 Colon and rectum cancer	**70.5**	**44.4**	**10.9**
13 Interpersonal violence	**-19.1**	**-31.4**	**-34.3**
14 Congenital anomalies	**-57.1**	**-63.7**	**-47.9**
15 Oesophageal cancer	**39.9**	**18.5**	-9.0
16 Falls	13.3	-4.0	**-14.3**
17 HIV/AIDS	**188.1**	**144.1**	**127.9**
18 Alcohol use disorders	**23.8**	**4.9**	**-12.9**
19 Diabetes mellitus	**51.8**	**28.6**	0.2
20 Chronic kidney disease	**28.3**	8.7	**-11.6**
21 Drowning			
22 Tuberculosis			
23 Pancreatic cancer			
24 Liver cancer			
47 Diarrhoeal diseases			

Leading causes 2019	Mean % change number of YLLs 2010-2019	Mean % change all-age YLL rate 2010-2019	Mean % change age-standardized YLL rate 2010-2019
1 Ischaemic heart disease	-1.1	-6.5	**-20.6**
2 Stroke	-1.8	-7.2	**-21.9**
3 Tracheal, bronchus, and lung cancer	9.1	3.1	**-13.4**
4 Road injuries	**-24.0**	**-28.1**	**-26.0**
5 Chronic obstructive pulmonary disease	-1.8	-7.2	**-24.5**
6 Cirrhosis and other chronic liver diseases	-7.7	**-12.8**	**-20.5**
7 Self-harm	**-14.8**	**-19.5**	**-18.4**
8 Stomach cancer	-4.2	-9.5	**-22.9**
9 Lower respiratory infections	**-11.8**	**-16.7**	**-27.8**
10 Colon and rectum cancer	**19.3**	**12.7**	-4.4
11 Neonatal disorders	**-38.6**	**-42.0**	**-36.5**
12 Cardiomyopathy and myocarditis	**-20.7**	**-25.1**	**-30.3**
13 Diabetes mellitus	**19.0**	**12.5**	-5.3
14 Interpersonal violence	**-18.0**	**-22.5**	**-17.7**
15 Oesophageal cancer	8.9	2.9	-12.9
16 Chronic kidney disease	**11.2**	5.1	**-9.9**
17 Falls	-4.1	-9.4	**-14.6**
18 Pancreatic cancer	**24.9**	**18.0**	0.3
19 Congenital anomalies	**-28.9**	**-32.8**	**-28.2**
20 Alcohol use disorders	**-11.3**	**-16.1**	**-20.2**
24 HIV/AIDS			
26 Drowning			

SR Figure 6j: Leading 20 GBD cause hierarchy level 3 causes of High SDI SDI YLLs for males for 1990, 2010, and 2019

Causes are connected by arrows between time periods. Communicable maternal, neonatal and nutritional disorders are shown in red, non-communicable causes in blue and injuries in green. For the time period 1990 to 2010 and for 2010 to 2019, three measures of change are shown: percent change in the number of YLLs, percent change in the all-age YLL rate and percent change in the age-standardized YLL rate. Numbers in bold highlight statistically significant changes.

Leading causes 1990	Leading causes 2010	Mean % change number of YLLs 1990-2010	Mean % change all-age YLL rate 1990-2010	Mean % change age-standardized YLL rate 1990-2010	Leading causes 2019	Mean % change number of YLLs 2010-2019	Mean % change all-age YLL rate 2010-2019	Mean % change age-standardized YLL rate 2010-2019
1 Ischaemic heart disease	1 Ischaemic heart disease	**-28.3**	**-38.9**	**-53.4**	1 Ischaemic heart disease	**8.0**	1.4	**-11.8**
2 Tracheal, bronchus, and lung cancer	2 Tracheal, bronchus, and lung cancer	**3.3**	**-11.9**	**-31.8**	2 Tracheal, bronchus, and lung cancer	**4.4**	-2.0	**-14.8**
3 Stroke	3 Stroke	**-22.1**	**-33.6**	**-50.0**	3 Stroke	**7.7**	1.2	**-12.7**
4 Road injuries	4 Self-harm	**10.0**	**-6.2**	**-9.5**	4 Self-harm	**-7.8**	**-13.5**	**-12.6**
5 Self-harm	5 Road injuries	**-34.5**	**-44.2**	**-43.8**	5 Chronic obstructive pulmonary disease	**21.9**	**14.4**	**-4.1**
6 Cirrhosis and other chronic liver diseases	6 Cirrhosis and other chronic liver diseases	1.0	**-13.9**	**-29.4**	6 Road injuries	-5.2	**-11.0**	**-10.4**
7 Chronic obstructive pulmonary disease	7 Colon and rectum cancer	**24.4**	**6.1**	**-18.1**	7 Colon and rectum cancer	**12.2**	**5.3**	**-7.7**
8 Neonatal disorders	8 Chronic obstructive pulmonary disease	**9.5**	**-6.7**	**-31.4**	8 Cirrhosis and other chronic liver diseases	1.9	**-4.3**	**-10.9**
9 Lower respiratory infections	9 Lower respiratory infections	-1.5	**-16.0**	**-38.4**	9 Lower respiratory infections	**13.8**	**6.8**	**-10.6**
10 Colon and rectum cancer	10 Prostate cancer	**16.8**	-0.4	**-28.5**	10 Drug use disorders	**91.9**	**80.2**	**87.4**
11 Stomach cancer	11 Diabetes mellitus	**40.9**	**20.1**	**-6.0**	11 Prostate cancer	**19.7**	**12.4**	**-7.0**
12 Congenital anomalies	12 Stomach cancer	**-19.3**	**-31.2**	**-46.6**	12 Chronic kidney disease	**25.9**	**18.3**	1.1
13 Prostate cancer	13 Chronic kidney disease	**78.5**	**52.2**	**14.6**	13 Diabetes mellitus	**11.7**	**4.9**	**-7.2**
14 HIV/AIDS	14 Pancreatic cancer	**59.2**	**35.8**	**5.3**	14 Pancreatic cancer	**19.3**	**12.0**	-1.6
15 Interpersonal violence	15 Liver cancer	**66.8**	**42.2**	**12.4**	15 Alzheimer's disease and other dementias	**34.1**	**25.9**	-0.3
16 Diabetes mellitus	16 Drug use disorders	**220.1**	**172.9**	**178.8**	16 Stomach cancer	**-5.2**	**-11.0**	**-22.3**
17 Falls	17 Alzheimer's disease and other dementias	**92.8**	**64.4**	**3.4**	17 Liver cancer	**9.2**	2.6	**-8.8**
18 Pancreatic cancer	18 Neonatal disorders	**-49.2**	**-56.7**	**-44.4**	18 Falls	**12.8**	**5.9**	-4.9
19 Cardiomyopathy and myocarditis	19 Falls	**17.4**	0.1	**-16.8**	19 Oesophageal cancer	**10.7**	**3.9**	**-7.9**
20 Leukaemia	20 Oesophageal cancer	**36.9**	**16.7**	**-8.8**	20 Leukaemia	**9.4**	2.7	**-7.9**
21 Chronic kidney disease	21 Interpersonal violence				21 Neonatal disorders			
22 Liver cancer	22 Congenital anomalies				23 Interpersonal violence			
23 Oesophageal cancer	23 Leukaemia							
27 Alzheimer's disease and other dementias	24 Cardiomyopathy and myocarditis							
38 Drug use disorders	41 HIV/AIDS							

Figure 7a. Global distribution of total YLDs by 23 GBD age groups for Level 2 causes of non−fatal burden in 1990 and 2019, females

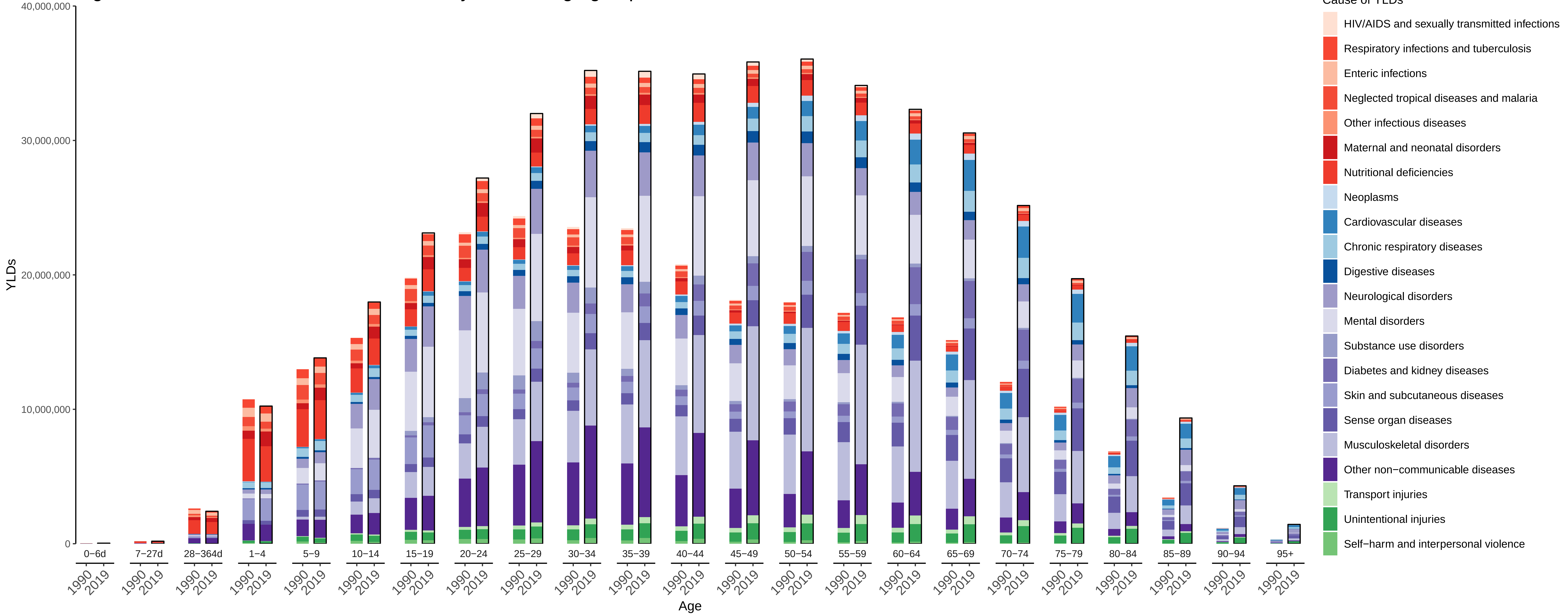

Composition of Level 2 causes of disability globally for females by age group showing difference in composition between 1990 and 2019. Number and rates of total YLDs due to Level 2 causes is indicated by height of bar; causes are color−coded to highlight the relative number of total YLDs due to a specific cause. YLD=years lived with disability.

Figure 7b. Global distribution of total YLDs by 23 GBD age groups for Level 2 causes of non−fatal burden in 1990 and 2019, males

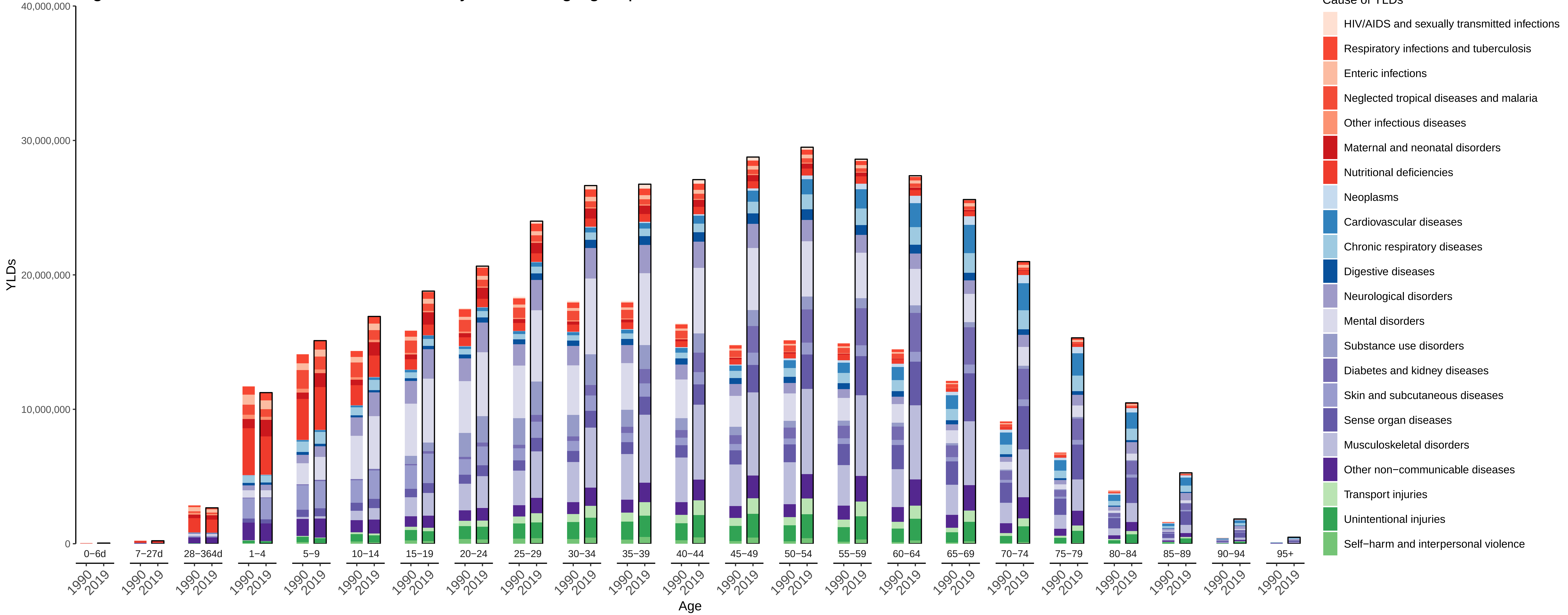

Composition of Level 2 causes of disability globally for males by age group showing difference in composition between 1990 and 2019. Number and rates of total YLDs due to Level 2 causes is indicated by height of bar; causes are color−coded to highlight the relative number of total YLDs due to a specific cause. YLD=years lived with disability.

SR Figure 8a: Leading 30 GBD cause hierarchy level 3 causes of Low SDI SDI DALYs for females for 1990, 2010, and 2019

Causes are connected by arrows between time periods. Communicable maternal, neonatal and nutritional disorders are shown in red, non-communicable causes in blue and injuries in green. For the time period 1990 to 2010 and for 2010 to 2019, two measures of change are shown: percent change in the number of DALYs, and percent change in the age-standardized DALY rate. Numbers in bold highlight statistically significant changes.

**Leading causes 1990**	**Leading causes 2010**	**Mean % change number of DALYs 1990-2010**	**Mean % change age-standardized DALY rate 1990-2010**	**Leading causes 2019**	**Mean % change number of DALYs 2010-2019**	**Mean % change age-standardized DALY rate 2010-2019**
1 Diarrhoeal diseases	1 Neonatal disorders	**17.4**	**-20.0**	1 Neonatal disorders	-7.4	**-14.3**
2 Neonatal disorders	2 Diarrhoeal diseases	-22.7	**-51.4**	2 Diarrhoeal diseases	**-23.7**	**-30.9**
3 Lower respiratory infections	3 Lower respiratory infections	**-17.8**	**-43.6**	3 Lower respiratory infections	**-19.3**	**-24.7**
4 Malaria	4 Malaria	21.0	-21.9	4 Malaria	**-34.3**	**-40.9**
5 Measles	5 HIV/AIDS	**137.9**	**54.3**	5 HIV/AIDS	**-50.5**	**-60.4**
6 Protein-energy malnutrition	6 Congenital anomalies	18.8	-22.3	6 Congenital anomalies	-2.4	-11.8
7 Tuberculosis	7 Tuberculosis	-15.0	**-46.6**	7 Tuberculosis	**-19.3**	**-32.7**
8 Congenital anomalies	8 Maternal disorders	**18.9**	**-33.3**	8 Maternal disorders	-9.8	**-30.6**
9 HIV/AIDS	9 Meningitis	-4.4	**-39.5**	9 Stroke	**24.4**	-6.5
10 Meningitis	10 Protein-energy malnutrition	**-40.2**	**-61.1**	10 Ischaemic heart disease	**28.8**	-3.7
11 Maternal disorders	11 Exposure to forces of nature	**35736.6**	**21088.2**	11 Dietary iron deficiency	**21.2**	**-2.6**
12 Whooping cough	12 Measles	**-68.2**	**-80.1**	12 Gynecological diseases	**31.2**	-0.3
13 Stroke	13 Stroke	**36.2**	**-17.8**	13 Meningitis	**-26.1**	**-33.5**
14 Tetanus	14 Ischaemic heart disease	**60.8**	-5.1	14 Protein-energy malnutrition	**-29.0**	**-35.0**
15 Road injuries	15 Dietary iron deficiency	**56.6**	**-7.1**	15 Depressive disorders	**29.2**	-2.2
16 Ischaemic heart disease	16 Whooping cough	-12.0	-44.1	16 Whooping cough	-12.1	-20.8
17 Dietary iron deficiency	17 Gynecological diseases	**68.5**	**-4.7**	17 Road injuries	3.4	**-12.6**
18 Conflict and terrorism	18 Road injuries	**23.3**	**-18.7**	18 Headache disorders	**30.4**	-0.1
19 Sexually transmitted infections excluding HIV	19 Depressive disorders	**67.9**	**-3.3**	19 Diabetes mellitus	**41.0**	5.3
20 Gynecological diseases	20 iNTS	**123.1**	**32.8**	20 Chronic obstructive pulmonary disease	**25.5**	-4.3
21 Asthma	21 Headache disorders	**75.7**	0.3	21 Low back pain	**29.1**	**-2.6**
22 Depressive disorders	22 Chronic obstructive pulmonary disease	**53.3**	-7.6	22 Measles	**-50.0**	**-54.3**
23 Drowning	23 Low back pain	**60.3**	**-6.7**	23 Hemoglobinopathies and hemolytic anaemias	**18.1**	-3.0
24 Cirrhosis and other chronic liver diseases	24 Diabetes mellitus	**84.8**	**11.5**	24 Cirrhosis and other chronic liver diseases	12.7	**-12.7**
25 Chronic obstructive pulmonary disease	25 Hemoglobinopathies and hemolytic anaemias	**41.2**	**-14.6**	25 Asthma	7.8	**-16.6**
26 Hemoglobinopathies and hemolytic anaemias	26 Cirrhosis and other chronic liver diseases	**35.1**	**-19.6**	26 Chronic kidney disease	**25.8**	-1.1
27 Typhoid and paratyphoid	27 Asthma	16.1	**-28.7**	27 iNTS	-15.1	**-27.4**
28 Low back pain	28 Sexually transmitted infections excluding HIV	-6.4	**-36.8**	28 Sexually transmitted infections excluding HIV	6.3	-4.2
29 Headache disorders	29 Chronic kidney disease	**50.7**	-5.8	29 Anxiety disorders	**30.4**	0.4
30 Leishmaniasis	30 Anxiety disorders	**79.2**	**2.2**	30 Age-related and other hearing loss	**28.5**	**-2.5**
31 Diabetes mellitus	31 Age-related and other hearing loss			125 Exposure to forces of nature		
32 Chronic kidney disease	32 Typhoid and paratyphoid					
33 iNTS	38 Drowning					
37 Age-related and other hearing loss	41 Conflict and terrorism					
41 Anxiety disorders	49 Tetanus					
150 Exposure to forces of nature	87 Leishmaniasis					

SR Figure 8b: Leading 30 GBD cause hierarchy level 3 causes of Low-middle SDI SDI DALYs for females for 1990, 2010, and 2019

Causes are connected by arrows between time periods. Communicable maternal, neonatal and nutritional disorders are shown in red, non-communicable causes in blue and injuries in green. For the time period 1990 to 2010 and for 2010 to 2019, two measures of change are shown: percent change in the number of DALYs, and percent change in the age-standardized DALY rate. Numbers in bold highlight statistically significant changes.

Leading causes 1990
1 Neonatal disorders
2 Diarrhoeal diseases
3 Lower respiratory infections
4 Tuberculosis
5 Measles
6 Protein-energy malnutrition
7 Congenital anomalies
8 Malaria
9 Stroke
10 Maternal disorders
11 Ischaemic heart disease
12 Meningitis
13 Chronic obstructive pulmonary disease
14 Tetanus
15 Dietary iron deficiency
16 Whooping cough
17 Self-harm
18 Drowning
19 Typhoid and paratyphoid
20 Road injuries
21 Gynecological diseases
22 Low back pain
23 Depressive disorders
24 Headache disorders
25 Asthma
26 Cirrhosis and other chronic liver diseases
27 Diabetes mellitus
28 Encephalitis
29 Other musculoskeletal disorders
30 Falls
31 Chronic kidney disease
33 HIV/AIDS
36 Age-related and other hearing loss
37 Anxiety disorders
42 Breast cancer

Leading causes 2010	Mean % change number of DALYs 1990-2010	Mean % change age-standardized DALY rate 1990-2010
1 Neonatal disorders	**-21.9**	**-21.2**
2 Diarrhoeal diseases	**-48.7**	**-57.1**
3 Lower respiratory infections	**-47.7**	**-49.9**
4 HIV/AIDS	**465.5**	**335.2**
5 Stroke	**36.9**	**-21.2**
6 Ischaemic heart disease	**56.3**	**-11.7**
7 Tuberculosis	**-39.4**	**-57.6**
8 Congenital anomalies	-21.1	-24.2
9 Chronic obstructive pulmonary disease	**37.2**	**-24.0**
10 Malaria	-25.7	**-36.5**
11 Dietary iron deficiency	**25.9**	**-9.9**
12 Gynecological diseases	**44.7**	**-8.5**
13 Depressive disorders	**45.3**	**-7.4**
14 Maternal disorders	**-32.2**	**-54.7**
15 Low back pain	**39.6**	**-13.4**
16 Headache disorders	**52.4**	0.5
17 Diabetes mellitus	**100.0**	**17.0**
18 Road injuries	**16.8**	**-14.4**
19 Self-harm	-2.6	**-33.9**
20 Other musculoskeletal disorders	**87.2**	**14.2**
21 Cirrhosis and other chronic liver diseases	**26.5**	**-20.2**
22 Chronic kidney disease	**57.8**	0.2
23 Meningitis	**-38.8**	**-46.6**
24 Age-related and other hearing loss	**60.9**	**-2.2**
25 Protein-energy malnutrition	**-75.1**	**-76.8**
26 Falls	**32.2**	-10.5
27 Anxiety disorders	**60.2**	**6.1**
28 Asthma	-2.0	**-38.7**
29 Breast cancer	**84.2**	8.4
30 Typhoid and paratyphoid	**-27.9**	**-41.8**
31 Measles		
32 Blindness and vision loss		
33 Hemoglobinopathies and hemolytic anaemias		
34 Endocrine, metabolic, blood, and immune disorders		
36 Whooping cough		
38 Drowning		
57 Encephalitis		
60 Tetanus		

Leading causes 2019	Mean % change number of DALYs 2010-2019	Mean % change age-standardized DALY rate 2010-2019
1 Neonatal disorders	**-24.3**	**-21.0**
2 Ischaemic heart disease	**35.4**	1.7
3 Stroke	**20.6**	-9.2
4 Lower respiratory infections	**-31.4**	**-31.4**
5 Diarrhoeal diseases	**-35.5**	**-39.4**
6 Chronic obstructive pulmonary disease	**25.9**	-6.5
7 HIV/AIDS	**-38.5**	**-46.4**
8 Diabetes mellitus	**46.2**	**11.2**
9 Tuberculosis	**-19.2**	**-31.2**
10 Gynecological diseases	**18.7**	0.2
11 Congenital anomalies	**-20.0**	-17.7
12 Depressive disorders	**19.8**	-1.2
13 Dietary iron deficiency	**3.2**	**-8.7**
14 Low back pain	**19.7**	**-3.3**
15 Headache disorders	**17.8**	0.5
16 Other musculoskeletal disorders	**32.4**	**7.3**
17 Malaria	-24.7	-26.3
18 Road injuries	0.4	**-13.1**
19 Chronic kidney disease	**26.2**	0.5
20 Maternal disorders	**-20.0**	**-30.1**
21 Self-harm	-5.7	**-17.6**
22 Breast cancer	**41.8**	10.7
23 Age-related and other hearing loss	**23.5**	**-2.7**
24 Cirrhosis and other chronic liver diseases	11.0	-10.0
25 Falls	**26.6**	2.4
26 Anxiety disorders	**14.0**	**-2.9**
27 Asthma	11.0	-12.2
28 Blindness and vision loss	**19.6**	**-9.3**
29 Endocrine, metabolic, blood, and immune disorders	**17.6**	**-2.0**
30 Hemoglobinopathies and hemolytic anaemias	3.8	**-7.3**
36 Typhoid and paratyphoid		
37 Meningitis		
38 Protein-energy malnutrition		
83 Measles		

SR Figure 8c: Leading 30 GBD cause hierarchy level 3 causes of Middle SDI SDI DALYs for females for 1990, 2010, and 2019

Causes are connected by arrows between time periods. Communicable maternal, neonatal and nutritional disorders are shown in red, non-communicable causes in blue and injuries in green. For the time period 1990 to 2010 and for 2010 to 2019, two measures of change are shown: percent change in the number of DALYs, and percent change in the age-standardized DALY rate. Numbers in bold highlight statistically significant changes.

Leading causes 1990
1 Neonatal disorders
2 Lower respiratory infections
3 Stroke
4 Diarrhoeal diseases
5 Congenital anomalies
6 Ischaemic heart disease
7 Chronic obstructive pulmonary disease
8 Tuberculosis
9 Road injuries
10 Low back pain
11 Gynecological diseases
12 Headache disorders
13 Self-harm
14 Depressive disorders
15 Diabetes mellitus
16 Measles
17 Malaria
18 Drowning
19 Dietary iron deficiency
20 Maternal disorders
21 Chronic kidney disease
22 Cirrhosis and other chronic liver diseases
23 Anxiety disorders
24 Protein-energy malnutrition
25 Age-related and other hearing loss
26 Other musculoskeletal disorders
27 Hypertensive heart disease
28 Meningitis
29 Asthma
30 Whooping cough
32 Endocrine, metabolic, blood, and immune disorders
33 Falls
34 Breast cancer
36 Neck pain
37 Blindness and vision loss
40 Oral disorders
44 Tracheal, bronchus, and lung cancer
81 HIV/AIDS

Leading causes 2010	Mean % change number of DALYs 1990-2010	Mean % change age-standardized DALY rate 1990-2010
1 Stroke	**24.8**	**-28.4**
2 Neonatal disorders	**-36.6**	**-28.9**
3 Ischaemic heart disease	**66.1**	-7.4
4 HIV/AIDS	**1982.1**	**1447.1**
5 Lower respiratory infections	**-62.8**	**-61.5**
6 Low back pain	**36.6**	**-14.3**
7 Chronic obstructive pulmonary disease	-4.6	**-47.3**
8 Diabetes mellitus	**95.3**	**10.8**
9 Headache disorders	**46.8**	3.2
10 Gynecological diseases	**36.5**	**-9.3**
11 Congenital anomalies	**-40.5**	**-35.2**
12 Depressive disorders	**40.2**	**-5.9**
13 Road injuries	-7.8	**-28.6**
14 Diarrhoeal diseases	**-58.4**	**-60.0**
15 Other musculoskeletal disorders	**95.6**	**19.7**
16 Chronic kidney disease	**62.6**	2.6
17 Age-related and other hearing loss	**58.0**	**-3.4**
18 Anxiety disorders	**44.1**	**4.1**
19 Breast cancer	**87.0**	5.1
20 Tuberculosis	**-46.0**	**-61.9**
21 Dietary iron deficiency	**-5.1**	**-27.1**
22 Cirrhosis and other chronic liver diseases	8.3	**-33.7**
23 Neck pain	**67.2**	0.5
24 Endocrine, metabolic, blood, and immune disorders	**40.4**	**-6.2**
25 Blindness and vision loss	**61.1**	**-8.8**
26 Malaria	-23.6	-24.9
27 Self-harm	**-37.1**	**-54.8**
28 Tracheal, bronchus, and lung cancer	**91.0**	9.1
29 Oral disorders	**59.8**	**-1.9**
30 Hypertensive heart disease	7.3	**-41.6**
31 Falls		
32 Alzheimer's disease and other dementias		
35 Asthma		
36 Maternal disorders		
48 Drowning		
49 Protein-energy malnutrition		
51 Meningitis		
62 Whooping cough		
72 Measles		

Leading causes 2019	Mean % change number of DALYs 2010-2019	Mean % change age-standardized DALY rate 2010-2019
1 Stroke	11.1	**-17.8**
2 Ischaemic heart disease	**23.3**	**-9.0**
3 Neonatal disorders	**-27.5**	**-27.0**
4 Diabetes mellitus	**37.3**	3.3
5 Low back pain	**17.9**	**-1.1**
6 Chronic obstructive pulmonary disease	7.5	**-21.5**
7 Headache disorders	**11.1**	1.4
8 Gynecological diseases	**11.4**	**2.2**
9 Depressive disorders	**17.9**	2.8
10 Other musculoskeletal disorders	**27.8**	**7.4**
11 Lower respiratory infections	**-27.0**	**-34.8**
12 Chronic kidney disease	**20.5**	-4.9
13 HIV/AIDS	**-38.8**	**-44.2**
14 Age-related and other hearing loss	**23.5**	**-2.0**
15 Road injuries	**-11.1**	**-21.7**
16 Breast cancer	**26.1**	0.7
17 Anxiety disorders	**8.3**	-1.4
18 Congenital anomalies	**-25.4**	**-24.9**
19 Diarrhoeal diseases	**-26.4**	**-34.9**
20 Neck pain	**16.7**	**-2.9**
21 Tracheal, bronchus, and lung cancer	**36.2**	2.3
22 Blindness and vision loss	**22.7**	**-7.2**
23 Endocrine, metabolic, blood, and immune disorders	**18.5**	-0.5
24 Falls	**33.6**	**7.1**
25 Cirrhosis and other chronic liver diseases	5.8	**-16.1**
26 Dietary iron deficiency	1.8	**-7.4**
27 Alzheimer's disease and other dementias	**43.9**	-0.5
28 Hypertensive heart disease	**26.3**	-7.6
29 Oral disorders	**23.8**	0.3
30 Tuberculosis	**-27.1**	**-37.8**
34 Self-harm		
38 Malaria		

SR Figure 8d: Leading 30 GBD cause hierarchy level 3 causes of High-middle SDI SDI DALYs for females for 1990, 2010, and 2019

Causes are connected by arrows between time periods. Communicable maternal, neonatal and nutritional disorders are shown in red, non-communicable causes in blue and injuries in green. For the time period 1990 to 2010 and for 2010 to 2019, two measures of change are shown: percent change in the number of DALYs, and percent change in the age-standardized DALY rate. Numbers in bold highlight statistically significant changes.

Leading causes 1990	Leading causes 2010	Mean % change number of DALYs 1990-2010	Mean % change age-standardized DALY rate 1990-2010	Leading causes 2019	Mean % change number of DALYs 2010-2019	Mean % change age-standardized DALY rate 2010-2019
1 Stroke	1 Ischaemic heart disease	**20.6**	**-22.9**	1 Ischaemic heart disease	1.4	**-19.8**
2 Ischaemic heart disease	2 Stroke	-0.6	**-35.1**	2 Stroke	-1.2	**-20.7**
3 Neonatal disorders	3 Low back pain	**17.7**	**-14.7**	3 Low back pain	**11.2**	**-1.0**
4 Lower respiratory infections	4 Headache disorders	**28.3**	0.9	4 Diabetes mellitus	**19.6**	-2.3
5 Chronic obstructive pulmonary disease	5 Gynecological diseases	**16.6**	**-11.4**	5 Headache disorders	**3.9**	-0.1
6 Low back pain	6 Diabetes mellitus	**57.2**	**7.2**	6 Gynecological diseases	**3.7**	-0.2
7 Congenital anomalies	7 Depressive disorders	**21.7**	**-8.3**	7 Depressive disorders	**9.3**	0.9
8 Gynecological diseases	8 Neonatal disorders	**-48.9**	**-40.6**	8 Chronic obstructive pulmonary disease	4.3	**-16.7**
9 Headache disorders	9 Chronic obstructive pulmonary disease	-18.6	**-46.4**	9 Age-related and other hearing loss	**18.6**	-0.2
10 Depressive disorders	10 Breast cancer	**31.5**	**-11.4**	10 Breast cancer	**9.1**	-8.0
11 Road injuries	11 Road injuries	-1.6	**-22.7**	11 Other musculoskeletal disorders	**22.0**	**9.1**
12 Diabetes mellitus	12 Age-related and other hearing loss	**40.8**	-2.1	12 Alzheimer's disease and other dementias	**31.4**	-2.1
13 Breast cancer	13 Other musculoskeletal disorders	**77.2**	**28.1**	13 Neonatal disorders	**-22.9**	**-26.7**
14 Diarrhoeal diseases	14 Anxiety disorders	**26.9**	**2.5**	14 Tracheal, bronchus, and lung cancer	**22.2**	-0.6
15 Age-related and other hearing loss	15 Alzheimer's disease and other dementias	**79.4**	3.8	15 Anxiety disorders	1.5	-2.7
16 Anxiety disorders	16 Tracheal, bronchus, and lung cancer	**66.6**	**13.3**	16 Falls	**19.6**	0.4
17 Self-harm	17 Lower respiratory infections	**-60.9**	**-67.0**	17 Road injuries	**-15.9**	**-23.9**
18 Stomach cancer	18 Falls	**19.1**	**-15.3**	18 Endocrine, metabolic, blood, and immune disorders	**12.9**	-0.9
19 Falls	19 Congenital anomalies	**-51.8**	**-43.5**	19 Oral disorders	**18.6**	1.5
20 Endocrine, metabolic, blood, and immune disorders	20 Endocrine, metabolic, blood, and immune disorders	**23.3**	**-7.2**	20 Neck pain	**15.0**	**2.1**
21 Dietary iron deficiency	21 Colon and rectum cancer	**36.4**	**-8.0**	21 Colon and rectum cancer	**12.8**	-7.8
22 Other musculoskeletal disorders	22 Chronic kidney disease	**39.5**	-3.2	22 Chronic kidney disease	**12.8**	**-6.7**
23 Oral disorders	23 Neck pain	**48.4**	**5.5**	23 Osteoarthritis	**22.4**	-0.5
24 Colon and rectum cancer	24 Oral disorders	**29.5**	**-7.6**	24 Lower respiratory infections	**-8.7**	**-30.3**
25 Cirrhosis and other chronic liver diseases	25 Cirrhosis and other chronic liver diseases	**29.0**	**-9.6**	25 Blindness and vision loss	**20.3**	**-1.3**
26 Tracheal, bronchus, and lung cancer	26 Osteoarthritis	**54.8**	**3.7**	26 Cirrhosis and other chronic liver diseases	**-9.6**	**-22.7**
27 Chronic kidney disease	27 Blindness and vision loss	**43.4**	-2.4	27 Congenital anomalies	**-23.7**	**-24.8**
28 Alzheimer's disease and other dementias	28 Stomach cancer	**-14.7**	**-42.0**	28 Hypertensive heart disease	**14.7**	**-11.2**
29 Neck pain	29 Hypertensive heart disease	**25.4**	**-19.2**	29 Stomach cancer	-8.2	**-24.4**
30 Asthma	30 Self-harm	**-32.0**	**-47.2**	30 Dietary iron deficiency	**-5.6**	**-10.1**
32 Osteoarthritis	31 Dietary iron deficiency			34 Self-harm		
33 Blindness and vision loss	35 Diarrhoeal diseases					
34 Hypertensive heart disease	39 Asthma					

SR Figure 8e: Leading 30 GBD cause hierarchy level 3 causes of High SDI SDI DALYs for females for 1990, 2010, and 2019

Causes are connected by arrows between time periods. Communicable maternal, neonatal and nutritional disorders are shown in red, non-communicable causes in blue and injuries in green. For the time period 1990 to 2010 and for 2010 to 2019, two measures of change are shown: percent change in the number of DALYs, and percent change in the age-standardized DALY rate. Numbers in bold highlight statistically significant changes.

Leading causes 1990	Leading causes 2010	Mean % change number of DALYs 1990-2010	Mean % change age-standardized DALY rate 1990-2010	Leading causes 2019	Mean % change number of DALYs 2010-2019	Mean % change age-standardized DALY rate 2010-2019
1 Ischaemic heart disease	1 Low back pain	**19.0**	**-6.0**	1 Low back pain	**4.9**	**-4.4**
2 Stroke	2 Ischaemic heart disease	**-33.3**	**-55.1**	2 Ischaemic heart disease	**5.3**	**-12.1**
3 Low back pain	3 Stroke	**-21.5**	**-45.4**	3 Stroke	**8.6**	**-8.8**
4 Headache disorders	4 Other musculoskeletal disorders	**51.0**	**21.3**	4 Alzheimer's disease and other dementias	**24.5**	-0.3
5 Breast cancer	5 Headache disorders	**15.2**	-0.7	5 Other musculoskeletal disorders	**12.9**	**3.9**
6 Gynecological diseases	6 Depressive disorders	**30.2**	**12.5**	6 Diabetes mellitus	**22.4**	**6.4**
7 Depressive disorders	7 Chronic obstructive pulmonary disease	**51.7**	5.3	7 Chronic obstructive pulmonary disease	**16.7**	-1.8
8 Other musculoskeletal disorders	8 Alzheimer's disease and other dementias	**73.6**	**4.2**	8 Headache disorders	**4.6**	0.9
9 Diabetes mellitus	9 Diabetes mellitus	**40.8**	3.6	9 Depressive disorders	2.2	-2.4
10 Chronic obstructive pulmonary disease	10 Breast cancer	**4.7**	**-24.4**	10 Tracheal, bronchus, and lung cancer	**11.9**	**-5.5**
11 Tracheal, bronchus, and lung cancer	11 Tracheal, bronchus, and lung cancer	**48.0**	**4.6**	11 Breast cancer	**4.8**	**-8.3**
12 Neonatal disorders	12 Gynecological diseases	**5.5**	**-7.9**	12 Falls	**23.5**	**8.1**
13 Road injuries	13 Falls	**32.4**	**-6.9**	13 Gynecological diseases	**6.0**	**2.2**
14 Anxiety disorders	14 Anxiety disorders	**20.2**	**3.8**	14 Osteoarthritis	**17.0**	0.5
15 Falls	15 Osteoarthritis	**53.5**	**9.8**	15 Anxiety disorders	**5.0**	-0.2
16 Alzheimer's disease and other dementias	16 Age-related and other hearing loss	**36.9**	**-2.0**	16 Drug use disorders	**49.1**	**44.9**
17 Colon and rectum cancer	17 Endocrine, metabolic, blood, and immune disorders	**21.6**	**-5.2**	17 Age-related and other hearing loss	**15.9**	-0.8
18 Endocrine, metabolic, blood, and immune disorders	18 Colon and rectum cancer	**9.3**	**-22.6**	18 Endocrine, metabolic, blood, and immune disorders	**10.4**	-0.1
19 Lower respiratory infections	19 Chronic kidney disease	**69.8**	**17.0**	19 Neck pain	**39.4**	**32.0**
20 Asthma	20 Drug use disorders	**123.5**	**101.5**	20 Colon and rectum cancer	**10.4**	**-5.9**
21 Osteoarthritis	21 Neck pain	**7.1**	**-16.3**	21 Chronic kidney disease	**19.8**	1.8
22 Congenital anomalies	22 Lower respiratory infections	**-7.6**	**-40.4**	22 Oral disorders	**31.0**	**13.6**
23 Age-related and other hearing loss	23 Neonatal disorders	**-30.2**	**-32.3**	23 Lower respiratory infections	**13.0**	**-8.0**
24 Neck pain	24 Road injuries	**-31.7**	**-43.8**	24 Road injuries	-0.4	**-7.3**
25 Oral disorders	25 Oral disorders	3.4	**-18.8**	25 Asthma	**6.6**	1.2
26 Self-harm	26 Asthma	**-15.7**	**-24.1**	26 Pancreatic cancer	**19.9**	0.8
27 Cirrhosis and other chronic liver diseases	27 Self-harm	**7.9**	**-7.8**	27 Neonatal disorders	**-10.7**	**-12.2**
28 Stomach cancer	28 Cirrhosis and other chronic liver diseases	**7.9**	**-21.5**	28 Cirrhosis and other chronic liver diseases	**7.2**	**-5.4**
29 Chronic kidney disease	29 Pancreatic cancer	**55.3**	**10.9**	29 Self-harm	**-8.5**	**-13.0**
30 Ovarian cancer	30 Schizophrenia	**27.9**	**5.9**	30 Ovarian cancer	**7.3**	**-7.0**
31 Schizophrenia	31 Congenital anomalies			31 Schizophrenia		
32 Drug use disorders	32 Ovarian cancer			34 Congenital anomalies		
33 Pancreatic cancer	35 Stomach cancer					

SR Figure 8f: Leading 30 GBD cause hierarchy level 3 causes of Low SDI SDI DALYs for males for 1990, 2010, and 2019

Causes are connected by arrows between time periods. Communicable maternal, neonatal and nutritional disorders are shown in red, non-communicable causes in blue and injuries in green. For the time period 1990 to 2010 and for 2010 to 2019, two measures of change are shown: percent change in the number of DALYs, and percent change in the age-standardized DALY rate. Numbers in bold highlight statistically significant changes.

Leading causes 1990	Leading causes 2010	Mean % change number of DALYs 1990-2010	Mean % change age-standardized DALY rate 1990-2010	Leading causes 2019	Mean % change number of DALYs 2010-2019	Mean % change age-standardized DALY rate 2010-2019
1 Neonatal disorders	1 Neonatal disorders	**26.6**	**-14.0**	1 Neonatal disorders	-7.0	**-13.9**
2 Diarrhoeal diseases	2 Diarrhoeal diseases	**-18.9**	**-45.8**	2 Diarrhoeal diseases	**-23.0**	**-30.7**
3 Lower respiratory infections	3 Lower respiratory infections	**-21.6**	**-44.3**	3 Lower respiratory infections	**-21.3**	**-26.3**
4 Malaria	4 Malaria	25.6	-16.9	4 Malaria	**-33.8**	**-41.0**
5 Measles	5 HIV/AIDS	**115.2**	**36.8**	5 Congenital anomalies	-3.4	-12.5
6 Tuberculosis	6 Tuberculosis	**-9.6**	**-43.4**	6 Tuberculosis	**-11.1**	**-28.7**
7 Protein-energy malnutrition	7 Congenital anomalies	**21.5**	-20.5	7 Ischaemic heart disease	**23.3**	-6.9
8 Congenital anomalies	8 Exposure to forces of nature	**33073.4**	**19044.4**	8 Road injuries	7.9	**-12.3**
9 Meningitis	9 Road injuries	**50.5**	-5.6	9 HIV/AIDS	**-40.7**	**-50.4**
10 HIV/AIDS	10 Meningitis	2.9	**-34.4**	10 Stroke	**18.5**	**-10.2**
11 Road injuries	11 Ischaemic heart disease	**56.0**	-3.9	11 Meningitis	**-23.8**	**-31.7**
12 Ischaemic heart disease	12 Protein-energy malnutrition	**-42.3**	**-62.0**	12 Cirrhosis and other chronic liver diseases	9.1	**-16.0**
13 Stroke	13 Measles	**-66.6**	**-79.2**	13 Dietary iron deficiency	**16.1**	**-4.8**
14 Conflict and terrorism	14 Stroke	**30.0**	**-17.9**	14 Chronic obstructive pulmonary disease	**17.4**	**-11.0**
15 Whooping cough	15 Cirrhosis and other chronic liver diseases	**43.5**	-13.7	15 Protein-energy malnutrition	**-35.9**	**-41.3**
16 Tetanus	16 Dietary iron deficiency	**55.4**	**-9.2**	16 Diabetes mellitus	**37.0**	3.3
17 Cirrhosis and other chronic liver diseases	17 Whooping cough	-8.1	-41.7	17 Interpersonal violence	**19.9**	-6.7
18 Sexually transmitted infections excluding HIV	18 Chronic obstructive pulmonary disease	**39.2**	**-12.4**	18 Whooping cough	-10.7	-19.5
19 Chronic obstructive pulmonary disease	19 iNTS	**132.2**	**39.5**	19 Measles	**-49.1**	**-53.3**
20 Leishmaniasis	20 Interpersonal violence	**76.4**	0.5	20 iNTS	-12.3	**-26.0**
21 Dietary iron deficiency	21 Diabetes mellitus	**71.0**	7.1	21 Low back pain	**29.3**	**-1.6**
22 Typhoid and paratyphoid	22 Sexually transmitted infections excluding HIV	-7.4	**-37.8**	22 Depressive disorders	**31.2**	-0.2
23 Drowning	23 Self-harm	**59.2**	-8.5	23 Chronic kidney disease	**23.0**	-5.0
24 Asthma	24 Chronic kidney disease	**58.9**	-1.8	24 Self-harm	**18.1**	-9.2
25 Interpersonal violence	25 Hemoglobinopathies and hemolytic anaemias	**32.0**	-17.1	25 Conflict and terrorism	**39.1**	**10.8**
26 Hemoglobinopathies and hemolytic anaemias	26 Low back pain	**65.6**	**-3.1**	26 Sexually transmitted infections excluding HIV	6.4	-3.2
27 Diabetes mellitus	27 Depressive disorders	**67.6**	-2.5	27 Hemoglobinopathies and hemolytic anaemias	11.5	-6.1
28 Self-harm	28 Typhoid and paratyphoid	3.4	**-41.2**	28 Headache disorders	**30.5**	0.8
29 iNTS	29 Asthma	**11.8**	**-32.3**	29 Asthma	6.6	**-17.9**
30 Chronic kidney disease	30 Conflict and terrorism	**-52.1**	**-68.8**	30 Age-related and other hearing loss	**28.6**	**-1.6**
31 Low back pain	31 Drowning			32 Typhoid and paratyphoid		
32 Depressive disorders	32 Headache disorders			34 Drowning		
144 Exposure to forces of nature	34 Age-related and other hearing loss			114 Exposure to forces of nature		
	36 Tetanus					
	73 Leishmaniasis					

SR Figure 8g: Leading 30 GBD cause hierarchy level 3 causes of Low-middle SDI SDI DALYs for males for 1990, 2010, and 2019

Causes are connected by arrows between time periods. Communicable maternal, neonatal and nutritional disorders are shown in red, non-communicable causes in blue and injuries in green. For the time period 1990 to 2010 and for 2010 to 2019, two measures of change are shown: percent change in the number of DALYs, and percent change in the age-standardized DALY rate. Numbers in bold highlight statistically significant changes.

Leading causes 1990
1 Neonatal disorders
2 Lower respiratory infections
3 Diarrhoeal diseases
4 Tuberculosis
5 Congenital anomalies
6 Measles
7 Ischaemic heart disease
8 Protein-energy malnutrition
9 Stroke
10 Road injuries
11 Malaria
12 Chronic obstructive pulmonary disease
13 Drowning
14 Tetanus
15 Cirrhosis and other chronic liver diseases
16 Meningitis
17 Typhoid and paratyphoid
18 Self-harm
19 Dietary iron deficiency
20 Interpersonal violence
21 Whooping cough
22 Falls
23 Asthma
24 Chronic kidney disease
25 Diabetes mellitus
26 Low back pain
27 Upper digestive system diseases
28 Depressive disorders
29 Headache disorders
30 Acute hepatitis
31 Age-related and other hearing loss
34 Alcohol use disorders
36 HIV/AIDS
43 Tracheal, bronchus, and lung cancer
44 Other musculoskeletal disorders

Leading causes 2010	Mean % change number of DALYs 1990-2010	Mean % change age-standardized DALY rate 1990-2010
1 Neonatal disorders	**-19.1**	**-18.7**
2 Lower respiratory infections	**-49.3**	**-50.0**
3 Ischaemic heart disease	**68.8**	1.3
4 Diarrhoeal diseases	**-50.2**	**-56.2**
5 Stroke	**43.5**	**-11.7**
6 Road injuries	**44.8**	3.3
7 Tuberculosis	**-24.4**	**-49.2**
8 Chronic obstructive pulmonary disease	**32.3**	**-20.9**
9 Congenital anomalies	-21.8	-24.8
10 HIV/AIDS	**430.3**	**313.8**
11 Cirrhosis and other chronic liver diseases	**39.8**	**-10.0**
12 Malaria	-20.8	**-31.2**
13 Self-harm	**30.2**	**-12.0**
14 Interpersonal violence	**58.9**	**7.7**
15 Diabetes mellitus	**104.5**	**25.1**
16 Chronic kidney disease	**65.8**	9.8
17 Falls	**25.4**	**-11.0**
18 Dietary iron deficiency	**7.3**	**-18.0**
19 Drowning	**-40.2**	**-48.7**
20 Meningitis	**-32.9**	**-40.9**
21 Low back pain	**38.7**	**-10.9**
22 Depressive disorders	**47.3**	-3.6
23 Typhoid and paratyphoid	**-31.7**	**-45.3**
24 Headache disorders	**48.0**	-0.6
25 Age-related and other hearing loss	**52.4**	**-2.5**
26 Asthma	-6.6	**-39.4**
27 Protein-energy malnutrition	**-75.1**	**-76.3**
28 Measles	**-76.2**	**-78.0**
29 Alcohol use disorders	**45.4**	-5.5
30 Other musculoskeletal disorders	**80.8**	**15.2**
31 Tracheal, bronchus, and lung cancer		
32 Blindness and vision loss		
33 Upper digestive system diseases		
36 Anxiety disorders		
39 Whooping cough		
43 Acute hepatitis		
59 Tetanus		

Leading causes 2019	Mean % change number of DALYs 2010-2019	Mean % change age-standardized DALY rate 2010-2019
1 Neonatal disorders	**-24.8**	**-21.2**
2 Ischaemic heart disease	**22.0**	-4.2
3 Stroke	**12.4**	**-12.3**
4 Lower respiratory infections	**-32.3**	**-31.7**
5 Road injuries	-1.6	**-14.5**
6 Chronic obstructive pulmonary disease	12.9	**-13.0**
7 Tuberculosis	**-15.3**	**-29.4**
8 Diarrhoeal diseases	**-40.1**	**-42.6**
9 Cirrhosis and other chronic liver diseases	7.9	**-11.6**
10 Diabetes mellitus	**44.1**	**12.2**
11 Congenital anomalies	-21.6	-19.1
12 HIV/AIDS	**-33.6**	**-41.3**
13 Self-harm	0.9	**-13.5**
14 Interpersonal violence	1.0	**-11.9**
15 Chronic kidney disease	**17.0**	-4.9
16 Malaria	-23.0	-25.3
17 Falls	**13.1**	-3.8
18 Depressive disorders	**20.4**	0.4
19 Low back pain	**16.8**	**-3.7**
20 Age-related and other hearing loss	**21.5**	**-1.9**
21 Headache disorders	**17.9**	1.7
22 Dietary iron deficiency	**-4.7**	**-11.3**
23 Other musculoskeletal disorders	**36.4**	**11.9**
24 Asthma	1.0	**-17.6**
25 Tracheal, bronchus, and lung cancer	**28.3**	-0.4
26 Alcohol use disorders	**10.0**	**-7.9**
27 Drowning	**-30.6**	**-34.1**
28 Blindness and vision loss	**17.0**	**-8.9**
29 Typhoid and paratyphoid	**-27.6**	**-30.1**
30 Anxiety disorders	**17.5**	1.2
32 Meningitis		
39 Protein-energy malnutrition		
85 Measles		

SR Figure 8h: Leading 30 GBD cause hierarchy level 3 causes of Middle SDI SDI DALYs for males for 1990, 2010, and 2019

Causes are connected by arrows between time periods. Communicable maternal, neonatal and nutritional disorders are shown in red, non-communicable causes in blue and injuries in green. For the time period 1990 to 2010 and for 2010 to 2019, two measures of change are shown: percent change in the number of DALYs, and percent change in the age-standardized DALY rate. Numbers in bold highlight statistically significant changes.

Leading causes 1990
1 Neonatal disorders
2 Lower respiratory infections
3 Road injuries
4 Stroke
5 Diarrhoeal diseases
6 Ischaemic heart disease
7 Congenital anomalies
8 Tuberculosis
9 Chronic obstructive pulmonary disease
10 Drowning
11 Cirrhosis and other chronic liver diseases
12 Interpersonal violence
13 Self-harm
14 Low back pain
15 Measles
16 Falls
17 Malaria
18 Tracheal, bronchus, and lung cancer
19 Stomach cancer
20 Diabetes mellitus
21 Meningitis
22 Liver cancer
23 Chronic kidney disease
24 Age-related and other hearing loss
25 Headache disorders
26 Protein-energy malnutrition
27 Depressive disorders
28 Asthma
29 Dietary iron deficiency
30 Hypertensive heart disease
32 Oesophageal cancer
34 Anxiety disorders
42 Neck pain
44 Other musculoskeletal disorders
56 Colon and rectum cancer
74 HIV/AIDS

Leading causes 2010	Mean % change number of DALYs 1990-2010	Mean % change age-standardized DALY rate 1990-2010
1 Ischaemic heart disease	**80.0**	0.9
2 Stroke	**46.5**	**-14.7**
3 Neonatal disorders	**-36.4**	**-28.8**
4 Road injuries	15.5	**-12.5**
5 Chronic obstructive pulmonary disease	12.7	**-36.5**
6 Lower respiratory infections	**-60.4**	**-58.7**
7 HIV/AIDS	**1144.4**	**802.6**
8 Cirrhosis and other chronic liver diseases	**27.7**	**-22.6**
9 Congenital anomalies	**-40.8**	**-36.0**
10 Diabetes mellitus	**113.0**	**22.5**
11 Tracheal, bronchus, and lung cancer	**94.9**	13.8
12 Tuberculosis	**-30.6**	**-54.2**
13 Interpersonal violence	2.7	**-22.8**
14 Diarrhoeal diseases	**-61.5**	**-62.7**
15 Low back pain	**36.2**	**-12.8**
16 Chronic kidney disease	**75.1**	**11.4**
17 Stomach cancer	**38.7**	**-20.1**
18 Age-related and other hearing loss	**55.4**	**-3.0**
19 Self-harm	2.9	**-26.5**
20 Falls	**19.6**	**-15.6**
21 Headache disorders	**45.1**	3.8
22 Depressive disorders	**46.7**	-0.1
23 Drowning	**-50.3**	**-53.8**
24 Other musculoskeletal disorders	**102.4**	**28.8**
25 Oesophageal cancer	**45.2**	-17.0
26 Malaria	-19.1	-23.0
27 Hypertensive heart disease	**18.8**	**-34.8**
28 Colon and rectum cancer	**154.6**	**48.0**
29 Liver cancer	-17.3	**-50.1**
30 Anxiety disorders	**34.5**	-0.9
32 Neck pain		
33 Blindness and vision loss		
34 Asthma		
38 Dietary iron deficiency		
47 Meningitis		
50 Protein-energy malnutrition		
77 Measles		

Leading causes 2019	Mean % change number of DALYs 2010-2019	Mean % change age-standardized DALY rate 2010-2019
1 Ischaemic heart disease	**20.8**	-6.6
2 Stroke	11.3	**-15.0**
3 Road injuries	**-12.2**	**-19.6**
4 Neonatal disorders	**-30.0**	**-28.6**
5 Chronic obstructive pulmonary disease	5.0	**-21.7**
6 Diabetes mellitus	**42.1**	**9.9**
7 Tracheal, bronchus, and lung cancer	**25.1**	-4.6
8 Cirrhosis and other chronic liver diseases	7.2	**-11.8**
9 Lower respiratory infections	**-25.5**	**-31.9**
10 Chronic kidney disease	**21.5**	-2.2
11 Low back pain	**12.4**	**-3.8**
12 HIV/AIDS	**-27.2**	**-34.2**
13 Interpersonal violence	-0.2	-5.2
14 Age-related and other hearing loss	**19.0**	**-3.2**
15 Congenital anomalies	**-26.5**	**-25.5**
16 Falls	**17.4**	1.0
17 Tuberculosis	**-21.9**	**-33.9**
18 Headache disorders	**11.1**	2.0
19 Stomach cancer	-3.9	**-25.7**
20 Self-harm	-2.8	**-11.6**
21 Depressive disorders	**15.9**	2.0
22 Diarrhoeal diseases	**-28.5**	**-34.7**
23 Other musculoskeletal disorders	**29.0**	**10.3**
24 Hypertensive heart disease	**31.5**	-0.7
25 Colon and rectum cancer	**32.0**	3.2
26 Liver cancer	**29.7**	3.5
27 Blindness and vision loss	**19.9**	**-7.2**
28 Anxiety disorders	**11.2**	**1.6**
29 Neck pain	**15.9**	**-1.6**
30 Oesophageal cancer	-2.1	**-25.1**
33 Drowning		
38 Malaria		

SR Figure 8i: Leading 30 GBD cause hierarchy level 3 causes of High-middle SDI SDI DALYs for males for 1990, 2010, and 2019

Causes are connected by arrows between time periods. Communicable maternal, neonatal and nutritional disorders are shown in red, non-communicable causes in blue and injuries in green. For the time period 1990 to 2010 and for 2010 to 2019, two measures of change are shown: percent change in the number of DALYs, and percent change in the age-standardized DALY rate. Numbers in bold highlight statistically significant changes.

Leading causes 1990	Leading causes 2010	Mean % change number of DALYs 1990-2010	Mean % change age-standardized DALY rate 1990-2010	Leading causes 2019	Mean % change number of DALYs 2010-2019	Mean % change age-standardized DALY rate 2010-2019
1 Ischaemic heart disease	1 Ischaemic heart disease	**27.2**	**-19.8**	1 Ischaemic heart disease	-0.5	**-20.2**
2 Stroke	2 Stroke	**16.7**	**-26.2**	2 Stroke	0.4	**-20.0**
3 Neonatal disorders	3 Road injuries	-2.5	**-23.2**	3 Tracheal, bronchus, and lung cancer	9.2	**-13.3**
4 Road injuries	4 Tracheal, bronchus, and lung cancer	**20.3**	**-19.6**	4 Road injuries	**-18.6**	**-22.9**
5 Lower respiratory infections	5 Chronic obstructive pulmonary disease	-13.9	**-45.2**	5 Chronic obstructive pulmonary disease	1.1	**-21.6**
6 Chronic obstructive pulmonary disease	6 Neonatal disorders	**-52.7**	**-44.1**	6 Diabetes mellitus	**22.6**	-1.3
7 Tracheal, bronchus, and lung cancer	7 Cirrhosis and other chronic liver diseases	**34.2**	**-10.1**	7 Low back pain	**6.7**	**-5.2**
8 Congenital anomalies	8 Self-harm	-0.1	**-24.2**	8 Cirrhosis and other chronic liver diseases	-7.5	**-20.3**
9 Self-harm	9 Low back pain	**21.1**	**-13.3**	9 Falls	**5.2**	**-7.7**
10 Stomach cancer	10 Diabetes mellitus	**84.8**	**21.1**	10 Self-harm	**-14.7**	**-18.4**
11 Low back pain	11 Falls	**14.1**	**-16.4**	11 Neonatal disorders	**-26.2**	**-28.7**
12 Cirrhosis and other chronic liver diseases	12 Lower respiratory infections	**-48.9**	**-56.5**	12 Stomach cancer	-3.9	**-22.6**
13 Falls	13 Stomach cancer	1.5	**-33.4**	13 Age-related and other hearing loss	**17.7**	**-1.9**
14 Interpersonal violence	14 Alcohol use disorders	**17.3**	**-15.0**	14 Colon and rectum cancer	**20.1**	-3.8
15 Drowning	15 Age-related and other hearing loss	**45.2**	-0.9	15 Lower respiratory infections	**-11.6**	**-27.6**
16 Alcohol use disorders	16 Interpersonal violence	**-16.2**	**-32.7**	16 Alcohol use disorders	-4.1	**-12.5**
17 Tuberculosis	17 Colon and rectum cancer	**72.8**	**12.4**	17 Headache disorders	**7.0**	1.6
18 Diarrhoeal diseases	18 Cardiomyopathy and myocarditis	**105.5**	30.8	18 Depressive disorders	**9.5**	0.1
19 Diabetes mellitus	19 Congenital anomalies	**-53.0**	**-45.1**	19 Chronic kidney disease	**11.8**	**-8.4**
20 Age-related and other hearing loss	20 Headache disorders	**29.9**	1.6	20 Interpersonal violence	**-15.3**	**-16.2**
21 Headache disorders	21 Depressive disorders	**28.3**	**-4.1**	21 Cardiomyopathy and myocarditis	**-20.2**	**-30.0**
22 Liver cancer	22 Chronic kidney disease	**37.7**	-5.3	22 Other musculoskeletal disorders	**22.7**	**9.0**
23 Depressive disorders	23 HIV/AIDS	**191.7**	**130.5**	23 Oesophageal cancer	9.0	-12.8
24 Exposure to mechanical forces	24 Oesophageal cancer	**40.1**	-8.9	24 Congenital anomalies	**-24.0**	**-24.9**
25 Colon and rectum cancer	25 Tuberculosis	**-29.7**	**-50.8**	25 Alzheimer's disease and other dementias	**33.5**	-3.1
26 Chronic kidney disease	26 Drug use disorders	**32.2**	**6.0**	26 Oral disorders	**19.2**	1.7
27 Cardiomyopathy and myocarditis	27 Other musculoskeletal disorders	**95.1**	**38.9**	27 Neck pain	**15.1**	**1.4**
28 Drug use disorders	28 Drowning	**-46.1**	**-50.7**	28 Pancreatic cancer	**24.9**	0.3
29 Oesophageal cancer	29 Exposure to mechanical forces	**-7.9**	**-30.4**	29 Drug use disorders	**-7.2**	**-8.7**
30 Leukaemia	30 Neck pain	**47.4**	**4.1**	30 Prostate cancer	**19.9**	**-8.9**
35 Oral disorders	31 Oral disorders			34 Exposure to mechanical forces		
37 Neck pain	33 Alzheimer's disease and other dementias			36 HIV/AIDS		
43 Other musculoskeletal disorders	34 Prostate cancer			39 Drowning		
58 HIV/AIDS	35 Pancreatic cancer			41 Tuberculosis		
	37 Liver cancer					
	41 Leukaemia					
	45 Diarrhoeal diseases					

SR Figure 8j: Leading 30 GBD cause hierarchy level 3 causes of High SDI SDI DALYs for males for 1990, 2010, and 2019

Causes are connected by arrows between time periods. Communicable maternal, neonatal and nutritional disorders are shown in red, non-communicable causes in blue and injuries in green. For the time period 1990 to 2010 and for 2010 to 2019, two measures of change are shown: percent change in the number of DALYs, and percent change in the age-standardized DALY rate. Numbers in bold highlight statistically significant changes.

Leading causes 1990
1 Ischaemic heart disease
2 Stroke
3 Tracheal, bronchus, and lung cancer
4 Road injuries
5 Low back pain
6 Self-harm
7 Chronic obstructive pulmonary disease
8 Neonatal disorders
9 Cirrhosis and other chronic liver diseases
10 Falls
11 Diabetes mellitus
12 Colon and rectum cancer
13 Lower respiratory infections
14 Stomach cancer
15 Congenital anomalies
16 Alcohol use disorders
17 Depressive disorders
18 Headache disorders
19 Age-related and other hearing loss
20 Prostate cancer
21 Interpersonal violence
22 Other musculoskeletal disorders
23 HIV/AIDS
24 Asthma
25 Exposure to mechanical forces
26 Drug use disorders
27 Anxiety disorders
28 Chronic kidney disease
29 Oral disorders
30 Neck pain
31 Endocrine, metabolic, blood, and immune disorders
33 Pancreatic cancer
34 Alzheimer's disease and other dementias
36 Osteoarthritis
38 Liver cancer

Leading causes 2010	Mean % change number of DALYs 1990-2010	Mean % change age-standardized DALY rate 1990-2010
1 Ischaemic heart disease	**-27.4**	**-52.8**
2 Tracheal, bronchus, and lung cancer	**3.6**	**-31.6**
3 Low back pain	**22.5**	**-5.9**
4 Stroke	**-14.6**	**-44.8**
5 Diabetes mellitus	**74.2**	**17.5**
6 Chronic obstructive pulmonary disease	**21.9**	**-22.8**
7 Self-harm	**10.2**	**-9.4**
8 Road injuries	**-29.7**	**-41.2**
9 Falls	**24.8**	**-9.8**
10 Drug use disorders	**115.7**	**92.5**
11 Colon and rectum cancer	**26.7**	**-16.7**
12 Cirrhosis and other chronic liver diseases	1.2	**-29.3**
13 Other musculoskeletal disorders	**67.3**	**32.3**
14 Age-related and other hearing loss	**45.7**	-1.6
15 Depressive disorders	**29.1**	**7.0**
16 Lower respiratory infections	-1.4	**-38.2**
17 Chronic kidney disease	**77.9**	**16.4**
18 Prostate cancer	**23.9**	**-24.0**
19 Alcohol use disorders	**11.8**	**-10.2**
20 Headache disorders	**19.0**	0.3
21 Neonatal disorders	**-35.1**	**-35.7**
22 Alzheimer's disease and other dementias	**90.6**	**2.8**
23 Stomach cancer	**-18.7**	**-46.2**
24 Anxiety disorders	**25.6**	**6.5**
25 Endocrine, metabolic, blood, and immune disorders	**45.2**	**9.0**
26 Pancreatic cancer	**59.4**	**5.4**
27 Osteoarthritis	**49.8**	-1.0
28 Liver cancer	**67.5**	**12.8**
29 Oral disorders	**11.0**	**-18.5**
30 Neck pain	**10.3**	**-17.3**
31 Interpersonal violence		
32 Exposure to mechanical forces		
33 Congenital anomalies		
35 Asthma		
61 HIV/AIDS		

Leading causes 2019	Mean % change number of DALYs 2010-2019	Mean % change age-standardized DALY rate 2010-2019
1 Ischaemic heart disease	**8.4**	**-11.5**
2 Tracheal, bronchus, and lung cancer	**4.4**	**-14.8**
3 Low back pain	**6.1**	**-4.7**
4 Stroke	**8.8**	**-11.6**
5 Diabetes mellitus	**26.5**	**6.4**
6 Chronic obstructive pulmonary disease	**18.5**	**-6.0**
7 Drug use disorders	**55.5**	**50.5**
8 Road injuries	-3.5	**-10.0**
9 Self-harm	**-7.6**	**-12.5**
10 Falls	**18.2**	**2.4**
11 Colon and rectum cancer	**12.7**	**-7.3**
12 Other musculoskeletal disorders	**15.6**	**5.3**
13 Age-related and other hearing loss	**17.4**	**-2.3**
14 Cirrhosis and other chronic liver diseases	2.0	**-10.9**
15 Chronic kidney disease	**23.7**	0.7
16 Lower respiratory infections	**13.7**	**-10.6**
17 Prostate cancer	**20.1**	**-6.3**
18 Depressive disorders	**6.2**	-0.5
19 Alzheimer's disease and other dementias	**34.5**	0.3
20 Headache disorders	**7.2**	1.7
21 Alcohol use disorders	**5.4**	**-2.9**
22 Pancreatic cancer	**19.3**	-1.6
23 Endocrine, metabolic, blood, and immune disorders	**13.7**	0.2
24 Osteoarthritis	**25.6**	**4.6**
25 Neck pain	**39.4**	**27.5**
26 Oral disorders	**32.2**	**13.6**
27 Neonatal disorders	**-11.6**	**-13.0**
28 Anxiety disorders	**5.2**	-1.0
29 Stomach cancer	**-5.1**	**-22.2**
30 Liver cancer	**9.4**	**-8.8**
35 Interpersonal violence		

SR Table 1: All-age DALYs counts and age standardized DALYs rates per 100,000 individuals for all causes and level 1 cause groups by location for 1990, 2010, and 2019, both sexes combined, Global							
		All-age DALYs			Age-standardized rate of DALYs per 100,000		
Cause Level	Cause	1990	2010	2019	1990	2010	2019
0-0	All causes	2,590,000,000 (2,440,000,000–2,770,000,000)	2,570,000,000 (2,370,000,000–2,790,000,000)	2,530,000,000 (2,280,000,000–2,810,000,000)	50,000 (46,900–53,500)	38,400 (35,400–41,600)	32,800 (29,600–36,300)
1-0	Communicable, maternal, neonatal, and nutritional disorders	1,200,000,000 (1,120,000,000–1,280,000,000)	873,000,000 (809,000,000–941,000,000)	666,000,000 (589,000,000–752,000,000)	19,900 (18,700–21,200)	13,000 (12,100–14,100)	9,440 (8,320–10,700)
1-1	HIV/AIDS and sexually transmitted infections	30,700,000 (24,400,000–41,100,000)	85,400,000 (79,200,000–93,700,000)	55,900,000 (50,500,000–63,800,000)	534 (435–694)	1,210 (1,120–1,330)	724 (646–846)
1-2	HIV/AIDS	21,100,000 (19,000,000–23,400,000)	77,000,000 (73,800,000–80,400,000)	47,400,000 (45,100,000–49,700,000)	384 (346–426)	1,080 (1,030–1,130)	598 (569–628)
1-3	HIV/AIDS - drug-susceptible tuberculosis	7,580,000 (6,310,000–8,660,000)	20,700,000 (15,200,000–25,600,000)	10,700,000 (7,930,000–13,400,000)	137 (114–156)	291 (213–359)	136 (100–170)
1-4	HIV/AIDS - multidrug-resistant tuberculosis without extensive drug resistance	42,400 (17,000–89,500)	1,740,000 (823,000–3,190,000)	1,040,000 (475,000–1,930,000)	0·8 (0·3–1·6)	24·4 (11·6–44·9)	13·2 (6·1–24·8)
1-5	HIV/AIDS - extensively drug-resistant Tuberculosis	0 (0–0)	47,500 (21,500–87,900)	41,300 (19,600–76,300)	0 (0–0)	0·7 (0·3–1·2)	0·5 (0·2–1)
1-6	HIV/AIDS resulting in other diseases	13,500,000 (11,900,000–15,700,000)	54,500,000 (48,900,000–60,800,000)	35,600,000 (32,100,000–39,200,000)	246 (218–287)	763 (685–852)	448 (403–494)
1-7	Sexually transmitted infections excluding HIV	9,570,000 (3,780,000–19,100,000)	8,470,000 (3,540,000–16,700,000)	8,490,000 (3,770,000–16,400,000)	151 (62·2–297)	127 (52·6–253)	127 (54·4–248)
1-8	Syphilis	8,710,000 (2,910,000–18,400,000)	7,380,000 (2,580,000–15,600,000)	7,280,000 (2,550,000–14,900,000)	134 (45·1–281)	112 (39·2–238)	112 (39·1–229)
1-9	Chlamydial infection	121,000 (89,800–165,000)	145,000 (105,000–203,000)	160,000 (114,000–223,000)	2·3 (1·7–3·2)	2 (1·4–2·8)	2 (1·4–2·8)
1-10	Gonococcal infection	155,000 (121,000–181,000)	158,000 (131,000–186,000)	163,000 (133,000–195,000)	3·1 (2·4–3·6)	2·2 (1·8–2·6)	2 (1·7–2·4)
1-11	Trichomoniasis	169,000 (65,000–345,000)	249,000 (96,400–509,000)	287,000 (113,000–592,000)	3·4 (1·3–6·9)	3·4 (1·3–7·1)	3·5 (1·4–7·3)
1-12	Genital herpes	142,000 (47,000–354,000)	214,000 (69,800–532,000)	253,000 (81,700–628,000)	3 (1–7·4)	3 (1–7·5)	3·1 (1–7·7)
1-13	Other sexually transmitted infections	276,000 (209,000–357,000)	323,000 (236,000–430,000)	345,000 (248,000–465,000)	5·4 (4·1–6·9)	4·4 (3·3–5·9)	4·3 (3·1–5·8)
1-14	Respiratory infections and tuberculosis	312,000,000 (283,000,000–349,000,000)	192,000,000 (176,000,000–210,000,000)	153,000,000 (136,000,000–171,000,000)	5,420 (4,960–6,010)	2,890 (2,650–3,160)	2,090 (1,850–2,360)
1-15	Tuberculosis	79,600,000 (72,800,000–86,400,000)	57,600,000 (53,500,000–61,500,000)	47,000,000 (42,800,000–51,400,000)	1,580 (1,450–1,710)	832 (773–888)	590 (537–646)
1-16	Latent tuberculosis infection	0 (0–0)	0 (0–0)	0 (0–0)	0 (0–0)	0 (0–0)	0 (0–0)
1-17	Drug-susceptible tuberculosis	78,900,000 (72,100,000–85,800,000)	52,600,000 (47,500,000–57,200,000)	42,500,000 (37,100,000–47,400,000)	1,570 (1,440–1,700)	760 (687–826)	534 (466–596)
1-18	Multidrug-resistant tuberculosis without extensive drug resistance	687,000 (241,000–1,590,000)	4,700,000 (2,280,000–8,050,000)	4,190,000 (1,800,000–7,830,000)	13·9 (4·9–32·5)	67·7 (32·9–116)	52·4 (22·6–97·6)
1-19	Extensively drug-resistant tuberculosis	0 (0–0)	291,000 (151,000–493,000)	310,000 (154,000–543,000)	0 (0–0)	4·2 (2·2–7·1)	3·8 (1·9–6·7)
1-20	Lower respiratory infections	223,000,000 (198,000,000–258,000,000)	126,000,000 (114,000,000–141,000,000)	96,800,000 (84,600,000–113,000,000)	3,680 (3,290–4,220)	1,940 (1,750–2,160)	1,380 (1,200–1,620)
1-21	Upper respiratory infections	6,490,000 (3,600,000–10,300,000)	6,130,000 (3,770,000–9,400,000)	6,380,000 (3,950,000–9,720,000)	115 (64·9–179)	88·3 (54·3–136)	84·3 (52–128)
1-22	Otitis media	2,250,000 (1,380,000–3,550,000)	2,370,000 (1,420,000–3,850,000)	2,440,000 (1,440,000–4,000,000)	39·8 (24·3–62·8)	34 (20·4–55·2)	32·6 (19·2–53·2)
1-23	Enteric infections	210,000,000 (173,000,000–246,000,000)	132,000,000 (112,000,000–155,000,000)	96,700,000 (79,000,000–119,000,000)	3,550 (2,910–4,190)	2,000 (1,700–2,340)	1,370 (1,120–1,680)
1-24	Diarrhoeal diseases	190,000,000 (155,000,000–225,000,000)	113,000,000 (95,000,000–133,000,000)	80,700,000 (65,200,000–103,000,000)	3,220 (2,580–3,840)	1,710 (1,440–2,020)	1,140 (927–1,430)
1-25	Typhoid and paratyphoid	16,100,000 (7,860,000–28,100,000)	12,200,000 (5,980,000–20,700,000)	9,690,000 (4,690,000–16,700,000)	268 (131–467)	179 (87·3–301)	135 (65·1–233)
1-26	Typhoid fever	13,700,000 (6,620,000–24,000,000)	10,300,000 (5,080,000–17,700,000)	8,050,000 (3,860,000–13,900,000)	226 (110–398)	152 (74·3–258)	113 (53·8–193)
1-27	Paratyphoid fever	2,480,000 (1,030,000–5,090,000)	1,850,000 (766,000–3,710,000)	1,640,000 (682,000–3,210,000)	41·3 (17·2–84·1)	27·1 (11·2–54·6)	22·7 (9·4–44·3)
1-28	Invasive non-typhoidal Salmonella (iNTS)	3,790,000 (2,090,000–6,120,000)	7,170,000 (4,150,000–11,400,000)	6,110,000 (3,320,000–9,700,000)	62·3 (34·4–101)	108 (62·4–172)	89·3 (48·5–141)
1-29	Other intestinal infectious diseases	314,000 (232,000–406,000)	161,000 (116,000–210,000)	112,000 (79,200–149,000)	5 (3·7–6·5)	2·4 (1·7–3·2)	1·6 (1·1–2·2)
1-30	Neglected tropical diseases and malaria	94,300,000 (63,800,000–135,000,000)	87,000,000 (55,600,000–124,000,000)	62,800,000 (38,600,000–95,800,000)	1,590 (1,100–2,260)	1,300 (823–1,860)	884 (537–1,350)
1-31	Malaria	65,600,000 (37,100,000–105,000,000)	68,500,000 (37,100,000–107,000,000)	46,400,000 (23,500,000–80,000,000)	1,070 (608–1,720)	1,030 (556–1,610)	667 (337–1,150)
1-32	Chagas disease	361,000 (154,000–451,000)	263,000 (175,000–381,000)	275,000 (184,000–459,000)	8·5 (3·7–10·5)	4 (2·7–5·7)	3·3 (2·2–5·6)
1-33	Leishmaniasis	4,430,000 (1,620,000–14,200,000)	1,150,000 (387,000–4,250,000)	697,000 (375,000–1,620,000)	74·1 (26·9–236)	16·8 (5·6–62·6)	9·4 (5–22·2)
1-34	Visceral leishmaniasis	4,300,000 (1,490,000–14,000,000)	916,000 (178,000–4,010,000)	403,000 (128,000–1,320,000)	71·5 (24·5–234)	13·5 (2·6–59·1)	5·7 (1·8–18·5)
1-35	Cutaneous and mucocutaneous leishmaniasis	129,000 (66,100–228,000)	233,000 (149,000–351,000)	293,000 (188,000–437,000)	2·6 (1·4–4·6)	3·3 (2·1–5)	3·7 (2·4–5·5)
1-36	African trypanosomiasis	1,010,000 (492,000–1,630,000)	329,000 (168,000–539,000)	82,600 (37,600–156,000)	18·1 (8·8–29·4)	4·6 (2·4–7·6)	1·1 (0·5–2·1)
1-37	Schistosomiasis	1,890,000 (1,280,000–2,900,000)	2,070,000 (1,280,000–3,370,000)	1,640,000 (1,040,000–2,640,000)	36·3 (25·1–54·9)	29·1 (18–47·3)	20·9 (13·2–33·7)
1-38	Cysticercosis	1,060,000 (681,000–1,520,000)	1,270,000 (806,000–1,860,000)	1,370,000 (874,000–1,960,000)	23·8 (15·1–34)	18·9 (11·9–27·2)	16·8 (10·7–23·9)
1-39	Cystic echinococcosis	210,000 (166,000–261,000)	130,000 (96,700–171,000)	122,000 (89,200–169,000)	3·8 (3–4·7)	1·9 (1·4–2·5)	1·6 (1·1–2·2)
1-40	Lymphatic filariasis	4,190,000 (2,790,000–6,060,000)	1,850,000 (1,120,000–2,970,000)	1,630,000 (959,000–2,710,000)	81·2 (54·3–117)	26·1 (15·8–41·9)	20·7 (12·2–34·7)
1-41	Onchocerciasis	1,350,000 (850,000–1,990,000)	1,090,000 (679,000–1,600,000)	1,230,000 (765,000–1,820,000)	26·2 (16·7–38·2)	15·4 (9·6–22·5)	15·7 (9·7–23·3)
1-42	Trachoma	288,000 (182,000–433,000)	187,000 (117,000–282,000)	181,000 (114,000–274,000)	7·3 (4·6–11)	2·9 (1·8–4·4)	2·2 (1·4–3·3)
1-43	Dengue	2,340,000 (542,000–3,700,000)	2,400,000 (952,000–3,010,000)	2,380,000 (827,000–3,270,000)	39·2 (9·3–61·9)	35·1 (13·7–44·1)	32·1 (11·1–44·1)
1-44	Yellow fever	843,000 (315,000–1,780,000)	446,000 (163,000–951,000)	290,000 (107,000–598,000)	14·5 (5·4–30·5)	6·4 (2·4–13·8)	4 (1·5–8·2)
1-45	Rabies	1,720,000 (616,000–2,780,000)	1,050,000 (401,000–1,480,000)	782,000 (320,000–1,080,000)	29·7 (10·8–47·6)	15·3 (5·9–21·8)	10·6 (4·4–14·7)
1-46	Intestinal nematode infections	5,280,000 (3,410,000–7,830,000)	2,840,000 (1,850,000–4,280,000)	1,970,000 (1,260,000–3,000,000)	91·2 (58·7–136)	40·9 (26·7–61·5)	26·6 (17–40·5)
1-47	Ascariasis	2,060,000 (1,380,000–3,000,000)	1,020,000 (688,000–1,510,000)	754,000 (479,000–1,140,000)	35·3 (23·2–51·9)	15 (10·2–22)	10·4 (6·6–15·6)
1-48	Trichuriasis	535,000 (295,000–904,000)	277,000 (152,000–469,000)	236,000 (126,000–402,000)	9·2 (5·1–15·6)	3·9 (2·1–6·6)	3·1 (1·7–5·3)

1-49	Hookworm disease	2,690,000 (1,690,000–4,050,000)	1,540,000 (972,000–2,330,000)	984,000 (626,000–1,470,000)	46·7 (29·4–70·5)	22 (13·8–33·2)	13·2 (8·4–19·6)
1-50	Food-borne trematodiases	1,090,000 (482,000–2,150,000)	738,000 (379,000–1,330,000)	780,000 (386,000–1,450,000)	22·5 (10–44·2)	10·6 (5·4–18·9)	9·5 (4·7–17·6)
1-51	Leprosy	44,300 (28,600–64,900)	28,800 (18,600–41,900)	28,800 (18,600–42,000)	0·9 (0·6–1·4)	0·4 (0·3–0·6)	0·4 (0·2–0·5)
1-52	Ebola virus disease	0 (0–0)	0 (0–0)	195,000 (160,000–231,000)	0 (0–0)	0 (0–0)	2·5 (2·1–3)
1-53	Zika virus disease	0 (0–0)	0 (0–0)	347 (256–455)	0 (0–0)	0 (0–0)	0 (0–0)
1-54	Guinea worm disease	24,900 (15,600–36,800)	30 (18–44)	1 (0–1)	0·5 (0·3–0·7)	0 (0–0)	0 (0–0)
1-55	Other neglected tropical diseases	2,550,000 (1,750,000–4,070,000)	2,740,000 (1,860,000–4,410,000)	2,800,000 (1,930,000–4,460,000)	43·6 (30·3–67·7)	40·7 (27·7–66)	39·4 (27–63·6)
1-56	Other infectious diseases	173,000,000 (124,000,000–250,000,000)	78,600,000 (61,600,000–102,000,000)	51,300,000 (40,600,000–65,900,000)	2,790 (2,020–4,010)	1,180 (924–1,540)	736 (578–953)
1-57	Meningitis	33,500,000 (28,800,000–38,600,000)	24,100,000 (20,900,000–27,400,000)	16,300,000 (13,700,000–19,600,000)	546 (471–627)	361 (312–411)	233 (195–282)
1-58	Encephalitis	8,460,000 (6,710,000–10,300,000)	5,180,000 (4,590,000–6,710,000)	4,790,000 (4,060,000–6,410,000)	143 (115–171)	76·6 (67·7–99)	65·2 (55–87·2)
1-59	Diphtheria	1,570,000 (1,140,000–2,200,000)	649,000 (449,000–902,000)	366,000 (243,000–523,000)	25·1 (18·3–35)	9·9 (6·8–13·7)	5·4 (3·6–7·8)
1-60	Whooping cough	22,400,000 (9,330,000–44,800,000)	13,200,000 (5,760,000–24,600,000)	10,200,000 (4,730,000–18,400,000)	351 (146–702)	201 (87·7–374)	153 (71·1–278)
1-61	Tetanus	21,600,000 (18,300,000–25,300,000)	4,450,000 (3,650,000–5,680,000)	2,300,000 (1,760,000–3,250,000)	343 (295–403)	66·8 (54·8–85·2)	33·5 (25·4–47·4)
1-62	Measles	68,800,000 (23,800,000–143,000,000)	18,100,000 (6,470,000–38,500,000)	7,020,000 (2,520,000–15,400,000)	1,090 (376–2,260)	276 (98·6–587)	105 (37·5–229)
1-63	Varicella and herpes zoster	1,200,000 (1,040,000–1,380,000)	1,030,000 (889,000–1,170,000)	948,000 (808,000–1,130,000)	21·1 (18·4–24·3)	15·6 (13·5–17·8)	13·3 (11·3–15·7)
1-64	Acute hepatitis	9,980,000 (8,440,000–11,400,000)	6,760,000 (5,970,000–7,670,000)	4,340,000 (3,560,000–5,210,000)	178 (149–202)	98·8 (87·1–112)	57·9 (47·3–69·9)
1-65	Acute hepatitis A	6,880,000 (5,310,000–8,380,000)	4,330,000 (3,270,000–5,460,000)	2,350,000 (1,700,000–3,090,000)	119 (91·9–146)	63·7 (47·9–80·5)	31·9 (23–42·4)
1-66	Acute hepatitis B	2,340,000 (1,420,000–3,840,000)	1,910,000 (1,300,000–2,730,000)	1,610,000 (1,170,000–2,200,000)	44·1 (27·5–69·7)	27·6 (18·7–39·6)	20·9 (15–28·8)
1-67	Acute hepatitis C	575,000 (299,000–1,020,000)	359,000 (187,000–580,000)	253,000 (145,000–375,000)	10·8 (5·7–18·6)	5·2 (2·7–8·5)	3·3 (1·9–4·9)
1-68	Acute hepatitis E	188,000 (113,000–292,000)	158,000 (103,000–227,000)	131,000 (84,500–191,000)	3·3 (2–5·1)	2·3 (1·5–3·3)	1·8 (1·1–2·6)
1-69	Other unspecified infectious diseases	5,340,000 (3,600,000–6,490,000)	5,110,000 (3,900,000–6,080,000)	5,080,000 (3,810,000–6,280,000)	93·1 (64·2–112)	75·6 (57·7–89·8)	69·6 (52·2–86·2)
1-70	Maternal and neonatal disorders	291,000,000 (272,000,000–311,000,000)	242,000,000 (221,000,000–263,000,000)	197,000,000 (171,000,000–230,000,000)	4,490 (4,210–4,810)	3,650 (3,340–3,970)	2,960 (2,560–3,460)
1-71	Maternal disorders	19,200,000 (17,500,000–21,300,000)	15,400,000 (13,900,000–16,700,000)	12,700,000 (11,100,000–14,400,000)	343 (312–381)	208 (188–226)	161 (140–182)
1-72	Maternal haemorrhage	6,120,000 (5,440,000–6,930,000)	4,220,000 (3,760,000–4,670,000)	3,090,000 (2,660,000–3,540,000)	109 (97–123)	57·1 (50·9–63·2)	39·1 (33·7–44·9)
1-73	Maternal sepsis and other pregnancy related infections	2,370,000 (2,050,000–2,710,000)	1,670,000 (1,450,000–1,870,000)	1,060,000 (896,000–1,260,000)	41·9 (36·4–47·8)	22·5 (19·6–25·2)	13·5 (11·4–16)
1-74	Maternal hypertensive disorders	2,580,000 (2,340,000–2,840,000)	2,190,000 (1,960,000–2,410,000)	1,820,000 (1,590,000–2,080,000)	45·1 (40·9–49·6)	29·5 (26·4–32·6)	23·2 (20·3–26·4)
1-75	Maternal obstructed labour and uterine rupture	926,000 (743,000–1,170,000)	871,000 (707,000–1,090,000)	1,000,000 (817,000–1,210,000)	17·1 (13·6–21·7)	11·9 (9·6–14·8)	12·6 (10·3–15·2)
1-76	Maternal abortive outcome	3,450,000 (3,000,000–3,950,000)	1,770,000 (1,550,000–1,990,000)	1,130,000 (948,000–1,340,000)	63·1 (54·9–72·1)	23·9 (20·9–27)	14·3 (12–16·9)
1-77	Ectopic pregnancy	339,000 (302,000–379,000)	404,000 (355,000–451,000)	378,000 (323,000–440,000)	6·2 (5·5–6·9)	5·5 (4·8–6·1)	4·8 (4·1–5·6)
1-78	Indirect maternal deaths	1,300,000 (1,150,000–1,470,000)	1,350,000 (1,210,000–1,490,000)	1,490,000 (1,280,000–1,720,000)	22·7 (20·1–25·7)	18·2 (16·4–20·2)	19 (16·3–21·9)
1-79	Late maternal deaths	581,000 (471,000–712,000)	750,000 (600,000–927,000)	767,000 (594,000–975,000)	10·4 (8·5–12·8)	10·2 (8·1–12·6)	9·7 (7·5–12·3)
1-80	Maternal deaths aggravated by HIV/AIDS	42,400 (25,600–58,600)	109,000 (69,500–151,000)	86,300 (51,300–125,000)	0·8 (0·5–1·1)	1·5 (0·9–2·1)	1·1 (0·6–1·6)
1-81	Other maternal disorders	1,530,000 (1,370,000–1,710,000)	2,030,000 (1,800,000–2,250,000)	1,850,000 (1,600,000–2,120,000)	27·3 (24·6–30·5)	27·4 (24·4–30·3)	23·4 (20·3–26·8)
1-82	Neonatal disorders	271,000,000 (253,000,000–291,000,000)	226,000,000 (207,000,000–247,000,000)	184,000,000 (159,000,000–216,000,000)	4,150 (3,870–4,450)	3,440 (3,140–3,750)	2,800 (2,420–3,290)
1-83	Neonatal preterm birth	116,000,000 (107,000,000–127,000,000)	86,300,000 (79,100,000–94,400,000)	67,900,000 (58,400,000–79,200,000)	1,790 (1,650–1,940)	1,310 (1,200–1,430)	1,030 (883–1,200)
1-84	Neonatal encephalopathy due to birth asphyxia and trauma	71,700,000 (64,300,000–80,100,000)	64,800,000 (57,300,000–72,200,000)	52,800,000 (44,600,000–62,100,000)	1,090 (981–1,220)	987 (872–1,100)	808 (683–953)
1-85	Neonatal sepsis and other neonatal infections	23,600,000 (19,100,000–27,100,000)	25,000,000 (21,600,000–28,400,000)	24,300,000 (20,600,000–29,200,000)	362 (294–416)	379 (328–430)	365 (309–439)
1-86	Haemolytic disease and other neonatal jaundice	11,700,000 (9,930,000–13,300,000)	7,890,000 (7,000,000–8,950,000)	5,370,000 (4,570,000–6,330,000)	179 (153–204)	120 (106–136)	81·2 (69·2–95·9)
1-87	Other neonatal disorders	47,900,000 (40,900,000–57,000,000)	42,400,000 (37,900,000–47,000,000)	33,500,000 (28,100,000–40,500,000)	730 (623–869)	646 (578–717)	517 (434–624)
1-88	Nutritional deficiencies	87,700,000 (69,800,000–112,000,000)	55,400,000 (43,600,000–71,400,000)	49,800,000 (36,900,000–65,800,000)	1,490 (1,180–1,900)	822 (649–1,060)	680 (507–894)
1-89	Protein-energy malnutrition	52,600,000 (40,100,000–70,300,000)	21,500,000 (18,500,000–24,900,000)	15,200,000 (12,500,000–18,300,000)	853 (653–1,140)	326 (281–377)	218 (179–262)
1-90	Iodine deficiency	2,500,000 (1,530,000–4,030,000)	2,530,000 (1,490,000–4,200,000)	2,440,000 (1,370,000–4,240,000)	46·8 (28·6–76)	35·3 (20·9–58·6)	30·7 (17·3–53·1)
1-91	Vitamin A deficiency	1,970,000 (1,360,000–2,800,000)	1,500,000 (1,020,000–2,090,000)	1,180,000 (805,000–1,640,000)	31·9 (22·1–45·3)	22·6 (15·3–31·5)	16·9 (11·5–23·5)
1-92	Dietary iron deficiency	25,100,000 (16,800,000–36,100,000)	27,300,000 (18,400,000–39,300,000)	28,500,000 (19,100,000–41,100,000)	458 (309–657)	400 (269–576)	383 (257–554)
1-93	Other nutritional deficiencies	5,560,000 (3,540,000–7,690,000)	2,550,000 (2,120,000–3,030,000)	2,370,000 (1,880,000–2,950,000)	104 (66·2–144)	37·7 (31·5–44·7)	30·8 (24·6–38·3)
1-0	Non-communicable diseases	1,120,000,000 (999,000,000–1,240,000,000)	1,430,000,000 (1,270,000,000–1,600,000,000)	1,620,000,000 (1,430,000,000–1,820,000,000)	25,000 (22,600–27,500)	21,500 (19,200–24,000)	20,200 (17,800–22,600)
1-1	Neoplasms	165,000,000 (157,000,000–172,000,000)	217,000,000 (208,000,000–224,000,000)	251,000,000 (236,000,000–266,000,000)	3,820 (3,640–3,990)	3,280 (3,150–3,380)	3,060 (2,870–3,240)
1-2	Lip and oral cavity cancer	2,850,000 (2,680,000–3,050,000)	4,500,000 (4,330,000–4,700,000)	5,510,000 (5,000,000–6,030,000)	67 (62·9–71·6)	66·7 (64·1–69·6)	66·1 (60·1–72·4)
1-3	Nasopharynx cancer	1,880,000 (1,720,000–2,050,000)	2,070,000 (1,960,000–2,200,000)	2,340,000 (2,140,000–2,540,000)	41·7 (38·1–45·3)	29·9 (28·3–31·7)	28 (25·7–30·4)
1-4	Other pharynx cancer	1,560,000 (1,450,000–1,710,000)	2,560,000 (2,440,000–2,680,000)	3,230,000 (2,910,000–3,570,000)	36·4 (33·8–39·8)	37·8 (36–39·6)	38·4 (34·5–42·4)
1-5	Oesophageal cancer	8,210,000 (6,330,000–9,070,000)	11,000,000 (9,010,000–11,800,000)	11,700,000 (10,400,000–12,900,000)	199 (154–220)	167 (137–179)	140 (124–155)
1-6	Stomach cancer	20,500,000 (19,200,000–21,700,000)	22,300,000 (21,200,000–23,500,000)	22,200,000 (20,300,000–24,100,000)	493 (464–524)	339 (322–356)	268 (246–291)
1-7	Colon and rectum cancer	12,400,000 (11,900,000–12,900,000)	19,700,000 (18,900,000–20,500,000)	24,300,000 (22,600,000–25,700,000)	308 (295–321)	304 (290–315)	296 (275–313)

1-8	Liver cancer	11,300,000 (10,100,000–12,700,000)	10,400,000 (9,870,000–10,900,000)	12,500,000 (11,400,000–13,700,000)	258 (231–290)	155 (148–163)	151 (138–165)
1-9	Liver cancer due to hepatitis B	6,480,000 (5,540,000–7,600,000)	4,890,000 (4,420,000–5,440,000)	5,800,000 (4,910,000–6,770,000)	146 (125–171)	70·9 (64·1–78·9)	69·2 (58·7–80·6)
1-10	Liver cancer due to hepatitis C	2,000,000 (1,730,000–2,320,000)	2,400,000 (2,070,000–2,710,000)	2,880,000 (2,440,000–3,320,000)	49·7 (43–57·4)	37·5 (32·4–42·3)	35 (29·7–40·3)
1-11	Liver cancer due to alcohol use	1,270,000 (1,020,000–1,560,000)	1,700,000 (1,390,000–2,040,000)	2,180,000 (1,760,000–2,650,000)	30·6 (24·5–37·6)	25·8 (21·1–30·9)	26·1 (21·1–31·6)
1-12	Liver cancer due to NASH	479,000 (393,000–578,000)	596,000 (497,000–713,000)	796,000 (657,000–976,000)	11·4 (9·3–13·7)	9·1 (7·6–10·9)	9·6 (8–11·8)
1-13	Gallbladder and biliary tract cancer	2,150,000 (1,930,000–2,460,000)	3,080,000 (2,670,000–3,250,000)	3,620,000 (3,100,000–3,970,000)	54·1 (48·7–61·3)	47·9 (41·4–50·5)	44 (37·6–48·2)
1-14	Pancreatic cancer	4,650,000 (4,470,000–4,810,000)	8,690,000 (8,350,000–8,990,000)	11,600,000 (10,800,000–12,300,000)	116 (111–120)	134 (128–139)	140 (130–149)
1-15	Larynx cancer	2,470,000 (2,350,000–2,590,000)	2,850,000 (2,740,000–2,980,000)	3,260,000 (3,030,000–3,510,000)	59 (56·1–61·9)	42·8 (41·1–44·6)	38·8 (36·1–41·8)
1-16	Tracheal, bronchus, and lung cancer	27,100,000 (25,900,000–28,500,000)	38,800,000 (37,200,000–40,400,000)	45,900,000 (42,300,000–49,400,000)	658 (628–691)	595 (570–619)	552 (509–593)
1-17	Malignant skin melanoma	1,030,000 (853,000–1,300,000)	1,520,000 (1,150,000–1,720,000)	1,710,000 (1,300,000–2,000,000)	23·6 (19·6–29·9)	22·7 (17·1–25·7)	20·8 (15·8–24·3)
1-18	Non-melanoma skin cancer	562,000 (519,000–599,000)	952,000 (897,000–1,000,000)	1,180,000 (1,090,000–1,260,000)	14·4 (13·3–15·4)	15·1 (14·1–15·9)	14·7 (13·4–15·7)
1-19	Non-melanoma skin cancer (squamous-cell carcinoma)	561,000 (518,000–599,000)	950,000 (896,000–1,000,000)	1,180,000 (1,080,000–1,260,000)	14·4 (13·3–15·4)	15 (14·1–15·9)	14·6 (13·4–15·6)
1-20	Non-melanoma skin cancer (basal-cell carcinoma)	562 (257–1,040)	1,390 (650–2,620)	1,700 (782–3,190)	0 (0–0)	0 (0–0)	0 (0–0)
1-21	Breast cancer	11,700,000 (11,200,000–12,300,000)	17,000,000 (16,300,000–17,700,000)	20,600,000 (19,000,000–22,200,000)	275 (263–289)	251 (240–262)	248 (229–266)
1-22	Cervical cancer	6,180,000 (5,440,000–7,320,000)	7,820,000 (6,850,000–8,680,000)	8,950,000 (7,550,000–9,980,000)	140 (124–166)	113 (99·2–125)	107 (90·5–119)
1-23	Uterine cancer	1,480,000 (1,320,000–1,610,000)	2,090,000 (1,760,000–2,220,000)	2,330,000 (2,090,000–2,560,000)	36·1 (32·3–39·2)	31·7 (27–33·7)	28 (25·1–30·8)
1-24	Ovarian cancer	2,730,000 (2,490,000–3,170,000)	4,200,000 (3,880,000–4,550,000)	5,360,000 (4,690,000–5,960,000)	64·7 (59·3–74·3)	62·7 (58·1–67·7)	64·3 (56·4–71·5)
1-25	Prostate cancer	4,360,000 (3,530,000–4,950,000)	6,850,000 (5,840,000–8,120,000)	8,640,000 (7,550,000–10,600,000)	119 (96·3–136)	112 (95·4–133)	108 (94–132)
1-26	Testicular cancer	391,000 (359,000–456,000)	490,000 (457,000–562,000)	562,000 (511,000–628,000)	7·3 (6·8–8·3)	6·8 (6·4–7·9)	7·1 (6·5–8)
1-27	Kidney cancer	2,040,000 (1,940,000–2,140,000)	3,350,000 (3,230,000–3,460,000)	4,050,000 (3,800,000–4,320,000)	47·3 (45·2–49·4)	51·1 (49·3–52·8)	49·6 (46·5–52·9)
1-28	Bladder cancer	2,570,000 (2,430,000–2,690,000)	3,610,000 (3,460,000–3,740,000)	4,390,000 (4,090,000–4,700,000)	66·6 (63–69·7)	57·5 (54·8–59·6)	54·2 (50·4–58)
1-29	Brain and nervous system cancer	6,160,000 (4,960,000–8,490,000)	7,750,000 (6,220,000–8,600,000)	8,660,000 (6,720,000–9,570,000)	122 (100–165)	114 (90·9–126)	109 (84·6–121)
1-30	Thyroid cancer	667,000 (614,000–733,000)	1,050,000 (943,000–1,100,000)	1,230,000 (1,110,000–1,330,000)	15·5 (14·4–17)	15·7 (14·1–16·5)	15 (13·6–16·1)
1-31	Mesothelioma	391,000 (343,000–443,000)	582,000 (531,000–621,000)	668,000 (608,000–717,000)	9·4 (8·4–10·6)	8·9 (8·1–9·4)	8·1 (7·4–8·7)
1-32	Hodgkin lymphoma	1,220,000 (929,000–1,340,000)	1,120,000 (967,000–1,300,000)	1,150,000 (977,000–1,320,000)	24·1 (18·6–26·5)	16 (13·8–18·5)	14·4 (12·3–16·7)
1-33	Non-Hodgkin's lymphoma	4,150,000 (3,810,000–4,450,000)	5,910,000 (5,640,000–6,200,000)	6,990,000 (6,570,000–7,450,000)	90·4 (84·4–96·2)	88·5 (84·3–92·7)	86·5 (81·3–92·3)
1-34	Multiple myeloma	1,220,000 (1,120,000–1,410,000)	1,980,000 (1,780,000–2,140,000)	2,500,000 (2,190,000–2,720,000)	30·5 (28–35)	30·7 (27·5–33·1)	30·3 (26·6–32·9)
1-35	Leukaemia	12,800,000 (10,700,000–15,400,000)	11,600,000 (10,600,000–12,600,000)	11,700,000 (10,500,000–12,700,000)	240 (206–283)	171 (156–187)	150 (136–164)
1-36	Acute lymphoid leukaemia	2,730,000 (1,990,000–3,960,000)	2,700,000 (2,230,000–3,180,000)	2,660,000 (2,190,000–3,010,000)	46·7 (34·7–66·6)	39·4 (32·5–46·6)	36 (29·5–41)
1-37	Chronic lymphoid leukaemia	492,000 (445,000–532,000)	769,000 (720,000–855,000)	948,000 (874,000–1,070,000)	12·5 (11·4–13·5)	12·1 (11·3–13·4)	11·7 (10·7–13·1)
1-38	Acute myeloid leukaemia	2,030,000 (1,720,000–2,530,000)	2,710,000 (2,410,000–2,940,000)	3,060,000 (2,770,000–3,370,000)	40 (34·8–48·7)	40 (35·6–43·4)	39 (35·2–43·1)
1-39	Chronic myeloid leukaemia	1,340,000 (900,000–2,110,000)	1,060,000 (890,000–1,320,000)	1,050,000 (924,000–1,190,000)	26·5 (19·1–39)	15·6 (13–19·4)	13·3 (11·6–15·2)
1-40	Other leukaemia	6,180,000 (4,060,000–7,770,000)	4,310,000 (3,630,000–4,960,000)	3,940,000 (3,370,000–4,470,000)	114 (79·1–141)	63·6 (53·5–73·1)	50·5 (43·3–57·5)
1-41	Other neoplasms	572,000 (485,000–755,000)	954,000 (818,000–1,160,000)	1,220,000 (1,040,000–1,470,000)	14·5 (12·2–19·3)	15·3 (13·1–18·6)	15·3 (13·1–18·4)
1-42	Myelodysplastic, myeloproliferative, and other hematopoietic neoplasms	572,000 (485,000–755,000)	954,000 (818,000–1,160,000)	1,220,000 (1,040,000–1,470,000)	14·5 (12·2–19·3)	15·3 (13·1–18·6)	15·3 (13·1–18·4)
1-43	Benign and in situ intestinal neoplasms	0 (0–0)	0 (0–0)	0 (0–0)	0 (0–0)	0 (0–0)	0 (0–0)
1-44	Benign and in situ cervical and uterine neoplasms	0 (0–0)	0 (0–0)	0 (0–0)	0 (0–0)	0 (0–0)	0 (0–0)
1-45	Other benign and in situ neoplasms	0 (0–0)	0 (0–0)	0 (0–0)	0 (0–0)	0 (0–0)	0 (0–0)
1-46	Cardiovascular diseases	280,000,000 (268,000,000–291,000,000)	349,000,000 (335,000,000–363,000,000)	393,000,000 (368,000,000–417,000,000)	7,080 (6,760–7,370)	5,470 (5,230–5,690)	4,860 (4,550–5,160)
1-47	Rheumatic heart disease	13,200,000 (11,900,000–14,600,000)	11,300,000 (10,100,000–12,500,000)	10,700,000 (9,210,000–12,100,000)	283 (256–315)	164 (147–181)	133 (115–150)
1-48	Ischaemic heart disease	121,000,000 (116,000,000–126,000,000)	159,000,000 (152,000,000–165,000,000)	182,000,000 (170,000,000–193,000,000)	3,140 (3,010–3,260)	2,490 (2,380–2,590)	2,240 (2,100–2,380)
1-49	Stroke	108,000,000 (102,000,000–115,000,000)	131,000,000 (124,000,000–137,000,000)	143,000,000 (133,000,000–153,000,000)	2,730 (2,580–2,900)	2,050 (1,940–2,140)	1,770 (1,640–1,890)
1-50	Ischaemic stroke	40,500,000 (37,600,000–43,700,000)	53,300,000 (49,400,000–56,900,000)	63,500,000 (57,800,000–69,000,000)	1,120 (1,040–1,200)	871 (808–929)	799 (728–867)
1-51	Intracerebral haemorrhage	54,700,000 (50,700,000–59,600,000)	67,000,000 (63,300,000–70,600,000)	68,600,000 (63,300,000–73,700,000)	1,310 (1,210–1,430)	1,020 (969–1,080)	833 (769–895)
1-52	Subarachnoid haemorrhage	13,000,000 (9,480,000–15,400,000)	10,400,000 (8,960,000–12,200,000)	11,200,000 (9,890,000–12,700,000)	298 (217–355)	154 (133–180)	136 (121–155)
1-53	Hypertensive heart disease	13,900,000 (11,300,000–15,600,000)	17,200,000 (14,000,000–18,900,000)	21,500,000 (16,400,000–23,900,000)	364 (298–406)	277 (225–303)	268 (205–298)
1-54	Non-rheumatic valvular heart disease	1,670,000 (1,490,000–1,850,000)	2,260,000 (2,050,000–2,510,000)	2,790,000 (2,520,000–3,130,000)	44·5 (40–49·2)	37·8 (33·9–41·6)	35·9 (32·3–40·2)
1-55	Non-rheumatic calcific aortic valvular heart disease	976,000 (872,000–1,110,000)	1,450,000 (1,310,000–1,610,000)	1,840,000 (1,640,000–2,030,000)	26·9 (24·1–30·3)	24·9 (22·1–27·6)	23·9 (21·1–26·5)
1-56	Non-rheumatic degenerative mitral valvular heart disease	626,000 (539,000–751,000)	737,000 (631,000–899,000)	883,000 (754,000–1,090,000)	16·1 (13·8–19·3)	11·8 (10·1–14·6)	11·1 (9·5–13·8)
1-57	Other non-rheumatic valvular heart diseases	65,000 (47,900–81,900)	73,200 (54,000–85,100)	72,600 (55,600–85,300)	1·5 (1·1–1·8)	1·1 (0·8–1·3)	0·9 (0·7–1·1)
1-58	Cardiomyopathy and myocarditis	7,050,000 (6,290,000–8,620,000)	9,760,000 (8,260,000–10,300,000)	9,130,000 (7,860,000–10,000,000)	161 (147–185)	146 (124–154)	115 (98·6–126)
1-59	Myocarditis	979,000 (706,000–1,570,000)	1,020,000 (800,000–1,130,000)	977,000 (803,000–1,130,000)	18·3 (13·8–27·5)	15·3 (11·9–17·1)	12·8 (10·5–14·7)
1-60	Alcoholic cardiomyopathy	1,760,000 (1,590,000–2,290,000)	3,630,000 (2,780,000–3,830,000)	2,440,000 (2,050,000–2,780,000)	40·2 (36·6–52·1)	51·7 (39·7–54·6)	29·2 (24·5–33·3)

1-61	Other cardiomyopathy	4,320,000 (3,830,000–4,970,000)	5,120,000 (4,420,000–5,510,000)	5,710,000 (4,890,000–6,330,000)	103 (90·4–113)	79·2 (68·8–85·3)	72·7 (62·2–80·4)
1-62	Atrial fibrillation and flutter	3,790,000 (2,960,000–4,830,000)	6,340,000 (5,070,000–7,890,000)	8,390,000 (6,690,000–10,500,000)	110 (87·6–139)	107 (86·5–133)	107 (86·2–134)
1-63	Aortic aneurysm	1,990,000 (1,820,000–2,190,000)	2,720,000 (2,540,000–2,910,000)	3,320,000 (3,110,000–3,530,000)	50·8 (46·5–55·7)	42·7 (39·7–45·6)	40·9 (38·2–43·4)
1-64	Peripheral vascular disease	776,000 (488,000–1,180,000)	1,200,000 (771,000–1,830,000)	1,540,000 (1,010,000–2,370,000)	22·4 (14·1–34·1)	20·2 (13–30·8)	19·6 (12·9–30·2)
1-65	Endocarditis	1,120,000 (836,000–1,320,000)	1,530,000 (1,220,000–1,690,000)	1,720,000 (1,350,000–1,930,000)	22·8 (18–27)	23·2 (18·2–25·6)	21·9 (17·2–24·6)
1-66	Chronic respiratory diseases	85,700,000 (74,900,000–92,600,000)	93,600,000 (86,200,000–100,000,000)	104,000,000 (94,800,000–112,000,000)	2,110 (1,840–2,270)	1,470 (1,350–1,570)	1,290 (1,180–1,400)
1-67	Chronic obstructive pulmonary disease	59,200,000 (51,200,000–63,600,000)	66,700,000 (62,200,000–70,600,000)	74,400,000 (68,200,000–80,200,000)	1,540 (1,330–1,650)	1,070 (996–1,130)	926 (849–998)
1-68	Pneumoconiosis	827,000 (657,000–979,000)	904,000 (759,000–1,070,000)	919,000 (762,000–1,120,000)	20 (15·9–23·6)	13·7 (11·5–16·2)	11·1 (9·2–13·5)
1-69	Silicosis	577,000 (446,000–709,000)	651,000 (525,000–806,000)	656,000 (519,000–828,000)	13·9 (10·8–17)	9·8 (7·9–12·1)	7·9 (6·2–10)
1-70	Asbestosis	38,100 (30,200–46,100)	63,500 (47,700–73,400)	71,200 (53,900–81,800)	0·9 (0·8–1·1)	1 (0·8–1·2)	0·9 (0·7–1)
1-71	Coal workers pneumoconiosis	117,000 (77,200–143,000)	78,800 (58,400–97,000)	75,300 (55,900–96,500)	2·9 (1·9–3·5)	1·2 (0·9–1·5)	0·9 (0·7–1·2)
1-72	Other pneumoconiosis	94,200 (64,900–131,000)	110,000 (86,600–141,000)	117,000 (93,600–148,000)	2·2 (1·5–3·1)	1·7 (1·3–2·1)	1·4 (1·1–1·8)
1-73	Asthma	22,300,000 (17,900,000–27,300,000)	20,400,000 (16,400,000–25,000,000)	21,500,000 (17,100,000–27,000,000)	476 (378–579)	304 (245–374)	274 (217–343)
1-74	Interstitial lung disease and pulmonary sarcoidosis	1,690,000 (1,380,000–2,120,000)	3,050,000 (2,340,000–3,620,000)	3,770,000 (2,860,000–4,470,000)	41·6 (33·9–51·9)	47·7 (36·4–56·8)	46·4 (35·1–55)
1-75	Other chronic respiratory diseases	1,620,000 (1,330,000–2,030,000)	2,510,000 (2,130,000–2,860,000)	2,860,000 (2,460,000–3,220,000)	32·1 (26·6–39·4)	35·9 (30·4–41·1)	36·5 (31·4–41·1)
1-76	Digestive diseases	72,000,000 (66,900,000–77,600,000)	83,600,000 (78,400,000–89,700,000)	89,000,000 (81,400,000–97,600,000)	1,570 (1,470–1,680)	1,230 (1,150–1,320)	1,100 (1,000–1,200)
1-77	Cirrhosis and other chronic liver diseases	34,700,000 (32,400,000–37,100,000)	44,100,000 (42,400,000–46,300,000)	46,200,000 (43,000,000–49,500,000)	766 (718–813)	640 (616–672)	560 (522–602)
1-78	Cirrhosis and other chronic liver diseases due to hepatitis B	9,810,000 (8,470,000–11,200,000)	11,700,000 (10,200,000–13,300,000)	10,800,000 (8,990,000–12,700,000)	220 (190–251)	169 (147–191)	130 (108–153)
1-79	Cirrhosis and other chronic liver diseases due to hepatitis C	8,070,000 (6,900,000–9,280,000)	11,000,000 (9,590,000–12,600,000)	12,200,000 (10,300,000–14,200,000)	182 (156–210)	159 (139–182)	146 (124–170)
1-80	Cirrhosis and other chronic liver diseases due to alcohol use	7,310,000 (6,170,000–8,540,000)	10,100,000 (8,600,000–11,700,000)	11,200,000 (9,450,000–13,100,000)	169 (142–198)	147 (126–170)	133 (113–156)
1-81	Cirrhosis and other chronic liver diseases due to NAFLD	2,230,000 (1,600,000–2,970,000)	2,990,000 (2,130,000–4,000,000)	3,620,000 (2,580,000–4,860,000)	51·9 (37·2–69·2)	44·3 (31·8–59)	43·7 (31·3–58·4)
1-82	Cirrhosis and other chronic liver diseases due to other causes	7,310,000 (5,890,000–8,890,000)	8,280,000 (7,020,000–9,780,000)	8,440,000 (6,930,000–10,200,000)	143 (116–173)	121 (102–142)	107 (88·8–130)
1-83	Upper digestive system diseases	13,800,000 (11,700,000–17,000,000)	13,900,000 (10,800,000–18,300,000)	14,900,000 (11,300,000–20,200,000)	309 (262–375)	204 (160–267)	183 (139–248)
1-84	Peptic ulcer disease	8,190,000 (7,580,000–8,960,000)	6,420,000 (6,030,000–7,000,000)	6,030,000 (5,590,000–6,640,000)	189 (176–206)	96·5 (90·6–105)	74·4 (68·9–81·9)
1-85	Gastritis and duodenitis	2,220,000 (1,690,000–2,920,000)	2,530,000 (1,930,000–3,360,000)	2,810,000 (2,090,000–3,790,000)	48·1 (36·8–63·4)	37 (28·4–48·9)	34·8 (25·9–46·8)
1-86	Gastro-oesophageal reflux disease (GERD)	3,400,000 (1,760,000–6,090,000)	4,980,000 (2,580,000–8,860,000)	6,030,000 (3,100,000–10,800,000)	71·7 (37–129)	70·8 (36·6–126)	73·6 (38–132)
1-87	Appendicitis	2,210,000 (1,560,000–2,840,000)	1,640,000 (1,380,000–1,910,000)	1,500,000 (1,240,000–1,710,000)	41·3 (29·7–52·4)	23·6 (19·9–27·5)	19·4 (15·9–22)
1-88	Paralytic ileus and intestinal obstruction	6,680,000 (5,800,000–7,590,000)	6,690,000 (5,480,000–7,460,000)	7,070,000 (5,640,000–8,240,000)	128 (112–144)	102 (83·6–113)	93·3 (74·3–110)
1-89	Inguinal, femoral, and abdominal hernia	3,070,000 (2,350,000–3,890,000)	3,040,000 (2,400,000–3,850,000)	3,350,000 (2,610,000–4,250,000)	62 (48·4–77·4)	45·3 (36–56·9)	42·7 (33·3–54)
1-90	Inflammatory bowel disease	1,250,000 (963,000–1,500,000)	1,470,000 (1,230,000–1,730,000)	1,620,000 (1,360,000–1,920,000)	27·2 (21·7–32·4)	22 (18·5–25·7)	20·2 (16·9–23·7)
1-91	Vascular intestinal disorders	1,090,000 (1,020,000–1,170,000)	1,580,000 (1,470,000–1,710,000)	1,870,000 (1,720,000–2,030,000)	28·6 (26·8–30·3)	25·6 (23·7–27·6)	23·5 (21·6–25·6)
1-92	Gallbladder and biliary diseases	4,600,000 (3,590,000–5,850,000)	5,590,000 (4,290,000–7,250,000)	6,350,000 (4,870,000–8,250,000)	107 (84·2–135)	83·9 (64·8–108)	78·2 (60·3–101)
1-93	Pancreatitis	2,440,000 (2,180,000–2,880,000)	3,370,000 (3,050,000–3,740,000)	3,640,000 (3,280,000–4,030,000)	53·9 (48·2–63·3)	48·9 (44·4–54)	44·4 (40·1–49·1)
1-94	Other digestive diseases	2,170,000 (1,520,000–2,860,000)	2,150,000 (1,910,000–2,460,000)	2,500,000 (2,120,000–2,800,000)	46·9 (34·3–59·6)	33·5 (29·8–38·4)	31·9 (27·1–35·7)
1-95	Neurological disorders	57,700,000 (32,000,000–94,000,000)	83,000,000 (46,500,000–135,000,000)	97,700,000 (55,900,000–159,000,000)	1,270 (743–2,050)	1,260 (735–2,050)	1,250 (720–2,040)
1-96	Alzheimer's disease and other dementias	9,810,000 (4,270,000–21,800,000)	18,600,000 (8,190,000–41,000,000)	25,300,000 (11,200,000–54,600,000)	331 (144–742)	341 (150–746)	339 (151–732)
1-97	Parkinson's disease	2,750,000 (2,540,000–2,990,000)	4,910,000 (4,550,000–5,290,000)	6,290,000 (5,770,000–6,830,000)	78·1 (72·2–84·6)	81·6 (75·6–87·8)	80 (73·2–86·6)
1-98	Idiopathic epilepsy	11,300,000 (8,610,000–14,100,000)	12,500,000 (9,780,000–15,600,000)	13,100,000 (9,990,000–16,700,000)	204 (158–254)	179 (140–224)	171 (130–218)
1-99	Multiple sclerosis	726,000 (622,000–868,000)	1,010,000 (878,000–1,210,000)	1,160,000 (1,000,000–1,380,000)	16·1 (13·8–19·3)	14·6 (12·6–17·3)	14 (12–16·6)
1-100	Motor neuron disease	624,000 (594,000–664,000)	835,000 (806,000–862,000)	1,030,000 (980,000–1,090,000)	13·2 (12·7–13·9)	12·6 (12·2–13·1)	12·7 (12–13·3)
1-101	Headache disorders	29,700,000 (6,040,000–64,200,000)	41,500,000 (8,650,000–89,200,000)	46,600,000 (9,770,000–100,000,000)	575 (125–1,230)	578 (121–1,240)	582 (120–1,260)
1-102	Migraine	26,900,000 (3,970,000–61,400,000)	37,500,000 (5,660,000–85,300,000)	42,100,000 (6,420,000–95,600,000)	518 (82–1,170)	522 (80–1,190)	526 (78·8–1,190)
1-103	Tension-type headache	2,880,000 (853,000–9,770,000)	4,010,000 (1,210,000–13,200,000)	4,540,000 (1,400,000–15,000,000)	57·7 (17·7–188)	56·4 (17·1–187)	56·2 (17–188)
1-104	Other neurological disorders	2,720,000 (2,240,000–3,280,000)	3,590,000 (2,960,000–4,370,000)	4,260,000 (3,460,000–5,170,000)	50·5 (42·5–60·2)	52·2 (43·2–63·4)	55·9 (45·2–68·3)
1-105	Mental disorders	80,800,000 (59,500,000–106,000,000)	111,000,000 (82,300,000–146,000,000)	125,000,000 (93,000,000–163,000,000)	1,580 (1,170–2,060)	1,570 (1,160–2,050)	1,570 (1,160–2,040)
1-106	Schizophrenia	9,130,000 (6,690,000–11,600,000)	13,300,000 (9,700,000–17,000,000)	15,100,000 (11,000,000–19,200,000)	185 (135–235)	185 (135–236)	184 (134–234)
1-107	Depressive disorders	29,100,000 (20,300,000–40,000,000)	40,500,000 (28,400,000–55,500,000)	46,900,000 (32,900,000–63,800,000)	589 (414–803)	572 (402–781)	578 (406–789)
1-108	Major depressive disorder	23,500,000 (16,000,000–32,500,000)	32,200,000 (22,200,000–44,000,000)	37,200,000 (25,700,000–51,200,000)	473 (325–652)	454 (315–621)	460 (315–635)
1-109	Dysthymia	5,630,000 (3,640,000–8,400,000)	8,360,000 (5,420,000–12,300,000)	9,660,000 (6,310,000–14,400,000)	116 (75·4–170)	118 (76·6–174)	118 (77·3–177)
1-110	Bipolar disorder	5,350,000 (3,270,000–8,220,000)	7,530,000 (4,580,000–11,600,000)	8,500,000 (5,200,000–13,000,000)	105 (64·5–160)	105 (64·2–161)	105 (64·3–162)
1-111	Anxiety disorders	18,700,000 (12,900,000–25,500,000)	25,900,000 (18,100,000–35,300,000)	28,700,000 (19,900,000–39,300,000)	360 (251–492)	364 (254–496)	360 (249–494)
1-112	Eating disorders	1,820,000 (1,140,000–2,700,000)	2,560,000 (1,620,000–3,820,000)	2,910,000 (1,830,000–4,350,000)	32·2 (20·2–47·9)	34·8 (22–52)	37·2 (23·5–55·7)
1-113	Anorexia nervosa	571,000 (341,000–900,000)	763,000 (458,000–1,200,000)	845,000 (511,000–1,320,000)	9·9 (6–15·5)	10·3 (6·2–16·2)	10·9 (6·6–17·2)

1-114	Bulimia nervosa	1,250,000 (723,000–1,940,000)	1,800,000 (1,040,000–2,810,000)	2,070,000 (1,190,000–3,200,000)	22·2 (12·8–34·6)	24·4 (14·1–38·2)	26·3 (15·2–40·6)
1-115	Autism spectrum disorders	3,110,000 (2,030,000–4,510,000)	3,960,000 (2,590,000–5,740,000)	4,310,000 (2,820,000–6,230,000)	56·7 (37–82·2)	56·5 (37–82·1)	56·3 (36·8–81·5)
1-116	Attention-deficit/hyperactivity disorder	881,000 (500,000–1,500,000)	1,020,000 (579,000–1,730,000)	1,030,000 (585,000–1,740,000)	15·1 (8·6–25·4)	14·4 (8·1–24·3)	13·8 (7·9–23·4)
1-117	Conduct disorder	3,960,000 (2,220,000–6,210,000)	4,590,000 (2,580,000–7,180,000)	4,870,000 (2,740,000–7,620,000)	65·2 (36·6–102)	66·4 (37·2–104)	67·9 (38·3–106)
1-118	Idiopathic developmental intellectual disability	3,790,000 (2,000,000–6,140,000)	4,430,000 (2,330,000–7,230,000)	4,390,000 (2,300,000–7,240,000)	67 (35·3–109)	63·3 (33·3–103)	58·2 (30·5–96)
1-119	Other mental disorders	5,010,000 (3,220,000–7,580,000)	7,440,000 (4,770,000–11,300,000)	8,650,000 (5,580,000–13,100,000)	106 (68·1–160)	106 (67·8–160)	106 (68–160)
1-120	Substance use disorders	22,700,000 (18,100,000–27,600,000)	30,900,000 (24,900,000–37,600,000)	35,100,000 (28,300,000–43,000,000)	444 (357–540)	427 (346–518)	432 (347–530)
1-121	Alcohol use disorders	12,600,000 (9,970,000–15,900,000)	16,200,000 (13,000,000–20,300,000)	17,000,000 (13,500,000–21,500,000)	256 (205–322)	226 (181–284)	207 (164–262)
1-122	Drug use disorders	10,100,000 (7,840,000–12,700,000)	14,700,000 (11,400,000–18,200,000)	18,100,000 (14,300,000–22,000,000)	188 (147–235)	201 (157–249)	225 (178–274)
1-123	Opioid use disorders	6,240,000 (4,740,000–8,150,000)	10,200,000 (7,780,000–12,900,000)	12,900,000 (10,000,000–16,200,000)	118 (90·8–151)	140 (107–177)	160 (124–202)
1-124	Cocaine use disorders	538,000 (372,000–746,000)	883,000 (670,000–1,140,000)	1,150,000 (933,000–1,430,000)	10 (6·9–13·8)	12·1 (9·2–15·7)	14·3 (11·5–17·8)
1-125	Amphetamine use disorders	1,310,000 (872,000–1,950,000)	1,260,000 (821,000–1,880,000)	1,400,000 (954,000–2,040,000)	23·3 (15·6–34·3)	17·1 (11·1–25·6)	17·5 (11·9–25·6)
1-126	Cannabis use disorders	498,000 (298,000–781,000)	623,000 (377,000–968,000)	690,000 (421,000–1,080,000)	8·8 (5·3–13·7)	8·4 (5·1–13·1)	8·8 (5·3–13·7)
1-127	Other drug use disorders	1,520,000 (1,130,000–2,000,000)	1,750,000 (1,340,000–2,280,000)	1,950,000 (1,540,000–2,480,000)	27·8 (21·1–36·4)	23·9 (18·3–31·1)	24·2 (19·1–31)
1-128	Diabetes and kidney diseases	50,800,000 (45,600,000–56,400,000)	87,700,000 (77,700,000–98,400,000)	113,000,000 (99,300,000–128,000,000)	1,190 (1,070–1,320)	1,330 (1,180–1,500)	1,380 (1,210–1,570)
1-129	Diabetes mellitus	28,600,000 (24,600,000–33,200,000)	52,700,000 (44,500,000–62,000,000)	70,900,000 (59,700,000–84,200,000)	690 (595–800)	804 (680–943)	859 (723–1,020)
1-130	Diabetes mellitus type 1	3,110,000 (2,560,000–3,570,000)	4,030,000 (3,460,000–4,710,000)	4,580,000 (3,900,000–5,380,000)	62·3 (51·3–71·6)	57·9 (49·6–67·6)	57·4 (49·1–67·2)
1-131	Diabetes mellitus type 2	25,500,000 (21,700,000–29,800,000)	48,700,000 (41,000,000–57,400,000)	66,300,000 (55,500,000–79,000,000)	628 (537–731)	746 (629–878)	802 (670–954)
1-132	Chronic kidney disease	21,500,000 (20,000,000–23,100,000)	34,600,000 (32,200,000–37,200,000)	41,500,000 (38,300,000–45,000,000)	484 (452–519)	524 (490–562)	515 (475–559)
1-133	Chronic kidney disease due to diabetes mellitus type 1	1,870,000 (1,350,000–2,460,000)	2,830,000 (2,050,000–3,730,000)	3,220,000 (2,280,000–4,370,000)	39·5 (28·4–52·7)	40 (29–52·7)	38·8 (27·7–52·3)
1-134	Chronic kidney disease due to diabetes mellitus type 2	4,080,000 (3,300,000–4,860,000)	7,830,000 (6,510,000–9,130,000)	9,870,000 (8,110,000–11,700,000)	102 (82·9–120)	122 (102–142)	120 (99·1–143)
1-135	Chronic kidney disease due to hypertension	4,420,000 (3,820,000–5,210,000)	7,890,000 (6,900,000–9,060,000)	9,960,000 (8,580,000–11,500,000)	111 (96·3–129)	124 (109–141)	123 (107–143)
1-136	Chronic kidney disease due to glomerulonephritis	4,150,000 (3,460,000–4,840,000)	5,930,000 (5,030,000–6,880,000)	6,900,000 (5,780,000–8,120,000)	84·8 (71·1–99·2)	86·2 (73·4–100)	86·2 (72·3–101)
1-137	Chronic kidney disease due to other and unspecified causes	6,970,000 (5,980,000–8,010,000)	10,100,000 (8,610,000–11,700,000)	11,600,000 (9,700,000–13,700,000)	147 (126–170)	152 (130–176)	146 (123–172)
1-138	Acute glomerulonephritis	695,000 (572,000–825,000)	370,000 (263,000–432,000)	307,000 (204,000–363,000)	13·5 (11·4–15·9)	5·5 (3·9–6·4)	3·9 (2·6–4·6)
1-139	Skin and subcutaneous diseases	30,700,000 (20,600,000–44,800,000)	39,100,000 (26,100,000–57,800,000)	42,900,000 (28,600,000–63,400,000)	573 (388–838)	564 (378–833)	559 (373–828)
1-140	Dermatitis	7,400,000 (4,310,000–11,700,000)	9,230,000 (5,450,000–14,500,000)	10,100,000 (5,960,000–15,800,000)	136 (79·9–214)	133 (78·6–210)	132 (77·6–207)
1-141	Atopic dermatitis	5,830,000 (3,090,000–9,780,000)	7,000,000 (3,750,000–11,800,000)	7,480,000 (3,990,000–12,600,000)	104 (55·3–175)	102 (54·4–171)	99·7 (53·1–167)
1-142	Contact dermatitis	1,380,000 (872,000–2,040,000)	1,960,000 (1,230,000–2,900,000)	2,280,000 (1,430,000–3,400,000)	28·1 (17·6–41·7)	27·9 (17·6–41·5)	28·1 (17·6–41·8)
1-143	Seborrhoeic dermatitis	195,000 (111,000–311,000)	269,000 (154,000–427,000)	308,000 (176,000–489,000)	3·8 (2·2–6·1)	3·9 (2·2–6·2)	3·9 (2·2–6·2)
1-144	Psoriasis	2,730,000 (1,940,000–3,640,000)	3,300,000 (2,350,000–4,370,000)	3,510,000 (2,500,000–4,640,000)	56·7 (40·5–75·4)	47·4 (33·7–62·8)	43·3 (30·9–57·4)
1-145	Bacterial skin diseases	1,600,000 (1,170,000–2,030,000)	1,910,000 (1,480,000–2,270,000)	2,200,000 (1,660,000–2,640,000)	31·6 (23·8–38·9)	28·8 (22·3–34·3)	28·4 (21·3–34·1)
1-146	Cellulitis	381,000 (242,000–545,000)	485,000 (347,000–590,000)	551,000 (378,000–660,000)	7·8 (5·2–10·8)	7·3 (5·2–8·9)	7 (4·7–8·4)
1-147	Pyoderma	1,220,000 (870,000–1,650,000)	1,420,000 (1,100,000–1,740,000)	1,640,000 (1,240,000–2,010,000)	23·8 (17·2–31·1)	21·5 (16·7–26·2)	21·4 (16·2–26·2)
1-148	Scabies	3,700,000 (2,030,000–5,910,000)	4,530,000 (2,500,000–7,250,000)	4,840,000 (2,680,000–7,730,000)	65·6 (36–105)	64·5 (35·6–103)	63·5 (35·2–101)
1-149	Fungal skin diseases	1,970,000 (799,000–4,150,000)	2,790,000 (1,140,000–5,860,000)	3,220,000 (1,320,000–6,780,000)	39·2 (16–82·9)	41·2 (16·8–86·7)	42 (17·2–88·2)
1-150	Viral skin diseases	3,810,000 (2,430,000–5,690,000)	4,430,000 (2,830,000–6,620,000)	4,730,000 (3,030,000–7,060,000)	66·9 (42·7–99·8)	64·6 (41·3–96·6)	63·9 (40·9–95·6)
1-151	Acne vulgaris	3,360,000 (2,020,000–5,330,000)	4,580,000 (2,750,000–7,240,000)	4,960,000 (2,980,000–7,850,000)	56·6 (34·1–89·6)	63·3 (38·1–100)	65·9 (39·6–104)
1-152	Alopecia areata	402,000 (252,000–595,000)	542,000 (342,000–803,000)	601,000 (378,000–891,000)	7·7 (4·9–11·5)	7·6 (4·8–11·2)	7·5 (4·7–11·1)
1-153	Pruritus	457,000 (220,000–816,000)	669,000 (321,000–1,200,000)	786,000 (376,000–1,410,000)	9·2 (4·4–16·5)	9·7 (4·6–17·4)	9·9 (4·7–17·7)
1-154	Urticaria	2,890,000 (1,880,000–4,200,000)	3,600,000 (2,350,000–5,180,000)	3,900,000 (2,550,000–5,580,000)	51·6 (33·8–74·2)	52 (34–74·8)	51·9 (34–75·1)
1-155	Decubitus ulcer	268,000 (211,000–360,000)	401,000 (313,000–489,000)	481,000 (374,000–583,000)	7·8 (6·2–10·4)	6·8 (5·3–8·2)	6·2 (4·8–7·5)
1-156	Other skin and subcutaneous diseases	2,160,000 (1,070,000–3,880,000)	3,070,000 (1,520,000–5,550,000)	3,600,000 (1,770,000–6,500,000)	44·7 (22·3–80·4)	45 (22·2–81·1)	45·2 (22·3–81·5)
1-157	Sense organ diseases	36,200,000 (25,200,000–50,500,000)	54,800,000 (37,600,000–77,000,000)	66,100,000 (45,100,000–93,000,000)	852 (594–1,180)	836 (574–1,170)	819 (561–1,150)
1-158	Blindness and vision loss	12,400,000 (8,640,000–17,400,000)	18,700,000 (12,900,000–26,400,000)	22,600,000 (15,600,000–31,700,000)	304 (212–421)	290 (200–405)	278 (192–392)
1-159	Glaucoma	442,000 (302,000–626,000)	607,000 (416,000–849,000)	748,000 (516,000–1,040,000)	12·8 (8·7–17·9)	10·3 (7·1–14·3)	9·5 (6·6–13·2)
1-160	Cataract	3,490,000 (2,480,000–4,720,000)	5,560,000 (3,960,000–7,510,000)	6,680,000 (4,760,000–9,010,000)	93·2 (66·1–125)	89·4 (63·6–120)	82·9 (59·1–112)
1-161	Age-related macular degeneration	297,000 (205,000–419,000)	451,000 (315,000–630,000)	564,000 (393,000–789,000)	8·3 (5·8–11·6)	7·5 (5·2–10·4)	7 (4·9–9·8)
1-162	Refraction disorders	4,080,000 (2,760,000–5,790,000)	5,670,000 (3,840,000–7,960,000)	6,570,000 (4,430,000–9,290,000)	88·9 (60·5–124)	84·1 (56·9–118)	81·5 (55–115)
1-163	Near vision loss	2,260,000 (1,040,000–4,440,000)	3,880,000 (1,770,000–7,700,000)	4,900,000 (2,260,000–9,670,000)	55·5 (25·7–109)	58·8 (27·1–116)	58·9 (27·1–116)
1-164	Other vision loss	1,870,000 (1,340,000–2,560,000)	2,570,000 (1,830,000–3,510,000)	3,100,000 (2,210,000–4,240,000)	44·8 (32·1–60·8)	39·7 (28·4–54·1)	38·3 (27·2–52·3)
1-165	Age-related and other hearing loss	22,000,000 (14,900,000–31,300,000)	33,300,000 (22,600,000–47,500,000)	40,200,000 (27,400,000–57,100,000)	509 (347–725)	505 (344–718)	499 (340–710)
1-166	Other sense organ diseases	1,800,000 (1,080,000–2,760,000)	2,740,000 (1,630,000–4,230,000)	3,320,000 (1,970,000–5,130,000)	39·8 (23·7–61·6)	40·9 (24·4–63·4)	41·2 (24·5–63·8)

1-167	Musculoskeletal disorders	84,600,000 (61,400,000–112,000,000)	125,000,000 (90,800,000–165,000,000)	150,000,000 (109,000,000–198,000,000)	1,850 (1,340–2,440)	1,820 (1,310–2,390)	1,830 (1,320–2,400)
1-168	Rheumatoid arthritis	1,660,000 (1,270,000–2,080,000)	2,670,000 (2,040,000–3,360,000)	3,260,000 (2,510,000–4,090,000)	39·1 (30·1–48·6)	40 (30·7–50·1)	39·6 (30·5–49·5)
1-169	Osteoarthritis	8,830,000 (4,500,000–17,400,000)	14,900,000 (7,510,000–29,500,000)	18,900,000 (9,570,000–37,700,000)	221 (112–434)	228 (115–451)	228 (115–453)
1-170	Low back pain	43,400,000 (30,500,000–57,900,000)	56,300,000 (39,700,000–75,200,000)	63,700,000 (45,000,000–85,200,000)	932 (659–1,250)	814 (575–1,090)	780 (549–1,050)
1-171	Neck pain	12,400,000 (8,130,000–17,700,000)	18,200,000 (11,900,000–26,000,000)	22,100,000 (14,500,000–31,700,000)	268 (177–383)	260 (170–370)	267 (176–384)
1-172	Gout	691,000 (437,000–994,000)	1,280,000 (817,000–1,830,000)	1,670,000 (1,070,000–2,390,000)	16·6 (10·4–23·8)	19·2 (12·4–27·5)	20·2 (12·9–28·9)
1-173	Other musculoskeletal disorders	17,700,000 (12,300,000–24,300,000)	32,000,000 (22,300,000–44,000,000)	40,400,000 (28,200,000–55,500,000)	375 (260–516)	456 (318–624)	490 (343–673)
1-174	Other non-communicable diseases	153,000,000 (116,000,000–194,000,000)	151,000,000 (121,000,000–184,000,000)	153,000,000 (124,000,000–187,000,000)	2,700 (2,060–3,390)	2,220 (1,780–2,690)	2,050 (1,670–2,480)
1-175	Congenital anomalies	83,600,000 (58,800,000–121,000,000)	62,700,000 (49,600,000–79,300,000)	52,500,000 (43,300,000–65,100,000)	1,310 (921–1,890)	947 (746–1,200)	784 (646–978)
1-176	Neural tube defects	14,500,000 (10,400,000–23,200,000)	9,380,000 (6,720,000–14,300,000)	7,690,000 (5,680,000–11,000,000)	224 (160–358)	143 (102–218)	117 (86·3–166)
1-177	Congenital heart anomalies	32,600,000 (23,000,000–48,700,000)	23,500,000 (19,300,000–29,400,000)	18,600,000 (15,100,000–22,500,000)	509 (360–759)	355 (291–444)	279 (226–339)
1-178	Orofacial clefts	1,240,000 (801,000–1,740,000)	610,000 (416,000–947,000)	528,000 (361,000–794,000)	19·5 (12·8–27·2)	9·1 (6·2–14·2)	7·5 (5·1–11·5)
1-179	Down syndrome	2,210,000 (1,490,000–4,590,000)	1,820,000 (1,360,000–3,150,000)	1,780,000 (1,370,000–2,730,000)	35 (23·7–72)	27·3 (20·3–47·5)	25·9 (19·7–40·6)
1-180	Turner syndrome	11,000 (5,070–18,900)	12,900 (5,990–22,200)	13,500 (6,230–23,200)	0·2 (0·1–0·3)	0·2 (0·1–0·3)	0·2 (0·1–0·3)
1-181	Klinefelter syndrome	1,250 (554–2,370)	1,270 (568–2,460)	1,290 (574–2,470)	0 (0–0)	0 (0–0)	0 (0–0)
1-182	Other chromosomal abnormalities	1,810,000 (1,060,000–4,770,000)	2,050,000 (1,400,000–3,810,000)	2,050,000 (1,470,000–3,330,000)	28·2 (16·6–74)	30·9 (21·1–57·8)	30·9 (22–50·4)
1-183	Congenital musculoskeletal and limb anomalies	3,310,000 (2,240,000–4,670,000)	3,470,000 (2,450,000–4,700,000)	3,510,000 (2,500,000–4,750,000)	55·3 (37·6–77·6)	50·9 (36–68·6)	49 (34·8–65·8)
1-184	Urogenital congenital anomalies	1,460,000 (913,000–2,220,000)	1,250,000 (913,000–1,660,000)	1,090,000 (826,000–1,400,000)	22·8 (14·3–34·5)	18·8 (13·7–25·1)	16·3 (12·3–20·9)
1-185	Digestive congenital anomalies	7,010,000 (3,320,000–14,000,000)	5,170,000 (3,140,000–9,020,000)	4,250,000 (2,740,000–6,670,000)	108 (51·1–216)	78·7 (47·7–137)	64·9 (41·8–102)
1-186	Other congenital anomalies	19,400,000 (10,000,000–36,300,000)	15,500,000 (10,000,000–25,200,000)	13,000,000 (9,350,000–19,200,000)	304 (158–567)	234 (150–382)	194 (140–286)
1-187	Urinary diseases and male infertility	5,580,000 (4,850,000–6,370,000)	7,680,000 (6,650,000–8,790,000)	9,340,000 (7,980,000–10,900,000)	125 (108–142)	118 (102–135)	117 (100–136)
1-188	Urinary tract infections and interstitial nephritis	3,080,000 (2,650,000–3,380,000)	4,310,000 (3,760,000–4,630,000)	5,200,000 (4,450,000–5,700,000)	67·7 (59·9–73·4)	66·6 (58·4–71·7)	66·2 (56·6–72·5)
1-189	Urolithiasis	517,000 (374,000–636,000)	521,000 (416,000–642,000)	604,000 (477,000–745,000)	11·7 (8·6–14·4)	7·7 (6·1–9·4)	7·4 (5·8–9)
1-190	Benign prostatic hyperplasia	884,000 (527,000–1,340,000)	1,420,000 (858,000–2,130,000)	1,860,000 (1,130,000–2,780,000)	22·6 (13·4–33·9)	22·5 (13·6–33·8)	22·6 (13·7–33·7)
1-191	Male infertility	182,000 (69,000–435,000)	257,000 (96,300–614,000)	322,000 (121,000–771,000)	3·4 (1·3–8)	3·5 (1·3–8·3)	4 (1·5–9·7)
1-192	Other urinary diseases	921,000 (732,000–1,080,000)	1,170,000 (933,000–1,330,000)	1,350,000 (1,050,000–1,580,000)	19·2 (15·3–22·2)	17·7 (14–20)	17·1 (13·4–20·1)
1-193	Gynaecological diseases	20,000,000 (13,800,000–28,000,000)	26,300,000 (18,000,000–36,500,000)	29,700,000 (20,400,000–41,300,000)	395 (274–550)	362 (249–503)	368 (253–511)
1-194	Uterine fibroids	861,000 (473,000–1,510,000)	1,210,000 (637,000–2,170,000)	1,380,000 (711,000–2,480,000)	18·2 (10–31·9)	16·8 (8·9–30·2)	16·7 (8·6–30)
1-195	Polycystic ovarian syndrome	302,000 (127,000–607,000)	490,000 (209,000–980,000)	577,000 (246,000–1,160,000)	5·6 (2·4–11·2)	6·6 (2·8–13·3)	7·3 (3·1–14·6)
1-196	Female infertility	353,000 (119,000–839,000)	502,000 (170,000–1,180,000)	659,000 (222,000–1,570,000)	6·6 (2·2–15·5)	6·8 (2·3–16·3)	8·3 (2·8–19·7)
1-197	Endometriosis	1,880,000 (1,120,000–3,000,000)	2,180,000 (1,310,000–3,440,000)	2,250,000 (1,360,000–3,540,000)	35·1 (21–55·6)	29·4 (17·8–46·6)	28·1 (16·9–44·4)
1-198	Genital prolapse	276,000 (155,000–475,000)	375,000 (207,000–656,000)	442,000 (245,000–764,000)	6·4 (3·5–11·1)	5·6 (3–9·7)	5·3 (3–9·3)
1-199	Premenstrual syndrome	5,440,000 (3,320,000–8,510,000)	7,340,000 (4,510,000–11,400,000)	7,970,000 (4,900,000–12,300,000)	100 (61·6–155)	99·7 (61·3–154)	101 (61·8–156)
1-200	Other gynaecological diseases	10,900,000 (7,110,000–15,400,000)	14,200,000 (9,220,000–20,300,000)	16,500,000 (10,700,000–23,400,000)	222 (145–316)	197 (129–280)	201 (131–286)
1-201	Haemoglobinopathies and haemolytic anaemias	11,600,000 (8,840,000–14,800,000)	12,400,000 (9,610,000–15,800,000)	12,800,000 (9,970,000–16,700,000)	206 (157–262)	180 (140–230)	172 (134–224)
1-202	Thalassaemias	1,360,000 (989,000–1,760,000)	881,000 (769,000–997,000)	776,000 (650,000–927,000)	22 (16·1–28·2)	13 (11·4–14·8)	11 (9·2–13·2)
1-203	Thalassaemias trait	3,020,000 (1,960,000–4,600,000)	3,170,000 (2,050,000–4,820,000)	3,230,000 (2,100,000–4,910,000)	54·3 (35·5–82·8)	45·5 (29·4–69·3)	42·7 (27·7–64·8)
1-204	Sickle cell disorders	2,950,000 (1,880,000–4,250,000)	3,350,000 (2,280,000–4,660,000)	3,420,000 (2,470,000–4,620,000)	47·9 (30·9–68·7)	49·5 (33·5–69·2)	48·6 (34·9–66)
1-205	Sickle cell trait	1,750,000 (1,150,000–2,540,000)	2,190,000 (1,440,000–3,210,000)	2,420,000 (1,580,000–3,540,000)	30·7 (20·1–44·6)	31·2 (20·5–45·7)	32·2 (21–47·1)
1-206	G6PD deficiency	459,000 (373,000–552,000)	649,000 (534,000–809,000)	679,000 (542,000–886,000)	8·5 (7–10·3)	9·2 (7·5–11·4)	8·6 (6·9–11·2)
1-207	G6PD trait	13,200 (8,800–18,900)	22,400 (14,800–32,300)	23,600 (15,700–34,200)	0·3 (0·2–0·4)	0·3 (0·2–0·4)	0·3 (0·2–0·4)
1-208	Other haemoglobinopathies and haemolytic anaemias	2,030,000 (1,550,000–2,660,000)	2,100,000 (1,580,000–2,810,000)	2,260,000 (1,700,000–3,020,000)	41·8 (32·4–54·2)	31·3 (23·6–41·7)	29 (21·7–38·9)
1-209	Endocrine, metabolic, blood, and immune disorders	14,600,000 (10,900,000–18,900,000)	19,700,000 (14,700,000–25,600,000)	22,900,000 (17,000,000–30,000,000)	300 (221–392)	288 (215–375)	286 (213–373)
1-210	Oral disorders	13,200,000 (7,820,000–20,900,000)	19,000,000 (11,300,000–30,300,000)	23,100,000 (13,600,000–37,400,000)	295 (176–461)	281 (168–441)	285 (168–459)
1-211	Caries of deciduous teeth	188,000 (82,600–398,000)	189,000 (83,400–400,000)	200,000 (86,900–426,000)	3 (1·3–6·4)	2·9 (1·3–6·1)	2·9 (1·3–6·3)
1-212	Caries of permanent teeth	1,370,000 (618,000–2,670,000)	1,820,000 (825,000–3,540,000)	2,000,000 (915,000–3,880,000)	26·2 (11·9–50·8)	25·5 (11·6–49·8)	25·3 (11·5–49)
1-213	Periodontal disease	3,570,000 (1,380,000–7,780,000)	5,490,000 (2,150,000–11,900,000)	7,090,000 (2,780,000–15,500,000)	78·6 (30·6–172)	78·5 (30·9–169)	85·5 (33·5–186)
1-214	Edentulism	5,320,000 (3,420,000–7,840,000)	7,770,000 (5,030,000–11,300,000)	9,620,000 (6,150,000–14,200,000)	134 (86·1–197)	120 (78–176)	118 (75·3–172)
1-215	Other oral disorders	2,760,000 (1,720,000–4,050,000)	3,760,000 (2,350,000–5,490,000)	4,220,000 (2,640,000–6,160,000)	53·2 (33·2–77·5)	53·2 (33·3–77·6)	53·3 (33·3–77·9)
1-216	Sudden infant death syndrome	4,920,000 (1,970,000–11,000,000)	3,090,000 (1,260,000–6,890,000)	2,430,000 (1,060,000–5,350,000)	75·8 (30·3–170)	46·9 (19·2–105)	37·4 (16·3–82·2)
1-0	Injuries	270,000,000 (252,000,000–289,000,000)	273,000,000 (251,000,000–294,000,000)	249,000,000 (226,000,000–275,000,000)	5,040 (4,690–5,420)	3,890 (3,580–4,200)	3,170 (2,880–3,490)
1-1	Transport injuries	76,100,000 (71,000,000–82,400,000)	83,600,000 (74,100,000–90,400,000)	77,600,000 (69,200,000–85,500,000)	1,420 (1,320–1,530)	1,180 (1,050–1,280)	978 (869–1,080)
1-2	Road injuries	71,200,000 (66,400,000–77,000,000)	78,600,000 (69,500,000–85,000,000)	72,900,000 (64,800,000–80,200,000)	1,330 (1,240–1,440)	1,110 (980–1,200)	918 (814–1,010)

1-3	Pedestrian road injuries	29,700,000<br>(25,300,000–34,200,000)	27,500,000<br>(23,400,000–30,100,000)	23,500,000<br>(20,500,000–26,300,000)	554<br>(475–633)	393<br>(336–429)	298<br>(260–332)
1-4	Cyclist road injuries	4,480,000<br>(3,690,000–5,480,000)	6,380,000<br>(5,270,000–7,700,000)	6,510,000<br>(5,250,000–7,910,000)	89·3<br>(73·2–109)	90·8<br>(74·8–109)	80·4<br>(65·1–97·5)
1-5	Motorcyclist road injuries	11,300,000<br>(9,850,000–13,300,000)	17,200,000<br>(14,700,000–19,000,000)	16,000,000<br>(13,900,000–18,000,000)	209<br>(182–246)	238<br>(203–264)	200<br>(174–224)
1-6	Motor vehicle road injuries	24,900,000<br>(21,900,000–28,100,000)	26,600,000<br>(24,000,000–29,100,000)	26,000,000<br>(23,000,000–29,000,000)	463<br>(410–520)	374<br>(336–408)	330<br>(292–369)
1-7	Other road injuries	734,000<br>(633,000–914,000)	854,000<br>(740,000–960,000)	846,000<br>(726,000–975,000)	13·9<br>(12–17)	12·1<br>(10·5–13·6)	10·6<br>(9·2–12·2)
1-8	Other transport injuries	4,920,000<br>(4,340,000–5,500,000)	5,080,000<br>(4,450,000–5,560,000)	4,740,000<br>(4,150,000–5,290,000)	92·2<br>(81·8–102)	71·5<br>(62·8–78·1)	60<br>(52·7–66·8)
1-9	Unintentional injuries	124,000,000<br>(111,000,000–136,000,000)	120,000,000<br>(107,000,000–133,000,000)	104,000,000<br>(88,900,000–120,000,000)	2,310<br>(2,070–2,560)	1,750<br>(1,570–1,940)	1,330<br>(1,140–1,540)
1-10	Falls	26,700,000<br>(22,700,000–31,800,000)	33,200,000<br>(27,800,000–39,800,000)	39,400,000<br>(32,500,000–47,500,000)	580<br>(491–687)	500<br>(419–600)	496<br>(411–597)
1-11	Drowning	33,100,000<br>(28,300,000–36,400,000)	17,700,000<br>(16,000,000–19,300,000)	13,000,000<br>(11,700,000–14,700,000)	553<br>(476–607)	259<br>(234–283)	176<br>(157–199)
1-12	Fire, heat, and hot substances	9,240,000<br>(6,970,000–11,500,000)	7,790,000<br>(6,280,000–9,280,000)	7,460,000<br>(5,790,000–9,470,000)	170<br>(130–209)	113<br>(90·9–134)	96·6<br>(75–123)
1-13	Poisonings	5,640,000<br>(4,870,000–6,270,000)	4,760,000<br>(4,010,000–5,210,000)	4,010,000<br>(3,400,000–4,550,000)	100<br>(87·3–110)	68·7<br>(57·8–75·3)	52·7<br>(44·6–60·3)
1-14	Poisoning by carbon monoxide	2,210,000<br>(1,810,000–2,550,000)	2,120,000<br>(1,700,000–2,400,000)	1,840,000<br>(1,460,000–2,100,000)	41<br>(34·2–47·1)	30·1<br>(24·1–34·2)	23·5<br>(18·6–27·2)
1-15	Poisoning by other means	3,440,000<br>(2,730,000–4,040,000)	2,640,000<br>(2,180,000–3,070,000)	2,170,000<br>(1,790,000–2,590,000)	59<br>(47·3–69·1)	38·6<br>(31·8–44·8)	29·3<br>(24·1–34·9)
1-16	Exposure to mechanical forces	11,500,000<br>(9,840,000–13,800,000)	11,600,000<br>(9,220,000–14,200,000)	11,700,000<br>(9,160,000–14,700,000)	222<br>(187–267)	166<br>(132–204)	148<br>(116–186)
1-17	Unintentional firearm injuries	1,830,000<br>(1,390,000–2,140,000)	1,400,000<br>(1,120,000–1,640,000)	1,360,000<br>(1,050,000–1,660,000)	33·2<br>(25·7–38·8)	19·7<br>(15·8–23·3)	17·6<br>(13·5–21·5)
1-18	Other exposure to mechanical forces	9,720,000<br>(8,120,000–11,700,000)	10,200,000<br>(7,900,000–12,700,000)	10,300,000<br>(7,960,000–13,100,000)	189<br>(156–231)	146<br>(113–182)	131<br>(101–166)
1-19	Adverse effects of medical treatment	4,770,000<br>(3,480,000–5,570,000)	4,000,000<br>(3,260,000–4,530,000)	3,840,000<br>(3,180,000–4,360,000)	88·9<br>(67·2–102)	59·6<br>(48·7–67·3)	50·4<br>(41·6–57·6)
1-20	Animal contact	6,740,000<br>(4,480,000–8,080,000)	5,710,000<br>(3,980,000–6,410,000)	5,220,000<br>(3,670,000–6,440,000)	121<br>(81·3–144)	83·1<br>(57·7–93·4)	68·5<br>(48·1–85·3)
1-21	Venomous animal contact	5,430,000<br>(3,420,000–6,650,000)	4,610,000<br>(2,970,000–5,230,000)	4,090,000<br>(2,680,000–5,160,000)	97·2<br>(61·4–118)	66·8<br>(42·9–75·8)	53·6<br>(35·1–68·2)
1-22	Non-venomous animal contact	1,300,000<br>(910,000–1,580,000)	1,110,000<br>(886,000–1,330,000)	1,130,000<br>(914,000–1,420,000)	23·8<br>(17·3–29)	16·3<br>(13–19·6)	15<br>(12·1–18·6)
1-23	Foreign body	9,640,000<br>(8,790,000–10,700,000)	6,740,000<br>(6,180,000–7,410,000)	6,460,000<br>(5,750,000–7,240,000)	164<br>(151–184)	100<br>(92–110)	87·4<br>(77·8–97·8)
1-24	Pulmonary aspiration and foreign body in airway	7,470,000<br>(6,830,000–8,270,000)	5,320,000<br>(5,000,000–5,690,000)	4,710,000<br>(4,260,000–5,230,000)	125<br>(115–138)	79·7<br>(74·8–85·2)	65·2<br>(58·7–72·6)
1-25	Foreign body in eyes	421,000<br>(216,000–746,000)	431,000<br>(219,000–756,000)	590,000<br>(312,000–1,030,000)	8·4<br>(4·5–14·5)	6·1<br>(3·2–10·6)	7·4<br>(3·9–13)
1-26	Foreign body in other body part	1,750,000<br>(1,170,000–2,300,000)	984,000<br>(761,000–1,230,000)	1,150,000<br>(892,000–1,460,000)	30·7<br>(21·2–40·2)	14·3<br>(11·1–18)	14·9<br>(11·6–18·9)
1-27	Environmental heat and cold exposure	3,110,000<br>(2,320,000–3,550,000)	2,930,000<br>(2,360,000–3,360,000)	2,550,000<br>(1,980,000–3,080,000)	62·1<br>(47·3–70·6)	42·3<br>(33·8–48·6)	32<br>(24·7–38·7)
1-28	Exposure to forces of nature	3,260,000<br>(2,960,000–3,590,000)	15,500,000<br>(14,100,000–17,100,000)	961,000<br>(777,000–1,210,000)	57·4<br>(52·1–63·1)	222<br>(201–244)	12·2<br>(9·9–15·4)
1-29	Other unintentional injuries	10,100,000<br>(8,460,000–11,800,000)	9,630,000<br>(7,580,000–11,200,000)	9,010,000<br>(7,360,000–10,900,000)	190<br>(158–224)	137<br>(108–159)	114<br>(94–137)
1-30	Self-harm and interpersonal violence	70,300,000<br>(65,900,000–74,300,000)	69,600,000<br>(66,100,000–73,300,000)	67,900,000<br>(63,400,000–72,800,000)	1,310<br>(1,230–1,390)	964<br>(915–1,020)	856<br>(800–919)
1-31	Self-harm	36,100,000<br>(32,100,000–38,700,000)	36,300,000<br>(33,300,000–39,100,000)	34,100,000<br>(30,800,000–37,500,000)	695<br>(618–743)	503<br>(462–542)	425<br>(383–467)
1-32	Self-harm by firearm	2,420,000<br>(2,140,000–2,730,000)	2,330,000<br>(2,080,000–2,720,000)	2,270,000<br>(2,000,000–2,680,000)	47·2<br>(41·8–52·8)	32·4<br>(28·9–37·8)	28·1<br>(24·8–33·2)
1-33	Self-harm by other specified means	33,700,000<br>(30,000,000–36,200,000)	34,000,000<br>(31,000,000–36,500,000)	31,900,000<br>(28,700,000–35,100,000)	648<br>(577–694)	471<br>(429–505)	397<br>(358–437)
1-34	Interpersonal violence	24,500,000<br>(23,300,000–25,700,000)	27,000,000<br>(25,700,000–28,500,000)	27,000,000<br>(24,900,000–29,200,000)	449<br>(427–472)	374<br>(355–394)	342<br>(317–372)
1-35	Physical violence by firearm	7,460,000<br>(7,200,000–7,800,000)	9,780,000<br>(9,460,000–10,200,000)	10,000,000<br>(9,280,000–10,900,000)	135<br>(130–141)	133<br>(129–138)	128<br>(118–139)
1-36	Physical violence by sharp object	5,980,000<br>(5,030,000–6,450,000)	5,960,000<br>(4,980,000–6,390,000)	5,600,000<br>(4,660,000–6,260,000)	110<br>(92·7–119)	82<br>(68·5–87·9)	70·7<br>(58·8–79·3)
1-37	Sexual violence	1,430,000<br>(947,000–2,030,000)	1,940,000<br>(1,300,000–2,770,000)	2,090,000<br>(1,400,000–2,980,000)	26·5<br>(17·6–37·7)	26·7<br>(17·9–38)	26·2<br>(17·6–37·4)
1-38	Physical violence by other means	9,590,000<br>(8,850,000–10,500,000)	9,350,000<br>(8,590,000–10,200,000)	9,250,000<br>(8,270,000–10,400,000)	178<br>(164–194)	131<br>(120–143)	118<br>(105–132)
1-39	Conflict and terrorism	9,390,000<br>(8,060,000–11,800,000)	5,840,000<br>(4,620,000–7,830,000)	6,260,000<br>(4,980,000–8,210,000)	162<br>(138–204)	81·7<br>(64·8–109)	82·1<br>(65·9–107)
1-40	Police conflict and executions	337,000<br>(159,000–396,000)	392,000<br>(363,000–431,000)	535,000<br>(469,000–603,000)	6·1<br>(2·9–7·2)	5·4<br>(5–5·9)	6·8<br>(6–7·7)

SR Table 1: All-age DALYs counts and age standardized DALYs rates per 100,000 individuals for all causes and level 1 cause groups by location for 1990, 2010, and 2019, both sexes combined, Cambodia

Cause Level	Cause	All-age DALYs			Age-standardized rate of DALYs per 100,000		
		1990	2010	2019	1990	2010	2019
0-0	All causes	7,870,000 (7,280,000–8,470,000)	5,520,000 (4,870,000–6,210,000)	5,360,000 (4,620,000–6,170,000)	69,800 (64,200–75,300)	44,600 (39,700–49,800)	37,500 (32,500–42,800)
1-0	Communicable, maternal, neonatal, and nutritional disorders	5,100,000 (4,600,000–5,680,000)	2,280,000 (1,970,000–2,610,000)	1,600,000 (1,350,000–1,910,000)	34,300 (31,200–37,900)	15,800 (13,800–17,800)	10,200 (8,710–12,000)
1-1	HIV/AIDS and sexually transmitted infections	5,800 (3,030–10,800)	190,000 (132,000–253,000)	78,500 (54,200–113,000)	41·9 (26·5–67·5)	1,400 (988–1,850)	486 (338–676)
1-2	HIV/AIDS	776 (438–1,310)	187,000 (130,000–250,000)	74,800 (50,500–110,000)	7 (4·1–12)	1,370 (964–1,830)	465 (317–655)
1-3	HIV/AIDS - drug-susceptible tuberculosis	381 (223–628)	57,500 (38,100–79,600)	22,700 (15,800–31,700)	3·5 (2·1–5·9)	424 (281–589)	142 (98·8–197)
1-4	HIV/AIDS - multidrug-resistant tuberculosis without extensive drug resistance	1 (0–3)	1,940 (537–5,050)	433 (46–1,560)	0 (0–0)	14·3 (4–36·9)	2·7 (0·3–9·8)
1-5	HIV/AIDS - extensively drug-resistant tuberculosis	0 (0–0)	237 (65–634)	81 (8–296)	0 (0–0)	1·7 (0·5–4·7)	0·5 (0·1–1·8)
1-6	HIV/AIDS resulting in other diseases	395 (211–686)	127,000 (77,500–193,000)	51,600 (30,200–86,300)	3·5 (1·8–6·3)	935 (577–1,410)	319 (192–520)
1-7	Sexually transmitted infections excluding HIV	5,030 (2,290–9,860)	3,050 (1,950–4,780)	3,670 (2,230–5,870)	34·9 (19·9–59·9)	20·9 (13·2–33·1)	21·8 (13·3–34·6)
1-8	Syphilis	3,800 (1,170–8,640)	1,110 (415–2,390)	1,380 (466–3,070)	19·3 (6·6–42·9)	6·5 (2·6–13·8)	7·9 (2·7–17·6)
1-9	Chlamydial infection	173 (108–257)	281 (181–422)	337 (213–514)	2·1 (1·3–3)	2 (1·3–3)	1·9 (1·3–2·9)
1-10	Gonococcal infection	164 (100–256)	254 (163–365)	272 (174–392)	2 (1·2–3)	1·8 (1·2–2·5)	1·6 (1·1–2·3)
1-11	Trichomoniasis	293 (112–602)	469 (184–978)	557 (215–1,170)	3·9 (1·5–8)	3·6 (1·4–7·6)	3·4 (1·3–7·1)
1-12	Genital herpes	264 (83–651)	463 (149–1,140)	584 (185–1,470)	3·7 (1·2–9·4)	3·7 (1·2–9·2)	3·7 (1·2–9·3)
1-13	Other sexually transmitted infections	337 (232–479)	478 (330–665)	546 (370–772)	4 (2·8–5·6)	3·3 (2·3–4·6)	3·2 (2·2–4·5)
1-14	Respiratory infections and tuberculosis	2,380,000 (2,010,000–2,780,000)	1,010,000 (855,000–1,180,000)	734,000 (614,000–880,000)	17,100 (14,800–19,500)	7,600 (6,490–8,780)	5,030 (4,250–5,920)
1-15	Tuberculosis	443,000 (324,000–556,000)	302,000 (235,000–379,000)	221,000 (165,000–289,000)	5,560 (4,120–6,910)	2,640 (2,050–3,280)	1,550 (1,170–2,030)
1-16	Latent tuberculosis infection	0 (0–0)	0 (0–0)	0 (0–0)	0 (0–0)	0 (0–0)	0 (0–0)
1-17	Drug-susceptible tuberculosis	442,000 (324,000–556,000)	293,000 (227,000–371,000)	217,000 (163,000–286,000)	5,550 (4,120–6,900)	2,560 (1,990–3,230)	1,530 (1,140–2,000)
1-18	Multidrug-resistant tuberculosis without extensive drug resistance	746 (65–3,380)	8,030 (2,370–20,100)	3,340 (366–12,700)	9·3 (0·8–42·4)	70·5 (21–176)	23·5 (2·6–89)
1-19	Extensively drug-resistant tuberculosis	0 (0–0)	975 (285–2,510)	621 (64–2,450)	0 (0–0)	8·6 (2·5–22·1)	4·4 (0·5–17·4)
1-20	Lower respiratory infections	1,920,000 (1,580,000–2,310,000)	693,000 (572,000–825,000)	494,000 (405,000–607,000)	11,400 (9,630–13,400)	4,850 (4,040–5,700)	3,370 (2,810–4,020)
1-21	Upper respiratory infections	10,200 (5,410–15,900)	10,800 (6,690–16,800)	11,900 (7,440–18,700)	84 (46·9–126)	71·3 (44·2–111)	70·3 (43·8–110)
1-22	Otitis media	6,230 (3,340–11,800)	5,850 (3,420–9,500)	5,930 (3,430–9,800)	49·2 (28–84·3)	37·9 (22·1–61·5)	34·7 (20·3–56·9)
1-23	Enteric infections	565,000 (380,000–818,000)	150,000 (112,000–203,000)	105,000 (76,200–146,000)	4,020 (2,740–5,630)	1,100 (827–1,490)	709 (518–1,010)
1-24	Diarrhoeal diseases	520,000 (338,000–772,000)	117,000 (84,600–163,000)	79,700 (56,000–112,000)	3,700 (2,430–5,250)	909 (650–1,280)	564 (396–826)
1-25	Typhoid and paratyphoid	38,700 (17,200–73,100)	29,400 (13,200–55,000)	22,800 (10,200–42,100)	281 (125–526)	172 (78·7–318)	129 (57·7–235)
1-26	Typhoid fever	36,700 (16,400–69,400)	28,900 (13,100–54,200)	20,500 (9,080–37,800)	266 (120–500)	169 (78–313)	116 (51·4–212)
1-27	Paratyphoid fever	2,030 (736–4,490)	467 (173–988)	2,330 (901–5,080)	14·4 (5·4–31·4)	2·7 (1–5·8)	13·1 (5·1–28·7)
1-28	Invasive non-typhoidal Salmonella (iNTS)	5,320 (2,870–8,980)	3,280 (1,800–5,650)	2,610 (1,370–4,230)	38·5 (20·8–63·7)	20·8 (11·5–35·2)	15·4 (8·2–24·9)
1-29	Other intestinal infectious diseases	1,010 (622–1,470)	296 (138–486)	191 (92–320)	5·9 (3·8–8·4)	1·8 (0·9–3)	1·1 (0·6–1·9)
1-30	Neglected tropical diseases and malaria	111,000 (70,600–177,000)	46,500 (31,400–71,200)	23,700 (16,800–31,900)	945 (628–1,460)	305 (209–460)	143 (102–191)
1-31	Malaria	53,000 (21,600–118,000)	22,900 (10,900–45,600)	4,690 (1,820–10,600)	407 (172–898)	145 (68·8–287)	27·3 (10·6–62·1)
1-32	Chagas disease	0 (0–0)	0 (0–0)	0 (0–0)	0 (0–0)	0 (0–0)	0 (0–0)
1-33	Leishmaniasis	0 (0–0)	0 (0–0)	0 (0–0)	0 (0–0)	0 (0–0)	0 (0–0)
1-34	Visceral leishmaniasis	0 (0–0)	0 (0–0)	0 (0–0)	0 (0–0)	0 (0–0)	0 (0–0)
1-35	Cutaneous and mucocutaneous leishmaniasis	0 (0–0)	0 (0–0)	0 (0–0)	0 (0–0)	0 (0–0)	0 (0–0)
1-36	African trypanosomiasis	0 (0–0)	0 (0–0)	0 (0–0)	0 (0–0)	0 (0–0)	0 (0–0)
1-37	Schistosomiasis	1,440 (923–2,340)	983 (609–1,440)	849 (494–1,290)	21·9 (14·3–33·4)	9 (5·6–13·2)	6·1 (3·7–9·1)
1-38	Cysticercosis	1,310 (713–2,120)	1,590 (836–2,710)	1,810 (930–3,140)	24·5 (13·2–39·3)	16·5 (8·9–28·3)	14·2 (7·3–24·2)
1-39	Cystic echinococcosis	3 (1–9)	5 (1–16)	33 (20–51)	0 (0–0·1)	0 (0–0·1)	0·2 (0·1–0·3)
1-40	Lymphatic filariasis	9,530 (5,770–14,800)	0 (0–0)	0 (0–0)	118 (72–179)	0 (0–0)	0 (0–0)
1-41	Onchocerciasis	0 (0–0)	0 (0–0)	0 (0–0)	0 (0–0)	0 (0–0)	0 (0–0)
1-42	Trachoma	610 (310–1,080)	68 (37–116)	61 (29–111)	15·6 (8·1–27·5)	0·9 (0·5–1·6)	0·6 (0·3–1)
1-43	Dengue	6,570 (2,230–15,400)	5,860 (3,230–10,500)	5,480 (3,210–8,970)	44·9 (18·3–99)	37·2 (20·7–65·7)	31·9 (18·9–51·3)
1-44	Yellow fever	0 (0–0)	0 (0–0)	0 (0–0)	0 (0–0)	0 (0–0)	0 (0–0)
1-45	Rabies	9,620 (194–20,900)	2,810 (67–5,840)	2,200 (54–4,190)	74·7 (1·6–153)	18·6 (0·5–37·2)	13·2 (0·3–25)
1-46	Intestinal nematode infections	21,700 (14,000–32,400)	5,890 (3,660–9,010)	2,700 (1,650–4,150)	188 (119–283)	36·7 (23–56·2)	15·3 (9·4–23·4)
1-47	Ascariasis	5,650 (3,720–8,420)	567 (328–895)	334 (156–565)	43·7 (28·3–66·9)	3·5 (2·1–5·5)	2 (0·9–3·3)
1-48	Trichuriasis	21 (10–38)	0 (0–0)	0 (0–0)	0·2 (0·1–0·3)	0 (0–0)	0 (0–0)

1·49	Hookworm disease	16,000 (9,730–24,300)	5,320 (3,070–8,380)	2,360 (1,330–3,760)	144 (88·5–219)	33·2 (19·1–52·2)	13·3 (7·5–21·3)
1·50	Food-borne trematodiases	0 (0–0)	0 (0–0)	0 (0–0)	0 (0–0)	0 (0–0)	0 (0–0)
1·51	Leprosy	110 (66–169)	60 (36–92)	61 (37–94)	1·8 (1·1–2·7)	0·6 (0·3–0·8)	0·4 (0·3–0·7)
1·52	Ebola virus disease	0 (0–0)	0 (0–0)	0 (0–0)	0 (0–0)	0 (0–0)	0 (0–0)
1·53	Zika virus disease	0 (0–0)	0 (0–0)	0 (0–0)	0 (0–0)	0 (0–0)	0 (0–0)
1·54	Guinea worm disease	0 (0–0)	0 (0–0)	0 (0–0)	0 (0–0)	0 (0–0)	0 (0–0)
1·55	Other neglected tropical diseases	6,640 (4,310–9,770)	6,270 (4,170–9,180)	5,790 (3,810–8,400)	48·5 (32·3–69·5)	40·4 (27·1–59)	34·2 (22·6–49·5)
1·56	Other infectious diseases	677,000 (425,000–1,060,000)	169,000 (118,000–247,000)	87,900 (64,700–129,000)	3,910 (2,600–5,930)	1,070 (764–1,530)	528 (395–759)
1·57	Meningitis	82,900 (58,200–117,000)	27,200 (18,600–38,900)	11,400 (7,930–16,800)	435 (313–606)	159 (109–226)	65·3 (45·5–95·4)
1·58	Encephalitis	21,000 (10,900–35,900)	11,700 (8,270–16,700)	10,800 (8,000–14,700)	131 (77·1–212)	74·3 (53·3–105)	63·7 (47·5–86·9)
1·59	Diphtheria	5,080 (2,090–10,100)	99 (47–175)	50 (27–83)	29·9 (13·6–56·8)	0·6 (0·3–1·1)	0·3 (0·2–0·5)
1·60	Whooping cough	102,000 (10,300–318,000)	24,800 (2,440–75,700)	15,400 (1,800–48,200)	537 (54–1,670)	144 (14·2–441)	86·7 (10·2–272)
1·61	Tetanus	170,000 (81,000–327,000)	11,800 (5,390–23,600)	5,550 (2,530–10,800)	1,030 (586–1,880)	82·5 (35·5–160)	35·2 (15·8–67·6)
1·62	Measles	258,000 (84,800–573,000)	52,200 (18,500–112,000)	10,400 (3,460–24,000)	1,400 (458–3,100)	307 (109–656)	58·7 (19·5–135)
1·63	Varicella and herpes zoster	4,310 (2,580–6,950)	2,570 (1,680–3,900)	2,090 (1,410–3,070)	35 (24·3–49·8)	19·2 (13·6–27·2)	13·9 (9·9–19·5)
1·64	Acute hepatitis	13,000 (9,940–16,300)	18,700 (15,000–23,300)	13,100 (9,350–19,100)	146 (112–180)	148 (120–183)	85·7 (61·6–125)
1·65	Acute hepatitis A	7,930 (4,730–10,800)	8,780 (4,210–13,700)	5,140 (2,450–8,860)	82·5 (45·9–113)	66·7 (30·2–103)	33·3 (15·6–57·7)
1·66	Acute hepatitis B	2,800 (1,600–4,890)	6,630 (3,410–10,300)	5,660 (2,860–9,190)	36·9 (21·2–61·6)	54·7 (28·4–85)	37·6 (19–61·8)
1·67	Acute hepatitis C	2,000 (1,090–3,630)	2,790 (1,510–5,150)	1,860 (972–3,590)	24·1 (13·1–41·4)	22·5 (12·1–41·9)	12·2 (6·5–23·6)
1·68	Acute hepatitis E	222 (115–409)	532 (280–868)	427 (210–737)	2·2 (1·1–3·8)	3·8 (1·9–6·1)	2·7 (1·3–4·6)
1·69	Other unspecified infectious diseases	20,200 (15,200–27,600)	20,000 (13,200–26,900)	19,100 (12,800–26,000)	171 (132–233)	140 (93–184)	118 (79·8–160)
1·70	Maternal and neonatal disorders	950,000 (773,000–1,130,000)	588,000 (486,000–698,000)	457,000 (357,000–592,000)	5,240 (4,340–6,190)	3,400 (2,820–4,040)	2,600 (2,030–3,370)
1·71	Maternal disorders	96,000 (74,800–123,000)	39,300 (30,700–48,400)	28,400 (20,900–38,100)	1,100 (860–1,410)	268 (209–331)	160 (117–215)
1·72	Maternal haemorrhage	10,600 (7,600–14,500)	9,980 (7,450–12,600)	7,540 (5,440–10,300)	118 (84·7–160)	68·6 (51·2–87)	42·4 (30·6–58·2)
1·73	Maternal sepsis and other pregnancy related infections	13,500 (9,580–19,000)	4,590 (3,350–6,000)	2,520 (1,720–3,450)	148 (106–206)	30·9 (22·3–40·6)	14·1 (9·8–19·3)
1·74	Maternal hypertensive disorders	5,180 (3,840–7,070)	5,060 (3,770–6,440)	4,000 (2,860–5,330)	53·1 (39·6–71·6)	32·6 (24·4–41·1)	21·6 (15·4–28·9)
1·75	Maternal obstructed labour and uterine rupture	142 (76–243)	265 (179–371)	456 (308–649)	1·5 (0·8–2·6)	1·8 (1·2–2·5)	2·6 (1·7–3·7)
1·76	Maternal abortive outcome	61,800 (46,400–80,600)	9,780 (7,200–12,900)	4,520 (3,080–6,410)	727 (545–954)	68·9 (50·6–92·1)	26·6 (18·1–38·3)
1·77	Ectopic pregnancy	401 (283–566)	459 (328–619)	414 (281–585)	4·3 (3·1–6)	3·1 (2·2–4·1)	2·3 (1·5–3·2)
1·78	Indirect maternal deaths	153 (102–218)	957 (680–1,270)	1,290 (861–1,810)	1·6 (1·1–2·3)	6·4 (4·6–8·5)	7·1 (4·8–10)
1·79	Late maternal deaths	809 (573–1,140)	560 (393–791)	546 (359–794)	9·2 (6·5–13·1)	3·8 (2·7–5·4)	3·1 (2–4·5)
1·80	Maternal deaths aggravated by HIV/AIDS	5 (2–9)	173 (83–277)	87 (37–159)	0 (0–0·1)	1·3 (0·6–2)	0·5 (0·2–1)
1·81	Other maternal disorders	3,370 (2,310–4,800)	7,450 (5,350–9,720)	7,020 (4,880–9,690)	37·3 (25·6–52·4)	51·1 (36·4–66·9)	39·7 (27·7–55·3)
1·82	Neonatal disorders	854,000 (687,000–1,030,000)	549,000 (452,000–652,000)	429,000 (331,000–558,000)	4,140 (3,350–4,980)	3,140 (2,580–3,720)	2,440 (1,880–3,180)
1·83	Neonatal preterm birth	455,000 (353,000–575,000)	286,000 (222,000–353,000)	214,000 (156,000–291,000)	2,220 (1,740–2,800)	1,630 (1,270–2,020)	1,220 (889–1,660)
1·84	Neonatal encephalopathy due to birth asphyxia and trauma	232,000 (151,000–328,000)	150,000 (103,000–215,000)	112,000 (71,200–167,000)	1,110 (726–1,570)	852 (587–1,220)	639 (406–949)
1·85	Neonatal sepsis and other neonatal infections	83,000 (46,900–131,000)	65,000 (41,400–100,000)	66,000 (45,100–99,600)	400 (227–631)	372 (238–574)	377 (258–569)
1·86	Haemolytic disease and other neonatal jaundice	13,300 (8,540–20,000)	6,470 (4,400–9,400)	4,620 (3,200–6,890)	68·1 (46·2–101)	37·9 (26·1–54·6)	26·4 (18·4–39·4)
1·87	Other neonatal disorders	71,200 (44,300–99,600)	42,300 (27,400–62,200)	32,300 (20,200–48,500)	343 (213–477)	241 (156–354)	184 (115–276)
1·88	Nutritional deficiencies	413,000 (198,000–645,000)	126,000 (92,000–171,000)	109,000 (77,100–151,000)	3,030 (1,550–4,460)	896 (663–1,200)	687 (495–941)
1·89	Protein-energy malnutrition	311,000 (98,100–531,000)	35,200 (26,100–49,000)	23,100 (17,800–28,900)	2,100 (678–3,450)	262 (201–346)	160 (126–197)
1·90	Iodine deficiency	5,580 (3,520–8,720)	1,450 (669–2,890)	2,350 (1,420–3,750)	66·9 (42·1–105)	10·8 (5–21·3)	14·4 (8·7–22·9)
1·91	Vitamin A deficiency	11,600 (7,460–17,300)	4,860 (3,040–7,500)	3,390 (2,010–5,380)	66·6 (42·7–99·4)	29 (18–44·8)	19·3 (11·4–30·5)
1·92	Dietary iron deficiency	76,100 (49,000–112,000)	81,300 (52,500–120,000)	76,600 (49,500–113,000)	683 (451–993)	562 (366–830)	468 (303–685)
1·93	Other nutritional deficiencies	9,170 (3,410–14,800)	3,630 (2,660–5,030)	3,320 (2,520–4,460)	114 (41·6–190)	33 (24·6–44·4)	25 (19·5–33·6)
1·0	Non-communicable diseases	2,140,000 (1,730,000–2,520,000)	2,690,000 (2,350,000–3,090,000)	3,240,000 (2,760,000–3,760,000)	29,800 (26,000–33,400)	24,900 (22,100–28,200)	24,000 (20,600–27,500)
1·1	Neoplasms	232,000 (180,000–281,000)	329,000 (283,000–383,000)	433,000 (348,000–518,000)	3,570 (2,970–4,150)	3,170 (2,750–3,660)	3,230 (2,610–3,830)
1·2	Lip and oral cavity cancer	3,340 (2,440–4,390)	6,110 (4,590–7,770)	8,740 (6,450–11,400)	64·5 (47·8–85·2)	64·2 (48·2–81·5)	68 (50·6–88·4)
1·3	Nasopharynx cancer	4,170 (2,980–5,280)	6,200 (4,860–7,750)	7,930 (5,900–10,300)	70·6 (52–88·1)	58·5 (46·4–72·8)	57·1 (42·4–73·7)
1·4	Other pharynx cancer	894 (680–1,260)	1,410 (1,120–1,800)	2,060 (1,550–2,650)	17·4 (13·3–24·4)	14·8 (11·9–18·8)	15·8 (12–20·2)
1·5	Oesophageal cancer	4,690 (3,330–6,370)	6,620 (5,340–8,730)	8,980 (6,710–12,400)	93·9 (68·3–125)	70·7 (58–94·6)	70·3 (53·5–97·2)
1·6	Stomach cancer	20,300 (15,400–24,800)	22,500 (18,500–26,900)	27,100 (21,400–33,900)	396 (305–481)	239 (198–282)	214 (169–261)
1·7	Colon and rectum cancer	11,600 (8,650–15,000)	27,400 (22,700–32,300)	42,100 (32,900–52,100)	226 (172–293)	289 (240–338)	329 (260–401)

1-8	Liver cancer	31,700 (22,000–49,500)	35,200 (28,000–43,700)	40,100 (30,500–51,200)	371 (291–482)	303 (250–363)	284 (218–356)
1-9	Liver cancer due to hepatitis B	9,470 (7,110–12,200)	13,100 (10,100–16,600)	14,200 (10,100–19,600)	135 (102–177)	110 (84–142)	96·1 (68·8–133)
1-10	Liver cancer due to hepatitis C	4,970 (3,350–6,700)	7,470 (5,380–9,970)	9,990 (6,690–13,700)	105 (73·5–141)	84·6 (62·1–111)	82·3 (56·8–110)
1-11	Liver cancer due to alcohol use	1,560 (989–2,320)	3,110 (1,940–4,570)	5,020 (3,130–7,470)	31·3 (20–46·7)	33·3 (20·9–48·5)	39·1 (24·7–57·1)
1-12	Liver cancer due to NASH	1,020 (680–1,490)	1,670 (1,150–2,360)	2,180 (1,430–3,200)	17·2 (11·7–25)	15·9 (10·9–22·4)	16·3 (10·8–23·9)
1-13	Gallbladder and biliary tract cancer	1,840 (1,090–2,840)	2,620 (1,980–4,290)	3,710 (2,660–6,250)	38·4 (23·5–59·8)	29·3 (22·4–48·9)	30·3 (22–51·3)
1-14	Pancreatic cancer	2,660 (1,970–3,510)	6,460 (5,380–7,770)	10,700 (8,380–13,200)	54·4 (40·2–70·9)	70·6 (59·3–84·6)	85·3 (67·5–104)
1-15	Larynx cancer	2,390 (1,880–2,940)	3,330 (2,660–4,140)	4,850 (3,600–6,360)	48·7 (38·7–59·7)	36·6 (29·4–45·9)	38·5 (28·8–50·4)
1-16	Tracheal, bronchus, and lung cancer	29,900 (22,900–38,300)	52,200 (43,200–63,700)	75,900 (58,600–94,200)	621 (477–781)	578 (481–712)	606 (474–749)
1-17	Malignant skin melanoma	507 (290–978)	834 (556–1,350)	1,140 (742–1,720)	8·3 (4·9–15·3)	7·9 (5·4–12·5)	8·4 (5·5–12·6)
1-18	Non-melanoma skin cancer	762 (588–1,080)	1,650 (1,290–2,060)	2,340 (1,700–2,950)	15·5 (11·7–21·5)	18·2 (13·9–22·7)	19·1 (13·8–23·9)
1-19	Non-melanoma skin cancer (squamous-cell carcinoma)	762 (588–1,080)	1,650 (1,290–2,060)	2,340 (1,700–2,950)	15·5 (11·7–21·5)	18·2 (13·9–22·7)	19·1 (13·7–23·9)
1-20	Non-melanoma skin cancer (basal-cell carcinoma)	0 (0–0)	0 (0–0)	0 (0–0)	0 (0–0)	0 (0–0)	0 (0–0)
1-21	Breast cancer	12,500 (9,520–16,600)	25,400 (20,500–30,900)	34,900 (26,300–44,300)	213 (161–281)	236 (192–284)	248 (189–311)
1-22	Cervical cancer	16,100 (8,770–22,800)	20,000 (13,600–28,800)	23,700 (16,700–35,600)	276 (154–394)	186 (129–275)	168 (119–255)
1-23	Uterine cancer	2,660 (1,520–4,050)	4,600 (2,670–6,070)	6,210 (3,670–8,410)	51·2 (30·4–77·6)	47·3 (28·9–61·7)	47 (29–62·8)
1-24	Ovarian cancer	2,770 (1,440–8,310)	8,250 (5,440–14,500)	12,500 (8,110–21,100)	48·1 (25·9–137)	77·4 (52·6–135)	89·4 (58·8–150)
1-25	Prostate cancer	3,700 (2,760–4,630)	8,120 (5,950–10,200)	12,300 (8,720–15,400)	98·3 (74·1–123)	110 (81·2–138)	120 (84·8–148)
1-26	Testicular cancer	310 (201–429)	598 (438–780)	785 (583–1,050)	3·4 (2·2–4·6)	4·3 (3·2–5·4)	4·7 (3·6–6·2)
1-27	Kidney cancer	1,640 (1,110–2,390)	2,850 (2,280–3,510)	4,130 (3,190–5,140)	22·1 (15·7–30·1)	26·9 (21·3–33·8)	30·6 (23·5–37·9)
1-28	Bladder cancer	1,800 (1,360–2,360)	2,980 (2,370–3,620)	4,410 (3,330–5,520)	40·8 (30·9–53·1)	35·7 (28·4–43·3)	38·3 (29·1–47·6)
1-29	Brain and nervous system cancer	9,690 (4,560–19,400)	12,400 (7,740–17,600)	15,600 (9,770–21,100)	98·7 (54–176)	95·9 (60·5–134)	101 (63·9–137)
1-30	Thyroid cancer	1,760 (1,090–2,340)	3,010 (1,940–3,900)	4,170 (2,680–5,480)	33 (21–42·7)	31·5 (20·6–40·4)	32·5 (20·9–42·2)
1-31	Mesothelioma	317 (169–589)	626 (427–903)	862 (576–1,210)	5·1 (2·9–8·7)	5·6 (3·9–7·8)	6 (4·1–8·2)
1-32	Hodgkin lymphoma	1,650 (1,250–2,340)	1,650 (1,030–2,940)	1,670 (1,010–3,090)	20·1 (15·3–28·8)	12·6 (8–22·2)	10·7 (6·6–19·6)
1-33	Non-Hodgkin's lymphoma	5,960 (3,690–8,620)	8,840 (7,240–10,900)	11,800 (9,370–14,500)	74·4 (53·9–99·4)	75·7 (63·4–91·4)	82·5 (65·9–100)
1-34	Multiple myeloma	1,020 (704–1,710)	1,930 (1,420–2,980)	3,000 (2,100–4,550)	21·1 (14·7–34·6)	21·1 (15·6–32·6)	23·7 (16·7–35·9)
1-35	Leukaemia	40,200 (19,600–67,000)	33,400 (23,900–44,300)	34,000 (25,900–43,100)	357 (202–555)	244 (181–317)	219 (170–276)
1-36	Acute lymphoid leukaemia	6,780 (2,300–15,100)	6,380 (3,710–9,940)	6,200 (4,060–9,200)	47·8 (18·4–101)	40 (23·8–61·8)	36·5 (24·2–53·8)
1-37	Chronic lymphoid leukaemia	192 (115–270)	395 (266–518)	612 (429–816)	3·9 (2·4–5·5)	4·3 (3–5·7)	5 (3·5–6·5)
1-38	Acute myeloid leukaemia	2,730 (1,310–6,530)	4,020 (2,560–6,870)	5,040 (3,240–8,170)	27 (14·8–56·7)	29·3 (18·8–49·9)	31·9 (20·8–51·8)
1-39	Chronic myeloid leukaemia	1,560 (464–3,290)	1,590 (851–2,380)	1,900 (1,080–2,750)	18·2 (7·2–33·6)	13·4 (7·5–19·3)	13·2 (7·7–18·8)
1-40	Other leukaemia	28,900 (9,760–50,900)	21,000 (12,700–29,600)	20,200 (13,800–26,800)	260 (114–425)	157 (99·2–215)	132 (92·2–173)
1-41	Other neoplasms	302 (229–517)	595 (455–860)	923 (674–1,320)	5·3 (3·8–8·8)	6·3 (4·8–9·2)	7·5 (5·4–10·7)
1-42	Myelodysplastic, myeloproliferative, and other haematopoietic neoplasms	302 (229–517)	595 (455–860)	923 (674–1,320)	5·3 (3·8–8·8)	6·3 (4·8–9·2)	7·5 (5·4–10·7)
1-43	Benign and in situ intestinal neoplasms	0 (0–0)	0 (0–0)	0 (0–0)	0 (0–0)	0 (0–0)	0 (0–0)
1-44	Benign and in situ cervical and uterine neoplasms	0 (0–0)	0 (0–0)	0 (0–0)	0 (0–0)	0 (0–0)	0 (0–0)
1-45	Other benign and in situ neoplasms	0 (0–0)	0 (0–0)	0 (0–0)	0 (0–0)	0 (0–0)	0 (0–0)
1-46	Cardiovascular diseases	409,000 (357,000–467,000)	574,000 (506,000–651,000)	734,000 (608,000–856,000)	8,110 (7,090–9,240)	6,550 (5,830–7,400)	6,260 (5,270–7,190)
1-47	Rheumatic heart disease	27,500 (19,400–36,000)	21,000 (15,500–26,900)	19,100 (13,400–26,100)	326 (236–425)	158 (117–202)	120 (84·5–164)
1-48	Ischaemic heart disease	122,000 (99,300–154,000)	194,000 (162,000–231,000)	262,000 (207,000–321,000)	2,620 (2,160–3,270)	2,260 (1,910–2,650)	2,250 (1,810–2,720)
1-49	Stroke	204,000 (175,000–241,000)	282,000 (246,000–325,000)	354,000 (292,000–419,000)	4,140 (3,570–4,800)	3,270 (2,860–3,750)	3,060 (2,530–3,580)
1-50	Ischaemic stroke	41,000 (34,100–50,100)	72,900 (60,500–86,200)	103,000 (84,300–121,000)	1,060 (876–1,320)	1,000 (841–1,180)	1,030 (854–1,200)
1-51	Intracerebral haemorrhage	146,000 (122,000–175,000)	189,000 (163,000–224,000)	230,000 (185,000–278,000)	2,790 (2,370–3,280)	2,070 (1,800–2,420)	1,870 (1,510–2,240)
1-52	Subarachnoid haemorrhage	17,500 (8,960–25,200)	19,400 (13,900–26,600)	21,800 (16,000–30,800)	291 (157–440)	188 (131–262)	161 (118–227)
1-53	Hypertensive heart disease	30,300 (17,100–41,400)	46,200 (28,700–58,900)	60,000 (36,800–78,000)	682 (395–913)	553 (352–698)	524 (327–667)
1-54	Non-rheumatic valvular heart disease	513 (336–763)	765 (577–1,150)	1,020 (755–1,530)	10·3 (7·1–15)	8·3 (6·4–12·5)	8·4 (6·2–12·5)
1-55	Non-rheumatic calcific aortic valvular heart disease	234 (135–404)	391 (286–632)	556 (402–870)	4·8 (2·9–8)	4·3 (3·2–6·8)	4·5 (3·3–7·1)
1-56	Non-rheumatic degenerative mitral valvular heart disease	212 (124–371)	288 (187–491)	371 (233–665)	4·3 (2·7–7)	3·2 (2·2–5·3)	3·1 (2–5·4)
1-57	Other non-rheumatic valvular heart diseases	67 (22–138)	86 (28–147)	97 (29–172)	1·2 (0·3–2·2)	0·8 (0·2–1·4)	0·7 (0·2–1·3)
1-58	Cardiomyopathy and myocarditis	8,370 (4,080–20,100)	9,560 (6,730–16,000)	11,300 (8,200–17,500)	96·6 (60·9–178)	88·2 (66·9–132)	85·9 (65·1–126)
1-59	Myocarditis	2,360 (679–7,840)	1,940 (1,110–3,810)	1,850 (1,140–3,150)	16·8 (7·2–44)	14·1 (9·1–24·8)	12·3 (8·2–19·2)
1-60	Alcoholic cardiomyopathy	257 (108–567)	363 (214–711)	473 (271–967)	4·3 (1·9–9·4)	3·3 (2–6·5)	3·3 (2–6·7)

1-61	Other cardiomyopathy	5,750 (2,890–13,500)	7,260 (5,100–12,100)	8,930 (6,500–13,900)	75·4 (47·7–138)	70·8 (52·9–108)	70·2 (53·1–104)
1-62	Atrial fibrillation and flutter	2,940 (2,120–3,890)	6,540 (4,900–8,600)	9,440 (7,040–12,400)	82·1 (60·6–108)	91·8 (69·7–119)	95·4 (72·3–124)
1-63	Aortic aneurysm	897 (587–1,370)	1,820 (1,410–2,490)	2,730 (2,060–3,720)	19·9 (13·4–29·4)	21·3 (16·7–28·8)	23·5 (17·8–31·7)
1-64	Peripheral vascular disease	398 (215–677)	766 (429–1,250)	1,070 (610–1,730)	10·9 (5·9–18·6)	10·7 (6–17·5)	10·5 (6–16·8)
1-65	Endocarditis	2,200 (1,330–3,780)	3,120 (2,110–4,500)	3,760 (2,470–5,570)	23·7 (14·5–36·4)	24·9 (16·8–36·3)	25 (16·5–37·4)
1-66	Chronic respiratory diseases	96,700 (81,300–111,000)	121,000 (107,000–137,000)	150,000 (126,000–172,000)	1,730 (1,480–1,970)	1,340 (1,170–1,510)	1,260 (1,060–1,440)
1-67	Chronic obstructive pulmonary disease	44,500 (37,800–51,300)	70,500 (61,200–79,400)	94,400 (78,400–110,000)	1,000 (839–1,160)	863 (741–972)	850 (705–977)
1-68	Pneumoconiosis	57 (39–76)	96 (73–125)	137 (102–183)	1·2 (0·8–1·6)	1·1 (0·8–1·4)	1·1 (0·8–1·5)
1-69	Silicosis	26 (14–39)	52 (34–77)	83 (53–125)	0·5 (0·3–0·8)	0·6 (0·4–0·8)	0·7 (0·4–1)
1-70	Asbestosis	7 (4–12)	11 (6–17)	15 (9–23)	0·1 (0·1–0·2)	0·1 (0·1–0·2)	0·1 (0·1–0·2)
1-71	Coal workers pneumoconiosis	5 (2–10)	7 (2–13)	8 (3–15)	0·1 (0–0·2)	0·1 (0–0·1)	0·1 (0–0·1)
1-72	Other pneumoconiosis	20 (9–33)	26 (15–40)	31 (18–49)	0·4 (0·2–0·7)	0·3 (0·2–0·5)	0·3 (0·2–0·4)
1-73	Asthma	49,400 (37,800–60,100)	45,600 (36,900–54,800)	48,900 (39,100–60,100)	696 (531–827)	436 (352–522)	368 (296–448)
1-74	Interstitial lung disease and pulmonary sarcoidosis	562 (293–1,110)	1,170 (615–2,290)	1,650 (841–3,350)	11·8 (5·8–23·6)	13·3 (6·8–26·8)	13·8 (6·9–28·3)
1-75	Other chronic respiratory diseases	2,140 (1,370–4,700)	3,880 (3,010–5,240)	4,960 (3,930–6,400)	21·9 (14·9–41·8)	27·2 (21·4–36·6)	29·9 (23·9–38·6)
1-76	Digestive diseases	332,000 (253,000–408,000)	372,000 (316,000–430,000)	407,000 (331,000–490,000)	4,690 (3,850–5,460)	3,380 (2,900–3,870)	2,930 (2,390–3,500)
1-77	Cirrhosis and other chronic liver diseases	189,000 (146,000–230,000)	246,000 (203,000–293,000)	283,000 (224,000–353,000)	2,850 (2,290–3,370)	2,200 (1,840–2,590)	1,980 (1,580–2,460)
1-78	Cirrhosis and other chronic liver diseases due to hepatitis B	59,900 (43,600–77,700)	83,500 (63,200–107,000)	66,000 (44,500–93,800)	985 (732–1,260)	758 (579–963)	460 (315–644)
1-79	Cirrhosis and other chronic liver diseases due to hepatitis C	45,000 (32,200–61,300)	61,900 (45,500–83,200)	84,800 (58,300–116,000)	738 (528–986)	562 (416–743)	594 (413–802)
1-80	Cirrhosis and other chronic liver diseases due to alcohol use	23,600 (15,800–33,500)	37,400 (25,500–52,700)	55,400 (36,700–79,600)	422 (281–596)	359 (245–500)	401 (265–576)
1-81	Cirrhosis and other chronic liver diseases due to NAFLD	11,400 (7,540–16,400)	16,500 (11,000–23,800)	24,100 (15,800–35,600)	196 (132–279)	159 (108–228)	179 (120–257)
1-82	Cirrhosis and other chronic liver diseases due to other causes	48,900 (32,000–71,000)	46,800 (35,800–60,100)	52,200 (38,300–68,800)	508 (360–686)	359 (269–456)	349 (259–455)
1-83	Upper digestive system diseases	68,900 (53,000–85,700)	63,000 (52,300–75,000)	62,700 (48,400–81,600)	1,160 (927–1,390)	666 (560–781)	510 (400–653)
1-84	Peptic ulcer disease	60,000 (45,600–74,900)	52,400 (43,200–62,900)	50,400 (38,100–67,900)	1,050 (828–1,260)	576 (480–681)	427 (329–562)
1-85	Gastritis and duodenitis	6,030 (3,240–9,630)	5,630 (3,700–7,510)	5,930 (4,110–8,170)	73·6 (43·9–102)	49·3 (32·3–65·1)	42·1 (29·3–56·7)
1-86	Gastro-oesophageal reflux disease (GERD)	2,840 (1,450–5,130)	5,020 (2,570–9,150)	6,310 (3,210–11,500)	40·9 (20·7–73·9)	41 (21–75·2)	40·9 (20·8–74·9)
1-87	Appendicitis	8,110 (2,390–13,800)	5,220 (3,060–7,170)	4,050 (2,540–5,600)	67·2 (24–106)	35·6 (22·1–48·6)	25 (15·9–34·1)
1-88	Paralytic ileus and intestinal obstruction	37,000 (18,400–65,300)	29,300 (19,100–41,700)	26,500 (17,200–38,700)	255 (156–397)	214 (145–292)	179 (123–254)
1-89	Inguinal, femoral, and abdominal hernia	11,300 (3,790–25,000)	6,720 (4,580–9,990)	6,630 (4,900–8,960)	85·7 (41·7–155)	53·8 (39·3–74·2)	46·5 (34·9–61)
1-90	Inflammatory bowel disease	1,170 (547–2,170)	1,130 (793–1,490)	1,220 (890–1,550)	14·5 (8·3–21·4)	10·2 (7·2–13)	9 (6·4–11·3)
1-91	Vascular intestinal disorders	530 (369–785)	993 (802–1,220)	1,330 (1,050–1,640)	11·8 (8·8–15·4)	12·6 (10·2–15·3)	12·6 (10–15·5)
1-92	Gallbladder and biliary diseases	7,760 (4,770–10,800)	9,770 (7,100–13,100)	11,400 (8,250–15,400)	124 (84·4–161)	96·7 (71·1–129)	87·3 (65·4–117)
1-93	Pancreatitis	4,000 (2,680–6,680)	5,290 (3,630–8,980)	6,150 (3,960–10,700)	60·3 (43–94)	46·7 (32·8–77·2)	42·8 (28·4–72·9)
1-94	Other digestive diseases	4,570 (2,020–9,230)	4,460 (3,440–5,530)	4,990 (3,720–6,540)	58 (35·7–87·1)	43·9 (35–54·5)	39·7 (29·9–51·6)
1-95	Neurological disorders	89,000 (39,200–163,000)	143,000 (61,000–268,000)	176,000 (78,800–320,000)	1,250 (625–2,200)	1,260 (627–2,240)	1,290 (652–2,280)
1-96	Alzheimer's disease and other dementias	9,700 (3,920–23,400)	22,400 (8,580–53,600)	33,800 (13,100–79,500)	337 (136–805)	373 (146–884)	404 (157–945)
1-97	Parkinson's disease	2,870 (2,220–3,630)	5,650 (4,540–6,660)	8,390 (6,660–9,970)	79·9 (62·1–102)	79·7 (63·9–93·7)	86·2 (68·8–102)
1-98	Idiopathic epilepsy	17,400 (7,580–29,900)	18,700 (8,280–32,400)	20,700 (9,220–37,500)	154 (74·6–262)	127 (56·1–219)	126 (55·7–228)
1-99	Multiple sclerosis	344 (244–576)	573 (448–823)	749 (569–1,060)	5·4 (3·8–8·7)	4·9 (3·8–7·1)	5 (3·8–7·1)
1-100	Motor neuron disease	209 (161–276)	280 (214–362)	394 (282–528)	2·8 (2·1–3·6)	2·4 (1·8–3·2)	2·7 (1·9–3·6)
1-101	Headache disorders	54,600 (7,330–128,000)	89,900 (12,700–210,000)	106,000 (15,300–244,000)	635 (96·8–1,460)	634 (95·9–1,460)	632 (96·2–1,450)
1-102	Migraine	50,500 (4,960–124,000)	83,100 (8,470–202,000)	97,400 (10,500–236,000)	585 (66·4–1,410)	583 (65·3–1,410)	581 (64·9–1,400)
1-103	Tension-type headache	4,040 (1,020–17,900)	6,780 (1,760–27,600)	8,150 (2,220–32,200)	50·1 (13·6–197)	50·3 (13·6–191)	50·3 (14–193)
1-104	Other neurological disorders	3,920 (2,350–6,420)	5,150 (2,880–8,420)	6,300 (3,380–10,800)	33·5 (20·9–52·7)	34·4 (20·4–54·1)	38·3 (21·3–64·1)
1-105	Mental disorders	132,000 (96,400–174,000)	203,000 (149,000–269,000)	236,000 (171,000–310,000)	1,520 (1,120–1,990)	1,460 (1,080–1,930)	1,440 (1,050–1,900)
1-106	Schizophrenia	12,500 (8,610–16,900)	22,600 (15,600–30,500)	29,100 (20,300–39,000)	164 (117–219)	171 (119–229)	176 (124–234)
1-107	Depressive disorders	40,400 (27,200–56,900)	61,800 (42,200–87,800)	73,100 (50,100–103,000)	514 (353–721)	464 (320–650)	456 (314–644)
1-108	Major depressive disorder	31,000 (20,200–44,500)	45,000 (30,100–64,000)	51,900 (34,600–73,900)	377 (253–532)	327 (220–466)	320 (216–451)
1-109	Dysthymia	9,480 (5,840–14,300)	16,800 (10,400–25,400)	21,200 (13,200–31,900)	138 (86·5–208)	137 (85·6–210)	137 (85·8–208)
1-110	Bipolar disorder	5,450 (3,210–8,620)	9,380 (5,470–14,700)	11,200 (6,590–17,700)	68·1 (41·2–107)	68·6 (40·5–108)	68·5 (40·7–107)
1-111	Anxiety disorders	35,900 (23,500–50,700)	59,600 (39,200–84,500)	71,200 (47,200–102,000)	419 (278–593)	429 (287–604)	436 (290–620)
1-112	Eating disorders	1,350 (821–2,010)	2,560 (1,570–3,810)	3,330 (2,030–5,040)	13·1 (7·8–19·5)	15·6 (9·6–23·2)	18·2 (11–27·3)
1-113	Anorexia nervosa	526 (289–839)	950 (532–1,610)	1,140 (672–1,870)	4·8 (2·6–7·7)	5·5 (3·2–9·2)	6·3 (3·7–10·2)

1-114	Bulimia nervosa	828<br>(464–1,330)	1,610<br>(909–2,580)	2,190<br>(1,240–3,550)	8·3<br>(4·6–13·3)	10·1<br>(5·7–16)	11·9<br>(6·8–19·2)
1-115	Autism spectrum disorders	5,190<br>(3,380–7,560)	7,120<br>(4,650–10,600)	8,020<br>(5,130–11,600)	45·5<br>(30–66·1)	46·6<br>(30·3–69·6)	46·9<br>(30·1–67·9)
1-116	Attention-deficit/hyperactivity disorder	1,370<br>(760–2,350)	1,880<br>(1,060–3,250)	1,990<br>(1,110–3,410)	10·9<br>(6·1–18·4)	11·1<br>(6·3–19·2)	11·2<br>(6·2–19·1)
1-117	Conduct disorder	9,140<br>(5,130–14,900)	12,000<br>(6,840–19,000)	11,700<br>(6,530–18,800)	65<br>(36·6–104)	65·9<br>(37·7–105)	66·1<br>(37–106)
1-118	Idiopathic developmental intellectual disability	13,200<br>(7,380–21,300)	12,600<br>(6,770–20,400)	9,090<br>(4,520–15,600)	113<br>(63·2–184)	82·2<br>(44·4–132)	52·7<br>(26·1–90)
1-119	Other mental disorders	7,370<br>(4,600–11,200)	13,200<br>(8,240–20,000)	16,800<br>(10,600–25,600)	106<br>(67·3–158)	107<br>(67·6–161)	107<br>(68·2–163)
1-120	Substance use disorders	16,900<br>(12,400–21,600)	26,400<br>(19,900–34,000)	32,500<br>(24,000–42,400)	214<br>(161–267)	188<br>(143–240)	191<br>(142–247)
1-121	Alcohol use disorders	9,970<br>(7,230–12,800)	14,600<br>(10,900–19,300)	18,200<br>(13,300–24,200)	140<br>(102–178)	111<br>(84·1–146)	111<br>(82·1–147)
1-122	Drug use disorders	6,970<br>(4,770–9,750)	11,800<br>(8,050–16,500)	14,300<br>(9,940–19,700)	74·9<br>(52·6–102)	76·7<br>(53·3–106)	79·9<br>(56·1–108)
1-123	Opioid use disorders	2,900<br>(2,070–3,990)	5,260<br>(3,660–7,300)	6,730<br>(4,640–9,420)	34·5<br>(25–45·8)	36·8<br>(25·8–50·1)	38·9<br>(27·1–53·8)
1-124	Cocaine use disorders	96<br>(63–149)	169<br>(113–250)	211<br>(144–296)	1·1<br>(0·7–1·7)	1·1<br>(0·8–1·7)	1·2<br>(0·8–1·7)
1-125	Amphetamine use disorders	1,830<br>(971–3,100)	3,010<br>(1,600–5,060)	3,490<br>(1,870–5,810)	17·8<br>(9·7–29·9)	18<br>(9·6–30·1)	18·4<br>(9·9–30·5)
1-126	Cannabis use disorders	724<br>(395–1,270)	1,030<br>(561–1,720)	1,150<br>(615–1,910)	7·1<br>(3·9–12·1)	6·3<br>(3·5–10·4)	6·3<br>(3·4–10·4)
1-127	Other drug use disorders	1,420<br>(788–2,320)	2,340<br>(1,370–3,870)	2,760<br>(1,580–4,460)	14·4<br>(8·4–23·1)	14·5<br>(8·7–23·6)	15<br>(8·8–23·9)
1-128	Diabetes and kidney diseases	118,000<br>(97,200–139,000)	170,000<br>(146,000–197,000)	239,000<br>(197,000–285,000)	1,880<br>(1,590–2,200)	1,710<br>(1,480–1,980)	1,850<br>(1,540–2,200)
1-129	Diabetes mellitus	56,600<br>(45,500–69,400)	99,300<br>(83,000–119,000)	154,000<br>(123,000–189,000)	1,060<br>(880–1,280)	1,070<br>(897–1,280)	1,230<br>(994–1,510)
1-130	Diabetes mellitus type 1	11,100<br>(4,450–16,000)	10,600<br>(8,100–13,700)	11,500<br>(8,630–16,200)	128<br>(52·1–182)	84·4<br>(62·9–118)	76·4<br>(56·6–112)
1-131	Diabetes mellitus type 2	45,500<br>(36,200–55,300)	88,800<br>(73,400–107,000)	142,000<br>(113,000–178,000)	930<br>(752–1,120)	986<br>(816–1,190)	1,160<br>(928–1,440)
1-132	Chronic kidney disease	52,800<br>(44,500–61,800)	67,900<br>(58,100–78,000)	83,300<br>(69,400–99,000)	760<br>(641–887)	621<br>(539–707)	608<br>(510–714)
1-133	Chronic kidney disease due to diabetes mellitus type 1	9,300<br>(6,700–12,400)	11,900<br>(8,500–15,900)	13,600<br>(9,270–18,800)	126<br>(89–173)	93·2<br>(66·9–126)	85·2<br>(58·3–119)
1-134	Chronic kidney disease due to diabetes mellitus type 2	11,000<br>(8,130–14,200)	17,000<br>(13,200–21,100)	22,800<br>(17,300–29,300)	214<br>(164–273)	181<br>(144–220)	181<br>(140–228)
1-135	Chronic kidney disease due to hypertension	11,700<br>(8,900–14,800)	17,800<br>(14,300–22,500)	23,100<br>(18,000–29,200)	217<br>(169–273)	184<br>(150–228)	183<br>(145–226)
1-136	Chronic kidney disease due to glomerulonephritis	6,500<br>(4,690–8,740)	7,260<br>(5,320–9,420)	8,080<br>(6,120–10,500)	65·9<br>(49·8–85·1)	52·2<br>(39·1–66·3)	50·3<br>(38·3–64·2)
1-137	Chronic kidney disease due to other and unspecified causes	14,300<br>(10,600–18,500)	14,000<br>(11,200–17,000)	15,800<br>(12,600–19,300)	136<br>(109–169)	111<br>(89·7–134)	108<br>(87·5–132)
1-138	Acute glomerulonephritis	8,110<br>(318–20,800)	3,040<br>(92–5,720)	1,990<br>(64–3,610)	65·2<br>(3–158)	21·6<br>(0·8–39·5)	12·8<br>(0·5–22·9)
1-139	Skin and subcutaneous diseases	64,400<br>(41,500–96,400)	91,000<br>(59,000–136,000)	103,000<br>(67,100–154,000)	635<br>(419–928)	631<br>(416–936)	632<br>(414–939)
1-140	Dermatitis	17,500<br>(10,100–28,200)	25,100<br>(14,500–40,100)	29,100<br>(16,700–46,200)	179<br>(104–284)	177<br>(103–281)	176<br>(102–278)
1-141	Atopic dermatitis	14,800<br>(7,970–25,000)	20,600<br>(10,900–34,900)	23,700<br>(12,500–39,700)	144<br>(78–242)	143<br>(76·7–242)	142<br>(75·7–238)
1-142	Contact dermatitis	2,360<br>(1,470–3,630)	4,010<br>(2,500–6,030)	4,870<br>(3,020–7,320)	30·9<br>(19·2–46·6)	31<br>(19·3–46·4)	30·9<br>(19·3–46·5)
1-143	Seborrhoeic dermatitis	295<br>(166–470)	453<br>(253–734)	539<br>(302–870)	3·4<br>(1·9–5·3)	3·4<br>(1·9–5·4)	3·4<br>(1·9–5·4)
1-144	Psoriasis	1,430<br>(1,020–1,940)	2,030<br>(1,440–2,760)	2,370<br>(1,650–3,240)	18·1<br>(13–24·5)	15·8<br>(11·3–21·5)	15·2<br>(10·7–20·7)
1-145	Bacterial skin diseases	2,880<br>(1,800–4,500)	3,290<br>(2,460–4,210)	3,620<br>(2,800–4,760)	41·8<br>(26·7–56·3)	31<br>(23·3–40·1)	27·7<br>(21·3–36·2)
1-146	Cellulitis	761<br>(369–1,160)	872<br>(501–1,200)	975<br>(607–1,340)	12·2<br>(6·3–17·2)	8·6<br>(5·1–11·8)	7·5<br>(4·8–10·5)
1-147	Pyoderma	2,120<br>(1,310–3,830)	2,410<br>(1,780–3,320)	2,650<br>(1,970–3,590)	29·6<br>(18·5–43·2)	22·4<br>(16·7–30·3)	20·1<br>(14·9–27·8)
1-148	Scabies	13,700<br>(7,550–21,900)	18,100<br>(10,000–29,000)	19,900<br>(10,800–31,800)	118<br>(66·1–190)	117<br>(64·6–187)	117<br>(64·3–187)
1-149	Fungal skin diseases	4,470<br>(1,760–9,640)	6,830<br>(2,690–14,800)	8,000<br>(3,210–17,300)	52·2<br>(21–112)	52<br>(20·9–113)	51·7<br>(20·8–112)
1-150	Viral skin diseases	7,260<br>(4,610–10,900)	8,530<br>(5,470–12,700)	9,220<br>(5,890–13,900)	53<br>(33·8–79·5)	53·2<br>(34·1–79·1)	53·2<br>(34–80·4)
1-151	Acne vulgaris	6,640<br>(4,000–11,000)	11,100<br>(6,580–17,700)	11,900<br>(7,130–19,000)	55·5<br>(33·7–91·3)	61·8<br>(36·8–98·4)	66·2<br>(39·9–106)
1-152	Alopecia areata	789<br>(498–1,180)	1,250<br>(791–1,890)	1,490<br>(946–2,260)	8·9<br>(5·7–13·2)	8·9<br>(5·6–13·4)	8·9<br>(5·6–13·3)
1-153	Pruritus	885<br>(422–1,590)	1,450<br>(686–2,620)	1,800<br>(844–3,290)	10·9<br>(5·2–20)	11·4<br>(5·4–20·4)	11·7<br>(5·5–21·2)
1-154	Urticaria	4,780<br>(3,080–6,870)	6,590<br>(4,190–9,560)	7,330<br>(4,720–10,400)	42·6<br>(28–60·6)	42·9<br>(27·7–61·8)	43<br>(27·8–61·1)
1-155	Decubitus ulcer	723<br>(352–1,000)	1,640<br>(793–2,260)	2,240<br>(1,100–3,030)	17·7<br>(9–24·4)	21·3<br>(10·5–28·9)	21·7<br>(11·1–29·4)
1-156	Other skin and subcutaneous diseases	3,390<br>(1,680–6,130)	5,190<br>(2,540–9,360)	6,290<br>(3,110–11,300)	37<br>(18·6–66·6)	38·5<br>(19–68·8)	39·6<br>(19·7–70·8)
1-157	Sense organ diseases	72,600<br>(49,700–101,000)	111,000<br>(76,200–155,000)	136,000<br>(93,100–189,000)	1,290<br>(907–1,750)	1,120<br>(781–1,550)	1,060<br>(737–1,460)
1-158	Blindness and vision loss	31,400<br>(21,800–43,400)	43,500<br>(30,400–60,400)	52,200<br>(36,300–72,400)	645<br>(454–872)	499<br>(352–679)	447<br>(317–612)
1-159	Glaucoma	516<br>(338–779)	650<br>(425–926)	885<br>(579–1,290)	15<br>(9·9–22·1)	9·6<br>(6·3–13·6)	9·2<br>(6·1–13·3)
1-160	Cataract	13,400<br>(9,480–18,600)	18,000<br>(12,800–24,500)	22,400<br>(15,800–30,300)	348<br>(245–474)	242<br>(172–326)	218<br>(154–294)
1-161	Age-related macular degeneration	301<br>(196–441)	440<br>(291–630)	634<br>(414–921)	7·8<br>(5·1–11·2)	5·9<br>(4–8·4)	5·9<br>(3·9–8·7)
1-162	Refraction disorders	10,300<br>(6,870–15,000)	14,600<br>(9,690–21,000)	15,500<br>(10,300–22,400)	135<br>(90·8–189)	129<br>(86·8–181)	107<br>(71·6–154)
1-163	Near vision loss	2,860<br>(1,300–5,710)	5,140<br>(2,350–10,300)	6,920<br>(3,110–13,900)	64·9<br>(29·6–129)	60·1<br>(28·2–117)	58·4<br>(27–115)
1-164	Other vision loss	3,930<br>(2,630–5,480)	4,790<br>(3,330–6,640)	5,840<br>(3,980–8,160)	74·1<br>(50·8–102)	52·6<br>(37·1–72·2)	48·9<br>(33·9–67·5)
1-165	Age-related and other hearing loss	38,900<br>(25,100–55,200)	63,200<br>(42,400–91,200)	78,400<br>(52,500–113,000)	608<br>(414–863)	586<br>(400–833)	572<br>(388–820)
1-166	Other sense organ diseases	2,330<br>(1,390–3,550)	3,900<br>(2,330–6,080)	4,980<br>(2,940–7,640)	34·4<br>(20·6–53·4)	35·3<br>(21–54·9)	35·6<br>(21·1–55·1)

1-167	Musculoskeletal disorders	117,000 (82,900–157,000)	214,000 (151,000–288,000)	274,000 (195,000–365,000)	1,790 (1,270–2,400)	1,840 (1,320–2,470)	1,850 (1,320–2,460)
1-168	Rheumatoid arthritis	890 (629–1,180)	1,810 (1,330–2,380)	2,500 (1,830–3,300)	14·7 (10·6–19·4)	16·9 (12·4–22)	18 (13·5–23·4)
1-169	Osteoarthritis	5,790 (2,880–11,500)	11,800 (5,820–24,100)	16,600 (8,200–33,500)	124 (61–246)	129 (64·1–262)	132 (65·6–265)
1-170	Low back pain	67,600 (47,200–90,500)	115,000 (80,800–155,000)	138,000 (95,400–185,000)	994 (706–1,320)	971 (682–1,310)	931 (647–1,240)
1-171	Neck pain	24,200 (16,000–35,300)	44,100 (29,100–65,000)	56,600 (37,100–82,700)	379 (251–554)	380 (252–553)	380 (251–551)
1-172	Gout	823 (511–1,180)	1,660 (1,040–2,450)	2,340 (1,420–3,370)	15·9 (9·9–22·5)	16·8 (10·5–24·4)	17·5 (10·6–25·3)
1-173	Other musculoskeletal disorders	17,300 (9,480–26,000)	39,600 (21,300–57,900)	57,300 (35,600–81,500)	266 (148–392)	330 (176–486)	370 (225–526)
1-174	Other non-communicable diseases	457,000 (232,000–788,000)	339,000 (237,000–460,000)	323,000 (248,000–417,000)	3,070 (1,860–4,740)	2,260 (1,650–3,000)	1,970 (1,530–2,530)
1-175	Congenital anomalies	335,000 (134,000–670,000)	193,000 (106,000–302,000)	158,000 (102,000–237,000)	1,750 (736–3,430)	1,120 (625–1,750)	898 (580–1,350)
1-176	Neural tube defects	64,300 (27,000–134,000)	21,700 (9,700–40,800)	14,900 (6,560–29,800)	316 (132–660)	124 (56·1–233)	84·9 (37·3–170)
1-177	Congenital heart anomalies	138,000 (43,500–286,000)	87,400 (44,200–145,000)	71,600 (42,900–108,000)	717 (237–1,460)	504 (256–831)	406 (243–615)
1-178	Orofacial clefts	2,590 (962–6,490)	1,510 (757–3,020)	1,090 (608–1,950)	13·4 (5·6–32·4)	8·9 (4·5–17·5)	6·3 (3·5–11·2)
1-179	Down syndrome	3,660 (1,240–11,900)	2,280 (1,270–4,160)	2,140 (1,340–3,360)	20·2 (7·2–62·7)	13·4 (7·5–24·1)	12·1 (7·6–19)
1-180	Turner syndrome	16 (7–30)	19 (9–35)	19 (8–35)	0·1 (0·1–0·2)	0·1 (0·1–0·2)	0·1 (0–0·2)
1-181	Klinefelter syndrome	2 (1–4)	2 (1–4)	2 (1–5)	0 (0–0)	0 (0–0)	0 (0–0)
1-182	Other chromosomal abnormalities	4,120 (1,390–18,500)	2,510 (1,350–6,650)	2,380 (1,410–5,050)	21 (7·5–92·3)	14·4 (7·8–38·1)	13·5 (8–28·6)
1-183	Congenital musculoskeletal and limb anomalies	8,600 (5,200–14,000)	7,470 (4,960–10,700)	7,280 (4,790–10,300)	54·4 (33·6–84·8)	45·5 (30·3–65)	41·9 (27·7–59·3)
1-184	Urogenital congenital anomalies	2,830 (939–6,880)	2,050 (995–3,870)	1,780 (999–3,100)	14·6 (5·3–34·4)	11·8 (5·8–22·2)	10·1 (5·7–17·6)
1-185	Digestive congenital anomalies	31,600 (5,360–104,000)	18,400 (6,940–37,400)	15,600 (6,150–27,100)	157 (26·7–518)	105 (39·7–214)	88·4 (34·9–154)
1-186	Other congenital anomalies	79,400 (21,600–227,000)	49,200 (22,500–105,000)	41,000 (22,400–80,100)	439 (137–1,170)	291 (137–610)	235 (128–457)
1-187	Urinary diseases and male infertility	10,400 (7,810–13,200)	14,100 (10,600–17,100)	17,900 (13,600–21,700)	158 (126–198)	142 (110–176)	140 (108–168)
1-188	Urinary tract infections and interstitial nephritis	6,500 (4,580–8,740)	8,200 (5,770–10,300)	9,980 (7,090–12,600)	90·3 (66–117)	79·3 (56·6–99·3)	77·1 (56·2–95·7)
1-189	Urolithiasis	1,070 (284–1,660)	1,490 (500–2,290)	1,820 (655–2,700)	18·2 (4·9–29·5)	14·9 (4·9–23·4)	13·8 (4·8–20·6)
1-190	Benign prostatic hyperplasia	1,090 (635–1,670)	2,040 (1,180–3,100)	2,920 (1,710–4,430)	26·6 (15·5–40·3)	25·7 (15–38·8)	25·6 (15–38·6)
1-191	Male infertility	199 (72–501)	212 (86–464)	460 (171–1,120)	2·3 (0·8–5·6)	1·5 (0·6–3·1)	2·6 (1–6·2)
1-192	Other urinary diseases	1,540 (807–2,870)	2,140 (1,500–3,430)	2,680 (1,850–4,290)	21·1 (13·3–36·3)	21 (15·2–32·8)	21 (14·8–32·7)
1-193	Gynaecological diseases	25,900 (18,000–36,600)	39,100 (26,900–55,500)	46,200 (32,100–65,800)	312 (219–435)	278 (192–392)	273 (190–386)
1-194	Uterine fibroids	862 (425–1,490)	1,340 (688–2,450)	1,640 (846–2,920)	11·5 (5·7–19·9)	10·4 (5·4–18·8)	10·1 (5·2–17·9)
1-195	Polycystic ovarian syndrome	439 (184–887)	1,050 (440–2,130)	1,410 (588–2,870)	4·8 (2–9·6)	6·8 (2·9–13·7)	7·9 (3·3–16·2)
1-196	Female infertility	684 (228–1,660)	461 (188–956)	1,250 (400–3,180)	8·2 (2·6–19·6)	3·3 (1·3–6·7)	7·1 (2·2–18·2)
1-197	Endometriosis	4,660 (2,720–7,480)	5,130 (3,050–8,030)	5,740 (3,420–9,230)	53·9 (31·9–85·3)	34·9 (21–55·1)	32·4 (19·5–51·4)
1-198	Genital prolapse	234 (120–449)	384 (201–688)	498 (270–892)	4·6 (2·3–8·7)	3·9 (2–7)	3·8 (2–6·8)
1-199	Premenstrual syndrome	10,200 (6,190–15,800)	16,700 (10,100–26,200)	18,700 (11,400–29,300)	111 (68·5–170)	109 (66·4–170)	106 (65–166)
1-200	Other gynaecological diseases	8,810 (5,700–12,600)	14,100 (9,030–20,400)	16,900 (10,800–24,700)	118 (78·1–167)	109 (70·9–158)	105 (68·2–151)
1-201	Haemoglobinopathies and haemolytic anaemias	29,400 (20,800–39,500)	31,100 (22,600–42,500)	31,500 (22,800–44,500)	273 (202–361)	214 (158–290)	191 (139–268)
1-202	Thalassaemias	6,910 (3,630–10,200)	4,490 (3,260–5,780)	3,810 (2,950–4,780)	43·2 (24·1–63·4)	26 (18·9–33·5)	21·4 (16·6–26·8)
1-203	Thalassaemias trait	15,200 (9,900–22,900)	17,800 (11,200–27,100)	18,700 (11,900–28,700)	138 (90·1–206)	116 (74·6–176)	109 (69·7–166)
1-204	Sickle cell disorders	9 (3–17)	4 (2–7)	3 (2–5)	0·1 (0–0·1)	0 (0–0)	0 (0–0)
1-205	Sickle cell trait	564 (350–852)	648 (396–1,020)	651 (402–1,040)	5·5 (3·4–8·3)	4·3 (2·6–6·6)	3·7 (2·3–5·9)
1-206	G6PD deficiency	1,680 (1,230–2,240)	2,670 (2,140–3,260)	2,720 (2,040–3,510)	20·4 (15·4–26·5)	20·5 (16·6–24·5)	17·3 (13–22·2)
1-207	G6PD trait	15 (8–23)	50 (29–80)	45 (25–75)	0·2 (0·1–0·4)	0·4 (0·2–0·6)	0·3 (0·2–0·5)
1-208	Other haemoglobinopathies and haemolytic anaemias	4,980 (3,550–6,790)	5,450 (4,000–7,460)	5,570 (4,070–7,600)	66 (49·9–86·3)	46·8 (34·8–62·6)	39·7 (29·7–53·1)
1-209	Endocrine, metabolic, blood, and immune disorders	20,000 (13,600–27,100)	25,000 (18,700–32,500)	28,900 (21,600–37,500)	229 (168–297)	202 (152–261)	192 (144–248)
1-210	Oral disorders	15,700 (9,660–23,900)	26,200 (16,100–40,600)	32,300 (19,800–49,800)	247 (154–370)	240 (149–368)	233 (143–358)
1-211	Caries of deciduous teeth	553 (245–1,120)	603 (262–1,160)	550 (236–1,080)	3·1 (1·4–6·4)	3·6 (1·6–7)	3·1 (1·3–6·1)
1-212	Caries of permanent teeth	2,610 (1,170–5,150)	4,210 (1,910–8,060)	4,630 (2,070–8,800)	28·4 (12·7–54·3)	28·7 (12·9–54·3)	27·7 (12·5–52·7)
1-213	Periodontal disease	1,500 (554–3,520)	3,130 (1,180–7,230)	4,520 (1,700–10,500)	27·8 (10·5–62·6)	30·7 (11·8–68)	33·1 (12·6–75)
1-214	Edentulism	6,140 (3,880–9,160)	10,800 (6,880–16,000)	13,800 (8,790–20,600)	134 (85·4–197)	124 (79·7–184)	116 (74·2–171)
1-215	Other oral disorders	4,890 (3,040–7,230)	7,450 (4,620–10,900)	8,780 (5,400–12,900)	53·2 (33·2–78)	53·3 (33–77·8)	53·3 (32·9–78·1)
1-216	Sudden infant death syndrome	20,700 (2,740–69,200)	11,100 (3,320–30,500)	7,930 (3,090–18,600)	102 (13·4–341)	63·2 (19–174)	44·9 (17·5–105)
1-0	Injuries	630,000 (491,000–759,000)	547,000 (462,000–633,000)	524,000 (433,000–625,000)	5,780 (4,740–6,900)	3,890 (3,300–4,520)	3,290 (2,720–3,910)
1-1	Transport injuries	178,000 (137,000–216,000)	180,000 (145,000–214,000)	179,000 (144,000–219,000)	1,720 (1,390–2,070)	1,230 (1,000–1,470)	1,080 (872–1,310)
1-2	Road injuries	167,000 (124,000–204,000)	169,000 (135,000–200,000)	167,000 (134,000–205,000)	1,620 (1,280–1,960)	1,150 (935–1,360)	1,010 (817–1,230)

1-3	Pedestrian road injuries	49,300 (31,800–74,100)	40,500 (29,400–54,900)	36,600 (27,500–48,200)	468 (318–681)	297 (221–393)	235 (178–307)
1-4	Cyclist road injuries	9,710 (5,810–14,600)	11,000 (7,700–14,500)	13,500 (9,630–17,900)	96 (63–133)	81·4 (58·2–107)	85·3 (61·3–112)
1-5	Motorcyclist road injuries	58,100 (41,300–78,100)	65,000 (49,100–84,500)	63,200 (47,400–80,000)	557 (412–738)	419 (317–536)	366 (277–463)
1-6	Motor vehicle road injuries	47,600 (26,300–69,800)	49,800 (35,800–71,200)	51,200 (37,800–72,300)	475 (283–686)	336 (250–480)	306 (229–430)
1-7	Other road injuries	1,880 (1,290–2,780)	2,340 (1,570–3,460)	2,520 (1,650–3,690)	19·2 (13·2–27·5)	16·3 (10·9–23·8)	15·3 (10·2–22·4)
1-8	Other transport injuries	11,200 (7,810–16,100)	11,900 (8,450–16,800)	11,700 (8,200–16,800)	108 (77·7–154)	81·5 (58·6–114)	70·6 (50–101)
1-9	Unintentional injuries	311,000 (221,000–382,000)	258,000 (218,000–302,000)	243,000 (203,000–290,000)	2,630 (2,030–3,140)	1,880 (1,600–2,180)	1,590 (1,330–1,880)
1-10	Falls	68,200 (51,000–92,700)	84,800 (71,200–101,000)	107,000 (88,300–131,000)	772 (632–942)	726 (619–855)	771 (644–928)
1-11	Drowning	136,000 (91,300–187,000)	76,000 (54,900–101,000)	62,000 (45,500–83,600)	968 (683–1,300)	479 (348–636)	362 (266–488)
1-12	Fire, heat, and hot substances	26,400 (10,600–38,800)	16,100 (11,000–23,300)	15,000 (11,200–21,000)	225 (111–313)	114 (79·7–160)	93·2 (69·9–128)
1-13	Poisonings	8,320 (4,570–12,000)	4,750 (3,570–6,180)	4,220 (3,230–5,470)	62 (39·4–83·8)	31 (23·8–39·8)	25 (19·2–32·1)
1-14	Poisoning by carbon monoxide	622 (297–1,180)	502 (313–780)	491 (320–732)	5·6 (3–9·6)	3·4 (2·2–5·1)	3 (2–4·4)
1-15	Poisoning by other means	7,700 (4,110–11,300)	4,250 (3,140–5,530)	3,730 (2,820–4,890)	56·4 (34·9–77·5)	27·7 (20·9–35·9)	22 (16·7–28·7)
1-16	Exposure to mechanical forces	18,200 (12,200–28,400)	39,300 (34,900–44,900)	18,600 (14,600–23,500)	181 (136–240)	267 (237–302)	116 (91·5–146)
1-17	Unintentional firearm injuries	3,830 (1,570–7,530)	2,450 (1,510–3,870)	2,200 (1,520–3,350)	37·4 (17·2–65·2)	17·3 (10·9–26·4)	13·5 (9·5–20·2)
1-18	Other exposure to mechanical forces	14,400 (9,710–22,500)	36,900 (32,700–42,200)	16,400 (12,600–20,900)	144 (106–194)	250 (222–283)	103 (78·7–129)
1-19	Adverse effects of medical treatment	11,000 (6,770–17,500)	6,700 (4,660–10,600)	6,080 (4,230–9,580)	97·1 (72·1–144)	50·5 (35·2–79·6)	40·2 (27·9–63·5)
1-20	Animal contact	8,230 (3,290–12,800)	6,040 (3,510–8,410)	5,360 (3,430–7,140)	69·1 (31·7–101)	42·3 (25·3–58·3)	33·2 (21·5–44·2)
1-21	Venomous animal contact	474 (309–724)	666 (457–961)	741 (512–1,010)	4·7 (3·2–6·9)	4·4 (3–6·2)	4·4 (3–5·9)
1-22	Non-venomous animal contact	7,750 (2,900–12,300)	5,370 (3,010–7,490)	4,620 (2,850–6,220)	64·4 (28·2–95·5)	37·9 (21·8–53·1)	28·9 (18·1–38·9)
1-23	Foreign body	21,800 (13,900–32,200)	12,600 (9,140–17,700)	11,100 (8,440–15,200)	139 (96·7–198)	82 (61·5–112)	68·2 (52·4–92·5)
1-24	Pulmonary aspiration and foreign body in airway	19,300 (12,200–28,800)	11,200 (7,860–16,000)	9,590 (7,020–13,700)	120 (82·8–169)	71·4 (51·8–99·8)	58·3 (43·6–82·5)
1-25	Foreign body in eyes	252 (109–458)	403 (178–728)	484 (221–868)	2·9 (1·4–5·1)	3 (1·4–5·2)	3 (1·4–5·2)
1-26	Foreign body in other body part	2,230 (739–5,430)	1,010 (716–1,440)	1,050 (783–1,360)	16·3 (7·8–32·9)	7·7 (5·6–10·5)	6·9 (5·2–8·9)
1-27	Environmental heat and cold exposure	946 (719–1,210)	1,150 (855–1,500)	1,330 (980–1,770)	10·7 (8·2–13·6)	8·5 (6·4–11·1)	8·3 (6·2–11)
1-28	Exposure to forces of nature	0 (0–0)	1,090 (880–1,380)	763 (528–1,070)	0 (0–0)	7·5 (6·1–9·4)	4·7 (3·3–6·5)
1-29	Other unintentional injuries	11,900 (8,020–16,700)	9,840 (7,200–13,100)	10,700 (7,790–13,900)	108 (77·1–143)	70·1 (51·7–91·7)	66·2 (48·4–86·3)
1-30	Self-harm and interpersonal violence	141,000 (97,500–238,000)	109,000 (80,700–165,000)	102,000 (75,700–148,000)	1,420 (988–2,360)	776 (562–1,230)	625 (459–931)
1-31	Self-harm	32,100 (24,600–40,100)	36,900 (29,500–45,200)	38,600 (28,900–49,000)	370 (283–457)	258 (208–312)	232 (174–292)
1-32	Self-harm by firearm	727 (397–1,290)	760 (489–1,590)	736 (468–1,490)	8·5 (5–14·9)	5·3 (3·5–11·1)	4·4 (2·9–8·8)
1-33	Self-harm by other specified means	31,300 (24,000–39,200)	36,100 (28,900–44,000)	37,900 (28,100–47,800)	362 (276–447)	253 (204–306)	227 (170–285)
1-34	Interpersonal violence	30,800 (25,200–37,300)	35,200 (28,000–44,200)	35,800 (27,700–45,700)	313 (259–377)	230 (186–282)	206 (160–262)
1-35	Physical violence by firearm	7,440 (4,920–11,400)	8,870 (6,280–13,100)	9,170 (5,950–14,400)	77·1 (52·3–116)	57·3 (41·3–85·1)	51·8 (34·2–81·7)
1-36	Physical violence by sharp object	13,100 (6,700–17,400)	15,400 (8,140–20,900)	14,900 (7,700–21,200)	135 (69·7–177)	99·1 (52·8–131)	84·3 (44·2–119)
1-37	Sexual violence	1,750 (1,140–2,470)	2,580 (1,690–3,630)	3,060 (2,040–4,320)	18·6 (12·3–26·1)	17·2 (11·4–24)	17·8 (11·9–25)
1-38	Physical violence by other means	8,600 (6,650–11,400)	8,350 (6,570–10,700)	8,670 (6,720–11,200)	83 (64·8–108)	56·6 (44·9–71·8)	51·8 (40·4–66·6)
1-39	Conflict and terrorism	78,400 (38,200–175,000)	35,700 (11,900–90,600)	27,500 (8,520–71,000)	739 (344–1,670)	282 (90·8–726)	186 (57·3–483)
1-40	Police conflict and executions	135 (58–250)	722 (606–908)	317 (153–647)	1·4 (0·5–2·5)	4·7 (3·9–5·8)	1·8 (0·9–3·5)

SR Table 1: All-age DALYs counts and age standardized DALYs rates per 100,000 individuals for all causes and level 1 cause groups by location for 1990, 2010, and 2019, both sexes combined, High-income North America

Cause Level	Cause	All-age DALYs			Age-standardized rate of DALYs per 100,000		
		1990	2010	2019	1990	2010	2019
0-0	All causes	90,700,000 (81,200,000–102,000,000)	104,000,000 (91,600,000–118,000,000)	121,000,000 (107,000,000–137,000,000)	28,800 (25,700–32,400)	24,800 (21,700–28,300)	25,400 (22,100–29,100)
1-0	Communicable, maternal, neonatal, and nutritional disorders	6,660,000 (6,220,000–7,140,000)	5,250,000 (4,760,000–5,820,000)	5,390,000 (4,840,000–6,050,000)	2,430 (2,270–2,600)	1,650 (1,500–1,820)	1,560 (1,400–1,750)
1-1	HIV/AIDS and sexually transmitted infections	1,580,000 (1,520,000–1,660,000)	557,000 (482,000–661,000)	485,000 (399,000–599,000)	503 (485–528)	139 (120–164)	115 (94·7–141)
1-2	HIV/AIDS	1,530,000 (1,480,000–1,600,000)	508,000 (444,000–595,000)	433,000 (362,000–529,000)	488 (472–508)	126 (110–147)	102 (85·9–124)
1-3	HIV/AIDS - drug-susceptible tuberculosis	42,200 (27,400–63,800)	7,670 (5,070–11,400)	7,160 (4,680–10,700)	13·4 (8·7–20·3)	1·9 (1·3–2·9)	1·7 (1·1–2·6)
1-4	HIV/AIDS - multidrug-resistant tuberculosis without extensive drug resistance	3,210 (1,130–7,090)	251 (102–517)	237 (66–613)	1 (0·4–2·3)	0·1 (0–0·1)	0·1 (0–0·1)
1-5	HIV/AIDS - extensively drug-resistant tuberculosis	0 (0–0)	42 (16–91)	61 (17–161)	0 (0–0)	0 (0–0)	0 (0–0)
1-6	HIV/AIDS resulting in other diseases	1,490,000 (1,430,000–1,550,000)	500,000 (436,000–587,000)	426,000 (354,000–522,000)	473 (457–494)	124 (108–145)	100 (83·9–122)
1-7	Sexually transmitted infections excluding HIV	47,000 (27,500–82,600)	49,000 (27,300–87,800)	52,000 (28,800–93,700)	15 (8·7–26·2)	13 (7·2–23·3)	12·8 (7–23·1)
1-8	Syphilis	5,330 (4,360–6,510)	4,030 (3,180–5,010)	4,880 (3,800–6,200)	1·7 (1·4–2·1)	1 (0·8–1·2)	1 (0·8–1·2)
1-9	Chlamydial infection	2,810 (1,950–4,200)	2,370 (1,670–3,470)	2,470 (1,750–3,530)	0·9 (0·6–1·3)	0·7 (0·5–1)	0·7 (0·5–1)
1-10	Gonococcal infection	3,590 (3,020–4,390)	2,710 (2,320–3,290)	2,880 (2,460–3,490)	1·1 (0·9–1·4)	0·7 (0·6–0·9)	0·7 (0·6–0·8)
1-11	Trichomoniasis	14,200 (5,590–29,300)	16,000 (6,370–33,300)	16,800 (6,660–34,700)	4·5 (1·8–9·3)	4·3 (1·7–8·8)	4·3 (1·7–8·8)
1-12	Genital herpes	12,500 (3,960–30,500)	14,400 (4,580–35,000)	15,100 (4,770–36,700)	4 (1·3–9·8)	3·6 (1·2–8·8)	3·4 (1·1–8·4)
1-13	Other sexually transmitted infections	8,540 (5,600–12,400)	9,590 (6,320–14,000)	9,830 (6,390–14,400)	2·7 (1·8–3·9)	2·8 (1·8–4·1)	2·7 (1·8–4·1)
1-14	Respiratory infections and tuberculosis	1,770,000 (1,610,000–1,980,000)	1,560,000 (1,380,000–1,790,000)	1,800,000 (1,600,000–2,050,000)	561 (505–638)	375 (323–447)	376 (322–450)
1-15	Tuberculosis	68,500 (64,800–72,200)	26,200 (24,500–28,200)	28,200 (26,200–30,400)	21 (19·9–22·2)	6 (5·6–6·5)	5·5 (5·1–6)
1-16	Latent tuberculosis infection	0 (0–0)	0 (0–0)	0 (0–0)	0 (0–0)	0 (0–0)	0 (0–0)
1-17	Drug-susceptible tuberculosis	64,400 (59,300–68,700)	25,500 (23,700–27,400)	27,400 (25,000–29,700)	19·7 (18·2–21·1)	5·8 (5·4–6·3)	5·3 (4·9–5·8)
1-18	Multidrug-resistant tuberculosis without extensive drug resistance	4,150 (1,700–8,260)	662 (328–1,220)	717 (252–1,710)	1·3 (0·5–2·5)	0·1 (0·1–0·3)	0·1 (0–0·3)
1-19	Extensively drug-resistant tuberculosis	0 (0–0)	107 (51–207)	181 (59–436)	0 (0–0)	0 (0–0)	0 (0–0·1)
1-20	Lower respiratory infections	1,340,000 (1,270,000–1,380,000)	1,140,000 (1,060,000–1,180,000)	1,340,000 (1,240,000–1,400,000)	404 (386–416)	243 (231–250)	240 (226–248)
1-21	Upper respiratory infections	313,000 (192,000–482,000)	347,000 (212,000–539,000)	379,000 (232,000–584,000)	116 (71–178)	109 (66·3–169)	114 (69·2–176)
1-22	Otitis media	51,400 (29,900–84,600)	53,100 (30,900–85,900)	55,400 (32,200–90,000)	19·6 (11·5–32·2)	16·9 (9·7–27·6)	17·3 (10–28·3)
1-23	Enteric infections	240,000 (172,000–323,000)	446,000 (364,000–546,000)	506,000 (409,000–618,000)	88·8 (63·7–120)	116 (91–146)	121 (93·7–154)
1-24	Diarrhoeal diseases	236,000 (168,000–319,000)	444,000 (362,000–544,000)	504,000 (407,000–615,000)	87·2 (62–119)	115 (90·3–145)	120 (93·1–153)
1-25	Typhoid and paratyphoid	53 (46–94)	57 (40–67)	65 (41–78)	0 (0–0)	0 (0–0)	0 (0–0)
1-26	Typhoid fever	15 (10–20)	16 (7–23)	17 (8–24)	0 (0–0)	0 (0–0)	0 (0–0)
1-27	Paratyphoid fever	38 (32–78)	41 (30–52)	48 (31–55)	0 (0–0)	0 (0–0)	0 (0–0)
1-28	Invasive non-typhoidal Salmonella (iNTS)	2,680 (1,240–2,980)	1,100 (902–2,410)	1,280 (1,060–2,550)	1 (0·5–1·1)	0·3 (0·3–0·8)	0·4 (0·3–0·7)
1-29	Other intestinal infectious diseases	1,230 (713–1,810)	928 (591–1,290)	930 (642–1,260)	0·5 (0·3–0·7)	0·3 (0·2–0·5)	0·3 (0·2–0·4)
1-30	Neglected tropical diseases and malaria	72,700 (47,200–105,000)	105,000 (66,700–156,000)	101,000 (64,300–150,000)	22·5 (14·9–32·3)	23·7 (15·6–34·4)	19·7 (13·1–28·3)
1-31	Malaria	0 (0–0)	0 (0–0)	0 (0–0)	0 (0–0)	0 (0–0)	0 (0–0)
1-32	Chagas disease	666 (448–950)	1,200 (865–1,560)	720 (455–1,060)	0·2 (0·1–0·3)	0·3 (0·2–0·3)	0·1 (0·1–0·2)
1-33	Leishmaniasis	0 (0–0)	0 (0–1)	0 (0–1)	0 (0–0)	0 (0–0)	0 (0–0)
1-34	Visceral leishmaniasis	0 (0–0)	0 (0–0)	0 (0–0)	0 (0–0)	0 (0–0)	0 (0–0)
1-35	Cutaneous and mucocutaneous leishmaniasis	0 (0–0)	0 (0–1)	0 (0–1)	0 (0–0)	0 (0–0)	0 (0–0)
1-36	African trypanosomiasis	0 (0–0)	0 (0–0)	0 (0–0)	0 (0–0)	0 (0–0)	0 (0–0)
1-37	Schistosomiasis	0 (0–0)	0 (0–0)	0 (0–0)	0 (0–0)	0 (0–0)	0 (0–0)
1-38	Cysticercosis	60,200 (35,600–92,000)	88,900 (51,900–139,000)	82,800 (47,900–130,000)	17·7 (10·3–27·2)	18·7 (10·9–28·9)	14·3 (8·2–22·5)
1-39	Cystic echinococcosis	202 (129–289)	117 (76–163)	154 (99–214)	0·1 (0–0·1)	0 (0–0)	0 (0–0)
1-40	Lymphatic filariasis	0 (0–0)	0 (0–0)	0 (0–0)	0 (0–0)	0 (0–0)	0 (0–0)
1-41	Onchocerciasis	0 (0–0)	0 (0–0)	0 (0–0)	0 (0–0)	0 (0–0)	0 (0–0)
1-42	Trachoma	0 (0–0)	0 (0–0)	0 (0–0)	0 (0–0)	0 (0–0)	0 (0–0)
1-43	Dengue	144 (81–236)	266 (164–412)	268 (165–425)	0·1 (0–0·1)	0·1 (0–0·1)	0·1 (0–0·1)
1-44	Yellow fever	0 (0–0)	0 (0–0)	0 (0–0)	0 (0–0)	0 (0–0)	0 (0–0)
1-45	Rabies	119 (111–128)	204 (193–216)	148 (134–162)	0 (0–0·1)	0·1 (0·1–0·1)	0·1 (0–0·1)
1-46	Intestinal nematode infections	0 (0–0)	0 (0–0)	0 (0–0)	0 (0–0)	0 (0–0)	0 (0–0)
1-47	Ascariasis	0 (0–0)	0 (0–0)	0 (0–0)	0 (0–0)	0 (0–0)	0 (0–0)
1-48	Trichuriasis	0 (0–0)	0 (0–0)	0 (0–0)	0 (0–0)	0 (0–0)	0 (0–0)

1-49	Hookworm disease	0 (0–0)	0 (0–0)	0 (0–0)	0 (0–0)	0 (0–0)	0 (0–0)
1-50	Food-borne trematodiases	0 (0–0)	0 (0–0)	0 (0–0)	0 (0–0)	0 (0–0)	0 (0–0)
1-51	Leprosy	0 (0–0)	0 (0–0)	0 (0–0)	0 (0–0)	0 (0–0)	0 (0–0)
1-52	Ebola virus disease	0 (0–0)	0 (0–0)	0 (0–0)	0 (0–0)	0 (0–0)	0 (0–0)
1-53	Zika virus disease	0 (0–0)	0 (0–0)	2 (0–11)	0 (0–0)	0 (0–0)	0 (0–0)
1-54	Guinea worm disease	0 (0–0)	0 (0–0)	0 (0–0)	0 (0–0)	0 (0–0)	0 (0–0)
1-55	Other neglected tropical diseases	11,400 (7,420–17,100)	14,500 (9,880–20,400)	16,900 (11,100–23,900)	4·4 (2·8–6·6)	4·5 (3·1–6·5)	5·1 (3·3–7·3)
1-56	Other infectious diseases	303,000 (256,000–333,000)	248,000 (218,000–307,000)	261,000 (229,000–324,000)	111 (95·5–122)	72·8 (64·3–89)	69·7 (61·3–85·4)
1-57	Meningitis	111,000 (105,000–119,000)	53,600 (52,000–55,300)	49,800 (47,700–52,000)	44 (41·6–47·4)	17·2 (16·6–17·9)	15·1 (14·4–15·9)
1-58	Encephalitis	18,500 (17,100–23,300)	26,900 (22,800–28,700)	27,600 (23,600–29,700)	6·7 (6·2–8·5)	8·1 (6·9–8·7)	7·7 (6·5–8·3)
1-59	Diphtheria	53 (50–56)	78 (75–82)	86 (79–92)	0 (0–0)	0 (0–0)	0 (0–0)
1-60	Whooping cough	1,870 (1,270–2,700)	3,640 (2,530–5,150)	3,530 (2,360–5,180)	0·8 (0·6–1·2)	1·6 (1·1–2·3)	1·7 (1·1–2·4)
1-61	Tetanus	491 (354–679)	167 (126–498)	166 (117–472)	0·2 (0·1–0·2)	0 (0–0·1)	0 (0–0·1)
1-62	Measles	1,940 (1,330–2,790)	24 (11–47)	69 (44–104)	0·9 (0·6–1·3)	0 (0–0)	0 (0–0)
1-63	Varicella and herpes zoster	15,900 (11,900–21,300)	18,800 (13,200–27,500)	19,500 (13,900–28,200)	5·4 (4–7·3)	4·7 (3·4–7)	4·3 (3·1–6·4)
1-64	Acute hepatitis	15,700 (12,800–19,500)	14,600 (11,000–19,300)	14,500 (10,900–19,300)	5·4 (4·4–6·8)	4·2 (3·1–5·6)	3·9 (2·9–5·3)
1-65	Acute hepatitis A	9,560 (6,980–12,600)	9,640 (6,730–13,300)	9,660 (6,740–13,500)	3·4 (2·4–4·5)	2·9 (2–4)	2·8 (1·9–3·9)
1-66	Acute hepatitis B	3,190 (2,360–4,050)	3,100 (2,210–3,880)	2,900 (2,120–3,570)	1·1 (0·8–1·4)	0·8 (0·6–1)	0·7 (0·5–0·8)
1-67	Acute hepatitis C	2,170 (879–2,820)	1,010 (715–1,700)	1,000 (703–1,620)	0·7 (0·3–0·9)	0·2 (0·2–0·4)	0·2 (0·2–0·4)
1-68	Acute hepatitis E	802 (573–1,160)	821 (572–1,200)	895 (628–1,280)	0·3 (0·2–0·4)	0·2 (0·2–0·3)	0·2 (0·2–0·3)
1-69	Other unspecified infectious diseases	138,000 (91,700–159,000)	130,000 (109,000–187,000)	146,000 (122,000–208,000)	47·7 (32·1–55·3)	36·8 (30·8–52·4)	36·9 (30·5–52·5)
1-70	Maternal and neonatal disorders	2,380,000 (2,230,000–2,550,000)	1,960,000 (1,790,000–2,130,000)	1,790,000 (1,610,000–1,980,000)	1,030 (973–1,100)	823 (762–880)	748 (681–821)
1-71	Maternal disorders	53,600 (45,300–63,600)	75,200 (68,500–83,800)	74,300 (66,600–84,100)	17·8 (15–21·2)	23·6 (21·4–26·3)	22·1 (19·7–25)
1-72	Maternal haemorrhage	8,730 (6,220–12,100)	7,420 (5,570–9,720)	7,170 (5,050–10,200)	2·9 (2–4)	2·4 (1·8–3·1)	2·1 (1·5–3)
1-73	Maternal sepsis and other pregnancy related infections	4,650 (2,730–7,670)	3,890 (2,390–6,070)	3,280 (2,010–5,290)	1·6 (0·9–2·7)	1·3 (0·8–2)	1 (0·6–1·6)
1-74	Maternal hypertensive disorders	10,000 (7,270–13,800)	11,100 (8,770–14,000)	10,700 (8,020–14,100)	3·4 (2·4–4·6)	3·6 (2·8–4·5)	3·2 (2·4–4·3)
1-75	Maternal obstructed labour and uterine rupture	3,440 (1,770–5,620)	2,410 (1,470–3,560)	2,500 (1,470–3,790)	1·1 (0·6–1·9)	0·8 (0·5–1·1)	0·8 (0·4–1·1)
1-76	Maternal abortive outcome	2,990 (2,350–3,740)	1,900 (1,460–2,400)	1,500 (1,180–1,910)	1 (0·8–1·3)	0·6 (0·4–0·7)	0·4 (0·4–0·6)
1-77	Ectopic pregnancy	2,670 (2,240–3,140)	1,550 (1,290–1,820)	1,280 (1,050–1,540)	0·9 (0·7–1)	0·5 (0·4–0·6)	0·4 (0·3–0·5)
1-78	Indirect maternal deaths	2,140 (1,770–2,550)	11,300 (9,620–13,000)	11,700 (10,100–13,700)	0·7 (0·6–0·8)	3·4 (2·9–4)	3·4 (2·9–4)
1-79	Late maternal deaths	6,120 (4,800–7,670)	11,900 (10,400–13,600)	12,800 (10,100–16,000)	2 (1·6–2·5)	3·7 (3·3–4·3)	3·8 (3–4·8)
1-80	Maternal deaths aggravated by HIV/AIDS	41 (23–64)	97 (53–152)	123 (68–197)	0 (0–0)	0 (0–0)	0 (0–0·1)
1-81	Other maternal disorders	12,800 (11,200–14,400)	23,600 (21,000–26,300)	23,200 (20,400–26,500)	4·2 (3·7–4·8)	7·3 (6·5–8·2)	6·9 (6–7·9)
1-82	Neonatal disorders	2,330,000 (2,180,000–2,490,000)	1,880,000 (1,710,000–2,050,000)	1,710,000 (1,540,000–1,900,000)	1,020 (956–1,080)	799 (738–856)	726 (658–797)
1-83	Neonatal preterm birth	1,550,000 (1,430,000–1,670,000)	1,240,000 (1,110,000–1,360,000)	1,110,000 (985,000–1,250,000)	669 (625–719)	509 (467–552)	455 (411–501)
1-84	Neonatal encephalopathy due to birth asphyxia and trauma	353,000 (313,000–402,000)	281,000 (238,000–337,000)	269,000 (233,000–312,000)	155 (139–173)	119 (104–137)	112 (98·9–127)
1-85	Neonatal sepsis and other neonatal infections	110,000 (97,500–128,000)	91,300 (80,200–109,000)	86,000 (75,800–98,900)	48·3 (43·3–55·1)	40·7 (36·4–46·5)	38·2 (34·1–43)
1-86	Haemolytic disease and other neonatal jaundice	9,850 (8,490–11,300)	8,140 (6,530–9,940)	8,080 (6,420–9,830)	4 (3·5–4·6)	2·8 (2·3–3·3)	2·7 (2·2–3·2)
1-87	Other neonatal disorders	308,000 (288,000–328,000)	268,000 (249,000–284,000)	239,000 (215,000–265,000)	140 (131–149)	128 (119–135)	118 (106–131)
1-88	Nutritional deficiencies	316,000 (223,000–429,000)	373,000 (270,000–499,000)	443,000 (322,000–601,000)	109 (75·1–150)	98·6 (69·8–134)	108 (76·5–152)
1-89	Protein-energy malnutrition	89,200 (68,700–118,000)	131,000 (101,000–168,000)	163,000 (129,000–205,000)	28·4 (21·2–38·5)	30·8 (22·9–41·1)	33·5 (24·9–44·2)
1-90	Iodine deficiency	7,300 (3,340–13,900)	9,070 (4,210–17,100)	9,590 (4,460–18,100)	2·5 (1·1–4·7)	2·5 (1·1–4·6)	2·4 (1·1–4·6)
1-91	Vitamin A deficiency	632 (350–1,060)	396 (208–649)	337 (171–602)	0·3 (0·2–0·5)	0·2 (0·1–0·3)	0·1 (0·1–0·3)
1-92	Dietary iron deficiency	180,000 (112,000–274,000)	196,000 (124,000–300,000)	229,000 (143,000–355,000)	64·5 (40·4–99·5)	55·6 (35·2–85·9)	62·7 (39–97·7)
1-93	Other nutritional deficiencies	39,400 (26,000–57,300)	36,700 (24,200–52,700)	40,500 (27,100–57,900)	13 (8·6–19·1)	9·5 (6·2–13·9)	9·6 (6·3–13·9)
1-0	Non-communicable diseases	73,300,000 (64,700,000–82,800,000)	88,800,000 (77,500,000–101,000,000)	105,000,000 (91,600,000–119,000,000)	22,700 (20,000–25,900)	20,500 (17,700–23,600)	21,200 (18,200–24,500)
1-1	Neoplasms	14,000,000 (13,600,000–14,300,000)	16,000,000 (15,400,000–16,500,000)	18,600,000 (17,800,000–19,300,000)	4,220 (4,100–4,320)	3,350 (3,240–3,460)	3,210 (3,090–3,330)
1-2	Lip and oral cavity cancer	172,000 (167,000–176,000)	173,000 (167,000–179,000)	204,000 (195,000–212,000)	52·7 (51·3–54·1)	35·9 (34·8–37)	35·2 (33·7–36·6)
1-3	Nasopharynx cancer	33,200 (32,400–34,100)	33,200 (32,200–34,200)	36,600 (35,000–38,200)	10·7 (10·4–11)	7·3 (7·1–7·6)	7·1 (6·8–7·4)
1-4	Other pharynx cancer	60,100 (58,300–62,400)	75,000 (72,500–77,500)	91,200 (86,300–96,000)	18·9 (18·3–19·6)	15·5 (15–16)	15·8 (15–16·7)
1-5	Oesophageal cancer	293,000 (286,000–300,000)	438,000 (426,000–447,000)	525,000 (504,000–544,000)	89 (87–91·2)	89·9 (87·5–91·6)	88·3 (85–91·4)
1-6	Stomach cancer	441,000 (428,000–450,000)	396,000 (381,000–406,000)	446,000 (427,000–461,000)	129 (126–132)	81·9 (79·1–83·7)	77·4 (74·4–79·9)
1-7	Colon and rectum cancer	1,510,000 (1,460,000–1,550,000)	1,670,000 (1,600,000–1,730,000)	1,990,000 (1,900,000–2,060,000)	441 (426–452)	342 (329–353)	340 (326–352)

1-8	Liver cancer	167,000 (163,000–171,000)	444,000 (430,000–457,000)	608,000 (544,000–664,000)	51 (49·8–51·9)	92·9 (90·3–95·6)	106 (94·5–115)
1-9	Liver cancer due to hepatitis B	25,400 (22,000–29,100)	60,500 (52,400–69,900)	77,100 (62,300–95,400)	7·9 (6·9–9·1)	13·1 (11·4–15·1)	14·4 (11·7–17·6)
1-10	Liver cancer due to hepatitis C	53,800 (48,000–59,200)	156,000 (139,000–173,000)	210,000 (177,000–243,000)	16 (14·2–17·6)	31·6 (28·1–35)	34·6 (29·3–40·3)
1-11	Liver cancer due to alcohol use	52,700 (46,700–58,700)	141,000 (125,000–158,000)	209,000 (171,000–247,000)	15·8 (14–17·6)	29·3 (25·9–32·6)	35·6 (29·2–42·2)
1-12	Liver cancer due to NASH	14,800 (12,700–17,300)	42,000 (36,500–48,500)	58,600 (47,500–70,800)	4·3 (3·7–5)	8·6 (7·5–9·9)	9·8 (8–11·8)
1-13	Gallbladder and biliary tract cancer	100,000 (85,500–107,000)	104,000 (94,900–122,000)	119,000 (110,000–143,000)	29 (24·7–31)	21·1 (19·3–24·5)	19·7 (18·2–23·6)
1-14	Pancreatic cancer	653,000 (632,000–667,000)	998,000 (960,000–1,020,000)	1,260,000 (1,200,000–1,310,000)	192 (186–196)	203 (196–207)	209 (199–216)
1-15	Larynx cancer	118,000 (114,000–121,000)	114,000 (110,000–117,000)	129,000 (123,000–135,000)	36·1 (35–37·1)	23·3 (22·5–24)	21·6 (20·6–22·6)
1-16	Tracheal, bronchus, and lung cancer	3,960,000 (3,870,000–4,020,000)	4,210,000 (4,060,000–4,290,000)	4,670,000 (4,460,000–4,820,000)	1,190 (1,160–1,210)	857 (830–873)	762 (729–787)
1-17	Malignant skin melanoma	232,000 (163,000–277,000)	304,000 (225,000–388,000)	342,000 (265,000–459,000)	72·4 (50·7–86·3)	66·7 (50·2–86·5)	64·2 (51·7–89·7)
1-18	Non-melanoma skin cancer	95,100 (79,400–116,000)	139,000 (115,000–171,000)	195,000 (157,000–246,000)	27·7 (23·3–33·1)	27·6 (22·9–33·8)	31·1 (25·2–39)
1-19	Non-melanoma skin cancer (squamous-cell carcinoma)	94,800 (79,200–115,000)	138,000 (114,000–170,000)	194,000 (156,000–244,000)	27·6 (23·2–33·1)	27·4 (22·7–33·6)	30·9 (25–38·8)
1-20	Non-melanoma skin cancer (basal-cell carcinoma)	296 (137–541)	927 (437–1,720)	1,110 (518–2,070)	0·1 (0–0·2)	0·2 (0·1–0·3)	0·2 (0·1–0·3)
1-21	Breast cancer	1,470,000 (1,410,000–1,530,000)	1,430,000 (1,360,000–1,520,000)	1,560,000 (1,460,000–1,670,000)	453 (436–472)	301 (288–318)	282 (266–302)
1-22	Cervical cancer	207,000 (178,000–216,000)	220,000 (199,000–228,000)	246,000 (212,000–259,000)	64·9 (55·4–67·7)	49·7 (45·2–51·6)	49·7 (43·2–52·4)
1-23	Uterine cancer	140,000 (133,000–146,000)	206,000 (193,000–219,000)	272,000 (254,000–291,000)	40·4 (38·5–42·4)	42·1 (39·6–44·7)	45·2 (42·3–48·3)
1-24	Ovarian cancer	379,000 (349,000–393,000)	431,000 (409,000–455,000)	473,000 (439,000–513,000)	115 (106–119)	89·8 (85·5–94·6)	82·2 (76·4–89·4)
1-25	Prostate cancer	782,000 (543,000–874,000)	815,000 (714,000–1,180,000)	1,030,000 (897,000–1,520,000)	212 (148–237)	158 (139–230)	159 (138–233)
1-26	Testicular cancer	30,600 (27,900–34,100)	30,100 (27,000–34,600)	29,200 (25,700–33,700)	10·1 (9·1–11·5)	8·8 (7·8–10·4)	8·1 (7–9·7)
1-27	Kidney cancer	299,000 (290,000–309,000)	404,000 (390,000–417,000)	481,000 (453,000–506,000)	91·5 (88·8–94·5)	84·3 (81·7–86·9)	83 (78·3–87·1)
1-28	Bladder cancer	255,000 (245,000–264,000)	351,000 (332,000–363,000)	434,000 (405,000–459,000)	72 (69·3–74·4)	68·9 (65·5–71·2)	68·4 (64·2–72·2)
1-29	Brain and nervous system cancer	451,000 (391,000–538,000)	554,000 (456,000–616,000)	633,000 (507,000–692,000)	150 (130–179)	135 (112–151)	134 (110–148)
1-30	Thyroid cancer	36,500 (34,000–39,100)	58,700 (53,700–63,900)	69,000 (62,800–74,900)	11·1 (10·3–11·9)	12·5 (11·4–13·6)	12·2 (11·1–13·3)
1-31	Mesothelioma	52,100 (49,100–56,300)	61,100 (57,400–65,000)	70,400 (65,400–75,300)	15 (14·1–16·2)	12·4 (11·6–13·2)	11·4 (10·5–12·2)
1-32	Hodgkin lymphoma	77,300 (58,600–83,800)	54,400 (49,100–69,500)	54,300 (48,100–73,100)	24·8 (18·7–26·8)	13·9 (12·5–18·1)	12·2 (10·6–17)
1-33	Non-Hodgkin's lymphoma	583,000 (565,000–597,000)	581,000 (557,000–602,000)	682,000 (645,000–715,000)	176 (172–180)	123 (118–127)	118 (113–124)
1-34	Multiple myeloma	235,000 (195,000–244,000)	315,000 (293,000–375,000)	389,000 (360,000–478,000)	68·6 (56·6–71·2)	63·5 (59·5–76)	63·2 (58·8–78·6)
1-35	Leukaemia	625,000 (606,000–637,000)	662,000 (636,000–679,000)	739,000 (703,000–770,000)	200 (195–204)	153 (148–157)	144 (138–149)
1-36	Acute lymphoid leukaemia	89,800 (76,900–93,500)	80,400 (72,100–86,300)	80,200 (71,700–87,700)	34·4 (29·5–35·8)	24·5 (22·2–26·1)	22·5 (20·4–24·6)
1-37	Chronic lymphoid leukaemia	88,300 (79,300–93,900)	105,000 (95,300–123,000)	118,000 (106,000–144,000)	25 (22·5–26·5)	20·4 (18·7–24·2)	18·4 (16·6–22·5)
1-38	Acute myeloid leukaemia	201,000 (186,000–228,000)	285,000 (250,000–298,000)	330,000 (293,000–353,000)	64 (59·6–72·5)	66 (58·1–69·4)	63·8 (57·5–68·6)
1-39	Chronic myeloid leukaemia	80,300 (75,300–85,600)	34,800 (32,700–38,900)	36,600 (33,600–41,700)	25·1 (23·6–26·8)	7·8 (7·3–8·6)	7 (6·5–7·8)
1-40	Other leukaemia	166,000 (151,000–174,000)	157,000 (145,000–171,000)	174,000 (158,000–194,000)	52 (47·7–54·9)	34·7 (32·2–37·7)	32·1 (29·5–35·3)
1-41	Other neoplasms	111,000 (86,200–150,000)	157,000 (122,000–207,000)	198,000 (155,000–263,000)	31·2 (24·3–42·1)	31·2 (24·2–41·3)	32·1 (25·2–42·4)
1-42	Myelodysplastic, myeloproliferative, and other haematopoietic neoplasms	111,000 (86,200–150,000)	157,000 (122,000–207,000)	198,000 (155,000–263,000)	31·2 (24·3–42·1)	31·2 (24·2–41·3)	32·1 (25·2–42·4)
1-43	Benign and in situ intestinal neoplasms	0 (0–0)	0 (0–0)	0 (0–0)	0 (0–0)	0 (0–0)	0 (0–0)
1-44	Benign and in situ cervical and uterine neoplasms	0 (0–0)	0 (0–0)	0 (0–0)	0 (0–0)	0 (0–0)	0 (0–0)
1-45	Other benign and in situ neoplasms	0 (0–0)	0 (0–0)	0 (0–0)	0 (0–0)	0 (0–0)	0 (0–0)
1-46	Cardiovascular diseases	17,900,000 (17,100,000–18,600,000)	16,200,000 (15,100,000–17,000,000)	18,700,000 (17,500,000–19,700,000)	5,160 (4,930–5,340)	3,230 (3,050–3,390)	3,100 (2,910–3,260)
1-47	Rheumatic heart disease	194,000 (184,000–205,000)	119,000 (108,000–130,000)	141,000 (127,000–157,000)	57·4 (54·4–60·4)	24·1 (21·9–26·4)	23·8 (21·5–26·4)
1-48	Ischaemic heart disease	11,300,000 (10,800,000–11,600,000)	8,580,000 (8,060,000–8,990,000)	9,680,000 (9,050,000–10,200,000)	3,240 (3,090–3,320)	1,680 (1,590–1,760)	1,570 (1,480–1,650)
1-49	Stroke	3,430,000 (3,180,000–3,650,000)	3,560,000 (3,240,000–3,850,000)	4,160,000 (3,770,000–4,520,000)	978 (908–1,040)	712 (648–775)	690 (626–752)
1-50	Ischaemic stroke	2,070,000 (1,860,000–2,260,000)	1,930,000 (1,680,000–2,170,000)	2,260,000 (1,960,000–2,540,000)	565 (509–617)	369 (318–417)	352 (303–400)
1-51	Intracerebral haemorrhage	902,000 (863,000–937,000)	1,090,000 (1,040,000–1,160,000)	1,270,000 (1,190,000–1,370,000)	268 (257–279)	227 (216–241)	220 (207–236)
1-52	Subarachnoid haemorrhage	457,000 (429,000–485,000)	527,000 (493,000–559,000)	623,000 (573,000–671,000)	145 (136–153)	116 (109–123)	118 (109–127)
1-53	Hypertensive heart disease	526,000 (452,000–582,000)	909,000 (630,000–988,000)	1,100,000 (735,000–1,200,000)	155 (133–172)	186 (128–202)	194 (126–210)
1-54	Non-rheumatic valvular heart disease	306,000 (280,000–342,000)	347,000 (309,000–391,000)	436,000 (385,000–499,000)	85·7 (78·7–95·5)	64·1 (57·6–72·4)	66·2 (59·2–75·7)
1-55	Non-rheumatic calcific aortic valvular heart disease	207,000 (190,000–225,000)	249,000 (218,000–270,000)	315,000 (274,000–346,000)	57·8 (53·4–62·8)	44·9 (40–48·5)	46·8 (41·5–51·2)
1-56	Non-rheumatic degenerative mitral valvular heart disease	97,900 (73,800–126,000)	96,600 (74,700–131,000)	119,000 (91,200–163,000)	27·5 (20·7–35·2)	18·9 (14·6–25·1)	19·1 (14·7–25·9)
1-57	Other non-rheumatic valvular heart diseases	1,510 (945–2,140)	1,250 (947–2,300)	1,390 (1,040–2,360)	0·5 (0·3–0·7)	0·3 (0·2–0·6)	0·3 (0·2–0·6)
1-58	Cardiomyopathy and myocarditis	755,000 (599,000–807,000)	757,000 (685,000–829,000)	839,000 (768,000–951,000)	237 (189–254)	175 (158–191)	166 (153–187)
1-59	Myocarditis	29,400 (24,400–41,200)	50,800 (35,100–58,500)	49,300 (35,700–56,700)	11 (9·2–15·4)	15·9 (11·1–18·2)	14·6 (10·7–16·6)
1-60	Alcoholic cardiomyopathy	204,000 (154,000–239,000)	165,000 (139,000–200,000)	187,000 (157,000–229,000)	63·9 (48·3–74·9)	35·8 (30·3–43·7)	34·5 (29·5–42·2)

1·61	Other cardiomyopathy	521,000 (408,000–565,000)	542,000 (493,000–603,000)	603,000 (551,000–689,000)	162 (128–176)	124 (113–137)	117 (107–132)
1·62	Atrial fibrillation and flutter	515,000 (397,000–675,000)	818,000 (644,000–1,010,000)	1,060,000 (838,000–1,340,000)	141 (108–184)	153 (121–190)	160 (126–203)
1·63	Aortic aneurysm	345,000 (332,000–356,000)	244,000 (231,000–254,000)	278,000 (258,000–293,000)	97·1 (93·6–100)	49·8 (47·6–52)	47·1 (43·9–49·7)
1·64	Peripheral vascular disease	131,000 (77,600–209,000)	207,000 (118,000–353,000)	260,000 (146,000–452,000)	36·2 (21·4–58·3)	39 (22·3–66·8)	39·2 (22–68·2)
1·65	Endocarditis	49,800 (40,400–82,700)	139,000 (74,000–162,000)	159,000 (85,900–188,000)	15 (12·2–24·7)	29·1 (16–33·7)	28·6 (15·8–33·4)
1·66	Chronic respiratory diseases	4,310,000 (3,810,000–4,870,000)	6,260,000 (5,530,000–6,950,000)	7,450,000 (6,540,000–8,270,000)	1,310 (1,140–1,510)	1,380 (1,200–1,560)	1,370 (1,180–1,570)
1·67	Chronic obstructive pulmonary disease	2,880,000 (2,670,000–3,100,000)	4,480,000 (4,050,000–4,750,000)	5,350,000 (4,810,000–5,690,000)	815 (752–875)	885 (805–938)	848 (766–903)
1·68	Pneumoconiosis	27,700 (25,400–30,300)	21,600 (19,500–24,100)	26,000 (23,100–28,900)	7·6 (7–8·3)	4·2 (3·8–4·7)	4·1 (3·6–4·6)
1·69	Silicosis	5,180 (4,380–6,330)	2,700 (2,340–3,780)	3,200 (2,690–4,500)	1·5 (1·2–1·8)	0·6 (0·5–0·8)	0·5 (0·5–0·7)
1·70	Asbestosis	7,950 (6,830–10,100)	12,200 (10,400–13,800)	14,900 (12,600–16,800)	2·2 (1·9–2·7)	2·4 (2–2·7)	2·3 (1·9–2·6)
1·71	Coal workers pneumoconiosis	13,100 (11,500–14,100)	5,080 (4,560–6,040)	5,560 (4,800–6,540)	3·5 (3·1–3·8)	1 (0·9–1·2)	0·9 (0·7–1)
1·72	Other pneumoconiosis	1,530 (1,140–2,040)	1,630 (1,250–2,140)	2,350 (1,740–3,130)	0·5 (0·3–0·6)	0·3 (0·3–0·5)	0·4 (0·3–0·6)
1·73	Asthma	1,120,000 (784,000–1,580,000)	1,260,000 (877,000–1,740,000)	1,480,000 (1,020,000–2,060,000)	402 (277–570)	379 (260–531)	413 (282–584)
1·74	Interstitial lung disease and pulmonary sarcoidosis	217,000 (186,000–295,000)	381,000 (256,000–449,000)	470,000 (310,000–560,000)	63·5 (54·5–85·1)	78·1 (53·3–91·5)	78·3 (53·5–92·3)
1·75	Other chronic respiratory diseases	62,600 (54,600–78,600)	114,000 (94,600–124,000)	124,000 (101,000–136,000)	21·8 (19–27·2)	30 (25·3–32·9)	30·2 (25–33·3)
1·76	Digestive diseases	2,800,000 (2,570,000–3,100,000)	3,490,000 (3,230,000–3,800,000)	4,000,000 (3,710,000–4,360,000)	868 (795–966)	770 (711–846)	772 (711–852)
1·77	Cirrhosis and other chronic liver diseases	1,180,000 (1,150,000–1,200,000)	1,700,000 (1,660,000–1,740,000)	1,960,000 (1,890,000–2,010,000)	372 (362–378)	365 (355–372)	371 (360–382)
1·78	Cirrhosis and other chronic liver diseases due to hepatitis B	54,800 (46,700–63,800)	56,700 (48,700–66,000)	54,100 (44,500–64,900)	17·5 (14·7–20·5)	12·2 (10·5–14·1)	10·5 (8·7–12·6)
1·79	Cirrhosis and other chronic liver diseases due to hepatitis C	473,000 (424,000–528,000)	779,000 (712,000–853,000)	898,000 (795,000–1,010,000)	149 (133–167)	168 (154–184)	174 (155–194)
1·80	Cirrhosis and other chronic liver diseases due to alcohol use	337,000 (291,000–386,000)	486,000 (431,000–548,000)	517,000 (433,000–616,000)	107 (91·7–123)	103 (91·2–116)	95·8 (80·5–113)
1·81	Cirrhosis and other chronic liver diseases due to NAFLD	101,000 (71,200–137,000)	133,000 (89,800–185,000)	178,000 (120,000–251,000)	31·5 (22–43·2)	28 (19·1–38·8)	33·2 (23–47)
1·82	Cirrhosis and other chronic liver diseases due to other causes	213,000 (168,000–265,000)	245,000 (185,000–319,000)	308,000 (231,000–401,000)	66·8 (52·8–83·7)	53·3 (40·7–68·7)	58·1 (44·4–74·9)
1·83	Upper digestive system diseases	536,000 (390,000–759,000)	445,000 (296,000–677,000)	527,000 (346,000–803,000)	164 (118–235)	104 (67·7–160)	110 (70·2–171)
1·84	Peptic ulcer disease	188,000 (177,000–199,000)	105,000 (96,700–115,000)	119,000 (109,000–132,000)	54·3 (51–57·3)	21·5 (19·7–23·5)	20·7 (18·7–22·8)
1·85	Gastritis and duodenitis	91,300 (61,700–131,000)	73,700 (50,900–101,000)	81,600 (55,300–113,000)	28·9 (19·4–41·6)	17·5 (12–24·5)	17·4 (11·6–24·5)
1·86	Gastro-oesophageal reflux disease (GERD)	257,000 (132,000–460,000)	266,000 (134,000–478,000)	326,000 (164,000–580,000)	81·1 (41·7–145)	64·6 (32·8–116)	71·6 (36·3–128)
1·87	Appendicitis	19,300 (15,900–23,600)	21,000 (17,100–25,000)	22,700 (18,400–27,400)	6·5 (5·3–7·9)	5·4 (4·4–6·4)	5·2 (4·3–6·3)
1·88	Paralytic ileus and intestinal obstruction	132,000 (119,000–160,000)	197,000 (166,000–217,000)	222,000 (185,000–245,000)	41·9 (38·1–50·3)	48·3 (42·2–52·5)	45·3 (39·1–49·4)
1·89	Inguinal, femoral, and abdominal hernia	76,200 (59,900–96,000)	80,800 (65,300–99,900)	92,200 (74,000–113,000)	24·4 (18·9–30·9)	19·5 (15·7–24·2)	19·5 (15·4–24·2)
1·90	Inflammatory bowel disease	209,000 (155,000–271,000)	220,000 (179,000–265,000)	245,000 (201,000–294,000)	65·6 (48·6–85)	52·2 (41·6–63·9)	51·3 (40·9–62·4)
1·91	Vascular intestinal disorders	150,000 (141,000–156,000)	186,000 (173,000–196,000)	212,000 (196,000–223,000)	42·8 (40·3–44·5)	37·2 (34·9–38·9)	34·8 (32·4–36·5)
1·92	Gallbladder and biliary diseases	270,000 (196,000–369,000)	310,000 (236,000–404,000)	340,000 (260,000–440,000)	82·6 (59–113)	70·4 (52·4–93·5)	66·7 (49·7–88·6)
1·93	Pancreatitis	100,000 (92,700–110,000)	133,000 (123,000–145,000)	145,000 (134,000–161,000)	30·8 (28·5–33·9)	29·8 (27·6–32·8)	28·6 (26·4–31·6)
1·94	Other digestive diseases	127,000 (101,000–158,000)	194,000 (165,000–261,000)	237,000 (200,000–326,000)	36·8 (29·5–46·1)	39·3 (33·4–52·9)	40 (33·5–54·9)
1·95	Neurological disorders	4,320,000 (2,450,000–7,060,000)	5,800,000 (3,490,000–9,200,000)	6,580,000 (4,020,000–10,400,000)	1,360 (752–2,230)	1,340 (765–2,160)	1,340 (758–2,180)
1·96	Alzheimer's disease and other dementias	1,240,000 (593,000–2,630,000)	1,880,000 (907,000–3,900,000)	2,280,000 (1,100,000–4,720,000)	331 (158–701)	320 (155–661)	319 (154–656)
1·97	Parkinson's disease	242,000 (223,000–261,000)	435,000 (399,000–462,000)	557,000 (511,000–590,000)	64·7 (59·7–69·6)	81·1 (74·8–86·1)	84 (77·5–89·1)
1·98	Idiopathic epilepsy	309,000 (209,000–452,000)	387,000 (247,000–572,000)	361,000 (236,000–531,000)	109 (73·1–160)	111 (69·5–162)	95·2 (62·1–139)
1·99	Multiple sclerosis	142,000 (117,000–171,000)	216,000 (176,000–251,000)	242,000 (196,000–279,000)	45·3 (37·3–54·5)	49·5 (40·3–57·5)	49·3 (40·2–57·4)
1·100	Motor neuron disease	116,000 (112,000–119,000)	202,000 (196,000–206,000)	253,000 (243,000–260,000)	37·3 (36·2–38·4)	45·5 (44·2–46·5)	46·9 (45·2–48·3)
1·101	Headache disorders	2,070,000 (407,000–4,470,000)	2,410,000 (519,000–5,180,000)	2,590,000 (558,000–5,580,000)	695 (134–1,520)	663 (134–1,430)	678 (133–1,470)
1·102	Migraine	1,850,000 (268,000–4,190,000)	2,150,000 (350,000–4,850,000)	2,310,000 (371,000–5,170,000)	625 (86·8–1,420)	593 (86·8–1,360)	609 (86–1,390)
1·103	Tension-type headache	213,000 (58,200–724,000)	264,000 (75,100–879,000)	282,000 (79,400–945,000)	70·5 (18·9–241)	69·7 (19–239)	69·4 (18·8–237)
1·104	Other neurological disorders	208,000 (189,000–233,000)	265,000 (235,000–302,000)	296,000 (265,000–333,000)	73·1 (66–82·7)	69·6 (60·2–81·2)	69 (60–80·5)
1·105	Mental disorders	5,700,000 (4,260,000–7,420,000)	7,800,000 (5,800,000–10,200,000)	7,980,000 (5,980,000–10,400,000)	1,920 (1,430–2,500)	2,170 (1,610–2,860)	2,100 (1,570–2,750)
1·106	Schizophrenia	825,000 (609,000–1,030,000)	1,070,000 (793,000–1,330,000)	1,070,000 (793,000–1,330,000)	265 (196–332)	279 (207–350)	262 (195–328)
1·107	Depressive disorders	1,880,000 (1,310,000–2,540,000)	2,860,000 (2,000,000–3,900,000)	2,860,000 (2,020,000–3,870,000)	624 (437–848)	796 (550–1,090)	754 (526–1,020)
1·108	Major depressive disorder	1,470,000 (1,020,000–2,010,000)	2,480,000 (1,710,000–3,370,000)	2,410,000 (1,660,000–3,280,000)	492 (339–676)	696 (475–954)	640 (441–874)
1·109	Dysthymia	405,000 (265,000–604,000)	379,000 (248,000–562,000)	458,000 (300,000–676,000)	133 (86·4–198)	99·7 (64·4–149)	114 (74·8–169)
1·110	Bipolar disorder	394,000 (250,000–573,000)	484,000 (311,000–702,000)	511,000 (329,000–744,000)	133 (84·5–194)	133 (84·8–192)	132 (84·2–192)
1·111	Anxiety disorders	1,460,000 (1,020,000–1,980,000)	1,950,000 (1,360,000–2,640,000)	2,030,000 (1,430,000–2,750,000)	486 (339–659)	533 (372–726)	522 (362–709)
1·112	Eating disorders	253,000 (160,000–375,000)	303,000 (192,000–449,000)	290,000 (185,000–427,000)	85·5 (54·3–127)	96·7 (61·4–143)	89·7 (57·2–132)
1·113	Anorexia nervosa	73,500 (45,200–114,000)	84,200 (52,100–131,000)	86,800 (53,300–135,000)	25·7 (15·7–40·1)	27·1 (16·6–42·7)	27·4 (16·7–42·8)

1-114	Bulimia nervosa	180,000 (104,000–280,000)	219,000 (130,000–344,000)	203,000 (119,000–317,000)	59·8 (35–93·9)	69·6 (41·3–110)	62·3 (37–97·4)
1-115	Autism spectrum disorders	217,000 (142,000–308,000)	304,000 (199,000–433,000)	326,000 (215,000–463,000)	79·9 (52·3–113)	94·3 (61·7–134)	96·9 (63·7–138)
1-116	Attention-deficit/hyperactivity disorder	57,100 (32,200–96,500)	72,400 (41,700–125,000)	73,300 (41,900–125,000)	22·8 (13–39·1)	25·3 (14·7–44·1)	25·5 (14·4–43·8)
1-117	Conduct disorder	142,000 (78,600–225,000)	161,000 (89,300–258,000)	175,000 (98,800–279,000)	63·6 (35·2–101)	61·5 (34–98·7)	66·6 (37·6–106)
1-118	Idiopathic developmental intellectual disability	71,300 (30,700–121,000)	75,700 (30,000–131,000)	73,700 (28,400–130,000)	25·9 (10·9–44·2)	23·1 (8·9–40·4)	21·5 (7·9–38·2)
1-119	Other mental disorders	410,000 (262,000–611,000)	520,000 (334,000–780,000)	563,000 (365,000–842,000)	131 (83·4–196)	132 (83·7–198)	131 (83·6–196)
1-120	Substance use disorders	2,510,000 (1,940,000–3,120,000)	4,990,000 (4,140,000–5,880,000)	7,790,000 (6,610,000–9,040,000)	825 (638–1,030)	1,410 (1,160–1,690)	2,120 (1,780–2,480)
1-121	Alcohol use disorders	1,110,000 (830,000–1,460,000)	1,280,000 (1,030,000–1,590,000)	1,390,000 (1,110,000–1,720,000)	363 (271–474)	337 (264–424)	337 (265–424)
1-122	Drug use disorders	1,400,000 (1,060,000–1,770,000)	3,710,000 (3,070,000–4,440,000)	6,400,000 (5,410,000–7,480,000)	462 (347–587)	1,080 (880–1,300)	1,780 (1,500–2,100)
1-123	Opioid use disorders	825,000 (628,000–1,070,000)	2,810,000 (2,270,000–3,400,000)	4,980,000 (4,150,000–5,930,000)	268 (203–349)	816 (652–999)	1,390 (1,150–1,680)
1-124	Cocaine use disorders	195,000 (130,000–278,000)	361,000 (289,000–453,000)	599,000 (510,000–711,000)	65·6 (43·4–93·5)	101 (79·7–129)	162 (136–196)
1-125	Amphetamine use disorders	88,200 (52,500–140,000)	138,000 (113,000–175,000)	270,000 (231,000–315,000)	29·4 (17·4–47)	39·9 (31·7–51·4)	74·3 (62·8–88·6)
1-126	Cannabis use disorders	83,100 (50,000–127,000)	93,100 (57,200–142,000)	92,700 (57,100–142,000)	29·8 (17·7–46)	29·4 (17·9–45·6)	28·7 (17·4–44)
1-127	Other drug use disorders	205,000 (146,000–282,000)	314,000 (251,000–388,000)	458,000 (382,000–545,000)	68·4 (48·2–94·5)	89·6 (71–112)	125 (104–150)
1-128	Diabetes and kidney diseases	3,190,000 (2,770,000–3,670,000)	5,860,000 (5,000,000–6,830,000)	7,200,000 (6,120,000–8,430,000)	948 (822–1,090)	1,220 (1,040–1,420)	1,250 (1,060–1,460)
1-129	Diabetes mellitus	2,250,000 (1,900,000–2,670,000)	3,920,000 (3,190,000–4,770,000)	4,770,000 (3,830,000–5,890,000)	674 (565–799)	819 (664–998)	834 (671–1,030)
1-130	Diabetes mellitus type 1	262,000 (214,000–322,000)	339,000 (267,000–422,000)	377,000 (293,000–474,000)	83·8 (68·5–104)	85·8 (68·4–106)	86·3 (67·7–108)
1-131	Diabetes mellitus type 2	1,990,000 (1,670,000–2,370,000)	3,580,000 (2,900,000–4,380,000)	4,390,000 (3,530,000–5,430,000)	590 (492–705)	733 (590–898)	748 (596–931)
1-132	Chronic kidney disease	934,000 (847,000–1,030,000)	1,930,000 (1,770,000–2,110,000)	2,420,000 (2,220,000–2,640,000)	274 (248–303)	397 (362–435)	414 (380–451)
1-133	Chronic kidney disease due to diabetes mellitus type 1	32,400 (22,200–44,400)	66,800 (45,000–91,900)	86,800 (58,900–123,000)	10·2 (6·9–14·1)	15·2 (10·3–20·8)	17·3 (11·5–23·9)
1-134	Chronic kidney disease due to diabetes mellitus type 2	187,000 (145,000–231,000)	547,000 (443,000–654,000)	706,000 (567,000–843,000)	53·8 (41·9–66·1)	108 (87·8–129)	114 (91·7–135)
1-135	Chronic kidney disease due to hypertension	282,000 (242,000–322,000)	590,000 (499,000–686,000)	763,000 (650,000–878,000)	79·1 (67·9–90)	113 (95·8–132)	121 (103–139)
1-136	Chronic kidney disease due to glomerulonephritis	164,000 (137,000–197,000)	270,000 (218,000–329,000)	316,000 (256,000–393,000)	50·6 (42·3–60·3)	61·6 (50·2–74·3)	62·5 (51·2–76)
1-137	Chronic kidney disease due to other and unspecified causes	268,000 (219,000–325,000)	462,000 (372,000–565,000)	553,000 (437,000–677,000)	80 (65·8–96·4)	98·8 (80·1–120)	99·6 (80·4–121)
1-138	Acute glomerulonephritis	1,770 (1,670–1,840)	1,560 (1,460–1,640)	4,110 (3,770–4,440)	0·5 (0·5–0·5)	0·3 (0·3–0·3)	0·7 (0·7–0·8)
1-139	Skin and subcutaneous diseases	1,900,000 (1,330,000–2,650,000)	2,260,000 (1,580,000–3,160,000)	2,430,000 (1,710,000–3,390,000)	682 (473–952)	656 (456–917)	656 (455–919)
1-140	Dermatitis	521,000 (314,000–807,000)	572,000 (344,000–890,000)	599,000 (360,000–924,000)	197 (117–309)	182 (107–285)	181 (107–284)
1-141	Atopic dermatitis	346,000 (185,000–579,000)	387,000 (207,000–649,000)	397,000 (213,000–661,000)	138 (74·3–232)	133 (70·7–223)	132 (70·6–222)
1-142	Contact dermatitis	160,000 (102,000–234,000)	166,000 (106,000–244,000)	182,000 (117,000–266,000)	53·2 (34·4–78·3)	43·8 (28·4–64·4)	43·5 (28·2–63·8)
1-143	Seborrhoeic dermatitis	15,700 (8,970–24,700)	18,300 (10,400–28,600)	20,400 (11,700–32,100)	5·1 (2·9–8·1)	4·8 (2·7–7·6)	4·8 (2·7–7·6)
1-144	Psoriasis	327,000 (232,000–432,000)	367,000 (261,000–483,000)	392,000 (283,000–516,000)	109 (77–144)	94·8 (67·6–125)	92 (66·2–122)
1-145	Bacterial skin diseases	44,200 (33,800–68,600)	111,000 (73,100–155,000)	133,000 (84,800–189,000)	14 (10·7–21·3)	24·8 (16·8–34·1)	25·3 (16·9–34·9)
1-146	Cellulitis	35,500 (26,900–53,600)	74,800 (49,600–99,100)	88,800 (56,100–117,000)	11·3 (8·5–16·9)	17·1 (11·7–22)	17·4 (11·4–22·3)
1-147	Pyoderma	8,720 (5,660–16,300)	36,200 (19,600–56,700)	44,100 (23,500–71,100)	2·7 (1·8–5)	7·8 (4·2–11·8)	7·9 (4·3–12·3)
1-148	Scabies	10,000 (5,430–16,000)	11,700 (6,680–18,600)	12,100 (6,830–19,100)	3·7 (2–5·8)	3·8 (2·1–6)	3·7 (2·1–5·8)
1-149	Fungal skin diseases	42,700 (17,600–89,600)	55,900 (23,000–117,000)	63,100 (26,000–132,000)	13·7 (5·6–28·7)	13·6 (5·6–28·7)	13·6 (5·5–28·5)
1-150	Viral skin diseases	362,000 (234,000–534,000)	424,000 (275,000–627,000)	444,000 (289,000–655,000)	137 (88·6–203)	134 (86·4–199)	133 (86·4–198)
1-151	Acne vulgaris	169,000 (101,000–265,000)	186,000 (112,000–293,000)	201,000 (121,000–315,000)	63·8 (38·2–100)	60·9 (36·6–96·1)	64·4 (39–101)
1-152	Alopecia areata	35,400 (22,400–52,700)	41,700 (26,300–61,700)	44,500 (28,200–65,700)	11·8 (7·4–17·6)	11·5 (7·3–17)	11·4 (7·2–16·9)
1-153	Pruritus	24,700 (11,900–44,700)	33,000 (16,000–59,900)	36,500 (17,800–66,500)	8·2 (4–14·8)	8·6 (4·2–15·6)	8·7 (4·2–15·7)
1-154	Urticaria	140,000 (92,700–197,000)	166,000 (110,000–232,000)	171,000 (114,000–240,000)	53 (35·1–74·9)	53·6 (35·7–75·6)	53·5 (35·5–75·6)
1-155	Decubitus ulcer	48,700 (35,500–67,600)	46,100 (35,400–63,200)	55,800 (42,500–77,300)	13·5 (9·9–19)	8·9 (6·8–12·1)	8·8 (6·7–12·2)
1-156	Other skin and subcutaneous diseases	180,000 (89,900–325,000)	246,000 (122,000–447,000)	281,000 (140,000–511,000)	58 (28·9–105)	60 (29·6–109)	60·2 (29·8–110)
1-157	Sense organ diseases	1,850,000 (1,290,000–2,570,000)	2,560,000 (1,790,000–3,540,000)	3,010,000 (2,120,000–4,160,000)	548 (382–760)	544 (381–754)	537 (376–742)
1-158	Blindness and vision loss	282,000 (195,000–391,000)	362,000 (252,000–503,000)	436,000 (303,000–602,000)	86·5 (59·4–121)	82·4 (56·4–116)	83·8 (57·7–117)
1-159	Glaucoma	17,300 (12,000–23,700)	22,500 (15,600–30,700)	30,600 (21,300–41,700)	4·6 (3·2–6·3)	4 (2·7–5·4)	4·4 (3·1–6)
1-160	Cataract	65,200 (46,300–88,500)	89,600 (63,800–121,000)	113,000 (80,400–152,000)	18·1 (12·8–24·7)	16·8 (11·9–22·9)	17·4 (12·3–23·8)
1-161	Age-related macular degeneration	10,800 (7,400–15,400)	13,400 (9,230–19,000)	18,200 (12,500–25,700)	2·9 (2–4·1)	2·4 (1·7–3·4)	2·6 (1·8–3·7)
1-162	Refraction disorders	118,000 (78,000–169,000)	143,000 (94,600–208,000)	161,000 (107,000–231,000)	39·6 (25·9–57·4)	39·3 (25·7–57·6)	39·1 (25·6–57·3)
1-163	Near vision loss	26,900 (12,300–52,900)	38,300 (17,700–75,200)	46,500 (21,200–91,400)	7·7 (3·6–15·1)	7·7 (3·5–15·1)	7·7 (3·5–15)
1-164	Other vision loss	44,500 (31,300–61,100)	55,300 (39,000–75,600)	67,500 (48,100–92,400)	13·6 (9·5–18·8)	12·2 (8·6–16·8)	12·6 (8·9–17·3)
1-165	Age-related and other hearing loss	1,450,000 (1,010,000–2,020,000)	2,040,000 (1,420,000–2,850,000)	2,390,000 (1,670,000–3,340,000)	426 (294–596)	426 (295–598)	418 (288–585)
1-166	Other sense organ diseases	112,000 (68,800–165,000)	157,000 (96,200–230,000)	184,000 (113,000–268,000)	34·8 (21·4–51·2)	35·8 (21·9–52·5)	35·8 (22–52·3)

1·167	Musculoskeletal disorders	9,640,000 (7,010,000–12,700,000)	12,600,000 (9,120,000–16,400,000)	15,300,000 (11,100,000–20,000,000)	3,070 (2,220–4,050)	3,010 (2,180–3,920)	3,260 (2,370–4,260)
1·168	Rheumatoid arthritis	158,000 (119,000–199,000)	244,000 (183,000–309,000)	289,000 (217,000–367,000)	47·7 (36–60·3)	53·3 (39·8–67·7)	53·5 (39·8–68·3)
1·169	Osteoarthritis	1,170,000 (598,000–2,360,000)	1,650,000 (847,000–3,320,000)	2,160,000 (1,120,000–4,320,000)	352 (179–709)	337 (173–679)	367 (189–739)
1·170	Low back pain	4,790,000 (3,390,000–6,430,000)	5,680,000 (4,100,000–7,470,000)	6,170,000 (4,450,000–8,120,000)	1,540 (1,090–2,060)	1,390 (997–1,830)	1,360 (976–1,800)
1·171	Neck pain	1,290,000 (853,000–1,840,000)	1,160,000 (804,000–1,600,000)	2,190,000 (1,490,000–3,090,000)	410 (270–586)	268 (185–368)	479 (323–678)
1·172	Gout	97,300 (62,000–137,000)	219,000 (147,000–300,000)	286,000 (187,000–395,000)	29·4 (18·6–41·6)	47·1 (31·3–64·8)	52·3 (34·4–72·3)
1·173	Other musculoskeletal disorders	2,130,000 (1,520,000–2,900,000)	3,640,000 (2,620,000–4,860,000)	4,160,000 (3,000,000–5,510,000)	692 (494–940)	911 (653–1,220)	946 (681–1,260)
1·174	Other non-communicable diseases	5,080,000 (4,190,000–6,230,000)	5,010,000 (4,050,000–6,180,000)	5,660,000 (4,520,000–7,060,000)	1,830 (1,540–2,200)	1,420 (1,180–1,720)	1,440 (1,180–1,760)
1·175	Congenital anomalies	1,290,000 (1,140,000–1,400,000)	981,000 (889,000–1,120,000)	930,000 (834,000–1,070,000)	556 (492–602)	403 (368–464)	380 (342–438)
1·176	Neural tube defects	79,700 (72,200–94,800)	59,100 (49,200–67,800)	70,800 (57,700–81,600)	35·5 (32·2–42·2)	26·2 (21·8–30·2)	32·5 (26·5–37·8)
1·177	Congenital heart anomalies	509,000 (413,000–547,000)	278,000 (252,000–337,000)	234,000 (207,000–284,000)	220 (178–237)	115 (104–140)	96·6 (84·6–119)
1·178	Orofacial clefts	4,210 (3,040–5,760)	3,080 (2,050–4,400)	3,490 (2,230–5,070)	1·7 (1·2–2·3)	1 (0·7–1·4)	1·1 (0·7–1·5)
1·179	Down syndrome	32,700 (28,900–38,800)	58,700 (47,400–64,100)	67,400 (55,300–74,700)	13·1 (11·6–15·4)	18 (14·9–19·9)	19·1 (15·9–21·4)
1·180	Turner syndrome	1,290 (607–2,200)	1,390 (653–2,310)	1,390 (645–2,380)	0·5 (0·2–0·8)	0·5 (0·2–0·8)	0·5 (0·2–0·8)
1·181	Klinefelter syndrome	301 (134–586)	318 (143–618)	328 (145–637)	0·1 (0–0·2)	0·1 (0–0·2)	0·1 (0–0·2)
1·182	Other chromosomal abnormalities	118,000 (98,700–150,000)	146,000 (121,000–174,000)	147,000 (126,000–178,000)	52 (43·5–66·2)	64·6 (54–78·3)	66·5 (57·2–82·1)
1·183	Congenital musculoskeletal and limb anomalies	127,000 (88,800–173,000)	138,000 (101,000–181,000)	145,000 (102,000–196,000)	49·5 (35–67·5)	47·9 (35·5–62·4)	48·7 (35·5–65·5)
1·184	Urogenital congenital anomalies	51,800 (38,500–75,200)	39,100 (29,300–50,700)	33,400 (24,200–43,000)	23·1 (17·1–33·7)	17·8 (13·2–23·3)	15·4 (11–20·3)
1·185	Digestive congenital anomalies	70,800 (57,300–95,900)	60,100 (44,700–73,700)	51,800 (39,000–64,000)	31·6 (25·4–42·9)	27 (19·9–33·4)	23·7 (17·6–29·5)
1·186	Other congenital anomalies	292,000 (236,000–326,000)	198,000 (176,000–247,000)	177,000 (151,000–222,000)	129 (103–143)	85·2 (76·9–108)	76·3 (66–96·8)
1·187	Urinary diseases and male infertility	292,000 (252,000–336,000)	400,000 (352,000–462,000)	497,000 (433,000–574,000)	84·6 (72·9–97·7)	81 (70·6–94·1)	83·9 (72·3–98·1)
1·188	Urinary tract infections and interstitial nephritis	189,000 (167,000–202,000)	276,000 (248,000–304,000)	338,000 (304,000–368,000)	53·2 (47·2–57·2)	54 (49·3–59)	54·9 (49·4–59·7)
1·189	Urolithiasis	19,700 (14,700–25,600)	18,300 (14,700–22,700)	23,400 (18,800–29,000)	6·2 (4·5–8·1)	4·1 (3·3–5·1)	4·5 (3·6–5·6)
1·190	Benign prostatic hyperplasia	52,500 (32,100–77,100)	80,200 (51,100–117,000)	101,000 (64,300–148,000)	15·4 (9·3–22·4)	16·1 (10·2–23·4)	16·1 (10·3–23·7)
1·191	Male infertility	7,830 (2,890–18,400)	6,290 (2,370–14,200)	11,500 (4,320–25,800)	2·5 (0·9–5·7)	1·9 (0·7–4·4)	3·4 (1·3–7·8)
1·192	Other urinary diseases	22,800 (17,800–27,100)	19,600 (16,300–29,300)	23,000 (19,200–34,000)	7·4 (5·9–8·7)	4·8 (4–7·1)	4·9 (4·1–7·1)
1·193	Gynaecological diseases	898,000 (617,000–1,270,000)	924,000 (639,000–1,280,000)	982,000 (675,000–1,370,000)	291 (200–410)	254 (175–355)	260 (179–363)
1·194	Uterine fibroids	45,300 (21,600–85,300)	64,500 (30,400–121,000)	67,500 (32,600–124,000)	14·6 (7–27·6)	16·2 (7·6–30·4)	16·3 (7·8–30·2)
1·195	Polycystic ovarian syndrome	37,500 (16,100–77,600)	32,700 (14,800–64,000)	45,300 (20,500–87,300)	12·5 (5·4–25·7)	9·9 (4·5–19·4)	13·4 (6·1–25·9)
1·196	Female infertility	5,140 (515–15,800)	3,410 (362–10,600)	10,300 (2,090–27,400)	1·6 (0·2–4·9)	1·1 (0·1–3·3)	3 (0·6–8·2)
1·197	Endometriosis	77,000 (43,700–126,000)	62,500 (40,600–90,600)	60,500 (37,900–92,700)	24·7 (14·1–40·2)	18·1 (11·7–26·6)	17·3 (10·8–26·5)
1·198	Genital prolapse	16,300 (8,010–30,600)	20,400 (10,000–36,900)	24,200 (11,800–44,200)	4·9 (2·4–9·3)	4·1 (2–7·5)	4·1 (2–7·5)
1·199	Premenstrual syndrome	249,000 (151,000–395,000)	306,000 (186,000–476,000)	322,000 (197,000–498,000)	84·2 (51–132)	89·6 (54·6–139)	94·5 (58·4–147)
1·200	Other gynaecological diseases	469,000 (299,000–678,000)	435,000 (291,000–603,000)	452,000 (300,000–636,000)	148 (95·1–214)	115 (76·6–159)	111 (73·5–158)
1·201	Haemoglobinopathies and haemolytic anaemias	145,000 (118,000–186,000)	108,000 (90,000–136,000)	121,000 (99,000–153,000)	50·8 (40·7–65·4)	30·2 (24·8–38·3)	30·9 (24·9–39·7)
1·202	Thalassaemias	3,170 (2,590–3,840)	5,990 (5,150–6,890)	5,570 (4,610–6,850)	1·3 (1·1–1·6)	2·2 (1·9–2·6)	2·1 (1·7–2·6)
1·203	Thalassaemias trait	19,600 (12,100–31,000)	18,800 (11,700–29,400)	21,900 (13,300–34,700)	6·9 (4·2–10·7)	5·7 (3·5–8·9)	6·2 (3·8–10)
1·204	Sickle cell disorders	44,400 (39,600–50,700)	22,500 (20,700–24,200)	21,200 (18,600–24,700)	17 (15·1–19·7)	6·8 (6·2–7·3)	6·1 (5·3–7·1)
1·205	Sickle cell trait	29,400 (18,000–45,300)	12,500 (7,790–19,500)	14,200 (8,260–23,100)	10·6 (6·5–16·6)	3·8 (2·4–6)	4·1 (2·4–6·7)
1·206	G6PD deficiency	8,590 (6,940–10,200)	10,200 (8,080–12,100)	15,100 (12,600–17,600)	2·9 (2·3–3·4)	2·9 (2·3–3·4)	3·9 (3·3–4·6)
1·207	G6PD trait	369 (224–569)	555 (352–829)	569 (339–887)	0·1 (0·1–0·2)	0·2 (0·1–0·2)	0·2 (0·1–0·3)
1·208	Other haemoglobinopathies and haemolytic anaemias	39,800 (34,600–46,900)	37,600 (32,100–45,400)	42,500 (35,000–52,300)	12 (10·3–14·3)	8·6 (7·2–10·5)	8·4 (6·8–10·6)
1·209	Endocrine, metabolic, blood, and immune disorders	994,000 (770,000–1,260,000)	1,450,000 (1,180,000–1,760,000)	1,650,000 (1,350,000–1,980,000)	322 (250–409)	347 (284–423)	346 (285–417)
1·210	Oral disorders	967,000 (592,000–1,480,000)	963,000 (590,000–1,480,000)	1,340,000 (813,000–2,040,000)	301 (182–462)	223 (138–344)	269 (163–418)
1·211	Caries of deciduous teeth	6,210 (2,680–13,300)	5,750 (2,440–12,200)	6,480 (2,800–13,700)	2·9 (1·2–6·2)	2·5 (1–5·3)	2·9 (1·3–6·2)
1·212	Caries of permanent teeth	65,700 (30,100–126,000)	75,300 (33,600–145,000)	81,300 (35,900–156,000)	22·5 (10·3–43·5)	21 (9·5–41)	21·3 (9·6–41·1)
1·213	Periodontal disease	210,000 (82,400–467,000)	210,000 (81,900–463,000)	316,000 (125,000–692,000)	66·2 (25·9–150)	46·3 (18·1–105)	63 (24·8–144)
1·214	Edentulism	531,000 (328,000–801,000)	484,000 (314,000–690,000)	729,000 (462,000–1,080,000)	156 (96·5–235)	102 (65·5–146)	129 (81·4–192)
1·215	Other oral disorders	155,000 (96,500–227,000)	188,000 (117,000–276,000)	205,000 (128,000–299,000)	53 (33·1–77·7)	52 (32·3–76·3)	52·9 (33·1–77·2)
1·216	Sudden infant death syndrome	499,000 (457,000–542,000)	181,000 (163,000–202,000)	139,000 (125,000–154,000)	224 (205–243)	84·9 (76·2–94·5)	68 (60·8–75·2)
1·0	Injuries	10,800,000 (9,910,000–12,000,000)	10,000,000 (9,080,000–11,200,000)	11,100,000 (9,830,000–12,600,000)	3,680 (3,390–4,050)	2,670 (2,450–2,950)	2,680 (2,430–3,000)
1·1	Transport injuries	3,560,000 (3,370,000–3,780,000)	2,810,000 (2,610,000–3,050,000)	2,840,000 (2,590,000–3,110,000)	1,240 (1,180–1,310)	786 (738–842)	734 (679–794)
1·2	Road injuries	3,250,000 (3,100,000–3,430,000)	2,570,000 (2,400,000–2,780,000)	2,540,000 (2,340,000–2,760,000)	1,140 (1,090–1,200)	721 (679–770)	664 (618–712)

1-3	Pedestrian road injuries	518,000 (493,000–546,000)	366,000 (341,000–397,000)	399,000 (368,000–434,000)	186 (178–195)	102 (96·1–109)	103 (96–110)
1-4	Cyclist road injuries	123,000 (107,000–141,000)	105,000 (87,500–125,000)	123,000 (102,000–148,000)	44·9 (39·8–50·8)	27·3 (23·1–32·1)	28·9 (24·4–34·1)
1-5	Motorcyclist road injuries	282,000 (262,000–307,000)	325,000 (297,000–359,000)	328,000 (295,000–368,000)	96 (89·5–104)	87·3 (80·8–95·1)	81·8 (74·4–90)
1-6	Motor vehicle road injuries	2,320,000 (2,220,000–2,430,000)	1,750,000 (1,640,000–1,880,000)	1,670,000 (1,540,000–1,810,000)	809 (777–846)	499 (472–530)	445 (414–476)
1-7	Other road injuries	13,400 (10,900–16,500)	20,300 (17,200–23,900)	22,400 (18,800–26,800)	4·6 (3·8–5·6)	5·5 (4·7–6·4)	5·6 (4·8–6·5)
1-8	Other transport injuries	310,000 (271,000–358,000)	243,000 (214,000–278,000)	296,000 (253,000–350,000)	103 (90·4–118)	64·2 (57·5–72·1)	70·1 (61·2–81·4)
1-9	Unintentional injuries	3,760,000 (3,100,000–4,640,000)	3,890,000 (3,190,000–4,790,000)	4,820,000 (3,880,000–6,050,000)	1,260 (1,040–1,540)	939 (780–1,150)	1,030 (833–1,280)
1-10	Falls	1,400,000 (1,090,000–1,800,000)	1,930,000 (1,550,000–2,420,000)	2,520,000 (2,010,000–3,190,000)	431 (335–558)	405 (324–512)	458 (361–582)
1-11	Drowning	291,000 (284,000–298,000)	215,000 (208,000–223,000)	200,000 (193,000–206,000)	113 (110–117)	71·3 (68·1–74·3)	64 (61·6–66·3)
1-12	Fire, heat, and hot substances	426,000 (351,000–538,000)	271,000 (208,000–367,000)	293,000 (217,000–411,000)	152 (128–189)	71·7 (56·6–94·4)	70·1 (53·5–95·6)
1-13	Poisonings	117,000 (99,500–138,000)	95,300 (86,600–106,000)	116,000 (100,000–136,000)	40·9 (34·9–48·4)	27·7 (25·2–30·8)	31·9 (27·4–37·3)
1-14	Poisoning by carbon monoxide	44,400 (42,700–46,500)	50,400 (49,000–52,000)	53,400 (48,400–57,000)	15·4 (14·8–16·1)	14·4 (14–14·9)	14·6 (13·2–15·5)
1-15	Poisoning by other means	72,400 (56,200–92,000)	45,000 (36,900–54,900)	63,000 (47,800–81,700)	25·6 (19·9–32·5)	13·3 (11–16·2)	17·3 (13·2–22·4)
1-16	Exposure to mechanical forces	731,000 (553,000–982,000)	517,000 (381,000–700,000)	625,000 (448,000–871,000)	246 (188–328)	130 (97·7–175)	144 (105–201)
1-17	Unintentional firearm injuries	118,000 (90,800–132,000)	49,100 (43,500–59,100)	49,900 (42,300–61,200)	43·1 (33·2–47·8)	14·5 (12·9–17·4)	13·6 (11·9–16·2)
1-18	Other exposure to mechanical forces	614,000 (441,000–856,000)	468,000 (337,000–646,000)	575,000 (403,000–813,000)	203 (147–282)	116 (84·3–160)	131 (92·1–186)
1-19	Adverse effects of medical treatment	141,000 (125,000–160,000)	169,000 (143,000–206,000)	188,000 (159,000–230,000)	45·7 (40·3–51·9)	40·2 (34·2–48·7)	39 (33·1–47·4)
1-20	Animal contact	42,200 (29,800–60,100)	40,600 (29,300–56,800)	61,800 (43,000–89,100)	14·3 (10·2–20·1)	10·4 (7·6–14·3)	14·7 (10·3–20·9)
1-21	Venomous animal contact	15,500 (11,200–20,500)	12,200 (9,310–15,600)	21,100 (15,200–27,800)	5·3 (3·8–7)	3·3 (2·5–4·2)	5·3 (3·8–7)
1-22	Non-venomous animal contact	26,700 (17,500–41,500)	28,300 (19,200–42,700)	40,700 (26,300–64,300)	9 (6–13·7)	7·1 (5–10·6)	9·4 (6·2–14·6)
1-23	Foreign body	255,000 (232,000–285,000)	310,000 (287,000–337,000)	373,000 (337,000–418,000)	92·6 (84·8–102)	99·6 (92·5–107)	107 (98·2–118)
1-24	Pulmonary aspiration and foreign body in airway	184,000 (179,000–189,000)	246,000 (235,000–257,000)	262,000 (250,000–273,000)	69·2 (67·1–71·5)	83·6 (78·8–88·6)	82·4 (77·5–87·3)
1-25	Foreign body in eyes	24,900 (14,200–41,000)	16,700 (9,770–26,600)	32,000 (19,000–51,700)	8·3 (4·6–14)	4·4 (2·5–7·3)	7·7 (4·3–12·9)
1-26	Foreign body in other body part	46,300 (33,600–62,900)	47,500 (34,500–63,700)	79,300 (57,200–107,000)	15·1 (11–20·4)	11·5 (8·4–15·5)	17·2 (12·5–23·2)
1-27	Environmental heat and cold exposure	74,600 (63,900–88,500)	71,700 (60,000–86,300)	87,900 (70,900–109,000)	23·8 (20·4–28·1)	17 (14·3–20·2)	18·7 (15·3–23)
1-28	Exposure to forces of nature	12,600 (11,500–13,900)	9,920 (8,910–11,100)	6,200 (5,460–7,110)	4·6 (4·2–5·1)	3 (2·7–3·3)	1·7 (1·5–2)
1-29	Other unintentional injuries	278,000 (211,000–363,000)	260,000 (186,000–358,000)	348,000 (243,000–484,000)	90·7 (69·3–118)	62·4 (44·6–84·8)	75·6 (52·6–105)
1-30	Self-harm and interpersonal violence	3,480,000 (3,410,000–3,570,000)	3,330,000 (3,250,000–3,420,000)	3,390,000 (3,280,000–3,500,000)	1,180 (1,160–1,210)	950 (929–974)	922 (892–949)
1-31	Self-harm	1,780,000 (1,750,000–1,800,000)	2,030,000 (2,000,000–2,060,000)	2,120,000 (2,070,000–2,170,000)	590 (583–599)	552 (544–561)	548 (534–561)
1-32	Self-harm by firearm	912,000 (820,000–975,000)	864,000 (814,000–992,000)	923,000 (867,000–1,050,000)	305 (274–326)	231 (215–264)	233 (219–266)
1-33	Self-harm by other specified means	866,000 (801,000–954,000)	1,170,000 (1,030,000–1,220,000)	1,190,000 (1,060,000–1,250,000)	286 (265–316)	321 (285–337)	314 (279–330)
1-34	Interpersonal violence	1,680,000 (1,630,000–1,760,000)	1,230,000 (1,170,000–1,310,000)	1,240,000 (1,150,000–1,330,000)	585 (567–608)	378 (362–397)	364 (341–384)
1-35	Physical violence by firearm	1,010,000 (993,000–1,020,000)	753,000 (740,000–766,000)	772,000 (717,000–799,000)	350 (345–356)	234 (230–238)	231 (215–239)
1-36	Physical violence by sharp object	261,000 (251,000–275,000)	145,000 (136,000–158,000)	136,000 (125,000–149,000)	87·7 (84·3–92)	41·7 (39·3–44·8)	36·8 (34·2–39·8)
1-37	Sexual violence	32,000 (21,000–44,900)	38,700 (25,500–54,400)	38,800 (25,600–54,100)	11·4 (7·4–15·9)	12 (7·9–16·9)	11·5 (7·6–16·1)
1-38	Physical violence by other means	381,000 (343,000–432,000)	298,000 (256,000–353,000)	296,000 (252,000–354,000)	136 (124–152)	90·7 (80·1–104)	84·7 (74·6–97·6)
1-39	Conflict and terrorism	1,610 (1,380–1,960)	36,100 (32,400–40,900)	6,070 (4,360–9,010)	0·6 (0·5–0·7)	11·4 (10·3–12·9)	1·6 (1·2–2·3)
1-40	Police conflict and executions	19,500 (18,400–20,700)	25,600 (24,300–27,000)	29,300 (25,300–33,600)	6·6 (6·2–7)	7·7 (7·3–8·1)	8·5 (7·3–9·8)

SR Table 1: All-age DALYs counts and age standardized DALYs rates per 100,000 individuals for all causes and level 1 cause groups by location for 1990, 2010, and 2019, both sexes combined, Canada

Cause Level	Cause	All-age DALYs			Age-standardized rate of DALYs per 100,000		
		1990	2010	2019	1990	2010	2019
0-0	All causes	7,260,000 (6,450,000–8,170,000)	8,620,000 (7,520,000–9,870,000)	9,980,000 (8,720,000–11,400,000)	24,200 (21,500–27,200)	19,900 (17,200–23,000)	19,700 (16,900–22,800)
1-0	Communicable, maternal, neonatal, and nutritional disorders	410,000 (372,000–454,000)	410,000 (362,000–463,000)	429,000 (375,000–490,000)	1,620 (1,470–1,780)	1,360 (1,210–1,530)	1,250 (1,100–1,430)
1-1	HIV/AIDS and sexually transmitted infections	46,700 (43,600–51,100)	23,700 (19,600–29,500)	22,100 (17,600–28,400)	148 (138–162)	59·3 (49·1–73·9)	52·6 (42·1–67·7)
1-2	HIV/AIDS	43,000 (40,600–46,300)	19,400 (16,800–23,600)	17,800 (14,600–22,200)	136 (128–146)	48·1 (41·6–58·4)	42 (34·7–52·7)
1-3	HIV/AIDS - drug-susceptible tuberculosis	3,950 (2,580–5,950)	1,130 (741–1,710)	1,170 (745–1,780)	12·5 (8·2–18·8)	2·8 (1·8–4·3)	2·9 (1·8–4·4)
1-4	HIV/AIDS - Multidrug-resistant Tuberculosis without extensive drug resistance	94 (19–274)	36 (14–80)	36 (9–99)	0·3 (0·1–0·9)	0·1 (0–0·2)	0·1 (0–0·2)
1-5	HIV/AIDS - Extensively drug-resistant Tuberculosis	0 (0–0)	6 (2–13)	9 (2–25)	0 (0–0)	0 (0–0)	0 (0–0·1)
1-6	HIV/AIDS resulting in other diseases	39,000 (35,900–42,600)	18,300 (15,600–22,400)	16,500 (13,400–21,100)	123 (113–135)	45·2 (38·6–55·6)	39 (31·5–49·7)
1-7	Sexually transmitted infections excluding HIV	3,670 (2,120–6,350)	4,230 (2,300–7,650)	4,360 (2,410–7,900)	11·8 (6·8–20·5)	11·2 (6·1–20·3)	10·6 (5·8–19·1)
1-8	Syphilis	314 (235–394)	303 (221–405)	353 (253–480)	1 (0·8–1·3)	0·6 (0·5–0·8)	0·6 (0·5–0·8)
1-9	Chlamydial infection	225 (150–341)	219 (147–332)	232 (156–356)	0·7 (0·5–1·1)	0·6 (0·4–1)	0·6 (0·4–1)
1-10	Gonococcal infection	269 (215–341)	241 (193–311)	267 (215–340)	0·9 (0·7–1·1)	0·6 (0·5–0·8)	0·6 (0·5–0·8)
1-11	Trichomoniasis	1,270 (483–2,610)	1,490 (587–3,140)	1,570 (610–3,260)	4·1 (1·6–8·4)	4 (1·6–8·4)	4 (1·5–8·3)
1-12	Genital herpes	681 (220–1,700)	1,080 (373–2,610)	1,010 (315–2,520)	2·2 (0·7–5·5)	2·6 (0·9–6·4)	2·2 (0·7–5·5)
1-13	Other sexually transmitted infections	916 (579–1,370)	899 (564–1,390)	931 (577–1,430)	2·9 (1·8–4·3)	2·6 (1·6–4·1)	2·6 (1·6–4)
1-14	Respiratory infections and tuberculosis	138,000 (124,000–158,000)	127,000 (110,000–150,000)	155,000 (133,000–180,000)	478 (423–553)	301 (250–373)	303 (248–377)
1-15	Tuberculosis	4,870 (4,460–5,290)	2,710 (2,450–2,980)	3,150 (2,760–3,530)	15·3 (14·1–16·6)	5·7 (5·1–6·4)	5·5 (4·8–6·2)
1-16	Latent tuberculosis infection	0 (0–0)	0 (0–0)	0 (0–0)	0 (0–0)	0 (0–0)	0 (0–0)
1-17	Drug-susceptible tuberculosis	4,780 (4,340–5,200)	2,630 (2,380–2,900)	3,050 (2,660–3,450)	15 (13·7–16·3)	5·6 (5–6·2)	5·4 (4·6–6·1)
1-18	Multidrug-resistant tuberculosis without extensive drug resistance	96 (21–289)	65 (31–125)	74 (21–178)	0·3 (0·1–0·9)	0·1 (0·1–0·3)	0·1 (0–0·3)
1-19	Extensively drug-resistant tuberculosis	0 (0–0)	10 (5–21)	18 (5–46)	0 (0–0)	0 (0–0)	0 (0–0·1)
1-20	Lower respiratory infections	99,800 (93,500–104,000)	85,400 (77,700–90,500)	111,000 (98,600–121,000)	333 (312–349)	169 (156–178)	172 (155–186)
1-21	Upper respiratory infections	28,900 (17,700–44,900)	33,500 (20,400–52,500)	35,800 (21,900–56,300)	111 (67·9–171)	110 (66·8–172)	109 (65·9–171)
1-22	Otitis media	4,580 (2,600–7,600)	4,990 (2,870–8,120)	5,240 (3,060–8,680)	18 (10·3–29·8)	16·6 (9·4–27·9)	16·5 (9·4–28·1)
1-23	Enteric infections	24,400 (17,200–33,500)	59,600 (47,900–73,500)	61,500 (48,300–77,600)	91·6 (64·2–126)	156 (120–200)	143 (106–189)
1-24	Diarrhoeal diseases	24,100 (16,900–33,200)	59,500 (47,800–73,400)	61,300 (48,200–77,500)	90·7 (63·3–124)	155 (120–199)	143 (106–188)
1-25	Typhoid and paratyphoid	2 (1–3)	3 (0–8)	3 (0–9)	0 (0–0)	0 (0–0)	0 (0–0)
1-26	Typhoid fever	2 (0–3)	2 (0–7)	2 (0–8)	0 (0–0)	0 (0–0)	0 (0–0)
1-27	Paratyphoid fever	0 (0–1)	1 (0–1)	1 (0–1)	0 (0–0)	0 (0–0)	0 (0–0)
1-28	iNTS	142 (19–190)	32 (18–42)	42 (20–55)	0·5 (0·1–0·7)	0·1 (0·1–0·1)	0·1 (0·1–0·1)
1-29	Other intestinal infectious diseases	97 (61–138)	76 (54–102)	78 (56–104)	0·4 (0·2–0·6)	0·3 (0·2–0·4)	0·3 (0·2–0·4)
1-30	Neglected tropical diseases and malaria	961 (594–1,490)	936 (623–1,400)	811 (501–1,260)	3·8 (2·3–5·9)	2·8 (1·8–4·4)	2·5 (1·5–4)
1-31	Malaria	0 (0–0)	0 (0–0)	0 (0–0)	0 (0–0)	0 (0–0)	0 (0–0)
1-32	Chagas disease	60 (37–96)	187 (101–310)	34 (21–49)	0·2 (0·1–0·3)	0·4 (0·2–0·6)	0·1 (0–0·1)
1-33	Leishmaniasis	0 (0–0)	0 (0–0)	0 (0–0)	0 (0–0)	0 (0–0)	0 (0–0)
1-34	Visceral leishmaniasis	0 (0–0)	0 (0–0)	0 (0–0)	0 (0–0)	0 (0–0)	0 (0–0)
1-35	Cutaneous and mucocutaneous leishmaniasis	0 (0–0)	0 (0–0)	0 (0–0)	0 (0–0)	0 (0–0)	0 (0–0)
1-36	African trypanosomiasis	0 (0–0)	0 (0–0)	0 (0–0)	0 (0–0)	0 (0–0)	0 (0–0)
1-37	Schistosomiasis	0 (0–0)	0 (0–0)	0 (0–0)	0 (0–0)	0 (0–0)	0 (0–0)
1-38	Cysticercosis	0 (0–0)	0 (0–0)	0 (0–0)	0 (0–0)	0 (0–0)	0 (0–0)
1-39	Cystic echinococcosis	28 (18–39)	18 (13–25)	17 (12–23)	0·1 (0·1–0·1)	0 (0–0·1)	0 (0–0·1)
1-40	Lymphatic filariasis	0 (0–0)	0 (0–0)	0 (0–0)	0 (0–0)	0 (0–0)	0 (0–0)
1-41	Onchocerciasis	0 (0–0)	0 (0–0)	0 (0–0)	0 (0–0)	0 (0–0)	0 (0–0)
1-42	Trachoma	0 (0–0)	0 (0–0)	0 (0–0)	0 (0–0)	0 (0–0)	0 (0–0)
1-43	Dengue	0 (0–0)	0 (0–0)	0 (0–0)	0 (0–0)	0 (0–0)	0 (0–0)
1-44	Yellow fever	0 (0–0)	0 (0–0)	0 (0–0)	0 (0–0)	0 (0–0)	0 (0–0)
1-45	Rabies	7 (3–13)	2 (1–3)	2 (2–4)	0 (0–0)	0 (0–0)	0 (0–0)
1-46	Intestinal nematode infections	0 (0–0)	0 (0–0)	0 (0–0)	0 (0–0)	0 (0–0)	0 (0–0)
1-47	Ascariasis	0 (0–0)	0 (0–0)	0 (0–0)	0 (0–0)	0 (0–0)	0 (0–0)
1-48	Trichuriasis	0 (0–0)	0 (0–0)	0 (0–0)	0 (0–0)	0 (0–0)	0 (0–0)

1-49	Hookworm disease	0 (0–0)	0 (0–0)	0 (0–0)	0 (0–0)	0 (0–0)	0 (0–0)
1-50	Food-borne trematodiases	0 (0–0)	0 (0–0)	0 (0–0)	0 (0–0)	0 (0–0)	0 (0–0)
1-51	Leprosy	0 (0–0)	0 (0–0)	0 (0–0)	0 (0–0)	0 (0–0)	0 (0–0)
1-52	Ebola virus disease	0 (0–0)	0 (0–0)	0 (0–0)	0 (0–0)	0 (0–0)	0 (0–0)
1-53	Zika virus disease	0 (0–0)	0 (0–0)	2 (0–10)	0 (0–0)	0 (0–0)	0 (0–0)
1-54	Guinea worm disease	0 (0–0)	0 (0–0)	0 (0–0)	0 (0–0)	0 (0–0)	0 (0–0)
1-55	Other neglected tropical diseases	866 (513–1,400)	729 (434–1,150)	755 (452–1,190)	3·5 (2–5·5)	2·4 (1·4–3·9)	2·4 (1·4–3·9)
1-56	Other infectious diseases	19,500 (17,500–22,300)	19,800 (17,400–23,100)	20,800 (18,000–24,400)	76·9 (69·2–87·3)	59·5 (52·5–69·4)	55 (47·7–64·5)
1-57	Meningitis	7,320 (6,760–7,960)	4,490 (4,140–4,850)	3,820 (3,450–4,230)	31·9 (29·2–35·1)	16·5 (15–18·2)	12·7 (11·1–14·6)
1-58	Encephalitis	2,030 (1,720–2,440)	2,990 (2,470–3,470)	3,100 (2,540–3,550)	7·5 (6·5–9·1)	8·7 (7·2–9·9)	8·1 (6·6–9·2)
1-59	Diphtheria	1 (1–2)	2 (2–2)	2 (2–2)	0 (0–0)	0 (0–0)	0 (0–0)
1-60	Whooping cough	381 (222–613)	403 (254–620)	411 (253–629)	1·9 (1·1–3·1)	2·1 (1·3–3·2)	2·1 (1·3–3·2)
1-61	Tetanus	4 (1–51)	7 (3–46)	6 (1–32)	0 (0–0·2)	0 (0–0·1)	0 (0–0·1)
1-62	Measles	6 (4–8)	3 (2–4)	5 (2–9)	0 (0–0)	0 (0–0)	0 (0–0)
1-63	Varicella and herpes zoster	1,310 (959–1,810)	1,930 (1,360–2,650)	2,230 (1,580–3,030)	4·8 (3·5–6·6)	4·7 (3·4–6·4)	4·5 (3·2–6·2)
1-64	Acute hepatitis	1,480 (1,140–1,900)	1,430 (1,060–1,940)	1,540 (1,100–2,090)	5·2 (4–6·7)	3·9 (2·8–5·3)	3·9 (2·7–5·3)
1-65	Acute hepatitis A	731 (506–987)	653 (430–940)	755 (491–1,090)	2·6 (1·8–3·5)	1·9 (1·2–2·7)	2·1 (1·3–3·1)
1-66	Acute hepatitis B	514 (367–705)	619 (444–859)	627 (443–859)	1·7 (1·2–2·4)	1·6 (1·1–2·2)	1·4 (1–2)
1-67	Acute hepatitis C	180 (105–239)	105 (75–174)	92 (62–159)	0·6 (0·4–0·8)	0·2 (0·2–0·4)	0·2 (0·1–0·3)
1-68	Acute hepatitis E	53 (36–76)	58 (38–88)	66 (44–99)	0·2 (0·1–0·3)	0·2 (0·1–0·3)	0·2 (0·1–0·3)
1-69	Other unspecified infectious diseases	6,950 (5,480–9,060)	8,520 (6,890–11,600)	9,660 (7,800–13,000)	25·5 (20·3–33·5)	23·6 (19·4–32·2)	23·6 (19·1–32)
1-70	Maternal and neonatal disorders	152,000 (138,000–168,000)	150,000 (134,000–165,000)	137,000 (120,000–156,000)	724 (663–797)	703 (638–760)	619 (548–696)
1-71	Maternal disorders	2,400 (1,850–3,100)	3,000 (2,420–3,750)	2,550 (2,060–3,180)	7·8 (6–10)	10 (8–12·5)	7·9 (6·4–9·8)
1-72	Maternal haemorrhage	744 (480–1,180)	651 (412–1,020)	502 (303–796)	2·4 (1·5–3·7)	2·2 (1·4–3·4)	1·5 (0·9–2·5)
1-73	Maternal sepsis and other pregnancy related infections	161 (89–269)	159 (87–272)	118 (62–208)	0·6 (0·3–0·9)	0·5 (0·3–0·9)	0·4 (0·2–0·7)
1-74	Maternal hypertensive disorders	309 (224–430)	379 (278–527)	203 (154–262)	1 (0·7–1·4)	1·3 (0·9–1·8)	0·6 (0·5–0·8)
1-75	Maternal obstructed labour and uterine rupture	324 (136–590)	317 (139–570)	320 (130–598)	1·1 (0·4–1·9)	1·1 (0·5–1·9)	1 (0·4–1·8)
1-76	Maternal abortive outcome	151 (116–194)	117 (72–179)	99 (58–157)	0·5 (0·4–0·6)	0·4 (0·2–0·6)	0·3 (0·2–0·5)
1-77	Ectopic pregnancy	67 (51–84)	58 (46–74)	51 (39–67)	0·2 (0·2–0·3)	0·2 (0·2–0·2)	0·2 (0·1–0·2)
1-78	Indirect maternal deaths	34 (26–43)	202 (158–250)	179 (136–227)	0·1 (0·1–0·1)	0·7 (0·5–0·8)	0·6 (0·4–0·7)
1-79	Late maternal deaths	190 (127–335)	219 (187–257)	281 (207–383)	0·6 (0·4–1·1)	0·7 (0·6–0·8)	0·9 (0·6–1·2)
1-80	Maternal deaths aggravated by HIV/AIDS	0 (0–0)	1 (1–2)	2 (1–3)	0 (0–0)	0 (0–0)	0 (0–0)
1-81	Other maternal disorders	420 (339–507)	895 (767–1,040)	799 (667–936)	1·4 (1·1–1·6)	2·9 (2·5–3·4)	2·5 (2–2·9)
1-82	Neonatal disorders	150,000 (136,000–166,000)	147,000 (131,000–162,000)	134,000 (118,000–154,000)	716 (656–789)	693 (628–750)	611 (540–688)
1-83	Neonatal preterm birth	98,400 (87,900–110,000)	86,600 (75,300–98,500)	80,100 (68,300–93,000)	462 (418–514)	388 (345–430)	340 (293–389)
1-84	Neonatal encephalopathy due to birth asphyxia and trauma	30,600 (26,400–35,000)	29,800 (25,900–34,300)	26,300 (22,200–30,900)	150 (131–172)	146 (128–163)	124 (105–146)
1-85	Neonatal sepsis and other neonatal infections	6,120 (5,080–7,470)	6,850 (5,570–8,890)	6,190 (5,010–7,670)	29·2 (24·5–35·1)	30·8 (25·8–37·1)	27·4 (22·3–33·4)
1-86	Hemolytic disease and other neonatal jaundice	747 (624–913)	554 (447–670)	530 (413–664)	3·4 (2·8–4·1)	2·1 (1·7–2·5)	1·9 (1·5–2·3)
1-87	Other neonatal disorders	14,000 (11,800–16,600)	23,300 (20,700–25,700)	21,400 (17,100–26,100)	71·6 (60·6–84·9)	127 (113–140)	117 (93·6–143)
1-88	Nutritional deficiencies	28,400 (19,400–39,900)	29,200 (20,600–40,500)	31,200 (21,800–43,000)	100 (68·1–141)	76·9 (52·7–111)	74·1 (49·4–104)
1-89	Protein-energy malnutrition	7,490 (5,360–10,100)	11,400 (8,230–15,300)	12,300 (8,560–16,400)	25·1 (17·8–33·7)	26·4 (18·4–36·8)	24·8 (16·8–34·7)
1-90	Iodine deficiency	728 (329–1,400)	881 (406–1,650)	935 (425–1,800)	2·5 (1·1–4·7)	2·4 (1·1–4·5)	2·3 (1·1–4·5)
1-91	Vitamin A deficiency	121 (57–231)	43 (18–87)	38 (15–78)	0·6 (0·3–1·2)	0·2 (0·1–0·4)	0·2 (0·1–0·4)
1-92	Dietary iron deficiency	15,900 (9,360–25,500)	12,900 (7,500–21,500)	13,900 (8,170–22,500)	58·3 (34·7–92·9)	38 (21·8–62·2)	37·9 (21·9–61·3)
1-93	Other nutritional deficiencies	4,110 (2,740–5,770)	4,000 (2,680–5,740)	4,050 (2,630–5,770)	13·8 (9·2–19·5)	9·9 (6·5–14·3)	9 (5·6–12·9)
1-0	Non-communicable diseases	5,980,000 (5,260,000–6,810,000)	7,360,000 (6,380,000–8,440,000)	8,630,000 (7,520,000–9,890,000)	19,600 (17,200–22,400)	16,400 (13,900–19,100)	16,400 (13,900–19,200)
1-1	Neoplasms	1,280,000 (1,250,000–1,310,000)	1,650,000 (1,570,000–1,700,000)	1,940,000 (1,840,000–2,040,000)	4,070 (3,950–4,160)	3,250 (3,110–3,340)	3,090 (2,930–3,230)
1-2	Lip and oral cavity cancer	21,500 (20,700–22,200)	20,900 (19,800–21,800)	23,000 (21,000–24,900)	68·4 (65·9–71)	41 (38·9–42·7)	36·8 (33·9–39·8)
1-3	Nasopharynx cancer	3,210 (2,990–3,450)	3,630 (3,310–3,970)	4,090 (3,580–4,670)	10·5 (9·8–11·3)	7·7 (7–8·4)	7·5 (6·7–8·6)
1-4	Other pharynx cancer	6,130 (5,790–6,460)	8,270 (7,750–8,790)	9,610 (8,400–10,900)	19·7 (18·6–20·8)	16 (15–17)	15·3 (13·3–17·3)
1-5	Oesophageal cancer	25,300 (24,200–26,200)	43,500 (41,500–45,400)	52,500 (47,300–57,600)	79·6 (76·4–82·4)	83·2 (79·4–86·8)	80·9 (73–88·5)
1-6	Stomach cancer	51,600 (49,600–53,400)	51,000 (48,400–53,300)	58,200 (53,900–62,700)	161 (155–166)	98·2 (93·5–102)	90·5 (84·2–97·3)
1-7	Colon and rectum cancer	138,000 (133,000–143,000)	186,000 (176,000–194,000)	226,000 (208,000–243,000)	431 (416–446)	354 (337–369)	346 (320–371)

1-8	Liver cancer	14,900 (14,300–15,500)	41,400 (39,300–43,200)	56,800 (50,600–63,100)	47·3 (45·4–49·2)	81·9 (77·8–85·4)	91 (81·1–101)
1-9	Liver cancer due to hepatitis B	1,400 (985–1,990)	3,300 (2,240–4,820)	4,260 (2,850–6,300)	4·5 (3·1–6·4)	6·8 (4·7–9·8)	7·5 (5·2–10·9)
1-10	Liver cancer due to hepatitis C	2,370 (1,600–3,290)	7,370 (5,100–10,200)	9,890 (6,660–13,800)	7·4 (5–10·2)	13·9 (9·6–19·2)	14·8 (10·1–20·5)
1-11	Liver cancer due to alcohol use	8,450 (7,280–9,600)	23,200 (19,600–26,500)	32,500 (26,100–38,600)	26·4 (22·7–30)	45 (38·1–51·5)	51 (41·1–60·3)
1-12	Liver cancer due to NASH	1,370 (997–1,890)	4,710 (3,490–6,320)	6,630 (4,680–9,570)	4·3 (3·1–5·9)	9·1 (6·8–12·1)	10·2 (7·3–14·4)
1-13	Gallbladder and biliary tract cancer	13,600 (11,500–14,600)	14,700 (13,200–17,100)	16,400 (14,600–20,200)	42·1 (35·5–45·1)	27·8 (24·9–32·1)	24·6 (22–30·4)
1-14	Pancreatic cancer	59,800 (57,600–61,800)	91,900 (86,700–95,800)	117,000 (107,000–127,000)	186 (179–193)	175 (166–182)	177 (162–192)
1-15	Larynx cancer	12,100 (11,600–12,700)	10,000 (9,470–10,600)	11,300 (10,100–12,500)	38·5 (36·6–40·4)	19·2 (18·1–20·4)	17·2 (15·4–19)
1-16	Tracheal, bronchus, and lung cancer	352,000 (343,000–361,000)	428,000 (409,000–441,000)	479,000 (448,000–507,000)	1,100 (1,080–1,130)	813 (780–837)	717 (673–756)
1-17	Malignant skin melanoma	18,900 (14,800–24,800)	29,400 (20,000–35,300)	33,300 (23,600–41,300)	60·7 (47·4–78·8)	61·8 (42·4–75)	59·7 (42·5–75·4)
1-18	Non-melanoma skin cancer	8,090 (6,180–10,600)	23,100 (17,000–31,400)	46,500 (31,700–66,200)	25·2 (19·3–32·9)	42·8 (31·5–58)	65·7 (44·9–94)
1-19	Non-melanoma skin cancer (squamous-cell carcinoma)	8,090 (6,180–10,600)	23,100 (17,000–31,300)	46,500 (31,700–66,200)	25·2 (19·3–32·9)	42·8 (31·5–57·9)	65·7 (44·9–94)
1-20	Non-melanoma skin cancer (basal-cell carcinoma)	5 (2–10)	9 (4–18)	12 (5–24)	0 (0–0)	0 (0–0)	0 (0–0)
1-21	Breast cancer	129,000 (123,000–135,000)	145,000 (136,000–154,000)	160,000 (145,000–176,000)	411 (392–430)	288 (272–304)	268 (244–294)
1-22	Cervical cancer	15,900 (14,900–16,900)	18,900 (17,100–20,100)	21,100 (17,800–23,400)	50·4 (47·1–53·9)	40·5 (36·6–43·3)	39·8 (34·2–44·7)
1-23	Uterine cancer	12,000 (11,200–12,700)	18,200 (16,800–19,700)	22,900 (19,900–25,900)	37 (34·6–39·4)	34·7 (32–37·6)	34·9 (30·5–39·4)
1-24	Ovarian cancer	35,600 (33,100–37,400)	41,200 (38,000–44,600)	46,400 (39,200–54,700)	113 (106–119)	81 (74·9–87·6)	75·2 (63·7–88·6)
1-25	Prostate cancer	71,700 (50,500–81,400)	85,500 (74,200–121,000)	107,000 (90,300–158,000)	215 (153–246)	154 (134–220)	148 (125–219)
1-26	Testicular cancer	2,630 (2,260–3,190)	2,650 (2,180–3,290)	2,660 (2,120–3,320)	8·9 (7·5–11·2)	8·3 (6·5–10·8)	7·8 (5·9–10·3)
1-27	Kidney cancer	20,300 (19,400–21,200)	40,400 (38,100–42,500)	46,800 (41,800–52,100)	65 (62·1–67·9)	78·7 (74·2–82·8)	74 (66·3–81·6)
1-28	Bladder cancer	32,000 (30,500–33,500)	41,400 (38,400–43,600)	49,600 (44,500–54,900)	98·4 (93·8–103)	76 (71–79·9)	71·1 (64–78·5)
1-29	Brain and nervous system cancer	44,500 (38,800–53,200)	57,800 (43,800–63,100)	67,400 (48,700–75,400)	153 (133–183)	138 (104–151)	139 (99·8–155)
1-30	Thyroid cancer	3,590 (3,330–3,890)	6,380 (5,700–6,950)	6,550 (5,740–7,360)	11·4 (10·5–12·3)	12·8 (11·4–14)	10·8 (9·5–12·3)
1-31	Mesothelioma	6,870 (6,160–7,710)	9,650 (9,000–10,200)	11,500 (10,500–12,500)	21·4 (19·3–24)	18·3 (17–19·3)	17·1 (15·5–18·5)
1-32	Hodgkin lymphoma	6,040 (4,790–6,770)	4,880 (4,200–5,750)	5,010 (4,200–6,130)	19·9 (15·7–22·3)	12·5 (10·7–14·8)	11·3 (9·5–14)
1-33	Non-Hodgkin's lymphoma	51,200 (48,000–54,500)	63,000 (58,200–67,500)	74,100 (61,900–88,000)	163 (153–174)	128 (118–136)	120 (102–141)
1-34	Multiple myeloma	20,400 (17,900–21,800)	31,400 (28,500–36,300)	38,700 (34,200–46,900)	63·2 (55·3–67·3)	59·1 (54·2–68·6)	57·6 (51·1–70·6)
1-35	Leukaemia	54,200 (52,200–55,900)	61,700 (58,400–64,400)	70,300 (64,500–76,400)	184 (178–190)	139 (133–145)	130 (120–140)
1-36	Acute lymphoid leukaemia	8,510 (6,980–9,270)	6,700 (5,910–8,760)	7,470 (6,370–9,260)	34·1 (27·9–37·4)	21·3 (18·8–27·5)	21·1 (17·9–26·2)
1-37	Chronic lymphoid leukaemia	7,630 (6,770–8,370)	11,000 (9,830–12,500)	13,500 (11,200–16,200)	23·4 (20·9–25·6)	20·2 (18·1–22·9)	19·2 (16·1–23·2)
1-38	Acute myeloid leukaemia	14,700 (13,700–17,900)	25,600 (21,100–27,400)	28,600 (24,200–31,800)	49·4 (46–60)	58 (47·4–61·9)	53·4 (45·2–58·6)
1-39	Chronic myeloid leukaemia	6,240 (5,640–6,700)	3,240 (2,940–3,630)	3,690 (3,120–4,340)	20·1 (18·3–21·6)	7 (6·4–7·8)	6·6 (5·7–7·7)
1-40	Other leukaemia	17,100 (15,400–18,100)	15,100 (13,900–16,900)	17,100 (14,900–19,800)	57·3 (52·1–60·9)	32·5 (30–35·9)	29·6 (26·2–33·5)
1-41	Other neoplasms	9,790 (7,630–14,500)	14,700 (11,800–20,700)	18,700 (14,800–26,900)	30·6 (23·8–44·8)	28 (22·6–39)	27·9 (22·1–39·5)
1-42	Myelodysplastic, myeloproliferative, and other hematopoietic neoplasms	9,790 (7,630–14,500)	14,700 (11,800–20,700)	18,700 (14,800–26,900)	30·6 (23·8–44·8)	28 (22·6–39)	27·9 (22·1–39·5)
1-43	Benign and in situ intestinal neoplasms	0 (0–0)	0 (0–0)	0 (0–0)	0 (0–0)	0 (0–0)	0 (0–0)
1-44	Benign and in situ cervical and uterine neoplasms	0 (0–0)	0 (0–0)	0 (0–0)	0 (0–0)	0 (0–0)	0 (0–0)
1-45	Other benign and in situ neoplasms	0 (0–0)	0 (0–0)	0 (0–0)	0 (0–0)	0 (0–0)	0 (0–0)
1-46	Cardiovascular diseases	1,430,000 (1,350,000–1,490,000)	1,200,000 (1,110,000–1,280,000)	1,400,000 (1,290,000–1,510,000)	4,460 (4,220–4,650)	2,250 (2,090–2,400)	2,080 (1,920–2,240)
1-47	Rheumatic heart disease	15,300 (13,900–16,700)	12,800 (11,400–14,300)	15,400 (13,000–18,300)	47·8 (43·3–52·3)	24·5 (21·9–27·4)	23·5 (19·8–27·7)
1-48	Ischaemic heart disease	911,000 (872,000–938,000)	652,000 (608,000–682,000)	736,000 (681,000–778,000)	2,840 (2,710–2,920)	1,200 (1,130–1,250)	1,070 (998–1,130)
1-49	Stroke	296,000 (272,000–318,000)	280,000 (252,000–305,000)	329,000 (290,000–364,000)	929 (853–998)	521 (470–569)	487 (428–541)
1-50	Ischaemic stroke	183,000 (164,000–202,000)	167,000 (146,000–189,000)	202,000 (173,000–230,000)	572 (509–631)	300 (258–339)	282 (240–323)
1-51	Intracerebral hemorrhage	69,900 (65,700–73,900)	70,100 (65,000–74,500)	80,400 (72,700–88,600)	218 (206–231)	134 (125–142)	122 (111–135)
1-52	Subarachnoid hemorrhage	42,900 (39,400–45,800)	42,100 (38,800–45,200)	47,000 (42,300–52,300)	139 (128–148)	87·1 (80·4–93·6)	82·4 (74·2–91·7)
1-53	Hypertensive heart disease	12,900 (11,400–15,600)	19,100 (16,100–21,800)	26,100 (18,700–29,700)	40·7 (35·7–48·8)	35·6 (30·5–40·9)	39·1 (29·3–44·4)
1-54	Non-rheumatic valvular heart disease	17,300 (16,100–18,800)	26,000 (23,100–28,500)	33,600 (29,400–37,300)	53·9 (49·9–58·2)	46·4 (41·6–50·6)	46·5 (41·2–51·7)
1-55	Non-rheumatic calcific aortic valve disease	11,900 (10,700–13,600)	20,600 (17,800–22,200)	27,200 (23,300–29,900)	37 (33·3–42·2)	36·3 (31·9–39·1)	37·2 (32·3–40·8)
1-56	Non-rheumatic degenerative mitral valve disease	5,410 (3,560–6,670)	5,370 (4,180–7,280)	6,360 (4,860–8,850)	16·8 (11·1–20·7)	10 (7·8–13·5)	9·3 (7·2–12·7)
1-57	Other non-rheumatic valve diseases	53 (26–73)	42 (29–63)	44 (31–68)	0·2 (0·1–0·2)	0·1 (0·1–0·2)	0·1 (0·1–0·2)
1-58	Cardiomyopathy and myocarditis	37,600 (33,400–43,100)	36,000 (32,100–45,700)	41,900 (37,100–52,900)	124 (111–141)	81·4 (73–100)	79 (70·3–95·6)
1-59	Myocarditis	2,290 (1,640–3,390)	3,710 (2,430–4,570)	3,800 (2,530–4,730)	8·6 (6·1–13)	11·1 (7·6–13·4)	10·3 (7·2–12·5)
1-60	Alcoholic cardiomyopathy	10,200 (8,620–11,900)	10,900 (8,630–13,800)	12,400 (9,660–15,300)	32·3 (27·6–38·1)	22·4 (17·9–28·4)	21·4 (17–26·4)

1-61	Other cardiomyopathy	25,200 (21,700–28,700)	21,400 (18,200–29,900)	25,700 (21,700–35,000)	83·6 (71·3–94·4)	47·9 (41·4–64·1)	47·3 (40·9–62·1)
1-62	Atrial fibrillation and flutter	47,800 (36,200–62,800)	79,800 (60,100–105,000)	104,000 (78,100–137,000)	148 (112–194)	142 (106–187)	143 (107–189)
1-63	Aortic aneurysm	36,100 (34,200–38,100)	25,800 (23,900–27,500)	31,000 (27,600–34,300)	110 (104–115)	48·1 (44·7–51·2)	45·7 (40·9–50·2)
1-64	Peripheral vascular disease	12,000 (7,020–18,900)	18,700 (11,000–31,900)	24,100 (14,000–41,500)	37 (21·6–58·2)	33·4 (19·5–57·1)	33·1 (19·3–56·7)
1-65	Endocarditis	2,960 (2,260–5,070)	9,140 (4,530–11,100)	10,800 (5,300–13,700)	9·5 (7·3–16·2)	18·4 (9·3–22·4)	17·9 (8·9–22·4)
1-66	Chronic respiratory diseases	269,000 (240,000–303,000)	368,000 (323,000–409,000)	447,000 (389,000–500,000)	868 (763–993)	780 (673–903)	745 (641–861)
1-67	Chronic obstructive pulmonary disease	179,000 (166,000–192,000)	258,000 (231,000–283,000)	324,000 (286,000–361,000)	547 (506–585)	469 (421–515)	458 (405–508)
1-68	Pneumoconiosis	1,430 (1,220–1,670)	2,310 (1,980–2,660)	2,750 (2,310–3,290)	4·3 (3·7–5)	4·3 (3·7–5)	4 (3·3–4·7)
1-69	Silicosis	700 (596–824)	632 (510–1,090)	658 (483–1,310)	2·1 (1·8–2·5)	1·2 (1–2)	1 (0·8–1·9)
1-70	Asbestosis	582 (440–747)	1,440 (877–1,730)	1,790 (1,090–2,240)	1·7 (1·3–2·2)	2·6 (1·6–3·1)	2·5 (1·5–3·1)
1-71	Coal workers pneumoconiosis	85 (62–114)	141 (98–229)	196 (135–317)	0·3 (0·2–0·3)	0·3 (0·2–0·4)	0·3 (0·2–0·5)
1-72	Other pneumoconiosis	60 (41–86)	90 (60–132)	107 (70–158)	0·2 (0·1–0·3)	0·2 (0·1–0·3)	0·2 (0·1–0·3)
1-73	Asthma	68,600 (49,200–95,600)	68,600 (46,800–98,500)	69,800 (47,200–101,000)	254 (179–361)	226 (148–332)	202 (133–301)
1-74	Interstitial lung disease and pulmonary sarcoidosis	15,800 (13,400–21,400)	33,900 (22,400–41,900)	42,900 (27,200–56,600)	49·2 (41·8–66·6)	65·1 (43·9–79·5)	65·1 (42·8–84·5)
1-75	Other chronic respiratory diseases	3,820 (3,220–4,450)	5,810 (4,890–6,760)	6,870 (5,690–7,960)	13·5 (11·5–15·8)	15 (12·7–17·6)	16·1 (13·4–19)
1-76	Digestive diseases	219,000 (200,000–242,000)	288,000 (260,000–320,000)	331,000 (299,000–367,000)	706 (643–781)	602 (539–677)	585 (521–659)
1-77	Cirrhosis and other chronic liver diseases	82,600 (79,900–85,300)	111,000 (106,000–117,000)	129,000 (119,000–140,000)	265 (256–274)	223 (214–233)	221 (205–238)
1-78	Cirrhosis and other chronic liver diseases due to hepatitis B	3,970 (2,680–5,630)	6,710 (4,520–9,610)	6,680 (4,420–9,710)	12·8 (8·6–18·3)	13·4 (9·2–19·2)	11·8 (8–16·8)
1-79	Cirrhosis and other chronic liver diseases due to hepatitis C	35,000 (27,600–43,000)	45,900 (35,500–57,400)	52,800 (39,900–67,400)	112 (87·5–138)	91·9 (71–114)	91·6 (70·7–115)
1-80	Cirrhosis and other chronic liver diseases due to alcohol use	25,400 (18,500–33,000)	35,200 (25,300–46,100)	41,700 (29,700–54,400)	81·5 (58·8–106)	69·5 (50·4–90·7)	70·1 (50·6–90·8)
1-81	Cirrhosis and other chronic liver diseases due to NAFLD	5,970 (4,030–8,350)	8,050 (5,450–11,400)	9,420 (6,310–13,400)	19 (12·8–26·6)	15·8 (10·7–22·3)	15·7 (10·5–22·5)
1-82	Cirrhosis and other chronic liver diseases due to other causes	12,100 (9,000–15,800)	15,500 (11,300–20,500)	18,600 (13,300–24,900)	39·4 (29·9–50·9)	31·8 (23·7–41·2)	31·8 (23·3–41·7)
1-83	Upper digestive system diseases	42,100 (32,000–57,700)	42,300 (28,900–63,300)	47,500 (32,500–70,700)	134 (102–184)	94·5 (62·8–144)	92·3 (61–141)
1-84	Peptic ulcer disease	18,100 (16,700–19,500)	10,900 (9,750–12,300)	12,300 (10,600–14,200)	56·8 (52·5–61·1)	21 (18·8–23·5)	19·1 (16·4–22·1)
1-85	Gastritis and duodenitis	6,810 (4,790–9,390)	7,940 (5,350–11,400)	9,050 (6,100–12,800)	22·1 (15·6–30·5)	18·1 (12–26)	17·9 (12–25·7)
1-86	GERD	17,200 (8,770–31,300)	23,400 (12,000–42,600)	26,200 (13,400–47,700)	55·4 (28·3–101)	55·4 (28·3–99·6)	55·3 (28·3–100)
1-87	Appendicitis	1,690 (1,380–2,110)	1,900 (1,510–2,340)	2,050 (1,630–2,570)	5·8 (4·7–7·3)	4·7 (3·7–6)	4·5 (3·4–5·7)
1-88	Paralytic ileus and intestinal obstruction	10,400 (9,030–11,900)	17,500 (12,900–19,900)	21,100 (15,300–24,500)	35·4 (30·9–40·6)	36 (28–40·3)	34·5 (26·8–39·3)
1-89	Inguinal, femoral, and abdominal hernia	7,900 (6,320–9,910)	10,400 (8,150–13,000)	11,900 (9,500–14,800)	26·5 (21·1–33·2)	22·6 (17·7–28·6)	21·6 (16·6–27·3)
1-90	Inflammatory bowel disease	22,700 (17,300–29,100)	30,000 (22,400–38,500)	29,900 (21,900–38,500)	74·3 (56·5–95·5)	72·4 (53·3–94)	64·9 (46·6–85·1)
1-91	Vascular intestinal disorders	12,100 (11,200–12,900)	18,400 (16,500–20,600)	21,800 (19,000–25,000)	37·6 (34·8–40·4)	34·2 (30·9–37·9)	31·9 (28·1–36·3)
1-92	Gallbladder and biliary diseases	21,800 (16,400–29,100)	28,400 (21,600–37,700)	33,700 (25,600–45,300)	69·9 (52·2–93·4)	60·4 (44·7–82·8)	60·8 (44·7–83·3)
1-93	Pancreatitis	7,890 (7,190–8,800)	10,600 (9,610–11,900)	12,200 (10,700–14,000)	25·2 (22·9–28)	22 (19·9–24·8)	21·2 (18·6–24·4)
1-94	Other digestive diseases	9,950 (6,920–11,500)	17,200 (11,900–19,800)	21,300 (14,800–25,400)	31·9 (22·2–36·8)	32·6 (22·5–37·7)	32·2 (22·3–38·7)
1-95	Neurological disorders	405,000 (225,000–664,000)	585,000 (349,000–929,000)	684,000 (418,000–1,090,000)	1,360 (744–2,260)	1,340 (732–2,210)	1,330 (739–2,190)
1-96	Alzheimer's disease and other dementias	102,000 (47,500–218,000)	179,000 (84,300–374,000)	240,000 (114,000–498,000)	329 (153–701)	302 (142–630)	307 (145–640)
1-97	Parkinson's disease	24,900 (23,200–26,600)	53,000 (48,000–57,700)	64,600 (58,400–70,400)	75·7 (70·4–80·9)	92·8 (84·2–101)	87·8 (79·5–95·5)
1-98	Idiopathic epilepsy	27,800 (16,200–45,800)	29,100 (14,800–52,400)	30,700 (16,000–54,600)	98·8 (56·8–163)	83 (41·8–151)	79·9 (41·6–142)
1-99	Multiple sclerosis	16,200 (13,300–19,100)	25,800 (19,600–30,800)	30,300 (22,800–36,200)	52·1 (42·9–61·4)	56·2 (43·4–67)	58·8 (45·2–70·7)
1-100	Motor neuron disease	12,000 (11,400–12,600)	22,100 (21,100–23,100)	27,700 (25,400–30,100)	40·2 (38·2–42·3)	46·2 (44·2–48·2)	47 (43·4–50·8)
1-101	Headache disorders	202,000 (38,200–442,000)	249,000 (51,100–540,000)	258,000 (54,400–555,000)	685 (125–1,500)	685 (124–1,500)	672 (124–1,460)
1-102	Migraine	182,000 (26,000–424,000)	223,000 (34,700–514,000)	230,000 (36,900–532,000)	618 (84·6–1,450)	618 (84–1,440)	605 (82·9–1,400)
1-103	Tension-type headache	20,000 (5,370–67,700)	25,700 (7,200–85,700)	27,800 (7,850–93,100)	67 (17·3–232)	67 (18–232)	67 (17·9–232)
1-104	Other neurological disorders	20,700 (18,700–23,600)	27,200 (24,400–31,300)	32,900 (29,100–38,400)	75·2 (67·3–86·6)	70·3 (61·4–84)	74 (63·1–89·6)
1-105	Mental disorders	510,000 (373,000–668,000)	614,000 (453,000–805,000)	665,000 (487,000–865,000)	1,740 (1,270–2,290)	1,720 (1,260–2,270)	1,760 (1,290–2,310)
1-106	Schizophrenia	57,400 (43,900–68,700)	72,900 (56,100–87,000)	78,000 (59,300–92,000)	186 (141–221)	185 (142–222)	185 (140–218)
1-107	Depressive disorders	165,000 (115,000–226,000)	190,000 (133,000–261,000)	211,000 (145,000–289,000)	557 (387–768)	527 (365–729)	556 (382–763)
1-108	Major depressive disorder	130,000 (87,600–177,000)	145,000 (101,000–200,000)	163,000 (112,000–224,000)	440 (296–604)	410 (282–568)	439 (297–614)
1-109	Dysthymia	35,600 (22,400–54,500)	45,200 (28,400–69,800)	48,100 (30,500–75,000)	117 (73·4–180)	118 (73·5–182)	117 (73·3–181)
1-110	Bipolar disorder	49,300 (30,100–74,800)	62,800 (39,300–95,600)	66,500 (40,500–101,000)	169 (103–258)	175 (107–268)	174 (105–266)
1-111	Anxiety disorders	125,000 (88,900–170,000)	150,000 (99,200–213,000)	161,000 (109,000–228,000)	425 (300–576)	409 (268–584)	413 (277–581)
1-112	Eating disorders	25,300 (15,700–37,300)	27,100 (17,000–40,600)	28,200 (18,000–41,900)	86·9 (53·9–129)	90·5 (57–135)	91·6 (58·4–136)
1-113	Anorexia nervosa	6,540 (3,890–10,400)	7,450 (4,580–11,600)	7,570 (4,650–11,900)	23·4 (13·9–37·3)	25·4 (15·4–39·9)	25·4 (15·6–40·4)

1-114	Bulimia nervosa	18,800 (10,900–29,000)	19,700 (11,700–30,800)	20,600 (12,300–32,500)	63·5 (36·9–98)	65 (38·7–102)	66·2 (39·3–103)
1-115	Autism spectrum disorders	24,200 (16,000–33,900)	28,600 (18,800–40,500)	31,100 (21,000–44,000)	91·6 (60·3–129)	91·5 (59·8–129)	92·9 (62·4–132)
1-116	Attention-deficit/hyperactivity disorder	5,850 (3,330–9,520)	6,560 (3,800–11,100)	6,800 (3,870–11,400)	23·9 (13·7–38·9)	24·8 (14·1–42)	24·8 (13·9–42·3)
1-117	Conduct disorder	12,300 (6,830–19,400)	14,100 (7,670–22,500)	14,200 (7,760–22,800)	57·2 (31·8–90·8)	61 (33·2–97·8)	61 (33·4–97·8)
1-118	Idiopathic developmental intellectual disability	4,100 (1,730–7,280)	8,230 (3,290–14,100)	8,360 (3,270–14,600)	15·1 (6·2–27·2)	25·8 (9·9–44·7)	24·6 (9–43)
1-119	Other mental disorders	41,300 (26,000–62,200)	54,100 (34,300–80,900)	59,400 (37,500–88,100)	134 (83·9–201)	134 (83·5–201)	134 (83·8–200)
1-120	Substance use disorders	163,000 (128,000–202,000)	290,000 (236,000–348,000)	390,000 (325,000–464,000)	545 (429–678)	826 (664–1,010)	1,060 (870–1,280)
1-121	Alcohol use disorders	73,900 (55,600–96,100)	99,100 (78,500–125,000)	110,000 (87,500–138,000)	244 (184–318)	257 (198–330)	262 (200–338)
1-122	Drug use disorders	88,900 (68,400–112,000)	191,000 (153,000–233,000)	280,000 (230,000–336,000)	301 (231–381)	569 (448–703)	799 (649–970)
1-123	Opioid use disorders	50,900 (39,500–64,400)	132,000 (106,000–160,000)	206,000 (167,000–250,000)	168 (130–212)	389 (310–481)	587 (472–718)
1-124	Cocaine use disorders	14,300 (9,140–21,000)	24,400 (17,900–33,200)	31,800 (24,900–40,900)	48·8 (31·1–71·9)	71·4 (50·7–98·2)	88·7 (68–116)
1-125	Amphetamine use disorders	2,310 (1,560–3,350)	5,060 (4,170–6,400)	8,570 (7,350–10,100)	7·7 (5·2–11·2)	14·8 (11·9–19·4)	23·7 (20·1–28·3)
1-126	Cannabis use disorders	9,470 (6,080–13,800)	11,200 (7,410–16,300)	10,300 (6,720–15,000)	35·9 (23·2–52·3)	38·1 (25·1–55·6)	35·1 (22·7–51)
1-127	Other drug use disorders	11,900 (7,960–17,200)	18,900 (13,600–25,900)	22,800 (17,100–30,100)	40·4 (26·8–59)	55·6 (39·9–76·9)	64·6 (48·3–87·3)
1-128	Diabetes and kidney diseases	195,000 (173,000–220,000)	346,000 (296,000–402,000)	444,000 (370,000–527,000)	609 (541–690)	674 (574–789)	698 (577–831)
1-129	Diabetes mellitus	134,000 (115,000–155,000)	238,000 (196,000–288,000)	308,000 (242,000–381,000)	416 (360–482)	465 (382–564)	489 (386–609)
1-130	Diabetes mellitus type 1	27,600 (22,000–33,900)	44,400 (33,800–56,600)	43,200 (32,500–55,900)	89·9 (71·3–111)	105 (80·6–133)	89·1 (67·4–115)
1-131	Diabetes mellitus type 2	106,000 (92,100–122,000)	193,000 (161,000–233,000)	265,000 (207,000–330,000)	326 (284–376)	360 (299–434)	400 (311–503)
1-132	Chronic kidney disease	61,100 (55,100–67,800)	108,000 (96,800–120,000)	136,000 (121,000–153,000)	193 (174–214)	209 (188–233)	210 (186–236)
1-133	Chronic kidney disease due to diabetes mellitus type 1	2,480 (1,680–3,460)	3,950 (2,620–5,390)	3,780 (2,580–5,250)	7·9 (5·4–11)	8·8 (5·9–12·2)	7·4 (4·9–10·3)
1-134	Chronic kidney disease due to diabetes mellitus type 2	13,300 (10,200–16,600)	21,600 (16,800–27,500)	28,500 (21,800–36,000)	40·7 (31·4–50·6)	39·4 (30·5–49·8)	40·7 (31·1–51·4)
1-135	Chronic kidney disease due to hypertension	19,100 (16,200–22,200)	37,400 (31,400–43,400)	48,900 (40,700–56,900)	59·8 (50·7–69·1)	66·8 (56·2–77·2)	67·7 (57–78·8)
1-136	Chronic kidney disease due to glomerulonephritis	9,170 (7,510–11,200)	15,300 (12,500–18,800)	18,600 (15,000–23,400)	29·7 (24·4–36·1)	33·5 (27·7–40)	33·6 (27·5–41·1)
1-137	Chronic kidney disease due to other and unspecified causes	17,000 (13,800–20,800)	29,600 (23,900–36,000)	36,700 (29,000–45,500)	55 (44·7–66·7)	60·9 (49·9–73·1)	60·1 (48·5–72·9)
1-138	Acute glomerulonephritis	38 (33–44)	29 (25–34)	60 (49–75)	0·1 (0·1–0·1)	0·1 (0·1–0·1)	0·1 (0·1–0·1)
1-139	Skin and subcutaneous diseases	168,000 (117,000–238,000)	210,000 (147,000–297,000)	232,000 (161,000–328,000)	631 (438–895)	636 (435–904)	634 (438–904)
1-140	Dermatitis	32,900 (19,800–51,000)	39,600 (23,900–60,800)	43,500 (26,300–65,700)	128 (76·4–203)	128 (77·1–202)	128 (76·6–199)
1-141	Atopic dermatitis	20,900 (11,200–34,900)	23,800 (12,900–39,600)	25,800 (13,800–43,300)	87·6 (46·7–146)	87·6 (47·3–146)	87·5 (46·7–148)
1-142	Contact dermatitis	10,700 (6,750–16,000)	14,100 (8,830–21,100)	15,700 (9,660–23,300)	36·5 (23–54·7)	36·5 (22·8–54·4)	36·4 (22·7–54·4)
1-143	Seborrhoeic dermatitis	1,280 (716–2,040)	1,690 (964–2,680)	1,930 (1,090–3,060)	4·4 (2·4–7)	4·4 (2·4–7)	4·4 (2·4–7)
1-144	Psoriasis	29,800 (21,300–39,500)	36,300 (25,600–48,600)	39,300 (28,100–51,600)	101 (71·6–134)	92 (64·4–123)	89·1 (63·4–118)
1-145	Bacterial skin diseases	3,090 (2,250–4,900)	8,470 (5,330–11,000)	10,800 (6,110–14,000)	10·6 (7·7–16·3)	18·3 (11·9–23·4)	19 (11·7–24·3)
1-146	Cellulitis	2,640 (1,910–4,090)	5,860 (3,520–7,470)	7,350 (4,130–9,580)	9 (6·4–13·5)	12·9 (8·2–16·8)	13·4 (8·3–17·2)
1-147	Pyoderma	445 (304–893)	2,610 (1,400–4,110)	3,450 (1,750–5,490)	1·6 (1·1–3)	5·3 (2·9–8·2)	5·6 (3–8·7)
1-148	Scabies	982 (540–1,570)	1,140 (630–1,840)	1,210 (667–1,970)	3·8 (2·1–6·1)	3·8 (2·1–6·2)	3·8 (2·1–6·2)
1-149	Fungal skin diseases	4,020 (1,630–8,700)	5,650 (2,290–12,200)	6,630 (2,680–14,300)	13·7 (5·5–29·8)	13·6 (5·4–29·6)	13·6 (5·5–29·6)
1-150	Viral skin diseases	42,100 (26,900–62,800)	48,300 (31,000–71,400)	52,000 (33,600–76,800)	172 (110–259)	172 (110–255)	172 (110–257)
1-151	Acne vulgaris	16,000 (9,670–25,300)	19,600 (11,700–31,200)	19,900 (11,900–31,600)	62 (37·2–98·3)	68·2 (41·1–109)	69·1 (41·3–110)
1-152	Alopecia areata	3,260 (2,070–4,910)	3,980 (2,510–5,900)	4,310 (2,720–6,420)	11 (6·9–16·5)	11 (6·8–16·2)	10·9 (6·9–16·2)
1-153	Pruritus	2,490 (1,190–4,490)	3,390 (1,590–6,110)	3,820 (1,840–6,910)	8·5 (4·1–15·3)	8·8 (4·2–15·8)	8·8 (4·2–15·7)
1-154	Urticaria	14,100 (9,250–20,000)	16,400 (10,900–23,300)	17,600 (11,500–25,100)	55·8 (36·3–79·5)	55·8 (36·6–80·7)	55·7 (36·4–79·6)
1-155	Decubitus ulcer	2,520 (1,880–3,400)	2,860 (2,120–3,740)	3,610 (2,640–4,780)	8 (6–10·8)	5·4 (4–7·1)	5·4 (3·9–7·1)
1-156	Other skin and subcutaneous diseases	16,800 (8,320–30,700)	24,600 (11,900–44,300)	28,800 (14,200–52,100)	56·4 (27·8–103)	58 (28–105)	58·4 (28·5–106)
1-157	Sense organ diseases	149,000 (103,000–210,000)	225,000 (157,000–314,000)	273,000 (191,000–378,000)	479 (331–673)	457 (318–640)	455 (315–634)
1-158	Blindness and vision loss	25,500 (17,500–35,300)	37,600 (26,000–51,700)	45,100 (31,600–61,600)	84·8 (58–118)	82·1 (56·2–116)	81·2 (55·9–113)
1-159	Glaucoma	1,470 (986–2,110)	2,490 (1,660–3,530)	3,250 (2,190–4,590)	4·6 (3·1–6·6)	4·3 (2·9–6·1)	4·3 (2·9–6)
1-160	Cataract	5,630 (3,920–7,690)	9,650 (6,720–13,200)	12,100 (8,460–16,600)	17·7 (12·4–24·3)	17·4 (12·1–23·9)	17·2 (12–23·6)
1-161	Age-related macular degeneration	874 (584–1,270)	1,460 (987–2,090)	1,900 (1,250–2,720)	2·7 (1·8–3·9)	2·6 (1·7–3·7)	2·5 (1·7–3·6)
1-162	Refraction disorders	10,800 (7,110–15,600)	14,000 (9,350–19,900)	15,700 (10,400–22,200)	38·1 (24·8–55·3)	37·3 (24·5–54·1)	37·1 (24·4–54·1)
1-163	Near vision loss	2,510 (1,160–4,610)	3,950 (1,830–7,240)	4,770 (2,230–8,680)	7·9 (3·7–14·6)	7·7 (3·5–14)	7·6 (3·5–13·9)
1-164	Other vision loss	4,190 (2,870–5,910)	6,100 (4,140–8,550)	7,260 (5,030–10,200)	13·8 (9·4–19·4)	12·9 (8·7–18·3)	12·7 (8·8–17·7)
1-165	Age-related and other hearing loss	111,000 (76,200–158,000)	168,000 (116,000–238,000)	205,000 (142,000–288,000)	354 (242–502)	334 (229–473)	332 (230–470)
1-166	Other sense organ diseases	12,400 (7,350–19,400)	18,800 (11,200–29,700)	22,700 (13,400–35,500)	40·4 (24–62·8)	41·2 (24·6–64·2)	41·5 (24·6–64·7)

1-167	Musculoskeletal disorders	758,000 (551,000–992,000)	1,100,000 (788,000–1,440,000)	1,300,000 (940,000–1,720,000)	2,470 (1,790–3,230)	2,510 (1,810–3,280)	2,650 (1,910–3,460)
1-168	Rheumatoid arthritis	15,000 (11,200–19,000)	25,400 (18,600–32,700)	31,100 (22,900–40,000)	47·3 (35·3–60·1)	52·4 (38·3–67·6)	53·7 (39·2–69·4)
1-169	Osteoarthritis	83,000 (41,700–169,000)	141,000 (70,700–291,000)	177,000 (90,000–360,000)	262 (132–536)	270 (135–556)	279 (141–568)
1-170	Low back pain	289,000 (206,000–378,000)	382,000 (273,000–500,000)	476,000 (336,000–642,000)	944 (675–1,240)	890 (636–1,170)	1,000 (697–1,360)
1-171	Neck pain	90,600 (59,600–130,000)	130,000 (84,800–187,000)	145,000 (95,200–210,000)	293 (192–423)	293 (193–424)	292 (191–420)
1-172	Gout	11,200 (7,110–16,000)	22,000 (13,900–31,900)	27,100 (16,800–39,600)	35·5 (22·4–50·9)	44·4 (28·1–63·7)	46·1 (28·9–66·1)
1-173	Other musculoskeletal disorders	270,000 (188,000–371,000)	395,000 (276,000–543,000)	447,000 (310,000–609,000)	886 (617–1,220)	957 (669–1,320)	974 (683–1,340)
1-174	Other non-communicable diseases	432,000 (351,000–542,000)	484,000 (376,000–626,000)	517,000 (401,000–667,000)	1,650 (1,380–2,010)	1,340 (1,070–1,680)	1,260 (1,010–1,610)
1-175	Congenital anomalies	118,000 (99,000–130,000)	80,200 (72,300–93,300)	78,200 (68,800–90,100)	561 (467–615)	357 (323–423)	335 (295–391)
1-176	Neural tube defects	9,110 (7,750–11,300)	3,770 (3,070–4,430)	4,590 (3,470–5,690)	44·3 (37·6–55·1)	18 (14·5–21·3)	22 (16·2–27·7)
1-177	Congenital heart anomalies	44,900 (30,500–51,000)	20,900 (17,000–24,400)	17,900 (13,900–20,900)	215 (143–246)	96 (76·9–114)	79·7 (60·6–95·7)
1-178	Orofacial clefts	244 (161–351)	277 (172–412)	276 (170–409)	1·1 (0·7–1·5)	1 (0·6–1·5)	0·9 (0·6–1·3)
1-179	Down's syndrome	3,780 (3,140–4,620)	7,570 (5,930–8,510)	8,910 (6,830–10,100)	15·7 (13·1–19·1)	25·7 (20·4–29·2)	28·2 (21·8–33·1)
1-180	Turner syndrome	91 (40–168)	89 (41–161)	89 (41–158)	0·3 (0·1–0·6)	0·3 (0·1–0·6)	0·3 (0·1–0·5)
1-181	Klinefelter syndrome	27 (12–53)	27 (12–53)	28 (12–55)	0·1 (0–0·2)	0·1 (0–0·2)	0·1 (0–0·2)
1-182	Other chromosomal abnormalities	10,300 (8,500–15,600)	12,000 (10,000–18,600)	12,700 (10,200–19,600)	49·6 (40·7–76·2)	59 (49–95·8)	61·8 (48·7–99)
1-183	Congenital musculoskeletal and limb anomalies	12,900 (9,260–17,300)	12,700 (8,770–17,500)	13,200 (9,000–18,500)	53·6 (39–71·6)	45·2 (31·9–61·7)	44·2 (31–61·6)
1-184	Urogenital congenital anomalies	3,130 (2,130–5,570)	2,510 (1,950–3,920)	2,060 (1,520–3,100)	15·5 (10·5–28)	12·9 (9·8–20·7)	10·4 (7·5–16·3)
1-185	Digestive congenital anomalies	6,640 (4,930–8,050)	5,070 (3,650–6,160)	4,460 (3,130–5,660)	33 (24·3–40·1)	25·7 (18·3–31·4)	22·1 (15·4–28·4)
1-186	Other congenital anomalies	27,100 (20,400–31,200)	15,200 (13,000–20,200)	14,000 (11,300–18,400)	132 (98·8–152)	72·9 (62·4–99)	65 (52·5–87)
1-187	Urinary diseases and male infertility	17,600 (14,300–22,100)	34,300 (28,500–40,600)	43,100 (35,600–51,600)	56·5 (45·9–70·4)	66·4 (55·2–79·7)	66·2 (54·4–80·4)
1-188	Urinary tract infections and interstitial nephritis	8,840 (7,750–11,700)	20,700 (17,400–22,700)	26,000 (21,600–29,100)	28·5 (25–37·5)	38·6 (32·8–42·3)	37·9 (32–42·3)
1-189	Urolithiasis	1,360 (1,030–1,780)	2,170 (1,690–2,790)	2,690 (2,080–3,420)	4·3 (3·3–5·7)	4·6 (3·5–6)	4·8 (3·7–6·2)
1-190	Benign prostatic hyperplasia	4,940 (2,790–7,560)	8,760 (5,070–13,400)	11,300 (6,570–17,500)	15·3 (8·6–23·5)	16·3 (9·4–24·9)	16·6 (9·6–25·7)
1-191	Male infertility	650 (240–1,500)	642 (248–1,440)	683 (254–1,550)	2 (0·7–4·7)	2·1 (0·8–4·7)	2·1 (0·8–4·7)
1-192	Other urinary diseases	1,860 (1,260–2,180)	1,990 (1,600–2,520)	2,440 (1,850–3,040)	6·3 (4·4–7·4)	4·8 (4–5·9)	4·9 (4–6)
1-193	Gynecological diseases	78,000 (53,500–109,000)	93,900 (63,300–131,000)	94,300 (64,000–131,000)	255 (175–358)	258 (174–360)	247 (168–346)
1-194	Uterine fibroids	2,510 (1,180–4,790)	3,760 (1,760–7,190)	3,470 (1,630–6,610)	8 (3·8–15·3)	9·2 (4·4–17·6)	8·1 (3·8–15·6)
1-195	Polycystic ovarian syndrome	1,810 (752–3,740)	2,500 (1,080–5,290)	2,600 (1,100–5,430)	6·1 (2·5–12·4)	7·7 (3·4–16·3)	7·9 (3·4–16·4)
1-196	Female infertility	483 (59–1,400)	450 (58–1,340)	482 (57–1,450)	1·5 (0·2–4·4)	1·5 (0·2–4·3)	1·5 (0·2–4·3)
1-197	Endometriosis	7,740 (4,530–12,300)	9,080 (5,640–13,900)	6,670 (3,920–10,700)	25 (14·7–39·6)	26·8 (16·5–41·1)	19·2 (11·2–30·9)
1-198	Genital prolapse	1,420 (670–2,680)	2,280 (1,090–4,310)	2,590 (1,260–4,970)	4·5 (2·1–8·5)	4·3 (2·1–8·1)	4 (2–7·5)
1-199	Premenstrual syndrome	28,900 (17,500–45,200)	32,600 (19,800–50,900)	32,300 (19,700–50,200)	98·2 (59·5–154)	96·6 (58·7–151)	95·9 (58·3–149)
1-200	Other gynecological diseases	35,100 (22,200–51,200)	43,200 (26,800–62,900)	46,300 (29,400–67,100)	112 (71–162)	112 (70·3–163)	110 (70–162)
1-201	Hemoglobinopathies and hemolytic anaemias	6,400 (5,560–7,610)	4,890 (4,170–5,910)	5,450 (4,600–6,630)	22·5 (19·6–26·8)	12·7 (10·7–15·4)	12·2 (10·1–15·1)
1-202	Thalassemias	267 (206–346)	134 (99–180)	176 (127–232)	1·3 (1–1·7)	0·6 (0·5–0·9)	0·8 (0·6–1·1)
1-203	Thalassaemias trait	408 (243–645)	330 (189–553)	349 (199–588)	1·4 (0·8–2·2)	1 (0·6–1·8)	1 (0·6–1·7)
1-204	Sickle cell disorders	223 (175–281)	128 (101–158)	53 (37–72)	1·1 (0·9–1·4)	0·6 (0·5–0·8)	0·2 (0·2–0·3)
1-205	Sickle cell trait	301 (168–506)	239 (127–415)	230 (116–392)	1 (0·6–1·7)	0·8 (0·4–1·4)	0·7 (0·3–1·2)
1-206	G6PD deficiency	1,260 (1,010–1,490)	1,040 (868–1,210)	1,080 (899–1,300)	4·7 (3·8–5·5)	3·1 (2·6–3·6)	3 (2·5–3·6)
1-207	G6PD trait	46 (20–82)	26 (7–59)	38 (15–72)	0·2 (0·1–0·3)	0·1 (0–0·2)	0·1 (0–0·2)
1-208	Other hemoglobinopathies and hemolytic anaemias	3,900 (3,370–4,620)	2,990 (2,550–3,630)	3,520 (2,960–4,270)	12·8 (11·1–15·3)	6·4 (5·3–8)	6·3 (5·1–7·9)
1-209	Endocrine, metabolic, blood, and immune disorders	91,900 (70,300–117,000)	133,000 (103,000–170,000)	153,000 (119,000–198,000)	308 (238–389)	312 (246–397)	311 (245–395)
1-210	Oral disorders	89,900 (52,700–144,000)	131,000 (78,400–203,000)	138,000 (80,600–223,000)	292 (172–469)	291 (174–452)	271 (156–436)
1-211	Caries of deciduous teeth	530 (224–1,110)	514 (220–1,090)	560 (238–1,200)	2·7 (1·1–5·6)	2·7 (1·1–5·6)	2·7 (1·1–5·8)
1-212	Caries of permanent teeth	6,520 (2,910–12,300)	7,960 (3,540–15,300)	8,510 (3,800–16,500)	22·5 (10·2–43)	22·3 (9·9–43·3)	22·1 (10·1–42·8)
1-213	Periodontal disease	28,800 (11,200–62,000)	42,400 (16,600–91,400)	48,800 (19,200–104,000)	92·4 (35·8–200)	93·1 (36·6–200)	95·4 (37·3–206)
1-214	Edentulism	38,800 (24,400–57,900)	60,500 (40,800–83,800)	59,800 (37,800–88,700)	121 (76·3–181)	120 (81–167)	97·7 (61·7–145)
1-215	Other oral disorders	15,200 (9,530–22,400)	19,200 (12,000–28,200)	20,800 (13,000–30,400)	53·2 (33·1–78·2)	53·2 (32·9–78)	53·1 (32·9–77·7)
1-216	Sudden infant death syndrome	30,400 (26,000–35,200)	7,460 (6,040–9,340)	4,120 (2,880–5,570)	154 (132–179)	40·1 (32·4–50·1)	22·4 (15·6–30·2)
1-0	Injuries	869,000 (791,000–972,000)	851,000 (742,000–986,000)	920,000 (793,000–1,080,000)	3,020 (2,770–3,360)	2,170 (1,920–2,470)	2,080 (1,830–2,390)
1-1	Transport injuries	291,000 (274,000–310,000)	193,000 (174,000–213,000)	183,000 (165,000–205,000)	1,050 (991–1,110)	542 (500–589)	474 (432–520)
1-2	Road injuries	264,000 (250,000–280,000)	159,000 (145,000–176,000)	150,000 (135,000–169,000)	953 (905–1,010)	452 (420–490)	394 (358–433)

1·3	Pedestrian road injuries	36,100<br>(34,100–38,500)	27,200<br>(25,200–29,500)	25,900<br>(23,500–28,600)	137<br>(129–146)	77·6<br>(72·6–83·5)	67·2<br>(61·5–73·5)
1·4	Cyclist road injuries	12,100<br>(10,300–14,300)	11,900<br>(9,640–14,600)	12,200<br>(9,790–14,900)	45·8<br>(39·5–52·9)	31·3<br>(26·1–37·6)	28·9<br>(23·8–34·6)
1·5	Motorcyclist road injuries	23,700<br>(21,300–26,400)	16,000<br>(14,100–18,400)	16,400<br>(14,000–19,400)	84·8<br>(76·1–94·2)	43·6<br>(38·8–49·2)	41·6<br>(35·2–48·9)
1·6	Motor vehicle road injuries	190,000<br>(180,000–201,000)	98,300<br>(90,000–108,000)	91,100<br>(82,300–102,000)	681<br>(647–717)	282<br>(263–306)	243<br>(222–266)
1·7	Other road injuries	1,360<br>(1,130–1,630)	5,860<br>(5,150–6,700)	4,780<br>(4,000–5,680)	4·7<br>(3·9–5·6)	17<br>(14·9–19·4)	13<br>(10·7–15·7)
1·8	Other transport injuries	27,000<br>(24,200–30,300)	33,400<br>(29,400–38,300)	33,000<br>(28,300–38,600)	92·6<br>(83·3–103)	90·3<br>(80·7–102)	80·3<br>(70·2–91·9)
1·9	Unintentional injuries	327,000<br>(268,000–404,000)	404,000<br>(320,000–514,000)	478,000<br>(375,000–612,000)	1,120<br>(932–1,380)	920<br>(733–1,170)	919<br>(724–1,170)
1·10	Falls	123,000<br>(97,300–157,000)	221,000<br>(177,000–281,000)	275,000<br>(218,000–348,000)	403<br>(319–517)	446<br>(355–570)	452<br>(355–581)
1·11	Drowning	23,200<br>(22,000–24,500)	14,600<br>(13,800–15,300)	14,200<br>(13,200–15,300)	92·5<br>(86·9–98·5)	49<br>(46·1–51·8)	45<br>(41·1–48·9)
1·12	Fire, heat, and hot substances	32,500<br>(27,200–40,000)	23,700<br>(17,800–31,800)	25,900<br>(19,000–36,000)	118<br>(100–144)	62<br>(48·4–81·6)	60·6<br>(45·8–81·7)
1·13	Poisonings	11,200<br>(10,000–12,600)	8,950<br>(7,630–10,500)	9,520<br>(8,030–11,300)	39·2<br>(35–44·2)	25·2<br>(21·5–29·6)	25<br>(21–29·6)
1·14	Poisoning by carbon monoxide	4,420<br>(4,050–4,790)	4,360<br>(4,040–4,700)	4,440<br>(3,910–4,960)	15·2<br>(14–16·5)	12·3<br>(11·4–13·4)	11·9<br>(10·5–13·4)
1·15	Poisoning by other means	6,810<br>(5,800–8,070)	4,590<br>(3,460–5,980)	5,080<br>(3,780–6,660)	24<br>(20·3–28·4)	12·8<br>(9·6–16·9)	13<br>(9·7–17·3)
1·16	Exposure to mechanical forces	56,100<br>(42,900–74,800)	54,700<br>(39,400–76,000)	62,100<br>(44,400–86,900)	190<br>(147–253)	135<br>(97·9–187)	136<br>(98·9–190)
1·17	Unintentional firearm injuries	5,160<br>(3,400–6,000)	2,920<br>(2,290–3,730)	3,270<br>(2,500–4,220)	18·9<br>(12·2–21·9)	7·8<br>(6·2–10·1)	7·9<br>(6·2–10·1)
1·18	Other exposure to mechanical forces	50,900<br>(37,900–69,300)	51,800<br>(36,900–72,700)	58,800<br>(41,600–83,000)	171<br>(129–232)	127<br>(91·4–178)	128<br>(91·8–181)
1·19	Adverse effects of medical treatment	10,400<br>(8,680–11,900)	10,300<br>(8,750–12,600)	11,600<br>(9,730–14,400)	35·5<br>(29·6–40·7)	24·4<br>(20·6–30)	23·4<br>(19·4–29)
1·20	Animal contact	3,040<br>(2,190–4,220)	3,710<br>(2,630–5,240)	4,130<br>(2,920–5,810)	10·5<br>(7·7–14·5)	9·5<br>(6·8–13·3)	9·4<br>(6·7–13·2)
1·21	Venomous animal contact	1,200<br>(840–1,620)	1,460<br>(1,040–1,990)	1,620<br>(1,160–2,200)	4·2<br>(2·9–5·6)	3·9<br>(2·8–5·3)	3·9<br>(2·8–5·4)
1·22	Non-venomous animal contact	1,830<br>(1,290–2,670)	2,240<br>(1,520–3,340)	2,510<br>(1,680–3,730)	6·4<br>(4·5–9·1)	5·6<br>(3·9–8·3)	5·5<br>(3·7–8·1)
1·23	Foreign body	19,400<br>(17,700–21,400)	17,800<br>(15,900–20,100)	20,300<br>(17,800–23,000)	74·5<br>(67·9–81·6)	52·1<br>(46·7–58·3)	52·1<br>(45·8–59·4)
1·24	Pulmonary aspiration and foreign body in airway	15,900<br>(14,900–17,100)	13,000<br>(12,300–13,800)	14,700<br>(13,500–15,900)	62·7<br>(58–68·2)	40·2<br>(37·1–44·1)	40·1<br>(35·7–45·2)
1·25	Foreign body in eyes	1,170<br>(571–2,050)	1,470<br>(756–2,520)	1,610<br>(843–2,700)	4·1<br>(1·9–7·2)	4·1<br>(1·9–7·2)	4·1<br>(1·9–7·2)
1·26	Foreign body in other body part	2,310<br>(1,670–3,120)	3,360<br>(2,430–4,540)	3,950<br>(2,850–5,310)	7·8<br>(5·7–10·4)	7·9<br>(5·8–10·6)	8<br>(5·8–10·6)
1·27	Environmental heat and cold exposure	6,980<br>(6,110–8,030)	7,050<br>(5,880–8,430)	7,690<br>(6,310–9,250)	23·4<br>(20·5–26·9)	16·9<br>(14·3–20)	16·2<br>(13·5–19·3)
1·28	Exposure to forces of nature	1,270<br>(1,150–1,400)	536<br>(483–595)	67<br>(48–91)	4·8<br>(4·3–5·3)	1·7<br>(1·5–1·9)	0·1<br>(0·1–0·2)
1·29	Other unintentional injuries	39,800<br>(28,300–55,300)	41,700<br>(27,600–60,100)	48,200<br>(31,900–69,500)	132<br>(94·6–183)	98<br>(65·3–141)	99·4<br>(66·2–143)
1·30	Self-harm and interpersonal violence	251,000<br>(243,000–259,000)	254,000<br>(245,000–264,000)	258,000<br>(244,000–272,000)	855<br>(829–883)	708<br>(683–732)	684<br>(648–719)
1·31	Self-harm	205,000<br>(199,000–211,000)	205,000<br>(199,000–212,000)	212,000<br>(201,000–223,000)	691<br>(671–712)	562<br>(545–579)	555<br>(526–585)
1·32	Self-harm by firearm	56,600<br>(49,000–66,500)	25,000<br>(21,300–33,800)	26,100<br>(22,200–34,000)	195<br>(169–228)	66·3<br>(55–88·4)	65·4<br>(54·4–86·2)
1·33	Self-harm by other specified means	148,000<br>(139,000–157,000)	180,000<br>(171,000–187,000)	186,000<br>(172,000–197,000)	496<br>(467–525)	496<br>(473–514)	490<br>(454–520)
1·34	Interpersonal violence	45,500<br>(42,200–49,100)	46,600<br>(42,200–51,200)	44,800<br>(39,600–50,300)	161<br>(150–174)	140<br>(129–152)	125<br>(112–139)
1·35	Physical violence by firearm	12,200<br>(11,500–13,000)	11,700<br>(11,000–12,300)	10,400<br>(9,390–11,500)	42·4<br>(40–45)	37·1<br>(34·9–39·3)	31·5<br>(28·2–35)
1·36	Physical violence by sharp object	12,400<br>(11,600–13,400)	12,400<br>(11,400–13,500)	11,700<br>(10,400–13,200)	42·7<br>(39·9–45·8)	36·9<br>(34·2–39·9)	32·9<br>(29·3–36·8)
1·37	Sexual violence	3,050<br>(1,990–4,290)	3,490<br>(2,300–4,910)	3,580<br>(2,360–5,010)	11·3<br>(7·4–15·9)	11·3<br>(7·4–15·9)	11·2<br>(7·4–15·9)
1·38	Physical violence by other means	17,800<br>(15,700–20,200)	19,000<br>(16,400–22,300)	19,000<br>(15,800–22,800)	64·8<br>(57·8–72·9)	54·4<br>(48·1–62·3)	49·1<br>(42·3–57·4)
1·39	Conflict and terrorism	222<br>(145–361)	1,120<br>(970–1,340)	311<br>(215–478)	0·8<br>(0·5–1·3)	3·6<br>(3·1–4·2)	0·7<br>(0·5–1·1)
1·40	Executions and police conflict	605<br>(486–754)	949<br>(771–1,180)	1,030<br>(690–1,530)	2·1<br>(1·7–2·7)	3·1<br>(2·4–3·8)	3·2<br>(2·1–4·8)

SR Table 1: All-age DALYs counts and age standardized DALYs rates per 100,000 individuals for all causes and level 1 cause groups by location for 1990, 2010, and 2019, both sexes combined, USA

Cause Level	Cause	All-age DALYs			Age-standardized rate of DALYs per 100,000		
		1990	2010	2019	1990	2010	2019
0-0	All causes	83,500,000 (74,700,000–93,400,000)	95,400,000 (84,000,000–108,000,000)	111,000,000 (97,800,000–126,000,000)	29,300 (26,200–32,900)	25,400 (22,200–28,900)	26,100 (22,700–29,800)
1-0	Communicable, maternal, neonatal, and nutritional disorders	6,250,000 (5,850,000–6,690,000)	4,840,000 (4,390,000–5,360,000)	4,960,000 (4,460,000–5,580,000)	2,510 (2,350–2,690)	1,680 (1,530–1,850)	1,590 (1,430–1,780)
1-1	HIV/AIDS and sexually transmitted infections	1,530,000 (1,480,000–1,610,000)	534,000 (462,000–632,000)	463,000 (381,000–572,000)	542 (523–568)	148 (128–175)	122 (101–150)
1-2	HIV/AIDS	1,490,000 (1,440,000–1,550,000)	489,000 (427,000–572,000)	416,000 (346,000–509,000)	527 (510–549)	135 (118–157)	109 (91·7–132)
1-3	HIV/AIDS - drug-susceptible tuberculosis	38,200 (24,800–57,500)	6,500 (4,300–9,680)	5,970 (3,910–8,980)	13·5 (8·8–20·4)	1·8 (1·2–2·7)	1·6 (1–2·4)
1-4	HIV/AIDS - Multidrug-resistant Tuberculosis without extensive drug resistance	3,110 (1,090–6,940)	214 (86–446)	200 (50–544)	1·1 (0·4–2·5)	0·1 (0–0·1)	0·1 (0–0·1)
1-5	HIV/AIDS - Extensively drug-resistant Tuberculosis	0 (0–0)	36 (14–79)	52 (13–140)	0 (0–0)	0 (0–0)	0 (0–0)
1-6	HIV/AIDS resulting in other diseases	1,450,000 (1,400,000–1,510,000)	482,000 (420,000–565,000)	409,000 (339,000–501,000)	512 (494–534)	133 (116–156)	107 (89·7–130)
1-7	Sexually transmitted infections excluding HIV	43,300 (25,200–76,300)	44,800 (24,900–80,800)	47,600 (26,400–86,500)	15·3 (8·9–26·9)	13·2 (7·4–23·6)	13 (7·1–23·6)
1-8	Syphilis	5,010 (4,100–6,160)	3,720 (2,940–4,610)	4,530 (3,530–5,720)	1·8 (1·5–2·2)	1 (0·8–1·2)	1 (0·8–1·3)
1-9	Chlamydial infection	2,580 (1,780–3,850)	2,160 (1,520–3,140)	2,240 (1,590–3,220)	0·9 (0·6–1·3)	0·7 (0·5–1)	0·7 (0·5–1)
1-10	Gonococcal infection	3,320 (2,800–4,060)	2,470 (2,120–2,970)	2,610 (2,240–3,150)	1·1 (1–1·4)	0·7 (0·6–0·9)	0·7 (0·6–0·8)
1-11	Trichomoniasis	12,900 (5,070–26,800)	14,500 (5,770–30,200)	15,300 (6,040–31,500)	4·6 (1·8–9·4)	4·3 (1·7–8·9)	4·3 (1·7–8·9)
1-12	Genital herpes	11,800 (3,750–28,800)	13,300 (4,190–32,500)	14,000 (4,470–34,200)	4·2 (1·3–10·2)	3·7 (1·2–9·1)	3·6 (1·1–8·8)
1-13	Other sexually transmitted infections	7,620 (5,030–11,000)	8,690 (5,760–12,700)	8,900 (5,770–13,100)	2·7 (1·8–3·9)	2·8 (1·8–4·1)	2·8 (1·8–4·1)
1-14	Respiratory infections and tuberculosis	1,630,000 (1,490,000–1,830,000)	1,440,000 (1,270,000–1,640,000)	1,650,000 (1,470,000–1,870,000)	570 (514–648)	383 (332–455)	385 (330–458)
1-15	Tuberculosis	63,500 (60,000–66,800)	23,500 (21,900–25,200)	25,000 (23,200–27,000)	21·6 (20·4–22·7)	6 (5·6–6·5)	5·5 (5–6)
1-16	Latent tuberculosis infection	0 (0–0)	0 (0–0)	0 (0–0)	0 (0–0)	0 (0–0)	0 (0–0)
1-17	Drug-susceptible tuberculosis	59,400 (54,400–63,500)	22,800 (21,200–24,500)	24,200 (22,100–26,400)	20·2 (18·5–21·6)	5·8 (5·4–6·3)	5·3 (4·8–5·8)
1-18	Multidrug-resistant tuberculosis without extensive drug resistance	4,050 (1,640–8,070)	593 (297–1,100)	640 (205–1,590)	1·4 (0·6–2·7)	0·1 (0·1–0·3)	0·1 (0–0·3)
1-19	Extensively drug-resistant tuberculosis	0 (0–0)	96 (45–188)	162 (49–405)	0 (0–0)	0 (0–0)	0 (0–0·1)
1-20	Lower respiratory infections	1,240,000 (1,170,000–1,280,000)	1,050,000 (983,000–1,090,000)	1,230,000 (1,140,000–1,280,000)	412 (394–424)	251 (239–259)	247 (234–256)
1-21	Upper respiratory infections	284,000 (174,000–438,000)	314,000 (192,000–486,000)	343,000 (210,000–527,000)	117 (71·4–179)	109 (66·2–169)	114 (69·4–177)
1-22	Otitis media	46,800 (27,400–76,700)	48,100 (28,000–77,900)	50,100 (29,200–81,400)	19·8 (11·6–32·5)	16·9 (9·8–27·7)	17·4 (10–28·5)
1-23	Enteric infections	216,000 (155,000–290,000)	387,000 (316,000–472,000)	445,000 (361,000–542,000)	88·4 (63·5–120)	111 (87·8–140)	118 (92·1–150)
1-24	Diarrhoeal diseases	212,000 (151,000–286,000)	385,000 (314,000–470,000)	442,000 (359,000–540,000)	86·8 (61·8–118)	111 (87·3–140)	118 (91·5–149)
1-25	Typhoid and paratyphoid	51 (44–93)	54 (40–64)	62 (41–71)	0 (0–0)	0 (0–0)	0 (0–0)
1-26	Typhoid fever	13 (9–20)	14 (7–17)	14 (8–18)	0 (0–0)	0 (0–0)	0 (0–0)
1-27	Paratyphoid fever	38 (32–77)	40 (30–52)	48 (30–55)	0 (0–0)	0 (0–0)	0 (0–0)
1-28	iNTS	2,540 (1,220–2,820)	1,070 (872–2,370)	1,230 (1,030–2,500)	1·1 (0·5–1·2)	0·4 (0·3–0·8)	0·4 (0·3–0·8)
1-29	Other intestinal infectious diseases	1,130 (653–1,670)	852 (536–1,190)	851 (583–1,160)	0·5 (0·3–0·8)	0·3 (0·2–0·5)	0·3 (0·2–0·4)
1-30	Neglected tropical diseases and malaria	71,700 (46,300–104,000)	104,000 (66,000–155,000)	100,000 (63,500–149,000)	24·4 (16–35·2)	26·2 (16·9–38)	21·7 (14·3–31·4)
1-31	Malaria	0 (0–0)	0 (0–0)	0 (0–0)	0 (0–0)	0 (0–0)	0 (0–0)
1-32	Chagas disease	606 (401–863)	1,010 (709–1,360)	686 (434–1,010)	0·2 (0·1–0·3)	0·2 (0·2–0·3)	0·2 (0·1–0·2)
1-33	Leishmaniasis	0 (0–0)	0 (0–1)	0 (0–1)	0 (0–0)	0 (0–0)	0 (0–0)
1-34	Visceral leishmaniasis	0 (0–0)	0 (0–0)	0 (0–0)	0 (0–0)	0 (0–0)	0 (0–0)
1-35	Cutaneous and mucocutaneous leishmaniasis	0 (0–0)	0 (0–1)	0 (0–1)	0 (0–0)	0 (0–0)	0 (0–0)
1-36	African trypanosomiasis	0 (0–0)	0 (0–0)	0 (0–0)	0 (0–0)	0 (0–0)	0 (0–0)
1-37	Schistosomiasis	0 (0–0)	0 (0–0)	0 (0–0)	0 (0–0)	0 (0–0)	0 (0–0)
1-38	Cysticercosis	60,100 (35,600–92,000)	88,900 (51,900–139,000)	82,800 (47,900–130,000)	19·5 (11·4–30·1)	20·9 (12·2–32·4)	16 (9·2–25·2)
1-39	Cystic echinococcosis	174 (110–250)	99 (62–140)	136 (86–190)	0·1 (0–0·1)	0 (0–0)	0 (0–0)
1-40	Lymphatic filariasis	0 (0–0)	0 (0–0)	0 (0–0)	0 (0–0)	0 (0–0)	0 (0–0)
1-41	Onchocerciasis	0 (0–0)	0 (0–0)	0 (0–0)	0 (0–0)	0 (0–0)	0 (0–0)
1-42	Trachoma	0 (0–0)	0 (0–0)	0 (0–0)	0 (0–0)	0 (0–0)	0 (0–0)
1-43	Dengue	144 (81–236)	266 (164–412)	268 (165–425)	0·1 (0–0·1)	0·1 (0·1–0·1)	0·1 (0–0·1)
1-44	Yellow fever	0 (0–0)	0 (0–0)	0 (0–0)	0 (0–0)	0 (0–0)	0 (0–0)
1-45	Rabies	112 (106–119)	202 (191–214)	145 (132–159)	0·1 (0–0·1)	0·1 (0·1–0·1)	0·1 (0·1–0·1)
1-46	Intestinal nematode infections	0 (0–0)	0 (0–0)	0 (0–0)	0 (0–0)	0 (0–0)	0 (0–0)
1-47	Ascariasis	0 (0–0)	0 (0–0)	0 (0–0)	0 (0–0)	0 (0–0)	0 (0–0)
1-48	Trichuriasis	0 (0–0)	0 (0–0)	0 (0–0)	0 (0–0)	0 (0–0)	0 (0–0)

1-49	Hookworm disease	0 (0–0)	0 (0–0)	0 (0–0)	0 (0–0)	0 (0–0)	0 (0–0)
1-50	Food-borne trematodiases	0 (0–0)	0 (0–0)	0 (0–0)	0 (0–0)	0 (0–0)	0 (0–0)
1-51	Leprosy	0 (0–0)	0 (0–0)	0 (0–0)	0 (0–0)	0 (0–0)	0 (0–0)
1-52	Ebola virus disease	0 (0–0)	0 (0–0)	0 (0–0)	0 (0–0)	0 (0–0)	0 (0–0)
1-53	Zika virus disease	0 (0–0)	0 (0–0)	0 (0–1)	0 (0–0)	0 (0–0)	0 (0–0)
1-54	Guinea worm disease	0 (0–0)	0 (0–0)	0 (0–0)	0 (0–0)	0 (0–0)	0 (0–0)
1-55	Other neglected tropical diseases	10,500 (6,840–15,700)	13,700 (9,380–19,300)	16,100 (10,500–22,800)	4·5 (2·9–6·7)	4·8 (3·3–6·8)	5·4 (3·5–7·8)
1-56	Other infectious diseases	283,000 (238,000–312,000)	228,000 (201,000–284,000)	240,000 (211,000–300,000)	115 (97·8–126)	74·2 (65·4–91·2)	71·3 (62·6–87·9)
1-57	Meningitis	104,000 (97,800–111,000)	49,100 (47,600–50,600)	45,900 (44,000–47,800)	45·2 (42·6–48·9)	17·3 (16·7–18)	15·4 (14·6–16·2)
1-58	Encephalitis	16,500 (15,300–20,900)	23,900 (20,300–25,500)	24,500 (21,100–26,400)	6·6 (6·2–8·5)	8·1 (6·8–8·6)	7·6 (6·5–8·3)
1-59	Diphtheria	51 (48–54)	77 (73–80)	84 (78–90)	0 (0–0)	0 (0–0)	0 (0–0)
1-60	Whooping cough	1,430 (985–2,050)	3,200 (2,220–4,510)	3,100 (2,080–4,530)	0·7 (0·5–1)	1·6 (1·1–2·2)	1·6 (1·1–2·3)
1-61	Tetanus	486 (353–643)	159 (123–458)	160 (114–441)	0·2 (0·1–0·2)	0 (0–0·1)	0 (0–0·1)
1-62	Measles	1,850 (1,260–2,630)	3 (2–4)	50 (33–75)	0·9 (0·6–1·3)	0 (0–0)	0 (0–0)
1-63	Varicella and herpes zoster	14,600 (10,900–19,500)	16,900 (11,700–25,000)	17,300 (12,200–25,400)	5·5 (4·1–7·4)	4·7 (3·3–7)	4·3 (3·1–6·4)
1-64	Acute hepatitis	14,200 (11,600–17,600)	13,100 (9,960–17,400)	12,900 (9,770–17,200)	5·5 (4·4–6·8)	4·2 (3·1–5·6)	3·9 (2·9–5·3)
1-65	Acute hepatitis A	8,830 (6,480–11,600)	8,980 (6,280–12,400)	8,900 (6,220–12,500)	3·5 (2·5–4·6)	3 (2–4·2)	2·8 (1·9–4)
1-66	Acute hepatitis B	2,670 (1,950–3,370)	2,480 (1,710–3,050)	2,270 (1,620–2,770)	1 (0·7–1·3)	0·7 (0·5–0·9)	0·6 (0·4–0·7)
1-67	Acute hepatitis C	1,990 (774–2,610)	904 (628–1,530)	908 (627–1,450)	0·7 (0·3–1)	0·2 (0·2–0·4)	0·2 (0·2–0·4)
1-68	Acute hepatitis E	749 (536–1,100)	763 (534–1,110)	829 (586–1,180)	0·3 (0·2–0·4)	0·2 (0·2–0·4)	0·2 (0·2–0·3)
1-69	Other unspecified infectious diseases	131,000 (86,000–151,000)	122,000 (102,000–176,000)	136,000 (114,000–195,000)	50·1 (33·3–58·1)	38·2 (31·9–54·6)	38·3 (31·7–54·7)
1-70	Maternal and neonatal disorders	2,230,000 (2,090,000–2,380,000)	1,810,000 (1,650,000–1,970,000)	1,650,000 (1,490,000–1,820,000)	1,060 (1,000–1,130)	834 (772–895)	761 (694–833)
1-71	Maternal disorders	51,200 (43,300–60,500)	72,200 (65,900–80,400)	71,700 (64,400–81,300)	18·9 (16–22·4)	25 (22·8–28)	23·6 (21·1–26·8)
1-72	Maternal haemorrhage	7,980 (5,750–11,100)	6,770 (5,110–8,780)	6,660 (4,700–9,410)	2·9 (2·1–4·1)	2·4 (1·8–3·1)	2·2 (1·6–3·1)
1-73	Maternal sepsis and other pregnancy related infections	4,490 (2,630–7,410)	3,730 (2,300–5,800)	3,170 (1,950–5,080)	1·7 (1–2·9)	1·3 (0·8–2·1)	1·1 (0·7–1·8)
1-74	Maternal hypertensive disorders	9,710 (7,010–13,400)	10,700 (8,470–13,600)	10,500 (7,830–13,900)	3·6 (2·6–5)	3·8 (3–4·8)	3·5 (2·6–4·6)
1-75	Maternal obstructed labour and uterine rupture	3,110 (1,600–5,150)	2,100 (1,310–3,020)	2,180 (1,320–3,210)	1·2 (0·6–1·9)	0·7 (0·5–1·1)	0·7 (0·4–1·1)
1-76	Maternal abortive outcome	2,840 (2,220–3,560)	1,780 (1,370–2,260)	1,400 (1,100–1,780)	1·1 (0·8–1·3)	0·6 (0·5–0·8)	0·5 (0·4–0·6)
1-77	Ectopic pregnancy	2,600 (2,180–3,070)	1,490 (1,230–1,760)	1,230 (1,010–1,480)	1 (0·8–1·1)	0·5 (0·4–0·6)	0·4 (0·3–0·5)
1-78	Indirect maternal deaths	2,110 (1,740–2,520)	11,100 (9,460–12,800)	11,500 (9,860–13,500)	0·8 (0·6–0·9)	3·7 (3·2–4·3)	3·7 (3·2–4·4)
1-79	Late maternal deaths	5,930 (4,630–7,490)	11,700 (10,200–13,300)	12,500 (9,870–15,700)	2·2 (1·7–2·7)	4·1 (3·5–4·7)	4·1 (3·2–5·2)
1-80	Maternal deaths aggravated by HIV/AIDS	40 (22–63)	96 (52–151)	122 (67–194)	0 (0–0)	0 (0–0·1)	0 (0–0·1)
1-81	Other maternal disorders	12,300 (10,800–14,000)	22,700 (20,100–25,300)	22,400 (19,600–25,700)	4·5 (4–5·1)	7·8 (6·9–8·7)	7·3 (6·4–8·4)
1-82	Neonatal disorders	2,170,000 (2,030,000–2,330,000)	1,740,000 (1,580,000–1,900,000)	1,580,000 (1,420,000–1,750,000)	1,050 (982–1,120)	809 (748–870)	737 (670–809)
1-83	Neonatal preterm birth	1,450,000 (1,340,000–1,560,000)	1,150,000 (1,030,000–1,270,000)	1,030,000 (916,000–1,150,000)	689 (643–741)	521 (476–566)	466 (422–515)
1-84	Neonatal encephalopathy due to birth asphyxia and trauma	322,000 (284,000–367,000)	251,000 (211,000–303,000)	243,000 (210,000–282,000)	155 (139–174)	116 (101–135)	111 (97·7–126)
1-85	Neonatal sepsis and other neonatal infections	104,000 (92,100–120,000)	84,500 (74,200–101,000)	79,700 (70,500–91,200)	50·2 (44·9–57·2)	41·6 (37·1–47·8)	39·3 (35·1–44·1)
1-86	Hemolytic disease and other neonatal jaundice	9,100 (7,830–10,500)	7,580 (6,070–9,270)	7,540 (5,970–9,240)	4·1 (3·6–4·7)	2·8 (2·3–3·4)	2·7 (2·2–3·3)
1-87	Other neonatal disorders	294,000 (274,000–313,000)	245,000 (227,000–259,000)	218,000 (196,000–241,000)	147 (137–156)	128 (119–136)	118 (106–131)
1-88	Nutritional deficiencies	288,000 (204,000–391,000)	343,000 (249,000–459,000)	412,000 (300,000–558,000)	110 (75·4–151)	101 (71·3–137)	112 (78·9–158)
1-89	Protein-energy malnutrition	81,700 (63,300–108,000)	119,000 (92,700–153,000)	151,000 (120,000–190,000)	28·7 (21·6–38·9)	31·3 (23·4–41·5)	34·5 (25·9–45·3)
1-90	Iodine deficiency	6,570 (3,010–12,500)	8,190 (3,800–15,500)	8,650 (4,030–16,300)	2·5 (1·1–4·7)	2·5 (1·1–4·7)	2·4 (1·1–4·6)
1-91	Vitamin A deficiency	511 (275–896)	353 (180–587)	298 (144–542)	0·3 (0·1–0·5)	0·2 (0·1–0·3)	0·1 (0·1–0·3)
1-92	Dietary iron deficiency	164,000 (102,000–250,000)	183,000 (115,000–281,000)	215,000 (134,000–334,000)	65·1 (40·5–99·2)	57·5 (36·1–88·2)	65·4 (40·3–103)
1-93	Other nutritional deficiencies	35,300 (23,300–51,600)	32,700 (21,500–47,100)	36,500 (24,400–52,400)	13 (8·5–19·1)	9·5 (6·1–14)	9·7 (6·3–14·2)
1-0	Non-communicable diseases	67,300,000 (59,400,000–76,000,000)	81,400,000 (71,100,000–92,700,000)	96,000,000 (83,900,000–109,000,000)	23,100 (20,300–26,300)	21,000 (18,100–24,100)	21,700 (18,600–25,100)
1-1	Neoplasms	12,700,000 (12,300,000–13,000,000)	14,300,000 (13,800,000–14,800,000)	16,600,000 (15,900,000–17,300,000)	4,240 (4,110–4,340)	3,360 (3,250–3,470)	3,230 (3,110–3,350)
1-2	Lip and oral cavity cancer	150,000 (146,000–155,000)	152,000 (147,000–157,000)	181,000 (172,000–188,000)	51 (49·5–52·5)	35·3 (34·1–36·4)	35 (33·4–36·4)
1-3	Nasopharynx cancer	29,900 (29,100–30,700)	29,400 (28,600–30,300)	32,400 (30,900–33,900)	10·7 (10·4–11)	7·3 (7–7·5)	7 (6·7–7·3)
1-4	Other pharynx cancer	53,900 (52,200–56,100)	66,700 (64,400–69,000)	81,500 (77,300–85,700)	18·7 (18·2–19·5)	15·4 (14·9–16)	15·9 (15·1–16·7)
1-5	Oesophageal cancer	267,000 (261,000–274,000)	395,000 (383,000–402,000)	472,000 (453,000–489,000)	90·1 (87·9–92·4)	90·6 (88·3–92·3)	89·1 (85·9–92·2)
1-6	Stomach cancer	390,000 (377,000–398,000)	345,000 (332,000–353,000)	388,000 (371,000–402,000)	126 (122–128)	79·9 (77·2–81·6)	75·7 (72·8–78·3)
1-7	Colon and rectum cancer	1,370,000 (1,320,000–1,410,000)	1,490,000 (1,420,000–1,530,000)	1,760,000 (1,680,000–1,830,000)	442 (427–453)	341 (328–351)	339 (325–351)

1-8	Liver cancer	152,000 (148,000–155,000)	402,000 (390,000–414,000)	551,000 (491,000–604,000)	51·3 (50·2–52·4)	94·2 (91·6–97)	107 (95·6–117)
1-9	Liver cancer due to hepatitis B	24,000 (21,000–27,400)	57,200 (49,900–65,700)	72,900 (58,800–89,800)	8·3 (7·2–9·5)	13·8 (12·1–15·7)	15·2 (12·4–18·5)
1-10	Liver cancer due to hepatitis C	51,400 (46,200–56,500)	149,000 (133,000–164,000)	200,000 (170,000–231,000)	16·9 (15·1–18·5)	33·7 (30·2–37·2)	37 (31·4–42·8)
1-11	Liver cancer due to alcohol use	44,200 (39,200–49,200)	118,000 (104,000–132,000)	177,000 (144,000–211,000)	14·7 (13–16·4)	27·4 (24·2–30·7)	33·8 (27·6–40·4)
1-12	Liver cancer due to NASH	13,400 (11,600–15,500)	37,300 (32,200–42,800)	52,000 (42,100–62,300)	4·3 (3·7–5)	8·6 (7·4–9·8)	9·8 (7·9–11·6)
1-13	Gallbladder and biliary tract cancer	86,700 (73,900–92,700)	89,500 (81,500–105,000)	103,000 (95,200–123,000)	27·6 (23·6–29·5)	20·3 (18·6–23·7)	19·1 (17·7–22·9)
1-14	Pancreatic cancer	593,000 (574,000–606,000)	906,000 (872,000–927,000)	1,150,000 (1,090,000–1,190,000)	192 (187–196)	206 (199–210)	212 (203–220)
1-15	Larynx cancer	106,000 (103,000–109,000)	103,000 (100,000–107,000)	118,000 (112,000–123,000)	35·9 (34·8–36·9)	23·8 (23–24·5)	22·1 (21·1–23·2)
1-16	Tracheal, bronchus, and lung cancer	3,600,000 (3,520,000–3,660,000)	3,780,000 (3,650,000–3,850,000)	4,190,000 (4,000,000–4,330,000)	1,200 (1,170–1,220)	862 (836–878)	767 (735–792)
1-17	Malignant skin melanoma	213,000 (148,000–253,000)	275,000 (204,000–351,000)	309,000 (241,000–419,000)	73·6 (51·3–87)	67·3 (51·2–88·3)	64·8 (52·8–91·5)
1-18	Non-melanoma skin cancer	87,000 (73,200–105,000)	116,000 (97,600–139,000)	149,000 (124,000–179,000)	28 (23·8–33·5)	25·8 (21·8–31)	26·8 (22·5–32·3)
1-19	Non-melanoma skin cancer (squamous-cell carcinoma)	86,700 (73,000–105,000)	115,000 (96,800–138,000)	147,000 (123,000–179,000)	27·9 (23·7–33·3)	25·6 (21·6–30·8)	26·6 (22·3–32)
1-20	Non-melanoma skin cancer (basal-cell carcinoma)	290 (134–531)	917 (433–1,710)	1,100 (513–2,040)	0·1 (0–0·2)	0·2 (0·1–0·4)	0·2 (0·1–0·4)
1-21	Breast cancer	1,340,000 (1,290,000–1,400,000)	1,290,000 (1,220,000–1,360,000)	1,400,000 (1,310,000–1,500,000)	458 (441–478)	303 (289–320)	284 (267–303)
1-22	Cervical cancer	191,000 (162,000–200,000)	201,000 (182,000–208,000)	225,000 (193,000–237,000)	66·5 (56–69·5)	50·8 (46·3–52·6)	50·9 (44·3–53·7)
1-23	Uterine cancer	128,000 (121,000–134,000)	188,000 (176,000–200,000)	250,000 (233,000–267,000)	40·8 (38·8–42·8)	43 (40·5–45·7)	46·5 (43·4–49·7)
1-24	Ovarian cancer	344,000 (314,000–356,000)	390,000 (371,000–414,000)	427,000 (397,000–462,000)	116 (106–120)	90·9 (86·5–96·4)	83 (77·6–90·2)
1-25	Prostate cancer	710,000 (491,000–793,000)	729,000 (638,000–1,060,000)	926,000 (800,000–1,360,000)	212 (146–237)	159 (139–231)	160 (139–235)
1-26	Testicular cancer	27,900 (25,500–31,200)	27,400 (24,700–31,400)	26,600 (23,500–30,500)	10·3 (9·2–11·7)	8·9 (7·9–10·5)	8·1 (7–9·8)
1-27	Kidney cancer	278,000 (270,000–288,000)	364,000 (352,000–375,000)	434,000 (409,000–457,000)	94·3 (91·5–97·6)	85 (82·3–87·5)	84 (79·3–88·2)
1-28	Bladder cancer	223,000 (214,000–231,000)	310,000 (293,000–321,000)	384,000 (359,000–407,000)	69·3 (66·6–71·6)	68 (64·7–70·3)	68·1 (63·9–72·1)
1-29	Brain and nervous system cancer	406,000 (351,000–485,000)	496,000 (411,000–556,000)	565,000 (459,000–619,000)	149 (129–178)	135 (112–152)	134 (110–149)
1-30	Thyroid cancer	32,900 (30,600–35,400)	52,300 (48,000–57,100)	62,500 (56,900–68,000)	11 (10·2–11·9)	12·4 (11·4–13·6)	12·4 (11·2–13·5)
1-31	Mesothelioma	45,200 (42,500–48,500)	51,500 (48,100–55,100)	58,900 (54,700–63,500)	14·3 (13·4–15·4)	11·7 (11–12·5)	10·7 (9·9–11·5)
1-32	Hodgkin lymphoma	71,300 (53,700–77,300)	49,500 (44,600–64,000)	49,300 (43,400–67,400)	25·3 (19–27·4)	14·1 (12·7–18·5)	12·3 (10·7–17·4)
1-33	Non-Hodgkin's lymphoma	532,000 (515,000–545,000)	518,000 (496,000–535,000)	608,000 (576,000–635,000)	178 (173–182)	122 (118–126)	118 (113–123)
1-34	Multiple myeloma	215,000 (177,000–223,000)	283,000 (265,000–340,000)	350,000 (326,000–431,000)	69·2 (56·9–71·9)	64 (60·3–77)	63·9 (59·6–79·4)
1-35	Leukaemia	570,000 (553,000–582,000)	600,000 (577,000–616,000)	668,000 (636,000–696,000)	202 (197–206)	155 (150–159)	145 (140–151)
1-36	Acute lymphoid leukaemia	81,300 (69,900–84,600)	73,700 (64,500–78,400)	72,700 (63,900–79,100)	34·4 (29·7–35·8)	24·8 (22·1–26·2)	22·7 (20·3–24·7)
1-37	Chronic lymphoid leukaemia	80,700 (72,400–85,800)	93,600 (85,100–111,000)	105,000 (93,600–129,000)	25·1 (22·6–26·7)	20·4 (18·7–24·3)	18·3 (16·5–22·5)
1-38	Acute myeloid leukaemia	186,000 (172,000–210,000)	259,000 (228,000–272,000)	301,000 (269,000–324,000)	65·5 (60·8–73·9)	66·9 (59·2–70·9)	65 (58·8–70·3)
1-39	Chronic myeloid leukaemia	74,000 (69,600–79,200)	31,600 (29,600–35,400)	32,900 (30,300–37,500)	25·6 (24·1–27·4)	7·9 (7·4–8·7)	7 (6·5–7·9)
1-40	Other leukaemia	148,000 (136,000–157,000)	142,000 (131,000–154,000)	157,000 (142,000–175,000)	51·5 (47·4–54·4)	35 (32·5–37·7)	32·4 (29·7–35·8)
1-41	Other neoplasms	101,000 (77,900–136,000)	142,000 (110,000–186,000)	180,000 (139,000–236,000)	31·3 (24·1–42)	31·6 (24·4–41·7)	32·6 (25·5–42·6)
1-42	Myelodysplastic, myeloproliferative, and other hematopoietic neoplasms	101,000 (77,900–136,000)	142,000 (110,000–186,000)	180,000 (139,000–236,000)	31·3 (24·1–42)	31·6 (24·4–41·7)	32·6 (25·5–42·6)
1-43	Benign and in situ intestinal neoplasms	0 (0–0)	0 (0–0)	0 (0–0)	0 (0–0)	0 (0–0)	0 (0–0)
1-44	Benign and in situ cervical and uterine neoplasms	0 (0–0)	0 (0–0)	0 (0–0)	0 (0–0)	0 (0–0)	0 (0–0)
1-45	Other benign and in situ neoplasms	0 (0–0)	0 (0–0)	0 (0–0)	0 (0–0)	0 (0–0)	0 (0–0)
1-46	Cardiovascular diseases	16,500,000 (15,700,000–17,100,000)	15,000,000 (14,000,000–15,700,000)	17,300,000 (16,200,000–18,200,000)	5,240 (5,000–5,420)	3,340 (3,160–3,500)	3,220 (3,030–3,380)
1-47	Rheumatic heart disease	179,000 (170,000–189,000)	106,000 (96,300–116,000)	126,000 (113,000–140,000)	58·5 (55·5–61·5)	24 (21·9–26·3)	23·8 (21·5–26·5)
1-48	Ischaemic heart disease	10,400,000 (9,890,000–10,700,000)	7,930,000 (7,440,000–8,310,000)	8,940,000 (8,380,000–9,400,000)	3,280 (3,130–3,360)	1,740 (1,640–1,820)	1,630 (1,540–1,710)
1-49	Stroke	3,130,000 (2,900,000–3,340,000)	3,270,000 (2,980,000–3,540,000)	3,830,000 (3,470,000–4,160,000)	984 (914–1,050)	735 (668–799)	714 (648–778)
1-50	Ischaemic stroke	1,880,000 (1,700,000–2,050,000)	1,770,000 (1,540,000–1,980,000)	2,060,000 (1,790,000–2,310,000)	565 (509–618)	377 (325–426)	360 (310–408)
1-51	Intracerebral hemorrhage	832,000 (797,000–864,000)	1,020,000 (969,000–1,090,000)	1,190,000 (1,120,000–1,290,000)	274 (262–284)	238 (226–254)	232 (218–249)
1-52	Subarachnoid hemorrhage	413,000 (389,000–439,000)	485,000 (453,000–514,000)	576,000 (529,000–621,000)	145 (137–154)	120 (112–127)	122 (113–131)
1-53	Hypertensive heart disease	513,000 (440,000–568,000)	889,000 (613,000–967,000)	1,080,000 (716,000–1,170,000)	167 (143–185)	204 (139–222)	212 (138–230)
1-54	Non-rheumatic valvular heart disease	289,000 (264,000–324,000)	321,000 (285,000–363,000)	402,000 (357,000–461,000)	88·9 (81·6–99·3)	66·1 (59·4–75·1)	68·6 (61·3–78·8)
1-55	Non-rheumatic calcific aortic valve disease	195,000 (179,000–212,000)	228,000 (201,000–248,000)	287,000 (250,000–318,000)	59·9 (55·2–65·1)	45·9 (40·7–49·6)	48 (42·6–52·8)
1-56	Non-rheumatic degenerative mitral valve disease	92,500 (69,800–120,000)	91,200 (70,100–123,000)	113,000 (86,100–155,000)	28·5 (21·5–36·6)	19·9 (15·4–26·5)	20·3 (15·5–27·4)
1-57	Other non-rheumatic valve diseases	1,460 (921–2,090)	1,210 (908–2,240)	1,340 (1,010–2,300)	0·5 (0·3–0·7)	0·4 (0·3–0·6)	0·4 (0·3–0·6)
1-58	Cardiomyopathy and myocarditis	717,000 (566,000–766,000)	721,000 (648,000–785,000)	797,000 (729,000–899,000)	249 (197–266)	186 (167–202)	176 (163–197)
1-59	Myocarditis	27,100 (22,700–37,700)	47,100 (32,600–54,100)	45,500 (33,000–52,100)	11·2 (9·5–15·7)	16·4 (11·4–18·8)	15 (11–17·1)
1-60	Alcoholic cardiomyopathy	194,000 (145,000–229,000)	154,000 (129,000–186,000)	174,000 (146,000–213,000)	67·4 (50·4–79·4)	37·4 (31·5–45·4)	36 (30·8–44)

1-61	Other cardiomyopathy	496,000 (385,000–537,000)	520,000 (470,000–571,000)	577,000 (526,000–654,000)	171 (133–185)	132 (120–145)	125 (114–141)
1-62	Atrial fibrillation and flutter	467,000 (360,000–611,000)	738,000 (585,000–907,000)	955,000 (755,000–1,200,000)	140 (108–183)	155 (122–191)	162 (127–204)
1-63	Aortic aneurysm	309,000 (297,000–320,000)	218,000 (206,000–228,000)	247,000 (229,000–260,000)	96 (92·4–99·3)	50 (47·7–52·4)	47·2 (43·9–50)
1-64	Peripheral vascular disease	119,000 (70,700–191,000)	188,000 (107,000–320,000)	236,000 (132,000–410,000)	36·1 (21·4–58·8)	39·6 (22·7–67·8)	40 (22·3–69·5)
1-65	Endocarditis	46,800 (38,100–77,500)	129,000 (69,400–151,000)	149,000 (80,500–175,000)	15·6 (12·7–25·7)	30·3 (16·8–35·1)	29·9 (16·6–34·9)
1-66	Chronic respiratory diseases	4,040,000 (3,570,000–4,570,000)	5,890,000 (5,190,000–6,530,000)	7,000,000 (6,140,000–7,770,000)	1,360 (1,180–1,570)	1,450 (1,260–1,640)	1,450 (1,240–1,650)
1-67	Chronic obstructive pulmonary disease	2,700,000 (2,500,000–2,920,000)	4,220,000 (3,810,000–4,480,000)	5,020,000 (4,510,000–5,340,000)	844 (779–908)	935 (849–991)	896 (807–954)
1-68	Pneumoconiosis	26,300 (24,200–28,600)	19,300 (17,400–21,500)	23,300 (20,600–26,000)	8 (7·3–8·7)	4·2 (3·8–4·7)	4·1 (3·6–4·6)
1-69	Silicosis	4,480 (3,750–5,530)	2,070 (1,780–2,710)	2,540 (2,130–3,120)	1·4 (1·2–1·7)	0·5 (0·4–0·6)	0·5 (0·4–0·6)
1-70	Asbestosis	7,360 (6,370–9,250)	10,800 (9,410–12,100)	13,100 (11,400–14,900)	2·2 (1·9–2·8)	2·3 (2–2·6)	2·2 (2–2·5)
1-71	Coal workers pneumoconiosis	13,000 (11,500–14,000)	4,930 (4,440–5,790)	5,370 (4,610–6,280)	3·9 (3·4–4·2)	1·1 (1–1·3)	0·9 (0·8–1·1)
1-72	Other pneumoconiosis	1,470 (1,100–1,950)	1,540 (1,180–2,000)	2,250 (1,660–2,990)	0·5 (0·4–0·6)	0·4 (0·3–0·5)	0·4 (0·3–0·6)
1-73	Asthma	1,050,000 (733,000–1,480,000)	1,190,000 (828,000–1,640,000)	1,410,000 (975,000–1,960,000)	418 (288–592)	395 (271–550)	436 (298–616)
1-74	Interstitial lung disease and pulmonary sarcoidosis	201,000 (172,000–274,000)	347,000 (234,000–408,000)	427,000 (283,000–505,000)	65 (55·9–87)	79·6 (54·5–92·9)	79·9 (54·8–93·7)
1-75	Other chronic respiratory diseases	58,800 (51,100–74,500)	108,000 (89,600–118,000)	118,000 (95,000–129,000)	22·7 (19·8–28·5)	31·7 (26·7–34·7)	31·8 (26·3–35·1)
1-76	Digestive diseases	2,580,000 (2,360,000–2,860,000)	3,200,000 (2,970,000–3,480,000)	3,670,000 (3,410,000–3,990,000)	886 (810–988)	790 (730–866)	794 (733–874)
1-77	Cirrhosis and other chronic liver diseases	1,100,000 (1,060,000–1,120,000)	1,590,000 (1,550,000–1,620,000)	1,830,000 (1,770,000–1,880,000)	384 (373–391)	382 (371–389)	389 (377–400)
1-78	Cirrhosis and other chronic liver diseases due to hepatitis B	50,900 (43,400–58,800)	50,000 (43,300–57,700)	47,400 (39,600–56,100)	18 (15·4–20·9)	12 (10·4–13·8)	10·4 (8·7–12·3)
1-79	Cirrhosis and other chronic liver diseases due to hepatitis C	438,000 (394,000–487,000)	733,000 (673,000–800,000)	845,000 (753,000–945,000)	154 (138–171)	177 (163–193)	183 (165–204)
1-80	Cirrhosis and other chronic liver diseases due to alcohol use	312,000 (271,000–356,000)	451,000 (402,000–509,000)	476,000 (399,000–563,000)	110 (94·7–126)	107 (95·8–120)	98·9 (83·1–117)
1-81	Cirrhosis and other chronic liver diseases due to NAFLD	95,200 (67,000–130,000)	125,000 (84,100–174,000)	169,000 (113,000–238,000)	32·9 (23–45·2)	29·4 (19·9–40·8)	35·3 (24·4–50)
1-82	Cirrhosis and other chronic liver diseases due to other causes	201,000 (158,000–251,000)	230,000 (174,000–297,000)	289,000 (217,000–378,000)	69·9 (55·3–87·1)	55·8 (42·8–71·5)	61·2 (46·5–79·1)
1-83	Upper digestive system diseases	494,000 (357,000–702,000)	402,000 (267,000–612,000)	479,000 (313,000–733,000)	168 (120–241)	105 (68·1–162)	112 (71–173)
1-84	Peptic ulcer disease	170,000 (159,000–180,000)	94,200 (86,500–103,000)	107,000 (96,900–118,000)	54 (50·7–57·1)	21·6 (19·8–23·6)	20·8 (18·8–23)
1-85	Gastritis and duodenitis	84,500 (56,600–122,000)	65,800 (45,500–90,900)	72,500 (49,200–101,000)	29·7 (19·8–43)	17·5 (12–24·4)	17·3 (11·6–24·4)
1-86	GERD	240,000 (123,000–428,000)	242,000 (123,000–436,000)	299,000 (151,000–532,000)	83·9 (43·2–150)	65·7 (33·4–118)	73·5 (37·2–132)
1-87	Appendicitis	17,600 (14,400–21,500)	19,100 (15,400–22,700)	20,600 (16,700–24,900)	6·6 (5·4–8)	5·5 (4·5–6·5)	5·3 (4·3–6·4)
1-88	Paralytic ileus and intestinal obstruction	121,000 (110,000–148,000)	180,000 (152,000–200,000)	201,000 (169,000–224,000)	42·6 (38·8–51·2)	49·5 (43·5–54·1)	46·4 (40·2–50·9)
1-89	Inguinal, femoral, and abdominal hernia	68,300 (53,300–86,300)	70,400 (56,700–86,800)	80,200 (64,200–99,400)	24·2 (18·8–30·7)	19·2 (15·4–23·6)	19·2 (15·2–23·9)
1-90	Inflammatory bowel disease	186,000 (138,000–244,000)	190,000 (155,000–228,000)	215,000 (176,000–256,000)	64·7 (47·5–84·9)	49·9 (40·4–60·6)	49·8 (40·2–60·2)
1-91	Vascular intestinal disorders	138,000 (129,000–144,000)	168,000 (156,000–176,000)	190,000 (176,000–200,000)	43·4 (40·7–45·1)	37·5 (35·2–39·3)	35·1 (32·8–36·8)
1-92	Gallbladder and biliary diseases	248,000 (180,000–339,000)	281,000 (214,000–366,000)	306,000 (233,000–396,000)	84·1 (59·9–116)	71·6 (53·1–94·9)	67·4 (50–89·4)
1-93	Pancreatitis	92,100 (85,400–101,000)	122,000 (113,000–133,000)	133,000 (123,000–148,000)	31·5 (29·2–34·6)	30·7 (28·5–33·7)	29·4 (27·3–32·6)
1-94	Other digestive diseases	117,000 (93,700–147,000)	177,000 (152,000–242,000)	216,000 (182,000–301,000)	37·3 (30·1–47·1)	40·1 (34·4–54·8)	40·9 (34·8–57·1)
1-95	Neurological disorders	3,920,000 (2,230,000–6,380,000)	5,220,000 (3,140,000–8,270,000)	5,890,000 (3,600,000–9,310,000)	1,360 (750–2,230)	1,340 (767–2,160)	1,340 (758–2,180)
1-96	Alzheimer's disease and other dementias	1,140,000 (545,000–2,410,000)	1,700,000 (821,000–3,500,000)	2,040,000 (984,000–4,210,000)	331 (158–701)	322 (157–665)	320 (156–659)
1-97	Parkinson's disease	217,000 (200,000–235,000)	382,000 (351,000–406,000)	492,000 (453,000–522,000)	63·7 (58·5–68·7)	79·7 (73·6–84·6)	83·6 (77·1–88·4)
1-98	Idiopathic epilepsy	281,000 (188,000–411,000)	358,000 (228,000–537,000)	330,000 (216,000–482,000)	110 (72·9–162)	114 (71·4–170)	96·8 (62·5–142)
1-99	Multiple sclerosis	126,000 (103,000–153,000)	191,000 (156,000–220,000)	211,000 (173,000–244,000)	44·6 (36·6–54·2)	48·7 (39·7–56·5)	48·2 (39·6–56·2)
1-100	Motor neuron disease	104,000 (100,000–107,000)	180,000 (175,000–184,000)	225,000 (217,000–231,000)	37 (35·8–38·1)	45·3 (44·1–46·4)	46·9 (45·2–48·3)
1-101	Headache disorders	1,860,000 (369,000–4,030,000)	2,160,000 (470,000–4,650,000)	2,330,000 (506,000–5,030,000)	696 (135–1,520)	660 (134–1,430)	679 (134–1,470)
1-102	Migraine	1,670,000 (242,000–3,780,000)	1,920,000 (315,000–4,330,000)	2,080,000 (334,000–4,660,000)	625 (87·7–1,430)	590 (87·1–1,340)	609 (86·1–1,390)
1-103	Tension-type headache	193,000 (52,800–657,000)	239,000 (67,800–796,000)	254,000 (71,300–855,000)	70·9 (19–243)	70 (19·1–240)	69·6 (18·9–238)
1-104	Other neurological disorders	187,000 (169,000–210,000)	238,000 (211,000–271,000)	263,000 (235,000–297,000)	72·9 (65·5–82·7)	69·5 (60–81·1)	68·4 (59·3–80·2)
1-105	Mental disorders	5,190,000 (3,870,000–6,760,000)	7,180,000 (5,340,000–9,400,000)	7,310,000 (5,490,000–9,500,000)	1,940 (1,450–2,520)	2,220 (1,650–2,920)	2,140 (1,600–2,790)
1-106	Schizophrenia	767,000 (563,000–971,000)	996,000 (734,000–1,250,000)	993,000 (733,000–1,240,000)	274 (201–346)	290 (213–365)	271 (200–341)
1-107	Depressive disorders	1,710,000 (1,200,000–2,320,000)	2,670,000 (1,860,000–3,640,000)	2,650,000 (1,870,000–3,590,000)	632 (441–859)	825 (569–1,120)	775 (540–1,060)
1-108	Major depressive disorder	1,340,000 (931,000–1,840,000)	2,340,000 (1,610,000–3,180,000)	2,240,000 (1,550,000–3,060,000)	497 (342–684)	727 (496–997)	662 (456–902)
1-109	Dysthymia	370,000 (243,000–550,000)	334,000 (220,000–493,000)	410,000 (270,000–599,000)	134 (87·7–200)	97·7 (63·7–145)	113 (75–168)
1-110	Bipolar disorder	344,000 (220,000–500,000)	421,000 (271,000–612,000)	444,000 (286,000–647,000)	129 (82·1–189)	128 (81·9–186)	127 (81·5–185)
1-111	Anxiety disorders	1,330,000 (932,000–1,820,000)	1,800,000 (1,270,000–2,430,000)	1,870,000 (1,310,000–2,530,000)	492 (344–672)	547 (381–743)	534 (373–725)
1-112	Eating disorders	228,000 (144,000–338,000)	276,000 (176,000–407,000)	261,000 (166,000–385,000)	85·3 (54–127)	97·4 (61·8–144)	89·5 (56·9–132)
1-113	Anorexia nervosa	66,900 (41,200–104,000)	76,700 (47,200–120,000)	79,200 (48,400–122,000)	26 (15·9–40·4)	27·3 (16·7–42·9)	27·6 (16·9–42·7)

1-114	Bulimia nervosa	161,000 (92,800–252,000)	199,000 (119,000–314,000)	182,000 (107,000–284,000)	59·4 (34·6–93·2)	70·1 (41·5–110)	61·9 (36·6–97)
1-115	Autism spectrum disorders	193,000 (125,000–275,000)	276,000 (180,000–394,000)	295,000 (195,000–420,000)	78·6 (51·3–112)	94·6 (62–135)	97·3 (64–139)
1-116	Attention-deficit/hyperactivity disorder	51,200 (29,100–86,700)	65,800 (37,800–113,000)	66,500 (37,900–115,000)	22·7 (12·9–38·9)	25·4 (14·7–44·3)	25·5 (14·4–44·2)
1-117	Conduct disorder	130,000 (71,600–205,000)	147,000 (81,500–235,000)	161,000 (90,600–256,000)	64·3 (35·5–102)	61·5 (34·1–98·8)	67·1 (37·7–107)
1-118	Idiopathic developmental intellectual disability	67,200 (29,000–114,000)	67,500 (26,700–117,000)	65,300 (24,900–115,000)	27·1 (11·4–46)	22·8 (8·8–40)	21·2 (7·6–37·7)
1-119	Other mental disorders	368,000 (236,000–552,000)	466,000 (300,000–699,000)	504,000 (325,000–756,000)	130 (83·3–196)	131 (83·7–198)	130 (83·6–196)
1-120	Substance use disorders	2,350,000 (1,810,000–2,910,000)	4,700,000 (3,890,000–5,540,000)	7,400,000 (6,280,000–8,570,000)	856 (660–1,070)	1,480 (1,210–1,760)	2,240 (1,880–2,610)
1-121	Alcohol use disorders	1,040,000 (775,000–1,360,000)	1,180,000 (939,000–1,460,000)	1,280,000 (1,020,000–1,580,000)	376 (281–492)	346 (272–435)	346 (272–433)
1-122	Drug use disorders	1,310,000 (989,000–1,660,000)	3,520,000 (2,910,000–4,220,000)	6,120,000 (5,170,000–7,160,000)	479 (361–609)	1,130 (922–1,370)	1,890 (1,590–2,230)
1-123	Opioid use disorders	774,000 (587,000–1,000,000)	2,680,000 (2,160,000–3,250,000)	4,780,000 (3,970,000–5,700,000)	279 (211–364)	862 (688–1,060)	1,480 (1,220–1,790)
1-124	Cocaine use disorders	181,000 (121,000–258,000)	336,000 (271,000–419,000)	567,000 (485,000–671,000)	67·4 (44·8–96·3)	104 (82·9–132)	170 (143–205)
1-125	Amphetamine use disorders	85,900 (50,800–137,000)	133,000 (109,000–168,000)	261,000 (223,000–305,000)	31·8 (18·7–50·8)	42·6 (33·8–54·7)	79·8 (67·3–95·4)
1-126	Cannabis use disorders	73,600 (43,400–115,000)	81,900 (49,300–127,000)	82,300 (50,000–127,000)	29·2 (16·9–45·9)	28·6 (17–44·7)	28·1 (16·7–43·9)
1-127	Other drug use disorders	193,000 (137,000–265,000)	295,000 (236,000–362,000)	435,000 (362,000–514,000)	71·5 (50·3–98·5)	93·3 (74·3–116)	132 (109–157)
1-128	Diabetes and kidney diseases	3,000,000 (2,600,000–3,450,000)	5,510,000 (4,710,000–6,430,000)	6,750,000 (5,750,000–7,910,000)	985 (852–1,130)	1,280 (1,090–1,500)	1,310 (1,110–1,540)
1-129	Diabetes mellitus	2,120,000 (1,780,000–2,520,000)	3,680,000 (2,990,000–4,480,000)	4,460,000 (3,590,000–5,520,000)	702 (588–835)	861 (697–1,050)	876 (703–1,080)
1-130	Diabetes mellitus type 1	234,000 (191,000–289,000)	294,000 (234,000–365,000)	334,000 (260,000–419,000)	83·2 (67·9–103)	83·5 (67·1–103)	85·8 (67·6–107)
1-131	Diabetes mellitus type 2	1,890,000 (1,580,000–2,250,000)	3,390,000 (2,740,000–4,140,000)	4,130,000 (3,310,000–5,110,000)	619 (516–741)	778 (626–952)	790 (629–981)
1-132	Chronic kidney disease	873,000 (792,000–966,000)	1,830,000 (1,670,000–1,990,000)	2,290,000 (2,100,000–2,490,000)	282 (256–313)	420 (383–459)	438 (402–477)
1-133	Chronic kidney disease due to diabetes mellitus type 1	29,900 (20,400–41,100)	62,900 (42,200–86,700)	83,000 (55,500–118,000)	10·5 (7·1–14·5)	16 (10·8–21·9)	18·5 (12·2–25·6)
1-134	Chronic kidney disease due to diabetes mellitus type 2	174,000 (135,000–215,000)	525,000 (426,000–627,000)	678,000 (543,000–808,000)	55·2 (42·9–68·3)	117 (94·7–139)	122 (99–146)
1-135	Chronic kidney disease due to hypertension	263,000 (225,000–300,000)	552,000 (467,000–644,000)	714,000 (606,000–824,000)	81·1 (69·5–92·2)	119 (101–138)	127 (108–147)
1-136	Chronic kidney disease due to glomerulonephritis	155,000 (129,000–186,000)	254,000 (204,000–312,000)	297,000 (240,000–371,000)	52·8 (44·1–63)	64·9 (52·8–78·5)	65·9 (53·8–80·3)
1-137	Chronic kidney disease due to other and unspecified causes	251,000 (204,000–303,000)	432,000 (348,000–528,000)	516,000 (407,000–633,000)	82·6 (68·1–99·5)	103 (83·6–125)	104 (83·9–127)
1-138	Acute glomerulonephritis	1,730 (1,640–1,800)	1,530 (1,430–1,610)	4,050 (3,710–4,380)	0·6 (0·5–0·6)	0·4 (0·3–0·4)	0·8 (0·7–0·8)
1-139	Skin and subcutaneous diseases	1,740,000 (1,210,000–2,410,000)	2,050,000 (1,440,000–2,860,000)	2,200,000 (1,550,000–3,070,000)	687 (477–960)	658 (458–922)	658 (457–922)
1-140	Dermatitis	488,000 (294,000–756,000)	532,000 (320,000–827,000)	555,000 (334,000–857,000)	204 (121–319)	187 (110–293)	186 (110–293)
1-141	Atopic dermatitis	325,000 (175,000–545,000)	364,000 (195,000–610,000)	371,000 (199,000–618,000)	144 (77·1–241)	138 (73·1–231)	137 (73·2–230)
1-142	Contact dermatitis	149,000 (95,400–218,000)	152,000 (97,200–223,000)	166,000 (106,000–243,000)	55 (35·7–80·7)	44·5 (29–65·4)	44·3 (28·7–64·8)
1-143	Seborrhoeic dermatitis	14,400 (8,210–22,600)	16,600 (9,440–25,900)	18,500 (10,600–29,000)	5·2 (3–8·2)	4·8 (2·7–7·6)	4·8 (2·7–7·6)
1-144	Psoriasis	297,000 (211,000–392,000)	330,000 (236,000–434,000)	353,000 (255,000–464,000)	109 (77·7–145)	95·1 (67·9–125)	92·3 (66·2–122)
1-145	Bacterial skin diseases	41,100 (31,600–64,000)	102,000 (67,600–143,000)	122,000 (78,300–175,000)	14·4 (11–21·8)	25·6 (17·4–35·2)	26 (17·3–36)
1-146	Cellulitis	32,800 (25,000–49,700)	68,900 (46,000–92,100)	81,500 (52,000–108,000)	11·5 (8·7–17·3)	17·5 (12·1–22·7)	17·9 (11·8–23)
1-147	Pyoderma	8,270 (5,340–15,400)	33,500 (18,100–53,300)	40,600 (21,600–66,100)	2·8 (1·9–5·1)	8 (4·3–12·3)	8·2 (4·4–12·9)
1-148	Scabies	9,030 (4,910–14,400)	10,600 (6,030–16,800)	10,900 (6,150–17,100)	3·6 (2–5·8)	3·8 (2·1–6)	3·7 (2·1–5·8)
1-149	Fungal skin diseases	38,700 (15,900–81,100)	50,300 (20,700–106,000)	56,500 (23,200–119,000)	13·7 (5·6–28·7)	13·7 (5·6–28·6)	13·6 (5·6–28·4)
1-150	Viral skin diseases	319,000 (207,000–471,000)	376,000 (244,000–555,000)	392,000 (256,000–578,000)	133 (86·3–197)	130 (84·3–193)	129 (84–192)
1-151	Acne vulgaris	153,000 (91,700–240,000)	166,000 (100,000–261,000)	181,000 (109,000–283,000)	64 (38·4–101)	60·1 (36·2–94·3)	64 (38·7–100)
1-152	Alopecia areata	32,100 (20,300–47,600)	37,700 (23,800–56,000)	40,200 (25,400–59,100)	11·9 (7·5–17·6)	11·5 (7·3–17·2)	11·5 (7·3–16·9)
1-153	Pruritus	22,200 (10,700–40,300)	29,600 (14,300–53,900)	32,700 (15,900–59,600)	8·2 (3·9–14·8)	8·6 (4·2–15·6)	8·7 (4·2–15·8)
1-154	Urticaria	125,000 (83,800–176,000)	149,000 (99,600–209,000)	153,000 (103,000–214,000)	52·7 (35·1–74·1)	53·4 (35·5–75·2)	53·3 (35·5–74·9)
1-155	Decubitus ulcer	46,200 (33,700–64,300)	43,200 (33,100–60,100)	52,200 (39,800–72,600)	14·1 (10·3–19·7)	9·3 (7–12·8)	9·3 (7–12·9)
1-156	Other skin and subcutaneous diseases	163,000 (81,500–295,000)	221,000 (110,000–403,000)	252,000 (125,000–459,000)	58·2 (29–105)	60·2 (29·7–110)	60·4 (29·8–110)
1-157	Sense organ diseases	1,700,000 (1,190,000–2,360,000)	2,340,000 (1,640,000–3,230,000)	2,740,000 (1,930,000–3,790,000)	555 (387–767)	555 (388–768)	547 (384–755)
1-158	Blindness and vision loss	257,000 (178,000–356,000)	325,000 (226,000–451,000)	391,000 (271,000–540,000)	86·7 (59·6–121)	82·3 (56·3–116)	84·1 (58–118)
1-159	Glaucoma	15,800 (11,000–21,600)	20,000 (13,900–27,300)	27,300 (19,200–37,200)	4·6 (3·2–6·3)	3·9 (2·7–5·4)	4·4 (3–6)
1-160	Cataract	59,500 (42,300–80,700)	79,900 (57,000–108,000)	101,000 (71,800–136,000)	18·1 (12·8–24·7)	16·7 (11·9–22·8)	17·5 (12·4–23·7)
1-161	Age-related macular degeneration	9,920 (6,810–14,200)	12,000 (8,230–16,800)	16,300 (11,300–23,000)	2·9 (2–4·2)	2·4 (1·6–3·4)	2·7 (1·8–3·7)
1-162	Refraction disorders	107,000 (70,800–153,000)	129,000 (85,400–188,000)	145,000 (96,600–208,000)	39·7 (26·1–57·7)	39·5 (25·8–57·9)	39·4 (25·8–57·7)
1-163	Near vision loss	24,400 (10,900–48,400)	34,300 (15,500–68,800)	41,700 (18,800–83,500)	7·7 (3·5–15·3)	7·7 (3·5–15·4)	7·7 (3·5–15·3)
1-164	Other vision loss	40,300 (28,300–55,400)	49,200 (34,800–67,400)	60,300 (43,000–82,500)	13·6 (9·5–18·8)	12·1 (8·5–16·7)	12·6 (8·9–17·4)
1-165	Age-related and other hearing loss	1,340,000 (933,000–1,870,000)	1,870,000 (1,300,000–2,620,000)	2,190,000 (1,520,000–3,050,000)	434 (300–606)	437 (303–613)	428 (296–599)
1-166	Other sense organ diseases	100,000 (61,200–147,000)	138,000 (85,200–201,000)	161,000 (99,900–235,000)	34·3 (21·2–50·4)	35·2 (21·7–51·5)	35·1 (21·8–51·2)

1-167	Musculoskeletal disorders	8,880,000 (6,460,000–11,700,000)	11,500,000 (8,340,000–15,000,000)	14,000,000 (10,200,000–18,200,000)	3,130 (2,270–4,120)	3,060 (2,230–4,000)	3,330 (2,430–4,350)
1-168	Rheumatoid arthritis	143,000 (108,000–180,000)	219,000 (164,000–278,000)	258,000 (193,000–329,000)	47·8 (35·9–60·5)	53·4 (39·8–67·8)	53·5 (39·6–68·3)
1-169	Osteoarthritis	1,090,000 (557,000–2,190,000)	1,510,000 (776,000–3,020,000)	1,990,000 (1,020,000–3,960,000)	362 (184–727)	345 (177–690)	378 (195–760)
1-170	Low back pain	4,500,000 (3,170,000–6,040,000)	5,300,000 (3,820,000–6,960,000)	5,700,000 (4,110,000–7,470,000)	1,600 (1,130–2,150)	1,450 (1,040–1,910)	1,400 (1,010–1,850)
1-171	Neck pain	1,200,000 (793,000–1,710,000)	1,030,000 (716,000–1,420,000)	2,040,000 (1,390,000–2,890,000)	422 (280–605)	265 (182–363)	500 (339–705)
1-172	Gout	86,100 (54,900–122,000)	197,000 (133,000–269,000)	258,000 (171,000–356,000)	28·7 (18·1–40·6)	47·4 (31·7–64·7)	53 (35–73·6)
1-173	Other musculoskeletal disorders	1,860,000 (1,330,000–2,550,000)	3,250,000 (2,330,000–4,350,000)	3,710,000 (2,690,000–4,910,000)	671 (481–913)	905 (647–1,210)	943 (678–1,260)
1-174	Other non-communicable diseases	4,650,000 (3,840,000–5,680,000)	4,520,000 (3,680,000–5,550,000)	5,140,000 (4,120,000–6,400,000)	1,850 (1,560–2,220)	1,430 (1,190–1,720)	1,460 (1,200–1,780)
1-175	Congenital anomalies	1,170,000 (1,040,000–1,280,000)	900,000 (813,000–1,030,000)	852,000 (763,000–976,000)	555 (494–604)	408 (372–468)	385 (346–443)
1-176	Neural tube defects	70,500 (63,600–83,900)	55,300 (46,300–63,700)	66,200 (54,600–76,400)	34·6 (31·2–41·1)	27 (22·4–31·2)	33·6 (27·5–39)
1-177	Congenital heart anomalies	464,000 (383,000–502,000)	257,000 (234,000–314,000)	216,000 (191,000–264,000)	220 (181–238)	117 (106–142)	98·3 (86·4–122)
1-178	Orofacial clefts	3,960 (2,870–5,420)	2,800 (1,870–4,000)	3,210 (2,040–4,680)	1·8 (1·3–2·4)	1 (0·7–1·4)	1·1 (0·7–1·6)
1-179	Down's syndrome	29,000 (25,600–34,300)	51,100 (41,800–55,900)	58,500 (48,200–64,700)	12·8 (11·3–15·1)	17·3 (14·3–19)	18·2 (15·3–20·3)
1-180	Turner syndrome	1,200 (570–2,040)	1,300 (617–2,150)	1,300 (604–2,230)	0·5 (0·2–0·8)	0·5 (0·2–0·8)	0·5 (0·2–0·8)
1-181	Klinefelter syndrome	274 (123–532)	291 (130–568)	300 (132–586)	0·1 (0–0·2)	0·1 (0–0·2)	0·1 (0–0·2)
1-182	Other chromosomal abnormalities	108,000 (88,800–134,000)	133,000 (107,000–156,000)	134,000 (113,000–160,000)	52·2 (43·2–65·3)	65·1 (52·6–77·1)	66·9 (56·8–81·2)
1-183	Congenital musculoskeletal and limb anomalies	114,000 (79,500–155,000)	125,000 (91,900–163,000)	132,000 (92,800–178,000)	49·1 (34·5–67·2)	48·1 (35·6–62·2)	49·2 (35·8–65·9)
1-184	Urogenital congenital anomalies	48,700 (35,900–69,200)	36,600 (27,100–47,100)	31,300 (22,500–40,000)	23·9 (17·5–34·1)	18·3 (13·4–23·8)	15·9 (11·3–20·7)
1-185	Digestive congenital anomalies	64,200 (52,600–88,100)	55,000 (41,300–67,700)	47,300 (35,900–58,700)	31·5 (25·7–43·1)	27·2 (20·2–33·7)	23·8 (17·8–29·8)
1-186	Other congenital anomalies	264,000 (215,000–298,000)	182,000 (162,000–227,000)	163,000 (139,000–204,000)	128 (104–144)	86·4 (77·8–108)	77·5 (67·1–97·8)
1-187	Urinary diseases and male infertility	274,000 (237,000–315,000)	366,000 (322,000–423,000)	454,000 (397,000–523,000)	87·4 (75·6–101)	82·7 (72·4–95·9)	86 (74·4–100)
1-188	Urinary tract infections and interstitial nephritis	180,000 (158,000–192,000)	255,000 (230,000–283,000)	312,000 (281,000–341,000)	55·6 (48·9–59·6)	55·9 (51–61·3)	56·9 (51·3–62·1)
1-189	Urolithiasis	18,400 (13,600–23,900)	16,100 (12,900–20,100)	20,700 (16,600–25,800)	6·4 (4·7–8·4)	4 (3·2–5·1)	4·5 (3·5–5·6)
1-190	Benign prostatic hyperplasia	47,600 (29,000–69,600)	71,400 (45,100–105,000)	89,400 (56,800–132,000)	15·4 (9·3–22·5)	16 (10·1–23·6)	16·1 (10·2–23·7)
1-191	Male infertility	7,180 (2,660–16,900)	5,640 (2,130–12,600)	10,800 (4,060–24,300)	2·5 (0·9–5·8)	1·9 (0·7–4·3)	3·6 (1·3–8·1)
1-192	Other urinary diseases	20,900 (16,500–25,000)	17,600 (14,500–27,000)	20,500 (17,100–31,000)	7·6 (6–8·9)	4·8 (4–7·2)	4·9 (4·1–7·3)
1-193	Gynecological diseases	820,000 (565,000–1,150,000)	830,000 (574,000–1,160,000)	887,000 (610,000–1,240,000)	295 (204–414)	254 (175–355)	261 (180–365)
1-194	Uterine fibroids	42,700 (20,400–80,400)	60,700 (28,700–114,000)	64,000 (31,000–118,000)	15·4 (7·3–29)	17 (8–31·9)	17·2 (8·2–32)
1-195	Polycystic ovarian syndrome	35,700 (15,300–74,000)	30,200 (13,800–59,500)	42,700 (19,400–82,300)	13·2 (5·7–27·2)	10·1 (4·6–20)	14 (6·4–27)
1-196	Female infertility	4,650 (461–14,400)	2,960 (301–9,310)	9,770 (2,030–26,400)	1·6 (0·2–5)	1 (0·1–3·2)	3·2 (0·7–8·7)
1-197	Endometriosis	69,200 (39,100–114,000)	53,400 (34,900–77,500)	53,800 (34,400–82,000)	24·7 (13·9–40·4)	17·2 (11·1–25·3)	17 (10·7–25·9)
1-198	Genital prolapse	14,900 (7,310–27,800)	18,100 (8,880–33,100)	21,600 (10,600–39,500)	4·9 (2·4–9·3)	4·1 (2–7·4)	4·1 (2–7·5)
1-199	Premenstrual syndrome	220,000 (133,000–350,000)	273,000 (166,000–425,000)	290,000 (177,000–448,000)	82·7 (49·9–130)	88·8 (54·2–138)	94·3 (58·3–147)
1-200	Other gynecological diseases	433,000 (278,000–627,000)	392,000 (264,000–539,000)	406,000 (271,000–572,000)	152 (97·7–221)	115 (76·9–159)	111 (73·8–159)
1-201	Hemoglobinopathies and hemolytic anaemias	139,000 (112,000–178,000)	103,000 (85,600–130,000)	116,000 (94,100–146,000)	53·8 (42·9–69·5)	32 (26·3–40·6)	32·9 (26·5–42·4)
1-202	Thalassemias	2,900 (2,340–3,530)	5,850 (5,030–6,730)	5,390 (4,470–6,640)	1·3 (1·1–1·6)	2·4 (2·1–2·8)	2·2 (1·9–2·7)
1-203	Thalassaemias trait	19,200 (11,800–30,400)	18,500 (11,500–28,900)	21,500 (13,100–34,200)	7·5 (4·6–11·7)	6·2 (3·8–9·7)	6·7 (4·1–10·9)
1-204	Sickle cell disorders	44,200 (39,400–50,500)	22,400 (20,600–24,000)	21,200 (18,600–24,600)	18·7 (16·6–21·6)	7·4 (6·8–8)	6·7 (5·9–7·8)
1-205	Sickle cell trait	29,100 (17,800–44,900)	12,300 (7,620–19,100)	14,000 (8,160–22,600)	11·6 (7·1–18·2)	4·2 (2·6–6·5)	4·5 (2·6–7·3)
1-206	G6PD deficiency	7,330 (5,860–8,740)	9,170 (7,210–11,000)	14,000 (11,500–16,400)	2·7 (2·1–3·2)	2·9 (2·3–3·5)	4·1 (3·3–4·8)
1-207	G6PD trait	322 (193–495)	528 (335–800)	531 (311–833)	0·1 (0·1–0·2)	0·2 (0·1–0·3)	0·2 (0·1–0·3)
1-208	Other hemoglobinopathies and hemolytic anaemias	35,900 (31,200–42,300)	34,600 (29,500–41,900)	39,000 (32,000–48,300)	11·9 (10·2–14·3)	8·8 (7·4–10·8)	8·6 (6·9–10·9)
1-209	Endocrine, metabolic, blood, and immune disorders	902,000 (697,000–1,150,000)	1,320,000 (1,080,000–1,590,000)	1,500,000 (1,230,000–1,800,000)	323 (250–412)	351 (289–425)	350 (289–422)
1-210	Oral disorders	877,000 (535,000–1,330,000)	832,000 (511,000–1,280,000)	1,200,000 (728,000–1,830,000)	302 (184–464)	216 (133–332)	269 (164–415)
1-211	Caries of deciduous teeth	5,680 (2,440–12,100)	5,230 (2,230–11,200)	5,910 (2,560–12,600)	2·9 (1·3–6·2)	2·5 (1–5·3)	3 (1·3–6·2)
1-212	Caries of permanent teeth	59,200 (27,100–114,000)	67,300 (30,000–130,000)	72,800 (32,100–140,000)	22·5 (10·3–43·5)	20·9 (9·5–40·7)	21·3 (9·6–40·9)
1-213	Periodontal disease	181,000 (71,000–406,000)	167,000 (64,800–371,000)	267,000 (105,000–589,000)	63·3 (24·7–144)	40·9 (15·7–93·3)	59·3 (23·2–137)
1-214	Edentulism	492,000 (304,000–742,000)	424,000 (273,000–614,000)	669,000 (424,000–985,000)	160 (98·5–241)	99·3 (63·8–144)	132 (84–197)
1-215	Other oral disorders	140,000 (87,100–205,000)	169,000 (105,000–247,000)	184,000 (115,000–268,000)	53 (33·2–77·8)	51·9 (32·3–76·1)	52·8 (33·1–77·3)
1-216	Sudden infant death syndrome	468,000 (426,000–510,000)	174,000 (155,000–194,000)	135,000 (121,000–150,000)	231 (210–252)	89·1 (79·3–99·3)	72·5 (64·8–80·5)
1-0	Injuries	9,930,000 (9,100,000–11,000,000)	9,180,000 (8,330,000–10,200,000)	10,100,000 (9,030,000–11,500,000)	3,750 (3,460–4,130)	2,730 (2,510–3,000)	2,750 (2,490–3,070)
1-1	Transport injuries	3,270,000 (3,100,000–3,470,000)	2,620,000 (2,440,000–2,840,000)	2,650,000 (2,420,000–2,910,000)	1,260 (1,200–1,340)	812 (763–869)	763 (704–823)
1-2	Road injuries	2,990,000 (2,850,000–3,150,000)	2,410,000 (2,250,000–2,600,000)	2,390,000 (2,200,000–2,600,000)	1,160 (1,110–1,220)	751 (708–801)	694 (644–743)

1-3	Pedestrian road injuries	482,000 (459,000–508,000)	339,000 (316,000–368,000)	373,000 (344,000–406,000)	191 (183–201)	105 (98·6–112)	107 (99·7–114)
1-4	Cyclist road injuries	111,000 (96,800–127,000)	93,100 (77,900–111,000)	110,000 (91,700–133,000)	44·9 (39·8–50·7)	26·9 (22·9–31·6)	28·9 (24·5–34·1)
1-5	Motorcyclist road injuries	258,000 (239,000–281,000)	309,000 (283,000–341,000)	312,000 (281,000–348,000)	97·3 (90·5–106)	92·2 (85·4–100)	86·3 (78·5–95·1)
1-6	Motor vehicle road injuries	2,130,000 (2,040,000–2,230,000)	1,650,000 (1,550,000–1,780,000)	1,580,000 (1,450,000–1,710,000)	822 (790–860)	522 (495–555)	467 (433–499)
1-7	Other road injuries	12,000 (9,730–14,900)	14,400 (11,900–17,600)	17,600 (14,600–21,500)	4·6 (3·8–5·6)	4·3 (3·6–5·1)	4·8 (4–5·7)
1-8	Other transport injuries	283,000 (247,000–328,000)	209,000 (185,000–240,000)	263,000 (224,000–311,000)	104 (90·8–119)	61·4 (55–69·1)	69 (60–80·2)
1-9	Unintentional injuries	3,440,000 (2,820,000–4,230,000)	3,490,000 (2,860,000–4,270,000)	4,340,000 (3,500,000–5,440,000)	1,270 (1,050–1,550)	940 (784–1,140)	1,040 (844–1,290)
1-10	Falls	1,270,000 (989,000–1,640,000)	1,710,000 (1,380,000–2,150,000)	2,240,000 (1,790,000–2,840,000)	435 (338–564)	401 (321–505)	459 (361–584)
1-11	Drowning	267,000 (260,000–275,000)	200,000 (193,000–207,000)	185,000 (180,000–191,000)	115 (112–119)	73·5 (70·1–76·7)	65·9 (63·5–68·3)
1-12	Fire, heat, and hot substances	394,000 (324,000–498,000)	248,000 (190,000–336,000)	267,000 (198,000–376,000)	156 (131–194)	72·7 (57·4–95·9)	71·2 (54·3–97·3)
1-13	Poisonings	106,000 (89,300–125,000)	86,300 (78,800–95,800)	107,000 (91,700–125,000)	41·1 (34·8–48·8)	28 (25·5–30·9)	32·6 (28·1–38·2)
1-14	Poisoning by carbon monoxide	39,900 (38,400–41,900)	46,000 (44,700–47,500)	48,900 (44,300–52,200)	15·3 (14·8–16·1)	14·6 (14·2–15·1)	14·9 (13·4–15·9)
1-15	Poisoning by other means	65,600 (50,400–83,900)	40,400 (33,500–49,100)	57,900 (44,000–75,200)	25·7 (19·9–32·9)	13·3 (11·1–16·1)	17·8 (13·6–22·9)
1-16	Exposure to mechanical forces	675,000 (511,000–907,000)	462,000 (342,000–625,000)	563,000 (403,000–784,000)	252 (193–336)	130 (97·5–174)	145 (105–203)
1-17	Unintentional firearm injuries	112,000 (87,400–126,000)	46,100 (41,000–55,900)	46,600 (39,800–57,000)	45·7 (35·4–50·6)	15·1 (13·5–18·2)	14·2 (12·4–17)
1-18	Other exposure to mechanical forces	563,000 (403,000–787,000)	416,000 (299,000–574,000)	516,000 (361,000–731,000)	206 (149–288)	115 (83·4–158)	131 (92·2–187)
1-19	Adverse effects of medical treatment	131,000 (116,000–149,000)	159,000 (134,000–193,000)	177,000 (149,000–216,000)	46·8 (41·4–53)	42 (35·8–51)	40·8 (34·7–49·5)
1-20	Animal contact	39,100 (27,600–55,800)	36,900 (26,700–51,700)	57,700 (40,100–83,100)	14·7 (10·4–20·7)	10·5 (7·7–14·5)	15·3 (10·7–21·7)
1-21	Venomous animal contact	14,300 (10,300–19,000)	10,800 (8,270–13,700)	19,500 (14,000–25,600)	5·4 (3·9–7·2)	3·2 (2·4–4·1)	5·5 (3·9–7·2)
1-22	Non-venomous animal contact	24,800 (16,200–38,800)	26,100 (17,600–39,300)	38,200 (24,600–60,600)	9·3 (6·1–14·2)	7·3 (5·1–10·8)	9·8 (6·5–15·3)
1-23	Foreign body	235,000 (214,000–263,000)	292,000 (271,000–316,000)	353,000 (319,000–395,000)	94·4 (86·3–105)	104 (97·1–112)	113 (103–125)
1-24	Pulmonary aspiration and foreign body in airway	168,000 (163,000–173,000)	233,000 (222,000–244,000)	247,000 (236,000–258,000)	69·8 (67·5–72·1)	87·9 (82·6–93·4)	86·8 (81·7–92)
1-25	Foreign body in eyes	23,700 (13,600–38,800)	15,300 (8,870–24,100)	30,400 (18,100–49,000)	8·7 (4·9–14·6)	4·5 (2·5–7·3)	8·1 (4·5–13·5)
1-26	Foreign body in other body part	44,000 (31,900–59,700)	44,100 (32,100–59,200)	75,300 (54,300–102,000)	15·9 (11·5–21·5)	11·9 (8·7–16·1)	18·2 (13·2–24·7)
1-27	Environmental heat and cold exposure	67,500 (57,600–80,200)	64,500 (54,000–77,800)	80,100 (64,800–99,500)	23·7 (20·3–28·2)	17 (14·3–20·3)	19 (15·5–23·5)
1-28	Exposure to forces of nature	11,300 (10,300–12,500)	9,380 (8,430–10,500)	6,130 (5,400–7,010)	4·6 (4·2–5·1)	3·1 (2·8–3·5)	1·9 (1·7–2·1)
1-29	Other unintentional injuries	238,000 (182,000–309,000)	219,000 (157,000–298,000)	300,000 (211,000–415,000)	86·1 (66·4–111)	58·3 (42·3–78·6)	72·9 (51–100)
1-30	Self-harm and interpersonal violence	3,230,000 (3,160,000–3,310,000)	3,070,000 (3,000,000–3,160,000)	3,130,000 (3,030,000–3,240,000)	1,220 (1,190–1,250)	974 (953–999)	946 (916–976)
1-31	Self-harm	1,570,000 (1,550,000–1,590,000)	1,820,000 (1,800,000–1,850,000)	1,900,000 (1,860,000–1,950,000)	578 (571–586)	551 (542–559)	547 (533–560)
1-32	Self-harm by firearm	855,000 (769,000–908,000)	838,000 (791,000–957,000)	897,000 (846,000–1,020,000)	316 (284–336)	249 (233–284)	252 (237–286)
1-33	Self-harm by other specified means	715,000 (657,000–796,000)	986,000 (873,000–1,040,000)	1,010,000 (886,000–1,050,000)	262 (241–292)	302 (268–319)	295 (260–310)
1-34	Interpersonal violence	1,640,000 (1,580,000–1,710,000)	1,190,000 (1,130,000–1,260,000)	1,200,000 (1,110,000–1,280,000)	631 (612–656)	403 (387–422)	389 (364–410)
1-35	Physical violence by firearm	997,000 (981,000–1,010,000)	741,000 (729,000–754,000)	761,000 (707,000–789,000)	384 (378–390)	255 (250–259)	252 (234–261)
1-36	Physical violence by sharp object	249,000 (239,000–261,000)	132,000 (124,000–144,000)	124,000 (115,000–136,000)	92·5 (88·9–97·1)	42·2 (39·8–45·4)	37·2 (34·6–40·3)
1-37	Sexual violence	29,000 (19,000–40,500)	35,200 (23,200–49,500)	35,200 (23,100–49,100)	11·4 (7·4–15·9)	12·1 (7·9–16·9)	11·5 (7·6–16·1)
1-38	Physical violence by other means	363,000 (327,000–411,000)	279,000 (239,000–331,000)	276,000 (236,000–331,000)	143 (130–161)	94·3 (83·3–109)	88·4 (77·8–102)
1-39	Conflict and terrorism	1,390 (1,230–1,620)	34,900 (31,400–39,600)	5,760 (4,120–8,560)	0·6 (0·5–0·6)	12·3 (11–13·8)	1·7 (1·2–2·5)
1-40	Executions and police conflict	18,900 (17,800–20,100)	24,700 (23,400–26,000)	28,300 (24,300–32,500)	7·1 (6·7–7·5)	8·2 (7·7–8·7)	9·1 (7·7–10·5)

SR Table 1: All-age DALYs counts and age standardized DALYs rates per 100,000 individuals for all causes and level 1 cause groups by location for 1990, 2010, and 2019, both sexes combined, Latin America and Caribbean

Cause Level	Cause	All-age DALYs			Age-standardized rate of DALYs per 100,000		
		1990	2010	2019	1990	2010	2019
0-0	All causes	147,000,000 (136,000,000–158,000,000)	168,000,000 (152,000,000–184,000,000)	166,000,000 (147,000,000–188,000,000)	41,700 (38,700–45,100)	33,600 (30,600–36,800)	28,500 (25,200–32,400)
1-0	Communicable, maternal, neonatal, and nutritional disorders	53,600,000 (49,900,000–57,900,000)	29,800,000 (27,200,000–32,400,000)	24,400,000 (21,400,000–27,800,000)	11,900 (11,100–12,800)	5,890 (5,380–6,400)	4,590 (3,990–5,260)
1-1	HIV/AIDS and sexually transmitted infections	1,370,000 (1,190,000–1,660,000)	2,800,000 (2,580,000–3,100,000)	2,380,000 (2,180,000–2,690,000)	346 (305–411)	519 (477–576)	395 (360–450)
1-2	HIV/AIDS	1,040,000 (946,000–1,210,000)	2,510,000 (2,340,000–2,740,000)	2,070,000 (1,940,000–2,320,000)	273 (250–318)	463 (433–505)	336 (314–378)
1-3	HIV/AIDS - drug-susceptible tuberculosis	297,000 (221,000–374,000)	274,000 (182,000–405,000)	205,000 (134,000–303,000)	77·7 (57·8–98·3)	50·4 (33·6–74·8)	33·3 (21·8–49·1)
1-4	HIV/AIDS - Multidrug-resistant Tuberculosis without extensive drug resistance	1,120 (355–2,810)	17,400 (6,820–36,000)	14,700 (5,060–35,900)	0·3 (0·1–0·7)	3·2 (1·2–6·6)	2·4 (0·8–5·8)
1-5	HIV/AIDS - Extensively drug-resistant Tuberculosis	0 (0–0)	1,850 (697–3,990)	2,390 (785–5,590)	0 (0–0)	0·3 (0·1–0·7)	0·4 (0·1–0·9)
1-6	HIV/AIDS resulting in other diseases	743,000 (626,000–921,000)	2,220,000 (2,000,000–2,470,000)	1,850,000 (1,680,000–2,100,000)	195 (165–242)	409 (370–455)	300 (273–343)
1-7	Sexually transmitted infections excluding HIV	328,000 (183,000–565,000)	291,000 (176,000–479,000)	306,000 (184,000–498,000)	72·5 (43·3–120)	56·3 (33·7–93·4)	58·4 (33·3–97·8)
1-8	Syphilis	255,000 (120,000–482,000)	180,000 (79,700–334,000)	183,000 (82,300–351,000)	51 (24·4–95·6)	35·8 (15·8–66·4)	38·6 (17·1–74·2)
1-9	Chlamydial infection	8,280 (5,940–11,600)	11,900 (8,580–16,800)	12,700 (9,110–17,700)	2·3 (1·7–3·2)	2·2 (1·6–3)	2 (1·5–2·8)
1-10	Gonococcal infection	7,970 (6,970–9,200)	10,700 (9,390–12,200)	11,500 (9,980–13,300)	2·3 (2–2·6)	2 (1·8–2·3)	1·9 (1·6–2·2)
1-11	Trichomoniasis	20,900 (8,120–43,200)	32,900 (12,900–67,300)	38,300 (15,100–79,000)	6·2 (2·4–12·8)	6·1 (2·4–12·5)	6·1 (2·4–12·6)
1-12	Genital herpes	18,100 (6,080–44,900)	30,900 (10,100–76,000)	34,700 (11,100–85,000)	5·7 (1·9–14·1)	5·9 (1·9–14·5)	5·6 (1·8–13·7)
1-13	Other sexually transmitted infections	17,800 (13,100–23,500)	24,200 (17,900–32,400)	25,500 (18,600–34,500)	5 (3·7–6·6)	4·4 (3·3–5·8)	4·1 (3–5·5)
1-14	Respiratory infections and tuberculosis	13,900,000 (12,900,000–15,100,000)	7,270,000 (6,690,000–7,840,000)	6,520,000 (5,740,000–7,410,000)	3,350 (3,120–3,600)	1,490 (1,380–1,600)	1,190 (1,040–1,350)
1-15	Tuberculosis	2,060,000 (1,930,000–2,190,000)	838,000 (784,000–900,000)	710,000 (627,000–805,000)	593 (561–625)	164 (154–176)	118 (104–134)
1-16	Latent tuberculosis infection	0 (0–0)	0 (0–0)	0 (0–0)	0 (0–0)	0 (0–0)	0 (0–0)
1-17	Drug-susceptible tuberculosis	2,040,000 (1,920,000–2,170,000)	782,000 (714,000–846,000)	659,000 (567,000–758,000)	588 (556–620)	153 (140–165)	110 (94·3–126)
1-18	Multidrug-resistant tuberculosis without extensive drug resistance	16,500 (5,180–44,400)	50,900 (24,900–91,900)	43,500 (18,300–88,000)	4·6 (1·5–12·3)	10 (4·9–18·1)	7·2 (3·1–14·6)
1-19	Extensively drug-resistant tuberculosis	0 (0–0)	5,350 (2,420–10,200)	6,970 (2,800–14,200)	0 (0–0)	1·1 (0·5–2)	1·2 (0·5–2·4)
1-20	Lower respiratory infections	11,100,000 (10,300,000–12,300,000)	5,720,000 (5,300,000–6,170,000)	5,070,000 (4,460,000–5,810,000)	2,590 (2,420–2,820)	1,190 (1,110–1,280)	940 (822–1,080)
1-21	Upper respiratory infections	530,000 (365,000–761,000)	545,000 (343,000–834,000)	566,000 (349,000–871,000)	124 (84·2–180)	102 (64·4–156)	98·8 (60·7–152)
1-22	Otitis media	158,000 (102,000–245,000)	163,000 (95,900–267,000)	169,000 (98,500–276,000)	36·4 (23·4–56·7)	30·4 (18–49·7)	29·9 (17·5–49)
1-23	Enteric infections	10,100,000 (9,240,000–11,300,000)	2,610,000 (2,250,000–3,030,000)	2,040,000 (1,660,000–2,470,000)	2,170 (1,980–2,390)	520 (450–605)	387 (313–470)
1-24	Diarrhoeal diseases	10,000,000 (9,160,000–11,200,000)	2,590,000 (2,240,000–3,010,000)	2,020,000 (1,650,000–2,460,000)	2,150 (1,960–2,370)	517 (447–600)	384 (311–467)
1-25	Typhoid and paratyphoid	47,300 (25,100–58,400)	6,680 (4,510–17,100)	5,480 (3,510–13,400)	11·5 (6·2–14)	1·3 (0·9–3·3)	1 (0·6–2·3)
1-26	Typhoid fever	46,500 (24,500–57,200)	6,470 (4,390–16,700)	5,220 (3,340–13,200)	11·3 (6·1–13·8)	1·3 (0·8–3·2)	0·9 (0·6–2·3)
1-27	Paratyphoid fever	863 (413–1,260)	210 (117–371)	259 (129–492)	0·2 (0·1–0·3)	0 (0–0·1)	0 (0–0·1)
1-28	iNTS	22,100 (13,200–28,200)	7,850 (4,400–14,100)	7,550 (3,970–13,600)	5·1 (2·9–6·5)	1·5 (0·9–2·7)	1·4 (0·7–2·6)
1-29	Other intestinal infectious diseases	10,700 (4,870–17,100)	4,820 (2,360–7,700)	3,660 (2,020–5,790)	2·2 (1–3·5)	0·9 (0·4–1·5)	0·7 (0·4–1·1)
1-30	Neglected tropical diseases and malaria	1,790,000 (1,290,000–2,360,000)	1,290,000 (1,010,000–1,630,000)	1,190,000 (905,000–1,520,000)	496 (356–632)	253 (199–319)	204 (155–259)
1-31	Malaria	570,000 (272,000–1,020,000)	176,000 (87,200–336,000)	87,200 (34,500–185,000)	129 (61·3–231)	32·5 (16·1–62·2)	15·1 (6–32·1)
1-32	Chagas disease	311,000 (115,000–396,000)	230,000 (145,000–325,000)	246,000 (163,000–402,000)	122 (45·9–153)	49·8 (31·1–70)	41·2 (27·3–67·2)
1-33	Leishmaniasis	88,200 (7,370–372,000)	90,700 (9,980–345,000)	79,200 (10,200–290,000)	20·1 (2·2–79·5)	17·2 (2–64·9)	14·2 (1·7–52·9)
1-34	Visceral leishmaniasis	78,100 (119–363,000)	78,100 (114–332,000)	66,400 (108–276,000)	17 (0–76·7)	14·7 (0–62·2)	12·1 (0–50·9)
1-35	Cutaneous and mucocutaneous leishmaniasis	10,100 (5,570–17,200)	12,700 (8,170–18,800)	12,800 (8,280–19,400)	3·1 (1·7–5·3)	2·5 (1·6–3·7)	2·2 (1·4–3·3)
1-36	African trypanosomiasis	0 (0–0)	0 (0–0)	0 (0–0)	0 (0–0)	0 (0–0)	0 (0–0)
1-37	Schistosomiasis	91,300 (61,800–137,000)	91,400 (57,100–153,000)	95,000 (55,700–163,000)	26·2 (18·7–37·8)	16·9 (10·7–28)	15·7 (9·2–26·8)
1-38	Cysticercosis	215,000 (141,000–300,000)	228,000 (148,000–322,000)	287,000 (182,000–406,000)	76·4 (51·1–108)	47·2 (30·4–66·4)	47·7 (30·5–67·4)
1-39	Cystic echinococcosis	996 (572–1,570)	404 (260–575)	572 (357–816)	0·3 (0·2–0·4)	0·1 (0·1–0·1)	0·1 (0·1–0·1)
1-40	Lymphatic filariasis	58,500 (39,000–83,100)	21,400 (13,300–33,300)	18,100 (11,200–29,000)	16·7 (11·2–23·7)	4 (2·5–6·2)	3 (1·9–4·9)
1-41	Onchocerciasis	15,100 (10,100–21,200)	1,380 (844–2,050)	473 (185–874)	4·3 (2·9–6·1)	0·3 (0·2–0·4)	0·1 (0–0·1)
1-42	Trachoma	1,080 (598–1,760)	893 (581–1,310)	1,090 (715–1,580)	0·5 (0·3–0·8)	0·2 (0·1–0·3)	0·2 (0·1–0·3)
1-43	Dengue	34,900 (24,000–49,700)	172,000 (141,000–220,000)	116,000 (87,000–139,000)	8·6 (6–12·2)	32·9 (26·9–41·7)	20·6 (15·5–24·6)
1-44	Yellow fever	16,700 (5,220–41,900)	7,310 (2,370–18,000)	4,830 (1,480–12,400)	3·8 (1·2–9·5)	1·3 (0·4–3·3)	0·8 (0·3–2·2)
1-45	Rabies	12,200 (9,390–15,700)	671 (514–972)	696 (421–1,010)	2·8 (2·2–3·5)	0·1 (0·1–0·2)	0·1 (0·1–0·2)
1-46	Intestinal nematode infections	170,000 (109,000–256,000)	130,000 (80,000–203,000)	104,000 (62,700–164,000)	38·3 (24·5–57·9)	23·7 (14·6–37)	18·1 (10·9–28·4)
1-47	Ascariasis	52,800 (38,200–75,200)	46,900 (28,600–74,700)	35,600 (21,000–58,800)	11·6 (8·3–16·8)	8·8 (5·4–14)	6·3 (3·8–10·4)
1-48	Trichuriasis	57,000 (30,500–96,000)	23,200 (12,600–39,200)	23,200 (12,600–38,800)	12·8 (6·9–21·5)	4·2 (2·3–7·1)	4 (2·2–6·7)

1-49	Hookworm disease	60,000 (37,400–90,900)	60,000 (37,100–91,700)	45,400 (27,900–68,500)	13·9 (8·7–21)	10·7 (6·6–16·4)	7·7 (4·8–11·7)
1-50	Food-borne trematodiases	24,500 (9,560–45,300)	29,400 (10,200–55,600)	34,900 (11,600–67,600)	7·4 (2·9–13·7)	5·6 (1·9–10·6)	5·7 (1·9–11)
1-51	Leprosy	1,550 (987–2,300)	2,620 (1,640–3,870)	2,920 (1,840–4,330)	0·6 (0·4–0·8)	0·5 (0·3–0·8)	0·5 (0·3–0·7)
1-52	Ebola virus disease	0 (0–0)	0 (0–0)	0 (0–0)	0 (0–0)	0 (0–0)	0 (0–0)
1-53	Zika virus disease	0 (0–0)	0 (0–0)	324 (235–429)	0 (0–0)	0 (0–0)	0·1 (0–0·1)
1-54	Guinea worm disease	0 (0–0)	0 (0–0)	0 (0–0)	0 (0–0)	0 (0–0)	0 (0–0)
1-55	Other neglected tropical diseases	178,000 (107,000–236,000)	109,000 (77,800–151,000)	111,000 (80,100–153,000)	39·3 (24·1–52·2)	20·9 (15–29·2)	20·7 (14·9–28·5)
1-56	Other infectious diseases	2,900,000 (2,530,000–3,410,000)	1,340,000 (1,160,000–1,590,000)	1,100,000 (890,000–1,360,000)	623 (548–725)	260 (225–310)	207 (166–259)
1-57	Meningitis	1,430,000 (1,320,000–1,570,000)	537,000 (485,000–595,000)	355,000 (294,000–420,000)	301 (278–328)	104 (94·1–116)	67·3 (55·2–80·4)
1-58	Encephalitis	226,000 (180,000–255,000)	192,000 (172,000–215,000)	187,000 (155,000–227,000)	49·3 (39·9–55·3)	37 (33–41·3)	34·7 (28·5–42·1)
1-59	Diphtheria	12,600 (9,450–17,300)	1,170 (755–1,840)	849 (594–1,250)	2·6 (1·9–3·5)	0·2 (0·1–0·4)	0·2 (0·1–0·2)
1-60	Whooping cough	529,000 (256,000–1,080,000)	206,000 (86,700–442,000)	181,000 (82,000–349,000)	105 (50·7–214)	40·6 (17·1–87·4)	37·4 (17–72·4)
1-61	Tetanus	227,000 (186,000–281,000)	36,100 (24,600–55,300)	23,300 (14,700–43,700)	52·8 (43·1–64)	7·2 (4·9–11)	4·5 (2·7–8·6)
1-62	Measles	72,500 (34,100–90,100)	34 (20–163)	79 (60–133)	14·4 (6·8–17·9)	0 (0–0)	0 (0–0)
1-63	Varicella and herpes zoster	73,100 (57,000–107,000)	65,800 (48,000–85,200)	54,200 (41,900–74,900)	16·8 (13·4–23·9)	13·1 (9·6–16·9)	10·1 (7·9–14·1)
1-64	Acute hepatitis	103,000 (88,100–119,000)	67,200 (56,300–80,100)	60,500 (50,100–73,300)	24·8 (21·4–28·4)	12·8 (10·8–15·2)	10·6 (8·7–12·8)
1-65	Acute hepatitis A	67,900 (57,400–81,200)	38,600 (30,300–47,100)	32,600 (24,900–41,300)	15·6 (13·2–18·5)	7·2 (5·7–8·8)	5·8 (4·4–7·3)
1-66	Acute hepatitis B	19,900 (15,700–26,500)	19,900 (16,000–24,500)	19,300 (15,200–23,700)	5·2 (4·1–6·8)	3·8 (3·1–4·7)	3·3 (2·6–4)
1-67	Acute hepatitis C	11,300 (7,700–15,700)	5,900 (4,400–9,370)	5,920 (4,400–8,480)	3·1 (2·1–4·2)	1·2 (0·9–1·8)	1 (0·7–1·5)
1-68	Acute hepatitis E	3,780 (2,750–5,280)	2,830 (2,150–3,980)	2,750 (2,090–3,780)	0·9 (0·6–1·2)	0·5 (0·4–0·8)	0·5 (0·4–0·7)
1-69	Other unspecified infectious diseases	228,000 (178,000–280,000)	231,000 (188,000–297,000)	237,000 (188,000–310,000)	56·2 (43·8–68·8)	44·7 (36·4–57·4)	42·3 (33·5–55·5)
1-70	Maternal and neonatal disorders	18,700,000 (17,300,000–20,300,000)	12,000,000 (10,700,000–13,300,000)	8,870,000 (7,340,000–10,700,000)	3,740 (3,450–4,060)	2,350 (2,100–2,610)	1,800 (1,480–2,170)
1-71	Maternal disorders	829,000 (784,000–880,000)	613,000 (569,000–666,000)	539,000 (470,000–622,000)	205 (194–218)	106 (98·5–116)	87 (75·8–100)
1-72	Maternal haemorrhage	193,000 (175,000–214,000)	123,000 (110,000–139,000)	99,200 (83,800–117,000)	48·3 (44–53·4)	21·5 (19·2–24·1)	16 (13·5–18·9)
1-73	Maternal sepsis and other pregnancy related infections	106,000 (95,900–118,000)	61,300 (52,200–72,700)	51,600 (42,000–63,100)	25·9 (23·6–28·8)	10·6 (9–12·6)	8·3 (6·8–10·2)
1-74	Maternal hypertensive disorders	184,000 (174,000–196,000)	134,000 (123,000–145,000)	113,000 (98,000–132,000)	44·5 (42–47·5)	23·1 (21·3–25·1)	18·4 (15·9–21·4)
1-75	Maternal obstructed labour and uterine rupture	10,400 (8,600–12,600)	12,100 (10,400–14,100)	12,400 (10,300–14,900)	2·6 (2·1–3·1)	2·1 (1·8–2·4)	2 (1·7–2·4)
1-76	Maternal abortive outcome	166,000 (148,000–186,000)	70,400 (59,300–84,000)	50,200 (40,900–61,200)	42·1 (37·7–47·3)	12·3 (10·3–14·7)	8·1 (6·6–9·8)
1-77	Ectopic pregnancy	11,000 (9,940–12,200)	11,300 (10,300–12,400)	10,800 (9,320–12,600)	2·7 (2·5–3)	2 (1·8–2·1)	1·7 (1·5–2)
1-78	Indirect maternal deaths	39,300 (36,100–43,100)	83,000 (77,600–88,900)	82,300 (71,400–96,000)	9·5 (8·7–10·4)	14·3 (13·4–15·3)	13·3 (11·5–15·5)
1-79	Late maternal deaths	38,600 (31,300–47,500)	32,400 (27,500–37,900)	36,800 (28,200–47,200)	9·4 (7·7–11·6)	5·6 (4·8–6·6)	5·9 (4·6–7·6)
1-80	Maternal deaths aggravated by HIV/AIDS	1,040 (460–2,040)	1,830 (975–2,850)	1,800 (979–2,750)	0·3 (0·1–0·5)	0·3 (0·2–0·5)	0·3 (0·2–0·4)
1-81	Other maternal disorders	80,000 (72,400–87,900)	83,000 (75,600–91,400)	80,800 (70,000–93,300)	19·8 (18–21·8)	14·4 (13·1–15·9)	13 (11·3–15)
1-82	Neonatal disorders	17,900,000 (16,500,000–19,400,000)	11,300,000 (10,100,000–12,600,000)	8,330,000 (6,850,000–10,100,000)	3,540 (3,260–3,840)	2,240 (2,000–2,500)	1,720 (1,410–2,080)
1-83	Neonatal preterm birth	9,470,000 (8,640,000–10,400,000)	5,040,000 (4,500,000–5,600,000)	3,370,000 (2,770,000–4,040,000)	1,870 (1,710–2,050)	995 (889–1,110)	693 (568–836)
1-84	Neonatal encephalopathy due to birth asphyxia and trauma	4,160,000 (3,760,000–4,600,000)	2,350,000 (2,060,000–2,670,000)	1,700,000 (1,370,000–2,090,000)	818 (740–905)	466 (409–529)	354 (285–437)
1-85	Neonatal sepsis and other neonatal infections	2,400,000 (2,170,000–2,680,000)	2,260,000 (1,960,000–2,620,000)	1,940,000 (1,560,000–2,370,000)	478 (430–536)	444 (386–513)	392 (315–478)
1-86	Hemolytic disease and other neonatal jaundice	301,000 (269,000–338,000)	165,000 (143,000–188,000)	133,000 (110,000–158,000)	61·5 (54·9–69·1)	31·7 (27·5–35·9)	24·7 (20·5–29·2)
1-87	Other neonatal disorders	1,570,000 (1,410,000–1,750,000)	1,520,000 (1,320,000–1,730,000)	1,190,000 (931,000–1,500,000)	308 (277–345)	304 (263–345)	253 (198–319)
1-88	Nutritional deficiencies	4,780,000 (4,130,000–5,630,000)	2,540,000 (2,020,000–3,240,000)	2,270,000 (1,740,000–2,980,000)	1,140 (988–1,350)	501 (402–634)	409 (315–531)
1-89	Protein-energy malnutrition	2,990,000 (2,710,000–3,300,000)	1,060,000 (952,000–1,160,000)	810,000 (696,000–947,000)	689 (624–750)	217 (196–238)	152 (129–178)
1-90	Iodine deficiency	24,300 (13,300–41,900)	25,600 (12,800–46,500)	33,900 (18,400–58,800)	6·4 (3·6–10·9)	4·7 (2·3–8·5)	5·6 (3–9·8)
1-91	Vitamin A deficiency	90,600 (58,700–131,000)	56,600 (36,900–81,000)	47,900 (31,500–69,400)	18·5 (12–26·6)	10·8 (7·1–15·6)	9·1 (6–13·1)
1-92	Dietary iron deficiency	1,430,000 (919,000–2,130,000)	1,300,000 (840,000–1,910,000)	1,290,000 (845,000–1,920,000)	362 (236–537)	248 (161–365)	227 (149–338)
1-93	Other nutritional deficiencies	243,000 (206,000–312,000)	101,000 (83,300–129,000)	90,700 (72,200–116,000)	66·5 (56·4–92·9)	20·4 (16·9–26·3)	15·6 (12·5–19·8)
1-0	Non-communicable diseases	70,400,000 (62,100,000–79,200,000)	99,400,000 (87,400,000–113,000,000)	117,000,000 (102,000,000–135,000,000)	24,000 (21,500–26,700)	20,700 (18,300–23,300)	19,900 (17,400–22,800)
1-1	Neoplasms	8,480,000 (8,210,000–8,820,000)	13,700,000 (13,100,000–14,000,000)	16,500,000 (15,100,000–18,000,000)	3,220 (3,110–3,350)	2,930 (2,810–3,000)	2,770 (2,540–3,030)
1-2	Lip and oral cavity cancer	123,000 (119,000–127,000)	211,000 (204,000–217,000)	253,000 (235,000–272,000)	50·8 (48·9–52·4)	45·5 (44–46·8)	42·1 (39–45·2)
1-3	Nasopharynx cancer	20,600 (19,800–21,400)	37,600 (36,300–38,900)	42,100 (38,200–46,300)	7·4 (7·1–7·6)	7·6 (7·4–7·9)	6·9 (6·3–7·6)
1-4	Other pharynx cancer	72,500 (70,300–74,800)	124,000 (120,000–127,000)	148,000 (138,000–158,000)	29·8 (28·9–30·8)	26·4 (25·6–27·2)	24·3 (22·7–25·8)
1-5	Oesophageal cancer	252,000 (244,000–260,000)	400,000 (388,000–412,000)	485,000 (457,000–515,000)	108 (104–111)	87·6 (84·7–90·2)	80·6 (75·8–85·6)
1-6	Stomach cancer	1,040,000 (1,000,000–1,060,000)	1,400,000 (1,350,000–1,440,000)	1,560,000 (1,400,000–1,750,000)	440 (423–452)	307 (294–317)	262 (235–292)
1-7	Colon and rectum cancer	484,000 (469,000–497,000)	1,130,000 (1,080,000–1,160,000)	1,500,000 (1,380,000–1,640,000)	204 (197–210)	248 (238–255)	252 (231–275)

1-8	Liver cancer	211,000 (203,000–218,000)	314,000 (300,000–328,000)	426,000 (386,000–473,000)	84·8 (81·4–87·9)	69·1 (65·8–72·3)	72·1 (65·4–80·2)
1-9	Liver cancer due to hepatitis B	60,100 (49,800–71,900)	73,900 (61,400–88,400)	95,500 (76,400–120,000)	22·9 (18·7–27·9)	15·5 (12·8–18·7)	15·7 (12·6–19·8)
1-10	Liver cancer due to hepatitis C	52,400 (42,400–62,400)	92,500 (78,200–107,000)	129,000 (107,000–154,000)	23·8 (19·5–28·2)	21·3 (18·1–24·6)	22·1 (18·2–26·3)
1-11	Liver cancer due to alcohol use	55,600 (44,200–67,900)	91,000 (75,400–107,000)	130,000 (105,000–159,000)	24·1 (19·1–29·3)	20·4 (16·9–24·1)	21·9 (17·8–26·8)
1-12	Liver cancer due to NASH	13,400 (10,600–16,800)	22,700 (18,500–27,900)	33,500 (26,900–42,400)	5·7 (4·4–7·2)	5·1 (4·1–6·3)	5·7 (4·6–7·2)
1-13	Gallbladder and biliary tract cancer	198,000 (161,000–207,000)	256,000 (238,000–299,000)	314,000 (281,000–376,000)	86·8 (70·4–91)	57·4 (53·2–67·2)	52·9 (47·3–63·3)
1-14	Pancreatic cancer	226,000 (220,000–231,000)	544,000 (525,000–559,000)	770,000 (701,000–840,000)	97·8 (94·7–100)	122 (117–125)	130 (118–141)
1-15	Larynx cancer	145,000 (141,000–149,000)	221,000 (213,000–227,000)	257,000 (239,000–275,000)	61·6 (59·6–63·6)	48·4 (46·7–49·8)	42·7 (39·7–45·8)
1-16	Tracheal, bronchus, and lung cancer	932,000 (905,000–956,000)	1,530,000 (1,480,000–1,580,000)	1,900,000 (1,740,000–2,090,000)	400 (387–411)	343 (330–352)	320 (292–351)
1-17	Malignant skin melanoma	55,700 (48,100–77,400)	109,000 (88,700–140,000)	134,000 (110,000–186,000)	20·6 (17·6–28·6)	22·8 (18·4–29·2)	22·3 (18·3–30·7)
1-18	Non-melanoma skin cancer	49,100 (47,100–50,800)	97,800 (90,800–101,000)	127,000 (115,000–139,000)	21·6 (20·4–22·3)	22·1 (20·4–22·9)	21·8 (19·6–23·8)
1-19	Non-melanoma skin cancer (squamous-cell carcinoma)	49,100 (47,100–50,800)	97,700 (90,700–101,000)	127,000 (115,000–139,000)	21·5 (20·4–22·3)	22·1 (20·4–22·9)	21·8 (19·6–23·8)
1-20	Non-melanoma skin cancer (basal-cell carcinoma)	43 (20–82)	93 (43–176)	119 (54–227)	0 (0–0)	0 (0–0)	0 (0–0)
1-21	Breast cancer	622,000 (605,000–640,000)	1,170,000 (1,130,000–1,210,000)	1,420,000 (1,290,000–1,560,000)	241 (234–248)	243 (235–251)	232 (211–255)
1-22	Cervical cancer	743,000 (699,000–807,000)	924,000 (871,000–1,040,000)	1,050,000 (919,000–1,210,000)	277 (262–299)	188 (177–212)	170 (149–197)
1-23	Uterine cancer	88,700 (84,200–92,700)	153,000 (145,000–165,000)	203,000 (183,000–229,000)	37·9 (36–39·6)	33·9 (32·2–36·5)	33·9 (30·7–38·2)
1-24	Ovarian cancer	139,000 (134,000–149,000)	314,000 (298,000–331,000)	408,000 (360,000–460,000)	52·7 (50·6–56·3)	65·6 (62·1–69)	67 (59·2–75·5)
1-25	Prostate cancer	378,000 (340,000–517,000)	831,000 (686,000–1,020,000)	1,070,000 (897,000–1,400,000)	190 (170–260)	202 (168–250)	189 (158–248)
1-26	Testicular cancer	40,900 (36,400–64,600)	77,300 (72,500–97,900)	90,400 (78,100–108,000)	10·4 (9·4–15·1)	13·9 (13–18)	14·9 (12·9–18·5)
1-27	Kidney cancer	143,000 (138,000–149,000)	256,000 (247,000–264,000)	341,000 (307,000–379,000)	49·6 (48·2–51·1)	54·8 (52·9–56·5)	57·6 (51·9–64·1)
1-28	Bladder cancer	95,500 (91,800–98,600)	173,000 (165,000–179,000)	221,000 (202,000–241,000)	44·3 (42·3–45·8)	40·3 (38·4–41·7)	38·2 (34·8–41·6)
1-29	Brain and nervous system cancer	338,000 (292,000–510,000)	648,000 (443,000–718,000)	712,000 (480,000–832,000)	94·3 (82·4–141)	129 (87·5–143)	121 (81·6–142)
1-30	Thyroid cancer	46,000 (44,000–47,900)	83,100 (77,400–87,000)	104,000 (93,000–116,000)	18·5 (17·6–19·3)	18 (16·7–18·9)	17·4 (15·6–19·4)
1-31	Mesothelioma	22,000 (18,400–27,400)	38,200 (34,200–40,600)	47,900 (41,500–53,700)	8·4 (7·1–9·9)	8 (7·1–8·4)	7·9 (6·8–8·8)
1-32	Hodgkin lymphoma	82,700 (74,200–95,200)	83,200 (73,700–95,500)	84,800 (74,300–101,000)	23·9 (21·4–27·5)	16 (14·1–18·3)	14 (12·3–16·7)
1-33	Non-Hodgkin's lymphoma	310,000 (297,000–322,000)	460,000 (444,000–474,000)	533,000 (482,000–592,000)	97·5 (94·3–101)	94·4 (91–97·3)	89·5 (80·9–99·4)
1-34	Multiple myeloma	71,200 (66,200–80,800)	160,000 (145,000–171,000)	221,000 (193,000–249,000)	30·6 (28·4–34·7)	35·7 (32·2–38)	37 (32·4–41·7)
1-35	Leukaemia	867,000 (811,000–925,000)	1,010,000 (951,000–1,060,000)	1,020,000 (905,000–1,140,000)	219 (207–232)	196 (185–206)	177 (157–199)
1-36	Acute lymphoid leukaemia	315,000 (285,000–359,000)	398,000 (345,000–428,000)	372,000 (302,000–429,000)	67·9 (61·9–77·2)	74·4 (64·5–80·3)	66·9 (54·6–77·5)
1-37	Chronic lymphoid leukaemia	13,900 (12,800–14,700)	28,300 (25,800–31,300)	37,000 (32,900–42,900)	6·1 (5·6–6·4)	6·5 (5·9–7·2)	6·4 (5·6–7·3)
1-38	Acute myeloid leukaemia	179,000 (160,000–201,000)	268,000 (234,000–287,000)	297,000 (257,000–335,000)	47·1 (42·7–52·4)	51·9 (45·3–55·5)	50·8 (43·7–57·5)
1-39	Chronic myeloid leukaemia	58,500 (53,700–66,700)	57,600 (53,800–63,700)	56,100 (49,400–63,900)	18·6 (17·3–20·5)	11·6 (10·9–12·8)	9·4 (8·2–10·8)
1-40	Other leukaemia	300,000 (260,000–342,000)	258,000 (232,000–292,000)	255,000 (219,000–297,000)	79·1 (70·3–88)	51·2 (46·2–57·9)	43·8 (37·6–51·3)
1-41	Other neoplasms	36,100 (29,600–50,700)	72,500 (57,800–86,600)	97,300 (74,700–116,000)	12·4 (10·2–17·5)	15·6 (12·4–18·6)	16·8 (12·9–20)
1-42	Myelodysplastic, myeloproliferative, and other hematopoietic neoplasms	36,100 (29,600–50,700)	72,500 (57,800–86,600)	97,300 (74,700–116,000)	12·4 (10·2–17·5)	15·6 (12·4–18·6)	16·8 (12·9–20)
1-43	Benign and in situ intestinal neoplasms	0 (0–0)	0 (0–0)	0 (0–0)	0 (0–0)	0 (0–0)	0 (0–0)
1-44	Benign and in situ cervical and uterine neoplasms	0 (0–0)	0 (0–0)	0 (0–0)	0 (0–0)	0 (0–0)	0 (0–0)
1-45	Other benign and in situ neoplasms	0 (0–0)	0 (0–0)	0 (0–0)	0 (0–0)	0 (0–0)	0 (0–0)
1-46	Cardiovascular diseases	14,000,000 (13,500,000–14,300,000)	17,200,000 (16,400,000–17,800,000)	20,400,000 (18,800,000–22,100,000)	5,970 (5,740–6,140)	3,860 (3,660–4,000)	3,500 (3,220–3,790)
1-47	Rheumatic heart disease	454,000 (401,000–520,000)	393,000 (321,000–485,000)	392,000 (309,000–491,000)	135 (121–152)	75·2 (62·3–91·9)	64·7 (51·3–81)
1-48	Ischaemic heart disease	6,160,000 (5,960,000–6,320,000)	8,090,000 (7,720,000–8,350,000)	9,840,000 (8,990,000–10,700,000)	2,780 (2,660–2,860)	1,840 (1,740–1,900)	1,680 (1,530–1,830)
1-49	Stroke	5,070,000 (4,890,000–5,240,000)	5,410,000 (5,130,000–5,640,000)	6,190,000 (5,710,000–6,690,000)	2,130 (2,040–2,210)	1,210 (1,140–1,260)	1,060 (976–1,140)
1-50	Ischaemic stroke	1,860,000 (1,750,000–1,960,000)	2,150,000 (1,980,000–2,280,000)	2,580,000 (2,350,000–2,800,000)	905 (846–954)	519 (478–549)	458 (417–496)
1-51	Intracerebral hemorrhage	2,500,000 (2,400,000–2,610,000)	2,360,000 (2,260,000–2,470,000)	2,610,000 (2,380,000–2,850,000)	991 (950–1,030)	512 (489–535)	437 (398–477)
1-52	Subarachnoid hemorrhage	714,000 (651,000–773,000)	901,000 (858,000–944,000)	1,000,000 (911,000–1,110,000)	238 (217–259)	182 (173–191)	165 (150–182)
1-53	Hypertensive heart disease	743,000 (585,000–785,000)	1,010,000 (923,000–1,220,000)	1,250,000 (1,110,000–1,580,000)	346 (274–367)	237 (215–284)	216 (192–273)
1-54	Non-rheumatic valvular heart disease	111,000 (107,000–115,000)	155,000 (148,000–161,000)	201,000 (183,000–221,000)	42·5 (40·8–44)	33·8 (32–35·1)	34 (30·9–37·3)
1-55	Non-rheumatic calcific aortic valve disease	70,900 (65,100–78,900)	101,000 (94,500–114,000)	134,000 (120,000–158,000)	28 (25·7–30·7)	22·5 (20·9–25·3)	22·8 (20·3–26·9)
1-56	Non-rheumatic degenerative mitral valve disease	38,500 (31,700–44,700)	51,200 (41,100–57,600)	63,800 (49,100–74,100)	13·9 (11·5–16·4)	10·8 (8·6–12·1)	10·6 (8·1–12·4)
1-57	Other non-rheumatic valve diseases	2,000 (1,250–2,520)	2,520 (1,800–3,680)	3,140 (2,180–4,490)	0·7 (0·4–0·8)	0·5 (0·4–0·7)	0·5 (0·4–0·7)
1-58	Cardiomyopathy and myocarditis	591,000 (497,000–638,000)	734,000 (643,000–787,000)	790,000 (709,000–888,000)	206 (172–219)	155 (136–167)	136 (121–153)
1-59	Myocarditis	36,700 (25,700–50,800)	44,000 (35,800–57,000)	44,800 (36,100–60,500)	9·1 (6·7–12·1)	8·8 (7·1–11·3)	8·1 (6·5–11)
1-60	Alcoholic cardiomyopathy	83,600 (67,300–103,000)	76,200 (63,700–94,900)	78,700 (63,200–95,900)	30·1 (24·3–37·4)	15·3 (12·8–19·1)	12·7 (10·2–15·5)

1-61	Other cardiomyopathy	470,000 (392,000–509,000)	614,000 (529,000–652,000)	667,000 (595,000–749,000)	166 (137–179)	131 (114–140)	115 (103–130)
1-62	Atrial fibrillation and flutter	159,000 (134,000–195,000)	361,000 (303,000–439,000)	497,000 (417,000–604,000)	85·9 (72·7–104)	89·5 (75·1–108)	89 (74·6–108)
1-63	Aortic aneurysm	131,000 (127,000–135,000)	271,000 (260,000–279,000)	356,000 (330,000–383,000)	55·8 (53·8–57·6)	60·7 (58·1–62·7)	60·4 (55·9–65·1)
1-64	Peripheral vascular disease	37,000 (21,400–59,100)	73,000 (42,800–118,000)	104,000 (60,800–172,000)	19 (11–30·2)	17·8 (10·5–28·6)	18·4 (10·8–30·4)
1-65	Endocarditis	85,400 (73,300–116,000)	134,000 (104,000–150,000)	148,000 (116,000–176,000)	24·2 (21·4–33·4)	27 (20·7–29·8)	25·1 (19·6–29·9)
1-66	Chronic respiratory diseases	3,280,000 (2,870,000–3,820,000)	4,030,000 (3,610,000–4,510,000)	4,770,000 (4,250,000–5,370,000)	1,160 (1,060–1,290)	887 (801–987)	837 (744–946)
1-67	Chronic obstructive pulmonary disease	1,550,000 (1,440,000–1,630,000)	2,450,000 (2,270,000–2,620,000)	3,060,000 (2,780,000–3,370,000)	709 (659–746)	573 (528–614)	534 (485–588)
1-68	Pneumoconiosis	21,300 (19,100–23,900)	29,300 (26,000–33,300)	33,600 (29,000–39,000)	8·5 (7·7–9·5)	6·3 (5·6–7·1)	5·6 (4·9–6·5)
1-69	Silicosis	13,700 (12,300–15,500)	16,500 (14,600–19,000)	18,500 (15,800–22,000)	5·6 (5–6·3)	3·5 (3·1–4·1)	3·1 (2·6–3·6)
1-70	Asbestosis	823 (662–959)	1,600 (1,180–1,810)	2,070 (1,500–2,360)	0·3 (0·3–0·4)	0·3 (0·3–0·4)	0·3 (0·3–0·4)
1-71	Coal workers pneumoconiosis	1,320 (1,150–1,490)	2,180 (1,930–2,450)	2,760 (2,260–3,210)	0·5 (0·5–0·6)	0·5 (0·4–0·5)	0·5 (0·4–0·5)
1-72	Other pneumoconiosis	5,400 (4,580–6,490)	8,980 (7,390–10,700)	10,200 (8,500–12,500)	2·1 (1·8–2·5)	2 (1·6–2·3)	1·7 (1·4–2·1)
1-73	Asthma	1,470,000 (1,090,000–1,960,000)	1,130,000 (827,000–1,570,000)	1,180,000 (836,000–1,690,000)	368 (284–480)	219 (161–301)	212 (148–305)
1-74	Interstitial lung disease and pulmonary sarcoidosis	101,000 (88,100–132,000)	243,000 (179,000–281,000)	303,000 (216,000–370,000)	40·5 (35·1–53·4)	54·6 (40–63·3)	52·1 (37·3–63·9)
1-75	Other chronic respiratory diseases	148,000 (112,000–188,000)	177,000 (153,000–200,000)	190,000 (163,000–218,000)	36·6 (28·8–45)	33·9 (29·1–38·3)	33 (28·2–37·7)
1-76	Digestive diseases	5,880,000 (5,490,000–6,340,000)	6,990,000 (6,460,000–7,690,000)	8,170,000 (7,320,000–9,100,000)	1,950 (1,840–2,100)	1,440 (1,340–1,580)	1,360 (1,220–1,520)
1-77	Cirrhosis and other chronic liver diseases	2,560,000 (2,480,000–2,650,000)	3,240,000 (3,120,000–3,380,000)	3,880,000 (3,490,000–4,310,000)	914 (885–940)	669 (642–697)	635 (573–706)
1-78	Cirrhosis and other chronic liver diseases due to hepatitis B	319,000 (276,000–367,000)	266,000 (223,000–317,000)	270,000 (221,000–328,000)	114 (99·6–132)	53·5 (45·1–63·6)	43·6 (35·8–53)
1-79	Cirrhosis and other chronic liver diseases due to hepatitis C	566,000 (496,000–646,000)	742,000 (645,000–861,000)	896,000 (743,000–1,090,000)	204 (178–234)	150 (131–175)	146 (121–176)
1-80	Cirrhosis and other chronic liver diseases due to alcohol use	872,000 (778,000–964,000)	1,290,000 (1,150,000–1,440,000)	1,560,000 (1,340,000–1,810,000)	333 (297–368)	269 (240–298)	255 (218–295)
1-81	Cirrhosis and other chronic liver diseases due to NAFLD	304,000 (226,000–399,000)	477,000 (353,000–626,000)	611,000 (445,000–797,000)	118 (87–154)	101 (74·9–133)	101 (73·6–131)
1-82	Cirrhosis and other chronic liver diseases due to other causes	503,000 (434,000–580,000)	463,000 (382,000–565,000)	534,000 (421,000–683,000)	145 (123–172)	94·9 (77·5–116)	90·6 (71·4–115)
1-83	Upper digestive system diseases	972,000 (758,000–1,300,000)	1,160,000 (816,000–1,680,000)	1,320,000 (917,000–1,950,000)	345 (277–453)	234 (169–338)	218 (152–321)
1-84	Peptic ulcer disease	438,000 (419,000–459,000)	343,000 (324,000–369,000)	362,000 (326,000–407,000)	172 (164–179)	75·1 (70·6–80·8)	61·5 (55·2–69·3)
1-85	Gastritis and duodenitis	144,000 (108,000–191,000)	172,000 (133,000–221,000)	193,000 (150,000–252,000)	49·1 (37·7–63·9)	35·6 (28–45·4)	32·4 (25·2–41·9)
1-86	GERD	389,000 (204,000–691,000)	642,000 (337,000–1,140,000)	763,000 (400,000–1,360,000)	124 (64·9–220)	124 (64·9–220)	124 (64·9–220)
1-87	Appendicitis	305,000 (263,000–368,000)	170,000 (152,000–200,000)	174,000 (149,000–206,000)	72·7 (63·4–86·3)	33 (29·6–38·9)	29·8 (25·6–35·3)
1-88	Paralytic ileus and intestinal obstruction	649,000 (556,000–732,000)	498,000 (439,000–544,000)	511,000 (423,000–592,000)	162 (142–180)	105 (92·1–114)	93·3 (77·6–109)
1-89	Inguinal, femoral, and abdominal hernia	373,000 (286,000–474,000)	384,000 (298,000–489,000)	441,000 (343,000–560,000)	109 (85·9–135)	78·8 (61·7–99·3)	75·8 (58·9–95·7)
1-90	Inflammatory bowel disease	64,000 (52,100–75,900)	81,500 (70,800–93,800)	98,000 (84,600–113,000)	20·4 (17·1–23·6)	16·6 (14·4–19)	16·4 (14·2–18·9)
1-91	Vascular intestinal disorders	109,000 (104,000–115,000)	162,000 (153,000–173,000)	205,000 (186,000–227,000)	46·3 (44·1–48·3)	37 (34·7–39·7)	35·5 (32–39·3)
1-92	Gallbladder and biliary diseases	421,000 (331,000–543,000)	708,000 (551,000–922,000)	825,000 (639,000–1,060,000)	152 (122–192)	145 (115–187)	137 (107–175)
1-93	Pancreatitis	225,000 (207,000–239,000)	325,000 (306,000–345,000)	381,000 (347,000–424,000)	75·6 (69·9–80·3)	65·3 (61·5–69·6)	62·6 (56·9–69·6)
1-94	Other digestive diseases	195,000 (164,000–254,000)	270,000 (214,000–313,000)	344,000 (264,000–414,000)	57·6 (50·2–74·8)	58 (45·5–67·2)	59·5 (45·7–71·3)
1-95	Neurological disorders	4,420,000 (2,420,000–7,350,000)	6,630,000 (3,620,000–11,000,000)	7,920,000 (4,430,000–13,000,000)	1,390 (807–2,250)	1,350 (761–2,210)	1,360 (769–2,210)
1-96	Alzheimer's disease and other dementias	575,000 (239,000–1,330,000)	1,390,000 (583,000–3,090,000)	1,950,000 (831,000–4,260,000)	350 (145–804)	358 (150–795)	356 (152–777)
1-97	Parkinson's disease	134,000 (125,000–143,000)	301,000 (276,000–323,000)	412,000 (372,000–452,000)	70·5 (65·4–74·9)	74·8 (68·5–80)	74·2 (67·2–81·4)
1-98	Idiopathic epilepsy	1,140,000 (860,000–1,510,000)	1,140,000 (845,000–1,510,000)	1,290,000 (945,000–1,720,000)	289 (218–382)	214 (159–283)	220 (161–295)
1-99	Multiple sclerosis	22,900 (19,200–30,000)	48,200 (41,100–56,400)	59,300 (49,900–72,000)	8 (6·7–10·6)	9·6 (8·2–11·3)	9·6 (8·1–11·7)
1-100	Motor neuron disease	27,700 (26,400–29,000)	62,500 (60,300–64,700)	89,200 (79,300–98,700)	8·8 (8·5–9·1)	13 (12·6–13·5)	14·9 (13·2–16·5)
1-101	Headache disorders	2,260,000 (358,000–5,010,000)	3,360,000 (566,000–7,510,000)	3,720,000 (683,000–8,210,000)	599 (110–1,300)	612 (107–1,350)	612 (111–1,350)
1-102	Migraine	2,070,000 (239,000–4,830,000)	3,080,000 (374,000–7,120,000)	3,390,000 (456,000–7,790,000)	545 (73·6–1,250)	559 (71·3–1,290)	557 (74·1–1,280)
1-103	Tension-type headache	188,000 (51,000–770,000)	283,000 (80,000–1,080,000)	332,000 (95,800–1,200,000)	54·1 (15·6–202)	53·1 (15–197)	54·3 (15·6–196)
1-104	Other neurological disorders	266,000 (214,000–338,000)	336,000 (276,000–418,000)	406,000 (325,000–507,000)	66·4 (54·5–82·6)	63·8 (52·8–78·4)	70·4 (55·9–88·4)
1-105	Mental disorders	6,310,000 (4,630,000–8,310,000)	10,100,000 (7,370,000–13,200,000)	11,200,000 (8,190,000–14,600,000)	1,770 (1,300–2,310)	1,880 (1,380–2,460)	1,830 (1,340–2,400)
1-106	Schizophrenia	577,000 (416,000–752,000)	948,000 (681,000–1,230,000)	1,110,000 (795,000–1,430,000)	175 (127–226)	177 (127–229)	178 (127–230)
1-107	Depressive disorders	2,080,000 (1,430,000–2,860,000)	3,270,000 (2,290,000–4,460,000)	3,720,000 (2,590,000–5,060,000)	619 (432–848)	620 (435–841)	607 (423–825)
1-108	Major depressive disorder	1,790,000 (1,220,000–2,500,000)	2,820,000 (1,940,000–3,850,000)	3,180,000 (2,200,000–4,360,000)	533 (366–740)	534 (371–724)	520 (360–712)
1-109	Dysthymia	286,000 (183,000–428,000)	454,000 (291,000–682,000)	535,000 (346,000–801,000)	86·5 (55·9–129)	85·8 (55·3–128)	86·7 (56–129)
1-110	Bipolar disorder	763,000 (462,000–1,200,000)	1,140,000 (692,000–1,770,000)	1,280,000 (779,000–1,980,000)	209 (127–324)	209 (127–322)	208 (126–321)
1-111	Anxiety disorders	1,630,000 (1,120,000–2,230,000)	3,020,000 (2,090,000–4,130,000)	3,190,000 (2,190,000–4,370,000)	450 (309–612)	560 (389–766)	524 (361–721)
1-112	Eating disorders	184,000 (114,000–277,000)	273,000 (171,000–409,000)	303,000 (188,000–457,000)	43·5 (27·1–64·9)	47·2 (29·7–70·8)	49·2 (30·7–74·1)
1-113	Anorexia nervosa	49,600 (29,100–80,100)	69,700 (41,000–111,000)	75,500 (45,000–120,000)	11·2 (6·6–17·8)	12 (7·1–18·9)	12·5 (7·4–19·8)

1-114	Bulimia nervosa	135,000 (78,300–215,000)	204,000 (119,000–321,000)	227,000 (131,000–358,000)	32·2 (18·7–50·9)	35·2 (20·6–55·4)	36·8 (21·2–58)
1-115	Autism spectrum disorders	220,000 (144,000–318,000)	288,000 (187,000–416,000)	309,000 (202,000–447,000)	53·4 (35–77·3)	53·5 (34·8–77·2)	53·5 (34·9–77·2)
1-116	Attention-deficit/hyperactivity disorder	101,000 (56,900–170,000)	121,000 (69,400–204,000)	127,000 (72,400–215,000)	22 (12·6–36·9)	21·6 (12·3–36·3)	22 (12·5–37·5)
1-117	Conduct disorder	356,000 (198,000–558,000)	393,000 (222,000–615,000)	382,000 (212,000–600,000)	70·2 (39·1–110)	69·2 (39·2–108)	69·8 (38·8–110)
1-118	Idiopathic developmental intellectual disability	86,900 (34,400–156,000)	103,000 (38,700–182,000)	95,800 (32,100–175,000)	20·9 (8·4–37·3)	18·9 (7·1–33·6)	16·5 (5·5–30·2)
1-119	Other mental disorders	318,000 (203,000–484,000)	532,000 (339,000–810,000)	636,000 (409,000–965,000)	103 (66·1–156)	103 (66–156)	103 (66·4–156)
1-120	Substance use disorders	1,850,000 (1,460,000–2,280,000)	2,750,000 (2,150,000–3,420,000)	3,040,000 (2,410,000–3,790,000)	543 (437–662)	506 (398–626)	489 (387–610)
1-121	Alcohol use disorders	1,330,000 (1,080,000–1,660,000)	1,900,000 (1,510,000–2,380,000)	2,070,000 (1,660,000–2,600,000)	409 (338–501)	356 (284–444)	332 (266–418)
1-122	Drug use disorders	521,000 (370,000–692,000)	849,000 (611,000–1,130,000)	970,000 (707,000–1,260,000)	134 (96·4–176)	151 (109–199)	157 (114–204)
1-123	Opioid use disorders	200,000 (135,000–283,000)	326,000 (220,000–453,000)	398,000 (274,000–544,000)	54·9 (37·3–75·5)	59·4 (40·6–81·7)	64·1 (44·2–87·5)
1-124	Cocaine use disorders	87,900 (60,400–123,000)	170,000 (121,000–232,000)	190,000 (143,000–249,000)	22·3 (15·7–30·7)	29·9 (21·4–40·6)	30·8 (23·1–40·5)
1-125	Amphetamine use disorders	83,000 (45,500–137,000)	117,000 (66,000–191,000)	130,000 (73,400–214,000)	19·9 (11–33·1)	20·3 (11·5–33·1)	21 (11·8–34·5)
1-126	Cannabis use disorders	43,100 (24,800–70,000)	59,400 (35,400–92,600)	63,400 (37,900–97,400)	10·3 (6–16·5)	10·3 (6·2–16)	10·3 (6·2–15·9)
1-127	Other drug use disorders	107,000 (70,400–156,000)	177,000 (117,000–249,000)	189,000 (128,000–267,000)	26·6 (17·7–38·3)	31 (20·6–43·5)	30·6 (20·7–43·3)
1-128	Diabetes and kidney diseases	4,930,000 (4,470,000–5,420,000)	10,100,000 (9,090,000–11,300,000)	13,700,000 (12,200,000–15,500,000)	1,980 (1,800–2,190)	2,220 (2,000–2,470)	2,310 (2,050–2,610)
1-129	Diabetes mellitus	3,170,000 (2,790,000–3,590,000)	6,010,000 (5,170,000–7,000,000)	8,320,000 (7,090,000–9,840,000)	1,340 (1,180–1,510)	1,330 (1,150–1,540)	1,400 (1,200–1,650)
1-130	Diabetes mellitus type 1	234,000 (202,000–273,000)	312,000 (263,000–363,000)	362,000 (299,000–432,000)	70 (59·6–84)	60·7 (51·5–70·8)	60·2 (49·8–71·7)
1-131	Diabetes mellitus type 2	2,930,000 (2,560,000–3,340,000)	5,700,000 (4,880,000–6,670,000)	7,960,000 (6,760,000–9,410,000)	1,270 (1,110–1,440)	1,270 (1,090–1,480)	1,340 (1,140–1,580)
1-132	Chronic kidney disease	1,740,000 (1,640,000–1,860,000)	4,120,000 (3,870,000–4,390,000)	5,380,000 (4,860,000–5,980,000)	642 (604–684)	889 (835–944)	906 (819–1,000)
1-133	Chronic kidney disease due to diabetes mellitus type 1	125,000 (89,500–167,000)	295,000 (200,000–405,000)	375,000 (252,000–526,000)	42·6 (29·6–57·6)	58·7 (39·6–81·3)	60·5 (40·5–84·8)
1-134	Chronic kidney disease due to diabetes mellitus type 2	297,000 (240,000–351,000)	883,000 (716,000–1,040,000)	1,220,000 (982,000–1,470,000)	133 (109–156)	201 (164–238)	206 (167–247)
1-135	Chronic kidney disease due to hypertension	279,000 (237,000–330,000)	817,000 (690,000–954,000)	1,100,000 (923,000–1,320,000)	125 (106–145)	186 (159–218)	189 (158–227)
1-136	Chronic kidney disease due to glomerulonephritis	466,000 (404,000–531,000)	977,000 (827,000–1,150,000)	1,230,000 (999,000–1,480,000)	151 (129–175)	200 (167–238)	204 (166–246)
1-137	Chronic kidney disease due to other and unspecified causes	575,000 (495,000–661,000)	1,150,000 (952,000–1,370,000)	1,460,000 (1,180,000–1,780,000)	191 (162–222)	242 (200–290)	247 (200–300)
1-138	Acute glomerulonephritis	22,100 (19,300–25,200)	5,810 (5,240–6,480)	10,200 (9,100–11,400)	5·1 (4·5–5·8)	1·2 (1–1·3)	1·8 (1·6–2)
1-139	Skin and subcutaneous diseases	2,120,000 (1,410,000–3,140,000)	2,890,000 (1,940,000–4,300,000)	3,220,000 (2,190,000–4,740,000)	545 (368–804)	554 (374–820)	559 (380–822)
1-140	Dermatitis	479,000 (279,000–761,000)	606,000 (364,000–949,000)	655,000 (395,000–1,020,000)	116 (68·8–181)	115 (69–180)	116 (68·8–181)
1-141	Atopic dermatitis	372,000 (201,000–622,000)	436,000 (235,000–730,000)	454,000 (246,000–762,000)	83·1 (45–139)	82·3 (44·4–138)	82·5 (44·7–138)
1-142	Contact dermatitis	95,400 (59,400–142,000)	153,000 (95,100–227,000)	181,000 (113,000–268,000)	29·4 (18·5–43·7)	29·6 (18·4–44)	29·6 (18·6–44·1)
1-143	Seborrhoeic dermatitis	12,000 (6,820–19,200)	17,600 (9,990–28,300)	20,100 (11,300–32,000)	3·4 (1·9–5·4)	3·4 (1·9–5·4)	3·4 (1·9–5·4)
1-144	Psoriasis	151,000 (107,000–201,000)	216,000 (153,000–287,000)	243,000 (172,000–323,000)	44·3 (31·5–58·7)	41·5 (29·5–55·2)	40·4 (28·6–53·6)
1-145	Bacterial skin diseases	95,800 (74,600–143,000)	186,000 (125,000–259,000)	266,000 (160,000–355,000)	28·5 (22–43·4)	39·2 (25·9–54·8)	46 (27·9–61·5)
1-146	Cellulitis	32,700 (22,000–51,200)	49,100 (31,500–72,600)	63,000 (36,300–86,700)	9·5 (6·4–15·6)	10·2 (6·5–14·9)	10·8 (6·3–14·8)
1-147	Pyoderma	63,100 (48,100–93,100)	137,000 (89,200–193,000)	203,000 (115,000–275,000)	19 (14·4–28·3)	29 (18·7–40·6)	35·2 (20·1–48·1)
1-148	Scabies	385,000 (213,000–616,000)	510,000 (283,000–811,000)	530,000 (294,000–839,000)	92·2 (51·5–148)	95 (53–152)	92·3 (51·3–147)
1-149	Fungal skin diseases	133,000 (52,900–282,000)	199,000 (80,000–420,000)	238,000 (95,800–502,000)	40·6 (16·5–86·1)	40 (16·2–85)	40·7 (16·4–86·4)
1-150	Viral skin diseases	212,000 (135,000–318,000)	244,000 (156,000–365,000)	252,000 (161,000–378,000)	46 (29·5–69)	46·1 (29·4–69)	46·3 (29·5–69·4)
1-151	Acne vulgaris	195,000 (116,000–315,000)	274,000 (163,000–435,000)	280,000 (167,000–447,000)	40·7 (24·3–65·3)	46·8 (28–74·4)	48·3 (28·8–77·1)
1-152	Alopecia areata	26,200 (16,500–39,200)	38,700 (24,400–57,500)	43,500 (27,500–64,500)	7·1 (4·5–10·6)	7·1 (4·5–10·6)	7·1 (4·5–10·6)
1-153	Pruritus	29,400 (14,100–52,500)	45,500 (21,900–81,300)	53,700 (25,800–95,800)	8·8 (4·2–15·7)	9 (4·3–16)	9 (4·3–16·2)
1-154	Urticaria	210,000 (137,000–309,000)	264,000 (172,000–383,000)	281,000 (183,000–407,000)	49·7 (32·6–72)	49·8 (32·5–72·4)	49·8 (32·5–72·4)
1-155	Decubitus ulcer	48,600 (32,400–72,800)	64,600 (44,100–94,700)	80,800 (55,200–110,000)	21·6 (14·6–31·6)	15 (10·3–21·4)	14·2 (9·7–19·3)
1-156	Other skin and subcutaneous diseases	158,000 (82,100–280,000)	247,000 (126,000–442,000)	295,000 (148,000–526,000)	49·6 (25·8–87·9)	49·7 (25·4–88·8)	49·8 (25–88·7)
1-157	Sense organ diseases	2,400,000 (1,650,000–3,370,000)	3,990,000 (2,730,000–5,710,000)	4,850,000 (3,310,000–6,900,000)	939 (649–1,310)	866 (593–1,230)	825 (564–1,170)
1-158	Blindness and vision loss	871,000 (611,000–1,200,000)	1,370,000 (944,000–1,900,000)	1,700,000 (1,180,000–2,340,000)	353 (251–478)	301 (209–415)	292 (203–402)
1-159	Glaucoma	34,700 (23,300–49,100)	54,800 (37,700–75,900)	73,800 (50,700–103,000)	18·8 (12·6–26·6)	13·7 (9·4–19·1)	13·3 (9·1–18·5)
1-160	Cataract	183,000 (130,000–247,000)	297,000 (212,000–396,000)	385,000 (275,000–511,000)	93·4 (66·2–125)	71·8 (51·1–95·2)	68·2 (48·6–90·7)
1-161	Age-related macular degeneration	10,200 (7,100–14,400)	18,700 (13,000–26,200)	26,100 (18,000–36,600)	5·2 (3·6–7·3)	4·6 (3·2–6·4)	4·6 (3·2–6·4)
1-162	Refraction disorders	349,000 (234,000–504,000)	515,000 (348,000–739,000)	593,000 (398,000–843,000)	109 (74·3–154)	102 (69·1–145)	99·6 (67·2–142)
1-163	Near vision loss	102,000 (47,600–200,000)	198,000 (92,700–387,000)	263,000 (123,000–511,000)	47 (22·2–91·3)	45·6 (21·3–88·9)	45·1 (21·2–87·4)
1-164	Other vision loss	191,000 (135,000–263,000)	282,000 (200,000–384,000)	356,000 (255,000–488,000)	79·9 (56·7–110)	63·1 (44·8–86)	61·3 (43·8–83·8)
1-165	Age-related and other hearing loss	1,410,000 (942,000–2,070,000)	2,440,000 (1,610,000–3,540,000)	2,910,000 (1,920,000–4,230,000)	546 (365–792)	526 (349–764)	492 (327–708)
1-166	Other sense organ diseases	115,000 (68,400–176,000)	193,000 (114,000–297,000)	238,000 (142,000–367,000)	39·4 (23·4–61)	40·2 (23·8–62)	40·4 (24·1–62·4)

1-167	Musculoskeletal disorders	5,490,000 (3,980,000–7,170,000)	9,430,000 (6,830,000–12,400,000)	11,700,000 (8,430,000–15,400,000)	1,840 (1,330–2,420)	1,870 (1,350–2,470)	1,910 (1,380–2,510)
1-168	Rheumatoid arthritis	138,000 (105,000–174,000)	252,000 (188,000–318,000)	322,000 (243,000–407,000)	51·1 (39·3–63·8)	51·7 (39·1–64·9)	52·9 (39·8–66·9)
1-169	Osteoarthritis	448,000 (226,000–892,000)	957,000 (482,000–1,900,000)	1,300,000 (656,000–2,590,000)	197 (99·3–392)	213 (108–423)	218 (110–433)
1-170	Low back pain	2,490,000 (1,750,000–3,330,000)	4,030,000 (2,840,000–5,370,000)	4,830,000 (3,390,000–6,440,000)	789 (557–1,050)	782 (550–1,050)	792 (558–1,060)
1-171	Neck pain	515,000 (338,000–741,000)	965,000 (635,000–1,390,000)	1,080,000 (713,000–1,560,000)	175 (115–253)	190 (124–274)	175 (116–252)
1-172	Gout	15,800 (10,000–22,900)	33,200 (20,900–47,600)	44,100 (27,800–63,500)	6·1 (3·9–8·8)	6·9 (4·4–10)	7·3 (4·6–10·4)
1-173	Other musculoskeletal disorders	1,880,000 (1,320,000–2,570,000)	3,200,000 (2,250,000–4,370,000)	4,080,000 (2,870,000–5,570,000)	624 (438–852)	627 (441–856)	661 (466–902)
1-174	Other non-communicable diseases	11,200,000 (8,500,000–13,900,000)	11,600,000 (9,780,000–13,800,000)	11,700,000 (9,460,000–14,400,000)	2,690 (2,060–3,340)	2,300 (1,940–2,730)	2,120 (1,720–2,590)
1-175	Congenital anomalies	7,160,000 (5,030,000–9,570,000)	5,490,000 (4,660,000–6,210,000)	4,280,000 (3,390,000–5,410,000)	1,430 (1,010–1,910)	1,080 (916–1,220)	876 (692–1,110)
1-176	Neural tube defects	761,000 (565,000–923,000)	418,000 (348,000–522,000)	365,000 (273,000–490,000)	150 (112–182)	82·6 (68·7–103)	76·1 (56·5–102)
1-177	Congenital heart anomalies	3,250,000 (2,150,000–4,590,000)	2,380,000 (1,930,000–2,740,000)	1,700,000 (1,260,000–2,240,000)	647 (429–910)	468 (379–540)	352 (258–464)
1-178	Orofacial clefts	79,900 (59,000–97,600)	24,800 (19,900–30,700)	20,200 (15,400–26,300)	16·1 (11·9–19·6)	4·8 (3·9–6)	3·9 (3–5)
1-179	Down's syndrome	238,000 (170,000–348,000)	223,000 (191,000–292,000)	206,000 (168,000–256,000)	48·3 (34·8–70·1)	43·4 (37–56·9)	40·9 (33–51·2)
1-180	Turner syndrome	1,150 (530–2,010)	1,430 (670–2,440)	1,490 (688–2,530)	0·3 (0·1–0·4)	0·3 (0·1–0·4)	0·3 (0·1–0·4)
1-181	Klinefelter syndrome	72 (32–135)	91 (40–172)	94 (41–178)	0 (0–0)	0 (0–0)	0 (0–0)
1-182	Other chromosomal abnormalities	113,000 (81,200–221,000)	175,000 (117,000–210,000)	180,000 (110,000–228,000)	22·7 (16·4–43·9)	34·3 (22·8–41·1)	36·9 (22·5–46·9)
1-183	Congenital musculoskeletal and limb anomalies	295,000 (211,000–401,000)	311,000 (219,000–420,000)	331,000 (229,000–458,000)	64·4 (46–86·8)	58·9 (41·5–79·3)	60·6 (42·1–83·5)
1-184	Urogenital congenital anomalies	194,000 (94,400–423,000)	149,000 (119,000–233,000)	117,000 (88,800–176,000)	38·5 (18·9–83·4)	29·3 (23·3–46)	24 (18–36·9)
1-185	Digestive congenital anomalies	803,000 (493,000–1,310,000)	630,000 (480,000–769,000)	460,000 (326,000–610,000)	159 (97·3–258)	125 (95·2–152)	96·9 (68·7–129)
1-186	Other congenital anomalies	1,420,000 (990,000–2,070,000)	1,170,000 (888,000–1,380,000)	893,000 (670,000–1,150,000)	284 (198–411)	231 (175–272)	184 (137–238)
1-187	Urinary diseases and male infertility	353,000 (309,000–413,000)	680,000 (576,000–779,000)	955,000 (767,000–1,100,000)	127 (110–152)	150 (127–172)	166 (132–190)
1-188	Urinary tract infections and interstitial nephritis	235,000 (217,000–276,000)	468,000 (387,000–502,000)	673,000 (499,000–748,000)	79·5 (73·5–96)	104 (84·9–111)	118 (87·1–131)
1-189	Urolithiasis	18,900 (15,400–23,100)	36,800 (30,700–45,400)	49,900 (40,300–65,500)	6·7 (5·5–8·1)	7·5 (6·3–9·3)	8·2 (6·6–10·8)
1-190	Benign prostatic hyperplasia	57,500 (35,000–86,400)	113,000 (68,600–168,000)	155,000 (94,500–231,000)	27·5 (16·8–41·2)	26·9 (16·4–40)	26·9 (16·4–39·9)
1-191	Male infertility	7,960 (3,060–18,400)	16,000 (6,080–36,700)	18,900 (7,070–43,600)	2·1 (0·8–4·9)	2·8 (1·1–6·5)	3 (1·1–7)
1-192	Other urinary diseases	33,700 (27,100–43,100)	46,300 (36,500–59,300)	57,600 (44,200–78,600)	11·3 (8·8–14·6)	9·6 (7·6–12·5)	9·8 (7·6–13·4)
1-193	Gynecological diseases	1,310,000 (899,000–1,840,000)	1,870,000 (1,280,000–2,610,000)	2,110,000 (1,440,000–2,960,000)	370 (255–520)	340 (232–476)	338 (232–475)
1-194	Uterine fibroids	74,600 (39,500–134,000)	128,000 (64,500–230,000)	157,000 (78,400–285,000)	24 (12·7–43)	24·1 (12·2–43·2)	25 (12·5–45·3)
1-195	Polycystic ovarian syndrome	28,000 (11,900–56,400)	43,600 (18,900–87,000)	52,100 (22,400–105,000)	7 (3–14)	7·6 (3·3–15·2)	8·4 (3·6–16·9)
1-196	Female infertility	10,200 (3,050–25,100)	26,300 (8,330–63,600)	32,400 (9,730–77,200)	2·7 (0·8–6·8)	4·7 (1·5–11·1)	5·1 (1·5–12·3)
1-197	Endometriosis	138,000 (81,200–225,000)	156,000 (93,600–247,000)	162,000 (97,400–257,000)	35·8 (21·5–57·5)	27·4 (16·5–43·5)	25·9 (15·6–41·1)
1-198	Genital prolapse	22,400 (11,700–40,400)	32,000 (16,100–59,100)	39,300 (19,500–73,000)	8·4 (4·4–15·4)	6·7 (3·4–12·3)	6·5 (3·2–12)
1-199	Premenstrual syndrome	410,000 (250,000–643,000)	592,000 (362,000–922,000)	644,000 (396,000–993,000)	104 (64·1–160)	104 (63·7–161)	104 (64–161)
1-200	Other gynecological diseases	622,000 (408,000–886,000)	892,000 (583,000–1,280,000)	1,020,000 (656,000–1,480,000)	188 (123–269)	166 (109–236)	163 (106–237)
1-201	Hemoglobinopathies and hemolytic anaemias	359,000 (291,000–444,000)	377,000 (312,000–461,000)	387,000 (315,000–475,000)	90·5 (73·1–113)	71·6 (59·4–87·2)	67 (54·5–82·2)
1-202	Thalassemias	6,550 (4,490–9,170)	7,610 (5,330–10,100)	6,040 (4,050–8,350)	1·3 (0·9–1·8)	1·5 (1–2)	1·2 (0·8–1·7)
1-203	Thalassaemias trait	30,200 (19,400–45,800)	31,700 (20,500–49,000)	33,400 (21,400–51,400)	7·5 (4·8–11·2)	5·8 (3·8–9)	5·7 (3·6–8·7)
1-204	Sickle cell disorders	83,300 (64,300–104,000)	84,100 (69,200–102,000)	84,500 (66,100–104,000)	17·7 (13·8–21·9)	15·6 (12·8–19)	15·4 (11·9–19·2)
1-205	Sickle cell trait	63,800 (40,900–94,900)	62,200 (40,400–92,300)	62,800 (39,900–94,200)	15·2 (9·8–22·6)	11·1 (7·2–16·5)	10·6 (6·7–15·8)
1-206	G6PD deficiency	41,700 (34,200–49,000)	63,600 (54,300–72,100)	62,300 (50,900–74,800)	10 (8·2–11·7)	11·8 (10·1–13·4)	10·5 (8·6–12·7)
1-207	G6PD trait	706 (460–1,020)	1,140 (729–1,680)	1,280 (814–1,890)	0·2 (0·1–0·3)	0·2 (0·1–0·3)	0·2 (0·1–0·3)
1-208	Other hemoglobinopathies and hemolytic anaemias	132,000 (105,000–171,000)	127,000 (100,000–165,000)	136,000 (107,000–175,000)	38·7 (31–49·4)	25·6 (20·5–32·9)	23·4 (18·4–30)
1-209	Endocrine, metabolic, blood, and immune disorders	856,000 (678,000–1,060,000)	1,290,000 (1,040,000–1,580,000)	1,570,000 (1,250,000–1,940,000)	259 (204–324)	263 (212–324)	268 (216–332)
1-210	Oral disorders	1,070,000 (639,000–1,660,000)	1,790,000 (1,070,000–2,810,000)	2,290,000 (1,370,000–3,550,000)	387 (234–588)	372 (224–571)	382 (230–586)
1-211	Caries of deciduous teeth	15,700 (6,880–33,100)	14,600 (6,390–30,800)	15,000 (6,500–31,700)	3·1 (1·4–6·6)	2·9 (1·3–6·1)	3 (1·3–6·4)
1-212	Caries of permanent teeth	96,700 (43,300–189,000)	139,000 (62,900–269,000)	155,000 (69,700–302,000)	25·4 (11·6–49·1)	25·6 (11·6–49·4)	25·7 (11·6–50·1)
1-213	Periodontal disease	268,000 (101,000–590,000)	484,000 (185,000–1,060,000)	588,000 (226,000–1,270,000)	89·9 (34·7–195)	93·8 (36·1–204)	94·5 (36·5–204)
1-214	Edentulism	491,000 (313,000–720,000)	865,000 (555,000–1,250,000)	1,220,000 (775,000–1,780,000)	215 (137–314)	196 (126–283)	206 (131–301)
1-215	Other oral disorders	196,000 (122,000–287,000)	284,000 (178,000–415,000)	320,000 (201,000–465,000)	53·3 (33·3–77·7)	53·3 (33·4–77·8)	53·4 (33·5–77·6)
1-216	Sudden infant death syndrome	122,000 (85,000–179,000)	115,000 (89,800–152,000)	97,600 (70,000–134,000)	24 (16·8–35·4)	22·8 (17·7–30)	20·6 (14·8–28·3)
1-0	Injuries	22,600,000 (21,600,000–23,700,000)	38,400,000 (36,500,000–40,600,000)	24,400,000 (22,100,000–26,800,000)	5,830 (5,550–6,160)	7,070 (6,710–7,490)	4,050 (3,660–4,450)
1-1	Transport injuries	6,150,000 (5,930,000–6,400,000)	6,570,000 (6,280,000–6,880,000)	6,180,000 (5,590,000–6,790,000)	1,620 (1,560–1,690)	1,210 (1,160–1,270)	1,020 (922–1,120)
1-2	Road injuries	5,860,000 (5,650,000–6,090,000)	6,240,000 (5,970,000–6,540,000)	5,870,000 (5,320,000–6,450,000)	1,540 (1,480–1,610)	1,150 (1,100–1,210)	969 (876–1,060)

1-3	Pedestrian road injuries	3,370,000 (3,220,000–3,520,000)	2,210,000 (2,100,000–2,320,000)	1,870,000 (1,660,000–2,090,000)	890 (852–930)	420 (399–440)	312 (277–350)
1-4	Cyclist road injuries	225,000 (198,000–255,000)	318,000 (286,000–358,000)	298,000 (262,000–341,000)	62·1 (54·1–71·1)	60 (53·9–67·8)	49·5 (43·5–56·4)
1-5	Motorcyclist road injuries	705,000 (671,000–746,000)	1,530,000 (1,460,000–1,620,000)	1,600,000 (1,480,000–1,740,000)	182 (172–194)	273 (260–289)	260 (240–282)
1-6	Motor vehicle road injuries	1,440,000 (1,350,000–1,550,000)	2,010,000 (1,920,000–2,110,000)	1,940,000 (1,750,000–2,140,000)	374 (351–401)	368 (351–385)	319 (288–353)
1-7	Other road injuries	126,000 (114,000–139,000)	171,000 (155,000–188,000)	170,000 (150,000–193,000)	34 (30·5–37·6)	31·8 (28·8–35·2)	28·1 (24·8–31·8)
1-8	Other transport injuries	291,000 (276,000–307,000)	324,000 (310,000–339,000)	310,000 (275,000–347,000)	76·5 (72·6–81·1)	59·4 (56·9–62·5)	51 (45·3–57·2)
1-9	Unintentional injuries	8,480,000 (7,810,000–9,320,000)	21,400,000 (19,700,000–23,200,000)	7,430,000 (6,380,000–8,700,000)	2,200 (2,000–2,450)	4,010 (3,700–4,360)	1,270 (1,090–1,490)
1-10	Falls	1,750,000 (1,500,000–2,070,000)	2,330,000 (1,930,000–2,840,000)	2,550,000 (2,110,000–3,100,000)	565 (485–669)	473 (393–574)	430 (356–521)
1-11	Drowning	1,690,000 (1,600,000–1,800,000)	1,110,000 (1,040,000–1,190,000)	904,000 (786,000–1,030,000)	379 (358–401)	206 (192–221)	159 (137–182)
1-12	Fire, heat, and hot substances	807,000 (663,000–969,000)	565,000 (442,000–732,000)	560,000 (429,000–738,000)	208 (171–252)	108 (85–141)	95·5 (73·4–124)
1-13	Poisonings	236,000 (215,000–259,000)	138,000 (122,000–157,000)	129,000 (108,000–152,000)	53·9 (49·4–59)	25·8 (22·8–29·6)	22·5 (18·9–26·8)
1-14	Poisoning by carbon monoxide	41,200 (37,400–46,900)	31,600 (28,300–36,500)	32,200 (28,000–37,800)	9·8 (9–10·9)	5·9 (5·3–6·8)	5·5 (4·8–6·5)
1-15	Poisoning by other means	194,000 (175,000–217,000)	106,000 (91,300–124,000)	96,900 (78,600–118,000)	44·1 (39·9–49)	19·9 (17·2–23·4)	17 (13·8–20·7)
1-16	Exposure to mechanical forces	872,000 (749,000–1,030,000)	933,000 (767,000–1,160,000)	1,030,000 (834,000–1,290,000)	238 (200–288)	176 (144–220)	171 (139–214)
1-17	Unintentional firearm injuries	332,000 (286,000–373,000)	286,000 (247,000–332,000)	286,000 (235,000–354,000)	83·8 (71·7–94·4)	51·6 (44·4–60)	47·3 (38·8–58·6)
1-18	Other exposure to mechanical forces	540,000 (433,000–697,000)	647,000 (500,000–861,000)	741,000 (572,000–978,000)	154 (122–201)	124 (95·9–166)	123 (95·3–162)
1-19	Adverse effects of medical treatment	256,000 (214,000–280,000)	218,000 (199,000–240,000)	230,000 (199,000–263,000)	70·5 (59–76)	44·5 (40·4–48·8)	40·6 (35·1–46·9)
1-20	Animal contact	177,000 (153,000–205,000)	126,000 (106,000–151,000)	128,000 (105,000–157,000)	44·5 (38·2–52·3)	24·5 (20·7–29·4)	22 (18·2–26·9)
1-21	Venomous animal contact	139,000 (122,000–157,000)	86,300 (73,500–102,000)	84,700 (70,000–102,000)	33·6 (29·5–38·2)	16·8 (14·4–19·7)	14·6 (12·1–17·6)
1-22	Non-venomous animal contact	38,500 (29,500–52,700)	39,300 (30,600–53,200)	43,400 (33,000–60,500)	10·9 (8·3–15)	7·7 (6–10·4)	7·4 (5·7–10·3)
1-23	Foreign body	1,510,000 (1,350,000–1,660,000)	987,000 (895,000–1,080,000)	886,000 (740,000–1,060,000)	328 (296–359)	192 (175–211)	166 (138–200)
1-24	Pulmonary aspiration and foreign body in airway	1,410,000 (1,260,000–1,560,000)	902,000 (821,000–989,000)	786,000 (646,000–955,000)	303 (273–331)	176 (160–193)	149 (122–183)
1-25	Foreign body in eyes	29,600 (15,100–51,200)	44,400 (23,700–75,000)	51,000 (27,900–84,500)	8·6 (4·7–14·2)	8·5 (4·6–14·1)	8·5 (4·6–14)
1-26	Foreign body in other body part	65,800 (47,400–89,700)	40,100 (31,300–51,400)	48,900 (37,400–63,000)	16·7 (12·4–22·2)	7·7 (6–9·9)	8·4 (6·4–10·7)
1-27	Environmental heat and cold exposure	143,000 (108,000–189,000)	147,000 (110,000–197,000)	193,000 (134,000–271,000)	44·8 (33·7–59·5)	29 (21·6–38·6)	32·2 (22·5–45)
1-28	Exposure to forces of nature	54,600 (45,700–65,800)	14,100,000 (12,800,000–15,600,000)	126,000 (89,000–176,000)	13·7 (11·4–16·5)	2,600 (2,360–2,860)	20·7 (14·7–29·1)
1-29	Other unintentional injuries	984,000 (914,000–1,070,000)	732,000 (656,000–827,000)	698,000 (601,000–816,000)	254 (234–279)	136 (122–155)	116 (100–136)
1-30	Self-harm and interpersonal violence	7,920,000 (7,740,000–8,140,000)	10,400,000 (10,200,000–10,700,000)	10,800,000 (9,830,000–11,800,000)	2,010 (1,960–2,060)	1,850 (1,800–1,890)	1,750 (1,600–1,920)
1-31	Self-harm	1,130,000 (1,100,000–1,180,000)	1,690,000 (1,630,000–1,740,000)	1,820,000 (1,650,000–2,000,000)	308 (300–321)	305 (294–313)	295 (267–324)
1-32	Self-harm by firearm	232,000 (198,000–261,000)	214,000 (192,000–274,000)	196,000 (166,000–264,000)	61·2 (52·7–69·6)	38·4 (34·5–49·3)	31·7 (26·8–42·7)
1-33	Self-harm by other specified means	899,000 (864,000–956,000)	1,480,000 (1,380,000–1,520,000)	1,620,000 (1,470,000–1,780,000)	246 (236–261)	267 (250–275)	263 (239–289)
1-34	Interpersonal violence	6,250,000 (6,110,000–6,380,000)	8,510,000 (8,290,000–8,720,000)	8,740,000 (7,980,000–9,600,000)	1,570 (1,540–1,610)	1,500 (1,460–1,540)	1,420 (1,300–1,560)
1-35	Physical violence by firearm	3,660,000 (3,590,000–3,730,000)	6,010,000 (5,880,000–6,140,000)	6,100,000 (5,540,000–6,720,000)	911 (892–927)	1,050 (1,030–1,070)	991 (899–1,090)
1-36	Physical violence by sharp object	992,000 (953,000–1,040,000)	1,240,000 (1,180,000–1,320,000)	1,300,000 (1,180,000–1,450,000)	257 (246–271)	220 (210–234)	212 (192–235)
1-37	Sexual violence	65,200 (43,100–92,500)	95,300 (63,800–134,000)	105,000 (70,000–148,000)	17·1 (11·4–24·2)	17·2 (11·5–24·2)	17·1 (11·4–24·2)
1-38	Physical violence by other means	1,530,000 (1,470,000–1,580,000)	1,170,000 (1,090,000–1,240,000)	1,230,000 (1,110,000–1,370,000)	390 (373–405)	214 (200–229)	205 (184–228)
1-39	Conflict and terrorism	535,000 (428,000–697,000)	152,000 (103,000–229,000)	96,700 (56,600–162,000)	126 (100–164)	28·1 (19–42·7)	15·5 (9·1–26)
1-40	Executions and police conflict	5,880 (5,370–6,510)	76,700 (70,400–83,900)	105,000 (74,300–133,000)	1·4 (1·3–1·5)	13·2 (12·1–14·4)	17·1 (12·2–21·8)

SR Table 1: All-age DALYs counts and age standardized DALYs rates per 100,000 individuals for all causes and level 1 cause groups by location for 1990, 2010, and 2019, both sexes combined, Caribbean

Cause Level	Cause	All-age DALYs			Age-standardized rate of DALYs per 100,000		
		1990	2010	2019	1990	2010	2019
0-0	All causes	14,600,000 (13,600,000–15,800,000)	29,500,000 (27,500,000–31,800,000)	16,600,000 (14,500,000–19,000,000)	43,200 (40,100–46,600)	68,200 (63,500–73,400)	34,800 (30,200–39,900)
1-0	Communicable, maternal, neonatal, and nutritional disorders	5,490,000 (5,040,000–6,030,000)	4,430,000 (4,020,000–4,910,000)	3,660,000 (3,140,000–4,250,000)	13,700 (12,600–15,000)	10,600 (9,590–11,700)	8,540 (7,290–9,980)
1-1	HIV/AIDS and sexually transmitted infections	381,000 (276,000–548,000)	900,000 (743,000–1,090,000)	556,000 (454,000–686,000)	1,040 (764–1,500)	2,040 (1,680–2,460)	1,190 (956–1,480)
1-2	HIV/AIDS	303,000 (218,000–468,000)	814,000 (670,000–987,000)	460,000 (385,000–564,000)	852 (617–1,310)	1,830 (1,510–2,230)	949 (794–1,170)
1-3	HIV/AIDS - drug-susceptible tuberculosis	57,900 (38,700–83,000)	58,500 (36,800–90,000)	36,000 (22,900–55,500)	161 (107–229)	132 (82·7–203)	74·3 (47·2–115)
1-4	HIV/AIDS - Multidrug-resistant Tuberculosis without extensive drug resistance	484 (98–1,580)	482 (94–1,580)	460 (96–1,520)	1·3 (0·3–4·4)	1·1 (0·2–3·6)	0·9 (0·2–3·2)
1-5	HIV/AIDS - Extensively drug-resistant Tuberculosis	0 (0–0)	52 (10–182)	75 (15–242)	0 (0–0)	0·1 (0–0·4)	0·2 (0–0·5)
1-6	HIV/AIDS resulting in other diseases	244,000 (162,000–410,000)	755,000 (613,000–925,000)	423,000 (348,000–530,000)	690 (464–1,150)	1,700 (1,370–2,090)	874 (717–1,100)
1-7	Sexually transmitted infections excluding HIV	78,200 (31,000–158,000)	85,700 (36,700–164,000)	95,900 (41,500–187,000)	187 (77·9–372)	208 (87·6–401)	239 (100–470)
1-8	Syphilis	71,000 (24,400–150,000)	75,500 (28,000–154,000)	85,000 (31,000–174,000)	165 (57·8–348)	186 (68·7–380)	217 (78·6–445)
1-9	Chlamydial infection	868 (607–1,240)	1,280 (929–1,750)	1,320 (959–1,820)	2·5 (1·8–3·6)	2·8 (2–3·8)	2·7 (2–3·7)
1-10	Gonococcal infection	995 (767–1,230)	1,530 (1,240–1,900)	1,670 (1,300–2,150)	2·9 (2·2–3·5)	3·3 (2·7–4·2)	3·4 (2·7–4·4)
1-11	Trichomoniasis	1,620 (639–3,290)	2,220 (862–4,580)	2,380 (938–4,940)	5 (1·9–10·2)	4·9 (1·9–10·2)	4·8 (1·9–9·9)
1-12	Genital herpes	1,680 (549–4,140)	2,380 (769–5,840)	2,630 (854–6,420)	5·4 (1·7–13·3)	5·3 (1·7–13)	5·2 (1·7–12·8)
1-13	Other sexually transmitted infections	2,050 (1,500–2,720)	2,780 (2,080–3,670)	2,900 (2,180–3,830)	6 (4·4–8)	6·1 (4·6–8)	6 (4·5–7·9)
1-14	Respiratory infections and tuberculosis	1,240,000 (1,080,000–1,430,000)	838,000 (731,000–960,000)	784,000 (658,000–929,000)	3,270 (2,880–3,710)	2,010 (1,760–2,320)	1,760 (1,470–2,110)
1-15	Tuberculosis	200,000 (177,000–226,000)	113,000 (93,800–135,000)	110,000 (88,600–138,000)	564 (500–633)	259 (215–311)	231 (186–292)
1-16	Latent tuberculosis infection	0 (0–0)	0 (0–0)	0 (0–0)	0 (0–0)	0 (0–0)	0 (0–0)
1-17	Drug-susceptible tuberculosis	199,000 (175,000–224,000)	112,000 (93,200–134,000)	108,000 (87,100–137,000)	560 (497–626)	257 (214–307)	228 (183–290)
1-18	Multidrug-resistant tuberculosis without extensive drug resistance	1,510 (339–4,730)	756 (194–2,300)	1,110 (250–3,500)	4·3 (1–13·3)	1·7 (0·4–5·3)	2·3 (0·5–7·4)
1-19	Extensively drug-resistant tuberculosis	0 (0–0)	80 (18–271)	180 (38–579)	0 (0–0)	0·2 (0–0·6)	0·4 (0·1–1·2)
1-20	Lower respiratory infections	996,000 (855,000–1,170,000)	672,000 (579,000–777,000)	620,000 (507,000–751,000)	2,580 (2,240–2,980)	1,630 (1,400–1,890)	1,410 (1,150–1,720)
1-21	Upper respiratory infections	36,500 (21,300–57,500)	38,200 (23,400–58,500)	40,200 (24,600–61,300)	98·2 (58·4–152)	87·6 (53·8–134)	87·7 (53·6–135)
1-22	Otitis media	12,500 (7,350–20,300)	14,500 (8,560–23,700)	14,800 (8,700–24,200)	33·7 (19·8–54·8)	33·3 (19·7–54·2)	32·4 (19–52·7)
1-23	Enteric infections	1,190,000 (944,000–1,470,000)	609,000 (444,000–794,000)	452,000 (279,000–689,000)	2,890 (2,320–3,540)	1,490 (1,080–1,940)	1,080 (652–1,680)
1-24	Diarrhoeal diseases	1,180,000 (941,000–1,470,000)	607,000 (441,000–792,000)	450,000 (277,000–687,000)	2,880 (2,310–3,530)	1,490 (1,070–1,940)	1,080 (648–1,670)
1-25	Typhoid and paratyphoid	1,470 (764–2,200)	727 (363–1,290)	629 (307–1,140)	3·9 (2–5·7)	1·7 (0·9–3·1)	1·5 (0·7–2·6)
1-26	Typhoid fever	1,390 (709–2,090)	648 (321–1,180)	461 (224–842)	3·7 (1·9–5·5)	1·5 (0·8–2·8)	1·1 (0·5–1·9)
1-27	Paratyphoid fever	72 (28–155)	79 (34–168)	168 (68–361)	0·2 (0·1–0·4)	0·2 (0·1–0·4)	0·4 (0·2–0·9)
1-28	iNTS	496 (278–810)	510 (277–858)	551 (276–965)	1·3 (0·7–2·1)	1·2 (0·7–2)	1·3 (0·6–2·3)
1-29	Other intestinal infectious diseases	1,520 (808–2,350)	775 (362–1,270)	694 (318–1,150)	3·7 (2–5·7)	1·9 (0·9–3·1)	1·7 (0·7–2·8)
1-30	Neglected tropical diseases and malaria	141,000 (98,100–193,000)	204,000 (129,000–333,000)	101,000 (64,300–166,000)	396 (279–535)	464 (293–761)	215 (137–356)
1-31	Malaria	44,000 (17,300–88,500)	125,000 (57,000–251,000)	28,600 (4,680–91,000)	115 (45·5–233)	285 (129–570)	62·1 (10·1–198)
1-32	Chagas disease	268 (182–374)	24 (16–36)	28 (20–45)	1·3 (0·9–1·8)	0·1 (0–0·1)	0·1 (0–0·1)
1-33	Leishmaniasis	126 (16–604)	37 (18–81)	42 (20–81)	0·3 (0–1·5)	0·1 (0–0·2)	0·1 (0–0·2)
1-34	Visceral leishmaniasis	103 (0–579)	10 (0–53)	9 (0–46)	0·3 (0–1·5)	0 (0–0·1)	0 (0–0·1)
1-35	Cutaneous and mucocutaneous leishmaniasis	23 (13–36)	27 (16–41)	33 (19–54)	0·1 (0–0·1)	0·1 (0–0·1)	0·1 (0–0·1)
1-36	African trypanosomiasis	0 (0–0)	0 (0–0)	0 (0–0)	0 (0–0)	0 (0–0)	0 (0–0)
1-37	Schistosomiasis	12,900 (6,920–23,500)	13,700 (6,840–25,800)	14,100 (7,120–26,600)	36·1 (19·8–64·3)	30·4 (15·3–57·1)	29·3 (14·6–55·3)
1-38	Cysticercosis	13,000 (8,510–18,600)	14,600 (9,450–21,200)	16,400 (10,700–24,000)	43·6 (28·5–62·2)	33·5 (21·8–48·3)	32·4 (21·2–47·7)
1-39	Cystic echinococcosis	33 (16–56)	20 (12–28)	22 (15–31)	0·1 (0–0·2)	0 (0–0·1)	0 (0–0·1)
1-40	Lymphatic filariasis	40,600 (27,200–57,500)	15,000 (9,150–23,800)	11,200 (6,680–18,900)	119 (80·3–168)	33·6 (20·4–53·3)	23·5 (13·9–39·5)
1-41	Onchocerciasis	0 (0–0)	0 (0–0)	0 (0–0)	0 (0–0)	0 (0–0)	0 (0–0)
1-42	Trachoma	0 (0–0)	0 (0–0)	0 (0–0)	0 (0–0)	0 (0–0)	0 (0–0)
1-43	Dengue	3,750 (2,280–5,880)	10,700 (5,660–14,200)	6,440 (3,270–9,990)	10·4 (6·4–16·2)	25·1 (12·9–33·5)	14·2 (7·2–21·9)
1-44	Yellow fever	39 (9–115)	14 (3–45)	9 (2–28)	0·1 (0–0·3)	0 (0–0·1)	0 (0–0·1)
1-45	Rabies	285 (86–503)	125 (36–213)	268 (46–438)	0·7 (0·2–1·3)	0·3 (0·1–0·5)	0·6 (0·1–1)
1-46	Intestinal nematode infections	13,700 (9,240–20,000)	12,500 (8,100–18,900)	10,500 (6,580–15,900)	35·2 (23·7–51·9)	28 (18·2–42·3)	22·9 (14·5–34·8)
1-47	Ascariasis	4,120 (2,810–5,950)	1,930 (1,230–2,790)	1,390 (892–2,100)	10·5 (7·2–15·1)	4·6 (2·9–6·7)	3·2 (2·1–4·8)
1-48	Trichuriasis	1,940 (981–3,500)	642 (320–1,120)	627 (304–1,130)	5 (2·5–9·1)	1·5 (0·7–2·5)	1·4 (0·7–2·5)

1-49	Hookworm disease	7,600<br>(4,770–11,600)	9,890<br>(6,070–15,000)	8,450<br>(5,130–13,000)	19·7<br>(12·4–30)	22<br>(13·4–33·3)	18·3<br>(11·1–28·2)
1-50	Food-borne trematodiases	0<br>(0–0)	0<br>(0–0)	0<br>(0–0)	0<br>(0–0)	0<br>(0–0)	0<br>(0–0)
1-51	Leprosy	102<br>(64–154)	127<br>(79–192)	142<br>(91–213)	0·3<br>(0·2–0·5)	0·3<br>(0·2–0·4)	0·3<br>(0·2–0·4)
1-52	Ebola virus disease	0<br>(0–0)	0<br>(0–0)	0<br>(0–0)	0<br>(0–0)	0<br>(0–0)	0<br>(0–0)
1-53	Zika virus disease	0<br>(0–0)	0<br>(0–0)	82<br>(44–138)	0<br>(0–0)	0<br>(0–0)	0·2<br>(0·1–0·3)
1-54	Guinea worm disease	0<br>(0–0)	0<br>(0–0)	0<br>(0–0)	0<br>(0–0)	0<br>(0–0)	0<br>(0–0)
1-55	Other neglected tropical diseases	12,400<br>(8,470–17,300)	11,700<br>(7,760–16,700)	12,700<br>(8,540–18,200)	33<br>(22·6–45·6)	27·8<br>(18·3–39·5)	29·2<br>(19·6–42)
1-56	Other infectious diseases	585,000<br>(447,000–870,000)	321,000<br>(232,000–486,000)	268,000<br>(179,000–414,000)	1,440<br>(1,110–2,120)	785<br>(563–1,190)	649<br>(426–1,010)
1-57	Meningitis	274,000<br>(228,000–326,000)	137,000<br>(109,000–176,000)	106,000<br>(77,600–143,000)	669<br>(558–794)	336<br>(266–432)	260<br>(188–353)
1-58	Encephalitis	33,000<br>(21,700–49,400)	19,100<br>(14,600–30,700)	21,600<br>(16,000–31,700)	83·7<br>(56·3–123)	45·4<br>(34·4–73·8)	50·1<br>(36·6–74·7)
1-59	Diphtheria	3,480<br>(1,450–7,090)	694<br>(316–1,370)	445<br>(228–799)	8·5<br>(3·6–17·1)	1·7<br>(0·8–3·4)	1·1<br>(0·5–2)
1-60	Whooping cough	149,000<br>(30,100–451,000)	98,400<br>(19,400–265,000)	80,500<br>(17,100–224,000)	355<br>(71·9–1,070)	245<br>(48·2–658)	203<br>(43·1–564)
1-61	Tetanus	73,300<br>(42,000–121,000)	23,000<br>(12,700–41,000)	14,700<br>(7,460–31,100)	182<br>(108–290)	56·1<br>(30·7–100)	35·8<br>(17·9–78·1)
1-62	Measles	4,440<br>(3,130–5,920)	1<br>(0–3)	3<br>(1–5)	10·7<br>(7·6–14·3)	0<br>(0–0)	0<br>(0–0)
1-63	Varicella and herpes zoster	8,340<br>(5,620–12,200)	6,910<br>(4,750–9,980)	6,520<br>(4,480–9,430)	22<br>(15·3–31·2)	16·7<br>(11·4–24·4)	15·1<br>(10·1–22·6)
1-64	Acute hepatitis	18,000<br>(12,600–24,100)	8,930<br>(5,920–12,200)	9,150<br>(6,570–11,800)	48·6<br>(35·2–63·7)	20·5<br>(13·6–28·2)	19·9<br>(14·1–25·9)
1-65	Acute hepatitis A	11,700<br>(7,200–17,200)	5,500<br>(3,070–8,270)	4,960<br>(2,780–7,090)	30·8<br>(19·5–44·4)	12·7<br>(7·1–19·2)	11<br>(6·1–16)
1-66	Acute hepatitis B	3,620<br>(2,240–7,360)	2,240<br>(1,260–4,170)	2,610<br>(1,570–4,270)	10<br>(6·3–19·6)	5·1<br>(2·9–9·6)	5·6<br>(3·3–9·3)
1-67	Acute hepatitis C	2,180<br>(1,510–3,610)	845<br>(585–1,390)	1,180<br>(727–1,820)	6·5<br>(4·6–10·5)	1·9<br>(1·3–3·2)	2·4<br>(1·5–3·8)
1-68	Acute hepatitis E	469<br>(320–848)	348<br>(240–555)	401<br>(275–618)	1·3<br>(0·9–2·2)	0·8<br>(0·5–1·3)	0·9<br>(0·6–1·4)
1-69	Other unspecified infectious diseases	22,100<br>(15,800–31,500)	27,100<br>(19,300–36,400)	29,000<br>(21,000–39,300)	61·9<br>(45·5–85·9)	63·2<br>(44·8–85·8)	63·4<br>(45·4–88)
1-70	Maternal and neonatal disorders	1,420,000<br>(1,280,000–1,570,000)	1,270,000<br>(1,110,000–1,440,000)	1,200,000<br>(958,000–1,490,000)	3,360<br>(3,010–3,700)	3,070<br>(2,690–3,510)	2,970<br>(2,350–3,700)
1-71	Maternal disorders	106,000<br>(88,900–130,000)	131,000<br>(102,000–165,000)	120,000<br>(92,800–154,000)	288<br>(240–355)	286<br>(224–361)	249<br>(193–320)
1-72	Maternal haemorrhage	12,800<br>(10,400–15,500)	18,100<br>(13,800–23,500)	16,800<br>(12,600–21,900)	34·3<br>(28–41·8)	39·8<br>(30·2–51·6)	34·9<br>(26·3–45·5)
1-73	Maternal sepsis and other pregnancy related infections	15,400<br>(11,400–20,800)	23,000<br>(16,400–31,600)	21,200<br>(14,500–29,200)	41·6<br>(30·8–56·8)	50·5<br>(35·9–69·4)	44·1<br>(30·2–60·8)
1-74	Maternal hypertensive disorders	14,000<br>(12,100–16,300)	21,300<br>(16,600–27,200)	21,000<br>(16,200–26,800)	36·3<br>(31·2–42·3)	46·7<br>(36·3–59·5)	43·7<br>(33·6–55·9)
1-75	Maternal obstructed labour and uterine rupture	251<br>(149–393)	745<br>(607–915)	849<br>(664–1,070)	0·6<br>(0·4–1)	1·6<br>(1·3–2)	1·8<br>(1·4–2·2)
1-76	Maternal abortive outcome	53,100<br>(41,400–68,400)	36,900<br>(26,700–49,600)	25,700<br>(17,800–35,100)	147<br>(114–191)	80·2<br>(58·3–108)	53·3<br>(36·8–72·7)
1-77	Ectopic pregnancy	546<br>(454–668)	2,800<br>(2,160–3,620)	2,970<br>(2,220–3,880)	1·4<br>(1·2–1·8)	6·2<br>(4·7–8)	6·2<br>(4·6–8·1)
1-78	Indirect maternal deaths	4,120<br>(3,600–4,660)	6,130<br>(5,140–7,360)	7,450<br>(5,760–9,470)	10·5<br>(9·2–11·9)	13·4<br>(11·2–16·1)	15·5<br>(12–19·7)
1-79	Late maternal deaths	2,490<br>(1,890–3,300)	4,040<br>(3,030–5,350)	4,290<br>(3,120–5,900)	6·6<br>(5–8·8)	8·8<br>(6·6–11·7)	8·9<br>(6·5–12·3)
1-80	Maternal deaths aggravated by HIV/AIDS	884<br>(335–1,820)	1,380<br>(716–2,230)	1,290<br>(680–2,070)	2·4<br>(0·9–4·9)	3<br>(1·6–4·9)	2·7<br>(1·4–4·3)
1-81	Other maternal disorders	2,670<br>(2,180–3,270)	16,300<br>(12,300–21,300)	18,400<br>(13,800–24,300)	7·1<br>(5·8–8·7)	35·5<br>(26·7–46·6)	38·3<br>(28·8–50·6)
1-82	Neonatal disorders	1,320,000<br>(1,170,000–1,460,000)	1,140,000<br>(987,000–1,300,000)	1,080,000<br>(844,000–1,360,000)	3,070<br>(2,730–3,410)	2,780<br>(2,410–3,200)	2,730<br>(2,110–3,440)
1-83	Neonatal preterm birth	582,000<br>(510,000–656,000)	441,000<br>(373,000–526,000)	392,000<br>(298,000–519,000)	1,360<br>(1,200–1,530)	1,080<br>(908–1,290)	984<br>(741–1,300)
1-84	Neonatal encephalopathy due to birth asphyxia and trauma	327,000<br>(262,000–407,000)	264,000<br>(205,000–334,000)	254,000<br>(182,000–340,000)	757<br>(607–944)	649<br>(505–823)	644<br>(460–866)
1-85	Neonatal sepsis and other neonatal infections	175,000<br>(142,000–216,000)	217,000<br>(176,000–265,000)	214,000<br>(160,000–284,000)	411<br>(332–511)	527<br>(430–644)	526<br>(391–705)
1-86	Hemolytic disease and other neonatal jaundice	26,900<br>(22,500–32,200)	16,000<br>(13,200–19,500)	14,100<br>(11,100–18,100)	63·7<br>(53·6–76·1)	38·3<br>(31·5–47)	33·8<br>(26·5–43·7)
1-87	Other neonatal disorders	205,000<br>(142,000–276,000)	199,000<br>(130,000–276,000)	210,000<br>(121,000–312,000)	474<br>(328–638)	492<br>(321–680)	538<br>(311–797)
1-88	Nutritional deficiencies	523,000<br>(412,000–668,000)	294,000<br>(225,000–386,000)	292,000<br>(220,000–380,000)	1,340<br>(1,060–1,700)	698<br>(534–911)	664<br>(499–867)
1-89	Protein-energy malnutrition	356,000<br>(267,000–485,000)	120,000<br>(87,800–165,000)	106,000<br>(75,600–151,000)	871<br>(660–1,180)	293<br>(214–406)	254<br>(178–365)
1-90	Iodine deficiency	4,830<br>(2,600–7,870)	5,500<br>(2,990–8,920)	5,150<br>(2,780–8,460)	13·9<br>(7·5–22·5)	12·2<br>(6·7–19·8)	10·7<br>(5·8–17·7)
1-91	Vitamin A deficiency	6,690<br>(4,400–9,640)	5,520<br>(3,540–8,170)	5,290<br>(3,440–7,940)	16·7<br>(11–24·1)	13·4<br>(8·6–19·9)	12·8<br>(8·3–19·1)
1-92	Dietary iron deficiency	144,000<br>(95,200–209,000)	157,000<br>(102,000–230,000)	168,000<br>(110,000–247,000)	405<br>(269–590)	364<br>(236–534)	371<br>(243–549)
1-93	Other nutritional deficiencies	11,400<br>(9,080–14,600)	6,870<br>(5,370–8,770)	7,330<br>(5,730–9,190)	33·1<br>(26·7–41·4)	15·8<br>(12·3–20·2)	15·3<br>(12–19·4)
1-0	Non-communicable diseases	7,340,000<br>(6,560,000–8,190,000)	9,330,000<br>(8,210,000–10,500,000)	10,900,000<br>(9,500,000–12,600,000)	24,400<br>(22,000–26,900)	21,600<br>(19,100–24,300)	22,000<br>(19,100–25,300)
1-1	Neoplasms	980,000<br>(888,000–1,040,000)	1,420,000<br>(1,310,000–1,550,000)	1,720,000<br>(1,480,000–1,990,000)	3,470<br>(3,180–3,660)	3,320<br>(3,050–3,610)	3,380<br>(2,910–3,930)
1-2	Lip and oral cavity cancer	17,000<br>(15,700–18,500)	23,600<br>(21,500–25,900)	30,100<br>(25,700–35,200)	63·4<br>(58·8–69)	55·2<br>(50·3–60·5)	58<br>(49·5–67·8)
1-3	Nasopharynx cancer	3,840<br>(3,400–4,220)	6,500<br>(5,770–7,310)	7,760<br>(6,530–9,120)	13·4<br>(12–14·7)	14·9<br>(13·3–16·8)	15·2<br>(12·7–17·8)
1-4	Other pharynx cancer	9,240<br>(8,710–10,200)	11,000<br>(9,990–12,300)	14,600<br>(12,200–17,300)	34·8<br>(32·7–38·4)	25·6<br>(23·2–28·6)	28·1<br>(23·5–33·2)
1-5	Oesophageal cancer	24,300<br>(22,600–26,500)	36,600<br>(34,000–40,300)	47,300<br>(40,100–54,700)	92·3<br>(86–101)	85·4<br>(79·1–94)	90·7<br>(76·9–105)
1-6	Stomach cancer	71,100<br>(63,800–76,200)	83,600<br>(73,700–93,700)	97,700<br>(82,700–113,000)	266<br>(239–284)	195<br>(172–218)	190<br>(160–220)
1-7	Colon and rectum cancer	75,900<br>(72,000–79,000)	132,000<br>(124,000–141,000)	172,000<br>(147,000–200,000)	286<br>(271–297)	311<br>(291–330)	333<br>(285–388)

1-8	Liver cancer	41,300 (38,200–44,100)	30,000 (26,300–33,900)	41,300 (33,600–50,600)	151 (140–161)	70·2 (61·7–79·3)	80·6 (65·6–99·2)
1-9	Liver cancer due to hepatitis B	13,400 (10,300–17,000)	9,380 (6,920–12,400)	12,700 (8,870–17,500)	48·3 (36·7–62·1)	21·6 (15·9–28·6)	24·8 (17·3–34·1)
1-10	Liver cancer due to hepatitis C	7,940 (5,450–10,700)	5,600 (3,830–7,650)	7,610 (4,940–11,100)	30·7 (21·1–41·4)	13·3 (9·1–18·2)	14·7 (9·6–21·4)
1-11	Liver cancer due to alcohol use	12,600 (9,230–16,100)	9,530 (6,970–12,300)	13,700 (9,780–18,700)	47·4 (34·9–61)	22·5 (16·5–29·1)	26·5 (18·9–36·1)
1-12	Liver cancer due to NASH	3,080 (2,230–4,170)	2,460 (1,790–3,360)	3,520 (2,420–4,980)	11·5 (8·2–15·6)	5·8 (4·2–8)	6·8 (4·7–9·7)
1-13	Gallbladder and biliary tract cancer	17,900 (11,300–20,200)	14,300 (12,000–18,900)	18,700 (15,300–23,400)	67·9 (43·2–76·5)	33·6 (28·2–44·4)	36·2 (29·7–45·2)
1-14	Pancreatic cancer	10,100 (9,580–10,500)	46,500 (43,900–49,300)	60,200 (50,200–70,500)	38·2 (36·3–39·9)	109 (103–116)	116 (96·7–136)
1-15	Larynx cancer	18,800 (17,500–20,000)	30,500 (28,400–32,800)	37,800 (32,100–44,100)	71·3 (66·4–75·7)	71·6 (66·7–77·1)	72·5 (61·5–84·3)
1-16	Tracheal, bronchus, and lung cancer	137,000 (130,000–146,000)	210,000 (199,000–223,000)	254,000 (216,000–299,000)	519 (493–551)	496 (469–525)	489 (416–575)
1-17	Malignant skin melanoma	3,850 (3,200–5,500)	5,770 (4,800–7,520)	7,250 (5,740–9,690)	13·4 (11·2–18·8)	13·2 (11–17·3)	14·3 (11·3–19·1)
1-18	Non-melanoma skin cancer	5,430 (5,090–5,800)	10,900 (9,870–11,800)	13,300 (11,200–15,400)	20·9 (19·5–22·3)	25·5 (23·2–27·6)	25·7 (21·7–29·8)
1-19	Non-melanoma skin cancer (squamous-cell carcinoma)	5,430 (5,080–5,800)	10,900 (9,870–11,800)	13,300 (11,200–15,400)	20·9 (19·5–22·3)	25·5 (23·2–27·6)	25·7 (21·7–29·8)
1-20	Non-melanoma skin cancer (basal-cell carcinoma)	1 (1–2)	2 (1–4)	2 (1–4)	0 (0–0)	0 (0–0)	0 (0–0)
1-21	Breast cancer	85,500 (80,000–91,800)	142,000 (128,000–159,000)	169,000 (140,000–200,000)	311 (292–332)	324 (292–361)	328 (272–389)
1-22	Cervical cancer	77,300 (58,600–90,600)	101,000 (77,900–123,000)	115,000 (86,700–145,000)	270 (206–314)	228 (176–276)	226 (170–287)
1-23	Uterine cancer	16,600 (15,300–18,300)	31,600 (28,800–34,900)	39,700 (33,600–46,800)	61·5 (56·3–67·6)	73·5 (67–81·1)	76·9 (65–90·7)
1-24	Ovarian cancer	5,990 (5,180–9,690)	24,900 (17,000–34,100)	29,300 (21,100–40,900)	21·3 (18·5–34)	56·9 (38·7–77·7)	56·8 (41–79·6)
1-25	Prostate cancer	69,900 (61,600–87,700)	136,000 (107,000–154,000)	167,000 (134,000–205,000)	278 (245–349)	328 (258–373)	323 (259–398)
1-26	Testicular cancer	739 (612–1,060)	1,990 (1,710–2,400)	2,270 (1,820–2,870)	2 (1·7–2·9)	4·5 (3·8–5·5)	4·7 (3·8–6·1)
1-27	Kidney cancer	19,700 (18,200–21,300)	20,700 (19,000–22,600)	28,300 (23,100–33,700)	65·7 (61·7–69·9)	48·3 (44·3–52·9)	56·5 (46·1–67·4)
1-28	Bladder cancer	14,300 (13,300–15,200)	23,300 (21,600–24,900)	28,600 (24,800–33,100)	55·5 (51·8–59)	55·5 (51·6–59·3)	55·3 (47·9–64)
1-29	Brain and nervous system cancer	14,700 (9,180–32,100)	41,000 (21,000–56,300)	44,500 (23,200–62,000)	42·7 (27·5–91·2)	95·2 (48·7–132)	93·3 (48·9–132)
1-30	Thyroid cancer	4,330 (3,850–4,790)	6,530 (5,750–7,360)	8,610 (7,130–10,100)	15·6 (14–17·2)	15·1 (13·4–17)	16·8 (13·9–19·8)
1-31	Mesothelioma	1,620 (1,330–2,110)	2,330 (1,950–2,970)	2,750 (2,170–3,580)	5·7 (4·7–7·3)	5·3 (4·4–6·7)	5·3 (4·2–7)
1-32	Hodgkin lymphoma	4,440 (3,870–5,870)	8,240 (5,350–9,600)	8,560 (5,990–10,400)	13·5 (11·8–17·7)	18·5 (12–21·6)	17·3 (12·2–20·9)
1-33	Non-Hodgkin's lymphoma	45,400 (40,000–50,900)	48,900 (44,100–54,700)	58,900 (49,200–69,500)	144 (129–158)	113 (102–126)	119 (99·1–141)
1-34	Multiple myeloma	12,500 (11,000–15,300)	22,500 (20,000–26,400)	28,900 (24,000–34,500)	47·6 (41·8–57·9)	53 (47·2–62·1)	55·5 (46·2–66·5)
1-35	Leukaemia	85,900 (64,800–119,000)	84,100 (66,800–110,000)	93,100 (71,600–119,000)	242 (189–324)	196 (155–259)	200 (151–261)
1-36	Acute lymphoid leukaemia	22,100 (15,500–34,100)	20,700 (14,800–29,000)	22,400 (15,600–31,600)	56·4 (40·1–85·7)	49 (34·8–69·4)	51·7 (35·3–73·9)
1-37	Chronic lymphoid leukaemia	2,300 (2,050–2,560)	3,520 (3,210–3,870)	4,340 (3,630–5,180)	8·7 (7·8–9·7)	8·4 (7·6–9·2)	8·4 (7–10)
1-38	Acute myeloid leukaemia	15,600 (13,200–19,800)	18,600 (15,700–22,500)	21,700 (17,400–26,600)	44·8 (38·5–55·5)	42·8 (36–52·1)	45·7 (36–56·3)
1-39	Chronic myeloid leukaemia	8,920 (6,190–15,900)	8,000 (6,120–12,000)	8,270 (6,170–12,000)	27·6 (20·5–44·8)	18·6 (14·1–28·6)	17 (12·4–25·8)
1-40	Other leukaemia	37,000 (20,900–59,000)	33,300 (21,800–46,900)	36,400 (24,000–50,200)	104 (63·6–159)	77·6 (50·3–110)	77·6 (49·7–109)
1-41	Other neoplasms	2,970 (2,490–3,950)	5,060 (4,120–5,860)	6,420 (5,180–7,740)	10 (8·5–13)	12 (9·7–13·9)	12·9 (10·4–15·6)
1-42	Myelodysplastic, myeloproliferative, and other hematopoietic neoplasms	2,970 (2,490–3,950)	5,060 (4,120–5,860)	6,420 (5,180–7,740)	10 (8·5–13)	12 (9·7–13·9)	12·9 (10·4–15·6)
1-43	Benign and in situ intestinal neoplasms	0 (0–0)	0 (0–0)	0 (0–0)	0 (0–0)	0 (0–0)	0 (0–0)
1-44	Benign and in situ cervical and uterine neoplasms	0 (0–0)	0 (0–0)	0 (0–0)	0 (0–0)	0 (0–0)	0 (0–0)
1-45	Other benign and in situ neoplasms	0 (0–0)	0 (0–0)	0 (0–0)	0 (0–0)	0 (0–0)	0 (0–0)
1-46	Cardiovascular diseases	1,840,000 (1,750,000–1,940,000)	2,180,000 (1,960,000–2,400,000)	2,650,000 (2,300,000–3,020,000)	6,870 (6,520–7,230)	5,110 (4,610–5,630)	5,170 (4,490–5,910)
1-47	Rheumatic heart disease	79,400 (63,800–97,300)	66,900 (50,000–87,900)	68,600 (50,900–90,800)	235 (190–284)	150 (112–197)	142 (105–190)
1-48	Ischaemic heart disease	900,000 (857,000–944,000)	1,010,000 (913,000–1,110,000)	1,240,000 (1,070,000–1,430,000)	3,490 (3,310–3,660)	2,380 (2,160–2,620)	2,410 (2,080–2,780)
1-49	Stroke	589,000 (548,000–633,000)	708,000 (626,000–808,000)	850,000 (726,000–984,000)	2,170 (2,010–2,330)	1,660 (1,480–1,890)	1,660 (1,420–1,920)
1-50	Ischaemic stroke	205,000 (186,000–228,000)	280,000 (253,000–315,000)	340,000 (295,000–390,000)	807 (733–892)	671 (607–755)	662 (575–760)
1-51	Intracerebral hemorrhage	317,000 (290,000–347,000)	350,000 (300,000–410,000)	418,000 (349,000–497,000)	1,140 (1,050–1,250)	815 (700–954)	817 (682–971)
1-52	Subarachnoid hemorrhage	67,100 (45,800–88,000)	78,200 (58,100–103,000)	91,600 (67,000–123,000)	222 (154–290)	179 (133–235)	184 (133–250)
1-53	Hypertensive heart disease	101,000 (86,700–120,000)	155,000 (131,000–182,000)	192,000 (158,000–229,000)	388 (335–467)	365 (308–429)	372 (306–444)
1-54	Non-rheumatic valvular heart disease	10,700 (9,220–12,100)	14,500 (12,700–16,800)	18,200 (14,800–22,000)	37·4 (32·5–41·9)	33·4 (29·3–38·6)	35·7 (29–43·1)
1-55	Non-rheumatic calcific aortic valve disease	6,310 (5,370–7,230)	8,920 (7,820–10,500)	11,300 (9,160–14,200)	22·6 (19·3–25·7)	20·8 (18·3–24·4)	22·1 (17·9–27·7)
1-56	Non-rheumatic degenerative mitral valve disease	4,150 (3,560–5,020)	5,280 (4,450–6,640)	6,550 (5,220–8,360)	14·1 (12·1–16·8)	12 (10·1–15)	12·9 (10·3–16·5)
1-57	Other non-rheumatic valve diseases	224 (162–342)	280 (194–439)	357 (256–538)	0·7 (0·5–1·1)	0·6 (0·4–1)	0·7 (0·5–1·1)
1-58	Cardiomyopathy and myocarditis	52,600 (39,000–76,300)	69,300 (55,100–90,700)	83,700 (64,500–109,000)	166 (128–231)	161 (128–212)	172 (130–228)
1-59	Myocarditis	7,020 (3,070–18,100)	6,960 (3,760–14,000)	7,620 (4,350–14,200)	18·7 (9–45)	16·5 (8·7–34·1)	17·1 (9·3–33·5)
1-60	Alcoholic cardiomyopathy	9,470 (7,100–13,000)	18,400 (15,800–23,100)	23,600 (18,900–28,900)	33·8 (25·6–46·6)	42 (36·1–52·5)	45·5 (36·4–55·8)

1-61	Other cardiomyopathy	36,100 (27,100–53,400)	43,900 (33,600–59,900)	52,500 (39,300–71,300)	113 (87·9–156)	103 (78·3–141)	109 (80·2–151)
1-62	Atrial fibrillation and flutter	18,700 (15,800–22,500)	33,100 (27,900–39,500)	43,200 (36,100–51,700)	79·3 (66·4–94·6)	79·5 (66·9–94·8)	83·2 (69·6–99·8)
1-63	Aortic aneurysm	16,000 (14,600–17,500)	23,700 (21,600–26,100)	29,500 (24,600–34,500)	61·1 (55·9–66·8)	56·3 (51·5–62·1)	57·3 (47·8–66·8)
1-64	Peripheral vascular disease	7,120 (4,170–12,100)	12,600 (7,430–20,900)	17,100 (9,850–28,100)	29 (17–49·2)	30·3 (17·9–50·1)	32·8 (19–54·2)
1-65	Endocarditis	9,700 (7,330–14,100)	13,000 (10,500–16,300)	15,400 (12,000–19,700)	28·5 (22·7–39·8)	29·9 (24·1–37·9)	31·9 (24·6–41·7)
1-66	Chronic respiratory diseases	347,000 (287,000–414,000)	413,000 (347,000–478,000)	466,000 (389,000–539,000)	1,080 (916–1,260)	972 (818–1,130)	960 (798–1,120)
1-67	Chronic obstructive pulmonary disease	109,000 (96,900–122,000)	194,000 (170,000–217,000)	238,000 (205,000–270,000)	411 (368–456)	461 (405–515)	463 (400–528)
1-68	Pneumoconiosis	458 (302–611)	467 (309–672)	563 (374–821)	1·6 (1·1–2·2)	1·1 (0·7–1·6)	1·1 (0·7–1·6)
1-69	Silicosis	138 (68–237)	134 (72–238)	160 (87–286)	0·5 (0·3–0·8)	0·3 (0·2–0·6)	0·3 (0·2–0·6)
1-70	Asbestosis	73 (51–104)	80 (56–120)	106 (69–154)	0·3 (0·2–0·4)	0·2 (0·1–0·3)	0·2 (0·1–0·3)
1-71	Coal workers pneumoconiosis	57 (28–98)	57 (30–97)	66 (33–113)	0·2 (0·1–0·4)	0·1 (0·1–0·2)	0·1 (0·1–0·2)
1-72	Other pneumoconiosis	191 (127–272)	196 (128–290)	232 (150–346)	0·7 (0·5–0·9)	0·5 (0·3–0·7)	0·5 (0·3–0·7)
1-73	Asthma	217,000 (163,000–276,000)	189,000 (142,000–243,000)	193,000 (146,000–250,000)	609 (462–764)	441 (330–570)	423 (316–553)
1-74	Interstitial lung disease and pulmonary sarcoidosis	6,060 (4,900–8,490)	11,800 (8,940–14,900)	14,500 (10,400–19,000)	20·9 (17·2–28)	27·7 (21–35)	28·9 (20·7–38·2)
1-75	Other chronic respiratory diseases	14,600 (7,260–29,600)	17,500 (11,500–30,000)	19,900 (13,600–31,100)	39·6 (21–76·5)	41·3 (26·3–72)	44·1 (29·1–70·9)
1-76	Digestive diseases	499,000 (411,000–589,000)	513,000 (423,000–606,000)	619,000 (502,000–736,000)	1,620 (1,370–1,860)	1,180 (975–1,400)	1,250 (1,000–1,490)
1-77	Cirrhosis and other chronic liver diseases	203,000 (173,000–232,000)	212,000 (174,000–251,000)	278,000 (219,000–341,000)	701 (606–785)	486 (400–577)	546 (429–672)
1-78	Cirrhosis and other chronic liver diseases due to hepatitis B	23,100 (16,100–32,000)	20,200 (13,300–29,700)	24,200 (15,300–36,000)	82·7 (57·2–114)	45·6 (29·9–66·7)	47 (29·6–69·9)
1-79	Cirrhosis and other chronic liver diseases due to hepatitis C	35,500 (25,500–47,800)	39,800 (27,300–55,000)	52,400 (36,000–76,000)	126 (90·7–171)	90·4 (62·7–125)	102 (69·3–147)
1-80	Cirrhosis and other chronic liver diseases due to alcohol use	67,500 (52,200–82,600)	81,400 (62,500–102,000)	111,000 (81,500–143,000)	248 (191–302)	187 (143–235)	214 (158–275)
1-81	Cirrhosis and other chronic liver diseases due to NAFLD	25,500 (18,000–35,000)	30,600 (21,200–43,100)	41,500 (28,000–60,000)	92·6 (64·8–126)	70·5 (49·2–99·4)	80·6 (54·5–116)
1-82	Cirrhosis and other chronic liver diseases due to other causes	51,500 (35,600–71,400)	39,900 (26,500–54,700)	49,400 (33,500–68,000)	152 (107–206)	93·1 (61·3–128)	102 (68·8–142)
1-83	Upper digestive system diseases	104,000 (80,500–140,000)	116,000 (84,000–162,000)	129,000 (93,200–179,000)	349 (274–463)	264 (193–368)	257 (184–355)
1-84	Peptic ulcer disease	50,900 (42,000–61,700)	40,600 (33,500–48,900)	44,100 (36,200–53,500)	176 (150–209)	94·5 (78–114)	87·4 (71·2–107)
1-85	Gastritis and duodenitis	15,000 (11,000–19,700)	20,100 (14,800–26,800)	23,400 (17,000–31,000)	49·9 (37·1–65·4)	46·1 (34·1–61·4)	46·7 (33·9–62·1)
1-86	GERD	38,400 (20,000–68,200)	54,900 (28,500–96,700)	61,600 (32,200–109,000)	123 (64·1–217)	123 (63·9–218)	123 (64–217)
1-87	Appendicitis	29,000 (17,500–46,500)	17,300 (10,800–26,200)	19,800 (14,200–27,800)	78·6 (49·3–123)	40·4 (25·1–62·5)	43·3 (30·3–61·7)
1-88	Paralytic ileus and intestinal obstruction	48,500 (34,000–73,800)	42,600 (31,600–62,800)	46,600 (34,200–65,900)	134 (99·9–193)	102 (75·2–152)	103 (73·3–150)
1-89	Inguinal, femoral, and abdominal hernia	45,000 (27,900–67,800)	40,900 (28,200–57,200)	44,200 (31,400–60,100)	124 (80·6–181)	95·4 (65·3–134)	94·8 (66·4–130)
1-90	Inflammatory bowel disease	7,450 (5,010–11,800)	7,790 (5,710–10,300)	8,900 (6,520–11,700)	23·4 (16·7–34·1)	18 (13–24·1)	18·2 (13·1–24·8)
1-91	Vascular intestinal disorders	7,870 (6,910–9,070)	10,000 (9,150–11,300)	12,500 (10,400–15,200)	29·6 (26·1–33·7)	23·8 (21·6–26·7)	24·4 (20·3–29·6)
1-92	Gallbladder and biliary diseases	27,500 (22,100–34,000)	29,300 (22,800–37,200)	33,900 (26,500–42,600)	94·5 (76·1–116)	67·1 (52·5–84·9)	67·1 (52·5–84·5)
1-93	Pancreatitis	13,200 (11,700–15,600)	17,400 (15,100–20,200)	20,300 (16,900–24,600)	44·5 (39·3–51·6)	39·4 (34·2–45·7)	40·1 (33·3–48·7)
1-94	Other digestive diseases	13,600 (8,460–23,100)	20,400 (15,500–28,000)	25,100 (19,100–32,900)	39·8 (26·4–64·7)	48·3 (36·6–67·4)	52·4 (39–71·2)
1-95	Neurological disorders	403,000 (233,000–652,000)	544,000 (318,000–891,000)	625,000 (371,000–1,010,000)	1,280 (760–2,060)	1,240 (737–2,030)	1,270 (753–2,040)
1-96	Alzheimer's disease and other dementias	67,600 (28,100–156,000)	126,000 (52,300–287,000)	158,000 (66,500–348,000)	303 (125–702)	303 (126–693)	304 (128–671)
1-97	Parkinson's disease	17,200 (16,000–18,700)	31,500 (28,600–34,400)	40,600 (35,800–45,800)	69·2 (63·9–75·1)	76·4 (69·3–83·3)	79 (69·7–89·1)
1-98	Idiopathic epilepsy	96,700 (68,000–131,000)	94,600 (67,800–126,000)	107,000 (75,800–146,000)	269 (191–361)	216 (154–288)	229 (160–310)
1-99	Multiple sclerosis	2,980 (2,560–3,600)	4,910 (3,990–5,900)	5,730 (4,630–7,080)	10 (8·7–12·1)	11 (8·9–13·2)	11·3 (9·1–14)
1-100	Motor neuron disease	2,400 (2,030–2,940)	6,000 (5,450–6,760)	8,450 (6,860–10,300)	7·6 (6·7–8·9)	14 (12·6–15·8)	16·9 (13·7–20·9)
1-101	Headache disorders	191,000 (34,600–425,000)	250,000 (49,100–552,000)	268,000 (53,700–587,000)	552 (109–1,210)	552 (109–1,210)	551 (108–1,210)
1-102	Migraine	174,000 (23,100–402,000)	226,000 (33,100–523,000)	242,000 (36,700–554,000)	500 (72·6–1,150)	500 (73·6–1,150)	499 (73·5–1,140)
1-103	Tension-type headache	17,200 (4,910–66,500)	23,500 (6,860–85,300)	25,700 (7,590–92,600)	52·1 (15·7–192)	52·2 (15·2–189)	52·2 (15·1–187)
1-104	Other neurological disorders	25,800 (20,600–31,500)	30,800 (24,900–38,400)	36,900 (29,200–46,900)	71·4 (57·7–85·9)	70·7 (57·3–88·1)	79·3 (62·3–102)
1-105	Mental disorders	624,000 (457,000–821,000)	793,000 (580,000–1,040,000)	870,000 (634,000–1,140,000)	1,850 (1,350–2,430)	1,760 (1,290–2,310)	1,780 (1,290–2,340)
1-106	Schizophrenia	55,600 (39,000–73,900)	78,500 (54,500–104,000)	86,600 (60,400–115,000)	174 (122–229)	174 (121–231)	174 (121–231)
1-107	Depressive disorders	241,000 (165,000–335,000)	292,000 (202,000–404,000)	327,000 (226,000–451,000)	737 (503–1,020)	650 (449–900)	657 (454–906)
1-108	Major depressive disorder	212,000 (143,000–302,000)	251,000 (171,000–347,000)	282,000 (190,000–392,000)	647 (435–907)	559 (382–772)	567 (382–792)
1-109	Dysthymia	29,100 (17,700–44,800)	40,500 (25,200–62,000)	44,800 (28,200–68,100)	90·2 (56·2–138)	90·1 (56·3–137)	90 (56·3–137)
1-110	Bipolar disorder	68,300 (39,500–106,000)	89,900 (52,300–141,000)	96,700 (56,400–149,000)	199 (116–310)	198 (115–310)	196 (115–303)
1-111	Anxiety disorders	140,000 (92,600–198,000)	186,000 (124,000–259,000)	204,000 (135,000–287,000)	409 (272–573)	412 (276–575)	420 (279–587)
1-112	Eating disorders	15,100 (9,230–22,500)	18,200 (11,300–27,500)	19,700 (11,900–29,800)	39·2 (23·8–58·8)	39·6 (24·6–59·7)	41·2 (25–62·2)
1-113	Anorexia nervosa	3,830 (2,230–6,180)	4,460 (2,670–7,060)	4,750 (2,780–7,640)	9·6 (5·6–15·3)	9·6 (5·8–15·2)	10·1 (5·9–16·1)

1-114	Bulimia nervosa	11,300 (6,490–18,000)	13,700 (7,910–21,800)	14,900 (8,510–23,500)	29·6 (16·9–46·6)	30 (17·2–47·5)	31·2 (17·7–49)
1-115	Autism spectrum disorders	19,100 (12,500–27,500)	23,100 (15,000–33,400)	24,300 (15,900–35,200)	52·5 (34·5–75·8)	52·5 (34–76)	52·4 (34·2–75·9)
1-116	Attention-deficit/hyperactivity disorder	14,400 (8,170–24,100)	16,600 (9,380–27,600)	16,900 (9,610–28,500)	37 (21·1–62)	37·1 (21·1–61·8)	37·2 (21·1–63)
1-117	Conduct disorder	27,500 (15,500–43,800)	30,000 (17,100–47,400)	29,400 (16,400–46,700)	67·4 (37·9–108)	67·8 (38·4–107)	67·9 (38–108)
1-118	Idiopathic developmental intellectual disability	8,570 (3,320–15,600)	11,100 (4,500–19,800)	10,800 (4,120–19,500)	23·1 (9–42·3)	25·3 (10·2–45)	23·6 (9–42·6)
1-119	Other mental disorders	33,600 (21,300–51,000)	48,300 (30,900–73,500)	54,100 (34,700–82,100)	108 (69–164)	108 (69·2–164)	108 (69·2–164)
1-120	Substance use disorders	117,000 (91,100–146,000)	163,000 (129,000–202,000)	176,000 (139,000–217,000)	356 (280–440)	360 (287–446)	353 (278–435)
1-121	Alcohol use disorders	75,900 (59,800–95,800)	109,000 (85,500–137,000)	121,000 (96,000–150,000)	241 (193–302)	241 (190–305)	240 (191–299)
1-122	Drug use disorders	41,300 (29,600–55,200)	54,300 (40,600–69,700)	55,200 (41,100–71,100)	114 (82·7–152)	119 (88·9–152)	113 (83·6–145)
1-123	Opioid use disorders	19,800 (13,500–27,700)	22,700 (16,200–30,200)	23,700 (16,800–31,700)	57·5 (39·8–78·5)	50 (35·7–66·8)	47·9 (34·1–64·1)
1-124	Cocaine use disorders	7,270 (4,700–10,500)	12,600 (9,100–16,800)	11,600 (8,040–16,100)	19·5 (13–28)	27·4 (19·8–36·5)	23·9 (16·5–33·2)
1-125	Amphetamine use disorders	2,410 (1,350–3,950)	3,480 (2,220–5,340)	3,530 (2,220–5,470)	6·3 (3·6–10·1)	7·6 (4·9–11·6)	7·2 (4·5–11·2)
1-126	Cannabis use disorders	5,100 (2,810–8,700)	5,850 (3,370–9,820)	6,240 (3,510–10,500)	13·1 (7·3–21·9)	12·6 (7·2–21)	13 (7·3–22)
1-127	Other drug use disorders	6,620 (4,380–9,420)	9,730 (6,770–13,100)	10,100 (7,070–13,800)	17·8 (12–25·1)	21·2 (14·8–28·6)	20·8 (14·5–28·4)
1-128	Diabetes and kidney diseases	584,000 (522,000–652,000)	948,000 (819,000–1,100,000)	1,210,000 (1,030,000–1,410,000)	2,110 (1,890–2,360)	2,210 (1,910–2,560)	2,360 (2,020–2,750)
1-129	Diabetes mellitus	411,000 (357,000–471,000)	632,000 (523,000–755,000)	815,000 (674,000–990,000)	1,520 (1,320–1,750)	1,480 (1,220–1,760)	1,580 (1,310–1,920)
1-130	Diabetes mellitus type 1	37,300 (29,000–43,400)	41,600 (34,500–49,000)	48,200 (38,600–58,200)	116 (90–135)	94·5 (78·4–111)	99·4 (79·6–120)
1-131	Diabetes mellitus type 2	374,000 (325,000–432,000)	590,000 (485,000–707,000)	767,000 (634,000–931,000)	1,410 (1,220–1,620)	1,380 (1,140–1,650)	1,480 (1,230–1,800)
1-132	Chronic kidney disease	172,000 (158,000–190,000)	316,000 (285,000–352,000)	392,000 (338,000–454,000)	587 (542–647)	730 (660–815)	775 (667–896)
1-133	Chronic kidney disease due to diabetes mellitus type 1	12,600 (8,770–17,200)	23,300 (15,500–33,200)	28,400 (18,700–40,700)	42·7 (29·2–59)	52·1 (35–74·4)	56·1 (37·5–79·9)
1-134	Chronic kidney disease due to diabetes mellitus type 2	31,700 (25,300–38,200)	65,900 (52,400–79,500)	85,200 (66,800–105,000)	121 (97–146)	155 (124–188)	164 (129–203)
1-135	Chronic kidney disease due to hypertension	28,000 (23,100–33,600)	60,000 (49,600–71,600)	76,500 (62,100–93,600)	106 (88–127)	141 (116–168)	148 (120–182)
1-136	Chronic kidney disease due to glomerulonephritis	43,500 (36,500–52,600)	73,500 (60,200–89,200)	89,100 (69,400–111,000)	139 (116–167)	168 (137–203)	179 (140–223)
1-137	Chronic kidney disease due to other and unspecified causes	55,800 (46,100–66,000)	92,700 (76,400–112,000)	113,000 (89,500–140,000)	178 (147–212)	214 (177–259)	227 (181–280)
1-138	Acute glomerulonephritis	1,080 (647–1,670)	752 (419–1,250)	782 (423–1,370)	3 (1·9–4·5)	1·8 (1–3)	1·7 (0·9–3·1)
1-139	Skin and subcutaneous diseases	194,000 (131,000–285,000)	243,000 (164,000–355,000)	264,000 (181,000–380,000)	555 (381–815)	557 (378–816)	562 (386–809)
1-140	Dermatitis	42,100 (24,600–67,000)	49,800 (29,800–78,400)	52,600 (31,200–82,400)	116 (68·3–182)	115 (68·8–182)	115 (67·9–182)
1-141	Atopic dermatitis	32,200 (17,300–53,700)	36,300 (19,500–61,500)	37,600 (20,200–62,900)	85 (45·8–142)	84·9 (45·5–144)	84·8 (45·7–142)
1-142	Contact dermatitis	8,690 (5,450–13,100)	12,000 (7,510–17,900)	13,500 (8,330–20,100)	27·1 (16·8–40·6)	27·1 (16·9–40·3)	27 (16·9–40·3)
1-143	Seborrhoeic dermatitis	1,130 (635–1,790)	1,490 (857–2,380)	1,640 (925–2,570)	3·4 (1·9–5·4)	3·4 (1·9–5·4)	3·4 (1·9–5·3)
1-144	Psoriasis	12,700 (8,990–16,900)	16,300 (11,500–21,600)	17,500 (12,500–23,500)	38·8 (27·8–51·3)	36·8 (26·1–48·9)	35·8 (25·5–47·9)
1-145	Bacterial skin diseases	12,300 (9,010–19,600)	18,500 (14,300–28,100)	23,100 (17,800–34,400)	36·7 (27·8–58·2)	43·4 (33·6–66·8)	47·9 (36·7–71·9)
1-146	Cellulitis	4,130 (2,250–7,910)	5,030 (3,280–8,780)	6,070 (3,970–10,200)	12·5 (7·1–23)	11·8 (7·7–20·8)	12·6 (8·1–21·4)
1-147	Pyoderma	8,120 (5,560–14,200)	13,400 (10,200–20,400)	17,000 (12,800–25,300)	24·1 (17·2–39·5)	31·6 (23·9–48·3)	35·4 (26·4–54·6)
1-148	Scabies	31,000 (16,900–49,600)	36,900 (20,200–58,600)	38,500 (21,200–61,300)	83·9 (46·2–134)	83·8 (46·1–133)	83·5 (45·8–133)
1-149	Fungal skin diseases	14,100 (5,640–29,900)	19,200 (7,750–40,500)	21,600 (8,700–45,700)	44·4 (17·9–93·9)	44·2 (17·8–93·5)	44·1 (17·7–93·5)
1-150	Viral skin diseases	18,200 (11,500–27,300)	20,200 (12,800–30,300)	20,800 (13,400–31,100)	47·5 (30·2–71·1)	47·5 (30·1–71·1)	47·4 (30·4–70·8)
1-151	Acne vulgaris	16,900 (10,100–26,900)	21,600 (12,900–34,800)	22,100 (13,100–35,700)	41·1 (24·5–65·1)	46·9 (27·9–75·5)	48·4 (28·6–78·2)
1-152	Alopecia areata	2,440 (1,550–3,630)	3,180 (2,030–4,760)	3,450 (2,170–5,110)	7·1 (4·5–10·6)	7·1 (4·5–10·6)	7·1 (4·5–10·5)
1-153	Pruritus	2,670 (1,280–4,740)	3,650 (1,740–6,510)	4,090 (1,970–7,310)	8·2 (3·9–14·7)	8·3 (4–14·9)	8·4 (4–14·9)
1-154	Urticaria	17,700 (11,500–25,800)	20,800 (13,500–30,100)	21,800 (14,400–31,400)	48·1 (31·5–69·8)	48 (31·2–69·3)	48 (31·5–69·1)
1-155	Decubitus ulcer	8,970 (6,400–11,600)	11,300 (8,380–14,600)	13,700 (10,000–17,600)	34·9 (24·7–44·2)	26·7 (20–34·7)	26·8 (19·6–34·4)
1-156	Other skin and subcutaneous diseases	15,300 (7,980–27,000)	21,300 (10,700–37,700)	24,200 (12,100–43,300)	48·9 (25·7–86·4)	48·8 (24·7–86·6)	49·1 (24·4–87·6)
1-157	Sense organ diseases	237,000 (162,000–336,000)	346,000 (235,000–494,000)	400,000 (271,000–567,000)	852 (582–1,210)	807 (547–1,150)	786 (532–1,110)
1-158	Blindness and vision loss	77,000 (53,500–107,000)	107,000 (73,500–151,000)	122,000 (84,000–172,000)	282 (196–390)	251 (172–353)	239 (165–338)
1-159	Glaucoma	4,750 (3,120–6,880)	6,230 (4,170–8,770)	7,210 (4,810–10,400)	19·6 (13–28·2)	15·1 (10·1–21·3)	13·9 (9·3–20·1)
1-160	Cataract	14,000 (9,860–19,200)	19,400 (13,600–26,000)	22,100 (15,500–30,200)	56·3 (39·6–76·8)	46·4 (32·6–62·1)	42·9 (30–58·4)
1-161	Age-related macular degeneration	505 (338–729)	724 (495–1,020)	887 (591–1,280)	2 (1·4–2·9)	1·7 (1·2–2·5)	1·7 (1·1–2·5)
1-162	Refraction disorders	22,700 (15,100–33,100)	29,500 (19,600–42,600)	32,000 (21,100–45,700)	71·1 (47·2–102)	67·2 (44·5–96·7)	65·2 (42·8–93·5)
1-163	Near vision loss	14,100 (6,500–27,500)	21,900 (10,200–43,400)	26,000 (12,100–50,900)	53·9 (25·1–106)	51·5 (24·1–102)	50·1 (23·4–98·4)
1-164	Other vision loss	21,100 (14,800–29,000)	29,400 (20,700–40,400)	33,500 (23,700–46,100)	78·9 (55·4–108)	69·2 (48·5–95·1)	65·3 (46·2–90·1)
1-165	Age-related and other hearing loss	149,000 (99,900–218,000)	223,000 (148,000–324,000)	260,000 (173,000–374,000)	535 (357–772)	520 (347–753)	510 (340–733)
1-166	Other sense organ diseases	10,700 (6,320–16,500)	15,600 (9,240–24,200)	18,100 (10,700–28,100)	35·9 (21·1–56)	36·2 (21·4–56·1)	36·3 (21·7–56)

1-167	Musculoskeletal disorders	436,000 (317,000–575,000)	666,000 (483,000–881,000)	772,000 (556,000–1,020,000)	1,450 (1,050–1,910)	1,510 (1,090–1,990)	1,530 (1,100–2,010)
1-168	Rheumatoid arthritis	9,080 (6,710–11,600)	15,300 (11,200–19,700)	18,400 (13,500–23,500)	31·7 (23·5–40·1)	34·8 (25·5–44·6)	36·1 (26·6–46·3)
1-169	Osteoarthritis	49,400 (24,700–98,900)	85,800 (42,900–173,000)	106,000 (53,400–214,000)	189 (94·8–378)	201 (100–405)	204 (103–410)
1-170	Low back pain	230,000 (162,000–306,000)	326,000 (229,000–435,000)	365,000 (257,000–486,000)	741 (524–986)	735 (515–980)	728 (513–965)
1-171	Neck pain	40,200 (26,800–58,000)	60,200 (39,800–87,100)	68,000 (45,100–98,500)	135 (89·7–194)	135 (89·2–195)	135 (89·5–194)
1-172	Gout	1,860 (1,170–2,680)	3,320 (2,070–4,740)	4,010 (2,510–5,760)	6·7 (4·2–9·6)	7·6 (4·7–10·9)	7·8 (4·9–11·3)
1-173	Other musculoskeletal disorders	106,000 (76,000–141,000)	176,000 (126,000–238,000)	211,000 (150,000–284,000)	346 (247–463)	394 (282–532)	418 (299–566)
1-174	Other non-communicable diseases	1,070,000 (925,000–1,250,000)	1,100,000 (911,000–1,340,000)	1,150,000 (913,000–1,450,000)	2,870 (2,450–3,350)	2,600 (2,150–3,140)	2,580 (2,030–3,270)
1-175	Congenital anomalies	574,000 (488,000–668,000)	499,000 (390,000–634,000)	469,000 (330,000–674,000)	1,360 (1,160–1,590)	1,220 (950–1,550)	1,160 (819–1,680)
1-176	Neural tube defects	80,700 (34,100–145,000)	58,900 (22,600–120,000)	58,700 (20,200–135,000)	190 (79·7–340)	145 (55·4–297)	148 (50·8–339)
1-177	Congenital heart anomalies	234,000 (161,000–329,000)	209,000 (132,000–284,000)	188,000 (106,000–280,000)	553 (382–774)	512 (322–698)	469 (261–697)
1-178	Orofacial clefts	2,560 (1,490–5,170)	1,940 (1,200–3,250)	2,010 (1,260–3,310)	6·3 (3·8–12·4)	4·6 (2·8–7·7)	4·5 (2·8–7·7)
1-179	Down's syndrome	18,000 (10,300–33,400)	20,600 (13,000–30,700)	21,200 (11,300–35,100)	45·2 (26·3–81·5)	49·1 (30·8–73·8)	49·4 (26–82·4)
1-180	Turner syndrome	54 (24–95)	60 (26–105)	61 (28–107)	0·1 (0·1–0·2)	0·1 (0·1–0·2)	0·1 (0·1–0·2)
1-181	Klinefelter syndrome	5 (2–9)	6 (3–11)	6 (3–11)	0 (0–0)	0 (0–0)	0 (0–0)
1-182	Other chromosomal abnormalities	15,000 (8,040–32,100)	18,900 (9,140–32,800)	19,400 (8,820–33,400)	35·2 (19–75·3)	46 (22·2–80·6)	48·3 (21·8–83·9)
1-183	Congenital musculoskeletal and limb anomalies	22,800 (15,900–30,900)	25,800 (17,700–35,600)	27,100 (18,500–38,000)	58·2 (40·5–79·5)	60·6 (41·5–83·5)	62·5 (42·6–87·4)
1-184	Urogenital congenital anomalies	9,370 (5,390–14,700)	7,920 (4,720–11,700)	7,570 (4,180–12,800)	22·1 (12·8–34·5)	19·3 (11·5–28·6)	18·9 (10·4–32·1)
1-185	Digestive congenital anomalies	77,100 (35,400–137,000)	60,200 (26,500–96,600)	54,600 (22,400–89,500)	180 (82·4–321)	149 (65·1–239)	139 (56·8–228)
1-186	Other congenital anomalies	115,000 (53,700–195,000)	95,800 (43,700–144,000)	90,200 (39,700–142,000)	274 (128–463)	234 (106–354)	224 (96·3–354)
1-187	Urinary diseases and male infertility	25,900 (21,000–31,900)	47,200 (38,100–56,200)	58,200 (45,900–70,900)	87·1 (70·9–108)	111 (89·7–132)	116 (91·6–142)
1-188	Urinary tract infections and interstitial nephritis	12,900 (11,000–15,600)	26,900 (21,000–30,700)	33,500 (25,600–39,800)	41·5 (36·4–51·3)	63·4 (49·4–72·2)	67·5 (51·6–80·2)
1-189	Urolithiasis	1,930 (1,480–2,480)	3,370 (2,610–4,190)	4,160 (3,210–5,320)	6·6 (5·1–8·3)	7·7 (6–9·6)	8·1 (6·3–10·4)
1-190	Benign prostatic hyperplasia	6,740 (3,960–10,500)	11,000 (6,430–17,000)	13,300 (7,800–20,600)	26·1 (15·4–40·6)	26·5 (15·5–41)	25·8 (15·1–39·8)
1-191	Male infertility	1,280 (487–2,910)	1,560 (610–3,560)	1,990 (752–4,840)	3·6 (1·4–8·3)	3·4 (1·3–7·8)	4·1 (1·6–10·1)
1-192	Other urinary diseases	3,100 (2,460–3,830)	4,360 (3,380–5,200)	5,230 (3,850–6,540)	9·4 (7·6–11·4)	10·1 (7·9–12·1)	10·8 (8–13·5)
1-193	Gynecological diseases	129,000 (89,300–179,000)	170,000 (117,000–238,000)	183,000 (127,000–259,000)	386 (269–536)	373 (258–524)	372 (258–526)
1-194	Uterine fibroids	8,170 (4,390–14,300)	12,000 (6,260–21,400)	13,000 (6,720–23,400)	26·9 (14·4–47·1)	26·2 (13·7–46·8)	26 (13·5–46·9)
1-195	Polycystic ovarian syndrome	1,780 (748–3,650)	2,770 (1,170–5,620)	2,980 (1,270–6,060)	4·9 (2–9·9)	6 (2·5–12·1)	6·2 (2·6–12·6)
1-196	Female infertility	2,310 (905–5,440)	2,710 (1,060–6,120)	3,820 (1,310–9,130)	6·6 (2·6–15·4)	6 (2·3–13·5)	8 (2·7–19)
1-197	Endometriosis	13,100 (7,780–21,300)	14,400 (8,460–23,000)	14,000 (8,300–22,600)	36·5 (21·8–58·7)	31·1 (18·3–50)	28·8 (16·9–46·2)
1-198	Genital prolapse	2,300 (1,230–4,110)	3,050 (1,580–5,590)	3,370 (1,790–6,210)	8·3 (4·4–14·8)	6·9 (3·6–12·7)	6·6 (3·5–12·1)
1-199	Premenstrual syndrome	36,500 (22,400–57,000)	46,200 (28,400–71,900)	48,200 (29,500–74,300)	101 (62·5–158)	100 (61·3–155)	100 (61·6–155)
1-200	Other gynecological diseases	64,400 (42,200–91,800)	88,900 (57,400–126,000)	97,900 (63,500–139,000)	202 (133–288)	197 (128–279)	197 (128–279)
1-201	Hemoglobinopathies and hemolytic anaemias	85,400 (62,500–110,000)	83,800 (64,000–105,000)	90,800 (68,500–115,000)	227 (170–289)	193 (146–243)	200 (150–253)
1-202	Thalassemias	143 (58–269)	133 (58–239)	109 (49–191)	0·3 (0·1–0·7)	0·3 (0·1–0·6)	0·3 (0·1–0·5)
1-203	Thalassaemias trait	2,490 (1,610–3,740)	3,250 (2,100–4,840)	3,600 (2,270–5,400)	6·7 (4·4–10·1)	7·4 (4·8–11)	7·8 (4·9–11·8)
1-204	Sickle cell disorders	44,200 (27,900–61,800)	41,100 (28,300–56,100)	42,600 (28,200–58,200)	111 (71·6–154)	96·2 (65·2–132)	97·7 (63·4–135)
1-205	Sickle cell trait	21,700 (14,000–31,800)	21,300 (13,800–31,400)	22,800 (14,900–33,900)	56·9 (36·9–83·5)	48 (31·2–70·8)	49 (31·9–73·1)
1-206	G6PD deficiency	4,630 (3,340–6,030)	5,430 (4,130–6,940)	7,210 (5,240–9,420)	13·5 (9·8–17·1)	12·3 (9·4–15·6)	14·9 (10·8–19·5)
1-207	G6PD trait	51 (31–80)	78 (47–120)	90 (56–137)	0·2 (0·1–0·3)	0·2 (0·1–0·3)	0·2 (0·1–0·3)
1-208	Other hemoglobinopathies and hemolytic anaemias	12,200 (9,280–16,000)	12,500 (9,300–16,700)	14,400 (10,800–19,000)	38·5 (30–50·3)	28·9 (21·5–38·6)	29·9 (22·3–39·7)
1-209	Endocrine, metabolic, blood, and immune disorders	139,000 (99,300–173,000)	141,000 (112,000–176,000)	173,000 (137,000–214,000)	410 (302–508)	329 (261–412)	365 (288–453)
1-210	Oral disorders	101,000 (58,900–163,000)	146,000 (85,100–231,000)	167,000 (99,300–266,000)	345 (203–554)	334 (196–530)	332 (196–527)
1-211	Caries of deciduous teeth	1,260 (544–2,630)	1,240 (533–2,600)	1,260 (552–2,650)	3·1 (1·4–6·6)	3·1 (1·3–6·5)	3·1 (1·4–6·5)
1-212	Caries of permanent teeth	9,750 (4,360–18,800)	12,700 (5,650–24,500)	13,100 (5,860–25,700)	27·9 (12·4–53·9)	28·2 (12·6–54·1)	27·1 (12·1–53·7)
1-213	Periodontal disease	32,000 (12,300–69,100)	47,800 (18,700–103,000)	53,900 (21,100–115,000)	107 (41–230)	107 (41·7–229)	107 (41·7–229)
1-214	Edentulism	40,100 (25,600–59,800)	60,200 (38,600–88,900)	73,300 (47,700–106,000)	154 (98–229)	143 (91·3–211)	142 (92·4–204)
1-215	Other oral disorders	18,100 (11,300–26,500)	23,600 (14,700–34,500)	25,700 (16,000–37,300)	53·2 (33·2–77·5)	53·1 (33–77·4)	53·1 (33·1–77·3)
1-216	Sudden infant death syndrome	19,400 (5,410–49,000)	14,000 (4,540–34,600)	12,500 (3,800–32,200)	45 (12·5–114)	34·5 (11·2–85·1)	32 (9·7–82)
1-0	Injuries	1,820,000 (1,690,000–1,960,000)	15,800,000 (14,500,000–17,300,000)	2,020,000 (1,760,000–2,310,000)	5,070 (4,720–5,460)	36,000 (33,100–39,400)	4,250 (3,690–4,860)
1-1	Transport injuries	452,000 (419,000–517,000)	398,000 (346,000–506,000)	417,000 (345,000–523,000)	1,270 (1,180–1,470)	891 (776–1,140)	866 (715–1,090)
1-2	Road injuries	432,000 (401,000–495,000)	375,000 (327,000–476,000)	393,000 (326,000–491,000)	1,220 (1,130–1,400)	841 (732–1,070)	817 (675–1,020)

1-3	Pedestrian road injuries	120,000 (103,000–142,000)	71,500 (60,200–99,000)	71,200 (57,300–95,000)	345 (298–406)	164 (138–228)	150 (120–204)
1-4	Cyclist road injuries	58,200 (49,900–69,700)	33,900 (27,700–43,100)	36,400 (29,300–46,400)	169 (145–201)	76·1 (62–98)	74·8 (59·7–96)
1-5	Motorcyclist road injuries	50,300 (43,200–65,400)	62,400 (50,900–97,400)	71,800 (56,900–110,000)	141 (121–185)	138 (113–216)	148 (117–227)
1-6	Motor vehicle road injuries	197,000 (175,000–241,000)	201,000 (172,000–242,000)	206,000 (167,000–260,000)	543 (486–658)	447 (385–539)	429 (346–544)
1-7	Other road injuries	5,920 (4,910–7,030)	6,890 (5,830–8,250)	7,570 (6,190–9,250)	17·8 (14·7–21)	15·4 (13·1–18·5)	15·3 (12·5–18·7)
1-8	Other transport injuries	20,000 (17,900–24,400)	22,100 (19,000–30,500)	23,300 (18,900–33,900)	56·3 (50·7–68·3)	49·6 (42·7–68·6)	49 (39·5–71·5)
1-9	Unintentional injuries	849,000 (693,000–959,000)	14,700,000 (13,400,000–16,200,000)	919,000 (761,000–1,080,000)	2,340 (1,960–2,640)	33,700 (30,700–36,900)	1,970 (1,620–2,310)
1-10	Falls	98,400 (83,000–115,000)	147,000 (125,000–176,000)	183,000 (152,000–218,000)	338 (289–393)	344 (292–409)	363 (300–433)
1-11	Drowning	212,000 (141,000–263,000)	149,000 (112,000–189,000)	155,000 (108,000–200,000)	541 (366–664)	352 (256–451)	355 (238–462)
1-12	Fire, heat, and hot substances	125,000 (73,200–169,000)	93,400 (61,200–121,000)	98,000 (66,700–125,000)	339 (207–453)	217 (140–284)	214 (143–275)
1-13	Poisonings	39,900 (31,400–52,500)	19,300 (14,000–28,700)	20,300 (14,800–32,400)	103 (83·2–133)	46 (33·3–68·4)	46·4 (33·6–72·5)
1-14	Poisoning by carbon monoxide	6,580 (4,560–10,700)	4,430 (2,900–8,290)	5,100 (3,320–10,400)	17·7 (12·7–27·8)	10·3 (6·6–19)	11·2 (7·2–22·5)
1-15	Poisoning by other means	33,400 (25,200–45,100)	14,800 (10,400–22,100)	15,200 (11,000–22,900)	85·6 (66·4–114)	35·7 (24·7–53·4)	35·2 (25·1–52·7)
1-16	Exposure to mechanical forces	99,300 (71,100–134,000)	128,000 (91,600–178,000)	141,000 (96,700–202,000)	285 (204–381)	290 (207–402)	297 (202–432)
1-17	Unintentional firearm injuries	48,100 (28,900–76,700)	61,700 (41,100–100,000)	64,200 (37,900–114,000)	133 (80·7–212)	138 (91·8–225)	136 (79·3–242)
1-18	Other exposure to mechanical forces	51,200 (37,300–74,700)	66,600 (46,800–99,900)	77,200 (53,300–114,000)	152 (111–220)	152 (106–230)	161 (110–241)
1-19	Adverse effects of medical treatment	44,500 (34,100–55,200)	40,300 (32,100–49,600)	41,900 (32,400–52,700)	126 (103–152)	94·9 (75·5–118)	90·8 (69·2–115)
1-20	Animal contact	5,990 (4,380–7,830)	9,420 (7,330–11,900)	10,100 (7,840–12,700)	17·9 (13·4–23·1)	21·6 (16·7–27·3)	21 (16·1–26·5)
1-21	Venomous animal contact	3,700 (2,630–4,870)	6,230 (4,830–7,840)	6,690 (5,170–8,420)	10·8 (7·8–14·2)	14·3 (11·1–18)	13·9 (10·7–17·8)
1-22	Non-venomous animal contact	2,290 (1,650–3,090)	3,190 (2,330–4,230)	3,430 (2,490–4,560)	7·1 (5·3–9·5)	7·3 (5·3–9·7)	7·1 (5–9·4)
1-23	Foreign body	124,000 (98,700–153,000)	74,800 (57,200–94,500)	79,900 (59,800–100,000)	312 (253–381)	180 (137–228)	186 (137–235)
1-24	Pulmonary aspiration and foreign body in airway	116,000 (91,800–144,000)	67,100 (50,300–85,800)	71,600 (53,000–91,300)	291 (233–355)	163 (121–209)	168 (123–216)
1-25	Foreign body in eyes	1,920 (897–3,400)	2,530 (1,250–4,410)	2,740 (1,390–4,740)	5·7 (2·9–9·9)	5·7 (2·8–9·9)	5·6 (2·8–9·8)
1-26	Foreign body in other body part	5,400 (3,560–9,080)	5,110 (3,780–7,230)	5,540 (4,140–7,460)	15·3 (10·5–24·1)	11·8 (8·7–17)	11·7 (8·7–15·9)
1-27	Environmental heat and cold exposure	22,100 (15,900–32,500)	24,600 (17,200–37,200)	27,600 (19,400–42,000)	66·2 (47·3–97·2)	56·2 (39·4–84·5)	57·2 (40·2–86·8)
1-28	Exposure to forces of nature	687 (532–904)	14,000,000 (12,700,000–15,400,000)	85,300 (57,600–125,000)	2 (1·5–2·6)	31,900 (29,000–35,100)	178 (120–259)
1-29	Other unintentional injuries	76,600 (64,200–93,100)	75,800 (62,000–93,500)	77,300 (62,200–95,500)	212 (177–260)	172 (141–211)	163 (131–202)
1-30	Self-harm and interpersonal violence	521,000 (454,000–566,000)	662,000 (530,000–766,000)	685,000 (521,000–826,000)	1,460 (1,270–1,580)	1,460 (1,170–1,690)	1,420 (1,070–1,710)
1-31	Self-harm	199,000 (190,000–212,000)	182,000 (166,000–202,000)	191,000 (165,000–225,000)	583 (556–620)	402 (367–447)	387 (332–455)
1-32	Self-harm by firearm	13,800 (11,500–18,400)	11,600 (9,370–16,300)	12,200 (9,240–18,800)	39 (32·6–52·4)	25·5 (20·7–36)	24·9 (18·8–38·3)
1-33	Self-harm by other specified means	185,000 (176,000–197,000)	170,000 (155,000–189,000)	179,000 (154,000–209,000)	544 (517–576)	376 (344–417)	362 (311–423)
1-34	Interpersonal violence	312,000 (244,000–355,000)	477,000 (348,000–572,000)	491,000 (332,000–615,000)	849 (657–968)	1,050 (767–1,260)	1,020 (692–1,280)
1-35	Physical violence by firearm	130,000 (95,200–153,000)	244,000 (185,000–291,000)	240,000 (167,000–307,000)	346 (254–408)	533 (405–636)	496 (345–636)
1-36	Physical violence by sharp object	84,800 (67,200–125,000)	113,000 (78,800–176,000)	121,000 (80,800–199,000)	231 (182–345)	247 (173–387)	250 (167–413)
1-37	Sexual violence	6,960 (4,580–9,830)	9,460 (6,330–13,300)	9,910 (6,610–14,000)	19·3 (12·8–27·2)	20·8 (13·9–29·1)	20·6 (13·7–29·1)
1-38	Physical violence by other means	90,500 (59,700–112,000)	110,000 (60,600–154,000)	120,000 (63,100–173,000)	252 (162–314)	249 (138–346)	256 (136–367)
1-39	Conflict and terrorism	8,570 (7,670–9,620)	1,800 (1,120–2,940)	1,510 (946–2,400)	22·6 (20·2–25·4)	4 (2·5–6·5)	3 (1·9–4·8)
1-40	Executions and police conflict	636 (415–1,110)	2,260 (1,830–2,800)	1,740 (1,100–2,580)	1·7 (1·1–3)	5 (4–6·1)	3·6 (2·3–5·4)

SR Table 1: All-age DALYs counts and age standardized DALYs rates per 100,000 individuals for all causes and level 1 cause groups by location for 1990, 2010, and 2019, both sexes combined, Antigua and Barbuda

Cause Level	Cause	All-age DALYs			Age-standardized rate of DALYs per 100,000		
		1990	2010	2019	1990	2010	2019
0-0	All causes	16,500 (14,900–18,300)	21,500 (19,100–24,400)	24,200 (21,000–28,000)	29,100 (26,400–32,300)	26,300 (23,500–29,600)	25,700 (22,200–29,800)
1-0	Communicable, maternal, neonatal, and nutritional disorders	2,750 (2,440–3,100)	2,580 (2,280–2,950)	2,380 (2,040–2,810)	4,500 (4,010–5,060)	3,480 (3,060–3,970)	3,160 (2,650–3,780)
1-1	HIV/AIDS and sexually transmitted infections	355 (345–367)	423 (406–446)	387 (370–407)	631 (613–652)	442 (425–466)	384 (368–405)
1-2	HIV/AIDS	340 (332–349)	404 (390–422)	367 (353–381)	605 (591–622)	422 (407–441)	364 (351–378)
1-3	HIV/AIDS - drug-susceptible tuberculosis	13 (9–19)	8 (6–11)	6 (4–8)	23·5 (16·5–33·4)	8·4 (6·1–11·4)	6 (4·2–8·4)
1-4	HIV/AIDS - Multidrug-resistant Tuberculosis without extensive drug resistance	0 (0–1)	0 (0–0)	0 (0–0)	0·4 (0–2·2)	0·1 (0–0·4)	0·1 (0–0·4)
1-5	HIV/AIDS - Extensively drug-resistant Tuberculosis	0 (0–0)	0 (0–0)	0 (0–0)	0 (0–0)	0 (0–0)	0 (0–0·1)
1-6	HIV/AIDS resulting in other diseases	327 (317–336)	396 (382–414)	360 (347–374)	581 (564–598)	413 (399–432)	358 (345–372)
1-7	Sexually transmitted infections excluding HIV	15 (10–23)	19 (12–33)	20 (12–34)	25·7 (17·1–40·4)	20·7 (12·6–35·1)	20·1 (12·1–33·7)
1-8	Syphilis	4 (3–5)	2 (2–2)	2 (1–2)	6·6 (5·1–8·4)	2·3 (2–2·9)	2·1 (1·6–2·9)
1-9	Chlamydial infection	1 (1–2)	2 (1–3)	2 (1–3)	2·1 (1·4–3·2)	2·1 (1·4–3·1)	2·1 (1·4–3·1)
1-10	Gonococcal infection	1 (1–1)	1 (1–2)	2 (1–2)	1·8 (1·3–2·3)	1·6 (1·2–2)	1·6 (1·2–2·2)
1-11	Trichomoniasis	3 (1–6)	5 (2–10)	5 (2–10)	5·1 (2–10·5)	5·1 (2–10·4)	4·8 (1·9–10·1)
1-12	Genital herpes	3 (1–7)	5 (2–13)	6 (2–14)	5·4 (1·7–13·1)	5·5 (1·7–13·5)	5·4 (1·7–13·1)
1-13	Other sexually transmitted infections	3 (2–4)	4 (3–6)	4 (3–6)	4·8 (3·4–6·6)	4·2 (2·9–5·9)	4·1 (2·8–5·7)
1-14	Respiratory infections and tuberculosis	569 (513–628)	669 (595–746)	687 (581–808)	961 (870–1,060)	907 (805–1,010)	840 (705–1,010)
1-15	Tuberculosis	36 (32–42)	27 (22–34)	21 (17–26)	62·1 (54·8–70·9)	30·9 (25–38·2)	22 (17·7–27·3)
1-16	Latent tuberculosis infection	0 (0–0)	0 (0–0)	0 (0–0)	0 (0–0)	0 (0–0)	0 (0–0)
1-17	Drug-susceptible tuberculosis	36 (31–42)	27 (22–33)	21 (17–26)	61·2 (53·4–70·3)	30·7 (24·8–38·2)	21·8 (17·6–27·1)
1-18	Multidrug-resistant tuberculosis without extensive drug resistance	1 (0–3)	0 (0–1)	0 (0–1)	0·9 (0·1–4·4)	0·2 (0–0·9)	0·2 (0–1)
1-19	Extensively drug-resistant tuberculosis	0 (0–0)	0 (0–0)	0 (0–0)	0 (0–0)	0 (0–0·1)	0 (0–0·1)
1-20	Lower respiratory infections	464 (419–511)	552 (497–614)	577 (486–688)	789 (716–865)	768 (684–860)	711 (589–862)
1-21	Upper respiratory infections	51 (31–80)	69 (42–108)	69 (42–108)	82·6 (50–129)	82·2 (50–130)	82 (50–128)
1-22	Otitis media	17 (10–28)	21 (12–34)	20 (12–34)	27·5 (15·7–45·5)	25·2 (14·5–42·1)	25 (14·6–41·8)
1-23	Enteric infections	174 (146–211)	177 (138–227)	191 (146–248)	287 (242–345)	234 (183–297)	237 (181–308)
1-24	Diarrhoeal diseases	174 (146–210)	177 (137–226)	191 (146–247)	286 (241–343)	233 (182–296)	237 (180–307)
1-25	Typhoid and paratyphoid	0 (0–0)	0 (0–0)	0 (0–0)	0·3 (0·1–0·4)	0·1 (0–0·1)	0·1 (0–0·1)
1-26	Typhoid fever	0 (0–0)	0 (0–0)	0 (0–0)	0·3 (0·1–0·4)	0 (0–0·1)	0 (0–0·1)
1-27	Paratyphoid fever	0 (0–0)	0 (0–0)	0 (0–0)	0 (0–0)	0 (0–0·1)	0·1 (0–0·1)
1-28	iNTS	0 (0–0)	0 (0–0)	0 (0–0)	0·2 (0·1–0·4)	0·2 (0·1–0·3)	0·2 (0·1–0·3)
1-29	Other intestinal infectious diseases	0 (0–1)	0 (0–1)	0 (0–0)	0·5 (0·3–0·8)	0·5 (0·3–0·8)	0·4 (0·3–0·7)
1-30	Neglected tropical diseases and malaria	78 (41–146)	98 (49–182)	90 (45–173)	126 (66·9–232)	114 (58·1–209)	102 (53·7–195)
1-31	Malaria	0 (0–0)	0 (0–0)	0 (0–0)	0 (0–0)	0 (0–0)	0 (0–0)
1-32	Chagas disease	0 (0–0)	0 (0–0)	0 (0–0)	0 (0–0)	0 (0–0)	0 (0–0)
1-33	Leishmaniasis	0 (0–0)	0 (0–0)	0 (0–0)	0 (0–0)	0 (0–0)	0 (0–0)
1-34	Visceral leishmaniasis	0 (0–0)	0 (0–0)	0 (0–0)	0 (0–0)	0 (0–0)	0 (0–0)
1-35	Cutaneous and mucocutaneous leishmaniasis	0 (0–0)	0 (0–0)	0 (0–0)	0 (0–0)	0 (0–0)	0 (0–0)
1-36	African trypanosomiasis	0 (0–0)	0 (0–0)	0 (0–0)	0 (0–0)	0 (0–0)	0 (0–0)
1-37	Schistosomiasis	50 (16–111)	61 (17–143)	58 (17–137)	79·8 (25·8–179)	68·1 (19·2–157)	62·3 (17·9–145)
1-38	Cysticercosis	1 (1–2)	1 (1–2)	1 (1–2)	2 (1–3·1)	1·1 (0·6–1·7)	1 (0·6–1·6)
1-39	Cystic echinococcosis	0 (0–0)	0 (0–0)	0 (0–0)	0 (0–0)	0 (0–0)	0 (0–0)
1-40	Lymphatic filariasis	0 (0–0)	0 (0–0)	0 (0–0)	0 (0–0)	0 (0–0)	0 (0–0)
1-41	Onchocerciasis	0 (0–0)	0 (0–0)	0 (0–0)	0 (0–0)	0 (0–0)	0 (0–0)
1-42	Trachoma	0 (0–0)	0 (0–0)	0 (0–0)	0 (0–0)	0 (0–0)	0 (0–0)
1-43	Dengue	5 (3–9)	15 (9–24)	9 (5–14)	8 (4·2–14·5)	18·1 (11·3–28·3)	10·5 (5·9–17·8)
1-44	Yellow fever	0 (0–0)	0 (0–0)	0 (0–0)	0 (0–0)	0 (0–0)	0 (0–0)
1-45	Rabies	0 (0–0)	0 (0–0)	0 (0–0)	0·1 (0·1–0·2)	0·2 (0·1–0·3)	0·3 (0·2–0·7)
1-46	Intestinal nematode infections	4 (2–7)	3 (2–5)	3 (2–4)	6·1 (3·5–10·3)	3·8 (2·1–6·2)	3·1 (1·8–5·1)
1-47	Ascariasis	0 (0–1)	0 (0–1)	0 (0–0)	0·5 (0·2–1)	0·3 (0·1–0·7)	0·2 (0·1–0·5)
1-48	Trichuriasis	0 (0–0)	0 (0–0)	0 (0–0)	0 (0–0)	0 (0–0)	0 (0–0)

1-49	Hookworm disease	4 (2–6)	3 (2–5)	3 (1–4)	5·6 (3–9·8)	3·4 (1·8–5·9)	2·9 (1·6–4·8)
1-50	Food-borne trematodiases	0 (0–0)	0 (0–0)	0 (0–0)	0 (0–0)	0 (0–0)	0 (0–0)
1-51	Leprosy	0 (0–0)	0 (0–0)	0 (0–0)	0 (0–0)	0 (0–0)	0 (0–0)
1-52	Ebola virus disease	0 (0–0)	0 (0–0)	0 (0–0)	0 (0–0)	0 (0–0)	0 (0–0)
1-53	Zika virus disease	0 (0–0)	0 (0–0)	3 (0–12)	0 (0–0)	0 (0–0)	3·3 (0–14)
1-54	Guinea worm disease	0 (0–0)	0 (0–0)	0 (0–0)	0 (0–0)	0 (0–0)	0 (0–0)
1-55	Other neglected tropical diseases	18 (12–28)	17 (11–26)	16 (10–24)	29·7 (19·9–44·5)	22·4 (14·1–33·2)	21·9 (14–32·8)
1-56	Other infectious diseases	189 (167–213)	125 (108–147)	113 (91–138)	312 (278–352)	162 (138–190)	143 (115–178)
1-57	Meningitis	106 (92–123)	49 (41–57)	39 (31–50)	173 (150–198)	67·8 (55–81·2)	55·2 (42·1–72·7)
1-58	Encephalitis	24 (18–28)	20 (16–26)	18 (14–24)	38·1 (29·5–45)	25·8 (21·3–33·5)	24·1 (18·1–32·8)
1-59	Diphtheria	0 (0–0)	0 (0–0)	0 (0–0)	0 (0–0)	0 (0–0)	0 (0–0)
1-60	Whooping cough	0 (0–0)	1 (0–1)	1 (0–1)	0·3 (0·2–0·6)	1·3 (0·7–1·9)	1·3 (0·8–2·1)
1-61	Tetanus	7 (3–10)	3 (2–7)	2 (1–7)	12·9 (5·5–19·2)	3·4 (2·1–7·6)	1·6 (0·8–6·7)
1-62	Measles	0 (0–0)	0 (0–0)	0 (0–0)	0 (0–0)	0 (0–0)	0 (0–0)
1-63	Varicella and herpes zoster	4 (3–6)	5 (4–7)	5 (4–7)	7·3 (5·2–9·8)	6·5 (4·9–8·9)	6·1 (4·4–8·5)
1-64	Acute hepatitis	18 (16–20)	7 (6–9)	6 (5–8)	29·9 (26·4–33·9)	8 (6·4–10)	7 (5·3–9)
1-65	Acute hepatitis A	9 (7–11)	4 (3–5)	3 (2–5)	15·6 (11·5–18·9)	4·4 (3–6·1)	3·9 (2·6–5·7)
1-66	Acute hepatitis B	6 (5–8)	2 (2–3)	2 (2–3)	11 (8·5–14·2)	2·6 (2·2–3·1)	2·1 (1·7–2·6)
1-67	Acute hepatitis C	1 (0–1)	0 (0–0)	0 (0–0)	1·5 (0·7–2·1)	0·2 (0·2–0·4)	0·2 (0·1–0·4)
1-68	Acute hepatitis E	1 (1–2)	1 (0–1)	1 (0–1)	1·8 (1·2–2·9)	0·7 (0·5–1)	0·7 (0·5–0·9)
1-69	Other unspecified infectious diseases	30 (22–39)	41 (29–53)	43 (29–56)	50·9 (37·6–66·5)	49 (34·8–63·2)	48·1 (33·5–62·6)
1-70	Maternal and neonatal disorders	1,030 (866–1,210)	745 (620–891)	588 (461–742)	1,580 (1,330–1,860)	1,190 (978–1,440)	1,040 (795–1,360)
1-71	Maternal disorders	32 (27–37)	32 (28–37)	30 (25–36)	47·1 (40·4–55)	35·1 (30·1–40·6)	32·4 (27·1–39)
1-72	Maternal haemorrhage	6 (5–8)	5 (4–7)	5 (3–6)	9·2 (6·8–12·4)	5·9 (4·2–8·1)	4·9 (3·5–6·7)
1-73	Maternal sepsis and other pregnancy related infections	7 (6–9)	4 (3–5)	4 (3–5)	10·9 (8·9–13·4)	4·5 (3·5–5·7)	4·2 (3·2–5·4)
1-74	Maternal hypertensive disorders	7 (6–9)	6 (5–7)	5 (4–6)	10·6 (8·9–12·7)	6·3 (5·2–7·7)	5·7 (4·6–7)
1-75	Maternal obstructed labour and uterine rupture	0 (0–1)	1 (1–2)	1 (1–1)	0·5 (0·3–0·8)	1·3 (1–1·7)	1·1 (0·8–1·4)
1-76	Maternal abortive outcome	6 (5–8)	3 (2–3)	2 (2–3)	9·1 (7·1–11·7)	2·9 (2·2–3·7)	2·3 (1·7–3)
1-77	Ectopic pregnancy	1 (1–1)	3 (2–3)	3 (2–3)	1 (0·8–1·2)	3·1 (2·5–3·7)	2·8 (2·2–3·6)
1-78	Indirect maternal deaths	0 (0–1)	1 (1–1)	1 (1–1)	0·7 (0·5–0·9)	0·8 (0·6–1)	0·8 (0·6–1)
1-79	Late maternal deaths	2 (1–2)	2 (2–3)	2 (2–3)	2·3 (1·6–3·1)	2·5 (1·9–3·5)	2·6 (1·9–3·6)
1-80	Maternal deaths aggravated by HIV/AIDS	0 (0–0)	0 (0–0)	0 (0–0)	0 (0–0)	0 (0–0)	0 (0–0)
1-81	Other maternal disorders	2 (1–2)	7 (6–9)	8 (6–9)	2·7 (2·1–3·4)	7·8 (6·4–9·4)	8 (6·4–9·9)
1-82	Neonatal disorders	994 (833–1,180)	713 (586–857)	558 (434–704)	1,530 (1,280–1,810)	1,160 (942–1,400)	1,010 (766–1,320)
1-83	Neonatal preterm birth	617 (510–737)	420 (345–505)	323 (251–408)	951 (786–1,140)	673 (546–820)	577 (434–748)
1-84	Neonatal encephalopathy due to birth asphyxia and trauma	176 (126–226)	103 (80–130)	79 (57–108)	271 (194–348)	171 (133–216)	144 (99·9–201)
1-85	Neonatal sepsis and other neonatal infections	91 (67–121)	89 (65–118)	78 (57–104)	141 (103–187)	139 (102–178)	131 (93·6–177)
1-86	Hemolytic disease and other neonatal jaundice	24 (16–34)	14 (10–18)	9 (6–13)	36·2 (24·5–52·8)	21·8 (16·1–29·1)	15·9 (10·6–23·7)
1-87	Other neonatal disorders	87 (61–122)	87 (63–114)	68 (46–95)	133 (93–187)	152 (110–199)	140 (95·4–198)
1-88	Nutritional deficiencies	363 (269–493)	345 (243–477)	328 (234–457)	603 (447–823)	431 (304–596)	408 (289–569)
1-89	Protein-energy malnutrition	114 (100–131)	84 (71–99)	83 (68–102)	190 (167–215)	116 (97·3–139)	109 (85·7–138)
1-90	Iodine deficiency	4 (2–7)	4 (2–6)	3 (2–6)	6·9 (3·4–11·5)	4·2 (2–7)	3·4 (1·7–5·8)
1-91	Vitamin A deficiency	5 (3–8)	4 (3–7)	4 (2–6)	8·2 (5·1–12·4)	5·7 (3·6–8·7)	5·4 (3·4–8·1)
1-92	Dietary iron deficiency	230 (144–355)	245 (151–370)	229 (142–347)	380 (239–584)	296 (183–448)	281 (174–427)
1-93	Other nutritional deficiencies	10 (7–14)	8 (6–12)	9 (6–12)	17·7 (12·5–24·9)	9·7 (6·8–13·5)	9·3 (6·4–13)
1-0	Non-communicable diseases	12,100 (10,800–13,600)	16,700 (14,700–19,000)	19,700 (16,900–22,900)	21,900 (19,700–24,400)	20,300 (18,000–22,900)	20,200 (17,400–23,500)
1-1	Neoplasms	1,740 (1,640–1,860)	2,600 (2,410–2,770)	3,250 (2,800–3,780)	3,290 (3,090–3,510)	3,240 (2,990–3,450)	3,240 (2,790–3,760)
1-2	Lip and oral cavity cancer	25 (23–28)	34 (30–38)	42 (34–49)	49·9 (44·9–55·8)	41·1 (36·7–46)	39·7 (32·9–46·6)
1-3	Nasopharynx cancer	5 (4–5)	9 (8–10)	11 (9–13)	9 (8·1–10)	10·5 (9·3–11·7)	10·2 (8·4–12·3)
1-4	Other pharynx cancer	19 (17–22)	21 (18–24)	27 (22–33)	39·4 (34·2–45·1)	25·2 (21·8–28·7)	25·2 (20·4–30·8)
1-5	Oesophageal cancer	40 (36–44)	55 (50–61)	71 (59–85)	78·8 (71·1–87·2)	68·1 (61·2–75·5)	67·9 (56·8–80·5)
1-6	Stomach cancer	193 (176–210)	197 (179–217)	231 (196–271)	371 (338–406)	246 (223–272)	226 (192–264)
1-7	Colon and rectum cancer	136 (125–147)	241 (219–265)	327 (278–381)	258 (238–280)	304 (277–334)	323 (276–375)

1-8	Liver cancer	102 (92–112)	51 (46–56)	67 (56–79)	197 (178–218)	62·9 (56·9–70)	64·6 (54·3–76·3)
1-9	Liver cancer due to hepatitis B	35 (27–46)	16 (12–21)	20 (14–28)	69·5 (52·7–91·6)	18·5 (13·4–24·9)	18·6 (13·5–25·6)
1-10	Liver cancer due to hepatitis C	20 (14–28)	10 (7–13)	13 (9–19)	38·6 (25·8–53·1)	12·7 (8·8–17·3)	13 (8·8–18·7)
1-11	Liver cancer due to alcohol use	31 (22–41)	17 (12–22)	23 (16–31)	60·8 (42·9–80·9)	21·3 (15·5–27·7)	22·1 (15·4–29·8)
1-12	Liver cancer due to NASH	7 (5–10)	4 (3–6)	6 (4–8)	13·7 (9·8–19·5)	5·2 (3·7–7·2)	5·7 (3·9–8·1)
1-13	Gallbladder and biliary tract cancer	41 (23–47)	25 (21–39)	31 (24–50)	79 (44·7–91·4)	31·2 (26·1–49·3)	29·7 (23·1–48·2)
1-14	Pancreatic cancer	17 (15–18)	100 (89–111)	138 (115–165)	32·8 (29·5–36·2)	125 (111–139)	133 (112–158)
1-15	Larynx cancer	21 (19–24)	32 (28–36)	40 (32–48)	42·5 (37·6–48)	39·9 (35·1–45)	37·1 (30·4–45)
1-16	Tracheal, bronchus, and lung cancer	120 (110–131)	176 (160–191)	220 (185–258)	236 (218–259)	219 (200–238)	210 (178–245)
1-17	Malignant skin melanoma	11 (9–15)	20 (16–26)	25 (19–34)	21·1 (16·7–28·7)	23·1 (18·6–30·9)	24·5 (18·4–32·8)
1-18	Non-melanoma skin cancer	2 (2–4)	14 (8–15)	17 (10–20)	4·3 (3·7–7·4)	17·5 (10·5–19·5)	17·6 (10·7–20·8)
1-19	Non-melanoma skin cancer (squamous-cell carcinoma)	2 (2–4)	14 (8–15)	17 (10–20)	4·3 (3·7–7·4)	17·5 (10·5–19·5)	17·6 (10·7–20·7)
1-20	Non-melanoma skin cancer (basal-cell carcinoma)	0 (0–0)	0 (0–0)	0 (0–0)	0 (0–0)	0 (0–0)	0 (0–0)
1-21	Breast cancer	189 (169–212)	347 (304–391)	461 (390–547)	375 (334–423)	398 (350–448)	430 (366–506)
1-22	Cervical cancer	105 (91–122)	146 (125–167)	174 (141–214)	204 (177–237)	163 (140–186)	162 (132–197)
1-23	Uterine cancer	22 (20–26)	51 (44–60)	72 (59–87)	43·9 (38·4–50·5)	62·8 (53·6–73·1)	68·5 (56·6–82·3)
1-24	Ovarian cancer	11 (10–21)	75 (46–87)	98 (60–120)	22·1 (18·6–41)	87 (52·3–102)	92·2 (55·8–112)
1-25	Prostate cancer	284 (240–378)	441 (341–518)	537 (412–672)	504 (425–672)	611 (474–723)	575 (440–713)
1-26	Testicular cancer	1 (1–1)	4 (4–5)	5 (4–7)	1·6 (1·3–2·2)	5·1 (4·3–6·5)	5·8 (4·5–7·3)
1-27	Kidney cancer	34 (31–37)	40 (36–45)	53 (44–63)	63·8 (57·7–71)	49·3 (44·5–54·8)	52·9 (43·6–63·8)
1-28	Bladder cancer	26 (23–28)	38 (34–43)	51 (42–60)	47·8 (43–52·7)	50·7 (45·3–56·4)	51·9 (43·4–61·2)
1-29	Brain and nervous system cancer	15 (10–31)	69 (32–88)	78 (34–109)	26 (17·4–54·3)	82·7 (38–107)	84·1 (36·3–120)
1-30	Thyroid cancer	8 (8–9)	14 (12–16)	20 (17–24)	16·3 (14·4–18·2)	17·1 (15·1–19·3)	19·2 (16·1–22·6)
1-31	Mesothelioma	2 (2–2)	2 (2–3)	2 (2–3)	3·9 (3·4–4·4)	2·5 (2·2–2·9)	2·3 (1·9–2·9)
1-32	Hodgkin lymphoma	3 (3–4)	4 (4–5)	4 (3–5)	5·8 (4·8–7·6)	4·8 (4·1–5·6)	4·3 (3·5–5·4)
1-33	Non-Hodgkin's lymphoma	69 (62–76)	91 (82–101)	106 (86–127)	125 (113–138)	108 (96·7–120)	108 (87·9–129)
1-34	Multiple myeloma	29 (26–33)	53 (47–59)	72 (59–86)	56·5 (50·2–65·7)	66·4 (58·6–75)	68·6 (56·7–81·5)
1-35	Leukaemia	91 (83–99)	124 (112–138)	131 (110–157)	155 (142–168)	153 (137–171)	147 (122–179)
1-36	Acute lymphoid leukaemia	15 (12–18)	19 (16–24)	18 (14–23)	22·7 (18·6–27·7)	25·3 (20·2–31·8)	24·4 (18·4–32·5)
1-37	Chronic lymphoid leukaemia	1 (1–1)	2 (1–2)	2 (2–3)	1·8 (1·3–2·6)	2·1 (1·7–2·5)	2·1 (1·7–2·7)
1-38	Acute myeloid leukaemia	18 (16–23)	35 (30–40)	38 (30–48)	30·4 (26–38·1)	42·1 (35·5–48·9)	43·4 (34·1–55)
1-39	Chronic myeloid leukaemia	12 (10–13)	12 (10–13)	13 (10–16)	20·9 (18·3–24·1)	14·1 (12–16)	12·9 (10·4–15·7)
1-40	Other leukaemia	45 (40–50)	57 (50–64)	60 (50–71)	78·7 (70·2–86·8)	69·8 (61·5–78·6)	64·4 (53·5–77·9)
1-41	Other neoplasms	3 (3–4)	6 (5–8)	8 (7–10)	6·2 (4·9–8·1)	8 (6·7–10·2)	8·7 (7–10·7)
1-42	Myelodysplastic, myeloproliferative, and other hematopoietic neoplasms	3 (3–4)	6 (5–8)	8 (7–10)	6·2 (4·9–8·1)	8 (6·7–10·2)	8·7 (7–10·7)
1-43	Benign and in situ intestinal neoplasms	0 (0–0)	0 (0–0)	0 (0–0)	0 (0–0)	0 (0–0)	0 (0–0)
1-44	Benign and in situ cervical and uterine neoplasms	0 (0–0)	0 (0–0)	0 (0–0)	0 (0–0)	0 (0–0)	0 (0–0)
1-45	Other benign and in situ neoplasms	0 (0–0)	0 (0–0)	0 (0–0)	0 (0–0)	0 (0–0)	0 (0–0)
1-46	Cardiovascular diseases	3,230 (3,050–3,420)	3,380 (3,160–3,600)	4,030 (3,530–4,580)	6,000 (5,660–6,330)	4,340 (4,050–4,620)	4,140 (3,650–4,680)
1-47	Rheumatic heart disease	70 (59–86)	73 (57–94)	72 (54–95)	121 (102–146)	80·4 (62·8–103)	73·7 (55·3–97·8)
1-48	Ischaemic heart disease	1,350 (1,260–1,430)	1,290 (1,190–1,380)	1,460 (1,260–1,660)	2,530 (2,370–2,690)	1,680 (1,550–1,800)	1,510 (1,300–1,710)
1-49	Stroke	1,150 (1,070–1,230)	1,090 (1,000–1,190)	1,310 (1,130–1,520)	2,120 (1,960–2,280)	1,400 (1,290–1,520)	1,340 (1,160–1,540)
1-50	Ischaemic stroke	450 (408–496)	423 (384–465)	519 (453–588)	784 (711–865)	579 (522–634)	573 (499–648)
1-51	Intracerebral hemorrhage	597 (547–648)	547 (499–600)	651 (552–766)	1,150 (1,050–1,250)	681 (622–744)	630 (536–740)
1-52	Subarachnoid hemorrhage	101 (88–113)	122 (109–137)	143 (119–172)	191 (166–214)	140 (126–157)	136 (114–164)
1-53	Hypertensive heart disease	293 (253–328)	391 (334–443)	526 (414–629)	545 (472–610)	506 (430–575)	536 (424–638)
1-54	Non-rheumatic valvular heart disease	21 (19–24)	29 (26–32)	36 (30–42)	39·6 (35·1–44·7)	35·3 (31·9–38·7)	36 (30·4–42)
1-55	Non-rheumatic calcific aortic valve disease	10 (8–11)	14 (12–16)	18 (14–22)	17·9 (14·8–21·1)	17·7 (15–20·2)	17·8 (14·6–22·4)
1-56	Non-rheumatic degenerative mitral valve disease	9 (7–11)	12 (10–14)	15 (12–19)	17·3 (13·8–20·1)	13·8 (11·4–16·6)	14·4 (11·3–17·8)
1-57	Other non-rheumatic valve diseases	3 (2–3)	3 (2–5)	4 (3–5)	4·3 (2·9–5·8)	3·8 (2·7–5·4)	3·9 (2·7–5·4)
1-58	Cardiomyopathy and myocarditis	72 (64–87)	116 (94–128)	146 (109–175)	132 (118–159)	140 (114–155)	147 (110–176)
1-59	Myocarditis	13 (10–16)	20 (16–26)	23 (18–30)	22·9 (17·6–27·8)	25·1 (20·2–30·9)	25·3 (19·8–33·1)
1-60	Alcoholic cardiomyopathy	4 (3–6)	11 (7–13)	16 (9–22)	7·9 (5·8–11·7)	12·4 (8·5–15·1)	14·4 (8·3–19·3)

1-61	Other cardiomyopathy	54 (48–68)	84 (68–94)	107 (77–130)	101 (89·3–126)	103 (82·4–115)	107 (77·2–130)
1-62	Atrial fibrillation and flutter	55 (46–66)	79 (68–94)	100 (84–121)	96·1 (81·5–115)	110 (94–131)	114 (96·7–139)
1-63	Aortic aneurysm	28 (24–31)	35 (31–39)	43 (36–51)	50·4 (44·9–56·5)	46·5 (41·3–52)	44·1 (36·8–52)
1-64	Peripheral vascular disease	16 (9–29)	26 (14–46)	35 (19–64)	29·5 (16–51·8)	35·5 (19·3–62·3)	37·8 (19·9–67·6)
1-65	Endocarditis	16 (13–23)	30 (23–37)	33 (25–43)	29·4 (23·8–41·4)	34·7 (26·6–42·2)	33·5 (24·7–42·6)
1-66	Chronic respiratory diseases	282 (227–357)	384 (312–475)	435 (354–534)	478 (391–595)	485 (394–601)	501 (399–628)
1-67	Chronic obstructive pulmonary disease	87 (78–98)	133 (117–150)	170 (143–199)	155 (138–174)	174 (154–197)	178 (150–207)
1-68	Pneumoconiosis	2 (1–2)	1 (1–2)	2 (1–2)	2·8 (2·3–3·3)	1·7 (1·4–2)	1·6 (1·3–2)
1-69	Silicosis	0 (0–0)	0 (0–0)	0 (0–0)	0·1 (0·1–0·1)	0·1 (0–0·1)	0 (0–0·1)
1-70	Asbestosis	0 (0–0)	0 (0–0)	0 (0–0)	0·5 (0·4–0·7)	0·4 (0·3–0·5)	0·4 (0·3–0·5)
1-71	Coal workers pneumoconiosis	0 (0–0)	0 (0–0)	0 (0–0)	0 (0–0)	0 (0–0)	0 (0–0)
1-72	Other pneumoconiosis	1 (1–1)	1 (1–1)	1 (1–1)	2·1 (1·8–2·6)	1·2 (1–1·5)	1·2 (0·9–1·5)
1-73	Asthma	173 (123–246)	210 (149–297)	216 (151–304)	283 (204–395)	260 (183–370)	271 (185–390)
1-74	Interstitial lung disease and pulmonary sarcoidosis	6 (4–8)	12 (9–14)	16 (11–20)	10·1 (8–14·4)	14·9 (11·1–18)	16·3 (11–21)
1-75	Other chronic respiratory diseases	15 (13–18)	28 (24–33)	31 (25–39)	27·2 (22·4–31·5)	33·9 (28·3–39·8)	34·6 (27·6–43)
1-76	Digestive diseases	563 (509–634)	725 (637–839)	845 (716–991)	1,060 (958–1,190)	854 (754–978)	841 (714–986)
1-77	Cirrhosis and other chronic liver diseases	245 (225–268)	287 (260–315)	349 (295–410)	485 (447–532)	336 (304–370)	331 (282–388)
1-78	Cirrhosis and other chronic liver diseases due to hepatitis B	30 (22–41)	20 (13–28)	23 (16–34)	61·7 (42·9–85)	22·1 (15·1–31·7)	21·5 (14·7–30·7)
1-79	Cirrhosis and other chronic liver diseases due to hepatitis C	47 (35–63)	56 (41–76)	67 (46–94)	93·7 (67·7–128)	64·3 (47·3–86·8)	62·2 (43·8–87·1)
1-80	Cirrhosis and other chronic liver diseases due to alcohol use	90 (69–109)	119 (95–145)	148 (113–189)	185 (140–225)	139 (111–170)	136 (104–172)
1-81	Cirrhosis and other chronic liver diseases due to NAFLD	35 (25–48)	45 (32–62)	58 (39–80)	69·4 (48·1–95·5)	53·9 (38·3–73·3)	55·1 (38·1–75·4)
1-82	Cirrhosis and other chronic liver diseases due to other causes	42 (34–53)	46 (36–59)	53 (38–73)	75·2 (59·5–95·9)	57 (44–71·7)	55·3 (40·7–74·5)
1-83	Upper digestive system diseases	133 (95–191)	182 (120–274)	203 (134–308)	243 (176–345)	206 (138–304)	199 (132–301)
1-84	Peptic ulcer disease	40 (34–47)	31 (27–36)	34 (28–41)	75·1 (62·8–88·3)	38·8 (33·3–45)	34 (27·7–40·8)
1-85	Gastritis and duodenitis	24 (18–31)	38 (28–50)	43 (31–58)	44·4 (33·7–57·9)	43·8 (32·5–58·5)	42·2 (31·1–56·8)
1-86	GERD	69 (36–124)	113 (58–199)	127 (66–224)	123 (64·1–218)	123 (64·1–217)	123 (63·8–217)
1-87	Appendicitis	14 (10–17)	14 (11–19)	15 (12–20)	24 (16·7–28·9)	16·8 (13·5–23)	17·1 (12·9–22·6)
1-88	Paralytic ileus and intestinal obstruction	36 (31–46)	52 (44–60)	59 (46–71)	63·3 (54·5–80·5)	69·6 (58–79·4)	68·3 (53·4–82·6)
1-89	Inguinal, femoral, and abdominal hernia	33 (24–44)	48 (34–66)	55 (40–75)	57·3 (41·9–76·3)	57 (41–77)	60·1 (43·2–82·1)
1-90	Inflammatory bowel disease	18 (13–20)	20 (17–25)	22 (18–28)	31·6 (24–36·8)	23·5 (20–29·5)	22·3 (17·9–29)
1-91	Vascular intestinal disorders	6 (5–7)	7 (6–8)	9 (7–11)	10·8 (9·5–12·2)	9·3 (8·3–10·5)	9·3 (7·8–11·1)
1-92	Gallbladder and biliary diseases	43 (34–54)	50 (38–66)	58 (45–76)	78·5 (61·6–99·6)	58·3 (45·5–75·6)	57·2 (44·2–74·8)
1-93	Pancreatitis	17 (14–19)	25 (22–30)	28 (23–34)	31·1 (27–36·3)	28·6 (25–32·8)	27·1 (22·3–32·7)
1-94	Other digestive diseases	18 (15–26)	38 (27–46)	46 (32–60)	32·4 (26·2–46·7)	48·6 (34·9–58·5)	50 (35·5–65·4)
1-95	Neurological disorders	865 (525–1,380)	1,210 (715–1,960)	1,310 (768–2,080)	1,460 (890–2,310)	1,430 (867–2,280)	1,420 (841–2,240)
1-96	Alzheimer's disease and other dementias	175 (73–404)	221 (91–504)	265 (109–600)	298 (124–692)	315 (130–728)	317 (132–724)
1-97	Parkinson's disease	46 (42–50)	70 (64–76)	88 (78–99)	79 (72·7–85·3)	97·5 (89·6–106)	98·8 (87·2–110)
1-98	Idiopathic epilepsy	258 (158–410)	305 (181–482)	311 (183–500)	431 (267–681)	350 (207–554)	338 (197–549)
1-99	Multiple sclerosis	8 (6–10)	16 (12–19)	18 (14–23)	14·3 (12–19·2)	16·7 (12·9–20·2)	16·9 (12·7–20·8)
1-100	Motor neuron disease	3 (2–3)	9 (8–10)	12 (10–14)	4·9 (4·5–5·4)	10·6 (9·6–11·7)	12·1 (9·8–14·7)
1-101	Headache disorders	333 (61–750)	519 (101–1,150)	539 (112–1,180)	558 (108–1,240)	560 (108–1,240)	554 (109–1,230)
1-102	Migraine	303 (41–703)	470 (68–1,080)	487 (76–1,120)	506 (74·1–1,170)	507 (73·1–1,160)	502 (74·7–1,160)
1-103	Tension-type headache	30 (9–114)	49 (15–172)	52 (16–178)	52·6 (15·6–189)	52·7 (15·6–190)	52·4 (15·5–188)
1-104	Other neurological disorders	43 (27–66)	69 (46–103)	76 (50–113)	71·1 (45·4–110)	81·7 (54·5–120)	87·5 (56·8–131)
1-105	Mental disorders	975 (709–1,290)	1,480 (1,090–1,960)	1,620 (1,190–2,130)	1,650 (1,200–2,180)	1,620 (1,190–2,140)	1,660 (1,220–2,190)
1-106	Schizophrenia	103 (71–136)	177 (124–237)	193 (132–255)	182 (125–242)	185 (129–247)	185 (126–246)
1-107	Depressive disorders	302 (203–417)	444 (301–619)	527 (359–725)	525 (359–724)	486 (331–678)	524 (354–721)
1-108	Major depressive disorder	251 (164–355)	360 (241–506)	435 (287–604)	434 (288–613)	395 (264–555)	434 (287–608)
1-109	Dysthymia	52 (32–80)	84 (52–129)	92 (57–140)	90·6 (56·5–138)	90·6 (56·4–139)	90·3 (56–138)
1-110	Bipolar disorder	122 (72–192)	192 (112–299)	204 (120–323)	205 (119–322)	205 (120–319)	205 (120–321)
1-111	Anxiety disorders	244 (164–349)	376 (250–536)	401 (266–567)	410 (277–581)	412 (275–581)	414 (276–592)
1-112	Eating disorders	33 (20–50)	53 (33–80)	53 (33–80)	50 (30·1–75·6)	57·6 (35·3–86·7)	57·7 (35·6–87·1)
1-113	Anorexia nervosa	8 (5–13)	12 (7–20)	12 (7–20)	11·9 (6·9–19)	13·5 (7·7–22·4)	13·6 (7·8–21·7)

1-114	Bulimia nervosa	25 (14–40)	41 (24–64)	41 (24–65)	38·1 (21·3–59)	44·1 (25·2–68·9)	44·1 (25·8–69·4)
1-115	Autism spectrum disorders	32 (21–47)	44 (29–65)	45 (30–66)	52·3 (33·6–75·9)	52·3 (34·4–76·9)	52·8 (35–77·1)
1-116	Attention-deficit/hyperactivity disorder	24 (14–40)	32 (18–53)	30 (17–50)	36·9 (21·3–62·1)	36·8 (20·6–61·5)	37·2 (21·4–62·6)
1-117	Conduct disorder	45 (25–72)	56 (32–89)	48 (27–76)	67·8 (38–108)	67·9 (38·3–108)	68·2 (38·5–108)
1-118	Idiopathic developmental intellectual disability	9 (3–16)	10 (3–18)	9 (2–18)	13·8 (4·1–25·9)	11·1 (3·1–21·5)	10·7 (2·9–20·6)
1-119	Other mental disorders	60 (38–91)	100 (63–152)	112 (71–167)	108 (68·7–162)	107 (68·5–163)	108 (69·1–162)
1-120	Substance use disorders	285 (231–349)	494 (407–596)	545 (444–669)	516 (423–625)	530 (436–636)	526 (429–648)
1-121	Alcohol use disorders	221 (183–268)	380 (314–460)	428 (348–531)	414 (344–496)	407 (337–492)	406 (328–507)
1-122	Drug use disorders	64 (45–86)	114 (84–147)	117 (87–151)	101 (71–136)	123 (90·1–158)	120 (89·1–157)
1-123	Opioid use disorders	28 (18–41)	49 (33–68)	53 (36–72)	46·2 (29·8–65·6)	52·8 (35·4–72·7)	53·2 (36·4–72·7)
1-124	Cocaine use disorders	11 (7–17)	26 (19–34)	24 (18–33)	17·6 (11·4–25·5)	27·5 (20·4–36·3)	25 (17·9–34·3)
1-125	Amphetamine use disorders	4 (2–7)	7 (5–11)	8 (5–11)	6·4 (3·6–10·4)	8 (5·1–12·1)	7·9 (5–12)
1-126	Cannabis use disorders	10 (5–17)	14 (7–23)	14 (8–23)	15·2 (8·1–25·5)	15·1 (8·1–25·2)	15·3 (8·1–25·6)
1-127	Other drug use disorders	10 (6–15)	18 (12–25)	18 (12–25)	15·7 (9·7–23·2)	19·2 (13·2–26·9)	18·7 (12·7–26·7)
1-128	Diabetes and kidney diseases	1,380 (1,250–1,520)	2,380 (2,110–2,690)	3,090 (2,660–3,550)	2,620 (2,370–2,890)	2,940 (2,610–3,300)	3,040 (2,630–3,480)
1-129	Diabetes mellitus	974 (868–1,100)	1,590 (1,370–1,840)	2,060 (1,710–2,430)	1,860 (1,650–2,100)	1,980 (1,720–2,280)	2,010 (1,680–2,360)
1-130	Diabetes mellitus type 1	80 (64–97)	97 (82–127)	107 (87–142)	143 (113–175)	112 (94·8–147)	107 (87·6–142)
1-131	Diabetes mellitus type 2	894 (793–1,010)	1,490 (1,270–1,740)	1,950 (1,610–2,330)	1,720 (1,520–1,950)	1,870 (1,610–2,170)	1,900 (1,570–2,250)
1-132	Chronic kidney disease	406 (370–442)	791 (717–870)	1,030 (886–1,200)	756 (689–829)	959 (874–1,050)	1,030 (888–1,180)
1-133	Chronic kidney disease due to diabetes mellitus type 1	29 (20–40)	60 (41–82)	73 (47–108)	56·6 (38·5–79·6)	64·1 (44·4–89·3)	67·8 (44·3–99·2)
1-134	Chronic kidney disease due to diabetes mellitus type 2	93 (75–111)	180 (145–214)	252 (196–311)	178 (142–216)	228 (185–272)	248 (194–305)
1-135	Chronic kidney disease due to hypertension	82 (67–99)	158 (131–187)	215 (172–265)	152 (124–184)	202 (167–239)	219 (177–267)
1-136	Chronic kidney disease due to glomerulonephritis	90 (74–107)	175 (143–214)	219 (170–282)	167 (136–202)	201 (165–247)	215 (168–274)
1-137	Chronic kidney disease due to other and unspecified causes	112 (92–135)	219 (178–265)	276 (219–343)	202 (165–247)	264 (214–319)	282 (226–346)
1-138	Acute glomerulonephritis	0 (0–0)	0 (0–0)	0 (0–0)	0·3 (0·2–0·3)	0·2 (0·1–0·2)	0·2 (0·1–0·2)
1-139	Skin and subcutaneous diseases	364 (252–529)	522 (369–745)	557 (395–777)	602 (419–869)	634 (450–896)	644 (456–902)
1-140	Dermatitis	72 (42–114)	95 (57–148)	94 (56–145)	116 (68·5–184)	116 (69·3–182)	116 (68·1–179)
1-141	Atopic dermatitis	54 (30–91)	67 (36–114)	63 (34–105)	85·3 (46·6–144)	85·2 (45·7–145)	85·1 (46·1–141)
1-142	Contact dermatitis	16 (10–24)	24 (15–37)	27 (17–40)	27·2 (17·2–41·3)	27·2 (17–40·8)	27·1 (17·1–40·6)
1-143	Seborrhoeic dermatitis	2 (1–3)	3 (2–5)	3 (2–5)	3·4 (1·9–5·3)	3·4 (1·9–5·4)	3·4 (1·9–5·3)
1-144	Psoriasis	20 (14–26)	28 (19–38)	30 (21–40)	34 (24–45·5)	31·2 (21·9–42·2)	30·5 (21·5–41)
1-145	Bacterial skin diseases	11 (7–22)	32 (18–60)	43 (24–76)	19·4 (12·7–38·5)	40 (22·9–73·8)	47·5 (26·4–81·4)
1-146	Cellulitis	5 (3–10)	10 (6–20)	13 (7–24)	7·8 (5–16·7)	12·7 (6·9–24·3)	14·2 (7·6–25·5)
1-147	Pyoderma	7 (4–13)	22 (12–39)	30 (16–52)	11·6 (6·8–22·9)	27·3 (14·8–48·3)	33·3 (17·6–56·5)
1-148	Scabies	52 (29–84)	70 (38–112)	69 (38–112)	83·6 (46·6–134)	83·1 (45·3–133)	83 (45·7–133)
1-149	Fungal skin diseases	26 (10–55)	37 (15–78)	40 (16–86)	44·2 (17·8–93·2)	43·9 (17·7–92·4)	43·8 (17·6–93·2)
1-150	Viral skin diseases	30 (19–46)	37 (24–56)	35 (22–52)	47·5 (30·1–71·5)	47·6 (30–71)	47·6 (30–72·3)
1-151	Acne vulgaris	32 (19–51)	47 (28–76)	45 (27–73)	47·4 (28·1–75·4)	53·7 (31·8–87·2)	55·3 (33·1–89)
1-152	Alopecia areata	4 (3–6)	7 (4–10)	7 (4–10)	7·2 (4·6–10·6)	7·2 (4·5–10·6)	7·1 (4·4–10·7)
1-153	Pruritus	5 (2–9)	8 (4–14)	8 (4–15)	8·6 (4·1–15·6)	8·8 (4·2–16)	8·8 (4·2–15·7)
1-154	Urticaria	30 (20–44)	40 (26–57)	39 (26–56)	48·3 (31·7–69·9)	48·3 (31·6–69·8)	48·2 (31·9–70·2)
1-155	Decubitus ulcer	50 (34–88)	74 (48–129)	92 (59–154)	90 (60·7–158)	97·3 (63·9–165)	99·7 (63·4–167)
1-156	Other skin and subcutaneous diseases	31 (17–53)	48 (26–82)	55 (30–94)	55·5 (30·1–94·4)	56·8 (31·4–96·3)	57·4 (32·1–97·3)
1-157	Sense organ diseases	432 (295–613)	608 (406–884)	721 (483–1,050)	787 (533–1,120)	742 (497–1,060)	729 (493–1,050)
1-158	Blindness and vision loss	125 (86–177)	160 (109–230)	188 (127–271)	226 (154–322)	200 (136–286)	194 (132–277)
1-159	Glaucoma	8 (6–12)	8 (5–11)	9 (6–14)	14·4 (9·6–20·7)	11·2 (7·5–16·2)	10·5 (7–15·1)
1-160	Cataract	24 (17–32)	26 (18–35)	30 (21–41)	41·8 (29·2–56·9)	34·3 (23·8–46·7)	32·7 (22·8–44·9)
1-161	Age-related macular degeneration	1 (1–1)	1 (1–1)	1 (1–2)	1·6 (1·1–2·4)	1·4 (0·9–2)	1·4 (0·9–2)
1-162	Refraction disorders	34 (22–49)	48 (31–68)	51 (34–73)	58·1 (38·2–83·6)	54·7 (36·1–78·4)	53·2 (35·3–76·6)
1-163	Near vision loss	27 (12–53)	39 (18–76)	48 (22–99)	51·4 (23·2–102)	48 (22·1–94·5)	47 (21·2–94·7)
1-164	Other vision loss	32 (22–44)	40 (28–55)	48 (34–65)	58·3 (41·1–80·3)	50·3 (35·4–68·6)	48·8 (34·6–67·4)
1-165	Age-related and other hearing loss	287 (193–417)	417 (274–617)	497 (326–735)	525 (349–763)	506 (334–739)	498 (330–727)
1-166	Other sense organ diseases	20 (12–32)	31 (18–48)	36 (22–56)	36·6 (21·5–56·8)	37 (21·9–57·5)	37 (22·2–57·1)

1-167	Musculoskeletal disorders	817 (591–1,080)	1,400 (1,020–1,860)	1,630 (1,170–2,140)	1,500 (1,080–1,970)	1,560 (1,130–2,060)	1,570 (1,130–2,060)
1-168	Rheumatoid arthritis	17 (12–22)	32 (23–42)	38 (28–50)	30·9 (22·4–40)	36 (26·4–46·9)	37·3 (27·5–48·8)
1-169	Osteoarthritis	97 (49–195)	166 (82–334)	215 (108–431)	191 (96·8–389)	201 (100–404)	203 (102–408)
1-170	Low back pain	405 (283–544)	652 (454–872)	726 (507–979)	725 (510–971)	722 (508–965)	713 (500–956)
1-171	Neck pain	72 (48–104)	126 (82–184)	142 (93–207)	136 (89·1–197)	135 (89·2–196)	135 (88·5–194)
1-172	Gout	4 (2–5)	7 (4–10)	8 (5–12)	6·6 (4·1–9·7)	7·6 (4·8–11)	7·9 (4·8–11·4)
1-173	Other musculoskeletal disorders	222 (158–301)	417 (292–572)	495 (347–669)	407 (289–554)	453 (319–619)	473 (333–640)
1-174	Other non-communicable diseases	1,150 (945–1,420)	1,560 (1,250–1,970)	1,640 (1,290–2,080)	1,940 (1,590–2,410)	1,930 (1,570–2,390)	1,900 (1,510–2,360)
1-175	Congenital anomalies	320 (264–398)	327 (268–402)	265 (206–348)	494 (408–614)	501 (404–623)	452 (344–606)
1-176	Neural tube defects	17 (12–24)	12 (9–16)	13 (9–19)	26·4 (18·1–37·6)	19·8 (14·5–26·3)	23·8 (15·7–35·2)
1-177	Congenital heart anomalies	154 (123–196)	148 (110–189)	109 (76–151)	237 (190–302)	235 (174–302)	198 (135–277)
1-178	Orofacial clefts	7 (3–16)	4 (3–6)	4 (3–6)	11 (5–23·8)	6·5 (4·4–9·2)	5·6 (3·8–8·5)
1-179	Down's syndrome	7 (6–9)	14 (11–16)	14 (10–18)	11·8 (9·7–14·4)	18·2 (14·6–22·4)	18·6 (13·8–24·7)
1-180	Turner syndrome	0 (0–0)	0 (0–0)	0 (0–0)	0·1 (0·1–0·2)	0·1 (0·1–0·2)	0·1 (0·1–0·2)
1-181	Klinefelter syndrome	0 (0–0)	0 (0–0)	0 (0–0)	0 (0–0)	0 (0–0)	0 (0–0)
1-182	Other chromosomal abnormalities	16 (11–29)	24 (17–32)	19 (14–26)	24·7 (17·5–43·7)	37·5 (27·2–51·4)	34·5 (24·4–49·2)
1-183	Congenital musculoskeletal and limb anomalies	32 (22–44)	41 (29–57)	40 (27–55)	50·3 (35·1–68·9)	52·8 (37·5–73·2)	53·2 (37·2–72·6)
1-184	Urogenital congenital anomalies	3 (2–5)	3 (2–4)	3 (2–4)	4·8 (3·5–7·3)	4·9 (3·6–6·6)	4·7 (3·3–6·5)
1-185	Digestive congenital anomalies	19 (13–30)	17 (12–25)	13 (9–20)	28·6 (20·2–46·5)	28·5 (20·2–41·9)	26 (17·2–39·3)
1-186	Other congenital anomalies	64 (49–83)	64 (50–82)	51 (37–70)	99·5 (76–128)	98·4 (75·5–128)	87·4 (62–121)
1-187	Urinary diseases and male infertility	44 (34–57)	101 (82–119)	130 (106–157)	78·2 (61·3–102)	128 (105–151)	135 (110–163)
1-188	Urinary tract infections and interstitial nephritis	21 (18–28)	63 (52–70)	82 (65–97)	36·7 (31·7–49·5)	80·8 (65·6–90·1)	87 (69·5–103)
1-189	Urolithiasis	2 (2–3)	5 (4–7)	6 (5–8)	4·5 (3·3–5·9)	5·6 (4·3–7·2)	6 (4·6–7·8)
1-190	Benign prostatic hyperplasia	13 (8–20)	19 (11–31)	27 (16–42)	24·2 (14·1–37·8)	26·3 (15·3–41·2)	26·7 (15·6–41·6)
1-191	Male infertility	3 (1–6)	4 (1–9)	4 (2–9)	4 (1·5–9·8)	4 (1·5–9·5)	4·1 (1·5–9·7)
1-192	Other urinary diseases	5 (4–8)	9 (6–12)	11 (7–14)	8·8 (6·8–13·7)	11·6 (7·7–15·1)	11·6 (7·3–14·9)
1-193	Gynecological diseases	226 (155–318)	370 (257–525)	374 (257–528)	386 (265–543)	384 (266–542)	369 (254–521)
1-194	Uterine fibroids	15 (8–26)	27 (15–48)	29 (15–51)	27·5 (15–48·9)	27·3 (14·7–48·7)	26·8 (14·3–48·3)
1-195	Polycystic ovarian syndrome	3 (1–6)	6 (3–13)	6 (3–13)	4·9 (2–10·1)	6·4 (2·6–13·1)	6·7 (2·8–13·6)
1-196	Female infertility	5 (2–13)	8 (3–20)	8 (3–19)	8·3 (2·9–19·6)	8·5 (3–20·1)	8·2 (2·9–20)
1-197	Endometriosis	20 (11–32)	27 (16–43)	25 (15–41)	31·3 (18·5–49·7)	28·2 (16·2–44·7)	25·8 (14·9–42·2)
1-198	Genital prolapse	4 (2–7)	5 (3–10)	6 (3–11)	7·2 (3·4–13·8)	6 (2·8–11·6)	5·4 (2·5–10·3)
1-199	Premenstrual syndrome	66 (40–102)	102 (62–159)	99 (60–153)	105 (64·6–162)	106 (64·3–165)	103 (62·5–160)
1-200	Other gynecological diseases	114 (74–162)	194 (125–280)	201 (128–291)	202 (130–287)	201 (131–289)	193 (123–277)
1-201	Hemoglobinopathies and hemolytic anaemias	116 (97–140)	134 (115–160)	136 (112–168)	187 (158–224)	161 (137–191)	159 (130–198)
1-202	Thalassemias	0 (0–0)	0 (0–0)	0 (0–0)	0·1 (0·1–0·3)	0·3 (0·2–0·6)	0·2 (0·1–0·5)
1-203	Thalassaemias trait	2 (1–4)	3 (2–5)	3 (2–5)	3·7 (2·3–5·9)	3·7 (2·2–5·8)	3·6 (2·1–5·6)
1-204	Sickle cell disorders	50 (43–58)	64 (56–74)	61 (48–79)	78·4 (67·7–91·2)	76·8 (66·6–89·9)	76·5 (59·2–99·5)
1-205	Sickle cell trait	26 (16–40)	24 (14–39)	20 (12–33)	39·4 (24·2–60·5)	28·7 (17–45·8)	24·2 (13·9–38)
1-206	G6PD deficiency	12 (8–16)	17 (12–21)	20 (14–26)	20·1 (14–26·8)	18·7 (13·6–24·2)	20·4 (14·4–27)
1-207	G6PD trait	0 (0–0)	0 (0–0)	0 (0–0)	0·2 (0·1–0·3)	0·2 (0·1–0·4)	0·2 (0·1–0·4)
1-208	Other hemoglobinopathies and hemolytic anaemias	26 (21–32)	26 (21–34)	32 (25–40)	44·8 (36·5–56·2)	32·5 (26–41·3)	34·1 (27·1–43·4)
1-209	Endocrine, metabolic, blood, and immune disorders	257 (196–306)	344 (280–459)	411 (328–536)	454 (344–541)	424 (344–566)	454 (363–594)
1-210	Oral disorders	186 (109–304)	281 (160–463)	324 (186–528)	337 (198–552)	324 (188–525)	321 (185–517)
1-211	Caries of deciduous teeth	2 (1–4)	2 (1–4)	2 (1–4)	3·1 (1·3–6·3)	3·1 (1·3–6·4)	3·1 (1·3–6·4)
1-212	Caries of permanent teeth	16 (7–30)	23 (10–44)	24 (10–47)	26 (11·5–50·1)	25·1 (11·2–48·4)	25 (11–48·7)
1-213	Periodontal disease	63 (24–132)	110 (43–231)	123 (48–260)	116 (45·4–245)	116 (45·5–243)	116 (45·1–245)
1-214	Edentulism	74 (47–111)	99 (63–148)	126 (80–188)	138 (87·6–210)	126 (80·1–189)	124 (78·5–186)
1-215	Other oral disorders	32 (20–46)	48 (30–70)	50 (32–74)	53·3 (33–78·1)	53·4 (33·2–78·2)	53·2 (33·2–78·3)
1-216	Sudden infant death syndrome	2 (1–3)	4 (2–6)	3 (2–5)	3 (2·1–4·3)	6·7 (4·3–10·1)	7·1 (4·3–11)
1-0	Injuries	1,640 (1,490–1,810)	2,170 (1,950–2,430)	2,170 (1,860–2,520)	2,720 (2,470–3,030)	2,480 (2,240–2,770)	2,340 (2,000–2,720)
1-1	Transport injuries	394 (359–433)	387 (340–454)	448 (374–520)	657 (600–724)	433 (382–510)	471 (392–548)
1-2	Road injuries	375 (342–414)	350 (307–411)	408 (340–473)	627 (571–692)	392 (345–460)	428 (356–500)

1-3	Pedestrian road injuries	59<br>(53–66)	49<br>(42–56)	55<br>(45–66)	102<br>(92·5–115)	57·2<br>(49·9–65·7)	59·1<br>(48·4–70·9)
1-4	Cyclist road injuries	41<br>(33–49)	43<br>(34–54)	50<br>(39–62)	69·9<br>(57·1–84·3)	47·5<br>(37·8–59·5)	51<br>(40·2–63·1)
1-5	Motorcyclist road injuries	57<br>(48–67)	74<br>(62–93)	96<br>(75–118)	92·3<br>(78·3–109)	81·4<br>(68–102)	99·9<br>(77·1–124)
1-6	Motor vehicle road injuries	212<br>(192–236)	175<br>(152–207)	196<br>(160–232)	351<br>(317–388)	196<br>(170–230)	206<br>(168–246)
1-7	Other road injuries	7<br>(5–8)	9<br>(8–12)	11<br>(9–14)	11·5<br>(9·2–14·1)	10·6<br>(8·5–13)	11·7<br>(9·3–14·4)
1-8	Other transport injuries	18<br>(16–20)	36<br>(31–44)	40<br>(32–48)	30·5<br>(27·3–34·1)	41·3<br>(35–49·3)	42·6<br>(33·6–51·2)
1-9	Unintentional injuries	1,030<br>(918–1,170)	1,350<br>(1,180–1,560)	1,310<br>(1,090–1,560)	1,720<br>(1,530–1,960)	1,570<br>(1,380–1,810)	1,440<br>(1,200–1,710)
1-10	Falls	84<br>(70–102)	144<br>(117–178)	179<br>(144–225)	147<br>(123–179)	173<br>(142–212)	188<br>(152–236)
1-11	Drowning	208<br>(186–233)	232<br>(206–260)	202<br>(169–243)	331<br>(296–369)	278<br>(246–315)	246<br>(202–301)
1-12	Fire, heat, and hot substances	157<br>(134–188)	149<br>(121–187)	144<br>(115–184)	266<br>(226–319)	176<br>(146–218)	160<br>(128–202)
1-13	Poisonings	41<br>(36–47)	17<br>(14–20)	15<br>(12–19)	66·6<br>(59·1–75·2)	21·1<br>(17·8–24·7)	18·4<br>(14·6–23·5)
1-14	Poisoning by carbon monoxide	12<br>(10–14)	8<br>(7–9)	7<br>(6–8)	19·4<br>(16·5–22·6)	9·5<br>(8–11·1)	8·1<br>(6·4–10·1)
1-15	Poisoning by other means	29<br>(26–34)	9<br>(7–11)	8<br>(6–11)	47·2<br>(41·2–54·3)	11·6<br>(9·2–14·3)	10·4<br>(7·7–13·8)
1-16	Exposure to mechanical forces	304<br>(260–358)	524<br>(444–629)	487<br>(398–596)	514<br>(438–609)	587<br>(498–702)	508<br>(417–623)
1-17	Unintentional firearm injuries	148<br>(128–170)	255<br>(221–288)	216<br>(179–259)	242<br>(210–277)	281<br>(244–316)	227<br>(186–272)
1-18	Other exposure to mechanical forces	156<br>(121–206)	269<br>(203–354)	270<br>(201–362)	273<br>(210–359)	306<br>(233–401)	282<br>(213–373)
1-19	Adverse effects of medical treatment	44<br>(38–49)	43<br>(38–49)	48<br>(39–58)	76·6<br>(65·5–85·1)	54·4<br>(47·7–61·2)	53·1<br>(43·4–64·5)
1-20	Animal contact	6<br>(5–8)	16<br>(14–19)	18<br>(14–21)	10·6<br>(7·8–14·2)	19·8<br>(16·9–23·3)	19·5<br>(16·2–23·1)
1-21	Venomous animal contact	4<br>(3–5)	10<br>(8–12)	11<br>(9–13)	6·8<br>(4·8–9·2)	11·7<br>(9·7–13·9)	11·8<br>(9·6–14·3)
1-22	Non-venomous animal contact	2<br>(2–3)	7<br>(6–8)	7<br>(6–9)	3·8<br>(2·8–5·3)	8·2<br>(7–9·7)	7·7<br>(6·4–9·5)
1-23	Foreign body	54<br>(47–62)	50<br>(44–59)	52<br>(43–63)	89·3<br>(78·6–101)	67·2<br>(57·2–79·3)	67·9<br>(53·6–85·3)
1-24	Pulmonary aspiration and foreign body in airway	44<br>(39–51)	37<br>(32–44)	38<br>(30–48)	72·8<br>(64·2–83)	51·6<br>(42·7–63·6)	52·4<br>(40·1–69·2)
1-25	Foreign body in eyes	3<br>(1–6)	5<br>(2–9)	5<br>(3–9)	5·4<br>(2·6–9·5)	5·4<br>(2·6–9·4)	5·4<br>(2·6–9·5)
1-26	Foreign body in other body part	7<br>(5–8)	9<br>(7–11)	9<br>(7–12)	11·2<br>(9–13·9)	10·2<br>(8·2–12·7)	10·1<br>(7·9–12·8)
1-27	Environmental heat and cold exposure	28<br>(20–38)	39<br>(28–53)	44<br>(32–61)	48·4<br>(34·9–65·5)	44·1<br>(31·8–60·2)	45·9<br>(33·3–62·5)
1-28	Exposure to forces of nature	1<br>(0–1)	6<br>(5–9)	5<br>(3–6)	1<br>(0·7–1·4)	7·6<br>(5·6–10)	4·7<br>(3·4–6·2)
1-29	Other unintentional injuries	100<br>(84–120)	125<br>(105–153)	118<br>(95–148)	166<br>(140–202)	142<br>(119–174)	127<br>(103–158)
1-30	Self-harm and interpersonal violence	216<br>(197–236)	435<br>(397–474)	410<br>(354–473)	347<br>(318–378)	477<br>(434–521)	432<br>(373–500)
1-31	Self-harm	63<br>(56–71)	120<br>(106–135)	130<br>(108–154)	104<br>(93·4–117)	129<br>(115–146)	132<br>(110–157)
1-32	Self-harm by firearm	2<br>(1–2)	3<br>(3–4)	3<br>(2–4)	3·3<br>(2·5–4·2)	3·5<br>(2·8–4·8)	3·1<br>(2·5–4·1)
1-33	Self-harm by other specified means	61<br>(54–69)	117<br>(103–131)	127<br>(105–150)	101<br>(90·4–113)	126<br>(111–142)	129<br>(107–153)
1-34	Interpersonal violence	153<br>(137–169)	312<br>(281–344)	279<br>(238–325)	242<br>(218–267)	344<br>(309–379)	298<br>(254–348)
1-35	Physical violence by firearm	45<br>(39–52)	122<br>(108–137)	106<br>(89–125)	67·7<br>(58·6–78·3)	134<br>(118–151)	114<br>(94·9–134)
1-36	Physical violence by sharp object	26<br>(22–30)	52<br>(46–58)	46<br>(39–53)	40·9<br>(34·5–47·5)	56·1<br>(49·4–62·7)	48·3<br>(41–56)
1-37	Sexual violence	11<br>(7–16)	16<br>(11–23)	17<br>(11–24)	17·9<br>(11·8–25·4)	18<br>(11·9–25·5)	17·8<br>(11·8–25·3)
1-38	Physical violence by other means	71<br>(63–79)	122<br>(110–134)	111<br>(93–130)	116<br>(104–128)	135<br>(123–149)	119<br>(99·5–141)
1-39	Conflict and terrorism	0<br>(0–0)	0<br>(0–0)	0<br>(0–0)	0<br>(0–0)	0·4<br>(0·3–0·6)	0·2<br>(0·1–0·3)
1-40	Executions and police conflict	0<br>(0–0)	3<br>(2–4)	1<br>(1–2)	0·6<br>(0·6–0·7)	3·4<br>(2·5–4·9)	1·2<br>(0·8–1·8)

SR Table 1: All-age DALYs counts and age standardized DALYs rates per 100,000 individuals for all causes and level 1 cause groups by location for 1990, 2010, and 2019, both sexes combined, The Bahamas

Cause Level	Cause	All-age DALYs			Age-standardized rate of DALYs per 100,000		
		1990	2010	2019	1990	2010	2019
0-0	All causes	73,600 (66,800–81,200)	104,000 (93,500–115,000)	122,000 (104,000–142,000)	35,100 (32,100–38,500)	31,300 (28,300–34,500)	31,300 (26,900–36,500)
1-0	Communicable, maternal, neonatal, and nutritional disorders	15,200 (13,800–16,800)	15,800 (14,600–17,200)	14,900 (13,300–16,600)	6,250 (5,670–6,860)	4,840 (4,440–5,280)	4,330 (3,830–4,920)
1-1	HIV/AIDS and sexually transmitted infections	3,510 (3,370–3,700)	6,310 (6,090–6,620)	5,810 (5,600–6,070)	1,390 (1,330–1,460)	1,600 (1,550–1,680)	1,350 (1,300–1,410)
1-2	HIV/AIDS	3,460 (3,320–3,640)	6,220 (6,020–6,530)	5,720 (5,530–5,970)	1,370 (1,310–1,440)	1,580 (1,530–1,660)	1,330 (1,280–1,390)
1-3	HIV/AIDS - drug-susceptible tuberculosis	273 (176–421)	198 (131–294)	176 (113–263)	108 (69–166)	50·5 (33·4–74·9)	41 (26·2–61)
1-4	HIV/AIDS - Multidrug-resistant Tuberculosis without extensive drug resistance	1 (0–3)	5 (1–19)	7 (1–28)	0·3 (0–1·1)	1·4 (0·2–4·8)	1·6 (0·2–6·5)
1-5	HIV/AIDS - Extensively drug-resistant Tuberculosis	0 (0–0)	1 (0–2)	1 (0–5)	0 (0–0)	0·1 (0–0·5)	0·3 (0–1·1)
1-6	HIV/AIDS resulting in other diseases	3,180 (2,990–3,390)	6,020 (5,800–6,320)	5,540 (5,330–5,780)	1,260 (1,180–1,340)	1,530 (1,480–1,610)	1,290 (1,240–1,350)
1-7	Sexually transmitted infections excluding HIV	54 (34–88)	81 (50–136)	86 (51–147)	21·6 (13·5–35·9)	21·1 (13·2–35·3)	20·4 (12·3–34·7)
1-8	Syphilis	6 (4–7)	5 (4–6)	5 (4–6)	2·3 (1·8–2·8)	1·4 (1·2–1·7)	1·3 (1–1·6)
1-9	Chlamydial infection	6 (4–9)	9 (6–14)	10 (7–14)	2·3 (1·6–3·3)	2·4 (1·7–3·4)	2·3 (1·6–3·3)
1-10	Gonococcal infection	5 (4–7)	9 (7–11)	9 (7–12)	2 (1·6–2·6)	2·3 (1·9–2·8)	2·2 (1·6–2·8)
1-11	Trichomoniasis	13 (5–26)	19 (8–40)	21 (8–43)	5·1 (2–10·5)	5 (2–10·3)	4·8 (1·9–9·9)
1-12	Genital herpes	12 (4–30)	20 (6–49)	23 (7–56)	5·4 (1·8–13·3)	5·5 (1·7–13·3)	5·4 (1·7–13·2)
1-13	Other sexually transmitted infections	12 (8–17)	18 (13–25)	19 (13–26)	4·5 (3·2–6·3)	4·6 (3·3–6·3)	4·4 (3·1–6)
1-14	Respiratory infections and tuberculosis	3,080 (2,770–3,420)	2,860 (2,590–3,160)	2,980 (2,470–3,590)	1,460 (1,330–1,610)	950 (861–1,050)	873 (717–1,040)
1-15	Tuberculosis	498 (447–556)	321 (288–355)	307 (245–379)	221 (199–246)	89·6 (80·8–99)	75 (60·3–92·3)
1-16	Latent tuberculosis infection	0 (0–0)	0 (0–0)	0 (0–0)	0 (0–0)	0 (0–0)	0 (0–0)
1-17	Drug-susceptible tuberculosis	497 (445–555)	313 (278–347)	296 (234–367)	221 (199–245)	87·5 (78–97)	72·4 (57·4–89)
1-18	Multidrug-resistant tuberculosis without extensive drug resistance	1 (0–5)	7 (1–21)	9 (1–37)	0·4 (0–2)	1·9 (0·4–6)	2·3 (0·3–9)
1-19	Extensively drug-resistant tuberculosis	0 (0–0)	1 (0–2)	1 (0–6)	0 (0–0)	0·2 (0–0·6)	0·4 (0–1·4)
1-20	Lower respiratory infections	2,280 (2,020–2,570)	2,160 (1,980–2,370)	2,290 (1,850–2,780)	1,130 (1,010–1,250)	753 (684–826)	690 (560–832)
1-21	Upper respiratory infections	234 (148–359)	293 (178–458)	303 (187–471)	88·4 (56·1–135)	83·5 (50·4–130)	83·8 (51·8–130)
1-22	Otitis media	65 (37–109)	83 (48–137)	85 (48–141)	24·3 (14·1–40·1)	23·8 (13·5–39·2)	24·2 (13·8–39·7)
1-23	Enteric infections	650 (519–810)	616 (473–798)	686 (517–891)	279 (226–345)	197 (153–252)	203 (153–261)
1-24	Diarrhoeal diseases	644 (515–805)	610 (466–791)	680 (511–885)	277 (224–343)	195 (151–250)	202 (152–259)
1-25	Typhoid and paratyphoid	0 (0–0)	0 (0–0)	0 (0–0)	0 (0–0)	0 (0–0)	0 (0–0)
1-26	Typhoid fever	0 (0–0)	0 (0–0)	0 (0–0)	0 (0–0)	0 (0–0)	0 (0–0)
1-27	Paratyphoid fever	0 (0–0)	0 (0–0)	0 (0–0)	0 (0–0)	0 (0–0)	0 (0–0)
1-28	iNTS	3 (2–8)	5 (3–7)	5 (3–7)	1·3 (0·9–3)	1·5 (0·9–2·1)	1·4 (0·9–2·2)
1-29	Other intestinal infectious diseases	2 (1–3)	2 (1–2)	1 (1–2)	0·7 (0·3–1·2)	0·5 (0·3–0·7)	0·4 (0·3–0·7)
1-30	Neglected tropical diseases and malaria	200 (133–283)	259 (171–376)	268 (175–399)	92·2 (60·1–134)	77·5 (51·5–112)	71·5 (47–104)
1-31	Malaria	0 (0–0)	0 (0–0)	0 (0–0)	0 (0–0)	0 (0–0)	0 (0–0)
1-32	Chagas disease	0 (0–0)	0 (0–0)	0 (0–0)	0 (0–0)	0 (0–0)	0 (0–0)
1-33	Leishmaniasis	0 (0–0)	0 (0–0)	0 (0–0)	0 (0–0)	0 (0–0)	0 (0–0)
1-34	Visceral leishmaniasis	0 (0–0)	0 (0–0)	0 (0–0)	0 (0–0)	0 (0–0)	0 (0–0)
1-35	Cutaneous and mucocutaneous leishmaniasis	0 (0–0)	0 (0–0)	0 (0–0)	0 (0–0)	0 (0–0)	0 (0–0)
1-36	African trypanosomiasis	0 (0–0)	0 (0–0)	0 (0–0)	0 (0–0)	0 (0–0)	0 (0–0)
1-37	Schistosomiasis	0 (0–0)	0 (0–0)	0 (0–0)	0 (0–0)	0 (0–0)	0 (0–0)
1-38	Cysticercosis	100 (48–170)	127 (60–233)	147 (70–267)	54·4 (26·4–91·7)	39·3 (18·7–70·6)	36·3 (17·5–65·1)
1-39	Cystic echinococcosis	0 (0–0)	0 (0–0)	0 (0–0)	0 (0–0)	0 (0–0)	0 (0–0)
1-40	Lymphatic filariasis	0 (0–0)	0 (0–0)	0 (0–0)	0 (0–0)	0 (0–0)	0 (0–0)
1-41	Onchocerciasis	0 (0–0)	0 (0–0)	0 (0–0)	0 (0–0)	0 (0–0)	0 (0–0)
1-42	Trachoma	0 (0–0)	0 (0–0)	0 (0–0)	0 (0–0)	0 (0–0)	0 (0–0)
1-43	Dengue	18 (9–32)	46 (27–74)	34 (19–56)	7·1 (3·7–12·3)	13 (7·9–20·8)	9·4 (5·3–15·3)
1-44	Yellow fever	0 (0–0)	0 (0–0)	0 (0–0)	0 (0–0)	0 (0–0)	0 (0–0)
1-45	Rabies	0 (0–0)	0 (0–0)	0 (0–0)	0 (0–0·1)	0 (0–0)	0 (0–0)
1-46	Intestinal nematode infections	10 (6–16)	13 (8–22)	12 (7–20)	3·5 (2·1–5·5)	3·6 (2·1–6)	3·3 (1·9–5·3)
1-47	Ascariasis	1 (0–2)	1 (0–1)	0 (0–1)	0·5 (0·2–0·9)	0·2 (0·1–0·3)	0·1 (0·1–0·3)
1-48	Trichuriasis	0 (0–0)	0 (0–0)	0 (0–0)	0 (0–0)	0 (0–0)	0 (0–0)

1-49	Hookworm disease	9 (5–15)	13 (7–22)	12 (7–20)	3 (1·7–5)	3·4 (1·9–5·8)	3·1 (1·7–5·1)
1-50	Food-borne trematodiases	0 (0–0)	0 (0–0)	0 (0–0)	0 (0–0)	0 (0–0)	0 (0–0)
1-51	Leprosy	0 (0–0)	0 (0–1)	1 (0–1)	0·1 (0·1–0·2)	0·1 (0·1–0·2)	0·1 (0·1–0·2)
1-52	Ebola virus disease	0 (0–0)	0 (0–0)	0 (0–0)	0 (0–0)	0 (0–0)	0 (0–0)
1-53	Zika virus disease	0 (0–0)	0 (0–0)	2 (0–10)	0 (0–0)	0 (0–0)	0·6 (0–2·8)
1-54	Guinea worm disease	0 (0–0)	0 (0–0)	0 (0–0)	0 (0–0)	0 (0–0)	0 (0–0)
1-55	Other neglected tropical diseases	71 (45–107)	73 (45–111)	72 (46–105)	27·1 (17·1–40·6)	21·4 (13·3–32·8)	21·8 (13·8–32·2)
1-56	Other infectious diseases	751 (660–853)	520 (453–587)	492 (401–599)	301 (265–342)	158 (138–180)	142 (116–173)
1-57	Meningitis	398 (330–474)	178 (155–201)	137 (107–173)	160 (133–190)	57·5 (49·6–66·4)	42·1 (32·2–53·5)
1-58	Encephalitis	163 (118–195)	119 (104–143)	118 (94–154)	62·4 (45·1–75)	36 (31·1–44·5)	35·9 (28·2–47·7)
1-59	Diphtheria	0 (0–0)	0 (0–0)	0 (0–0)	0 (0–0)	0 (0–0)	0 (0–0)
1-60	Whooping cough	2 (1–2)	4 (2–6)	3 (2–5)	0·6 (0·4–0·9)	1·5 (0·9–2·4)	1·4 (0·8–2·2)
1-61	Tetanus	8 (6–12)	3 (2–5)	2 (1–5)	4·1 (2·9–5·8)	0·9 (0·7–1·4)	0·6 (0·3–1·2)
1-62	Measles	0 (0–0)	0 (0–0)	0 (0–0)	0·1 (0·1–0·1)	0 (0–0)	0 (0–0)
1-63	Varicella and herpes zoster	16 (10–26)	20 (13–30)	22 (14–32)	7·1 (4·3–10·8)	6·1 (4–9·4)	5·9 (3·9–9·2)
1-64	Acute hepatitis	60 (51–70)	28 (23–36)	29 (23–37)	23·5 (20·2–27·3)	7·8 (6·2–10)	7·6 (5·9–9·9)
1-65	Acute hepatitis A	36 (27–46)	17 (12–23)	16 (11–23)	13·8 (10·4–17·5)	4·6 (3·3–6·3)	4·4 (3–6·1)
1-66	Acute hepatitis B	18 (13–24)	9 (7–10)	9 (7–12)	7·2 (5·3–10)	2·4 (1·9–2·8)	2·3 (1·7–3)
1-67	Acute hepatitis C	3 (1–5)	1 (1–2)	1 (1–2)	1·4 (0·6–2·2)	0·3 (0·2–0·5)	0·3 (0·2–0·5)
1-68	Acute hepatitis E	3 (2–4)	2 (1–3)	2 (2–3)	1·1 (0·7–1·7)	0·6 (0·4–0·8)	0·6 (0·4–0·9)
1-69	Other unspecified infectious diseases	104 (78–142)	168 (113–216)	181 (124–237)	43·4 (33·2–58·3)	48·7 (33·1–62·5)	48·3 (33·7–63)
1-70	Maternal and neonatal disorders	5,600 (4,760–6,450)	3,710 (3,250–4,260)	3,040 (2,500–3,750)	2,140 (1,810–2,460)	1,400 (1,210–1,620)	1,250 (998–1,560)
1-71	Maternal disorders	214 (185–244)	284 (250–321)	233 (182–298)	70·3 (61–80·5)	74 (65–83·8)	58·1 (45·3–74·2)
1-72	Maternal haemorrhage	31 (22–41)	24 (17–32)	19 (13–26)	10 (7·3–13·3)	6·2 (4·5–8·4)	4·7 (3·3–6·6)
1-73	Maternal sepsis and other pregnancy related infections	19 (14–24)	11 (9–15)	10 (7–13)	6·1 (4·8–7·7)	3 (2·3–4)	2·4 (1·7–3·3)
1-74	Maternal hypertensive disorders	45 (38–54)	37 (31–44)	30 (23–39)	14·8 (12·5–17·4)	9·8 (8·1–11·6)	7·5 (5·7–9·8)
1-75	Maternal obstructed labour and uterine rupture	2 (1–3)	7 (5–9)	6 (4–8)	0·7 (0·5–0·9)	1·9 (1·4–2·4)	1·4 (1–1·9)
1-76	Maternal abortive outcome	49 (39–60)	21 (16–27)	16 (11–22)	16·4 (13–20·2)	5·4 (4–7)	3·9 (2·8–5·5)
1-77	Ectopic pregnancy	7 (6–9)	28 (22–34)	24 (17–32)	2·4 (1·9–3)	7·3 (5·8–9)	5·9 (4·2–8·1)
1-78	Indirect maternal deaths	23 (18–29)	34 (28–41)	28 (20–38)	7·4 (5·9–9·2)	9 (7·3–11)	7·1 (5·1–9·7)
1-79	Late maternal deaths	10 (7–14)	20 (15–26)	17 (11–23)	3·3 (2·5–4·4)	5·2 (3·8–6·9)	4·2 (2·8–5·8)
1-80	Maternal deaths aggravated by HIV/AIDS	1 (0–1)	2 (1–2)	2 (1–3)	0·2 (0·1–0·3)	0·4 (0·2–0·6)	0·5 (0·2–0·8)
1-81	Other maternal disorders	27 (22–34)	100 (83–119)	83 (62–109)	9·1 (7·3–11·2)	25·8 (21·5–30·9)	20·5 (15·3–27·1)
1-82	Neonatal disorders	5,380 (4,550–6,220)	3,420 (2,980–3,970)	2,810 (2,300–3,450)	2,070 (1,750–2,390)	1,330 (1,140–1,540)	1,190 (946–1,490)
1-83	Neonatal preterm birth	2,370 (1,950–2,830)	1,380 (1,170–1,620)	1,060 (867–1,310)	906 (746–1,080)	520 (441–610)	432 (342–546)
1-84	Neonatal encephalopathy due to birth asphyxia and trauma	1,170 (835–1,510)	623 (515–758)	492 (358–667)	450 (322–585)	252 (207–306)	222 (156–310)
1-85	Neonatal sepsis and other neonatal infections	904 (682–1,160)	795 (624–998)	762 (581–995)	345 (261–444)	302 (241–374)	307 (231–403)
1-86	Hemolytic disease and other neonatal jaundice	64 (50–79)	63 (48–79)	62 (46–79)	23·8 (18·6–29·6)	18·1 (13·9–22·7)	17·4 (13·1–22)
1-87	Other neonatal disorders	880 (618–1,240)	561 (449–681)	425 (307–575)	341 (239–481)	234 (188–285)	211 (152–286)
1-88	Nutritional deficiencies	1,450 (1,030–2,040)	1,530 (1,040–2,200)	1,580 (1,090–2,220)	593 (432–824)	447 (307–637)	440 (304–609)
1-89	Protein-energy malnutrition	373 (306–457)	313 (244–409)	325 (244–424)	163 (136–197)	98·6 (77·9–127)	94·1 (71·3–122)
1-90	Iodine deficiency	11 (5–20)	12 (6–21)	12 (6–22)	4·4 (2·1–7·5)	3·4 (1·6–5·8)	3·1 (1·5–5·3)
1-91	Vitamin A deficiency	17 (10–26)	17 (10–26)	16 (10–24)	6·2 (3·8–9·3)	5 (3·1–7·6)	5·1 (3·2–7·6)
1-92	Dietary iron deficiency	959 (591–1,480)	1,090 (668–1,700)	1,120 (698–1,680)	378 (233–583)	313 (191–481)	312 (194–468)
1-93	Other nutritional deficiencies	91 (68–123)	94 (67–131)	101 (70–138)	40·8 (30·6–55)	27·1 (19·6–37·4)	25·6 (17·9–35·1)
1-0	Non-communicable diseases	47,400 (42,200–53,000)	72,300 (64,100–81,700)	87,300 (73,200–104,000)	24,600 (22,200–27,200)	22,200 (19,900–24,900)	22,000 (18,500–26,100)
1-1	Neoplasms	7,730 (7,140–8,340)	12,700 (11,900–13,600)	15,800 (12,800–19,400)	4,370 (4,040–4,690)	3,950 (3,700–4,220)	3,890 (3,160–4,770)
1-2	Lip and oral cavity cancer	164 (146–181)	243 (222–265)	313 (250–392)	96·6 (86·6–107)	74·6 (68·1–81)	74·2 (59·5–92·4)
1-3	Nasopharynx cancer	28 (26–32)	54 (49–60)	68 (54–85)	15·1 (13·5–16·8)	15·7 (14·3–17·3)	15·8 (12·6–19·9)
1-4	Other pharynx cancer	111 (98–126)	136 (121–152)	181 (142–229)	65·9 (58·3–74·6)	41·3 (36·9–45·9)	41·9 (32·8–52·9)
1-5	Oesophageal cancer	280 (248–315)	420 (376–468)	550 (434–691)	170 (151–191)	130 (117–145)	130 (103–163)
1-6	Stomach cancer	573 (523–626)	709 (653–769)	870 (699–1,080)	341 (311–372)	224 (206–244)	213 (172–263)
1-7	Colon and rectum cancer	677 (622–738)	1,310 (1,210–1,410)	1,710 (1,390–2,100)	406 (374–441)	421 (390–453)	424 (346–515)

1-8	Liver cancer	378 (331–424)	245 (223–267)	338 (274–419)	219 (192–246)	76 (69·4–82·9)	81·8 (66·8–101)
1-9	Liver cancer due to hepatitis B	136 (103–175)	92 (71–117)	116 (82–160)	73 (55·1–96·5)	26·4 (20·4–33·9)	26·7 (19·1–36·9)
1-10	Liver cancer due to hepatitis C	57 (39–79)	39 (26–54)	58 (37–83)	37·1 (25·2–51·5)	13·5 (9·1–18·5)	14·8 (9·5–20·8)
1-11	Liver cancer due to alcohol use	128 (94–168)	73 (52–96)	109 (74–153)	78·4 (56·5–102)	23·5 (17–30·4)	26·4 (18·2–36·7)
1-12	Liver cancer due to NASH	26 (18–35)	20 (14–27)	29 (20–44)	15·2 (10·7–21·1)	6·5 (4·7–8·9)	7·3 (5–10·6)
1-13	Gallbladder and biliary tract cancer	169 (105–193)	132 (115–196)	169 (130–265)	104 (64·6–119)	42·4 (37–63·4)	41·4 (31·8–64·8)
1-14	Pancreatic cancer	71 (64–78)	339 (310–371)	452 (360–563)	44·3 (39·7–49)	108 (98–118)	110 (88–137)
1-15	Larynx cancer	119 (107–134)	201 (180–223)	266 (211–336)	73·1 (65·6–81·8)	62·9 (56·7–69·6)	62·9 (50·1–78·7)
1-16	Tracheal, bronchus, and lung cancer	707 (637–782)	1,190 (1,080–1,300)	1,480 (1,190–1,860)	431 (387–475)	374 (341–410)	355 (287–443)
1-17	Malignant skin melanoma	52 (40–67)	95 (71–128)	121 (86–167)	26·8 (20·7–35·1)	27·3 (20·6–37·1)	28·9 (20·7–39·8)
1-18	Non-melanoma skin cancer	23 (20–26)	43 (39–48)	56 (45–69)	13·6 (12·1–15·6)	13·9 (12·6–15·3)	14·2 (11·6–17·2)
1-19	Non-melanoma skin cancer (squamous-cell carcinoma)	23 (20–26)	43 (39–48)	56 (45–69)	13·6 (12·1–15·6)	13·9 (12·6–15·3)	14·2 (11·6–17·2)
1-20	Non-melanoma skin cancer (basal-cell carcinoma)	0 (0–0)	0 (0–0)	0 (0–0)	0 (0–0)	0 (0–0)	0 (0–0)
1-21	Breast cancer	1,120 (997–1,250)	2,180 (1,970–2,410)	2,520 (2,010–3,160)	604 (540–676)	621 (562–684)	587 (473–733)
1-22	Cervical cancer	499 (434–569)	703 (615–801)	813 (619–1,050)	254 (222–288)	195 (170–220)	188 (143–241)
1-23	Uterine cancer	122 (109–136)	253 (225–284)	313 (249–391)	70·9 (63·8–79)	78·3 (69·9–87·2)	75·2 (60·1–93·6)
1-24	Ovarian cancer	75 (65–119)	394 (255–447)	495 (337–637)	40·3 (35–65)	115 (75·5–130)	116 (79·9–149)
1-25	Prostate cancer	676 (568–925)	1,300 (1,070–1,680)	1,820 (1,400–2,450)	477 (400–655)	503 (418–662)	512 (397–693)
1-26	Testicular cancer	2 (1–2)	5 (4–5)	5 (4–6)	0·6 (0·5–0·7)	1·3 (1·1–1·5)	1·3 (0·9–1·6)
1-27	Kidney cancer	155 (139–172)	204 (187–224)	254 (202–317)	80·8 (72·8–89·2)	61·3 (56·3–67·3)	62·1 (49·6–77·5)
1-28	Bladder cancer	58 (53–65)	105 (96–116)	139 (112–171)	38·2 (34·3–42·4)	36·7 (33·4–40·7)	36·2 (29·3–44)
1-29	Brain and nervous system cancer	83 (59–164)	295 (149–361)	330 (162–461)	36·4 (25·9–72·4)	84·1 (42·5–103)	83·5 (40·4–117)
1-30	Thyroid cancer	33 (30–37)	58 (53–64)	74 (60–93)	18·6 (16·7–20·5)	17·6 (16–19·3)	17·8 (14·4–22·2)
1-31	Mesothelioma	17 (15–20)	25 (23–28)	30 (24–38)	9·1 (7·9–10·6)	7·2 (6·4–8)	6·9 (5·5–8·7)
1-32	Hodgkin lymphoma	46 (37–56)	52 (43–63)	57 (43–75)	18·7 (15·2–22·4)	14·2 (11·8–17·4)	14 (10·6–18·2)
1-33	Non-Hodgkin's lymphoma	340 (302–384)	480 (432–533)	550 (437–698)	159 (142–179)	138 (125–152)	134 (107–170)
1-34	Multiple myeloma	178 (156–221)	358 (321–401)	486 (391–621)	111 (97–137)	116 (104–130)	118 (94·9–150)
1-35	Leukaemia	373 (339–409)	505 (467–545)	539 (438–668)	159 (145–174)	146 (135–157)	140 (114–173)
1-36	Acute lymphoid leukaemia	85 (72–100)	94 (82–108)	90 (71–114)	30·4 (25·6–35·8)	27·1 (23·4–31·2)	26·4 (20·5–33·5)
1-37	Chronic lymphoid leukaemia	20 (17–23)	38 (34–42)	48 (39–60)	12·3 (10·6–13·9)	12·7 (11·4–14·1)	12·1 (9·9–15)
1-38	Acute myeloid leukaemia	127 (109–146)	199 (175–221)	218 (171–276)	51·4 (44·6–59·1)	55·8 (49·4–62)	55·7 (43·6–70·3)
1-39	Chronic myeloid leukaemia	48 (41–56)	62 (55–70)	65 (51–81)	22·7 (19·5–26)	17·8 (15·9–19·8)	15·7 (12·4–19·5)
1-40	Other leukaemia	92 (82–104)	112 (101–126)	119 (94–149)	41·9 (37·2–46·8)	32·6 (29·5–36·1)	30·1 (24–37·6)
1-41	Other neoplasms	35 (28–54)	68 (54–92)	86 (64–119)	18·4 (14·7–29·2)	22 (17·5–29·9)	22·6 (16·9–30·8)
1-42	Myelodysplastic, myeloproliferative, and other hematopoietic neoplasms	35 (28–54)	68 (54–92)	86 (64–119)	18·4 (14·7–29·2)	22 (17·5–29·9)	22·6 (16·9–30·8)
1-43	Benign and in situ intestinal neoplasms	0 (0–0)	0 (0–0)	0 (0–0)	0 (0–0)	0 (0–0)	0 (0–0)
1-44	Benign and in situ cervical and uterine neoplasms	0 (0–0)	0 (0–0)	0 (0–0)	0 (0–0)	0 (0–0)	0 (0–0)
1-45	Other benign and in situ neoplasms	0 (0–0)	0 (0–0)	0 (0–0)	0 (0–0)	0 (0–0)	0 (0–0)
1-46	Cardiovascular diseases	11,000 (10,300–11,800)	16,300 (15,300–17,300)	20,500 (16,900–25,100)	6,760 (6,310–7,230)	5,370 (5,030–5,690)	5,210 (4,340–6,350)
1-47	Rheumatic heart disease	271 (216–340)	301 (235–387)	311 (228–415)	110 (88·6–136)	80·2 (62·9–104)	75·3 (55·5–101)
1-48	Ischaemic heart disease	4,460 (4,120–4,810)	5,720 (5,300–6,130)	7,390 (6,110–9,080)	2,850 (2,630–3,060)	1,910 (1,770–2,050)	1,870 (1,560–2,300)
1-49	Stroke	3,000 (2,730–3,270)	4,130 (3,820–4,460)	5,170 (4,280–6,290)	1,860 (1,690–2,020)	1,370 (1,270–1,480)	1,320 (1,110–1,610)
1-50	Ischaemic stroke	1,020 (918–1,140)	1,400 (1,280–1,520)	1,800 (1,520–2,130)	708 (640–790)	534 (484–578)	513 (437–604)
1-51	Intracerebral hemorrhage	1,680 (1,520–1,870)	2,270 (2,080–2,490)	2,820 (2,290–3,500)	997 (895–1,100)	709 (652–774)	683 (558–843)
1-52	Subarachnoid hemorrhage	302 (249–349)	456 (401–510)	550 (433–684)	150 (125–175)	128 (114–143)	129 (102–159)
1-53	Hypertensive heart disease	1,710 (1,510–2,010)	3,390 (2,880–3,760)	4,260 (3,370–5,400)	1,090 (966–1,280)	1,140 (977–1,260)	1,090 (866–1,360)
1-54	Non-rheumatic valvular heart disease	64 (56–72)	117 (107–129)	145 (117–181)	33·4 (29·5–37·6)	35·2 (32–38·6)	35·5 (28·7–44)
1-55	Non-rheumatic calcific aortic valve disease	34 (28–40)	69 (60–81)	86 (69–110)	19 (15·9–22)	21·5 (19–25)	21·5 (17·4–27·2)
1-56	Non-rheumatic degenerative mitral valve disease	24 (20–29)	41 (33–51)	50 (37–66)	12 (9·9–14·5)	11·6 (9·3–14·3)	11·8 (8·8–15·6)
1-57	Other non-rheumatic valve diseases	5 (3–7)	7 (5–11)	9 (6–13)	2·4 (1·4–3·2)	2·1 (1·4–3·1)	2·2 (1·4–3·3)
1-58	Cardiomyopathy and myocarditis	539 (463–644)	977 (785–1,100)	1,160 (896–1,460)	273 (233–327)	285 (234–319)	280 (220–349)
1-59	Myocarditis	19 (14–26)	32 (23–40)	37 (25–49)	8·3 (6·2–11·2)	9·3 (6·8–11·6)	9·5 (6·5–12·7)
1-60	Alcoholic cardiomyopathy	104 (78–146)	251 (181–308)	299 (214–391)	55·6 (41·8–78·1)	69·6 (51·1–84·1)	67·5 (48·5–88·6)

1-61	Other cardiomyopathy	416 (359–490)	694 (564–782)	820 (634–1,040)	209 (180–247)	206 (171–231)	203 (159–255)
1-62	Atrial fibrillation and flutter	132 (109–159)	261 (223–318)	354 (292–441)	99·9 (83·3–120)	104 (88·8–126)	104 (86·1–129)
1-63	Aortic aneurysm	118 (105–133)	220 (200–243)	293 (236–369)	75·1 (66·3–85)	75·4 (68–83·2)	75·8 (61·4–94·9)
1-64	Peripheral vascular disease	53 (28–93)	113 (60–196)	158 (83–281)	38·9 (21·2–67·3)	43·4 (23·4–76)	44·4 (23·7–79)
1-65	Endocarditis	115 (87–149)	192 (142–226)	218 (157–285)	52·3 (40·6–68·6)	53·9 (40·4–63·4)	52·7 (38·7–68·7)
1-66	Chronic respiratory diseases	1,330 (1,090–1,660)	1,930 (1,620–2,340)	2,210 (1,820–2,690)	635 (540–750)	599 (507–720)	602 (491–746)
1-67	Chronic obstructive pulmonary disease	364 (321–410)	660 (586–738)	854 (713–1,020)	230 (202–259)	228 (203–256)	226 (188–268)
1-68	Pneumoconiosis	4 (3–4)	5 (4–5)	6 (4–7)	2 (1·7–2·4)	1·4 (1·2–1·6)	1·4 (1·1–1·8)
1-69	Silicosis	2 (1–2)	3 (2–3)	3 (2–4)	1 (0·8–1·2)	0·9 (0·7–1)	0·8 (0·6–1·1)
1-70	Asbestosis	1 (0–1)	1 (1–1)	1 (1–1)	0·3 (0·2–0·4)	0·2 (0·2–0·3)	0·2 (0·2–0·3)
1-71	Coal workers pneumoconiosis	0 (0–0)	0 (0–0)	0 (0–0)	0·1 (0·1–0·1)	0 (0–0)	0 (0–0)
1-72	Other pneumoconiosis	1 (1–1)	1 (1–1)	1 (1–2)	0·7 (0·5–0·8)	0·3 (0·3–0·4)	0·3 (0·2–0·4)
1-73	Asthma	810 (593–1,120)	979 (707–1,350)	1,010 (724–1,380)	328 (249–438)	284 (204–392)	288 (203–409)
1-74	Interstitial lung disease and pulmonary sarcoidosis	61 (48–84)	140 (103–167)	176 (124–230)	35 (27·2–49·4)	44·8 (33·6–53·5)	44·9 (32–58·4)
1-75	Other chronic respiratory diseases	91 (74–109)	147 (123–174)	168 (130–212)	39 (31·5–47·1)	41 (34·3–48·3)	42·6 (33·1–54·5)
1-76	Digestive diseases	2,960 (2,670–3,290)	3,890 (3,490–4,360)	4,610 (3,810–5,620)	1,520 (1,380–1,670)	1,140 (1,020–1,270)	1,120 (928–1,350)
1-77	Cirrhosis and other chronic liver diseases	1,340 (1,210–1,470)	1,610 (1,450–1,770)	1,950 (1,550–2,450)	719 (651–788)	459 (417–504)	453 (361–565)
1-78	Cirrhosis and other chronic liver diseases due to hepatitis B	171 (117–240)	216 (149–299)	147 (91–225)	90·7 (62·4–128)	59 (41·4–80·7)	33·1 (20·7–49·9)
1-79	Cirrhosis and other chronic liver diseases due to hepatitis C	256 (184–341)	312 (227–425)	398 (269–576)	136 (98·4–183)	86·9 (63·5–118)	91 (62·4–129)
1-80	Cirrhosis and other chronic liver diseases due to alcohol use	503 (389–617)	618 (480–765)	820 (598–1,100)	283 (219–346)	176 (137–218)	186 (137–246)
1-81	Cirrhosis and other chronic liver diseases due to NAFLD	179 (125–251)	226 (157–316)	303 (199–443)	99·8 (69·2–139)	66·4 (47–90·4)	71·5 (47·7–102)
1-82	Cirrhosis and other chronic liver diseases due to other causes	229 (181–285)	238 (185–307)	285 (202–386)	109 (83·6–138)	70·7 (54·8–90)	71·2 (51·7–94·9)
1-83	Upper digestive system diseases	586 (433–817)	810 (570–1,190)	948 (659–1,380)	289 (218–396)	232 (166–335)	228 (159–331)
1-84	Peptic ulcer disease	224 (188–263)	231 (199–269)	268 (205–345)	125 (106–148)	70·7 (61·2–81·7)	65·9 (50·7–84·8)
1-85	Gastritis and duodenitis	82 (61–110)	132 (95–178)	160 (116–218)	40·9 (30·9–53·8)	38·1 (27·9–51·4)	38·8 (28·2–52·4)
1-86	GERD	280 (145–499)	448 (232–782)	520 (270–919)	123 (63·8–220)	123 (64·2–217)	123 (64–218)
1-87	Appendicitis	66 (49–80)	69 (55–90)	75 (56–99)	27·5 (20·2–34)	19·5 (15·7–25)	19 (14·5–25·4)
1-88	Paralytic ileus and intestinal obstruction	231 (192–280)	280 (235–322)	318 (244–410)	113 (95·1–135)	93·1 (79·4–108)	89·8 (69·9–116)
1-89	Inguinal, femoral, and abdominal hernia	187 (140–247)	247 (181–332)	273 (203–365)	82·1 (62·4–106)	72·2 (53·5–95·7)	71·1 (53–94·1)
1-90	Inflammatory bowel disease	49 (41–57)	68 (58–80)	78 (63–98)	23·2 (19·8–26·8)	19·4 (16·7–22·8)	19 (15·3–23·9)
1-91	Vascular intestinal disorders	62 (53–71)	89 (79–99)	115 (91–147)	37 (31·8–42·6)	29·2 (25·9–32·6)	29·4 (23·4–37·1)
1-92	Gallbladder and biliary diseases	202 (158–256)	258 (200–327)	309 (242–397)	106 (83·6–131)	76·6 (60·5–95·9)	75·3 (59·2–96·1)
1-93	Pancreatitis	155 (132–185)	249 (216–288)	287 (223–371)	74·8 (63·7–88·7)	68·8 (60·7–79)	67·5 (52·9–86·8)
1-94	Other digestive diseases	87 (68–135)	207 (138–251)	251 (157–337)	44·1 (34·5–70·3)	65·2 (44–78·6)	65·5 (41·3–87·3)
1-95	Neurological disorders	2,860 (1,490–4,850)	4,250 (2,380–7,130)	4,920 (2,750–8,090)	1,320 (746–2,170)	1,300 (753–2,120)	1,300 (752–2,170)
1-96	Alzheimer's disease and other dementias	359 (148–825)	700 (294–1,580)	959 (406–2,210)	303 (125–705)	307 (128–706)	307 (129–705)
1-97	Parkinson's disease	116 (106–126)	225 (207–244)	302 (256–353)	84 (76·8–91·6)	91·1 (83·3–98·8)	89·4 (75·9–104)
1-98	Idiopathic epilepsy	711 (359–1,150)	835 (420–1,400)	893 (441–1,460)	282 (143–457)	233 (115–394)	230 (111–380)
1-99	Multiple sclerosis	27 (23–36)	55 (44–64)	65 (50–81)	12·6 (10·7–16·8)	14·5 (11·8–16·8)	14·8 (11·5–18·5)
1-100	Motor neuron disease	15 (13–17)	51 (47–56)	66 (52–84)	7·5 (6·7–8·4)	15·3 (14–16·7)	16·4 (12·8–20·8)
1-101	Headache disorders	1,440 (256–3,260)	2,090 (403–4,620)	2,290 (457–5,000)	557 (107–1,230)	557 (108–1,230)	556 (106–1,220)
1-102	Migraine	1,320 (173–3,070)	1,890 (272–4,370)	2,070 (315–4,750)	504 (72·6–1,160)	504 (74·4–1,160)	503 (74·1–1,160)
1-103	Tension-type headache	128 (36–489)	195 (57–702)	219 (65–761)	52·7 (15·5–189)	52·7 (15·4–189)	52·6 (15·4–188)
1-104	Other neurological disorders	193 (135–271)	298 (215–412)	346 (240–477)	77·1 (56–106)	84·5 (61·2–116)	91·7 (62·4–127)
1-105	Mental disorders	4,190 (3,040–5,600)	6,010 (4,380–7,900)	6,820 (4,970–9,020)	1,660 (1,220–2,200)	1,630 (1,190–2,150)	1,660 (1,210–2,200)
1-106	Schizophrenia	438 (301–593)	700 (475–935)	788 (549–1,050)	183 (128–244)	184 (125–244)	183 (127–244)
1-107	Depressive disorders	1,280 (861–1,790)	1,830 (1,250–2,570)	2,210 (1,500–3,110)	540 (368–753)	503 (347–704)	531 (360–737)
1-108	Major depressive disorder	1,070 (708–1,520)	1,500 (1,000–2,090)	1,830 (1,210–2,570)	449 (298–628)	412 (279–577)	441 (294–616)
1-109	Dysthymia	215 (131–332)	335 (206–517)	382 (241–588)	90·5 (56·4–138)	90·5 (56·8–138)	90·4 (56·8–139)
1-110	Bipolar disorder	505 (288–794)	733 (431–1,140)	817 (477–1,260)	195 (114–304)	195 (114–302)	195 (113–300)
1-111	Anxiety disorders	1,040 (680–1,490)	1,510 (1,010–2,140)	1,700 (1,110–2,420)	409 (270–582)	409 (276–576)	416 (273–591)
1-112	Eating disorders	189 (116–287)	242 (150–365)	248 (153–372)	62 (38·2–93·4)	63·6 (39·6–96·3)	62·1 (38·5–93·7)
1-113	Anorexia nervosa	45 (26–73)	56 (32–87)	57 (32–90)	14·5 (8·6–23·1)	14·7 (8·5–23·1)	14·5 (8·2–23·1)

1·114	Bulimia nervosa	144 (83–228)	186 (110–297)	191 (111–299)	47·5 (27·8–74·9)	48·9 (29–77·9)	47·6 (27·7–74·7)
1·115	Autism spectrum disorders	138 (90–202)	184 (119–268)	190 (125–278)	51·8 (33·8–75·9)	51·8 (33·8–75·6)	51·9 (34·2–75·7)
1·116	Attention-deficit/hyperactivity disorder	110 (62–188)	135 (76–228)	135 (77–228)	37·1 (20·9–63·1)	37 (20·8–62·1)	37 (21·1–63)
1·117	Conduct disorder	208 (117–331)	245 (137–392)	232 (130–371)	67·9 (38·1–108)	67·8 (38–109)	67·6 (37·9–108)
1·118	Idiopathic developmental intellectual disability	26 (7–52)	34 (8–66)	36 (9–70)	9·7 (2·4–18·9)	9·5 (2·3–18·4)	9·8 (2·5–18·9)
1·119	Other mental disorders	245 (154–370)	396 (250–600)	457 (289–692)	108 (68·1–163)	108 (68·6–163)	108 (68·3–163)
1·120	Substance use disorders	1,060 (823–1,320)	1,830 (1,460–2,270)	2,020 (1,590–2,480)	436 (347–540)	483 (385–597)	472 (370–583)
1·121	Alcohol use disorders	720 (567–917)	1,320 (1,060–1,650)	1,480 (1,150–1,840)	316 (254–394)	350 (281–435)	341 (267–427)
1·122	Drug use disorders	337 (237–455)	504 (367–657)	541 (396–699)	120 (86·1–159)	133 (96·7–174)	131 (96–170)
1·123	Opioid use disorders	163 (103–240)	252 (171–351)	271 (186–371)	61·2 (39·7–87·1)	66·6 (45·6–93·3)	64·7 (44·1–89·5)
1·124	Cocaine use disorders	46 (29–70)	76 (53–106)	81 (56–113)	16·2 (10·5–23·7)	19·8 (13·8–27·9)	19·7 (13·6–27·7)
1·125	Amphetamine use disorders	20 (11–33)	27 (17–43)	30 (19–47)	6·5 (3·7–10·8)	7·2 (4·4–11·3)	7·5 (4·5–11·5)
1·126	Cannabis use disorders	59 (33–95)	74 (43–115)	74 (41–118)	19·2 (10·8–30·9)	19·6 (11·4–30·9)	18·6 (10·3–30)
1·127	Other drug use disorders	48 (31–70)	76 (54–104)	85 (61–116)	16·8 (11·2–24·4)	19·9 (14–27·3)	20·7 (14·7–28·2)
1·128	Diabetes and kidney diseases	4,190 (3,780–4,690)	7,920 (7,010–9,000)	10,500 (8,640–12,600)	2,430 (2,190–2,710)	2,500 (2,220–2,830)	2,590 (2,150–3,110)
1·129	Diabetes mellitus	2,770 (2,430–3,160)	4,940 (4,170–5,860)	6,690 (5,380–8,260)	1,670 (1,470–1,910)	1,580 (1,350–1,870)	1,650 (1,340–2,030)
1·130	Diabetes mellitus type 1	267 (219–323)	370 (317–481)	416 (325–553)	121 (97·1–150)	103 (87·7–133)	102 (79·3–135)
1·131	Diabetes mellitus type 2	2,500 (2,170–2,890)	4,570 (3,830–5,480)	6,270 (5,020–7,790)	1,550 (1,350–1,780)	1,480 (1,250–1,770)	1,550 (1,250–1,920)
1·132	Chronic kidney disease	1,420 (1,280–1,570)	2,970 (2,720–3,270)	3,780 (3,150–4,550)	756 (686–834)	909 (831–994)	933 (781–1,120)
1·133	Chronic kidney disease due to diabetes mellitus type 1	131 (92–182)	275 (191–374)	329 (214–487)	62·7 (42·6–88·2)	72·5 (50·5–98·7)	74·9 (49–110)
1·134	Chronic kidney disease due to diabetes mellitus type 2	270 (214–326)	602 (479–733)	821 (621–1,040)	169 (135–203)	200 (161–242)	205 (158–256)
1·135	Chronic kidney disease due to hypertension	231 (190–284)	521 (432–625)	697 (542–891)	143 (118–173)	175 (146–209)	180 (140–228)
1·136	Chronic kidney disease due to glomerulonephritis	366 (302–439)	733 (609–871)	894 (684–1,160)	174 (142–212)	208 (172–247)	214 (164–275)
1·137	Chronic kidney disease due to other and unspecified causes	417 (342–507)	842 (691–1,020)	1,040 (812–1,330)	207 (168–255)	253 (208–308)	260 (204–330)
1·138	Acute glomerulonephritis	8 (6–11)	9 (8–10)	11 (8–14)	3·6 (2·7–4·7)	2·6 (2·3–3)	2·8 (2·1–3·7)
1·139	Skin and subcutaneous diseases	1,540 (1,070–2,220)	2,170 (1,520–3,130)	2,390 (1,690–3,360)	649 (465–920)	651 (460–931)	655 (464–924)
1·140	Dermatitis	303 (175–485)	399 (238–626)	406 (244–634)	116 (68·7–182)	116 (68·9–183)	116 (68·3–182)
1·141	Atopic dermatitis	232 (126–393)	290 (157–489)	281 (151–478)	85·3 (46·3–145)	85·4 (46·1–144)	85·3 (45·6–144)
1·142	Contact dermatitis	63 (40–96)	97 (60–145)	112 (70–167)	27·2 (17·2–41·2)	27·2 (16·8–40·8)	27·2 (17·3–40·6)
1·143	Seborrhoeic dermatitis	8 (5–13)	12 (7–19)	13 (7–21)	3·4 (1·9–5·4)	3·4 (1·9–5·4)	3·4 (1·9–5·4)
1·144	Psoriasis	77 (54–103)	110 (78–147)	123 (87–167)	32·6 (23·2–43·8)	30·5 (21·6–40·8)	30·2 (21·4–41·1)
1·145	Bacterial skin diseases	50 (34–78)	93 (62–145)	114 (67–184)	22·1 (14·7–34·2)	28·7 (19·2–44·6)	31·2 (18·7–50)
1·146	Cellulitis	15 (9–24)	22 (14–35)	26 (16–44)	6·3 (3·9–10·3)	6·7 (4·2–10·7)	7·1 (4·4–11·7)
1·147	Pyoderma	36 (24–58)	72 (45–110)	89 (52–138)	15·8 (10·4–25·8)	22 (13·7–33·4)	24·1 (14·1–38·6)
1·148	Scabies	224 (121–359)	292 (160–471)	300 (165–479)	83 (45·4–133)	82·9 (45·9–134)	82·8 (45·4–133)
1·149	Fungal skin diseases	98 (38–211)	146 (58–312)	167 (68–354)	44·5 (17·9–94·2)	44·3 (17·8–94·1)	44·2 (17·9–94·2)
1·150	Viral skin diseases	132 (84–198)	162 (103–244)	155 (99–233)	47·6 (30·6–71·5)	47·6 (30·3–71·8)	47·6 (30·3–72·3)
1·151	Acne vulgaris	145 (86–237)	199 (119–323)	205 (121–336)	46·6 (27·6–76·3)	53·8 (32·1–87·1)	55·1 (32·4–90·3)
1·152	Alopecia areata	18 (12–28)	27 (17–40)	29 (18–44)	7·2 (4·6–10·8)	7·2 (4·5–10·7)	7·2 (4·6–10·7)
1·153	Pruritus	20 (10–37)	31 (15–55)	35 (16–63)	8·8 (4·2–16)	8·9 (4·3–15·9)	8·9 (4·2–16·1)
1·154	Urticaria	127 (82–183)	166 (109–241)	168 (111–242)	48·3 (31·3–69·5)	48·3 (31·6–69·7)	48·3 (31·7–69·9)
1·155	Decubitus ulcer	202 (139–332)	329 (236–546)	432 (295–724)	127 (89·3–209)	116 (85·2–189)	118 (80·7–197)
1·156	Other skin and subcutaneous diseases	143 (86–232)	221 (129–366)	255 (148–422)	66·2 (39·2–108)	65·9 (38·5–108)	65·5 (37·8–107)
1·157	Sense organ diseases	1,370 (920–1,970)	2,260 (1,510–3,280)	2,810 (1,870–4,100)	769 (521–1,100)	734 (496–1,050)	722 (485–1,040)
1·158	Blindness and vision loss	372 (251–530)	591 (401–854)	732 (496–1,050)	219 (150–309)	198 (134–284)	193 (132–278)
1·159	Glaucoma	18 (12–27)	27 (18–39)	36 (24–52)	14·2 (9·5–20·4)	11·3 (7·5–16)	10·8 (7·2–15·5)
1·160	Cataract	56 (38–76)	88 (62–119)	114 (79–155)	40·1 (27·8–54·5)	34·1 (23·7–46·2)	32·8 (22·6–44·7)
1·161	Age-related macular degeneration	2 (1–3)	4 (2–6)	5 (3–7)	1·7 (1·1–2·5)	1·5 (1–2·1)	1·4 (0·9–2)
1·162	Refraction disorders	129 (84–189)	186 (123–272)	210 (138–307)	57·4 (37·9–83)	54 (35·6–78·2)	53 (34·7–77·3)
1·163	Near vision loss	76 (35–150)	139 (63–272)	181 (84–360)	48·5 (22·3–94·5)	46·5 (21·3–90·9)	45·7 (21·1–90·3)
1·164	Other vision loss	91 (64–127)	147 (103–201)	186 (131–256)	57·1 (40·4–78·4)	50·6 (35·7–69·9)	49·3 (35–67·6)
1·165	Age-related and other hearing loss	920 (611–1,370)	1,550 (1,010–2,310)	1,930 (1,250–2,890)	513 (341–754)	498 (331–727)	492 (322–723)
1·166	Other sense organ diseases	73 (44–113)	121 (72–188)	146 (86–224)	37·1 (22–57·2)	37·4 (22·2–58·1)	37·3 (22–57·6)

1-167	Musculoskeletal disorders	3,380 (2,480–4,400)	5,820 (4,270–7,650)	6,940 (5,060–9,080)	1,610 (1,180–2,090)	1,650 (1,210–2,170)	1,650 (1,210–2,150)
1-168	Rheumatoid arthritis	66 (48–86)	128 (92–169)	155 (112–207)	34·5 (25·7–44)	37·4 (27·5–48·9)	37·4 (27·3–49·5)
1-169	Osteoarthritis	315 (159–635)	645 (320–1,300)	863 (429–1,750)	201 (101–405)	210 (104–420)	211 (104–430)
1-170	Low back pain	1,610 (1,120–2,140)	2,570 (1,780–3,480)	2,970 (2,090–4,010)	732 (512–975)	723 (505–970)	714 (502–962)
1-171	Neck pain	285 (185–412)	494 (320–721)	580 (375–845)	136 (89·7–195)	136 (89·5–196)	135 (88·9–195)
1-172	Gout	13 (8–18)	26 (16–38)	33 (20–48)	7 (4·4–10·1)	7·8 (4·9–11·4)	8 (5–11·7)
1-173	Other musculoskeletal disorders	1,100 (802–1,430)	1,960 (1,410–2,590)	2,330 (1,680–3,080)	497 (362–655)	539 (391–709)	547 (394–724)
1-174	Other non-communicable diseases	5,780 (4,900–6,900)	7,180 (5,930–8,910)	7,810 (6,230–9,710)	2,460 (2,080–2,940)	2,190 (1,820–2,690)	2,170 (1,740–2,660)
1-175	Congenital anomalies	1,550 (1,270–1,930)	1,370 (1,170–1,660)	1,170 (924–1,480)	593 (487–740)	493 (415–606)	455 (351–596)
1-176	Neural tube defects	78 (54–108)	57 (41–73)	48 (34–66)	29·5 (20·3–41)	21 (14·6–27·8)	19·8 (13·1–28·4)
1-177	Congenital heart anomalies	750 (594–954)	530 (420–662)	417 (303–565)	289 (228–368)	197 (155–252)	174 (123–243)
1-178	Orofacial clefts	7 (4–10)	10 (6–15)	11 (7–17)	2·6 (1·6–3·8)	2·9 (1·7–4·3)	3·1 (1·9–4·8)
1-179	Down's syndrome	49 (39–60)	88 (75–103)	89 (67–113)	19·1 (15·4–23·9)	27·4 (22·9–32·3)	27·4 (20·8–35·8)
1-180	Turner syndrome	0 (0–1)	0 (0–1)	0 (0–1)	0·1 (0·1–0·2)	0·1 (0·1–0·2)	0·1 (0·1–0·2)
1-181	Klinefelter syndrome	0 (0–0)	0 (0–0)	0 (0–0)	0 (0–0)	0 (0–0)	0 (0–0)
1-182	Other chromosomal abnormalities	87 (62–150)	159 (126–226)	134 (100–192)	32·9 (23·4–57·4)	60·5 (47·2–87·9)	57·8 (41·8–86)
1-183	Congenital musculoskeletal and limb anomalies	132 (93–184)	170 (116–240)	173 (116–239)	49·4 (34·9–69)	51·7 (36·1–71·6)	53 (36·4–72·7)
1-184	Urogenital congenital anomalies	28 (19–44)	22 (16–34)	18 (13–28)	10·5 (7·1–17·1)	8·1 (5·9–12·8)	7·3 (5–12·4)
1-185	Digestive congenital anomalies	95 (67–144)	65 (48–93)	48 (33–75)	36·7 (26–56·1)	26 (19·1–37·7)	22·9 (15·2–35·9)
1-186	Other congenital anomalies	322 (253–420)	272 (220–345)	229 (172–301)	123 (95·9–161)	98·1 (78·1–126)	90·2 (66·1–119)
1-187	Urinary diseases and male infertility	217 (182–278)	545 (453–618)	687 (540–841)	119 (101–153)	178 (148–203)	180 (142–218)
1-188	Urinary tract infections and interstitial nephritis	136 (119–186)	393 (315–433)	494 (379–618)	75·1 (65·7–104)	129 (103–142)	131 (99·8–162)
1-189	Urolithiasis	10 (8–13)	22 (17–28)	27 (20–35)	5 (3·8–6·5)	6·1 (4·8–7·6)	6·2 (4·7–8·1)
1-190	Benign prostatic hyperplasia	34 (20–53)	69 (39–108)	94 (55–149)	23·6 (13·7–36·7)	25·4 (15–39·8)	25 (14·6–39·3)
1-191	Male infertility	12 (4–29)	16 (6–39)	17 (6–41)	4·1 (1·6–10)	4·1 (1·6–9·9)	4·1 (1·6–9·8)
1-192	Other urinary diseases	24 (19–33)	45 (33–57)	55 (37–74)	11·7 (9·3–16·4)	13·5 (9·9–17·1)	14·1 (9·5–18·9)
1-193	Gynecological diseases	978 (676–1,370)	1,470 (1,020–2,060)	1,590 (1,100–2,230)	387 (271–538)	378 (263–530)	374 (259–525)
1-194	Uterine fibroids	61 (33–110)	106 (55–193)	118 (60–214)	27·2 (14·4–48·9)	26·6 (13·6–49)	26·6 (13·8–48·5)
1-195	Polycystic ovarian syndrome	18 (8–38)	30 (12–60)	32 (13–64)	6·3 (2·6–13·1)	7·6 (3·1–15·2)	7·7 (3·2–15·6)
1-196	Female infertility	23 (8–55)	33 (11–78)	34 (12–80)	8·2 (2·8–19·7)	8·3 (2·9–19·7)	8·2 (2·8–19·5)
1-197	Endometriosis	85 (49–137)	104 (61–168)	103 (60–164)	29·9 (17·6–47·5)	26·5 (15·6–42·9)	24·5 (14·4–39·3)
1-198	Genital prolapse	14 (6–26)	21 (10–40)	23 (11–44)	7·5 (3·6–14·3)	6·1 (2·9–11·6)	5·4 (2·6–10·2)
1-199	Premenstrual syndrome	284 (171–449)	400 (246–620)	422 (258–654)	100 (60·5–158)	102 (62·3–158)	102 (62·2–159)
1-200	Other gynecological diseases	493 (321–703)	772 (504–1,110)	857 (556–1,220)	207 (136–293)	202 (131–287)	199 (130–282)
1-201	Hemoglobinopathies and hemolytic anaemias	537 (444–651)	593 (504–707)	636 (509–803)	204 (170–245)	167 (142–198)	169 (136–213)
1-202	Thalassemias	0 (0–1)	1 (0–1)	0 (0–0)	0·1 (0·1–0·2)	0·2 (0·1–0·3)	0·1 (0–0·1)
1-203	Thalassaemias trait	10 (6–16)	14 (8–22)	15 (9–24)	3·8 (2·3–6)	3·9 (2·3–6·1)	3·9 (2·3–6·3)
1-204	Sickle cell disorders	267 (231–305)	306 (269–345)	314 (244–401)	94·6 (81·6–108)	84 (74–94·5)	84·9 (66·2–108)
1-205	Sickle cell trait	127 (78–195)	110 (66–173)	116 (64–186)	44 (26·9–67·3)	31·3 (18·7–49)	32·2 (18·2–51·1)
1-206	G6PD deficiency	41 (29–54)	61 (44–82)	71 (49–98)	16·9 (12–21·9)	16·5 (11·8–22)	16·9 (11·7–23·4)
1-207	G6PD trait	0 (0–1)	1 (0–2)	1 (1–2)	0·2 (0·1–0·3)	0·3 (0·1–0·4)	0·3 (0·2–0·5)
1-208	Other hemoglobinopathies and hemolytic anaemias	91 (70–120)	101 (76–135)	118 (90–155)	43·9 (34·6–55·9)	31 (23·9–40·9)	31·1 (23·7–40·4)
1-209	Endocrine, metabolic, blood, and immune disorders	1,720 (1,240–2,020)	2,050 (1,750–2,610)	2,380 (1,930–3,150)	778 (570–917)	625 (534–810)	644 (524–859)
1-210	Oral disorders	661 (371–1,080)	1,080 (606–1,800)	1,290 (734–2,130)	328 (190–527)	321 (186–525)	319 (184–519)
1-211	Caries of deciduous teeth	8 (4–18)	9 (4–20)	8 (3–16)	3·1 (1·3–6·6)	3·1 (1·3–6·4)	3·1 (1·3–6·2)
1-212	Caries of permanent teeth	67 (30–131)	92 (41–181)	101 (45–195)	25·3 (11·5–49)	24·9 (11–48·6)	25 (11·3–48·4)
1-213	Periodontal disease	256 (96–553)	437 (168–927)	505 (194–1,070)	118 (45·2–248)	118 (45·3–247)	116 (44·6–245)
1-214	Edentulism	197 (125–295)	350 (222–528)	470 (296–710)	129 (81·9–194)	122 (76·8–184)	121 (76·9–183)
1-215	Other oral disorders	133 (82–193)	193 (120–283)	212 (132–309)	53·4 (32·8–76·9)	53·3 (33·1–77·9)	53·3 (33·4–77·7)
1-216	Sudden infant death syndrome	126 (80–187)	70 (50–98)	50 (31–76)	49·8 (31·6–73·8)	29 (20·6–40·3)	24·7 (15·3–37·4)
1-0	Injuries	10,900 (10,100–11,900)	15,500 (14,500–16,600)	19,600 (17,100–22,700)	4,230 (3,890–4,600)	4,230 (3,960–4,540)	4,970 (4,340–5,730)
1-1	Transport injuries	3,720 (3,360–4,080)	3,880 (3,560–4,230)	5,230 (4,560–6,080)	1,430 (1,300–1,570)	1,050 (961–1,140)	1,290 (1,130–1,500)
1-2	Road injuries	3,460 (3,130–3,810)	3,340 (3,060–3,660)	3,330 (2,760–4,030)	1,340 (1,210–1,470)	903 (829–987)	817 (678–988)

1-3	Pedestrian road injuries	761 (610–889)	610 (557–668)	624 (515–761)	320 (257–371)	169 (155–185)	155 (128–189)
1-4	Cyclist road injuries	235 (173–291)	193 (156–236)	218 (171–273)	93·9 (69·5–116)	52·5 (42·4–64·1)	52·6 (41·2–66)
1-5	Motorcyclist road injuries	471 (357–565)	674 (599–753)	688 (570–829)	173 (133–207)	180 (160–201)	167 (139–201)
1-6	Motor vehicle road injuries	1,970 (1,720–2,330)	1,830 (1,670–2,010)	1,760 (1,440–2,140)	737 (642–869)	492 (449–540)	432 (353–528)
1-7	Other road injuries	26 (20–34)	35 (27–45)	40 (30–52)	11·5 (8·7–15·1)	9·7 (7·5–12·5)	9·7 (7·3–12·5)
1-8	Other transport injuries	253 (223–285)	537 (479–606)	1,910 (1,740–2,100)	98·5 (87–110)	144 (129–162)	474 (432–522)
1-9	Unintentional injuries	4,180 (3,760–4,690)	4,690 (4,200–5,320)	7,420 (6,580–8,480)	1,710 (1,540–1,920)	1,350 (1,210–1,530)	1,970 (1,760–2,250)
1-10	Falls	400 (341–476)	736 (617–891)	920 (750–1,140)	206 (177–243)	229 (194–274)	239 (196–293)
1-11	Drowning	1,330 (1,200–1,480)	1,370 (1,240–1,490)	1,310 (1,060–1,620)	487 (439–537)	374 (341–408)	346 (280–428)
1-12	Fire, heat, and hot substances	840 (728–982)	744 (623–896)	792 (637–1,000)	344 (298–404)	210 (177–253)	204 (165–256)
1-13	Poisonings	112 (98–126)	50 (42–58)	53 (43–66)	43 (38·2–48·6)	13·9 (11·8–16·3)	14 (11·2–17·3)
1-14	Poisoning by carbon monoxide	37 (30–44)	24 (20–28)	25 (20–31)	13·8 (11·5–16·5)	6·5 (5·5–7·5)	6·4 (5–8)
1-15	Poisoning by other means	75 (65–86)	26 (20–33)	28 (21–37)	29·2 (25·4–33·5)	7·4 (5·8–9·2)	7·6 (5·6–9·8)
1-16	Exposure to mechanical forces	313 (250–407)	465 (363–605)	540 (404–729)	128 (101–168)	128 (100–167)	134 (100–179)
1-17	Unintentional firearm injuries	49 (39–63)	60 (48–74)	70 (53–90)	19·3 (15·1–24·4)	16·5 (13–20·3)	17·2 (13·1–22)
1-18	Other exposure to mechanical forces	264 (206–354)	405 (312–535)	470 (346–641)	109 (84·4–147)	112 (85·9–148)	116 (86–158)
1-19	Adverse effects of medical treatment	228 (187–256)	252 (219–279)	287 (228–363)	107 (90–119)	77·4 (68·3–85·9)	76·4 (60·6–95·9)
1-20	Animal contact	23 (17–31)	53 (43–64)	60 (47–73)	9·8 (7·2–13·1)	15·4 (12·7–18·5)	15·2 (12–18·7)
1-21	Venomous animal contact	16 (11–21)	34 (27–41)	38 (30–48)	6·5 (4·7–8·7)	9·6 (7·7–11·8)	9·7 (7·7–12·1)
1-22	Non-venomous animal contact	7 (5–11)	19 (16–24)	21 (16–27)	3·3 (2·4–4·7)	5·8 (4·8–7·1)	5·5 (4·3–6·9)
1-23	Foreign body	567 (482–664)	402 (361–450)	416 (340–507)	235 (202–275)	128 (114–145)	126 (102–157)
1-24	Pulmonary aspiration and foreign body in airway	527 (445–625)	353 (317–394)	360 (290–447)	218 (186–257)	114 (102–130)	111 (88·7–140)
1-25	Foreign body in eyes	13 (6–24)	20 (9–35)	22 (10–38)	5·4 (2·6–9·5)	5·4 (2·6–9·5)	5·4 (2·6–9·5)
1-26	Foreign body in other body part	26 (21–33)	30 (23–38)	34 (27–45)	11·3 (9·1–14)	8·6 (6·8–11)	9 (7–11·6)
1-27	Environmental heat and cold exposure	107 (83–139)	142 (107–187)	165 (122–218)	46·6 (36·1–60·1)	40·4 (30·6–52·9)	41·2 (30·7–54·1)
1-28	Exposure to forces of nature	0 (0–0)	166 (151–183)	2,540 (2,300–2,790)	0 (0–0)	46·9 (42·6–51·8)	695 (632–765)
1-29	Other unintentional injuries	263 (218–326)	318 (261–402)	339 (263–439)	104 (85·4–130)	87·2 (71·6–110)	85·1 (66·3–110)
1-30	Self-harm and interpersonal violence	3,030 (2,770–3,300)	6,900 (6,390–7,430)	6,920 (5,700–8,470)	1,090 (996–1,180)	1,830 (1,700–1,970)	1,700 (1,400–2,080)
1-31	Self-harm	337 (302–376)	512 (462–566)	547 (444–689)	134 (121–148)	136 (123–150)	131 (106–164)
1-32	Self-harm by firearm	28 (22–42)	39 (31–54)	40 (30–53)	11·1 (8·9–16·5)	10·3 (8·2–14·2)	9·5 (7·2–12·8)
1-33	Self-harm by other specified means	309 (276–345)	473 (428–525)	507 (410–636)	122 (110–135)	125 (113–139)	121 (98·3–152)
1-34	Interpersonal violence	2,690 (2,450–2,950)	6,330 (5,840–6,850)	6,330 (5,220–7,740)	954 (870–1,040)	1,680 (1,550–1,810)	1,560 (1,290–1,910)
1-35	Physical violence by firearm	1,680 (1,490–1,870)	4,580 (4,210–4,980)	4,570 (3,740–5,640)	576 (512–642)	1,210 (1,110–1,320)	1,120 (919–1,380)
1-36	Physical violence by sharp object	590 (486–702)	1,120 (1,010–1,230)	1,100 (884–1,370)	213 (178–251)	293 (265–322)	269 (216–334)
1-37	Sexual violence	49 (32–70)	67 (44–95)	72 (48–103)	17·8 (11·8–25·3)	17·9 (11·8–25·4)	17·8 (11·8–25·3)
1-38	Physical violence by other means	373 (330–419)	560 (515–609)	583 (474–722)	148 (131–165)	155 (142–168)	150 (122–186)
1-39	Conflict and terrorism	2 (1–3)	8 (5–11)	6 (4–9)	0·6 (0·4–1)	2 (1·3–2·9)	1·4 (0·9–2)
1-40	Executions and police conflict	2 (2–3)	52 (39–69)	36 (18–64)	0·8 (0·7–1)	13·8 (10·2–18·5)	9 (4·5–16)

SR Table 1: All-age DALYs counts and age standardized DALYs rates per 100,000 individuals for all causes and level 1 cause groups by location for 1990, 2010, and 2019, both sexes combined, Barbados

Cause Level	Cause	All-age DALYs			Age-standardized rate of DALYs per 100,000		
		1990	2010	2019	1990	2010	2019
0-0	All causes	78,500 (71,600–86,500)	85,600 (77,200–95,800)	99,600 (84,900–115,000)	29,600 (27,000–32,700)	26,200 (23,600–29,400)	26,100 (22,000–30,400)
1-0	Communicable, maternal, neonatal, and nutritional disorders	11,800 (10,800–13,100)	9,820 (8,750–11,000)	9,470 (8,080–11,100)	5,080 (4,600–5,660)	4,030 (3,520–4,580)	3,700 (3,050–4,510)
1-1	HIV/AIDS and sexually transmitted infections	1,950 (1,890–2,030)	1,610 (1,550–1,680)	1,280 (1,210–1,360)	763 (740–792)	518 (499–543)	395 (376–420)
1-2	HIV/AIDS	1,880 (1,820–1,940)	1,530 (1,490–1,580)	1,210 (1,160–1,260)	734 (714–758)	493 (479–510)	372 (358–388)
1-3	HIV/AIDS - drug-susceptible tuberculosis	67 (45–99)	27 (19–40)	22 (15–32)	26·2 (17·4–38·5)	8·7 (6·1–12·7)	6·7 (4·6–9·9)
1-4	HIV/AIDS - Multidrug-resistant Tuberculosis without extensive drug resistance	2 (0–9)	0 (0–1)	0 (0–1)	0·6 (0–3·5)	0·1 (0–0·3)	0·1 (0–0·3)
1-5	HIV/AIDS - Extensively drug-resistant Tuberculosis	0 (0–0)	0 (0–0)	0 (0–0)	0 (0–0)	0 (0–0)	0 (0–0)
1-6	HIV/AIDS resulting in other diseases	1,810 (1,750–1,880)	1,500 (1,460–1,550)	1,180 (1,140–1,230)	707 (683–732)	484 (470–500)	365 (351–381)
1-7	Sexually transmitted infections excluding HIV	74 (52–113)	76 (49–124)	74 (46–125)	29·3 (20·6–44·6)	25·2 (16·7–39·9)	23·1 (14·8–37·6)
1-8	Syphilis	18 (14–22)	7 (6–9)	6 (5–8)	8·2 (6·4–10·3)	3·2 (2·6–4·1)	2·6 (1·9–3·5)
1-9	Chlamydial infection	8 (5–12)	10 (7–14)	9 (6–13)	2·9 (2–4·3)	3·2 (2·2–4·6)	3 (2–4·3)
1-10	Gonococcal infection	6 (5–8)	8 (7–10)	8 (6–10)	2·3 (1·8–2·9)	2·7 (2·2–3·2)	2·3 (1·8–3)
1-11	Trichomoniasis	13 (5–27)	16 (6–34)	16 (6–35)	5·1 (2–10·7)	5 (1·9–10·4)	4·8 (1·9–10)
1-12	Genital herpes	14 (5–36)	18 (6–44)	20 (6–50)	5·4 (1·8–13·7)	5·5 (1·8–13·3)	5·4 (1·7–13·4)
1-13	Other sexually transmitted infections	14 (10–19)	17 (12–22)	15 (11–21)	5·4 (3·9–7·3)	5·5 (4·1–7·4)	5 (3·7–6·9)
1-14	Respiratory infections and tuberculosis	2,270 (2,080–2,500)	2,600 (2,380–2,850)	2,920 (2,440–3,440)	874 (792–970)	828 (748–920)	784 (639–937)
1-15	Tuberculosis	142 (128–158)	73 (63–85)	66 (54–80)	53·4 (48·1–59·4)	23·2 (20–27·5)	18·6 (15·3–22·7)
1-16	Latent tuberculosis infection	0 (0–0)	0 (0–0)	0 (0–0)	0 (0–0)	0 (0–0)	0 (0–0)
1-17	Drug-susceptible tuberculosis	139 (123–156)	73 (63–84)	65 (54–79)	52·4 (46·1–58·8)	23·1 (19·8–27·3)	18·5 (15·1–22·5)
1-18	Multidrug-resistant tuberculosis without extensive drug resistance	3 (0–14)	0 (0–1)	0 (0–2)	1 (0–5·6)	0·1 (0–0·4)	0·1 (0–0·6)
1-19	Extensively drug-resistant tuberculosis	0 (0–0)	0 (0–0)	0 (0–0)	0 (0–0)	0 (0–0)	0 (0–0·1)
1-20	Lower respiratory infections	1,860 (1,710–2,020)	2,240 (2,050–2,440)	2,560 (2,080–3,040)	708 (648–777)	695 (632–763)	656 (526–798)
1-21	Upper respiratory infections	210 (129–328)	218 (132–343)	223 (137–352)	84·4 (51·8–132)	82·4 (49·8–130)	82·2 (49·9–129)
1-22	Otitis media	67 (38–112)	68 (40–112)	69 (40–115)	27·5 (15·8–45·3)	26·6 (15·5–44)	26·8 (15·3–44·3)
1-23	Enteric infections	614 (505–747)	606 (456–784)	721 (534–946)	249 (206–302)	215 (162–279)	231 (172–305)
1-24	Diarrhoeal diseases	601 (495–731)	603 (453–781)	718 (532–944)	244 (202–296)	214 (161–278)	230 (172–304)
1-25	Typhoid and paratyphoid	10 (3–14)	1 (1–2)	1 (1–2)	3·9 (1–5·1)	0·3 (0·2–0·7)	0·2 (0·2–0·6)
1-26	Typhoid fever	10 (3–14)	1 (1–2)	1 (0–2)	3·9 (1–5·1)	0·3 (0·2–0·6)	0·2 (0·1–0·6)
1-27	Paratyphoid fever	0 (0–0)	0 (0–0)	0 (0–0)	0 (0–0)	0 (0–0·1)	0 (0–0·1)
1-28	iNTS	1 (1–2)	1 (0–1)	1 (0–1)	0·4 (0·3–0·7)	0·4 (0·2–0·5)	0·3 (0·2–0·5)
1-29	Other intestinal infectious diseases	1 (1–2)	1 (1–1)	1 (1–1)	0·6 (0·3–0·9)	0·4 (0·3–0·6)	0·4 (0·3–0·6)
1-30	Neglected tropical diseases and malaria	251 (165–367)	318 (213–457)	300 (205–429)	96·4 (63·7–139)	108 (75·6–152)	91·7 (65–125)
1-31	Malaria	0 (0–0)	0 (0–0)	0 (0–0)	0 (0–0)	0 (0–0)	0 (0–0)
1-32	Chagas disease	0 (0–0)	0 (0–0)	0 (0–0)	0 (0–0)	0 (0–0)	0 (0–0)
1-33	Leishmaniasis	0 (0–0)	0 (0–0)	0 (0–0)	0 (0–0)	0 (0–0)	0 (0–0)
1-34	Visceral leishmaniasis	0 (0–0)	0 (0–0)	0 (0–0)	0 (0–0)	0 (0–0)	0 (0–0)
1-35	Cutaneous and mucocutaneous leishmaniasis	0 (0–0)	0 (0–0)	0 (0–0)	0 (0–0)	0 (0–0)	0 (0–0)
1-36	African trypanosomiasis	0 (0–0)	0 (0–0)	0 (0–0)	0 (0–0)	0 (0–0)	0 (0–0)
1-37	Schistosomiasis	0 (0–0)	0 (0–0)	0 (0–0)	0 (0–0)	0 (0–0)	0 (0–0)
1-38	Cysticercosis	144 (74–249)	138 (65–242)	150 (73–272)	52·4 (26·4–91·3)	37·9 (18–66·3)	33·9 (16·1–61·3)
1-39	Cystic echinococcosis	0 (0–0)	0 (0–0)	0 (0–0)	0 (0–0)	0 (0–0)	0 (0–0)
1-40	Lymphatic filariasis	0 (0–0)	0 (0–0)	0 (0–0)	0 (0–0)	0 (0–0)	0 (0–0)
1-41	Onchocerciasis	0 (0–0)	0 (0–0)	0 (0–0)	0 (0–0)	0 (0–0)	0 (0–0)
1-42	Trachoma	0 (0–0)	0 (0–0)	0 (0–0)	0 (0–0)	0 (0–0)	0 (0–0)
1-43	Dengue	32 (18–56)	127 (82–209)	99 (62–132)	12·4 (7·1–21·8)	47·1 (30·7–76·5)	35·3 (21·2–48·5)
1-44	Yellow fever	0 (0–0)	0 (0–0)	0 (0–0)	0 (0–0)	0 (0–0)	0 (0–0)
1-45	Rabies	0 (0–0)	0 (0–0)	0 (0–0)	0·1 (0·1–0·2)	0·1 (0·1–0·2)	0·1 (0·1–0·2)
1-46	Intestinal nematode infections	12 (7–19)	8 (5–14)	7 (4–12)	4·3 (2·5–6·9)	3·3 (1·8–5·5)	2·7 (1·4–4·6)
1-47	Ascariasis	1 (0–2)	0 (0–1)	0 (0–0)	0·4 (0·1–0·8)	0·2 (0·1–0·3)	0·1 (0–0·2)
1-48	Trichuriasis	0 (0–0)	0 (0–0)	0 (0–0)	0 (0–0)	0 (0–0)	0 (0–0)

1-49	Hookworm disease	11 (6–18)	8 (4–14)	7 (4–12)	3·9 (2·1–6·5)	3·1 (1·7–5·4)	2·6 (1·3–4·5)
1-50	Food-borne trematodiases	0 (0–0)	0 (0–0)	0 (0–0)	0 (0–0)	0 (0–0)	0 (0–0)
1-51	Leprosy	0 (0–0)	0 (0–0)	0 (0–1)	0·1 (0·1–0·2)	0·1 (0·1–0·1)	0·1 (0·1–0·1)
1-52	Ebola virus disease	0 (0–0)	0 (0–0)	0 (0–0)	0 (0–0)	0 (0–0)	0 (0–0)
1-53	Zika virus disease	0 (0–0)	0 (0–0)	3 (0–13)	0 (0–0)	0 (0–0)	1·2 (0–5·3)
1-54	Guinea worm disease	0 (0–0)	0 (0–0)	0 (0–0)	0 (0–0)	0 (0–0)	0 (0–0)
1-55	Other neglected tropical diseases	63 (42–90)	44 (28–66)	40 (26–61)	27 (18·3–39)	19·1 (11·8–29·1)	18·4 (11·5–28·5)
1-56	Other infectious diseases	947 (852–1,050)	520 (438–592)	513 (398–638)	396 (353–443)	193 (165–221)	178 (140–223)
1-57	Meningitis	437 (388–492)	131 (116–148)	122 (97–153)	188 (165–215)	53·7 (46–62·7)	46·9 (36–60·8)
1-58	Encephalitis	204 (160–234)	115 (97–161)	113 (87–162)	86·1 (67·3–99·5)	47·3 (39·1–68·5)	45·9 (34·3–68·4)
1-59	Diphtheria	0 (0–0)	0 (0–0)	0 (0–0)	0 (0–0)	0 (0–0)	0 (0–0)
1-60	Whooping cough	2 (1–4)	4 (2–6)	2 (1–3)	1·2 (0·7–1·9)	2·1 (1·2–3·4)	1·4 (0·9–2·3)
1-61	Tetanus	64 (44–88)	22 (14–45)	17 (9–37)	24·8 (17·2–33·6)	6·4 (4·3–13·5)	4·4 (2·4–9·2)
1-62	Measles	0 (0–1)	0 (0–0)	0 (0–0)	0·2 (0·1–0·3)	0 (0–0)	0 (0–0)
1-63	Varicella and herpes zoster	18 (14–27)	19 (15–27)	19 (14–28)	7·4 (5·6–10·7)	6·7 (5·1–9·5)	6·1 (4·4–8·9)
1-64	Acute hepatitis	61 (54–68)	20 (16–24)	19 (15–24)	23·7 (21·3–26·7)	7·2 (5·6–9·1)	6·7 (5·1–8·8)
1-65	Acute hepatitis A	41 (33–49)	12 (9–16)	11 (8–15)	16·1 (13–19)	4·7 (3·4–6·4)	4·4 (3–6)
1-66	Acute hepatitis B	16 (12–22)	5 (4–6)	6 (4–7)	6·1 (4·5–8·6)	1·8 (1·4–2)	1·7 (1·3–2·2)
1-67	Acute hepatitis C	2 (1–3)	1 (0–1)	1 (0–1)	0·8 (0·4–1·2)	0·2 (0·1–0·3)	0·2 (0·1–0·3)
1-68	Acute hepatitis E	2 (1–3)	1 (1–2)	1 (1–2)	0·7 (0·5–1·1)	0·5 (0·3–0·7)	0·5 (0·3–0·7)
1-69	Other unspecified infectious diseases	160 (112–202)	211 (127–264)	221 (131–295)	64·4 (45·7–81·6)	69·9 (43·6–86·9)	66·6 (41·8–88·3)
1-70	Maternal and neonatal disorders	4,660 (4,010–5,400)	3,360 (2,770–4,000)	2,960 (2,320–3,770)	2,230 (1,910–2,580)	1,850 (1,510–2,230)	1,720 (1,310–2,270)
1-71	Maternal disorders	140 (124–157)	138 (123–154)	106 (85–131)	49·2 (43·9–55·4)	52·5 (46·8–58·7)	40 (32·3–49·4)
1-72	Maternal haemorrhage	32 (26–40)	22 (17–27)	16 (12–21)	11·3 (9·2–14)	8·2 (6·5–10·2)	6 (4·5–7·8)
1-73	Maternal sepsis and other pregnancy related infections	29 (24–35)	15 (12–18)	12 (9–15)	10·2 (8·3–12·3)	5·6 (4·5–7)	4·4 (3·3–5·8)
1-74	Maternal hypertensive disorders	26 (22–31)	18 (15–22)	14 (11–17)	9·3 (7·8–10·9)	7 (5·8–8·3)	5·3 (4·1–6·7)
1-75	Maternal obstructed labour and uterine rupture	1 (1–2)	4 (3–5)	3 (2–4)	0·4 (0·3–0·7)	1·5 (1·1–1·9)	1·1 (0·8–1·5)
1-76	Maternal abortive outcome	22 (17–28)	9 (7–11)	7 (5–9)	7·9 (6·2–9·8)	3·4 (2·5–4·4)	2·5 (1·8–3·3)
1-77	Ectopic pregnancy	4 (3–5)	16 (13–18)	12 (9–15)	1·5 (1·2–1·8)	5·8 (4·8–6·9)	4·4 (3·2–5·8)
1-78	Indirect maternal deaths	6 (5–8)	8 (7–10)	7 (5–9)	2·2 (1·8–2·7)	3·3 (2·7–4·1)	2·6 (1·9–3·4)
1-79	Late maternal deaths	8 (6–10)	12 (9–16)	10 (7–13)	2·7 (2–3·6)	4·6 (3·4–6)	3·7 (2·5–5)
1-80	Maternal deaths aggravated by HIV/AIDS	0 (0–0)	0 (0–1)	0 (0–1)	0 (0–0·1)	0·1 (0·1–0·2)	0·1 (0·1–0·2)
1-81	Other maternal disorders	10 (8–13)	34 (28–41)	27 (21–34)	3·6 (2·9–4·4)	12·9 (10·7–15·4)	10 (7·7–12·8)
1-82	Neonatal disorders	4,520 (3,880–5,250)	3,230 (2,630–3,870)	2,860 (2,230–3,640)	2,180 (1,860–2,530)	1,800 (1,460–2,180)	1,680 (1,280–2,220)
1-83	Neonatal preterm birth	2,020 (1,700–2,430)	1,210 (991–1,480)	1,010 (769–1,320)	968 (810–1,170)	676 (545–830)	607 (443–822)
1-84	Neonatal encephalopathy due to birth asphyxia and trauma	1,280 (975–1,650)	740 (583–940)	588 (398–827)	632 (479–816)	445 (349–560)	391 (257–560)
1-85	Neonatal sepsis and other neonatal infections	703 (523–922)	875 (644–1,160)	928 (692–1,220)	327 (244–427)	446 (334–572)	466 (343–626)
1-86	Hemolytic disease and other neonatal jaundice	115 (84–160)	77 (59–97)	73 (56–93)	51·6 (37·2–73·3)	32·7 (25·4–41·2)	30·4 (22·9–39)
1-87	Other neonatal disorders	403 (290–553)	321 (233–417)	263 (172–380)	200 (144–275)	200 (145–261)	190 (124–275)
1-88	Nutritional deficiencies	1,140 (842–1,550)	802 (563–1,140)	778 (532–1,100)	470 (344–636)	312 (215–445)	297 (198–440)
1-89	Protein-energy malnutrition	269 (224–311)	149 (123–176)	156 (122–191)	111 (92·6–128)	55 (44·8–65·6)	51·4 (39·4–65·3)
1-90	Iodine deficiency	25 (12–42)	24 (12–41)	26 (13–44)	9·6 (4·8–16·2)	8 (4–13·7)	8·1 (3·9–13·8)
1-91	Vitamin A deficiency	12 (7–18)	9 (5–14)	8 (5–12)	5·2 (3·2–8·1)	4 (2·5–6·2)	3·9 (2·4–5·9)
1-92	Dietary iron deficiency	731 (464–1,100)	558 (341–871)	523 (317–817)	305 (194–456)	225 (138–346)	216 (129–343)
1-93	Other nutritional deficiencies	103 (82–135)	62 (48–76)	65 (47–82)	38·9 (31·2–50·9)	19·5 (15·2–24·2)	17·6 (13·1–22·6)
1-0	Non-communicable diseases	59,400 (53,400–65,900)	69,100 (61,800–77,500)	83,000 (70,500–96,400)	21,800 (19,500–24,300)	19,900 (17,600–22,500)	20,100 (17,100–23,600)
1-1	Neoplasms	10,400 (9,830–10,900)	14,500 (13,600–15,200)	18,000 (15,000–21,300)	3,820 (3,620–4,020)	3,920 (3,690–4,120)	3,950 (3,280–4,710)
1-2	Lip and oral cavity cancer	145 (134–156)	177 (163–193)	225 (184–271)	54·6 (50·5–59)	46·5 (42·9–50·8)	47·8 (38·9–57·5)
1-3	Nasopharynx cancer	34 (31–36)	60 (54–66)	73 (59–90)	13·2 (12·1–14·3)	16·5 (15–18)	16·6 (13·4–20·6)
1-4	Other pharynx cancer	119 (107–131)	120 (108–134)	159 (125–197)	46·4 (41·8–51·5)	31·5 (28·3–35·1)	33·4 (26·3–41·4)
1-5	Oesophageal cancer	365 (333–401)	426 (386–466)	553 (442–680)	137 (125–151)	111 (101–121)	115 (92·3–142)
1-6	Stomach cancer	1,000 (927–1,080)	864 (798–934)	1,040 (849–1,240)	358 (332–384)	228 (210–246)	219 (180–264)
1-7	Colon and rectum cancer	1,060 (986–1,120)	1,850 (1,720–1,980)	2,390 (1,970–2,860)	380 (354–404)	485 (452–518)	500 (413–601)

1·8	Liver cancer	196 (147–252)	218 (204–235)	295 (242–354)	72·9 (54·1–93·2)	59·9 (55·7–64·6)	65·1 (53·3–79)
1·9	Liver cancer due to hepatitis B	57 (37–85)	59 (45–78)	74 (52–105)	22·3 (14·1–32·7)	16·1 (12·3–21)	16·6 (11·8–23·1)
1·10	Liver cancer due to hepatitis C	39 (24–58)	45 (31–60)	60 (39–85)	13·3 (8·3–20·2)	11·4 (7·9–15·4)	12 (8–17·1)
1·11	Liver cancer due to alcohol use	61 (38–93)	72 (53–92)	109 (74–146)	22·2 (13·8–34)	18·8 (13·9–24)	22·3 (15·4–29·6)
1·12	Liver cancer due to NASH	16 (11–24)	21 (15–29)	29 (20–42)	5·7 (3·7–8·4)	5·4 (3·9–7·5)	6·1 (4·1–8·7)
1·13	Gallbladder and biliary tract cancer	242 (150–271)	133 (112–233)	166 (125–283)	87·3 (54·2–97·7)	34·6 (29–60·4)	34·3 (25·8–58·2)
1·14	Pancreatic cancer	92 (84–100)	625 (576–682)	840 (675–1,010)	33·7 (31–36·4)	163 (150–177)	174 (140–211)
1·15	Larynx cancer	86 (80–94)	122 (111–133)	162 (131–196)	32·6 (30–35·5)	31·8 (28·9–34·7)	33·4 (27·1–40·4)
1·16	Tracheal, bronchus, and lung cancer	626 (576–676)	829 (764–890)	1,050 (848–1,260)	232 (214–252)	217 (200–233)	217 (176–261)
1·17	Malignant skin melanoma	30 (24–41)	47 (36–62)	59 (43–79)	11·4 (9·2–15·6)	13·2 (10·1–17·2)	13·9 (10–18·4)
1·18	Non-melanoma skin cancer	22 (20–24)	32 (28–34)	40 (33–47)	7·6 (6·9–8·4)	8·3 (7·6–9)	8·5 (7·1–10·1)
1·19	Non-melanoma skin cancer (squamous-cell carcinoma)	22 (20–24)	32 (28–34)	40 (33–47)	7·6 (6·9–8·4)	8·3 (7·6–9)	8·5 (7·1–10·1)
1·20	Non-melanoma skin cancer (basal-cell carcinoma)	0 (0–0)	0 (0–0)	0 (0–0)	0 (0–0)	0 (0–0)	0 (0–0)
1·21	Breast cancer	1,270 (1,160–1,380)	2,140 (1,950–2,340)	2,430 (1,960–2,900)	502 (457–548)	572 (520–623)	540 (434–649)
1·22	Cervical cancer	735 (657–805)	813 (725–905)	882 (702–1,090)	288 (256–316)	223 (199–248)	206 (164–257)
1·23	Uterine cancer	201 (182–222)	405 (364–447)	477 (388–580)	74·9 (67·9–82·7)	107 (96·1–118)	101 (81·5–123)
1·24	Ovarian cancer	58 (50–103)	371 (174–418)	429 (211–537)	22·4 (19·6–40·3)	101 (47·3–113)	96·3 (47·2–120)
1·25	Prostate cancer	1,380 (1,120–1,760)	2,020 (1,500–2,290)	2,930 (2,140–3,590)	430 (351–549)	513 (380–581)	578 (424–707)
1·26	Testicular cancer	3 (2–3)	9 (8–11)	9 (7–11)	1 (0·8–1·3)	3·3 (2·7–4·1)	3·1 (2·3–3·9)
1·27	Kidney cancer	256 (238–276)	255 (236–276)	328 (262–399)	98·9 (91·9–106)	72·1 (66·6–77·9)	76 (60·6–92·5)
1·28	Bladder cancer	134 (122–147)	184 (168–202)	246 (201–294)	45·3 (41·4–49·3)	47·4 (43·4–51·7)	49·8 (40·6–59·6)
1·29	Brain and nervous system cancer	65 (47–128)	283 (105–358)	307 (114–444)	26·2 (18·9–51·4)	90·5 (33·8–115)	86·5 (32·4–126)
1·30	Thyroid cancer	44 (40–48)	63 (58–68)	77 (64–93)	16·3 (15–17·8)	16·8 (15·4–18·3)	16·9 (13·8–20·5)
1·31	Mesothelioma	15 (13–17)	21 (19–24)	23 (18–28)	5·9 (5·1–6·6)	5·6 (5–6·4)	5·3 (4·2–6·6)
1·32	Hodgkin lymphoma	48 (40–59)	40 (34–48)	42 (33–54)	17·8 (14·9–21·5)	12·8 (11–15·5)	12 (9·3–15·4)
1·33	Non-Hodgkin's lymphoma	557 (502–612)	627 (567–694)	715 (572–882)	211 (191–232)	186 (168–206)	180 (145–224)
1·34	Multiple myeloma	306 (274–370)	512 (466–580)	680 (552–834)	112 (100–135)	133 (122–151)	140 (113–171)
1·35	Leukaemia	519 (486–554)	567 (530–605)	616 (507–747)	201 (188–215)	186 (173–200)	177 (146–217)
1·36	Acute lymphoid leukaemia	62 (51–78)	53 (45–63)	50 (39–65)	25·8 (21·1–32·5)	22 (18·5–26·6)	20·9 (16·1–27·1)
1·37	Chronic lymphoid leukaemia	22 (18–25)	34 (29–39)	46 (36–57)	7·5 (6·4–8·8)	8·8 (7·6–10·2)	9·6 (7·6–11·7)
1·38	Acute myeloid leukaemia	181 (163–210)	229 (204–251)	247 (199–304)	71·2 (63·7–82)	77·7 (69·3–85·6)	75·3 (60·6–93·7)
1·39	Chronic myeloid leukaemia	46 (40–53)	52 (46–58)	57 (44–71)	17·3 (15–19·7)	15·3 (13·5–17·1)	14·2 (11·2–17·8)
1·40	Other leukaemia	208 (186–226)	200 (181–216)	216 (173–264)	78·9 (70·4–86)	61·8 (56·1–67)	56·9 (45·7–70·5)
1·41	Other neoplasms	29 (24–42)	47 (40–60)	58 (46–78)	10·5 (8·8–14·7)	13·3 (11·4–16·6)	13·6 (10·8–17·6)
1·42	Myelodysplastic, myeloproliferative, and other hematopoietic neoplasms	29 (24–42)	47 (40–60)	58 (46–78)	10·5 (8·8–14·7)	13·3 (11·4–16·6)	13·6 (10·8–17·6)
1·43	Benign and in situ intestinal neoplasms	0 (0–0)	0 (0–0)	0 (0–0)	0 (0–0)	0 (0–0)	0 (0–0)
1·44	Benign and in situ cervical and uterine neoplasms	0 (0–0)	0 (0–0)	0 (0–0)	0 (0–0)	0 (0–0)	0 (0–0)
1·45	Other benign and in situ neoplasms	0 (0–0)	0 (0–0)	0 (0–0)	0 (0–0)	0 (0–0)	0 (0–0)
1·46	Cardiovascular diseases	15,200 (14,300–15,900)	13,200 (12,400–13,900)	16,600 (14,100–19,200)	5,250 (4,970–5,490)	3,480 (3,260–3,670)	3,510 (3,000–4,060)
1·47	Rheumatic heart disease	301 (249–368)	250 (198–322)	252 (192–328)	116 (95·6–142)	80·6 (62·9–105)	75·2 (56·1–100)
1·48	Ischaemic heart disease	6,440 (6,040–6,780)	4,720 (4,360–5,020)	6,040 (5,120–7,050)	2,230 (2,100–2,350)	1,220 (1,130–1,300)	1,240 (1,050–1,450)
1·49	Stroke	5,650 (5,270–5,990)	4,660 (4,290–4,990)	5,820 (4,880–6,850)	1,920 (1,790–2,040)	1,220 (1,120–1,310)	1,220 (1,030–1,440)
1·50	Ischaemic stroke	3,060 (2,760–3,340)	2,390 (2,180–2,590)	3,040 (2,580–3,520)	966 (871–1,050)	612 (556–661)	621 (528–717)
1·51	Intracerebral hemorrhage	2,110 (1,910–2,330)	1,780 (1,630–1,920)	2,210 (1,810–2,680)	771 (698–845)	471 (431–506)	467 (381–568)
1·52	Subarachnoid hemorrhage	478 (412–552)	487 (439–540)	567 (455–697)	186 (161–214)	136 (123–151)	135 (108–166)
1·53	Hypertensive heart disease	832 (716–947)	1,010 (870–1,160)	1,260 (1,010–1,560)	286 (246–322)	261 (226–299)	260 (210–323)
1·54	Non-rheumatic valvular heart disease	82 (73–92)	109 (99–119)	136 (111–163)	29·9 (26·6–33·6)	29·9 (27·1–32·4)	30·4 (25–36·6)
1·55	Non-rheumatic calcific aortic valve disease	62 (53–70)	84 (75–93)	107 (87–129)	22 (18·9–25·3)	22·8 (20·5–25)	23·5 (19·2–28·4)
1·56	Non-rheumatic degenerative mitral valve disease	17 (13–21)	20 (16–25)	23 (17–31)	6·4 (4·9–7·9)	5·6 (4·4–7)	5·5 (4·1–7·3)
1·57	Other non-rheumatic valve diseases	4 (3–6)	5 (3–8)	6 (4–9)	1·5 (1–2·3)	1·4 (1–2·2)	1·4 (0·9–2·3)
1·58	Cardiomyopathy and myocarditis	484 (426–568)	658 (553–725)	786 (625–964)	184 (162–215)	184 (155–202)	183 (145–225)
1·59	Myocarditis	24 (19–31)	30 (23–38)	35 (25–45)	9·3 (7·3–12·1)	9·6 (7·4–12)	9·7 (7·2–12·7)
1·60	Alcoholic cardiomyopathy	110 (87–144)	200 (155–234)	238 (172–305)	44·9 (35·6–58·2)	54·1 (41·9–63·3)	53·7 (38·5–68·7)

1·61	Other cardiomyopathy	350 (308–409)	427 (362–475)	513 (411–629)	130 (114–151)	120 (102–133)	120 (95·7–148)
1·62	Atrial fibrillation and flutter	257 (213–314)	343 (290–409)	456 (375–561)	83·6 (69·5–101)	87·5 (73·8–104)	92·2 (76–113)
1·63	Aortic aneurysm	140 (123–157)	171 (153–189)	241 (194–292)	47·1 (42–52·6)	44·4 (39·8–49)	49·2 (39·6–59·8)
1·64	Peripheral vascular disease	257 (134–465)	366 (192–647)	506 (256–901)	85·1 (44·2–154)	93·2 (48·7–165)	102 (51·6–182)
1·65	Endocarditis	129 (105–162)	174 (140–209)	195 (150–256)	49·2 (39·9–61·9)	50·8 (40·8–61)	49·4 (38–64·7)
1·66	Chronic respiratory diseases	1,540 (1,260–1,850)	1,820 (1,520–2,180)	2,060 (1,700–2,480)	570 (461–699)	584 (470–730)	576 (457–731)
1·67	Chronic obstructive pulmonary disease	467 (413–524)	638 (568–718)	842 (706–983)	154 (136–174)	168 (150–188)	177 (150–207)
1·68	Pneumoconiosis	1 (1–1)	1 (1–1)	1 (1–2)	0·4 (0·3–0·5)	0·3 (0·2–0·3)	0·3 (0·2–0·4)
1·69	Silicosis	0 (0–0)	0 (0–0)	0 (0–0)	0·1 (0·1–0·1)	0·1 (0–0·1)	0·1 (0–0·1)
1·70	Asbestosis	0 (0–0)	0 (0–0)	0 (0–0)	0·1 (0–0·1)	0 (0–0·1)	0·1 (0–0·1)
1·71	Coal workers pneumoconiosis	0 (0–0)	0 (0–0)	0 (0–0)	0 (0–0)	0 (0–0)	0 (0–0)
1·72	Other pneumoconiosis	1 (1–1)	1 (0–1)	1 (1–1)	0·2 (0·2–0·3)	0·2 (0·1–0·2)	0·2 (0·1–0·2)
1·73	Asthma	968 (727–1,280)	1,010 (740–1,350)	999 (731–1,340)	379 (283–506)	364 (258–502)	345 (240–483)
1·74	Interstitial lung disease and pulmonary sarcoidosis	60 (47–87)	114 (85–138)	141 (102–179)	21·2 (16·5–30·4)	30·9 (23·2–37·2)	31·1 (22·6–39·6)
1·75	Other chronic respiratory diseases	39 (33–48)	63 (53–75)	74 (58–90)	15·6 (13–19)	20·7 (17·7–24·7)	22·6 (18·1–27·6)
1·76	Digestive diseases	2,640 (2,400–2,990)	2,910 (2,600–3,310)	3,420 (2,850–4,090)	1,020 (921–1,150)	830 (739–953)	828 (690–991)
1·77	Cirrhosis and other chronic liver diseases	972 (894–1,060)	995 (903–1,090)	1,200 (950–1,470)	387 (356–421)	270 (245–296)	272 (215–335)
1·78	Cirrhosis and other chronic liver diseases due to hepatitis B	121 (86–164)	92 (63–130)	86 (56–126)	49·6 (34·5–68·7)	24·7 (17–34·9)	19·8 (12·9–28·8)
1·79	Cirrhosis and other chronic liver diseases due to hepatitis C	180 (133–244)	187 (135–261)	220 (144–319)	72·6 (52·9–99·9)	50·8 (36·9–70)	50·6 (33·6–72·2)
1·80	Cirrhosis and other chronic liver diseases due to alcohol use	376 (301–450)	429 (339–515)	546 (402–712)	152 (121–184)	114 (90·5–137)	120 (89·2–157)
1·81	Cirrhosis and other chronic liver diseases due to NAFLD	141 (101–189)	153 (107–212)	194 (130–277)	54·3 (38·4–74·3)	41·1 (28·6–56·6)	42·8 (28·7–61·1)
1·82	Cirrhosis and other chronic liver diseases due to other causes	154 (118–196)	134 (101–176)	158 (112–225)	58·1 (45·3–74·6)	39·5 (30·4–51·1)	38·8 (28·1–53·7)
1·83	Upper digestive system diseases	690 (513–956)	734 (515–1,060)	834 (581–1,230)	257 (189–357)	212 (146–314)	209 (141–312)
1·84	Peptic ulcer disease	293 (256–331)	229 (200–260)	261 (207–325)	106 (92·3–120)	61·5 (53·8–69·7)	57·1 (45·8–71·2)
1·85	Gastritis and duodenitis	71 (52–96)	94 (65–131)	114 (79–160)	27·4 (19·7–37·9)	27·3 (18·8–38·3)	28·6 (19·6–40·3)
1·86	GERD	326 (170–577)	411 (214–730)	459 (237–817)	124 (64·1–219)	123 (63·9–218)	123 (64·2–218)
1·87	Appendicitis	63 (46–76)	45 (37–61)	48 (37–68)	24·1 (17·7–29·3)	14·9 (12·2–20)	14·3 (11–20·1)
1·88	Paralytic ileus and intestinal obstruction	305 (266–363)	341 (295–406)	401 (316–506)	114 (99·5–136)	100 (87·3–118)	98·8 (77–124)
1·89	Inguinal, femoral, and abdominal hernia	162 (123–214)	193 (145–257)	217 (160–287)	64·4 (48·3–85)	62·4 (46·1–84·1)	62·7 (45·9–84·7)
1·90	Inflammatory bowel disease	58 (47–71)	71 (58–87)	81 (62–101)	22·2 (18·1–27·2)	20·4 (16·5–24·9)	20 (15·5–25·3)
1·91	Vascular intestinal disorders	44 (37–51)	46 (41–52)	60 (47–74)	15·4 (12·9–17·8)	12·2 (10·7–13·7)	12·7 (10–15·6)
1·92	Gallbladder and biliary diseases	172 (133–224)	169 (126–224)	194 (143–253)	64·6 (49·6–84·4)	48·1 (35·5–64·7)	47·3 (34·6–63·4)
1·93	Pancreatitis	101 (87–116)	124 (109–140)	145 (113–180)	38·5 (33·1–44·8)	35·3 (31·1–40)	35·7 (27·8–44·7)
1·94	Other digestive diseases	76 (60–120)	190 (106–240)	239 (131–324)	28·3 (22·2–44·5)	53·6 (30·4–67·4)	55·7 (30·8–76)
1·95	Neurological disorders	3,630 (2,170–5,820)	4,380 (2,660–6,970)	5,010 (3,010–7,790)	1,320 (780–2,110)	1,330 (785–2,190)	1,340 (770–2,140)
1·96	Alzheimer's disease and other dementias	912 (385–2,130)	1,250 (522–2,890)	1,570 (648–3,540)	291 (122–664)	317 (132–728)	317 (131–717)
1·97	Parkinson's disease	227 (210–246)	336 (305–366)	437 (374–502)	68·1 (62·7–73·6)	83·1 (75·3–90·6)	87·1 (74·6–100)
1·98	Idiopathic epilepsy	756 (405–1,260)	677 (363–1,140)	706 (369–1,140)	290 (156–486)	233 (124–396)	226 (117–371)
1·99	Multiple sclerosis	40 (34–54)	70 (56–82)	79 (60–97)	15·9 (13·6–21·7)	20·3 (16–23·3)	20·1 (15·2–25)
1·100	Motor neuron disease	16 (14–17)	104 (96–112)	138 (105–175)	6·5 (6–7)	30·3 (28–32·7)	33 (24·8–42·1)
1·101	Headache disorders	1,460 (279–3,220)	1,670 (358–3,610)	1,770 (394–3,820)	559 (108–1,230)	557 (110–1,220)	556 (107–1,220)
1·102	Migraine	1,320 (190–3,040)	1,510 (247–3,440)	1,590 (270–3,630)	506 (74·7–1,160)	505 (75·2–1,160)	503 (72·7–1,170)
1·103	Tension-type headache	138 (40–496)	166 (51–556)	182 (56–596)	52·8 (15·7–190)	52·7 (15·6–190)	52·6 (15·4–188)
1·104	Other neurological disorders	222 (167–301)	262 (205–350)	305 (228–404)	87·3 (65·5–119)	92·1 (70·2–126)	99·3 (70·2–137)
1·105	Mental disorders	4,380 (3,190–5,760)	5,070 (3,690–6,670)	5,580 (4,100–7,390)	1,670 (1,220–2,200)	1,660 (1,210–2,190)	1,690 (1,230–2,230)
1·106	Schizophrenia	470 (327–630)	593 (409–791)	637 (446–849)	182 (126–242)	182 (125–245)	182 (128–243)
1·107	Depressive disorders	1,450 (989–2,000)	1,710 (1,180–2,390)	1,980 (1,380–2,770)	551 (377–763)	532 (366–740)	557 (382–772)
1·108	Major depressive disorder	1,210 (821–1,700)	1,410 (955–1,980)	1,660 (1,120–2,320)	460 (310–644)	441 (298–620)	466 (314–659)
1·109	Dysthymia	238 (148–360)	294 (184–447)	322 (205–500)	90·9 (56·8–139)	90·8 (57·1–138)	90·6 (56·4–138)
1·110	Bipolar disorder	535 (309–841)	621 (367–961)	665 (390–1,020)	203 (118–318)	203 (117–319)	202 (118–314)
1·111	Anxiety disorders	1,070 (704–1,520)	1,240 (818–1,750)	1,350 (899–1,900)	410 (269–581)	410 (273–585)	415 (275–583)
1·112	Eating disorders	139 (84–212)	137 (84–208)	135 (83–202)	49·7 (30·1–75·6)	52·1 (32·1–79·7)	51·2 (31·3–76·3)
1·113	Anorexia nervosa	33 (19–54)	31 (18–49)	31 (18–50)	11·9 (6·8–19·2)	12·3 (7·2–19·6)	12·1 (7·1–19·5)

1-114	Bulimia nervosa	107 (62–171)	105 (61–168)	104 (61–163)	37·8 (22·1–60·6)	39·7 (23·3–63·4)	39·1 (22·7–61·3)
1-115	Autism spectrum disorders	132 (85–194)	141 (92–204)	146 (95–212)	52·7 (33·9–77·4)	52·9 (34·1–76·3)	52·9 (34·7–76·4)
1-116	Attention-deficit/hyperactivity disorder	95 (54–162)	91 (52–152)	90 (51–151)	37·2 (20·9–63·7)	37·2 (21·1–63·2)	37·3 (21·1–63·8)
1-117	Conduct disorder	168 (95–270)	147 (84–234)	143 (80–226)	68 (38·4–109)	68 (38·8–108)	68·2 (38·4–108)
1-118	Idiopathic developmental intellectual disability	33 (9–64)	32 (9–62)	33 (8–66)	13·3 (3·6–25·7)	12·3 (3·3–23·7)	12·5 (3·2–24·3)
1-119	Other mental disorders	283 (180–429)	361 (232–544)	401 (257–603)	108 (68·2–164)	108 (69·3–165)	108 (68·2–163)
1-120	Substance use disorders	820 (606–1,050)	1,010 (787–1,280)	1,030 (806–1,320)	310 (231–398)	325 (248–413)	309 (237–395)
1-121	Alcohol use disorders	481 (355–636)	653 (507–844)	673 (518–867)	186 (138–245)	200 (153–259)	189 (144–246)
1-122	Drug use disorders	339 (235–467)	360 (259–481)	361 (260–481)	124 (86–170)	126 (89·2–169)	120 (85·5–161)
1-123	Opioid use disorders	214 (138–314)	218 (145–304)	220 (145–309)	78·9 (51·1–115)	73 (48·1–103)	69·5 (45·4–98·7)
1-124	Cocaine use disorders	18 (12–27)	28 (20–37)	27 (20–36)	6·6 (4·4–9·8)	9·7 (7–13·5)	9·1 (6·5–12·7)
1-125	Amphetamine use disorders	16 (9–28)	17 (10–28)	18 (11–28)	5·8 (3·1–9·7)	6·5 (3·6–10·5)	6·4 (3·7–10·3)
1-126	Cannabis use disorders	66 (36–109)	66 (39–100)	64 (38–98)	24 (13·2–39·5)	25·4 (14·8–39)	24 (14–37·2)
1-127	Other drug use disorders	25 (16–36)	31 (21–43)	32 (23–44)	9 (5·8–13·1)	11 (7·4–15·5)	10·8 (7·5–15·2)
1-128	Diabetes and kidney diseases	7,200 (6,510–7,930)	9,640 (8,610–10,800)	12,500 (10,600–14,500)	2,560 (2,300–2,830)	2,570 (2,300–2,890)	2,710 (2,280–3,150)
1-129	Diabetes mellitus	5,690 (5,090–6,340)	7,060 (6,170–8,090)	9,240 (7,700–11,000)	2,010 (1,790–2,250)	1,860 (1,620–2,140)	1,960 (1,630–2,330)
1-130	Diabetes mellitus type 1	350 (281–438)	337 (287–436)	384 (299–517)	132 (106–164)	104 (90–132)	103 (81·5–137)
1-131	Diabetes mellitus type 2	5,340 (4,750–5,970)	6,730 (5,850–7,760)	8,860 (7,320–10,500)	1,880 (1,660–2,110)	1,760 (1,520–2,030)	1,860 (1,540–2,210)
1-132	Chronic kidney disease	1,510 (1,380–1,650)	2,580 (2,330–2,830)	3,250 (2,740–3,800)	554 (505–605)	714 (646–784)	744 (627–875)
1-133	Chronic kidney disease due to diabetes mellitus type 1	101 (70–133)	182 (122–256)	205 (130–307)	40·7 (27·7–54·9)	51·9 (36–71·4)	52·1 (34·2–74·9)
1-134	Chronic kidney disease due to diabetes mellitus type 2	356 (293–419)	632 (517–756)	844 (664–1,040)	127 (104–151)	164 (135–197)	174 (138–214)
1-135	Chronic kidney disease due to hypertension	315 (260–374)	545 (447–648)	721 (573–881)	109 (90·3–131)	144 (119–170)	153 (122–186)
1-136	Chronic kidney disease due to glomerulonephritis	318 (264–379)	527 (432–641)	629 (481–823)	122 (101–146)	154 (127–186)	158 (124–204)
1-137	Chronic kidney disease due to other and unspecified causes	420 (343–509)	691 (554–844)	847 (660–1,070)	155 (126–187)	199 (162–240)	206 (163–258)
1-138	Acute glomerulonephritis	0 (0–0)	0 (0–0)	0 (0–0)	0·1 (0·1–0·2)	0·1 (0·1–0·1)	0·1 (0·1–0·1)
1-139	Skin and subcutaneous diseases	2,190 (1,640–3,000)	2,470 (1,860–3,410)	2,830 (2,110–3,870)	820 (617–1,110)	801 (592–1,080)	809 (596–1,100)
1-140	Dermatitis	286 (170–443)	303 (182–468)	312 (187–484)	116 (68·4–182)	116 (68·4–182)	116 (68·4–183)
1-141	Atopic dermatitis	204 (110–347)	204 (110–342)	201 (107–340)	85·2 (46–145)	85·2 (46·2–143)	85·1 (45·5–144)
1-142	Contact dermatitis	73 (46–109)	88 (54–132)	100 (62–152)	27·3 (17·1–41·1)	27·2 (16·8–40·9)	27·2 (17–41·1)
1-143	Seborrhoeic dermatitis	9 (5–14)	10 (6–16)	11 (7–18)	3·4 (1·9–5·4)	3·4 (1·9–5·4)	3·4 (1·9–5·4)
1-144	Psoriasis	81 (58–108)	94 (66–127)	103 (72–137)	31·7 (22·4–42·3)	30 (20·9–40·3)	29·5 (20·6–39·6)
1-145	Bacterial skin diseases	257 (165–473)	361 (224–639)	473 (284–889)	98·4 (62·4–180)	111 (69·2–189)	123 (72·7–222)
1-146	Cellulitis	84 (46–179)	99 (52–211)	122 (65–276)	33·1 (18·1–67·6)	32·1 (16·6–63·3)	33·8 (17·8–70·3)
1-147	Pyoderma	174 (111–310)	262 (159–446)	351 (204–613)	65·3 (42·1–115)	78·8 (49·1–132)	88·9 (53·3–153)
1-148	Scabies	207 (114–331)	217 (120–349)	222 (123–354)	82·5 (45·2–132)	82·5 (45·3–132)	82·2 (45·1–131)
1-149	Fungal skin diseases	121 (49–256)	141 (57–298)	161 (66–342)	44·3 (17·8–93·9)	43·9 (17·7–93)	43·9 (17·6–93)
1-150	Viral skin diseases	113 (72–169)	111 (70–165)	107 (69–161)	47·6 (30·5–71·3)	47·6 (30·1–70·8)	47·6 (30·3–70·8)
1-151	Acne vulgaris	115 (68–185)	113 (68–181)	118 (70–191)	43·3 (25·5–69·4)	47·8 (28·6–76·7)	49·5 (29·2–79·9)
1-152	Alopecia areata	19 (12–28)	21 (14–31)	23 (14–34)	7·2 (4·5–10·8)	7·2 (4·6–10·6)	7·2 (4·6–10·7)
1-153	Pruritus	23 (11–41)	28 (13–50)	31 (15–56)	8·6 (4·1–15·7)	8·7 (4·2–15·8)	8·7 (4·1–15·9)
1-154	Urticaria	118 (77–170)	124 (81–177)	126 (83–181)	48·4 (31·7–70)	48·3 (31·4–69·7)	48·3 (31·7–70·5)
1-155	Decubitus ulcer	717 (513–1,170)	791 (577–1,370)	959 (679–1,630)	241 (173–395)	207 (151–352)	203 (143–343)
1-156	Other skin and subcutaneous diseases	136 (72–239)	169 (87–301)	193 (101–346)	51·5 (27·3–90·7)	51·3 (26·4–91·8)	51·1 (26·5–91·5)
1-157	Sense organ diseases	1,900 (1,270–2,720)	2,390 (1,580–3,490)	2,840 (1,880–4,120)	678 (450–978)	656 (435–958)	646 (428–941)
1-158	Blindness and vision loss	368 (241–549)	440 (281–678)	524 (335–819)	129 (84·1–193)	119 (75·6–184)	116 (74·6–179)
1-159	Glaucoma	22 (14–31)	20 (14–29)	24 (16–34)	6·7 (4·5–9·6)	5·2 (3·4–7·3)	4·8 (3·2–6·8)
1-160	Cataract	55 (38–75)	62 (43–86)	73 (50–101)	17·4 (12·2–23·6)	15·7 (11–21·6)	14·9 (10·3–20·5)
1-161	Age-related macular degeneration	2 (1–3)	2 (1–3)	3 (2–4)	0·6 (0·4–0·8)	0·5 (0·3–0·8)	0·5 (0·3–0·7)
1-162	Refraction disorders	79 (53–114)	90 (59–130)	99 (65–142)	29·7 (19·8–43·1)	28·3 (18·7–41·1)	27·7 (18·4–40·3)
1-163	Near vision loss	140 (63–271)	183 (85–363)	227 (105–451)	50 (22·5–98·2)	47·7 (22–94·2)	47·3 (21·9–93·1)
1-164	Other vision loss	72 (50–98)	82 (57–112)	99 (68–136)	24·9 (17·4–34·4)	21·8 (15·2–30·1)	21 (14·6–28·9)
1-165	Age-related and other hearing loss	1,430 (957–2,070)	1,820 (1,210–2,660)	2,170 (1,430–3,190)	512 (343–746)	500 (330–730)	492 (325–724)
1-166	Other sense organ diseases	101 (59–157)	128 (74–200)	151 (89–236)	36·9 (21·6–57·3)	37·2 (21·8–57·9)	37·2 (22·1–56·6)

1-167	Musculoskeletal disorders	4,230 (3,100–5,540)	5,740 (4,190–7,500)	6,620 (4,840–8,670)	1,610 (1,180–2,110)	1,660 (1,220–2,170)	1,660 (1,220–2,170)
1-168	Rheumatoid arthritis	114 (85–146)	176 (129–228)	207 (151–269)	42·8 (31·9–54·3)	48·9 (35·8–63·4)	48·9 (35·6–64·2)
1-169	Osteoarthritis	541 (274–1,090)	810 (401–1,620)	1,020 (512–2,030)	200 (101–405)	210 (104–419)	211 (106–420)
1-170	Low back pain	1,930 (1,350–2,590)	2,450 (1,720–3,290)	2,760 (1,930–3,710)	733 (512–985)	729 (512–979)	723 (505–963)
1-171	Neck pain	349 (231–500)	472 (307–687)	531 (347–777)	136 (90·4–197)	136 (89·3–197)	136 (89–196)
1-172	Gout	18 (11–26)	29 (18–42)	35 (22–51)	6·8 (4·2–9·9)	7·8 (4·9–11·5)	8·1 (5·1–11·7)
1-173	Other musculoskeletal disorders	1,270 (926–1,670)	1,800 (1,310–2,390)	2,070 (1,480–2,750)	490 (356–643)	529 (389–694)	537 (385–706)
1-174	Other non-communicable diseases	5,360 (4,430–6,590)	6,000 (4,950–7,430)	6,590 (5,230–8,230)	2,180 (1,810–2,650)	2,110 (1,750–2,560)	2,090 (1,660–2,590)
1-175	Congenital anomalies	1,430 (1,180–1,700)	1,140 (909–1,380)	980 (719–1,300)	671 (552–802)	614 (482–759)	576 (409–787)
1-176	Neural tube defects	40 (30–54)	28 (21–36)	25 (17–36)	19 (13·9–25·8)	15·6 (11·4–20·6)	15·3 (10–22·8)
1-177	Congenital heart anomalies	681 (541–836)	466 (353–579)	381 (255–535)	324 (255–399)	262 (196–330)	239 (156–341)
1-178	Orofacial clefts	7 (4–14)	8 (5–12)	9 (5–13)	3 (1·8–6·6)	2·9 (1·8–4·5)	3·2 (2–5)
1-179	Down's syndrome	52 (43–63)	70 (55–82)	67 (48–89)	23·1 (19–28)	29·8 (23·1–35·9)	28·9 (20·2–38·9)
1-180	Turner syndrome	0 (0–1)	0 (0–0)	0 (0–0)	0·1 (0·1–0·2)	0·1 (0·1–0·2)	0·1 (0–0·2)
1-181	Klinefelter syndrome	0 (0–0)	0 (0–0)	0 (0–0)	0 (0–0)	0 (0–0)	0 (0–0)
1-182	Other chromosomal abnormalities	69 (52–108)	95 (71–125)	82 (56–119)	32·7 (24·4–51·8)	54·7 (40·4–73·2)	53·3 (35·6–78)
1-183	Congenital musculoskeletal and limb anomalies	124 (89–165)	125 (89–170)	123 (85–169)	53·1 (38·6–71·2)	53·5 (38·9–71·3)	52·8 (37–71·4)
1-184	Urogenital congenital anomalies	65 (45–102)	49 (34–70)	41 (26–60)	30·9 (21·7–49·5)	28·4 (19·8–41·1)	26·1 (16·4–40·1)
1-185	Digestive congenital anomalies	139 (100–199)	100 (70–141)	81 (53–120)	68·4 (49–98·2)	60·9 (42·6–86·4)	56·6 (36·7–84·7)
1-186	Other congenital anomalies	248 (193–320)	196 (146–248)	171 (119–240)	116 (89·6–150)	106 (77·8–136)	101 (67·1–143)
1-187	Urinary diseases and male infertility	378 (317–523)	928 (687–1,050)	1,200 (882–1,450)	133 (112–183)	253 (189–287)	266 (196–322)
1-188	Urinary tract infections and interstitial nephritis	258 (227–373)	750 (535–833)	972 (692–1,190)	90·8 (80·3–130)	204 (147–226)	216 (155–264)
1-189	Urolithiasis	16 (12–21)	27 (20–34)	34 (25–43)	6·3 (4·9–8·3)	7·5 (5·6–9·3)	7·8 (5·8–9·9)
1-190	Benign prostatic hyperplasia	71 (41–111)	98 (57–153)	133 (76–206)	23·6 (13·6–36·9)	25·4 (14·6–39·9)	25·9 (14·9–40·2)
1-191	Male infertility	12 (4–28)	12 (4–28)	12 (4–28)	4·2 (1·6–9·9)	4·2 (1·6–9·7)	4·2 (1·5–10·1)
1-192	Other urinary diseases	22 (17–38)	41 (23–50)	52 (28–70)	8·3 (6·4–13·7)	12·2 (7·2–15)	12·9 (7·3–17)
1-193	Gynecological diseases	994 (685–1,390)	1,160 (809–1,640)	1,200 (823–1,690)	379 (262–532)	370 (254–520)	366 (252–516)
1-194	Uterine fibroids	68 (37–125)	92 (48–167)	96 (49–172)	27·6 (14·9–50·7)	27 (14·1–48·9)	26·7 (13·8–48)
1-195	Polycystic ovarian syndrome	17 (7–34)	20 (9–42)	21 (9–42)	6·2 (2·6–12·6)	7·3 (3–14·9)	7·5 (3·2–15)
1-196	Female infertility	23 (8–54)	23 (8–54)	23 (8–55)	8·1 (2·7–19·3)	8·1 (2·8–19·4)	8·1 (2·8–19·1)
1-197	Endometriosis	79 (47–129)	78 (47–124)	73 (43–116)	29·3 (17·7–46·6)	26·8 (16–43·7)	24·9 (14·5–40·4)
1-198	Genital prolapse	19 (10–36)	23 (12–42)	25 (13–47)	7·5 (3·9–13·7)	6·2 (3·1–11·4)	5·6 (2·9–10·5)
1-199	Premenstrual syndrome	273 (167–430)	290 (176–450)	290 (177–450)	102 (62·4–159)	102 (62·8–160)	102 (62–160)
1-200	Other gynecological diseases	514 (333–735)	638 (410–909)	678 (434–969)	199 (128–283)	193 (124–276)	191 (124–275)
1-201	Hemoglobinopathies and hemolytic anaemias	372 (315–444)	304 (264–360)	306 (247–378)	144 (122–172)	111 (95·2–133)	105 (84·3–132)
1-202	Thalassemias	0 (0–0)	0 (0–0)	0 (0–0)	0·1 (0·1–0·2)	0·1 (0·1–0·2)	0·1 (0–0·2)
1-203	Thalassaemias trait	7 (4–12)	7 (4–11)	7 (4–12)	2·9 (1·7–4·5)	2·7 (1·6–4·4)	2·7 (1·6–4·5)
1-204	Sickle cell disorders	167 (144–192)	140 (122–156)	123 (94–160)	66·8 (57·5–76·2)	54·7 (48–61·4)	49·8 (38·1–64·5)
1-205	Sickle cell trait	69 (41–110)	45 (25–74)	44 (24–71)	26·3 (15·7–41·6)	17·6 (9·9–28·9)	16·6 (9·1–27·1)
1-206	G6PD deficiency	38 (26–50)	39 (29–50)	45 (31–59)	14·6 (10·2–19·3)	13 (9·4–16·8)	13·2 (9·1–17·9)
1-207	G6PD trait	0 (0–1)	1 (0–1)	1 (0–1)	0·1 (0·1–0·2)	0·2 (0·1–0·4)	0·2 (0·1–0·3)
1-208	Other hemoglobinopathies and hemolytic anaemias	89 (73–113)	72 (59–91)	86 (68–109)	33·3 (26·9–42·4)	22·5 (17·7–29·2)	22·6 (17·3–29·4)
1-209	Endocrine, metabolic, blood, and immune disorders	1,250 (961–1,480)	1,310 (1,080–1,740)	1,550 (1,230–2,080)	492 (380–581)	415 (343–562)	433 (339–595)
1-210	Oral disorders	900 (527–1,470)	1,130 (660–1,840)	1,320 (776–2,110)	335 (194–542)	326 (187–527)	324 (186–529)
1-211	Caries of deciduous teeth	7 (3–14)	6 (3–12)	5 (2–10)	3·1 (1·3–6·6)	3·1 (1·3–6·3)	3·1 (1·3–6·5)
1-212	Caries of permanent teeth	68 (31–134)	75 (33–148)	79 (36–156)	26·1 (11·7–50·5)	25·6 (11·4–49)	25·6 (11·4–49·2)
1-213	Periodontal disease	288 (111–606)	393 (154–838)	434 (168–905)	114 (43·6–241)	114 (44·8–248)	113 (43·7–239)
1-214	Edentulism	400 (254–600)	498 (314–748)	629 (392–954)	139 (87·6–209)	130 (81·8–194)	129 (80·5–193)
1-215	Other oral disorders	137 (84–200)	159 (99–232)	171 (107–249)	53·4 (33·1–78·1)	53·4 (32·8–78)	53·3 (33·1–77·4)
1-216	Sudden infant death syndrome	44 (30–64)	28 (18–40)	21 (12–33)	21·8 (14·7–31·8)	17·1 (11·4–24·4)	15·1 (8·8–23·9)
1-0	Injuries	7,260 (6,710–7,900)	6,720 (6,130–7,490)	7,170 (6,060–8,450)	2,740 (2,530–2,990)	2,280 (2,090–2,520)	2,250 (1,900–2,660)
1-1	Transport injuries	2,160 (1,990–2,350)	1,630 (1,480–1,810)	1,780 (1,420–2,140)	817 (752–886)	549 (500–606)	554 (440–672)
1-2	Road injuries	2,100 (1,930–2,280)	1,570 (1,420–1,730)	1,710 (1,370–2,050)	793 (731–860)	527 (480–581)	532 (422–642)

1-3	Pedestrian road injuries	388 (336–432)	249 (224–278)	277 (220–338)	150 (130–168)	80·3 (72·6–89·1)	79·6 (63·2–98·6)
1-4	Cyclist road injuries	167 (134–205)	143 (110–179)	171 (131–215)	63·7 (51·1–78)	45·3 (35·5–56·4)	49·1 (38·1–61·1)
1-5	Motorcyclist road injuries	272 (230–317)	247 (213–288)	270 (212–333)	101 (85·5–117)	83·8 (72·6–97·5)	86·5 (67·1–108)
1-6	Motor vehicle road injuries	1,250 (1,140–1,350)	903 (827–986)	966 (739–1,190)	469 (429–507)	310 (284–337)	309 (234–384)
1-7	Other road injuries	24 (18–32)	26 (19–35)	30 (22–40)	9·3 (6·8–12·2)	8 (5·9–10·5)	8·1 (5·9–10·7)
1-8	Other transport injuries	61 (53–70)	66 (57–75)	70 (56–85)	23·5 (20·3–27)	22·2 (19·4–25·4)	21·9 (17·5–26·8)
1-9	Unintentional injuries	3,030 (2,660–3,470)	2,760 (2,370–3,290)	3,020 (2,460–3,680)	1,160 (1,020–1,340)	914 (790–1,080)	898 (732–1,090)
1-10	Falls	407 (337–494)	587 (484–727)	744 (596–924)	146 (120–178)	169 (138–211)	183 (147–230)
1-11	Drowning	824 (757–899)	562 (512–615)	513 (415–630)	321 (294–350)	215 (194–236)	191 (154–236)
1-12	Fire, heat, and hot substances	475 (387–582)	351 (270–458)	376 (284–502)	186 (152–227)	116 (90·3–150)	113 (85·7–150)
1-13	Poisonings	117 (104–129)	34 (28–42)	36 (28–45)	46 (41–51·3)	12·2 (10–14·8)	12·2 (9·5–15·2)
1-14	Poisoning by carbon monoxide	9 (7–11)	6 (5–8)	6 (5–9)	3·2 (2·5–4·1)	2·1 (1·6–2·7)	2·1 (1·5–2·8)
1-15	Poisoning by other means	108 (97–120)	28 (23–35)	30 (23–38)	42·7 (38·2–47·8)	10·1 (8·3–12·4)	10·1 (7·8–12·5)
1-16	Exposure to mechanical forces	401 (315–520)	496 (382–651)	531 (401–713)	153 (120–199)	161 (126–210)	157 (120–208)
1-17	Unintentional firearm injuries	147 (127–167)	155 (136–178)	147 (121–177)	54·9 (47·3–62·6)	52·7 (46·3–60·3)	47 (38·7–57·1)
1-18	Other exposure to mechanical forces	254 (179–361)	341 (238–481)	384 (269–549)	98·2 (69·2–140)	108 (76·9–153)	110 (78·3–155)
1-19	Adverse effects of medical treatment	253 (217–279)	233 (204–260)	259 (209–321)	95·6 (82·3–105)	71·8 (63·1–81)	68·6 (55·3–85·9)
1-20	Animal contact	25 (18–35)	30 (22–41)	35 (25–47)	9·7 (6·8–13·5)	9·8 (7·2–13·2)	10 (7·2–13·4)
1-21	Venomous animal contact	17 (12–24)	20 (14–28)	23 (16–31)	6·7 (4·5–9·1)	6·6 (4·7–8·9)	6·7 (4·7–9·1)
1-22	Non-venomous animal contact	8 (5–12)	10 (7–15)	12 (8–17)	3·1 (2·1–4·6)	3·2 (2·3–4·6)	3·2 (2·3–4·8)
1-23	Foreign body	194 (172–218)	130 (114–149)	147 (120–178)	79·3 (70·1–90·1)	48·2 (41·5–55·9)	49·9 (39·5–61·6)
1-24	Pulmonary aspiration and foreign body in airway	155 (139–171)	90 (82–101)	103 (83–129)	64·3 (57·1–72·5)	35·2 (30·6–40·3)	36·6 (28·3–47·5)
1-25	Foreign body in eyes	14 (7–25)	16 (8–28)	18 (9–30)	5·4 (2·6–9·5)	5·4 (2·6–9·5)	5·4 (2·6–9·5)
1-26	Foreign body in other body part	25 (20–32)	23 (18–30)	27 (20–35)	9·6 (7·5–12·1)	7·6 (5·7–10)	8 (6–10·4)
1-27	Environmental heat and cold exposure	101 (72–138)	110 (78–153)	127 (89–177)	38·4 (27·3–52·4)	34·2 (24·2–47·8)	35·3 (24·8–49·5)
1-28	Exposure to forces of nature	0 (0–0)	0 (0–1)	1 (0–1)	0 (0–0)	0·1 (0·1–0·2)	0·2 (0·1–0·3)
1-29	Other unintentional injuries	231 (180–305)	229 (174–310)	251 (187–341)	88·9 (68·8–117)	76·8 (59–102)	77·6 (58·6–105)
1-30	Self-harm and interpersonal violence	2,070 (1,920–2,210)	2,320 (2,180–2,480)	2,370 (1,990–2,840)	762 (712–815)	817 (766–870)	796 (670–959)
1-31	Self-harm	537 (496–585)	526 (483–572)	556 (457–678)	200 (186–218)	171 (156–187)	166 (137–203)
1-32	Self-harm by firearm	36 (28–43)	30 (23–37)	30 (22–38)	13·2 (10·3–15·9)	10·1 (7·7–12·2)	9·2 (6·8–11·8)
1-33	Self-harm by other specified means	501 (462–545)	496 (455–539)	526 (432–641)	187 (173–203)	161 (147–176)	157 (129–192)
1-34	Interpersonal violence	1,520 (1,400–1,650)	1,790 (1,660–1,920)	1,800 (1,510–2,170)	560 (516–606)	642 (595–689)	628 (524–761)
1-35	Physical violence by firearm	482 (424–563)	709 (648–770)	689 (571–841)	172 (151–201)	262 (240–284)	249 (207–304)
1-36	Physical violence by sharp object	542 (472–614)	565 (519–614)	579 (480–704)	196 (171–222)	202 (185–220)	202 (167–247)
1-37	Sexual violence	48 (32–68)	51 (34–72)	53 (36–75)	17·8 (11·8–25·3)	17·8 (11·8–25·3)	17·8 (11·8–25·3)
1-38	Physical violence by other means	452 (410–494)	461 (426–498)	480 (400–579)	174 (159–190)	160 (148–173)	159 (132–194)
1-39	Conflict and terrorism	4 (2–6)	8 (5–13)	7 (5–11)	1·3 (0·8–2)	2·5 (1·6–3·9)	1·8 (1·2–2·9)
1-40	Executions and police conflict	2 (2–2)	2 (2–2)	2 (1–2)	0·7 (0·6–0·7)	0·7 (0·6–0·7)	0·7 (0·5–0·8)

SR Table 1: All-age DALYs counts and age standardized DALYs rates per 100,000 individuals for all causes and level 1 cause groups by location for 1990, 2010, and 2019, both sexes combined, Belize

Cause Level	Cause	All-age DALYs			Age-standardized rate of DALYs per 100,000		
		1990	2010	2019	1990	2010	2019
0-0	All causes	55,300 (50,200–61,100)	84,800 (76,300–93,700)	107,000 (94,200–121,000)	33,300 (30,200–36,700)	31,500 (28,600–34,600)	30,100 (26,600–34,000)
1-0	Communicable, maternal, neonatal, and nutritional disorders	20,500 (18,500–22,900)	19,300 (17,300–21,500)	19,700 (17,500–22,100)	8,900 (8,140–9,850)	6,130 (5,540–6,780)	5,240 (4,640–5,850)
1-1	HIV/AIDS and sexually transmitted infections	1,720 (1,680–1,750)	4,210 (4,060–4,400)	4,420 (4,260–4,630)	1,070 (1,050–1,100)	1,360 (1,310–1,420)	1,080 (1,040–1,130)
1-2	HIV/AIDS	1,680 (1,650–1,720)	4,150 (4,010–4,330)	4,340 (4,200–4,540)	1,050 (1,030–1,070)	1,340 (1,300–1,400)	1,060 (1,030–1,110)
1-3	HIV/AIDS - drug-susceptible tuberculosis	318 (206–492)	351 (224–528)	319 (204–479)	201 (130–310)	113 (72·1–170)	78 (49·9–117)
1-4	HIV/AIDS - Multidrug-resistant Tuberculosis without extensive drug resistance	4 (0–18)	3 (0–12)	4 (0–17)	2·4 (0·2–11)	0·9 (0·1–3·8)	1 (0·1–4·1)
1-5	HIV/AIDS - Extensively drug-resistant Tuberculosis	0 (0–0)	0 (0–1)	1 (0–3)	0 (0–0)	0·1 (0–0·4)	0·2 (0–0·7)
1-6	HIV/AIDS resulting in other diseases	1,360 (1,190–1,480)	3,800 (3,570–4,010)	4,020 (3,800–4,250)	845 (739–919)	1,230 (1,150–1,290)	982 (929–1,040)
1-7	Sexually transmitted infections excluding HIV	31 (20–49)	58 (34–98)	76 (45–131)	19·9 (11·9–33·9)	19 (11·1–33)	18·7 (11–32·1)
1-8	Syphilis	6 (4–7)	3 (2–4)	3 (2–4)	2·3 (1·8–2·9)	0·9 (0·7–1·1)	0·8 (0·6–1)
1-9	Chlamydial infection	3 (2–5)	7 (5–11)	9 (6–14)	2·2 (1·4–3·2)	2·2 (1·5–3·3)	2·2 (1·5–3·2)
1-10	Gonococcal infection	2 (1–3)	5 (4–7)	7 (5–9)	1·2 (0·9–1·8)	1·5 (1·2–2)	1·5 (1·2–2)
1-11	Trichomoniasis	7 (3–14)	15 (6–30)	19 (7–40)	4·8 (1·9–9·8)	4·9 (1·9–10·1)	4·8 (1·8–10·1)
1-12	Genital herpes	7 (2–17)	15 (5–37)	20 (7–51)	5·3 (1·8–13)	5·4 (1·8–13·3)	5·4 (1·8–13·3)
1-13	Other sexually transmitted infections	6 (4–9)	13 (9–19)	17 (12–25)	4·1 (2·7–5·8)	4·1 (2·8–5·8)	4·1 (2·8–5·8)
1-14	Respiratory infections and tuberculosis	4,500 (3,930–5,150)	4,020 (3,610–4,480)	4,410 (3,810–5,030)	2,260 (2,030–2,500)	1,490 (1,360–1,620)	1,310 (1,130–1,490)
1-15	Tuberculosis	691 (615–764)	622 (569–679)	652 (555–761)	436 (390–480)	224 (205–242)	173 (148–201)
1-16	Latent tuberculosis infection	0 (0–0)	0 (0–0)	0 (0–0)	0 (0–0)	0 (0–0)	0 (0–0)
1-17	Drug-susceptible tuberculosis	684 (608–758)	618 (563–673)	645 (550–754)	432 (385–476)	222 (204–240)	171 (147–200)
1-18	Multidrug-resistant tuberculosis without extensive drug resistance	7 (1–30)	4 (0–16)	6 (1–26)	4·1 (0·4–19·1)	1·4 (0·1–5·9)	1·7 (0·2–7·1)
1-19	Extensively drug-resistant tuberculosis	0 (0–0)	0 (0–2)	1 (0–4)	0 (0–0)	0·2 (0–0·6)	0·3 (0–1·1)
1-20	Lower respiratory infections	3,550 (3,040–4,110)	3,000 (2,660–3,370)	3,280 (2,790–3,800)	1,700 (1,500–1,900)	1,150 (1,040–1,260)	1,020 (877–1,180)
1-21	Upper respiratory infections	193 (124–291)	291 (180–457)	350 (214–547)	92·1 (58·5–139)	84·4 (51·9–131)	83·7 (51·3–130)
1-22	Otitis media	71 (41–116)	108 (63–182)	128 (74–210)	34·1 (19·9–56·1)	30·7 (17·8–50·9)	30·4 (17·6–49·3)
1-23	Enteric infections	2,240 (1,830–2,690)	1,130 (933–1,340)	1,170 (933–1,460)	906 (757–1,080)	365 (303–429)	321 (258–398)
1-24	Diarrhoeal diseases	2,220 (1,820–2,690)	1,120 (927–1,330)	1,160 (928–1,450)	901 (754–1,070)	363 (301–428)	320 (257–396)
1-25	Typhoid and paratyphoid	5 (1–8)	0 (0–1)	0 (0–1)	2·3 (0·3–3·8)	0·1 (0·1–0·3)	0·1 (0·1–0·2)
1-26	Typhoid fever	5 (1–8)	0 (0–1)	0 (0–1)	2·3 (0·3–3·8)	0·1 (0·1–0·3)	0·1 (0–0·2)
1-27	Paratyphoid fever	0 (0–0)	0 (0–0)	0 (0–0)	0 (0–0)	0 (0–0)	0 (0–0)
1-28	iNTS	3 (2–5)	3 (2–4)	3 (2–4)	1·1 (0·7–1·9)	0·8 (0·5–1·2)	0·7 (0·4–1·1)
1-29	Other intestinal infectious diseases	4 (1–9)	3 (1–4)	2 (1–4)	1·7 (0·5–3·2)	0·7 (0·4–1·2)	0·6 (0·3–0·9)
1-30	Neglected tropical diseases and malaria	341 (185–793)	405 (268–593)	421 (275–605)	189 (107–405)	135 (88·9–195)	111 (72·5–158)
1-31	Malaria	114 (8–544)	8 (5–12)	2 (0–4)	53·7 (3·6–256)	2·1 (1·3–3·3)	0·4 (0–0·8)
1-32	Chagas disease	3 (1–5)	2 (1–4)	3 (2–6)	3·7 (1·3–5·2)	1·2 (0·8–2·7)	1·1 (0·7–2·6)
1-33	Leishmaniasis	0 (0–0)	0 (0–0)	0 (0–0)	0 (0–0·1)	0 (0–0)	0 (0–0·1)
1-34	Visceral leishmaniasis	0 (0–0)	0 (0–0)	0 (0–0)	0 (0–0)	0 (0–0)	0 (0–0)
1-35	Cutaneous and mucocutaneous leishmaniasis	0 (0–0)	0 (0–0)	0 (0–0)	0 (0–0·1)	0 (0–0)	0 (0–0·1)
1-36	African trypanosomiasis	0 (0–0)	0 (0–0)	0 (0–0)	0 (0–0)	0 (0–0)	0 (0–0)
1-37	Schistosomiasis	0 (0–0)	0 (0–0)	0 (0–0)	0 (0–0)	0 (0–0)	0 (0–0)
1-38	Cysticercosis	76 (45–119)	134 (76–215)	170 (96–271)	64·8 (38·3–100)	56·2 (31·1–88·2)	50·4 (28·3–80·4)
1-39	Cystic echinococcosis	0 (0–0)	0 (0–0)	0 (0–0)	0 (0–0)	0 (0–0)	0 (0–0)
1-40	Lymphatic filariasis	0 (0–0)	0 (0–0)	0 (0–0)	0 (0–0)	0 (0–0)	0 (0–0)
1-41	Onchocerciasis	0 (0–0)	0 (0–0)	0 (0–0)	0 (0–0)	0 (0–0)	0 (0–0)
1-42	Trachoma	0 (0–0)	0 (0–0)	0 (0–0)	0 (0–0)	0 (0–0)	0 (0–0)
1-43	Dengue	20 (12–34)	118 (51–238)	104 (38–223)	10·4 (5·8–17·1)	35·4 (14·9–71·4)	24·9 (9·1–52)
1-44	Yellow fever	0 (0–0)	0 (0–0)	0 (0–0)	0 (0–0)	0 (0–0)	0 (0–0)
1-45	Rabies	1 (1–2)	1 (1–2)	1 (1–2)	0·5 (0·4–0·7)	0·4 (0·3–0·6)	0·3 (0·2–0·4)
1-46	Intestinal nematode infections	52 (30–82)	46 (26–76)	31 (19–52)	24·3 (14·4–38·8)	12·9 (7·5–20·9)	7 (4·2–11·6)
1-47	Ascariasis	11 (3–24)	8 (4–13)	6 (3–10)	4·3 (1·5–9)	2·2 (1·2–3·8)	1·4 (0·8–2·4)
1-48	Trichuriasis	2 (1–4)	1 (0–1)	1 (0–2)	1 (0·4–1·9)	0·2 (0·1–0·4)	0·2 (0·1–0·4)

1-49	Hookworm disease	38 (22–66)	38 (20–65)	24 (14–42)	19 (10·8–32·4)	10·5 (5·7–17·6)	5·3 (3–9·2)
1-50	Food-borne trematodiases	0 (0–0)	0 (0–0)	0 (0–0)	0 (0–0)	0 (0–0)	0 (0–0)
1-51	Leprosy	1 (0–1)	1 (0–1)	1 (1–2)	0·5 (0·3–0·8)	0·3 (0·2–0·5)	0·3 (0·2–0·5)
1-52	Ebola virus disease	0 (0–0)	0 (0–0)	0 (0–0)	0 (0–0)	0 (0–0)	0 (0–0)
1-53	Zika virus disease	0 (0–0)	0 (0–0)	3 (0–12)	0 (0–0)	0 (0–0)	0·7 (0·1–2·9)
1-54	Guinea worm disease	0 (0–0)	0 (0–0)	0 (0–0)	0 (0–0)	0 (0–0)	0 (0–0)
1-55	Other neglected tropical diseases	74 (47–111)	95 (61–141)	107 (66–160)	31·5 (20·4–46·8)	26·3 (16·9–38·3)	25·8 (16·3–38·5)
1-56	Other infectious diseases	1,010 (839–1,180)	521 (451–600)	543 (460–641)	420 (357–481)	156 (136–179)	137 (116–162)
1-57	Meningitis	540 (435–652)	178 (149–214)	160 (133–194)	210 (173–250)	52·8 (44·5–62·8)	41 (33·9–50·1)
1-58	Encephalitis	219 (110–279)	138 (118–173)	150 (124–191)	86·8 (48·1–109)	39·4 (33·8–49·2)	36·8 (30·1–47)
1-59	Diphtheria	0 (0–0)	0 (0–0)	0 (0–1)	0·1 (0·1–0·2)	0·1 (0·1–0·1)	0·1 (0–0·1)
1-60	Whooping cough	24 (16–34)	13 (8–19)	12 (8–18)	8·4 (5·5–12·1)	3·5 (2·3–5·2)	3·2 (2–4·9)
1-61	Tetanus	20 (13–32)	9 (6–16)	5 (2–15)	12 (7·6–20·3)	3·2 (2·1–6·4)	1·4 (0·7–4·3)
1-62	Measles	26 (4–45)	0 (0–0)	0 (0–0)	9·2 (1·4–16)	0 (0–0)	0 (0–0)
1-63	Varicella and herpes zoster	21 (12–34)	23 (15–36)	26 (18–39)	9·9 (6·1–15·6)	7·4 (5–11·2)	6·9 (4·8–10·5)
1-64	Acute hepatitis	82 (67–101)	36 (30–44)	38 (30–47)	41·7 (35·5–49·2)	10·7 (9–12·8)	9 (7·2–11·1)
1-65	Acute hepatitis A	56 (41–72)	20 (15–27)	21 (14–28)	26·3 (19·7–32·5)	5·6 (4·2–7·3)	4·6 (3·2–6·3)
1-66	Acute hepatitis B	17 (10–26)	11 (8–13)	12 (9–14)	9·7 (6·4–13·3)	3·5 (2·8–4·1)	2·9 (2·2–3·6)
1-67	Acute hepatitis C	6 (3–11)	2 (2–4)	2 (2–4)	4·1 (2·2–6·2)	0·8 (0·6–1·4)	0·7 (0·4–1·1)
1-68	Acute hepatitis E	3 (2–6)	3 (2–4)	3 (2–5)	1·6 (1–2·9)	0·8 (0·6–1·2)	0·8 (0·5–1·1)
1-69	Other unspecified infectious diseases	81 (57–109)	124 (90–164)	151 (112–202)	41·1 (30–54)	39·1 (28·2–51)	38·6 (28·5–50·7)
1-70	Maternal and neonatal disorders	8,450 (7,400–9,550)	7,060 (5,960–8,340)	6,620 (5,570–7,790)	3,070 (2,700–3,460)	2,040 (1,720–2,410)	1,730 (1,450–2,040)
1-71	Maternal disorders	150 (125–174)	361 (324–402)	373 (316–434)	79·5 (66·1–91·7)	100 (90·2–112)	80·5 (68–93·7)
1-72	Maternal haemorrhage	44 (34–58)	72 (58–90)	72 (56–92)	23·8 (18·5–30·9)	20·2 (16·3–25)	15·7 (12·2–19·8)
1-73	Maternal sepsis and other pregnancy related infections	23 (18–30)	33 (26–41)	35 (26–44)	12·3 (9·6–15·4)	9·2 (7·3–11·4)	7·5 (5·7–9·5)
1-74	Maternal hypertensive disorders	35 (28–42)	73 (63–85)	72 (60–86)	18·3 (14·8–21·8)	19·8 (17–22·9)	15·4 (12·8–18·3)
1-75	Maternal obstructed labour and uterine rupture	2 (1–3)	14 (10–17)	13 (10–18)	1 (0·5–1·6)	3·8 (3–4·9)	2·9 (2·2–3·8)
1-76	Maternal abortive outcome	26 (20–33)	28 (21–35)	24 (19–32)	13·8 (10·4–17·3)	7·7 (6–9·7)	5·2 (4–6·9)
1-77	Ectopic pregnancy	1 (1–1)	9 (7–10)	9 (7–11)	0·6 (0·4–0·7)	2·5 (2–3)	1·9 (1·5–2·5)
1-78	Indirect maternal deaths	4 (3–5)	16 (13–20)	18 (14–23)	1·9 (1·4–2·4)	4·5 (3·6–5·4)	3·9 (3–5)
1-79	Late maternal deaths	5 (3–7)	20 (14–26)	22 (15–29)	2·5 (1·8–3·5)	5·4 (4–7)	4·6 (3·3–6·3)
1-80	Maternal deaths aggravated by HIV/AIDS	0 (0–0)	1 (1–2)	2 (1–3)	0·1 (0–0·1)	0·3 (0·2–0·5)	0·4 (0·2–0·6)
1-81	Other maternal disorders	10 (8–13)	95 (80–112)	105 (84–129)	5·4 (4·2–6·7)	26·8 (22·3–31·5)	22·9 (18·4–28·1)
1-82	Neonatal disorders	8,300 (7,260–9,390)	6,700 (5,620–7,980)	6,250 (5,240–7,410)	2,990 (2,620–3,370)	1,940 (1,620–2,310)	1,650 (1,380–1,960)
1-83	Neonatal preterm birth	4,580 (3,870–5,410)	2,980 (2,460–3,580)	2,790 (2,270–3,380)	1,650 (1,400–1,950)	859 (710–1,030)	733 (594–888)
1-84	Neonatal encephalopathy due to birth asphyxia and trauma	1,910 (1,550–2,340)	1,560 (1,250–1,930)	1,360 (1,050–1,710)	679 (548–832)	453 (364–560)	363 (278–456)
1-85	Neonatal sepsis and other neonatal infections	848 (650–1,080)	1,240 (988–1,550)	1,280 (1,010–1,600)	308 (237–394)	357 (285–447)	332 (262–418)
1-86	Hemolytic disease and other neonatal jaundice	384 (278–530)	206 (159–259)	180 (140–230)	141 (103–193)	59·6 (46·2–75)	46·3 (35·7–59·6)
1-87	Other neonatal disorders	577 (432–746)	717 (570–898)	645 (486–858)	204 (153–264)	208 (165–260)	175 (132–233)
1-88	Nutritional deficiencies	2,200 (1,780–2,740)	1,930 (1,400–2,590)	2,150 (1,530–2,940)	990 (797–1,240)	590 (438–787)	552 (398–743)
1-89	Protein-energy malnutrition	1,190 (958–1,440)	525 (438–628)	524 (417–656)	489 (404–580)	172 (146–202)	149 (120–185)
1-90	Iodine deficiency	6 (3–9)	8 (4–12)	9 (5–15)	3·6 (2–5·8)	2·4 (1·3–4)	2·2 (1·2–3·7)
1-91	Vitamin A deficiency	48 (28–73)	36 (22–54)	35 (22–53)	17·5 (10·6–26·7)	9·2 (5·8–14)	8·3 (5·1–12·6)
1-92	Dietary iron deficiency	885 (545–1,340)	1,310 (823–1,930)	1,540 (956–2,310)	443 (278–658)	390 (248–570)	380 (235–566)
1-93	Other nutritional deficiencies	71 (49–110)	45 (36–59)	46 (33–64)	37·7 (27·9–56·6)	16·5 (12·5–21·4)	12·8 (9·3–17·7)
1-0	Non-communicable diseases	26,500 (23,100–30,200)	49,500 (43,200–56,500)	67,600 (58,400–78,300)	20,000 (17,800–22,600)	20,400 (18,100–22,900)	20,200 (17,500–23,100)
1-1	Neoplasms	2,670 (2,480–2,890)	6,110 (5,800–6,400)	8,650 (7,560–9,820)	2,380 (2,210–2,580)	2,780 (2,640–2,920)	2,750 (2,400–3,110)
1-2	Lip and oral cavity cancer	28 (25–30)	69 (64–74)	106 (89–123)	28·4 (26–31·1)	33·4 (30·9–35·9)	34·2 (28·9–39·7)
1-3	Nasopharynx cancer	7 (7–8)	25 (23–27)	36 (31–43)	6·4 (5·8–7)	10·6 (9·9–11·4)	10·7 (9·1–12·6)
1-4	Other pharynx cancer	16 (14–18)	33 (30–36)	52 (44–62)	16·9 (14·9–19)	16·2 (14·7–17·8)	16·8 (14·1–19·8)
1-5	Oesophageal cancer	45 (40–51)	118 (108–129)	184 (156–215)	48·4 (43·3–54·2)	59·4 (54·2–64·8)	60·8 (51·7–70·8)
1-6	Stomach cancer	272 (245–299)	512 (478–546)	720 (620–828)	284 (256–313)	252 (235–269)	238 (205–274)
1-7	Colon and rectum cancer	144 (132–158)	454 (426–483)	716 (622–829)	150 (138–164)	226 (212–240)	240 (209–276)

1-8	Liver cancer	157 (142–172)	149 (139–159)	234 (199–271)	153 (139–167)	72·2 (67–77·3)	77·6 (66·2–89·7)
1-9	Liver cancer due to hepatitis B	50 (39–64)	49 (38–63)	78 (58–104)	48 (36–62)	21·9 (16·6–28·5)	23·7 (17·6–31·9)
1-10	Liver cancer due to hepatitis C	30 (21–41)	25 (18–34)	40 (26–57)	33 (23·1–44·6)	14 (9·7–19·1)	14·7 (9·7–20·9)
1-11	Liver cancer due to alcohol use	42 (31–56)	44 (32–57)	74 (53–98)	45·2 (32·5–59·3)	23·4 (17–30)	25·7 (18·5–33·9)
1-12	Liver cancer due to NASH	12 (8–16)	13 (10–18)	21 (14–28)	12 (8·4–16·5)	6·7 (4·8–9·2)	7·1 (4·9–9·9)
1-13	Gallbladder and biliary tract cancer	70 (42–80)	69 (60–110)	97 (77–155)	74·7 (45·3–85·7)	35·5 (30·6–56·3)	32·9 (26·3–53)
1-14	Pancreatic cancer	34 (31–38)	285 (265–308)	436 (363–509)	36·7 (33–41)	145 (134–158)	147 (122–172)
1-15	Larynx cancer	28 (25–31)	83 (76–90)	129 (110–150)	29·6 (26·6–32·9)	42·3 (38·7–45·8)	42·9 (36·8–50)
1-16	Tracheal, bronchus, and lung cancer	237 (213–261)	648 (599–699)	979 (834–1,140)	251 (226–278)	328 (304–354)	328 (281–379)
1-17	Malignant skin melanoma	8 (6–10)	26 (20–36)	41 (30–58)	6·6 (5·1–9·3)	10·7 (8·5–15·3)	11·9 (8·9–17·1)
1-18	Non-melanoma skin cancer	15 (11–17)	37 (34–39)	56 (48–65)	14·9 (11·6–17·7)	18·3 (17–19·7)	19 (16·3–21·9)
1-19	Non-melanoma skin cancer (squamous-cell carcinoma)	15 (11–17)	37 (34–39)	56 (48–65)	14·9 (11·6–17·7)	18·3 (17–19·7)	19 (16·3–21·9)
1-20	Non-melanoma skin cancer (basal-cell carcinoma)	0 (0–0)	0 (0–0)	0 (0–0)	0 (0–0)	0 (0–0)	0 (0–0)
1-21	Breast cancer	146 (130–164)	445 (404–491)	612 (512–726)	148 (132–166)	196 (178–214)	184 (154–218)
1-22	Cervical cancer	319 (276–370)	783 (672–868)	1,010 (821–1,190)	304 (265–350)	320 (275–353)	288 (236–340)
1-23	Uterine cancer	46 (40–57)	135 (122–149)	189 (161–220)	48·1 (41·7–58·5)	64·5 (58·6–71·1)	60·7 (51·7–70·4)
1-24	Ovarian cancer	14 (12–18)	95 (63–106)	140 (98–169)	13 (11·3–17)	41·1 (27·6–45·7)	41·8 (29·5–50·4)
1-25	Prostate cancer	195 (156–325)	515 (422–670)	803 (631–1,040)	218 (174–364)	315 (259–411)	329 (258–430)
1-26	Testicular cancer	2 (1–4)	16 (14–20)	23 (18–30)	1·1 (0·8–1·7)	4·8 (4·1–6)	5·5 (4·3–6·9)
1-27	Kidney cancer	86 (75–98)	129 (120–138)	187 (158–219)	59·1 (53·1–64·8)	53·1 (49·3–56·7)	55·6 (46·9–65·3)
1-28	Bladder cancer	30 (26–32)	76 (70–82)	114 (98–132)	32·1 (28·6–35·4)	41·8 (38·1–45·3)	41·9 (35·9–48·5)
1-29	Brain and nervous system cancer	48 (33–103)	233 (111–290)	294 (134–390)	27·1 (19·1–54)	78·2 (37·6–95·7)	77·6 (35·6–103)
1-30	Thyroid cancer	8 (7–8)	21 (19–23)	32 (28–37)	7·4 (6·6–8·1)	9·5 (8·8–10·3)	10·1 (8·6–11·6)
1-31	Mesothelioma	12 (10–14)	29 (26–32)	47 (39–55)	11 (9·4–12·7)	12·5 (11·3–13·8)	13·8 (11·7–16·2)
1-32	Hodgkin lymphoma	31 (25–38)	63 (54–74)	85 (68–106)	20 (16·2–25·5)	20·9 (18–24·5)	20·9 (16·9–26·2)
1-33	Non-Hodgkin's lymphoma	90 (79–102)	181 (165–199)	247 (206–289)	58·6 (52·3–65·3)	67 (61·6–73)	67·4 (56·9–79)
1-34	Multiple myeloma	15 (11–18)	42 (38–46)	65 (55–79)	16 (11·5–19·6)	21·6 (19·7–24·4)	22·2 (18·8–27·1)
1-35	Leukaemia	278 (244–316)	429 (397–466)	503 (438–572)	141 (127–156)	139 (130–149)	130 (113–147)
1-36	Acute lymphoid leukaemia	84 (65–105)	119 (101–138)	126 (106–149)	33·1 (26·5–40·4)	31·1 (26·8–36)	28·7 (24·1–34·1)
1-37	Chronic lymphoid leukaemia	4 (3–5)	11 (10–13)	17 (14–21)	3·5 (2·6–5)	5·8 (5·1–6·7)	5·7 (4·7–7)
1-38	Acute myeloid leukaemia	17 (12–21)	36 (31–43)	48 (39–59)	8·7 (6·7–10·8)	11·3 (9·7–13·1)	11·9 (9·7–14·5)
1-39	Chronic myeloid leukaemia	8 (7–10)	15 (13–17)	20 (16–24)	5·3 (4·2–6·7)	5·6 (4·9–6·2)	5·4 (4·5–6·5)
1-40	Other leukaemia	166 (142–192)	248 (228–271)	292 (249–335)	90·3 (80·3–99·6)	85·5 (79·5–91·7)	78·2 (66·9–89·4)
1-41	Other neoplasms	9 (6–13)	24 (19–30)	35 (27–45)	6·7 (4·8–10·1)	10·1 (8·3–13·6)	10·9 (8·6–14·5)
1-42	Myelodysplastic, myeloproliferative, and other hematopoietic neoplasms	9 (6–13)	24 (19–30)	35 (27–45)	6·7 (4·8–10·1)	10·1 (8·3–13·6)	10·9 (8·6–14·5)
1-43	Benign and in situ intestinal neoplasms	0 (0–0)	0 (0–0)	0 (0–0)	0 (0–0)	0 (0–0)	0 (0–0)
1-44	Benign and in situ cervical and uterine neoplasms	0 (0–0)	0 (0–0)	0 (0–0)	0 (0–0)	0 (0–0)	0 (0–0)
1-45	Other benign and in situ neoplasms	0 (0–0)	0 (0–0)	0 (0–0)	0 (0–0)	0 (0–0)	0 (0–0)
1-46	Cardiovascular diseases	5,090 (4,720–5,430)	7,940 (7,530–8,330)	11,100 (9,870–12,600)	5,120 (4,750–5,460)	4,030 (3,820–4,240)	3,860 (3,420–4,350)
1-47	Rheumatic heart disease	174 (139–217)	271 (212–347)	330 (247–429)	110 (89·9–136)	87·2 (69·6–109)	80·3 (60·9–102)
1-48	Ischaemic heart disease	2,420 (2,240–2,610)	3,340 (3,160–3,520)	4,600 (4,050–5,220)	2,610 (2,420–2,810)	1,790 (1,690–1,890)	1,650 (1,460–1,870)
1-49	Stroke	1,490 (1,360–1,620)	2,360 (2,210–2,520)	3,300 (2,880–3,760)	1,510 (1,370–1,640)	1,210 (1,130–1,290)	1,150 (1,010–1,310)
1-50	Ischaemic stroke	517 (459–575)	799 (732–868)	1,110 (964–1,260)	558 (494–621)	460 (420–500)	436 (379–495)
1-51	Intracerebral hemorrhage	810 (730–892)	1,300 (1,210–1,390)	1,830 (1,580–2,110)	816 (729–899)	643 (598–690)	611 (531–705)
1-52	Subarachnoid hemorrhage	164 (137–187)	264 (240–292)	362 (309–422)	134 (112–154)	107 (97·8–119)	105 (89·7–122)
1-53	Hypertensive heart disease	333 (291–402)	694 (610–777)	1,090 (887–1,290)	360 (314–435)	373 (331–425)	393 (322–464)
1-54	Non-rheumatic valvular heart disease	26 (20–36)	65 (59–71)	98 (82–115)	23·6 (18·6–32·4)	28·5 (26·1–30·8)	30 (25·3–35·1)
1-55	Non-rheumatic calcific aortic valve disease	9 (6–14)	24 (20–29)	35 (28–46)	8·7 (5·3–12·8)	11·2 (9·2–13·2)	11·4 (9·3–14·5)
1-56	Non-rheumatic degenerative mitral valve disease	15 (11–21)	38 (32–43)	57 (46–70)	13·3 (9·9–18·3)	15·8 (13·5–18·2)	16·8 (13·8–20·7)
1-57	Other non-rheumatic valve diseases	2 (1–3)	4 (3–6)	6 (4–9)	1·6 (0·9–2·2)	1·5 (1–2·3)	1·8 (1·1–2·6)
1-58	Cardiomyopathy and myocarditis	236 (192–282)	418 (352–456)	599 (469–703)	167 (127–207)	173 (146–189)	179 (142–209)
1-59	Myocarditis	23 (14–31)	39 (30–48)	47 (37–64)	11·8 (7·9–15·9)	13·3 (10·2–16·7)	12·7 (10–17·4)
1-60	Alcoholic cardiomyopathy	10 (6–16)	32 (23–39)	55 (36–69)	9·4 (5·4–15·1)	14 (10·1–17·4)	16·4 (10·9–20·7)

1-61	Other cardiomyopathy	204 (167–242)	348 (293–381)	497 (386–585)	146 (111–181)	146 (123–159)	150 (119–176)
1-62	Atrial fibrillation and flutter	63 (50–80)	135 (113–164)	201 (167–246)	71·7 (57·1–90·7)	83·4 (69·9–101)	83·3 (69·3–101)
1-63	Aortic aneurysm	26 (21–33)	59 (54–64)	88 (73–103)	27·1 (22·3–34·9)	31·9 (28·8–35)	31·9 (26·7–37·6)
1-64	Peripheral vascular disease	10 (6–16)	22 (13–35)	33 (20–55)	11·2 (6·4–18·1)	13·4 (8–21·4)	13·5 (8·1–22·3)
1-65	Endocarditis	16 (12–21)	40 (32–48)	56 (44–71)	10·7 (7·7–15·2)	15 (12·2–18·1)	15·4 (12·2–19·9)
1-66	Chronic respiratory diseases	1,250 (1,020–1,530)	2,360 (2,000–2,810)	3,130 (2,620–3,730)	804 (690–931)	935 (821–1,070)	936 (794–1,090)
1-67	Chronic obstructive pulmonary disease	336 (294–380)	858 (777–948)	1,280 (1,110–1,470)	338 (294–384)	452 (408–501)	458 (395–530)
1-68	Pneumoconiosis	4 (3–6)	9 (8–11)	14 (12–18)	3·6 (2·7–4·7)	3·8 (3·3–4·4)	4·2 (3·4–5·1)
1-69	Silicosis	0 (0–0)	1 (0–1)	1 (1–2)	0·2 (0·1–0·3)	0·3 (0·2–0·4)	0·3 (0·2–0·5)
1-70	Asbestosis	0 (0–0)	0 (0–1)	1 (0–1)	0·1 (0·1–0·1)	0·1 (0·1–0·2)	0·2 (0·1–0·3)
1-71	Coal workers pneumoconiosis	3 (2–5)	7 (6–9)	11 (9–14)	2·8 (2·1–3·9)	3·1 (2·6–3·6)	3·2 (2·6–4)
1-72	Other pneumoconiosis	1 (0–1)	1 (1–1)	1 (1–2)	0·5 (0·3–0·6)	0·3 (0·3–0·4)	0·4 (0·3–0·6)
1-73	Asthma	752 (551–1,020)	1,100 (799–1,540)	1,290 (924–1,790)	362 (274–480)	325 (242–440)	314 (228–430)
1-74	Interstitial lung disease and pulmonary sarcoidosis	83 (64–116)	269 (205–314)	391 (287–493)	65·7 (49·1–94·7)	117 (89·7–137)	122 (89·4–153)
1-75	Other chronic respiratory diseases	72 (49–96)	123 (102–146)	155 (127–190)	35 (25·7–43·2)	37·8 (31·7–45)	38·4 (31·4–47·2)
1-76	Digestive diseases	1,690 (1,510–1,900)	3,010 (2,720–3,360)	4,270 (3,720–4,890)	1,250 (1,130–1,390)	1,230 (1,130–1,360)	1,250 (1,090–1,430)
1-77	Cirrhosis and other chronic liver diseases	648 (585–705)	1,390 (1,300–1,480)	2,150 (1,860–2,480)	565 (514–616)	608 (568–648)	643 (556–739)
1-78	Cirrhosis and other chronic liver diseases due to hepatitis B	66 (47–90)	110 (74–157)	166 (108–245)	64·7 (45·4–88·5)	47·4 (32·1–66·4)	48·6 (32·1–71·1)
1-79	Cirrhosis and other chronic liver diseases due to hepatitis C	106 (77–141)	277 (204–366)	440 (312–598)	103 (73·8–138)	120 (87·7–160)	130 (92·5–176)
1-80	Cirrhosis and other chronic liver diseases due to alcohol use	189 (147–232)	522 (418–626)	853 (662–1,070)	195 (151–240)	241 (190–288)	261 (204–326)
1-81	Cirrhosis and other chronic liver diseases due to NAFLD	80 (58–107)	206 (146–284)	325 (219–459)	80·5 (57·3–110)	95·2 (67·2–130)	101 (68·9–141)
1-82	Cirrhosis and other chronic liver diseases due to other causes	207 (169–248)	274 (221–336)	365 (281–467)	122 (97·7–150)	104 (80·9–131)	103 (78·4–133)
1-83	Upper digestive system diseases	307 (218–442)	587 (397–865)	800 (541–1,200)	248 (181–353)	231 (162–335)	225 (155–331)
1-84	Peptic ulcer disease	82 (72–95)	121 (106–137)	158 (129–191)	74·7 (64·4–86·3)	56 (48·7–64·4)	50·4 (40·9–60·8)
1-85	Gastritis and duodenitis	65 (49–86)	130 (95–175)	179 (130–241)	50 (38–65·3)	51·9 (38·8–68·3)	51·2 (37·4–68)
1-86	GERD	159 (83–283)	336 (174–593)	463 (240–818)	123 (64·2–219)	123 (63·8–218)	123 (63·8–218)
1-87	Appendicitis	66 (48–86)	68 (56–85)	84 (67–106)	31·7 (24·3–39·7)	21·7 (17·8–27·1)	21·1 (16·7–26·7)
1-88	Paralytic ileus and intestinal obstruction	265 (200–343)	262 (223–313)	306 (251–374)	136 (110–165)	101 (87·5–121)	93·6 (77·3–116)
1-89	Inguinal, femoral, and abdominal hernia	165 (118–223)	211 (156–281)	267 (199–353)	84·6 (62·6–110)	71·4 (54·1–93)	71·5 (54–93)
1-90	Inflammatory bowel disease	40 (29–50)	60 (52–71)	77 (64–97)	26·2 (19·1–32·4)	22·9 (20·1–27·5)	21·8 (18–27·3)
1-91	Vascular intestinal disorders	10 (9–12)	23 (20–25)	32 (27–38)	9·7 (8·1–12)	11·3 (10·1–12·5)	11·1 (9·3–13)
1-92	Gallbladder and biliary diseases	107 (83–134)	187 (144–238)	258 (198–327)	92·1 (72–113)	78·5 (62·2–98·1)	76·5 (59·7–96)
1-93	Pancreatitis	34 (24–45)	89 (78–100)	124 (103–151)	27·9 (19·4–37·5)	34·7 (30·9–39)	34·5 (28·7–41·4)
1-94	Other digestive diseases	50 (37–76)	135 (90–161)	175 (117–223)	28·5 (22·1–43·4)	55 (36·5–65·4)	53·6 (36·1–68)
1-95	Neurological disorders	1,770 (919–3,000)	3,420 (1,790–5,710)	4,600 (2,440–7,670)	1,210 (669–2,020)	1,270 (735–2,070)	1,280 (745–2,050)
1-96	Alzheimer's disease and other dementias	264 (110–602)	479 (196–1,080)	706 (296–1,580)	307 (128–703)	317 (130–707)	313 (132–707)
1-97	Parkinson's disease	59 (53–65)	140 (128–152)	202 (179–227)	66·1 (59·8–73·1)	86·5 (79–94·1)	85·9 (75·8–96·4)
1-98	Idiopathic epilepsy	431 (240–711)	772 (422–1,220)	994 (546–1,630)	229 (130–379)	238 (131–376)	241 (132–392)
1-99	Multiple sclerosis	7 (5–9)	18 (15–24)	27 (22–37)	5·7 (4·7–7·7)	6·9 (5·8–9·3)	7·4 (6–10·1)
1-100	Motor neuron disease	7 (6–8)	21 (19–23)	33 (27–38)	5·4 (4·9–6)	8·1 (7·5–8·8)	9·5 (7·9–11·2)
1-101	Headache disorders	898 (147–2,000)	1,770 (309–3,950)	2,330 (421–5,240)	548 (108–1,210)	550 (106–1,210)	552 (107–1,220)
1-102	Migraine	821 (100–1,940)	1,620 (207–3,770)	2,120 (286–4,930)	496 (72·7–1,150)	498 (72·2–1,150)	499 (74·1–1,140)
1-103	Tension-type headache	77 (20–329)	156 (44–616)	209 (60–792)	51·9 (15·4–199)	52 (15·9–190)	52·2 (15·4–188)
1-104	Other neurological disorders	106 (69–159)	219 (144–323)	304 (193–461)	52·7 (35·4–76·7)	66 (45·4–94·2)	73·3 (48·5–110)
1-105	Mental disorders	2,620 (1,900–3,490)	5,180 (3,760–6,860)	6,900 (5,000–9,150)	1,650 (1,210–2,180)	1,660 (1,220–2,190)	1,680 (1,230–2,230)
1-106	Schizophrenia	229 (157–307)	500 (347–670)	686 (479–936)	172 (119–228)	173 (121–229)	174 (122–236)
1-107	Depressive disorders	817 (549–1,150)	1,680 (1,150–2,370)	2,310 (1,570–3,240)	565 (384–783)	572 (396–803)	584 (402–810)
1-108	Major depressive disorder	692 (461–1,000)	1,420 (951–2,010)	1,960 (1,320–2,760)	475 (317–668)	482 (324–676)	494 (331–693)
1-109	Dysthymia	125 (75–192)	260 (158–402)	354 (218–541)	90·2 (56·7–138)	90·1 (55·9–137)	90·1 (56·9–137)
1-110	Bipolar disorder	307 (178–491)	618 (356–962)	824 (482–1,290)	197 (115–307)	197 (116–304)	197 (117–303)
1-111	Anxiety disorders	636 (416–902)	1,250 (815–1,770)	1,660 (1,100–2,360)	390 (259–548)	393 (264–557)	400 (267–569)
1-112	Eating disorders	66 (40–101)	148 (90–226)	195 (120–295)	34·7 (21·4–52·5)	40·7 (24·7–62·2)	41·7 (25·9–63·7)
1-113	Anorexia nervosa	18 (10–30)	38 (21–62)	49 (28–80)	8·7 (5–14)	10 (5·8–16·1)	10·3 (5·9–16·7)

1-114	Bulimia nervosa	48 (28–78)	110 (63–176)	145 (83–229)	26 (14·8–41·5)	30·7 (17·7–49)	31·5 (18–49·5)
1-115	Autism spectrum disorders	104 (69–153)	179 (117–260)	219 (143–323)	52·5 (34·7–76·7)	52·2 (34·1–75·6)	52 (34·2–76·7)
1-116	Attention-deficit/hyperactivity disorder	85 (48–146)	144 (81–248)	172 (96–291)	37·4 (21·3–63·3)	37·2 (21–62·9)	37·1 (20·7–62·6)
1-117	Conduct disorder	182 (102–293)	291 (165–458)	335 (187–536)	67·9 (38·1–109)	67·7 (38·5–107)	67·8 (37·9–109)
1-118	Idiopathic developmental intellectual disability	55 (23–96)	72 (27–129)	84 (29–151)	27·2 (11·6–47·2)	20·5 (7·7–36·6)	19·6 (6·9–35·2)
1-119	Other mental disorders	140 (88–212)	297 (184–451)	409 (259–618)	110 (69·7–166)	109 (68·7–164)	108 (69·3–164)
1-120	Substance use disorders	436 (325–565)	1,080 (846–1,350)	1,490 (1,150–1,860)	298 (228–382)	368 (291–457)	370 (290–460)
1-121	Alcohol use disorders	271 (201–362)	746 (582–940)	1,050 (820–1,330)	204 (157–265)	268 (214–336)	271 (214–340)
1-122	Drug use disorders	165 (114–227)	338 (242–454)	439 (312–582)	94·8 (66·7–128)	99·9 (72–133)	99·3 (70·9–131)
1-123	Opioid use disorders	62 (38–92)	125 (82–180)	170 (111–241)	40·2 (25·6–58·2)	39·7 (26·4–56·1)	40·6 (26·9–57·1)
1-124	Cocaine use disorders	26 (15–41)	63 (41–93)	82 (54–121)	14·4 (8·5–21·8)	18·2 (12·3–26·8)	18·2 (12·1–26·4)
1-125	Amphetamine use disorders	11 (5–19)	22 (12–37)	29 (16–48)	5·9 (3·1–10)	6·3 (3·4–10·5)	6·4 (3·7–10·6)
1-126	Cannabis use disorders	42 (24–65)	75 (43–116)	85 (48–136)	20·3 (11·8–31·7)	19·8 (11·5–30·6)	17·8 (10·2–28·4)
1-127	Other drug use disorders	25 (15–39)	54 (34–79)	72 (46–107)	13·9 (8·6–21·1)	15·9 (10·2–22·8)	16·2 (10·5–23·7)
1-128	Diabetes and kidney diseases	2,160 (1,950–2,400)	6,660 (6,080–7,300)	9,810 (8,550–11,200)	2,040 (1,840–2,270)	3,150 (2,880–3,450)	3,170 (2,760–3,600)
1-129	Diabetes mellitus	1,350 (1,190–1,530)	3,970 (3,540–4,480)	5,830 (5,010–6,780)	1,350 (1,190–1,530)	1,980 (1,780–2,220)	1,950 (1,680–2,260)
1-130	Diabetes mellitus type 1	152 (121–179)	295 (255–386)	390 (324–517)	99·1 (78·4–120)	104 (89·6–140)	102 (84·8–138)
1-131	Diabetes mellitus type 2	1,200 (1,050–1,370)	3,680 (3,260–4,160)	5,440 (4,640–6,380)	1,250 (1,100–1,420)	1,880 (1,680–2,110)	1,850 (1,580–2,150)
1-132	Chronic kidney disease	801 (722–884)	2,680 (2,470–2,890)	3,960 (3,440–4,550)	679 (614–749)	1,170 (1,080–1,260)	1,210 (1,060–1,390)
1-133	Chronic kidney disease due to diabetes mellitus type 1	52 (37–71)	251 (176–342)	365 (251–514)	46 (31·1–63·9)	96·5 (66·2–134)	99·6 (67–142)
1-134	Chronic kidney disease due to diabetes mellitus type 2	139 (111–168)	509 (402–610)	794 (632–979)	149 (119–179)	266 (215–317)	275 (220–337)
1-135	Chronic kidney disease due to hypertension	127 (104–155)	433 (354–519)	667 (532–817)	131 (108–159)	224 (185–268)	232 (187–281)
1-136	Chronic kidney disease due to glomerulonephritis	205 (170–242)	690 (571–809)	998 (792–1,230)	152 (126–182)	266 (218–317)	277 (218–347)
1-137	Chronic kidney disease due to other and unspecified causes	278 (232–325)	795 (663–951)	1,140 (906–1,410)	201 (164–241)	318 (261–388)	331 (261–410)
1-138	Acute glomerulonephritis	14 (9–22)	13 (11–16)	16 (12–20)	7·2 (4·7–10·4)	4·1 (3·5–4·8)	4 (3·1–5)
1-139	Skin and subcutaneous diseases	1,020 (671–1,500)	1,760 (1,160–2,630)	2,180 (1,430–3,230)	531 (354–790)	540 (363–807)	541 (362–804)
1-140	Dermatitis	243 (140–394)	401 (237–643)	477 (279–753)	116 (68·3–180)	115 (68·9–181)	116 (68·3–182)
1-141	Atopic dermatitis	200 (108–337)	315 (170–529)	360 (194–607)	84·9 (45·5–144)	85 (46–144)	85 (45·8–143)
1-142	Contact dermatitis	38 (24–58)	76 (47–115)	104 (65–157)	27·1 (17–40·3)	27·1 (17·2–40·6)	27·1 (16·8–40·6)
1-143	Seborrhoeic dermatitis	5 (3–9)	10 (6–16)	13 (8–21)	3·4 (1·9–5·4)	3·4 (1·9–5·4)	3·4 (1·9–5·4)
1-144	Psoriasis	66 (46–89)	108 (76–145)	139 (98–185)	43·4 (30·7–57·6)	36·8 (26–48·9)	35·8 (25–47·2)
1-145	Bacterial skin diseases	32 (21–51)	76 (51–113)	105 (68–159)	16·8 (11·5–26·1)	28·6 (18·9–45·3)	30·9 (19·8–46·8)
1-146	Cellulitis	13 (7–20)	21 (13–35)	28 (17–47)	6 (3·7–9·8)	7·3 (4·7–13)	7·7 (4·6–13·4)
1-147	Pyoderma	20 (12–32)	55 (36–82)	78 (48–116)	10·8 (6·9–17·2)	21·3 (13·5–32·6)	23·1 (14·2–35·7)
1-148	Scabies	176 (95–284)	298 (164–478)	359 (196–576)	84·7 (46·5–135)	84·6 (46·9–135)	84·2 (46·5–135)
1-149	Fungal skin diseases	67 (26–142)	122 (48–263)	159 (63–344)	44·3 (17·7–94·4)	44·2 (17·8–93·2)	44·1 (17·7–93·4)
1-150	Viral skin diseases	115 (73–176)	180 (116–272)	203 (128–306)	47·6 (30·5–72·4)	47·6 (30·6–71·8)	47·6 (29·9–71·3)
1-151	Acne vulgaris	95 (56–156)	187 (111–301)	237 (141–385)	39·8 (23·7–65·3)	45·7 (27·2–73·7)	47·4 (28·1–76·6)
1-152	Alopecia areata	11 (7–17)	22 (14–34)	29 (18–44)	7·1 (4·5–10·7)	7·1 (4·5–10·6)	7·1 (4·5–10·7)
1-153	Pruritus	12 (6–22)	24 (11–43)	31 (15–57)	8·1 (3·8–14·6)	8·3 (4–15·1)	8·4 (4–15)
1-154	Urticaria	102 (66–149)	168 (109–248)	199 (130–289)	48 (31·3–70·1)	48·1 (31·3–70·6)	48·1 (31·3–69·9)
1-155	Decubitus ulcer	27 (18–44)	42 (31–72)	59 (41–98)	26·3 (17·8–43·8)	23 (17·3–38·8)	22·1 (15·8–36·6)
1-156	Other skin and subcutaneous diseases	71 (37–126)	133 (69–237)	179 (93–326)	49·8 (26·2–89·1)	50·1 (26·3–88·9)	50·2 (26·3–90·6)
1-157	Sense organ diseases	946 (647–1,340)	1,700 (1,160–2,450)	2,370 (1,600–3,420)	854 (582–1,210)	784 (529–1,120)	767 (520–1,090)
1-158	Blindness and vision loss	281 (191–394)	462 (310–665)	634 (428–916)	262 (180–366)	221 (150–312)	213 (144–304)
1-159	Glaucoma	16 (11–24)	22 (14–31)	30 (20–43)	18·9 (12·4–27·2)	13·8 (9–20)	12·9 (8·5–18·7)
1-160	Cataract	48 (33–65)	67 (47–91)	92 (64–125)	52·6 (36·6–72·6)	39·5 (27·7–53·9)	37·2 (26·1–51·1)
1-161	Age-related macular degeneration	2 (1–2)	2 (2–4)	4 (2–5)	1·8 (1·1–2·6)	1·5 (1–2·2)	1·5 (1–2·1)
1-162	Refraction disorders	89 (58–130)	159 (104–235)	206 (135–302)	60·7 (40–86·8)	56·2 (37·5–81·8)	55 (36·3–79·7)
1-163	Near vision loss	55 (25–107)	99 (46–196)	146 (68–291)	58·3 (27–114)	52·6 (24·4–106)	51·5 (23·9–104)
1-164	Other vision loss	72 (50–99)	112 (79–155)	156 (108–216)	69·8 (49–96·5)	57·4 (40·5–78·7)	55·1 (38·6–76·2)
1-165	Age-related and other hearing loss	620 (417–904)	1,150 (767–1,700)	1,610 (1,070–2,370)	557 (371–807)	527 (354–768)	518 (346–748)
1-166	Other sense organ diseases	45 (27–70)	88 (53–134)	121 (72–186)	35·3 (21–55·3)	36 (21·5–56)	36 (21·2–56·1)

1-167	Musculoskeletal disorders	1,780 (1,280–2,340)	3,940 (2,860–5,200)	5,590 (4,050–7,360)	1,460 (1,050–1,940)	1,540 (1,110–2,030)	1,560 (1,130–2,070)
1-168	Rheumatoid arthritis	37 (27–48)	91 (66–119)	133 (97–176)	32·1 (23·8–41·6)	37·6 (27·9–48·7)	38·6 (28·5–50·5)
1-169	Osteoarthritis	179 (90–359)	405 (203–827)	613 (305–1,230)	193 (97·4–387)	212 (107–425)	212 (106–422)
1-170	Low back pain	972 (678–1,310)	1,990 (1,390–2,660)	2,720 (1,910–3,620)	740 (521–990)	737 (517–988)	733 (515–982)
1-171	Neck pain	161 (106–235)	349 (226–509)	489 (318–702)	135 (88·8–195)	135 (88·7–195)	135 (90·4–195)
1-172	Gout	7 (5–11)	19 (12–27)	28 (17–40)	7·1 (4·4–10·4)	8·5 (5·3–12·2)	8·6 (5·4–12·6)
1-173	Other musculoskeletal disorders	428 (302–589)	1,090 (776–1,500)	1,600 (1,120–2,140)	349 (244–480)	411 (290–552)	433 (303–581)
1-174	Other non-communicable diseases	5,030 (3,900–6,100)	6,330 (5,210–7,730)	7,440 (6,040–9,210)	2,440 (1,940–2,980)	2,100 (1,720–2,580)	2,000 (1,620–2,460)
1-175	Congenital anomalies	2,450 (1,470–3,210)	2,220 (1,740–2,670)	2,090 (1,650–2,610)	894 (557–1,170)	632 (496–759)	541 (424–679)
1-176	Neural tube defects	168 (102–237)	121 (86–155)	132 (93–189)	60·2 (37·2–84·7)	34·2 (24·4–44)	34·5 (24·3–49·8)
1-177	Congenital heart anomalies	1,090 (548–1,510)	873 (634–1,080)	752 (549–984)	391 (202–537)	248 (180–308)	196 (142–258)
1-178	Orofacial clefts	19 (8–39)	17 (12–23)	15 (10–22)	7·3 (3·4–14·6)	5 (3·5–6·8)	3·8 (2·5–5·5)
1-179	Down's syndrome	79 (51–140)	122 (94–146)	138 (101–173)	31·7 (22·6–53·7)	35·6 (27·6–42·4)	34·8 (25–43·9)
1-180	Turner syndrome	0 (0–1)	1 (0–1)	1 (0–1)	0·1 (0·1–0·2)	0·1 (0·1–0·2)	0·1 (0·1–0·2)
1-181	Klinefelter syndrome	0 (0–0)	0 (0–0)	0 (0–0)	0 (0–0)	0 (0–0)	0 (0–0)
1-182	Other chromosomal abnormalities	59 (42–104)	85 (62–108)	83 (59–109)	21·4 (15·2–37·4)	24 (17·4–30·5)	21·2 (15–27·9)
1-183	Congenital musculoskeletal and limb anomalies	126 (88–175)	192 (132–261)	226 (155–314)	53·1 (37·2–73·6)	53·9 (37·2–72·9)	54·5 (37·6–74·8)
1-184	Urogenital congenital anomalies	57 (29–101)	53 (39–76)	49 (35–68)	20·5 (10·8–36·1)	14·9 (11–21·6)	12·6 (9–18)
1-185	Digestive congenital anomalies	321 (161–505)	266 (194–355)	235 (163–332)	114 (57·5–179)	76·5 (56–102)	63·4 (44–89·6)
1-186	Other congenital anomalies	532 (371–750)	492 (367–604)	462 (342–594)	195 (137–273)	140 (105–171)	119 (88·1–154)
1-187	Urinary diseases and male infertility	110 (90–140)	369 (317–420)	542 (444–636)	90·5 (73·4–117)	171 (145–197)	178 (145–211)
1-188	Urinary tract infections and interstitial nephritis	55 (46–73)	240 (201–265)	353 (282–413)	42·6 (36·7–57·4)	110 (91·3–121)	116 (91·5–135)
1-189	Urolithiasis	6 (4–8)	20 (16–24)	29 (23–37)	5·1 (3·8–6·9)	8·2 (6·4–10)	8·6 (6·7–10·6)
1-190	Benign prostatic hyperplasia	25 (14–39)	50 (29–79)	75 (44–116)	27·2 (15·6–42·8)	30·3 (17·6–47·6)	29·5 (17·3–46)
1-191	Male infertility	3 (1–8)	9 (3–21)	12 (4–27)	2·1 (0·8–4·6)	2·7 (1–6·4)	2·7 (1–6·2)
1-192	Other urinary diseases	21 (17–28)	50 (38–63)	72 (49–91)	13·4 (10·4–18·6)	20 (14·6–25·6)	21·5 (14·2–27)
1-193	Gynecological diseases	532 (365–742)	1,130 (774–1,590)	1,510 (1,040–2,120)	366 (254–513)	367 (253–512)	367 (254–513)
1-194	Uterine fibroids	27 (13–50)	67 (32–128)	93 (45–175)	21·9 (10·6–41)	23·7 (11·3–45·1)	24 (11·4–45)
1-195	Polycystic ovarian syndrome	8 (3–16)	25 (11–51)	33 (14–67)	4·7 (1·9–9·4)	7·2 (3–14·5)	7·3 (3–14·8)
1-196	Female infertility	4 (1–9)	14 (4–35)	18 (5–44)	2·4 (0·9–5·4)	4·2 (1·2–10·4)	4·1 (1·2–10·2)
1-197	Endometriosis	67 (39–109)	104 (61–169)	124 (72–197)	40·9 (24·3–65)	30·9 (18·2–49·4)	28·1 (16·5–44·5)
1-198	Genital prolapse	9 (5–18)	15 (8–28)	20 (11–38)	9·3 (4·8–17·3)	6·8 (3·5–12·5)	6·3 (3·2–11·5)
1-199	Premenstrual syndrome	160 (97–252)	338 (204–530)	447 (273–698)	93·2 (56·5–144)	97·7 (60·5–152)	99·9 (60·8–156)
1-200	Other gynecological diseases	257 (167–369)	570 (370–823)	778 (503–1,110)	194 (126–273)	196 (128–280)	197 (128–280)
1-201	Hemoglobinopathies and hemolytic anaemias	324 (244–424)	464 (358–603)	583 (445–766)	164 (123–216)	137 (106–178)	138 (106–181)
1-202	Thalassemias	0 (0–0)	0 (0–0)	0 (0–0)	0·1 (0–0·1)	0·1 (0–0·1)	0 (0–0·1)
1-203	Thalassaemias trait	9 (5–13)	16 (10–25)	20 (12–30)	4·5 (2·7–6·8)	4·6 (2·8–7·2)	4·6 (2·8–7)
1-204	Sickle cell disorders	115 (96–133)	163 (144–183)	192 (159–234)	48·5 (41·2–56·2)	44·5 (39·6–49·9)	43·4 (35·8–52·8)
1-205	Sickle cell trait	132 (79–201)	179 (113–274)	227 (133–348)	66·7 (40–101)	50·7 (32–77·2)	51·9 (30·7–79·1)
1-206	G6PD deficiency	12 (7–18)	23 (16–31)	38 (26–51)	7·7 (4·7–11·4)	7·8 (5·7–10·1)	9·5 (6·7–12·6)
1-207	G6PD trait	0 (0–0)	0 (0–1)	1 (0–1)	0·2 (0·1–0·3)	0·1 (0·1–0·2)	0·2 (0·1–0·3)
1-208	Other hemoglobinopathies and hemolytic anaemias	56 (38–79)	82 (56–116)	106 (73–150)	35·9 (25·5–49·2)	29·1 (20·6–40·2)	28·9 (20·5–39·9)
1-209	Endocrine, metabolic, blood, and immune disorders	904 (505–1,120)	1,150 (960–1,490)	1,460 (1,200–1,890)	483 (322–588)	412 (342–541)	408 (335–529)
1-210	Oral disorders	409 (235–665)	798 (453–1,300)	1,110 (628–1,800)	338 (198–552)	327 (190–536)	325 (187–529)
1-211	Caries of deciduous teeth	9 (4–19)	12 (5–26)	13 (6–26)	3·1 (1·4–6·5)	3·1 (1·3–6·4)	3·1 (1·4–6·5)
1-212	Caries of permanent teeth	48 (21–94)	87 (40–170)	111 (50–218)	27·5 (12·3–53·5)	26·4 (11·9–50·9)	26·2 (11·9–51·3)
1-213	Periodontal disease	125 (48–269)	285 (109–616)	398 (149–870)	106 (41·2–223)	108 (41·9–234)	108 (41–234)
1-214	Edentulism	139 (88–209)	247 (157–368)	370 (232–559)	149 (93·7–222)	135 (85·9–204)	134 (84·6–204)
1-215	Other oral disorders	88 (55–129)	166 (103–246)	215 (135–316)	53·2 (33–77·8)	53·2 (33–78)	53·2 (33·3–77·7)
1-216	Sudden infant death syndrome	301 (197–472)	194 (135–268)	140 (87–216)	107 (69·5–167)	55·8 (38·8–77·1)	38 (23·7–58·4)
1-0	Injuries	8,360 (7,710–9,090)	16,000 (15,200–16,900)	19,700 (17,600–21,900)	4,360 (4,020–4,720)	4,940 (4,670–5,240)	4,710 (4,200–5,250)
1-1	Transport injuries	2,410 (2,190–2,650)	4,000 (3,750–4,270)	5,300 (4,580–6,010)	1,340 (1,220–1,480)	1,250 (1,170–1,340)	1,260 (1,090–1,430)
1-2	Road injuries	2,290 (2,090–2,520)	3,620 (3,390–3,880)	4,830 (4,180–5,450)	1,280 (1,160–1,410)	1,130 (1,050–1,210)	1,150 (996–1,300)

1-3	Pedestrian road injuries	511 (446–587)	597 (549–646)	727 (623–832)	271 (236–310)	196 (182–212)	186 (159–211)
1-4	Cyclist road injuries	292 (242–379)	407 (351–473)	530 (442–626)	175 (145–229)	130 (111–152)	129 (107–152)
1-5	Motorcyclist road injuries	237 (180–276)	681 (618–749)	1,040 (903–1,190)	137 (105–160)	207 (188–227)	240 (208–273)
1-6	Motor vehicle road injuries	1,230 (1,100–1,440)	1,890 (1,760–2,030)	2,470 (2,110–2,840)	681 (607–815)	583 (543–623)	581 (496–666)
1-7	Other road injuries	21 (16–25)	41 (34–48)	57 (47–68)	13·1 (10·2–15·9)	13·7 (11·4–16·2)	14·4 (11·8–17·2)
1-8	Other transport injuries	114 (98–134)	382 (343–421)	473 (389–555)	61·8 (53·8–72·5)	118 (107–130)	113 (92·9–132)
1-9	Unintentional injuries	4,000 (3,550–4,480)	5,190 (4,690–5,750)	6,280 (5,440–7,230)	1,990 (1,780–2,210)	1,670 (1,500–1,860)	1,590 (1,380–1,840)
1-10	Falls	237 (201–281)	556 (467–669)	823 (678–997)	175 (148–208)	224 (190–268)	241 (200–290)
1-11	Drowning	1,110 (983–1,250)	1,370 (1,250–1,500)	1,530 (1,320–1,760)	474 (424–525)	381 (348–413)	349 (301–398)
1-12	Fire, heat, and hot substances	409 (344–481)	429 (350–528)	543 (434–692)	213 (177–255)	138 (111–172)	136 (109–174)
1-13	Poisonings	339 (281–408)	100 (89–113)	109 (90–127)	146 (124–171)	29·9 (26·7–33·5)	26·4 (21·8–30·9)
1-14	Poisoning by carbon monoxide	46 (34–63)	51 (45–59)	57 (46–69)	21·1 (16·8–27·4)	15·3 (13·5–17·4)	13·8 (11·2–16·6)
1-15	Poisoning by other means	293 (243–351)	49 (42–57)	51 (42–61)	124 (106–146)	14·6 (12·6–16·9)	12·6 (10·2–15)
1-16	Exposure to mechanical forces	566 (487–657)	1,270 (1,100–1,480)	1,510 (1,280–1,790)	332 (282–396)	410 (350–485)	372 (312–448)
1-17	Unintentional firearm injuries	283 (239–332)	684 (609–758)	768 (652–892)	160 (136–185)	209 (187–230)	180 (153–208)
1-18	Other exposure to mechanical forces	284 (222–366)	585 (453–774)	738 (560–997)	173 (132–229)	201 (153–267)	192 (145–261)
1-19	Adverse effects of medical treatment	365 (277–427)	414 (364–455)	523 (439–616)	205 (176–232)	153 (136–167)	148 (126–173)
1-20	Animal contact	29 (23–37)	133 (118–152)	166 (142–192)	18 (14–22·9)	47·4 (42·3–53·6)	45·1 (38·9–52·3)
1-21	Venomous animal contact	20 (15–25)	88 (77–101)	112 (95–130)	11·9 (9·1–15·2)	31·2 (27·6–35·5)	30·2 (25·8–35)
1-22	Non-venomous animal contact	10 (7–13)	45 (39–53)	54 (45–66)	6·1 (4·7–8·2)	16·3 (14·1–18·8)	14·9 (12·5–18)
1-23	Foreign body	697 (561–843)	458 (395–531)	502 (423–598)	283 (235–335)	141 (124–162)	132 (111–157)
1-24	Pulmonary aspiration and foreign body in airway	672 (538–819)	418 (356–488)	450 (375–541)	268 (220–320)	128 (110–149)	118 (98·7–143)
1-25	Foreign body in eyes	9 (4–16)	17 (7–31)	22 (10–39)	5·5 (2·7–9·7)	5·5 (2·6–9·6)	5·5 (2·6–9·6)
1-26	Foreign body in other body part	16 (12–20)	23 (18–30)	31 (23–41)	9·4 (7·2–12)	7·7 (5·8–10)	7·9 (6–10·4)
1-27	Environmental heat and cold exposure	59 (42–80)	103 (74–141)	142 (101–195)	40·1 (28·8–53·7)	35·9 (25·9–48·5)	37·3 (26·8–50·7)
1-28	Exposure to forces of nature	0 (0–0)	20 (14–29)	19 (13–27)	0 (0–0)	6·5 (4·5–9·2)	4·9 (3·4–6·9)
1-29	Other unintentional injuries	190 (157–232)	332 (277–402)	412 (331–521)	106 (85·3–132)	104 (85·7–127)	101 (80–128)
1-30	Self-harm and interpersonal violence	1,950 (1,820–2,100)	6,810 (6,490–7,130)	8,140 (7,210–9,150)	1,030 (958–1,110)	2,020 (1,930–2,120)	1,860 (1,640–2,090)
1-31	Self-harm	370 (329–414)	1,010 (925–1,100)	1,360 (1,160–1,570)	242 (218–268)	326 (301–354)	328 (281–378)
1-32	Self-harm by firearm	33 (25–47)	122 (102–161)	156 (122–206)	20·5 (16·1–29)	38 (32–49·3)	36·7 (29·1–47·8)
1-33	Self-harm by other specified means	338 (300–377)	888 (811–969)	1,200 (1,030–1,400)	222 (199–246)	288 (265–313)	291 (250–337)
1-34	Interpersonal violence	1,100 (988–1,220)	5,740 (5,440–6,050)	6,730 (5,950–7,570)	587 (530–649)	1,680 (1,590–1,770)	1,520 (1,340–1,710)
1-35	Physical violence by firearm	415 (364–477)	3,260 (3,050–3,450)	3,890 (3,410–4,420)	226 (198–262)	933 (878–988)	858 (752–973)
1-36	Physical violence by sharp object	298 (240–340)	1,540 (1,440–1,650)	1,760 (1,520–2,000)	170 (136–192)	450 (422–482)	398 (344–453)
1-37	Sexual violence	31 (20–44)	60 (39–85)	78 (51–110)	17·6 (11·6–25)	17·7 (11·7–25·2)	17·8 (11·7–25·3)
1-38	Physical violence by other means	355 (300–407)	884 (820–950)	1,000 (875–1,160)	174 (150–195)	275 (256–296)	243 (212–281)
1-39	Conflict and terrorism	481 (436–535)	48 (32–72)	43 (29–65)	198 (179–220)	16·4 (11·2–24·8)	11·8 (8·1–17·5)
1-40	Executions and police conflict	1 (1–1)	17 (12–23)	7 (5–10)	0·6 (0·6–0·7)	4·7 (3·5–6·3)	1·5 (1·1–2·2)

SR Table 1: All-age DALYs counts and age standardized DALYs rates per 100,000 individuals for all causes and level 1 cause groups by location for 1990, 2010, and 2019, both sexes combined, Cuba

Cause Level	Cause	All-age DALYs			Age-standardized rate of DALYs per 100,000		
		1990	2010	2019	1990	2010	2019
0-0	All causes	2,980,000 (2,690,000–3,310,000)	3,150,000 (2,810,000–3,550,000)	3,480,000 (2,960,000–4,060,000)	28,200 (25,500–31,200)	22,600 (20,000–25,700)	22,400 (19,100–26,400)
1-0	Communicable, maternal, neonatal, and nutritional disorders	321,000 (292,000–354,000)	239,000 (210,000–274,000)	250,000 (213,000–291,000)	3,280 (3,010–3,610)	2,150 (1,870–2,460)	2,080 (1,760–2,430)
1-1	HIV/AIDS and sexually transmitted infections	5,350 (4,430–6,950)	15,000 (13,500–17,100)	20,300 (18,400–22,800)	46·5 (38·4–60·4)	115 (104–130)	163 (149–183)
1-2	HIV/AIDS	3,350 (3,260–3,470)	11,700 (11,200–12,500)	17,400 (16,300–19,100)	29 (28·2–30)	91·1 (86·8–97·1)	141 (132–154)
1-3	HIV/AIDS - drug-susceptible tuberculosis	276 (188–401)	256 (180–357)	257 (176–371)	2·4 (1·6–3·5)	2 (1·4–2·8)	2·1 (1·4–3)
1-4	HIV/AIDS - Multidrug-resistant Tuberculosis without extensive drug resistance	11 (2–33)	8 (3–18)	14 (2–46)	0·1 (0–0·3)	0·1 (0–0·1)	0·1 (0–0·4)
1-5	HIV/AIDS - Extensively drug-resistant Tuberculosis	0 (0–0)	1 (0–2)	2 (0–7)	0 (0–0)	0 (0–0)	0 (0–0·1)
1-6	HIV/AIDS resulting in other diseases	3,070 (2,910–3,210)	11,500 (10,900–12,300)	17,200 (16,000–18,800)	26·5 (25·2–27·8)	89·1 (84·7–95)	139 (130–152)
1-7	Sexually transmitted infections excluding HIV	2,000 (1,090–3,590)	3,230 (2,100–5,270)	2,840 (1,760–4,720)	17·5 (9·5–31·5)	23·8 (15·5–38·6)	22·2 (14·1–35·8)
1-8	Syphilis	59 (47–78)	130 (113–151)	117 (91–149)	0·6 (0·5–0·7)	1 (0·9–1·2)	0·9 (0·7–1·2)
1-9	Chlamydial infection	239 (149–370)	410 (304–551)	330 (237–454)	2 (1·3–3·1)	3·1 (2·2–4·1)	2·8 (2–3·9)
1-10	Gonococcal infection	129 (84–193)	449 (391–517)	373 (296–463)	1 (0·7–1·5)	3·3 (2·9–3·9)	3 (2·4–3·8)
1-11	Trichomoniasis	547 (216–1,130)	645 (248–1,360)	612 (240–1,280)	4·8 (1·9–9·9)	4·7 (1·8–10)	4·6 (1·8–9·6)
1-12	Genital herpes	590 (192–1,480)	753 (239–1,860)	761 (239–1,870)	5·3 (1·7–13·3)	5·4 (1·7–13·4)	5·3 (1·7–13)
1-13	Other sexually transmitted infections	438 (281–647)	845 (639–1,130)	647 (479–862)	3·8 (2·4–5·5)	6·3 (4·7–8·4)	5·5 (4·1–7·3)
1-14	Respiratory infections and tuberculosis	88,800 (82,200–96,300)	98,300 (90,700–107,000)	117,000 (98,200–138,000)	891 (828–964)	727 (665–803)	732 (610–861)
1-15	Tuberculosis	3,650 (3,090–4,310)	2,010 (1,670–2,420)	1,570 (1,270–1,920)	33·4 (28·5–39)	14·9 (12–18·4)	10·7 (8·5–13·3)
1-16	Latent tuberculosis infection	0 (0–0)	0 (0–0)	0 (0–0)	0 (0–0)	0 (0–0)	0 (0–0)
1-17	Drug-susceptible tuberculosis	3,550 (2,970–4,200)	1,960 (1,620–2,370)	1,500 (1,190–1,850)	32·5 (27·4–38·2)	14·5 (11·7–18)	10·3 (8·1–12·8)
1-18	Multidrug-resistant tuberculosis without extensive drug resistance	100 (22–287)	46 (22–91)	61 (10–174)	0·9 (0·2–2·7)	0·3 (0·2–0·6)	0·4 (0·1–1·1)
1-19	Extensively drug-resistant tuberculosis	0 (0–0)	4 (2–9)	9 (2–27)	0 (0–0)	0 (0–0·1)	0·1 (0–0·2)
1-20	Lower respiratory infections	72,500 (69,000–76,000)	84,100 (77,900–88,200)	104,000 (85,400–125,000)	738 (700–774)	596 (556–627)	607 (502–724)
1-21	Upper respiratory infections	9,370 (5,850–14,400)	8,920 (5,400–14,000)	8,570 (5,270–13,400)	88 (55·3–134)	83·7 (50·8–133)	83·3 (50·3–132)
1-22	Otitis media	3,270 (1,900–5,460)	3,350 (1,950–5,480)	3,060 (1,760–5,120)	30·9 (17·6–51·3)	32·1 (18·5–52·8)	30·4 (17·6–49·9)
1-23	Enteric infections	31,900 (26,800–37,700)	26,500 (20,400–34,100)	28,600 (21,600–37,400)	324 (273–383)	218 (169–281)	218 (164–289)
1-24	Diarrhoeal diseases	31,700 (26,700–37,600)	26,400 (20,400–34,000)	28,600 (21,500–37,300)	322 (272–382)	218 (168–280)	217 (163–288)
1-25	Typhoid and paratyphoid	9 (2–14)	14 (1–32)	10 (1–27)	0·1 (0–0·1)	0·1 (0–0·2)	0·1 (0–0·1)
1-26	Typhoid fever	9 (1–13)	13 (1–32)	9 (1–27)	0·1 (0–0·1)	0·1 (0–0·2)	0·1 (0–0·1)
1-27	Paratyphoid fever	0 (0–1)	0 (0–1)	0 (0–1)	0 (0–0)	0 (0–0)	0 (0–0)
1-28	iNTS	36 (11–49)	25 (7–35)	15 (5–23)	0·4 (0·1–0·5)	0·4 (0·1–0·5)	0·2 (0·1–0·4)
1-29	Other intestinal infectious diseases	63 (35–96)	37 (25–51)	33 (24–45)	0·6 (0·3–1)	0·4 (0·2–0·6)	0·4 (0·2–0·5)
1-30	Neglected tropical diseases and malaria	4,670 (3,050–6,830)	3,800 (2,320–5,840)	2,960 (1,670–4,770)	45·2 (29·5–66·2)	37·8 (23·2–57·8)	30·8 (17·3–49·2)
1-31	Malaria	0 (0–0)	0 (0–0)	0 (0–0)	0 (0–0)	0 (0–0)	0 (0–0)
1-32	Chagas disease	57 (23–110)	0 (0–0)	0 (0–0)	0·6 (0·2–1·1)	0 (0–0)	0 (0–0)
1-33	Leishmaniasis	0 (0–0)	0 (0–0)	0 (0–0)	0 (0–0)	0 (0–0)	0 (0–0)
1-34	Visceral leishmaniasis	0 (0–0)	0 (0–0)	0 (0–0)	0 (0–0)	0 (0–0)	0 (0–0)
1-35	Cutaneous and mucocutaneous leishmaniasis	0 (0–0)	0 (0–0)	0 (0–0)	0 (0–0)	0 (0–0)	0 (0–0)
1-36	African trypanosomiasis	0 (0–0)	0 (0–0)	0 (0–0)	0 (0–0)	0 (0–0)	0 (0–0)
1-37	Schistosomiasis	0 (0–0)	0 (0–0)	0 (0–0)	0 (0–0)	0 (0–0)	0 (0–0)
1-38	Cysticercosis	0 (0–0)	0 (0–0)	0 (0–0)	0 (0–0)	0 (0–0)	0 (0–0)
1-39	Cystic echinococcosis	1 (0–1)	1 (0–1)	2 (1–2)	0 (0–0)	0 (0–0)	0 (0–0)
1-40	Lymphatic filariasis	0 (0–0)	0 (0–0)	0 (0–0)	0 (0–0)	0 (0–0)	0 (0–0)
1-41	Onchocerciasis	0 (0–0)	0 (0–0)	0 (0–0)	0 (0–0)	0 (0–0)	0 (0–0)
1-42	Trachoma	0 (0–0)	0 (0–0)	0 (0–0)	0 (0–0)	0 (0–0)	0 (0–0)
1-43	Dengue	977 (434–1,870)	856 (286–1,910)	708 (143–1,840)	9 (3·9–17·3)	7·6 (2·4–17·1)	6·4 (1·3–16·6)
1-44	Yellow fever	0 (0–0)	0 (0–0)	0 (0–0)	0 (0–0)	0 (0–0)	0 (0–0)
1-45	Rabies	44 (28–71)	6 (4–9)	15 (10–25)	0·4 (0·3–0·7)	0·1 (0–0·1)	0·1 (0·1–0·2)
1-46	Intestinal nematode infections	1,400 (792–2,300)	1,470 (806–2,400)	944 (519–1,590)	12·7 (7·1–20·9)	14·4 (7·9–23·6)	9·7 (5·4–16·3)
1-47	Ascariasis	556 (287–1,060)	580 (274–1,090)	354 (163–647)	5·5 (2·8–10·3)	5·8 (2·7–10·9)	3·7 (1·7–6·8)
1-48	Trichuriasis	77 (35–145)	49 (22–96)	44 (19–84)	0·7 (0·3–1·4)	0·5 (0·2–1)	0·5 (0·2–0·9)

1-49	Hookworm disease	765 (413–1,310)	842 (446–1,450)	546 (283–947)	6·5 (3·5–11·1)	8·1 (4·3–13·9)	5·5 (2·9–9·6)
1-50	Food-borne trematodiases	0 (0–0)	0 (0–0)	0 (0–0)	0 (0–0)	0 (0–0)	0 (0–0)
1-51	Leprosy	11 (7–16)	18 (11–26)	18 (11–27)	0·1 (0·1–0·2)	0·1 (0·1–0·2)	0·1 (0·1–0·2)
1-52	Ebola virus disease	0 (0–0)	0 (0–0)	0 (0–0)	0 (0–0)	0 (0–0)	0 (0–0)
1-53	Zika virus disease	0 (0–0)	0 (0–0)	3 (0–12)	0 (0–0)	0 (0–0)	0 (0–0·1)
1-54	Guinea worm disease	0 (0–0)	0 (0–0)	0 (0–0)	0 (0–0)	0 (0–0)	0 (0–0)
1-55	Other neglected tropical diseases	2,180 (1,390–3,220)	1,450 (861–2,270)	1,270 (750–1,970)	22·5 (14·2–33·6)	15·6 (9·1–24)	14·4 (8·4–22·9)
1-56	Other infectious diseases	46,400 (43,300–49,300)	12,800 (11,400–14,500)	11,500 (9,450–13,700)	475 (444–505)	127 (112–143)	113 (93·2–134)
1-57	Meningitis	32,700 (30,500–35,100)	4,770 (4,390–5,180)	4,010 (3,310–4,790)	342 (319–367)	50·4 (45·2–56·2)	41·3 (33·8–49·6)
1-58	Encephalitis	6,940 (3,480–8,110)	2,540 (2,270–3,160)	2,310 (1,880–3,020)	68·6 (34–80·8)	26·7 (23·5–33)	25·2 (20·2–33·2)
1-59	Diphtheria	1 (1–1)	1 (1–1)	1 (0–1)	0 (0–0)	0 (0–0)	0 (0–0)
1-60	Whooping cough	135 (80–210)	69 (41–107)	32 (19–51)	1·5 (0·9–2·3)	1·1 (0·6–1·7)	0·6 (0·3–0·9)
1-61	Tetanus	47 (8–154)	24 (4–59)	13 (2–38)	0·5 (0·1–1·5)	0·2 (0–0·5)	0·1 (0–0·3)
1-62	Measles	1 (1–2)	0 (0–0)	1 (1–2)	0 (0–0)	0 (0–0)	0 (0–0)
1-63	Varicella and herpes zoster	753 (531–1,090)	742 (529–1,010)	691 (504–954)	7·4 (5·2–10·8)	6·8 (4·6–8·8)	6 (4·2–8·2)
1-64	Acute hepatitis	2,170 (1,930–2,460)	632 (475–827)	533 (385–710)	19·9 (17·6–22·5)	5·9 (4·4–7·9)	5·3 (3·8–7·3)
1-65	Acute hepatitis A	1,520 (1,240–1,820)	431 (305–591)	355 (238–497)	13·9 (11·3–16·7)	4·3 (3–6)	3·9 (2·6–5·5)
1-66	Acute hepatitis B	425 (263–604)	123 (94–149)	109 (76–142)	3·9 (2·4–5·5)	0·9 (0·7–1·1)	0·8 (0·6–1·1)
1-67	Acute hepatitis C	169 (87–292)	32 (22–55)	26 (17–45)	1·5 (0·8–2·6)	0·3 (0·2–0·4)	0·2 (0·1–0·4)
1-68	Acute hepatitis E	57 (37–86)	46 (28–70)	43 (27–66)	0·5 (0·3–0·8)	0·4 (0·2–0·6)	0·4 (0·2–0·6)
1-69	Other unspecified infectious diseases	3,640 (2,790–4,780)	4,040 (2,930–5,080)	3,870 (2,810–5,080)	35·3 (27·1–46·5)	35·7 (25–45·5)	34 (24·5–44·6)
1-70	Maternal and neonatal disorders	105,000 (95,700–114,000)	50,500 (43,000–58,100)	40,400 (33,200–47,900)	1,120 (1,030–1,210)	631 (530–713)	557 (448–660)
1-71	Maternal disorders	6,940 (6,330–7,590)	4,710 (4,290–5,180)	3,760 (2,990–4,670)	53·9 (49·3–58·8)	43·1 (39·1–47·7)	38·5 (30·6–47·6)
1-72	Maternal haemorrhage	839 (607–1,160)	729 (556–956)	528 (383–715)	6·5 (4·8–8·9)	6·7 (5·1–8·9)	5·4 (3·9–7·3)
1-73	Maternal sepsis and other pregnancy related infections	849 (683–1,040)	320 (244–421)	221 (156–304)	6·7 (5·4–8·2)	2·9 (2·2–3·9)	2·3 (1·6–3·1)
1-74	Maternal hypertensive disorders	959 (818–1,130)	483 (398–593)	377 (283–480)	7·3 (6·2–8·6)	4·6 (3·7–5·6)	4 (3–5)
1-75	Maternal obstructed labour and uterine rupture	42 (22–71)	75 (54–99)	52 (36–71)	0·3 (0·2–0·5)	0·7 (0·5–0·9)	0·5 (0·4–0·7)
1-76	Maternal abortive outcome	1,590 (1,280–1,970)	428 (333–535)	283 (203–380)	12·5 (10·1–15·4)	3·8 (2·9–4·8)	2·9 (2·1–3·9)
1-77	Ectopic pregnancy	45 (38–54)	243 (204–288)	183 (135–242)	0·4 (0·3–0·4)	2·2 (1·8–2·6)	1·8 (1·4–2·4)
1-78	Indirect maternal deaths	1,820 (1,520–2,130)	1,020 (874–1,200)	864 (645–1,140)	14 (11·8–16·3)	9·5 (8·1–11·2)	8·9 (6·6–11·7)
1-79	Late maternal deaths	581 (432–779)	523 (437–627)	518 (364–704)	4·5 (3·4–6)	4·8 (4–5·7)	5·3 (3·7–7·2)
1-80	Maternal deaths aggravated by HIV/AIDS	0 (0–1)	1 (1–3)	3 (1–5)	0 (0–0)	0 (0–0)	0 (0–0·1)
1-81	Other maternal disorders	221 (177–275)	883 (732–1,040)	734 (558–937)	1·7 (1·4–2·1)	7·9 (6·5–9·4)	7·4 (5·7–9·4)
1-82	Neonatal disorders	97,900 (89,100–107,000)	45,800 (38,200–53,400)	36,600 (29,700–43,900)	1,070 (977–1,160)	588 (490–672)	518 (414–616)
1-83	Neonatal preterm birth	44,400 (38,100–51,400)	17,300 (14,400–20,200)	14,600 (11,600–17,800)	479 (410–557)	204 (170–236)	188 (149–228)
1-84	Neonatal encephalopathy due to birth asphyxia and trauma	27,700 (19,600–34,500)	8,590 (6,720–10,300)	6,320 (4,830–7,820)	312 (220–390)	118 (91·9–139)	96 (70·9–121)
1-85	Neonatal sepsis and other neonatal infections	14,700 (10,300–20,600)	11,600 (8,430–16,300)	9,700 (6,970–13,000)	152 (108–209)	136 (103–181)	120 (89·1–155)
1-86	Hemolytic disease and other neonatal jaundice	2,650 (1,870–3,860)	730 (594–879)	538 (412–676)	29·5 (20·7–43·1)	9·1 (7·3–11)	7·1 (5·3–9·2)
1-87	Other neonatal disorders	8,470 (5,510–12,700)	7,650 (5,910–8,690)	5,470 (3,740–7,080)	96·7 (62·7–145)	121 (93·3–137)	107 (72·5–139)
1-88	Nutritional deficiencies	39,000 (26,200–56,500)	32,000 (20,600–47,400)	28,900 (18,400–42,500)	378 (252–551)	291 (187–428)	269 (168–398)
1-89	Protein-energy malnutrition	4,520 (3,430–5,830)	4,270 (3,030–5,860)	4,480 (3,060–6,120)	43·8 (33·6–55·7)	36·8 (26–50·6)	36·8 (25–51·1)
1-90	Iodine deficiency	1,190 (629–1,980)	1,210 (646–1,950)	851 (453–1,390)	10·5 (5·5–17·2)	9·7 (5·2–15·9)	6·9 (3·6–11·4)
1-91	Vitamin A deficiency	276 (137–471)	135 (67–245)	89 (43–166)	3·2 (1·5–5·4)	2 (1–3·6)	1·4 (0·7–2·7)
1-92	Dietary iron deficiency	31,000 (18,900–47,200)	24,700 (14,800–38,100)	21,900 (12,800–34,600)	303 (187–462)	230 (137–359)	211 (121–334)
1-93	Other nutritional deficiencies	2,050 (1,400–2,890)	1,590 (1,070–2,240)	1,560 (1,010–2,210)	18·4 (12·7–25·6)	12·9 (8·6–18·2)	12·4 (8–17·8)
1-0	Non-communicable diseases	2,180,000 (1,930,000–2,460,000)	2,590,000 (2,300,000–2,930,000)	2,900,000 (2,460,000–3,390,000)	20,700 (18,500–23,200)	18,000 (15,800–20,500)	17,900 (15,100–21,100)
1-1	Neoplasms	371,000 (360,000–384,000)	528,000 (501,000–544,000)	616,000 (505,000–743,000)	3,580 (3,470–3,700)	3,490 (3,300–3,600)	3,480 (2,840–4,180)
1-2	Lip and oral cavity cancer	6,990 (6,620–7,380)	10,700 (10,200–11,300)	13,300 (10,600–16,600)	67·9 (64·2–71·6)	69·2 (65·5–72·9)	72·1 (57·8–89·8)
1-3	Nasopharynx cancer	1,250 (1,160–1,350)	2,110 (1,950–2,280)	2,550 (2,010–3,260)	11·9 (11·1–12·8)	14·1 (13–15·2)	14·6 (11·5–18·4)
1-4	Other pharynx cancer	3,760 (3,510–4,030)	4,220 (3,930–4,540)	5,610 (4,370–7,080)	36·8 (34·2–39·5)	27·3 (25·3–29·3)	30·3 (23·5–38·1)
1-5	Oesophageal cancer	8,960 (8,500–9,450)	18,200 (17,300–19,200)	23,800 (18,800–29,700)	87·6 (82·9–92·2)	117 (111–123)	128 (101–160)
1-6	Stomach cancer	17,800 (17,100–18,600)	21,500 (20,400–22,500)	23,100 (18,600–28,500)	172 (165–180)	138 (131–145)	126 (101–155)
1-7	Colon and rectum cancer	34,700 (33,200–36,000)	52,800 (50,200–55,200)	68,500 (56,100–83,700)	336 (321–349)	337 (321–352)	369 (301–452)

1-8	Liver cancer	16,400 (15,600–17,200)	7,100 (6,720–7,520)	9,740 (7,740–12,100)	158 (150–166)	46·5 (44–49·1)	53·5 (42·7–66·4)
1-9	Liver cancer due to hepatitis B	5,250 (4,040–6,720)	1,980 (1,460–2,610)	2,640 (1,790–3,820)	50·4 (38·6–65·1)	13 (9·6–17·1)	14·8 (10·2–21·3)
1-10	Liver cancer due to hepatitis C	3,410 (2,370–4,490)	1,480 (1,030–1,990)	1,990 (1,270–2,910)	33·1 (23–43·9)	9·4 (6·5–12·6)	10·4 (6·6–15·2)
1-11	Liver cancer due to alcohol use	5,100 (3,770–6,510)	2,510 (1,870–3,220)	3,620 (2,490–5,000)	49·3 (36·3–63·1)	16·1 (11·9–20·6)	19·3 (13·3–26·8)
1-12	Liver cancer due to NASH	1,310 (939–1,770)	642 (453–908)	927 (602–1,400)	12·5 (9–17·1)	4·1 (2·9–5·8)	5 (3·3–7·5)
1-13	Gallbladder and biliary tract cancer	7,760 (4,420–8,610)	4,270 (3,720–6,890)	4,590 (3,410–7,900)	75·5 (42·9–83·8)	27·3 (23·8–44)	24·6 (18·3–42·4)
1-14	Pancreatic cancer	4,030 (3,770–4,300)	20,000 (19,000–21,000)	25,300 (20,400–31,200)	39·2 (36·6–41·8)	128 (122–134)	136 (111–168)
1-15	Larynx cancer	10,200 (9,650–10,700)	19,300 (18,300–20,400)	23,000 (18,300–28,600)	99·6 (94·3–105)	124 (118–131)	123 (98–153)
1-16	Tracheal, bronchus, and lung cancer	80,200 (77,200–83,100)	122,000 (117,000–126,000)	143,000 (115,000–175,000)	779 (749–806)	784 (755–812)	765 (618–937)
1-17	Malignant skin melanoma	1,420 (1,180–1,990)	2,070 (1,660–2,770)	2,640 (1,930–3,630)	13·4 (11·2–18·9)	13·9 (11·2–18·5)	15·9 (11·7–21·8)
1-18	Non-melanoma skin cancer	3,240 (2,960–3,530)	5,500 (5,030–5,840)	6,490 (5,180–8,010)	32 (29·3–34·7)	34·9 (32·1–37)	34·5 (27·5–42·6)
1-19	Non-melanoma skin cancer (squamous-cell carcinoma)	3,240 (2,960–3,530)	5,500 (5,030–5,840)	6,490 (5,180–8,000)	32 (29·3–34·7)	34·9 (32·1–37)	34·5 (27·5–42·6)
1-20	Non-melanoma skin cancer (basal-cell carcinoma)	1 (0–1)	1 (0–2)	1 (0–2)	0 (0–0)	0 (0–0)	0 (0–0)
1-21	Breast cancer	29,700 (28,300–31,200)	41,300 (39,300–43,600)	44,800 (36,000–55,500)	288 (275–303)	265 (252–280)	252 (202–312)
1-22	Cervical cancer	19,000 (16,400–20,500)	21,600 (18,100–23,100)	20,300 (15,600–25,500)	180 (156–194)	141 (118–151)	124 (94·3–155)
1-23	Uterine cancer	6,970 (6,440–7,540)	13,700 (12,700–14,700)	15,600 (12,500–19,500)	67·4 (62·3–72·9)	88·3 (81·9–95)	86·7 (69·4–109)
1-24	Ovarian cancer	1,890 (1,700–2,970)	8,790 (4,890–9,560)	9,700 (5,500–12,300)	18·1 (16·3–28·5)	57·4 (32·1–62·3)	56·1 (32·4–70·9)
1-25	Prostate cancer	24,700 (20,700–32,700)	46,000 (29,700–51,100)	56,200 (33,400–72,500)	240 (202–321)	288 (186–319)	287 (171–371)
1-26	Testicular cancer	231 (194–317)	716 (606–811)	767 (543–981)	2 (1·7–2·8)	5·9 (4·9–6·8)	6·4 (4·5–8·2)
1-27	Kidney cancer	7,770 (7,330–8,260)	7,180 (6,790–7,600)	9,040 (7,200–11,200)	75·4 (71·1–80)	49·7 (47–52·6)	53·9 (43–66·9)
1-28	Bladder cancer	7,300 (6,890–7,710)	12,100 (11,400–12,800)	14,600 (11,600–18,100)	70·9 (66·8–74·8)	76·9 (72·1–81·4)	76·6 (61·2–94·8)
1-29	Brain and nervous system cancer	3,570 (2,510–7,920)	16,800 (5,740–20,900)	17,200 (5,670–24,700)	34·5 (24·2–76·4)	129 (45·7–161)	124 (41·4–176)
1-30	Thyroid cancer	1,320 (1,220–1,440)	2,030 (1,860–2,200)	2,630 (2,100–3,290)	12·7 (11·7–13·8)	13·3 (12·2–14·5)	14·9 (11·9–18·7)
1-31	Mesothelioma	467 (395–545)	647 (590–737)	605 (479–766)	4·4 (3·7–5)	4·3 (3·9–4·9)	3·6 (2·8–4·6)
1-32	Hodgkin lymphoma	871 (594–1,900)	3,640 (1,400–4,500)	3,350 (1,380–4,630)	7·5 (5·1–16·5)	27·2 (10·5–33·7)	24·3 (10–33·4)
1-33	Non-Hodgkin's lymphoma	17,500 (16,500–18,600)	14,900 (14,100–15,700)	17,200 (13,800–21,200)	163 (154–173)	108 (102–114)	112 (90–138)
1-34	Multiple myeloma	4,640 (3,880–5,280)	7,430 (6,740–8,100)	9,290 (7,320–11,900)	45 (37·7–51·4)	47·7 (43·1–52·1)	49·9 (39·3–64·2)
1-35	Leukaemia	22,000 (20,900–23,100)	18,400 (17,600–19,300)	18,600 (15,200–22,800)	207 (197–217)	150 (143–158)	141 (115–170)
1-36	Acute lymphoid leukaemia	6,480 (5,400–7,360)	4,040 (3,680–4,480)	3,640 (2,930–4,450)	62·7 (52·4–70·9)	43 (38·6–48)	38·9 (31·3–48)
1-37	Chronic lymphoid leukaemia	1,390 (1,220–1,590)	1,850 (1,680–2,120)	2,210 (1,720–2,880)	13·4 (11·7–15·3)	12 (10·9–13·6)	11·9 (9·3–15·5)
1-38	Acute myeloid leukaemia	6,520 (5,670–8,060)	6,230 (5,680–7,100)	6,750 (5,370–8,430)	60 (52·2–74·4)	50·4 (45·2–56·6)	51·8 (41·2–63·6)
1-39	Chronic myeloid leukaemia	3,590 (3,250–3,970)	3,150 (2,870–3,390)	3,020 (2,370–3,790)	33·3 (30·1–36·8)	21·9 (19·9–23·5)	18·6 (14·8–23·2)
1-40	Other leukaemia	3,990 (3,570–4,410)	3,140 (2,890–3,390)	3,030 (2,410–3,720)	37·5 (33·7–41·5)	23·1 (21·1–24·9)	19·8 (15·8–24·2)
1-41	Other neoplasms	788 (626–1,160)	1,140 (881–1,470)	1,510 (1,090–2,030)	7·5 (6–11)	8 (6–10)	9·2 (6·5–12·2)
1-42	Myelodysplastic, myeloproliferative, and other hematopoietic neoplasms	788 (626–1,160)	1,140 (881–1,470)	1,510 (1,090–2,030)	7·5 (6–11)	8 (6–10)	9·2 (6·5–12·2)
1-43	Benign and in situ intestinal neoplasms	0 (0–0)	0 (0–0)	0 (0–0)	0 (0–0)	0 (0–0)	0 (0–0)
1-44	Benign and in situ cervical and uterine neoplasms	0 (0–0)	0 (0–0)	0 (0–0)	0 (0–0)	0 (0–0)	0 (0–0)
1-45	Other benign and in situ neoplasms	0 (0–0)	0 (0–0)	0 (0–0)	0 (0–0)	0 (0–0)	0 (0–0)
1-46	Cardiovascular diseases	596,000 (573,000–612,000)	619,000 (589,000–642,000)	698,000 (588,000–833,000)	5,810 (5,580–5,970)	3,950 (3,770–4,090)	3,730 (3,130–4,450)
1-47	Rheumatic heart disease	15,800 (13,600–18,500)	12,400 (10,200–15,200)	10,400 (7,910–13,400)	141 (122–164)	93·1 (75·3–116)	79·4 (59·5–104)
1-48	Ischaemic heart disease	371,000 (355,000–382,000)	330,000 (312,000–347,000)	371,000 (310,000–446,000)	3,650 (3,480–3,750)	2,080 (1,970–2,190)	1,940 (1,620–2,340)
1-49	Stroke	156,000 (149,000–162,000)	177,000 (167,000–186,000)	194,000 (161,000–231,000)	1,510 (1,440–1,570)	1,130 (1,060–1,190)	1,040 (858–1,240)
1-50	Ischaemic stroke	70,700 (65,200–76,300)	97,000 (90,300–103,000)	110,000 (92,300–130,000)	693 (639–749)	608 (567–644)	566 (472–672)
1-51	Intracerebral hemorrhage	70,100 (65,900–74,700)	66,200 (63,000–69,900)	69,800 (56,300–84,600)	676 (636–720)	427 (405–451)	383 (309–464)
1-52	Subarachnoid hemorrhage	15,000 (12,700–16,900)	13,500 (12,700–15,200)	14,700 (11,700–18,200)	142 (121–160)	91·5 (85·7–103)	89·3 (71·7–111)
1-53	Hypertensive heart disease	12,100 (11,000–16,100)	32,900 (18,800–36,200)	40,000 (21,600–50,300)	119 (107–158)	208 (119–228)	210 (114–264)
1-54	Non-rheumatic valvular heart disease	3,840 (3,330–4,230)	5,360 (5,040–5,720)	6,390 (5,060–7,970)	36 (31·4–39·5)	35·4 (33·3–37·8)	37·1 (29·4–46·3)
1-55	Non-rheumatic calcific aortic valve disease	2,440 (2,070–2,780)	3,510 (3,200–4,060)	4,180 (3,160–5,640)	22·9 (19·5–26)	23 (21–26·7)	23·7 (17·9–32)
1-56	Non-rheumatic degenerative mitral valve disease	1,390 (1,140–1,700)	1,830 (1,350–2,080)	2,170 (1,460–2,830)	12·9 (10·6–15·8)	12·2 (9–13·9)	13·1 (8·9–16·9)
1-57	Other non-rheumatic valve diseases	15 (10–30)	27 (16–39)	44 (18–72)	0·1 (0·1–0·3)	0·2 (0·1–0·3)	0·3 (0·1–0·5)
1-58	Cardiomyopathy and myocarditis	9,950 (8,480–14,100)	18,700 (17,300–22,400)	23,200 (18,200–28,500)	96 (81·9–135)	130 (121–154)	140 (110–172)
1-59	Myocarditis	961 (560–1,280)	760 (632–1,000)	788 (575–1,190)	9·5 (5·5–12·6)	7 (5·5–8·6)	6·9 (5·3–9·4)
1-60	Alcoholic cardiomyopathy	3,150 (2,600–4,400)	10,500 (8,930–12,800)	13,800 (10,300–17,400)	30·2 (25·1–42·2)	68·3 (58·3–83·8)	78·9 (58·5–99)

1-61	Other cardiomyopathy	5,830 (4,710–8,850)	7,470 (6,760–9,000)	8,580 (6,700–10,900)	56·3 (45·5–85·1)	54·7 (50–64·6)	54·5 (43·3–67·7)
1-62	Atrial fibrillation and flutter	7,190 (5,870–8,890)	11,700 (9,790–14,100)	15,300 (12,300–19,000)	73·3 (60–90·4)	71·5 (59·9–86·4)	75·8 (61–94·5)
1-63	Aortic aneurysm	8,330 (7,150–9,450)	11,700 (10,900–12,600)	13,600 (10,800–16,800)	80 (68·8–90·5)	74·3 (69·4–79·7)	71·4 (57·1–88·3)
1-64	Peripheral vascular disease	3,850 (2,040–7,170)	6,650 (3,520–11,700)	9,120 (4,620–16,500)	38·2 (20·3–70·9)	41·4 (21·8–73·1)	46·3 (23·3–83·4)
1-65	Endocarditis	1,480 (1,140–2,200)	2,080 (1,620–2,470)	2,310 (1,710–3,000)	13·7 (10·6–20·3)	14·8 (11·6–17·6)	15·1 (11·3–19·6)
1-66	Chronic respiratory diseases	81,800 (67,800–98,800)	114,000 (96,200–130,000)	131,000 (106,000–154,000)	778 (646–941)	840 (698–1,000)	853 (684–1,040)
1-67	Chronic obstructive pulmonary disease	37,000 (33,800–39,900)	74,600 (62,200–81,300)	93,500 (74,500–112,000)	359 (328–388)	477 (399–521)	498 (398–592)
1-68	Pneumoconiosis	37 (31–43)	55 (47–64)	67 (52–86)	0·4 (0·3–0·4)	0·4 (0·3–0·4)	0·4 (0·3–0·5)
1-69	Silicosis	19 (16–24)	28 (24–32)	34 (26–46)	0·2 (0·2–0·2)	0·2 (0·1–0·2)	0·2 (0·1–0·2)
1-70	Asbestosis	3 (2–4)	5 (3–6)	7 (3–9)	0 (0–0)	0 (0–0)	0 (0–0·1)
1-71	Coal workers pneumoconiosis	2 (1–4)	2 (2–3)	2 (2–3)	0 (0–0)	0 (0–0)	0 (0–0)
1-72	Other pneumoconiosis	12 (8–16)	21 (15–28)	24 (16–33)	0·1 (0·1–0·2)	0·1 (0·1–0·2)	0·1 (0·1–0·2)
1-73	Asthma	42,100 (30,000–57,700)	35,500 (25,500–48,800)	33,000 (23,500–45,700)	393 (279–550)	335 (232–479)	326 (222–471)
1-74	Interstitial lung disease and pulmonary sarcoidosis	985 (783–1,240)	1,510 (1,280–1,950)	1,780 (1,370–2,290)	9·5 (7·5–12)	10·9 (9–13·5)	11·4 (8·6–14·5)
1-75	Other chronic respiratory diseases	1,740 (1,340–2,030)	1,950 (1,620–2,250)	2,110 (1,690–2,550)	16·2 (12·9–18·7)	16·4 (13·7–19·3)	17·4 (13·9–21)
1-76	Digestive diseases	95,400 (85,900–109,000)	118,000 (106,000–135,000)	137,000 (114,000–164,000)	904 (817–1,030)	808 (720–933)	835 (693–1,000)
1-77	Cirrhosis and other chronic liver diseases	30,800 (29,300–33,000)	43,900 (41,400–47,800)	56,100 (44,200–69,100)	295 (280–316)	287 (270–311)	322 (254–395)
1-78	Cirrhosis and other chronic liver diseases due to hepatitis B	3,610 (2,570–4,880)	3,100 (2,090–4,480)	3,950 (2,450–6,060)	34·6 (24·6–46·9)	19·9 (13·5–28·8)	22·8 (14·4–34·5)
1-79	Cirrhosis and other chronic liver diseases due to hepatitis C	5,630 (4,220–7,550)	8,680 (6,230–12,000)	10,700 (7,020–15,600)	53·8 (40–72·7)	56·1 (40·5–77·5)	61·6 (40·6–89)
1-80	Cirrhosis and other chronic liver diseases due to alcohol use	11,100 (8,810–13,400)	19,300 (15,300–23,200)	25,500 (18,600–33,800)	108 (85·5–130)	124 (98·6–149)	143 (105–189)
1-81	Cirrhosis and other chronic liver diseases due to NAFLD	4,630 (3,370–6,180)	6,930 (4,920–9,620)	8,960 (5,800–13,400)	44·3 (31·7–59·4)	44·7 (31·8–61·9)	50·3 (32·9–74·7)
1-82	Cirrhosis and other chronic liver diseases due to other causes	5,860 (4,640–7,190)	5,860 (4,370–7,640)	6,960 (4,670–9,890)	54·5 (42·9–67·1)	41·8 (32–53·2)	43·8 (30·6–60·8)
1-83	Upper digestive system diseases	27,300 (19,900–38,700)	30,600 (21,000–44,500)	32,100 (22,100–47,200)	254 (186–357)	212 (145–313)	206 (139–312)
1-84	Peptic ulcer disease	9,280 (8,450–10,300)	7,570 (6,850–8,620)	7,920 (6,230–10,100)	89·5 (81·4–99·1)	48·9 (44·1–55·6)	43·3 (34·1–55·3)
1-85	Gastritis and duodenitis	4,330 (3,190–5,830)	5,840 (4,230–7,860)	6,430 (4,580–8,750)	41·1 (30·2–55·4)	40·2 (29·2–54·4)	39·8 (28·1–54·1)
1-86	GERD	13,700 (7,150–24,200)	17,200 (8,860–30,300)	17,800 (9,140–31,500)	123 (64–217)	123 (64·2–218)	123 (64·2–218)
1-87	Appendicitis	3,460 (2,560–4,020)	2,580 (2,080–3,210)	2,790 (2,020–3,680)	31·6 (23·6–36·8)	19·4 (15·9–24·2)	19·7 (14·7–25·8)
1-88	Paralytic ileus and intestinal obstruction	6,770 (5,230–7,610)	7,960 (5,800–8,990)	8,970 (6,040–11,700)	66·4 (51·6–74·7)	56·7 (42·8–63·5)	55·6 (39·3–70·5)
1-89	Inguinal, femoral, and abdominal hernia	7,570 (5,740–9,740)	9,420 (7,090–12,200)	10,100 (7,720–13,200)	73·2 (55·8–94)	72·8 (54·1–95·6)	71 (53–94·7)
1-90	Inflammatory bowel disease	1,520 (1,150–1,820)	1,530 (1,260–1,840)	1,680 (1,340–2,090)	14·2 (10·7–17)	10·3 (8·5–12·5)	10·5 (8·4–12·9)
1-91	Vascular intestinal disorders	4,270 (3,700–4,930)	5,770 (5,270–6,470)	6,970 (5,510–8,860)	41·3 (35·9–47·6)	36·8 (33·6–41·2)	37·3 (29·6–47·2)
1-92	Gallbladder and biliary diseases	9,100 (6,710–11,300)	8,190 (6,480–10,400)	8,860 (6,760–11,400)	85·8 (63·1–106)	55·5 (43·5–70·9)	54·2 (40·6–70·2)
1-93	Pancreatitis	3,380 (3,000–3,790)	5,470 (4,140–5,960)	5,920 (4,500–7,550)	31·1 (27·7–34·7)	36·8 (27·8–40)	37 (28–47)
1-94	Other digestive diseases	1,190 (915–1,800)	2,970 (1,570–3,610)	3,760 (1,950–5,020)	11·7 (8·9–17·5)	20·7 (11·4–25·2)	22·6 (12·1–29·9)
1-95	Neurological disorders	123,000 (65,200–207,000)	153,000 (86,400–255,000)	167,000 (95,900–267,000)	1,140 (610–1,900)	1,120 (616–1,870)	1,130 (625–1,890)
1-96	Alzheimer's disease and other dementias	27,600 (11,500–63,600)	47,200 (19,900–106,000)	58,300 (25,400–131,000)	288 (121–660)	285 (120–640)	281 (122–638)
1-97	Parkinson's disease	6,410 (5,930–6,900)	11,400 (10,500–12,400)	14,700 (12,500–17,100)	61·6 (57–66·4)	70·8 (65–76·6)	74 (62·8–86·5)
1-98	Idiopathic epilepsy	16,500 (8,710–27,800)	12,600 (6,120–21,700)	13,200 (5,940–24,400)	148 (77·3–250)	110 (51·5–194)	117 (51·9–222)
1-99	Multiple sclerosis	1,300 (1,100–1,590)	1,920 (1,510–2,260)	2,100 (1,500–2,640)	12 (10·2–14·8)	13 (10·1–15·3)	13·6 (9·7–17·1)
1-100	Motor neuron disease	655 (607–703)	2,420 (2,260–2,590)	3,200 (2,570–3,950)	6·4 (6–6·9)	17·3 (16·2–18·5)	20·1 (16·3–24·5)
1-101	Headache disorders	63,000 (12,300–139,000)	69,000 (14,700–149,000)	67,000 (14,900–143,000)	550 (110–1,200)	550 (107–1,210)	549 (108–1,210)
1-102	Migraine	57,100 (8,090–132,000)	62,200 (10,100–142,000)	60,100 (10,400–137,000)	498 (72·7–1,140)	498 (73–1,150)	497 (72·5–1,140)
1-103	Tension-type headache	5,860 (1,710–21,400)	6,890 (2,100–23,500)	6,930 (2,150–22,700)	52·1 (15·3–191)	52·2 (15·1–184)	52·3 (15·1–187)
1-104	Other neurological disorders	7,700 (6,240–9,520)	8,040 (6,740–9,890)	9,060 (7,000–11,700)	71·2 (57·8–88)	70·6 (57·7–89·3)	78·2 (58·2–104)
1-105	Mental disorders	231,000 (167,000–306,000)	231,000 (169,000–304,000)	234,000 (171,000–308,000)	2,030 (1,470–2,690)	1,790 (1,310–2,360)	1,810 (1,320–2,390)
1-106	Schizophrenia	20,200 (14,100–26,900)	24,600 (17,100–32,700)	24,500 (17,200–32,300)	180 (124–237)	179 (124–240)	180 (126–241)
1-107	Depressive disorders	105,000 (70,400–149,000)	92,900 (63,900–129,000)	97,300 (66,800–136,000)	916 (615–1,280)	678 (465–949)	685 (470–953)
1-108	Major depressive disorder	94,700 (62,700–135,000)	80,600 (54,700–112,000)	84,900 (57,500–118,000)	826 (551–1,180)	588 (400–818)	595 (405–825)
1-109	Dysthymia	10,200 (6,290–15,500)	12,300 (7,650–18,900)	12,400 (7,900–19,300)	90·1 (56·2–138)	90·2 (56·5–138)	90·1 (56·5–139)
1-110	Bipolar disorder	24,100 (13,900–37,300)	26,500 (15,400–40,800)	26,000 (15,300–39,700)	206 (119–317)	206 (119–321)	206 (120–318)
1-111	Anxiety disorders	45,500 (30,200–65,000)	50,600 (33,300–71,700)	50,500 (33,800–71,400)	402 (265–569)	403 (268–573)	407 (268–579)
1-112	Eating disorders	5,040 (3,020–7,690)	4,130 (2,480–6,290)	4,000 (2,400–6,020)	40 (23·9–60·9)	37·5 (22·5–57·5)	40·9 (24·8–61·4)
1-113	Anorexia nervosa	1,240 (732–2,040)	976 (566–1,580)	935 (541–1,530)	9·8 (5·8–15·8)	9·2 (5·4–14·9)	10 (5·7–16·4)

1-114	Bulimia nervosa	3,800 (2,160–6,120)	3,150 (1,780–5,010)	3,060 (1,720–4,830)	30·2 (17·4–48)	28·3 (15·9–44·8)	30·9 (17·5–48·5)
1-115	Autism spectrum disorders	5,830 (3,750–8,570)	5,870 (3,790–8,610)	5,670 (3,710–8,310)	53·9 (34·7–79·2)	54 (34·7–79·8)	54 (35·2–79·2)
1-116	Attention-deficit/hyperactivity disorder	4,160 (2,360–7,040)	3,680 (2,100–6,110)	3,340 (1,900–5,620)	37·5 (21·2–63·6)	37·6 (21·2–63·2)	37·7 (21·3–64·3)
1-117	Conduct disorder	6,990 (3,940–11,200)	5,670 (3,170–8,960)	4,870 (2,740–7,660)	68·3 (38·7–108)	68·5 (38·2–108)	68·6 (38·8–109)
1-118	Idiopathic developmental intellectual disability	2,000 (652–3,750)	2,290 (783–4,240)	1,920 (637–3,520)	18·5 (6·1–34·5)	21·5 (7·5–39·5)	18·9 (6·4–34·4)
1-119	Other mental disorders	12,100 (7,660–18,400)	15,300 (9,730–23,100)	15,700 (9,940–23,700)	109 (68·9–166)	109 (68·9–165)	109 (68·9–166)
1-120	Substance use disorders	35,700 (26,200–46,100)	47,000 (37,200–58,200)	49,900 (39,300–61,400)	305 (227–391)	345 (268–432)	357 (276–444)
1-121	Alcohol use disorders	20,200 (15,000–26,800)	34,900 (28,000–43,900)	38,000 (29,900–47,100)	179 (134–238)	245 (193–313)	254 (196–320)
1-122	Drug use disorders	15,400 (10,500–20,800)	12,100 (8,490–16,000)	11,900 (8,550–15,700)	126 (86·7–168)	100 (69·5–134)	102 (72·7–138)
1-123	Opioid use disorders	9,100 (5,820–13,200)	6,330 (4,200–9,050)	6,200 (4,100–8,470)	75·9 (48·5–109)	50 (32·6–71·2)	50·1 (32·3–70·6)
1-124	Cocaine use disorders	1,840 (1,110–2,780)	1,740 (1,080–2,550)	1,790 (1,170–2,600)	14·6 (8·9–21·8)	14·8 (8·9–22·2)	16 (10·1–23·9)
1-125	Amphetamine use disorders	778 (401–1,330)	672 (368–1,100)	668 (393–1,100)	5·9 (3·2–10·1)	6 (3·1–10·1)	6·3 (3·5–10·6)
1-126	Cannabis use disorders	1,940 (985–3,430)	1,670 (910–2,860)	1,530 (829–2,610)	15 (7·8–26·3)	15·1 (8–26·4)	15·1 (8–26·2)
1-127	Other drug use disorders	1,780 (1,100–2,680)	1,670 (1,070–2,430)	1,690 (1,100–2,480)	14·1 (8·9–21·1)	14·2 (8·9–21)	15 (9·5–22·7)
1-128	Diabetes and kidney diseases	141,000 (120,000–165,000)	192,000 (157,000–232,000)	243,000 (194,000–301,000)	1,350 (1,150–1,580)	1,260 (1,030–1,530)	1,380 (1,110–1,710)
1-129	Diabetes mellitus	108,000 (89,500–131,000)	126,000 (95,300–162,000)	163,000 (122,000–214,000)	1,040 (860–1,260)	816 (617–1,050)	912 (679–1,200)
1-130	Diabetes mellitus type 1	6,350 (4,190–7,270)	3,650 (2,920–4,580)	3,640 (2,790–4,700)	56 (37–64·1)	26·6 (21·4–33·2)	26·3 (20·4–33·8)
1-131	Diabetes mellitus type 2	102,000 (83,200–124,000)	122,000 (92,100–157,000)	160,000 (119,000–210,000)	987 (804–1,200)	790 (594–1,020)	886 (656–1,170)
1-132	Chronic kidney disease	32,300 (29,400–35,800)	66,200 (60,600–72,400)	79,300 (66,100–94,300)	303 (275–335)	447 (409–491)	472 (395–562)
1-133	Chronic kidney disease due to diabetes mellitus type 1	2,230 (1,560–3,050)	4,090 (2,670–5,790)	4,410 (2,690–6,580)	20·8 (14·4–28·8)	27·4 (18·2–38·8)	28·3 (18·2–41·4)
1-134	Chronic kidney disease due to diabetes mellitus type 2	6,120 (4,930–7,340)	14,700 (11,700–17,800)	18,800 (14,300–24,100)	59·4 (47·8–71·4)	94·5 (75·2–114)	101 (76·6–130)
1-135	Chronic kidney disease due to hypertension	5,800 (4,810–7,010)	14,400 (11,900–17,000)	18,000 (14,200–22,300)	55·5 (46·2–67·3)	92·9 (76·8–110)	98·7 (78–123)
1-136	Chronic kidney disease due to glomerulonephritis	7,950 (6,700–9,310)	14,100 (11,500–17,000)	15,900 (12,100–20,700)	72·5 (61–85·1)	99·4 (82·5–119)	104 (80·8–132)
1-137	Chronic kidney disease due to other and unspecified causes	10,200 (8,320–12,300)	18,900 (15,200–23,200)	22,300 (17,300–27,900)	95·1 (77·5–114)	133 (108–162)	141 (110–175)
1-138	Acute glomerulonephritis	171 (127–225)	61 (53–70)	29 (22–36)	1·6 (1·2–2·2)	0·6 (0·5–0·6)	0·3 (0·2–0·3)
1-139	Skin and subcutaneous diseases	55,800 (37,200–83,300)	59,000 (39,400–87,900)	60,400 (41,200–89,500)	525 (351–784)	527 (349–790)	531 (356–790)
1-140	Dermatitis	12,000 (7,090–18,800)	11,900 (7,180–18,400)	11,800 (7,060–17,900)	115 (68·2–182)	115 (68·6–182)	115 (67·7–181)
1-141	Atopic dermatitis	8,570 (4,600–14,400)	7,850 (4,180–13,300)	7,530 (4,100–12,500)	85 (45·7–143)	84·9 (45·1–144)	84·8 (46·3–141)
1-142	Contact dermatitis	3,020 (1,870–4,540)	3,650 (2,270–5,500)	3,800 (2,360–5,740)	27·1 (16·7–40·8)	27·1 (17–40·1)	27·1 (16·7–40·3)
1-143	Seborrhoeic dermatitis	372 (209–587)	425 (244–687)	434 (243–685)	3·4 (1·9–5·4)	3·4 (1·9–5·5)	3·4 (1·9–5·4)
1-144	Psoriasis	3,950 (2,800–5,320)	4,640 (3,280–6,190)	4,570 (3,200–6,090)	36·3 (25·8–49)	35·7 (24·8–47·5)	34·2 (23·8–46)
1-145	Bacterial skin diseases	3,420 (2,200–5,220)	4,490 (3,320–8,360)	5,940 (4,020–11,100)	33·1 (21·3–50·3)	34·3 (24·3–58·1)	39·9 (26·8–66·8)
1-146	Cellulitis	1,540 (860–2,480)	1,660 (1,170–3,710)	2,150 (1,390–4,830)	15 (8·3–23·9)	13 (8·9–24·7)	14·8 (9·3–28·2)
1-147	Pyoderma	1,870 (1,210–3,000)	2,820 (1,980–4,990)	3,800 (2,570–6,790)	18·1 (11·8–28·9)	21·4 (14·5–35·2)	25·1 (16·7–41·8)
1-148	Scabies	9,080 (4,940–14,500)	8,810 (4,890–14,100)	8,470 (4,760–13,600)	83·7 (45·5–133)	83·4 (46–133)	82·9 (45·4–132)
1-149	Fungal skin diseases	4,690 (1,870–10,100)	5,720 (2,310–12,100)	6,150 (2,500–13,000)	43·8 (17·7–92·4)	43·5 (17·5–92·1)	43·4 (17·4–92·1)
1-150	Viral skin diseases	4,740 (3,030–7,110)	4,260 (2,750–6,400)	4,040 (2,610–6,050)	47·6 (30·3–71·2)	47·6 (30·8–71·1)	47·6 (30·7–71·8)
1-151	Acne vulgaris	4,860 (2,870–7,730)	4,400 (2,590–7,160)	4,010 (2,440–6,490)	40·5 (24–64·1)	46·2 (27·4–75·4)	48 (28·7–77·7)
1-152	Alopecia areata	807 (509–1,220)	878 (559–1,310)	856 (536–1,280)	7·1 (4·5–10·7)	7·1 (4·5–10·6)	7·1 (4·4–10·6)
1-153	Pruritus	895 (425–1,590)	1,080 (508–1,940)	1,140 (540–2,020)	8·3 (3·9–14·7)	8·3 (4–14·7)	8·3 (4–14·7)
1-154	Urticaria	5,010 (3,280–7,190)	4,910 (3,220–7,050)	4,750 (3,120–6,830)	48·1 (31·8–69·1)	48 (31·2–70·1)	48 (31·1–69·4)
1-155	Decubitus ulcer	1,160 (735–1,980)	1,470 (1,030–2,720)	1,660 (1,120–2,900)	11·4 (7·4–19·5)	9·1 (6·4–17)	8·4 (5·7–14·5)
1-156	Other skin and subcutaneous diseases	5,250 (2,710–9,290)	6,470 (3,310–11,500)	7,020 (3,530–12,500)	49·4 (25·6–87)	47·9 (24·3–85·9)	48·1 (24·1–86·1)
1-157	Sense organ diseases	90,600 (62,100–128,000)	124,000 (84,900–176,000)	139,000 (94,800–195,000)	870 (597–1,230)	835 (568–1,190)	810 (552–1,140)
1-158	Blindness and vision loss	31,700 (22,100–43,600)	42,000 (29,300–58,300)	46,100 (32,200–64,100)	306 (214–419)	280 (194–391)	266 (184–369)
1-159	Glaucoma	2,140 (1,390–3,110)	2,740 (1,880–3,820)	3,150 (2,100–4,520)	21·2 (13·9–31)	16·9 (11·6–23·6)	15·7 (10·5–22·6)
1-160	Cataract	6,700 (4,720–9,230)	9,200 (6,500–12,400)	10,200 (7,190–13,900)	66·2 (46·8–91·1)	57·1 (40·3–76·9)	52·5 (36·9–71·5)
1-161	Age-related macular degeneration	219 (146–315)	294 (195–422)	368 (241–536)	2·2 (1·4–3·1)	1·8 (1·2–2·6)	1·9 (1·2–2·7)
1-162	Refraction disorders	8,450 (5,610–12,200)	9,840 (6,550–14,000)	9,950 (6,650–14,100)	77·9 (51·6–112)	75·2 (49·8–108)	72·8 (48–104)
1-163	Near vision loss	5,360 (2,460–10,400)	7,950 (3,660–15,500)	9,130 (4,290–18,100)	52·3 (24–102)	50·7 (23·4–100)	49 (22·9–96·6)
1-164	Other vision loss	8,820 (6,180–12,200)	11,900 (8,360–16,300)	13,200 (9,360–18,200)	86 (60·3–118)	78·4 (54·6–107)	74·1 (52·1–102)
1-165	Age-related and other hearing loss	55,100 (36,800–80,100)	77,400 (51,800–112,000)	87,200 (58,100–125,000)	528 (353–764)	518 (346–749)	508 (336–731)
1-166	Other sense organ diseases	3,810 (2,240–5,940)	5,100 (3,010–7,970)	5,690 (3,340–8,910)	36·1 (21·1–56·2)	36·3 (21·3–56·3)	36·5 (21·8–56·3)

1-167	Musculoskeletal disorders	160,000 (116,000–212,000)	220,000 (160,000–290,000)	245,000 (177,000–323,000)	1,470 (1,060–1,960)	1,540 (1,110–2,020)	1,590 (1,150–2,100)
1-168	Rheumatoid arthritis	3,700 (2,710–4,810)	5,630 (4,100–7,370)	6,390 (4,640–8,270)	34·8 (25·5–45)	38·2 (27·6–50·2)	40 (28·8–52·2)
1-169	Osteoarthritis	19,200 (9,520–38,200)	31,400 (15,700–63,600)	38,000 (19,100–76,500)	188 (93·2–374)	200 (99·8–405)	204 (103–408)
1-170	Low back pain	86,400 (61,000–116,000)	108,000 (76,600–144,000)	112,000 (79,700–149,000)	786 (556–1,050)	771 (548–1,030)	753 (540–1,000)
1-171	Neck pain	14,500 (9,640–20,800)	19,700 (12,900–28,700)	20,600 (13,500–30,000)	135 (89·6–194)	135 (88·7–196)	135 (88·8–196)
1-172	Gout	702 (430–1,020)	1,140 (709–1,670)	1,320 (823–1,940)	6·7 (4·1–9·7)	7·5 (4·6–10·9)	7·8 (4·9–11·4)
1-173	Other musculoskeletal disorders	35,400 (25,600–46,900)	54,500 (38,600–72,600)	67,200 (47,000–91,800)	321 (232–426)	385 (275–512)	455 (320–615)
1-174	Other non-communicable diseases	197,000 (161,000–245,000)	180,000 (135,000–238,000)	178,000 (134,000–236,000)	1,920 (1,590–2,360)	1,500 (1,160–1,930)	1,440 (1,110–1,870)
1-175	Congenital anomalies	74,300 (63,900–80,900)	33,100 (28,900–45,000)	26,100 (21,100–35,300)	790 (689–860)	424 (372–608)	366 (291–537)
1-176	Neural tube defects	1,040 (807–1,300)	984 (795–1,210)	1,450 (1,080–1,940)	10·5 (8·1–13·2)	12·8 (10·2–16·3)	22·6 (16·2–31·2)
1-177	Congenital heart anomalies	35,500 (26,700–40,600)	12,200 (9,870–18,200)	8,200 (6,130–12,900)	384 (287–439)	168 (135–260)	129 (93·5–214)
1-178	Orofacial clefts	251 (156–371)	296 (183–471)	312 (190–477)	2·4 (1·5–3·5)	2·7 (1·7–4·2)	2·9 (1·8–4·4)
1-179	Down's syndrome	1,880 (1,530–2,330)	2,050 (1,810–2,500)	2,050 (1,600–2,610)	18·4 (15–23)	19·4 (16·8–23·7)	18·8 (14·4–24·1)
1-180	Turner syndrome	12 (6–22)	11 (5–19)	10 (5–17)	0·1 (0–0·2)	0·1 (0–0·2)	0·1 (0–0·2)
1-181	Klinefelter syndrome	1 (1–2)	1 (1–2)	1 (0–2)	0 (0–0)	0 (0–0)	0 (0–0)
1-182	Other chromosomal abnormalities	1,370 (969–2,220)	1,500 (1,190–1,970)	1,120 (802–1,440)	14·3 (10–23·9)	20·4 (16–27)	17·5 (12–23·7)
1-183	Congenital musculoskeletal and limb anomalies	4,370 (3,030–5,960)	4,080 (2,720–5,750)	3,940 (2,680–5,570)	42·3 (29·5–57·8)	40·6 (27·5–56·8)	40·8 (28–57·7)
1-184	Urogenital congenital anomalies	1,100 (791–1,610)	547 (400–944)	411 (285–763)	11·9 (8·5–17·8)	7·3 (5·3–13·8)	6·2 (4·2–12·9)
1-185	Digestive congenital anomalies	11,900 (8,740–15,500)	3,620 (2,790–5,870)	2,560 (1,730–4,350)	134 (98·2–174)	55·5 (42·7–90·5)	47·6 (31·7–81·8)
1-186	Other congenital anomalies	16,800 (14,200–21,800)	7,800 (6,370–10,900)	6,000 (4,500–8,400)	173 (142–231)	96·2 (76·8–140)	80·8 (58·3–120)
1-187	Urinary diseases and male infertility	6,840 (5,110–9,030)	11,500 (9,100–14,700)	14,300 (10,800–18,000)	64·8 (48·5–85·5)	77·9 (61·2–99·3)	83·9 (63·9–107)
1-188	Urinary tract infections and interstitial nephritis	2,020 (1,650–2,730)	4,590 (3,540–5,140)	6,160 (4,420–7,720)	19·1 (15·6–26·1)	31·5 (24·3–35·5)	36·6 (26·5–45·5)
1-189	Urolithiasis	736 (592–924)	1,250 (1,010–1,560)	1,590 (1,200–2,120)	7 (5·6–8·8)	8·3 (6·7–10·4)	9·4 (7·1–12·3)
1-190	Benign prostatic hyperplasia	2,730 (1,600–4,260)	4,100 (2,390–6,380)	4,800 (2,790–7,400)	26·4 (15·5–41)	26 (15·2–40·6)	25·2 (14·5–38·8)
1-191	Male infertility	517 (193–1,260)	540 (205–1,250)	461 (179–1,120)	4·3 (1·6–10·4)	4·4 (1·7–10·7)	4·4 (1·7–10·8)
1-192	Other urinary diseases	841 (640–1,000)	1,050 (834–1,400)	1,250 (898–1,780)	8 (6·1–9·6)	7·7 (6·1–9·8)	8·3 (6·1–11·2)
1-193	Gynecological diseases	43,500 (30,200–60,800)	49,300 (34,200–69,100)	46,100 (31,700–63,800)	380 (266–530)	368 (253–520)	361 (251–507)
1-194	Uterine fibroids	2,980 (1,590–5,300)	3,970 (2,080–7,130)	3,750 (1,920–6,810)	27·7 (14·7–49·1)	26·7 (13·9–48·2)	26·3 (13·4–48·4)
1-195	Polycystic ovarian syndrome	637 (269–1,320)	784 (326–1,600)	739 (307–1,500)	5·2 (2·2–10·7)	6·5 (2·7–13·2)	6·9 (2·9–14·2)
1-196	Female infertility	938 (323–2,280)	929 (292–2,180)	786 (266–1,860)	7·9 (2·7–19)	7·5 (2·5–17·8)	7·6 (2·6–18)
1-197	Endometriosis	4,200 (2,490–6,810)	3,880 (2,330–6,110)	3,290 (1,930–5,110)	34·2 (20·4–54·9)	31·1 (18·4–49·2)	29·1 (16·9–46·5)
1-198	Genital prolapse	743 (396–1,330)	1,000 (508–1,880)	1,050 (546–1,930)	7·2 (3·8–12·9)	6·5 (3·3–12)	6·1 (3·1–11·2)
1-199	Premenstrual syndrome	12,100 (7,410–19,000)	12,100 (7,430–18,900)	10,500 (6,430–16,500)	101 (62·1–157)	98·4 (59·9–155)	98·3 (60·1–152)
1-200	Other gynecological diseases	21,900 (14,200–31,300)	26,600 (17,000–38,400)	26,000 (16,700–37,200)	196 (127–280)	191 (123–271)	187 (121–268)
1-201	Hemoglobinopathies and hemolytic anaemias	9,330 (7,240–11,900)	7,130 (5,550–9,190)	6,750 (5,140–8,900)	83·1 (64·5–106)	63·2 (48·6–81·7)	60·5 (45·7–80·4)
1-202	Thalassemias	9 (5–18)	8 (4–13)	4 (2–7)	0·1 (0–0·2)	0·1 (0·1–0·2)	0·1 (0–0·1)
1-203	Thalassaemias trait	207 (117–325)	202 (113–330)	187 (105–300)	1·8 (1–2·8)	1·9 (1–3·1)	1·9 (1–3)
1-204	Sickle cell disorders	2,460 (1,820–3,040)	1,750 (1,540–1,990)	1,470 (1,110–1,910)	22·2 (16·5–27·2)	17 (15–19·2)	16·1 (12·5–20·6)
1-205	Sickle cell trait	2,730 (1,680–4,220)	1,900 (1,120–2,920)	1,790 (1,030–2,920)	22·7 (14–35)	18·7 (11–29)	17·5 (9·9–28·3)
1-206	G6PD deficiency	909 (645–1,200)	815 (609–1,020)	872 (621–1,160)	7·9 (5·7–10·4)	6·4 (4·8–8)	7·1 (5–9·3)
1-207	G6PD trait	20 (8–36)	21 (10–38)	20 (10–36)	0·2 (0·1–0·3)	0·1 (0·1–0·2)	0·2 (0·1–0·4)
1-208	Other hemoglobinopathies and hemolytic anaemias	2,990 (2,260–3,970)	2,430 (1,780–3,290)	2,410 (1,780–3,240)	28·1 (21·2–37·5)	18·9 (13·4–26·3)	17·7 (12·7–24·6)
1-209	Endocrine, metabolic, blood, and immune disorders	24,500 (18,600–31,500)	28,600 (21,200–37,600)	31,500 (23,300–42,600)	238 (180–306)	216 (164–280)	220 (166–287)
1-210	Oral disorders	37,300 (22,000–60,300)	49,800 (29,200–79,400)	53,100 (32,000–81,900)	350 (206–559)	346 (202–550)	336 (199–533)
1-211	Caries of deciduous teeth	273 (119–569)	207 (88–435)	195 (83–417)	3·1 (1·4–6·5)	3·1 (1·3–6·6)	3·1 (1·3–6·6)
1-212	Caries of permanent teeth	3,380 (1,470–6,430)	3,990 (1,780–7,790)	3,530 (1,560–6,930)	29·8 (13·3–56·9)	32·7 (14·4–63·7)	29·4 (12·9–58·3)
1-213	Periodontal disease	11,700 (4,520–24,700)	15,400 (5,990–32,800)	16,200 (6,320–34,700)	108 (41·6–231)	106 (40·9–225)	108 (41·7–232)
1-214	Edentulism	16,100 (10,200–24,100)	23,700 (15,000–35,300)	26,600 (17,600–37,000)	156 (99·4–234)	152 (96–225)	143 (94·6–199)
1-215	Other oral disorders	5,890 (3,650–8,620)	6,550 (4,100–9,540)	6,550 (4,110–9,450)	53·2 (32·9–77·7)	53·1 (33·2–77·2)	53·1 (33·1–77·5)
1-216	Sudden infant death syndrome	914 (511–1,370)	429 (314–559)	341 (216–498)	10·1 (5·7–15·2)	6·7 (4·9–8·7)	6·6 (4·2–9·6)
1-0	Injuries	477,000 (452,000–508,000)	321,000 (289,000–361,000)	332,000 (279,000–394,000)	4,190 (3,960–4,470)	2,460 (2,220–2,740)	2,420 (2,040–2,860)
1-1	Transport injuries	159,000 (152,000–168,000)	69,900 (64,100–76,500)	64,300 (53,500–76,700)	1,380 (1,310–1,460)	556 (514–605)	510 (426–607)
1-2	Road injuries	153,000 (146,000–161,000)	63,600 (58,100–69,700)	60,100 (50,100–71,800)	1,330 (1,260–1,400)	506 (466–550)	476 (396–570)

1-3	Pedestrian road injuries	42,400 (35,700–48,400)	15,600 (14,600–16,800)	15,000 (12,400–18,200)	388 (330–440)	124 (116–133)	113 (93·1–136)
1-4	Cyclist road injuries	38,300 (32,600–44,600)	12,900 (10,900–15,200)	11,900 (9,430–14,600)	331 (285–385)	100 (85·7–117)	90·6 (73–111)
1-5	Motorcyclist road injuries	19,400 (17,500–22,400)	11,000 (9,720–12,500)	10,400 (8,650–12,400)	162 (146–186)	86·4 (77·3–97·4)	83·5 (69·7–99)
1-6	Motor vehicle road injuries	49,100 (42,400–53,700)	20,100 (18,700–21,800)	18,700 (14,500–23,300)	414 (358–453)	164 (154–178)	158 (122–197)
1-7	Other road injuries	3,530 (2,930–4,240)	3,960 (3,460–4,490)	4,140 (3,340–5,080)	31·1 (26–36·9)	30·5 (26·8–34·7)	31·7 (25·8–38·4)
1-8	Other transport injuries	6,340 (5,360–7,280)	6,370 (5,770–7,030)	4,180 (3,400–5,040)	55·1 (47·1–63)	50·5 (45·7–55·9)	33·1 (26·8–40)
1-9	Unintentional injuries	157,000 (140,000–178,000)	156,000 (131,000–189,000)	174,000 (141,000–213,000)	1,460 (1,300–1,660)	1,170 (987–1,410)	1,180 (954–1,450)
1-10	Falls	45,600 (39,900–52,700)	72,700 (62,600–85,900)	90,000 (74,300–107,000)	440 (385–508)	478 (411–566)	503 (415–606)
1-11	Drowning	28,100 (26,500–29,600)	14,000 (13,300–14,700)	12,100 (9,970–14,400)	254 (240–269)	133 (126–142)	120 (98·8–142)
1-12	Fire, heat, and hot substances	18,000 (14,700–22,500)	13,600 (9,240–19,600)	14,000 (9,320–20,700)	164 (134–204)	106 (72·4–150)	106 (70·5–155)
1-13	Poisonings	2,770 (2,170–3,290)	1,450 (1,120–1,850)	1,490 (1,110–1,950)	26·6 (20·7–32)	12·6 (9·8–16)	12·8 (9·6–16·7)
1-14	Poisoning by carbon monoxide	312 (248–391)	379 (306–479)	389 (297–506)	2·7 (2·2–3·4)	3·2 (2·6–4)	3·3 (2·5–4·3)
1-15	Poisoning by other means	2,450 (1,890–2,940)	1,070 (777–1,430)	1,100 (778–1,470)	23·9 (18·3–29)	9·5 (7–12·4)	9·5 (6·8–12·8)
1-16	Exposure to mechanical forces	17,800 (14,300–22,800)	19,100 (13,900–26,200)	21,500 (15,200–30,500)	160 (128–205)	148 (109–202)	160 (115–226)
1-17	Unintentional firearm injuries	2,810 (2,000–3,450)	2,380 (1,810–3,060)	2,790 (2,050–3,700)	24 (17·1–29·3)	18·2 (14–23·1)	20·3 (14·9–26·6)
1-18	Other exposure to mechanical forces	15,000 (11,800–19,500)	16,700 (12,100–23,400)	18,700 (13,100–26,800)	136 (107–177)	129 (94·6–180)	140 (98·8–200)
1-19	Adverse effects of medical treatment	4,360 (2,860–4,760)	2,420 (2,160–3,390)	2,490 (2,000–3,400)	41·8 (27·5–45·6)	18·5 (16·4–26)	17·6 (14·1–24·4)
1-20	Animal contact	1,860 (1,450–2,370)	2,980 (2,370–3,790)	3,040 (2,390–3,820)	17·1 (13·3–21·7)	21·9 (17·3–28·2)	20·7 (16–26·6)
1-21	Venomous animal contact	885 (610–1,200)	1,730 (1,320–2,220)	1,790 (1,370–2,270)	8·1 (5·6–11)	12·8 (9·5–16·6)	12·2 (9·1–15·9)
1-22	Non-venomous animal contact	975 (795–1,200)	1,250 (1,000–1,630)	1,260 (964–1,640)	9 (7·4–11·1)	9·2 (7·3–11·9)	8·5 (6·5–11·1)
1-23	Foreign body	14,600 (13,500–15,900)	6,720 (5,950–7,580)	7,010 (5,840–8,270)	147 (136–161)	62·7 (55·7–70·6)	60·7 (50–72·8)
1-24	Pulmonary aspiration and foreign body in airway	12,900 (12,000–14,000)	4,600 (4,260–4,930)	4,840 (3,990–5,790)	132 (122–144)	46·2 (41·5–50·7)	44·6 (36·2–54·3)
1-25	Foreign body in eyes	606 (288–1,080)	684 (344–1,200)	680 (356–1,150)	5·5 (2·6–9·6)	5·4 (2·6–9·5)	5·4 (2·6–9·6)
1-26	Foreign body in other body part	1,060 (838–1,350)	1,430 (1,110–1,840)	1,480 (1,140–1,920)	9·9 (7·8–12·5)	11·1 (8·5–14·3)	10·7 (8·2–14·1)
1-27	Environmental heat and cold exposure	3,720 (2,650–5,170)	4,090 (2,880–5,700)	4,410 (3,110–6,170)	34 (24·3–47·3)	30·7 (21·5–42·9)	31·5 (22·1–44)
1-28	Exposure to forces of nature	227 (205–252)	221 (197–247)	298 (268–331)	2·1 (1·9–2·3)	2 (1·8–2·2)	2·8 (2·5–3·1)
1-29	Other unintentional injuries	19,600 (16,600–23,600)	19,000 (15,400–24,400)	17,300 (13,200–22,600)	175 (148–210)	156 (129–198)	140 (109–181)
1-30	Self-harm and interpersonal violence	162,000 (157,000–166,000)	94,600 (91,700–98,100)	94,100 (77,300–114,000)	1,350 (1,310–1,380)	731 (707–760)	735 (607–887)
1-31	Self-harm	102,000 (99,100–106,000)	59,400 (57,200–61,700)	58,000 (47,000–71,300)	868 (842–894)	435 (419–453)	417 (340–510)
1-32	Self-harm by firearm	4,520 (3,290–5,620)	1,450 (1,010–1,680)	1,190 (833–1,520)	35·2 (26·1–42·8)	11·9 (7·9–13·8)	9·8 (6·4–12·7)
1-33	Self-harm by other specified means	97,700 (94,600–101,000)	57,900 (55,800–60,400)	56,900 (46,100–69,900)	832 (806–860)	423 (407–441)	408 (332–498)
1-34	Interpersonal violence	56,700 (54,200–59,600)	34,900 (33,200–36,900)	35,700 (29,500–42,900)	462 (441–485)	294 (278–309)	315 (262–379)
1-35	Physical violence by firearm	6,240 (5,040–7,640)	1,840 (1,690–2,010)	1,960 (1,590–2,410)	48·8 (39·9–59·5)	16·5 (15·1–18)	18·5 (14·8–22·7)
1-36	Physical violence by sharp object	36,300 (33,200–39,600)	22,900 (21,800–24,200)	23,500 (19,100–28,400)	288 (263–314)	192 (183–203)	212 (173–257)
1-37	Sexual violence	2,100 (1,370–2,990)	2,110 (1,400–2,990)	1,990 (1,330–2,810)	17·7 (11·7–25·1)	17·6 (11·6–25)	17·6 (11·6–25)
1-38	Physical violence by other means	12,100 (10,700–13,600)	8,070 (7,500–8,670)	8,300 (6,880–10,000)	108 (95·6–121)	67 (62·4–71·9)	67·9 (56–81·5)
1-39	Conflict and terrorism	2,340 (2,100–2,630)	261 (174–411)	214 (143–332)	17·9 (16–20·1)	1·7 (1·1–2·7)	1·2 (0·8–1·9)
1-40	Executions and police conflict	309 (180–513)	67 (64–70)	63 (52–76)	2·6 (1·6–4·2)	0·7 (0·7–0·7)	0·7 (0·6–0·8)

SR Table 1: All-age DALYs counts and age standardized DALYs rates per 100,000 individuals for all causes and level 1 cause groups by location for 1990, 2010, and 2019, both sexes combined, Indonesia

Cause Level	Cause	All-age DALYs			Age-standardized rate of DALYs per 100,000		
		1990	2010	2019	1990	2010	2019
0-0	All causes	85,400,000 (79,800,000–91,400,000)	77,200,000 (70,100,000–85,700,000)	77,400,000 (68,400,000–86,900,000)	52,200 (48,600–56,100)	38,300 (35,100–42,100)	34,000 (30,300–37,800)
1-0	Communicable, maternal, neonatal, and nutritional disorders	44,000,000 (40,900,000–47,500,000)	22,800,000 (20,600,000–25,400,000)	16,000,000 (14,100,000–18,000,000)	22,600 (21,000–24,400)	10,600 (9,560–11,700)	7,550 (6,610–8,530)
1-1	HIV/AIDS and sexually transmitted infections	346,000 (123,000–755,000)	714,000 (440,000–1,180,000)	760,000 (536,000–1,150,000)	158 (59·6–337)	301 (179–512)	337 (222–546)
1-2	HIV/AIDS	1,080 (775–1,360)	289,000 (253,000–328,000)	407,000 (362,000–461,000)	0·5 (0·3–0·6)	111 (97·6–126)	154 (137–174)
1-3	HIV/AIDS - drug-susceptible tuberculosis	0 (0–0)	171,000 (141,000–202,000)	225,000 (192,000–259,000)	0 (0–0)	65·8 (54·4–77·9)	84·2 (72·2–96·9)
1-4	HIV/AIDS - Multidrug-resistant Tuberculosis without extensive drug resistance	0 (0–0)	2,810 (283–13,300)	1,820 (182–7,020)	0 (0–0)	1·1 (0·1–5·1)	0·7 (0·1–2·6)
1-5	HIV/AIDS - Extensively drug-resistant Tuberculosis	0 (0–0)	293 (28–1,400)	308 (28–1,240)	0 (0–0)	0·1 (0–0·5)	0·1 (0–0·5)
1-6	HIV/AIDS resulting in other diseases	1,080 (775–1,360)	115,000 (105,000–128,000)	180,000 (161,000–207,000)	0·5 (0·3–0·6)	44·4 (40·7–49·3)	69·1 (61·8–79·2)
1-7	Sexually transmitted infections excluding HIV	345,000 (122,000–754,000)	425,000 (155,000–888,000)	353,000 (134,000–729,000)	157 (59·1–337)	190 (68·5–399)	183 (66–388)
1-8	Syphilis	320,000 (96,600–730,000)	391,000 (119,000–860,000)	317,000 (97,800–698,000)	142 (43·5–322)	176 (53·8–388)	170 (52·4–376)
1-9	Chlamydial infection	3,320 (2,240–4,750)	4,480 (2,960–6,700)	4,760 (3,090–7,110)	2 (1·4–2·8)	1·7 (1·2–2·5)	1·7 (1·1–2·5)
1-10	Gonococcal infection	4,060 (2,820–5,260)	4,450 (3,130–5,980)	4,430 (3,180–6,030)	2·5 (1·6–3·1)	1·9 (1·3–2·4)	1·7 (1·2–2·2)
1-11	Trichomoniasis	5,840 (2,240–12,100)	8,860 (3,460–18,400)	9,790 (3,860–20,200)	3·7 (1·4–7·6)	3·5 (1·4–7·3)	3·5 (1·4–7·1)
1-12	Genital herpes	4,830 (1,610–11,800)	7,660 (2,430–19,000)	8,940 (2,850–22,300)	3·2 (1·1–8)	3·2 (1–8)	3·2 (1–8·1)
1-13	Other sexually transmitted infections	7,140 (4,780–9,500)	7,860 (5,400–10,800)	7,920 (5,490–11,000)	4·1 (2·9–5·4)	3·1 (2·2–4·2)	2·8 (2–3·9)
1-14	Respiratory infections and tuberculosis	12,900,000 (11,400,000–15,200,000)	6,480,000 (5,840,000–7,190,000)	4,550,000 (4,000,000–5,200,000)	7,230 (6,520–8,170)	3,120 (2,850–3,440)	2,050 (1,820–2,320)
1-15	Tuberculosis	5,890,000 (5,210,000–6,550,000)	3,760,000 (3,390,000–4,200,000)	2,660,000 (2,290,000–3,120,000)	3,840 (3,410–4,290)	1,800 (1,640–1,990)	1,110 (957–1,280)
1-16	Latent tuberculosis infection	0 (0–0)	0 (0–0)	0 (0–0)	0 (0–0)	0 (0–0)	0 (0–0)
1-17	Drug-susceptible tuberculosis	5,860,000 (5,180,000–6,530,000)	3,680,000 (3,250,000–4,120,000)	2,640,000 (2,250,000–3,100,000)	3,830 (3,380–4,280)	1,770 (1,570–1,960)	1,100 (939–1,270)
1-18	Multidrug-resistant tuberculosis without extensive drug resistance	23,100 (1,520–110,000)	65,500 (6,420–288,000)	21,600 (2,060–89,500)	15·1 (1–73·2)	31·7 (3·1–139)	9 (0·9–37·4)
1-19	Extensively drug-resistant tuberculosis	0 (0–0)	8,030 (685–37,000)	4,070 (354–17,400)	0 (0–0)	3·9 (0·3–17·9)	1·7 (0·1–7·3)
1-20	Lower respiratory infections	6,750,000 (5,530,000–8,760,000)	2,410,000 (2,070,000–2,810,000)	1,560,000 (1,330,000–1,840,000)	3,250 (2,700–4,100)	1,190 (1,040–1,370)	818 (696–962)
1-21	Upper respiratory infections	198,000 (123,000–305,000)	232,000 (141,000–364,000)	248,000 (151,000–394,000)	101 (63·4–157)	94·9 (58·1–149)	96·6 (58·7–152)
1-22	Otitis media	76,900 (45,000–126,000)	82,000 (47,600–135,000)	79,200 (46,500–131,000)	37·6 (22·1–61·1)	33·4 (19·6–55·1)	31·2 (18·4–51·5)
1-23	Enteric infections	8,780,000 (7,370,000–10,800,000)	3,710,000 (2,990,000–4,660,000)	2,600,000 (1,980,000–3,380,000)	4,810 (3,920–6,050)	1,840 (1,470–2,350)	1,280 (972–1,660)
1-24	Diarrhoeal diseases	7,700,000 (6,350,000–9,530,000)	3,110,000 (2,510,000–4,010,000)	2,140,000 (1,600,000–2,870,000)	4,340 (3,460–5,560)	1,610 (1,250–2,100)	1,100 (816–1,450)
1-25	Typhoid and paratyphoid	1,040,000 (489,000–1,860,000)	563,000 (267,000–995,000)	435,000 (203,000–781,000)	458 (217–812)	225 (107–398)	172 (79·6–309)
1-26	Typhoid fever	1,020,000 (481,000–1,820,000)	553,000 (260,000–981,000)	403,000 (186,000–732,000)	448 (212–795)	221 (104–391)	159 (73·5–286)
1-27	Paratyphoid fever	23,000 (9,250–48,200)	10,200 (4,260–21,100)	31,900 (12,100–65,900)	9·9 (4–20·8)	4·1 (1·7–8·4)	12·7 (4·9–26·1)
1-28	iNTS	36,600 (20,100–62,300)	30,900 (16,900–53,000)	23,300 (12,400–39,700)	17·1 (9·4–28·6)	13·1 (7·3–22·4)	10 (5·3–17·1)
1-29	Other intestinal infectious diseases	6,810 (3,250–11,200)	2,370 (1,070–3,940)	1,540 (784–2,540)	3·1 (1·5–4·9)	1 (0·5–1·7)	0·7 (0·3–1·2)
1-30	Neglected tropical diseases and malaria	2,150,000 (955,000–3,280,000)	1,400,000 (851,000–1,760,000)	934,000 (501,000–1,200,000)	1,050 (508–1,590)	587 (354–739)	400 (199–518)
1-31	Malaria	223,000 (46,500–1,030,000)	348,000 (181,000–576,000)	44,400 (18,900–104,000)	116 (23·1–536)	143 (74·1–240)	17·1 (7·2–40·4)
1-32	Chagas disease	0 (0–0)	0 (0–0)	0 (0–0)	0 (0–0)	0 (0–0)	0 (0–0)
1-33	Leishmaniasis	0 (0–0)	0 (0–0)	0 (0–0)	0 (0–0)	0 (0–0)	0 (0–0)
1-34	Visceral leishmaniasis	0 (0–0)	0 (0–0)	0 (0–0)	0 (0–0)	0 (0–0)	0 (0–0)
1-35	Cutaneous and mucocutaneous leishmaniasis	0 (0–0)	0 (0–0)	0 (0–0)	0 (0–0)	0 (0–0)	0 (0–0)
1-36	African trypanosomiasis	0 (0–0)	0 (0–0)	0 (0–0)	0 (0–0)	0 (0–0)	0 (0–0)
1-37	Schistosomiasis	737 (460–1,270)	815 (500–1,460)	506 (306–840)	0·5 (0·3–0·7)	0·3 (0·2–0·6)	0·2 (0·1–0·3)
1-38	Cysticercosis	663 (398–1,070)	756 (435–1,230)	838 (466–1,380)	0·6 (0·3–1)	0·4 (0·2–0·7)	0·4 (0·2–0·6)
1-39	Cystic echinococcosis	169 (36–538)	115 (26–337)	1,650 (1,040–2,370)	0·1 (0–0·3)	0 (0–0·1)	0·7 (0·4–1)
1-40	Lymphatic filariasis	317,000 (206,000–474,000)	161,000 (97,400–259,000)	132,000 (74,600–223,000)	194 (127–286)	67·1 (40·8–107)	49·4 (28·1–83·3)
1-41	Onchocerciasis	0 (0–0)	0 (0–0)	0 (0–0)	0 (0–0)	0 (0–0)	0 (0–0)
1-42	Trachoma	0 (0–0)	0 (0–0)	0 (0–0)	0 (0–0)	0 (0–0)	0 (0–0)
1-43	Dengue	1,320,000 (227,000–2,220,000)	764,000 (224,000–976,000)	647,000 (205,000–878,000)	599 (109–996)	326 (93·9–419)	290 (85·9–397)
1-44	Yellow fever	0 (0–0)	0 (0–0)	0 (0–0)	0 (0–0)	0 (0–0)	0 (0–0)
1-45	Rabies	32,500 (1,380–49,500)	9,570 (561–14,100)	6,600 (462–9,880)	16·1 (0·7–24)	4·1 (0·2–5·9)	2·8 (0·2–4·1)
1-46	Intestinal nematode infections	180,000 (108,000–282,000)	59,800 (34,300–97,200)	56,200 (32,000–93,900)	87·6 (52·3–138)	23·9 (13·7–38·9)	21·6 (12·3–36·2)
1-47	Ascariasis	106,000 (63,700–173,000)	23,400 (12,500–41,900)	20,500 (10,600–37,400)	51·7 (30·9–84·8)	9·6 (5·2–17·1)	8·2 (4·3–14·8)
1-48	Trichuriasis	20,900 (10,100–36,800)	7,420 (3,400–14,300)	8,700 (4,050–15,700)	10·1 (4·9–17·7)	2·9 (1·3–5·6)	3·3 (1·5–5·9)

1-49	Hookworm disease	52,600 (28,500–84,500)	28,900 (16,200–48,700)	27,100 (14,600–45,700)	25·8 (14·1–41·5)	11·4 (6·4–19·2)	10·2 (5·5–17·3)
1-50	Food-borne trematodiases	0 (0–0)	0 (0–0)	0 (0–0)	0 (0–0)	0 (0–0)	0 (0–0)
1-51	Leprosy	975 (619–1,420)	1,280 (810–1,910)	1,170 (741–1,700)	0·8 (0·5–1·1)	0·6 (0·4–0·9)	0·5 (0·3–0·7)
1-52	Ebola virus disease	0 (0–0)	0 (0–0)	0 (0–0)	0 (0–0)	0 (0–0)	0 (0–0)
1-53	Zika virus disease	0 (0–0)	0 (0–0)	0 (0–0)	0 (0–0)	0 (0–0)	0 (0–0)
1-54	Guinea worm disease	0 (0–0)	0 (0–0)	0 (0–0)	0 (0–0)	0 (0–0)	0 (0–0)
1-55	Other neglected tropical diseases	77,100 (50,600–115,000)	50,900 (34,100–73,800)	44,200 (29,400–64,600)	37·7 (25·1–55·5)	21·7 (14·7–31·3)	18 (12·1–26·1)
1-56	Other infectious diseases	6,790,000 (5,010,000–9,300,000)	2,260,000 (1,700,000–3,180,000)	1,330,000 (976,000–1,910,000)	3,200 (2,400–4,300)	984 (746–1,380)	610 (434–894)
1-57	Meningitis	1,610,000 (1,290,000–2,060,000)	661,000 (540,000–814,000)	327,000 (264,000–400,000)	720 (583–919)	286 (233–353)	153 (123–191)
1-58	Encephalitis	258,000 (73,400–379,000)	184,000 (73,800–242,000)	148,000 (66,100–198,000)	118 (38–170)	79·1 (31·9–105)	68·1 (28–93·9)
1-59	Diphtheria	33,100 (23,400–49,000)	3,830 (2,990–4,830)	1,630 (1,270–2,070)	14·6 (10·4–21·5)	1·6 (1·2–2)	0·7 (0·6–0·9)
1-60	Whooping cough	941,000 (104,000–2,720,000)	485,000 (57,300–1,410,000)	308,000 (38,300–862,000)	413 (45·7–1,190)	209 (24·8–606)	155 (19·4–430)
1-61	Tetanus	1,310,000 (927,000–1,830,000)	261,000 (171,000–329,000)	127,000 (68,700–165,000)	674 (500–923)	119 (78·3–150)	55·3 (32·4–71·3)
1-62	Measles	1,840,000 (619,000–4,020,000)	250,000 (82,300–549,000)	61,600 (19,400–144,000)	800 (270–1,750)	107 (35·1–234)	30·2 (9·5–70·4)
1-63	Varicella and herpes zoster	46,000 (36,700–58,100)	33,900 (28,500–40,900)	27,600 (22,600–34,100)	25·2 (20·9–30·5)	16·2 (13·8–19·2)	13·1 (10·8–15·9)
1-64	Acute hepatitis	569,000 (320,000–916,000)	250,000 (221,000–286,000)	222,000 (185,000–266,000)	337 (187–557)	110 (97·6–125)	84·8 (71·1–101)
1-65	Acute hepatitis A	329,000 (169,000–690,000)	100,000 (50,100–175,000)	75,000 (34,400–141,000)	187 (92·7–411)	44 (21·6–77·9)	29·3 (13·4–54·3)
1-66	Acute hepatitis B	153,000 (63,100–296,000)	113,000 (56,200–163,000)	114,000 (54,100–157,000)	97·8 (41·4–181)	50·4 (24·7–72·4)	43·4 (20·6–59·4)
1-67	Acute hepatitis C	68,000 (30,400–138,000)	25,700 (15,000–52,000)	22,200 (13,000–43,400)	41·7 (18·6–87·4)	11·2 (6·6–22·2)	8·3 (4·9–16)
1-68	Acute hepatitis E	18,600 (9,550–34,500)	10,600 (5,860–15,700)	10,100 (5,280–14,900)	10·1 (5–18·9)	4·4 (2·4–6·5)	3·8 (2–5·6)
1-69	Other unspecified infectious diseases	185,000 (93,800–260,000)	128,000 (82,100–160,000)	112,000 (77,300–143,000)	95·1 (51·6–128)	56·6 (35–71)	49·8 (30·6–65·4)
1-70	Maternal and neonatal disorders	10,200,000 (8,960,000–11,800,000)	6,560,000 (5,440,000–7,840,000)	4,190,000 (3,430,000–5,080,000)	4,600 (4,020–5,290)	2,900 (2,410–3,480)	2,130 (1,710–2,600)
1-71	Maternal disorders	1,110,000 (927,000–1,320,000)	562,000 (464,000–655,000)	327,000 (267,000–421,000)	584 (489–692)	208 (172–243)	115 (94·1–149)
1-72	Maternal haemorrhage	478,000 (384,000–592,000)	238,000 (190,000–290,000)	132,000 (104,000–173,000)	252 (204–312)	88·1 (70·5–108)	46·4 (36·8–60·6)
1-73	Maternal sepsis and other pregnancy related infections	72,600 (55,000–96,600)	25,400 (19,500–32,500)	11,700 (8,830–15,600)	37·1 (28·2–48·6)	9·4 (7·2–12)	4·2 (3·1–5·6)
1-74	Maternal hypertensive disorders	207,000 (168,000–253,000)	145,000 (117,000–173,000)	89,500 (70,400–115,000)	105 (85·2–128)	54 (43·5–64·5)	31·7 (24·9–40·9)
1-75	Maternal obstructed labour and uterine rupture	6,120 (4,610–8,020)	8,330 (6,160–11,100)	4,820 (3,470–6,700)	3·2 (2·4–4·3)	3·1 (2·3–4·1)	1·7 (1·2–2·3)
1-76	Maternal abortive outcome	227,000 (169,000–297,000)	33,100 (24,100–44,900)	13,900 (9,880–19,600)	123 (92·4–159)	12·3 (8·9–16·6)	4·9 (3·5–6·9)
1-77	Ectopic pregnancy	10,500 (8,230–13,400)	7,410 (5,720–9,340)	4,380 (3,320–5,820)	5·7 (4·4–7·3)	2·8 (2·1–3·5)	1·5 (1·2–2)
1-78	Indirect maternal deaths	11,600 (8,940–15,000)	14,600 (11,100–18,900)	11,900 (8,640–16,300)	6·1 (4·7–7·8)	5·4 (4·1–7)	4·2 (3–5·7)
1-79	Late maternal deaths	13,800 (10,000–18,900)	9,780 (7,250–13,200)	7,000 (5,060–10,100)	7·2 (5·2–9·7)	3·6 (2·7–4·9)	2·5 (1·8–3·6)
1-80	Maternal deaths aggravated by HIV/AIDS	6 (0–13)	166 (86–278)	173 (87–294)	0 (0–0)	0·1 (0–0·1)	0·1 (0–0·1)
1-81	Other maternal disorders	87,100 (65,600–115,000)	79,700 (60,600–101,000)	51,100 (38,700–66,700)	45·3 (34·3–59·3)	29·4 (22·4–37·4)	18 (13·6–23·5)
1-82	Neonatal disorders	9,130,000 (7,820,000–10,600,000)	6,000,000 (4,930,000–7,300,000)	3,870,000 (3,110,000–4,700,000)	4,010 (3,440–4,660)	2,700 (2,210–3,280)	2,020 (1,610–2,470)
1-83	Neonatal preterm birth	4,460,000 (3,730,000–5,230,000)	2,840,000 (2,260,000–3,540,000)	1,780,000 (1,420,000–2,240,000)	1,960 (1,640–2,290)	1,270 (1,010–1,590)	932 (733–1,180)
1-84	Neonatal encephalopathy due to birth asphyxia and trauma	2,840,000 (2,040,000–3,500,000)	1,850,000 (1,290,000–2,370,000)	1,060,000 (753,000–1,410,000)	1,240 (894–1,530)	836 (580–1,070)	570 (398–759)
1-85	Neonatal sepsis and other neonatal infections	1,030,000 (727,000–1,340,000)	810,000 (627,000–1,030,000)	687,000 (536,000–860,000)	455 (321–590)	361 (280–456)	338 (263–424)
1-86	Hemolytic disease and other neonatal jaundice	106,000 (83,000–131,000)	61,400 (49,900–73,700)	43,300 (35,400–51,400)	47 (36·9–58·1)	26·8 (21·8–32·4)	20·1 (16·3–24·2)
1-87	Other neonatal disorders	699,000 (448,000–978,000)	444,000 (297,000–579,000)	288,000 (185,000–387,000)	307 (197–430)	200 (134–261)	155 (99·6–209)
1-88	Nutritional deficiencies	2,750,000 (2,150,000–3,570,000)	1,680,000 (1,290,000–2,170,000)	1,620,000 (1,240,000–2,100,000)	1,560 (1,240–1,980)	826 (660–1,040)	731 (577–922)
1-89	Protein-energy malnutrition	1,480,000 (1,120,000–1,930,000)	731,000 (622,000–854,000)	691,000 (568,000–822,000)	852 (659–1,070)	402 (355–456)	356 (300–415)
1-90	Iodine deficiency	37,900 (25,100–55,000)	10,700 (4,880–21,000)	10,700 (5,340–20,100)	22·1 (14·8–32)	4·3 (2–8·5)	3·9 (1·9–7·2)
1-91	Vitamin A deficiency	123,000 (81,000–182,000)	57,300 (36,900–84,600)	42,500 (27,000–62,800)	54·4 (36·1–80·1)	23·7 (15·3–34·8)	17·6 (11·2–25·7)
1-92	Dietary iron deficiency	993,000 (656,000–1,490,000)	779,000 (503,000–1,150,000)	747,000 (479,000–1,110,000)	555 (367–820)	346 (225–508)	300 (193–445)
1-93	Other nutritional deficiencies	117,000 (87,600–152,000)	100,000 (76,300–128,000)	126,000 (92,100–166,000)	75·2 (57·2–95·3)	49·8 (39·8–62·4)	54·4 (41–69·5)
1-0	Non-communicable diseases	34,000,000 (29,600,000–38,400,000)	48,700,000 (43,000,000–55,100,000)	56,000,000 (48,600,000–63,500,000)	25,600 (22,900–28,500)	25,300 (22,600–28,100)	24,300 (21,300–27,300)
1-1	Neoplasms	3,410,000 (2,830,000–3,870,000)	5,540,000 (4,810,000–6,300,000)	6,750,000 (5,630,000–7,980,000)	2,620 (2,210–2,920)	2,830 (2,470–3,200)	2,830 (2,350–3,310)
1-2	Lip and oral cavity cancer	51,800 (43,500–60,900)	90,300 (72,500–114,000)	113,000 (83,200–153,000)	45·5 (38·1–52·9)	49·2 (39·6–62)	48·8 (36·1–65·9)
1-3	Nasopharynx cancer	60,100 (48,600–70,800)	89,200 (73,300–108,000)	101,000 (78,100–131,000)	46·1 (37·9–54·4)	43·5 (36–52·4)	39·6 (31–51)
1-4	Other pharynx cancer	15,800 (12,200–22,300)	25,000 (18,700–38,700)	32,900 (22,800–56,700)	13·8 (10·8–19·4)	13·4 (10·2–20·6)	13·7 (9·6–23·5)
1-5	Oesophageal cancer	71,000 (59,600–85,000)	109,000 (92,000–150,000)	133,000 (106,000–190,000)	64·3 (54·9–77·6)	59·9 (50·7–82·8)	57·3 (45·9–81·3)
1-6	Stomach cancer	247,000 (199,000–281,000)	310,000 (260,000–353,000)	333,000 (274,000–391,000)	220 (179–251)	170 (144–192)	147 (122–170)
1-7	Colon and rectum cancer	244,000 (184,000–292,000)	591,000 (431,000–725,000)	807,000 (556,000–1,040,000)	217 (166–258)	321 (232–391)	350 (240–449)

1-8	Liver cancer	85,200 (71,700–99,900)	106,000 (95,800–118,000)	126,000 (106,000–146,000)	68·2 (58·3–77·2)	58·5 (52·9–64·5)	56·5 (47·9–64·7)
1-9	Liver cancer due to hepatitis B	21,500 (17,700–25,500)	26,200 (21,900–31,400)	30,900 (23,900–38,500)	15·7 (13–18·9)	12·7 (10·6–15·2)	12·3 (9·5–15·3)
1-10	Liver cancer due to hepatitis C	26,500 (22,100–31,800)	39,100 (33,800–44,700)	47,800 (38,900–56,900)	26·9 (22·5–32·4)	24·3 (21·3–27·4)	23·1 (19–27)
1-11	Liver cancer due to alcohol use	11,500 (9,130–14,200)	17,300 (14,000–21,400)	22,200 (16,900–28,000)	10·7 (8·5–13·4)	9·8 (7·9–12·1)	9·8 (7·5–12·3)
1-12	Liver cancer due to NASH	6,490 (5,210–7,830)	9,380 (7,800–11,100)	11,800 (9,370–14,500)	5·5 (4·4–6·8)	5·3 (4·4–6·2)	5·3 (4·3–6·4)
1-13	Gallbladder and biliary tract cancer	26,900 (18,200–41,100)	40,000 (29,200–70,000)	48,500 (36,700–83,000)	25·7 (17·5–39·4)	23·2 (17·1–40·9)	22 (16·6–38·2)
1-14	Pancreatic cancer	61,000 (50,200–71,700)	176,000 (129,000–231,000)	271,000 (180,000–381,000)	56·3 (46·4–65·8)	98 (71·3–129)	119 (78·4–166)
1-15	Larynx cancer	30,500 (25,500–36,300)	45,800 (37,200–58,600)	55,400 (42,500–74,300)	27·9 (23·3–33·5)	25·7 (21–32·7)	24·2 (18·6–32·3)
1-16	Tracheal, bronchus, and lung cancer	472,000 (401,000–538,000)	950,000 (768,000–1,120,000)	1,280,000 (926,000–1,600,000)	437 (372–496)	531 (432–622)	555 (403–685)
1-17	Malignant skin melanoma	7,810 (5,540–12,800)	12,200 (9,330–17,200)	14,100 (10,900–20,100)	6 (4·4–9·5)	6·2 (4·8–8·7)	5·9 (4·6–8·3)
1-18	Non-melanoma skin cancer	14,100 (10,800–17,200)	30,600 (23,600–36,500)	39,400 (30,100–48,100)	12·9 (9·8–15·5)	17·4 (13·5–20·4)	17·9 (13·7–21·6)
1-19	Non-melanoma skin cancer (squamous-cell carcinoma)	14,100 (10,800–17,200)	30,600 (23,600–36,500)	39,400 (30,100–48,100)	12·9 (9·8–15·5)	17·4 (13·5–20·4)	17·9 (13·7–21·6)
1-20	Non-melanoma skin cancer (basal-cell carcinoma)	1 (0–1)	1 (1–2)	2 (1–3)	0 (0–0)	0 (0–0)	0 (0–0)
1-21	Breast cancer	434,000 (369,000–519,000)	836,000 (694,000–992,000)	981,000 (763,000–1,260,000)	318 (274–378)	369 (309–437)	358 (279–457)
1-22	Cervical cancer	210,000 (126,000–311,000)	283,000 (184,000–428,000)	292,000 (190,000–466,000)	161 (98·2–243)	131 (85·9–202)	110 (72·8–180)
1-23	Uterine cancer	43,400 (27,200–54,900)	80,400 (47,200–103,000)	96,100 (54,500–129,000)	37·8 (24·7–47·2)	41·8 (26·2–52·8)	39·2 (23·4–51·8)
1-24	Ovarian cancer	65,700 (47,200–125,000)	147,000 (99,900–267,000)	203,000 (127,000–381,000)	51 (37·7–95·4)	70·1 (48·4–128)	78·1 (49·3–147)
1-25	Prostate cancer	62,300 (48,400–75,400)	152,000 (110,000–183,000)	215,000 (149,000–269,000)	77·4 (60·2–93·9)	110 (80·9–131)	120 (83·8–149)
1-26	Testicular cancer	6,080 (4,660–7,780)	10,900 (8,940–13,400)	12,600 (9,690–17,000)	3·4 (2·6–4·2)	4·5 (3·7–5·5)	4·8 (3·7–6·4)
1-27	Kidney cancer	30,800 (23,300–44,000)	64,100 (49,500–89,200)	88,700 (63,200–128,000)	21·5 (17·3–28·6)	32·7 (25·4–44·8)	37·5 (27–54·1)
1-28	Bladder cancer	30,200 (24,400–36,400)	53,300 (43,000–69,600)	70,900 (52,900–101,000)	31·5 (25·5–37·8)	33·2 (27·1–43)	34·5 (26–48·9)
1-29	Brain and nervous system cancer	142,000 (91,500–228,000)	186,000 (133,000–227,000)	202,000 (137,000–263,000)	81·2 (56·4–123)	82·4 (58·7–100)	79·7 (55·1–103)
1-30	Thyroid cancer	25,500 (18,300–30,200)	45,900 (32,900–53,700)	55,300 (40,100–67,300)	21·9 (16·1–25·6)	24·9 (18–28·7)	23·8 (17·5–28·5)
1-31	Mesothelioma	4,260 (2,590–6,470)	9,040 (6,230–11,700)	10,400 (7,500–13,600)	3 (1·9–4·4)	4·2 (3–5·3)	4 (2·9–5·2)
1-32	Hodgkin lymphoma	21,900 (16,600–32,700)	21,200 (13,700–38,500)	20,400 (12,800–38,400)	13·2 (9·9–19·8)	9·3 (6·1–16·6)	7·9 (5–14·5)
1-33	Non-Hodgkin's lymphoma	105,000 (75,300–131,000)	159,000 (128,000–193,000)	193,000 (150,000–245,000)	66·9 (51·7–81·3)	76·1 (62·2–91·5)	78·7 (61·6–98·9)
1-34	Multiple myeloma	16,000 (13,000–24,000)	29,500 (24,100–41,500)	38,400 (29,000–54,300)	14·9 (12·1–22·1)	16·5 (13·5–23·3)	16·6 (12·6–23·5)
1-35	Leukaemia	489,000 (316,000–683,000)	448,000 (358,000–547,000)	438,000 (352,000–539,000)	259 (177–344)	200 (162–242)	180 (145–221)
1-36	Acute lymphoid leukaemia	94,300 (53,000–185,000)	85,700 (63,400–126,000)	80,200 (61,500–110,000)	42·9 (25–83·1)	35·7 (26·5–52·2)	33·2 (25·6–45·4)
1-37	Chronic lymphoid leukaemia	2,870 (2,210–3,990)	7,040 (5,480–9,660)	9,870 (7,270–14,000)	2·7 (2·1–3·5)	4·1 (3·2–5·4)	4·5 (3·4–6·3)
1-38	Acute myeloid leukaemia	54,100 (34,800–110,000)	77,200 (55,200–128,000)	88,500 (57,600–148,000)	29·8 (20·3–58·3)	33·7 (24·3–55·7)	35·2 (23·1–58·5)
1-39	Chronic myeloid leukaemia	17,100 (9,160–24,800)	18,200 (11,900–25,700)	19,100 (13,000–27,800)	10·7 (6·4–14·8)	8·5 (5·8–11·9)	7·8 (5·4–11·1)
1-40	Other leukaemia	321,000 (166,000–467,000)	260,000 (195,000–324,000)	240,000 (181,000–296,000)	173 (100–240)	118 (90·1–146)	99·6 (76·4–122)
1-41	Other neoplasms	5,970 (4,850–8,450)	11,700 (9,110–16,900)	16,400 (11,200–25,400)	5·1 (4·2–7)	6·7 (5·2–9·7)	7·6 (5·2–11·8)
1-42	Myelodysplastic, myeloproliferative, and other hematopoietic neoplasms	5,970 (4,850–8,450)	11,700 (9,110–16,900)	16,400 (11,200–25,400)	5·1 (4·2–7)	6·7 (5·2–9·7)	7·6 (5·2–11·8)
1-43	Benign and in situ intestinal neoplasms	0 (0–0)	0 (0–0)	0 (0–0)	0 (0–0)	0 (0–0)	0 (0–0)
1-44	Benign and in situ cervical and uterine neoplasms	0 (0–0)	0 (0–0)	0 (0–0)	0 (0–0)	0 (0–0)	0 (0–0)
1-45	Other benign and in situ neoplasms	0 (0–0)	0 (0–0)	0 (0–0)	0 (0–0)	0 (0–0)	0 (0–0)
1-46	Cardiovascular diseases	8,030,000 (7,310,000–8,740,000)	13,800,000 (12,400,000–15,100,000)	16,500,000 (14,000,000–18,700,000)	7,630 (6,900–8,320)	8,140 (7,440–8,810)	7,760 (6,650–8,590)
1-47	Rheumatic heart disease	124,000 (102,000–145,000)	83,500 (73,700–94,900)	65,000 (55,100–76,500)	79 (66·9–92)	37·7 (33·7–42·3)	26 (22·2–30·5)
1-48	Ischaemic heart disease	2,690,000 (2,370,000–3,010,000)	5,040,000 (4,510,000–5,590,000)	6,130,000 (5,120,000–7,090,000)	2,550 (2,260–2,850)	2,900 (2,630–3,180)	2,800 (2,380–3,180)
1-49	Stroke	4,200,000 (3,800,000–4,580,000)	7,040,000 (6,410,000–7,730,000)	8,390,000 (7,140,000–9,440,000)	4,020 (3,630–4,410)	4,220 (3,860–4,570)	4,000 (3,440–4,410)
1-50	Ischaemic stroke	1,060,000 (879,000–1,250,000)	2,230,000 (1,810,000–2,570,000)	2,950,000 (2,290,000–3,470,000)	1,260 (1,050–1,480)	1,590 (1,310–1,810)	1,650 (1,320–1,910)
1-51	Intracerebral hemorrhage	2,810,000 (2,510,000–3,160,000)	4,380,000 (3,850,000–5,010,000)	4,970,000 (4,110,000–5,850,000)	2,510 (2,220–2,840)	2,420 (2,130–2,790)	2,160 (1,780–2,550)
1-52	Subarachnoid hemorrhage	330,000 (222,000–492,000)	424,000 (304,000–619,000)	471,000 (300,000–671,000)	253 (176–390)	206 (148–308)	187 (119–266)
1-53	Hypertensive heart disease	594,000 (366,000–745,000)	979,000 (584,000–1,180,000)	1,160,000 (649,000–1,430,000)	621 (396–774)	615 (379–733)	574 (333–693)
1-54	Non-rheumatic valvular heart disease	12,800 (10,200–16,700)	19,300 (16,300–26,500)	23,700 (18,300–33,100)	11·5 (9·5–14·7)	11 (9·3–14·7)	10·9 (8·5–15)
1-55	Non-rheumatic calcific aortic valve disease	5,930 (4,620–8,430)	10,400 (7,800–14,200)	13,300 (9,000–18,800)	5·5 (4·3–7·7)	5·9 (4·4–8)	6·1 (4·1–8·5)
1-56	Non-rheumatic degenerative mitral valve disease	5,300 (3,560–8,120)	7,020 (5,010–11,300)	8,260 (5,840–14,000)	4·8 (3·5–7·1)	4·1 (3–6·4)	3·9 (2·8–6·5)
1-57	Other non-rheumatic valve diseases	1,580 (458–2,850)	1,970 (525–3,160)	2,080 (519–3,570)	1·2 (0·3–2)	1 (0·2–1·6)	0·9 (0·2–1·5)
1-58	Cardiomyopathy and myocarditis	121,000 (84,200–226,000)	174,000 (132,000–256,000)	201,000 (142,000–299,000)	93·9 (72–147)	96·5 (75·1–134)	93·7 (68·2–130)
1-59	Myocarditis	22,100 (11,600–55,100)	22,800 (16,100–37,000)	22,300 (15,400–36,300)	12·9 (8–27·1)	11·4 (8·5–16·8)	10·3 (7·5–15·2)
1-60	Alcoholic cardiomyopathy	6,250 (3,650–10,000)	9,570 (6,050–15,800)	10,900 (6,070–20,600)	4·8 (2·8–7·6)	4·4 (2·8–7·3)	4·2 (2·3–7·7)

1-61	Other cardiomyopathy	92,500 (66,600–169,000)	142,000 (104,000–210,000)	168,000 (113,000–249,000)	76·2 (58·9–121)	80·6 (60·3–112)	79·2 (56·1–111)
1-62	Atrial fibrillation and flutter	74,600 (54,800–98,600)	144,000 (108,000–186,000)	193,000 (143,000–249,000)	96·4 (72·5–127)	109 (83·5–140)	113 (86·4–144)
1-63	Aortic aneurysm	21,500 (17,200–28,900)	45,400 (35,500–60,400)	61,000 (43,000–86,000)	22 (17·7–28·9)	27·9 (22·1–37)	29·9 (21·5–42)
1-64	Peripheral vascular disease	8,530 (4,860–13,800)	17,400 (10,300–27,600)	23,100 (13,700–36,300)	11 (6·2–18·1)	12·8 (7·5–20·4)	13 (7·8–20·5)
1-65	Endocarditis	45,900 (35,000–67,000)	63,500 (46,200–91,700)	67,300 (46,000–100,000)	28·9 (21·2–42)	28·6 (20·7–41·4)	26·7 (18·4–39·2)
1-66	Chronic respiratory diseases	2,340,000 (2,060,000–2,570,000)	3,000,000 (2,680,000–3,310,000)	3,180,000 (2,760,000–3,570,000)	2,060 (1,810–2,280)	1,750 (1,590–1,910)	1,510 (1,320–1,690)
1-67	Chronic obstructive pulmonary disease	1,140,000 (990,000–1,270,000)	1,770,000 (1,560,000–1,940,000)	1,980,000 (1,690,000–2,260,000)	1,210 (1,050–1,350)	1,130 (1,010–1,230)	996 (851–1,120)
1-68	Pneumoconiosis	2,010 (1,500–2,710)	3,070 (2,300–4,050)	4,090 (2,940–5,500)	1·8 (1·4–2·4)	1·7 (1·3–2·1)	1·7 (1·3–2·3)
1-69	Silicosis	562 (365–816)	1,120 (732–1,650)	1,690 (1,110–2,500)	0·5 (0·3–0·8)	0·6 (0·4–0·9)	0·7 (0·5–1)
1-70	Asbestosis	178 (106–250)	306 (197–420)	343 (211–492)	0·2 (0·1–0·2)	0·2 (0·1–0·2)	0·2 (0·1–0·2)
1-71	Coal workers pneumoconiosis	97 (40–187)	142 (56–302)	143 (57–312)	0·1 (0–0·2)	0·1 (0–0·2)	0·1 (0–0·1)
1-72	Other pneumoconiosis	1,170 (788–1,770)	1,510 (1,040–2,190)	1,910 (1,290–2,820)	1 (0·7–1·5)	0·8 (0·6–1·1)	0·8 (0·6–1·2)
1-73	Asthma	1,120,000 (914,000–1,290,000)	1,090,000 (900,000–1,320,000)	1,040,000 (852,000–1,260,000)	798 (661–955)	560 (467–669)	455 (373–548)
1-74	Interstitial lung disease and pulmonary sarcoidosis	18,400 (10,400–35,700)	34,800 (18,700–69,300)	42,900 (21,700–88,300)	17·7 (9·6–35·1)	20·1 (10·4–41)	19·5 (9·6–40·4)
1-75	Other chronic respiratory diseases	55,300 (36,600–113,000)	90,400 (70,600–129,000)	101,000 (80,100–140,000)	30·9 (21–59·2)	37·1 (29·1–53·1)	37·6 (29·7–52)
1-76	Digestive diseases	3,540,000 (2,930,000–4,110,000)	3,990,000 (3,650,000–4,430,000)	4,030,000 (3,470,000–4,640,000)	2,510 (2,180–2,820)	2,010 (1,840–2,210)	1,730 (1,510–1,960)
1-77	Cirrhosis and other chronic liver diseases	2,210,000 (1,850,000–2,550,000)	2,710,000 (2,420,000–3,060,000)	2,700,000 (2,250,000–3,220,000)	1,610 (1,390–1,820)	1,330 (1,190–1,490)	1,110 (936–1,300)
1-78	Cirrhosis and other chronic liver diseases due to hepatitis B	524,000 (436,000–624,000)	577,000 (481,000–700,000)	581,000 (462,000–731,000)	398 (336–471)	276 (232–332)	231 (186–287)
1-79	Cirrhosis and other chronic liver diseases due to hepatitis C	647,000 (540,000–756,000)	912,000 (775,000–1,080,000)	913,000 (732,000–1,120,000)	497 (419–582)	444 (380–517)	370 (300–447)
1-80	Cirrhosis and other chronic liver diseases due to alcohol use	265,000 (213,000–332,000)	390,000 (317,000–477,000)	409,000 (319,000–525,000)	221 (177–275)	199 (163–241)	167 (131–213)
1-81	Cirrhosis and other chronic liver diseases due to NAFLD	155,000 (109,000–211,000)	245,000 (174,000–330,000)	258,000 (178,000–358,000)	130 (93·1–177)	132 (94·5–176)	115 (81·4–155)
1-82	Cirrhosis and other chronic liver diseases due to other causes	619,000 (436,000–808,000)	585,000 (478,000–707,000)	541,000 (424,000–674,000)	360 (263–456)	277 (226–335)	231 (183–288)
1-83	Upper digestive system diseases	176,000 (132,000–238,000)	199,000 (137,000–294,000)	218,000 (147,000–328,000)	129 (99·2–169)	97·4 (70·7–138)	89·2 (62·4–130)
1-84	Peptic ulcer disease	71,000 (60,200–87,600)	51,300 (45,000–57,700)	44,300 (37,300–50,900)	60·6 (51·9–70·5)	32·9 (28·8–36·5)	23·8 (19·5–27·3)
1-85	Gastritis and duodenitis	42,000 (27,800–61,200)	50,000 (31,900–74,600)	59,800 (37,500–89,700)	25·5 (17·1–36·7)	21·8 (14·1–32·2)	22·7 (14·4–33·7)
1-86	GERD	62,800 (32,000–112,000)	97,900 (49,800–174,000)	114,000 (57,800–204,000)	42·6 (21·6–76·2)	42·7 (21·6–76·4)	42·7 (21·6–76·5)
1-87	Appendicitis	97,000 (50,500–171,000)	59,900 (42,800–80,100)	49,300 (33,500–61,800)	51 (29·6–83·5)	26·7 (19·4–34·7)	20·5 (14–25·4)
1-88	Paralytic ileus and intestinal obstruction	465,000 (295,000–676,000)	412,000 (350,000–474,000)	397,000 (328,000–477,000)	280 (195–382)	217 (192–262)	196 (165–237)
1-89	Inguinal, femoral, and abdominal hernia	239,000 (149,000–396,000)	203,000 (167,000–244,000)	215,000 (170,000–256,000)	157 (111–214)	109 (91·7–128)	100 (79·8–118)
1-90	Inflammatory bowel disease	26,000 (17,000–34,400)	23,700 (18,700–29,500)	24,900 (18,200–32,200)	19·5 (11·7–24·6)	12·9 (9·5–16·1)	11·6 (8·1–14·9)
1-91	Vascular intestinal disorders	15,300 (12,400–18,800)	26,000 (23,100–29,500)	33,000 (27,300–39,200)	17·5 (14·3–21)	18·8 (16·7–21·3)	19·3 (15·9–22·6)
1-92	Gallbladder and biliary diseases	141,000 (96,500–175,000)	173,000 (126,000–213,000)	190,000 (136,000–235,000)	120 (85·2–150)	98·2 (72·9–118)	89 (64·9–108)
1-93	Pancreatitis	86,100 (63,000–141,000)	101,000 (74,300–169,000)	106,000 (78,000–178,000)	63·7 (47·1–101)	49·4 (37·7–81·1)	44·3 (33·3–71·6)
1-94	Other digestive diseases	85,500 (56,900–139,000)	87,200 (72,200–102,000)	96,300 (75,800–117,000)	66·5 (50·8–86·1)	52·6 (43·7–61)	49·6 (38·4–60·3)
1-95	Neurological disorders	1,640,000 (639,000–3,150,000)	2,420,000 (998,000–4,540,000)	2,810,000 (1,240,000–5,150,000)	1,150 (533–2,070)	1,210 (594–2,140)	1,240 (609–2,180)
1-96	Alzheimer's disease and other dementias	180,000 (76,100–421,000)	372,000 (146,000–892,000)	494,000 (194,000–1,170,000)	289 (123–672)	346 (135–827)	358 (139–843)
1-97	Parkinson's disease	52,400 (42,600–60,900)	111,000 (92,000–128,000)	144,000 (116,000–171,000)	69·6 (56·6–80·6)	84·9 (70·2–98)	87 (69·8–102)
1-98	Idiopathic epilepsy	196,000 (129,000–277,000)	222,000 (145,000–317,000)	253,000 (161,000–380,000)	102 (66·7–143)	93·6 (61·3–133)	101 (64·2–152)
1-99	Multiple sclerosis	5,670 (4,480–7,870)	9,440 (7,090–12,900)	11,000 (7,650–16,300)	4 (3·2–5·6)	4·1 (3·1–5·6)	4 (2·8–5·9)
1-100	Motor neuron disease	4,580 (3,620–5,990)	6,560 (4,950–8,600)	8,790 (6,190–12,100)	3·1 (2·4–4)	3·1 (2·3–4·1)	3·4 (2·4–4·6)
1-101	Headache disorders	1,140,000 (166,000–2,630,000)	1,620,000 (257,000–3,720,000)	1,810,000 (296,000–4,130,000)	648 (105–1,480)	649 (105–1,480)	648 (106–1,480)
1-102	Migraine	1,050,000 (112,000–2,540,000)	1,490,000 (170,000–3,570,000)	1,660,000 (198,000–3,950,000)	594 (71·3–1,420)	596 (71·2–1,420)	595 (71·4–1,420)
1-103	Tension-type headache	88,600 (23,400–358,000)	130,000 (36,200–491,000)	147,000 (41,300–536,000)	53·4 (14·9–202)	53·7 (15·1–197)	53·7 (15·1–197)
1-104	Other neurological disorders	61,400 (43,500–81,500)	77,000 (54,700–103,000)	95,200 (66,500–132,000)	31·5 (22·8–41)	33 (23·7–43·7)	38·5 (27–52·9)
1-105	Mental disorders	2,150,000 (1,600,000–2,840,000)	2,980,000 (2,220,000–3,930,000)	3,380,000 (2,530,000–4,440,000)	1,250 (931–1,640)	1,230 (918–1,610)	1,240 (929–1,630)
1-106	Schizophrenia	292,000 (214,000–375,000)	460,000 (336,000–591,000)	536,000 (396,000–684,000)	180 (132–229)	184 (135–235)	189 (140–241)
1-107	Depressive disorders	564,000 (384,000–782,000)	799,000 (552,000–1,110,000)	956,000 (658,000–1,330,000)	351 (244–487)	336 (233–469)	350 (243–488)
1-108	Major depressive disorder	367,000 (243,000–520,000)	488,000 (329,000–692,000)	592,000 (400,000–830,000)	219 (148–308)	204 (138–286)	218 (146–305)
1-109	Dysthymia	197,000 (128,000–288,000)	311,000 (203,000–454,000)	365,000 (236,000–531,000)	132 (85·5–192)	133 (85·4–193)	132 (85·5–193)
1-110	Bipolar disorder	118,000 (72,100–182,000)	174,000 (107,000–268,000)	197,000 (122,000–301,000)	71 (43·9–108)	71·3 (44·1–109)	71·2 (43·9–109)
1-111	Anxiety disorders	561,000 (389,000–770,000)	802,000 (565,000–1,100,000)	920,000 (648,000–1,270,000)	323 (227–441)	330 (235–450)	339 (240–465)
1-112	Eating disorders	35,900 (22,000–53,100)	54,500 (33,900–80,600)	67,100 (41,800–99,300)	17·6 (10·9–25·9)	20·4 (12·7–30·2)	23·9 (14·8–35·4)
1-113	Anorexia nervosa	13,100 (7,510–20,800)	18,400 (10,900–29,000)	22,100 (13,100–34,300)	6·2 (3·6–9·8)	7 (4·1–11)	8 (4·7–12·3)

1-114	Bulimia nervosa	22,800 (13,200–35,900)	36,100 (20,700–56,700)	44,900 (26,100–70,900)	11·4 (6·6–17·9)	13·4 (7·7–21·2)	16 (9·3–25)
1-115	Autism spectrum disorders	91,900 (60,000–135,000)	117,000 (76,200–171,000)	123,000 (80,100–179,000)	47 (30·7–69)	47·5 (31–69·6)	47·5 (30·9–69·1)
1-116	Attention-deficit/hyperactivity disorder	25,500 (14,400–44,200)	29,600 (16,800–50,800)	30,600 (17,500–52,400)	11·5 (6·5–19·6)	11·5 (6·5–19·8)	11·6 (6·6–20)
1-117	Conduct disorder	174,000 (98,900–276,000)	183,000 (103,000–289,000)	187,000 (106,000–293,000)	71·7 (40·6–113)	72·2 (40·9–114)	72·2 (40·9–113)
1-118	Idiopathic developmental intellectual disability	146,000 (78,000–237,000)	125,000 (65,900–208,000)	93,900 (45,700–160,000)	73·1 (39·2–118)	50·6 (26·7–84)	35·8 (17·3–61·1)
1-119	Other mental disorders	146,000 (93,000–224,000)	234,000 (150,000–359,000)	274,000 (176,000–418,000)	100 (64–152)	101 (64·9–153)	101 (65·2–153)
1-120	Substance use disorders	235,000 (170,000–309,000)	346,000 (251,000–456,000)	383,000 (280,000–501,000)	132 (96·7–172)	135 (99·3–178)	138 (101–180)
1-121	Alcohol use disorders	96,200 (68,600–129,000)	146,000 (103,000–198,000)	163,000 (115,000–223,000)	57·5 (41·5–76·4)	58·4 (41·6–78·3)	58·2 (41·6–79·4)
1-122	Drug use disorders	139,000 (96,300–193,000)	200,000 (140,000–274,000)	220,000 (157,000–297,000)	74·3 (52·4–101)	77 (54·2–105)	79·4 (56·8–106)
1-123	Opioid use disorders	64,900 (43,800–90,000)	96,500 (67,600–132,000)	108,000 (75,000–146,000)	37·3 (25·5–50·4)	38 (26·8–51·5)	38·8 (27·1–52·3)
1-124	Cocaine use disorders	1,680 (1,180–2,460)	3,020 (2,310–3,980)	3,550 (2,680–4,730)	0·9 (0·7–1·3)	1·2 (0·9–1·5)	1·3 (1–1·7)
1-125	Amphetamine use disorders	33,900 (17,900–56,500)	46,100 (24,800–76,400)	48,600 (26,300–80,400)	16·7 (9·1–27·6)	17·3 (9·3–28·5)	17·6 (9·5–29·1)
1-126	Cannabis use disorders	12,600 (7,170–20,100)	17,400 (10,000–27,600)	20,700 (12,200–32,900)	6·4 (3·7–10)	6·6 (3·8–10·6)	7·4 (4·3–11·8)
1-127	Other drug use disorders	25,700 (15,100–40,600)	36,500 (21,900–57,200)	39,600 (24,300–60,700)	13 (7·8–20·4)	13·9 (8·4–21·5)	14·4 (8·8–22)
1-128	Diabetes and kidney diseases	2,320,000 (2,110,000–2,570,000)	4,080,000 (3,640,000–4,560,000)	5,510,000 (4,730,000–6,270,000)	1,840 (1,660–2,050)	2,130 (1,910–2,360)	2,290 (1,970–2,570)
1-129	Diabetes mellitus	1,270,000 (1,120,000–1,420,000)	2,600,000 (2,310,000–2,930,000)	3,860,000 (3,290,000–4,400,000)	1,100 (975–1,240)	1,400 (1,260–1,580)	1,600 (1,370–1,810)
1-130	Diabetes mellitus type 1	182,000 (136,000–235,000)	264,000 (198,000–386,000)	302,000 (226,000–468,000)	122 (88–165)	116 (86·2–172)	112 (83·4–173)
1-131	Diabetes mellitus type 2	1,090,000 (941,000–1,230,000)	2,330,000 (2,050,000–2,650,000)	3,550,000 (3,000,000–4,060,000)	982 (857–1,110)	1,290 (1,140–1,450)	1,490 (1,270–1,690)
1-132	Chronic kidney disease	1,010,000 (899,000–1,120,000)	1,450,000 (1,310,000–1,610,000)	1,640,000 (1,420,000–1,890,000)	714 (648–788)	711 (644–784)	673 (587–764)
1-133	Chronic kidney disease due to diabetes mellitus type 1	169,000 (126,000–217,000)	245,000 (180,000–321,000)	253,000 (183,000–340,000)	106 (77·4–138)	99·7 (72·7–130)	89·5 (64·9–119)
1-134	Chronic kidney disease due to diabetes mellitus type 2	203,000 (157,000–250,000)	353,000 (276,000–432,000)	428,000 (329,000–539,000)	178 (142–215)	192 (156–231)	183 (144–226)
1-135	Chronic kidney disease due to hypertension	232,000 (190,000–279,000)	392,000 (326,000–477,000)	459,000 (376,000–568,000)	198 (165–236)	211 (179–251)	198 (164–239)
1-136	Chronic kidney disease due to glomerulonephritis	133,000 (97,500–175,000)	163,000 (127,000–206,000)	176,000 (138,000–220,000)	75·3 (58·4–95·1)	69·5 (55·3–86·1)	66·8 (52·6–83·3)
1-137	Chronic kidney disease due to other and unspecified causes	271,000 (208,000–325,000)	295,000 (239,000–352,000)	322,000 (260,000–389,000)	157 (126–185)	139 (113–164)	136 (110–162)
1-138	Acute glomerulonephritis	49,900 (1,780–87,300)	31,100 (919–50,800)	21,800 (678–35,600)	25·5 (1–44)	14 (0·5–22·8)	9·5 (0·3–15·4)
1-139	Skin and subcutaneous diseases	1,200,000 (767,000–1,790,000)	1,570,000 (1,000,000–2,340,000)	1,710,000 (1,100,000–2,530,000)	645 (415–958)	658 (423–977)	668 (432–987)
1-140	Dermatitis	357,000 (205,000–571,000)	481,000 (277,000–770,000)	523,000 (301,000–836,000)	199 (115–314)	200 (116–319)	200 (116–320)
1-141	Atopic dermatitis	298,000 (158,000–498,000)	392,000 (210,000–658,000)	421,000 (225,000–706,000)	161 (86·1–268)	162 (86·7–272)	162 (86·7–272)
1-142	Contact dermatitis	53,900 (33,600–80,200)	80,900 (49,900–120,000)	93,300 (57,400–139,000)	34·8 (21·8–51·6)	34·9 (21·7–51·8)	34·9 (21·6–51·9)
1-143	Seborrhoeic dermatitis	5,680 (3,230–9,040)	7,890 (4,440–12,700)	8,750 (4,930–14,000)	3·4 (1·9–5·3)	3·4 (1·9–5·4)	3·4 (1·9–5·4)
1-144	Psoriasis	12,200 (8,670–16,400)	16,400 (11,600–22,000)	18,700 (13,100–25,100)	7·7 (5·5–10·4)	7·1 (5–9·5)	7 (4·9–9·5)
1-145	Bacterial skin diseases	50,700 (30,300–66,400)	71,700 (46,400–81,600)	91,900 (57,000–110,000)	37·1 (25·7–48·2)	37·5 (27–41·9)	42·1 (27·1–48·8)
1-146	Cellulitis	13,800 (6,760–19,300)	19,500 (10,900–25,000)	24,700 (12,800–31,500)	10·7 (5·5–14·8)	10·4 (5·8–13·1)	11·3 (6·1–14·2)
1-147	Pyoderma	36,900 (22,400–54,100)	52,200 (35,200–63,300)	67,200 (44,000–82,500)	26·5 (18·7–35·4)	27 (20·1–32·3)	30·9 (21·1–37·1)
1-148	Scabies	248,000 (136,000–399,000)	304,000 (168,000–487,000)	318,000 (175,000–511,000)	125 (69·2–200)	124 (69·1–199)	124 (68·6–199)
1-149	Fungal skin diseases	89,800 (35,300–195,000)	124,000 (49,300–269,000)	139,000 (55,800–298,000)	54·4 (22–117)	54·4 (21·9–117)	54·3 (21·9–117)
1-150	Viral skin diseases	114,000 (72,600–171,000)	129,000 (82,500–194,000)	130,000 (82,700–193,000)	52·7 (33·8–78·7)	52·9 (33·7–79·3)	52·9 (33·8–79·1)
1-151	Acne vulgaris	139,000 (83,500–222,000)	183,000 (110,000–291,000)	204,000 (124,000–325,000)	61·6 (37·1–98·3)	71 (42·9–113)	75·4 (45·8–120)
1-152	Alopecia areata	15,600 (9,850–23,200)	22,100 (13,800–32,900)	24,200 (15,200–36,200)	8·8 (5·6–13·1)	8·9 (5·5–13·2)	8·8 (5·5–13·2)
1-153	Pruritus	18,800 (8,940–33,500)	27,900 (13,300–49,900)	32,400 (15,300–57,900)	11·9 (5·7–21·2)	12·3 (5·9–22·2)	12·7 (6·1–22·7)
1-154	Urticaria	89,400 (59,100–129,000)	111,000 (72,800–158,000)	117,000 (77,000–167,000)	45·3 (29·8–65·3)	45·5 (29·9–65·1)	45·5 (30·1–65·1)
1-155	Decubitus ulcer	2,990 (1,820–4,950)	5,090 (3,430–7,310)	5,840 (3,970–7,400)	3 (1·8–5·5)	3·5 (2·4–4·9)	3·3 (2·3–4·1)
1-156	Other skin and subcutaneous diseases	65,300 (32,500–118,000)	92,700 (45,700–167,000)	106,000 (52,000–191,000)	38·3 (19·3–68·5)	40·2 (20–72·1)	41·3 (20·5–74·6)
1-157	Sense organ diseases	1,510,000 (1,070,000–2,040,000)	2,200,000 (1,550,000–3,010,000)	2,550,000 (1,790,000–3,500,000)	1,350 (972–1,800)	1,230 (878–1,650)	1,160 (827–1,570)
1-158	Blindness and vision loss	693,000 (498,000–933,000)	992,000 (715,000–1,330,000)	1,130,000 (810,000–1,520,000)	720 (521–957)	619 (448–826)	559 (404–748)
1-159	Glaucoma	13,000 (8,590–18,900)	18,500 (12,100–26,800)	22,900 (15,000–33,100)	17·6 (11·6–25·4)	14·2 (9·3–20·4)	13·5 (8·9–19·4)
1-160	Cataract	397,000 (281,000–543,000)	588,000 (420,000–803,000)	642,000 (454,000–874,000)	463 (328–634)	395 (280–536)	339 (240–460)
1-161	Age-related macular degeneration	7,570 (4,860–11,200)	11,700 (7,540–17,500)	15,500 (10,000–22,800)	9·2 (6–13·3)	8·2 (5·3–12)	8·3 (5·4–12)
1-162	Refraction disorders	103,000 (66,900–151,000)	131,000 (85,700–192,000)	149,000 (96,900–218,000)	67·4 (44·8–97·3)	59·9 (39·6–87)	59·2 (39–86·5)
1-163	Near vision loss	40,600 (18,400–81,500)	65,500 (29,900–131,000)	82,700 (37,200–166,000)	43·8 (20·4–84·3)	40·9 (19–79)	39·8 (18·2–77·1)
1-164	Other vision loss	132,000 (93,000–183,000)	177,000 (124,000–247,000)	215,000 (151,000–298,000)	120 (84·7–165)	102 (72–140)	99·4 (70·6–136)
1-165	Age-related and other hearing loss	758,000 (511,000–1,080,000)	1,120,000 (745,000–1,610,000)	1,320,000 (878,000–1,900,000)	583 (399–823)	565 (385–808)	558 (379–792)
1-166	Other sense organ diseases	56,000 (33,700–84,900)	86,600 (51,500–133,000)	104,000 (60,700–161,000)	41·5 (24·6–63·7)	42·6 (25·1–65·5)	43·1 (25·6–66·8)

1-167	Musculoskeletal disorders	2,680,000 (1,930,000–3,570,000)	4,500,000 (3,230,000–5,980,000)	5,230,000 (3,750,000–6,960,000)	1,910 (1,380–2,540)	2,040 (1,460–2,690)	1,990 (1,430–2,630)
1-168	Rheumatoid arthritis	17,400 (12,600–22,700)	30,400 (22,300–39,100)	37,400 (27,900–48,200)	13·2 (9·6–17)	14·6 (10·9–18·6)	14·8 (11·1–18·9)
1-169	Osteoarthritis	131,000 (64,900–263,000)	242,000 (119,000–487,000)	327,000 (163,000–658,000)	125 (62·2–251)	134 (66·2–269)	139 (68·7–280)
1-170	Low back pain	1,420,000 (995,000–1,890,000)	2,250,000 (1,570,000–3,010,000)	2,430,000 (1,710,000–3,260,000)	994 (700–1,310)	1,020 (720–1,350)	936 (658–1,250)
1-171	Neck pain	600,000 (390,000–863,000)	974,000 (636,000–1,410,000)	1,160,000 (757,000–1,690,000)	426 (280–614)	428 (281–617)	428 (280–614)
1-172	Gout	20,100 (12,800–29,000)	39,100 (25,000–56,400)	51,100 (32,200–74,700)	17·4 (11–25·1)	19·9 (12·7–28·8)	20·7 (13·1–30)
1-173	Other musculoskeletal disorders	491,000 (339,000–685,000)	966,000 (674,000–1,340,000)	1,220,000 (842,000–1,670,000)	339 (232–469)	419 (294–575)	449 (311–615)
1-174	Other non-communicable diseases	4,930,000 (3,420,000–7,330,000)	4,330,000 (3,420,000–5,550,000)	3,980,000 (3,200,000–4,990,000)	2,550 (1,830–3,610)	1,930 (1,530–2,450)	1,730 (1,410–2,170)
1-175	Congenital anomalies	2,850,000 (1,610,000–5,240,000)	2,010,000 (1,480,000–2,950,000)	1,490,000 (1,180,000–2,030,000)	1,260 (722–2,320)	884 (648–1,300)	743 (581–1,020)
1-176	Neural tube defects	476,000 (302,000–830,000)	227,000 (155,000–326,000)	131,000 (87,200–180,000)	209 (132–365)	101 (69·2–146)	69·6 (45·7–95·8)
1-177	Congenital heart anomalies	1,220,000 (580,000–2,060,000)	912,000 (639,000–1,280,000)	667,000 (506,000–879,000)	537 (256–907)	399 (278–563)	336 (252–447)
1-178	Orofacial clefts	18,100 (10,300–37,300)	13,500 (8,840–21,900)	11,000 (7,230–16,200)	8·3 (4·8–16·7)	5·9 (3·8–9·7)	5·2 (3·3–7·9)
1-179	Down's syndrome	36,000 (20,100–80,700)	39,900 (29,600–58,800)	36,500 (28,000–47,300)	16 (9–35·8)	17 (12·5–25·4)	17·1 (12·9–22·6)
1-180	Turner syndrome	226 (102–405)	266 (119–472)	258 (117–456)	0·1 (0–0·2)	0·1 (0–0·2)	0·1 (0–0·2)
1-181	Klinefelter syndrome	29 (13–55)	33 (15–64)	34 (15–65)	0 (0–0)	0 (0–0)	0 (0–0)
1-182	Other chromosomal abnormalities	34,900 (19,600–121,000)	36,600 (24,300–81,100)	33,500 (23,400–61,400)	15·4 (8·6–53·3)	15·9 (10·5–35·7)	16·6 (11·4–31·3)
1-183	Congenital musculoskeletal and limb anomalies	102,000 (66,600–149,000)	102,000 (68,900–142,000)	96,500 (64,600–136,000)	47·7 (31·4–68·9)	42·3 (28·8–59·1)	40·7 (27·8–57·1)
1-184	Urogenital congenital anomalies	26,900 (14,500–48,400)	22,300 (15,400–32,500)	17,400 (12,900–23,200)	11·9 (6·5–21·4)	9·8 (6·7–14·3)	8·6 (6·3–11·6)
1-185	Digestive congenital anomalies	266,000 (72,200–731,000)	167,000 (82,200–330,000)	117,000 (64,700–199,000)	117 (31·7–322)	74·6 (36·7–147)	62 (34·2–105)
1-186	Other congenital anomalies	674,000 (273,000–1,790,000)	495,000 (302,000–1,070,000)	379,000 (267,000–744,000)	302 (124–795)	218 (132–470)	188 (130–379)
1-187	Urinary diseases and male infertility	213,000 (173,000–250,000)	287,000 (226,000–339,000)	339,000 (266,000–405,000)	174 (140–205)	156 (126–184)	150 (121–178)
1-188	Urinary tract infections and interstitial nephritis	110,000 (79,400–128,000)	151,000 (112,000–175,000)	171,000 (125,000–205,000)	81 (59·1–93·2)	79·6 (61·5–90·9)	74·8 (57·1–88·5)
1-189	Urolithiasis	22,900 (8,190–33,800)	29,100 (9,310–42,700)	32,300 (10,400–47,400)	18·7 (6·5–27·9)	15 (4·6–22·1)	13·3 (4·1–19·5)
1-190	Benign prostatic hyperplasia	44,100 (26,300–66,400)	51,800 (30,800–77,900)	69,700 (41,200–105,000)	48·4 (29–72·5)	33·4 (20–50·1)	33·8 (20·3–50·6)
1-191	Male infertility	6,830 (2,540–16,400)	11,600 (4,100–28,600)	16,400 (6,020–39,100)	3·7 (1·4–9·1)	4·3 (1·5–10·5)	5·7 (2·1–13·7)
1-192	Other urinary diseases	29,400 (21,000–52,800)	42,700 (32,500–69,700)	49,500 (35,600–82,300)	22·5 (16·4–39·3)	23·3 (17·9–37·3)	22·1 (16·2–36·7)
1-193	Gynecological diseases	525,000 (362,000–741,000)	668,000 (463,000–954,000)	727,000 (503,000–1,040,000)	301 (210–423)	258 (179–366)	256 (178–365)
1-194	Uterine fibroids	17,200 (9,560–28,300)	21,500 (11,500–36,900)	23,900 (12,400–42,200)	10·4 (5·8–17·3)	8·6 (4·7–14·7)	8·4 (4·5–14·8)
1-195	Polycystic ovarian syndrome	11,400 (4,830–23,000)	26,400 (11,300–53,300)	33,600 (14,400–68,800)	6 (2·5–12)	9·9 (4·2–19·9)	11·7 (5–24)
1-196	Female infertility	14,600 (5,180–35,300)	25,000 (8,020–62,200)	35,400 (12,400–86,000)	8·1 (2·9–19·9)	9·1 (2·9–22·7)	12·2 (4·3–29·4)
1-197	Endometriosis	80,600 (48,300–129,000)	108,000 (64,700–172,000)	106,000 (63,400–168,000)	44·1 (26·8–69·9)	40·1 (24·1–64)	36·8 (21·7–58·3)
1-198	Genital prolapse	4,780 (2,730–8,690)	7,500 (4,310–13,500)	8,840 (5,190–15,400)	3·9 (2·2–7)	3·8 (2·1–6·8)	3·5 (2·1–6·2)
1-199	Premenstrual syndrome	220,000 (134,000–339,000)	295,000 (180,000–454,000)	314,000 (194,000–483,000)	115 (71·1–176)	111 (67·9–170)	110 (67·6–170)
1-200	Other gynecological diseases	176,000 (117,000–252,000)	185,000 (124,000–265,000)	204,000 (136,000–293,000)	114 (75·8–160)	76 (51·7–107)	74·2 (50–106)
1-201	Hemoglobinopathies and hemolytic anaemias	424,000 (318,000–545,000)	325,000 (252,000–429,000)	287,000 (220,000–385,000)	226 (175–288)	144 (113–189)	119 (92–157)
1-202	Thalassemias	154,000 (95,700–212,000)	83,300 (69,800–99,200)	57,400 (47,900–68,700)	67·5 (42–92·6)	34·8 (29–41·4)	26 (21·4–31·4)
1-203	Thalassaemias trait	145,000 (91,900–225,000)	127,000 (80,100–200,000)	119,000 (74,100–189,000)	74·8 (48·1–117)	53 (33·6–83·4)	46·6 (29–73·7)
1-204	Sickle cell disorders	731 (333–1,280)	425 (259–682)	300 (188–494)	0·3 (0·1–0·6)	0·2 (0·1–0·3)	0·1 (0·1–0·2)
1-205	Sickle cell trait	7,980 (5,130–12,100)	7,970 (5,070–12,400)	7,420 (4,580–11,500)	4·1 (2·7–6·3)	3·1 (2–4·9)	2·8 (1·7–4·3)
1-206	G6PD deficiency	30,800 (24,800–37,300)	26,700 (21,500–32,400)	25,000 (19,700–31,100)	17·2 (14–20·4)	11·1 (9–13·4)	9·2 (7·2–11·5)
1-207	G6PD trait	452 (287–671)	795 (521–1,170)	814 (511–1,210)	0·3 (0·2–0·5)	0·3 (0·2–0·4)	0·3 (0·2–0·4)
1-208	Other hemoglobinopathies and hemolytic anaemias	85,000 (65,200–113,000)	78,500 (59,000–102,000)	76,800 (57,300–103,000)	62 (49·2–79·7)	41·4 (32·1–52·9)	34·1 (25·9–44·5)
1-209	Endocrine, metabolic, blood, and immune disorders	275,000 (199,000–362,000)	281,000 (208,000–366,000)	312,000 (229,000–409,000)	172 (125–226)	123 (91·9–159)	120 (90·4–156)
1-210	Oral disorders	404,000 (233,000–644,000)	625,000 (357,000–1,010,000)	734,000 (420,000–1,180,000)	307 (182–489)	303 (177–483)	296 (172–473)
1-211	Caries of deciduous teeth	7,800 (3,440–16,100)	7,840 (3,450–16,500)	7,050 (3,090–14,800)	3·3 (1·5–6·8)	3·3 (1·4–6·9)	3·3 (1·4–6·8)
1-212	Caries of permanent teeth	46,300 (20,800–90,800)	62,800 (28,600–122,000)	68,300 (31,100–135,000)	25·8 (11·7–49·6)	25·7 (11·7–49·5)	25·3 (11·5–49·1)
1-213	Periodontal disease	120,000 (45,900–263,000)	205,000 (77,800–449,000)	250,000 (95,800–543,000)	88·5 (34·1–191)	90·3 (34·8–193)	91·3 (35·4–195)
1-214	Edentulism	135,000 (86,100–199,000)	220,000 (140,000–324,000)	265,000 (167,000–394,000)	136 (87·5–201)	130 (82·8–192)	123 (78·5–182)
1-215	Other oral disorders	93,700 (58,200–138,000)	130,000 (80,800–191,000)	143,000 (89,200–211,000)	53·4 (33·4–78·4)	53·5 (33·4–78·6)	53·5 (33·4–78·5)
1-216	Sudden infant death syndrome	242,000 (53,800–562,000)	131,000 (48,700–254,000)	91,400 (37,400–179,000)	107 (23·8–249)	58·5 (21·8–113)	48·9 (20·1–96)
1-0	Injuries	7,420,000 (6,140,000–8,360,000)	5,730,000 (5,130,000–6,420,000)	5,350,000 (4,670,000–6,170,000)	3,970 (3,310–4,450)	2,470 (2,210–2,760)	2,120 (1,860–2,430)
1-1	Transport injuries	3,630,000 (2,900,000–4,210,000)	2,610,000 (2,320,000–3,100,000)	2,430,000 (2,060,000–3,030,000)	1,900 (1,540–2,170)	1,090 (966–1,290)	924 (789–1,140)
1-2	Road injuries	3,360,000 (2,670,000–3,900,000)	2,410,000 (2,130,000–2,850,000)	2,250,000 (1,900,000–2,790,000)	1,760 (1,420–2,010)	1,000 (892–1,180)	856 (729–1,050)

1-3	Pedestrian road injuries	1,150,000 (736,000–1,550,000)	589,000 (474,000–775,000)	515,000 (401,000–673,000)	611 (412–803)	261 (212–338)	207 (163–267)
1-4	Cyclist road injuries	153,000 (101,000–225,000)	148,000 (102,000–196,000)	152,000 (105,000–202,000)	83·4 (59·1–117)	63·6 (44·2–84·2)	57·8 (40·1–77·3)
1-5	Motorcyclist road injuries	999,000 (743,000–1,280,000)	984,000 (750,000–1,220,000)	941,000 (718,000–1,190,000)	510 (389–635)	396 (303–491)	348 (267–438)
1-6	Motor vehicle road injuries	1,010,000 (743,000–1,260,000)	649,000 (524,000–949,000)	609,000 (473,000–910,000)	528 (400–651)	268 (217–390)	230 (180–343)
1-7	Other road injuries	42,700 (29,100–64,500)	37,300 (27,900–51,400)	36,400 (27,000–52,400)	22·7 (15·7–32·9)	15·6 (11·7–21·4)	13·9 (10·3–20)
1-8	Other transport injuries	270,000 (199,000–330,000)	201,000 (154,000–280,000)	177,000 (131,000–255,000)	140 (105–170)	83·2 (64·4–115)	67·5 (50·1–97·4)
1-9	Unintentional injuries	3,170,000 (2,510,000–3,630,000)	2,480,000 (2,120,000–2,820,000)	2,290,000 (1,910,000–2,660,000)	1,740 (1,380–1,990)	1,120 (954–1,270)	969 (805–1,120)
1-10	Falls	768,000 (623,000–928,000)	824,000 (711,000–950,000)	909,000 (754,000–1,070,000)	555 (438–656)	419 (359–482)	404 (330–470)
1-11	Drowning	929,000 (620,000–1,140,000)	469,000 (354,000–555,000)	324,000 (259,000–384,000)	415 (281–504)	196 (147–231)	141 (108–169)
1-12	Fire, heat, and hot substances	179,000 (111,000–249,000)	149,000 (112,000–195,000)	147,000 (108,000–203,000)	96·7 (63·4–131)	64·4 (48·3–84·2)	58·2 (42·7–78·9)
1-13	Poisonings	70,600 (49,300–83,300)	59,900 (35,100–72,300)	56,700 (30,800–70,800)	34·7 (24·9–40·3)	24·2 (14·4–29·1)	21·4 (11·9–26·7)
1-14	Poisoning by carbon monoxide	8,950 (4,610–12,300)	11,300 (4,770–16,400)	12,900 (4,990–20,000)	4·7 (2·5–6·3)	4·5 (2–6·5)	4·8 (1·9–7·4)
1-15	Poisoning by other means	61,700 (43,500–74,000)	48,600 (29,600–59,500)	43,800 (24,900–55,800)	30 (21·8–35·7)	19·7 (12·2–24·1)	16·7 (9·7–21·3)
1-16	Exposure to mechanical forces	303,000 (165,000–421,000)	231,000 (151,000–293,000)	238,000 (163,000–311,000)	163 (94·1–220)	99·6 (64·4–126)	96·1 (62·7–126)
1-17	Unintentional firearm injuries	43,200 (12,700–78,500)	22,900 (10,900–36,100)	21,800 (12,400–31,900)	21·3 (7·5–36·8)	10 (4·7–15·9)	9·2 (4·8–14·2)
1-18	Other exposure to mechanical forces	260,000 (151,000–363,000)	208,000 (139,000–264,000)	216,000 (150,000–284,000)	142 (86–192)	89·6 (59·7–114)	86·9 (57·5–114)
1-19	Adverse effects of medical treatment	106,000 (59,400–170,000)	69,200 (40,900–113,000)	58,900 (33,000–99,500)	57·6 (35·2–91·1)	32·1 (19–52·9)	25·3 (14·3–42·5)
1-20	Animal contact	107,000 (66,200–133,000)	73,000 (60,500–85,500)	65,500 (53,600–79,100)	55·2 (37–67·7)	32·7 (26·6–38·1)	27·1 (22–32·1)
1-21	Venomous animal contact	50,100 (30,700–64,000)	33,500 (25,500–38,900)	28,600 (22,600–34,000)	25·6 (16·1–31·9)	15·2 (10·9–17·6)	12 (8·9–14·1)
1-22	Non-venomous animal contact	56,600 (34,400–71,500)	39,400 (32,000–48,400)	36,800 (29,600–46,200)	29·6 (19·2–37·3)	17·4 (14·2–21·3)	15·1 (12·3–18·6)
1-23	Foreign body	274,000 (225,000–348,000)	176,000 (150,000–209,000)	141,000 (117,000–168,000)	136 (112–169)	80·2 (68·5–94·6)	64·9 (54–77·9)
1-24	Pulmonary aspiration and foreign body in airway	228,000 (186,000–285,000)	131,000 (111,000–157,000)	90,000 (73,400–112,000)	108 (89·1–133)	60·3 (50·7–72)	45·1 (36·5–56·5)
1-25	Foreign body in eyes	9,520 (5,040–16,000)	14,000 (7,600–23,300)	15,900 (8,890–26,200)	6 (3·4–9·7)	6 (3·4–9·6)	6 (3·4–9·6)
1-26	Foreign body in other body part	37,300 (23,300–59,200)	30,900 (22,800–40,500)	35,100 (25,800–46,000)	21·9 (14·9–32·9)	13·9 (10·3–18·2)	13·8 (10·2–18·1)
1-27	Environmental heat and cold exposure	24,700 (17,700–33,000)	23,100 (16,100–30,800)	30,800 (21,300–42,300)	14·7 (10·7–19·8)	9·9 (7–13·2)	11·9 (8·2–16·2)
1-28	Exposure to forces of nature	15,800 (14,200–17,600)	144,000 (121,000–174,000)	93,600 (76,300–118,000)	8·2 (7·3–9·1)	58·6 (49·5–71·1)	34·8 (28·6–43·3)
1-29	Other unintentional injuries	388,000 (214,000–501,000)	260,000 (172,000–316,000)	224,000 (165,000–277,000)	201 (114–260)	107 (71·7–129)	83·9 (62·3–103)
1-30	Self-harm and interpersonal violence	627,000 (546,000–729,000)	640,000 (553,000–742,000)	628,000 (525,000–753,000)	338 (292–391)	256 (222–295)	230 (194–275)
1-31	Self-harm	353,000 (278,000–419,000)	383,000 (309,000–451,000)	372,000 (289,000–471,000)	195 (154–229)	153 (124–179)	135 (105–171)
1-32	Self-harm by firearm	8,000 (5,160–14,800)	8,170 (5,550–16,500)	7,260 (4,550–13,900)	4·5 (2·9–8·4)	3·3 (2·2–6·6)	2·6 (1·7–5)
1-33	Self-harm by other specified means	345,000 (272,000–407,000)	375,000 (299,000–439,000)	365,000 (283,000–461,000)	190 (150–223)	150 (120–174)	132 (103–166)
1-34	Interpersonal violence	229,000 (197,000–268,000)	230,000 (203,000–264,000)	229,000 (194,000–268,000)	120 (104–138)	92·6 (81·8–106)	85·5 (73–99·6)
1-35	Physical violence by firearm	11,100 (8,800–17,000)	9,990 (8,360–13,400)	9,180 (7,550–12,600)	5·8 (4·6–8·5)	4 (3·3–5·3)	3·4 (2·8–4·7)
1-36	Physical violence by sharp object	82,400 (58,600–95,600)	74,500 (55,100–88,300)	69,600 (49,900–87,800)	41·5 (30·1–47·9)	29·1 (21·5–34·4)	25·4 (18·2–31·9)
1-37	Sexual violence	36,000 (23,700–50,800)	49,100 (32,500–69,300)	53,400 (35,700–75,500)	19·3 (12·9–27·2)	19·2 (12·8–27·1)	19·2 (12·8–27)
1-38	Physical violence by other means	99,400 (81,400–143,000)	96,500 (82,900–117,000)	96,700 (80,800–119,000)	53·1 (43·8–73·1)	40·3 (34·7–48·6)	37·5 (31·5–45·3)
1-39	Conflict and terrorism	42,200 (19,100–98,600)	23,600 (8,320–60,000)	21,600 (9,280–50,200)	22 (9·7–52·2)	9·7 (3·4–25)	7·8 (3·4–18·3)
1-40	Executions and police conflict	2,480 (1,110–3,310)	3,150 (1,450–4,130)	5,530 (3,830–6,860)	1·3 (0·5–1·7)	1·2 (0·6–1·6)	2 (1·4–2·5)

SR Table 1: All-age DALYs counts and age standardized DALYs rates per 100,000 individuals for all causes and level 1 cause groups by location for 1990, 2010, and 2019, both sexes combined, Dominica

Cause Level	Cause	All-age DALYs			Age-standardized rate of DALYs per 100,000		
		1990	2010	2019	1990	2010	2019
0-0	All causes	24,600 (22,200–27,100)	23,900 (21,200–26,600)	25,000 (21,200–29,400)	34,300 (30,900–38,100)	32,800 (28,900–36,700)	33,200 (27,900–39,300)
1-0	Communicable, maternal, neonatal, and nutritional disorders	4,360 (3,860–4,920)	3,140 (2,730–3,580)	3,120 (2,550–3,830)	5,700 (5,070–6,420)	5,330 (4,580–6,140)	5,760 (4,560–7,260)
1-1	HIV/AIDS and sexually transmitted infections	433 (354–569)	372 (325–444)	335 (291–414)	640 (543–808)	573 (477–726)	538 (434–724)
1-2	HIV/AIDS	302 (294–309)	293 (283–306)	261 (251–272)	476 (464–488)	416 (402–435)	374 (360–389)
1-3	HIV/AIDS - drug-susceptible tuberculosis	77 (50–116)	36 (24–55)	33 (21–49)	121 (77·9–183)	51·9 (34–77·5)	46·8 (30·3–69·5)
1-4	HIV/AIDS - Multidrug-resistant Tuberculosis without extensive drug resistance	2 (0–11)	0 (0–2)	1 (0–2)	3·5 (0·2–17·8)	0·5 (0–2·3)	0·7 (0·1–3·3)
1-5	HIV/AIDS - Extensively drug-resistant Tuberculosis	0 (0–0)	0 (0–0)	0 (0–0)	0 (0–0)	0·1 (0–0·2)	0·1 (0–0·6)
1-6	HIV/AIDS resulting in other diseases	222 (181–250)	256 (236–274)	227 (209–244)	351 (288–396)	364 (335–389)	326 (299–351)
1-7	Sexually transmitted infections excluding HIV	131 (53–269)	79 (35–153)	74 (32–150)	165 (69–333)	157 (64·7–317)	165 (61·9–351)
1-8	Syphilis	118 (39–255)	66 (23–139)	60 (19–136)	144 (49·1–311)	138 (46·9–296)	146 (43–334)
1-9	Chlamydial infection	1 (1–2)	1 (1–2)	1 (1–2)	2·1 (1·4–3·2)	2·1 (1·3–3)	2·1 (1·3–3·1)
1-10	Gonococcal infection	2 (1–2)	1 (1–2)	1 (1–2)	2·2 (1·6–2·9)	1·8 (1·4–2·4)	1·8 (1·3–2·5)
1-11	Trichomoniasis	3 (1–6)	3 (1–7)	3 (1–7)	4·9 (2–10·2)	4·7 (1·8–9·8)	4·5 (1·8–9·4)
1-12	Genital herpes	3 (1–8)	4 (1–9)	4 (1–10)	5·4 (1·7–13)	5·3 (1·7–13)	5·3 (1·7–12·9)
1-13	Other sexually transmitted infections	4 (3–5)	3 (2–5)	3 (2–5)	5·7 (4·1–7·6)	4·7 (3·4–6·3)	4·7 (3·4–6·5)
1-14	Respiratory infections and tuberculosis	973 (866–1,090)	775 (677–878)	778 (632–948)	1,320 (1,180–1,480)	1,100 (954–1,260)	1,130 (901–1,410)
1-15	Tuberculosis	219 (193–248)	105 (91–120)	101 (81–125)	322 (282–365)	145 (126–166)	134 (108–166)
1-16	Latent tuberculosis infection	0 (0–0)	0 (0–0)	0 (0–0)	0 (0–0)	0 (0–0)	0 (0–0)
1-17	Drug-susceptible tuberculosis	214 (184–243)	104 (90–119)	99 (79–123)	315 (268–360)	144 (125–165)	132 (106–164)
1-18	Multidrug-resistant tuberculosis without extensive drug resistance	5 (0–27)	1 (0–4)	1 (0–6)	7·4 (0·4–39·4)	1·2 (0·1–5)	1·6 (0·1–7·9)
1-19	Extensively drug-resistant tuberculosis	0 (0–0)	0 (0–0)	0 (0–1)	0 (0–0)	0·1 (0–0·5)	0·3 (0–1·3)
1-20	Lower respiratory infections	658 (580–750)	591 (510–676)	603 (480–744)	871 (770–992)	837 (718–968)	882 (680–1,130)
1-21	Upper respiratory infections	71 (46–106)	59 (37–90)	56 (36–87)	92·9 (59·8–139)	87·6 (54·8–133)	87·6 (55·9–135)
1-22	Otitis media	24 (14–40)	20 (12–33)	19 (11–30)	31·9 (18·6–52·1)	29·3 (17·1–48·1)	29·1 (16·6–47·6)
1-23	Enteric infections	278 (225–350)	170 (132–218)	170 (126–223)	356 (287–449)	257 (203–326)	266 (198–351)
1-24	Diarrhoeal diseases	275 (221–347)	168 (130–216)	168 (125–222)	352 (283–445)	254 (200–323)	263 (196–348)
1-25	Typhoid and paratyphoid	1 (1–2)	1 (0–1)	0 (0–1)	1·6 (0·7–3)	0·9 (0·4–1·8)	0·7 (0·3–1·5)
1-26	Typhoid fever	1 (0–2)	1 (0–1)	0 (0–1)	1·5 (0·6–2·8)	0·8 (0·4–1·6)	0·6 (0·2–1·1)
1-27	Paratyphoid fever	0 (0–0)	0 (0–0)	0 (0–0)	0·1 (0–0·3)	0·1 (0–0·2)	0·2 (0·1–0·4)
1-28	iNTS	1 (1–2)	1 (0–2)	1 (0–1)	1·8 (0·9–3)	1·4 (0·7–2·4)	1·2 (0·5–2·2)
1-29	Other intestinal infectious diseases	1 (0–1)	0 (0–1)	0 (0–1)	0·7 (0·4–1·2)	0·7 (0·3–1·1)	0·7 (0·3–1·2)
1-30	Neglected tropical diseases and malaria	102 (69–141)	92 (57–137)	79 (51–110)	143 (96·7–201)	129 (80·3–190)	107 (71·1–147)
1-31	Malaria	0 (0–0)	0 (0–0)	0 (0–0)	0 (0–0)	0 (0–0)	0 (0–0)
1-32	Chagas disease	0 (0–0)	0 (0–0)	0 (0–0)	0 (0–0)	0 (0–0)	0 (0–0)
1-33	Leishmaniasis	0 (0–0)	0 (0–0)	0 (0–0)	0 (0–0)	0 (0–0)	0 (0–0)
1-34	Visceral leishmaniasis	0 (0–0)	0 (0–0)	0 (0–0)	0 (0–0)	0 (0–0)	0 (0–0)
1-35	Cutaneous and mucocutaneous leishmaniasis	0 (0–0)	0 (0–0)	0 (0–0)	0 (0–0)	0 (0–0)	0 (0–0)
1-36	African trypanosomiasis	0 (0–0)	0 (0–0)	0 (0–0)	0 (0–0)	0 (0–0)	0 (0–0)
1-37	Schistosomiasis	0 (0–0)	0 (0–0)	0 (0–0)	0 (0–0)	0 (0–0)	0 (0–0)
1-38	Cysticercosis	51 (27–84)	45 (22–78)	45 (22–74)	76·6 (41–125)	58·4 (28·5–102)	53·5 (26·5–88·9)
1-39	Cystic echinococcosis	0 (0–0)	0 (0–0)	0 (0–0)	0 (0–0)	0 (0–0)	0 (0–0)
1-40	Lymphatic filariasis	0 (0–0)	0 (0–0)	0 (0–0)	0 (0–0)	0 (0–0)	0 (0–0)
1-41	Onchocerciasis	0 (0–0)	0 (0–0)	0 (0–0)	0 (0–0)	0 (0–0)	0 (0–0)
1-42	Trachoma	0 (0–0)	0 (0–0)	0 (0–0)	0 (0–0)	0 (0–0)	0 (0–0)
1-43	Dengue	12 (5–25)	27 (12–52)	10 (5–15)	15·8 (6·1–33·4)	39·9 (19·1–76)	15·2 (7·7–24·6)
1-44	Yellow fever	0 (0–0)	0 (0–0)	0 (0–0)	0 (0–0)	0 (0–0)	0 (0–0)
1-45	Rabies	0 (0–0)	0 (0–0)	0 (0–0)	0·2 (0·1–0·3)	0·3 (0·1–0·4)	0·4 (0·1–0·7)
1-46	Intestinal nematode infections	13 (7–22)	4 (2–6)	3 (2–5)	16·9 (9·5–28·2)	5·4 (3·1–9·2)	4·2 (2·5–6·8)
1-47	Ascariasis	0 (0–1)	0 (0–0)	0 (0–0)	0·6 (0·2–1)	0·3 (0·1–0·7)	0·3 (0·1–0·6)
1-48	Trichuriasis	0 (0–1)	0 (0–0)	0 (0–0)	0·4 (0·2–0·7)	0 (0–0)	0 (0–0)

1-49	Hookworm disease	13 (7–21)	4 (2–6)	3 (2–4)	16 (8·7–27·3)	5·1 (2·8–8·7)	3·9 (2·2–6·5)
1-50	Food-borne trematodiases	0 (0–0)	0 (0–0)	0 (0–0)	0 (0–0)	0 (0–0)	0 (0–0)
1-51	Leprosy	0 (0–1)	0 (0–1)	1 (0–1)	0·6 (0·4–0·9)	0·5 (0·3–0·8)	0·6 (0·4–0·9)
1-52	Ebola virus disease	0 (0–0)	0 (0–0)	0 (0–0)	0 (0–0)	0 (0–0)	0 (0–0)
1-53	Zika virus disease	0 (0–0)	0 (0–0)	4 (0–14)	0 (0–0)	0 (0–0)	5·9 (0–22·3)
1-54	Guinea worm disease	0 (0–0)	0 (0–0)	0 (0–0)	0 (0–0)	0 (0–0)	0 (0–0)
1-55	Other neglected tropical diseases	25 (18–35)	16 (11–24)	17 (12–23)	33·1 (24·2–46·3)	24·5 (17·2–35·7)	27·6 (19·2–38·1)
1-56	Other infectious diseases	315 (272–365)	187 (144–259)	173 (126–244)	412 (358–476)	304 (225–447)	305 (211–468)
1-57	Meningitis	137 (116–163)	46 (38–57)	44 (33–60)	175 (149–207)	77·7 (62·3–98·5)	83·4 (60·9–115)
1-58	Encephalitis	71 (52–86)	40 (32–51)	36 (26–49)	90 (66·3–109)	62·7 (50·5–81·5)	62·3 (44·4–87·8)
1-59	Diphtheria	0 (0–0)	0 (0–0)	0 (0–0)	0·1 (0·1–0·2)	0·2 (0·1–0·4)	0·2 (0·1–0·3)
1-60	Whooping cough	12 (1–39)	37 (4–106)	30 (3–88)	14·1 (1·5–46·2)	72·4 (8–207)	69·8 (8–206)
1-61	Tetanus	22 (14–29)	6 (4–12)	5 (3–11)	31·3 (20·3–42·2)	8·1 (6–16·9)	6 (3·7–14·3)
1-62	Measles	3 (1–5)	0 (0–0)	0 (0–0)	3·8 (1·6–6·4)	0 (0–0)	0 (0–0)
1-63	Varicella and herpes zoster	8 (6–12)	6 (4–8)	6 (4–8)	10·8 (7·5–15·3)	8·9 (6·2–12·6)	9·4 (6·4–13·5)
1-64	Acute hepatitis	24 (21–28)	6 (5–8)	5 (4–7)	34 (29·1–39·1)	9 (7·3–11·2)	8·1 (6·3–10·4)
1-65	Acute hepatitis A	14 (10–17)	3 (2–5)	3 (2–4)	18·6 (13·7–22·8)	5 (3·6–6·6)	4·5 (3·2–6·2)
1-66	Acute hepatitis B	9 (7–13)	2 (2–3)	2 (1–3)	13·4 (9·9–18·2)	3·2 (2·6–3·9)	2·7 (2–3·6)
1-67	Acute hepatitis C	0 (0–0)	0 (0–0)	0 (0–0)	0·4 (0·2–0·6)	0·1 (0·1–0·3)	0·1 (0·1–0·2)
1-68	Acute hepatitis E	1 (1–2)	1 (0–1)	1 (0–1)	1·6 (1·1–2·4)	0·7 (0·5–1)	0·8 (0·5–1)
1-69	Other unspecified infectious diseases	37 (30–46)	46 (29–56)	46 (28–62)	52·8 (42·7–66·3)	64·8 (40·7–80·2)	65·7 (40·6–86·8)
1-70	Maternal and neonatal disorders	1,840 (1,540–2,150)	1,270 (1,020–1,530)	1,300 (983–1,740)	2,260 (1,900–2,650)	2,560 (2,040–3,110)	2,980 (2,190–4,020)
1-71	Maternal disorders	72 (58–88)	66 (52–82)	57 (42–77)	95 (77·7–116)	97·2 (76·7–122)	84·6 (62–114)
1-72	Maternal haemorrhage	21 (16–27)	19 (14–24)	17 (12–23)	28·3 (22–36·3)	27·8 (20·9–35·7)	24·7 (17·7–34·2)
1-73	Maternal sepsis and other pregnancy related infections	17 (13–23)	12 (9–15)	10 (7–14)	23 (17·1–30·6)	17·1 (13–22·8)	15·2 (10·6–21·2)
1-74	Maternal hypertensive disorders	3 (2–4)	2 (1–3)	2 (1–2)	3·6 (2·4–5·1)	2·9 (2·1–4·1)	2·6 (1·8–3·6)
1-75	Maternal obstructed labour and uterine rupture	0 (0–1)	1 (1–2)	1 (1–1)	0·6 (0·3–0·9)	1·6 (1·2–2·2)	1·3 (0·9–1·8)
1-76	Maternal abortive outcome	23 (17–30)	11 (7–15)	8 (6–12)	30·2 (22·9–40)	15·5 (10·9–21·5)	12·3 (8·1–17·9)
1-77	Ectopic pregnancy	1 (1–2)	10 (7–13)	8 (6–12)	1·8 (1·4–2·5)	14·2 (10·4–19)	12·5 (8·4–17·6)
1-78	Indirect maternal deaths	0 (0–1)	1 (1–1)	1 (1–1)	0·6 (0·4–0·8)	1·4 (1–1·9)	1·3 (0·9–1·9)
1-79	Late maternal deaths	3 (2–5)	4 (3–6)	3 (2–5)	4·4 (3·1–6·4)	5·8 (3·9–8·5)	5 (3·1–7·4)
1-80	Maternal deaths aggravated by HIV/AIDS	0 (0–0)	0 (0–0)	0 (0–0)	0 (0–0·1)	0·1 (0–0·1)	0·1 (0–0·1)
1-81	Other maternal disorders	2 (1–2)	7 (5–10)	7 (5–9)	2·4 (1·9–3·1)	10·7 (7·9–14·1)	9·9 (7–13·7)
1-82	Neonatal disorders	1,760 (1,470–2,080)	1,200 (954–1,460)	1,250 (939–1,670)	2,170 (1,810–2,550)	2,460 (1,940–3,010)	2,890 (2,120–3,920)
1-83	Neonatal preterm birth	651 (496–822)	384 (303–484)	388 (279–529)	802 (612–1,010)	769 (597–983)	894 (622–1,250)
1-84	Neonatal encephalopathy due to birth asphyxia and trauma	547 (401–763)	398 (298–515)	422 (276–599)	668 (489–931)	846 (629–1,100)	1,030 (661–1,460)
1-85	Neonatal sepsis and other neonatal infections	369 (268–481)	287 (213–385)	302 (220–408)	455 (332–593)	572 (425–758)	648 (463–905)
1-86	Hemolytic disease and other neonatal jaundice	54 (38–74)	28 (22–35)	26 (19–36)	67·2 (47·6–91·3)	52·9 (40·7–68)	55·2 (38·5–78·4)
1-87	Other neonatal disorders	144 (98–214)	103 (75–137)	108 (69–159)	175 (119–260)	220 (160–294)	268 (171–396)
1-88	Nutritional deficiencies	420 (321–565)	280 (202–381)	284 (206–389)	568 (433–764)	410 (298–555)	430 (314–579)
1-89	Protein-energy malnutrition	140 (117–167)	64 (54–77)	65 (50–84)	181 (153–214)	100 (81·4–122)	109 (79·9–148)
1-90	Iodine deficiency	8 (4–14)	5 (3–8)	4 (2–7)	11·7 (6·2–19·2)	7·1 (3·6–11·8)	5·8 (2·9–9·7)
1-91	Vitamin A deficiency	6 (4–9)	3 (2–5)	3 (2–4)	7·4 (4·7–11·4)	5·1 (3·2–7·6)	5·1 (3·1–7·7)
1-92	Dietary iron deficiency	235 (148–365)	187 (117–280)	192 (122–288)	325 (206–509)	270 (168–406)	284 (177–429)
1-93	Other nutritional deficiencies	31 (25–38)	20 (17–24)	20 (15–24)	42·8 (35·1–52·1)	27·6 (22·6–33·2)	26·7 (20·7–33·8)
1-0	Non-communicable diseases	17,600 (15,800–19,600)	18,100 (16,000–20,400)	19,600 (16,700–22,900)	25,100 (22,300–28,000)	23,700 (20,900–26,700)	24,200 (20,500–28,500)
1-1	Neoplasms	3,260 (2,930–3,620)	3,560 (3,130–4,030)	3,980 (3,320–4,850)	4,730 (4,220–5,290)	4,540 (3,990–5,150)	4,670 (3,860–5,700)
1-2	Lip and oral cavity cancer	52 (45–60)	57 (48–66)	66 (52–82)	78·2 (67·4–90·2)	72·2 (61·5–84·1)	74·4 (59·3–92·3)
1-3	Nasopharynx cancer	8 (7–9)	12 (11–15)	14 (11–18)	12·4 (10·6–14·4)	16·3 (13·9–19·3)	16·8 (13·3–21·5)
1-4	Other pharynx cancer	40 (34–47)	36 (30–44)	45 (34–58)	61·8 (52·2–72·9)	46·8 (39·1–56·6)	50·4 (38·2–65·6)
1-5	Oesophageal cancer	81 (70–93)	87 (74–104)	106 (84–133)	122 (104–141)	111 (94·5–132)	119 (94·1–149)
1-6	Stomach cancer	439 (388–490)	356 (309–409)	388 (313–475)	642 (562–727)	448 (390–515)	441 (355–544)
1-7	Colon and rectum cancer	182 (160–204)	240 (212–276)	277 (227–341)	262 (230–297)	300 (266–346)	314 (256–387)

1-8	Liver cancer	151 (131–171)	58 (50–68)	70 (56–88)	222 (191–253)	74·9 (64·3–88)	80·7 (63·9–101)
1-9	Liver cancer due to hepatitis B	46 (34–61)	18 (13–24)	20 (14–29)	71·9 (53–97)	23·7 (17·6–32)	24·1 (16·4–33·7)
1-10	Liver cancer due to hepatitis C	32 (22–44)	11 (8–16)	13 (9–20)	44·6 (30·7–61·7)	13·9 (9·5–19·3)	14·8 (9·6–21·8)
1-11	Liver cancer due to alcohol use	48 (34–64)	19 (14–26)	25 (17–35)	70·4 (49·8–93·9)	24·6 (17·5–32·8)	28·1 (19·1–39·1)
1-12	Liver cancer due to NASH	12 (8–17)	5 (4–7)	6 (4–9)	16·9 (11·9–23·3)	6·4 (4·5–8·9)	7·1 (4·8–10·3)
1-13	Gallbladder and biliary tract cancer	80 (48–94)	35 (28–63)	40 (30–72)	117 (70·5–137)	44·2 (35·2–79·4)	44·5 (33·4–81·4)
1-14	Pancreatic cancer	29 (26–33)	134 (117–155)	165 (130–204)	42·8 (37·3–48·6)	169 (148–197)	186 (146–229)
1-15	Larynx cancer	35 (30–41)	43 (36–52)	52 (40–65)	53·1 (45·3–62)	55·4 (45·7–66·4)	57·8 (45–72·4)
1-16	Tracheal, bronchus, and lung cancer	266 (233–305)	309 (268–358)	357 (286–439)	396 (344–456)	393 (341–456)	402 (322–496)
1-17	Malignant skin melanoma	8 (6–11)	10 (7–13)	11 (7–15)	11·8 (9·4–16·4)	13 (9·3–16·6)	13·3 (8·9–17·7)
1-18	Non-melanoma skin cancer	7 (6–9)	10 (8–11)	11 (9–14)	10·8 (9·1–12·6)	11·7 (10–13·6)	12·4 (10–15·4)
1-19	Non-melanoma skin cancer (squamous-cell carcinoma)	7 (6–9)	10 (8–11)	11 (9–14)	10·8 (9·1–12·6)	11·7 (10–13·6)	12·4 (10–15·4)
1-20	Non-melanoma skin cancer (basal-cell carcinoma)	0 (0–0)	0 (0–0)	0 (0–0)	0 (0–0)	0 (0–0)	0 (0–0)
1-21	Breast cancer	283 (240–325)	328 (275–386)	362 (287–457)	454 (386–527)	422 (354–497)	431 (340–547)
1-22	Cervical cancer	234 (192–283)	208 (169–254)	213 (161–276)	379 (308–461)	274 (222–334)	263 (197–341)
1-23	Uterine cancer	35 (29–49)	44 (34–54)	49 (36–64)	53·1 (43·4–72·6)	56·7 (43–69·1)	56·3 (41·8–73·2)
1-24	Ovarian cancer	12 (10–19)	34 (20–44)	39 (21–53)	19·6 (15·3–29·9)	44·9 (26·3–56·8)	46·5 (25·1–62·8)
1-25	Prostate cancer	481 (411–627)	704 (519–848)	818 (609–1,020)	627 (535–816)	830 (617–998)	887 (661–1,110)
1-26	Testicular cancer	1 (1–1)	3 (2–3)	3 (2–4)	1·1 (0·9–1·3)	4·1 (3·3–5)	4·3 (3–6)
1-27	Kidney cancer	54 (47–61)	49 (42–57)	59 (46–74)	79·2 (68·8–90·1)	65·6 (56·5–76·3)	72·8 (56·4–92·4)
1-28	Bladder cancer	47 (41–53)	56 (48–64)	64 (53–79)	64·5 (56·8–72·4)	67·9 (59–78·6)	71·1 (58·7–87·8)
1-29	Brain and nervous system cancer	17 (13–26)	41 (19–54)	42 (18–61)	23·8 (18·4–37·8)	58·4 (26·8–77·1)	59·4 (25·3–85·8)
1-30	Thyroid cancer	11 (9–12)	13 (11–15)	15 (12–19)	16·2 (14–18·6)	16·4 (13·9–19·3)	17·3 (13·7–21·7)
1-31	Mesothelioma	5 (4–6)	7 (6–8)	9 (7–11)	7·3 (6–8·9)	8·7 (7·3–10·7)	10 (7·8–12·8)
1-32	Hodgkin lymphoma	17 (13–22)	16 (12–20)	16 (12–21)	24·6 (19·1–30·7)	21·8 (17·4–27·9)	20·9 (15·5–27·9)
1-33	Non-Hodgkin's lymphoma	169 (148–190)	173 (151–199)	184 (146–231)	244 (212–276)	236 (204–270)	236 (187–298)
1-34	Multiple myeloma	83 (64–108)	101 (83–125)	119 (94–152)	121 (93·5–158)	128 (105–158)	133 (105–170)
1-35	Leukaemia	174 (151–201)	181 (153–211)	173 (136–218)	234 (202–271)	253 (214–296)	245 (190–308)
1-36	Acute lymphoid leukaemia	56 (45–74)	61 (49–74)	52 (39–67)	68·6 (54·7–91·5)	89·5 (71·9–109)	83·9 (62·4–111)
1-37	Chronic lymphoid leukaemia	5 (4–6)	6 (5–7)	8 (6–10)	6·9 (5·4–8·7)	7·6 (6·1–9·5)	8·7 (6·5–11·5)
1-38	Acute myeloid leukaemia	30 (23–37)	35 (26–43)	35 (26–46)	41·1 (31·6–50·8)	49·4 (37–61·2)	50·3 (36–65·9)
1-39	Chronic myeloid leukaemia	11 (8–14)	12 (9–15)	13 (9–17)	15·9 (12·4–20·2)	16·1 (12·2–20·3)	16·1 (11·2–21·6)
1-40	Other leukaemia	72 (59–88)	67 (54–81)	66 (50–85)	101 (82·6–124)	90·7 (72·6–110)	85·9 (64·6–113)
1-41	Other neoplasms	12 (10–16)	17 (14–20)	19 (15–24)	16·4 (13·4–21·7)	21·8 (18·5–26·5)	23·2 (18·4–29·1)
1-42	Myelodysplastic, myeloproliferative, and other hematopoietic neoplasms	12 (10–16)	17 (14–20)	19 (15–24)	16·4 (13·4–21·7)	21·8 (18·5–26·5)	23·2 (18·4–29·1)
1-43	Benign and in situ intestinal neoplasms	0 (0–0)	0 (0–0)	0 (0–0)	0 (0–0)	0 (0–0)	0 (0–0)
1-44	Benign and in situ cervical and uterine neoplasms	0 (0–0)	0 (0–0)	0 (0–0)	0 (0–0)	0 (0–0)	0 (0–0)
1-45	Other benign and in situ neoplasms	0 (0–0)	0 (0–0)	0 (0–0)	0 (0–0)	0 (0–0)	0 (0–0)
1-46	Cardiovascular diseases	4,730 (4,300–5,140)	4,060 (3,630–4,540)	4,440 (3,810–5,300)	6,620 (5,990–7,220)	4,970 (4,450–5,590)	5,040 (4,300–6,030)
1-47	Rheumatic heart disease	116 (99–137)	82 (68–98)	77 (59–96)	170 (145–201)	112 (92·6–135)	103 (79·1–131)
1-48	Ischaemic heart disease	1,850 (1,670–2,020)	1,290 (1,140–1,460)	1,400 (1,180–1,670)	2,570 (2,310–2,830)	1,560 (1,370–1,760)	1,560 (1,300–1,860)
1-49	Stroke	1,250 (1,120–1,380)	1,130 (1,010–1,270)	1,220 (1,020–1,440)	1,740 (1,550–1,920)	1,370 (1,230–1,540)	1,360 (1,140–1,620)
1-50	Ischaemic stroke	570 (504–629)	576 (503–641)	606 (515–707)	743 (662–822)	666 (584–741)	665 (565–774)
1-51	Intracerebral hemorrhage	580 (504–657)	461 (404–534)	506 (412–621)	844 (731–962)	581 (510–674)	573 (467–705)
1-52	Subarachnoid hemorrhage	103 (85–125)	94 (80–112)	104 (81–132)	157 (130–192)	123 (105–147)	125 (97·5–159)
1-53	Hypertensive heart disease	691 (605–778)	639 (552–747)	732 (597–908)	958 (844–1,080)	775 (668–905)	816 (666–1,010)
1-54	Non-rheumatic valvular heart disease	25 (20–30)	30 (25–36)	34 (27–44)	35·9 (29·2–43·4)	38·2 (31·4–46·5)	39·9 (31·6–50·9)
1-55	Non-rheumatic calcific aortic valve disease	14 (11–17)	17 (14–21)	19 (15–24)	19·7 (15·5–24·2)	21·3 (17–26·1)	21·8 (16·7–28·2)
1-56	Non-rheumatic degenerative mitral valve disease	10 (8–13)	12 (10–16)	15 (11–19)	15·3 (11·5–19·5)	16·2 (12·7–21)	17·3 (12·9–22·8)
1-57	Other non-rheumatic valve diseases	1 (0–1)	1 (0–1)	1 (0–1)	1 (0·7–1·2)	0·7 (0·5–0·9)	0·8 (0·5–1·1)
1-58	Cardiomyopathy and myocarditis	351 (286–473)	410 (331–502)	467 (338–601)	514 (417–689)	534 (433–654)	560 (410–718)
1-59	Myocarditis	17 (12–24)	16 (12–20)	16 (12–22)	23·6 (17·4–33·6)	21·2 (16·4–27·5)	21·6 (15·8–29·2)
1-60	Alcoholic cardiomyopathy	11 (8–16)	16 (11–22)	21 (14–30)	17·6 (12·2–25·8)	21·9 (14·5–29·2)	24·8 (15·6–35)

1-61	Other cardiomyopathy	324 (263–436)	378 (305–462)	429 (312–550)	472 (383–640)	491 (396–600)	514 (377–662)
1-62	Atrial fibrillation and flutter	82 (70–95)	101 (88–117)	109 (92–131)	110 (93·4–128)	114 (99–133)	119 (99·9–142)
1-63	Aortic aneurysm	65 (54–76)	69 (61–80)	77 (63–94)	88·4 (73·4–103)	84·1 (73·4–96·7)	85·4 (70–104)
1-64	Peripheral vascular disease	18 (14–23)	24 (20–30)	28 (22–35)	24·3 (19–30·9)	28 (22·7–35·2)	30·8 (24·6–38·8)
1-65	Endocarditis	32 (24–43)	37 (29–47)	39 (29–52)	46·6 (35·6–63·4)	48·8 (39·2–62·2)	48·7 (36·5–64·9)
1-66	Chronic respiratory diseases	588 (496–708)	635 (529–744)	645 (524–768)	797 (673–948)	842 (698–1,000)	831 (674–1,010)
1-67	Chronic obstructive pulmonary disease	224 (198–262)	277 (233–314)	304 (247–365)	304 (269–356)	337 (280–381)	340 (277–408)
1-68	Pneumoconiosis	1 (0–1)	1 (0–1)	1 (0–1)	0·9 (0·7–1·1)	0·6 (0·5–0·8)	0·6 (0·5–0·8)
1-69	Silicosis	0 (0–0)	0 (0–0)	0 (0–0)	0·1 (0·1–0·2)	0·1 (0·1–0·2)	0·1 (0·1–0·2)
1-70	Asbestosis	0 (0–0)	0 (0–0)	0 (0–0)	0·3 (0·2–0·5)	0·2 (0·2–0·3)	0·2 (0·2–0·3)
1-71	Coal workers pneumoconiosis	0 (0–0)	0 (0–0)	0 (0–0)	0·1 (0–0·1)	0 (0–0·1)	0 (0–0·1)
1-72	Other pneumoconiosis	0 (0–0)	0 (0–0)	0 (0–0)	0·3 (0·2–0·4)	0·2 (0·2–0·3)	0·2 (0·2–0·3)
1-73	Asthma	324 (248–428)	300 (227–389)	280 (214–362)	436 (337–566)	427 (320–563)	410 (309–540)
1-74	Interstitial lung disease and pulmonary sarcoidosis	14 (11–20)	23 (17–28)	26 (18–34)	20·4 (15·9–28·7)	29·6 (21·2–36·2)	30·5 (20·8–39·6)
1-75	Other chronic respiratory diseases	25 (20–32)	34 (26–43)	34 (25–46)	36 (28·2–45·5)	48·2 (36·5–61)	48·9 (35·3–64·9)
1-76	Digestive diseases	820 (724–929)	757 (663–868)	796 (657–967)	1,220 (1,070–1,390)	1,010 (879–1,150)	989 (813–1,200)
1-77	Cirrhosis and other chronic liver diseases	313 (269–359)	272 (234–319)	291 (229–368)	488 (418–562)	356 (306–416)	345 (272–436)
1-78	Cirrhosis and other chronic liver diseases due to hepatitis B	36 (25–51)	33 (23–46)	20 (13–28)	59·2 (40·4–83·9)	43·4 (30–60·8)	23·4 (14·9–33·8)
1-79	Cirrhosis and other chronic liver diseases due to hepatitis C	57 (41–79)	49 (35–68)	55 (37–78)	91·6 (65·7–127)	64·1 (45·6–89·3)	65·6 (44–92·7)
1-80	Cirrhosis and other chronic liver diseases due to alcohol use	113 (87–141)	106 (80–133)	123 (91–163)	181 (140–228)	138 (104–174)	143 (105–189)
1-81	Cirrhosis and other chronic liver diseases due to NAFLD	47 (33–65)	40 (28–54)	47 (31–68)	72·6 (49·8–100)	51·5 (36·4–70·5)	54·9 (36–78)
1-82	Cirrhosis and other chronic liver diseases due to other causes	60 (46–76)	44 (33–57)	46 (32–64)	83·5 (63·4–108)	58·9 (45·1–75·2)	57·9 (41·3–79·7)
1-83	Upper digestive system diseases	189 (144–261)	172 (124–247)	181 (128–260)	284 (214–392)	229 (163–333)	225 (157–327)
1-84	Peptic ulcer disease	74 (62–85)	50 (42–58)	51 (39–66)	108 (91·4–124)	63·1 (52·9–73·8)	59·3 (45·1–76·6)
1-85	Gastritis and duodenitis	35 (27–45)	32 (24–42)	34 (25–47)	53 (40·8–68·2)	42·7 (31·9–56·5)	42·6 (31–58·1)
1-86	GERD	80 (42–142)	90 (47–158)	95 (50–170)	123 (64·1–218)	123 (63·9–218)	123 (63·8–217)
1-87	Appendicitis	22 (18–26)	15 (12–18)	15 (11–19)	30·2 (25–35·4)	20·5 (17·3–24·6)	20·4 (15·8–26·4)
1-88	Paralytic ileus and intestinal obstruction	93 (78–119)	80 (67–95)	84 (63–109)	126 (105–163)	111 (92·5–132)	113 (84·8–152)
1-89	Inguinal, femoral, and abdominal hernia	48 (36–62)	47 (35–62)	49 (36–65)	66·6 (50·3–87·4)	66·1 (49·3–87·4)	68·8 (50·1–91·8)
1-90	Inflammatory bowel disease	23 (19–28)	20 (17–25)	20 (16–27)	34·2 (27·5–41·2)	27·4 (22·9–33·5)	26 (20·2–34·3)
1-91	Vascular intestinal disorders	26 (22–30)	26 (22–30)	27 (22–33)	36·4 (31·5–42·2)	31·6 (27–36·8)	30·5 (24·5–38)
1-92	Gallbladder and biliary diseases	48 (38–60)	39 (30–51)	40 (30–53)	72·2 (56·6–91·5)	51·4 (38·9–67·6)	49·9 (37·3–66·5)
1-93	Pancreatitis	36 (29–46)	36 (30–45)	37 (28–50)	55·3 (44–70·9)	48 (39·4–60·5)	46 (34·6–61·2)
1-94	Other digestive diseases	22 (18–33)	51 (30–60)	52 (32–66)	29·8 (25·2–46)	65·7 (38·8–78)	64·4 (39·4–83·2)
1-95	Neurological disorders	1,080 (659–1,670)	1,150 (714–1,790)	1,170 (728–1,760)	1,490 (911–2,310)	1,510 (925–2,360)	1,520 (946–2,300)
1-96	Alzheimer's disease and other dementias	235 (97–558)	288 (118–660)	295 (120–680)	314 (129–722)	317 (130–725)	320 (130–737)
1-97	Parkinson's disease	68 (59–76)	83 (70–94)	89 (74–105)	84·8 (74·3–94·6)	94·1 (79·5–107)	95·9 (79·5–113)
1-98	Idiopathic epilepsy	324 (198–491)	299 (186–459)	289 (175–428)	449 (277–671)	423 (264–652)	412 (249–609)
1-99	Multiple sclerosis	4 (4–5)	5 (5–6)	6 (5–8)	6·9 (5·8–8·6)	7·3 (6·1–8·8)	7·5 (5·9–9·5)
1-100	Motor neuron disease	5 (4–5)	16 (13–18)	20 (14–26)	7·2 (6·1–8·4)	22 (18·4–26·1)	25·9 (18·7–34·3)
1-101	Headache disorders	388 (72–859)	390 (78–853)	395 (80–853)	552 (107–1,210)	546 (107–1,190)	545 (106–1,190)
1-102	Migraine	352 (49–817)	352 (54–809)	356 (56–815)	500 (73·4–1,150)	494 (73·7–1,130)	493 (71·6–1,130)
1-103	Tension-type headache	36 (10–137)	38 (11–135)	39 (12–136)	52·4 (15·4–192)	52 (15·2–189)	51·9 (15·2–188)
1-104	Other neurological disorders	57 (37–84)	72 (49–102)	77 (52–107)	76·1 (50·3–111)	103 (70·7–147)	115 (76·4–162)
1-105	Mental disorders	1,140 (842–1,510)	1,150 (842–1,510)	1,210 (890–1,590)	1,630 (1,210–2,160)	1,600 (1,180–2,110)	1,640 (1,210–2,160)
1-106	Schizophrenia	110 (76–147)	126 (88–167)	132 (93–176)	174 (120–232)	175 (122–232)	176 (123–236)
1-107	Depressive disorders	364 (247–501)	367 (252–511)	408 (282–563)	534 (366–742)	503 (345–704)	536 (368–741)
1-108	Major depressive disorder	304 (205–425)	302 (203–425)	340 (230–471)	443 (296–621)	414 (277–584)	446 (301–619)
1-109	Dysthymia	60 (37–92)	65 (40–100)	68 (43–104)	90·4 (56·2–138)	89·8 (55·7–139)	89·6 (56·5–138)
1-110	Bipolar disorder	135 (79–211)	139 (82–216)	144 (86–224)	196 (115–302)	195 (115–302)	195 (116–303)
1-111	Anxiety disorders	287 (192–410)	289 (191–407)	298 (200–426)	406 (270–570)	403 (266–570)	409 (273–580)
1-112	Eating disorders	29 (18–44)	29 (18–44)	30 (18–44)	37·8 (23·2–57·1)	42·9 (25·9–64·6)	43·8 (26·7–65·4)
1-113	Anorexia nervosa	7 (4–12)	7 (4–12)	7 (4–12)	9·3 (5·4–15·3)	10·4 (6–16·6)	10·7 (6·1–17·4)

1-114	Bulimia nervosa	21 (12–34)	22 (13–35)	22 (13–36)	28·5 (16·2–45)	32·6 (18·7–52·2)	33·2 (18·8–52·6)
1-115	Autism spectrum disorders	40 (26–58)	36 (24–52)	35 (23–51)	52·5 (34·6–76·7)	52·9 (34·9–76·6)	53 (35–77·4)
1-116	Attention-deficit/hyperactivity disorder	31 (17–53)	26 (15–44)	24 (14–41)	37·8 (21–64·7)	37·6 (21·2–64·2)	37·6 (21·2–63·9)
1-117	Conduct disorder	62 (36–99)	48 (27–77)	42 (24–66)	68·6 (39·1–109)	68·2 (38·6–108)	68·2 (38·5–108)
1-118	Idiopathic developmental intellectual disability	16 (5–29)	11 (3–22)	11 (3–20)	20·5 (6·9–38·2)	16·7 (5·1–31·5)	16·1 (4·8–30·1)
1-119	Other mental disorders	70 (45–105)	80 (51–121)	84 (54–128)	108 (68·8–164)	109 (69·3–166)	109 (69·6–165)
1-120	Substance use disorders	276 (218–346)	369 (292–449)	386 (302–474)	420 (333–522)	512 (406–625)	512 (402–632)
1-121	Alcohol use disorders	196 (154–244)	270 (213–334)	287 (222–360)	310 (245–386)	370 (291–458)	373 (288–469)
1-122	Drug use disorders	80 (57–109)	100 (75–128)	99 (74–127)	109 (78·3–147)	143 (108–184)	140 (104–180)
1-123	Opioid use disorders	24 (16–34)	31 (22–42)	34 (23–45)	35·5 (23·9–50·1)	44·7 (30·7–60·5)	46·4 (31·7–63·2)
1-124	Cocaine use disorders	18 (12–27)	29 (22–39)	29 (21–39)	24·7 (16·7–35·2)	42 (31·6–56)	41·3 (29·7–56·3)
1-125	Amphetamine use disorders	5 (3–8)	5 (3–8)	5 (3–8)	6·4 (3·5–10·9)	7·2 (4·4–11·3)	7·5 (4·5–11·8)
1-126	Cannabis use disorders	20 (10–33)	17 (9–27)	14 (7–22)	23·9 (13–39·1)	23·8 (13·3–38·7)	19·7 (10·6–32·8)
1-127	Other drug use disorders	14 (9–20)	17 (12–25)	17 (12–25)	18·5 (11·8–27·4)	24·8 (16·6–36·4)	24·6 (16·2–35·4)
1-128	Diabetes and kidney diseases	2,090 (1,860–2,330)	2,720 (2,360–3,090)	3,110 (2,610–3,660)	3,020 (2,670–3,390)	3,460 (3,010–3,940)	3,610 (3,030–4,250)
1-129	Diabetes mellitus	1,400 (1,230–1,600)	1,730 (1,470–2,040)	1,990 (1,630–2,410)	2,020 (1,760–2,310)	2,190 (1,850–2,580)	2,270 (1,860–2,750)
1-130	Diabetes mellitus type 1	85 (72–99)	81 (69–96)	83 (66–105)	125 (105–145)	111 (95·1–131)	111 (87·8–139)
1-131	Diabetes mellitus type 2	1,320 (1,150–1,510)	1,650 (1,390–1,950)	1,910 (1,560–2,320)	1,900 (1,650–2,170)	2,080 (1,740–2,450)	2,160 (1,760–2,630)
1-132	Chronic kidney disease	686 (613–762)	982 (867–1,100)	1,110 (923–1,340)	1,000 (886–1,120)	1,270 (1,130–1,430)	1,340 (1,110–1,620)
1-133	Chronic kidney disease due to diabetes mellitus type 1	47 (33–66)	74 (50–103)	81 (51–122)	77·4 (53·1–109)	99·3 (68–138)	103 (66·6–150)
1-134	Chronic kidney disease due to diabetes mellitus type 2	160 (131–193)	227 (183–276)	270 (210–341)	231 (186–280)	283 (228–345)	302 (234–380)
1-135	Chronic kidney disease due to hypertension	140 (115–171)	201 (165–238)	232 (184–295)	197 (163–244)	248 (202–294)	262 (208–333)
1-136	Chronic kidney disease due to glomerulonephritis	150 (122–184)	215 (175–263)	239 (180–316)	224 (180–275)	290 (238–353)	302 (229–394)
1-137	Chronic kidney disease due to other and unspecified causes	188 (151–229)	265 (216–327)	292 (225–378)	270 (215–332)	351 (286–432)	366 (284–470)
1-138	Acute glomerulonephritis	0 (0–1)	0 (0–0)	0 (0–1)	0·6 (0·4–0·8)	0·6 (0·5–0·7)	0·7 (0·5–0·9)
1-139	Skin and subcutaneous diseases	518 (379–716)	501 (372–682)	505 (374–677)	684 (503–944)	688 (507–945)	700 (511–954)
1-140	Dermatitis	90 (52–142)	79 (47–124)	74 (44–115)	116 (67·8–181)	115 (67·9–182)	115 (67·9–180)
1-141	Atopic dermatitis	68 (37–115)	56 (30–95)	51 (27–86)	85 (46–143)	85 (45·7–144)	84·9 (45·4–143)
1-142	Contact dermatitis	19 (12–28)	20 (12–30)	21 (13–31)	27·2 (17–41·1)	27 (17–40·3)	27 (16·8–40·3)
1-143	Seborrhoeic dermatitis	2 (1–4)	2 (1–4)	2 (1–4)	3·4 (1·9–5·5)	3·4 (1·9–5·4)	3·4 (1·9–5·4)
1-144	Psoriasis	24 (17–33)	23 (16–31)	23 (16–30)	35·2 (24·9–46·9)	31·2 (22·3–42·4)	30·6 (22–40·7)
1-145	Bacterial skin diseases	55 (38–75)	61 (46–75)	70 (48–93)	75·9 (53·1–103)	85 (63·8–105)	94·6 (66·5–127)
1-146	Cellulitis	15 (9–22)	15 (10–21)	16 (10–24)	21·2 (12·6–30·8)	21·4 (13·7–29·1)	22·9 (14–32·9)
1-147	Pyoderma	40 (27–56)	46 (34–57)	54 (36–73)	54·7 (36·8–75·9)	63·7 (47·4–78·4)	71·7 (49·4–97·8)
1-148	Scabies	65 (36–105)	58 (32–92)	54 (30–88)	84·1 (46·2–135)	83·4 (46–133)	83·1 (46–134)
1-149	Fungal skin diseases	32 (13–68)	33 (13–70)	34 (14–72)	44·3 (17·9–93·5)	44·3 (17·8–93·8)	44·4 (17·9–93·6)
1-150	Viral skin diseases	39 (25–58)	31 (20–48)	28 (18–42)	47·6 (30·4–71·5)	47·6 (30–72·1)	47·6 (30·1–71·3)
1-151	Acne vulgaris	37 (22–59)	34 (20–55)	33 (19–52)	41·6 (24·8–67·4)	47·8 (28·6–76·4)	49·1 (29·2–78·1)
1-152	Alopecia areata	5 (3–7)	5 (3–7)	5 (3–8)	7·1 (4·5–10·7)	7 (4·4–10·4)	7 (4·5–10·6)
1-153	Pruritus	6 (3–11)	6 (3–11)	6 (3–11)	8·3 (3·9–14·9)	8·4 (4–15·2)	8·4 (4–15)
1-154	Urticaria	37 (24–54)	32 (21–46)	30 (20–43)	48·2 (31·6–69·9)	48·1 (31·3–70)	48 (31·5–69·8)
1-155	Decubitus ulcer	92 (70–123)	100 (83–129)	106 (84–140)	125 (95·8–169)	117 (97·7–150)	120 (94·4–158)
1-156	Other skin and subcutaneous diseases	36 (18–65)	39 (20–69)	41 (21–72)	51·3 (26·1–92·6)	52·7 (26·5–93·2)	52·2 (26·5–92·3)
1-157	Sense organ diseases	559 (384–800)	589 (401–843)	626 (422–905)	794 (542–1,140)	748 (506–1,070)	735 (495–1,060)
1-158	Blindness and vision loss	166 (114–235)	165 (112–234)	173 (117–244)	231 (159–331)	206 (140–293)	201 (137–285)
1-159	Glaucoma	12 (8–17)	11 (7–16)	11 (7–16)	15·4 (10·1–22·1)	12·3 (8–17·7)	11·8 (7·8–16·8)
1-160	Cataract	33 (23–46)	31 (22–42)	31 (22–43)	43·8 (30·9–60)	35·9 (25·2–48·8)	34·4 (24·2–46·7)
1-161	Age-related macular degeneration	1 (1–2)	1 (1–2)	1 (1–2)	1·7 (1·1–2·5)	1·5 (1–2·2)	1·5 (0·9–2·1)
1-162	Refraction disorders	42 (28–60)	40 (27–58)	41 (27–59)	58·5 (39·1–83·9)	54·6 (35·9–78·9)	53·6 (35·6–77·8)
1-163	Near vision loss	36 (16–70)	39 (18–76)	43 (20–84)	51·7 (24–103)	49 (22·6–95·3)	48·3 (22·2–94·6)
1-164	Other vision loss	43 (30–59)	43 (30–58)	45 (32–62)	60 (42·3–82·9)	52·8 (37·4–71·7)	51·3 (36·2–70·6)
1-165	Age-related and other hearing loss	367 (246–533)	397 (266–575)	424 (283–623)	526 (349–758)	505 (338–740)	497 (332–727)
1-166	Other sense organ diseases	26 (15–40)	28 (17–43)	30 (17–46)	36·4 (21·4–56·2)	36·7 (21·7–56·9)	36·6 (22–56·6)

1-167	Musculoskeletal disorders	998 (720–1,320)	1,170 (845–1,550)	1,260 (909–1,670)	1,520 (1,100–2,000)	1,560 (1,130–2,060)	1,560 (1,130–2,070)
1-168	Rheumatoid arthritis	23 (17–29)	28 (21–35)	30 (22–38)	34·7 (26·2–44·2)	35·9 (27–45·8)	36·3 (26·9–47·1)
1-169	Osteoarthritis	129 (65–259)	158 (79–319)	180 (91–362)	193 (97–388)	200 (100–403)	202 (102–406)
1-170	Low back pain	501 (354–678)	542 (381–724)	567 (400–756)	745 (522–1,010)	731 (513–969)	720 (508–965)
1-171	Neck pain	86 (57–124)	100 (66–145)	107 (70–153)	136 (89–197)	134 (88·7–194)	134 (88·5–194)
1-172	Gout	4 (3–6)	6 (4–9)	7 (4–10)	6·5 (4–9·5)	7·7 (4·8–11·2)	7·8 (4·9–11·6)
1-173	Other musculoskeletal disorders	255 (178–346)	334 (234–451)	366 (258–492)	400 (280–547)	454 (319–613)	464 (328–625)
1-174	Other non-communicable diseases	1,560 (1,300–1,890)	1,480 (1,210–1,810)	1,510 (1,190–1,890)	2,140 (1,770–2,620)	2,290 (1,890–2,790)	2,410 (1,900–2,990)
1-175	Congenital anomalies	505 (420–617)	421 (338–519)	399 (296–537)	621 (516–757)	806 (637–1,010)	881 (633–1,200)
1-176	Neural tube defects	30 (19–48)	18 (13–25)	23 (14–36)	36·9 (23·5–58·3)	36·4 (25·6–50·2)	53·1 (31·6–84·7)
1-177	Congenital heart anomalies	188 (144–240)	149 (113–189)	136 (91–194)	230 (177–294)	290 (216–374)	310 (203–446)
1-178	Orofacial clefts	7 (3–15)	4 (3–6)	3 (2–5)	8·5 (4·1–18)	6·7 (4·4–10·2)	6 (3·7–10·1)
1-179	Down's syndrome	15 (11–20)	19 (15–24)	19 (14–26)	20 (15·1–26·3)	32·4 (25·1–41·7)	35·2 (24·7–49·3)
1-180	Turner syndrome	0 (0–0)	0 (0–0)	0 (0–0)	0·1 (0·1–0·2)	0·1 (0·1–0·2)	0·1 (0·1–0·2)
1-181	Klinefelter syndrome	0 (0–0)	0 (0–0)	0 (0–0)	0 (0–0)	0 (0–0)	0 (0–0)
1-182	Other chromosomal abnormalities	38 (26–59)	45 (32–61)	40 (26–59)	46·6 (31·1–71·2)	89·1 (63–122)	91·3 (58·3–139)
1-183	Congenital musculoskeletal and limb anomalies	56 (42–75)	50 (37–65)	48 (35–63)	70·9 (52·3–94·2)	85·1 (63·3–110)	90·8 (65·8–120)
1-184	Urogenital congenital anomalies	4 (3–5)	3 (2–4)	3 (2–4)	4·3 (3·2–5·9)	5·1 (3·8–6·8)	5·6 (3·9–7·7)
1-185	Digestive congenital anomalies	37 (26–54)	30 (21–41)	30 (18–45)	44·8 (30·9–65·8)	62·4 (44·3–86·8)	72·4 (44·1–111)
1-186	Other congenital anomalies	129 (100–166)	104 (80–136)	98 (68–138)	159 (123–204)	198 (150–263)	217 (149–312)
1-187	Urinary diseases and male infertility	45 (34–60)	70 (55–87)	81 (62–103)	61·7 (46·3–82·6)	89·9 (70·4–112)	95·6 (73·8–122)
1-188	Urinary tract infections and interstitial nephritis	17 (14–22)	33 (24–39)	38 (26–47)	23·1 (19·2–31·2)	41·5 (31·2–48·8)	45·4 (32·1–57·3)
1-189	Urolithiasis	2 (2–3)	3 (2–4)	4 (3–5)	3·6 (2·5–5)	4·3 (3·1–5·9)	4·6 (3·3–6·2)
1-190	Benign prostatic hyperplasia	17 (10–27)	22 (13–33)	25 (15–39)	23·1 (13·4–36·1)	27·4 (16·1–41·8)	27·8 (16·6–43·1)
1-191	Male infertility	3 (1–7)	3 (1–7)	3 (1–7)	4·4 (1·7–10·8)	4·3 (1·6–10·6)	4·3 (1·6–10·5)
1-192	Other urinary diseases	5 (4–7)	9 (7–12)	11 (7–15)	7·6 (5·9–10·2)	12·3 (8·9–16·2)	13·5 (9·1–18·5)
1-193	Gynecological diseases	244 (168–339)	255 (177–356)	256 (177–356)	375 (262–525)	356 (246–502)	352 (244–493)
1-194	Uterine fibroids	15 (8–27)	18 (9–32)	18 (9–32)	25·8 (13·8–46·1)	23·9 (12·2–44·2)	24·1 (12·2–43·3)
1-195	Polycystic ovarian syndrome	3 (1–7)	4 (2–9)	4 (2–9)	4·6 (1·9–9·5)	6·3 (2·7–12·8)	6·5 (2·8–13·2)
1-196	Female infertility	5 (2–12)	5 (2–13)	5 (2–13)	7·6 (2·6–18·7)	7·8 (2·7–18·7)	7·8 (2·7–19·1)
1-197	Endometriosis	21 (12–34)	18 (11–29)	17 (10–27)	31·2 (18·4–49·3)	26·2 (15·5–42·3)	24·6 (14·3–39·4)
1-198	Genital prolapse	6 (4–11)	5 (3–9)	5 (3–9)	9·8 (5·3–17·1)	6·9 (3·7–12·3)	6·4 (3·4–11·3)
1-199	Premenstrual syndrome	67 (41–105)	68 (41–106)	67 (41–103)	95·9 (58·7–150)	96·9 (59–151)	97·7 (60·2–152)
1-200	Other gynecological diseases	125 (82–178)	136 (88–193)	139 (90–196)	200 (131–286)	188 (122–268)	185 (120–264)
1-201	Hemoglobinopathies and hemolytic anaemias	189 (161–224)	168 (144–194)	159 (128–196)	250 (214–296)	243 (206–281)	237 (190–293)
1-202	Thalassemias	0 (0–0)	0 (0–0)	0 (0–0)	0·2 (0·1–0·4)	0·3 (0·1–0·5)	0·2 (0·1–0·5)
1-203	Thalassaemias trait	3 (2–4)	3 (2–4)	3 (2–4)	3·6 (2·1–5·6)	3·7 (2·2–5·7)	3·8 (2·4–6·1)
1-204	Sickle cell disorders	101 (84–120)	96 (81–113)	83 (63–108)	129 (108–152)	143 (121–168)	136 (102–178)
1-205	Sickle cell trait	32 (20–50)	24 (14–37)	20 (12–32)	41·4 (26–64·7)	35·4 (21·8–55·1)	30·2 (18·3–47·3)
1-206	G6PD deficiency	16 (10–21)	16 (11–21)	19 (13–26)	22·3 (15–30·8)	22·5 (15·7–29·9)	25·6 (17·3–35·4)
1-207	G6PD trait	0 (0–0)	0 (0–0)	0 (0–0)	0·1 (0·1–0·2)	0·2 (0·1–0·4)	0·3 (0·1–0·4)
1-208	Other hemoglobinopathies and hemolytic anaemias	38 (32–47)	30 (24–36)	33 (27–41)	53·9 (44·6–66·2)	38·3 (31·1–47·4)	40·9 (32·6–51)
1-209	Endocrine, metabolic, blood, and immune disorders	338 (256–411)	315 (256–407)	344 (268–457)	478 (364–581)	452 (365–584)	490 (374–654)
1-210	Oral disorders	231 (136–375)	246 (142–404)	261 (154–428)	340 (198–556)	325 (189–537)	322 (188–529)
1-211	Caries of deciduous teeth	3 (1–6)	2 (1–4)	1 (1–3)	3·1 (1·4–6·7)	3·1 (1·3–6·6)	3·1 (1·3–6·5)
1-212	Caries of permanent teeth	20 (9–38)	19 (8–36)	19 (8–36)	27 (12·1–51·1)	26·1 (11·7–50·8)	26 (11·6–50·2)
1-213	Periodontal disease	66 (26–141)	81 (32–173)	86 (33–184)	108 (41·4–234)	110 (42·9–236)	110 (42–236)
1-214	Edentulism	105 (67–158)	106 (68–158)	117 (74–176)	148 (94·3–222)	133 (84·8–199)	130 (82·7–198)
1-215	Other oral disorders	38 (23–55)	38 (23–55)	38 (24–56)	53·3 (33·1–77·5)	53·1 (33·2–77·2)	53 (33·1–77·4)
1-216	Sudden infant death syndrome	12 (6–23)	10 (4–20)	11 (4–23)	14·5 (7·6–27·4)	22·1 (9·4–42·3)	28 (10–58)
1-0	Injuries	2,600 (2,330–2,890)	2,580 (2,310–2,880)	2,290 (1,900–2,730)	3,580 (3,200–4,010)	3,690 (3,290–4,110)	3,260 (2,680–3,880)
1-1	Transport injuries	884 (764–1,000)	754 (653–873)	697 (564–858)	1,230 (1,050–1,410)	1,070 (925–1,240)	977 (787–1,210)
1-2	Road injuries	854 (737–972)	718 (622–832)	663 (536–818)	1,190 (1,020–1,360)	1,020 (880–1,180)	928 (747–1,150)

1-3	Pedestrian road injuries	199 (158–249)	146 (125–175)	139 (110–178)	278 (220–352)	204 (175–246)	194 (152–250)
1-4	Cyclist road injuries	62 (50–77)	59 (49–71)	55 (44–67)	88·9 (71·1–110)	82·1 (68–99·2)	74 (59·3–90·9)
1-5	Motorcyclist road injuries	172 (134–218)	190 (157–226)	177 (138–224)	241 (189–302)	271 (227–324)	249 (193–317)
1-6	Motor vehicle road injuries	413 (327–486)	315 (267–379)	284 (220–359)	570 (450–673)	447 (379–538)	401 (307–508)
1-7	Other road injuries	8 (6–10)	8 (6–10)	8 (6–10)	11·7 (9·2–14·7)	10·7 (8·4–13·4)	10·9 (8·3–13·8)
1-8	Other transport injuries	30 (24–36)	36 (30–42)	34 (26–43)	41·6 (34·3–50·7)	50·7 (42·3–60·6)	48·4 (37·6–62·2)
1-9	Unintentional injuries	1,240 (1,080–1,400)	1,150 (1,010–1,300)	981 (811–1,180)	1,690 (1,470–1,910)	1,640 (1,450–1,850)	1,400 (1,150–1,690)
1-10	Falls	122 (102–145)	156 (128–188)	171 (137–210)	174 (145–208)	204 (167–248)	214 (171–262)
1-11	Drowning	297 (252–350)	258 (215–308)	213 (161–273)	385 (327–455)	377 (313–448)	330 (249–427)
1-12	Fire, heat, and hot substances	170 (138–205)	133 (108–168)	133 (105–168)	238 (194–288)	190 (154–240)	192 (150–242)
1-13	Poisonings	216 (119–264)	50 (39–96)	47 (33–92)	293 (162–356)	76·7 (58·9–145)	74·7 (51·4–143)
1-14	Poisoning by carbon monoxide	12 (8–20)	6 (4–8)	6 (4–9)	16·5 (11·5–27·5)	8·7 (5·4–12)	9·2 (5·7–13·5)
1-15	Poisoning by other means	203 (109–251)	44 (34–89)	40 (28–83)	276 (147–340)	67·9 (51·1–135)	65·5 (44·1–131)
1-16	Exposure to mechanical forces	109 (86–137)	127 (98–163)	128 (97–165)	156 (123–197)	177 (137–225)	174 (133–226)
1-17	Unintentional firearm injuries	30 (22–40)	28 (21–35)	26 (19–34)	41·7 (31–55·3)	39·9 (29·1–49·7)	36·3 (26·1–47·9)
1-18	Other exposure to mechanical forces	79 (59–106)	99 (74–131)	102 (76–135)	114 (84·4–152)	137 (103–182)	138 (103–181)
1-19	Adverse effects of medical treatment	84 (66–98)	67 (54–81)	67 (51–88)	116 (91·5–137)	92·3 (74·3–112)	91·2 (68·5–119)
1-20	Animal contact	9 (6–12)	12 (8–15)	12 (8–15)	12·1 (8·6–16·7)	16·6 (11·5–21)	16 (11·4–20·3)
1-21	Venomous animal contact	6 (4–8)	7 (5–9)	7 (5–9)	8 (5·4–10·9)	9·7 (7·1–12·3)	9·7 (7–12·4)
1-22	Non-venomous animal contact	3 (2–4)	5 (3–7)	5 (3–6)	4·1 (2·9–6)	6·9 (4–8·8)	6·3 (3·8–8·4)
1-23	Foreign body	99 (83–118)	63 (54–76)	66 (51–87)	130 (109–152)	98·7 (82·3–122)	110 (81·7–151)
1-24	Pulmonary aspiration and foreign body in airway	88 (72–106)	53 (44–65)	55 (42–76)	114 (93·8–136)	84·7 (69·1–107)	96·2 (68·4–135)
1-25	Foreign body in eyes	4 (2–7)	4 (2–7)	4 (2–7)	5·5 (2·6–9·6)	5·5 (2·6–9·6)	5·5 (2·6–9·7)
1-26	Foreign body in other body part	7 (6–9)	6 (5–8)	6 (5–8)	10·2 (8–12·9)	8·5 (6·6–11)	8·9 (6·8–11·4)
1-27	Environmental heat and cold exposure	32 (24–42)	32 (24–42)	35 (26–45)	46·3 (34·4–60·5)	43·9 (32·5–57·7)	45·4 (33·7–59)
1-28	Exposure to forces of nature	1 (0–1)	152 (138–168)	18 (12–24)	0·8 (0·6–1·1)	221 (201–244)	21·1 (15·2–28·9)
1-29	Other unintentional injuries	98 (79–120)	99 (77–123)	93 (70–120)	137 (110–167)	141 (109–174)	132 (99·1–169)
1-30	Self-harm and interpersonal violence	476 (414–554)	681 (580–787)	616 (483–761)	661 (573–770)	983 (839–1,130)	878 (683–1,090)
1-31	Self-harm	141 (118–176)	161 (124–196)	162 (121–205)	204 (170–253)	229 (175–278)	222 (165–280)
1-32	Self-harm by firearm	6 (4–9)	6 (3–8)	5 (3–8)	8·1 (5·3–13)	8 (4·7–11·7)	7·4 (4·6–11·2)
1-33	Self-harm by other specified means	135 (113–168)	156 (119–189)	157 (117–198)	196 (162–241)	221 (169–268)	215 (160–271)
1-34	Interpersonal violence	326 (276–386)	514 (432–599)	449 (343–566)	444 (377–527)	746 (629–864)	650 (493–822)
1-35	Physical violence by firearm	87 (62–116)	169 (131–209)	142 (99–189)	116 (82·3–153)	246 (190–303)	206 (144–276)
1-36	Physical violence by sharp object	134 (103–178)	209 (162–254)	183 (130–241)	184 (142–246)	303 (234–367)	263 (187–347)
1-37	Sexual violence	13 (8–18)	12 (8–18)	12 (8–18)	17·6 (11·6–25)	17·6 (11·6–25)	17·6 (11·6–24·9)
1-38	Physical violence by other means	92 (72–115)	124 (100–147)	112 (83–144)	127 (101–160)	180 (145–215)	163 (120–211)
1-39	Conflict and terrorism	9 (6–13)	5 (4–8)	4 (3–6)	12·6 (8·1–19·2)	7·2 (4·8–10·7)	4·9 (3·3–7·2)
1-40	Executions and police conflict	0 (0–1)	0 (0–0)	0 (0–0)	0·6 (0·5–0·6)	0·6 (0·5–0·7)	0·6 (0·5–0·7)

SR Table 1: All-age DALYs counts and age standardized DALYs rates per 100,000 individuals for all causes and level 1 cause groups by location for 1990, 2010, and 2019, both sexes combined, Dominican Republic

Cause Level	Cause	All-age DALYs			Age-standardized rate of DALYs per 100,000		
		1990	2010	2019	1990	2010	2019
0-0	All causes	2,730,000 (2,500,000–2,980,000)	2,890,000 (2,480,000–3,320,000)	3,300,000 (2,720,000–3,980,000)	37,500 (34,100–41,400)	32,000 (27,600–36,800)	32,100 (26,400–38,500)
1-0	Communicable, maternal, neonatal, and nutritional disorders	1,310,000 (1,190,000–1,450,000)	791,000 (691,000–902,000)	710,000 (565,000–882,000)	13,200 (12,000–14,500)	7,860 (6,870–8,960)	6,550 (5,210–8,130)
1-1	HIV/AIDS and sexually transmitted infections	38,800 (22,900–65,400)	117,000 (93,400–152,000)	82,400 (58,500–116,000)	455 (272–773)	1,210 (978–1,570)	751 (536–1,050)
1-2	HIV/AIDS	21,700 (11,400–41,700)	105,000 (83,800–137,000)	63,400 (44,800–96,100)	289 (147–580)	1,090 (883–1,420)	577 (413–867)
1-3	HIV/AIDS - drug-susceptible tuberculosis	8,360 (4,790–13,500)	13,100 (8,530–19,800)	10,100 (6,640–15,600)	110 (61·5–178)	137 (89·1–206)	92·4 (60·7–142)
1-4	HIV/AIDS - Multidrug-resistant Tuberculosis without extensive drug resistance	90 (6–381)	112 (10–512)	138 (11–670)	1·2 (0·1–5)	1·2 (0·1–5·4)	1·3 (0·1–6·1)
1-5	HIV/AIDS - Extensively drug-resistant Tuberculosis	0 (0–0)	12 (1–60)	23 (2–111)	0 (0–0)	0·1 (0–0·6)	0·2 (0–1)
1-6	HIV/AIDS resulting in other diseases	13,300 (6,550–29,600)	91,500 (69,500–124,000)	53,200 (35,000–84,900)	178 (85·7–416)	955 (733–1,290)	484 (318–769)
1-7	Sexually transmitted infections excluding HIV	17,100 (6,640–35,300)	12,400 (5,340–23,900)	19,000 (8,020–37,900)	166 (73·3–325)	120 (52·5–230)	173 (73–345)
1-8	Syphilis	15,500 (5,030–33,800)	10,500 (3,570–21,800)	16,600 (5,690–35,000)	141 (48·6–301)	99·7 (34·5–207)	151 (52–320)
1-9	Chlamydial infection	188 (133–267)	236 (158–340)	288 (200–404)	2·8 (2–4)	2·3 (1·6–3·4)	2·5 (1·8–3·5)
1-10	Gonococcal infection	277 (191–352)	241 (184–323)	352 (243–487)	4·2 (2·9–5·2)	2·4 (1·9–3·2)	3·2 (2·2–4·4)
1-11	Trichomoniasis	305 (119–618)	458 (177–954)	515 (199–1,060)	5·1 (2–10·5)	4·8 (1·9–9·9)	4·6 (1·8–9·5)
1-12	Genital herpes	302 (100–748)	493 (164–1,200)	576 (194–1,400)	5·4 (1·8–13·4)	5·4 (1·8–13·2)	5·3 (1·8–13)
1-13	Other sexually transmitted infections	516 (356–668)	520 (382–697)	685 (499–913)	7·9 (5·6–10·2)	5·3 (3·9–7·1)	6·1 (4·5–8·2)
1-14	Respiratory infections and tuberculosis	230,000 (195,000–268,000)	119,000 (100,000–141,000)	109,000 (86,000–138,000)	2,640 (2,310–3,020)	1,270 (1,060–1,490)	1,040 (825–1,310)
1-15	Tuberculosis	65,400 (57,800–73,300)	29,800 (24,700–36,600)	34,100 (25,900–45,700)	914 (821–1,010)	318 (261–388)	312 (238–417)
1-16	Latent tuberculosis infection	0 (0–0)	0 (0–0)	0 (0–0)	0 (0–0)	0 (0–0)	0 (0–0)
1-17	Drug-susceptible tuberculosis	64,900 (57,200–72,800)	29,600 (24,500–36,400)	33,700 (25,500–44,900)	906 (809–1,010)	315 (260–385)	309 (234–412)
1-18	Multidrug-resistant tuberculosis without extensive drug resistance	573 (42–2,450)	200 (22–853)	365 (31–1,790)	8 (0·6–35)	2·1 (0·2–9·1)	3·4 (0·3–16·4)
1-19	Extensively drug-resistant tuberculosis	0 (0–0)	21 (2–98)	60 (5–290)	0 (0–0)	0·2 (0–1)	0·5 (0–2·7)
1-20	Lower respiratory infections	156,000 (126,000–190,000)	79,300 (64,800–95,800)	63,300 (47,400–82,700)	1,620 (1,350–1,930)	846 (696–1,010)	625 (474–807)
1-21	Upper respiratory infections	5,890 (3,610–9,180)	7,350 (4,420–11,600)	8,250 (4,980–13,000)	75·7 (46·7–119)	73·2 (44·3–116)	75·1 (45·4–118)
1-22	Otitis media	2,580 (1,480–4,210)	2,980 (1,720–4,880)	3,020 (1,750–4,970)	32·9 (19·1–53·7)	29·5 (17·1–47·8)	27·6 (15·9–45·1)
1-23	Enteric infections	266,000 (201,000–337,000)	52,800 (39,300–68,600)	49,800 (33,500–72,300)	2,560 (1,960–3,220)	541 (404–698)	471 (320–681)
1-24	Diarrhoeal diseases	266,000 (200,000–336,000)	52,400 (39,000–68,100)	49,400 (33,000–71,800)	2,560 (1,960–3,210)	537 (401–693)	468 (316–677)
1-25	Typhoid and paratyphoid	174 (75–352)	167 (75–330)	169 (73–327)	2·2 (1–4·3)	1·7 (0·8–3·3)	1·6 (0·7–3)
1-26	Typhoid fever	160 (68–325)	150 (66–301)	125 (52–246)	2 (0·9–4)	1·5 (0·7–3)	1·1 (0·5–2·3)
1-27	Paratyphoid fever	14 (5–32)	17 (6–36)	44 (17–97)	0·2 (0·1–0·4)	0·2 (0·1–0·4)	0·4 (0·2–0·9)
1-28	iNTS	188 (90–329)	153 (73–271)	164 (71–306)	2·3 (1·2–4)	1·5 (0·8–2·7)	1·5 (0·7–2·8)
1-29	Other intestinal infectious diseases	283 (85–513)	102 (46–174)	88 (46–147)	2·8 (1–5)	1 (0·5–1·7)	0·8 (0·4–1·3)
1-30	Neglected tropical diseases and malaria	29,600 (20,400–42,100)	26,000 (17,900–37,600)	22,900 (15,200–34,200)	462 (329–638)	268 (186–385)	212 (142–314)
1-31	Malaria	436 (181–855)	2,300 (970–4,760)	272 (63–987)	5·8 (2·4–11·4)	22·6 (9·5–46·8)	2·4 (0·6–8·7)
1-32	Chagas disease	11 (7–16)	9 (6–13)	8 (5–11)	0·3 (0·2–0·4)	0·1 (0·1–0·2)	0·1 (0–0·1)
1-33	Leishmaniasis	0 (0–3)	0 (0–3)	1 (0–3)	0 (0–0)	0 (0–0)	0 (0–0)
1-34	Visceral leishmaniasis	0 (0–0)	0 (0–0)	0 (0–0)	0 (0–0)	0 (0–0)	0 (0–0)
1-35	Cutaneous and mucocutaneous leishmaniasis	0 (0–3)	0 (0–3)	1 (0–3)	0 (0–0)	0 (0–0)	0 (0–0)
1-36	African trypanosomiasis	0 (0–0)	0 (0–0)	0 (0–0)	0 (0–0)	0 (0–0)	0 (0–0)
1-37	Schistosomiasis	9,250 (4,990–16,300)	10,100 (5,110–19,100)	10,900 (5,510–20,300)	133 (76·8–228)	101 (51·9–188)	97·6 (49·3–183)
1-38	Cysticercosis	2,540 (1,450–4,000)	3,520 (1,890–5,780)	4,100 (2,170–6,800)	52·5 (29·5–82)	42·9 (23·2–69·9)	41·5 (22–67·7)
1-39	Cystic echinococcosis	0 (0–1)	0 (0–1)	2 (1–3)	0 (0–0)	0 (0–0)	0 (0–0)
1-40	Lymphatic filariasis	12,900 (8,390–19,300)	3,440 (1,840–6,260)	4,020 (2,130–7,240)	216 (140–320)	35·6 (19·1–64·7)	37·1 (19·6–66·6)
1-41	Onchocerciasis	0 (0–0)	0 (0–0)	0 (0–0)	0 (0–0)	0 (0–0)	0 (0–0)
1-42	Trachoma	0 (0–0)	0 (0–0)	0 (0–0)	0 (0–0)	0 (0–0)	0 (0–0)
1-43	Dengue	651 (247–1,390)	4,450 (1,330–6,220)	1,240 (563–2,410)	8·4 (3·2–17·8)	43·7 (13·5–61·3)	11·4 (5·2–22·3)
1-44	Yellow fever	0 (0–0)	0 (0–0)	0 (0–0)	0 (0–0)	0 (0–0)	0 (0–0)
1-45	Rabies	74 (4–120)	50 (2–82)	178 (2–325)	1 (0–1·5)	0·6 (0–1·1)	1·7 (0–3·1)
1-46	Intestinal nematode infections	1,270 (792–1,960)	457 (269–720)	330 (190–532)	15·2 (9·7–23·4)	4·4 (2·6–6·9)	2·9 (1·7–4·7)
1-47	Ascariasis	420 (199–734)	100 (28–209)	56 (18–116)	4·3 (2·2–7·3)	1 (0·3–2·1)	0·5 (0·2–1·1)
1-48	Trichuriasis	0 (0–0)	0 (0–0)	0 (0–0)	0 (0–0)	0 (0–0)	0 (0–0)

1·49	Hookworm disease	848 (464–1,430)	357 (192–614)	274 (142–477)	10·9 (6–18·3)	3·4 (1·8–5·8)	2·4 (1·2–4·2)
1·50	Food-borne trematodiases	0 (0–0)	0 (0–0)	0 (0–0)	0 (0–0)	0 (0–0)	0 (0–0)
1·51	Leprosy	22 (14–35)	20 (12–30)	24 (14–36)	0·5 (0·3–0·7)	0·2 (0·1–0·4)	0·2 (0·1–0·3)
1·52	Ebola virus disease	0 (0–0)	0 (0–0)	0 (0–0)	0 (0–0)	0 (0–0)	0 (0–0)
1·53	Zika virus disease	0 (0–0)	0 (0–0)	18 (2–54)	0 (0–0)	0 (0–0)	0·2 (0–0·5)
1·54	Guinea worm disease	0 (0–0)	0 (0–0)	0 (0–0)	0 (0–0)	0 (0–0)	0 (0–0)
1·55	Other neglected tropical diseases	2,410 (1,560–3,490)	1,670 (1,030–2,570)	1,830 (1,170–2,760)	29·7 (19·5–42·9)	16·8 (10·6–25·8)	17 (10·8–25·6)
1·56	Other infectious diseases	118,000 (79,600–193,000)	48,100 (30,700–80,800)	44,500 (25,400–75,600)	1,180 (824–1,870)	475 (308–788)	409 (236–692)
1·57	Meningitis	58,100 (45,600–72,200)	18,600 (14,800–23,400)	13,400 (9,120–19,200)	561 (446–688)	183 (146–228)	124 (84·5–177)
1·58	Encephalitis	6,310 (3,560–8,710)	3,750 (2,890–4,960)	5,160 (3,360–7,630)	67·7 (41·2–90·1)	37·1 (28·8–49)	47·3 (30·8–69·4)
1·59	Diphtheria	348 (138–720)	88 (46–164)	80 (41–147)	3·5 (1·4–6·9)	0·9 (0·4–1·6)	0·7 (0·4–1·3)
1·60	Whooping cough	38,800 (4,280–114,000)	18,400 (2,420–51,600)	18,000 (2,320–47,200)	364 (40·4–1,070)	178 (23·3–496)	163 (21–430)
1·61	Tetanus	3,100 (2,350–4,950)	1,100 (698–1,800)	820 (442–1,950)	41·2 (31·7–77·4)	11·9 (7·7–20·7)	7·8 (4·3–18·6)
1·62	Measles	2,230 (1,570–3,070)	0 (0–0)	0 (0–0)	21·5 (15·1–29·5)	0 (0–0)	0 (0–0)
1·63	Varicella and herpes zoster	1,660 (1,020–2,570)	965 (646–1,430)	928 (595–1,380)	20·5 (14–29·4)	10·2 (7–14·7)	8·9 (5·9–13·2)
1·64	Acute hepatitis	3,750 (3,010–4,530)	1,500 (1,110–1,930)	2,360 (1,360–3,710)	47·8 (39·8–56·1)	15·2 (11·2–19·7)	21·7 (12·5–34)
1·65	Acute hepatitis A	1,700 (1,130–2,280)	625 (437–831)	738 (458–1,070)	19·4 (13–25·2)	6 (4·2–7·9)	6·6 (4·1–9·6)
1·66	Acute hepatitis B	997 (625–1,370)	417 (286–584)	770 (399–1,330)	11·7 (8·1–15·6)	4·2 (2·9–5·8)	7 (3·6–12)
1·67	Acute hepatitis C	931 (620–1,520)	384 (238–594)	739 (325–1,340)	15·1 (11·1–21·7)	4·3 (2·7–6·4)	7 (3·2–12·7)
1·68	Acute hepatitis E	116 (72–191)	76 (51–108)	115 (68–181)	1·5 (0·9–2·2)	0·8 (0·5–1·1)	1·1 (0·6–1·7)
1·69	Other unspecified infectious diseases	3,900 (2,890–5,120)	3,650 (2,830–4,690)	3,700 (2,660–5,110)	54·6 (41·7–68·2)	39·3 (30·5–50)	35·2 (25·4–48·2)
1·70	Maternal and neonatal disorders	501,000 (428,000–581,000)	383,000 (322,000–448,000)	354,000 (264,000–471,000)	4,500 (3,860–5,190)	3,640 (3,060–4,250)	3,220 (2,400–4,280)
1·71	Maternal disorders	14,300 (12,200–16,700)	13,600 (10,900–17,100)	15,300 (10,900–20,600)	181 (155–211)	128 (102–161)	129 (91·4–173)
1·72	Maternal haemorrhage	3,350 (2,700–4,170)	2,200 (1,650–2,870)	2,140 (1,520–2,920)	44·1 (35·5–54·7)	20·9 (15·7–27·2)	18·1 (12·9–24·8)
1·73	Maternal sepsis and other pregnancy related infections	1,090 (845–1,390)	705 (519–951)	753 (516–1,070)	13·6 (10·6–17·4)	6·5 (4·8–8·8)	6·3 (4·3–8·9)
1·74	Maternal hypertensive disorders	4,980 (4,140–5,960)	3,830 (2,960–4,870)	4,020 (2,810–5,630)	62 (51·5–74)	35·7 (27·5–45·4)	33·8 (23·6–47·3)
1·75	Maternal obstructed labour and uterine rupture	61 (25–116)	166 (114–234)	215 (140–315)	0·7 (0·3–1·4)	1·6 (1·1–2·2)	1·8 (1·2–2·6)
1·76	Maternal abortive outcome	2,110 (1,590–2,750)	1,170 (838–1,620)	1,210 (777–1,770)	27·5 (20·8–35·1)	11·1 (7·9–15·3)	10·2 (6·6–15)
1·77	Ectopic pregnancy	76 (60–96)	181 (136–240)	225 (152–326)	1 (0·8–1·2)	1·7 (1·3–2·3)	1·9 (1·3–2·7)
1·78	Indirect maternal deaths	1,720 (1,320–2,170)	2,900 (2,190–3,820)	3,660 (2,410–5,180)	21·1 (16·3–26·4)	27 (20·2–35·6)	30·7 (20·3–43·4)
1·79	Late maternal deaths	453 (327–616)	810 (560–1,130)	1,010 (627–1,520)	5·7 (4·2–7·7)	7·6 (5·3–10·7)	8·5 (5·3–12·7)
1·80	Maternal deaths aggravated by HIV/AIDS	5 (2–12)	56 (26–96)	65 (28–112)	0·1 (0–0·2)	0·5 (0·3–0·9)	0·6 (0·2–1)
1·81	Other maternal disorders	457 (353–578)	1,630 (1,200–2,160)	2,000 (1,360–2,820)	5·7 (4·4–7·2)	15·3 (11·3–20·4)	16·8 (11·5–23·6)
1·82	Neonatal disorders	486,000 (414,000–566,000)	369,000 (310,000–432,000)	339,000 (253,000–451,000)	4,320 (3,680–5,000)	3,510 (2,950–4,100)	3,090 (2,300–4,120)
1·83	Neonatal preterm birth	235,000 (191,000–286,000)	176,000 (144,000–212,000)	152,000 (107,000–208,000)	2,100 (1,710–2,540)	1,670 (1,370–2,020)	1,390 (971–1,900)
1·84	Neonatal encephalopathy due to birth asphyxia and trauma	96,500 (61,900–131,000)	49,400 (36,100–64,200)	43,400 (25,800–67,200)	846 (545–1,140)	469 (343–610)	397 (236–614)
1·85	Neonatal sepsis and other neonatal infections	89,200 (64,500–119,000)	112,000 (88,000–139,000)	111,000 (75,700–156,000)	794 (573–1,050)	1,060 (837–1,320)	1,010 (689–1,420)
1·86	Hemolytic disease and other neonatal jaundice	9,520 (7,210–12,400)	4,090 (3,260–4,970)	3,130 (2,360–4,060)	87·7 (67·4–113)	39·2 (31·4–47·6)	28·4 (21·4–36·9)
1·87	Other neonatal disorders	56,000 (40,700–74,000)	28,100 (21,800–34,800)	28,700 (18,100–43,100)	491 (358–648)	267 (207–331)	262 (166–394)
1·88	Nutritional deficiencies	130,000 (103,000–162,000)	44,600 (33,400–58,300)	47,400 (34,800–63,400)	1,430 (1,150–1,760)	461 (346–605)	446 (328–594)
1·89	Protein-energy malnutrition	99,300 (75,500–128,000)	17,400 (13,300–23,200)	19,000 (12,900–27,200)	996 (772–1,260)	178 (138–233)	181 (124–256)
1·90	Iodine deficiency	982 (492–1,640)	894 (437–1,500)	741 (361–1,270)	14·5 (7·4–23·9)	9·1 (4·5–15·3)	6·7 (3·3–11·5)
1·91	Vitamin A deficiency	1,490 (913–2,250)	810 (508–1,250)	735 (444–1,120)	16 (9·9–24)	7·9 (4·9–12·2)	6·8 (4·1–10·4)
1·92	Dietary iron deficiency	26,700 (16,900–39,800)	24,400 (15,300–37,400)	25,300 (15,700–38,800)	380 (247–559)	254 (162–389)	236 (146–359)
1·93	Other nutritional deficiencies	1,430 (1,110–1,880)	1,130 (880–1,410)	1,640 (1,190–2,260)	21·6 (17·3–27·3)	12·5 (9·7–15·6)	15·8 (11·5–21·8)
1·0	Non-communicable diseases	1,120,000 (979,000–1,270,000)	1,690,000 (1,430,000–1,970,000)	2,150,000 (1,770,000–2,580,000)	20,200 (17,800–23,000)	20,000 (17,000–23,300)	21,500 (17,800–25,800)
1·1	Neoplasms	107,000 (96,100–120,000)	209,000 (170,000–253,000)	283,000 (208,000–368,000)	2,270 (2,020–2,550)	2,620 (2,130–3,180)	2,900 (2,140–3,750)
1·2	Lip and oral cavity cancer	1,980 (1,740–2,240)	4,210 (3,280–5,310)	6,190 (4,360–8,470)	49·1 (42·6–55·9)	54·9 (42·7–69)	64·7 (45·9–88)
1·3	Nasopharynx cancer	420 (368–481)	993 (780–1,230)	1,110 (810–1,470)	9·1 (7·9–10·4)	12·3 (9·6–15·3)	11·3 (8·3–14·9)
1·4	Other pharynx cancer	1,050 (890–1,210)	2,380 (1,810–3,040)	3,620 (2,560–5,130)	26·5 (22·4–30·6)	31·2 (23·8–39·7)	37·9 (26·7–53·4)
1·5	Oesophageal cancer	1,680 (1,430–2,060)	3,930 (2,950–5,450)	5,950 (3,930–8,370)	41·7 (35·4–52·5)	51·7 (38·6–70·8)	61·7 (41·2–86·8)
1·6	Stomach cancer	7,550 (6,620–8,600)	15,300 (12,300–18,900)	20,000 (14,800–26,900)	181 (158–206)	196 (158–241)	206 (153–276)
1·7	Colon and rectum cancer	6,700 (5,930–7,570)	18,100 (14,600–22,000)	26,700 (18,800–35,200)	165 (146–186)	234 (190–283)	278 (198–367)

1-8	Liver cancer	4,920 (4,320–5,570)	6,710 (5,050–9,130)	12,000 (7,930–18,700)	108 (94·4–122)	84·2 (63·5–115)	123 (81·6–191)
1-9	Liver cancer due to hepatitis B	1,740 (1,320–2,200)	2,200 (1,450–3,290)	3,980 (2,290–6,720)	36·5 (27·4–47·2)	26·3 (17·5–39·5)	39·2 (22·5–66·2)
1-10	Liver cancer due to hepatitis C	731 (499–1,000)	1,110 (733–1,640)	1,970 (1,180–3,220)	19·7 (13·7–27·1)	15·2 (10–22·4)	21·4 (13–34·9)
1-11	Liver cancer due to alcohol use	1,300 (938–1,720)	2,050 (1,340–3,090)	3,910 (2,270–6,890)	32·5 (23·5–43·2)	27 (17·7–41·1)	41 (24·2–70·9)
1-12	Liver cancer due to NASH	297 (210–411)	507 (341–739)	948 (566–1,510)	7 (5–9·9)	6·5 (4·3–9·6)	9·9 (6–15·7)
1-13	Gallbladder and biliary tract cancer	2,040 (1,170–2,440)	3,180 (2,430–3,990)	5,830 (3,280–8,020)	51·3 (29·8–61·2)	41·6 (31·9–51·9)	60·9 (34·7–83·3)
1-14	Pancreatic cancer	1,340 (1,170–1,520)	6,420 (5,110–7,890)	8,220 (5,470–11,300)	34·2 (29·9–38·9)	84·9 (67·6–104)	86·3 (57·7–119)
1-15	Larynx cancer	1,600 (1,380–1,850)	3,040 (2,380–3,840)	4,800 (3,560–6,370)	40·2 (34·6–46·4)	39·8 (31·4–50·3)	50 (37·4–66)
1-16	Tracheal, bronchus, and lung cancer	11,200 (9,820–12,700)	24,300 (19,400–29,700)	35,000 (24,700–48,000)	279 (245–317)	320 (256–391)	366 (259–501)
1-17	Malignant skin melanoma	262 (211–376)	464 (353–657)	600 (433–895)	5·3 (4·3–7·6)	5·6 (4·3–7·8)	6 (4·3–8·8)
1-18	Non-melanoma skin cancer	801 (683–939)	2,620 (2,090–3,180)	3,390 (2,520–4,430)	19·2 (16·4–22·5)	33·4 (26·8–40·5)	35·2 (26·3–45·7)
1-19	Non-melanoma skin cancer (squamous-cell carcinoma)	800 (682–939)	2,620 (2,090–3,180)	3,390 (2,520–4,430)	19·1 (16·4–22·5)	33·4 (26·8–40·5)	35·2 (26·3–45·7)
1-20	Non-melanoma skin cancer (basal-cell carcinoma)	0 (0–0)	0 (0–0)	0 (0–1)	0 (0–0)	0 (0–0)	0 (0–0)
1-21	Breast cancer	7,690 (6,640–8,800)	20,700 (16,800–25,600)	25,000 (17,800–33,600)	171 (147–195)	248 (201–305)	247 (177–330)
1-22	Cervical cancer	9,120 (7,630–11,900)	16,500 (12,900–21,200)	20,900 (14,400–29,400)	195 (164–254)	196 (154–250)	204 (142–289)
1-23	Uterine cancer	2,270 (1,690–2,710)	4,410 (3,270–5,590)	7,140 (4,470–10,000)	53·8 (40·8–64·2)	55·6 (41·5–70·4)	73·1 (46·5–102)
1-24	Ovarian cancer	722 (573–1,030)	2,150 (1,550–2,840)	2,570 (1,650–3,880)	15·7 (12·4–22·8)	26·1 (19·1–34·5)	25·4 (16·5–38·5)
1-25	Prostate cancer	7,910 (5,440–14,100)	23,800 (18,500–32,300)	28,700 (18,800–47,500)	240 (164–426)	342 (266–465)	326 (215–538)
1-26	Testicular cancer	112 (82–180)	200 (144–290)	257 (159–429)	1·6 (1·2–2·3)	2·1 (1·5–3)	2·4 (1·5–3·9)
1-27	Kidney cancer	2,910 (2,500–3,360)	3,930 (2,970–4,990)	7,350 (5,020–9,990)	49 (42·8–56·4)	46·4 (35·1–58·8)	72·7 (49·9–98·3)
1-28	Bladder cancer	757 (656–864)	1,700 (1,330–2,160)	2,520 (1,830–3,440)	20·7 (17·8–23·6)	23·2 (18·2–29·5)	27·5 (20–37·4)
1-29	Brain and nervous system cancer	2,160 (1,390–4,950)	3,970 (3,040–5,730)	3,660 (2,300–6,630)	30·8 (20·1–64·3)	43·1 (33·1–61·2)	34·8 (21·9–62·7)
1-30	Thyroid cancer	581 (437–678)	1,170 (890–1,480)	1,870 (1,220–2,600)	13·2 (9·8–15·5)	14·5 (11–18·5)	19 (12·5–26·2)
1-31	Mesothelioma	137 (93–186)	233 (172–296)	352 (224–508)	2·8 (1·9–3·6)	2·7 (2–3·4)	3·4 (2·2–4·9)
1-32	Hodgkin lymphoma	343 (220–625)	472 (312–754)	488 (261–881)	5·4 (3·5–9·9)	5 (3·3–8)	4·5 (2·5–8·1)
1-33	Non-Hodgkin's lymphoma	3,760 (3,260–4,300)	5,800 (4,580–7,310)	8,780 (6,050–11,900)	61·2 (53·5–69·5)	65·7 (52·2–83·2)	84·8 (58·9–114)
1-34	Multiple myeloma	922 (723–1,120)	2,330 (1,740–2,960)	3,510 (2,390–4,880)	23·4 (18·5–29)	30·8 (23·1–38·8)	36·8 (25·3–50·7)
1-35	Leukaemia	12,600 (10,500–15,700)	13,600 (10,500–17,400)	16,600 (12,000–22,800)	166 (145–196)	144 (113–184)	157 (115–216)
1-36	Acute lymphoid leukaemia	2,840 (2,010–4,680)	3,090 (2,230–4,340)	3,650 (2,520–5,200)	31·2 (22·9–50)	30·1 (21·7–41·9)	33·6 (23·2–47·8)
1-37	Chronic lymphoid leukaemia	88 (68–112)	283 (213–375)	451 (310–647)	2·2 (1·6–2·7)	3·8 (2·8–5)	4·8 (3·3–6·8)
1-38	Acute myeloid leukaemia	1,550 (1,190–2,140)	2,940 (2,150–3,770)	3,910 (2,600–5,620)	20·4 (16·1–27·6)	30·7 (22·4–39·4)	36·4 (24·3–52·1)
1-39	Chronic myeloid leukaemia	289 (229–408)	557 (408–761)	721 (476–1,060)	4·7 (3·8–5·9)	6·2 (4·6–8·3)	6·9 (4·6–10·1)
1-40	Other leukaemia	7,870 (5,680–9,890)	6,680 (5,120–8,900)	7,900 (5,350–11,000)	108 (83·9–128)	73·6 (56·7–97)	75·7 (51·5–106)
1-41	Other neoplasms	515 (396–652)	955 (761–1,180)	1,250 (939–1,630)	8·8 (7·2–11·1)	11·3 (9·1–14·2)	12·6 (9·5–16·5)
1-42	Myelodysplastic, myeloproliferative, and other hematopoietic neoplasms	515 (396–652)	955 (761–1,180)	1,250 (939–1,630)	8·8 (7·2–11·1)	11·3 (9·1–14·2)	12·6 (9·5–16·5)
1-43	Benign and in situ intestinal neoplasms	0 (0–0)	0 (0–0)	0 (0–0)	0 (0–0)	0 (0–0)	0 (0–0)
1-44	Benign and in situ cervical and uterine neoplasms	0 (0–0)	0 (0–0)	0 (0–0)	0 (0–0)	0 (0–0)	0 (0–0)
1-45	Other benign and in situ neoplasms	0 (0–0)	0 (0–0)	0 (0–0)	0 (0–0)	0 (0–0)	0 (0–0)
1-46	Cardiovascular diseases	222,000 (200,000–245,000)	429,000 (356,000–509,000)	601,000 (469,000–769,000)	5,370 (4,830–5,920)	5,630 (4,670–6,660)	6,370 (4,960–8,100)
1-47	Rheumatic heart disease	9,470 (8,010–11,300)	8,640 (6,590–11,200)	9,700 (7,100–13,000)	138 (118–162)	89·5 (68·9–114)	89·1 (65·3–119)
1-48	Ischaemic heart disease	103,000 (91,400–114,000)	226,000 (186,000–272,000)	327,000 (251,000–424,000)	2,660 (2,370–2,950)	3,000 (2,470–3,590)	3,470 (2,690–4,480)
1-49	Stroke	73,500 (65,800–81,800)	135,000 (111,000–162,000)	186,000 (141,000–244,000)	1,770 (1,580–1,970)	1,780 (1,460–2,130)	1,970 (1,510–2,560)
1-50	Ischaemic stroke	21,700 (19,100–24,300)	49,300 (41,200–57,600)	67,100 (53,400–85,400)	608 (540–684)	685 (573–800)	750 (596–949)
1-51	Intracerebral hemorrhage	44,400 (38,400–50,100)	73,100 (58,500–89,600)	102,000 (73,700–137,000)	1,030 (890–1,160)	943 (754–1,160)	1,060 (767–1,420)
1-52	Subarachnoid hemorrhage	7,480 (6,140–9,710)	12,700 (10,000–15,600)	16,600 (11,800–23,000)	140 (115–183)	148 (118–182)	162 (115–223)
1-53	Hypertensive heart disease	13,700 (12,000–15,900)	27,900 (22,600–33,700)	36,500 (26,100–49,200)	377 (329–436)	380 (310–460)	399 (290–534)
1-54	Non-rheumatic valvular heart disease	1,240 (963–1,450)	1,820 (1,460–2,280)	2,660 (1,970–3,480)	25·9 (20·8–30·2)	22·3 (17·9–27·8)	26·9 (20·1–35)
1-55	Non-rheumatic calcific aortic valve disease	751 (574–892)	1,170 (918–1,470)	1,730 (1,290–2,300)	16·4 (13·1–19·4)	14·7 (11·7–18·5)	17·9 (13·4–23·6)
1-56	Non-rheumatic degenerative mitral valve disease	485 (356–589)	644 (493–838)	913 (633–1,230)	9·4 (6·9–11·4)	7·5 (5·8–9·8)	8·9 (6·2–12)
1-57	Other non-rheumatic valve diseases	7 (5–10)	10 (5–15)	11 (7–17)	0·1 (0·1–0·2)	0·1 (0·1–0·2)	0·1 (0·1–0·2)
1-58	Cardiomyopathy and myocarditis	4,510 (3,410–6,900)	6,120 (4,290–7,770)	7,920 (5,140–10,700)	69·3 (56·5–95·1)	71 (50·5–89·6)	78·4 (51–105)
1-59	Myocarditis	520 (309–1,550)	430 (284–650)	509 (315–764)	6·9 (4·7–15·3)	4·8 (3–6·7)	5 (3–7·4)
1-60	Alcoholic cardiomyopathy	357 (250–525)	1,020 (655–1,380)	1,490 (855–2,190)	7·6 (5·2–11·4)	12·4 (7·9–16·7)	14·7 (8·3–21·4)

1-61	Other cardiomyopathy	3,630 (2,720–5,350)	4,660 (3,290–5,900)	5,920 (3,740–8,020)	54·8 (44·3–75·2)	53·8 (38–68·1)	58·7 (37·5–79·1)
1-62	Atrial fibrillation and flutter	2,320 (1,890–2,780)	5,780 (4,700–6,980)	7,670 (6,120–9,370)	76 (62·5–90·2)	83·2 (67·6–100)	90 (72–109)
1-63	Aortic aneurysm	1,150 (994–1,320)	2,650 (2,130–3,220)	3,970 (2,980–5,190)	29 (25·1–33·2)	34·9 (27·9–42·4)	42 (31·6–55)
1-64	Peripheral vascular disease	278 (174–431)	617 (415–921)	847 (575–1,220)	8·7 (5·4–13·3)	8·9 (6–13·3)	9·8 (6·6–14·1)
1-65	Endocarditis	2,080 (1,510–2,910)	2,430 (1,820–3,360)	3,210 (2,190–4,550)	27·4 (21·7–37·3)	26·3 (20·1–36·3)	30·4 (21·2–43)
1-66	Chronic respiratory diseases	49,600 (40,700–59,000)	54,200 (44,700–64,600)	67,100 (52,600–83,100)	796 (681–912)	637 (529–749)	677 (531–833)
1-67	Chronic obstructive pulmonary disease	10,100 (8,850–11,700)	23,300 (19,400–27,300)	31,200 (24,100–39,200)	255 (223–298)	309 (257–363)	336 (262–421)
1-68	Pneumoconiosis	27 (21–34)	34 (27–43)	43 (31–63)	0·7 (0·5–0·9)	0·4 (0·3–0·6)	0·4 (0·3–0·6)
1-69	Silicosis	12 (5–18)	10 (7–16)	13 (7–22)	0·3 (0·1–0·4)	0·1 (0·1–0·2)	0·1 (0·1–0·2)
1-70	Asbestosis	4 (3–6)	6 (4–8)	9 (5–14)	0·1 (0·1–0·2)	0·1 (0·1–0·1)	0·1 (0·1–0·2)
1-71	Coal workers pneumoconiosis	2 (1–4)	2 (2–4)	3 (2–5)	0·1 (0–0·1)	0 (0–0)	0 (0–0·1)
1-72	Other pneumoconiosis	8 (6–12)	15 (11–20)	19 (13–26)	0·2 (0·2–0·3)	0·2 (0·1–0·3)	0·2 (0·1–0·3)
1-73	Asthma	36,600 (27,900–45,500)	27,400 (21,200–35,600)	31,400 (23,300–40,700)	502 (397–608)	290 (228–370)	298 (221–385)
1-74	Interstitial lung disease and pulmonary sarcoidosis	551 (447–762)	1,180 (774–1,540)	1,670 (1,010–2,410)	11·1 (8·9–15)	14·3 (9·4–18·5)	16·8 (10·2–24·2)
1-75	Other chronic respiratory diseases	2,290 (1,030–3,900)	2,310 (1,570–3,010)	2,880 (1,980–3,910)	27 (14·4–42·9)	23·3 (16–30·3)	26·5 (18·1–35·8)
1-76	Digestive diseases	114,000 (101,000–130,000)	97,300 (80,700–116,000)	142,000 (108,000–181,000)	1,930 (1,720–2,160)	1,140 (948–1,370)	1,400 (1,070–1,780)
1-77	Cirrhosis and other chronic liver diseases	53,200 (47,100–59,500)	50,600 (39,400–63,100)	82,900 (56,500–114,000)	1,080 (960–1,210)	623 (487–775)	833 (573–1,150)
1-78	Cirrhosis and other chronic liver diseases due to hepatitis B	5,420 (3,840–7,620)	4,390 (2,850–6,510)	6,560 (3,740–10,200)	121 (86·1–170)	53·6 (34·9–78·5)	65·1 (37·3–102)
1-79	Cirrhosis and other chronic liver diseases due to hepatitis C	8,670 (6,390–11,400)	9,480 (6,530–13,300)	15,700 (9,310–24,200)	192 (141–255)	116 (81–165)	156 (93·9–243)
1-80	Cirrhosis and other chronic liver diseases due to alcohol use	15,200 (11,900–18,700)	18,500 (13,200–24,700)	31,600 (19,900–45,200)	362 (282–444)	236 (167–314)	321 (205–458)
1-81	Cirrhosis and other chronic liver diseases due to NAFLD	6,180 (4,390–8,390)	7,460 (5,120–10,600)	12,500 (7,410–19,000)	146 (103–196)	95 (64·7–135)	129 (77·4–193)
1-82	Cirrhosis and other chronic liver diseases due to other causes	17,700 (14,100–21,800)	10,700 (7,800–14,100)	16,500 (11,300–23,200)	258 (210–314)	122 (88·7–160)	162 (111–226)
1-83	Upper digestive system diseases	16,600 (12,700–22,400)	21,100 (14,900–30,500)	25,400 (17,800–36,900)	312 (240–419)	243 (174–344)	244 (174–353)
1-84	Peptic ulcer disease	7,760 (6,800–9,130)	6,890 (5,460–8,410)	8,300 (5,460–11,300)	153 (134–179)	85·2 (67·6–104)	83·8 (55·3–113)
1-85	Gastritis and duodenitis	1,970 (1,390–2,760)	3,040 (2,120–4,320)	3,880 (2,680–5,530)	35·6 (25·4–49·6)	34·6 (24·4–48·8)	37·4 (25·9–52·9)
1-86	GERD	6,860 (3,530–12,200)	11,200 (5,820–19,800)	13,200 (6,840–23,400)	123 (64·2–219)	123 (64·1–219)	123 (64–218)
1-87	Appendicitis	8,750 (4,310–12,100)	3,040 (1,860–4,020)	4,620 (2,180–7,380)	99·8 (52·5–132)	30·6 (18·9–40·3)	42·6 (20·5–68·2)
1-88	Paralytic ileus and intestinal obstruction	14,400 (9,310–21,500)	5,910 (4,430–7,780)	7,110 (4,490–11,700)	143 (95·8–206)	61·7 (46·9–79·9)	67·2 (42·9–108)
1-89	Inguinal, femoral, and abdominal hernia	11,000 (7,040–16,400)	6,110 (4,390–8,220)	7,290 (5,180–9,840)	124 (84·6–173)	63·9 (46·1–85·3)	68·2 (48·6–91·9)
1-90	Inflammatory bowel disease	732 (527–1,060)	888 (683–1,100)	1,110 (820–1,450)	12·1 (9·5–15·9)	10·2 (7·8–12·6)	10·7 (8–14)
1-91	Vascular intestinal disorders	662 (565–811)	982 (794–1,190)	1,490 (1,050–1,990)	15·3 (13·2–18·6)	12·9 (10·4–15·6)	15·8 (11·2–21·2)
1-92	Gallbladder and biliary diseases	3,410 (2,550–4,420)	4,140 (3,040–5,670)	5,440 (3,960–7,350)	66 (49·3–84·2)	48·2 (36–65·2)	53·3 (38·8–71·6)
1-93	Pancreatitis	1,590 (1,250–1,970)	1,890 (1,450–2,390)	2,670 (1,710–3,780)	29·1 (22·8–36·2)	21·9 (16·9–27·6)	25·9 (16·8–36·3)
1-94	Other digestive diseases	4,070 (2,650–5,720)	2,640 (1,970–3,460)	3,710 (2,550–5,310)	52·2 (39·9–68·8)	29·3 (22·2–38)	35·9 (24·9–50·9)
1-95	Neurological disorders	68,300 (34,900–120,000)	107,000 (55,100–182,000)	130,000 (68,800–214,000)	1,200 (661–1,990)	1,220 (661–2,060)	1,290 (702–2,080)
1-96	Alzheimer's disease and other dementias	8,680 (3,530–20,100)	22,700 (9,270–51,700)	29,100 (11,500–66,200)	308 (127–707)	333 (135–765)	355 (141–808)
1-97	Parkinson's disease	2,000 (1,740–2,280)	5,390 (4,510–6,260)	7,420 (6,000–9,040)	62·6 (54·4–71·4)	78 (65–90·5)	85·7 (69·4–104)
1-98	Idiopathic epilepsy	15,900 (9,010–24,900)	19,200 (10,300–33,100)	23,900 (12,600–39,800)	212 (118–334)	195 (105–336)	219 (115–365)
1-99	Multiple sclerosis	294 (242–357)	607 (483–761)	799 (590–1,080)	5·8 (4·8–6·9)	6·9 (5·5–8·6)	7·6 (5·6–10·3)
1-100	Motor neuron disease	425 (337–530)	591 (448–763)	1,310 (818–2,060)	7·2 (5·9–8·5)	7·2 (5·4–9·3)	12·9 (8·1–20·1)
1-101	Headache disorders	37,500 (6,410–84,400)	54,500 (10,000–121,000)	61,000 (11,600–134,000)	556 (109–1,230)	551 (107–1,210)	550 (107–1,200)
1-102	Migraine	34,200 (4,200–80,700)	49,600 (6,740–115,000)	55,300 (7,810–127,000)	503 (72·3–1,160)	499 (72·7–1,150)	497 (72–1,140)
1-103	Tension-type headache	3,240 (907–13,300)	4,950 (1,430–18,800)	5,680 (1,620–20,800)	52·3 (15·8–194)	52·1 (15·3–192)	52·2 (15–188)
1-104	Other neurological disorders	3,580 (2,230–5,170)	4,470 (2,550–7,370)	6,070 (3,470–9,780)	45·6 (29·7–65·2)	45·2 (26·9–73·1)	56 (32·4–89·5)
1-105	Mental disorders	116,000 (84,400–154,000)	176,000 (128,000–232,000)	200,000 (145,000–264,000)	1,800 (1,310–2,380)	1,840 (1,340–2,410)	1,830 (1,330–2,410)
1-106	Schizophrenia	10,000 (6,920–13,500)	16,500 (11,500–22,000)	19,600 (13,700–26,400)	173 (120–229)	176 (122–233)	179 (125–239)
1-107	Depressive disorders	40,900 (27,700–57,300)	65,300 (44,900–89,800)	74,300 (50,200–104,000)	682 (466–949)	707 (493–972)	691 (466–966)
1-108	Major depressive disorder	35,500 (23,300–50,600)	56,800 (38,800–79,000)	64,500 (42,600–91,300)	591 (395–826)	617 (422–850)	601 (398–846)
1-109	Dysthymia	5,350 (3,210–8,300)	8,440 (5,170–13,100)	9,800 (5,990–15,100)	90·5 (56·1–138)	90·3 (56·2–138)	90·1 (55·7–138)
1-110	Bipolar disorder	13,000 (7,420–20,500)	19,400 (11,200–30,300)	22,000 (12,900–34,200)	198 (115–307)	198 (116–306)	198 (116–306)
1-111	Anxiety disorders	28,500 (18,700–40,400)	41,800 (27,900–59,100)	47,300 (31,300–66,100)	429 (286–606)	429 (288–606)	431 (286–606)
1-112	Eating disorders	2,970 (1,820–4,470)	4,660 (2,900–7,050)	5,750 (3,560–8,770)	37·2 (22·7–55·4)	43·5 (26·9–65·8)	48·9 (30·1–74·2)
1-113	Anorexia nervosa	779 (437–1,280)	1,150 (662–1,850)	1,380 (781–2,230)	9·3 (5·3–15)	10·5 (6·1–16·9)	11·8 (6·7–19)

1-114	Bulimia nervosa	2,190 (1,280–3,520)	3,510 (2,020–5,690)	4,370 (2,540–7,010)	28 (16·4–44·2)	33 (19·1–53·1)	37·1 (21·6–59·4)
1-115	Autism spectrum disorders	3,940 (2,550–5,730)	5,250 (3,420–7,640)	5,750 (3,730–8,340)	51·6 (33·7–74·7)	52·2 (33·9–76)	52·3 (33·9–75·8)
1-116	Attention-deficit/hyperactivity disorder	3,100 (1,740–5,280)	4,000 (2,270–6,750)	4,190 (2,350–7,070)	36·8 (20·8–61·3)	37·4 (21·3–63)	37·4 (21·1–63·2)
1-117	Conduct disorder	6,300 (3,560–10,100)	7,620 (4,290–12,100)	7,390 (4,180–11,700)	67·3 (38–108)	68 (38·5–108)	68·1 (38·4–108)
1-118	Idiopathic developmental intellectual disability	1,670 (569–3,090)	1,600 (460–3,060)	1,450 (379–2,820)	21·4 (7·4–39·6)	15·7 (4·5–30·1)	13·1 (3·4–25·5)
1-119	Other mental disorders	5,980 (3,700–9,090)	9,910 (6,230–15,000)	11,700 (7,400–17,700)	108 (68·9–165)	109 (68·8–165)	109 (69·1–164)
1-120	Substance use disorders	16,900 (12,400–21,900)	25,800 (19,200–33,700)	32,300 (24,400–41,100)	268 (202–343)	267 (199–346)	290 (220–367)
1-121	Alcohol use disorders	12,000 (8,700–16,000)	17,300 (12,500–23,300)	20,500 (14,900–27,300)	199 (148–261)	183 (133–245)	186 (136–245)
1-122	Drug use disorders	4,870 (3,400–6,660)	8,470 (6,060–11,200)	11,800 (8,730–15,300)	68·4 (49·1–91·4)	84 (60·3–110)	104 (77·1–134)
1-123	Opioid use disorders	1,600 (1,080–2,250)	3,600 (2,420–4,950)	5,270 (3,760–7,170)	24·8 (17·2–34·1)	37 (24·9–50·5)	47 (33·8–63·5)
1-124	Cocaine use disorders	1,040 (593–1,650)	1,540 (920–2,390)	2,090 (1,340–3,050)	14 (8·3–21·4)	14·9 (9·1–22·6)	18·4 (11·8–26·5)
1-125	Amphetamine use disorders	454 (224–786)	655 (360–1,080)	852 (501–1,340)	5·7 (3–9·8)	6·2 (3·5–10·2)	7·3 (4·4–11·4)
1-126	Cannabis use disorders	658 (356–1,080)	918 (512–1,480)	1,310 (682–2,240)	8·5 (4·8–13·5)	8·7 (4·9–13·8)	11·2 (5·9–19·1)
1-127	Other drug use disorders	1,120 (703–1,680)	1,760 (1,180–2,580)	2,310 (1,610–3,190)	15·3 (10·1–22·6)	17·2 (11·7–24·9)	20·2 (14·2–27·7)
1-128	Diabetes and kidney diseases	59,400 (52,200–66,500)	137,000 (115,000–163,000)	193,000 (155,000–236,000)	1,270 (1,120–1,430)	1,710 (1,440–2,020)	1,960 (1,590–2,400)
1-129	Diabetes mellitus	34,000 (29,300–39,000)	77,900 (64,800–92,700)	117,000 (92,900–145,000)	786 (673–904)	994 (824–1,180)	1,210 (958–1,490)
1-130	Diabetes mellitus type 1	5,590 (4,800–6,390)	7,130 (5,790–8,750)	9,410 (7,040–12,200)	85·5 (74·4–98·4)	77·6 (62·9–94·9)	88·7 (66·5–115)
1-131	Diabetes mellitus type 2	28,400 (24,200–32,900)	70,800 (58,200–85,100)	108,000 (85,000–133,000)	700 (594–812)	916 (756–1,100)	1,120 (883–1,380)
1-132	Chronic kidney disease	25,200 (22,400–28,000)	59,000 (49,100–70,600)	75,400 (58,000–97,200)	484 (432–541)	718 (599–854)	758 (584–978)
1-133	Chronic kidney disease due to diabetes mellitus type 1	1,700 (1,170–2,360)	4,490 (2,880–6,630)	5,710 (3,520–9,190)	33 (22·1–46·7)	51·1 (32·7–76·1)	54 (33·2–87·5)
1-134	Chronic kidney disease due to diabetes mellitus type 2	3,390 (2,630–4,160)	10,900 (8,170–14,200)	14,400 (10,300–20,100)	88·4 (69·4–108)	146 (108–189)	153 (110–211)
1-135	Chronic kidney disease due to hypertension	3,360 (2,730–4,130)	10,900 (8,490–13,700)	14,000 (10,300–19,100)	86·3 (71–105)	144 (112–181)	149 (110–203)
1-136	Chronic kidney disease due to glomerulonephritis	7,210 (5,950–8,600)	14,900 (11,400–18,900)	18,900 (13,300–26,100)	120 (98·7–144)	171 (130–219)	181 (127–251)
1-137	Chronic kidney disease due to other and unspecified causes	9,560 (8,000–11,200)	17,800 (14,000–22,300)	22,400 (16,700–29,600)	156 (131–187)	207 (162–262)	220 (163–290)
1-138	Acute glomerulonephritis	155 (18–274)	11 (9–15)	8 (6–13)	1·8 (0·2–3·1)	0·1 (0·1–0·2)	0·1 (0·1–0·1)
1-139	Skin and subcutaneous diseases	37,700 (24,600–56,200)	49,600 (32,200–75,400)	54,600 (35,400–82,300)	513 (336–774)	504 (328–764)	507 (329–764)
1-140	Dermatitis	8,990 (5,170–14,400)	11,500 (6,790–18,100)	12,500 (7,290–19,800)	116 (68–183)	116 (68·8–181)	116 (67·6–183)
1-141	Atopic dermatitis	7,180 (3,840–12,100)	8,670 (4,640–14,700)	9,240 (4,940–15,500)	85·2 (45·7–143)	85·1 (45·5–144)	85·1 (45·7–143)
1-142	Contact dermatitis	1,590 (982–2,420)	2,490 (1,540–3,760)	2,890 (1,800–4,330)	27·2 (16·7–41·2)	27·1 (16·8–40·6)	27·1 (17–40·7)
1-143	Seborrhoeic dermatitis	217 (122–349)	320 (183–515)	363 (205–576)	3·4 (1·9–5·4)	3·4 (1·9–5·4)	3·4 (1·9–5·4)
1-144	Psoriasis	2,620 (1,820–3,510)	3,530 (2,500–4,760)	3,890 (2,750–5,240)	42·7 (30·1–56·9)	37·9 (26·9–50·9)	36·4 (25·7–48·7)
1-145	Bacterial skin diseases	1,540 (1,000–2,080)	1,210 (898–1,740)	1,550 (1,130–2,120)	22·8 (15·5–29·3)	13·5 (10·1–19)	15·1 (11·1–20·7)
1-146	Cellulitis	169 (93–264)	166 (111–236)	191 (130–268)	2·5 (1·3–3·7)	1·8 (1·2–2·5)	1·8 (1·2–2·6)
1-147	Pyoderma	1,370 (854–1,910)	1,050 (770–1,540)	1,360 (970–1,900)	20·4 (14–26·6)	11·7 (8·7–17·1)	13·3 (9·6–18·7)
1-148	Scabies	6,620 (3,600–10,600)	8,550 (4,670–13,800)	9,220 (5,050–14,800)	84·5 (46·4–135)	84·3 (46·5–135)	84 (46–135)
1-149	Fungal skin diseases	2,630 (1,030–5,600)	3,950 (1,580–8,410)	4,530 (1,810–9,660)	44·7 (18–94·5)	44·2 (17·8–93·9)	44·2 (17·7–94·1)
1-150	Viral skin diseases	4,080 (2,580–6,130)	4,890 (3,090–7,380)	5,170 (3,280–7,760)	47·5 (30·1–71·3)	47·6 (30·1–71·8)	47·6 (30·1–71·4)
1-151	Acne vulgaris	3,630 (2,150–5,910)	5,310 (3,170–8,520)	5,480 (3,220–8,710)	40·1 (23·8–65·2)	46·9 (28–75·3)	48·3 (28·4–76·9)
1-152	Alopecia areata	478 (303–715)	691 (441–1,040)	785 (500–1,170)	7·2 (4·5–10·7)	7·1 (4·5–10·7)	7·1 (4·5–10·5)
1-153	Pruritus	496 (240–894)	771 (369–1,380)	900 (430–1,610)	8·2 (3·9–14·7)	8·4 (4·1–15·1)	8·6 (4·1–15·5)
1-154	Urticaria	3,820 (2,470–5,550)	4,820 (3,130–6,960)	5,240 (3,440–7,610)	48·2 (31·2–69·6)	48·1 (31·2–69·2)	48·1 (31·6–69·8)
1-155	Decubitus ulcer	233 (108–308)	310 (237–389)	437 (321–549)	6 (2·9–7·8)	4·1 (3·1–5·2)	4·9 (3·6–6·1)
1-156	Other skin and subcutaneous diseases	2,580 (1,240–4,720)	4,110 (1,960–7,440)	4,870 (2,340–8,870)	45·5 (22–82·5)	46·5 (22·2–84·1)	47·2 (22·8–85·9)
1-157	Sense organ diseases	40,000 (27,200–56,700)	66,800 (45,200–95,700)	78,600 (52,900–112,000)	906 (617–1,270)	836 (568–1,190)	814 (550–1,160)
1-158	Blindness and vision loss	13,500 (9,300–18,900)	21,200 (14,400–30,100)	24,400 (16,700–34,400)	319 (224–443)	270 (185–379)	257 (177–362)
1-159	Glaucoma	720 (474–1,050)	1,100 (741–1,580)	1,250 (833–1,770)	23·1 (15·2–33·5)	16·1 (10·8–23·1)	14·7 (9·8–20·8)
1-160	Cataract	1,650 (1,150–2,290)	2,700 (1,850–3,680)	3,090 (2,150–4,230)	49·7 (34·4–69)	38 (26·2–51·8)	35·3 (24·5–48·3)
1-161	Age-related macular degeneration	84 (54–127)	144 (92–211)	170 (108–252)	2·5 (1·6–3·8)	2·1 (1·3–3)	1·9 (1·2–2·9)
1-162	Refraction disorders	5,050 (3,350–7,360)	7,360 (4,900–10,700)	8,330 (5,500–11,900)	88·4 (59·4–126)	81·8 (54·7–117)	80 (53·1–114)
1-163	Near vision loss	2,180 (1,020–4,320)	3,980 (1,820–8,120)	4,850 (2,160–9,660)	57·7 (27–114)	53·2 (24·3–108)	51·8 (23·2–103)
1-164	Other vision loss	3,850 (2,690–5,310)	5,890 (4,130–8,170)	6,720 (4,750–9,210)	98 (68·5–134)	78·5 (55·1–108)	73·1 (51·6–100)
1-165	Age-related and other hearing loss	24,600 (16,500–35,700)	42,500 (28,400–61,800)	50,500 (33,500–73,700)	551 (366–797)	531 (356–775)	521 (346–757)
1-166	Other sense organ diseases	1,820 (1,080–2,750)	3,050 (1,800–4,690)	3,620 (2,140–5,650)	35·5 (20·9–54·6)	36·1 (21·3–56·1)	36·1 (21·4–56·7)

1-167	Musculoskeletal disorders	71,000 (50,800–93,600)	126,000 (90,400–167,000)	155,000 (112,000–205,000)	1,380 (988–1,830)	1,440 (1,040–1,910)	1,490 (1,080–1,990)
1-168	Rheumatoid arthritis	1,100 (805–1,450)	2,060 (1,470–2,790)	2,710 (1,940–3,600)	24·9 (18·1–32·2)	24·2 (17·5–32·4)	26·9 (19·4–35·6)
1-169	Osteoarthritis	7,080 (3,510–14,000)	15,100 (7,570–30,400)	19,500 (9,730–39,300)	186 (92·5–369)	201 (100–400)	206 (103–416)
1-170	Low back pain	37,500 (26,300–50,400)	61,100 (42,800–81,900)	73,600 (51,500–99,000)	681 (479–917)	682 (478–905)	699 (490–940)
1-171	Neck pain	6,940 (4,550–10,100)	11,900 (7,790–17,400)	14,200 (9,370–20,400)	135 (89·6–195)	135 (88·3–197)	135 (89·8–194)
1-172	Gout	286 (180–413)	637 (394–918)	807 (498–1,150)	6·6 (4·1–9·4)	7·8 (4·8–11·3)	8·1 (5–11·6)
1-173	Other musculoskeletal disorders	18,100 (12,500–24,800)	35,000 (24,500–48,500)	44,000 (31,000–60,000)	344 (240–472)	395 (276–547)	416 (296–567)
1-174	Other non-communicable diseases	214,000 (173,000–256,000)	210,000 (169,000–259,000)	210,000 (157,000–284,000)	2,550 (2,070–3,100)	2,160 (1,730–2,670)	1,960 (1,470–2,640)
1-175	Congenital anomalies	106,000 (83,000–140,000)	97,700 (68,800–125,000)	70,800 (44,800–117,000)	976 (773–1,280)	931 (657–1,190)	645 (408–1,070)
1-176	Neural tube defects	9,780 (6,040–15,500)	4,080 (2,850–5,780)	2,960 (1,560–5,640)	87·1 (53·9–137)	38·8 (27–54·9)	27 (14·2–51·3)
1-177	Congenital heart anomalies	43,200 (32,000–64,900)	47,500 (34,500–62,100)	31,300 (18,000–53,300)	396 (295–589)	452 (328–592)	285 (163–485)
1-178	Orofacial clefts	662 (434–1,060)	386 (266–544)	418 (276–610)	6·7 (4·5–10·3)	3·8 (2·6–5·5)	3·8 (2·5–5·6)
1-179	Down's syndrome	5,780 (3,660–8,710)	5,870 (3,850–7,900)	4,660 (2,830–7,520)	55·1 (36–81·3)	56·2 (37·1–75·5)	42·2 (25·5–68·2)
1-180	Turner syndrome	14 (6–24)	14 (6–25)	15 (7–26)	0·2 (0·1–0·3)	0·1 (0·1–0·2)	0·1 (0·1–0·2)
1-181	Klinefelter syndrome	1 (0–2)	1 (1–3)	1 (1–3)	0 (0–0)	0 (0–0)	0 (0–0)
1-182	Other chromosomal abnormalities	1,710 (1,100–2,960)	1,750 (1,090–2,340)	1,540 (990–2,350)	16·1 (10·6–27·2)	16·6 (10·3–22·1)	13·9 (8·9–21·3)
1-183	Congenital musculoskeletal and limb anomalies	4,300 (2,910–6,070)	4,820 (3,200–6,790)	5,090 (3,340–7,350)	48·7 (32·7–68·7)	47·1 (31·4–66·5)	46·4 (30·5–66·8)
1-184	Urogenital congenital anomalies	1,070 (703–1,660)	1,120 (644–1,560)	913 (534–1,620)	10 (6·6–15)	10·6 (6·1–14·8)	8·3 (4·9–14·7)
1-185	Digestive congenital anomalies	17,700 (9,100–25,800)	13,300 (7,220–18,000)	10,100 (5,060–18,400)	157 (80·7–229)	126 (68·5–171)	91·8 (46·1–168)
1-186	Other congenital anomalies	21,600 (8,250–32,800)	18,800 (7,130–27,100)	13,800 (5,930–26,600)	199 (80·2–297)	179 (68·1–259)	126 (54–242)
1-187	Urinary diseases and male infertility	2,860 (2,220–3,670)	4,860 (3,630–6,300)	6,310 (4,640–8,300)	59·3 (44·4–78·1)	61·4 (45·8–80·2)	65 (47·5–85·5)
1-188	Urinary tract infections and interstitial nephritis	1,260 (1,050–1,480)	1,890 (1,480–2,360)	2,430 (1,810–3,140)	21·3 (18·2–25·3)	22·4 (17·5–27·9)	24·5 (18·1–31·7)
1-189	Urolithiasis	190 (130–260)	312 (214–434)	380 (258–533)	3·7 (2·6–5·1)	3·6 (2·5–5)	3·7 (2·5–5·1)
1-190	Benign prostatic hyperplasia	930 (546–1,450)	1,880 (1,100–2,950)	2,320 (1,350–3,600)	26·5 (15·4–41·6)	26·8 (15·6–42·2)	25·9 (15–40)
1-191	Male infertility	135 (53–293)	183 (74–397)	420 (154–1,010)	2 (0·8–4·2)	1·8 (0·7–3·9)	3·6 (1·3–8·7)
1-192	Other urinary diseases	352 (274–440)	596 (421–763)	750 (507–1,010)	5·8 (4·3–7·2)	6·8 (4·7–8·8)	7·3 (4·9–9·9)
1-193	Gynecological diseases	23,900 (16,400–33,400)	34,500 (23,600–49,100)	39,900 (27,300–56,500)	382 (264–536)	356 (244–506)	357 (244–507)
1-194	Uterine fibroids	1,290 (639–2,400)	2,220 (1,080–4,080)	2,640 (1,280–4,830)	23·8 (11·9–44·3)	24 (11·8–44)	24·3 (11·9–44·2)
1-195	Polycystic ovarian syndrome	312 (129–630)	636 (268–1,270)	766 (318–1,550)	4·2 (1·7–8·5)	6·1 (2·5–12·2)	6·6 (2·7–13·4)
1-196	Female infertility	176 (71–388)	186 (69–415)	732 (223–1,770)	2·5 (1–5·4)	1·8 (0·7–4)	6·3 (1·9–15·3)
1-197	Endometriosis	2,800 (1,600–4,530)	3,260 (1,900–5,270)	3,440 (1,990–5,560)	39·5 (22·9–63·3)	31·7 (18·5–51·2)	29·5 (17·2–47·5)
1-198	Genital prolapse	432 (234–767)	617 (312–1,120)	742 (388–1,360)	9·2 (4·7–16·8)	7·4 (3·7–13·5)	7·3 (3·8–13·6)
1-199	Premenstrual syndrome	7,340 (4,500–11,400)	10,100 (6,140–15,800)	11,100 (6,780–17,400)	102 (62·9–158)	97·5 (59·5–150)	96·7 (58·9–150)
1-200	Other gynecological diseases	11,600 (7,600–16,600)	17,500 (11,200–25,200)	20,400 (13,100–29,400)	200 (130–287)	187 (120–268)	187 (120–268)
1-201	Hemoglobinopathies and hemolytic anaemias	20,500 (16,500–25,300)	14,600 (11,700–18,500)	15,200 (11,700–19,100)	248 (199–307)	145 (116–184)	138 (107–174)
1-202	Thalassemias	50 (27–88)	24 (14–40)	19 (11–30)	0·5 (0·3–0·9)	0·2 (0·1–0·4)	0·2 (0·1–0·3)
1-203	Thalassaemias trait	805 (502–1,260)	840 (533–1,290)	894 (542–1,370)	10·8 (6·7–16·8)	8·3 (5·3–12·7)	8 (4·9–12·3)
1-204	Sickle cell disorders	11,900 (9,390–14,900)	6,960 (5,720–8,480)	6,760 (5,110–8,690)	126 (102–156)	66·8 (54·9–81·4)	60·5 (45·9–77·8)
1-205	Sickle cell trait	4,950 (3,000–7,470)	3,970 (2,400–6,210)	4,150 (2,400–6,340)	62·2 (38·1–93·1)	38·3 (23·4–59·5)	36·6 (21·2–55·7)
1-206	G6PD deficiency	774 (543–1,050)	796 (554–1,070)	1,100 (738–1,560)	11·3 (8–15·2)	8·3 (5·9–11·2)	10·1 (6·8–14·2)
1-207	G6PD trait	9 (5–16)	18 (9–30)	19 (10–31)	0·2 (0·1–0·3)	0·2 (0·1–0·3)	0·2 (0·1–0·3)
1-208	Other hemoglobinopathies and hemolytic anaemias	2,060 (1,500–2,900)	2,020 (1,460–2,830)	2,280 (1,670–3,160)	36·7 (27·3–50·4)	23·1 (17–32)	22·5 (16·6–30·9)
1-209	Endocrine, metabolic, blood, and immune disorders	36,000 (15,300–51,100)	25,100 (19,200–32,300)	39,300 (25,100–55,000)	460 (239–625)	280 (214–358)	378 (243–526)
1-210	Oral disorders	18,000 (10,300–28,300)	29,900 (17,400–47,500)	35,500 (20,700–57,300)	368 (218–574)	356 (210–566)	352 (208–567)
1-211	Caries of deciduous teeth	304 (132–641)	318 (138–672)	333 (143–705)	3·1 (1·4–6·6)	3·1 (1·4–6·6)	3·1 (1·3–6·6)
1-212	Caries of permanent teeth	1,880 (842–3,600)	2,610 (1,180–5,040)	2,850 (1,280–5,620)	27·1 (12·2–50·9)	26·3 (11·9–51)	25·8 (11·6–50·6)
1-213	Periodontal disease	5,420 (2,010–11,600)	9,590 (3,670–20,400)	11,600 (4,510–24,700)	104 (39·6–222)	107 (41·1–228)	109 (42·2–229)
1-214	Edentulism	6,800 (4,300–10,100)	12,300 (7,860–18,300)	14,900 (9,360–22,200)	181 (114–266)	166 (106–249)	161 (102–240)
1-215	Other oral disorders	3,560 (2,200–5,240)	5,130 (3,180–7,570)	5,800 (3,600–8,510)	53·3 (32·9–78·4)	53·2 (33–78·2)	53·1 (33–77·7)
1-216	Sudden infant death syndrome	6,360 (1,350–17,400)	3,100 (1,330–6,100)	2,770 (1,100–5,510)	56·2 (11·9–154)	29·4 (12·6–57·7)	25·1 (10–49·9)
1-0	Injuries	298,000 (270,000–338,000)	407,000 (345,000–478,000)	446,000 (358,000–542,000)	4,040 (3,660–4,520)	4,150 (3,520–4,870)	4,020 (3,250–4,890)
1-1	Transport injuries	103,000 (92,900–126,000)	144,000 (120,000–170,000)	157,000 (119,000–198,000)	1,470 (1,320–1,710)	1,470 (1,230–1,740)	1,410 (1,080–1,780)
1-2	Road injuries	98,200 (87,900–120,000)	141,000 (118,000–168,000)	153,000 (116,000–194,000)	1,390 (1,250–1,630)	1,440 (1,210–1,710)	1,380 (1,050–1,740)

1-3	Pedestrian road injuries	17,900 (11,800–22,500)	12,100 (9,790–15,900)	13,600 (10,000–18,400)	255 (168–319)	131 (107–169)	128 (94·9–173)
1-4	Cyclist road injuries	3,810 (2,930–4,860)	4,850 (3,700–6,130)	5,990 (4,600–7,650)	63·7 (48·5–81·8)	52·4 (39·8–66·3)	55·6 (42·6–71)
1-5	Motorcyclist road injuries	7,520 (5,470–10,200)	17,000 (13,600–21,200)	21,900 (15,200–30,000)	112 (82·6–150)	173 (140–216)	196 (137–265)
1-6	Motor vehicle road injuries	68,300 (59,100–89,400)	106,000 (86,800–127,000)	111,000 (82,200–142,000)	950 (826–1,210)	1,080 (880–1,290)	989 (737–1,270)
1-7	Other road injuries	724 (553–912)	958 (713–1,240)	1,130 (851–1,460)	12·3 (9·3–15·8)	10·6 (7·8–13·8)	10·6 (8–13·8)
1-8	Other transport injuries	5,320 (4,840–5,970)	2,490 (2,030–3,020)	3,580 (2,640–4,930)	75·4 (69·1–83·4)	25·7 (21–31·3)	32·5 (24·1–44·6)
1-9	Unintentional injuries	129,000 (113,000–148,000)	124,000 (103,000–146,000)	141,000 (113,000–173,000)	1,670 (1,470–1,900)	1,300 (1,090–1,540)	1,310 (1,060–1,610)
1-10	Falls	10,200 (8,330–12,100)	16,500 (13,300–20,300)	22,500 (17,600–28,100)	189 (155–224)	193 (156–236)	224 (176–276)
1-11	Drowning	20,900 (17,300–27,200)	15,400 (11,900–21,700)	18,300 (12,500–25,400)	237 (201–305)	151 (116–211)	164 (113–228)
1-12	Fire, heat, and hot substances	19,800 (14,700–25,200)	14,200 (11,100–18,400)	16,000 (12,100–21,100)	258 (199–322)	148 (116–192)	148 (112–195)
1-13	Poisonings	5,800 (4,040–7,680)	2,500 (1,940–3,200)	3,410 (2,520–4,560)	62·9 (46–80·7)	25 (19·4–32)	31 (22·8–41·4)
1-14	Poisoning by carbon monoxide	565 (414–958)	584 (417–799)	792 (508–1,170)	7·2 (5·4–11·4)	5·8 (4·2–8)	7·1 (4·6–10·6)
1-15	Poisoning by other means	5,230 (3,490–7,030)	1,920 (1,480–2,510)	2,610 (1,910–3,560)	55·7 (38·3–73·3)	19·2 (14·9–25)	23·9 (17·4–32·5)
1-16	Exposure to mechanical forces	12,100 (9,360–15,800)	23,800 (16,200–31,100)	22,900 (16,900–30,100)	186 (143–242)	248 (171–323)	210 (156–278)
1-17	Unintentional firearm injuries	5,110 (3,780–7,970)	13,000 (7,150–17,900)	9,640 (6,550–13,900)	73 (54·9–111)	130 (73·4–178)	86·1 (59·2–123)
1-18	Other exposure to mechanical forces	6,990 (5,220–9,240)	10,800 (7,660–15,400)	13,200 (9,360–18,600)	113 (83–151)	118 (83·3–167)	124 (88·1–175)
1-19	Adverse effects of medical treatment	7,860 (6,110–11,000)	8,560 (5,540–11,200)	8,250 (5,440–11,700)	106 (89·6–138)	96·1 (60·9–127)	80 (53·2–114)
1-20	Animal contact	858 (600–1,190)	1,060 (746–1,470)	1,130 (794–1,560)	13·6 (9·7–18·9)	11·4 (8–15·8)	10·6 (7·4–14·6)
1-21	Venomous animal contact	600 (415–829)	745 (520–1,010)	796 (543–1,080)	9·4 (6·5–12·9)	7·9 (5·6–10·8)	7·4 (5·1–10)
1-22	Non-venomous animal contact	258 (171–387)	314 (209–471)	336 (223–511)	4·2 (2·8–6·3)	3·4 (2·3–5·1)	3·2 (2·1–4·8)
1-23	Foreign body	29,600 (23,600–36,300)	15,200 (12,400–18,600)	20,100 (14,700–26,300)	314 (258–377)	154 (127–188)	187 (138–245)
1-24	Pulmonary aspiration and foreign body in airway	28,400 (22,500–35,100)	13,800 (11,100–17,100)	18,400 (13,200–24,600)	296 (239–358)	139 (112–173)	171 (123–229)
1-25	Foreign body in eyes	357 (156–653)	525 (243–943)	596 (278–1,060)	5·5 (2·7–9·6)	5·5 (2·7–9·7)	5·5 (2·6–9·6)
1-26	Foreign body in other body part	845 (635–1,080)	883 (679–1,130)	1,070 (825–1,350)	12·6 (9·6–15·9)	9·5 (7·3–12·1)	10·2 (7·9–12·8)
1-27	Environmental heat and cold exposure	2,910 (2,160–3,790)	3,580 (2,560–4,800)	4,300 (3,110–5,750)	48·1 (35·3–62·9)	38·9 (27·8–52·1)	40·5 (29·2–54)
1-28	Exposure to forces of nature	20 (14–29)	905 (755–1,100)	356 (248–506)	0·3 (0·2–0·4)	9·1 (7·6–11)	3·3 (2·3–4·6)
1-29	Other unintentional injuries	18,600 (15,800–22,300)	22,300 (16,800–27,600)	23,600 (14,900–31,900)	253 (217–300)	227 (172–281)	213 (136–286)
1-30	Self-harm and interpersonal violence	65,700 (58,800–74,200)	139,000 (115,000–169,000)	148,000 (111,000–191,000)	902 (805–1,020)	1,380 (1,140–1,670)	1,300 (977–1,670)
1-31	Self-harm	14,300 (12,200–20,100)	27,600 (21,700–35,400)	30,800 (21,700–43,700)	216 (185–308)	286 (225–365)	277 (196–392)
1-32	Self-harm by firearm	2,360 (1,710–4,670)	4,340 (2,680–7,020)	4,680 (2,540–8,620)	34·9 (25·6–69·5)	44·3 (27–70·6)	41·5 (22·7–76·9)
1-33	Self-harm by other specified means	11,900 (9,930–15,800)	23,300 (18,300–29,700)	26,200 (18,400–36,000)	181 (154–245)	242 (190–309)	235 (166–326)
1-34	Interpersonal violence	51,100 (44,700–58,500)	112,000 (91,300–133,000)	117,000 (85,900–153,000)	681 (599–778)	1,090 (896–1,300)	1,020 (752–1,330)
1-35	Physical violence by firearm	25,600 (17,700–31,100)	67,300 (53,900–82,400)	72,000 (50,700–97,500)	345 (240–418)	655 (523–800)	621 (438–842)
1-36	Physical violence by sharp object	7,960 (5,660–17,500)	24,500 (18,500–31,700)	25,400 (17,900–36,900)	112 (81–242)	241 (183–311)	221 (156–320)
1-37	Sexual violence	1,310 (859–1,860)	1,790 (1,190–2,500)	2,040 (1,350–2,900)	18·3 (12·1–26)	17·6 (11·8–24·8)	18 (12–25·6)
1-38	Physical violence by other means	16,200 (12,900–19,200)	18,000 (14,700–21,800)	17,600 (13,500–22,900)	205 (160–242)	180 (146–217)	159 (122–206)
1-39	Conflict and terrorism	277 (236–328)	55 (36–85)	44 (29–66)	3·8 (3·2–4·6)	0·6 (0·4–0·9)	0·4 (0·3–0·6)
1-40	Executions and police conflict	49 (45–54)	60 (50–71)	70 (54–88)	0·6 (0·6–0·7)	0·6 (0·5–0·7)	0·6 (0·5–0·8)

SR Table 1: All-age DALYs counts and age standardized DALYs rates per 100,000 individuals for all causes and level 1 cause groups by location for 1990, 2010, and 2019, both sexes combined, Grenada

Cause Level	Cause	All-age DALYs			Age-standardized rate of DALYs per 100,000		
		1990	2010	2019	1990	2010	2019
0-0	All causes	29,300 (26,300–32,500)	30,900 (27,900–34,300)	32,400 (28,800–36,200)	38,000 (34,100–42,000)	31,700 (28,800–34,900)	30,500 (27,100–34,200)
1-0	Communicable, maternal, neonatal, and nutritional disorders	5,930 (5,100–6,860)	4,100 (3,620–4,680)	3,690 (3,180–4,290)	6,680 (5,810–7,670)	4,530 (3,980–5,180)	4,230 (3,570–5,020)
1-1	HIV/AIDS and sexually transmitted infections	360 (349–372)	357 (341–381)	296 (279–316)	517 (501–534)	325 (310–346)	268 (253–287)
1-2	HIV/AIDS	339 (332–347)	334 (322–351)	274 (263–288)	489 (478–501)	304 (293–319)	249 (238–262)
1-3	HIV/AIDS - drug-susceptible tuberculosis	22 (15–32)	9 (7–13)	6 (4–9)	32 (21·7–46·7)	8·4 (6–11·8)	5·8 (4–8)
1-4	HIV/AIDS - Multidrug-resistant Tuberculosis without extensive drug resistance	0 (0–1)	0 (0–0)	0 (0–1)	0·3 (0–1·3)	0·1 (0–0·3)	0·1 (0–0·5)
1-5	HIV/AIDS - Extensively drug-resistant Tuberculosis	0 (0–0)	0 (0–0)	0 (0–0)	0 (0–0)	0 (0–0)	0 (0–0·1)
1-6	HIV/AIDS resulting in other diseases	317 (305–328)	325 (312–340)	268 (256–281)	457 (439–473)	295 (284–308)	243 (232–255)
1-7	Sexually transmitted infections excluding HIV	20 (14–31)	24 (15–39)	21 (13–37)	27·7 (19·2–42·3)	21·4 (13·5–35·5)	19·3 (11·6–33·1)
1-8	Syphilis	6 (5–8)	3 (2–3)	2 (2–2)	7·5 (6–9·3)	2·8 (2·3–3·3)	1·8 (1·5–2·3)
1-9	Chlamydial infection	2 (1–3)	3 (2–4)	2 (1–3)	2·3 (1·5–3·5)	2·2 (1·5–3·3)	2·1 (1·4–3·2)
1-10	Gonococcal infection	2 (1–2)	2 (2–3)	2 (1–2)	2·1 (1·6–2·6)	1·9 (1·5–2·4)	1·5 (1·1–2·1)
1-11	Trichomoniasis	3 (1–7)	5 (2–11)	5 (2–11)	5 (2–10·5)	4·8 (1·9–9·7)	4·6 (1·8–9·5)
1-12	Genital herpes	4 (1–9)	6 (2–14)	6 (2–15)	5·4 (1·8–13·1)	5·4 (1·7–13·2)	5·3 (1·7–13·1)
1-13	Other sexually transmitted infections	4 (3–5)	5 (4–7)	4 (3–6)	5·3 (3·9–7·3)	4·4 (3·2–6·1)	4 (2·7–5·8)
1-14	Respiratory infections and tuberculosis	1,640 (1,410–1,880)	1,280 (1,160–1,410)	1,200 (1,060–1,350)	1,920 (1,680–2,190)	1,450 (1,310–1,600)	1,270 (1,110–1,450)
1-15	Tuberculosis	67 (59–76)	39 (33–46)	29 (24–34)	88·9 (78·9–99·7)	36·4 (31·3–42·6)	26·1 (22–31·2)
1-16	Latent tuberculosis infection	0 (0–0)	0 (0–0)	0 (0–0)	0 (0–0)	0 (0–0)	0 (0–0)
1-17	Drug-susceptible tuberculosis	66 (59–76)	38 (33–45)	28 (24–34)	88·2 (78·5–99·2)	36·2 (31·1–42·3)	25·7 (21·5–30·9)
1-18	Multidrug-resistant tuberculosis without extensive drug resistance	1 (0–2)	0 (0–1)	0 (0–2)	0·7 (0·1–2·9)	0·2 (0–1)	0·3 (0–1·7)
1-19	Extensively drug-resistant tuberculosis	0 (0–0)	0 (0–0)	0 (0–0)	0 (0–0)	0 (0–0·1)	0 (0–0·3)
1-20	Lower respiratory infections	1,460 (1,250–1,680)	1,120 (1,020–1,240)	1,060 (928–1,200)	1,710 (1,480–1,960)	1,300 (1,180–1,440)	1,130 (983–1,310)
1-21	Upper respiratory infections	80 (51–123)	90 (55–139)	82 (50–130)	89·1 (56·2–136)	85·4 (52·5–131)	82·9 (49·7–130)
1-22	Otitis media	30 (17–49)	30 (17–50)	27 (16–44)	32·3 (18·5–53·4)	28·5 (16·6–46·9)	27·7 (15·9–45·4)
1-23	Enteric infections	341 (276–418)	238 (180–309)	242 (181–318)	375 (306–454)	247 (189–319)	252 (189–332)
1-24	Diarrhoeal diseases	340 (275–416)	237 (179–308)	242 (181–317)	373 (304–451)	246 (188–318)	251 (188–330)
1-25	Typhoid and paratyphoid	0 (0–0)	0 (0–0)	0 (0–0)	0·3 (0·1–0·3)	0·1 (0–0·1)	0·1 (0–0·1)
1-26	Typhoid fever	0 (0–0)	0 (0–0)	0 (0–0)	0·2 (0·1–0·3)	0 (0–0)	0 (0–0)
1-27	Paratyphoid fever	0 (0–0)	0 (0–0)	0 (0–0)	0 (0–0·1)	0 (0–0·1)	0 (0–0·1)
1-28	iNTS	0 (0–1)	0 (0–0)	0 (0–0)	0·4 (0·3–0·8)	0·2 (0·2–0·4)	0·1 (0·1–0·3)
1-29	Other intestinal infectious diseases	1 (0–2)	1 (0–1)	1 (0–1)	1·2 (0·5–2)	0·7 (0·4–1·1)	0·6 (0·3–0·9)
1-30	Neglected tropical diseases and malaria	152 (108–202)	115 (80–161)	107 (71–153)	180 (126–242)	115 (80–160)	104 (70·8–146)
1-31	Malaria	0 (0–0)	0 (0–0)	0 (0–0)	0 (0–0)	0 (0–0)	0 (0–0)
1-32	Chagas disease	0 (0–0)	0 (0–0)	0 (0–0)	0 (0–0)	0·1 (0·1–0·2)	0·2 (0·1–0·3)
1-33	Leishmaniasis	0 (0–0)	0 (0–0)	0 (0–0)	0 (0–0)	0 (0–0)	0 (0–0)
1-34	Visceral leishmaniasis	0 (0–0)	0 (0–0)	0 (0–0)	0 (0–0)	0 (0–0)	0 (0–0)
1-35	Cutaneous and mucocutaneous leishmaniasis	0 (0–0)	0 (0–0)	0 (0–0)	0 (0–0)	0 (0–0)	0 (0–0)
1-36	African trypanosomiasis	0 (0–0)	0 (0–0)	0 (0–0)	0 (0–0)	0 (0–0)	0 (0–0)
1-37	Schistosomiasis	0 (0–0)	0 (0–0)	0 (0–0)	0 (0–0)	0 (0–0)	0 (0–0)
1-38	Cysticercosis	47 (26–78)	50 (25–86)	54 (27–94)	68 (37·2–112)	49·8 (24·9–85·6)	48 (23·9–83·5)
1-39	Cystic echinococcosis	0 (0–0)	0 (0–0)	0 (0–0)	0 (0–0)	0 (0–0)	0 (0–0)
1-40	Lymphatic filariasis	0 (0–0)	0 (0–0)	0 (0–0)	0 (0–0)	0 (0–0)	0 (0–0)
1-41	Onchocerciasis	0 (0–0)	0 (0–0)	0 (0–0)	0 (0–0)	0 (0–0)	0 (0–0)
1-42	Trachoma	0 (0–0)	0 (0–0)	0 (0–0)	0 (0–0)	0 (0–0)	0 (0–0)
1-43	Dengue	9 (4–16)	18 (9–31)	14 (7–23)	10·1 (5·2–17·7)	16·8 (8·6–29·2)	13·9 (6·6–22·9)
1-44	Yellow fever	0 (0–0)	0 (0–0)	0 (0–0)	0 (0–0)	0 (0–0)	0 (0–0)
1-45	Rabies	0 (0–0)	0 (0–0)	0 (0–0)	0·1 (0·1–0·2)	0·2 (0·1–0·2)	0·1 (0·1–0·2)
1-46	Intestinal nematode infections	31 (19–50)	10 (6–17)	6 (4–10)	34·7 (20·6–54·7)	9·1 (5·1–15)	6·1 (3·5–10·1)
1-47	Ascariasis	14 (7–25)	1 (1–2)	1 (0–2)	15·7 (7·7–28·3)	1·3 (0·7–2·1)	0·9 (0·5–1·6)
1-48	Trichuriasis	6 (3–11)	0 (0–0)	0 (0–0)	6 (2·8–11·5)	0 (0–0·1)	0 (0–0·1)

1-49	Hookworm disease	12 (7–19)	9 (5–15)	5 (3–9)	13 (7·4–20·9)	7·7 (4·1–13·1)	5·2 (2·7–8·6)
1-50	Food-borne trematodiases	0 (0–0)	0 (0–0)	0 (0–0)	0 (0–0)	0 (0–0)	0 (0–0)
1-51	Leprosy	1 (0–1)	1 (0–1)	1 (0–1)	0·9 (0·6–1·4)	0·6 (0·4–0·9)	0·5 (0·3–0·8)
1-52	Ebola virus disease	0 (0–0)	0 (0–0)	0 (0–0)	0 (0–0)	0 (0–0)	0 (0–0)
1-53	Zika virus disease	0 (0–0)	0 (0–0)	3 (0–13)	0 (0–0)	0 (0–0)	3·2 (0·1–12·9)
1-54	Guinea worm disease	0 (0–0)	0 (0–0)	0 (0–0)	0 (0–0)	0 (0–0)	0 (0–0)
1-55	Other neglected tropical diseases	64 (39–84)	36 (26–54)	28 (19–44)	65·8 (40·1–85·9)	38·3 (27·1–56·4)	32 (21·3–50)
1-56	Other infectious diseases	423 (359–493)	192 (166–224)	139 (115–175)	480 (412–556)	193 (167–224)	141 (116–177)
1-57	Meningitis	199 (162–241)	50 (43–58)	25 (22–30)	215 (177–258)	53·1 (45·3–62·7)	27·8 (22·9–34·1)
1-58	Encephalitis	80 (57–100)	39 (33–52)	26 (21–42)	85·1 (60·4–105)	39·1 (32·4–53·7)	28·5 (21·6–46·6)
1-59	Diphtheria	0 (0–0)	0 (0–0)	0 (0–0)	0 (0–0)	0 (0–0)	0 (0–0)
1-60	Whooping cough	2 (1–3)	1 (1–2)	1 (1–2)	2 (1·2–3·2)	1·6 (1–2·5)	1·9 (1·1–2·9)
1-61	Tetanus	29 (20–42)	7 (5–12)	3 (2–12)	39·1 (25·3–59·1)	7·1 (5–11·3)	2·9 (1·4–10)
1-62	Measles	1 (0–1)	0 (0–0)	0 (0–0)	0·7 (0·1–1·3)	0 (0–0)	0 (0–0)
1-63	Varicella and herpes zoster	13 (8–18)	11 (7–14)	6 (5–12)	14·4 (9·6–20·3)	11·3 (7·6–15)	6·6 (4·7–13)
1-64	Acute hepatitis	36 (32–41)	10 (8–12)	8 (6–10)	45·4 (39·7–51·1)	9·3 (7·6–11·3)	7·5 (5·8–9·6)
1-65	Acute hepatitis A	19 (14–23)	5 (3–7)	4 (3–6)	22·3 (15·7–27·8)	4·5 (3·1–6·1)	3·9 (2·6–5·6)
1-66	Acute hepatitis B	14 (11–19)	4 (4–5)	3 (2–3)	18·9 (14·1–25)	3·8 (3·3–4·3)	2·6 (2·1–3·2)
1-67	Acute hepatitis C	2 (1–2)	0 (0–0)	0 (0–0)	2·2 (1–3·4)	0·3 (0·2–0·5)	0·2 (0·1–0·4)
1-68	Acute hepatitis E	2 (1–3)	1 (1–1)	1 (0–1)	2 (1·2–3·7)	0·7 (0·5–1·1)	0·7 (0·4–0·9)
1-69	Other unspecified infectious diseases	64 (45–80)	74 (52–93)	68 (50–92)	78·2 (54·3–97·5)	71·9 (50·4–90·8)	66·1 (49·4–88·4)
1-70	Maternal and neonatal disorders	2,340 (1,870–2,910)	1,460 (1,190–1,760)	1,300 (1,010–1,710)	2,470 (1,980–3,060)	1,720 (1,400–2,110)	1,760 (1,340–2,350)
1-71	Maternal disorders	80 (69–92)	83 (74–94)	56 (47–66)	94·2 (81·9–109)	72·4 (64·5–81·1)	51·9 (43·6–60·9)
1-72	Maternal haemorrhage	17 (14–22)	13 (10–17)	8 (6–11)	20·7 (16·3–25·9)	11·6 (9·1–14·7)	7·5 (5·6–10)
1-73	Maternal sepsis and other pregnancy related infections	4 (3–6)	2 (2–3)	1 (1–2)	4·7 (3·4–6·4)	2 (1·4–2·9)	1·3 (0·8–2·1)
1-74	Maternal hypertensive disorders	33 (28–39)	31 (27–35)	20 (17–24)	38·5 (32·5–45·6)	26·4 (23–29·9)	18·7 (15·4–22·5)
1-75	Maternal obstructed labour and uterine rupture	1 (0–1)	2 (2–3)	1 (1–2)	0·9 (0·6–1·3)	1·9 (1·4–2·4)	1·3 (0·9–1·7)
1-76	Maternal abortive outcome	14 (11–18)	6 (4–7)	3 (2–4)	16·7 (13·1–21·1)	4·8 (3·7–6·1)	2·6 (1·9–3·4)
1-77	Ectopic pregnancy	1 (1–2)	5 (4–6)	2 (2–3)	1·6 (1·2–1·9)	4 (3·3–4·8)	2·1 (1·6–2·6)
1-78	Indirect maternal deaths	3 (2–4)	5 (4–6)	2 (2–3)	3·3 (2·6–4·2)	4 (3·3–4·9)	2·2 (1·7–2·9)
1-79	Late maternal deaths	3 (2–4)	5 (4–7)	4 (2–5)	3·5 (2·5–4·7)	4·3 (3·1–5·7)	3·3 (2·3–4·5)
1-80	Maternal deaths aggravated by HIV/AIDS	0 (0–0)	0 (0–0)	0 (0–0)	0 (0–0·1)	0 (0–0·1)	0 (0–0)
1-81	Other maternal disorders	4 (3–4)	15 (12–19)	14 (11–17)	4·2 (3·4–5·2)	13·5 (11–16·2)	12·8 (10·3–15·8)
1-82	Neonatal disorders	2,260 (1,790–2,810)	1,370 (1,110–1,680)	1,250 (958–1,660)	2,380 (1,890–2,950)	1,650 (1,330–2,030)	1,710 (1,290–2,290)
1-83	Neonatal preterm birth	1,090 (864–1,350)	577 (470–708)	504 (381–671)	1,140 (911–1,420)	687 (557–857)	692 (509–943)
1-84	Neonatal encephalopathy due to birth asphyxia and trauma	686 (517–900)	396 (307–510)	332 (238–463)	720 (544–944)	491 (379–638)	475 (338–665)
1-85	Neonatal sepsis and other neonatal infections	270 (194–366)	262 (197–343)	278 (206–370)	284 (205–385)	306 (230–400)	354 (260–478)
1-86	Hemolytic disease and other neonatal jaundice	31 (24–41)	23 (18–28)	20 (16–25)	34·3 (26–44·6)	22·5 (17·6–28·1)	21·7 (16·8–27·1)
1-87	Other neonatal disorders	189 (126–273)	115 (83–153)	111 (76–161)	197 (131–285)	146 (105–192)	166 (113–241)
1-88	Nutritional deficiencies	674 (511–885)	463 (326–643)	405 (283–561)	734 (560–957)	474 (335–652)	433 (303–602)
1-89	Protein-energy malnutrition	227 (185–282)	98 (82–116)	88 (73–104)	248 (205–306)	108 (90·6–128)	94 (76·6–114)
1-90	Iodine deficiency	10 (5–16)	7 (4–12)	5 (3–9)	12·2 (6·4–20·1)	6·3 (3·2–10·7)	4·8 (2·4–8)
1-91	Vitamin A deficiency	18 (11–28)	8 (5–12)	6 (4–9)	17·1 (10·5–25·9)	8 (5–12·1)	7·2 (4·5–10·8)
1-92	Dietary iron deficiency	391 (254–570)	334 (210–502)	292 (180–438)	420 (274–618)	335 (212–504)	314 (193–476)
1-93	Other nutritional deficiencies	28 (21–41)	16 (13–22)	14 (10–19)	35·7 (26·8–51·1)	16·4 (12·7–21·8)	13·1 (9·6–17·8)
1-0	Non-communicable diseases	20,300 (18,200–22,500)	23,600 (21,100–26,500)	25,800 (22,900–28,900)	27,500 (24,700–30,400)	24,200 (21,800–27,000)	23,600 (20,900–26,400)
1-1	Neoplasms	3,220 (2,950–3,520)	4,180 (3,900–4,420)	4,770 (4,290–5,240)	4,590 (4,210–4,990)	4,350 (4,040–4,600)	4,220 (3,810–4,630)
1-2	Lip and oral cavity cancer	57 (52–64)	74 (66–82)	81 (70–94)	86·8 (77·3–97·7)	73·9 (66·7–81·6)	68·7 (59·5–78·7)
1-3	Nasopharynx cancer	12 (10–13)	20 (18–22)	19 (16–22)	17·3 (15·4–19·4)	19 (17·4–20·8)	16·1 (13·9–18·3)
1-4	Other pharynx cancer	30 (26–34)	32 (28–36)	44 (37–51)	46·3 (40·2–52·8)	31·3 (27·7–35)	36·4 (31–42·4)
1-5	Oesophageal cancer	120 (107–135)	164 (147–182)	193 (169–220)	184 (163–206)	165 (149–183)	162 (142–184)
1-6	Stomach cancer	260 (235–286)	239 (220–259)	256 (228–288)	380 (341–417)	252 (234–274)	226 (201–253)
1-7	Colon and rectum cancer	223 (204–244)	348 (323–374)	467 (419–517)	321 (292–353)	372 (344–399)	415 (374–458)

1·8	Liver cancer	160 (143–178)	70 (64–76)	85 (74–98)	235 (208–261)	72·2 (65·9–79·1)	74 (64·8–84·5)
1·9	Liver cancer due to hepatitis B	50 (38–65)	21 (16–28)	25 (18–34)	76·5 (57·8–99·8)	20·4 (15·4–26·9)	20·9 (15–28·5)
1·10	Liver cancer due to hepatitis C	34 (23–45)	13 (9–18)	16 (10–22)	47·1 (32·2–64·2)	14·5 (9·9–19·5)	14 (9·4–19·4)
1·11	Liver cancer due to alcohol use	51 (37–66)	24 (18–31)	32 (23–42)	75·7 (55·1–99·2)	25·2 (18·8–32·2)	27·3 (19·9–35·5)
1·12	Liver cancer due to NASH	11 (8–16)	5 (4–8)	7 (5–9)	15·8 (11·1–22)	5·9 (4·2–8·2)	6 (4·2–8·3)
1·13	Gallbladder and biliary tract cancer	82 (42–96)	39 (34–58)	44 (36–68)	120 (61·5–142)	41·5 (35·9–61·8)	38·3 (31·8–58·8)
1·14	Pancreatic cancer	28 (25–31)	188 (172–207)	248 (217–284)	41·2 (36·6–45·8)	197 (180–217)	216 (189–245)
1·15	Larynx cancer	28 (24–31)	39 (35–44)	36 (31–41)	41·9 (37·1–47·3)	39·7 (35·6–44·3)	30·3 (26·4–34·7)
1·16	Tracheal, bronchus, and lung cancer	251 (226–278)	344 (314–373)	398 (352–450)	376 (337–418)	353 (324–382)	339 (301–379)
1·17	Malignant skin melanoma	12 (9–16)	19 (16–28)	19 (14–33)	17·9 (12·6–23·1)	19 (15·6–27·6)	16·5 (13–28·9)
1·18	Non-melanoma skin cancer	3 (3–6)	14 (9–16)	15 (11–17)	4·6 (4–7·9)	15·4 (9·3–17·2)	13·5 (9·5–15·3)
1·19	Non-melanoma skin cancer (squamous-cell carcinoma)	3 (3–6)	14 (9–16)	15 (11–17)	4·6 (4–7·9)	15·4 (9·3–17·2)	13·5 (9·5–15·3)
1·20	Non-melanoma skin cancer (basal-cell carcinoma)	0 (0–0)	0 (0–0)	0 (0–0)	0 (0–0)	0 (0–0)	0 (0–0)
1·21	Breast cancer	279 (248–311)	426 (385–473)	506 (441–578)	436 (384–489)	418 (378–464)	439 (383–500)
1·22	Cervical cancer	256 (222–295)	283 (244–321)	302 (248–353)	399 (345–461)	273 (236–308)	262 (213–305)
1·23	Uterine cancer	73 (64–84)	130 (114–147)	165 (141–191)	109 (95·3–126)	134 (118–150)	145 (124–166)
1·24	Ovarian cancer	19 (16–33)	113 (70–129)	126 (80–150)	29·2 (24·7–50·4)	111 (68·9–125)	109 (68·5–128)
1·25	Prostate cancer	383 (325–540)	563 (372–644)	735 (491–854)	496 (420–694)	693 (442–792)	677 (454–785)
1·26	Testicular cancer	1 (1–2)	6 (5–7)	3 (3–4)	1·7 (1·4–2·4)	5 (4·3–6·3)	3·2 (2·6–3·9)
1·27	Kidney cancer	42 (37–46)	45 (41–49)	64 (55–72)	57·6 (51·8–63·8)	45 (41·3–48·8)	55·8 (48·6–63·3)
1·28	Bladder cancer	39 (34–44)	51 (46–57)	67 (59–76)	53·6 (47–60·9)	58·5 (52·5–65·2)	61 (53·5–69·1)
1·29	Brain and nervous system cancer	26 (16–63)	101 (49–123)	97 (47–125)	32·3 (20·1–77·8)	97·1 (47·3–119)	89·9 (43·6–116)
1·30	Thyroid cancer	22 (20–25)	31 (28–34)	28 (24–33)	32·5 (28·7–36·2)	31 (27·7–34)	24·3 (21·1–28·5)
1·31	Mesothelioma	9 (7–11)	13 (11–14)	9 (7–12)	13·7 (11–16·6)	12·3 (10·8–13·8)	7·3 (6·1–10)
1·32	Hodgkin lymphoma	12 (10–15)	12 (10–15)	13 (10–16)	16·4 (13·4–20·1)	11·2 (9·5–13·5)	11·5 (9–14·4)
1·33	Non-Hodgkin's lymphoma	292 (256–333)	321 (285–359)	292 (252–339)	395 (347–449)	311 (276–349)	260 (224–301)
1·34	Multiple myeloma	44 (33–54)	78 (62–87)	77 (67–94)	63·8 (48·5–78·9)	80·8 (63·6–89·9)	66·2 (57·3–80·9)
1·35	Leukaemia	181 (158–205)	184 (170–199)	157 (137–177)	215 (191–242)	181 (168–196)	150 (132–168)
1·36	Acute lymphoid leukaemia	37 (28–48)	29 (25–35)	20 (16–26)	36·5 (27·6–45·7)	28·7 (23·8–34·6)	21·3 (17–28·3)
1·37	Chronic lymphoid leukaemia	10 (8–12)	14 (12–16)	11 (9–13)	14·3 (11·3–17·7)	15·3 (13·2–17·5)	9·3 (7·7–11·2)
1·38	Acute myeloid leukaemia	25 (20–32)	39 (33–45)	36 (30–42)	29·3 (22·8–36·5)	37·3 (30·9–42·8)	34·1 (28·6–39·9)
1·39	Chronic myeloid leukaemia	20 (17–24)	19 (17–22)	14 (11–16)	27·3 (23·2–32·2)	18·7 (16·8–20·9)	12·3 (10·4–14·3)
1·40	Other leukaemia	88 (75–102)	82 (74–89)	77 (66–87)	107 (93·8–124)	80·9 (73·9–88·5)	72·6 (62·3–82·2)
1·41	Other neoplasms	13 (9–20)	22 (19–31)	31 (24–39)	16·2 (11·4–26·8)	24·1 (20·2–32·9)	29·3 (22·6–35·7)
1·42	Myelodysplastic, myeloproliferative, and other hematopoietic neoplasms	13 (9–20)	22 (19–31)	31 (24–39)	16·2 (11·4–26·8)	24·1 (20·2–32·9)	29·3 (22·6–35·7)
1·43	Benign and in situ intestinal neoplasms	0 (0–0)	0 (0–0)	0 (0–0)	0 (0–0)	0 (0–0)	0 (0–0)
1·44	Benign and in situ cervical and uterine neoplasms	0 (0–0)	0 (0–0)	0 (0–0)	0 (0–0)	0 (0–0)	0 (0–0)
1·45	Other benign and in situ neoplasms	0 (0–0)	0 (0–0)	0 (0–0)	0 (0–0)	0 (0–0)	0 (0–0)
1·46	Cardiovascular diseases	6,300 (5,870–6,730)	5,170 (4,900–5,440)	5,760 (5,290–6,250)	8,690 (8,090–9,300)	5,710 (5,420–6,000)	5,300 (4,890–5,730)
1·47	Rheumatic heart disease	200 (172–230)	158 (134–188)	135 (110–166)	274 (233–317)	145 (124–172)	121 (98·1–149)
1·48	Ischaemic heart disease	2,640 (2,440–2,840)	1,940 (1,820–2,050)	2,210 (2,010–2,410)	3,670 (3,400–3,950)	2,160 (2,040–2,290)	2,020 (1,840–2,190)
1·49	Stroke	2,380 (2,190–2,580)	1,800 (1,680–1,920)	1,950 (1,760–2,140)	3,280 (3,000–3,550)	2,010 (1,880–2,150)	1,810 (1,640–1,990)
1·50	Ischaemic stroke	1,190 (1,040–1,320)	806 (746–865)	905 (811–996)	1,520 (1,320–1,690)	1,010 (925–1,080)	904 (808–995)
1·51	Intracerebral hemorrhage	1,020 (903–1,170)	807 (750–871)	862 (767–966)	1,510 (1,340–1,720)	831 (773–899)	746 (667–834)
1·52	Subarachnoid hemorrhage	166 (136–197)	184 (166–204)	182 (155–213)	245 (202–292)	175 (158–193)	157 (134–184)
1·53	Hypertensive heart disease	389 (333–463)	437 (367–488)	531 (430–614)	537 (458–642)	493 (412–547)	491 (395–565)
1·54	Non-rheumatic valvular heart disease	37 (30–46)	53 (48–58)	65 (56–74)	52·3 (42·7–65·3)	53·4 (48·4–58·9)	56·8 (49·6–64·5)
1·55	Non-rheumatic calcific aortic valve disease	16 (11–21)	23 (19–27)	28 (23–36)	22·9 (15·4–30·4)	24·2 (20·2–28·6)	24·7 (20·4–32·1)
1·56	Non-rheumatic degenerative mitral valve disease	20 (16–24)	29 (25–34)	36 (29–43)	28·9 (22·9–35·5)	28·7 (24·6–34·2)	31·5 (25–37·4)
1·57	Other non-rheumatic valve diseases	0 (0–1)	0 (0–1)	1 (0–1)	0·5 (0·3–0·8)	0·5 (0·3–0·8)	0·7 (0·3–1)
1·58	Cardiomyopathy and myocarditis	213 (188–250)	275 (225–301)	315 (237–365)	295 (261–346)	277 (228–303)	282 (214–326)
1·59	Myocarditis	16 (11–21)	20 (15–24)	19 (15–28)	19·7 (13·1–26·2)	20·7 (15·5–24·8)	18·9 (14·5–26·8)
1·60	Alcoholic cardiomyopathy	29 (21–44)	59 (43–71)	72 (44–91)	46·3 (33·5–70)	55·1 (40·5–66·3)	59·5 (36·7–75·3)

1-61	Other cardiomyopathy	168 (147–195)	195 (163–215)	223 (175–260)	229 (200–265)	201 (168–221)	204 (160–236)
1-62	Atrial fibrillation and flutter	75 (62–90)	80 (69–96)	94 (80–116)	93·4 (77·1–112)	102 (87·7–122)	98·2 (83·6–122)
1-63	Aortic aneurysm	64 (54–79)	89 (80–98)	99 (86–113)	87·7 (73·7–106)	100 (90·3–112)	89·8 (78·7–102)
1-64	Peripheral vascular disease	27 (15–49)	37 (20–64)	49 (26–87)	34·9 (18·7–63·3)	44·3 (24·2–77·8)	48·4 (26·2–85·2)
1-65	Endocarditis	55 (40–67)	82 (65–97)	72 (58–105)	74·9 (55·3–92·8)	80·1 (63·1–95·2)	65·5 (52·4–95·8)
1-66	Chronic respiratory diseases	671 (555–810)	768 (639–930)	782 (654–941)	824 (693–972)	797 (671–955)	755 (623–918)
1-67	Chronic obstructive pulmonary disease	244 (216–277)	305 (271–341)	355 (312–404)	326 (288–370)	345 (308–384)	327 (288–372)
1-68	Pneumoconiosis	5 (4–7)	6 (5–7)	3 (3–4)	7·2 (5·7–9)	6·3 (5·3–7·5)	3·1 (2·6–3·9)
1-69	Silicosis	0 (0–0)	0 (0–0)	0 (0–0)	0·3 (0·2–0·4)	0·3 (0·2–0·3)	0·2 (0·1–0·2)
1-70	Asbestosis	0 (0–0)	0 (0–0)	0 (0–0)	0·2 (0·1–0·2)	0·2 (0·1–0·2)	0·1 (0·1–0·2)
1-71	Coal workers pneumoconiosis	4 (3–6)	5 (4–6)	3 (2–3)	6·1 (4·7–7·9)	5·4 (4·5–6·5)	2·4 (1·9–3)
1-72	Other pneumoconiosis	0 (0–1)	0 (0–1)	0 (0–1)	0·6 (0·5–0·8)	0·5 (0·4–0·6)	0·4 (0·3–0·5)
1-73	Asthma	361 (262–492)	362 (258–497)	329 (229–469)	414 (308–546)	351 (251–485)	338 (230–487)
1-74	Interstitial lung disease and pulmonary sarcoidosis	17 (13–24)	31 (24–38)	36 (26–43)	23·1 (18·2–33·2)	33·3 (25·2–40·8)	32·4 (23·7–39·6)
1-75	Other chronic respiratory diseases	43 (35–53)	64 (55–75)	59 (49–73)	54·4 (43·4–65·8)	61·1 (52·1–71·4)	54·8 (45·7–66·4)
1-76	Digestive diseases	1,060 (962–1,180)	1,170 (1,050–1,310)	1,300 (1,150–1,460)	1,520 (1,370–1,690)	1,150 (1,050–1,280)	1,150 (1,020–1,290)
1-77	Cirrhosis and other chronic liver diseases	445 (399–493)	466 (427–507)	537 (470–611)	685 (614–760)	448 (412–486)	455 (400–518)
1-78	Cirrhosis and other chronic liver diseases due to hepatitis B	54 (39–75)	34 (23–48)	38 (26–57)	88·5 (60·6–123)	31·9 (22·1–44·8)	32 (21·8–46·4)
1-79	Cirrhosis and other chronic liver diseases due to hepatitis C	84 (60–113)	94 (68–128)	105 (74–148)	133 (94–182)	88·7 (65·3–120)	88·5 (63·8–124)
1-80	Cirrhosis and other chronic liver diseases due to alcohol use	163 (128–199)	201 (162–243)	243 (190–297)	263 (205–321)	192 (155–230)	202 (159–247)
1-81	Cirrhosis and other chronic liver diseases due to NAFLD	60 (43–82)	68 (47–96)	80 (54–114)	92·6 (64–127)	67·6 (47·8–92·4)	68·2 (47·5–95·1)
1-82	Cirrhosis and other chronic liver diseases due to other causes	83 (66–103)	69 (53–89)	72 (53–94)	108 (84–138)	68·3 (52·4–87·6)	64·2 (48·7–83)
1-83	Upper digestive system diseases	246 (197–321)	269 (195–383)	287 (211–406)	353 (280–460)	260 (192–364)	253 (186–357)
1-84	Peptic ulcer disease	111 (95–129)	80 (69–94)	86 (71–103)	160 (135–187)	83·7 (72–97·8)	76·2 (63·6–90·7)
1-85	Gastritis and duodenitis	48 (37–60)	55 (42–71)	61 (46–78)	69·5 (52·6–87·4)	53·4 (41·2–67·8)	53·9 (41·2–68·9)
1-86	GERD	86 (45–153)	134 (70–236)	140 (72–249)	123 (64·1–218)	123 (64·1–218)	123 (63·8–217)
1-87	Appendicitis	21 (16–26)	16 (13–20)	16 (13–20)	25·6 (19·4–30·9)	15·3 (12·6–19·2)	15·2 (12·4–19·1)
1-88	Paralytic ileus and intestinal obstruction	118 (99–141)	114 (97–133)	121 (99–144)	145 (122–173)	123 (104–143)	118 (97·1–140)
1-89	Inguinal, femoral, and abdominal hernia	62 (47–80)	74 (55–98)	80 (59–104)	75·2 (58·5–96·9)	72·4 (54·5–94·9)	74·6 (55·9–97·3)
1-90	Inflammatory bowel disease	33 (27–39)	39 (34–47)	43 (36–52)	44·3 (36·6–52·7)	38·7 (34·3–45·7)	38·4 (32·6–46·6)
1-91	Vascular intestinal disorders	9 (8–11)	11 (10–13)	15 (13–18)	12·6 (10·7–15·1)	12·6 (11·1–14·5)	13·8 (11·9–16·2)
1-92	Gallbladder and biliary diseases	73 (56–90)	70 (55–88)	75 (58–96)	104 (79·9–128)	69·6 (55·1–87·5)	66·6 (51·4–86)
1-93	Pancreatitis	22 (19–26)	30 (26–34)	32 (27–38)	32·9 (28·2–38·9)	28·8 (25·6–32·8)	27·9 (23·5–32·8)
1-94	Other digestive diseases	34 (26–56)	76 (50–92)	91 (59–116)	43·2 (32·8–72·1)	80·8 (53·3–97·6)	85·2 (55·4–107)
1-95	Neurological disorders	1,070 (619–1,720)	1,300 (731–2,140)	1,340 (798–2,130)	1,320 (765–2,120)	1,320 (769–2,140)	1,300 (782–2,060)
1-96	Alzheimer's disease and other dementias	258 (107–597)	231 (95–523)	257 (109–578)	301 (125–693)	316 (130–733)	298 (125–675)
1-97	Parkinson's disease	59 (54–64)	69 (64–75)	86 (79–95)	71·9 (65·8–78·1)	91·7 (84·1–99·4)	87 (79·3–95·5)
1-98	Idiopathic epilepsy	254 (147–401)	277 (158–458)	273 (145–440)	312 (186–483)	258 (146–427)	258 (134–416)
1-99	Multiple sclerosis	10 (8–13)	18 (15–22)	17 (14–24)	15·2 (12·8–20·5)	16·6 (14·1–20·3)	14·6 (11·9–20·2)
1-100	Motor neuron disease	4 (4–5)	16 (15–18)	21 (18–24)	5·9 (5·2–6·6)	15·7 (14·4–17·3)	18·4 (15·8–20·9)
1-101	Headache disorders	433 (78–967)	617 (119–1,360)	603 (119–1,330)	554 (107–1,220)	548 (108–1,200)	545 (105–1,200)
1-102	Migraine	393 (53–915)	559 (81–1,290)	544 (84–1,240)	502 (73·1–1,170)	496 (72·9–1,140)	493 (72·3–1,130)
1-103	Tension-type headache	39 (11–156)	58 (17–213)	58 (17–209)	52·3 (15·7–194)	52·1 (15·3–189)	51·9 (15·1–187)
1-104	Other neurological disorders	52 (34–77)	74 (50–109)	81 (54–117)	60·2 (40·8–87·6)	70·2 (48·4–102)	78·3 (51·3–114)
1-105	Mental disorders	1,300 (946–1,710)	1,850 (1,350–2,440)	1,860 (1,370–2,450)	1,670 (1,230–2,190)	1,670 (1,220–2,190)	1,680 (1,240–2,220)
1-106	Schizophrenia	119 (82–157)	198 (138–263)	204 (144–271)	173 (121–231)	178 (123–236)	179 (125–239)
1-107	Depressive disorders	421 (285–583)	616 (420–868)	641 (437–889)	568 (388–780)	556 (382–780)	567 (387–786)
1-108	Major depressive disorder	356 (238–501)	516 (344–738)	539 (359–748)	478 (324–670)	466 (312–660)	477 (318–660)
1-109	Dysthymia	65 (40–98)	100 (61–153)	102 (63–156)	90·4 (56·3–139)	90 (55·9–138)	89·7 (55·7–137)
1-110	Bipolar disorder	151 (87–235)	227 (133–354)	225 (131–349)	200 (116–309)	199 (117–310)	199 (115–308)
1-111	Anxiety disorders	320 (213–454)	450 (300–637)	450 (300–636)	407 (274–579)	405 (270–572)	409 (272–580)
1-112	Eating disorders	32 (20–48)	53 (32–80)	52 (32–78)	37·3 (22·8–55·9)	45·4 (27·5–68·6)	47·5 (29·7–71·5)
1-113	Anorexia nervosa	8 (5–14)	13 (7–22)	12 (7–20)	9·3 (5·4–15·1)	11 (6·3–17·9)	11·4 (6·6–18·7)

1-114	Bulimia nervosa	24 (14–38)	40 (23–64)	39 (23–62)	28 (16·1–44·6)	34·5 (20·1–55)	36·1 (21–57·2)
1-115	Autism spectrum disorders	47 (31–68)	57 (37–82)	54 (35–78)	52·5 (34·6–76·2)	53 (34·3–77·2)	53·4 (34·5–77·4)
1-116	Attention-deficit/hyperactivity disorder	36 (20–62)	41 (24–70)	38 (22–63)	37·3 (20·9–63·4)	37·5 (21·5–63·4)	37·8 (21·7–63·9)
1-117	Conduct disorder	76 (43–120)	73 (41–116)	62 (35–98)	68·1 (38·5–108)	68·1 (37·6–109)	68·6 (39·1–109)
1-118	Idiopathic developmental intellectual disability	20 (7–36)	17 (5–31)	14 (4–27)	21·6 (7·9–39·9)	15·6 (4·7–28·9)	14·2 (4·3–27)
1-119	Other mental disorders	75 (48–114)	118 (74–180)	124 (78–186)	108 (68·4–163)	108 (68·4–165)	108 (68·9–164)
1-120	Substance use disorders	257 (200–323)	455 (362–565)	478 (388–586)	361 (286–448)	403 (322–500)	417 (338–514)
1-121	Alcohol use disorders	168 (132–214)	288 (228–365)	308 (249–387)	249 (199–313)	259 (206–327)	266 (213–334)
1-122	Drug use disorders	89 (64–121)	166 (126–214)	169 (128–215)	112 (80·8–149)	144 (110–185)	151 (115–192)
1-123	Opioid use disorders	35 (23–50)	60 (42–81)	61 (43–80)	46·5 (31·3–65·3)	52·6 (37·1–70·5)	53·8 (38·4–71·8)
1-124	Cocaine use disorders	18 (12–27)	47 (36–61)	48 (36–63)	23 (15·7–32·6)	40·7 (31·5–52·7)	43·4 (33–56·4)
1-125	Amphetamine use disorders	6 (3–10)	10 (6–15)	10 (7–15)	7 (4–11·6)	8·4 (5·4–12·8)	8·8 (6–13·2)
1-126	Cannabis use disorders	14 (8–25)	20 (10–33)	18 (10–30)	16·2 (8·7–26·6)	16·3 (8·7–27·3)	16·4 (9–27·1)
1-127	Other drug use disorders	16 (10–23)	30 (21–42)	32 (23–44)	19·2 (13–27·8)	26·4 (18·5–36)	28·8 (20·6–40)
1-128	Diabetes and kidney diseases	2,230 (2,000–2,460)	3,560 (3,190–3,950)	4,220 (3,720–4,770)	3,190 (2,850–3,530)	3,720 (3,360–4,110)	3,750 (3,320–4,220)
1-129	Diabetes mellitus	1,520 (1,340–1,710)	2,320 (2,020–2,650)	2,730 (2,320–3,190)	2,180 (1,930–2,470)	2,460 (2,160–2,790)	2,410 (2,060–2,820)
1-130	Diabetes mellitus type 1	108 (83–132)	111 (96–144)	108 (89–143)	146 (111–179)	106 (91·3–139)	98·6 (81·2–130)
1-131	Diabetes mellitus type 2	1,410 (1,240–1,590)	2,200 (1,910–2,540)	2,620 (2,230–3,080)	2,040 (1,790–2,310)	2,350 (2,050–2,680)	2,310 (1,980–2,700)
1-132	Chronic kidney disease	711 (644–778)	1,240 (1,140–1,350)	1,500 (1,320–1,670)	1,000 (907–1,100)	1,260 (1,160–1,370)	1,340 (1,190–1,490)
1-133	Chronic kidney disease due to diabetes mellitus type 1	52 (36–72)	109 (75–151)	121 (80–170)	82·7 (56·9–115)	101 (70·5–138)	104 (69·2–146)
1-134	Chronic kidney disease due to diabetes mellitus type 2	160 (129–193)	280 (223–337)	365 (291–448)	230 (184–278)	298 (240–356)	322 (260–391)
1-135	Chronic kidney disease due to hypertension	139 (114–169)	229 (189–273)	293 (237–360)	193 (158–236)	251 (208–296)	268 (220–324)
1-136	Chronic kidney disease due to glomerulonephritis	162 (133–195)	289 (239–345)	332 (263–412)	231 (187–281)	275 (229–328)	292 (234–358)
1-137	Chronic kidney disease due to other and unspecified causes	198 (162–240)	333 (271–404)	387 (308–478)	268 (216–331)	334 (272–403)	352 (280–432)
1-138	Acute glomerulonephritis	0 (0–0)	0 (0–0)	0 (0–0)	0·3 (0·3–0·4)	0·2 (0·2–0·2)	0·1 (0·1–0·2)
1-139	Skin and subcutaneous diseases	581 (412–814)	703 (506–982)	718 (518–995)	669 (478–931)	703 (511–968)	711 (513–981)
1-140	Dermatitis	108 (63–172)	119 (70–187)	112 (68–174)	116 (68·4–181)	115 (68·3–181)	115 (68·7–180)
1-141	Atopic dermatitis	85 (46–142)	86 (46–145)	78 (42–130)	85 (46–143)	85 (45·4–144)	84·7 (46·1–142)
1-142	Contact dermatitis	20 (13–30)	29 (18–44)	30 (19–46)	27·2 (16·9–40·1)	27·1 (16·8–40·3)	27 (17–40·1)
1-143	Seborrhoeic dermatitis	3 (2–4)	4 (2–6)	4 (2–6)	3·4 (1·9–5·5)	3·4 (1·9–5·4)	3·4 (1·9–5·4)
1-144	Psoriasis	30 (21–40)	36 (25–48)	35 (25–46)	38·8 (27·5–52)	33·2 (23·2–44·2)	32·1 (22·8–42·1)
1-145	Bacterial skin diseases	52 (33–92)	105 (65–177)	124 (72–210)	64·6 (39·5–113)	111 (67·1–185)	118 (70·1–196)
1-146	Cellulitis	13 (7–25)	19 (10–35)	14 (8–36)	16·3 (8·9–31·4)	18·9 (10·5–34·8)	13 (7·8–33·1)
1-147	Pyoderma	39 (25–71)	86 (54–145)	110 (62–181)	48·2 (29·7–88·6)	91·6 (57·7–153)	105 (60·7–175)
1-148	Scabies	77 (42–125)	89 (49–143)	83 (46–135)	84 (46–134)	83·1 (45·5–133)	82·9 (45·5–133)
1-149	Fungal skin diseases	36 (14–77)	44 (18–95)	46 (18–98)	44·2 (17·7–93·1)	44 (17·6–93·2)	44 (17·6–93·2)
1-150	Viral skin diseases	48 (31–72)	48 (31–72)	43 (28–64)	47·5 (30·3–71·2)	47·5 (30·3–71·3)	47·5 (30·4–71·1)
1-151	Acne vulgaris	42 (24–67)	56 (33–90)	51 (30–82)	41 (24·1–66·6)	48·7 (29·1–77·6)	50 (29·7–80·3)
1-152	Alopecia areata	6 (4–8)	8 (5–12)	8 (5–11)	7·1 (4·5–10·6)	7·1 (4·4–10·5)	7 (4·4–10·5)
1-153	Pruritus	6 (3–12)	9 (4–16)	9 (4–17)	8·3 (3·9–14·9)	8·5 (4·1–15·2)	8·5 (4·1–15·5)
1-154	Urticaria	44 (29–65)	49 (32–72)	46 (30–66)	48·1 (31·2–69·4)	48 (31·5–69·7)	47·9 (31·2–70·2)
1-155	Decubitus ulcer	88 (58–152)	84 (61–145)	106 (72–172)	113 (75–200)	102 (73·9–173)	107 (74–165)
1-156	Other skin and subcutaneous diseases	43 (24–72)	56 (30–97)	55 (28–99)	56·3 (31·8–95)	54·9 (29·9–94·2)	50·7 (26–90·1)
1-157	Sense organ diseases	617 (424–872)	735 (494–1,070)	814 (548–1,190)	829 (566–1,180)	764 (518–1,090)	746 (505–1,070)
1-158	Blindness and vision loss	190 (131–264)	196 (132–281)	216 (146–310)	250 (173–351)	210 (143–298)	202 (137–289)
1-159	Glaucoma	14 (9–21)	9 (6–13)	11 (7–16)	17·4 (11·3–25·2)	12·1 (8–17·6)	11·4 (7·6–16·4)
1-160	Cataract	39 (27–54)	30 (21–42)	34 (24–47)	49·7 (34·7–68·2)	37·6 (26–51·5)	34·8 (24·4–47·4)
1-161	Age-related macular degeneration	1 (1–2)	1 (1–2)	1 (1–2)	1·7 (1·1–2·5)	1·5 (1–2·2)	1·4 (0·9–2·1)
1-162	Refraction disorders	47 (31–68)	58 (38–84)	58 (38–85)	59·9 (39·7–85·4)	55·4 (36·7–80·3)	53·9 (35·2–78·7)
1-163	Near vision loss	39 (18–76)	48 (22–98)	56 (26–112)	55·8 (26–110)	50·2 (23·1–101)	49·6 (22·7–97·4)
1-164	Other vision loss	49 (34–67)	49 (34–68)	54 (38–75)	65·7 (46·4–90·2)	53·1 (37·2–73·2)	50·6 (35·4–69·8)
1-165	Age-related and other hearing loss	400 (269–570)	503 (333–749)	559 (368–822)	543 (363–785)	517 (346–753)	508 (339–740)
1-166	Other sense organ diseases	27 (16–41)	36 (21–56)	40 (23–61)	35·7 (21–55)	36·5 (21·4–56·5)	36·4 (21·5–55·8)

1-167	Musculoskeletal disorders	1,030 (751–1,360)	1,640 (1,190–2,170)	1,760 (1,270–2,330)	1,470 (1,080–1,950)	1,550 (1,120–2,030)	1,540 (1,120–2,030)
1-168	Rheumatoid arthritis	14 (10–19)	25 (17–33)	28 (19–37)	20·1 (14·2–27·4)	23·6 (16·7–31·5)	24·7 (17·2–32·7)
1-169	Osteoarthritis	126 (64–254)	195 (97–388)	230 (116–463)	186 (93·6–373)	198 (99·3–393)	198 (101–396)
1-170	Low back pain	534 (376–715)	784 (547–1,050)	812 (571–1,090)	742 (521–1,000)	732 (512–982)	718 (505–961)
1-171	Neck pain	91 (60–130)	145 (95–212)	154 (101–225)	136 (89·4–196)	135 (89·4–194)	134 (89·8–193)
1-172	Gout	4 (3–6)	8 (5–11)	9 (5–13)	6·3 (3·9–9·2)	7·4 (4·6–10·6)	7·6 (4·8–11)
1-173	Other musculoskeletal disorders	262 (191–347)	485 (348–655)	527 (378–708)	384 (278–511)	449 (324–599)	460 (333–614)
1-174	Other non-communicable diseases	1,960 (1,630–2,350)	2,040 (1,670–2,520)	1,990 (1,600–2,490)	2,360 (1,940–2,850)	2,070 (1,720–2,520)	2,000 (1,620–2,480)
1-175	Congenital anomalies	697 (558–887)	506 (411–632)	399 (312–515)	721 (577–918)	577 (463–730)	520 (394–687)
1-176	Neural tube defects	42 (29–59)	26 (20–34)	29 (19–43)	43·2 (30·2–60·7)	30·7 (22·8–40·2)	40·7 (25·8–61·3)
1-177	Congenital heart anomalies	430 (330–565)	281 (217–362)	206 (150–279)	442 (340–579)	327 (249–427)	278 (197–383)
1-178	Orofacial clefts	3 (2–5)	3 (2–5)	3 (2–5)	3·6 (2·2–5·8)	3·1 (2–4·6)	3·2 (1·9–4·9)
1-179	Down's syndrome	15 (10–24)	21 (17–26)	19 (14–23)	16·4 (11·9–26·6)	21·6 (17·1–27·2)	20·4 (15·2–26·2)
1-180	Turner syndrome	0 (0–0)	0 (0–0)	0 (0–0)	0·1 (0·1–0·3)	0·1 (0·1–0·2)	0·1 (0·1–0·2)
1-181	Klinefelter syndrome	0 (0–0)	0 (0–0)	0 (0–0)	0 (0–0)	0 (0–0)	0 (0–0)
1-182	Other chromosomal abnormalities	27 (18–41)	31 (24–45)	24 (18–38)	27·6 (18·2–42·2)	36·5 (27·3–53·6)	33·1 (23·2–53·1)
1-183	Congenital musculoskeletal and limb anomalies	46 (32–65)	50 (34–70)	46 (31–65)	49·2 (33·9–68·7)	50·2 (34·3–69)	49·6 (34·1–70·1)
1-184	Urogenital congenital anomalies	6 (4–10)	5 (4–7)	5 (3–6)	6·4 (4·5–10·2)	5·8 (4·2–8·2)	6 (4·3–8·4)
1-185	Digestive congenital anomalies	43 (29–68)	27 (19–40)	20 (13–32)	44·4 (29·7–69·6)	33·2 (23–49·6)	28·6 (18·4–45·9)
1-186	Other congenital anomalies	84 (62–113)	61 (47–80)	48 (35–67)	87·9 (64·5–118)	68·9 (52·1–90·8)	60·7 (43·7–85·5)
1-187	Urinary diseases and male infertility	70 (57–92)	148 (120–172)	190 (149–220)	93·1 (75·7–122)	161 (130–184)	177 (139–204)
1-188	Urinary tract infections and interstitial nephritis	34 (29–48)	90 (73–100)	118 (91–136)	44·4 (37·5–62·2)	101 (81·1–112)	112 (87–129)
1-189	Urolithiasis	5 (4–6)	12 (9–15)	15 (11–19)	7·6 (5·9–9·7)	11·8 (9–14·4)	12·7 (9·7–16)
1-190	Benign prostatic hyperplasia	17 (10–27)	23 (13–36)	30 (17–47)	23·3 (13·7–36·6)	24·4 (13·8–38·4)	26·7 (15·4–41·4)
1-191	Male infertility	3 (1–8)	5 (2–12)	5 (2–11)	4·2 (1·6–10)	4·4 (1·7–10·6)	4·4 (1·6–10·5)
1-192	Other urinary diseases	10 (8–17)	19 (13–24)	22 (14–29)	13·6 (10·3–23)	19·4 (13·2–25·2)	20·6 (12·6–26·3)
1-193	Gynecological diseases	283 (197–392)	409 (282–575)	400 (279–561)	396 (279–552)	368 (254–518)	359 (251–504)
1-194	Uterine fibroids	15 (8–28)	26 (13–47)	26 (13–48)	24·4 (12·5–44·4)	23·3 (11·6–42·7)	22·8 (11·2–42·8)
1-195	Polycystic ovarian syndrome	3 (1–7)	6 (3–13)	6 (3–13)	3·9 (1·7–8·1)	5·5 (2·3–11·2)	5·7 (2·4–11·6)
1-196	Female infertility	6 (2–14)	9 (3–20)	8 (3–20)	7·9 (2·7–18·9)	7·8 (2·8–18·4)	7·8 (2·7–18·7)
1-197	Endometriosis	26 (15–44)	32 (19–51)	29 (17–47)	35 (20·2–56·5)	27·6 (16·2–44)	25·9 (15·4–42·1)
1-198	Genital prolapse	7 (4–12)	7 (4–14)	8 (4–14)	10·7 (6·2–18·7)	7·4 (4–13·3)	6·7 (3·7–12)
1-199	Premenstrual syndrome	78 (47–122)	112 (68–175)	105 (63–165)	99·4 (60·5–154)	97·9 (59·2–152)	97·1 (58·6–152)
1-200	Other gynecological diseases	147 (99–202)	217 (143–307)	218 (145–303)	215 (145–299)	198 (130–278)	194 (129–272)
1-201	Hemoglobinopathies and hemolytic anaemias	259 (214–303)	232 (203–271)	190 (158–229)	296 (246–345)	220 (192–256)	188 (156–226)
1-202	Thalassemias	0 (0–0)	0 (0–0)	0 (0–0)	0·2 (0·1–0·4)	0·2 (0·1–0·4)	0·1 (0·1–0·2)
1-203	Thalassaemias trait	4 (2–6)	4 (2–7)	4 (2–6)	4 (2·4–6·3)	4 (2·3–6·4)	3·9 (2·3–6·2)
1-204	Sickle cell disorders	139 (111–168)	125 (111–138)	91 (74–111)	151 (120–181)	116 (104–129)	92·1 (75·6–112)
1-205	Sickle cell trait	51 (33–76)	42 (25–67)	32 (19–52)	57·1 (36·6–84·7)	38·9 (23·2–62·1)	32·5 (19·2–51·4)
1-206	G6PD deficiency	18 (13–25)	24 (17–30)	25 (18–32)	24·9 (17·3–33·1)	21·6 (15·8–27·7)	22·3 (16·1–28·8)
1-207	G6PD trait	0 (0–0)	0 (0–0)	0 (0–0)	0·1 (0·1–0·2)	0·1 (0·1–0·2)	0·2 (0·1–0·3)
1-208	Other hemoglobinopathies and hemolytic anaemias	47 (38–57)	37 (30–47)	38 (30–47)	59 (48·3–71·5)	39·5 (32·4–49·2)	36·7 (29·5–46·4)
1-209	Endocrine, metabolic, blood, and immune disorders	396 (274–477)	404 (333–527)	440 (360–581)	499 (350–601)	410 (339–536)	427 (350–562)
1-210	Oral disorders	244 (144–391)	333 (192–540)	360 (208–588)	338 (198–544)	326 (191–529)	322 (187–527)
1-211	Caries of deciduous teeth	4 (2–7)	3 (1–6)	2 (1–5)	3·2 (1·4–6·6)	3·1 (1·3–6·6)	3·1 (1·3–6·5)
1-212	Caries of permanent teeth	22 (10–43)	29 (13–55)	28 (12–54)	27·2 (12·2–53·5)	25·8 (11·7–49·2)	25·5 (11·4–48·8)
1-213	Periodontal disease	70 (27–148)	121 (46–257)	129 (49–274)	107 (41·4–229)	111 (42·4–236)	112 (42·5–239)
1-214	Edentulism	106 (67–159)	123 (78–186)	143 (92–215)	148 (93·3–221)	133 (84·4–200)	129 (82·5–193)
1-215	Other oral disorders	42 (26–62)	58 (36–85)	57 (36–84)	53·3 (32·9–77·6)	53·1 (32·9–78)	52·9 (32·9–78·4)
1-216	Sudden infant death syndrome	16 (9–26)	10 (6–15)	8 (5–13)	16·3 (9·5–26·5)	12·2 (7·9–18·3)	11·6 (6·9–18·7)
1-0	Injuries	3,090 (2,790–3,410)	3,230 (2,980–3,530)	2,920 (2,570–3,290)	3,830 (3,480–4,220)	3,000 (2,770–3,270)	2,710 (2,390–3,050)
1-1	Transport injuries	765 (690–851)	754 (680–828)	668 (582–756)	976 (882–1,080)	682 (614–747)	604 (526–682)
1-2	Road injuries	722 (651–804)	669 (600–736)	594 (517–675)	922 (832–1,020)	604 (543–664)	537 (467–608)

1·3	Pedestrian road injuries	136 (117–154)	93 (84–104)	84 (73–95)	174 (150–195)	89·1 (80–98·7)	76·7 (66·8–86·6)
1·4	Cyclist road injuries	75 (62–89)	82 (69–96)	77 (65–94)	97·9 (81–117)	74 (62·3–86·6)	69·4 (58·1–83·5)
1·5	Motorcyclist road injuries	102 (90–115)	128 (111–145)	116 (99–135)	131 (116–149)	112 (98·3–128)	104 (88·7–121)
1·6	Motor vehicle road injuries	399 (356–445)	355 (316–395)	307 (263–353)	506 (453–563)	318 (284–353)	277 (239–318)
1·7	Other road injuries	11 (9–13)	11 (9–13)	11 (8–13)	13·7 (11–16·9)	10·1 (8·1–12·4)	9·5 (7·4–12)
1·8	Other transport injuries	42 (37–48)	85 (75–95)	73 (62–84)	53·6 (47·1–60·5)	77·6 (68·5–86·5)	67 (56·3–76·7)
1·9	Unintentional injuries	1,850 (1,630–2,070)	1,770 (1,590–2,000)	1,640 (1,410–1,890)	2,240 (1,980–2,510)	1,690 (1,520–1,900)	1,550 (1,340–1,790)
1·10	Falls	178 (155–206)	257 (219–306)	310 (259–373)	234 (203–272)	263 (226–309)	290 (245–348)
1·11	Drowning	503 (445–570)	417 (382–454)	299 (258–339)	569 (506–638)	387 (354–422)	293 (253–333)
1·12	Fire, heat, and hot substances	251 (214–296)	187 (156–230)	177 (144–221)	306 (259–361)	178 (150–217)	167 (136–208)
1·13	Poisonings	116 (99–136)	24 (22–28)	18 (15–21)	133 (115–155)	23·7 (21–27·2)	17·3 (14·4–20·6)
1·14	Poisoning by carbon monoxide	12 (9–15)	7 (6–8)	6 (4–7)	14·5 (11·5–18·9)	6·7 (5·9–7·7)	5·3 (4·3–6·4)
1·15	Poisoning by other means	104 (88–123)	17 (15–20)	12 (10–15)	118 (102–138)	17 (14·6–19·8)	12 (9·8–14·8)
1·16	Exposure to mechanical forces	280 (242–329)	426 (365–507)	383 (314–475)	363 (313–431)	388 (331–461)	348 (286–429)
1·17	Unintentional firearm injuries	100 (83–117)	121 (104–138)	92 (75–108)	130 (108–153)	106 (90·8–121)	82·5 (67·2–97·2)
1·18	Other exposure to mechanical forces	179 (147–227)	306 (247–384)	291 (229–377)	233 (189–296)	282 (229–353)	265 (210–342)
1·19	Adverse effects of medical treatment	145 (121–166)	115 (104–128)	102 (88–120)	185 (154–209)	119 (107–133)	97·7 (84·5–114)
1·20	Animal contact	12 (9–16)	28 (24–33)	30 (26–35)	15·4 (11·8–20·1)	27·3 (23·6–31·6)	28·1 (23·8–32·8)
1·21	Venomous animal contact	6 (4–9)	9 (7–12)	10 (7–12)	8·1 (5·5–11·1)	9 (6·8–11·4)	9 (6·9–11·5)
1·22	Non-venomous animal contact	6 (5–7)	19 (17–21)	21 (18–24)	7·3 (5·9–9·5)	18·4 (16·5–20·8)	19 (16·4–21·8)
1·23	Foreign body	179 (149–216)	105 (92–120)	117 (102–136)	199 (168–237)	111 (96·3–128)	126 (106–150)
1·24	Pulmonary aspiration and foreign body in airway	168 (138–203)	92 (80–105)	104 (89–122)	185 (155–222)	98·1 (85·1–115)	113 (94·1–138)
1·25	Foreign body in eyes	4 (2–8)	6 (3–10)	6 (3–10)	5·5 (2·6–9·6)	5·5 (2·6–9·6)	5·5 (2·6–9·7)
1·26	Foreign body in other body part	7 (5–9)	8 (6–10)	8 (6–10)	8·6 (6·6–11·1)	7·1 (5·2–9·4)	7·1 (5·3–9·5)
1·27	Environmental heat and cold exposure	35 (25–47)	45 (32–61)	47 (34–65)	45·8 (32·9–61·4)	42·2 (30·1–57·3)	43·1 (30·8–59·1)
1·28	Exposure to forces of nature	0 (0–0)	5 (3–7)	4 (3–5)	0 (0–0)	4·3 (3–6·1)	3·2 (2·2–4·6)
1·29	Other unintentional injuries	150 (128–179)	159 (136–190)	149 (122–184)	188 (160–223)	146 (124–175)	138 (113–169)
1·30	Self-harm and interpersonal violence	479 (425–535)	707 (655–761)	618 (532–701)	618 (548–688)	626 (582–674)	558 (481–632)
1·31	Self-harm	204 (182–228)	236 (214–259)	218 (186–254)	279 (249–311)	212 (193–232)	193 (165–225)
1·32	Self-harm by firearm	4 (2–4)	3 (2–3)	2 (2–3)	4·8 (3·4–5·8)	2·3 (1·8–2·8)	2 (1·5–2·5)
1·33	Self-harm by other specified means	201 (179–225)	233 (212–257)	215 (184–251)	274 (245–307)	210 (191–230)	190 (163–222)
1·34	Interpersonal violence	217 (191–243)	443 (405–486)	382 (323–440)	268 (237–298)	389 (356–426)	348 (295–400)
1·35	Physical violence by firearm	20 (16–24)	55 (49–62)	45 (37–54)	24 (19·4–27·9)	46·4 (41·4–52·4)	40·9 (33·6–48·5)
1·36	Physical violence by sharp object	24 (20–28)	50 (45–56)	42 (35–50)	29·8 (25·1–34·6)	43·2 (38·9–48·6)	38·2 (31·9–44·7)
1·37	Sexual violence	14 (9–20)	20 (13–28)	19 (13–27)	17·7 (11·7–25·2)	17·6 (11·6–25)	17·5 (11·6–24·9)
1·38	Physical violence by other means	158 (138–178)	319 (291–349)	275 (232–318)	196 (173–220)	282 (257–308)	251 (213–289)
1·39	Conflict and terrorism	57 (29–106)	27 (14–48)	18 (10–32)	70·6 (35·2–133)	25 (13·2–44·9)	16·5 (8·8–29·1)
1·40	Executions and police conflict	1 (1–1)	1 (1–1)	1 (1–1)	0·7 (0·6–0·8)	0·7 (0·7–0·8)	0·7 (0·6–0·8)

SR Table 1: All-age DALYs counts and age standardized DALYs rates per 100,000 individuals for all causes and level 1 cause groups by location for 1990, 2010, and 2019, both sexes combined, Guyana

Cause Level	Cause	All-age DALYs 1990	All-age DALYs 2010	All-age DALYs 2019	Age-standardized rate of DALYs per 100,000 1990	Age-standardized rate of DALYs per 100,000 2010	Age-standardized rate of DALYs per 100,000 2019
0-0	All causes	354,000 (321,000–391,000)	302,000 (270,000–336,000)	309,000 (254,000–371,000)	56,300 (50,900–62,300)	47,200 (42,400–52,300)	43,600 (36,000–52,200)
1-0	Communicable, maternal, neonatal, and nutritional disorders	136,000 (123,000–153,000)	72,500 (63,300–82,000)	59,400 (48,700–72,200)	15,700 (14,100–17,700)	10,500 (9,170–11,800)	8,260 (6,780–10,100)
1-1	HIV/AIDS and sexually transmitted infections	5,200 (5,040–5,410)	11,300 (10,900–11,800)	10,100 (9,690–10,500)	716 (693–747)	1,540 (1,480–1,600)	1,300 (1,250–1,360)
1-2	HIV/AIDS	4,820 (4,690–4,980)	11,100 (10,700–11,500)	9,840 (9,480–10,300)	668 (651–693)	1,500 (1,450–1,560)	1,270 (1,220–1,320)
1-3	HIV/AIDS - drug-susceptible tuberculosis	1,620 (1,060–2,140)	1,840 (1,190–2,770)	1,200 (750–1,810)	224 (147–296)	250 (161–375)	154 (96·6–233)
1-4	HIV/AIDS - Multidrug-resistant Tuberculosis without extensive drug resistance	11 (1–47)	20 (2–99)	22 (1–124)	1·5 (0·1–6·5)	2·7 (0·2–13·4)	2·8 (0·2–16)
1-5	HIV/AIDS - Extensively drug-resistant Tuberculosis	0 (0–0)	2 (0–11)	4 (0–21)	0 (0–0)	0·3 (0–1·5)	0·5 (0–2·6)
1-6	HIV/AIDS resulting in other diseases	3,190 (2,640–3,760)	9,200 (8,160–10,000)	8,620 (7,880–9,250)	443 (365–522)	1,250 (1,110–1,360)	1,110 (1,020–1,190)
1-7	Sexually transmitted infections excluding HIV	382 (292–509)	251 (188–360)	234 (165–345)	47·8 (36·9–64·3)	34·7 (25·9–49·8)	30·1 (21·3–44·5)
1-8	Syphilis	217 (156–306)	57 (44–72)	38 (26–54)	23·3 (17·2–31·8)	8·1 (6·4–10·3)	5·2 (3·5–7·5)
1-9	Chlamydial infection	21 (15–30)	26 (20–34)	26 (19–36)	2·9 (2·1–4)	3·4 (2·6–4·5)	3·2 (2·4–4·4)
1-10	Gonococcal infection	28 (22–34)	38 (32–46)	37 (27–48)	4 (3·3–4·9)	5·2 (4·3–6·2)	4·6 (3·5–6)
1-11	Trichomoniasis	33 (13–69)	36 (14–74)	38 (15–77)	5 (1·9–10·4)	4·9 (1·9–10·3)	4·8 (1·9–10)
1-12	Genital herpes	32 (11–79)	37 (12–95)	41 (13–101)	5·3 (1·7–13)	5·4 (1·8–13·6)	5·4 (1·7–13·2)
1-13	Other sexually transmitted infections	51 (39–66)	57 (46–72)	55 (42–71)	7·3 (5·7–9·4)	7·6 (6–9·4)	6·9 (5·3–8·9)
1-14	Respiratory infections and tuberculosis	18,000 (15,600–20,500)	13,200 (11,600–15,000)	11,500 (8,980–14,600)	2,690 (2,370–3,000)	2,090 (1,860–2,360)	1,700 (1,330–2,140)
1-15	Tuberculosis	4,550 (3,900–5,160)	3,510 (3,060–4,010)	2,570 (1,980–3,320)	732 (636–831)	510 (445–580)	341 (262–438)
1-16	Latent tuberculosis infection	0 (0–0)	0 (0–0)	0 (0–0)	0 (0–0)	0 (0–0)	0 (0–0)
1-17	Drug-susceptible tuberculosis	4,530 (3,880–5,150)	3,470 (3,000–3,980)	2,530 (1,920–3,280)	728 (632–827)	505 (438–576)	336 (256–434)
1-18	Multidrug-resistant tuberculosis without extensive drug resistance	25 (2–111)	30 (3–141)	37 (3–204)	4·1 (0·3–17·9)	4·4 (0·4–20·7)	4·9 (0·4–27)
1-19	Extensively drug-resistant tuberculosis	0 (0–0)	3 (0–15)	6 (0–31)	0 (0–0)	0·5 (0–2·3)	0·8 (0·1–4·1)
1-20	Lower respiratory infections	12,500 (10,700–14,400)	8,800 (7,610–10,200)	8,080 (6,000–10,500)	1,840 (1,600–2,070)	1,470 (1,290–1,680)	1,250 (950–1,600)
1-21	Upper respiratory infections	688 (427–1,070)	633 (388–973)	635 (389–991)	83·3 (51·4–128)	82·8 (50·7–127)	81·9 (50·2–125)
1-22	Otitis media	302 (176–492)	256 (149–421)	242 (141–395)	36·4 (21·4–59·3)	33·1 (19·1–53·8)	31·1 (18·2–50·6)
1-23	Enteric infections	17,700 (14,700–21,000)	5,080 (4,170–6,130)	4,010 (3,090–5,130)	1,960 (1,650–2,300)	762 (631–915)	576 (447–737)
1-24	Diarrhoeal diseases	17,100 (14,100–20,200)	5,030 (4,130–6,070)	3,970 (3,050–5,100)	1,880 (1,580–2,190)	755 (626–905)	572 (442–733)
1-25	Typhoid and paratyphoid	611 (151–845)	34 (21–96)	23 (13–61)	79·2 (19·8–108)	4·8 (3–13·3)	3·1 (1·7–8)
1-26	Typhoid fever	610 (150–845)	32 (19–96)	22 (12–61)	79·2 (19·8–108)	4·5 (2·6–13·2)	2·9 (1·5–7·9)
1-27	Paratyphoid fever	0 (0–1)	2 (0–3)	1 (0–2)	0 (0–0·1)	0·3 (0–0·5)	0·1 (0–0·3)
1-28	iNTS	9 (5–14)	7 (4–10)	5 (3–9)	1 (0·6–1·6)	0·9 (0·6–1·4)	0·7 (0·4–1·2)
1-29	Other intestinal infectious diseases	16 (6–28)	9 (4–14)	7 (3–11)	1·7 (0·7–3)	1·2 (0·6–1·9)	0·9 (0·4–1·4)
1-30	Neglected tropical diseases and malaria	16,200 (9,710–29,300)	7,730 (4,990–12,300)	4,980 (3,330–7,780)	2,160 (1,340–3,800)	1,040 (678–1,640)	644 (436–1,000)
1-31	Malaria	10,300 (4,230–23,500)	4,020 (1,770–8,650)	1,110 (261–3,840)	1,260 (510–2,910)	527 (232–1,140)	140 (33·4–482)
1-32	Chagas disease	12 (4–17)	5 (4–11)	6 (4–12)	4·1 (1·4–6)	1·3 (0·9–2·8)	1·2 (0·8–2·6)
1-33	Leishmaniasis	2 (0–9)	1 (0–5)	4 (0–20)	0·2 (0–1·2)	0·2 (0–0·7)	0·6 (0–2·7)
1-34	Visceral leishmaniasis	0 (0–0)	0 (0–0)	0 (0–0)	0 (0–0)	0 (0–0)	0 (0–0)
1-35	Cutaneous and mucocutaneous leishmaniasis	2 (0–9)	1 (0–5)	4 (0–20)	0·2 (0–1·2)	0·2 (0–0·7)	0·6 (0–2·7)
1-36	African trypanosomiasis	0 (0–0)	0 (0–0)	0 (0–0)	0 (0–0)	0 (0–0)	0 (0–0)
1-37	Schistosomiasis	0 (0–0)	0 (0–0)	0 (0–0)	0 (0–0)	0 (0–0)	0 (0–0)
1-38	Cysticercosis	338 (181–573)	336 (184–546)	376 (198–643)	67·8 (37·5–110)	55·9 (31·7–89·2)	54·4 (28·4–91·5)
1-39	Cystic echinococcosis	5 (3–7)	3 (2–4)	2 (1–3)	0·8 (0·5–1·1)	0·4 (0·3–0·6)	0·3 (0·2–0·4)
1-40	Lymphatic filariasis	4,680 (3,180–6,660)	2,600 (1,740–3,630)	2,820 (1,890–3,940)	718 (483–1,030)	356 (238–494)	361 (243–503)
1-41	Onchocerciasis	0 (0–0)	0 (0–0)	0 (0–0)	0 (0–0)	0 (0–0)	0 (0–0)
1-42	Trachoma	0 (0–0)	0 (0–0)	0 (0–0)	0 (0–0)	0 (0–0)	0 (0–0)
1-43	Dengue	85 (42–160)	296 (197–421)	223 (137–323)	10·9 (5·6–20·3)	39·3 (26–56)	29·2 (18·2–42·9)
1-44	Yellow fever	18 (4–49)	6 (2–17)	4 (1–11)	2·1 (0·5–5·5)	0·7 (0·2–2·1)	0·5 (0·1–1·4)
1-45	Rabies	0 (0–0)	0 (0–0)	0 (0–0)	0 (0–0)	0 (0–0)	0 (0–0)
1-46	Intestinal nematode infections	184 (115–291)	90 (53–141)	58 (35–94)	20·9 (13·2–32·9)	11·3 (6·7–17·6)	7·3 (4·4–11·8)
1-47	Ascariasis	36 (15–68)	25 (13–42)	16 (9–27)	4 (1·8–7·1)	3·2 (1·8–5·4)	2·1 (1·1–3·6)
1-48	Trichuriasis	19 (9–36)	2 (1–4)	2 (1–4)	2·1 (1–4)	0·3 (0·1–0·5)	0·2 (0·1–0·5)

1·49	Hookworm disease	129 (74–210)	64 (35–105)	40 (22–68)	14·8 (8·7–24·2)	7·9 (4·3–13)	4·9 (2·7–8·2)
1·50	Food-borne trematodiases	0 (0–0)	0 (0–0)	0 (0–0)	0 (0–0)	0 (0–0)	0 (0–0)
1·51	Leprosy	6 (3–9)	6 (4–10)	7 (4–10)	1·2 (0·7–1·7)	1 (0·7–1·5)	0·9 (0·6–1·4)
1·52	Ebola virus disease	0 (0–0)	0 (0–0)	0 (0–0)	0 (0–0)	0 (0–0)	0 (0–0)
1·53	Zika virus disease	0 (0–0)	0 (0–0)	3 (0–14)	0 (0–0)	0 (0–0)	0·4 (0–1·8)
1·54	Guinea worm disease	0 (0–0)	0 (0–0)	0 (0–0)	0 (0–0)	0 (0–0)	0 (0–0)
1·55	Other neglected tropical diseases	597 (404–811)	363 (251–513)	364 (255–499)	71·2 (48·5–96·5)	48 (33·5–67·6)	49·1 (34·2–67·2)
1·56	Other infectious diseases	4,400 (3,770–5,030)	1,900 (1,610–2,210)	1,670 (1,280–2,110)	530 (456–600)	260 (222–303)	222 (170–280)
1·57	Meningitis	1,750 (1,480–2,050)	569 (471–694)	357 (265–472)	201 (171–231)	78·8 (65·1–96·4)	48·1 (35·8–63·8)
1·58	Encephalitis	1,220 (962–1,460)	589 (489–745)	584 (431–782)	135 (108–160)	78·4 (65·1–100)	77 (56·8–104)
1·59	Diphtheria	2 (1–4)	1 (0–1)	1 (0–2)	0·2 (0·1–0·4)	0·1 (0–0·2)	0·1 (0–0·3)
1·60	Whooping cough	40 (25–59)	16 (10–24)	16 (10–27)	3·8 (2·4–5·7)	2·2 (1·4–3·4)	2·3 (1·3–3·8)
1·61	Tetanus	119 (82–182)	31 (20–54)	20 (10–41)	19·5 (12·6–32·1)	4·6 (3·1–8)	2·8 (1·3–5·6)
1·62	Measles	113 (12–223)	0 (0–0)	0 (0–0)	11·2 (1·2–21·9)	0 (0–0)	0 (0–0)
1·63	Varicella and herpes zoster	27 (18–52)	30 (21–41)	30 (21–42)	3·8 (2·6–6·7)	4·3 (3·1–6·1)	4·1 (2·9–5·8)
1·64	Acute hepatitis	525 (439–610)	123 (103–147)	108 (79–143)	71·7 (60·3–83·1)	16·2 (13·7–19·2)	13·7 (10·1–18·1)
1·65	Acute hepatitis A	330 (247–404)	60 (47–78)	48 (34–64)	42·2 (31·3–51·2)	7·6 (5·9–9·7)	5·9 (4·2–8)
1·66	Acute hepatitis B	92 (54–184)	44 (31–54)	42 (28–62)	13·1 (7·8–25·6)	6·1 (4·3–7·4)	5·4 (3·6–7·9)
1·67	Acute hepatitis C	73 (30–107)	9 (6–19)	9 (5–17)	12·3 (4·9–17·9)	1·3 (0·9–2·7)	1·2 (0·7–2·2)
1·68	Acute hepatitis E	29 (16–44)	9 (6–13)	9 (6–13)	4 (2·3–6·1)	1·2 (0·9–1·7)	1·2 (0·8–1·7)
1·69	Other unspecified infectious diseases	603 (428–788)	539 (386–685)	552 (385–723)	83·5 (59·6–107)	75·3 (54–95·4)	73·7 (51·7–96·1)
1·70	Maternal and neonatal disorders	59,000 (50,600–68,400)	26,000 (20,700–32,200)	21,000 (15,400–27,800)	5,640 (4,850–6,540)	3,740 (2,970–4,670)	2,960 (2,160–3,950)
1·71	Maternal disorders	1,950 (1,560–2,380)	1,760 (1,420–2,100)	1,570 (1,130–2,110)	226 (180–276)	226 (182–273)	183 (131–246)
1·72	Maternal haemorrhage	601 (468–759)	385 (301–483)	311 (218–427)	70·9 (54·7–89)	50·1 (39–63)	36·8 (25·7–50·7)
1·73	Maternal sepsis and other pregnancy related infections	161 (120–209)	80 (60–105)	58 (40–80)	18·6 (13·9–23·9)	10·3 (7·7–13·5)	6·7 (4·6–9·2)
1·74	Maternal hypertensive disorders	375 (288–468)	258 (206–316)	224 (158–307)	42·4 (32·7–52·7)	32·8 (26·1–40·3)	25·8 (18·3–35·3)
1·75	Maternal obstructed labour and uterine rupture	17 (12–23)	96 (70–128)	107 (69–162)	1·9 (1·4–2·6)	12·5 (9–16·9)	12·6 (8·1–19·1)
1·76	Maternal abortive outcome	483 (355–637)	173 (125–226)	121 (81–174)	56·4 (41·5–74·6)	22·2 (16–29)	14·1 (9·4–20·3)
1·77	Ectopic pregnancy	36 (26–47)	133 (101–170)	128 (86–182)	4·2 (3·1–5·4)	17·4 (13·2–22·3)	14·9 (10–21·4)
1·78	Indirect maternal deaths	111 (82–146)	154 (119–200)	159 (107–228)	12·5 (9·2–16·4)	19·8 (15·1–25·8)	18·2 (12·3–26·4)
1·79	Late maternal deaths	56 (38–81)	68 (48–93)	73 (45–109)	6·5 (4·4–9·3)	8·7 (6·2–12)	8·5 (5·3–12·6)
1·80	Maternal deaths aggravated by HIV/AIDS	3 (1–5)	16 (8–24)	14 (7–24)	0·3 (0·1–0·6)	2·1 (1·1–3·2)	1·7 (0·9–2·9)
1·81	Other maternal disorders	110 (82–145)	392 (297–498)	372 (255–526)	12·9 (9·6–16·9)	50·5 (38·3–64·4)	43·7 (29·9–61·8)
1·82	Neonatal disorders	57,000 (48,700–66,300)	24,200 (19,000–30,400)	19,400 (14,200–25,900)	5,410 (4,630–6,290)	3,520 (2,740–4,430)	2,770 (2,020–3,720)
1·83	Neonatal preterm birth	30,600 (25,400–36,500)	11,000 (8,610–14,000)	8,890 (6,380–12,200)	2,910 (2,430–3,470)	1,580 (1,240–2,020)	1,270 (907–1,740)
1·84	Neonatal encephalopathy due to birth asphyxia and trauma	13,300 (10,600–16,700)	5,740 (4,290–7,520)	4,220 (2,820–5,950)	1,260 (1,000–1,570)	841 (629–1,100)	609 (402–860)
1·85	Neonatal sepsis and other neonatal infections	5,230 (3,970–6,970)	4,030 (3,010–5,210)	3,610 (2,540–5,020)	497 (378–662)	584 (436–757)	512 (358–717)
1·86	Hemolytic disease and other neonatal jaundice	1,700 (1,230–2,380)	424 (325–549)	308 (223–416)	164 (120–228)	59·4 (45·5–77·8)	42·1 (30·3–57·6)
1·87	Other neonatal disorders	6,130 (4,400–8,280)	3,070 (2,210–4,120)	2,350 (1,520–3,530)	578 (415–780)	450 (324–606)	342 (220–513)
1·88	Nutritional deficiencies	15,900 (13,000–19,300)	7,310 (5,590–9,380)	6,230 (4,650–8,320)	1,980 (1,630–2,380)	1,040 (804–1,330)	856 (643–1,130)
1·89	Protein-energy malnutrition	8,890 (7,180–10,700)	2,670 (2,160–3,240)	2,000 (1,510–2,720)	1,030 (841–1,230)	406 (332–489)	292 (222–395)
1·90	Iodine deficiency	139 (74–224)	100 (52–166)	76 (40–125)	18·7 (10·1–30)	13·3 (6·9–22)	9·6 (5·1–15·9)
1·91	Vitamin A deficiency	202 (121–313)	93 (57–140)	69 (41–103)	20·7 (12·5–32·1)	11·6 (7·1–17·6)	9·3 (5·5–13·9)
1·92	Dietary iron deficiency	5,620 (3,670–8,460)	4,140 (2,660–5,940)	3,840 (2,420–5,590)	739 (485–1,100)	561 (361–801)	510 (320–738)
1·93	Other nutritional deficiencies	1,080 (742–1,680)	304 (225–437)	241 (160–365)	165 (116–260)	47·3 (34–66·1)	34·4 (22·7–51·5)
1·0	Non-communicable diseases	170,000 (151,000–192,000)	181,000 (159,000–204,000)	200,000 (165,000–240,000)	34,300 (30,700–38,300)	30,100 (26,800–33,600)	29,200 (24,100–34,900)
1·1	Neoplasms	15,800 (13,700–17,900)	19,800 (17,600–22,300)	22,800 (17,600–29,100)	3,430 (2,990–3,890)	3,350 (2,990–3,740)	3,350 (2,600–4,220)
1·2	Lip and oral cavity cancer	209 (179–242)	252 (216–291)	301 (224–390)	49·3 (42·2–57)	43 (37·2–49·5)	43·4 (32·7–55·5)
1·3	Nasopharynx cancer	42 (35–48)	66 (56–76)	75 (55–98)	8·4 (7·1–9·9)	10·2 (8·8–11·9)	10·2 (7·6–13·3)
1·4	Other pharynx cancer	102 (85–121)	101 (85–119)	128 (95–172)	24·3 (20·3–28·9)	17·1 (14·4–20)	18·1 (13·4–24·1)
1·5	Oesophageal cancer	236 (197–277)	298 (251–347)	380 (284–498)	58·2 (48·5–68·2)	51·9 (44·5–60·2)	55·1 (41·9–71·6)
1·6	Stomach cancer	1,470 (1,270–1,670)	1,340 (1,170–1,520)	1,440 (1,110–1,830)	353 (307–400)	235 (208–265)	213 (166–269)
1·7	Colon and rectum cancer	1,220 (1,050–1,390)	1,790 (1,570–2,020)	2,300 (1,770–2,940)	294 (255–336)	317 (281–355)	345 (269–438)

1-8	Liver cancer	774 (664–884)	404 (351–460)	543 (411–702)	176 (151–201)	70·4 (61·6–79·9)	80 (61·1–103)
1-9	Liver cancer due to hepatitis B	268 (205–354)	143 (106–189)	177 (118–257)	55·8 (41·4–75·2)	22·9 (17–30·3)	24·5 (16·4–35·4)
1-10	Liver cancer due to hepatitis C	127 (84–183)	68 (45–95)	95 (58–142)	33·7 (22·6–48·4)	13·4 (9·1–18·5)	15·2 (9·5–22·3)
1-11	Liver cancer due to alcohol use	241 (169–321)	121 (85–161)	175 (115–250)	59·2 (41·6–77·8)	22·1 (15·9–29·3)	26·4 (17·8–37·2)
1-12	Liver cancer due to NASH	50 (36–72)	30 (22–42)	45 (29–67)	12 (8·2–17)	5·6 (4–7·8)	6·8 (4·5–10·2)
1-13	Gallbladder and biliary tract cancer	365 (208–438)	200 (165–296)	232 (169–345)	90·8 (51·9–108)	36·1 (29·8–53·7)	34·6 (25·5–52)
1-14	Pancreatic cancer	164 (141–189)	728 (634–837)	979 (734–1,260)	41·6 (35·6–48)	130 (114–149)	146 (110–187)
1-15	Larynx cancer	140 (117–166)	190 (160–224)	223 (165–293)	34·4 (28·9–40·7)	33·2 (28·1–38·9)	32·1 (24–41·8)
1-16	Tracheal, bronchus, and lung cancer	881 (756–1,010)	1,200 (1,050–1,370)	1,500 (1,160–1,930)	216 (186–247)	213 (187–242)	222 (172–281)
1-17	Malignant skin melanoma	53 (40–72)	79 (59–106)	100 (68–138)	10·5 (8–14·6)	12·4 (9·4–16·8)	14 (9·6–19·3)
1-18	Non-melanoma skin cancer	20 (17–30)	83 (57–96)	99 (67–128)	5·2 (4·3–7·6)	15·1 (10·8–17·4)	15·2 (10·5–19·4)
1-19	Non-melanoma skin cancer (squamous-cell carcinoma)	20 (17–30)	83 (57–96)	99 (67–128)	5·2 (4·3–7·6)	15·1 (10·8–17·4)	15·2 (10·5–19·4)
1-20	Non-melanoma skin cancer (basal-cell carcinoma)	0 (0–0)	0 (0–0)	0 (0–0)	0 (0–0)	0 (0–0)	0 (0–0)
1-21	Breast cancer	1,640 (1,370–1,950)	2,580 (2,150–3,010)	2,880 (2,140–3,760)	359 (302–426)	408 (342–472)	403 (303–526)
1-22	Cervical cancer	2,300 (1,850–2,800)	2,550 (2,090–3,080)	2,570 (1,880–3,470)	465 (379–563)	391 (322–465)	354 (261–474)
1-23	Uterine cancer	328 (271–389)	545 (454–654)	664 (501–865)	77·2 (63·8–90·8)	94·2 (79·1–112)	96·8 (73·4–125)
1-24	Ovarian cancer	162 (128–293)	802 (500–969)	929 (628–1,250)	34 (27·1–62·2)	127 (80·9–153)	128 (87·5–171)
1-25	Prostate cancer	1,420 (1,140–2,070)	2,020 (1,630–2,640)	2,430 (1,800–3,420)	428 (346–616)	459 (374–606)	444 (333–618)
1-26	Testicular cancer	16 (12–27)	52 (41–68)	62 (43–84)	2 (1·6–3·1)	7·1 (5·6–9·1)	7·8 (5·4–10·5)
1-27	Kidney cancer	393 (334–455)	364 (318–414)	459 (344–607)	73·2 (62·7–84·6)	57·6 (50·4–65·6)	63·9 (48–84·3)
1-28	Bladder cancer	145 (123–167)	197 (172–226)	242 (186–312)	38·7 (33·2–44·5)	38·7 (33·7–44·2)	38·8 (30–49·5)
1-29	Brain and nervous system cancer	165 (114–322)	378 (215–468)	395 (209–575)	24·1 (16·9–46·2)	53·2 (30·2–65·8)	53 (27·8–77)
1-30	Thyroid cancer	83 (70–96)	117 (101–134)	141 (107–183)	18·4 (15·7–21·2)	19·3 (16·8–22·1)	20·1 (15·3–25·8)
1-31	Mesothelioma	57 (42–70)	75 (62–90)	82 (59–112)	11·2 (8·5–13·6)	11·5 (9·6–13·7)	11·1 (8–15)
1-32	Hodgkin lymphoma	74 (47–151)	145 (112–176)	151 (106–203)	11·2 (7·2–22·6)	20·4 (15·8–24·6)	19·1 (13·5–25·6)
1-33	Non-Hodgkin's lymphoma	610 (504–713)	649 (560–750)	738 (546–984)	102 (85·3–118)	95·9 (82·7–110)	99 (73·5–132)
1-34	Multiple myeloma	73 (62–93)	147 (119–170)	197 (144–256)	18·4 (15·4–23·7)	26·7 (22·2–30·8)	29·2 (21·6–37·7)
1-35	Leukaemia	1,170 (995–1,340)	1,180 (1,030–1,330)	1,170 (903–1,510)	162 (139–184)	163 (143–183)	156 (120–200)
1-36	Acute lymphoid leukaemia	478 (381–586)	516 (434–605)	477 (366–613)	52·2 (42–64·7)	64·2 (54·1–74·9)	61·1 (46·8–78·7)
1-37	Chronic lymphoid leukaemia	12 (10–16)	39 (32–46)	51 (37–67)	2·9 (2·4–4)	7·3 (5·9–8·6)	7·7 (5·7–10·1)
1-38	Acute myeloid leukaemia	82 (59–102)	87 (72–111)	101 (72–138)	11·4 (8·1–14)	11·9 (9·8–15·2)	13·2 (9·2–18)
1-39	Chronic myeloid leukaemia	183 (152–217)	154 (131–180)	161 (119–216)	31·8 (26·7–37·2)	23·5 (20·1–27·2)	21·9 (16·3–29·1)
1-40	Other leukaemia	415 (346–488)	380 (327–437)	383 (286–498)	63·7 (54·1–74·3)	55·9 (48·4–63·6)	52·2 (39·4–67·8)
1-41	Other neoplasms	41 (32–62)	60 (47–80)	75 (55–104)	7·9 (6–12·3)	10·1 (8·1–13·9)	11·2 (8·3–15·5)
1-42	Myelodysplastic, myeloproliferative, and other hematopoietic neoplasms	41 (32–62)	60 (47–80)	75 (55–104)	7·9 (6–12·3)	10·1 (8·1–13·9)	11·2 (8·3–15·5)
1-43	Benign and in situ intestinal neoplasms	0 (0–0)	0 (0–0)	0 (0–0)	0 (0–0)	0 (0–0)	0 (0–0)
1-44	Benign and in situ cervical and uterine neoplasms	0 (0–0)	0 (0–0)	0 (0–0)	0 (0–0)	0 (0–0)	0 (0–0)
1-45	Other benign and in situ neoplasms	0 (0–0)	0 (0–0)	0 (0–0)	0 (0–0)	0 (0–0)	0 (0–0)
1-46	Cardiovascular diseases	60,200 (52,800–67,100)	54,100 (48,300–60,200)	60,100 (47,300–74,600)	14,800 (13,100–16,400)	10,100 (9,110–11,200)	9,420 (7,550–11,600)
1-47	Rheumatic heart disease	1,470 (1,220–1,740)	1,080 (897–1,290)	963 (732–1,230)	217 (182–255)	146 (122–173)	123 (93·5–157)
1-48	Ischaemic heart disease	22,400 (19,600–25,200)	22,900 (20,400–25,500)	24,800 (19,500–31,100)	5,730 (5,050–6,370)	4,370 (3,900–4,840)	3,920 (3,100–4,860)
1-49	Stroke	25,600 (22,400–28,600)	18,500 (16,500–20,700)	20,400 (15,900–25,900)	6,330 (5,540–7,070)	3,540 (3,180–3,930)	3,230 (2,540–4,030)
1-50	Ischaemic stroke	7,720 (6,800–8,660)	6,560 (5,890–7,240)	7,350 (5,870–9,040)	2,170 (1,910–2,430)	1,440 (1,290–1,580)	1,340 (1,070–1,640)
1-51	Intracerebral hemorrhage	16,100 (14,000–18,300)	10,500 (9,280–11,800)	11,400 (8,660–14,600)	3,840 (3,320–4,330)	1,880 (1,670–2,100)	1,680 (1,290–2,130)
1-52	Subarachnoid hemorrhage	1,720 (1,380–2,030)	1,470 (1,270–1,700)	1,640 (1,220–2,160)	320 (262–374)	224 (194–256)	223 (167–291)
1-53	Hypertensive heart disease	6,060 (4,560–6,990)	5,950 (5,160–6,930)	7,260 (5,520–9,500)	1,610 (1,210–1,840)	1,130 (984–1,330)	1,160 (895–1,490)
1-54	Non-rheumatic valvular heart disease	586 (496–680)	461 (395–534)	568 (424–736)	119 (101–138)	75·4 (65·3–86·8)	80·7 (61·1–104)
1-55	Non-rheumatic calcific aortic valve disease	327 (260–396)	254 (212–307)	318 (235–425)	69·7 (56·4–83·3)	43·5 (36·2–52)	46·5 (35·1–61·5)
1-56	Non-rheumatic degenerative mitral valve disease	228 (150–286)	178 (142–220)	217 (155–294)	43·8 (28·8–54)	27·7 (22·5–33·8)	29·7 (21·4–40·2)
1-57	Other non-rheumatic valve diseases	30 (19–48)	28 (17–40)	33 (21–51)	5·1 (3·2–8)	4·2 (2·6–6)	4·5 (2·9–6·9)
1-58	Cardiomyopathy and myocarditis	1,870 (1,580–2,240)	2,170 (1,710–2,530)	2,500 (1,780–3,280)	334 (280–403)	343 (272–398)	352 (255–458)
1-59	Myocarditis	219 (112–437)	437 (292–551)	517 (315–731)	29·5 (14·9–59·6)	63·9 (42·6–80·4)	71 (43–99·6)
1-60	Alcoholic cardiomyopathy	456 (353–565)	448 (323–564)	512 (324–713)	94·4 (73·9–117)	70·3 (51·1–87·6)	69·7 (44·2–96·7)

1·61	Other cardiomyopathy	1,200 (961–1,440)	1,290 (1,040–1,530)	1,470 (1,070–1,930)	210 (173–254)	209 (171–246)	212 (157–276)
1·62	Atrial fibrillation and flutter	314 (267–398)	431 (363–523)	558 (446–701)	101 (86–126)	102 (86–124)	109 (88·1–137)
1·63	Aortic aneurysm	234 (202–268)	420 (367–476)	577 (441–733)	59·9 (51·8–68·8)	81 (71·3–91·3)	91·7 (70·4–115)
1·64	Peripheral vascular disease	54 (29–91)	85 (48–143)	116 (65–205)	16·6 (9·2–27·6)	19·2 (10·9–32·1)	21·3 (12–37·9)
1·65	Endocarditis	255 (188–411)	482 (346–576)	575 (395–778)	41·6 (30·6–68·6)	71·5 (52·6–84·8)	77·8 (54·5–104)
1·66	Chronic respiratory diseases	5,010 (4,090–6,140)	5,150 (4,280–6,110)	5,540 (4,390–6,820)	873 (740–1,020)	810 (686–938)	796 (636–975)
1·67	Chronic obstructive pulmonary disease	1,190 (1,020–1,380)	1,700 (1,480–1,940)	2,090 (1,650–2,540)	312 (268–360)	333 (292–379)	337 (269–406)
1·68	Pneumoconiosis	6 (5–7)	5 (4–7)	7 (5–9)	1·3 (1·1–1·6)	0·9 (0·8–1·1)	1 (0·7–1·2)
1·69	Silicosis	2 (2–3)	3 (2–4)	3 (2–5)	0·4 (0·3–0·6)	0·5 (0·4–0·6)	0·5 (0·3–0·6)
1·70	Asbestosis	1 (1–1)	1 (1–1)	1 (1–2)	0·2 (0·1–0·3)	0·2 (0·1–0·2)	0·2 (0·1–0·3)
1·71	Coal workers pneumoconiosis	1 (0–1)	0 (0–0)	0 (0–1)	0·1 (0·1–0·2)	0 (0–0)	0 (0–0·1)
1·72	Other pneumoconiosis	2 (2–3)	1 (1–2)	2 (1–2)	0·5 (0·4–0·7)	0·2 (0·2–0·3)	0·3 (0·2–0·3)
1·73	Asthma	3,490 (2,670–4,570)	3,020 (2,330–3,930)	2,950 (2,180–3,920)	503 (403–634)	412 (322–526)	390 (290–518)
1·74	Interstitial lung disease and pulmonary sarcoidosis	121 (83–158)	196 (144–244)	239 (161–329)	26·7 (19–34·6)	33·4 (24·8–41·3)	35·5 (24·4–48)
1·75	Other chronic respiratory diseases	200 (131–252)	225 (187–271)	256 (198–333)	29·2 (18·6–36·4)	30·6 (25·4–36·7)	33·3 (25·7–43·2)
1·76	Digestive diseases	14,600 (12,800–16,500)	14,200 (12,600–16,000)	15,000 (11,900–18,500)	2,780 (2,440–3,120)	2,210 (1,970–2,490)	2,080 (1,660–2,560)
1·77	Cirrhosis and other chronic liver diseases	7,790 (6,680–8,920)	7,930 (6,910–9,130)	8,420 (6,470–10,800)	1,570 (1,350–1,790)	1,230 (1,080–1,410)	1,150 (895–1,470)
1·78	Cirrhosis and other chronic liver diseases due to hepatitis B	1,010 (700–1,430)	1,090 (737–1,550)	640 (389–983)	206 (141–289)	163 (112–227)	86·6 (54–134)
1·79	Cirrhosis and other chronic liver diseases due to hepatitis C	1,520 (1,090–2,060)	1,560 (1,100–2,170)	1,740 (1,150–2,520)	308 (221–421)	238 (169–330)	237 (158–340)
1·80	Cirrhosis and other chronic liver diseases due to alcohol use	2,860 (2,190–3,610)	3,120 (2,380–3,950)	3,580 (2,540–4,850)	619 (477–780)	492 (380–621)	492 (353–660)
1·81	Cirrhosis and other chronic liver diseases due to NAFLD	940 (646–1,330)	1,010 (670–1,470)	1,210 (755–1,840)	202 (137–283)	162 (111–232)	170 (108–256)
1·82	Cirrhosis and other chronic liver diseases due to other causes	1,460 (1,130–1,840)	1,160 (886–1,500)	1,240 (876–1,770)	238 (178–312)	173 (132–225)	169 (120–238)
1·83	Upper digestive system diseases	2,640 (2,150–3,300)	2,350 (1,860–3,060)	2,480 (1,830–3,310)	521 (434–638)	375 (300–477)	347 (261–457)
1·84	Peptic ulcer disease	1,580 (1,340–1,830)	1,140 (987–1,320)	1,140 (847–1,510)	340 (290–393)	194 (169–224)	168 (126–221)
1·85	Gastritis and duodenitis	322 (250–414)	375 (290–480)	409 (304–537)	59·7 (46·7–75·2)	58·5 (46·2–74·5)	57·1 (42·9–74·8)
1·86	GERD	739 (383–1,310)	831 (432–1,470)	925 (481–1,630)	122 (63·6–216)	122 (63·5–216)	122 (63·6–216)
1·87	Appendicitis	633 (445–783)	444 (356–582)	408 (297–554)	90·4 (63–112)	61·6 (49·2–81)	54 (39·8–72·8)
1·88	Paralytic ileus and intestinal obstruction	929 (741–1,120)	781 (642–934)	774 (563–1,020)	140 (116–168)	126 (106–150)	115 (85·7–150)
1·89	Inguinal, femoral, and abdominal hernia	903 (697–1,140)	743 (572–957)	720 (543–936)	131 (104–160)	109 (85·6–138)	99 (75–128)
1·90	Inflammatory bowel disease	239 (189–291)	224 (193–270)	257 (191–336)	40·7 (32·9–48·4)	34·2 (29·6–41·3)	35·4 (26·5–46·1)
1·91	Vascular intestinal disorders	100 (83–116)	122 (107–141)	150 (114–197)	23·8 (19·9–27·6)	22 (19·2–25·3)	23 (17·6–29·9)
1·92	Gallbladder and biliary diseases	668 (527–844)	592 (465–741)	639 (487–824)	137 (109–168)	95·5 (76·3–119)	90·8 (70·1–116)
1·93	Pancreatitis	419 (348–508)	544 (463–642)	584 (436–753)	76·4 (63·7–92·9)	81·7 (70–96·1)	79·4 (59·3–102)
1·94	Other digestive diseases	262 (197–416)	493 (317–606)	555 (346–779)	46 (34·9–75·3)	80·7 (52·9–99·5)	82 (51·2–114)
1·95	Neurological disorders	8,430 (4,630–13,600)	8,850 (5,150–14,300)	9,730 (5,540–15,600)	1,370 (833–2,160)	1,360 (838–2,120)	1,400 (829–2,210)
1·96	Alzheimer's disease and other dementias	747 (311–1,730)	1,050 (430–2,440)	1,290 (536–2,980)	284 (117–648)	290 (119–668)	296 (122–668)
1·97	Parkinson's disease	281 (252–310)	398 (356–442)	482 (401–579)	87·6 (78·9–96·1)	92·6 (83·2–103)	93·2 (77·9–111)
1·98	Idiopathic epilepsy	2,720 (1,750–4,060)	2,420 (1,500–3,650)	2,630 (1,610–4,010)	366 (239–542)	325 (200–491)	337 (205–514)
1·99	Multiple sclerosis	30 (24–44)	43 (36–59)	50 (38–71)	5·5 (4·3–7·9)	6·3 (5·2–8·7)	6·7 (5–9·3)
1·100	Motor neuron disease	29 (25–34)	85 (73–98)	109 (83–142)	5·3 (4·6–6·1)	13·1 (11·5–15·1)	15·1 (11·4–19·5)
1·101	Headache disorders	4,020 (691–8,930)	4,150 (767–9,200)	4,370 (823–9,710)	545 (107–1,200)	545 (106–1,200)	546 (107–1,200)
1·102	Migraine	3,680 (469–8,630)	3,780 (513–8,740)	3,960 (562–9,110)	493 (72·7–1,130)	494 (72·9–1,130)	494 (72·2–1,130)
1·103	Tension-type headache	348 (95–1,420)	375 (109–1,460)	403 (117–1,480)	51·5 (15·1–193)	51·7 (15·6–189)	51·8 (15·3–187)
1·104	Other neurological disorders	595 (410–845)	695 (496–960)	800 (529–1,130)	75·8 (54·2–105)	92·4 (67·5–125)	103 (68·9–146)
1·105	Mental disorders	13,600 (9,880–18,000)	15,000 (10,800–19,800)	15,800 (11,400–20,800)	1,920 (1,410–2,530)	2,030 (1,480–2,680)	2,000 (1,460–2,630)
1·106	Schizophrenia	1,030 (718–1,380)	1,170 (806–1,570)	1,300 (907–1,730)	160 (112–212)	164 (113–218)	166 (116–221)
1·107	Depressive disorders	5,680 (3,830–8,040)	6,770 (4,610–9,690)	7,170 (4,850–10,100)	846 (580–1,170)	944 (640–1,350)	915 (622–1,270)
1·108	Major depressive disorder	5,110 (3,410–7,210)	6,140 (4,160–8,700)	6,480 (4,310–9,100)	756 (510–1,070)	855 (581–1,210)	826 (557–1,160)
1·109	Dysthymia	576 (349–900)	631 (395–962)	692 (428–1,060)	89·2 (55·6–135)	89·3 (55·8–136)	89·3 (55·9–136)
1·110	Bipolar disorder	1,350 (775–2,120)	1,390 (814–2,170)	1,510 (877–2,370)	186 (110–289)	186 (110–290)	186 (110–293)
1·111	Anxiety disorders	2,970 (1,950–4,230)	3,080 (2,040–4,340)	3,270 (2,150–4,610)	410 (272–577)	411 (275–575)	414 (273–584)
1·112	Eating disorders	273 (165–406)	283 (172–421)	338 (209–502)	30·9 (18·7–46)	35·5 (21·5–53·4)	39·6 (24·4–58·7)
1·113	Anorexia nervosa	73 (41–121)	73 (42–118)	85 (48–138)	7·9 (4·5–12·8)	8·8 (5–14·2)	9·8 (5·6–15·7)

1-114	Bulimia nervosa	200 (111–320)	209 (122–326)	253 (147–402)	23 (13–36·6)	26·7 (15·5–41·3)	29·8 (17·4–46·7)
1-115	Autism spectrum disorders	418 (273–610)	395 (257–576)	401 (260–589)	51·1 (33·7–74·6)	51·4 (33·5–75·1)	51·4 (33·3–75·5)
1-116	Attention-deficit/hyperactivity disorder	337 (191–570)	314 (179–534)	301 (172–514)	36·8 (20·9–61·8)	37·1 (21·3–63)	37 (21·1–63·4)
1-117	Conduct disorder	680 (384–1,070)	645 (359–1,020)	534 (299–840)	66·9 (37·8–105)	67·7 (37·5–107)	67·6 (37·9–106)
1-118	Idiopathic developmental intellectual disability	246 (94–447)	178 (61–324)	151 (48–277)	29·2 (11·5–53)	22·9 (7·9–41·6)	19·3 (6·1–35·2)
1-119	Other mental disorders	644 (401–973)	733 (467–1,110)	807 (516–1,220)	107 (68·1–161)	107 (68·9–162)	107 (68·4–161)
1-120	Substance use disorders	2,430 (1,860–3,090)	3,060 (2,400–3,800)	3,390 (2,620–4,260)	374 (295–469)	427 (336–528)	429 (333–536)
1-121	Alcohol use disorders	1,760 (1,360–2,250)	2,330 (1,830–2,910)	2,500 (1,900–3,190)	288 (228–362)	329 (260–411)	322 (246–409)
1-122	Drug use disorders	679 (473–920)	734 (521–969)	890 (645–1,160)	86 (60·9–115)	97·4 (69·6–128)	107 (77·8–139)
1-123	Opioid use disorders	306 (203–427)	354 (244–483)	440 (311–598)	42·2 (28·5–57)	48·6 (33·7–65·9)	54·3 (38·7–73)
1-124	Cocaine use disorders	105 (57–168)	123 (77–184)	148 (96–215)	12·4 (7·1–19·4)	15·7 (10·1–23·6)	17·5 (11·4–25·1)
1-125	Amphetamine use disorders	53 (25–94)	48 (27–81)	59 (32–96)	6 (3–10·6)	6·4 (3·6–10·8)	6·7 (3·8–10·9)
1-126	Cannabis use disorders	101 (54–163)	93 (53–149)	104 (54–182)	11·4 (6·3–18·2)	11·4 (6·5–18·4)	11·9 (6·3–20·6)
1-127	Other drug use disorders	115 (70–175)	117 (73–174)	139 (91–203)	13·9 (8·8–20·8)	15·3 (9·7–22·4)	16·5 (10·9–24)
1-128	Diabetes and kidney diseases	15,800 (14,000–17,800)	26,700 (23,600–30,200)	31,700 (25,700–38,500)	3,590 (3,170–4,030)	4,690 (4,150–5,270)	4,680 (3,840–5,650)
1-129	Diabetes mellitus	11,600 (10,100–13,200)	18,500 (16,000–21,300)	21,900 (17,600–26,400)	2,720 (2,390–3,080)	3,320 (2,890–3,810)	3,270 (2,650–3,940)
1-130	Diabetes mellitus type 1	1,220 (947–1,510)	1,220 (1,010–1,660)	1,260 (941–1,750)	198 (147–247)	176 (146–245)	167 (125–233)
1-131	Diabetes mellitus type 2	10,300 (8,960–11,900)	17,300 (14,900–20,000)	20,600 (16,500–25,000)	2,520 (2,200–2,880)	3,150 (2,730–3,620)	3,100 (2,500–3,750)
1-132	Chronic kidney disease	4,280 (3,750–4,850)	8,200 (7,260–9,220)	9,820 (7,840–12,200)	872 (768–987)	1,360 (1,210–1,530)	1,420 (1,130–1,760)
1-133	Chronic kidney disease due to diabetes mellitus type 1	382 (261–532)	832 (555–1,140)	920 (573–1,420)	70·1 (47–99·4)	120 (80·2–167)	122 (75·4–188)
1-134	Chronic kidney disease due to diabetes mellitus type 2	775 (607–971)	1,710 (1,330–2,130)	2,170 (1,580–2,890)	197 (157–243)	317 (250–385)	330 (245–435)
1-135	Chronic kidney disease due to hypertension	660 (526–821)	1,340 (1,090–1,630)	1,700 (1,270–2,230)	166 (132–204)	254 (207–305)	266 (201–344)
1-136	Chronic kidney disease due to glomerulonephritis	1,140 (918–1,380)	2,050 (1,620–2,500)	2,370 (1,720–3,250)	200 (160–246)	310 (244–382)	320 (232–439)
1-137	Chronic kidney disease due to other and unspecified causes	1,330 (1,080–1,610)	2,260 (1,830–2,800)	2,660 (1,990–3,500)	240 (193–298)	360 (291–449)	376 (283–492)
1-138	Acute glomerulonephritis	2 (1–2)	18 (15–21)	14 (10–18)	0·3 (0·2–0·3)	2·4 (2·1–2·9)	1·9 (1·4–2·5)
1-139	Skin and subcutaneous diseases	3,890 (2,510–5,830)	3,890 (2,570–5,760)	3,980 (2,640–5,910)	498 (325–753)	525 (351–780)	529 (352–778)
1-140	Dermatitis	944 (554–1,510)	874 (514–1,380)	873 (517–1,380)	115 (68·2–180)	115 (68·2–179)	115 (68·1–181)
1-141	Atopic dermatitis	751 (401–1,270)	664 (356–1,110)	644 (348–1,080)	84·6 (45·5–143)	84·6 (45·4–142)	84·6 (45·7–141)
1-142	Contact dermatitis	171 (108–260)	186 (117–282)	203 (129–309)	26·9 (16·8–40·1)	26·9 (17–40·6)	26·8 (17·1–40·4)
1-143	Seborrhoeic dermatitis	23 (13–37)	24 (14–39)	25 (14–39)	3·4 (1·9–5·4)	3·4 (1·9–5·4)	3·4 (1·9–5·2)
1-144	Psoriasis	239 (165–324)	236 (166–318)	240 (171–324)	35·8 (25·2–47·9)	33 (23·4–44·1)	31·5 (22·3–42·3)
1-145	Bacterial skin diseases	63 (35–123)	151 (98–203)	191 (111–269)	8·4 (4·6–16·7)	23·5 (14·6–32·1)	27·8 (16–39·6)
1-146	Cellulitis	25 (16–48)	31 (20–47)	35 (22–53)	3·2 (2·1–6·6)	4·5 (2·9–7·3)	4·9 (3–7·4)
1-147	Pyoderma	39 (16–85)	120 (70–165)	155 (86–223)	5·2 (2·1–11·6)	19 (11·1–26·1)	22·9 (12·5–33·1)
1-148	Scabies	698 (382–1,120)	651 (358–1,040)	655 (355–1,060)	83·5 (46·1–134)	83·8 (46·3–134)	83·6 (45·9–134)
1-149	Fungal skin diseases	278 (109–594)	291 (116–620)	311 (125–666)	44·4 (17·9–93·8)	44·3 (17·9–94·3)	44·2 (17·8–93·1)
1-150	Viral skin diseases	427 (268–641)	375 (241–562)	361 (232–538)	47·2 (29·7–70·6)	47·3 (30·4–71)	47·3 (30·3–70·9)
1-151	Acne vulgaris	397 (234–643)	423 (250–691)	401 (235–655)	40·2 (23·8–64·9)	46·1 (27·3–75·2)	47·6 (27·9–77·4)
1-152	Alopecia areata	52 (33–76)	52 (33–77)	56 (35–83)	7·1 (4·5–10·5)	7 (4·4–10·6)	7·1 (4·5–10·5)
1-153	Pruritus	52 (25–93)	56 (27–100)	61 (29–109)	8 (3·8–14·2)	8·2 (3·9–14·6)	8·3 (4–14·8)
1-154	Urticaria	400 (260–582)	363 (238–524)	366 (239–532)	47·8 (31·3–68·6)	47·8 (31·5–68·9)	47·8 (31·2–69·7)
1-155	Decubitus ulcer	68 (42–170)	88 (61–139)	103 (66–162)	16·2 (9·9–40·7)	18·2 (13–29)	18·4 (12·3–28)
1-156	Other skin and subcutaneous diseases	271 (133–503)	332 (177–579)	367 (196–638)	45 (22–83)	50·7 (27·1–88·2)	51 (27·3–88·9)
1-157	Sense organ diseases	3,770 (2,580–5,350)	4,370 (2,960–6,360)	4,920 (3,290–7,100)	825 (564–1,160)	778 (531–1,110)	757 (512–1,070)
1-158	Blindness and vision loss	1,100 (754–1,560)	1,210 (809–1,730)	1,330 (906–1,900)	256 (178–359)	224 (154–317)	213 (146–302)
1-159	Glaucoma	58 (38–83)	60 (39–86)	66 (44–96)	19·1 (12·5–27·6)	14·8 (9·7–21·1)	13·3 (8·8–19·3)
1-160	Cataract	170 (118–235)	183 (127–248)	200 (140–273)	51·6 (35·7–71·2)	41·3 (28·8–56·2)	38 (26·7–51·8)
1-161	Age-related macular degeneration	6 (4–9)	7 (4–10)	8 (5–12)	1·8 (1·2–2·7)	1·6 (1–2·3)	1·5 (1–2·2)
1-162	Refraction disorders	369 (245–543)	378 (248–552)	398 (264–584)	59 (38·7–84·4)	55·4 (36·8–79·9)	54·1 (36–78·2)
1-163	Near vision loss	213 (97–425)	272 (124–541)	318 (140–625)	55·1 (25·5–109)	52 (23·7–101)	50·2 (22·4–97·7)
1-164	Other vision loss	283 (197–390)	306 (214–421)	339 (237–473)	69·3 (48·6–94·4)	59·4 (41·7–81·5)	55·7 (39·2–77·2)
1-165	Age-related and other hearing loss	2,480 (1,640–3,610)	2,950 (1,960–4,340)	3,340 (2,220–4,920)	534 (356–773)	518 (348–750)	508 (341–731)
1-166	Other sense organ diseases	191 (115–287)	220 (129–338)	248 (146–385)	35·2 (20·9–54·5)	35·8 (21·3–55·9)	36 (21·3–55·4)

1-167	Musculoskeletal disorders	7,830 (5,660–10,300)	9,630 (6,980–12,700)	10,900 (7,970–14,300)	1,400 (1,010–1,840)	1,460 (1,070–1,930)	1,490 (1,090–1,960)
1-168	Rheumatoid arthritis	98 (66–134)	147 (105–192)	180 (127–236)	17·6 (12·3–23·7)	22·8 (16·5–29·4)	24·9 (18–32·4)
1-169	Osteoarthritis	715 (361–1,450)	1,050 (524–2,110)	1,300 (650–2,580)	184 (92·8–369)	195 (98–388)	198 (99·6–392)
1-170	Low back pain	4,300 (2,990–5,770)	4,890 (3,420–6,530)	5,310 (3,750–7,130)	722 (511–966)	723 (509–964)	714 (505–954)
1-171	Neck pain	740 (480–1,080)	898 (587–1,310)	988 (645–1,430)	133 (88·6–193)	133 (89·2–194)	133 (88·8–192)
1-172	Gout	30 (19–43)	44 (27–65)	51 (31–75)	6·4 (4–9·1)	7·4 (4·6–10·6)	7·5 (4·6–10·9)
1-173	Other musculoskeletal disorders	1,950 (1,370–2,690)	2,600 (1,810–3,490)	3,090 (2,200–4,160)	336 (236–459)	382 (265–512)	409 (289–553)
1-174	Other non-communicable diseases	18,800 (16,000–22,700)	16,300 (13,400–20,000)	16,500 (12,900–20,800)	2,440 (2,030–2,970)	2,360 (1,940–2,890)	2,260 (1,780–2,860)
1-175	Congenital anomalies	7,770 (6,280–10,900)	5,390 (4,170–6,670)	4,540 (3,230–6,210)	763 (624–1,060)	756 (581–936)	632 (446–867)
1-176	Neural tube defects	515 (357–761)	275 (195–369)	230 (151–342)	49·4 (34·4–72·6)	38·7 (27·5–52·5)	32·3 (21–48·5)
1-177	Congenital heart anomalies	3,460 (2,670–5,170)	2,480 (1,780–3,210)	2,000 (1,310–2,890)	338 (262–500)	349 (249–455)	280 (184–406)
1-178	Orofacial clefts	76 (36–147)	44 (31–60)	35 (23–50)	7·7 (3·9–14·6)	6·1 (4·3–8·4)	4·7 (3·1–6·8)
1-179	Down's syndrome	72 (57–100)	87 (69–106)	85 (58–114)	8·2 (6·6–11·1)	11·7 (9·3–14·3)	11·2 (7·6–15·1)
1-180	Turner syndrome	2 (1–3)	1 (1–2)	1 (1–2)	0·2 (0·1–0·3)	0·2 (0·1–0·3)	0·1 (0·1–0·3)
1-181	Klinefelter syndrome	0 (0–0)	0 (0–0)	0 (0–0)	0 (0–0)	0 (0–0)	0 (0–0)
1-182	Other chromosomal abnormalities	81 (61–106)	74 (54–94)	70 (50–94)	8 (6–10·4)	10·1 (7·3–12·9)	9·6 (6·7–12·8)
1-183	Congenital musculoskeletal and limb anomalies	468 (312–663)	439 (296–625)	423 (284–602)	51·2 (34·1–72·2)	56·8 (38·6–80·4)	55·6 (37·4–78·8)
1-184	Urogenital congenital anomalies	269 (165–540)	173 (121–255)	145 (93–224)	26 (16·2–51·6)	24·4 (17–36·6)	20·4 (12·9–31·7)
1-185	Digestive congenital anomalies	1,110 (762–1,850)	643 (441–940)	529 (332–824)	105 (72·4–175)	93·3 (63·7–137)	76·2 (47·7–119)
1-186	Other congenital anomalies	1,710 (1,320–2,380)	1,180 (898–1,530)	1,020 (697–1,470)	169 (132–234)	165 (124–216)	142 (95·8–204)
1-187	Urinary diseases and male infertility	502 (404–656)	930 (749–1,090)	1,150 (884–1,430)	104 (84–136)	165 (133–193)	175 (135–218)
1-188	Urinary tract infections and interstitial nephritis	246 (205–334)	589 (466–681)	732 (539–946)	50·7 (42·7–70·1)	104 (81·8–120)	114 (84·8–146)
1-189	Urolithiasis	39 (30–52)	73 (56–91)	84 (62–110)	7·9 (6·1–10·6)	11·5 (8·7–14·2)	11·6 (8·6–15·1)
1-190	Benign prostatic hyperplasia	92 (53–146)	127 (74–199)	158 (92–249)	26 (15·2–40·9)	27·3 (15·7–42·7)	26·3 (15·3–41·1)
1-191	Male infertility	42 (16–100)	26 (10–57)	44 (16–106)	5·4 (2–12·9)	3·5 (1·4–7·6)	5·3 (2–12·9)
1-192	Other urinary diseases	84 (67–116)	115 (87–151)	129 (87–174)	14·4 (11·4–21·5)	18·3 (13·5–23·7)	18·3 (12·2–24·6)
1-193	Gynecological diseases	2,710 (1,880–3,740)	2,760 (1,930–3,870)	2,980 (2,060–4,160)	390 (273–540)	372 (260–522)	378 (265–528)
1-194	Uterine fibroids	171 (99–295)	209 (119–362)	216 (119–375)	28·7 (16·6–49·6)	28·6 (16·2–49·8)	28·3 (15·5–49·3)
1-195	Polycystic ovarian syndrome	34 (14–67)	45 (19–91)	51 (21–104)	4·1 (1·7–8·2)	5·7 (2·4–11·3)	6·1 (2·5–12·5)
1-196	Female infertility	87 (31–213)	43 (17–90)	90 (32–222)	11·3 (4–26·7)	5·8 (2·3–12·1)	11·2 (4–27·7)
1-197	Endometriosis	274 (161–436)	226 (134–356)	233 (135–379)	34·9 (20·8–54·7)	29·7 (17·7–46·6)	27·9 (16·3–45·2)
1-198	Genital prolapse	40 (21–71)	41 (21–76)	44 (23–82)	8·8 (4·9–15·5)	6·8 (3·5–12·4)	6·3 (3·2–11·6)
1-199	Premenstrual syndrome	801 (481–1,260)	785 (480–1,220)	827 (502–1,280)	100 (60·8–156)	98·8 (59·9–155)	100 (61–156)
1-200	Other gynecological diseases	1,300 (855–1,850)	1,410 (914–2,000)	1,520 (990–2,170)	202 (133–286)	197 (128–276)	198 (130–282)
1-201	Hemoglobinopathies and hemolytic anaemias	2,500 (2,040–3,030)	1,970 (1,650–2,340)	1,910 (1,510–2,370)	312 (257–378)	263 (219–311)	244 (194–304)
1-202	Thalassemias	2 (1–3)	2 (1–3)	1 (1–2)	0·2 (0·1–0·3)	0·2 (0·1–0·4)	0·1 (0·1–0·3)
1-203	Thalassaemias trait	47 (28–72)	42 (25–64)	42 (25–64)	5·8 (3·5–8·8)	5·5 (3·2–8·4)	5·3 (3·2–8·1)
1-204	Sickle cell disorders	1,150 (954–1,350)	980 (840–1,130)	889 (656–1,190)	128 (106–150)	123 (105–142)	110 (81·8–148)
1-205	Sickle cell trait	627 (396–934)	412 (259–608)	397 (250–612)	71·8 (45·5–106)	54·1 (34·1–79·6)	49·1 (30·9–75)
1-206	G6PD deficiency	203 (137–282)	198 (140–264)	221 (147–307)	29·5 (20·6–39·8)	27·2 (19·4–35·9)	28·4 (19·1–39·2)
1-207	G6PD trait	2 (1–3)	2 (1–3)	2 (1–3)	0·4 (0·2–0·6)	0·3 (0·1–0·4)	0·2 (0·1–0·4)
1-208	Other hemoglobinopathies and hemolytic anaemias	462 (344–624)	335 (249–444)	354 (261–468)	76·9 (59·6–99·8)	52·8 (40·2–68·9)	50·3 (37·4–65·6)
1-209	Endocrine, metabolic, blood, and immune disorders	3,500 (2,500–4,190)	3,110 (2,550–3,970)	3,590 (2,780–4,690)	524 (383–625)	468 (384–596)	499 (387–647)
1-210	Oral disorders	1,770 (1,010–2,820)	2,030 (1,160–3,300)	2,270 (1,310–3,680)	336 (198–541)	326 (192–532)	323 (190–524)
1-211	Caries of deciduous teeth	31 (13–64)	24 (10–49)	23 (10–47)	3·2 (1·3–6·5)	3·1 (1·4–6·4)	3·1 (1·4–6·5)
1-212	Caries of permanent teeth	210 (94–407)	204 (91–393)	209 (93–398)	27·8 (12·4–53·1)	26·7 (11·9–51·3)	26·2 (11·9–50·1)
1-213	Periodontal disease	570 (219–1,230)	709 (270–1,530)	793 (302–1,720)	101 (39·5–218)	104 (40·3–220)	106 (41·1–229)
1-214	Edentulism	576 (367–862)	705 (452–1,060)	838 (537–1,260)	151 (97·2–226)	140 (89–210)	135 (86·9–202)
1-215	Other oral disorders	381 (234–556)	387 (240–566)	411 (257–603)	52·8 (32·6–76·4)	52·8 (32·8–77)	52·8 (32·8–77·3)
1-216	Sudden infant death syndrome	109 (70–166)	66 (43–97)	62 (36–100)	10·2 (6·5–15·5)	9·6 (6·2–14·1)	8·9 (5·2–14·4)
1-0	Injuries	47,000 (41,000–52,500)	48,700 (43,800–54,300)	48,900 (39,400–59,600)	6,310 (5,530–7,060)	6,600 (5,940–7,360)	6,170 (4,990–7,530)
1-1	Transport injuries	9,880 (8,470–11,200)	9,840 (8,730–11,000)	10,200 (8,050–12,800)	1,320 (1,150–1,500)	1,310 (1,160–1,470)	1,270 (999–1,580)
1-2	Road injuries	9,220 (7,900–10,500)	8,750 (7,750–9,840)	9,050 (7,080–11,300)	1,240 (1,070–1,400)	1,170 (1,030–1,310)	1,120 (879–1,390)

1-3	Pedestrian road injuries	2,990 (2,580–3,400)	1,780 (1,570–2,000)	1,650 (1,270–2,070)	417 (363–471)	243 (215–272)	214 (167–269)
1-4	Cyclist road injuries	885 (737–1,060)	834 (715–967)	927 (724–1,190)	120 (101–141)	111 (95·2–129)	116 (91·2–148)
1-5	Motorcyclist road injuries	1,040 (880–1,200)	1,610 (1,380–1,850)	1,750 (1,380–2,160)	135 (115–154)	213 (184–246)	211 (167–260)
1-6	Motor vehicle road injuries	4,220 (3,570–4,840)	4,450 (3,910–5,030)	4,640 (3,530–5,890)	551 (472–627)	586 (513–663)	566 (432–719)
1-7	Other road injuries	90 (73–110)	81 (66–98)	90 (70–112)	12·6 (10·3–15·5)	11·2 (9·1–13·6)	11·6 (9·1–14·4)
1-8	Other transport injuries	657 (563–754)	1,090 (952–1,260)	1,180 (905–1,490)	87·8 (75·8–100)	145 (127–167)	148 (113–187)
1-9	Unintentional injuries	19,300 (16,900–21,700)	16,800 (14,800–18,800)	16,000 (13,000–19,400)	2,650 (2,340–2,970)	2,350 (2,070–2,650)	2,130 (1,750–2,570)
1-10	Falls	2,720 (2,380–3,110)	2,950 (2,560–3,440)	3,310 (2,730–3,980)	508 (447–578)	482 (419–557)	487 (404–580)
1-11	Drowning	4,170 (3,500–4,820)	4,270 (3,750–4,850)	3,650 (2,840–4,580)	499 (423–572)	538 (472–611)	452 (352–566)
1-12	Fire, heat, and hot substances	2,570 (2,210–2,960)	1,690 (1,440–2,000)	1,570 (1,280–1,930)	342 (295–395)	231 (197–274)	205 (167–252)
1-13	Poisonings	633 (532–741)	153 (130–180)	148 (116–183)	76·2 (64·5–88·3)	20·6 (17·5–24·2)	19·1 (15–23·6)
1-14	Poisoning by carbon monoxide	69 (55–85)	54 (46–64)	57 (43–74)	8·8 (7·2–10·6)	7·3 (6·2–8·7)	7·3 (5·4–9·4)
1-15	Poisoning by other means	564 (471–662)	99 (82–118)	91 (70–114)	67·4 (56·9–78·4)	13·3 (11–15·9)	11·8 (9·2–14·8)
1-16	Exposure to mechanical forces	1,470 (1,230–1,760)	1,710 (1,440–2,040)	1,750 (1,400–2,180)	203 (169–248)	233 (196–281)	224 (177–279)
1-17	Unintentional firearm injuries	381 (305–481)	366 (298–439)	354 (271–456)	49 (39·8–60·6)	48·2 (39·4–58)	43·2 (32·9–55)
1-18	Other exposure to mechanical forces	1,090 (884–1,360)	1,350 (1,110–1,680)	1,390 (1,100–1,770)	154 (124–196)	185 (151–231)	180 (142–229)
1-19	Adverse effects of medical treatment	1,020 (796–1,180)	776 (666–885)	829 (624–1,100)	159 (130–183)	120 (104–137)	116 (87·9–152)
1-20	Animal contact	235 (195–280)	762 (668–866)	736 (585–923)	36·1 (30·3–43·2)	114 (101–129)	103 (82·1–128)
1-21	Venomous animal contact	201 (168–237)	686 (602–779)	658 (523–829)	30·8 (26–36·2)	103 (90·9–116)	92·1 (73·2–115)
1-22	Non-venomous animal contact	34 (25–48)	76 (62–92)	78 (60–99)	5·3 (3·9–7·4)	11·3 (9·2–13·7)	10·8 (8·4–13·8)
1-23	Foreign body	2,660 (2,280–3,080)	1,580 (1,310–1,890)	1,470 (1,110–1,950)	320 (280–364)	223 (186–267)	202 (152–269)
1-24	Pulmonary aspiration and foreign body in airway	2,380 (2,030–2,780)	1,400 (1,160–1,690)	1,290 (940–1,740)	280 (242–323)	198 (163–239)	177 (130–240)
1-25	Foreign body in eyes	39 (17–71)	40 (18–71)	42 (20–75)	5·5 (2·7–9·6)	5·5 (2·6–9·6)	5·4 (2·6–9·6)
1-26	Foreign body in other body part	241 (200–291)	139 (113–170)	144 (114–178)	35·3 (29·7–41·8)	20·1 (16·5–24·4)	19·8 (15·7–24·2)
1-27	Environmental heat and cold exposure	543 (424–677)	495 (389–630)	504 (389–643)	83·7 (66·5–104)	72·2 (57–91·2)	68·2 (52·8–86·7)
1-28	Exposure to forces of nature	0 (0–0)	10 (7–15)	8 (5–11)	0 (0–0)	1·4 (0·9–1·9)	1 (0·7–1·4)
1-29	Other unintentional injuries	3,260 (2,790–3,740)	2,370 (2,050–2,730)	2,000 (1,590–2,470)	422 (364–487)	313 (270–361)	253 (201–312)
1-30	Self-harm and interpersonal violence	17,900 (15,200–20,200)	22,100 (19,500–24,900)	22,600 (17,700–28,500)	2,340 (2,000–2,650)	2,940 (2,600–3,300)	2,770 (2,170–3,500)
1-31	Self-harm	10,100 (8,620–11,600)	12,600 (11,000–14,400)	12,300 (9,430–15,700)	1,380 (1,180–1,590)	1,690 (1,480–1,930)	1,520 (1,170–1,940)
1-32	Self-harm by firearm	151 (114–212)	189 (148–304)	156 (108–248)	21·1 (16·4–28·7)	25·5 (20–40·3)	19·2 (13·5–30·5)
1-33	Self-harm by other specified means	9,970 (8,480–11,400)	12,400 (10,800–14,100)	12,100 (9,320–15,500)	1,360 (1,160–1,560)	1,670 (1,460–1,890)	1,500 (1,150–1,910)
1-34	Interpersonal violence	7,680 (6,510–8,740)	9,370 (8,250–10,500)	10,300 (7,920–13,000)	945 (802–1,080)	1,230 (1,080–1,380)	1,240 (963–1,570)
1-35	Physical violence by firearm	2,110 (1,760–2,480)	4,600 (3,980–5,270)	4,740 (3,660–6,000)	250 (208–293)	597 (516–685)	564 (436–714)
1-36	Physical violence by sharp object	1,010 (844–1,180)	2,430 (2,120–2,730)	2,900 (2,190–3,690)	124 (104–144)	317 (276–357)	348 (263–442)
1-37	Sexual violence	140 (92–199)	136 (90–193)	145 (95–206)	17·8 (11·7–25·3)	17·7 (11·7–25·2)	17·8 (11·7–25·2)
1-38	Physical violence by other means	4,420 (3,730–5,020)	2,210 (1,950–2,470)	2,500 (1,920–3,200)	553 (471–629)	295 (261–329)	314 (243–402)
1-39	Conflict and terrorism	65 (43–97)	77 (51–116)	57 (38–84)	9·1 (6·1–13·6)	10·4 (7–15·5)	7·4 (5–10·8)
1-40	Executions and police conflict	5 (5–6)	48 (36–64)	36 (18–66)	0·6 (0·5–0·7)	6·2 (4·6–8·2)	4·3 (2·2–7·6)

SR Table 1: All-age DALYs counts and age standardized DALYs rates per 100,000 individuals for all causes and level 1 cause groups by location for 1990, 2010, and 2019, both sexes combined, Haiti

Cause Level	Cause	All-age DALYs			Age-standardized rate of DALYs per 100,000		
		1990	2010	2019	1990	2010	2019
0-0	All causes	5,500,000 (5,040,000–5,960,000)	19,300,000 (17,900,000–20,900,000)	5,860,000 (5,010,000–6,820,000)	79,800 (73,000–86,500)	184,000 (171,000–200,000)	53,000 (45,200–62,200)
1-0	Communicable, maternal, neonatal, and nutritional disorders	2,990,000 (2,620,000–3,420,000)	2,790,000 (2,490,000–3,170,000)	2,180,000 (1,850,000–2,540,000)	32,700 (28,800–37,300)	23,400 (20,900–26,500)	16,000 (13,600–18,600)
1-1	HIV/AIDS and sexually transmitted infections	237,000 (144,000–393,000)	641,000 (499,000–808,000)	359,000 (273,000–478,000)	3,310 (2,030–5,800)	6,380 (5,100–8,010)	2,950 (2,300–3,820)
1-2	HIV/AIDS	184,000 (103,000–339,000)	577,000 (440,000–742,000)	291,000 (224,000–388,000)	2,820 (1,630–5,230)	5,940 (4,730–7,480)	2,510 (1,970–3,260)
1-3	HIV/AIDS - drug-susceptible tuberculosis	39,400 (24,700–60,000)	38,700 (23,000–61,400)	21,100 (13,100–32,900)	600 (380–911)	399 (238–634)	184 (114–286)
1-4	HIV/AIDS - Multidrug-resistant Tuberculosis without extensive drug resistance	303 (25–1,320)	274 (20–1,160)	226 (17–915)	4·6 (0·4–20)	2·8 (0·2–11·9)	2 (0·1–7·9)
1-5	HIV/AIDS - Extensively drug-resistant Tuberculosis	0 (0–0)	30 (2–127)	37 (3–149)	0 (0–0)	0·3 (0–1·3)	0·3 (0–1·3)
1-6	HIV/AIDS resulting in other diseases	144,000 (65,600–298,000)	538,000 (406,000–698,000)	270,000 (201,000–368,000)	2,220 (1,050–4,650)	5,540 (4,310–7,080)	2,320 (1,790–3,100)
1-7	Sexually transmitted infections excluding HIV	53,200 (18,900–114,000)	64,000 (25,000–129,000)	67,800 (26,700–135,000)	483 (183–998)	443 (181–882)	440 (176–866)
1-8	Syphilis	51,500 (17,400–112,000)	61,400 (22,200–127,000)	64,600 (23,700–132,000)	450 (158–968)	414 (152–855)	412 (153–837)
1-9	Chlamydial infection	203 (143–275)	334 (231–475)	401 (267–610)	3·8 (2·7–5·1)	3·5 (2·4–5)	3·3 (2·2–5)
1-10	Gonococcal infection	389 (270–518)	569 (361–865)	670 (416–1,110)	7·7 (5·3–10·3)	6·3 (3·9–10)	5·9 (3·6–10·1)
1-11	Trichomoniasis	267 (105–549)	480 (186–994)	598 (238–1,250)	5·3 (2·1–10·9)	5·3 (2·1–10·8)	5·1 (2–10·6)
1-12	Genital herpes	223 (73–551)	398 (133–950)	506 (168–1,250)	4·7 (1·5–11·7)	4·7 (1·6–11·5)	4·7 (1·5–11·6)
1-13	Other sexually transmitted infections	599 (422–804)	822 (577–1,160)	984 (678–1,450)	11·7 (8·1–15·7)	9 (6·2–13)	8·4 (5·8–12·8)
1-14	Respiratory infections and tuberculosis	773,000 (637,000–940,000)	512,000 (416,000–617,000)	454,000 (354,000–567,000)	8,870 (7,510–10,500)	4,480 (3,700–5,390)	3,620 (2,850–4,490)
1-15	Tuberculosis	112,000 (92,100–134,000)	69,800 (54,800–87,500)	63,900 (48,100–86,300)	1,800 (1,490–2,110)	750 (583–944)	567 (423–756)
1-16	Latent tuberculosis infection	0 (0–0)	0 (0–0)	0 (0–0)	0 (0–0)	0 (0–0)	0 (0–0)
1-17	Drug-susceptible tuberculosis	111,000 (91,700–133,000)	69,300 (53,500–86,800)	63,300 (47,400–85,400)	1,790 (1,480–2,100)	746 (577–942)	561 (419–750)
1-18	Multidrug-resistant tuberculosis without extensive drug resistance	693 (68–2,940)	396 (34–1,710)	551 (48–2,380)	11·1 (1·1–47·2)	4·3 (0·4–18·7)	4·9 (0·4–20·3)
1-19	Extensively drug-resistant tuberculosis	0 (0–0)	42 (4–195)	90 (8–402)	0 (0–0)	0·5 (0–2·1)	0·8 (0·1–3·7)
1-20	Lower respiratory infections	646,000 (520,000–805,000)	425,000 (340,000–523,000)	371,000 (284,000–468,000)	6,880 (5,670–8,230)	3,580 (2,890–4,370)	2,910 (2,240–3,670)
1-21	Upper respiratory infections	11,800 (4,820–25,000)	11,900 (6,720–19,200)	13,600 (8,040–21,600)	146 (67·5–278)	105 (60·8–166)	103 (62·2–164)
1-22	Otitis media	3,340 (1,880–5,910)	4,900 (2,920–8,020)	5,540 (3,290–8,900)	45·2 (26·1–74·1)	44 (26·1–72·8)	42·4 (25–69·2)
1-23	Enteric infections	793,000 (587,000–1,050,000)	482,000 (329,000–652,000)	330,000 (179,000–535,000)	8,010 (5,970–10,500)	3,670 (2,600–4,850)	2,340 (1,340–3,660)
1-24	Diarrhoeal diseases	791,000 (585,000–1,050,000)	481,000 (328,000–651,000)	329,000 (178,000–534,000)	7,990 (5,950–10,500)	3,660 (2,590–4,840)	2,330 (1,320–3,650)
1-25	Typhoid and paratyphoid	593 (256–1,140)	477 (204–893)	397 (167–775)	6·9 (3–13·1)	3·7 (1·6–6·9)	2·7 (1·1–5·2)
1-26	Typhoid fever	540 (233–1,040)	423 (180–801)	283 (109–561)	6·3 (2·8–11·7)	3·3 (1·4–6·2)	1·9 (0·8–3·8)
1-27	Paratyphoid fever	54 (19–121)	54 (19–126)	114 (41–260)	0·6 (0·2–1·4)	0·4 (0·2–1)	0·8 (0·3–1·8)
1-28	iNTS	214 (105–399)	277 (135–504)	324 (142–590)	2·8 (1·4–4·8)	2·4 (1·2–4·2)	2·4 (1·1–4·2)
1-29	Other intestinal infectious diseases	1,030 (544–1,650)	561 (224–964)	508 (198–881)	10·4 (5·6–16·4)	4·2 (1·8–7)	3·4 (1·4–5·9)
1-30	Neglected tropical diseases and malaria	70,500 (46,000–104,000)	146,000 (81,200–261,000)	54,100 (28,600–113,000)	1,100 (738–1,580)	1,360 (765–2,420)	434 (234–920)
1-31	Malaria	28,600 (8,590–58,500)	114,000 (52,000–228,000)	26,200 (3,790–84,200)	394 (118–803)	1,040 (476–2,090)	206 (30·4–658)
1-32	Chagas disease	0 (0–0)	0 (0–0)	0 (0–0)	0 (0–0)	0 (0–0)	0 (0–0)
1-33	Leishmaniasis	0 (0–0)	0 (0–0)	0 (0–0)	0 (0–0)	0 (0–0)	0 (0–0)
1-34	Visceral leishmaniasis	0 (0–0)	0 (0–0)	0 (0–0)	0 (0–0)	0 (0–0)	0 (0–0)
1-35	Cutaneous and mucocutaneous leishmaniasis	0 (0–0)	0 (0–0)	0 (0–0)	0 (0–0)	0 (0–0)	0 (0–0)
1-36	African trypanosomiasis	0 (0–0)	0 (0–0)	0 (0–0)	0 (0–0)	0 (0–0)	0 (0–0)
1-37	Schistosomiasis	0 (0–0)	0 (0–0)	0 (0–0)	0 (0–0)	0 (0–0)	0 (0–0)
1-38	Cysticercosis	5,430 (3,390–7,840)	5,790 (3,550–8,770)	6,510 (3,940–9,710)	118 (73·2–168)	76·8 (47·4–115)	68·1 (42–99·7)
1-39	Cystic echinococcosis	22 (8–42)	10 (5–17)	8 (4–14)	0·3 (0·1–0·5)	0·1 (0·1–0·2)	0·1 (0–0·1)
1-40	Lymphatic filariasis	21,600 (14,400–30,400)	8,470 (4,990–13,600)	4,020 (2,190–7,170)	394 (264–551)	94·6 (57–149)	33·7 (18·5–60)
1-41	Onchocerciasis	0 (0–0)	0 (0–0)	0 (0–0)	0 (0–0)	0 (0–0)	0 (0–0)
1-42	Trachoma	0 (0–0)	0 (0–0)	0 (0–0)	0 (0–0)	0 (0–0)	0 (0–0)
1-43	Dengue	885 (365–1,810)	2,030 (832–3,980)	2,160 (607–4,250)	12·1 (5·7–23·6)	17·7 (7·8–32·1)	16·2 (4·7–30·8)
1-44	Yellow fever	0 (0–0)	0 (0–0)	0 (0–0)	0 (0–0)	0 (0–0)	0 (0–0)
1-45	Rabies	153 (26–352)	55 (13–128)	56 (15–125)	1·7 (0·3–3·7)	0·4 (0·1–1)	0·4 (0·1–0·9)
1-46	Intestinal nematode infections	9,230 (6,030–13,400)	8,990 (5,710–13,700)	7,860 (4,900–11,900)	125 (81·9–186)	77·4 (49·1–119)	57·1 (35·8–86·2)
1-47	Ascariasis	2,830 (1,740–4,260)	1,070 (658–1,630)	862 (520–1,330)	31·5 (20–47·5)	8·6 (5·4–12·8)	6·1 (3·8–9·3)
1-48	Trichuriasis	1,360 (638–2,480)	69 (30–135)	76 (34–144)	18·8 (8·9–34·4)	0·6 (0·3–1·2)	0·6 (0·3–1·1)

1-49	Hookworm disease	5,040 (3,010–7,910)	7,850 (4,720–12,200)	6,920 (4,090–10,700)	74·9 (44·9–118)	68·2 (40·8–106)	50·4 (29·9–78)
1-50	Food-borne trematodiases	0 (0–0)	0 (0–0)	0 (0–0)	0 (0–0)	0 (0–0)	0 (0–0)
1-51	Leprosy	50 (30–77)	66 (40–105)	75 (46–116)	1·1 (0·7–1·7)	0·8 (0·5–1·3)	0·7 (0·5–1·1)
1-52	Ebola virus disease	0 (0–0)	0 (0–0)	0 (0–0)	0 (0–0)	0 (0–0)	0 (0–0)
1-53	Zika virus disease	0 (0–0)	0 (0–0)	3 (0–12)	0 (0–0)	0 (0–0)	0 (0–0·1)
1-54	Guinea worm disease	0 (0–0)	0 (0–0)	0 (0–0)	0 (0–0)	0 (0–0)	0 (0–0)
1-55	Other neglected tropical diseases	4,500 (2,820–6,900)	6,130 (4,040–8,760)	7,130 (4,770–10,500)	56·9 (36·9–83·5)	51·4 (34·3–72·9)	51·6 (34·8–75·6)
1-56	Other infectious diseases	369,000 (254,000–615,000)	233,000 (151,000–390,000)	188,000 (112,000–326,000)	3,740 (2,630–6,080)	1,740 (1,150–2,830)	1,290 (791–2,180)
1-57	Meningitis	159,000 (118,000–205,000)	105,000 (78,300–140,000)	81,700 (55,800–115,000)	1,560 (1,170–2,000)	758 (575–1,000)	546 (376–759)
1-58	Encephalitis	10,900 (5,940–28,100)	8,780 (4,880–18,500)	10,100 (5,830–19,300)	120 (68·7–284)	70·6 (39·8–141)	72·1 (42·4–132)
1-59	Diphtheria	3,010 (1,080–6,520)	577 (211–1,220)	346 (141–685)	30·3 (11·5–65)	4·3 (1·7–8·8)	2·3 (1–4·6)
1-60	Whooping cough	104,000 (9,540–371,000)	75,900 (6,280–229,000)	59,400 (6,380–187,000)	981 (90·3–3,560)	533 (44·2–1,610)	386 (41·5–1,210)
1-61	Tetanus	67,000 (36,800–113,000)	20,900 (11,100–38,200)	13,200 (6,300–29,400)	712 (423–1,110)	164 (89–285)	94·8 (46·9–197)
1-62	Measles	1,890 (1,240–2,790)	0 (0–0)	0 (0–0)	18·4 (12–27·2)	0 (0–0)	0 (0–0)
1-63	Varicella and herpes zoster	5,110 (2,720–8,800)	4,500 (2,570–7,500)	4,190 (2,380–6,900)	62·9 (39·1–97·8)	39·7 (25·2–60·7)	33 (20·8–50·9)
1-64	Acute hepatitis	9,040 (4,140–14,900)	5,600 (2,660–8,710)	5,150 (2,630–7,710)	125 (63·6–194)	52·7 (24·6–78·3)	41 (20·8–60·2)
1-65	Acute hepatitis A	6,900 (2,640–12,300)	3,760 (1,520–6,340)	3,270 (1,380–5,270)	91·8 (38·7–150)	34·1 (13·5–56·3)	25·3 (10·4–40·4)
1-66	Acute hepatitis B	1,370 (224–4,840)	1,350 (409–3,210)	1,390 (477–3,030)	20·5 (4·2–65·7)	13·3 (4·4–29·5)	11·5 (4–24·2)
1-67	Acute hepatitis C	612 (213–1,820)	343 (127–840)	322 (134–720)	10·4 (3·9–32·3)	3·8 (1·4–9·1)	2·9 (1·2–6·8)
1-68	Acute hepatitis E	152 (52–436)	147 (77–351)	165 (90–344)	2·2 (0·9–5·9)	1·4 (0·7–3·3)	1·4 (0·7–2·9)
1-69	Other unspecified infectious diseases	9,070 (4,760–16,500)	12,100 (6,650–20,700)	13,800 (8,000–23,500)	127 (75·4–204)	113 (67·1–178)	111 (66–178)
1-70	Maternal and neonatal disorders	475,000 (364,000–600,000)	614,000 (502,000–746,000)	630,000 (500,000–783,000)	4,740 (3,690–5,860)	4,410 (3,640–5,330)	4,120 (3,290–5,100)
1-71	Maternal disorders	73,700 (57,200–96,000)	100,000 (74,400–133,000)	90,600 (64,900–121,000)	1,270 (983–1,660)	955 (702–1,270)	684 (485–921)
1-72	Maternal haemorrhage	6,260 (4,480–8,510)	13,300 (9,360–18,400)	12,500 (8,980–17,100)	107 (76·8–146)	127 (89–176)	94·3 (67–128)
1-73	Maternal sepsis and other pregnancy related infections	12,200 (8,470–17,300)	20,900 (14,500–29,100)	19,200 (12,900–26,800)	207 (143–296)	196 (136–276)	144 (95·8–202)
1-74	Maternal hypertensive disorders	5,570 (4,010–7,460)	14,900 (10,600–20,300)	14,700 (10,600–19,800)	91·9 (66–124)	136 (96·4–186)	109 (78·4–146)
1-75	Maternal obstructed labour and uterine rupture	61 (30–104)	257 (180–359)	346 (241–491)	1 (0·5–1·7)	2·4 (1·7–3·4)	2·6 (1·8–3·7)
1-76	Maternal abortive outcome	45,900 (34,600–61,000)	33,500 (23,700–45,800)	22,900 (15,500–31,900)	800 (604–1,070)	326 (230–446)	177 (120–248)
1-77	Ectopic pregnancy	275 (192–386)	1,870 (1,270–2,650)	2,120 (1,410–2,950)	4·6 (3·2–6·5)	17·3 (11·6–24·6)	15·7 (10·5–22·1)
1-78	Indirect maternal deaths	189 (129–269)	1,590 (1,090–2,250)	2,260 (1,540–3,140)	3·1 (2·1–4·5)	14·4 (9·9–20·7)	16·7 (11·4–23·3)
1-79	Late maternal deaths	1,050 (718–1,540)	2,030 (1,340–2,990)	2,190 (1,440–3,190)	17·9 (12·3–26)	19·1 (12·6–28)	16·5 (10·8–24)
1-80	Maternal deaths aggravated by HIV/AIDS	841 (311–1,740)	1,240 (635–2,020)	1,140 (592–1,880)	14·1 (5·2–29·6)	12·3 (6·3–19·8)	8·9 (4·6–14·7)
1-81	Other maternal disorders	1,350 (926–1,910)	11,000 (7,270–15,800)	13,100 (8,790–18,700)	23·1 (16–32·4)	105 (69·7–152)	100 (66·6–142)
1-82	Neonatal disorders	402,000 (295,000–530,000)	514,000 (402,000–640,000)	540,000 (410,000–684,000)	3,470 (2,560–4,560)	3,460 (2,710–4,300)	3,440 (2,620–4,350)
1-83	Neonatal preterm birth	116,000 (71,100–177,000)	149,000 (89,800–220,000)	149,000 (88,000–221,000)	1,030 (645–1,540)	1,010 (621–1,490)	952 (569–1,410)
1-84	Neonatal encephalopathy due to birth asphyxia and trauma	136,000 (88,900–197,000)	167,000 (115,000–230,000)	172,000 (115,000–244,000)	1,160 (760–1,680)	1,110 (767–1,530)	1,100 (735–1,550)
1-85	Neonatal sepsis and other neonatal infections	34,800 (18,400–59,000)	51,500 (28,200–83,600)	55,500 (31,900–88,000)	297 (158–503)	344 (188–557)	356 (206–562)
1-86	Hemolytic disease and other neonatal jaundice	7,750 (4,920–11,900)	8,190 (5,800–11,300)	8,070 (5,740–11,400)	75·7 (51·1–113)	60·3 (44·2–81·4)	54·6 (39·2–75·3)
1-87	Other neonatal disorders	107,000 (52,400–171,000)	138,000 (73,000–211,000)	155,000 (72,400–248,000)	916 (450–1,460)	925 (489–1,410)	982 (459–1,570)
1-88	Nutritional deficiencies	268,000 (186,000–395,000)	166,000 (120,000–224,000)	165,000 (120,000–219,000)	2,960 (2,140–4,150)	1,390 (1,030–1,860)	1,230 (909–1,610)
1-89	Protein-energy malnutrition	212,000 (132,000–330,000)	83,900 (54,400–125,000)	69,600 (44,000–106,000)	2,120 (1,370–3,250)	638 (424–927)	490 (318–735)
1-90	Iodine deficiency	1,610 (922–2,560)	2,530 (1,410–4,050)	2,800 (1,550–4,570)	28·2 (16·2–44·9)	25·9 (14·7–41·2)	23 (12·9–37·2)
1-91	Vitamin A deficiency	3,820 (2,390–5,520)	3,910 (2,450–5,940)	3,870 (2,450–5,860)	40·5 (25·3–58·4)	29·8 (18·6–45·1)	26·1 (16·7–39·4)
1-92	Dietary iron deficiency	46,700 (30,100–68,400)	73,000 (46,900–108,000)	86,500 (56,800–128,000)	708 (470–1,030)	676 (439–989)	675 (449–996)
1-93	Other nutritional deficiencies	4,440 (2,750–6,880)	2,270 (1,410–3,430)	2,290 (1,480–3,320)	64·3 (45·2–91·1)	23·6 (15·7–33·1)	20·5 (13·9–28·7)
1-0	Non-communicable diseases	1,880,000 (1,590,000–2,150,000)	2,400,000 (2,050,000–2,770,000)	2,870,000 (2,380,000–3,470,000)	38,300 (34,000–42,500)	31,700 (27,100–36,600)	30,700 (25,500–37,100)
1-1	Neoplasms	209,000 (128,000–264,000)	276,000 (178,000–357,000)	345,000 (227,000–457,000)	4,770 (3,080–5,710)	4,030 (2,670–5,100)	3,960 (2,650–5,180)
1-2	Lip and oral cavity cancer	2,580 (1,520–3,800)	3,350 (2,050–4,910)	4,370 (2,620–6,620)	70·2 (40·7–103)	55·6 (34–81·7)	55·6 (33·2–84·2)
1-3	Nasopharynx cancer	1,030 (614–1,350)	1,590 (966–2,140)	2,040 (1,230–2,840)	24·1 (15·3–30·7)	22·7 (14·4–29·9)	22·6 (14–31·4)
1-4	Other pharynx cancer	1,340 (1,010–2,260)	1,630 (1,090–2,600)	2,090 (1,330–3,390)	36·9 (27·7–61·7)	27·1 (18·2–43)	26·6 (16·8–43)
1-5	Oesophageal cancer	4,120 (2,730–5,870)	5,310 (3,570–7,970)	6,830 (4,460–10,300)	116 (78–168)	91·4 (61·9–137)	89·8 (59·4–135)
1-6	Stomach cancer	21,200 (14,100–25,500)	24,200 (15,400–31,500)	29,100 (18,200–39,800)	587 (401–703)	406 (260–526)	374 (238–506)
1-7	Colon and rectum cancer	8,510 (5,600–10,600)	13,700 (9,450–18,000)	18,300 (12,600–25,400)	238 (156–294)	233 (162–303)	240 (166–329)

1·8	Liver cancer	6,550 (4,050–8,890)	6,990 (4,290–10,100)	8,490 (5,110–12,700)	175 (109–239)	115 (69·8–167)	107 (64·8–160)
1·9	Liver cancer due to hepatitis B	2,160 (1,260–3,250)	2,360 (1,320–3,820)	2,900 (1,630–4,820)	55·5 (33·1–85·9)	36·4 (20·3–60·8)	34·1 (18·9–56·6)
1·10	Liver cancer due to hepatitis C	1,220 (647–2,010)	1,280 (677–2,020)	1,510 (792–2,460)	38·3 (21·1–62·4)	24·4 (13–38·5)	22·1 (11·9–35·9)
1·11	Liver cancer due to alcohol use	1,820 (1,050–2,850)	1,990 (1,090–3,200)	2,450 (1,330–4,200)	53 (30·8–83·7)	35·5 (19·6–58·7)	33·6 (18–57·7)
1·12	Liver cancer due to NASH	370 (200–589)	419 (226–668)	514 (265–859)	10·8 (5·9–17·2)	7·3 (3·9–11·8)	6·9 (3·5–11·5)
1·13	Gallbladder and biliary tract cancer	3,520 (2,020–4,870)	3,370 (1,840–5,070)	4,050 (2,190–6,390)	100 (59–137)	58·5 (32·6–87·2)	53·9 (29·6–83·6)
1·14	Pancreatic cancer	1,060 (792–1,340)	2,970 (2,050–4,050)	4,410 (2,990–6,310)	30 (22·9–37·6)	51·6 (36–69·6)	58·7 (39·7–83)
1·15	Larynx cancer	2,780 (1,550–3,620)	3,500 (2,080–4,800)	4,520 (2,650–6,610)	78 (43·8–100)	60·8 (36·1–82·7)	59·9 (35·4–86·7)
1·16	Tracheal, bronchus, and lung cancer	12,700 (7,570–20,300)	16,500 (10,100–25,600)	21,500 (13,200–34,800)	355 (213–570)	285 (176–448)	284 (176–462)
1·17	Malignant skin melanoma	995 (502–1,900)	1,490 (776–2,680)	1,920 (1,000–3,390)	23·7 (12·8–41·6)	21·6 (11·7–37·1)	21·4 (11·5–36·4)
1·18	Non-melanoma skin cancer	431 (321–565)	886 (618–1,200)	1,180 (790–1,640)	13 (9·5–16·4)	16·2 (11·3–21·4)	16·5 (11·1–22·5)
1·19	Non-melanoma skin cancer (squamous-cell carcinoma)	431 (321–565)	886 (618–1,200)	1,180 (790–1,640)	13 (9·5–16·4)	16·2 (11·3–21·4)	16·5 (11·1–22·5)
1·20	Non-melanoma skin cancer (basal-cell carcinoma)	0 (0–0)	0 (0–0)	0 (0–0)	0 (0–0)	0 (0–0)	0 (0–0)
1·21	Breast cancer	17,500 (13,000–23,600)	31,900 (20,600–45,900)	42,700 (27,100–63,500)	439 (339–568)	469 (314–653)	474 (303–690)
1·22	Cervical cancer	29,600 (11,400–41,000)	39,000 (16,700–57,100)	46,400 (21,200–69,700)	696 (287–944)	536 (236–772)	484 (222–724)
1·23	Uterine cancer	3,220 (2,190–4,470)	5,240 (3,350–7,430)	6,630 (4,330–9,750)	86 (59·7–121)	83·9 (55·2–117)	81·2 (53–118)
1·24	Ovarian cancer	1,130 (572–4,740)	3,270 (1,560–12,100)	4,710 (2,340–15,200)	27·6 (14·3–112)	47·9 (23·3–173)	53·1 (26·7–167)
1·25	Prostate cancer	11,400 (8,130–14,800)	19,700 (13,600–27,600)	25,300 (17,300–35,600)	408 (287–533)	445 (306–612)	444 (308–608)
1·26	Testicular cancer	166 (98–324)	357 (218–580)	494 (303–781)	2·5 (1·6–4·2)	3·4 (2·1–5·1)	3·9 (2·4–5·9)
1·27	Kidney cancer	2,870 (1,880–4,130)	3,110 (2,300–4,160)	3,990 (2,850–5,440)	50·9 (39·3–64)	37·7 (28·5–51)	39·6 (28–54·5)
1·28	Bladder cancer	2,060 (1,380–2,750)	2,970 (1,840–4,250)	3,820 (2,350–5,620)	64·4 (43·3–85·1)	57·2 (36·1–80·5)	56·6 (35·3–82·8)
1·29	Brain and nervous system cancer	5,620 (2,370–14,600)	10,900 (4,360–23,400)	13,800 (5,790–27,600)	78·7 (39·3–187)	108 (46·5–211)	114 (50·7–216)
1·30	Thyroid cancer	1,010 (600–1,400)	1,430 (863–2,050)	1,800 (1,120–2,660)	25·9 (16–35·3)	22·2 (13·8–31·4)	21·6 (13·6–31·6)
1·31	Mesothelioma	431 (229–862)	675 (367–1,260)	884 (494–1,570)	10·7 (6–19·9)	9·9 (5·5–17·2)	10 (5·7–17·1)
1·32	Hodgkin lymphoma	1,450 (1,130–1,800)	2,150 (1,510–2,870)	2,720 (1,820–3,750)	29 (23·1–36·5)	24·9 (17·3–34·1)	24·7 (16·6–35·1)
1·33	Non-Hodgkin's lymphoma	9,780 (5,230–14,900)	10,600 (7,100–14,900)	13,400 (9,380–18,600)	167 (108–230)	123 (88·4–164)	126 (88·4–171)
1·34	Multiple myeloma	2,340 (1,500–4,530)	3,690 (2,230–7,020)	4,790 (2,780–9,010)	67·7 (42·8–128)	64·9 (39–120)	64·5 (37·7–118)
1·35	Leukaemia	32,300 (12,600–63,600)	32,100 (16,600–57,200)	36,800 (20,200–61,600)	425 (201–778)	300 (167–506)	294 (168–476)
1·36	Acute lymphoid leukaemia	7,690 (2,380–18,400)	9,510 (4,390–17,000)	11,400 (5,580–20,000)	86 (29·7–196)	75 (36·2–132)	78·8 (39·5–136)
1·37	Chronic lymphoid leukaemia	210 (135–363)	343 (210–551)	466 (289–768)	6·1 (3·9–10·9)	6·2 (3·7–10·1)	6·4 (4–10·6)
1·38	Acute myeloid leukaemia	3,060 (1,410–6,430)	4,180 (2,240–7,830)	5,310 (2,870–9,370)	42·5 (22·6–79·8)	39·4 (22·5–68·5)	42·3 (23·1–71·7)
1·39	Chronic myeloid leukaemia	3,230 (767–9,710)	2,660 (980–6,590)	2,840 (1,210–6,260)	45·5 (16·9–114)	27 (12·6–58·4)	24·5 (11·6–49·8)
1·40	Other leukaemia	18,100 (4,160–38,900)	15,400 (4,830–28,400)	16,800 (5,750–29,600)	245 (73·9–471)	153 (54·9–262)	142 (54·5–236)
1·41	Other neoplasms	525 (344–1,120)	786 (548–1,450)	1,010 (684–1,770)	10·7 (8–19·4)	10·8 (7·3–18·3)	11·2 (7·4–18·9)
1·42	Myelodysplastic, myeloproliferative, and other hematopoietic neoplasms	525 (344–1,120)	786 (548–1,450)	1,010 (684–1,770)	10·7 (8–19·4)	10·8 (7·3–18·3)	11·2 (7·4–18·9)
1·43	Benign and in situ intestinal neoplasms	0 (0–0)	0 (0–0)	0 (0–0)	0 (0–0)	0 (0–0)	0 (0–0)
1·44	Benign and in situ cervical and uterine neoplasms	0 (0–0)	0 (0–0)	0 (0–0)	0 (0–0)	0 (0–0)	0 (0–0)
1·45	Other benign and in situ neoplasms	0 (0–0)	0 (0–0)	0 (0–0)	0 (0–0)	0 (0–0)	0 (0–0)
1·46	Cardiovascular diseases	488,000 (422,000–563,000)	592,000 (460,000–756,000)	718,000 (527,000–966,000)	13,000 (11,100–15,300)	10,000 (7,880–12,900)	9,490 (7,050–12,700)
1·47	Rheumatic heart disease	40,900 (27,600–57,100)	36,200 (23,100–55,200)	38,600 (24,300–59,600)	738 (501–1,000)	404 (253–604)	342 (213–524)
1·48	Ischaemic heart disease	168,000 (140,000–204,000)	214,000 (160,000–282,000)	269,000 (189,000–376,000)	5,090 (4,250–6,180)	4,000 (3,040–5,200)	3,850 (2,770–5,320)
1·49	Stroke	190,000 (154,000–225,000)	227,000 (161,000–309,000)	271,000 (182,000–387,000)	5,050 (3,830–6,130)	3,840 (2,680–5,240)	3,590 (2,440–5,110)
1·50	Ischaemic stroke	43,300 (33,400–57,200)	61,000 (44,300–88,600)	76,500 (54,300–116,000)	1,470 (1,050–1,930)	1,280 (904–1,860)	1,260 (884–1,890)
1·51	Intracerebral hemorrhage	119,000 (96,300–145,000)	132,000 (92,300–181,000)	154,000 (100,000–222,000)	2,990 (2,320–3,680)	2,110 (1,430–2,890)	1,910 (1,250–2,750)
1·52	Subarachnoid hemorrhage	27,500 (9,370–45,300)	34,300 (15,300–58,000)	40,500 (19,500–70,900)	590 (214–980)	454 (210–781)	420 (201–744)
1·53	Hypertensive heart disease	30,600 (19,900–48,900)	42,700 (24,800–68,100)	52,900 (30,900–82,800)	947 (626–1,640)	801 (471–1,310)	762 (450–1,180)
1·54	Non-rheumatic valvular heart disease	2,290 (1,320–3,440)	3,170 (1,750–5,040)	4,140 (2,310–6,680)	59·8 (36·6–85)	49·2 (29·2–73·6)	49·2 (29–76·5)
1·55	Non-rheumatic calcific aortic valve disease	1,030 (527–1,630)	1,470 (767–2,430)	1,960 (1,040–3,320)	29·1 (15·7–42·8)	25 (13·8–38·3)	25·5 (14·3–40·7)
1·56	Non-rheumatic degenerative mitral valve disease	1,130 (648–1,830)	1,530 (830–2,580)	1,970 (1,080–3,220)	27·7 (16·7–42·3)	21·9 (12·6–34·8)	21·6 (12·4–33·8)
1·57	Other non-rheumatic valve diseases	127 (69–237)	165 (82–318)	207 (108–386)	3 (1·8–5·1)	2·2 (1·2–4)	2·1 (1·2–3·7)
1·58	Cardiomyopathy and myocarditis	21,900 (10,100–41,200)	26,700 (14,200–46,000)	32,200 (17,000–55,000)	392 (215–627)	319 (184–498)	316 (179–513)
1·59	Myocarditis	4,200 (797–14,400)	4,200 (1,440–10,900)	4,580 (1,730–10,600)	52·4 (17·4–148)	38·1 (16·3–83·5)	36·3 (15·9–75·2)
1·60	Alcoholic cardiomyopathy	3,320 (1,330–5,460)	3,540 (1,750–6,020)	4,390 (2,170–7,490)	82 (34·9–137)	52·4 (26·5–88·7)	49·6 (25·3–85·1)

1-61	Other cardiomyopathy	14,300 (6,790–28,700)	19,000 (9,690–32,900)	23,200 (12,100–39,800)	258 (141–420)	229 (126–364)	230 (127–380)
1-62	Atrial fibrillation and flutter	2,530 (1,940–3,340)	4,120 (3,100–5,670)	5,240 (3,900–7,240)	106 (79·1–139)	101 (77·5–138)	98·4 (74·6–135)
1-63	Aortic aneurysm	2,280 (1,420–3,390)	3,300 (2,010–5,020)	4,290 (2,520–6,530)	71·4 (46·1–104)	63·3 (39·5–94·1)	62·5 (37·6–94·7)
1-64	Peripheral vascular disease	635 (437–952)	1,030 (712–1,620)	1,370 (923–2,140)	23·8 (16·2–35·3)	23·2 (16·1–35·3)	23·7 (16·2–36·2)
1-65	Endocarditis	3,500 (1,850–6,660)	4,240 (2,660–7,300)	5,080 (3,250–8,570)	53·5 (36·5–89)	45 (30·4–70·7)	45·1 (29·7–71·9)
1-66	Chronic respiratory diseases	126,000 (86,400–163,000)	140,000 (101,000–178,000)	156,000 (113,000–200,000)	2,300 (1,690–2,910)	1,780 (1,270–2,330)	1,630 (1,180–2,140)
1-67	Chronic obstructive pulmonary disease	29,600 (19,200–40,900)	43,600 (28,200–60,000)	52,100 (34,300–70,700)	865 (558–1,190)	810 (523–1,120)	754 (500–1,040)
1-68	Pneumoconiosis	235 (99–376)	262 (111–450)	313 (132–566)	6·3 (2·6–10·2)	4·4 (1·8–7·7)	4 (1·6–7·3)
1-69	Silicosis	73 (13–164)	75 (15–178)	88 (18–213)	2 (0·4–4·7)	1·3 (0·2–3·1)	1·2 (0·2–2·9)
1-70	Asbestosis	30 (12–57)	38 (16–76)	48 (19–94)	0·8 (0·3–1·5)	0·6 (0·2–1·2)	0·6 (0·2–1·2)
1-71	Coal workers pneumoconiosis	36 (10–74)	35 (9–73)	41 (10–87)	1 (0·3–2)	0·6 (0·2–1·3)	0·5 (0·1–1·1)
1-72	Other pneumoconiosis	97 (46–171)	114 (53–205)	135 (62–241)	2·6 (1·2–4·5)	1·9 (0·9–3·4)	1·7 (0·8–3)
1-73	Asthma	87,000 (57,100–119,000)	83,000 (58,000–111,000)	88,600 (63,400–116,000)	1,280 (913–1,690)	829 (580–1,130)	737 (525–979)
1-74	Interstitial lung disease and pulmonary sarcoidosis	1,920 (1,040–3,790)	3,470 (2,040–5,720)	4,400 (2,630–7,110)	46·4 (27·9–72·7)	52·5 (31·2–82·7)	52 (30·2–80·1)
1-75	Other chronic respiratory diseases	7,540 (1,940–21,000)	9,600 (4,150–21,500)	11,000 (5,510–21,300)	97·8 (33·3–238)	86·8 (41·9–178)	85·9 (45·3–155)
1-76	Digestive diseases	155,000 (83,100–230,000)	165,000 (100,000–229,000)	191,000 (118,000–267,000)	3,040 (1,930–3,960)	2,150 (1,380–2,800)	2,000 (1,260–2,720)
1-77	Cirrhosis and other chronic liver diseases	53,900 (25,900–80,000)	60,300 (31,300–86,600)	73,300 (37,000–109,000)	1,230 (678–1,680)	872 (469–1,220)	823 (428–1,200)
1-78	Cirrhosis and other chronic liver diseases due to hepatitis B	5,830 (2,820–9,320)	6,990 (3,460–11,500)	8,840 (4,370–14,300)	147 (74·9–233)	104 (52·5–168)	99·5 (48·3–161)
1-79	Cirrhosis and other chronic liver diseases due to hepatitis C	8,980 (4,340–13,800)	10,800 (5,130–17,100)	13,400 (6,580–21,300)	226 (115–344)	162 (80·6–255)	152 (74·3–242)
1-80	Cirrhosis and other chronic liver diseases due to alcohol use	16,700 (8,800–23,600)	20,300 (10,300–29,800)	25,600 (12,900–39,200)	438 (236–615)	321 (164–467)	306 (156–465)
1-81	Cirrhosis and other chronic liver diseases due to NAFLD	6,030 (3,080–9,660)	7,530 (3,700–12,200)	9,390 (4,550–15,600)	160 (87·4–249)	120 (60·7–191)	114 (55·4–188)
1-82	Cirrhosis and other chronic liver diseases due to other causes	16,400 (4,510–33,200)	14,600 (5,750–25,400)	16,000 (6,300–27,300)	264 (102–454)	167 (74–265)	152 (65·8–246)
1-83	Upper digestive system diseases	32,700 (22,600–44,800)	33,700 (23,800–45,500)	38,800 (27,500–52,400)	739 (556–942)	474 (346–629)	424 (302–564)
1-84	Peptic ulcer disease	22,300 (14,000–31,900)	17,600 (11,500–25,000)	18,700 (12,400–26,100)	518 (365–678)	272 (187–374)	227 (155–315)
1-85	Gastritis and duodenitis	4,630 (2,630–7,100)	5,910 (3,920–8,030)	7,090 (4,850–9,640)	98·7 (60·7–143)	79·8 (53–110)	75·4 (52·7–104)
1-86	GERD	5,740 (2,980–10,100)	10,200 (5,320–18,100)	13,000 (6,740–23,200)	122 (63·7–217)	122 (63·9–217)	122 (63·9–216)
1-87	Appendicitis	13,000 (2,120–29,500)	9,240 (2,890–17,700)	9,810 (3,550–17,600)	176 (36·4–354)	90·2 (32·6–159)	80·8 (32·6–138)
1-88	Paralytic ileus and intestinal obstruction	17,300 (5,710–40,200)	19,100 (8,470–38,700)	20,300 (9,330–38,600)	233 (99·2–447)	200 (111–338)	185 (100–309)
1-89	Inguinal, femoral, and abdominal hernia	17,800 (6,510–35,200)	16,400 (8,680–28,400)	17,400 (9,920–28,100)	229 (107–392)	161 (94·2–247)	149 (88·7–229)
1-90	Inflammatory bowel disease	3,030 (946–6,880)	3,220 (1,510–5,420)	3,780 (1,880–6,100)	52 (22·4–92·4)	38·9 (20·4–58·1)	36·9 (19·9–54·2)
1-91	Vascular intestinal disorders	1,030 (635–1,580)	1,260 (840–1,840)	1,580 (1,020–2,370)	29·5 (20·5–39·3)	22·3 (15·8–30·3)	21·6 (14·5–30·6)
1-92	Gallbladder and biliary diseases	7,320 (4,570–10,100)	9,060 (5,790–12,500)	10,700 (7,070–14,700)	172 (115–225)	128 (82·1–175)	119 (77·1–161)
1-93	Pancreatitis	3,630 (2,320–5,540)	4,630 (3,030–6,890)	5,880 (3,860–8,890)	82·1 (57·7–121)	63·1 (41·8–95·9)	61·4 (40·4–93·4)
1-94	Other digestive diseases	5,310 (1,610–13,400)	8,000 (3,670–16,900)	9,550 (4,770–18,700)	88·1 (42·4–173)	99·3 (57·7–170)	97·9 (57·2–166)
1-95	Neurological disorders	82,500 (46,100–129,000)	115,000 (65,200–188,000)	141,000 (76,000–231,000)	1,610 (974–2,450)	1,410 (854–2,250)	1,390 (808–2,190)
1-96	Alzheimer's disease and other dementias	6,750 (2,570–16,400)	11,200 (4,390–27,200)	13,800 (5,590–34,400)	360 (138–887)	328 (128–800)	309 (125–738)
1-97	Parkinson's disease	2,570 (1,810–3,490)	4,260 (2,970–5,720)	5,300 (3,660–7,460)	98·1 (71·2–131)	97·8 (69·4–131)	96 (66·9–134)
1-98	Idiopathic epilepsy	33,800 (14,300–51,900)	34,400 (19,000–52,200)	40,500 (21,800–60,200)	486 (233–734)	324 (187–484)	321 (181–472)
1-99	Multiple sclerosis	519 (358–937)	940 (627–1,680)	1,280 (843–2,200)	11·6 (8·1–20·1)	12 (8·2–20·7)	12·5 (8·3–20·9)
1-100	Motor neuron disease	698 (394–1,230)	1,040 (672–1,660)	1,380 (893–2,200)	10·4 (7·3–15·3)	11·6 (8·1–16·2)	13 (8·6–18·8)
1-101	Headache disorders	31,100 (5,310–69,800)	54,600 (9,460–122,000)	67,100 (11,900–150,000)	550 (107–1,210)	552 (108–1,200)	551 (107–1,200)
1-102	Migraine	28,400 (3,500–66,300)	49,800 (6,300–117,000)	61,100 (8,140–142,000)	498 (71·1–1,140)	500 (72·8–1,150)	500 (72·6–1,150)
1-103	Tension-type headache	2,710 (755–11,400)	4,750 (1,340–19,400)	5,930 (1,660–23,500)	51·7 (15·3–198)	51·9 (15·5–194)	51·9 (15·4–191)
1-104	Other neurological disorders	6,980 (4,010–10,400)	8,920 (5,370–13,100)	11,400 (6,930–16,800)	90·7 (55·1–130)	79·6 (50·4–114)	87·3 (55·5–127)
1-105	Mental disorders	96,500 (70,200–127,000)	166,000 (121,000–221,000)	206,000 (150,000–271,000)	1,750 (1,290–2,290)	1,730 (1,270–2,290)	1,750 (1,290–2,280)
1-106	Schizophrenia	7,660 (5,310–10,300)	13,600 (9,430–18,300)	17,400 (12,300–23,400)	155 (108–205)	153 (107–204)	153 (108–203)
1-107	Depressive disorders	34,000 (22,900–47,800)	57,700 (38,700–81,500)	72,100 (48,500–102,000)	664 (453–931)	639 (435–889)	644 (442–893)
1-108	Major depressive disorder	29,500 (19,600–42,200)	49,800 (32,600–71,400)	62,100 (41,400–87,900)	575 (383–816)	549 (365–772)	555 (375–775)
1-109	Dysthymia	4,450 (2,680–6,890)	7,930 (4,850–12,200)	10,000 (6,060–15,700)	89·6 (56–136)	89·8 (56·4–137)	89·8 (56·3–137)
1-110	Bipolar disorder	9,880 (5,680–15,700)	17,700 (10,200–28,200)	21,700 (12,500–34,300)	181 (106–284)	182 (107–285)	181 (106–280)
1-111	Anxiety disorders	23,600 (15,500–33,400)	41,300 (27,000–58,500)	52,400 (34,300–73,700)	422 (278–594)	426 (285–596)	438 (291–614)
1-112	Eating disorders	1,710 (1,040–2,570)	2,930 (1,780–4,480)	3,580 (2,160–5,390)	26·7 (16·2–40·2)	25·3 (15·4–38·5)	25·8 (15·5–38·8)
1-113	Anorexia nervosa	474 (270–776)	794 (444–1,300)	952 (550–1,570)	7 (4·1–11·4)	6·6 (3·7–10·6)	6·8 (4–11·2)

1-114	Bulimia nervosa	1,230 (715–1,930)	2,140 (1,220–3,520)	2,620 (1,480–4,140)	19·7 (11·5–30·9)	18·7 (10·7–30·4)	19 (10·8–30)
1-115	Autism spectrum disorders	3,450 (2,250–4,960)	5,520 (3,580–7,970)	6,560 (4,290–9,540)	50·6 (33–72·6)	50·6 (32·7–72·8)	50·5 (33·2–73·3)
1-116	Attention-deficit/hyperactivity disorder	2,680 (1,510–4,540)	4,360 (2,500–7,400)	5,130 (2,910–8,720)	36·4 (20·7–61·5)	36·5 (20·7–61·9)	36·6 (20·8–61·9)
1-117	Conduct disorder	5,580 (3,160–8,840)	8,590 (4,940–13,700)	9,950 (5,580–15,700)	66·6 (37·6–106)	67·2 (38·6–107)	67·2 (37·7–106)
1-118	Idiopathic developmental intellectual disability	2,960 (1,310–5,180)	5,380 (2,590–9,080)	5,700 (2,560–9,980)	42·6 (19·4–74·6)	48·2 (23·4–81·2)	43 (19·3–75·4)
1-119	Other mental disorders	5,000 (3,150–7,580)	8,850 (5,540–13,300)	11,300 (7,100–17,100)	107 (68·3–163)	107 (67·7–161)	106 (67·4–161)
1-120	Substance use disorders	20,800 (15,700–26,300)	35,000 (26,700–43,800)	43,400 (33,000–54,500)	425 (325–532)	400 (305–500)	389 (295–488)
1-121	Alcohol use disorders	15,800 (11,700–20,300)	25,300 (18,500–32,700)	31,100 (22,800–40,400)	339 (251–434)	309 (224–394)	293 (214–377)
1-122	Drug use disorders	5,010 (3,580–6,890)	9,680 (6,920–13,000)	12,300 (8,910–16,300)	85·8 (61·9–116)	90·9 (66·3–121)	95·8 (69·8–126)
1-123	Opioid use disorders	1,380 (944–1,890)	2,670 (1,830–3,730)	3,600 (2,530–4,980)	25·7 (17·8–34·9)	27·5 (18·9–37·7)	29·6 (21·1–39·9)
1-124	Cocaine use disorders	1,420 (868–2,160)	2,810 (1,780–4,210)	3,530 (2,290–5,190)	23·7 (14·8–35)	25·7 (16·7–37·6)	27·2 (18–39·3)
1-125	Amphetamine use disorders	388 (211–636)	739 (404–1,240)	912 (535–1,460)	6·4 (3·6–10·4)	6·5 (3·7–10·9)	6·8 (4·1–10·8)
1-126	Cannabis use disorders	640 (337–1,100)	1,170 (617–2,010)	1,380 (731–2,340)	10·2 (5·6–17)	10·2 (5·5–17·1)	10·2 (5·6–17·3)
1-127	Other drug use disorders	1,180 (753–1,770)	2,290 (1,470–3,410)	2,850 (1,870–4,180)	19·8 (12·9–29·1)	21 (13·8–30·7)	22 (14·6–31·7)
1-128	Diabetes and kidney diseases	146,000 (121,000–173,000)	215,000 (172,000–274,000)	278,000 (216,000–363,000)	3,690 (3,080–4,440)	3,390 (2,710–4,330)	3,410 (2,640–4,510)
1-129	Diabetes mellitus	96,300 (76,600–118,000)	141,000 (109,000–182,000)	184,000 (140,000–239,000)	2,610 (2,090–3,180)	2,340 (1,800–3,010)	2,360 (1,800–3,090)
1-130	Diabetes mellitus type 1	12,900 (7,830–18,000)	16,900 (10,900–22,700)	20,000 (13,300–26,800)	245 (142–345)	193 (123–265)	184 (121–250)
1-131	Diabetes mellitus type 2	83,400 (66,500–102,000)	124,000 (95,400–161,000)	164,000 (123,000–214,000)	2,360 (1,900–2,870)	2,140 (1,650–2,740)	2,180 (1,650–2,860)
1-132	Chronic kidney disease	48,700 (38,900–62,500)	73,500 (57,700–104,000)	92,800 (69,300–130,000)	1,070 (863–1,430)	1,050 (804–1,510)	1,040 (770–1,510)
1-133	Chronic kidney disease due to diabetes mellitus type 1	3,950 (2,530–6,120)	5,990 (3,630–9,830)	7,890 (4,690–13,400)	87·8 (55–138)	78·3 (45·9–130)	78·4 (45·2–133)
1-134	Chronic kidney disease due to diabetes mellitus type 2	7,600 (5,290–11,200)	12,600 (8,590–19,100)	16,400 (10,900–25,600)	222 (157–330)	225 (155–344)	225 (153–346)
1-135	Chronic kidney disease due to hypertension	6,140 (4,320–9,030)	11,000 (7,810–16,400)	14,100 (9,750–21,500)	183 (128–277)	198 (140–299)	195 (136–301)
1-136	Chronic kidney disease due to glomerulonephritis	13,500 (9,950–18,400)	19,400 (14,200–27,400)	24,400 (17,100–35,300)	263 (198–366)	241 (173–359)	241 (169–359)
1-137	Chronic kidney disease due to other and unspecified causes	17,400 (12,300–23,200)	24,400 (18,900–32,500)	30,000 (22,600–40,700)	319 (247–425)	304 (232–419)	303 (226–419)
1-138	Acute glomerulonephritis	648 (287–1,230)	584 (264–1,070)	625 (277–1,190)	9·5 (4·9–15·8)	5·8 (2·7–10·2)	5·2 (2·5–9·7)
1-139	Skin and subcutaneous diseases	39,500 (26,900–55,500)	62,200 (42,800–87,800)	74,100 (51,200–104,000)	643 (463–894)	637 (447–878)	634 (448–882)
1-140	Dermatitis	8,130 (4,740–13,000)	12,600 (7,320–20,400)	14,900 (8,680–23,700)	115 (68·5–180)	115 (68·3–183)	115 (68–180)
1-141	Atopic dermatitis	6,620 (3,590–11,100)	9,980 (5,350–16,700)	11,600 (6,210–19,500)	84·6 (45·8–141)	84·6 (45·3–141)	84·5 (45·5–142)
1-142	Contact dermatitis	1,330 (834–1,990)	2,330 (1,470–3,490)	2,910 (1,830–4,350)	27 (17–39·9)	27 (17·2–39·8)	27 (16·8–40·3)
1-143	Seborrhoeic dermatitis	187 (105–297)	314 (177–498)	384 (216–614)	3·4 (1·9–5·3)	3·4 (1·9–5·4)	3·4 (1·9–5·3)
1-144	Psoriasis	2,580 (1,840–3,420)	4,030 (2,820–5,440)	4,850 (3,380–6,530)	48·5 (34·6–64·1)	44·8 (31·6–60·1)	43·4 (30·5–58·2)
1-145	Bacterial skin diseases	4,230 (2,030–10,100)	6,430 (3,540–12,200)	7,640 (4,450–13,300)	62 (34·4–113)	70·4 (41–107)	71 (42·8–105)
1-146	Cellulitis	1,320 (323–4,300)	1,790 (631–3,950)	2,050 (809–4,360)	20 (6·9–46·6)	19·6 (7·9–34)	18·9 (8–34)
1-147	Pyoderma	2,910 (1,160–8,100)	4,650 (2,510–9,720)	5,580 (3,220–10,700)	42 (23·1–89·2)	50·8 (30–88)	52·1 (31·5–87·8)
1-148	Scabies	5,860 (3,170–9,470)	9,370 (5,110–15,000)	11,000 (5,970–17,400)	84·4 (46·5–135)	84·3 (46·5–134)	83·9 (45·9–134)
1-149	Fungal skin diseases	2,320 (915–4,990)	3,810 (1,500–8,200)	4,630 (1,830–9,790)	45·3 (18·3–96·4)	45 (18·1–95·8)	44·7 (18·1–94·9)
1-150	Viral skin diseases	3,790 (2,400–5,720)	5,680 (3,580–8,530)	6,590 (4,240–9,890)	47·2 (30·2–70·9)	47·2 (29·9–70·8)	47·2 (30·4–70·7)
1-151	Acne vulgaris	2,980 (1,750–4,830)	5,720 (3,420–9,120)	6,820 (4,020–11,200)	39·8 (23·4–64·1)	45·7 (27·3–72·7)	47·4 (28–77·7)
1-152	Alopecia areata	402 (255–603)	701 (443–1,040)	869 (556–1,290)	7·1 (4·5–10·6)	7·1 (4·5–10·5)	7·1 (4·6–10·6)
1-153	Pruritus	412 (196–733)	686 (331–1,230)	849 (408–1,530)	7·8 (3·7–14)	7·8 (3·7–14·1)	7·8 (3·8–14)
1-154	Urticaria	3,470 (2,250–5,090)	5,370 (3,480–7,880)	6,320 (4,170–9,130)	47·9 (31·5–69·8)	47·9 (31·1–69·3)	47·9 (31·7–68·7)
1-155	Decubitus ulcer	2,770 (1,810–4,080)	3,630 (2,390–5,010)	4,480 (2,880–6,430)	87·6 (54·6–127)	71·6 (45·9–102)	68 (42·5–97·6)
1-156	Other skin and subcutaneous diseases	2,550 (1,290–4,690)	4,160 (2,160–7,300)	5,150 (2,630–9,010)	50·4 (26·7–86·5)	50·6 (26·3–88·3)	50·5 (26·1–88·5)
1-157	Sense organ diseases	33,800 (22,900–47,500)	53,700 (36,600–76,300)	66,400 (44,900–94,600)	909 (626–1,270)	849 (582–1,200)	824 (562–1,160)
1-158	Blindness and vision loss	10,900 (7,590–15,200)	16,500 (11,400–22,900)	19,800 (13,500–27,700)	314 (222–433)	276 (193–380)	260 (181–362)
1-159	Glaucoma	639 (412–934)	864 (560–1,260)	1,000 (656–1,470)	26·6 (17·2–38·6)	21·1 (13·8–30·7)	19·2 (12·5–27·9)
1-160	Cataract	1,950 (1,340–2,740)	2,660 (1,830–3,690)	3,110 (2,160–4,330)	73·1 (50·4–102)	57·1 (39·8–78·5)	52 (36·4–71·8)
1-161	Age-related macular degeneration	59 (38–88)	88 (57–131)	107 (68–161)	2·2 (1·4–3·2)	1·9 (1·3–2·8)	1·8 (1·2–2·7)
1-162	Refraction disorders	3,100 (2,070–4,540)	5,010 (3,300–7,410)	6,060 (3,980–8,890)	62 (42–88·2)	58·4 (39–84·4)	57 (37·4–81·2)
1-163	Near vision loss	1,980 (881–4,020)	3,150 (1,430–6,350)	3,980 (1,800–7,880)	60·6 (27·8–121)	57·5 (26·2–113)	55·8 (26–111)
1-164	Other vision loss	3,150 (2,200–4,350)	4,710 (3,270–6,530)	5,550 (3,860–7,690)	89·9 (63–125)	79·5 (55·7–110)	74 (52·4–102)
1-165	Age-related and other hearing loss	21,400 (14,300–30,900)	34,600 (23,300–50,200)	43,300 (29,100–63,100)	560 (377–803)	538 (364–772)	529 (356–764)
1-166	Other sense organ diseases	1,550 (935–2,370)	2,600 (1,570–4,020)	3,280 (1,970–5,060)	34·6 (20·4–53·6)	35 (20·8–54·2)	35·1 (20·9–54·7)

1-167	Musculoskeletal disorders	61,100 (44,200–81,000)	109,000 (78,500–146,000)	136,000 (97,000–182,000)	1,370 (997–1,810)	1,410 (1,020–1,860)	1,380 (983–1,820)
1-168	Rheumatoid arthritis	1,360 (1,020–1,750)	2,600 (1,880–3,380)	3,470 (2,470–4,560)	34·3 (26–44·6)	36·9 (27·4–47·4)	37·9 (27·3–48·9)
1-169	Osteoarthritis	5,740 (2,910–11,600)	9,970 (4,950–20,200)	13,200 (6,550–26,900)	172 (87·1–347)	178 (89·1–363)	180 (90·6–368)
1-170	Low back pain	33,900 (23,900–45,400)	59,900 (41,900–80,600)	74,700 (51,700–100,000)	725 (514–963)	733 (516–985)	723 (513–967)
1-171	Neck pain	5,950 (3,910–8,630)	10,400 (6,750–15,100)	13,500 (8,660–19,700)	134 (88·7–194)	134 (88·5–194)	134 (87·3–193)
1-172	Gout	224 (139–332)	432 (264–631)	577 (365–859)	5·9 (3·7–8·7)	6·6 (4–9·6)	6·8 (4·2–9·9)
1-173	Other musculoskeletal disorders	13,900 (9,840–19,100)	26,200 (17,700–36,300)	30,900 (19,300–44,100)	300 (209–413)	323 (217–447)	294 (183–424)
1-174	Other non-communicable diseases	427,000 (338,000–521,000)	469,000 (370,000–589,000)	516,000 (391,000–673,000)	4,800 (3,960–5,740)	3,890 (3,080–4,850)	3,820 (2,910–4,990)
1-175	Congenital anomalies	303,000 (235,000–377,000)	301,000 (215,000–419,000)	316,000 (210,000–466,000)	2,810 (2,230–3,490)	2,110 (1,500–2,970)	2,060 (1,360–3,070)
1-176	Neural tube defects	62,300 (19,000–124,000)	49,100 (14,300–108,000)	50,200 (13,800–121,000)	566 (170–1,120)	338 (97·8–751)	323 (88·2–777)
1-177	Congenital heart anomalies	114,000 (49,400–195,000)	121,000 (55,600–186,000)	126,000 (56,100–201,000)	1,060 (456–1,770)	848 (388–1,300)	820 (365–1,320)
1-178	Orofacial clefts	1,130 (352–3,370)	826 (352–2,000)	847 (426–1,800)	10·8 (3·8–30·5)	6·3 (3–14·3)	6 (3·1–12·4)
1-179	Down's syndrome	7,520 (1,810–19,900)	9,170 (2,520–18,500)	11,200 (3,120–22,700)	84·2 (21·3–200)	74·1 (20·3–144)	80·2 (22·5–165)
1-180	Turner syndrome	13 (6–25)	19 (8–36)	22 (9–40)	0·2 (0·1–0·3)	0·2 (0·1–0·3)	0·2 (0·1–0·3)
1-181	Klinefelter syndrome	1 (0–2)	2 (1–3)	2 (1–4)	0 (0–0)	0 (0–0)	0 (0–0)
1-182	Other chromosomal abnormalities	8,800 (2,910–23,800)	12,100 (3,940–25,100)	13,900 (4,680–27,200)	80·2 (27–215)	83·5 (27·1–172)	89·5 (30·3–174)
1-183	Congenital musculoskeletal and limb anomalies	8,760 (5,130–12,900)	11,300 (6,970–16,600)	12,600 (7,990–18,700)	92·5 (55·7–133)	86·4 (54·6–126)	86·8 (55·5–128)
1-184	Urogenital congenital anomalies	4,620 (1,870–9,520)	4,760 (2,150–8,170)	5,040 (2,200–9,400)	41·9 (17·4–85·3)	32·9 (14·8–56)	32·5 (14·3–60·9)
1-185	Digestive congenital anomalies	36,900 (7,870–89,300)	35,800 (9,840–69,400)	36,100 (10,100–70,300)	327 (67·7–798)	243 (66·1–471)	231 (64·4–449)
1-186	Other congenital anomalies	58,600 (13,900–124,000)	57,100 (17,700–102,000)	60,100 (19,100–104,000)	548 (132–1,140)	402 (125–712)	393 (124–677)
1-187	Urinary diseases and male infertility	8,050 (6,340–10,200)	12,200 (8,970–15,800)	15,200 (11,000–20,000)	161 (132–204)	173 (126–238)	174 (124–245)
1-188	Urinary tract infections and interstitial nephritis	5,400 (3,970–6,980)	8,350 (5,920–11,100)	10,200 (6,810–14,300)	102 (81·8–133)	114 (76·2–165)	115 (75·7–171)
1-189	Urolithiasis	538 (272–922)	830 (452–1,260)	1,030 (575–1,590)	11·6 (6–18·7)	11·7 (6·3–19·4)	11·3 (6·2–19)
1-190	Benign prostatic hyperplasia	798 (460–1,280)	1,330 (769–2,090)	1,660 (966–2,630)	25·8 (14·9–40·5)	27·1 (15·7–42·1)	26·1 (15·2–41)
1-191	Male infertility	173 (70–363)	297 (118–646)	549 (206–1,310)	3·1 (1·3–6·5)	2·8 (1·1–6·2)	4·1 (1·6–9·9)
1-192	Other urinary diseases	1,140 (715–1,800)	1,430 (951–2,020)	1,730 (1,150–2,460)	18·5 (11·9–25·9)	17·1 (10·9–24·3)	17·3 (10·7–25·2)
1-193	Gynecological diseases	21,900 (15,400–30,300)	37,700 (26,200–52,500)	47,700 (33,300–67,200)	417 (294–571)	398 (277–556)	397 (276–556)
1-194	Uterine fibroids	1,400 (701–2,480)	2,210 (1,140–3,970)	2,850 (1,490–5,100)	29·6 (15·3–52·3)	26·5 (13·7–47·8)	25·8 (13·5–46)
1-195	Polycystic ovarian syndrome	184 (77–383)	402 (167–830)	529 (221–1,080)	3·1 (1·3–6·4)	3·7 (1·5–7·7)	4 (1·7–8·1)
1-196	Female infertility	325 (135–664)	579 (237–1,210)	1,210 (418–2,950)	5·9 (2·5–11·9)	5·5 (2·2–11·5)	9·2 (3·1–21·9)
1-197	Endometriosis	2,520 (1,450–4,050)	3,520 (2,050–5,850)	3,880 (2,240–6,300)	44·2 (25·6–70·1)	33·5 (19·6–54·9)	29·9 (17·5–48·2)
1-198	Genital prolapse	403 (211–734)	549 (280–1,000)	668 (341–1,220)	10·5 (5·5–19)	8·2 (4·3–14·9)	7·6 (3·9–13·8)
1-199	Premenstrual syndrome	5,910 (3,620–9,170)	11,100 (6,770–17,400)	13,800 (8,410–21,100)	98·9 (60·6–155)	104 (64·2–163)	105 (63·9–160)
1-200	Other gynecological diseases	11,200 (7,420–15,800)	19,300 (12,700–27,200)	24,700 (16,200–34,900)	225 (150–318)	217 (144–304)	216 (143–304)
1-201	Hemoglobinopathies and hemolytic anaemias	37,000 (21,400–53,900)	45,100 (30,400–61,300)	51,600 (35,700–68,800)	509 (333–699)	404 (287–531)	393 (280–514)
1-202	Thalassemias	71 (14–163)	88 (25–179)	76 (24–150)	0·7 (0·2–1·6)	0·6 (0·2–1·3)	0·5 (0·2–1)
1-203	Thalassaemias trait	1,010 (659–1,510)	1,700 (1,070–2,550)	2,000 (1,270–2,950)	15·4 (10·1–22·7)	15·5 (9·7–23·2)	15·2 (9·7–22·6)
1-204	Sickle cell disorders	21,900 (8,650–37,100)	24,700 (13,000–38,600)	26,900 (14,600–40,900)	252 (111–414)	200 (109–306)	188 (104–284)
1-205	Sickle cell trait	8,580 (5,570–12,600)	11,400 (7,180–16,700)	13,200 (8,530–19,600)	134 (87·5–196)	102 (65–151)	97·9 (63·4–146)
1-206	G6PD deficiency	1,790 (1,170–2,580)	2,370 (1,610–3,270)	3,340 (2,220–4,930)	30·4 (21·1–41)	25·6 (17·8–36·2)	29·3 (19·4–43·7)
1-207	G6PD trait	5 (3–8)	8 (4–13)	18 (10–30)	0·1 (0·1–0·2)	0·1 (0–0·2)	0·2 (0·1–0·3)
1-208	Other hemoglobinopathies and hemolytic anaemias	3,640 (2,700–4,840)	4,880 (3,480–6,630)	6,100 (4,330–8,300)	77 (58·9–99·7)	61·2 (45–81·3)	61·9 (45·1–82)
1-209	Endocrine, metabolic, blood, and immune disorders	32,900 (20,300–50,400)	39,400 (28,700–52,200)	47,100 (35,100–63,800)	483 (351–655)	408 (306–524)	412 (311–536)
1-210	Oral disorders	14,400 (8,350–23,000)	24,100 (13,900–38,200)	30,500 (17,500–48,500)	339 (201–546)	332 (198–535)	330 (196–523)
1-211	Caries of deciduous teeth	310 (133–642)	424 (181–874)	476 (210–970)	3·2 (1·4–6·7)	3·2 (1·4–6·5)	3·2 (1·4–6·5)
1-212	Caries of permanent teeth	1,670 (754–3,280)	2,830 (1,260–5,540)	3,450 (1,550–6,750)	28·5 (12·7–54·7)	28·2 (12·5–55·5)	28·1 (12·6–55·3)
1-213	Periodontal disease	4,350 (1,650–9,550)	7,480 (2,840–16,400)	9,720 (3,710–21,500)	97·5 (37·5–210)	95·2 (37–205)	94·6 (36·8–206)
1-214	Edentulism	5,040 (3,200–7,550)	8,220 (5,180–12,200)	10,600 (6,670–15,800)	157 (99·5–233)	153 (96·2–226)	151 (95·1–226)
1-215	Other oral disorders	3,060 (1,910–4,490)	5,170 (3,220–7,630)	6,320 (3,940–9,280)	53 (33–76·8)	53 (32·9–77·6)	53 (33·1–77·4)
1-216	Sudden infant death syndrome	9,530 (1,080–31,900)	8,940 (1,460–27,500)	8,240 (1,410–25,700)	84 (9·5–282)	60·3 (9·8–186)	52·7 (9–165)
1-0	Injuries	627,000 (529,000–734,000)	14,100,000 (12,900,000–15,500,000)	806,000 (661,000–969,000)	8,730 (7,540–10,100)	129,000 (118,000–142,000)	6,380 (5,220–7,700)
1-1	Transport injuries	79,400 (60,700–132,000)	85,200 (61,800–190,000)	101,000 (71,100–217,000)	1,160 (887–2,170)	807 (582–1,870)	802 (567–1,740)
1-2	Road injuries	75,100 (57,300–124,000)	78,200 (56,600–173,000)	93,100 (65,400–198,000)	1,100 (838–2,040)	740 (533–1,730)	738 (521–1,580)

1·3	Pedestrian road injuries	28,400 (17,300–44,800)	23,300 (14,600–49,400)	24,300 (15,600–49,400)	384 (238–670)	218 (139–489)	194 (124–408)
1·4	Cyclist road injuries	7,060 (4,280–15,200)	7,980 (4,790–15,800)	10,100 (6,230–19,100)	109 (70·6–227)	78·9 (49·1–154)	82·6 (52·5–154)
1·5	Motorcyclist road injuries	12,400 (7,540–24,500)	17,200 (8,030–51,400)	22,900 (10,900–64,500)	193 (119–428)	165 (78·8–506)	181 (87·8–512)
1·6	Motor vehicle road injuries	26,700 (14,600–49,000)	28,900 (17,300–56,500)	34,900 (21,400–67,700)	402 (230–743)	271 (165–562)	274 (170–542)
1·7	Other road injuries	556 (328–949)	710 (480–1,570)	930 (647–1,950)	8·8 (5·6–15)	7·1 (5–15·8)	7·7 (5·5–16·2)
1·8	Other transport injuries	4,250 (3,000–7,650)	7,020 (4,850–14,600)	8,000 (5,190–18,400)	62 (44·2–117)	66·9 (46·5–144)	63·6 (42–151)
1·9	Unintentional injuries	406,000 (271,000–498,000)	13,800,000 (12,600,000–15,200,000)	445,000 (350,000–550,000)	5,160 (3,870–6,190)	126,000 (115,000–139,000)	3,530 (2,810–4,370)
1·10	Falls	17,400 (10,200–26,700)	21,600 (14,700–29,400)	27,500 (19,200–36,200)	340 (229–449)	283 (204–360)	291 (214–369)
1·11	Drowning	132,000 (64,200–181,000)	98,200 (62,200–136,000)	105,000 (63,600–144,000)	1,500 (806–2,000)	786 (526–1,070)	736 (448–1,010)
1·12	Fire, heat, and hot substances	66,800 (20,000–107,000)	48,700 (22,100–70,500)	50,400 (25,800–69,700)	827 (325–1,260)	429 (216–604)	386 (210–526)
1·13	Poisonings	21,500 (13,900–33,300)	13,000 (8,340–22,000)	13,100 (8,430–24,900)	238 (163–350)	105 (67·9–188)	93·7 (60·3–188)
1·14	Poisoning by carbon monoxide	3,310 (1,470–7,370)	2,790 (1,430–6,570)	3,250 (1,680–8,560)	41·7 (20·9–85·5)	24·7 (13·1–65·2)	24·5 (12·8–68·6)
1·15	Poisoning by other means	18,200 (10,900–29,400)	10,200 (6,260–17,000)	9,880 (6,210–17,100)	196 (131–301)	80·1 (49·9–135)	69·2 (43·5–121)
1·16	Exposure to mechanical forces	48,500 (25,900–79,300)	57,500 (28,200–102,000)	67,800 (32,700–119,000)	754 (404–1,210)	564 (280–967)	552 (274–952)
1·17	Unintentional firearm injuries	32,500 (14,800–58,300)	35,600 (14,100–75,700)	41,200 (15,600–89,500)	499 (224–951)	340 (136–708)	325 (124–717)
1·18	Other exposure to mechanical forces	16,000 (9,050–34,500)	21,900 (12,200–44,400)	26,600 (15,400–53,100)	256 (150–516)	224 (132–434)	226 (134–427)
1·19	Adverse effects of medical treatment	23,400 (14,500–33,200)	20,400 (14,500–28,200)	21,400 (15,300–29,200)	327 (245–422)	206 (156–270)	186 (136–247)
1·20	Animal contact	1,870 (1,010–2,750)	2,840 (1,650–4,090)	3,300 (2,020–4,690)	28·6 (17·8–38·9)	29·3 (17·7–41·3)	28·7 (18·1–39·7)
1·21	Venomous animal contact	1,300 (698–1,940)	1,950 (1,180–2,870)	2,250 (1,460–3,260)	19·7 (12·8–27·2)	20 (12·7–28·9)	19·4 (13–27·8)
1·22	Non-venomous animal contact	576 (207–928)	892 (368–1,410)	1,060 (472–1,630)	9 (4·2–13·3)	9·3 (4·4–13·8)	9·3 (4·5–13·8)
1·23	Foreign body	59,400 (37,100–85,600)	41,300 (25,600–58,700)	41,100 (26,000–57,500)	604 (396–850)	313 (201–435)	285 (186–390)
1·24	Pulmonary aspiration and foreign body in airway	57,200 (35,600–82,500)	39,300 (23,700–55,800)	38,900 (24,000–55,200)	575 (372–813)	293 (185–412)	267 (169–371)
1·25	Foreign body in eyes	308 (135–561)	520 (227–948)	639 (285–1,160)	5·5 (2·7–9·6)	5·5 (2·7–9·6)	5·5 (2·7–9·5)
1·26	Foreign body in other body part	1,910 (664–5,260)	1,490 (875–2,920)	1,630 (1,040–2,810)	23·6 (10·7–56·2)	14 (9–24·5)	13·3 (8·8–20·9)
1·27	Environmental heat and cold exposure	11,000 (7,020–19,900)	11,900 (7,230–22,100)	13,200 (8,060–24,600)	197 (116–380)	137 (78·9–270)	124 (73·3–237)
1·28	Exposure to forces of nature	224 (146–332)	13,500,000 (12,300,000–14,900,000)	78,600 (52,300–114,000)	3·8 (2·5–5·5)	123,000 (112,000–136,000)	658 (445–959)
1·29	Other unintentional injuries	24,200 (16,500–37,400)	21,600 (14,000–36,400)	23,800 (15,300–39,500)	338 (221–562)	200 (128–347)	187 (120–313)
1·30	Self-harm and interpersonal violence	141,000 (77,700–181,000)	214,000 (97,300–296,000)	260,000 (121,000–364,000)	2,410 (1,280–3,120)	2,070 (931–2,900)	2,050 (948–2,900)
1·31	Self-harm	31,700 (24,700–40,000)	41,800 (31,000–54,100)	51,700 (37,000–70,400)	580 (442–726)	435 (321–558)	433 (308–585)
1·32	Self-harm by firearm	2,600 (1,490–3,900)	2,540 (1,500–4,290)	3,110 (1,830–5,530)	47·3 (27·1–71·7)	26·2 (15·6–44·6)	25·7 (15·3–45·3)
1·33	Self-harm by other specified means	29,100 (22,500–36,600)	39,200 (29,000–51,000)	48,600 (35,000–65,800)	533 (405–669)	408 (300–526)	407 (289–551)
1·34	Interpersonal violence	108,000 (45,600–146,000)	171,000 (54,800–250,000)	207,000 (69,300–307,000)	1,810 (696–2,480)	1,630 (503–2,400)	1,610 (525–2,410)
1·35	Physical violence by firearm	35,600 (8,830–56,400)	59,300 (11,400–98,100)	74,800 (15,000–120,000)	581 (139–916)	535 (99·3–884)	559 (109–895)
1·36	Physical violence by sharp object	25,900 (8,950–64,200)	42,400 (12,400–104,000)	51,300 (14,700–128,000)	448 (149–1,130)	408 (117–1,010)	399 (113–995)
1·37	Sexual violence	1,710 (1,140–2,430)	3,410 (2,260–4,810)	3,680 (2,450–5,240)	28·9 (19·3–41·1)	32 (21·5–45·2)	28·9 (19·3–41·1)
1·38	Physical violence by other means	45,100 (18,800–65,500)	65,500 (19,200–106,000)	77,100 (22,900–125,000)	756 (260–1,120)	652 (178–1,060)	623 (178–1,030)
1·39	Conflict and terrorism	1,080 (965–1,220)	986 (540–1,670)	848 (473–1,430)	15·5 (13·9–17·5)	9·8 (5·4–16·5)	7·3 (4·2–12·2)
1·40	Executions and police conflict	42 (35–52)	87 (67–121)	350 (309–402)	0·6 (0·5–0·7)	0·8 (0·6–1·1)	2·6 (2·3–3)

SR Table 1: All-age DALYs counts and age standardized DALYs rates per 100,000 individuals for all causes and level 1 cause groups by location for 1990, 2010, and 2019, both sexes combined, Jamaica

Cause Level	Cause	All-age DALYs			Age-standardized rate of DALYs per 100,000		
		1990	2010	2019	1990	2010	2019
0-0	All causes	597,000 (535,000–666,000)	698,000 (623,000–781,000)	763,000 (638,000–901,000)	28,200 (25,300–31,400)	26,900 (24,100–30,000)	26,800 (22,300–31,800)
1-0	Communicable, maternal, neonatal, and nutritional disorders	159,000 (141,000–178,000)	122,000 (108,000–140,000)	105,000 (88,800–125,000)	6,300 (5,620–7,030)	5,170 (4,510–6,010)	4,540 (3,740–5,560)
1-1	HIV/AIDS and sexually transmitted infections	10,200 (9,860–10,700)	31,300 (30,500–32,400)	21,500 (20,700–22,500)	499 (482–520)	1,140 (1,110–1,180)	727 (701–760)
1-2	HIV/AIDS	9,710 (9,460–10,000)	30,700 (30,000–31,700)	20,900 (20,200–21,600)	474 (462–491)	1,120 (1,090–1,150)	706 (684–732)
1-3	HIV/AIDS - drug-susceptible tuberculosis	618 (413–910)	418 (289–598)	303 (203–441)	30·1 (20·1–44·4)	15·2 (10·5–21·8)	10·2 (6·9–14·9)
1-4	HIV/AIDS - Multidrug-resistant Tuberculosis without extensive drug resistance	7 (0–35)	9 (1–32)	11 (1–40)	0·3 (0–1·7)	0·3 (0–1·2)	0·4 (0–1·3)
1-5	HIV/AIDS - Extensively drug-resistant Tuberculosis	0 (0–0)	1 (0–3)	2 (0–6)	0 (0–0)	0 (0–0·1)	0·1 (0–0·2)
1-6	HIV/AIDS resulting in other diseases	9,080 (8,710–9,450)	30,300 (29,500–31,300)	20,500 (19,900–21,300)	444 (426–462)	1,100 (1,070–1,130)	695 (673–720)
1-7	Sexually transmitted infections excluding HIV	501 (322–830)	610 (371–1,030)	653 (398–1,090)	24·2 (15·2–40·4)	22 (13·4–37·4)	21·7 (13·3–36·3)
1-8	Syphilis	76 (59–96)	52 (39–62)	46 (33–62)	3·2 (2·5–3·9)	2·1 (1·6–2·6)	1·7 (1·3–2·3)
1-9	Chlamydial infection	50 (33–73)	66 (45–97)	72 (50–104)	2·4 (1·6–3·5)	2·3 (1·6–3·4)	2·4 (1·6–3·4)
1-10	Gonococcal infection	42 (32–57)	58 (46–76)	67 (51–87)	2 (1·7–2·6)	2 (1·6–2·6)	2·2 (1·7–2·9)
1-11	Trichomoniasis	100 (39–204)	138 (53–289)	145 (56–297)	5 (1·9–10·3)	4·9 (1·9–10·4)	4·7 (1·8–9·7)
1-12	Genital herpes	135 (47–332)	168 (54–414)	184 (60–455)	6·9 (2·3–16·4)	6·1 (2–15)	6·1 (2–15)
1-13	Other sexually transmitted infections	98 (69–136)	129 (92–181)	138 (98–192)	4·7 (3·3–6·5)	4·5 (3·2–6·3)	4·6 (3·3–6·4)
1-14	Respiratory infections and tuberculosis	20,600 (18,100–23,600)	12,200 (10,600–14,000)	12,900 (10,300–15,800)	871 (776–985)	475 (411–546)	474 (373–586)
1-15	Tuberculosis	978 (871–1,090)	509 (443–592)	466 (366–585)	49 (43·8–54·4)	18·7 (16·3–21·7)	15·6 (12·3–19·5)
1-16	Latent tuberculosis infection	0 (0–0)	0 (0–0)	0 (0–0)	0 (0–0)	0 (0–0)	0 (0–0)
1-17	Drug-susceptible tuberculosis	969 (862–1,080)	500 (430–584)	452 (351–571)	48·6 (43·2–54)	18·4 (15·9–21·4)	15·1 (11·7–19)
1-18	Multidrug-resistant tuberculosis without extensive drug resistance	8 (1–44)	8 (1–26)	12 (2–44)	0·4 (0–2·2)	0·3 (0–1)	0·4 (0·1–1·5)
1-19	Extensively drug-resistant tuberculosis	0 (0–0)	1 (0–3)	2 (0–7)	0 (0–0)	0 (0–0·1)	0·1 (0–0·2)
1-20	Lower respiratory infections	16,800 (14,800–19,300)	8,580 (7,640–9,590)	9,380 (7,360–11,800)	709 (632–802)	344 (302–390)	346 (264–444)
1-21	Upper respiratory infections	2,020 (1,210–3,230)	2,230 (1,330–3,480)	2,220 (1,330–3,550)	81·9 (49·3–129)	81·5 (48·8–127)	81·4 (48·9–130)
1-22	Otitis media	798 (456–1,310)	834 (481–1,400)	815 (464–1,350)	31·7 (18·2–52·1)	30·5 (17·7–50·5)	30·3 (17·3–49·9)
1-23	Enteric infections	18,600 (15,500–22,400)	4,490 (3,430–5,710)	5,130 (3,850–6,620)	724 (606–864)	176 (136–224)	197 (149–254)
1-24	Diarrhoeal diseases	18,600 (15,400–22,300)	4,480 (3,410–5,690)	5,120 (3,840–6,600)	723 (604–862)	176 (135–223)	197 (148–253)
1-25	Typhoid and paratyphoid	4 (1–6)	1 (1–1)	0 (0–1)	0·2 (0–0·2)	0 (0–0)	0 (0–0)
1-26	Typhoid fever	4 (1–6)	1 (0–1)	0 (0–0)	0·2 (0–0·2)	0 (0–0)	0 (0–0)
1-27	Paratyphoid fever	0 (0–0)	0 (0–0)	0 (0–0)	0 (0–0)	0 (0–0)	0 (0–0)
1-28	iNTS	9 (1–14)	5 (1–8)	2 (1–3)	0·3 (0–0·5)	0·2 (0–0·3)	0·1 (0–0·1)
1-29	Other intestinal infectious diseases	23 (8–44)	10 (6–16)	9 (6–14)	0·9 (0·3–1·6)	0·4 (0·2–0·6)	0·4 (0·2–0·6)
1-30	Neglected tropical diseases and malaria	2,560 (1,730–3,540)	2,580 (1,670–3,580)	2,650 (1,770–3,770)	114 (76·5–156)	95·9 (62·2–134)	94·9 (62·8–135)
1-31	Malaria	0 (0–0)	0 (0–0)	0 (0–0)	0 (0–0)	0 (0–0)	0 (0–0)
1-32	Chagas disease	58 (23–109)	0 (0–0)	0 (0–0)	3·3 (1·3–6·1)	0 (0–0)	0 (0–0)
1-33	Leishmaniasis	0 (0–0)	0 (0–0)	0 (0–0)	0 (0–0)	0 (0–0)	0 (0–0)
1-34	Visceral leishmaniasis	0 (0–0)	0 (0–0)	0 (0–0)	0 (0–0)	0 (0–0)	0 (0–0)
1-35	Cutaneous and mucocutaneous leishmaniasis	0 (0–0)	0 (0–0)	0 (0–0)	0 (0–0)	0 (0–0)	0 (0–0)
1-36	African trypanosomiasis	0 (0–0)	0 (0–0)	0 (0–0)	0 (0–0)	0 (0–0)	0 (0–0)
1-37	Schistosomiasis	0 (0–0)	0 (0–0)	0 (0–0)	0 (0–0)	0 (0–0)	0 (0–0)
1-38	Cysticercosis	931 (468–1,560)	990 (491–1,720)	1,110 (545–1,970)	50·2 (25·2–85·5)	37·7 (18·8–65·8)	37 (18·1–66)
1-39	Cystic echinococcosis	0 (0–0)	0 (0–0)	0 (0–1)	0 (0–0)	0 (0–0)	0 (0–0)
1-40	Lymphatic filariasis	0 (0–0)	0 (0–0)	0 (0–0)	0 (0–0)	0 (0–0)	0 (0–0)
1-41	Onchocerciasis	0 (0–0)	0 (0–0)	0 (0–0)	0 (0–0)	0 (0–0)	0 (0–0)
1-42	Trachoma	0 (0–0)	0 (0–0)	0 (0–0)	0 (0–0)	0 (0–0)	0 (0–0)
1-43	Dengue	188 (68–417)	256 (117–486)	263 (103–548)	8 (3·1–17·2)	9·3 (4·3–17·5)	9·4 (3·7–19·6)
1-44	Yellow fever	0 (0–0)	0 (0–0)	0 (0–0)	0 (0–0)	0 (0–0)	0 (0–0)
1-45	Rabies	0 (0–0)	0 (0–0)	0 (0–0)	0 (0–0)	0 (0–0)	0 (0–0)
1-46	Intestinal nematode infections	720 (409–1,190)	811 (433–1,340)	754 (402–1,290)	27·4 (15·6–45·5)	28·4 (15·2–47·2)	27 (14·3–46·2)
1-47	Ascariasis	76 (37–128)	68 (33–126)	39 (19–73)	2·9 (1·4–5)	2·5 (1·2–4·6)	1·5 (0·7–2·8)
1-48	Trichuriasis	342 (162–683)	497 (230–883)	480 (218–897)	12·8 (6·1–25·7)	17·4 (8–31)	17·6 (8–32·7)

1-49	Hookworm disease	302 (160–494)	246 (132–430)	234 (123–394)	11·7 (6·3–19·1)	8·5 (4·5–14·7)	7·9 (4·2–13·3)
1-50	Food-borne trematodiases	0 (0–0)	0 (0–0)	0 (0–0)	0 (0–0)	0 (0–0)	0 (0–0)
1-51	Leprosy	2 (1–3)	2 (2–4)	3 (2–5)	0·1 (0·1–0·1)	0·1 (0·1–0·1)	0·1 (0·1–0·2)
1-52	Ebola virus disease	0 (0–0)	0 (0–0)	0 (0–0)	0 (0–0)	0 (0–0)	0 (0–0)
1-53	Zika virus disease	0 (0–0)	0 (0–0)	3 (0–13)	0 (0–0)	0 (0–0)	0·1 (0–0·5)
1-54	Guinea worm disease	0 (0–0)	0 (0–0)	0 (0–0)	0 (0–0)	0 (0–0)	0 (0–0)
1-55	Other neglected tropical diseases	656 (414–968)	517 (317–782)	519 (325–782)	25·4 (16·2–37·3)	20·4 (12·5–31·1)	21·4 (13·2–32·6)
1-56	Other infectious diseases	7,570 (6,590–8,740)	4,260 (3,230–5,140)	4,260 (2,990–5,680)	300 (263–344)	166 (127–199)	161 (115–212)
1-57	Meningitis	4,190 (3,540–4,980)	1,160 (1,020–1,330)	1,080 (839–1,370)	163 (139–191)	47·5 (41·1–55·8)	43 (33·2–55·7)
1-58	Encephalitis	1,980 (1,530–2,370)	1,010 (832–1,580)	995 (748–1,510)	76·2 (58·7–90·4)	40·1 (32·3–63·9)	39·6 (28·9–62·4)
1-59	Diphtheria	0 (0–0)	0 (0–1)	0 (0–1)	0 (0–0)	0 (0–0)	0 (0–0)
1-60	Whooping cough	48 (27–78)	30 (18–49)	17 (10–27)	1·7 (1–2·8)	1·4 (0·8–2·3)	0·9 (0·5–1·4)
1-61	Tetanus	181 (74–279)	15 (9–36)	11 (5–27)	8·3 (3·5–12·1)	0·6 (0·3–1·4)	0·4 (0·2–0·9)
1-62	Measles	11 (6–17)	0 (0–0)	0 (0–0)	0·4 (0·2–0·6)	0 (0–0)	0 (0–0)
1-63	Varicella and herpes zoster	76 (48–109)	77 (48–116)	82 (52–122)	3·6 (2·3–5·1)	2·9 (1·8–4·4)	2·8 (1·8–4·2)
1-64	Acute hepatitis	255 (209–312)	187 (142–240)	183 (136–239)	10·9 (9·2–13·1)	6·6 (5–8·4)	6·4 (4·7–8·3)
1-65	Acute hepatitis A	139 (102–185)	115 (77–160)	112 (74–157)	5·6 (4·2–7·3)	4 (2·7–5·5)	4 (2·6–5·6)
1-66	Acute hepatitis B	87 (65–104)	51 (42–59)	50 (35–69)	4 (3·2–4·6)	1·9 (1·5–2·2)	1·7 (1·2–2·3)
1-67	Acute hepatitis C	14 (9–22)	7 (5–11)	9 (5–13)	0·7 (0·4–1·1)	0·3 (0·2–0·4)	0·3 (0·2–0·5)
1-68	Acute hepatitis E	15 (10–22)	13 (8–20)	13 (8–19)	0·6 (0·4–0·9)	0·5 (0·3–0·7)	0·4 (0·3–0·7)
1-69	Other unspecified infectious diseases	835 (581–1,170)	1,780 (841–2,550)	1,890 (866–2,950)	36 (25–51·8)	67·2 (32·4–96)	67·7 (32·2–104)
1-70	Maternal and neonatal disorders	78,600 (66,600–91,400)	56,900 (45,900–70,900)	47,700 (36,800–62,300)	2,940 (2,490–3,410)	2,710 (2,160–3,410)	2,480 (1,860–3,290)
1-71	Maternal disorders	1,660 (1,430–1,870)	2,100 (1,890–2,350)	1,830 (1,380–2,390)	67 (58·4–75·9)	70·9 (63·6–79·1)	58·3 (44·1–76·3)
1-72	Maternal haemorrhage	518 (411–640)	265 (203–343)	202 (145–275)	21·5 (17·2–26·1)	9 (6·9–11·7)	6·4 (4·6–8·7)
1-73	Maternal sepsis and other pregnancy related infections	180 (136–229)	60 (41–86)	50 (33–75)	7·3 (5·6–9·3)	2 (1·4–2·8)	1·6 (1–2·3)
1-74	Maternal hypertensive disorders	510 (429–595)	448 (377–532)	426 (311–577)	19·6 (16·6–22·8)	14·9 (12·5–17·6)	13·4 (9·8–18·1)
1-75	Maternal obstructed labour and uterine rupture	13 (6–22)	34 (25–45)	26 (18–37)	0·5 (0·2–0·8)	1·2 (0·9–1·6)	0·8 (0·6–1·2)
1-76	Maternal abortive outcome	296 (231–370)	151 (117–198)	134 (90–192)	12·5 (9·8–15·6)	5·1 (3·9–6·7)	4·3 (2·9–6·2)
1-77	Ectopic pregnancy	8 (6–9)	67 (53–84)	57 (38–81)	0·3 (0·2–0·4)	2·2 (1·8–2·8)	1·8 (1·2–2·5)
1-78	Indirect maternal deaths	1 (1–2)	10 (8–13)	11 (8–16)	0·1 (0–0·1)	0·4 (0·3–0·4)	0·4 (0·2–0·5)
1-79	Late maternal deaths	73 (55–98)	246 (182–336)	164 (106–242)	3 (2·2–4)	8·3 (6·1–11·2)	5·2 (3·4–7·6)
1-80	Maternal deaths aggravated by HIV/AIDS	1 (0–2)	6 (3–10)	7 (4–13)	0 (0–0·1)	0·2 (0·1–0·3)	0·2 (0·1–0·4)
1-81	Other maternal disorders	55 (43–70)	815 (696–947)	754 (534–1,000)	2·2 (1·7–2·8)	27·7 (23·6–32·2)	24·1 (17·3–32·1)
1-82	Neonatal disorders	77,000 (65,000–89,800)	54,800 (43,800–68,800)	45,900 (35,300–60,000)	2,870 (2,430–3,350)	2,640 (2,080–3,340)	2,420 (1,810–3,220)
1-83	Neonatal preterm birth	48,500 (40,500–57,000)	31,000 (24,500–38,800)	24,100 (17,900–32,000)	1,810 (1,510–2,130)	1,510 (1,180–1,910)	1,310 (953–1,780)
1-84	Neonatal encephalopathy due to birth asphyxia and trauma	13,700 (10,400–18,000)	9,240 (7,140–11,800)	7,460 (5,140–10,400)	510 (388–668)	456 (352–586)	407 (274–572)
1-85	Neonatal sepsis and other neonatal infections	7,500 (5,530–9,970)	10,400 (7,990–13,400)	10,700 (8,160–13,900)	282 (207–373)	475 (364–608)	506 (375–666)
1-86	Hemolytic disease and other neonatal jaundice	2,110 (1,530–2,930)	694 (549–855)	554 (425–696)	79·9 (58·7–111)	28·9 (23–35·8)	22·8 (17·5–28·9)
1-87	Other neonatal disorders	5,180 (3,680–7,070)	3,440 (2,590–4,550)	3,070 (2,110–4,390)	191 (136–261)	173 (130–228)	175 (120–250)
1-88	Nutritional deficiencies	21,000 (16,700–26,200)	10,500 (7,180–15,000)	10,800 (7,450–15,200)	846 (673–1,060)	407 (281–575)	409 (282–578)
1-89	Protein-energy malnutrition	11,400 (9,270–13,900)	2,020 (1,690–2,420)	1,930 (1,520–2,390)	440 (362–535)	82·3 (68–99)	74·8 (57·8–94·5)
1-90	Iodine deficiency	262 (138–427)	235 (123–387)	207 (106–340)	11·6 (6·1–18·8)	8·3 (4·3–13·6)	6·9 (3·6–11·4)
1-91	Vitamin A deficiency	274 (166–420)	156 (96–236)	137 (86–211)	9·8 (6·1–15)	6·1 (3·8–9·3)	5·8 (3·6–8·9)
1-92	Dietary iron deficiency	8,510 (5,170–12,800)	7,800 (4,740–12,000)	8,160 (5,070–12,400)	357 (220–534)	298 (180–460)	310 (193–470)
1-93	Other nutritional deficiencies	625 (450–899)	326 (244–442)	347 (246–475)	28·2 (20·8–39·5)	12·1 (9·1–16·5)	11·8 (8·4–16·2)
1-0	Non-communicable diseases	390,000 (344,000–441,000)	510,000 (449,000–581,000)	595,000 (497,000–704,000)	19,800 (17,600–22,200)	19,400 (17,100–22,000)	20,100 (16,800–23,900)
1-1	Neoplasms	50,900 (48,900–53,000)	87,900 (82,100–91,300)	106,000 (84,300–133,000)	2,810 (2,700–2,920)	3,440 (3,220–3,580)	3,590 (2,850–4,490)
1-2	Lip and oral cavity cancer	615 (572–664)	633 (587–687)	726 (560–919)	35·4 (32·8–38·4)	24·9 (23–27·1)	24·3 (18·7–30·7)
1-3	Nasopharynx cancer	173 (156–192)	436 (393–481)	511 (390–654)	9·1 (8·2–10·2)	16·5 (14·9–18·2)	17·1 (13·1–21·9)
1-4	Other pharynx cancer	325 (291–363)	433 (388–478)	487 (364–639)	19·2 (17·1–21·6)	17·3 (15·4–19·2)	16·2 (12·1–21·2)
1-5	Oesophageal cancer	1,200 (1,080–1,330)	1,560 (1,430–1,720)	1,790 (1,360–2,310)	68·7 (62–76·4)	63·5 (57·8–70)	60·7 (46·2–78·2)
1-6	Stomach cancer	5,420 (5,060–5,780)	5,680 (5,300–6,070)	6,300 (4,880–7,970)	308 (287–329)	226 (212–242)	212 (164–269)
1-7	Colon and rectum cancer	3,970 (3,720–4,210)	8,350 (7,840–8,910)	11,300 (8,950–14,200)	224 (210–238)	331 (311–353)	378 (301–477)

1·8	Liver cancer	1,790 (1,680–1,910)	1,510 (1,390–1,630)	1,940 (1,520–2,450)	101 (94·3–108)	59·7 (55–64·2)	66 (51·5–83·7)
1·9	Liver cancer due to hepatitis B	586 (450–750)	508 (395–646)	605 (416–842)	33·7 (25·7–43·6)	19·8 (15·2–25·4)	20·3 (13·8–28·3)
1·10	Liver cancer due to hepatitis C	354 (245–474)	282 (192–381)	376 (239–558)	20 (13·7–27)	11·2 (7·5–15·2)	12·7 (8·1–18·9)
1·11	Liver cancer due to alcohol use	539 (399–694)	455 (337–587)	617 (417–866)	30·9 (22·8–40)	18·4 (13·5–23·8)	21 (14·2–29·5)
1·12	Liver cancer due to NASH	129 (92–181)	129 (92–176)	178 (118–259)	7·2 (5·1–10·2)	5 (3·5–7)	6 (4–8·7)
1·13	Gallbladder and biliary tract cancer	850 (691–940)	833 (737–1,180)	1,050 (804–1,490)	48·9 (39·8–54·1)	33·2 (29·3–47·2)	35·4 (27·1–50·1)
1·14	Pancreatic cancer	546 (499–598)	2,530 (2,330–2,730)	3,460 (2,650–4,390)	31·4 (28·7–34·5)	101 (92·8–109)	117 (89·4–148)
1·15	Larynx cancer	421 (381–467)	816 (740–913)	912 (696–1,180)	24·5 (22·1–27·2)	33·2 (30–37·2)	31 (23·6–40·2)
1·16	Tracheal, bronchus, and lung cancer	6,630 (6,260–7,020)	12,000 (11,300–12,600)	14,000 (10,800–17,600)	390 (369–413)	485 (459–512)	472 (365–592)
1·17	Malignant skin melanoma	151 (108–181)	237 (201–392)	318 (227–512)	8·4 (6–10)	9·1 (7·8–15·1)	10·7 (7·7–17·2)
1·18	Non-melanoma skin cancer	107 (95–119)	211 (194–228)	245 (194–306)	6 (5·3–6·7)	8·1 (7·4–8·7)	8·1 (6·4–10·1)
1·19	Non-melanoma skin cancer (squamous-cell carcinoma)	107 (95–119)	211 (194–228)	245 (194–306)	6 (5·3–6·7)	8·1 (7·4–8·7)	8·1 (6·4–10·1)
1·20	Non-melanoma skin cancer (basal-cell carcinoma)	0 (0–0)	0 (0–0)	0 (0–0)	0 (0–0)	0 (0–0)	0 (0–0)
1·21	Breast cancer	5,310 (4,950–5,710)	10,600 (9,960–11,300)	14,100 (10,900–17,800)	315 (294–340)	404 (380–432)	470 (366–597)
1·22	Cervical cancer	4,830 (4,190–5,260)	6,460 (5,580–6,980)	7,770 (5,780–10,200)	283 (244–308)	246 (211–266)	260 (193–342)
1·23	Uterine cancer	639 (567–712)	1,880 (1,710–2,050)	2,490 (1,940–3,120)	36·7 (32·6–41)	76·8 (69·6–84)	84·3 (65·6–106)
1·24	Ovarian cancer	344 (302–549)	1,980 (1,100–2,210)	2,390 (1,390–3,090)	19·7 (17·2–31·1)	76·8 (42·6–85·6)	79·8 (46·5–103)
1·25	Prostate cancer	4,750 (3,820–6,050)	12,600 (7,240–14,200)	14,100 (7,840–18,300)	257 (207–326)	498 (283–564)	478 (263–622)
1·26	Testicular cancer	21 (17–41)	75 (62–104)	85 (62–118)	0·9 (0·7–1·7)	2·7 (2·2–4·2)	2·9 (2·1–4·4)
1·27	Kidney cancer	998 (897–1,110)	848 (775–929)	1,050 (780–1,370)	50·5 (45·7–55·5)	33·1 (30·3–36·4)	35·9 (27–46·6)
1·28	Bladder cancer	891 (807–980)	1,150 (1,040–1,260)	1,380 (1,070–1,740)	49·7 (45·2–54·6)	45·7 (41·2–50·3)	46·4 (35·9–58·5)
1·29	Brain and nervous system cancer	518 (410–865)	1,480 (690–1,860)	1,650 (719–2,370)	23 (18·4–37·9)	56·9 (26·7–71·4)	59·5 (25·9–85·5)
1·30	Thyroid cancer	185 (169–202)	429 (388–467)	528 (407–662)	10·2 (9·3–11·2)	16·7 (15·1–18·3)	17·7 (13·6–22·3)
1·31	Mesothelioma	86 (76–101)	91 (82–116)	142 (108–186)	4·8 (4·3–5·7)	3·5 (3·2–4·4)	4·8 (3·6–6·2)
1·32	Hodgkin lymphoma	188 (126–227)	210 (156–254)	244 (166–336)	8·8 (6–10·4)	7·6 (5·7–9·1)	8·1 (5·5–11)
1·33	Non-Hodgkin's lymphoma	2,540 (2,280–2,810)	4,320 (3,960–4,730)	5,250 (4,010–6,800)	128 (114–141)	163 (150–179)	177 (135–229)
1·34	Multiple myeloma	662 (598–778)	2,000 (1,300–2,200)	2,450 (1,530–3,190)	38·1 (34·4–44·7)	81 (52·2–89·2)	82·9 (51·9–108)
1·35	Leukaemia	2,850 (2,550–3,200)	4,330 (3,930–4,720)	4,850 (3,750–6,180)	122 (111–135)	163 (148–178)	171 (132–217)
1·36	Acute lymphoid leukaemia	884 (596–1,130)	803 (660–958)	703 (533–936)	31·4 (21·3–40·2)	30·3 (24·7–36·7)	28·4 (21·3–37·9)
1·37	Chronic lymphoid leukaemia	30 (23–38)	199 (129–234)	212 (149–282)	1·7 (1·3–2·1)	8·2 (5·3–9·6)	7·2 (5–9·5)
1·38	Acute myeloid leukaemia	459 (367–608)	894 (773–1,060)	1,120 (819–1,450)	18·8 (15·5–24·6)	33·4 (28·7–39·7)	39·9 (29·3–51·8)
1·39	Chronic myeloid leukaemia	127 (107–159)	397 (350–450)	411 (305–529)	6·4 (5·3–8·1)	15 (13·2–16·8)	13·7 (10·2–17·6)
1·40	Other leukaemia	1,350 (1,180–1,610)	2,040 (1,830–2,240)	2,400 (1,780–3,090)	63·7 (56·4–73·1)	76·5 (68·7–84·4)	81·4 (60·3–105)
1·41	Other neoplasms	184 (141–245)	483 (325–550)	552 (368–729)	9·5 (7·2–12·5)	18·7 (12·6–21·4)	18·9 (12·6–24·9)
1·42	Myelodysplastic, myeloproliferative, and other hematopoietic neoplasms	184 (141–245)	483 (325–550)	552 (368–729)	9·5 (7·2–12·5)	18·7 (12·6–21·4)	18·9 (12·6–24·9)
1·43	Benign and in situ intestinal neoplasms	0 (0–0)	0 (0–0)	0 (0–0)	0 (0–0)	0 (0–0)	0 (0–0)
1·44	Benign and in situ cervical and uterine neoplasms	0 (0–0)	0 (0–0)	0 (0–0)	0 (0–0)	0 (0–0)	0 (0–0)
1·45	Other benign and in situ neoplasms	0 (0–0)	0 (0–0)	0 (0–0)	0 (0–0)	0 (0–0)	0 (0–0)
1·46	Cardiovascular diseases	93,100 (88,700–96,600)	95,600 (89,900–100,000)	120,000 (98,700–144,000)	5,060 (4,810–5,250)	3,670 (3,470–3,840)	3,970 (3,260–4,800)
1·47	Rheumatic heart disease	3,950 (3,400–4,570)	2,370 (1,840–3,030)	2,610 (1,930–3,420)	165 (141–191)	82·4 (64·4–105)	87·4 (64·9–114)
1·48	Ischaemic heart disease	26,100 (24,300–28,400)	29,100 (27,100–30,800)	37,300 (30,300–45,100)	1,460 (1,360–1,590)	1,120 (1,050–1,180)	1,220 (998–1,480)
1·49	Stroke	42,200 (39,800–45,400)	41,000 (38,300–43,300)	51,900 (42,400–62,900)	2,300 (2,170–2,480)	1,590 (1,490–1,670)	1,730 (1,410–2,100)
1·50	Ischaemic stroke	17,700 (15,400–19,600)	17,800 (16,000–19,100)	21,900 (18,100–26,000)	936 (813–1,040)	662 (601–709)	717 (595–850)
1·51	Intracerebral hemorrhage	19,600 (17,700–21,500)	19,000 (17,800–20,100)	24,900 (19,700–31,000)	1,110 (1,010–1,220)	760 (714–809)	840 (665–1,050)
1·52	Subarachnoid hemorrhage	4,950 (4,290–5,580)	4,260 (3,910–4,610)	5,150 (4,000–6,530)	253 (220–286)	165 (151–178)	175 (136–223)
1·53	Hypertensive heart disease	13,000 (8,250–14,300)	11,400 (10,400–14,500)	13,700 (10,800–18,000)	721 (461–791)	439 (399–548)	450 (357–594)
1·54	Non-rheumatic valvular heart disease	206 (179–236)	370 (339–404)	418 (326–528)	10·8 (9·4–12·3)	13·9 (12·8–15·2)	13·8 (10·7–17·5)
1·55	Non-rheumatic calcific aortic valve disease	110 (83–130)	205 (178–235)	225 (174–298)	5·6 (4·3–6·6)	7·7 (6·7–8·8)	7·4 (5·7–9·8)
1·56	Non-rheumatic degenerative mitral valve disease	94 (78–113)	163 (136–191)	190 (143–246)	5 (4·2–6)	6·2 (5·1–7·2)	6·3 (4·8–8·2)
1·57	Other non-rheumatic valve diseases	2 (1–3)	2 (2–3)	3 (2–4)	0·1 (0·1–0·1)	0·1 (0·1–0·1)	0·1 (0·1–0·1)
1·58	Cardiomyopathy and myocarditis	2,320 (1,900–2,670)	2,830 (2,490–3,130)	3,360 (2,570–4,270)	117 (94·4–136)	109 (96·3–120)	114 (87·4–145)
1·59	Myocarditis	139 (93–179)	125 (101–160)	145 (104–203)	5·7 (3·8–7·3)	4·8 (3·9–6·2)	5·4 (3·8–7·4)
1·60	Alcoholic cardiomyopathy	122 (84–192)	400 (324–554)	480 (335–696)	7·2 (4·9–11·5)	15·9 (12·9–22·1)	16·1 (11·2–23·2)

1-61	Other cardiomyopathy	2,060 (1,660–2,380)	2,300 (1,990–2,510)	2,730 (2,100–3,490)	104 (82·1–121)	87·9 (76·2–95·9)	92·9 (71·8–118)
1-62	Atrial fibrillation and flutter	1,420 (1,170–1,720)	2,370 (1,960–3,010)	2,980 (2,380–3,850)	78·1 (64·6–94·8)	85·5 (70·9–109)	92·9 (73·9–120)
1-63	Aortic aneurysm	532 (478–601)	1,110 (1,020–1,220)	1,390 (1,100–1,730)	29·6 (26·6–33·3)	43·8 (40·1–48)	46·7 (36·8–58·2)
1-64	Peripheral vascular disease	505 (243–984)	937 (480–1,700)	1,180 (592–2,230)	27·9 (13·4–54·3)	35·3 (18–64·3)	38·1 (19·2–73·9)
1-65	Endocarditis	495 (362–596)	995 (591–1,190)	1,230 (673–1,720)	24·5 (17·7–29·6)	36·8 (22·2–44)	40·8 (22·8–57·2)
1-66	Chronic respiratory diseases	16,700 (13,700–20,300)	19,900 (17,000–23,200)	21,000 (17,100–25,500)	760 (640–902)	759 (651–882)	741 (597–906)
1-67	Chronic obstructive pulmonary disease	5,930 (5,380–6,600)	10,500 (9,570–11,800)	11,600 (9,280–14,300)	323 (292–359)	410 (373–459)	390 (311–478)
1-68	Pneumoconiosis	52 (40–68)	13 (11–15)	14 (11–19)	2·9 (2·2–3·7)	0·5 (0·4–0·6)	0·5 (0·4–0·6)
1-69	Silicosis	11 (7–18)	2 (2–3)	2 (1–3)	0·6 (0·4–1)	0·1 (0·1–0·1)	0·1 (0–0·1)
1-70	Asbestosis	12 (5–18)	3 (3–4)	6 (4–8)	0·6 (0·3–1)	0·1 (0·1–0·2)	0·2 (0·1–0·3)
1-71	Coal workers pneumoconiosis	3 (1–6)	0 (0–1)	0 (0–1)	0·2 (0·1–0·4)	0 (0–0)	0 (0–0)
1-72	Other pneumoconiosis	26 (18–37)	6 (5–8)	6 (4–8)	1·5 (1–2)	0·2 (0·2–0·3)	0·2 (0·1–0·3)
1-73	Asthma	9,800 (7,170–13,200)	8,120 (5,890–11,300)	7,960 (5,660–10,900)	396 (297–521)	302 (220–417)	301 (211–425)
1-74	Interstitial lung disease and pulmonary sarcoidosis	260 (205–340)	524 (389–634)	634 (424–864)	13·5 (10·8–17·7)	20·4 (15·1–24·7)	21·5 (14·4–29·3)
1-75	Other chronic respiratory diseases	643 (446–827)	701 (593–888)	782 (606–1,040)	26 (19·1–32·4)	26·1 (21·9–33·2)	28·2 (21·8–37·3)
1-76	Digestive diseases	17,100 (15,300–19,600)	17,700 (15,400–20,900)	20,500 (16,500–25,000)	859 (772–988)	674 (589–793)	694 (561–847)
1-77	Cirrhosis and other chronic liver diseases	4,870 (4,520–5,300)	4,250 (3,920–4,660)	5,080 (3,810–6,550)	260 (241–282)	164 (151–180)	170 (128–219)
1-78	Cirrhosis and other chronic liver diseases due to hepatitis B	491 (349–666)	493 (347–672)	331 (211–500)	29 (20·4–40)	19 (13·4–26)	11 (7–16·6)
1-79	Cirrhosis and other chronic liver diseases due to hepatitis C	774 (572–1,030)	758 (576–1,010)	972 (643–1,390)	44·9 (32·6–60·9)	29·3 (22·1–39·3)	32·4 (21·4–46·2)
1-80	Cirrhosis and other chronic liver diseases due to alcohol use	1,460 (1,120–1,780)	1,510 (1,190–1,830)	1,950 (1,370–2,690)	87·3 (66·6–107)	60 (46·7–72·7)	65·3 (45·7–90·1)
1-81	Cirrhosis and other chronic liver diseases due to NAFLD	582 (418–787)	616 (446–830)	825 (533–1,200)	33·4 (23·4–45·7)	23·9 (17·1–32·8)	27·5 (17·8–40·1)
1-82	Cirrhosis and other chronic liver diseases due to other causes	1,570 (1,300–1,870)	876 (697–1,090)	1,000 (704–1,400)	65·2 (53·9–77·8)	32·2 (25·6–40·4)	34·3 (24–47·7)
1-83	Upper digestive system diseases	5,290 (3,950–7,300)	6,290 (4,470–9,130)	7,090 (4,990–10,400)	280 (211–383)	235 (168–339)	235 (166–342)
1-84	Peptic ulcer disease	2,230 (2,000–2,460)	2,000 (1,790–2,230)	2,250 (1,720–2,860)	125 (111–138)	77·4 (69–86·2)	74·9 (57·2–95·3)
1-85	Gastritis and duodenitis	632 (444–883)	923 (658–1,300)	1,100 (780–1,530)	31·8 (22·2–44·7)	34·9 (24·7–49)	37 (26·4–51·7)
1-86	GERD	2,430 (1,260–4,310)	3,370 (1,760–5,940)	3,740 (1,950–6,630)	123 (64·2–219)	123 (64·2–218)	123 (64·2–217)
1-87	Appendicitis	666 (516–849)	386 (305–562)	465 (346–649)	27 (20·8–34·2)	14 (11–20·8)	16·1 (12–22·7)
1-88	Paralytic ileus and intestinal obstruction	2,100 (1,730–2,540)	1,490 (1,290–1,890)	1,640 (1,230–2,200)	91·7 (77·6–110)	59·8 (51·5–75·7)	60·4 (44·9–80·8)
1-89	Inguinal, femoral, and abdominal hernia	1,500 (1,110–1,980)	1,770 (1,300–2,360)	1,950 (1,440–2,560)	66·5 (49·4–87·5)	67·6 (50·3–90·1)	69·9 (51·7–92·6)
1-90	Inflammatory bowel disease	304 (231–367)	306 (251–375)	343 (264–431)	14·9 (11·5–17·7)	11·5 (9·4–14·1)	11·5 (8·8–14·5)
1-91	Vascular intestinal disorders	260 (221–300)	288 (259–321)	352 (268–450)	14·1 (12–16·1)	11·2 (10–12·5)	11·8 (9–15·2)
1-92	Gallbladder and biliary diseases	1,070 (816–1,420)	1,190 (867–1,620)	1,400 (1,010–1,840)	56·8 (43·6–74·3)	44·2 (32·5–59·7)	46·5 (33·5–61·4)
1-93	Pancreatitis	360 (300–420)	432 (384–483)	543 (415–695)	18·9 (15·7–22·3)	16·4 (14·6–18·3)	18·1 (13·8–23·2)
1-94	Other digestive diseases	644 (511–935)	1,280 (764–1,570)	1,600 (945–2,220)	28·8 (23–42·7)	49·1 (29·5–60·3)	54·8 (32·7–75·6)
1-95	Neurological disorders	25,500 (13,900–42,400)	34,400 (19,300–57,600)	37,900 (20,700–62,100)	1,190 (663–2,000)	1,200 (681–2,010)	1,240 (677–2,020)
1-96	Alzheimer's disease and other dementias	5,380 (2,290–12,400)	9,290 (3,920–20,800)	10,500 (4,390–23,200)	293 (125–671)	313 (131–701)	312 (132–694)
1-97	Parkinson's disease	1,160 (1,060–1,260)	2,000 (1,810–2,180)	2,300 (1,930–2,720)	62 (56·5–67·2)	75·2 (68·7–81·6)	76·4 (64–90·8)
1-98	Idiopathic epilepsy	5,210 (2,810–8,650)	5,280 (2,580–9,010)	6,090 (3,040–10,200)	221 (119–365)	190 (93·5–324)	212 (105–357)
1-99	Multiple sclerosis	106 (80–126)	175 (144–206)	212 (164–265)	6 (4·5–7·1)	6·5 (5·4–7·7)	7·1 (5·5–8·8)
1-100	Motor neuron disease	110 (101–122)	295 (265–328)	383 (285–496)	5·8 (5·4–6·3)	12 (10·7–13·5)	13·9 (10·1–18·2)
1-101	Headache disorders	12,500 (2,200–28,000)	15,900 (3,050–35,200)	16,600 (3,280–36,500)	556 (110–1,230)	554 (110–1,220)	552 (108–1,220)
1-102	Migraine	11,400 (1,430–26,600)	14,400 (2,030–33,300)	15,000 (2,220–34,300)	504 (73·2–1,160)	502 (73–1,150)	500 (73–1,140)
1-103	Tension-type headache	1,110 (310–4,390)	1,470 (428–5,430)	1,570 (466–5,710)	52·4 (15·5–194)	52·4 (15·5–187)	52·3 (15·4–189)
1-104	Other neurological disorders	1,100 (681–1,710)	1,470 (928–2,210)	1,800 (1,090–2,710)	46·8 (30·4–70·2)	53·6 (34·4–80·1)	64·4 (38·6–98·4)
1-105	Mental disorders	35,500 (26,000–47,300)	44,900 (32,600–59,400)	48,800 (35,700–64,400)	1,610 (1,190–2,130)	1,590 (1,160–2,100)	1,630 (1,190–2,140)
1-106	Schizophrenia	3,490 (2,420–4,760)	4,930 (3,430–6,570)	5,410 (3,750–7,230)	178 (124–237)	178 (124–237)	178 (123–236)
1-107	Depressive disorders	11,100 (7,440–15,400)	13,800 (9,400–19,200)	16,200 (11,000–22,400)	527 (360–730)	493 (338–687)	532 (361–735)
1-108	Major depressive disorder	9,230 (6,080–13,000)	11,300 (7,590–15,900)	13,400 (8,890–18,800)	437 (287–614)	403 (270–560)	442 (293–620)
1-109	Dysthymia	1,840 (1,130–2,870)	2,510 (1,540–3,860)	2,740 (1,700–4,200)	90·6 (56·5–139)	90·3 (56–138)	90·2 (55·8–137)
1-110	Bipolar disorder	4,520 (2,580–7,080)	5,880 (3,400–9,040)	6,270 (3,680–9,640)	206 (119–318)	205 (120–316)	205 (120–316)
1-111	Anxiety disorders	8,430 (5,550–12,100)	10,900 (7,220–15,400)	11,600 (7,650–16,500)	377 (249–531)	383 (253–540)	388 (257–553)
1-112	Eating disorders	997 (604–1,520)	1,230 (736–1,860)	1,280 (769–1,960)	39·1 (23·7–59·9)	40·8 (24·6–61·8)	41·1 (24·7–62·2)
1-113	Anorexia nervosa	259 (146–431)	306 (171–493)	310 (178–501)	9·6 (5·6–15·5)	9·9 (5·6–15·8)	10·1 (5·8–16·2)

1-114	Bulimia nervosa	738 (421–1,210)	922 (517–1,450)	972 (558–1,560)	29·4 (16·6–47·8)	30·9 (17·4–48·9)	31 (17·9–49·6)
1-115	Autism spectrum disorders	1,300 (858–1,900)	1,470 (961–2,120)	1,470 (948–2,120)	52·9 (35·1–77·1)	53·1 (34·8–76·6)	53·2 (34·1–76·8)
1-116	Attention-deficit/hyperactivity disorder	1,010 (569–1,710)	1,100 (621–1,880)	1,040 (595–1,760)	37·1 (21·1–62·6)	37·3 (21·1–63·7)	37·3 (21·2–63·4)
1-117	Conduct disorder	2,060 (1,170–3,300)	2,110 (1,210–3,350)	1,760 (968–2,800)	67·9 (38·4–109)	68·2 (39·3–107)	68·2 (37·9–108)
1-118	Idiopathic developmental intellectual disability	504 (170–940)	512 (163–947)	503 (156–933)	20·2 (6·8–37·6)	18·4 (5·8–34·1)	18·2 (5·6–33·7)
1-119	Other mental disorders	2,110 (1,320–3,200)	2,970 (1,890–4,490)	3,300 (2,100–5,000)	108 (68–165)	108 (69·3–163)	108 (69–165)
1-120	Substance use disorders	5,240 (3,660–7,030)	7,070 (5,030–9,400)	7,300 (5,150–9,660)	232 (163–310)	245 (174–325)	235 (166–311)
1-121	Alcohol use disorders	2,520 (1,620–3,650)	3,450 (2,320–4,890)	3,560 (2,400–5,060)	118 (77·8–169)	123 (82·8–173)	115 (78·3–163)
1-122	Drug use disorders	2,720 (1,850–3,730)	3,620 (2,510–4,910)	3,740 (2,620–5,050)	113 (78·6–153)	122 (85·1–165)	120 (83·8–161)
1-123	Opioid use disorders	880 (557–1,290)	1,210 (779–1,700)	1,370 (904–1,920)	40·6 (26·2–57·7)	42·7 (27·5–58·9)	44 (29·1–61·3)
1-124	Cocaine use disorders	663 (387–1,040)	877 (502–1,370)	821 (475–1,280)	26·6 (15·8–41)	29 (16·7–45·4)	26·3 (15·2–40·8)
1-125	Amphetamine use disorders	146 (70–250)	176 (92–298)	198 (107–331)	5·8 (2·9–9·7)	5·9 (3·2–9·9)	6·1 (3·3–10·2)
1-126	Cannabis use disorders	481 (254–825)	626 (355–983)	621 (351–1,000)	18·2 (10·1–30·5)	20·1 (11·4–31·5)	20 (11·3–31·9)
1-127	Other drug use disorders	549 (336–847)	732 (444–1,090)	726 (447–1,090)	22·3 (13·7–33·9)	24·4 (14·9–36·4)	23·2 (14·2–34·8)
1-128	Diabetes and kidney diseases	44,000 (40,300–47,900)	76,000 (68,100–84,300)	97,100 (80,600–115,000)	2,450 (2,240–2,670)	2,970 (2,660–3,290)	3,250 (2,690–3,870)
1-129	Diabetes mellitus	32,200 (29,100–35,600)	54,600 (47,800–62,100)	69,600 (57,200–83,700)	1,820 (1,640–2,010)	2,150 (1,880–2,440)	2,340 (1,920–2,810)
1-130	Diabetes mellitus type 1	1,900 (1,390–2,190)	1,920 (1,600–2,860)	2,280 (1,700–3,530)	98·4 (70·6–114)	71·8 (59·6–108)	77 (57·3–119)
1-131	Diabetes mellitus type 2	30,300 (27,300–33,700)	52,700 (46,200–60,000)	67,300 (55,500–80,800)	1,720 (1,550–1,910)	2,080 (1,820–2,370)	2,260 (1,860–2,710)
1-132	Chronic kidney disease	11,800 (10,900–12,800)	21,400 (19,700–23,200)	27,500 (22,400–33,600)	629 (582–684)	817 (755–884)	918 (747–1,120)
1-133	Chronic kidney disease due to diabetes mellitus type 1	725 (503–987)	1,730 (1,180–2,350)	2,310 (1,470–3,460)	41·4 (27·5–57·1)	64·6 (43·5–88·4)	77·4 (49·2–116)
1-134	Chronic kidney disease due to diabetes mellitus type 2	2,620 (2,140–3,100)	4,900 (4,010–5,790)	6,500 (4,990–8,360)	148 (120–175)	193 (158–228)	217 (166–278)
1-135	Chronic kidney disease due to hypertension	2,380 (1,980–2,830)	4,210 (3,510–4,960)	5,360 (4,190–6,790)	131 (109–155)	162 (135–191)	177 (138–226)
1-136	Chronic kidney disease due to glomerulonephritis	2,580 (2,180–3,060)	4,660 (3,820–5,580)	6,000 (4,430–7,860)	133 (110–160)	176 (143–212)	201 (148–264)
1-137	Chronic kidney disease due to other and unspecified causes	3,500 (2,910–4,100)	5,900 (4,830–7,160)	7,320 (5,690–9,420)	176 (146–210)	222 (181–269)	245 (190–315)
1-138	Acute glomerulonephritis	2 (1–2)	3 (2–3)	2 (2–3)	0·1 (0–0·1)	0·1 (0·1–0·1)	0·1 (0·1–0·1)
1-139	Skin and subcutaneous diseases	13,600 (9,320–19,800)	15,900 (10,900–23,100)	16,500 (11,500–23,600)	573 (398–830)	576 (398–835)	588 (408–840)
1-140	Dermatitis	2,910 (1,700–4,700)	3,120 (1,850–4,920)	3,060 (1,820–4,760)	116 (68·2–184)	116 (68·6–182)	115 (68·2–182)
1-141	Atopic dermatitis	2,270 (1,230–3,850)	2,280 (1,240–3,830)	2,150 (1,150–3,640)	85·3 (46·1–144)	85 (46·1–142)	85 (46–144)
1-142	Contact dermatitis	565 (354–867)	748 (463–1,120)	816 (515–1,210)	27·2 (16·9–41·2)	27·1 (16·9–40·6)	27·1 (17·1–40·2)
1-143	Seborrhoeic dermatitis	76 (42–122)	94 (52–149)	99 (56–159)	3·4 (1·9–5·4)	3·4 (1·9–5·4)	3·4 (1·9–5·4)
1-144	Psoriasis	805 (558–1,080)	923 (647–1,240)	958 (676–1,290)	37·8 (26·5–50·6)	33·7 (23·8–45)	32·7 (23–43·9)
1-145	Bacterial skin diseases	401 (257–781)	665 (397–1,120)	876 (480–1,390)	18·4 (12–32·8)	25·1 (15·4–43)	30·2 (17·3–49)
1-146	Cellulitis	104 (65–231)	144 (81–225)	175 (86–262)	4·8 (3·1–9·4)	5·5 (3·1–8·6)	6·1 (3·2–9·5)
1-147	Pyoderma	297 (182–563)	521 (304–896)	701 (377–1,150)	13·5 (8·4–23·9)	19·6 (11·6–34·6)	24·1 (13·7–40·9)
1-148	Scabies	2,120 (1,180–3,420)	2,330 (1,270–3,700)	2,280 (1,260–3,660)	83·8 (46·4–134)	83·3 (45·3–133)	82·9 (46·2–132)
1-149	Fungal skin diseases	955 (379–2,010)	1,200 (484–2,560)	1,290 (520–2,720)	44·1 (17·7–93·3)	43·9 (17·7–93·4)	44 (17·7–93·3)
1-150	Viral skin diseases	1,290 (810–1,940)	1,270 (812–1,930)	1,190 (754–1,780)	47·6 (29·9–71·4)	47·6 (30·5–72·1)	47·5 (30·2–71·4)
1-151	Acne vulgaris	1,210 (715–1,960)	1,520 (902–2,430)	1,410 (848–2,250)	41·5 (24·5–66·9)	47·7 (28·2–76·1)	49·1 (29·4–78)
1-152	Alopecia areata	159 (99–240)	200 (126–300)	213 (136–320)	7·2 (4·5–10·7)	7·1 (4·5–10·7)	7·1 (4·5–10·7)
1-153	Pruritus	177 (85–323)	227 (108–413)	243 (116–438)	8·3 (4–15)	8·4 (4–15·2)	8·4 (4–15·1)
1-154	Urticaria	1,210 (793–1,760)	1,290 (839–1,870)	1,260 (834–1,810)	48·3 (31·6–70·6)	48·1 (31·4–70·1)	48·1 (31·6–69·7)
1-155	Decubitus ulcer	1,360 (731–1,770)	1,860 (1,170–2,780)	2,310 (1,340–3,320)	73 (39·1–95)	68·8 (43·1–103)	74·7 (43·2–107)
1-156	Other skin and subcutaneous diseases	979 (494–1,760)	1,260 (622–2,280)	1,390 (696–2,480)	47·4 (23·8–85·5)	47·2 (23·3–85·7)	47·9 (24–85·2)
1-157	Sense organ diseases	15,200 (10,400–21,600)	20,000 (13,500–28,500)	22,100 (14,900–31,700)	807 (545–1,150)	752 (506–1,080)	736 (496–1,060)
1-158	Blindness and vision loss	4,440 (3,040–6,230)	5,430 (3,690–7,790)	5,910 (4,010–8,530)	235 (161–332)	203 (138–292)	196 (132–283)
1-159	Glaucoma	288 (188–414)	317 (211–457)	340 (225–485)	15·5 (10·2–22·2)	11·6 (7·7–16·7)	10·9 (7·2–15·6)
1-160	Cataract	808 (556–1,100)	956 (668–1,310)	1,020 (713–1,390)	44·1 (30·6–60·1)	34·9 (24·3–47·8)	32·9 (22·7–44·7)
1-161	Age-related macular degeneration	30 (20–43)	37 (24–53)	41 (27–59)	1·6 (1·1–2·4)	1·4 (0·9–2·1)	1·4 (0·9–1·9)
1-162	Refraction disorders	1,250 (823–1,810)	1,500 (990–2,170)	1,570 (1,030–2,280)	58·9 (39–84·7)	55 (36·1–79·1)	53·6 (35·2–78)
1-163	Near vision loss	952 (432–1,850)	1,290 (585–2,520)	1,470 (680–2,910)	54·3 (25·1–106)	50·3 (22·7–98·6)	49·1 (22·6–97·2)
1-164	Other vision loss	1,110 (784–1,530)	1,330 (940–1,810)	1,460 (1,030–2,000)	60·3 (42·6–83·1)	50 (35·5–68·6)	47·9 (33·5–65·7)
1-165	Age-related and other hearing loss	10,100 (6,740–14,500)	13,600 (9,050–19,700)	15,100 (10,100–22,100)	536 (353–777)	513 (341–746)	504 (336–738)
1-166	Other sense organ diseases	719 (426–1,100)	963 (569–1,490)	1,070 (628–1,640)	36·2 (21·2–55·9)	36·6 (21·8–56·6)	36·6 (21·7–56)

1-167	Musculoskeletal disorders	29,500 (21,500–38,600)	43,300 (31,600–57,000)	48,700 (35,800–63,800)	1,540 (1,110–2,020)	1,610 (1,170–2,110)	1,620 (1,190–2,120)
1-168	Rheumatoid arthritis	443 (310–590)	775 (549–1,030)	927 (656–1,270)	23·7 (16·7–31·7)	28·7 (20·5–38·5)	30·4 (21·5–41·3)
1-169	Osteoarthritis	3,240 (1,640–6,420)	5,100 (2,540–10,300)	6,060 (3,050–12,000)	189 (95·5–373)	201 (100–405)	203 (102–401)
1-170	Low back pain	14,900 (10,600–20,000)	20,600 (14,400–27,800)	22,300 (15,700–29,700)	750 (528–1,010)	754 (526–1,020)	741 (524–993)
1-171	Neck pain	2,510 (1,640–3,620)	3,650 (2,400–5,270)	4,050 (2,660–5,820)	136 (88·5–196)	135 (88·9–195)	135 (89·4–194)
1-172	Gout	118 (74–175)	205 (126–295)	238 (147–348)	6·6 (4·2–9·8)	7·8 (4·8–11·2)	7·9 (4·9–11·6)
1-173	Other musculoskeletal disorders	8,240 (5,750–11,000)	13,000 (9,270–17,200)	15,100 (10,900–20,200)	434 (306–580)	481 (343–636)	500 (360–666)
1-174	Other non-communicable diseases	44,200 (34,600–55,000)	47,700 (37,600–60,100)	49,200 (38,400–63,100)	1,920 (1,500–2,430)	1,890 (1,470–2,360)	1,850 (1,440–2,350)
1-175	Congenital anomalies	18,000 (11,100–22,800)	13,000 (8,620–16,600)	10,800 (7,170–15,000)	667 (414–843)	600 (388–777)	545 (352–768)
1-176	Neural tube defects	891 (613–1,240)	435 (296–589)	391 (261–582)	33 (22·7–45·9)	20·5 (13·7–28·3)	20·5 (13·3–31·1)
1-177	Congenital heart anomalies	8,820 (4,730–12,500)	5,690 (3,470–7,380)	4,490 (2,890–6,400)	324 (176–460)	267 (160–349)	232 (147–335)
1-178	Orofacial clefts	95 (61–153)	82 (51–121)	88 (57–135)	3·8 (2·4–5·9)	3·1 (2–4·6)	3·3 (2·2–5·1)
1-179	Down's syndrome	333 (241–555)	616 (464–777)	553 (391–754)	12·7 (9·4–20·8)	26·5 (19·5–34)	24·6 (17–34·4)
1-180	Turner syndrome	3 (1–6)	3 (2–6)	3 (2–6)	0·1 (0·1–0·2)	0·1 (0·1–0·2)	0·1 (0·1–0·2)
1-181	Klinefelter syndrome	0 (0–1)	0 (0–1)	0 (0–1)	0 (0–0)	0 (0–0)	0 (0–0)
1-182	Other chromosomal abnormalities	544 (248–794)	768 (315–1,090)	632 (270–949)	20 (9·1–29·2)	36·4 (14·1–52·4)	33·5 (13·5–51·3)
1-183	Congenital musculoskeletal and limb anomalies	1,160 (791–1,590)	1,130 (743–1,620)	1,160 (761–1,650)	45·6 (31·2–61·8)	42·9 (28·6–60·8)	45·2 (30·1–64·3)
1-184	Urogenital congenital anomalies	560 (276–1,000)	332 (189–604)	280 (167–465)	20·6 (10·3–37·1)	15·6 (8·7–29·5)	14·5 (8·4–25·4)
1-185	Digestive congenital anomalies	2,500 (973–3,910)	1,590 (633–2,330)	1,210 (485–1,860)	91·7 (35·9–143)	78·3 (30·9–114)	67·3 (26·9–104)
1-186	Other congenital anomalies	3,100 (1,890–4,340)	2,340 (1,260–3,280)	2,020 (1,060–3,030)	115 (70·7–161)	109 (57·3–155)	104 (52·8–157)
1-187	Urinary diseases and male infertility	1,490 (1,170–1,940)	3,350 (2,500–3,990)	4,060 (2,960–5,070)	76·5 (60·1–99·3)	129 (96·4–154)	136 (99·7–171)
1-188	Urinary tract infections and interstitial nephritis	737 (639–990)	2,140 (1,570–2,400)	2,610 (1,810–3,320)	37·1 (32·3–50·3)	81·5 (59·4–90·9)	87·1 (60·4–111)
1-189	Urolithiasis	62 (42–86)	121 (88–161)	163 (120–220)	3·3 (2·2–4·7)	4·6 (3·4–6·2)	5·5 (4–7·4)
1-190	Benign prostatic hyperplasia	466 (274–731)	683 (398–1,070)	821 (479–1,280)	25·8 (15·1–40·4)	28·2 (16·3–44·5)	28·2 (16·4–44)
1-191	Male infertility	106 (40–245)	139 (52–325)	149 (56–358)	4·7 (1·8–11·4)	4·8 (1·8–11·3)	4·8 (1·8–11·4)
1-192	Other urinary diseases	117 (92–156)	261 (139–341)	320 (150–452)	5·6 (4·3–7·4)	9·8 (5·3–13)	10·8 (5·3–15·3)
1-193	Gynecological diseases	7,970 (5,510–11,200)	10,700 (7,420–15,000)	11,400 (7,890–16,000)	384 (263–538)	378 (262–532)	373 (259–524)
1-194	Uterine fibroids	461 (240–843)	797 (453–1,400)	883 (496–1,510)	25·5 (13·2–46)	28·9 (16·3–50·6)	29·5 (16·6–50·6)
1-195	Polycystic ovarian syndrome	110 (46–227)	186 (77–374)	199 (82–407)	4·7 (1·9–9·4)	6·2 (2·6–12·5)	6·5 (2·7–13·2)
1-196	Female infertility	215 (77–521)	283 (100–665)	298 (108–690)	9·7 (3·5–22·9)	9·9 (3·5–23·2)	9·7 (3·5–22·9)
1-197	Endometriosis	799 (450–1,340)	895 (523–1,440)	871 (501–1,410)	35·2 (20·4–57·9)	30·6 (17·9–49·1)	27·9 (16·1–45·2)
1-198	Genital prolapse	143 (74–261)	185 (104–328)	196 (112–341)	8·4 (4·3–15·3)	7·1 (4–12·4)	6·5 (3·7–11·3)
1-199	Premenstrual syndrome	2,370 (1,460–3,750)	3,020 (1,860–4,690)	3,130 (1,930–4,880)	102 (62·9–160)	102 (61·9–160)	103 (63·1–159)
1-200	Other gynecological diseases	3,870 (2,470–5,540)	5,330 (3,420–7,640)	5,810 (3,730–8,330)	198 (128–284)	193 (124–277)	191 (122–273)
1-201	Hemoglobinopathies and hemolytic anaemias	4,900 (3,930–6,070)	4,850 (4,050–5,830)	5,090 (4,000–6,360)	198 (160–245)	177 (149–213)	181 (142–226)
1-202	Thalassemias	3 (1–5)	2 (1–4)	2 (1–4)	0·1 (0–0·2)	0·1 (0·1–0·2)	0·1 (0·1–0·2)
1-203	Thalassaemias trait	115 (71–172)	126 (75–200)	131 (80–206)	4·7 (2·9–7)	4·6 (2·7–7·2)	4·7 (2·9–7·4)
1-204	Sickle cell disorders	2,360 (2,020–2,710)	2,540 (2,280–2,800)	2,610 (1,960–3,410)	90·1 (78–102)	91·8 (82·3–102)	93·8 (71–123)
1-205	Sickle cell trait	1,480 (889–2,270)	1,080 (627–1,820)	834 (474–1,360)	58·6 (35·3–90·1)	39·4 (23·5–64·3)	30·4 (17·6–47·3)
1-206	G6PD deficiency	232 (157–324)	372 (281–483)	620 (414–858)	10·9 (7·6–15·1)	13·8 (10·5–17·8)	20·8 (13·8–28·7)
1-207	G6PD trait	3 (2–7)	10 (4–17)	11 (6–18)	0·2 (0·1–0·4)	0·3 (0·1–0·6)	0·4 (0·2–0·6)
1-208	Other hemoglobinopathies and hemolytic anaemias	703 (511–953)	719 (532–965)	884 (656–1,170)	33·6 (25·1–45)	27·2 (20·3–36·4)	30·6 (22·6–40·8)
1-209	Endocrine, metabolic, blood, and immune disorders	5,030 (3,900–6,370)	6,970 (5,540–8,760)	8,040 (6,230–10,300)	246 (189–313)	269 (214–338)	283 (219–364)
1-210	Oral disorders	6,520 (3,810–10,600)	8,750 (5,090–14,300)	9,740 (5,640–15,800)	339 (199–550)	330 (193–540)	328 (190–531)
1-211	Caries of deciduous teeth	90 (39–186)	72 (31–148)	64 (27–135)	3·1 (1·4–6·4)	3·1 (1·3–6·4)	3·1 (1·3–6·7)
1-212	Caries of permanent teeth	628 (279–1,220)	756 (338–1,450)	781 (342–1,510)	27·1 (12–52·7)	26·3 (11·8–50·5)	26·3 (11·6–50·7)
1-213	Periodontal disease	1,980 (756–4,170)	2,940 (1,110–6,310)	3,270 (1,250–6,900)	108 (41·9–229)	109 (41–232)	108 (41·2–228)
1-214	Edentulism	2,630 (1,670–3,930)	3,510 (2,210–5,270)	4,080 (2,570–6,100)	148 (93·9–221)	139 (87·3–210)	137 (86·1–205)
1-215	Other oral disorders	1,190 (734–1,740)	1,470 (912–2,160)	1,550 (964–2,270)	53·4 (33·2–78·1)	53·2 (33–78)	53·2 (33·1–78·4)
1-216	Sudden infant death syndrome	249 (142–417)	90 (57–131)	86 (48–145)	9·2 (5·2–15·4)	4·5 (2·9–6·6)	4·9 (2·8–8·3)
1-0	Injuries	47,500 (43,000–52,500)	65,300 (60,000–71,800)	63,300 (51,900–76,100)	2,110 (1,900–2,360)	2,340 (2,150–2,580)	2,110 (1,730–2,540)
1-1	Transport injuries	8,170 (7,380–9,030)	11,100 (10,200–12,200)	11,100 (9,000–13,500)	358 (322–400)	403 (367–440)	372 (301–452)
1-2	Road injuries	7,680 (6,960–8,480)	10,500 (9,610–11,500)	10,400 (8,430–12,600)	336 (304–374)	380 (347–415)	349 (281–424)

1·3	Pedestrian road injuries	1,950 (1,750–2,180)	2,400 (2,180–2,630)	2,110 (1,720–2,580)	85·2 (76·6–94·5)	90·3 (82–98·7)	73·8 (59·8–90·4)
1·4	Cyclist road injuries	1,000 (796–1,250)	1,320 (1,100–1,580)	1,380 (1,090–1,700)	47·6 (37·6–59·5)	48 (39·8–57·5)	46 (36·2–56·7)
1·5	Motorcyclist road injuries	1,190 (925–1,360)	1,600 (1,430–1,780)	1,650 (1,320–2,000)	52 (41·2–59·8)	56·1 (50·1–62·8)	53·7 (43·1–65·4)
1·6	Motor vehicle road injuries	3,380 (3,040–3,910)	5,010 (4,640–5,440)	5,040 (3,950–6,270)	144 (129–166)	179 (165–194)	168 (131–208)
1·7	Other road injuries	162 (122–209)	196 (149–254)	217 (164–280)	8 (5·9–10·5)	7·2 (5·5–9·4)	7·3 (5·5–9·4)
1·8	Other transport injuries	489 (408–615)	613 (535–703)	688 (553–841)	21·9 (18·3–27·6)	22·2 (19·3–25·4)	23·3 (18·7–28·4)
1·9	Unintentional injuries	21,900 (18,500–26,100)	23,000 (18,900–28,100)	25,200 (20,100–31,400)	994 (830–1,190)	840 (694–1,030)	854 (683–1,060)
1·10	Falls	2,810 (2,270–3,500)	4,600 (3,740–5,720)	5,650 (4,480–7,050)	138 (111–172)	168 (137–209)	187 (148–234)
1·11	Drowning	1,460 (1,300–1,630)	674 (603–755)	744 (595–919)	55·9 (50·2–61·9)	24·3 (21·8–27·5)	26·2 (20·8–32·3)
1·12	Fire, heat, and hot substances	3,020 (2,350–3,850)	2,530 (1,840–3,460)	2,930 (2,120–4,030)	136 (105–175)	92·2 (67·5–126)	99·9 (73–137)
1·13	Poisonings	413 (337–514)	336 (267–414)	329 (251–419)	17 (13·7–21·4)	12·2 (9·8–15)	11·5 (8·7–14·6)
1·14	Poisoning by carbon monoxide	64 (50–84)	82 (67–103)	87 (66–111)	2·6 (2–3·4)	2·9 (2·4–3·7)	3 (2·3–3·8)
1·15	Poisoning by other means	349 (280–436)	254 (196–324)	243 (178–318)	14·3 (11·5–18·1)	9·3 (7·2–11·8)	8·5 (6·2–11·1)
1·16	Exposure to mechanical forces	6,060 (4,930–7,630)	7,810 (6,410–9,720)	8,840 (7,030–11,000)	304 (246–383)	282 (230–353)	292 (232–365)
1·17	Unintentional firearm injuries	3,100 (2,760–3,480)	4,080 (3,690–4,470)	4,600 (3,690–5,590)	157 (139–177)	145 (130–159)	150 (120–182)
1·18	Other exposure to mechanical forces	2,960 (2,050–4,260)	3,730 (2,570–5,390)	4,240 (2,940–6,170)	147 (102–211)	137 (94·9–199)	142 (99·1–207)
1·19	Adverse effects of medical treatment	684 (596–933)	1,670 (1,070–1,860)	1,860 (1,130–2,420)	31·3 (27·3–44·5)	63·8 (41·8–71·6)	65·3 (40·2–85·4)
1·20	Animal contact	293 (202–406)	315 (227–432)	335 (236–464)	13·4 (9·4–18·8)	11·4 (8·3–15·7)	11·4 (8·1–15·9)
1·21	Venomous animal contact	202 (134–277)	208 (142–288)	224 (155–306)	9·2 (6·1–12·7)	7·5 (5·1–10·4)	7·6 (5·3–10·5)
1·22	Non-venomous animal contact	91 (61–138)	107 (76–153)	111 (78–163)	4·3 (2·9–6·5)	3·9 (2·8–5·6)	3·8 (2·7–5·6)
1·23	Foreign body	4,120 (3,570–4,760)	1,110 (937–1,320)	1,100 (867–1,350)	159 (139–183)	43·8 (36·9–52)	42·7 (33·4–52·7)
1·24	Pulmonary aspiration and foreign body in airway	3,570 (3,050–4,180)	716 (619–830)	705 (557–891)	136 (117–158)	29·5 (25–34·9)	28·9 (22·4–37·1)
1·25	Foreign body in eyes	120 (54–217)	151 (71–268)	160 (78–281)	5·4 (2·6–9·5)	5·4 (2·6–9·5)	5·4 (2·6–9·5)
1·26	Foreign body in other body part	431 (333–554)	243 (177–324)	239 (175–319)	18·1 (13·9–23·3)	8·9 (6·5–11·8)	8·4 (6·2–11·3)
1·27	Environmental heat and cold exposure	797 (569–1,090)	957 (679–1,330)	1,090 (770–1,510)	38·1 (27·2–51·6)	34·8 (24·7–48·3)	36·7 (25·9–51)
1·28	Exposure to forces of nature	36 (26–50)	828 (750–913)	36 (26–50)	1·6 (1·1–2·1)	29·9 (27·1–33)	1·2 (0·9–1·7)
1·29	Other unintentional injuries	2,250 (1,730–3,000)	2,120 (1,560–2,930)	2,320 (1,660–3,240)	100 (76·8–135)	77·1 (56·7–106)	79·6 (57·2–111)
1·30	Self-harm and interpersonal violence	17,400 (16,400–18,400)	31,200 (29,900–32,600)	27,000 (21,300–33,700)	757 (717–800)	1,100 (1,050–1,150)	882 (700–1,100)
1·31	Self-harm	1,670 (1,530–1,830)	3,170 (2,950–3,410)	4,060 (3,170–5,120)	77·1 (71·1–84)	112 (104–120)	132 (103–167)
1·32	Self-harm by firearm	44 (37–67)	533 (456–762)	795 (591–1,100)	2·2 (1·8–3·1)	18·9 (16·2–26·8)	25·7 (19·1–35·7)
1·33	Self-harm by other specified means	1,630 (1,490–1,790)	2,630 (2,420–2,840)	3,270 (2,560–4,130)	75 (69·1–81·8)	92·8 (85·6–100)	107 (83·2–134)
1·34	Interpersonal violence	14,100 (13,100–15,000)	27,900 (26,600–29,100)	22,800 (18,000–28,600)	616 (576–656)	980 (934–1,030)	746 (589–937)
1·35	Physical violence by firearm	8,690 (8,140–9,260)	19,400 (18,500–20,300)	16,200 (12,600–20,500)	381 (358–405)	678 (647–710)	526 (409–669)
1·36	Physical violence by sharp object	3,270 (2,740–3,580)	6,000 (5,620–6,350)	4,160 (3,240–5,280)	145 (122–159)	211 (198–223)	137 (107–174)
1·37	Sexual violence	421 (275–597)	518 (340–733)	539 (354–766)	17·8 (11·8–25·3)	17·8 (11·7–25·3)	17·7 (11·7–25·2)
1·38	Physical violence by other means	1,700 (1,510–1,930)	1,970 (1,760–2,200)	1,900 (1,530–2,330)	72·6 (64·6–81·8)	73·7 (65·7–82·1)	65·6 (52·8–80·8)
1·39	Conflict and terrorism	1,610 (1,450–1,790)	101 (67–155)	81 (54–122)	63·1 (56·9–70·2)	3·7 (2·4–5·6)	2·7 (1·8–4)
1·40	Executions and police conflict	16 (15–18)	101 (80–129)	27 (18–40)	0·6 (0·6–0·7)	3·5 (2·8–4·4)	1 (0·7–1·4)

SR Table 1: All-age DALYs counts and age standardized DALYs rates per 100,000 individuals for all causes and level 1 cause groups by location for 1990, 2010, and 2019, both sexes combined, Saint Lucia

Cause Level	Cause	All-age DALYs			Age-standardized rate of DALYs per 100,000		
		1990	2010	2019	1990	2010	2019
0-0	All causes	41,400 (37,600–45,500)	46,700 (41,700–52,300)	55,000 (47,500–63,600)	36,900 (33,700–40,600)	28,700 (25,800–32,100)	28,700 (24,700–33,300)
1-0	Communicable, maternal, neonatal, and nutritional disorders	9,550 (8,370–10,900)	6,100 (5,320–7,030)	5,580 (4,700–6,700)	6,360 (5,650–7,120)	4,490 (3,840–5,230)	4,080 (3,320–5,080)
1-1	HIV/AIDS and sexually transmitted infections	464 (448–483)	464 (439–495)	420 (393–455)	406 (392–422)	257 (243–274)	212 (199–230)
1-2	HIV/AIDS	409 (400–418)	415 (399–435)	371 (356–392)	364 (356–373)	228 (220–239)	186 (179–198)
1-3	HIV/AIDS - drug-susceptible tuberculosis	96 (62–143)	43 (28–63)	36 (24–53)	85·3 (55·2–127)	23·4 (15·6–34·4)	18 (12–26·5)
1-4	HIV/AIDS - Multidrug-resistant Tuberculosis without extensive drug resistance	2 (0–11)	0 (0–2)	0 (0–2)	1·9 (0·1–10·1)	0·2 (0–0·9)	0·2 (0–0·8)
1-5	HIV/AIDS - Extensively drug-resistant Tuberculosis	0 (0–0)	0 (0–0)	0 (0–0)	0 (0–0)	0 (0–0·1)	0 (0–0·2)
1-6	HIV/AIDS resulting in other diseases	311 (262–346)	372 (347–396)	334 (312–357)	277 (234–309)	205 (191–218)	168 (157–180)
1-7	Sexually transmitted infections excluding HIV	55 (43–74)	49 (34–75)	49 (33–77)	41·3 (31·7–56·8)	28·5 (20·4–43·1)	26·1 (17·8–39·5)
1-8	Syphilis	29 (23–37)	9 (7–11)	7 (5–9)	18·1 (14·6–22·3)	6·6 (5·4–8·4)	5·1 (3·6–7·1)
1-9	Chlamydial infection	3 (2–5)	5 (4–7)	5 (4–7)	2·7 (1·9–3·8)	2·7 (1·9–3·8)	2·6 (1·9–3·6)
1-10	Gonococcal infection	4 (3–5)	6 (5–7)	6 (5–7)	3·5 (2·9–4·1)	3·2 (2·7–3·8)	3 (2·4–3·6)
1-11	Trichomoniasis	6 (2–12)	9 (3–18)	10 (4–20)	5·1 (1·9–10·5)	4·9 (1·9–10·1)	4·7 (1·9–9·7)
1-12	Genital herpes	6 (2–14)	10 (3–24)	11 (3–27)	5·4 (1·7–13·1)	5·4 (1·7–13·2)	5·3 (1·7–13)
1-13	Other sexually transmitted infections	8 (6–10)	10 (8–14)	10 (8–14)	6·6 (5–8·6)	5·7 (4·4–7·4)	5·3 (4–7·1)
1-14	Respiratory infections and tuberculosis	1,660 (1,490–1,840)	1,230 (1,100–1,370)	1,390 (1,180–1,640)	1,390 (1,270–1,510)	813 (728–913)	794 (665–954)
1-15	Tuberculosis	334 (306–363)	171 (156–188)	155 (130–186)	302 (277–328)	98·8 (89·9–109)	77·4 (64·9–93)
1-16	Latent tuberculosis infection	0 (0–0)	0 (0–0)	0 (0–0)	0 (0–0)	0 (0–0)	0 (0–0)
1-17	Drug-susceptible tuberculosis	328 (297–358)	170 (154–187)	153 (129–184)	297 (269–326)	98 (89–108)	76·6 (64·5–92·1)
1-18	Multidrug-resistant tuberculosis without extensive drug resistance	6 (0–28)	1 (0–5)	1 (0–6)	5·3 (0·3–25·9)	0·7 (0·1–2·8)	0·7 (0·1–2·9)
1-19	Extensively drug-resistant tuberculosis	0 (0–0)	0 (0–1)	0 (0–1)	0 (0–0)	0·1 (0–0·3)	0·1 (0–0·5)
1-20	Lower respiratory infections	1,140 (1,010–1,290)	871 (788–960)	1,050 (867–1,260)	962 (876–1,060)	601 (539–671)	604 (491–739)
1-21	Upper respiratory infections	135 (87–205)	140 (84–214)	137 (83–214)	91·1 (58·3–139)	84·8 (51·9–130)	83·8 (51–131)
1-22	Otitis media	46 (27–76)	47 (27–78)	45 (26–74)	30·7 (17·6–51)	28·7 (16·5–47)	28·2 (16·2–46·7)
1-23	Enteric infections	753 (636–894)	429 (335–547)	482 (371–621)	510 (438–596)	284 (225–359)	296 (224–379)
1-24	Diarrhoeal diseases	750 (633–890)	428 (334–545)	481 (370–620)	508 (437–594)	284 (224–358)	295 (223–378)
1-25	Typhoid and paratyphoid	1 (0–1)	0 (0–0)	0 (0–0)	0·5 (0·2–0·6)	0·1 (0–0·1)	0·1 (0–0·1)
1-26	Typhoid fever	1 (0–1)	0 (0–0)	0 (0–0)	0·5 (0·2–0·6)	0 (0–0)	0 (0–0)
1-27	Paratyphoid fever	0 (0–0)	0 (0–0)	0 (0–0)	0 (0–0·1)	0·1 (0–0·1)	0·1 (0–0·1)
1-28	iNTS	1 (0–1)	0 (0–1)	0 (0–1)	0·4 (0·3–0·7)	0·3 (0·1–0·4)	0·2 (0·1–0·4)
1-29	Other intestinal infectious diseases	2 (1–3)	1 (1–1)	1 (0–1)	0·9 (0·4–1·6)	0·6 (0·3–0·9)	0·5 (0·3–0·8)
1-30	Neglected tropical diseases and malaria	272 (189–370)	207 (144–285)	214 (146–303)	212 (147–287)	126 (89·1–173)	119 (83·5–165)
1-31	Malaria	0 (0–0)	0 (0–0)	0 (0–0)	0 (0–0)	0 (0–0)	0 (0–0)
1-32	Chagas disease	0 (0–0)	0 (0–0)	0 (0–0)	0 (0–0)	0 (0–0)	0 (0–0)
1-33	Leishmaniasis	0 (0–0)	0 (0–0)	0 (0–0)	0 (0–0)	0 (0–0)	0 (0–0)
1-34	Visceral leishmaniasis	0 (0–0)	0 (0–0)	0 (0–0)	0 (0–0)	0 (0–0)	0 (0–0)
1-35	Cutaneous and mucocutaneous leishmaniasis	0 (0–0)	0 (0–0)	0 (0–0)	0 (0–0)	0 (0–0)	0 (0–0)
1-36	African trypanosomiasis	0 (0–0)	0 (0–0)	0 (0–0)	0 (0–0)	0 (0–0)	0 (0–0)
1-37	Schistosomiasis	1 (0–1)	1 (0–2)	1 (0–2)	0·7 (0·3–1·2)	0·5 (0·2–1)	0·6 (0·2–1·1)
1-38	Cysticercosis	84 (42–137)	93 (44–168)	111 (54–191)	86·3 (43·2–142)	55·2 (26·5–98·8)	52·6 (25·8–90·4)
1-39	Cystic echinococcosis	0 (0–0)	0 (0–0)	0 (0–0)	0 (0–0)	0 (0–0)	0 (0–0)
1-40	Lymphatic filariasis	0 (0–0)	0 (0–0)	0 (0–0)	0 (0–0)	0 (0–0)	0 (0–0)
1-41	Onchocerciasis	0 (0–0)	0 (0–0)	0 (0–0)	0 (0–0)	0 (0–0)	0 (0–0)
1-42	Trachoma	0 (0–0)	0 (0–0)	0 (0–0)	0 (0–0)	0 (0–0)	0 (0–0)
1-43	Dengue	16 (9–27)	47 (33–66)	38 (24–54)	11·4 (6·4–19·2)	29·2 (20·6–41)	23·7 (14·7–33·7)
1-44	Yellow fever	0 (0–0)	0 (0–0)	0 (0–0)	0 (0–0)	0 (0–0)	0 (0–0)
1-45	Rabies	0 (0–0)	0 (0–0)	0 (0–1)	0·2 (0·1–0·3)	0·2 (0·2–0·3)	0·2 (0·1–0·4)
1-46	Intestinal nematode infections	102 (55–164)	19 (11–32)	14 (8–23)	67·8 (36·7–109)	11·1 (6·3–18·3)	8·3 (4·8–13·8)
1-47	Ascariasis	14 (7–27)	4 (2–7)	3 (2–6)	9·7 (5–18·7)	2·5 (1·3–4·5)	2 (1–3·5)
1-48	Trichuriasis	61 (30–107)	2 (1–3)	2 (1–3)	40·5 (19·6–71·1)	1 (0·5–1·9)	0·9 (0·4–1·9)

1-49	Hookworm disease	27 (14–43)	13 (7–23)	9 (5–16)	17·7 (9·4–28·4)	7·6 (4·2–13)	5·4 (3–9·2)
1-50	Food-borne trematodiases	0 (0–0)	0 (0–0)	0 (0–0)	0 (0–0)	0 (0–0)	0 (0–0)
1-51	Leprosy	1 (0–1)	1 (0–1)	1 (1–1)	0·6 (0·3–0·9)	0·4 (0·3–0·7)	0·4 (0·3–0·7)
1-52	Ebola virus disease	0 (0–0)	0 (0–0)	0 (0–0)	0 (0–0)	0 (0–0)	0 (0–0)
1-53	Zika virus disease	0 (0–0)	0 (0–0)	3 (0–12)	0 (0–0)	0 (0–0)	1·8 (0–7·4)
1-54	Guinea worm disease	0 (0–0)	0 (0–0)	0 (0–0)	0 (0–0)	0 (0–0)	0 (0–0)
1-55	Other neglected tropical diseases	69 (47–99)	45 (31–67)	45 (31–64)	44·9 (30·8–62·5)	29·6 (20·1–43·6)	31 (21·1–45·4)
1-56	Other infectious diseases	662 (578–757)	305 (265–353)	307 (247–378)	446 (394–501)	196 (170–230)	190 (152–235)
1-57	Meningitis	349 (295–416)	84 (74–97)	77 (62–95)	222 (192–258)	58·5 (50·3–69·5)	52·6 (40·8–67·7)
1-58	Encephalitis	148 (109–180)	71 (60–100)	68 (52–94)	93·5 (69·5–111)	46·8 (38·5–66·7)	46·1 (34·6–66·8)
1-59	Diphtheria	0 (0–0)	0 (0–0)	0 (0–0)	0·1 (0–0·1)	0 (0–0)	0 (0–0)
1-60	Whooping cough	5 (3–7)	3 (2–4)	2 (2–4)	2·6 (1·6–4)	2·1 (1·3–3·2)	2·5 (1·5–4)
1-61	Tetanus	23 (18–31)	6 (5–13)	6 (3–16)	21·4 (16·2–28·8)	3·9 (2·8–7·7)	3 (1·5–8·1)
1-62	Measles	1 (0–1)	0 (0–0)	0 (0–0)	0·4 (0·1–0·7)	0 (0–0)	0 (0–0)
1-63	Varicella and herpes zoster	16 (10–29)	14 (9–23)	15 (10–24)	12 (7·4–20·6)	9·1 (5·9–15)	8·7 (5·6–14·5)
1-64	Acute hepatitis	24 (20–28)	11 (8–14)	10 (7–13)	17·8 (15·7–20·4)	6·2 (4·6–8·2)	5·9 (4·3–7·9)
1-65	Acute hepatitis A	13 (10–16)	7 (4–9)	6 (4–9)	8·9 (6·8–11·2)	3·8 (2·5–5·5)	3·7 (2·4–5·3)
1-66	Acute hepatitis B	8 (6–10)	3 (2–3)	3 (2–4)	6·6 (5·3–8·1)	1·6 (1·4–1·9)	1·5 (1·1–1·8)
1-67	Acute hepatitis C	1 (1–2)	0 (0–1)	0 (0–1)	1·2 (0·5–2)	0·2 (0·1–0·4)	0·2 (0·1–0·4)
1-68	Acute hepatitis E	1 (1–2)	1 (1–1)	1 (1–1)	1·1 (0·7–1·7)	0·5 (0·4–0·8)	0·5 (0·4–0·8)
1-69	Other unspecified infectious diseases	95 (67–121)	115 (80–148)	129 (85–168)	75·3 (52·7–95·7)	69·5 (48·4–88·7)	70·8 (47·7–92·1)
1-70	Maternal and neonatal disorders	4,620 (3,860–5,450)	2,700 (2,200–3,280)	2,020 (1,540–2,650)	2,600 (2,180–3,050)	2,330 (1,880–2,860)	2,010 (1,490–2,710)
1-71	Maternal disorders	128 (113–144)	136 (123–150)	102 (84–122)	90·9 (80·4–102)	74·6 (67·2–82·6)	55·9 (46·2–67·1)
1-72	Maternal haemorrhage	38 (30–46)	23 (18–28)	16 (12–20)	27·2 (22·1–32·9)	12·5 (9·9–15·6)	8·5 (6·6–11·2)
1-73	Maternal sepsis and other pregnancy related infections	9 (7–11)	4 (3–6)	3 (2–4)	6 (4·6–7·7)	2·2 (1·6–3·1)	1·7 (1·2–2·4)
1-74	Maternal hypertensive disorders	23 (19–27)	14 (12–17)	10 (8–12)	15·5 (13·2–18·4)	7·8 (6·6–9·3)	5·6 (4·5–6·9)
1-75	Maternal obstructed labour and uterine rupture	2 (2–3)	10 (7–13)	7 (5–10)	1·7 (1·2–2·3)	5·3 (4–7)	3·8 (2·7–5·2)
1-76	Maternal abortive outcome	21 (17–27)	6 (5–8)	4 (3–5)	15·2 (12·1–18·8)	3·3 (2·5–4·3)	2 (1·5–2·7)
1-77	Ectopic pregnancy	1 (1–1)	1 (1–1)	1 (1–1)	0·5 (0·4–0·6)	0·6 (0·5–0·7)	0·5 (0·4–0·6)
1-78	Indirect maternal deaths	12 (10–15)	14 (11–17)	11 (8–14)	8·3 (6·7–10·2)	7·7 (6·1–9·4)	6·1 (4·5–8)
1-79	Late maternal deaths	5 (4–7)	9 (7–12)	8 (5–11)	3·6 (2·7–4·7)	5·2 (3·8–6·8)	4·2 (2·9–5·8)
1-80	Maternal deaths aggravated by HIV/AIDS	0 (0–0)	0 (0–0)	0 (0–0)	0 (0–0)	0 (0–0)	0 (0–0)
1-81	Other maternal disorders	18 (14–22)	55 (47–64)	43 (34–54)	12·9 (10·4–16)	29·9 (25·5–35)	23·4 (18·7–29·2)
1-82	Neonatal disorders	4,500 (3,730–5,320)	2,560 (2,070–3,140)	1,920 (1,460–2,530)	2,510 (2,090–2,970)	2,260 (1,810–2,790)	1,950 (1,440–2,650)
1-83	Neonatal preterm birth	2,520 (2,080–3,040)	1,290 (1,030–1,610)	930 (695–1,240)	1,410 (1,170–1,700)	1,140 (901–1,440)	964 (696–1,320)
1-84	Neonatal encephalopathy due to birth asphyxia and trauma	1,100 (850–1,390)	561 (426–723)	375 (256–522)	609 (471–767)	512 (389–665)	403 (267–579)
1-85	Neonatal sepsis and other neonatal infections	417 (306–543)	384 (284–511)	362 (265–481)	236 (174–308)	312 (234–405)	311 (225–423)
1-86	Hemolytic disease and other neonatal jaundice	42 (32–55)	32 (24–40)	29 (22–37)	26·7 (20·5–34·3)	20·4 (15·9–25·6)	19 (14·3–24·1)
1-87	Other neonatal disorders	413 (294–560)	291 (211–397)	221 (142–331)	227 (161–308)	273 (198–373)	257 (164–386)
1-88	Nutritional deficiencies	1,120 (849–1,490)	769 (538–1,110)	754 (533–1,050)	797 (617–1,050)	484 (344–697)	460 (327–648)
1-89	Protein-energy malnutrition	412 (339–504)	161 (137–186)	165 (134–201)	288 (246–339)	111 (93·6–130)	102 (81·3–128)
1-90	Iodine deficiency	5 (3–9)	5 (3–9)	5 (3–9)	4·3 (2·4–7)	3·1 (1·6–5)	2·8 (1·5–4·5)
1-91	Vitamin A deficiency	19 (12–30)	10 (6–15)	8 (5–13)	11 (6·7–17)	6·7 (4·1–10·2)	6·3 (3·8–9·7)
1-92	Dietary iron deficiency	620 (382–953)	563 (344–874)	542 (336–825)	440 (279–664)	345 (212–546)	332 (207–513)
1-93	Other nutritional deficiencies	61 (48–80)	30 (22–39)	33 (24–43)	53·6 (43·1–72·5)	18·5 (13·8–23·7)	17·3 (12·6–22·5)
1-0	Non-communicable diseases	26,700 (23,900–29,900)	33,800 (29,700–38,400)	43,200 (37,300–50,100)	26,500 (24,000–29,300)	20,300 (17,900–23,000)	21,300 (18,400–24,800)
1-1	Neoplasms	3,920 (3,710–4,150)	5,270 (4,980–5,590)	7,330 (6,210–8,610)	4,170 (3,930–4,420)	3,170 (2,990–3,370)	3,470 (2,940–4,070)
1-2	Lip and oral cavity cancer	78 (72–84)	103 (94–112)	152 (125–183)	88·1 (81·2–95)	61·5 (56·3–67·2)	69·1 (57·2–82·9)
1-3	Nasopharynx cancer	18 (17–20)	32 (30–35)	44 (36–53)	19·3 (17·6–21)	19·1 (17·6–20·6)	20·4 (16·9–24·5)
1-4	Other pharynx cancer	37 (34–41)	42 (37–46)	62 (50–76)	42·7 (38·7–47·5)	24·9 (22·3–27·6)	28 (22·6–34·1)
1-5	Oesophageal cancer	114 (102–125)	156 (141–172)	240 (198–287)	131 (118–143)	94 (85·1–104)	108 (89·1–130)
1-6	Stomach cancer	421 (389–452)	415 (387–447)	577 (484–685)	475 (440–510)	250 (233–269)	267 (224–316)
1-7	Colon and rectum cancer	230 (213–247)	355 (328–380)	559 (467–663)	260 (241–279)	215 (199–231)	260 (218–308)

1-8	Liver cancer	143 (131–155)	76 (69–82)	115 (94–139)	156 (143–170)	46 (42·2–50·3)	53·6 (44·2–64·7)
1-9	Liver cancer due to hepatitis B	45 (34–57)	24 (19–31)	33 (24–46)	48·5 (37·1–63)	14·3 (10·9–18·5)	15·4 (10·9–21)
1-10	Liver cancer due to hepatitis C	26 (18–36)	12 (8–17)	20 (13–30)	29·9 (20·8–40·8)	7·8 (5·3–10·6)	9·5 (6·2–13·7)
1-11	Liver cancer due to alcohol use	49 (37–63)	27 (20–35)	44 (32–58)	55 (41·2–70·5)	16·6 (12·2–21·2)	20·2 (14·6–26·6)
1-12	Liver cancer due to NASH	10 (7–14)	6 (4–8)	9 (6–14)	10·6 (7·5–14·9)	3·4 (2·5–4·7)	4·4 (3–6·3)
1-13	Gallbladder and biliary tract cancer	68 (41–76)	36 (31–60)	52 (40–84)	76·9 (46·8–87·1)	22 (18·8–36·6)	23·8 (18·6–38·9)
1-14	Pancreatic cancer	38 (35–42)	229 (211–246)	370 (307–440)	43·8 (40·3–47·7)	139 (128–149)	170 (142–202)
1-15	Larynx cancer	51 (47–56)	79 (72–86)	116 (96–139)	58·6 (53·4–64·3)	48 (43·7–52·5)	52·4 (43·5–62·5)
1-16	Tracheal, bronchus, and lung cancer	299 (276–326)	422 (390–456)	636 (535–757)	340 (313–369)	256 (236–276)	290 (244–343)
1-17	Malignant skin melanoma	19 (15–24)	30 (25–42)	42 (32–59)	19·7 (15·2–26)	17·4 (14·7–24·6)	20·1 (15·4–28)
1-18	Non-melanoma skin cancer	6 (5–7)	14 (13–15)	19 (16–22)	7·1 (6·2–8·5)	8·4 (7·7–9·1)	9·1 (7·7–10·7)
1-19	Non-melanoma skin cancer (squamous-cell carcinoma)	6 (5–7)	14 (13–15)	19 (16–22)	7·1 (6·2–8·5)	8·4 (7·7–9·1)	9·1 (7·7–10·7)
1-20	Non-melanoma skin cancer (basal-cell carcinoma)	0 (0–0)	0 (0–0)	0 (0–0)	0 (0–0)	0 (0–0)	0 (0–0)
1-21	Breast cancer	400 (362–441)	550 (496–606)	740 (614–885)	454 (410–501)	315 (285–346)	336 (280–401)
1-22	Cervical cancer	369 (327–410)	392 (348–440)	473 (381–584)	401 (357–446)	221 (196–248)	218 (176–268)
1-23	Uterine cancer	58 (52–64)	90 (81–100)	132 (109–160)	65·1 (58·7–71·9)	53·3 (47·9–59·2)	60·9 (50·3–73·3)
1-24	Ovarian cancer	29 (25–43)	148 (99–166)	198 (134–250)	31·3 (27·5–46·7)	85·3 (57–95·6)	91·1 (61·8–115)
1-25	Prostate cancer	422 (357–567)	765 (654–1,070)	1,140 (916–1,530)	486 (413–650)	489 (418–680)	543 (439–732)
1-26	Testicular cancer	4 (3–6)	18 (15–21)	19 (15–24)	2·9 (2·4–4·1)	10·2 (8·5–12·7)	10·6 (8–13·5)
1-27	Kidney cancer	66 (61–72)	68 (62–73)	96 (78–118)	64·2 (59–69·6)	40·9 (37·5–44·4)	46·7 (38–56·6)
1-28	Bladder cancer	63 (57–68)	90 (83–99)	137 (114–162)	72·6 (66·1–78·7)	56·6 (51·7–62·1)	64·2 (53·6–76·2)
1-29	Brain and nervous system cancer	32 (22–63)	114 (50–141)	128 (56–177)	25·8 (18·4–51·8)	68·5 (30·6–85·3)	68·9 (30·1–95·5)
1-30	Thyroid cancer	23 (21–25)	28 (26–30)	41 (34–49)	24·8 (22·5–27·1)	16·5 (15·2–18)	18·9 (15·9–22·5)
1-31	Mesothelioma	7 (6–9)	8 (7–10)	11 (9–14)	8 (6·1–9·6)	4·8 (4·2–5·8)	5·1 (4·1–6·5)
1-32	Hodgkin lymphoma	29 (24–38)	36 (30–44)	40 (31–50)	25·2 (20·7–32·3)	20·5 (17·2–24·6)	20·2 (15·6–25·5)
1-33	Non-Hodgkin's lymphoma	250 (227–278)	280 (251–310)	351 (284–427)	228 (208–253)	164 (148–181)	174 (141–212)
1-34	Multiple myeloma	67 (59–82)	130 (118–149)	203 (167–248)	76·4 (67·4–93·4)	79·2 (72–91·2)	92·8 (76·3–113)
1-35	Leukaemia	244 (223–270)	255 (236–276)	279 (232–329)	191 (177–207)	154 (142–167)	154 (127–182)
1-36	Acute lymphoid leukaemia	64 (52–78)	56 (48–65)	49 (39–61)	38·4 (31·7–46·2)	35·2 (30·1–41·4)	34 (26·7–42·8)
1-37	Chronic lymphoid leukaemia	12 (10–14)	18 (16–21)	27 (22–34)	12·9 (10·8–15·8)	11 (9·6–12·6)	12·7 (10·1–15·8)
1-38	Acute myeloid leukaemia	45 (37–56)	66 (58–75)	73 (59–88)	33·2 (27·9–41·4)	39·1 (34·1–44·2)	40·5 (32·5–49·5)
1-39	Chronic myeloid leukaemia	26 (23–30)	24 (21–27)	28 (22–34)	25 (22–28·4)	14 (12·2–15·7)	13·6 (10·9–16·6)
1-40	Other leukaemia	97 (86–108)	92 (83–99)	103 (84–122)	81·8 (73·8–89·8)	54·5 (49·2–59·2)	53·2 (43·3–63·1)
1-41	Other neoplasms	13 (9–22)	30 (22–39)	40 (30–55)	12 (8·9–21·5)	18·3 (14–23·9)	20·4 (14·9–27·4)
1-42	Myelodysplastic, myeloproliferative, and other hematopoietic neoplasms	13 (9–22)	30 (22–39)	40 (30–55)	12 (8·9–21·5)	18·3 (14–23·9)	20·4 (14·9–27·4)
1-43	Benign and in situ intestinal neoplasms	0 (0–0)	0 (0–0)	0 (0–0)	0 (0–0)	0 (0–0)	0 (0–0)
1-44	Benign and in situ cervical and uterine neoplasms	0 (0–0)	0 (0–0)	0 (0–0)	0 (0–0)	0 (0–0)	0 (0–0)
1-45	Other benign and in situ neoplasms	0 (0–0)	0 (0–0)	0 (0–0)	0 (0–0)	0 (0–0)	0 (0–0)
1-46	Cardiovascular diseases	6,610 (6,260–6,920)	6,340 (5,990–6,680)	8,880 (7,700–10,300)	7,600 (7,190–7,960)	3,930 (3,710–4,130)	4,220 (3,670–4,870)
1-47	Rheumatic heart disease	240 (205–282)	196 (159–241)	199 (155–255)	217 (188–251)	110 (89·3–134)	100 (78–129)
1-48	Ischaemic heart disease	2,250 (2,110–2,380)	1,810 (1,690–1,930)	2,540 (2,190–2,960)	2,690 (2,520–2,850)	1,140 (1,060–1,210)	1,200 (1,030–1,390)
1-49	Stroke	2,630 (2,450–2,810)	2,350 (2,180–2,500)	3,340 (2,860–3,880)	3,030 (2,830–3,230)	1,460 (1,350–1,550)	1,580 (1,360–1,830)
1-50	Ischaemic stroke	1,170 (1,050–1,270)	1,030 (951–1,110)	1,530 (1,310–1,750)	1,410 (1,270–1,540)	666 (612–716)	745 (640–853)
1-51	Intracerebral hemorrhage	1,260 (1,140–1,430)	1,090 (1,010–1,170)	1,530 (1,280–1,810)	1,410 (1,280–1,590)	663 (612–713)	706 (593–836)
1-52	Subarachnoid hemorrhage	197 (167–226)	221 (201–247)	282 (234–338)	204 (172–235)	128 (116–144)	132 (109–157)
1-53	Hypertensive heart disease	511 (428–574)	615 (541–724)	892 (738–1,090)	610 (509–684)	386 (340–452)	421 (347–512)
1-54	Non-rheumatic valvular heart disease	32 (27–38)	42 (39–46)	60 (49–71)	34·3 (29·2–40·7)	25·1 (23–27·4)	28·1 (23·3–33·5)
1-55	Non-rheumatic calcific aortic valve disease	20 (16–24)	29 (26–32)	41 (34–51)	22·3 (18·3–26·7)	17·4 (15·4–19·3)	19·6 (16·1–24·1)
1-56	Non-rheumatic degenerative mitral valve disease	11 (9–14)	13 (10–16)	18 (13–22)	11·5 (8·9–14·2)	7·5 (6–9·2)	8·2 (6·3–10·5)
1-57	Other non-rheumatic valve diseases	1 (0–1)	1 (0–1)	1 (0–1)	0·5 (0·3–0·7)	0·3 (0·2–0·6)	0·3 (0·2–0·6)
1-58	Cardiomyopathy and myocarditis	304 (262–371)	456 (375–506)	609 (484–734)	309 (266–380)	272 (224–301)	290 (232–350)
1-59	Myocarditis	32 (25–41)	46 (37–58)	59 (45–76)	27·8 (20·9–34·8)	28·1 (22·4–35·2)	31 (23·5–39·5)
1-60	Alcoholic cardiomyopathy	62 (47–89)	137 (101–162)	179 (130–227)	69·1 (51·6–97·1)	79·5 (58·6–94·6)	80·8 (58·2–102)

1-61	Other cardiomyopathy	209 (179–256)	273 (230–306)	371 (297–452)	212 (182–259)	164 (138–184)	178 (142–217)
1-62	Atrial fibrillation and flutter	89 (75–106)	143 (121–174)	222 (185–271)	118 (101–140)	95·5 (81·3–115)	110 (92·2–134)
1-63	Aortic aneurysm	104 (92–117)	148 (133–163)	230 (189–274)	120 (106–135)	92·4 (83·3–102)	109 (89·4–130)
1-64	Peripheral vascular disease	41 (22–73)	75 (40–135)	120 (64–218)	50·4 (27·2–91·1)	49·2 (26·7–87·5)	58 (30·7–105)
1-65	Endocarditis	58 (48–80)	116 (93–144)	150 (115–195)	56 (46·1–78)	67·1 (53·7–83·7)	72·7 (55·6–94·1)
1-66	Chronic respiratory diseases	1,200 (1,020–1,430)	1,500 (1,300–1,730)	1,830 (1,540–2,130)	1,090 (951–1,250)	923 (799–1,070)	955 (794–1,130)
1-67	Chronic obstructive pulmonary disease	426 (386–474)	692 (621–769)	981 (819–1,140)	484 (438–537)	432 (387–480)	466 (390–542)
1-68	Pneumoconiosis	3 (2–3)	1 (1–2)	2 (2–2)	2·9 (2·2–3·5)	0·8 (0·7–0·9)	0·9 (0·8–1·2)
1-69	Silicosis	1 (0–1)	0 (0–0)	0 (0–0)	0·6 (0·4–0·8)	0·2 (0·1–0·2)	0·2 (0·1–0·2)
1-70	Asbestosis	0 (0–1)	0 (0–0)	1 (0–1)	0·4 (0·3–0·7)	0·2 (0·2–0·3)	0·3 (0·2–0·4)
1-71	Coal workers pneumoconiosis	0 (0–0)	0 (0–0)	0 (0–0)	0·3 (0·1–0·4)	0·1 (0–0·1)	0·1 (0·1–0·1)
1-72	Other pneumoconiosis	1 (1–2)	1 (0–1)	1 (1–1)	1·6 (1·2–2)	0·4 (0·3–0·4)	0·4 (0·3–0·5)
1-73	Asthma	689 (523–903)	661 (504–872)	658 (498–860)	531 (419–674)	401 (303–532)	391 (289–523)
1-74	Interstitial lung disease and pulmonary sarcoidosis	32 (26–44)	74 (56–88)	101 (70–128)	33·3 (26·9–45·2)	44·8 (33·8–53·3)	49·2 (33·9–62·4)
1-75	Other chronic respiratory diseases	54 (41–66)	75 (64–92)	86 (68–108)	42·3 (32·9–50·1)	45·2 (38·5–55·6)	47·9 (38–61)
1-76	Digestive diseases	1,430 (1,300–1,580)	1,560 (1,390–1,760)	2,000 (1,690–2,350)	1,430 (1,310–1,570)	919 (823–1,040)	966 (818–1,130)
1-77	Cirrhosis and other chronic liver diseases	704 (652–763)	689 (635–742)	940 (776–1,120)	760 (703–826)	402 (370–432)	433 (357–515)
1-78	Cirrhosis and other chronic liver diseases due to hepatitis B	83 (59–115)	88 (60–124)	67 (44–100)	92·9 (65·9–129)	49·9 (34·6–69·9)	30·3 (19·9–44·5)
1-79	Cirrhosis and other chronic liver diseases due to hepatitis C	129 (96–172)	127 (94–171)	182 (128–256)	143 (104–192)	73·2 (54·2–98·5)	83 (58·8–115)
1-80	Cirrhosis and other chronic liver diseases due to alcohol use	254 (201–304)	272 (220–327)	410 (314–525)	289 (228–345)	159 (129–190)	184 (142–236)
1-81	Cirrhosis and other chronic liver diseases due to NAFLD	97 (70–131)	97 (69–133)	148 (99–206)	108 (77·3–148)	57 (40·5–78·5)	68·1 (45·9–93·6)
1-82	Cirrhosis and other chronic liver diseases due to other causes	141 (114–172)	104 (83–135)	133 (98–180)	127 (99–160)	62·2 (49·5–80·4)	66·8 (50–88·5)
1-83	Upper digestive system diseases	286 (213–394)	364 (248–535)	440 (301–655)	285 (215–387)	210 (144–308)	212 (144–317)
1-84	Peptic ulcer disease	111 (97–126)	85 (75–97)	109 (88–135)	121 (105–137)	51·2 (44·8–58·5)	51·7 (42–63·9)
1-85	Gastritis and duodenitis	43 (32–56)	61 (44–85)	77 (54–107)	41·3 (30·8–54·2)	35·8 (25·8–49)	37·4 (26·4–51·4)
1-86	GERD	132 (69–233)	217 (112–381)	253 (131–448)	123 (63·8–217)	123 (63·7–217)	123 (63·8–217)
1-87	Appendicitis	49 (37–59)	36 (30–46)	38 (30–51)	39 (29·1–46·3)	20·9 (17·6–27·3)	20·7 (16–27·3)
1-88	Paralytic ileus and intestinal obstruction	98 (81–120)	101 (88–123)	120 (95–155)	78·3 (66·2–98·2)	66·2 (57·2–79)	66·2 (52–84·7)
1-89	Inguinal, femoral, and abdominal hernia	90 (68–119)	101 (74–136)	116 (86–156)	69·7 (53–90·8)	61·5 (45·2–83)	63·2 (46·2–85·1)
1-90	Inflammatory bowel disease	36 (29–41)	36 (31–44)	45 (36–56)	32·6 (26·6–37·3)	21 (18·1–25·8)	22·3 (17·9–28·2)
1-91	Vascular intestinal disorders	24 (21–28)	30 (27–34)	45 (36–55)	27·2 (23·4–31·1)	18·4 (16·4–20·5)	21·4 (17·4–26·1)
1-92	Gallbladder and biliary diseases	78 (60–98)	86 (65–112)	107 (80–139)	79·5 (61–100)	49·9 (37·9–65·4)	51·2 (38·4–67·2)
1-93	Pancreatitis	19 (16–22)	28 (24–32)	35 (28–43)	19·2 (16·4–22·4)	16 (13·9–18·1)	16·5 (13·3–20·3)
1-94	Other digestive diseases	42 (34–67)	86 (52–104)	116 (68–154)	38 (30·5–61·8)	53·6 (32·8–64·5)	59·5 (35·5–78·1)
1-95	Neurological disorders	1,640 (924–2,620)	2,220 (1,240–3,560)	2,610 (1,530–4,120)	1,450 (864–2,280)	1,310 (753–2,110)	1,360 (790–2,180)
1-96	Alzheimer's disease and other dementias	220 (90–522)	403 (176–911)	584 (248–1,330)	305 (124–716)	272 (118–611)	294 (125–674)
1-97	Parkinson's disease	79 (74–85)	127 (115–139)	194 (169–222)	95·3 (88·4–102)	81 (73·3–89)	94·1 (82·2–108)
1-98	Idiopathic epilepsy	521 (313–797)	532 (308–832)	572 (330–887)	404 (246–608)	307 (176–483)	311 (178–491)
1-99	Multiple sclerosis	8 (7–11)	16 (13–20)	20 (16–25)	8·1 (6·9–11·1)	8·7 (7·4–11·2)	9·1 (7·5–11·8)
1-100	Motor neuron disease	7 (6–7)	30 (27–32)	45 (36–55)	6·4 (5·9–7)	18·1 (16·5–19·8)	22·2 (17·9–27·4)
1-101	Headache disorders	705 (122–1,570)	991 (192–2,200)	1,050 (217–2,300)	555 (109–1,210)	550 (106–1,200)	548 (106–1,200)
1-102	Migraine	643 (83–1,510)	898 (129–2,050)	949 (147–2,180)	502 (74·5–1,160)	498 (72·5–1,140)	496 (72·9–1,140)
1-103	Tension-type headache	62 (17–252)	93 (28–337)	103 (31–356)	52·3 (15·5–193)	52·1 (15·4–191)	52 (15·2–187)
1-104	Other neurological disorders	100 (64–148)	127 (84–185)	146 (98–210)	72·3 (48·4–104)	75 (49·8–110)	84·2 (54·3–124)
1-105	Mental disorders	2,090 (1,510–2,770)	2,970 (2,170–3,930)	3,280 (2,420–4,330)	1,680 (1,220–2,210)	1,670 (1,220–2,210)	1,700 (1,250–2,250)
1-106	Schizophrenia	190 (131–259)	319 (219–426)	364 (256–482)	174 (121–233)	176 (122–235)	177 (124–235)
1-107	Depressive disorders	672 (451–928)	995 (679–1,380)	1,170 (807–1,600)	575 (394–791)	559 (383–774)	582 (402–797)
1-108	Major depressive disorder	570 (379–806)	835 (564–1,160)	984 (666–1,370)	485 (325–675)	469 (317–654)	493 (333–687)
1-109	Dysthymia	102 (62–157)	160 (100–246)	182 (114–280)	90·1 (56·6–137)	89·9 (56·3–138)	89·8 (55·9–136)
1-110	Bipolar disorder	248 (143–388)	362 (212–569)	393 (232–613)	200 (116–308)	200 (117–314)	200 (117–311)
1-111	Anxiety disorders	513 (333–739)	722 (475–1,020)	786 (522–1,100)	406 (269–578)	406 (269–572)	411 (275–577)
1-112	Eating disorders	60 (37–90)	82 (50–126)	84 (50–126)	41·2 (25·3–60·8)	45·1 (27·3–69)	46·6 (28·2–70·3)
1-113	Anorexia nervosa	16 (9–26)	20 (12–33)	20 (11–31)	10·1 (5·9–16·3)	10·9 (6·4–17·9)	11·3 (6·4–18·1)

1-114	Bulimia nervosa	44 (26–69)	62 (36–100)	64 (37–101)	31·1 (18·2–48·4)	34·2 (19·5–54·6)	35·3 (20·4–55·4)
1-115	Autism spectrum disorders	75 (49–110)	88 (58–128)	88 (58–130)	51·8 (33·9–75·5)	52·5 (34·6–76·2)	52·7 (34·3–78·1)
1-116	Attention-deficit/hyperactivity disorder	60 (34–102)	65 (36–110)	59 (34–99)	36·9 (21·1–63·7)	37·2 (21–63)	37·4 (21·4–63·4)
1-117	Conduct disorder	124 (69–197)	118 (66–189)	94 (53–149)	67·6 (37·9–107)	67·9 (38·5–108)	68·1 (38·1–109)
1-118	Idiopathic developmental intellectual disability	30 (11–53)	29 (9–52)	26 (9–48)	20·3 (7·6–36·2)	16·9 (5·6–30·8)	15·8 (5·1–28·5)
1-119	Other mental disorders	114 (72–172)	191 (122–290)	223 (143–338)	107 (68·1–162)	108 (68·5–163)	108 (68·1–163)
1-120	Substance use disorders	483 (378–600)	787 (633–962)	894 (722–1,100)	430 (346–524)	436 (352–532)	440 (354–543)
1-121	Alcohol use disorders	338 (270–418)	566 (457–698)	653 (530–810)	324 (265–396)	315 (255–388)	312 (252–389)
1-122	Drug use disorders	145 (102–198)	221 (162–289)	241 (178–314)	106 (75·7–142)	121 (88·9–159)	127 (93·3–167)
1-123	Opioid use disorders	54 (34–80)	91 (60–127)	104 (70–142)	44·5 (28·7–63)	50·3 (33·3–70·1)	53·1 (35·6–72·9)
1-124	Cocaine use disorders	21 (12–32)	38 (27–52)	42 (31–57)	14·9 (9·2–22·1)	20·8 (14·9–28·6)	22·3 (16·1–30·6)
1-125	Amphetamine use disorders	9 (4–15)	12 (7–20)	14 (8–22)	6·1 (3·2–10·2)	6·8 (3·9–10·9)	7·3 (4·3–11·6)
1-126	Cannabis use disorders	42 (23–68)	49 (27–79)	47 (26–75)	26·3 (14·3–42·2)	26·5 (14·7–42·5)	26·6 (14·5–42·7)
1-127	Other drug use disorders	20 (12–31)	30 (20–43)	34 (23–48)	14·2 (9–21·5)	16·7 (11–23·8)	17·7 (12·1–25·4)
1-128	Diabetes and kidney diseases	3,230 (2,900–3,580)	5,130 (4,430–5,890)	7,190 (6,090–8,360)	3,540 (3,180–3,920)	3,100 (2,690–3,550)	3,370 (2,850–3,920)
1-129	Diabetes mellitus	2,390 (2,100–2,700)	3,600 (2,980–4,280)	5,090 (4,180–6,140)	2,670 (2,340–3,010)	2,190 (1,820–2,610)	2,370 (1,950–2,850)
1-130	Diabetes mellitus type 1	147 (117–176)	160 (137–213)	189 (152–258)	132 (102–159)	91·9 (79·2–122)	95·5 (77–129)
1-131	Diabetes mellitus type 2	2,240 (1,960–2,550)	3,440 (2,840–4,120)	4,900 (4,000–5,930)	2,540 (2,210–2,880)	2,100 (1,740–2,510)	2,270 (1,870–2,740)
1-132	Chronic kidney disease	836 (767–906)	1,520 (1,390–1,660)	2,100 (1,800–2,450)	874 (805–948)	907 (831–986)	999 (858–1,170)
1-133	Chronic kidney disease due to diabetes mellitus type 1	68 (48–93)	132 (91–179)	167 (106–242)	71·2 (48·6–98·3)	73·1 (50·5–99·1)	77·3 (50·2–110)
1-134	Chronic kidney disease due to diabetes mellitus type 2	179 (144–213)	324 (264–383)	488 (381–606)	206 (166–244)	200 (164–235)	226 (177–280)
1-135	Chronic kidney disease due to hypertension	150 (123–184)	282 (235–338)	419 (337–524)	172 (142–208)	174 (145–208)	198 (160–246)
1-136	Chronic kidney disease due to glomerulonephritis	199 (166–237)	359 (299–429)	466 (356–594)	193 (158–233)	207 (172–248)	224 (174–284)
1-137	Chronic kidney disease due to other and unspecified causes	239 (198–286)	426 (353–518)	557 (438–707)	232 (189–282)	253 (210–307)	274 (217–342)
1-138	Acute glomerulonephritis	1 (1–1)	0 (0–0)	0 (0–1)	0·7 (0·6–0·9)	0·3 (0·2–0·3)	0·3 (0·2–0·3)
1-139	Skin and subcutaneous diseases	813 (559–1,180)	1,010 (706–1,440)	1,100 (781–1,530)	608 (426–874)	610 (428–876)	628 (447–886)
1-140	Dermatitis	173 (100–279)	186 (110–294)	183 (110–282)	116 (67·9–183)	115 (68·1–183)	115 (68·2–184)
1-141	Atopic dermatitis	137 (74–231)	133 (72–226)	122 (66–207)	85·1 (45·9–143)	85 (45·9–144)	85 (46·1–144)
1-142	Contact dermatitis	31 (20–47)	47 (30–71)	54 (34–81)	27·1 (17–40·8)	27 (17–41)	26·9 (16·9–40·2)
1-143	Seborrhoeic dermatitis	4 (2–7)	6 (3–9)	6 (4–10)	3·4 (1·9–5·4)	3·4 (1·9–5·4)	3·4 (1·9–5·4)
1-144	Psoriasis	45 (31–60)	57 (40–77)	62 (44–83)	37·2 (26·1–49·5)	33 (23·3–44·6)	32·1 (22·6–42·9)
1-145	Bacterial skin diseases	39 (26–70)	92 (56–152)	130 (76–216)	30·8 (20·6–57·2)	56·7 (34·7–93·5)	67·5 (39·9–110)
1-146	Cellulitis	18 (11–32)	31 (19–58)	42 (26–79)	14·4 (9·1–27·2)	19·2 (11·5–35·3)	22·3 (13·3–39·7)
1-147	Pyoderma	20 (12–39)	61 (34–102)	88 (47–147)	16·4 (10·1–31·9)	37·5 (20·9–62·6)	45·2 (24·8–74·2)
1-148	Scabies	125 (68–200)	139 (77–223)	136 (75–216)	83·5 (45·8–134)	83·1 (45·7–132)	82·8 (45·6–132)
1-149	Fungal skin diseases	52 (21–111)	73 (30–155)	83 (33–176)	44·1 (17·7–93·6)	43·8 (17·6–92·8)	43·8 (17·6–92·5)
1-150	Viral skin diseases	78 (49–117)	74 (48–110)	67 (42–99)	47·5 (29·9–70·9)	47·6 (30·7–70·9)	47·5 (30·2–71·3)
1-151	Acne vulgaris	74 (43–121)	90 (53–146)	80 (47–130)	42·8 (25–69·6)	48·8 (28·8–79·5)	50·4 (29·8–81·5)
1-152	Alopecia areata	9 (6–13)	12 (8–19)	13 (8–20)	7·2 (4·5–10·5)	7·1 (4·5–10·6)	7·1 (4·5–10·4)
1-153	Pruritus	10 (5–18)	14 (7–26)	16 (8–29)	8·3 (4–15)	8·5 (4·1–15)	8·5 (4·1–15·3)
1-154	Urticaria	72 (47–106)	77 (50–112)	75 (50–108)	48·2 (31·5–70·1)	48·1 (31·5–70·1)	48 (31·3–69·4)
1-155	Decubitus ulcer	76 (52–136)	102 (74–177)	148 (102–247)	89·3 (61·9–156)	65·6 (47·1–112)	72·6 (50·3–120)
1-156	Other skin and subcutaneous diseases	61 (33–105)	88 (46–153)	103 (55–179)	53·2 (28·9–91·8)	52·3 (27·7–90·9)	52·5 (27·9–91·2)
1-157	Sense organ diseases	781 (531–1,120)	1,240 (838–1,780)	1,540 (1,030–2,210)	810 (547–1,160)	756 (511–1,080)	742 (501–1,060)
1-158	Blindness and vision loss	226 (154–321)	337 (229–481)	415 (283–597)	240 (165–339)	208 (141–295)	201 (138–289)
1-159	Glaucoma	13 (9–19)	19 (12–27)	23 (16–33)	16·2 (10·6–23·5)	12·1 (8·1–17·4)	11·4 (7·6–16·3)
1-160	Cataract	38 (26–52)	57 (40–77)	71 (50–97)	46·2 (31·9–62·8)	36·3 (25·3–49·1)	34·5 (23·9–46·8)
1-161	Age-related macular degeneration	1 (1–2)	2 (1–3)	3 (2–4)	1·7 (1·1–2·4)	1·5 (1–2·2)	1·4 (0·9–2)
1-162	Refraction disorders	69 (46–101)	94 (63–135)	105 (69–149)	59·2 (39·2–85·6)	55·1 (36·5–79·1)	54·1 (35·6–78)
1-163	Near vision loss	47 (21–94)	81 (36–162)	107 (49–211)	54 (24·8–107)	50 (22·5–99·1)	49·3 (22·4–97·1)
1-164	Other vision loss	57 (40–78)	84 (60–116)	105 (74–145)	62·3 (44–84·9)	52·5 (37·4–72·6)	50·7 (35·5–69·9)
1-165	Age-related and other hearing loss	516 (346–749)	847 (559–1,230)	1,050 (691–1,540)	534 (358–778)	513 (341–741)	504 (334–733)
1-166	Other sense organ diseases	38 (23–59)	60 (36–94)	73 (43–113)	35·8 (21–55·9)	36·3 (21·5–56·3)	36·4 (21·6–56·6)

1-167	Musculoskeletal disorders	1,570 (1,140–2,050)	2,730 (1,980–3,590)	3,300 (2,410–4,360)	1,520 (1,110–1,990)	1,570 (1,140–2,070)	1,580 (1,150–2,080)
1-168	Rheumatoid arthritis	33 (24–43)	64 (48–83)	82 (61–107)	35·3 (26·6–46·3)	37·6 (28–48·5)	39·1 (29–50·9)
1-169	Osteoarthritis	163 (82–327)	328 (165–661)	446 (222–895)	188 (94·5–380)	200 (101–400)	203 (102–409)
1-170	Low back pain	826 (577–1,100)	1,300 (906–1,740)	1,500 (1,060–2,030)	761 (532–1,020)	744 (519–994)	734 (515–984)
1-171	Neck pain	135 (89–196)	238 (157–346)	284 (187–414)	135 (88·7–194)	134 (89·8–193)	134 (89·1–194)
1-172	Gout	6 (4–9)	13 (8–19)	17 (10–25)	6·5 (4–9·3)	7·7 (4·7–11·1)	7·9 (4·9–11·3)
1-173	Other musculoskeletal disorders	410 (302–546)	787 (558–1,060)	972 (682–1,300)	398 (292–535)	447 (317–605)	464 (326–616)
1-174	Other non-communicable diseases	2,940 (2,440–3,520)	3,000 (2,390–3,750)	3,240 (2,530–4,150)	2,170 (1,790–2,670)	1,920 (1,570–2,380)	1,900 (1,510–2,390)
1-175	Congenital anomalies	1,100 (892–1,350)	682 (555–836)	548 (420–723)	624 (511–766)	553 (442–687)	508 (375–692)
1-176	Neural tube defects	86 (61–117)	40 (29–55)	32 (22–45)	48 (34·4–65·1)	34·2 (24·1–47·3)	32·7 (21·4–47·3)
1-177	Congenital heart anomalies	447 (343–579)	243 (186–306)	180 (122–249)	251 (194–324)	204 (153–261)	177 (116–252)
1-178	Orofacial clefts	4 (2–6)	5 (3–7)	5 (3–8)	2·7 (1·7–4)	2·8 (1·8–4·4)	3 (1·9–4·6)
1-179	Down's syndrome	25 (20–32)	32 (26–39)	34 (27–44)	16·1 (13–20·4)	22·1 (17·6–27·3)	24·1 (18–32)
1-180	Turner syndrome	0 (0–0)	0 (0–0)	0 (0–0)	0·1 (0·1–0·3)	0·1 (0·1–0·2)	0·1 (0·1–0·2)
1-181	Klinefelter syndrome	0 (0–0)	0 (0–0)	0 (0–0)	0 (0–0)	0 (0–0)	0 (0–0)
1-182	Other chromosomal abnormalities	20 (15–28)	21 (15–27)	18 (13–25)	11·2 (8·5–15·7)	16·8 (11·9–21·9)	17·6 (12–24·3)
1-183	Congenital musculoskeletal and limb anomalies	84 (59–116)	80 (55–110)	75 (52–106)	52·4 (36·9–72·3)	52·6 (37·2–72)	52·1 (36·6–73·7)
1-184	Urogenital congenital anomalies	39 (26–64)	25 (18–37)	19 (12–28)	21·6 (14·9–35·9)	21·2 (14·9–32·2)	19·1 (12·3–29·2)
1-185	Digestive congenital anomalies	110 (78–162)	59 (40–87)	41 (26–62)	60·3 (43–88·9)	53·9 (36·4–79·8)	46·2 (28·9–70·3)
1-186	Other congenital anomalies	282 (218–361)	178 (138–229)	143 (105–200)	161 (126–206)	145 (111–191)	135 (95·2–193)
1-187	Urinary diseases and male infertility	93 (76–119)	216 (168–256)	311 (240–374)	94·5 (77·3–121)	133 (103–159)	152 (117–182)
1-188	Urinary tract infections and interstitial nephritis	42 (36–57)	124 (92–141)	189 (135–230)	43 (36·7–58·9)	77·2 (57·2–87·1)	92·8 (66·6–112)
1-189	Urolithiasis	6 (5–7)	13 (10–16)	17 (13–22)	6·1 (4·8–7·7)	7·3 (5·7–9·1)	8 (6·1–10·2)
1-190	Benign prostatic hyperplasia	22 (13–35)	42 (24–65)	56 (33–87)	24·3 (14·2–38·4)	26·5 (15·6–41·7)	26·4 (15·5–41)
1-191	Male infertility	5 (2–13)	8 (3–18)	8 (3–19)	4·1 (1·5–10·2)	4·2 (1·6–10)	4·3 (1·6–10·4)
1-192	Other urinary diseases	18 (14–25)	30 (19–38)	41 (25–54)	17 (13·3–24·9)	18·1 (11·6–22·7)	20·5 (12·5–26·8)
1-193	Gynecological diseases	449 (307–629)	664 (457–929)	717 (492–1,010)	387 (268–540)	363 (250–509)	359 (246–504)
1-194	Uterine fibroids	25 (13–46)	45 (22–83)	51 (25–94)	25·3 (13–46·1)	24·2 (12·2–45·3)	24·1 (11·9–44·3)
1-195	Polycystic ovarian syndrome	6 (3–13)	11 (5–22)	12 (5–24)	4·5 (1·9–9·2)	6 (2·5–12·1)	6·1 (2·6–12·7)
1-196	Female infertility	10 (4–24)	15 (5–35)	15 (5–35)	8·2 (2·8–19·7)	8 (2·8–19·3)	8 (2·8–18·9)
1-197	Endometriosis	44 (25–73)	51 (30–81)	50 (30–78)	34·6 (20·1–55·2)	27·6 (16·1–44·1)	25·7 (15·2–41·2)
1-198	Genital prolapse	7 (3–14)	9 (4–18)	11 (5–20)	7·9 (3·7–15·2)	5·4 (2·6–10·4)	4·9 (2·3–9·3)
1-199	Premenstrual syndrome	135 (81–212)	189 (115–293)	190 (115–297)	102 (61·9–158)	101 (62·2–158)	101 (61–158)
1-200	Other gynecological diseases	222 (145–314)	344 (221–496)	389 (252–555)	205 (135–291)	191 (122–272)	189 (122–269)
1-201	Hemoglobinopathies and hemolytic anaemias	361 (302–435)	318 (273–375)	314 (260–383)	259 (217–309)	190 (163–224)	183 (150–224)
1-202	Thalassemias	0 (0–0)	0 (0–1)	0 (0–0)	0·1 (0·1–0·3)	0·2 (0·1–0·4)	0·2 (0·1–0·3)
1-203	Thalassaemias trait	6 (4–9)	7 (4–11)	7 (4–11)	4·2 (2·6–6·5)	4 (2·4–6·3)	4 (2·4–6·3)
1-204	Sickle cell disorders	179 (152–206)	162 (143–183)	145 (113–184)	117 (99·1–134)	97 (85·3–110)	91 (71·2–116)
1-205	Sickle cell trait	89 (55–134)	53 (31–83)	47 (27–75)	60·2 (37·5–91·8)	31·8 (19–50)	28·6 (16·7–45·4)
1-206	G6PD deficiency	27 (18–37)	39 (28–50)	46 (33–61)	22·9 (16–30·5)	21·8 (15·7–28·2)	23·3 (16·3–30·8)
1-207	G6PD trait	0 (0–0)	1 (0–1)	0 (0–1)	0·2 (0·1–0·3)	0·3 (0·1–0·4)	0·3 (0·1–0·4)
1-208	Other hemoglobinopathies and hemolytic anaemias	60 (47–77)	56 (44–75)	69 (54–88)	54·6 (43·9–68·1)	34·4 (26·5–45·2)	35·9 (28·1–46·4)
1-209	Endocrine, metabolic, blood, and immune disorders	502 (362–600)	537 (431–718)	658 (516–864)	422 (307–502)	331 (266–445)	351 (274–461)
1-210	Oral disorders	345 (202–556)	551 (317–894)	664 (383–1,090)	336 (197–546)	325 (189–526)	323 (187–528)
1-211	Caries of deciduous teeth	6 (2–12)	4 (2–9)	3 (1–7)	3·1 (1·3–6·5)	3·1 (1·3–6·6)	3·1 (1·3–6·4)
1-212	Caries of permanent teeth	35 (16–68)	46 (21–88)	48 (22–93)	26·7 (11·8–50·3)	25·8 (11·7–49)	25·8 (11·6–50·3)
1-213	Periodontal disease	109 (41–233)	196 (75–416)	234 (91–498)	109 (41·4–232)	110 (42·4–232)	110 (42·8–236)
1-214	Edentulism	128 (80–190)	212 (135–316)	279 (175–420)	144 (91–215)	133 (84·3–198)	131 (81·9–196)
1-215	Other oral disorders	68 (42–100)	92 (58–134)	99 (62–145)	53·2 (33·1–77·6)	53·1 (33·3–77·5)	53 (33·3–77·3)
1-216	Sudden infant death syndrome	90 (60–131)	30 (20–45)	22 (13–35)	49 (32·5–71·3)	28·2 (18·1–41·7)	25·1 (14·5–40·2)
1-0	Injuries	5,160 (4,810–5,520)	6,850 (6,450–7,330)	6,210 (5,390–7,110)	4,000 (3,720–4,300)	3,920 (3,690–4,190)	3,310 (2,860–3,800)
1-1	Transport injuries	1,640 (1,520–1,770)	1,530 (1,400–1,650)	1,620 (1,380–1,860)	1,290 (1,190–1,390)	864 (792–936)	850 (724–981)
1-2	Road injuries	1,580 (1,460–1,700)	1,400 (1,280–1,510)	1,470 (1,260–1,700)	1,240 (1,150–1,340)	790 (724–857)	774 (660–898)

1·3	Pedestrian road injuries	328 (292–361)	214 (195–235)	221 (188–260)	267 (239–292)	125 (114–137)	117 (99·2–138)
1·4	Cyclist road injuries	138 (115–164)	124 (103–148)	138 (110–170)	112 (93·6–132)	69·8 (58·1–83·4)	70·7 (56·5–87·4)
1·5	Motorcyclist road injuries	171 (153–189)	269 (241–298)	292 (245–344)	133 (119–148)	149 (134–166)	152 (128–179)
1·6	Motor vehicle road injuries	924 (849–1,010)	765 (703–829)	792 (671–927)	713 (660–774)	430 (395–466)	418 (354–490)
1·7	Other road injuries	20 (16–24)	27 (22–32)	30 (24–37)	16·4 (13·6–19·6)	15·3 (12·7–18·2)	15·4 (12·4–19)
1·8	Other transport injuries	64 (57–71)	130 (116–144)	144 (117–180)	50 (45·2–55·5)	73·9 (65·9–81·9)	76·4 (61·9–95·4)
1·9	Unintentional injuries	2,150 (1,920–2,390)	2,800 (2,540–3,120)	2,230 (1,870–2,650)	1,640 (1,470–1,820)	1,660 (1,510–1,840)	1,210 (1,020–1,440)
1·10	Falls	187 (160–221)	315 (259–385)	416 (339–506)	183 (156–215)	188 (155–228)	208 (170–253)
1·11	Drowning	639 (579–705)	513 (471–562)	479 (399–572)	432 (396–472)	300 (274–330)	282 (233–340)
1·12	Fire, heat, and hot substances	344 (294–401)	252 (206–318)	266 (210–340)	265 (226–312)	149 (122–186)	144 (115–182)
1·13	Poisonings	76 (67–88)	22 (18–27)	23 (18–29)	53·6 (47·4–60·1)	13·3 (11–15·9)	13·3 (10·6–16·6)
1·14	Poisoning by carbon monoxide	16 (13–20)	10 (9–12)	10 (8–13)	12·2 (10·2–14·7)	5·8 (5–6·7)	5·9 (4·6–7·3)
1·15	Poisoning by other means	60 (52–70)	12 (9–16)	13 (9–17)	41·4 (36·5–47·1)	7·5 (5·7–9·7)	7·4 (5·4–9·8)
1·16	Exposure to mechanical forces	285 (241–344)	448 (375–553)	470 (377–587)	232 (193–284)	257 (214–317)	246 (198–305)
1·17	Unintentional firearm injuries	99 (86–115)	154 (132–175)	143 (118–171)	77·6 (67–89·6)	86·4 (73·7–97·6)	75·6 (62·2–90·7)
1·18	Other exposure to mechanical forces	186 (146–240)	294 (227–390)	327 (248–436)	155 (120–201)	170 (132–225)	170 (130–225)
1·19	Adverse effects of medical treatment	134 (114–149)	115 (104–130)	134 (111–164)	116 (98·5–128)	71·3 (64·2–80·9)	71·8 (58·9–88·4)
1·20	Animal contact	15 (11–20)	29 (24–36)	33 (26–41)	12·4 (9·1–16·6)	17·2 (14·2–21)	17·2 (13·8–21·3)
1·21	Venomous animal contact	11 (8–14)	21 (18–26)	24 (19–29)	8·7 (6·2–11·5)	12·5 (10·3–15·2)	12·6 (10–15·4)
1·22	Non-venomous animal contact	4 (3–6)	8 (6–10)	9 (7–12)	3·7 (2·6–5·4)	4·7 (3·6–6·2)	4·7 (3·6–6·3)
1·23	Foreign body	239 (204–282)	122 (108–138)	132 (111–158)	162 (143–185)	81·2 (70·6–93·2)	83·2 (68–103)
1·24	Pulmonary aspiration and foreign body in airway	221 (187–264)	100 (89–113)	108 (89–132)	148 (129–170)	68·4 (59·4–79·2)	70·4 (55·8–89·3)
1·25	Foreign body in eyes	7 (3–12)	9 (5–17)	10 (5–18)	5·4 (2·6–9·5)	5·4 (2·6–9·6)	5·4 (2·6–9·6)
1·26	Foreign body in other body part	11 (9–15)	12 (9–16)	14 (10–18)	8·9 (6·9–11·4)	7·3 (5·5–9·7)	7·4 (5·6–9·8)
1·27	Environmental heat and cold exposure	49 (36–65)	63 (46–85)	73 (52–99)	42 (31·3–55·4)	36·3 (26·6–49·4)	37·4 (27–50·5)
1·28	Exposure to forces of nature	3 (2–4)	726 (658–801)	8 (6–11)	2·1 (1·4–3)	433 (393–478)	3·9 (2·7–5·5)
1·29	Other unintentional injuries	176 (148–212)	199 (165–248)	196 (157–251)	137 (114–166)	114 (94·2–142)	105 (84–133)
1·30	Self-harm and interpersonal violence	1,370 (1,270–1,460)	2,520 (2,370–2,670)	2,360 (2,010–2,740)	1,070 (1,000–1,140)	1,400 (1,320–1,490)	1,250 (1,060–1,450)
1·31	Self-harm	397 (363–434)	505 (460–549)	564 (474–663)	340 (312–371)	280 (255–305)	283 (238–332)
1·32	Self-harm by firearm	18 (14–22)	21 (16–27)	24 (17–33)	15·6 (11·8–19·1)	11·8 (9–14·7)	12 (8·5–16·6)
1·33	Self-harm by other specified means	379 (348–414)	483 (440–525)	541 (454–635)	325 (299–353)	268 (244–291)	271 (228–318)
1·34	Interpersonal violence	910 (830–998)	1,990 (1,860–2,130)	1,780 (1,510–2,070)	677 (623–742)	1,110 (1,040–1,180)	956 (814–1,110)
1·35	Physical violence by firearm	392 (355–432)	1,130 (1,050–1,210)	964 (810–1,130)	286 (260–315)	622 (578–667)	519 (437–607)
1·36	Physical violence by sharp object	306 (273–338)	591 (544–642)	541 (461–637)	234 (211–259)	325 (299–354)	286 (244–336)
1·37	Sexual violence	24 (16–34)	32 (21–45)	33 (22–47)	17·9 (11·8–25·4)	17·7 (11·7–25·2)	17·7 (11·7–25·1)
1·38	Physical violence by other means	188 (169–209)	245 (225–270)	239 (202–284)	140 (127–154)	144 (132–159)	133 (111–160)
1·39	Conflict and terrorism	58 (52–65)	8 (5–12)	7 (5–10)	55·7 (50·2–62)	4·3 (3–6·5)	3 (2–4·3)
1·40	Executions and police conflict	1 (1–1)	14 (11–20)	9 (5–16)	0·7 (0·6–0·7)	8·1 (5·9–11·1)	5 (2·7–8·6)

SR Table 1: All-age DALYs counts and age standardized DALYs rates per 100,000 individuals for all causes and level 1 cause groups by location for 1990, 2010, and 2019, both sexes combined, Saint Vincent and the Grenadines

Cause Level	Cause	All-age DALYs			Age-standardized rate of DALYs per 100,000		
		1990	2010	2019	1990	2010	2019
0-0	All causes	34,000 (30,600–37,400)	34,300 (30,800–38,000)	38,600 (33,800–44,100)	37,000 (33,800–40,400)	31,700 (28,600–35,000)	31,700 (27,700–36,200)
1-0	Communicable, maternal, neonatal, and nutritional disorders	9,110 (7,770–10,600)	5,700 (5,140–6,370)	4,910 (4,240–5,720)	8,040 (6,980–9,220)	5,700 (5,100–6,400)	4,910 (4,140–5,870)
1-1	HIV/AIDS and sexually transmitted infections	863 (837–892)	1,160 (1,110–1,220)	1,010 (981–1,060)	943 (916–974)	996 (960–1,050)	848 (820–888)
1-2	HIV/AIDS	787 (770–807)	1,130 (1,080–1,190)	984 (954–1,030)	871 (852–896)	967 (932–1,020)	822 (797–858)
1-3	HIV/AIDS - drug-susceptible tuberculosis	88 (56–131)	55 (36–83)	49 (32–73)	97·7 (62·8–146)	47·1 (31·3–71·1)	40·5 (26·5–60·7)
1-4	HIV/AIDS - Multidrug-resistant Tuberculosis without extensive drug resistance	3 (0–15)	1 (0–2)	1 (0–3)	3 (0·1–16·5)	0·4 (0–2·1)	0·6 (0–2·4)
1-5	HIV/AIDS - Extensively drug-resistant Tuberculosis	0 (0–0)	0 (0–0)	0 (0–0)	0 (0–0)	0 (0–0·2)	0·1 (0–0·4)
1-6	HIV/AIDS resulting in other diseases	696 (649–733)	1,070 (1,020–1,130)	935 (896–978)	770 (717–812)	920 (879–972)	781 (749–818)
1-7	Sexually transmitted infections excluding HIV	76 (56–98)	31 (22–47)	30 (20–47)	72·5 (52·6–92·6)	28·2 (20·3–42·1)	26 (18–39·8)
1-8	Syphilis	53 (38–71)	9 (7–11)	7 (5–9)	46·8 (33·2–61·7)	8·9 (7·4–10·8)	6·9 (5·2–9·2)
1-9	Chlamydial infection	2 (1–3)	3 (2–4)	3 (2–4)	2·3 (1·6–3·5)	2·2 (1·5–3·3)	2·2 (1·5–3·2)
1-10	Gonococcal infection	4 (2–5)	2 (2–3)	2 (2–3)	3·9 (2·4–4·7)	2 (1·6–2·6)	2 (1·6–2·6)
1-11	Trichomoniasis	4 (2–9)	5 (2–12)	6 (2–12)	4·9 (1·9–10·2)	4·7 (1·8–9·9)	4·6 (1·8–9·5)
1-12	Genital herpes	5 (2–11)	6 (2–15)	7 (2–16)	5·4 (1·8–13·2)	5·3 (1·7–13·1)	5·3 (1·7–12·9)
1-13	Other sexually transmitted infections	8 (5–11)	6 (4–8)	6 (4–8)	9·1 (5·9–11·4)	5 (3·7–6·8)	4·9 (3·6–6·6)
1-14	Respiratory infections and tuberculosis	1,400 (1,230–1,600)	1,010 (912–1,110)	1,050 (902–1,220)	1,420 (1,270–1,580)	978 (883–1,090)	931 (794–1,100)
1-15	Tuberculosis	214 (193–238)	105 (95–116)	98 (83–115)	236 (212–262)	93·2 (84·5–103)	78·9 (66·8–92·2)
1-16	Latent tuberculosis infection	0 (0–0)	0 (0–0)	0 (0–0)	0 (0–0)	0 (0–0)	0 (0–0)
1-17	Drug-susceptible tuberculosis	209 (180–236)	104 (94–116)	97 (82–114)	230 (199–258)	92·5 (83·6–102)	77·9 (65·8–91·2)
1-18	Multidrug-resistant tuberculosis without extensive drug resistance	5 (0–30)	1 (0–3)	1 (0–4)	5·8 (0·3–33·2)	0·7 (0·1–3)	0·8 (0·1–3·4)
1-19	Extensively drug-resistant tuberculosis	0 (0–0)	0 (0–0)	0 (0–1)	0 (0–0)	0·1 (0–0·3)	0·1 (0–0·5)
1-20	Lower respiratory infections	1,040 (888–1,220)	772 (695–859)	824 (701–977)	1,060 (932–1,200)	767 (690–859)	736 (616–886)
1-21	Upper respiratory infections	107 (68–162)	97 (61–149)	94 (60–145)	91·3 (58·7–137)	88·5 (56·2–136)	87·7 (55·8–133)
1-22	Otitis media	39 (22–66)	32 (18–53)	31 (18–50)	32·8 (19–54·5)	29·3 (16·8–48·3)	28·7 (16·2–47·1)
1-23	Enteric infections	831 (670–1,020)	332 (273–407)	328 (260–409)	723 (596–872)	327 (268–398)	312 (246–387)
1-24	Diarrhoeal diseases	828 (667–1,010)	331 (272–406)	327 (258–408)	720 (594–870)	325 (267–396)	310 (244–386)
1-25	Typhoid and paratyphoid	0 (0–1)	0 (0–0)	0 (0–0)	0·4 (0·2–0·6)	0·1 (0·1–0·1)	0·1 (0–0·2)
1-26	Typhoid fever	0 (0–1)	0 (0–0)	0 (0–0)	0·4 (0·1–0·5)	0 (0–0·1)	0 (0–0·1)
1-27	Paratyphoid fever	0 (0–0)	0 (0–0)	0 (0–0)	0 (0–0·1)	0·1 (0–0·1)	0·1 (0–0·2)
1-28	iNTS	1 (1–2)	1 (0–1)	1 (0–1)	0·9 (0·5–1·2)	0·6 (0·3–0·8)	0·5 (0·3–0·8)
1-29	Other intestinal infectious diseases	2 (1–3)	1 (0–1)	1 (0–1)	1·2 (0·5–2·2)	0·7 (0·4–1·1)	0·6 (0·3–0·9)
1-30	Neglected tropical diseases and malaria	160 (116–216)	128 (86–178)	126 (85–179)	156 (112–211)	117 (77·8–160)	107 (73·6–150)
1-31	Malaria	0 (0–0)	0 (0–0)	0 (0–0)	0 (0–0)	0 (0–0)	0 (0–0)
1-32	Chagas disease	0 (0–0)	0 (0–0)	0 (0–0)	0 (0–0)	0 (0–0)	0 (0–0)
1-33	Leishmaniasis	0 (0–0)	0 (0–0)	0 (0–0)	0 (0–0)	0 (0–0)	0 (0–0)
1-34	Visceral leishmaniasis	0 (0–0)	0 (0–0)	0 (0–0)	0 (0–0)	0 (0–0)	0 (0–0)
1-35	Cutaneous and mucocutaneous leishmaniasis	0 (0–0)	0 (0–0)	0 (0–0)	0 (0–0)	0 (0–0)	0 (0–0)
1-36	African trypanosomiasis	0 (0–0)	0 (0–0)	0 (0–0)	0 (0–0)	0 (0–0)	0 (0–0)
1-37	Schistosomiasis	0 (0–0)	0 (0–0)	0 (0–0)	0 (0–0)	0 (0–0)	0 (0–0)
1-38	Cysticercosis	52 (27–88)	61 (29–101)	70 (36–117)	66·8 (35·2–110)	55·4 (26·4–91·9)	53·1 (27·8–88·7)
1-39	Cystic echinococcosis	0 (0–0)	0 (0–0)	0 (0–0)	0 (0–0)	0 (0–0)	0 (0–0)
1-40	Lymphatic filariasis	0 (0–0)	0 (0–0)	0 (0–0)	0 (0–0)	0 (0–0)	0 (0–0)
1-41	Onchocerciasis	0 (0–0)	0 (0–0)	0 (0–0)	0 (0–0)	0 (0–0)	0 (0–0)
1-42	Trachoma	0 (0–0)	0 (0–0)	0 (0–0)	0 (0–0)	0 (0–0)	0 (0–0)
1-43	Dengue	19 (7–37)	21 (11–36)	12 (6–21)	16·4 (6·3–32·1)	19 (10·3–32·3)	10·5 (5·5–19·4)
1-44	Yellow fever	0 (0–0)	0 (0–0)	0 (0–0)	0 (0–0)	0 (0–0)	0 (0–0)
1-45	Rabies	0 (0–1)	0 (0–0)	0 (0–1)	0·3 (0·2–0·4)	0·3 (0·2–0·4)	0·4 (0·3–0·6)
1-46	Intestinal nematode infections	35 (21–57)	12 (7–19)	9 (5–15)	28·2 (17–46)	10·2 (5·8–17·1)	8·1 (4·6–13·6)
1-47	Ascariasis	4 (2–7)	2 (1–2)	1 (1–2)	3·3 (1·6–5·3)	1·4 (0·7–2·4)	1 (0·6–1·7)
1-48	Trichuriasis	12 (6–22)	0 (0–0)	0 (0–0)	9·7 (4·6–18·2)	0·1 (0–0·1)	0·1 (0–0·1)

1-49	Hookworm disease	19 (10–32)	10 (5–17)	8 (4–14)	15·2 (8·1–25·7)	8·8 (4·7–15·2)	7·1 (3·8–12·2)
1-50	Food-borne trematodiases	0 (0–0)	0 (0–0)	0 (0–0)	0 (0–0)	0 (0–0)	0 (0–0)
1-51	Leprosy	0 (0–1)	0 (0–1)	0 (0–1)	0·5 (0·3–0·7)	0·4 (0·2–0·6)	0·4 (0·2–0·6)
1-52	Ebola virus disease	0 (0–0)	0 (0–0)	0 (0–0)	0 (0–0)	0 (0–0)	0 (0–0)
1-53	Zika virus disease	0 (0–0)	0 (0–0)	3 (0–14)	0 (0–0)	0 (0–0)	2·8 (0–12·5)
1-54	Guinea worm disease	0 (0–0)	0 (0–0)	0 (0–0)	0 (0–0)	0 (0–0)	0 (0–0)
1-55	Other neglected tropical diseases	53 (36–75)	33 (23–48)	32 (22–45)	43·7 (30·2–60·4)	31·4 (21·4–45·1)	31·8 (21·8–45·1)
1-56	Other infectious diseases	586 (497–685)	246 (207–287)	229 (179–281)	513 (439–589)	233 (195–271)	210 (165–260)
1-57	Meningitis	347 (288–421)	82 (70–96)	65 (52–81)	292 (246–348)	81·4 (68·4–96·7)	65·1 (51·2–84)
1-58	Encephalitis	90 (60–112)	38 (32–46)	33 (26–42)	73·5 (49·5–90·1)	35·8 (30·2–44·3)	32·4 (25·3–42·6)
1-59	Diphtheria	3 (2–5)	0 (0–1)	1 (0–1)	2·2 (1·3–3·7)	0·3 (0·2–0·5)	0·8 (0·4–1·5)
1-60	Whooping cough	2 (1–4)	2 (1–2)	1 (1–2)	1·9 (0·9–3·4)	1·8 (1·1–2·6)	1·6 (1–2·4)
1-61	Tetanus	2 (1–5)	1 (1–2)	0 (0–2)	2·6 (1·5–5·7)	0·7 (0·5–2·1)	0·4 (0·1–1·8)
1-62	Measles	0 (0–0)	0 (0–0)	0 (0–0)	0·2 (0–0·3)	0 (0–0)	0 (0–0)
1-63	Varicella and herpes zoster	7 (5–11)	7 (5–9)	6 (5–9)	6·7 (4·9–10·6)	6·1 (4·6–8·6)	5·7 (4·2–8·2)
1-64	Acute hepatitis	21 (18–25)	8 (6–10)	7 (5–9)	19·2 (16·7–22)	7 (5·4–9)	6·2 (4·6–8·2)
1-65	Acute hepatitis A	11 (7–15)	5 (3–7)	4 (3–6)	9·5 (5·8–12·7)	4·3 (2·9–5·9)	3·9 (2·6–5·5)
1-66	Acute hepatitis B	7 (5–11)	2 (2–3)	2 (1–2)	6·9 (5–11)	1·9 (1·6–2·3)	1·6 (1·2–1·9)
1-67	Acute hepatitis C	1 (0–1)	0 (0–0)	0 (0–0)	0·6 (0·2–1)	0·1 (0·1–0·3)	0·1 (0·1–0·3)
1-68	Acute hepatitis E	2 (1–3)	1 (0–1)	1 (0–1)	2·1 (1–2·9)	0·6 (0·4–0·9)	0·6 (0·4–0·8)
1-69	Other unspecified infectious diseases	112 (69–144)	110 (73–138)	115 (75–146)	114 (69·8–145)	99·7 (66·8–126)	97·9 (64·3–125)
1-70	Maternal and neonatal disorders	4,110 (3,270–5,060)	2,170 (1,830–2,550)	1,510 (1,160–1,980)	3,240 (2,580–3,990)	2,430 (2,040–2,860)	1,900 (1,420–2,550)
1-71	Maternal disorders	96 (84–108)	95 (84–106)	82 (68–98)	81·5 (71·4–91·9)	82·2 (72·9–91·7)	73·4 (60·5–87·6)
1-72	Maternal haemorrhage	12 (8–17)	9 (6–12)	7 (5–10)	10·1 (7–14·3)	7·4 (5·2–10·3)	6·3 (4·4–8·9)
1-73	Maternal sepsis and other pregnancy related infections	3 (2–5)	2 (1–3)	2 (1–3)	2·3 (1·4–3·6)	1·6 (1–2·6)	1·4 (0·9–2·3)
1-74	Maternal hypertensive disorders	32 (28–37)	25 (22–29)	21 (17–26)	26·9 (23·3–30·9)	22 (19·2–25)	19·1 (15·4–23·1)
1-75	Maternal obstructed labour and uterine rupture	3 (3–4)	5 (4–6)	4 (3–5)	3 (2·3–3·9)	4 (3–5·1)	3·2 (2·4–4·4)
1-76	Maternal abortive outcome	15 (12–18)	7 (5–8)	5 (3–6)	12·9 (10·3–15·9)	5·6 (4·3–7·2)	4·2 (3·1–5·7)
1-77	Ectopic pregnancy	6 (5–7)	10 (8–11)	8 (6–10)	5·2 (4·3–6·3)	8·3 (6·8–10)	7·1 (5·5–9·1)
1-78	Indirect maternal deaths	10 (8–13)	14 (12–17)	13 (10–16)	8·3 (6·7–10·4)	12·2 (10–14·7)	11·8 (9–14·8)
1-79	Late maternal deaths	4 (3–5)	5 (4–7)	5 (3–6)	3·3 (2·4–4·3)	4·6 (3·4–6·1)	4·2 (2·9–5·7)
1-80	Maternal deaths aggravated by HIV/AIDS	0 (0–0)	0 (0–0)	0 (0–0)	0·1 (0–0·1)	0·1 (0·1–0·2)	0·1 (0–0·2)
1-81	Other maternal disorders	11 (9–13)	19 (16–23)	18 (14–22)	9·3 (7·5–11·3)	16·4 (13·5–19·9)	15·8 (12·5–20)
1-82	Neonatal disorders	4,010 (3,180–4,960)	2,080 (1,740–2,450)	1,430 (1,090–1,880)	3,160 (2,500–3,910)	2,340 (1,960–2,780)	1,820 (1,360–2,460)
1-83	Neonatal preterm birth	2,250 (1,780–2,790)	1,060 (874–1,290)	658 (482–878)	1,770 (1,400–2,200)	1,200 (981–1,460)	846 (607–1,140)
1-84	Neonatal encephalopathy due to birth asphyxia and trauma	915 (691–1,180)	327 (258–410)	219 (154–308)	720 (544–929)	374 (294–469)	285 (197–404)
1-85	Neonatal sepsis and other neonatal infections	397 (293–529)	455 (362–571)	370 (271–492)	314 (232–419)	502 (401–619)	452 (326–614)
1-86	Hemolytic disease and other neonatal jaundice	129 (94–181)	34 (27–42)	26 (20–32)	103 (75·3–144)	34·7 (27·7–42·8)	27 (21·3–34·1)
1-87	Other neonatal disorders	319 (228–444)	202 (155–260)	156 (105–222)	250 (179–348)	234 (179–302)	213 (144–304)
1-88	Nutritional deficiencies	1,160 (933–1,450)	662 (492–876)	651 (484–873)	1,050 (851–1,290)	624 (465–830)	604 (447–810)
1-89	Protein-energy malnutrition	621 (502–759)	212 (179–251)	209 (169–259)	555 (460–662)	213 (177–253)	200 (158–257)
1-90	Iodine deficiency	13 (7–22)	8 (4–13)	7 (3–11)	12·7 (6·5–20·8)	6·9 (3·4–11·3)	5·5 (2·8–9·1)
1-91	Vitamin A deficiency	21 (13–33)	9 (5–13)	7 (4–11)	15·4 (9·3–24)	8·2 (5–12·3)	7·6 (4·7–11·4)
1-92	Dietary iron deficiency	463 (286–703)	403 (253–608)	400 (247–602)	414 (262–622)	369 (229–557)	368 (228–556)
1-93	Other nutritional deficiencies	45 (34–73)	30 (21–40)	29 (20–43)	47·6 (36·7–76·9)	27·3 (19·6–36·7)	24 (16·9–34·4)
1-0	Non-communicable diseases	20,900 (18,700–23,400)	24,700 (22,000–27,800)	29,800 (26,100–34,000)	25,200 (22,800–27,800)	22,600 (20,200–25,300)	23,500 (20,500–26,900)
1-1	Neoplasms	3,030 (2,820–3,270)	3,950 (3,670–4,220)	5,100 (4,470–5,830)	3,900 (3,630–4,210)	3,590 (3,330–3,840)	3,860 (3,380–4,410)
1-2	Lip and oral cavity cancer	70 (64–77)	95 (87–103)	134 (115–156)	97·3 (88·8–107)	85·6 (78·3–93)	97·4 (83·7–113)
1-3	Nasopharynx cancer	12 (11–13)	19 (18–21)	25 (21–29)	15·2 (13·8–16·7)	17·3 (15·7–18·9)	18·4 (15·9–21·5)
1-4	Other pharynx cancer	32 (29–36)	37 (33–42)	54 (45–64)	46·1 (41–51·9)	33·6 (30·1–37·6)	38·7 (32·8–45·5)
1-5	Oesophageal cancer	40 (36–45)	57 (51–63)	84 (72–99)	57·2 (50·9–64·6)	51·6 (46·5–57·1)	60·7 (51·6–71·1)
1-6	Stomach cancer	274 (250–301)	271 (247–293)	344 (298–399)	378 (345–415)	247 (226–267)	255 (221–295)
1-7	Colon and rectum cancer	184 (170–200)	292 (268–317)	422 (367–487)	254 (234–276)	268 (246–291)	314 (274–361)

1-8	Liver cancer	139 (127–150)	74 (68–80)	105 (89–122)	186 (170–202)	67·4 (61·6–73·1)	77 (65·8–89·6)
1-9	Liver cancer due to hepatitis B	48 (37–61)	26 (20–33)	32 (23–44)	63·9 (49·1–82·8)	22·9 (17·5–29·5)	23·8 (17·3–31·9)
1-10	Liver cancer due to hepatitis C	26 (18–36)	12 (9–17)	18 (12–25)	36·8 (25·4–49·8)	11·7 (8·1–15·9)	13·2 (8·8–18·5)
1-11	Liver cancer due to alcohol use	42 (30–54)	24 (18–31)	39 (28–51)	57·2 (41·7–74)	22·1 (16·4–28·5)	28·1 (20·5–36·6)
1-12	Liver cancer due to NASH	10 (7–13)	6 (4–8)	8 (6–12)	12·8 (9·1–17·8)	5·2 (3·7–7)	6·2 (4·3–8·7)
1-13	Gallbladder and biliary tract cancer	48 (27–55)	28 (24–41)	36 (29–53)	66·6 (38·1–76·5)	25·8 (22·4–38·2)	26·2 (21·5–38·7)
1-14	Pancreatic cancer	28 (25–31)	132 (121–144)	198 (170–230)	38·8 (35·2–42·7)	121 (111–132)	145 (125–167)
1-15	Larynx cancer	41 (36–45)	68 (62–75)	99 (85–117)	57·5 (51·5–64·2)	61·8 (56·2–68·1)	71·3 (61·3–83·2)
1-16	Tracheal, bronchus, and lung cancer	167 (152–183)	244 (224–266)	341 (295–396)	233 (212–256)	224 (205–243)	247 (214–287)
1-17	Malignant skin melanoma	13 (10–18)	19 (15–25)	25 (19–33)	16·7 (13·1–23·2)	17 (13·4–22·6)	19 (15–25·6)
1-18	Non-melanoma skin cancer	4 (3–7)	22 (16–24)	28 (20–32)	5·4 (4·7–9·9)	20·1 (15–22)	21·6 (14·9–25)
1-19	Non-melanoma skin cancer (squamous-cell carcinoma)	4 (3–7)	22 (16–24)	28 (20–32)	5·4 (4·7–9·9)	20·1 (15–22)	21·6 (14·9–24·9)
1-20	Non-melanoma skin cancer (basal-cell carcinoma)	0 (0–0)	0 (0–0)	0 (0–0)	0 (0–0)	0 (0–0)	0 (0–0)
1-21	Breast cancer	325 (292–360)	466 (422–516)	593 (505–698)	459 (413–511)	414 (376–456)	442 (377–520)
1-22	Cervical cancer	339 (299–382)	371 (327–415)	420 (346–505)	460 (404–519)	325 (288–364)	318 (262–382)
1-23	Uterine cancer	64 (57–70)	103 (93–114)	135 (116–158)	88·6 (79·4–97·9)	93·9 (85·1–104)	99·8 (85·9–117)
1-24	Ovarian cancer	20 (17–36)	92 (68–104)	116 (87–138)	27·2 (23·2–48·9)	82·2 (61·5–92·4)	86·5 (64·3–102)
1-25	Prostate cancer	308 (258–462)	583 (457–714)	808 (632–1,010)	423 (356–634)	556 (436–678)	617 (482–768)
1-26	Testicular cancer	1 (1–2)	5 (4–6)	5 (4–7)	1·3 (1·1–2·1)	4·4 (3·7–5·5)	4·6 (3·6–6·1)
1-27	Kidney cancer	60 (54–65)	49 (45–53)	65 (55–77)	70·1 (64·1–76·2)	44·4 (40·8–48·3)	50 (42·1–58·9)
1-28	Bladder cancer	34 (30–38)	50 (46–54)	70 (61–82)	47·1 (42·5–52·1)	46·8 (42·9–51·3)	52·7 (45·8–61·1)
1-29	Brain and nervous system cancer	31 (21–64)	80 (44–96)	88 (46–114)	30·3 (21·1–61·9)	71·5 (39·7–86·3)	72·2 (38·2–94·5)
1-30	Thyroid cancer	18 (16–20)	24 (22–26)	33 (28–39)	23·9 (21·7–26·1)	21·6 (19·7–23·7)	24·8 (21·1–28·9)
1-31	Mesothelioma	4 (3–5)	6 (5–7)	7 (6–9)	5·3 (4·6–6·1)	5·4 (4·8–6)	5·5 (4·7–6·6)
1-32	Hodgkin lymphoma	11 (7–26)	24 (20–28)	25 (20–31)	11·4 (7–27·5)	21 (17·3–24·5)	20·3 (16·1–25·4)
1-33	Non-Hodgkin's lymphoma	229 (195–263)	241 (210–276)	287 (240–344)	250 (216–287)	215 (188–245)	226 (189–270)
1-34	Multiple myeloma	23 (20–27)	40 (36–46)	56 (47–68)	31·6 (28·3–38)	36·6 (32·8–42·2)	40·7 (34·3–49)
1-35	Leukaemia	220 (199–245)	197 (180–215)	204 (175–239)	206 (188–225)	178 (162–194)	173 (147–204)
1-36	Acute lymphoid leukaemia	36 (28–53)	38 (31–44)	31 (24–38)	26·7 (21·1–38·7)	34·5 (28·6–40·6)	30·2 (23·6–37·6)
1-37	Chronic lymphoid leukaemia	7 (6–9)	15 (13–17)	20 (16–24)	9·8 (8–12·1)	13·6 (11·8–15·6)	14·8 (12·2–17·8)
1-38	Acute myeloid leukaemia	28 (23–37)	40 (35–46)	44 (36–53)	24 (20–31·4)	35·7 (30·9–41·1)	38 (31–46·4)
1-39	Chronic myeloid leukaemia	39 (34–44)	16 (14–18)	18 (15–21)	42·7 (37·4–47·9)	14·3 (12·6–16·1)	14·1 (11·6–16·9)
1-40	Other leukaemia	110 (94–125)	89 (80–97)	92 (78–108)	102 (90·1–115)	79·4 (71·5–86·9)	76 (64·2–89·1)
1-41	Other neoplasms	16 (12–26)	26 (21–37)	35 (27–49)	18·5 (14–30·4)	23·9 (19·2–34·3)	27·5 (21·2–38·6)
1-42	Myelodysplastic, myeloproliferative, and other hematopoietic neoplasms	16 (12–26)	26 (21–37)	35 (27–49)	18·5 (14–30·4)	23·9 (19·2–34·3)	27·5 (21·2–38·6)
1-43	Benign and in situ intestinal neoplasms	0 (0–0)	0 (0–0)	0 (0–0)	0 (0–0)	0 (0–0)	0 (0–0)
1-44	Benign and in situ cervical and uterine neoplasms	0 (0–0)	0 (0–0)	0 (0–0)	0 (0–0)	0 (0–0)	0 (0–0)
1-45	Other benign and in situ neoplasms	0 (0–0)	0 (0–0)	0 (0–0)	0 (0–0)	0 (0–0)	0 (0–0)
1-46	Cardiovascular diseases	5,300 (4,990–5,630)	5,730 (5,360–6,090)	7,070 (6,280–7,930)	7,270 (6,830–7,710)	5,370 (5,010–5,700)	5,430 (4,830–6,080)
1-47	Rheumatic heart disease	171 (143–203)	131 (106–161)	128 (100–159)	176 (149–208)	113 (92·7–139)	105 (82·1–132)
1-48	Ischaemic heart disease	2,410 (2,240–2,570)	2,410 (2,230–2,550)	2,940 (2,600–3,320)	3,400 (3,170–3,640)	2,270 (2,110–2,420)	2,260 (2,000–2,540)
1-49	Stroke	1,660 (1,530–1,780)	1,820 (1,690–1,960)	2,210 (1,940–2,500)	2,270 (2,090–2,440)	1,700 (1,570–1,820)	1,680 (1,480–1,900)
1-50	Ischaemic stroke	963 (879–1,040)	815 (747–882)	971 (851–1,100)	1,340 (1,230–1,450)	781 (716–846)	763 (670–858)
1-51	Intracerebral hemorrhage	537 (485–595)	877 (806–949)	1,090 (938–1,260)	729 (656–807)	802 (736–869)	808 (694–929)
1-52	Subarachnoid hemorrhage	158 (137–202)	129 (116–142)	148 (125–172)	195 (169–251)	114 (103–126)	113 (95·3–131)
1-53	Hypertensive heart disease	496 (434–574)	657 (571–749)	879 (734–1,040)	704 (613–813)	617 (537–702)	670 (557–788)
1-54	Non-rheumatic valvular heart disease	47 (41–53)	44 (40–49)	58 (49–68)	59·7 (52·2–67·4)	40·2 (36·5–44·3)	44·5 (37·5–51·9)
1-55	Non-rheumatic calcific aortic valve disease	27 (21–32)	23 (20–26)	30 (25–38)	35·2 (27·9–40·9)	21·1 (17·8–24)	23·1 (19·1–29)
1-56	Non-rheumatic degenerative mitral valve disease	15 (12–19)	18 (15–21)	24 (19–30)	19·1 (15·3–23·9)	16·2 (13–19)	18·4 (14·3–22·7)
1-57	Other non-rheumatic valve diseases	5 (2–6)	3 (2–5)	4 (3–5)	5·4 (2·5–7·5)	2·9 (2–4·1)	3·1 (2–4·3)
1-58	Cardiomyopathy and myocarditis	91 (80–116)	128 (109–143)	164 (131–197)	105 (92·7–136)	117 (99·2–130)	128 (103–153)
1-59	Myocarditis	16 (13–23)	19 (15–23)	20 (15–26)	15·7 (12·3–22·6)	17·7 (13·7–21·1)	17·3 (13·2–22·8)
1-60	Alcoholic cardiomyopathy	12 (10–18)	36 (28–44)	53 (38–67)	17·2 (13·9–24·3)	31·8 (24·5–38·7)	38·6 (27·8–48·2)

1·61	Other cardiomyopathy	62 (54–80)	73 (62–82)	91 (72–110)	72·5 (63·3–93·4)	67·1 (57·4–75)	72 (56·8–86·8)
1·62	Atrial fibrillation and flutter	68 (56–80)	101 (86–121)	132 (111–164)	104 (85·3–121)	99·6 (84·8–120)	108 (91·1–134)
1·63	Aortic aneurysm	38 (34–44)	58 (52–64)	79 (67–91)	52·2 (46·3–59)	54·1 (48·2–60·5)	59·2 (50·2–68·3)
1·64	Peripheral vascular disease	16 (9–27)	28 (16–47)	38 (21–68)	23·6 (12·6–39·1)	27·1 (15·3–45·7)	30·1 (16·8–52·4)
1·65	Endocarditis	14 (11–22)	26 (20–32)	29 (23–37)	15·6 (12·3–24·3)	23·5 (18·5–28·3)	23·6 (18·3–29·7)
1·66	Chronic respiratory diseases	575 (461–731)	621 (520–751)	727 (608–863)	593 (492–714)	572 (479–690)	610 (502–731)
1·67	Chronic obstructive pulmonary disease	138 (122–157)	232 (206–260)	316 (270–363)	186 (165–212)	218 (194–245)	241 (207–277)
1·68	Pneumoconiosis	2 (2–3)	3 (3–3)	4 (3–5)	2·7 (2·3–3·2)	2·7 (2·3–3·2)	3·2 (2·6–3·8)
1·69	Silicosis	0 (0–0)	0 (0–1)	1 (0–1)	0·3 (0·2–0·3)	0·4 (0·3–0·6)	0·5 (0·4–0·6)
1·70	Asbestosis	0 (0–0)	0 (0–1)	1 (0–1)	0·3 (0·2–0·4)	0·4 (0·3–0·5)	0·5 (0·3–0·6)
1·71	Coal workers pneumoconiosis	0 (0–0)	0 (0–0)	0 (0–0)	0·4 (0·3–0·6)	0·1 (0·1–0·2)	0·1 (0·1–0·2)
1·72	Other pneumoconiosis	1 (1–2)	2 (2–2)	3 (2–3)	1·7 (1·4–2)	1·8 (1·5–2·1)	2·1 (1·7–2·6)
1·73	Asthma	400 (293–550)	331 (247–449)	340 (253–456)	366 (280–486)	302 (225–412)	312 (224–428)
1·74	Interstitial lung disease and pulmonary sarcoidosis	9 (5–18)	25 (18–30)	33 (23–43)	12·1 (6·9–22·5)	22·7 (16·5–27·2)	25·4 (17·7–32·8)
1·75	Other chronic respiratory diseases	26 (18–32)	30 (25–36)	34 (27–41)	25·3 (17·9–31)	26·9 (22·5–31·9)	28·7 (23·2–35·5)
1·76	Digestive diseases	1,100 (991–1,230)	1,180 (1,050–1,340)	1,460 (1,260–1,680)	1,350 (1,220–1,510)	1,060 (946–1,200)	1,120 (966–1,300)
1·77	Cirrhosis and other chronic liver diseases	377 (341–414)	409 (369–453)	564 (470–668)	506 (456–557)	362 (327–401)	415 (347–491)
1·78	Cirrhosis and other chronic liver diseases due to hepatitis B	47 (33–64)	55 (37–79)	40 (26–60)	66 (45·8–90·8)	47·9 (32·5–68·2)	29·6 (18·9–43·6)
1·79	Cirrhosis and other chronic liver diseases due to hepatitis C	72 (53–96)	78 (56–105)	112 (77–157)	99·3 (72·4–135)	68·3 (49·1–91·3)	82·4 (57·3–115)
1·80	Cirrhosis and other chronic liver diseases due to alcohol use	134 (103–165)	165 (128–204)	256 (195–327)	191 (146–235)	146 (114–179)	185 (142–235)
1·81	Cirrhosis and other chronic liver diseases due to NAFLD	49 (35–67)	55 (38–78)	85 (55–121)	68·3 (47·6–93·8)	49·4 (34·8–69·2)	62·5 (41–87·8)
1·82	Cirrhosis and other chronic liver diseases due to other causes	76 (60–93)	55 (42–72)	71 (52–98)	81·4 (63·2–104)	49·8 (38–64·9)	55·9 (41·7–75·8)
1·83	Upper digestive system diseases	313 (249–408)	335 (250–455)	383 (289–523)	391 (314–500)	299 (223–404)	295 (222–408)
1·84	Peptic ulcer disease	141 (120–163)	109 (92–128)	125 (100–153)	186 (158–215)	98·5 (83·3–115)	94·3 (75·6–115)
1·85	Gastritis and duodenitis	65 (52–82)	86 (68–109)	103 (80–130)	81·5 (64·8–103)	77·3 (61·1–97·7)	78·4 (61·6–99·2)
1·86	GERD	106 (55–188)	140 (72–244)	155 (80–275)	123 (64–218)	123 (63·6–216)	123 (63·9–218)
1·87	Appendicitis	29 (21–36)	19 (15–25)	21 (16–28)	27·4 (19·9–33·7)	16·8 (13·6–21·9)	18 (13·5–23·3)
1·88	Paralytic ileus and intestinal obstruction	93 (76–116)	75 (65–90)	85 (69–105)	97·2 (81·6–120)	72·4 (62·6–86·8)	72·3 (57·9–89·6)
1·89	Inguinal, femoral, and abdominal hernia	89 (68–115)	88 (66–114)	99 (75–128)	88·3 (68·9–112)	80·1 (61·1–104)	82·4 (62·3–107)
1·90	Inflammatory bowel disease	38 (32–45)	42 (37–50)	49 (40–62)	44·2 (37·3–51·6)	38 (33·3–44·8)	38·6 (31·5–48·1)
1·91	Vascular intestinal disorders	8 (7–9)	8 (7–9)	11 (9–13)	10·3 (9·1–11·7)	7·3 (6·4–8·2)	8·1 (6·6–9·6)
1·92	Gallbladder and biliary diseases	70 (54–87)	69 (54–88)	82 (64–105)	88·5 (68·4–110)	61·6 (48–79·2)	63·4 (49·6–80·8)
1·93	Pancreatitis	39 (34–45)	50 (44–57)	60 (49–73)	49·3 (42·5–56·8)	43·9 (38·5–49·7)	46·1 (37·4–56)
1·94	Other digestive diseases	42 (33–66)	83 (58–100)	107 (75–141)	47 (36·8–74·8)	76·3 (53·4–92)	85 (59·7–111)
1·95	Neurological disorders	1,300 (741–2,120)	1,450 (862–2,340)	1,600 (967–2,550)	1,400 (841–2,230)	1,320 (797–2,120)	1,350 (807–2,150)
1·96	Alzheimer's disease and other dementias	207 (83–493)	291 (121–676)	351 (147–800)	330 (132–783)	294 (123–681)	298 (124–680)
1·97	Parkinson's disease	53 (48–57)	83 (76–90)	111 (99–124)	74 (68·1–80·1)	79·1 (72–85·7)	86·8 (77·6–96·9)
1·98	Idiopathic epilepsy	356 (220–556)	328 (182–531)	343 (202–546)	336 (211–516)	289 (160–469)	293 (172–475)
1·99	Multiple sclerosis	6 (5–8)	10 (8–11)	12 (9–14)	7·7 (6·5–9·9)	8·4 (6·7–9·9)	8·8 (6·9–10·6)
1·100	Motor neuron disease	6 (5–7)	14 (13–16)	22 (18–26)	6·9 (6·3–7·6)	13·1 (11·9–14·4)	16·8 (14–20·2)
1·101	Headache disorders	567 (97–1,270)	632 (122–1,390)	652 (133–1,410)	551 (108–1,210)	546 (106–1,190)	545 (105–1,180)
1·102	Migraine	517 (64–1,220)	572 (82–1,320)	588 (90–1,340)	499 (73·7–1,160)	494 (70·7–1,140)	493 (72·5–1,130)
1·103	Tension-type headache	50 (13–204)	60 (17–222)	64 (19–223)	52·2 (15–194)	51·9 (15·2–192)	51·8 (15–186)
1·104	Other neurological disorders	110 (83–146)	96 (68–136)	111 (79–156)	99·2 (77·3–129)	86·2 (60·7–121)	97 (67·6–139)
1·105	Mental disorders	1,680 (1,220–2,220)	1,920 (1,410–2,550)	2,060 (1,510–2,700)	1,670 (1,220–2,200)	1,670 (1,230–2,220)	1,700 (1,240–2,250)
1·106	Schizophrenia	150 (104–201)	202 (142–269)	219 (153–291)	172 (121–229)	175 (123–233)	175 (122–234)
1·107	Depressive disorders	537 (362–748)	653 (447–906)	737 (505–1,030)	573 (393–793)	571 (392–792)	594 (405–828)
1·108	Major depressive disorder	455 (303–639)	550 (368–783)	625 (421–871)	483 (323–676)	481 (322–681)	505 (338–708)
1·109	Dysthymia	82 (50–125)	103 (64–157)	112 (70–172)	90·3 (56·4–139)	89·7 (56·2–137)	89·6 (55·8–138)
1·110	Bipolar disorder	197 (113–306)	228 (132–350)	239 (139–372)	197 (115–307)	197 (114–305)	196 (114–309)
1·111	Anxiety disorders	415 (271–588)	463 (308–658)	491 (326–697)	406 (268–570)	403 (268–570)	410 (271–579)
1·112	Eating disorders	44 (26–66)	51 (31–76)	50 (31–77)	36·7 (22·2–55·1)	43·7 (26·7–65·9)	45·3 (27·8–69·3)
1·113	Anorexia nervosa	12 (6–19)	12 (7–20)	12 (7–20)	9·1 (5·1–14·7)	10·5 (6–17·1)	11 (6·3–18)

1-114	Bulimia nervosa	32 (18–51)	38 (22–61)	38 (22–60)	27·6 (15·9–43·6)	33·2 (19·3–53)	34·3 (20·1–54·1)
1-115	Autism spectrum disorders	61 (40–90)	59 (39–85)	58 (38–84)	52·3 (34·1–77·1)	52·9 (34·8–76·7)	52·9 (34·7–76·8)
1-116	Attention-deficit/hyperactivity disorder	49 (28–84)	43 (24–73)	40 (23–67)	37·4 (21·2–63·1)	37·6 (21·3–63·8)	37·5 (21·5–63·3)
1-117	Conduct disorder	104 (60–166)	80 (45–127)	68 (39–109)	68 (38·8–108)	68·1 (38·3–108)	68·3 (38·3–109)
1-118	Idiopathic developmental intellectual disability	26 (10–48)	18 (6–34)	17 (5–32)	22·3 (8–40·7)	16·6 (5·2–30·8)	15·7 (4·7–28·9)
1-119	Other mental disorders	92 (58–140)	124 (78–188)	137 (87–206)	108 (68·6–164)	109 (68·4–164)	109 (68·9–163)
1-120	Substance use disorders	376 (297–469)	593 (479–721)	659 (531–805)	424 (342–524)	513 (414–624)	523 (418–641)
1-121	Alcohol use disorders	271 (218–345)	464 (377–564)	528 (430–645)	327 (267–407)	401 (326–488)	410 (332–506)
1-122	Drug use disorders	105 (72–144)	129 (94–171)	132 (95–172)	97·2 (69–131)	111 (80·7–147)	112 (80·5–147)
1-123	Opioid use disorders	45 (29–65)	55 (38–75)	59 (40–80)	45·9 (30·8–64·5)	47·5 (32·6–64·9)	49·2 (33–67·8)
1-124	Cocaine use disorders	18 (10–26)	25 (17–36)	24 (16–35)	15·4 (9·4–22·9)	21·8 (14·6–31)	21 (13·8–30)
1-125	Amphetamine use disorders	7 (3–12)	8 (4–12)	8 (5–12)	5·7 (3–9·7)	6·5 (3·7–10·6)	6·8 (3·9–11)
1-126	Cannabis use disorders	19 (10–34)	20 (11–34)	20 (11–33)	15·5 (8·1–26·8)	17·5 (9·4–29·1)	17·6 (9·3–29·3)
1-127	Other drug use disorders	16 (10–25)	21 (13–31)	21 (14–30)	14·6 (9·1–22·1)	18 (11·5–26·5)	18 (11·8–26·1)
1-128	Diabetes and kidney diseases	2,470 (2,230–2,730)	3,760 (3,350–4,210)	5,070 (4,420–5,810)	3,310 (2,980–3,660)	3,450 (3,070–3,860)	3,800 (3,310–4,350)
1-129	Diabetes mellitus	1,880 (1,680–2,100)	2,750 (2,390–3,150)	3,690 (3,160–4,330)	2,570 (2,290–2,880)	2,530 (2,210–2,890)	2,740 (2,350–3,210)
1-130	Diabetes mellitus type 1	148 (117–177)	143 (120–181)	158 (130–208)	162 (126–199)	126 (106–160)	129 (105–166)
1-131	Diabetes mellitus type 2	1,730 (1,540–1,960)	2,600 (2,260–3,010)	3,530 (3,010–4,140)	2,410 (2,130–2,720)	2,400 (2,090–2,770)	2,610 (2,220–3,060)
1-132	Chronic kidney disease	589 (534–641)	1,010 (922–1,110)	1,380 (1,190–1,600)	744 (676–813)	919 (836–1,000)	1,060 (918–1,230)
1-133	Chronic kidney disease due to diabetes mellitus type 1	46 (32–62)	90 (62–122)	116 (76–166)	59·7 (40·8–80·7)	77·2 (53·3–105)	89·2 (59·9–125)
1-134	Chronic kidney disease due to diabetes mellitus type 2	122 (98–147)	224 (181–268)	332 (264–407)	171 (137–206)	208 (170–248)	246 (196–300)
1-135	Chronic kidney disease due to hypertension	103 (84–125)	187 (155–223)	268 (218–334)	141 (117–172)	174 (145–207)	204 (167–253)
1-136	Chronic kidney disease due to glomerulonephritis	141 (117–167)	231 (191–275)	303 (234–380)	166 (135–199)	205 (169–244)	236 (184–293)
1-137	Chronic kidney disease due to other and unspecified causes	177 (147–212)	282 (227–341)	363 (289–453)	207 (171–250)	255 (206–306)	287 (230–355)
1-138	Acute glomerulonephritis	3 (2–4)	2 (2–2)	2 (2–3)	2·4 (1·7–3·3)	1·7 (1·4–2·1)	1·7 (1·3–2·2)
1-139	Skin and subcutaneous diseases	609 (398–902)	680 (478–967)	721 (516–1,000)	548 (358–812)	619 (436–878)	634 (453–886)
1-140	Dermatitis	138 (82–220)	126 (74–197)	123 (74–191)	116 (69·2–182)	115 (67·9–180)	115 (68·1–182)
1-141	Atopic dermatitis	110 (59–184)	92 (49–154)	86 (46–144)	85 (46–142)	84·9 (45·7–142)	84·8 (45·3–142)
1-142	Contact dermatitis	25 (16–38)	31 (19–46)	34 (21–51)	27·1 (17·2–40·7)	27 (16·9–40·5)	27 (16·8–40·2)
1-143	Seborrhoeic dermatitis	3 (2–5)	4 (2–6)	4 (2–6)	3·4 (1·9–5·4)	3·4 (1·9–5·4)	3·4 (1·9–5·4)
1-144	Psoriasis	37 (26–50)	39 (27–52)	41 (29–55)	38·8 (27–51·9)	34·6 (24·1–46·4)	33·4 (23·5–44·7)
1-145	Bacterial skin diseases	15 (7–42)	57 (34–91)	78 (43–127)	13·1 (6·5–38·9)	53·1 (31·2–84·7)	65·1 (36·4–104)
1-146	Cellulitis	6 (4–14)	13 (8–24)	17 (10–31)	5·2 (3·2–12·5)	12·2 (7·4–21·7)	14·3 (8·1–25)
1-147	Pyoderma	9 (3–30)	44 (25–70)	61 (32–99)	7·9 (2·7–27)	40·9 (23·3–65)	50·8 (27·3–81·4)
1-148	Scabies	101 (55–162)	93 (51–148)	90 (50–144)	84·1 (46·6–134)	83·5 (46·1–133)	83 (45·4–132)
1-149	Fungal skin diseases	42 (17–90)	49 (20–102)	53 (21–112)	44·1 (17·8–93·4)	44·1 (17·7–93·3)	44·1 (17·8–93·3)
1-150	Viral skin diseases	63 (40–94)	51 (32–77)	47 (30–70)	47·5 (30·5–71·2)	47·5 (30–71·7)	47·5 (30·2–70·7)
1-151	Acne vulgaris	58 (35–96)	55 (33–88)	52 (30–81)	40·8 (24·4–67·1)	46·1 (27·5–74·2)	47·8 (27·8–75·5)
1-152	Alopecia areata	7 (5–11)	8 (5–12)	8 (5–12)	7·1 (4·5–10·6)	7 (4·5–10·5)	7 (4·4–10·5)
1-153	Pruritus	8 (4–14)	9 (4–17)	10 (5–18)	8·2 (3·9–14·8)	8·4 (4–15)	8·4 (4–15·2)
1-154	Urticaria	57 (37–84)	52 (34–75)	50 (33–72)	48·1 (31·6–69·6)	48 (31·4–69·3)	47·9 (31·4–69·4)
1-155	Decubitus ulcer	41 (22–125)	90 (59–153)	111 (71–186)	54·3 (28·9–170)	84·9 (56·8–146)	87·8 (56·6–146)
1-156	Other skin and subcutaneous diseases	42 (21–76)	51 (25–93)	58 (28–105)	45·7 (22·5–82·9)	46·5 (22·6–84)	46·7 (22·7–84·7)
1-157	Sense organ diseases	644 (436–915)	836 (563–1,200)	976 (656–1,390)	821 (556–1,170)	772 (521–1,100)	754 (509–1,080)
1-158	Blindness and vision loss	187 (128–265)	229 (155–332)	265 (179–382)	243 (166–346)	213 (144–309)	206 (140–296)
1-159	Glaucoma	11 (7–17)	13 (9–19)	15 (10–22)	16·4 (10·8–23·9)	12·8 (8·4–18·6)	12·2 (8–17·7)
1-160	Cataract	33 (23–45)	39 (27–54)	45 (31–61)	47·4 (32·9–65·2)	37·5 (26·2–51·3)	35·4 (24·8–48·1)
1-161	Age-related macular degeneration	1 (1–2)	2 (1–2)	2 (1–3)	1·7 (1·1–2·5)	1·5 (1–2·1)	1·4 (0·9–2·1)
1-162	Refraction disorders	56 (37–82)	62 (40–90)	66 (44–95)	59·5 (39·5–84·7)	55·5 (36–79·8)	54·1 (35·7–78·1)
1-163	Near vision loss	39 (18–77)	56 (25–112)	69 (32–139)	55·2 (25·5–108)	52 (23·5–104)	51 (23·4–102)
1-164	Other vision loss	47 (33–65)	57 (40–79)	68 (47–93)	63·1 (43·8–86·8)	53·9 (38·2–74·4)	52·3 (36·9–72)
1-165	Age-related and other hearing loss	426 (286–614)	568 (378–821)	665 (444–980)	542 (363–780)	522 (349–752)	512 (341–750)
1-166	Other sense organ diseases	31 (18–47)	40 (23–62)	46 (27–71)	35·7 (21·2–55·5)	36·1 (21·2–56·6)	36·2 (21·5–55·8)

1-167	Musculoskeletal disorders	1,210 (868–1,580)	1,710 (1,240–2,240)	1,970 (1,430–2,600)	1,460 (1,050–1,920)	1,510 (1,100–1,980)	1,530 (1,110–2,010)
1-168	Rheumatoid arthritis	19 (13–25)	28 (20–37)	34 (24–45)	23·6 (17·2–31·4)	24·7 (17·8–32·6)	26·1 (18·7–34·6)
1-169	Osteoarthritis	130 (66–263)	214 (108–426)	276 (139–555)	187 (94–376)	198 (99·8–394)	200 (101–403)
1-170	Low back pain	633 (446–847)	814 (569–1,090)	902 (635–1,200)	726 (515–975)	719 (504–962)	711 (499–950)
1-171	Neck pain	108 (70–156)	153 (101–224)	174 (114–253)	135 (88·3–196)	134 (88·7–195)	134 (88·3–194)
1-172	Gout	5 (3–7)	9 (5–12)	10 (6–16)	6·4 (4–9·2)	7·7 (4·8–11)	7·9 (5–11·7)
1-173	Other musculoskeletal disorders	311 (219–422)	489 (347–660)	577 (405–781)	378 (267–513)	431 (305–581)	450 (318–608)
1-174	Other non-communicable diseases	2,580 (2,130–3,090)	2,300 (1,880–2,840)	2,390 (1,930–2,960)	2,430 (2,010–2,920)	2,190 (1,810–2,670)	2,160 (1,740–2,670)
1-175	Congenital anomalies	1,060 (809–1,380)	614 (507–743)	480 (362–636)	832 (636–1,080)	661 (541–805)	578 (427–780)
1-176	Neural tube defects	111 (72–152)	46 (35–60)	39 (27–59)	86·1 (55·5–119)	50·3 (37·9–66·1)	49·6 (33·1–75·8)
1-177	Congenital heart anomalies	396 (279–599)	251 (190–316)	179 (123–250)	309 (217–467)	275 (206–347)	223 (150–316)
1-178	Orofacial clefts	58 (32–96)	6 (4–9)	4 (3–6)	45·3 (25·1–74·7)	6·4 (4·5–8·8)	4·3 (2·8–6·2)
1-179	Down's syndrome	22 (15–29)	25 (20–30)	26 (19–34)	18 (13·2–23·5)	24·4 (19–30·3)	26·2 (19·1–36)
1-180	Turner syndrome	0 (0–0)	0 (0–0)	0 (0–0)	0·1 (0·1–0·2)	0·1 (0·1–0·2)	0·1 (0·1–0·2)
1-181	Klinefelter syndrome	0 (0–0)	0 (0–0)	0 (0–0)	0 (0–0)	0 (0–0)	0 (0–0)
1-182	Other chromosomal abnormalities	15 (11–21)	14 (11–19)	13 (10–18)	11·5 (8·4–16·5)	15·3 (11·7–19·9)	15·8 (11·5–21·6)
1-183	Congenital musculoskeletal and limb anomalies	63 (44–88)	57 (40–79)	55 (38–76)	51·3 (36·1–70·4)	54·6 (39·1–74·9)	55·7 (38·6–76·3)
1-184	Urogenital congenital anomalies	39 (23–66)	19 (14–29)	13 (9–20)	30·3 (17·9–51·4)	20·5 (14·9–32·3)	16·7 (10·9–26·8)
1-185	Digestive congenital anomalies	74 (44–171)	59 (43–82)	43 (27–64)	57·2 (33·9–133)	67·1 (49–94·4)	57·2 (35·4–85·9)
1-186	Other congenital anomalies	285 (200–371)	137 (109–174)	108 (79–148)	224 (157–290)	147 (116–189)	130 (93·1–182)
1-187	Urinary diseases and male infertility	82 (67–102)	170 (143–197)	231 (191–273)	101 (83·9–127)	158 (133–184)	181 (150–214)
1-188	Urinary tract infections and interstitial nephritis	48 (42–62)	119 (101–131)	165 (134–194)	58·9 (52·2–76·1)	111 (94·2–122)	131 (107–154)
1-189	Urolithiasis	5 (4–6)	7 (5–10)	10 (8–13)	6·3 (4·8–8·4)	6·6 (4·8–8·5)	7·8 (5·8–9·9)
1-190	Benign prostatic hyperplasia	18 (10–29)	30 (18–48)	41 (24–64)	24·7 (14·1–39)	29·2 (16·9–46)	30·2 (17·7–46·9)
1-191	Male infertility	4 (2–10)	5 (2–12)	5 (2–12)	4·3 (1·6–10·4)	4·3 (1·6–10·4)	4·3 (1·6–10·2)
1-192	Other urinary diseases	6 (5–8)	8 (6–10)	10 (7–13)	6·9 (5–8·7)	7·1 (5·3–9·2)	8 (5·6–10·3)
1-193	Gynecological diseases	351 (243–492)	419 (290–586)	433 (300–602)	379 (264–534)	360 (249–504)	359 (248–502)
1-194	Uterine fibroids	18 (9–33)	28 (14–51)	30 (15–56)	22·7 (11·4–41·4)	23·7 (12·1–43·6)	24·1 (12–43·9)
1-195	Polycystic ovarian syndrome	4 (2–9)	7 (3–14)	7 (3–14)	3·9 (1·7–8)	5·8 (2·5–11·7)	6·2 (2·6–12·4)
1-196	Female infertility	8 (3–19)	9 (3–21)	9 (3–21)	7·8 (2·7–18·6)	7·8 (2·7–18·6)	7·9 (2·7–18·7)
1-197	Endometriosis	34 (19–56)	32 (19–52)	30 (18–49)	32·7 (19·2–53·1)	27·5 (16·4–44)	26 (15–42·5)
1-198	Genital prolapse	6 (3–11)	7 (3–13)	8 (4–15)	8·3 (4–15·7)	6·2 (3–11·7)	5·7 (2·8–10·8)
1-199	Premenstrual syndrome	106 (64–168)	115 (71–178)	114 (70–178)	99·9 (61·1–156)	97·4 (60·1–152)	98·7 (60·3–154)
1-200	Other gynecological diseases	175 (116–246)	221 (144–314)	235 (153–336)	204 (135–287)	191 (125–270)	191 (125–271)
1-201	Hemoglobinopathies and hemolytic anaemias	254 (211–310)	231 (196–275)	231 (190–277)	222 (185–271)	207 (175–247)	206 (169–249)
1-202	Thalassemias	0 (0–0)	0 (0–0)	0 (0–0)	0·1 (0·1–0·2)	0·2 (0·1–0·3)	0·1 (0·1–0·2)
1-203	Thalassaemias trait	5 (3–7)	5 (3–8)	5 (3–8)	4·1 (2·5–6·5)	4·3 (2·6–6·8)	4·5 (2·7–6·9)
1-204	Sickle cell disorders	122 (105–140)	114 (100–129)	104 (83–131)	96·9 (84·1–111)	102 (89–115)	98·3 (77·8–123)
1-205	Sickle cell trait	72 (44–109)	49 (30–75)	44 (26–70)	59·5 (37·4–90·8)	44·1 (26·7–67·7)	40·5 (23·9–63·8)
1-206	G6PD deficiency	15 (10–20)	22 (16–28)	28 (20–37)	15·6 (11·1–20·2)	19 (13·8–24·6)	22·6 (15·9–29·6)
1-207	G6PD trait	0 (0–0)	0 (0–0)	0 (0–0)	0·1 (0·1–0·3)	0·2 (0·1–0·3)	0·2 (0·1–0·3)
1-208	Other hemoglobinopathies and hemolytic anaemias	41 (31–54)	40 (31–52)	49 (39–63)	45·2 (35·5–58·6)	37·4 (29–48·6)	40·3 (31·2–51·7)
1-209	Endocrine, metabolic, blood, and immune disorders	500 (348–602)	493 (407–650)	585 (474–766)	514 (372–618)	460 (382–609)	501 (404–668)
1-210	Oral disorders	293 (170–468)	365 (211–597)	419 (245–677)	349 (203–563)	330 (192–538)	327 (190–528)
1-211	Caries of deciduous teeth	6 (3–12)	3 (1–7)	3 (1–6)	4·4 (2–8·6)	3·3 (1·4–6·7)	3·3 (1·5–6·8)
1-212	Caries of permanent teeth	41 (18–80)	36 (16–71)	37 (16–73)	36·8 (16–70)	31·4 (13·6–61·4)	31·3 (13·9–61·4)
1-213	Periodontal disease	84 (32–180)	127 (49–269)	142 (55–302)	107 (41·5–229)	110 (43–234)	110 (42·2–235)
1-214	Edentulism	107 (68–160)	140 (89–208)	174 (110–263)	148 (94·5–220)	132 (83·6–197)	129 (81·7–195)
1-215	Other oral disorders	54 (34–80)	60 (37–87)	63 (39–91)	53·2 (33·2–77·9)	53 (32·6–77·2)	53 (32·7–77·4)
1-216	Sudden infant death syndrome	39 (25–59)	14 (9–20)	9 (5–15)	30·7 (19·6–45·7)	15·8 (10·7–22·9)	12·1 (7·3–19·7)
1-0	Injuries	4,010 (3,690–4,320)	3,820 (3,530–4,170)	3,910 (3,420–4,450)	3,800 (3,510–4,110)	3,360 (3,110–3,670)	3,320 (2,890–3,790)
1-1	Transport injuries	817 (745–897)	637 (581–695)	621 (526–717)	789 (718–862)	555 (506–606)	518 (439–599)
1-2	Road injuries	783 (713–862)	584 (532–640)	569 (484–660)	755 (688–826)	508 (464–557)	474 (403–550)

1-3	Pedestrian road injuries	407 (369–446)	126 (115–138)	123 (106–143)	395 (360–431)	111 (101–121)	102 (86·7–119)
1-4	Cyclist road injuries	80 (68–96)	72 (60–86)	74 (60–89)	79·9 (67·2–95·8)	62·5 (52·3–74·9)	60·6 (49·4–72·6)
1-5	Motorcyclist road injuries	63 (55–72)	95 (84–106)	98 (84–115)	60·6 (52·7–69·4)	82·1 (72·8–92·1)	82·5 (70·1–96·7)
1-6	Motor vehicle road injuries	225 (202–250)	282 (258–307)	263 (218–307)	211 (190–233)	244 (224–266)	221 (183–260)
1-7	Other road injuries	8 (7–10)	10 (8–12)	11 (8–14)	9 (7·1–11·5)	8·6 (6·7–10·9)	8·5 (6·6–10·8)
1-8	Other transport injuries	34 (31–39)	53 (48–59)	52 (42–62)	33·2 (29·8–37·3)	46·3 (41·8–51·4)	43·7 (35·6–51·8)
1-9	Unintentional injuries	2,000 (1,780–2,240)	1,640 (1,460–1,880)	1,690 (1,430–1,980)	1,880 (1,680–2,100)	1,470 (1,310–1,690)	1,430 (1,220–1,670)
1-10	Falls	218 (189–254)	315 (270–374)	397 (334–472)	255 (222–298)	285 (245–338)	318 (268–378)
1-11	Drowning	608 (540–680)	389 (352–432)	346 (290–406)	505 (453–558)	344 (310–383)	312 (259–367)
1-12	Fire, heat, and hot substances	374 (320–433)	229 (191–277)	222 (181–274)	355 (304–414)	206 (172–248)	189 (155–233)
1-13	Poisonings	48 (42–57)	14 (11–16)	13 (10–16)	42·8 (37·4–49·2)	12·4 (10·2–14·8)	11·6 (9·3–14·4)
1-14	Poisoning by carbon monoxide	6 (5–8)	3 (3–4)	3 (3–4)	5·7 (4·8–6·9)	3 (2·5–3·7)	2·9 (2·3–3·6)
1-15	Poisoning by other means	42 (36–49)	10 (8–13)	10 (8–12)	37·1 (32·2–42·8)	9·3 (7·5–11·4)	8·8 (6·9–11)
1-16	Exposure to mechanical forces	279 (241–331)	337 (284–409)	329 (269–409)	279 (238–334)	296 (249–360)	273 (224–337)
1-17	Unintentional firearm injuries	105 (91–135)	122 (105–137)	104 (86–122)	101 (88·2–131)	106 (90·7–119)	87·7 (72·5–103)
1-18	Other exposure to mechanical forces	174 (133–224)	215 (167–281)	226 (172–299)	178 (135–231)	190 (148–249)	185 (142–244)
1-19	Adverse effects of medical treatment	71 (61–80)	59 (51–66)	63 (53–74)	74·5 (65·9–85·3)	54·8 (47·7–60·8)	52·9 (43·9–62·9)
1-20	Animal contact	13 (10–18)	24 (21–29)	26 (22–31)	13·5 (9·9–18)	22·4 (19·1–26·6)	22·1 (18·4–26·4)
1-21	Venomous animal contact	8 (5–11)	12 (10–16)	14 (11–17)	8 (5·5–10·9)	11·4 (9·2–14)	11·5 (9–14·2)
1-22	Non-venomous animal contact	5 (4–7)	12 (10–14)	13 (11–15)	5·5 (4·2–7·5)	11 (9·6–13·2)	10·7 (8·8–12·9)
1-23	Foreign body	214 (179–257)	107 (94–123)	112 (92–134)	185 (158–218)	104 (89·8–120)	106 (85·2–129)
1-24	Pulmonary aspiration and foreign body in airway	180 (149–221)	90 (79–105)	94 (76–115)	153 (129–183)	88·7 (76·3–104)	90·9 (70·8–114)
1-25	Foreign body in eyes	5 (2–10)	6 (3–11)	7 (3–11)	5·5 (2·6–9·6)	5·5 (2·6–9·7)	5·5 (2·6–9·6)
1-26	Foreign body in other body part	28 (20–33)	10 (8–13)	11 (9–14)	26·8 (18·8–31·7)	9·5 (7·4–12·1)	9·6 (7·5–12·2)
1-27	Environmental heat and cold exposure	44 (33–59)	48 (36–64)	55 (40–73)	47·3 (35·3–62·2)	42·8 (31·8–56·8)	44·9 (33·1–59·6)
1-28	Exposure to forces of nature	0 (0–0)	3 (2–5)	6 (4–8)	0 (0–0)	3 (2·2–4·2)	4·8 (3·4–6·7)
1-29	Other unintentional injuries	130 (110–156)	117 (98–147)	118 (94–149)	125 (105–152)	103 (85·4–129)	99·3 (79·6–126)
1-30	Self-harm and interpersonal violence	1,190 (1,080–1,290)	1,540 (1,440–1,660)	1,600 (1,390–1,830)	1,130 (1,030–1,230)	1,340 (1,240–1,430)	1,370 (1,190–1,570)
1-31	Self-harm	288 (258–322)	320 (292–353)	337 (289–394)	304 (273–338)	276 (252–304)	275 (234–320)
1-32	Self-harm by firearm	8 (6–10)	8 (6–10)	8 (6–10)	8·6 (6–10·4)	7 (5·4–8·5)	6·7 (5·1–8·3)
1-33	Self-harm by other specified means	280 (251–312)	312 (284–344)	329 (282–384)	295 (265–328)	270 (246–297)	268 (229–312)
1-34	Interpersonal violence	896 (806–984)	1,210 (1,110–1,300)	1,260 (1,080–1,440)	821 (740–897)	1,040 (962–1,130)	1,090 (938–1,250)
1-35	Physical violence by firearm	440 (390–490)	544 (498–588)	588 (496–682)	391 (348–434)	468 (429–506)	512 (432–594)
1-36	Physical violence by sharp object	62 (56–70)	407 (372–445)	409 (350–475)	58·9 (52·2–65·9)	350 (321–383)	351 (300–408)
1-37	Sexual violence	19 (12–27)	20 (13–29)	20 (14–29)	17·7 (11·7–25·1)	17·6 (11·6–25)	17·6 (11·6–25)
1-38	Physical violence by other means	375 (338–415)	235 (214–256)	238 (203–279)	353 (320–389)	208 (190–228)	206 (175–243)
1-39	Conflict and terrorism	4 (2–5)	4 (3–5)	3 (2–4)	4 (2·7–5·9)	3·3 (2·3–4·8)	2·3 (1·6–3·2)
1-40	Executions and police conflict	1 (1–1)	15 (11–19)	4 (2–5)	0·7 (0·6–0·7)	12·6 (9·4–16·2)	3·2 (2–4·9)

SR Table 1: All-age DALYs counts and age standardized DALYs rates per 100,000 individuals for all causes and level 1 cause groups by location for 1990, 2010, and 2019, both sexes combined, Suriname

Cause Level	Cause	All-age DALYs			Age-standardized rate of DALYs per 100,000		
		1990	2010	2019	1990	2010	2019
0-0	All causes	144,000 (133,000–156,000)	177,000 (161,000–195,000)	195,000 (167,000–226,000)	41,500 (38,300–44,900)	35,200 (32,100–38,700)	33,900 (28,900–39,500)
1-0	Communicable, maternal, neonatal, and nutritional disorders	49,000 (42,900–59,100)	40,500 (36,000–45,600)	34,100 (28,400–41,100)	12,000 (10,600–14,600)	8,120 (7,180–9,180)	6,760 (5,540–8,290)
1-1	HIV/AIDS and sexually transmitted infections	4,170 (3,580–5,220)	7,420 (6,880–8,250)	5,830 (5,380–6,540)	1,110 (974–1,350)	1,320 (1,210–1,500)	1,010 (916–1,170)
1-2	HIV/AIDS	3,250 (3,190–3,320)	6,670 (6,460–6,950)	5,170 (5,000–5,400)	896 (878–915)	1,170 (1,130–1,220)	869 (841–908)
1-3	HIV/AIDS - drug-susceptible tuberculosis	499 (327–754)	397 (260–598)	312 (205–468)	138 (90·2–208)	69·5 (45·5–105)	52·4 (34·4–78·7)
1-4	HIV/AIDS - Multidrug-resistant Tuberculosis without extensive drug resistance	5 (0–22)	3 (0–13)	3 (0–12)	1·4 (0·1–5·9)	0·5 (0–2·3)	0·5 (0–2)
1-5	HIV/AIDS - Extensively drug-resistant Tuberculosis	0 (0–0)	0 (0–1)	0 (0–2)	0 (0–0)	0·1 (0–0·2)	0·1 (0–0·3)
1-6	HIV/AIDS resulting in other diseases	2,750 (2,490–2,940)	6,270 (5,980–6,570)	4,850 (4,630–5,100)	757 (686–811)	1,100 (1,050–1,150)	816 (779–859)
1-7	Sexually transmitted infections excluding HIV	916 (324–1,950)	753 (288–1,590)	659 (271–1,370)	215 (78·4–453)	156 (57·8–335)	144 (55·1–308)
1-8	Syphilis	844 (254–1,880)	644 (192–1,490)	544 (158–1,220)	194 (58·9–431)	137 (40·6–316)	125 (35·9–282)
1-9	Chlamydial infection	9 (6–13)	14 (9–20)	14 (9–20)	2·5 (1·6–3·6)	2·4 (1·6–3·5)	2·4 (1·6–3·5)
1-10	Gonococcal infection	9 (6–12)	12 (9–15)	12 (9–16)	2·4 (1·8–3·1)	2·1 (1·6–2·7)	2·1 (1·5–2·8)
1-11	Trichomoniasis	17 (7–35)	28 (11–58)	29 (11–61)	5 (1·9–10·3)	4·9 (1·9–10·3)	4·8 (1·9–10)
1-12	Genital herpes	18 (6–43)	29 (10–72)	33 (11–81)	5·3 (1·7–13·2)	5·4 (1·7–13·2)	5·4 (1·7–13·2)
1-13	Other sexually transmitted infections	19 (13–26)	26 (19–37)	27 (20–38)	5·2 (3·7–7·1)	4·6 (3·3–6·4)	4·6 (3·3–6·3)
1-14	Respiratory infections and tuberculosis	5,750 (4,730–6,610)	5,440 (4,800–6,120)	5,310 (4,380–6,460)	1,570 (1,330–1,770)	1,140 (1,000–1,280)	991 (812–1,220)
1-15	Tuberculosis	686 (545–771)	456 (414–501)	413 (337–490)	198 (163–220)	85·9 (78·2–93·8)	69·2 (56·7–82·5)
1-16	Latent tuberculosis infection	0 (0–0)	0 (0–0)	0 (0–0)	0 (0–0)	0 (0–0)	0 (0–0)
1-17	Drug-susceptible tuberculosis	680 (540–766)	453 (411–499)	409 (333–489)	196 (161–219)	85·3 (77·6–93·5)	68·6 (56–82)
1-18	Multidrug-resistant tuberculosis without extensive drug resistance	5 (1–22)	3 (0–12)	3 (0–13)	1·6 (0·1–6·3)	0·5 (0·1–2·3)	0·5 (0·1–2·1)
1-19	Extensively drug-resistant tuberculosis	0 (0–0)	0 (0–1)	1 (0–2)	0 (0–0)	0·1 (0–0·2)	0·1 (0–0·3)
1-20	Lower respiratory infections	4,600 (3,750–5,400)	4,390 (3,870–5,000)	4,270 (3,440–5,280)	1,260 (1,050–1,440)	941 (826–1,070)	812 (648–1,020)
1-21	Upper respiratory infections	341 (210–531)	445 (275–693)	463 (283–723)	84·5 (52–132)	82·5 (51·1–128)	82 (49·9–129)
1-22	Otitis media	125 (70–206)	154 (89–255)	156 (90–255)	30·3 (17·1–50·1)	28·6 (16·6–47·3)	27·8 (15·9–45·6)
1-23	Enteric infections	6,340 (4,980–7,670)	2,740 (2,190–3,450)	2,550 (1,890–3,430)	1,560 (1,230–1,890)	567 (454–714)	490 (357–660)
1-24	Diarrhoeal diseases	6,330 (4,970–7,660)	2,720 (2,180–3,430)	2,540 (1,880–3,410)	1,550 (1,230–1,890)	564 (452–711)	487 (354–656)
1-25	Typhoid and paratyphoid	5 (2–9)	5 (2–9)	4 (2–8)	1·1 (0·5–2·1)	0·9 (0·4–1·7)	0·7 (0·3–1·4)
1-26	Typhoid fever	4 (2–8)	4 (2–8)	3 (1–6)	1 (0·4–2)	0·8 (0·4–1·5)	0·5 (0·2–1·1)
1-27	Paratyphoid fever	0 (0–1)	0 (0–1)	1 (0–2)	0·1 (0–0·2)	0·1 (0–0·2)	0·2 (0·1–0·4)
1-28	iNTS	6 (3–12)	7 (3–12)	7 (3–12)	1·6 (0·8–2·8)	1·4 (0·7–2·4)	1·2 (0·5–2·2)
1-29	Other intestinal infectious diseases	6 (2–11)	4 (2–7)	4 (2–6)	1·4 (0·5–2·6)	0·9 (0·4–1·5)	0·7 (0·4–1·2)
1-30	Neglected tropical diseases and malaria	4,220 (1,130–17,000)	1,960 (1,420–2,660)	1,680 (1,180–2,370)	1,060 (304–4,250)	370 (270–497)	295 (205–414)
1-31	Malaria	3,090 (165–15,800)	274 (122–583)	39 (8–220)	754 (39·6–3,930)	50·6 (22·5–107)	6·8 (1·4–38·6)
1-32	Chagas disease	6 (2–9)	3 (2–7)	4 (3–10)	2·5 (1–3·6)	0·9 (0·6–2)	0·8 (0·5–2)
1-33	Leishmaniasis	120 (15–582)	34 (16–76)	36 (18–72)	28·8 (4–133)	6·6 (3·2–14·7)	6·3 (3·1–13·2)
1-34	Visceral leishmaniasis	100 (0–559)	10 (0–52)	8 (0–44)	23·4 (0–126)	1·9 (0–9·8)	1·6 (0–8·4)
1-35	Cutaneous and mucocutaneous leishmaniasis	20 (12–31)	24 (15–36)	27 (17–41)	5·5 (3·3–8·4)	4·7 (2·9–6·9)	4·8 (2·9–7·2)
1-36	African trypanosomiasis	0 (0–0)	0 (0–0)	0 (0–0)	0 (0–0)	0 (0–0)	0 (0–0)
1-37	Schistosomiasis	463 (248–819)	627 (319–1,170)	625 (319–1,160)	122 (67·4–212)	114 (58·2–209)	106 (53·7–200)
1-38	Cysticercosis	201 (104–330)	286 (134–472)	317 (162–539)	67·7 (35·5–108)	57·7 (28–94·4)	52·4 (27·1–87·8)
1-39	Cystic echinococcosis	1 (0–1)	0 (0–1)	0 (0–1)	0·2 (0·1–0·3)	0·1 (0·1–0·1)	0·1 (0–0·1)
1-40	Lymphatic filariasis	0 (0–0)	0 (0–0)	0 (0–0)	0 (0–0)	0 (0–0)	0 (0–0)
1-41	Onchocerciasis	0 (0–0)	0 (0–0)	0 (0–0)	0 (0–0)	0 (0–0)	0 (0–0)
1-42	Trachoma	0 (0–0)	0 (0–0)	0 (0–0)	0 (0–0)	0 (0–0)	0 (0–0)
1-43	Dengue	93 (54–227)	493 (160–637)	423 (110–622)	23·4 (13·6–57)	95·5 (30–124)	78·5 (20–118)
1-44	Yellow fever	13 (3–37)	6 (1–18)	4 (1–11)	3 (0·7–8·6)	1·1 (0·2–3·3)	0·7 (0·1–2)
1-45	Rabies	2 (1–6)	6 (2–9)	6 (1–12)	0·4 (0·2–1·3)	1·1 (0·3–1·6)	1·2 (0·3–2·3)
1-46	Intestinal nematode infections	52 (30–82)	46 (28–74)	33 (19–54)	12·4 (7·1–19·3)	8·4 (5–13·5)	5·8 (3·3–9·6)
1-47	Ascariasis	11 (3–24)	5 (2–10)	3 (1–6)	2·6 (0·7–5·5)	1 (0·3–2·1)	0·6 (0·2–1·1)
1-48	Trichuriasis	0 (0–0)	0 (0–0)	0 (0–0)	0 (0–0)	0 (0–0)	0 (0–0)

1-49	Hookworm disease	41 (21–70)	41 (22–69)	30 (16–50)	9·7 (5·1–16·6)	7·4 (4–12·5)	5·2 (2·9–8·9)
1-50	Food-borne trematodiases	0 (0–0)	0 (0–0)	0 (0–0)	0 (0–0)	0 (0–0)	0 (0–0)
1-51	Leprosy	2 (1–3)	4 (2–6)	4 (2–6)	0·8 (0·5–1·1)	0·7 (0·4–1·1)	0·6 (0·4–0·9)
1-52	Ebola virus disease	0 (0–0)	0 (0–0)	0 (0–0)	0 (0–0)	0 (0–0)	0 (0–0)
1-53	Zika virus disease	0 (0–0)	0 (0–0)	3 (0–13)	0 (0–0)	0 (0–0)	0·6 (0–2·3)
1-54	Guinea worm disease	0 (0–0)	0 (0–0)	0 (0–0)	0 (0–0)	0 (0–0)	0 (0–0)
1-55	Other neglected tropical diseases	186 (126–255)	177 (122–251)	186 (129–258)	44·9 (30·8–61·8)	34 (23·4–48)	34·3 (23·7–47·7)
1-56	Other infectious diseases	3,750 (2,510–5,840)	2,030 (1,580–2,840)	1,700 (1,310–2,210)	903 (624–1,380)	405 (311–579)	320 (243–424)
1-57	Meningitis	1,380 (1,070–1,680)	527 (428–653)	426 (319–560)	330 (259–399)	107 (86–133)	84·6 (62·5–113)
1-58	Encephalitis	562 (277–769)	354 (293–432)	343 (255–458)	132 (67·1–180)	69·1 (56·8–85)	66 (48·3–88·8)
1-59	Diphtheria	5 (3–10)	1 (1–2)	1 (0–1)	1·3 (0·6–2·2)	0·2 (0·1–0·4)	0·1 (0·1–0·2)
1-60	Whooping cough	1,080 (116–3,230)	397 (38–1,210)	157 (18–507)	244 (26·4–733)	83·9 (8·1–255)	34·3 (3·9–111)
1-61	Tetanus	123 (86–158)	42 (33–75)	33 (23–62)	35·4 (25·1–46·4)	8·3 (6·6–14·7)	5·6 (3·9–10·4)
1-62	Measles	12 (7–18)	0 (0–0)	0 (0–0)	2·7 (1·5–4·1)	0 (0–0)	0 (0–0)
1-63	Varicella and herpes zoster	60 (39–90)	58 (40–84)	53 (37–74)	16 (10·9–22·8)	12·2 (8·4–17·6)	10·3 (6·9–14·6)
1-64	Acute hepatitis	164 (125–192)	58 (48–70)	58 (46–72)	42·6 (33·2–49·3)	10·6 (8·9–12·7)	10 (7·9–12·5)
1-65	Acute hepatitis A	56 (35–72)	23 (16–32)	23 (15–31)	13·4 (8·4–17·3)	4·2 (2·9–5·8)	4 (2·7–5·6)
1-66	Acute hepatitis B	90 (68–115)	29 (25–33)	29 (22–36)	24·1 (18·3–30·4)	5·3 (4·6–6·1)	4·9 (3·8–6·2)
1-67	Acute hepatitis C	11 (6–17)	2 (2–3)	3 (2–4)	3·2 (1·7–4·9)	0·4 (0·3–0·6)	0·4 (0·3–0·7)
1-68	Acute hepatitis E	7 (5–11)	4 (3–5)	4 (3–5)	1·9 (1·3–2·7)	0·7 (0·5–0·9)	0·7 (0·5–0·9)
1-69	Other unspecified infectious diseases	365 (284–455)	591 (376–720)	625 (387–827)	98·7 (78·7–123)	114 (72·1–138)	109 (67·5–144)
1-70	Maternal and neonatal disorders	21,300 (18,800–24,000)	17,900 (15,000–21,200)	14,200 (10,700–18,800)	4,910 (4,330–5,530)	3,740 (3,110–4,430)	3,150 (2,350–4,210)
1-71	Maternal disorders	522 (360–660)	692 (588–802)	695 (535–897)	124 (87–154)	121 (102–140)	122 (93·8–158)
1-72	Maternal haemorrhage	129 (91–166)	140 (112–174)	129 (96–168)	31·3 (22·8–40·1)	24·6 (19·6–30·5)	22·7 (16·9–29·6)
1-73	Maternal sepsis and other pregnancy related infections	35 (22–47)	34 (27–44)	29 (21–39)	8·2 (5·4–11)	6 (4·6–7·7)	5·2 (3·7–6·9)
1-74	Maternal hypertensive disorders	140 (91–183)	141 (116–170)	137 (103–179)	32·6 (21·3–42)	24·7 (20·3–29·7)	24·2 (18·2–31·6)
1-75	Maternal obstructed labour and uterine rupture	10 (7–14)	18 (14–24)	20 (13–28)	2·5 (1·6–3·3)	3·2 (2·4–4·2)	3·4 (2·4–4·9)
1-76	Maternal abortive outcome	51 (32–72)	31 (23–40)	22 (16–32)	12·2 (7·8–16·9)	5·4 (4–7·1)	4 (2·8–5·6)
1-77	Ectopic pregnancy	26 (16–35)	53 (42–66)	54 (38–73)	6·3 (3·9–8·4)	9·2 (7·3–11·6)	9·5 (6·7–12·9)
1-78	Indirect maternal deaths	58 (36–80)	106 (82–134)	123 (86–169)	13·5 (8·6–18·3)	18·6 (14·4–23·5)	21·8 (15·2–29·8)
1-79	Late maternal deaths	10 (6–14)	30 (20–47)	34 (22–58)	2·3 (1·4–3·4)	5·2 (3·6–8·2)	6·1 (3·9–10·1)
1-80	Maternal deaths aggravated by HIV/AIDS	0 (0–1)	3 (2–5)	3 (2–6)	0·1 (0–0·2)	0·5 (0·3–0·9)	0·6 (0·3–1)
1-81	Other maternal disorders	63 (40–84)	136 (108–171)	143 (104–193)	15·3 (10·1–20)	23·8 (18·8–29·8)	25 (18·2–33·9)
1-82	Neonatal disorders	20,800 (18,300–23,500)	17,200 (14,300–20,500)	13,500 (10,100–18,000)	4,790 (4,210–5,420)	3,620 (3,000–4,310)	3,030 (2,250–4,070)
1-83	Neonatal preterm birth	12,100 (10,100–14,000)	8,970 (7,280–11,000)	6,520 (4,640–9,020)	2,780 (2,330–3,230)	1,880 (1,530–2,310)	1,470 (1,030–2,050)
1-84	Neonatal encephalopathy due to birth asphyxia and trauma	4,620 (3,480–5,900)	3,630 (2,770–4,640)	2,780 (1,890–4,020)	1,060 (800–1,360)	768 (586–984)	632 (425–918)
1-85	Neonatal sepsis and other neonatal infections	2,800 (1,980–3,830)	3,370 (2,550–4,290)	3,210 (2,210–4,440)	645 (456–884)	704 (531–893)	696 (473–977)
1-86	Hemolytic disease and other neonatal jaundice	282 (213–375)	194 (154–235)	151 (116–195)	65·8 (49·9–87·3)	38·8 (30·9–47·1)	30·5 (23·1–40·3)
1-87	Other neonatal disorders	1,020 (716–1,440)	1,080 (808–1,390)	869 (567–1,290)	234 (164–332)	229 (172–295)	200 (130–297)
1-88	Nutritional deficiencies	3,450 (2,600–4,440)	2,940 (2,110–4,080)	2,790 (1,970–3,900)	874 (669–1,120)	567 (411–785)	503 (356–694)
1-89	Protein-energy malnutrition	1,510 (1,140–1,910)	695 (561–846)	574 (437–748)	376 (288–468)	145 (117–177)	112 (83·7–150)
1-90	Iodine deficiency	36 (19–59)	35 (18–58)	29 (15–48)	9·6 (5–15·8)	6·3 (3·3–10·4)	4·9 (2·5–8·1)
1-91	Vitamin A deficiency	71 (43–109)	55 (34–84)	50 (31–73)	16 (9·7–24·2)	10·5 (6·5–16·2)	9·3 (5·8–13·9)
1-92	Dietary iron deficiency	1,750 (1,090–2,620)	2,100 (1,330–3,160)	2,080 (1,300–3,110)	450 (284–677)	395 (250–595)	366 (228–552)
1-93	Other nutritional deficiencies	81 (62–105)	52 (41–64)	56 (43–72)	23 (18·1–29)	10·2 (8·2–12·7)	9·8 (7·5–12·7)
1-0	Non-communicable diseases	75,400 (66,000–84,900)	112,000 (100,000–126,000)	137,000 (117,000–160,000)	24,400 (21,600–27,200)	22,700 (20,400–25,300)	23,000 (19,800–26,800)
1-1	Neoplasms	8,560 (7,450–9,260)	14,300 (13,400–15,100)	19,100 (16,000–22,600)	2,930 (2,610–3,160)	2,930 (2,750–3,100)	3,140 (2,650–3,720)
1-2	Lip and oral cavity cancer	102 (89–113)	167 (150–185)	228 (182–285)	36·9 (32·2–40·7)	34·4 (31–38)	36·4 (29·1–45)
1-3	Nasopharynx cancer	61 (51–68)	129 (118–141)	161 (129–199)	19·9 (17–22·3)	25·3 (23·1–27·8)	25·6 (20·6–31·4)
1-4	Other pharynx cancer	52 (45–59)	68 (61–77)	95 (75–118)	18·6 (16·2–21)	13·8 (12·3–15·7)	14·8 (11·8–18·4)
1-5	Oesophageal cancer	93 (77–105)	152 (135–174)	220 (173–270)	34·4 (28·6–38·5)	31·5 (28·2–36·3)	35 (27·5–42·7)
1-6	Stomach cancer	652 (570–713)	798 (732–863)	1,030 (847–1,250)	240 (212–261)	167 (154–180)	168 (139–203)
1-7	Colon and rectum cancer	755 (674–819)	1,560 (1,440–1,670)	2,240 (1,840–2,690)	279 (250–301)	331 (306–355)	367 (303–440)

1-8	Liver cancer	486 (423–549)	300 (269–331)	415 (329–527)	173 (152–194)	62·3 (56–68·6)	67·5 (53·8–85)
1-9	Liver cancer due to hepatitis B	171 (129–222)	105 (80–137)	132 (90–187)	57·8 (43·7–75·5)	20·6 (15·6–27·2)	20·9 (14·4–29·3)
1-10	Liver cancer due to hepatitis C	85 (57–119)	52 (36–71)	75 (48–109)	33 (22·4–45)	11·5 (8–15·8)	12·4 (8·2–18·1)
1-11	Liver cancer due to alcohol use	146 (104–192)	93 (68–122)	140 (97–199)	54·6 (38·8–71·7)	19·8 (14·5–25·8)	22·6 (15·8–31·8)
1-12	Liver cancer due to NASH	33 (24–46)	23 (17–32)	36 (24–52)	12·3 (8·6–17·1)	5 (3·6–6·9)	5·9 (4–8·5)
1-13	Gallbladder and biliary tract cancer	187 (109–213)	130 (110–220)	179 (139–303)	69·8 (40·8–79·6)	27·6 (23·4–46·9)	29·1 (22·6–49·5)
1-14	Pancreatic cancer	105 (95–116)	672 (617–731)	1,040 (851–1,250)	39·5 (35·5–43·4)	142 (130–155)	168 (138–203)
1-15	Larynx cancer	55 (48–63)	102 (89–116)	138 (110–171)	20 (17·5–22·8)	21·3 (18·6–24·1)	21·8 (17·4–26·9)
1-16	Tracheal, bronchus, and lung cancer	905 (814–994)	1,640 (1,520–1,760)	2,310 (1,880–2,810)	333 (302–364)	345 (319–369)	370 (302–450)
1-17	Malignant skin melanoma	34 (25–47)	63 (45–76)	80 (54–103)	11·5 (8·7–15·8)	12·4 (8·8–15·1)	13 (8·8–16·6)
1-18	Non-melanoma skin cancer	19 (17–21)	45 (41–49)	59 (48–72)	7·1 (6·2–8)	9·7 (8·7–10·5)	10 (8·1–12·1)
1-19	Non-melanoma skin cancer (squamous-cell carcinoma)	19 (17–21)	45 (41–49)	59 (48–72)	7·1 (6·2–8)	9·7 (8·7–10·5)	10 (8·1–12·1)
1-20	Non-melanoma skin cancer (basal-cell carcinoma)	0 (0–0)	0 (0–0)	0 (0–0)	0 (0–0)	0 (0–0)	0 (0–0)
1-21	Breast cancer	717 (612–830)	1,300 (1,150–1,460)	1,860 (1,480–2,320)	251 (217–289)	256 (229–286)	297 (238–365)
1-22	Cervical cancer	984 (783–1,140)	1,380 (1,190–1,630)	1,760 (1,360–2,230)	333 (270–384)	262 (226–309)	282 (218–359)
1-23	Uterine cancer	78 (64–103)	157 (121–184)	226 (163–285)	28·3 (23·3–37·5)	32·5 (25·3–38·4)	36·6 (26·4–46·5)
1-24	Ovarian cancer	82 (61–144)	430 (237–517)	581 (303–757)	27·9 (20·9–49)	84·9 (46·7–102)	93·2 (48·3–122)
1-25	Prostate cancer	562 (472–767)	1,210 (940–1,370)	1,670 (1,240–2,090)	236 (198–325)	290 (227–329)	296 (220–368)
1-26	Testicular cancer	11 (7–15)	51 (37–68)	53 (34–79)	2·7 (1·8–3·8)	9·3 (6·9–12·3)	9·3 (6–13·8)
1-27	Kidney cancer	196 (161–221)	232 (212–256)	321 (256–394)	61·2 (52–68·3)	46·6 (42·6–51·4)	52·5 (41·9–64·5)
1-28	Bladder cancer	84 (75–92)	159 (144–174)	222 (183–267)	32·9 (29·8–36·3)	35·6 (32·2–39)	37·3 (30·8–44·8)
1-29	Brain and nervous system cancer	152 (90–315)	732 (302–937)	823 (327–1,170)	41·7 (25·5–86·6)	140 (57·7–179)	140 (55·2–199)
1-30	Thyroid cancer	43 (36–50)	72 (64–81)	100 (81–121)	15·2 (12·7–17·4)	14·7 (13·1–16·4)	16·2 (13·1–19·6)
1-31	Mesothelioma	17 (12–22)	27 (22–32)	34 (25–46)	5·6 (4·1–7·1)	5·1 (4·2–6)	5·4 (4–7·3)
1-32	Hodgkin lymphoma	78 (57–104)	122 (90–141)	128 (90–159)	21·7 (16·1–29·2)	22·4 (16·6–25·9)	21·4 (15–26·6)
1-33	Non-Hodgkin's lymphoma	447 (354–501)	619 (574–669)	770 (623–934)	132 (108–146)	119 (111–128)	128 (104–155)
1-34	Multiple myeloma	105 (86–133)	212 (170–243)	304 (226–390)	39·2 (32·3–49·6)	45 (36·2–51·8)	48·8 (36·4–62·5)
1-35	Leukaemia	586 (429–690)	744 (668–838)	827 (660–1,010)	155 (118–179)	143 (128–161)	144 (115–176)
1-36	Acute lymphoid leukaemia	120 (67–159)	136 (111–167)	136 (103–182)	27 (15·5–35·6)	25·6 (20·7–31·7)	25 (18·8–33·8)
1-37	Chronic lymphoid leukaemia	9 (7–11)	22 (18–26)	34 (27–44)	3·3 (2·6–4·1)	4·7 (3·9–5·7)	5·7 (4·4–7·2)
1-38	Acute myeloid leukaemia	113 (78–145)	171 (145–201)	198 (152–248)	28·7 (20·7–35·8)	32·1 (27·2–37·8)	34·4 (26·4–43·6)
1-39	Chronic myeloid leukaemia	42 (31–50)	49 (42–57)	56 (43–72)	12·4 (9·7–14·7)	9·4 (8·1–10·9)	9·3 (7·2–11·8)
1-40	Other leukaemia	303 (223–366)	367 (311–434)	403 (316–508)	83·4 (64·3–99·4)	70·9 (60·4–84·3)	69·1 (54·3–86·8)
1-41	Other neoplasms	24 (20–32)	55 (48–63)	72 (57–88)	8·1 (6·6–10·5)	11·5 (10–13·3)	12·4 (9·9–15·2)
1-42	Myelodysplastic, myeloproliferative, and other hematopoietic neoplasms	24 (20–32)	55 (48–63)	72 (57–88)	8·1 (6·6–10·5)	11·5 (10–13·3)	12·4 (9·9–15·2)
1-43	Benign and in situ intestinal neoplasms	0 (0–0)	0 (0–0)	0 (0–0)	0 (0–0)	0 (0–0)	0 (0–0)
1-44	Benign and in situ cervical and uterine neoplasms	0 (0–0)	0 (0–0)	0 (0–0)	0 (0–0)	0 (0–0)	0 (0–0)
1-45	Other benign and in situ neoplasms	0 (0–0)	0 (0–0)	0 (0–0)	0 (0–0)	0 (0–0)	0 (0–0)
1-46	Cardiovascular diseases	20,000 (18,200–21,200)	25,100 (23,900–26,200)	32,400 (27,500–38,100)	7,430 (6,840–7,840)	5,400 (5,150–5,650)	5,410 (4,610–6,330)
1-47	Rheumatic heart disease	457 (359–549)	461 (371–578)	468 (362–590)	125 (100–148)	83·1 (67·4–104)	78·5 (60·6–99·4)
1-48	Ischaemic heart disease	9,570 (8,680–10,200)	10,000 (9,500–10,600)	13,500 (11,300–15,900)	3,640 (3,310–3,860)	2,170 (2,050–2,290)	2,230 (1,880–2,620)
1-49	Stroke	7,070 (6,330–7,570)	10,400 (9,790–10,900)	13,000 (10,800–15,400)	2,620 (2,370–2,800)	2,250 (2,120–2,370)	2,170 (1,820–2,550)
1-50	Ischaemic stroke	2,190 (2,000–2,380)	3,390 (3,130–3,640)	4,480 (3,760–5,200)	902 (822–978)	800 (736–861)	797 (673–922)
1-51	Intracerebral hemorrhage	4,220 (3,710–4,560)	6,110 (5,730–6,470)	7,480 (6,170–9,090)	1,510 (1,350–1,630)	1,280 (1,200–1,360)	1,210 (1,000–1,460)
1-52	Subarachnoid hemorrhage	655 (531–770)	875 (782–1,010)	998 (794–1,280)	204 (168–242)	169 (151–195)	162 (129–208)
1-53	Hypertensive heart disease	1,370 (1,200–1,520)	1,880 (1,720–2,100)	2,590 (2,090–3,140)	533 (466–589)	416 (378–470)	437 (352–530)
1-54	Non-rheumatic valvular heart disease	130 (106–148)	200 (181–220)	255 (209–310)	43·6 (36·2–49·3)	40·3 (36·7–44·2)	42·3 (34·7–51·4)
1-55	Non-rheumatic calcific aortic valve disease	49 (39–59)	83 (72–97)	110 (87–141)	17·2 (14·2–20·6)	17·5 (15·2–20·2)	18·5 (14·8–23·7)
1-56	Non-rheumatic degenerative mitral valve disease	78 (61–91)	112 (98–130)	140 (111–176)	25·2 (20·4–29·5)	22 (19·2–25·5)	22·9 (18·2–28·8)
1-57	Other non-rheumatic valve diseases	4 (2–5)	4 (3–6)	5 (3–7)	1·2 (0·8–1·6)	0·8 (0·6–1·1)	0·8 (0·6–1·2)
1-58	Cardiomyopathy and myocarditis	554 (438–717)	802 (642–940)	941 (700–1,190)	170 (138–223)	161 (130–189)	158 (118–201)
1-59	Myocarditis	53 (32–83)	58 (42–72)	62 (41–84)	14·1 (9·3–20·7)	11·5 (8·4–14·1)	11 (7·2–14·9)
1-60	Alcoholic cardiomyopathy	19 (14–27)	37 (24–48)	48 (30–66)	6·4 (4·6–9)	7 (4·5–9·2)	7·4 (4·5–10·1)

1·61	Other cardiomyopathy	482 (381–624)	707 (563–833)	831 (623–1,060)	150 (120–199)	142 (114–168)	140 (105–178)
1·62	Atrial fibrillation and flutter	201 (171–234)	344 (295–402)	495 (409–597)	84·5 (72·1–98·6)	85·1 (73·2–99·8)	90 (74·1–108)
1·63	Aortic aneurysm	124 (109–138)	197 (181–213)	268 (220–326)	47·7 (42·1–52·8)	43·6 (40·1–47·1)	45·3 (37·3–54·8)
1·64	Peripheral vascular disease	20 (13–30)	35 (23–54)	51 (34–76)	8·4 (5·3–13)	8·5 (5·5–13·2)	9 (6–13·6)
1·65	Endocarditis	65 (49–91)	104 (79–123)	122 (90–158)	18·8 (14·4–25·8)	20 (15·3–23·5)	20·6 (15·2–26·6)
1·66	Chronic respiratory diseases	2,690 (2,230–3,230)	3,660 (3,160–4,260)	4,270 (3,550–5,070)	833 (712–968)	749 (653–866)	742 (614–882)
1·67	Chronic obstructive pulmonary disease	953 (852–1,070)	1,560 (1,390–1,700)	2,010 (1,710–2,360)	368 (328–412)	348 (309–380)	344 (292–403)
1·68	Pneumoconiosis	33 (26–42)	24 (20–29)	30 (22–38)	11·5 (9·2–14·3)	5 (4·2–5·9)	5 (3·8–6·5)
1·69	Silicosis	3 (2–6)	3 (2–4)	3 (2–5)	1·1 (0·7–1·8)	0·6 (0·3–0·8)	0·5 (0·3–0·8)
1·70	Asbestosis	6 (4–9)	8 (5–10)	11 (6–15)	1·9 (1·2–3)	1·7 (1·2–2·2)	1·8 (1·1–2·5)
1·71	Coal workers pneumoconiosis	1 (1–2)	1 (0–1)	1 (1–2)	0·3 (0·2–0·6)	0·2 (0·1–0·2)	0·2 (0·1–0·3)
1·72	Other pneumoconiosis	23 (18–29)	12 (10–16)	15 (11–20)	8·1 (6·4–10·2)	2·6 (2·1–3·3)	2·5 (1·8–3·4)
1·73	Asthma	1,380 (1,020–1,870)	1,550 (1,160–2,080)	1,640 (1,190–2,220)	362 (276–479)	293 (219–392)	292 (210–402)
1·74	Interstitial lung disease and pulmonary sarcoidosis	78 (61–109)	183 (133–215)	223 (157–285)	27·3 (21·8–38·3)	37·9 (27·5–44·4)	37·5 (26·4–47·9)
1·75	Other chronic respiratory diseases	237 (159–316)	348 (291–425)	371 (296–472)	64·1 (44·7–83·8)	65·6 (54·6–80·5)	63·6 (50·7–81·5)
1·76	Digestive diseases	5,720 (4,890–6,340)	7,420 (6,830–8,140)	8,500 (7,090–10,100)	1,820 (1,590–2,000)	1,480 (1,360–1,610)	1,410 (1,180–1,670)
1·77	Cirrhosis and other chronic liver diseases	2,350 (1,990–2,570)	3,040 (2,810–3,340)	3,660 (2,920–4,500)	787 (676–860)	603 (560–657)	584 (466–714)
1·78	Cirrhosis and other chronic liver diseases due to hepatitis B	282 (196–394)	386 (271–539)	252 (163–368)	96 (67·8–133)	74·2 (53–103)	39·5 (25·9–57·4)
1·79	Cirrhosis and other chronic liver diseases due to hepatitis C	433 (312–590)	568 (417–760)	718 (489–1,020)	147 (107–200)	111 (81·1–149)	114 (77·4–159)
1·80	Cirrhosis and other chronic liver diseases due to alcohol use	827 (637–1,000)	1,170 (923–1,440)	1,540 (1,120–2,010)	290 (224–353)	234 (184–283)	240 (177–312)
1·81	Cirrhosis and other chronic liver diseases due to NAFLD	311 (215–428)	425 (304–581)	577 (376–822)	110 (75·8–150)	86·5 (61·9–118)	93 (61·7–131)
1·82	Cirrhosis and other chronic liver diseases due to other causes	495 (367–610)	489 (384–616)	577 (419–794)	144 (108–180)	97·7 (76·2–124)	97·3 (71–132)
1·83	Upper digestive system diseases	1,130 (889–1,490)	1,490 (1,130–2,030)	1,650 (1,230–2,290)	371 (297–476)	291 (224–392)	273 (202–378)
1·84	Peptic ulcer disease	587 (501–662)	602 (536–667)	646 (515–794)	204 (175–230)	125 (110–137)	107 (86–132)
1·85	Gastritis and duodenitis	140 (105–184)	223 (167–294)	261 (194–351)	44·2 (33·7–57·8)	43·4 (33·1–56·8)	42·8 (32–57·1)
1·86	GERD	407 (213–717)	661 (343–1,160)	746 (387–1,320)	123 (63·9–217)	123 (63·9–217)	122 (63·6–216)
1·87	Appendicitis	161 (114–200)	122 (105–140)	126 (100–161)	42·5 (31·2–51·3)	23·1 (19·9–26·5)	21·7 (17·3–27·6)
1·88	Paralytic ileus and intestinal obstruction	800 (597–1,030)	965 (806–1,130)	988 (753–1,280)	223 (170–294)	204 (172–237)	181 (137–237)
1·89	Inguinal, femoral, and abdominal hernia	462 (347–601)	517 (397–660)	555 (425–721)	127 (97·7–161)	102 (78·7–129)	95·7 (72·7–125)
1·90	Inflammatory bowel disease	118 (85–146)	118 (103–143)	138 (109–175)	35·6 (26·8–43)	23 (20·1–27·9)	23 (18·2–29·1)
1·91	Vascular intestinal disorders	32 (28–36)	50 (45–54)	66 (54–81)	12 (10·4–13·3)	10·9 (9·9–12)	11·4 (9·2–13·8)
1·92	Gallbladder and biliary diseases	353 (278–431)	415 (332–519)	490 (381–621)	118 (95–144)	82·7 (67·1–103)	80·8 (63·1–102)
1·93	Pancreatitis	176 (139–224)	288 (238–337)	327 (242–426)	56·4 (45·6–71·6)	54·4 (45·1–63·8)	53·2 (39·7–69·2)
1·94	Other digestive diseases	141 (108–209)	417 (265–469)	493 (312–624)	42·4 (34–65·9)	87·2 (56·1–97·9)	85·2 (54·2–107)
1·95	Neurological disorders	4,700 (2,670–7,620)	6,780 (3,710–10,900)	7,850 (4,620–12,500)	1,390 (829–2,230)	1,340 (765–2,130)	1,370 (813–2,180)
1·96	Alzheimer's disease and other dementias	755 (306–1,720)	1,090 (457–2,490)	1,580 (651–3,470)	330 (134–753)	291 (122–665)	302 (125–669)
1·97	Parkinson's disease	147 (133–166)	296 (265–322)	430 (369–496)	63·4 (57·4–72)	72·7 (65–79·1)	78·3 (67–89·9)
1·98	Idiopathic epilepsy	1,390 (785–2,170)	1,730 (996–2,730)	1,840 (1,060–2,930)	360 (206–555)	318 (182–504)	317 (181–502)
1·99	Multiple sclerosis	19 (16–25)	37 (32–46)	46 (36–59)	6·1 (5–7·9)	6·8 (5·9–8·4)	7·3 (5·8–9·4)
1·100	Motor neuron disease	20 (17–24)	66 (57–76)	89 (66–117)	6·3 (5·3–7·3)	13·2 (11·4–15·3)	14·9 (11–19·8)
1·101	Headache disorders	2,060 (367–4,540)	3,070 (582–6,780)	3,280 (662–7,190)	548 (107–1,200)	548 (108–1,200)	549 (108–1,210)
1·102	Migraine	1,870 (250–4,340)	2,780 (398–6,400)	2,960 (439–6,850)	496 (71·9–1,140)	496 (72·8–1,140)	497 (72–1,150)
1·103	Tension-type headache	185 (52–730)	286 (83–1,060)	314 (94–1,110)	52 (15·5–193)	52 (15·2–187)	52 (15·4–187)
1·104	Other neurological disorders	316 (206–450)	491 (347–693)	584 (396–814)	79·3 (53–111)	91·9 (65·4–128)	102 (68·7–143)
1·105	Mental disorders	7,010 (5,070–9,250)	10,800 (7,830–14,300)	11,700 (8,570–15,400)	1,910 (1,390–2,530)	1,960 (1,420–2,600)	1,960 (1,430–2,580)
1·106	Schizophrenia	592 (408–796)	976 (678–1,300)	1,070 (751–1,430)	174 (119–232)	174 (120–230)	176 (123–235)
1·107	Depressive disorders	2,940 (1,990–4,150)	4,810 (3,270–6,720)	5,240 (3,580–7,340)	827 (568–1,160)	880 (601–1,230)	870 (596–1,220)
1·108	Major depressive disorder	2,630 (1,760–3,760)	4,320 (2,900–6,060)	4,700 (3,170–6,610)	737 (496–1,050)	790 (532–1,100)	780 (528–1,100)
1·109	Dysthymia	310 (190–482)	493 (303–757)	544 (338–829)	89·9 (55·8–138)	89·8 (55·9–138)	89·6 (55·3–136)
1·110	Bipolar disorder	719 (408–1,140)	1,080 (632–1,680)	1,160 (677–1,820)	194 (112–301)	194 (112–302)	193 (112–302)
1·111	Anxiety disorders	1,460 (970–2,040)	2,160 (1,440–3,040)	2,350 (1,570–3,310)	392 (261–554)	390 (262–545)	394 (263–557)
1·112	Eating disorders	175 (106–261)	260 (158–391)	274 (165–412)	41·5 (25·2–62·3)	45·4 (27·5–68·4)	48·2 (28·9–72·5)
1·113	Anorexia nervosa	44 (25–72)	63 (36–101)	66 (38–108)	10·1 (5·8–16·3)	10·9 (6·4–17·6)	11·5 (6·7–19)

1-114	Bulimia nervosa	131 (75–208)	198 (115–307)	209 (121–326)	31·4 (18·2–49·1)	34·5 (20–53·6)	36·7 (21·2–57·3)
1-115	Autism spectrum disorders	210 (137–305)	282 (183–412)	295 (193–433)	52·2 (34·2–76·2)	52 (33·7–76·1)	51·9 (34·1–76·4)
1-116	Attention-deficit/hyperactivity disorder	165 (92–281)	210 (119–359)	211 (121–357)	37·5 (21–63·4)	37·4 (21·2–63·8)	37·3 (21·4–63·2)
1-117	Conduct disorder	317 (177–498)	385 (217–617)	378 (212–602)	68 (37·9–107)	68·2 (38·4–109)	68·2 (38·3–108)
1-118	Idiopathic developmental intellectual disability	72 (22–136)	85 (24–159)	81 (23–149)	17·5 (5·3–33·2)	15·5 (4·4–29·2)	14·2 (4·1–26·4)
1-119	Other mental disorders	358 (226–540)	585 (368–882)	655 (419–993)	108 (68·9–164)	108 (68·2–162)	107 (68·8–163)
1-120	Substance use disorders	1,110 (836–1,430)	2,060 (1,600–2,570)	2,230 (1,720–2,770)	312 (239–394)	368 (286–460)	367 (283–456)
1-121	Alcohol use disorders	763 (572–998)	1,390 (1,070–1,770)	1,500 (1,150–1,930)	225 (172–289)	251 (194–319)	243 (187–314)
1-122	Drug use disorders	350 (244–478)	666 (493–860)	730 (543–932)	87·5 (62·1–118)	117 (86·6–152)	124 (91·9–159)
1-123	Opioid use disorders	131 (84–189)	260 (175–358)	301 (204–411)	35·1 (23·4–49·3)	46·2 (31·2–63·1)	50·8 (34·7–69·5)
1-124	Cocaine use disorders	70 (43–106)	164 (121–221)	176 (130–234)	17·2 (10·8–25·2)	28·8 (21·2–38·8)	30 (22·1–40·2)
1-125	Amphetamine use disorders	28 (14–46)	51 (32–76)	54 (36–80)	6·5 (3·5–10·6)	8·9 (5·6–13·2)	9·3 (6·2–13·7)
1-126	Cannabis use disorders	54 (28–96)	68 (38–114)	69 (39–114)	12·5 (6·6–21·8)	11·9 (6·7–19·9)	11·9 (6·7–19·9)
1-127	Other drug use disorders	66 (42–99)	122 (84–170)	129 (92–176)	16·2 (10·5–23·8)	21·5 (14·7–29·8)	22·1 (15·5–30·2)
1-128	Diabetes and kidney diseases	6,580 (5,720–7,370)	14,700 (12,800–16,700)	20,500 (17,300–24,000)	2,310 (2,040–2,590)	3,040 (2,670–3,440)	3,350 (2,840–3,920)
1-129	Diabetes mellitus	3,990 (3,390–4,690)	8,910 (7,310–10,700)	12,600 (10,300–15,300)	1,450 (1,240–1,710)	1,870 (1,540–2,230)	2,050 (1,680–2,490)
1-130	Diabetes mellitus type 1	285 (217–328)	375 (330–438)	434 (354–527)	82·9 (65·1–95·1)	70·5 (62·3–82·2)	73·4 (59·8–89·2)
1-131	Diabetes mellitus type 2	3,710 (3,140–4,380)	8,530 (6,980–10,300)	12,200 (9,910–14,800)	1,370 (1,160–1,620)	1,800 (1,470–2,150)	1,980 (1,610–2,400)
1-132	Chronic kidney disease	2,570 (2,240–2,830)	5,750 (5,340–6,190)	7,830 (6,660–9,180)	858 (757–939)	1,170 (1,090–1,260)	1,300 (1,110–1,520)
1-133	Chronic kidney disease due to diabetes mellitus type 1	208 (139–289)	494 (338–671)	648 (416–967)	66·7 (44·6–92·3)	90·5 (62·2–123)	103 (67–150)
1-134	Chronic kidney disease due to diabetes mellitus type 2	491 (386–598)	1,230 (987–1,460)	1,820 (1,400–2,280)	185 (147–223)	267 (215–318)	299 (232–372)
1-135	Chronic kidney disease due to hypertension	426 (346–518)	1,040 (848–1,240)	1,490 (1,170–1,870)	160 (131–195)	228 (188–273)	251 (199–314)
1-136	Chronic kidney disease due to glomerulonephritis	665 (531–791)	1,380 (1,140–1,640)	1,790 (1,360–2,350)	203 (163–244)	266 (220–320)	292 (226–379)
1-137	Chronic kidney disease due to other and unspecified causes	785 (621–937)	1,600 (1,310–1,930)	2,090 (1,630–2,650)	244 (193–294)	321 (262–388)	351 (276–442)
1-138	Acute glomerulonephritis	16 (11–23)	13 (11–16)	14 (10–19)	4·2 (3–5·7)	2·6 (2·1–3)	2·5 (1·8–3·3)
1-139	Skin and subcutaneous diseases	2,400 (1,710–3,400)	3,460 (2,530–4,780)	3,870 (2,820–5,290)	646 (465–913)	674 (495–922)	681 (494–934)
1-140	Dermatitis	467 (273–745)	610 (363–960)	645 (385–1,010)	115 (68·4–182)	115 (68·5–181)	115 (68·5–182)
1-141	Atopic dermatitis	363 (196–601)	448 (242–755)	463 (251–784)	84·9 (45·9–141)	84·7 (45·8–143)	84·8 (46–143)
1-142	Contact dermatitis	92 (58–140)	144 (90–213)	163 (102–245)	27·1 (16·8–40·7)	27 (16·8–40·3)	27 (17–40·9)
1-143	Seborrhoeic dermatitis	12 (7–19)	18 (10–28)	20 (11–31)	3·4 (1·9–5·4)	3·4 (1·9–5·4)	3·4 (1·9–5·3)
1-144	Psoriasis	123 (86–165)	171 (122–229)	185 (131–248)	34·7 (24·6–46·4)	31·8 (22·6–42·5)	30·7 (21·7–41·2)
1-145	Bacterial skin diseases	238 (172–341)	486 (348–580)	557 (386–719)	64·7 (48·4–89·9)	98·4 (69·1–118)	98·6 (67·8–128)
1-146	Cellulitis	53 (27–87)	70 (46–97)	77 (47–114)	13·9 (7·6–22·3)	14 (8·9–19·4)	13·6 (8·3–20)
1-147	Pyoderma	185 (128–282)	415 (286–501)	480 (333–623)	50·8 (36·3–74·3)	84·5 (57–102)	85 (58·1–110)
1-148	Scabies	343 (188–556)	450 (245–717)	469 (259–751)	83·6 (46·5–135)	83·2 (45·5–133)	82·9 (46–133)
1-149	Fungal skin diseases	151 (60–324)	224 (90–475)	255 (103–539)	44·6 (18–94·4)	44·3 (17·8–93·9)	44·2 (17·7–93·5)
1-150	Viral skin diseases	207 (131–311)	252 (160–374)	258 (166–389)	47·4 (30·1–71·1)	47·5 (30·1–70·4)	47·5 (30·5–71·5)
1-151	Acne vulgaris	184 (109–301)	253 (150–409)	263 (155–425)	40·4 (23·8–65·9)	44·6 (26·5–72·3)	46·5 (27·4–75·2)
1-152	Alopecia areata	26 (16–39)	39 (25–59)	42 (27–63)	7 (4·5–10·4)	7·1 (4·5–10·6)	7·1 (4·5–10·6)
1-153	Pruritus	29 (14–52)	44 (21–78)	50 (24–90)	8·3 (4–14·6)	8·5 (4–15·1)	8·5 (4·1–15·3)
1-154	Urticaria	195 (127–283)	255 (167–368)	267 (174–387)	48 (31·4–69·4)	48 (31·4–69·3)	48 (31·3–69·7)
1-155	Decubitus ulcer	274 (219–384)	413 (352–561)	574 (446–789)	102 (82·6–145)	94 (79·6–126)	101 (78·8–138)
1-156	Other skin and subcutaneous diseases	165 (83–295)	261 (131–463)	305 (153–545)	50·2 (25·4–88·9)	51·1 (25·7–90·7)	51·3 (25·8–91·3)
1-157	Sense organ diseases	2,490 (1,700–3,530)	3,880 (2,650–5,530)	4,720 (3,200–6,710)	875 (600–1,240)	819 (562–1,160)	796 (543–1,130)
1-158	Blindness and vision loss	833 (577–1,150)	1,220 (843–1,720)	1,470 (1,020–2,060)	302 (211–416)	264 (183–369)	251 (174–352)
1-159	Glaucoma	51 (34–75)	68 (47–97)	86 (59–122)	21·9 (14·5–32·1)	17·2 (11·8–24·4)	15·7 (10·8–22·2)
1-160	Cataract	194 (136–268)	277 (195–374)	339 (234–459)	79·4 (56–110)	65 (45·7–88·5)	60·2 (41·7–80·9)
1-161	Age-related macular degeneration	8 (5–12)	14 (9–19)	18 (12–25)	3·3 (2·1–4·7)	3·2 (2·2–4·6)	3·1 (2·1–4·3)
1-162	Refraction disorders	205 (136–305)	289 (189–426)	314 (205–458)	58·6 (38·9–85·7)	54·2 (35·6–79·3)	53·1 (34·7–77·3)
1-163	Near vision loss	143 (65–283)	235 (106–463)	301 (135–609)	54·3 (24·9–107)	51 (23·3–99·5)	49·6 (22·6–98·4)
1-164	Other vision loss	232 (162–319)	342 (239–470)	408 (284–562)	84·2 (59–116)	73·9 (51·8–101)	69·2 (49–95·3)
1-165	Age-related and other hearing loss	1,550 (1,030–2,240)	2,480 (1,630–3,640)	3,040 (2,010–4,430)	537 (361–782)	519 (345–754)	509 (338–739)
1-166	Other sense organ diseases	111 (66–169)	179 (106–277)	215 (127–333)	35·7 (21·4–54·7)	36·1 (21·5–55·8)	36·3 (21·3–56·3)

1-167	Musculoskeletal disorders	4,630 (3,330–6,170)	8,000 (5,820–10,500)	9,590 (7,020–12,500)	1,460 (1,050–1,950)	1,520 (1,100–2,010)	1,560 (1,150–2,040)
1-168	Rheumatoid arthritis	61 (43–82)	112 (78–150)	144 (102–190)	19·6 (14·1–26)	21·5 (15·3–28·3)	23·5 (16·8–31·3)
1-169	Osteoarthritis	505 (256–1,020)	952 (477–1,910)	1,280 (640–2,560)	190 (96·3–382)	202 (102–405)	206 (103–415)
1-170	Low back pain	2,370 (1,660–3,190)	3,830 (2,680–5,130)	4,410 (3,110–5,930)	722 (510–972)	722 (508–965)	725 (511–976)
1-171	Neck pain	423 (278–615)	721 (476–1,050)	832 (543–1,210)	135 (89·3–195)	134 (89·3–194)	134 (88·4–195)
1-172	Gout	19 (12–28)	39 (24–57)	49 (30–71)	6·7 (4·2–9·8)	7·8 (4·9–11·5)	8 (4·9–11·6)
1-173	Other musculoskeletal disorders	1,250 (896–1,680)	2,350 (1,680–3,160)	2,870 (2,070–3,830)	390 (280–525)	436 (315–586)	467 (340–617)
1-174	Other non-communicable diseases	9,440 (7,810–11,300)	12,000 (9,960–14,500)	12,100 (9,710–15,100)	2,470 (2,040–2,970)	2,390 (1,990–2,870)	2,250 (1,790–2,810)
1-175	Congenital anomalies	4,420 (3,500–5,370)	4,590 (3,620–5,600)	3,690 (2,690–5,010)	1,020 (806–1,230)	951 (746–1,170)	803 (577–1,100)
1-176	Neural tube defects	858 (557–1,220)	558 (380–751)	423 (266–664)	197 (128–280)	117 (78·9–158)	94 (57·9–149)
1-177	Congenital heart anomalies	1,620 (1,140–2,160)	1,800 (1,270–2,270)	1,400 (930–1,990)	372 (262–494)	374 (262–474)	308 (202–444)
1-178	Orofacial clefts	15 (10–22)	26 (18–36)	24 (16–34)	3·7 (2·5–5·4)	5·1 (3·6–6·9)	4·6 (3·1–6·5)
1-179	Down's syndrome	67 (47–107)	115 (87–162)	117 (87–164)	16·3 (11·6–25·4)	22·4 (16·6–32·2)	22·1 (16·1–31·7)
1-180	Turner syndrome	1 (0–1)	1 (0–1)	1 (0–1)	0·2 (0·1–0·3)	0·1 (0·1–0·3)	0·1 (0·1–0·3)
1-181	Klinefelter syndrome	0 (0–0)	0 (0–0)	0 (0–0)	0 (0–0)	0 (0–0)	0 (0–0)
1-182	Other chromosomal abnormalities	57 (39–85)	89 (61–120)	84 (57–120)	12·9 (8·8–19·4)	18·3 (12·4–24·7)	17·9 (12–26·1)
1-183	Congenital musculoskeletal and limb anomalies	214 (147–305)	299 (205–415)	296 (201–417)	50·7 (34·6–72)	57·4 (39·6–79·2)	55·8 (38·2–78·2)
1-184	Urogenital congenital anomalies	77 (51–113)	92 (65–125)	78 (52–119)	17·6 (11·8–25·9)	19·1 (13·4–25·9)	17·1 (11·2–26·3)
1-185	Digestive congenital anomalies	967 (619–1,400)	1,020 (740–1,320)	777 (500–1,190)	222 (142–322)	217 (157–280)	178 (114–273)
1-186	Other congenital anomalies	542 (337–748)	588 (432–765)	493 (344–696)	125 (78·2–172)	121 (88·3–158)	106 (72·6–151)
1-187	Urinary diseases and male infertility	368 (298–492)	990 (714–1,130)	1,280 (910–1,550)	125 (102–169)	212 (151–241)	220 (156–267)
1-188	Urinary tract infections and interstitial nephritis	231 (191–344)	715 (476–804)	937 (602–1,160)	76·8 (64·1–115)	153 (102–172)	163 (105–202)
1-189	Urolithiasis	22 (16–31)	59 (41–79)	75 (49–102)	7·4 (5·4–10·4)	11·6 (8·2–15·6)	11·9 (7·7–16·2)
1-190	Benign prostatic hyperplasia	65 (38–101)	114 (66–176)	151 (88–238)	26·2 (15·4–40·6)	26·5 (15·3–40·9)	25·7 (14·9–40·1)
1-191	Male infertility	16 (6–39)	25 (9–61)	24 (9–58)	4·3 (1·6–10·3)	4·3 (1·6–10·4)	4·2 (1·6–10)
1-192	Other urinary diseases	34 (23–44)	78 (53–113)	93 (60–134)	10·4 (6·9–14)	15·8 (10·5–23·3)	15·9 (10·2–23·1)
1-193	Gynecological diseases	1,310 (903–1,840)	2,080 (1,440–2,950)	2,230 (1,530–3,130)	372 (258–521)	367 (254–520)	371 (256–522)
1-194	Uterine fibroids	75 (37–141)	137 (66–252)	154 (75–287)	23·6 (11·5–43·9)	24 (11·6–44·4)	24·7 (12·1–46·1)
1-195	Polycystic ovarian syndrome	18 (8–38)	35 (15–70)	39 (16–81)	4·6 (1·9–9·5)	6 (2·5–12·1)	6·7 (2·8–13·9)
1-196	Female infertility	29 (10–69)	45 (16–108)	46 (16–110)	7·8 (2·7–18·6)	7·8 (2·7–18·7)	8 (2·7–19·3)
1-197	Endometriosis	136 (80–223)	186 (107–302)	179 (105–290)	35·2 (20·6–57·2)	31·9 (18·3–51·8)	30·5 (17·9–49·5)
1-198	Genital prolapse	23 (12–42)	34 (17–62)	39 (20–74)	7·9 (3·9–14·6)	6·6 (3·2–12)	6·3 (3·2–11·8)
1-199	Premenstrual syndrome	379 (232–596)	568 (346–875)	580 (357–910)	97·9 (60·2–153)	97·6 (59·4–150)	99·4 (61·1–157)
1-200	Other gynecological diseases	651 (421–921)	1,080 (695–1,550)	1,190 (772–1,690)	195 (126–275)	193 (125–275)	195 (128–278)
1-201	Hemoglobinopathies and hemolytic anaemias	728 (548–927)	869 (709–1,090)	886 (704–1,120)	181 (138–230)	162 (132–202)	156 (124–200)
1-202	Thalassemias	0 (0–1)	1 (0–1)	0 (0–1)	0·1 (0–0·2)	0·1 (0·1–0·2)	0·1 (0–0·1)
1-203	Thalassaemias trait	18 (11–28)	27 (16–42)	27 (16–42)	4·5 (2·7–7·1)	4·9 (3–7·6)	4·8 (2·9–7·5)
1-204	Sickle cell disorders	300 (208–376)	354 (310–401)	328 (261–404)	69·4 (48·7–86·5)	65·1 (56·9–73·8)	59·7 (47·3–73·8)
1-205	Sickle cell trait	227 (137–351)	254 (154–386)	267 (159–420)	54·1 (32·8–83·4)	46·5 (28·2–70·5)	47·4 (28·2–74·5)
1-206	G6PD deficiency	46 (31–62)	70 (51–92)	80 (56–109)	12·5 (8·7–16·6)	12·8 (9·4–16·6)	13·3 (9·2–18)
1-207	G6PD trait	1 (0–1)	1 (0–1)	1 (0–2)	0·2 (0·1–0·4)	0·1 (0·1–0·2)	0·2 (0·1–0·3)
1-208	Other hemoglobinopathies and hemolytic anaemias	136 (98–188)	163 (118–226)	182 (136–245)	40·5 (30·2–55·4)	32·4 (23·7–44)	31·1 (23·3–42)
1-209	Endocrine, metabolic, blood, and immune disorders	1,390 (988–1,730)	1,700 (1,370–2,170)	1,990 (1,550–2,590)	395 (289–487)	337 (272–432)	347 (270–447)
1-210	Oral disorders	1,000 (581–1,640)	1,620 (923–2,630)	1,920 (1,110–3,130)	328 (192–538)	320 (186–520)	318 (184–516)
1-211	Caries of deciduous teeth	14 (6–30)	15 (7–33)	15 (7–33)	3·1 (1·4–6·6)	3·1 (1·3–6·6)	3·1 (1·3–6·6)
1-212	Caries of permanent teeth	79 (35–149)	107 (47–202)	114 (51–218)	20·1 (9–37·8)	19·1 (8·5–35·9)	19·4 (8·6–37)
1-213	Periodontal disease	347 (133–747)	601 (233–1,280)	689 (269–1,470)	109 (41·9–233)	110 (42·7–233)	111 (43·5–234)
1-214	Edentulism	366 (232–550)	604 (382–904)	784 (501–1,180)	142 (89·6–213)	135 (85·2–202)	132 (83·7–198)
1-215	Other oral disorders	197 (122–290)	289 (180–420)	313 (195–457)	53·1 (33–77·7)	53 (33–76·9)	53 (33·1–77·4)
1-216	Sudden infant death syndrome	218 (70–480)	180 (69–324)	128 (50–247)	49·9 (16·1–110)	38·2 (14·7–68·7)	29·3 (11·6–56·7)
1-0	Injuries	19,500 (15,100–21,600)	24,200 (22,700–25,900)	24,100 (20,700–28,200)	5,080 (4,020–5,630)	4,430 (4,150–4,760)	4,140 (3,540–4,820)
1-1	Transport injuries	5,380 (4,040–6,050)	6,240 (5,710–6,940)	6,250 (5,220–7,580)	1,400 (1,080–1,570)	1,130 (1,030–1,260)	1,060 (886–1,280)
1-2	Road injuries	5,230 (3,920–5,870)	5,910 (5,410–6,580)	5,910 (4,930–7,150)	1,360 (1,050–1,520)	1,070 (978–1,190)	1,000 (838–1,210)

1-3	Pedestrian road injuries	949 (681–1,160)	823 (716–980)	849 (667–1,070)	254 (189–308)	154 (134–185)	144 (113–182)
1-4	Cyclist road injuries	552 (423–670)	563 (470–663)	593 (471–724)	148 (114–178)	102 (85·4–121)	99·2 (78·9–121)
1-5	Motorcyclist road injuries	760 (560–981)	1,380 (1,130–1,590)	1,350 (1,040–1,670)	196 (148–248)	246 (201–284)	229 (176–283)
1-6	Motor vehicle road injuries	2,930 (2,130–3,370)	3,100 (2,790–3,680)	3,060 (2,500–3,840)	754 (562–860)	557 (502–663)	520 (425–654)
1-7	Other road injuries	32 (24–41)	48 (36–61)	57 (43–73)	9·4 (6·9–12·2)	8·9 (6·7–11·4)	9·4 (7·1–12·1)
1-8	Other transport injuries	153 (115–180)	330 (278–381)	348 (267–442)	39·8 (31·1–46·4)	59·9 (50·6–69)	59·2 (45·5–75·2)
1-9	Unintentional injuries	7,560 (6,070–8,550)	8,000 (7,130–8,960)	8,330 (6,990–9,820)	1,980 (1,620–2,230)	1,530 (1,370–1,720)	1,460 (1,230–1,720)
1-10	Falls	669 (557–786)	1,140 (943–1,360)	1,430 (1,180–1,730)	214 (181–251)	233 (194–276)	247 (203–298)
1-11	Drowning	1,740 (1,170–2,080)	1,690 (1,490–1,960)	1,570 (1,270–1,960)	411 (285–488)	310 (271–358)	278 (225–349)
1-12	Fire, heat, and hot substances	1,110 (871–1,350)	1,050 (860–1,290)	1,060 (850–1,320)	292 (232–354)	197 (162–244)	183 (146–228)
1-13	Poisonings	342 (189–434)	112 (90–184)	117 (90–185)	84·4 (46·8–106)	21·2 (17·1–34·4)	20·9 (16–33)
1-14	Poisoning by carbon monoxide	12 (8–17)	12 (8–16)	14 (10–18)	3 (2·1–4·3)	2·1 (1·6–2·8)	2·4 (1·7–3·2)
1-15	Poisoning by other means	331 (180–421)	101 (80–170)	103 (79–170)	81·5 (44·8–102)	19·1 (15·1–31·9)	18·5 (13·9–30·3)
1-16	Exposure to mechanical forces	719 (562–890)	1,110 (887–1,360)	1,170 (916–1,490)	195 (153–242)	204 (162–250)	198 (155–252)
1-17	Unintentional firearm injuries	215 (153–310)	334 (241–404)	304 (215–393)	55·1 (40·5–79)	59·5 (43·3–71·9)	51·7 (36·5–67·2)
1-18	Other exposure to mechanical forces	504 (385–642)	778 (596–1,010)	868 (658–1,160)	140 (106–180)	144 (110–187)	146 (111–195)
1-19	Adverse effects of medical treatment	895 (684–1,060)	904 (746–1,060)	951 (742–1,200)	254 (200–294)	182 (150–214)	168 (130–211)
1-20	Animal contact	60 (45–94)	217 (120–253)	233 (129–288)	16·9 (12·6–26·6)	42·6 (23·1–49·4)	40·4 (22–50)
1-21	Venomous animal contact	46 (34–76)	180 (95–209)	192 (103–241)	12·9 (9·5–21·1)	35·3 (18·3–40·7)	33·3 (17·6–41·6)
1-22	Non-venomous animal contact	14 (10–21)	37 (22–48)	41 (24–53)	4 (2·8–6)	7·3 (4·3–9·3)	7·1 (4·2–9·1)
1-23	Foreign body	1,400 (1,060–1,750)	953 (796–1,170)	897 (689–1,190)	343 (264–422)	192 (160–238)	174 (130–234)
1-24	Pulmonary aspiration and foreign body in airway	1,320 (983–1,660)	834 (683–1,050)	770 (576–1,050)	320 (242–398)	170 (138–214)	152 (111–211)
1-25	Foreign body in eyes	20 (9–36)	30 (14–53)	32 (16–56)	5·5 (2·7–9·6)	5·5 (2·6–9·6)	5·4 (2·6–9·6)
1-26	Foreign body in other body part	65 (49–87)	89 (72–110)	94 (74–117)	17·7 (13·6–22·9)	17·2 (14–21·2)	16·4 (13–20·5)
1-27	Environmental heat and cold exposure	187 (134–247)	260 (188–349)	298 (214–403)	53·6 (38·7–70·8)	48·9 (35·4–65·3)	49·9 (35·8–67·6)
1-28	Exposure to forces of nature	0 (0–0)	0 (0–0)	0 (0–0)	0 (0–0)	0 (0–0)	0 (0–0)
1-29	Other unintentional injuries	434 (337–530)	562 (456–692)	598 (461–762)	114 (89·4–139)	103 (83·4–127)	102 (79·2–130)
1-30	Self-harm and interpersonal violence	6,580 (4,920–7,630)	9,930 (9,000–10,900)	9,560 (7,990–11,400)	1,700 (1,310–1,990)	1,770 (1,610–1,960)	1,620 (1,350–1,920)
1-31	Self-harm	4,320 (3,110–4,970)	6,760 (5,730–7,600)	6,580 (5,300–7,980)	1,150 (861–1,330)	1,210 (1,020–1,360)	1,100 (892–1,330)
1-32	Self-harm by firearm	250 (148–371)	299 (193–470)	295 (193–473)	65·9 (40·4–99·4)	53·3 (34·8–83·9)	49·2 (32·1–79·1)
1-33	Self-harm by other specified means	4,070 (2,970–4,670)	6,460 (5,490–7,190)	6,280 (5,060–7,610)	1,080 (819–1,240)	1,150 (978–1,290)	1,050 (850–1,270)
1-34	Interpersonal violence	2,090 (1,490–2,460)	3,030 (2,720–3,390)	2,880 (2,340–3,530)	514 (375–603)	539 (483–602)	499 (406–613)
1-35	Physical violence by firearm	615 (388–790)	1,020 (852–1,230)	957 (715–1,250)	145 (93·4–185)	178 (149–214)	166 (123–217)
1-36	Physical violence by sharp object	539 (354–737)	796 (624–943)	747 (563–987)	133 (89·8–178)	140 (110–165)	128 (96·9–170)
1-37	Sexual violence	69 (45–98)	100 (66–141)	104 (69–147)	17·6 (11·7–25)	17·7 (11·7–25·1)	17·8 (11·7–25·2)
1-38	Physical violence by other means	866 (626–1,040)	1,120 (988–1,290)	1,070 (849–1,350)	218 (162–261)	204 (181–234)	187 (148–235)
1-39	Conflict and terrorism	25 (16–39)	20 (13–30)	16 (10–24)	6·1 (3·9–9·3)	3·5 (2·3–5·2)	2·5 (1·7–3·7)
1-40	Executions and police conflict	142 (17–542)	114 (21–408)	89 (14–326)	35·7 (4·3–134)	20·2 (3·7–72·1)	13·7 (2·2–50·1)

SR Table 1: All-age DALYs counts and age standardized DALYs rates per 100,000 individuals for all causes and level 1 cause groups by location for 1990, 2010, and 2019, both sexes combined, Trinidad and Tobago

Cause Level	Cause	All-age DALYs			Age-standardized rate of DALYs per 100,000		
		1990	2010	2019	1990	2010	2019
0-0	All causes	376,000 (346,000–413,000)	431,000 (391,000–478,000)	461,000 (375,000–557,000)	37,600 (34,800–41,000)	31,100 (28,100–34,300)	29,200 (23,700–35,300)
1-0	Communicable, maternal, neonatal, and nutritional disorders	71,300 (63,800–80,000)	51,700 (46,700–57,500)	45,400 (38,700–53,800)	6,340 (5,690–7,070)	4,430 (3,960–5,000)	3,940 (3,260–4,780)
1-1	HIV/AIDS and sexually transmitted infections	5,740 (5,520–6,090)	12,200 (11,800–12,800)	10,900 (10,500–11,500)	486 (466–515)	811 (786–848)	710 (683–745)
1-2	HIV/AIDS	5,540 (5,350–5,810)	11,900 (11,600–12,400)	10,700 (10,300–11,100)	468 (452–492)	793 (772–822)	692 (669–720)
1-3	HIV/AIDS - drug-susceptible tuberculosis	605 (400–907)	577 (383–864)	498 (329–752)	51 (33·7–76·4)	38·3 (25·4–57·4)	32·4 (21·3–48·8)
1-4	HIV/AIDS - Multidrug-resistant Tuberculosis without extensive drug resistance	8 (1–34)	5 (1–23)	6 (1–28)	0·6 (0·1–2·9)	0·4 (0–1·5)	0·4 (0–1·8)
1-5	HIV/AIDS - Extensively drug-resistant Tuberculosis	0 (0–0)	1 (0–2)	1 (0–5)	0 (0–0)	0 (0–0·2)	0·1 (0–0·3)
1-6	HIV/AIDS resulting in other diseases	4,930 (4,590–5,260)	11,400 (10,900–11,800)	10,200 (9,730–10,600)	416 (388–445)	754 (727–786)	659 (631–689)
1-7	Sexually transmitted infections excluding HIV	203 (114–354)	274 (154–478)	284 (160–502)	17·9 (9·9–31·5)	18·1 (10·2–31·6)	18 (10·3–31·4)
1-8	Syphilis	6 (5–8)	8 (7–10)	10 (7–12)	0·6 (0·5–0·7)	0·6 (0·5–0·7)	0·6 (0·5–0·8)
1-9	Chlamydial infection	25 (16–39)	32 (21–48)	32 (21–47)	2·1 (1·3–3·2)	2·1 (1·4–3·2)	2·1 (1·4–3·1)
1-10	Gonococcal infection	14 (10–21)	21 (16–28)	23 (17–30)	1·2 (0·8–1·7)	1·4 (1·1–1·9)	1·5 (1·1–2·1)
1-11	Trichomoniasis	55 (22–115)	74 (28–153)	74 (28–156)	4·9 (1·9–10·2)	4·8 (1·8–9·9)	4·6 (1·8–9·7)
1-12	Genital herpes	57 (19–141)	82 (26–199)	88 (28–214)	5·3 (1·7–13·2)	5·4 (1·7–13·1)	5·3 (1·7–13·1)
1-13	Other sexually transmitted infections	46 (30–65)	57 (38–84)	57 (38–80)	3·8 (2·5–5·4)	3·8 (2·5–5·6)	3·8 (2·5–5·3)
1-14	Respiratory infections and tuberculosis	14,000 (12,800–15,300)	8,580 (7,790–9,460)	8,320 (6,510–10,600)	1,370 (1,260–1,480)	683 (617–757)	599 (466–766)
1-15	Tuberculosis	1,040 (949–1,140)	803 (720–886)	693 (524–894)	104 (95·1–113)	54·4 (48·9–59·9)	41·3 (31·6–52·9)
1-16	Latent tuberculosis infection	0 (0–0)	0 (0–0)	0 (0–0)	0 (0–0)	0 (0–0)	0 (0–0)
1-17	Drug-susceptible tuberculosis	1,030 (935–1,140)	797 (711–883)	685 (519–889)	103 (93·4–113)	54 (48·2–59·5)	40·9 (31·2–52·5)
1-18	Multidrug-resistant tuberculosis without extensive drug resistance	10 (1–44)	6 (1–24)	7 (1–29)	1·1 (0·1–4·4)	0·4 (0–1·6)	0·4 (0–1·7)
1-19	Extensively drug-resistant tuberculosis	0 (0–0)	1 (0–3)	1 (0–5)	0 (0–0)	0 (0–0·2)	0·1 (0–0·3)
1-20	Lower respiratory infections	11,600 (10,600–12,700)	6,410 (5,890–6,930)	6,260 (4,710–8,130)	1,150 (1,070–1,240)	522 (474–576)	452 (336–595)
1-21	Upper respiratory infections	1,030 (618–1,620)	1,060 (644–1,690)	1,060 (643–1,660)	82·3 (49·4–128)	81·7 (48·6–129)	81·4 (48·8–128)
1-22	Otitis media	362 (207–603)	309 (179–505)	303 (174–501)	28·4 (16·3–46·8)	24·8 (14·3–40·7)	24·4 (14–40·8)
1-23	Enteric infections	3,660 (3,150–4,240)	2,420 (1,940–3,000)	2,560 (1,940–3,330)	325 (280–376)	203 (163–250)	199 (150–257)
1-24	Diarrhoeal diseases	3,650 (3,130–4,220)	2,410 (1,930–3,000)	2,560 (1,940–3,320)	324 (279–375)	202 (162–249)	198 (150–256)
1-25	Typhoid and paratyphoid	1 (0–3)	0 (0–0)	0 (0–0)	0·1 (0–0·3)	0 (0–0)	0 (0–0)
1-26	Typhoid fever	1 (0–2)	0 (0–0)	0 (0–0)	0·1 (0–0·3)	0 (0–0)	0 (0–0)
1-27	Paratyphoid fever	0 (0–0)	0 (0–0)	0 (0–0)	0 (0–0)	0 (0–0)	0 (0–0)
1-28	iNTS	0 (0–1)	1 (0–1)	0 (0–1)	0 (0–0·1)	0·1 (0–0·1)	0·1 (0–0·1)
1-29	Other intestinal infectious diseases	10 (4–16)	6 (3–9)	5 (3–8)	0·8 (0·4–1·2)	0·5 (0·3–0·8)	0·5 (0·2–0·7)
1-30	Neglected tropical diseases and malaria	1,240 (847–1,760)	1,690 (1,200–2,290)	1,530 (1,000–2,210)	114 (75·2–164)	123 (88–165)	101 (68·5–141)
1-31	Malaria	0 (0–0)	0 (0–0)	0 (0–0)	0 (0–0)	0 (0–0)	0 (0–0)
1-32	Chagas disease	1 (0–1)	0 (0–1)	0 (0–1)	0·1 (0–0·1)	0 (0–0)	0 (0–0)
1-33	Leishmaniasis	0 (0–0)	0 (0–0)	0 (0–0)	0 (0–0)	0 (0–0)	0 (0–0)
1-34	Visceral leishmaniasis	0 (0–0)	0 (0–0)	0 (0–0)	0 (0–0)	0 (0–0)	0 (0–0)
1-35	Cutaneous and mucocutaneous leishmaniasis	0 (0–0)	0 (0–0)	0 (0–0)	0 (0–0)	0 (0–0)	0 (0–0)
1-36	African trypanosomiasis	0 (0–0)	0 (0–0)	0 (0–0)	0 (0–0)	0 (0–0)	0 (0–0)
1-37	Schistosomiasis	0 (0–0)	0 (0–0)	0 (0–0)	0 (0–0)	0 (0–0)	0 (0–0)
1-38	Cysticercosis	583 (293–1,000)	713 (345–1,280)	796 (358–1,380)	61·9 (31·9–105)	48·7 (23·5–86·4)	44·7 (20·1–77·1)
1-39	Cystic echinococcosis	0 (0–0)	0 (0–0)	0 (0–0)	0 (0–0)	0 (0–0)	0 (0–0)
1-40	Lymphatic filariasis	0 (0–0)	0 (0–0)	0 (0–0)	0 (0–0)	0 (0–0)	0 (0–0)
1-41	Onchocerciasis	0 (0–0)	0 (0–0)	0 (0–0)	0 (0–0)	0 (0–0)	0 (0–0)
1-42	Trachoma	0 (0–0)	0 (0–0)	0 (0–0)	0 (0–0)	0 (0–0)	0 (0–0)
1-43	Dengue	168 (96–280)	637 (345–852)	411 (198–595)	14·2 (8·1–23·1)	46·2 (25·8–61)	29·7 (14·3–43·5)
1-44	Yellow fever	7 (1–26)	2 (0–7)	1 (0–5)	0·5 (0·1–2)	0·2 (0–0·6)	0·1 (0–0·4)
1-45	Rabies	0 (0–0)	0 (0–0)	0 (0–0)	0 (0–0)	0 (0–0)	0 (0–0)
1-46	Intestinal nematode infections	101 (56–161)	58 (34–96)	49 (27–81)	7·7 (4·4–12·4)	4·3 (2·5–7)	3·8 (2·1–6·3)
1-47	Ascariasis	11 (3–24)	4 (2–8)	3 (1–5)	0·8 (0·3–1·7)	0·4 (0·1–0·7)	0·2 (0·1–0·5)
1-48	Trichuriasis	0 (0–1)	0 (0–0)	0 (0–0)	0 (0–0)	0 (0–0)	0 (0–0)

1-49	Hookworm disease	90 (47–153)	54 (30–93)	46 (24–78)	6·9 (3·7–11·6)	3·9 (2·2–6·7)	3·6 (1·9–6)
1-50	Food-borne trematodiases	0 (0–0)	0 (0–0)	0 (0–0)	0 (0–0)	0 (0–0)	0 (0–0)
1-51	Leprosy	2 (1–3)	3 (2–4)	4 (2–5)	0·2 (0·1–0·3)	0·2 (0·1–0·3)	0·2 (0·1–0·3)
1-52	Ebola virus disease	0 (0–0)	0 (0–0)	0 (0–0)	0 (0–0)	0 (0–0)	0 (0–0)
1-53	Zika virus disease	0 (0–0)	0 (0–0)	5 (1–16)	0 (0–0)	0 (0–0)	0·6 (0·1–1·6)
1-54	Guinea worm disease	0 (0–0)	0 (0–0)	0 (0–0)	0 (0–0)	0 (0–0)	0 (0–0)
1-55	Other neglected tropical diseases	381 (239–567)	272 (169–414)	260 (159–396)	29·4 (19–43·4)	23 (14·1–35·2)	22·2 (13·5–34)
1-56	Other infectious diseases	4,530 (4,030–4,990)	2,020 (1,740–2,330)	1,890 (1,460–2,420)	379 (338–416)	159 (137–185)	144 (112–186)
1-57	Meningitis	2,080 (1,880–2,300)	657 (597–725)	505 (369–671)	174 (158–192)	53·3 (47·6–60)	39·1 (28·5–51·8)
1-58	Encephalitis	1,160 (791–1,360)	378 (297–637)	379 (263–635)	92·3 (62·9–108)	32·3 (24·9–55·7)	33·2 (22·4–56·4)
1-59	Diphtheria	0 (0–0)	0 (0–0)	0 (0–0)	0 (0–0)	0 (0–0)	0 (0–0)
1-60	Whooping cough	58 (34–92)	27 (16–43)	23 (14–37)	4·5 (2·6–7·2)	2·9 (1·7–4·6)	2·8 (1·6–4·5)
1-61	Tetanus	62 (21–89)	2 (1–10)	2 (0–7)	6·4 (2·2–9·2)	0·2 (0–0·7)	0·2 (0–0·5)
1-62	Measles	1 (1–2)	0 (0–0)	0 (0–0)	0·1 (0·1–0·2)	0 (0–0)	0 (0–0)
1-63	Varicella and herpes zoster	28 (18–42)	40 (26–61)	43 (27–65)	2·7 (1·7–4·1)	2·9 (1·9–4·3)	2·7 (1·7–4·1)
1-64	Acute hepatitis	462 (418–512)	92 (72–119)	90 (67–118)	40·8 (37·1–44·9)	6·8 (5·2–8·8)	6·6 (4·9–8·7)
1-65	Acute hepatitis A	136 (105–191)	51 (35–72)	47 (31–68)	11·1 (8·5–16·1)	3·9 (2·6–5·5)	3·8 (2·5–5·5)
1-66	Acute hepatitis B	232 (183–271)	28 (24–34)	29 (21–39)	20·8 (16·5–24·2)	1·9 (1·6–2·3)	1·9 (1·3–2·5)
1-67	Acute hepatitis C	75 (29–100)	7 (3–10)	7 (3–11)	7·1 (2·8–9·8)	0·5 (0·2–0·7)	0·5 (0·2–0·7)
1-68	Acute hepatitis E	19 (11–27)	7 (4–10)	6 (4–9)	1·7 (1–2·5)	0·5 (0·3–0·7)	0·5 (0·3–0·7)
1-69	Other unspecified infectious diseases	676 (501–871)	827 (529–1,050)	846 (538–1,160)	58·2 (43·3–73·1)	60·5 (39·9–76·7)	59·6 (38·6–81·7)
1-70	Maternal and neonatal disorders	34,900 (29,700–40,800)	19,500 (16,600–23,100)	15,000 (11,500–19,600)	3,050 (2,580–3,570)	2,040 (1,710–2,430)	1,790 (1,330–2,410)
1-71	Maternal disorders	1,070 (964–1,180)	844 (758–940)	514 (363–708)	83·2 (74·8–91·5)	57·3 (51·4–63·7)	36·5 (25·9–49·8)
1-72	Maternal haemorrhage	137 (100–180)	157 (123–195)	104 (74–142)	10·5 (7·7–13·7)	10·7 (8·4–13·2)	7·3 (5·2–9·9)
1-73	Maternal sepsis and other pregnancy related infections	96 (75–122)	33 (24–46)	22 (14–33)	7·4 (5·8–9·3)	2·2 (1·6–3)	1·6 (1–2·4)
1-74	Maternal hypertensive disorders	258 (216–301)	137 (116–161)	85 (58–118)	19·9 (16·7–23·1)	9·3 (7·9–10·9)	6 (4·1–8·4)
1-75	Maternal obstructed labour and uterine rupture	5 (3–9)	11 (8–15)	8 (5–11)	0·4 (0·2–0·7)	0·8 (0·6–1)	0·6 (0·4–0·8)
1-76	Maternal abortive outcome	469 (396–550)	87 (66–110)	48 (32–72)	36·6 (31·2–42·8)	5·9 (4·5–7·4)	3·4 (2·3–5)
1-77	Ectopic pregnancy	9 (7–10)	61 (50–74)	34 (21–50)	0·7 (0·5–0·8)	4·1 (3·4–4·9)	2·3 (1·5–3·5)
1-78	Indirect maternal deaths	1 (1–2)	7 (6–9)	5 (3–7)	0·1 (0·1–0·1)	0·5 (0·4–0·6)	0·3 (0·2–0·5)
1-79	Late maternal deaths	44 (32–59)	52 (37–71)	32 (18–51)	3·4 (2·5–4·5)	3·5 (2·6–4·8)	2·3 (1·3–3·5)
1-80	Maternal deaths aggravated by HIV/AIDS	1 (0–2)	4 (2–6)	3 (1–5)	0·1 (0–0·2)	0·3 (0·1–0·4)	0·2 (0·1–0·3)
1-81	Other maternal disorders	53 (42–67)	295 (247–344)	174 (117–251)	4·1 (3·2–5·1)	20·1 (16·9–23·4)	12·4 (8·4–17·6)
1-82	Neonatal disorders	33,800 (28,600–39,800)	18,700 (15,800–22,300)	14,500 (11,100–19,000)	2,960 (2,500–3,490)	1,980 (1,660–2,370)	1,760 (1,300–2,360)
1-83	Neonatal preterm birth	16,300 (13,500–19,600)	8,390 (7,010–9,980)	6,350 (4,720–8,440)	1,430 (1,180–1,720)	877 (729–1,050)	762 (552–1,040)
1-84	Neonatal encephalopathy due to birth asphyxia and trauma	9,710 (7,500–12,000)	3,620 (2,920–4,490)	2,700 (1,840–3,720)	858 (662–1,060)	392 (315–487)	337 (224–473)
1-85	Neonatal sepsis and other neonatal infections	3,420 (2,550–4,540)	2,500 (1,950–3,180)	2,410 (1,800–3,180)	296 (221–390)	257 (204–320)	263 (192–357)
1-86	Hemolytic disease and other neonatal jaundice	848 (644–1,120)	493 (396–609)	329 (251–418)	73·2 (55·7–97·1)	46·8 (37·3–58·8)	31·2 (23·5–40·8)
1-87	Other neonatal disorders	3,480 (2,580–4,580)	3,660 (2,870–4,580)	2,710 (1,830–3,970)	306 (226–405)	407 (320–511)	364 (245–533)
1-88	Nutritional deficiencies	7,210 (5,180–9,990)	5,300 (3,610–7,570)	5,200 (3,470–7,460)	619 (459–842)	414 (279–600)	393 (256–571)
1-89	Protein-energy malnutrition	1,580 (1,380–1,790)	753 (655–879)	755 (585–955)	156 (138–175)	59·9 (51·9–69·9)	53·1 (40·8–67·1)
1-90	Iodine deficiency	162 (82–278)	96 (46–166)	90 (43–155)	13·7 (7–23·4)	6·6 (3·1–11·4)	6 (2·8–10·5)
1-91	Vitamin A deficiency	110 (66–170)	60 (37–91)	58 (36–87)	8 (4·9–12·2)	5·4 (3·4–8·1)	5·2 (3·2–7·9)
1-92	Dietary iron deficiency	5,140 (3,250–7,720)	4,280 (2,660–6,430)	4,180 (2,520–6,370)	420 (273–627)	334 (207–510)	321 (191–495)
1-93	Other nutritional deficiencies	213 (159–295)	114 (79–158)	119 (80–169)	21·1 (15·8–30·5)	8·1 (5·7–11·1)	7·9 (5·3–11)
1-0	Non-communicable diseases	258,000 (235,000–287,000)	315,000 (281,000–353,000)	365,000 (295,000–442,000)	27,300 (25,100–30,000)	22,200 (19,800–24,900)	21,700 (17,600–26,400)
1-1	Neoplasms	31,600 (30,200–32,900)	42,700 (40,300–44,600)	49,500 (37,300–64,800)	3,430 (3,270–3,570)	2,960 (2,780–3,090)	2,800 (2,120–3,650)
1-2	Lip and oral cavity cancer	422 (395–451)	525 (493–562)	588 (434–783)	47·5 (44·4–50·7)	35 (32·9–37·3)	32·3 (23·7–42·8)
1-3	Nasopharynx cancer	92 (86–99)	180 (165–195)	180 (132–240)	9·4 (8·8–10·1)	11·9 (11–12·9)	10·6 (7·7–14·2)
1-4	Other pharynx cancer	249 (226–276)	270 (243–298)	320 (230–435)	28·7 (26–31·7)	17·4 (15·7–19·2)	17·3 (12·4–23·5)
1-5	Oesophageal cancer	511 (469–553)	535 (486–589)	719 (520–968)	59 (54·1–63·9)	35·6 (32·5–39)	38·7 (28·2–51·6)
1-6	Stomach cancer	2,160 (2,010–2,310)	1,510 (1,410–1,610)	1,760 (1,320–2,320)	252 (234–269)	104 (97·8–112)	95·9 (72·1–126)
1-7	Colon and rectum cancer	2,880 (2,730–3,040)	4,710 (4,460–4,970)	5,800 (4,360–7,590)	332 (315–350)	324 (306–341)	316 (238–412)

1-8	Liver cancer	1,520 (1,430–1,610)	892 (830–958)	1,160 (859–1,540)	171 (160–181)	61·2 (57·1–65·5)	63·3 (47·1–84·3)
1-9	Liver cancer due to hepatitis B	498 (383–639)	289 (221–373)	334 (216–493)	54·2 (41–70·7)	19·1 (14·7–24·7)	18·3 (12–26·9)
1-10	Liver cancer due to hepatitis C	292 (202–392)	168 (115–232)	229 (141–349)	35·1 (24·3–47)	11·9 (8·2–16·3)	12·3 (7·7–18·4)
1-11	Liver cancer due to alcohol use	456 (339–594)	281 (204–362)	393 (253–569)	52·6 (38·5–68·7)	19·2 (14·1–24·6)	20·8 (13·4–30)
1-12	Liver cancer due to NASH	123 (89–169)	82 (57–113)	117 (74–180)	14·1 (10–19·4)	5·7 (4–7·8)	6·4 (4·1–9·8)
1-13	Gallbladder and biliary tract cancer	713 (470–787)	393 (327–716)	498 (339–886)	83·2 (55–91·9)	26·8 (22·2–48·9)	26·6 (18·2–47·5)
1-14	Pancreatic cancer	280 (257–304)	1,840 (1,700–1,990)	2,420 (1,780–3,200)	32·6 (30–35·3)	127 (117–137)	130 (96–171)
1-15	Larynx cancer	303 (281–327)	387 (355–420)	436 (321–576)	35·2 (32·6–37·9)	25·8 (23·8–27·9)	23·5 (17·4–30·9)
1-16	Tracheal, bronchus, and lung cancer	2,600 (2,440–2,760)	4,160 (3,890–4,430)	4,740 (3,530–6,240)	298 (279–317)	277 (259–294)	253 (189–333)
1-17	Malignant skin melanoma	72 (54–87)	100 (85–142)	119 (82–173)	7·7 (5·6–9·2)	6·9 (5·8–9·6)	6·9 (4·8–10·1)
1-18	Non-melanoma skin cancer	70 (63–77)	116 (106–125)	142 (107–186)	8·1 (7·3–8·9)	8 (7·2–8·6)	8 (6–10·4)
1-19	Non-melanoma skin cancer (squamous-cell carcinoma)	70 (63–77)	116 (105–125)	142 (107–186)	8 (7·3–8·9)	8 (7·2–8·6)	8 (6–10·4)
1-20	Non-melanoma skin cancer (basal-cell carcinoma)	0 (0–0)	0 (0–0)	0 (0–0)	0 (0–0)	0 (0–0)	0 (0–0)
1-21	Breast cancer	3,750 (3,480–4,000)	5,880 (5,440–6,350)	6,460 (4,760–8,580)	416 (387–444)	385 (356–416)	355 (261–472)
1-22	Cervical cancer	2,520 (2,300–2,870)	2,990 (2,710–3,320)	3,090 (2,230–4,190)	274 (251–302)	198 (179–218)	172 (124–234)
1-23	Uterine cancer	564 (519–618)	1,200 (1,090–1,330)	1,490 (1,110–1,940)	66·4 (60·9–72·7)	82·5 (74·7–90·8)	77·8 (57·8–101)
1-24	Ovarian cancer	188 (166–319)	1,670 (644–1,890)	1,750 (754–2,420)	20·4 (18–34·4)	110 (42·6–124)	97·2 (42·1–135)
1-25	Prostate cancer	3,270 (2,300–3,700)	5,340 (3,780–6,030)	7,090 (4,670–9,520)	401 (286–454)	402 (284–455)	388 (256–518)
1-26	Testicular cancer	28 (22–48)	96 (74–130)	85 (55–117)	2·1 (1·7–3·8)	7·1 (5·4–10·5)	7 (4·5–9·9)
1-27	Kidney cancer	925 (866–990)	754 (705–806)	902 (662–1,210)	93·2 (87·2–99·6)	52·7 (49·3–56·2)	53·8 (39·7–71·4)
1-28	Bladder cancer	360 (337–389)	545 (501–592)	650 (479–865)	42·6 (39·8–45·9)	38·1 (35–41·5)	35·1 (26–46·5)
1-29	Brain and nervous system cancer	305 (248–487)	870 (374–1,080)	880 (367–1,320)	26·2 (21·3–43)	66·5 (27–83·9)	62·7 (24·3–94·5)
1-30	Thyroid cancer	150 (140–161)	213 (196–232)	252 (187–331)	16·6 (15·4–17·8)	14·4 (13·3–15·6)	13·9 (10·3–18·2)
1-31	Mesothelioma	41 (36–47)	84 (73–94)	70 (50–96)	4·4 (3·9–5·1)	5·5 (4·7–6·1)	4 (2·8–5·4)
1-32	Hodgkin lymphoma	127 (102–154)	205 (106–255)	170 (98–246)	11·2 (9·2–13·4)	13·7 (7·1–17)	11·1 (6·3–16·2)
1-33	Non-Hodgkin's lymphoma	1,710 (1,540–1,900)	1,800 (1,610–2,010)	1,910 (1,340–2,590)	160 (145–177)	123 (110–137)	118 (83·5–160)
1-34	Multiple myeloma	424 (308–489)	848 (628–926)	1,130 (761–1,540)	50 (35·9–57·8)	57·7 (42·8–63·1)	59·6 (40·2–80·5)
1-35	Leukaemia	2,540 (2,330–2,760)	2,120 (1,960–2,290)	2,100 (1,580–2,730)	213 (197–228)	160 (148–176)	151 (113–195)
1-36	Acute lymphoid leukaemia	867 (664–1,030)	483 (389–558)	449 (323–601)	63 (48·6–74·6)	41·8 (33·6–49·1)	40·4 (29·1–54)
1-37	Chronic lymphoid leukaemia	43 (36–50)	72 (54–82)	91 (63–124)	5 (4·1–5·7)	4·8 (3·7–5·5)	4·9 (3·5–6·7)
1-38	Acute myeloid leukaemia	752 (637–899)	780 (691–915)	801 (593–1,050)	62·2 (53·4–73·1)	58·4 (51·9–68·4)	57·5 (42·6–75·8)
1-39	Chronic myeloid leukaemia	213 (187–249)	180 (160–216)	176 (128–241)	20·6 (18·2–23·7)	12·4 (11–14·8)	10·5 (7·6–14·5)
1-40	Other leukaemia	669 (602–781)	606 (550–707)	587 (430–784)	62 (56·3–70·9)	43·1 (39·2–50·7)	37·5 (27·4–50)
1-41	Other neoplasms	142 (101–168)	283 (173–338)	348 (192–502)	15·5 (10·8–18·2)	20 (12·3–23·8)	19·9 (11·2–28·5)
1-42	Myelodysplastic, myeloproliferative, and other hematopoietic neoplasms	142 (101–168)	283 (173–338)	348 (192–502)	15·5 (10·8–18·2)	20 (12·3–23·8)	19·9 (11·2–28·5)
1-43	Benign and in situ intestinal neoplasms	0 (0–0)	0 (0–0)	0 (0–0)	0 (0–0)	0 (0–0)	0 (0–0)
1-44	Benign and in situ cervical and uterine neoplasms	0 (0–0)	0 (0–0)	0 (0–0)	0 (0–0)	0 (0–0)	0 (0–0)
1-45	Other benign and in situ neoplasms	0 (0–0)	0 (0–0)	0 (0–0)	0 (0–0)	0 (0–0)	0 (0–0)
1-46	Cardiovascular diseases	72,000 (69,500–74,400)	70,600 (67,500–73,700)	83,800 (65,100–108,000)	8,520 (8,220–8,800)	4,970 (4,740–5,180)	4,650 (3,630–5,960)
1-47	Rheumatic heart disease	1,720 (1,440–2,050)	1,170 (911–1,500)	1,100 (806–1,460)	150 (127–177)	79·9 (62–103)	73·9 (54–98·4)
1-48	Ischaemic heart disease	37,500 (36,100–39,100)	37,200 (35,200–38,900)	43,700 (33,300–56,800)	4,510 (4,340–4,700)	2,590 (2,440–2,710)	2,370 (1,810–3,080)
1-49	Stroke	21,900 (20,800–23,100)	19,900 (18,600–21,200)	24,000 (18,700–30,900)	2,590 (2,460–2,730)	1,430 (1,330–1,520)	1,340 (1,040–1,720)
1-50	Ischaemic stroke	10,500 (9,650–11,200)	9,720 (8,950–10,400)	12,300 (9,760–15,400)	1,310 (1,210–1,400)	735 (676–785)	698 (555–874)
1-51	Intracerebral hemorrhage	9,450 (8,790–10,200)	7,890 (7,310–8,490)	9,140 (6,780–12,200)	1,080 (1,000–1,170)	536 (497–575)	493 (367–652)
1-52	Subarachnoid hemorrhage	1,940 (1,700–2,380)	2,320 (2,090–2,530)	2,550 (1,870–3,420)	202 (176–245)	155 (140–169)	147 (108–197)
1-53	Hypertensive heart disease	6,010 (4,350–6,520)	4,610 (4,060–7,770)	5,850 (4,280–9,810)	737 (531–800)	326 (287–554)	323 (237–539)
1-54	Non-rheumatic valvular heart disease	284 (252–323)	328 (299–358)	386 (287–515)	29·3 (25·8–33·4)	22·4 (20·4–24·4)	22·7 (16·9–30·2)
1-55	Non-rheumatic calcific aortic valve disease	138 (114–167)	206 (161–235)	245 (180–331)	15·1 (12·4–18·2)	14·2 (11·3–16·1)	14·1 (10·4–19)
1-56	Non-rheumatic degenerative mitral valve disease	145 (111–174)	120 (102–159)	139 (96–197)	14·1 (10·7–17)	8 (6·8–10·6)	8·5 (5·9–12)
1-57	Other non-rheumatic valve diseases	1 (1–2)	2 (1–2)	2 (1–3)	0·1 (0·1–0·2)	0·1 (0·1–0·2)	0·1 (0·1–0·2)
1-58	Cardiomyopathy and myocarditis	1,770 (1,540–2,000)	2,080 (1,880–2,430)	2,380 (1,740–3,180)	176 (152–202)	145 (132–169)	145 (106–192)
1-59	Myocarditis	295 (172–391)	266 (191–322)	294 (191–409)	26·1 (14·9–34·6)	19·8 (14·7–23·9)	20·6 (13·7–28·4)
1-60	Alcoholic cardiomyopathy	155 (103–263)	271 (216–429)	338 (224–533)	17·1 (11·3–29·6)	17·6 (14·1–27·8)	18·1 (12–28·4)

1·61	Other cardiomyopathy	1,320 (1,130–1,540)	1,550 (1,370–1,750)	1,750 (1,270–2,340)	132 (113–156)	108 (96–122)	106 (77·1–142)
1·62	Atrial fibrillation and flutter	570 (461–689)	1,060 (828–1,280)	1,460 (1,120–1,830)	78·1 (63·9–94)	81 (63·3–96·8)	82·8 (63·3–104)
1·63	Aortic aneurysm	791 (704–895)	1,180 (1,070–1,290)	1,610 (1,170–2,130)	92·1 (81·7–104)	83 (75·1–90·5)	87·6 (64·3–116)
1·64	Peripheral vascular disease	115 (62–192)	203 (116–332)	282 (158–497)	15·1 (8–25·4)	15·2 (8·7–24·9)	15·6 (8·7–27·2)
1·65	Endocarditis	262 (154–326)	372 (233–439)	412 (226–595)	26·1 (14·7–33·1)	26 (16–30·8)	24·9 (14·1–35·6)
1·66	Chronic respiratory diseases	7,900 (6,960–9,090)	7,750 (6,810–8,980)	8,980 (7,100–11,300)	779 (702–873)	577 (501–673)	584 (458–732)
1·67	Chronic obstructive pulmonary disease	2,710 (2,490–2,940)	3,490 (3,150–4,050)	4,270 (3,270–5,470)	318 (292–344)	250 (225–289)	239 (184–304)
1·68	Pneumoconiosis	5 (4–6)	5 (4–6)	6 (5–8)	0·5 (0·4–0·6)	0·4 (0·3–0·4)	0·4 (0·3–0·5)
1·69	Silicosis	1 (1–2)	1 (1–1)	1 (1–2)	0·1 (0·1–0·2)	0·1 (0–0·1)	0·1 (0–0·1)
1·70	Asbestosis	1 (1–2)	1 (1–2)	2 (1–2)	0·1 (0·1–0·2)	0·1 (0·1–0·1)	0·1 (0·1–0·1)
1·71	Coal workers pneumoconiosis	0 (0–0)	0 (0–0)	0 (0–0)	0 (0–0)	0 (0–0)	0 (0–0)
1·72	Other pneumoconiosis	2 (1–3)	3 (2–4)	3 (2–4)	0·2 (0·1–0·3)	0·2 (0·1–0·3)	0·2 (0·1–0·3)
1·73	Asthma	4,620 (3,770–5,760)	3,370 (2,630–4,360)	3,670 (2,710–4,900)	406 (339–494)	264 (202–348)	280 (202–389)
1·74	Interstitial lung disease and pulmonary sarcoidosis	362 (292–459)	624 (437–756)	746 (477–1,050)	38·7 (31·5–50)	44·5 (30·8–54)	43·2 (27·7–60·2)
1·75	Other chronic respiratory diseases	196 (147–234)	258 (202–301)	289 (208–373)	16·5 (12·6–19·6)	18·5 (14·8–21·6)	20·5 (15·1–26·3)
1·76	Digestive diseases	13,100 (12,100–14,600)	14,700 (13,300–16,600)	16,200 (12,800–20,400)	1,350 (1,250–1,490)	1,010 (914–1,140)	957 (756–1,200)
1·77	Cirrhosis and other chronic liver diseases	5,000 (4,730–5,310)	5,020 (4,690–5,450)	5,580 (4,100–7,480)	524 (494–556)	330 (307–359)	315 (230–420)
1·78	Cirrhosis and other chronic liver diseases due to hepatitis B	630 (444–867)	643 (452–904)	433 (254–686)	66·5 (46·7–91·9)	41·2 (29·4–57·4)	24·3 (14·3–38·5)
1·79	Cirrhosis and other chronic liver diseases due to hepatitis C	955 (717–1,270)	930 (685–1,260)	1,070 (682–1,600)	100 (74·7–135)	60·1 (44·5–81·2)	60·1 (38·3–88·8)
1·80	Cirrhosis and other chronic liver diseases due to alcohol use	1,780 (1,400–2,130)	1,960 (1,520–2,360)	2,370 (1,600–3,370)	195 (153–233)	126 (98·9–152)	129 (87·3–184)
1·81	Cirrhosis and other chronic liver diseases due to NAFLD	659 (469–905)	718 (502–1,000)	884 (558–1,330)	71·3 (50·4–97·6)	47·1 (33·5–64·9)	49 (31·1–73·4)
1·82	Cirrhosis and other chronic liver diseases due to other causes	981 (807–1,180)	772 (600–981)	822 (551–1,190)	89·9 (71·7–110)	55·1 (43·5–69)	52·6 (36·2–74·9)
1·83	Upper digestive system diseases	3,900 (3,140–5,090)	4,570 (3,450–6,190)	4,880 (3,540–6,640)	408 (334–520)	309 (234–416)	284 (205–387)
1·84	Peptic ulcer disease	1,560 (1,390–1,770)	1,470 (1,300–1,690)	1,560 (1,110–2,140)	177 (159–200)	103 (91·7–119)	87·5 (62·4–119)
1·85	Gastritis and duodenitis	1,040 (800–1,250)	1,240 (974–1,550)	1,290 (967–1,680)	107 (82·7–129)	82·7 (65·2–104)	73·9 (55·3–95·5)
1·86	GERD	1,310 (680–2,320)	1,860 (965–3,280)	2,030 (1,050–3,600)	123 (64·1–218)	123 (63·8–217)	123 (63·8–218)
1·87	Appendicitis	366 (253–441)	232 (191–332)	228 (161–349)	32·4 (22·4–38·5)	17·1 (14–24·1)	15·5 (11–23·2)
1·88	Paralytic ileus and intestinal obstruction	1,040 (827–1,180)	986 (844–1,320)	1,120 (791–1,630)	104 (84·1–118)	77·2 (65·8–102)	75·2 (53·4–108)
1·89	Inguinal, femoral, and abdominal hernia	1,040 (805–1,340)	1,180 (898–1,520)	1,230 (918–1,600)	97 (76·4–122)	86·8 (66·7–112)	81·5 (60·7–107)
1·90	Inflammatory bowel disease	143 (110–169)	199 (158–237)	215 (162–279)	14·3 (10·9–16·8)	13·4 (10·8–15·9)	12·5 (9·5–16·1)
1·91	Vascular intestinal disorders	224 (197–254)	212 (189–238)	284 (207–377)	26·2 (22·9–29·8)	15·2 (13·5–17)	16 (11·7–21·1)
1·92	Gallbladder and biliary diseases	800 (628–1,000)	902 (698–1,150)	1,020 (731–1,330)	83·8 (65·8–104)	61·1 (47·4–78·2)	58·6 (42·8–77·4)
1·93	Pancreatitis	357 (317–399)	515 (433–577)	559 (399–759)	35·1 (31·3–39·1)	34·3 (29–38·5)	33 (23·7–44·9)
1·94	Other digestive diseases	239 (176–415)	910 (364–1,190)	1,090 (442–1,680)	24·4 (18·2–42·3)	66·3 (26·7–86·5)	65·6 (27–100)
1·95	Neurological disorders	14,100 (7,740–22,800)	18,300 (10,600–28,800)	19,900 (11,800–31,500)	1,350 (793–2,150)	1,320 (775–2,080)	1,300 (752–2,090)
1·96	Alzheimer's disease and other dementias	1,910 (792–4,450)	3,710 (1,550–8,390)	4,940 (2,100–11,200)	292 (121–687)	296 (124–676)	291 (124–660)
1·97	Parkinson's disease	563 (525–606)	943 (864–1,030)	1,260 (1,020–1,550)	72·2 (67·3–77·5)	72·9 (66·8–79·2)	71·6 (58–87·6)
1·98	Idiopathic epilepsy	4,050 (2,270–6,390)	4,130 (2,300–6,510)	4,050 (2,130–6,540)	343 (193–539)	295 (162–466)	281 (145–450)
1·99	Multiple sclerosis	62 (51–74)	127 (82–155)	138 (86–199)	6·2 (5·1–7·3)	8·3 (5·3–10·1)	8·2 (5–11·9)
1·100	Motor neuron disease	53 (49–58)	180 (165–196)	209 (150–278)	5·5 (5·1–5·9)	11·8 (10·8–12·8)	11·3 (8·2–15)
1·101	Headache disorders	6,460 (1,150–14,500)	8,000 (1,620–17,600)	8,130 (1,710–17,500)	550 (108–1,210)	548 (107–1,210)	548 (106–1,200)
1·102	Migraine	5,870 (789–13,700)	7,230 (1,100–16,800)	7,310 (1,170–16,600)	498 (73–1,150)	496 (72–1,150)	495 (72·1–1,130)
1·103	Tension-type headache	584 (166–2,270)	771 (234–2,730)	814 (245–2,760)	52·1 (15·4–190)	52·2 (15·4–188)	52·1 (14·8–187)
1·104	Other neurological disorders	978 (682–1,370)	1,170 (833–1,600)	1,190 (778–1,670)	82·2 (58·9–113)	86·6 (60·9–120)	87·6 (55·7–125)
1·105	Mental disorders	20,700 (15,100–27,500)	25,600 (18,600–33,600)	26,900 (19,900–35,300)	1,790 (1,310–2,370)	1,750 (1,270–2,300)	1,780 (1,300–2,340)
1·106	Schizophrenia	1,950 (1,350–2,590)	2,800 (1,940–3,720)	2,980 (2,040–3,970)	177 (123–233)	182 (126–243)	183 (125–244)
1·107	Depressive disorders	8,180 (5,600–11,600)	10,100 (6,860–14,200)	10,800 (7,510–15,000)	732 (503–1,030)	677 (460–951)	691 (476–964)
1·108	Major depressive disorder	7,190 (4,840–10,200)	8,720 (5,860–12,200)	9,380 (6,380–13,100)	642 (438–907)	587 (395–823)	602 (407–847)
1·109	Dysthymia	987 (603–1,520)	1,360 (856–2,080)	1,440 (916–2,210)	90 (56·4–137)	89·9 (56·9–137)	89·8 (56–136)
1·110	Bipolar disorder	2,280 (1,310–3,550)	2,950 (1,690–4,520)	2,980 (1,750–4,560)	197 (115–303)	197 (113–302)	197 (116–300)
1·111	Anxiety disorders	4,200 (2,770–6,010)	5,200 (3,460–7,350)	5,510 (3,660–7,790)	360 (240–508)	360 (240–509)	370 (245–522)
1·112	Eating disorders	602 (364–917)	850 (515–1,290)	805 (490–1,210)	46 (27·9–70)	58·2 (35·3–88·8)	59·9 (36·5–89·6)
1·113	Anorexia nervosa	147 (85–233)	192 (112–313)	179 (104–285)	11·1 (6·5–17·5)	13·5 (7·8–21·9)	14·1 (8·1–22·9)

1-114	Bulimia nervosa	455 (258–722)	657 (381–1,050)	626 (357–975)	34·9 (19·9–55·8)	44·7 (25·9–71·2)	45·9 (26·5–71·4)
1-115	Autism spectrum disorders	656 (428–954)	696 (453–1,000)	699 (455–1,010)	52·7 (34·3–76·5)	52·9 (34·3–76·2)	52·9 (34·4–76·3)
1-116	Attention-deficit/hyperactivity disorder	503 (287–844)	476 (273–791)	452 (255–753)	37·4 (21·2–62·2)	37·5 (21·4–63·1)	37·5 (21–63·3)
1-117	Conduct disorder	962 (546–1,520)	738 (408–1,170)	714 (406–1,130)	68 (38·5–107)	68·2 (37·9–108)	68·1 (38·7–107)
1-118	Idiopathic developmental intellectual disability	186 (53–357)	132 (30–262)	124 (27–250)	14·7 (4·2–28·1)	10·1 (2·2–19·8)	9·5 (2–19)
1-119	Other mental disorders	1,150 (721–1,730)	1,640 (1,060–2,500)	1,790 (1,140–2,720)	108 (68·7–164)	108 (69·8–165)	108 (69·3–164)
1-120	Substance use disorders	3,340 (2,470–4,310)	5,320 (4,160–6,670)	5,150 (3,930–6,510)	295 (223–379)	349 (272–438)	330 (249–418)
1-121	Alcohol use disorders	2,150 (1,610–2,830)	3,420 (2,620–4,380)	3,290 (2,450–4,280)	198 (149–257)	223 (171–287)	204 (150–266)
1-122	Drug use disorders	1,180 (814–1,610)	1,900 (1,400–2,460)	1,860 (1,390–2,370)	97 (68·1–130)	126 (92·5–162)	127 (94·2–164)
1-123	Opioid use disorders	538 (342–792)	812 (562–1,120)	820 (562–1,110)	45·5 (29·6–65·9)	53·2 (36·9–73·5)	53·8 (36·6–74·4)
1-124	Cocaine use disorders	192 (115–292)	466 (355–624)	460 (329–612)	15·6 (9·7–23·5)	30·7 (23·2–41·4)	30·7 (21·7–41·5)
1-125	Amphetamine use disorders	77 (39–134)	112 (69–174)	105 (67–160)	5·9 (3·1–10·1)	7·3 (4·5–11·3)	7·5 (4·5–11·7)
1-126	Cannabis use disorders	193 (99–347)	217 (121–355)	194 (110–314)	15 (7·9–26·5)	15 (8·3–24·5)	14·9 (8·3–24·6)
1-127	Other drug use disorders	185 (115–284)	293 (191–414)	282 (190–397)	14·9 (9·4–22·7)	19·6 (12·8–27·7)	19·8 (13–28·4)
1-128	Diabetes and kidney diseases	38,300 (35,200–41,700)	59,400 (53,700–66,000)	77,700 (61,700–95,800)	4,410 (4,060–4,800)	4,060 (3,680–4,510)	4,220 (3,350–5,190)
1-129	Diabetes mellitus	32,300 (29,500–35,600)	44,700 (39,500–51,100)	59,800 (47,400–74,100)	3,770 (3,440–4,140)	3,060 (2,710–3,480)	3,200 (2,540–3,970)
1-130	Diabetes mellitus type 1	1,530 (1,180–1,900)	1,530 (1,280–2,050)	1,700 (1,240–2,450)	150 (116–195)	104 (88·2–140)	103 (75·4–145)
1-131	Diabetes mellitus type 2	30,800 (27,900–34,000)	43,100 (38,100–49,400)	58,100 (46,000–72,000)	3,620 (3,290–3,990)	2,950 (2,620–3,370)	3,100 (2,460–3,830)
1-132	Chronic kidney disease	5,960 (5,520–6,470)	14,700 (13,800–15,800)	17,900 (14,000–22,700)	637 (592–689)	1,010 (941–1,080)	1,010 (793–1,280)
1-133	Chronic kidney disease due to diabetes mellitus type 1	453 (318–615)	1,140 (752–1,590)	1,270 (734–2,100)	45·3 (31–63·1)	73·2 (49·3–101)	72·9 (42·9–118)
1-134	Chronic kidney disease due to diabetes mellitus type 2	1,170 (936–1,410)	3,230 (2,550–3,920)	4,230 (3,040–5,670)	139 (111–167)	221 (176–267)	225 (163–304)
1-135	Chronic kidney disease due to hypertension	1,070 (894–1,300)	2,910 (2,410–3,450)	3,750 (2,770–4,980)	127 (106–152)	204 (169–241)	205 (152–269)
1-136	Chronic kidney disease due to glomerulonephritis	1,490 (1,240–1,760)	3,460 (2,840–4,190)	3,960 (2,760–5,600)	146 (121–176)	232 (191–280)	232 (165–322)
1-137	Chronic kidney disease due to other and unspecified causes	1,780 (1,480–2,120)	3,980 (3,260–4,900)	4,710 (3,470–6,290)	180 (148–216)	276 (227–336)	279 (208–370)
1-138	Acute glomerulonephritis	1 (0–1)	3 (3–4)	2 (2–3)	0 (0–0·1)	0·2 (0·2–0·3)	0·1 (0·1–0·2)
1-139	Skin and subcutaneous diseases	6,450 (4,270–9,640)	7,110 (4,850–10,500)	7,540 (5,100–10,900)	546 (370–806)	546 (370–805)	548 (367–799)
1-140	Dermatitis	1,460 (852–2,340)	1,440 (869–2,220)	1,480 (889–2,290)	116 (68·1–183)	115 (68·6–181)	115 (68·3–183)
1-141	Atopic dermatitis	1,130 (613–1,890)	997 (542–1,680)	992 (535–1,670)	85·1 (46·3–142)	84·9 (46·1–143)	84·9 (45·8–143)
1-142	Contact dermatitis	293 (185–441)	399 (250–598)	435 (270–659)	27·1 (17·1–40·8)	27·1 (17·1–40·5)	27 (17·1–40·4)
1-143	Seborrhoeic dermatitis	39 (21–61)	47 (27–75)	51 (29–80)	3·4 (1·9–5·3)	3·4 (1·9–5·3)	3·4 (1·9–5·4)
1-144	Psoriasis	355 (249–474)	413 (289–560)	438 (311–586)	31·8 (22·6–42·1)	28·7 (20–39·1)	28·1 (19·8–37·9)
1-145	Bacterial skin diseases	406 (264–594)	659 (430–975)	842 (498–1,290)	41·5 (26·7–61·5)	47·2 (31·2–70·2)	50·7 (30·6–77·5)
1-146	Cellulitis	202 (108–296)	282 (144–438)	360 (162–561)	20·7 (10·4–30·6)	20 (10·5–31·7)	21·4 (10–33·1)
1-147	Pyoderma	204 (139–334)	377 (259–600)	482 (314–785)	20·8 (14·3–35·4)	27·3 (18·8–44)	29·3 (19·3–47·5)
1-148	Scabies	1,050 (571–1,670)	1,070 (593–1,710)	1,060 (589–1,680)	83·2 (45·9–133)	83 (46–132)	82·7 (45·6–132)
1-149	Fungal skin diseases	476 (188–1,000)	605 (244–1,280)	680 (275–1,450)	44·5 (17·8–94)	44·2 (17·8–93·7)	44·2 (17·7–93·7)
1-150	Viral skin diseases	643 (409–963)	549 (354–829)	543 (348–803)	47·6 (30·3–71·3)	47·5 (30·3–71·8)	47·6 (30·6–70·5)
1-151	Acne vulgaris	565 (335–910)	583 (346–934)	548 (327–876)	41·8 (24·8–67·3)	46·5 (27·5–74·6)	48 (28·4–76·9)
1-152	Alopecia areata	84 (53–126)	103 (65–154)	106 (67–159)	7·1 (4·5–10·6)	7·1 (4·5–10·6)	7·1 (4·5–10·6)
1-153	Pruritus	93 (44–168)	123 (59–223)	137 (65–245)	8·4 (4–15)	8·8 (4·2–15·8)	8·8 (4·2–15·8)
1-154	Urticaria	607 (395–891)	602 (390–869)	606 (395–863)	48·1 (31·3–70·1)	48·1 (31·2–69·9)	48 (31·4–69·4)
1-155	Decubitus ulcer	196 (64–267)	244 (92–317)	290 (107–422)	26·7 (8·2–36·6)	18·7 (7–24·2)	16·7 (6·2–24·2)
1-156	Other skin and subcutaneous diseases	521 (267–919)	718 (367–1,280)	812 (411–1,450)	49·7 (25·6–87·5)	50·9 (26·1–90·7)	50·6 (25·6–89·9)
1-157	Sense organ diseases	7,200 (4,830–10,300)	10,600 (7,120–15,500)	12,800 (8,540–18,600)	788 (532–1,120)	749 (504–1,090)	736 (495–1,070)
1-158	Blindness and vision loss	2,070 (1,410–2,950)	2,950 (1,990–4,330)	3,520 (2,380–5,170)	232 (160–329)	210 (142–308)	203 (138–297)
1-159	Glaucoma	114 (76–166)	144 (98–204)	184 (122–259)	15·2 (10·2–22·2)	11·2 (7·5–15·8)	10·4 (7–14·6)
1-160	Cataract	323 (222–440)	438 (309–601)	552 (388–754)	41·7 (28·8–56·6)	33 (23·2–45·5)	31·4 (22·1–42·7)
1-161	Age-related macular degeneration	13 (8–19)	19 (13–28)	25 (16–36)	1·7 (1·1–2·4)	1·4 (1–2·1)	1·4 (0·9–2)
1-162	Refraction disorders	684 (447–994)	829 (547–1,200)	902 (597–1,300)	63·9 (41·6–92·7)	58·7 (38·7–84·9)	57·6 (38·3–83·7)
1-163	Near vision loss	431 (193–848)	833 (368–1,610)	1,020 (468–1,920)	50·9 (23–99·8)	56·2 (25·1–108)	55·3 (25·5–104)
1-164	Other vision loss	501 (348–697)	688 (487–950)	841 (596–1,170)	58·8 (41·2–81·3)	49·2 (34·9–67·8)	47 (33·3–65·1)
1-165	Age-related and other hearing loss	4,770 (3,150–7,030)	7,180 (4,690–10,700)	8,650 (5,650–12,700)	520 (346–760)	502 (334–736)	496 (328–723)
1-166	Other sense organ diseases	363 (215–560)	520 (302–805)	619 (370–969)	36·4 (21·5–56·7)	36·9 (21·6–56·8)	37 (22·4–57·5)

1-167	Musculoskeletal disorders	15,700 (11,500–20,400)	24,200 (17,600–31,900)	27,600 (20,000–36,100)	1,550 (1,130–2,030)	1,620 (1,180–2,120)	1,620 (1,180–2,120)
1-168	Rheumatoid arthritis	404 (305–526)	697 (519–911)	834 (612–1,120)	44·3 (33·8–58)	47 (35·1–61·3)	47·8 (35·1–64·1)
1-169	Osteoarthritis	1,640 (821–3,300)	3,040 (1,520–6,110)	3,890 (1,970–7,890)	194 (97·2–388)	205 (103–409)	208 (105–421)
1-170	Low back pain	7,670 (5,330–10,300)	10,800 (7,590–14,400)	11,900 (8,390–16,000)	734 (517–983)	728 (515–970)	720 (508–963)
1-171	Neck pain	1,360 (898–1,950)	2,050 (1,350–2,990)	2,290 (1,500–3,330)	135 (89·7–193)	135 (88·8–195)	134 (87·9–195)
1-172	Gout	64 (40–93)	118 (72–171)	144 (91–211)	6·9 (4·3–10)	7·9 (4·9–11·5)	8·1 (5·1–11·9)
1-173	Other musculoskeletal disorders	4,530 (3,280–6,010)	7,480 (5,340–10,000)	8,540 (6,040–11,500)	435 (315–580)	493 (353–657)	506 (361–681)
1-174	Other non-communicable diseases	28,000 (23,800–33,400)	28,400 (23,300–35,200)	29,000 (22,500–36,900)	2,500 (2,130–3,000)	2,270 (1,870–2,740)	2,200 (1,710–2,770)
1-175	Congenital anomalies	11,300 (9,670–13,300)	8,700 (6,750–10,500)	6,940 (4,800–9,380)	954 (811–1,120)	885 (681–1,080)	797 (542–1,090)
1-176	Neural tube defects	483 (341–699)	295 (224–391)	202 (132–298)	41·9 (29·3–60·9)	31·3 (23·5–42·1)	24·8 (15·6–37·3)
1-177	Congenital heart anomalies	5,930 (4,650–9,190)	4,430 (3,640–5,630)	3,330 (2,330–4,750)	496 (388–776)	456 (368–587)	392 (272–561)
1-178	Orofacial clefts	31 (19–46)	36 (22–54)	40 (24–60)	2·5 (1·6–3·8)	2·7 (1·7–4·1)	3 (1·8–4·6)
1-179	Down's syndrome	500 (410–619)	580 (470–678)	594 (410–816)	40·7 (33·6–50·6)	49·6 (40–59·3)	53·3 (37·2–74·2)
1-180	Turner syndrome	2 (1–3)	2 (1–3)	2 (1–3)	0·1 (0·1–0·2)	0·1 (0·1–0·2)	0·1 (0·1–0·2)
1-181	Klinefelter syndrome	0 (0–0)	0 (0–0)	0 (0–0)	0 (0–0)	0 (0–0)	0 (0–0)
1-182	Other chromosomal abnormalities	237 (125–326)	289 (113–396)	278 (116–413)	19·7 (10·2–27·4)	30·3 (11·2–42·2)	34·2 (13·1–52)
1-183	Congenital musculoskeletal and limb anomalies	543 (359–785)	593 (402–830)	578 (385–823)	43·1 (28·7–61·6)	49·6 (33·7–69·4)	49·6 (33·4–70·8)
1-184	Urogenital congenital anomalies	311 (91–506)	199 (62–306)	134 (48–222)	26·8 (7·6–44·1)	21·1 (6·2–32·9)	16·4 (5·4–28)
1-185	Digestive congenital anomalies	915 (542–1,170)	782 (376–1,060)	565 (265–866)	78·6 (46·8–101)	85·5 (40·9–116)	73·2 (34·4–113)
1-186	Other congenital anomalies	2,380 (743–3,490)	1,500 (519–2,230)	1,220 (404–2,000)	204 (62·2–300)	159 (51·5–238)	150 (45·2–251)
1-187	Urinary diseases and male infertility	982 (825–1,170)	2,010 (1,440–2,390)	2,440 (1,740–3,140)	107 (90·6–128)	142 (102–168)	139 (100–178)
1-188	Urinary tract infections and interstitial nephritis	518 (432–577)	1,050 (716–1,200)	1,310 (846–1,790)	56·5 (46·5–62·5)	75·2 (51·5–85·2)	75·4 (49·8–102)
1-189	Urolithiasis	100 (79–138)	305 (246–415)	332 (237–489)	11 (8·6–15·4)	20·1 (16·2–27·3)	18 (13–26·1)
1-190	Benign prostatic hyperplasia	212 (122–332)	363 (212–565)	499 (288–791)	25·2 (14·5–39·3)	26 (15·3–40·5)	25·9 (15·1–40·8)
1-191	Male infertility	46 (18–96)	75 (28–176)	74 (28–176)	3·6 (1·4–7·7)	5 (1·8–11·9)	5 (1·9–11·9)
1-192	Other urinary diseases	107 (73–141)	214 (89–290)	234 (97–344)	10·9 (7·1–15·1)	15·6 (6·5–21·1)	14·3 (6·3–20·7)
1-193	Gynecological diseases	4,320 (3,010–6,100)	5,470 (3,770–7,680)	5,590 (3,900–7,810)	377 (263–529)	361 (250–508)	359 (251–502)
1-194	Uterine fibroids	322 (195–533)	435 (246–760)	464 (253–809)	30·3 (18–50·4)	28·3 (16–49·3)	27·9 (15·3–48·5)
1-195	Polycystic ovarian syndrome	59 (24–121)	102 (42–209)	99 (40–201)	4·7 (1·9–9·6)	6·9 (2·9–14·1)	7·1 (2·9–14·4)
1-196	Female infertility	72 (30–149)	132 (49–311)	135 (46–313)	5·8 (2·4–11·9)	8·9 (3·2–21)	9 (3·2–21·3)
1-197	Endometriosis	404 (238–655)	427 (251–699)	381 (226–620)	32·6 (19·5–52·3)	27·8 (16·2–45·5)	26·4 (15·6–43·6)
1-198	Genital prolapse	70 (37–123)	86 (44–158)	98 (51–181)	7·7 (4·2–13·6)	5·7 (2·9–10·4)	5·4 (2·8–10)
1-199	Premenstrual syndrome	1,240 (760–1,950)	1,440 (887–2,260)	1,380 (833–2,150)	100 (61·6–156)	98·3 (60–153)	98·3 (59·8–152)
1-200	Other gynecological diseases	2,150 (1,390–3,080)	2,840 (1,850–4,060)	3,020 (1,930–4,360)	196 (127–278)	185 (120–265)	185 (119–265)
1-201	Hemoglobinopathies and hemolytic anaemias	2,330 (1,900–2,890)	1,940 (1,590–2,390)	1,800 (1,370–2,310)	191 (156–235)	143 (118–177)	134 (102–173)
1-202	Thalassemias	1 (1–2)	1 (0–2)	1 (0–1)	0·1 (0–0·1)	0·1 (0–0·2)	0·1 (0–0·1)
1-203	Thalassaemias trait	50 (30–80)	53 (32–82)	53 (32–82)	4 (2·4–6·4)	3·9 (2·3–6·1)	4 (2·4–6·2)
1-204	Sickle cell disorders	1,000 (883–1,120)	797 (705–901)	660 (467–894)	76·6 (67·6–85·7)	60·8 (53·8–68·8)	55 (39·1–74·1)
1-205	Sickle cell trait	664 (404–1,040)	477 (276–750)	411 (239–648)	51·5 (31·3–79·7)	34·8 (20·6–54·2)	31·8 (18·6–50)
1-206	G6PD deficiency	162 (111–218)	204 (149–262)	227 (151–315)	14·3 (9·9–18·9)	14 (10·2–17·9)	14·6 (9·7–20·4)
1-207	G6PD trait	2 (1–4)	4 (2–6)	4 (2–6)	0·2 (0·1–0·3)	0·3 (0·2–0·4)	0·3 (0·1–0·4)
1-208	Other hemoglobinopathies and hemolytic anaemias	453 (347–597)	402 (303–528)	448 (328–592)	44·4 (34·9–56·9)	29·5 (22·1–39·4)	28·8 (20·8–38·5)
1-209	Endocrine, metabolic, blood, and immune disorders	5,480 (4,300–6,330)	5,490 (4,410–7,540)	6,710 (5,050–9,220)	524 (411–609)	398 (320–564)	436 (326–623)
1-210	Oral disorders	3,300 (1,890–5,350)	4,680 (2,700–7,690)	5,420 (3,160–8,870)	333 (195–546)	320 (186–518)	318 (185–521)
1-211	Caries of deciduous teeth	41 (18–86)	22 (9–46)	21 (9–44)	2·9 (1·2–6)	2·3 (1–4·8)	2·3 (0·9–4·7)
1-212	Caries of permanent teeth	315 (141–611)	359 (163–694)	362 (162–698)	26·2 (11·8–50·8)	25·2 (11·4–48)	25 (11·4–48·5)
1-213	Periodontal disease	1,150 (441–2,460)	1,790 (688–3,760)	1,980 (773–4,140)	112 (42·9–237)	116 (44·7–246)	116 (45·1–245)
1-214	Edentulism	1,180 (750–1,760)	1,760 (1,110–2,650)	2,270 (1,450–3,480)	139 (88·8–208)	124 (78·3–187)	122 (77·8–185)
1-215	Other oral disorders	618 (382–913)	755 (471–1,100)	786 (491–1,140)	53·1 (32·8–78·1)	53·1 (33·1–77·4)	53 (33·1–77·4)
1-216	Sudden infant death syndrome	204 (124–316)	161 (109–237)	101 (60–160)	17·6 (10·7–27·2)	17·8 (12–26·1)	13·3 (7·9–21·1)
1-0	Injuries	46,700 (44,300–49,500)	65,100 (61,800–68,700)	50,700 (40,300–62,900)	3,920 (3,710–4,180)	4,480 (4,250–4,740)	3,530 (2,810–4,380)
1-1	Transport injuries	11,800 (11,100–12,700)	14,100 (13,100–15,100)	11,100 (8,770–14,000)	992 (930–1,060)	967 (902–1,040)	765 (600–960)
1-2	Road injuries	11,500 (10,800–12,300)	13,600 (12,700–14,600)	10,700 (8,440–13,400)	965 (905–1,030)	936 (871–1,000)	738 (579–926)

1-3	Pedestrian road injuries	3,920 (3,570–4,250)	2,580 (2,390–2,810)	2,030 (1,590–2,550)	336 (308–362)	183 (169–198)	134 (105–169)
1-4	Cyclist road injuries	801 (651–967)	907 (738–1,110)	868 (668–1,100)	70·6 (56·8–85·8)	62·1 (50·7–75·2)	55·9 (43·2–71)
1-5	Motorcyclist road injuries	638 (552–737)	1,370 (1,210–1,550)	1,080 (867–1,330)	54·1 (46·6–62·9)	91 (80·4–103)	72·4 (57·9–88·7)
1-6	Motor vehicle road injuries	6,090 (5,650–6,580)	8,640 (8,090–9,230)	6,620 (5,100–8,470)	496 (461–536)	591 (554–631)	467 (357–599)
1-7	Other road injuries	93 (66–124)	131 (94–173)	135 (96–180)	8·8 (6·2–11·7)	8·9 (6·5–11·7)	8·3 (6–11)
1-8	Other transport injuries	306 (267–370)	461 (413–509)	409 (316–532)	26·9 (23·6–32)	31·6 (28·3–35·1)	27·4 (21–35·7)
1-9	Unintentional injuries	16,900 (15,400–18,900)	14,200 (12,300–16,400)	13,700 (10,800–17,000)	1,460 (1,320–1,630)	1,040 (907–1,190)	946 (748–1,170)
1-10	Falls	2,820 (2,460–3,260)	3,320 (2,790–3,990)	3,540 (2,790–4,400)	280 (245–324)	233 (196–278)	222 (175–277)
1-11	Drowning	4,580 (4,270–4,970)	2,580 (2,350–2,810)	1,990 (1,470–2,610)	358 (335–386)	187 (171–204)	152 (113–200)
1-12	Fire, heat, and hot substances	2,510 (2,130–2,980)	1,950 (1,570–2,420)	1,890 (1,470–2,440)	213 (180–253)	146 (119–179)	133 (104–169)
1-13	Poisonings	508 (450–571)	172 (143–207)	170 (130–214)	40·9 (36·5–45·7)	12·8 (10·7–15·4)	12·4 (9·5–15·7)
1-14	Poisoning by carbon monoxide	133 (113–157)	45 (38–54)	45 (33–58)	10·3 (8·8–12·1)	3·3 (2·8–3·9)	3·3 (2·5–4·3)
1-15	Poisoning by other means	375 (329–424)	127 (102–157)	125 (93–158)	30·6 (26·9–34·3)	9·5 (7·7–11·7)	9·1 (6·8–11·5)
1-16	Exposure to mechanical forces	1,800 (1,480–2,220)	1,890 (1,470–2,480)	2,020 (1,500–2,690)	157 (127–197)	132 (102–172)	132 (98·5–174)
1-17	Unintentional firearm injuries	321 (262–445)	349 (275–415)	343 (264–425)	27·3 (22·2–37·7)	24·3 (19–28·7)	23 (17·8–28·5)
1-18	Other exposure to mechanical forces	1,480 (1,180–1,870)	1,540 (1,150–2,080)	1,670 (1,230–2,300)	130 (103–167)	107 (80·4–144)	109 (80–150)
1-19	Adverse effects of medical treatment	985 (859–1,090)	1,060 (874–1,160)	1,120 (803–1,500)	96·7 (84·4–106)	79·2 (65·2–86·9)	73·7 (53–98·8)
1-20	Animal contact	110 (78–151)	182 (146–224)	184 (139–232)	9·8 (6·9–13·4)	13 (10·4–15·9)	12·2 (9·2–15·3)
1-21	Venomous animal contact	77 (53–105)	129 (103–158)	130 (97–166)	6·8 (4·6–9·3)	9·1 (7·3–11·2)	8·7 (6·4–11·1)
1-22	Non-venomous animal contact	33 (22–49)	53 (41–70)	53 (39–74)	3 (2–4·5)	3·8 (3–5)	3·5 (2·6–4·8)
1-23	Foreign body	1,810 (1,580–2,070)	1,160 (1,020–1,320)	1,090 (825–1,390)	152 (133–174)	102 (87·5–117)	95·7 (71·1–125)
1-24	Pulmonary aspiration and foreign body in airway	1,650 (1,440–1,910)	985 (861–1,130)	903 (657–1,200)	138 (120–159)	89·1 (76·1–104)	83·1 (59·7–112)
1-25	Foreign body in eyes	63 (28–115)	78 (38–137)	82 (42–141)	5·5 (2·6–9·6)	5·5 (2·6–9·6)	5·5 (2·6–9·6)
1-26	Foreign body in other body part	96 (73–124)	96 (72–127)	104 (77–138)	8·3 (6·4–10·8)	7 (5·2–9·3)	7·1 (5·3–9·4)
1-27	Environmental heat and cold exposure	483 (343–656)	573 (404–787)	641 (450–887)	43·9 (31·3–59·5)	39·7 (28–54·4)	40·8 (28·7–56·5)
1-28	Exposure to forces of nature	0 (0–0)	254 (230–280)	6 (4–8)	0 (0–0)	19·2 (17·4–21·2)	0·4 (0·3–0·5)
1-29	Other unintentional injuries	1,290 (1,070–1,580)	1,040 (814–1,360)	1,060 (783–1,430)	108 (89·1–133)	74·3 (58·5–96·9)	72·2 (54·2–96·7)
1-30	Self-harm and interpersonal violence	17,900 (17,200–18,600)	36,800 (35,400–38,100)	25,800 (20,000–33,300)	1,470 (1,410–1,530)	2,480 (2,390–2,570)	1,820 (1,410–2,340)
1-31	Self-harm	8,980 (8,460–9,490)	8,420 (7,870–8,970)	7,290 (5,450–9,590)	742 (700–782)	562 (526–597)	493 (369–645)
1-32	Self-harm by firearm	133 (105–230)	139 (112–244)	104 (74–170)	11·4 (9·1–19·1)	9·1 (7·4–16)	6·7 (4·7–11·1)
1-33	Self-harm by other specified means	8,850 (8,320–9,360)	8,280 (7,740–8,830)	7,190 (5,360–9,470)	730 (689–770)	553 (517–588)	486 (364–637)
1-34	Interpersonal violence	6,640 (6,210–7,110)	26,700 (25,500–27,800)	17,500 (13,600–22,600)	538 (504–575)	1,810 (1,730–1,880)	1,250 (971–1,620)
1-35	Physical violence by firearm	3,400 (3,130–3,680)	19,300 (18,400–20,200)	12,100 (9,240–15,600)	268 (246–289)	1,290 (1,230–1,350)	878 (672–1,130)
1-36	Physical violence by sharp object	1,530 (1,390–1,680)	3,950 (3,710–4,200)	2,760 (1,970–3,760)	126 (116–138)	268 (251–285)	192 (136–264)
1-37	Sexual violence	217 (142–306)	256 (169–364)	251 (167–356)	17·7 (11·7–25·1)	17·6 (11·7–25·1)	17·6 (11·6–25)
1-38	Physical violence by other means	1,500 (1,390–1,630)	3,190 (2,980–3,400)	2,390 (1,830–3,110)	127 (117–138)	229 (214–246)	167 (127–216)
1-39	Conflict and terrorism	2,270 (2,060–2,520)	116 (79–173)	96 (65–140)	187 (170–208)	7·6 (5·1–11·2)	5·3 (3·6–7·8)
1-40	Executions and police conflict	17 (13–21)	1,570 (1,200–2,060)	965 (408–1,690)	1·3 (1–1·6)	104 (79–135)	70·7 (30–127)

SR Table 1: All-age DALYs counts and age standardized DALYs rates per 100,000 individuals for all causes and level 1 cause groups by location for 1990, 2010, and 2019, both sexes combined, Laos

Cause Level	Cause	All-age DALYs			Age-standardized rate of DALYs per 100,000		
		1990	2010	2019	1990	2010	2019
0-0	All causes	3,910,000 (3,550,000–4,300,000)	2,690,000 (2,340,000–3,060,000)	2,370,000 (1,970,000–2,820,000)	83,900 (74,500–93,500)	49,200 (43,100–55,800)	39,400 (33,100–46,400)
1-0	Communicable, maternal, neonatal, and nutritional disorders	2,620,000 (2,300,000–2,990,000)	1,220,000 (1,030,000–1,430,000)	794,000 (628,000–988,000)	44,100 (38,700–50,000)	17,800 (15,000–20,900)	11,000 (8,800–13,700)
1-1	HIV/AIDS and sexually transmitted infections	4,270 (1,640–8,910)	27,200 (4,420–126,000)	25,000 (4,220–139,000)	65·1 (30·1–124)	446 (69–2,260)	348 (57·8–1,970)
1-2	HIV/AIDS	3 (0–17)	22,900 (1,640–123,000)	21,200 (1,480–135,000)	0 (0–0·3)	388 (28·1–2,220)	298 (21·3–1,930)
1-3	HIV/AIDS - drug-susceptible tuberculosis	0 (0–0)	9,070 (1,240–43,700)	7,190 (1,130–31,700)	0 (0–0)	153 (22–747)	101 (16·2–448)
1-4	HIV/AIDS - Multidrug-resistant Tuberculosis without extensive drug resistance	0 (0–0)	219 (4–1,600)	87 (2–559)	0 (0–0)	3·7 (0·1–28·1)	1·2 (0–7·9)
1-5	HIV/AIDS - Extensively drug-resistant Tuberculosis	0 (0–0)	26 (0–214)	16 (0–106)	0 (0–0)	0·4 (0–3·8)	0·2 (0–1·5)
1-6	HIV/AIDS resulting in other diseases	3 (0–17)	13,500 (203–80,700)	13,900 (176–102,000)	0 (0–0·3)	230 (3·4–1,470)	196 (2·4–1,480)
1-7	Sexually transmitted infections excluding HIV	4,270 (1,630–8,910)	4,310 (1,820–8,760)	3,890 (1,760–7,930)	65·1 (30·1–124)	58·3 (26·9–114)	49·7 (23·3–99·8)
1-8	Syphilis	3,750 (1,120–8,400)	3,450 (1,030–7,740)	2,880 (840–6,740)	49·2 (15·2–108)	43·1 (13·1–96·1)	35·5 (10·5–82·4)
1-9	Chlamydial infection	74 (48–109)	128 (84–193)	151 (95–228)	2·2 (1·4–3·2)	2·1 (1·4–3·2)	2 (1·3–3)
1-10	Gonococcal infection	73 (44–117)	125 (82–178)	129 (88–183)	2·2 (1·3–3·6)	2·1 (1·4–2·9)	1·8 (1·3–2·5)
1-11	Trichomoniasis	115 (45–239)	192 (75–387)	231 (88–468)	3·7 (1·4–7·6)	3·5 (1·3–7·1)	3·3 (1·3–6·8)
1-12	Genital herpes	110 (36–278)	190 (61–470)	241 (79–604)	3·7 (1·2–9·4)	3·7 (1·2–9·2)	3·7 (1·2–9·4)
1-13	Other sexually transmitted infections	145 (100–204)	229 (160–317)	249 (175–346)	4·2 (3–5·9)	3·8 (2·7–5·2)	3·4 (2·4–4·7)
1-14	Respiratory infections and tuberculosis	1,020,000 (813,000–1,310,000)	457,000 (364,000–578,000)	270,000 (202,000–347,000)	18,500 (15,100–23,100)	7,280 (5,910–8,920)	4,160 (3,220–5,260)
1-15	Tuberculosis	225,000 (172,000–283,000)	124,000 (94,100–154,000)	81,500 (58,100–105,000)	6,520 (4,910–8,110)	2,510 (1,890–3,090)	1,390 (1,010–1,780)
1-16	Latent tuberculosis infection	0 (0–0)	0 (0–0)	0 (0–0)	0 (0–0)	0 (0–0)	0 (0–0)
1-17	Drug-susceptible tuberculosis	224,000 (172,000–282,000)	121,000 (91,000–151,000)	80,500 (57,500–104,000)	6,500 (4,910–8,070)	2,450 (1,840–3,030)	1,370 (998–1,750)
1-18	Multidrug-resistant tuberculosis without extensive drug resistance	867 (54–4,060)	2,390 (223–9,440)	808 (82–3,310)	25 (1·5–114)	48·2 (4·5–189)	13·9 (1·4–55·6)
1-19	Extensively drug-resistant tuberculosis	0 (0–0)	294 (27–1,170)	152 (14–647)	0 (0–0)	5·9 (0·6–23·3)	2·6 (0·2–11·3)
1-20	Lower respiratory infections	791,000 (607,000–1,050,000)	325,000 (246,000–433,000)	180,000 (130,000–240,000)	11,900 (9,310–15,400)	4,650 (3,640–6,040)	2,660 (1,970–3,450)
1-21	Upper respiratory infections	4,980 (2,640–7,900)	6,010 (3,660–9,350)	6,320 (3,840–9,940)	104 (57·9–160)	89 (54·8–139)	85·2 (52–132)
1-22	Otitis media	3,560 (1,300–10,500)	2,490 (1,450–4,020)	2,350 (1,370–3,920)	62 (27·3–152)	36 (21–58·3)	31·4 (18·3–52)
1-23	Enteric infections	365,000 (241,000–513,000)	131,000 (78,200–203,000)	72,100 (43,500–114,000)	6,680 (4,210–9,510)	2,020 (1,300–2,950)	1,090 (685–1,640)
1-24	Diarrhoeal diseases	347,000 (224,000–498,000)	113,000 (65,000–185,000)	60,300 (34,000–102,000)	6,350 (3,870–9,190)	1,800 (1,100–2,690)	938 (563–1,490)
1-25	Typhoid and paratyphoid	15,800 (6,800–29,500)	15,200 (6,750–28,000)	10,600 (4,780–20,100)	290 (127–538)	194 (87·5–354)	133 (60·6–253)
1-26	Typhoid fever	15,000 (6,460–28,200)	15,000 (6,640–27,600)	9,500 (4,220–18,300)	274 (120–518)	191 (85·6–350)	119 (53·2–228)
1-27	Paratyphoid fever	832 (309–1,900)	244 (89–546)	1,070 (412–2,310)	14·9 (5·6–33·4)	3·1 (1·1–6·8)	13·4 (5·2–29·1)
1-28	iNTS	1,710 (910–2,910)	1,570 (856–2,780)	1,090 (536–1,930)	31·5 (17–53)	21·7 (12·1–38·1)	14·6 (7·3–25·5)
1-29	Other intestinal infectious diseases	550 (269–885)	204 (69–375)	106 (38–209)	8·4 (4·3–13·2)	2·7 (1–4·8)	1·4 (0·5–2·6)
1-30	Neglected tropical diseases and malaria	37,600 (26,000–50,400)	38,800 (27,500–53,500)	20,600 (14,900–28,400)	964 (676–1,300)	621 (444–844)	297 (216–406)
1-31	Malaria	7,860 (3,520–16,500)	11,200 (4,800–21,700)	1,730 (662–3,980)	167 (74·6–352)	166 (71·4–323)	23·2 (8·8–53·2)
1-32	Chagas disease	0 (0–0)	0 (0–0)	0 (0–0)	0 (0–0)	0 (0–0)	0 (0–0)
1-33	Leishmaniasis	0 (0–0)	0 (0–0)	0 (0–0)	0 (0–0)	0 (0–0)	0 (0–0)
1-34	Visceral leishmaniasis	0 (0–0)	0 (0–0)	0 (0–0)	0 (0–0)	0 (0–0)	0 (0–0)
1-35	Cutaneous and mucocutaneous leishmaniasis	0 (0–0)	0 (0–0)	0 (0–0)	0 (0–0)	0 (0–0)	0 (0–0)
1-36	African trypanosomiasis	0 (0–0)	0 (0–0)	0 (0–0)	0 (0–0)	0 (0–0)	0 (0–0)
1-37	Schistosomiasis	919 (557–1,440)	561 (330–864)	416 (214–686)	31·9 (19·2–50)	12·3 (7·2–18·8)	7·5 (4–12·3)
1-38	Cysticercosis	909 (495–1,450)	923 (503–1,520)	984 (544–1,650)	37·2 (20·7–58·5)	24 (13·3–38·5)	19·9 (11·2–33)
1-39	Cystic echinococcosis	144 (67–242)	47 (24–74)	29 (17–45)	3·3 (1·8–5·2)	0·8 (0·5–1·3)	0·4 (0·3–0·7)
1-40	Lymphatic filariasis	2,970 (1,720–4,820)	2,000 (1,080–3,610)	2,100 (1,120–3,870)	87·8 (51·7–139)	32·8 (17·8–59)	29·7 (16–54·8)
1-41	Onchocerciasis	0 (0–0)	0 (0–0)	0 (0–0)	0 (0–0)	0 (0–0)	0 (0–0)
1-42	Trachoma	356 (202–563)	90 (52–148)	48 (26–80)	21·9 (12·7–35)	3·3 (1·8–5·4)	1·3 (0·7–2·3)
1-43	Dengue	1,990 (458–5,570)	2,350 (1,060–4,540)	1,960 (1,030–3,400)	35 (10·5–88·9)	33·6 (15·6–63·9)	26·2 (13·9–45·2)
1-44	Yellow fever	0 (0–0)	0 (0–0)	0 (0–0)	0 (0–0)	0 (0–0)	0 (0–0)
1-45	Rabies	4,100 (79–9,180)	2,320 (58–4,960)	1,160 (34–2,250)	79·4 (1·7–165)	33·3 (0·9–67·6)	16·1 (0·5–30·3)
1-46	Intestinal nematode infections	7,660 (4,900–11,500)	12,300 (7,600–19,100)	7,190 (4,310–11,400)	162 (103–246)	176 (109–274)	94·1 (56·3–151)
1-47	Ascariasis	1,960 (1,280–2,890)	707 (402–1,150)	529 (294–931)	36 (23·3–54·3)	10·2 (5·9–16·8)	7·1 (4–12·5)
1-48	Trichuriasis	1,240 (637–2,290)	855 (417–1,570)	1,030 (479–1,870)	25·3 (12·9–46·6)	12·1 (5·9–22·2)	13·2 (6·1–23·9)

1-49	Hookworm disease	4,450 (2,590–6,880)	10,700 (6,530–16,900)	5,630 (3,310–9,110)	101 (59·7–157)	154 (94·6–244)	73·8 (43·3–120)
1-50	Food-borne trematodiases	8,460 (4,490–14,000)	5,180 (2,760–8,660)	3,170 (1,640–5,190)	293 (153–474)	110 (56·3–181)	53·6 (27·4–87·9)
1-51	Leprosy	132 (82–201)	43 (26–65)	41 (24–64)	4·8 (3–7·2)	1 (0·6–1·4)	0·7 (0·4–1·1)
1-52	Ebola virus disease	0 (0–0)	0 (0–0)	0 (0–0)	0 (0–0)	0 (0–0)	0 (0–0)
1-53	Zika virus disease	0 (0–0)	0 (0–0)	0 (0–0)	0 (0–0)	0 (0–0)	0 (0–0)
1-54	Guinea worm disease	0 (0–0)	0 (0–0)	0 (0–0)	0 (0–0)	0 (0–0)	0 (0–0)
1-55	Other neglected tropical diseases	2,060 (1,330–3,010)	1,860 (1,200–2,730)	1,780 (1,130–2,650)	40·2 (26·5–57·6)	27·4 (18·1–39·6)	24·5 (15·8–36·3)
1-56	Other infectious diseases	416,000 (266,000–621,000)	108,000 (66,200–181,000)	65,200 (36,800–112,000)	6,470 (4,320–9,440)	1,440 (905–2,360)	840 (486–1,420)
1-57	Meningitis	56,200 (38,200–81,900)	28,500 (19,500–39,900)	12,400 (8,110–18,400)	850 (597–1,210)	369 (257–513)	158 (104–232)
1-58	Encephalitis	6,650 (3,610–13,600)	5,800 (3,810–9,140)	4,890 (3,330–7,270)	113 (69·5–219)	80·6 (54·5–123)	64·8 (44·6–95·9)
1-59	Diphtheria	11,900 (4,810–24,200)	539 (245–1,050)	162 (80–307)	182 (79·4–360)	7 (3·3–13·3)	2·1 (1·1–3·9)
1-60	Whooping cough	61,200 (6,020–196,000)	35,400 (3,870–101,000)	23,300 (2,830–66,100)	867 (85·6–2,790)	445 (48·7–1,270)	288 (35–816)
1-61	Tetanus	116,000 (49,000–205,000)	12,000 (5,650–20,200)	4,070 (2,110–7,100)	1,840 (945–2,990)	180 (98·4–287)	58·5 (31·2–97·7)
1-62	Measles	142,000 (45,700–314,000)	16,700 (5,500–38,100)	13,100 (4,230–29,700)	2,060 (666–4,570)	210 (69·3–481)	163 (52·7–370)
1-63	Varicella and herpes zoster	2,400 (1,330–3,970)	1,670 (1,030–2,650)	1,240 (759–1,890)	48·7 (32·1–72·6)	27 (18·4–40)	18·8 (12·4–26·8)
1-64	Acute hepatitis	16,000 (6,270–31,500)	3,250 (1,920–5,120)	2,130 (1,440–3,110)	415 (167–810)	57·4 (33·1–88·6)	32·1 (21·5–46·9)
1-65	Acute hepatitis A	10,000 (2,540–23,400)	1,600 (809–2,800)	882 (476–1,420)	236 (61·1–528)	26·4 (12·1–47·5)	12·8 (6·6–20·7)
1-66	Acute hepatitis B	4,310 (1,590–8,810)	1,280 (716–2,020)	993 (648–1,460)	132 (46·2–276)	24·2 (13·3–38·7)	15·4 (10–22·8)
1-67	Acute hepatitis C	1,440 (436–3,000)	288 (110–554)	184 (84–344)	41·8 (12·2–86·9)	5·4 (2–10·4)	2·9 (1·3–5·3)
1-68	Acute hepatitis E	236 (71–625)	88 (48–155)	72 (42–118)	5·3 (1·8–12·5)	1·4 (0·7–2·4)	1 (0·6–1·6)
1-69	Other unspecified infectious diseases	4,310 (2,420–9,320)	4,250 (2,710–7,470)	3,890 (2,610–6,420)	92·3 (52·8–204)	66·4 (42·4–118)	54·4 (36·4–91·9)
1-70	Maternal and neonatal disorders	635,000 (510,000–774,000)	411,000 (345,000–481,000)	301,000 (239,000–368,000)	8,900 (7,290–10,800)	5,160 (4,330–6,030)	3,680 (2,930–4,510)
1-71	Maternal disorders	58,100 (43,500–77,900)	27,700 (19,900–36,100)	19,400 (13,500–25,600)	1,530 (1,140–2,080)	412 (296–534)	241 (168–318)
1-72	Maternal haemorrhage	19,200 (13,700–26,300)	9,540 (6,740–12,600)	5,880 (3,960–7,970)	518 (369–711)	145 (101–190)	73·8 (50·1–99·7)
1-73	Maternal sepsis and other pregnancy related infections	6,810 (4,770–9,770)	1,960 (1,350–2,760)	696 (454–939)	173 (121–248)	28·2 (19·6–39·3)	8·5 (5·6–11·4)
1-74	Maternal hypertensive disorders	1,770 (1,330–2,390)	1,250 (899–1,610)	921 (644–1,190)	45·8 (34·3–61·4)	18·4 (13·4–23·7)	11·5 (8·1–14·9)
1-75	Maternal obstructed labour and uterine rupture	1,480 (1,000–2,120)	1,580 (1,040–2,270)	1,640 (1,030–2,390)	40·3 (27·4–57·2)	23·7 (15·9–33·8)	20·5 (13·1–29·6)
1-76	Maternal abortive outcome	16,700 (11,900–23,700)	3,490 (2,360–5,050)	1,030 (649–1,490)	446 (317–624)	53 (36–76·4)	13·1 (8·4–18·9)
1-77	Ectopic pregnancy	105 (75–151)	107 (74–141)	85 (56–115)	2·8 (2–4·1)	1·6 (1·1–2·1)	1·1 (0·7–1·5)
1-78	Indirect maternal deaths	8,370 (6,000–11,600)	6,520 (4,490–8,960)	6,620 (4,350–9,150)	216 (154–301)	94·9 (65·3–130)	81·2 (53·1–112)
1-79	Late maternal deaths	437 (301–619)	326 (215–483)	298 (189–438)	11·4 (7·9–16·3)	4·8 (3·2–7)	3·7 (2·4–5·4)
1-80	Maternal deaths aggravated by HIV/AIDS	0 (0–0)	16 (0–81)	12 (0–52)	0 (0–0)	0·2 (0–1·2)	0·2 (0–0·6)
1-81	Other maternal disorders	3,160 (2,210–4,570)	2,900 (1,990–4,060)	2,260 (1,510–3,170)	81·5 (56·7–120)	42·7 (29·2–59·8)	28 (18·7–38·9)
1-82	Neonatal disorders	577,000 (446,000–708,000)	384,000 (319,000–449,000)	281,000 (224,000–346,000)	7,360 (5,670–9,030)	4,740 (3,940–5,550)	3,440 (2,740–4,220)
1-83	Neonatal preterm birth	320,000 (253,000–397,000)	199,000 (159,000–242,000)	139,000 (105,000–177,000)	4,090 (3,240–5,080)	2,460 (1,970–2,990)	1,700 (1,280–2,160)
1-84	Neonatal encephalopathy due to birth asphyxia and trauma	154,000 (87,200–220,000)	104,000 (69,900–142,000)	74,500 (50,200–103,000)	1,960 (1,100–2,800)	1,290 (863–1,750)	909 (614–1,260)
1-85	Neonatal sepsis and other neonatal infections	43,100 (22,200–71,900)	42,300 (27,200–63,600)	40,400 (25,900–59,200)	551 (285–917)	524 (338–787)	498 (321–728)
1-86	Hemolytic disease and other neonatal jaundice	8,490 (5,430–12,400)	4,360 (3,050–5,870)	2,860 (2,060–4,020)	112 (72·6–161)	55·2 (38·6–74)	35·6 (25·7–49·7)
1-87	Other neonatal disorders	50,500 (19,800–72,900)	34,100 (19,800–49,700)	24,900 (14,500–37,700)	647 (252–934)	421 (245–614)	304 (177–460)
1-88	Nutritional deficiencies	132,000 (72,900–222,000)	50,000 (35,700–67,900)	40,000 (28,400–54,900)	2,430 (1,450–3,800)	808 (593–1,070)	600 (431–816)
1-89	Protein-energy malnutrition	100,000 (44,100–187,000)	21,200 (13,700–32,500)	11,400 (7,860–15,500)	1,670 (759–3,010)	327 (223–478)	176 (127–234)
1-90	Iodine deficiency	894 (588–1,290)	282 (126–548)	562 (322–922)	26 (17·5–37·2)	4·9 (2·3–9·5)	8·1 (4·8–13·2)
1-91	Vitamin A deficiency	3,210 (2,060–4,730)	1,980 (1,200–3,030)	1,220 (714–1,930)	49 (31·4–71·9)	25 (15·3–38·6)	15·4 (9·1–24·5)
1-92	Dietary iron deficiency	24,600 (15,700–36,800)	24,700 (15,800–36,800)	25,300 (16,000–38,000)	591 (384–864)	415 (269–612)	373 (237–558)
1-93	Other nutritional deficiencies	3,240 (1,670–5,910)	1,810 (1,250–2,700)	1,570 (1,130–2,160)	88·5 (46·9–145)	35·9 (26–51·2)	27·2 (20·3–36·6)
1-0	Non-communicable diseases	993,000 (757,000–1,230,000)	1,210,000 (1,030,000–1,410,000)	1,360,000 (1,130,000–1,610,000)	32,900 (27,300–38,500)	27,300 (23,700–31,100)	25,300 (21,400–29,400)
1-1	Neoplasms	109,000 (73,600–143,000)	129,000 (99,400–160,000)	156,000 (118,000–197,000)	3,950 (2,860–5,090)	3,080 (2,400–3,750)	2,990 (2,300–3,710)
1-2	Lip and oral cavity cancer	1,730 (1,140–2,390)	2,090 (1,550–2,640)	2,700 (2,010–3,540)	72·9 (48·9–100)	55·5 (41·3–68·9)	55·1 (41·1–71·8)
1-3	Nasopharynx cancer	2,090 (1,400–2,850)	2,260 (1,640–2,920)	2,590 (1,800–3,480)	79·8 (54·8–108)	52·8 (38–68·3)	47·1 (33·4–62·5)
1-4	Other pharynx cancer	482 (342–680)	510 (379–673)	679 (481–916)	20·4 (14·7–28·5)	13·7 (10·2–18)	13·7 (9·8–18·3)
1-5	Oesophageal cancer	2,440 (1,570–3,330)	2,400 (1,640–3,400)	2,920 (1,950–4,190)	106 (69·9–142)	65·6 (45·4–94)	60·5 (41·4–88)
1-6	Stomach cancer	10,300 (7,650–13,000)	8,600 (6,520–10,600)	9,260 (6,890–11,900)	438 (330–548)	230 (175–280)	191 (144–242)
1-7	Colon and rectum cancer	6,130 (4,010–8,310)	10,700 (7,870–13,600)	15,400 (10,900–20,800)	262 (175–353)	285 (212–358)	315 (224–420)

1-8	Liver cancer	6,820 (4,540–9,690)	7,570 (5,520–9,880)	9,130 (6,560–12,000)	282 (192–392)	195 (142–252)	180 (132–234)
1-9	Liver cancer due to hepatitis B	3,330 (2,080–5,020)	3,780 (2,610–5,140)	4,320 (2,890–6,070)	132 (82·2–196)	89·8 (61·5–123)	78·3 (52·6–111)
1-10	Liver cancer due to hepatitis C	1,390 (760–2,190)	1,400 (870–2,100)	1,740 (1,070–2,520)	65·6 (37·6–102)	42·9 (27·2–62·6)	40·1 (25·2–57·2)
1-11	Liver cancer due to alcohol use	1,230 (720–2,010)	1,430 (858–2,190)	1,970 (1,190–3,040)	54·2 (31·7–87·9)	40·3 (24·1–60·5)	41·3 (25·5–62·7)
1-12	Liver cancer due to NASH	411 (234–675)	492 (309–728)	630 (391–936)	18 (10·4–29·4)	13·5 (8·5–20·2)	13·2 (8·2–19·5)
1-13	Gallbladder and biliary tract cancer	954 (538–1,520)	958 (650–1,620)	1,200 (863–2,040)	42·8 (25·1–68·6)	27·5 (18·9–47·4)	26·2 (19·2–45·2)
1-14	Pancreatic cancer	1,370 (939–1,970)	2,500 (2,000–3,080)	3,830 (2,900–4,970)	60·1 (42–85·1)	69·8 (56·3–85·2)	81 (62·3–105)
1-15	Larynx cancer	1,300 (942–1,720)	1,120 (823–1,480)	1,370 (960–1,870)	56·5 (41·6–74·8)	31·7 (23·4–41·9)	29·2 (20·6–39·7)
1-16	Tracheal, bronchus, and lung cancer	15,300 (10,300–21,600)	19,700 (15,000–25,700)	26,500 (19,100–35,100)	675 (466–944)	559 (428–720)	566 (418–737)
1-17	Malignant skin melanoma	283 (138–587)	372 (233–616)	456 (294–706)	10·6 (5·4–20·9)	8·7 (5·5–13·8)	8·4 (5·6–12·6)
1-18	Non-melanoma skin cancer	347 (250–496)	573 (444–728)	756 (556–964)	15·3 (11·5–21·1)	15·8 (12·1–19·7)	15·9 (11·9–20·1)
1-19	Non-melanoma skin cancer (squamous-cell carcinoma)	347 (250–496)	573 (444–728)	755 (556–964)	15·3 (11·5–21·1)	15·8 (12·1–19·7)	15·9 (11·9–20·1)
1-20	Non-melanoma skin cancer (basal-cell carcinoma)	0 (0–0)	0 (0–0)	0 (0–0)	0 (0–0)	0 (0–0)	0 (0–0)
1-21	Breast cancer	9,020 (5,700–13,500)	14,200 (9,750–21,400)	18,600 (13,000–26,900)	358 (232–533)	335 (237–497)	334 (239–478)
1-22	Cervical cancer	7,700 (3,870–11,500)	7,610 (4,290–11,400)	8,380 (5,150–12,500)	299 (157–452)	174 (98·5–265)	147 (91·4–222)
1-23	Uterine cancer	1,340 (746–2,060)	1,750 (963–2,470)	2,180 (1,240–3,040)	56·6 (32·2–85·8)	45·4 (27–62·8)	42·7 (25·8–58·9)
1-24	Ovarian cancer	1,370 (668–4,370)	3,150 (1,950–5,680)	4,550 (2,890–7,330)	53·5 (26·3–162)	73 (46·8–129)	81·2 (53–130)
1-25	Prostate cancer	1,740 (1,220–2,250)	2,790 (2,020–3,510)	3,920 (2,800–4,940)	94·9 (66·8–122)	101 (73·5–125)	108 (77·6–134)
1-26	Testicular cancer	135 (86–197)	245 (170–327)	313 (211–412)	3·7 (2·4–5·2)	4·1 (2·9–5·4)	4·4 (3–5·7)
1-27	Kidney cancer	854 (505–1,270)	1,310 (986–1,740)	1,670 (1,210–2,320)	25·8 (17·1–36·6)	29 (22·5–37·8)	31·1 (23–42·7)
1-28	Bladder cancer	971 (676–1,300)	1,160 (837–1,480)	1,540 (1,100–1,980)	46·6 (32·3–62)	35·7 (25·7–45·2)	36·3 (26·4–45·4)
1-29	Brain and nervous system cancer	4,870 (2,000–10,800)	5,730 (3,340–8,820)	6,170 (3,910–8,640)	119 (57·4–238)	100 (60·7–150)	94·8 (60·6–132)
1-30	Thyroid cancer	871 (515–1,280)	1,120 (710–1,470)	1,370 (882–1,840)	35·9 (21·7–51·6)	29·4 (18·9–38·2)	27·7 (18·2–36·5)
1-31	Mesothelioma	119 (56–233)	197 (122–310)	244 (150–364)	4·3 (2·2–8·1)	4·3 (2·8–6·3)	4·1 (2·6–5·9)
1-32	Hodgkin lymphoma	742 (528–1,100)	715 (436–1,280)	704 (389–1,340)	21·9 (15·7–32·2)	13 (8–23)	10·7 (6·1–20)
1-33	Non-Hodgkin's lymphoma	3,080 (1,560–4,790)	4,030 (2,770–5,330)	4,820 (3,300–6,660)	89·7 (52·4–134)	80·8 (57·8–104)	82 (58–111)
1-34	Multiple myeloma	539 (311–1,030)	692 (482–1,090)	932 (640–1,380)	23·8 (14·1–44·8)	19·5 (13·6–30·5)	19·7 (13·8–29·3)
1-35	Leukaemia	18,700 (8,060–31,900)	16,300 (10,600–22,400)	14,300 (9,960–18,900)	406 (205–657)	264 (179–350)	214 (153–281)
1-36	Acute lymphoid leukaemia	3,070 (931–8,130)	3,420 (1,860–6,100)	2,790 (1,680–4,420)	54·9 (18·5–141)	46·2 (25·8–81·8)	37 (22·8–58·3)
1-37	Chronic lymphoid leukaemia	84 (50–126)	127 (89–171)	179 (125–251)	3·7 (2·3–5·5)	3·5 (2·6–4·7)	3·8 (2·7–5·2)
1-38	Acute myeloid leukaemia	1,280 (561–3,340)	1,950 (1,200–3,530)	2,260 (1,400–3,890)	30·4 (15·6–69·9)	32·2 (20·6–56·1)	33·5 (21–57·3)
1-39	Chronic myeloid leukaemia	861 (257–1,760)	670 (351–989)	661 (389–980)	23·3 (9·1–41·9)	12·8 (7·3–18·3)	11 (6·8–16·1)
1-40	Other leukaemia	13,400 (3,900–25,300)	10,200 (5,550–15,100)	8,380 (5,330–11,600)	293 (112–514)	169 (97·8–242)	129 (83·3–175)
1-41	Other neoplasms	150 (107–257)	239 (188–348)	336 (249–473)	5·8 (4·2–9·5)	6·2 (4·9–9·1)	7·1 (5·4–10)
1-42	Myelodysplastic, myeloproliferative, and other hematopoietic neoplasms	150 (107–257)	239 (188–348)	336 (249–473)	5·8 (4·2–9·5)	6·2 (4·9–9·1)	7·1 (5·4–10)
1-43	Benign and in situ intestinal neoplasms	0 (0–0)	0 (0–0)	0 (0–0)	0 (0–0)	0 (0–0)	0 (0–0)
1-44	Benign and in situ cervical and uterine neoplasms	0 (0–0)	0 (0–0)	0 (0–0)	0 (0–0)	0 (0–0)	0 (0–0)
1-45	Other benign and in situ neoplasms	0 (0–0)	0 (0–0)	0 (0–0)	0 (0–0)	0 (0–0)	0 (0–0)
1-46	Cardiovascular diseases	214,000 (174,000–259,000)	306,000 (260,000–358,000)	353,000 (286,000–431,000)	9,670 (7,920–11,700)	8,650 (7,410–10,100)	7,860 (6,480–9,350)
1-47	Rheumatic heart disease	11,500 (8,030–15,300)	14,000 (10,500–17,500)	11,700 (8,390–15,400)	330 (236–436)	235 (178–291)	168 (122–220)
1-48	Ischaemic heart disease	76,500 (60,200–98,300)	113,000 (93,400–137,000)	137,000 (107,000–169,000)	3,630 (2,890–4,600)	3,320 (2,780–4,010)	3,110 (2,520–3,750)
1-49	Stroke	102,000 (82,800–125,000)	144,000 (120,000–173,000)	165,000 (133,000–208,000)	4,590 (3,730–5,630)	4,120 (3,490–5,010)	3,690 (3,010–4,560)
1-50	Ischaemic stroke	23,800 (19,100–29,200)	37,000 (29,900–47,700)	46,800 (37,700–58,700)	1,300 (1,030–1,610)	1,290 (1,050–1,680)	1,270 (1,030–1,570)
1-51	Intracerebral hemorrhage	70,500 (56,400–86,600)	96,900 (79,300–117,000)	108,000 (84,600–137,000)	3,010 (2,400–3,710)	2,600 (2,150–3,100)	2,230 (1,780–2,800)
1-52	Subarachnoid hemorrhage	7,310 (3,400–12,300)	10,100 (6,670–17,200)	10,800 (7,300–18,200)	280 (138–492)	230 (151–404)	194 (132–332)
1-53	Hypertensive heart disease	15,500 (7,970–23,600)	19,400 (10,500–27,300)	22,200 (11,800–31,400)	770 (411–1,170)	604 (337–844)	531 (292–744)
1-54	Non-rheumatic valvular heart disease	242 (147–386)	386 (286–565)	494 (365–718)	11 (7·1–16·6)	10·4 (8–14·7)	10·3 (7·9–14·8)
1-55	Non-rheumatic calcific aortic valve disease	115 (61–217)	195 (139–305)	261 (185–387)	5·2 (3–9·7)	5·3 (3·9–8·2)	5·5 (4–8)
1-56	Non-rheumatic degenerative mitral valve disease	98 (55–170)	144 (93–241)	180 (117–311)	4·6 (2·8–7·3)	4 (2·7–6·3)	3·9 (2·7–6·4)
1-57	Other non-rheumatic valve diseases	29 (9–60)	47 (14–85)	53 (14–96)	1·2 (0·3–2·3)	1·1 (0·3–2)	1 (0·2–1·8)
1-58	Cardiomyopathy and myocarditis	2,750 (1,350–7,700)	4,600 (2,870–8,540)	5,180 (3,430–8,280)	92·8 (55·5–199)	104 (72·2–167)	98·5 (70·2–146)
1-59	Myocarditis	613 (163–2,190)	932 (469–2,270)	856 (470–1,630)	13 (5·4–35·5)	15·7 (9·1–31·7)	13·4 (8·4–23·1)
1-60	Alcoholic cardiomyopathy	213 (72–435)	246 (135–432)	290 (164–519)	8·3 (2·9–16·9)	5·5 (3·1–9·3)	4·9 (2·9–8·8)

1-61	Other cardiomyopathy	1,930 (983–5,200)	3,420 (2,160–6,410)	4,040 (2,690–6,390)	71·5 (43·5–149)	82·4 (56·9–132)	80·2 (57·8–116)
1-62	Atrial fibrillation and flutter	1,450 (1,030–1,980)	2,570 (1,950–3,360)	3,500 (2,640–4,550)	88·9 (65·2–118)	96·8 (74·4–125)	100 (77·3–128)
1-63	Aortic aneurysm	483 (292–785)	908 (649–1,230)	1,260 (922–1,660)	23·6 (14·6–36·9)	27·1 (19·8–36)	28·9 (21·6–37)
1-64	Peripheral vascular disease	205 (112–341)	322 (188–515)	425 (253–671)	12 (6·7–20·1)	11·9 (6·9–19·2)	11·8 (7–18·7)
1-65	Endocarditis	826 (533–1,420)	1,690 (1,210–2,460)	1,980 (1,330–2,960)	24·2 (16·9–38·2)	31·6 (22·7–45·1)	31·1 (21–46·1)
1-66	Chronic respiratory diseases	91,300 (71,000–113,000)	80,000 (66,200–95,400)	84,500 (69,400–103,000)	3,400 (2,730–4,080)	2,120 (1,760–2,520)	1,830 (1,510–2,200)
1-67	Chronic obstructive pulmonary disease	37,500 (29,000–46,300)	42,400 (34,700–51,100)	50,300 (41,600–60,500)	1,780 (1,390–2,170)	1,310 (1,070–1,590)	1,200 (1,000–1,440)
1-68	Pneumoconiosis	42 (28–56)	51 (37–66)	64 (46–87)	1·9 (1·3–2·5)	1·4 (1–1·9)	1·4 (1–1·9)
1-69	Silicosis	17 (9–27)	25 (16–38)	37 (23–56)	0·8 (0·4–1·2)	0·7 (0·5–1·1)	0·8 (0·5–1·2)
1-70	Asbestosis	6 (3–9)	8 (4–12)	9 (5–14)	0·2 (0·1–0·4)	0·2 (0·1–0·3)	0·2 (0·1–0·3)
1-71	Coal workers pneumoconiosis	5 (2–10)	5 (2–9)	5 (2–8)	0·2 (0·1–0·4)	0·1 (0–0·2)	0·1 (0–0·2)
1-72	Other pneumoconiosis	14 (7–22)	13 (8–20)	14 (8–21)	0·6 (0·3–1)	0·4 (0·2–0·6)	0·3 (0·2–0·5)
1-73	Asthma	51,700 (36,900–67,600)	34,300 (26,800–42,200)	30,200 (22,600–38,800)	1,550 (1,150–1,930)	749 (581–949)	562 (422–729)
1-74	Interstitial lung disease and pulmonary sarcoidosis	464 (224–1,010)	732 (358–1,520)	953 (463–2,080)	20·8 (9·7–45·1)	20·7 (9·8–44·6)	20·7 (9·9–45·8)
1-75	Other chronic respiratory diseases	1,630 (810–4,140)	2,580 (1,870–3,950)	3,030 (2,250–4,250)	40·1 (22–89·6)	42 (30·6–63·3)	42·9 (32–60·8)
1-76	Digestive diseases	77,500 (55,700–97,700)	80,500 (61,500–99,600)	86,400 (64,300–113,000)	2,880 (2,200–3,490)	1,870 (1,450–2,360)	1,610 (1,200–2,110)
1-77	Cirrhosis and other chronic liver diseases	33,200 (21,300–44,700)	38,000 (27,200–49,900)	43,900 (31,100–59,600)	1,230 (840–1,620)	866 (626–1,130)	783 (552–1,070)
1-78	Cirrhosis and other chronic liver diseases due to hepatitis B	12,000 (7,400–16,800)	13,900 (9,170–19,100)	13,000 (8,020–19,300)	466 (296–642)	321 (216–439)	230 (144–341)
1-79	Cirrhosis and other chronic liver diseases due to hepatitis C	7,810 (4,770–11,600)	9,200 (5,820–12,900)	12,200 (7,510–17,900)	303 (191–441)	213 (137–298)	217 (135–322)
1-80	Cirrhosis and other chronic liver diseases due to alcohol use	5,320 (3,090–8,000)	6,350 (3,900–9,420)	8,710 (5,240–13,400)	215 (128–320)	156 (98·1–234)	162 (97·9–250)
1-81	Cirrhosis and other chronic liver diseases due to NAFLD	1,960 (1,160–3,130)	2,380 (1,470–3,650)	3,280 (1,900–5,140)	79·5 (47·6–124)	58·8 (35·9–90·1)	62·7 (37·2–96·7)
1-82	Cirrhosis and other chronic liver diseases due to other causes	6,110 (2,820–10,100)	6,230 (4,320–8,820)	6,700 (4,430–9,730)	170 (92·4–257)	118 (83·4–168)	112 (75·6–161)
1-83	Upper digestive system diseases	29,500 (23,100–37,100)	24,400 (18,200–31,900)	23,600 (16,900–31,800)	1,180 (921–1,480)	626 (464–825)	480 (348–648)
1-84	Peptic ulcer disease	26,300 (20,000–33,300)	20,100 (14,500–26,800)	18,500 (13,000–25,600)	1,080 (828–1,370)	538 (390–722)	397 (282–545)
1-85	Gastritis and duodenitis	2,010 (1,240–2,870)	2,250 (1,490–3,100)	2,410 (1,600–3,360)	64·1 (40·7–86·7)	46·6 (31–65·3)	41·6 (27·4–58·3)
1-86	GERD	1,200 (612–2,180)	2,060 (1,050–3,770)	2,600 (1,330–4,740)	40·8 (20·8–75)	40·9 (20·8–74·9)	40·8 (20·9–74·8)
1-87	Appendicitis	2,350 (671–3,990)	2,070 (1,050–3,130)	1,540 (858–2,440)	55·3 (18·6–86·9)	32·2 (16·6–49·4)	22 (12·5–35·3)
1-88	Paralytic ileus and intestinal obstruction	3,990 (2,100–7,250)	5,670 (3,550–8,280)	5,730 (3,270–8,290)	109 (69·5–160)	112 (70·2–149)	101 (60·2–144)
1-89	Inguinal, femoral, and abdominal hernia	1,750 (862–3,220)	2,110 (1,420–3,070)	2,290 (1,620–3,040)	50·2 (30·2–72·6)	44·4 (31·6–59·2)	41·5 (30–54·5)
1-90	Inflammatory bowel disease	385 (177–639)	365 (223–543)	387 (234–610)	13 (6·6–19·2)	7·9 (4·9–12·1)	7 (4·1–11·2)
1-91	Vascular intestinal disorders	225 (139–345)	353 (255–449)	463 (329–622)	10·9 (7·4–15·5)	11·1 (8·1–14·4)	11·4 (8·2–15·1)
1-92	Gallbladder and biliary diseases	3,010 (1,910–4,130)	3,810 (2,540–5,330)	4,450 (2,900–6,520)	116 (77·3–153)	91·9 (62·1–130)	84·8 (56·3–127)
1-93	Pancreatitis	1,740 (1,020–2,860)	2,210 (1,420–3,560)	2,530 (1,600–4,010)	62·8 (39·4–99·5)	47·8 (31·2–75·2)	43·1 (27·4–67·3)
1-94	Other digestive diseases	1,370 (634–2,560)	1,480 (957–2,010)	1,600 (1,090–2,250)	46·3 (26·1–70·8)	35·1 (24·2–46·3)	31·7 (21·8–44·7)
1-95	Neurological disorders	38,700 (17,500–69,300)	59,500 (24,300–112,000)	71,000 (29,200–132,000)	1,270 (644–2,200)	1,220 (601–2,170)	1,240 (610–2,190)
1-96	Alzheimer's disease and other dementias	4,020 (1,630–9,520)	7,210 (2,920–17,100)	9,870 (4,020–23,700)	315 (130–759)	326 (133–768)	340 (140–818)
1-97	Parkinson's disease	1,490 (1,170–1,910)	2,190 (1,830–2,570)	2,930 (2,410–3,420)	87·3 (69·5–112)	81·9 (69·2–95·6)	85·6 (71·5–99)
1-98	Idiopathic epilepsy	8,500 (3,630–13,900)	9,070 (4,360–15,700)	9,690 (4,050–16,900)	187 (87·8–302)	140 (66·3–244)	136 (55·9–238)
1-99	Multiple sclerosis	177 (116–325)	251 (188–365)	316 (232–447)	6·4 (4·3–11·4)	5·2 (4–7·8)	5·1 (3·8–7·3)
1-100	Motor neuron disease	102 (76–149)	124 (92–169)	162 (110–226)	3·1 (2·2–4·3)	2·5 (1·8–3·5)	2·7 (1·8–3·8)
1-101	Headache disorders	22,400 (3,080–52,400)	38,000 (5,310–88,500)	45,100 (6,590–105,000)	630 (97·5–1,460)	629 (96–1,440)	628 (97·2–1,450)
1-102	Migraine	20,700 (2,020–51,100)	35,200 (3,510–85,800)	41,600 (4,330–101,000)	580 (64·9–1,400)	578 (65·4–1,390)	578 (64–1,390)
1-103	Tension-type headache	1,680 (418–7,270)	2,860 (732–11,900)	3,440 (907–13,900)	50 (13·8–199)	50·2 (13·7–192)	50·2 (13·8–196)
1-104	Other neurological disorders	2,020 (1,200–3,030)	2,580 (1,430–4,240)	2,990 (1,460–4,980)	41·9 (26–61·7)	38·4 (22·7–60·5)	41·3 (21·3–66·7)
1-105	Mental disorders	51,800 (37,800–69,100)	82,600 (60,000–110,000)	98,200 (71,500–131,000)	1,450 (1,060–1,910)	1,390 (1,020–1,840)	1,400 (1,030–1,850)
1-106	Schizophrenia	5,290 (3,660–7,090)	9,620 (6,690–12,900)	12,600 (8,670–17,000)	168 (117–223)	175 (123–234)	181 (127–244)
1-107	Depressive disorders	15,000 (10,100–21,000)	22,800 (15,500–32,300)	28,500 (19,500–40,500)	456 (314–640)	406 (275–571)	418 (287–590)
1-108	Major depressive disorder	11,000 (7,170–15,700)	16,000 (10,600–23,200)	19,900 (13,200–28,200)	320 (215–452)	270 (181–382)	282 (190–399)
1-109	Dysthymia	3,980 (2,480–6,000)	6,830 (4,220–10,200)	8,670 (5,360–13,200)	136 (86–206)	136 (84·6–205)	136 (84·6–205)
1-110	Bipolar disorder	2,310 (1,320–3,660)	4,020 (2,320–6,470)	4,880 (2,890–7,760)	69·8 (41·3–110)	70·1 (41·4–111)	70·2 (41·7–110)
1-111	Anxiety disorders	14,300 (9,470–20,200)	25,000 (16,800–34,800)	30,500 (20,500–42,700)	402 (268–569)	419 (282–584)	437 (296–606)
1-112	Eating disorders	592 (360–891)	1,250 (752–1,880)	1,730 (1,060–2,590)	14·3 (8·7–21·4)	17·6 (10·7–26·6)	21·3 (13–31·8)
1-113	Anorexia nervosa	227 (129–374)	458 (255–745)	589 (345–966)	5·2 (3–8·4)	6·1 (3·5–9·9)	7·2 (4·2–11·7)

1-114	Bulimia nervosa	365 (205–578)	795 (446–1,270)	1,140 (647–1,800)	9·2 (5·2–14·4)	11·5 (6·5–18·1)	14·1 (8–22·2)
1-115	Autism spectrum disorders	2,090 (1,340–3,040)	3,190 (2,070–4,660)	3,530 (2,300–5,110)	46·5 (30·3–67·3)	47·4 (30·6–69·1)	47·5 (31–68·7)
1-116	Attention-deficit/hyperactivity disorder	550 (303–957)	854 (482–1,460)	888 (500–1,540)	11 (6·1–18·9)	11·1 (6·3–19)	11·2 (6·3–19·3)
1-117	Conduct disorder	3,740 (2,090–5,980)	5,570 (3,110–8,910)	5,290 (3,000–8,580)	65·3 (36·9–105)	65·7 (36·7–105)	65·8 (37·3–107)
1-118	Idiopathic developmental intellectual disability	4,820 (2,680–7,720)	4,770 (2,530–7,680)	3,140 (1,500–5,360)	104 (58·1–167)	70 (37·2–112)	41·5 (19·9–70·5)
1-119	Other mental disorders	3,150 (1,990–4,780)	5,520 (3,430–8,370)	7,030 (4,420–10,600)	107 (68·4–162)	108 (68·3–165)	109 (69·7–163)
1-120	Substance use disorders	8,730 (6,220–11,300)	12,500 (9,230–16,200)	14,700 (11,000–19,100)	276 (196–360)	216 (161–279)	205 (153–263)
1-121	Alcohol use disorders	6,000 (4,030–8,160)	7,540 (5,390–10,100)	8,820 (6,280–12,000)	202 (134–276)	140 (99·6–185)	129 (93·2–173)
1-122	Drug use disorders	2,730 (1,970–3,690)	4,970 (3,510–6,700)	5,910 (4,190–8,020)	74·1 (54·4–98·6)	76·1 (54·6–101)	75·8 (54·5–102)
1-123	Opioid use disorders	1,280 (931–1,710)	2,320 (1,610–3,230)	2,910 (2,000–4,090)	37·3 (27·8–49·6)	38·3 (26·8–51·8)	39·1 (27·3–53·4)
1-124	Cocaine use disorders	45 (30–72)	82 (55–126)	103 (69–149)	1·3 (0·8–2·1)	1·3 (0·9–2)	1·4 (0·9–2)
1-125	Amphetamine use disorders	588 (311–999)	1,100 (576–1,860)	1,220 (631–2,080)	14·7 (7·9–24·8)	15·4 (8·3–26)	14·7 (7·7–24·9)
1-126	Cannabis use disorders	331 (167–585)	586 (309–1,050)	660 (347–1,160)	8·1 (4·3–13·9)	8·1 (4·4–14)	8·2 (4·3–14·1)
1-127	Other drug use disorders	489 (289–764)	886 (526–1,420)	1,010 (601–1,580)	12·8 (7·9–19·6)	13 (8–20·4)	12·6 (7·6–19·3)
1-128	Diabetes and kidney diseases	77,600 (63,700–94,900)	106,000 (87,800–125,000)	130,000 (105,000–155,000)	2,950 (2,390–3,620)	2,670 (2,240–3,170)	2,610 (2,130–3,120)
1-129	Diabetes mellitus	33,400 (26,200–43,500)	51,300 (41,900–62,600)	68,300 (53,900–84,400)	1,390 (1,090–1,780)	1,400 (1,150–1,700)	1,440 (1,150–1,790)
1-130	Diabetes mellitus type 1	5,710 (2,610–8,240)	5,230 (3,720–6,990)	5,130 (3,570–7,470)	158 (77·9–231)	95·5 (68·2–136)	80·5 (54·7–124)
1-131	Diabetes mellitus type 2	27,700 (21,400–36,000)	46,000 (37,500–56,400)	63,200 (49,700–78,600)	1,230 (967–1,570)	1,300 (1,070–1,590)	1,360 (1,090–1,690)
1-132	Chronic kidney disease	40,900 (32,700–50,300)	52,400 (42,700–64,700)	60,300 (46,600–75,600)	1,500 (1,200–1,840)	1,250 (1,020–1,540)	1,150 (913–1,440)
1-133	Chronic kidney disease due to diabetes mellitus type 1	6,970 (4,700–10,000)	9,430 (6,430–13,200)	10,400 (6,790–14,800)	237 (155–345)	185 (122–264)	161 (104–232)
1-134	Chronic kidney disease due to diabetes mellitus type 2	10,600 (7,580–14,000)	14,300 (10,600–19,100)	17,700 (12,900–23,900)	471 (340–614)	403 (305–529)	380 (284–499)
1-135	Chronic kidney disease due to hypertension	10,500 (7,660–14,300)	14,000 (10,800–18,600)	17,000 (12,700–22,500)	462 (342–625)	388 (303–508)	364 (278–472)
1-136	Chronic kidney disease due to glomerulonephritis	4,020 (2,780–5,520)	5,000 (3,640–6,620)	5,280 (3,870–7,080)	107 (77·4–143)	88·4 (65·8–115)	80·5 (59·9–107)
1-137	Chronic kidney disease due to other and unspecified causes	8,810 (5,760–11,200)	9,630 (7,640–11,800)	9,900 (7,580–12,400)	219 (164–277)	182 (146–222)	168 (131–210)
1-138	Acute glomerulonephritis	3,220 (110–6,100)	1,870 (47–2,990)	1,010 (29–1,580)	63·4 (2·4–112)	28·2 (0·8–43·5)	14·9 (0·5–22·8)
1-139	Skin and subcutaneous diseases	26,000 (16,900–38,600)	39,900 (25,900–59,700)	44,500 (28,800–66,600)	637 (423–930)	634 (418–942)	634 (416–940)
1-140	Dermatitis	6,990 (4,000–11,400)	10,800 (6,230–17,400)	12,400 (7,240–19,800)	177 (103–284)	176 (103–280)	176 (103–278)
1-141	Atopic dermatitis	5,890 (3,130–9,950)	8,950 (4,830–14,900)	10,100 (5,420–17,100)	143 (75·7–240)	141 (75·9–236)	141 (75·7–237)
1-142	Contact dermatitis	986 (612–1,490)	1,670 (1,040–2,540)	2,030 (1,260–3,080)	30·9 (19·2–46)	30·9 (19·4–46·2)	30·9 (19·3–46·2)
1-143	Seborrhoeic dermatitis	121 (68–190)	194 (109–315)	227 (128–363)	3·4 (1·9–5·3)	3·4 (1·9–5·4)	3·4 (1·9–5·4)
1-144	Psoriasis	623 (433–843)	897 (630–1,220)	1,020 (716–1,380)	18·9 (13·1–25·4)	16·6 (11·7–22·8)	15·7 (11·1–21·1)
1-145	Bacterial skin diseases	1,270 (776–2,230)	1,650 (1,100–2,320)	1,730 (1,190–2,360)	42·1 (26·3–62·3)	37·3 (23·7–50·3)	32·7 (22–44·6)
1-146	Cellulitis	353 (138–650)	413 (212–618)	432 (244–636)	12·7 (5·4–19·8)	9·8 (5·2–14·6)	8·5 (4·6–12·5)
1-147	Pyoderma	914 (526–1,880)	1,240 (814–1,900)	1,300 (874–1,810)	29·4 (18·3–48·3)	27·4 (17·4–39·5)	24·3 (16·2–34·3)
1-148	Scabies	5,440 (3,010–8,830)	8,080 (4,460–12,900)	8,790 (4,780–14,100)	119 (66·2–191)	118 (66–188)	118 (64·4–188)
1-149	Fungal skin diseases	1,860 (741–4,020)	2,960 (1,170–6,370)	3,420 (1,360–7,420)	52·9 (21·4–114)	52·6 (21·3–114)	52·4 (21·1–113)
1-150	Viral skin diseases	2,830 (1,820–4,270)	3,920 (2,490–5,880)	4,070 (2,600–6,120)	53·1 (34·1–79·9)	53·3 (33·8–79·8)	53·3 (34–80·2)
1-151	Acne vulgaris	2,570 (1,550–4,170)	4,590 (2,710–7,440)	5,030 (2,980–8,080)	52·3 (31·5–84·9)	57·3 (33·9–92·9)	61·8 (36·5–98·9)
1-152	Alopecia areata	319 (204–474)	529 (336–802)	637 (402–950)	8·9 (5·7–13·1)	8·8 (5·6–13·2)	8·8 (5·6–13·2)
1-153	Pruritus	370 (174–667)	622 (296–1,130)	767 (358–1,390)	11 (5·3–19·6)	11·5 (5·5–20·9)	11·9 (5·6–21·4)
1-154	Urticaria	1,920 (1,240–2,790)	2,930 (1,910–4,220)	3,210 (2,090–4,560)	42·9 (27·7–62)	43·1 (28·5–61·4)	43·2 (28·3–61·5)
1-155	Decubitus ulcer	398 (200–569)	650 (309–967)	787 (382–1,150)	21 (10·8–30·6)	21·4 (10·4–31·7)	20·5 (10·5–29·2)
1-156	Other skin and subcutaneous diseases	1,390 (692–2,510)	2,250 (1,110–4,080)	2,700 (1,320–4,940)	37·3 (18·8–66·7)	38·7 (19·2–69·7)	40·1 (19·8–72·9)
1-157	Sense organ diseases	24,100 (16,400–34,000)	37,200 (25,000–53,000)	44,800 (30,300–63,800)	945 (654–1,320)	906 (626–1,280)	872 (600–1,230)
1-158	Blindness and vision loss	6,380 (4,320–9,140)	9,520 (6,470–13,700)	11,400 (7,700–16,600)	299 (206–416)	279 (191–391)	260 (178–368)
1-159	Glaucoma	143 (94–203)	212 (141–300)	240 (160–341)	9·5 (6·4–13·3)	8·6 (5·7–12)	7·4 (5–10·3)
1-160	Cataract	921 (641–1,270)	1,440 (1,010–1,990)	1,750 (1,220–2,410)	58 (40·5–78·6)	54·1 (37·6–74·3)	50·2 (35–68·7)
1-161	Age-related macular degeneration	176 (117–252)	276 (186–394)	345 (234–491)	10 (6·7–14·2)	10 (6·8–14·2)	9·4 (6·3–13·2)
1-162	Refraction disorders	2,200 (1,430–3,240)	3,260 (2,140–4,810)	3,770 (2,460–5,500)	74 (49·9–107)	69·6 (45·7–100)	66·2 (43·8–94·8)
1-163	Near vision loss	1,310 (596–2,590)	1,950 (890–3,920)	2,510 (1,140–5,070)	64·5 (29·6–126)	59·8 (27·6–120)	57·5 (26·7–113)
1-164	Other vision loss	1,640 (1,150–2,250)	2,370 (1,660–3,280)	2,790 (1,930–3,870)	83 (58–113)	77·1 (54·7–106)	69·8 (48·4–96·3)
1-165	Age-related and other hearing loss	16,700 (11,200–24,000)	26,100 (17,300–37,300)	31,400 (20,900–45,200)	612 (415–870)	592 (401–845)	576 (391–819)
1-166	Other sense organ diseases	990 (594–1,510)	1,600 (942–2,460)	1,990 (1,170–3,090)	34·4 (20·3–54·2)	35·2 (20·7–54·6)	35·6 (20·8–55·3)

1-167	Musculoskeletal disorders	49,100 (35,500–65,300)	85,900 (61,800–115,000)	112,000 (80,500–148,000)	1,760 (1,280–2,330)	1,810 (1,310–2,410)	1,860 (1,350–2,470)
1-168	Rheumatoid arthritis	384 (273–510)	692 (494–913)	929 (671–1,250)	14·5 (10·5–19·2)	15·9 (11·5–20·6)	16·7 (12·2–21·9)
1-169	Osteoarthritis	2,650 (1,300–5,320)	4,590 (2,250–9,360)	6,360 (3,130–12,800)	123 (60·8–246)	131 (64·5–265)	135 (66·7–272)
1-170	Low back pain	25,900 (18,300–34,500)	42,700 (30,100–57,400)	54,200 (37,800–72,400)	898 (632–1,200)	877 (623–1,170)	892 (627–1,180)
1-171	Neck pain	10,400 (6,940–15,100)	17,900 (11,800–25,800)	23,000 (15,100–33,700)	378 (252–549)	379 (250–551)	379 (251–551)
1-172	Gout	397 (248–574)	728 (457–1,050)	1,010 (602–1,440)	17 (10·6–24·7)	18·8 (11·9–27·3)	19·6 (11·9–28·5)
1-173	Other musculoskeletal disorders	9,390 (6,510–13,000)	19,200 (13,200–26,400)	26,500 (18,000–36,600)	325 (224–448)	390 (269–532)	420 (289–578)
1-174	Other non-communicable diseases	226,000 (95,600–401,000)	194,000 (118,000–280,000)	161,000 (115,000–218,000)	3,700 (1,890–5,990)	2,760 (1,790–3,840)	2,180 (1,600–2,910)
1-175	Congenital anomalies	172,000 (55,300–349,000)	130,000 (59,700–216,000)	94,000 (51,700–145,000)	2,380 (790–4,770)	1,630 (756–2,710)	1,160 (644–1,790)
1-176	Neural tube defects	33,600 (13,600–73,800)	17,900 (7,980–37,000)	10,200 (4,540–19,100)	439 (176–970)	221 (99·1–458)	125 (55·6–234)
1-177	Congenital heart anomalies	69,900 (15,900–164,000)	58,200 (23,200–110,000)	43,300 (22,400–74,100)	966 (228–2,240)	726 (292–1,370)	532 (276–909)
1-178	Orofacial clefts	1,050 (361–2,770)	750 (353–1,590)	546 (292–992)	14·4 (5·4–36·4)	9·7 (4·7–19·9)	6·9 (3·7–12·3)
1-179	Down's syndrome	1,560 (383–5,380)	1,340 (607–2,960)	1,120 (627–1,910)	22·7 (6·1–75·7)	17·1 (7·8–37·4)	13·9 (7·8–23·5)
1-180	Turner syndrome	6 (3–11)	9 (4–17)	9 (4–17)	0·1 (0–0·2)	0·1 (0·1–0·2)	0·1 (0–0·2)
1-181	Klinefelter syndrome	1 (0–1)	1 (0–2)	1 (0–2)	0 (0–0)	0 (0–0)	0 (0–0)
1-182	Other chromosomal abnormalities	1,790 (466–8,990)	1,430 (635–4,380)	1,250 (679–2,780)	24·2 (6·7–120)	17·8 (7·9–54·3)	15·3 (8·3–34)
1-183	Congenital musculoskeletal and limb anomalies	3,820 (2,130–6,710)	3,990 (2,480–6,010)	3,710 (2,420–5,330)	61·7 (35·7–100)	52·8 (33·6–79·3)	47·4 (31–68·4)
1-184	Urogenital congenital anomalies	1,430 (329–3,790)	1,250 (471–2,770)	986 (489–1,850)	19·4 (4·9–50·8)	15·6 (6–34·5)	12·2 (6·1–22·7)
1-185	Digestive congenital anomalies	18,900 (2,090–63,800)	14,300 (3,110–38,300)	9,880 (3,080–20,100)	253 (27·7–856)	177 (38·7–476)	121 (37·7–246)
1-186	Other congenital anomalies	40,300 (7,560–120,000)	30,800 (10,500–72,600)	23,000 (10,600–48,300)	578 (126–1,660)	394 (141–920)	287 (135–597)
1-187	Urinary diseases and male infertility	5,250 (3,830–6,910)	6,220 (4,590–7,790)	6,730 (4,980–8,460)	179 (137–232)	151 (113–191)	136 (101–169)
1-188	Urinary tract infections and interstitial nephritis	3,240 (2,120–4,580)	3,620 (2,350–4,760)	3,660 (2,410–4,850)	102 (70·9–139)	81·4 (54·8–106)	70·1 (48·3–91)
1-189	Urolithiasis	565 (131–945)	606 (194–969)	653 (240–1,030)	21·4 (5·1–38·4)	14·9 (4·6–24·4)	12·6 (4·4–20·1)
1-190	Benign prostatic hyperplasia	585 (338–894)	920 (539–1,390)	1,260 (721–1,930)	29·5 (17·2–45)	30·7 (17·9–46·4)	31·2 (18–47·8)
1-191	Male infertility	77 (30–188)	142 (54–344)	181 (68–443)	2·2 (0·8–5·3)	2·3 (0·9–5·4)	2·3 (0·9–5·5)
1-192	Other urinary diseases	778 (413–1,470)	939 (649–1,480)	982 (666–1,520)	24 (15·4–43·2)	21·7 (15·4–32·5)	19·3 (13·4–29·1)
1-193	Gynecological diseases	9,930 (6,910–14,000)	16,200 (11,100–23,000)	19,600 (13,400–27,800)	290 (203–408)	269 (187–378)	267 (184–378)
1-194	Uterine fibroids	337 (170–600)	553 (283–984)	697 (352–1,250)	10·8 (5·4–19·1)	10·2 (5·2–18·3)	10·1 (5·1–18)
1-195	Polycystic ovarian syndrome	198 (85–395)	544 (229–1,110)	771 (329–1,560)	5·2 (2·3–10·5)	8·2 (3·4–16·6)	9·8 (4·2–20)
1-196	Female infertility	205 (68–498)	335 (108–815)	412 (128–1,010)	6 (2–14·8)	5·6 (1·8–13·4)	5·4 (1·7–13·2)
1-197	Endometriosis	1,720 (1,000–2,800)	2,190 (1,280–3,440)	2,520 (1,480–4,040)	49 (29–79·1)	35 (20·7–55·2)	32·6 (19·3–51·9)
1-198	Genital prolapse	97 (49–186)	137 (69–253)	179 (91–323)	4·3 (2·1–8·2)	3·6 (1·8–6·7)	3·5 (1·7–6·4)
1-199	Premenstrual syndrome	3,990 (2,410–6,180)	6,860 (4,220–10,800)	7,990 (4,840–12,500)	106 (65·2–164)	104 (64·3–163)	103 (62·9–161)
1-200	Other gynecological diseases	3,390 (2,180–4,850)	5,570 (3,610–8,080)	7,020 (4,550–10,300)	109 (70·1–156)	103 (66·5–148)	102 (66–148)
1-201	Hemoglobinopathies and hemolytic anaemias	11,000 (7,730–14,900)	12,100 (8,820–16,000)	11,500 (8,200–15,700)	258 (188–340)	192 (141–255)	163 (118–220)
1-202	Thalassemias	3,330 (1,500–5,330)	2,630 (1,760–3,670)	1,940 (1,380–2,590)	53·3 (25·2–83·8)	33 (22·2–46·2)	24 (17·1–32)
1-203	Thalassaemias trait	4,840 (3,170–7,290)	6,320 (4,070–9,280)	6,250 (3,970–9,330)	113 (73·8–169)	94·5 (60·6–139)	83·8 (53–125)
1-204	Sickle cell disorders	5 (2–11)	3 (2–5)	2 (1–3)	0·1 (0–0·2)	0 (0–0·1)	0 (0–0)
1-205	Sickle cell trait	204 (126–317)	279 (171–437)	294 (183–459)	5 (3·1–7·8)	4·2 (2·6–6·5)	3·8 (2·4–5·9)
1-206	G6PD deficiency	593 (389–844)	846 (606–1,120)	821 (585–1,090)	17·5 (11·8–24·4)	15·5 (11·4–20)	12·3 (8·7–16·4)
1-207	G6PD trait	5 (3–8)	14 (8–23)	15 (8–23)	0·2 (0·1–0·3)	0·3 (0·2–0·4)	0·2 (0·1–0·4)
1-208	Other hemoglobinopathies and hemolytic anaemias	2,070 (1,520–2,760)	2,030 (1,470–2,730)	2,140 (1,560–2,900)	69·2 (52·4–89)	44·6 (33·7–58·4)	38·6 (29–51)
1-209	Endocrine, metabolic, blood, and immune disorders	8,940 (5,470–13,100)	10,900 (7,780–14,300)	12,100 (8,990–15,900)	238 (163–318)	206 (149–267)	193 (144–250)
1-210	Oral disorders	5,440 (3,290–8,550)	8,650 (5,240–13,500)	10,300 (6,210–16,100)	190 (117–294)	185 (113–287)	179 (108–280)
1-211	Caries of deciduous teeth	208 (92–418)	283 (121–555)	245 (105–504)	3·2 (1·4–6·4)	3·6 (1·5–7)	3·1 (1·3–6·4)
1-212	Caries of permanent teeth	989 (443–1,970)	1,660 (751–3,230)	1,790 (806–3,430)	26 (11·8–50·4)	26·1 (11·6–50·7)	24·8 (11–47·8)
1-213	Periodontal disease	578 (212–1,340)	1,070 (402–2,450)	1,530 (578–3,490)	24·5 (9·2–55)	27·2 (10·3–61·3)	29·4 (11·2–66·4)
1-214	Edentulism	1,680 (1,060–2,560)	2,450 (1,550–3,700)	2,980 (1,880–4,510)	82·9 (52·9–125)	74·9 (47·6–112)	68·7 (43·7–103)
1-215	Other oral disorders	1,980 (1,230–2,900)	3,190 (1,980–4,720)	3,740 (2,310–5,490)	53·1 (33–77·6)	53·2 (33–77·7)	53·2 (32·9–77·6)
1-216	Sudden infant death syndrome	12,700 (1,250–41,000)	9,740 (1,820–27,800)	6,640 (1,760–15,700)	169 (16·5–547)	121 (22·6–345)	81·1 (21·5–191)
1-0	Injuries	303,000 (237,000–362,000)	259,000 (209,000–303,000)	220,000 (170,000–267,000)	6,920 (5,600–8,230)	4,090 (3,330–4,780)	3,110 (2,430–3,750)
1-1	Transport injuries	83,900 (55,900–107,000)	82,400 (63,800–101,000)	73,200 (55,000–91,900)	2,000 (1,420–2,520)	1,300 (1,020–1,580)	1,030 (782–1,290)
1-2	Road injuries	78,600 (50,300–101,000)	77,000 (59,700–93,800)	68,700 (51,500–86,200)	1,880 (1,310–2,360)	1,220 (958–1,480)	964 (733–1,200)

1-3	Pedestrian road injuries	27,500 (15,800–43,300)	22,500 (15,200–32,500)	16,000 (11,200–22,600)	636 (387–985)	368 (256–523)	241 (172–333)
1-4	Cyclist road injuries	4,970 (3,170–7,710)	4,850 (3,480–6,240)	4,860 (3,530–6,270)	120 (83·4–170)	83·3 (61·4–105)	73·2 (54·3–93·5)
1-5	Motorcyclist road injuries	24,400 (15,900–35,100)	27,500 (20,300–35,400)	26,000 (18,900–33,200)	586 (404–812)	419 (313–535)	349 (256–443)
1-6	Motor vehicle road injuries	20,800 (11,200–33,400)	21,100 (14,700–30,800)	20,700 (14,100–30,800)	512 (301–798)	330 (237–480)	286 (200–422)
1-7	Other road injuries	878 (580–1,390)	1,100 (723–1,490)	1,050 (675–1,490)	21·9 (15·1–32·5)	17·7 (11·9–23·8)	14·9 (9·7–20·6)
1-8	Other transport injuries	5,240 (3,520–8,060)	5,410 (3,840–7,480)	4,590 (3,150–6,350)	124 (85·9–180)	85·3 (61·6–117)	63·9 (44·8–87·8)
1-9	Unintentional injuries	138,000 (95,200–173,000)	105,000 (84,100–126,000)	87,600 (69,500–107,000)	2,840 (2,140–3,470)	1,650 (1,350–1,970)	1,280 (1,030–1,550)
1-10	Falls	17,700 (10,500–26,700)	21,100 (16,500–26,600)	23,200 (18,300–28,600)	491 (336–683)	415 (336–503)	396 (319–481)
1-11	Drowning	60,700 (38,600–84,900)	36,500 (26,000–48,700)	24,300 (16,900–33,200)	1,090 (725–1,470)	503 (360–674)	322 (225–439)
1-12	Fire, heat, and hot substances	13,400 (4,950–20,100)	8,510 (5,210–12,100)	7,030 (4,800–9,670)	282 (131–403)	134 (86·3–189)	100 (69·9–136)
1-13	Poisonings	5,370 (2,920–8,280)	3,140 (2,210–4,300)	1,840 (1,280–2,550)	101 (65·2–146)	45·5 (32·3–61·7)	24·8 (17·6–34·2)
1-14	Poisoning by carbon monoxide	530 (230–1,120)	469 (278–820)	336 (209–493)	11·9 (5·9–22·3)	7·1 (4·3–12·4)	4·6 (2·9–6·7)
1-15	Poisoning by other means	4,840 (2,410–7,670)	2,670 (1,850–3,760)	1,510 (1,030–2,100)	89 (52·7–132)	38·3 (27–52·9)	20·2 (14·1–28·1)
1-16	Exposure to mechanical forces	7,190 (4,870–11,000)	7,330 (5,660–9,980)	7,270 (5,520–9,710)	181 (133–242)	124 (97·1–161)	107 (81·7–139)
1-17	Unintentional firearm injuries	1,510 (736–3,130)	1,020 (623–1,840)	901 (563–1,570)	36·8 (19·2–67·4)	16·7 (10·5–28·1)	12·8 (8·2–21·5)
1-18	Other exposure to mechanical forces	5,680 (3,690–8,790)	6,310 (4,780–8,560)	6,370 (4,770–8,510)	144 (103–195)	107 (82·6–139)	93·8 (71–123)
1-19	Adverse effects of medical treatment	5,510 (2,910–8,820)	3,510 (2,210–5,640)	2,670 (1,710–4,430)	116 (75·9–180)	57·8 (38·7–93·6)	40·6 (26·5–66·7)
1-20	Animal contact	5,790 (2,250–10,000)	4,570 (2,330–7,220)	3,450 (1,850–5,610)	120 (53–201)	71·2 (37·9–111)	49·9 (27·1–81·2)
1-21	Venomous animal contact	3,650 (1,300–6,850)	2,770 (1,290–4,680)	1,960 (886–3,350)	73·7 (28·5–133)	41·8 (19·8–69·2)	27·5 (12·7–47)
1-22	Non-venomous animal contact	2,140 (822–4,000)	1,800 (958–2,990)	1,490 (856–2,490)	46 (21·6–77·2)	29·4 (16·8–48·8)	22·4 (12·8–37·1)
1-23	Foreign body	11,000 (6,700–17,000)	7,540 (5,020–11,400)	5,480 (3,600–8,050)	175 (115–258)	104 (72·2–153)	73·5 (49·5–105)
1-24	Pulmonary aspiration and foreign body in airway	9,640 (5,880–14,500)	6,820 (4,430–10,400)	4,800 (3,000–7,340)	151 (98·6–223)	91·9 (61·4–139)	63·2 (40·5–94)
1-25	Foreign body in eyes	106 (48–191)	176 (77–318)	209 (94–376)	3 (1·5–5·3)	3 (1·5–5·3)	3 (1·5–5·3)
1-26	Foreign body in other body part	1,220 (324–3,300)	543 (310–1,010)	470 (338–665)	21·4 (8·1–51·1)	9·1 (5·8–15)	7·3 (5·3–10·1)
1-27	Environmental heat and cold exposure	1,170 (562–1,880)	986 (543–1,440)	889 (530–1,220)	29·1 (15·2–42·8)	17·1 (9·7–24·2)	13·4 (8–18·3)
1-28	Exposure to forces of nature	9 (6–14)	90 (60–131)	1,130 (999–1,260)	0·2 (0·2–0·4)	1·6 (1·1–2·2)	15·3 (13·6–17·2)
1-29	Other unintentional injuries	10,700 (7,010–14,500)	11,400 (7,130–17,100)	10,400 (6,450–16,000)	254 (172–346)	176 (112–260)	142 (89·7–218)
1-30	Self-harm and interpersonal violence	81,100 (59,500–108,000)	71,900 (52,900–94,300)	59,000 (42,200–74,400)	2,080 (1,540–2,730)	1,140 (835–1,480)	796 (573–1,010)
1-31	Self-harm	25,400 (18,300–33,200)	23,900 (17,600–32,000)	23,400 (16,100–31,900)	693 (503–902)	377 (282–506)	314 (218–430)
1-32	Self-harm by firearm	633 (285–1,120)	520 (279–1,110)	459 (258–990)	17·7 (8·5–31·1)	8·4 (4·7–18·4)	6·2 (3·6–13·6)
1-33	Self-harm by other specified means	24,800 (17,900–32,400)	23,400 (17,000–31,300)	23,000 (15,800–31,100)	675 (488–878)	368 (274–490)	308 (214–415)
1-34	Interpersonal violence	38,400 (23,800–55,700)	47,500 (32,000–67,800)	35,100 (23,900–46,100)	1,000 (634–1,460)	755 (514–1,080)	474 (323–620)
1-35	Physical violence by firearm	10,100 (4,870–18,800)	13,400 (7,620–20,300)	9,410 (5,390–15,100)	274 (134–496)	214 (122–319)	125 (72·2–199)
1-36	Physical violence by sharp object	16,400 (9,020–25,500)	21,400 (11,400–33,900)	16,000 (8,690–22,500)	442 (239–687)	339 (179–538)	214 (116–300)
1-37	Sexual violence	743 (483–1,050)	1,250 (815–1,770)	1,430 (945–2,040)	19·3 (12·7–27·4)	19·1 (12·6–27·1)	19 (12·5–27·1)
1-38	Physical violence by other means	11,100 (6,900–18,900)	11,500 (7,900–16,700)	8,220 (5,940–11,200)	268 (173–426)	183 (129–255)	116 (85·6–155)
1-39	Conflict and terrorism	17,300 (7,470–34,900)	393 (213–698)	329 (180–584)	381 (165–769)	6·6 (3·6–11·9)	4·9 (2·7–8·8)
1-40	Executions and police conflict	57 (24–110)	87 (44–151)	175 (122–264)	1·4 (0·5–2·8)	1·3 (0·6–2·3)	2·3 (1·6–3·4)

SR Table 1: All-age DALYs counts and age standardized DALYs rates per 100,000 individuals for all causes and level 1 cause groups by location for 1990, 2010, and 2019, both sexes combined, Andean Latin America

Cause Level	Cause	All-age DALYs			Age-standardized rate of DALYs per 100,000		
		1990	2010	2019	1990	2010	2019
0-0	All causes	16,500,000 (15,300,000–17,800,000)	14,700,000 (13,200,000–16,500,000)	15,400,000 (13,100,000–18,100,000)	43,800 (40,500–47,500)	29,500 (26,500–33,000)	25,500 (21,600–29,900)
1-0	Communicable, maternal, neonatal, and nutritional disorders	7,810,000 (7,290,000–8,370,000)	3,830,000 (3,440,000–4,250,000)	3,170,000 (2,620,000–3,820,000)	16,400 (15,300–17,600)	7,000 (6,310–7,750)	5,100 (4,230–6,140)
1-1	HIV/AIDS and sexually transmitted infections	140,000 (65,700–263,000)	241,000 (178,000–376,000)	242,000 (174,000–481,000)	287 (151–528)	436 (326–679)	375 (268–745)
1-2	HIV/AIDS	31,600 (20,800–74,100)	178,000 (139,000–301,000)	181,000 (137,000–407,000)	89·2 (59–208)	328 (258–550)	279 (210–632)
1-3	HIV/AIDS - drug-susceptible tuberculosis	12,900 (9,520–18,700)	32,500 (20,600–47,800)	22,600 (14,200–34,900)	36·4 (27–53·4)	60 (38–88·3)	34·7 (21·8–53·7)
1-4	HIV/AIDS - Multidrug-resistant Tuberculosis without extensive drug resistance	240 (55–693)	5,790 (2,310–12,100)	3,630 (1,220–8,300)	0·7 (0·2–2)	10·7 (4·3–22·4)	5·6 (1·9–12·7)
1-5	HIV/AIDS - Extensively drug-resistant Tuberculosis	0 (0–0)	615 (232–1,330)	589 (187–1,400)	0 (0–0)	1·1 (0·4–2·5)	0·9 (0·3–2·1)
1-6	HIV/AIDS resulting in other diseases	18,500 (11,100–52,400)	139,000 (96,400–261,000)	155,000 (106,000–379,000)	52·1 (31·4–148)	256 (179–474)	238 (164–587)
1-7	Sexually transmitted infections excluding HIV	109,000 (37,600–231,000)	63,200 (26,400–121,000)	60,700 (26,300–119,000)	198 (73·7–412)	108 (46·3–204)	95·9 (41·4–188)
1-8	Syphilis	103,000 (31,000–224,000)	54,000 (18,300–111,000)	50,100 (16,400–108,000)	180 (55–392)	90·5 (30·7–185)	79·3 (26–171)
1-9	Chlamydial infection	734 (500–1,030)	1,120 (778–1,600)	1,270 (851–1,820)	2·1 (1·5–3)	2·1 (1·4–2·9)	1·9 (1·3–2·8)
1-10	Gonococcal infection	587 (475–742)	827 (654–1,010)	832 (628–1,050)	1·8 (1·5–2·3)	1·6 (1·3–1·9)	1·3 (1–1·7)
1-11	Trichomoniasis	1,190 (467–2,450)	1,880 (721–3,910)	2,240 (874–4,660)	3·8 (1·5–7·9)	3·6 (1·4–7·5)	3·5 (1·4–7·3)
1-12	Genital herpes	1,770 (581–4,370)	2,980 (980–7,400)	3,710 (1,190–9,010)	5·8 (1·9–14·4)	5·9 (1·9–14·7)	5·9 (1·9–14·3)
1-13	Other sexually transmitted infections	1,630 (1,170–2,230)	2,350 (1,700–3,240)	2,640 (1,860–3,700)	4·9 (3·5–6·6)	4·4 (3·2–6)	4 (2·9–5·6)
1-14	Respiratory infections and tuberculosis	3,120,000 (2,820,000–3,470,000)	1,310,000 (1,170,000–1,480,000)	1,030,000 (831,000–1,250,000)	7,180 (6,560–7,850)	2,570 (2,280–2,890)	1,710 (1,390–2,090)
1-15	Tuberculosis	788,000 (697,000–878,000)	230,000 (202,000–259,000)	165,000 (132,000–205,000)	2,240 (1,990–2,500)	454 (400–511)	266 (213–330)
1-16	Latent tuberculosis infection	0 (0–0)	0 (0–0)	0 (0–0)	0 (0–0)	0 (0–0)	0 (0–0)
1-17	Drug-susceptible tuberculosis	775,000 (683,000–868,000)	200,000 (168,000–232,000)	144,000 (109,000–183,000)	2,210 (1,950–2,470)	396 (332–457)	232 (175–295)
1-18	Multidrug-resistant tuberculosis without extensive drug resistance	12,900 (2,870–38,800)	26,700 (12,700–49,400)	18,600 (7,150–38,500)	36·5 (8·2–108)	52·9 (25–98)	30 (11·6–62·7)
1-19	Extensively drug-resistant tuberculosis	0 (0–0)	2,810 (1,250–5,560)	2,980 (1,040–6,350)	0 (0–0)	5·6 (2·5–11)	4·8 (1·7–10·3)
1-20	Lower respiratory infections	2,270,000 (2,000,000–2,570,000)	1,010,000 (893,000–1,140,000)	783,000 (623,000–976,000)	4,780 (4,300–5,340)	1,990 (1,770–2,230)	1,320 (1,060–1,650)
1-21	Upper respiratory infections	51,500 (29,400–80,600)	52,800 (33,000–81,500)	59,400 (36,500–91,600)	117 (69·4–177)	95·1 (59·3–147)	92·8 (57·2–142)
1-22	Otitis media	13,300 (7,740–21,900)	16,500 (9,470–27,100)	17,900 (10,400–29,600)	31·8 (18·6–52·6)	29·7 (17·3–48·5)	28 (16·3–46·5)
1-23	Enteric infections	984,000 (819,000–1,180,000)	231,000 (179,000–297,000)	220,000 (168,000–292,000)	1,930 (1,610–2,320)	427 (331–547)	355 (272–469)
1-24	Diarrhoeal diseases	980,000 (815,000–1,170,000)	227,000 (176,000–293,000)	217,000 (165,000–288,000)	1,920 (1,600–2,310)	421 (326–540)	350 (267–464)
1-25	Typhoid and paratyphoid	350 (157–666)	268 (127–505)	264 (122–503)	0·9 (0·4–1·6)	0·5 (0·2–0·9)	0·4 (0·2–0·8)
1-26	Typhoid fever	343 (154–657)	264 (125–496)	260 (120–491)	0·8 (0·4–1·6)	0·5 (0·2–0·9)	0·4 (0·2–0·8)
1-27	Paratyphoid fever	7 (2–14)	4 (2–9)	4 (1–9)	0 (0–0)	0 (0–0)	0 (0–0)
1-28	iNTS	2,560 (1,360–4,090)	2,400 (1,230–3,960)	2,370 (1,120–4,130)	5·5 (3–8·9)	4·3 (2·2–7)	3·7 (1·8–6·5)
1-29	Other intestinal infectious diseases	1,370 (628–2,140)	533 (248–880)	395 (208–640)	2·6 (1·2–4)	0·9 (0·4–1·5)	0·6 (0·3–1)
1-30	Neglected tropical diseases and malaria	241,000 (156,000–371,000)	117,000 (84,100–160,000)	120,000 (82,800–166,000)	651 (443–969)	234 (169–322)	196 (135–271)
1-31	Malaria	108,000 (32,900–231,000)	2,700 (1,750–4,050)	2,370 (1,020–6,980)	248 (75·6–545)	4·9 (3·1–7·3)	3·7 (1·6–10·8)
1-32	Chagas disease	17,000 (9,090–23,800)	19,600 (10,500–29,200)	21,300 (10,700–33,900)	71·8 (36·7–101)	44·5 (23·3–67·4)	36·7 (18·1–58·9)
1-33	Leishmaniasis	10,400 (3,410–33,600)	6,940 (4,050–11,000)	7,200 (4,200–11,200)	29·5 (11·4–76·2)	14·3 (8·4–22·8)	11·9 (6·9–18·4)
1-34	Visceral leishmaniasis	4,680 (5–28,200)	492 (0–2,920)	466 (0–2,790)	9·8 (0–57·1)	0·9 (0–5·1)	0·7 (0–4·3)
1-35	Cutaneous and mucocutaneous leishmaniasis	5,700 (2,850–9,820)	6,450 (3,890–10,200)	6,730 (4,070–10,600)	19·7 (9·7–34·1)	13·5 (8·1–21·5)	11·1 (6·7–17·5)
1-36	African trypanosomiasis	0 (0–0)	0 (0–0)	0 (0–0)	0 (0–0)	0 (0–0)	0 (0–0)
1-37	Schistosomiasis	0 (0–0)	0 (0–0)	0 (0–0)	0 (0–0)	0 (0–0)	0 (0–0)
1-38	Cysticercosis	25,200 (16,600–36,000)	24,700 (14,900–36,900)	26,400 (16,000–40,000)	93·4 (61·5–135)	53·4 (32·2–80·7)	44·6 (27–67·2)
1-39	Cystic echinococcosis	393 (193–630)	131 (72–201)	85 (48–133)	1 (0·6–1·5)	0·3 (0·1–0·4)	0·1 (0·1–0·2)
1-40	Lymphatic filariasis	0 (0–0)	0 (0–0)	0 (0–0)	0 (0–0)	0 (0–0)	0 (0–0)
1-41	Onchocerciasis	1,150 (764–1,670)	0 (0–0)	0 (0–0)	3·6 (2·4–5·1)	0 (0–0)	0 (0–0)
1-42	Trachoma	28 (14–51)	13 (6–23)	10 (5–19)	0·1 (0·1–0·3)	0 (0–0·1)	0 (0–0)
1-43	Dengue	2,080 (1,090–3,670)	6,490 (4,690–9,040)	5,020 (3,400–7,280)	5·3 (2·9–9·1)	11·7 (8·5–16·4)	7·8 (5·3–11·3)
1-44	Yellow fever	12,400 (3,160–34,800)	5,410 (1,350–14,700)	3,600 (853–10,000)	28·7 (7·4–80·7)	9·5 (2·4–26·1)	5·5 (1·3–15·5)
1-45	Rabies	1,770 (251–2,330)	188 (110–614)	171 (81–618)	4·6 (0·9–5·8)	0·4 (0·2–1·3)	0·3 (0·1–1)
1-46	Intestinal nematode infections	17,800 (11,600–26,700)	9,350 (5,630–14,700)	7,680 (4,430–12,200)	40·6 (26·3–61·6)	16·6 (9·9–26·3)	11·8 (6·8–18·8)
1-47	Ascariasis	10,900 (7,180–16,300)	5,050 (2,870–8,010)	3,460 (1,920–5,740)	24·2 (15·4–36·7)	9·1 (5·2–14·4)	5·4 (3–8·9)
1-48	Trichuriasis	2,380 (1,240–4,030)	2,190 (1,110–3,890)	2,470 (1,270–4,290)	5·5 (2·9–9·3)	3·8 (1·9–6·8)	3·8 (1·9–6·5)

1-49	Hookworm disease	4,470 (2,730–6,970)	2,110 (1,270–3,380)	1,750 (1,010–2,770)	11 (6·7–17)	3·7 (2·2–5·9)	2·7 (1·5–4·2)
1-50	Food-borne trematodiases	24,500 (9,560–45,300)	29,400 (10,200–55,600)	34,900 (11,600–67,600)	79·2 (31·1–146)	57·8 (20–110)	55·6 (18·5–107)
1-51	Leprosy	60 (37–91)	41 (25–61)	43 (26–65)	0·2 (0·1–0·3)	0·1 (0·1–0·1)	0·1 (0–0·1)
1-52	Ebola virus disease	0 (0–0)	0 (0–0)	0 (0–0)	0 (0–0)	0 (0–0)	0 (0–0)
1-53	Zika virus disease	0 (0–0)	0 (0–0)	39 (19–75)	0 (0–0)	0 (0–0)	0·1 (0–0·1)
1-54	Guinea worm disease	0 (0–0)	0 (0–0)	0 (0–0)	0 (0–0)	0 (0–0)	0 (0–0)
1-55	Other neglected tropical diseases	20,400 (13,500–27,700)	11,500 (7,610–16,500)	11,300 (7,470–16,600)	44·7 (29·6–60·8)	20·4 (13·5–29·5)	17·8 (11·8–26·1)
1-56	Other infectious diseases	446,000 (279,000–748,000)	181,000 (127,000–280,000)	154,000 (107,000–232,000)	875 (573–1,420)	318 (226–488)	243 (168–365)
1-57	Meningitis	102,000 (88,400–120,000)	38,000 (32,400–44,700)	23,300 (18,500–29,400)	203 (176–236)	67·1 (57·4–78·8)	36·7 (29·1–46·4)
1-58	Encephalitis	47,400 (35,200–60,100)	36,800 (31,000–44,200)	37,100 (27,000–49,700)	95·5 (73·1–118)	65·3 (55–78·5)	58·2 (42·5–78)
1-59	Diphtheria	3,370 (1,780–6,120)	282 (182–419)	212 (137–318)	6·4 (3·4–11·4)	0·5 (0·3–0·7)	0·3 (0·2–0·5)
1-60	Whooping cough	225,000 (64,600–529,000)	72,700 (18,700–172,000)	62,300 (21,600–139,000)	408 (117–957)	124 (32·1–294)	97·7 (33·8–218)
1-61	Tetanus	20,300 (12,700–30,900)	2,540 (1,770–4,680)	1,730 (1,110–3,630)	48·9 (30·7–69)	4·9 (3·4–8·9)	2·8 (1·8–5·8)
1-62	Measles	987 (745–1,280)	0 (0–0)	0 (0–1)	1·8 (1·4–2·4)	0 (0–0)	0 (0–0)
1-63	Varicella and herpes zoster	11,000 (7,940–14,900)	5,730 (4,400–7,450)	4,890 (3,540–6,480)	25·3 (19·4–32·9)	10·9 (8·5–14)	8 (5·8–10·5)
1-64	Acute hepatitis	16,200 (12,500–19,200)	5,330 (4,340–6,500)	5,200 (4,020–6,630)	40·6 (31·8–48)	9·8 (8–11·8)	8·2 (6·3–10·4)
1-65	Acute hepatitis A	11,200 (8,300–14,400)	3,600 (2,820–4,580)	3,410 (2,530–4,460)	27·1 (20·2–34·4)	6·5 (5·1–8·2)	5·3 (4–7)
1-66	Acute hepatitis B	2,970 (1,650–4,270)	1,050 (834–1,330)	1,140 (861–1,500)	7·9 (4·5–10·9)	2 (1·6–2·6)	1·8 (1·4–2·4)
1-67	Acute hepatitis C	1,710 (846–2,780)	415 (309–596)	371 (245–586)	4·9 (2·5–7·5)	0·8 (0·6–1·2)	0·6 (0·4–0·9)
1-68	Acute hepatitis E	312 (219–435)	260 (178–371)	279 (191–400)	0·8 (0·6–1·1)	0·5 (0·3–0·7)	0·4 (0·3–0·6)
1-69	Other unspecified infectious diseases	19,600 (14,800–25,700)	19,100 (13,100–24,500)	19,400 (13,700–25,300)	45·4 (34·5–59·2)	35 (24–44·8)	30·8 (21·7–40·1)
1-70	Maternal and neonatal disorders	2,220,000 (2,020,000–2,440,000)	1,470,000 (1,310,000–1,670,000)	1,170,000 (919,000–1,460,000)	4,050 (3,710–4,430)	2,490 (2,210–2,820)	1,830 (1,440–2,290)
1-71	Maternal disorders	163,000 (147,000–184,000)	111,000 (94,500–128,000)	84,500 (65,000–108,000)	438 (393–493)	193 (165–224)	126 (96·8–161)
1-72	Maternal haemorrhage	45,900 (39,600–53,800)	28,400 (23,800–33,800)	19,800 (15,600–25,300)	125 (108–147)	50·1 (42·1–59·8)	29·7 (23·3–37·8)
1-73	Maternal sepsis and other pregnancy related infections	23,300 (19,700–27,600)	8,600 (6,930–10,500)	5,640 (4,120–7,410)	61·4 (51·9–72·8)	14·9 (12·1–18·2)	8·4 (6·1–11)
1-74	Maternal hypertensive disorders	41,900 (36,600–47,500)	30,500 (25,700–35,900)	21,600 (16,000–28,100)	109 (95·1–124)	53 (44·6–62·5)	32·1 (23·8–41·9)
1-75	Maternal obstructed labour and uterine rupture	555 (264–951)	1,680 (1,310–2,120)	1,900 (1,390–2,470)	1·4 (0·7–2·4)	2·9 (2·3–3·7)	2·8 (2·1–3·7)
1-76	Maternal abortive outcome	36,700 (31,000–43,200)	9,330 (7,400–11,800)	5,930 (4,340–7,900)	102 (85·4–120)	16·6 (13·1–21)	8·9 (6·5–11·9)
1-77	Ectopic pregnancy	538 (450–656)	2,100 (1,750–2,480)	1,730 (1,290–2,270)	1·4 (1·2–1·7)	3·6 (3–4·3)	2·6 (1·9–3·4)
1-78	Indirect maternal deaths	1,390 (1,160–1,640)	6,850 (5,740–8,110)	7,020 (5,230–9,100)	3·5 (3–4·2)	11·8 (9·9–13·9)	10·4 (7·7–13·5)
1-79	Late maternal deaths	10,600 (7,540–14,000)	10,100 (7,450–13,500)	9,420 (6,180–13,600)	28 (20–37)	17·6 (12·9–23·4)	14 (9·2–20·2)
1-80	Maternal deaths aggravated by HIV/AIDS	33 (12–93)	82 (36–166)	93 (40–187)	0·1 (0–0·2)	0·1 (0·1–0·3)	0·1 (0·1–0·3)
1-81	Other maternal disorders	2,560 (2,080–3,100)	12,900 (10,700–15,500)	11,400 (8,560–14,900)	6·7 (5·4–8·1)	22·7 (18·9–27·2)	17 (12·8–22·2)
1-82	Neonatal disorders	2,060,000 (1,870,000–2,280,000)	1,360,000 (1,210,000–1,560,000)	1,080,000 (853,000–1,350,000)	3,610 (3,280–3,990)	2,290 (2,030–2,620)	1,710 (1,350–2,130)
1-83	Neonatal preterm birth	996,000 (870,000–1,130,000)	599,000 (514,000–689,000)	417,000 (315,000–537,000)	1,750 (1,530–1,990)	1,010 (864–1,160)	660 (498–850)
1-84	Neonatal encephalopathy due to birth asphyxia and trauma	526,000 (422,000–639,000)	325,000 (272,000–388,000)	235,000 (175,000–309,000)	914 (732–1,110)	545 (456–650)	372 (276–489)
1-85	Neonatal sepsis and other neonatal infections	341,000 (265,000–435,000)	331,000 (266,000–412,000)	339,000 (253,000–436,000)	601 (465–768)	559 (448–696)	534 (398–687)
1-86	Hemolytic disease and other neonatal jaundice	26,300 (22,000–31,200)	16,900 (14,100–19,800)	14,800 (12,000–18,000)	51 (42·6–59·9)	29·5 (24·6–34·6)	23·2 (18·7–28·1)
1-87	Other neonatal disorders	171,000 (132,000–209,000)	92,000 (76,500–111,000)	75,400 (55,800–99,800)	298 (230–364)	154 (128–186)	119 (88·3–158)
1-88	Nutritional deficiencies	652,000 (545,000–779,000)	283,000 (224,000–361,000)	241,000 (180,000–317,000)	1,440 (1,210–1,720)	531 (422–674)	388 (290–509)
1-89	Protein-energy malnutrition	425,000 (351,000–518,000)	117,000 (98,000–140,000)	83,800 (64,900–105,000)	878 (739–1,050)	224 (191–264)	139 (108–173)
1-90	Iodine deficiency	451 (244–789)	590 (297–1,100)	645 (322–1,210)	1·4 (0·7–2·4)	1·1 (0·6–2·1)	1 (0·5–1·9)
1-91	Vitamin A deficiency	9,780 (6,470–14,100)	6,630 (4,340–9,650)	5,830 (3,830–8,560)	19·3 (12·7–27·7)	11·6 (7·6–16·9)	9·1 (6–13·3)
1-92	Dietary iron deficiency	198,000 (128,000–294,000)	147,000 (95,500–217,000)	140,000 (87,300–210,000)	488 (320–716)	269 (176–397)	221 (138–332)
1-93	Other nutritional deficiencies	19,300 (14,600–25,000)	12,200 (10,600–14,100)	10,800 (8,670–13,200)	52·4 (42·2–64·9)	24·9 (21·8–28·8)	18 (14·5–22)
1-0	Non-communicable diseases	6,330,000 (5,570,000–7,130,000)	8,790,000 (7,620,000–10,100,000)	10,300,000 (8,710,000–12,200,000)	21,600 (19,100–24,200)	18,600 (16,200–21,300)	17,400 (14,600–20,500)
1-1	Neoplasms	833,000 (755,000–919,000)	1,400,000 (1,260,000–1,550,000)	1,650,000 (1,340,000–2,030,000)	3,320 (3,020–3,650)	3,140 (2,820–3,440)	2,830 (2,320–3,490)
1-2	Lip and oral cavity cancer	5,940 (5,230–6,680)	10,500 (9,280–11,700)	12,700 (10,200–15,700)	25·8 (22·7–29)	23·9 (21·2–26·7)	22 (17·7–27)
1-3	Nasopharynx cancer	1,420 (1,270–1,590)	2,260 (1,990–2,520)	2,550 (2,030–3,160)	5·5 (4·9–6·1)	4·9 (4·3–5·5)	4·3 (3·4–5·3)
1-4	Other pharynx cancer	2,360 (2,090–2,680)	2,960 (2,610–3,350)	3,610 (2,850–4,530)	10·4 (9·2–11·8)	6·8 (6–7·7)	6·3 (4·9–7·9)
1-5	Oesophageal cancer	9,880 (8,310–11,200)	15,500 (13,500–18,000)	18,800 (15,100–23,600)	46·7 (39·4–53)	36·9 (32·3–43)	33·4 (26·8–41·9)
1-6	Stomach cancer	156,000 (142,000–171,000)	247,000 (222,000–273,000)	263,000 (213,000–322,000)	716 (650–783)	576 (516–635)	461 (374–562)
1-7	Colon and rectum cancer	38,000 (33,900–42,300)	97,000 (85,300–109,000)	126,000 (102,000–152,000)	174 (154–194)	227 (200–254)	221 (179–266)

1-8	Liver cancer	30,600 (27,000–34,400)	34,900 (30,700–39,400)	44,300 (35,800–54,400)	130 (114–146)	79·8 (70·2–90·1)	77·2 (62·6–94·7)
1-9	Liver cancer due to hepatitis B	15,200 (12,500–18,400)	16,400 (13,100–19,900)	20,100 (14,800–26,100)	63·9 (51·4–78·4)	36·9 (29–45·2)	34·5 (25·2–44·9)
1-10	Liver cancer due to hepatitis C	1,550 (957–2,350)	2,080 (1,310–3,140)	2,730 (1,650–4,220)	7·8 (4·8–11·8)	5·2 (3·3–7·7)	5 (3–7·7)
1-11	Liver cancer due to alcohol use	7,510 (5,210–10,000)	9,300 (6,620–12,300)	12,800 (8,620–17,800)	36 (25·1–48·3)	22·4 (15·9–29·7)	22·8 (15·4–31·7)
1-12	Liver cancer due to NASH	1,940 (1,380–2,690)	2,830 (2,010–3,920)	3,900 (2,690–5,530)	9·1 (6·3–12·8)	6·7 (4·8–9·3)	7 (4·8–9·9)
1-13	Gallbladder and biliary tract cancer	25,700 (21,600–29,400)	43,100 (36,600–50,800)	50,500 (39,300–64,500)	122 (102–139)	103 (87·6–121)	89·7 (69·9–114)
1-14	Pancreatic cancer	10,200 (8,980–11,500)	49,800 (43,300–56,000)	66,400 (53,700–80,700)	47·9 (42·3–54·1)	118 (104–133)	118 (95·2–142)
1-15	Larynx cancer	6,280 (5,580–7,030)	7,490 (6,590–8,510)	8,820 (7,090–10,900)	29·2 (25·9–32·7)	17·8 (15·6–20·2)	15·6 (12·6–19·2)
1-16	Tracheal, bronchus, and lung cancer	70,000 (60,800–79,400)	113,000 (97,400–127,000)	139,000 (111,000–172,000)	323 (280–366)	265 (230–299)	245 (195–303)
1-17	Malignant skin melanoma	5,640 (4,210–8,510)	9,870 (7,740–12,900)	12,000 (8,570–17,200)	22·7 (17·3–32·7)	22·1 (17·2–28·6)	20·5 (14·7–29·2)
1-18	Non-melanoma skin cancer	3,150 (2,780–3,680)	7,930 (6,850–8,800)	10,200 (8,180–12,300)	14·8 (13·1–17·2)	18·9 (16·3–21)	18·1 (14·5–21·9)
1-19	Non-melanoma skin cancer (squamous-cell carcinoma)	3,150 (2,770–3,680)	7,920 (6,840–8,790)	10,200 (8,170–12,300)	14·8 (13·1–17·2)	18·9 (16·3–21)	18·1 (14·5–21·8)
1-20	Non-melanoma skin cancer (basal-cell carcinoma)	2 (1–4)	5 (2–9)	5 (2–10)	0 (0–0)	0 (0–0)	0 (0–0)
1-21	Breast cancer	48,100 (43,200–53,800)	91,300 (80,000–104,000)	114,000 (92,500–142,000)	200 (180–223)	202 (178–229)	193 (156–240)
1-22	Cervical cancer	79,200 (66,300–92,900)	121,000 (98,000–142,000)	130,000 (99,400–165,000)	322 (269–378)	264 (214–311)	217 (167–277)
1-23	Uterine cancer	12,700 (10,200–14,600)	21,200 (17,200–26,200)	25,300 (19,700–33,200)	57·9 (46·4–66·6)	49·5 (40·4–61·6)	44·3 (34·5–58·2)
1-24	Ovarian cancer	10,600 (8,510–15,700)	29,800 (22,700–35,700)	40,900 (29,300–52,500)	42·7 (34·2–62·6)	65·4 (49·7–78·7)	68·9 (49·3–88·2)
1-25	Prostate cancer	32,600 (28,000–42,100)	77,700 (60,200–89,600)	101,000 (78,100–129,000)	178 (152–230)	198 (153–228)	188 (145–240)
1-26	Testicular cancer	5,650 (3,910–9,250)	8,990 (7,710–10,600)	10,800 (7,740–14,300)	14·4 (10·2–22·1)	16·3 (14–19·1)	16·5 (11·9–21·7)
1-27	Kidney cancer	13,700 (12,400–15,300)	23,600 (21,000–26,500)	31,100 (24,700–38,600)	50·9 (46·1–56·5)	53·1 (47–59·5)	53·6 (42·6–66·4)
1-28	Bladder cancer	6,720 (6,030–7,520)	12,600 (11,100–14,000)	16,300 (13,200–19,800)	33·5 (30·1–37·4)	30·7 (27·1–34·2)	29·5 (24–35·8)
1-29	Brain and nervous system cancer	35,500 (26,900–57,700)	66,200 (41,000–79,200)	74,500 (42,700–99,400)	98·6 (75·7–154)	130 (80·1–156)	121 (69·1–161)
1-30	Thyroid cancer	5,510 (4,820–6,630)	12,700 (10,300–14,400)	15,300 (11,900–18,800)	23·7 (20·7–28·3)	29·2 (23·3–33·1)	26·7 (20·8–32·7)
1-31	Mesothelioma	2,420 (1,410–3,330)	2,560 (2,110–2,960)	3,100 (2,300–4,010)	10·4 (6·1–14·3)	5·8 (4·7–6·7)	5·3 (3·9–6·8)
1-32	Hodgkin lymphoma	7,150 (5,630–8,340)	7,470 (5,960–9,080)	7,450 (5,720–9,510)	21·4 (17–24·9)	14·7 (11·6–17·5)	12 (9·1–15·3)
1-33	Non-Hodgkin's lymphoma	32,000 (28,000–35,900)	64,100 (56,900–71,300)	76,300 (61,500–94,700)	109 (96·3–122)	137 (121–152)	128 (103–159)
1-34	Multiple myeloma	6,990 (5,590–9,920)	14,300 (12,100–17,500)	18,900 (14,700–24,100)	32·7 (26·2–46·3)	34 (28·5–41·4)	33·3 (25·8–42·3)
1-35	Leukaemia	94,500 (80,700–119,000)	130,000 (100,000–149,000)	130,000 (94,600–167,000)	237 (205–296)	244 (190–279)	208 (152–267)
1-36	Acute lymphoid leukaemia	34,400 (26,800–47,500)	49,500 (35,900–57,400)	48,800 (32,800–63,600)	73·3 (57·8–99·6)	86·8 (62·8–100)	76·3 (51·3–99·4)
1-37	Chronic lymphoid leukaemia	865 (704–1,100)	2,300 (1,780–2,710)	3,050 (2,320–3,860)	3·7 (3–4·6)	5·3 (4·1–6·2)	5·3 (4·1–6·7)
1-38	Acute myeloid leukaemia	13,700 (10,800–18,700)	26,500 (18,900–30,900)	30,300 (20,400–38,900)	35·9 (28·9–48·1)	50·6 (36·4–59)	48·7 (33–62·4)
1-39	Chronic myeloid leukaemia	3,630 (2,710–4,960)	5,760 (4,460–6,770)	5,980 (4,580–7,580)	11·5 (8·9–14·8)	11·7 (9·2–13·8)	9·8 (7·5–12·4)
1-40	Other leukaemia	41,900 (29,100–57,200)	45,800 (33,100–53,700)	41,900 (29,300–54,400)	113 (82·6–147)	89·7 (65·3–105)	68·2 (47·8–88·3)
1-41	Other neoplasms	2,200 (1,730–2,690)	3,500 (2,940–4,090)	4,920 (3,870–5,970)	8 (6·3–9·6)	7·7 (6·5–9·1)	8·5 (6·7–10·3)
1-42	Myelodysplastic, myeloproliferative, and other hematopoietic neoplasms	2,200 (1,730–2,690)	3,500 (2,940–4,090)	4,920 (3,870–5,970)	8 (6·3–9·6)	7·7 (6·5–9·1)	8·5 (6·7–10·3)
1-43	Benign and in situ intestinal neoplasms	0 (0–0)	0 (0–0)	0 (0–0)	0 (0–0)	0 (0–0)	0 (0–0)
1-44	Benign and in situ cervical and uterine neoplasms	0 (0–0)	0 (0–0)	0 (0–0)	0 (0–0)	0 (0–0)	0 (0–0)
1-45	Other benign and in situ neoplasms	0 (0–0)	0 (0–0)	0 (0–0)	0 (0–0)	0 (0–0)	0 (0–0)
1-46	Cardiovascular diseases	1,010,000 (919,000–1,100,000)	1,210,000 (1,090,000–1,360,000)	1,400,000 (1,170,000–1,660,000)	4,400 (4,000–4,800)	2,810 (2,520–3,140)	2,460 (2,070–2,920)
1-47	Rheumatic heart disease	40,300 (33,200–48,600)	40,300 (31,500–52,200)	42,200 (31,300–56,100)	131 (111–157)	79·1 (63–101)	67·4 (50·2–88·8)
1-48	Ischaemic heart disease	416,000 (372,000–464,000)	529,000 (471,000–593,000)	642,000 (533,000–771,000)	2,010 (1,790–2,230)	1,270 (1,130–1,420)	1,150 (954–1,380)
1-49	Stroke	398,000 (363,000–435,000)	425,000 (380,000–486,000)	471,000 (393,000–573,000)	1,670 (1,510–1,820)	964 (860–1,110)	820 (686–996)
1-50	Ischaemic stroke	104,000 (90,100–118,000)	139,000 (124,000–159,000)	173,000 (146,000–205,000)	525 (457–593)	343 (306–392)	316 (268–375)
1-51	Intracerebral hemorrhage	207,000 (176,000–233,000)	186,000 (162,000–216,000)	193,000 (156,000–238,000)	841 (728–946)	414 (361–480)	331 (268–409)
1-52	Subarachnoid hemorrhage	87,300 (66,000–111,000)	101,000 (87,200–116,000)	105,000 (84,500–129,000)	300 (226–388)	207 (179–241)	172 (139–212)
1-53	Hypertensive heart disease	47,100 (40,400–53,900)	83,200 (71,500–93,200)	90,900 (74,500–108,000)	232 (199–265)	202 (173–227)	164 (135–196)
1-54	Non-rheumatic valvular heart disease	5,380 (4,270–6,530)	8,290 (7,230–9,490)	10,500 (8,590–12,800)	21·3 (17–25·6)	18·3 (15·9–20·8)	18 (14·7–21·8)
1-55	Non-rheumatic calcific aortic valve disease	3,300 (2,470–4,280)	5,510 (4,720–6,530)	7,260 (5,880–8,870)	13·7 (10·4–17·4)	12·4 (10·6–14·6)	12·5 (10·2–15·3)
1-56	Non-rheumatic degenerative mitral valve disease	1,900 (1,540–2,300)	2,620 (2,140–3,110)	3,090 (2,340–3,880)	7·1 (5·7–8·5)	5·6 (4·5–6·6)	5·2 (3·9–6·5)
1-57	Other non-rheumatic valve diseases	178 (110–245)	162 (123–213)	177 (124–235)	0·6 (0·3–0·8)	0·3 (0·2–0·4)	0·3 (0·2–0·4)
1-58	Cardiomyopathy and myocarditis	23,400 (15,100–30,900)	26,100 (20,300–30,800)	26,400 (20,400–34,000)	66·5 (45·8–84)	53·1 (41·2–62·4)	44·1 (34–56·2)
1-59	Myocarditis	2,730 (1,200–4,230)	2,030 (1,450–2,530)	2,120 (1,600–2,840)	8 (4·1–11·2)	4·1 (2·9–5)	3·5 (2·6–4·7)
1-60	Alcoholic cardiomyopathy	684 (438–954)	669 (512–886)	795 (568–1,090)	2·9 (1·8–4·1)	1·5 (1·1–2)	1·3 (1–1·8)

1-61	Other cardiomyopathy	20,000 (13,100–27,000)	23,400 (18,000–27,600)	23,500 (17,700–30,200)	55·6 (38·9–72)	47·5 (36·7–56)	39·2 (29·7–50·1)
1-62	Atrial fibrillation and flutter	11,200 (9,580–13,100)	24,800 (21,100–28,800)	33,900 (28,100–40,100)	64·3 (55·1–74·2)	64·5 (55·1–75)	63·6 (52·7–75·2)
1-63	Aortic aneurysm	6,680 (5,570–7,770)	13,000 (11,600–14,900)	15,800 (13,000–19,100)	30·7 (25·8–35·6)	30·5 (27·1–35)	27·9 (22·9–33·7)
1-64	Peripheral vascular disease	1,160 (681–1,850)	2,270 (1,400–3,620)	3,140 (1,980–4,920)	6·5 (3·8–10·2)	5·8 (3·6–9·4)	5·9 (3·7–9·2)
1-65	Endocarditis	11,500 (7,570–15,800)	11,100 (8,960–13,700)	11,300 (8,600–14,600)	30·2 (21·2–38·8)	21·9 (17·5–27)	18·5 (14·1–23·9)
1-66	Chronic respiratory diseases	348,000 (286,000–411,000)	348,000 (299,000–406,000)	394,000 (323,000–472,000)	1,030 (902–1,180)	764 (661–878)	680 (560–812)
1-67	Chronic obstructive pulmonary disease	96,100 (80,400–110,000)	135,000 (117,000–154,000)	156,000 (130,000–184,000)	408 (351–457)	327 (281–372)	282 (236–335)
1-68	Pneumoconiosis	2,790 (2,170–3,460)	3,360 (2,650–4,050)	3,030 (2,310–3,950)	11·8 (9·1–14·6)	7·9 (6·2–9·5)	5·3 (4·1–7)
1-69	Silicosis	874 (391–1,220)	626 (442–924)	603 (408–914)	3·9 (1·7–5·3)	1·5 (1–2·2)	1·1 (0·7–1·6)
1-70	Asbestosis	103 (72–143)	129 (94–164)	147 (106–205)	0·4 (0·3–0·6)	0·3 (0·2–0·4)	0·3 (0·2–0·4)
1-71	Coal workers pneumoconiosis	104 (60–151)	99 (63–134)	86 (58–123)	0·4 (0·2–0·6)	0·2 (0·1–0·3)	0·1 (0·1–0·2)
1-72	Other pneumoconiosis	1,710 (1,270–2,270)	2,510 (1,850–3,050)	2,190 (1,600–2,890)	7·1 (5·3–9·6)	5·9 (4·4–7·2)	3·9 (2·8–5·2)
1-73	Asthma	184,000 (131,000–246,000)	109,000 (74,000–159,000)	122,000 (78,600–186,000)	396 (290–522)	196 (136–285)	192 (125–292)
1-74	Interstitial lung disease and pulmonary sarcoidosis	31,200 (24,200–45,700)	80,000 (60,400–93,300)	93,400 (68,200–118,000)	142 (110–212)	194 (146–226)	169 (123–213)
1-75	Other chronic respiratory diseases	34,100 (18,100–58,100)	20,300 (16,500–25,400)	20,200 (15,900–25,800)	76·2 (42·2–123)	38·1 (31–47·7)	31·8 (25·1–40·8)
1-76	Digestive diseases	918,000 (806,000–1,030,000)	716,000 (634,000–804,000)	802,000 (662,000–972,000)	2,670 (2,380–2,950)	1,540 (1,370–1,720)	1,350 (1,120–1,640)
1-77	Cirrhosis and other chronic liver diseases	246,000 (211,000–294,000)	324,000 (285,000–364,000)	373,000 (294,000–465,000)	928 (802–1,090)	727 (639–818)	639 (505–795)
1-78	Cirrhosis and other chronic liver diseases due to hepatitis B	25,900 (18,700–35,400)	29,500 (20,400–41,100)	26,600 (17,000–38,500)	106 (75·8–146)	65·3 (45–92·2)	44·8 (28·8–65·3)
1-79	Cirrhosis and other chronic liver diseases due to hepatitis C	27,200 (18,900–38,400)	40,500 (28,600–55,900)	47,400 (31,600–68,900)	111 (77·3–157)	90·1 (63·7–125)	80·3 (53·8–117)
1-80	Cirrhosis and other chronic liver diseases due to alcohol use	77,200 (58,000–98,800)	127,000 (97,900–157,000)	153,000 (111,000–201,000)	335 (253–431)	291 (224–361)	264 (191–346)
1-81	Cirrhosis and other chronic liver diseases due to NAFLD	33,500 (23,700–46,700)	57,600 (40,800–77,600)	70,000 (47,300–98,900)	145 (101–200)	133 (94·1–179)	122 (82·4–172)
1-82	Cirrhosis and other chronic liver diseases due to other causes	82,100 (63,000–107,000)	69,800 (54,800–87,700)	75,500 (55,300–102,000)	230 (178–294)	148 (114–188)	128 (93·2–173)
1-83	Upper digestive system diseases	102,000 (80,400–132,000)	125,000 (90,000–178,000)	141,000 (97,800–208,000)	370 (298–474)	265 (195–370)	233 (164–341)
1-84	Peptic ulcer disease	50,100 (42,500–58,700)	36,700 (31,800–42,800)	33,700 (27,000–41,800)	193 (166–222)	84·1 (72·9–97·9)	59·1 (47·4–73·3)
1-85	Gastritis and duodenitis	15,600 (11,200–20,900)	26,800 (20,800–34,200)	30,200 (22,700–39,600)	53·8 (39·4–73)	58 (45·6–74)	50·7 (38·3–66·2)
1-86	GERD	36,000 (18,800–63,700)	61,400 (32,000–109,000)	76,900 (40,000–136,000)	123 (63·9–218)	123 (64·1–218)	123 (64–218)
1-87	Appendicitis	131,000 (95,800–172,000)	27,200 (22,700–35,000)	24,900 (19,700–31,900)	282 (212–362)	50·9 (42·7–65·4)	39·5 (31·3–50·6)
1-88	Paralytic ileus and intestinal obstruction	213,000 (157,000–271,000)	70,700 (59,400–82,900)	72,300 (53,900–94,900)	453 (345–563)	144 (120–168)	122 (90·4–159)
1-89	Inguinal, femoral, and abdominal hernia	79,800 (54,400–111,000)	46,200 (34,300–61,100)	52,200 (38,800–70,100)	182 (130–243)	89·6 (67·1–118)	84·5 (63·1–113)
1-90	Inflammatory bowel disease	10,800 (5,530–17,300)	5,170 (4,330–6,100)	5,940 (4,660–7,390)	26·3 (15·8–39)	10·7 (9–12·5)	9·8 (7·8–12·2)
1-91	Vascular intestinal disorders	7,010 (5,020–9,750)	8,210 (7,120–9,840)	10,500 (8,620–12,700)	25·8 (20·3–32·8)	19·5 (16·9–23·3)	18·8 (15·5–22·8)
1-92	Gallbladder and biliary diseases	40,300 (33,400–48,600)	43,200 (36,300–53,900)	50,500 (40,700–62,100)	158 (132–189)	96·5 (81·3–121)	86·8 (70–106)
1-93	Pancreatitis	41,700 (30,700–49,400)	39,700 (33,900–51,600)	43,600 (34,000–57,100)	145 (108–173)	84 (71·8–110)	72·5 (56·5–95·9)
1-94	Other digestive diseases	47,200 (29,800–72,000)	26,500 (21,700–31,600)	28,300 (20,700–36,800)	101 (68·7–149)	54·8 (43·8–64·6)	47·4 (34·9–61)
1-95	Neurological disorders	363,000 (222,000–571,000)	548,000 (317,000–866,000)	671,000 (404,000–1,060,000)	1,210 (779–1,830)	1,140 (692–1,790)	1,120 (682–1,760)
1-96	Alzheimer's disease and other dementias	52,400 (21,400–123,000)	118,000 (48,700–268,000)	165,000 (69,800–369,000)	322 (132–754)	315 (130–719)	315 (134–703)
1-97	Parkinson's disease	14,400 (12,800–16,500)	34,900 (30,400–38,700)	44,800 (37,500–52,100)	79·9 (71–91)	89·3 (77·8–99)	84 (70·5–97·9)
1-98	Idiopathic epilepsy	126,000 (82,700–177,000)	126,000 (77,000–188,000)	137,000 (81,600–211,000)	329 (215–465)	232 (140–345)	216 (128–333)
1-99	Multiple sclerosis	1,490 (1,250–1,840)	2,960 (2,450–3,440)	3,730 (2,950–4,620)	5·7 (4·8–7)	6·3 (5·2–7·3)	6·1 (4·9–7·6)
1-100	Motor neuron disease	1,620 (1,430–1,820)	3,310 (2,900–3,760)	4,720 (3,760–5,890)	5·8 (5·2–6·6)	7·3 (6·4–8·3)	8 (6·4–10)
1-101	Headache disorders	145,000 (33,000–302,000)	236,000 (55,500–499,000)	281,000 (66,600–592,000)	413 (109–846)	437 (109–924)	437 (106–919)
1-102	Migraine	129,000 (22,600–290,000)	210,000 (36,800–460,000)	250,000 (45,800–548,000)	364 (71·6–798)	388 (73·1–851)	388 (73·3–850)
1-103	Tension-type headache	15,900 (4,740–65,600)	25,600 (7,810–97,800)	31,200 (9,620–117,000)	48·8 (15·4–183)	49·4 (15·3–181)	49·3 (15·4–182)
1-104	Other neurological disorders	22,500 (14,600–31,700)	27,400 (17,500–39,900)	34,900 (21,800–52,600)	57·2 (38·5–79·3)	50·6 (33·1–72·7)	55·2 (34·8–82·7)
1-105	Mental disorders	583,000 (424,000–774,000)	896,000 (653,000–1,180,000)	1,090,000 (788,000–1,450,000)	1,710 (1,250–2,260)	1,690 (1,230–2,230)	1,700 (1,240–2,270)
1-106	Schizophrenia	53,200 (36,900–70,600)	90,900 (63,200–121,000)	114,000 (79,000–151,000)	174 (121–228)	177 (123–234)	178 (124–236)
1-107	Depressive disorders	153,000 (104,000–214,000)	234,000 (162,000–327,000)	291,000 (199,000–403,000)	486 (337–678)	458 (320–633)	462 (318–640)
1-108	Major depressive disorder	125,000 (83,300–176,000)	188,000 (129,000–260,000)	233,000 (157,000–324,000)	396 (267–552)	368 (253–509)	372 (251–513)
1-109	Dysthymia	28,000 (16,900–43,000)	46,300 (28,300–70,800)	57,200 (35,300–87,200)	90·2 (55·7–137)	90·4 (56·4–138)	90·4 (56·5–138)
1-110	Bipolar disorder	67,300 (39,600–105,000)	106,000 (63,000–167,000)	128,000 (74,800–199,000)	198 (118–307)	198 (116–309)	198 (116–307)
1-111	Anxiety disorders	179,000 (118,000–252,000)	277,000 (185,000–391,000)	337,000 (224,000–474,000)	516 (345–723)	520 (346–728)	527 (351–742)
1-112	Eating disorders	19,800 (11,900–30,100)	34,100 (21,000–51,300)	40,500 (24,500–63,100)	49 (29·4–74·5)	58·3 (35·9–87·6)	60·1 (36·3–93·6)
1-113	Anorexia nervosa	3,860 (2,200–6,360)	5,760 (3,270–9,410)	7,080 (4,130–11,400)	9 (5·2–14·6)	9·7 (5·5–15·9)	10·5 (6·1–16·8)

1-114	Bulimia nervosa	15,900 (9,190–25,400)	28,400 (17,000–44,100)	33,500 (19,700–53,400)	40 (23–63·3)	48·6 (29·1–75·5)	49·5 (29·2–78·9)
1-115	Autism spectrum disorders	21,000 (13,700–30,700)	28,900 (19,000–42,100)	33,600 (22,100–48,700)	51·9 (33·9–75·6)	52·3 (34·3–76·1)	52·3 (34·4–75·9)
1-116	Attention-deficit/hyperactivity disorder	11,500 (6,520–19,400)	15,100 (8,500–25,000)	16,800 (9,540–27,900)	25·5 (14·7–42·7)	25·7 (14·5–42·5)	25·7 (14·6–42·7)
1-117	Conduct disorder	35,000 (19,700–54,600)	42,600 (23,800–67,100)	44,900 (24,700–71,000)	68·8 (38·6–107)	69·4 (38·6–109)	69·6 (38·5–110)
1-118	Idiopathic developmental intellectual disability	11,300 (5,110–19,400)	12,500 (5,180–21,800)	12,100 (4,650–21,500)	27·8 (12·7–47·6)	22·5 (9·4–39)	18·7 (7·2–33·4)
1-119	Other mental disorders	31,600 (19,900–47,900)	54,100 (34,400–81,800)	67,900 (43,100–103,000)	108 (69·2–164)	109 (69·4–164)	109 (69·3–165)
1-120	Substance use disorders	134,000 (103,000–169,000)	192,000 (147,000–242,000)	211,000 (159,000–270,000)	415 (322–511)	360 (278–450)	326 (247–415)
1-121	Alcohol use disorders	73,900 (57,100–90,800)	89,500 (70,700–114,000)	92,900 (70,900–121,000)	253 (198–303)	177 (141–224)	147 (113–191)
1-122	Drug use disorders	60,200 (41,600–83,900)	103,000 (73,000–140,000)	118,000 (82,900–161,000)	162 (112–224)	184 (131–247)	179 (125–242)
1-123	Opioid use disorders	18,600 (12,200–26,000)	35,500 (24,200–49,000)	43,600 (29,800–60,100)	55·1 (37·3–75·5)	66·6 (46–90·8)	67·3 (46·3–92·3)
1-124	Cocaine use disorders	6,400 (4,650–8,780)	13,000 (9,910–16,900)	13,300 (9,810–17,700)	16·9 (12·5–22·6)	22·9 (17·7–29·7)	20·2 (14·9–26·8)
1-125	Amphetamine use disorders	16,900 (8,910–27,600)	26,100 (14,300–42,500)	29,700 (16,500–48,600)	43·1 (23·3–71·3)	45 (24·8–73·4)	43·9 (24·4–71·9)
1-126	Cannabis use disorders	2,840 (1,610–4,540)	4,120 (2,390–6,370)	4,770 (2,760–7,500)	7·1 (4·1–11·2)	7·1 (4·1–10·9)	7·1 (4·2–11·2)
1-127	Other drug use disorders	15,500 (9,380–23,200)	24,200 (15,400–35,900)	26,900 (16,800–40,200)	39·8 (24·8–59·6)	42·1 (26·9–62)	40·1 (25·1–59·8)
1-128	Diabetes and kidney diseases	332,000 (302,000–367,000)	785,000 (704,000–876,000)	997,000 (844,000–1,150,000)	1,380 (1,250–1,530)	1,820 (1,630–2,030)	1,750 (1,480–2,020)
1-129	Diabetes mellitus	151,000 (130,000–176,000)	378,000 (324,000–440,000)	517,000 (429,000–618,000)	696 (602–812)	893 (765–1,040)	912 (757–1,090)
1-130	Diabetes mellitus type 1	13,300 (11,800–15,400)	21,100 (17,500–25,100)	23,800 (19,000–29,400)	43 (37·8–50·1)	42·6 (35–50·4)	38·7 (30·8–47·8)
1-131	Diabetes mellitus type 2	138,000 (118,000–161,000)	357,000 (305,000–417,000)	493,000 (408,000–592,000)	653 (563–763)	850 (728–996)	873 (722–1,040)
1-132	Chronic kidney disease	180,000 (166,000–196,000)	407,000 (371,000–446,000)	480,000 (404,000–565,000)	680 (626–742)	926 (840–1,010)	836 (704–981)
1-133	Chronic kidney disease due to diabetes mellitus type 1	11,500 (7,930–15,800)	26,900 (17,700–37,600)	30,100 (19,200–44,900)	43·2 (28·9–60·4)	57·3 (37–81·7)	49·7 (31·4–75·4)
1-134	Chronic kidney disease due to diabetes mellitus type 2	28,300 (22,000–34,600)	86,500 (68,800–104,000)	110,000 (84,000–141,000)	139 (108–170)	211 (167–254)	199 (152–254)
1-135	Chronic kidney disease due to hypertension	27,200 (22,400–32,900)	84,400 (68,500–102,000)	104,000 (82,300–129,000)	134 (110–162)	208 (168–252)	188 (148–235)
1-136	Chronic kidney disease due to glomerulonephritis	49,300 (41,400–58,700)	94,900 (77,700–114,000)	105,000 (79,400–135,000)	161 (133–196)	201 (163–247)	176 (132–227)
1-137	Chronic kidney disease due to other and unspecified causes	63,700 (54,200–73,500)	114,000 (93,800–140,000)	131,000 (103,000–166,000)	203 (168–242)	248 (201–306)	224 (175–286)
1-138	Acute glomerulonephritis	1,220 (603–2,170)	262 (192–368)	240 (162–333)	2·8 (1·5–4·6)	0·5 (0·4–0·7)	0·4 (0·3–0·6)
1-139	Skin and subcutaneous diseases	232,000 (153,000–348,000)	315,000 (207,000–481,000)	369,000 (242,000–563,000)	599 (398–906)	582 (381–884)	584 (382–893)
1-140	Dermatitis	47,700 (28,000–75,300)	62,800 (37,400–99,100)	73,700 (44,000–116,000)	115 (68·9–180)	115 (68·7–180)	116 (69·6–182)
1-141	Atopic dermatitis	37,200 (20,100–62,900)	45,900 (24,700–76,400)	52,900 (28,000–88,700)	82·2 (44·5–138)	81·5 (43·8–136)	82·9 (43·9–139)
1-142	Contact dermatitis	9,290 (6,000–13,900)	15,100 (9,530–22,800)	18,600 (11,600–27,800)	29·8 (18·7–44·2)	29·8 (18·6–44·5)	29·8 (18·7–44·3)
1-143	Seborrhoeic dermatitis	1,150 (653–1,860)	1,760 (995–2,780)	2,130 (1,190–3,380)	3·4 (1·9–5·4)	3·4 (1·9–5·3)	3·4 (1·9–5·4)
1-144	Psoriasis	24,200 (17,100–32,500)	34,200 (24,200–45,900)	38,800 (27,400–51,300)	74·1 (52·5–99·1)	66·7 (47·1–89·2)	62·1 (44–82)
1-145	Bacterial skin diseases	8,100 (5,820–12,000)	7,250 (5,680–10,100)	9,360 (7,120–12,500)	21·5 (15·8–29·9)	14·8 (11·8–20·3)	15·6 (11·9–20·6)
1-146	Cellulitis	2,700 (1,040–4,340)	1,530 (1,180–2,020)	1,970 (1,490–2,580)	7·4 (3·2–11·1)	3·1 (2·4–4·1)	3·3 (2·5–4·2)
1-147	Pyoderma	5,400 (3,100–9,650)	5,720 (4,240–8,330)	7,390 (5,350–10,100)	14 (8·6–23·3)	11·7 (8·8–16·6)	12·4 (9–16·8)
1-148	Scabies	34,400 (18,500–55,000)	45,900 (25,300–73,400)	52,700 (28,900–83,800)	82·7 (45·1–132)	82·2 (45·4–132)	82·1 (45·3–131)
1-149	Fungal skin diseases	23,500 (9,350–50,600)	35,900 (14,500–77,400)	43,600 (17,600–94,700)	72 (29·2–155)	71·2 (28·8–154)	71 (28·8–153)
1-150	Viral skin diseases	23,900 (15,300–36,000)	29,500 (18,800–44,000)	33,300 (21,100–50,100)	51·9 (33·2–77·9)	52·1 (33·3–77·7)	52 (33·1–78·3)
1-151	Acne vulgaris	29,200 (17,300–46,300)	41,700 (24,800–66,100)	47,300 (27,900–74,800)	61·4 (36·5–97·3)	67·4 (40–107)	71·5 (42·1–113)
1-152	Alopecia areata	2,480 (1,570–3,720)	3,810 (2,410–5,640)	4,570 (2,910–6,780)	7·1 (4·5–10·7)	7·1 (4·5–10·5)	7·1 (4·5–10·5)
1-153	Pruritus	2,640 (1,270–4,720)	4,220 (2,010–7,580)	5,250 (2,520–9,390)	8·2 (3·9–14·6)	8·4 (4–15·1)	8·5 (4·1–15·2)
1-154	Urticaria	20,300 (13,300–29,700)	26,600 (17,200–39,200)	30,700 (20,000–44,500)	48 (31·5–69·2)	48·2 (31·2–70·4)	48·1 (31·4–69·8)
1-155	Decubitus ulcer	2,170 (615–3,150)	1,210 (965–1,620)	1,770 (1,380–2,290)	10·2 (3·2–14·6)	3 (2·4–4·1)	3·2 (2·5–4·2)
1-156	Other skin and subcutaneous diseases	13,900 (6,830–25,000)	22,500 (10,900–41,100)	28,400 (13,800–51,000)	46 (22·9–82·2)	45·9 (22·4–83·9)	46·7 (22·7–83·7)
1-157	Sense organ diseases	229,000 (157,000–318,000)	401,000 (276,000–566,000)	499,000 (342,000–709,000)	972 (675–1,360)	913 (632–1,290)	865 (594–1,230)
1-158	Blindness and vision loss	91,800 (64,600–126,000)	153,000 (107,000–212,000)	182,000 (126,000–254,000)	406 (290–548)	355 (249–491)	320 (222–445)
1-159	Glaucoma	4,050 (2,670–5,820)	6,510 (4,350–9,250)	7,850 (5,270–11,100)	23·1 (15·2–33·2)	16·9 (11·3–24)	14·8 (9·9–20·8)
1-160	Cataract	26,100 (18,300–35,100)	45,600 (32,100–61,300)	52,100 (36,900–70,000)	140 (99–188)	115 (81–154)	96·2 (68·3–129)
1-161	Age-related macular degeneration	1,710 (1,160–2,430)	3,470 (2,360–4,820)	4,390 (3,020–6,180)	9·2 (6·2–13·1)	8·8 (6–12·2)	8·1 (5·6–11·4)
1-162	Refraction disorders	34,000 (22,800–49,200)	54,200 (36,100–77,700)	63,800 (42,400–92,200)	113 (75·3–160)	111 (74·1–158)	104 (69·5–151)
1-163	Near vision loss	9,960 (4,640–19,600)	19,600 (9,040–38,400)	26,200 (12,100–51,100)	49 (23·1–94·4)	47·3 (22–91·6)	46·8 (21·6–91·1)
1-164	Other vision loss	16,000 (11,100–22,300)	23,700 (16,600–32,500)	28,100 (19,600–38,900)	71·2 (49·3–98·7)	55·5 (39·1–76)	49·7 (34·7–69)
1-165	Age-related and other hearing loss	127,000 (85,300–186,000)	231,000 (155,000–335,000)	295,000 (197,000–429,000)	530 (356–765)	522 (352–752)	508 (341–738)
1-166	Other sense organ diseases	9,850 (5,910–15,100)	17,000 (10,000–26,000)	21,700 (12,800–33,800)	35·7 (21·3–55)	36·3 (21·4–56·1)	36·5 (21·5–56·9)

1-167	Musculoskeletal disorders	413,000 (296,000–542,000)	768,000 (554,000–1,020,000)	990,000 (712,000–1,310,000)	1,510 (1,080–2,010)	1,600 (1,150–2,140)	1,630 (1,170–2,150)
1-168	Rheumatoid arthritis	8,740 (6,590–11,200)	20,000 (14,700–25,600)	27,400 (20,200–35,400)	35·3 (27·2–44·3)	43·8 (32·8–55·7)	46·2 (34·3–59·1)
1-169	Osteoarthritis	40,900 (20,700–82,000)	90,200 (45,300–183,000)	124,000 (62,400–248,000)	195 (99·1–390)	214 (108–430)	218 (110–434)
1-170	Low back pain	197,000 (138,000–264,000)	336,000 (235,000–451,000)	416,000 (293,000–559,000)	677 (478–909)	682 (479–915)	674 (476–910)
1-171	Neck pain	37,100 (23,900–53,900)	65,500 (42,800–94,300)	83,300 (55,100–120,000)	135 (88·5–195)	135 (89–194)	135 (89·6–195)
1-172	Gout	1,740 (1,100–2,530)	3,990 (2,530–5,770)	5,470 (3,380–7,890)	7·4 (4·6–10·8)	8·9 (5·6–12·8)	9·3 (5·8–13·5)
1-173	Other musculoskeletal disorders	128,000 (88,100–177,000)	252,000 (176,000–350,000)	334,000 (229,000–461,000)	462 (318–636)	520 (358–721)	543 (370–749)
1-174	Other non-communicable diseases	936,000 (756,000–1,210,000)	1,210,000 (988,000–1,470,000)	1,270,000 (1,000,000–1,620,000)	2,350 (1,870–2,970)	2,270 (1,850–2,780)	2,050 (1,620–2,620)
1-175	Congenital anomalies	446,000 (339,000–733,000)	543,000 (394,000–636,000)	465,000 (310,000–635,000)	805 (612–1,310)	916 (665–1,070)	732 (488–1,000)
1-176	Neural tube defects	47,100 (29,700–71,000)	35,900 (24,000–49,600)	28,800 (17,900–44,300)	83·6 (52·9–126)	60·6 (40·5–83·6)	45·5 (28·1–70·1)
1-177	Congenital heart anomalies	181,000 (132,000–266,000)	231,000 (161,000–278,000)	196,000 (125,000–279,000)	326 (238–475)	389 (271–469)	308 (196–440)
1-178	Orofacial clefts	7,310 (4,420–12,600)	3,030 (2,150–4,130)	2,900 (2,010–3,950)	13·5 (8·3–22·8)	5·3 (3·8–7·2)	4·6 (3·2–6·2)
1-179	Down's syndrome	19,900 (13,700–32,500)	22,700 (16,300–29,100)	19,300 (13,300–26,500)	36 (24·9–58·6)	38·3 (27·5–49·2)	30·4 (20·9–41·6)
1-180	Turner syndrome	64 (28–115)	74 (33–129)	76 (34–130)	0·1 (0·1–0·2)	0·1 (0·1–0·2)	0·1 (0·1–0·2)
1-181	Klinefelter syndrome	5 (2–9)	5 (2–10)	6 (2–11)	0 (0–0)	0 (0–0)	0 (0–0)
1-182	Other chromosomal abnormalities	9,700 (6,280–23,200)	15,600 (11,300–20,200)	15,800 (9,910–22,000)	17·5 (11·5–41·2)	26·3 (19–34)	24·9 (15·6–34·6)
1-183	Congenital musculoskeletal and limb anomalies	18,800 (12,900–26,100)	24,600 (17,500–33,200)	26,900 (18,700–37,300)	39·9 (27·6–55·2)	43·2 (30·7–58·3)	42·1 (29·2–58·4)
1-184	Urogenital congenital anomalies	5,440 (3,650–10,200)	8,380 (6,070–10,600)	7,360 (4,830–10,800)	9·9 (6·7–18·2)	14·1 (10·2–17·9)	11·6 (7·6–17)
1-185	Digestive congenital anomalies	52,500 (31,200–105,000)	61,300 (44,700–80,700)	51,500 (31,500–75,300)	92·2 (54·9–185)	103 (74·9–135)	81·4 (49·8–119)
1-186	Other congenital anomalies	104,000 (63,300–217,000)	141,000 (97,300–173,000)	116,000 (68,100–171,000)	187 (114–384)	237 (164–291)	184 (108–270)
1-187	Urinary diseases and male infertility	31,900 (27,300–38,200)	60,900 (47,700–72,900)	71,300 (55,500–88,800)	115 (96·3–140)	139 (108–167)	125 (97·1–157)
1-188	Urinary tract infections and interstitial nephritis	22,000 (19,500–25,900)	39,200 (28,900–44,600)	43,400 (31,500–54,300)	71 (63·1–87·2)	87·7 (63·7–99·6)	75·9 (54·8–95·1)
1-189	Urolithiasis	1,720 (1,190–2,320)	3,100 (2,270–4,110)	3,930 (2,830–5,240)	6·3 (4·5–8·5)	6·5 (4·8–8·5)	6·4 (4·6–8·4)
1-190	Benign prostatic hyperplasia	5,960 (3,460–9,360)	14,400 (8,510–21,600)	18,800 (11,300–28,600)	31·1 (18·1–48·9)	36 (21·3–54·3)	34·5 (20·6–52·1)
1-191	Male infertility	343 (137–747)	712 (281–1,570)	995 (387–2,210)	1 (0·4–2·2)	1·3 (0·5–2·9)	1·5 (0·6–3·3)
1-192	Other urinary diseases	1,860 (1,390–2,350)	3,520 (2,440–4,470)	4,150 (2,990–5,310)	6·1 (4·5–7·6)	7·4 (5–9·4)	6·9 (4·9–8·9)
1-193	Gynecological diseases	157,000 (108,000–220,000)	239,000 (163,000–334,000)	286,000 (196,000–399,000)	479 (332–666)	450 (309–629)	442 (304–618)
1-194	Uterine fibroids	11,500 (6,400–20,300)	17,600 (9,020–32,500)	22,100 (10,900–41,200)	39·1 (21·7–69·2)	34·9 (17·8–64·2)	34·8 (17·2–64·8)
1-195	Polycystic ovarian syndrome	3,800 (1,650–7,600)	7,540 (3,200–14,800)	9,330 (4,020–18,400)	10 (4·3–20)	13·2 (5·6–26)	14 (6–27·7)
1-196	Female infertility	70 (23–181)	285 (55–901)	514 (77–1,660)	0·2 (0·1–0·6)	0·5 (0·1–1·6)	0·8 (0·1–2·5)
1-197	Endometriosis	15,000 (8,760–24,300)	18,000 (10,700–29,100)	19,800 (12,000–31,400)	41·6 (24·7–66)	32·2 (19·2–51·7)	29·6 (18–47·1)
1-198	Genital prolapse	2,590 (1,460–4,520)	3,570 (1,820–6,610)	4,550 (2,330–8,320)	10·7 (5·8–19)	8 (4–14·9)	7·7 (3·9–14·2)
1-199	Premenstrual syndrome	36,200 (22,100–56,500)	55,500 (33,900–86,200)	64,600 (39,300–101,000)	96·8 (59·7–151)	98·2 (60–152)	97·4 (59·4–152)
1-200	Other gynecological diseases	87,400 (57,100–124,000)	136,000 (88,900–195,000)	165,000 (107,000–239,000)	281 (183–401)	263 (171–377)	258 (167–372)
1-201	Hemoglobinopathies and hemolytic anaemias	32,600 (25,900–41,500)	27,000 (20,900–35,300)	26,700 (20,400–35,500)	86·5 (68·4–110)	50·4 (39·4–65·7)	42·3 (32·3–56)
1-202	Thalassemias	913 (637–1,270)	536 (385–729)	340 (223–492)	1·7 (1·2–2·3)	0·9 (0·7–1·3)	0·5 (0·3–0·8)
1-203	Thalassaemias trait	3,610 (2,350–5,300)	3,880 (2,490–5,780)	3,960 (2,510–5,870)	8·9 (5·8–13·2)	6·9 (4·5–10·3)	6·1 (3·9–9)
1-204	Sickle cell disorders	3,280 (2,510–4,190)	2,560 (2,010–3,180)	1,810 (1,290–2,460)	6·6 (5·1–8·5)	4·3 (3·4–5·3)	2·8 (2–3·7)
1-205	Sickle cell trait	4,800 (3,010–7,200)	4,670 (2,890–7,060)	4,680 (2,870–7,150)	12·2 (7·6–18·1)	8·1 (5·1–12·3)	7·1 (4·3–10·8)
1-206	G6PD deficiency	2,890 (2,160–3,710)	2,700 (2,040–3,390)	4,090 (3,080–5,250)	7·1 (5·4–9)	4·9 (3·8–6·2)	6·4 (4·8–8·2)
1-207	G6PD trait	44 (27–71)	74 (44–112)	80 (48–123)	0·2 (0·1–0·3)	0·1 (0·1–0·2)	0·1 (0·1–0·2)
1-208	Other hemoglobinopathies and hemolytic anaemias	17,000 (13,400–22,200)	12,600 (9,660–16,700)	11,800 (8,850–16,100)	49·8 (39·6–63·8)	25·1 (19·5–33·1)	19·3 (14·6–26·2)
1-209	Endocrine, metabolic, blood, and immune disorders	128,000 (83,200–162,000)	119,000 (86,200–159,000)	150,000 (110,000–200,000)	361 (252–458)	246 (179–330)	250 (184–333)
1-210	Oral disorders	119,000 (72,900–181,000)	206,000 (125,000–313,000)	264,000 (160,000–402,000)	466 (287–706)	446 (274–667)	445 (272–680)
1-211	Caries of deciduous teeth	1,660 (713–3,480)	1,760 (776–3,600)	1,960 (837–4,070)	3·1 (1·3–6·6)	3·1 (1·4–6·3)	3·1 (1·3–6·4)
1-212	Caries of permanent teeth	12,800 (5,660–25,600)	18,700 (8,120–37,400)	21,800 (9,480–42,800)	34·7 (15·2–67·7)	34·4 (14·9–68·3)	34·2 (14·9–67·2)
1-213	Periodontal disease	24,400 (9,230–52,300)	44,200 (16,900–96,300)	56,700 (21,500–123,000)	87·5 (33·2–187)	90·2 (34·6–194)	91 (34·7–195)
1-214	Edentulism	61,700 (39,700–89,900)	113,000 (73,500–161,000)	150,000 (95,400–219,000)	287 (185–415)	266 (174–380)	263 (168–382)
1-215	Other oral disorders	18,700 (11,600–27,500)	28,200 (17,600–41,200)	33,900 (21,200–49,300)	53·2 (33·2–77·7)	53·2 (33·2–77·5)	53·3 (33·3–77·4)
1-216	Sudden infant death syndrome	21,300 (6,970–50,300)	14,000 (6,860–23,700)	11,200 (6,070–17,800)	37·2 (12·2–88)	23·4 (11·4–39·4)	17·6 (9·5–28)
1-0	Injuries	2,360,000 (2,190,000–2,550,000)	2,110,000 (1,930,000–2,310,000)	1,930,000 (1,620,000–2,270,000)	5,810 (5,400–6,250)	3,920 (3,580–4,290)	3,030 (2,550–3,560)
1-1	Transport injuries	531,000 (476,000–599,000)	697,000 (591,000–766,000)	634,000 (515,000–760,000)	1,430 (1,290–1,630)	1,300 (1,100–1,430)	991 (804–1,190)
1-2	Road injuries	490,000 (440,000–555,000)	638,000 (539,000–704,000)	591,000 (481,000–709,000)	1,320 (1,190–1,510)	1,190 (1,000–1,310)	924 (751–1,110)

1-3	Pedestrian road injuries	257,000 (216,000–313,000)	309,000 (250,000–344,000)	239,000 (191,000–295,000)	691 (586–831)	581 (471–650)	379 (303–466)
1-4	Cyclist road injuries	13,800 (11,300–17,100)	25,600 (21,700–29,900)	25,900 (20,700–31,300)	40·8 (33·2–50·2)	49·2 (41·7–57·7)	41·1 (33–49·7)
1-5	Motorcyclist road injuries	30,800 (24,900–39,600)	66,000 (49,600–78,500)	79,400 (49,900–99,000)	81·5 (66·2–105)	118 (89·1–139)	120 (76·2–150)
1-6	Motor vehicle road injuries	181,000 (142,000–226,000)	223,000 (190,000–257,000)	233,000 (182,000–288,000)	485 (385–602)	412 (351–474)	362 (284–448)
1-7	Other road injuries	7,060 (5,540–9,530)	14,300 (11,700–16,800)	13,800 (10,400–17,300)	20·1 (15·9–26)	26·9 (21·9–31·5)	21·7 (16·4–27·2)
1-8	Other transport injuries	40,500 (34,800–46,200)	59,000 (50,600–65,200)	42,900 (33,400–52,500)	111 (96·6–126)	110 (94·1–121)	66·9 (52–81·7)
1-9	Unintentional injuries	1,390,000 (1,250,000–1,540,000)	925,000 (823,000–1,040,000)	879,000 (722,000–1,040,000)	3,240 (2,930–3,570)	1,740 (1,540–1,970)	1,400 (1,150–1,660)
1-10	Falls	120,000 (102,000–139,000)	184,000 (156,000–218,000)	217,000 (177,000–262,000)	394 (337–455)	377 (319–446)	357 (291–431)
1-11	Drowning	183,000 (151,000–214,000)	123,000 (107,000–144,000)	99,000 (78,300–128,000)	405 (342–467)	218 (191–256)	154 (122–199)
1-12	Fire, heat, and hot substances	103,000 (78,300–126,000)	67,800 (54,800–84,200)	68,400 (52,800–90,300)	256 (200–312)	128 (104–159)	109 (84–143)
1-13	Poisonings	42,400 (30,800–52,600)	21,500 (18,700–24,900)	19,500 (15,600–24,300)	93·2 (70·3–112)	38·6 (33·6–44·8)	30·4 (24·4–37·8)
1-14	Poisoning by carbon monoxide	1,320 (943–2,090)	2,760 (1,330–3,620)	2,840 (1,450–3,870)	3·4 (2·5–5·3)	5 (2·4–6·6)	4·4 (2·3–6)
1-15	Poisoning by other means	41,100 (29,600–51,200)	18,700 (16,000–22,300)	16,700 (13,100–21,300)	89·8 (67·2–109)	33·6 (29–40)	26 (20·5–33·2)
1-16	Exposure to mechanical forces	91,100 (76,200–108,000)	105,000 (86,100–129,000)	111,000 (86,700–140,000)	266 (222–315)	201 (165–248)	175 (137–222)
1-17	Unintentional firearm injuries	40,000 (31,900–47,300)	39,700 (28,700–47,400)	32,600 (24,200–42,400)	109 (86·7–128)	72·5 (52·9–86·7)	50·2 (37·5–65·3)
1-18	Other exposure to mechanical forces	51,000 (38,300–66,300)	65,100 (50,600–85,300)	77,900 (58,600–103,000)	157 (120–204)	128 (99·4–169)	125 (93·9–166)
1-19	Adverse effects of medical treatment	40,900 (27,600–50,000)	22,200 (18,700–27,900)	20,400 (15,700–27,200)	99·6 (69·8–119)	42·7 (36·3–53·6)	33·2 (25·7–44·2)
1-20	Animal contact	7,380 (5,700–9,270)	12,400 (7,980–15,100)	12,200 (8,550–15,200)	19·8 (15·3–25·4)	23·6 (15·3–28·9)	19·5 (13·6–24·4)
1-21	Venomous animal contact	5,500 (4,250–6,880)	9,160 (5,790–11,200)	8,820 (6,260–11,100)	14·7 (11·3–18·6)	17·4 (11·1–21·2)	14·1 (10–17·6)
1-22	Non-venomous animal contact	1,880 (1,340–2,540)	3,200 (2,100–4,160)	3,350 (2,250–4,540)	5·2 (3·8–7·1)	6·2 (4–8·1)	5·4 (3·6–7·3)
1-23	Foreign body	630,000 (517,000–753,000)	235,000 (204,000–271,000)	195,000 (149,000–253,000)	1,230 (1,040–1,450)	417 (363–479)	309 (236–399)
1-24	Pulmonary aspiration and foreign body in airway	601,000 (493,000–721,000)	224,000 (193,000–259,000)	183,000 (137,000–240,000)	1,170 (975–1,390)	395 (342–454)	288 (217–379)
1-25	Foreign body in eyes	1,890 (829–3,460)	2,880 (1,330–5,140)	3,460 (1,630–6,150)	5·5 (2·7–9·7)	5·5 (2·6–9·6)	5·4 (2·6–9·6)
1-26	Foreign body in other body part	26,900 (15,500–46,500)	8,750 (6,740–11,200)	9,260 (6,930–11,900)	58·6 (36·4–95·2)	16·4 (12·6–21)	14·7 (11–18·9)
1-27	Environmental heat and cold exposure	15,700 (12,200–19,900)	31,800 (27,000–37,700)	20,300 (15,000–26,400)	47·9 (36·9–61·1)	63·9 (54·4–75·3)	32·8 (24·2–42·6)
1-28	Exposure to forces of nature	23,000 (20,300–26,200)	20,000 (17,600–22,700)	12,500 (10,800–14,700)	58·3 (51·3–66·4)	36·9 (32·4–42)	19·8 (16·9–23·2)
1-29	Other unintentional injuries	132,000 (114,000–149,000)	103,000 (87,200–125,000)	104,000 (82,500–130,000)	362 (314–409)	194 (164–235)	164 (130–203)
1-30	Self-harm and interpersonal violence	444,000 (415,000–484,000)	492,000 (445,000–535,000)	418,000 (347,000–496,000)	1,140 (1,070–1,250)	879 (794–958)	638 (528–756)
1-31	Self-harm	85,600 (75,400–107,000)	174,000 (128,000–196,000)	178,000 (124,000–220,000)	235 (207–291)	311 (228–350)	272 (190–336)
1-32	Self-harm by firearm	7,970 (4,970–10,900)	9,980 (5,940–13,500)	8,850 (5,040–12,800)	22·4 (13·8–30·3)	18·2 (10·8–24·5)	13·6 (7·7–19·6)
1-33	Self-harm by other specified means	77,600 (68,600–98,500)	164,000 (120,000–185,000)	169,000 (118,000–209,000)	212 (186–271)	293 (212–330)	258 (179–319)
1-34	Interpersonal violence	182,000 (164,000–202,000)	296,000 (270,000–327,000)	223,000 (183,000–270,000)	485 (438–536)	527 (481–581)	339 (277–409)
1-35	Physical violence by firearm	78,700 (67,700–92,600)	158,000 (139,000–180,000)	99,900 (77,800–125,000)	212 (184–248)	279 (246–317)	150 (117–188)
1-36	Physical violence by sharp object	39,500 (29,900–48,600)	64,600 (55,100–83,100)	51,700 (40,200–74,300)	109 (82·6–132)	115 (98·7–148)	78·4 (60·9–112)
1-37	Sexual violence	6,900 (4,540–9,760)	10,500 (7,030–14,800)	12,300 (8,160–17,400)	19·2 (12·7–27·3)	19·1 (12·8–26·9)	18·8 (12·6–26·7)
1-38	Physical violence by other means	57,100 (48,700–67,400)	63,700 (56,200–72,700)	59,100 (47,000–72,300)	145 (123–172)	114 (101–130)	91 (72·7–112)
1-39	Conflict and terrorism	176,000 (160,000–198,000)	20,600 (13,000–33,100)	16,500 (9,940–27,000)	422 (382–478)	39·9 (25–63·9)	26·8 (16·2–43·8)
1-40	Executions and police conflict	240 (222–260)	332 (300–368)	826 (738–925)	0·6 (0·5–0·6)	0·6 (0·5–0·7)	1·3 (1·1–1·4)

SR Table 1: All-age DALYs counts and age standardized DALYs rates per 100,000 individuals for all causes and level 1 cause groups by location for 1990, 2010, and 2019, both sexes combined, Bolivia

Cause Level	Cause	All-age DALYs			Age-standardized rate of DALYs per 100,000		
		1990	2010	2019	1990	2010	2019
0-0	All causes	4,040,000 (3,750,000–4,340,000)	3,500,000 (3,030,000–3,990,000)	3,640,000 (3,080,000–4,280,000)	59,200 (54,500–64,300)	37,700 (32,700–42,900)	33,600 (28,400–39,300)
1-0	Communicable, maternal, neonatal, and nutritional disorders	2,300,000 (2,090,000–2,560,000)	1,230,000 (1,050,000–1,450,000)	1,010,000 (831,000–1,280,000)	25,800 (23,400–28,700)	10,700 (9,180–12,600)	7,830 (6,420–9,820)
1-1	HIV/AIDS and sexually transmitted infections	46,600 (17,500–107,000)	62,900 (20,100–185,000)	64,800 (16,000–294,000)	515 (192–1,290)	574 (170–1,700)	489 (121–2,270)
1-2	HIV/AIDS	12,000 (1,540–54,700)	42,400 (5,120–167,000)	44,000 (3,330–269,000)	200 (24·1–902)	419 (54·7–1,560)	350 (28·1–2,120)
1-3	HIV/AIDS - drug-susceptible tuberculosis	3,800 (671–9,620)	4,340 (1,570–7,210)	2,460 (896–4,730)	63·6 (10·8–160)	43·7 (16·9–71·4)	20·2 (7·5–37·6)
1-4	HIV/AIDS - Multidrug-resistant Tuberculosis without extensive drug resistance	63 (2–346)	350 (34–1,200)	312 (36–1,070)	1 (0–6)	3·5 (0·4–12·1)	2·6 (0·3–9·1)
1-5	HIV/AIDS - Extensively drug-resistant Tuberculosis	0 (0–0)	38 (4–134)	50 (5–168)	0 (0–0)	0·4 (0–1·4)	0·4 (0–1·3)
1-6	HIV/AIDS resulting in other diseases	8,100 (844–42,100)	37,600 (3,230–161,000)	41,200 (2,030–265,000)	136 (12·7–696)	371 (34·6–1,500)	327 (17·2–2,080)
1-7	Sexually transmitted infections excluding HIV	34,600 (11,000–76,200)	20,600 (8,060–40,200)	20,700 (8,350–39,800)	315 (108–674)	155 (63·7–297)	139 (59·1–265)
1-8	Syphilis	33,500 (9,990–74,900)	18,900 (6,660–38,800)	18,700 (6,510–37,600)	293 (88·3–653)	136 (48·4–280)	121 (42·4–244)
1-9	Chlamydial infection	144 (102–208)	210 (146–296)	258 (172–376)	2·7 (1·9–3·8)	2·2 (1·5–3)	2·2 (1·5–3·2)
1-10	Gonococcal infection	157 (114–220)	197 (135–280)	213 (147–305)	3·2 (2·3–4·4)	2·2 (1·5–3·2)	2 (1·4–2·8)
1-11	Trichomoniasis	196 (75–404)	335 (129–693)	400 (153–834)	3·9 (1·5–8)	3·6 (1·4–7·5)	3·5 (1·4–7·4)
1-12	Genital herpes	283 (97–698)	521 (171–1,290)	648 (212–1,610)	5·9 (1·9–14·6)	5·9 (1·9–14·6)	5·9 (1·9–14·6)
1-13	Other sexually transmitted infections	340 (249–470)	445 (313–617)	539 (375–753)	6·4 (4·8–8·9)	4·7 (3·3–6·5)	4·6 (3·3–6·5)
1-14	Respiratory infections and tuberculosis	1,000,000 (830,000–1,190,000)	414,000 (335,000–509,000)	315,000 (250,000–389,000)	12,100 (10,400–14,300)	4,010 (3,310–4,810)	2,740 (2,220–3,330)
1-15	Tuberculosis	238,000 (186,000–295,000)	81,600 (61,800–103,000)	59,900 (43,700–76,700)	4,020 (3,140–4,940)	903 (687–1,140)	553 (405–706)
1-16	Latent tuberculosis infection	0 (0–0)	0 (0–0)	0 (0–0)	0 (0–0)	0 (0–0)	0 (0–0)
1-17	Drug-susceptible tuberculosis	235,000 (183,000–291,000)	76,100 (54,500–98,800)	53,500 (35,600–71,300)	3,960 (3,090–4,870)	842 (606–1,090)	494 (329–653)
1-18	Multidrug-resistant tuberculosis without extensive drug resistance	3,380 (251–15,600)	4,990 (576–18,900)	5,460 (798–17,200)	57 (4·2–268)	55·3 (6·5–212)	50·5 (7·2–160)
1-19	Extensively drug-resistant tuberculosis	0 (0–0)	532 (62–1,940)	883 (129–2,940)	0 (0–0)	5·9 (0·7–21·8)	8·2 (1·2–27·5)
1-20	Lower respiratory infections	748,000 (598,000–919,000)	319,000 (255,000–401,000)	240,000 (189,000–302,000)	7,940 (6,590–9,600)	2,980 (2,450–3,640)	2,070 (1,660–2,530)
1-21	Upper respiratory infections	11,800 (4,780–26,100)	10,100 (6,080–15,400)	10,900 (6,780–16,800)	139 (64·6–274)	91·9 (56·6–139)	86·2 (54–132)
1-22	Otitis media	2,580 (1,490–4,380)	3,500 (2,030–5,730)	3,870 (2,230–6,310)	36·1 (21·1–59·4)	32·8 (19–53·8)	30·9 (17·9–51·3)
1-23	Enteric infections	229,000 (105,000–408,000)	70,500 (43,700–112,000)	58,800 (39,400–87,900)	2,440 (1,280–4,140)	635 (412–971)	464 (325–676)
1-24	Diarrhoeal diseases	228,000 (103,000–407,000)	69,700 (42,700–111,000)	58,000 (38,900–87,000)	2,430 (1,260–4,130)	628 (406–965)	458 (317–670)
1-25	Typhoid and paratyphoid	90 (41–175)	76 (35–143)	70 (32–133)	1·3 (0·6–2·4)	0·7 (0·3–1·4)	0·6 (0·3–1·1)
1-26	Typhoid fever	88 (40–172)	74 (35–141)	69 (31–131)	1·3 (0·6–2·4)	0·7 (0·3–1·3)	0·6 (0·3–1·1)
1-27	Paratyphoid fever	2 (1–4)	1 (0–3)	1 (0–2)	0 (0–0·1)	0 (0–0)	0 (0–0)
1-28	iNTS	559 (291–909)	553 (281–950)	559 (282–939)	6·7 (3·5–10·8)	4·9 (2·5–8·5)	4·3 (2·1–7·2)
1-29	Other intestinal infectious diseases	486 (157–882)	205 (73–375)	150 (59–262)	4·8 (1·7–8·6)	1·7 (0·7–3)	1·1 (0·5–1·8)
1-30	Neglected tropical diseases and malaria	51,600 (31,000–96,400)	40,100 (27,100–55,700)	42,600 (28,100–61,100)	965 (592–1,550)	486 (316–676)	415 (266–597)
1-31	Malaria	12,000 (1,770–53,600)	1,190 (678–2,130)	180 (46–458)	154 (23·8–688)	11·3 (6·4–20·4)	1·4 (0·3–3·5)
1-32	Chagas disease	12,500 (5,670–18,900)	15,300 (6,050–24,500)	16,800 (6,140–29,100)	340 (143–531)	214 (80·7–355)	183 (64·5–328)
1-33	Leishmaniasis	9,640 (2,680–32,800)	5,850 (3,160–9,610)	6,150 (3,500–9,810)	166 (58·6–428)	71·6 (39–118)	60·2 (34·4–96·6)
1-34	Visceral leishmaniasis	4,680 (5–28,200)	492 (0–2,920)	466 (0–2,790)	55·6 (0·1–321)	4·3 (0–24·9)	3·4 (0–20·4)
1-35	Cutaneous and mucocutaneous leishmaniasis	4,960 (2,250–9,060)	5,360 (3,010–8,770)	5,680 (3,330–9,100)	110 (50·3–199)	67·3 (37·5–110)	56·8 (32·7–91·4)
1-36	African trypanosomiasis	0 (0–0)	0 (0–0)	0 (0–0)	0 (0–0)	0 (0–0)	0 (0–0)
1-37	Schistosomiasis	0 (0–0)	0 (0–0)	0 (0–0)	0 (0–0)	0 (0–0)	0 (0–0)
1-38	Cysticercosis	4,830 (3,100–7,050)	4,040 (2,440–6,080)	4,710 (2,800–7,270)	108 (67·3–158)	50·9 (30·8–77·3)	47·2 (28·3–72·1)
1-39	Cystic echinococcosis	74 (26–141)	29 (12–51)	19 (9–32)	1 (0·5–1·8)	0·3 (0·1–0·5)	0·2 (0·1–0·3)
1-40	Lymphatic filariasis	0 (0–0)	0 (0–0)	0 (0–0)	0 (0–0)	0 (0–0)	0 (0–0)
1-41	Onchocerciasis	0 (0–0)	0 (0–0)	0 (0–0)	0 (0–0)	0 (0–0)	0 (0–0)
1-42	Trachoma	0 (0–0)	0 (0–0)	0 (0–0)	0 (0–0)	0 (0–0)	0 (0–0)
1-43	Dengue	298 (88–629)	1,860 (769–3,880)	1,350 (586–2,740)	4·4 (1·5–9·1)	17·8 (7–37·6)	11·1 (4·9–22·6)
1-44	Yellow fever	908 (191–2,760)	338 (71–1,060)	247 (50–773)	12·3 (2·6–37·8)	3·1 (0·6–9·6)	1·9 (0·4–6·1)
1-45	Rabies	309 (75–472)	43 (20–156)	40 (14–178)	5·8 (1·7–8·7)	0·6 (0·3–1·9)	0·4 (0·1–1·7)
1-46	Intestinal nematode infections	2,510 (1,700–3,540)	1,010 (606–1,550)	730 (438–1,140)	31·6 (21–44·9)	9 (5·5–13·8)	5·7 (3·4–8·8)
1-47	Ascariasis	1,410 (888–2,150)	404 (180–721)	256 (106–461)	15·2 (9·9–22·5)	3·4 (1·6–6·1)	1·9 (0·8–3·3)
1-48	Trichuriasis	35 (18–62)	46 (20–87)	48 (21–97)	0·5 (0·2–0·9)	0·4 (0·2–0·8)	0·4 (0·2–0·8)

1-49	Hookworm disease	1,070 (580–1,740)	562 (304–926)	426 (230–718)	15·9 (8·7–25·6)	5·2 (2·8–8·5)	3·4 (1·8–5·8)
1-50	Food-borne trematodiases	4,510 (1,080–9,160)	6,480 (1,490–13,200)	7,950 (1,800–16,400)	90·6 (21·8–184)	71·7 (16·2–146)	71·7 (16·5–148)
1-51	Leprosy	38 (23–57)	22 (13–32)	21 (13–32)	0·9 (0·5–1·3)	0·3 (0·2–0·4)	0·2 (0·1–0·3)
1-52	Ebola virus disease	0 (0–0)	0 (0–0)	0 (0–0)	0 (0–0)	0 (0–0)	0 (0–0)
1-53	Zika virus disease	0 (0–0)	0 (0–0)	14 (5–34)	0 (0–0)	0 (0–0)	0·1 (0–0·2)
1-54	Guinea worm disease	0 (0–0)	0 (0–0)	0 (0–0)	0 (0–0)	0 (0–0)	0 (0–0)
1-55	Other neglected tropical diseases	3,980 (2,670–5,470)	4,000 (2,600–5,760)	4,320 (2,800–6,370)	50·6 (34·5–69·7)	35·4 (23·2–50·8)	32·1 (20·8–47·3)
1-56	Other infectious diseases	160,000 (79,100–326,000)	56,900 (33,800–107,000)	44,000 (26,000–79,600)	1,640 (881–3,210)	474 (292–854)	319 (197–553)
1-57	Meningitis	36,700 (26,500–50,400)	15,900 (11,900–21,000)	8,350 (6,020–11,100)	380 (284–514)	132 (100–173)	62·8 (45·9–82·3)
1-58	Encephalitis	8,540 (5,700–14,700)	7,860 (5,440–11,100)	7,970 (5,400–11,200)	93·5 (64·4–154)	68·1 (48–95·5)	59·7 (41·1–82·6)
1-59	Diphtheria	2,370 (847–4,940)	81 (31–154)	50 (22–101)	23·5 (9–48·1)	0·7 (0·3–1·2)	0·3 (0·2–0·7)
1-60	Whooping cough	87,400 (7,800–259,000)	22,900 (2,100–69,100)	17,700 (1,950–54,200)	818 (73·5–2,430)	177 (16·3–536)	117 (12·8–357)
1-61	Tetanus	12,100 (6,330–21,400)	1,230 (643–2,180)	692 (368–1,280)	157 (91·2–256)	12 (6·2–21·6)	5·8 (3–11)
1-62	Measles	426 (297–595)	0 (0–0)	0 (0–0)	4·1 (2·8–5·7)	0 (0–0)	0 (0–0)
1-63	Varicella and herpes zoster	3,710 (2,010–6,210)	2,120 (1,260–3,400)	1,780 (1,080–2,840)	46·4 (29·8–70·2)	19·9 (13–30·3)	14·1 (9·3–21·3)
1-64	Acute hepatitis	3,240 (1,290–4,930)	1,380 (922–1,860)	1,300 (909–1,750)	45·7 (19·2–68)	13·9 (9·2–18·6)	11·1 (7·8–14·9)
1-65	Acute hepatitis A	2,570 (942–3,990)	933 (606–1,300)	859 (579–1,170)	35·1 (13·5–53·7)	9·1 (5·9–12·7)	7·1 (4·7–9·7)
1-66	Acute hepatitis B	345 (145–721)	271 (168–412)	273 (182–399)	5·2 (2·4–9·9)	2·9 (1·8–4·3)	2·4 (1·6–3·6)
1-67	Acute hepatitis C	266 (89–587)	123 (65–212)	110 (63–202)	4·5 (1·6–9·4)	1·3 (0·7–2·3)	1 (0·6–1·8)
1-68	Acute hepatitis E	60 (32–111)	57 (39–81)	61 (42–87)	0·9 (0·5–1·5)	0·6 (0·4–0·8)	0·5 (0·3–0·7)
1-69	Other unspecified infectious diseases	5,340 (3,750–7,660)	5,470 (3,850–7,270)	6,120 (4,520–8,080)	70·6 (52–96·3)	50·7 (36–67)	48·3 (35·7–63·3)
1-70	Maternal and neonatal disorders	621,000 (537,000–711,000)	478,000 (400,000–560,000)	398,000 (315,000–495,000)	5,760 (5,020–6,580)	3,560 (2,990–4,170)	2,670 (2,130–3,320)
1-71	Maternal disorders	43,900 (35,900–54,100)	39,100 (27,900–52,400)	33,600 (22,400–45,500)	746 (613–929)	382 (271–510)	274 (182–372)
1-72	Maternal haemorrhage	9,990 (7,800–12,800)	10,700 (7,490–14,400)	8,510 (5,760–11,700)	171 (133–221)	105 (74·3–142)	69·8 (47·3–95·6)
1-73	Maternal sepsis and other pregnancy related infections	8,000 (6,160–10,400)	3,930 (2,680–5,570)	2,750 (1,730–3,830)	134 (102–177)	37·8 (26–53·5)	22·3 (14–30·9)
1-74	Maternal hypertensive disorders	8,410 (6,540–10,700)	11,200 (7,650–15,300)	9,270 (5,960–12,800)	138 (108–176)	107 (73·5–146)	75·2 (48·7–104)
1-75	Maternal obstructed labour and uterine rupture	107 (48–192)	366 (245–521)	530 (336–756)	1·7 (0·8–3)	3·5 (2·4–5)	4·3 (2·7–6·2)
1-76	Maternal abortive outcome	15,500 (11,800–19,900)	4,530 (2,930–6,600)	3,030 (1,890–4,530)	268 (204–348)	45·4 (29·5–66·4)	25·2 (15·8–37·8)
1-77	Ectopic pregnancy	102 (78–129)	637 (426–909)	646 (414–905)	1·7 (1·3–2·1)	6 (4·1–8·5)	5·2 (3·3–7·3)
1-78	Indirect maternal deaths	184 (134–239)	1,610 (1,050–2,270)	2,140 (1,310–3,050)	3 (2·2–3·9)	15·2 (9·9–21·5)	17·1 (10·4–24·4)
1-79	Late maternal deaths	1,150 (818–1,610)	1,980 (1,220–2,970)	2,160 (1,220–3,230)	19·3 (14–26·9)	19·1 (11·7–28·6)	17·5 (10–26·3)
1-80	Maternal deaths aggravated by HIV/AIDS	17 (2–68)	30 (5–103)	38 (6–113)	0·3 (0–1·1)	0·3 (0–1)	0·3 (0–0·9)
1-81	Other maternal disorders	518 (397–674)	4,200 (2,880–5,890)	4,570 (2,980–6,520)	8·6 (6·6–11·2)	41·2 (28·3–57·6)	37·5 (24·4–53·5)
1-82	Neonatal disorders	577,000 (495,000–665,000)	439,000 (366,000–514,000)	365,000 (286,000–456,000)	5,020 (4,310–5,800)	3,180 (2,660–3,710)	2,400 (1,890–3,000)
1-83	Neonatal preterm birth	295,000 (236,000–363,000)	199,000 (152,000–249,000)	151,000 (109,000–201,000)	2,570 (2,060–3,150)	1,440 (1,100–1,800)	989 (719–1,310)
1-84	Neonatal encephalopathy due to birth asphyxia and trauma	143,000 (101,000–185,000)	100,000 (74,900–136,000)	81,100 (58,200–113,000)	1,230 (867–1,600)	722 (542–974)	535 (387–745)
1-85	Neonatal sepsis and other neonatal infections	74,900 (45,400–112,000)	98,200 (57,600–144,000)	97,300 (60,100–139,000)	651 (395–965)	710 (421–1,040)	644 (401–915)
1-86	Hemolytic disease and other neonatal jaundice	8,120 (6,240–10,400)	4,720 (3,750–5,800)	4,170 (3,290–5,180)	78·6 (61·5–98·9)	38·6 (30·7–46·7)	30·8 (24·4–38·1)
1-87	Other neonatal disorders	55,900 (36,000–78,300)	37,600 (26,500–51,000)	31,000 (20,500–44,600)	485 (311–678)	271 (191–367)	201 (133–288)
1-88	Nutritional deficiencies	195,000 (140,000–262,000)	104,000 (78,100–136,000)	89,700 (66,500–120,000)	2,310 (1,740–2,980)	977 (745–1,250)	728 (549–962)
1-89	Protein-energy malnutrition	145,000 (94,000–211,000)	47,000 (32,800–66,900)	32,800 (22,700–44,900)	1,560 (1,080–2,190)	440 (326–597)	278 (206–364)
1-90	Iodine deficiency	190 (104–317)	217 (120–353)	227 (125–378)	3·6 (2–5·9)	2·3 (1·3–3·8)	2 (1·1–3·3)
1-91	Vitamin A deficiency	1,650 (1,040–2,440)	1,840 (1,110–2,690)	1,720 (1,080–2,590)	18 (11·3–26·4)	15·5 (9·4–22·6)	12·5 (7·8–18·7)
1-92	Dietary iron deficiency	43,500 (28,800–63,200)	51,800 (32,700–76,100)	51,900 (32,600–77,600)	646 (425–931)	484 (305–710)	407 (256–606)
1-93	Other nutritional deficiencies	5,060 (3,070–7,720)	3,190 (2,290–4,260)	3,070 (2,230–4,040)	79 (54·2–111)	35·9 (26·7–46·9)	29·4 (21·6–38·2)
1-0	Non-communicable diseases	1,290,000 (1,100,000–1,490,000)	1,880,000 (1,600,000–2,160,000)	2,250,000 (1,880,000–2,660,000)	27,100 (23,900–30,500)	23,100 (19,800–26,400)	22,400 (18,900–26,400)
1-1	Neoplasms	173,000 (140,000–202,000)	286,000 (230,000–347,000)	365,000 (285,000–455,000)	4,330 (3,540–4,990)	3,850 (3,140–4,640)	3,850 (3,030–4,790)
1-2	Lip and oral cavity cancer	1,210 (919–1,500)	2,180 (1,690–2,760)	2,910 (2,110–3,870)	33·6 (25·3–41·7)	30·4 (23·4–38·3)	31·2 (22·6–41·2)
1-3	Nasopharynx cancer	335 (259–415)	542 (421–687)	656 (489–860)	8·2 (6·4–10·1)	7 (5·5–8·8)	6·7 (5–8·8)
1-4	Other pharynx cancer	439 (340–549)	709 (552–918)	935 (679–1,240)	12·3 (9·6–15·4)	9·9 (7·8–12·8)	10 (7·4–13·2)
1-5	Oesophageal cancer	2,070 (1,510–2,760)	3,530 (2,790–4,360)	4,690 (3,590–6,040)	62·3 (45·3–82·3)	51·9 (41·3–64·1)	52·6 (40·3–67)
1-6	Stomach cancer	38,200 (30,800–44,900)	55,200 (44,500–66,600)	67,300 (50,800–85,900)	1,120 (911–1,310)	800 (653–957)	746 (571–943)
1-7	Colon and rectum cancer	6,750 (4,870–8,460)	15,800 (11,700–20,300)	23,000 (16,100–30,500)	197 (141–246)	229 (169–292)	255 (178–338)

1-8	Liver cancer	4,920 (3,710–6,500)	8,050 (6,120–10,200)	10,500 (7,770–14,000)	130 (98·1–171)	111 (84·9–141)	113 (83·3–148)
1-9	Liver cancer due to hepatitis B	2,420 (1,740–3,270)	3,760 (2,580–5,120)	4,840 (3,350–6,780)	65·1 (46·5–88)	51·4 (35·3–69·8)	51 (35·2–70·6)
1-10	Liver cancer due to hepatitis C	280 (149–484)	522 (291–867)	689 (371–1,160)	9·1 (5–15·4)	8·1 (4·6–13·4)	8·1 (4·5–13·6)
1-11	Liver cancer due to alcohol use	1,090 (666–1,610)	2,080 (1,390–2,980)	2,910 (1,890–4,280)	32·9 (20·4–49·3)	30·7 (20·5–44)	32·5 (21·4–47·7)
1-12	Liver cancer due to NASH	308 (188–522)	630 (395–968)	871 (534–1,380)	9·3 (5·6–15·8)	9·4 (5·8–14·5)	10 (6·2–15·7)
1-13	Gallbladder and biliary tract cancer	6,320 (4,230–8,230)	10,200 (7,550–13,100)	13,200 (9,620–17,500)	190 (128–247)	150 (112–192)	147 (109–194)
1-14	Pancreatic cancer	1,950 (1,420–2,490)	6,590 (4,850–8,620)	10,200 (7,070–13,800)	58·1 (42·6–74·1)	96·1 (70·7–126)	113 (77·9–152)
1-15	Larynx cancer	1,380 (1,070–1,710)	1,960 (1,560–2,450)	2,550 (1,940–3,360)	40·8 (31·5–50·5)	28·5 (22·8–35·4)	28·2 (21·5–36·7)
1-16	Tracheal, bronchus, and lung cancer	11,000 (7,370–14,200)	20,400 (14,500–26,400)	28,100 (18,600–38,400)	323 (216–419)	295 (209–383)	310 (204–425)
1-17	Malignant skin melanoma	1,480 (758–2,600)	2,650 (1,680–3,900)	3,390 (2,130–5,130)	37·2 (20–61·6)	35·8 (22·8–52)	35·8 (22·6–54)
1-18	Non-melanoma skin cancer	620 (488–810)	1,630 (1,250–2,090)	2,240 (1,660–2,890)	19·3 (15·6–24·9)	25·3 (19·7–32·3)	26·9 (20·2–34·3)
1-19	Non-melanoma skin cancer (squamous-cell carcinoma)	620 (488–809)	1,630 (1,250–2,090)	2,240 (1,660–2,890)	19·3 (15·5–24·9)	25·3 (19·6–32·3)	26·8 (20·2–34·3)
1-20	Non-melanoma skin cancer (basal-cell carcinoma)	0 (0–1)	1 (0–1)	1 (0–1)	0 (0–0)	0 (0–0)	0 (0–0)
1-21	Breast cancer	10,200 (7,740–13,300)	20,500 (15,500–26,300)	27,000 (20,000–35,900)	267 (206–346)	276 (211–350)	279 (208–367)
1-22	Cervical cancer	20,800 (13,200–26,600)	28,900 (20,300–38,100)	34,200 (23,600–47,500)	534 (344–678)	382 (274–502)	350 (244–482)
1-23	Uterine cancer	2,490 (1,830–3,400)	4,550 (3,490–6,330)	6,030 (4,440–8,640)	72·2 (53·5–98·9)	65·2 (50·5–90·6)	65·7 (49·2–94)
1-24	Ovarian cancer	1,550 (965–3,760)	4,650 (3,190–8,150)	6,560 (4,400–10,500)	40·1 (24·9–96·3)	61·1 (42–109)	66·7 (44·7–108)
1-25	Prostate cancer	6,160 (4,900–7,660)	15,200 (11,900–18,900)	22,300 (16,300–28,900)	219 (175–271)	252 (197–313)	279 (206–359)
1-26	Testicular cancer	712 (504–1,100)	1,620 (1,100–2,220)	2,020 (1,210–2,870)	10·8 (7·9–14·9)	15·6 (10·7–21·3)	16·4 (10·1–23·2)
1-27	Kidney cancer	1,840 (1,490–2,340)	3,670 (2,900–4,640)	5,260 (3,940–6,860)	39·4 (32·3–49·1)	48·2 (38·1–60·3)	54·4 (41·2–70·5)
1-28	Bladder cancer	1,510 (1,200–1,840)	2,970 (2,410–3,610)	4,090 (3,170–5,180)	48·3 (38·7–59·2)	45·8 (37·2–55·6)	48·1 (37·6–60·4)
1-29	Brain and nervous system cancer	5,260 (3,260–10,900)	11,500 (7,140–16,000)	14,100 (8,360–19,200)	84·7 (54·6–168)	122 (77·2–168)	124 (74·3–169)
1-30	Thyroid cancer	1,300 (945–1,730)	2,690 (2,020–3,440)	3,540 (2,600–4,620)	35·9 (26·1–47·3)	37·8 (28·7–47·9)	38·3 (28·3–49·7)
1-31	Mesothelioma	331 (207–514)	687 (463–941)	896 (583–1,290)	9·1 (5·7–13·7)	9·5 (6·4–13)	9·6 (6·3–13·7)
1-32	Hodgkin lymphoma	1,840 (1,200–2,490)	1,970 (1,390–2,710)	2,030 (1,380–2,700)	33·8 (23–44·2)	22·1 (15·8–30)	18·8 (12·9–24·9)
1-33	Non-Hodgkin's lymphoma	5,520 (4,150–6,890)	10,400 (8,400–12,800)	13,700 (10,600–17,200)	115 (90·3–140)	130 (105–159)	137 (107–172)
1-34	Multiple myeloma	1,380 (966–2,320)	2,920 (2,230–3,960)	4,140 (3,040–5,520)	40·7 (28·9–68·9)	42·4 (32·7–57·5)	45·3 (33·5–60·4)
1-35	Leukaemia	22,700 (15,400–32,700)	27,600 (21,100–36,200)	29,300 (21,300–38,000)	326 (235–452)	278 (215–361)	253 (188–323)
1-36	Acute lymphoid leukaemia	8,110 (4,800–12,500)	10,600 (7,480–14,600)	10,900 (7,570–14,800)	96·7 (59·9–144)	94·8 (67·1–130)	84·9 (59·6–115)
1-37	Chronic lymphoid leukaemia	153 (108–214)	420 (299–559)	633 (437–858)	4·4 (3·2–6·2)	6·1 (4·3–8·2)	7·1 (4·9–9·6)
1-38	Acute myeloid leukaemia	2,720 (1,760–4,290)	5,110 (3,390–7,180)	6,130 (4,130–8,260)	41·3 (28·1–62·3)	52·1 (34·5–71·6)	53·2 (36·2–71·4)
1-39	Chronic myeloid leukaemia	1,160 (657–2,160)	1,440 (931–1,990)	1,660 (1,110–2,280)	21·8 (13·7–36·2)	17·2 (11·4–23·6)	16·2 (10·9–22·4)
1-40	Other leukaemia	10,500 (5,300–16,700)	9,990 (6,860–13,900)	9,990 (7,030–13,600)	162 (91·1–242)	108 (75·4–147)	91·5 (65–125)
1-41	Other neoplasms	412 (291–603)	706 (555–880)	959 (726–1,220)	8·6 (6·6–11·3)	9·2 (7·2–11·3)	10 (7·5–12·6)
1-42	Myelodysplastic, myeloproliferative, and other hematopoietic neoplasms	412 (291–603)	706 (555–880)	959 (726–1,220)	8·6 (6·6–11·3)	9·2 (7·2–11·3)	10 (7·5–12·6)
1-43	Benign and in situ intestinal neoplasms	0 (0–0)	0 (0–0)	0 (0–0)	0 (0–0)	0 (0–0)	0 (0–0)
1-44	Benign and in situ cervical and uterine neoplasms	0 (0–0)	0 (0–0)	0 (0–0)	0 (0–0)	0 (0–0)	0 (0–0)
1-45	Other benign and in situ neoplasms	0 (0–0)	0 (0–0)	0 (0–0)	0 (0–0)	0 (0–0)	0 (0–0)
1-46	Cardiovascular diseases	218,000 (172,000–260,000)	284,000 (217,000–353,000)	346,000 (255,000–443,000)	6,130 (4,920–7,280)	4,140 (3,200–5,080)	3,960 (2,920–5,010)
1-47	Rheumatic heart disease	11,300 (8,480–14,600)	10,700 (7,840–14,000)	10,800 (8,030–14,100)	241 (182–312)	125 (93·5–163)	103 (76·8–132)
1-48	Ischaemic heart disease	81,200 (58,500–108,000)	116,000 (82,600–151,000)	151,000 (104,000–202,000)	2,580 (1,900–3,430)	1,800 (1,290–2,310)	1,790 (1,260–2,380)
1-49	Stroke	90,800 (71,300–110,000)	107,000 (80,400–140,000)	125,000 (92,100–165,000)	2,460 (1,950–2,980)	1,510 (1,150–1,980)	1,390 (1,030–1,820)
1-50	Ischaemic stroke	20,700 (15,400–26,300)	30,100 (23,000–39,200)	39,900 (30,000–52,100)	693 (521–879)	491 (375–639)	502 (382–654)
1-51	Intracerebral hemorrhage	48,600 (35,400–62,100)	49,500 (35,800–67,600)	55,500 (39,500–75,800)	1,280 (964–1,630)	683 (502–931)	596 (427–812)
1-52	Subarachnoid hemorrhage	21,600 (12,100–30,900)	27,300 (18,000–38,300)	29,500 (19,700–41,200)	484 (263–701)	339 (222–474)	292 (196–408)
1-53	Hypertensive heart disease	11,600 (7,030–15,800)	19,300 (13,600–25,600)	23,800 (16,500–32,900)	380 (234–513)	306 (216–404)	291 (205–398)
1-54	Non-rheumatic valvular heart disease	1,210 (760–1,780)	2,050 (1,440–2,850)	2,570 (1,810–3,550)	31·5 (20·1–45·5)	27·6 (19·5–38·1)	27·4 (19·5–37·7)
1-55	Non-rheumatic calcific aortic valve disease	682 (390–1,070)	1,290 (879–1,870)	1,700 (1,180–2,390)	18·7 (10·8–28·9)	17·9 (12·3–25·7)	18·5 (12·8–26)
1-56	Non-rheumatic degenerative mitral valve disease	483 (313–726)	699 (463–1,010)	811 (523–1,190)	11·8 (7·9–17·1)	9 (6–12·8)	8·4 (5·4–12·2)
1-57	Other non-rheumatic valve diseases	48 (24–83)	56 (28–91)	56 (30–89)	1 (0·5–1·7)	0·7 (0·3–1)	0·6 (0·3–0·9)
1-58	Cardiomyopathy and myocarditis	5,770 (2,910–9,280)	6,880 (4,480–9,530)	7,220 (4,740–9,710)	92·5 (52–138)	75·8 (50·3–104)	68 (44·3–91·4)
1-59	Myocarditis	583 (170–1,150)	574 (306–873)	574 (340–831)	9·9 (3·8–16·2)	6·6 (3·7–9·8)	5·6 (3·3–7·9)
1-60	Alcoholic cardiomyopathy	187 (87–322)	195 (121–296)	227 (138–337)	5·2 (2·4–9·4)	2·7 (1·7–4·1)	2·4 (1·5–3·5)

1-61	Other cardiomyopathy	5,000 (2,560–8,260)	6,110 (3,970–8,580)	6,420 (4,180–8,690)	77·3 (43·6–120)	66·6 (44·6–91·9)	60·1 (39·2–80·7)
1-62	Atrial fibrillation and flutter	1,970 (1,470–2,730)	4,820 (3,730–6,440)	6,690 (5,070–8,920)	77·6 (58·7–106)	87 (67·3–114)	92·1 (70·2–121)
1-63	Aortic aneurysm	1,270 (856–1,790)	2,620 (1,800–3,520)	3,540 (2,360–4,850)	38·2 (25·9–53·5)	38·8 (26·9–51·5)	40·4 (27·1–55·1)
1-64	Peripheral vascular disease	203 (117–323)	408 (249–646)	554 (342–852)	7·6 (4·4–12)	7 (4·3–11)	7·1 (4·5–11)
1-65	Endocarditis	2,490 (1,470–4,130)	2,960 (2,040–4,200)	3,050 (2,080–4,210)	35·6 (24·4–53)	31·1 (22·1–42·6)	27·6 (19·4–37·8)
1-66	Chronic respiratory diseases	86,300 (63,500–109,000)	85,300 (70,900–100,000)	97,900 (79,300–118,000)	1,620 (1,320–1,910)	1,140 (950–1,330)	1,060 (862–1,270)
1-67	Chronic obstructive pulmonary disease	29,200 (21,600–36,600)	39,700 (32,600–47,400)	47,300 (37,500–58,600)	820 (652–966)	627 (515–749)	586 (464–720)
1-68	Pneumoconiosis	422 (287–596)	563 (395–756)	645 (451–879)	11·9 (8·1–16·6)	8·1 (5·7–10·9)	7·2 (5·1–9·7)
1-69	Silicosis	192 (86–319)	191 (100–309)	204 (114–317)	5·5 (2·5–9·2)	2·7 (1·4–4·4)	2·2 (1·3–3·5)
1-70	Asbestosis	28 (15–46)	42 (24–66)	51 (30–80)	0·8 (0·4–1·3)	0·6 (0·3–0·9)	0·6 (0·3–0·9)
1-71	Coal workers pneumoconiosis	34 (14–60)	31 (14–51)	32 (15–58)	0·9 (0·4–1·6)	0·4 (0·2–0·7)	0·3 (0·2–0·6)
1-72	Other pneumoconiosis	167 (108–258)	299 (205–428)	358 (242–512)	4·7 (3·1–7·2)	4·4 (3–6·2)	4·1 (2·8–5·7)
1-73	Asthma	45,300 (29,300–64,100)	27,400 (19,500–37,700)	28,500 (19,500–40,900)	556 (390–751)	257 (189–347)	228 (160–320)
1-74	Interstitial lung disease and pulmonary sarcoidosis	4,830 (3,200–8,040)	12,600 (9,520–16,700)	16,200 (11,800–21,300)	147 (96·7–243)	195 (148–257)	193 (142–253)
1-75	Other chronic respiratory diseases	6,600 (2,670–15,700)	5,090 (3,300–7,960)	5,200 (3,560–7,590)	84·7 (40–183)	50·4 (33·8–76·7)	43·5 (30·8–61·8)
1-76	Digestive diseases	163,000 (108,000–222,000)	167,000 (133,000–203,000)	190,000 (149,000–234,000)	3,040 (2,210–3,910)	2,080 (1,680–2,530)	1,910 (1,500–2,370)
1-77	Cirrhosis and other chronic liver diseases	51,200 (32,300–72,800)	73,000 (55,200–93,400)	89,200 (65,700–118,000)	1,220 (815–1,670)	983 (751–1,240)	935 (690–1,230)
1-78	Cirrhosis and other chronic liver diseases due to hepatitis B	5,510 (3,250–8,640)	5,400 (3,450–8,130)	5,950 (3,670–8,900)	144 (83·9–226)	72·5 (46·6–110)	61·6 (38–92·2)
1-79	Cirrhosis and other chronic liver diseases due to hepatitis C	5,650 (3,300–8,830)	9,150 (6,050–13,500)	11,400 (6,880–17,400)	148 (87·6–233)	123 (80·9–184)	119 (72·1–181)
1-80	Cirrhosis and other chronic liver diseases due to alcohol use	15,900 (9,750–22,800)	28,300 (19,800–38,300)	36,400 (24,400–51,300)	438 (272–634)	393 (274–531)	386 (261–540)
1-81	Cirrhosis and other chronic liver diseases due to NAFLD	7,050 (4,080–11,100)	13,100 (8,460–19,500)	16,700 (10,400–24,800)	196 (114–306)	185 (120–270)	181 (112–268)
1-82	Cirrhosis and other chronic liver diseases due to other causes	17,200 (8,350–29,000)	17,000 (11,500–23,400)	18,700 (12,700–26,400)	294 (162–456)	208 (142–288)	188 (126–267)
1-83	Upper digestive system diseases	22,000 (16,100–28,800)	25,600 (19,100–35,600)	29,300 (21,300–41,300)	518 (389–660)	329 (251–442)	296 (220–406)
1-84	Peptic ulcer disease	13,400 (9,000–18,200)	10,400 (7,780–13,300)	10,500 (7,510–14,000)	334 (236–443)	149 (113–188)	119 (85·9–157)
1-85	Gastritis and duodenitis	2,840 (1,850–4,060)	4,550 (3,270–6,020)	5,450 (3,970–7,240)	61·8 (41·2–86·7)	57·1 (41·9–75·2)	54·8 (40·1–72·3)
1-86	GERD	5,750 (2,990–10,200)	10,700 (5,570–18,900)	13,400 (6,940–23,700)	123 (63·5–217)	123 (63·8–217)	123 (63·9–218)
1-87	Appendicitis	19,500 (8,170–34,800)	8,140 (5,390–12,000)	7,020 (5,130–9,290)	248 (111–415)	81·3 (55·3–118)	60·3 (44·5–79·8)
1-88	Paralytic ileus and intestinal obstruction	28,400 (13,900–49,400)	19,600 (13,800–27,000)	19,400 (13,800–26,200)	340 (193–541)	208 (153–275)	177 (129–236)
1-89	Inguinal, femoral, and abdominal hernia	15,200 (8,320–25,000)	11,800 (8,510–15,800)	12,200 (9,010–16,200)	198 (120–300)	120 (87·6–157)	106 (78·5–138)
1-90	Inflammatory bowel disease	1,550 (546–3,370)	1,240 (838–1,800)	1,350 (984–1,770)	22·2 (10·3–41·4)	14·2 (10·1–19·1)	12·7 (9·5–16·3)
1-91	Vascular intestinal disorders	1,330 (692–2,220)	1,760 (1,330–2,330)	2,290 (1,680–2,990)	31·6 (19·8–46·3)	26·3 (20·2–34)	27 (19·9–34·7)
1-92	Gallbladder and biliary diseases	7,330 (4,770–10,300)	9,750 (7,320–13,100)	11,500 (8,470–15,500)	187 (122–261)	133 (100–178)	124 (90·9–167)
1-93	Pancreatitis	7,910 (5,040–10,800)	9,180 (6,720–12,400)	10,500 (7,410–14,500)	176 (119–250)	116 (86·9–159)	106 (75·9–146)
1-94	Other digestive diseases	8,190 (2,710–18,200)	6,520 (4,120–10,100)	7,030 (4,940–9,520)	102 (42·9–206)	72·2 (48·3–105)	65·6 (46·6–87)
1-95	Neurological disorders	65,700 (39,600–103,000)	100,000 (56,500–161,000)	123,000 (70,800–196,000)	1,350 (840–2,060)	1,250 (724–2,000)	1,270 (753–2,000)
1-96	Alzheimer's disease and other dementias	7,450 (2,910–17,600)	19,900 (7,600–47,400)	27,700 (10,300–64,300)	333 (133–782)	388 (149–909)	411 (154–955)
1-97	Parkinson's disease	2,800 (2,370–3,270)	6,790 (5,740–7,900)	9,410 (7,800–11,200)	104 (87·6–121)	115 (97·4–134)	123 (102–146)
1-98	Idiopathic epilepsy	25,400 (13,700–39,700)	23,400 (12,700–36,200)	26,000 (13,000–43,500)	387 (202–611)	231 (124–359)	220 (108–366)
1-99	Multiple sclerosis	335 (249–463)	681 (529–865)	888 (669–1,150)	8 (6–11)	8·5 (6·6–10·7)	8·7 (6·7–11·3)
1-100	Motor neuron disease	390 (294–522)	752 (542–1,020)	1,110 (764–1,590)	8·6 (6·6–11·6)	9·8 (7–13·4)	11·3 (7·8–16·3)
1-101	Headache disorders	25,200 (5,390–53,900)	43,800 (9,730–92,600)	51,700 (12,000–109,000)	444 (108–924)	444 (105–929)	444 (107–926)
1-102	Migraine	22,700 (3,560–50,100)	39,200 (6,560–85,900)	46,200 (8,080–102,000)	396 (71·2–868)	395 (71·8–864)	395 (72·6–871)
1-103	Tension-type headache	2,550 (738–10,500)	4,550 (1,380–18,000)	5,490 (1,690–21,100)	48·5 (15·4–181)	48·7 (15·3–180)	48·6 (15·4–179)
1-104	Other neurological disorders	4,100 (2,090–6,650)	5,010 (2,790–7,850)	6,410 (3,370–10,600)	60·4 (32·6–96·1)	49·4 (29·2–75·2)	54·2 (30–87·1)
1-105	Mental disorders	103,000 (75,100–136,000)	176,000 (128,000–234,000)	211,000 (155,000–280,000)	1,880 (1,380–2,480)	1,840 (1,340–2,440)	1,840 (1,350–2,450)
1-106	Schizophrenia	8,170 (5,720–11,000)	15,400 (10,500–20,900)	19,300 (13,300–25,900)	166 (116–219)	170 (118–229)	171 (118–229)
1-107	Depressive disorders	32,400 (22,000–45,200)	54,700 (37,400–76,500)	66,000 (45,400–91,800)	655 (450–912)	609 (418–853)	600 (416–834)
1-108	Major depressive disorder	27,900 (18,700–39,400)	46,500 (31,500–65,900)	55,900 (37,800–78,400)	565 (384–786)	519 (355–729)	510 (348–712)
1-109	Dysthymia	4,470 (2,720–6,940)	8,180 (4,970–12,700)	10,100 (6,120–15,400)	89·9 (56·2–138)	90 (55·3–138)	90·1 (56·2–138)
1-110	Bipolar disorder	10,500 (6,070–16,400)	18,700 (11,000–29,500)	22,400 (13,000–34,800)	192 (114–302)	192 (113–303)	192 (114–299)
1-111	Anxiety disorders	30,100 (19,800–42,500)	52,600 (34,600–74,300)	63,500 (41,900–90,800)	537 (355–755)	540 (356–754)	549 (363–782)
1-112	Eating disorders	2,610 (1,600–3,960)	5,020 (3,000–7,810)	6,410 (3,870–9,910)	40·5 (24·7–61·5)	45·4 (27·6–69·9)	50·5 (30·6–78·3)
1-113	Anorexia nervosa	525 (297–860)	947 (535–1,570)	1,140 (637–1,860)	7·6 (4·3–12·6)	8·2 (4·7–13·4)	8·9 (5–14·5)

1-114	Bulimia nervosa	2,090 (1,230–3,270)	4,070 (2,340–6,600)	5,270 (3,060–8,410)	32·9 (19·1–51·9)	37·2 (21·4–60·1)	41·5 (24·1–66)
1-115	Autism spectrum disorders	3,530 (2,330–5,190)	5,500 (3,580–8,020)	6,440 (4,230–9,440)	51·3 (33·8–75·4)	52 (33·9–76·1)	52 (34·2–76·1)
1-116	Attention-deficit/hyperactivity disorder	1,910 (1,070–3,240)	2,930 (1,660–4,930)	3,250 (1,840–5,510)	25·4 (14·4–42·4)	25·6 (14·6–43)	25·6 (14·5–43·2)
1-117	Conduct disorder	5,910 (3,270–9,300)	8,590 (4,870–13,600)	8,780 (4,920–13,900)	68·8 (37·8–108)	69·1 (39·1–109)	69·2 (38·6–110)
1-118	Idiopathic developmental intellectual disability	2,760 (1,390–4,640)	3,250 (1,500–5,640)	3,000 (1,200–5,370)	40·3 (20·5–67·5)	30·5 (14·3–53)	24·1 (9·7–43·1)
1-119	Other mental disorders	5,020 (3,180–7,590)	9,400 (5,870–14,200)	11,800 (7,380–17,900)	108 (68·1–162)	108 (68·3–164)	108 (69–164)
1-120	Substance use disorders	24,800 (18,800–30,500)	38,700 (29,900–49,100)	44,200 (32,900–55,300)	500 (382–609)	416 (328–520)	390 (292–488)
1-121	Alcohol use disorders	14,500 (10,800–18,200)	20,700 (16,100–26,200)	23,400 (17,500–30,200)	323 (236–402)	242 (189–305)	218 (163–280)
1-122	Drug use disorders	10,200 (7,080–13,900)	18,000 (12,600–24,400)	20,800 (14,700–27,600)	177 (125–238)	174 (124–235)	172 (124–226)
1-123	Opioid use disorders	4,370 (2,870–6,280)	6,380 (4,220–9,090)	7,930 (5,300–11,100)	81·7 (55·4–115)	67·3 (45·9–93·1)	68·9 (46·6–95·3)
1-124	Cocaine use disorders	1,350 (896–1,950)	2,940 (1,980–4,220)	3,120 (2,070–4,450)	22·2 (15·4–31·5)	27·6 (18·9–39·6)	26 (17·5–36·5)
1-125	Amphetamine use disorders	1,940 (1,050–3,170)	3,610 (2,010–5,950)	4,170 (2,340–6,930)	31·1 (16·9–50·9)	33·1 (18·6–54·2)	32·8 (18·7–54)
1-126	Cannabis use disorders	440 (250–683)	811 (462–1,270)	920 (546–1,430)	7 (4–10·8)	7·3 (4·3–11·3)	7·4 (4·4–11·5)
1-127	Other drug use disorders	2,150 (1,330–3,200)	4,230 (2,640–6,260)	4,620 (2,870–6,860)	34·8 (22·1–51·6)	39·1 (24·9–57·7)	37·2 (23·4–54·8)
1-128	Diabetes and kidney diseases	84,500 (73,900–97,600)	174,000 (147,000–207,000)	237,000 (192,000–289,000)	2,230 (1,930–2,620)	2,460 (2,080–2,900)	2,600 (2,110–3,150)
1-129	Diabetes mellitus	41,400 (34,500–50,900)	88,300 (72,300–106,000)	125,000 (98,800–153,000)	1,210 (1,010–1,490)	1,280 (1,060–1,540)	1,380 (1,100–1,700)
1-130	Diabetes mellitus type 1	3,580 (2,830–4,440)	5,250 (3,960–6,660)	5,990 (4,410–7,830)	70·2 (55·6–89)	60·2 (45·8–77)	55·8 (41·3–72·7)
1-131	Diabetes mellitus type 2	37,900 (31,400–46,600)	83,000 (67,900–100,000)	119,000 (94,300–147,000)	1,140 (953–1,400)	1,220 (1,000–1,470)	1,330 (1,060–1,640)
1-132	Chronic kidney disease	42,500 (36,100–51,300)	85,600 (69,300–105,000)	112,000 (86,500–141,000)	1,010 (855–1,230)	1,180 (958–1,440)	1,220 (948–1,530)
1-133	Chronic kidney disease due to diabetes mellitus type 1	2,910 (1,920–4,190)	5,820 (3,650–8,970)	7,570 (4,450–11,900)	69 (44·3–104)	73·8 (46·2–115)	74·8 (43·6–119)
1-134	Chronic kidney disease due to diabetes mellitus type 2	7,280 (5,450–9,560)	18,900 (14,200–24,800)	27,000 (19,500–36,100)	229 (172–298)	289 (219–376)	313 (226–416)
1-135	Chronic kidney disease due to hypertension	6,170 (4,750–7,950)	17,000 (12,800–22,500)	23,300 (17,300–31,400)	198 (154–255)	266 (201–349)	279 (206–370)
1-136	Chronic kidney disease due to glomerulonephritis	11,300 (8,720–14,700)	19,800 (14,600–26,700)	24,700 (17,400–34,900)	231 (179–302)	247 (179–335)	248 (173–352)
1-137	Chronic kidney disease due to other and unspecified causes	14,800 (11,800–18,300)	24,000 (18,500–31,000)	29,700 (21,700–39,300)	286 (228–360)	305 (230–396)	307 (224–409)
1-138	Acute glomerulonephritis	531 (165–1,320)	125 (63–220)	97 (54–170)	6·4 (2·6–14·3)	1·3 (0·7–2·1)	0·9 (0·5–1·4)
1-139	Skin and subcutaneous diseases	38,800 (25,700–57,100)	60,600 (40,000–90,500)	70,500 (46,800–106,000)	594 (397–892)	592 (393–887)	591 (392–894)
1-140	Dermatitis	8,290 (4,870–13,100)	12,300 (7,300–19,500)	14,500 (8,610–22,800)	116 (70·1–182)	117 (69·9–183)	117 (69·8–182)
1-141	Atopic dermatitis	6,660 (3,620–11,300)	9,390 (5,090–15,900)	10,900 (5,930–18,400)	84·2 (45·9–141)	84·4 (45·8–143)	84·4 (45·7–142)
1-142	Contact dermatitis	1,440 (911–2,150)	2,610 (1,640–3,940)	3,180 (1,990–4,790)	28·8 (18·1–42·8)	28·9 (18·1–43·6)	28·9 (18·2–43·3)
1-143	Seborrhoeic dermatitis	189 (106–305)	320 (180–517)	385 (217–612)	3·4 (1·9–5·4)	3·4 (1·9–5·4)	3·4 (1·9–5·4)
1-144	Psoriasis	4,690 (3,320–6,360)	7,090 (5,030–9,510)	8,190 (5,780–10,900)	87·8 (62·3–119)	76·7 (54·1–102)	73 (51·8–96·6)
1-145	Bacterial skin diseases	1,260 (770–2,050)	1,700 (1,270–2,270)	1,980 (1,470–2,650)	18·9 (12·2–27·1)	19·6 (14·8–25·8)	19·1 (14·2–25·1)
1-146	Cellulitis	244 (121–413)	332 (224–459)	391 (264–548)	4·3 (2·1–6·7)	3·8 (2·6–5·3)	3·7 (2·4–5·2)
1-147	Pyoderma	1,020 (553–1,810)	1,360 (969–1,910)	1,590 (1,130–2,200)	14·7 (8·8–22·8)	15·8 (11·6–21·4)	15·4 (11·1–20·8)
1-148	Scabies	5,830 (3,160–9,360)	8,860 (4,830–14,200)	10,200 (5,580–16,300)	82·7 (45·1–132)	82·3 (45·4–132)	82 (45–131)
1-149	Fungal skin diseases	3,780 (1,500–8,130)	6,370 (2,530–13,700)	7,660 (3,080–16,500)	71·1 (28·8–153)	70·6 (28·4–152)	70·3 (28·5–151)
1-150	Viral skin diseases	4,150 (2,640–6,220)	5,790 (3,720–8,740)	6,730 (4,290–10,100)	51·5 (32·9–77·3)	51·6 (33·3–77·8)	51·7 (33–77·6)
1-151	Acne vulgaris	4,140 (2,430–6,570)	7,720 (4,570–12,400)	8,220 (4,920–13,000)	53·3 (31·5–84·5)	62·4 (37–100)	64·9 (38·9–103)
1-152	Alopecia areata	404 (258–603)	692 (440–1,040)	836 (539–1,260)	7·1 (4·6–10·7)	7·1 (4·5–10·6)	7·1 (4·6–10·7)
1-153	Pruritus	423 (199–760)	742 (357–1,340)	920 (439–1,650)	8 (3·8–14·2)	8·2 (3·9–14·7)	8·3 (3·9–14·8)
1-154	Urticaria	3,510 (2,260–5,140)	5,170 (3,360–7,530)	6,080 (3,950–8,840)	47·9 (31–69·6)	48·1 (31·3–69·6)	48 (31·3–69·7)
1-155	Decubitus ulcer	137 (77–210)	212 (145–278)	290 (197–383)	4·6 (2·7–7·2)	3·5 (2·4–4·7)	3·7 (2·5–4·9)
1-156	Other skin and subcutaneous diseases	2,230 (1,090–4,000)	3,960 (1,940–7,190)	4,950 (2,430–8,880)	45 (22·5–80·1)	45·6 (22·7–82·2)	46·2 (22·8–82·7)
1-157	Sense organ diseases	36,800 (25,400–51,100)	66,900 (46,300–94,600)	83,100 (57,600–117,000)	993 (692–1,380)	918 (638–1,290)	889 (617–1,260)
1-158	Blindness and vision loss	14,300 (10,100–19,700)	24,500 (17,100–33,900)	29,600 (20,600–41,200)	408 (292–553)	348 (245–474)	327 (230–450)
1-159	Glaucoma	643 (418–932)	1,060 (695–1,530)	1,250 (838–1,780)	25·1 (16·3–36)	18·5 (12·2–26·5)	16·4 (11–23·2)
1-160	Cataract	3,900 (2,770–5,360)	6,700 (4,700–9,100)	8,100 (5,720–11,000)	141 (100–191)	111 (78·2–151)	102 (71·7–138)
1-161	Age-related macular degeneration	191 (127–278)	360 (241–518)	457 (304–659)	6·8 (4·6–9·9)	5·9 (4–8·4)	5·6 (3·7–8)
1-162	Refraction disorders	5,040 (3,310–7,300)	8,490 (5,590–12,300)	10,200 (6,720–14,800)	102 (68·3–145)	96·5 (64–138)	93·9 (62·2–135)
1-163	Near vision loss	1,620 (751–3,140)	3,250 (1,490–6,360)	4,250 (1,930–8,500)	51·2 (23·8–98)	49 (22·6–94·8)	48·4 (22·3–96)
1-164	Other vision loss	2,940 (2,040–4,110)	4,670 (3,250–6,440)	5,440 (3,780–7,550)	82·1 (57·4–114)	66·7 (46·3–91·7)	60·7 (42·4–84·5)
1-165	Age-related and other hearing loss	20,800 (14,100–30,400)	39,500 (26,700–57,400)	49,800 (33,300–72,500)	550 (375–794)	534 (362–768)	526 (357–758)
1-166	Other sense organ diseases	1,580 (942–2,430)	2,910 (1,750–4,470)	3,680 (2,210–5,650)	35·1 (20·7–54·2)	35·8 (21·3–55·4)	36 (21·6–55·5)

1-167	Musculoskeletal disorders	66,600 (48,000–87,100)	134,000 (96,500–176,000)	170,000 (122,000–224,000)	1,520 (1,100–2,010)	1,620 (1,170–2,140)	1,630 (1,180–2,150)
1-168	Rheumatoid arthritis	1,520 (1,160–1,900)	3,600 (2,710–4,590)	4,940 (3,680–6,410)	40·5 (31·5–49·8)	48·3 (37–60·9)	51·5 (39·3–65·8)
1-169	Osteoarthritis	6,270 (3,140–12,400)	14,100 (7,080–28,100)	19,000 (9,550–38,200)	190 (95·7–378)	204 (103–409)	209 (105–419)
1-170	Low back pain	33,200 (23,300–44,700)	62,100 (43,600–83,800)	75,200 (52,500–100,000)	713 (503–955)	720 (508–974)	704 (492–941)
1-171	Neck pain	5,940 (3,860–8,670)	11,200 (7,240–16,300)	14,200 (9,490–20,500)	134 (89·3–195)	135 (87·6–194)	135 (89·6–194)
1-172	Gout	257 (158–374)	608 (384–898)	834 (511–1,220)	6·8 (4·2–9·8)	8·2 (5·1–12·1)	8·6 (5·3–12·6)
1-173	Other musculoskeletal disorders	19,400 (13,400–26,800)	42,200 (29,400–58,100)	55,500 (38,600–75,900)	435 (302–601)	503 (350–691)	523 (362–717)
1-174	Other non-communicable diseases	232,000 (155,000–318,000)	305,000 (241,000–373,000)	311,000 (247,000–388,000)	2,950 (2,110–3,850)	2,770 (2,230–3,380)	2,460 (1,960–3,060)
1-175	Congenital anomalies	147,000 (78,500–230,000)	178,000 (128,000–231,000)	160,000 (111,000–212,000)	1,340 (728–2,080)	1,320 (954–1,720)	1,060 (741–1,400)
1-176	Neural tube defects	17,900 (8,360–34,100)	14,200 (6,530–27,500)	10,900 (4,830–22,200)	160 (74·8–304)	105 (48·6–202)	71·8 (32·2–145)
1-177	Congenital heart anomalies	60,400 (29,000–108,000)	75,200 (49,200–107,000)	66,800 (44,900–91,800)	553 (267–985)	559 (365–795)	441 (297–603)
1-178	Orofacial clefts	2,020 (785–4,430)	921 (480–1,660)	760 (451–1,240)	18·5 (7·7–39·3)	7·1 (3·9–12·5)	5·3 (3·3–8·4)
1-179	Down's syndrome	6,300 (2,850–14,700)	7,890 (4,660–13,000)	7,600 (4,660–11,800)	58·5 (27·1–134)	59·3 (35·2–98)	50·6 (31·1–77·8)
1-180	Turner syndrome	14 (5–27)	18 (8–34)	18 (8–33)	0·2 (0·1–0·3)	0·2 (0·1–0·3)	0·1 (0·1–0·2)
1-181	Klinefelter syndrome	1 (0–2)	1 (0–2)	1 (1–2)	0 (0–0)	0 (0–0)	0 (0–0)
1-182	Other chromosomal abnormalities	2,480 (1,210–6,920)	5,160 (3,210–8,430)	5,470 (3,360–8,590)	22·8 (11·4–62·3)	38·1 (23·9–62·1)	36·1 (22·2–56·2)
1-183	Congenital musculoskeletal and limb anomalies	4,380 (2,820–6,370)	6,390 (4,440–8,870)	6,930 (4,740–9,500)	49·1 (31·9–69·9)	53·2 (36·9–73·9)	50·9 (34·8–70·2)
1-184	Urogenital congenital anomalies	1,740 (835–3,320)	2,630 (1,620–4,050)	2,470 (1,500–4,190)	15·9 (7·8–29·7)	19·4 (12–29·5)	16·4 (10–27·5)
1-185	Digestive congenital anomalies	16,800 (6,120–37,500)	20,900 (12,800–32,800)	18,300 (11,400–28,200)	149 (54·1–332)	152 (92·9–238)	119 (74·1–183)
1-186	Other congenital anomalies	35,100 (12,700–77,400)	45,100 (29,300–64,300)	41,000 (26,400–59,000)	318 (118–704)	332 (215–473)	270 (173–385)
1-187	Urinary diseases and male infertility	6,330 (5,320–7,560)	10,300 (8,260–12,700)	12,900 (10,100–16,100)	138 (115–165)	140 (112–175)	140 (110–174)
1-188	Urinary tract infections and interstitial nephritis	4,540 (3,770–5,460)	6,790 (5,250–8,320)	8,300 (6,150–10,400)	89·4 (75·5–107)	90·4 (68·7–112)	91·1 (66·9–114)
1-189	Urolithiasis	376 (240–527)	622 (422–855)	799 (545–1,090)	8·9 (5·6–12·8)	7·8 (5·2–10·6)	7·8 (5·2–10·6)
1-190	Benign prostatic hyperplasia	904 (519–1,430)	2,090 (1,210–3,290)	2,720 (1,580–4,280)	29·9 (17·2–46·8)	32·3 (18·9–50·8)	31·3 (18·3–49·1)
1-191	Male infertility	66 (26–145)	93 (37–199)	208 (75–485)	1·2 (0·5–2·7)	1 (0·4–2)	1·7 (0·6–4)
1-192	Other urinary diseases	443 (277–646)	662 (452–899)	829 (559–1,120)	8·6 (5·4–11·8)	8·1 (5·4–11)	8·3 (5·5–11·2)
1-193	Gynecological diseases	25,600 (17,900–35,500)	42,600 (29,300–60,200)	51,600 (35,400–72,000)	489 (344–676)	454 (313–636)	447 (307–619)
1-194	Uterine fibroids	1,980 (1,080–3,480)	3,130 (1,660–5,760)	3,950 (2,010–7,240)	41·8 (22·9–74·5)	36 (18·9–66·4)	35·8 (18·2–65·5)
1-195	Polycystic ovarian syndrome	552 (237–1,110)	1,120 (485–2,250)	1,530 (652–3,040)	9·1 (3·9–18·2)	10·6 (4·6–21·5)	12·4 (5·3–24·6)
1-196	Female infertility	25 (6–65)	15 (5–44)	175 (15–606)	0·5 (0·1–1·3)	0·2 (0·1–0·5)	1·4 (0·1–4·9)
1-197	Endometriosis	2,850 (1,630–4,540)	3,570 (2,060–5,950)	4,060 (2,390–6,640)	49·6 (28·7–78·5)	35·1 (20·6–58·2)	33·2 (19·5–53·8)
1-198	Genital prolapse	424 (224–775)	675 (359–1,240)	843 (430–1,530)	11·3 (6–20·7)	9 (4·8–16·8)	8·7 (4·4–15·9)
1-199	Premenstrual syndrome	5,710 (3,450–9,030)	10,000 (6,100–15,600)	11,600 (7,050–18,100)	94·8 (57·9–149)	96·2 (59·3–149)	95·3 (58–148)
1-200	Other gynecological diseases	14,100 (9,200–20,000)	24,100 (15,700–34,900)	29,400 (19,000–42,400)	282 (186–398)	266 (173–381)	260 (168–372)
1-201	Hemoglobinopathies and hemolytic anaemias	7,810 (6,110–10,000)	8,640 (6,470–11,400)	8,750 (6,460–11,800)	125 (97·4–159)	86·3 (65·4–112)	73·9 (55·6–99·1)
1-202	Thalassemias	318 (175–515)	213 (127–329)	149 (86–231)	3·1 (1·7–5)	1·7 (1·2–2·6)	1 (0·6–1·6)
1-203	Thalassaemias trait	954 (623–1,430)	1,370 (866–2,050)	1,450 (918–2,190)	14·5 (9·5–22)	12·7 (8·1–19·1)	11·5 (7·2–17·3)
1-204	Sickle cell disorders	920 (621–1,300)	752 (513–1,060)	514 (336–739)	10·3 (7–14·3)	6·3 (4·3–8·9)	3·9 (2·5–5·6)
1-205	Sickle cell trait	952 (586–1,420)	1,250 (743–1,910)	1,300 (784–1,950)	14·9 (9·2–22)	11·5 (6·9–17·4)	10·2 (6·1–15·3)
1-206	G6PD deficiency	577 (387–814)	730 (501–987)	1,120 (772–1,510)	8·5 (5·9–11·8)	7·3 (5·1–9·9)	9·5 (6·6–12·9)
1-207	G6PD trait	6 (3–10)	18 (10–30)	19 (10–32)	0·1 (0·1–0·2)	0·2 (0·1–0·4)	0·2 (0·1–0·3)
1-208	Other hemoglobinopathies and hemolytic anaemias	4,080 (3,120–5,290)	4,310 (3,090–5,790)	4,200 (3,010–5,850)	73·3 (57·3–94·4)	46·5 (34·8–61·2)	37·6 (27·9–51)
1-209	Endocrine, metabolic, blood, and immune disorders	20,200 (15,100–25,700)	24,600 (18,500–32,500)	28,900 (21,300–38,500)	343 (264–431)	280 (210–368)	268 (198–356)
1-210	Oral disorders	19,100 (11,700–28,800)	35,400 (21,700–54,000)	44,400 (27,000–67,200)	465 (287–698)	453 (278–692)	444 (273–672)
1-211	Caries of deciduous teeth	310 (134–630)	374 (159–787)	443 (190–927)	3·1 (1·4–6·4)	3·1 (1·3–6·7)	3·1 (1·3–6·5)
1-212	Caries of permanent teeth	2,130 (920–4,220)	3,530 (1,530–7,080)	4,080 (1,800–8,220)	35·4 (15·2–68·5)	35·1 (15·3–69·6)	34·8 (15·5–69·6)
1-213	Periodontal disease	3,760 (1,430–8,140)	7,250 (2,750–15,600)	9,430 (3,580–20,600)	83·7 (31·9–177)	85·9 (32·7–185)	87·7 (33·6–190)
1-214	Edentulism	9,800 (6,250–14,300)	19,100 (12,300–27,700)	24,200 (15,600–35,600)	290 (186–420)	275 (177–402)	265 (171–385)
1-215	Other oral disorders	3,090 (1,910–4,570)	5,170 (3,200–7,540)	6,220 (3,870–9,150)	53 (32·9–78·1)	53·1 (33·1–77·3)	53·1 (33·1–77·8)
1-216	Sudden infant death syndrome	5,560 (1,400–14,800)	4,840 (1,620–10,800)	3,990 (1,610–8,090)	49 (12·3–130)	35 (11·7–78)	25·7 (10·4–52)
1-0	Injuries	440,000 (371,000–503,000)	394,000 (322,000–472,000)	384,000 (297,000–467,000)	6,270 (5,450–7,020)	3,920 (3,220–4,660)	3,280 (2,560–3,980)
1-1	Transport injuries	124,000 (98,700–146,000)	114,000 (89,600–140,000)	108,000 (79,500–136,000)	1,890 (1,560–2,180)	1,170 (924–1,420)	937 (704–1,190)
1-2	Road injuries	118,000 (94,200–140,000)	102,000 (80,000–126,000)	99,700 (73,700–126,000)	1,800 (1,480–2,080)	1,050 (828–1,290)	868 (653–1,100)

1-3	Pedestrian road injuries	47,400 (30,800–66,200)	34,000 (25,300–43,600)	26,600 (19,100–35,500)	701 (478–951)	352 (266–441)	234 (171–313)
1-4	Cyclist road injuries	2,230 (1,730–3,030)	2,150 (1,730–2,640)	2,420 (1,870–3,040)	37·4 (30·2–47·2)	23·4 (19–28·8)	22·1 (17·1–27·8)
1-5	Motorcyclist road injuries	8,950 (6,570–12,800)	8,690 (6,370–11,600)	9,630 (6,570–13,000)	138 (106–185)	86·1 (63·8–114)	81·1 (56–109)
1-6	Motor vehicle road injuries	58,400 (43,000–74,800)	56,100 (42,900–70,800)	59,600 (43,000–77,700)	900 (686–1,120)	574 (443–725)	518 (375–677)
1-7	Other road injuries	1,270 (953–1,950)	1,360 (1,060–1,700)	1,450 (1,000–1,930)	20·6 (15·9–28·5)	14·4 (11·4–17·8)	12·9 (9·2–17)
1-8	Other transport injuries	5,690 (3,990–7,410)	11,300 (8,990–13,900)	7,900 (5,490–10,600)	93·1 (67·3–120)	116 (91·8–142)	69 (48·6–92·2)
1-9	Unintentional injuries	265,000 (214,000–312,000)	205,000 (170,000–244,000)	200,000 (161,000–240,000)	3,530 (2,970–4,050)	2,020 (1,690–2,370)	1,700 (1,380–2,030)
1-10	Falls	25,400 (19,800–32,200)	36,800 (29,900–44,300)	44,500 (35,400–54,200)	499 (395–613)	437 (357–518)	432 (345–520)
1-11	Drowning	54,900 (34,800–76,500)	33,200 (24,200–44,600)	27,800 (19,700–37,800)	646 (439–858)	297 (221–395)	216 (156–290)
1-12	Fire, heat, and hot substances	27,500 (17,100–38,300)	18,400 (14,300–22,900)	17,300 (13,700–21,700)	374 (257–501)	182 (144–224)	146 (117–182)
1-13	Poisonings	9,880 (5,680–14,200)	5,310 (3,880–6,970)	4,580 (3,380–6,110)	119 (77·8–164)	48·7 (36·1–63·4)	36·7 (27·5–48·5)
1-14	Poisoning by carbon monoxide	414 (229–702)	675 (301–1,080)	706 (324–1,070)	6·2 (3·6–9·8)	6·5 (3–10·2)	5·9 (2·8–8·7)
1-15	Poisoning by other means	9,460 (5,310–13,800)	4,630 (3,330–6,270)	3,870 (2,760–5,370)	113 (72–157)	42·2 (30·6–56·9)	30·8 (22·1–42·5)
1-16	Exposure to mechanical forces	18,900 (14,700–25,100)	20,500 (15,700–26,200)	22,500 (17,400–28,900)	323 (259–401)	217 (168–278)	199 (154–252)
1-17	Unintentional firearm injuries	8,520 (5,840–11,700)	7,120 (4,880–10,000)	6,580 (4,540–9,370)	138 (96–181)	71·3 (49·4–99·3)	55·7 (38·9–78·5)
1-18	Other exposure to mechanical forces	10,300 (7,030–15,100)	13,300 (9,980–17,600)	15,900 (11,900–21,200)	185 (129–252)	146 (109–192)	143 (108–189)
1-19	Adverse effects of medical treatment	9,230 (6,640–11,900)	6,340 (4,760–8,300)	5,880 (4,350–7,970)	126 (93·1–153)	64 (48·7–83·9)	51·2 (38·1–68·5)
1-20	Animal contact	1,650 (1,140–2,130)	2,780 (1,690–3,530)	2,830 (1,810–3,620)	25·6 (18·8–32·5)	28·4 (17·7–35·8)	24·6 (16·1–31·1)
1-21	Venomous animal contact	1,270 (873–1,660)	2,010 (1,210–2,570)	2,010 (1,300–2,560)	19·4 (14·2–24·7)	20·5 (12·7–25·8)	17·4 (11·4–22·2)
1-22	Non-venomous animal contact	387 (246–561)	765 (457–1,050)	823 (525–1,100)	6·3 (4·3–8·7)	7·9 (4·9–10·7)	7·2 (4·6–9·6)
1-23	Foreign body	87,500 (64,200–115,000)	53,800 (40,100–70,600)	45,200 (32,300–60,900)	925 (705–1,170)	454 (347–588)	338 (246–448)
1-24	Pulmonary aspiration and foreign body in airway	84,400 (62,100–112,000)	51,400 (38,100–68,100)	42,700 (30,200–58,400)	883 (676–1,130)	430 (324–561)	317 (227–426)
1-25	Foreign body in eyes	311 (136–570)	527 (237–959)	632 (288–1,130)	5·5 (2·7–9·6)	5·5 (2·7–9·6)	5·5 (2·6–9·6)
1-26	Foreign body in other body part	2,790 (1,380–5,870)	1,830 (1,340–2,550)	1,830 (1,370–2,380)	36·6 (20·8–66·8)	18 (13·5–24·7)	15·5 (11·6–20·3)
1-27	Environmental heat and cold exposure	2,770 (2,170–3,490)	3,610 (2,770–4,580)	3,890 (2,910–5,030)	50·9 (39·9–64)	40·3 (31–51)	35·7 (26·9–45·9)
1-28	Exposure to forces of nature	142 (95–215)	1,920 (1,690–2,170)	3,730 (3,350–4,160)	2·4 (1·6–3·6)	18·6 (16·3–21·1)	30·6 (27·3–34·1)
1-29	Other unintentional injuries	26,900 (22,100–32,400)	22,100 (16,900–28,600)	22,000 (16,500–28,100)	440 (365–526)	229 (177–292)	192 (144–243)
1-30	Self-harm and interpersonal violence	51,900 (41,300–62,900)	75,800 (57,000–97,300)	76,700 (54,500–98,200)	854 (686–1,040)	735 (554–937)	639 (456–819)
1-31	Self-harm	20,900 (16,100–26,000)	34,700 (24,100–46,000)	36,100 (23,700–48,300)	368 (279–463)	346 (240–458)	309 (204–414)
1-32	Self-harm by firearm	2,120 (1,080–3,060)	2,180 (1,250–3,370)	2,140 (1,170–3,290)	38·7 (20–56·2)	22·5 (12·5–34)	18·7 (10·3–28·1)
1-33	Self-harm by other specified means	18,700 (14,400–23,500)	32,500 (22,800–43,200)	34,000 (22,100–45,300)	330 (252–416)	323 (225–427)	291 (192–390)
1-34	Interpersonal violence	30,500 (22,000–38,600)	41,000 (31,300–52,800)	40,000 (28,800–51,600)	479 (347–604)	388 (296–499)	325 (234–419)
1-35	Physical violence by firearm	12,200 (8,060–17,300)	17,400 (12,000–24,400)	17,300 (11,100–23,800)	199 (133–279)	166 (114–230)	141 (90·8–192)
1-36	Physical violence by sharp object	6,760 (4,340–9,770)	9,330 (6,060–12,900)	8,770 (5,780–12,500)	112 (73·5–160)	89·5 (58·9–124)	72·2 (47·8–102)
1-37	Sexual violence	1,510 (998–2,150)	2,730 (1,840–3,810)	3,050 (2,030–4,350)	26 (17·4–37·1)	27·1 (18·4–38)	25·6 (17·1–36·6)
1-38	Physical violence by other means	10,000 (7,050–13,800)	11,500 (8,610–15,000)	10,900 (7,850–14,200)	142 (102–193)	106 (80·5–138)	86·7 (63·9–112)
1-39	Conflict and terrorism	422 (380–468)	34 (22–52)	28 (18–43)	6 (5·4–6·7)	0·4 (0·2–0·6)	0·3 (0·2–0·4)
1-40	Executions and police conflict	40 (35–47)	63 (49–81)	525 (481–575)	0·6 (0·5–0·7)	0·6 (0·5–0·7)	4·2 (3·9–4·6)

SR Table 1: All-age DALYs counts and age standardized DALYs rates per 100,000 individuals for all causes and level 1 cause groups by location for 1990, 2010, and 2019, both sexes combined, Ecuador

Cause Level	Cause	All-age DALYs			Age-standardized rate of DALYs per 100,000		
		1990	2010	2019	1990	2010	2019
0-0	All causes	3,240,000 (2,980,000–3,520,000)	4,140,000 (3,710,000–4,610,000)	4,370,000 (3,710,000–5,130,000)	35,600 (32,700–38,700)	30,600 (27,500–34,000)	26,600 (22,600–31,300)
1-0	Communicable, maternal, neonatal, and nutritional disorders	1,290,000 (1,180,000–1,410,000)	827,000 (723,000–941,000)	675,000 (566,000–806,000)	10,800 (9,980–11,800)	5,550 (4,870–6,270)	4,030 (3,380–4,800)
1-1	HIV/AIDS and sexually transmitted infections	27,100 (12,100–54,100)	77,900 (65,600–97,700)	83,500 (71,200–103,000)	212 (108–400)	518 (440–642)	475 (402–593)
1-2	HIV/AIDS	4,170 (3,880–4,470)	58,500 (56,300–61,300)	65,800 (61,000–70,900)	45 (41·9–48·2)	394 (380–413)	370 (344–400)
1-3	HIV/AIDS - drug-susceptible tuberculosis	2,080 (1,890–2,300)	15,300 (9,160–22,200)	11,400 (6,540–17,700)	22·5 (20·4–24·9)	103 (61·7–150)	64·3 (36·8–99·4)
1-4	HIV/AIDS - Multidrug-resistant Tuberculosis without extensive drug resistance	12 (1–51)	2,180 (473–5,900)	1,520 (218–4,840)	0·1 (0–0·5)	14·7 (3·2–39·8)	8·6 (1·2–27·3)
1-5	HIV/AIDS - Extensively drug-resistant Tuberculosis	0 (0–0)	232 (48–646)	245 (34–722)	0 (0–0)	1·6 (0·3–4·4)	1·4 (0·2–4·1)
1-6	HIV/AIDS resulting in other diseases	2,080 (1,960–2,210)	40,800 (32,800–47,400)	52,600 (44,800–59,400)	22·4 (21·1–23·8)	275 (221–320)	296 (252–334)
1-7	Sexually transmitted infections excluding HIV	23,000 (7,910–50,200)	19,400 (7,520–39,900)	17,700 (7,030–36,800)	168 (61·6–357)	123 (48·7–251)	104 (41·1–218)
1-8	Syphilis	21,500 (6,490–48,300)	17,000 (5,280–37,200)	14,800 (4,250–34,000)	150 (45·7–337)	106 (33·1–233)	88 (25·2–202)
1-9	Chlamydial infection	177 (115–266)	296 (196–438)	340 (226–509)	1·9 (1·3–2·9)	2 (1·3–2·9)	1·9 (1·3–2·8)
1-10	Gonococcal infection	100 (80–129)	163 (133–204)	182 (138–235)	1·1 (0·9–1·4)	1·1 (0·9–1·4)	1 (0·8–1·3)
1-11	Trichomoniasis	313 (123–654)	515 (200–1,070)	612 (234–1,290)	3·8 (1·4–7·8)	3·6 (1·4–7·6)	3·5 (1·4–7·3)
1-12	Genital herpes	465 (154–1,130)	815 (268–2,000)	1,010 (330–2,470)	5·9 (1·9–14·3)	5·9 (1·9–14·5)	5·9 (1·9–14·3)
1-13	Other sexually transmitted infections	380 (259–546)	626 (437–893)	701 (475–1,010)	4·2 (2·9–6·1)	4·2 (3–6·1)	3·9 (2·7–5·6)
1-14	Respiratory infections and tuberculosis	367,000 (331,000–404,000)	256,000 (221,000–298,000)	200,000 (163,000–244,000)	3,550 (3,290–3,830)	1,840 (1,600–2,100)	1,250 (1,020–1,520)
1-15	Tuberculosis	98,000 (91,100–105,000)	39,300 (35,800–43,300)	28,900 (23,100–36,500)	1,150 (1,080–1,220)	285 (260–314)	170 (137–215)
1-16	Latent tuberculosis infection	0 (0–0)	0 (0–0)	0 (0–0)	0 (0–0)	0 (0–0)	0 (0–0)
1-17	Drug-susceptible tuberculosis	97,500 (90,600–104,000)	34,900 (28,000–40,100)	25,700 (18,000–33,400)	1,140 (1,070–1,210)	253 (203–289)	152 (106–196)
1-18	Multidrug-resistant tuberculosis without extensive drug resistance	522 (56–2,120)	3,980 (904–9,660)	2,730 (441–8,520)	6·1 (0·7–25)	28·9 (6·6–70·2)	16·2 (2·6–50·4)
1-19	Extensively drug-resistant tuberculosis	0 (0–0)	418 (90–1,080)	435 (68–1,340)	0 (0–0)	3 (0·6–7·8)	2·6 (0·4–7·9)
1-20	Lower respiratory infections	253,000 (222,000–285,000)	198,000 (165,000–236,000)	150,000 (119,000–188,000)	2,270 (2,040–2,500)	1,430 (1,220–1,670)	960 (767–1,200)
1-21	Upper respiratory infections	12,300 (7,670–18,300)	14,200 (8,820–21,700)	15,900 (9,750–24,600)	109 (69·5–164)	92·5 (57·7–141)	89·7 (55·2–138)
1-22	Otitis media	3,380 (1,920–5,610)	4,580 (2,670–7,640)	5,010 (2,890–8,340)	30·7 (17·7–50·8)	29·6 (17·4–49·4)	28·2 (16·3–46·7)
1-23	Enteric infections	225,000 (187,000–265,000)	55,100 (43,100–70,600)	46,200 (34,200–61,100)	1,760 (1,470–2,070)	372 (288–483)	274 (202–366)
1-24	Diarrhoeal diseases	225,000 (187,000–264,000)	54,300 (42,300–69,700)	45,400 (33,600–60,300)	1,750 (1,460–2,070)	367 (284–479)	270 (199–362)
1-25	Typhoid and paratyphoid	73 (32–143)	59 (27–113)	63 (29–120)	0·7 (0·3–1·3)	0·4 (0·2–0·7)	0·4 (0·2–0·7)
1-26	Typhoid fever	72 (32–141)	58 (26–111)	62 (28–119)	0·7 (0·3–1·3)	0·4 (0·2–0·7)	0·4 (0·2–0·7)
1-27	Paratyphoid fever	1 (0–3)	1 (0–2)	1 (0–2)	0 (0–0)	0 (0–0)	0 (0–0)
1-28	iNTS	608 (319–994)	635 (323–1,070)	635 (310–1,080)	5·1 (2·7–8·2)	4 (2·1–6·8)	3·6 (1·8–6·2)
1-29	Other intestinal infectious diseases	187 (66–346)	123 (61–204)	92 (51–147)	1·4 (0·6–2·6)	0·8 (0·4–1·3)	0·5 (0·3–0·8)
1-30	Neglected tropical diseases and malaria	87,900 (50,900–144,000)	25,300 (17,500–35,400)	24,000 (16,500–34,300)	841 (503–1,330)	182 (126–254)	142 (97·3–203)
1-31	Malaria	55,800 (22,000–111,000)	279 (172–426)	224 (91–546)	484 (192–961)	1·8 (1·1–2·8)	1·2 (0·5–3)
1-32	Chagas disease	1,560 (1,090–2,100)	1,530 (1,030–2,240)	1,530 (1,020–2,340)	24·8 (17·1–33)	12·4 (8·4–18·3)	9·7 (6·5–14·9)
1-33	Leishmaniasis	148 (19–443)	177 (98–288)	151 (44–338)	1·7 (0·2–5)	1·3 (0·7–2)	0·9 (0·3–2)
1-34	Visceral leishmaniasis	0 (0–0)	0 (0–0)	0 (0–0)	0 (0–0)	0 (0–0)	0 (0–0)
1-35	Cutaneous and mucocutaneous leishmaniasis	148 (19–443)	177 (98–288)	151 (44–338)	1·7 (0·2–5)	1·3 (0·7–2)	0·9 (0·3–2)
1-36	African trypanosomiasis	0 (0–0)	0 (0–0)	0 (0–0)	0 (0–0)	0 (0–0)	0 (0–0)
1-37	Schistosomiasis	0 (0–0)	0 (0–0)	0 (0–0)	0 (0–0)	0 (0–0)	0 (0–0)
1-38	Cysticercosis	5,760 (3,510–8,720)	7,060 (3,640–11,400)	7,420 (3,910–12,100)	85 (51–130)	56·5 (29·2–91·4)	46·4 (24·5–75·4)
1-39	Cystic echinococcosis	14 (6–23)	9 (5–14)	6 (3–9)	0·1 (0·1–0·2)	0·1 (0–0·1)	0 (0–0·1)
1-40	Lymphatic filariasis	0 (0–0)	0 (0–0)	0 (0–0)	0 (0–0)	0 (0–0)	0 (0–0)
1-41	Onchocerciasis	1,150 (764–1,670)	0 (0–0)	0 (0–0)	13·5 (9–19·4)	0 (0–0)	0 (0–0)
1-42	Trachoma	0 (0–0)	0 (0–0)	0 (0–0)	0 (0–0)	0 (0–0)	0 (0–0)
1-43	Dengue	676 (352–1,210)	2,620 (1,050–3,570)	1,600 (909–2,510)	6·6 (3·6–11·7)	17 (6·8–23·2)	9·1 (5·1–14·2)
1-44	Yellow fever	1,110 (258–3,100)	454 (100–1,250)	301 (68–854)	9·8 (2·3–27·6)	2·9 (0·6–7·9)	1·7 (0·4–4·6)
1-45	Rabies	1,020 (76–1,430)	34 (8–237)	31 (5–259)	9·4 (1–12·8)	0·3 (0·1–1·8)	0·2 (0–1·5)
1-46	Intestinal nematode infections	8,500 (5,070–13,900)	5,030 (2,700–8,460)	4,230 (2,220–7,240)	75·6 (44·9–124)	32·5 (17·4–54·4)	23·5 (12·4–40·3)
1-47	Ascariasis	5,900 (3,350–9,650)	3,460 (1,740–5,800)	2,510 (1,240–4,420)	52·3 (29·1–86·2)	22·5 (11·3–37·8)	14·1 (7–24·8)
1-48	Trichuriasis	1,280 (602–2,290)	1,270 (584–2,410)	1,420 (667–2,600)	11·2 (5·3–19·9)	8 (3·7–15·1)	7·7 (3·6–14·2)

1·49	Hookworm disease	1,330 (709–2,260)	308 (157–519)	298 (146–510)	12·1 (6·4–20·8)	2 (1–3·3)	1·6 (0·8–2·8)
1·50	Food-borne trematodiases	7,580 (2,300–15,800)	6,580 (2,270–13,200)	6,990 (2,530–14,300)	91·8 (28·4–188)	47·2 (16·3–95·5)	40·6 (14·5–83·5)
1·51	Leprosy	10 (6–15)	9 (6–14)	11 (6–16)	0·1 (0·1–0·2)	0·1 (0–0·1)	0·1 (0–0·1)
1·52	Ebola virus disease	0 (0–0)	0 (0–0)	0 (0–0)	0 (0–0)	0 (0–0)	0 (0–0)
1·53	Zika virus disease	0 (0–0)	0 (0–0)	7 (1–18)	0 (0–0)	0 (0–0)	0 (0–0·1)
1·54	Guinea worm disease	0 (0–0)	0 (0–0)	0 (0–0)	0 (0–0)	0 (0–0)	0 (0–0)
1·55	Other neglected tropical diseases	4,630 (2,400–6,460)	1,480 (974–2,230)	1,460 (968–2,200)	38·7 (20·3–53·4)	9·7 (6·5–14·7)	8·5 (5·7–12·8)
1·56	Other infectious diseases	83,200 (45,700–169,000)	34,200 (23,100–55,400)	36,800 (21,500–69,500)	648 (380–1,260)	220 (152–352)	214 (125–403)
1·57	Meningitis	20,000 (17,300–22,800)	9,870 (8,180–11,900)	6,690 (5,240–8,560)	158 (139–179)	63·8 (53·3–76·6)	38·7 (30·2–49·3)
1·58	Encephalitis	6,980 (5,860–8,870)	6,560 (5,410–9,050)	6,620 (4,930–10,200)	57·1 (48·8–72)	42·4 (35–57·6)	38 (28·2–58·7)
1·59	Diphtheria	238 (105–459)	42 (22–70)	62 (33–101)	1·8 (0·8–3·4)	0·3 (0·1–0·4)	0·4 (0·2–0·6)
1·60	Whooping cough	42,900 (4,750–128,000)	10,700 (1,030–31,300)	16,700 (2,070–48,500)	306 (33·8–915)	66·2 (6·4–195)	97·2 (12·1–282)
1·61	Tetanus	3,530 (2,060–4,390)	499 (358–1,050)	412 (258–1,040)	35·8 (19·5–44·3)	3·5 (2·5–7·4)	2·4 (1·5–6)
1·62	Measles	371 (273–490)	0 (0–0)	0 (0–0)	2·7 (2–3·5)	0 (0–0)	0 (0–0)
1·63	Varicella and herpes zoster	2,390 (1,420–3,880)	1,340 (881–1,980)	1,190 (836–1,710)	22 (14·7–33·1)	9·3 (6·4–13·4)	7·2 (5·1–10·2)
1·64	Acute hepatitis	3,190 (2,740–3,650)	1,300 (1,040–1,620)	1,270 (960–1,640)	30·8 (27·1–34·4)	8·5 (6·9–10·5)	7·1 (5·4–9·2)
1·65	Acute hepatitis A	2,240 (1,790–2,700)	940 (724–1,200)	900 (654–1,200)	20·9 (17·1–25·1)	6 (4·7–7·5)	5 (3·6–6·6)
1·66	Acute hepatitis B	454 (310–629)	208 (162–272)	231 (171–300)	4·7 (3·3–6·4)	1·5 (1·2–1·9)	1·4 (1–1·8)
1·67	Acute hepatitis C	406 (183–749)	84 (59–124)	66 (39–115)	4·3 (2·1–7·5)	0·6 (0·4–0·9)	0·4 (0·2–0·7)
1·68	Acute hepatitis E	84 (56–116)	69 (47–101)	75 (50–108)	0·8 (0·6–1·1)	0·5 (0·3–0·7)	0·4 (0·3–0·6)
1·69	Other unspecified infectious diseases	3,650 (2,690–5,010)	3,890 (2,940–4,660)	3,880 (2,920–4,850)	33·5 (24·8–45·8)	26·5 (19·7–31·7)	22·7 (17–28·4)
1·70	Maternal and neonatal disorders	396,000 (342,000–461,000)	332,000 (281,000–388,000)	246,000 (186,000–322,000)	2,860 (2,480–3,310)	2,080 (1,760–2,430)	1,440 (1,080–1,890)
1·71	Maternal disorders	22,900 (20,800–25,200)	21,300 (18,400–24,500)	18,500 (14,200–24,300)	225 (205–246)	136 (117–156)	98·8 (76–129)
1·72	Maternal haemorrhage	7,460 (6,370–8,740)	5,550 (4,550–6,670)	4,100 (3,050–5,350)	75 (64·1–87·8)	35·6 (29·1–42·7)	22 (16·3–28·8)
1·73	Maternal sepsis and other pregnancy related infections	3,110 (2,520–3,800)	1,470 (1,130–1,880)	1,190 (830–1,640)	29·9 (24·3–36·3)	9·2 (7·2–11·8)	6·3 (4·4–8·7)
1·74	Maternal hypertensive disorders	8,000 (6,970–9,140)	6,630 (5,630–7,840)	5,280 (4,020–6,930)	77·5 (67·7–87·7)	42·2 (36–49·8)	28·2 (21·5–36·9)
1·75	Maternal obstructed labour and uterine rupture	132 (54–255)	559 (408–741)	590 (421–816)	1·2 (0·5–2·4)	3·5 (2·6–4·7)	3·1 (2·2–4·3)
1·76	Maternal abortive outcome	3,010 (2,340–3,790)	1,310 (992–1,660)	1,030 (738–1,440)	29·8 (23·3–37·5)	8·4 (6·3–10·5)	5·5 (3·9–7·7)
1·77	Ectopic pregnancy	50 (40–62)	443 (349–547)	407 (294–566)	0·5 (0·4–0·6)	2·8 (2·2–3·5)	2·2 (1·6–3)
1·78	Indirect maternal deaths	279 (226–347)	1,880 (1,460–2,360)	2,180 (1,500–3,070)	2·6 (2·1–3·2)	11·7 (9·1–14·8)	11·5 (7·9–16·2)
1·79	Late maternal deaths	602 (458–799)	1,090 (796–1,590)	1,530 (987–2,380)	5·8 (4·4–7·7)	6·9 (5·1–10)	8·1 (5·2–12·6)
1·80	Maternal deaths aggravated by HIV/AIDS	2 (1–3)	15 (7–27)	22 (10–39)	0 (0–0)	0·1 (0–0·2)	0·1 (0·1–0·2)
1·81	Other maternal disorders	263 (201–340)	2,370 (1,890–2,960)	2,230 (1,640–3,030)	2·5 (1·9–3·2)	15·1 (12·1–18·9)	11·9 (8·8–16·1)
1·82	Neonatal disorders	373,000 (318,000–436,000)	310,000 (260,000–367,000)	228,000 (167,000–305,000)	2,640 (2,260–3,080)	1,940 (1,630–2,300)	1,340 (980–1,800)
1·83	Neonatal preterm birth	182,000 (151,000–218,000)	167,000 (139,000–197,000)	115,000 (83,400–157,000)	1,290 (1,070–1,540)	1,050 (870–1,240)	677 (490–928)
1·84	Neonatal encephalopathy due to birth asphyxia and trauma	78,900 (59,700–108,000)	56,800 (44,300–73,200)	41,600 (28,500–59,800)	553 (418–754)	356 (278–458)	244 (167–352)
1·85	Neonatal sepsis and other neonatal infections	64,200 (48,300–82,600)	48,700 (38,600–59,900)	40,100 (29,000–54,800)	458 (343–592)	305 (242–375)	235 (170–323)
1·86	Hemolytic disease and other neonatal jaundice	5,530 (4,350–7,000)	5,180 (4,160–6,260)	4,370 (3,370–5,480)	43·4 (34·6–54·3)	33·1 (26·6–40)	24·9 (19·2–31·3)
1·87	Other neonatal disorders	41,900 (32,000–54,900)	32,700 (26,300–40,600)	27,100 (18,300–38,600)	292 (224–383)	204 (164–254)	160 (108–229)
1·88	Nutritional deficiencies	103,000 (86,300–122,000)	46,800 (38,400–57,700)	38,300 (30,200–48,200)	927 (786–1,100)	338 (282–411)	238 (190–296)
1·89	Protein-energy malnutrition	64,400 (54,700–74,400)	23,700 (19,400–28,700)	17,400 (13,900–21,600)	561 (489–636)	176 (149–207)	113 (91·4–138)
1·90	Iodine deficiency	82 (36–171)	125 (54–251)	142 (61–286)	0·9 (0·4–1·8)	0·9 (0·4–1·7)	0·8 (0·4–1·6)
1·91	Vitamin A deficiency	1,800 (1,090–2,820)	1,010 (622–1,570)	937 (586–1,460)	13·7 (8·4–21·4)	6·3 (3·9–9·8)	5·3 (3·3–8·2)
1·92	Dietary iron deficiency	32,300 (20,100–49,100)	18,000 (11,500–27,100)	16,400 (10,500–25,400)	307 (196–459)	125 (80·7–187)	97 (62·5–149)
1·93	Other nutritional deficiencies	3,900 (2,970–5,110)	3,860 (3,300–4,510)	3,370 (2,700–4,130)	43·8 (35·4–54·4)	30·3 (26·2–34·9)	21·7 (17·5–26·5)
1·0	Non-communicable diseases	1,490,000 (1,310,000–1,700,000)	2,520,000 (2,190,000–2,900,000)	3,000,000 (2,510,000–3,580,000)	20,100 (17,900–22,500)	19,700 (17,300–22,500)	18,700 (15,700–22,200)
1·1	Neoplasms	182,000 (173,000–194,000)	376,000 (343,000–409,000)	448,000 (356,000–566,000)	2,840 (2,710–2,990)	3,070 (2,790–3,330)	2,850 (2,260–3,580)
1·2	Lip and oral cavity cancer	1,230 (1,140–1,320)	2,810 (2,520–3,130)	3,530 (2,760–4,530)	20·5 (19–22·1)	23·5 (21·2–26·1)	22·7 (17·8–29·2)
1·3	Nasopharynx cancer	334 (307–361)	642 (570–728)	733 (564–956)	4·9 (4·5–5·3)	5 (4·5–5·7)	4·6 (3·5–5·9)
1·4	Other pharynx cancer	513 (471–560)	739 (649–835)	922 (701–1,210)	8·7 (8–9·6)	6·2 (5·5–7)	5·9 (4·5–7·7)
1·5	Oesophageal cancer	2,610 (2,120–2,830)	3,960 (3,470–5,170)	4,940 (3,720–6,920)	47·8 (38·3–51·9)	34·6 (30·4–44·5)	32·6 (24·8–45·2)
1·6	Stomach cancer	39,600 (37,700–41,600)	66,600 (60,600–72,800)	69,400 (54,300–89,300)	702 (667–738)	570 (519–622)	452 (355–581)
1·7	Colon and rectum cancer	8,840 (8,370–9,320)	27,100 (24,800–29,600)	36,500 (28,800–46,600)	155 (147–164)	232 (211–252)	238 (189–301)

1-8	Liver cancer	4,560 (4,110–5,020)	9,050 (8,240–9,990)	12,500 (9,780–16,100)	73·8 (66·2–81·4)	76·6 (69·7–84·2)	80·6 (63·6–104)
1-9	Liver cancer due to hepatitis B	2,310 (1,910–2,740)	4,290 (3,490–5,190)	5,660 (3,990–7,600)	37·4 (30·2–44·9)	35·6 (28·7–43·4)	36 (25·5–48·5)
1-10	Liver cancer due to hepatitis C	250 (156–367)	574 (369–838)	827 (495–1,270)	4·9 (3–7·2)	5·3 (3·5–7·8)	5·7 (3·4–8·6)
1-11	Liver cancer due to alcohol use	930 (650–1,240)	2,310 (1,630–3,110)	3,390 (2,250–4,910)	17·1 (12–23)	20·4 (14·4–27·3)	22·4 (14·8–32·2)
1-12	Liver cancer due to NASH	359 (256–491)	864 (614–1,190)	1,300 (866–1,900)	6·5 (4·5–9)	7·7 (5·5–10·6)	8·7 (5·8–12·8)
1-13	Gallbladder and biliary tract cancer	6,290 (5,380–6,960)	9,550 (8,390–13,500)	11,300 (8,590–17,100)	115 (98·4–127)	83·5 (73·4–118)	74·4 (57·2–113)
1-14	Pancreatic cancer	2,350 (2,210–2,510)	13,100 (11,900–14,200)	18,400 (14,600–23,500)	42·7 (40–45·6)	114 (103–124)	121 (95·5–153)
1-15	Larynx cancer	1,240 (1,140–1,340)	2,020 (1,770–2,330)	2,410 (1,850–3,150)	22·1 (20·2–24)	17·4 (15·3–19·9)	15·7 (12·2–20·6)
1-16	Tracheal, bronchus, and lung cancer	11,900 (11,300–12,600)	27,300 (24,900–29,900)	34,000 (26,500–43,700)	211 (200–222)	236 (215–258)	222 (174–285)
1-17	Malignant skin melanoma	1,700 (1,430–2,450)	3,020 (2,420–4,610)	3,630 (2,490–5,790)	26·4 (22·3–37·4)	24·8 (20·1–37·1)	23·1 (16·1–36·4)
1-18	Non-melanoma skin cancer	875 (799–958)	2,320 (2,080–2,540)	3,110 (2,460–3,950)	16·3 (14·9–18)	21·7 (19·4–23·8)	22 (17·6–27·7)
1-19	Non-melanoma skin cancer (squamous-cell carcinoma)	874 (798–957)	2,320 (2,070–2,540)	3,110 (2,460–3,940)	16·3 (14·9–17·9)	21·7 (19·4–23·8)	22 (17·6–27·7)
1-20	Non-melanoma skin cancer (basal-cell carcinoma)	1 (0–1)	2 (1–3)	2 (1–3)	0 (0–0)	0 (0–0)	0 (0–0)
1-21	Breast cancer	9,750 (9,120–10,400)	23,700 (20,900–26,800)	31,700 (24,200–41,400)	155 (145–166)	192 (169–216)	198 (152–257)
1-22	Cervical cancer	17,300 (14,800–20,200)	28,800 (24,200–34,100)	31,900 (23,900–43,500)	270 (229–314)	230 (193–271)	198 (148–270)
1-23	Uterine cancer	4,000 (2,500–4,540)	6,360 (4,730–7,380)	7,500 (5,500–10,300)	70·6 (43·6–79·9)	54·3 (40·4–62·9)	48·5 (35·7–66·4)
1-24	Ovarian cancer	1,630 (1,300–2,780)	8,060 (5,280–9,630)	11,400 (6,730–15,400)	24·8 (19·6–42·8)	64·5 (41·9–76·8)	70·6 (41·6–95·8)
1-25	Prostate cancer	8,410 (7,380–10,900)	21,700 (16,400–25,700)	26,700 (20,100–36,300)	179 (156–230)	208 (155–246)	189 (141–256)
1-26	Testicular cancer	500 (390–645)	2,650 (2,150–3,240)	3,260 (2,300–4,490)	5·1 (4–6·5)	17·6 (14·4–21·5)	18·1 (12·9–24·7)
1-27	Kidney cancer	2,810 (2,580–3,050)	6,390 (5,770–7,050)	8,360 (6,550–10,700)	39·5 (36·8–42·5)	51·5 (46·4–56·6)	52·6 (41·3–67·2)
1-28	Bladder cancer	1,540 (1,430–1,660)	3,150 (2,820–3,490)	4,370 (3,420–5,550)	29·4 (27·2–31·8)	28·5 (25·5–31·5)	29·7 (23·4–37·6)
1-29	Brain and nervous system cancer	3,210 (2,280–6,060)	16,700 (7,280–21,500)	18,300 (7,570–26,100)	34·8 (25–65·7)	119 (50·9–152)	108 (44·2–155)
1-30	Thyroid cancer	1,180 (1,020–1,880)	4,220 (3,390–4,760)	5,190 (3,860–6,720)	19·3 (16·6–30·7)	35·4 (27·9–40)	33·5 (24·9–43·5)
1-31	Mesothelioma	248 (215–318)	581 (509–666)	708 (534–932)	3·9 (3·4–5·1)	4·7 (4·1–5·4)	4·4 (3·3–5·8)
1-32	Hodgkin lymphoma	2,400 (1,950–2,990)	2,780 (2,220–3,570)	2,720 (2,030–3,840)	26·4 (21·9–33·6)	19·6 (15·5–24·7)	15·8 (12–21·9)
1-33	Non-Hodgkin's lymphoma	6,410 (5,950–6,890)	17,900 (16,200–19,700)	21,500 (17,100–27,800)	82·2 (77·1–87·5)	137 (124–150)	132 (105–170)
1-34	Multiple myeloma	1,320 (1,070–1,930)	3,670 (3,150–4,450)	4,960 (3,630–6,640)	23·7 (19–34·7)	31·5 (27–38·1)	32·1 (23·6–42·8)
1-35	Leukaemia	22,200 (19,500–27,600)	38,600 (31,400–43,600)	40,300 (30,000–50,500)	219 (195–269)	260 (212–292)	232 (173–290)
1-36	Acute lymphoid leukaemia	8,730 (6,920–12,500)	16,500 (12,300–19,400)	17,600 (11,500–22,300)	72·4 (58·3–104)	103 (76·9–120)	97·7 (64·2–124)
1-37	Chronic lymphoid leukaemia	158 (131–194)	533 (448–621)	789 (581–1,040)	2·8 (2·3–3·4)	4·6 (3·9–5·4)	5·2 (3·9–6·8)
1-38	Acute myeloid leukaemia	3,380 (2,670–4,800)	8,360 (6,100–9,680)	10,300 (6,760–13,300)	34·6 (28·2–50)	57·5 (41·8–66)	59·6 (39·4–77·2)
1-39	Chronic myeloid leukaemia	847 (709–1,050)	1,880 (1,580–2,200)	2,010 (1,510–2,660)	10·7 (9–13·1)	13·9 (11·8–16·2)	12·1 (9–16)
1-40	Other leukaemia	9,120 (7,160–11,100)	11,300 (8,810–13,400)	9,600 (7,210–13,000)	98·2 (79·2–118)	80·7 (62·5–95·3)	57 (42·8–76·3)
1-41	Other neoplasms	767 (625–1,050)	1,570 (1,330–2,020)	2,280 (1,840–2,880)	10·2 (8·2–14·6)	12·4 (10·4–16·3)	14·4 (11·6–18·3)
1-42	Myelodysplastic, myeloproliferative, and other hematopoietic neoplasms	767 (625–1,050)	1,570 (1,330–2,020)	2,280 (1,840–2,880)	10·2 (8·2–14·6)	12·4 (10·4–16·3)	14·4 (11·6–18·3)
1-43	Benign and in situ intestinal neoplasms	0 (0–0)	0 (0–0)	0 (0–0)	0 (0–0)	0 (0–0)	0 (0–0)
1-44	Benign and in situ cervical and uterine neoplasms	0 (0–0)	0 (0–0)	0 (0–0)	0 (0–0)	0 (0–0)	0 (0–0)
1-45	Other benign and in situ neoplasms	0 (0–0)	0 (0–0)	0 (0–0)	0 (0–0)	0 (0–0)	0 (0–0)
1-46	Cardiovascular diseases	251,000 (237,000–263,000)	381,000 (348,000–417,000)	455,000 (370,000–566,000)	4,300 (4,050–4,490)	3,310 (3,020–3,610)	3,040 (2,480–3,780)
1-47	Rheumatic heart disease	11,100 (9,390–13,400)	11,500 (9,130–14,600)	11,900 (8,780–15,900)	139 (121–162)	82·5 (66·8–103)	69·6 (51·8–91·2)
1-48	Ischaemic heart disease	101,000 (95,700–106,000)	165,000 (150,000–183,000)	211,000 (168,000–268,000)	1,860 (1,750–1,950)	1,470 (1,340–1,620)	1,430 (1,150–1,800)
1-49	Stroke	96,200 (87,100–102,000)	121,000 (110,000–133,000)	143,000 (117,000–179,000)	1,590 (1,420–1,680)	1,020 (929–1,120)	939 (769–1,170)
1-50	Ischaemic stroke	29,000 (25,600–33,000)	43,300 (38,700–48,200)	54,800 (44,900–66,700)	557 (490–632)	405 (363–451)	386 (318–470)
1-51	Intracerebral hemorrhage	44,700 (37,900–49,400)	46,600 (42,200–52,100)	53,400 (42,100–68,400)	724 (613–792)	381 (345–425)	339 (269–436)
1-52	Subarachnoid hemorrhage	22,600 (18,400–28,000)	30,600 (27,600–34,700)	35,200 (27,700–44,400)	308 (248–385)	230 (208–259)	213 (168–268)
1-53	Hypertensive heart disease	19,900 (18,200–21,900)	45,800 (34,900–50,800)	45,200 (35,100–56,000)	382 (349–419)	427 (324–473)	319 (249–393)
1-54	Non-rheumatic valvular heart disease	1,720 (1,490–2,020)	3,460 (3,050–3,950)	4,720 (3,640–6,030)	25·5 (22·3–29·9)	27·8 (24·6–31·6)	29·7 (23–37·9)
1-55	Non-rheumatic calcific aortic valve disease	848 (683–1,060)	2,140 (1,840–2,530)	3,070 (2,330–3,950)	13·5 (10·9–16·8)	17·7 (15·2–20·6)	19·7 (15–25·3)
1-56	Non-rheumatic degenerative mitral valve disease	814 (667–988)	1,280 (1,070–1,490)	1,590 (1,140–2,120)	11·4 (9·3–13·9)	9·9 (8·3–11·5)	9·7 (7–13)
1-57	Other non-rheumatic valve diseases	59 (33–89)	45 (32–75)	60 (39–87)	0·7 (0·4–1)	0·3 (0·2–0·5)	0·4 (0·2–0·5)
1-58	Cardiomyopathy and myocarditis	3,970 (3,160–5,980)	8,000 (5,940–9,340)	7,850 (5,920–10,300)	48·5 (39·5–71·3)	60·6 (44·7–70·4)	48·6 (36·7–63·5)
1-59	Myocarditis	454 (325–662)	501 (339–669)	530 (394–731)	5·4 (3·8–8)	3·8 (2·5–4·9)	3·2 (2·4–4·4)
1-60	Alcoholic cardiomyopathy	132 (91–198)	142 (102–195)	174 (124–242)	2·1 (1·4–3)	1·1 (0·8–1·5)	1·1 (0·8–1·5)

1-61	Other cardiomyopathy	3,390 (2,600–5,270)	7,360 (5,460–8,590)	7,140 (5,370–9,310)	41 (32·4–61·7)	55·7 (40·7–64·5)	44·3 (33·4–57·5)
1-62	Atrial fibrillation and flutter	2,500 (2,190–2,960)	6,760 (5,930–7,640)	9,560 (7,760–11,600)	55 (48·1–65·7)	69·7 (61·2–79·2)	72·8 (59·5–88·3)
1-63	Aortic aneurysm	2,020 (1,810–2,240)	4,970 (4,480–5,610)	5,660 (4,450–7,320)	35·5 (31·6–39·6)	43 (38·6–48·6)	37·3 (29·4–48·2)
1-64	Peripheral vascular disease	300 (170–503)	623 (373–1,000)	879 (547–1,370)	6·4 (3·6–10·6)	6 (3·6–9·6)	6·3 (3·9–9·7)
1-65	Endocarditis	1,800 (1,320–2,220)	3,000 (2,370–3,650)	3,010 (2,280–3,970)	19·4 (14·2–24·1)	21·4 (17–25·9)	18 (13·7–23·7)
1-66	Chronic respiratory diseases	81,100 (63,400–98,800)	79,700 (67,700–95,000)	97,500 (80,400–118,000)	943 (794–1,080)	659 (572–760)	644 (536–775)
1-67	Chronic obstructive pulmonary disease	25,100 (20,700–27,700)	37,300 (33,000–41,900)	46,300 (37,700–55,600)	443 (377–481)	357 (311–402)	335 (274–403)
1-68	Pneumoconiosis	199 (163–242)	278 (235–337)	349 (269–465)	3·4 (2·8–4·1)	2·4 (2–2·8)	2·3 (1·8–3)
1-69	Silicosis	93 (64–120)	124 (95–161)	117 (80–182)	1·6 (1·1–2·1)	1·1 (0·8–1·3)	0·8 (0·5–1·2)
1-70	Asbestosis	28 (20–38)	31 (24–45)	43 (29–69)	0·5 (0·3–0·6)	0·3 (0·2–0·4)	0·3 (0·2–0·4)
1-71	Coal workers pneumoconiosis	28 (18–38)	20 (14–30)	26 (18–40)	0·4 (0·3–0·6)	0·2 (0·1–0·2)	0·2 (0·1–0·3)
1-72	Other pneumoconiosis	51 (39–69)	104 (82–131)	163 (117–218)	0·9 (0·7–1·2)	0·9 (0·7–1·1)	1·1 (0·8–1·4)
1-73	Asthma	46,500 (31,800–62,300)	27,400 (17,900–42,000)	30,700 (19,600–47,900)	382 (268–501)	175 (116–265)	175 (112–271)
1-74	Interstitial lung disease and pulmonary sarcoidosis	3,910 (2,820–6,220)	11,200 (7,980–13,000)	16,000 (10,200–21,700)	67·3 (47·7–109)	100 (71·3–117)	109 (68·4–148)
1-75	Other chronic respiratory diseases	5,460 (3,320–8,340)	3,590 (2,930–4,920)	4,090 (3,190–5,510)	47·6 (30·5–69·1)	24·2 (19·9–32·8)	23·4 (18·3–31·6)
1-76	Digestive diseases	154,000 (142,000–167,000)	192,000 (173,000–214,000)	217,000 (176,000–269,000)	1,970 (1,840–2,120)	1,530 (1,380–1,690)	1,360 (1,110–1,680)
1-77	Cirrhosis and other chronic liver diseases	50,200 (46,800–56,100)	88,000 (79,400–97,200)	101,000 (77,300–130,000)	743 (695–834)	723 (653–796)	641 (492–828)
1-78	Cirrhosis and other chronic liver diseases due to hepatitis B	5,980 (4,210–8,220)	8,070 (5,660–11,200)	6,580 (4,140–10,200)	92·6 (65·2–128)	65·4 (46·1–90·5)	41·3 (25·9–63·8)
1-79	Cirrhosis and other chronic liver diseases due to hepatitis C	6,150 (4,430–8,280)	11,000 (7,860–15,000)	12,800 (8,400–19,100)	95·2 (68·5–129)	89·7 (64–123)	80·7 (53·2–120)
1-80	Cirrhosis and other chronic liver diseases due to alcohol use	15,900 (12,300–19,700)	33,600 (26,600–41,300)	40,400 (28,800–55,200)	263 (204–327)	281 (222–347)	257 (185–352)
1-81	Cirrhosis and other chronic liver diseases due to NAFLD	7,590 (5,450–10,100)	16,600 (11,700–22,100)	20,600 (13,400–29,000)	125 (89·2–168)	140 (99·1–187)	133 (87·7–187)
1-82	Cirrhosis and other chronic liver diseases due to other causes	14,500 (12,000–17,300)	18,700 (14,800–23,300)	20,400 (14,600–27,800)	167 (133–204)	146 (114–184)	128 (91·1–174)
1-83	Upper digestive system diseases	25,400 (20,000–33,600)	31,800 (22,800–45,800)	35,500 (23,900–52,400)	371 (301–477)	249 (183–352)	218 (149–316)
1-84	Peptic ulcer disease	12,900 (11,900–14,000)	10,000 (8,960–11,600)	8,900 (6,890–11,600)	208 (191–224)	87·5 (78–102)	59·9 (46·5–78·1)
1-85	Gastritis and duodenitis	3,020 (2,060–4,250)	4,990 (3,650–6,630)	5,640 (4,180–7,460)	39·7 (27·2–55·8)	38·7 (28·7–50·6)	34·9 (26–45·6)
1-86	GERD	9,510 (4,950–16,900)	16,800 (8,660–29,700)	21,000 (10,900–37,300)	123 (63·7–217)	123 (63·7–218)	123 (63·9–218)
1-87	Appendicitis	14,500 (12,200–17,000)	6,780 (5,880–8,430)	6,690 (5,160–8,540)	133 (115–155)	46·3 (40·4–57·3)	38·5 (29·8–48·9)
1-88	Paralytic ileus and intestinal obstruction	23,500 (18,500–29,000)	15,800 (13,400–18,800)	15,800 (12,200–20,300)	220 (173–265)	121 (103–141)	100 (76·9–128)
1-89	Inguinal, femoral, and abdominal hernia	11,700 (9,020–15,000)	12,100 (9,110–15,700)	14,400 (10,800–19,100)	117 (90·5–148)	87·2 (66·3–112)	86·3 (65·2–114)
1-90	Inflammatory bowel disease	982 (705–1,300)	1,150 (968–1,350)	1,480 (1,120–1,820)	10·9 (8·2–13·9)	8·7 (7·4–10·2)	9 (6·8–11·1)
1-91	Vascular intestinal disorders	1,650 (1,410–1,920)	3,460 (3,070–4,070)	4,440 (3,490–5,820)	26·7 (23·4–30·6)	31 (27·4–36·8)	30·3 (23·9–39·4)
1-92	Gallbladder and biliary diseases	8,250 (6,970–9,530)	12,300 (10,400–14,500)	15,500 (11,800–19,300)	128 (107–146)	102 (86·5–119)	100 (76–125)
1-93	Pancreatitis	12,200 (8,270–15,600)	12,300 (10,300–17,300)	13,300 (9,720–20,100)	169 (112–217)	95·6 (80·1–134)	81·7 (59·6–123)
1-94	Other digestive diseases	5,480 (4,220–7,720)	8,510 (5,900–9,670)	9,040 (6,280–11,400)	51·4 (41·3–72·9)	65·9 (44–74·8)	57 (39·2–71·7)
1-95	Neurological disorders	109,000 (60,800–172,000)	175,000 (96,500–284,000)	208,000 (120,000–337,000)	1,340 (792–2,110)	1,320 (755–2,080)	1,280 (757–2,050)
1-96	Alzheimer's disease and other dementias	13,200 (5,470–30,100)	30,500 (12,600–69,000)	40,800 (17,400–96,500)	315 (130–714)	324 (133–735)	315 (132–725)
1-97	Parkinson's disease	2,960 (2,700–3,320)	9,140 (7,340–10,200)	11,900 (9,480–14,600)	62·4 (56·7–70·2)	87·7 (70·4–97·2)	85·8 (68·3–105)
1-98	Idiopathic epilepsy	40,800 (22,400–65,400)	50,000 (24,100–83,400)	51,600 (25,100–87,500)	414 (220–669)	335 (160–559)	293 (142–496)
1-99	Multiple sclerosis	361 (311–424)	768 (654–883)	980 (764–1,230)	5·2 (4·5–6·2)	5·9 (5–6·8)	5·9 (4·6–7·4)
1-100	Motor neuron disease	382 (347–420)	1,150 (1,020–1,300)	1,860 (1,420–2,430)	5·3 (4·8–5·8)	9·2 (8·2–10·4)	11·6 (8·9–15·2)
1-101	Headache disorders	44,900 (8,780–96,300)	73,200 (15,100–158,000)	87,900 (18,300–191,000)	483 (108–1,020)	494 (107–1,050)	493 (106–1,060)
1-102	Migraine	41,000 (5,970–92,300)	66,400 (10,200–151,000)	79,600 (12,400–178,000)	437 (72·4–972)	446 (73·6–1,000)	446 (72·5–998)
1-103	Tension-type headache	3,920 (1,230–15,500)	6,730 (2,140–26,000)	8,230 (2,680–30,400)	45·9 (15·2–164)	47·3 (15·5–176)	47·2 (15·6–172)
1-104	Other neurological disorders	5,850 (2,750–10,000)	10,300 (5,220–17,100)	12,800 (6,810–21,100)	57·4 (28·7–95·6)	68 (36–112)	72·6 (39·3–118)
1-105	Mental disorders	160,000 (117,000–214,000)	260,000 (189,000–345,000)	317,000 (230,000–423,000)	1,770 (1,290–2,330)	1,790 (1,310–2,370)	1,800 (1,310–2,380)
1-106	Schizophrenia	14,100 (9,720–18,900)	24,800 (17,000–33,000)	30,900 (21,400–41,400)	175 (121–231)	177 (120–234)	178 (123–238)
1-107	Depressive disorders	49,200 (33,200–69,200)	82,800 (57,200–116,000)	102,000 (70,300–142,000)	579 (398–798)	591 (412–823)	588 (405–816)
1-108	Major depressive disorder	41,800 (27,700–59,200)	70,100 (48,200–97,800)	86,500 (57,800–122,000)	489 (326–681)	501 (344–696)	498 (335–695)
1-109	Dysthymia	7,400 (4,450–11,600)	12,700 (7,740–19,300)	15,700 (9,650–24,100)	90·3 (56·3–140)	90·3 (56·3–139)	90·3 (56·4–139)
1-110	Bipolar disorder	17,800 (10,400–28,000)	28,900 (16,600–45,200)	35,200 (20,400–54,600)	197 (116–306)	198 (114–306)	197 (114–306)
1-111	Anxiety disorders	45,000 (29,500–63,700)	72,200 (47,800–101,000)	88,800 (59,200–125,000)	490 (327–685)	492 (324–694)	501 (335–703)
1-112	Eating disorders	5,580 (3,340–8,580)	8,920 (5,470–13,600)	11,400 (6,880–17,600)	52 (31·3–80·2)	55·9 (34·3–85·7)	60·4 (36·5–92·6)
1-113	Anorexia nervosa	1,070 (600–1,740)	1,600 (925–2,640)	1,990 (1,140–3,280)	9·4 (5·4–15·1)	9·8 (5·6–16·2)	10·5 (6·1–17·2)

1-114	Bulimia nervosa	4,520 (2,620–7,250)	7,320 (4,240–11,700)	9,410 (5,560–14,900)	42·6 (24·7–68·5)	46·1 (26·6–73·6)	49·9 (29·3–79)
1-115	Autism spectrum disorders	5,510 (3,600–8,000)	7,960 (5,190–11,600)	9,270 (6,090–13,400)	51·9 (33·7–75·7)	52 (34–75·3)	52·1 (34·2–75·4)
1-116	Attention-deficit/hyperactivity disorder	3,030 (1,730–5,130)	4,190 (2,360–6,980)	4,740 (2,670–7,990)	25·5 (14·8–42·7)	25·6 (14·5–42·4)	25·7 (14·5–43·1)
1-117	Conduct disorder	9,270 (5,160–14,800)	12,000 (6,880–19,100)	13,000 (7,270–20,500)	68·9 (38·5–110)	69·3 (39·6–110)	69·6 (39–110)
1-118	Idiopathic developmental intellectual disability	2,570 (1,100–4,480)	3,210 (1,300–5,710)	3,270 (1,250–5,850)	24 (10·5–41·8)	20·8 (8·5–36·9)	18·3 (7–32·6)
1-119	Other mental disorders	8,360 (5,230–12,700)	14,700 (9,410–22,500)	18,500 (11,700–28,100)	109 (69·4–165)	108 (69·4–166)	108 (68·9–165)
1-120	Substance use disorders	36,900 (29,200–44,200)	51,300 (40,300–63,700)	53,000 (40,500–67,200)	462 (360–546)	360 (287–447)	300 (230–382)
1-121	Alcohol use disorders	26,900 (20,600–32,000)	32,300 (25,800–40,800)	30,100 (22,700–41,200)	358 (270–421)	233 (188–294)	173 (131–236)
1-122	Drug use disorders	9,970 (6,960–13,600)	19,000 (13,700–25,200)	22,900 (16,600–30,100)	104 (74·5–141)	127 (91·8–167)	126 (92·1–164)
1-123	Opioid use disorders	4,280 (2,790–6,200)	9,140 (6,110–12,900)	11,800 (7,890–16,400)	49·1 (32·7–68·4)	63·6 (43·2–88·5)	66·4 (45·2–91·4)
1-124	Cocaine use disorders	1,280 (880–1,810)	2,390 (1,720–3,240)	2,930 (2,130–3,960)	13 (9·4–17·8)	15·6 (11·4–20·8)	16 (11·7–21·5)
1-125	Amphetamine use disorders	1,560 (806–2,680)	2,770 (1,450–4,570)	2,920 (1,580–4,880)	14·7 (7·9–25·1)	17·4 (9·2–28·5)	15·4 (8·4–25·5)
1-126	Cannabis use disorders	907 (497–1,460)	1,340 (758–2,120)	1,600 (910–2,560)	8·5 (4·8–13·6)	8·5 (4·9–13·3)	8·5 (4·9–13·6)
1-127	Other drug use disorders	1,950 (1,260–2,950)	3,400 (2,250–4,980)	3,700 (2,400–5,400)	19·1 (12·7–28·5)	21·8 (14·6–31·8)	19·9 (13·1–28·8)
1-128	Diabetes and kidney diseases	82,800 (75,400–91,200)	290,000 (261,000–322,000)	354,000 (290,000–428,000)	1,350 (1,230–1,490)	2,480 (2,230–2,750)	2,310 (1,900–2,800)
1-129	Diabetes mellitus	44,600 (38,800–51,400)	142,000 (121,000–165,000)	184,000 (149,000–225,000)	790 (686–914)	1,230 (1,050–1,420)	1,200 (979–1,470)
1-130	Diabetes mellitus type 1	3,580 (3,140–4,160)	6,470 (5,520–7,490)	7,010 (5,500–8,850)	44·4 (38·8–52·5)	46·6 (40–54·3)	41·1 (32·2–51·4)
1-131	Diabetes mellitus type 2	41,000 (35,500–47,600)	136,000 (115,000–157,000)	177,000 (144,000–216,000)	746 (646–865)	1,180 (1,010–1,370)	1,160 (946–1,420)
1-132	Chronic kidney disease	37,900 (35,300–40,900)	148,000 (136,000–161,000)	170,000 (137,000–212,000)	560 (524–602)	1,250 (1,150–1,360)	1,110 (900–1,380)
1-133	Chronic kidney disease due to diabetes mellitus type 1	2,630 (1,820–3,540)	10,400 (6,780–14,800)	10,900 (6,710–16,800)	37·1 (25·2–51·4)	80·7 (52·1–116)	66·7 (40·9–103)
1-134	Chronic kidney disease due to diabetes mellitus type 2	6,200 (4,910–7,430)	32,400 (25,400–39,400)	40,400 (29,800–52,900)	116 (92·8–139)	293 (232–356)	273 (204–357)
1-135	Chronic kidney disease due to hypertension	5,980 (4,980–7,190)	30,500 (24,700–37,400)	36,900 (28,500–48,000)	112 (93·1–133)	283 (231–347)	256 (198–329)
1-136	Chronic kidney disease due to glomerulonephritis	10,300 (8,700–12,100)	34,600 (28,200–41,800)	36,900 (27,400–49,400)	131 (110–156)	270 (217–331)	228 (169–307)
1-137	Chronic kidney disease due to other and unspecified causes	12,800 (10,800–15,000)	39,800 (32,500–48,800)	44,800 (34,400–58,600)	164 (137–197)	321 (260–398)	286 (218–373)
1-138	Acute glomerulonephritis	286 (125–530)	40 (30–51)	54 (35–73)	2·5 (1·2–4·3)	0·3 (0·2–0·4)	0·3 (0·2–0·4)
1-139	Skin and subcutaneous diseases	59,400 (38,500–90,700)	88,100 (56,700–134,000)	104,000 (67,300–158,000)	577 (374–882)	584 (379–893)	591 (384–898)
1-140	Dermatitis	13,600 (7,810–21,500)	19,200 (11,200–30,300)	22,000 (13,000–34,900)	125 (73·5–194)	125 (73·7–196)	125 (74–198)
1-141	Atopic dermatitis	10,900 (5,850–18,500)	14,700 (7,810–24,400)	16,500 (8,720–27,600)	92·3 (49·6–155)	92·7 (49·4–154)	92·7 (49·1–155)
1-142	Contact dermatitis	2,370 (1,490–3,550)	4,030 (2,510–6,110)	4,980 (3,100–7,430)	29 (18·2–43·4)	29 (18–43·8)	29 (18·1–43·5)
1-143	Seborrhoeic dermatitis	303 (169–494)	484 (273–788)	585 (329–924)	3·4 (1·9–5·5)	3·4 (1·9–5·5)	3·4 (1·9–5·4)
1-144	Psoriasis	5,850 (4,150–7,860)	8,480 (5,950–11,300)	9,630 (6,810–12,800)	67·8 (48·3–90·5)	60 (42·4–79·7)	55·9 (39·7–74·5)
1-145	Bacterial skin diseases	1,010 (717–1,510)	1,730 (1,270–2,470)	2,880 (1,860–3,830)	11 (7·9–16·1)	12·9 (9·8–17·9)	18·2 (11·5–24)
1-146	Cellulitis	183 (125–259)	284 (199–390)	511 (270–733)	2 (1·4–2·7)	2 (1·4–2·7)	3·1 (1·6–4·4)
1-147	Pyoderma	825 (561–1,280)	1,440 (1,040–2,150)	2,370 (1,550–3,270)	9 (6·2–13·9)	10·8 (8–15·6)	15·1 (9·7–20·4)
1-148	Scabies	9,010 (4,900–14,400)	12,700 (6,940–20,300)	14,700 (8,040–23,400)	82·5 (45–132)	82·2 (45·1–131)	82·1 (45·2–130)
1-149	Fungal skin diseases	6,040 (2,380–13,000)	9,680 (3,890–20,900)	11,800 (4,730–25,400)	71 (28·7–153)	70·2 (28·3–151)	70 (28·3–150)
1-150	Viral skin diseases	6,220 (3,920–9,420)	8,270 (5,220–12,500)	9,230 (5,920–13,900)	51·7 (32·8–78)	51·9 (32·8–77·9)	51·8 (33·2–78·2)
1-151	Acne vulgaris	7,360 (4,350–11,700)	12,000 (7,080–19,100)	14,200 (8,250–22,200)	58·4 (34·5–93·2)	70·4 (41·4–112)	74 (43·1–115)
1-152	Alopecia areata	655 (414–987)	1,050 (655–1,580)	1,260 (800–1,890)	7·1 (4·5–10·7)	7·1 (4·5–10·8)	7·1 (4·5–10·7)
1-153	Pruritus	701 (337–1,270)	1,170 (556–2,090)	1,440 (692–2,560)	8·3 (4–14·9)	8·4 (4–15·1)	8·5 (4·1–15·2)
1-154	Urticaria	5,320 (3,490–7,870)	7,420 (4,780–10,900)	8,520 (5,520–12,400)	48·2 (31·7–69·9)	48·2 (31–70·5)	48·3 (31·2–70·5)
1-155	Decubitus ulcer	89 (66–116)	210 (156–273)	440 (240–568)	1·7 (1·2–2·2)	1·9 (1·4–2·5)	3·1 (1·7–4)
1-156	Other skin and subcutaneous diseases	3,590 (1,710–6,550)	6,200 (2,980–11,400)	7,860 (3,810–14,000)	45·2 (21·9–82·1)	46 (22·3–84·1)	47·2 (22·9–84·2)
1-157	Sense organ diseases	56,600 (38,900–80,200)	105,000 (71,800–148,000)	128,000 (87,400–182,000)	907 (622–1,280)	866 (596–1,220)	820 (560–1,160)
1-158	Blindness and vision loss	17,200 (11,800–24,000)	30,100 (20,600–42,400)	37,300 (25,500–53,100)	292 (204–403)	256 (176–360)	244 (167–345)
1-159	Glaucoma	743 (485–1,080)	1,190 (784–1,710)	1,440 (956–2,030)	16·2 (10·6–23·4)	11·6 (7·6–16·5)	10·3 (6·8–14·6)
1-160	Cataract	4,070 (2,860–5,510)	7,450 (5,190–10,100)	9,360 (6,540–12,700)	83·7 (59–114)	69·7 (48·8–94·8)	65·5 (45·8–88·6)
1-161	Age-related macular degeneration	137 (91–199)	260 (173–373)	340 (226–494)	2·8 (1·9–4·1)	2·4 (1·6–3·5)	2·4 (1·6–3·4)
1-162	Refraction disorders	5,830 (3,900–8,490)	9,450 (6,320–13,600)	11,400 (7,560–16,400)	71·9 (48·4–102)	68·9 (46·3–98·1)	67·3 (44·7–96·2)
1-163	Near vision loss	2,580 (1,170–5,040)	5,270 (2,410–10,200)	6,860 (3,080–13,600)	48·6 (22·4–94·1)	46·7 (21·4–89·6)	45·5 (20·6–89·8)
1-164	Other vision loss	3,890 (2,700–5,390)	6,490 (4,570–8,870)	7,970 (5,600–10,900)	68·7 (47·9–94·5)	56·7 (39·9–77·6)	53 (37·3–72·6)
1-165	Age-related and other hearing loss	36,800 (24,700–52,600)	69,800 (47,600–100,000)	84,700 (57,400–122,000)	579 (390–835)	573 (392–820)	539 (366–775)
1-166	Other sense organ diseases	2,590 (1,560–3,990)	4,680 (2,780–7,210)	5,950 (3,540–9,180)	35·8 (21·2–56·1)	36·4 (21·5–56·2)	36·7 (21·8–56·6)

1-167	Musculoskeletal disorders	111,000 (80,000–147,000)	211,000 (153,000–277,000)	274,000 (198,000–362,000)	1,550 (1,100–2,060)	1,610 (1,160–2,110)	1,650 (1,200–2,180)
1-168	Rheumatoid arthritis	2,930 (2,200–3,750)	6,930 (5,200–8,750)	9,310 (6,810–11,900)	46·1 (35·6–58·7)	56·6 (42·8–71·1)	58·8 (43·7–75)
1-169	Osteoarthritis	12,000 (6,070–23,800)	28,100 (14,300–55,400)	38,400 (19,300–75,600)	220 (112–436)	243 (124–478)	249 (125–491)
1-170	Low back pain	51,200 (35,800–68,700)	85,100 (60,500–114,000)	107,000 (75,700–142,000)	669 (469–900)	633 (452–844)	635 (450–843)
1-171	Neck pain	9,750 (6,310–14,200)	17,900 (11,800–25,900)	22,600 (14,900–32,700)	135 (89–194)	135 (89·3–196)	135 (89·1–196)
1-172	Gout	492 (303–710)	1,220 (759–1,740)	1,650 (1,040–2,390)	8 (4·9–11·6)	10 (6·3–14·3)	10·4 (6·6–14·9)
1-173	Other musculoskeletal disorders	34,900 (24,300–48,300)	71,400 (49,900–97,800)	95,200 (66,700–130,000)	473 (331–657)	535 (371–728)	566 (397–775)
1-174	Other non-communicable diseases	211,000 (175,000–253,000)	312,000 (250,000–383,000)	342,000 (264,000–437,000)	2,100 (1,700–2,590)	2,150 (1,720–2,660)	2,030 (1,570–2,600)
1-175	Congenital anomalies	99,700 (81,900–123,000)	140,000 (94,900–172,000)	126,000 (74,100–175,000)	716 (590–875)	872 (594–1,080)	734 (431–1,030)
1-176	Neural tube defects	6,000 (4,110–8,240)	6,710 (5,170–8,620)	7,160 (4,860–10,300)	42·7 (29·5–58·5)	42 (32·4–54)	41·9 (28·4–60·7)
1-177	Congenital heart anomalies	48,600 (38,300–61,400)	67,500 (48,300–85,700)	59,100 (34,900–83,700)	346 (274–436)	420 (300–533)	345 (204–490)
1-178	Orofacial clefts	1,950 (781–4,560)	351 (243–509)	305 (205–445)	13·9 (5·7–32·1)	2·2 (1·6–3·2)	1·8 (1·2–2·6)
1-179	Down's syndrome	6,480 (4,670–9,130)	8,240 (6,050–10,800)	6,460 (4,390–9,030)	46·5 (33·6–65·4)	51·4 (37·8–67·1)	37·6 (25·3–52·6)
1-180	Turner syndrome	16 (7–29)	20 (9–36)	21 (9–37)	0·1 (0·1–0·2)	0·1 (0·1–0·2)	0·1 (0·1–0·2)
1-181	Klinefelter syndrome	1 (1–2)	2 (1–3)	2 (1–3)	0 (0–0)	0 (0–0)	0 (0–0)
1-182	Other chromosomal abnormalities	1,730 (1,190–3,490)	3,720 (2,350–4,790)	4,070 (2,040–5,870)	12·5 (8·6–24·9)	23·1 (14·7–29·8)	23·7 (11·9–34·4)
1-183	Congenital musculoskeletal and limb anomalies	4,560 (3,100–6,340)	5,930 (4,100–8,050)	7,110 (4,820–9,950)	37·7 (25·7–52·1)	37·8 (26·1–51·4)	40·3 (27·4–56·3)
1-184	Urogenital congenital anomalies	1,210 (873–1,680)	2,400 (1,010–3,350)	2,210 (853–3,610)	8·7 (6·3–12)	15 (6·3–21)	13 (4·9–21·3)
1-185	Digestive congenital anomalies	8,300 (5,870–11,800)	14,600 (6,640–19,700)	13,700 (4,540–22,100)	58·2 (41·2–82·6)	91·3 (41·5–123)	81·3 (26·8–131)
1-186	Other congenital anomalies	20,800 (15,700–28,500)	30,200 (18,100–38,100)	25,400 (13,000–37,700)	150 (113–205)	189 (113–238)	149 (76·2–222)
1-187	Urinary diseases and male infertility	5,360 (4,260–6,720)	12,600 (9,750–16,300)	16,900 (12,600–21,800)	82·7 (64·6–106)	106 (82·3–138)	110 (82·5–142)
1-188	Urinary tract infections and interstitial nephritis	2,720 (2,340–3,230)	5,640 (4,690–6,560)	7,830 (5,430–9,740)	37·4 (32·7–43·4)	46·3 (38·1–53·3)	51·8 (35·4–64·4)
1-189	Urolithiasis	429 (302–578)	920 (672–1,220)	1,230 (890–1,610)	6 (4·3–8)	7 (5·2–9·2)	7·4 (5·4–9·7)
1-190	Benign prostatic hyperplasia	1,590 (938–2,500)	4,860 (2,850–7,430)	6,280 (3,730–9,770)	31·8 (18·7–49·8)	44·4 (26·1–68)	42·1 (25·1–65·4)
1-191	Male infertility	112 (45–242)	269 (97–632)	327 (120–784)	1·2 (0·5–2·6)	1·8 (0·7–4·2)	1·8 (0·7–4·3)
1-192	Other urinary diseases	514 (389–651)	943 (714–1,270)	1,190 (900–1,570)	6·4 (4·8–7·9)	7·1 (5·4–9·7)	7·2 (5·5–9·8)
1-193	Gynecological diseases	41,300 (28,500–57,400)	66,700 (45,800–93,200)	77,800 (53,200–108,000)	479 (331–663)	459 (317–640)	440 (302–609)
1-194	Uterine fibroids	3,190 (1,790–5,580)	4,990 (2,540–9,070)	6,040 (2,980–11,100)	41·3 (22·9–72·3)	36·3 (18·5–65·8)	35·2 (17·4–64·8)
1-195	Polycystic ovarian syndrome	1,220 (508–2,480)	2,710 (1,170–5,230)	2,970 (1,270–5,910)	12 (5·1–24·4)	17·5 (7·5–33·6)	16 (6·9–31·9)
1-196	Female infertility	22 (6–68)	184 (16–660)	243 (19–833)	0·2 (0·1–0·7)	1·2 (0·1–4·5)	1·4 (0·1–4·7)
1-197	Endometriosis	3,980 (2,310–6,460)	4,780 (2,930–7,400)	5,270 (3,210–8,190)	41·3 (24·2–66·4)	31·4 (19·3–48·3)	28·8 (17·5–44·4)
1-198	Genital prolapse	698 (391–1,230)	932 (478–1,640)	1,190 (605–2,190)	10·6 (5·6–18·8)	7·6 (3·9–13·5)	7·5 (3·8–13·7)
1-199	Premenstrual syndrome	9,630 (5,790–15,300)	15,300 (9,340–24,100)	18,000 (11,000–28,200)	97 (59·7–152)	99·2 (60·6–155)	98·1 (59·6–153)
1-200	Other gynecological diseases	22,600 (14,700–32,600)	37,800 (25,100–54,200)	44,100 (28,800–63,200)	276 (178–398)	266 (179–380)	253 (165–361)
1-201	Hemoglobinopathies and hemolytic anaemias	7,830 (6,500–9,530)	6,760 (5,670–8,210)	6,650 (5,260–8,250)	80·9 (67·6–97·9)	46·7 (39·3–56·6)	38·6 (30·7–47·6)
1-202	Thalassemias	293 (210–397)	217 (157–294)	122 (79–179)	2·1 (1·5–2·9)	1·3 (1–1·8)	0·7 (0·5–1)
1-203	Thalassaemias trait	805 (520–1,200)	717 (450–1,110)	733 (458–1,150)	7·4 (4·8–11)	4·7 (2·9–7·3)	4·1 (2·5–6·4)
1-204	Sickle cell disorders	904 (707–1,130)	1,080 (869–1,330)	815 (587–1,070)	7·1 (5·6–8·8)	6·5 (5·2–8)	4·4 (3·1–5·8)
1-205	Sickle cell trait	918 (588–1,350)	962 (597–1,460)	1,020 (608–1,610)	8·7 (5·6–12·8)	6·2 (3·8–9·4)	5·5 (3·3–8·7)
1-206	G6PD deficiency	1,010 (739–1,320)	1,040 (780–1,310)	1,530 (1,130–2,020)	9·5 (7–12·3)	6·8 (5·1–8·6)	8·6 (6·4–11·3)
1-207	G6PD trait	15 (8–26)	17 (9–28)	18 (8–31)	0·2 (0·1–0·4)	0·1 (0·1–0·2)	0·1 (0–0·2)
1-208	Other hemoglobinopathies and hemolytic anaemias	3,880 (3,240–4,710)	2,730 (2,280–3,320)	2,410 (1,900–3,070)	45·9 (38·8–55)	21·1 (17·8–25·5)	15·3 (12·1–19·2)
1-209	Endocrine, metabolic, blood, and immune disorders	23,900 (17,900–29,400)	32,100 (24,700–40,800)	44,500 (34,300–56,800)	278 (209–348)	245 (188–312)	273 (211–349)
1-210	Oral disorders	29,700 (17,800–45,600)	51,200 (30,900–78,000)	67,800 (40,800–104,000)	439 (268–670)	402 (248–609)	419 (253–645)
1-211	Caries of deciduous teeth	428 (184–924)	495 (218–963)	539 (227–1,120)	3·1 (1·3–6·7)	3 (1·3–5·9)	3·1 (1·3–6·5)
1-212	Caries of permanent teeth	3,350 (1,480–6,730)	5,150 (2,260–10,200)	6,090 (2,650–12,100)	34·4 (15·1–67·8)	34·4 (14·9–67·5)	34·2 (15–67·4)
1-213	Periodontal disease	6,700 (2,540–14,400)	12,700 (4,930–27,800)	15,800 (5,980–34,200)	91·7 (35–196)	95·5 (37–207)	93·9 (35·6–201)
1-214	Edentulism	14,300 (9,200–20,700)	25,100 (16,800–35,100)	36,000 (23,000–52,900)	257 (166–374)	216 (145–302)	234 (149–345)
1-215	Other oral disorders	4,940 (3,040–7,260)	7,770 (4,830–11,400)	9,340 (5,780–13,600)	53·2 (32·8–77·9)	53·3 (32·9–77·9)	53·3 (33·1–77·6)
1-216	Sudden infant death syndrome	3,290 (1,580–7,160)	3,350 (1,610–6,020)	2,770 (1,350–4,870)	23 (11–49·9)	20·9 (10–37·5)	16·3 (8–28·7)
1-0	Injuries	454,000 (425,000–482,000)	795,000 (721,000–867,000)	698,000 (585,000–841,000)	4,730 (4,430–5,030)	5,320 (4,830–5,810)	3,940 (3,300–4,740)
1-1	Transport injuries	148,000 (136,000–170,000)	286,000 (240,000–316,000)	262,000 (207,000–321,000)	1,560 (1,450–1,790)	1,910 (1,610–2,110)	1,470 (1,160–1,800)
1-2	Road injuries	143,000 (132,000–165,000)	268,000 (225,000–296,000)	248,000 (196,000–304,000)	1,510 (1,400–1,730)	1,790 (1,500–1,980)	1,390 (1,100–1,710)

1-3	Pedestrian road injuries	95,500 (81,900–108,000)	134,000 (116,000–153,000)	98,700 (78,800–131,000)	1,010 (874–1,140)	909 (789–1,030)	566 (452–746)
1-4	Cyclist road injuries	3,820 (2,900–4,740)	10,500 (8,770–12,800)	10,200 (8,170–12,900)	44·2 (33·5–54·2)	72·8 (60·8–87·9)	59 (47·4–74)
1-5	Motorcyclist road injuries	9,090 (5,780–15,300)	37,600 (24,400–49,700)	50,600 (25,000–68,300)	92·4 (60–152)	241 (159–317)	273 (137–366)
1-6	Motor vehicle road injuries	32,200 (24,600–46,800)	79,700 (52,900–94,300)	83,600 (55,500–108,000)	340 (264–491)	528 (355–624)	464 (310–602)
1-7	Other road injuries	1,980 (1,410–2,530)	5,980 (4,820–8,010)	5,270 (3,960–6,800)	22 (16–27·8)	40·3 (32·5–53·6)	29·7 (22·5–38·1)
1-8	Other transport injuries	4,960 (4,390–6,060)	18,100 (15,600–20,500)	13,400 (9,740–17,100)	52·4 (46·7–64·5)	121 (105–136)	74·9 (54·8–94·7)
1-9	Unintentional injuries	206,000 (185,000–226,000)	241,000 (210,000–280,000)	236,000 (194,000–284,000)	2,140 (1,910–2,370)	1,670 (1,450–1,920)	1,380 (1,130–1,650)
1-10	Falls	40,100 (34,300–45,700)	69,100 (58,100–82,700)	74,600 (60,400–92,200)	507 (434–579)	516 (436–615)	452 (366–557)
1-11	Drowning	39,200 (34,000–44,000)	43,200 (37,400–51,600)	33,000 (26,000–43,500)	348 (297–386)	275 (238–326)	183 (144–240)
1-12	Fire, heat, and hot substances	22,200 (18,000–26,400)	19,300 (15,500–24,200)	19,700 (15,200–25,500)	221 (181–264)	132 (106–167)	114 (88·3–148)
1-13	Poisonings	6,210 (5,090–7,260)	4,960 (4,330–5,780)	4,690 (3,850–5,770)	57 (47–65·3)	32·5 (28·4–37·8)	26·3 (21·7–32·4)
1-14	Poisoning by carbon monoxide	290 (187–576)	976 (428–1,320)	992 (465–1,400)	2·9 (1·9–5·7)	6·4 (2·8–8·6)	5·5 (2·6–7·8)
1-15	Poisoning by other means	5,920 (4,720–6,990)	3,980 (3,330–5,190)	3,700 (2,930–5,000)	54·1 (43·2–62·6)	26 (21·8–33·6)	20·8 (16·5–28·1)
1-16	Exposure to mechanical forces	20,000 (16,400–23,900)	22,900 (18,200–29,500)	25,400 (19,200–33,200)	224 (182–270)	160 (127–206)	147 (111–192)
1-17	Unintentional firearm injuries	6,760 (4,190–8,600)	4,970 (3,970–7,640)	4,160 (3,080–6,940)	68·8 (43·4–86·6)	33 (26·5–50·2)	23·3 (17·4–38·1)
1-18	Other exposure to mechanical forces	13,300 (10,400–16,800)	17,900 (14,000–23,700)	21,300 (16,100–28,400)	156 (122–197)	127 (99·1–168)	124 (93·6–165)
1-19	Adverse effects of medical treatment	5,520 (3,920–6,640)	4,290 (3,570–5,830)	4,210 (3,320–5,580)	56·6 (39·5–66·5)	30·5 (25·6–41·3)	25·3 (20·2–33·2)
1-20	Animal contact	1,220 (844–1,810)	4,220 (2,140–5,130)	3,850 (2,320–4,800)	13·8 (9·6–19·9)	29·3 (14·8–35·5)	22·4 (13·4–27·9)
1-21	Venomous animal contact	903 (620–1,420)	3,380 (1,640–4,030)	2,900 (1,730–3,620)	10 (6·9–15·1)	23·3 (11·3–27·8)	16·8 (10–21)
1-22	Non-venomous animal contact	319 (206–488)	847 (489–1,120)	950 (579–1,270)	3·8 (2·5–5·7)	6 (3·5–7·9)	5·6 (3·4–7·5)
1-23	Foreign body	38,000 (33,600–42,700)	35,600 (29,700–43,000)	35,200 (27,900–43,600)	342 (307–377)	234 (197–281)	204 (162–253)
1-24	Pulmonary aspiration and foreign body in airway	35,300 (31,200–39,900)	32,900 (27,100–40,400)	32,000 (24,800–40,300)	314 (282–347)	216 (180–263)	186 (144–234)
1-25	Foreign body in eyes	499 (216–912)	788 (364–1,410)	950 (443–1,690)	5·5 (2·7–9·7)	5·4 (2·6–9·5)	5·4 (2·6–9·6)
1-26	Foreign body in other body part	2,180 (1,660–2,770)	1,900 (1,410–2,500)	2,240 (1,650–2,960)	22·8 (17·6–28·7)	13 (9·7–17·1)	12·9 (9·6–17)
1-27	Environmental heat and cold exposure	2,830 (2,030–3,760)	4,020 (2,870–5,270)	4,860 (3,460–6,480)	34·3 (24·9–45·2)	29·2 (20·9–38·2)	28·7 (20·4–38·1)
1-28	Exposure to forces of nature	4,060 (2,770–5,790)	5,370 (4,400–6,720)	3,400 (2,580–4,530)	41·9 (29·3–58·6)	37·3 (30·3–46·7)	20·2 (15·3–26·8)
1-29	Other unintentional injuries	27,000 (22,400–30,600)	27,700 (23,200–34,500)	26,800 (21,200–35,100)	291 (239–333)	189 (158–233)	152 (120–200)
1-30	Self-harm and interpersonal violence	99,700 (93,100–109,000)	269,000 (238,000–298,000)	201,000 (159,000–252,000)	1,030 (958–1,120)	1,750 (1,540–1,930)	1,090 (870–1,380)
1-31	Self-harm	28,200 (25,600–37,200)	86,800 (59,400–98,600)	90,900 (57,700–118,000)	282 (257–376)	558 (382–633)	492 (312–644)
1-32	Self-harm by firearm	3,690 (2,520–5,850)	5,430 (3,130–7,810)	4,570 (2,780–7,210)	38·4 (26·3–61·5)	35·9 (20·8–51·8)	25·2 (15·3–39·3)
1-33	Self-harm by other specified means	24,500 (21,800–32,500)	81,400 (55,900–92,800)	86,300 (53,900–113,000)	243 (218–327)	522 (358–594)	467 (292–612)
1-34	Interpersonal violence	71,100 (64,500–76,600)	182,000 (161,000–205,000)	109,000 (83,900–140,000)	738 (674–790)	1,190 (1,050–1,330)	601 (461–767)
1-35	Physical violence by firearm	40,200 (33,900–45,300)	113,000 (95,800–130,000)	54,000 (40,500–70,400)	415 (349–464)	736 (623–844)	294 (220–383)
1-36	Physical violence by sharp object	18,300 (14,200–23,700)	39,800 (33,500–55,300)	28,500 (20,500–49,900)	193 (150–252)	261 (221–361)	157 (113–272)
1-37	Sexual violence	1,980 (1,300–2,820)	3,130 (2,040–4,450)	3,740 (2,450–5,320)	20·8 (13·7–29·6)	20·8 (13·7–29·6)	20·8 (13·7–29·5)
1-38	Physical violence by other means	10,600 (9,050–12,900)	25,600 (22,400–29,200)	23,200 (18,100–29,700)	109 (93·9–133)	168 (147–191)	129 (102–164)
1-39	Conflict and terrorism	383 (340–432)	103 (68–161)	90 (60–139)	4·4 (3·9–4·9)	0·7 (0·5–1·1)	0·5 (0·4–0·8)
1-40	Executions and police conflict	62 (57–67)	88 (81–98)	101 (83–124)	0·6 (0·5–0·6)	0·6 (0·5–0·6)	0·6 (0·5–0·7)

SR Table 1: All-age DALYs counts and age standardized DALYs rates per 100,000 individuals for all causes and level 1 cause groups by location for 1990, 2010, and 2019, both sexes combined, Peru

Cause Level	Cause	All-age DALYs			Age-standardized rate of DALYs per 100,000		
		1990	2010	2019	1990	2010	2019
0-0	All causes	9,230,000 (8,430,000–10,100,000)	7,100,000 (6,240,000–8,160,000)	7,420,000 (6,000,000–8,950,000)	42,800 (38,900–46,900)	26,300 (23,100–30,200)	22,300 (18,000–27,000)
1-0	Communicable, maternal, neonatal, and nutritional disorders	4,220,000 (3,820,000–4,690,000)	1,780,000 (1,580,000–2,000,000)	1,480,000 (1,180,000–1,840,000)	15,900 (14,500–17,500)	6,270 (5,590–7,030)	4,550 (3,620–5,680)
1-1	HIV/AIDS and sexually transmitted infections	66,500 (33,300–122,000)	100,000 (86,600–121,000)	94,000 (80,000–116,000)	247 (140–421)	340 (295–409)	273 (230–345)
1-2	HIV/AIDS	15,400 (14,500–16,400)	76,800 (73,300–80,900)	71,700 (65,600–78,600)	76·7 (72·3–81·6)	262 (250–275)	203 (186–223)
1-3	HIV/AIDS - drug-susceptible tuberculosis	6,990 (6,450–7,560)	12,800 (7,990–19,400)	8,700 (5,410–13,200)	34·7 (32–37·6)	43·6 (27·2–66·2)	24·7 (15·3–37·4)
1-4	HIV/AIDS - Multidrug-resistant Tuberculosis without extensive drug resistance	165 (31–528)	3,250 (1,410–6,050)	1,790 (623–3,880)	0·8 (0·2–2·6)	11·1 (4·8–20·6)	5·1 (1·8–11)
1-5	HIV/AIDS - Extensively drug-resistant Tuberculosis	0 (0–0)	345 (141–705)	293 (96–695)	0 (0–0)	1·2 (0·5–2·4)	0·8 (0·3–2)
1-6	HIV/AIDS resulting in other diseases	8,270 (7,760–8,890)	60,400 (51,100–67,200)	60,900 (53,100–68,800)	41·1 (38·6–44·2)	206 (174–229)	173 (151–195)
1-7	Sexually transmitted infections excluding HIV	51,100 (17,900–106,000)	23,200 (10,500–43,100)	22,300 (10,300–43,600)	170 (63·9–346)	78·2 (35·6–145)	70 (31·3–139)
1-8	Syphilis	47,800 (14,300–103,000)	18,100 (6,050–38,100)	16,600 (5,070–38,000)	152 (46·1–325)	60·7 (20·3–128)	53·6 (16·4–123)
1-9	Chlamydial infection	412 (280–581)	611 (420–895)	670 (446–990)	2·1 (1·4–2·9)	2 (1·4–3)	1·9 (1·2–2·8)
1-10	Gonococcal infection	330 (258–428)	467 (348–593)	438 (314–571)	1·7 (1·4–2·3)	1·6 (1·2–2·1)	1·3 (0·9–1·6)
1-11	Trichomoniasis	681 (269–1,390)	1,030 (395–2,160)	1,230 (487–2,550)	3·8 (1·5–7·8)	3·6 (1·4–7·4)	3·5 (1·4–7·2)
1-12	Genital herpes	1,020 (341–2,530)	1,640 (542–4,100)	2,050 (654–4,970)	5·8 (1·9–14·2)	5·9 (1·9–14·8)	5·9 (1·9–14·3)
1-13	Other sexually transmitted infections	910 (652–1,240)	1,280 (903–1,790)	1,400 (962–1,950)	4·7 (3·4–6·5)	4·3 (3·1–6)	3·9 (2·7–5·4)
1-14	Respiratory infections and tuberculosis	1,760,000 (1,550,000–1,990,000)	638,000 (566,000–725,000)	511,000 (395,000–662,000)	7,220 (6,470–8,050)	2,380 (2,100–2,700)	1,560 (1,200–2,020)
1-15	Tuberculosis	451,000 (395,000–510,000)	109,000 (93,700–126,000)	76,300 (57,800–101,000)	2,220 (1,950–2,520)	394 (338–457)	224 (169–296)
1-16	Latent tuberculosis infection	0 (0–0)	0 (0–0)	0 (0–0)	0 (0–0)	0 (0–0)	0 (0–0)
1-17	Drug-susceptible tuberculosis	442,000 (385,000–502,000)	89,400 (72,700–106,000)	64,300 (46,600–87,200)	2,180 (1,890–2,470)	323 (262–385)	188 (136–256)
1-18	Multidrug-resistant tuberculosis without extensive drug resistance	9,000 (1,670–28,500)	17,700 (8,880–30,000)	10,400 (3,760–21,700)	44·4 (8·2–140)	64·2 (32·2–109)	30·5 (11–63·8)
1-19	Extensively drug-resistant tuberculosis	0 (0–0)	1,860 (884–3,360)	1,660 (557–3,520)	0 (0–0)	6·7 (3·2–12·2)	4·9 (1·6–10·3)
1-20	Lower respiratory infections	1,270,000 (1,090,000–1,470,000)	492,000 (432,000–559,000)	393,000 (289,000–523,000)	4,850 (4,250–5,530)	1,860 (1,630–2,110)	1,210 (892–1,620)
1-21	Upper respiratory infections	27,400 (16,400–42,600)	28,500 (17,300–44,700)	32,600 (20,000–50,300)	113 (68·3–174)	97·1 (58·8–152)	96·4 (59–149)
1-22	Otitis media	7,310 (4,210–12,200)	8,370 (4,830–13,800)	9,060 (5,240–15,100)	30·9 (17·8–50·8)	28·6 (16·6–47)	26·9 (15·5–44·5)
1-23	Enteric infections	530,000 (376,000–732,000)	105,000 (82,200–133,000)	115,000 (84,300–155,000)	1,840 (1,330–2,510)	374 (293–478)	354 (259–480)
1-24	Diarrhoeal diseases	527,000 (373,000–730,000)	104,000 (81,200–132,000)	114,000 (83,300–154,000)	1,840 (1,320–2,500)	368 (288–473)	349 (256–473)
1-25	Typhoid and paratyphoid	186 (81–355)	133 (58–254)	131 (57–258)	0·8 (0·4–1·5)	0·5 (0·2–0·9)	0·4 (0·2–0·8)
1-26	Typhoid fever	183 (79–350)	131 (57–250)	129 (56–253)	0·8 (0·4–1·5)	0·5 (0·2–0·9)	0·4 (0·2–0·8)
1-27	Paratyphoid fever	4 (1–8)	2 (1–5)	2 (1–4)	0 (0–0)	0 (0–0)	0 (0–0)
1-28	iNTS	1,400 (726–2,270)	1,210 (614–1,990)	1,180 (526–2,080)	5·4 (2·8–8·6)	4·2 (2·1–6·8)	3·6 (1·6–6·3)
1-29	Other intestinal infectious diseases	699 (330–1,120)	204 (107–324)	153 (90–243)	2·4 (1·2–3·8)	0·7 (0·4–1·1)	0·5 (0·3–0·8)
1-30	Neglected tropical diseases and malaria	102,000 (58,200–206,000)	51,100 (33,200–75,200)	53,600 (33,200–79,300)	480 (284–920)	185 (121–272)	159 (98·5–233)
1-31	Malaria	40,100 (6,710–142,000)	1,230 (828–1,750)	1,970 (752–6,330)	168 (28·2–594)	4·2 (2·8–5·9)	5·8 (2·2–18·4)
1-32	Chagas disease	2,960 (1,940–4,000)	2,830 (2,060–3,870)	2,930 (2,050–4,120)	21·5 (13·7–29)	11·3 (8·3–15·4)	8·8 (6·2–12·4)
1-33	Leishmaniasis	593 (300–956)	917 (562–1,410)	899 (473–1,590)	3·1 (1·6–5)	3·3 (2–5·1)	2·7 (1·4–4·8)
1-34	Visceral leishmaniasis	0 (0–0)	0 (0–0)	0 (0–0)	0 (0–0)	0 (0–0)	0 (0–0)
1-35	Cutaneous and mucocutaneous leishmaniasis	593 (300–956)	917 (562–1,410)	899 (473–1,590)	3·1 (1·6–5)	3·3 (2–5·1)	2·7 (1·4–4·8)
1-36	African trypanosomiasis	0 (0–0)	0 (0–0)	0 (0–0)	0 (0–0)	0 (0–0)	0 (0–0)
1-37	Schistosomiasis	0 (0–0)	0 (0–0)	0 (0–0)	0 (0–0)	0 (0–0)	0 (0–0)
1-38	Cysticercosis	14,600 (8,840–22,100)	13,600 (7,250–23,000)	14,300 (7,220–24,500)	93 (56·7–141)	52·7 (28·1–88·2)	42·9 (21·8–73·2)
1-39	Cystic echinococcosis	306 (159–470)	94 (52–139)	60 (33–94)	1·4 (0·8–2)	0·3 (0·2–0·5)	0·2 (0·1–0·3)
1-40	Lymphatic filariasis	0 (0–0)	0 (0–0)	0 (0–0)	0 (0–0)	0 (0–0)	0 (0–0)
1-41	Onchocerciasis	0 (0–0)	0 (0–0)	0 (0–0)	0 (0–0)	0 (0–0)	0 (0–0)
1-42	Trachoma	28 (14–51)	13 (6–23)	10 (5–19)	0·2 (0·1–0·5)	0·1 (0–0·1)	0 (0–0·1)
1-43	Dengue	1,100 (536–2,110)	2,000 (1,160–3,270)	2,080 (1,160–3,430)	5 (2·5–9·4)	6·9 (4–11·2)	6·1 (3·4–10)
1-44	Yellow fever	10,400 (2,180–30,900)	4,620 (956–13,800)	3,060 (586–9,280)	42·3 (8·9–125)	15·4 (3·2–45·8)	8·9 (1·7–27·1)
1-45	Rabies	447 (96–622)	110 (62–201)	100 (51–210)	2 (0·6–2·7)	0·4 (0·2–0·8)	0·3 (0·1–0·6)
1-46	Intestinal nematode infections	6,740 (4,420–10,000)	3,310 (1,980–5,270)	2,730 (1,530–4,550)	27 (17·4–40·4)	11 (6·6–17·5)	7·9 (4·4–13·3)
1-47	Ascariasis	3,590 (2,350–5,370)	1,180 (672–2,070)	694 (349–1,230)	13·6 (8·7–20·6)	4 (2·3–7·1)	2·1 (1–3·6)
1-48	Trichuriasis	1,070 (473–2,010)	878 (397–1,610)	1,000 (476–1,900)	4·3 (1·9–8·2)	2·9 (1·3–5·3)	2·9 (1·4–5·5)

1-49	Hookworm disease	2,070 (1,110–3,500)	1,240 (707–2,150)	1,030 (529–1,750)	9 (4·9–15·3)	4 (2·3–7)	3 (1·5–5·1)
1-50	Food-borne trematodiases	12,400 (3,520–24,700)	16,300 (4,530–32,200)	20,000 (5,490–40,000)	70·4 (20–141)	58·7 (16·4–115)	57·9 (15·9–116)
1-51	Leprosy	12 (8–19)	10 (6–15)	11 (7–16)	0·1 (0·1–0·1)	0 (0–0·1)	0 (0–0)
1-52	Ebola virus disease	0 (0–0)	0 (0–0)	0 (0–0)	0 (0–0)	0 (0–0)	0 (0–0)
1-53	Zika virus disease	0 (0–0)	0 (0–0)	19 (6–50)	0 (0–0)	0 (0–0)	0·1 (0–0·1)
1-54	Guinea worm disease	0 (0–0)	0 (0–0)	0 (0–0)	0 (0–0)	0 (0–0)	0 (0–0)
1-55	Other neglected tropical diseases	11,800 (7,950–16,400)	6,000 (3,720–8,970)	5,500 (3,430–8,370)	45·9 (31·2–63·9)	20·6 (12·8–30·8)	16·9 (10·5–25·8)
1-56	Other infectious diseases	203,000 (115,000–387,000)	89,500 (54,100–163,000)	73,300 (43,900–129,000)	719 (429–1,320)	306 (186–556)	226 (134–402)
1-57	Meningitis	45,700 (38,100–56,200)	12,200 (10,400–14,200)	8,240 (5,900–11,200)	164 (138–198)	41·7 (35·5–48·6)	24·9 (17·7–33·9)
1-58	Encephalitis	31,900 (21,200–42,500)	22,400 (17,500–27,300)	22,500 (14,700–32,400)	114 (79·6–148)	76·4 (59·6–93·3)	68·6 (44·7–99·3)
1-59	Diphtheria	764 (295–1,490)	159 (80–275)	101 (52–178)	2·6 (1·1–5)	0·5 (0·3–0·9)	0·3 (0·2–0·6)
1-60	Whooping cough	94,900 (7,890–287,000)	39,200 (4,420–113,000)	27,900 (3,410–82,100)	311 (25·9–942)	134 (15·1–383)	88·8 (10·8–262)
1-61	Tetanus	4,600 (3,250–6,860)	812 (572–1,730)	628 (354–1,480)	20·5 (13·8–29·5)	2·9 (2·1–6·1)	1·9 (1·1–4·4)
1-62	Measles	190 (140–250)	0 (0–0)	0 (0–1)	0·6 (0·5–0·8)	0 (0–0)	0 (0–0)
1-63	Varicella and herpes zoster	4,920 (3,010–7,750)	2,270 (1,580–3,200)	1,930 (1,290–2,730)	20·4 (13·9–29·8)	8·3 (5·8–11·5)	5·9 (3·9–8·4)
1-64	Acute hepatitis	9,750 (7,940–11,900)	2,640 (2,090–3,340)	2,630 (1,990–3,410)	43·3 (35·5–52·2)	9 (7·2–11·4)	7·7 (5·9–10)
1-65	Acute hepatitis A	6,370 (4,850–8,420)	1,730 (1,320–2,240)	1,660 (1,190–2,230)	27·3 (21–36·1)	5·8 (4·5–7·5)	4·9 (3·5–6·6)
1-66	Acute hepatitis B	2,170 (1,060–3,200)	570 (445–728)	635 (453–878)	10 (5·1–14·4)	2 (1·6–2·6)	1·9 (1·3–2·6)
1-67	Acute hepatitis C	1,040 (491–1,930)	208 (148–288)	195 (117–320)	5·2 (2·6–8·7)	0·7 (0·5–1)	0·6 (0·3–0·9)
1-68	Acute hepatitis E	167 (113–243)	134 (88–195)	143 (95–211)	0·7 (0·5–1)	0·5 (0·3–0·7)	0·4 (0·3–0·6)
1-69	Other unspecified infectious diseases	10,600 (7,700–14,500)	9,740 (6,260–12,800)	9,430 (6,000–13,100)	43·2 (31·5–58·2)	33·8 (21·7–44·4)	28·4 (18·1–39·5)
1-70	Maternal and neonatal disorders	1,210,000 (1,040,000–1,390,000)	665,000 (570,000–768,000)	521,000 (402,000–674,000)	3,990 (3,450–4,570)	2,220 (1,900–2,560)	1,640 (1,250–2,120)
1-71	Maternal disorders	96,600 (81,000–113,000)	50,100 (41,300–60,400)	32,300 (22,600–45,000)	450 (378–530)	161 (132–194)	89·9 (63–125)
1-72	Maternal haemorrhage	28,400 (22,900–35,000)	12,200 (9,670–15,300)	7,240 (5,180–9,810)	136 (109–168)	39·3 (31·3–49·3)	20·1 (14·4–27·2)
1-73	Maternal sepsis and other pregnancy related infections	12,200 (9,440–15,400)	3,200 (2,440–4,160)	1,700 (1,120–2,430)	55·7 (43·3–70·9)	10·2 (7·9–13·2)	4·7 (3·1–6·7)
1-74	Maternal hypertensive disorders	25,500 (20,800–30,700)	12,700 (10,200–15,500)	7,010 (4,650–10,100)	115 (93·3–138)	40·5 (32·6–49·6)	19·5 (13–28)
1-75	Maternal obstructed labour and uterine rupture	317 (138–557)	758 (518–1,060)	776 (504–1,130)	1·4 (0·6–2·4)	2·4 (1·7–3·4)	2·2 (1·4–3·1)
1-76	Maternal abortive outcome	18,200 (14,100–23,700)	3,480 (2,610–4,540)	1,870 (1,240–2,710)	87·6 (67·3–113)	11·3 (8·5–14·9)	5·2 (3·4–7·6)
1-77	Ectopic pregnancy	387 (308–491)	1,020 (793–1,280)	682 (461–1,000)	1·7 (1·4–2·2)	3·2 (2·5–4·1)	1·9 (1·3–2·8)
1-78	Indirect maternal deaths	925 (705–1,170)	3,360 (2,610–4,300)	2,710 (1,710–3,950)	4·1 (3·2–5·2)	10·6 (8·3–13·7)	7·5 (4·7–11)
1-79	Late maternal deaths	8,870 (6,120–12,100)	7,050 (4,960–9,610)	5,730 (3,420–8,910)	40·7 (28·3–55·5)	22·5 (15·8–30·6)	15·9 (9·5–24·7)
1-80	Maternal deaths aggravated by HIV/AIDS	14 (6–28)	38 (19–62)	34 (15–61)	0·1 (0–0·1)	0·1 (0·1–0·2)	0·1 (0–0·2)
1-81	Other maternal disorders	1,780 (1,400–2,220)	6,370 (4,920–8,070)	4,590 (3,150–6,650)	8·1 (6·4–10·1)	20·5 (15·9–26·1)	12·7 (8·8–18·5)
1-82	Neonatal disorders	1,110,000 (941,000–1,290,000)	615,000 (521,000–714,000)	489,000 (377,000–630,000)	3,540 (3,010–4,120)	2,060 (1,740–2,390)	1,550 (1,190–2,010)
1-83	Neonatal preterm birth	518,000 (411,000–649,000)	234,000 (189,000–286,000)	152,000 (108,000–206,000)	1,660 (1,320–2,070)	783 (634–957)	482 (339–658)
1-84	Neonatal encephalopathy due to birth asphyxia and trauma	304,000 (215,000–407,000)	168,000 (127,000–209,000)	112,000 (77,200–164,000)	962 (680–1,280)	562 (427–700)	361 (246–530)
1-85	Neonatal sepsis and other neonatal infections	202,000 (140,000–282,000)	184,000 (142,000–235,000)	201,000 (149,000–270,000)	646 (451–900)	617 (477–786)	628 (462–848)
1-86	Hemolytic disease and other neonatal jaundice	12,600 (9,690–16,500)	7,000 (5,540–8,520)	6,300 (4,920–7,950)	44·6 (34·7–57)	23·7 (18·7–28·8)	18·9 (14·7–23·8)
1-87	Other neonatal disorders	73,500 (50,100–99,500)	21,700 (17,000–27,400)	17,400 (12,400–24,300)	232 (159–314)	73·1 (57·3–92·2)	56·1 (39·9–78·5)
1-88	Nutritional deficiencies	355,000 (287,000–444,000)	132,000 (99,600–175,000)	113,000 (81,400–152,000)	1,400 (1,140–1,730)	471 (357–617)	343 (247–464)
1-89	Protein-energy malnutrition	215,000 (169,000–280,000)	46,200 (38,500–54,300)	33,600 (24,200–46,300)	798 (648–1,000)	170 (143–198)	103 (74·3–144)
1-90	Iodine deficiency	179 (79–360)	249 (110–497)	276 (118–543)	0·9 (0·4–1·8)	0·9 (0·4–1·8)	0·8 (0·3–1·6)
1-91	Vitamin A deficiency	6,330 (4,120–9,210)	3,790 (2,440–5,620)	3,170 (2,030–4,790)	22·3 (14·6–32·3)	12·9 (8·3–19·1)	9·7 (6·2–14·6)
1-92	Dietary iron deficiency	122,000 (78,300–182,000)	77,000 (48,200–115,000)	71,400 (44,700–109,000)	527 (337–782)	268 (169–401)	216 (135–331)
1-93	Other nutritional deficiencies	10,300 (7,620–14,200)	5,140 (4,300–6,100)	4,350 (3,230–5,730)	48·7 (37·7–63·1)	19·2 (16·1–22·7)	13·1 (9·7–17·2)
1-0	Non-communicable diseases	3,540,000 (3,080,000–4,050,000)	4,390,000 (3,730,000–5,170,000)	5,090,000 (4,130,000–6,210,000)	20,700 (18,100–23,500)	16,800 (14,300–19,800)	15,300 (12,400–18,600)
1-1	Neoplasms	478,000 (418,000–544,000)	741,000 (630,000–854,000)	834,000 (628,000–1,090,000)	3,270 (2,850–3,730)	2,970 (2,530–3,430)	2,550 (1,910–3,330)
1-2	Lip and oral cavity cancer	3,500 (2,940–4,060)	5,480 (4,560–6,440)	6,300 (4,680–8,460)	26·2 (22–30·5)	22·3 (18·7–26·3)	19·2 (14·3–25·8)
1-3	Nasopharynx cancer	751 (637–882)	1,070 (881–1,280)	1,160 (837–1,540)	5 (4·2–5·9)	4·2 (3·5–5)	3·5 (2·5–4·6)
1-4	Other pharynx cancer	1,400 (1,170–1,670)	1,510 (1,220–1,830)	1,760 (1,270–2,420)	10·7 (8·9–12·7)	6·2 (5–7·5)	5·4 (3·9–7·4)
1-5	Oesophageal cancer	5,200 (4,360–6,130)	8,010 (6,590–9,630)	9,210 (6,810–12,200)	42 (35·3–49·4)	33·8 (27·8–40·7)	28·6 (21·1–38)
1-6	Stomach cancer	78,600 (67,900–91,100)	125,000 (105,000–148,000)	126,000 (93,600–168,000)	614 (531–712)	517 (432–612)	388 (289–515)
1-7	Colon and rectum cancer	22,400 (19,200–26,000)	54,000 (45,000–63,200)	66,100 (49,400–86,800)	176 (151–204)	225 (187–263)	204 (153–268)

1·8	Liver cancer	21,100 (18,200–24,400)	17,800 (14,600–21,100)	21,300 (15,600–28,200)	155 (133–178)	72·3 (59·5–86·1)	65·8 (48·2–87)
1·9	Liver cancer due to hepatitis B	10,500 (8,320–12,900)	8,320 (6,380–10,500)	9,590 (6,500–13,400)	75·5 (59–95)	33·3 (25·2–42·1)	29·1 (19·7–40·7)
1·10	Liver cancer due to hepatitis C	1,030 (628–1,570)	984 (615–1,470)	1,220 (721–2,000)	8·9 (5·4–13·6)	4·3 (2·6–6·4)	3·8 (2·2–6·4)
1·11	Liver cancer due to alcohol use	5,490 (3,830–7,480)	4,910 (3,420–6,690)	6,460 (4,150–9,360)	45·4 (31·4–61·6)	21 (14·6–28·8)	20·3 (13–29·2)
1·12	Liver cancer due to NASH	1,270 (886–1,810)	1,340 (924–1,850)	1,730 (1,080–2,540)	10·2 (7–14·6)	5·6 (3·8–7·8)	5·4 (3·4–7·9)
1·13	Gallbladder and biliary tract cancer	13,100 (11,100–15,700)	23,300 (18,300–27,600)	26,000 (18,300–35,800)	106 (90·2–128)	99·4 (78–118)	81·3 (57·4–112)
1·14	Pancreatic cancer	5,890 (5,020–6,890)	30,100 (25,000–35,400)	37,700 (28,000–50,200)	47·5 (40·6–55·7)	128 (106–150)	118 (87·1–156)
1·15	Larynx cancer	3,660 (3,100–4,270)	3,510 (2,850–4,270)	3,870 (2,810–5,260)	29·3 (24·8–34·2)	14·8 (12·1–18)	12·1 (8·8–16·4)
1·16	Tracheal, bronchus, and lung cancer	47,100 (40,400–55,200)	64,900 (53,800–75,500)	76,900 (57,000–102,000)	374 (320–437)	272 (226–315)	238 (177–317)
1·17	Malignant skin melanoma	2,460 (1,840–3,810)	4,200 (2,900–5,370)	4,960 (3,120–7,550)	17·1 (13·1–25·3)	16·9 (11·6–21·3)	15 (9·5–22·8)
1·18	Non-melanoma skin cancer	1,660 (1,370–2,050)	3,970 (3,230–4,690)	4,840 (3,540–6,390)	13·2 (10·9–16·1)	16·4 (13·3–19·4)	14·7 (10·8–19·5)
1·19	Non-melanoma skin cancer (squamous-cell carcinoma)	1,660 (1,370–2,050)	3,970 (3,220–4,680)	4,840 (3,530–6,390)	13·2 (10·9–16·1)	16·4 (13·2–19·4)	14·7 (10·8–19·5)
1·20	Non-melanoma skin cancer (basal-cell carcinoma)	1 (0–2)	2 (1–5)	3 (1–5)	0 (0–0)	0 (0–0)	0 (0–0)
1·21	Breast cancer	28,100 (23,800–32,700)	47,000 (38,700–56,200)	55,700 (40,900–74,000)	201 (171–233)	186 (153–222)	166 (123–220)
1·22	Cervical cancer	41,100 (33,900–50,900)	62,900 (48,800–76,200)	63,400 (43,400–87,800)	287 (238–355)	246 (190–297)	189 (129–261)
1·23	Uterine cancer	6,230 (5,020–7,600)	10,300 (7,960–12,900)	11,800 (8,370–16,300)	48·3 (39·2–59·2)	42·8 (33·3–53·9)	36·3 (25·9–50·3)
1·24	Ovarian cancer	7,460 (5,540–10,300)	17,100 (13,400–20,600)	23,000 (15,800–31,500)	51·5 (38·9–70·1)	67·4 (52·8–81·2)	69 (47·4–94)
1·25	Prostate cancer	18,100 (14,700–24,400)	40,700 (30,900–49,700)	52,000 (37,500–71,200)	167 (136–227)	180 (137–220)	165 (119–227)
1·26	Testicular cancer	4,440 (2,770–7,740)	4,730 (3,690–6,140)	5,480 (3,280–8,250)	19·8 (12·7–32·7)	15·8 (12·4–20·5)	15·6 (9·5–23·6)
1·27	Kidney cancer	9,100 (7,900–10,400)	13,600 (11,400–16,000)	17,500 (12,600–23,400)	59·2 (51·4–67·7)	55·2 (46·2–65·4)	53·9 (39–72·4)
1·28	Bladder cancer	3,680 (3,140–4,310)	6,460 (5,350–7,620)	7,830 (5,860–10,400)	31·4 (26·9–36·8)	27·8 (23–32·8)	24·5 (18·4–32·5)
1·29	Brain and nervous system cancer	27,000 (20,400–41,700)	37,900 (26,000–45,500)	42,100 (26,100–58,000)	132 (101–197)	138 (94·2–167)	126 (77·9–173)
1·30	Thyroid cancer	3,030 (2,520–3,580)	5,770 (4,460–6,860)	6,610 (4,730–8,910)	22·4 (18·6–26·6)	23·8 (18·2–28·3)	20·3 (14·6–27·3)
1·31	Mesothelioma	1,840 (929–2,630)	1,290 (971–1,610)	1,500 (973–2,220)	13·8 (7–19·4)	5·2 (3·9–6·5)	4·5 (3–6·7)
1·32	Hodgkin lymphoma	2,910 (2,300–3,590)	2,720 (2,090–3,430)	2,710 (1,910–3,680)	15·6 (12·1–18·9)	9·9 (7·4–12·3)	8 (5·6–10·8)
1·33	Non-Hodgkin's lymphoma	20,000 (17,100–23,200)	35,800 (30,100–41,700)	41,100 (30,300–54,800)	119 (102–138)	139 (117–162)	124 (92·1–165)
1·34	Multiple myeloma	4,300 (3,300–5,920)	7,750 (6,190–9,780)	9,790 (6,890–13,500)	34·6 (26·7–47·2)	32·8 (26·2–41·6)	30·5 (21·4–42)
1·35	Leukaemia	49,500 (41,800–64,500)	63,600 (43,700–75,600)	60,600 (39,900–84,900)	218 (186–277)	224 (155–267)	181 (119–253)
1·36	Acute lymphoid leukaemia	17,500 (13,200–24,600)	22,300 (14,900–27,000)	20,400 (12,900–29,500)	66·1 (50·3–91·6)	75·1 (50–90·6)	61·1 (38·5–88·9)
1·37	Chronic lymphoid leukaemia	554 (428–726)	1,350 (959–1,690)	1,620 (1,100–2,280)	3·9 (3–5·1)	5·4 (3·9–6·8)	4·9 (3·3–6·9)
1·38	Acute myeloid leukaemia	7,600 (5,630–10,500)	13,000 (8,750–15,700)	13,900 (9,030–19,700)	34·9 (26·7–46·5)	46·6 (31·2–56)	41·6 (26·9–58·6)
1·39	Chronic myeloid leukaemia	1,620 (1,170–2,220)	2,440 (1,750–3,030)	2,310 (1,530–3,250)	8·8 (6·5–11·7)	9 (6·5–11·2)	6·8 (4·5–9·6)
1·40	Other leukaemia	22,200 (15,500–32,300)	24,400 (15,600–29,800)	22,400 (12,700–31,900)	104 (77–143)	88·2 (56·7–108)	66·4 (38·1–95·2)
1·41	Other neoplasms	1,020 (683–1,360)	1,220 (897–1,500)	1,680 (1,160–2,270)	6·8 (4·4–9·1)	5 (3·6–6·2)	5·2 (3·6–7·1)
1·42	Myelodysplastic, myeloproliferative, and other hematopoietic neoplasms	1,020 (683–1,360)	1,220 (897–1,500)	1,680 (1,160–2,270)	6·8 (4·4–9·1)	5 (3·6–6·2)	5·2 (3·6–7·1)
1·43	Benign and in situ intestinal neoplasms	0 (0–0)	0 (0–0)	0 (0–0)	0 (0–0)	0 (0–0)	0 (0–0)
1·44	Benign and in situ cervical and uterine neoplasms	0 (0–0)	0 (0–0)	0 (0–0)	0 (0–0)	0 (0–0)	0 (0–0)
1·45	Other benign and in situ neoplasms	0 (0–0)	0 (0–0)	0 (0–0)	0 (0–0)	0 (0–0)	0 (0–0)
1·46	Cardiovascular diseases	538,000 (477,000–609,000)	546,000 (468,000–638,000)	598,000 (460,000–765,000)	3,990 (3,530–4,530)	2,220 (1,900–2,600)	1,820 (1,400–2,330)
1·47	Rheumatic heart disease	17,900 (14,100–22,400)	18,100 (13,500–24,300)	19,400 (13,500–27,100)	97·7 (78·9–119)	64·1 (48·1–84·8)	56·3 (39·4–78·3)
1·48	Ischaemic heart disease	234,000 (204,000–268,000)	248,000 (209,000–292,000)	280,000 (209,000–361,000)	1,930 (1,680–2,210)	1,040 (877–1,220)	860 (640–1,110)
1·49	Stroke	211,000 (186,000–239,000)	197,000 (168,000–236,000)	202,000 (157,000–266,000)	1,490 (1,310–1,690)	789 (669–946)	614 (475–810)
1·50	Ischaemic stroke	54,600 (47,000–63,000)	65,200 (55,600–78,600)	78,000 (61,000–100,000)	469 (401–543)	279 (238–336)	241 (189–310)
1·51	Intracerebral hemorrhage	113,000 (96,700–131,000)	89,500 (74,700–109,000)	84,300 (62,700–113,000)	776 (663–893)	353 (294–430)	254 (190–340)
1·52	Subarachnoid hemorrhage	43,100 (34,500–55,100)	42,700 (35,800–51,200)	40,100 (29,100–55,200)	247 (196–325)	157 (131–190)	118 (85·4–162)
1·53	Hypertensive heart disease	15,600 (12,900–18,300)	18,100 (15,000–22,400)	21,900 (16,600–29,400)	130 (108–153)	76·4 (63·5–95)	67·5 (51–90·1)
1·54	Non-rheumatic valvular heart disease	2,450 (1,840–3,190)	2,780 (2,290–3,390)	3,250 (2,400–4,360)	16·8 (12·7–21·5)	11 (9–13·5)	9·8 (7·3–13·1)
1·55	Non-rheumatic calcific aortic valve disease	1,770 (1,280–2,430)	2,070 (1,690–2,540)	2,490 (1,810–3,360)	12·4 (9·1–16·6)	8·3 (6·7–10·2)	7·6 (5·5–10·2)
1·56	Non-rheumatic degenerative mitral valve disease	605 (435–818)	647 (502–814)	693 (487–960)	3·9 (2·8–5·2)	2·5 (1·9–3·2)	2·1 (1·5–2·9)
1·57	Other non-rheumatic valve diseases	70 (45–106)	61 (44–82)	60 (38–88)	0·4 (0·3–0·6)	0·2 (0·2–0·3)	0·2 (0·1–0·3)
1·58	Cardiomyopathy and myocarditis	13,600 (8,010–18,300)	11,300 (9,030–13,800)	11,400 (8,090–16,300)	66·9 (42·3–85·7)	42 (33·8–52·1)	34·4 (24·6–49)
1·59	Myocarditis	1,690 (566–2,900)	953 (758–1,260)	1,020 (703–1,530)	8·6 (3·5–13·2)	3·5 (2·8–4·7)	3 (2·1–4·5)
1·60	Alcoholic cardiomyopathy	365 (228–542)	332 (254–484)	394 (265–614)	2·6 (1·6–3·9)	1·3 (1–1·9)	1·2 (0·8–1·8)

1-61	Other cardiomyopathy	11,600 (7,150–15,600)	9,970 (8,010–12,200)	9,960 (7,090–14,200)	55·7 (36·2–71)	37·1 (29·9–45·9)	30·2 (21·5–43)
1-62	Atrial fibrillation and flutter	6,770 (5,660–7,990)	13,200 (11,000–15,900)	17,600 (13,800–22,000)	64·8 (54·3–75·9)	58·1 (48·2–70·1)	54·6 (42·6–68·3)
1-63	Aortic aneurysm	3,390 (2,630–4,070)	5,410 (4,470–6,490)	6,600 (4,850–8,780)	26·6 (21·1–31·7)	22·4 (18·5–26·8)	20·3 (14·9–27)
1-64	Peripheral vascular disease	656 (395–1,030)	1,240 (759–1,980)	1,710 (1,060–2,740)	6·2 (3·7–9·7)	5·5 (3·4–8·9)	5·4 (3·3–8·7)
1-65	Endocarditis	7,250 (4,190–10,400)	5,140 (4,140–6,770)	5,200 (3,710–7,400)	33·3 (21·1–44·8)	18·9 (15·2–24·9)	15·6 (11·1–22)
1-66	Chronic respiratory diseases	180,000 (148,000–219,000)	183,000 (151,000–218,000)	198,000 (157,000–247,000)	920 (782–1,090)	720 (596–853)	608 (479–759)
1-67	Chronic obstructive pulmonary disease	41,800 (34,000–50,600)	58,500 (48,200–68,500)	62,000 (49,500–77,300)	289 (241–345)	244 (200–287)	190 (151–238)
1-68	Pneumoconiosis	2,160 (1,590–2,800)	2,520 (1,930–3,110)	2,030 (1,460–2,800)	15·6 (11·3–20·2)	10·5 (8–12·9)	6·3 (4·5–8·7)
1-69	Silicosis	589 (212–870)	311 (201–556)	282 (166–504)	4·4 (1·7–6·6)	1·3 (0·8–2·3)	0·9 (0·5–1·6)
1-70	Asbestosis	47 (29–78)	56 (37–81)	53 (31–88)	0·3 (0·2–0·5)	0·2 (0·1–0·3)	0·2 (0·1–0·3)
1-71	Coal workers pneumoconiosis	42 (21–76)	49 (22–75)	28 (15–50)	0·3 (0·1–0·6)	0·2 (0·1–0·3)	0·1 (0–0·2)
1-72	Other pneumoconiosis	1,490 (1,070–2,010)	2,100 (1,540–2,610)	1,670 (1,180–2,280)	10·6 (7·6–14·5)	8·8 (6·4–10·9)	5·2 (3·7–7·1)
1-73	Asthma	91,900 (63,900–128,000)	53,800 (35,600–81,300)	62,300 (39,500–96,700)	353 (253–482)	186 (124–280)	190 (120–295)
1-74	Interstitial lung disease and pulmonary sarcoidosis	22,500 (17,500–32,500)	56,200 (40,500–67,200)	61,200 (42,300–79,700)	175 (137–258)	239 (171–286)	190 (131–249)
1-75	Other chronic respiratory diseases	22,000 (11,100–37,300)	11,700 (9,210–13,800)	10,900 (7,980–14,400)	86·9 (46·3–141)	40·8 (32·1–48·6)	31·8 (23·4–42·4)
1-76	Digestive diseases	602,000 (522,000–688,000)	357,000 (306,000–415,000)	395,000 (303,000–520,000)	2,900 (2,550–3,260)	1,390 (1,190–1,610)	1,190 (913–1,570)
1-77	Cirrhosis and other chronic liver diseases	144,000 (122,000–176,000)	163,000 (135,000–192,000)	183,000 (130,000–251,000)	930 (790–1,110)	655 (543–769)	555 (395–760)
1-78	Cirrhosis and other chronic liver diseases due to hepatitis B	14,400 (10,500–20,000)	16,000 (10,700–22,700)	14,000 (8,370–21,800)	101 (74–139)	63·3 (42·4–90·4)	41·9 (25·1–65·1)
1-79	Cirrhosis and other chronic liver diseases due to hepatitis C	15,400 (10,600–21,800)	20,300 (13,900–28,700)	23,100 (14,400–35,400)	108 (73·8–154)	80·7 (54·8–114)	69·3 (43–106)
1-80	Cirrhosis and other chronic liver diseases due to alcohol use	45,400 (33,800–58,500)	64,900 (48,000–82,800)	76,600 (50,900–109,000)	340 (253–437)	266 (196–338)	233 (155–333)
1-81	Cirrhosis and other chronic liver diseases due to NAFLD	18,800 (13,200–26,300)	27,900 (19,200–38,200)	32,800 (21,100–49,600)	140 (97·7–194)	114 (78·6–157)	100 (64–151)
1-82	Cirrhosis and other chronic liver diseases due to other causes	50,400 (38,600–69,900)	34,100 (25,900–44,700)	36,400 (24,800–51,900)	242 (189–314)	131 (98·5–172)	110 (75–158)
1-83	Upper digestive system diseases	54,300 (41,900–71,300)	67,500 (48,000–97,700)	76,000 (51,700–114,000)	330 (255–430)	257 (186–367)	224 (153–333)
1-84	Peptic ulcer disease	23,800 (19,600–28,800)	16,300 (13,300–20,400)	14,400 (10,500–19,500)	149 (123–177)	65·9 (53·7–82·2)	43·7 (31·7–59·3)
1-85	Gastritis and duodenitis	9,700 (7,000–13,100)	17,200 (13,500–22,100)	19,100 (14,000–25,500)	58 (42·7–77)	67·7 (53·2–85·8)	57·3 (42·2–76·4)
1-86	GERD	20,800 (10,800–36,700)	34,000 (17,700–60,500)	42,600 (22,100–75,300)	123 (64–218)	123 (64·3–219)	123 (64·1–219)
1-87	Appendicitis	97,100 (63,000–132,000)	12,300 (10,300–15,900)	11,200 (8,000–15,400)	363 (244–483)	42·8 (35·7–55·3)	32·9 (23·5–45·6)
1-88	Paralytic ileus and intestinal obstruction	161,000 (110,000–221,000)	35,200 (26,500–42,000)	37,000 (24,200–54,200)	598 (419–792)	134 (100–160)	114 (74·5–167)
1-89	Inguinal, femoral, and abdominal hernia	52,800 (34,800–75,800)	22,200 (16,200–30,200)	25,500 (18,200–34,500)	207 (145–284)	80·3 (59–109)	76·5 (54·5–104)
1-90	Inflammatory bowel disease	8,230 (3,970–13,900)	2,770 (2,300–3,330)	3,120 (2,310–4,200)	34·8 (19·6–53·8)	10·4 (8·7–12·6)	9·3 (6·9–12·6)
1-91	Vascular intestinal disorders	4,030 (2,680–6,040)	2,980 (2,440–3,650)	3,740 (2,740–5,030)	24 (17·9–31·8)	12·4 (10·1–15·2)	11·6 (8·5–15·6)
1-92	Gallbladder and biliary diseases	24,700 (20,300–29,700)	21,100 (17,000–27,300)	23,500 (17,500–31,600)	165 (135–196)	83·9 (67·6–109)	70·8 (52·7–95·7)
1-93	Pancreatitis	21,600 (15,200–27,100)	18,200 (14,700–23,300)	19,900 (13,900–27,100)	125 (92·1–155)	69 (55·6–89·2)	58·8 (41–80·3)
1-94	Other digestive diseases	33,600 (19,900–54,500)	11,400 (9,320–13,400)	12,200 (8,110–18,400)	125 (79·2–194)	43·6 (35·4–51)	37·2 (24·7–56·1)
1-95	Neurological disorders	189,000 (109,000–292,000)	272,000 (159,000–439,000)	340,000 (197,000–548,000)	1,110 (684–1,710)	1,020 (620–1,640)	1,010 (590–1,620)
1-96	Alzheimer's disease and other dementias	31,800 (13,100–74,900)	67,400 (28,600–151,000)	96,400 (42,200–213,000)	320 (132–746)	299 (127–670)	296 (129–657)
1-97	Parkinson's disease	8,620 (7,510–10,100)	19,000 (16,300–21,800)	23,500 (18,400–28,900)	81·6 (71–95·5)	83·6 (71·7–95·9)	74·2 (58·4–91·7)
1-98	Idiopathic epilepsy	59,700 (31,500–96,600)	52,200 (23,400–93,400)	59,500 (24,600–112,000)	272 (141–440)	180 (79·9–322)	175 (71·8–328)
1-99	Multiple sclerosis	799 (647–985)	1,520 (1,210–1,840)	1,870 (1,410–2,480)	5·3 (4·3–6·5)	5·8 (4·6–6·9)	5·5 (4·1–7·3)
1-100	Motor neuron disease	851 (704–1,020)	1,400 (1,140–1,690)	1,750 (1,230–2,430)	5·3 (4·3–6·3)	5·6 (4·5–6·8)	5·3 (3·7–7·4)
1-101	Headache disorders	74,800 (18,700–157,000)	119,000 (30,600–246,000)	141,000 (36,700–292,000)	372 (106–766)	407 (110–834)	406 (106–842)
1-102	Migraine	65,300 (13,000–145,000)	104,000 (20,000–226,000)	124,000 (24,800–270,000)	322 (71·3–706)	356 (72·4–776)	356 (72–775)
1-103	Tension-type headache	9,440 (2,700–39,500)	14,400 (4,290–55,300)	17,500 (5,250–64,100)	50·2 (15·3–193)	50·6 (15·4–188)	50·6 (15·3–185)
1-104	Other neurological disorders	12,500 (7,330–19,500)	12,100 (6,220–20,700)	15,700 (7,720–28,000)	56·1 (35·1–84·6)	42·1 (22·6–70·5)	46·6 (23·1–82·9)
1-105	Mental disorders	319,000 (231,000–424,000)	460,000 (334,000–612,000)	557,000 (403,000–742,000)	1,630 (1,190–2,150)	1,590 (1,160–2,110)	1,610 (1,160–2,140)
1-106	Schizophrenia	30,900 (21,400–41,300)	50,700 (35,600–67,800)	63,400 (44,300–84,000)	176 (124–231)	179 (126–238)	181 (127–238)
1-107	Depressive disorders	71,600 (48,200–100,000)	96,800 (66,800–135,000)	123,000 (82,700–170,000)	397 (270–552)	345 (238–481)	355 (240–493)
1-108	Major depressive disorder	55,500 (36,800–78,900)	71,300 (48,600–98,800)	91,100 (60,100–127,000)	307 (204–434)	254 (175–350)	265 (175–369)
1-109	Dysthymia	16,100 (9,790–24,800)	25,500 (15,700–38,800)	31,500 (19,500–47,700)	90·2 (55·5–136)	90·5 (56·6–138)	90·5 (56·7–137)
1-110	Bipolar disorder	39,100 (22,900–60,900)	58,500 (33,600–92,600)	70,200 (40,900–109,000)	200 (119–308)	200 (116–314)	200 (117–310)
1-111	Anxiety disorders	104,000 (68,600–147,000)	153,000 (102,000–216,000)	185,000 (123,000–260,000)	522 (348–733)	527 (350–742)	533 (355–747)
1-112	Eating disorders	11,600 (6,840–17,700)	20,200 (12,400–30,200)	22,700 (13,600–35,600)	50 (29·6–76)	64 (39·4–95·6)	63·2 (38·1–99·2)
1-113	Anorexia nervosa	2,270 (1,290–3,700)	3,210 (1,830–5,220)	3,940 (2,290–6,380)	9·3 (5·3–15·1)	10·1 (5·8–16·3)	11·1 (6·4–18)

1·114	Bulimia nervosa	9,300 (5,340–14,800)	17,000 (10,000–26,200)	18,800 (11,000–30,600)	40·7 (22·9–64·4)	53·9 (31·8–82·7)	52·1 (30·6–84·8)
1·115	Autism spectrum disorders	11,900 (7,830–17,500)	15,400 (10,100–22,700)	17,900 (11,700–26,200)	52 (34·2–76·4)	52·6 (34·4–77·4)	52·6 (34·6–77·3)
1·116	Attention-deficit/hyperactivity disorder	6,530 (3,700–11,000)	7,940 (4,470–13,300)	8,830 (5,060–14,800)	25·5 (14·7–42·9)	25·7 (14·5–42·7)	25·8 (14·7–43·3)
1·117	Conduct disorder	19,800 (11,100–31,300)	22,000 (12,200–34,900)	23,100 (12,700–36,800)	68·8 (38·6–109)	69·6 (38·5–111)	69·8 (38·2–111)
1·118	Idiopathic developmental intellectual disability	5,970 (2,620–10,300)	6,050 (2,390–10,700)	5,800 (2,200–10,300)	25·9 (11·5–44·8)	20·5 (8·1–36·2)	17 (6·4–30·1)
1·119	Other mental disorders	18,200 (11,300–27,600)	30,000 (19,000–45,200)	37,600 (23,700–57,200)	109 (69·3–164)	109 (68·8–164)	109 (68·5–165)
1·120	Substance use disorders	72,400 (53,800–95,700)	102,000 (75,500–134,000)	114,000 (82,800–152,000)	371 (278–480)	344 (256–449)	322 (234–429)
1·121	Alcohol use disorders	32,400 (24,500–41,500)	36,500 (26,700–49,000)	39,400 (28,700–53,200)	186 (143–234)	129 (95·3–171)	113 (82·4–152)
1·122	Drug use disorders	40,000 (26,300–56,800)	66,000 (45,800–91,100)	74,600 (51,000–104,000)	185 (124–261)	216 (152–296)	209 (143–292)
1·123	Opioid use disorders	9,920 (6,620–13,900)	20,000 (13,700–27,600)	23,900 (16,200–33,000)	50·6 (34·3–69·1)	68·1 (47·1–93)	67·6 (46·1–93·1)
1·124	Cocaine use disorders	3,780 (2,730–5,090)	7,680 (5,980–9,930)	7,280 (5,330–9,710)	17·2 (12·7–22·8)	25 (19·5–32·1)	20·6 (15·1–27·4)
1·125	Amphetamine use disorders	13,400 (7,040–22,200)	19,700 (10,700–32,100)	22,600 (12,400–37,300)	59·6 (31·8–99·2)	63 (34·7–102)	62·5 (34·6–103)
1·126	Cannabis use disorders	1,490 (811–2,460)	1,960 (1,110–3,090)	2,250 (1,250–3,550)	6·5 (3·6–10·7)	6·3 (3·6–9·9)	6·3 (3·5–10)
1·127	Other drug use disorders	11,400 (6,670–17,700)	16,600 (10,100–25,400)	18,600 (11,000–29,000)	50·8 (30·4–78·9)	53·2 (32·5–81·7)	51·7 (30·7–80·6)
1·128	Diabetes and kidney diseases	165,000 (145,000–185,000)	322,000 (277,000–373,000)	406,000 (323,000–495,000)	1,160 (1,010–1,310)	1,320 (1,130–1,530)	1,250 (994–1,520)
1·129	Diabetes mellitus	65,000 (54,100–77,000)	148,000 (123,000–179,000)	208,000 (165,000–259,000)	515 (428–611)	619 (514–749)	645 (510–801)
1·130	Diabetes mellitus type 1	6,180 (5,230–7,370)	9,400 (7,290–11,700)	10,800 (8,220–14,100)	34·6 (29–41·6)	34·9 (27–43·4)	32·1 (24·3–41·7)
1·131	Diabetes mellitus type 2	58,900 (48,800–70,100)	138,000 (115,000–167,000)	197,000 (156,000–245,000)	480 (397–571)	584 (483–709)	613 (484–761)
1·132	Chronic kidney disease	99,400 (88,900–111,000)	174,000 (151,000–198,000)	198,000 (155,000–251,000)	642 (572–723)	702 (605–801)	604 (471–767)
1·133	Chronic kidney disease due to diabetes mellitus type 1	5,970 (4,020–8,340)	10,700 (6,960–15,400)	11,600 (7,160–17,800)	38·8 (25·6–55·5)	40·7 (25·6–59·8)	34·1 (20·9–53)
1·134	Chronic kidney disease due to diabetes mellitus type 2	14,800 (11,200–18,700)	35,200 (27,000–43,700)	42,800 (31,000–58,000)	125 (94·8–156)	152 (116–189)	134 (97·4–181)
1·135	Chronic kidney disease due to hypertension	15,000 (12,000–18,900)	37,000 (29,200–46,300)	43,400 (32,700–57,000)	126 (102–158)	158 (124–199)	134 (101–177)
1·136	Chronic kidney disease due to glomerulonephritis	27,600 (22,700–33,500)	40,400 (32,100–48,800)	43,200 (31,300–59,400)	155 (126–191)	154 (121–189)	129 (93·6–177)
1·137	Chronic kidney disease due to other and unspecified causes	36,000 (30,300–42,200)	50,600 (40,600–62,200)	56,800 (42,600–74,800)	197 (163–238)	197 (156–245)	172 (129–227)
1·138	Acute glomerulonephritis	402 (202–721)	97 (79–120)	89 (61–124)	1·8 (1–2·9)	0·4 (0·3–0·5)	0·3 (0·2–0·4)
1·139	Skin and subcutaneous diseases	134,000 (88,800–200,000)	167,000 (109,000–253,000)	195,000 (127,000–299,000)	609 (407–918)	577 (378–874)	579 (378–887)
1·140	Dermatitis	25,800 (15,100–40,800)	31,300 (18,800–49,100)	37,200 (22,200–58,100)	111 (65·9–172)	108 (64·9–170)	111 (66·4–174)
1·141	Atopic dermatitis	19,700 (10,600–33,200)	21,800 (11,600–36,300)	25,600 (13,800–42,900)	77 (41·5–130)	74·4 (39·7–124)	77·1 (41·5–130)
1·142	Contact dermatitis	5,490 (3,490–8,190)	8,510 (5,360–12,800)	10,500 (6,570–15,600)	30·5 (18·9–45·1)	30·5 (19·2–45·6)	30·5 (19·1–45·5)
1·143	Seborrhoeic dermatitis	658 (376–1,060)	957 (541–1,520)	1,160 (647–1,840)	3·4 (1·9–5·4)	3·4 (1·9–5·4)	3·4 (1·9–5·4)
1·144	Psoriasis	13,700 (9,730–18,400)	18,600 (13,000–24,800)	21,000 (14,700–27,800)	73·1 (52–98·2)	66·9 (46·9–89·1)	61·6 (43·1–81·8)
1·145	Bacterial skin diseases	5,830 (4,000–8,940)	3,830 (2,920–5,480)	4,500 (3,290–6,330)	27 (19·1–38·7)	14·3 (11–20·2)	13·6 (9·9–19·1)
1·146	Cellulitis	2,280 (747–3,830)	918 (691–1,270)	1,070 (758–1,560)	10·9 (4·1–17)	3·4 (2·6–4·8)	3·2 (2·3–4·7)
1·147	Pyoderma	3,550 (1,790–7,000)	2,910 (2,060–4,310)	3,430 (2,320–5,080)	16·1 (8·8–29·3)	10·9 (7·8–16)	10·3 (7–15·3)
1·148	Scabies	19,500 (10,400–31,400)	24,300 (13,400–38,700)	27,800 (15,400–44,600)	82·8 (45·4–132)	82·3 (45·7–131)	82·2 (45·5–131)
1·149	Fungal skin diseases	13,600 (5,490–29,400)	19,800 (8,000–42,900)	24,100 (9,810–52,600)	72·7 (29·5–157)	72·1 (29·3–156)	71·8 (29·1–156)
1·150	Viral skin diseases	13,500 (8,660–20,200)	15,400 (9,820–23,000)	17,300 (11,000–26,100)	52·2 (33·6–77·6)	52·3 (33·3–77·8)	52·3 (33·2–78·8)
1·151	Acne vulgaris	17,700 (10,500–27,700)	21,900 (13,000–34,600)	24,900 (14,800–39,600)	65·1 (38·8–102)	67·6 (40·3–107)	72·5 (43–115)
1·152	Alopecia areata	1,420 (914–2,150)	2,070 (1,300–3,080)	2,480 (1,570–3,670)	7·1 (4·6–10·7)	7·1 (4·5–10·5)	7·1 (4·5–10·5)
1·153	Pruritus	1,520 (724–2,720)	2,310 (1,100–4,200)	2,880 (1,380–5,270)	8·2 (4–14·7)	8·4 (4–15·3)	8·5 (4·1–15·5)
1·154	Urticaria	11,500 (7,520–16,700)	14,000 (9,090–20,700)	16,100 (10,400–23,200)	48 (31·7–68·6)	48·1 (31·2–70·5)	48·1 (31·2–69·5)
1·155	Decubitus ulcer	1,950 (442–2,880)	792 (596–1,200)	1,040 (739–1,600)	15·4 (3·9–22·3)	3·4 (2·5–5·2)	3·2 (2·3–4·9)
1·156	Other skin and subcutaneous diseases	8,100 (4,030–14,500)	12,300 (6,030–22,400)	15,600 (7,540–28,400)	46·7 (23·5–82·8)	46 (22·7–83·5)	46·7 (22·6–84·9)
1·157	Sense organ diseases	135,000 (93,200–187,000)	230,000 (159,000–324,000)	288,000 (197,000–407,000)	995 (694–1,380)	932 (644–1,310)	878 (603–1,240)
1·158	Blindness and vision loss	60,300 (42,700–82,400)	98,400 (69,000–136,000)	115,000 (80,500–160,000)	456 (324–619)	404 (284–558)	354 (247–491)
1·159	Glaucoma	2,670 (1,750–3,830)	4,270 (2,870–6,010)	5,170 (3,470–7,250)	25·6 (16·8–36·9)	19 (12·8–26·7)	16·3 (10·9–22·9)
1·160	Cataract	18,100 (12,800–24,500)	31,500 (22,200–42,300)	34,600 (24,600–46,700)	165 (116–222)	137 (96·5–184)	108 (76·7–146)
1·161	Age-related macular degeneration	1,380 (934–1,950)	2,850 (1,940–3,950)	3,600 (2,460–5,000)	12·6 (8·5–18)	12·7 (8·6–17·6)	11·5 (7·9–16)
1·162	Refraction disorders	23,200 (15,500–33,600)	36,300 (24,200–51,800)	42,300 (28,200–61,100)	134 (90·8–191)	136 (91–194)	126 (84–182)
1·163	Near vision loss	5,770 (2,640–11,300)	11,000 (5,120–21,500)	15,100 (6,820–29,500)	48·6 (22·3–95·4)	47·1 (22–92·4)	47 (21·3–91·9)
1·164	Other vision loss	9,180 (6,300–12,800)	12,500 (8,670–17,200)	14,700 (10,200–20,400)	69·6 (48–97·1)	51·7 (36·2–70·9)	45 (31·2–62·7)
1·165	Age-related and other hearing loss	69,300 (46,300–102,000)	122,000 (80,800–179,000)	160,000 (107,000–238,000)	503 (338–728)	492 (329–720)	488 (326–717)
1·166	Other sense organ diseases	5,680 (3,410–8,670)	9,410 (5,540–14,400)	12,100 (7,050–18,900)	35·8 (21·3–55·2)	36·4 (21·5–56·5)	36·6 (21·4–57·2)

1-167	Musculoskeletal disorders	235,000 (168,000–309,000)	423,000 (304,000–568,000)	546,000 (391,000–725,000)	1,490 (1,060–1,980)	1,600 (1,140–2,130)	1,610 (1,150–2,140)
1-168	Rheumatoid arthritis	4,290 (3,130–5,610)	9,460 (6,810–12,500)	13,200 (9,550–17,300)	29·1 (21·5–37·8)	36·7 (26·7–48)	39·1 (28·6–51·4)
1-169	Osteoarthritis	22,600 (11,500–45,300)	48,000 (24,100–96,500)	66,500 (33,500–134,000)	185 (94·1–374)	203 (101–408)	206 (104–414)
1-170	Low back pain	112,000 (78,300–150,000)	189,000 (131,000–254,000)	233,000 (164,000–314,000)	670 (471–906)	696 (489–934)	685 (483–922)
1-171	Neck pain	21,400 (13,700–31,200)	36,400 (23,600–52,400)	46,400 (30,400–66,800)	135 (88–196)	136 (89·1–196)	136 (89·1–195)
1-172	Gout	995 (610–1,470)	2,160 (1,380–3,120)	2,980 (1,820–4,410)	7·3 (4·5–10·7)	8·6 (5·5–12·5)	8·9 (5·5–13·3)
1-173	Other musculoskeletal disorders	73,300 (49,800–102,000)	138,000 (94,800–192,000)	184,000 (125,000–254,000)	464 (315–642)	517 (352–717)	537 (363–745)
1-174	Other non-communicable diseases	493,000 (401,000–682,000)	590,000 (475,000–741,000)	621,000 (477,000–809,000)	2,260 (1,780–2,950)	2,110 (1,710–2,660)	1,880 (1,440–2,440)
1-175	Congenital anomalies	199,000 (157,000–404,000)	225,000 (161,000–272,000)	179,000 (115,000–270,000)	653 (518–1,310)	754 (540–911)	569 (363–863)
1-176	Neural tube defects	23,200 (13,300–38,900)	15,000 (9,560–20,500)	10,700 (5,860–18,100)	74·6 (43·2–125)	50·3 (32–68·8)	34·3 (18·5–58·1)
1-177	Congenital heart anomalies	72,200 (51,100–124,000)	88,000 (57,700–110,000)	69,900 (38,500–117,000)	236 (168–403)	294 (193–369)	222 (122–371)
1-178	Orofacial clefts	3,340 (1,880–6,340)	1,760 (1,240–2,370)	1,830 (1,230–2,590)	11·4 (6·7–20·9)	6 (4·2–8)	5·6 (3·7–7·9)
1-179	Down's syndrome	7,090 (4,360–11,300)	6,550 (3,660–8,750)	5,290 (2,730–8,340)	23·4 (14·7–37)	21·9 (12·2–29·2)	16·6 (8·5–26·5)
1-180	Turner syndrome	34 (14–60)	36 (16–63)	36 (16–63)	0·1 (0·1–0·2)	0·1 (0·1–0·2)	0·1 (0–0·2)
1-181	Klinefelter syndrome	3 (1–5)	3 (1–5)	3 (1–6)	0 (0–0)	0 (0–0)	0 (0–0)
1-182	Other chromosomal abnormalities	5,480 (3,050–12,500)	6,730 (4,860–8,880)	6,310 (3,610–9,670)	17·9 (10·1–40·2)	22·5 (16·3–29·7)	20·1 (11·4–30·8)
1-183	Congenital musculoskeletal and limb anomalies	9,870 (6,640–14,000)	12,300 (8,510–16,800)	12,900 (8,680–17,800)	37·7 (25·2–53·9)	41·7 (28·8–56·9)	38·9 (26·3–53·8)
1-184	Urogenital congenital anomalies	2,490 (1,500–6,780)	3,350 (2,490–4,600)	2,680 (1,660–4,160)	8·2 (5–21·9)	11·2 (8·3–15·4)	8·5 (5·2–13·3)
1-185	Digestive congenital anomalies	27,400 (16,400–72,400)	25,800 (19,500–38,600)	19,500 (10,300–32,600)	87·4 (52·5–231)	86·3 (65·1–129)	62·7 (33·2–105)
1-186	Other congenital anomalies	48,300 (29,000–128,000)	65,500 (44,500–81,300)	49,900 (27,300–80,100)	156 (94·4–410)	219 (149–272)	160 (87·6–258)
1-187	Urinary diseases and male infertility	20,200 (17,000–24,300)	38,000 (28,200–45,700)	41,600 (31,300–53,000)	124 (102–152)	155 (115–187)	128 (96·1–164)
1-188	Urinary tract infections and interstitial nephritis	14,800 (12,600–18,100)	26,700 (18,300–31,100)	27,300 (19,000–36,200)	81·1 (69·4–106)	107 (72·8–125)	83·4 (57·8–111)
1-189	Urolithiasis	920 (611–1,300)	1,560 (1,070–2,160)	1,910 (1,270–2,670)	5·8 (3·9–8·1)	5·8 (4–8)	5·5 (3·7–7·8)
1-190	Benign prostatic hyperplasia	3,470 (2,040–5,440)	7,420 (4,370–11,500)	9,840 (5,760–15,200)	31·1 (18·3–48·9)	33 (19·4–51·3)	31·7 (18·5–49·2)
1-191	Male infertility	164 (64–358)	349 (136–760)	461 (181–988)	0·8 (0·3–1·8)	1·1 (0·4–2·5)	1·3 (0·5–2·7)
1-192	Other urinary diseases	899 (675–1,180)	1,920 (1,170–2,540)	2,130 (1,400–2,920)	5·2 (3·9–6·8)	7·4 (4·4–9·8)	6·4 (4·1–8·8)
1-193	Gynecological diseases	89,700 (62,200–126,000)	129,000 (88,600–182,000)	156,000 (106,000–219,000)	477 (331–664)	445 (305–623)	442 (301–619)
1-194	Uterine fibroids	6,350 (3,440–11,500)	9,480 (4,630–18,000)	12,100 (5,950–23,100)	37·3 (20·3–68·1)	33·8 (16·6–64·3)	34·3 (16·9–65·6)
1-195	Polycystic ovarian syndrome	2,030 (864–4,030)	3,700 (1,550–7,450)	4,830 (2,040–9,610)	9·3 (4–18·6)	12 (5–24·1)	13·5 (5·7–26·8)
1-196	Female infertility	23 (9–55)	85 (22–244)	96 (25–290)	0·1 (0–0·3)	0·3 (0·1–0·8)	0·3 (0·1–0·8)
1-197	Endometriosis	8,200 (4,760–13,300)	9,660 (5,650–15,800)	10,400 (6,170–16,900)	39·6 (23·2–62·4)	31·6 (18·5–51·4)	28·8 (17·1–46·8)
1-198	Genital prolapse	1,470 (821–2,590)	1,970 (971–3,710)	2,520 (1,270–4,600)	10·5 (5·7–18·8)	7·8 (3·9–14·7)	7·5 (3·8–13·8)
1-199	Premenstrual syndrome	20,800 (12,600–32,400)	30,200 (18,200–46,600)	35,000 (21,400–54,500)	97·3 (60·3–151)	98·3 (59·1–152)	97·7 (59·9–152)
1-200	Other gynecological diseases	50,800 (33,200–71,800)	74,200 (47,400–108,000)	91,300 (58,400–134,000)	283 (184–402)	261 (168–376)	259 (166–378)
1-201	Hemoglobinopathies and hemolytic anaemias	16,900 (12,800–22,000)	11,600 (8,470–16,100)	11,300 (8,170–15,700)	78·3 (59·4–102)	40·3 (29·5–55·4)	33·5 (24–46·3)
1-202	Thalassemias	302 (191–455)	106 (67–154)	70 (38–118)	1 (0·6–1·5)	0·4 (0·2–0·5)	0·2 (0·1–0·4)
1-203	Thalassaemias trait	1,850 (1,150–2,750)	1,800 (1,120–2,770)	1,780 (1,090–2,660)	8·1 (5·1–12)	6 (3·7–9·3)	5·2 (3·2–7·7)
1-204	Sickle cell disorders	1,450 (1,070–1,890)	729 (544–937)	486 (316–714)	5·2 (3·8–6·8)	2·4 (1·8–3)	1·4 (0·9–2·1)
1-205	Sickle cell trait	2,930 (1,770–4,410)	2,450 (1,490–3,720)	2,360 (1,440–3,640)	13·1 (7·9–19·7)	8 (4·9–12·1)	6·8 (4·1–10·4)
1-206	G6PD deficiency	1,300 (925–1,720)	928 (684–1,190)	1,440 (1,040–1,930)	5·6 (4–7·3)	3·1 (2·3–4)	4·2 (3–5·6)
1-207	G6PD trait	23 (13–38)	39 (21–64)	43 (22–72)	0·1 (0·1–0·2)	0·1 (0·1–0·2)	0·1 (0·1–0·2)
1-208	Other hemoglobinopathies and hemolytic anaemias	9,090 (6,750–12,100)	5,550 (4,090–7,540)	5,170 (3,630–7,250)	45·3 (34·3–60·3)	20·3 (15–27·5)	15·6 (10·9–21·8)
1-209	Endocrine, metabolic, blood, and immune disorders	83,900 (49,700–111,000)	61,700 (42,500–86,200)	76,800 (52,000–106,000)	406 (261–528)	236 (162–329)	231 (157–321)
1-210	Oral disorders	70,500 (43,200–108,000)	119,000 (72,300–179,000)	152,000 (93,000–230,000)	478 (297–720)	466 (286–708)	457 (279–695)
1-211	Caries of deciduous teeth	923 (392–1,890)	892 (387–1,870)	976 (416–2,060)	3·1 (1·3–6·4)	3·1 (1·3–6·5)	3·1 (1·3–6·5)
1-212	Caries of permanent teeth	7,300 (3,250–14,600)	10,000 (4,340–20,000)	11,700 (5,100–22,700)	34·6 (15·2–67·4)	34·3 (14·8–67·6)	34 (14·9–65·8)
1-213	Periodontal disease	14,000 (5,220–29,900)	24,300 (9,200–52,800)	31,500 (11,900–67,800)	86·6 (33–185)	88·9 (34·2–193)	90·6 (34·7–195)
1-214	Edentulism	37,700 (24,200–54,500)	68,400 (43,900–98,700)	89,300 (56,900–129,000)	300 (194–435)	287 (184–414)	276 (176–402)
1-215	Other oral disorders	10,700 (6,630–15,800)	15,200 (9,540–22,400)	18,300 (11,400–26,800)	53·2 (32·9–77·9)	53·3 (33·4–77·8)	53·3 (33·3–77·9)
1-216	Sudden infant death syndrome	12,400 (3,400–29,500)	5,840 (3,090–10,100)	4,450 (2,370–7,430)	39·4 (10·8–93·7)	19·3 (10·2–33·3)	14·3 (7·6–23·8)
1-0	Injuries	1,470,000 (1,330,000–1,620,000)	924,000 (821,000–1,040,000)	849,000 (677,000–1,050,000)	6,160 (5,630–6,770)	3,200 (2,840–3,590)	2,480 (1,970–3,070)
1-1	Transport injuries	259,000 (227,000–311,000)	297,000 (239,000–342,000)	265,000 (197,000–346,000)	1,220 (1,070–1,460)	1,030 (830–1,190)	766 (571–1,000)
1-2	Road injuries	229,000 (200,000–276,000)	268,000 (214,000–309,000)	243,000 (180,000–319,000)	1,080 (942–1,310)	928 (742–1,070)	704 (522–925)

1-3	Pedestrian road injuries	114,000 (91,800–152,000)	141,000 (101,000–168,000)	114,000 (79,900–157,000)	536 (434–707)	491 (353–586)	333 (234–456)
1-4	Cyclist road injuries	7,720 (6,080–9,990)	13,000 (10,400–15,400)	13,200 (9,850–17,100)	40 (31·7–51·2)	45·9 (36·9–54·8)	38·3 (28·7–49·6)
1-5	Motorcyclist road injuries	12,700 (9,680–16,900)	19,700 (15,100–23,600)	19,100 (13,800–25,700)	59·6 (46·5–78·2)	66·4 (50·8–79·3)	54·6 (39·5–73·5)
1-6	Motor vehicle road injuries	90,600 (65,100–115,000)	87,400 (72,500–114,000)	89,500 (65,100–123,000)	427 (310–541)	300 (250–391)	258 (188–353)
1-7	Other road injuries	3,810 (2,860–5,460)	6,950 (5,220–8,290)	7,090 (4,920–9,780)	18·9 (14·5–26)	24·4 (18·4–29)	20·6 (14·3–28·4)
1-8	Other transport injuries	29,900 (25,900–34,200)	29,600 (24,700–34,100)	21,600 (15,900–28,200)	142 (125–162)	102 (85·2–118)	62·1 (46–81·1)
1-9	Unintentional injuries	918,000 (791,000–1,050,000)	480,000 (423,000–550,000)	443,000 (348,000–550,000)	3,660 (3,220–4,130)	1,680 (1,480–1,930)	1,320 (1,030–1,630)
1-10	Falls	54,900 (46,100–65,500)	78,300 (64,100–95,900)	98,000 (75,600–123,000)	312 (262–370)	292 (240–357)	290 (223–363)
1-11	Drowning	88,900 (74,700–109,000)	46,400 (39,400–57,600)	38,200 (27,600–54,000)	351 (299–420)	157 (133–196)	113 (81·4–159)
1-12	Fire, heat, and hot substances	52,900 (40,400–64,700)	30,100 (23,700–38,700)	31,400 (23,100–43,200)	235 (184–285)	106 (83·7–137)	92·4 (67·7–127)
1-13	Poisonings	26,400 (18,100–34,300)	11,200 (9,600–13,000)	10,200 (7,700–13,300)	101 (72·2–129)	38 (32·6–44)	30·1 (22·7–39·4)
1-14	Poisoning by carbon monoxide	613 (422–1,040)	1,110 (562–1,500)	1,140 (600–1,660)	2·7 (1·9–4·5)	3·8 (1·9–5)	3·3 (1·7–4·9)
1-15	Poisoning by other means	25,800 (17,500–33,900)	10,100 (8,560–12,000)	9,090 (6,800–12,000)	98·7 (69·5–126)	34·3 (29·1–40·8)	26·8 (20–35·6)
1-16	Exposure to mechanical forces	52,100 (43,500–62,500)	61,500 (47,300–77,900)	62,600 (48,200–81,200)	267 (220–321)	216 (168–273)	182 (140–236)
1-17	Unintentional firearm injuries	24,700 (19,900–30,400)	27,600 (17,600–34,800)	21,900 (15,000–29,500)	118 (95·4–144)	92·6 (60–116)	62·1 (42·5–83·6)
1-18	Other exposure to mechanical forces	27,400 (20,500–35,700)	33,800 (25,600–45,600)	40,700 (29,600–54,800)	149 (112–195)	123 (93–165)	119 (87–160)
1-19	Adverse effects of medical treatment	26,200 (16,600–33,600)	11,600 (9,620–14,200)	10,300 (7,430–14,700)	111 (72·6–139)	41·6 (34·6–51·2)	31·1 (22·3–44·3)
1-20	Animal contact	4,500 (3,490–5,610)	5,360 (4,040–6,780)	5,490 (4,060–7,190)	20·9 (16·4–26·4)	19 (14·3–24·1)	16·2 (12·1–21·4)
1-21	Venomous animal contact	3,330 (2,600–4,180)	3,770 (2,850–4,770)	3,910 (2,900–5,080)	15·4 (11·9–19·3)	13·3 (10·1–16·9)	11·5 (8·5–15)
1-22	Non-venomous animal contact	1,170 (823–1,580)	1,590 (1,120–2,160)	1,580 (1,090–2,220)	5·5 (4–7·5)	5·7 (4–7·7)	4·7 (3·2–6·6)
1-23	Foreign body	505,000 (390,000–618,000)	146,000 (123,000–173,000)	115,000 (80,700–158,000)	1,750 (1,380–2,120)	497 (421–587)	351 (245–488)
1-24	Pulmonary aspiration and foreign body in airway	482,000 (370,000–594,000)	139,000 (117,000–166,000)	108,000 (74,300–151,000)	1,660 (1,290–2,030)	474 (398–565)	330 (226–465)
1-25	Foreign body in eyes	1,080 (477–1,980)	1,560 (733–2,790)	1,870 (894–3,300)	5·5 (2·7–9·6)	5·5 (2·6–9·6)	5·4 (2·6–9·6)
1-26	Foreign body in other body part	22,000 (11,800–41,200)	5,030 (3,910–6,390)	5,190 (3,870–6,690)	82·5 (48–145)	17·6 (13·7–22·4)	15·4 (11·5–19·8)
1-27	Environmental heat and cold exposure	10,100 (7,910–12,900)	24,200 (21,200–27,700)	11,600 (8,440–15,200)	53·2 (41·7–67·6)	88·5 (77·8–101)	34 (24·9–44·5)
1-28	Exposure to forces of nature	18,800 (17,100–20,800)	12,700 (11,400–14,100)	5,390 (4,730–6,160)	82·1 (74·4–90·6)	43·4 (39–48·2)	15·8 (13·9–18·1)
1-29	Other unintentional injuries	78,000 (66,200–90,800)	52,900 (43,500–67,000)	55,200 (41,700–71,100)	371 (314–431)	184 (152–234)	160 (121–206)
1-30	Self-harm and interpersonal violence	292,000 (268,000–321,000)	147,000 (127,000–169,000)	141,000 (110,000–178,000)	1,280 (1,170–1,410)	491 (423–566)	401 (313–504)
1-31	Self-harm	36,500 (30,100–47,200)	52,800 (39,700–62,800)	51,100 (35,800–69,200)	176 (146–225)	176 (132–208)	145 (102–196)
1-32	Self-harm by firearm	2,160 (1,250–3,150)	2,370 (1,350–3,080)	2,140 (1,250–3,250)	10·5 (5·9–14·9)	8 (4·6–10·3)	6·1 (3·6–9·2)
1-33	Self-harm by other specified means	34,300 (28,300–44,700)	50,400 (37,700–59,900)	49,000 (34,400–66,600)	165 (137–215)	168 (125–199)	139 (97·4–188)
1-34	Interpersonal violence	80,600 (69,400–92,800)	73,600 (62,100–87,900)	73,600 (56,000–96,800)	368 (317–425)	243 (206–290)	208 (158–274)
1-35	Physical violence by firearm	26,200 (19,900–35,800)	26,700 (21,000–35,300)	28,600 (19,500–39,600)	122 (93·2–166)	87·4 (69·1–114)	80 (54·4–111)
1-36	Physical violence by sharp object	14,500 (9,050–19,000)	15,500 (10,800–19,500)	14,400 (9,270–20,300)	68·8 (43·4–88·1)	50·8 (35·6–63·7)	40·6 (26·2–57·1)
1-37	Sexual violence	3,410 (2,230–4,820)	4,660 (3,100–6,580)	5,500 (3,670–7,760)	16·6 (11–23·5)	15·6 (10·5–22)	15·6 (10·4–22)
1-38	Physical violence by other means	36,500 (29,200–43,500)	26,700 (22,800–31,900)	25,100 (18,200–33,300)	161 (129–193)	89·1 (76–106)	72·2 (52·8–95·6)
1-39	Conflict and terrorism	175,000 (159,000–197,000)	20,400 (12,900–32,900)	16,300 (9,850–26,800)	735 (666–832)	71·9 (45·1–115)	47·5 (28·8–77·7)
1-40	Executions and police conflict	137 (124–153)	180 (158–206)	200 (145–267)	0·6 (0·5–0·6)	0·6 (0·5–0·7)	0·6 (0·4–0·8)

SR Table 1: All-age DALYs counts and age standardized DALYs rates per 100,000 individuals for all causes and level 1 cause groups by location for 1990, 2010, and 2019, both sexes combined, Central Latin America

Cause Level	Cause	All-age DALYs			Age-standardized rate of DALYs per 100,000		
		1990	2010	2019	1990	2010	2019
0-0	All causes	54,600,000 (50,600,000–59,400,000)	59,400,000 (53,500,000–66,000,000)	66,700,000 (58,000,000–77,000,000)	37,700 (34,800–41,000)	28,800 (26,000–31,800)	27,400 (23,800–31,500)
1-0	Communicable, maternal, neonatal, and nutritional disorders	19,500,000 (17,900,000–21,000,000)	9,990,000 (9,090,000–10,900,000)	8,240,000 (7,030,000–9,610,000)	9,740 (9,000–10,400)	4,480 (4,090–4,860)	3,570 (3,030–4,180)
1-1	HIV/AIDS and sexually transmitted infections	268,000 (252,000–291,000)	719,000 (690,000–760,000)	668,000 (632,000–720,000)	176 (166–192)	315 (302–333)	258 (244–277)
1-2	HIV/AIDS	217,000 (214,000–221,000)	666,000 (650,000–691,000)	609,000 (588,000–635,000)	145 (143–148)	291 (284–302)	234 (226–244)
1-3	HIV/AIDS - drug-susceptible tuberculosis	72,800 (57,500–89,300)	64,400 (42,900–94,900)	55,600 (36,600–82,100)	48·7 (38·5–59·6)	28·1 (18·7–41·5)	21·4 (14·1–31·7)
1-4	HIV/AIDS - Multidrug-resistant Tuberculosis without extensive drug resistance	221 (57–619)	4,580 (1,790–9,670)	4,050 (1,320–9,510)	0·1 (0–0·4)	2 (0·8–4·2)	1·6 (0·5–3·7)
1-5	HIV/AIDS - Extensively drug-resistant Tuberculosis	0 (0–0)	487 (186–1,070)	657 (212–1,610)	0 (0–0)	0·2 (0·1–0·5)	0·3 (0·1–0·6)
1-6	HIV/AIDS resulting in other diseases	144,000 (128,000–159,000)	596,000 (560,000–629,000)	549,000 (514,000–580,000)	96·1 (85·4–106)	261 (245–275)	211 (198–223)
1-7	Sexually transmitted infections excluding HIV	50,400 (35,900–73,500)	53,500 (34,000–88,900)	59,300 (36,800–99,400)	30·9 (21·2–47·2)	23·8 (15·2–39·6)	23·4 (14·9–38·7)
1-8	Syphilis	22,300 (16,400–32,000)	9,670 (7,340–13,400)	8,840 (5,910–13,700)	10·4 (8–14·5)	4·3 (3·3–6)	3·9 (2·6–6·2)
1-9	Chlamydial infection	2,820 (2,020–4,000)	4,260 (3,050–6,030)	4,860 (3,480–6,870)	1·9 (1·4–2·7)	1·8 (1·3–2·6)	1·9 (1·3–2·6)
1-10	Gonococcal infection	2,820 (2,460–3,250)	3,840 (3,380–4,390)	4,510 (3,770–5,340)	2 (1·8–2·3)	1·7 (1·5–1·9)	1·7 (1·5–2·1)
1-11	Trichomoniasis	10,200 (3,980–20,900)	16,900 (6,580–34,700)	19,200 (7,410–39,300)	7·4 (2·9–15·2)	7·5 (2·9–15·5)	7·3 (2·8–15)
1-12	Genital herpes	6,480 (2,160–15,900)	10,700 (3,470–26,500)	12,700 (4,090–31,100)	5·1 (1·7–12·6)	4·9 (1·6–12·2)	5 (1·6–12·1)
1-13	Other sexually transmitted infections	5,750 (4,280–7,540)	8,120 (6,040–10,800)	9,250 (6,770–12,500)	4 (3–5·3)	3·5 (2·6–4·7)	3·5 (2·6–4·8)
1-14	Respiratory infections and tuberculosis	4,680,000 (4,280,000–5,050,000)	2,420,000 (2,200,000–2,640,000)	2,150,000 (1,800,000–2,550,000)	2,600 (2,410–2,770)	1,130 (1,030–1,230)	923 (768–1,100)
1-15	Tuberculosis	577,000 (555,000–599,000)	225,000 (214,000–237,000)	213,000 (182,000–251,000)	446 (430–461)	108 (103–114)	85 (72·7–100)
1-16	Latent tuberculosis infection	0 (0–0)	0 (0–0)	0 (0–0)	0 (0–0)	0 (0–0)	0 (0–0)
1-17	Drug-susceptible tuberculosis	576,000 (553,000–597,000)	212,000 (196,000–226,000)	199,000 (167,000–236,000)	445 (428–460)	102 (94·4–108)	79·6 (66·6–94·2)
1-18	Multidrug-resistant tuberculosis without extensive drug resistance	1,530 (440–4,090)	12,000 (5,580–22,700)	11,700 (4,110–27,100)	1·2 (0·3–3·1)	5·8 (2·7–11)	4·7 (1·7–10·9)
1-19	Extensively drug-resistant tuberculosis	0 (0–0)	1,260 (530–2,540)	1,860 (635–4,330)	0 (0–0)	0·6 (0·3–1·2)	0·7 (0·3–1·7)
1-20	Lower respiratory infections	3,790,000 (3,440,000–4,110,000)	1,890,000 (1,730,000–2,070,000)	1,640,000 (1,350,000–2,010,000)	1,980 (1,830–2,120)	891 (817–969)	715 (585–879)
1-21	Upper respiratory infections	245,000 (179,000–338,000)	228,000 (145,000–349,000)	228,000 (140,000–351,000)	130 (93·7–182)	98 (62·4–148)	92 (56·7–142)
1-22	Otitis media	66,500 (41,800–104,000)	72,800 (42,500–120,000)	74,300 (42,700–122,000)	35·9 (22·4–56·4)	30·8 (17·9–50·5)	30·2 (17·4–49·6)
1-23	Enteric infections	4,270,000 (3,850,000–4,680,000)	962,000 (840,000–1,090,000)	778,000 (641,000–946,000)	2,100 (1,910–2,280)	435 (382–491)	338 (278–411)
1-24	Diarrhoeal diseases	4,210,000 (3,800,000–4,620,000)	953,000 (834,000–1,080,000)	771,000 (633,000–938,000)	2,070 (1,890–2,240)	431 (379–487)	335 (276–407)
1-25	Typhoid and paratyphoid	42,200 (20,600–51,500)	5,000 (3,460–14,800)	4,150 (2,530–12,100)	24·4 (12·4–29·5)	2·3 (1·6–6·8)	1·7 (1–4·9)
1-26	Typhoid fever	41,700 (20,400–50,800)	4,930 (3,410–14,700)	4,100 (2,470–12,000)	24·1 (12·3–29·1)	2·2 (1·5–6·8)	1·7 (1–4·9)
1-27	Paratyphoid fever	521 (179–651)	70 (49–110)	52 (33–96)	0·3 (0·1–0·4)	0 (0–0·1)	0 (0–0)
1-28	iNTS	12,900 (6,770–15,300)	2,020 (1,030–6,790)	1,810 (891–5,970)	7·2 (3·4–8·5)	0·9 (0·5–2·9)	0·8 (0·4–2·5)
1-29	Other intestinal infectious diseases	3,940 (1,610–6,640)	1,730 (817–2,760)	1,310 (711–2,120)	1·8 (0·8–3)	0·7 (0·3–1·2)	0·6 (0·3–0·9)
1-30	Neglected tropical diseases and malaria	501,000 (375,000–654,000)	400,000 (318,000–496,000)	430,000 (327,000–543,000)	323 (249–410)	185 (148–228)	175 (134–221)
1-31	Malaria	111,000 (47,300–228,000)	19,300 (8,950–36,900)	45,100 (14,000–114,000)	58·5 (25·1–120)	8·3 (3·8–15·8)	18 (5·6–45·2)
1-32	Chagas disease	36,400 (21,700–45,800)	39,400 (29,100–54,200)	48,900 (34,800–73,000)	39·1 (22·2–48·7)	21·3 (15·6–29·5)	20·3 (14·4–30·4)
1-33	Leishmaniasis	4,190 (774–23,900)	4,260 (2,070–11,300)	3,460 (1,410–9,890)	2·3 (0·5–11·7)	1·9 (1–4·9)	1·4 (0·6–4·2)
1-34	Visceral leishmaniasis	2,720 (6–22,600)	996 (2–7,910)	986 (2–7,470)	1·4 (0–10·9)	0·4 (0–3·3)	0·4 (0–3·2)
1-35	Cutaneous and mucocutaneous leishmaniasis	1,460 (688–3,360)	3,260 (1,950–5,020)	2,470 (1,270–4,880)	1 (0·5–2·3)	1·5 (0·9–2·3)	1 (0·5–2)
1-36	African trypanosomiasis	0 (0–0)	0 (0–0)	0 (0–0)	0 (0–0)	0 (0–0)	0 (0–0)
1-37	Schistosomiasis	10,700 (5,690–19,500)	13,000 (6,210–25,700)	12,400 (6,030–24,400)	6·9 (3·9–12·3)	5·6 (2·7–10·8)	4·9 (2·4–9·4)
1-38	Cysticercosis	104,000 (68,800–147,000)	129,000 (81,700–183,000)	159,000 (102,000–224,000)	92·3 (60·5–129)	64·9 (42–91·5)	64 (41·2–90)
1-39	Cystic echinococcosis	220 (114–341)	111 (69–160)	133 (83–194)	0·1 (0·1–0·2)	0·1 (0–0·1)	0·1 (0–0·1)
1-40	Lymphatic filariasis	0 (0–0)	0 (0–0)	0 (0–0)	0 (0–0)	0 (0–0)	0 (0–0)
1-41	Onchocerciasis	14,000 (9,290–19,500)	1,380 (844–2,050)	473 (185–874)	10 (6·7–13·9)	0·6 (0·4–0·9)	0·2 (0·1–0·3)
1-42	Trachoma	922 (497–1,540)	792 (512–1,170)	956 (626–1,390)	1·1 (0·6–1·7)	0·4 (0·3–0·6)	0·4 (0·2–0·6)
1-43	Dengue	14,400 (10,400–19,800)	80,300 (67,400–96,000)	62,400 (49,400–74,500)	8·5 (6·1–11·6)	34·7 (29·1–41·4)	26 (20·5–30·9)
1-44	Yellow fever	1,920 (564–4,830)	896 (263–2,250)	592 (173–1,520)	1 (0·3–2·6)	0·4 (0·1–0·9)	0·2 (0·1–0·6)
1-45	Rabies	4,570 (3,980–5,080)	205 (112–280)	178 (81–272)	2·5 (2·2–2·7)	0·1 (0·1–0·1)	0·1 (0–0·1)
1-46	Intestinal nematode infections	110,000 (67,600–170,000)	77,000 (45,400–122,000)	61,800 (36,500–100,000)	58·4 (35·7–90·6)	32·1 (19–51·1)	24·7 (14·6–40)
1-47	Ascariasis	29,600 (20,700–44,300)	32,900 (19,300–54,000)	25,700 (14,800–43,700)	15·1 (10·4–23)	14 (8·2–22·9)	10·5 (6–17·8)
1-48	Trichuriasis	50,200 (26,700–83,600)	14,900 (7,630–25,600)	14,700 (7,900–25,000)	26·6 (14·2–44·2)	6·1 (3·1–10·5)	5·8 (3·1–9·8)

1-49	Hookworm disease	30,100 (18,300–46,400)	29,200 (17,600–44,900)	21,400 (12,800–34,200)	16·6 (10·1–25·6)	12 (7·3–18·6)	8·4 (5–13·4)
1-50	Food-borne trematodiases	0 (0–0)	0 (0–0)	0 (0–0)	0 (0–0)	0 (0–0)	0 (0–0)
1-51	Leprosy	154 (96–232)	145 (89–218)	172 (107–259)	0·1 (0·1–0·2)	0·1 (0–0·1)	0·1 (0–0·1)
1-52	Ebola virus disease	0 (0–0)	0 (0–0)	0 (0–0)	0 (0–0)	0 (0–0)	0 (0–0)
1-53	Zika virus disease	0 (0–0)	0 (0–0)	104 (64–161)	0 (0–0)	0 (0–0)	0 (0–0·1)
1-54	Guinea worm disease	0 (0–0)	0 (0–0)	0 (0–0)	0 (0–0)	0 (0–0)	0 (0–0)
1-55	Other neglected tropical diseases	88,000 (39,700–112,000)	34,500 (24,200–52,800)	34,700 (24,600–49,500)	42·4 (19·5–54·1)	14·9 (10·5–22·8)	15 (10·7–21·6)
1-56	Other infectious diseases	860,000 (762,000–967,000)	385,000 (345,000–443,000)	344,000 (279,000–420,000)	418 (375–467)	169 (152–194)	146 (117–179)
1-57	Meningitis	365,000 (337,000–395,000)	123,000 (113,000–135,000)	91,400 (73,900–112,000)	175 (163–188)	53·9 (49·7–59)	38·4 (30·8–47·4)
1-58	Encephalitis	109,000 (91,500–120,000)	107,000 (92,800–122,000)	100,000 (82,800–121,000)	53·8 (45·9–59·4)	46·5 (40·1–53)	42 (34·5–50·7)
1-59	Diphtheria	1,630 (1,350–2,020)	72 (52–95)	73 (48–102)	0·8 (0·6–0·9)	0 (0–0)	0 (0–0)
1-60	Whooping cough	124,000 (70,800–223,000)	22,600 (12,600–57,500)	27,000 (13,100–59,900)	53·4 (30·6–96·6)	9·7 (5·4–24·9)	12·4 (6–27·7)
1-61	Tetanus	53,400 (42,900–61,300)	3,830 (2,910–8,090)	2,660 (1,600–7,510)	29·1 (22·9–33·9)	1·8 (1·4–3·9)	1·1 (0·7–3·1)
1-62	Measles	57,000 (28,100–72,100)	0 (0–0)	10 (7–22)	25·1 (12·3–31·7)	0 (0–0)	0 (0–0)
1-63	Varicella and herpes zoster	27,800 (19,900–42,300)	23,400 (17,300–35,500)	20,700 (15,600–32,800)	14·9 (10·9–22)	10·6 (7·9–15·9)	8·8 (6·6–14)
1-64	Acute hepatitis	37,600 (33,300–42,900)	27,900 (23,600–32,900)	24,100 (19,900–29,200)	20·2 (18·1–22·6)	12·3 (10·5–14·3)	9·9 (8·2–12)
1-65	Acute hepatitis A	26,100 (22,100–30,600)	18,400 (14,600–22,600)	14,500 (11,100–18,300)	13·3 (11·3–15·5)	7·9 (6·3–9·6)	6 (4·6–7·5)
1-66	Acute hepatitis B	4,680 (3,570–6,050)	5,690 (3,770–7,140)	5,660 (3,720–7,360)	2·7 (2·1–3·5)	2·6 (1·7–3·3)	2·4 (1·5–3)
1-67	Acute hepatitis C	5,060 (2,960–7,100)	2,630 (1,960–5,100)	2,760 (1,960–4,410)	3·2 (2–4·5)	1·2 (0·9–2·3)	1·1 (0·8–1·8)
1-68	Acute hepatitis E	1,760 (941–2,250)	1,160 (855–1,730)	1,190 (896–1,650)	0·9 (0·5–1·1)	0·5 (0·4–0·8)	0·5 (0·4–0·7)
1-69	Other unspecified infectious diseases	84,500 (61,700–102,000)	77,100 (63,200–103,000)	77,600 (59,700–107,000)	46·1 (33·8–55·5)	34·3 (28·2–45·6)	32·8 (25·1–45·8)
1-70	Maternal and neonatal disorders	7,260,000 (6,470,000–8,060,000)	4,230,000 (3,770,000–4,680,000)	3,100,000 (2,510,000–3,750,000)	3,150 (2,810–3,480)	1,840 (1,640–2,040)	1,400 (1,130–1,700)
1-71	Maternal disorders	308,000 (293,000–327,000)	208,000 (196,000–223,000)	197,000 (163,000–238,000)	181 (172–192)	84·2 (79·4–90·1)	74 (61·4–89·3)
1-72	Maternal haemorrhage	79,700 (71,600–89,200)	48,000 (42,900–53,900)	40,100 (32,500–48,800)	47·6 (42·9–53)	19·6 (17·5–22)	15·1 (12·3–18·4)
1-73	Maternal sepsis and other pregnancy related infections	37,300 (33,500–41,500)	13,700 (11,500–16,700)	12,400 (9,450–15,900)	21·5 (19·4–23·9)	5·5 (4·6–6·7)	4·6 (3·5–6)
1-74	Maternal hypertensive disorders	68,100 (63,400–73,200)	47,500 (43,900–51,200)	43,100 (35,300–52,700)	38·9 (36·3–41·8)	19·1 (17·7–20·6)	16·2 (13·3–19·8)
1-75	Maternal obstructed labour and uterine rupture	6,130 (4,920–7,560)	6,750 (5,690–8,070)	6,970 (5,540–8,720)	3·6 (2·9–4·4)	2·7 (2·3–3·3)	2·6 (2·1–3·3)
1-76	Maternal abortive outcome	48,000 (43,100–53,000)	13,900 (12,000–16,000)	11,200 (8,950–13,900)	28·5 (25·7–31·5)	5·6 (4·9–6·5)	4·2 (3·4–5·2)
1-77	Ectopic pregnancy	4,330 (3,890–4,830)	3,310 (3,010–3,640)	3,270 (2,690–4,030)	2·5 (2·3–2·8)	1·3 (1·2–1·5)	1·2 (1–1·5)
1-78	Indirect maternal deaths	15,400 (13,800–17,100)	37,800 (34,700–40,900)	38,300 (30,800–47,300)	8·9 (7·9–9·8)	15·2 (14–16·5)	14·4 (11·6–17·8)
1-79	Late maternal deaths	10,200 (8,360–12,600)	8,900 (7,770–10,200)	13,100 (9,570–17,900)	6 (5–7·3)	3·6 (3·1–4·1)	4·9 (3·6–6·7)
1-80	Maternal deaths aggravated by HIV/AIDS	35 (18–59)	131 (68–212)	171 (85–291)	0 (0–0)	0·1 (0–0·1)	0·1 (0–0·1)
1-81	Other maternal disorders	38,800 (34,400–43,400)	28,400 (25,500–31,400)	28,200 (23,300–34,300)	23·1 (20·5–25·8)	11·5 (10·4–12·7)	10·6 (8·7–12·9)
1-82	Neonatal disorders	6,950,000 (6,170,000–7,740,000)	4,020,000 (3,570,000–4,460,000)	2,900,000 (2,340,000–3,510,000)	2,970 (2,640–3,300)	1,760 (1,560–1,950)	1,330 (1,060–1,620)
1-83	Neonatal preterm birth	3,640,000 (3,210,000–4,050,000)	1,940,000 (1,720,000–2,150,000)	1,270,000 (1,010,000–1,550,000)	1,550 (1,370–1,730)	849 (754–941)	581 (462–712)
1-84	Neonatal encephalopathy due to birth asphyxia and trauma	1,750,000 (1,490,000–2,000,000)	708,000 (626,000–801,000)	489,000 (380,000–617,000)	740 (629–844)	311 (275–352)	228 (177–289)
1-85	Neonatal sepsis and other neonatal infections	835,000 (725,000–954,000)	883,000 (757,000–1,050,000)	773,000 (623,000–946,000)	363 (312–418)	384 (330–455)	348 (279–424)
1-86	Hemolytic disease and other neonatal jaundice	117,000 (103,000–131,000)	65,500 (55,600–75,900)	56,700 (46,000–68,500)	53·7 (47·2–60)	28·2 (23·9–32·7)	23·6 (19·3–28·3)
1-87	Other neonatal disorders	612,000 (541,000–687,000)	423,000 (372,000–478,000)	314,000 (243,000–398,000)	258 (228–290)	186 (163–210)	148 (114–188)
1-88	Nutritional deficiencies	1,640,000 (1,450,000–1,880,000)	872,000 (730,000–1,060,000)	768,000 (612,000–963,000)	981 (873–1,120)	405 (342–485)	326 (260–406)
1-89	Protein-energy malnutrition	1,090,000 (967,000–1,200,000)	479,000 (433,000–528,000)	375,000 (318,000–446,000)	639 (556–695)	228 (207–249)	162 (137–194)
1-90	Iodine deficiency	17,000 (9,050–29,600)	17,100 (7,860–32,300)	25,500 (13,500–44,300)	10·8 (5·9–18·4)	7·3 (3·4–13·7)	9·9 (5·3–17·2)
1-91	Vitamin A deficiency	31,900 (21,100–45,700)	20,600 (13,500–30,000)	18,000 (11,900–26,000)	15 (9·9–21·3)	8·7 (5·7–12·7)	7·6 (5·1–11)
1-92	Dietary iron deficiency	367,000 (240,000–545,000)	308,000 (202,000–456,000)	305,000 (202,000–445,000)	218 (143–324)	138 (90·4–204)	128 (84·6–186)
1-93	Other nutritional deficiencies	139,000 (114,000–194,000)	47,600 (38,200–63,200)	44,500 (33,900–56,900)	97·7 (80·7–150)	23·1 (18·5–31)	18·1 (13·9–23·2)
1-0	Non-communicable diseases	25,500,000 (22,500,000–28,700,000)	39,000,000 (34,100,000–44,600,000)	48,000,000 (41,500,000–56,000,000)	22,000 (19,600–24,500)	19,700 (17,500–22,400)	19,700 (17,100–23,000)
1-1	Neoplasms	3,020,000 (2,950,000–3,110,000)	4,850,000 (4,690,000–4,980,000)	6,100,000 (5,240,000–7,070,000)	2,900 (2,830–2,990)	2,550 (2,450–2,620)	2,520 (2,160–2,910)
1-2	Lip and oral cavity cancer	28,000 (27,200–28,600)	44,800 (43,100–46,100)	59,300 (51,000–69,400)	30·6 (29·6–31·5)	24·5 (23·5–25·3)	24·6 (21·2–28·8)
1-3	Nasopharynx cancer	6,830 (6,640–7,020)	11,000 (10,600–11,300)	13,200 (11,100–15,800)	6·3 (6·2–6·5)	5·6 (5·4–5·8)	5·3 (4·5–6·3)
1-4	Other pharynx cancer	9,610 (9,280–9,930)	13,900 (13,400–14,400)	18,400 (15,300–22,200)	10·6 (10·3–11)	7·6 (7·3–7·9)	7·6 (6·3–9·1)
1-5	Oesophageal cancer	48,200 (46,800–49,500)	69,500 (66,800–71,400)	90,700 (76,700–107,000)	56 (54·1–57·5)	39·2 (37·5–40·3)	37·9 (32·1–44·7)
1-6	Stomach cancer	388,000 (377,000–397,000)	551,000 (531,000–566,000)	647,000 (550,000–763,000)	434 (420–445)	301 (289–310)	268 (228–315)
1-7	Colon and rectum cancer	148,000 (144,000–151,000)	371,000 (358,000–379,000)	539,000 (465,000–626,000)	162 (157–166)	203 (195–208)	224 (193–260)

1-8	Liver cancer	84,900 (79,500–89,000)	138,000 (126,000–148,000)	197,000 (171,000–231,000)	89·2 (83·1–93·7)	76·5 (69·7–81·9)	82·7 (72·1–96·9)
1-9	Liver cancer due to hepatitis B	17,700 (13,800–22,400)	24,000 (18,700–30,400)	33,000 (24,700–44,900)	17·2 (13·3–22·1)	12·5 (9·6–16·1)	13·4 (9·9–18·2)
1-10	Liver cancer due to hepatitis C	26,600 (21,200–32,100)	47,600 (39,100–56,100)	68,700 (54,700–85,400)	32·1 (25·8–38·6)	27·9 (23–32·8)	29·3 (23·4–36·3)
1-11	Liver cancer due to alcohol use	21,900 (16,800–27,000)	38,900 (30,900–46,500)	60,000 (46,400–76,200)	25 (19·4–30·9)	22·1 (17·5–26·5)	25·2 (19·5–31·9)
1-12	Liver cancer due to NASH	5,600 (4,380–7,240)	11,400 (9,210–14,400)	17,400 (13,600–22,500)	6·1 (4·7–7·9)	6·4 (5·1–8·1)	7·3 (5·7–9·4)
1-13	Gallbladder and biliary tract cancer	81,900 (62,200–86,600)	97,800 (91,100–124,000)	123,000 (104,000–165,000)	95·2 (72·4–101)	55·4 (51·5–70·5)	51·6 (43·6–69)
1-14	Pancreatic cancer	89,900 (87,600–92,000)	203,000 (195,000–208,000)	296,000 (255,000–343,000)	103 (100–106)	114 (109–117)	124 (107–143)
1-15	Larynx cancer	39,400 (38,000–41,000)	50,200 (48,400–52,500)	61,600 (51,800–73,400)	45·5 (43·7–47·2)	28·5 (27·3–29·7)	25·8 (21·7–30·6)
1-16	Tracheal, bronchus, and lung cancer	303,000 (296,000–309,000)	478,000 (460,000–492,000)	620,000 (525,000–734,000)	345 (335–352)	269 (259–278)	260 (220–307)
1-17	Malignant skin melanoma	14,900 (13,200–22,000)	32,300 (26,300–43,100)	44,400 (34,500–61,900)	14·3 (12·6–21·4)	16·8 (13·7–22·3)	18·2 (14·1–25·2)
1-18	Non-melanoma skin cancer	21,100 (19,800–21,800)	38,600 (36,100–39,900)	50,600 (43,600–58,300)	24·2 (22·6–25·1)	22 (20·3–22·8)	21·5 (18·4–24·8)
1-19	Non-melanoma skin cancer (squamous-cell carcinoma)	21,100 (19,800–21,800)	38,600 (36,100–39,800)	50,500 (43,600–58,300)	24·2 (22·6–25·1)	22 (20·3–22·7)	21·5 (18·4–24·7)
1-20	Non-melanoma skin cancer (basal-cell carcinoma)	14 (6–26)	28 (12–53)	37 (17–72)	0 (0–0)	0 (0–0)	0 (0–0)
1-21	Breast cancer	195,000 (190,000–200,000)	396,000 (384,000–407,000)	521,000 (441,000–615,000)	194 (188–199)	203 (197–209)	209 (177–246)
1-22	Cervical cancer	324,000 (302,000–338,000)	378,000 (362,000–427,000)	437,000 (362,000–538,000)	314 (289–328)	190 (181–214)	174 (144–214)
1-23	Uterine cancer	23,200 (22,400–24,200)	41,200 (39,100–44,000)	61,400 (52,100–72,100)	26·1 (25·1–27·2)	22·8 (21·6–24·4)	25·3 (21·5–29·7)
1-24	Ovarian cancer	55,800 (54,100–58,400)	137,000 (131,000–142,000)	184,000 (152,000–220,000)	54·2 (52·3–57·7)	70 (66·9–72·7)	73·9 (61·1–88·5)
1-25	Prostate cancer	117,000 (101,000–158,000)	277,000 (226,000–351,000)	378,000 (296,000–494,000)	158 (136–214)	172 (141–218)	167 (131–218)
1-26	Testicular cancer	21,800 (19,400–36,800)	43,600 (40,600–59,000)	52,900 (43,400–68,500)	13·2 (12–19·6)	18·3 (17–25·1)	20·3 (16·7–27·2)
1-27	Kidney cancer	57,300 (55,300–59,400)	111,000 (108,000–114,000)	154,000 (132,000–180,000)	51·1 (49·7–52·5)	58·6 (56·7–60·2)	63·6 (54·6–74·1)
1-28	Bladder cancer	26,500 (25,500–27,600)	50,100 (47,700–51,900)	67,300 (57,700–78,800)	32·4 (30·9–33·8)	29·3 (27·7–30·4)	28·8 (24·7–33·7)
1-29	Brain and nervous system cancer	106,000 (95,800–152,000)	193,000 (133,000–213,000)	215,000 (147,000–262,000)	72·1 (65·5–103)	90·3 (62·1–99·4)	87·1 (59·4–107)
1-30	Thyroid cancer	19,100 (17,800–19,800)	35,000 (32,400–36,800)	48,300 (41,400–56,000)	20·2 (18·8–21)	19·1 (17·6–20·1)	20 (17·1–23·1)
1-31	Mesothelioma	5,290 (4,600–5,960)	11,700 (10,300–12,200)	14,600 (12,400–17,000)	5·3 (4·6–5·9)	6·1 (5·4–6·4)	5·9 (5–6·9)
1-32	Hodgkin lymphoma	41,400 (37,000–48,100)	37,900 (34,500–46,800)	41,100 (34,200–50,800)	29·5 (26·2–34·3)	17·7 (16–21·5)	16·3 (13·6–20·2)
1-33	Non-Hodgkin's lymphoma	111,000 (107,000–115,000)	173,000 (168,000–178,000)	208,000 (179,000–243,000)	85·4 (82·7–88·2)	85·8 (83–88·2)	84·4 (73·1–98·6)
1-34	Multiple myeloma	22,000 (20,000–24,100)	52,800 (47,500–56,500)	76,700 (62,500–91,200)	24·7 (22·8–27·3)	29·3 (26·6–31·4)	31·7 (26–37·7)
1-35	Leukaemia	382,000 (364,000–399,000)	468,000 (440,000–498,000)	470,000 (404,000–543,000)	223 (214–231)	206 (195–219)	190 (164–220)
1-36	Acute lymphoid leukaemia	160,000 (146,000–179,000)	225,000 (192,000–246,000)	212,000 (169,000–247,000)	80·3 (74·1–90·4)	94 (80–103)	86·3 (68·5–101)
1-37	Chronic lymphoid leukaemia	4,560 (4,070–4,850)	9,520 (8,750–10,700)	12,600 (10,500–15,500)	5·1 (4·6–5·4)	5·4 (5–6·1)	5·4 (4·4–6·6)
1-38	Acute myeloid leukaemia	65,400 (59,400–76,700)	109,000 (96,100–123,000)	121,000 (101,000–146,000)	40·3 (37·4–47·2)	49 (43·2–55)	48·7 (40·7–58·8)
1-39	Chronic myeloid leukaemia	19,900 (18,500–20,900)	22,800 (21,600–26,500)	24,100 (20,200–29,500)	15·8 (14·6–16·5)	11·2 (10·6–12·8)	9·7 (8·1–11·8)
1-40	Other leukaemia	132,000 (118,000–141,000)	102,000 (94,300–121,000)	99,900 (82,700–124,000)	81·2 (74·3–85·4)	46·8 (43·5–55·2)	40·4 (33·5–50·2)
1-41	Other neoplasms	20,100 (16,000–29,400)	40,000 (31,700–49,200)	52,600 (40,600–67,000)	16·4 (13·1–24·5)	20·6 (16·4–25·7)	22 (17–28)
1-42	Myelodysplastic, myeloproliferative, and other hematopoietic neoplasms	20,100 (16,000–29,400)	40,000 (31,700–49,200)	52,600 (40,600–67,000)	16·4 (13·1–24·5)	20·6 (16·4–25·7)	22 (17–28)
1-43	Benign and in situ intestinal neoplasms	0 (0–0)	0 (0–0)	0 (0–0)	0 (0–0)	0 (0–0)	0 (0–0)
1-44	Benign and in situ cervical and uterine neoplasms	0 (0–0)	0 (0–0)	0 (0–0)	0 (0–0)	0 (0–0)	0 (0–0)
1-45	Other benign and in situ neoplasms	0 (0–0)	0 (0–0)	0 (0–0)	0 (0–0)	0 (0–0)	0 (0–0)
1-46	Cardiovascular diseases	4,000,000 (3,860,000–4,110,000)	5,660,000 (5,380,000–5,870,000)	7,310,000 (6,390,000–8,420,000)	4,540 (4,340–4,680)	3,220 (3,030–3,330)	3,110 (2,720–3,570)
1-47	Rheumatic heart disease	134,000 (122,000–149,000)	89,100 (74,000–110,000)	91,800 (72,900–117,000)	107 (98·8–116)	42 (35·4–50·6)	36·4 (29–46)
1-48	Ischaemic heart disease	2,000,000 (1,930,000–2,060,000)	3,130,000 (2,980,000–3,220,000)	4,140,000 (3,620,000–4,780,000)	2,400 (2,290–2,480)	1,800 (1,710–1,860)	1,760 (1,540–2,030)
1-49	Stroke	1,270,000 (1,220,000–1,310,000)	1,570,000 (1,490,000–1,640,000)	1,940,000 (1,700,000–2,230,000)	1,390 (1,320–1,440)	877 (826–916)	820 (717–941)
1-50	Ischaemic stroke	471,000 (439,000–501,000)	598,000 (547,000–643,000)	771,000 (676,000–874,000)	602 (558–640)	362 (331–388)	340 (297–385)
1-51	Intracerebral hemorrhage	600,000 (577,000–623,000)	672,000 (642,000–705,000)	813,000 (699,000–944,000)	621 (596–644)	366 (349–383)	338 (290–392)
1-52	Subarachnoid hemorrhage	197,000 (182,000–215,000)	299,000 (283,000–312,000)	355,000 (301,000–420,000)	164 (152–182)	149 (139–155)	143 (121–169)
1-53	Hypertensive heart disease	225,000 (161,000–238,000)	283,000 (257,000–370,000)	392,000 (329,000–503,000)	289 (208–307)	171 (155–222)	171 (144–219)
1-54	Non-rheumatic valvular heart disease	28,100 (27,000–29,000)	45,200 (43,400–46,800)	65,400 (54,600–77,900)	28·6 (27·5–29·7)	24·5 (23·4–25·4)	27·1 (22·7–32·3)
1-55	Non-rheumatic calcific aortic valve disease	18,100 (16,400–20,000)	30,800 (28,700–34,100)	44,900 (36,800–55,500)	18·9 (17·2–20·9)	16·9 (15·7–18·7)	18·7 (15·4–23·1)
1-56	Non-rheumatic degenerative mitral valve disease	9,020 (7,490–10,800)	13,700 (10,200–15,400)	19,500 (13,300–24,900)	9 (7·3–10·7)	7·3 (5·4–8·1)	8 (5·4–10·2)
1-57	Other non-rheumatic valve diseases	919 (349–1,310)	700 (546–1,240)	966 (675–1,600)	0·8 (0·3–1·2)	0·4 (0·3–0·6)	0·4 (0·3–0·6)
1-58	Cardiomyopathy and myocarditis	80,800 (71,300–91,200)	118,000 (98,200–129,000)	130,000 (105,000–157,000)	64·5 (57·3–70·1)	58·6 (49·2–63·5)	54·5 (43·9–65·6)
1-59	Myocarditis	8,340 (6,380–10,500)	11,900 (9,060–15,000)	12,500 (9,440–16,100)	4·9 (3·8–5·9)	5·4 (4·1–6·7)	5·2 (3·9–6·8)
1-60	Alcoholic cardiomyopathy	9,030 (6,850–10,500)	7,490 (6,220–9,210)	9,540 (7,310–12,000)	8·9 (6·6–10·5)	3·8 (3·1–4·6)	3·8 (2·9–4·8)

1-61	Other cardiomyopathy	63,500 (56,900–72,900)	98,800 (79,700–108,000)	108,000 (86,000–131,000)	50·8 (45·9–56·7)	49·4 (40·6–53·6)	45·5 (36·1–55)
1-62	Atrial fibrillation and flutter	56,000 (47,200–68,800)	127,000 (107,000–155,000)	185,000 (153,000–228,000)	80·5 (68·2–98)	80·4 (67·7–96·8)	82·8 (68·2–102)
1-63	Aortic aneurysm	30,400 (29,200–31,600)	54,700 (52,300–56,600)	75,900 (62,400–91,700)	34·3 (32·8–35·7)	31 (29·5–32·1)	32·2 (26·6–38·8)
1-64	Peripheral vascular disease	10,100 (6,060–15,900)	19,800 (12,200–31,400)	28,900 (17,700–48,100)	14·1 (8·5–22·2)	12·4 (7·6–19·7)	12·9 (7·9–21·4)
1-65	Endocarditis	20,300 (17,500–27,100)	37,900 (26,500–41,700)	42,500 (29,400–52,900)	14·2 (12·5–19·6)	18·1 (12·7–19·9)	17·3 (12–21·6)
1-66	Chronic respiratory diseases	1,130,000 (987,000–1,310,000)	1,500,000 (1,340,000–1,670,000)	1,830,000 (1,600,000–2,080,000)	1,030 (935–1,130)	817 (738–896)	784 (686–890)
1-67	Chronic obstructive pulmonary disease	503,000 (464,000–532,000)	930,000 (853,000–1,000,000)	1,210,000 (1,060,000–1,390,000)	614 (567–649)	555 (505–596)	529 (460–605)
1-68	Pneumoconiosis	8,180 (7,350–9,110)	7,820 (6,790–9,120)	9,970 (8,230–12,000)	9·2 (8·3–10·2)	4·3 (3·8–5)	4·2 (3·4–5)
1-69	Silicosis	5,680 (5,090–6,360)	4,220 (3,660–4,970)	5,490 (4,380–6,790)	6·5 (5·9–7·3)	2·3 (2–2·7)	2·3 (1·8–2·8)
1-70	Asbestosis	386 (242–448)	523 (384–607)	611 (487–758)	0·4 (0·3–0·5)	0·3 (0·2–0·3)	0·3 (0·2–0·3)
1-71	Coal workers pneumoconiosis	599 (508–683)	783 (691–897)	1,040 (819–1,290)	0·6 (0·5–0·7)	0·4 (0·4–0·5)	0·4 (0·3–0·5)
1-72	Other pneumoconiosis	1,510 (1,220–1,820)	2,290 (1,800–2,870)	2,830 (2,230–3,630)	1·6 (1·3–1·9)	1·3 (1–1·6)	1·2 (0·9–1·5)
1-73	Asthma	529,000 (405,000–700,000)	393,000 (283,000–553,000)	404,000 (283,000–575,000)	338 (271–429)	174 (128–242)	166 (116–237)
1-74	Interstitial lung disease and pulmonary sarcoidosis	35,600 (30,800–47,200)	91,200 (62,000–108,000)	121,000 (78,600–154,000)	36·2 (31·3–47·6)	49·9 (33·5–59·3)	50·6 (32·9–64·7)
1-75	Other chronic respiratory diseases	55,500 (43,900–63,500)	76,000 (63,700–86,300)	82,200 (68,300–95,900)	32·7 (26·3–36·8)	33·5 (28–38·1)	33·5 (27·5–39·2)
1-76	Digestive diseases	2,500,000 (2,350,000–2,690,000)	3,160,000 (2,930,000–3,440,000)	3,870,000 (3,410,000–4,400,000)	2,150 (2,030–2,300)	1,600 (1,490–1,730)	1,570 (1,390–1,790)
1-77	Cirrhosis and other chronic liver diseases	1,200,000 (1,170,000–1,220,000)	1,570,000 (1,520,000–1,630,000)	2,030,000 (1,740,000–2,340,000)	1,110 (1,090–1,130)	806 (779–836)	816 (701–940)
1-78	Cirrhosis and other chronic liver diseases due to hepatitis B	67,400 (58,000–78,500)	59,900 (50,100–72,300)	74,400 (56,000–96,100)	62·3 (53·6–72·5)	29·8 (24·9–35·8)	29·5 (22·3–37·8)
1-79	Cirrhosis and other chronic liver diseases due to hepatitis C	328,000 (289,000–370,000)	427,000 (372,000–492,000)	553,000 (443,000–689,000)	306 (269–348)	214 (186–247)	220 (176–273)
1-80	Cirrhosis and other chronic liver diseases due to alcohol use	389,000 (343,000–434,000)	568,000 (499,000–639,000)	741,000 (598,000–896,000)	390 (344–435)	295 (259–332)	297 (241–359)
1-81	Cirrhosis and other chronic liver diseases due to NAFLD	182,000 (136,000–237,000)	293,000 (220,000–379,000)	379,000 (275,000–504,000)	183 (136–238)	155 (115–200)	154 (112–205)
1-82	Cirrhosis and other chronic liver diseases due to other causes	230,000 (199,000–269,000)	227,000 (185,000–279,000)	284,000 (220,000–370,000)	169 (139–204)	113 (89·9–141)	116 (89·8–151)
1-83	Upper digestive system diseases	388,000 (309,000–515,000)	469,000 (335,000–681,000)	546,000 (382,000–805,000)	361 (296–462)	235 (172–335)	220 (156–322)
1-84	Peptic ulcer disease	186,000 (178,000–193,000)	146,000 (137,000–155,000)	161,000 (139,000–189,000)	193 (184–200)	80·7 (75·1–85·7)	67·9 (58·5–79·3)
1-85	Gastritis and duodenitis	47,800 (39,700–58,500)	59,500 (50,100–71,300)	70,800 (58,200–86,300)	44·7 (38·1–53·5)	31·5 (26·7–37·2)	29·3 (24·2–35·6)
1-86	GERD	154,000 (80,600–273,000)	263,000 (138,000–467,000)	314,000 (163,000–559,000)	123 (63·8–218)	123 (63·9–219)	123 (63·8–219)
1-87	Appendicitis	104,000 (90,300–122,000)	75,200 (64,200–87,900)	79,600 (63,700–97,600)	61·6 (52·5–71·7)	34·6 (29·2–40·4)	32·2 (25·8–39·4)
1-88	Paralytic ileus and intestinal obstruction	235,000 (198,000–266,000)	208,000 (173,000–237,000)	211,000 (163,000–258,000)	134 (118–148)	102 (84·7–115)	91·6 (71·3–113)
1-89	Inguinal, femoral, and abdominal hernia	145,000 (113,000–184,000)	156,000 (122,000–198,000)	186,000 (144,000–237,000)	112 (89–139)	77·7 (61·3–97·5)	76·6 (59·6–97·4)
1-90	Inflammatory bowel disease	18,100 (15,500–20,800)	28,400 (24,400–32,900)	36,200 (30,200–43,000)	14·8 (12·7–17·3)	14·2 (12·2–16·4)	14·7 (12·2–17·4)
1-91	Vascular intestinal disorders	38,600 (36,900–40,200)	68,700 (64,600–73,100)	91,200 (78,100–105,000)	43·3 (41·1–45)	40·1 (37·5–42·8)	39·3 (33·6–45·2)
1-92	Gallbladder and biliary diseases	207,000 (156,000–276,000)	341,000 (256,000–457,000)	393,000 (297,000–517,000)	186 (144–242)	170 (130–225)	158 (121–207)
1-93	Pancreatitis	80,000 (74,400–84,100)	114,000 (108,000–120,000)	142,000 (122,000–165,000)	67 (63·3–70·8)	55·6 (52·9–58·7)	56·5 (48·7–65·7)
1-94	Other digestive diseases	83,600 (70,200–104,000)	121,000 (89,000–139,000)	156,000 (113,000–189,000)	60·3 (51·3–76·1)	62·9 (46–72)	65·5 (47·4–79·1)
1-95	Neurological disorders	1,790,000 (1,030,000–2,900,000)	2,710,000 (1,540,000–4,380,000)	3,250,000 (1,850,000–5,260,000)	1,370 (826–2,150)	1,320 (768–2,120)	1,330 (771–2,130)
1-96	Alzheimer's disease and other dementias	212,000 (86,200–493,000)	513,000 (209,000–1,160,000)	745,000 (304,000–1,670,000)	337 (136–782)	338 (138–765)	341 (138–766)
1-97	Parkinson's disease	48,600 (45,400–51,800)	115,000 (105,000–123,000)	163,000 (144,000–184,000)	68·1 (63·1–72·3)	72·5 (66·3–77·7)	73·2 (64·6–82·5)
1-98	Idiopathic epilepsy	540,000 (399,000–730,000)	565,000 (409,000–776,000)	625,000 (448,000–849,000)	325 (239–433)	247 (179–338)	250 (179–340)
1-99	Multiple sclerosis	7,920 (6,630–11,300)	19,500 (15,800–22,500)	24,500 (20,000–29,800)	7 (5·8–10)	9·4 (7·7–10·9)	9·6 (7·9–11·7)
1-100	Motor neuron disease	9,290 (8,820–9,810)	21,100 (20,300–22,000)	31,300 (26,100–37,000)	7·3 (7·1–7·7)	10·7 (10·3–11·1)	12·7 (10·6–15)
1-101	Headache disorders	852,000 (150,000–1,870,000)	1,320,000 (252,000–2,890,000)	1,470,000 (296,000–3,230,000)	559 (114–1,220)	569 (115–1,250)	568 (115–1,240)
1-102	Migraine	776,000 (101,000–1,810,000)	1,200,000 (167,000–2,740,000)	1,330,000 (195,000–3,040,000)	505 (76–1,150)	515 (75·9–1,180)	514 (76·2–1,170)
1-103	Tension-type headache	75,800 (21,400–311,000)	121,000 (35,800–452,000)	139,000 (42,000–496,000)	53·9 (16·1–198)	54·2 (16·3–193)	54·2 (16·3–193)
1-104	Other neurological disorders	120,000 (93,100–155,000)	158,000 (123,000–201,000)	189,000 (146,000–245,000)	69·2 (54·7–88·3)	68·8 (54·7–86·6)	76·1 (58·4–98·3)
1-105	Mental disorders	2,230,000 (1,640,000–2,970,000)	3,630,000 (2,650,000–4,760,000)	4,140,000 (3,050,000–5,430,000)	1,520 (1,120–2,000)	1,600 (1,170–2,100)	1,610 (1,190–2,110)
1-106	Schizophrenia	234,000 (168,000–306,000)	398,000 (282,000–518,000)	465,000 (333,000–603,000)	178 (127–230)	179 (127–232)	179 (128–232)
1-107	Depressive disorders	690,000 (475,000–946,000)	1,230,000 (850,000–1,680,000)	1,450,000 (1,010,000–1,980,000)	517 (362–709)	561 (390–763)	564 (393–771)
1-108	Major depressive disorder	577,000 (390,000–806,000)	1,040,000 (714,000–1,440,000)	1,230,000 (830,000–1,710,000)	432 (297–595)	476 (327–656)	478 (326–664)
1-109	Dysthymia	113,000 (71,500–170,000)	186,000 (119,000–282,000)	219,000 (141,000–331,000)	84·8 (55–128)	84·5 (54·7–127)	85·1 (55·1–128)
1-110	Bipolar disorder	275,000 (166,000–429,000)	426,000 (255,000–659,000)	482,000 (291,000–741,000)	185 (112–284)	185 (112–284)	185 (112–284)
1-111	Anxiety disorders	511,000 (345,000–710,000)	848,000 (583,000–1,170,000)	968,000 (659,000–1,340,000)	336 (229–462)	368 (254–506)	376 (258–521)
1-112	Eating disorders	77,900 (47,300–119,000)	116,000 (71,100–175,000)	127,000 (78,500–193,000)	43·9 (27–66·1)	46·6 (28·7–70)	48 (29·6–72·5)
1-113	Anorexia nervosa	18,900 (11,000–31,300)	26,900 (15,700–43,000)	29,700 (17,400–47,200)	10·1 (5·9–15·9)	10·6 (6·2–16·9)	11·2 (6·6–17·9)

1·114	Bulimia nervosa	59,000 (34,300–94,100)	88,700 (52,100–142,000)	97,600 (56,100–156,000)	33·8 (19·5–53·6)	35·9 (21–56·5)	36·7 (21·1–58·5)
1·115	Autism spectrum disorders	93,700 (61,400–135,000)	125,000 (82,000–182,000)	134,000 (87,300–195,000)	53·7 (35·1–77·3)	53·6 (35–78·1)	53·6 (35–78·2)
1·116	Attention-deficit/hyperactivity disorder	33,100 (18,600–58,000)	42,700 (23,900–73,800)	43,300 (24,600–74,600)	16·8 (9·5–29·2)	17·1 (9·6–29·4)	17·1 (9·7–29·5)
1·117	Conduct disorder	156,000 (86,900–245,000)	182,000 (102,000–286,000)	174,000 (97,400–272,000)	70·4 (39·3–111)	70·2 (39·3–110)	70·3 (39·3–110)
1·118	Idiopathic developmental intellectual disability	31,700 (10,700–59,100)	41,500 (15,100–74,500)	36,900 (11,300–69,000)	18 (6–33·6)	17·6 (6·4–31·5)	14·7 (4·5–27·6)
1·119	Other mental disorders	128,000 (81,200–194,000)	221,000 (141,000–336,000)	265,000 (171,000–401,000)	104 (66·4–157)	104 (66·8–157)	104 (66·9–157)
1·120	Substance use disorders	774,000 (637,000–933,000)	948,000 (749,000–1,180,000)	1,090,000 (869,000–1,360,000)	581 (488–689)	421 (336–520)	419 (334–521)
1·121	Alcohol use disorders	596,000 (498,000–716,000)	681,000 (549,000–846,000)	774,000 (623,000–976,000)	468 (400–554)	308 (251–381)	298 (241–376)
1·122	Drug use disorders	178,000 (130,000–234,000)	267,000 (194,000–347,000)	318,000 (236,000–410,000)	113 (84·1–145)	113 (82·9–146)	121 (89·7–156)
1·123	Opioid use disorders	79,800 (53,900–112,000)	124,000 (85,600–172,000)	149,000 (104,000–202,000)	53·8 (37·3–73·2)	54·4 (37·9–74·1)	56·9 (40–76·8)
1·124	Cocaine use disorders	40,800 (29,500–55,100)	56,900 (40,200–77,400)	65,500 (47,800–88,000)	24·9 (18·4–33)	23·4 (16·7–31·5)	24·8 (18·1–33·4)
1·125	Amphetamine use disorders	14,100 (8,370–22,300)	19,400 (11,900–30,500)	27,300 (16,700–43,000)	8·4 (5·2–13·1)	7·9 (4·9–12·5)	10·2 (6·3–16·1)
1·126	Cannabis use disorders	11,400 (6,610–18,300)	18,600 (11,400–28,300)	20,700 (12,800–31,100)	6·6 (3·9–10·4)	7·5 (4·6–11·4)	7·8 (4·8–11·7)
1·127	Other drug use disorders	32,000 (21,600–45,300)	48,100 (33,400–65,500)	55,800 (39,800–75,700)	19·5 (13·5–26·8)	19·8 (13·8–26·8)	21·1 (15·1–28·5)
1·128	Diabetes and kidney diseases	2,250,000 (2,030,000–2,490,000)	5,420,000 (4,880,000–5,990,000)	7,620,000 (6,670,000–8,710,000)	2,380 (2,140–2,640)	2,950 (2,660–3,260)	3,150 (2,770–3,600)
1·129	Diabetes mellitus	1,470,000 (1,290,000–1,680,000)	2,980,000 (2,540,000–3,490,000)	4,340,000 (3,700,000–5,160,000)	1,640 (1,430–1,860)	1,640 (1,410–1,920)	1,800 (1,540–2,140)
1·130	Diabetes mellitus type 1	85,200 (73,400–104,000)	115,000 (95,000–136,000)	137,000 (111,000–170,000)	60·6 (51·7–77·1)	52·6 (43·7–63·2)	54·2 (44·1–67·1)
1·131	Diabetes mellitus type 2	1,390,000 (1,210,000–1,590,000)	2,870,000 (2,440,000–3,360,000)	4,210,000 (3,580,000–5,000,000)	1,580 (1,380–1,800)	1,590 (1,360–1,860)	1,750 (1,480–2,070)
1·132	Chronic kidney disease	778,000 (720,000–840,000)	2,440,000 (2,290,000–2,590,000)	3,270,000 (2,920,000–3,700,000)	739 (689–794)	1,300 (1,230–1,380)	1,350 (1,200–1,520)
1·133	Chronic kidney disease due to diabetes mellitus type 1	55,500 (40,000–73,200)	182,000 (123,000–249,000)	241,000 (156,000–345,000)	47·9 (32·8–64·7)	89·1 (59·5–125)	94·6 (61–135)
1·134	Chronic kidney disease due to diabetes mellitus type 2	139,000 (113,000–163,000)	542,000 (439,000–640,000)	763,000 (606,000–925,000)	164 (136–192)	309 (252–364)	320 (256–388)
1·135	Chronic kidney disease due to hypertension	126,000 (106,000–148,000)	479,000 (403,000–567,000)	660,000 (539,000–800,000)	148 (126–173)	274 (230–322)	279 (228–338)
1·136	Chronic kidney disease due to glomerulonephritis	206,000 (179,000–236,000)	580,000 (487,000–687,000)	756,000 (607,000–921,000)	168 (143–194)	292 (242–350)	304 (244–372)
1·137	Chronic kidney disease due to other and unspecified causes	252,000 (214,000–292,000)	655,000 (538,000–792,000)	854,000 (683,000–1,050,000)	212 (177–250)	339 (277–412)	350 (282–430)
1·138	Acute glomerulonephritis	2,930 (2,570–3,250)	1,580 (1,490–1,670)	6,800 (5,870–7,750)	2 (1·8–2·2)	0·8 (0·7–0·8)	2·8 (2·5–3·2)
1·139	Skin and subcutaneous diseases	754,000 (496,000–1,120,000)	1,060,000 (699,000–1,580,000)	1,160,000 (772,000–1,710,000)	454 (305–673)	467 (311–696)	469 (314–696)
1·140	Dermatitis	169,000 (100,000–267,000)	222,000 (133,000–347,000)	237,000 (144,000–368,000)	96·4 (58·4–150)	96·4 (57·8–150)	96·5 (58·2–150)
1·141	Atopic dermatitis	126,000 (68,200–212,000)	152,000 (81,600–257,000)	155,000 (84,200–260,000)	64·1 (34·7–108)	64·1 (34·3–108)	64·1 (35–108)
1·142	Contact dermatitis	37,700 (23,700–57,000)	62,300 (38,800–92,800)	73,600 (46,300–110,000)	28·9 (18·1–43·1)	29 (18·1–43·4)	29 (18·3–43·2)
1·143	Seborrhoeic dermatitis	4,960 (2,800–7,920)	7,480 (4,210–12,000)	8,490 (4,790–13,500)	3·4 (1·9–5·4)	3·4 (1·9–5·4)	3·4 (1·9–5·4)
1·144	Psoriasis	18,700 (13,200–25,000)	26,400 (18,800–35,100)	29,300 (20,600–39,000)	13·1 (9·3–17·4)	11·9 (8·5–15·9)	11·6 (8·1–15·4)
1·145	Bacterial skin diseases	32,500 (23,500–46,000)	63,600 (42,800–91,200)	92,000 (56,400–133,000)	22·9 (16·5–34)	32·4 (21·5–47·1)	38·3 (23·4–55·6)
1·146	Cellulitis	12,200 (8,310–18,700)	21,700 (13,400–32,100)	28,900 (16,400–41,200)	9·1 (6·1–15·2)	10·9 (6·5–16·3)	11·8 (6·7–17)
1·147	Pyoderma	20,200 (14,200–30,200)	41,900 (27,200–64,300)	63,100 (37,200–92,700)	13·9 (9·8–20·7)	21·5 (13·7–32·7)	26·5 (15·6–39·1)
1·148	Scabies	130,000 (70,800–209,000)	191,000 (104,000–306,000)	187,000 (102,000–297,000)	72·3 (39·5–116)	80·7 (44·4–129)	75·1 (41–120)
1·149	Fungal skin diseases	37,000 (14,800–78,900)	56,300 (22,700–120,000)	69,400 (28,000–148,000)	27·5 (11·1–58·6)	27·1 (10·9–57·9)	28·5 (11·4–60·9)
1·150	Viral skin diseases	87,000 (55,100–132,000)	104,000 (65,400–156,000)	105,000 (66,700–157,000)	43·3 (27·5–65)	43·3 (27·4–65·2)	43·7 (27·8–65·7)
1·151	Acne vulgaris	81,000 (48,200–130,000)	118,000 (70,300–188,000)	121,000 (72,400–197,000)	39·1 (23·4–62·8)	45 (27–72)	46·8 (27·9–75·8)
1·152	Alopecia areata	10,800 (6,830–16,100)	16,400 (10,400–24,500)	18,500 (11,700–27,400)	7·1 (4·5–10·6)	7·2 (4·6–10·7)	7·2 (4·5–10·6)
1·153	Pruritus	12,300 (5,910–21,900)	19,600 (9,380–35,000)	23,200 (11,100–41,500)	9·1 (4·4–16·3)	9·3 (4·4–16·6)	9·3 (4·5–16·8)
1·154	Urticaria	90,000 (58,500–132,000)	116,000 (75,600–169,000)	121,000 (79,100–176,000)	49·6 (32·3–72)	49·7 (32·5–71·9)	49·7 (32·4–72·3)
1·155	Decubitus ulcer	22,400 (14,400–32,900)	25,800 (19,600–42,500)	31,200 (23,100–48,700)	25·1 (16·7–36·4)	15 (11·5–24·3)	13·5 (10–21·3)
1·156	Other skin and subcutaneous diseases	63,200 (32,400–113,000)	102,000 (51,800–184,000)	122,000 (60,800–218,000)	48·8 (25–87·3)	49·3 (25–88·6)	49·3 (24·6–88·2)
1·157	Sense organ diseases	918,000 (631,000–1,290,000)	1,600,000 (1,090,000–2,280,000)	2,000,000 (1,370,000–2,830,000)	926 (642–1,290)	857 (584–1,220)	836 (573–1,180)
1·158	Blindness and vision loss	328,000 (228,000–452,000)	542,000 (372,000–757,000)	674,000 (465,000–940,000)	350 (248–478)	298 (206–412)	285 (197–395)
1·159	Glaucoma	11,800 (7,940–16,600)	19,300 (13,300–26,800)	25,300 (17,500–35,100)	16·9 (11·5–23·7)	12·3 (8·4–17·2)	11·4 (7·8–15·7)
1·160	Cataract	67,300 (47,700–90,800)	115,000 (81,700–153,000)	147,000 (104,000–197,000)	90·6 (64·3–122)	70·3 (50·2–94·2)	64·7 (46–87·2)
1·161	Age-related macular degeneration	3,860 (2,640–5,530)	7,020 (4,820–10,100)	9,520 (6,520–13,500)	5·3 (3·6–7·5)	4·4 (3–6·2)	4·2 (2·9–6)
1·162	Refraction disorders	130,000 (88,400–187,000)	200,000 (135,000–283,000)	231,000 (156,000–325,000)	106 (72–147)	96·9 (65·4–135)	93·2 (62·8–131)
1·163	Near vision loss	45,100 (20,800–89,400)	92,800 (43,700–183,000)	127,000 (59,300–248,000)	54·7 (25·8–108)	53·8 (25·1–106)	53·9 (25·3–105)
1·164	Other vision loss	70,100 (49,200–95,700)	109,000 (77,300–149,000)	135,000 (96,500–186,000)	77·1 (54·7–105)	60·8 (43·3–82·9)	57·4 (40·8–78·6)
1·165	Age-related and other hearing loss	545,000 (364,000–794,000)	976,000 (646,000–1,420,000)	1,230,000 (820,000–1,800,000)	537 (361–769)	519 (347–748)	511 (342–741)
1·166	Other sense organ diseases	45,400 (27,100–69,300)	78,500 (46,600–120,000)	96,600 (57,600–150,000)	38·8 (22·9–60·1)	39·5 (23·4–61)	39·7 (23·7–61·2)

1-167	Musculoskeletal disorders	2,180,000 (1,570,000–2,850,000)	3,890,000 (2,800,000–5,110,000)	4,960,000 (3,580,000–6,530,000)	1,860 (1,350–2,440)	1,880 (1,350–2,470)	1,970 (1,420–2,590)
1-168	Rheumatoid arthritis	71,400 (55,500–90,300)	130,000 (99,600–162,000)	169,000 (129,000–214,000)	72·3 (56·8–90·7)	67·8 (52·3–84·3)	68·7 (52·7–86·7)
1-169	Osteoarthritis	182,000 (91,900–357,000)	409,000 (207,000–801,000)	561,000 (285,000–1,100,000)	212 (107–418)	229 (116–451)	233 (119–460)
1-170	Low back pain	976,000 (679,000–1,310,000)	1,600,000 (1,120,000–2,130,000)	1,950,000 (1,370,000–2,600,000)	766 (538–1,020)	747 (525–1,000)	769 (541–1,020)
1-171	Neck pain	171,000 (112,000–249,000)	307,000 (202,000–444,000)	373,000 (247,000–540,000)	146 (97·3–212)	147 (97·1–212)	147 (97·3–212)
1-172	Gout	5,270 (3,260–7,640)	10,700 (6,760–15,400)	14,600 (9,230–20,900)	5·1 (3·2–7·4)	5·5 (3·5–7·8)	5·9 (3·7–8·5)
1-173	Other musculoskeletal disorders	777,000 (544,000–1,060,000)	1,430,000 (1,010,000–1,950,000)	1,890,000 (1,340,000–2,570,000)	654 (457–896)	685 (486–939)	743 (524–1,010)
1-174	Other non-communicable diseases	3,960,000 (3,320,000–5,170,000)	4,540,000 (3,750,000–5,520,000)	4,630,000 (3,650,000–5,810,000)	2,250 (1,850–2,850)	2,070 (1,720–2,530)	1,950 (1,540–2,430)
1-175	Congenital anomalies	2,420,000 (2,050,000–3,510,000)	2,150,000 (1,710,000–2,470,000)	1,690,000 (1,240,000–2,190,000)	1,060 (900–1,520)	932 (740–1,070)	764 (560–991)
1-176	Neural tube defects	303,000 (247,000–347,000)	122,000 (103,000–141,000)	117,000 (85,600–155,000)	130 (106–148)	53 (44·7–61·3)	53·6 (39–71·4)
1-177	Congenital heart anomalies	988,000 (819,000–1,630,000)	983,000 (686,000–1,150,000)	703,000 (448,000–953,000)	428 (356–701)	426 (297–501)	322 (205–436)
1-178	Orofacial clefts	30,600 (24,000–38,300)	10,800 (8,530–13,600)	8,930 (6,460–11,900)	13·5 (10·6–16·8)	4·7 (3·7–5·9)	3·9 (2·8–5·2)
1-179	Down's syndrome	63,300 (55,600–78,200)	66,400 (54,600–76,800)	63,900 (45,900–82,500)	28·4 (25–34·8)	28·5 (23·4–32·9)	28·1 (20·1–36·4)
1-180	Turner syndrome	746 (347–1,310)	961 (451–1,650)	1,010 (470–1,740)	0·4 (0·2–0·7)	0·4 (0·2–0·7)	0·4 (0·2–0·7)
1-181	Klinefelter syndrome	25 (11–48)	30 (13–56)	29 (13–56)	0 (0–0)	0 (0–0)	0 (0–0)
1-182	Other chromosomal abnormalities	39,100 (28,600–82,000)	52,500 (43,100–66,300)	51,100 (36,000–65,600)	17·3 (12·7–35·5)	22·6 (18·5–28·5)	22·9 (16·1–29·7)
1-183	Congenital musculoskeletal and limb anomalies	153,000 (104,000–213,000)	164,000 (111,000–228,000)	180,000 (120,000–255,000)	76·8 (52·8–107)	69·5 (47·3–96·6)	74·6 (49·9–106)
1-184	Urogenital congenital anomalies	92,400 (42,800–254,000)	74,800 (53,100–144,000)	59,900 (41,200–111,000)	40 (18·9–109)	32·3 (22·8–62·9)	27 (18·1–51·6)
1-185	Digestive congenital anomalies	239,000 (190,000–450,000)	233,000 (172,000–294,000)	170,000 (114,000–235,000)	102 (80·6–191)	102 (75·1–128)	79·4 (53·2–110)
1-186	Other congenital anomalies	513,000 (430,000–722,000)	446,000 (317,000–523,000)	334,000 (231,000–451,000)	223 (186–314)	194 (137–227)	153 (106–207)
1-187	Urinary diseases and male infertility	134,000 (116,000–159,000)	242,000 (207,000–284,000)	352,000 (293,000–423,000)	130 (110–156)	133 (113–156)	149 (124–179)
1-188	Urinary tract infections and interstitial nephritis	76,100 (69,900–89,700)	143,000 (127,000–153,000)	214,000 (174,000–246,000)	66·8 (61·8–78·9)	76·8 (67·9–82·4)	90·3 (73·4–104)
1-189	Urolithiasis	8,830 (7,400–10,500)	14,200 (12,100–17,000)	20,600 (16,700–26,300)	8·2 (6·9–9·8)	7·1 (6·1–8·5)	8·2 (6·7–10·6)
1-190	Benign prostatic hyperplasia	30,400 (18,600–45,600)	61,400 (37,500–90,900)	86,200 (52,700–128,000)	39·3 (24–58·7)	37·3 (22·8–54·9)	37·5 (23–55·3)
1-191	Male infertility	2,890 (1,120–6,760)	6,590 (2,500–15,200)	8,050 (3,000–19,200)	1·9 (0·7–4·4)	2·8 (1·1–6·5)	3 (1·1–7·3)
1-192	Other urinary diseases	15,600 (11,600–19,100)	17,400 (14,600–25,300)	23,300 (18,100–33,400)	13·6 (9·5–16·5)	8·9 (7·4–13·5)	9·7 (7·6–14)
1-193	Gynecological diseases	530,000 (364,000–747,000)	757,000 (521,000–1,070,000)	875,000 (602,000–1,230,000)	374 (260–525)	330 (227–466)	335 (231–471)
1-194	Uterine fibroids	36,900 (19,300–67,400)	62,600 (31,100–114,000)	74,900 (37,000–137,000)	30·5 (15·9–55·1)	28·7 (14·2–52·4)	28·8 (14·3–52·6)
1-195	Polycystic ovarian syndrome	18,700 (8,040–38,100)	28,000 (12,300–56,400)	33,400 (14,400–67,200)	11·3 (4·8–22·9)	11·5 (5–23·1)	12·6 (5·4–25·4)
1-196	Female infertility	3,690 (940–9,500)	11,500 (3,440–28,900)	14,300 (4,040–36,300)	2·5 (0·6–6·3)	4·9 (1·4–12)	5·4 (1·5–13·7)
1-197	Endometriosis	58,800 (35,000–94,400)	61,900 (36,500–98,400)	62,300 (36,800–98,400)	37·2 (22·3–59·6)	25·8 (15·3–40·8)	23·5 (13·9–37·1)
1-198	Genital prolapse	7,400 (3,940–13,300)	11,400 (5,650–21,000)	15,200 (7,470–28,600)	8 (4·2–14·5)	6·2 (3·1–11·5)	6·2 (3·1–11·7)
1-199	Premenstrual syndrome	168,000 (102,000–263,000)	254,000 (155,000–396,000)	277,000 (169,000–428,000)	103 (63–158)	104 (63·6–163)	105 (64·1–162)
1-200	Other gynecological diseases	236,000 (154,000–338,000)	328,000 (213,000–471,000)	398,000 (253,000–582,000)	182 (118–260)	148 (96·2–212)	153 (97·5–224)
1-201	Hemoglobinopathies and hemolytic anaemias	88,600 (75,500–106,000)	87,400 (75,800–103,000)	91,100 (75,900–108,000)	55·8 (47·4–67·1)	39·5 (34·3–46·4)	37 (30·9–44)
1-202	Thalassemias	1,510 (841–2,400)	2,250 (1,370–3,300)	2,040 (1,190–3,080)	0·7 (0·4–1·1)	1 (0·6–1·4)	0·9 (0·5–1·4)
1-203	Thalassaemias trait	5,090 (2,880–8,300)	5,430 (3,160–8,660)	5,860 (3,410–9,580)	3 (1·7–4·8)	2·3 (1·3–3·6)	2·3 (1·3–3·8)
1-204	Sickle cell disorders	9,270 (7,690–11,200)	7,010 (5,720–8,520)	6,820 (4,910–9,160)	4·3 (3·6–5·2)	2·9 (2·4–3·5)	2·9 (2·1–3·9)
1-205	Sickle cell trait	9,020 (5,600–13,600)	6,990 (4,230–10,500)	6,180 (3,660–9,380)	5·2 (3·2–7·8)	2·9 (1·8–4·3)	2·4 (1·4–3·6)
1-206	G6PD deficiency	20,000 (16,400–23,200)	25,200 (21,500–28,400)	24,000 (18,400–30,100)	11·1 (9·2–12·8)	10·7 (9·2–12)	9·4 (7·2–11·9)
1-207	G6PD trait	208 (134–313)	351 (219–513)	375 (231–578)	0·2 (0·1–0·3)	0·2 (0·1–0·2)	0·1 (0·1–0·2)
1-208	Other hemoglobinopathies and hemolytic anaemias	43,500 (36,100–53,000)	40,200 (33,300–49,000)	45,900 (37,900–55,700)	31·4 (26·3–38·2)	19·6 (16·4–23·7)	18·9 (15·6–22·9)
1-209	Endocrine, metabolic, blood, and immune disorders	326,000 (259,000–405,000)	552,000 (438,000–692,000)	698,000 (549,000–886,000)	251 (196–319)	274 (217–347)	287 (226–364)
1-210	Oral disorders	405,000 (234,000–640,000)	686,000 (395,000–1,090,000)	869,000 (514,000–1,380,000)	358 (214–563)	339 (199–542)	350 (208–557)
1-211	Caries of deciduous teeth	6,670 (2,880–14,000)	6,830 (2,990–14,500)	6,350 (2,750–13,500)	3 (1·3–6·2)	2·9 (1·3–6·1)	2·8 (1·2–6)
1-212	Caries of permanent teeth	39,500 (17,600–77,800)	61,300 (27,600–118,000)	67,000 (29,900–130,000)	25·3 (11·5–48·9)	26·6 (12–51)	26·1 (11·6–50·6)
1-213	Periodontal disease	128,000 (48,300–277,000)	234,000 (89,800–502,000)	278,000 (108,000–598,000)	107 (41·4–230)	110 (42·6–238)	109 (42·1–234)
1-214	Edentulism	150,000 (95,200–221,000)	263,000 (166,000–388,000)	382,000 (242,000–560,000)	169 (107–247)	146 (92·2–215)	159 (101–234)
1-215	Other oral disorders	81,000 (50,300–119,000)	121,000 (75,400–176,000)	136,000 (85,300–197,000)	53·3 (33·4–77·7)	53·4 (33·4–77·7)	53·4 (33·6–77·6)
1-216	Sudden infant death syndrome	58,100 (48,200–70,200)	57,900 (47,700–70,100)	52,000 (36,300–70,700)	24·6 (20·4–29·7)	25·2 (20·8–30·5)	24·3 (17–33·1)
1-0	Injuries	9,630,000 (9,200,000–10,100,000)	10,500,000 (9,880,000–11,200,000)	10,500,000 (9,110,000–12,000,000)	6,030 (5,730–6,400)	4,540 (4,260–4,850)	4,080 (3,540–4,660)
1-1	Transport injuries	2,420,000 (2,330,000–2,520,000)	2,580,000 (2,430,000–2,730,000)	2,520,000 (2,170,000–2,910,000)	1,550 (1,490–1,630)	1,130 (1,060–1,200)	981 (843–1,130)
1-2	Road injuries	2,320,000 (2,230,000–2,420,000)	2,440,000 (2,300,000–2,590,000)	2,370,000 (2,030,000–2,730,000)	1,490 (1,430–1,560)	1,070 (1,010–1,140)	923 (792–1,060)

1-3	Pedestrian road injuries	1,360,000 (1,300,000–1,420,000)	1,030,000 (982,000–1,090,000)	947,000 (809,000–1,100,000)	871 (840–910)	463 (440–487)	375 (320–435)
1-4	Cyclist road injuries	93,200 (80,600–109,000)	139,000 (118,000–163,000)	129,000 (109,000–153,000)	62·8 (53·6–73·9)	62·5 (53·1–73·7)	51·1 (43–60·4)
1-5	Motorcyclist road injuries	225,000 (213,000–238,000)	411,000 (388,000–440,000)	451,000 (380,000–530,000)	140 (131–149)	172 (162–186)	172 (145–201)
1-6	Motor vehicle road injuries	576,000 (544,000–610,000)	759,000 (715,000–811,000)	743,000 (639,000–847,000)	369 (347–395)	328 (308–353)	287 (247–327)
1-7	Other road injuries	70,100 (62,700–77,600)	99,600 (90,200–111,000)	99,000 (86,200–114,000)	46·4 (41·1–51·9)	44 (39·5–49·2)	38·6 (33·6–44·4)
1-8	Other transport injuries	98,100 (93,100–105,000)	136,000 (131,000–143,000)	151,000 (128,000–177,000)	63 (59·7–67·1)	59 (56·4–62·1)	58·6 (49·5–68·5)
1-9	Unintentional injuries	3,370,000 (3,080,000–3,730,000)	3,040,000 (2,670,000–3,520,000)	2,900,000 (2,430,000–3,440,000)	2,120 (1,920–2,380)	1,380 (1,200–1,600)	1,170 (976–1,380)
1-10	Falls	746,000 (647,000–873,000)	915,000 (750,000–1,130,000)	944,000 (775,000–1,140,000)	596 (516–699)	443 (365–544)	382 (315–463)
1-11	Drowning	563,000 (535,000–597,000)	367,000 (343,000–394,000)	292,000 (250,000–342,000)	297 (284–312)	154 (144–165)	117 (99·5–137)
1-12	Fire, heat, and hot substances	323,000 (268,000–399,000)	248,000 (183,000–338,000)	247,000 (180,000–341,000)	200 (163–249)	111 (81·7–152)	98·4 (72·2–135)
1-13	Poisonings	118,000 (108,000–128,000)	78,800 (69,000–89,900)	73,500 (62,200–87,400)	63·9 (58·9–69·9)	33·9 (29·7–38·7)	29·3 (24·7–34·8)
1-14	Poisoning by carbon monoxide	27,800 (25,700–30,300)	20,400 (18,900–22,300)	19,800 (16,900–22,800)	15·4 (14·4–16·6)	8·8 (8·2–9·6)	7·9 (6·7–9·1)
1-15	Poisoning by other means	89,700 (81,800–98,800)	58,400 (49,800–68,700)	53,700 (43,600–65,400)	48·5 (44·2–53·6)	25·1 (21·4–29·6)	21·4 (17·4–26·1)
1-16	Exposure to mechanical forces	455,000 (395,000–542,000)	476,000 (385,000–607,000)	504,000 (404,000–641,000)	302 (256–366)	212 (170–272)	197 (158–252)
1-17	Unintentional firearm injuries	174,000 (138,000–184,000)	136,000 (123,000–149,000)	144,000 (119,000–170,000)	106 (83·3–113)	57·1 (51·7–62·6)	55·3 (45·7–65·2)
1-18	Other exposure to mechanical forces	282,000 (224,000–365,000)	339,000 (254,000–468,000)	359,000 (269,000–488,000)	196 (153–255)	154 (115–213)	142 (107–193)
1-19	Adverse effects of medical treatment	93,400 (65,600–102,000)	70,700 (63,900–81,900)	75,900 (64,000–91,200)	62·8 (45·3–67·6)	34 (30·7–39·3)	31·7 (26·7–38·2)
1-20	Animal contact	117,000 (102,000–136,000)	70,100 (58,000–85,300)	70,500 (56,700–88,000)	66·4 (57·7–77·5)	31·8 (26·4–38·9)	28·6 (23·1–35·6)
1-21	Venomous animal contact	94,300 (81,400–108,000)	49,000 (41,300–58,600)	47,300 (38,500–57,800)	51·2 (44·8–58·3)	22·1 (18·7–26·3)	19·2 (15·7–23·4)
1-22	Non-venomous animal contact	22,600 (17,800–29,600)	21,100 (15,600–29,800)	23,200 (16,800–33,700)	15·2 (11·8–20·2)	9·8 (7·2–13·7)	9·4 (6·8–13·6)
1-23	Foreign body	524,000 (483,000–563,000)	418,000 (374,000–464,000)	375,000 (309,000–451,000)	266 (247–284)	183 (164–203)	159 (130–192)
1-24	Pulmonary aspiration and foreign body in airway	492,000 (454,000–528,000)	382,000 (342,000–425,000)	331,000 (268,000–407,000)	245 (228–261)	167 (150–186)	141 (113–175)
1-25	Foreign body in eyes	11,500 (5,660–20,000)	17,600 (9,180–29,900)	20,100 (10,700–33,900)	8·1 (4·4–13·5)	8 (4·2–13·3)	7·9 (4·2–13·3)
1-26	Foreign body in other body part	20,000 (15,200–25,800)	18,600 (14,100–24,500)	24,000 (18,000–31,700)	12·9 (9·8–16·5)	8·3 (6·3–10·9)	9·6 (7·2–12·6)
1-27	Environmental heat and cold exposure	40,000 (32,500–50,300)	26,500 (20,500–34,100)	36,900 (27,300–49,400)	29·2 (23·8–36·4)	12·4 (9·6–16)	14·8 (10·9–19·7)
1-28	Exposure to forces of nature	28,100 (22,200–35,900)	94,500 (82,500–108,000)	23,600 (17,000–33,100)	17·5 (13·8–22·1)	41 (35·7–46·9)	9·3 (6·7–12·9)
1-29	Other unintentional injuries	360,000 (334,000–393,000)	279,000 (252,000–314,000)	260,000 (220,000–307,000)	224 (207–246)	120 (109–136)	101 (85·7–120)
1-30	Self-harm and interpersonal violence	3,840,000 (3,730,000–4,000,000)	4,870,000 (4,740,000–5,000,000)	5,090,000 (4,370,000–5,900,000)	2,360 (2,280–2,450)	2,030 (1,980–2,090)	1,940 (1,660–2,240)
1-31	Self-harm	375,000 (365,000–387,000)	718,000 (700,000–739,000)	797,000 (688,000–920,000)	238 (232–245)	301 (294–310)	303 (262–350)
1-32	Self-harm by firearm	92,800 (74,200–101,000)	120,000 (110,000–155,000)	113,000 (90,300–154,000)	58·7 (47·2–63·7)	50·7 (46–65·3)	42·8 (34·5–58·7)
1-33	Self-harm by other specified means	282,000 (271,000–304,000)	598,000 (565,000–619,000)	685,000 (591,000–791,000)	179 (172–193)	250 (236–259)	260 (225–301)
1-34	Interpersonal violence	3,120,000 (3,060,000–3,190,000)	3,990,000 (3,890,000–4,090,000)	4,180,000 (3,560,000–4,870,000)	1,920 (1,880–1,970)	1,660 (1,620–1,710)	1,590 (1,360–1,850)
1-35	Physical violence by firearm	2,030,000 (1,990,000–2,070,000)	2,940,000 (2,870,000–3,000,000)	2,970,000 (2,510,000–3,490,000)	1,240 (1,220–1,260)	1,210 (1,180–1,240)	1,120 (949–1,320)
1-36	Physical violence by sharp object	459,000 (442,000–481,000)	490,000 (468,000–516,000)	587,000 (500,000–689,000)	290 (279–304)	207 (198–219)	224 (190–262)
1-37	Sexual violence	24,300 (16,100–34,600)	36,500 (24,500–51,500)	40,300 (27,000–57,400)	15·5 (10·4–22·1)	15·5 (10·5–21·9)	15·5 (10·3–22)
1-38	Physical violence by other means	606,000 (580,000–636,000)	526,000 (492,000–566,000)	584,000 (507,000–671,000)	380 (362–398)	227 (212–245)	228 (198–262)
1-39	Conflict and terrorism	348,000 (258,000–485,000)	129,000 (88,600–195,000)	78,400 (45,500–132,000)	195 (144–273)	57 (38·7–85·9)	30·6 (17·8–51·4)
1-40	Executions and police conflict	2,500 (2,220–2,820)	28,800 (24,900–33,400)	32,000 (19,300–48,600)	1·4 (1·2–1·5)	11·6 (10·1–13·4)	12 (7·3–18·1)

SR Table 1: All-age DALYs counts and age standardized DALYs rates per 100,000 individuals for all causes and level 1 cause groups by location for 1990, 2010, and 2019, both sexes combined, Colombia

Cause Level	Cause	All-age DALYs			Age-standardized rate of DALYs per 100,000		
		1990	2010	2019	1990	2010	2019
0-0	All causes	10,400,000 (9,550,000–11,400,000)	11,200,000 (10,000,000–12,500,000)	11,600,000 (9,540,000–13,900,000)	36,200 (33,300–39,500)	26,300 (23,700–29,200)	23,200 (19,100–27,800)
1-0	Communicable, maternal, neonatal, and nutritional disorders	2,840,000 (2,560,000–3,160,000)	1,710,000 (1,520,000–1,930,000)	1,330,000 (1,090,000–1,630,000)	7,230 (6,560–7,990)	4,040 (3,580–4,560)	3,050 (2,470–3,760)
1-1	HIV/AIDS and sexually transmitted infections	39,100 (35,800–44,000)	155,000 (149,000–164,000)	147,000 (137,000–158,000)	124 (113–141)	344 (330–363)	290 (271–313)
1-2	HIV/AIDS	26,000 (25,000–27,000)	143,000 (139,000–149,000)	135,000 (128,000–143,000)	85·9 (82·9–89·4)	318 (307–331)	267 (252–282)
1-3	HIV/AIDS - drug-susceptible tuberculosis	9,500 (6,270–11,800)	10,600 (7,060–16,000)	8,750 (5,460–13,300)	31·4 (20·7–39·2)	23·5 (15·6–35·4)	17·3 (10·8–26·2)
1-4	HIV/AIDS - Multidrug-resistant Tuberculosis without extensive drug resistance	54 (5–224)	1,010 (281–2,440)	941 (155–2,830)	0·2 (0–0·7)	2·2 (0·6–5·4)	1·9 (0·3–5·6)
1-5	HIV/AIDS - Extensively drug-resistant Tuberculosis	0 (0–0)	106 (28–262)	152 (24–470)	0 (0–0)	0·2 (0·1–0·6)	0·3 (0–0·9)
1-6	HIV/AIDS resulting in other diseases	16,400 (14,000–19,600)	132,000 (125,000–138,000)	125,000 (116,000–133,000)	54·3 (46·3–65)	292 (276–306)	247 (230–263)
1-7	Sexually transmitted infections excluding HIV	13,100 (9,980–18,200)	11,900 (7,740–19,400)	11,700 (7,030–20,000)	37·9 (27·5–55·8)	26·9 (17·6–43·7)	23·7 (14·6–39·8)
1-8	Syphilis	6,640 (5,500–7,940)	2,930 (2,330–3,640)	1,940 (1,360–2,710)	15·9 (13·3–18·8)	7 (5·6–8·7)	4·6 (3·2–6·6)
1-9	Chlamydial infection	618 (432–898)	807 (549–1,170)	846 (575–1,230)	2 (1·4–2·8)	1·8 (1·2–2·5)	1·7 (1·1–2·4)
1-10	Gonococcal infection	608 (518–737)	704 (598–838)	689 (532–873)	2 (1·8–2·4)	1·5 (1·3–1·8)	1·4 (1–1·7)
1-11	Trichomoniasis	2,170 (849–4,480)	3,310 (1,300–7,020)	3,640 (1,430–7,410)	7·3 (2·8–15·2)	7·3 (2·9–15·5)	7·1 (2·8–14·5)
1-12	Genital herpes	1,830 (639–4,420)	2,610 (855–6,470)	3,040 (959–7,520)	6·7 (2·3–16·3)	5·9 (1·9–14·6)	5·9 (1·9–14·6)
1-13	Other sexually transmitted infections	1,240 (924–1,650)	1,550 (1,120–2,080)	1,560 (1,130–2,120)	4 (3–5·4)	3·4 (2·4–4·5)	3·1 (2·2–4·2)
1-14	Respiratory infections and tuberculosis	593,000 (529,000–666,000)	375,000 (334,000–425,000)	319,000 (250,000–400,000)	1,720 (1,570–1,890)	892 (795–1,010)	690 (537–874)
1-15	Tuberculosis	70,200 (65,900–74,400)	37,800 (35,600–40,500)	30,800 (24,200–39,100)	262 (248–276)	88·8 (83·5–94·9)	61·1 (47·8–77·5)
1-16	Latent tuberculosis infection	0 (0–0)	0 (0–0)	0 (0–0)	0 (0–0)	0 (0–0)	0 (0–0)
1-17	Drug-susceptible tuberculosis	69,900 (65,500–74,000)	34,900 (30,800–38,500)	28,100 (20,500–36,800)	261 (246–274)	81·9 (72·3–90·3)	55·6 (40·6–72·7)
1-18	Multidrug-resistant tuberculosis without extensive drug resistance	326 (33–1,380)	2,650 (860–5,770)	2,400 (380–7,220)	1·2 (0·1–5·2)	6·2 (2–13·6)	4·8 (0·8–14·2)
1-19	Extensively drug-resistant tuberculosis	0 (0–0)	275 (84–624)	380 (55–1,170)	0 (0–0)	0·6 (0·2–1·5)	0·8 (0·1–2·3)
1-20	Lower respiratory infections	469,000 (410,000–533,000)	271,000 (241,000–309,000)	225,000 (168,000–296,000)	1,300 (1,170–1,450)	656 (583–746)	492 (362–655)
1-21	Upper respiratory infections	40,900 (26,700–61,600)	52,300 (31,700–83,400)	50,000 (30,300–78,300)	117 (76–176)	117 (71·1–187)	108 (65·3–167)
1-22	Otitis media	13,200 (8,290–20,500)	13,700 (8,040–22,700)	13,100 (7,360–21,600)	37 (23·3–57·4)	30·4 (17·9–50·4)	28·8 (16·2–47·2)
1-23	Enteric infections	407,000 (350,000–474,000)	107,000 (87,700–129,000)	90,200 (69,600–116,000)	1,020 (891–1,180)	252 (207–304)	204 (157–261)
1-24	Diarrhoeal diseases	403,000 (347,000–469,000)	106,000 (86,900–129,000)	89,600 (69,100–115,000)	1,010 (878–1,160)	250 (205–302)	202 (156–258)
1-25	Typhoid and paratyphoid	3,010 (1,020–3,990)	245 (166–563)	168 (88–382)	8·8 (3·3–11·3)	0·6 (0·4–1·3)	0·3 (0·2–0·8)
1-26	Typhoid fever	2,970 (1,000–3,940)	213 (138–547)	147 (75–366)	8·7 (3·2–11·2)	0·5 (0·3–1·3)	0·3 (0·2–0·8)
1-27	Paratyphoid fever	46 (21–62)	32 (5–62)	21 (3–55)	0·2 (0·1–0·2)	0·1 (0–0·1)	0 (0–0·1)
1-28	iNTS	849 (542–1,380)	305 (187–984)	256 (137–797)	2·3 (1·4–3·5)	0·7 (0·4–2·3)	0·6 (0·3–1·9)
1-29	Other intestinal infectious diseases	488 (256–754)	297 (153–454)	211 (118–327)	1·2 (0·7–1·9)	0·7 (0·3–1·1)	0·5 (0·3–0·8)
1-30	Neglected tropical diseases and malaria	138,000 (78,500–243,000)	82,400 (65,400–102,000)	64,300 (50,300–79,900)	392 (232–667)	189 (150–234)	141 (112–173)
1-31	Malaria	89,600 (33,000–198,000)	13,400 (5,560–26,400)	3,830 (992–9,450)	238 (88·4–528)	30·1 (12·5–59·6)	8·5 (2·1–21)
1-32	Chagas disease	2,620 (1,970–4,190)	5,230 (2,470–6,620)	5,430 (2,530–8,180)	12·3 (9·1–21·3)	12·7 (6·1–15·9)	10·3 (4·8–15·5)
1-33	Leishmaniasis	1,370 (5–8,350)	2,090 (868–5,190)	1,160 (58–3,680)	3·9 (0–21·6)	4·8 (2–11·9)	2·5 (0·1–8·4)
1-34	Visceral leishmaniasis	989 (2–8,020)	442 (1–3,490)	328 (1–2,470)	2·6 (0–20·5)	1 (0–7·9)	0·8 (0–6)
1-35	Cutaneous and mucocutaneous leishmaniasis	377 (0–1,950)	1,650 (795–2,880)	833 (21–3,110)	1·3 (0–6·8)	3·8 (1·8–6·7)	1·7 (0–6·5)
1-36	African trypanosomiasis	0 (0–0)	0 (0–0)	0 (0–0)	0 (0–0)	0 (0–0)	0 (0–0)
1-37	Schistosomiasis	0 (0–0)	0 (0–0)	0 (0–0)	0 (0–0)	0 (0–0)	0 (0–0)
1-38	Cysticercosis	12,100 (7,450–18,400)	12,300 (6,890–20,200)	13,600 (7,140–22,800)	49·7 (30–76)	29·4 (16·4–48·1)	26·1 (13·7–43·7)
1-39	Cystic echinococcosis	50 (26–74)	25 (15–36)	18 (11–27)	0·2 (0·1–0·2)	0·1 (0–0·1)	0 (0–0·1)
1-40	Lymphatic filariasis	0 (0–0)	0 (0–0)	0 (0–0)	0 (0–0)	0 (0–0)	0 (0–0)
1-41	Onchocerciasis	58 (13–103)	0 (0–0)	0 (0–0)	0·2 (0–0·4)	0 (0–0)	0 (0–0)
1-42	Trachoma	237 (128–412)	141 (74–237)	129 (71–222)	1·5 (0·8–2·6)	0·4 (0·2–0·6)	0·2 (0·1–0·4)
1-43	Dengue	4,480 (2,670–7,620)	29,700 (24,000–38,400)	24,600 (19,000–29,800)	12·9 (7·5–22·3)	67·7 (54·7–86·9)	57·7 (44·3–69·9)
1-44	Yellow fever	622 (133–1,770)	276 (60–795)	171 (35–509)	1·7 (0·4–4·9)	0·6 (0·1–1·7)	0·4 (0·1–1·1)
1-45	Rabies	136 (107–171)	36 (27–51)	22 (14–37)	0·4 (0·3–0·5)	0·1 (0·1–0·1)	0 (0–0·1)
1-46	Intestinal nematode infections	14,900 (8,580–23,700)	13,400 (7,190–22,600)	9,770 (5,450–16,500)	40·7 (23·6–64·9)	29·7 (16·1–50·2)	21·5 (12–36·3)
1-47	Ascariasis	6,630 (3,840–11,000)	8,200 (4,160–15,100)	5,450 (2,720–9,770)	18 (10·4–30)	18·4 (9·3–33·7)	12·3 (6·2–22)
1-48	Trichuriasis	4,490 (2,180–8,110)	1,180 (556–2,170)	1,130 (524–2,090)	12·1 (5·9–21·8)	2·6 (1·2–4·7)	2·5 (1·2–4·6)

1-49	Hookworm disease	3,770 (1,970–6,450)	4,000 (2,050–6,850)	3,180 (1,620–5,600)	10·6 (5·6–18·3)	8·8 (4·5–15·1)	6·7 (3·4–11·7)
1-50	Food-borne trematodiases	0 (0–0)	0 (0–0)	0 (0–0)	0 (0–0)	0 (0–0)	0 (0–0)
1-51	Leprosy	27 (17–42)	41 (26–63)	47 (29–72)	0·1 (0·1–0·2)	0·1 (0·1–0·1)	0·1 (0·1–0·1)
1-52	Ebola virus disease	0 (0–0)	0 (0–0)	0 (0–0)	0 (0–0)	0 (0–0)	0 (0–0)
1-53	Zika virus disease	0 (0–0)	0 (0–0)	22 (7–57)	0 (0–0)	0 (0–0)	0·1 (0–0·1)
1-54	Guinea worm disease	0 (0–0)	0 (0–0)	0 (0–0)	0 (0–0)	0 (0–0)	0 (0–0)
1-55	Other neglected tropical diseases	11,500 (7,500–15,300)	5,820 (3,980–10,400)	5,480 (3,680–9,230)	30·4 (20–40·6)	13·5 (9·2–24·4)	13·1 (8·7–22·4)
1-56	Other infectious diseases	151,000 (132,000–171,000)	79,500 (70,600–89,600)	63,700 (49,600–80,300)	387 (343–435)	184 (163–208)	144 (112–182)
1-57	Meningitis	90,800 (78,500–107,000)	33,000 (29,000–37,800)	20,200 (14,900–26,500)	228 (200–265)	76·5 (67·2–88)	45·9 (33·6–60·8)
1-58	Encephalitis	14,300 (12,400–17,300)	19,600 (16,900–22,200)	17,400 (13,100–22,400)	37·9 (33·2–46)	44·9 (38·6–51)	39·5 (29·5–50·9)
1-59	Diphtheria	410 (317–532)	14 (8–27)	13 (5–24)	1·1 (0·8–1·4)	0 (0–0·1)	0 (0–0)
1-60	Whooping cough	5,230 (1,470–7,500)	2,030 (1,140–2,940)	2,270 (891–4,270)	12 (3·4–17·1)	4·8 (2·7–7)	5·9 (2·3–11)
1-61	Tetanus	12,500 (9,780–15,300)	1,440 (1,070–2,520)	724 (372–1,700)	32·4 (25·7–39·1)	3·4 (2·5–5·9)	1·6 (0·8–3·6)
1-62	Measles	3,730 (98–5,820)	0 (0–0)	4 (3–10)	8·8 (0·2–13·8)	0 (0–0)	0 (0–0)
1-63	Varicella and herpes zoster	3,270 (2,430–6,940)	4,110 (3,090–6,800)	4,190 (2,930–6,440)	9·8 (7·4–19·2)	9·6 (7·2–16)	9·2 (6·4–14·7)
1-64	Acute hepatitis	3,670 (2,850–4,690)	3,310 (2,410–4,480)	2,950 (2,110–4,040)	10·1 (7·9–12·8)	7·3 (5·4–9·9)	6·4 (4·5–8·7)
1-65	Acute hepatitis A	2,490 (1,900–3,140)	2,160 (1,540–2,960)	1,870 (1,300–2,620)	6·5 (5–8·2)	4·7 (3·4–6·4)	4·1 (2·9–5·8)
1-66	Acute hepatitis B	736 (470–1,100)	886 (576–1,370)	842 (547–1,280)	2·3 (1·5–3·5)	2 (1·3–3·1)	1·7 (1·1–2·6)
1-67	Acute hepatitis C	347 (151–507)	164 (111–267)	130 (81–217)	1 (0·5–1·5)	0·4 (0·3–0·6)	0·3 (0·2–0·5)
1-68	Acute hepatitis E	100 (62–149)	106 (69–159)	108 (71–164)	0·3 (0·2–0·4)	0·2 (0·2–0·4)	0·2 (0·1–0·3)
1-69	Other unspecified infectious diseases	16,900 (11,200–21,200)	16,100 (12,400–21,500)	15,900 (11,400–22,300)	47·3 (32·3–58·5)	37·3 (28·7–50)	35·7 (25·3–50·1)
1-70	Maternal and neonatal disorders	1,300,000 (1,160,000–1,460,000)	783,000 (662,000–915,000)	543,000 (408,000–710,000)	2,980 (2,650–3,340)	1,870 (1,580–2,190)	1,340 (998–1,770)
1-71	Maternal disorders	51,000 (45,700–56,700)	32,800 (30,600–35,500)	31,800 (24,400–40,900)	143 (129–158)	70 (65·3–75·7)	63·3 (48·5–81·2)
1-72	Maternal haemorrhage	11,300 (9,410–13,700)	5,550 (4,570–6,700)	4,340 (3,250–5,730)	32 (26·8–38·5)	12 (9·9–14·5)	8·6 (6·5–11·4)
1-73	Maternal sepsis and other pregnancy related infections	5,320 (4,330–6,570)	2,660 (2,110–3,330)	2,110 (1,480–2,910)	14·7 (11·9–18·1)	5·6 (4·5–7)	4·2 (2·9–5·8)
1-74	Maternal hypertensive disorders	13,400 (11,600–15,300)	6,940 (6,210–7,720)	5,910 (4,320–7,760)	37 (32·2–42·1)	14·8 (13·2–16·4)	11·8 (8·6–15·5)
1-75	Maternal obstructed labour and uterine rupture	788 (566–1,070)	816 (610–1,060)	807 (571–1,140)	2·2 (1·6–2·9)	1·7 (1·3–2·3)	1·6 (1·1–2·3)
1-76	Maternal abortive outcome	6,670 (5,160–8,500)	2,010 (1,510–2,600)	1,550 (1,070–2,120)	18·7 (14·6–23·6)	4·3 (3·2–5·5)	3·1 (2·1–4·2)
1-77	Ectopic pregnancy	830 (672–1,010)	619 (515–738)	596 (414–816)	2·3 (1·9–2·7)	1·3 (1·1–1·6)	1·2 (0·8–1·6)
1-78	Indirect maternal deaths	7,010 (5,790–8,420)	8,810 (7,790–9,900)	8,840 (6,330–12,000)	19·5 (16·2–23·3)	18·8 (16·6–21·1)	17·6 (12·6–23·7)
1-79	Late maternal deaths	1,850 (1,370–2,450)	2,140 (1,750–2,600)	4,500 (2,740–8,070)	5·2 (3·9–6·8)	4·6 (3·8–5·5)	8·9 (5·4–16)
1-80	Maternal deaths aggravated by HIV/AIDS	2 (1–4)	15 (7–25)	26 (12–47)	0 (0–0)	0 (0–0·1)	0·1 (0–0·1)
1-81	Other maternal disorders	3,890 (3,100–4,870)	3,240 (2,650–3,890)	3,160 (2,270–4,270)	11 (8·9–13·7)	6·9 (5·7–8·3)	6·3 (4·5–8·5)
1-82	Neonatal disorders	1,250,000 (1,110,000–1,410,000)	750,000 (629,000–881,000)	511,000 (381,000–671,000)	2,840 (2,510–3,200)	1,800 (1,500–2,110)	1,280 (946–1,690)
1-83	Neonatal preterm birth	632,000 (552,000–720,000)	342,000 (282,000–405,000)	206,000 (149,000–280,000)	1,430 (1,250–1,630)	820 (674–974)	516 (369–708)
1-84	Neonatal encephalopathy due to birth asphyxia and trauma	391,000 (333,000–453,000)	118,000 (95,000–145,000)	73,600 (49,000–106,000)	876 (745–1,020)	285 (230–351)	190 (124–275)
1-85	Neonatal sepsis and other neonatal infections	93,300 (77,800–112,000)	164,000 (129,000–210,000)	148,000 (108,000–195,000)	217 (179–264)	387 (307–490)	356 (258–470)
1-86	Hemolytic disease and other neonatal jaundice	33,400 (27,900–39,400)	14,300 (11,600–17,400)	11,600 (8,940–14,800)	79·9 (66·9–93·9)	32·8 (26·5–39·6)	25·8 (19·7–32·8)
1-87	Other neonatal disorders	104,000 (86,000–125,000)	112,000 (91,500–138,000)	72,300 (48,400–104,000)	233 (193–280)	272 (222–336)	190 (126–271)
1-88	Nutritional deficiencies	209,000 (172,000–255,000)	132,000 (108,000–160,000)	108,000 (82,000–137,000)	609 (498–740)	312 (255–378)	242 (185–309)
1-89	Protein-energy malnutrition	120,000 (104,000–138,000)	78,500 (66,500–93,000)	56,600 (41,100–75,300)	329 (290–371)	188 (159–222)	133 (94·2–179)
1-90	Iodine deficiency	3,390 (1,660–5,550)	2,550 (1,160–4,920)	4,580 (2,260–7,620)	10·7 (5·4–17·4)	5·6 (2·5–10·7)	9·3 (4·6–15·4)
1-91	Vitamin A deficiency	3,610 (2,210–5,600)	2,080 (1,290–3,130)	1,880 (1,150–2,830)	9·2 (5·7–14·3)	4·7 (2·9–7·1)	4·3 (2·6–6·5)
1-92	Dietary iron deficiency	76,300 (46,200–116,000)	46,200 (28,500–70,500)	41,800 (25,600–64,400)	239 (150–360)	108 (66·4–165)	90·2 (55·2–140)
1-93	Other nutritional deficiencies	6,230 (5,030–8,940)	2,830 (2,230–3,810)	2,560 (1,820–3,520)	20·6 (16·8–31·7)	6·6 (5·2–8·9)	5·3 (3·7–7·2)
1-0	Non-communicable diseases	4,880,000 (4,270,000–5,570,000)	7,080,000 (6,110,000–8,190,000)	8,180,000 (6,750,000–9,940,000)	20,900 (18,700–23,400)	17,000 (14,800–19,600)	16,000 (13,200–19,500)
1-1	Neoplasms	704,000 (684,000–733,000)	1,070,000 (1,030,000–1,110,000)	1,240,000 (963,000–1,550,000)	3,340 (3,240–3,450)	2,600 (2,500–2,710)	2,400 (1,870–3,010)
1-2	Lip and oral cavity cancer	7,520 (7,160–7,870)	9,870 (9,320–10,400)	11,900 (9,070–15,200)	39·5 (37·5–41·4)	24·7 (23·3–26)	22·5 (17·2–28·9)
1-3	Nasopharynx cancer	2,040 (1,890–2,180)	2,310 (2,140–2,480)	2,480 (1,850–3,260)	9·1 (8·4–9·7)	5·5 (5·1–6)	4·8 (3·6–6·2)
1-4	Other pharynx cancer	2,230 (2,060–2,390)	2,590 (2,400–2,800)	3,220 (2,390–4,250)	11·6 (10·7–12·4)	6·4 (6–7)	6·1 (4·5–8)
1-5	Oesophageal cancer	17,100 (16,300–17,900)	18,400 (17,300–19,300)	21,700 (16,300–28,100)	95·4 (90·7–100)	47 (44·2–49·3)	41·2 (30·8–53·5)
1-6	Stomach cancer	125,000 (121,000–130,000)	148,000 (142,000–154,000)	154,000 (118,000–197,000)	671 (646–695)	368 (350–382)	294 (225–375)
1-7	Colon and rectum cancer	39,900 (38,400–41,400)	92,900 (88,500–96,600)	117,000 (90,000–150,000)	209 (200–218)	231 (219–240)	222 (171–285)

1-8	Liver cancer	14,800 (14,100–15,600)	22,300 (21,100–23,300)	31,000 (23,400–40,300)	73·4 (69·8–77·1)	56·2 (53·3–58·9)	59·8 (45·2–77·5)
1-9	Liver cancer due to hepatitis B	3,710 (2,880–4,730)	4,780 (3,570–6,270)	6,200 (3,960–9,330)	16·9 (12·7–22·3)	11·6 (8·6–15·5)	11·8 (7·5–17·7)
1-10	Liver cancer due to hepatitis C	4,310 (3,220–5,400)	6,990 (5,230–8,860)	10,200 (6,880–14,500)	24·7 (18·7–30·9)	18·2 (13·5–23·1)	19·3 (13–27·4)
1-11	Liver cancer due to alcohol use	3,850 (2,790–4,950)	6,540 (4,820–8,430)	9,620 (6,270–13,700)	20·6 (14·8–26·5)	16·7 (12·3–21·5)	18·4 (12–26·2)
1-12	Liver cancer due to NASH	811 (581–1,120)	1,490 (1,060–2,070)	2,260 (1,440–3,420)	4·2 (2·9–6)	3·8 (2·7–5·3)	4·3 (2·7–6·5)
1-13	Gallbladder and biliary tract cancer	18,000 (15,700–20,600)	23,300 (21,200–30,200)	27,300 (20,500–37,700)	98·8 (86·4–113)	59·1 (53·6–76·6)	51·7 (38·8–71·3)
1-14	Pancreatic cancer	22,900 (21,900–24,000)	39,200 (37,300–41,000)	54,500 (41,800–69,800)	124 (118–130)	98·8 (93·9–103)	103 (79·2–132)
1-15	Larynx cancer	10,400 (9,730–11,000)	10,600 (9,950–11,400)	11,800 (8,880–15,500)	56·2 (52·8–59·4)	27·1 (25·4–29·1)	22·4 (16·9–29·4)
1-16	Tracheal, bronchus, and lung cancer	69,900 (67,300–72,500)	112,000 (107,000–117,000)	136,000 (106,000–176,000)	378 (363–392)	286 (272–297)	259 (202–334)
1-17	Malignant skin melanoma	4,860 (4,200–7,860)	9,120 (8,020–14,300)	11,600 (7,970–19,300)	22·2 (19·4–35·2)	22 (19·3–34·4)	22·3 (15·4–37·5)
1-18	Non-melanoma skin cancer	4,320 (3,990–4,560)	8,010 (7,330–8,470)	10,000 (7,800–12,800)	23·9 (21·7–25·3)	20·5 (18·6–21·7)	18·7 (14·5–23·9)
1-19	Non-melanoma skin cancer (squamous-cell carcinoma)	4,310 (3,990–4,560)	8,010 (7,320–8,460)	10,000 (7,790–12,800)	23·9 (21·7–25·3)	20·5 (18·6–21·7)	18·7 (14·5–23·9)
1-20	Non-melanoma skin cancer (basal-cell carcinoma)	3 (1–6)	6 (3–12)	8 (4–16)	0 (0–0)	0 (0–0)	0 (0–0)
1-21	Breast cancer	47,400 (45,400–49,700)	86,000 (81,800–89,900)	102,000 (78,300–133,000)	226 (216–236)	203 (194–212)	194 (149–252)
1-22	Cervical cancer	55,500 (51,800–61,600)	69,700 (65,600–75,100)	73,600 (55,300–96,600)	253 (237–280)	163 (154–176)	141 (106–185)
1-23	Uterine cancer	4,550 (4,240–4,860)	8,050 (7,430–8,680)	11,100 (8,540–14,300)	24·2 (22·4–25·9)	20·3 (18·7–21·8)	21 (16·2–27·1)
1-24	Ovarian cancer	15,900 (15,000–17,000)	29,000 (27,300–35,500)	38,200 (29,200–52,300)	74·6 (70·2–79·1)	69 (64·7–84·9)	72·9 (55·9–100)
1-25	Prostate cancer	28,100 (22,900–36,300)	58,100 (49,400–84,600)	75,400 (54,200–117,000)	181 (148–236)	156 (133–228)	143 (103–220)
1-26	Testicular cancer	3,850 (3,350–5,090)	6,200 (5,500–7,640)	7,370 (5,370–9,980)	11·5 (10·1–14·3)	13·6 (12·1–17)	14·7 (10·6–20)
1-27	Kidney cancer	8,140 (7,560–8,750)	16,300 (15,400–17,300)	20,200 (15,300–26,300)	34·4 (32·3–36·7)	39·8 (37·5–42·1)	40·1 (30·5–51·8)
1-28	Bladder cancer	6,880 (6,460–7,350)	10,900 (10,200–11,500)	13,400 (10,200–17,400)	40·1 (37·5–43·1)	28·4 (26·4–29·9)	25·4 (19·4–32·8)
1-29	Brain and nervous system cancer	27,500 (24,800–38,100)	45,900 (32,800–50,700)	49,000 (31,300–66,400)	95·6 (86·9–130)	106 (75·4–117)	101 (65·2–137)
1-30	Thyroid cancer	5,340 (5,010–5,690)	7,590 (7,060–8,640)	9,420 (7,230–12,000)	26·7 (25–28·4)	18·9 (17·6–21·4)	17·9 (13·8–23)
1-31	Mesothelioma	1,080 (946–1,280)	2,080 (1,870–2,280)	2,580 (1,930–3,450)	5·1 (4·4–6)	5 (4·5–5·5)	4·9 (3·7–6·5)
1-32	Hodgkin lymphoma	7,760 (6,880–9,340)	6,970 (6,130–8,530)	6,670 (4,860–8,970)	26·5 (23·5–31·7)	15·9 (14–19·4)	13·3 (9·7–17·9)
1-33	Non-Hodgkin's lymphoma	26,000 (24,600–27,600)	40,300 (38,300–42,500)	45,200 (35,100–58,200)	101 (95·4–106)	95·7 (90·9–101)	89·4 (69·5–115)
1-34	Multiple myeloma	5,300 (4,940–6,570)	12,300 (11,400–16,200)	17,300 (12,900–24,400)	28·3 (26·3–35·1)	30·8 (28·4–40·6)	32·9 (24·5–46·2)
1-35	Leukaemia	75,300 (70,700–80,400)	93,500 (87,700–99,400)	89,200 (69,100–112,000)	232 (220–245)	213 (200–226)	190 (148–238)
1-36	Acute lymphoid leukaemia	30,400 (26,800–34,600)	42,500 (37,900–46,800)	38,000 (28,500–48,800)	81·1 (72–91·9)	94·5 (84·4–104)	85·6 (64·1–110)
1-37	Chronic lymphoid leukaemia	1,300 (1,130–1,440)	2,430 (2,190–2,760)	2,880 (2,090–3,860)	7 (6·1–7·8)	6·2 (5·6–7·1)	5·5 (4–7·4)
1-38	Acute myeloid leukaemia	11,600 (10,300–15,600)	23,300 (20,100–26,100)	24,800 (18,600–32,500)	36·8 (33·1–49·2)	53·1 (45·4–59·4)	51·8 (38·9–67·8)
1-39	Chronic myeloid leukaemia	4,150 (3,620–4,520)	5,850 (5,410–6,650)	5,410 (4,030–7,140)	16·5 (14·5–18)	13·7 (12·7–15·6)	10·6 (7·9–14)
1-40	Other leukaemia	27,800 (23,400–30,000)	19,400 (17,900–22,800)	18,100 (13,700–23,700)	90·4 (78·2–96·5)	45·3 (41·7–53)	36·8 (27·8–48·3)
1-41	Other neoplasms	3,450 (2,560–6,340)	9,480 (7,330–11,500)	12,100 (8,710–15,900)	14·4 (10·7–26·4)	23·1 (17·9–28)	24·1 (17·3–31·7)
1-42	Myelodysplastic, myeloproliferative, and other hematopoietic neoplasms	3,450 (2,560–6,340)	9,480 (7,330–11,500)	12,100 (8,710–15,900)	14·4 (10·7–26·4)	23·1 (17·9–28)	24·1 (17·3–31·7)
1-43	Benign and in situ intestinal neoplasms	0 (0–0)	0 (0–0)	0 (0–0)	0 (0–0)	0 (0–0)	0 (0–0)
1-44	Benign and in situ cervical and uterine neoplasms	0 (0–0)	0 (0–0)	0 (0–0)	0 (0–0)	0 (0–0)	0 (0–0)
1-45	Other benign and in situ neoplasms	0 (0–0)	0 (0–0)	0 (0–0)	0 (0–0)	0 (0–0)	0 (0–0)
1-46	Cardiovascular diseases	995,000 (962,000–1,020,000)	1,190,000 (1,120,000–1,240,000)	1,350,000 (1,080,000–1,680,000)	5,480 (5,250–5,640)	3,040 (2,850–3,170)	2,540 (2,040–3,170)
1-47	Rheumatic heart disease	17,300 (15,700–19,100)	8,040 (7,220–8,980)	6,220 (4,710–8,120)	69 (63·2–75·7)	19 (17–21·2)	12·1 (9·2–15·7)
1-48	Ischaemic heart disease	496,000 (477,000–516,000)	640,000 (603,000–665,000)	717,000 (568,000–902,000)	2,840 (2,710–2,980)	1,650 (1,550–1,720)	1,350 (1,060–1,690)
1-49	Stroke	305,000 (292,000–319,000)	319,000 (300,000–336,000)	349,000 (277,000–436,000)	1,620 (1,530–1,700)	806 (754–849)	661 (524–827)
1-50	Ischaemic stroke	112,000 (104,000–120,000)	116,000 (104,000–126,000)	137,000 (110,000–167,000)	689 (639–738)	309 (276–333)	257 (207–313)
1-51	Intracerebral hemorrhage	139,000 (133,000–146,000)	118,000 (111,000–124,000)	118,000 (91,600–151,000)	707 (670–743)	295 (277–310)	224 (174–286)
1-52	Subarachnoid hemorrhage	54,100 (50,900–58,900)	85,400 (79,600–90,000)	93,600 (71,600–122,000)	222 (208–246)	203 (188–214)	180 (138–235)
1-53	Hypertensive heart disease	74,200 (42,200–81,100)	63,900 (55,800–101,000)	82,200 (62,200–131,000)	461 (260–506)	171 (148–267)	153 (115–245)
1-54	Non-rheumatic valvular heart disease	11,200 (10,700–11,800)	16,100 (15,200–16,900)	24,000 (18,200–30,700)	54·4 (51·6–57·3)	39·8 (37·6–42)	45·8 (34·7–58·7)
1-55	Non-rheumatic calcific aortic valve disease	7,200 (6,360–7,860)	9,850 (8,990–11,600)	14,500 (10,400–20,400)	35·3 (31·6–39·1)	24·5 (22·3–29)	27·7 (19·8–38·9)
1-56	Non-rheumatic degenerative mitral valve disease	3,990 (3,120–4,750)	6,090 (4,290–6,840)	9,190 (5,380–12,800)	18·9 (14·5–22·3)	15 (10·4–16·9)	17·5 (10·3–24·4)
1-57	Other non-rheumatic valve diseases	37 (24–73)	131 (56–186)	246 (78–445)	0·2 (0·1–0·3)	0·3 (0·1–0·5)	0·5 (0·2–0·9)
1-58	Cardiomyopathy and myocarditis	22,500 (19,700–25,400)	29,100 (24,800–33,200)	28,400 (21,500–37,900)	87·2 (77·2–97·9)	70·7 (59·8–80·5)	57·7 (43·2–77·2)
1-59	Myocarditis	2,430 (1,620–3,170)	3,980 (2,460–5,160)	3,620 (2,290–5,070)	6·6 (4·7–8·3)	9·3 (5·7–12·1)	8·1 (4·9–11·6)
1-60	Alcoholic cardiomyopathy	2,440 (1,750–2,780)	479 (400–719)	569 (385–871)	11 (7·8–12·5)	1·1 (0·9–1·7)	1·1 (0·7–1·7)

1-61	Other cardiomyopathy	17,600 (15,600–20,500)	24,600 (21,400–29,200)	24,200 (18,400–33,000)	69·7 (62·6–79·6)	60·3 (51·8–71·1)	48·5 (36·5–66)
1-62	Atrial fibrillation and flutter	10,200 (8,240–13,200)	26,300 (21,700–32,200)	38,500 (30,500–48,100)	69·8 (57–90·5)	71·5 (59–87·6)	70·5 (55·8–88·4)
1-63	Aortic aneurysm	13,500 (12,800–14,300)	22,700 (21,300–24,000)	30,200 (22,800–39,200)	72 (68·1–76·3)	57·7 (54–61·1)	57·6 (43·6–74·9)
1-64	Peripheral vascular disease	1,950 (1,150–3,100)	4,150 (2,520–6,660)	6,340 (3,690–11,000)	13·1 (7·8–20·9)	11·2 (6·8–18)	11·8 (6·9–20·2)
1-65	Endocarditis	5,690 (4,750–8,510)	12,900 (7,950–14,800)	14,400 (7,730–19,700)	20·3 (17–30·7)	30·5 (18·6–34·9)	28·7 (15·5–39·4)
1-66	Chronic respiratory diseases	222,000 (193,000–259,000)	337,000 (295,000–375,000)	388,000 (319,000–468,000)	1,050 (935–1,170)	854 (751–946)	755 (621–916)
1-67	Chronic obstructive pulmonary disease	119,000 (104,000–128,000)	236,000 (209,000–255,000)	285,000 (228,000–347,000)	708 (615–760)	623 (551–674)	532 (425–649)
1-68	Pneumoconiosis	1,690 (1,510–1,870)	1,700 (1,510–1,930)	1,990 (1,520–2,560)	9 (8–10)	4·2 (3·8–4·8)	3·8 (2·9–4·9)
1-69	Silicosis	982 (871–1,110)	855 (745–987)	965 (710–1,270)	5·2 (4·6–5·9)	2·1 (1·8–2·4)	1·8 (1·4–2·4)
1-70	Asbestosis	88 (48–116)	60 (46–84)	80 (54–125)	0·5 (0·3–0·7)	0·2 (0·1–0·2)	0·2 (0·1–0·2)
1-71	Coal workers pneumoconiosis	308 (243–366)	451 (377–531)	507 (331–720)	1·6 (1·2–1·9)	1·1 (0·9–1·3)	1 (0·6–1·4)
1-72	Other pneumoconiosis	315 (240–385)	338 (257–440)	441 (323–592)	1·7 (1·2–2·1)	0·8 (0·6–1·1)	0·8 (0·6–1·1)
1-73	Asthma	90,100 (65,400–125,000)	70,900 (49,200–102,000)	68,800 (45,600–101,000)	298 (228–395)	160 (112–230)	153 (101–230)
1-74	Interstitial lung disease and pulmonary sarcoidosis	2,510 (1,700–3,960)	11,000 (5,630–14,800)	13,200 (6,570–20,300)	12·1 (8–19·6)	26·7 (13·8–35·9)	25·5 (12·7–39·7)
1-75	Other chronic respiratory diseases	7,840 (6,560–10,700)	17,000 (14,300–19,700)	19,300 (14,100–24,500)	24·7 (21·1–33·4)	39·3 (32·5–45·6)	40·4 (29·7–51·4)
1-76	Digestive diseases	272,000 (244,000–308,000)	367,000 (325,000–422,000)	404,000 (322,000–495,000)	1,150 (1,050–1,290)	885 (788–1,010)	787 (629–966)
1-77	Cirrhosis and other chronic liver diseases	67,800 (63,400–71,600)	90,200 (84,800–98,600)	96,300 (72,900–125,000)	310 (292–326)	221 (208–242)	185 (140–240)
1-78	Cirrhosis and other chronic liver diseases due to hepatitis B	3,750 (2,610–5,130)	3,430 (2,400–4,810)	3,090 (1,960–4,670)	17·7 (12·3–24·3)	8·2 (5·8–11·6)	5·9 (3·7–8·9)
1-79	Cirrhosis and other chronic liver diseases due to hepatitis C	15,300 (11,800–19,400)	21,700 (16,600–28,000)	23,100 (15,600–33,000)	72·5 (55·3–93·2)	52·7 (40·2–68·4)	44 (29·7–62·9)
1-80	Cirrhosis and other chronic liver diseases due to alcohol use	21,600 (17,400–25,900)	32,600 (26,000–39,000)	36,100 (25,300–50,400)	109 (88·4–131)	80·4 (63·8–97)	68·5 (48–95·1)
1-81	Cirrhosis and other chronic liver diseases due to NAFLD	9,940 (7,200–13,200)	16,000 (11,600–21,100)	18,200 (12,100–26,400)	50·7 (36·7–68)	40·1 (29·2–53)	34·6 (23·1–50·1)
1-82	Cirrhosis and other chronic liver diseases due to other causes	17,100 (14,500–20,000)	16,500 (13,300–20,300)	15,800 (11,300–21,500)	59·8 (49–71·6)	39·8 (31·9–49·3)	31·9 (23·2–43·1)
1-83	Upper digestive system diseases	72,200 (55,100–99,100)	83,600 (55,900–127,000)	93,500 (60,400–145,000)	320 (255–422)	197 (134–296)	180 (116–279)
1-84	Peptic ulcer disease	33,800 (31,300–36,000)	20,200 (18,300–22,600)	19,200 (14,700–25,200)	172 (161–183)	51 (46–57·2)	36·4 (27·9–47·9)
1-85	Gastritis and duodenitis	5,680 (4,300–7,490)	9,410 (7,120–12,200)	10,500 (7,840–13,900)	24·7 (19·3–31·8)	22·8 (17·4–29·1)	20·3 (15·1–26·9)
1-86	GERD	32,700 (17,000–58,000)	54,000 (28,100–95,200)	63,800 (33,200–113,000)	123 (63·9–218)	123 (64·2–218)	123 (63·9–218)
1-87	Appendicitis	9,380 (7,800–12,800)	12,800 (10,200–14,800)	11,700 (8,400–15,400)	32·3 (27·2–42·6)	29·5 (23·4–34)	23·9 (17·2–31·5)
1-88	Paralytic ileus and intestinal obstruction	32,900 (28,200–38,600)	37,000 (30,900–41,600)	36,900 (27,800–48,000)	104 (92·3–124)	90·8 (75·4–102)	77·1 (58–100)
1-89	Inguinal, femoral, and abdominal hernia	22,800 (17,300–29,200)	28,500 (21,600–37,700)	32,300 (23,700–42,800)	91·2 (71·6–115)	67·9 (51·8–88·7)	64·6 (47·5–85·4)
1-90	Inflammatory bowel disease	3,220 (2,690–3,800)	5,580 (4,720–6,530)	6,420 (4,990–7,990)	12·9 (10·9–15·2)	13·3 (11·3–15·5)	12·5 (9·8–15·6)
1-91	Vascular intestinal disorders	7,800 (7,240–8,340)	15,800 (14,500–17,500)	19,000 (14,500–24,400)	41·9 (38·7–44·7)	41 (37·4–45·5)	36·3 (27·7–46·7)
1-92	Gallbladder and biliary diseases	37,500 (27,900–50,500)	62,000 (46,900–82,600)	70,900 (52,000–94,300)	162 (124–213)	147 (112–194)	136 (100–182)
1-93	Pancreatitis	10,000 (9,250–10,900)	13,100 (12,100–15,100)	14,700 (11,200–19,000)	42·7 (39·6–46)	31·4 (29·1–36)	28·3 (21·7–36·8)
1-94	Other digestive diseases	8,780 (7,240–11,900)	18,300 (11,900–22,200)	22,200 (13,900–31,300)	37·9 (30·4–50·4)	45·4 (29·1–54·9)	43·2 (27·3–61·1)
1-95	Neurological disorders	345,000 (179,000–592,000)	538,000 (287,000–898,000)	650,000 (358,000–1,050,000)	1,300 (728–2,180)	1,270 (703–2,110)	1,270 (686–2,050)
1-96	Alzheimer's disease and other dementias	42,900 (17,600–99,600)	122,000 (51,700–271,000)	188,000 (80,800–410,000)	329 (136–753)	340 (144–749)	336 (143–739)
1-97	Parkinson's disease	9,260 (8,570–9,970)	23,700 (21,400–25,800)	32,300 (26,100–39,500)	63·1 (58·1–67·7)	64·1 (57·9–69·7)	60·9 (49·2–74·8)
1-98	Idiopathic epilepsy	92,800 (44,900–166,000)	96,900 (46,000–175,000)	103,000 (46,600–186,000)	275 (132–500)	216 (102–389)	214 (96·7–390)
1-99	Multiple sclerosis	1,320 (1,130–1,810)	2,880 (2,220–3,490)	3,400 (2,430–4,560)	5·5 (4·7–7·5)	6·6 (5·1–8)	6·5 (4·7–8·7)
1-100	Motor neuron disease	1,870 (1,740–2,030)	5,290 (4,990–5,590)	7,270 (5,470–9,420)	7·5 (7–8)	12·7 (12–13·4)	14·3 (10·8–18·5)
1-101	Headache disorders	176,000 (29,800–395,000)	261,000 (48,300–581,000)	286,000 (55,700–633,000)	562 (107–1,230)	570 (107–1,260)	570 (108–1,260)
1-102	Migraine	161,000 (20,300–376,000)	237,000 (33,000–552,000)	259,000 (37,900–600,000)	510 (73·8–1,180)	518 (74·2–1,200)	517 (73·8–1,200)
1-103	Tension-type headache	15,200 (4,160–62,200)	23,500 (6,950–88,700)	26,600 (8,050–95,500)	52·2 (15·4–194)	52·5 (15·8–193)	52·5 (15·7–189)
1-104	Other neurological disorders	20,900 (12,100–34,300)	26,300 (15,900–42,500)	30,500 (17,600–49,200)	62·1 (38·3–99)	58·7 (36·2–93·7)	65·1 (36·9–107)
1-105	Mental disorders	452,000 (328,000–600,000)	633,000 (464,000–840,000)	733,000 (540,000–966,000)	1,480 (1,080–1,960)	1,400 (1,030–1,860)	1,460 (1,080–1,930)
1-106	Schizophrenia	49,900 (34,100–67,000)	80,300 (55,400–107,000)	92,700 (63,800–123,000)	177 (123–235)	179 (124–238)	180 (124–239)
1-107	Depressive disorders	114,000 (78,700–160,000)	168,000 (115,000–233,000)	188,000 (130,000–259,000)	406 (282–559)	377 (259–523)	367 (253–504)
1-108	Major depressive disorder	91,900 (61,600–131,000)	133,000 (91,600–183,000)	147,000 (101,000–202,000)	326 (221–458)	299 (205–411)	286 (196–394)
1-109	Dysthymia	22,600 (13,900–34,800)	34,700 (21,200–54,300)	41,600 (26,400–63,400)	80·5 (50·9–123)	78 (47·9–121)	81 (51·4–123)
1-110	Bipolar disorder	49,400 (29,700–76,500)	73,400 (44,900–113,000)	81,900 (49,900–127,000)	160 (98·2–248)	161 (98·8–250)	161 (97·8–250)
1-111	Anxiety disorders	138,000 (88,400–198,000)	174,000 (120,000–240,000)	225,000 (148,000–318,000)	441 (285–631)	383 (266–528)	452 (296–633)
1-112	Eating disorders	14,200 (8,520–21,500)	19,900 (12,300–30,000)	23,600 (14,400–35,900)	38·6 (23·4–58·5)	42·5 (26·2–63·7)	47·2 (28·8–71·5)
1-113	Anorexia nervosa	3,570 (2,050–5,900)	4,870 (2,800–7,910)	5,580 (3,190–9,190)	9·5 (5·5–15·5)	10·2 (5·9–16·4)	11·3 (6·5–18·5)

1-114	Bulimia nervosa	10,600 (6,000–17,100)	15,100 (8,820–23,900)	18,000 (10,400–28,600)	29·1 (16·6–46·4)	32·3 (18·8–51·1)	35·9 (20·7–56·8)
1-115	Autism spectrum disorders	17,600 (11,600–25,600)	23,200 (15,100–33,400)	24,100 (15,700–34,900)	51·3 (33·8–74·4)	51·4 (33·6–73·9)	51·5 (33·5–75·1)
1-116	Attention-deficit/hyperactivity disorder	5,810 (3,240–9,980)	7,280 (4,150–12,400)	7,100 (4,050–12,000)	15·4 (8·7–26·1)	15·5 (8·8–26·6)	15·6 (8·8–26·4)
1-117	Conduct disorder	27,100 (15,200–42,700)	31,800 (17,800–50,200)	28,000 (15,700–44,100)	67·2 (37·6–106)	65·8 (36·7–104)	65·9 (37·1–103)
1-118	Idiopathic developmental intellectual disability	6,760 (2,300–12,600)	8,340 (3,190–14,900)	6,340 (1,680–12,200)	19·5 (6·8–36·1)	18·4 (7–32·9)	13·6 (3·6–26·2)
1-119	Other mental disorders	28,600 (18,100–43,600)	47,400 (30,300–72,000)	55,900 (36,100–84,700)	108 (69·6–165)	108 (69·2–163)	108 (69·7–164)
1-120	Substance use disorders	111,000 (79,000–149,000)	158,000 (111,000–210,000)	176,000 (123,000–233,000)	356 (254–474)	345 (244–462)	344 (240–455)
1-121	Alcohol use disorders	68,600 (44,100–99,200)	98,300 (66,200–138,000)	113,000 (75,600–158,000)	227 (150–324)	218 (147–306)	221 (148–309)
1-122	Drug use disorders	42,900 (31,700–55,700)	59,200 (42,000–79,000)	62,600 (44,800–81,800)	129 (96·6–166)	128 (90·7–170)	123 (87·6–160)
1-123	Opioid use disorders	19,200 (12,600–27,500)	26,100 (17,100–37,400)	31,000 (20,500–43,900)	60·8 (41–85·2)	57·6 (37·6–82·3)	60·3 (39·7–85·7)
1-124	Cocaine use disorders	10,500 (7,600–14,300)	12,500 (8,280–18,000)	10,500 (6,730–15,200)	30·7 (22·6–40·9)	26·4 (17·5–37·8)	20·7 (13·2–30·1)
1-125	Amphetamine use disorders	2,720 (1,630–4,320)	2,990 (1,660–4,880)	3,400 (1,960–5,560)	7·7 (4·8–11·9)	6·4 (3·6–10·4)	6·6 (3·8–10·8)
1-126	Cannabis use disorders	3,170 (1,860–4,940)	7,200 (4,480–10,800)	8,400 (5,310–12,600)	8·8 (5·2–13·5)	15 (9·4–22·6)	16·8 (10·6–25·1)
1-127	Other drug use disorders	7,290 (4,780–10,500)	10,400 (6,750–15,100)	9,320 (5,990–13,200)	21·1 (14·2–30)	22·1 (14·4–32)	18·3 (11·8–26)
1-128	Diabetes and kidney diseases	318,000 (278,000–361,000)	558,000 (466,000–665,000)	739,000 (593,000–910,000)	1,570 (1,360–1,800)	1,390 (1,170–1,650)	1,410 (1,130–1,740)
1-129	Diabetes mellitus	185,000 (152,000–223,000)	354,000 (276,000–452,000)	478,000 (361,000–613,000)	973 (795–1,180)	888 (691–1,130)	907 (685–1,160)
1-130	Diabetes mellitus type 1	11,700 (9,870–13,600)	11,900 (9,790–14,800)	12,700 (9,760–16,400)	38·3 (31·9–45·3)	27·1 (22·3–33·9)	25·8 (19·8–33)
1-131	Diabetes mellitus type 2	174,000 (140,000–211,000)	343,000 (265,000–438,000)	465,000 (351,000–596,000)	935 (758–1,140)	861 (667–1,100)	882 (666–1,130)
1-132	Chronic kidney disease	132,000 (122,000–142,000)	203,000 (184,000–223,000)	261,000 (212,000–321,000)	596 (554–639)	504 (457–552)	501 (407–617)
1-133	Chronic kidney disease due to diabetes mellitus type 1	9,520 (6,650–13,000)	11,100 (7,400–15,500)	13,600 (8,370–20,900)	38·7 (26·5–53·4)	25·7 (17·1–36·6)	26·2 (16·3–40)
1-134	Chronic kidney disease due to diabetes mellitus type 2	23,000 (18,300–27,500)	43,900 (34,800–52,600)	61,800 (47,100–80,000)	131 (105–156)	113 (90·1–136)	116 (88·9–152)
1-135	Chronic kidney disease due to hypertension	21,500 (17,900–26,200)	44,200 (36,800–53,100)	58,800 (45,400–76,000)	122 (101–147)	114 (95–137)	110 (85–143)
1-136	Chronic kidney disease due to glomerulonephritis	35,900 (30,300–41,700)	44,300 (37,000–52,400)	52,900 (39,400–70,100)	136 (114–160)	105 (87·4–125)	104 (77·3–137)
1-137	Chronic kidney disease due to other and unspecified causes	42,100 (35,400–49,300)	59,700 (49,100–71,600)	74,200 (57,400–94,400)	169 (141–201)	145 (120–175)	145 (113–184)
1-138	Acute glomerulonephritis	634 (537–752)	209 (183–237)	271 (201–358)	2 (1·7–2·3)	0·5 (0·4–0·6)	0·6 (0·4–0·8)
1-139	Skin and subcutaneous diseases	148,000 (98,100–220,000)	209,000 (140,000–308,000)	222,000 (149,000–326,000)	462 (313–676)	473 (321–694)	470 (316–689)
1-140	Dermatitis	32,200 (19,100–51,100)	42,000 (25,000–64,700)	43,300 (26,100–66,500)	94·3 (56·6–146)	94·5 (56·4–146)	94·4 (56·3–147)
1-141	Atopic dermatitis	23,800 (12,800–40,600)	28,500 (15,400–47,400)	27,700 (14,800–46,200)	63·8 (34·4–109)	63·9 (34·5–106)	63·8 (34·5–106)
1-142	Contact dermatitis	7,430 (4,650–11,100)	11,900 (7,380–18,100)	13,900 (8,600–20,800)	27·1 (17–40·1)	27·2 (16·8–40·9)	27·2 (17–40·8)
1-143	Seborrhoeic dermatitis	1,000 (563–1,620)	1,490 (827–2,440)	1,680 (947–2,700)	3·4 (1·9–5·4)	3·4 (1·9–5·6)	3·4 (1·9–5·5)
1-144	Psoriasis	3,490 (2,370–4,770)	4,880 (3,420–6,580)	5,250 (3,640–7,110)	12 (8·2–16·3)	11 (7·7–14·8)	10·5 (7·3–14·3)
1-145	Bacterial skin diseases	7,440 (4,920–11,200)	19,100 (10,700–27,700)	24,000 (13,000–40,300)	25·8 (16·7–40·9)	46·8 (26–68)	47·4 (26·1–79·2)
1-146	Cellulitis	2,390 (1,540–4,310)	5,890 (2,830–8,620)	6,710 (3,450–11,100)	8·7 (5·5–17·2)	14·2 (6·9–20·6)	13·3 (6·8–21·6)
1-147	Pyoderma	5,040 (3,230–7,320)	13,300 (7,310–20,900)	17,300 (9,310–29,500)	17·2 (10·8–25·5)	32·6 (17·7–51·2)	34·2 (18·6–57·7)
1-148	Scabies	25,800 (13,900–41,200)	33,200 (18,100–53,500)	34,000 (18,600–54,300)	73·7 (40·6–118)	73·3 (40·1–118)	73·1 (40·2–117)
1-149	Fungal skin diseases	7,430 (2,930–15,600)	11,500 (4,650–24,300)	13,600 (5,490–28,800)	27·7 (11·1–58·8)	27·2 (10·9–57·8)	27·1 (10·9–57·7)
1-150	Viral skin diseases	16,600 (10,500–24,800)	19,500 (12,300–29,600)	18,600 (11,900–28,100)	43·8 (27·9–65·1)	43·8 (27·7–66·5)	43·8 (27·9–66·2)
1-151	Acne vulgaris	14,100 (8,230–22,200)	21,200 (12,600–33,900)	21,000 (12,500–33,800)	35·9 (21–56·6)	43·3 (25·8–69)	45 (26·7–72·6)
1-152	Alopecia areata	2,220 (1,400–3,330)	3,230 (2,040–4,780)	3,580 (2,300–5,370)	7·1 (4·5–10·6)	7·2 (4·6–10·6)	7·2 (4·6–10·7)
1-153	Pruritus	2,370 (1,130–4,280)	3,790 (1,810–6,840)	4,470 (2,150–8,120)	8·6 (4·2–15·4)	8·8 (4·2–15·9)	8·9 (4·3–16·3)
1-154	Urticaria	16,900 (10,900–24,700)	21,500 (14,100–31,300)	21,900 (14,400–31,300)	48·2 (31·2–69·1)	48·3 (31·7–70·5)	48·3 (31·6–69·7)
1-155	Decubitus ulcer	7,120 (4,160–10,800)	7,760 (5,180–12,800)	8,100 (5,460–15,100)	37·1 (22·7–54)	19·8 (13·5–32·4)	15·2 (10·1–28·5)
1-156	Other skin and subcutaneous diseases	12,700 (6,560–22,500)	20,700 (10,300–37,600)	24,600 (12,300–44,100)	48·2 (24·7–85·1)	48·5 (24·1–87·8)	48·9 (24·4–87·7)
1-157	Sense organ diseases	188,000 (130,000–268,000)	344,000 (234,000–491,000)	433,000 (296,000–617,000)	920 (632–1,300)	852 (583–1,210)	828 (565–1,180)
1-158	Blindness and vision loss	69,700 (48,500–96,600)	122,000 (84,000–170,000)	153,000 (106,000–215,000)	358 (254–491)	307 (212–424)	292 (201–411)
1-159	Glaucoma	2,480 (1,660–3,560)	4,660 (3,160–6,590)	6,160 (4,190–8,700)	17·3 (11·6–24·6)	12·7 (8·6–18)	11·4 (7·8–16·1)
1-160	Cataract	13,100 (9,210–17,700)	24,700 (17,300–33,500)	32,400 (22,500–43,900)	84·9 (59·7–116)	65·8 (46–89)	60·1 (41·8–81·6)
1-161	Age-related macular degeneration	718 (480–1,040)	1,480 (997–2,140)	2,030 (1,380–2,880)	4·7 (3·2–6·7)	4 (2·7–5·8)	3·8 (2·6–5·4)
1-162	Refraction disorders	26,800 (17,800–38,600)	41,600 (27,700–58,900)	47,800 (31,900–66,800)	105 (71·2–148)	97·4 (65–137)	94·1 (62·7–132)
1-163	Near vision loss	9,950 (4,590–19,800)	21,700 (10,000–42,600)	29,700 (13,800–58,400)	56·9 (26·7–113)	55·7 (25·9–108)	56·1 (25·9–110)
1-164	Other vision loss	16,700 (11,500–23,000)	28,200 (19,800–38,900)	35,200 (24,800–48,200)	88·9 (61·5–122)	71·6 (50·7–98·3)	66·1 (46·4–90·8)
1-165	Age-related and other hearing loss	110,000 (73,000–162,000)	207,000 (137,000–301,000)	261,000 (174,000–381,000)	526 (352–758)	509 (337–738)	500 (331–727)
1-166	Other sense organ diseases	8,490 (5,020–13,100)	15,100 (8,900–23,400)	18,600 (11,100–28,700)	35·5 (20·9–55)	36·3 (21·2–56·1)	36·6 (21·9–56·1)

1·167	Musculoskeletal disorders	465,000 (336,000–613,000)	858,000 (623,000–1,130,000)	1,050,000 (760,000–1,380,000)	1,850 (1,330–2,430)	1,980 (1,430–2,590)	2,020 (1,470–2,670)
1·168	Rheumatoid arthritis	9,950 (7,620–12,800)	19,700 (14,500–25,600)	24,500 (18,000–32,000)	44·5 (34·5–56·7)	46·4 (34·7–59·9)	46·9 (34·4–61·2)
1·169	Osteoarthritis	34,000 (17,000–69,200)	79,600 (40,000–160,000)	108,000 (54,300–219,000)	187 (94·9–377)	199 (100–400)	204 (102–411)
1·170	Low back pain	231,000 (160,000–312,000)	396,000 (279,000–532,000)	455,000 (317,000–609,000)	866 (608–1,170)	901 (632–1,210)	885 (618–1,190)
1·171	Neck pain	33,300 (21,800–48,900)	59,100 (38,500–85,300)	70,200 (46,600–102,000)	135 (87·8–195)	136 (88·3–196)	136 (90–197)
1·172	Gout	1,010 (619–1,490)	2,180 (1,360–3,200)	2,860 (1,740–4,100)	4·6 (2·8–6·8)	5·2 (3·2–7·6)	5·4 (3·3–7·8)
1·173	Other musculoskeletal disorders	155,000 (107,000–211,000)	301,000 (211,000–415,000)	387,000 (270,000–532,000)	610 (424–834)	693 (485–959)	746 (521–1,020)
1·174	Other non-communicable diseases	655,000 (553,000–796,000)	824,000 (629,000–1,040,000)	803,000 (603,000–1,050,000)	1,930 (1,580–2,400)	1,940 (1,480–2,420)	1,730 (1,290–2,280)
1·175	Congenital anomalies	370,000 (309,000–449,000)	367,000 (237,000–455,000)	270,000 (168,000–384,000)	858 (718–1,040)	873 (561–1,080)	681 (418–976)
1·176	Neural tube defects	22,400 (17,500–27,000)	15,400 (12,300–19,800)	13,700 (9,430–19,500)	51·2 (40–61·6)	36·5 (29·3–47·3)	34·7 (23·5–49·7)
1·177	Congenital heart anomalies	188,000 (154,000–238,000)	182,000 (111,000–230,000)	118,000 (68,100–175,000)	431 (354–544)	435 (264–549)	302 (173–448)
1·178	Orofacial clefts	2,850 (2,140–3,630)	1,040 (671–1,530)	1,090 (681–1,610)	6·8 (5·1–8·7)	2·4 (1·5–3·5)	2·4 (1·5–3·5)
1·179	Down's syndrome	8,620 (6,730–10,400)	4,840 (3,920–6,020)	5,770 (3,910–7,900)	20·2 (15·9–24·4)	11·3 (9·1–14)	14·3 (9·6–19·7)
1·180	Turner syndrome	64 (29–111)	68 (33–118)	66 (30–114)	0·2 (0·1–0·3)	0·1 (0·1–0·3)	0·1 (0·1–0·2)
1·181	Klinefelter syndrome	5 (2–9)	5 (2–10)	5 (2–10)	0 (0–0)	0 (0–0)	0 (0–0)
1·182	Other chromosomal abnormalities	5,380 (4,200–7,220)	10,100 (4,420–13,200)	8,880 (3,500–13,300)	12·6 (9·9–16·8)	23·9 (10·3–31·4)	22·5 (8·7–33·6)
1·183	Congenital musculoskeletal and limb anomalies	25,300 (17,400–34,700)	26,600 (17,100–38,500)	26,200 (16,900–37,800)	66·7 (45·9–92·2)	59·5 (38·3–86·1)	58·7 (37·4–84·8)
1·184	Urogenital congenital anomalies	8,950 (5,010–12,800)	10,300 (4,320–14,100)	7,020 (3,180–10,900)	20·5 (11·5–29·1)	24·6 (10·3–33·8)	17·9 (8–27·9)
1·185	Digestive congenital anomalies	40,300 (31,100–60,100)	40,900 (24,300–51,500)	29,300 (16,000–44,600)	90·9 (70·2–135)	98·6 (58·7–124)	76·1 (41·7–116)
1·186	Other congenital anomalies	68,300 (53,200–85,200)	75,900 (38,900–101,000)	59,600 (27,500–93,300)	158 (125–198)	182 (92·3–241)	152 (69·1–240)
1·187	Urinary diseases and male infertility	23,300 (19,700–28,700)	49,800 (41,600–58,300)	62,200 (49,200–77,600)	110 (91·6–138)	126 (105–149)	121 (95·2–151)
1·188	Urinary tract infections and interstitial nephritis	13,900 (12,400–18,100)	33,200 (26,800–35,700)	39,300 (30,000–50,400)	59·7 (53·7–79·4)	83·5 (67–90)	76·4 (58·1–97·6)
1·189	Urolithiasis	855 (645–1,110)	1,550 (1,190–2,010)	2,060 (1,540–2,750)	3·5 (2·7–4·6)	3·6 (2·8–4·6)	4 (3–5·3)
1·190	Benign prostatic hyperplasia	5,650 (3,280–8,810)	12,300 (7,220–19,000)	17,400 (10,100–26,900)	34·8 (20·3–54·4)	33 (19·3–50·8)	33·3 (19·3–51·4)
1·191	Male infertility	345 (137–745)	512 (208–1,130)	1,090 (399–2,600)	1·1 (0·4–2·3)	1·1 (0·5–2·5)	2·2 (0·8–5·1)
1·192	Other urinary diseases	2,600 (1,580–3,090)	2,200 (1,750–3,590)	2,390 (1,750–3,860)	11·2 (6·3–13·1)	5·3 (4·2–8·8)	4·9 (3·6–7·7)
1·193	Gynecological diseases	99,900 (68,300–142,000)	144,000 (98,600–204,000)	161,000 (110,000–227,000)	330 (227–464)	317 (216–448)	315 (216–446)
1·194	Uterine fibroids	6,760 (3,330–12,500)	11,800 (5,610–22,500)	13,500 (6,450–25,200)	26·2 (12·9–48·4)	26·1 (12·4–49·8)	26·2 (12·6–49·1)
1·195	Polycystic ovarian syndrome	2,360 (1,010–4,760)	3,990 (1,680–8,040)	4,670 (1,970–9,340)	6·9 (3–13·9)	8·5 (3·6–17·2)	9·3 (3·9–18·7)
1·196	Female infertility	187 (40–491)	310 (67–763)	1,670 (401–4,280)	0·6 (0·1–1·5)	0·7 (0·1–1·7)	3·3 (0·8–8·5)
1·197	Endometriosis	11,700 (6,830–19,000)	12,100 (7,060–19,400)	11,800 (6,830–18,800)	34·9 (20·7–55·4)	25·9 (15·1–41·8)	23·3 (13·5–37)
1·198	Genital prolapse	1,470 (737–2,690)	2,540 (1,200–4,840)	3,130 (1,520–5,940)	7·6 (3·8–13·9)	6·2 (3–11·8)	5·9 (2·9–11·2)
1·199	Premenstrual syndrome	34,300 (20,800–53,700)	48,100 (29,300–74,800)	51,000 (31,200–79,000)	102 (62·5–158)	103 (62·7–159)	102 (62·7–158)
1·200	Other gynecological diseases	43,100 (27,400–63,700)	65,700 (41,900–97,100)	74,800 (46,500–111,000)	152 (97–221)	147 (93·5–216)	145 (90·7–214)
1·201	Hemoglobinopathies and hemolytic anaemias	14,500 (11,600–18,300)	15,500 (13,200–18,400)	15,000 (11,700–18,900)	46·6 (37–58·9)	35·5 (30·3–42·1)	31·5 (24·6–39·8)
1·202	Thalassemias	107 (62–174)	249 (138–388)	168 (86–284)	0·3 (0·1–0·4)	0·6 (0·3–0·9)	0·4 (0·2–0·7)
1·203	Thalassaemias trait	1,100 (657–1,700)	996 (579–1,610)	971 (568–1,560)	3·2 (2–4·9)	2·2 (1·3–3·6)	2 (1·2–3·2)
1·204	Sickle cell disorders	1,520 (1,220–1,830)	1,840 (1,460–2,310)	1,270 (859–1,810)	3·6 (2·8–4·3)	4·3 (3·4–5·4)	3·2 (2·2–4·5)
1·205	Sickle cell trait	1,820 (1,110–2,750)	1,300 (737–2,010)	1,240 (683–2,010)	5·2 (3·2–7·9)	2·9 (1·6–4·4)	2·5 (1·4–4)
1·206	G6PD deficiency	3,260 (2,660–3,910)	4,670 (3,900–5,340)	5,380 (3,970–7,170)	9·3 (7·7–11·2)	10·2 (8·5–11·7)	11·4 (8·4–15·2)
1·207	G6PD trait	52 (26–88)	47 (14–95)	63 (16–197)	0·2 (0·1–0·3)	0·1 (0–0·2)	0·1 (0–0·4)
1·208	Other hemoglobinopathies and hemolytic anaemias	6,680 (5,000–8,960)	6,380 (5,100–8,100)	5,930 (4,460–7,660)	24·8 (18·8–32·9)	15·2 (12·2–19·2)	11·8 (8·9–15·3)
1·209	Endocrine, metabolic, blood, and immune disorders	59,300 (45,700–75,800)	98,800 (75,100–129,000)	122,000 (91,100–161,000)	233 (176–305)	235 (179–306)	245 (184–322)
1·210	Oral disorders	79,700 (44,900–130,000)	143,000 (81,100–233,000)	169,000 (97,300–278,000)	330 (192–530)	334 (193–547)	328 (189–540)
1·211	Caries of deciduous teeth	1,190 (515–2,460)	1,040 (454–2,060)	859 (367–1,810)	2·9 (1·2–6)	2·4 (1·1–4·8)	2·3 (1–4·9)
1·212	Caries of permanent teeth	9,450 (4,200–18,400)	17,700 (7,710–33,900)	19,100 (8,350–36,900)	30·2 (13·4–58·6)	39·1 (16·9–74·7)	38·3 (16·7–74)
1·213	Periodontal disease	28,200 (10,700–60,400)	51,100 (19,900–110,000)	59,600 (23,300–128,000)	112 (42·8–239)	116 (45·1–248)	115 (44·7–247)
1·214	Edentulism	24,500 (15,500–36,200)	48,900 (30,500–72,500)	62,900 (40,000–94,300)	132 (83·6–195)	123 (77·4–184)	119 (75·8–178)
1·215	Other oral disorders	16,400 (10,200–24,100)	23,900 (14,800–34,700)	26,300 (16,300–38,300)	53·2 (33·1–78·1)	53·3 (33·1–77·7)	53·3 (33–77·9)
1·216	Sudden infant death syndrome	8,550 (6,750–10,500)	6,120 (4,530–8,210)	4,740 (2,740–7,530)	19·2 (15·2–23·7)	14·7 (10·9–19·7)	12·3 (7·1–19·5)
1·0	Injuries	2,670,000 (2,570,000–2,780,000)	2,360,000 (2,240,000–2,490,000)	2,060,000 (1,690,000–2,500,000)	8,020 (7,680–8,420)	5,190 (4,920–5,500)	4,120 (3,380–4,990)
1·1	Transport injuries	448,000 (422,000–479,000)	505,000 (467,000–550,000)	457,000 (365,000–561,000)	1,440 (1,350–1,540)	1,130 (1,040–1,230)	913 (728–1,120)
1·2	Road injuries	436,000 (411,000–466,000)	488,000 (450,000–532,000)	439,000 (349,000–540,000)	1,400 (1,310–1,500)	1,090 (1,000–1,190)	876 (697–1,080)

1-3	Pedestrian road injuries	274,000 (259,000–290,000)	161,000 (151,000–173,000)	131,000 (103,000–163,000)	880 (836–929)	370 (345–396)	265 (208–332)
1-4	Cyclist road injuries	20,500 (16,900–24,600)	43,200 (35,800–51,900)	34,800 (27,700–42,700)	69 (56·4–83·6)	97·4 (80·6–117)	69·9 (55·7–85·4)
1-5	Motorcyclist road injuries	44,100 (40,800–47,800)	178,000 (166,000–193,000)	181,000 (140,000–227,000)	135 (124–148)	389 (361–422)	356 (276–446)
1-6	Motor vehicle road injuries	92,400 (85,700–99,900)	91,100 (81,000–103,000)	79,600 (64,600–96,900)	298 (275–325)	204 (180–230)	159 (129–193)
1-7	Other road injuries	5,480 (4,200–6,970)	13,900 (11,300–16,700)	13,000 (10,400–16,100)	20·3 (15·4–26·2)	31·6 (25·9–38·1)	26 (20·8–32·2)
1-8	Other transport injuries	12,300 (11,300–13,500)	17,200 (15,700–18,700)	18,300 (14,800–22,700)	39·1 (36–42·6)	38 (34·6–41·3)	36·7 (29·5–45·5)
1-9	Unintentional injuries	595,000 (536,000–667,000)	502,000 (439,000–578,000)	429,000 (343,000–527,000)	1,860 (1,660–2,110)	1,140 (1,000–1,320)	883 (706–1,080)
1-10	Falls	127,000 (110,000–149,000)	129,000 (106,000–158,000)	143,000 (113,000–179,000)	480 (412–565)	300 (248–367)	282 (222–353)
1-11	Drowning	114,000 (105,000–123,000)	89,000 (80,500–98,600)	58,400 (44,000–74,800)	300 (278–324)	200 (180–221)	131 (98·9–167)
1-12	Fire, heat, and hot substances	78,500 (64,400–96,100)	42,100 (31,100–57,100)	38,900 (27,100–55,100)	237 (193–293)	94·9 (70·4–128)	79·9 (56·3–113)
1-13	Poisonings	18,500 (16,300–21,000)	9,660 (7,970–11,700)	8,120 (6,010–10,500)	52·2 (45·9–59·1)	21·6 (17·8–26·1)	17 (12·7–22)
1-14	Poisoning by carbon monoxide	2,180 (1,860–2,570)	2,480 (2,170–2,840)	2,010 (1,530–2,590)	6·2 (5·3–7·2)	5·5 (4·8–6·3)	4·2 (3·2–5·4)
1-15	Poisoning by other means	16,400 (14,300–18,600)	7,180 (5,620–9,060)	6,110 (4,330–8,270)	46 (40·3–52·5)	16·1 (12·6–20·3)	12·8 (9·1–17·4)
1-16	Exposure to mechanical forces	84,200 (71,900–101,000)	77,800 (63,500–98,700)	71,200 (53,700–94,900)	273 (229–332)	174 (142–222)	142 (107–190)
1-17	Unintentional firearm injuries	26,900 (22,100–29,600)	9,980 (8,620–14,100)	8,660 (6,710–12,000)	80·2 (64·8–88·5)	21·9 (18·8–30·8)	17·3 (13·4–23·9)
1-18	Other exposure to mechanical forces	57,300 (45,800–73,800)	67,800 (53,900–88,200)	62,500 (46,700–85,500)	193 (152–249)	152 (121–199)	125 (93·5–171)
1-19	Adverse effects of medical treatment	9,120 (7,320–10,000)	7,850 (7,140–10,200)	9,070 (6,970–11,800)	32·9 (26·3–35·6)	18·7 (17–24·3)	18·5 (14·2–24·2)
1-20	Animal contact	17,800 (15,500–20,400)	13,100 (10,900–16,000)	12,700 (9,830–15,800)	57·8 (50·5–66·8)	29·9 (24·9–36·4)	26·1 (20·3–32·6)
1-21	Venomous animal contact	13,400 (11,600–15,300)	8,990 (7,420–11,000)	8,490 (6,580–10,800)	42·8 (37·4–48·7)	20·4 (16·9–24·8)	17·5 (13·6–22·4)
1-22	Non-venomous animal contact	4,440 (3,610–5,510)	4,130 (3,310–5,490)	4,180 (3,150–5,620)	15 (12·2–18·6)	9·5 (7·6–12·7)	8·6 (6·5–11·6)
1-23	Foreign body	47,900 (41,900–55,400)	49,900 (42,500–57,800)	36,000 (26,600–46,800)	124 (110–142)	117 (99·6–136)	83·5 (60·9–110)
1-24	Pulmonary aspiration and foreign body in airway	44,000 (38,200–51,200)	44,700 (37,800–52,400)	30,300 (21,500–40,800)	111 (97·7–128)	105 (88·7–124)	71·8 (50·2–97·3)
1-25	Foreign body in eyes	1,650 (720–3,020)	2,410 (1,140–4,290)	2,670 (1,300–4,690)	5·5 (2·7–9·6)	5·4 (2·6–9·5)	5·4 (2·6–9·5)
1-26	Foreign body in other body part	2,340 (1,770–3,120)	2,820 (2,090–3,820)	3,040 (2,240–4,100)	7·7 (5·8–10·3)	6·4 (4·7–8·7)	6·2 (4·6–8·4)
1-27	Environmental heat and cold exposure	4,820 (3,890–6,040)	4,080 (3,000–5,430)	4,630 (3,340–6,240)	17·1 (13·8–21·3)	9·4 (6·9–12·5)	9·2 (6·6–12·4)
1-28	Exposure to forces of nature	16,100 (11,900–22,000)	33,900 (29,500–38,800)	8,710 (6,400–11,900)	50·9 (37·7–68·3)	75·8 (66–87)	17 (12·5–23·1)
1-29	Other unintentional injuries	78,000 (70,500–87,900)	45,800 (39,600–55,400)	38,000 (29,600–49,100)	240 (215–271)	102 (87·5–123)	77·1 (60·3–98·9)
1-30	Self-harm and interpersonal violence	1,630,000 (1,590,000–1,660,000)	1,350,000 (1,320,000–1,390,000)	1,170,000 (939,000–1,460,000)	4,720 (4,620–4,820)	2,920 (2,850–2,990)	2,320 (1,860–2,880)
1-31	Self-harm	79,400 (75,100–83,900)	149,000 (141,000–156,000)	140,000 (107,000–180,000)	239 (228–251)	320 (305–335)	278 (211–354)
1-32	Self-harm by firearm	31,100 (22,400–34,500)	32,700 (29,200–43,200)	24,200 (17,200–34,600)	91·9 (66·7–101)	70·6 (63·1–93·1)	47·5 (33·9–68)
1-33	Self-harm by other specified means	48,400 (44,900–56,500)	116,000 (104,000–123,000)	116,000 (87,700–150,000)	147 (137–171)	250 (224–264)	230 (174–296)
1-34	Interpersonal violence	1,450,000 (1,420,000–1,480,000)	1,130,000 (1,100,000–1,160,000)	995,000 (798,000–1,250,000)	4,210 (4,120–4,300)	2,440 (2,380–2,500)	1,970 (1,580–2,470)
1-35	Physical violence by firearm	1,130,000 (1,110,000–1,160,000)	853,000 (835,000–871,000)	722,000 (570,000–925,000)	3,260 (3,190–3,320)	1,830 (1,800–1,870)	1,430 (1,130–1,830)
1-36	Physical violence by sharp object	208,000 (199,000–217,000)	176,000 (167,000–185,000)	182,000 (136,000–236,000)	619 (592–648)	380 (361–400)	359 (267–465)
1-37	Sexual violence	5,150 (3,430–7,380)	7,270 (4,870–10,200)	7,920 (5,270–11,300)	15·9 (10·6–22·6)	15·8 (10·6–22·3)	15·8 (10·5–22·6)
1-38	Physical violence by other means	105,000 (98,100–113,000)	95,200 (87,800–104,000)	82,700 (63,500–105,000)	317 (294–343)	212 (195–232)	169 (130–214)
1-39	Conflict and terrorism	97,100 (87,400–109,000)	54,300 (45,400–68,800)	21,900 (14,300–34,900)	268 (241–302)	119 (99·4–150)	42·6 (27·9–67·9)
1-40	Executions and police conflict	632 (498–821)	16,800 (13,400–20,900)	14,300 (7,130–26,400)	1·7 (1·4–2·2)	35·9 (28·8–44·7)	28·2 (14·1–51·7)

SR Table 1: All-age DALYs counts and age standardized DALYs rates per 100,000 individuals for all causes and level 1 cause groups by location for 1990, 2010, and 2019, both sexes combined, Costa Rica

Cause Level	Cause	All-age DALYs			Age-standardized rate of DALYs per 100,000		
		1990	2010	2019	1990	2010	2019
0-0	All causes	642,000 (574,000–724,000)	937,000 (823,000–1,070,000)	1,090,000 (903,000–1,300,000)	25,800 (23,100–29,000)	22,700 (20,100–25,700)	22,000 (18,200–26,200)
1-0	Communicable, maternal, neonatal, and nutritional disorders	123,000 (112,000–135,000)	92,700 (82,900–105,000)	84,800 (69,600–102,000)	3,640 (3,340–4,000)	2,370 (2,120–2,670)	2,020 (1,650–2,460)
1-1	HIV/AIDS and sexually transmitted infections	4,080 (3,770–4,540)	9,050 (8,480–9,850)	8,570 (7,860–9,500)	139 (128–156)	199 (187–217)	169 (155–187)
1-2	HIV/AIDS	3,390 (3,230–3,600)	8,030 (7,710–8,390)	7,500 (7,070–7,960)	115 (109–122)	176 (169–184)	148 (140–157)
1-3	HIV/AIDS - drug-susceptible tuberculosis	709 (463–1,070)	430 (280–637)	347 (225–531)	23·9 (15·7–36)	9·5 (6·1–14)	6·8 (4·4–10·5)
1-4	HIV/AIDS - Multidrug-resistant Tuberculosis without extensive drug resistance	2 (0–6)	21 (5–60)	18 (2–66)	0·1 (0–0·2)	0·5 (0·1–1·3)	0·4 (0–1·3)
1-5	HIV/AIDS - Extensively drug-resistant Tuberculosis	0 (0–0)	2 (0–7)	3 (0–11)	0 (0–0)	0·1 (0–0·1)	0·1 (0–0·2)
1-6	HIV/AIDS resulting in other diseases	2,680 (2,250–3,020)	7,570 (7,190–7,960)	7,130 (6,680–7,600)	90·7 (76–102)	166 (158–175)	140 (132–150)
1-7	Sexually transmitted infections excluding HIV	693 (453–1,120)	1,020 (621–1,760)	1,070 (621–1,910)	24·5 (15·6–40·8)	22·9 (14·2–39·1)	21·1 (12·6–37·4)
1-8	Syphilis	185 (153–225)	159 (135–190)	122 (90–160)	5·5 (4·7–6·5)	4·2 (3·5–5·1)	2·9 (2·1–3·9)
1-9	Chlamydial infection	47 (31–70)	76 (49–111)	80 (54–119)	1·6 (1·1–2·4)	1·6 (1·1–2·4)	1·5 (1·1–2·3)
1-10	Gonococcal infection	32 (25–43)	60 (49–73)	58 (44–75)	1·2 (1–1·5)	1·3 (1·1–1·6)	1·1 (0·9–1·5)
1-11	Trichomoniasis	195 (77–402)	334 (131–685)	372 (141–757)	7·1 (2·8–14·7)	7·2 (2·8–14·7)	7·1 (2·7–14·5)
1-12	Genital herpes	145 (50–351)	247 (81–614)	287 (91–705)	5·8 (2–14)	5·5 (1·8–13·7)	5·5 (1·8–13·6)
1-13	Other sexually transmitted infections	88 (61–125)	144 (102–197)	148 (106–208)	3·2 (2·3–4·4)	3·1 (2·2–4·2)	2·9 (2·1–4)
1-14	Respiratory infections and tuberculosis	25,400 (22,300–29,500)	18,100 (15,900–21,200)	21,200 (16,900–26,400)	882 (794–994)	454 (398–524)	453 (358–564)
1-15	Tuberculosis	3,250 (3,010–3,510)	1,760 (1,610–1,940)	1,510 (1,170–1,930)	149 (139–161)	42·4 (38·9–46·5)	29·5 (22·8–37·6)
1-16	Latent tuberculosis infection	0 (0–0)	0 (0–0)	0 (0–0)	0 (0–0)	0 (0–0)	0 (0–0)
1-17	Drug-susceptible tuberculosis	3,240 (3,010–3,510)	1,690 (1,510–1,880)	1,440 (1,100–1,850)	149 (138–161)	40·6 (36·3–44·9)	28·2 (21·6–36·1)
1-18	Multidrug-resistant tuberculosis without extensive drug resistance	5 (0–26)	66 (16–177)	59 (7–216)	0·3 (0–1·2)	1·6 (0·4–4·3)	1·2 (0·1–4·2)
1-19	Extensively drug-resistant tuberculosis	0 (0–0)	7 (2–20)	9 (1–33)	0 (0–0)	0·2 (0–0·5)	0·2 (0–0·6)
1-20	Lower respiratory infections	18,400 (15,800–22,200)	11,500 (10,600–12,900)	14,700 (11,400–18,700)	618 (550–713)	299 (274–337)	313 (240–402)
1-21	Upper respiratory infections	2,690 (1,620–4,270)	3,620 (2,160–5,720)	3,780 (2,230–5,990)	83·2 (50·1–131)	82·8 (49·3–130)	82·7 (49·1–131)
1-22	Otitis media	1,040 (598–1,700)	1,260 (717–2,100)	1,230 (701–2,030)	31·6 (18·2–51·6)	29 (16·5–48·6)	27·8 (15·7–45·8)
1-23	Enteric infections	12,700 (10,400–15,500)	7,340 (5,810–9,110)	8,150 (6,320–10,400)	393 (327–470)	186 (148–229)	184 (142–236)
1-24	Diarrhoeal diseases	12,700 (10,400–15,500)	7,330 (5,800–9,090)	8,130 (6,310–10,400)	392 (326–469)	186 (148–228)	184 (142–236)
1-25	Typhoid and paratyphoid	0 (0–0)	0 (0–0)	0 (0–0)	0 (0–0)	0 (0–0)	0 (0–0)
1-26	Typhoid fever	0 (0–0)	0 (0–0)	0 (0–0)	0 (0–0)	0 (0–0)	0 (0–0)
1-27	Paratyphoid fever	0 (0–0)	0 (0–0)	0 (0–0)	0 (0–0)	0 (0–0)	0 (0–0)
1-28	iNTS	9 (2–13)	3 (2–4)	2 (1–4)	0·2 (0·1–0·3)	0·1 (0–0·1)	0·1 (0–0·1)
1-29	Other intestinal infectious diseases	23 (11–40)	15 (9–22)	14 (9–20)	0·6 (0·3–1)	0·4 (0·2–0·6)	0·3 (0·2–0·5)
1-30	Neglected tropical diseases and malaria	3,430 (2,350–4,750)	4,190 (2,610–6,180)	4,120 (2,590–6,220)	126 (86·3–177)	98·6 (62–144)	84·1 (53·3–125)
1-31	Malaria	105 (75–148)	24 (19–32)	0 (0–0)	3·2 (2·4–4·5)	0·5 (0·4–0·7)	0 (0–0)
1-32	Chagas disease	190 (123–271)	210 (135–300)	237 (153–341)	9·1 (6–12·7)	5·1 (3·3–7·2)	4·6 (3–6·6)
1-33	Leishmaniasis	120 (24–327)	157 (86–259)	200 (59–417)	4·1 (0·9–10·3)	3·8 (2·1–6·3)	4·3 (1·3–9)
1-34	Visceral leishmaniasis	24 (0–199)	11 (0–88)	10 (0–77)	0·7 (0–5·3)	0·3 (0–2·2)	0·2 (0–1·9)
1-35	Cutaneous and mucocutaneous leishmaniasis	96 (20–246)	146 (82–234)	190 (58–412)	3·4 (0·7–8·6)	3·5 (2–5·6)	4·1 (1·2–8·8)
1-36	African trypanosomiasis	0 (0–0)	0 (0–0)	0 (0–0)	0 (0–0)	0 (0–0)	0 (0–0)
1-37	Schistosomiasis	0 (0–0)	0 (0–0)	0 (0–0)	0 (0–0)	0 (0–0)	0 (0–0)
1-38	Cysticercosis	1,230 (609–2,180)	1,800 (871–3,250)	2,220 (1,020–3,960)	56·7 (28·1–97·8)	43·7 (21·3–78·5)	42·9 (19·7–76·3)
1-39	Cystic echinococcosis	2 (1–3)	2 (1–2)	1 (1–2)	0·1 (0–0·1)	0 (0–0·1)	0 (0–0)
1-40	Lymphatic filariasis	0 (0–0)	0 (0–0)	0 (0–0)	0 (0–0)	0 (0–0)	0 (0–0)
1-41	Onchocerciasis	0 (0–0)	0 (0–0)	0 (0–0)	0 (0–0)	0 (0–0)	0 (0–0)
1-42	Trachoma	0 (0–0)	0 (0–0)	0 (0–0)	0 (0–0)	0 (0–0)	0 (0–0)
1-43	Dengue	328 (175–554)	843 (261–1,920)	456 (248–770)	10·8 (5·7–18·1)	18·9 (6·1–42·6)	9·6 (5·2–16)
1-44	Yellow fever	0 (0–0)	0 (0–0)	0 (0–0)	0 (0–0)	0 (0–0)	0 (0–0)
1-45	Rabies	3 (2–7)	0 (0–0)	0 (0–0)	0·2 (0·1–0·4)	0 (0–0)	0 (0–0)
1-46	Intestinal nematode infections	789 (461–1,300)	773 (439–1,330)	654 (366–1,120)	23·5 (13·7–38·6)	17·2 (9·8–29·8)	14·2 (8–24·2)
1-47	Ascariasis	186 (99–323)	291 (142–517)	221 (108–406)	5·4 (2·9–9·5)	6·8 (3·4–12·1)	5 (2·4–9·1)
1-48	Trichuriasis	166 (80–294)	50 (22–94)	50 (22–97)	5 (2·4–8·8)	1·1 (0·5–2)	1·1 (0·5–2)

1-49	Hookworm disease	437 (235–726)	432 (219–756)	383 (200–679)	13·1 (7·1–21·7)	9·3 (4·7–16·2)	8·1 (4·2–14·4)
1-50	Food-borne trematodiases	0 (0–0)	0 (0–0)	0 (0–0)	0 (0–0)	0 (0–0)	0 (0–0)
1-51	Leprosy	1 (1–2)	2 (1–3)	3 (2–4)	0 (0–0·1)	0·1 (0–0·1)	0·1 (0–0·1)
1-52	Ebola virus disease	0 (0–0)	0 (0–0)	0 (0–0)	0 (0–0)	0 (0–0)	0 (0–0)
1-53	Zika virus disease	0 (0–0)	0 (0–0)	7 (2–17)	0 (0–0)	0 (0–0)	0·2 (0–0·4)
1-54	Guinea worm disease	0 (0–0)	0 (0–0)	0 (0–0)	0 (0–0)	0 (0–0)	0 (0–0)
1-55	Other neglected tropical diseases	659 (373–966)	372 (226–572)	340 (202–533)	18·2 (10·6–26·3)	9·2 (5·6–14·2)	8·3 (4·8–13·3)
1-56	Other infectious diseases	7,760 (7,020–8,630)	4,970 (4,420–5,750)	4,670 (3,700–5,800)	234 (213–258)	121 (107–142)	106 (83·3–133)
1-57	Meningitis	3,370 (2,930–3,920)	1,910 (1,700–2,220)	1,610 (1,230–2,050)	100 (89·1–114)	47·2 (41·8–55·4)	36·3 (27·3–46·9)
1-58	Encephalitis	2,420 (1,640–2,810)	1,290 (1,110–1,800)	1,330 (1,020–1,860)	69·3 (47·4–79·7)	30·7 (26·2–44)	30·5 (23·2–43·3)
1-59	Diphtheria	0 (0–0)	0 (0–1)	0 (0–1)	0 (0–0)	0 (0–0)	0 (0–0)
1-60	Whooping cough	92 (59–142)	77 (49–114)	34 (21–51)	2·3 (1·5–3·6)	2·1 (1·4–3·2)	1 (0·6–1·5)
1-61	Tetanus	12 (5–66)	4 (0–34)	3 (0–24)	0·5 (0·2–2·6)	0·1 (0–0·8)	0·1 (0–0·5)
1-62	Measles	4 (2–6)	0 (0–0)	0 (0–1)	0·1 (0–0·2)	0 (0–0)	0 (0–0)
1-63	Varicella and herpes zoster	181 (120–234)	176 (128–252)	178 (123–259)	6·3 (4·1–8)	4·2 (3·1–6·2)	3·7 (2·6–5·4)
1-64	Acute hepatitis	838 (746–938)	295 (226–384)	300 (225–386)	28·9 (26·1–31·8)	6·7 (5·2–8·6)	6·4 (4·8–8·3)
1-65	Acute hepatitis A	310 (229–394)	169 (112–241)	170 (113–240)	9·9 (7·1–12·4)	3·8 (2·5–5·4)	3·8 (2·5–5·4)
1-66	Acute hepatitis B	285 (169–366)	63 (49–83)	69 (51–93)	9·7 (5·7–12·1)	1·4 (1·1–1·9)	1·4 (1–1·9)
1-67	Acute hepatitis C	211 (152–364)	48 (31–62)	45 (29–63)	8·3 (6·1–13·5)	1·1 (0·7–1·4)	0·9 (0·6–1·3)
1-68	Acute hepatitis E	32 (22–54)	15 (10–20)	16 (11–22)	1·1 (0·7–1·7)	0·3 (0·2–0·5)	0·3 (0·2–0·5)
1-69	Other unspecified infectious diseases	852 (628–1,230)	1,220 (851–1,510)	1,220 (844–1,610)	26 (19·5–37·1)	30 (21–36·9)	28·1 (19·7–37)
1-70	Maternal and neonatal disorders	59,900 (51,800–69,000)	41,300 (36,900–47,600)	30,600 (23,500–39,100)	1,580 (1,370–1,820)	1,120 (1,000–1,300)	861 (649–1,110)
1-71	Maternal disorders	1,600 (1,420–1,810)	1,490 (1,320–1,670)	1,460 (1,120–1,860)	49 (43·7–55·2)	30·6 (27·1–34·4)	28·5 (22·1–36·4)
1-72	Maternal haemorrhage	373 (285–480)	264 (201–357)	237 (171–325)	11·4 (8·7–14·5)	5·5 (4·2–7·3)	4·6 (3·3–6·3)
1-73	Maternal sepsis and other pregnancy related infections	269 (212–335)	94 (63–141)	82 (54–124)	8·2 (6·5–10·2)	1·9 (1·3–2·8)	1·6 (1·1–2·4)
1-74	Maternal hypertensive disorders	381 (330–437)	215 (183–250)	193 (148–251)	11·4 (9·9–13·1)	4·4 (3·7–5·1)	3·8 (2·9–4·9)
1-75	Maternal obstructed labour and uterine rupture	22 (9–42)	26 (16–41)	24 (15–37)	0·7 (0·3–1·2)	0·5 (0·3–0·8)	0·5 (0·3–0·7)
1-76	Maternal abortive outcome	369 (298–445)	122 (93–160)	103 (72–141)	11·6 (9·5–14)	2·5 (1·9–3·2)	2 (1·4–2·8)
1-77	Ectopic pregnancy	16 (13–19)	13 (11–16)	11 (8–15)	0·5 (0·4–0·6)	0·3 (0·2–0·3)	0·2 (0·2–0·3)
1-78	Indirect maternal deaths	18 (14–22)	281 (235–336)	311 (220–423)	0·5 (0·4–0·7)	5·8 (4·8–6·9)	6 (4·3–8·2)
1-79	Late maternal deaths	68 (51–90)	82 (61–107)	89 (58–130)	2·1 (1·6–2·8)	1·7 (1·2–2·2)	1·7 (1·1–2·5)
1-80	Maternal deaths aggravated by HIV/AIDS	0 (0–0)	0 (0–1)	1 (0–1)	0 (0–0)	0 (0–0)	0 (0–0)
1-81	Other maternal disorders	84 (67–103)	390 (330–453)	406 (298–543)	2·5 (2–3·1)	8 (6·8–9·3)	8 (5·9–10·6)
1-82	Neonatal disorders	58,300 (50,200–67,200)	39,800 (35,400–46,100)	29,100 (22,200–37,200)	1,530 (1,310–1,760)	1,090 (970–1,270)	833 (625–1,080)
1-83	Neonatal preterm birth	34,000 (29,100–39,100)	22,900 (20,400–26,400)	17,000 (12,900–21,600)	890 (761–1,030)	629 (558–725)	483 (362–633)
1-84	Neonatal encephalopathy due to birth asphyxia and trauma	10,500 (8,350–12,800)	6,680 (5,600–8,250)	4,850 (3,460–6,510)	270 (215–330)	188 (158–234)	144 (101–195)
1-85	Neonatal sepsis and other neonatal infections	6,610 (4,880–9,060)	3,940 (2,840–5,720)	2,900 (2,140–3,830)	182 (133–255)	98 (71·7–137)	72·6 (53·5–96)
1-86	Hemolytic disease and other neonatal jaundice	348 (277–433)	399 (318–484)	351 (269–440)	10·2 (8·1–12·6)	9·7 (7·7–11·7)	8 (6·2–10)
1-87	Other neonatal disorders	6,900 (5,030–9,140)	5,870 (4,890–7,170)	4,010 (2,710–5,690)	176 (128–234)	168 (140–206)	125 (84–177)
1-88	Nutritional deficiencies	9,280 (6,510–13,000)	7,710 (5,060–11,200)	7,490 (4,900–10,800)	290 (206–403)	184 (122–268)	165 (108–238)
1-89	Protein-energy malnutrition	2,400 (1,910–2,990)	1,470 (1,060–1,970)	1,570 (1,080–2,170)	72·9 (59·2–88·7)	35·5 (26·3–46·9)	33·9 (23·8–46·4)
1-90	Iodine deficiency	300 (155–496)	231 (105–440)	241 (108–461)	10·2 (5·3–16·7)	5 (2·3–9·6)	4·9 (2·2–9·3)
1-91	Vitamin A deficiency	270 (160–413)	183 (111–284)	171 (106–261)	7·2 (4·4–11)	4·5 (2·7–6·9)	4·1 (2·5–6·2)
1-92	Dietary iron deficiency	5,900 (3,640–9,270)	5,380 (3,240–8,320)	5,010 (2,990–7,870)	184 (114–283)	129 (77·6–199)	112 (66·8–178)
1-93	Other nutritional deficiencies	408 (293–561)	450 (285–667)	503 (317–741)	15·6 (11·3–21·6)	10 (6·4–14·7)	10·1 (6·4–15)
1-0	Non-communicable diseases	429,000 (374,000–499,000)	712,000 (613,000–824,000)	858,000 (710,000–1,040,000)	19,000 (16,800–21,700)	17,400 (15,100–20,100)	17,100 (14,100–20,600)
1-1	Neoplasms	64,600 (61,800–67,500)	115,000 (110,000–119,000)	145,000 (114,000–183,000)	3,230 (3,070–3,370)	2,920 (2,770–3,030)	2,840 (2,240–3,590)
1-2	Lip and oral cavity cancer	733 (680–787)	1,130 (1,050–1,220)	1,430 (1,090–1,830)	38·6 (35·7–41·6)	28·5 (26·4–30·7)	27·7 (21·1–35·3)
1-3	Nasopharynx cancer	289 (265–315)	540 (483–603)	677 (507–895)	13·1 (12–14·3)	12·5 (11·2–13·9)	13·3 (10–17·5)
1-4	Other pharynx cancer	321 (293–353)	492 (442–542)	679 (502–899)	17·5 (15·9–19·2)	12·5 (11·1–13·7)	13 (9·6–17·1)
1-5	Oesophageal cancer	1,110 (1,030–1,190)	1,490 (1,360–1,630)	1,900 (1,450–2,460)	62·7 (57·9–67·6)	39·4 (35·9–43)	36·9 (28·3–47·8)
1-6	Stomach cancer	14,900 (14,100–15,600)	18,100 (17,100–19,100)	21,300 (16,500–27,200)	818 (773–858)	467 (439–493)	412 (320–525)
1-7	Colon and rectum cancer	3,920 (3,700–4,130)	12,300 (11,600–13,000)	17,600 (13,700–22,400)	212 (200–224)	317 (298–334)	340 (265–432)

1-8	Liver cancer	2,510 (2,340–2,680)	4,200 (3,900–4,510)	6,200 (4,740–7,940)	134 (124–143)	109 (101–117)	121 (92·6–154)
1-9	Liver cancer due to hepatitis B	527 (399–690)	797 (572–1,070)	1,120 (727–1,700)	25·7 (18·8–34·4)	19·4 (13·9–26·2)	21·5 (14–32·3)
1-10	Liver cancer due to hepatitis C	769 (573–991)	1,330 (984–1,710)	1,990 (1,320–2,810)	44·5 (33·2–57·5)	36 (26·4–46)	38·8 (25·7–54·9)
1-11	Liver cancer due to alcohol use	803 (590–1,020)	1,400 (1,050–1,760)	2,150 (1,440–3,090)	44·7 (32·7–57)	37 (27·3–46·5)	41·6 (27·8–59·9)
1-12	Liver cancer due to NASH	167 (119–227)	338 (240–466)	530 (344–794)	8·9 (6·2–12·5)	8·8 (6·2–12·2)	10·4 (6·8–15·5)
1-13	Gallbladder and biliary tract cancer	1,440 (1,100–1,590)	1,710 (1,510–2,420)	2,010 (1,450–3,180)	81·5 (62·2–90·1)	44·7 (39·3–63·5)	38·9 (28·1–61·6)
1-14	Pancreatic cancer	1,250 (1,160–1,360)	5,420 (5,070–5,760)	7,850 (6,000–10,000)	70·7 (65–76·9)	143 (133–152)	152 (116–193)
1-15	Larynx cancer	712 (650–774)	1,040 (943–1,130)	1,240 (937–1,600)	40·1 (36·5–43·6)	27·3 (24·8–29·8)	24 (18·2–30·9)
1-16	Tracheal, bronchus, and lung cancer	4,610 (4,340–4,850)	8,340 (7,870–8,780)	10,500 (8,130–13,500)	260 (245–274)	221 (208–232)	205 (158–262)
1-17	Malignant skin melanoma	489 (378–634)	1,160 (872–1,500)	1,600 (1,080–2,210)	23·7 (18·5–31·1)	28·5 (21·4–37)	30·8 (21–42·7)
1-18	Non-melanoma skin cancer	362 (303–401)	625 (556–677)	897 (685–1,120)	21·1 (17–23·5)	16·7 (14·8–18·1)	17·4 (13·3–21·7)
1-19	Non-melanoma skin cancer (squamous-cell carcinoma)	362 (303–400)	624 (555–676)	896 (685–1,120)	21·1 (17–23·4)	16·7 (14·8–18·1)	17·4 (13·3–21·7)
1-20	Non-melanoma skin cancer (basal-cell carcinoma)	0 (0–1)	1 (0–2)	1 (0–2)	0 (0–0)	0 (0–0)	0 (0–0)
1-21	Breast cancer	4,440 (4,150–4,750)	10,100 (9,430–10,800)	12,200 (9,400–15,600)	226 (211–241)	247 (231–264)	233 (180–299)
1-22	Cervical cancer	4,760 (4,170–5,140)	5,220 (4,740–6,320)	6,150 (4,550–8,250)	231 (202–249)	123 (112–149)	118 (87·2–158)
1-23	Uterine cancer	402 (362–445)	1,160 (1,040–1,280)	1,670 (1,270–2,150)	22·6 (20·2–25)	30·5 (27·3–33·4)	32 (24·4–41·2)
1-24	Ovarian cancer	688 (628–766)	2,420 (1,850–2,640)	2,980 (2,200–3,900)	34·9 (31·8–38·5)	59·3 (44·7–65)	57·3 (42·2–74·8)
1-25	Prostate cancer	2,450 (2,040–3,310)	7,260 (4,500–8,250)	9,500 (5,960–12,800)	150 (125–202)	205 (126–233)	190 (119–255)
1-26	Testicular cancer	241 (203–352)	731 (600–896)	753 (521–1,020)	7·7 (6·5–10·6)	15·6 (12·8–19·8)	14·9 (10·4–20·7)
1-27	Kidney cancer	738 (688–796)	2,030 (1,860–2,200)	2,900 (2,190–3,800)	36·2 (33·6–39·1)	51·4 (47·2–55·8)	56·4 (42·7–74)
1-28	Bladder cancer	703 (645–761)	1,430 (1,300–1,550)	1,900 (1,450–2,440)	41·2 (37·7–44·7)	38·9 (35·4–42·3)	37·4 (28·6–48·1)
1-29	Brain and nervous system cancer	1,440 (1,210–2,180)	4,250 (2,300–4,990)	4,820 (2,490–6,760)	54·4 (46·1–82·5)	101 (54·7–120)	98·5 (51·1–137)
1-30	Thyroid cancer	391 (357–424)	642 (584–720)	891 (676–1,160)	19·5 (17·8–21·1)	16 (14·5–18)	17·3 (13·1–22·5)
1-31	Mesothelioma	62 (54–69)	97 (86–107)	139 (105–180)	3·1 (2·7–3·4)	2·4 (2·1–2·6)	2·7 (2–3·4)
1-32	Hodgkin lymphoma	1,040 (730–1,240)	1,170 (917–1,370)	1,330 (955–1,780)	37·9 (28·1–44·2)	26·5 (21·1–30·8)	26·2 (18·9–35)
1-33	Non-Hodgkin's lymphoma	2,820 (2,590–3,080)	5,140 (4,690–5,600)	6,420 (4,900–8,290)	123 (113–135)	126 (115–137)	126 (96·6–163)
1-34	Multiple myeloma	619 (557–712)	1,870 (1,470–2,050)	2,680 (1,920–3,520)	34·7 (31·2–39·7)	49·3 (37·9–53·9)	51·9 (37–68·1)
1-35	Leukaemia	6,410 (6,020–6,890)	8,230 (7,720–8,790)	8,820 (6,860–11,200)	225 (211–239)	194 (181–207)	184 (143–231)
1-36	Acute lymphoid leukaemia	3,040 (2,710–3,400)	3,310 (2,970–3,630)	3,340 (2,550–4,280)	89·6 (80·2–99·4)	75·1 (67·5–83)	73·1 (56·4–93·2)
1-37	Chronic lymphoid leukaemia	135 (102–158)	316 (259–377)	406 (291–550)	7·7 (5·7–9·1)	8·5 (6·9–10·2)	8 (5·8–11)
1-38	Acute myeloid leukaemia	1,720 (1,490–2,010)	2,730 (2,450–3,100)	3,090 (2,340–4,060)	60·9 (53·2–70·3)	64·2 (57·5–72·6)	63·3 (48·2–83·3)
1-39	Chronic myeloid leukaemia	585 (504–673)	734 (652–848)	710 (529–945)	27·7 (24–32)	17·9 (16–20·8)	13·9 (10·4–18·5)
1-40	Other leukaemia	939 (835–1,060)	1,140 (1,030–1,310)	1,270 (948–1,670)	38·7 (34·6–43·3)	27·7 (24·9–32)	25·3 (19–33·2)
1-41	Other neoplasms	330 (256–470)	888 (657–1,040)	1,160 (826–1,520)	15·8 (12–22·1)	22·8 (16·8–26·7)	23·2 (16·5–30·4)
1-42	Myelodysplastic, myeloproliferative, and other hematopoietic neoplasms	330 (256–470)	888 (657–1,040)	1,160 (826–1,520)	15·8 (12–22·1)	22·8 (16·8–26·7)	23·2 (16·5–30·4)
1-43	Benign and in situ intestinal neoplasms	0 (0–0)	0 (0–0)	0 (0–0)	0 (0–0)	0 (0–0)	0 (0–0)
1-44	Benign and in situ cervical and uterine neoplasms	0 (0–0)	0 (0–0)	0 (0–0)	0 (0–0)	0 (0–0)	0 (0–0)
1-45	Other benign and in situ neoplasms	0 (0–0)	0 (0–0)	0 (0–0)	0 (0–0)	0 (0–0)	0 (0–0)
1-46	Cardiovascular diseases	73,900 (69,900–77,500)	107,000 (100,000–112,000)	131,000 (106,000–162,000)	4,060 (3,820–4,260)	2,790 (2,610–2,930)	2,550 (2,070–3,160)
1-47	Rheumatic heart disease	3,200 (2,650–3,940)	3,770 (2,930–4,870)	3,820 (2,790–5,130)	123 (105–149)	83·8 (65·9–107)	75·6 (55–102)
1-48	Ischaemic heart disease	40,600 (38,400–42,600)	56,000 (52,500–59,000)	68,300 (55,000–85,800)	2,340 (2,200–2,460)	1,480 (1,390–1,560)	1,320 (1,070–1,670)
1-49	Stroke	18,100 (16,900–19,200)	22,600 (20,900–24,100)	28,800 (23,400–35,300)	991 (922–1,050)	585 (540–624)	563 (458–690)
1-50	Ischaemic stroke	7,450 (6,640–8,110)	9,680 (8,560–10,600)	13,300 (10,700–16,000)	440 (392–480)	263 (233–288)	264 (213–319)
1-51	Intracerebral hemorrhage	7,500 (6,900–8,130)	8,330 (7,710–9,100)	10,100 (7,950–12,800)	405 (371–439)	214 (198–233)	196 (154–247)
1-52	Subarachnoid hemorrhage	3,170 (2,780–3,500)	4,560 (3,930–4,930)	5,390 (4,120–6,860)	146 (124–162)	108 (93–118)	104 (79·5–132)
1-53	Hypertensive heart disease	3,290 (2,730–3,650)	7,640 (5,860–8,440)	8,510 (6,700–10,800)	196 (160–218)	207 (158–229)	166 (131–211)
1-54	Non-rheumatic valvular heart disease	548 (496–606)	1,410 (1,290–1,520)	1,800 (1,380–2,330)	28·4 (25·6–31·5)	35·8 (32·8–38·7)	34·9 (26·7–44·9)
1-55	Non-rheumatic calcific aortic valve disease	422 (360–476)	1,130 (1,020–1,250)	1,470 (1,120–1,890)	22·3 (19–25·3)	29·1 (26·3–32·2)	28·4 (21·7–36·5)
1-56	Non-rheumatic degenerative mitral valve disease	123 (101–163)	272 (205–337)	324 (229–455)	5·9 (4·9–8·1)	6·6 (5–8·4)	6·3 (4·4–8·8)
1-57	Other non-rheumatic valve diseases	2 (2–3)	5 (3–6)	7 (3–11)	0·1 (0·1–0·1)	0·1 (0·1–0·1)	0·1 (0·1–0·2)
1-58	Cardiomyopathy and myocarditis	3,480 (3,060–3,840)	5,090 (4,600–5,730)	5,590 (4,240–7,430)	149 (129–166)	125 (114–143)	111 (84·1–147)
1-59	Myocarditis	397 (226–527)	540 (365–659)	526 (367–726)	12·8 (7·4–16·7)	12·5 (8·6–15·1)	10·9 (7·7–14·9)
1-60	Alcoholic cardiomyopathy	250 (187–316)	225 (178–308)	372 (249–525)	11·8 (8·7–15·1)	5·3 (4·2–7·1)	7·1 (4·7–10)

1-61	Other cardiomyopathy	2,830 (2,540–3,250)	4,320 (3,910–4,980)	4,690 (3,550–6,240)	124 (110–140)	108 (97·3–126)	92·7 (70–124)
1-62	Atrial fibrillation and flutter	1,090 (882–1,330)	2,560 (2,060–3,110)	3,730 (2,930–4,700)	67·9 (55–83)	70·6 (56·5–85·7)	73 (57·4–92·4)
1-63	Aortic aneurysm	730 (660–809)	2,130 (1,940–2,320)	2,790 (2,100–3,620)	39 (34·9–43·5)	55·3 (50·3–60·5)	54·6 (41·1–70·9)
1-64	Peripheral vascular disease	153 (90–243)	347 (207–558)	488 (296–827)	9·5 (5·6–15)	9·7 (5·8–15·6)	9·6 (5·8–16·2)
1-65	Endocarditis	549 (426–659)	1,290 (806–1,510)	1,680 (916–2,360)	23·5 (17–27·7)	31 (19·3–36·3)	32·9 (18·2–46·1)
1-66	Chronic respiratory diseases	19,800 (16,000–24,500)	30,700 (26,200–36,100)	35,900 (29,300–43,200)	869 (742–1,020)	785 (677–911)	737 (601–892)
1-67	Chronic obstructive pulmonary disease	7,900 (7,110–8,670)	15,900 (14,200–17,500)	20,100 (16,300–24,300)	457 (409–500)	433 (386–475)	398 (322–480)
1-68	Pneumoconiosis	16 (13–21)	59 (48–71)	76 (59–98)	0·8 (0·7–1·1)	1·5 (1·2–1·8)	1·5 (1·1–1·9)
1-69	Silicosis	5 (4–7)	24 (20–31)	32 (23–44)	0·3 (0·2–0·4)	0·6 (0·5–0·8)	0·6 (0·4–0·8)
1-70	Asbestosis	1 (1–1)	4 (3–5)	4 (3–6)	0 (0–0·1)	0·1 (0·1–0·1)	0·1 (0·1–0·1)
1-71	Coal workers pneumoconiosis	0 (0–0)	1 (1–2)	1 (0–2)	0 (0–0)	0 (0–0)	0 (0–0)
1-72	Other pneumoconiosis	10 (7–14)	30 (21–40)	39 (27–54)	0·5 (0·3–0·7)	0·7 (0·5–1)	0·8 (0·5–1·1)
1-73	Asthma	10,400 (7,080–15,000)	11,200 (7,610–16,200)	11,200 (7,630–16,200)	346 (244–482)	261 (176–376)	248 (166–365)
1-74	Interstitial lung disease and pulmonary sarcoidosis	1,150 (856–1,370)	2,760 (2,210–3,280)	3,540 (2,590–4,660)	54·7 (41·3–67·2)	71·8 (56·6–84·4)	70·3 (51·4–92·2)
1-75	Other chronic respiratory diseases	325 (267–432)	782 (619–908)	883 (676–1,110)	10·4 (8·7–13·6)	17·5 (14·1–20·3)	18·6 (14·5–23·3)
1-76	Digestive diseases	25,100 (22,400–28,400)	45,500 (41,100–51,200)	56,600 (45,900–69,200)	1,160 (1,050–1,300)	1,110 (1,010–1,240)	1,100 (893–1,340)
1-77	Cirrhosis and other chronic liver diseases	10,200 (9,600–10,700)	20,600 (19,400–21,900)	26,100 (20,000–33,300)	500 (472–529)	507 (476–541)	500 (384–638)
1-78	Cirrhosis and other chronic liver diseases due to hepatitis B	607 (418–850)	712 (482–1,040)	872 (548–1,300)	29·6 (20·6–40·8)	17 (11·5–24·1)	16·6 (10·5–24·7)
1-79	Cirrhosis and other chronic liver diseases due to hepatitis C	2,650 (2,050–3,340)	5,400 (4,050–7,040)	6,670 (4,500–9,530)	130 (98·8–166)	130 (97·7–172)	127 (85·6–181)
1-80	Cirrhosis and other chronic liver diseases due to alcohol use	3,610 (2,900–4,290)	7,970 (6,390–9,560)	10,300 (7,300–14,100)	186 (148–222)	196 (156–237)	196 (139–267)
1-81	Cirrhosis and other chronic liver diseases due to NAFLD	1,620 (1,190–2,180)	3,710 (2,690–5,000)	4,860 (3,180–7,230)	83·6 (60·3–112)	93·2 (67·8–125)	93·3 (61·3–138)
1-82	Cirrhosis and other chronic liver diseases due to other causes	1,670 (1,360–2,050)	2,810 (2,170–3,640)	3,400 (2,330–4,770)	70·2 (54·6–89·6)	70 (53·5–91·4)	67·6 (46·7–94·3)
1-83	Upper digestive system diseases	4,950 (3,410–7,380)	7,800 (5,050–12,100)	9,160 (5,820–14,200)	221 (158–320)	182 (120–279)	177 (113–274)
1-84	Peptic ulcer disease	1,480 (1,310–1,690)	1,740 (1,550–1,960)	2,060 (1,530–2,740)	79·4 (69·5–91·4)	45 (39·9–50·8)	40·2 (29·9–53·4)
1-85	Gastritis and duodenitis	416 (294–571)	593 (406–835)	693 (469–1,000)	18·8 (13·5–25·5)	14·2 (9·8–19·8)	13·6 (9·3–19·8)
1-86	GERD	3,050 (1,590–5,400)	5,470 (2,830–9,580)	6,400 (3,330–11,400)	123 (64·1–218)	123 (64·3–218)	123 (64·1–218)
1-87	Appendicitis	819 (640–975)	829 (681–1,050)	940 (695–1,230)	29·2 (23·4–34·4)	19·4 (16·1–24·6)	19·5 (14·5–25·3)
1-88	Paralytic ileus and intestinal obstruction	1,390 (1,190–1,650)	1,820 (1,480–2,020)	2,150 (1,530–2,790)	53·2 (43·4–60·6)	47·6 (38·7–52·7)	45·6 (33·3–58·9)
1-89	Inguinal, femoral, and abdominal hernia	1,680 (1,230–2,240)	2,500 (1,860–3,310)	3,020 (2,220–3,990)	65·9 (49–86·3)	60·9 (45·7–80·6)	61·6 (45·4–81·2)
1-90	Inflammatory bowel disease	190 (150–238)	421 (332–521)	524 (399–669)	8·6 (6·8–10·8)	10 (8–12·3)	10·1 (7·7–12·9)
1-91	Vascular intestinal disorders	983 (874–1,100)	2,330 (2,060–2,650)	3,080 (2,340–3,910)	57 (50·3–63·8)	63·3 (55·9–72·2)	61·1 (46·4–77·6)
1-92	Gallbladder and biliary diseases	3,010 (2,180–4,120)	4,950 (3,630–6,720)	6,190 (4,420–8,350)	139 (103–187)	118 (87·7–158)	120 (86·2–162)
1-93	Pancreatitis	1,420 (1,090–1,590)	2,380 (1,980–2,620)	2,870 (2,150–3,680)	66·8 (50·3–74·7)	57·4 (47·6–63·3)	55·5 (41·7–71·2)
1-94	Other digestive diseases	455 (335–739)	1,870 (970–2,320)	2,490 (1,260–3,570)	20·3 (15·2–33·7)	47·9 (24·8–59·4)	49·3 (25·2–70)
1-95	Neurological disorders	31,000 (15,300–51,700)	54,500 (30,400–90,100)	65,400 (35,600–108,000)	1,250 (690–2,050)	1,290 (742–2,120)	1,310 (712–2,170)
1-96	Alzheimer's disease and other dementias	5,110 (2,120–11,500)	12,600 (5,340–27,700)	17,700 (7,530–38,200)	333 (138–753)	349 (148–774)	345 (146–747)
1-97	Parkinson's disease	1,100 (994–1,210)	2,610 (2,350–2,830)	3,610 (2,960–4,370)	68·2 (61·2–74·8)	73·1 (65·9–79·3)	73 (59·7–88·2)
1-98	Idiopathic epilepsy	6,350 (2,860–11,400)	8,220 (3,590–15,100)	9,560 (4,020–17,400)	215 (99·1–386)	187 (80·9–344)	200 (82·1–364)
1-99	Multiple sclerosis	158 (137–196)	344 (292–425)	445 (345–563)	7·2 (6·2–9)	8 (6·8–9·8)	8·5 (6·6–10·7)
1-100	Motor neuron disease	136 (125–150)	1,030 (958–1,100)	1,470 (1,110–1,890)	6 (5·6–6·5)	25·8 (24–27·6)	28·2 (21·2–36·4)
1-101	Headache disorders	16,000 (2,710–35,500)	25,900 (4,840–56,800)	28,200 (5,640–61,600)	556 (106–1,220)	560 (107–1,220)	562 (110–1,230)
1-102	Migraine	14,600 (1,870–34,100)	23,600 (3,290–54,000)	25,500 (3,870–58,900)	504 (72·2–1,160)	508 (73·1–1,170)	509 (75·1–1,180)
1-103	Tension-type headache	1,400 (390–5,580)	2,380 (692–8,730)	2,670 (799–9,380)	52·2 (15·4–192)	52·5 (15·4–190)	52·5 (15·4–187)
1-104	Other neurological disorders	2,100 (1,440–3,060)	3,790 (2,840–5,260)	4,480 (3,070–6,340)	68·3 (48·7–96·5)	87·8 (66·3–121)	97·3 (65·7–140)
1-105	Mental disorders	44,800 (32,200–59,600)	74,700 (54,500–99,000)	83,300 (60,700–110,000)	1,610 (1,170–2,120)	1,640 (1,200–2,180)	1,660 (1,210–2,190)
1-106	Schizophrenia	4,710 (3,230–6,350)	8,400 (5,870–11,200)	9,600 (6,640–12,700)	181 (125–242)	184 (129–243)	184 (127–243)
1-107	Depressive disorders	14,000 (9,550–19,300)	25,400 (17,400–35,300)	29,200 (19,900–40,300)	542 (374–748)	564 (388–782)	569 (390–785)
1-108	Major depressive disorder	11,800 (7,950–16,700)	21,600 (14,400–30,100)	24,800 (16,500–34,700)	457 (310–640)	479 (322–665)	484 (323–675)
1-109	Dysthymia	2,190 (1,340–3,380)	3,820 (2,340–6,000)	4,370 (2,770–6,770)	84·5 (52·5–130)	84·6 (53·1–132)	84·7 (53·7–131)
1-110	Bipolar disorder	5,340 (3,080–8,390)	8,830 (5,140–13,700)	9,680 (5,600–14,900)	189 (110–291)	190 (111–294)	190 (109–291)
1-111	Anxiety disorders	11,200 (7,330–16,000)	18,000 (11,900–25,500)	20,000 (13,200–28,300)	387 (256–547)	393 (260–553)	402 (268–568)
1-112	Eating disorders	1,360 (821–2,040)	2,280 (1,370–3,470)	2,540 (1,550–3,910)	40·6 (24·5–61·3)	46·5 (28–70·6)	50·6 (31–77·6)
1-113	Anorexia nervosa	325 (187–534)	534 (305–876)	575 (334–933)	9·5 (5·5–15·5)	10·8 (6·2–17·6)	11·8 (6·9–19·3)

1-114	Bulimia nervosa	1,040 (591–1,640)	1,750 (997–2,750)	1,960 (1,140–3,190)	31·1 (17·9–49·2)	35·7 (20·4–56·1)	38·7 (22·4–62·7)
1-115	Autism spectrum disorders	1,690 (1,100–2,440)	2,320 (1,510–3,330)	2,420 (1,560–3,530)	52·9 (34·4–76·2)	52·7 (34·1–75·8)	52·4 (34–76·3)
1-116	Attention-deficit/hyperactivity disorder	660 (366–1,150)	865 (487–1,500)	842 (479–1,440)	18·9 (10·5–32·9)	18·8 (10·6–32·5)	18·8 (10·6–32·6)
1-117	Conduct disorder	2,520 (1,420–4,030)	3,080 (1,700–4,860)	2,740 (1,520–4,290)	67·7 (38–108)	67·7 (37·3–106)	67·6 (37·3–106)
1-118	Idiopathic developmental intellectual disability	605 (208–1,120)	767 (302–1,360)	697 (255–1,250)	18·7 (6·3–34·4)	17·2 (6·7–30·8)	14·9 (5·4–27)
1-119	Other mental disorders	2,680 (1,670–4,060)	4,810 (3,040–7,350)	5,610 (3,530–8,440)	108 (68·8–163)	108 (68·8–165)	108 (67·8–162)
1-120	Substance use disorders	9,430 (7,050–12,200)	16,700 (12,500–21,700)	18,800 (14,000–24,300)	345 (264–438)	359 (270–466)	363 (270–470)
1-121	Alcohol use disorders	6,510 (4,750–8,620)	11,200 (8,250–15,000)	12,600 (9,270–16,800)	247 (186–320)	243 (180–325)	243 (178–324)
1-122	Drug use disorders	2,920 (2,100–3,950)	5,510 (3,990–7,270)	6,210 (4,620–8,100)	97·5 (70·4–129)	116 (84·1–153)	121 (89·8–158)
1-123	Opioid use disorders	1,430 (918–2,100)	2,860 (1,870–4,050)	3,410 (2,310–4,710)	49·8 (32·6–70·7)	61·4 (40·6–86·3)	65·6 (44·4–90·6)
1-124	Cocaine use disorders	571 (409–789)	1,040 (765–1,420)	1,140 (854–1,490)	18·7 (13·7–25·1)	21·5 (15·9–29·1)	22·4 (16·7–29·3)
1-125	Amphetamine use disorders	208 (109–354)	357 (211–572)	415 (259–641)	6·3 (3·5–10·6)	7·3 (4·3–11·6)	8 (5–12·4)
1-126	Cannabis use disorders	292 (153–512)	468 (270–740)	467 (272–737)	9·1 (4·9–15·5)	9·5 (5·5–15)	9·5 (5·5–15)
1-127	Other drug use disorders	424 (269–633)	781 (535–1,120)	781 (533–1,080)	13·6 (8·9–19·9)	16 (11·1–22·8)	15·4 (10·5–21·2)
1-128	Diabetes and kidney diseases	25,400 (21,500–29,500)	61,500 (51,900–72,300)	86,300 (69,100–106,000)	1,330 (1,130–1,560)	1,560 (1,320–1,830)	1,670 (1,340–2,050)
1-129	Diabetes mellitus	15,100 (12,200–18,500)	30,500 (22,900–39,600)	46,900 (34,900–61,500)	824 (668–1,010)	779 (584–1,010)	905 (674–1,180)
1-130	Diabetes mellitus type 1	703 (540–846)	822 (646–1,040)	1,030 (791–1,340)	27·1 (20·6–32·7)	18·8 (14·7–23·7)	20·5 (15·7–26·5)
1-131	Diabetes mellitus type 2	14,400 (11,500–17,800)	29,700 (22,200–38,600)	45,900 (34,100–60,200)	797 (642–985)	760 (569–991)	884 (658–1,160)
1-132	Chronic kidney disease	10,300 (9,020–11,600)	31,000 (28,100–33,800)	39,400 (31,700–48,300)	510 (451–574)	781 (710–849)	766 (617–936)
1-133	Chronic kidney disease due to diabetes mellitus type 1	568 (392–778)	2,040 (1,330–2,930)	2,350 (1,440–3,700)	26·3 (17·6–37)	47·4 (30·8–68·8)	44·9 (27·6–71)
1-134	Chronic kidney disease due to diabetes mellitus type 2	2,140 (1,720–2,580)	7,210 (5,730–8,650)	9,600 (7,240–12,400)	122 (98·6–148)	190 (152–228)	185 (141–240)
1-135	Chronic kidney disease due to hypertension	2,000 (1,630–2,430)	6,670 (5,500–8,070)	8,710 (6,770–11,300)	110 (90·8–133)	175 (145–212)	169 (131–218)
1-136	Chronic kidney disease due to glomerulonephritis	2,250 (1,820–2,690)	6,660 (5,480–7,990)	8,130 (6,010–10,900)	98·3 (79·6–119)	159 (131–193)	158 (118–212)
1-137	Chronic kidney disease due to other and unspecified causes	3,310 (2,680–3,990)	8,420 (6,860–10,300)	10,600 (8,040–13,400)	153 (125–186)	209 (171–255)	209 (159–263)
1-138	Acute glomerulonephritis	7 (5–9)	4 (4–5)	5 (4–6)	0·3 (0·2–0·4)	0·1 (0·1–0·1)	0·1 (0·1–0·1)
1-139	Skin and subcutaneous diseases	13,400 (8,700–20,300)	19,200 (12,700–29,000)	20,700 (13,600–30,700)	438 (290–662)	446 (295–668)	448 (295–666)
1-140	Dermatitis	3,050 (1,800–4,820)	4,040 (2,400–6,240)	4,250 (2,560–6,590)	94·4 (56·7–145)	94·5 (56·2–147)	94·6 (56·5–148)
1-141	Atopic dermatitis	2,260 (1,210–3,840)	2,690 (1,440–4,550)	2,700 (1,440–4,630)	63·9 (34·3–108)	64 (34·3–109)	64 (34·3–110)
1-142	Contact dermatitis	693 (438–1,050)	1,200 (746–1,800)	1,390 (877–2,080)	27·1 (17·1–40·4)	27·2 (17–40·8)	27·2 (17·2–40·7)
1-143	Seborrhoeic dermatitis	94 (53–150)	148 (83–237)	166 (95–260)	3·4 (1·9–5·4)	3·4 (1·9–5·4)	3·4 (1·9–5·4)
1-144	Psoriasis	319 (222–439)	475 (332–635)	517 (360–694)	11·8 (8·3–16·2)	10·8 (7·5–14·4)	10·4 (7·2–13·9)
1-145	Bacterial skin diseases	432 (301–645)	918 (638–1,390)	1,250 (862–1,920)	17·4 (12·3–26·3)	22·7 (15·9–34·8)	25·2 (17·5–38·2)
1-146	Cellulitis	233 (166–382)	506 (342–822)	732 (471–1,200)	10·1 (6·9–17)	12·4 (8·4–20·8)	14·4 (9·3–23·6)
1-147	Pyoderma	198 (114–352)	411 (266–664)	522 (339–842)	7·3 (4·3–12·8)	10·2 (6·7–16·4)	10·7 (6·9–17·1)
1-148	Scabies	2,400 (1,300–3,830)	3,220 (1,760–5,200)	3,290 (1,790–5,240)	73·3 (40·4–118)	72·9 (39·9–117)	72·5 (38·9–116)
1-149	Fungal skin diseases	698 (275–1,470)	1,130 (456–2,380)	1,320 (532–2,790)	27·3 (10·9–57·7)	27·1 (10·9–57·5)	27 (10·8–57·2)
1-150	Viral skin diseases	1,580 (994–2,390)	1,820 (1,160–2,730)	1,820 (1,170–2,720)	43·7 (27·5–66·2)	43·8 (27·8–66)	43·8 (28·1–65·4)
1-151	Acne vulgaris	1,410 (830–2,300)	2,260 (1,330–3,590)	2,140 (1,280–3,460)	39·8 (23·5–64·9)	46·5 (27·4–73·6)	48 (28·7–77·4)
1-152	Alopecia areata	205 (130–308)	325 (207–486)	361 (227–538)	7·1 (4·5–10·6)	7·1 (4·6–10·6)	7·2 (4·5–10·7)
1-153	Pruritus	224 (106–403)	377 (181–673)	444 (212–794)	8·6 (4·2–15·4)	8·9 (4·2–15·9)	9 (4·3–16·1)
1-154	Urticaria	1,590 (1,020–2,330)	2,060 (1,330–2,960)	2,150 (1,410–3,080)	48·2 (31–69·5)	48·4 (31·2–69·7)	48·3 (31·7–70)
1-155	Decubitus ulcer	289 (167–386)	501 (293–664)	667 (389–889)	16·7 (9·4–21·6)	13·6 (7·9–17·8)	13·1 (7·7–17·4)
1-156	Other skin and subcutaneous diseases	1,220 (629–2,170)	2,080 (1,050–3,710)	2,440 (1,220–4,390)	49·5 (25·8–87·5)	49·6 (25·1–88·5)	49·2 (24·6–88·4)
1-157	Sense organ diseases	17,300 (11,900–24,800)	32,100 (21,700–46,200)	40,400 (27,500–57,600)	861 (590–1,220)	808 (549–1,160)	793 (540–1,130)
1-158	Blindness and vision loss	6,000 (4,100–8,350)	10,700 (7,320–15,100)	13,500 (9,250–18,800)	308 (215–429)	273 (188–386)	266 (182–371)
1-159	Glaucoma	209 (139–297)	363 (246–510)	481 (325–673)	13·1 (8·8–18·8)	10·2 (6·9–14·3)	9·5 (6·4–13·3)
1-160	Cataract	1,350 (945–1,830)	2,540 (1,760–3,420)	3,330 (2,340–4,500)	81·7 (57·1–110)	69·1 (48–92·8)	65·7 (46·1–88·6)
1-161	Age-related macular degeneration	64 (43–91)	128 (86–181)	177 (120–254)	3·9 (2·6–5·6)	3·6 (2·4–5·1)	3·5 (2·4–5)
1-162	Refraction disorders	2,210 (1,460–3,190)	3,560 (2,380–5,110)	4,080 (2,700–5,800)	89·3 (59·8–127)	83·4 (56–120)	81·5 (54·1–117)
1-163	Near vision loss	970 (434–1,900)	2,030 (938–4,010)	2,810 (1,230–5,500)	55·4 (25·1–109)	53·6 (25·1–105)	54·5 (23·9–107)
1-164	Other vision loss	1,200 (822–1,680)	2,060 (1,430–2,850)	2,620 (1,830–3,580)	64·3 (44·5–89·5)	53·2 (37–73·4)	50·9 (35·3–69·8)
1-165	Age-related and other hearing loss	10,500 (7,010–15,400)	19,900 (13,200–29,300)	25,100 (16,600–37,200)	517 (347–752)	498 (332–727)	490 (327–723)
1-166	Other sense organ diseases	821 (491–1,260)	1,490 (882–2,320)	1,840 (1,080–2,870)	35·9 (21·3–56·4)	36·6 (21·8–57·1)	36·8 (21·8–56·7)

1-167	Musculoskeletal disorders	43,400 (31,400–56,600)	83,300 (60,200–109,000)	101,000 (73,200–132,000)	1,860 (1,350–2,410)	1,930 (1,400–2,520)	1,950 (1,410–2,540)
1-168	Rheumatoid arthritis	1,020 (734–1,320)	2,520 (1,850–3,230)	3,430 (2,550–4,400)	49·3 (36–63·4)	61·1 (45·1–78)	65·8 (48·9–84·4)
1-169	Osteoarthritis	3,390 (1,700–6,760)	7,930 (3,950–16,000)	10,800 (5,420–21,600)	192 (96·5–383)	204 (102–412)	208 (104–414)
1-170	Low back pain	19,500 (13,600–26,200)	33,900 (23,700–45,700)	39,100 (27,300–52,900)	787 (555–1,060)	769 (538–1,030)	762 (532–1,030)
1-171	Neck pain	3,110 (2,010–4,560)	5,900 (3,900–8,570)	7,030 (4,640–10,200)	135 (88·6–195)	135 (89–196)	135 (89·3–196)
1-172	Gout	100 (60–148)	231 (141–338)	295 (183–433)	4·8 (2·9–7·1)	5·5 (3·4–8·1)	5·6 (3·5–8·3)
1-173	Other musculoskeletal disorders	16,200 (11,600–22,000)	32,800 (23,000–45,000)	40,200 (28,100–54,800)	689 (486–933)	753 (526–1,030)	770 (539–1,050)
1-174	Other non-communicable diseases	61,400 (47,800–74,300)	71,700 (55,300–91,100)	74,400 (56,300–96,500)	1,950 (1,540–2,430)	1,790 (1,380–2,230)	1,650 (1,240–2,120)
1-175	Congenital anomalies	36,000 (25,800–41,300)	27,400 (18,700–31,600)	21,000 (13,700–28,600)	928 (669–1,060)	748 (508–865)	600 (380–828)
1-176	Neural tube defects	3,460 (2,600–4,430)	1,900 (1,570–2,310)	1,890 (1,360–2,620)	88·9 (67–114)	51·5 (42·3–63·4)	54·3 (38·4–76·2)
1-177	Congenital heart anomalies	13,900 (8,020–17,200)	9,230 (5,050–11,100)	5,760 (3,150–8,180)	354 (206–437)	256 (138–309)	169 (89·8–243)
1-178	Orofacial clefts	41 (26–61)	58 (35–87)	61 (37–92)	1·3 (0·8–1·9)	1·4 (0·8–2)	1·3 (0·8–2)
1-179	Down's syndrome	1,430 (1,000–2,050)	772 (629–935)	692 (484–949)	36·7 (25·9–52·3)	20·5 (16·6–25)	19 (13·2–26·3)
1-180	Turner syndrome	5 (2–9)	6 (3–10)	6 (3–10)	0·2 (0·1–0·3)	0·1 (0·1–0·2)	0·1 (0·1–0·2)
1-181	Klinefelter syndrome	0 (0–1)	0 (0–1)	0 (0–1)	0 (0–0)	0 (0–0)	0 (0–0)
1-182	Other chromosomal abnormalities	1,360 (514–1,880)	1,870 (465–2,460)	1,830 (432–2,890)	35 (13·3–48·3)	52·2 (12·5–68·8)	55 (12·4–87·9)
1-183	Congenital musculoskeletal and limb anomalies	2,120 (1,460–2,870)	2,850 (1,980–3,900)	2,880 (1,960–4,060)	61 (42·4–82·6)	68·8 (47·7–94·1)	68·5 (46·6–95·9)
1-184	Urogenital congenital anomalies	987 (272–1,560)	803 (267–1,170)	566 (210–911)	25·5 (7·1–40)	22·3 (7·1–32·8)	16·6 (5·8–27·2)
1-185	Digestive congenital anomalies	3,960 (1,980–5,130)	2,600 (1,410–3,130)	1,870 (929–2,740)	101 (50·5–130)	73 (39·7–88·2)	56·7 (28·2–83·1)
1-186	Other congenital anomalies	8,730 (6,480–10,500)	7,310 (5,340–8,500)	5,460 (3,680–7,590)	225 (168–272)	202 (147–235)	160 (106–224)
1-187	Urinary diseases and male infertility	1,390 (1,040–1,870)	3,650 (2,900–4,550)	5,340 (4,070–6,800)	68·7 (50·5–93·7)	95·8 (75·9–120)	106 (80·6–134)
1-188	Urinary tract infections and interstitial nephritis	552 (470–746)	1,930 (1,540–2,120)	3,020 (2,070–3,880)	24·3 (20·7–34·1)	49·7 (39·2–54·7)	59·7 (41·3–76·7)
1-189	Urolithiasis	52 (33–74)	117 (80–160)	136 (94–189)	2·2 (1·4–3·2)	2·7 (1·9–3·7)	2·6 (1·8–3·6)
1-190	Benign prostatic hyperplasia	582 (343–899)	1,220 (719–1,900)	1,720 (1,010–2,660)	35 (20·6–54)	34·5 (20·3–53·6)	33·9 (19·8–52·1)
1-191	Male infertility	90 (33–216)	138 (51–326)	151 (56–358)	2·9 (1·1–7)	2·9 (1·1–6·9)	2·9 (1·1–6·9)
1-192	Other urinary diseases	111 (87–143)	246 (153–328)	313 (181–463)	4·2 (3·3–6)	6·1 (3·7–8·2)	6·4 (3·8–9·2)
1-193	Gynecological diseases	9,200 (6,310–12,900)	15,100 (10,300–21,400)	16,700 (11,400–23,700)	330 (228–461)	322 (221–458)	324 (221–462)
1-194	Uterine fibroids	642 (322–1,180)	1,240 (592–2,310)	1,440 (691–2,770)	27·1 (13·6–49·9)	27·2 (13–50·9)	27·6 (13·3–53·2)
1-195	Polycystic ovarian syndrome	267 (111–547)	534 (225–1,080)	603 (247–1,240)	8·5 (3·5–17·3)	11 (4·6–22·3)	12 (4·9–24·7)
1-196	Female infertility	161 (49–396)	245 (73–615)	278 (83–691)	5·3 (1·6–12·7)	5·2 (1·5–13·3)	5·3 (1·6–13·2)
1-197	Endometriosis	978 (562–1,580)	1,160 (676–1,830)	1,140 (674–1,820)	31·9 (18·9–50·8)	24 (14–38·5)	22·2 (13·1–35·3)
1-198	Genital prolapse	135 (66–261)	217 (102–409)	268 (124–515)	7·3 (3·5–14·2)	5·4 (2·6–10·3)	5·1 (2·4–9·8)
1-199	Premenstrual syndrome	3,060 (1,870–4,800)	4,890 (2,980–7,590)	5,160 (3,130–8,090)	99 (60·9–154)	101 (61·8–157)	103 (62·4–160)
1-200	Other gynecological diseases	3,960 (2,510–5,810)	6,770 (4,240–9,950)	7,780 (4,930–11,400)	151 (95·3–220)	148 (93·3–216)	149 (94·2–218)
1-201	Hemoglobinopathies and hemolytic anaemias	1,380 (1,190–1,610)	1,720 (1,520–1,990)	1,970 (1,550–2,490)	49·2 (42·8–57·3)	40·5 (35·8–46·6)	40·6 (32·2–51·1)
1-202	Thalassemias	16 (8–28)	24 (14–40)	26 (13–43)	0·4 (0·2–0·7)	0·6 (0·3–1)	0·7 (0·4–1·1)
1-203	Thalassaemias trait	53 (30–86)	72 (41–118)	73 (41–118)	1·6 (0·9–2·7)	1·6 (0·9–2·5)	1·5 (0·8–2·4)
1-204	Sickle cell disorders	10 (5–18)	12 (7–18)	8 (4–15)	0·3 (0·1–0·5)	0·3 (0·2–0·5)	0·2 (0·1–0·4)
1-205	Sickle cell trait	19 (7–35)	35 (17–57)	22 (8–42)	0·6 (0·2–1·1)	0·7 (0·4–1·2)	0·4 (0·2–0·9)
1-206	G6PD deficiency	479 (398–559)	704 (602–805)	812 (606–1,060)	15 (12·5–17·4)	15·8 (13·5–18)	16·9 (12·6–22)
1-207	G6PD trait	3 (1–5)	7 (3–12)	8 (4–14)	0·1 (0–0·2)	0·2 (0·1–0·3)	0·2 (0·1–0·3)
1-208	Other hemoglobinopathies and hemolytic anaemias	801 (657–986)	868 (712–1,080)	1,020 (790–1,310)	31·3 (26–37·8)	21·3 (17·6–26·2)	20·7 (16·1–26·6)
1-209	Endocrine, metabolic, blood, and immune disorders	4,980 (3,720–6,560)	9,050 (6,790–12,000)	11,500 (8,570–15,400)	210 (154–280)	223 (167–296)	234 (175–311)
1-210	Oral disorders	8,010 (4,610–12,800)	14,500 (8,430–23,000)	17,600 (10,400–28,100)	355 (212–562)	346 (204–547)	343 (202–548)
1-211	Caries of deciduous teeth	115 (48–246)	104 (45–216)	101 (44–210)	2·9 (1·2–6·1)	2·8 (1·2–5·8)	2·8 (1·2–5·8)
1-212	Caries of permanent teeth	807 (363–1,600)	1,220 (544–2,370)	1,290 (580–2,520)	27·7 (12·4–54·3)	26·7 (12–51·5)	26·2 (11·9–51·1)
1-213	Periodontal disease	2,600 (999–5,520)	4,950 (1,910–10,500)	5,920 (2,300–12,600)	110 (43–234)	112 (43·2–236)	113 (44–241)
1-214	Edentulism	2,970 (1,880–4,360)	5,860 (3,710–8,620)	7,640 (4,880–11,300)	161 (103–238)	151 (96·3–221)	147 (94·1–219)
1-215	Other oral disorders	1,530 (941–2,230)	2,380 (1,470–3,460)	2,620 (1,620–3,820)	53·2 (33·1–78)	53·3 (33·1–77·7)	53·3 (33–77·8)
1-216	Sudden infant death syndrome	515 (330–790)	365 (255–559)	256 (151–424)	13 (8·3–19·9)	10·3 (7·2–15·7)	7·8 (4·6–13)
1-0	Injuries	89,700 (83,700–97,100)	132,000 (123,000–143,000)	146,000 (121,000–176,000)	3,180 (2,930–3,460)	2,910 (2,700–3,160)	2,910 (2,410–3,500)
1-1	Transport injuries	32,000 (30,200–33,900)	43,100 (40,500–45,900)	49,800 (40,200–60,700)	1,130 (1,060–1,200)	949 (889–1,010)	985 (797–1,200)
1-2	Road injuries	30,300 (28,600–32,100)	39,900 (37,400–42,500)	45,500 (36,800–55,300)	1,070 (1,010–1,140)	880 (822–938)	902 (731–1,100)

1·3	Pedestrian road injuries	15,100 (14,200–16,000)	20,100 (19,000–21,100)	20,900 (16,700–25,800)	550 (518–581)	454 (431–478)	415 (333–513)
1·4	Cyclist road injuries	2,290 (1,950–2,690)	2,190 (1,850–2,590)	2,220 (1,740–2,740)	83·6 (71·1–98·2)	49·6 (41·8–58·6)	44·3 (34·8–54·9)
1·5	Motorcyclist road injuries	4,050 (3,560–4,590)	6,890 (6,280–7,570)	9,130 (7,240–11,400)	133 (117–149)	143 (131–158)	180 (142–224)
1·6	Motor vehicle road injuries	8,180 (7,520–8,950)	9,630 (8,760–10,400)	11,800 (9,500–14,400)	280 (257–308)	207 (188–225)	233 (188–285)
1·7	Other road injuries	690 (570–820)	1,150 (982–1,340)	1,500 (1,180–1,860)	25·5 (21·1–30·4)	25·6 (21·9–29·8)	29·7 (23·3–37)
1·8	Other transport injuries	1,680 (1,500–1,950)	3,190 (2,920–3,500)	4,230 (3,140–5,380)	59·1 (53·1–67·8)	69·2 (63·3–75·5)	83·6 (62·2–106)
1·9	Unintentional injuries	38,800 (34,600–44,300)	43,400 (37,200–51,500)	47,400 (37,800–58,400)	1,410 (1,240–1,610)	1,010 (866–1,190)	958 (765–1,180)
1·10	Falls	9,110 (7,420–11,300)	14,200 (11,600–17,500)	18,100 (14,400–22,500)	415 (346–504)	344 (282–424)	357 (284–444)
1·11	Drowning	11,000 (10,200–11,800)	7,580 (7,080–8,160)	7,120 (5,550–9,010)	333 (311–356)	168 (156–180)	150 (117–189)
1·12	Fire, heat, and hot substances	3,620 (2,760–4,800)	3,540 (2,460–5,030)	3,690 (2,490–5,370)	124 (93·5–166)	80·9 (56·7–114)	75·5 (51·3–109)
1·13	Poisonings	756 (598–971)	832 (650–1,040)	1,830 (1,590–2,120)	25·9 (20·6–33·6)	18·6 (14·5–23·2)	37·7 (32·8–43·7)
1·14	Poisoning by carbon monoxide	72 (56–92)	130 (107–159)	168 (130–214)	2·4 (1·8–3·1)	2·9 (2·4–3·6)	3·4 (2·6–4·4)
1·15	Poisoning by other means	685 (535–895)	702 (532–904)	1,660 (1,450–1,940)	23·6 (18·7–31)	15·7 (11·9–20·2)	34·3 (29·8–39·8)
1·16	Exposure to mechanical forces	3,800 (2,900–5,100)	4,850 (3,490–6,810)	5,920 (4,220–8,410)	140 (106–188)	110 (79·4–155)	119 (84·6–168)
1·17	Unintentional firearm injuries	425 (346–516)	557 (451–674)	653 (509–812)	15 (12·1–18·4)	12·4 (10·1–15·1)	13·1 (10·2–16·2)
1·18	Other exposure to mechanical forces	3,370 (2,540–4,650)	4,290 (3,000–6,230)	5,260 (3,670–7,690)	125 (92·9–172)	97·9 (68·5–142)	106 (73·6–154)
1·19	Adverse effects of medical treatment	2,630 (2,110–2,880)	2,260 (2,070–2,740)	2,640 (2,040–3,410)	108 (79·4–118)	54·9 (50·2–67·2)	53·1 (41–69)
1·20	Animal contact	843 (658–1,060)	1,050 (826–1,330)	1,180 (910–1,480)	30·4 (23·9–38·2)	24·1 (19–30·4)	23·9 (18·5–30·2)
1·21	Venomous animal contact	502 (372–652)	621 (464–812)	702 (528–914)	18·3 (13·9–23·5)	14·3 (10·7–18·5)	14·3 (10·7–18·7)
1·22	Non-venomous animal contact	341 (270–441)	433 (344–560)	473 (359–623)	12·1 (9·6–15·9)	9·9 (7·9–12·8)	9·6 (7·3–12·6)
1·23	Foreign body	3,500 (3,060–4,110)	1,660 (1,450–1,960)	1,840 (1,440–2,270)	111 (99·5–127)	40·7 (35·5–47·8)	40 (31·3–49·5)
1·24	Pulmonary aspiration and foreign body in airway	3,100 (2,690–3,690)	1,150 (1,050–1,310)	1,280 (1,010–1,610)	97·3 (86·6–112)	29·1 (26·4–33·6)	28·6 (22·2–36·4)
1·25	Foreign body in eyes	153 (67–279)	241 (113–431)	265 (129–466)	5·4 (2·6–9·5)	5·4 (2·6–9·5)	5·4 (2·6–9·4)
1·26	Foreign body in other body part	248 (194–319)	270 (198–366)	294 (216–397)	8·5 (6·6–11)	6·2 (4·6–8·4)	6·1 (4·4–8·2)
1·27	Environmental heat and cold exposure	268 (198–354)	417 (311–548)	483 (359–637)	10·4 (7·8–13·6)	9·6 (7·2–12·6)	9·7 (7·2–12·7)
1·28	Exposure to forces of nature	77 (68–86)	2,780 (2,530–3,070)	78 (56–110)	2·4 (2·2–2·8)	62·9 (57·1–69·4)	1·5 (1·1–2·1)
1·29	Other unintentional injuries	3,180 (2,670–3,890)	4,260 (3,540–5,260)	4,480 (3,500–5,760)	108 (89·9–133)	93·9 (77·9–117)	90·1 (70·7–116)
1·30	Self-harm and interpersonal violence	18,900 (17,900–19,900)	45,300 (43,700–46,800)	49,200 (39,400–60,500)	637 (607–670)	953 (921–987)	969 (775–1,190)
1·31	Self-harm	8,770 (8,260–9,350)	17,200 (16,300–18,100)	19,100 (14,900–24,200)	302 (286–322)	364 (345–382)	374 (293–474)
1·32	Self-harm by firearm	1,720 (1,440–2,200)	2,590 (2,260–3,740)	2,720 (1,990–3,950)	62·6 (53·4–80·3)	56 (48·9–80)	52·6 (38·5–76·1)
1·33	Self-harm by other specified means	7,050 (6,500–7,590)	14,600 (13,500–15,500)	16,400 (12,700–20,800)	240 (221–258)	308 (284–326)	322 (249–409)
1·34	Interpersonal violence	9,980 (9,300–10,700)	28,000 (26,800–29,300)	30,100 (23,700–36,800)	332 (309–355)	588 (562–615)	594 (470–729)
1·35	Physical violence by firearm	4,720 (4,380–5,080)	18,300 (17,500–19,200)	20,000 (15,300–25,200)	154 (142–164)	378 (361–396)	395 (300–498)
1·36	Physical violence by sharp object	2,820 (2,600–3,060)	5,280 (4,910–5,670)	5,330 (4,270–6,600)	95·5 (88·2–103)	111 (104–119)	104 (83·6–130)
1·37	Sexual violence	451 (297–641)	715 (476–1,010)	771 (513–1,090)	15·1 (10·1–21·4)	15·3 (10·2–21·6)	15·3 (10·2–21·8)
1·38	Physical violence by other means	1,980 (1,750–2,230)	3,720 (3,400–4,100)	3,930 (3,150–4,750)	67·6 (60–75·7)	84 (76·8–92·5)	79·7 (64·1–96·6)
1·39	Conflict and terrorism	78 (70–88)	11 (7–17)	9 (6–14)	2·2 (1·9–2·5)	0·2 (0·2–0·4)	0·2 (0·1–0·3)
1·40	Executions and police conflict	23 (22–25)	30 (28–32)	30 (24–38)	0·7 (0·6–0·7)	0·7 (0·6–0·7)	0·7 (0·5–0·9)

SR Table 1: All-age DALYs counts and age standardized DALYs rates per 100,000 individuals for all causes and level 1 cause groups by location for 1990, 2010, and 2019, both sexes combined, El Salvador

Cause Level	Cause	All-age DALYs			Age-standardized rate of DALYs per 100,000		
		1990	2010	2019	1990	2010	2019
0-0	All causes	2,120,000 (1,960,000–2,300,000)	1,650,000 (1,490,000–1,830,000)	1,740,000 (1,430,000–2,090,000)	42,300 (39,000–46,000)	30,100 (27,200–33,300)	28,400 (23,300–34,100)
1-0	Communicable, maternal, neonatal, and nutritional disorders	725,000 (648,000–803,000)	240,000 (213,000–270,000)	193,000 (157,000–236,000)	10,700 (9,720–11,800)	4,250 (3,770–4,760)	3,260 (2,650–4,010)
1-1	HIV/AIDS and sexually transmitted infections	8,430 (6,660–11,400)	24,200 (22,800–26,000)	21,200 (19,700–23,000)	160 (135–202)	439 (414–470)	347 (322–377)
1-2	HIV/AIDS	5,350 (5,080–5,660)	22,300 (21,300–23,400)	19,300 (18,200–20,500)	112 (106–118)	405 (386–424)	314 (296–334)
1-3	HIV/AIDS - drug-susceptible tuberculosis	2,000 (1,350–2,410)	860 (553–1,290)	708 (468–1,050)	41·7 (28·3–50·2)	15·7 (10·1–23·5)	11·6 (7·7–17·2)
1-4	HIV/AIDS - Multidrug-resistant Tuberculosis without extensive drug resistance	9 (1–42)	45 (4–177)	34 (4–142)	0·2 (0–0·9)	0·8 (0·1–3·2)	0·6 (0·1–2·3)
1-5	HIV/AIDS - Extensively drug-resistant Tuberculosis	0 (0–0)	5 (0–19)	6 (1–23)	0 (0–0)	0·1 (0–0·4)	0·1 (0–0·4)
1-6	HIV/AIDS resulting in other diseases	3,340 (2,870–4,020)	21,400 (20,200–22,500)	18,500 (17,400–19,800)	69·9 (59·9–84·6)	388 (368–408)	302 (283–323)
1-7	Sexually transmitted infections excluding HIV	3,080 (1,370–5,950)	1,930 (1,070–3,380)	1,930 (1,060–3,280)	48·7 (25–89·7)	34·5 (19–60·5)	32·6 (17·6–56·2)
1-8	Syphilis	2,270 (698–5,210)	900 (297–1,910)	781 (252–1,770)	29·5 (9·4–67)	16 (5·3–33·9)	14·3 (4·5–32·5)
1-9	Chlamydial infection	76 (50–114)	85 (54–128)	94 (60–141)	1·7 (1·1–2·5)	1·5 (0·9–2·2)	1·5 (0·9–2·2)
1-10	Gonococcal infection	50 (37–68)	48 (35–66)	52 (36–72)	1·1 (0·9–1·4)	0·8 (0·6–1·1)	0·8 (0·6–1·1)
1-11	Trichomoniasis	319 (128–656)	435 (176–905)	477 (186–985)	7·6 (3–15·5)	7·8 (3·1–16·2)	7·6 (3–15·8)
1-12	Genital herpes	223 (76–548)	310 (100–754)	353 (116–867)	5·6 (1·9–13·8)	5·7 (1·8–14·1)	5·7 (1·9–14·1)
1-13	Other sexually transmitted infections	144 (99–205)	155 (103–224)	169 (112–245)	3·2 (2·2–4·6)	2·7 (1·8–3·8)	2·7 (1·8–3·9)
1-14	Respiratory infections and tuberculosis	130,000 (112,000–151,000)	67,500 (60,900–75,400)	60,600 (46,900–77,000)	2,250 (2,010–2,560)	1,230 (1,110–1,360)	1,000 (771–1,280)
1-15	Tuberculosis	17,700 (16,100–19,400)	3,360 (3,100–3,670)	3,080 (2,400–3,910)	408 (378–438)	62·4 (57·6–68·1)	50·5 (39·2–63·9)
1-16	Latent tuberculosis infection	0 (0–0)	0 (0–0)	0 (0–0)	0 (0–0)	0 (0–0)	0 (0–0)
1-17	Drug-susceptible tuberculosis	17,700 (16,100–19,300)	3,210 (2,750–3,560)	2,950 (2,250–3,810)	407 (376–438)	59·7 (51–65·9)	48·4 (36·8–62·5)
1-18	Multidrug-resistant tuberculosis without extensive drug resistance	68 (5–309)	134 (14–544)	113 (13–429)	1·6 (0·1–7·3)	2·5 (0·3–10·2)	1·9 (0·2–7)
1-19	Extensively drug-resistant tuberculosis	0 (0–0)	14 (1–56)	18 (2–67)	0 (0–0)	0·3 (0–1·1)	0·3 (0–1·1)
1-20	Lower respiratory infections	105,000 (88,300–125,000)	57,000 (50,900–63,900)	50,400 (37,600–66,300)	1,720 (1,500–1,990)	1,050 (941–1,170)	836 (622–1,100)
1-21	Upper respiratory infections	5,090 (3,170–7,760)	5,060 (3,030–7,950)	5,150 (3,110–8,080)	88·9 (55·4–136)	82·4 (49·7–129)	82·3 (49·6–129)
1-22	Otitis media	2,080 (1,210–3,360)	2,020 (1,150–3,340)	1,950 (1,120–3,210)	35·8 (20·9–58·3)	32·3 (18·6–53·6)	31·1 (17·9–50·9)
1-23	Enteric infections	198,000 (159,000–237,000)	24,600 (19,300–32,100)	20,300 (14,900–27,700)	2,840 (2,300–3,370)	431 (336–568)	342 (249–463)
1-24	Diarrhoeal diseases	197,000 (159,000–237,000)	24,500 (19,100–31,900)	20,200 (14,800–27,600)	2,830 (2,300–3,360)	428 (334–565)	340 (248–461)
1-25	Typhoid and paratyphoid	86 (39–161)	21 (11–40)	18 (8–36)	1·6 (0·8–2·8)	0·4 (0·2–0·7)	0·3 (0·1–0·6)
1-26	Typhoid fever	86 (39–160)	21 (11–40)	18 (8–36)	1·6 (0·7–2·8)	0·4 (0·2–0·7)	0·3 (0·1–0·6)
1-27	Paratyphoid fever	0 (0–0)	0 (0–0)	0 (0–0)	0 (0–0)	0 (0–0)	0 (0–0)
1-28	iNTS	157 (75–273)	100 (47–175)	84 (38–155)	2·5 (1·3–4·4)	1·6 (0·8–2·8)	1·4 (0·6–2·5)
1-29	Other intestinal infectious diseases	180 (57–327)	46 (24–74)	33 (19–51)	2·5 (0·9–4·5)	0·8 (0·4–1·2)	0·5 (0·3–0·8)
1-30	Neglected tropical diseases and malaria	10,100 (7,280–12,900)	6,950 (4,840–9,460)	7,130 (4,980–9,930)	204 (151–262)	122 (85·3–165)	116 (80·9–160)
1-31	Malaria	378 (275–519)	40 (34–46)	27 (20–37)	6·7 (5–8·9)	0·7 (0·6–0·8)	0·4 (0·3–0·6)
1-32	Chagas disease	919 (595–1,290)	718 (519–1,180)	759 (489–1,310)	26·9 (17·5–39·5)	14·2 (10·2–23·7)	12·7 (8·2–22)
1-33	Leishmaniasis	96 (2–776)	17 (1–125)	24 (1–173)	1·5 (0–11·4)	0·3 (0–2)	0·4 (0–2·8)
1-34	Visceral leishmaniasis	92 (0–771)	16 (0–124)	23 (0–171)	1·4 (0–11·4)	0·3 (0–2)	0·4 (0–2·8)
1-35	Cutaneous and mucocutaneous leishmaniasis	4 (1–11)	1 (0–3)	2 (1–4)	0·1 (0–0·2)	0 (0–0)	0 (0–0·1)
1-36	African trypanosomiasis	0 (0–0)	0 (0–0)	0 (0–0)	0 (0–0)	0 (0–0)	0 (0–0)
1-37	Schistosomiasis	0 (0–0)	0 (0–0)	0 (0–0)	0 (0–0)	0 (0–0)	0 (0–0)
1-38	Cysticercosis	2,640 (1,510–4,150)	2,540 (1,330–4,260)	2,990 (1,510–5,100)	70·4 (39·9–110)	48·5 (25·3–81·1)	49 (24·8–83)
1-39	Cystic echinococcosis	38 (19–60)	14 (9–21)	11 (6–17)	0·7 (0·4–1·1)	0·3 (0·2–0·4)	0·2 (0·1–0·3)
1-40	Lymphatic filariasis	0 (0–0)	0 (0–0)	0 (0–0)	0 (0–0)	0 (0–0)	0 (0–0)
1-41	Onchocerciasis	0 (0–0)	0 (0–0)	0 (0–0)	0 (0–0)	0 (0–0)	0 (0–0)
1-42	Trachoma	0 (0–0)	0 (0–0)	0 (0–0)	0 (0–0)	0 (0–0)	0 (0–0)
1-43	Dengue	612 (324–1,030)	912 (454–1,680)	1,230 (773–2,030)	10·8 (6–17·9)	14·9 (7·4–27·2)	19·8 (12·6–32·3)
1-44	Yellow fever	0 (0–0)	0 (0–0)	0 (0–0)	0 (0–0)	0 (0–0)	0 (0–0)
1-45	Rabies	321 (12–496)	5 (2–21)	5 (2–22)	5·3 (0·2–7·8)	0·1 (0–0·4)	0·1 (0–0·4)
1-46	Intestinal nematode infections	3,270 (1,980–5,080)	1,840 (1,030–3,010)	1,330 (730–2,190)	54·5 (32·4–85·2)	29·1 (16·4–47·7)	20·4 (11·3–33·4)
1-47	Ascariasis	553 (307–892)	120 (57–203)	68 (36–109)	7·9 (4·5–12·3)	1·9 (1–3·2)	1·1 (0·6–1·8)
1-48	Trichuriasis	579 (310–1,020)	86 (41–162)	91 (42–172)	9·9 (5·3–17·4)	1·4 (0·7–2·6)	1·4 (0·6–2·6)

1-49	Hookworm disease	2,140 (1,190–3,610)	1,630 (863–2,790)	1,170 (607–1,980)	36·7 (20·1–61·9)	25·8 (13·6–43·9)	18 (9·4–30·3)
1-50	Food-borne trematodiases	0 (0–0)	0 (0–0)	0 (0–0)	0 (0–0)	0 (0–0)	0 (0–0)
1-51	Leprosy	5 (3–7)	3 (2–5)	4 (2–6)	0·1 (0·1–0·2)	0·1 (0–0·1)	0·1 (0–0·1)
1-52	Ebola virus disease	0 (0–0)	0 (0–0)	0 (0–0)	0 (0–0)	0 (0–0)	0 (0–0)
1-53	Zika virus disease	0 (0–0)	0 (0–0)	7 (2–18)	0 (0–0)	0 (0–0)	0·1 (0–0·3)
1-54	Guinea worm disease	0 (0–0)	0 (0–0)	0 (0–0)	0 (0–0)	0 (0–0)	0 (0–0)
1-55	Other neglected tropical diseases	1,800 (914–2,580)	863 (525–1,350)	747 (454–1,160)	27 (14·4–38·6)	13·8 (8·5–21·5)	12·5 (7·6–19·3)
1-56	Other infectious diseases	55,600 (31,700–106,000)	9,470 (6,980–14,100)	9,600 (5,830–17,000)	799 (482–1,470)	162 (119–242)	162 (96·3–291)
1-57	Meningitis	16,700 (13,600–20,200)	3,080 (2,710–3,530)	2,340 (1,730–3,120)	246 (206–293)	53·1 (46·9–60·8)	38·7 (28·5–51·8)
1-58	Encephalitis	4,580 (2,470–5,930)	1,220 (938–2,180)	1,250 (882–2,170)	70 (38·8–88·3)	20·3 (15·6–36·3)	20·5 (14·4–36·1)
1-59	Diphtheria	334 (157–629)	10 (6–16)	10 (6–17)	4·7 (2·3–8·6)	0·2 (0·1–0·3)	0·2 (0·1–0·3)
1-60	Whooping cough	27,300 (3,470–79,700)	2,420 (285–6,810)	3,460 (367–10,000)	361 (46–1,060)	41·6 (4·9–117)	60·8 (6·4–176)
1-61	Tetanus	2,460 (1,330–3,300)	89 (61–271)	86 (51–233)	46·1 (21·4–58·3)	1·6 (1·1–4·8)	1·4 (0·8–3·9)
1-62	Measles	0 (0–0)	0 (0–0)	0 (0–0)	0 (0–0)	0 (0–0)	0 (0–0)
1-63	Varicella and herpes zoster	1,210 (711–1,930)	445 (312–632)	383 (259–546)	20·1 (13·2–30)	7·8 (5·5–11)	6·3 (4·2–9·1)
1-64	Acute hepatitis	509 (402–639)	515 (417–637)	466 (354–608)	9 (7·3–11·1)	8·6 (7·1–10·5)	7·5 (5·7–9·7)
1-65	Acute hepatitis A	286 (200–392)	276 (192–381)	254 (174–361)	4·6 (3·4–6·2)	4·3 (3–5·9)	4 (2·7–5·6)
1-66	Acute hepatitis B	148 (109–202)	167 (141–197)	143 (104–193)	2·9 (2·2–3·7)	3 (2·6–3·6)	2·4 (1·7–3·2)
1-67	Acute hepatitis C	46 (28–72)	42 (31–58)	38 (25–58)	0·9 (0·6–1·4)	0·8 (0·6–1)	0·6 (0·4–1)
1-68	Acute hepatitis E	30 (20–45)	31 (24–43)	30 (22–41)	0·6 (0·4–0·8)	0·5 (0·4–0·7)	0·5 (0·4–0·7)
1-69	Other unspecified infectious diseases	2,540 (2,030–3,660)	1,690 (1,360–2,140)	1,600 (1,180–2,080)	42·2 (34·1–58·4)	28·8 (23·3–36·1)	26·4 (19·3–34·5)
1-70	Maternal and neonatal disorders	281,000 (239,000–330,000)	86,000 (71,200–105,000)	56,000 (41,200–74,300)	3,750 (3,220–4,360)	1,500 (1,240–1,830)	995 (726–1,330)
1-71	Maternal disorders	19,700 (17,300–22,000)	4,660 (4,030–5,350)	3,050 (2,180–4,200)	373 (329–417)	73·7 (63·6–84·6)	45·6 (32·2–62·7)
1-72	Maternal haemorrhage	3,820 (3,060–4,700)	889 (704–1,080)	566 (395–778)	74·8 (60·2–92·2)	14·3 (11·4–17·4)	8·5 (5·9–11·9)
1-73	Maternal sepsis and other pregnancy related infections	4,130 (3,330–5,060)	428 (329–548)	252 (171–363)	78·2 (63·5–95·3)	6·7 (5·2–8·6)	3·7 (2·5–5·3)
1-74	Maternal hypertensive disorders	3,780 (3,150–4,460)	919 (765–1,090)	559 (388–791)	68·4 (57·7–80·1)	14·2 (11·9–16·7)	8·3 (5·7–11·7)
1-75	Maternal obstructed labour and uterine rupture	92 (61–136)	111 (82–145)	94 (63–136)	1·7 (1·2–2·5)	1·8 (1·3–2·3)	1·4 (0·9–2)
1-76	Maternal abortive outcome	5,730 (4,540–6,980)	552 (418–714)	275 (181–410)	111 (88·4–134)	8·8 (6·7–11·4)	4·1 (2·7–6·2)
1-77	Ectopic pregnancy	137 (110–167)	145 (115–178)	101 (67–155)	2·6 (2·1–3·1)	2·3 (1·8–2·8)	1·5 (1–2·3)
1-78	Indirect maternal deaths	554 (431–697)	659 (518–816)	498 (327–724)	10·2 (8–12·7)	10·3 (8·1–12·8)	7·4 (4·9–10·8)
1-79	Late maternal deaths	411 (300–552)	166 (121–227)	121 (70–182)	7·8 (5·7–10·3)	2·6 (1·9–3·6)	1·8 (1–2·7)
1-80	Maternal deaths aggravated by HIV/AIDS	3 (1–6)	7 (3–11)	4 (2–8)	0·1 (0–0·1)	0·1 (0·1–0·2)	0·1 (0–0·1)
1-81	Other maternal disorders	1,000 (791–1,270)	786 (627–983)	583 (386–837)	19 (15–23·9)	12·5 (10–15·5)	8·8 (5·8–12·8)
1-82	Neonatal disorders	262,000 (219,000–310,000)	81,400 (66,200–99,900)	52,900 (38,800–70,600)	3,370 (2,830–3,990)	1,420 (1,160–1,750)	949 (690–1,270)
1-83	Neonatal preterm birth	148,000 (118,000–183,000)	49,000 (40,000–59,700)	30,100 (21,900–40,200)	1,910 (1,530–2,350)	858 (699–1,050)	538 (388–723)
1-84	Neonatal encephalopathy due to birth asphyxia and trauma	52,200 (39,700–66,800)	12,000 (9,130–16,500)	7,630 (5,210–11,200)	667 (507–852)	213 (161–293)	138 (93·9–204)
1-85	Neonatal sepsis and other neonatal infections	34,400 (25,600–44,800)	12,300 (9,680–15,800)	9,430 (6,550–13,300)	445 (332–577)	214 (168–272)	169 (116–240)
1-86	Hemolytic disease and other neonatal jaundice	1,370 (1,080–1,700)	1,160 (873–1,490)	1,140 (859–1,460)	22·6 (17·6–27·7)	18·9 (14·4–24·2)	18·2 (13·8–23·3)
1-87	Other neonatal disorders	25,800 (19,200–33,500)	6,830 (5,320–8,650)	4,680 (3,060–6,870)	331 (246–429)	121 (93·9–153)	85·3 (55·7–126)
1-88	Nutritional deficiencies	42,400 (34,500–51,800)	21,400 (16,600–28,300)	18,100 (13,600–24,300)	721 (595–883)	370 (290–481)	299 (224–400)
1-89	Protein-energy malnutrition	26,600 (21,700–32,100)	9,480 (8,410–10,800)	7,490 (5,700–9,510)	433 (367–504)	172 (153–196)	124 (93·5–158)
1-90	Iodine deficiency	719 (368–1,180)	381 (173–734)	681 (343–1,140)	14·5 (7·6–23·4)	6·3 (2·9–11·9)	10·7 (5·4–18)
1-91	Vitamin A deficiency	1,330 (803–2,040)	587 (357–896)	472 (289–721)	18·9 (11·5–29·1)	9·3 (5·7–14)	7·8 (4·8–11·9)
1-92	Dietary iron deficiency	13,400 (8,330–21,000)	10,600 (6,570–16,700)	9,130 (5,440–14,200)	246 (157–373)	176 (110–276)	151 (90·6–237)
1-93	Other nutritional deficiencies	368 (250–518)	341 (229–480)	369 (242–525)	8·6 (5·8–11·9)	6·1 (4·1–8·6)	5·9 (3·9–8·4)
1-0	Non-communicable diseases	903,000 (793,000–1,020,000)	1,050,000 (913,000–1,200,000)	1,200,000 (979,000–1,440,000)	22,100 (19,700–24,700)	19,700 (17,400–22,500)	19,700 (16,100–23,800)
1-1	Neoplasms	83,200 (78,600–92,900)	123,000 (117,000–128,000)	147,000 (111,000–189,000)	2,290 (2,180–2,510)	2,420 (2,300–2,510)	2,470 (1,870–3,180)
1-2	Lip and oral cavity cancer	1,050 (979–1,120)	1,160 (1,080–1,240)	1,500 (1,120–1,950)	33·1 (30·6–35·4)	23·6 (22–25·3)	25·5 (18·9–33·1)
1-3	Nasopharynx cancer	217 (203–233)	295 (274–316)	330 (247–431)	6 (5·6–6·5)	5·8 (5·4–6·2)	5·5 (4·1–7·3)
1-4	Other pharynx cancer	481 (436–524)	375 (343–408)	468 (345–619)	15·3 (13·9–16·7)	7·8 (7·1–8·4)	8 (5·9–10·6)
1-5	Oesophageal cancer	1,040 (955–1,250)	1,770 (1,580–1,920)	2,140 (1,610–2,790)	34·3 (31·2–41)	36·6 (32·8–39·7)	36·4 (27·3–47·7)
1-6	Stomach cancer	12,800 (12,100–13,500)	18,700 (17,700–19,700)	21,700 (16,400–28,300)	407 (382–430)	383 (361–402)	367 (277–480)
1-7	Colon and rectum cancer	3,700 (3,470–3,930)	9,480 (8,950–10,000)	12,600 (9,640–16,500)	116 (108–123)	191 (181–202)	213 (162–279)

1-8	Liver cancer	2,980 (2,780–3,190)	2,140 (1,990–2,290)	2,780 (2,090–3,650)	90 (83·4–96·7)	43·7 (40·7–46·9)	47·5 (35·6–62·4)
1-9	Liver cancer due to hepatitis B	677 (514–874)	394 (289–540)	499 (318–760)	19·4 (14·5–25·7)	8 (5·8–11·1)	8·5 (5·4–13)
1-10	Liver cancer due to hepatitis C	968 (719–1,220)	721 (539–909)	958 (633–1,350)	32·8 (24·5–41·5)	15·1 (11·1–19·1)	16·3 (10·8–22·9)
1-11	Liver cancer due to alcohol use	682 (482–905)	628 (458–816)	860 (553–1,260)	22·3 (15·9–29·6)	13·2 (9·6–17·1)	14·8 (9·5–21·6)
1-12	Liver cancer due to NASH	204 (148–281)	179 (130–249)	244 (153–373)	6·3 (4·4–8·8)	3·6 (2·6–5·2)	4·1 (2·6–6·3)
1-13	Gallbladder and biliary tract cancer	1,870 (1,650–2,180)	2,630 (2,340–3,230)	3,360 (2,470–4,540)	61·9 (54·5–73)	54·8 (48·6–67·4)	57·4 (42·2–77·6)
1-14	Pancreatic cancer	1,230 (1,150–1,330)	4,990 (4,680–5,280)	6,840 (5,060–8,930)	40·8 (38–44)	104 (97·4–110)	117 (86·4–153)
1-15	Larynx cancer	622 (574–683)	885 (820–949)	1,080 (804–1,430)	20·1 (18·5–22·1)	18·4 (17–19·7)	18·5 (13·7–24·4)
1-16	Tracheal, bronchus, and lung cancer	6,970 (6,570–7,400)	10,800 (10,200–11,400)	14,100 (10,500–18,200)	224 (211–238)	224 (211–235)	240 (179–311)
1-17	Malignant skin melanoma	199 (158–267)	268 (212–344)	331 (227–466)	5·5 (4·4–7·4)	5·2 (4·1–6·7)	5·5 (3·8–7·8)
1-18	Non-melanoma skin cancer	201 (185–217)	563 (503–606)	750 (561–968)	6·6 (6·1–7·2)	11·3 (10·1–12·2)	12·4 (9·2–16)
1-19	Non-melanoma skin cancer (squamous-cell carcinoma)	201 (185–216)	562 (502–606)	749 (560–967)	6·6 (6·1–7·1)	11·3 (10·1–12·2)	12·4 (9·2–16)
1-20	Non-melanoma skin cancer (basal-cell carcinoma)	0 (0–1)	1 (0–1)	1 (0–2)	0 (0–0)	0 (0–0)	0 (0–0)
1-21	Breast cancer	4,380 (3,910–4,900)	8,550 (7,860–9,260)	11,100 (8,050–14,900)	133 (118–148)	172 (158–187)	190 (138–254)
1-22	Cervical cancer	10,000 (8,940–12,900)	14,300 (12,000–15,900)	15,600 (11,000–21,200)	296 (264–382)	285 (238–316)	265 (188–361)
1-23	Uterine cancer	1,160 (896–1,320)	1,480 (1,300–2,100)	1,870 (1,340–2,770)	38 (29·3–43·2)	31 (27·1–43·8)	32·1 (23–47·6)
1-24	Ovarian cancer	933 (798–1,370)	3,200 (2,260–3,580)	3,940 (2,470–5,460)	27·2 (23–41·1)	63·7 (45·4–71·3)	66·8 (42–92·4)
1-25	Prostate cancer	2,930 (2,430–4,220)	6,750 (5,240–7,750)	8,670 (6,240–11,400)	106 (87·3–152)	138 (108–159)	144 (103–189)
1-26	Testicular cancer	217 (163–298)	448 (335–573)	519 (334–785)	4 (3·1–5·5)	7·4 (5·6–9·3)	7·9 (5·2–11·8)
1-27	Kidney cancer	1,140 (1,040–1,250)	1,770 (1,660–1,890)	2,290 (1,700–2,980)	28·8 (26·8–31)	35·3 (33·1–37·7)	38·9 (28·8–50·7)
1-28	Bladder cancer	555 (514–599)	952 (877–1,020)	1,240 (943–1,600)	18·9 (17·5–20·5)	19·6 (18–21)	20·8 (15·7–26·8)
1-29	Brain and nervous system cancer	2,660 (1,840–5,660)	6,210 (3,670–7,290)	6,610 (3,580–9,570)	54·8 (38·7–112)	112 (65·7–132)	109 (59·3–158)
1-30	Thyroid cancer	941 (508–1,080)	693 (633–841)	854 (639–1,150)	29·5 (15·3–33·9)	14·1 (12·9–16·9)	14·5 (10·8–19·6)
1-31	Mesothelioma	43 (37–52)	66 (56–73)	89 (62–122)	1·3 (1·1–1·5)	1·3 (1·1–1·5)	1·5 (1·1–2·1)
1-32	Hodgkin lymphoma	1,580 (1,270–1,910)	1,000 (837–1,450)	1,030 (697–1,570)	35 (27·8–41·5)	17·8 (15–25·6)	16·5 (11·2–25·2)
1-33	Non-Hodgkin's lymphoma	2,200 (2,030–2,380)	3,640 (3,440–3,850)	4,520 (3,420–5,920)	51·8 (48·6–55·1)	68·3 (64·4–72·3)	74·8 (56·4–98·2)
1-34	Multiple myeloma	404 (330–542)	725 (620–843)	1,000 (714–1,340)	13·2 (10·7–17·7)	15·2 (13–17·6)	17·2 (12·3–23·1)
1-35	Leukaemia	13,400 (11,700–16,900)	12,200 (10,600–13,300)	11,700 (8,530–15,400)	239 (215–295)	205 (178–222)	189 (137–248)
1-36	Acute lymphoid leukaemia	1,610 (1,000–3,840)	2,590 (1,890–3,090)	2,690 (1,690–3,750)	24·4 (15·7–57·3)	39·5 (29–46·9)	42·7 (26·8–59·6)
1-37	Chronic lymphoid leukaemia	42 (34–55)	126 (100–144)	188 (130–253)	1·3 (1–1·6)	2·5 (2–2·9)	3·1 (2·2–4·2)
1-38	Acute myeloid leukaemia	602 (434–1,090)	1,140 (861–1,330)	1,520 (884–2,100)	11·2 (8·4–18·9)	19·5 (14·6–22·5)	24·5 (14·3–33·9)
1-39	Chronic myeloid leukaemia	187 (149–259)	292 (244–339)	346 (243–469)	4·2 (3·4–5·6)	5·6 (4·6–6·4)	5·8 (4·1–7·8)
1-40	Other leukaemia	10,900 (9,060–12,700)	8,040 (6,690–9,020)	7,000 (5,130–9,140)	198 (171–230)	138 (115–154)	113 (82·3–147)
1-41	Other neoplasms	371 (293–451)	388 (329–484)	507 (386–648)	8·9 (7·1–10·4)	7·4 (6·3–9·3)	8·4 (6·4–10·8)
1-42	Myelodysplastic, myeloproliferative, and other hematopoietic neoplasms	371 (293–451)	388 (329–484)	507 (386–648)	8·9 (7·1–10·4)	7·4 (6·3–9·3)	8·4 (6·4–10·8)
1-43	Benign and in situ intestinal neoplasms	0 (0–0)	0 (0–0)	0 (0–0)	0 (0–0)	0 (0–0)	0 (0–0)
1-44	Benign and in situ cervical and uterine neoplasms	0 (0–0)	0 (0–0)	0 (0–0)	0 (0–0)	0 (0–0)	0 (0–0)
1-45	Other benign and in situ neoplasms	0 (0–0)	0 (0–0)	0 (0–0)	0 (0–0)	0 (0–0)	0 (0–0)
1-46	Cardiovascular diseases	157,000 (147,000–165,000)	147,000 (136,000–160,000)	179,000 (141,000–226,000)	4,720 (4,450–4,960)	2,920 (2,710–3,180)	2,950 (2,310–3,720)
1-47	Rheumatic heart disease	3,900 (3,040–5,060)	3,420 (2,420–4,780)	3,560 (2,450–4,980)	86·8 (70·1–110)	58·7 (42–80·8)	56·5 (39–78·8)
1-48	Ischaemic heart disease	77,600 (73,100–81,600)	90,100 (83,400–95,300)	111,000 (86,600–141,000)	2,490 (2,340–2,620)	1,810 (1,690–1,920)	1,830 (1,430–2,340)
1-49	Stroke	59,800 (52,400–64,200)	36,700 (33,900–45,300)	44,500 (34,500–57,800)	1,710 (1,540–1,830)	721 (665–896)	733 (568–956)
1-50	Ischaemic stroke	13,700 (12,000–15,300)	13,400 (11,900–15,400)	17,500 (13,900–21,700)	460 (409–514)	264 (236–306)	283 (224–352)
1-51	Intracerebral hemorrhage	33,600 (28,500–37,200)	16,800 (15,600–20,400)	19,700 (14,800–26,700)	951 (846–1,040)	334 (310–409)	330 (248–448)
1-52	Subarachnoid hemorrhage	12,600 (9,990–15,800)	6,550 (5,970–8,570)	7,200 (5,340–9,740)	298 (240–380)	123 (112–162)	120 (88·4–162)
1-53	Hypertensive heart disease	4,560 (4,120–5,340)	5,420 (4,890–6,220)	6,650 (5,130–8,490)	152 (136–182)	109 (98·4–126)	109 (83·8–139)
1-54	Non-rheumatic valvular heart disease	341 (288–386)	436 (395–481)	546 (412–713)	10 (8·5–11·3)	8·6 (7·8–9·4)	9·1 (6·9–11·9)
1-55	Non-rheumatic calcific aortic valve disease	243 (200–284)	340 (304–377)	429 (318–564)	7·1 (5·8–8·3)	6·7 (6–7·5)	7·2 (5·3–9·5)
1-56	Non-rheumatic degenerative mitral valve disease	94 (74–115)	94 (79–115)	113 (82–153)	2·8 (2·2–3·5)	1·8 (1·5–2·2)	1·9 (1·3–2·5)
1-57	Other non-rheumatic valve diseases	3 (2–5)	3 (2–3)	3 (2–4)	0·1 (0·1–0·1)	0 (0–0·1)	0·1 (0–0·1)
1-58	Cardiomyopathy and myocarditis	1,340 (953–1,840)	1,190 (964–1,460)	1,340 (946–1,800)	30·6 (21·9–38·8)	22·4 (18–27·4)	22·3 (15·6–30)
1-59	Myocarditis	185 (122–381)	166 (130–210)	178 (119–244)	3·7 (2·6–6·2)	3 (2·3–3·6)	2·9 (1·9–4)
1-60	Alcoholic cardiomyopathy	55 (36–75)	38 (28–47)	45 (30–64)	1·6 (1–2·2)	0·7 (0·5–0·9)	0·7 (0·5–1·1)

1-61	Other cardiomyopathy	1,100 (786–1,470)	988 (794–1,220)	1,120 (771–1,510)	25·3 (18·1–31·4)	18·7 (14·9–23)	18·6 (12·8–25·2)
1-62	Atrial fibrillation and flutter	1,840 (1,510–2,190)	3,540 (2,930–4,230)	4,660 (3,690–5,690)	67·2 (55·1–79·8)	69·3 (57·3–83)	73·2 (57·8–90·3)
1-63	Aortic aneurysm	579 (511–660)	865 (792–949)	1,130 (844–1,460)	18·3 (16·1–20·9)	17·4 (15·8–19·1)	18·8 (14·1–24·4)
1-64	Peripheral vascular disease	182 (100–301)	296 (169–488)	368 (216–601)	6·6 (3·7–11·1)	6 (3·4–9·9)	6 (3·5–9·8)
1-65	Endocarditis	1,060 (668–1,420)	670 (571–909)	694 (491–1,020)	19·8 (14·3–24·3)	12 (10·2–16·5)	11·4 (8·1–16·7)
1-66	Chronic respiratory diseases	53,700 (43,000–65,600)	37,200 (31,000–45,000)	41,300 (33,400–50,500)	1,160 (981–1,350)	678 (576–808)	667 (536–816)
1-67	Chronic obstructive pulmonary disease	13,200 (11,700–14,600)	16,700 (14,900–18,500)	20,800 (16,500–25,200)	420 (370–470)	329 (296–365)	336 (266–409)
1-68	Pneumoconiosis	28 (22–36)	39 (28–52)	48 (34–65)	0·9 (0·7–1·2)	0·8 (0·6–1)	0·8 (0·6–1·1)
1-69	Silicosis	12 (10–16)	12 (9–15)	14 (10–18)	0·4 (0·3–0·5)	0·2 (0·2–0·3)	0·2 (0·2–0·3)
1-70	Asbestosis	1 (1–1)	2 (1–2)	2 (1–3)	0 (0–0)	0 (0–0)	0 (0–0)
1-71	Coal workers pneumoconiosis	0 (0–1)	0 (0–1)	1 (0–1)	0 (0–0)	0 (0–0)	0 (0–0)
1-72	Other pneumoconiosis	14 (9–20)	25 (16–36)	32 (20–46)	0·4 (0·3–0·6)	0·5 (0·3–0·7)	0·5 (0·3–0·8)
1-73	Asthma	35,700 (25,800–47,300)	17,200 (12,200–24,700)	16,600 (11,600–23,500)	644 (486–825)	288 (207–405)	268 (188–377)
1-74	Interstitial lung disease and pulmonary sarcoidosis	1,260 (988–1,790)	2,020 (1,360–2,410)	2,550 (1,570–3,550)	33·8 (27–47·3)	40·2 (26·8–47·9)	42·3 (26·2–59)
1-75	Other chronic respiratory diseases	3,570 (1,970–5,840)	1,230 (1,010–1,570)	1,260 (994–1,660)	58·3 (31·5–90·9)	20·6 (17·2–26)	20·2 (15·8–26·7)
1-76	Digestive diseases	95,100 (87,300–104,000)	69,000 (63,200–76,400)	81,600 (65,400–102,000)	2,300 (2,150–2,480)	1,330 (1,220–1,470)	1,360 (1,090–1,700)
1-77	Cirrhosis and other chronic liver diseases	35,600 (33,100–41,500)	35,100 (32,300–37,100)	43,500 (32,400–57,200)	965 (904–1,100)	695 (640–737)	735 (547–967)
1-78	Cirrhosis and other chronic liver diseases due to hepatitis B	2,070 (1,440–2,910)	1,260 (864–1,790)	1,470 (902–2,320)	59·1 (41–83·2)	25 (17–35)	24·9 (15·2–39·4)
1-79	Cirrhosis and other chronic liver diseases due to hepatitis C	8,970 (6,940–11,300)	9,570 (7,350–12,200)	11,900 (7,970–17,100)	256 (197–322)	189 (146–243)	201 (134–290)
1-80	Cirrhosis and other chronic liver diseases due to alcohol use	10,600 (8,440–12,900)	12,300 (9,670–14,900)	15,800 (10,900–21,800)	321 (254–392)	250 (196–303)	270 (186–374)
1-81	Cirrhosis and other chronic liver diseases due to NAFLD	5,130 (3,680–6,970)	6,390 (4,660–8,470)	8,170 (5,330–12,100)	152 (110–207)	128 (92·9–171)	138 (89·7–205)
1-82	Cirrhosis and other chronic liver diseases due to other causes	8,850 (7,030–12,500)	5,530 (4,360–6,820)	6,160 (4,210–8,750)	177 (140–233)	102 (79·5–128)	101 (68·6–143)
1-83	Upper digestive system diseases	16,800 (14,100–20,800)	12,200 (8,790–17,500)	13,800 (9,790–20,200)	475 (405–576)	233 (169–332)	226 (160–327)
1-84	Peptic ulcer disease	11,000 (10,200–12,000)	4,700 (4,340–5,200)	5,150 (3,890–6,700)	325 (300–352)	93 (85·8–103)	84·9 (63·8–110)
1-85	Gastritis and duodenitis	973 (714–1,310)	877 (624–1,230)	1,070 (764–1,480)	26·6 (19·7–34·7)	16·7 (12–23·3)	17·7 (12·7–24·5)
1-86	GERD	4,840 (2,520–8,570)	6,630 (3,440–11,700)	7,590 (3,950–13,300)	123 (64·1–217)	123 (63·6–218)	123 (64–217)
1-87	Appendicitis	9,840 (6,300–12,500)	1,420 (1,230–1,820)	1,310 (985–1,750)	158 (104–196)	23·4 (20·4–30·1)	20·9 (15·7–28·1)
1-88	Paralytic ileus and intestinal obstruction	8,740 (6,410–11,200)	3,020 (2,620–3,580)	3,150 (2,280–4,230)	143 (110–177)	55 (48·1–65·5)	52·7 (38·2–71·7)
1-89	Inguinal, femoral, and abdominal hernia	5,990 (4,500–7,720)	3,710 (2,810–4,890)	4,090 (3,060–5,380)	122 (95·2–151)	68·7 (52·3–90)	68·2 (50·8–89·4)
1-90	Inflammatory bowel disease	661 (461–881)	380 (303–489)	437 (328–574)	15·1 (11·2–18·8)	7·3 (5·8–9·4)	7·2 (5·4–9·5)
1-91	Vascular intestinal disorders	500 (441–558)	827 (729–916)	1,080 (806–1,400)	16·1 (14·3–17·9)	16·8 (14·8–18·6)	17·9 (13·3–23·3)
1-92	Gallbladder and biliary diseases	7,610 (5,720–9,970)	7,070 (5,150–9,550)	8,230 (5,930–11,000)	213 (160–276)	136 (99·3–182)	136 (98·1–183)
1-93	Pancreatitis	3,120 (2,370–3,850)	2,350 (2,060–3,070)	2,730 (1,930–3,840)	82·3 (62·2–103)	44·9 (39·6–58·2)	45·1 (31·8–63·7)
1-94	Other digestive diseases	6,160 (4,520–8,250)	2,880 (2,580–3,210)	3,290 (2,430–4,410)	112 (90·6–141)	54·1 (48·6–60·5)	54·3 (40·2–73)
1-95	Neurological disorders	52,000 (26,600–88,100)	69,300 (35,700–117,000)	79,900 (42,300–132,000)	1,220 (659–2,010)	1,210 (642–2,040)	1,250 (662–2,060)
1-96	Alzheimer's disease and other dementias	8,590 (3,600–19,200)	17,900 (7,550–39,700)	23,500 (9,730–50,400)	322 (135–724)	339 (142–755)	356 (145–772)
1-97	Parkinson's disease	1,750 (1,590–1,910)	3,510 (3,060–3,840)	4,600 (3,710–5,620)	64·3 (58·4–70·2)	70·8 (61·9–77·4)	74·6 (59·9–91·2)
1-98	Idiopathic epilepsy	11,900 (6,030–21,100)	10,800 (5,060–19,700)	11,700 (4,950–21,700)	219 (110–383)	178 (84·2–324)	186 (78·6–342)
1-99	Multiple sclerosis	170 (141–197)	270 (214–313)	332 (243–430)	4·8 (3·9–5·5)	5·2 (4·2–6·1)	5·6 (4·1–7·2)
1-100	Motor neuron disease	183 (163–205)	164 (150–180)	227 (168–295)	4·6 (4·1–5·1)	3·2 (2·9–3·5)	3·9 (2·8–5)
1-101	Headache disorders	26,900 (4,460–59,600)	33,900 (6,070–74,900)	36,200 (6,720–79,300)	559 (108–1,220)	569 (110–1,250)	570 (108–1,240)
1-102	Migraine	24,600 (3,030–56,900)	30,900 (4,150–71,400)	32,900 (4,710–76,100)	507 (73·8–1,170)	517 (74·8–1,190)	517 (74·6–1,190)
1-103	Tension-type headache	2,310 (629–9,620)	3,010 (862–11,600)	3,300 (966–12,200)	52·1 (15·2–193)	52·7 (15·5–193)	52·7 (15·5–190)
1-104	Other neurological disorders	2,400 (1,260–4,060)	2,740 (1,540–4,750)	3,280 (1,630–5,700)	41·8 (23·3–69·3)	44·4 (25·6–75·1)	52·2 (26·1–90·5)
1-105	Mental disorders	80,800 (59,200–107,000)	98,900 (71,700–131,000)	106,000 (77,800–140,000)	1,720 (1,270–2,260)	1,690 (1,220–2,220)	1,680 (1,230–2,220)
1-106	Schizophrenia	6,780 (4,740–9,050)	9,320 (6,480–12,400)	10,600 (7,370–14,100)	167 (117–221)	171 (119–226)	171 (118–227)
1-107	Depressive disorders	29,600 (20,300–41,900)	35,000 (24,100–49,200)	38,300 (26,500–53,600)	671 (463–942)	616 (426–865)	607 (418–847)
1-108	Major depressive disorder	26,100 (17,600–37,700)	30,300 (20,300–43,500)	33,000 (22,300–46,800)	586 (396–834)	532 (360–751)	522 (353–743)
1-109	Dysthymia	3,520 (2,170–5,450)	4,700 (2,900–7,220)	5,280 (3,300–8,140)	84·2 (53·6–131)	84·7 (53–130)	84·8 (53·3–130)
1-110	Bipolar disorder	8,220 (4,720–12,800)	10,500 (6,160–16,300)	11,400 (6,710–17,900)	177 (104–272)	178 (106–278)	178 (105–278)
1-111	Anxiety disorders	19,500 (12,700–27,900)	24,900 (16,500–35,300)	26,800 (17,900–38,300)	401 (267–574)	416 (276–588)	421 (283–601)
1-112	Eating disorders	1,810 (1,110–2,740)	2,510 (1,530–3,780)	2,870 (1,760–4,330)	33 (20·1–49·4)	39·2 (23·7–59·4)	42·2 (25·9–63·5)
1-113	Anorexia nervosa	475 (262–777)	634 (359–1,020)	699 (399–1,140)	8 (4·5–13)	9·4 (5·4–15·2)	10·2 (5·8–16·6)

1-114	Bulimia nervosa	1,340 (758–2,140)	1,880 (1,080–3,030)	2,170 (1,240–3,420)	25 (14·2–39·4)	29·7 (17·1–47·9)	32 (18·3–50·2)
1-115	Autism spectrum disorders	2,830 (1,850–4,110)	3,090 (2,030–4,530)	3,150 (2,040–4,570)	50·4 (33–73·6)	50 (32·9–73·2)	50 (32·3–72·3)
1-116	Attention-deficit/hyperactivity disorder	1,180 (655–2,070)	1,270 (698–2,230)	1,220 (681–2,120)	18·6 (10·5–32·1)	18·6 (10·4–32·5)	18·7 (10·4–32·5)
1-117	Conduct disorder	4,970 (2,760–7,910)	5,190 (2,850–8,270)	4,350 (2,430–6,910)	66·8 (37·3–106)	67·6 (37·3–107)	67·9 (37·7–107)
1-118	Idiopathic developmental intellectual disability	1,650 (678–2,960)	1,470 (610–2,620)	1,140 (368–2,160)	29 (12·1–51·6)	23·8 (9·9–42·1)	18·1 (5·8–34·1)
1-119	Other mental disorders	4,180 (2,590–6,300)	5,700 (3,550–8,540)	6,510 (4,130–9,870)	107 (67·5–161)	106 (66·1–158)	106 (67·3–160)
1-120	Substance use disorders	62,500 (44,200–72,100)	55,200 (45,500–65,200)	62,300 (48,400–77,900)	1,600 (1,130–1,830)	1,020 (851–1,210)	1,010 (786–1,270)
1-121	Alcohol use disorders	58,800 (40,500–67,900)	50,300 (41,800–59,400)	56,600 (44,000–71,600)	1,520 (1,040–1,740)	940 (789–1,100)	927 (722–1,170)
1-122	Drug use disorders	3,680 (2,590–4,900)	4,870 (3,420–6,490)	5,720 (4,010–7,610)	78·5 (56·1–104)	83·2 (58·7–111)	87·6 (61·9–116)
1-123	Opioid use disorders	2,150 (1,400–3,090)	3,100 (2,040–4,360)	3,740 (2,510–5,200)	48·6 (32·2–67·4)	54·6 (36·2–75·6)	58·2 (38·9–80·2)
1-124	Cocaine use disorders	415 (259–623)	488 (301–753)	567 (360–851)	8·3 (5·5–12)	7·8 (4·9–11·6)	8·5 (5·5–12·6)
1-125	Amphetamine use disorders	324 (165–533)	377 (203–641)	432 (237–725)	6·1 (3·3–9·9)	6·1 (3·3–10·3)	6·2 (3·4–10·2)
1-126	Cannabis use disorders	236 (133–392)	278 (160–443)	261 (146–423)	4·4 (2·5–7·1)	4·3 (2·6–6·8)	3·9 (2·2–6·2)
1-127	Other drug use disorders	546 (357–793)	635 (416–948)	722 (486–1,040)	11 (7·5–15·5)	10·4 (6·9–15·4)	10·8 (7·4–15·4)
1-128	Diabetes and kidney diseases	50,000 (44,700–55,800)	166,000 (151,000–181,000)	205,000 (164,000–253,000)	1,490 (1,330–1,670)	3,340 (3,050–3,650)	3,460 (2,770–4,290)
1-129	Diabetes mellitus	25,700 (21,700–30,200)	68,000 (57,600–80,000)	91,400 (72,100–112,000)	811 (681–951)	1,390 (1,180–1,640)	1,560 (1,220–1,920)
1-130	Diabetes mellitus type 1	2,440 (2,190–2,740)	3,150 (2,430–3,620)	3,400 (2,430–4,430)	55·3 (49·2–62·2)	57·2 (43·3–65·9)	55·6 (39·6–72·7)
1-131	Diabetes mellitus type 2	23,300 (19,300–27,600)	64,900 (54,600–76,500)	88,000 (69,400–108,000)	756 (628–893)	1,340 (1,120–1,580)	1,500 (1,180–1,850)
1-132	Chronic kidney disease	24,300 (22,300–26,500)	97,700 (91,800–104,000)	114,000 (88,600–146,000)	676 (623–737)	1,940 (1,830–2,060)	1,910 (1,480–2,460)
1-133	Chronic kidney disease due to diabetes mellitus type 1	1,890 (1,300–2,570)	8,520 (5,800–11,800)	9,980 (6,210–15,600)	52·5 (35·6–72·9)	167 (113–233)	169 (104–266)
1-134	Chronic kidney disease due to diabetes mellitus type 2	4,510 (3,560–5,460)	22,700 (18,300–27,100)	27,500 (19,900–36,900)	149 (118–181)	475 (382–565)	470 (342–632)
1-135	Chronic kidney disease due to hypertension	3,950 (3,270–4,880)	18,800 (15,600–22,400)	22,400 (16,400–30,200)	127 (105–156)	385 (318–457)	376 (275–511)
1-136	Chronic kidney disease due to glomerulonephritis	6,450 (5,390–7,540)	23,400 (19,300–28,200)	26,700 (18,700–36,900)	160 (133–191)	449 (364–549)	445 (309–616)
1-137	Chronic kidney disease due to other and unspecified causes	7,460 (6,230–8,910)	24,300 (19,800–29,800)	27,100 (19,800–36,100)	186 (152–226)	466 (383–577)	449 (328–600)
1-138	Acute glomerulonephritis	6 (3–9)	2 (2–3)	2 (2–3)	0·1 (0·1–0·2)	0 (0–0·1)	0 (0–0·1)
1-139	Skin and subcutaneous diseases	24,100 (15,800–35,700)	27,400 (18,100–41,000)	28,200 (18,700–41,700)	444 (296–664)	448 (298–666)	450 (300–665)
1-140	Dermatitis	5,360 (3,140–8,530)	5,800 (3,430–9,080)	5,870 (3,520–9,120)	94 (57–146)	94·4 (56·7–147)	94·4 (56·5–147)
1-141	Atopic dermatitis	4,060 (2,180–6,770)	4,080 (2,200–6,920)	3,970 (2,150–6,670)	63·6 (34·3–106)	63·9 (34·4–108)	63·9 (34·7–107)
1-142	Contact dermatitis	1,140 (718–1,710)	1,520 (955–2,280)	1,690 (1,060–2,530)	27 (16·8–40·4)	27·2 (16·9–41·1)	27·2 (16·9–40·9)
1-143	Seborrhoeic dermatitis	158 (89–250)	195 (109–307)	211 (117–332)	3·4 (1·9–5·3)	3·4 (1·9–5·3)	3·4 (1·9–5·3)
1-144	Psoriasis	630 (443–870)	699 (489–969)	729 (499–994)	13·9 (9·8–19)	12·3 (8·7–17)	11·8 (8·1–16·1)
1-145	Bacterial skin diseases	1,320 (1,070–1,650)	1,550 (1,310–1,870)	1,740 (1,320–2,270)	33·1 (25·9–40·8)	29·4 (24·9–35·2)	28·7 (21·7–37·5)
1-146	Cellulitis	119 (78–174)	123 (84–175)	127 (87–178)	2·3 (1·6–3·3)	2·1 (1·5–3)	2·1 (1·4–3)
1-147	Pyoderma	1,200 (971–1,520)	1,420 (1,210–1,740)	1,620 (1,200–2,130)	30·8 (23·9–38·3)	27·2 (22·9–32·8)	26·6 (19·8–35)
1-148	Scabies	4,320 (2,350–6,940)	4,630 (2,490–7,390)	4,640 (2,520–7,410)	74·3 (40·9–119)	73·3 (39·7–118)	73 (39·6–116)
1-149	Fungal skin diseases	1,240 (492–2,600)	1,550 (620–3,250)	1,690 (678–3,570)	27·9 (11·2–59·4)	27·3 (11–57·8)	27·2 (10·9–57·6)
1-150	Viral skin diseases	2,850 (1,820–4,340)	2,820 (1,790–4,220)	2,710 (1,730–4,080)	43·6 (27·8–66·1)	43·7 (27·8–65·6)	43·7 (27·9–65·7)
1-151	Acne vulgaris	2,650 (1,540–4,370)	3,420 (2,020–5,490)	3,210 (1,860–5,180)	39·1 (22·7–64·2)	46·1 (27·2–73·8)	47·7 (27·6–76·2)
1-152	Alopecia areata	337 (215–500)	422 (266–628)	459 (288–679)	7·2 (4·5–10·6)	7·3 (4·6–10·8)	7·3 (4·6–10·7)
1-153	Pruritus	368 (176–663)	484 (231–871)	538 (257–978)	8·4 (4–15·1)	8·7 (4·1–15·6)	8·8 (4·2–15·9)
1-154	Urticaria	2,800 (1,830–4,080)	2,960 (1,940–4,300)	3,000 (1,940–4,380)	48 (31·5–69·1)	48·4 (31·8–70·2)	48·4 (31·3–70·6)
1-155	Decubitus ulcer	331 (245–416)	604 (409–714)	821 (507–1,080)	10·2 (7·4–13)	11·7 (7·9–13·8)	13·1 (8·1–17·3)
1-156	Other skin and subcutaneous diseases	1,880 (896–3,430)	2,500 (1,190–4,560)	2,810 (1,350–5,070)	44·8 (21·5–81·3)	45·8 (21·9–83·5)	46·3 (22·2–83·3)
1-157	Sense organ diseases	33,700 (23,100–46,700)	46,200 (31,500–65,200)	53,000 (36,400–74,600)	999 (692–1,380)	897 (613–1,280)	873 (599–1,230)
1-158	Blindness and vision loss	14,100 (9,950–19,100)	18,100 (12,500–25,000)	20,500 (14,200–28,200)	430 (304–582)	351 (242–487)	334 (232–461)
1-159	Glaucoma	586 (386–837)	709 (473–988)	794 (535–1,110)	21·6 (14·3–31)	14·1 (9·5–19·8)	12·7 (8·6–17·8)
1-160	Cataract	2,640 (1,870–3,640)	3,280 (2,330–4,450)	3,700 (2,590–4,970)	94·3 (66·5–130)	65·1 (46·2–88·3)	59·5 (41·8–80·1)
1-161	Age-related macular degeneration	160 (107–231)	221 (149–318)	262 (176–380)	5·8 (3·9–8·3)	4·5 (3·1–6·5)	4·3 (2·9–6·3)
1-162	Refraction disorders	4,840 (3,230–6,980)	5,950 (4,000–8,530)	6,490 (4,360–9,200)	120 (81–168)	108 (72·9–153)	106 (70·9–150)
1-163	Near vision loss	1,760 (811–3,430)	2,750 (1,250–5,380)	3,400 (1,590–6,550)	59·7 (27·4–116)	56·7 (25·5–111)	57·3 (26·8–111)
1-164	Other vision loss	4,110 (2,870–5,640)	5,170 (3,650–7,060)	5,810 (4,090–7,860)	128 (89·6–177)	102 (71·6–140)	95·2 (67–130)
1-165	Age-related and other hearing loss	18,200 (12,400–26,400)	26,200 (17,500–38,000)	30,400 (20,400–43,900)	534 (359–770)	511 (340–738)	502 (334–731)
1-166	Other sense organ diseases	1,360 (814–2,060)	1,910 (1,140–2,900)	2,190 (1,300–3,400)	35·2 (20·9–54·1)	36·1 (21·6–55·5)	36·3 (21·5–56·4)

1-167	Musculoskeletal disorders	59,600 (42,200–79,200)	91,400 (64,800–123,000)	109,000 (77,000–144,000)	1,590 (1,130–2,110)	1,720 (1,220–2,310)	1,780 (1,270–2,370)
1-168	Rheumatoid arthritis	1,080 (799–1,400)	1,740 (1,250–2,270)	2,150 (1,520–2,880)	30 (22·3–38·3)	33·5 (24·2–43·9)	35·7 (25·1–47·9)
1-169	Osteoarthritis	5,620 (2,810–11,100)	9,770 (4,920–19,600)	12,100 (6,060–24,100)	188 (94·4–374)	204 (102–408)	206 (104–412)
1-170	Low back pain	31,200 (21,800–41,700)	42,900 (30,300–57,600)	48,200 (33,800–64,800)	769 (540–1,030)	783 (552–1,050)	782 (552–1,050)
1-171	Neck pain	4,990 (3,300–7,270)	7,100 (4,650–10,200)	8,150 (5,370–11,800)	135 (89·1–194)	135 (88·3–194)	135 (89–196)
1-172	Gout	152 (95–223)	265 (164–389)	321 (202–467)	4·5 (2·8–6·6)	5·2 (3·2–7·7)	5·4 (3·4–7·8)
1-173	Other musculoskeletal disorders	16,600 (10,200–24,000)	29,600 (18,400–43,000)	37,700 (23,900–52,700)	464 (291–665)	563 (348–816)	619 (392–866)
1-174	Other non-communicable diseases	152,000 (115,000–189,000)	116,000 (86,400–142,000)	103,000 (77,700–135,000)	2,530 (1,950–3,130)	2,070 (1,550–2,550)	1,730 (1,300–2,280)
1-175	Congenital anomalies	94,000 (61,700–128,000)	57,600 (30,600–69,900)	37,700 (21,600–54,100)	1,230 (814–1,670)	992 (525–1,210)	666 (373–962)
1-176	Neural tube defects	18,700 (11,200–28,600)	5,610 (4,090–7,330)	3,030 (1,920–4,530)	240 (144–366)	98·2 (71·3–128)	54·8 (34·2–82·2)
1-177	Congenital heart anomalies	46,600 (26,500–70,900)	30,100 (12,500–37,600)	18,600 (8,370–28,400)	605 (347–920)	521 (216–654)	333 (149–509)
1-178	Orofacial clefts	462 (213–1,020)	188 (121–302)	176 (115–255)	6·5 (3·2–13·7)	3·2 (2·1–5·2)	2·9 (1·9–4·3)
1-179	Down's syndrome	637 (422–1,080)	1,210 (695–1,520)	1,020 (556–1,490)	8·7 (5·9–14·5)	20·1 (11·5–25·4)	17·4 (9·4–25·6)
1-180	Turner syndrome	12 (5–23)	11 (5–20)	11 (5–19)	0·2 (0·1–0·4)	0·2 (0·1–0·3)	0·2 (0·1–0·3)
1-181	Klinefelter syndrome	1 (0–2)	1 (0–2)	1 (0–2)	0 (0–0)	0 (0–0)	0 (0–0)
1-182	Other chromosomal abnormalities	1,250 (613–2,280)	868 (512–1,180)	864 (472–1,270)	16·5 (8·4–29·8)	14·6 (8·4–19·9)	15 (7·9–22·1)
1-183	Congenital musculoskeletal and limb anomalies	4,110 (2,600–6,100)	4,010 (2,490–5,870)	3,930 (2,480–5,810)	63·7 (41–93·1)	64 (40·2–93)	63·9 (40·4–94·2)
1-184	Urogenital congenital anomalies	1,180 (451–2,260)	2,380 (314–3,700)	1,340 (266–2,580)	15·5 (6·2–29·1)	42 (5·3–65·5)	24·4 (4·5–46·9)
1-185	Digestive congenital anomalies	2,620 (1,380–5,120)	1,800 (657–2,450)	1,330 (435–2,340)	33·8 (17·8–66)	31·5 (11·5–43·1)	24 (7·8–42·6)
1-186	Other congenital anomalies	18,400 (9,300–33,200)	11,500 (7,170–14,500)	7,420 (4,330–11,600)	242 (124–432)	197 (123–252)	131 (76·1–208)
1-187	Urinary diseases and male infertility	5,620 (4,930–6,480)	6,900 (5,910–7,960)	8,050 (6,220–10,200)	156 (134–181)	136 (116–158)	134 (103–170)
1-188	Urinary tract infections and interstitial nephritis	4,240 (3,820–4,860)	4,860 (4,170–5,260)	5,550 (4,110–7,210)	112 (99·4–127)	94·3 (80·3–102)	91·8 (67·7–120)
1-189	Urolithiasis	98 (66–139)	129 (89–179)	154 (105–213)	2·7 (1·8–3·9)	2·5 (1·7–3·4)	2·6 (1·7–3·6)
1-190	Benign prostatic hyperplasia	947 (567–1,470)	1,530 (915–2,330)	1,860 (1,080–2,870)	33·6 (20·1–52·4)	32·5 (19·3–49·3)	31·8 (18·4–49·3)
1-191	Male infertility	59 (24–131)	49 (19–105)	101 (37–242)	1·3 (0·5–2·8)	0·8 (0·3–1·8)	1·6 (0·6–3·7)
1-192	Other urinary diseases	277 (190–365)	322 (217–404)	389 (234–539)	6·6 (4·7–9·5)	6·1 (4–7·6)	6·4 (3·9–8·9)
1-193	Gynecological diseases	15,000 (10,300–21,100)	19,100 (13,000–27,100)	21,200 (14,500–30,000)	341 (235–480)	337 (231–475)	338 (231–478)
1-194	Uterine fibroids	1,130 (629–1,980)	1,600 (817–2,910)	1,820 (927–3,260)	30·7 (17·1–54)	30·5 (15·6–55·7)	30·6 (15·5–54·6)
1-195	Polycystic ovarian syndrome	356 (153–713)	602 (254–1,220)	697 (292–1,420)	7 (3–14)	9·8 (4·1–19·8)	10·6 (4·4–21·7)
1-196	Female infertility	60 (19–140)	28 (6–74)	154 (26–413)	1·3 (0·4–3·1)	0·5 (0·1–1·3)	2·4 (0·4–6·6)
1-197	Endometriosis	1,680 (980–2,750)	1,500 (882–2,390)	1,560 (921–2,430)	35·1 (21–56·2)	25·4 (15·2–40·5)	23·8 (14·1–36·9)
1-198	Genital prolapse	270 (141–489)	333 (170–605)	381 (196–697)	8·3 (4·2–15·2)	6·7 (3·4–12·3)	6·4 (3·3–11·8)
1-199	Premenstrual syndrome	5,130 (3,090–7,900)	6,590 (4,000–10,200)	7,010 (4,250–10,900)	102 (62·2–158)	108 (65·6–167)	108 (64·8–168)
1-200	Other gynecological diseases	6,360 (4,090–9,320)	8,480 (5,350–12,500)	9,580 (6,110–14,200)	156 (99·5–230)	156 (97·9–230)	156 (98·6–229)
1-201	Hemoglobinopathies and hemolytic anaemias	2,680 (2,280–3,220)	1,470 (1,160–1,900)	1,370 (1,040–1,770)	54·3 (46·2–65·1)	25·7 (20·5–33)	22·3 (16·9–28·9)
1-202	Thalassemias	44 (22–73)	13 (6–22)	10 (5–17)	0·6 (0·3–1)	0·2 (0·1–0·3)	0·2 (0·1–0·3)
1-203	Thalassaemias trait	120 (65–198)	123 (69–202)	116 (63–189)	2·2 (1·2–3·6)	2 (1·1–3·2)	1·8 (1–2·9)
1-204	Sickle cell disorders	154 (100–226)	22 (15–33)	16 (9–27)	2 (1·3–2·9)	0·4 (0·3–0·6)	0·3 (0·2–0·5)
1-205	Sickle cell trait	41 (15–82)	34 (13–65)	31 (11–62)	0·8 (0·3–1·5)	0·6 (0·2–1·1)	0·5 (0·2–1)
1-206	G6PD deficiency	732 (583–899)	359 (300–419)	311 (226–417)	12·8 (10·3–15·5)	6 (4·9–7)	5 (3·6–6·7)
1-207	G6PD trait	6 (3–10)	11 (3–27)	10 (5–17)	0·2 (0·1–0·3)	0·2 (0·1–0·5)	0·2 (0·1–0·3)
1-208	Other hemoglobinopathies and hemolytic anaemias	1,580 (1,250–2,000)	911 (675–1,250)	878 (657–1,170)	35·7 (29·1–44·5)	16·4 (12·3–22·3)	14·5 (10·8–19·2)
1-209	Endocrine, metabolic, blood, and immune disorders	19,200 (8,080–27,000)	11,200 (8,260–15,100)	12,700 (9,170–17,000)	362 (187–490)	214 (156–288)	214 (154–289)
1-210	Oral disorders	13,200 (7,680–20,800)	18,300 (10,800–28,900)	20,900 (12,400–33,300)	357 (212–562)	350 (208–552)	347 (206–551)
1-211	Caries of deciduous teeth	212 (91–442)	179 (77–377)	168 (71–359)	2·9 (1·2–6)	2·9 (1·2–6)	2·8 (1·2–6)
1-212	Caries of permanent teeth	1,420 (618–2,830)	1,650 (729–3,230)	1,720 (769–3,350)	28·6 (12·6–56)	27·4 (12·2–54)	27 (12·1–52·3)
1-213	Periodontal disease	3,830 (1,470–8,230)	5,580 (2,120–12,000)	6,490 (2,510–13,900)	103 (40–223)	106 (41·1–229)	108 (41·4–229)
1-214	Edentulism	5,160 (3,280–7,630)	7,800 (4,980–11,500)	9,230 (5,830–13,600)	169 (107–250)	160 (102–238)	156 (98–230)
1-215	Other oral disorders	2,550 (1,590–3,760)	3,100 (1,910–4,530)	3,340 (2,090–4,880)	53·1 (33·3–77·7)	53·4 (32·9–77·9)	53·4 (33·5–78·1)
1-216	Sudden infant death syndrome	2,080 (957–4,860)	963 (559–1,500)	705 (323–1,240)	26·8 (12·3–62·5)	17 (9·9–26·5)	12·8 (5·9–22·5)
1-0	Injuries	496,000 (461,000–539,000)	363,000 (342,000–388,000)	351,000 (286,000–428,000)	9,540 (8,870–10,400)	6,110 (5,750–6,580)	5,480 (4,450–6,680)
1-1	Transport injuries	97,600 (89,800–119,000)	68,000 (62,000–88,900)	70,500 (54,400–96,000)	1,970 (1,820–2,370)	1,190 (1,090–1,520)	1,120 (869–1,510)
1-2	Road injuries	86,000 (79,100–105,000)	60,500 (55,000–79,400)	62,600 (48,100–84,700)	1,730 (1,600–2,090)	1,060 (966–1,350)	995 (768–1,330)

1-3	Pedestrian road injuries	49,100 (42,100–59,900)	32,900 (29,700–42,900)	32,800 (24,100–45,300)	1,000 (864–1,200)	583 (530–744)	529 (387–730)
1-4	Cyclist road injuries	3,100 (2,400–4,010)	2,310 (1,840–2,850)	2,120 (1,630–2,680)	61·8 (48·8–78·6)	40·5 (32·3–49·9)	34·6 (26·7–43·8)
1-5	Motorcyclist road injuries	5,030 (3,700–6,580)	3,450 (2,740–4,360)	4,070 (2,860–5,550)	93·2 (69·1–120)	56·2 (45–70·4)	60·6 (43–81·9)
1-6	Motor vehicle road injuries	26,100 (19,900–35,600)	19,800 (17,100–29,100)	21,300 (15,800–31,300)	524 (407–712)	340 (296–490)	335 (250–486)
1-7	Other road injuries	2,690 (1,980–3,660)	2,030 (1,680–2,400)	2,270 (1,640–3,110)	56·7 (41·9–74·4)	36 (29·6–42·1)	36·2 (26·2–49·4)
1-8	Other transport injuries	11,600 (9,650–14,800)	7,540 (6,580–10,300)	7,970 (5,830–11,400)	235 (199–293)	131 (115–174)	126 (92·6–181)
1-9	Unintentional injuries	98,400 (85,700–111,000)	60,500 (52,000–70,200)	60,500 (48,400–73,800)	1,890 (1,630–2,130)	1,040 (897–1,220)	967 (773–1,180)
1-10	Falls	19,900 (16,300–23,400)	20,400 (16,900–24,600)	23,800 (18,700–29,300)	474 (393–554)	375 (312–450)	384 (304–474)
1-11	Drowning	13,100 (11,200–19,700)	13,500 (11,000–14,800)	11,700 (8,450–15,300)	218 (190–326)	213 (171–233)	181 (131–237)
1-12	Fire, heat, and hot substances	12,900 (9,860–16,000)	7,710 (6,160–9,770)	6,130 (4,360–8,440)	237 (184–295)	131 (105–167)	97·9 (69·7–134)
1-13	Poisonings	3,520 (3,200–3,870)	1,000 (785–1,280)	1,120 (830–1,440)	68·4 (62·5–74·8)	16·9 (13·3–21·5)	17·8 (13·2–22·8)
1-14	Poisoning by carbon monoxide	143 (103–184)	142 (98–183)	159 (105–213)	2·8 (2–3·6)	2·3 (1·6–3)	2·5 (1·7–3·3)
1-15	Poisoning by other means	3,380 (3,070–3,710)	859 (650–1,130)	960 (691–1,250)	65·6 (59·8–71·9)	14·5 (11·1–18·9)	15·3 (11·1–20)
1-16	Exposure to mechanical forces	7,200 (5,500–9,190)	5,300 (3,750–7,470)	6,310 (4,440–9,020)	147 (114–191)	92·6 (65·8–130)	101 (71·2–145)
1-17	Unintentional firearm injuries	1,560 (971–2,050)	500 (359–770)	585 (425–843)	28·7 (18·7–37)	8·7 (6·3–13·1)	9·4 (6·8–13·3)
1-18	Other exposure to mechanical forces	5,630 (4,230–7,400)	4,800 (3,350–6,900)	5,730 (3,980–8,320)	119 (89·8–158)	83·8 (58·6–120)	92·1 (64–133)
1-19	Adverse effects of medical treatment	3,240 (1,730–4,430)	984 (792–1,440)	979 (678–1,500)	60·7 (33·6–79·6)	17·8 (14·3–26·2)	16·1 (11·2–24·7)
1-20	Animal contact	1,070 (733–1,580)	1,330 (1,030–1,710)	1,330 (996–1,740)	22·2 (15·3–31·7)	23 (17·9–29·5)	21·5 (16·2–28·1)
1-21	Venomous animal contact	762 (516–1,160)	992 (776–1,250)	995 (742–1,300)	15·5 (10·6–22·7)	17·3 (13·6–21·7)	16·1 (12–20·9)
1-22	Non-venomous animal contact	311 (197–481)	333 (229–497)	334 (225–502)	6·7 (4·3–10·2)	5·8 (4–8·6)	5·4 (3·7–8·1)
1-23	Foreign body	22,300 (16,500–25,800)	3,250 (2,840–4,630)	3,280 (2,540–4,350)	367 (254–418)	55·8 (48·8–78·4)	53·4 (41·2–71·1)
1-24	Pulmonary aspiration and foreign body in airway	21,400 (15,700–24,900)	2,570 (2,280–3,930)	2,560 (1,900–3,560)	349 (235–398)	44·2 (39·1–67·7)	42 (31·2–58·8)
1-25	Foreign body in eyes	256 (112–469)	309 (141–561)	333 (158–592)	5·4 (2·7–9·5)	5·3 (2·6–9·4)	5·3 (2·6–9·3)
1-26	Foreign body in other body part	625 (471–794)	371 (275–501)	384 (280–514)	12 (9·3–15·1)	6·3 (4·7–8·5)	6·2 (4·5–8·2)
1-27	Environmental heat and cold exposure	591 (434–810)	603 (447–778)	636 (473–825)	13·2 (9·8–18)	10·6 (7·9–13·7)	10·2 (7·6–13·3)
1-28	Exposure to forces of nature	888 (599–1,290)	2,170 (1,830–2,590)	754 (538–1,060)	17·8 (12·3–25·4)	36·6 (30·7–43·8)	12·3 (8·8–17·2)
1-29	Other unintentional injuries	13,700 (7,990–16,100)	4,260 (3,480–5,520)	4,490 (3,330–5,980)	262 (155–305)	71·7 (58·4–92·3)	70·8 (52·7–94·2)
1-30	Self-harm and interpersonal violence	300,000 (272,000–338,000)	234,000 (215,000–251,000)	220,000 (175,000–274,000)	5,690 (5,180–6,440)	3,880 (3,560–4,200)	3,390 (2,700–4,220)
1-31	Self-harm	33,800 (28,700–38,600)	25,700 (23,100–31,000)	27,200 (19,600–36,000)	642 (548–739)	420 (372–506)	416 (302–550)
1-32	Self-harm by firearm	1,190 (534–1,650)	425 (316–873)	527 (325–960)	22·6 (10·6–30·3)	7 (5·3–14·5)	8 (5–14·6)
1-33	Self-harm by other specified means	32,600 (27,900–37,300)	25,300 (22,600–30,200)	26,700 (19,300–35,300)	619 (535–716)	413 (365–493)	408 (297–538)
1-34	Interpersonal violence	142,000 (131,000–152,000)	184,000 (167,000–193,000)	174,000 (135,000–223,000)	2,810 (2,620–3,010)	3,010 (2,740–3,150)	2,650 (2,030–3,410)
1-35	Physical violence by firearm	99,500 (89,300–109,000)	138,000 (121,000–146,000)	129,000 (97,100–168,000)	1,960 (1,760–2,140)	2,240 (1,970–2,370)	1,950 (1,470–2,540)
1-36	Physical violence by sharp object	20,500 (15,600–27,600)	20,800 (16,200–24,800)	21,200 (15,100–29,700)	416 (322–550)	340 (269–411)	324 (230–452)
1-37	Sexual violence	663 (434–942)	890 (594–1,240)	891 (588–1,270)	13·6 (9–19·3)	14·8 (9·9–20·8)	13·9 (9·2–19·7)
1-38	Physical violence by other means	20,900 (17,600–25,500)	24,300 (20,500–26,500)	23,600 (17,900–30,300)	420 (357–509)	408 (346–443)	370 (282–473)
1-39	Conflict and terrorism	124,000 (98,900–159,000)	24,100 (14,700–38,700)	18,600 (11,400–29,600)	2,230 (1,770–2,880)	456 (279–726)	319 (196–505)
1-40	Executions and police conflict	36 (33–38)	35 (32–38)	35 (26–45)	0·6 (0·6–0·6)	0·6 (0·5–0·6)	0·6 (0·4–0·7)

SR Table 1: All-age DALYs counts and age standardized DALYs rates per 100,000 individuals for all causes and level 1 cause groups by location for 1990, 2010, and 2019, both sexes combined, Guatemala

Cause Level	Cause	All-age DALYs			Age-standardized rate of DALYs per 100,000		
		1990	2010	2019	1990	2010	2019
0-0	All causes	4,820,000 (4,410,000–5,250,000)	4,670,000 (4,150,000–5,220,000)	5,100,000 (4,210,000–6,130,000)	60,100 (54,600–65,400)	38,500 (34,300–42,800)	33,800 (28,000–40,400)
1-0	Communicable, maternal, neonatal, and nutritional disorders	2,780,000 (2,530,000–3,050,000)	1,400,000 (1,220,000–1,590,000)	1,130,000 (893,000–1,420,000)	26,900 (24,600–29,200)	9,320 (8,260–10,400)	6,630 (5,280–8,250)
1-1	HIV/AIDS and sexually transmitted infections	15,400 (14,600–16,400)	43,200 (41,100–46,000)	42,300 (39,300–46,600)	240 (227–257)	337 (320–360)	248 (230–274)
1-2	HIV/AIDS	13,500 (13,000–14,000)	40,200 (38,900–42,100)	38,600 (36,400–42,200)	211 (203–219)	313 (302–327)	226 (213–245)
1-3	HIV/AIDS - drug-susceptible tuberculosis	6,280 (5,990–6,570)	9,230 (5,970–13,600)	7,370 (4,590–11,100)	98·7 (94–103)	71·6 (46·4–105)	42·9 (26·8–64·8)
1-4	HIV/AIDS - Multidrug-resistant Tuberculosis without extensive drug resistance	28 (3–103)	685 (135–2,060)	489 (57–1,710)	0·4 (0–1·6)	5·3 (1–15·9)	2·8 (0·3–9·9)
1-5	HIV/AIDS - Extensively drug-resistant Tuberculosis	0 (0–0)	73 (13–232)	80 (10–279)	0 (0–0)	0·6 (0·1–1·8)	0·5 (0·1–1·6)
1-6	HIV/AIDS resulting in other diseases	7,180 (6,930–7,420)	30,200 (25,000–34,100)	30,700 (25,900–34,700)	112 (108–116)	235 (195–265)	179 (152–202)
1-7	Sexually transmitted infections excluding HIV	1,910 (1,360–2,880)	2,970 (1,810–4,970)	3,670 (2,160–6,410)	28·8 (19·6–45·9)	24·1 (14·4–41·2)	22·3 (13·1–39·3)
1-8	Syphilis	543 (452–645)	229 (194–271)	207 (163–260)	5·8 (4·8–6·8)	1·8 (1·5–2·1)	1·3 (1–1·6)
1-9	Chlamydial infection	140 (100–192)	275 (200–380)	347 (241–493)	2·2 (1·6–3)	2·1 (1·6–3)	2 (1·4–2·8)
1-10	Gonococcal infection	165 (134–202)	302 (247–371)	341 (261–439)	2·7 (2·3–3·2)	2·4 (2–2·9)	2·1 (1·6–2·6)
1-11	Trichomoniasis	442 (177–914)	984 (391–2,000)	1,250 (480–2,590)	7·4 (3–15·4)	7·9 (3·2–16·4)	7·5 (2·9–15·6)
1-12	Genital herpes	306 (98–764)	640 (209–1,570)	872 (291–2,110)	5·5 (1·8–13·7)	5·7 (1·8–13·8)	5·6 (1·8–13·9)
1-13	Other sexually transmitted infections	310 (235–416)	540 (411–711)	656 (484–885)	5·1 (3·9–6·8)	4·2 (3·2–5·6)	3·8 (2·8–5·1)
1-14	Respiratory infections and tuberculosis	794,000 (713,000–875,000)	525,000 (453,000–605,000)	427,000 (331,000–553,000)	8,700 (7,930–9,500)	3,720 (3,300–4,180)	2,700 (2,110–3,430)
1-15	Tuberculosis	104,000 (92,900–115,000)	20,900 (18,600–23,300)	18,900 (14,900–23,900)	1,500 (1,340–1,670)	174 (154–195)	122 (95·8–154)
1-16	Latent tuberculosis infection	0 (0–0)	0 (0–0)	0 (0–0)	0 (0–0)	0 (0–0)	0 (0–0)
1-17	Drug-susceptible tuberculosis	104,000 (92,500–115,000)	19,600 (16,700–22,300)	17,800 (13,500–22,800)	1,500 (1,340–1,660)	163 (138–187)	115 (87·2–147)
1-18	Multidrug-resistant tuberculosis without extensive drug resistance	390 (41–1,490)	1,170 (246–3,250)	941 (115–3,040)	5·6 (0·6–21·7)	9·8 (2–27·4)	6·1 (0·7–19·7)
1-19	Extensively drug-resistant tuberculosis	0 (0–0)	124 (25–365)	152 (20–509)	0 (0–0)	1 (0·2–3·1)	1 (0·1–3·3)
1-20	Lower respiratory infections	672,000 (600,000–745,000)	486,000 (413,000–563,000)	388,000 (295,000–509,000)	7,020 (6,380–7,700)	3,430 (3,020–3,880)	2,460 (1,910–3,170)
1-21	Upper respiratory infections	14,600 (10,200–19,400)	13,000 (8,460–19,600)	14,700 (9,330–22,400)	139 (102–182)	84 (55·1–126)	79·9 (51·1–120)
1-22	Otitis media	3,160 (1,840–5,120)	5,230 (2,980–8,610)	5,990 (3,440–9,820)	34·6 (20·1–57·5)	32·7 (18·8–53·3)	31·8 (18·4–52·2)
1-23	Enteric infections	766,000 (682,000–850,000)	247,000 (210,000–288,000)	176,000 (136,000–226,000)	7,230 (6,540–7,920)	1,600 (1,390–1,820)	1,030 (804–1,300)
1-24	Diarrhoeal diseases	752,000 (669,000–835,000)	245,000 (208,000–285,000)	174,000 (135,000–223,000)	7,080 (6,390–7,760)	1,580 (1,380–1,810)	1,020 (797–1,290)
1-25	Typhoid and paratyphoid	13,500 (8,550–21,700)	1,700 (695–2,480)	1,450 (511–2,310)	147 (95·3–244)	12 (4·9–18·2)	8·4 (3·1–13·3)
1-26	Typhoid fever	13,500 (8,550–21,700)	1,700 (695–2,470)	1,450 (511–2,310)	147 (95·3–244)	12 (4·9–18·2)	8·4 (3·1–13·3)
1-27	Paratyphoid fever	0 (0–1)	1 (0–2)	1 (0–1)	0 (0–0)	0 (0–0)	0 (0–0)
1-28	iNTS	64 (16–97)	54 (7–87)	34 (6–60)	0·5 (0·1–0·7)	0·3 (0–0·4)	0·2 (0–0·3)
1-29	Other intestinal infectious diseases	726 (369–1,110)	270 (121–459)	199 (93–340)	5·5 (3–8·3)	1·5 (0·7–2·5)	1 (0·5–1·7)
1-30	Neglected tropical diseases and malaria	67,800 (43,200–88,700)	41,000 (32,200–52,800)	31,200 (22,100–42,600)	770 (541–995)	292 (229–374)	187 (132–252)
1-31	Malaria	3,310 (1,870–5,780)	374 (231–552)	270 (151–455)	39·8 (22·1–71·6)	2·3 (1·4–3·4)	1·4 (0·8–2·4)
1-32	Chagas disease	1,170 (816–1,590)	1,080 (767–1,490)	1,250 (877–1,750)	26·6 (18·5–35·1)	11·7 (8·4–16)	10 (7·1–14·1)
1-33	Leishmaniasis	368 (1–2,950)	257 (129–470)	462 (258–771)	3·4 (0–25·8)	1·8 (1–3·2)	2·7 (1·6–4·5)
1-34	Visceral leishmaniasis	359 (0–2,940)	26 (0–211)	32 (0–251)	3·3 (0–25·8)	0·2 (0–1·2)	0·2 (0–1·3)
1-35	Cutaneous and mucocutaneous leishmaniasis	10 (0–53)	231 (125–395)	430 (251–710)	0·1 (0–0·7)	1·7 (0·9–2·9)	2·6 (1·5–4·3)
1-36	African trypanosomiasis	0 (0–0)	0 (0–0)	0 (0–0)	0 (0–0)	0 (0–0)	0 (0–0)
1-37	Schistosomiasis	0 (0–0)	0 (0–0)	0 (0–0)	0 (0–0)	0 (0–0)	0 (0–0)
1-38	Cysticercosis	7,670 (4,690–11,200)	9,020 (5,170–14,300)	10,400 (5,990–17,100)	140 (85·7–202)	84·9 (48·9–132)	73·5 (42·1–120)
1-39	Cystic echinococcosis	42 (16–76)	17 (9–26)	13 (7–21)	0·5 (0·2–0·7)	0·1 (0·1–0·2)	0·1 (0–0·1)
1-40	Lymphatic filariasis	0 (0–0)	0 (0–0)	0 (0–0)	0 (0–0)	0 (0–0)	0 (0–0)
1-41	Onchocerciasis	5,600 (3,660–7,760)	18 (11–26)	0 (0–0)	87·9 (58·8–121)	0·1 (0·1–0·2)	0 (0–0)
1-42	Trachoma	314 (174–536)	73 (35–146)	59 (29–117)	7·9 (4·6–13)	0·7 (0·4–1·3)	0·4 (0·2–0·8)
1-43	Dengue	781 (420–1,380)	13,100 (11,000–15,100)	2,900 (1,950–4,210)	9·4 (4·7–17·1)	84·5 (71·8–97·5)	16·1 (10·9–23·1)
1-44	Yellow fever	0 (0–0)	0 (0–0)	0 (0–0)	0 (0–0)	0 (0–0)	0 (0–0)
1-45	Rabies	708 (531–944)	11 (8–15)	14 (6–24)	7·7 (6–9·9)	0·1 (0·1–0·1)	0·1 (0–0·2)
1-46	Intestinal nematode infections	24,200 (15,100–36,900)	13,000 (7,790–21,100)	11,200 (6,480–18,100)	267 (165–417)	82·1 (48·7–133)	57·9 (33·6–95·2)
1-47	Ascariasis	5,970 (3,880–8,680)	2,320 (1,390–3,670)	1,970 (1,070–3,380)	54·5 (35·1–82)	14·8 (8·7–23·7)	10·6 (5·8–18·3)
1-48	Trichuriasis	7,660 (3,960–12,900)	1,630 (737–3,120)	1,890 (866–3,600)	86·5 (44·7–146)	10·3 (4·7–19·7)	9·6 (4·4–18·3)

1-49	Hookworm disease	10,500 (6,120–17,500)	9,090 (5,110–15,300)	7,290 (3,980–12,200)	126 (73–210)	57 (32–95·6)	37·7 (20·7–63·2)
1-50	Food-borne trematodiases	0 (0–0)	0 (0–0)	0 (0–0)	0 (0–0)	0 (0–0)	0 (0–0)
1-51	Leprosy	59 (35–89)	16 (10–25)	18 (11–27)	1·2 (0·7–1·8)	0·2 (0·1–0·2)	0·1 (0·1–0·2)
1-52	Ebola virus disease	0 (0–0)	0 (0–0)	0 (0–0)	0 (0–0)	0 (0–0)	0 (0–0)
1-53	Zika virus disease	0 (0–0)	0 (0–0)	29 (10–71)	0 (0–0)	0 (0–0)	0·2 (0·1–0·4)
1-54	Guinea worm disease	0 (0–0)	0 (0–0)	0 (0–0)	0 (0–0)	0 (0–0)	0 (0–0)
1-55	Other neglected tropical diseases	23,600 (4,550–33,900)	4,000 (2,220–8,730)	4,650 (2,660–8,880)	179 (38·7–252)	23·1 (12·9–49·2)	24·3 (13·9–46·1)
1-56	Other infectious diseases	80,400 (66,200–107,000)	36,400 (30,200–60,500)	34,700 (25,800–58,500)	678 (564–832)	231 (196–355)	192 (144–308)
1-57	Meningitis	22,600 (20,100–25,500)	18,300 (15,700–21,300)	13,600 (10,400–17,700)	205 (185–228)	117 (103–135)	76·9 (58·9–99·5)
1-58	Encephalitis	7,400 (5,820–12,200)	5,520 (4,400–8,490)	6,580 (4,860–9,760)	64·3 (53–96·1)	34·5 (28·2–50·8)	35·6 (26·6–51·5)
1-59	Diphtheria	215 (157–302)	1 (1–1)	1 (1–1)	1·8 (1·4–2·4)	0 (0–0)	0 (0–0)
1-60	Whooping cough	18,700 (11,900–58,800)	2,820 (854–24,500)	3,850 (1,380–25,100)	128 (84·6–384)	15·1 (4·7–130)	19·4 (7–126)
1-61	Tetanus	3,850 (2,800–5,210)	320 (201–863)	376 (155–1,250)	54·3 (39·1–71·8)	2·7 (1·7–6·9)	2·5 (1–7·9)
1-62	Measles	19,200 (1,130–28,400)	0 (0–0)	0 (0–0)	145 (8·4–211)	0 (0–0)	0 (0–0)
1-63	Varicella and herpes zoster	2,430 (850–4,530)	1,430 (631–1,890)	1,310 (702–1,830)	19·1 (7·8–33·9)	9·1 (4·5–11·9)	7·4 (4·2–10·2)
1-64	Acute hepatitis	1,060 (877–1,270)	1,570 (1,290–1,930)	1,440 (1,100–1,840)	11·6 (9·8–13·6)	10·5 (8·8–12·6)	8 (6·2–10·2)
1-65	Acute hepatitis A	447 (248–842)	1,190 (846–1,510)	1,010 (681–1,360)	4·4 (2·4–8·5)	7·6 (5·3–9·5)	5·4 (3·6–7·2)
1-66	Acute hepatitis B	130 (79–220)	195 (125–297)	243 (156–347)	1·8 (1·1–2·7)	1·5 (0·9–2·2)	1·4 (0·9–2·1)
1-67	Acute hepatitis C	452 (112–660)	139 (80–356)	130 (77–275)	5·1 (1·5–7·1)	1·1 (0·6–2·7)	0·8 (0·5–1·7)
1-68	Acute hepatitis E	30 (19–49)	44 (28–75)	52 (36–78)	0·4 (0·2–0·6)	0·3 (0·2–0·5)	0·3 (0·2–0·4)
1-69	Other unspecified infectious diseases	4,950 (3,800–6,240)	6,450 (4,980–8,220)	7,480 (5,490–9,900)	48·5 (37·5–61·4)	41·5 (32·4–52·4)	41·7 (30·9–54·5)
1-70	Maternal and neonatal disorders	775,000 (694,000–869,000)	370,000 (306,000–439,000)	314,000 (233,000–416,000)	5,230 (4,670–5,850)	2,040 (1,690–2,410)	1,590 (1,180–2,110)
1-71	Maternal disorders	45,200 (37,700–53,000)	27,300 (22,700–32,500)	31,500 (23,900–40,700)	632 (525–744)	185 (152–220)	159 (120–206)
1-72	Maternal haemorrhage	12,700 (10,100–15,600)	8,010 (6,430–10,000)	8,650 (6,450–11,300)	181 (144–223)	55·5 (44·4–69·2)	44·3 (33–58·2)
1-73	Maternal sepsis and other pregnancy related infections	4,970 (3,760–6,280)	1,820 (1,410–2,340)	1,820 (1,320–2,490)	69·2 (52·9–87·6)	12·1 (9·4–15·4)	9·1 (6·5–12·3)
1-74	Maternal hypertensive disorders	9,910 (7,980–12,200)	6,100 (4,850–7,470)	7,510 (5,530–10,000)	133 (107–163)	39·8 (31·8–48·8)	37·5 (27·6–50)
1-75	Maternal obstructed labour and uterine rupture	209 (136–305)	570 (420–773)	930 (649–1,280)	2·9 (1·9–4·2)	3·9 (2·9–5·2)	4·7 (3·3–6·6)
1-76	Maternal abortive outcome	11,000 (8,570–14,100)	2,870 (2,150–3,810)	2,350 (1,640–3,230)	157 (122–201)	19·8 (14·9–26·1)	12·1 (8·4–16·7)
1-77	Ectopic pregnancy	237 (182–299)	247 (192–319)	301 (215–413)	3·3 (2·5–4·2)	1·6 (1·3–2·1)	1·5 (1·1–2)
1-78	Indirect maternal deaths	1,010 (775–1,310)	1,870 (1,420–2,420)	2,680 (1,880–3,670)	14·2 (10·7–18·4)	12·7 (9·6–16·2)	13·5 (9·5–18·5)
1-79	Late maternal deaths	444 (321–602)	416 (298–563)	569 (379–827)	6·2 (4·5–8·4)	2·8 (2–3·8)	2·9 (1·9–4·2)
1-80	Maternal deaths aggravated by HIV/AIDS	7 (3–12)	24 (11–46)	33 (14–67)	0·1 (0–0·2)	0·2 (0·1–0·3)	0·2 (0·1–0·3)
1-81	Other maternal disorders	4,660 (3,470–5,980)	5,380 (4,140–6,910)	6,650 (4,850–8,960)	65·2 (49·1–83·9)	36·5 (28·2–46·6)	33·6 (24·6–45·5)
1-82	Neonatal disorders	730,000 (654,000–820,000)	343,000 (280,000–411,000)	283,000 (209,000–376,000)	4,590 (4,120–5,150)	1,850 (1,510–2,210)	1,430 (1,060–1,900)
1-83	Neonatal preterm birth	486,000 (420,000–559,000)	159,000 (129,000–191,000)	123,000 (90,900–162,000)	3,060 (2,650–3,510)	862 (700–1,030)	624 (461–822)
1-84	Neonatal encephalopathy due to birth asphyxia and trauma	90,000 (71,100–113,000)	72,200 (56,800–88,600)	52,800 (37,700–73,100)	560 (443–702)	387 (304–475)	268 (191–370)
1-85	Neonatal sepsis and other neonatal infections	77,800 (58,900–98,900)	62,100 (49,000–76,400)	56,400 (39,700–77,800)	487 (369–617)	334 (264–412)	286 (202–394)
1-86	Hemolytic disease and other neonatal jaundice	4,910 (3,800–6,410)	5,920 (4,800–7,150)	5,830 (4,530–7,200)	40·9 (32·3–51·8)	35·1 (28·6–42·3)	30·6 (23·8–37·6)
1-87	Other neonatal disorders	71,900 (49,700–93,700)	43,800 (35,000–54,200)	45,000 (31,200–63,100)	448 (310–584)	234 (187–290)	227 (157–318)
1-88	Nutritional deficiencies	285,000 (256,000–317,000)	141,000 (120,000–165,000)	106,000 (83,100–135,000)	4,020 (3,650–4,410)	1,100 (971–1,270)	683 (545–844)
1-89	Protein-energy malnutrition	225,000 (185,000–256,000)	93,000 (81,000–107,000)	55,200 (42,300–70,600)	3,250 (2,680–3,640)	777 (684–868)	391 (307–488)
1-90	Iodine deficiency	802 (417–1,300)	807 (363–1,550)	1,900 (963–3,170)	11·4 (6·2–18·4)	5·7 (2·6–10·8)	10·7 (5·5–17·6)
1-91	Vitamin A deficiency	2,600 (1,640–3,870)	1,880 (1,170–2,820)	1,820 (1,150–2,770)	20·5 (13·1–30·4)	10·4 (6·6–15·4)	9·3 (5·8–14)
1-92	Dietary iron deficiency	31,700 (20,500–47,500)	37,600 (23,600–57,900)	43,400 (27,400–65,300)	365 (239–539)	245 (156–371)	243 (154–366)
1-93	Other nutritional deficiencies	25,700 (13,300–61,100)	7,260 (5,590–10,600)	4,140 (2,590–5,500)	375 (197–896)	65·6 (49·7–108)	29·2 (17·8–38·8)
1-0	Non-communicable diseases	1,410,000 (1,230,000–1,600,000)	2,360,000 (2,040,000–2,720,000)	3,010,000 (2,490,000–3,620,000)	24,700 (21,900–27,800)	22,600 (19,700–25,800)	21,800 (18,100–26,000)
1-1	Neoplasms	129,000 (115,000–151,000)	298,000 (266,000–331,000)	355,000 (281,000–446,000)	2,760 (2,460–3,170)	3,140 (2,800–3,500)	2,790 (2,210–3,490)
1-2	Lip and oral cavity cancer	1,180 (1,040–1,330)	2,120 (1,880–2,390)	2,680 (2,100–3,360)	28·9 (25·5–32·4)	24·4 (21·6–27·5)	22·4 (17·7–28)
1-3	Nasopharynx cancer	262 (233–294)	861 (751–981)	885 (687–1,110)	5·1 (4·5–5·8)	8·7 (7·6–10)	6·7 (5·2–8·4)
1-4	Other pharynx cancer	571 (497–655)	850 (742–985)	1,000 (777–1,280)	14·1 (12·2–16)	9·8 (8·5–11·4)	8·4 (6·6–10·8)
1-5	Oesophageal cancer	1,560 (1,360–1,770)	4,620 (4,030–5,300)	5,530 (4,260–7,080)	39·9 (34·9–45)	54·9 (47·9–62·8)	47·9 (37·1–61·3)
1-6	Stomach cancer	28,700 (25,400–31,900)	70,600 (62,600–79,300)	73,200 (57,200–92,400)	716 (638–792)	814 (723–917)	616 (483–770)
1-7	Colon and rectum cancer	4,710 (4,150–5,290)	17,500 (15,400–19,500)	24,500 (19,300–31,000)	116 (103–129)	200 (176–222)	203 (160–256)

1-8	Liver cancer	10,300 (9,050–11,500)	8,570 (7,560–9,680)	13,500 (10,500–17,200)	240 (212–269)	95·7 (84·6–108)	113 (88·3–144)
1-9	Liver cancer due to hepatitis B	2,510 (1,870–3,300)	1,950 (1,440–2,590)	2,840 (1,930–4,070)	52·8 (38·8–70·8)	20·3 (14·6–27·7)	21·8 (14·4–32)
1-10	Liver cancer due to hepatitis C	3,360 (2,480–4,290)	2,850 (2,120–3,690)	4,680 (3,290–6,510)	92·1 (69·6–116)	35·7 (26·7–46·2)	42·7 (30·1–59)
1-11	Liver cancer due to alcohol use	2,260 (1,560–3,070)	1,940 (1,340–2,630)	3,200 (2,070–4,710)	57·2 (39·5–77)	23 (15·9–31·1)	28 (18–41·5)
1-12	Liver cancer due to NASH	664 (481–933)	663 (468–922)	1,140 (746–1,640)	16·1 (11·3–23)	7·5 (5·2–10·6)	9·5 (6·1–13·9)
1-13	Gallbladder and biliary tract cancer	4,590 (3,080–5,290)	4,320 (3,650–6,320)	4,870 (3,500–7,990)	120 (80·9–137)	52·4 (44·3–76·6)	43 (31–70)
1-14	Pancreatic cancer	1,410 (1,220–1,610)	8,890 (7,860–10,100)	12,000 (9,180–15,100)	36·3 (31·4–41·5)	106 (94·2–120)	105 (80·5–132)
1-15	Larynx cancer	1,280 (1,120–1,470)	1,770 (1,530–2,050)	1,980 (1,560–2,520)	32·3 (28·2–36·9)	20·6 (17·8–23·9)	16·9 (13·3–21·5)
1-16	Tracheal, bronchus, and lung cancer	9,510 (8,390–10,700)	17,900 (15,700–20,200)	21,700 (16,700–27,500)	232 (206–261)	206 (181–232)	185 (142–234)
1-17	Malignant skin melanoma	256 (202–447)	924 (609–1,140)	1,270 (833–1,680)	5·1 (4·1–9·2)	9·8 (6·1–11·8)	10 (6·2–13·3)
1-18	Non-melanoma skin cancer	902 (760–1,090)	2,250 (1,970–2,560)	3,390 (2,620–4,300)	24 (20·4–28·8)	27·6 (24·1–31·2)	29·3 (22·9–36·8)
1-19	Non-melanoma skin cancer (squamous-cell carcinoma)	901 (760–1,090)	2,250 (1,970–2,560)	3,390 (2,620–4,300)	24 (20·4–28·8)	27·6 (24·1–31·2)	29·3 (22·9–36·7)
1-20	Non-melanoma skin cancer (basal-cell carcinoma)	1 (0–1)	1 (1–2)	2 (1–3)	0 (0–0)	0 (0–0)	0 (0–0)
1-21	Breast cancer	6,150 (5,180–7,120)	14,900 (12,800–17,400)	20,800 (16,000–26,700)	135 (115–156)	167 (143–194)	170 (132–218)
1-22	Cervical cancer	11,600 (9,350–20,900)	34,300 (23,400–40,800)	39,400 (28,100–51,900)	245 (200–434)	369 (248–438)	308 (217–405)
1-23	Uterine cancer	1,710 (1,450–1,970)	3,580 (3,050–4,210)	4,290 (3,290–5,450)	42·6 (36·5–48·8)	42·8 (36·6–49·9)	37·8 (29–47·8)
1-24	Ovarian cancer	662 (547–807)	4,440 (2,740–5,220)	6,720 (3,990–8,850)	13·8 (11·4–16·9)	46·1 (29·1–54·2)	52·6 (31·4–68·9)
1-25	Prostate cancer	3,920 (2,680–8,820)	15,900 (12,200–20,800)	20,400 (15,200–28,100)	138 (96·2–309)	219 (170–280)	197 (146–267)
1-26	Testicular cancer	224 (154–499)	2,440 (1,950–3,380)	3,580 (2,570–4,710)	3 (2·2–5·2)	16·9 (13·5–22·1)	18·9 (13·6–24·6)
1-27	Kidney cancer	1,910 (1,700–2,130)	4,400 (3,920–4,950)	5,740 (4,430–7,430)	33·2 (29·6–37·1)	43·8 (38·7–49·5)	43·9 (34·1–56·6)
1-28	Bladder cancer	791 (695–899)	1,720 (1,510–1,960)	2,300 (1,790–2,930)	22·9 (20·1–25·9)	21·5 (18·8–24·5)	20·8 (16·2–26·3)
1-29	Brain and nervous system cancer	2,950 (1,650–8,460)	11,600 (5,980–14,300)	12,300 (6,580–17,200)	38·5 (22·8–101)	92·4 (45·8–115)	78·8 (41·1–110)
1-30	Thyroid cancer	809 (711–919)	1,420 (1,250–1,630)	2,050 (1,590–2,590)	18·8 (16·5–21·2)	15·9 (13·9–18·1)	16·9 (13·2–21·3)
1-31	Mesothelioma	138 (101–183)	242 (203–287)	358 (269–470)	2·8 (2·1–3·7)	2·4 (2·1–2·9)	2·6 (2–3·4)
1-32	Hodgkin lymphoma	1,830 (1,530–2,370)	2,000 (1,690–2,540)	2,160 (1,560–3,100)	26·9 (22·2–35·3)	15·9 (13·4–20)	13·4 (9·9–19)
1-33	Non-Hodgkin's lymphoma	2,760 (2,420–3,130)	7,260 (6,380–8,220)	8,680 (6,680–11,100)	45·5 (39·9–51·5)	63·9 (56·3–72·4)	60·2 (46·6–77·1)
1-34	Multiple myeloma	546 (443–699)	1,330 (1,150–1,610)	1,880 (1,440–2,450)	13·7 (11·1–17·4)	15·6 (13·5–19·2)	16·3 (12·4–21·2)
1-35	Leukaemia	14,600 (13,000–17,000)	33,700 (29,800–37,400)	37,100 (28,900–46,500)	175 (156–206)	232 (207–259)	211 (164–264)
1-36	Acute lymphoid leukaemia	1,490 (837–3,860)	11,300 (8,780–12,900)	13,900 (9,340–18,200)	15·1 (9·1–37·8)	67·7 (51·3–77·4)	72·1 (47·6–94·5)
1-37	Chronic lymphoid leukaemia	60 (44–79)	260 (214–307)	337 (251–437)	1·4 (1–1·9)	2·8 (2·3–3·3)	2·7 (2–3·5)
1-38	Acute myeloid leukaemia	572 (406–1,050)	3,830 (2,730–4,410)	5,190 (3,480–6,850)	7·3 (5·4–12·4)	26·9 (19·5–31)	29·9 (20–39·1)
1-39	Chronic myeloid leukaemia	257 (208–328)	628 (543–739)	748 (567–966)	4·4 (3·6–5·8)	5·9 (5·1–6·9)	5·3 (4–6·8)
1-40	Other leukaemia	12,200 (10,300–14,000)	17,700 (15,600–20,600)	16,900 (12,900–21,900)	147 (125–165)	129 (113–150)	101 (77·6–129)
1-41	Other neoplasms	545 (350–933)	1,110 (878–1,360)	1,660 (1,180–2,150)	8·9 (6–14·2)	10 (7·9–12·5)	11·7 (8·5–15·1)
1-42	Myelodysplastic, myeloproliferative, and other hematopoietic neoplasms	545 (350–933)	1,110 (878–1,360)	1,660 (1,180–2,150)	8·9 (6–14·2)	10 (7·9–12·5)	11·7 (8·5–15·1)
1-43	Benign and in situ intestinal neoplasms	0 (0–0)	0 (0–0)	0 (0–0)	0 (0–0)	0 (0–0)	0 (0–0)
1-44	Benign and in situ cervical and uterine neoplasms	0 (0–0)	0 (0–0)	0 (0–0)	0 (0–0)	0 (0–0)	0 (0–0)
1-45	Other benign and in situ neoplasms	0 (0–0)	0 (0–0)	0 (0–0)	0 (0–0)	0 (0–0)	0 (0–0)
1-46	Cardiovascular diseases	207,000 (186,000–228,000)	275,000 (247,000–306,000)	360,000 (295,000–439,000)	5,110 (4,600–5,610)	3,290 (2,940–3,640)	3,110 (2,570–3,770)
1-47	Rheumatic heart disease	5,250 (4,020–6,880)	7,470 (5,190–10,700)	9,250 (6,210–13,400)	80·9 (63·2–104)	56·2 (40·5–76·7)	53·6 (37·1–76)
1-48	Ischaemic heart disease	113,000 (101,000–125,000)	139,000 (124,000–156,000)	187,000 (151,000–228,000)	3,050 (2,740–3,360)	1,800 (1,600–2,010)	1,710 (1,390–2,070)
1-49	Stroke	62,500 (55,800–69,400)	91,900 (82,000–102,000)	115,000 (94,100–141,000)	1,450 (1,290–1,600)	1,050 (936–1,160)	964 (789–1,180)
1-50	Ischaemic stroke	14,900 (12,100–17,600)	25,200 (22,400–28,100)	34,500 (28,300–41,400)	481 (395–563)	353 (314–391)	331 (274–396)
1-51	Intracerebral hemorrhage	39,900 (34,900–45,700)	52,500 (46,600–58,700)	62,900 (50,300–79,200)	844 (733–976)	573 (508–643)	512 (409–645)
1-52	Subarachnoid hemorrhage	7,780 (5,890–9,750)	14,200 (12,600–16,100)	18,100 (14,100–23,100)	124 (92·9–158)	122 (106–139)	120 (92·9–154)
1-53	Hypertensive heart disease	6,710 (5,050–7,650)	8,410 (7,200–12,100)	12,400 (9,630–17,500)	189 (157–214)	114 (97·1–167)	117 (91·8–170)
1-54	Non-rheumatic valvular heart disease	662 (515–840)	933 (814–1,070)	1,340 (1,030–1,740)	15·8 (12·5–19·7)	10·1 (8·8–11·5)	10·6 (8·1–13·5)
1-55	Non-rheumatic calcific aortic valve disease	351 (233–477)	606 (508–706)	877 (666–1,140)	8·5 (5·9–11·2)	6·7 (5·7–7·8)	7·1 (5·4–9·1)
1-56	Non-rheumatic degenerative mitral valve disease	218 (160–298)	304 (242–371)	433 (311–577)	5·1 (3·9–6·9)	3·2 (2·5–3·9)	3·3 (2·3–4·3)
1-57	Other non-rheumatic valve diseases	93 (12–196)	23 (11–53)	34 (16–77)	2·2 (0·3–4·5)	0·2 (0·1–0·5)	0·2 (0·1–0·5)
1-58	Cardiomyopathy and myocarditis	2,540 (1,750–3,980)	3,710 (3,230–4,470)	5,180 (3,800–6,730)	37·5 (28·1–52)	32·5 (28·3–37·6)	36·1 (25·9–46·6)
1-59	Myocarditis	331 (216–633)	545 (416–898)	698 (476–1,110)	4 (2·9–6)	4·1 (3·2–5·9)	4·3 (3–6·3)
1-60	Alcoholic cardiomyopathy	163 (103–246)	151 (109–183)	205 (134–271)	3·7 (2·2–5·6)	1·6 (1·1–1·9)	1·6 (1–2·1)

1-61	Other cardiomyopathy	2,050 (1,370–3,200)	3,010 (2,600–3,520)	4,280 (2,980–5,630)	29·8 (21·7–42·8)	26·8 (23·4–30·9)	30·2 (21–39·3)
1-62	Atrial fibrillation and flutter	1,920 (1,530–2,360)	4,400 (3,510–5,410)	6,480 (5,130–8,070)	72 (57·2–86·8)	67·3 (54·3–81·5)	67·2 (53–83·1)
1-63	Aortic aneurysm	797 (660–968)	1,650 (1,430–1,920)	2,280 (1,780–2,930)	21·2 (17·7–25·4)	19·7 (17·1–22·8)	19·6 (15·4–25·1)
1-64	Peripheral vascular disease	200 (104–337)	463 (257–769)	654 (363–1,110)	6·9 (3·6–11·4)	6·4 (3·6–10·7)	6·4 (3·6–11)
1-65	Endocarditis	1,010 (645–1,800)	2,570 (1,690–3,250)	2,950 (2,040–4,010)	12·3 (8·2–22·6)	18·5 (13·4–22·2)	18 (13–24·1)
1-66	Chronic respiratory diseases	91,700 (72,500–107,000)	66,400 (56,000–80,000)	82,700 (67,100–101,000)	1,320 (1,140–1,490)	648 (564–761)	622 (506–759)
1-67	Chronic obstructive pulmonary disease	18,100 (15,700–21,000)	27,000 (23,700–33,100)	38,200 (30,900–47,700)	482 (424–552)	351 (308–431)	348 (282–439)
1-68	Pneumoconiosis	348 (262–431)	86 (67–111)	107 (79–142)	6·9 (5·3–8·4)	1 (0·7–1·3)	0·9 (0·6–1·2)
1-69	Silicosis	22 (13–74)	16 (11–22)	21 (14–29)	0·5 (0·3–1·5)	0·2 (0·1–0·3)	0·2 (0·1–0·3)
1-70	Asbestosis	21 (10–38)	4 (3–5)	4 (3–6)	0·3 (0·2–0·6)	0 (0–0)	0 (0–0)
1-71	Coal workers pneumoconiosis	22 (11–64)	4 (3–5)	4 (2–5)	0·3 (0·2–1)	0 (0–0)	0 (0–0)
1-72	Other pneumoconiosis	282 (207–354)	62 (45–84)	79 (55–109)	5·7 (4·2–7)	0·7 (0·5–1)	0·6 (0·5–0·9)
1-73	Asthma	61,800 (45,400–76,500)	30,100 (22,300–41,600)	31,600 (22,400–44,400)	680 (522–817)	215 (166–279)	184 (136–250)
1-74	Interstitial lung disease and pulmonary sarcoidosis	2,660 (1,890–4,440)	4,130 (3,250–5,090)	6,300 (4,260–8,520)	56·1 (42–79·1)	46·4 (35·5–56·5)	52·4 (34·3–71·7)
1-75	Other chronic respiratory diseases	8,780 (4,950–12,700)	5,040 (4,200–6,110)	6,490 (5,040–8,380)	96·7 (56·9–131)	35·1 (29·1–42·3)	36·5 (28·2–47·4)
1-76	Digestive diseases	243,000 (219,000–268,000)	273,000 (244,000–305,000)	339,000 (274,000–416,000)	3,760 (3,390–4,150)	2,620 (2,340–2,940)	2,440 (1,970–3,010)
1-77	Cirrhosis and other chronic liver diseases	96,300 (85,700–108,000)	146,000 (128,000–166,000)	192,000 (151,000–241,000)	1,780 (1,580–2,010)	1,480 (1,300–1,680)	1,420 (1,120–1,800)
1-78	Cirrhosis and other chronic liver diseases due to hepatitis B	5,580 (3,720–8,100)	9,640 (6,510–13,900)	7,320 (4,490–11,400)	112 (75·4–160)	98·1 (65·9–142)	53·3 (33·3–82·1)
1-79	Cirrhosis and other chronic liver diseases due to hepatitis C	24,000 (18,300–31,400)	40,000 (30,500–51,400)	55,100 (39,000–75,400)	480 (370–630)	405 (308–524)	402 (280–556)
1-80	Cirrhosis and other chronic liver diseases due to alcohol use	26,600 (20,100–33,400)	46,200 (34,900–59,000)	63,900 (45,100–86,000)	569 (432–710)	499 (380–631)	498 (355–668)
1-81	Cirrhosis and other chronic liver diseases due to NAFLD	13,300 (9,250–18,300)	23,700 (16,800–32,500)	33,600 (22,200–49,400)	281 (200–387)	251 (177–339)	258 (170–380)
1-82	Cirrhosis and other chronic liver diseases due to other causes	26,800 (21,900–33,000)	26,600 (21,100–33,900)	31,700 (22,600–43,500)	340 (274–421)	221 (171–284)	209 (148–288)
1-83	Upper digestive system diseases	32,600 (28,000–39,100)	46,000 (37,900–57,900)	50,700 (38,800–66,400)	661 (569–781)	468 (394–574)	375 (291–484)
1-84	Peptic ulcer disease	23,100 (20,400–26,200)	27,100 (24,000–30,700)	25,600 (19,900–32,800)	483 (423–544)	293 (259–330)	204 (160–259)
1-85	Gastritis and duodenitis	2,840 (2,160–3,520)	5,130 (4,180–6,320)	6,240 (4,840–7,900)	55·4 (43·4–67)	52·6 (43·5–63·5)	47·9 (37·6–60·8)
1-86	GERD	6,650 (3,470–11,700)	13,800 (7,180–24,600)	18,800 (9,830–33,500)	122 (63·4–216)	123 (64–218)	123 (63·9–216)
1-87	Appendicitis	30,200 (21,500–36,700)	9,310 (7,720–11,900)	8,950 (6,590–12,400)	290 (211–344)	64·1 (52·9–83·7)	50·8 (37·4–70·7)
1-88	Paralytic ileus and intestinal obstruction	35,400 (26,600–45,500)	18,800 (15,400–22,300)	19,700 (14,700–25,700)	301 (244–370)	137 (116–159)	124 (94·5–163)
1-89	Inguinal, femoral, and abdominal hernia	15,200 (11,300–19,800)	12,400 (9,760–15,600)	14,600 (11,300–18,600)	186 (147–231)	104 (82·8–128)	98·8 (77·3–124)
1-90	Inflammatory bowel disease	866 (522–1,510)	1,340 (1,130–1,570)	1,760 (1,380–2,210)	11·8 (8·4–18·2)	11·8 (9·9–13·7)	12 (9·5–14·9)
1-91	Vascular intestinal disorders	954 (658–1,290)	1,210 (1,070–1,370)	1,610 (1,270–2,050)	18·7 (15·3–22·5)	14·1 (12·4–16·1)	13·8 (10·8–17·3)
1-92	Gallbladder and biliary diseases	12,700 (9,580–16,300)	16,300 (12,100–22,100)	22,200 (16,000–30,200)	248 (192–319)	160 (121–213)	160 (118–215)
1-93	Pancreatitis	10,700 (8,130–12,900)	13,000 (11,000–15,000)	16,500 (12,600–21,300)	178 (141–212)	116 (101–136)	108 (83·1–140)
1-94	Other digestive diseases	8,130 (4,330–11,000)	8,480 (5,220–10,500)	11,200 (6,030–15,400)	85·2 (60·2–115)	73·7 (48·4–89·8)	77·8 (44·5–104)
1-95	Neurological disorders	86,000 (48,700–141,000)	158,000 (86,200–264,000)	204,000 (110,000–342,000)	1,460 (873–2,310)	1,370 (784–2,200)	1,350 (767–2,210)
1-96	Alzheimer's disease and other dementias	7,780 (3,050–18,600)	20,800 (8,430–48,000)	32,200 (13,000–74,000)	361 (141–881)	353 (141–820)	353 (141–817)
1-97	Parkinson's disease	2,130 (1,890–2,360)	4,860 (4,260–5,450)	6,770 (5,620–8,130)	79·5 (71·2–87·2)	69·4 (61·2–77·3)	66 (55–79·1)
1-98	Idiopathic epilepsy	29,800 (18,600–46,400)	46,000 (26,800–73,200)	52,800 (29,800–83,400)	373 (229–578)	313 (182–504)	292 (164–466)
1-99	Multiple sclerosis	275 (227–358)	694 (542–821)	998 (733–1,270)	5·5 (4·6–7·1)	6·8 (5·4–8·1)	7·2 (5·2–9·1)
1-100	Motor neuron disease	311 (273–353)	339 (297–382)	566 (444–717)	5·2 (4·6–5·9)	3·5 (3–4)	4·5 (3·5–5·7)
1-101	Headache disorders	37,600 (6,250–83,300)	76,800 (13,000–171,000)	98,800 (17,000–218,000)	552 (106–1,210)	563 (107–1,230)	563 (107–1,230)
1-102	Migraine	34,400 (4,220–79,900)	70,200 (8,660–164,000)	90,200 (11,600–210,000)	501 (74·3–1,150)	510 (73·8–1,170)	510 (71·9–1,170)
1-103	Tension-type headache	3,210 (859–13,500)	6,590 (1,790–27,000)	8,630 (2,450–33,900)	51·5 (15·1–197)	52·3 (15·3–192)	52·3 (15·7–191)
1-104	Other neurological disorders	8,120 (5,390–11,500)	8,940 (4,680–15,000)	11,600 (6,000–19,000)	84 (57·8–119)	59·5 (34·1–95·7)	64·4 (35·4–103)
1-105	Mental disorders	111,000 (80,800–146,000)	227,000 (165,000–303,000)	288,000 (209,000–383,000)	1,710 (1,250–2,250)	1,760 (1,290–2,330)	1,710 (1,250–2,260)
1-106	Schizophrenia	9,440 (6,560–12,600)	19,500 (13,400–26,000)	26,800 (18,600–36,300)	165 (115–220)	168 (116–221)	167 (116–223)
1-107	Depressive disorders	39,100 (26,500–54,600)	83,300 (57,600–116,000)	104,000 (70,700–146,000)	681 (472–951)	719 (502–994)	658 (451–920)
1-108	Major depressive disorder	34,200 (22,800–49,000)	73,200 (49,700–102,000)	90,500 (60,000–129,000)	597 (406–848)	635 (441–883)	574 (390–814)
1-109	Dysthymia	4,860 (3,020–7,440)	10,000 (6,090–15,500)	13,500 (8,180–20,900)	83·6 (52·5–129)	84·3 (53·3–130)	84·4 (53–129)
1-110	Bipolar disorder	11,100 (6,420–17,200)	22,900 (13,000–36,000)	30,200 (17,600–47,100)	172 (100–266)	173 (101–267)	173 (101–267)
1-111	Anxiety disorders	26,700 (17,400–37,500)	54,900 (36,300–77,200)	71,200 (47,200–102,000)	386 (255–538)	399 (265–562)	406 (272–578)
1-112	Eating disorders	2,700 (1,630–4,060)	5,990 (3,580–9,150)	8,170 (4,990–12,600)	35·3 (21·5–53)	37·9 (22·8–57·4)	39·7 (24·2–61)
1-113	Anorexia nervosa	698 (389–1,150)	1,550 (886–2,540)	2,060 (1,190–3,370)	8·4 (4·8–13·6)	9·2 (5·3–14·9)	9·8 (5·7–15·9)

1-114	Bulimia nervosa	2,000 (1,150–3,160)	4,430 (2,510–7,090)	6,110 (3,490–9,970)	26·8 (15·4–42·2)	28·6 (16·2–45·8)	29·9 (17·2–48·3)
1-115	Autism spectrum disorders	4,360 (2,800–6,410)	7,780 (5,040–11,400)	9,350 (6,080–13,700)	50·4 (32·7–74)	50·1 (32·5–73·1)	50·2 (32·5–73·4)
1-116	Attention-deficit/hyperactivity disorder	1,750 (979–3,080)	3,290 (1,800–5,740)	3,840 (2,130–6,700)	18·6 (10·4–32·5)	18·7 (10·4–32·3)	18·7 (10·5–32·7)
1-117	Conduct disorder	7,350 (4,100–11,700)	13,700 (7,680–21,900)	14,500 (8,080–23,100)	66·7 (37·4–106)	67·7 (38·1–108)	67·7 (37·7–108)
1-118	Idiopathic developmental intellectual disability	2,280 (933–4,080)	3,790 (1,570–6,670)	3,610 (1,210–6,650)	26·4 (10·8–46·8)	24·5 (10·3–43·1)	19·1 (6·4–35·3)
1-119	Other mental disorders	5,790 (3,640–8,750)	11,800 (7,460–18,200)	16,300 (10,400–24,700)	107 (67·8–161)	106 (67·8–162)	106 (67·9–161)
1-120	Substance use disorders	96,400 (82,400–112,000)	127,000 (105,000–151,000)	160,000 (128,000–198,000)	1,700 (1,460–1,980)	1,100 (912–1,300)	983 (791–1,220)
1-121	Alcohol use disorders	87,700 (74,600–102,000)	111,000 (91,500–133,000)	132,000 (106,000–165,000)	1,570 (1,330–1,830)	982 (816–1,160)	832 (661–1,040)
1-122	Drug use disorders	8,710 (6,880–10,800)	16,000 (12,100–20,100)	28,000 (22,100–35,200)	135 (109–166)	116 (88–144)	151 (120–189)
1-123	Opioid use disorders	5,000 (3,650–6,680)	8,960 (6,310–12,000)	14,200 (10,200–19,000)	76·6 (56·9–101)	66·2 (47·6–87)	77·5 (56·8–101)
1-124	Cocaine use disorders	1,460 (1,110–1,840)	2,320 (1,850–2,900)	7,050 (5,380–8,940)	24 (18·2–30)	16·6 (13·5–20)	37·9 (28·9–47·7)
1-125	Amphetamine use disorders	595 (368–930)	1,130 (642–1,750)	1,990 (1,330–2,950)	9 (5·8–13·3)	7·7 (4·6–11·6)	10·5 (7·4–15)
1-126	Cannabis use disorders	301 (166–484)	628 (350–1,030)	689 (392–1,120)	4·1 (2·3–6·4)	4·1 (2·3–6·5)	3·6 (2·1–5·7)
1-127	Other drug use disorders	1,360 (1,030–1,770)	2,920 (2,250–3,690)	4,070 (3,100–5,180)	21·7 (16·7–27·8)	21·1 (16·5–26·6)	22 (16·9–27·7)
1-128	Diabetes and kidney diseases	79,800 (70,700–89,100)	324,000 (284,000–367,000)	458,000 (376,000–550,000)	1,710 (1,500–1,930)	3,620 (3,170–4,100)	3,770 (3,100–4,520)
1-129	Diabetes mellitus	33,000 (27,100–39,600)	190,000 (162,000–221,000)	269,000 (217,000–325,000)	805 (661–974)	2,190 (1,870–2,550)	2,270 (1,830–2,740)
1-130	Diabetes mellitus type 1	3,000 (2,520–4,020)	12,000 (8,180–14,100)	15,700 (10,200–20,900)	45·8 (36·9–60·2)	99·9 (65·6–119)	100 (61·9–134)
1-131	Diabetes mellitus type 2	30,000 (24,500–36,600)	178,000 (151,000–207,000)	254,000 (205,000–306,000)	760 (618–921)	2,090 (1,780–2,440)	2,170 (1,750–2,620)
1-132	Chronic kidney disease	46,800 (42,400–52,000)	134,000 (119,000–149,000)	189,000 (153,000–233,000)	906 (819–1,010)	1,430 (1,260–1,590)	1,500 (1,210–1,840)
1-133	Chronic kidney disease due to diabetes mellitus type 1	3,290 (2,260–4,470)	11,200 (7,630–15,400)	16,300 (10,400–24,300)	63·6 (42·9–87·8)	111 (73·1–157)	118 (73·2–180)
1-134	Chronic kidney disease due to diabetes mellitus type 2	7,100 (5,540–8,820)	28,600 (22,300–35,200)	42,100 (31,600–54,700)	194 (154–239)	358 (279–440)	381 (288–496)
1-135	Chronic kidney disease due to hypertension	6,190 (4,960–7,780)	23,000 (18,300–28,000)	33,100 (25,400–43,900)	174 (142–213)	291 (234–352)	298 (228–393)
1-136	Chronic kidney disease due to glomerulonephritis	13,500 (11,000–16,000)	33,700 (27,500–40,600)	46,400 (35,000–60,800)	214 (175–255)	310 (249–380)	325 (240–438)
1-137	Chronic kidney disease due to other and unspecified causes	16,800 (13,900–19,600)	37,500 (30,900–45,100)	51,200 (39,700–65,700)	260 (215–310)	356 (290–436)	375 (290–487)
1-138	Acute glomerulonephritis	34 (19–58)	11 (9–14)	9 (7–12)	0·4 (0·2–0·6)	0·1 (0·1–0·1)	0·1 (0–0·1)
1-139	Skin and subcutaneous diseases	38,100 (25,800–56,000)	67,200 (44,100–99,500)	81,400 (53,800–120,000)	469 (324–689)	453 (302–674)	461 (310–679)
1-140	Dermatitis	8,370 (4,880–13,200)	14,600 (8,550–23,100)	17,200 (10,200–26,900)	93·7 (56·4–144)	94·1 (56·4–145)	94·2 (56·9–146)
1-141	Atopic dermatitis	6,570 (3,540–11,000)	11,000 (5,930–18,400)	12,400 (6,710–20,800)	63·4 (34·2–106)	63·7 (34·6–106)	63·7 (34·6–107)
1-142	Contact dermatitis	1,570 (993–2,360)	3,240 (2,040–4,890)	4,280 (2,650–6,410)	26·9 (16·8–40·4)	27 (17–40·5)	27·1 (17–40·3)
1-143	Seborrhoeic dermatitis	227 (125–362)	442 (252–707)	563 (319–893)	3·4 (1·9–5·4)	3·4 (1·9–5·4)	3·4 (1·9–5·3)
1-144	Psoriasis	1,010 (692–1,380)	1,680 (1,170–2,280)	2,060 (1,410–2,810)	15·4 (10·8–20·9)	13·2 (9·3–18)	12·7 (8·7–17·1)
1-145	Bacterial skin diseases	2,190 (1,330–3,710)	4,120 (2,880–7,020)	6,160 (3,980–9,800)	24·7 (14·9–45·9)	37·2 (25·8–66·1)	44·3 (28·4–72)
1-146	Cellulitis	888 (461–1,460)	1,110 (773–2,060)	1,510 (936–2,540)	9·8 (5·3–17·7)	9·3 (6·3–18·4)	10·1 (6·2–18·1)
1-147	Pyoderma	1,300 (775–2,510)	3,010 (2,030–5,260)	4,650 (2,930–7,710)	14·9 (8·9–29)	28 (18·5–49·4)	34·2 (21·6–57·2)
1-148	Scabies	6,660 (3,600–10,800)	11,900 (6,430–19,000)	13,900 (7,480–22,500)	74·9 (40·9–120)	74·1 (40·4–118)	73·6 (40–118)
1-149	Fungal skin diseases	1,780 (708–3,720)	3,340 (1,330–7,000)	4,240 (1,690–8,920)	28·2 (11·4–59)	27·5 (11·1–58·1)	27·5 (11·1–58·4)
1-150	Viral skin diseases	4,650 (2,980–7,090)	7,690 (4,890–11,600)	8,610 (5,510–13,000)	43·5 (28–65·4)	43·7 (27·9–65·9)	43·7 (27·9–66·1)
1-151	Acne vulgaris	3,410 (2,000–5,540)	7,880 (4,640–12,700)	9,370 (5,540–15,200)	35·7 (21·1–57·6)	41·4 (24·7–66·7)	43·5 (25·7–70·4)
1-152	Alopecia areata	480 (305–721)	963 (601–1,450)	1,250 (791–1,880)	7·1 (4·6–10·6)	7·2 (4·5–10·8)	7·2 (4·5–10·8)
1-153	Pruritus	530 (255–955)	1,050 (499–1,900)	1,360 (646–2,460)	8·4 (4·1–14·9)	8·6 (4·1–15·4)	8·7 (4·1–15·8)
1-154	Urticaria	4,480 (2,870–6,560)	7,640 (4,980–11,300)	8,970 (5,830–13,100)	47·8 (31–69·2)	48·3 (31·4–70)	48·3 (31·5–70·2)
1-155	Decubitus ulcer	1,870 (799–2,760)	979 (688–2,270)	1,290 (874–2,630)	44·5 (20·8–63·2)	12 (8·4–27·2)	11·2 (7·6–22·8)
1-156	Other skin and subcutaneous diseases	2,700 (1,310–4,930)	5,370 (2,620–9,780)	6,920 (3,310–12,500)	45 (21·9–82·1)	46 (22·6–83·4)	46·3 (22·3–83·5)
1-157	Sense organ diseases	45,200 (31,100–63,300)	88,100 (60,500–125,000)	116,000 (79,700–163,000)	1,050 (733–1,450)	949 (649–1,320)	925 (641–1,290)
1-158	Blindness and vision loss	18,800 (13,100–25,700)	35,500 (24,400–49,600)	46,500 (32,000–64,200)	465 (329–626)	394 (276–546)	380 (265–521)
1-159	Glaucoma	657 (438–950)	1,100 (742–1,560)	1,460 (984–2,060)	24·6 (16·3–35·3)	15·9 (10·8–22·3)	14·5 (9·8–20·4)
1-160	Cataract	3,030 (2,140–4,120)	5,660 (3,960–7,700)	7,810 (5,470–10,500)	103 (72·8–138)	78·4 (54·9–106)	74·5 (52·4–99·8)
1-161	Age-related macular degeneration	172 (112–259)	324 (212–473)	455 (297–669)	5·7 (3·7–8·3)	4·4 (2·9–6·4)	4·4 (2·9–6·4)
1-162	Refraction disorders	6,890 (4,590–9,880)	13,000 (8,610–18,800)	16,300 (10,900–23,400)	121 (81·8–167)	110 (73·8–155)	106 (71·2–150)
1-163	Near vision loss	2,250 (977–4,600)	4,700 (2,140–9,160)	6,490 (2,970–12,800)	62·1 (28·6–124)	58·3 (27–115)	58·5 (27–115)
1-164	Other vision loss	5,810 (4,010–8,020)	10,700 (7,610–14,500)	14,000 (9,940–19,000)	149 (104–206)	127 (91–172)	121 (86·4–164)
1-165	Age-related and other hearing loss	24,600 (16,300–35,700)	48,800 (32,500–71,200)	64,500 (43,000–93,500)	550 (369–791)	520 (348–747)	510 (340–737)
1-166	Other sense organ diseases	1,840 (1,090–2,830)	3,800 (2,300–5,720)	5,000 (2,950–7,600)	34·4 (20·2–53·4)	35·5 (21·3–54·3)	35·8 (21·2–54·8)

1-167	Musculoskeletal disorders	84,300 (59,800–112,000)	189,000 (136,000–249,000)	262,000 (188,000–347,000)	1,620 (1,160–2,160)	1,770 (1,270–2,320)	1,810 (1,300–2,400)
1-168	Rheumatoid arthritis	1,470 (993–2,210)	4,530 (3,400–5,750)	6,430 (4,800–8,390)	31·2 (21·6–45·2)	47·9 (35·6–60·4)	49·5 (36·5–64·7)
1-169	Osteoarthritis	6,790 (3,410–13,500)	15,700 (7,880–31,700)	21,800 (11,000–43,400)	178 (89·5–357)	191 (96·3–385)	194 (98–387)
1-170	Low back pain	44,600 (31,400–60,100)	91,800 (63,800–124,000)	123,000 (85,300–165,000)	795 (560–1,070)	799 (556–1,060)	801 (562–1,070)
1-171	Neck pain	6,870 (4,410–10,100)	14,100 (9,280–20,500)	19,300 (12,700–28,200)	134 (87·9–193)	134 (89·9–195)	134 (88·7–195)
1-172	Gout	192 (117–287)	461 (290–687)	664 (421–968)	4·3 (2·6–6·3)	4·9 (3·1–7·2)	5·1 (3·2–7·4)
1-173	Other musculoskeletal disorders	24,300 (15,100–35,100)	62,700 (41,100–87,400)	91,400 (61,500–128,000)	483 (306–683)	590 (389–819)	623 (418–878)
1-174	Other non-communicable diseases	194,000 (152,000–293,000)	265,000 (213,000–323,000)	308,000 (232,000–400,000)	2,060 (1,610–2,790)	1,870 (1,500–2,340)	1,840 (1,390–2,380)
1-175	Congenital anomalies	108,000 (77,800–208,000)	131,000 (94,500–161,000)	130,000 (81,100–183,000)	738 (535–1,410)	712 (516–871)	660 (412–925)
1-176	Neural tube defects	15,900 (10,900–22,800)	12,800 (8,170–16,400)	13,400 (7,720–20,400)	102 (70·6–146)	68·5 (43·8–87·7)	67·2 (38·7–102)
1-177	Congenital heart anomalies	31,200 (17,500–86,000)	45,600 (30,300–57,600)	42,500 (21,900–63,200)	211 (122–576)	246 (163–310)	214 (110–319)
1-178	Orofacial clefts	5,780 (2,410–11,900)	1,110 (752–1,520)	1,010 (646–1,480)	36·9 (15·8–75·1)	6·3 (4·3–8·5)	5·2 (3·4–7·7)
1-179	Down's syndrome	1,670 (1,020–3,810)	8,260 (5,350–10,600)	8,150 (4,600–11,600)	12·3 (7·8–27·3)	45·2 (29·2–58)	41·1 (23·1–58·7)
1-180	Turner syndrome	21 (9–41)	34 (15–61)	38 (17–68)	0·2 (0·1–0·4)	0·2 (0·1–0·4)	0·2 (0·1–0·3)
1-181	Klinefelter syndrome	1 (1–3)	2 (1–4)	3 (1–5)	0 (0–0)	0 (0–0)	0 (0–0)
1-182	Other chromosomal abnormalities	2,900 (1,610–8,040)	3,050 (2,260–5,320)	3,650 (2,580–5,770)	20·2 (11·7–53·2)	16·6 (12·3–28·5)	18·3 (12·9–28·8)
1-183	Congenital musculoskeletal and limb anomalies	8,530 (5,460–12,600)	12,600 (8,150–18,600)	14,700 (9,420–21,500)	75·2 (48·1–110)	73·2 (47·5–106)	75·7 (49–111)
1-184	Urogenital congenital anomalies	3,320 (2,000–5,630)	3,360 (1,720–4,530)	3,300 (1,450–5,040)	22 (13·5–37·2)	18·1 (9·2–24·4)	16·6 (7·3–25·4)
1-185	Digestive congenital anomalies	9,930 (5,700–27,100)	14,500 (8,400–18,900)	11,900 (6,520–18,100)	63·4 (36·4–175)	77·6 (44·8–101)	60 (32·8–91·3)
1-186	Other congenital anomalies	28,600 (17,500–58,000)	29,300 (21,100–37,500)	31,800 (17,700–46,500)	194 (120–391)	159 (115–203)	161 (90·1–235)
1-187	Urinary diseases and male infertility	4,960 (4,140–6,020)	10,500 (8,380–12,800)	15,100 (11,500–19,100)	96·8 (77·9–121)	108 (86·4–133)	120 (91–151)
1-188	Urinary tract infections and interstitial nephritis	2,680 (2,270–3,260)	6,030 (4,730–6,890)	9,160 (6,340–11,700)	39·5 (33·5–51·6)	57 (45·9–64·8)	68·8 (48·1–87·6)
1-189	Urolithiasis	358 (265–466)	478 (380–598)	669 (503–886)	6·5 (4·9–8·2)	4·8 (3·8–5·9)	5 (3·7–6·6)
1-190	Benign prostatic hyperplasia	1,210 (699–1,880)	2,790 (1,630–4,310)	3,710 (2,160–5,680)	36·1 (21·2–55·8)	35 (20·3–53·9)	34·7 (20·2–53·1)
1-191	Male infertility	106 (42–232)	296 (105–719)	429 (151–1,060)	1·7 (0·7–3·8)	2·2 (0·8–5·4)	2·3 (0·8–5·6)
1-192	Other urinary diseases	609 (412–802)	883 (575–1,100)	1,160 (692–1,560)	12·9 (6·8–17·5)	9·2 (5·2–11·3)	9 (4·8–12·2)
1-193	Gynecological diseases	23,300 (16,800–32,200)	42,300 (28,900–60,200)	56,600 (38,700–80,100)	372 (268–512)	337 (232–470)	333 (228–468)
1-194	Uterine fibroids	1,970 (1,180–3,180)	2,890 (1,430–5,340)	4,070 (2,010–7,400)	36·4 (21·8–58·7)	27·6 (13·6–51·7)	27·7 (13·7–51)
1-195	Polycystic ovarian syndrome	406 (168–823)	1,170 (502–2,370)	1,640 (688–3,310)	5·7 (2·4–11·6)	8·1 (3·4–16·2)	8·6 (3·6–17·2)
1-196	Female infertility	169 (66–367)	621 (167–1,530)	868 (237–2,160)	2·8 (1·1–6·2)	4·9 (1·3–11·9)	4·8 (1·3–11·8)
1-197	Endometriosis	3,080 (1,810–5,030)	3,630 (2,060–5,850)	4,410 (2,480–7,060)	46 (27·4–74·5)	26·4 (15·5–42·2)	23·6 (13·6–37·2)
1-198	Genital prolapse	451 (246–802)	534 (251–1,020)	707 (340–1,360)	10·1 (5·4–18·1)	6·2 (2·9–12·1)	6 (2·8–11·5)
1-199	Premenstrual syndrome	6,690 (4,060–10,400)	15,100 (9,110–23,500)	19,800 (12,100–30,400)	94·6 (58·6–147)	106 (63·9–163)	105 (63·5–161)
1-200	Other gynecological diseases	10,500 (7,210–14,800)	18,400 (11,700–27,000)	25,000 (15,900–37,000)	176 (119–247)	158 (101–232)	158 (101–230)
1-201	Hemoglobinopathies and hemolytic anaemias	9,100 (7,910–10,400)	4,720 (3,770–6,100)	5,990 (4,610–7,620)	124 (108–142)	35·8 (28·7–45·9)	36·4 (28·3–46·3)
1-202	Thalassemias	181 (92–317)	60 (30–101)	79 (40–134)	1·3 (0·7–2·3)	0·3 (0·2–0·5)	0·4 (0·2–0·7)
1-203	Thalassaemias trait	228 (129–367)	401 (224–643)	492 (288–780)	2·9 (1·6–4·7)	2·6 (1·5–4·2)	2·6 (1·5–4·1)
1-204	Sickle cell disorders	426 (286–595)	92 (61–131)	110 (66–178)	2·8 (1·9–4)	0·5 (0·3–0·7)	0·5 (0·3–0·9)
1-205	Sickle cell trait	81 (28–154)	113 (39–225)	136 (47–267)	1·1 (0·4–2)	0·8 (0·3–1·5)	0·7 (0·3–1·4)
1-206	G6PD deficiency	3,170 (2,500–3,830)	1,300 (1,080–1,540)	1,660 (1,230–2,230)	36·3 (28·6–43·9)	8·9 (7·4–10·5)	9·1 (6·7–12·2)
1-207	G6PD trait	6 (3–10)	15 (8–26)	20 (10–34)	0·1 (0·1–0·2)	0·1 (0·1–0·3)	0·1 (0·1–0·2)
1-208	Other hemoglobinopathies and hemolytic anaemias	5,010 (4,080–6,110)	2,740 (2,010–3,770)	3,490 (2,560–4,720)	79·3 (66·1–95)	22·6 (17·1–30·3)	23 (17·3–30·7)
1-209	Endocrine, metabolic, blood, and immune disorders	21,200 (15,800–25,800)	28,000 (21,500–36,500)	38,000 (29,300–49,400)	295 (230–369)	248 (190–328)	268 (205–348)
1-210	Oral disorders	19,000 (11,200–29,800)	38,400 (22,500–60,400)	51,200 (30,200–80,300)	379 (229–587)	373 (223–588)	370 (224–582)
1-211	Caries of deciduous teeth	380 (160–800)	550 (236–1,150)	569 (245–1,170)	2·9 (1·2–6)	2·9 (1·2–6)	2·8 (1·2–5·8)
1-212	Caries of permanent teeth	1,980 (867–3,930)	3,840 (1,690–7,650)	4,810 (2,160–9,420)	28·2 (12·6–55·8)	27·4 (12·4–53·2)	27·2 (12–52·5)
1-213	Periodontal disease	5,340 (2,020–11,400)	11,000 (4,260–23,500)	15,400 (5,840–32,600)	103 (40·6–218)	104 (40·7–223)	105 (40·9–222)
1-214	Edentulism	7,580 (4,800–11,200)	15,900 (10,000–23,200)	21,300 (13,700–31,400)	193 (122–280)	186 (118–273)	182 (117–265)
1-215	Other oral disorders	3,740 (2,280–5,430)	7,170 (4,450–10,600)	9,110 (5,670–13,400)	52·9 (32·9–76·7)	53·2 (33·2–78·2)	53·2 (33·3–78)
1-216	Sudden infant death syndrome	9,010 (5,460–14,500)	11,000 (8,290–14,100)	10,500 (6,800–15,400)	56·6 (34·3–91)	58·4 (44–74·7)	52·7 (34·2–77·3)
1-0	Injuries	630,000 (562,000–714,000)	907,000 (815,000–1,010,000)	956,000 (789,000–1,150,000)	8,490 (7,520–9,590)	6,580 (5,870–7,310)	5,380 (4,440–6,430)
1-1	Transport injuries	62,600 (56,200–69,600)	124,000 (109,000–139,000)	172,000 (137,000–213,000)	903 (803–1,010)	932 (822–1,050)	980 (784–1,210)
1-2	Road injuries	60,800 (54,500–67,600)	116,000 (102,000–131,000)	158,000 (126,000–196,000)	878 (779–980)	874 (769–987)	899 (720–1,110)

1-3	Pedestrian road injuries	40,200 (35,100–45,300)	57,700 (50,900–65,100)	72,100 (56,600–90,300)	576 (504–650)	449 (396–506)	428 (337–536)
1-4	Cyclist road injuries	2,200 (1,700–2,770)	5,020 (4,230–5,890)	6,660 (5,390–8,060)	33·5 (26·1–41·7)	38·8 (32·6–45·6)	40·2 (32·5–48·4)
1-5	Motorcyclist road injuries	5,920 (5,050–7,100)	17,300 (14,800–20,100)	23,800 (18,900–29,600)	84·6 (72·3–101)	122 (105–140)	124 (99·3–153)
1-6	Motor vehicle road injuries	11,500 (9,700–14,400)	32,000 (28,000–36,300)	49,300 (39,400–61,100)	167 (142–209)	236 (207–267)	271 (218–336)
1-7	Other road injuries	1,030 (816–1,270)	3,750 (3,180–4,460)	6,140 (4,720–7,910)	16·1 (12·8–19·8)	28·8 (24·2–33·9)	35·2 (27·2–44·7)
1-8	Other transport injuries	1,810 (1,590–2,060)	7,810 (6,690–9,010)	14,300 (11,400–17,900)	25·2 (22–28·9)	58·4 (50·1–67·1)	80·6 (64·5–100)
1-9	Unintentional injuries	242,000 (218,000–269,000)	295,000 (263,000–330,000)	342,000 (284,000–410,000)	3,080 (2,740–3,440)	2,230 (1,980–2,500)	2,040 (1,680–2,440)
1-10	Falls	37,800 (32,800–43,500)	62,000 (52,700–72,700)	77,800 (63,200–94,300)	677 (586–776)	572 (488–668)	535 (436–648)
1-11	Drowning	20,700 (18,400–23,100)	25,100 (22,300–28,400)	23,400 (18,400–29,500)	255 (225–287)	166 (148–187)	125 (98·8–157)
1-12	Fire, heat, and hot substances	29,100 (24,100–34,600)	22,900 (18,300–28,500)	26,100 (20,600–33,200)	365 (300–435)	165 (132–207)	151 (119–192)
1-13	Poisonings	7,830 (6,910–8,840)	14,700 (13,000–16,600)	15,700 (12,400–19,400)	96·2 (85·1–109)	101 (89·3–115)	85·1 (67·9–106)
1-14	Poisoning by carbon monoxide	101 (72–144)	627 (517–745)	776 (585–990)	1·3 (0·9–1·9)	4·4 (3·7–5·2)	4·4 (3·3–5·6)
1-15	Poisoning by other means	7,730 (6,830–8,720)	14,100 (12,500–15,900)	14,900 (11,800–18,500)	94·9 (84–107)	96·9 (85·4–110)	80·7 (64·4–100)
1-16	Exposure to mechanical forces	19,200 (16,200–23,000)	46,800 (39,800–55,300)	84,600 (69,600–103,000)	285 (240–347)	359 (301–430)	490 (399–598)
1-17	Unintentional firearm injuries	8,390 (6,780–9,840)	24,200 (19,100–27,900)	46,800 (32,400–59,000)	121 (99·3–141)	174 (136–200)	254 (175–320)
1-18	Other exposure to mechanical forces	10,800 (8,150–14,200)	22,600 (17,000–30,700)	37,800 (27,800–55,000)	165 (124–220)	185 (138–252)	236 (172–334)
1-19	Adverse effects of medical treatment	14,200 (8,400–16,400)	6,900 (5,770–10,500)	7,190 (5,210–12,700)	178 (121–202)	54·7 (46–85·5)	46·3 (33·7–82·4)
1-20	Animal contact	1,850 (1,380–2,420)	3,760 (3,020–4,600)	4,100 (3,160–5,210)	25·1 (18·4–33·5)	27·6 (22·1–33·8)	24·8 (19·1–31·4)
1-21	Venomous animal contact	1,350 (1,010–1,760)	2,720 (2,190–3,330)	2,950 (2,250–3,750)	17·9 (13·1–23·4)	20·1 (16·3–24·7)	17·8 (13·7–22·6)
1-22	Non-venomous animal contact	495 (328–742)	1,030 (766–1,390)	1,150 (827–1,620)	7·3 (4·8–10·8)	7·5 (5·5–10·2)	6·9 (5–9·8)
1-23	Foreign body	81,600 (71,900–93,100)	49,100 (43,600–54,900)	50,700 (40,100–64,500)	782 (706–873)	328 (293–364)	284 (225–361)
1-24	Pulmonary aspiration and foreign body in airway	77,300 (68,200–88,200)	45,600 (40,200–51,300)	46,600 (36,300–59,800)	733 (658–819)	305 (270–339)	261 (203–335)
1-25	Foreign body in eyes	373 (161–687)	713 (309–1,310)	914 (405–1,680)	5·5 (2·7–9·6)	5·4 (2·6–9·5)	5·4 (2·6–9·5)
1-26	Foreign body in other body part	3,980 (2,760–5,590)	2,720 (2,200–3,380)	3,170 (2,470–4,010)	43·3 (31·8–57·5)	18·2 (14·6–22·5)	17·6 (13·8–22·2)
1-27	Environmental heat and cold exposure	898 (680–1,150)	3,680 (3,070–4,470)	4,040 (3,230–4,940)	13·8 (10·4–17·7)	28·4 (23·7–34·3)	25·3 (20·3–30·9)
1-28	Exposure to forces of nature	304 (204–445)	11,200 (10,200–12,400)	718 (500–1,020)	4·3 (3–6·1)	73·7 (66·6–81·5)	4·6 (3·2–6·4)
1-29	Other unintentional injuries	28,500 (25,100–32,300)	48,400 (43,000–54,600)	47,800 (38,900–58,300)	396 (346–451)	351 (310–396)	270 (220–330)
1-30	Self-harm and interpersonal violence	326,000 (275,000–397,000)	488,000 (435,000–549,000)	441,000 (357,000–537,000)	4,510 (3,840–5,380)	3,420 (3,020–3,850)	2,360 (1,900–2,870)
1-31	Self-harm	24,000 (20,700–27,400)	50,900 (45,000–57,700)	53,300 (41,800–67,100)	364 (314–417)	360 (317–407)	282 (222–354)
1-32	Self-harm by firearm	4,300 (3,140–6,330)	5,720 (4,150–7,000)	3,410 (2,130–4,490)	66·6 (49–99·5)	42·2 (30·8–51·2)	18·7 (11·8–24·7)
1-33	Self-harm by other specified means	19,700 (16,400–22,900)	45,200 (40,000–51,100)	49,900 (39,000–62,900)	297 (245–344)	317 (280–359)	263 (206–331)
1-34	Interpersonal violence	221,000 (197,000–249,000)	417,000 (370,000–468,000)	370,000 (295,000–458,000)	3,180 (2,800–3,580)	2,870 (2,540–3,230)	1,940 (1,540–2,410)
1-35	Physical violence by firearm	81,100 (67,000–93,100)	336,000 (295,000–382,000)	299,000 (236,000–372,000)	1,180 (967–1,350)	2,290 (2,010–2,610)	1,550 (1,220–1,930)
1-36	Physical violence by sharp object	26,000 (20,900–31,700)	30,700 (27,300–34,400)	32,100 (25,600–39,900)	392 (314–476)	223 (198–252)	178 (142–220)
1-37	Sexual violence	951 (628–1,350)	1,760 (1,150–2,460)	2,280 (1,500–3,210)	13·8 (9·2–19·5)	12·8 (8·4–17·9)	12·7 (8·4–17·8)
1-38	Physical violence by other means	113,000 (100,000–128,000)	48,900 (43,700–54,700)	36,700 (29,500–44,700)	1,600 (1,400–1,810)	338 (302–378)	200 (162–245)
1-39	Conflict and terrorism	80,100 (41,700–145,000)	20,500 (9,560–38,900)	17,500 (8,210–32,900)	971 (504–1,760)	191 (89–358)	134 (62·8–252)
1-40	Executions and police conflict	73 (65–84)	112 (100–126)	135 (105–170)	0·8 (0·7–1)	0·7 (0·6–0·8)	0·7 (0·5–0·9)

SR Table 1: All-age DALYs counts and age standardized DALYs rates per 100,000 individuals for all causes and level 1 cause groups by location for 1990, 2010, and 2019, both sexes combined, Honduras

Cause Level	Cause	All-age DALYs			Age-standardized rate of DALYs per 100,000		
		1990	2010	2019	1990	2010	2019
0-0	All causes	1,910,000 (1,740,000–2,090,000)	2,240,000 (1,910,000–2,600,000)	2,550,000 (2,150,000–3,040,000)	42,100 (37,900–46,500)	35,200 (30,100–40,800)	32,700 (28,000–38,300)
1-0	Communicable, maternal, neonatal, and nutritional disorders	849,000 (766,000–938,000)	502,000 (415,000–606,000)	436,000 (353,000–526,000)	12,400 (11,200–13,700)	5,860 (4,900–6,990)	4,420 (3,630–5,330)
1-1	HIV/AIDS and sexually transmitted infections	8,480 (6,090–12,200)	8,110 (6,430–10,600)	9,710 (7,240–13,100)	174 (134–224)	108 (85·6–138)	101 (76·8–136)
1-2	HIV/AIDS	4,750 (3,820–5,690)	4,980 (4,160–6,130)	5,790 (4,240–7,790)	116 (89–144)	67·9 (56·2–86·6)	61·5 (46·4–80·1)
1-3	HIV/AIDS - drug-susceptible tuberculosis	2,180 (1,760–2,610)	1,990 (1,590–2,470)	2,190 (1,580–2,910)	53·2 (41·1–66)	27 (21·6–34·8)	23·1 (17·1–29·9)
1-4	HIV/AIDS - Multidrug-resistant Tuberculosis without extensive drug resistance	5 (1–23)	127 (27–368)	143 (20–487)	0·1 (0–0·6)	1·7 (0·4–5·1)	1·5 (0·2–5)
1-5	HIV/AIDS - Extensively drug-resistant Tuberculosis	0 (0–0)	13 (3–42)	23 (3–74)	0 (0–0)	0·2 (0–0·6)	0·2 (0–0·8)
1-6	HIV/AIDS resulting in other diseases	2,570 (2,060–3,090)	2,850 (2,370–3,520)	3,430 (2,510–4,790)	62·3 (47·3–77·7)	39 (32–49·4)	36·6 (27·8–49·3)
1-7	Sexually transmitted infections excluding HIV	3,730 (1,570–7,460)	3,130 (1,750–5,330)	3,920 (2,110–6,850)	58·2 (30·6–104)	40 (23·1–68)	39·7 (22·1–68·3)
1-8	Syphilis	2,970 (912–6,620)	1,550 (531–3,210)	2,040 (650–4,630)	35·6 (11·7–78·3)	16·2 (5·9–32·9)	18·4 (6·1–41·3)
1-9	Chlamydial infection	74 (50–111)	131 (86–192)	167 (111–243)	2·1 (1·4–3·1)	1·9 (1·3–2·7)	1·8 (1·2–2·6)
1-10	Gonococcal infection	67 (48–129)	110 (72–182)	138 (90–206)	2 (1·5–3·6)	1·8 (1·1–2·7)	1·7 (1·1–2·5)
1-11	Trichomoniasis	255 (101–514)	512 (201–1,060)	677 (263–1,410)	7·5 (2·9–15·3)	7·5 (2·9–15·6)	7·4 (2·9–15·4)
1-12	Genital herpes	216 (73–526)	577 (195–1,390)	586 (192–1,440)	6·8 (2·2–16·9)	8·9 (3–21·4)	6·9 (2·2–16·8)
1-13	Other sexually transmitted infections	146 (102–218)	250 (174–354)	313 (218–451)	4·2 (3–6·2)	3·7 (2·6–5·1)	3·5 (2·5–5)
1-14	Respiratory infections and tuberculosis	145,000 (121,000–173,000)	78,000 (61,500–97,300)	72,100 (56,100–91,500)	2,490 (2,160–2,880)	1,090 (886–1,350)	877 (721–1,090)
1-15	Tuberculosis	24,500 (21,300–28,000)	18,000 (13,900–24,200)	18,600 (14,000–26,400)	690 (591–792)	314 (239–423)	254 (195–352)
1-16	Latent tuberculosis infection	0 (0–0)	0 (0–0)	0 (0–0)	0 (0–0)	0 (0–0)	0 (0–0)
1-17	Drug-susceptible tuberculosis	24,500 (21,300–28,000)	17,000 (12,600–22,500)	17,500 (12,800–25,100)	688 (591–791)	296 (221–395)	239 (178–337)
1-18	Multidrug-resistant tuberculosis without extensive drug resistance	50 (5–219)	895 (185–2,610)	953 (125–3,210)	1·4 (0·1–6·2)	15·7 (3·2–45·4)	13·1 (1·7–44·1)
1-19	Extensively drug-resistant tuberculosis	0 (0–0)	95 (19–277)	155 (19–538)	0 (0–0)	1·7 (0·3–4·9)	2·1 (0·3–7·5)
1-20	Lower respiratory infections	110,000 (90,700–136,000)	49,000 (34,900–66,600)	40,700 (28,400–58,100)	1,630 (1,380–1,940)	651 (491–876)	499 (380–702)
1-21	Upper respiratory infections	8,050 (3,470–16,200)	7,920 (4,840–12,100)	9,120 (5,620–14,100)	132 (64·5–238)	92·8 (57·4–142)	89·1 (54·9–137)
1-22	Otitis media	2,000 (1,150–3,240)	3,080 (1,780–5,020)	3,580 (2,060–5,840)	37·3 (21·2–61·3)	35·4 (20·4–57·7)	34·4 (19·7–55·8)
1-23	Enteric infections	274,000 (216,000–337,000)	94,900 (65,500–131,000)	75,700 (52,300–108,000)	3,820 (3,030–4,740)	1,150 (800–1,640)	799 (549–1,170)
1-24	Diarrhoeal diseases	274,000 (216,000–336,000)	94,500 (65,200–131,000)	75,400 (52,100–108,000)	3,820 (3,020–4,730)	1,140 (795–1,630)	796 (546–1,170)
1-25	Typhoid and paratyphoid	51 (23–98)	36 (17–70)	28 (13–56)	1 (0·5–1·9)	0·5 (0·2–0·9)	0·3 (0·1–0·6)
1-26	Typhoid fever	51 (23–97)	36 (17–70)	28 (13–56)	1 (0·5–1·9)	0·5 (0·2–0·9)	0·3 (0·1–0·6)
1-27	Paratyphoid fever	0 (0–0)	0 (0–0)	0 (0–0)	0 (0–0)	0 (0–0)	0 (0–0)
1-28	iNTS	200 (93–351)	197 (90–359)	172 (78–307)	3·3 (1·6–5·8)	2·3 (1·1–4)	1·7 (0·8–3·1)
1-29	Other intestinal infectious diseases	274 (90–484)	97 (45–175)	74 (38–130)	3·7 (1·4–6·2)	1 (0·5–1·8)	0·7 (0·4–1·2)
1-30	Neglected tropical diseases and malaria	22,300 (14,600–29,700)	29,200 (22,000–36,900)	32,500 (24,300–41,200)	447 (315–578)	365 (278–459)	334 (248–424)
1-31	Malaria	1,970 (1,150–3,220)	387 (251–572)	147 (22–313)	36·3 (21·1–60·6)	4·5 (2·9–6·7)	1·4 (0·2–3)
1-32	Chagas disease	607 (384–842)	779 (446–1,130)	1,000 (537–1,520)	24·5 (14·6–33·8)	16·1 (8·8–24·2)	15·7 (7·8–24·6)
1-33	Leishmaniasis	316 (69–1,420)	273 (118–835)	323 (122–838)	6·1 (1·6–22·3)	3·5 (1·6–9·4)	3·4 (1·4–8·2)
1-34	Visceral leishmaniasis	154 (0–1,290)	81 (0–631)	78 (0–602)	2·4 (0–19·2)	0·9 (0–6·5)	0·7 (0–5·4)
1-35	Cutaneous and mucocutaneous leishmaniasis	162 (52–339)	192 (109–305)	246 (115–423)	3·7 (1·2–7·6)	2·6 (1·5–4·1)	2·7 (1·3–4·7)
1-36	African trypanosomiasis	0 (0–0)	0 (0–0)	0 (0–0)	0 (0–0)	0 (0–0)	0 (0–0)
1-37	Schistosomiasis	0 (0–0)	0 (0–0)	0 (0–0)	0 (0–0)	0 (0–0)	0 (0–0)
1-38	Cysticercosis	4,200 (2,600–6,370)	5,390 (2,980–8,720)	6,210 (3,300–9,920)	134 (83·5–203)	91·6 (50·5–143)	80·5 (43·8–129)
1-39	Cystic echinococcosis	18 (7–32)	9 (4–13)	7 (4–12)	0·4 (0·2–0·6)	0·1 (0·1–0·2)	0·1 (0·1–0·1)
1-40	Lymphatic filariasis	0 (0–0)	0 (0–0)	0 (0–0)	0 (0–0)	0 (0–0)	0 (0–0)
1-41	Onchocerciasis	0 (0–0)	0 (0–0)	0 (0–0)	0 (0–0)	0 (0–0)	0 (0–0)
1-42	Trachoma	0 (0–0)	0 (0–0)	0 (0–0)	0 (0–0)	0 (0–0)	0 (0–0)
1-43	Dengue	579 (339–959)	10,600 (7,730–15,000)	14,000 (11,000–17,300)	12·8 (7·6–20·8)	119 (85·4–169)	132 (100–163)
1-44	Yellow fever	0 (0–0)	0 (0–0)	0 (0–0)	0 (0–0)	0 (0–0)	0 (0–0)
1-45	Rabies	49 (3–111)	94 (1–170)	91 (1–169)	0·8 (0·1–1·5)	1·2 (0–2·1)	1 (0–1·8)
1-46	Intestinal nematode infections	7,110 (4,410–11,100)	6,190 (3,640–9,990)	5,730 (3,290–9,600)	133 (82–207)	71·3 (41·9–115)	54·4 (31·4–91·5)
1-47	Ascariasis	3,020 (1,900–4,750)	2,580 (1,450–4,320)	2,630 (1,360–4,680)	52·6 (32·3–85·2)	30·4 (16·8–51·4)	25·7 (13·4–45·8)
1-48	Trichuriasis	1,400 (680–2,570)	1,370 (713–2,320)	1,500 (740–2,690)	27·4 (13·3–49·9)	15·4 (8–26·1)	13·9 (6·8–24·9)

1-49	Hookworm disease	2,690 (1,540–4,280)	2,250 (1,230–3,710)	1,600 (885–2,770)	53·2 (30·7–84·6)	25·4 (14–41·8)	14·9 (8·2–25·6)
1-50	Food-borne trematodiases	0 (0–0)	0 (0–0)	0 (0–0)	0 (0–0)	0 (0–0)	0 (0–0)
1-51	Leprosy	13 (8–21)	11 (7–17)	12 (8–19)	0·5 (0·3–0·7)	0·2 (0·1–0·3)	0·2 (0·1–0·3)
1-52	Ebola virus disease	0 (0–0)	0 (0–0)	0 (0–0)	0 (0–0)	0 (0–0)	0 (0–0)
1-53	Zika virus disease	0 (0–0)	0 (0–0)	6 (2–17)	0 (0–0)	0 (0–0)	0·1 (0–0·2)
1-54	Guinea worm disease	0 (0–0)	0 (0–0)	0 (0–0)	0 (0–0)	0 (0–0)	0 (0–0)
1-55	Other neglected tropical diseases	7,490 (2,250–12,500)	5,500 (2,190–9,960)	4,880 (2,100–8,790)	98·6 (33·1–161)	57·5 (23·5–102)	45·3 (19·8–79·9)
1-56	Other infectious diseases	66,900 (43,500–116,000)	32,400 (23,500–48,100)	31,900 (20,900–52,000)	935 (646–1,540)	376 (280–537)	320 (216–503)
1-57	Meningitis	15,400 (12,300–19,700)	8,500 (5,820–11,700)	5,450 (3,730–7,340)	232 (192–285)	102 (72·4–136)	59·3 (41–78·5)
1-58	Encephalitis	7,610 (4,700–10,700)	8,310 (4,750–13,100)	8,010 (4,380–12,800)	111 (71·8–147)	92·6 (52·2–142)	77·8 (42·4–121)
1-59	Diphtheria	96 (43–183)	20 (10–39)	22 (11–41)	1·3 (0·6–2·4)	0·2 (0·1–0·4)	0·2 (0·1–0·4)
1-60	Whooping cough	22,300 (2,260–72,500)	5,720 (647–18,000)	8,820 (842–28,400)	269 (27·3–881)	56·9 (6·4–179)	77·8 (7·4–250)
1-61	Tetanus	1,240 (704–2,070)	467 (219–974)	392 (166–930)	21·7 (12·1–37·2)	6·2 (2·9–12·1)	4·4 (1·9–9·9)
1-62	Measles	8,720 (6,040–12,300)	0 (0–0)	0 (0–0)	107 (73·8–151)	0 (0–0)	0 (0–0)
1-63	Varicella and herpes zoster	1,780 (958–2,880)	1,020 (603–1,720)	972 (588–1,530)	29·2 (18·6–42·6)	13·1 (8·6–19·6)	10·7 (7–15·6)
1-64	Acute hepatitis	3,010 (1,920–4,430)	1,570 (1,120–2,130)	1,420 (1,040–1,870)	48·1 (32·2–65·4)	20·2 (14·7–26·6)	15·4 (11·5–20·2)
1-65	Acute hepatitis A	2,310 (1,390–3,540)	1,040 (704–1,430)	889 (629–1,190)	35·9 (22·5–52·8)	12·6 (8·7–17·3)	9·1 (6·5–12·1)
1-66	Acute hepatitis B	321 (134–806)	306 (180–550)	312 (198–493)	5·7 (2·8–12·6)	4·3 (2·7–7·4)	3·7 (2·4–5·6)
1-67	Acute hepatitis C	298 (99–848)	172 (89–378)	168 (92–364)	5·3 (2·1–13·4)	2·5 (1·3–5·6)	2 (1·1–4·3)
1-68	Acute hepatitis E	83 (31–238)	52 (31–104)	52 (33–88)	1·3 (0·5–3·2)	0·7 (0·4–1·2)	0·6 (0·4–0·9)
1-69	Other unspecified infectious diseases	6,810 (4,890–9,510)	6,790 (4,760–9,160)	6,840 (4,720–9,170)	115 (84–151)	85·2 (58–112)	74·2 (50·5–97·4)
1-70	Maternal and neonatal disorders	284,000 (249,000–323,000)	228,000 (185,000–277,000)	181,000 (140,000–225,000)	3,710 (3,310–4,190)	2,360 (1,910–2,880)	1,630 (1,270–2,030)
1-71	Maternal disorders	29,900 (24,500–36,500)	20,000 (14,400–27,400)	15,800 (9,730–23,100)	702 (576–861)	250 (179–342)	152 (94·1–223)
1-72	Maternal haemorrhage	6,960 (5,300–9,030)	6,800 (4,800–9,400)	4,970 (3,050–7,260)	166 (126–216)	87·3 (61·2–122)	48·9 (29·9–72)
1-73	Maternal sepsis and other pregnancy related infections	7,150 (5,500–9,110)	1,590 (1,090–2,280)	1,100 (669–1,610)	166 (128–210)	19·2 (13·2–27·6)	10·3 (6·3–15·2)
1-74	Maternal hypertensive disorders	4,690 (3,690–5,800)	4,720 (3,360–6,370)	3,590 (2,170–5,320)	107 (83·9–132)	57·6 (40·6–79·1)	34·1 (20·6–50·9)
1-75	Maternal obstructed labour and uterine rupture	58 (30–99)	395 (262–573)	405 (248–621)	1·3 (0·7–2·2)	5 (3·3–7·3)	3·9 (2·4–6)
1-76	Maternal abortive outcome	7,700 (5,730–10,000)	815 (542–1,200)	512 (314–780)	184 (136–240)	10·2 (6·7–15·2)	4·9 (3–7·6)
1-77	Ectopic pregnancy	389 (299–495)	154 (108–217)	130 (78–196)	9·3 (7·2–11·9)	2 (1·4–2·8)	1·3 (0·8–2)
1-78	Indirect maternal deaths	261 (197–344)	3,360 (2,310–4,780)	3,110 (1,820–4,920)	6 (4·6–7·9)	41·1 (28–58·5)	29·5 (17·2–47·4)
1-79	Late maternal deaths	502 (355–694)	475 (307–704)	412 (230–665)	11·8 (8·4–16·4)	5·9 (3·8–8·7)	4 (2·2–6·4)
1-80	Maternal deaths aggravated by HIV/AIDS	6 (3–10)	12 (6–19)	10 (5–19)	0·1 (0·1–0·2)	0·1 (0·1–0·2)	0·1 (0–0·2)
1-81	Other maternal disorders	2,140 (1,590–2,870)	1,700 (1,180–2,440)	1,540 (934–2,310)	51·2 (38·1–67·9)	21·3 (14·6–30·5)	14·9 (9–22·5)
1-82	Neonatal disorders	254,000 (220,000–292,000)	208,000 (168,000–253,000)	165,000 (129,000–205,000)	3,010 (2,610–3,460)	2,110 (1,710–2,570)	1,480 (1,150–1,830)
1-83	Neonatal preterm birth	140,000 (111,000–168,000)	114,000 (86,500–144,000)	81,700 (60,500–105,000)	1,670 (1,330–2,000)	1,160 (881–1,460)	730 (543–939)
1-84	Neonatal encephalopathy due to birth asphyxia and trauma	36,500 (25,900–50,100)	27,700 (18,600–40,500)	21,000 (13,500–30,200)	426 (302–586)	280 (188–408)	187 (121–270)
1-85	Neonatal sepsis and other neonatal infections	38,900 (25,500–57,000)	36,500 (23,900–53,500)	37,800 (26,100–53,500)	458 (301–670)	372 (244–543)	340 (236–480)
1-86	Hemolytic disease and other neonatal jaundice	4,220 (3,120–5,660)	3,080 (2,390–3,930)	2,820 (2,180–3,550)	56·2 (42·8–73·6)	33·5 (26·1–42·1)	26·5 (20·4–33·4)
1-87	Other neonatal disorders	34,200 (21,900–48,200)	26,900 (16,700–40,800)	21,800 (12,800–33,700)	398 (256–562)	271 (168–411)	193 (113–299)
1-88	Nutritional deficiencies	47,700 (37,300–61,200)	31,500 (22,100–42,000)	32,700 (22,900–44,900)	808 (640–1,020)	405 (291–535)	356 (254–482)
1-89	Protein-energy malnutrition	26,300 (19,200–34,700)	9,090 (5,990–13,400)	8,070 (5,490–11,800)	400 (307–507)	128 (89·4–181)	99·8 (71·7–144)
1-90	Iodine deficiency	666 (360–1,090)	608 (269–1,200)	1,350 (697–2,240)	15·9 (8·8–25·4)	7·8 (3·5–14·9)	13·7 (7·1–22·4)
1-91	Vitamin A deficiency	1,170 (740–1,800)	807 (502–1,230)	773 (476–1,190)	15·7 (10–23·9)	8·3 (5·2–12·6)	7 (4·4–10·8)
1-92	Dietary iron deficiency	17,700 (11,000–26,500)	20,100 (12,800–30,200)	21,600 (13,400–32,700)	338 (216–497)	248 (160–371)	224 (140–332)
1-93	Other nutritional deficiencies	1,840 (1,300–2,620)	867 (610–1,200)	950 (662–1,330)	39·2 (29–51·7)	14·1 (9·8–19·6)	12·3 (8·7–17·2)
1-0	Non-communicable diseases	782,000 (688,000–885,000)	1,340,000 (1,130,000–1,570,000)	1,730,000 (1,470,000–2,050,000)	23,300 (20,500–26,200)	23,900 (20,300–27,800)	24,000 (20,800–27,900)
1-1	Neoplasms	80,300 (69,900–90,400)	155,000 (124,000–189,000)	213,000 (172,000–267,000)	2,670 (2,290–3,030)	3,010 (2,410–3,670)	3,150 (2,600–3,910)
1-2	Lip and oral cavity cancer	707 (576–843)	1,580 (1,110–2,220)	2,320 (1,630–3,280)	29·6 (23·6–35·2)	33 (23·1–46·5)	35·7 (25·1–50·3)
1-3	Nasopharynx cancer	133 (111–157)	238 (175–310)	308 (220–407)	4·7 (3·8–5·6)	4·4 (3·2–5·8)	4·4 (3·1–5·8)
1-4	Other pharynx cancer	265 (218–314)	623 (456–828)	914 (683–1,250)	11·4 (9·3–13·6)	13·2 (9·7–17·5)	14·1 (10·6–19·1)
1-5	Oesophageal cancer	639 (477–777)	1,600 (1,090–2,160)	2,410 (1,660–3,200)	29·5 (21·7–35·8)	35·9 (24·5–47·9)	39·1 (27·2–51·6)
1-6	Stomach cancer	8,240 (6,780–9,680)	16,100 (12,100–20,500)	22,200 (17,000–29,600)	342 (283–401)	341 (260–429)	348 (274–454)
1-7	Colon and rectum cancer	2,700 (2,220–3,150)	6,900 (4,910–9,680)	10,600 (7,360–15,000)	114 (93–133)	148 (104–207)	167 (116–237)

1-8	Liver cancer	6,570 (2,820–9,330)	15,700 (6,970–22,900)	22,700 (10,400–34,200)	283 (113–409)	337 (146–497)	358 (165–536)
1-9	Liver cancer due to hepatitis B	1,650 (656–2,700)	3,360 (1,410–5,580)	4,650 (1,910–8,060)	65·3 (23·6–108)	66·6 (27·3–113)	68·2 (27·5–117)
1-10	Liver cancer due to hepatitis C	2,110 (736–3,380)	5,450 (2,150–8,790)	8,040 (3,250–13,300)	103 (34·9–165)	127 (49·6–205)	134 (54·4–220)
1-11	Liver cancer due to alcohol use	1,590 (527–2,620)	4,180 (1,620–6,830)	6,370 (2,620–10,700)	74 (23·8–122)	92·9 (35·5–152)	102 (42–170)
1-12	Liver cancer due to NASH	408 (151–685)	1,220 (461–2,090)	1,820 (703–3,140)	17·9 (6·1–31·3)	26·4 (9·5–45·9)	28·9 (11·2–50·2)
1-13	Gallbladder and biliary tract cancer	912 (725–1,220)	2,110 (1,600–2,990)	3,100 (2,420–4,250)	42 (33–56)	47·5 (36·2–66·1)	50·8 (40·5–68·5)
1-14	Pancreatic cancer	1,010 (806–1,190)	3,540 (2,240–5,030)	5,890 (3,570–8,600)	46·9 (37·3–55·5)	79·6 (50–113)	96·1 (58·3–140)
1-15	Larynx cancer	597 (477–713)	1,270 (916–1,640)	1,850 (1,400–2,360)	26·4 (21·1–31·6)	27·8 (20·3–35·6)	29·7 (22·7–37·4)
1-16	Tracheal, bronchus, and lung cancer	7,960 (6,360–9,500)	22,600 (15,000–31,600)	34,800 (22,700–50,100)	355 (281–423)	492 (324–686)	551 (361–790)
1-17	Malignant skin melanoma	167 (129–223)	318 (238–442)	418 (309–593)	5·8 (4·5–7·9)	6 (4·5–8·6)	6 (4·5–8·6)
1-18	Non-melanoma skin cancer	267 (218–316)	633 (464–856)	909 (681–1,270)	11·7 (9·7–13·9)	13·9 (10·3–18·7)	14·6 (11·1–20·2)
1-19	Non-melanoma skin cancer (squamous-cell carcinoma)	267 (217–316)	632 (463–856)	908 (680–1,260)	11·7 (9·7–13·9)	13·8 (10·3–18·7)	14·5 (11–20·2)
1-20	Non-melanoma skin cancer (basal-cell carcinoma)	0 (0–1)	1 (0–1)	1 (0–2)	0 (0–0)	0 (0–0)	0 (0–0)
1-21	Breast cancer	3,750 (3,030–4,590)	9,320 (6,220–13,500)	13,700 (9,670–19,200)	149 (120–182)	186 (124–271)	202 (145–278)
1-22	Cervical cancer	5,980 (4,550–7,640)	10,100 (6,380–15,300)	13,200 (7,950–20,700)	214 (164–276)	189 (120–284)	182 (111–281)
1-23	Uterine cancer	967 (730–1,500)	2,530 (1,510–4,260)	3,720 (2,170–6,270)	40·3 (29·9–65·8)	53·2 (31·7–90·4)	57 (33·2–97·7)
1-24	Ovarian cancer	902 (676–1,400)	2,750 (1,330–5,930)	4,220 (1,980–9,170)	35·9 (27–53·3)	54·1 (26·2–116)	60·9 (29·4–129)
1-25	Prostate cancer	2,250 (1,650–3,080)	5,970 (4,050–8,630)	9,120 (6,710–13,300)	124 (90·4–173)	154 (106–222)	169 (124–250)
1-26	Testicular cancer	215 (144–336)	315 (197–483)	381 (222–618)	4·7 (3·2–6·9)	4·1 (2·6–6·4)	3·9 (2·3–6·4)
1-27	Kidney cancer	872 (725–1,030)	2,030 (1,530–2,640)	2,830 (2,070–3,850)	25·8 (21·5–30)	38·2 (28·5–51·2)	41·8 (30·9–58·5)
1-28	Bladder cancer	429 (349–510)	1,130 (841–1,570)	1,710 (1,290–2,460)	21·2 (17·2–25·2)	26·9 (20·1–37·4)	29·5 (22·3–42·5)
1-29	Brain and nervous system cancer	3,840 (3,100–5,040)	5,670 (4,010–7,710)	6,830 (4,200–9,820)	79·7 (66–110)	78·7 (57·4–105)	77·9 (52·3–111)
1-30	Thyroid cancer	1,160 (473–1,470)	3,400 (1,060–4,850)	4,900 (1,520–7,060)	48·6 (18·4–61·2)	71·3 (21·6–101)	75·9 (23·1–108)
1-31	Mesothelioma	130 (70–204)	371 (205–540)	532 (290–787)	5·1 (2·8–7·7)	7·5 (4·1–10·6)	8 (4·4–11·4)
1-32	Hodgkin lymphoma	262 (162–336)	347 (197–497)	393 (204–597)	7·7 (4·4–10·3)	5·7 (3–8·3)	5·1 (2·6–7·6)
1-33	Non-Hodgkin's lymphoma	1,850 (1,560–2,180)	3,400 (2,440–4,620)	4,520 (3,260–6,380)	54·4 (45·9–63·5)	60·6 (44·4–82·6)	62·9 (47–87·8)
1-34	Multiple myeloma	560 (385–746)	1,580 (1,100–2,220)	2,350 (1,670–3,270)	25·3 (16·9–33·4)	34·7 (24·1–48·2)	37·3 (26·7–51·1)
1-35	Leukaemia	16,500 (13,600–22,600)	18,300 (13,400–23,900)	18,600 (13,400–24,800)	309 (258–409)	247 (192–311)	215 (160–280)
1-36	Acute lymphoid leukaemia	6,690 (4,680–10,800)	7,910 (5,120–11,000)	7,020 (4,400–10,400)	105 (76·7–166)	90·3 (60·3–124)	71·1 (47·1–102)
1-37	Chronic lymphoid leukaemia	105 (80–136)	349 (247–473)	547 (363–724)	4·7 (3·5–6·1)	7·9 (5·6–10·6)	9·1 (6·1–12·1)
1-38	Acute myeloid leukaemia	2,330 (1,610–3,490)	3,460 (2,400–5,100)	4,100 (2,760–5,920)	47·6 (35·2–65·4)	47·8 (34·4–67·6)	46·9 (31·7–65·4)
1-39	Chronic myeloid leukaemia	871 (633–1,180)	1,190 (838–1,650)	1,390 (924–1,970)	24·3 (18·5–31·2)	21·1 (14·8–29)	19·4 (13·1–27·1)
1-40	Other leukaemia	6,470 (3,760–9,870)	5,410 (3,610–8,200)	5,540 (3,660–8,390)	127 (80·9–185)	79·9 (53·4–119)	68·9 (46–103)
1-41	Other neoplasms	513 (393–801)	983 (715–1,350)	1,320 (972–1,840)	14·5 (10·9–20·8)	17·9 (12·8–24·7)	19 (14–26·5)
1-42	Myelodysplastic, myeloproliferative, and other hematopoietic neoplasms	513 (393–801)	983 (715–1,350)	1,320 (972–1,840)	14·5 (10·9–20·8)	17·9 (12·8–24·7)	19 (14–26·5)
1-43	Benign and in situ intestinal neoplasms	0 (0–0)	0 (0–0)	0 (0–0)	0 (0–0)	0 (0–0)	0 (0–0)
1-44	Benign and in situ cervical and uterine neoplasms	0 (0–0)	0 (0–0)	0 (0–0)	0 (0–0)	0 (0–0)	0 (0–0)
1-45	Other benign and in situ neoplasms	0 (0–0)	0 (0–0)	0 (0–0)	0 (0–0)	0 (0–0)	0 (0–0)
1-46	Cardiovascular diseases	121,000 (104,000–144,000)	236,000 (190,000–284,000)	325,000 (273,000–391,000)	5,030 (4,270–6,220)	5,270 (4,280–6,340)	5,420 (4,590–6,460)
1-47	Rheumatic heart disease	2,420 (1,730–3,410)	4,180 (2,770–6,030)	5,220 (3,480–7,400)	68·8 (51·8–91·7)	60·8 (42·4–82·8)	58·5 (40·4–80·3)
1-48	Ischaemic heart disease	50,600 (42,100–64,100)	104,000 (82,500–131,000)	150,000 (123,000–187,000)	2,370 (1,970–3,050)	2,470 (1,960–3,090)	2,620 (2,130–3,250)
1-49	Stroke	50,800 (43,600–58,800)	94,700 (74,400–117,000)	126,000 (99,800–161,000)	1,890 (1,620–2,330)	2,020 (1,590–2,530)	2,030 (1,630–2,600)
1-50	Ischaemic stroke	13,800 (11,400–18,800)	31,600 (25,500–39,600)	46,100 (38,100–58,400)	654 (534–926)	781 (636–997)	841 (694–1,080)
1-51	Intracerebral hemorrhage	26,000 (21,000–31,500)	44,500 (33,300–57,900)	57,100 (43,600–77,300)	920 (744–1,160)	914 (682–1,200)	883 (682–1,190)
1-52	Subarachnoid hemorrhage	11,000 (7,020–15,500)	18,600 (12,700–25,300)	22,300 (15,200–30,900)	318 (210–458)	329 (220–446)	306 (212–421)
1-53	Hypertensive heart disease	6,290 (5,100–8,780)	12,200 (8,520–18,600)	16,700 (11,300–26,200)	291 (234–441)	281 (198–461)	283 (190–460)
1-54	Non-rheumatic valvular heart disease	441 (344–557)	945 (719–1,200)	1,350 (1,050–1,740)	19·2 (15·2–24·7)	21 (16·1–26·7)	21·9 (17·4–27·7)
1-55	Non-rheumatic calcific aortic valve disease	275 (209–359)	659 (495–855)	963 (736–1,250)	12·2 (9·4–16·1)	14·8 (11·2–19·1)	15·8 (12·4–20·2)
1-56	Non-rheumatic degenerative mitral valve disease	149 (111–198)	269 (192–366)	367 (263–500)	6·3 (4·7–8·3)	5·8 (4·1–7·6)	5·8 (4·3–7·8)
1-57	Other non-rheumatic valve diseases	17 (5–32)	16 (7–28)	20 (8–35)	0·7 (0·2–1·2)	0·3 (0·1–0·6)	0·3 (0·1–0·5)
1-58	Cardiomyopathy and myocarditis	3,950 (2,750–5,040)	6,980 (4,390–9,560)	9,020 (5,690–12,700)	136 (95·8–178)	139 (87·2–192)	137 (86·2–191)
1-59	Myocarditis	362 (232–556)	482 (286–690)	555 (331–837)	9·6 (6·2–14·1)	8·5 (4·8–12·6)	7·8 (4·4–12)
1-60	Alcoholic cardiomyopathy	408 (209–608)	590 (293–912)	765 (360–1,180)	16·4 (8·3–24·3)	12 (6–18·2)	11·4 (5·5–17·3)

1-61	Other cardiomyopathy	3,180 (2,240–4,160)	5,910 (3,720–8,140)	7,700 (4,840–10,800)	110 (78·7–147)	119 (74·9–166)	118 (74·2–163)
1-62	Atrial fibrillation and flutter	1,180 (876–1,670)	3,170 (2,440–4,100)	4,470 (3,630–5,600)	69·1 (49·8–101)	89·1 (67·5–118)	91·2 (73–116)
1-63	Aortic aneurysm	398 (272–566)	1,020 (730–1,350)	1,520 (1,130–1,980)	18·9 (12·9–27·7)	23·9 (17·2–31·8)	25·9 (19·6–33·4)
1-64	Peripheral vascular disease	198 (120–299)	415 (266–617)	581 (380–842)	11·3 (6·8–17·1)	11·2 (7·2–16·4)	11·3 (7·4–16·3)
1-65	Endocarditis	401 (261–691)	709 (503–1,020)	924 (614–1,350)	11·2 (8–18·4)	12·9 (9·4–18·3)	13·1 (9·2–19)
1-66	Chronic respiratory diseases	63,000 (49,700–74,900)	80,600 (62,800–97,900)	99,100 (73,900–126,000)	1,660 (1,400–1,880)	1,530 (1,150–1,880)	1,490 (1,060–1,900)
1-67	Chronic obstructive pulmonary disease	21,100 (16,800–25,300)	44,200 (31,300–54,900)	60,400 (40,900–76,900)	861 (672–1,020)	1,020 (707–1,270)	1,040 (684–1,320)
1-68	Pneumoconiosis	178 (98–250)	283 (160–428)	363 (217–532)	7·2 (3·8–10·1)	6 (3·3–9·1)	5·7 (3·4–8·5)
1-69	Silicosis	95 (27–155)	124 (42–211)	156 (54–268)	4·1 (1·2–6·6)	2·7 (1–4·7)	2·5 (0·9–4·3)
1-70	Asbestosis	12 (6–23)	23 (11–40)	30 (15–54)	0·5 (0·2–0·9)	0·5 (0·2–0·9)	0·5 (0·2–0·8)
1-71	Coal workers pneumoconiosis	19 (7–37)	29 (9–61)	36 (11–78)	0·7 (0·3–1·4)	0·6 (0·2–1·2)	0·6 (0·2–1·2)
1-72	Other pneumoconiosis	53 (32–97)	108 (64–179)	141 (85–234)	2 (1·2–3·7)	2·2 (1·3–3·6)	2·2 (1·3–3·5)
1-73	Asthma	35,100 (25,700–45,400)	26,800 (19,700–35,800)	27,700 (19,800–37,500)	647 (492–806)	355 (264–463)	306 (221–402)
1-74	Interstitial lung disease and pulmonary sarcoidosis	1,700 (1,180–2,190)	4,310 (2,500–6,430)	5,630 (3,070–9,030)	60·2 (42·4–78·2)	87·9 (49·6–136)	86·7 (46·6–140)
1-75	Other chronic respiratory diseases	4,850 (2,020–9,110)	5,000 (2,710–7,820)	4,940 (2,860–7,410)	80·3 (36·2–135)	60·4 (32·7–91·6)	51·2 (29·3–76·4)
1-76	Digestive diseases	155,000 (132,000–182,000)	171,000 (140,000–207,000)	198,000 (156,000–245,000)	3,670 (3,210–4,160)	2,930 (2,360–3,580)	2,710 (2,120–3,410)
1-77	Cirrhosis and other chronic liver diseases	45,800 (37,600–55,900)	66,900 (48,400–89,700)	84,800 (56,900–120,000)	1,420 (1,160–1,710)	1,280 (906–1,730)	1,240 (832–1,740)
1-78	Cirrhosis and other chronic liver diseases due to hepatitis B	2,030 (1,330–3,060)	2,070 (1,160–3,430)	2,530 (1,410–4,230)	76·6 (50·7–115)	40·4 (23–66·7)	36·7 (20·5–60)
1-79	Cirrhosis and other chronic liver diseases due to hepatitis C	9,120 (6,450–12,500)	16,600 (10,400–25,500)	21,500 (13,000–33,900)	344 (243–474)	327 (203–506)	314 (189–495)
1-80	Cirrhosis and other chronic liver diseases due to alcohol use	10,000 (7,120–13,500)	20,600 (12,800–30,300)	28,100 (17,000–42,500)	414 (296–556)	425 (263–622)	425 (259–639)
1-81	Cirrhosis and other chronic liver diseases due to NAFLD	5,610 (3,810–7,930)	12,100 (7,350–18,800)	16,500 (9,240–26,100)	226 (153–322)	253 (154–395)	253 (145–398)
1-82	Cirrhosis and other chronic liver diseases due to other causes	19,000 (14,400–25,300)	15,500 (11,200–20,700)	16,100 (10,800–23,200)	361 (282–457)	238 (165–329)	210 (138–301)
1-83	Upper digestive system diseases	17,200 (14,200–21,100)	23,500 (17,700–31,000)	29,000 (21,700–38,600)	562 (465–685)	449 (341–584)	417 (312–543)
1-84	Peptic ulcer disease	11,100 (9,110–13,200)	12,200 (8,990–16,100)	14,300 (10,400–19,200)	362 (300–425)	254 (186–333)	228 (166–300)
1-85	Gastritis and duodenitis	2,320 (1,620–3,000)	3,600 (2,530–4,890)	4,350 (3,010–6,050)	77·9 (54·2–98·7)	72·5 (50·4–98·3)	66·2 (45·5–92·1)
1-86	GERD	3,810 (1,960–6,760)	7,680 (4,010–13,700)	10,300 (5,330–18,300)	123 (63·6–217)	123 (63·9–217)	123 (63·8–217)
1-87	Appendicitis	10,300 (4,970–16,400)	7,690 (5,260–10,600)	6,950 (4,850–9,810)	182 (104–274)	103 (70·8–140)	80·7 (56·8–112)
1-88	Paralytic ileus and intestinal obstruction	34,800 (19,000–55,900)	22,000 (10,800–42,400)	18,400 (9,220–37,100)	439 (263–680)	238 (134–438)	177 (93·9–336)
1-89	Inguinal, femoral, and abdominal hernia	8,420 (4,690–18,300)	8,450 (5,900–12,400)	9,820 (7,170–13,100)	187 (126–302)	141 (103–190)	132 (99·7–172)
1-90	Inflammatory bowel disease	1,240 (711–1,930)	1,530 (995–2,100)	1,810 (1,190–2,500)	28·5 (19–38·6)	25·6 (16·7–33·6)	24·3 (15·9–32·5)
1-91	Vascular intestinal disorders	1,700 (1,350–2,130)	3,310 (2,490–4,190)	4,470 (3,180–5,820)	64·8 (51·4–78·7)	76·6 (58·2–97·7)	77·3 (55·7–99·8)
1-92	Gallbladder and biliary diseases	8,900 (7,000–11,200)	16,000 (12,100–20,600)	20,800 (15,900–27,400)	307 (246–385)	299 (231–382)	292 (223–378)
1-93	Pancreatitis	4,180 (2,850–5,350)	6,580 (4,660–8,850)	7,940 (5,380–11,600)	128 (89·7–165)	117 (83·5–157)	109 (74·9–156)
1-94	Other digestive diseases	21,900 (10,300–32,400)	14,800 (9,400–24,000)	14,000 (9,080–21,900)	348 (224–472)	199 (141–288)	165 (112–240)
1-95	Neurological disorders	53,600 (30,300–86,300)	94,700 (51,200–152,000)	119,000 (63,500–195,000)	1,520 (898–2,390)	1,580 (938–2,580)	1,570 (916–2,540)
1-96	Alzheimer's disease and other dementias	5,920 (2,330–13,900)	16,200 (6,020–38,100)	21,900 (8,000–53,100)	379 (152–894)	499 (182–1,180)	491 (178–1,170)
1-97	Parkinson's disease	1,470 (1,160–1,700)	4,170 (3,160–4,990)	6,500 (5,040–7,690)	83·2 (65·2–96·8)	111 (83·8–132)	126 (95·8–148)
1-98	Idiopathic epilepsy	19,700 (11,700–30,200)	24,700 (13,000–40,900)	27,200 (13,600–45,300)	416 (246–635)	320 (172–531)	289 (147–473)
1-99	Multiple sclerosis	171 (136–207)	514 (274–980)	644 (371–1,090)	6 (4·8–7·1)	7·9 (5–12·4)	7·9 (5–12·4)
1-100	Motor neuron disease	499 (366–699)	981 (631–1,420)	1,350 (839–1,980)	12·1 (9·4–15·4)	16·5 (10·6–23·8)	18·2 (11·1–26·9)
1-101	Headache disorders	22,300 (3,540–50,000)	42,500 (7,020–94,100)	54,300 (9,470–121,000)	557 (107–1,220)	560 (106–1,220)	562 (108–1,230)
1-102	Migraine	20,400 (2,370–47,400)	38,900 (4,840–90,400)	49,500 (6,450–116,000)	505 (72·3–1,170)	508 (73·6–1,180)	510 (75·3–1,170)
1-103	Tension-type headache	1,880 (512–7,990)	3,650 (1,020–14,900)	4,730 (1,320–18,600)	51·9 (15·5–201)	52·1 (15·3–194)	52·2 (15·5–192)
1-104	Other neurological disorders	3,570 (2,080–5,590)	5,570 (3,080–9,230)	6,850 (3,630–11,200)	68·2 (41·2–104)	70·1 (42·3–111)	71·6 (41·6–112)
1-105	Mental disorders	59,300 (43,300–79,100)	115,000 (83,600–152,000)	147,000 (107,000–196,000)	1,550 (1,140–2,020)	1,590 (1,160–2,090)	1,590 (1,150–2,100)
1-106	Schizophrenia	5,400 (3,760–7,180)	10,900 (7,530–14,600)	14,700 (10,300–19,700)	166 (117–219)	167 (118–223)	167 (119–222)
1-107	Depressive disorders	17,400 (11,700–24,300)	35,800 (24,500–50,300)	47,100 (31,900–66,400)	529 (364–730)	554 (385–780)	552 (376–763)
1-108	Major depressive disorder	14,600 (9,640–20,500)	30,200 (20,300–42,200)	39,700 (26,400–55,700)	444 (297–617)	470 (317–655)	468 (313–652)
1-109	Dysthymia	2,810 (1,720–4,340)	5,600 (3,370–8,550)	7,410 (4,540–11,400)	84·3 (53·4–132)	84·4 (53·1–130)	84·5 (53·4–130)
1-110	Bipolar disorder	6,610 (3,840–10,400)	13,000 (7,470–20,600)	16,800 (9,850–26,300)	176 (102–275)	176 (104–273)	176 (104–270)
1-111	Anxiety disorders	15,100 (9,800–21,600)	29,100 (19,100–41,800)	37,700 (24,600–53,400)	371 (246–519)	381 (253–542)	391 (257–551)
1-112	Eating disorders	1,440 (872–2,180)	2,940 (1,790–4,540)	3,950 (2,410–6,010)	31·4 (19–47·7)	33·6 (20·6–51·3)	35·7 (21·8–54·1)
1-113	Anorexia nervosa	385 (219–634)	776 (441–1,280)	1,010 (574–1,650)	7·7 (4·5–12·5)	8·3 (4·7–13·8)	8·9 (5·1–14·3)

1-114	Bulimia nervosa	1,050 (612–1,680)	2,170 (1,230–3,380)	2,930 (1,640–4,730)	23·7 (13·6–38·3)	25·3 (14·5–39·7)	26·8 (15·1–42·6)
1-115	Autism spectrum disorders	2,610 (1,690–3,810)	4,300 (2,790–6,200)	5,190 (3,360–7,630)	50·9 (33·1–74·1)	50·7 (32·9–73·2)	50·6 (32·7–74·4)
1-116	Attention-deficit/hyperactivity disorder	1,080 (593–1,890)	1,800 (998–3,140)	2,110 (1,190–3,680)	18·7 (10·5–32·2)	18·7 (10·6–32·2)	18·7 (10·5–32·4)
1-117	Conduct disorder	4,650 (2,600–7,460)	7,440 (4,160–11,700)	8,190 (4,550–13,000)	67·2 (37·3–108)	67·5 (37·8–106)	67·6 (37·5–107)
1-118	Idiopathic developmental intellectual disability	1,640 (685–2,920)	2,630 (1,150–4,530)	2,470 (854–4,570)	31·5 (13·4–56·3)	30·9 (13·6–53)	23·7 (8·3–43·7)
1-119	Other mental disorders	3,320 (2,080–5,070)	6,680 (4,220–10,100)	8,960 (5,670–13,500)	108 (68·8–164)	108 (68·9–164)	107 (68·7–161)
1-120	Substance use disorders	17,800 (13,900–22,100)	34,500 (26,600–44,200)	45,000 (34,500–57,500)	554 (438–678)	542 (418–689)	531 (411–671)
1-121	Alcohol use disorders	13,800 (10,600–17,500)	26,300 (19,800–34,400)	33,700 (25,500–44,100)	445 (347–552)	425 (322–544)	407 (308–526)
1-122	Drug use disorders	4,010 (3,050–5,060)	8,270 (6,300–10,400)	11,300 (8,470–14,300)	109 (85·5–135)	117 (90·3–146)	124 (95·4–154)
1-123	Opioid use disorders	1,820 (1,290–2,530)	3,660 (2,590–4,980)	5,100 (3,600–6,750)	52·3 (37·7–70)	54·5 (39·6–71·7)	57·6 (41·6–74·6)
1-124	Cocaine use disorders	893 (681–1,150)	1,880 (1,380–2,470)	2,560 (1,820–3,480)	24·3 (18·8–30·7)	27 (20–35)	29 (20·9–38·7)
1-125	Amphetamine use disorders	336 (198–530)	711 (449–1,090)	955 (596–1,460)	8·5 (5·3–12·9)	9·3 (6·2–13·7)	9·8 (6·4–14·3)
1-126	Cannabis use disorders	348 (193–598)	676 (381–1,160)	854 (467–1,440)	8 (4·7–13·1)	8 (4·6–13)	7·9 (4·5–13·1)
1-127	Other drug use disorders	612 (423–845)	1,340 (926–1,840)	1,790 (1,260–2,380)	15·9 (11·6–21·2)	18·5 (13·2–24·6)	19·7 (14·4–25·4)
1-128	Diabetes and kidney diseases	42,700 (36,300–50,200)	116,000 (94,600–143,000)	172,000 (142,000–211,000)	1,640 (1,370–1,970)	2,380 (1,930–2,940)	2,610 (2,160–3,200)
1-129	Diabetes mellitus	20,000 (15,800–24,900)	52,300 (39,800–66,500)	79,700 (61,200–102,000)	844 (657–1,060)	1,090 (827–1,380)	1,210 (931–1,540)
1-130	Diabetes mellitus type 1	1,760 (1,090–2,240)	2,730 (1,640–3,660)	3,180 (1,860–4,560)	42·9 (26·1–54)	41·3 (23·2–56·8)	38·9 (21·4–56·5)
1-131	Diabetes mellitus type 2	18,300 (14,100–22,900)	49,500 (37,500–63,300)	76,600 (58,400–97,700)	801 (615–1,010)	1,050 (794–1,340)	1,170 (898–1,490)
1-132	Chronic kidney disease	22,500 (19,100–27,100)	63,900 (50,600–79,500)	92,200 (74,700–118,000)	790 (656–1,010)	1,290 (1,010–1,630)	1,400 (1,150–1,770)
1-133	Chronic kidney disease due to diabetes mellitus type 1	1,760 (1,180–2,530)	5,310 (3,190–8,140)	7,350 (4,170–11,800)	61·6 (40·3–90·1)	97·8 (57·8–155)	101 (56·7–165)
1-134	Chronic kidney disease due to diabetes mellitus type 2	3,720 (2,710–5,240)	14,800 (10,500–20,100)	23,100 (17,000–30,600)	175 (126–248)	335 (241–457)	379 (280–503)
1-135	Chronic kidney disease due to hypertension	3,200 (2,410–4,460)	12,000 (8,800–16,100)	18,200 (13,700–24,500)	149 (110–215)	274 (203–373)	303 (230–407)
1-136	Chronic kidney disease due to glomerulonephritis	6,320 (5,020–7,880)	15,100 (11,000–20,000)	20,500 (14,500–28,500)	186 (145–246)	271 (197–373)	284 (198–396)
1-137	Chronic kidney disease due to other and unspecified causes	7,460 (6,080–9,030)	16,800 (12,800–22,300)	23,100 (17,300–30,900)	219 (173–281)	313 (231–428)	333 (246–446)
1-138	Acute glomerulonephritis	217 (48–330)	107 (24–150)	112 (19–165)	4·5 (0·8–6·3)	1·7 (0·3–2·5)	1·5 (0·2–2·2)
1-139	Skin and subcutaneous diseases	22,200 (14,800–32,900)	37,200 (24,700–55,100)	45,100 (29,700–67,000)	455 (311–670)	467 (318–683)	468 (316–695)
1-140	Dermatitis	5,020 (2,920–8,080)	8,020 (4,700–12,800)	9,580 (5,700–15,000)	94·1 (56·7–147)	94·2 (56·7–148)	94·3 (56·9–146)
1-141	Atopic dermatitis	3,970 (2,120–6,730)	5,980 (3,240–9,990)	6,920 (3,700–11,700)	63·7 (34–108)	63·8 (34·4–106)	63·8 (34·1–108)
1-142	Contact dermatitis	916 (574–1,390)	1,790 (1,120–2,730)	2,350 (1,480–3,520)	27·1 (16·9–40·4)	27·1 (16·9–40·4)	27·1 (16·9–40·8)
1-143	Seborrhoeic dermatitis	134 (76–216)	244 (136–387)	308 (172–496)	3·4 (1·9–5·3)	3·4 (1·9–5·3)	3·4 (1·9–5·4)
1-144	Psoriasis	578 (400–777)	955 (652–1,290)	1,180 (820–1,610)	15·2 (10·6–20·3)	13·6 (9·4–18·3)	13·1 (9·3–17·7)
1-145	Bacterial skin diseases	1,160 (853–1,550)	2,110 (1,580–2,690)	2,690 (2,020–3,460)	29·2 (20·8–38·1)	39·1 (27·5–49·9)	38·7 (28·2–48·9)
1-146	Cellulitis	356 (181–533)	599 (310–876)	761 (394–1,140)	9·9 (4·7–14·6)	10·8 (5·2–16·3)	10·6 (5·1–16·1)
1-147	Pyoderma	809 (520–1,220)	1,510 (1,080–2,030)	1,930 (1,420–2,580)	19·3 (13·4–26)	28·3 (19·3–37·6)	28·1 (20·7–36·6)
1-148	Scabies	3,930 (2,120–6,300)	6,430 (3,490–10,300)	7,650 (4,180–12,200)	73·9 (40·6–118)	73·4 (41–117)	73 (40·1–117)
1-149	Fungal skin diseases	1,050 (414–2,210)	1,830 (727–3,820)	2,300 (912–4,830)	28·1 (11·3–59·3)	27·7 (11·2–58·4)	27·6 (11–58·4)
1-150	Viral skin diseases	2,830 (1,780–4,290)	4,190 (2,680–6,290)	4,820 (3,080–7,270)	43·7 (27·5–65·8)	43·7 (28–65·6)	43·8 (28–66·3)
1-151	Acne vulgaris	2,200 (1,300–3,590)	4,410 (2,610–7,200)	5,260 (3,060–8,490)	37·3 (22·1–60·3)	42·3 (25·2–68·7)	43·8 (25·6–70·7)
1-152	Alopecia areata	282 (174–421)	531 (338–795)	685 (432–1,030)	7·1 (4·5–10·7)	7·2 (4·5–10·7)	7·2 (4·5–10·8)
1-153	Pruritus	305 (147–559)	567 (271–1,030)	733 (352–1,330)	8·3 (4–15·1)	8·4 (4–15·4)	8·5 (4·1–15·4)
1-154	Urticaria	2,660 (1,710–3,970)	4,170 (2,710–6,110)	4,980 (3,240–7,330)	48·1 (31·3–69·9)	48·2 (31·2–69·7)	48·3 (31·4–70·7)
1-155	Decubitus ulcer	483 (283–678)	882 (516–1,200)	1,200 (710–1,630)	21·3 (12·2–30·3)	20·7 (12–28·8)	21 (12·2–28·1)
1-156	Other skin and subcutaneous diseases	1,720 (903–3,040)	3,100 (1,600–5,520)	4,000 (2,030–7,020)	49 (25·9–86·7)	48·8 (25·3–86·6)	48·8 (25·1–84·8)
1-157	Sense organ diseases	23,500 (16,100–33,100)	43,700 (29,700–62,400)	57,300 (39,300–81,700)	935 (639–1,320)	859 (586–1,230)	841 (578–1,190)
1-158	Blindness and vision loss	8,410 (5,790–11,800)	14,700 (10,000–20,800)	19,000 (12,900–27,000)	352 (248–489)	299 (206–422)	289 (198–411)
1-159	Glaucoma	374 (247–542)	577 (390–817)	714 (475–1,010)	21·9 (14·5–31·5)	15·7 (10·5–22·1)	14·1 (9·4–19·8)
1-160	Cataract	1,130 (786–1,580)	1,750 (1,220–2,420)	2,220 (1,550–3,040)	62·1 (42·8–86·2)	45 (31·7–62·1)	41·8 (29·4–57·3)
1-161	Age-related macular degeneration	79 (52–113)	143 (96–207)	191 (127–276)	4·4 (2·9–6·2)	3·7 (2·5–5·3)	3·6 (2·4–5·1)
1-162	Refraction disorders	3,230 (2,140–4,760)	5,740 (3,770–8,400)	7,340 (4,820–10,800)	99·2 (66·4–140)	90·8 (60·4–129)	89·4 (59·1–128)
1-163	Near vision loss	1,260 (582–2,540)	2,590 (1,180–5,140)	3,620 (1,630–7,120)	61·7 (28·7–123)	59·5 (27·4–116)	60 (27·6–117)
1-164	Other vision loss	2,330 (1,630–3,220)	3,900 (2,760–5,380)	4,960 (3,480–6,820)	103 (72·2–141)	84·6 (60·2–116)	80 (56·7–109)
1-165	Age-related and other hearing loss	14,100 (9,440–20,200)	26,900 (17,900–39,500)	35,600 (23,900–51,800)	548 (368–790)	525 (351–760)	516 (346–743)
1-166	Other sense organ diseases	1,080 (642–1,650)	2,070 (1,230–3,160)	2,730 (1,610–4,220)	34·7 (20·6–53·7)	35·4 (21·3–54·7)	35·6 (20·9–55·6)

1-167	Musculoskeletal disorders	52,600 (38,400–68,300)	111,000 (80,100–145,000)	151,000 (108,000–197,000)	1,770 (1,290–2,320)	1,870 (1,350–2,440)	1,900 (1,370–2,470)
1-168	Rheumatoid arthritis	1,780 (1,350–2,260)	4,360 (3,210–5,560)	6,260 (4,640–8,040)	69·7 (53·1–87·9)	84·9 (62·5–107)	89·9 (66·4–114)
1-169	Osteoarthritis	3,830 (1,930–7,710)	8,740 (4,400–17,600)	12,300 (6,260–24,600)	182 (91·4–366)	194 (98·2–391)	197 (99–394)
1-170	Low back pain	25,400 (17,800–34,200)	50,600 (35,200–68,100)	66,500 (46,400–89,600)	786 (551–1,050)	791 (553–1,060)	790 (549–1,070)
1-171	Neck pain	3,900 (2,530–5,710)	7,900 (5,090–11,500)	10,700 (6,920–15,600)	135 (89·1–195)	135 (88·1–195)	135 (88·3–195)
1-172	Gout	109 (67–162)	264 (164–384)	375 (224–549)	4·3 (2·6–6·4)	5·1 (3·1–7·3)	5·2 (3·2–7·6)
1-173	Other musculoskeletal disorders	17,600 (12,600–24,200)	39,100 (27,500–53,500)	54,500 (37,600–74,900)	595 (418–810)	660 (466–904)	682 (477–934)
1-174	Other non-communicable diseases	90,800 (73,700–117,000)	144,000 (109,000–185,000)	156,000 (120,000–206,000)	1,840 (1,470–2,320)	1,900 (1,480–2,420)	1,720 (1,340–2,220)
1-175	Congenital anomalies	39,500 (30,300–65,000)	65,800 (44,200–94,400)	57,900 (37,900–88,700)	505 (394–805)	675 (455–963)	520 (342–796)
1-176	Neural tube defects	6,270 (3,160–11,000)	7,410 (3,590–13,200)	5,230 (2,400–10,000)	75·4 (38·8–131)	74·5 (36·2–133)	46·4 (21·2–89)
1-177	Congenital heart anomalies	14,000 (9,680–25,400)	26,400 (16,700–40,900)	22,600 (12,900–37,900)	174 (123–307)	269 (170–416)	202 (116–340)
1-178	Orofacial clefts	553 (251–1,140)	424 (253–703)	409 (247–651)	7·2 (3·6–14·1)	4·6 (2·8–7·4)	3·9 (2·4–6)
1-179	Down's syndrome	1,050 (654–1,840)	1,820 (1,070–2,730)	1,790 (1,050–2,890)	14 (9·2–23·5)	19·2 (11·2–28·6)	16·4 (9·5–26·3)
1-180	Turner syndrome	13 (5–25)	19 (9–34)	22 (9–40)	0·2 (0·1–0·4)	0·2 (0·1–0·4)	0·2 (0·1–0·4)
1-181	Klinefelter syndrome	1 (0–2)	1 (1–2)	1 (1–3)	0 (0–0)	0 (0–0)	0 (0–0)
1-182	Other chromosomal abnormalities	777 (510–1,780)	1,520 (971–2,320)	1,640 (1,040–2,490)	10·1 (6·7–21·9)	15·4 (9·8–23·4)	14·5 (9·1–22·1)
1-183	Congenital musculoskeletal and limb anomalies	4,610 (2,940–6,730)	7,210 (4,700–10,300)	8,180 (5,370–11,700)	71 (45·6–102)	77 (50·6–110)	75·5 (49·4–108)
1-184	Urogenital congenital anomalies	887 (602–1,340)	1,540 (929–2,500)	1,400 (798–2,480)	11·1 (7·7–16·5)	15·7 (9·4–25·3)	12·5 (7·1–22)
1-185	Digestive congenital anomalies	3,280 (1,870–5,570)	6,070 (3,100–10,300)	4,940 (2,270–9,240)	38·8 (22·1–65·6)	61·2 (31·1–104)	43·9 (20·1–82)
1-186	Other congenital anomalies	8,020 (5,030–18,100)	13,500 (8,590–20,300)	11,700 (6,710–19,500)	103 (64·7–219)	138 (88–205)	105 (60·6–174)
1-187	Urinary diseases and male infertility	3,880 (3,160–4,970)	6,240 (4,330–8,360)	8,470 (5,720–11,300)	138 (111–171)	130 (88·6–172)	134 (88·5–176)
1-188	Urinary tract infections and interstitial nephritis	2,440 (1,940–3,690)	3,660 (2,240–5,810)	4,920 (2,820–7,500)	77·2 (59·4–108)	73 (42·1–114)	76·7 (41·9–115)
1-189	Urolithiasis	240 (110–347)	374 (212–533)	483 (284–716)	8·6 (3·9–12·6)	7·1 (4–10·3)	6·9 (4–10·4)
1-190	Benign prostatic hyperplasia	683 (400–1,060)	1,500 (878–2,330)	2,030 (1,200–3,130)	35·8 (20·9–55·2)	36·3 (21·3–56·1)	35·2 (20·9–54·1)
1-191	Male infertility	96 (35–235)	116 (46–251)	250 (91–605)	2·6 (1–6·3)	1·5 (0·6–3·3)	2·5 (0·9–6·1)
1-192	Other urinary diseases	414 (236–571)	590 (365–827)	789 (484–1,110)	14·1 (7·3–20·5)	11·8 (6·9–17·1)	12·2 (7·1–17·4)
1-193	Gynecological diseases	12,500 (8,710–17,400)	23,300 (16,000–33,200)	31,200 (21,300–44,200)	350 (246–490)	331 (230–465)	336 (234–474)
1-194	Uterine fibroids	846 (449–1,530)	1,710 (860–3,130)	2,340 (1,150–4,350)	28·7 (15·4–51·7)	28·7 (14·6–52·6)	28·7 (14·3–53)
1-195	Polycystic ovarian syndrome	230 (98–466)	654 (276–1,330)	923 (389–1,870)	5·5 (2·3–11)	8 (3·4–16·3)	8·9 (3·7–17·9)
1-196	Female infertility	192 (54–487)	162 (62–347)	508 (146–1,250)	5·2 (1·4–13)	2·1 (0·8–4·5)	5·2 (1·5–13·1)
1-197	Endometriosis	1,710 (985–2,920)	2,250 (1,300–3,620)	2,740 (1,580–4,450)	44 (25·8–73)	29·2 (17·3–46·7)	27·1 (15·7–43·8)
1-198	Genital prolapse	221 (117–396)	324 (166–602)	424 (215–785)	9·7 (5·1–17·5)	6·8 (3·5–12·5)	6·4 (3·3–11·8)
1-199	Premenstrual syndrome	4,090 (2,440–6,360)	8,280 (5,050–13,000)	10,800 (6,560–16,800)	97·9 (60·1–152)	103 (63·1–161)	105 (64·2–163)
1-200	Other gynecological diseases	5,230 (3,400–7,560)	9,950 (6,430–14,700)	13,500 (8,600–19,700)	159 (105–228)	153 (98–225)	155 (99·5–223)
1-201	Hemoglobinopathies and hemolytic anaemias	6,360 (5,450–7,470)	5,700 (4,590–7,020)	5,890 (4,710–7,250)	128 (110–149)	81·5 (65–98·6)	71·2 (56·6–87)
1-202	Thalassemias	217 (107–383)	215 (96–413)	191 (83–375)	2·7 (1·3–4·8)	2·1 (0·9–4)	1·7 (0·7–3·3)
1-203	Thalassaemias trait	121 (65–201)	208 (121–337)	245 (138–399)	2·5 (1·4–4·2)	2·5 (1·5–4)	2·4 (1·3–3·8)
1-204	Sickle cell disorders	185 (96–298)	119 (56–226)	108 (46–209)	2·2 (1·2–3·6)	1·2 (0·6–2·3)	1 (0·4–1·8)
1-205	Sickle cell trait	36 (13–70)	52 (20–101)	61 (23–119)	0·8 (0·3–1·5)	0·6 (0·2–1·2)	0·6 (0·2–1·1)
1-206	G6PD deficiency	2,490 (1,910–3,150)	2,350 (1,770–3,100)	2,030 (1,420–2,790)	41·7 (32·5–51·8)	28·5 (21·4–36·5)	21·2 (14·9–29·2)
1-207	G6PD trait	3 (1–5)	9 (5–15)	10 (5–18)	0·1 (0–0·2)	0·1 (0·1–0·2)	0·1 (0·1–0·2)
1-208	Other hemoglobinopathies and hemolytic anaemias	3,300 (2,670–4,050)	2,750 (2,130–3,460)	3,250 (2,530–4,060)	77·5 (64·3–93·4)	46·5 (36·3–58·1)	44·3 (34·2–55·2)
1-209	Endocrine, metabolic, blood, and immune disorders	15,800 (10,300–20,700)	21,000 (15,500–26,900)	24,000 (18,200–31,100)	335 (253–418)	315 (240–395)	296 (228–379)
1-210	Oral disorders	10,400 (6,070–16,600)	20,200 (11,700–32,200)	26,700 (15,700–42,500)	360 (217–565)	352 (209–557)	349 (209–557)
1-211	Caries of deciduous teeth	231 (101–477)	296 (129–627)	329 (139–683)	2·9 (1·3–6)	2·9 (1·2–6·1)	2·9 (1·2–6)
1-212	Caries of permanent teeth	1,200 (526–2,410)	2,180 (968–4,280)	2,680 (1,170–5,350)	28·7 (12·8–56·8)	28·1 (12·7–54·5)	27·6 (12·2–54·4)
1-213	Periodontal disease	2,980 (1,110–6,420)	6,080 (2,300–13,300)	8,310 (3,130–18,000)	103 (40·2–220)	103 (40·2–222)	104 (40·2–220)
1-214	Edentulism	3,770 (2,400–5,510)	7,630 (4,850–11,200)	10,400 (6,590–15,400)	173 (110–253)	165 (105–241)	162 (102–240)
1-215	Other oral disorders	2,200 (1,360–3,230)	3,960 (2,480–5,860)	5,020 (3,140–7,320)	53·2 (33–77·8)	53·2 (33·1–78·1)	53·2 (33·3–77·5)
1-216	Sudden infant death syndrome	2,480 (1,020–6,090)	1,740 (942–3,010)	1,480 (809–2,770)	29·1 (11·9–71·5)	17·5 (9·5–30·3)	13·1 (7·2–24·5)
1-0	Injuries	278,000 (250,000–311,000)	404,000 (337,000–477,000)	392,000 (297,000–510,000)	6,390 (5,650–7,130)	5,410 (4,500–6,440)	4,250 (3,280–5,460)
1-1	Transport injuries	51,000 (44,200–60,100)	65,300 (52,900–80,500)	67,900 (50,200–89,600)	1,220 (1,040–1,420)	905 (729–1,120)	764 (576–992)
1-2	Road injuries	47,300 (40,700–55,900)	61,500 (49,600–76,100)	62,600 (46,000–82,500)	1,130 (956–1,320)	853 (689–1,060)	707 (530–921)

1-3	Pedestrian road injuries	26,700 (20,400–33,100)	30,600 (23,300–40,500)	30,400 (21,300–42,500)	621 (480–770)	436 (332–572)	358 (259–489)
1-4	Cyclist road injuries	2,280 (1,690–3,070)	3,370 (2,520–4,500)	3,530 (2,640–4,800)	55·6 (41·9–73)	47·8 (36·2–63·3)	40·9 (31·1–54·4)
1-5	Motorcyclist road injuries	5,210 (3,550–7,400)	7,540 (5,040–10,500)	7,650 (4,790–11,300)	125 (86·1–176)	96·7 (66·3–134)	77·9 (51–112)
1-6	Motor vehicle road injuries	11,700 (7,620–15,700)	18,000 (12,800–23,400)	18,800 (12,700–26,700)	290 (195–387)	244 (176–315)	205 (142–288)
1-7	Other road injuries	1,370 (890–2,090)	2,020 (1,490–2,750)	2,180 (1,530–2,990)	35 (23·5–49·9)	29 (21·4–38·6)	24·9 (18·1–34)
1-8	Other transport injuries	3,740 (3,390–4,190)	3,770 (2,870–5,040)	5,310 (4,150–6,790)	88·2 (79·9–98)	51·2 (38·5–67·8)	57·2 (44·8–73·3)
1-9	Unintentional injuries	116,000 (101,000–132,000)	123,000 (105,000–143,000)	123,000 (98,500–152,000)	2,390 (2,090–2,710)	1,700 (1,430–1,980)	1,410 (1,150–1,740)
1-10	Falls	12,700 (9,470–15,600)	19,600 (14,200–25,300)	24,700 (18,400–31,900)	347 (273–424)	307 (237–381)	305 (237–385)
1-11	Drowning	17,000 (13,700–26,800)	11,600 (8,010–20,500)	11,100 (7,100–19,200)	309 (254–466)	147 (103–242)	119 (77·5–193)
1-12	Fire, heat, and hot substances	9,540 (7,520–11,900)	10,200 (7,570–13,400)	11,900 (8,750–15,900)	201 (157–252)	132 (99·3–175)	126 (94–167)
1-13	Poisonings	5,170 (4,110–6,630)	3,270 (2,480–4,380)	3,440 (2,470–4,610)	90·5 (75–110)	43·5 (33–56·6)	38 (27·4–49·9)
1-14	Poisoning by carbon monoxide	316 (202–506)	405 (252–628)	484 (287–752)	7·1 (4·6–10·4)	5·8 (3·4–8·5)	5·6 (3·1–8·2)
1-15	Poisoning by other means	4,850 (3,810–6,200)	2,870 (2,150–3,870)	2,950 (2,130–4,030)	83·4 (68·4–102)	37·8 (28·2–49·1)	32·5 (23·4–43)
1-16	Exposure to mechanical forces	14,600 (12,100–17,600)	17,100 (13,400–21,900)	19,300 (14,500–25,100)	360 (296–434)	244 (191–311)	218 (164–285)
1-17	Unintentional firearm injuries	5,950 (3,850–8,130)	5,180 (3,230–7,620)	4,930 (2,940–7,280)	138 (87·8–185)	69·5 (43·1–101)	53·1 (31·9–79·1)
1-18	Other exposure to mechanical forces	8,660 (6,310–11,400)	11,900 (8,940–15,800)	14,300 (10,700–19,500)	222 (161–296)	174 (129–228)	165 (124–222)
1-19	Adverse effects of medical treatment	6,210 (2,800–8,590)	6,310 (2,530–9,860)	6,590 (2,550–11,000)	128 (61·3–173)	95·8 (39·5–153)	83·1 (32·8–140)
1-20	Animal contact	1,910 (1,500–2,360)	2,220 (1,670–2,920)	2,570 (1,900–3,410)	43·6 (34·2–54·3)	32·3 (24–42)	30·4 (22·2–40·3)
1-21	Venomous animal contact	1,450 (1,140–1,800)	1,620 (1,220–2,130)	1,870 (1,360–2,470)	32·1 (25·3–39·9)	23·4 (17·3–30·4)	22 (16·1–28·9)
1-22	Non-venomous animal contact	464 (340–636)	601 (426–873)	703 (485–1,030)	11·5 (8·4–15·6)	8·9 (6·3–12·8)	8·4 (5·9–12·3)
1-23	Foreign body	31,100 (25,000–39,400)	22,400 (17,300–30,000)	21,800 (16,000–29,500)	503 (417–612)	292 (230–381)	248 (189–328)
1-24	Pulmonary aspiration and foreign body in airway	30,300 (24,300–38,700)	21,300 (16,200–28,900)	20,500 (14,700–28,200)	486 (403–594)	276 (213–365)	233 (176–312)
1-25	Foreign body in eyes	219 (95–404)	397 (174–729)	502 (225–919)	5·5 (2·7–9·7)	5·4 (2·7–9·6)	5·4 (2·6–9·5)
1-26	Foreign body in other body part	510 (369–709)	711 (542–905)	837 (632–1,060)	11·5 (8·6–14·9)	9·8 (7·6–12·5)	9·4 (7·1–11·8)
1-27	Environmental heat and cold exposure	623 (476–803)	841 (621–1,100)	1,040 (758–1,370)	16·5 (12·5–21)	12·6 (9·3–16·4)	12·3 (9–16)
1-28	Exposure to forces of nature	392 (347–442)	13,600 (11,500–16,200)	4,280 (2,950–6,130)	8 (7·1–9·1)	171 (145–204)	50·6 (34·9–72)
1-29	Other unintentional injuries	16,400 (13,400–19,600)	15,900 (11,700–20,500)	16,400 (11,200–22,500)	380 (312–454)	219 (161–284)	182 (126–246)
1-30	Self-harm and interpersonal violence	112,000 (94,400–130,000)	215,000 (173,000–260,000)	201,000 (141,000–278,000)	2,780 (2,350–3,240)	2,810 (2,250–3,420)	2,080 (1,480–2,840)
1-31	Self-harm	9,060 (7,110–13,000)	9,710 (6,970–18,000)	10,600 (6,710–19,200)	234 (186–336)	139 (100–255)	120 (80·6–214)
1-32	Self-harm by firearm	2,100 (1,360–3,360)	1,410 (875–2,950)	1,420 (820–3,260)	54·6 (36–89·4)	20·8 (13·2–43·6)	16·7 (10·4–37·1)
1-33	Self-harm by other specified means	6,960 (5,340–10,200)	8,310 (5,950–14,800)	9,150 (5,830–16,300)	180 (138–258)	118 (84·1–208)	104 (69·5–180)
1-34	Interpersonal violence	102,000 (85,900–119,000)	205,000 (165,000–249,000)	190,000 (132,000–265,000)	2,540 (2,120–2,970)	2,670 (2,120–3,260)	1,950 (1,360–2,680)
1-35	Physical violence by firearm	55,400 (42,400–69,500)	117,000 (91,500–147,000)	109,000 (70,900–157,000)	1,380 (1,050–1,740)	1,510 (1,160–1,910)	1,100 (716–1,570)
1-36	Physical violence by sharp object	20,200 (13,700–30,700)	39,900 (29,200–54,700)	37,400 (24,400–54,500)	522 (355–774)	534 (394–728)	393 (261–570)
1-37	Sexual violence	685 (448–971)	1,260 (842–1,760)	1,690 (1,110–2,430)	16·9 (11·2–24·3)	16·4 (11·1–23)	17·1 (11·3–24·7)
1-38	Physical violence by other means	25,800 (19,700–33,400)	46,800 (36,500–59,100)	42,400 (30,300–58,300)	617 (478–801)	609 (472–768)	448 (324–604)
1-39	Conflict and terrorism	516 (423–639)	262 (170–397)	234 (155–350)	11·4 (9·4–14)	3·9 (2·6–5·8)	2·9 (1·9–4·3)
1-40	Executions and police conflict	51 (35–80)	150 (57–276)	186 (56–379)	1 (0·6–1·5)	2·1 (0·6–3·7)	2·1 (0·5–4·3)

SR Table 1: All-age DALYs counts and age standardized DALYs rates per 100,000 individuals for all causes and level 1 cause groups by location for 1990, 2010, and 2019, both sexes combined, Malaysia

Cause Level	Cause	All-age DALYs			Age-standardized rate of DALYs per 100,000		
		1990	2010	2019	1990	2010	2019
0-0	All causes	4,450,000 (4,040,000–4,920,000)	6,330,000 (5,650,000–7,090,000)	7,730,000 (6,510,000–9,090,000)	33,000 (30,200–36,100)	27,600 (24,900–30,600)	26,800 (22,600–31,400)
1-0	Communicable, maternal, neonatal, and nutritional disorders	1,170,000 (1,060,000–1,300,000)	1,080,000 (968,000–1,200,000)	1,200,000 (1,000,000–1,420,000)	6,480 (5,920–7,200)	4,650 (4,120–5,080)	4,300 (3,560–5,070)
1-1	HIV/AIDS and sexually transmitted infections	25,300 (22,600–29,200)	106,000 (100,000–111,000)	90,000 (82,900–97,900)	150 (137–169)	373 (355–393)	268 (248–292)
1-2	HIV/AIDS	20,200 (18,800–21,600)	100,000 (95,800–105,000)	84,100 (77,800–90,900)	124 (116–133)	353 (337–371)	249 (231–269)
1-3	HIV/AIDS - drug-susceptible tuberculosis	8,900 (7,050–9,840)	18,100 (11,800–26,800)	15,000 (9,930–22,300)	54·7 (43·3–60·3)	63·7 (41·6–94·4)	44·4 (29·4–66·2)
1-4	HIV/AIDS - Multidrug-resistant Tuberculosis without extensive drug resistance	14 (1–62)	506 (104–1,560)	317 (58–1,010)	0·1 (0–0·4)	1·8 (0·4–5·5)	0·9 (0·2–3)
1-5	HIV/AIDS - Extensively drug-resistant Tuberculosis	0 (0–0)	62 (12–201)	59 (10–193)	0 (0–0)	0·2 (0–0·7)	0·2 (0–0·6)
1-6	HIV/AIDS resulting in other diseases	11,200 (10,200–13,500)	81,600 (71,600–89,500)	68,700 (59,100–77,600)	69·1 (62·9–82·8)	287 (252–314)	204 (176–229)
1-7	Sexually transmitted infections excluding HIV	5,110 (2,870–8,860)	5,330 (3,340–8,340)	5,910 (3,640–9,470)	26·4 (15·7–43·8)	19·5 (12·2–31)	18·7 (11·5–30·4)
1-8	Syphilis	2,880 (916–6,350)	1,530 (555–3,130)	1,620 (588–3,550)	12·3 (4·1–26·9)	6·4 (2·3–13·2)	6·1 (2·2–13·5)
1-9	Chlamydial infection	335 (213–499)	567 (354–862)	639 (389–993)	2 (1·3–2·9)	1·9 (1·2–2·8)	1·8 (1·1–2·8)
1-10	Gonococcal infection	275 (188–389)	426 (287–611)	466 (306–673)	1·7 (1·2–2·3)	1·4 (1–2)	1·4 (0·9–2)
1-11	Trichomoniasis	519 (199–1,100)	910 (355–1,860)	1,030 (398–2,130)	3·3 (1·3–7·1)	3·2 (1·2–6·5)	3 (1·2–6·3)
1-12	Genital herpes	531 (172–1,310)	999 (316–2,480)	1,200 (386–2,980)	3·7 (1·2–9)	3·7 (1·2–9·1)	3·7 (1·2–9·1)
1-13	Other sexually transmitted infections	571 (389–813)	901 (622–1,280)	962 (650–1,380)	3·4 (2·3–4·7)	3 (2·1–4·2)	2·8 (1·9–4)
1-14	Respiratory infections and tuberculosis	286,000 (255,000–324,000)	442,000 (344,000–478,000)	562,000 (398,000–701,000)	2,110 (1,930–2,440)	2,200 (1,670–2,380)	2,140 (1,490–2,640)
1-15	Tuberculosis	69,600 (62,300–76,000)	69,500 (64,100–75,000)	65,700 (53,500–80,800)	581 (520–636)	305 (281–328)	226 (184–277)
1-16	Latent tuberculosis infection	0 (0–0)	0 (0–0)	0 (0–0)	0 (0–0)	0 (0–0)	0 (0–0)
1-17	Drug-susceptible tuberculosis	69,500 (62,300–75,900)	67,800 (61,700–73,600)	64,500 (52,600–79,400)	580 (519–635)	297 (271–323)	221 (180–272)
1-18	Multidrug-resistant tuberculosis without extensive drug resistance	85 (7–385)	1,510 (327–4,360)	1,070 (226–3,300)	0·7 (0·1–3·3)	6·7 (1·4–19·5)	3·7 (0·8–11·6)
1-19	Extensively drug-resistant tuberculosis	0 (0–0)	181 (35–532)	196 (37–637)	0 (0–0)	0·8 (0·2–2·4)	0·7 (0·1–2·2)
1-20	Lower respiratory infections	195,000 (168,000–231,000)	341,000 (244,000–368,000)	463,000 (305,000–581,000)	1,410 (1,260–1,730)	1,790 (1,260–1,940)	1,800 (1,180–2,250)
1-21	Upper respiratory infections	15,900 (9,660–25,000)	23,900 (14,200–37,900)	25,600 (15,400–40,700)	84·3 (51·6–132)	83·5 (50–132)	82·3 (49·5–131)
1-22	Otitis media	5,650 (3,310–9,330)	7,250 (4,170–12,100)	7,240 (4,170–12,000)	29·3 (17·4–48·1)	25·5 (14·8–41·4)	23·9 (13·9–39·3)
1-23	Enteric infections	92,900 (68,200–127,000)	85,500 (64,000–114,000)	92,100 (67,700–123,000)	544 (404–739)	369 (280–484)	335 (247–449)
1-24	Diarrhoeal diseases	57,400 (42,700–77,500)	55,700 (42,700–75,100)	68,200 (49,700–91,800)	377 (272–540)	268 (202–360)	256 (188–349)
1-25	Typhoid and paratyphoid	33,900 (14,900–64,800)	28,500 (12,500–52,400)	22,700 (9,230–44,200)	159 (69·4–304)	95·7 (42·2–175)	74·8 (30·5–144)
1-26	Typhoid fever	32,200 (13,900–61,600)	28,000 (12,300–51,800)	20,400 (8,200–39,600)	151 (65·6–291)	94·2 (41·4–173)	67·3 (27–130)
1-27	Paratyphoid fever	1,750 (675–3,700)	448 (170–932)	2,270 (809–4,820)	8·1 (3·1–17)	1·5 (0·6–3·1)	7·5 (2·7–16·1)
1-28	iNTS	1,500 (778–2,530)	1,260 (616–2,240)	1,160 (533–2,140)	7·5 (3·9–12·4)	4·6 (2·3–8)	4 (1·9–7·4)
1-29	Other intestinal infectious diseases	140 (69–227)	88 (55–123)	91 (62–129)	0·7 (0·4–1·1)	0·3 (0·2–0·5)	0·3 (0·2–0·4)
1-30	Neglected tropical diseases and malaria	76,200 (54,200–107,000)	44,700 (31,600–63,400)	50,300 (35,800–70,600)	420 (300–586)	156 (111–221)	159 (113–224)
1-31	Malaria	10,300 (4,810–23,600)	1,260 (817–2,010)	691 (387–1,280)	54·9 (25·2–124)	4·3 (2·8–6·9)	2·1 (1·2–3·9)
1-32	Chagas disease	0 (0–0)	0 (0–0)	0 (0–0)	0 (0–0)	0 (0–0)	0 (0–0)
1-33	Leishmaniasis	0 (0–0)	0 (0–0)	0 (0–0)	0 (0–0)	0 (0–0)	0 (0–0)
1-34	Visceral leishmaniasis	0 (0–0)	0 (0–0)	0 (0–0)	0 (0–0)	0 (0–0)	0 (0–0)
1-35	Cutaneous and mucocutaneous leishmaniasis	0 (0–0)	0 (0–0)	0 (0–0)	0 (0–0)	0 (0–0)	0 (0–0)
1-36	African trypanosomiasis	0 (0–0)	0 (0–0)	0 (0–0)	0 (0–0)	0 (0–0)	0 (0–0)
1-37	Schistosomiasis	0 (0–0)	0 (0–0)	0 (0–0)	0 (0–0)	0 (0–0)	0 (0–0)
1-38	Cysticercosis	135 (69–240)	189 (92–362)	218 (99–406)	1·3 (0·7–2·3)	0·9 (0·4–1·7)	0·8 (0·4–1·4)
1-39	Cystic echinococcosis	95 (57–137)	58 (36–86)	50 (29–77)	0·7 (0·4–1)	0·2 (0·1–0·3)	0·2 (0·1–0·3)
1-40	Lymphatic filariasis	16,900 (10,000–26,600)	10,400 (5,420–19,100)	10,400 (5,530–19,100)	110 (66·1–171)	36·5 (19·2–67·5)	32·5 (17·3–59·5)
1-41	Onchocerciasis	0 (0–0)	0 (0–0)	0 (0–0)	0 (0–0)	0 (0–0)	0 (0–0)
1-42	Trachoma	0 (0–0)	0 (0–0)	0 (0–0)	0 (0–0)	0 (0–0)	0 (0–0)
1-43	Dengue	13,400 (7,070–20,600)	13,500 (9,320–24,500)	17,600 (11,200–29,000)	70·3 (35·8–104)	48 (33·6–87·5)	55·9 (35·9–93·7)
1-44	Yellow fever	0 (0–0)	0 (0–0)	0 (0–0)	0 (0–0)	0 (0–0)	0 (0–0)
1-45	Rabies	18 (1–42)	15 (1–25)	9 (1–19)	0·1 (0–0·2)	0·1 (0–0·1)	0 (0–0·1)
1-46	Intestinal nematode infections	29,000 (16,000–48,700)	13,400 (6,730–23,500)	15,900 (8,060–28,300)	151 (83·2–253)	44·3 (22·4–77·2)	48·8 (24·7–86·9)
1-47	Ascariasis	10,200 (5,420–17,400)	2,530 (1,250–4,710)	1,960 (920–3,700)	53·8 (28·5–91·8)	8·8 (4·3–16·3)	6·3 (3–12)
1-48	Trichuriasis	18,100 (9,400–31,100)	9,960 (4,480–19,000)	12,700 (6,040–23,300)	92·9 (48·4–160)	32·4 (14·6–61·8)	38·7 (18·5–71·3)

1-49	Hookworm disease	787 (428–1,310)	917 (512–1,540)	1,260 (668–2,100)	4·1 (2·3–6·9)	3 (1·7–5)	3·8 (2–6·3)
1-50	Food-borne trematodiases	0 (0–0)	0 (0–0)	0 (0–0)	0 (0–0)	0 (0–0)	0 (0–0)
1-51	Leprosy	7 (4–11)	9 (6–14)	12 (7–18)	0·1 (0–0·1)	0 (0–0·1)	0 (0–0·1)
1-52	Ebola virus disease	0 (0–0)	0 (0–0)	0 (0–0)	0 (0–0)	0 (0–0)	0 (0–0)
1-53	Zika virus disease	0 (0–0)	0 (0–0)	0 (0–0)	0 (0–0)	0 (0–0)	0 (0–0)
1-54	Guinea worm disease	0 (0–0)	0 (0–0)	0 (0–0)	0 (0–0)	0 (0–0)	0 (0–0)
1-55	Other neglected tropical diseases	6,330 (4,150–9,300)	5,940 (3,740–8,840)	5,360 (3,320–7,980)	31·8 (20·6–46·6)	21·4 (13·5–31·9)	18·3 (11·3–27·3)
1-56	Other infectious diseases	190,000 (125,000–271,000)	89,100 (68,200–111,000)	89,200 (63,600–117,000)	904 (626–1,240)	344 (263–432)	298 (214–393)
1-57	Meningitis	28,200 (22,700–34,500)	14,800 (13,400–16,200)	14,800 (11,900–18,500)	146 (121–174)	56·2 (50·9–61·5)	48·7 (39–60·8)
1-58	Encephalitis	13,900 (11,000–17,200)	17,900 (14,700–19,800)	19,900 (14,700–25,600)	79 (64·2–94·8)	66·5 (55·1–73·4)	64·7 (48·6–82·8)
1-59	Diphtheria	169 (90–296)	35 (21–53)	29 (18–44)	0·8 (0·5–1·4)	0·1 (0·1–0·2)	0·1 (0·1–0·1)
1-60	Whooping cough	39,100 (4,380–105,000)	13,900 (2,010–34,700)	9,830 (1,250–25,800)	164 (18·4–442)	55·9 (8·1–140)	37·3 (4·8–97·6)
1-61	Tetanus	3,060 (2,040–5,060)	979 (576–1,630)	847 (441–1,370)	20·1 (12·8–33·5)	3·9 (2·3–6·5)	2·7 (1·5–4·5)
1-62	Measles	86,700 (34,800–163,000)	8,500 (2,860–18,200)	5,720 (1,710–13,000)	366 (146–690)	33·4 (11·2–71·4)	21·7 (6·5–49·7)
1-63	Varicella and herpes zoster	2,170 (1,440–3,210)	1,650 (1,180–2,240)	1,650 (1,130–2,260)	12·7 (9·1–17·2)	7 (5·1–9·4)	5·9 (4·1–8·1)
1-64	Acute hepatitis	7,590 (5,960–9,560)	8,050 (7,170–8,980)	7,870 (6,100–10,200)	60 (46·4–75·8)	32·3 (28·9–35·9)	25·7 (19·9–33·4)
1-65	Acute hepatitis A	972 (665–1,320)	1,080 (718–1,530)	1,080 (699–1,550)	5·2 (3·5–7·1)	3·5 (2·4–5)	3·3 (2·2–4·8)
1-66	Acute hepatitis B	6,190 (4,730–7,930)	6,660 (6,010–7,390)	6,470 (4,770–8,610)	51·9 (39·7–66)	27·6 (25–30·7)	21·3 (15·7–28·2)
1-67	Acute hepatitis C	336 (170–662)	210 (135–448)	220 (129–378)	2·4 (1·2–5·3)	0·8 (0·5–1·8)	0·7 (0·4–1·2)
1-68	Acute hepatitis E	86 (56–121)	101 (67–143)	109 (71–156)	0·5 (0·3–0·7)	0·4 (0·2–0·5)	0·3 (0·2–0·5)
1-69	Other unspecified infectious diseases	9,450 (6,630–14,900)	23,300 (12,600–28,700)	28,600 (14,000–40,300)	55·1 (39·7–86·1)	88·1 (46·6–108)	90·9 (44·8–127)
1-70	Maternal and neonatal disorders	372,000 (323,000–428,000)	193,000 (175,000–214,000)	200,000 (167,000–239,000)	1,640 (1,430–1,870)	753 (688–834)	701 (583–839)
1-71	Maternal disorders	30,200 (25,500–35,500)	17,300 (15,100–19,500)	17,500 (13,300–23,000)	168 (142–197)	55·8 (48·7–62·8)	48·3 (36·7–63·6)
1-72	Maternal haemorrhage	8,610 (6,950–10,500)	3,550 (2,820–4,470)	3,260 (2,350–4,430)	47·7 (38·6–58·5)	11·5 (9·1–14·4)	8·9 (6·5–12·1)
1-73	Maternal sepsis and other pregnancy related infections	3,480 (2,750–4,410)	1,020 (765–1,330)	843 (579–1,190)	19·1 (15·1–24·1)	3·3 (2·4–4·2)	2·3 (1·6–3·3)
1-74	Maternal hypertensive disorders	3,560 (2,870–4,390)	2,590 (2,100–3,220)	2,660 (1,940–3,580)	19·4 (15·7–23·7)	8·3 (6·7–10·3)	7·3 (5·4–9·8)
1-75	Maternal obstructed labour and uterine rupture	849 (615–1,130)	824 (631–1,040)	883 (595–1,260)	4·7 (3·4–6·3)	2·7 (2–3·4)	2·4 (1·6–3·5)
1-76	Maternal abortive outcome	2,510 (1,850–3,300)	651 (500–843)	546 (384–780)	14·5 (10·6–19·1)	2·1 (1·6–2·8)	1·6 (1·1–2·3)
1-77	Ectopic pregnancy	579 (448–750)	415 (330–518)	464 (317–666)	3·1 (2·4–4)	1·3 (1·1–1·7)	1·3 (0·9–1·8)
1-78	Indirect maternal deaths	2,820 (2,140–3,690)	2,770 (2,280–3,360)	3,040 (2,090–4,290)	15·2 (11·6–20)	8·8 (7·2–10·7)	8·3 (5·7–11·7)
1-79	Late maternal deaths	543 (400–746)	522 (387–693)	638 (408–966)	3 (2·2–4·1)	1·7 (1·2–2·2)	1·8 (1·1–2·7)
1-80	Maternal deaths aggravated by HIV/AIDS	1 (1–2)	11 (5–17)	13 (7–22)	0 (0–0)	0 (0–0·1)	0 (0–0·1)
1-81	Other maternal disorders	7,250 (5,740–9,110)	4,950 (4,080–5,910)	5,140 (3,760–7,070)	41 (32·5–51·2)	16·2 (13·4–19·3)	14·3 (10·4–19·6)
1-82	Neonatal disorders	342,000 (294,000–396,000)	175,000 (158,000–197,000)	182,000 (152,000–219,000)	1,470 (1,260–1,700)	698 (632–777)	653 (544–782)
1-83	Neonatal preterm birth	164,000 (131,000–207,000)	83,700 (73,300–94,900)	85,500 (70,200–103,000)	711 (572–890)	325 (285–368)	300 (245–366)
1-84	Neonatal encephalopathy due to birth asphyxia and trauma	71,400 (52,300–96,800)	27,600 (23,300–33,200)	28,400 (19,800–38,400)	302 (221–408)	114 (96·7–137)	105 (73–143)
1-85	Neonatal sepsis and other neonatal infections	52,600 (37,800–71,600)	36,200 (28,700–47,400)	40,500 (30,100–53,600)	231 (166–317)	141 (115–180)	142 (106–188)
1-86	Hemolytic disease and other neonatal jaundice	2,040 (1,570–2,740)	1,560 (1,220–1,900)	1,550 (1,210–1,930)	9·5 (7·4–12·5)	5·6 (4·4–6·8)	5 (4–6·3)
1-87	Other neonatal disorders	51,900 (36,800–73,700)	26,300 (22,500–30,800)	26,100 (18,900–34,600)	217 (154–308)	111 (95·1–130)	100 (72·2–133)
1-88	Nutritional deficiencies	127,000 (86,000–182,000)	123,000 (83,200–176,000)	120,000 (80,800–173,000)	720 (492–1,020)	456 (312–652)	401 (272–573)
1-89	Protein-energy malnutrition	17,600 (12,700–23,300)	19,200 (13,400–25,500)	21,900 (15,400–29,200)	99·3 (75·2–127)	81·5 (59·2–106)	79·7 (57–106)
1-90	Iodine deficiency	11,600 (6,290–20,500)	4,400 (2,010–8,850)	4,580 (2,480–7,560)	71·5 (38·9–128)	15·4 (7·1–30·6)	13·7 (7·4–22·3)
1-91	Vitamin A deficiency	867 (475–1,370)	209 (111–347)	126 (64–231)	3·7 (2–5·8)	0·8 (0·4–1·3)	0·5 (0·2–0·9)
1-92	Dietary iron deficiency	93,400 (60,900–137,000)	94,800 (60,800–142,000)	88,500 (56,300–135,000)	521 (342–752)	344 (221–515)	292 (185–438)
1-93	Other nutritional deficiencies	3,760 (2,510–5,390)	3,950 (2,540–5,710)	4,690 (2,940–6,890)	25 (17·3–35·2)	14·5 (9·4–20·7)	14·9 (9·4–21·7)
1-0	Non-communicable diseases	2,730,000 (2,420,000–3,090,000)	4,510,000 (3,940,000–5,160,000)	5,690,000 (4,770,000–6,750,000)	23,200 (20,900–25,700)	20,300 (18,000–22,900)	19,900 (16,800–23,500)
1-1	Neoplasms	349,000 (325,000–370,000)	616,000 (581,000–653,000)	853,000 (680,000–1,050,000)	3,100 (2,900–3,260)	2,830 (2,670–3,010)	2,990 (2,400–3,670)
1-2	Lip and oral cavity cancer	7,640 (6,700–8,680)	11,300 (10,200–12,400)	17,900 (13,600–23,000)	73·8 (64·8–84·2)	52·8 (48–58·5)	62·9 (48·5–80·8)
1-3	Nasopharynx cancer	27,900 (23,500–31,500)	31,400 (28,300–34,900)	46,900 (35,600–60,300)	228 (194–258)	127 (115–140)	152 (116–195)
1-4	Other pharynx cancer	2,570 (2,210–2,970)	3,930 (3,480–4,490)	6,250 (4,640–8,200)	25 (21·4–29·2)	18·1 (16·2–20·6)	21·6 (16·1–28·2)
1-5	Oesophageal cancer	6,460 (5,190–7,410)	10,700 (9,730–12,700)	16,300 (12,500–20,800)	67·2 (53·2–77·2)	53·4 (48·2–61)	59 (45·6–75)
1-6	Stomach cancer	23,200 (21,200–25,300)	30,900 (28,400–33,700)	39,700 (31,500–49,500)	234 (215–255)	156 (142–170)	146 (117–181)
1-7	Colon and rectum cancer	34,700 (31,100–38,500)	68,100 (63,100–73,800)	114,000 (88,500–143,000)	356 (316–397)	346 (318–376)	421 (331–524)

1-8	Liver cancer	15,300 (13,100–17,900)	38,100 (33,800–42,700)	43,400 (32,700–56,800)	146 (125–171)	175 (154–195)	151 (115–198)
1-9	Liver cancer due to hepatitis B	9,880 (7,830–12,000)	24,200 (20,200–28,400)	26,700 (19,100–35,800)	90·3 (71·8–110)	105 (86·3–124)	90·3 (64·5–120)
1-10	Liver cancer due to hepatitis C	1,930 (1,210–2,770)	4,770 (3,100–6,690)	5,620 (3,550–8,670)	21·6 (13·6–30·8)	25·4 (17·1–34·8)	21·4 (13·5–31·8)
1-11	Liver cancer due to alcohol use	1,730 (1,130–2,540)	4,680 (3,030–6,830)	5,520 (3,380–8,660)	18 (11·7–26·5)	22·5 (14·7–32·6)	19·6 (12·1–30·4)
1-12	Liver cancer due to NASH	1,090 (748–1,530)	3,320 (2,340–4,610)	4,190 (2,670–6,340)	11·3 (7·8–16·2)	16·6 (11·6–22·9)	15·4 (9·9–23)
1-13	Gallbladder and biliary tract cancer	2,980 (2,410–3,400)	5,050 (4,430–5,750)	7,300 (5,550–9,420)	31·4 (25·5–35·7)	25·6 (22·5–29·4)	26·8 (20·6–34·4)
1-14	Pancreatic cancer	4,230 (3,660–4,840)	16,200 (14,900–18,000)	22,600 (17,200–28,800)	44 (37·8–50·9)	80·6 (73·6–89·1)	81·9 (63·1–104)
1-15	Larynx cancer	4,060 (3,350–4,650)	5,240 (4,670–5,810)	8,320 (6,300–10,700)	41·5 (34·5–47·5)	25·7 (22·9–28·5)	29·8 (22·8–38)
1-16	Tracheal, bronchus, and lung cancer	39,600 (34,800–45,300)	99,100 (90,800–109,000)	125,000 (95,700–159,000)	415 (364–476)	490 (447–539)	452 (349–573)
1-17	Malignant skin melanoma	1,180 (726–1,540)	2,000 (1,330–2,400)	2,720 (1,590–3,820)	10·1 (6–13·3)	8·7 (5·8–10·4)	9·3 (5·5–12·9)
1-18	Non-melanoma skin cancer	965 (834–1,100)	2,320 (2,100–2,580)	3,260 (2,530–4,140)	10·1 (8·6–11·5)	11·9 (10·7–13·2)	12·3 (9·7–15·4)
1-19	Non-melanoma skin cancer (squamous-cell carcinoma)	965 (833–1,100)	2,320 (2,100–2,570)	3,260 (2,530–4,140)	10·1 (8·6–11·5)	11·9 (10·7–13·2)	12·3 (9·7–15·4)
1-20	Non-melanoma skin cancer (basal-cell carcinoma)	0 (0–0)	0 (0–1)	0 (0–1)	0 (0–0)	0 (0–0)	0 (0–0)
1-21	Breast cancer	38,900 (34,500–44,200)	77,600 (71,200–85,300)	111,000 (85,600–141,000)	339 (301–385)	330 (302–362)	374 (288–470)
1-22	Cervical cancer	21,400 (14,500–24,700)	25,600 (22,200–31,300)	35,400 (25,200–47,100)	191 (128–220)	112 (96·8–134)	120 (85·8–159)
1-23	Uterine cancer	3,280 (2,410–4,050)	6,440 (4,770–7,600)	9,050 (6,060–12,000)	33·4 (24·6–41·2)	30·4 (23·2–35·9)	32 (21·5–42·4)
1-24	Ovarian cancer	5,640 (4,330–8,510)	16,700 (14,700–19,700)	20,100 (14,800–27,400)	50·6 (39·3–75·1)	71·2 (62·6–82·6)	67·5 (50·3–91·2)
1-25	Prostate cancer	6,070 (4,700–7,320)	12,600 (10,000–14,800)	21,000 (14,500–27,700)	76·7 (59–92·5)	78 (60·7–90·8)	88·7 (61·3–118)
1-26	Testicular cancer	1,040 (729–1,480)	1,840 (1,420–2,410)	2,460 (1,620–3,550)	6·1 (4·3–8·6)	6·2 (4·9–8)	7·2 (4·8–10·3)
1-27	Kidney cancer	2,620 (2,230–3,010)	7,010 (6,230–7,830)	11,000 (8,040–14,200)	24·6 (20·7–28·5)	33 (29·4–37)	39·1 (28·8–50·4)
1-28	Bladder cancer	4,910 (4,080–5,660)	8,340 (7,410–9,320)	13,900 (10,800–17,600)	56·6 (46·2–65·5)	46·9 (41·6–52·1)	55·3 (43·3–69·3)
1-29	Brain and nervous system cancer	12,000 (7,140–15,400)	16,100 (11,800–18,400)	21,200 (13,300–28,800)	76·6 (47·6–98·2)	61·6 (45·8–70·2)	68·9 (43·6–93)
1-30	Thyroid cancer	2,670 (2,240–3,100)	4,820 (4,300–5,620)	6,580 (5,080–8,480)	24·7 (21–28·9)	22·9 (20·4–26·9)	23·1 (17·8–29·8)
1-31	Mesothelioma	287 (222–387)	710 (612–821)	925 (679–1,230)	2·3 (1·9–3·1)	3 (2·5–3·4)	3·1 (2·3–4)
1-32	Hodgkin lymphoma	2,310 (1,460–2,920)	2,570 (1,830–2,970)	3,020 (1,870–4,110)	14·9 (9·6–18·5)	9·3 (6·7–10·7)	9·3 (5·8–12·6)
1-33	Non-Hodgkin's lymphoma	14,000 (12,600–15,500)	28,600 (26,800–30,600)	38,400 (30,100–47,900)	112 (102–123)	123 (115–132)	130 (102–161)
1-34	Multiple myeloma	1,850 (1,520–2,710)	4,180 (3,640–5,170)	5,670 (4,310–7,620)	19·1 (15·7–28·3)	20·2 (17·5–25·1)	20·1 (15·3–27·2)
1-35	Leukaemia	40,500 (32,500–49,000)	46,100 (41,500–53,100)	54,600 (42,800–69,700)	238 (201–280)	180 (163–206)	181 (142–232)
1-36	Acute lymphoid leukaemia	16,500 (12,300–21,600)	15,100 (12,300–18,200)	17,600 (12,800–23,100)	80·3 (61·2–103)	52·6 (43·2–63·1)	57·4 (42–75·7)
1-37	Chronic lymphoid leukaemia	941 (751–1,360)	2,550 (2,130–3,310)	3,410 (2,480–5,130)	10·5 (8·3–15·7)	14·4 (11·9–19·2)	13·3 (9·7–20·3)
1-38	Acute myeloid leukaemia	9,160 (7,150–11,700)	15,600 (13,500–19,500)	19,500 (14,600–25,500)	59·9 (48·5–73·7)	61·1 (52·9–73·3)	63·2 (47·7–82·3)
1-39	Chronic myeloid leukaemia	3,260 (2,380–4,120)	4,560 (3,580–5,430)	5,260 (3,800–6,980)	24·2 (19·1–29·5)	18·6 (15·2–21·9)	17 (12·5–22·6)
1-40	Other leukaemia	10,600 (6,770–14,600)	8,280 (6,690–11,000)	8,960 (6,410–12,600)	63·4 (43·6–82·3)	33·7 (27·7–44·9)	30 (21·5–43·1)
1-41	Other neoplasms	1,430 (1,160–1,900)	3,710 (3,160–4,740)	5,410 (4,050–7,430)	14·2 (11·4–18·7)	19·4 (16·3–24·4)	20·6 (15·5–28·2)
1-42	Myelodysplastic, myeloproliferative, and other hematopoietic neoplasms	1,430 (1,160–1,900)	3,710 (3,160–4,740)	5,410 (4,050–7,430)	14·2 (11·4–18·7)	19·4 (16·3–24·4)	20·6 (15·5–28·2)
1-43	Benign and in situ intestinal neoplasms	0 (0–0)	0 (0–0)	0 (0–0)	0 (0–0)	0 (0–0)	0 (0–0)
1-44	Benign and in situ cervical and uterine neoplasms	0 (0–0)	0 (0–0)	0 (0–0)	0 (0–0)	0 (0–0)	0 (0–0)
1-45	Other benign and in situ neoplasms	0 (0–0)	0 (0–0)	0 (0–0)	0 (0–0)	0 (0–0)	0 (0–0)
1-46	Cardiovascular diseases	743,000 (709,000–775,000)	1,130,000 (1,060,000–1,210,000)	1,460,000 (1,190,000–1,770,000)	7,690 (7,350–8,020)	5,790 (5,420–6,240)	5,460 (4,440–6,580)
1-47	Rheumatic heart disease	25,000 (21,900–28,400)	16,500 (13,200–20,900)	16,900 (12,600–21,700)	168 (150–189)	60·3 (49·7–74·8)	52·6 (39·6–67·4)
1-48	Ischaemic heart disease	306,000 (287,000–325,000)	612,000 (574,000–656,000)	784,000 (626,000–968,000)	3,360 (3,140–3,560)	3,150 (2,940–3,370)	2,930 (2,340–3,580)
1-49	Stroke	342,000 (319,000–363,000)	389,000 (359,000–433,000)	512,000 (420,000–629,000)	3,530 (3,300–3,750)	2,030 (1,870–2,290)	1,940 (1,600–2,370)
1-50	Ischaemic stroke	94,300 (84,200–104,000)	138,000 (122,000–156,000)	198,000 (162,000–241,000)	1,100 (994–1,210)	812 (720–924)	810 (664–986)
1-51	Intracerebral hemorrhage	220,000 (203,000–235,000)	223,000 (208,000–248,000)	281,000 (225,000–347,000)	2,180 (2,020–2,330)	1,090 (1,010–1,220)	1,020 (820–1,250)
1-52	Subarachnoid hemorrhage	27,400 (21,800–33,500)	28,700 (25,700–34,700)	33,100 (25,900–43,600)	240 (190–295)	127 (115–151)	112 (87·7–147)
1-53	Hypertensive heart disease	10,300 (8,090–12,100)	11,200 (10,100–13,200)	14,900 (11,700–18,700)	112 (88·5–132)	59·2 (52·5–74)	57·2 (45–72·1)
1-54	Non-rheumatic valvular heart disease	1,410 (1,210–1,650)	3,310 (2,930–3,670)	4,200 (3,310–5,260)	13·4 (11·5–15·4)	16·6 (14·5–18·7)	15·4 (12·2–19·1)
1-55	Non-rheumatic calcific aortic valve disease	847 (706–1,020)	2,120 (1,860–2,420)	2,740 (2,100–3,560)	8·3 (6·9–9·8)	11 (9·6–12·5)	10·2 (7·9–13·2)
1-56	Non-rheumatic degenerative mitral valve disease	521 (412–708)	1,150 (899–1,330)	1,420 (1,060–1,800)	4·8 (3·9–6·3)	5·4 (4·2–6·5)	5 (3·8–6·4)
1-57	Other non-rheumatic valve diseases	40 (15–67)	45 (22–66)	41 (22–64)	0·3 (0·1–0·4)	0·2 (0·1–0·2)	0·1 (0·1–0·2)
1-58	Cardiomyopathy and myocarditis	14,400 (11,000–17,800)	21,900 (16,700–24,800)	26,100 (18,800–33,500)	108 (85·9–135)	93·7 (73·2–106)	89·8 (66·1–115)
1-59	Myocarditis	1,890 (669–3,280)	1,460 (822–1,940)	1,360 (876–1,890)	9·7 (4·1–15·8)	5·6 (3·3–7·3)	4·6 (3–6·4)
1-60	Alcoholic cardiomyopathy	226 (167–321)	253 (192–339)	308 (216–426)	2 (1·4–2·9)	1 (0·8–1·4)	1 (0·7–1·4)

1-61	Other cardiomyopathy	12,300 (9,820–15,600)	20,100 (15,600–22,900)	24,500 (17,600–31,400)	96·6 (78·6–124)	87·2 (68·8–100)	84·2 (61·9–108)
1-62	Atrial fibrillation and flutter	7,760 (5,890–10,100)	17,200 (13,300–21,900)	25,100 (19,000–32,300)	98·5 (76·1–128)	111 (87·1–142)	110 (84·4–140)
1-63	Aortic aneurysm	6,820 (5,790–8,070)	13,500 (12,300–14,800)	18,900 (14,900–23,700)	77·4 (65·6–91·6)	76·9 (69·5–85·7)	74·7 (59·2–93·7)
1-64	Peripheral vascular disease	764 (421–1,260)	1,620 (956–2,610)	2,300 (1,380–3,680)	9·8 (5·4–16·5)	10·2 (6–16·5)	9·7 (5·9–15·3)
1-65	Endocarditis	8,420 (5,960–10,500)	11,600 (8,680–13,400)	14,000 (9,860–18,200)	55·1 (41·1–67·4)	44 (33·8–51·1)	43·6 (31·5–56·9)
1-66	Chronic respiratory diseases	159,000 (137,000–179,000)	200,000 (178,000–230,000)	243,000 (204,000–296,000)	1,590 (1,360–1,810)	1,010 (913–1,180)	922 (778–1,120)
1-67	Chronic obstructive pulmonary disease	86,600 (74,300–98,700)	128,000 (116,000–146,000)	169,000 (142,000–205,000)	1,010 (864–1,160)	721 (656–842)	669 (564–814)
1-68	Pneumoconiosis	71 (52–95)	158 (108–229)	235 (152–355)	0·7 (0·6–1)	0·8 (0·5–1·1)	0·8 (0·6–1·3)
1-69	Silicosis	61 (41–84)	137 (88–205)	206 (126–324)	0·6 (0·4–0·9)	0·7 (0·4–1)	0·7 (0·5–1·2)
1-70	Asbestosis	3 (2–6)	7 (5–11)	9 (6–14)	0 (0–0·1)	0 (0–0)	0 (0–0)
1-71	Coal workers pneumoconiosis	1 (1–3)	3 (2–5)	4 (2–6)	0 (0–0)	0 (0–0)	0 (0–0)
1-72	Other pneumoconiosis	5 (3–11)	11 (8–17)	16 (11–23)	0·1 (0–0·1)	0·1 (0–0·1)	0·1 (0–0·1)
1-73	Asthma	66,000 (53,600–78,900)	57,500 (46,200–73,300)	56,900 (43,100–73,500)	531 (440–624)	233 (190–295)	193 (146–251)
1-74	Interstitial lung disease and pulmonary sarcoidosis	2,210 (1,780–3,020)	5,400 (4,460–7,290)	6,890 (5,100–10,300)	22·6 (17·8–32)	27·5 (22·5–38·2)	25·2 (18·5–38·3)
1-75	Other chronic respiratory diseases	4,350 (3,270–5,600)	8,870 (7,320–10,300)	10,800 (8,620–13,400)	27·6 (20·2–35·2)	31·7 (26·1–36·6)	33·7 (26·8–42·1)
1-76	Digestive diseases	119,000 (109,000–130,000)	231,000 (213,000–249,000)	288,000 (231,000–355,000)	1,070 (969–1,170)	1,110 (1,020–1,200)	1,030 (831–1,260)
1-77	Cirrhosis and other chronic liver diseases	53,700 (48,400–59,400)	106,000 (97,800–114,000)	136,000 (106,000–171,000)	474 (423–525)	472 (433–510)	465 (362–584)
1-78	Cirrhosis and other chronic liver diseases due to hepatitis B	24,900 (20,600–29,400)	44,200 (36,700–52,400)	55,200 (40,600–72,900)	221 (183–262)	194 (160–228)	187 (138–246)
1-79	Cirrhosis and other chronic liver diseases due to hepatitis C	11,600 (8,420–15,300)	24,900 (18,700–32,300)	32,100 (21,900–44,400)	103 (73·5–136)	110 (84–141)	109 (75–149)
1-80	Cirrhosis and other chronic liver diseases due to alcohol use	7,760 (5,370–10,800)	18,100 (12,600–24,800)	23,800 (15,300–35,600)	71·8 (49·2–100)	80·7 (57·1–110)	81 (52–120)
1-81	Cirrhosis and other chronic liver diseases due to NAFLD	3,050 (2,020–4,360)	7,160 (5,330–9,500)	10,700 (6,900–15,600)	29 (19·2–41·8)	35·1 (26·1–46·1)	38·4 (24·9–55·2)
1-82	Cirrhosis and other chronic liver diseases due to other causes	6,400 (5,040–8,040)	11,400 (8,930–14,200)	14,100 (10,100–19,500)	49·6 (37·9–65·1)	52·1 (40–65·7)	49·9 (35·7–69·4)
1-83	Upper digestive system diseases	22,900 (18,600–28,800)	38,500 (31,300–49,600)	45,100 (34,600–58,000)	213 (178–258)	190 (159–235)	164 (127–205)
1-84	Peptic ulcer disease	14,100 (12,300–16,300)	22,700 (20,400–26,600)	26,000 (19,700–33,000)	151 (131–178)	130 (115–156)	104 (78·8–132)
1-85	Gastritis and duodenitis	3,060 (1,990–4,500)	4,940 (3,200–7,270)	5,920 (3,760–8,710)	20·8 (13·8–30)	18·9 (12·3–27·6)	19·1 (12·2–28)
1-86	GERD	5,750 (2,940–10,400)	10,900 (5,520–19,900)	13,100 (6,670–24,300)	40·8 (20·8–74·5)	40·8 (20·8–74·6)	40·8 (20·9–75·5)
1-87	Appendicitis	2,530 (1,660–3,230)	3,010 (2,160–3,880)	3,200 (2,160–4,390)	16·3 (10·8–20·3)	11·6 (8·5–14·8)	10·5 (7·2–14·3)
1-88	Paralytic ileus and intestinal obstruction	10,800 (9,010–13,300)	23,500 (17,100–26,900)	27,300 (18,500–36,200)	90·1 (74·8–108)	129 (89·8–152)	108 (73·2–141)
1-89	Inguinal, femoral, and abdominal hernia	5,500 (4,260–7,020)	11,400 (8,830–14,500)	14,200 (10,900–18,300)	50·7 (40–63·3)	55·6 (43·8–68·9)	51·9 (40·2–66·1)
1-90	Inflammatory bowel disease	612 (503–742)	1,150 (982–1,380)	1,430 (1,090–1,810)	5 (4·1–6·1)	5·1 (4·3–6·2)	4·9 (3·7–6·2)
1-91	Vascular intestinal disorders	1,540 (1,300–1,840)	4,620 (4,100–5,390)	6,490 (4,920–8,480)	16·2 (13·7–19·4)	27·5 (23·7–32·7)	26·5 (20–35)
1-92	Gallbladder and biliary diseases	9,400 (7,510–11,700)	18,500 (15,600–22,100)	23,500 (18,100–29,600)	89·3 (72·3–111)	94·6 (80·4–111)	87·7 (67·7–109)
1-93	Pancreatitis	7,810 (6,410–10,800)	14,600 (12,700–19,100)	18,300 (13,300–25,300)	67·1 (56·1–86·7)	66·4 (57·4–81)	62·6 (45·7–83·6)
1-94	Other digestive diseases	4,650 (3,620–5,650)	9,650 (8,390–11,100)	12,200 (9,150–15,500)	46·3 (36–56·4)	54·4 (45·7–63·5)	48·2 (36·4–61·8)
1-95	Neurological disorders	155,000 (72,200–280,000)	284,000 (131,000–510,000)	340,000 (162,000–595,000)	1,150 (583–1,990)	1,260 (653–2,140)	1,210 (629–2,070)
1-96	Alzheimer's disease and other dementias	21,600 (9,130–49,900)	51,600 (20,300–119,000)	69,800 (28,500–163,000)	321 (137–728)	412 (161–957)	351 (145–812)
1-97	Parkinson's disease	5,590 (4,980–6,310)	12,200 (10,900–13,700)	19,200 (15,800–23,000)	72·4 (64·4–81·9)	81 (71·8–91·9)	85·3 (70·7–102)
1-98	Idiopathic epilepsy	27,100 (13,200–47,900)	38,100 (16,700–70,900)	42,900 (18,600–76,500)	154 (74·4–269)	140 (60·8–258)	140 (60·1–250)
1-99	Multiple sclerosis	480 (370–563)	941 (785–1,060)	1,180 (890–1,520)	3·7 (2·8–4·3)	3·7 (3·1–4·1)	3·7 (2·8–4·8)
1-100	Motor neuron disease	266 (222–320)	438 (384–496)	560 (437–721)	2 (1·6–2·5)	1·7 (1·5–1·9)	1·8 (1·4–2·4)
1-101	Headache disorders	92,900 (14,400–213,000)	169,000 (27,100–388,000)	192,000 (31,600–440,000)	562 (96·5–1,260)	575 (97·2–1,330)	574 (95·6–1,320)
1-102	Migraine	85,100 (9,580–206,000)	154,000 (17,900–373,000)	175,000 (21,000–420,000)	512 (64·5–1,220)	525 (64–1,260)	524 (63·9–1,250)
1-103	Tension-type headache	7,840 (2,000–31,000)	14,200 (3,820–55,900)	16,500 (4,540–63,000)	50 (13·8–192)	50·4 (13·8–192)	50·4 (13·7–189)
1-104	Other neurological disorders	6,990 (4,390–11,000)	12,100 (7,680–18,900)	15,200 (9,450–23,100)	39·4 (26·2–59·4)	45 (29·6–68·3)	50·4 (31·6–76·7)
1-105	Mental disorders	239,000 (173,000–318,000)	445,000 (326,000–585,000)	525,000 (383,000–696,000)	1,500 (1,090–1,980)	1,560 (1,140–2,040)	1,610 (1,170–2,140)
1-106	Schizophrenia	29,800 (20,600–40,300)	57,000 (39,500–76,800)	69,400 (49,000–93,900)	193 (134–256)	199 (140–266)	205 (146–276)
1-107	Depressive disorders	78,500 (53,000–111,000)	158,000 (108,000–221,000)	199,000 (135,000–277,000)	527 (364–740)	561 (384–783)	612 (417–854)
1-108	Major depressive disorder	59,500 (39,700–83,800)	122,000 (82,700–172,000)	155,000 (105,000–220,000)	392 (264–552)	426 (291–595)	477 (323–673)
1-109	Dysthymia	19,000 (11,800–28,700)	36,300 (22,600–54,900)	43,500 (27,200–66,500)	135 (84·5–204)	135 (83·9–204)	135 (84·5–206)
1-110	Bipolar disorder	11,500 (6,660–17,800)	21,000 (12,200–33,200)	24,400 (14,300–38,300)	73·3 (43·3–114)	73·5 (43·2–114)	73·3 (43–116)
1-111	Anxiety disorders	68,700 (44,900–97,600)	127,000 (89,200–172,000)	143,000 (95,200–204,000)	422 (282–596)	441 (311–596)	440 (295–623)
1-112	Eating disorders	4,570 (2,810–6,810)	9,340 (5,670–14,000)	11,500 (7,170–17,000)	23·7 (14·5–35·2)	29·1 (17·6–43·9)	32·4 (20·2–47·8)
1-113	Anorexia nervosa	1,580 (914–2,550)	3,110 (1,760–5,030)	3,640 (2,150–5,840)	8 (4·7–12·8)	9·5 (5·5–15·4)	10·5 (6·1–16·8)

1-114	Bulimia nervosa	2,990 (1,700–4,770)	6,230 (3,570–9,830)	7,860 (4,540–12,100)	15·7 (9–24·9)	19·5 (11·2–31·1)	21·9 (12·7–33·6)
1-115	Autism spectrum disorders	9,060 (5,920–13,100)	14,200 (9,180–20,500)	15,600 (10,200–22,900)	48·8 (31·6–69·8)	49·5 (32·1–71·1)	49·6 (32·4–72·9)
1-116	Attention-deficit/hyperactivity disorder	1,040 (567–1,830)	1,620 (897–2,910)	1,650 (922–2,950)	5 (2·7–8·8)	5·2 (2·9–9·4)	5·3 (2·9–9·4)
1-117	Conduct disorder	14,200 (7,760–23,000)	20,500 (11,500–33,000)	19,500 (11,000–31,300)	64·4 (35·1–104)	66·2 (37·4–107)	66·2 (37·2–106)
1-118	Idiopathic developmental intellectual disability	6,370 (2,760–11,200)	7,200 (3,220–12,600)	5,340 (1,800–9,780)	33·6 (14·7–59·5)	24·7 (11–43·2)	17 (5·6–31·1)
1-119	Other mental disorders	15,500 (9,770–23,300)	29,500 (18,700–44,400)	35,500 (22,400–53,800)	108 (69·3–164)	109 (69·6–164)	109 (69·3–165)
1-120	Substance use disorders	33,600 (24,800–44,100)	62,100 (45,700–80,800)	77,000 (56,400–99,400)	204 (153–264)	207 (153–266)	222 (164–284)
1-121	Alcohol use disorders	13,500 (9,690–18,200)	20,500 (15,200–27,500)	24,400 (17,600–33,000)	90·8 (66·1–121)	72·4 (54·3–96·4)	72·5 (52·9–97·7)
1-122	Drug use disorders	20,100 (14,100–27,100)	41,500 (29,000–55,800)	52,700 (37,100–70,000)	113 (79·9–152)	134 (95·1–180)	149 (106–197)
1-123	Opioid use disorders	12,200 (8,230–17,000)	27,700 (18,900–37,500)	36,600 (25,400–50,300)	71·6 (49·1–97·5)	91·7 (63·7–122)	104 (73–142)
1-124	Cocaine use disorders	250 (181–353)	615 (464–802)	931 (664–1,260)	1·5 (1·1–2·1)	2·1 (1·6–2·7)	2·7 (1·9–3·7)
1-125	Amphetamine use disorders	3,450 (1,770–5,750)	6,150 (3,240–10,400)	7,040 (3,870–11,500)	17·8 (9·3–29·6)	18·6 (9·9–31·1)	19·2 (10·6–31·3)
1-126	Cannabis use disorders	1,330 (709–2,310)	2,040 (1,120–3,380)	2,230 (1,230–3,710)	7 (3·8–11·8)	6·3 (3·5–10·4)	6·3 (3·5–10·6)
1-127	Other drug use disorders	2,840 (1,660–4,480)	5,030 (3,010–8,010)	5,880 (3,600–9,130)	15·2 (9·1–23·5)	15·6 (9·5–24·6)	16·3 (10·1–25·3)
1-128	Diabetes and kidney diseases	181,000 (164,000–201,000)	319,000 (273,000–369,000)	438,000 (359,000–526,000)	1,750 (1,590–1,940)	1,520 (1,310–1,750)	1,570 (1,290–1,890)
1-129	Diabetes mellitus	106,000 (91,300–122,000)	174,000 (139,000–217,000)	245,000 (189,000–315,000)	1,070 (921–1,240)	835 (667–1,040)	880 (678–1,130)
1-130	Diabetes mellitus type 1	13,200 (10,300–15,300)	14,900 (13,300–18,200)	18,000 (14,100–23,300)	112 (85·3–132)	64·2 (57·1–77·7)	60·2 (47·5–78·4)
1-131	Diabetes mellitus type 2	92,600 (78,800–108,000)	159,000 (125,000–200,000)	227,000 (173,000–292,000)	957 (819–1,110)	771 (605–965)	819 (625–1,050)
1-132	Chronic kidney disease	75,200 (69,100–81,600)	145,000 (132,000–158,000)	193,000 (160,000–234,000)	682 (627–738)	685 (623–744)	690 (575–828)
1-133	Chronic kidney disease due to diabetes mellitus type 1	12,100 (9,020–15,600)	18,600 (13,300–24,300)	23,300 (15,800–33,300)	88 (62·4–117)	71·2 (50·1–94·5)	72·9 (48·8–105)
1-134	Chronic kidney disease due to diabetes mellitus type 2	22,200 (18,100–26,400)	45,900 (37,900–53,700)	63,600 (49,700–80,300)	232 (189–271)	233 (196–271)	235 (186–294)
1-135	Chronic kidney disease due to hypertension	21,700 (18,000–25,800)	43,800 (37,100–51,900)	59,900 (46,800–75,600)	222 (185–261)	225 (192–263)	224 (176–281)
1-136	Chronic kidney disease due to glomerulonephritis	6,530 (5,090–8,140)	12,100 (9,230–15,300)	14,900 (11,300–19,300)	44·6 (35·1–54·5)	46·8 (36·5–58)	47·7 (36·4–61·3)
1-137	Chronic kidney disease due to other and unspecified causes	12,600 (10,300–15,100)	24,600 (19,400–29,800)	31,500 (24,200–39,100)	95·7 (78·2–116)	109 (87·2–133)	111 (86·2–137)
1-138	Acute glomerulonephritis	48 (20–66)	73 (27–101)	81 (27–118)	0·5 (0·1–0·7)	0·4 (0·1–0·5)	0·3 (0·1–0·5)
1-139	Skin and subcutaneous diseases	111,000 (72,600–166,000)	194,000 (132,000–279,000)	223,000 (152,000–313,000)	653 (440–962)	739 (524–1,040)	740 (513–1,030)
1-140	Dermatitis	30,100 (17,300–48,300)	48,700 (28,400–78,700)	54,800 (31,700–87,100)	175 (102–278)	174 (101–279)	174 (101–276)
1-141	Atopic dermatitis	25,000 (13,400–41,900)	39,400 (21,300–66,800)	43,800 (23,500–74,200)	141 (75·6–236)	140 (75·7–236)	140 (75·3–236)
1-142	Contact dermatitis	4,590 (2,840–6,910)	8,410 (5,230–12,700)	9,920 (6,140–14,800)	30·8 (19·2–46)	30·9 (19·4–45·8)	30·8 (19·3–46·1)
1-143	Seborrhoeic dermatitis	539 (306–856)	919 (520–1,450)	1,060 (592–1,710)	3·4 (1·9–5·3)	3·4 (1·9–5·3)	3·4 (1·9–5·5)
1-144	Psoriasis	1,900 (1,290–2,560)	2,940 (2,030–3,970)	3,340 (2,340–4,550)	12·6 (8·7–17)	10·9 (7·5–14·7)	10·6 (7·4–14·4)
1-145	Bacterial skin diseases	4,330 (3,280–5,570)	15,100 (10,500–17,900)	20,600 (14,300–28,600)	39·5 (27·9–51·1)	74·4 (49·7–90)	74·9 (51·4–104)
1-146	Cellulitis	1,390 (861–2,270)	4,700 (2,570–5,850)	6,370 (3,500–9,360)	13·1 (7·6–20·5)	23·8 (12·6–29·8)	23·2 (12·5–34)
1-147	Pyoderma	2,950 (2,030–3,810)	10,400 (7,300–13,700)	14,300 (9,690–21,900)	26·4 (16·8–34·8)	50·6 (33·2–69·2)	51·7 (34·8–81·4)
1-148	Scabies	22,000 (12,100–34,700)	33,300 (18,300–53,000)	35,800 (19,800–57,300)	116 (64·3–183)	115 (64–184)	115 (64·4–182)
1-149	Fungal skin diseases	8,070 (3,210–17,400)	13,700 (5,430–29,700)	15,800 (6,350–34,200)	52·1 (21·1–112)	51·8 (20·8–113)	51·7 (20·8–112)
1-150	Viral skin diseases	11,000 (7,040–16,500)	15,200 (9,560–23,000)	15,700 (9,960–23,500)	53·3 (34·2–79·3)	53·4 (33·6–81)	53·4 (33·9–80·4)
1-151	Acne vulgaris	11,900 (6,990–19,400)	22,000 (13,300–35,700)	23,300 (14,000–37,100)	57·8 (34·2–94·2)	68·3 (41·5–111)	71·6 (43–114)
1-152	Alopecia areata	1,480 (923–2,230)	2,550 (1,620–3,780)	2,920 (1,850–4,370)	8·8 (5·5–13·1)	8·8 (5·6–13)	8·7 (5·6–13·1)
1-153	Pruritus	1,780 (832–3,230)	3,240 (1,540–5,910)	3,910 (1,840–7,140)	11·8 (5·6–21·1)	12·3 (5·8–22·2)	12·6 (5·9–23·1)
1-154	Urticaria	8,060 (5,210–11,500)	12,500 (8,160–18,100)	13,600 (8,790–19,400)	43·1 (28·1–61·2)	43·3 (28·4–62·2)	43·2 (28·3–62·2)
1-155	Decubitus ulcer	3,340 (2,590–4,950)	13,000 (6,500–15,200)	18,700 (8,780–25,500)	38·3 (29·7–55·3)	79·5 (39·2–93·7)	77·9 (37·7–105)
1-156	Other skin and subcutaneous diseases	6,980 (3,670–12,300)	12,200 (6,490–21,400)	14,300 (7,640–25,000)	44·7 (24·4–76·7)	46·6 (25·5–81·1)	46·8 (25·3–81·1)
1-157	Sense organ diseases	117,000 (80,700–164,000)	200,000 (137,000–286,000)	245,000 (168,000–349,000)	1,040 (729–1,440)	930 (641–1,300)	882 (609–1,250)
1-158	Blindness and vision loss	41,900 (29,600–57,600)	66,200 (45,900–92,000)	81,400 (56,200–114,000)	435 (307–590)	347 (243–474)	314 (220–435)
1-159	Glaucoma	541 (360–771)	1,050 (689–1,520)	1,350 (897–1,940)	7·3 (4·9–10·4)	7 (4·6–10)	6 (4–8·6)
1-160	Cataract	18,000 (12,700–24,500)	27,800 (19,600–37,400)	35,300 (25,200–47,800)	217 (154–292)	166 (117–223)	147 (105–198)
1-161	Age-related macular degeneration	1,720 (1,130–2,440)	2,180 (1,480–3,140)	2,840 (1,900–4,130)	21·1 (13·9–29·9)	12·7 (8·7–18·5)	11·4 (7·7–16·3)
1-162	Refraction disorders	11,000 (7,270–15,800)	17,200 (11,500–24,800)	19,000 (12,600–27,300)	76·3 (51·5–107)	68·7 (46·3–97·3)	64 (42·6–91·3)
1-163	Near vision loss	5,090 (2,320–10,100)	9,940 (4,330–20,000)	13,200 (6,050–26,200)	55·3 (25·4–108)	50·8 (22·7–99·1)	49·4 (22·8–97·9)
1-164	Other vision loss	5,620 (3,810–7,870)	8,070 (5,470–11,400)	9,630 (6,630–13,500)	57·4 (39·4–79·1)	41·9 (28·7–58·5)	36·9 (25·7–51·4)
1-165	Age-related and other hearing loss	70,500 (47,300–101,000)	125,000 (84,100–183,000)	153,000 (103,000–222,000)	573 (394–822)	546 (368–787)	531 (359–761)
1-166	Other sense organ diseases	4,560 (2,720–6,970)	8,590 (5,090–13,400)	10,700 (6,330–16,700)	35·6 (21–55·4)	36·6 (21·7–57)	36·7 (21·9–56·9)

1-167	Musculoskeletal disorders	229,000 (164,000–301,000)	458,000 (329,000–608,000)	576,000 (417,000–758,000)	1,710 (1,240–2,270)	1,790 (1,300–2,360)	1,850 (1,340–2,430)
1-168	Rheumatoid arthritis	1,660 (1,160–2,250)	3,510 (2,460–4,740)	4,540 (3,150–6,180)	12·9 (9·1–17·8)	14 (10–19)	14·7 (10·2–20)
1-169	Osteoarthritis	12,900 (6,430–26,300)	30,500 (15,000–61,300)	41,800 (20,400–84,000)	132 (65·2–267)	144 (70·8–290)	149 (73–300)
1-170	Low back pain	120,000 (84,100–160,000)	215,000 (151,000–287,000)	268,000 (190,000–355,000)	876 (619–1,170)	839 (594–1,120)	864 (612–1,150)
1-171	Neck pain	35,000 (22,900–49,900)	77,600 (51,000–114,000)	94,600 (62,800–139,000)	267 (176–377)	300 (199–439)	300 (200–437)
1-172	Gout	2,070 (1,310–3,020)	4,860 (3,010–7,000)	6,650 (4,190–9,840)	18·9 (12·1–27·3)	21·6 (13·4–31·3)	22·8 (14·3–33·6)
1-173	Other musculoskeletal disorders	57,200 (40,100–79,300)	126,000 (89,100–172,000)	161,000 (110,000–221,000)	407 (284–559)	476 (336–648)	497 (345–681)
1-174	Other non-communicable diseases	296,000 (229,000–364,000)	371,000 (297,000–462,000)	427,000 (336,000–542,000)	1,690 (1,330–2,080)	1,510 (1,220–1,850)	1,460 (1,150–1,840)
1-175	Congenital anomalies	148,000 (103,000–193,000)	111,000 (96,700–126,000)	111,000 (88,500–141,000)	648 (455–840)	444 (380–498)	404 (318–518)
1-176	Neural tube defects	14,400 (7,330–25,000)	9,460 (7,000–12,300)	7,640 (4,780–11,500)	60·7 (31·2–105)	39 (28·6–51)	28·4 (17·5–43)
1-177	Congenital heart anomalies	58,500 (36,800–85,100)	36,400 (31,700–45,800)	37,500 (27,800–51,100)	253 (162–365)	145 (126–181)	136 (101–187)
1-178	Orofacial clefts	1,410 (722–2,800)	1,230 (712–2,270)	1,230 (711–2,110)	6·4 (3·4–12·1)	4·8 (2·7–8·9)	4·3 (2·4–7·6)
1-179	Down's syndrome	2,580 (1,690–3,960)	1,570 (1,280–1,920)	1,680 (1,220–2,290)	11·4 (7·6–17·3)	6 (4·9–7·4)	5·9 (4·2–8·1)
1-180	Turner syndrome	16 (7–28)	27 (12–48)	28 (13–49)	0·1 (0–0·1)	0·1 (0–0·2)	0·1 (0–0·2)
1-181	Klinefelter syndrome	3 (1–5)	4 (2–8)	4 (2–8)	0 (0–0)	0 (0–0)	0 (0–0)
1-182	Other chromosomal abnormalities	12,200 (7,260–21,000)	14,700 (8,150–17,800)	15,300 (8,970–21,800)	51·4 (30·8–88·4)	61·2 (33·3–74·3)	57·8 (33·1–82·3)
1-183	Congenital musculoskeletal and limb anomalies	10,600 (6,950–15,400)	11,500 (8,120–15,500)	11,800 (8,270–16,100)	49·8 (33·2–71·9)	43 (30·6–56·5)	40·3 (28·4–55)
1-184	Urogenital congenital anomalies	1,680 (905–3,080)	1,940 (901–2,620)	1,790 (925–2,750)	7·3 (4–13·1)	7·7 (3·3–10·5)	6·5 (3·2–10·1)
1-185	Digestive congenital anomalies	7,460 (4,420–11,900)	5,000 (2,840–8,200)	5,190 (2,760–9,290)	31·4 (18·7–50·1)	20·7 (11·7–34·3)	19·6 (10·4–35·3)
1-186	Other congenital anomalies	39,200 (23,700–62,100)	29,600 (21,500–38,400)	29,200 (20,900–39,800)	177 (108–279)	117 (82·4–149)	105 (73·2–143)
1-187	Urinary diseases and male infertility	14,700 (12,500–17,400)	46,400 (25,300–53,500)	65,700 (33,400–85,300)	152 (127–184)	253 (129–294)	256 (124–331)
1-188	Urinary tract infections and interstitial nephritis	5,180 (3,920–6,420)	11,600 (7,920–22,000)	17,100 (9,570–37,300)	50·5 (39–63·6)	59·7 (38·7–121)	64·5 (35–145)
1-189	Urolithiasis	619 (415–860)	1,270 (810–1,880)	1,690 (1,060–2,640)	5·4 (3·5–7·4)	5·4 (3·5–8·4)	5·7 (3·5–9·3)
1-190	Benign prostatic hyperplasia	2,590 (1,510–3,980)	5,910 (3,470–9,100)	8,570 (4,960–13,200)	30·8 (17·9–46·7)	32·5 (19·1–49·7)	32·8 (19–50·3)
1-191	Male infertility	377 (135–899)	755 (262–1,840)	903 (312–2,250)	2·1 (0·8–5·1)	2·5 (0·9–6)	2·5 (0·9–6·2)
1-192	Other urinary diseases	5,940 (4,900–7,380)	26,900 (9,540–34,800)	37,400 (12,200–56,000)	62·8 (50·5–78·9)	153 (47·6–202)	151 (45·1–223)
1-193	Gynecological diseases	44,500 (30,300–63,600)	75,600 (52,100–108,000)	85,800 (58,700–122,000)	264 (182–374)	253 (175–361)	250 (171–356)
1-194	Uterine fibroids	1,440 (711–2,710)	2,770 (1,300–5,190)	3,260 (1,540–6,040)	9·5 (4·6–17·8)	9·8 (4·6–18·3)	9·7 (4·6–17·9)
1-195	Polycystic ovarian syndrome	1,790 (770–3,650)	4,990 (2,140–10,200)	6,050 (2,510–12,600)	9·8 (4·2–20)	15·8 (6·8–32·4)	17·3 (7·2–36)
1-196	Female infertility	1,230 (375–3,130)	1,850 (522–4,750)	2,110 (563–5,500)	7 (2·1–17·4)	6·2 (1·7–15·7)	5·9 (1·6–15·4)
1-197	Endometriosis	6,260 (3,620–10,100)	9,080 (5,470–14,300)	10,000 (5,850–15,900)	35·4 (20·7–56·5)	29·2 (17·6–45·9)	28·2 (16·6–44·2)
1-198	Genital prolapse	337 (168–634)	613 (298–1,170)	789 (392–1,470)	3·3 (1·6–6·1)	2·8 (1·3–5·3)	2·8 (1·4–5·1)
1-199	Premenstrual syndrome	18,200 (11,200–28,400)	30,300 (18,200–47,600)	33,000 (20,000–51,500)	101 (61·7–156)	96·9 (58·6–151)	95·1 (58·5–149)
1-200	Other gynecological diseases	15,200 (9,630–22,300)	26,000 (16,300–38,300)	30,500 (19,000–44,800)	98·9 (63·3–144)	92·7 (59–135)	90·8 (57·1–133)
1-201	Hemoglobinopathies and hemolytic anaemias	18,400 (14,200–24,000)	20,500 (15,500–27,600)	21,100 (15,800–28,400)	110 (85·8–143)	76·6 (58·5–103)	68·3 (51·9–91)
1-202	Thalassemias	2,550 (1,930–3,250)	1,510 (1,150–2,030)	1,930 (1,450–2,570)	11·4 (8·6–14·5)	5·2 (3·9–7)	6·4 (4·8–8·6)
1-203	Thalassaemias trait	5,250 (3,450–7,780)	6,760 (4,340–10,300)	7,310 (4,620–11,100)	28·6 (18·8–42·6)	23·4 (15·1–35·5)	22·4 (14·3–34)
1-204	Sickle cell disorders	26 (16–40)	15 (9–23)	20 (11–33)	0·1 (0·1–0·2)	0 (0–0·1)	0·1 (0–0·1)
1-205	Sickle cell trait	645 (407–969)	778 (478–1,170)	765 (458–1,180)	3·5 (2·2–5·2)	2·6 (1·6–3·9)	2·3 (1·4–3·5)
1-206	G6PD deficiency	2,810 (2,260–3,380)	3,640 (3,080–4,280)	2,340 (1,680–3,080)	16·2 (13·2–19·4)	12·8 (10·9–15·1)	7·1 (5·1–9·2)
1-207	G6PD trait	104 (63–157)	133 (77–212)	75 (38–138)	0·6 (0·4–0·9)	0·4 (0·2–0·7)	0·2 (0·1–0·4)
1-208	Other hemoglobinopathies and hemolytic anaemias	7,050 (5,240–9,580)	7,610 (5,490–10,500)	8,620 (6,410–11,400)	50·1 (38·3–65·9)	32·1 (23·9–42·6)	29·9 (22·6–39)
1-209	Endocrine, metabolic, blood, and immune disorders	29,000 (21,300–37,900)	50,100 (36,900–65,500)	60,000 (44,400–78,500)	200 (147–262)	195 (146–254)	194 (145–252)
1-210	Oral disorders	34,600 (21,400–52,400)	62,300 (38,800–95,000)	78,700 (47,800–121,000)	289 (179–436)	268 (170–405)	270 (166–411)
1-211	Caries of deciduous teeth	725 (315–1,480)	808 (349–1,690)	802 (337–1,660)	3·1 (1·3–6·3)	3 (1·3–6·4)	3·1 (1·3–6·3)
1-212	Caries of permanent teeth	3,620 (1,610–7,060)	6,080 (2,710–11,500)	6,510 (2,880–12,800)	20·8 (9·4–40·3)	20·6 (9·1–39)	19·8 (8·8–38·8)
1-213	Periodontal disease	4,880 (1,830–11,500)	11,100 (4,160–25,900)	14,100 (5,320–32,600)	41·9 (15·8–95·5)	46·2 (17·6–109)	46·5 (17·6–107)
1-214	Edentulism	16,600 (10,700–24,200)	29,300 (19,300–41,900)	40,200 (25,500–59,600)	170 (110–248)	145 (96·3–206)	148 (93·9–217)
1-215	Other oral disorders	8,850 (5,550–13,100)	15,000 (9,300–22,200)	17,100 (10,600–24,900)	53·1 (32·9–77·8)	53·1 (32·8–78·3)	53·1 (33–77·4)
1-216	Sudden infant death syndrome	6,880 (3,490–12,300)	4,430 (2,180–8,870)	4,610 (2,030–9,180)	29 (14·7–51·9)	18·6 (9·1–37·2)	17·4 (7·7–34·7)
1-0	Injuries	550,000 (513,000–590,000)	738,000 (686,000–801,000)	832,000 (696,000–982,000)	3,390 (3,150–3,650)	2,670 (2,470–2,920)	2,590 (2,170–3,050)
1-1	Transport injuries	288,000 (260,000–313,000)	408,000 (366,000–441,000)	454,000 (369,000–549,000)	1,750 (1,580–1,910)	1,430 (1,280–1,550)	1,380 (1,130–1,680)
1-2	Road injuries	261,000 (237,000–286,000)	371,000 (332,000–400,000)	413,000 (337,000–499,000)	1,590 (1,440–1,730)	1,300 (1,160–1,410)	1,260 (1,030–1,520)

1·3	Pedestrian road injuries	60,600 (49,300–76,300)	74,600 (66,300–92,500)	80,900 (62,500–106,000)	398 (327–489)	289 (259–350)	262 (205–341)
1·4	Cyclist road injuries	11,800 (9,970–14,100)	17,700 (15,300–20,600)	21,300 (16,800–26,800)	79·1 (66·9–95·1)	66·5 (57·5–77·6)	66·9 (52·5–84)
1·5	Motorcyclist road injuries	74,200 (59,700–87,800)	105,000 (83,600–121,000)	116,000 (88,900–147,000)	427 (347–506)	348 (282–400)	344 (265–432)
1·6	Motor vehicle road injuries	109,000 (90,000–126,000)	165,000 (137,000–182,000)	185,000 (145,000–230,000)	652 (544–752)	571 (476–628)	558 (439–690)
1·7	Other road injuries	5,320 (4,060–6,870)	7,910 (6,580–9,270)	9,290 (6,870–12,700)	33·1 (25·4–42·2)	28·2 (23·8–32·6)	28·6 (21·1–38·8)
1·8	Other transport injuries	26,900 (21,800–32,200)	37,600 (31,800–42,700)	40,600 (30,800–51,700)	163 (134–192)	132 (111–148)	123 (94·2–157)
1·9	Unintentional injuries	188,000 (167,000–207,000)	217,000 (193,000–245,000)	251,000 (208,000–299,000)	1,170 (1,050–1,300)	850 (754–961)	825 (684–985)
1·10	Falls	41,500 (35,200–48,600)	67,500 (57,000–79,400)	87,800 (70,900–106,000)	324 (271–378)	299 (248–351)	305 (245–370)
1·11	Drowning	45,800 (36,500–55,700)	42,600 (36,600–46,800)	42,200 (32,500–53,900)	232 (190–278)	146 (126–159)	133 (102–170)
1·12	Fire, heat, and hot substances	16,600 (13,200–20,600)	17,500 (14,400–21,800)	19,100 (15,100–24,300)	104 (82·9–127)	66·4 (54·9–81·1)	61·2 (48·6–77·5)
1·13	Poisonings	8,590 (7,080–10,400)	9,190 (7,530–10,200)	9,750 (7,410–12,700)	52 (41·7–62·1)	32·4 (26·5–35·9)	29·5 (22·7–38·2)
1·14	Poisoning by carbon monoxide	1,040 (687–1,470)	1,420 (941–1,780)	1,570 (1,040–2,200)	6·2 (4–8·7)	5·1 (3·3–6·2)	4·8 (3·2–6·6)
1·15	Poisoning by other means	7,550 (6,120–9,170)	7,770 (6,310–8,730)	8,180 (6,080–10,700)	45·8 (36·1–55·2)	27·4 (22·1–30·7)	24·7 (18·5–32)
1·16	Exposure to mechanical forces	16,800 (13,700–20,700)	21,100 (16,900–27,200)	24,600 (18,800–31,900)	110 (90·2–138)	79·5 (63·4–103)	78·1 (59·6–101)
1·17	Unintentional firearm injuries	2,080 (1,550–2,660)	2,300 (1,730–2,890)	2,560 (1,870–3,290)	13·3 (9·9–17·1)	8·4 (6·4–10·6)	8 (5·9–10·3)
1·18	Other exposure to mechanical forces	14,700 (11,800–18,400)	18,800 (15,000–24,300)	22,100 (16,700–28,800)	97·2 (78·5–122)	71·1 (56–91·7)	70·1 (53·1–91·6)
1·19	Adverse effects of medical treatment	6,690 (4,850–8,200)	6,360 (5,230–7,170)	6,810 (5,140–8,780)	46·8 (33–59·5)	26·5 (21·6–30)	23·1 (17·5–29·6)
1·20	Animal contact	4,280 (3,280–5,190)	4,690 (4,060–5,400)	5,120 (4,050–6,420)	27·9 (21·6–33)	18·5 (16–21·2)	16·9 (13·4–21·2)
1·21	Venomous animal contact	3,280 (2,450–4,030)	3,540 (3,100–3,950)	3,860 (3,020–4,910)	21·2 (16·1–25·3)	14 (12–15·6)	12·8 (10–16·4)
1·22	Non-venomous animal contact	1,000 (739–1,320)	1,160 (866–1,590)	1,260 (927–1,770)	6·7 (5–8·8)	4·5 (3·4–6·1)	4·1 (3·1–5·8)
1·23	Foreign body	18,300 (14,600–23,000)	17,600 (15,900–19,700)	20,600 (16,600–25,400)	100 (83·8–120)	72·4 (65·6–80)	71·4 (57·2–88·2)
1·24	Pulmonary aspiration and foreign body in airway	16,700 (13,200–21,100)	15,300 (13,900–16,900)	17,900 (14,100–22,700)	89·8 (74·7–109)	63·4 (57·6–70·1)	62·5 (49·1–79·2)
1·25	Foreign body in eyes	487 (209–880)	852 (385–1,520)	986 (455–1,740)	3 (1·5–5·3)	3·1 (1·5–5·4)	3·1 (1·5–5·4)
1·26	Foreign body in other body part	1,070 (806–1,430)	1,520 (1,130–1,990)	1,750 (1,300–2,290)	7·1 (5·5–9·3)	6 (4·4–7·8)	5·8 (4·3–7·5)
1·27	Environmental heat and cold exposure	1,640 (1,260–2,090)	2,090 (1,560–2,750)	2,350 (1,720–3,080)	10·5 (8·1–13·6)	7·7 (5·9–10·2)	7·5 (5·5–9·8)
1·28	Exposure to forces of nature	28 (19–40)	559 (454–693)	368 (280–486)	0·2 (0·1–0·2)	2 (1·6–2·4)	1·1 (0·9–1·5)
1·29	Other unintentional injuries	27,400 (22,800–33,900)	27,900 (23,400–33,700)	32,000 (24,800–40,000)	166 (138–204)	99·4 (84·2–120)	98·5 (76·5–122)
1·30	Self-harm and interpersonal violence	74,300 (67,700–88,600)	112,000 (102,000–137,000)	127,000 (101,000–164,000)	461 (422–533)	391 (357–470)	378 (303–484)
1·31	Self-harm	47,800 (42,100–59,700)	61,500 (54,700–84,600)	80,500 (61,500–110,000)	308 (272–368)	218 (195–290)	240 (185–326)
1·32	Self-harm by firearm	1,190 (754–2,010)	1,680 (994–2,880)	2,100 (1,040–3,170)	7·2 (4·6–11·5)	5·7 (3·3–9·3)	6·1 (3·1–9·2)
1·33	Self-harm by other specified means	46,600 (41,100–58,300)	59,800 (53,200–81,900)	78,400 (59,800–107,000)	301 (266–360)	213 (190–282)	234 (180–319)
1·34	Interpersonal violence	26,300 (23,400–29,200)	50,000 (44,300–57,400)	46,000 (36,300–59,000)	152 (135–170)	170 (152–194)	136 (108–172)
1·35	Physical violence by firearm	3,000 (2,230–4,040)	7,170 (5,620–8,850)	6,870 (4,700–10,200)	16·6 (12·5–21·7)	23·4 (18·6–28·5)	19·6 (13·5–28·8)
1·36	Physical violence by sharp object	6,200 (4,870–8,100)	11,500 (9,530–15,900)	9,750 (7,000–15,500)	35·2 (28–45·1)	38·3 (32·1–52·2)	28·3 (20·4–44·3)
1·37	Sexual violence	3,380 (2,220–4,810)	5,730 (3,780–8,130)	6,340 (4,150–9,060)	19 (12·5–27·1)	18·9 (12·4–26·8)	18·8 (12·4–26·8)
1·38	Physical violence by other means	13,700 (11,700–15,800)	25,600 (22,100–29,600)	23,000 (17,900–29,800)	80·8 (69–93·4)	89·5 (77·5–103)	68·9 (54–88·4)
1·39	Conflict and terrorism	116 (29–429)	154 (78–418)	163 (90–371)	0·6 (0·2–2·4)	0·5 (0·3–1·5)	0·5 (0·3–1·2)
1·40	Executions and police conflict	138 (105–193)	582 (225–755)	539 (219–877)	0·8 (0·6–1·1)	2 (0·7–2·5)	1·6 (0·7–2·5)

SR Table 1: All-age DALYs counts and age standardized DALYs rates per 100,000 individuals for all causes and level 1 cause groups by location for 1990, 2010, and 2019, both sexes combined, Mexico

Cause Level	Cause	All-age DALYs			Age-standardized rate of DALYs per 100,000		
		1990	2010	2019	1990	2010	2019
0-0	All causes	27,400,000 (25,100,000–29,900,000)	29,200,000 (26,200,000–32,400,000)	34,000,000 (30,100,000–38,800,000)	37,300 (34,300–40,500)	28,600 (25,800–31,700)	28,000 (24,900–31,900)
1-0	Communicable, maternal, neonatal, and nutritional disorders	9,660,000 (8,530,000–10,700,000)	4,410,000 (3,990,000–4,850,000)	3,650,000 (3,230,000–4,120,000)	9,510 (8,550–10,400)	4,030 (3,650–4,420)	3,230 (2,850–3,660)
1-1	HIV/AIDS and sexually transmitted infections	134,000 (128,000–144,000)	326,000 (312,000–346,000)	292,000 (275,000–316,000)	177 (168–192)	281 (269–299)	223 (210–241)
1-2	HIV/AIDS	115,000 (113,000–117,000)	302,000 (295,000–314,000)	265,000 (257,000–277,000)	151 (149–154)	260 (254–270)	202 (196–211)
1-3	HIV/AIDS - drug-susceptible tuberculosis	39,000 (32,500–45,700)	29,700 (19,600–43,900)	24,700 (15,800–37,200)	51·1 (42·6–60·1)	25·5 (16·9–37·7)	18·8 (12·1–28·3)
1-4	HIV/AIDS - Multidrug-resistant Tuberculosis without extensive drug resistance	61 (5–289)	2,050 (736–4,430)	1,850 (311–5,730)	0·1 (0–0·4)	1·8 (0·6–3·8)	1·4 (0·2–4·4)
1-5	HIV/AIDS - Extensively drug-resistant Tuberculosis	0 (0–0)	218 (75–491)	299 (49–951)	0 (0–0)	0·2 (0·1–0·4)	0·2 (0–0·7)
1-6	HIV/AIDS resulting in other diseases	75,600 (68,600–82,100)	271,000 (253,000–286,000)	238,000 (222,000–252,000)	100 (90·7–109)	233 (218–246)	182 (170–192)
1-7	Sexually transmitted infections excluding HIV	19,700 (14,000–29,700)	23,300 (14,100–39,400)	27,400 (16,400–46,500)	25·9 (17·6–40·5)	20·6 (12·5–35)	20·9 (12·6–35·6)
1-8	Syphilis	5,580 (4,880–6,310)	1,520 (1,350–1,720)	1,600 (1,350–1,870)	5·7 (5·1–6·3)	1·4 (1·3–1·6)	1·3 (1·1–1·5)
1-9	Chlamydial infection	1,470 (1,070–2,030)	2,250 (1,640–3,180)	2,660 (1,930–3,770)	2 (1·5–2·7)	1·9 (1·4–2·7)	2 (1·5–2·8)
1-10	Gonococcal infection	1,640 (1,440–1,900)	2,190 (1,940–2,520)	2,740 (2,240–3,370)	2·3 (2·1–2·6)	2 (1·8–2·2)	2·1 (1·7–2·6)
1-11	Trichomoniasis	5,270 (2,010–10,800)	8,640 (3,390–17,700)	9,870 (3,950–20,200)	7·6 (3–15·5)	7·5 (3–15·4)	7·5 (3–15·3)
1-12	Genital herpes	2,660 (867–6,510)	4,340 (1,400–10,700)	5,330 (1,720–13,100)	4·1 (1·3–10·2)	4 (1·3–9·8)	4·1 (1·3–10·1)
1-13	Other sexually transmitted infections	3,090 (2,360–3,990)	4,340 (3,230–5,810)	5,180 (3,810–6,950)	4·2 (3·2–5·4)	3·7 (2·8–4·9)	3·9 (2·9–5·2)
1-14	Respiratory infections and tuberculosis	2,510,000 (2,200,000–2,800,000)	1,010,000 (902,000–1,130,000)	934,000 (820,000–1,070,000)	2,680 (2,410–2,920)	956 (859–1,060)	808 (709–925)
1-15	Tuberculosis	287,000 (275,000–297,000)	103,000 (98,900–107,000)	98,600 (84,900–114,000)	454 (437–467)	98·7 (95–103)	77·9 (67·2–90·1)
1-16	Latent tuberculosis infection	0 (0–0)	0 (0–0)	0 (0–0)	0 (0–0)	0 (0–0)	0 (0–0)
1-17	Drug-susceptible tuberculosis	286,000 (274,000–297,000)	96,900 (90,600–103,000)	92,100 (74,900–109,000)	453 (436–467)	92·9 (87–98·2)	72·7 (59·2–85·8)
1-18	Multidrug-resistant tuberculosis without extensive drug resistance	378 (31–1,900)	5,430 (2,420–10,000)	5,610 (968–17,100)	0·6 (0–3)	5·2 (2·3–9·7)	4·4 (0·8–13·6)
1-19	Extensively drug-resistant tuberculosis	0 (0–0)	572 (225–1,140)	893 (149–2,690)	0 (0–0)	0·6 (0·2–1·1)	0·7 (0·1–2·1)
1-20	Lower respiratory infections	2,030,000 (1,750,000–2,280,000)	754,000 (670,000–853,000)	685,000 (597,000–788,000)	2,030 (1,790–2,250)	727 (652–815)	607 (527–706)
1-21	Upper respiratory infections	151,000 (113,000–201,000)	116,000 (74,400–174,000)	114,000 (70,800–175,000)	151 (113–204)	99·6 (64·7–149)	92·3 (57·9–141)
1-22	Otitis media	36,400 (23,200–56,400)	36,100 (20,700–59,700)	37,000 (21,500–60,800)	37·3 (23·7–58)	30·7 (17·7–50·5)	30·3 (17·6–49·6)
1-23	Enteric infections	2,090,000 (1,770,000–2,380,000)	332,000 (281,000–392,000)	280,000 (236,000–334,000)	2,030 (1,760–2,280)	308 (263–361)	248 (208–294)
1-24	Diarrhoeal diseases	2,050,000 (1,740,000–2,340,000)	327,000 (278,000–386,000)	276,000 (233,000–329,000)	1,980 (1,730–2,230)	304 (260–356)	244 (205–291)
1-25	Typhoid and paratyphoid	25,500 (9,780–32,700)	2,930 (1,660–12,800)	2,420 (1,360–10,400)	29·5 (12·4–36·6)	2·8 (1·6–12·1)	2 (1·1–8·7)
1-26	Typhoid fever	25,000 (9,610–32,200)	2,890 (1,630–12,700)	2,390 (1,340–10,400)	28·9 (12·1–36)	2·7 (1·6–12·1)	2 (1·1–8·6)
1-27	Paratyphoid fever	473 (156–600)	36 (24–80)	29 (20–69)	0·6 (0·2–0·7)	0 (0–0·1)	0 (0–0·1)
1-28	iNTS	11,400 (5,780–13,500)	1,150 (448–5,320)	1,080 (423–4,710)	12·5 (5·5–14·7)	1 (0·4–4·6)	0·9 (0·4–4·1)
1-29	Other intestinal infectious diseases	1,790 (576–3,340)	742 (331–1,230)	567 (300–907)	1·6 (0·6–2·9)	0·6 (0·3–1·1)	0·5 (0·2–0·8)
1-30	Neglected tropical diseases and malaria	154,000 (115,000–198,000)	137,000 (101,000–182,000)	161,000 (117,000–212,000)	208 (156–265)	132 (96·6–175)	131 (95·9–171)
1-31	Malaria	3,150 (2,440–4,060)	251 (204–308)	110 (82–151)	3·5 (2·8–4·4)	0·2 (0·2–0·3)	0·1 (0·1–0·1)
1-32	Chagas disease	6,930 (4,480–9,990)	9,130 (6,060–12,900)	12,500 (8,300–17,500)	12·6 (8·3–17·9)	9·4 (6·2–13·1)	10·2 (6·8–14·3)
1-33	Leishmaniasis	280 (28–1,340)	231 (38–1,220)	192 (80–635)	0·3 (0–1·3)	0·2 (0–1)	0·2 (0·1–0·5)
1-34	Visceral leishmaniasis	144 (0–1,210)	144 (0–1,140)	64 (0–508)	0·1 (0–1·1)	0·1 (0–0·9)	0·1 (0–0·4)
1-35	Cutaneous and mucocutaneous leishmaniasis	136 (17–414)	87 (27–209)	127 (74–206)	0·2 (0–0·5)	0·1 (0–0·2)	0·1 (0·1–0·2)
1-36	African trypanosomiasis	0 (0–0)	0 (0–0)	0 (0–0)	0 (0–0)	0 (0–0)	0 (0–0)
1-37	Schistosomiasis	0 (0–0)	0 (0–0)	0 (0–0)	0 (0–0)	0 (0–0)	0 (0–0)
1-38	Cysticercosis	62,000 (39,300–90,100)	80,200 (49,600–116,000)	103,000 (65,300–149,000)	110 (70·1–158)	81·2 (51·1–116)	83·2 (53–120)
1-39	Cystic echinococcosis	41 (19–96)	23 (12–45)	63 (39–99)	0·1 (0–0·1)	0 (0–0)	0·1 (0–0·1)
1-40	Lymphatic filariasis	0 (0–0)	0 (0–0)	0 (0–0)	0 (0–0)	0 (0–0)	0 (0–0)
1-41	Onchocerciasis	0 (0–0)	0 (0–0)	0 (0–0)	0 (0–0)	0 (0–0)	0 (0–0)
1-42	Trachoma	371 (163–682)	578 (374–846)	768 (505–1,110)	0·8 (0·4–1·6)	0·5 (0·4–0·8)	0·6 (0·4–0·9)
1-43	Dengue	4,360 (2,740–6,800)	8,590 (5,550–11,700)	10,500 (7,700–13,800)	5·2 (3·4–7·7)	7·6 (4·9–10·2)	8·4 (6·1–11·1)
1-44	Yellow fever	0 (0–0)	0 (0–0)	0 (0–0)	0 (0–0)	0 (0–0)	0 (0–0)
1-45	Rabies	3,180 (2,860–3,530)	26 (24–29)	12 (10–14)	3·2 (3–3·5)	0 (0–0)	0 (0–0)
1-46	Intestinal nematode infections	40,400 (23,700–64,100)	24,600 (14,400–39,000)	19,800 (11,000–33,200)	40·5 (23·7–64·3)	20·6 (12–32·7)	16 (8·9–26·8)
1-47	Ascariasis	9,100 (5,970–14,600)	11,200 (5,980–19,400)	8,580 (4,410–15,500)	8·8 (5·7–14·2)	9·6 (5·1–16·5)	7·1 (3·7–12·8)
1-48	Trichuriasis	24,700 (12,600–41,700)	5,930 (2,830–10,600)	6,380 (3,000–12,000)	24·7 (12·6–41·7)	4·9 (2·3–8·8)	5·1 (2·4–9·6)

1-49	Hookworm disease	6,630 (3,760–11,000)	7,470 (4,260–12,200)	4,820 (2,710–8,230)	7 (4–11·6)	6·2 (3·5–10·1)	3·8 (2·1–6·5)
1-50	Food-borne trematodiases	0 (0–0)	0 (0–0)	0 (0–0)	0 (0–0)	0 (0–0)	0 (0–0)
1-51	Leprosy	27 (16–40)	36 (22–54)	45 (27–68)	0 (0–0·1)	0 (0–0·1)	0 (0–0·1)
1-52	Ebola virus disease	0 (0–0)	0 (0–0)	0 (0–0)	0 (0–0)	0 (0–0)	0 (0–0)
1-53	Zika virus disease	0 (0–0)	0 (0–0)	5 (3–11)	0 (0–0)	0 (0–0)	0 (0–0)
1-54	Guinea worm disease	0 (0–0)	0 (0–0)	0 (0–0)	0 (0–0)	0 (0–0)	0 (0–0)
1-55	Other neglected tropical diseases	33,500 (17,600–43,100)	13,400 (9,020–21,900)	14,300 (10,300–20,700)	32 (16·8–41)	11·7 (7·9–19·1)	12·4 (8·9–18·5)
1-56	Other infectious diseases	348,000 (301,000–391,000)	162,000 (141,000–189,000)	147,000 (125,000–177,000)	332 (290–369)	144 (126–168)	125 (106–151)
1-57	Meningitis	135,000 (118,000–154,000)	31,700 (28,000–35,900)	28,100 (24,100–32,500)	126 (111–142)	28·2 (24·9–31·8)	23·6 (20·1–27·4)
1-58	Encephalitis	54,100 (45,100–62,100)	59,400 (48,800–69,400)	53,000 (44,300–62,100)	51·2 (43·8–58·4)	52 (42·5–60·7)	44·9 (37·4–53·1)
1-59	Diphtheria	366 (299–439)	14 (12–16)	17 (14–22)	0·3 (0·3–0·4)	0 (0–0)	0 (0–0)
1-60	Whooping cough	26,300 (20,300–41,300)	5,110 (3,300–15,800)	5,720 (3,660–13,700)	22·4 (17·3–34·9)	4·5 (2·9–14)	5·4 (3·5–13·1)
1-61	Tetanus	31,900 (25,200–38,400)	1,040 (797–2,780)	673 (356–2,620)	33·2 (25·8–41·3)	1 (0·7–2·7)	0·6 (0·3–2·2)
1-62	Measles	21,800 (15,500–30,600)	0 (0–0)	1 (0–1)	18·6 (13·2–26)	0 (0–0)	0 (0–0)
1-63	Varicella and herpes zoster	13,700 (8,860–19,900)	12,300 (8,030–16,600)	9,750 (7,300–15,000)	13·8 (9·4–20·1)	11·1 (7·4–15)	8·4 (6·3–12·9)
1-64	Acute hepatitis	19,700 (16,800–23,000)	17,800 (15,300–20,900)	14,800 (12,700–17,600)	19·2 (16·7–22)	16 (13·8–18·6)	12·5 (10·7–14·8)
1-65	Acute hepatitis A	14,300 (11,800–17,200)	11,600 (9,200–14,300)	8,480 (6,700–10,700)	13·5 (11·2–16·1)	10·2 (8·1–12·6)	7·1 (5·7–9)
1-66	Acute hepatitis B	1,800 (1,290–2,860)	3,640 (1,850–4,750)	3,610 (1,920–4,840)	1·9 (1·4–2·9)	3·4 (1·7–4·4)	3·1 (1·6–4·2)
1-67	Acute hepatitis C	2,240 (987–3,320)	1,720 (1,230–3,590)	1,900 (1,240–3,160)	2·5 (1·2–3·6)	1·6 (1·2–3·4)	1·6 (1–2·6)
1-68	Acute hepatitis E	1,350 (586–1,760)	814 (593–1,330)	834 (606–1,220)	1·3 (0·6–1·6)	0·7 (0·5–1·2)	0·7 (0·5–1)
1-69	Other unspecified infectious diseases	44,400 (29,300–54,200)	34,700 (26,800–51,100)	34,900 (25,900–52,000)	47 (31·4–57)	31·2 (24·2–46·1)	29·9 (22–45)
1-70	Maternal and neonatal disorders	3,560,000 (2,860,000–4,300,000)	2,010,000 (1,740,000–2,280,000)	1,430,000 (1,180,000–1,760,000)	3,050 (2,470–3,670)	1,800 (1,560–2,040)	1,350 (1,090–1,660)
1-71	Maternal disorders	123,000 (117,000–132,000)	85,600 (80,400–91,800)	73,600 (60,200–89,600)	141 (134–150)	68·7 (64·6–73·6)	55·5 (45·4–67·5)
1-72	Maternal haemorrhage	35,200 (30,200–41,000)	18,900 (16,000–21,900)	14,500 (11,300–18,200)	40·9 (35·2–47·2)	15·3 (12·9–17·7)	10·9 (8·5–13·7)
1-73	Maternal sepsis and other pregnancy related infections	8,240 (6,560–10,200)	4,210 (3,150–5,520)	3,760 (2,570–5,470)	9·1 (7·3–11·2)	3·4 (2·5–4·4)	2·8 (1·9–4·1)
1-74	Maternal hypertensive disorders	27,500 (24,300–31,000)	19,900 (17,800–22,100)	15,600 (12,200–19,200)	30·5 (27·1–34·3)	15·9 (14·3–17·7)	11·7 (9·2–14·5)
1-75	Maternal obstructed labour and uterine rupture	4,730 (3,760–5,990)	3,630 (2,880–4,630)	3,340 (2,460–4,430)	5·4 (4·3–6·8)	2·9 (2·3–3·7)	2·5 (1·9–3·3)
1-76	Maternal abortive outcome	8,770 (6,980–11,000)	4,490 (3,540–5,540)	3,610 (2,690–4,780)	10·1 (8·1–12·7)	3·6 (2·8–4·4)	2·7 (2–3·6)
1-77	Ectopic pregnancy	2,580 (2,200–3,030)	1,490 (1,270–1,740)	1,410 (1,080–1,820)	2·9 (2·5–3·4)	1·2 (1–1·4)	1·1 (0·8–1·4)
1-78	Indirect maternal deaths	3,970 (3,260–4,710)	15,300 (13,400–17,600)	13,800 (10,700–17,800)	4·4 (3·6–5·2)	12·2 (10·7–14)	10·4 (8–13·4)
1-79	Late maternal deaths	6,260 (4,820–8,230)	4,810 (4,050–5,770)	6,170 (4,190–8,820)	7·2 (5·6–9·3)	3·9 (3·3–4·6)	4·7 (3·2–6·6)
1-80	Maternal deaths aggravated by HIV/AIDS	14 (7–23)	35 (18–55)	41 (20–68)	0 (0–0)	0 (0–0)	0 (0–0·1)
1-81	Other maternal disorders	26,300 (22,400–30,800)	12,900 (10,800–15,200)	11,400 (8,640–14,600)	30·4 (26·1–35·5)	10·3 (8·7–12·2)	8·6 (6·5–11)
1-82	Neonatal disorders	3,430,000 (2,740,000–4,180,000)	1,920,000 (1,660,000–2,190,000)	1,360,000 (1,090,000–1,680,000)	2,910 (2,330–3,540)	1,730 (1,490–1,970)	1,300 (1,030–1,610)
1-83	Neonatal preterm birth	1,680,000 (1,340,000–2,050,000)	925,000 (792,000–1,060,000)	585,000 (470,000–717,000)	1,420 (1,140–1,730)	832 (711–950)	559 (444–688)
1-84	Neonatal encephalopathy due to birth asphyxia and trauma	977,000 (765,000–1,210,000)	355,000 (301,000–412,000)	247,000 (191,000–312,000)	823 (645–1,010)	320 (271–372)	239 (184–305)
1-85	Neonatal sepsis and other neonatal infections	408,000 (326,000–492,000)	424,000 (360,000–505,000)	365,000 (294,000–452,000)	351 (278–427)	378 (322–450)	342 (277–425)
1-86	Hemolytic disease and other neonatal jaundice	59,100 (48,800–70,200)	32,600 (27,300–38,000)	28,200 (22,900–33,900)	52·9 (44–62·3)	28·4 (23·9–33·1)	23·7 (19·5–28·3)
1-87	Other neonatal disorders	309,000 (250,000–371,000)	188,000 (162,000–217,000)	135,000 (105,000–172,000)	260 (210–312)	169 (147–196)	133 (104–170)
1-88	Nutritional deficiencies	881,000 (756,000–1,010,000)	437,000 (361,000–535,000)	403,000 (327,000–503,000)	1,040 (910–1,170)	416 (348–502)	346 (283–429)
1-89	Protein-energy malnutrition	581,000 (484,000–666,000)	237,000 (210,000–269,000)	205,000 (179,000–233,000)	678 (560–756)	234 (209–263)	180 (157–206)
1-90	Iodine deficiency	8,850 (4,610–15,800)	10,600 (4,830–19,900)	12,900 (6,380–23,500)	10·7 (5·6–18·7)	9 (4·1–16·9)	10 (4·9–18·2)
1-91	Vitamin A deficiency	20,200 (13,100–29,200)	12,900 (8,440–18,800)	11,100 (7,360–16,200)	18·2 (11·9–26·2)	11 (7·2–16)	9·5 (6·3–13·8)
1-92	Dietary iron deficiency	170,000 (113,000–247,000)	143,000 (93,600–210,000)	141,000 (93,700–205,000)	189 (125–274)	128 (84·3–188)	118 (78·5–172)
1-93	Other nutritional deficiencies	100,000 (85,600–137,000)	33,900 (26,700–45,400)	33,900 (25,700–44,300)	140 (120–219)	33·2 (26·4–45·4)	28 (21·4–36·1)
1-0	Non-communicable diseases	13,400,000 (11,800,000–15,200,000)	20,400,000 (17,900,000–23,200,000)	25,500,000 (22,100,000–29,400,000)	22,500 (20,100–25,100)	20,700 (18,400–23,400)	21,000 (18,300–24,200)
1-1	Neoplasms	1,480,000 (1,440,000–1,520,000)	2,240,000 (2,170,000–2,310,000)	2,870,000 (2,500,000–3,270,000)	2,720 (2,640–2,790)	2,350 (2,260–2,420)	2,370 (2,070–2,690)
1-2	Lip and oral cavity cancer	12,000 (11,700–12,200)	19,800 (19,300–20,300)	27,200 (23,400–31,200)	25·5 (24·8–26·1)	21·8 (21–22·3)	22·6 (19·4–25·8)
1-3	Nasopharynx cancer	2,820 (2,760–2,870)	4,150 (4,050–4,230)	5,190 (4,350–6,110)	5·1 (5–5·2)	4·2 (4·1–4·3)	4·2 (3·5–4·9)
1-4	Other pharynx cancer	3,470 (3,370–3,560)	5,000 (4,850–5,140)	6,870 (5,740–8,090)	7·6 (7·3–7·8)	5·5 (5·4–5·7)	5·7 (4·8–6·7)
1-5	Oesophageal cancer	19,900 (19,400–20,400)	29,700 (28,800–30,400)	40,500 (33,900–47,700)	44·8 (43·3–46)	33·3 (32·1–34·1)	33·8 (28·4–39·7)
1-6	Stomach cancer	139,000 (136,000–142,000)	196,000 (190,000–200,000)	245,000 (211,000–283,000)	305 (296–312)	214 (206–219)	203 (174–234)
1-7	Colon and rectum cancer	66,800 (65,100–68,100)	166,000 (161,000–169,000)	260,000 (224,000–299,000)	142 (138–146)	181 (176–185)	215 (185–247)

1-8	Liver cancer	27,900 (27,000–28,800)	69,500 (65,900–72,700)	98,000 (84,100–112,000)	56 (54·3–57·5)	77·6 (73·7–81)	82·7 (71·1–94·6)
1-9	Liver cancer due to hepatitis B	4,480 (3,820–5,190)	9,690 (8,130–11,500)	13,500 (10,800–16,700)	8·6 (7·2–10·1)	10·1 (8·4–12·2)	10·9 (8·7–13·6)
1-10	Liver cancer due to hepatitis C	9,010 (8,070–9,950)	25,600 (22,800–28,300)	35,500 (29,500–42,400)	21·5 (19·3–23·7)	30·2 (27–33·3)	30·5 (25·5–36·5)
1-11	Liver cancer due to alcohol use	6,510 (5,660–7,420)	19,100 (16,500–21,700)	29,900 (23,800–36,900)	14·6 (12·7–16·7)	21·7 (18·7–24·7)	25·1 (20·1–30·9)
1-12	Liver cancer due to NASH	2,050 (1,780–2,360)	6,410 (5,470–7,430)	9,560 (7,860–11,700)	4·4 (3·8–5·1)	7·2 (6·1–8·4)	8·1 (6·6–9·9)
1-13	Gallbladder and biliary tract cancer	44,500 (32,000–47,400)	51,900 (47,900–65,800)	66,200 (54,900–87,500)	101 (72·8–108)	59·1 (54·2–74·9)	55·6 (46·2–73·3)
1-14	Pancreatic cancer	56,800 (55,200–58,200)	109,000 (105,000–112,000)	160,000 (137,000–185,000)	127 (123–131)	123 (118–126)	134 (114–154)
1-15	Larynx cancer	18,300 (17,600–19,100)	20,800 (20,100–21,900)	25,500 (21,300–30,700)	41·7 (40·1–43·5)	23·9 (23–25·1)	21·6 (18–25·9)
1-16	Tracheal, bronchus, and lung cancer	147,000 (144,000–150,000)	189,000 (182,000–192,000)	247,000 (211,000–285,000)	330 (321–337)	215 (207–220)	208 (178–241)
1-17	Malignant skin melanoma	7,140 (6,150–10,400)	16,400 (12,400–21,000)	23,600 (17,100–31,900)	13·4 (11·4–19·8)	17·1 (12·9–21·8)	19·2 (13·9–25·9)
1-18	Non-melanoma skin cancer	11,400 (10,600–11,700)	18,700 (17,600–19,200)	23,900 (20,400–27,700)	25·5 (23·8–26·2)	21·4 (20–22·2)	20·7 (17·7–23·9)
1-19	Non-melanoma skin cancer (squamous-cell carcinoma)	11,400 (10,600–11,700)	18,600 (17,600–19,200)	23,800 (20,400–27,600)	25·5 (23·8–26·2)	21·4 (19·9–22·2)	20·7 (17·6–23·9)
1-20	Non-melanoma skin cancer (basal-cell carcinoma)	7 (3–13)	14 (6–27)	19 (8–36)	0 (0–0)	0 (0–0)	0 (0–0)
1-21	Breast cancer	95,900 (93,200–98,700)	192,000 (187,000–198,000)	256,000 (213,000–313,000)	184 (179–189)	195 (189–201)	203 (169–247)
1-22	Cervical cancer	181,000 (156,000–190,000)	165,000 (158,000–215,000)	189,000 (151,000–255,000)	349 (293–366)	167 (159–214)	149 (120–201)
1-23	Uterine cancer	8,990 (8,710–9,270)	15,900 (15,300–16,500)	25,600 (21,200–30,900)	19·7 (19·1–20·4)	17·4 (16·8–18·1)	20·9 (17·4–25·2)
1-24	Ovarian cancer	33,300 (32,300–34,400)	74,700 (71,500–77,100)	102,000 (81,400–126,000)	62·5 (60·5–65·6)	76·2 (73–78·8)	80·9 (64·9–100)
1-25	Prostate cancer	56,000 (45,900–73,100)	119,000 (96,900–158,000)	162,000 (123,000–219,000)	148 (123–195)	149 (122–198)	146 (112–198)
1-26	Testicular cancer	16,500 (14,500–29,700)	30,000 (27,700–42,500)	36,600 (28,500–49,800)	19·1 (17·3–30·3)	25 (23–36·2)	28·3 (22–40·1)
1-27	Kidney cancer	33,200 (31,700–35,100)	65,300 (63,300–67,500)	94,000 (79,400–110,000)	58·5 (56·5–60·4)	69·9 (67·7–72)	77·5 (65·6–90·3)
1-28	Bladder cancer	12,900 (12,400–13,400)	24,700 (23,600–25,600)	33,700 (28,700–39,200)	30·8 (29·4–32)	29 (27·6–30·2)	29·2 (24·9–33·8)
1-29	Brain and nervous system cancer	59,800 (53,800–80,800)	92,300 (68,300–102,000)	105,000 (77,500–123,000)	77·9 (71·2–106)	86·5 (64·2–95)	85·2 (62·2–100)
1-30	Thyroid cancer	8,670 (8,380–8,980)	16,800 (16,000–17,400)	24,000 (20,700–27,700)	18·1 (17·4–18·7)	18·5 (17·5–19·1)	19·9 (17·3–23)
1-31	Mesothelioma	3,350 (2,930–3,800)	7,860 (6,940–8,210)	9,670 (8,050–11,500)	6·7 (5·8–7·5)	8·3 (7·4–8·7)	7·9 (6·5–9·3)
1-32	Hodgkin lymphoma	24,400 (21,400–28,600)	20,200 (18,500–26,300)	22,600 (18,600–30,000)	33·4 (29·3–39·3)	19 (17·2–24·5)	18 (14·9–23·7)
1-33	Non-Hodgkin's lymphoma	53,900 (51,000–57,200)	85,300 (82,900–88,200)	103,000 (89,700–118,000)	78·1 (75·2–81·3)	85 (82·5–87·8)	84·1 (73·1–95·8)
1-34	Multiple myeloma	10,700 (9,380–11,300)	25,300 (20,900–26,200)	37,600 (28,900–44,800)	23·2 (20·8–25·2)	27·9 (23·2–28·9)	30·9 (24–36·8)
1-35	Leukaemia	200,000 (186,000–214,000)	240,000 (220,000–261,000)	239,000 (210,000–269,000)	219 (207–233)	211 (195–229)	194 (171–218)
1-36	Acute lymphoid leukaemia	90,500 (81,100–101,000)	130,000 (106,000–145,000)	120,000 (95,600–137,000)	86·5 (78·5–95·6)	109 (88·9–123)	98·2 (78·1–111)
1-37	Chronic lymphoid leukaemia	1,950 (1,750–2,080)	4,290 (3,970–4,980)	5,860 (4,780–7,490)	4·3 (3·8–4·6)	4·9 (4·5–5·7)	5 (4·1–6·4)
1-38	Acute myeloid leukaemia	36,200 (30,900–40,700)	56,400 (49,700–65,900)	61,500 (51,800–75,400)	42·4 (37·5–47·9)	50·4 (44·4–58·6)	49·5 (41·8–61)
1-39	Chronic myeloid leukaemia	9,190 (8,500–9,670)	9,930 (9,380–12,200)	10,900 (9,010–13,700)	13·8 (12·8–14·4)	9·6 (9·1–11·6)	8·7 (7·2–10·9)
1-40	Other leukaemia	61,800 (54,600–67,300)	40,100 (36,700–51,200)	40,200 (33,700–52,400)	72·1 (63·8–76·8)	37·1 (34·2–46·8)	32·7 (27·5–42·5)
1-41	Other neoplasms	11,900 (9,360–17,200)	19,300 (16,000–26,300)	26,100 (21,000–35,800)	18·3 (14·5–27·4)	20 (16·7–27·5)	22 (17·8–30·2)
1-42	Myelodysplastic, myeloproliferative, and other hematopoietic neoplasms	11,900 (9,360–17,200)	19,300 (16,000–26,300)	26,100 (21,000–35,800)	18·3 (14·5–27·4)	20 (16·7–27·5)	22 (17·8–30·2)
1-43	Benign and in situ intestinal neoplasms	0 (0–0)	0 (0–0)	0 (0–0)	0 (0–0)	0 (0–0)	0 (0–0)
1-44	Benign and in situ cervical and uterine neoplasms	0 (0–0)	0 (0–0)	0 (0–0)	0 (0–0)	0 (0–0)	0 (0–0)
1-45	Other benign and in situ neoplasms	0 (0–0)	0 (0–0)	0 (0–0)	0 (0–0)	0 (0–0)	0 (0–0)
1-46	Cardiovascular diseases	1,700,000 (1,630,000–1,750,000)	2,580,000 (2,460,000–2,670,000)	3,430,000 (3,000,000–3,890,000)	3,800 (3,630–3,920)	2,960 (2,800–3,060)	2,970 (2,610–3,360)
1-47	Rheumatic heart disease	85,100 (78,000–94,300)	51,100 (43,100–61,900)	51,100 (41,400–64,400)	136 (126–146)	49 (42·2–58)	40·6 (33·1–51·1)
1-48	Ischaemic heart disease	822,000 (793,000–843,000)	1,450,000 (1,380,000–1,490,000)	2,020,000 (1,750,000–2,310,000)	1,980 (1,880–2,040)	1,690 (1,590–1,740)	1,750 (1,520–2,000)
1-49	Stroke	549,000 (525,000–571,000)	698,000 (661,000–732,000)	860,000 (752,000–974,000)	1,190 (1,130–1,240)	787 (743–824)	738 (646–834)
1-50	Ischaemic stroke	243,000 (227,000–258,000)	301,000 (277,000–323,000)	381,000 (334,000–434,000)	605 (562–638)	368 (338–393)	343 (302–390)
1-51	Intracerebral hemorrhage	235,000 (225,000–243,000)	276,000 (264,000–294,000)	333,000 (286,000–384,000)	471 (451–485)	300 (287–317)	277 (239–319)
1-52	Subarachnoid hemorrhage	70,900 (67,400–78,100)	121,000 (112,000–126,000)	146,000 (123,000–169,000)	114 (108–132)	119 (110–125)	118 (98·9–135)
1-53	Hypertensive heart disease	73,000 (54,900–78,000)	108,000 (94,300–125,000)	151,000 (126,000–178,000)	190 (141–204)	134 (115–152)	137 (113–160)
1-54	Non-rheumatic valvular heart disease	10,500 (10,200–10,900)	17,400 (16,800–18,000)	25,500 (21,300–29,700)	20·9 (20·1–21·7)	19 (18·2–19·8)	21·3 (17·9–24·8)
1-55	Non-rheumatic calcific aortic valve disease	6,740 (6,280–7,600)	13,000 (11,800–13,900)	19,400 (16,100–23,200)	13·7 (12·7–15·5)	14·4 (13–15·4)	16·3 (13·5–19·4)
1-56	Non-rheumatic degenerative mitral valve disease	3,060 (2,490–3,780)	3,980 (3,120–4,910)	5,560 (4,020–7,370)	6 (4·8–7·4)	4·3 (3·3–5·2)	4·6 (3·3–6)
1-57	Other non-rheumatic valve diseases	698 (249–996)	401 (245–864)	492 (257–1,080)	1·2 (0·4–1·8)	0·4 (0·2–0·9)	0·4 (0·2–0·9)
1-58	Cardiomyopathy and myocarditis	29,000 (26,400–36,000)	52,100 (38,500–59,200)	53,900 (42,200–62,900)	40·1 (36·9–53·3)	49·7 (38·4–55·8)	45·7 (35·7–53·3)
1-59	Myocarditis	3,260 (2,730–4,710)	5,090 (3,980–7,770)	5,600 (4,180–7,450)	3·8 (3·2–5·2)	4·7 (3·6–7)	4·8 (3·6–6·4)
1-60	Alcoholic cardiomyopathy	2,580 (2,130–3,340)	3,370 (2,900–4,380)	4,660 (3,510–6,460)	4·7 (3·9–6·1)	3·4 (2·9–4·3)	3·7 (2·8–5·1)

1·61	Other cardiomyopathy	23,100 (20,800–28,900)	43,600 (30,100–49,900)	43,600 (33,000–51,500)	31·6 (28·7–42·9)	41·7 (30·4–47·3)	37·2 (28·2–44·1)
1·62	Atrial fibrillation and flutter	31,800 (26,900–38,700)	68,600 (58,000–83,900)	99,100 (81,800–123,000)	90·1 (76·6–110)	87·9 (74·2–108)	91·9 (75·8–114)
1·63	Aortic aneurysm	9,250 (8,970–9,560)	15,800 (15,100–16,300)	22,700 (18,700–26,900)	20·3 (19·5–21)	18 (17·1–18·6)	19·4 (16–22·9)
1·64	Peripheral vascular disease	6,010 (3,510–9,510)	11,400 (6,930–18,500)	16,400 (9,550–27,800)	16·5 (9·7–25·9)	14·5 (8·8–23·6)	15·1 (8·8–25·6)
1·65	Endocarditis	8,470 (7,210–11,800)	15,700 (11,000–17,800)	16,800 (12,600–19,900)	11 (9·4–16·5)	14·8 (10·5–16·7)	13·8 (10·3–16·3)
1·66	Chronic respiratory diseases	539,000 (478,000–618,000)	739,000 (670,000–818,000)	922,000 (806,000–1,050,000)	1,040 (956–1,130)	825 (753–904)	808 (707–919)
1·67	Chronic obstructive pulmonary disease	274,000 (251,000–290,000)	474,000 (437,000–514,000)	623,000 (541,000–716,000)	669 (610–707)	574 (526–622)	560 (488–641)
1·68	Pneumoconiosis	5,670 (5,010–6,420)	5,290 (4,500–6,260)	6,920 (5,580–8,530)	12·6 (11·2–14·3)	5·9 (5–6·9)	5·8 (4·7–7·1)
1·69	Silicosis	4,470 (3,950–5,100)	3,090 (2,580–3,740)	4,180 (3,250–5,310)	10·1 (9–11·5)	3·4 (2·9–4·2)	3·5 (2·7–4·4)
1·70	Asbestosis	246 (146–281)	381 (275–442)	434 (334–551)	0·5 (0·3–0·6)	0·4 (0·3–0·5)	0·4 (0·3–0·5)
1·71	Coal workers pneumoconiosis	235 (199–265)	286 (250–343)	482 (384–591)	0·5 (0·4–0·6)	0·3 (0·3–0·4)	0·4 (0·3–0·5)
1·72	Other pneumoconiosis	722 (581–907)	1,530 (1,200–1,900)	1,830 (1,430–2,330)	1·5 (1·2–1·8)	1·7 (1·3–2·1)	1·5 (1·2–1·9)
1·73	Asthma	210,000 (160,000–281,000)	161,000 (116,000–228,000)	172,000 (120,000–250,000)	285 (232–358)	145 (106–202)	143 (100–207)
1·74	Interstitial lung disease and pulmonary sarcoidosis	22,800 (19,800–30,300)	58,000 (38,500–68,500)	76,800 (48,000–100,000)	46·4 (40·3–61)	64·3 (42·3–76·8)	65 (40·4–85·2)
1·75	Other chronic respiratory diseases	26,300 (22,500–33,600)	41,200 (34,600–48,800)	42,500 (36,500–50,100)	31·4 (27·2–38·9)	36·4 (30·6–43·1)	34·7 (29·7–40·7)
1·76	Digestive diseases	1,450,000 (1,370,000–1,560,000)	1,890,000 (1,770,000–2,050,000)	2,360,000 (2,040,000–2,710,000)	2,570 (2,450–2,720)	1,920 (1,800–2,060)	1,910 (1,660–2,190)
1·77	Cirrhosis and other chronic liver diseases	846,000 (825,000–861,000)	1,070,000 (1,050,000–1,100,000)	1,390,000 (1,170,000–1,640,000)	1,550 (1,520–1,580)	1,090 (1,060–1,120)	1,110 (933–1,310)
1·78	Cirrhosis and other chronic liver diseases due to hepatitis B	48,000 (42,700–53,500)	35,300 (31,400–39,700)	51,700 (39,400–66,700)	86 (76·9–95·5)	34·7 (30·9–38·8)	40·3 (30·7–51·6)
1·79	Cirrhosis and other chronic liver diseases due to hepatitis C	244,000 (218,000–272,000)	296,000 (263,000–331,000)	383,000 (306,000–476,000)	443 (397–495)	293 (261–330)	301 (241–374)
1·80	Cirrhosis and other chronic liver diseases due to alcohol use	284,000 (256,000–313,000)	392,000 (352,000–434,000)	508,000 (408,000–623,000)	553 (499–610)	403 (363–447)	404 (325–495)
1·81	Cirrhosis and other chronic liver diseases due to NAFLD	132,000 (97,900–171,000)	206,000 (152,000–265,000)	263,000 (187,000–353,000)	259 (192–338)	218 (163–280)	213 (152–283)
1·82	Cirrhosis and other chronic liver diseases due to other causes	137,000 (114,000–163,000)	139,000 (112,000–174,000)	186,000 (138,000–244,000)	210 (172–261)	141 (111–179)	152 (114–198)
1·83	Upper digestive system diseases	198,000 (157,000–262,000)	230,000 (163,000–337,000)	273,000 (190,000–405,000)	368 (303–472)	232 (169–333)	221 (156–324)
1·84	Peptic ulcer disease	89,500 (85,500–92,600)	65,000 (61,000–68,300)	76,500 (64,800–88,400)	190 (180–196)	74·5 (69·2–78·7)	66·1 (56·2–76·3)
1·85	Gastritis and duodenitis	29,600 (25,000–35,600)	31,800 (27,800–37,000)	38,300 (32,300–46,700)	55·7 (48·1–65·1)	34·5 (30·3–39·7)	32·3 (27·3–39·2)
1·86	GERD	79,100 (41,600–140,000)	133,000 (69,700–237,000)	158,000 (82,600–284,000)	123 (64–220)	123 (64·2–221)	123 (64·2–221)
1·87	Appendicitis	27,000 (23,400–36,800)	34,100 (26,800–39,000)	40,100 (30,000–47,500)	35·8 (30·3–47·1)	32·3 (25·2–36·9)	32·6 (24·3–38·5)
1·88	Paralytic ileus and intestinal obstruction	93,300 (79,300–105,000)	101,000 (84,800–115,000)	104,000 (82,500–121,000)	113 (99·3–128)	101 (84·9–114)	91·6 (73·2–107)
1·89	Inguinal, femoral, and abdominal hernia	71,400 (54,500–91,700)	77,600 (60,700–97,500)	96,000 (74,300–123,000)	116 (91·3–145)	79·3 (62·8–98·6)	79·7 (62·3–101)
1·90	Inflammatory bowel disease	9,620 (8,180–11,400)	16,000 (13,700–18,600)	21,100 (17,400–25,300)	16·1 (13·6–19·1)	16·1 (13·8–18·6)	17·1 (14·1–20·4)
1·91	Vascular intestinal disorders	21,500 (20,500–22,300)	34,500 (32,600–36,300)	47,200 (40,500–54,800)	47·7 (45·3–49·6)	40·9 (38·3–43·3)	41·4 (35·4–48)
1·92	Gallbladder and biliary diseases	111,000 (83,300–148,000)	197,000 (146,000–266,000)	218,000 (166,000–290,000)	194 (150–254)	195 (147–258)	176 (135–231)
1·93	Pancreatitis	43,500 (41,000–45,700)	65,400 (62,300–68,800)	82,900 (70,500–98,300)	71·5 (68·4–76·5)	63·4 (60·7–66·8)	65·7 (55·9–77·8)
1·94	Other digestive diseases	33,200 (26,900–45,100)	64,300 (46,900–77,400)	89,000 (63,500–113,000)	56·6 (46·5–76·5)	69·2 (49·5–82·3)	75·3 (53·6–94·9)
1·95	Neurological disorders	965,000 (570,000–1,540,000)	1,370,000 (793,000–2,190,000)	1,640,000 (960,000–2,660,000)	1,420 (865–2,200)	1,330 (790–2,130)	1,360 (815–2,190)
1·96	Alzheimer's disease and other dementias	108,000 (43,300–251,000)	237,000 (95,100–542,000)	340,000 (136,000–794,000)	336 (133–789)	320 (130–736)	330 (132–763)
1·97	Parkinson's disease	26,100 (24,200–27,800)	58,800 (54,100–63,100)	83,500 (72,700–94,800)	71·1 (65·9–75·3)	75·3 (69–80·7)	77·1 (66·9–87·5)
1·98	Idiopathic epilepsy	307,000 (227,000–420,000)	297,000 (212,000–404,000)	332,000 (238,000–450,000)	356 (264–476)	262 (186–355)	266 (191–360)
1·99	Multiple sclerosis	4,680 (3,710–7,190)	12,100 (9,940–14,200)	15,400 (12,700–18,900)	8 (6·2–12·6)	11·6 (9·5–13·6)	12 (9·8–14·7)
1·100	Motor neuron disease	5,280 (4,950–5,640)	10,500 (10,100–10,900)	16,300 (13,700–18,900)	8 (7·7–8·4)	10·5 (10·1–10·9)	13·2 (11·1–15·2)
1·101	Headache disorders	446,000 (80,500–977,000)	666,000 (132,000–1,450,000)	746,000 (155,000–1,620,000)	563 (119–1,230)	574 (120–1,240)	573 (119–1,240)
1·102	Migraine	405,000 (54,600–944,000)	603,000 (87,000–1,380,000)	674,000 (102,000–1,530,000)	507 (78·4–1,150)	518 (79·1–1,180)	517 (79–1,170)
1·103	Tension-type headache	40,600 (11,500–164,000)	62,800 (18,800–228,000)	72,400 (21,900–252,000)	55·6 (17–200)	55·9 (16·9–195)	56 (16·8–195)
1·104	Other neurological disorders	68,000 (52,400–89,000)	89,100 (70,900–112,000)	108,000 (85,500–136,000)	75·5 (59·5–96·9)	78·4 (63–97·4)	86·8 (68·7–110)
1·105	Mental disorders	1,130,000 (820,000–1,480,000)	1,880,000 (1,370,000–2,460,000)	2,140,000 (1,570,000–2,800,000)	1,490 (1,090–1,940)	1,660 (1,220–2,170)	1,650 (1,210–2,170)
1·106	Schizophrenia	122,000 (89,200–156,000)	204,000 (149,000–259,000)	238,000 (173,000–304,000)	180 (132–229)	181 (132–230)	181 (132–231)
1·107	Depressive disorders	361,000 (250,000–492,000)	678,000 (471,000–922,000)	815,000 (569,000–1,110,000)	527 (370–717)	618 (431–837)	631 (441–856)
1·108	Major depressive disorder	301,000 (205,000–417,000)	582,000 (400,000–800,000)	702,000 (480,000–971,000)	440 (303–603)	531 (367–729)	544 (374–750)
1·109	Dysthymia	59,600 (38,500–87,700)	96,400 (62,800–142,000)	113,000 (74,000–166,000)	86·9 (56·8–128)	87·1 (57–128)	87 (57–128)
1·110	Bipolar disorder	153,000 (92,100–238,000)	229,000 (140,000–353,000)	259,000 (159,000–397,000)	198 (121–304)	198 (122–302)	198 (121–303)
1·111	Anxiety disorders	216,000 (151,000–296,000)	408,000 (285,000–561,000)	434,000 (305,000–596,000)	274 (194–377)	352 (247–482)	336 (235–459)
1·112	Eating disorders	44,400 (27,000–67,100)	63,500 (38,900–95,900)	68,300 (41,900–103,000)	48·1 (29·7–72·2)	50·9 (31–76·9)	51·6 (31·7–77·6)
1·113	Anorexia nervosa	10,500 (6,080–17,200)	14,200 (8,280–22,600)	15,600 (9,060–25,000)	10·7 (6·2–17)	11·2 (6·6–17·7)	11·8 (6·9–19)

1-114	Bulimia nervosa	33,900 (19,700–54,500)	49,400 (28,800–78,500)	52,700 (30,200–84,100)	37·5 (21·9–59·4)	39·7 (23·1–63)	39·8 (22·8–63·3)
1-115	Autism spectrum disorders	51,600 (33,900–74,200)	66,100 (43,200–96,200)	70,700 (46,200–102,000)	56·6 (37·1–81·6)	56·7 (37·1–82·3)	56·8 (37·1–82)
1-116	Attention-deficit/hyperactivity disorder	14,000 (7,770–24,200)	16,400 (8,940–28,200)	17,200 (9,480–29,200)	13·4 (7·4–22·8)	13·2 (7·2–22·7)	13·6 (7·5–23·2)
1-117	Conduct disorder	87,000 (48,600–138,000)	93,500 (52,500–148,000)	90,800 (51,500–143,000)	73·3 (40·9–116)	73·5 (41·1–117)	73·6 (41·5–116)
1-118	Idiopathic developmental intellectual disability	14,000 (4,170–26,300)	17,300 (5,740–31,500)	16,400 (4,800–30,600)	15·4 (4·7–28·7)	14·7 (4·9–26·8)	13·1 (3·8–24·4)
1-119	Other mental disorders	62,400 (39,800–94,700)	107,000 (68,600–163,000)	128,000 (82,200–195,000)	99·8 (63·8–152)	99·8 (64·1–152)	99·7 (64–152)
1-120	Substance use disorders	397,000 (334,000–468,000)	436,000 (351,000–531,000)	502,000 (406,000–618,000)	593 (508–689)	387 (313–468)	384 (311–472)
1-121	Alcohol use disorders	305,000 (263,000–356,000)	299,000 (247,000–365,000)	335,000 (276,000–417,000)	481 (424–552)	272 (226–329)	258 (212–320)
1-122	Drug use disorders	92,400 (67,500–121,000)	137,000 (99,700–177,000)	167,000 (124,000–217,000)	112 (81·9–145)	115 (84·1–148)	127 (93·9–164)
1-123	Opioid use disorders	34,200 (23,500–48,200)	55,800 (38,400–76,200)	67,300 (47,200–91,100)	44·8 (31·2–60·6)	48·8 (33·9–65·8)	51·5 (36·1–69·5)
1-124	Cocaine use disorders	25,300 (17,600–35,100)	35,300 (24,600–49,400)	40,000 (28,300–55,200)	29·3 (21–40)	28·9 (20·3–40·1)	30·3 (21·5–41·8)
1-125	Amphetamine use disorders	8,260 (4,850–13,300)	11,200 (6,860–17,600)	17,400 (10,300–28,100)	9·5 (5·7–15·1)	9·1 (5·7–14·3)	13·1 (7·7–21·1)
1-126	Cannabis use disorders	5,290 (2,970–8,880)	6,810 (4,060–10,700)	7,590 (4,530–11,800)	5·8 (3·4–9·5)	5·5 (3·3–8·6)	5·7 (3·4–8·9)
1-127	Other drug use disorders	19,400 (13,000–27,100)	27,600 (18,400–38,400)	34,300 (23,600–47,900)	22·5 (15·3–30·8)	22·6 (15·2–31·4)	26 (17·9–36·2)
1-128	Diabetes and kidney diseases	1,480,000 (1,340,000–1,630,000)	3,510,000 (3,200,000–3,850,000)	4,950,000 (4,320,000–5,660,000)	3,090 (2,800–3,400)	3,830 (3,500–4,200)	4,100 (3,580–4,680)
1-129	Diabetes mellitus	1,030,000 (922,000–1,160,000)	1,910,000 (1,660,000–2,200,000)	2,800,000 (2,390,000–3,290,000)	2,250 (2,000–2,520)	2,120 (1,850–2,430)	2,330 (2,000–2,730)
1-130	Diabetes mellitus type 1	56,000 (48,000–71,700)	71,500 (58,100–87,200)	85,500 (67,500–112,000)	78·3 (66·8–104)	64·9 (53·3–81·9)	67·2 (53·5–87·6)
1-131	Diabetes mellitus type 2	978,000 (868,000–1,100,000)	1,840,000 (1,590,000–2,130,000)	2,720,000 (2,320,000–3,200,000)	2,170 (1,930–2,440)	2,050 (1,790–2,360)	2,260 (1,940–2,660)
1-132	Chronic kidney disease	448,000 (415,000–485,000)	1,600,000 (1,510,000–1,700,000)	2,140,000 (1,870,000–2,440,000)	841 (783–909)	1,720 (1,620–1,810)	1,770 (1,540–2,010)
1-133	Chronic kidney disease due to diabetes mellitus type 1	31,400 (22,800–41,300)	120,000 (81,000–166,000)	159,000 (102,000–227,000)	52·3 (36·6–70·6)	117 (77·8–165)	123 (79·2–176)
1-134	Chronic kidney disease due to diabetes mellitus type 2	80,000 (65,600–94,200)	351,000 (284,000–416,000)	487,000 (384,000–605,000)	186 (156–218)	402 (328–475)	411 (325–508)
1-135	Chronic kidney disease due to hypertension	73,400 (62,400–86,200)	312,000 (262,000–367,000)	430,000 (346,000–528,000)	170 (145–197)	358 (299–418)	366 (294–448)
1-136	Chronic kidney disease due to glomerulonephritis	118,000 (102,000–134,000)	388,000 (324,000–461,000)	506,000 (404,000–629,000)	190 (163–221)	393 (324–476)	406 (324–507)
1-137	Chronic kidney disease due to other and unspecified causes	145,000 (123,000–168,000)	430,000 (350,000–523,000)	560,000 (445,000–695,000)	243 (202–285)	447 (359–547)	460 (366–569)
1-138	Acute glomerulonephritis	1,530 (1,420–1,660)	1,090 (1,040–1,140)	6,270 (5,380–7,210)	2·3 (2·2–2·5)	1·1 (1·1–1·2)	5·3 (4·5–6·1)
1-139	Skin and subcutaneous diseases	398,000 (262,000–591,000)	543,000 (358,000–809,000)	594,000 (395,000–882,000)	461 (309–680)	481 (320–720)	484 (324–720)
1-140	Dermatitis	89,400 (52,700–141,000)	113,000 (68,100–177,000)	121,000 (72,800–189,000)	98·5 (59·3–154)	98·4 (59·7–154)	98·6 (59·4–154)
1-141	Atopic dermatitis	66,300 (35,800–111,000)	76,000 (40,700–128,000)	77,200 (41,800–129,000)	64·4 (34·7–108)	64·3 (34·5–108)	64·5 (34·9–108)
1-142	Contact dermatitis	20,500 (12,800–30,800)	33,100 (20,300–49,300)	39,200 (24,200–58,600)	30·7 (19·1–46)	30·7 (19·1–46·1)	30·7 (19·1–46·1)
1-143	Seborrhoeic dermatitis	2,580 (1,460–4,130)	3,750 (2,120–6,030)	4,250 (2,400–6,770)	3·4 (1·9–5·4)	3·4 (1·9–5·4)	3·4 (1·9–5·4)
1-144	Psoriasis	10,000 (7,100–13,400)	13,700 (9,640–18,100)	15,100 (10,600–20,100)	13·5 (9·6–17·9)	12·3 (8·7–16·4)	11·9 (8·4–15·8)
1-145	Bacterial skin diseases	17,000 (11,800–24,500)	31,700 (20,600–47,100)	51,100 (28,500–70,200)	24·1 (16·6–36·3)	32·3 (20·8–48·3)	42·6 (23·8–58·9)
1-146	Cellulitis	7,550 (5,030–11,800)	12,600 (7,640–19,600)	17,900 (9,540–25,400)	11·4 (7·5–19·1)	12·7 (7·6–20)	14·6 (7·8–20·8)
1-147	Pyoderma	9,480 (6,240–14,800)	19,100 (12,000–29,800)	33,200 (17,500–47,600)	12·7 (8·2–20)	19·6 (12·2–30·5)	28 (14·5–40·3)
1-148	Scabies	66,900 (36,500–107,000)	104,000 (56,900–165,000)	95,600 (52,100–152,000)	70·8 (38·9–113)	88 (48·6–140)	77 (42·3–122)
1-149	Fungal skin diseases	19,200 (7,610–41,100)	27,900 (11,200–59,700)	35,900 (14,500–76,800)	27·3 (11–58·3)	26·9 (10·9–57·9)	29·7 (12–63·7)
1-150	Viral skin diseases	45,100 (28,600–67,800)	50,900 (32,300–76,300)	51,800 (32,900–77,400)	42·9 (27·2–64·3)	42·9 (27·2–64·4)	43·5 (27·6–65·3)
1-151	Acne vulgaris	44,400 (26,700–72,000)	58,200 (35,000–93,700)	61,000 (36,800–98,300)	40 (24·1–64·6)	45·4 (27·3–72·9)	47·3 (28·4–76·1)
1-152	Alopecia areata	5,610 (3,530–8,340)	8,290 (5,250–12,400)	9,280 (5,840–13,700)	7·2 (4·5–10·6)	7·2 (4·6–10·7)	7·2 (4·5–10·6)
1-153	Pruritus	6,670 (3,190–11,800)	10,300 (4,930–18,400)	12,200 (5,830–21,800)	9·6 (4·6–17·2)	9·8 (4·7–17·6)	9·8 (4·7–17·6)
1-154	Urticaria	48,100 (31,400–70,900)	59,300 (38,700–86,000)	62,200 (40,500–90,200)	51 (33·4–73·8)	51·1 (33·4–74·1)	51 (33·3–74·2)
1-155	Decubitus ulcer	11,500 (7,630–17,800)	13,300 (10,200–23,100)	16,500 (11,700–26,300)	25·9 (17·6–39·6)	15·9 (12·3–26·6)	14·8 (10·8–23·4)
1-156	Other skin and subcutaneous diseases	33,800 (17,500–60,100)	52,900 (27,200–94,500)	62,800 (31,900–112,000)	50·2 (26·1–89·4)	50·9 (26·3–90·8)	50·8 (25·8–90·4)
1-157	Sense organ diseases	474,000 (324,000–666,000)	795,000 (543,000–1,130,000)	991,000 (674,000–1,400,000)	922 (635–1,280)	854 (584–1,210)	832 (568–1,180)
1-158	Blindness and vision loss	163,000 (113,000–225,000)	256,000 (175,000–358,000)	314,000 (216,000–438,000)	336 (237–460)	284 (196–393)	269 (185–374)
1-159	Glaucoma	5,760 (3,950–8,040)	9,040 (6,290–12,500)	11,700 (8,110–16,200)	16·2 (11·1–22·4)	11·7 (8·1–16·1)	10·9 (7·6–15)
1-160	Cataract	36,000 (25,700–48,600)	60,300 (42,900–81,700)	76,100 (54,000–104,000)	94·5 (67·8–128)	74·9 (53·5–102)	69 (49·1–94·7)
1-161	Age-related macular degeneration	2,200 (1,510–3,200)	3,850 (2,630–5,490)	5,160 (3,530–7,380)	5·9 (4–8·4)	4·9 (3·3–6·9)	4·7 (3·2–6·7)
1-162	Refraction disorders	68,000 (46,000–96,000)	99,600 (67,500–139,000)	114,000 (77,000–158,000)	107 (73·3–148)	96·6 (65·7–134)	91·8 (62·3–128)
1-163	Near vision loss	21,700 (10,100–43,200)	43,800 (20,500–86,100)	59,500 (28,000–117,000)	51·9 (24·2–101)	51·4 (23·8–100)	51·4 (24·2–100)
1-164	Other vision loss	28,800 (20,400–39,400)	39,900 (28,400–54,700)	48,200 (34,300–66,100)	60·6 (43·3–82·8)	44·3 (31·7–60·5)	41·4 (29·5–56·8)
1-165	Age-related and other hearing loss	286,000 (192,000–415,000)	496,000 (330,000–720,000)	624,000 (416,000–911,000)	544 (366–786)	527 (353–761)	520 (349–752)
1-166	Other sense organ diseases	25,500 (15,300–38,800)	42,700 (25,300–65,600)	52,400 (31,200–81,400)	42 (24·8–65·1)	42·9 (25·4–66·6)	43 (25·7–66·6)
[truncated: 873,117 more chars]
