# Supplementary material for: Global burden of 369 diseases and injuries in 204 countries and territories, 1990–2019: a systematic analysis for the Global Burden of Disease Study 2019
Source: Lancet. 2020 Oct 17;396(10258):1204–22. doi: 10.1016/S0140-6736(20)30925-9 (PMC7567026; doi:10.1016/S0140-6736(20)30925-9)
Supplement: Supplementary appendix 2 [file mmc2b.pdf]

THE LANCET

Supplementary appendix 2b (Part 2 of 5)

This appendix formed part of the original submission and has been peer reviewed. We post it as supplied by the authors.

Supplement to: GBD 2019 Diseases and Injuries Collaborators. Global burden of 369 diseases and injuries in 204 countries and territories, 1990–2019: a systematic analysis for the Global Burden of Disease Study 2019. *Lancet* 2020; **396:** 1204–22.

Appendix 2B: supplementary results appendix to “Global burden of 369 diseases and injuries in 204 countries and territories, 1990–2019: a systematic analysis for the Global Burden of Disease Study 2019”

This supplement provides additional figures and more detailed results for “Global burden of 369 diseases and injuries in 204 countries and territories, 1990–2019: a systematic analysis for the Global Burden of Disease Study 2019."

List of supplementary results appendix figures and tables:

Appendix 2A

Figure S1: Leading GBD Level 3 causes of age-standardised YLDs rates by location for a) females and b) males, 2019. 1

Figure S2: Leading GBD Level 3 causes of age-standardised prevalence rates by location for a) females and b) males, 2019. 3

Figure S3: Trends of age-standardised YLD rates per 100,000 for the top eight Level 3 causes of non-fatal burden in 2019 by SDI quintile for both sexes, from 1990 to 2019. 5

Figure S4: Death counts due to fatal discontinuities by type, 1980–2019. 6

Figure S5: Leading level 3 causes of all-age DALY counts by location for females in 1990 (A) males in 1990 (B) females in 2019 (C) males in 2019 (D) 10

Figure S6: Leading 20 GBD cause hierarchy Level 3 causes of YLLs by SDI grouping and sex for 1990, 2010, and 2019, with percentage change in number of YLLs, and all-age and age-standardised rates. 14

Figure S7: Global distribution of total YLDs by 23 GBD age groups for Level 2 causes of non-fatal burden in 1990 and 2019 for a) females and b) males. 24

Figure S8: Global leading 30 level 3 causes of DALYs for 1990, 2010, and 2019, with percentage change in number of DALYs, and age-standardised rates, by sex and SDI quintile 26

Table S1: All-age DALYs counts and age standardised DALY rates per 100,000 individuals for all causes and level 1 cause groups by location for 1990, 2010, 2019 , both sexes combined. 36

Appendix 2B

Table S2a: Global DALYS by cause and age group for males with 95% uncertainty intervals, by location for 2019. 4734

Appendix 2C

Table S2b: Global DALYS by cause and age group for females with 95% uncertainty intervals, by location for 2019. 6708

Appendix 2D

Table S2c: Global DALYS by cause and age group for both sexes combined with 95% uncertainty intervals, by location for 2019.   8680

Appendix 2E

Table S3: Global deaths and YLL numbers, age-standardised rates per 100,000, and percentage change between 2010 and 2019 for males for all GBD causes and Levels 1 through 4 of the cause hierarchy 10630

Table S4: Global deaths by cause and age group for females (1990, 2010, 2019) 10635

Table S5: Global deaths by cause and age group for males (1990, 2010, 2019) 10642

Table S6: Global deaths by cause and age group for both sexes combined (1990, 2010, 2019) 10648

Table S7: Global YLLs by cause and age group for females. 10654

Table S8: Global YLLs by cause and age group for males. 10656

Table S9: Global YLLs by cause and age group for both sexes combined 10658

Table S10: Deaths due to fatal discontinuities by country, all ages, for both sexes combined 10660

Table S11: Diarrhoeal diseases and lower respiratory infection aetiologies deaths, mortality rates, and annual percentage change from 1990 to 2019. 10676

Table S12: Deaths by location, cause, and age group for males. 10677

Table S13: Deaths by location, cause, and age group for females. 13007

Table S14: Deaths by location, cause, and age group for both sexes combined. 15354

Table S15: Distribution of percentage change in age-standardised deaths for Level 1 causes by location from 1990-2010 and 2010-2019, both sexes. 17543

Table S16: Global deaths and YLL numbers, age-standardised rates per 100,000, and percentage change between 2010 and 2019 for females for all GBD causes and Levels 1 through 4 of the cause hierarchy. 17561

Table S17: Global prevalence, incidence and YLDs for 2019, percentage change of YLDs counts, and percentage change of YLD rates between 2010 and 2019 by cause and by age 17566

Table S18: Prevalence, incidence, and YLDs for 2019, percentage change of YLD counts, and percentage change of age-standardised YLD rates between 2010 and 2019 by cause and GBD location. 17707

SR Table 2a: Global DALYS by cause and age group for males with 95% uncertainty intervals, 2019

Cause	Early Neonatal	Late Neonatal	Post Neonatal	1 to 4	5 to 9	10 to 14	15 to 19	20 to 24	25 to 29	30 to 34	35 to 39	40 to 44	45 to 49	50 to 54	55 to 59	60 to 64	65 to 69	70 to 74	75 to 79	80 to 84	85 to 89	90 to 94	95 plus	Total
All causes	97,500,000 (82,200,000 to 116,000,000)	23,000,000 (19,400,000 to 27,400,000)	63,500,000 (52,500,000 to 77,400,000)	68,700,000 (58,700,000 to 81,400,000)	32,200,000 (27,500,000 to 37,800,000)	29,900,000 (24,700,000 to 35,700,000)	40,400,000 (34,900,000 to 46,400,000)	49,700,000 (43,700,000 to 56,200,000)	54,500,000 (48,000,000 to 61,800,000)	61,700,000 (54,200,000 to 69,600,000)	65,100,000 (57,700,000 to 73,500,000)	69,600,000 (61,800,000 to 78,000,000)	78,400,000 (69,600,000 to 87,900,000)	88,300,000 (79,100,000 to 98,000,000)	94,200,000 (84,900,000 to 104,000,000)	97,600,000 (88,600,000 to 107,000,000)	95,600,000 (87,100,000 to 104,000,000)	83,600,000 (76,400,000 to 90,300,000)	64,800,000 (59,700,000 to 69,600,000)	48,700,000 (45,400,000 to 51,900,000)	26,900,000 (25,400,000 to 28,400,000)	9,340,000 (8,880,000 to 9,830,000)	2,430,000 (2,310,000 to 2,540,000)	1,350,000,000
Communicable, maternal, neonatal, and nutritional diseases	87,600,000 (73,900,000 to 104,000,000)	19,400,000 (16,300,000 to 23,200,000)	47,800,000 (39,300,000 to 59,500,000)	48,900,000 (41,100,000 to 59,200,000)	15,400,000 (13,000,000 to 18,000,000)	9,750,000 (8,300,000 to 11,400,000)	8,590,000 (7,420,000 to 9,940,000)	8,530,000 (7,500,000 to 9,720,000)	9,160,000 (8,180,000 to 10,300,000)	10,600,000 (9,570,000 to 11,700,000)	11,300,000 (10,300,000 to 12,400,000)	11,000,000 (9,970,000 to 12,000,000)	10,400,000 (9,420,000 to 11,400,000)	9,280,000 (8,380,000 to 10,300,000)	8,370,000 (7,560,000 to 9,340,000)	7,550,000 (6,820,000 to 8,540,000)	6,760,000 (6,100,000 to 7,730,000)	5,990,000 (5,410,000 to 6,980,000)	4,600,000 (4,160,000 to 5,310,000)	3,500,000 (3,130,000 to 4,050,000)	2,030,000 (1,790,000 to 2,320,000)	817,000 (683,000 to 933,000)	242,000 (191,000 to 279,000)	348,000,000
HIV/AIDS and sexually transmitted infections	2,230,000 (747,000 to 4,650,000)	671,000 (228,000 to 1,390,000)	1,730,000 (1,060,000 to 2,810,000)	1,360,000 (1,210,000 to 1,530,000)	542,000 (503,000 to 580,000)	623,000 (592,000 to 655,000)	983,000 (904,000 to 1,070,000)	901,000 (837,000 to 984,000)	1,680,000 (1,540,000 to 1,840,000)	2,880,000 (2,670,000 to 3,120,000)	3,640,000 (3,400,000 to 3,910,000)	3,440,000 (3,260,000 to 3,650,000)	2,810,000 (2,670,000 to 2,970,000)	1,860,000 (1,770,000 to 1,950,000)	1,040,000 (987,000 to 1,110,000)	554,000 (518,000 to 593,000)	301,000 (279,000 to 328,000)	131,000 (118,000 to 146,000)	58,900 (53,200 to 65,800)	16,900 (14,900 to 19,600)	6,190 (5,300 to 7,360)	1,630 (1,300 to 2,150)	452 (338 to 599)	27,500,000
HIV/AIDS	82·5 (57·5 to 114)	286 (195 to 390)	733,000 (642,000 to 849,000)	1,320,000 (1,180,000 to 1,480,000)	524,000 (490,000 to 560,000)	614,000 (584,000 to 645,000)	959,000 (880,000 to 1,040,000)	864,000 (804,000 to 938,000)	1,640,000 (1,500,000 to 1,790,000)	2,820,000 (2,620,000 to 3,050,000)	3,590,000 (3,360,000 to 3,850,000)	3,410,000 (3,230,000 to 3,620,000)	2,780,000 (2,640,000 to 2,930,000)	1,830,000 (1,740,000 to 1,930,000)	1,030,000 (973,000 to 1,080,000)	542,000 (508,000 to 580,000)	292,000 (272,000 to 318,000)	125,000 (113,000 to 139,000)	54,800 (49,600 to 61,200)	14,600 (12,900 to 16,600)	4,810 (4,200 to 5,540)	1,050 (898 to 1,220)	242 (209 to 281)	23,100,000
HIV/AIDS - Drug-susceptible Tuberculosis	2·2 (1·3 to 3·3)	23·2 (14·8 to 32·2)	180,000 (132,000 to 231,000)	346,000 (256,000 to 425,000)	141,000 (110,000 to 170,000)	167,000 (129,000 to 204,000)	241,000 (178,000 to 307,000)	201,000 (149,000 to 252,000)	343,000 (253,000 to 438,000)	562,000 (407,000 to 724,000)	724,000 (524,000 to 931,000)	724,000 (527,000 to 924,000)	594,000 (435,000 to 764,000)	400,000 (297,000 to 511,000)	218,000 (162,000 to 278,000)	111,000 (82,700 to 141,000)	54,100 (40,500 to 68,300)	22,900 (17,300 to 28,700)	9,810 (7,350 to 12,400)	2,200 (1,660 to 2,770)	718 (539 to 913)	150 (111 to 190)	30·6 (22·9 to 39·7)	5,040,000
HIV/AIDS - Multidrug-resistant Tuberculosis wi	0·1 (0·1 to 0·2)	1·1 (0·6 to 1·8)	21,900 (9,450 to 40,500)	40,300 (16,200 to 77,300)	14,600 (6,510 to 27,000)	16,300 (7,340 to 31,000)	24,800 (10,900 to 47,100)	19,800 (8,920 to 35,900)	34,100 (15,300 to 64,500)	57,100 (26,000 to 108,000)	71,500 (33,400 to 134,000)	69,300 (32,100 to 130,000)	55,600 (25,500 to 104,000)	37,300 (17,100 to 68,300)	20,200 (9,210 to 37,200)	10,100 (4,630 to 18,600)	4,880 (2,260 to 8,980)	2,060 (967 to 3,820)	869 (399 to 1,610)	189 (83·1 to 364)	59·7 (26·4 to 114)	12 (5·7 to 22·1)	2·4 (1·1 to 4·2)	501,000
HIV/AIDS - Extensively drug-resistant Tuberc	0 (0 to 0)	0 (0 to 0)	637 (284 to 1,190)	732 (301 to 1,420)	341 (152 to 643)	344 (152 to 643)	573 (249 to 1,090)	853 (371 to 1,630)	2,180 (1,010 to 4,090)	4,260 (2,050 to 7,730)	5,090 (2,480 to 9,160)	3,990 (1,890 to 7,340)	2,530 (1,180 to 4,770)	1,530 (705 to 2,860)	799 (370 to 1,550)	413 (185 to 801)	230 (98·1 to 475)	80·2 (31·2 to 173)	34·2 (12·9 to 72·6)	11·3 (3·5 to 28·8)	3·6 (1·2 to 8·9)	0·6 (0·2 to 1·2)	0·2 (0·1 to 0·4)	24,600
HIV/AIDS resulting in other diseases	80·2 (55·5 to 110)	261 (179 to 359)	530,000 (454,000 to 641,000)	932,000 (801,000 to 1,120,000)	369,000 (333,000 to 411,000)	430,000 (385,000 to 478,000)	692,000 (604,000 to 795,000)	642,000 (570,000 to 730,000)	1,260,000 (1,100,000 to 1,440,000)	2,200,000 (1,940,000 to 2,470,000)	2,790,000 (2,490,000 to 3,110,000)	2,610,000 (2,340,000 to 2,880,000)	2,130,000 (1,910,000 to 2,360,000)	1,390,000 (1,250,000 to 1,530,000)	786,000 (706,000 to 866,000)	420,000 (377,000 to 467,000)	233,000 (210,000 to 259,000)	99,900 (87,800 to 114,000)	44,100 (38,700 to 50,200)	12,200 (10,400 to 14,300)	4,030 (3,410 to 4,770)	888 (741 to 1,060)	209 (176 to 249)	17,600,000
Sexually transmitted infections excluding HIV	2,230,000 (747,000 to 4,650,000)	671,000 (228,000 to 1,390,000)	1,000,000 (346,000 to 2,070,000)	38,700 (13,000 to 84,400)	17,100 (5,520 to 37,500)	9,150 (6,350 to 13,400)	23,900 (15,500 to 35,800)	37,500 (23,600 to 56,600)	45,400 (28,400 to 71,200)	55,200 (33,800 to 87,200)	43,400 (25,900 to 69,000)	35,600 (21,500 to 57,200)	30,500 (18,000 to 50,900)	24,000 (14,200 to 40,900)	18,400 (10,600 to 30,700)	12,600 (7,400 to 22,000)	8,290 (4,770 to 15,500)	5,870 (3,600 to 10,400)	4,050 (2,630 to 6,780)	2,330 (1,530 to 3,790)	1,380 (851 to 2,180)	582 (310 to 1,050)	210 (108 to 345)	4,320,000
Syphilis	2,230,000 (747,000 to 4,650,000)	671,000 (228,000 to 1,390,000)	1,000,000 (346,000 to 2,070,000)	38,700 (13,000 to 84,400)	17,100 (5,520 to 37,500)	2,820 (1,850 to 3,950)	3,520 (2,270 to 4,650)	3,340 (2,100 to 5,100)	4,310 (2,900 to 6,640)	6,790 (4,040 to 10,200)	6,600 (3,920 to 9,200)	5,040 (3,390 to 6,840)	4,490 (2,930 to 6,010)	5,640 (3,280 to 7,960)	6,560 (3,510 to 9,280)	4,850 (3,230 to 6,620)	2,940 (2,280 to 4,040)	2,390 (1,860 to 3,210)	1,620 (1,280 to 2,220)	885 (686 to 1,110)	508 (361 to 678)	200 (130 to 309)	70·9 (46·3 to 98·8)	4,020,000
Chlamydial infection	0 (0 to 0)	0 (0 to 0)	0 (0 to 0)	0 (0 to 0)	0 (0 to 0)	84 (37·2 to 155)	1,560 (771 to 2,890)	5,910 (3,150 to 10,800)	11,300 (5,570 to 20,100)	13,200 (6,500 to 23,100)	10,400 (5,010 to 18,400)	8,270 (4,150 to 14,400)	5,790 (2,800 to 9,720)	2,510 (1,160 to 4,630)	643 (305 to 1,150)	111 (54·2 to 204)	30·4 (14·3 to 56·8)	0·4 (0·2 to 0·8)	0 (0 to 0)	0 (0 to 0)	0 (0 to 0)	0 (0 to 0)	0 (0 to 0)	59,900
Gonococcal infection	0 (0 to 0)	0 (0 to 0)	0 (0 to 0)	0 (0 to 0)	0 (0 to 0)	1,530 (803 to 2,740)	7,690 (4,360 to 12,200)	12,400 (7,140 to 20,100)	8,820 (4,540 to 15,400)	5,630 (2,880 to 9,830)	3,230 (1,740 to 5,630)	2,740 (1,640 to 4,490)	2,320 (1,300 to 4,010)	1,670 (912 to 2,960)	929 (532 to 1,480)	463 (282 to 660)	231 (168 to 364)	138 (98·1 to 226)	105 (73·7 to 177)	61·4 (34·5 to 88·2)	47·2 (18·1 to 81·7)	7·5 (2·2 to 16·7)	1·5 (0·8 to 2·4)	48,000
Trichomoniasis	0 (0 to 0)	0 (0 to 0)	0 (0 to 0)	0 (0 to 0)	0 (0 to 0)	5 (1·4 to 13·8)	71·4 (17·9 to 192)	375 (115 to 920)	1,030 (307 to 2,350)	1,670 (529 to 3,770)	1,770 (586 to 4,020)	1,730 (565 to 4,050)	1,530 (496 to 3,660)	1,070 (348 to 2,510)	554 (186 to 1,330)	270 (100 to 604)	184 (71·7 to 411)	108 (42·1 to 245)	56·1 (22·8 to 129)	26·4 (10·1 to 60·2)	9·1 (3·5 to 20·8)	1·9 (0·7 to 4·3)	0·2 (0·1 to 0·4)	10,400
Genital herpes	0 (0 to 0)	0 (0 to 0)	0 (0 to 0)	0 (0 to 0)	0 (0 to 0)	543 (171 to 1,300)	2,310 (770 to 5,530)	4,750 (1,570 to 11,500)	7,720 (2,660 to 18,800)	10,400 (3,450 to 25,300)	11,200 (3,630 to 27,600)	11,000 (3,580 to 27,700)	10,700 (3,400 to 27,000)	9,700 (3,020 to 23,900)	7,960 (2,420 to 19,700)	6,250 (1,890 to 15,400)	4,670 (1,400 to 11,500)	3,040 (914 to 7,620)	1,820 (546 to 4,520)	1,020 (306 to 2,550)	437 (130 to 1,090)	134 (40·4 to 337)	30·9 (9·4 to 75)	93,700
Other sexually transmitted infections	0 (0 to 0)	0 (0 to 0)	0 (0 to 0)	0 (0 to 0)	0 (0 to 0)	4,170 (2,700 to 6,370)	8,790 (5,220 to 13,600)	10,800 (6,580 to 16,800)	12,200 (7,380 to 19,500)	17,500 (10,200 to 27,000)	10,200 (5,670 to 16,500)	6,770 (4,000 to 10,900)	5,660 (3,460 to 8,530)	3,440 (2,170 to 5,240)	1,740 (964 to 2,470)	618 (375 to 886)	234 (166 to 379)	188 (133 to 307)	447 (315 to 757)	336 (189 to 483)	373 (142 to 646)	238 (95·8 to 536)	107 (31·7 to 209)	83,800
Respiratory infections and tuberculosis	5,720,000 (4,550,000 to 7,180,000)	3,510,000 (2,860,000 to 4,340,000)	14,700,000 (11,800,000 to 18,600,000)	8,920,000 (6,950,000 to 11,100,000)	2,080,000 (1,710,000 to 2,490,000)	1,360,000 (1,110,000 to 1,670,000)	1,740,000 (1,460,000 to 2,050,000)	2,570,000 (2,220,000 to 2,920,000)	3,110,000 (2,770,000 to 3,500,000)	3,590,000 (3,220,000 to 3,990,000)	3,840,000 (3,430,000 to 4,270,000)	3,870,000 (3,470,000 to 4,340,000)	4,060,000 (3,640,000 to 4,530,000)	4,120,000 (3,720,000 to 4,560,000)	4,160,000 (3,770,000 to 4,580,000)	4,020,000 (3,660,000 to 4,400,000)	3,730,000 (3,440,000 to 4,050,000)	3,430,000 (3,170,000 to 3,720,000)	2,750,000 (2,550,000 to 2,980,000)	2,240,000 (2,000,000 to 2,420,000)	1,370,000 (1,200,000 to 1,480,000)	573,000 (475,000 to 627,000)	176,000 (137,000 to 196,000)	85,700,000
Tuberculosis	0·4 (0·2 to 0·6)	12·9 (6·1 to 23·2)	1,110,000 (866,000 to 1,420,000)	1,160,000 (901,000 to 1,480,000)	362,000 (302,000 to 432,000)	301,000 (253,000 to 357,000)	774,000 (663,000 to 906,000)	1,580,000 (1,350,000 to 1,800,000)	2,100,000 (1,850,000 to 2,360,000)	2,500,000 (2,230,000 to 2,780,000)	2,660,000 (2,360,000 to 2,980,000)	2,640,000 (2,350,000 to 2,990,000)	2,700,000 (2,390,000 to 3,050,000)	2,610,000 (2,310,000 to 2,950,000)	2,470,000 (2,190,000 to 2,770,000)	2,040,000 (1,810,000 to 2,270,000)	1,700,000 (1,530,000 to 1,900,000)	1,370,000 (1,230,000 to 1,520,000)	868,000 (781,000 to 960,000)	540,000 (480,000 to 594,000)	221,000 (195,000 to 243,000)	62,000 (51,700 to 68,600)	11,400 (9,090 to 12,900)	29,800,000
Latent tuberculosis infection	0 (0 to 0)	0 (0 to 0)	0 (0 to 0)	0 (0 to 0)	[illegible]	[illegible]	[illegible]	[illegible]	[illegible]	[illegible]	[illegible]	[illegible]	[illegible]	[illegible]	[illegible]	[illegible]	[illegible]	[illegible]	[illegible]	[illegible]	[illegible]	[illegible]	[illegible]	[illegible]
Drug-susceptible tuberculosis	0·3 (0·2 to 0·6)	12·4 (5·8 to 22·4)	1,010,000 (776,000 to 1,310,000)	1,070,000 (821,000 to 1,370,000)	[illegible]	[illegible]	[illegible]	[illegible]	[illegible]	[illegible]	[illegible]	[illegible]	[illegible]	[illegible]	[illegible]	[illegible]	[illegible]	[illegible]	[illegible]	[illegible]	[illegible]	[illegible]	[illegible]	[illegible]
Multidrug-resistant tuberculosis without extens	0 (0 to 0)	0·6 (0·2 to 1·1)	92,000 (39,900 to 167,000)	86,400 (39,500 to 161,000)	[illegible]	[illegible]	[illegible]	[illegible]	[illegible]	[illegible]	[illegible]	[illegible]	[illegible]	[illegible]	[illegible]	[illegible]	[illegible]	[illegible]	[illegible]	[illegible]	[illegible]	[illegible]	[illegible]	[illegible]
Extensively drug-resistant tuberculosis	0 (0 to 0)	0 (0 to 0)	4,440 (2,090 to 7,930)	3,300 (1,550 to 5,990)	[illegible]	[illegible]	[illegible]	[illegible]	[illegible]	[illegible]	[illegible]	[illegible]	[illegible]	[illegible]	[illegible]	[illegible]	[illegible]	[illegible]	[illegible]	[illegible]	[illegible]	[illegible]	[illegible]	[illegible]
Lower respiratory infections	5,710,000 (4,530,000 to 7,160,000)	3,500,000 (2,850,000 to 4,330,000)	13,500,000 (10,700,000 to 16,900,000)	7,200,000 (5,610,000 to 9,080,000)	[illegible]	[illegible]	[illegible]	[illegible]	[illegible]	[illegible]	[illegible]	[illegible]	[illegible]	[illegible]	[illegible]	[illegible]	[illegible]	[illegible]	[illegible]	[illegible]	[illegible]	[illegible]	[illegible]	[illegible]

Anorexia nervosa	0 (0 to 0)	0 (0 to 0)	0 (0 to 0)	0 (0 to 0)	3,120 (954 to 6,850)	[illegible]
Bulimia nervosa	0 (0 to 0)	0 (0 to 0)	0 (0 to 0)	0 (0 to 0)	609 (184 to 1,410)	[illegible]
Autism spectrum disorders	1,370 (899 to 1,980)	4,020 (2,630 to 5,800)	64,400 (42,300 to 92,900)	277,000 (182,000 to 398,000)	331,000 (216,000 to 480,000)	[illegible]
Attention-deficit/hyperactivity disorder	0 (0 to 0)	0 (0 to 0)	0 (0 to 0)	12,500 (6,630 to 21,800)	128,000 (68,700 to 226,000)	[illegible]
Conduct disorder	0 (0 to 0)	0 (0 to 0)	0 (0 to 0)	0 (0 to 0)	551,000 (296,000 to 950,000)	[illegible]
Idiopathic developmental intellectual disability	785 (345 to 1,430)	2,410 (1,110 to 4,320)	44,700 (21,900 to 77,200)	224,000 (116,000 to 367,000)	292,000 (156,000 to 479,000)	[illegible]
Other mental disorders	0 (0 to 0)	0 (0 to 0)	0 (0 to 0)	0 (0 to 0)	0 (0 to 0)	[illegible]
Substance use disorders	5·4 (3·2 to 8·5)	16·3 (9·7 to 26)	278 (168 to 445)	1,410 (857 to 2,220)	1,740 (1,060 to 2,700)	[illegible]
Alcohol use disorders	5·4 (3·2 to 8·5)	16·3 (9·7 to 26)	278 (168 to 445)	1,410 (857 to 2,220)	1,740 (1,060 to 2,700)	[illegible]
Drug use disorders	0 (0 to 0)	0 (0 to 0)	0 (0 to 0)	0 (0 to 0)	0 (0 to 0)	[illegible]
Opioid use disorders	0 (0 to 0)	0 (0 to 0)	0 (0 to 0)	0 (0 to 0)	0 (0 to 0)	[illegible]
Cocaine use disorders	0 (0 to 0)	0 (0 to 0)	0 (0 to 0)	0 (0 to 0)	0 (0 to 0)	[illegible]
Amphetamine use disorders	0 (0 to 0)	0 (0 to 0)	0 (0 to 0)	0 (0 to 0)	0 (0 to 0)	[illegible]
Cannabis use disorders	0 (0 to 0)	0 (0 to 0)	0 (0 to 0)	0 (0 to 0)	0 (0 to 0)	[illegible]
Other drug use disorders	0 (0 to 0)	0 (0 to 0)	0 (0 to 0)	0 (0 to 0)	0 (0 to 0)	[illegible]
Diabetes and kidney diseases	2,540 (1,890 to 3,360)	6,430 (4,820 to 8,590)	255,000 (200,000 to 327,000)	332,000 (264,000 to 413,000)	343,000 (282,000 to 412,000)	[illegible]
Diabetes mellitus	2,530 (1,890 to 3,360)	6,400 (4,790 to 8,560)	71,400 (50,900 to 100,000)	57,000 (39,600 to 83,800)	56,400 (44,800 to 73,900)	[illegible]
Diabetes mellitus type 1	2,530 (1,890 to 3,360)	6,400 (4,790 to 8,560)	71,400 (50,900 to 100,000)	57,000 (39,600 to 83,800)	56,400 (44,800 to 73,900)	[illegible]
Diabetes mellitus type 2	0 (0 to 0)	0 (0 to 0)	0 (0 to 0)	0 (0 to 0)	0 (0 to 0)	[illegible]
Chronic kidney disease	1·8 (0·9 to 3·2)	31·8 (17·6 to 53·4)	178,000 (138,000 to 231,000)	269,000 (212,000 to 341,000)	276,000 (220,000 to 338,000)	[illegible]
Chronic kidney disease due to diabetes mellit	0 (0 to 0)	0 (0 to 0)	1,240 (643 to 2,310)	4,400 (2,850 to 6,520)	7,250 (4,460 to 11,200)	[illegible]
Chronic kidney disease due to diabetes mellit	0 (0 to 0)	0 (0 to 0)	0 (0 to 0)	0 (0 to 0)	0 (0 to 0)	[illegible]
Chronic kidney disease due to hypertension	0 (0 to 0)	0 (0 to 0)	0 (0 to 0)	0 (0 to 0)	0 (0 to 0)	[illegible]
Chronic kidney disease due to glomerulonephri	0·7 (0·3 to 1·3)	[illegible]	[illegible]	[illegible]	[illegible]	[illegible]
Chronic kidney disease due to other and unsp	1·1 (0·5 to 2)	[illegible]	[illegible]	[illegible]	[illegible]	[illegible]
Acute glomerulonephritis	0 (0 to 0)	[illegible]	[illegible]	[illegible]	[illegible]	[illegible]
Skin and subcutaneous diseases	87·6 (50·7 to 151)	[illegible]	[illegible]	[illegible]	[illegible]	[illegible]
Dermatitis	0 (0 to 0)	[illegible]	[illegible]	[illegible]	[illegible]	[illegible]
Atopic dermatitis	0 (0 to 0)	[illegible]	[illegible]	[illegible]	[illegible]	[illegible]
Contact dermatitis	0 (0 to 0)	[illegible]	[illegible]	[illegible]	[illegible]	[illegible]
Seborrhoeic dermatitis	0 (0 to 0)	[illegible]	[illegible]	[illegible]	[illegible]	[illegible]
Psoriasis	0 (0 to 0)	[illegible]	[illegible]	[illegible]	[illegible]	[illegible]
Bacterial skin diseases	13·7 (6·3 to 26·5)	[illegible]	[illegible]	[illegible]	[illegible]	[illegible]
Cellulitis	2·1 (1·4 to 3·1)	[illegible]	[illegible]	[illegible]	[illegible]	[illegible]
Pyoderma	11·5 (4·7 to 24·2)	[illegible]	[illegible]	[illegible]	[illegible]	[illegible]
Scabies	27·7 (14·1 to 48·8)	[illegible]	[illegible]	[illegible]	[illegible]	[illegible]
Fungal skin diseases	11·3 (4·3 to 25·1)	[illegible]	[illegible]	[illegible]	[illegible]	[illegible]
Viral skin diseases	0 (0 to 0)	[illegible]	[illegible]	[illegible]	[illegible]	[illegible]
Acne vulgaris	0 (0 to 0)	[illegible]	[illegible]	[illegible]	[illegible]	[illegible]
Alopecia areata	0 (0 to 0)	[illegible]	[illegible]	[illegible]	[illegible]	[illegible]
Pruritus	0·5 (0·2 to 1)	[illegible]	[illegible]	[illegible]	[illegible]	[illegible]
Urticaria	26·9 (16·1 to 42·3)	[illegible]	[illegible]	[illegible]	[illegible]	[illegible]
Decubitus ulcer	0 (0 to 0)	[illegible]	[illegible]	[illegible]	[illegible]	[illegible]
Other skin and subcutaneous diseases	7·5 (3·5 to 14·1)	[illegible]	[illegible]	[illegible]	[illegible]	[illegible]
Sense organ diseases	431 (264 to 666)	[illegible]	[illegible]	[illegible]	[illegible]	[illegible]
Blindness and vision loss	197 (104 to 329)	[illegible]	[illegible]	[illegible]	[illegible]	[illegible]
Glaucoma	0 (0 to 0)	[illegible]	[illegible]	[illegible]	[illegible]	[illegible]
Cataract	0 (0 to 0)	[illegible]	[illegible]	[illegible]	[illegible]	[illegible]
Age-related macular degeneration	0 (0 to 0)	[illegible]	[illegible]	[illegible]	[illegible]	[illegible]
Refraction disorders	10·9 (4·7 to 21·7)	[illegible]	[illegible]	[illegible]	[illegible]	[illegible]
Near vision loss	0 (0 to 0)	[illegible]	[illegible]	[illegible]	[illegible]	[illegible]
Other vision loss	186 (100 to 310)	[illegible]	[illegible]	[illegible]	[illegible]	[illegible]
Age-related and other hearing loss	142 (62·2 to 284)	[illegible]	[illegible]	[illegible]	[illegible]	[illegible]
Other sense organ diseases	90·8 (43·2 to 165)	[illegible]	[illegible]	[illegible]	[illegible]	[illegible]
Musculoskeletal disorders	0 (0 to 0)	[illegible]	[illegible]	[illegible]	[illegible]	[illegible]
Rheumatoid arthritis	0 (0 to 0)	[illegible]	[illegible]	[illegible]	[illegible]	[illegible]
Osteoarthritis	0 (0 to 0)	[illegible]	[illegible]	[illegible]	[illegible]	[illegible]
Osteoarthritis hip	0 (0 to 0)	[illegible]	[illegible]	[illegible]	[illegible]	[illegible]
Osteoarthritis knee	0 (0 to 0)	[illegible]	[illegible]	[illegible]	[illegible]	[illegible]
Osteoarthritis hand	0 (0 to 0)	[illegible]	[illegible]	[illegible]	[illegible]	[illegible]
Osteoarthritis other	0 (0 to 0)	[illegible]	[illegible]	[illegible]	[illegible]	[illegible]
Low back pain	0 (0 to 0)	[illegible]	[illegible]	[illegible]	[illegible]	[illegible]
Neck pain	0 (0 to 0)	[illegible]	[illegible]	[illegible]	[illegible]	[illegible]
Gout	0 (0 to 0)	[illegible]	[illegible]	[illegible]	[illegible]	[illegible]
Other musculoskeletal disorders	0 (0 to 0)	[illegible]	[illegible]	[illegible]	[illegible]	[illegible]
Other non-communicable diseases	8,930,000 (7,180,000 to 11,200,000)	[illegible]	[illegible]	[illegible]	[illegible]	[illegible]
Congenital birth defects	8,800,000 (7,070,000 to 11,100,000)	[illegible]	[illegible]	[illegible]	[illegible]	[illegible]
Neural tube defects	1,500,000 (987,000 to 2,220,000)	[illegible]	[illegible]	[illegible]	[illegible]	[illegible]
Congenital heart anomalies	2,640,000 (1,920,000 to 3,490,000)	[illegible]	[illegible]	[illegible]	[illegible]	[illegible]
Orofacial clefts	52,000 (27,200 to 129,000)	[illegible]	[illegible]	[illegible]	[illegible]	[illegible]
Down syndrome	139,000 (102,000 to 234,000)	[illegible]	[illegible]	[illegible]	[illegible]	[illegible]
Turner syndrome	0 (0 to 0)	[illegible]	[illegible]	[illegible]	[illegible]	[illegible]
Klinefelter syndrome	0·8 (0·3 to 1·6)	[illegible]	[illegible]	[illegible]	[illegible]	[illegible]
Other chromosomal abnormalities	337,000 (184,000 to 614,000)	[illegible]	[illegible]	[illegible]	[illegible]	[illegible]
Congenital musculoskeletal and limb anomalie	241,000 (156,000 to 363,000)	[illegible]	[illegible]	[illegible]	[illegible]	[illegible]
Urogenital congenital anomalies	316,000 (182,000 to 489,000)	[illegible]	[illegible]	[illegible]	[illegible]	[illegible]
Digestive congenital anomalies	830,000 (473,000 to 1,260,000)	[illegible]	[illegible]	[illegible]	[illegible]	[illegible]
Other congenital birth defects	2,740,000 (1,870,000 to 4,200,000)	[illegible]	[illegible]	[illegible]	[illegible]	[illegible]
Urinary diseases and male infertility	32,200 (23,000 to 44,400)	[illegible]	[illegible]	[illegible]	[illegible]	[illegible]
Urolithiasis	0 (0 to 0)	[illegible]	[illegible]	[illegible]	[illegible]	[illegible]
Benign prostatic hyperplasia	0 (0 to 0)	[illegible]	[illegible]	[illegible]	[illegible]	[illegible]
Male infertility	0 (0 to 0)	[illegible]	[illegible]	[illegible]	[illegible]	[illegible]
Other urinary diseases	24,200 (16,200 to 34,400)	[illegible]	[illegible]	[illegible]	[illegible]	[illegible]
Gynecological diseases	0 (0 to 0)	[illegible]	[illegible]	[illegible]	[illegible]	[illegible]
Uterine fibroids	0 (0 to 0)	[illegible]	[illegible]	[illegible]	[illegible]	[illegible]
Polycystic ovarian syndrome	0 (0 to 0)	[illegible]	[illegible]	[illegible]	[illegible]	[illegible]
Female infertility	0 (0 to 0)	[illegible]	[illegible]	[illegible]	[illegible]	[illegible]
Endometriosis	0 (0 to 0)	[illegible]	[illegible]	[illegible]	[illegible]	[illegible]
Genital prolapse	0 (0 to 0)	[illegible]	[illegible]	[illegible]	[illegible]	[illegible]
Premenstrual syndrome	0 (0 to 0)	[illegible]	[illegible]	[illegible]	[illegible]	[illegible]
Other gynecological diseases	0 (0 to 0)	[illegible]	[illegible]	[illegible]	[illegible]	[illegible]
Hemoglobinopathies and hemolytic anemias	38,800 (28,500 to 57,400)	[illegible]	[illegible]	[illegible]	[illegible]	[illegible]
Thalassemias	14,800 (10,900 to 22,000)	[illegible]	[illegible]	[illegible]	[illegible]	[illegible]
Thalassemias trait	806 (498 to 1,280)	[illegible]	[illegible]	[illegible]	[illegible]	[illegible]
Sickle cell disorders	20,300 (11,700 to 37,800)	[illegible]	[illegible]	[illegible]	[illegible]	[illegible]
Sickle cell trait	1,140 (711 to 1,700)	[illegible]	[illegible]	[illegible]	[illegible]	[illegible]
G6PD deficiency	779 (456 to 1,210)	[illegible]	[illegible]	[illegible]	[illegible]	[illegible]
G6PD trait	0 (0 to 0)	[illegible]	[illegible]	[illegible]	[illegible]	[illegible]
Other hemoglobinopathies and hemolytic anem	885 (643 to 1,230)	[illegible]	[illegible]	[illegible]	[illegible]	[illegible]
Endocrine, metabolic, blood, and immune disorders	60,600 (40,300 to 75,700)	[illegible]	[illegible]	[illegible]	[illegible]	[illegible]
Oral disorders	0 (0 to 0)	[illegible]	[illegible]	[illegible]	[illegible]	[illegible]
Caries of deciduous teeth	0 (0 to 0)	[illegible]	[illegible]	[illegible]	[illegible]	[illegible]
Caries of permanent teeth	0 (0 to 0)	[illegible]	[illegible]	[illegible]	[illegible]	[illegible]
Periodontal diseases	0 (0 to 0)	[illegible]	[illegible]	[illegible]	[illegible]	[illegible]
Other oral disorders	0 (0 to 0)	[illegible]	[illegible]	[illegible]	[illegible]	[illegible]
Sudden infant death syndrome	–	[illegible]	[illegible]	[illegible]	[illegible]	[illegible]
Injuries	586,000 (465,000 to 720,000)	[illegible]	[illegible]	[illegible]	[illegible]	[illegible]
Transport injuries	45,300 (29,200 to 59,800)	[illegible]	[illegible]	[illegible]	[illegible]	[illegible]
Road injuries	40,900 (26,100 to 54,000)	[illegible]	[illegible]	[illegible]	[illegible]	[illegible]
Pedestrian road injuries	15,000 (9,370 to 20,500)	[illegible]	[illegible]	[illegible]	[illegible]	[illegible]
Cyclist road injuries	0 (0 to 0)	[illegible]	[illegible]	[illegible]	[illegible]	[illegible]
Motorcyclist road injuries	4,090 (2,640 to 5,900)	[illegible]	[illegible]	[illegible]	[illegible]	[illegible]
Motor vehicle road injuries	21,300 (13,600 to 28,700)	[illegible]	[illegible]	[illegible]	[illegible]	[illegible]
Other road injuries	464 (300 to 725)	[illegible]	[illegible]	[illegible]	[illegible]	[illegible]
Other transport injuries	4,380 (2,870 to 6,020)	[illegible]	[illegible]	[illegible]	[illegible]	[illegible]
Unintentional injuries	327,000 (230,000 to 439,000)	[illegible]	[illegible]	[illegible]	[illegible]	[illegible]
Falls	46,800 (27,000 to 68,500)	[illegible]	[illegible]	[illegible]	[illegible]	[illegible]
Drowning	20,100 (14,700 to 26,800)	[illegible]	[illegible]	[illegible]	[illegible]	[illegible]
Fire, heat, and hot substances	17,000 (10,300 to 25,700)	[illegible]	[illegible]	[illegible]	[illegible]	[illegible]
Poisonings	16,800 (10,700 to 23,600)	[illegible]	[illegible]	[illegible]	[illegible]	[illegible]
Poisoning by carbon monoxide	2,820 (1,310 to 4,590)	[illegible]	[illegible]	[illegible]	[illegible]	[illegible]
Poisoning by other means	13,900 (9,090 to 19,500)	[illegible]	[illegible]	[illegible]	[illegible]	[illegible]
Exposure to mechanical forces	37,100 (13,100 to 70,300)	[illegible]	[illegible]	[illegible]	[illegible]	[illegible]
Unintentional firearm injuries	4,130 (905 to 9,350)	[illegible]	[illegible]	[illegible]	[illegible]	[illegible]
Other exposure to mechanical forces	33,000 (11,700 to 63,700)	[illegible]	[illegible]	[illegible]	[illegible]	[illegible]
Adverse effects of medical treatment	35,400 (24,900 to 49,500)	[illegible]	[illegible]	[illegible]	[illegible]	[illegible]
Animal contact	17,700 (9,880 to 25,800)	[illegible]	[illegible]	[illegible]	[illegible]	[illegible]
Venomous animal contact	14,700 (6,990 to 22,300)	[illegible]	[illegible]	[illegible]	[illegible]	[illegible]
Non-venomous animal contact	3,020 (2,270 to 4,290)	[illegible]	[illegible]	[illegible]	[illegible]	[illegible]
Foreign body	102,000 (79,400 to 127,000)	[illegible]	[illegible]	[illegible]	[illegible]	[illegible]
Pulmonary aspiration and foreign body in airw	97,800 (76,400 to 122,000)	[illegible]	[illegible]	[illegible]	[illegible]	[illegible]
Foreign body in eyes	30·1 (8·2 to 67)	[illegible]	[illegible]	[illegible]	[illegible]	[illegible]
Foreign body in other body part	4,310 (2,590 to 6,200)	[illegible]	[illegible]	[illegible]	[illegible]	[illegible]
Environmental heat and cold exposure	6,940 (1,690 to 11,700)	[illegible]	[illegible]	[illegible]	[illegible]	[illegible]
Exposure to forces of nature	981 (890 to 1,080)	[illegible]	[illegible]	[illegible]	[illegible]	[illegible]
Other unintentional injuries	26,000 (11,400 to 39,800)	[illegible]	[illegible]	[illegible]	[illegible]	[illegible]
Self-harm and interpersonal violence	214,000 (195,000 to 235,000)	[illegible]	[illegible]	[illegible]	[illegible]	[illegible]
Self-harm	0 (0 to 0)	[illegible]	[illegible]	[illegible]	[illegible]	[illegible]
Self-harm by firearm	0 (0 to 0)	[illegible]	[illegible]	[illegible]	[illegible]	[illegible]
Self-harm by other specified means	0 (0 to 0)	[illegible]	[illegible]	[illegible]	[illegible]	[illegible]
Interpersonal violence	56,100 (44,400 to 71,400)	[illegible]	[illegible]	[illegible]	[illegible]	[illegible]
Physical violence by firearm	11,500 (8,590 to 14,900)	[illegible]	[illegible]	[illegible]	[illegible]	[illegible]
Physical violence by sharp object	6,110 (3,800 to 8,470)	[illegible]	[illegible]	[illegible]	[illegible]	[illegible]
Sexual violence	0 (0 to 0)	[illegible]	[illegible]	[illegible]	[illegible]	[illegible]
Physical violence by other means	38,500 (30,400 to 48,500)	[illegible]	[illegible]	[illegible]	[illegible]	[illegible]
Conflict and terrorism	157,000 (143,000 to 173,000)	[illegible]	[illegible]	[illegible]	[illegible]	[illegible]

SR Table 2a: Lao People's Democratic Republic DALYS by cause and age group for males with 95% uncertainty intervals, 2019

Cause	Early Neonatal	Late Neonatal	Post Neonatal	1 to 4	5 to 9	10 to 14	15 to 19	20 to 24	25 to 29	30 to 34	35 to 39	40 to 44	45 to 49	50 to 54	55 to 59	60 to 64	65 to 69	70 to 74	75 to 79	80 to 84	85 to 89	90 to 94	95 plus	Total
All causes	139,000 (103,000 to 179,000)	33,200 (19,100 to 52,500)	143,000 (105,000 to 189,000)	47,300 (35,800 to 60,600)	35,800 (28,800 to 44,100)	33,000 (26,800 to 39,700)	50,000 (41,200 to 59,100)	62,500 (50,300 to 73,200)	63,200 (49,200 to 77,800)	63,400 (44,000 to 82,900)	61,200 (43,100 to 81,300)	63,600 (45,600 to 82,800)	67,400 (51,700 to 85,300)	74,900 (60,400 to 92,500)	79,900 (65,000 to 97,400)	79,200 (65,000 to 94,000)	66,300 (55,600 to 77,300)	51,500 (44,500 to 58,100)	37,200 (33,300 to 40,700)	23,300 (21,000 to 24,800)	10,500 (9,520 to 11,100)	3,260 (2,940 to 3,450)	676 (617 to 712)	1,290,000
Communicable, maternal, neonatal, and nutritional diseases	122,000 (89,300 to 158,000)	28,000 (16,400 to 44,200)	109,000 (75,000 to 148,000)	29,600 (20,800 to 40,700)	13,600 (10,100 to 18,300)	8,260 (6,360 to 10,700)	7,540 (5,690 to 10,000)	8,930 (6,540 to 12,500)	11,000 (7,340 to 20,700)	11,600 (6,750 to 27,600)	10,900 (6,250 to 26,500)	10,200 (6,070 to 23,900)	9,580 (6,070 to 19,300)	9,390 (6,310 to 15,800)	8,740 (5,980 to 13,200)	8,920 (6,290 to 12,900)	7,770 (5,550 to 11,200)	6,190 (4,550 to 8,310)	4,710 (3,560 to 6,130)	3,320 (2,510 to 4,220)	1,610 (1,240 to 2,070)	541 (410 to 685)	122 (89·5 to 155)	431,000
HIV/AIDS and sexually transmitted infections	866 (238 to 2,120)	280 (72·2 to 709)	713 (122 to 2,890)	1,290 (14·4 to 9,910)	184 (14·1 to 1,020)	159 (19·7 to 972)	411 (68·8 to 2,130)	788 (136 to 4,040)	1,770 (184 to 11,400)	2,710 (216 to 18,000)	2,610 (196 to 17,700)	2,150 (141 to 14,800)	1,550 (106 to 10,500)	923 (65·8 to 6,370)	535 (43 to 4,060)	429 (36·8 to 3,680)	339 (30 to 3,050)	165 (15·6 to 1,430)	51·2 (7·4 to 347)	14·4 (2·3 to 84·1)	5·4 (0·9 to 29·6)	1·5 (0·3 to 7·3)	0·3 (0·1 to 1·4)	17,900
HIV/AIDS	0 (0 to 0·2)	0·1 (0 to 0·6)	330 (2 to 2,440)	1,280 (8·3 to 9,910)	178 (10 to 1,010)	146 (9·8 to 964)	369 (32·1 to 2,100)	721 (84·3 to 3,970)	1,690 (120 to 11,300)	2,640 (147 to 17,900)	2,550 (137 to 17,700)	2,110 (107 to 14,800)	1,510 (76·5 to 10,500)	902 (47·3 to 6,360)	521 (29·7 to 4,050)	417 (26 to 3,660)	332 (23·3 to 3,040)	158 (10·1 to 1,420)	48 (5·1 to 344)	13·1 (1·2 to 83·2)	4·8 (0·5 to 29·1)	1·2 (0·1 to 7·1)	0·2 (0 to 1·2)	15,900
HIV/AIDS - Drug-susceptible Tuberculosis	0 (0 to 0)	0 (0 to 0)	103 (1·8 to 658)	409 (7·3 to 2,580)	66 (8·5 to 298)	52·2 (8·6 to 246)	149 (20·5 to 636)	299 (62·7 to 1,090)	613 (92·8 to 2,450)	885 (123 to 3,900)	851 (110 to 4,070)	707 (84 to 3,810)	506 (61·1 to 2,810)	305 (35·1 to 1,740)	172 (24·1 to 1,040)	133 (21·8 to 838)	96·7 (19·6 to 629)	44 (8·6 to 301)	15·9 (4·4 to 81·4)	4·4 (1 to 21·6)	1·6 (0·4 to 7·1)	0·4 (0·1 to 1·6)	0·1 (0 to 0·3)	5,410
HIV/AIDS - Multidrug-resistant Tuberculosis without exten	0 (0 to 0)	0 (0 to 0)	1·5 (0 to 13·2)	5·8 (0 to 51·4)	0·8 (0 to 5·9)	0·6 (0 to 4·7)	1·8 (0·1 to 11·1)	3·4 (0·1 to 21·3)	7 (0·2 to 50·7)	10·3 (0·2 to 74·5)	9·6 (0·2 to 67·1)	8·2 (0·1 to 54·4)	6·2 (0·1 to 42·4)	3·9 (0·1 to 26·3)	2·2 (0 to 15·6)	1·6 (0 to 12·4)	1·1 (0 to 9·2)	0·5 (0 to 4)	0·2 (0 to 1·4)	0·1 (0 to 0·4)	0 (0 to 0·1)	0 (0 to 0)	0 (0 to 0)	64·8
HIV/AIDS - Extensively drug-resistant Tuberculosis	0 (0 to 0)	0 (0 to 0)	0·3 (0 to 2·6)	1·1 (0 to 10·3)	0·2 (0 to 1·2)	0·1 (0 to 0·9)	0·3 (0 to 2·2)	0·6 (0 to 4)	1·3 (0 to 9·1)	1·9 (0 to 13·9)	1·7 (0 to 12·4)	1·5 (0 to 10·8)	1·1 (0 to 8·2)	0·7 (0 to 4·8)	0·4 (0 to 2·8)	0·3 (0 to 2·2)	0·2 (0 to 1·6)	0·1 (0 to 0·8)	0 (0 to 0·2)	0 (0 to 0·1)	0 (0 to 0)	0 (0 to 0)	0 (0 to 0)	11·7
HIV/AIDS resulting in other diseases	0 (0 to 0·2)	0·1 (0 to 0·5)	226 (0·1 to 1,860)	861 (0·2 to 7,730)	111 (0·1 to 719)	92·8 (0·1 to 689)	218 (7·9 to 1,550)	419 (13 to 2,800)	1,070 (11·4 to 9,380)	1,740 (12·9 to 14,400)	1,690 (10·2 to 13,900)	1,390 (7 to 12,000)	1,000 (4·2 to 8,150)	592 (3·3 to 4,710)	347 (0·6 to 3,050)	282 (0·5 to 2,690)	234 (0·2 to 2,450)	114 (0·1 to 1,180)	31·9 (0·1 to 267)	8·6 (0 to 58)	3·1 (0 to 21·4)	0·8 (0 to 5·3)	0·1 (0 to 0·9)	10,400
Sexually transmitted infections excluding HIV	866 (238 to 2,120)	279 (72·1 to 709)	383 (99·6 to 915)	9·2 (2·4 to 21·4)	6·3 (1·7 to 14·8)	13·7 (6·5 to 26·3)	41·7 (18·3 to 81·8)	66·3 (32·7 to 118)	75·6 (37·5 to 133)	77·1 (36·7 to 136)	60 (31·4 to 105)	39·2 (19·1 to 69·1)	31·8 (15 to 57)	21·6 (10·5 to 41·4)	14·1 (7·1 to 26·1)	11·6 (5·2 to 26)	7·4 (3·1 to 16·5)	6·7 (2·7 to 16)	3·2 (1·2 to 7·5)	1·3 (0·5 to 2·9)	0·7 (0·2 to 1·8)	0·3 (0·1 to 0·8)	0·1 (0 to 0·3)	2,020
Syphilis	866 (238 to 2,120)	279 (72·1 to 709)	383 (99·6 to 915)	9·2 (2·4 to 21·4)	6·3 (1·7 to 14·8)	2·8 (1·2 to 7·8)	2·7 (1·9 to 5·1)	2·2 (1·4 to 4)	2·4 (1·3 to 5)	2·4 (1·2 to 4·8)	6·9 (1·7 to 23·2)	1·8 (0·9 to 3·9)	3 (1 to 8·3)	3·4 (0·9 to 10·2)	3·2 (1 to 8·3)	4·8 (1·4 to 14·9)	3·2 (1 to 8·9)	3·8 (1·1 to 10·9)	1·2 (0·3 to 3·6)	0·4 (0·1 to 1·1)	0·2 (0 to 0·5)	0·1 (0 to 0·2)	0 (0 to 0)	1,590
Chlamydial infection	0 (0 to 0)	0 (0 to 0)	0 (0 to 0)	0 (0 to 0)	0 (0 to 0)	0·2 (0·1 to 0·3)	4·1 (2 to 7·6)	15·2 (4·2 to 32·7)	24·9 (9 to 50·4)	23·6 (7·3 to 47·4)	16·8 (5·3 to 33·9)	11·5 (3·7 to 22·6)	7·3 (1·9 to 16·8)	3 (0·2 to 7·5)	0·9 (0·4 to 1·5)	0·1 (0·1 to 0·2)	0 (0 to 0·1)	0 (0 to 0)	0 (0 to 0)	0 (0 to 0)	0 (0 to 0)	0 (0 to 0)	0 (0 to 0)	108
Gonococcal infection	0 (0 to 0)	0 (0 to 0)	0 (0 to 0)	0 (0 to 0)	0 (0 to 0)	3 (1·1 to 6·8)	14·1 (3·1 to 32·9)	20·2 (5·7 to 42)	13·2 (2·3 to 31·2)	7·8 (0·5 to 20)	4·8 (0·4 to 12·3)	3·3 (0·2 to 8·9)	2·9 (0·3 to 7·8)	2·1 (0·9 to 4·5)	1·1 (0·5 to 2·4)	0·7 (0·3 to 1·8)	0·5 (0·2 to 1·3)	0·4 (0·1 to 1·3)	0·2 (0 to 0·5)	0·1 (0 to 0·2)	0 (0 to 0·1)	0 (0 to 0)	0 (0 to 0)	74·5
Trichomoniasis	0 (0 to 0)	0 (0 to 0)	0 (0 to 0)	0 (0 to 0)	0 (0 to 0)	0 (0 to 0)	0·1 (0 to 0·3)	0·5 (0 to 1·4)	1·3 (0·3 to 3·2)	1·7 (0·4 to 4·1)	1·6 (0·4 to 4)	1·4 (0·4 to 3·5)	1·1 (0·3 to 2·5)	0·7 (0·2 to 1·9)	0·4 (0·1 to 1)	0·2 (0 to 0·5)	0·1 (0 to 0·3)	0·1 (0 to 0·1)	0 (0 to 0·1)	0 (0 to 0)	0 (0 to 0)	0 (0 to 0)	0 (0 to 0)	9·1
Genital herpes	0 (0 to 0)	0 (0 to 0)	0 (0 to 0)	0 (0 to 0)	0 (0 to 0)	0·8 (0·2 to 1·9)	3·5 (1 to 8·9)	7·3 (2·1 to 18·1)	11·5 (3·6 to 27·6)	14·3 (4·6 to 35·7)	13·8 (4·3 to 33·9)	12·1 (3·9 to 30·4)	10·4 (3·1 to 26·7)	8·5 (2·7 to 21·6)	6·8 (2 to 17·1)	4·9 (1·5 to 12·1)	3·1 (0·9 to 7·7)	1·8 (0·5 to 4·6)	1·1 (0·3 to 2·6)	0·5 (0·2 to 1·3)	0·2 (0·1 to 0·5)	0·1 (0 to 0·1)	0 (0 to 0)	101
Other sexually transmitted infections	0 (0 to 0)	0 (0 to 0)	0 (0 to 0)	0 (0 to 0)	0 (0 to 0)	6·9 (3 to 13·7)	17·3 (6·4 to 36·1)	20·9 (9·7 to 39·3)	22·2 (9·8 to 40·3)	27·3 (11·2 to 50·1)	16·1 (6·7 to 30·1)	9 (3·6 to 16·4)	7·1 (2·7 to 13·7)	3·9 (1·6 to 7·4)	1·7 (0·9 to 3·1)	0·9 (0·4 to 2·4)	0·5 (0·2 to 1·4)	0·6 (0·2 to 1·8)	0·7 (0·2 to 2·2)	0·3 (0·1 to 1·1)	0·3 (0·1 to 1)	0·1 (0 to 0·6)	0·1 (0 to 0·2)	136
Respiratory infections and tuberculosis	8,150 (3,920 to 14,600)	5,660 (2,450 to 10,800)	60,600 (36,900 to 89,100)	7,920 (4,560 to 11,900)	3,350 (2,420 to 4,510)	2,130 (1,530 to 2,800)	2,350 (1,640 to 3,200)	4,060 (2,730 to 5,580)	5,530 (3,620 to 8,060)	5,270 (2,800 to 7,510)	5,190 (2,780 to 7,560)	5,100 (3,010 to 7,580)	5,370 (3,260 to 7,800)	5,790 (3,800 to 8,530)	5,790 (3,850 to 8,360)	6,250 (4,240 to 8,770)	5,600 (3,730 to 7,610)	4,570 (3,210 to 6,310)	3,420 (2,410 to 4,590)	2,450 (1,760 to 3,200)	1,170 (827 to 1,580)	384 (273 to 496)	87·6 (60·7 to 118)	156,000
Tuberculosis	0 (0 to 0)	0 (0 to 0)	3,620 (1,770 to 6,290)	797 (415 to 1,390)	1,010 (616 to 1,610)	727 (436 to 1,100)	851 (411 to 1,530)	2,510 (1,460 to 4,010)	4,130 (2,400 to 6,420)	3,870 (1,860 to 5,990)	3,890 (1,850 to 6,040)	3,760 (2,070 to 6,060)	3,930 (2,240 to 6,010)	4,120 (2,410 to 6,520)	3,940 (2,390 to 6,060)	3,930 (2,430 to 5,960)	3,420 (2,090 to 5,110)	2,410 (1,420 to 3,660)	1,530 (964 to 2,340)	914 (553 to 1,370)	337 (202 to 517)	91·8 (60·2 to 133)	18·7 (11·7 to 28·2)	49,800
Latent tuberculosis infection	0 (0 to 0)	0 (0 to 0)	0 (0 to 0)	0 (0 to 0)	0 (0 to 0)	0 (0 to 0)	0 (0 to 0)	0 (0 to 0)	0 (0 to 0)	0 (0 to 0)	0 (0 to 0)	0 (0 to 0)	0 (0 to 0)	0 (0 to 0)	0 (0 to 0)	0 (0 to 0)	0 (0 to 0)	0 (0 to 0)	0 (0 to 0)	0 (0 to 0)	0 (0 to 0)	0 (0 to 0)	0 (0 to 0)	0
Drug-susceptible tuberculosis	0 (0 to 0)	0 (0 to 0)	3,570 (1,760 to 6,170)	788 (411 to 1,380)	1,000 (613 to 1,590)	719 (427 to 1,090)	840 (410 to 1,510)	2,480 (1,460 to 3,930)	4,090 (2,390 to 6,340)	3,830 (1,850 to 5,940)	3,850 (1,840 to 6,020)	3,720 (2,040 to 6,010)	3,880 (2,210 to 5,950)	4,070 (2,390 to 6,400)	3,890 (2,350 to 5,950)	3,880 (2,400 to 5,900)	3,380 (2,070 to 5,010)	2,380 (1,400 to 3,580)	1,510 (951 to 2,290)	902 (550 to 1,350)	333 (199 to 513)	90·6 (59·7 to 132)	18·4 (11·6 to 27·9)	49,200
Multidrug-resistant tuberculosis without extensive drug res	0 (0 to 0)	0 (0 to 0)	38·8 (3·1 to 171)	8·2 (0·8 to 36·4)	9·7 (0·9 to 38·9)	7·2 (0·7 to 30·1)	8·9 (0·8 to 36·5)	24 (2·1 to 95·9)	40·1 (3·8 to 178)	36·3 (3·2 to 150)	36·2 (3·1 to 171)	35·4 (3·1 to 145)	39·4 (3·5 to 162)	41·8 (3·8 to 174)	41 (4 to 179)	39·8 (3·5 to 175)	34·1 (3·1 to 140)	24·4 (2·2 to 97·9)	15·5 (1·4 to 60·8)	9·4 (0·8 to 38)	3·5 (0·3 to 14·3)	1 (0·1 to 3·8)	0·2 (0 to 0·8)	495
Extensively drug-resistant tuberculosis	0 (0 to 0)	0 (0 to 0)	7·5 (0·6 to 33·8)	1·6 (0·1 to 6·8)	1·8 (0·2 to 7·9)	1·3 (0·1 to 5·8)	1·7 (0·1 to 7·3)	4·5 (0·3 to 19·5)	7·5 (0·7 to 30·9)	6·8 (0·5 to 30·8)	6·8 (0·6 to 32·8)	6·7 (0·6 to 28·9)	7·5 (0·6 to 31·6)	8 (0·7 to 35·1)	7·8 (0·6 to 37·4)	7·6 (0·6 to 33·5)	6·5 (0·5 to 27·8)	4·6 (0·4 to 20·2)	2·9 (0·2 to 12)	1·8 (0·2 to 7·7)	0·6 (0·1 to 2·7)	0·2 (0 to 0·8)	0 (0 to 0·2)	93·7
Lower respiratory infections	8,140 (3,910 to 14,600)	5,650 (2,450 to 10,800)	56,800 (34,200 to 84,600)	6,510 (3,380 to 10,300)	1,700 (1,040 to 2,550)	917 (574 to 1,360)	1,030 (634 to 1,550)	1,120 (618 to 1,770)	1,010 (589 to 1,550)	1,080 (529 to 1,760)	1,030 (504 to 1,660)	1,130 (593 to 1,810)	1,270 (672 to 2,000)	1,530 (925 to 2,410)	1,750 (1,040 to 2,750)	2,250 (1,360 to 3,490)	2,130 (1,280 to 3,310)	2,130 (1,360 to 3,270)	1,880 (1,210 to 2,790)	1,530 (1,010 to 2,180)	826 (532 to 1,210)	291 (195 to 392)	68·7 (45·1 to 97·2)	102,000
Upper respiratory infections	1 (0·5 to 1·6)	6·2 (3·5 to 10·5)	149 (77·7 to 277)	437 (253 to 707)	400 (212 to 678)	332 (172 to 575)	325 (169 to 566)	304 (167 to 522)	285 (157 to 473)	246 (128 to 418)	203 (104 to 338)	160 (85·7 to 280)	125 (67·5 to 216)	99·1 (55·3 to 166)	77·6 (42·2 to 130)	58·8 (31·9 to 97·5)	38·3 (20·8 to 65·7)	21·8 (11·6 to 36·7)	13·7 (7·2 to 22·8)	6·2 (3·2 to 10·1)	2·6 (1·5 to 4·4)	0·8 (0·5 to 1·2)	0·2 (0·1 to 0·3)	3,290
Otitis media	11·9 (0·4 to 51·7)	0·4 (0·2 to 1·1)	16·5 (8·9 to 29)	180 (103 to 301)	237 (128 to 400)	151 (78·9 to 271)	139 (72·3 to 241)	132 (54 to 254)	104 (48·3 to 196)	74·5 (32·2 to 146)	61·1 (29·1 to 115)	53·9 (23·3 to 102)	47 (22·8 to 83)	39·7 (18 to 72·4)	30·5 (14·6 to 56·2)	20·8 (9·4 to 40·8)	11·3 (5·7 to 20·3)	6 (2·6 to 11·6)	3·7 (1·7 to 6·7)	2 (0·9 to 3·8)	0·8 (0·4 to 1·5)	0·2 (0·1 to 0·5)	0 (0 to 0·1)	1,320
Enteric infections	136 (38 to 381)	668 (169 to 1,820)	12,100 (2,810 to 32,900)	6,370 (2,990 to 12,000)	2,850 (1,590 to 4,660)	2,140 (1,240 to 3,540)	1,680 (879 to 2,950)	1,030 (543 to 1,770)	981 (556 to 1,590)	880 (466 to 1,510)	861 (442 to 1,500)	816 (428 to 1,440)	887 (470 to 1,680)	988 (513 to 1,880)	975 (512 to 1,970)	1,050 (533 to 2,050)	987 (507 to 1,920)	857 (429 to 1,700)	790 (409 to 1,590)	579 (291 to 1,150)	292 (141 to 605)	103 (48·2 to 215)	19·5 (8·4 to 41·6)	38,100
Diarrheal diseases	136 (38 to 381)	665 (167 to 1,810)	12,000 (2,780 to 32,900)	5,290 (2,170 to 10,900)	1,440 (672 to 2,860)	809 (406 to 1,440)	580 (285 to 1,060)	457 (227 to 817)	667 (343 to 1,160)	700 (354 to 1,300)	762 (377 to 1,410)	750 (377 to 1,360)	833 (427 to 1,620)	941 (476 to 1,830)	936 (472 to 1,940)	1,020 (505 to 2,030)	968 (489 to 1,900)	846 (419 to 1,690)	783 (401 to 1,580)	575 (287 to 1,150)	291 (140 to 604)	103 (48 to 214)	19·5 (8·4 to 41·5)	31,600
Typhoid and paratyphoid	0 (0 to 0)	0 (0 to 0)	10·2 (3·3 to 25·6)	948 (288 to 2,100)	1,280 (472 to 2,800)	1,250 (468 to 2,520)	1,050 (364 to 2,200)	539 (191 to 1,210)	293 (113 to 632)	164 (51·9 to 358)	88·4 (29·5 to 186)	57·2 (19·1 to 125)	45·5 (15·9 to 101)	37·4 (13·7 to 81·2)	29·4 (10 to 68·3)	20·2 (6·9 to 45·2)	11 (4·3 to 23·9)	5·6 (2·1 to 12·2)	2·9 (1·1 to 5·8)	1·4 (0·5 to 2·8)	0·5 (0·2 to 0·9)	0·1 (0 to 0·2)	0 (0 to 0)	5,830
Typhoid fever	0 (0 to 0)	0 (0 to 0)	8·6 (2·7 to 21·5)	801 (235 to 1,820)	1,080 (396 to 2,310)	1,060 (384 to 2,190)	894 (303 to 1,870)	461 (163 to 1,050)	251 (95·1 to 549)	141 (45·7 to 311)	75·8 (25·7 to 164)	49·1 (16·3 to 107)	38·9 (13·4 to 87·2)	32·1 (11·4 to 69·7)	25·3 (8·6 to 58·6)	17·4 (6 to 38·9)	9·5 (3·6 to 20)	4·8 (1·9 to 10·4)	2·5 (0·9 to 4·9)	1·2 (0·4 to 2·4)	0·4 (0·2 to 0·8)	0·1 (0 to 0·2)	0 (0 to 0)	4,960
Paratyphoid fever	0 (0 to 0)	0 (0 to 0)	1·6 (0·4 to 4·1)	147 (37·9 to 380)	198 (63·4 to 478)	188 (65·4 to 433)	154 (47·6 to 356)	77·7 (23·3 to 197)	41·7 (12·6 to 101)	23·3 (6·1 to 58·8)	12·6 (3·6 to 29·8)	8·2 (2·4 to 19·9)	6·5 (1·9 to 16·7)	5·4 (1·6 to 13·1)	4·2 (1·2 to 10·3)	2·9 (0·8 to 6·7)	1·6 (0·5 to 3·8)	0·8 (0·2 to 2)	0·4 (0·1 to 0·9)	0·2 (0·1 to 0·4)	0·1 (0 to 0·1)	0 (0 to 0)	0 (0 to 0)	873
Invasive Non-typhoidal Salmonella (iNTS)	0 (0 to 0)	3 (1·4 to 6)	48·7 (23·4 to 90·7)	124 (54·1 to 230)	124 (55·5 to 232)	74·5 (31·8 to 143)	53·8 (20·6 to 111)	34 (11·9 to 72·9)	20·5 (6·7 to 44·3)	13·8 (3·8 to 30·6)	9·9 (3·1 to 21·6)	8·4 (2·8 to 17·4)	7·8 (2·8 to 16)	8·5 (3·3 to 17·2)	9·7 (3·7 to 19·2)	10 (4 to 19·7)	7·7 (3 to 15·4)	5·5 (2·3 to 10)	3·4 (1·4 to 6·4)	1·8 (0·7 to 3·6)	0·6 (0·3 to 1·2)	0·2 (0 to 0·3)	0 (0 to 0·1)	570
Other intestinal infectious diseases	0 (0 to 0)	0 (0 to 0)	21·9 (0·3 to 80·4)	8·5 (1·2 to 29·7)	5·4 (0·3 to 20·2)	3·3 (0·2 to 12·4)	2·2 (0·1 to 7·7)	2·1 (0·1 to 7·3)	0·8 (0·1 to 3)	1·8 (0·2 to 6·1)	0·8 (0·1 to 2·7)	0·8 (0·1 to 2·8)	0·5 (0·1 to 1·8)	0·5 (0 to 1·6)	0·3 (0 to 1·2)	0·3 (0 to 1)	0·4 (0 to 1·3)	0·2 (0 to 0·7)	0·1 (0 to 0·4)	0·1 (0 to 0·3)	0·1 (0 to 0·2)	0 (0 to 0·1)	0 (0 to 0)	50
Neglected tropical diseases and malaria	2·8 (1 to 6·5)	20·5 (10·8 to 36·7)	253 (115 to 500)	716 (408 to 1,210)	1,230 (714 to 2,050)	933 (537 to 1,550)	856 (549 to 1,290)	946 (619 to 1,430)	692 (442 to 1,020)	789 (485 to 1,210)	496 (314 to 749)	668 (392 to 1,030)	396 (251 to 586)	537 (309 to 846)	327 (214 to 483)	347 (209 to 535)	243 (155 to 364)	166 (103 to 253)	101 (61 to 150)	53·3 (34·1 to 78·1)	21·3 (13·4 to 32·2)	6 (3·8 to 9·2)	1·2 (0·7 to 1·9)	9,800
Malaria	0·1 (0 to 0·2)	5·6 (1·5 to 15·1)	80·6 (21·2 to 228)	80·5 (25·4 to 187)	44·4 (16·1 to 118)	22·5 (12·5 to 43·6)	130 (41·8 to 326)	125 (39·3 to 321)	72·3 (26 to 174)	47·1 (18·7 to 110)	29·8 (13·1 to 68·6)	34·4 (13·4 to 82·1)	33·1 (11·8 to 80·6)	28·1 (9·7 to 69·3)	23·2 (8 to 57·2)	16·2 (5·6 to 39·9)	13·7 (4·1 to 35·4)	11·3 (3·2 to 29·4)	3·8 (1·1 to 9·3)	1·4 (0·5 to 3·5)	0·2 (0·1 to 0·5)	0 (0 to 0·1)	0 (0 to 0)	803
Chagas disease	0 (0 to 0)	0 (0 to 0)	0 (0 to 0)	0 (0 to 0)	0 (0 to 0)	0 (0 to 0)	0 (0 to 0)	0 (0 to 0)	0 (0 to 0)	0 (0 to 0)	0 (0 to 0)	0 (0 to 0)	0 (0 to 0)	0 (0 to 0)	0 (0 to 0)	0 (0 to 0)	0 (0 to 0)	0 (0 to 0)	0 (0 to 0)	0 (0 to 0)	0 (0 to 0)	0 (0 to 0)	0 (0 to 0)	0
Leishmaniasis	0 (0 to 0)	0 (0 to 0)	0 (0 to 0)	0 (0 to 0)	0 (0 to 0)	0 (0 to 0)	0 (0 to 0)	0 (0 to 0)	0 (0 to 0)	0 (0 to 0)	0 (0 to 0)	0 (0 to 0)	0 (0 to 0)	0 (0 to 0)	0 (0 to 0)	0 (0 to 0)	0 (0 to 0)	0 (0 to 0)	0 (0 to 0)	0 (0 to 0)	0 (0 to 0)	0 (0 to 0)	0 (0 to 0)	0
Visceral leishmaniasis	0 (0 to 0)	0 (0 to 0)	0 (0 to 0)	0 (0 to 0)	0 (0 to 0)	0 (0 to 0)	0 (0 to 0)	0 (0 to 0)	0 (0 to 0)	0 (0 to 0)	0 (0 to 0)	0 (0 to 0)	0 (0 to 0)	0 (0 to 0)	0 (0 to 0)	0 (0 to 0)	0 (0 to 0)	0 (0 to 0)	0 (0 to 0)	0 (0 to 0)	0 (0 to 0)	0 (0 to 0)	0 (0 to 0)	0
Cutaneous and mucocutaneous leishmaniasis	0 (0 to 0)	0 (0 to 0)	0 (0 to 0)	0 (0 to 0)	0 (0 to 0)	0 (0 to 0)	0 (0 to 0)	0 (0 to 0)	0 (0 to 0)	0 (0 to 0)	0 (0 to 0)	0 (0 to 0)	0 (0 to 0)	0 (0 to 0)	0 (0 to 0)	0 (0 to 0)	0 (0 to 0)	0 (0 to 0)	0 (0 to 0)	0 (0 to 0)	0 (0 to 0)	0 (0 to 0)	0 (0 to 0)	0
African trypanosomiasis	0 (0 to 0)	0 (0 to 0)	0 (0 to 0)	0 (0 to 0)	0 (0 to 0)	0 (0 to 0)	0 (0 to 0)	0 (0 to 0)	0 (0 to 0)	0 (0 to 0)	0 (0 to 0)	0 (0 to 0)	0 (0 to 0)	0 (0 to 0)	0 (0 to 0)	0 (0 to 0)	0 (0 to 0)	0 (0 to 0)	0 (0 to 0)	0 (0 to 0)	0 (0 to 0)	0 (0 to 0)	0 (0 to 0)	0
Schistosomiasis	0 (0 to 0)	0 (0 to 0)	0 (0 to 0)	0·4 (0 to 2·8)	2·2 (0 to 13·5)	3·8 (0 to 21·6)	6·8 (0·1 to 37·8)	11·7 (0·2 to 60·9)	15·2 (0·3 to 74)	18 (0·3 to 95·5)	20·4 (0·3 to 102)	22·2 (0·3 to 104)	23·1 (0·2 to 101)	25·1 (0·2 to 115)	25·7 (0·2 to 106)	21·5 (0·1 to 85·2)	14·1 (0·1 to 66·4)	11·2 (0 to 48·9)	7 (0 to 33·3)	3·2 (0 to 17·2)	1·2 (0 to 6·7)	0·2 (0 to 1·4)	0 (0 to 0·2)	233
Cysticercosis	0 (0 to 0)	0 (0 to 0)	0 (0 to 0)	0·1 (0 to 0·3)	0·9 (0 to 3·2)	1·8 (0·3 to 4·9)	6 (1·5 to 13·1)	16·3 (0 to 47·5)	27 (2·1 to 69·9)	31·2 (5·3 to 79·4)	32·2 (5·7 to 80·3)	33·7 (8·3 to 77·9)	37·3 (10 to 82·3)	39·8 (11·4 to 92·2)	48·4 (15·6 to 109)	51·3 (17·7 to 113)	46·1 (16·1 to 97·5)	35·1 (13·1 to 74·2)	24·9 (9·7 to 50·3)	14·1 (5·2 to 29·2)	5·5 (2·2 to 11·5)	1·7 (0·6 to 3·8)	0·5 (0·1 to 1·1)	454
Cystic echinococcosis	0 (0 to 0)	0 (0 to 0)	0 (0 to 0)	0·7 (0 to 3·1)	1·8 (0 to 7·4)	1·2 (0 to 4·3)	1 (0 to 2·9)	1·2 (0 to 3·8)	1·5 (0 to 5)	1·5 (0 to 5·5)	1·3 (0 to 4·8)	1·1 (0 to 4·1)	1 (0 to 3·9)	0·9 (0 to 3·4)	0·9 (0 to 3·2)	0·6 (0 to 2·4)	0·5 (0 to 1·7)	0·3 (0 to 1·1)	0·2 (0 to 0·7)	0·1 (0 to 0·4)	0 (0 to 0·2)	0 (0 to 0·1)	0 (0 to 0)	15·6
Lymphatic filariasis	0 (0 to 0)	0 (0 to 0)	0 (0 to 0)	0 (0 to 0)	158 (64 to 345)	158 (65·9 to 330)	161 (66·5 to 356)	162 (67·8 to 347)	160 (67·6 to 352)	147 (61·6 to 304)	122 (53·8 to 261)	101 (43·7 to 219)	84·1 (35·4 to 182)	70·3 (28·1 to 146)	58·1 (24·6 to 121)	43·9 (18·7 to 92·9)	29·6 (12·5 to 60·6)	18·5 (8·1 to 38·1)	11·4 (4·8 to 23·4)	6 (2·6 to 12·4)	2·4 (1 to 5·2)	0·7 (0·3 to 1·4)	0·1 (0·1 to 0·3)	1,490
Onchocerciasis	0 (0 to 0)	0 (0 to 0)	0 (0 to 0)	0 (0 to 0)	0 (0 to 0)	0 (0 to 0)	0 (0 to 0)	0 (0 to 0)	0 (0 to 0)	0 (0 to 0)	0 (0 to 0)	0 (0 to 0)	0 (0 to 0)	0 (0 to 0)	0 (0 to 0)	0 (0 to 0)	0 (0 to 0)	0 (0 to 0)	0 (0 to 0)	0 (0 to 0)	0 (0 to 0)	0 (0 to 0)	0 (0 to 0)	0
Trachoma	0 (0 to 0)	0 (0 to 0)	0 (0 to 0)	0 (0 to 0)	0 (0 to 0)	0 (0 to 0)	0 (0 to 0)	0 (0 to 0)	0 (0 to 0·1)	0·1 (0 to 0·1)	0·2 (0·1 to 0·4)	0·5 (0·2 to 0·9)	0·9 (0·5 to 1·7)	1·2 (0·6 to 2·3)	1·4 (0·7 to 2·7)	1·6 (0·7 to 3)	1·6 (0·3 to 4·1)	1·6 (0·4 to 3·7)	1·4 (0·4 to 3)	1 (0·3 to 2·1)	0·6 (0·2 to 1·2)	0·2 (0·1 to 0·4)	0 (0 to 0·1)	12·4
Dengue	0 (0 to 0)	2·3 (0·1 to 12·4)	64 (7·8 to 269)	159 (27·1 to 549)	158 (41·9 to 438)	113 (32·2 to 291)	93·5 (28·8 to 246)	86·1 (24·5 to 227)	86·5 (22·9 to 246)	53·9 (15·2 to 141)	39·3 (10·8 to 98·1)	30·6 (9·7 to 79)	25 (7·2 to 68·7)	20·9 (6·4 to 55·4)	17·3 (5·3 to 44·5)	14·1 (4·2 to 35·9)	9 (2·9 to 22·8)	6 (2 to 15·6)	4 (1·3 to 9·4)	2·5 (0·7 to 7)	1·4 (0·4 to 3·6)	0·5 (0·2 to 1·4)	0·2 (0·1 to 0·5)	988
Yellow fever	0 (0 to 0)	0 (0 to 0)	0 (0 to 0)	0 (0 to 0)	0 (0 to 0)	0 (0 to 0)	0 (0 to 0)	0 (0 to 0)	0 (0 to 0)	0 (0 to 0)	0 (0 to 0)	0 (0 to 0)	0 (0 to 0)	0 (0 to 0)	0 (0 to 0)	0 (0 to 0)	0 (0 to 0)	0 (0 to 0)	0 (0 to 0)	0 (0 to 0)	0 (0 to 0)	0 (0 to 0)	0 (0 to 0)	0
Rabies	0 (0 to 0)	0 (0 to 0)	12·3 (0·2 to 41·5)	29·1 (0·4 to 116)	203 (3·7 to 696)	178 (2·4 to 677)	37·1 (0·5 to 124)	50·6 (0·9 to 150)	35·4 (0·7 to 110)	44·5 (0·8 to 158)	38·1 (0·8 to 114)	33·7 (0·8 to 94·6)	33 (0·7 to 105)	24·1 (0·5 to 74·7)	23·8 (0·5 to 80·7)	14·1 (0·3 to 48·6)	7·6 (0·2 to 23·7)	5 (0·2 to 18·2)	2·1 (0·1 to 6·7)	1·1 (0·1 to 3·2)	0·6 (0 to 1·9)	0·1 (0 to 0·3)	0 (0 to 0·1)	772
Intestinal nematode infections	0 (0 to 0)	0 (0 to 0)	25·9 (7·1 to 78·3)	272 (149 to 450)	449 (226 to 758)	301 (157 to 539)	355 (188 to 616)	286 (151 to 495)	209 (112 to 356)	157 (83·5 to 271)	129 (68·3 to 223)	102 (53·3 to 178)	80·6 (44·3 to 143)	64·9 (34·4 to 110)	56·8 (30·3 to 96·9)	43 (23·3 to 72·7)	28·7 (15·5 to 48·5)	18·6 (10·1 to 31·4)	12·5 (7 to 21·6)	6·6 (3·6 to 11)	2·8 (1·6 to 4·5)	0·8 (0·4 to 1·3)	0·2 (0·1 to 0·2)	2,600
Ascariasis	0 (0 to 0)	0 (0 to 0)	15·4 (0·5 to 66·5)	48·1 (17 to 104)	39·6 (8·8 to 115)	14·1 (4·1 to 33·9)	26·4 (10·8 to 50·2)	22·1 (9·4 to 43·9)	20·3 (8·6 to 40·3)	18 (7·5 to 34·5)	15·2 (6·6 to 29·7)	11·9 (5·5 to 23·2)	8·9 (3·9 to 17·7)	6·8 (3 to 13·4)	5·5 (2·3 to 11)	4·4 (1·6 to 8·7)	2·5 (1·1 to 4·9)	1·7 (0·6 to 3·5)	1·1 (0·4 to 2·4)	0·6 (0·2 to 1·2)	0·2 (0·1 to 0·5)	0·1 (0 to 0·2)	0 (0 to 0)	263
Trichuriasis	0 (0 to 0)	0 (0 to 0)	1·9 (0·7 to 3·6)	47·6 (22·5 to 86·7)	48·4 (22·2 to 89·9)	34·5 (15·6 to 65·3)	95·5 (43·6 to 175)	88·3 (41 to 165)	65·4 (31·2 to 120)	45·3 (20·5 to 82·9)	30·4 (14·2 to 57·3)	20·6 (9·6 to 38·6)	14·6 (6·4 to 27·2)	10·6 (4·7 to 20)	7·9 (3·6 to 14·7)	5·1 (2·2 to 9·7)	2·9 (1·3 to 5·5)	1·6 (0·7 to 2·9)	0·9 (0·4 to 1·7)	0·4 (0·2 to 0·8)	0·1 (0·1 to 0·3)	0 (0 to 0·1)	0 (0 to 0)	522
Hookworm disease	0 (0 to 0)	0 (0 to 0)	8·6 (4·3 to 15·2)	176 (90·3 to 320)	361 (172 to 633)	252 (126 to 462)	233 (117 to 414)	176 (87·6 to 323)	123 (60·6 to 221)	93·2 (47 to 171)	83·5 (41·8 to 150)	69·7 (34·8 to 124)	57 (29·8 to 106)	47·5 (24·1 to 84)	43·4 (22·8 to 77·6)	33·5 (17 to 60·6)	23·3 (12·1 to 40·1)	15·3 (8 to 26·8)	10·5 (5·5 to 18·5)	5·6 (2·8 to 9·6)	2·4 (1·3 to 4)	0·7 (0·4 to 1·2)	0·1 (0·1 to 0·2)	1,820
Food-borne trematodiases	0 (0 to 0)	0 (0 to 0)	0 (0 to 0)	3·2 (0·8 to 7·6)	10·7 (2·6 to 25·3)	80·8 (10·6 to 218)	44·3 (6·5 to 111)	189 (39·2 to 463)	62·3 (11·5 to 150)	267 (74·4 to 562)	62·2 (12·4 to 148)	288 (87·7 to 574)	59·4 (14·5 to 133)	245 (71·4 to 498)	47·9 (9·9 to 109)	129 (36 to 256)	84·4 (23·1 to 170)	50·3 (13·1 to 102)	28·7 (7·8 to 58·7)	14·1 (4·1 to 28·7)	5·2 (1·4 to 10·7)	1·3 (0·3 to 2·7)	0 (0 to 0)	1,670
Leprosy	0 (0 to 0)	0 (0 to 0)	0 (0 to 0)	0 (0 to 0)	0 (0 to 0)	0 (0 to 0)	0·8 (0·5 to 1·3)	1·3 (0·8 to 2)	1·9 (1·1 to 3)	2·3 (0 to 6)	2·5 (0 to 6·2)	2·6 (0·5 to 5·9)	2·8 (0·6 to 5·8)	2·7 (0·6 to 5·8)	2·7 (0·8 to 5·4)	2·3 (0·8 to 4·5)	1·8 (0·7 to 3·4)	1·2 (0·5 to 2·2)	0·8 (0·3 to 1·6)	0·5 (0·2 to 0·9)	0·2 (0·1 to 0·4)	0·1 (0 to 0·1)	0 (0 to 0)	26·7
Ebola	0 (0 to 0)	0 (0 to 0)	0 (0 to 0)	0 (0 to 0)	0 (0 to 0)	0 (0 to 0)	0 (0 to 0)	0 (0 to 0)	0 (0 to 0)	0 (0 to 0)	0 (0 to 0)	0 (0 to 0)	0 (0 to 0)	0 (0 to 0)	0 (0 to 0)	0 (0 to 0)	0 (0 to 0)	0 (0 to 0)	0 (0 to 0)	0 (0 to 0)	0 (0 to 0)	0 (0 to 0)	0 (0 to 0)	0
Zika virus	0 (0 to 0)	0 (0 to 0)	0 (0 to 0)	0 (0 to 0)	0 (0 to 0)	0 (0 to 0)	0 (0 to 0)	0 (0 to 0)	0 (0 to 0)	0 (0 to 0)	0 (0 to 0)	0 (0 to 0)	0 (0 to 0)	0 (0 to 0)	0 (0 to 0)	0 (0 to 0)	0 (0 to 0)	0 (0 to 0)	0 (0 to 0)	0 (0 to 0)	0 (0 to 0)	0 (0 to 0)	0 (0 to 0)	0
Guinea worm disease	0 (0 to 0)	0 (0 to 0)	0 (0 to 0)	0 (0 to 0)	0 (0 to 0)	0 (0 to 0)	0 (0 to 0)	0 (0 to 0)	0 (0 to 0)	0 (0 to 0)	0 (0 to 0)	0 (0 to 0)	0 (0 to 0)	0 (0 to 0)	0 (0 to 0)	0 (0 to 0)	0 (0 to 0)	0 (0 to 0)	0 (0 to 0)	0 (0 to 0)	0 (0 to 0)	0 (0 to 0)	0 (0 to 0)	0
Other neglected tropical diseases	2·8 (1 to 6·4)	12·6 (6·8 to 19·7)	70·3 (27·2 to 138)	171 (65·8 to 336)	201 (50 to 469)	74·1 (18·9 to 182)	20 (5·8 to 51·8)	17 (4·6 to 42·3)	20·3 (5·5 to 50)	19·8 (5·5 to 49)	18·4 (5 to 47·8)	17·5 (4·9 to 38·7)	16 (4·6 to 38·5)	14·7 (4·1 to 34·7)	21·5 (8·5 to 43·1)	9·4 (4 to 18)	6·2 (2·2 to 13·1)	6·4 (2·8 to 11·5)	3·6 (1·5 to 6·7)	2·6 (1·1 to 4·7)	1·2 (0·6 to 2·1)	0·3 (0·2 to 0·6)	0·1 (0·1 to 0·2)	727
Other infectious diseases	511 (149 to 1,360)	836 (263 to 2,140)	13,300 (6,100 to 27,200)	9,170 (4,090 to 16,900)	2,660 (1,580 to 4,220)	1,100 (707 to 1,580)	862 (577 to 1,250)	837 (557 to 1,160)	712 (453 to 1,030)	792 (421 to 1,160)	685 (355 to 1,040)	611 (354 to 959)	596 (360 to 920)	458 (278 to 703)	425 (259 to 665)	294 (179 to 471)	187 (113 to 295)	124 (76 to 191)	85·7 (53 to 130)	37·2 (23·9 to 59·1)	20·6 (12·4 to 32·9)	6·5 (3·4 to 13·2)	2 (0·9 to 4·4)	34,300
Meningitis	84·3 (29·6 to 195)	207 (70·9 to 465)	3,760 (1,650 to 7,890)	632 (280 to 1,180)	589 (306 to 1,030)	341 (186 to 555)	250 (136 to 418)	292 (154 to 493)	193 (107 to 317)	194 (101 to 324)	147 (74 to 251)	112 (61·1 to 186)	99·3 (53·8 to 169)	69·2 (37 to 116)	63 (33·1 to 106)	39·6 (21·6 to 66·9)	25·2 (13·4 to 44·5)	18·3 (10 to 31·8)	12·3 (6·6 to 21·4)	5·6 (3 to 9·5)	2·6 (1·5 to 4·2)	0·7 (0·4 to 1·2)	0·2 (0·1 to 0·3)	7,140
Encephalitis	9·5 (3·1 to 23·4)	24 (6·3 to 73·1)	592 (189 to 1,600)	656 (202 to 1,610)	350 (135 to 813)	213 (96·7 to 426)	197 (85·5 to 405)	138 (64·2 to 248)	122 (52·1 to 242)	133 (53·3 to 256)	98·5 (38·8 to 192)	80·8 (33 to 151)	78·5 (32·5 to 159)	53·7 (24·3 to 105)	45·6 (19·4 to 92·8)	42·1 (16 to 102)	23·9 (9·7 to 58·6)	19·1 (7·1 to 50·2)	14·2 (5 to 37)	4 (1·6 to 9·6)	2·3 (0·9 to 5·6)	0·5 (0·2 to 1·3)	0·2 (0·1 to 0·4)	2,900
Diphtheria	0 (0 to 0)	0 (0 to 0)	34·2 (1·2 to 120)	15·3 (0·6 to 53·7)	16·2 (0·7 to 59·4)	6·5 (0·2 to 24·6)	3·3 (0·1 to 11·4)	2·1 (0·1 to 7·6)	1·6 (0·1 to 5·9)	1·6 (0·1 to 6·1)	1·6 (0·1 to 5·9)	1·8 (0·1 to 6·6)	2·9 (0·1 to 10·5)	2·2 (0·1 to 7·9)	1·4 (0 to 4·8)	0 (0 to 0)	0 (0 to 0)	0 (0 to 0)	0 (0 to 0)	0 (0 to 0)	0 (0 to 0)	0 (0 to 0)	0 (0 to 0)	90·7
Tetanus	357 (36·7 to 1,210)	520 (43·8 to 1,820)	182 (19·4 to 612)	137 (8·3 to 725)	130 (21·3 to 366)	93·8 (16·3 to 266)	90·4 (18·1 to 288)	92·8 (18·9 to 227)	75·9 (14 to 199)	140 (19·8 to 337)	141 (20 to 338)	157 (25 to 389)	166 (27·7 to 381)	136 (25 to 310)	135 (23·5 to 318)	86·1 (16·9 to 225)	63·5 (10·9 to 151)	38·7 (7·1 to 86·6)	25·8 (4·7 to 61·5)	8·1 (1·8 to 21·9)	3·7 (0·9 to 8·7)	1 (0·2 to 2·3)	0·3 (0 to 0·6)	2,780

Non-rheumatic degenerative mitral valve disease	0 (0 to 0)	0 (0 to 0)	0 (0 to 0)	0 (0 to 0)	0 (0 to 0)	0 (0 to 0)	3 (0·9 to 7·3)	4·1 (1 to 11)	2·4 (0·8 to 5·9)	3·9 (1·2 to 9·6)	3·1 (0·9 to 8·2)	3 (1 to 7·4)	6·3 (2·4 to 14·7)	6·1 (2·8 to 13·1)	9·6 (4·4 to 18·7)	7·8 (3·9 to 14·7)	8·6 (4·6 to 15·3)	6·9 (3·4 to 12·6)	5·5 (2·9 to 10·2)	4·1 (2·1 to 7·5)	1·8 (1 to 3·4)	0·6 (0·3 to 1·1)	0·1 (0·1 to 0·2)	76·9
Other non-rheumatic valve diseases	0 (0 to 0)	0 (0 to 0)	0 (0 to 0)	0 (0 to 0)	0 (0 to 0)	0 (0 to 0)	1·7 (0·4 to 4·8)	2·5 (0·6 to 6·9)	1 (0·2 to 3·1)	1·7 (0·3 to 5·1)	3·6 (0·8 to 9·7)	1·9 (0·3 to 5·9)	2·1 (0·4 to 6·5)	2 (0·3 to 6)	1·4 (0·2 to 4·7)	2·7 (0·4 to 8·9)	1·8 (0·2 to 6·1)	1·1 (0·1 to 4)	1·4 (0·2 to 4·6)	0·9 (0·1 to 3)	0·4 (0·1 to 1·5)	0·1 (0 to 0·3)	0 (0 to 0·1)	26·3
Cardiomyopathy and myocarditis	25·6 (4·8 to 88·2)	24·3 (5·3 to 68·9)	353 (58·9 to 1,050)	53·9 (16·9 to 132)	34·4 (11·6 to 92·1)	44·6 (14·1 to 112)	91 (38·9 to 181)	120 (51·4 to 238)	136 (55·2 to 262)	155 (65·6 to 294)	201 (80·2 to 415)	220 (94·5 to 440)	232 (96·4 to 463)	258 (115 to 524)	286 (139 to 550)	263 (126 to 495)	208 (110 to 376)	177 (96·1 to 294)	140 (81·3 to 227)	104 (53·3 to 186)	51·7 (23·3 to 102)	19·9 (8·3 to 38·5)	4·7 (1·8 to 9·6)	3,200
Myocarditis	6·6 (0·9 to 22·5)	9·2 (1·7 to 27)	138 (17·8 to 502)	25·5 (6·5 to 61·2)	18·6 (5·4 to 49·4)	17·5 (4·8 to 45·3)	22·2 (7·8 to 48·2)	24·9 (9·3 to 55·5)	23·9 (8·4 to 52·2)	19·6 (7·1 to 42·1)	21·6 (7·4 to 50·2)	21·7 (7·1 to 52·6)	21·2 (7·3 to 54·7)	19·8 (6·4 to 54·6)	22 (7·4 to 63·8)	22·5 (7 to 65·4)	13·2 (4·7 to 31·8)	11·6 (4·5 to 26·6)	11·3 (4·7 to 23·9)	10·3 (3·2 to 25·1)	5·8 (1·3 to 16·2)	2·7 (0·6 to 7·4)	0·7 (0·1 to 2·1)	490
Alcoholic cardiomyopathy	0 (0 to 0)	0 (0 to 0)	0 (0 to 0)	0 (0 to 0)	0 (0 to 0)	0 (0 to 0)	3·6 (1·1 to 8·7)	6·7 (1·7 to 17)	10·2 (2·7 to 27·9)	20·5 (6 to 44·4)	33 (11·1 to 71·7)	36·8 (11·7 to 87·6)	32·9 (11·2 to 76·6)	27·3 (8·2 to 65·2)	29·1 (10·7 to 69)	14·7 (4·7 to 35·5)	8·3 (2·7 to 18·9)	6·1 (1·8 to 15)	2·9 (1 to 6·9)	1·3 (0·4 to 2·8)	0·8 (0·3 to 1·9)	0·6 (0·2 to 1·4)	0·2 (0·1 to 0·6)	235
Other cardiomyopathy	18·9 (3·7 to 69·4)	15·2 (3·2 to 44)	215 (36·2 to 688)	28·4 (7·2 to 82·2)	15·8 (4·5 to 44·9)	27·1 (7·6 to 70·4)	65·2 (27·6 to 127)	88·1 (37·5 to 177)	102 (42·2 to 198)	115 (46·9 to 233)	146 (55·9 to 305)	162 (64·8 to 327)	178 (72·5 to 370)	211 (95·2 to 452)	235 (113 to 454)	226 (110 to 429)	186 (98·4 to 341)	160 (86·3 to 266)	126 (72·2 to 207)	92·5 (48 to 165)	45·2 (21 to 88·3)	16·6 (7·2 to 30·8)	3·8 (1·5 to 7·4)	2,480
Atrial fibrillation and flutter	0 (0 to 0)	0 (0 to 0)	0 (0 to 0)	0 (0 to 0)	0 (0 to 0)	0 (0 to 0)	0 (0 to 0)	0 (0 to 0)	0 (0 to 0)	4·3 (2 to 7·2)	17·8 (7·4 to 34)	36·2 (18·4 to 60·9)	69 (40·2 to 112)	114 (67·9 to 178)	167 (104 to 253)	220 (143 to 319)	242 (163 to 343)	236 (160 to 337)	217 (154 to 298)	184 (133 to 245)	104 (77·3 to 137)	41·9 (30 to 55·9)	8·7 (5·4 to 12·6)	1,660
Aortic aneurysm	--	--	--	--	--	--	22·2 (8·8 to 42·3)	19·4 (8·1 to 38·7)	25·4 (11·8 to 47·2)	26·3 (10·7 to 48·2)	37·1 (16·3 to 64·5)	41·2 (19·2 to 74·1)	51·7 (25·1 to 91·1)	64·1 (34 to 103)	99·1 (58·2 to 160)	113 (70·6 to 165)	113 (73·7 to 164)	96·7 (62·1 to 139)	76·9 (51·9 to 108)	48·8 (31·1 to 67·4)	21·8 (13·8 to 31·1)	6·2 (3·7 to 9·4)	0·7 (0·3 to 1·2)	NA
Endocarditis	1 (0·2 to 2·6)	1·8 (0·4 to 5·3)	102 (24·6 to 286)	10·7 (3·4 to 24·7)	10 (3·6 to 22)	12 (4·1 to 28·7)	59·3 (24·5 to 128)	89·7 (37·2 to 177)	129 (53·1 to 252)	107 (42·2 to 207)	102 (38·1 to 220)	117 (43·4 to 249)	87·3 (36·6 to 179)	72·7 (30·4 to 152)	64·3 (28·5 to 124)	45·9 (21·5 to 90)	40·3 (20 to 75·7)	26 (13·7 to 47·6)	18·8 (10·1 to 32·8)	10·2 (5·3 to 18)	5·1 (2·6 to 9·1)	1·7 (0·9 to 3)	0·3 (0·1 to 0·4)	1,110
Other cardiovascular and circulatory diseases	10·2 (2·4 to 29·1)	16·4 (4·9 to 43·7)	263 (69·3 to 609)	76·3 (35·2 to 153)	105 (55·9 to 183)	71·7 (32·6 to 135)	101 (57·1 to 163)	170 (93·4 to 286)	145 (79·1 to 241)	133 (67·8 to 225)	132 (64·3 to 227)	159 (87·3 to 272)	159 (88·4 to 287)	177 (104 to 324)	180 (106 to 324)	172 (103 to 304)	134 (81·3 to 240)	103 (63·1 to 174)	77·4 (48·6 to 135)	57·1 (36·3 to 93·2)	25·4 (16·7 to 41·6)	8·7 (5·4 to 14·4)	1·7 (1 to 2·8)	2,480
Chronic respiratory diseases	0 (0 to 0)	0 (0 to 0)	0·8 (0·6 to 1)	1,630 (944 to 2,670)	1,310 (907 to 1,900)	1,160 (819 to 1,550)	1,220 (828 to 1,700)	1,440 (1,030 to 1,980)	1,440 (1,010 to 1,990)	1,510 (990 to 2,090)	1,570 (1,010 to 2,220)	1,820 (1,170 to 2,550)	2,440 (1,730 to 3,390)	3,410 (2,410 to 4,700)	4,690 (3,260 to 6,410)	6,080 (4,140 to 8,500)	6,160 (4,220 to 8,400)	5,610 (3,880 to 7,610)	4,420 (3,140 to 5,970)	2,850 (1,990 to 3,760)	1,280 (918 to 1,710)	373 (261 to 501)	75·1 (52·8 to 102)	50,500
Chronic obstructive pulmonary disease	0 (0 to 0)	0 (0 to 0)	0·8 (0·6 to 1)	63·8 (33·2 to 124)	109 (59·9 to 176)	134 (81·2 to 209)	300 (186 to 454)	519 (332 to 763)	577 (401 to 804)	699 (478 to 980)	773 (512 to 1,090)	958 (648 to 1,360)	1,500 (1,060 to 2,080)	2,320 (1,640 to 3,170)	3,190 (2,180 to 4,450)	4,470 (3,070 to 6,410)	4,680 (3,190 to 6,500)	4,280 (2,990 to 5,910)	3,350 (2,320 to 4,560)	2,290 (1,610 to 3,080)	1,030 (729 to 1,380)	303 (214 to 409)	62·6 (44·1 to 84·3)	31,600
Pneumoconiosis	0 (0 to 0)	0 (0 to 0)	0 (0 to 0)	0 (0 to 0)	0 (0 to 0)	0 (0 to 0)	0·6 (0·3 to 1·3)	0·6 (0·2 to 1·3)	0·8 (0·4 to 1·6)	0·7 (0·4 to 1·2)	2 (1·1 to 3·4)	3·2 (1·7 to 5·5)	4·8 (2·7 to 8)	7·1 (4·2 to 11·5)	8·8 (5·4 to 14)	7·1 (2·6 to 14·2)	7 (3·1 to 12·4)	5 (2·3 to 9)	3·7 (1·7 to 6·7)	2 (0·9 to 3·5)	1·1 (0·6 to 2)	0·3 (0·2 to 0·6)	0·1 (0 to 0·1)	54·9
Silicosis	0 (0 to 0)	0 (0 to 0)	0 (0 to 0)	0 (0 to 0)	0 (0 to 0)	0 (0 to 0)	0·1 (0 to 0·2)	0·1 (0 to 0·3)	0·2 (0·1 to 0·4)	0·4 (0·2 to 0·7)	1·3 (0·6 to 2·2)	2·5 (1·2 to 4·4)	3·7 (2 to 6·2)	5 (2·7 to 8·3)	5·7 (3·3 to 8·9)	4·8 (1·1 to 10·9)	4·5 (1·4 to 9·2)	3·2 (1 to 6·3)	2 (0·7 to 4·2)	1·1 (0·4 to 2·1)	0·5 (0·2 to 1)	0·1 (0·1 to 0·3)	0 (0 to 0·1)	35·3
Asbestosis	0 (0 to 0)	0 (0 to 0)	0 (0 to 0)	0 (0 to 0)	0 (0 to 0)	0 (0 to 0)	0·2 (0 to 0·5)	0·2 (0 to 0·5)	0·2 (0·1 to 0·6)	0·1 (0 to 0·3)	0·2 (0·1 to 0·6)	0·2 (0 to 0·7)	0·3 (0 to 0·8)	0·8 (0·1 to 2·6)	0·9 (0·2 to 2·7)	0·7 (0·2 to 2)	0·8 (0·2 to 2·5)	0·8 (0·1 to 2·1)	0·3 (0 to 1·1)	0·2 (0 to 0·7)	0·1 (0 to 0·3)	0·1 (0 to 0·2)	0 (0 to 0)	6·2
Coal workers pneumoconiosis	0 (0 to 0)	0 (0 to 0)	0 (0 to 0)	0 (0 to 0)	0 (0 to 0)	0 (0 to 0)	0·1 (0 to 0·2)	0·1 (0 to 0·5)	0·1 (0 to 0·3)	0·1 (0 to 0·2)	0·2 (0 to 0·7)	0·2 (0 to 0·6)	0·3 (0 to 1·1)	0·5 (0·1 to 1·7)	0·8 (0·1 to 2·4)	0·4 (0 to 1·3)	0·4 (0 to 1·2)	0·3 (0 to 1)	0·3 (0 to 1)	0·1 (0 to 0·3)	0·1 (0 to 0·2)	0 (0 to 0·1)	0 (0 to 0)	3·8
Other pneumoconiosis	0 (0 to 0)	0 (0 to 0)	0 (0 to 0)	0 (0 to 0)	0 (0 to 0)	0 (0 to 0)	0·3 (0·1 to 0·7)	0·1 (0 to 0·4)	0·3 (0·1 to 0·7)	0·1 (0 to 0·3)	0·3 (0·1 to 0·8)	0·4 (0·1 to 1·1)	0·6 (0·1 to 1·7)	0·7 (0·2 to 2)	1·4 (0·4 to 3·6)	1·1 (0·3 to 2·9)	1·3 (0·4 to 3·2)	0·8 (0·2 to 2)	1 (0·3 to 2·4)	0·5 (0·2 to 1·2)	0·4 (0·1 to 0·8)	0·1 (0 to 0·3)	0 (0 to 0·1)	9·5
Asthma	0 (0 to 0)	0 (0 to 0)	0 (0 to 0)	1,440 (820 to 2,340)	1,070 (720 to 1,590)	860 (570 to 1,220)	790 (508 to 1,140)	738 (480 to 1,090)	709 (429 to 1,060)	646 (360 to 986)	649 (356 to 1,010)	695 (362 to 1,090)	770 (450 to 1,210)	928 (528 to 1,520)	1,260 (694 to 2,080)	1,520 (781 to 2,550)	1,400 (699 to 2,330)	1,260 (644 to 2,140)	1,010 (502 to 1,740)	534 (266 to 921)	237 (123 to 399)	66·8 (33·9 to 111)	11·9 (5·4 to 22·1)	16,600
Interstitial lung disease and pulmonary sarcoidosis	0 (0 to 0)	0 (0 to 0)	0 (0 to 0)	5·9 (2·1 to 13·9)	3·4 (1·4 to 7·4)	4·2 (1·7 to 8·5)	8·6 (2·4 to 19·9)	11·1 (3·4 to 27·8)	11·7 (3·8 to 28·7)	13·1 (4·5 to 31·5)	10 (3·8 to 22·4)	34 (10·5 to 90·3)	37·2 (12·2 to 96·1)	55·9 (17·3 to 150)	78·3 (23·4 to 217)	70·9 (21·8 to 194)	61·4 (16·8 to 171)	57·6 (15·3 to 168)	42·6 (11·7 to 120)	18·5 (4·9 to 51·6)	10·8 (2·6 to 31·9)	2·1 (0·5 to 5·8)	0·3 (0·1 to 1·1)	538
Other chronic respiratory diseases	0 (0 to 0)	0 (0 to 0)	0 (0 to 0)	127 (47·7 to 287)	123 (61·5 to 212)	164 (89·1 to 260)	120 (62·5 to 211)	170 (99·9 to 277)	145 (92·9 to 230)	155 (93·9 to 246)	134 (74·8 to 241)	127 (70·5 to 223)	122 (73 to 197)	104 (59 to 183)	149 (70·5 to 265)	17·6 (10·1 to 30·7)	14·7 (7·7 to 26·9)	7·5 (4·4 to 12·6)	4·5 (2·6 to 8·1)	5 (2·5 to 9·6)	1·5 (0·9 to 2·4)	1·2 (0·5 to 2·2)	0·2 (0·1 to 0·3)	1,690
Digestive diseases	33·6 (16·7 to 64·6)	57·8 (25·6 to 120)	825 (226 to 2,100)	814 (365 to 1,740)	818 (499 to 1,230)	591 (383 to 894)	1,050 (671 to 1,580)	1,590 (989 to 2,410)	2,290 (1,440 to 3,450)	3,440 (1,910 to 5,110)	4,450 (2,300 to 7,150)	5,570 (3,070 to 8,750)	6,180 (3,820 to 9,650)	6,700 (4,150 to 10,500)	6,620 (4,200 to 10,200)	5,710 (3,610 to 9,110)	4,120 (2,530 to 6,370)	2,720 (1,740 to 4,390)	1,760 (1,110 to 2,800)	984 (631 to 1,540)	417 (281 to 626)	126 (83·5 to 190)	23·8 (15·2 to 35·1)	56,900
Cirrhosis and other chronic liver diseases	0 (0 to 0)	0 (0 to 0)	0·5 (0·3 to 0·9)	177 (53·6 to 445)	195 (80·9 to 356)	147 (71·2 to 264)	373 (188 to 641)	712 (382 to 1,190)	1,110 (581 to 1,840)	2,050 (988 to 3,260)	2,870 (1,360 to 4,750)	3,760 (1,910 to 6,080)	4,230 (2,490 to 6,780)	4,430 (2,630 to 7,090)	4,120 (2,460 to 6,700)	3,170 (1,860 to 5,320)	1,980 (1,100 to 3,180)	1,060 (570 to 1,890)	546 (313 to 930)	272 (159 to 443)	98·4 (54·4 to 166)	25·6 (14·2 to 41·8)	4·5 (2·3 to 7·2)	31,300
Cirrhosis and other chronic liver diseases due to hepatitis	0 (0 to 0)	0 (0 to 0)	0 (0 to 0)	1·4 (0·3 to 4·1)	4·3 (1·4 to 10)	6·1 (2 to 13·7)	24 (7·5 to 51)	146 (54·2 to 305)	328 (124 to 666)	711 (299 to 1,230)	1,070 (424 to 2,000)	1,400 (670 to 2,500)	1,500 (757 to 2,610)	1,470 (698 to 2,670)	1,270 (546 to 2,400)	913 (397 to 1,790)	543 (249 to 1,040)	294 (127 to 590)	160 (72·3 to 304)	82·6 (37 to 155)	30 (14·7 to 55·3)	7·8 (3·5 to 14·4)	1·4 (0·5 to 2·7)	9,960
Cirrhosis and other chronic liver diseases due to hepatitis	0 (0 to 0)	0 (0 to 0)	0 (0 to 0)	1·8 (0·4 to 5)	5·5 (1·7 to 12·7)	7·6 (2·9 to 17·7)	30·2 (10 to 65)	157 (61·3 to 296)	340 (145 to 662)	664 (269 to 1,220)	904 (375 to 1,730)	1,170 (519 to 2,160)	1,280 (592 to 2,380)	1,300 (606 to 2,400)	1,160 (536 to 2,230)	868 (381 to 1,720)	554 (252 to 994)	300 (139 to 573)	152 (67·7 to 288)	75·8 (34·3 to 140)	27·9 (12·8 to 51·3)	7·3 (3·3 to 13·6)	1·3 (0·5 to 2·5)	9,000
Cirrhosis and other chronic liver diseases due to alcohol u	0 (0 to 0)	0 (0 to 0)	0 (0 to 0)	0 (0 to 0)	0 (0 to 0)	0 (0 to 0)	1·9 (0·4 to 5·7)	35·4 (11·4 to 82·7)	90·1 (30 to 210)	267 (107 to 500)	504 (169 to 1,030)	753 (313 to 1,430)	948 (422 to 1,740)	1,100 (493 to 2,100)	1,130 (511 to 2,120)	893 (403 to 1,770)	523 (222 to 972)	252 (100 to 507)	110 (47·6 to 218)	47 (18·7 to 94·3)	15·6 (6·9 to 29·8)	3·8 (1·6 to 7·9)	0·6 (0·2 to 1·4)	6,670
Cirrhosis and other chronic liver diseases due to NAFLD	0 (0 to 0)	0 (0 to 0)	0 (0 to 0)	0 (0 to 0)	0 (0 to 0)	0 (0 to 0)	12·5 (3·9 to 28·1)	42·8 (16·3 to 88·9)	82·7 (31·6 to 178)	137 (52 to 276)	159 (57·5 to 339)	204 (75 to 412)	251 (88·8 to 550)	278 (105 to 610)	265 (102 to 564)	225 (84 to 479)	159 (70·5 to 301)	96·6 (41·1 to 196)	54·6 (22 to 111)	29·4 (12·7 to 59·9)	11 (4·8 to 21·6)	3 (1·2 to 6)	0·5 (0·2 to 1·2)	2,010
Cirrhosis and other chronic liver diseases due to other ca	0 (0 to 0)	0 (0 to 0)	0·5 (0·3 to 0·9)	174 (52·9 to 436)	186 (75·5 to 340)	133 (64·7 to 240)	304 (154 to 536)	332 (156 to 611)	266 (111 to 508)	269 (98·9 to 544)	228 (81·4 to 479)	233 (82 to 491)	251 (97·7 to 528)	279 (109 to 575)	303 (113 to 608)	275 (114 to 566)	198 (74·7 to 427)	122 (49·2 to 264)	70·1 (29·1 to 144)	37·6 (15·6 to 77·1)	14 (6 to 26·8)	3·7 (1·5 to 7·4)	0·7 (0·2 to 1·4)	3,680
Upper digestive system diseases	0 (0 to 0)	0 (0 to 0)	0 (0 to 0)	178 (73·3 to 389)	138 (74 to 225)	140 (78·7 to 236)	293 (174 to 475)	484 (293 to 767)	581 (358 to 936)	771 (436 to 1,290)	861 (427 to 1,450)	1,020 (555 to 1,740)	1,170 (622 to 1,970)	1,400 (762 to 2,460)	1,560 (865 to 2,600)	1,660 (917 to 2,810)	1,420 (781 to 2,360)	1,110 (642 to 1,880)	833 (483 to 1,370)	475 (280 to 758)	209 (131 to 330)	66 (40·5 to 103)	13·3 (7·9 to 20·5)	14,400
Peptic ulcer disease	0 (0 to 0)	0 (0 to 0)	0 (0 to 0)	82·2 (18·7 to 213)	77·1 (31·4 to 150)	92·4 (43 to 177)	198 (101 to 351)	309 (156 to 561)	364 (182 to 654)	548 (249 to 1,020)	637 (252 to 1,170)	811 (392 to 1,480)	962 (463 to 1,710)	1,210 (610 to 2,210)	1,390 (722 to 2,420)	1,520 (812 to 2,640)	1,320 (711 to 2,230)	1,030 (582 to 1,780)	785 (451 to 1,300)	446 (263 to 715)	200 (124 to 319)	62·4 (37·5 to 98·4)	12·5 (7·4 to 19·5)	12,100
Gastritis and duodenitis	0 (0 to 0)	0 (0 to 0)	0 (0 to 0)	95·9 (37·7 to 214)	60 (26·3 to 107)	36·5 (15·6 to 67·1)	38·7 (16 to 75·8)	50·1 (19·7 to 94·1)	56·6 (22·8 to 105)	58·4 (25·4 to 107)	83·1 (37·7 to 156)	88·1 (43·6 to 164)	98·4 (46·4 to 187)	90·3 (42·4 to 181)	90·9 (43·6 to 178)	75·9 (35·6 to 153)	49·7 (21·7 to 104)	43·9 (15·4 to 101)	27·5 (9·1 to 62·9)	18·3 (5·6 to 43·2)	5·3 (1·6 to 11·9)	2·5 (0·8 to 5·5)	0·6 (0·2 to 1·4)	1,070
Gastroesophageal reflux disease	0 (0 to 0)	0 (0 to 0)	0 (0 to 0)	0 (0 to 0)	0·6 (0·2 to 1·3)	11·3 (4·6 to 22·6)	56·9 (27·5 to 110)	124 (59·7 to 246)	161 (73·6 to 308)	164 (74·7 to 307)	142 (63·8 to 262)	123 (57·7 to 231)	108 (52·5 to 208)	93 (44·4 to 172)	78·3 (34·6 to 156)	62·5 (29·1 to 121)	46·1 (22·7 to 87·5)	31·4 (15·2 to 58·7)	20 (9·7 to 37·1)	10·4 (5 to 19·6)	3·8 (1·8 to 7·1)	1 (0·5 to 2)	0·2 (0·1 to 0·4)	1,240
Appendicitis	0 (0 to 0)	0 (0 to 0)	0 (0 to 0)	48·5 (13·4 to 126)	130 (54 to 251)	112 (52·2 to 195)	109 (47·4 to 197)	92·8 (40·7 to 176)	78·8 (27·8 to 157)	42·9 (16 to 85·9)	37·6 (12·3 to 82·3)	36·3 (12 to 77·8)	40·7 (13·5 to 85·8)	31·3 (10·7 to 68·6)	40·3 (13·5 to 87·2)	32·6 (11·4 to 74·2)	19·8 (7·3 to 42)	12 (4·5 to 24·9)	7·9 (2·8 to 17·7)	5·5 (2·1 to 12)	2·5 (0·9 to 5·4)	0·9 (0·3 to 1·8)	0·1 (0 to 0·3)	882
Paralytic ileus and intestinal obstruction	23·7 (12·1 to 45·7)	49·5 (22·3 to 102)	701 (186 to 1,800)	232 (64·9 to 675)	212 (81·9 to 416)	86·3 (31·1 to 173)	147 (48·3 to 310)	83·1 (23·1 to 184)	140 (41·6 to 308)	94·1 (24·7 to 213)	141 (40·9 to 310)	162 (51·6 to 344)	168 (60·4 to 357)	220 (83·3 to 482)	210 (82·3 to 439)	238 (102 to 506)	199 (85·9 to 380)	160 (70·3 to 329)	123 (57·9 to 253)	85·2 (41 to 164)	40·8 (20·4 to 73·1)	13 (6·8 to 22·8)	2·5 (1·1 to 4·7)	3,530
Inguinal, femoral, and abdominal hernia	9·9 (2·6 to 23·7)	8·3 (1·7 to 24·7)	123 (14·9 to 462)	89·2 (40·2 to 170)	70·9 (30·5 to 129)	48·5 (19·7 to 87·8)	38·5 (13·2 to 75·5)	55·2 (25·4 to 99)	81·6 (38·9 to 140)	84·5 (41·9 to 144)	98·8 (50·7 to 171)	113 (62·6 to 187)	133 (73·9 to 226)	176 (97·5 to 283)	206 (127 to 325)	180 (110 to 272)	165 (101 to 257)	120 (72 to 198)	73·5 (46·2 to 120)	41·6 (23·7 to 73)	19·5 (11·1 to 35·8)	5·6 (2·9 to 10·6)	1 (0·5 to 1·9)	1,940
Inflammatory bowel disease	0 (0 to 0)	0 (0 to 0)	0 (0 to 0)	11·6 (2·1 to 42·3)	7·7 (2·6 to 18·9)	5·6 (2·1 to 12·6)	6·5 (2·8 to 14·5)	11·5 (5 to 22·9)	20·8 (8·3 to 44·5)	11·6 (5·2 to 24·3)	11·1 (5 to 22·7)	17·4 (7·5 to 36·6)	15 (6·7 to 33·2)	14·6 (6·4 to 32·4)	17·5 (7·7 to 40)	16 (6·5 to 37·7)	16·9 (7 to 39·4)	9·7 (3·8 to 23)	7·5 (3 to 16·8)	5·8 (2·3 to 13·4)	1·6 (0·7 to 3·7)	0·6 (0·2 to 1·4)	0·1 (0 to 0·2)	209
Vascular intestinal disorders	0 (0 to 0)	0 (0 to 0)	0 (0 to 0)	1·8 (0·7 to 4·4)	0·9 (0·4 to 1·8)	1 (0·5 to 2·1)	2 (0·9 to 3·7)	2·8 (1·1 to 5·7)	3·7 (1·8 to 7·2)	6·6 (2·7 to 12·9)	8 (3 to 15·6)	11·1 (4·6 to 21·4)	16·7 (7·7 to 31·7)	19·7 (9·2 to 37·1)	25·8 (13·2 to 46·2)	26·5 (13·3 to 48·1)	32·6 (16·1 to 57·6)	26·6 (13·5 to 51·3)	19·6 (9·9 to 34·7)	15·1 (8·2 to 25·5)	5·8 (3·3 to 9·8)	2·1 (1·1 to 3·6)	0·2 (0·1 to 0·4)	229
Gallbladder and biliary diseases	0 (0 to 0)	0 (0 to 0)	0 (0 to 0)	11·7 (3·7 to 33·2)	15·5 (7·5 to 28·9)	17·4 (9·8 to 30·1)	23·7 (11·7 to 41·6)	40·4 (14·5 to 78·1)	76·5 (31·4 to 140)	101 (42·9 to 182)	112 (50·7 to 211)	137 (65·9 to 247)	121 (60·1 to 220)	147 (70·8 to 270)	174 (84·5 to 323)	177 (89·1 to 341)	123 (62·8 to 236)	100 (52·6 to 190)	68·7 (37·3 to 129)	38·7 (22·9 to 68·8)	18·3 (10·9 to 31·5)	5·8 (3·3 to 9·9)	0·9 (0·5 to 1·6)	1,510
Pancreatitis	0 (0 to 0)	0 (0 to 0)	0 (0 to 0)	2·1 (0·7 to 5·1)	10 (3·7 to 22·4)	5·7 (2·4 to 11·4)	35·7 (15·4 to 68·7)	88·6 (38·7 to 177)	168 (74·8 to 326)	242 (96 to 463)	254 (101 to 512)	247 (108 to 488)	220 (99·7 to 443)	197 (93·8 to 372)	184 (91·8 to 348)	133 (63·1 to 251)	99·8 (46·8 to 187)	65·3 (33 to 125)	37·2 (18·7 to 72·7)	15·5 (8·2 to 26·3)	6·2 (3·3 to 11·2)	1·7 (0·9 to 3)	0·2 (0·1 to 0·4)	2,010
Other digestive diseases	0 (0 to 0)	0 (0 to 0)	0 (0 to 0)	62·3 (24·7 to 139)	37·5 (15·9 to 75·8)	27·7 (13·8 to 51·3)	20·7 (9·8 to 38·9)	23·8 (10·7 to 50·1)	36·2 (15·4 to 75·2)	41·7 (15·9 to 82·5)	54·2 (20·9 to 108)	60 (25·8 to 118)	68 (31·3 to 132)	72·1 (31·8 to 144)	90·2 (42 to 177)	72·7 (34·8 to 137)	72·2 (35·2 to 146)	52·6 (24·5 to 108)	39 (18 to 74·1)	30·2 (14·4 to 58)	14·7 (7·3 to 27·5)	4·7 (2·3 to 9·1)	0·9 (0·4 to 1·7)	881
Neurological disorders	1·4 (0·2 to 2·9)	3·9 (0·7 to 8·6)	424 (235 to 658)	685 (237 to 1,310)	1,080 (420 to 2,010)	2,160 (758 to 4,360)	2,750 (891 to 5,730)	2,880 (913 to 5,790)	2,890 (850 to 5,860)	2,670 (801 to 5,390)	2,290 (694 to 4,570)	1,970 (708 to 3,840)	1,670 (688 to 3,200)	1,410 (626 to 2,560)	1,270 (632 to 2,150)	1,190 (673 to 1,980)	1,090 (646 to 1,920)	1,040 (658 to 1,890)	1,020 (644 to 1,900)	1,060 (593 to 2,190)	671 (324 to 1,430)	267 (119 to 594)	67·1 (29·8 to 155)	30,600
Alzheimer's disease and other dementias	0 (0 to 0)	0 (0 to 0)	0 (0 to 0)	0 (0 to 0)	0 (0 to 0)	0 (0 to 0)	0 (0 to 0)	0 (0 to 0)	0 (0 to 0)	0 (0 to 0)	0 (0 to 0)	13·9 (3·4 to 42·8)	68·8 (20·4 to 189)	131 (43·3 to 329)	238 (82·4 to 596)	390 (137 to 999)	487 (180 to 1,230)	543 (222 to 1,350)	579 (239 to 1,470)	742 (293 to 1,870)	539 (192 to 1,300)	238 (87·6 to 565)	61·8 (24 to 151)	4,030
Parkinson's disease	0 (0 to 0)	0 (0 to 0)	0 (0 to 0)	0 (0 to 0)	0 (0 to 0)	0 (0 to 0)	0 (0 to 0)	0·5 (0·3 to 0·8)	1·7 (0·7 to 3·1)	8·3 (4·5 to 13·2)	6·5 (3·6 to 10·5)	40·7 (24·7 to 59·7)	57·4 (37·8 to 80·4)	75·8 (51·5 to 104)	96·8 (69·9 to 129)	150 (108 to 193)	197 (149 to 249)	267 (210 to 328)	304 (245 to 376)	255 (203 to 317)	107 (84 to 133)	21·9 (16·5 to 28·1)	3·6 (2·4 to 4·6)	1,590
Idiopathic epilepsy	1·3 (0·2 to 2·9)	3·8 (0·7 to 8·6)	238 (110 to 407)	515 (184 to 992)	606 (202 to 1,200)	561 (185 to 1,100)	538 (203 to 1,050)	542 (233 to 1,020)	477 (211 to 898)	374 (149 to 694)	317 (137 to 580)	264 (112 to 476)	218 (99·7 to 401)	168 (68·6 to 313)	150 (58·6 to 268)	129 (48·6 to 243)	97·5 (34·1 to 188)	71·1 (27·2 to 133)	46·9 (17·1 to 86·2)	27·5 (10 to 50·7)	11·5 (3·8 to 21·7)	3·7 (1·2 to 7·2)	1·1 (0·4 to 2)	5,360
Multiple sclerosis	0 (0 to 0)	0 (0 to 0)	0 (0 to 0)	0 (0 to 0)	0·2 (0 to 1·2)	0·3 (0·1 to 0·7)	1·2 (0·6 to 2·1)	5 (2·7 to 9·1)	7·2 (4·4 to 11·5)	11 (6·6 to 16·9)	13·9 (8·3 to 21·5)	19·3 (12 to 30·2)	19·1 (12·3 to 29·9)	16·7 (10·9 to 26·3)	16 (10·7 to 25·5)	11·1 (7·2 to 17·9)	5·6 (3·8 to 8·5)	3 (2 to 4·7)	1·7 (1·2 to 2·7)	0·9 (0·6 to 1·4)	0·3 (0·2 to 0·5)	0·1 (0 to 0·1)	0 (0 to 0)	133
Motor neuron disease	0 (0 to 0·1)	0 (0 to 0·1)	9·3 (1·5 to 29·3)	1·2 (0·6 to 2·7)	1·1 (0·7 to 1·6)	2 (1·3 to 3·3)	2·8 (1·6 to 5)	2 (1·2 to 2·9)	2·4 (1·4 to 4·2)	2·9 (1·6 to 5)	5 (2·5 to 9·1)	4·9 (2·1 to 10)	8·3 (3·9 to 15·8)	9 (3 to 21·6)	12·4 (5·9 to 22·8)	9·9 (4·7 to 18·3)	7·1 (3·4 to 12·8)	3·1 (1·5 to 5·8)	1·1 (0·5 to 2)	0·3 (0·2 to 0·6)	0·1 (0 to 0·1)	0 (0 to 0)	0 (0 to 0)	84·8
Headache disorders	0 (0 to 0)	0 (0 to 0)	0 (0 to 0)	0 (0 to 0)	281 (6·1 to 758)	1,350 (59·2 to 3,340)	1,980 (189 to 4,800)	2,170 (262 to 5,090)	2,310 (322 to 5,240)	2,210 (376 to 4,920)	1,890 (353 to 4,190)	1,570 (319 to 3,450)	1,230 (282 to 2,740)	957 (213 to 2,090)	705 (168 to 1,500)	467 (122 to 1,020)	271 (74·8 to 579)	140 (39·3 to 310)	71·2 (19·9 to 157)	32·1 (8·3 to 70·1)	11·3 (2·9 to 25)	2·6 (0·6 to 6)	0·4 (0·1 to 1)	17,700
Migraine	0 (0 to 0)	0 (0 to 0)	0 (0 to 0)	0 (0 to 0)	248 (4 to 700)	1,250 (35·4 to 3,290)	1,830 (131 to 4,720)	2,000 (196 to 4,930)	2,120 (224 to 4,900)	2,020 (247 to 4,680)	1,710 (238 to 4,010)	1,430 (216 to 3,270)	1,120 (179 to 2,550)	859 (150 to 1,970)	631 (119 to 1,440)	409 (82·3 to 942)	233 (48·9 to 527)	117 (26·5 to 277)	58·7 (12·8 to 138)	26·6 (6 to 60·4)	9·2 (1·9 to 21·7)	2·1 (0·4 to 5)	0·3 (0 to 0·7)	16,100
Tension-type headache	0 (0 to 0)	0 (0 to 0)	0 (0 to 0)	0 (0 to 0)	32·9 (0·7 to 222)	106 (3 to 846)	150 (17·7 to 927)	170 (27·9 to 829)	192 (34·6 to 904)	190 (37·7 to 807)	177 (37·3 to 730)	147 (38·1 to 585)	116 (32·7 to 384)	98·2 (25·1 to 340)	74 (20·4 to 234)	57·7 (14·8 to 178)	38·7 (9 to 132)	23·4 (4·5 to 87·8)	12·5 (1·8 to 52·6)	5·5 (0·8 to 22·7)	2 (0·3 to 8·7)	0·5 (0 to 2·9)	0·1 (0 to 0·6)	1,590
Other neurological disorders	0 (0 to 0)	0 (0 to 0)	177 (89·8 to 309)	169 (52·1 to 331)	191 (58·9 to 387)	248 (95·8 to 474)	236 (109 to 406)	160 (87·7 to 266)	92·1 (45·2 to 154)	60·3 (27·4 to 112)	57·5 (26·9 to 103)	54·9 (27·6 to 99·9)	59·9 (32·9 to 98·6)	53·1 (33·3 to 79·1)	48·5 (32·5 to 69·3)	35·2 (23·7 to 49·3)	25 (17·9 to 34·5)	16·2 (11·8 to 21)	11·8 (8·3 to 16·7)	4·6 (3 to 6)	2·4 (1·7 to 3·4)	0·6 (0·4 to 0·8)	0·2 (0·1 to 0·3)	1,700
Mental disorders	1·7 (1 to 2·5)	4·9 (3 to 7·4)	86·8 (53·4 to 131)	458 (290 to 692)	1,740 (1,160 to 2,540)	4,070 (2,750 to 5,860)	5,340 (3,680 to 7,310)	5,630 (3,860 to 7,750)	5,790 (4,050 to 7,860)	5,380 (3,790 to 7,360)	4,580 (3,320 to 6,130)	3,770 (2,710 to 5,100)	3,140 (2,290 to 4,160)	2,580 (1,880 to 3,540)	2,100 (1,530 to 2,810)	1,530 (1,120 to 2,050)	975 (704 to 1,290)	568 (409 to 745)	323 (230 to 428)	158 (114 to 207)	60·4 (43·6 to 80·2)	16·5 (11·5 to 22·5)	3 (2 to 4·3)	48,300
Schizophrenia	0 (0 to 0)	0 (0 to 0)	0 (0 to 0)	0 (0 to 0)	0 (0 to 0)	29·2 (12·7 to 56·9)	218 (88·8 to 398)	672 (357 to 1,140)	1,070 (608 to 1,650)	1,140 (691 to 1,650)	988 (620 to 1,440)	791 (504 to 1,100)	619 (399 to 862)	469 (305 to 664)	339 (219 to 479)	213 (137 to 305)	117 (73·7 to 169)	58 (35·9 to 84·6)	26·8 (15·1 to 40·1)	10·1 (5·3 to 15·6)	2·8 (1·3 to 4·8)	0·5 (0·2 to 0·9)	0·1 (0 to 0·1)	6,760
Depressive disorders	0 (0 to 0)	0 (0 to 0)	0 (0 to 0)	0·6 (0·2 to 1·3)	40·6 (16 to 81·6)	466 (222 to 846)	1,570 (908 to 2,490)	2,270 (1,280 to 3,580)	1,950 (1,180 to 3,010)	1,620 (1,000 to 2,520)	1,400 (893 to 2,050)	1,200 (753 to 1,830)	1,060 (686 to 1,580)	911 (598 to 1,380)	775 (504 to 1,160)	583 (370 to 898)	372 (238 to 546)	217 (143 to 312)	123 (77 to 181)	62·2 (38·6 to 89·6)	25·5 (16·4 to 37·3)	7·6 (4·8 to 11·6)	1·5 (0·8 to 2·5)	14,700
Major depressive disorder	0 (0 to 0)	0 (0 to 0)	0 (0 to 0)	0·5 (0·1 to 1·2)	32·2 (9·7 to 70·8)	413 (178 to 788)	1,420 (813 to 2,310)	2,010 (1,110 to 3,250)	1,580 (899 to 2,530)	1,190 (680 to 1,960)	968 (583 to 1,450)	796 (473 to 1,280)	691 (432 to 1,070)	595 (367 to 919)	509 (303 to 794)	383 (224 to 603)	245 (153 to 367)	147 (94·2 to 213)	86·2 (49·7 to 133)	45·8 (25·5 to 70)	19·7 (12·3 to 29·6)	6·1 (3·6 to 9·7)	1·2 (0·6 to 2·1)	11,100
Dysthymia	0 (0 to 0)	0 (0 to 0)	0 (0 to 0)	0·1 (0 to 0·3)	8·4 (0 to 22·9)	53·5 (18·3 to 110)	150 (67·1 to 267)	266 (120 to 485)	369 (187 to 656)	430 (240 to 692)	430 (239 to 717)	407 (217 to 683)	366 (199 to 613)	316 (181 to 517)	266 (153 to 431)	200 (108 to 330)	127 (67·8 to 210)	70·2 (38·7 to 117)	37 (20·6 to 62·1)	16·4 (8·9 to 28·6)	5·9 (3·1 to 10·1)	1·6 (0·8 to 2·6)	0·3 (0·1 to 0·5)	3,520
Bipolar disorder	0 (0 to 0)	0 (0 to 0)	0 (0 to 0)	0 (0 to 0)	0 (0 to 0)	75·9 (18·5 to 163)	272 (116 to 506)	331 (147 to 586)	334 (156 to 580)	295 (145 to 531)	249 (126 to 430)	210 (106 to 355)	176 (88·2 to 310)	145 (69·2 to 256)	123 (63·7 to 213)	93·9 (47·6 to 159)	61·9 (29·1 to 107)	34·9 (17·3 to 59·5)	18·7 (9·3 to 33·1)	8·2 (3·9 to 14·2)	2·8 (1·3 to 5)	0·7 (0·3 to 1·2)	0·1 (0 to 0·2)	2,430
Anxiety disorders	0 (0 to 0)	0 (0 to 0)	0 (0 to 0)	33·7 (6·8 to 74·4)	451 (220 to 805)	1,100 (583 to 1,810)	1,370 (832 to 2,140)	1,370 (806 to 2,230)	1,340 (766 to 2,190)	1,230 (725 to 1,970)	1,040 (612 to 1,650)	863 (491 to 1,430)	728 (416 to 1,170)	610 (370 to 924)	510 (316 to 768)	383 (225 to 601)	255 (149 to 405)	154 (91·5 to 240)	90·5 (55 to 141)	44·5 (26·3 to 68·8)	15·8 (9·1 to 24·9)	3·8 (2 to 6·1)	0·6 (0·3 to 0·9)	11,600
Eating disorders	0 (0 to 0)	0 (0 to 0)	0 (0 to 0)	0 (0 to 0)	3·3 (1·1 to 7·1)	43·7 (13·7 to 89·7)	112 (50·2 to 211)	136 (59·1 to 258)	138 (68·5 to 243)	136 (62·4 to 244)	84·8 (38·3 to 152)	28·6 (10·5 to 56)	8·1 (2·6 to 17)	0 (0 to 0)	0 (0 to 0)	0 (0 to 0)	0 (0 to 0)	0 (0 to 0)	0 (0 to 0)	0 (0 to 0)	0 (0 to 0)	0 (0 to 0)	0 (0 to 0)	690
Anorexia nervosa	0 (0 to 0)	0 (0 to 0)	0 (0 to 0)	0 (0 to 0)	2·8 (0·8 to 6·5)	17·6 (3 to 40·9)	41·6 (14 to 88·4)	36·9 (10·7 to 75·3)	24·6 (5·1 to 54·7)	14·3 (2·6 to 33·4)	9·1 (0 to 22·4)	6 (0 to 15·4)	3·6 (1·9 to 6·1)	0 (0 to 0)	0 (0 to 0)	0 (0 to 0)	0 (0 to 0)	0 (0 to 0)	0 (0 to 0)	0 (0 to 0)	0 (0 to 0)	0 (0 to 0)	0 (0 to 0)	157
Bulimia nervosa	0 (0 to 0)	0 (0 to 0)	0 (0 to 0)	0 (0 to 0)	0·5 (0·1 to 1·1)	26·1 (3·9 to 64·9)	70·1 (22·4 to 145)	99·2 (32·9 to 202)	113 (48·6 to 205)	121 (53·4 to 227)	75·7 (31·5 to 140)	22·6 (6 to 46·7)	4·5 (0 to 12·8)	0 (0 to 0)	0 (0 to 0)	0 (0 to 0)	0 (0 to 0)	0 (0 to 0)	0 (0 to 0)	0 (0 to 0)	0 (0 to 0)	0 (0 to 0)	0 (0 to 0)	533
Autism spectrum disorders	1·6 (1 to 2·3)	4·5 (2·8 to 6·7)	71·2 (45 to 108)	295 (186 to 454)	328 (204 to 491)	305 (185 to 468)	289 (172 to 435)	269 (164 to 406)	249 (153 to 387)	215 (137 to 326)	170 (105 to 248)	133 (82·7 to 198)	106 (64·7 to 159)	83·9 (50·8 to 131)	66·4 (40·8 to 99·6)	47·5 (28·4 to 73·4)	30·7 (18·8 to 45·8)	18·4 (11 to 28·2)	11·1 (6·7 to 16·7)	5·6 (3·3 to 8·6)	2·2 (1·3 to 3·4)	0·6 (0·4 to 0·9)	0·1 (0·1 to 0·2)	2,700
Attention-deficit/hyperactivity disorder	0 (0 to 0)	0 (0 to 0)	0 (0 to 0)	12·3 (5 to 24·2)	122 (62·6 to 219)	155 (80·6 to 283)	116 (61·9 to 212)	75·9 (39 to 137)	53·3 (26·2 to 96)	37·5 (17·5 to 68·3)	24·8 (11·2 to 45·6)	16·3 (7·4 to 29·7)	10·9 (4·8 to 20·9)	7 (2·6 to 13·8)	3·9 (1·3 to 7·9)	1·8 (0·5 to 3·8)	0·6 (0·1 to 1·5)	0·2 (0 to 0·6)	0 (0 to 0·1)	0 (0 to 0)	0 (0 to 0)	0 (0 to 0)	0 (0 to 0)	638
Conduct disorder	0 (0 to 0)	0 (0 to 0)	0 (0 to 0)	0 (0 to 0)	617 (316 to 1,090)	1,710 (926 to 2,870)	1,090 (556 to 1,790)	0 (0 to 0)	0 (0 to 0)	0 (0 to 0)	0 (0 to 0)	0 (0 to 0)	0 (0 to 0)	0 (0 to 0)	0 (0 to 0)	0 (0 to 0)	0 (0 to 0)	0 (0 to 0)	0 (0 to 0)	0 (0 to 0)	0 (0 to 0)	0 (0 to 0)	0 (0 to 0)	3,410
Idiopathic developmental intellectual disability	0·1 (0 to 0·4)	0·4 (0 to 1·5)	15·6 (1·7 to 36·2)	116 (37·2 to 220)	175 (73·2 to 316)	163 (69·2 to 284)	153 (64·9 to 272)	139 (62·1 to 254)	123 (49·4 to 217)	102 (41·7 to 182)	77·8 (33·1 to 141)	57·4 (22·5 to 107)	44·1 (16·7 to 81·2)	32·9 (12·1 to 60·9)	24·6 (9·6 to 45·9)	16·6 (6·3 to 30·1)	10 (3·4 to 18·8)	5·4 (2 to 10)	2·9 (0·9 to 5·4)	1·4 (0·5 to 2·6)	0·4 (0·1 to 0·8)	0·1 (0 to 0·2)	0 (0 to 0)	1,260
Other mental disorders	0 (0 to 0)	0 (0 to 0)	0 (0 to 0)	0 (0 to 0)	0 (0 to 0)	23·2 (10·5 to 40·5)	155 (72·3 to 279)	362 (173 to 602)	534 (287 to 841)	603 (343 to 946)	551 (329 to 857)	466 (280 to 734)	391 (235 to 614)	323 (188 to 503)	261 (163 to 404)	193 (120 to 288)	128 (80·8 to 196)	80·1 (50·3 to 119)	49·5 (31·7 to 73·4)	26·5 (17 to 38·6)	11 (7·1 to 15·9)	3·3 (2·1 to 4·8)	0·7 (0·4 to 1)	4,160
Substance use disorders	0 (0 to 0)	0 (0 to 0)	0·3 (0·2 to 0·5)	1·3 (0·7 to 2·4)	1·7 (0·8 to 3·2)	31·9 (14·1 to 55·8)	586 (371 to 842)	1,560 (1,030 to 2,220)	1,820 (1,250 to 2,530)	1,610 (1,080 to 2,290)	1,350 (936 to 1,910)	1,120 (730 to 1,570)	895 (605 to 1,300)	629 (416 to 897)	472 (306 to 667)	327 (210 to 462)	184 (122 to 255)	96·7 (64 to 134)	47·4 (31·7 to 65·5)	22 (14·8 to 30·6)	8·7 (5·9 to 12)	2·4 (1·6 to 3·4)	0·5 (0·3 to 0·8)	10,800
Alcohol use disorders	0 (0 to 0)	0 (0 to 0)	0·3 (0·2 to 0·5)	1·3 (0·7 to 2·4)	1·7 (0·8 to 3·2)	20 (7·4 to 40·1)	276 (153 to 455)	765 (421 to 1,230)	1,050 (657 to 1,590)	1,140 (700 to 1,750)	1,070 (697 to 1,590)	940 (574 to 1,370)	782 (509 to 1,170)	551 (354 to 814)	421 (265 to 615)	297 (187 to 431)	165 (107 to 235)	86·2 (54·6 to 121)	40·7 (26·5 to 58·3)	18 (11·4 to 25·7)	7·3 (4·7 to 10·4)	2 (1·3 to 2·9)	0·4 (0·2 to 0·6)	7,630
Drug use disorders	0 (0 to 0)	0 (0 to 0)	0 (0 to 0)	0 (0 to 0)	0 (0 to 0)	11·9 (2·9 to 27·1)	310 (177 to 490)	798 (492 to 1,170)	774 (479 to 1,130)	468 (295 to 677)	279 (179 to 411)	180 (111 to 264)	113 (67·6 to 172)	77·5 (48 to 118)	50·4 (28·9 to 79)	29·7 (16·2 to 49·9)	18·7 (9·6 to 31)	10·5 (5·2 to 17·4)	6·7 (3·5 to 11)	3·9 (2·1 to 6·4)	1·4 (0·7 to 2·2)	0·4 (0·2 to 0·7)	0·1 (0·1 to 0·2)	3,130
Opioid use disorders	0 (0 to 0)	0 (0 to 0)	0 (0 to 0)	0 (0 to 0)	0 (0 to 0)	0 (0 to 0)	98·8 (47·5 to 177)	341 (182 to 546)	376 (215 to 582)	278 (157 to 439)	185 (103 to 294)	124 (64·2 to 197)	76·6 (38·4 to 129)	53·4 (26·5 to 90)	36·5 (17·2 to 63·9)	22·8 (10·8 to 41·6)	14·6 (6·6 to 26·5)	8 (3 to 15)	5·3 (2·3 to 9·5)	3·2 (1·5 to 5·6)	1·1 (0·4 to 1·9)	0·3 (0·1 to 0·6)	0·1 (0 to 0·2)	1,630
Cocaine use disorders	0 (0 to 0)	0 (0 to 0)	0 (0 to 0)	0 (0 to 0)	0 (0 to 0)	0·1 (0 to 0·3)	7·8 (3·4 to 14·1)	11·9 (6·5 to 20·5)	11·4 (5·9 to 20·3)	11·3 (4·9 to 20·7)	8·2 (3·5 to 16·1)	5·8 (2·5 to 11·5)	3·7 (1·3 to 8·5)	3·7 (1·4 to 7·6)	2·8 (1 to 5·8)	1·5 (0·6 to 3·3)	1 (0·4 to 2·3)	0·6 (0·2 to 1·2)	0·2 (0·1 to 0·4)	0 (0 to 0)	0 (0 to 0)	0 (0 to 0)	0 (0 to 0)	69·9
Amphetamine use disorders	0 (0 to 0)	0 (0 to 0)	0 (0 to 0)	0 (0 to 0)	0 (0 to 0)	0 (0 to 0)	44·6 (9 to 101)	193 (74·3 to 374)	180 (77·1 to 338)	75·7 (25·8 to 150)	28·5 (7·5 to 61·3)	13·8 (7·1 to 24·6)	7·4 (3·8 to 12·7)	4·4 (2·3 to 7·9)	2·6 (1·3 to 4·6)	1·4 (0·7 to 2·5)	0·8 (0·4 to 1·5)	0·4 (0·2 to 0·7)	0·2 (0·1 to 0·3)	0 (0 to 0·1)	0 (0 to 0·1)	0 (0 to 0)	0 (0 to 0)	553
Cannabis use disorders	0 (0 to 0)	0 (0 to 0)	0 (0 to 0)	0 (0 to 0)	0 (0 to 0)	11·7 (2·7 to 26·7)	122 (40·7 to 257)	111 (46·9 to 212)	66·4 (25·6 to 132)	37·4 (13·3 to 72·3)	22·6 (7·2 to 46·8)	14·6 (4·7 to 30·7)	10·4 (2·9 to 21·4)	6·8 (3·2 to 12·6)	4 (1·7 to 7·2)	2 (0·9 to 3·8)	0·9 (0·4 to 1·7)	0·4 (0·1 to 0·8)	0·2 (0·1 to 0·3)	0·1 (0 to 0·1)	0 (0 to 0)	0 (0 to 0)	0 (0 to 0)	410
Other drug use disorders	0 (0 to 0)	0 (0 to 0)	0 (0 to 0)	0 (0 to 0)	0 (0 to 0)	0·1 (0 to 0·2)	36·9 (12·5 to 74·4)	142 (62 to 257)	139 (65 to 251)	65·8 (30·7 to 119)	34·8 (16 to 59·1)	21·4 (12·1 to 33·6)	14·8 (8·1 to 24)	9·2 (4·8 to 15·2)	4·6 (2·2 to 8)	2 (1·1 to 3·4)	1·4 (0·8 to 2·3)	1·2 (0·6 to 2)	0·8 (0·4 to 1·4)	0·6 (0·3 to 1)	0·2 (0·1 to 0·4)	0·1 (0 to 0·1)	0 (0 to 0)	474
Diabetes and kidney diseases	2·7 (1·4 to 4·7)	8·9 (3·2 to 19·5)	606 (292 to 1,050)	399 (232 to 680)	651 (412 to 988)	560 (381 to 787)	1,160 (808 to 1,600)	1,860 (1,260 to 2,600)	2,210 (1,520 to 3,030)	2,800 (1,830 to 3,780)	3,260 (2,130 to 4,480)	4,150 (2,800 to 5,670)	5,160 (3,610 to 7,140)	6,620 (4,790 to 9,150)	7,670 (5,670 to 10,500)	7,550 (5,420 to 10,100)	6,260 (4,730 to 8,210)	4,590 (3,470 to 5,870)	3,220 (2,550 to 4,100)	1,850 (1,460 to 2,370)	774 (598 to 988)	227 (178 to 287)	44·2 (33·4 to 57·4)	61,600
Diabetes mellitus	2·7 (1·4 to 4·7)	8·8 (3·1 to 19·5)	95·9 (25·1 to 224)	74·2 (17·6 to 197)	92·6 (30 to 193)	101 (31·5 to 224)	297 (199 to 438)	523 (328 to 852)	798 (508 to 1,180)	1,150 (727 to 1,700)	1,480 (953 to 2,150)	2,010 (1,330 to 2,840)	2,650 (1,800 to 3,770)	3,540 (2,490 to 5,020)	4,190 (2,980 to 5,940)	4,220 (2,950 to 5,970)	3,580 (2,590 to 4,800)	2,660 (1,910 to 3,570)	1,850 (1,340 to 2,480)	1,010 (741 to 1,330)	412 (301 to 552)	116 (83·2 to 155)	21·8 (15·6 to 31·1)	30,900
Diabetes mellitus type 1	2·7 (1·4 to 4·7)	8·8 (3·1 to 19·5)	95·9 (25·1 to 224)	74·2 (17·6 to 197)	92·6 (30 to 193)	101 (31·5 to 224)	124 (66·7 to 209)	184 (91·3 to 342)	223 (109 to 414)	158 (63·9 to 334)	183 (75 to 369)	224 (83·9 to 490)	94·9 (39·8 to 224)	113 (47·3 to 292)	132 (48·6 to 344)	99·1 (39·1 to 263)	82·4 (32·4 to 207)	49·6 (19 to 124)	28·7 (11·6 to 69·8)	12·3 (5·3 to 28·5)	5·5 (2·3 to 12·7)	1·7 (0·7 to 4·1)	0·3 (0·1 to 0·8)	2,090
Diabetes mellitus type 2	0 (0 to 0)	0 (0 to 0)	0 (0 to 0)	0 (0 to 0)	0 (0 to 0)	0 (0 to 0)	173 (110 to 267)	340 (215 to 518)	574 (362 to 847)	992 (634 to 1,460)	1,300 (840 to 1,880)	1,790 (1,200 to 2,560)	2,550 (1,730 to 3,620)	3,430 (2,410 to 4,840)	4,060 (2,890 to 5,690)	4,120 (2,890 to 5,860)	3,500 (2,540 to 4,700)	2,610 (1,880 to 3,520)	1,820 (1,330 to 2,440)	1,000 (733 to 1,310)	406 (297 to 545)	114 (81·4 to 153)	21·4 (15·4 to 30·6)	28,800
Chronic kidney disease	0 (0 to 0)	0·1 (0 to 0·1)	432 (168 to 786)	291 (176 to 490)	429 (287 to 614)	413 (281 to 569)	854 (563 to 1,240)	1,330 (841 to 1,980)	1,390 (864 to 2,060)	1,640 (964 to 2,380)	1,770 (1,040 to 2,690)	2,120 (1,230 to 3,240)	2,500 (1,520 to 3,880)	3,050 (1,920 to 4,840)	3,460 (2,170 to 5,440)	3,320 (2,110 to 5,000)	2,660 (1,770 to 3,910)	1,920 (1,280 to 2,750)	1,370 (962 to 2,000)	837 (600 to 1,220)	359 (259 to 509)	111 (79·6 to 158)	22·3 (14·4 to 32·2)	30,300
Chronic kidney disease due to diabetes mellitus type 1	0 (0 to 0)	0 (0 to 0)	8·2 (2·4 to 20·6)	9·8 (4·1 to 20·3)	23·9 (8·7 to 54·2)	43 (16·4 to 92·2)	129 (49·5 to 252)	300 (127 to 570)	435 (204 to 747)	619 (283 to 1,010)	679 (301 to 1,170)	748 (335 to 1,320)	701 (309 to 1,260)	593 (228 to 1,200)	431 (132 to 940)	288 (93·2 to 690)	168 (61·8 to 380)	88·7 (33 to 197)	40·6 (15·3 to 89)	12·4 (4·4 to 28·6)	1·9 (0·6 to 4·8)	0·1 (0 to 0·3)	0 (0 to 0)	5,320
Chronic kidney disease due to diabetes mellitus type 2	0 (0 to 0)	0 (0 to 0)	0 (0 to 0)	0 (0 to 0)	0 (0 to 0)	0 (0 to 0)	62·7 (17·9 to 150)	126 (41·6 to 283)	153 (55·8 to 314)	215 (74·8 to 429)	283 (104 to 602)	449 (174 to 873)	724 (329 to 1,310)	1,120 (576 to 1,960)	1,440 (780 to 2,520)	1,420 (806 to 2,300)	1,130 (696 to 1,720)	792 (488 to 1,230)	546 (340 to 842)	321 (208 to 494)	131 (82·5 to 197)	37·8 (23·4 to 56·6)	7·1 (3·7 to 11·2)	8,960
Chronic kidney disease due to hypertension	0 (0 to 0)	0 (0 to 0)	0 (0 to 0)	0 (0 to 0)	0 (0 to 0)	0 (0 to 0)	149 (76·8 to 267)	273 (134 to 489)	286 (141 to 500)	323 (155 to 584)	369 (179 to 657)	495 (258 to 816)	667 (372 to 1,120)	932 (513 to 1,550)	1,180 (668 to 1,970)	1,240 (720 to 2,040)	1,060 (651 to 1,690)	787 (496 to 1,220)	580 (377 to 913)	365 (244 to 555)	162 (109 to 236)	51·3 (34·4 to 76)	10·4 (6·4 to 16·4)	8,930
Chronic kidney disease due to glomerulonephritis	0 (0 to 0)	0 (0 to 0)	73·1 (24 to 166)	64·5 (32·5 to 113)	110 (52·7 to 197)	131 (64·4 to 233)	221 (113 to 391)	296 (146 to 553)	253 (129 to 439)	234 (120 to 411)	212 (108 to 372)	207 (101 to 362)	187 (96·2 to 332)	176 (87·5 to 316)	162 (80·9 to 298)	130 (61·9 to 240)	91·2 (42·3 to 170)	63 (30·5 to 116)	45·8 (20·2 to 86·2)	29·2 (12·4 to 61·7)	12·4 (5·4 to 24·1)	3·8 (1·7 to 7·4)	0·7 (0·3 to 1·6)	2,700
Chronic kidney disease due to other and unspecified caus	0 (0 to 0)	0 (0 to 0·1)	350 (133 to 659)	217 (124 to 377)	294 (183 to 446)	239 (141 to 374)	292 (149 to 507)	330 (166 to 564)	266 (136 to 440)	250 (121 to 439)	226 (110 to 391)	223 (111 to 377)	224 (119 to 368)	230 (119 to 392)	244 (126 to 417)	238 (126 to 403)	220 (122 to 370)	187 (110 to 315)	154 (90 to 252)	108 (63·8 to 185)	53 (31·3 to 84·1)	17·8 (11 to 27·3)	4 (2·3 to 6·7)	4,370
Acute glomerulonephritis	0 (0 to 0)	0 (0 to 0)	78·7 (1·1 to 254)	33·9 (0·6 to 108)	130 (1·7 to 366)	46·9 (0·7 to 137)	10·2 (0·2 to 28·3)	10 (0·2 to 27·9)	24·5 (0·3 to 66·3)	13·8 (0·2 to 34·7)	10·3 (0·2 to 24·5)	16·1 (0·3 to 38·1)	10 (0·3 to 26·7)	20·8 (0·7 to 54·3)	11·3 (0·4 to 29·1)	12·1 (0·4 to 33·3)	13·5 (0·5 to 33·1)	7·7 (0·3 to 18·1)	7·8 (0·3 to 19·5)	4·6 (0·2 to 12·9)	2·7 (0·2 to 7·1)	0·7 (0 to 1·8)	0·1 (0 to 0·4)	465
Skin and subcutaneous diseases	0·1 (0·1 to 0·3)	1·8 (1 to 3·2)	229 (121 to 402)	2,010 (1,220 to 3,050)	2,350 (1,440 to 3,550)	2,390 (1,490 to 3,660)	2,600 (1,620 to 3,960)	1,920 (1,200 to 2,940)	1,610 (986 to 2,470)	1,380 (841 to 2,090)	1,120 (701 to 1,690)	943 (604 to 1,420)	815 (524 to 1,240)	721 (461 to 1,080)	657 (434 to 957)	549 (363 to 775)	401 (265 to 577)	293 (192 to 430)	215 (143 to 317)	143 (94 to 207)	63·9 (41·3 to 95)	21·2 (13·3 to 32)	4·3 (2·8 to 6·4)	20,400
Dermatitis	0 (0 to 0)	0 (0 to 0)	0 (0 to 0)	588 (312 to 1,030)	631 (331 to 1,060)	447 (235 to 754)	385 (212 to 633)	360 (200 to 583)	354 (196 to 563)	327 (176 to 527)	272 (148 to 436)	232 (131 to 376)	209 (118 to 341)	194 (105 to 323)	180 (102 to 290)	146 (81 to 230)	107 (59·6 to 166)	69·5 (41 to 108)	44·1 (26·5 to 68·4)	24 (14 to 38·2)	9·5 (5·5 to 15·4)	2·6 (1·6 to 4·3)	0·5 (0·3 to 0·8)	4,580
Atopic dermatitis	0 (0 to 0)	0 (0 to 0)	0 (0 to 0)	582 (308 to 1,020)	612 (320 to 1,040)	387 (194 to 663)	288 (145 to 504)	250 (122 to 438)	229 (117 to 402)	201 (102 to 358)	167 (84·1 to 289)	148 (73·7 to 259)	137 (73·4 to 239)	126 (64·8 to 221)	119 (59·8 to 209)	97·3 (49 to 171)	70 (37·1 to 118)	45·4 (24·2 to 75·2)	29·8 (16 to 50)	16·9 (8·9 to 28·3)	6·9 (3·7 to 11·9)	2 (1·1 to 3·4)	0·4 (0·2 to 0·6)	3,520
Contact dermatitis	0 (0 to 0)	0 (0 to 0)	0 (0 to 0)	0 (0 to 0)	11·7 (4·1 to 24·2)	50·7 (19·8 to 100)	86·5 (34·3 to 167)	99·3 (41·5 to 190)	112 (48·3 to 216)	113 (46·4 to 216)	94·3 (37·5 to 189)	75·8 (34·9 to 152)	65·6 (27·7 to 130)	62·6 (27·1 to 117)	56·6 (24·2 to 112)	45·5 (18·6 to 82·3)	33·3 (15·2 to 67·1)	21·6 (9·8 to 42·3)	12·5 (5·9 to 22·9)	6·1 (2·7 to 11·3)	2·2 (0·9 to 4·2)	0·6 (0·2 to 1·1)	0·1 (0 to 0·2)	950
Seborrhoeic dermatitis	0 (0 to 0)	0 (0 to 0)	0 (0 to 0)	6·7 (2·7 to 12·8)	6·8 (2·5 to 13·5)	9 (3·9 to 16·5)	10·6 (4·6 to 20·3)	11·1 (4·8 to 20·9)	12·8 (6·1 to 22)	12·7 (5·9 to 23)	10·3 (4·9 to 17·6)	8 (3·6 to 13·9)	6·2 (2·9 to 11·4)	5·2 (2·5 to 9·4)	4·3 (2 to 7·6)	3·6 (1·7 to 6·5)	3·4 (1·7 to 6)	2·6 (1·2 to 4·3)	1·8 (0·9 to 3)	1 (0·5 to 1·6)	0·4 (0·2 to 0·7)	0·1 (0·1 to 0·2)	0 (0 to 0)	117
Psoriasis	0 (0 to 0)	0 (0 to 0)	0 (0 to 0)	11·2 (7·3 to 16)	20 (13·2 to 28·3)	25·6 (17·1 to 35·6)	31 (18 to 48·1)	34·4 (20·6 to 51·5)	37·4 (12·5 to 70·8)	42·8 (15·2 to 80·9)	44·9 (18·3 to 81)	45·7 (20·4 to 78)	44·9 (22·9 to 75·8)	41·5 (21·7 to 69·1)	36·7 (19·7 to 59)	28 (15·1 to 44·9)	17·9 (9·8 to 28·8)	10·6 (5·5 to 17)	5·9 (2·8 to 9·8)	2·7 (1·4 to 4·6)	0·9 (0·4 to 1·6)	0·2 (0·1 to 0·4)	0 (0 to 0·1)	483
Bacterial skin diseases	0 (0 to 0)	0·2 (0·1 to 0·3)	74 (12 to 232)	39·3 (15·4 to 82·4)	24·9 (10 to 52)	29·1 (13 to 55·2)	48 (21·4 to 98·7)	53·5 (24·1 to 100)	55·3 (26·4 to 105)	39·3 (13·5 to 89·5)	54·2 (20·6 to 111)	64·8 (24·3 to 138)	64·5 (26·3 to 124)	67·9 (27·8 to 135)	83·1 (32·6 to 170)	81·3 (27·7 to 169)	46·7 (18 to 91·1)	39·6 (12·6 to 86·2)	35·5 (11·2 to 76·4)	26·5 (8·5 to 56·5)	11·4 (3·3 to 26·7)	4·1 (1·2 to 8·7)	0·6 (0·2 to 1·4)	944
Cellulitis	0 (0 to 0)	0 (0 to 0·1)	8·7 (1·4 to 33·1)	6·9 (3·5 to 14·6)	5·8 (2·5 to 13)	15·7 (6·2 to 34·8)	6·7 (2·9 to 16·4)	17·1 (5·5 to 41·2)	5·9 (2·5 to 14·7)	6·7 (2·4 to 17·3)	11 (3·5 to 28·5)	18·5 (5·3 to 45·3)	17·2 (5·1 to 40·2)	21·2 (6·6 to 47·7)	20·2 (5·9 to 49·2)	20·9 (5·8 to 51·3)	19·3 (5·7 to 47·3)	14·2 (3·5 to 34·1)	8·8 (1·7 to 23·3)	5·9 (1·3 to 15·7)	2·8 (0·5 to 7·7)	1·1 (0·2 to 2·8)	0·1 (0 to 0·4)	235
Pyoderma	0 (0 to 0)	0·1 (0·1 to 0·3)	65·3 (8·9 to 214)	32·4 (10·7 to 71·3)	19·2 (6·4 to 43·7)	13·4 (3·9 to 32·7)	41·3 (16·6 to 85·6)	36·4 (14·2 to 76)	49·4 (22·8 to 97·4)	32·5 (9·9 to 75·1)	43·2 (15·6 to 88·6)	46·3 (16·7 to 101)	47·3 (19·1 to 93·3)	46·7 (17·1 to 93·7)	62·9 (23·4 to 134)	60·4 (19·2 to 131)	27·4 (10·1 to 60·4)	25·4 (8 to 59·7)	26·7 (8·4 to 57·3)	20·6 (6·4 to 45·3)	8·6 (2·4 to 20·9)	3 (0·9 to 6·7)	0·5 (0·1 to 1·2)	709
Scabies	0·1 (0 to 0·2)	1·2 (0·6 to 2)	99·8 (51·9 to 173)	569 (286 to 1,010)	571 (275 to 1,020)	508 (250 to 878)	544 (273 to 967)	515 (257 to 901)	400 (195 to 694)	299 (145 to 541)	233 (116 to 399)	188 (96·6 to 327)	148 (73·3 to 254)	118 (59·1 to 202)	91·6 (47·9 to 163)	64·9 (33·4 to 114)	44·3 (22·4 to 78·5)	28·4 (14·5 to 49·7)	19·5 (9·9 to 33·9)	11·8 (6 to 21·2)	5·5 (2·8 to 9·1)	1·8 (1 to 3·2)	0·4 (0·2 to 0·7)	4,460
Fungal skin diseases	0 (0 to 0)	0·1 (0 to 0·3)	10·7 (4 to 22·9)	78·5 (28·7 to 173)	121 (45·5 to 261)	157 (57·7 to 352)	182 (65·2 to 411)	171 (62·6 to 369)	164 (62·1 to 372)	154 (58 to 346)	138 (54·4 to 311)	123 (47·1 to 267)	111 (42·8 to 241)	97·2 (37·1 to 210)	80·5 (31·5 to 177)	61 (23·4 to 133)	48·5 (19·5 to 104)	40·4 (15·7 to 85·3)	30·2 (11·2 to 65·5)	20·7 (7·9 to 44·6)	10·9 (4·4 to 23·2)	3·7 (1·5 to 7·7)	0·8 (0·3 to 1·6)	1,800
Viral skin diseases	0 (0 to 0)	0 (0 to 0)	0 (0 to 0)	423 (267 to 663)	609 (371 to 926)	324 (199 to 499)	189 (117 to 289)	123 (75·3 to 192)	88·5 (53·6 to 141)	70·3 (40·1 to 113)	55·4 (32 to 88·6)	44·2 (26·3 to 69·5)	36·7 (21·2 to 58·5)	30·7 (17·6 to 50·7)	25·4 (15·2 to 40·5)	21·4 (12·6 to 34)	15·2 (8·9 to 23·4)	9·2 (5·4 to 14·2)	5·2 (3 to 7·8)	2·4 (1·4 to 4)	0·9 (0·5 to 1·4)	0·2 (0·1 to 0·3)	0 (0 to 0·1)	2,070
Acne vulgaris	0 (0 to 0)	0 (0 to 0)	0 (0 to 0)	0 (0 to 0)	55 (26·9 to 96·7)	530 (296 to 860)	809 (461 to 1,340)	277 (150 to 458)	145 (75·3 to 244)	91·9 (48·5 to 158)	56·1 (28 to 97·8)	35·4 (16·9 to 61·9)	21·9 (12·4 to 36·5)	12·3 (6·8 to 20·3)	7·7 (4·2 to 12·5)	4·6 (2·5 to 7·9)	2·7 (1·5 to 4·6)	1·5 (0·8 to 2·5)	0·8 (0·4 to 1·3)	0·3 (0·2 to 0·6)	0·1 (0·1 to 0·2)	0 (0 to 0)	0 (0 to 0)	2,050
Alopecia areata	0 (0 to 0)	0 (0 to 0)	0 (0 to 0)	7·3 (2·6 to 14·5)	9·4 (3·5 to 19)	12 (4·9 to 22·1)	19·6 (9·8 to 34·5)	30·1 (16·4 to 50·5)	40 (22·4 to 64·1)	38·8 (22·4 to 63·9)	29·4 (16·5 to 48·3)	20·3 (11·4 to 33·3)	14·7 (7·8 to 24·8)	10·8 (5·6 to 17·9)	8·5 (4·4 to 14·2)	6·5 (3·4 to 11·2)	4·1 (2·2 to 7·1)	2·4 (1·2 to 3·9)	1·2 (0·6 to 2)	0·5 (0·2 to 0·9)	0·2 (0·1 to 0·3)	0 (0 to 0·1)	0 (0 to 0)	256
Pruritus	0 (0 to 0)	0 (0 to 0)	1·3 (0·5 to 2·7)	14·7 (6 to 30·1)	20 (8·5 to 41·6)	21·1 (8·9 to 44·6)	26·2 (11·3 to 52·8)	31·8 (13·6 to 62·8)	36·4 (15·1 to 70·9)	36·6 (15·8 to 71·8)	29·6 (12·9 to 57·9)	23·8 (10·4 to 45·5)	20·8 (9·1 to 42·3)	18·9 (8·1 to 37·8)	17·2 (7·1 to 35·3)	15·4 (6·8 to 31)	13·1 (5·7 to 25·1)	9·4 (4 to 18·5)	6 (2·6 to 11·7)	3·2 (1·4 to 6·3)	1·3 (0·6 to 2·7)	0·4 (0·2 to 0·7)	0·1 (0 to 0·1)	347
Urticaria	0 (0 to 0)	0·2 (0 to 0·4)	19·8 (10·4 to 33·8)	144 (82·7 to 232)	190 (106 to 312)	216 (115 to 372)	217 (120 to 373)	189 (99·5 to 314)	162 (84·9 to 270)	137 (73·6 to 229)	101 (54·7 to 167)	72·5 (36·9 to 119)	55·6 (29·8 to 95·7)	45·7 (23·6 to 76·8)	40·2 (20·5 to 66·8)	33·2 (17·6 to 56)	23·3 (12·5 to 39·6)	14·2 (7·3 to 23·1)	8·2 (4·2 to 13·3)	3·9 (2·1 to 6·3)	1·3 (0·7 to 2·2)	0·3 (0·2 to 0·5)	0·1 (0 to 0·1)	1,680
Decubitus ulcer	0 (0 to 0)	0 (0 to 0)	0 (0 to 0)	0·4 (0·1 to 1·6)	1·6 (0·1 to 6·2)	3·2 (0·3 to 11·9)	8·9 (0·5 to 30·1)	10·2 (0·7 to 32·8)	7·3 (0·6 to 27·2)	30·9 (5·4 to 81·6)	20·9 (4·3 to 54)	20 (4·1 to 50·8)	22·7 (5·5 to 56·6)	26·3 (7·1 to 65·6)	32·8 (9·5 to 74·9)	40 (12·1 to 92·3)	41·8 (15·4 to 86·4)	42·3 (16·1 to 86·7)	41·7 (15·4 to 88·1)	37·1 (14·5 to 75·9)	18·3 (7·5 to 37·2)	6·7 (2·6 to 13·1)	1·6 (0·7 to 3)	415
Other skin and subcutaneous diseases	0 (0 to 0)	0·2 (0·1 to 0·4)	23·7 (11·2 to 43·2)	138 (63·6 to 255)	99·9 (47·8 to 187)	112 (53·5 to 213)	136 (64·3 to 249)	126 (59·7 to 239)	121 (56·9 to 225)	109 (51·2 to 209)	89·5 (41·6 to 164)	73·9 (36·4 to 135)	64·5 (32·5 to 117)	57·7 (28·3 to 107)	53·2 (25·8 to 97)	46·5 (23·4 to 83·7)	36·4 (18·2 to 66·3)	25·3 (12·4 to 44·4)	16·9 (8·8 to 29·4)	9·9 (5·3 to 17·2)	3·7 (1·9 to 6·6)	1 (0·5 to 1·8)	0·2 (0·1 to 0·4)	1,350
Sense organ diseases	0·6 (0·3 to 1·1)	1·9 (1 to 3·3)	43·3 (25 to 67·9)	418 (238 to 641)	808 (467 to 1,230)	933 (555 to 1,370)	1,020 (601 to 1,520)	1,100 (635 to 1,640)	1,280 (781 to 1,930)	1,390 (873 to 2,080)	1,300 (806 to 1,970)	1,280 (781 to 1,960)	1,480 (910 to 2,330)	1,700 (1,090 to 2,620)	1,880 (1,220 to 2,870)	1,880 (1,240 to 2,790)	1,710 (1,150 to 2,500)	1,400 (958 to 1,980)	1,110 (759 to 1,540)	706 (491 to 968)	324 (228 to 430)	104 (74·4 to 139)	22·1 (15·7 to 29·3)	21,900
Blindness and vision loss	0·1 (0 to 0·2)	0·3 (0·1 to 0·6)	5·6 (2·6 to 9·9)	69·3 (38·9 to 115)	123 (66·4 to 197)	133 (74·8 to 220)	126 (72·3 to 208)	114 (64·1 to 181)	114 (65·8 to 182)	128 (75·5 to 198)	164 (101 to 254)	223 (138 to 350)	309 (191 to 488)	407 (254 to 645)	515 (328 to 830)	577 (377 to 886)	595 (388 to 877)	520 (346 to 750)	413 (277 to 592)	267 (183 to 377)	127 (87·6 to 175)	43·5 (30·1 to 59·3)	10·3 (7·1 to 14·2)	4,980
Glaucoma	0 (0 to 0)	0 (0 to 0)	0 (0 to 0)	0 (0 to 0)	0 (0 to 0)	0 (0 to 0)	0 (0 to 0)	0 (0 to 0)	0 (0 to 0)	0 (0 to 0)	0 (0 to 0)	0 (0 to 0)	0·5 (0·2 to 1)	3·7 (1·9 to 6·2)	8·9 (3·2 to 16·8)	13·7 (6·6 to 23·4)	17 (9·3 to 27·3)	18·3 (11·1 to 28·4)	19·3 (11·8 to 29·1)	15·6 (10 to 23·5)	9·2 (5·7 to 13·7)	2·7 (1·6 to 4·1)	0·6 (0·3 to 0·9)	110
Cataract	0 (0 to 0)	0 (0 to 0)	0 (0 to 0)	0 (0 to 0)	0 (0 to 0)	0 (0 to 0)	0 (0 to 0)	0·4 (0·2 to 0·7)	2·5 (1·2 to 4·5)	6·3 (2·9 to 11·4)	11·9 (5·2 to 22·4)	19·8 (8·2 to 36·1)	33·6 (16·6 to 58·6)	43·5 (23 to 73·1)	53·9 (30·6 to 87·8)	63·3 (37·5 to 98·7)	78·8 (44·7 to 124)	95·5 (59 to 147)	107 (69·3 to 159)	85·2 (55·6 to 124)	44·7 (29·4 to 62·7)	12·2 (7·9 to 17·4)	2·3 (1·4 to 3·5)	661
Age-related macular degeneration	0 (0 to 0)	0 (0 to 0)	0 (0 to 0)	0 (0 to 0)	0 (0 to 0)	0 (0 to 0)	0 (0 to 0)	0 (0 to 0)	0 (0 to 0)	0 (0 to 0)	0 (0 to 0)	0 (0 to 0)	0·7 (0·3 to 1·2)	6 (3·3 to 9·9)	17 (8·1 to 28·5)	26·8 (14·8 to 41)	28·1 (16·6 to 44·4)	25 (15·4 to 38·1)	21·2 (13·5 to 32·2)	13·3 (8·5 to 19·5)	6 (3·8 to 8·8)	1·4 (0·9 to 2·1)	0·2 (0·1 to 0·4)	146
Refraction disorders	0 (0 to 0)	0·1 (0 to 0·2)	4·1 (1·8 to 7·5)	62·9 (35·1 to 106)	115 (62·2 to 186)	125 (69·5 to 209)	117 (66·6 to 196)	101 (54·9 to 160)	91·4 (51·5 to 145)	87·5 (48·3 to 139)	98·5 (56·7 to 155)	114 (66·3 to 179)	139 (85·5 to 215)	145 (88 to 226)	141 (87 to 216)	122 (74·5 to 187)	105 (64·2 to 161)	89·7 (55·1 to 136)	75·4 (47·8 to 112)	46·5 (28·6 to 70)	19·7 (12·5 to 28·9)	4·4 (2·8 to 6·7)	0·6 (0·4 to 1)	1,800
Near vision loss	0 (0 to 0)	0 (0 to 0)	0 (0 to 0)	0·2 (0·1 to 0·6)	0·7 (0 to 2)	1·1 (0·2 to 3·1)	1·5 (0·3 to 3·9)	3·9 (1·2 to 9·3)	8·1 (2·6 to 18)	19·4 (7·1 to 43·6)	32·9 (11·5 to 73·5)	58·7 (22·7 to 127)	90·1 (36·3 to 196)	128 (52·1 to 272)	166 (66·4 to 353)	177 (73·9 to 374)	160 (66 to 327)	124 (52·6 to 249)	90·3 (37·6 to 184)	53·5 (23·1 to 105)	23 (10·1 to 44·9)	6·9 (2·9 to 13·8)	1·4 (0·5 to 2·8)	1,150
Other vision loss	0·1 (0 to 0·2)	0·2 (0·1 to 0·5)	1·5 (0·5 to 3)	6·1 (2·5 to 11·7)	7·2 (3 to 13·8)	6·9 (3 to 13·2)	7·5 (3·4 to 13·7)	8·9 (1·2 to 22)	11·8 (2·2 to 28·5)	15·1 (4 to 32·3)	20·8 (7·6 to 40·1)	30·3 (13·6 to 56·2)	45·2 (23 to 74·6)	80·3 (47·3 to 127)	128 (77·3 to 195)	175 (112 to 254)	206 (131 to 306)	167 (109 to 254)	99·1 (63 to 146)	53·3 (33·6 to 78·4)	24·9 (15·6 to 37·1)	15·8 (10·7 to 22·3)	5·2 (3·5 to 7·3)	1,120
Age-related and other hearing loss	0·4 (0·2 to 0·8)	1·2 (0·5 to 2·4)	29·1 (13·8 to 49·6)	306 (147 to 510)	643 (342 to 1,030)	762 (424 to 1,160)	846 (487 to 1,310)	942 (527 to 1,430)	1,120 (662 to 1,710)	1,200 (726 to 1,820)	1,070 (649 to 1,660)	995 (580 to 1,560)	1,120 (657 to 1,790)	1,220 (768 to 1,910)	1,300 (813 to 2,000)	1,230 (779 to 1,840)	1,050 (688 to 1,540)	836 (564 to 1,190)	659 (446 to 935)	419 (283 to 577)	189 (131 to 253)	58·8 (41·4 to 79·4)	11·3 (8·1 to 15·1)	16,000
Other sense organ diseases	0·1 (0 to 0·2)	0·4 (0·2 to 0·8)	8·6 (4·2 to 15·2)	41·8 (19·4 to 74·8)	41·6 (19 to 75)	38·3 (16·3 to 69·9)	42·7 (18·7 to 84)	44·5 (17·8 to 85·9)	53·2 (22·3 to 104)	67·3 (27·6 to 133)	67·7 (29·7 to 129)	59·2 (26·3 to 121)	58·7 (24·9 to 113)	68·8 (32·5 to 135)	67·1 (31 to 131)	69·5 (32·3 to 128)	64·1 (29·6 to 123)	48·1 (22·6 to 90·4)	35·6 (16 to 63·3)	19·6 (9·7 to 37·1)	6·9 (3·2 to 12·7)	1·7 (0·8 to 3·1)	0·5 (0·2 to 0·8)	906
Musculoskeletal disorders	0 (0 to 0)	0 (0 to 0)	0 (0 to 0)	0 (0 to 0)	265 (134 to 460)	1,120 (700 to 1,780)	2,080 (1,360 to 3,070)	3,050 (2,030 to 4,430)	4,370 (2,750 to 6,300)	5,150 (3,280 to 7,320)	5,210 (3,460 to 7,450)	5,020 (3,280 to 7,090)	4,890 (3,300 to 7,020)	4,510 (3,060 to 6,550)	4,000 (2,670 to 5,540)	3,270 (2,230 to 4,590)	2,400 (1,670 to 3,430)	1,600 (1,120 to 2,210)	1,010 (697 to 1,410)	548 (379 to 755)	212 (142 to 291)	54·8 (37·1 to 77·1)	10 (6·8 to 14·2)	48,800
Rheumatoid arthritis	0 (0 to 0)	0 (0 to 0)	0 (0 to 0)	0 (0 to 0)	2·3 (0·9 to 4·8)	10·3 (4·6 to 18·8)	9·2 (5 to 15·3)	12·9 (5·6 to 23·6)	17·7 (8·6 to 30·5)	24·1 (13·7 to 37·9)	23 (12·6 to 35·3)	27·4 (15·1 to 45·2)	27·3 (15·1 to 44)	28·7 (17 to 45·5)	29·9 (16·2 to 48·6)	28·4 (16·9 to 44·6)	21·2 (12·5 to 32·2)	13·5 (8·1 to 20)	7·6 (4·4 to 12·1)	3·2 (2 to 4·9)	1·9 (1·1 to 3·1)	0·3 (0·2 to 0·6)	0 (0 to 0·1)	289
Osteoarthritis	0 (0 to 0)	0 (0 to 0)	0 (0 to 0)	0 (0 to 0)	0 (0 to 0)	0 (0 to 0)	0 (0 to 0)	0 (0 to 0)	0 (0 to 0)	16·5 (6 to 35·6)	82·8 (38·6 to 167)	190 (91·6 to 395)	313 (149 to 631)	415 (195 to 836)	451 (215 to 907)	401 (195 to 802)	300 (148 to 588)	202 (101 to 401)	134 (66·6 to 269)	76·2 (38·8 to 154)	31·8 (16·1 to 64·2)	9·3 (4·7 to 18·8)	1·8 (0·9 to 3·6)	2,620
Osteoarthritis hip	0 (0 to 0)	0 (0 to 0)	0 (0 to 0)	0 (0 to 0)	0 (0 to 0)	0 (0 to 0)	0 (0 to 0)	0 (0 to 0)	0 (0 to 0)	6·5 (1·4 to 15·4)	8·6 (2·5 to 19·2)	11·1 (3·5 to 26·8)	15·7 (5·7 to 37·5)	20·8 (7·5 to 49·7)	25·6 (10·9 to 54·1)	26·5 (11·8 to 56·4)	22·5 (9·7 to 48·4)	16·5 (7·6 to 34·2)	11·1 (5·3 to 23·2)	6·1 (2·8 to 12·4)	2·5 (1·2 to 5·2)	0·7 (0·3 to 1·5)	0·1 (0·1 to 0·3)	174
Osteoarthritis knee	0 (0 to 0)	0 (0 to 0)	0 (0 to 0)	0 (0 to 0)	0 (0 to 0)	0 (0 to 0)	0 (0 to 0)	0 (0 to 0)	0 (0 to 0)	8·2 (2·5 to 19·4)	63 (28·2 to 130)	150 (70·2 to 327)	247 (115 to 511)	323 (148 to 659)	338 (158 to 703)	287 (139 to 582)	205 (98·4 to 407)	132 (64·8 to 269)	84·6 (41·1 to 172)	46·8 (22·9 to 95)	18·8 (9·3 to 38)	5·2 (2·5 to 10·6)	1 (0·5 to 1·9)	1,910
Osteoarthritis hand	0 (0 to 0)	0 (0 to 0)	0 (0 to 0)	0 (0 to 0)	0 (0 to 0)	0 (0 to 0)	0 (0 to 0)	0 (0 to 0)	0 (0 to 0)	0·3 (0 to 1·1)	1·7 (0·3 to 4·9)	3·1 (0·5 to 8)	4·3 (0·7 to 11·2)	5·4 (1·3 to 14·1)	6·1 (1·5 to 15·9)	6·1 (1·8 to 15·1)	5·2 (1·6 to 12·5)	3·9 (1·3 to 9·1)	2·9 (1·1 to 6·7)	1·9 (0·8 to 4·3)	0·9 (0·4 to 2·1)	0·3 (0·1 to 0·7)	0·1 (0 to 0·2)	42·2
Osteoarthritis other	0 (0 to 0)	0 (0 to 0)	0 (0 to 0)	0 (0 to 0)	0 (0 to 0)	0 (0 to 0)	0 (0 to 0)	0 (0 to 0)	0 (0 to 0)	1·4 (0·4 to 3·8)	9·5 (2·6 to 22·8)	26 (9·3 to 61·2)	45·8 (18·3 to 103)	65·8 (27·9 to 141)	80·9 (36·4 to 173)	81·8 (36·8 to 174)	67·7 (31·6 to 143)	49·9 (24·1 to 105)	35·6 (18·1 to 71·6)	21·4 (10·7 to 44·1)	9·5 (4·7 to 19·8)	3 (1·5 to 6)	0·6 (0·3 to 1·2)	499
Low back pain	0 (0 to 0)	0 (0 to 0)	0 (0 to 0)	0 (0 to 0)	223 (101 to 419)	911 (525 to 1,520)	1,420 (840 to 2,210)	1,790 (1,050 to 2,760)	2,290 (1,300 to 3,540)	2,440 (1,380 to 3,760)	2,420 (1,420 to 3,760)	2,300 (1,390 to 3,470)	2,150 (1,280 to 3,360)	1,980 (1,210 to 3,100)	1,760 (1,070 to 2,650)	1,460 (916 to 2,220)	1,110 (701 to 1,680)	775 (487 to 1,180)	527 (329 to 806)	317 (202 to 469)	128 (78·3 to 182)	33·7 (21·5 to 49·3)	6·2 (4 to 9·3)	24,000
Neck pain	0 (0 to 0)	0 (0 to 0)	0 (0 to 0)	0 (0 to 0)	26·6 (8·3 to 61·1)	144 (63·5 to 294)	316 (141 to 609)	509 (234 to 1,000)	812 (386 to 1,580)	1,190 (560 to 2,190)	1,330 (696 to 2,290)	1,300 (664 to 2,250)	1,300 (669 to 2,300)	1,100 (555 to 2,070)	905 (431 to 1,640)	697 (353 to 1,210)	493 (265 to 836)	311 (175 to 527)	181 (92·3 to 321)	81·5 (41·6 to 144)	28·2 (14·9 to 47·9)	6·8 (3·5 to 11·4)	1·2 (0·6 to 2)	10,700
Gout	0 (0 to 0)	0 (0 to 0)	0 (0 to 0)	0 (0 to 0)	0 (0 to 0)	0 (0 to 0)	0·8 (0·2 to 1·8)	7·8 (4 to 13·5)	27·2 (7·2 to 58)	51·1 (20·7 to 95·4)	70·1 (30·9 to 126)	84·5 (39·8 to 153)	91·5 (45·2 to 154)	94·2 (43·4 to 167)	96·1 (49·4 to 164)	87·5 (43·3 to 156)	69·1 (37 to 117)	49·1 (24·7 to 78·6)	32·1 (17·3 to 49·6)	17·3 (8·8 to 28·9)	7 (3·9 to 11·6)	2·1 (1·1 to 3·4)	0·5 (0·2 to 0·8)	788
Other musculoskeletal disorders	0 (0 to 0)	0 (0 to 0)	0 (0 to 0)	0 (0 to 0)	12·9 (5·9 to 22·6)	53·8 (20·1 to 102)	335 (155 to 577)	728 (428 to 1,130)	1,220 (710 to 1,920)	1,430 (849 to 2,260)	1,280 (760 to 1,960)	1,120 (644 to 1,780)	1,010 (593 to 1,590)	891 (497 to 1,450)	765 (432 to 1,190)	599 (327 to 963)	404 (230 to 642)	245 (125 to 417)	129 (69 to 209)	52·6 (19·1 to 94)	15·1 (5 to 28)	2·6 (0·5 to 6·1)	0·3 (0·1 to 1)	10,300
Other non-communicable diseases	16,800 (7,450 to 29,600)	4,740 (2,190 to 8,640)	24,700 (9,420 to 46,300)	4,070 (2,670 to 6,060)	2,960 (2,030 to 4,260)	2,070 (1,500 to 2,810)	1,750 (1,280 to 2,400)	1,560 (1,150 to 2,130)	1,450 (1,050 to 1,970)	1,360 (964 to 1,850)	1,240 (861 to 1,760)	1,190 (823 to 1,640)	1,160 (831 to 1,580)	1,220 (882 to 1,670)	1,330 (954 to 1,820)	1,320 (949 to 1,770)	1,130 (812 to 1,540)	856 (613 to 1,160)	591 (428 to 801)	361 (264 to 483)	152 (111 to 207)	47 (34 to 61·6)	10·3 (7·2 to 14·2)	72,000
Congenital birth defects	16,700 (7,400 to 29,400)	4,290 (1,850 to 7,960)	20,100 (6,220 to 40,700)	3,090 (1,830 to 4,810)	1,840 (1,120 to 2,950)	1,290 (873 to 1,880)	1,030 (684 to 1,510)	740 (479 to 1,090)	568 (366 to 825)	411 (261 to 589)	299 (193 to 444)	246 (160 to 357)	182 (117 to 278)	141 (87·6 to 225)	115 (72·1 to 177)	84·9 (53·3 to 131)	53·7 (34·1 to 82·7)	24·9 (13 to 42·8)	14·7 (7·5 to 25·1)	7·6 (4 to 13)	3 (1·5 to 5·2)	0·8 (0·4 to 1·4)	0·2 (0·1 to 0·3)	51,200
Neural tube defects	3,440 (950 to 7,910)	344 (83·7 to 936)	1,320 (210 to 4,190)	223 (42·7 to 809)	64·1 (30·3 to 138)	43·8 (23·9 to 81·8)	23·8 (15·1 to 35·2)	17·5 (11·2 to 26)	11·9 (7·7 to 16·8)	8·2 (5·3 to 11·6)	5·3 (3·5 to 7·5)	3·5 (2·4 to 5)	2·6 (1·7 to 3·8)	1·8 (1·1 to 2·5)	1·3 (0·8 to 2·1)	0·9 (0·5 to 1·4)	0·5 (0·3 to 0·7)	0·1 (0·1 to 0·2)	0·1 (0 to 0·1)	0 (0 to 0)	0 (0 to 0)	0 (0 to 0)	0 (0 to 0)	5,520
Congenital heart anomalies	5,860 (2,110 to 11,600)	2,370 (990 to 4,500)	11,300 (2,940 to 26,100)	1,450 (695 to 2,550)	845 (407 to 1,480)	635 (352 to 1,060)	485 (263 to 826)	321 (156 to 554)	248 (131 to 407)	131 (64·7 to 209)	78·1 (40·3 to 127)	69 (38·5 to 110)	32·4 (18·2 to 52)	22·2 (13 to 36·3)	19 (10·9 to 31·7)	15 (8·9 to 23·8)	7·7 (4·4 to 12·7)	1 (0·3 to 2·2)	0·6 (0·2 to 1·3)	0·4 (0·1 to 0·8)	0·1 (0 to 0·3)	0 (0 to 0·1)	0 (0 to 0)	23,900
Orofacial clefts	81·6 (12·8 to 263)	31·7 (5·2 to 92·8)	110 (11·9 to 386)	15·5 (6·8 to 29)	9·3 (2·5 to 19·7)	8·2 (1·8 to 17·4)	7·8 (1·5 to 16·5)	7·2 (1·2 to 15·6)	7·2 (1·5 to 16)	6·5 (1·3 to 14·4)	5·3 (1 to 11·7)	4·2 (0·6 to 9·4)	3·5 (0·5 to 7·6)	3 (0·6 to 6·5)	2·4 (0·6 to 5·1)	1·7 (0·3 to 3·8)	1·2 (0·2 to 2·6)	0·7 (0·1 to 1·6)	0·4 (0·1 to 1)	0·2 (0 to 0·5)	0·1 (0 to 0·2)	0 (0 to 0·1)	0 (0 to 0)	308
Down syndrome	69·1 (22·2 to 172)	30·9 (9·2 to 76·1)	231 (57·5 to 744)	62·8 (24·4 to 144)	40·6 (20·7 to 81·6)	22·5 (12·9 to 38·7)	22·3 (12·2 to 42·5)	21·7 (11·3 to 40·3)	13 (6·5 to 25·5)	8·4 (3·6 to 17·2)	6·4 (2·6 to 12·9)	4·6 (1·9 to 9·2)	4·8 (1·9 to 9·7)	3 (1 to 7·3)	1·2 (0·4 to 2·7)	0·5 (0·2 to 1·2)	0·2 (0·1 to 0·5)	0 (0 to 0)	0 (0 to 0)	0 (0 to 0)	0 (0 to 0)	0 (0 to 0)	0 (0 to 0)	543
Turner syndrome	0 (0 to 0)	0 (0 to 0)	0 (0 to 0)	0 (0 to 0)	0 (0 to 0)	0 (0 to 0)	0 (0 to 0)	0 (0 to 0)	0 (0 to 0)	0 (0 to 0)	0 (0 to 0)	0 (0 to 0)	0 (0 to 0)	0 (0 to 0)	0 (0 to 0)	0 (0 to 0)	0 (0 to 0)	0 (0 to 0)	0 (0 to 0)	0 (0 to 0)	0 (0 to 0)	0 (0 to 0)	0 (0 to 0)	0
Klinefelter syndrome	0 (0 to 0)	0 (0 to 0)	0·1 (0 to 0·2)	0·3 (0·1 to 0·6)	0·2 (0·1 to 0·4)	0·1 (0 to 0·2)	0·1 (0 to 0·1)	0·1 (0·1 to 0·3)	0·1 (0 to 0·2)	0 (0 to 0·1)	0 (0 to 0)	0 (0 to 0)	0 (0 to 0)	0 (0 to 0)	0 (0 to 0)	0 (0 to 0)	0 (0 to 0)	0 (0 to 0)	0 (0 to 0)	0 (0 to 0)	0 (0 to 0)	0 (0 to 0)	0 (0 to 0)	1·1
Other chromosomal abnormalities	208 (56·6 to 619)	66·4 (20·1 to 176)	187 (58·9 to 591)	50·8 (29·6 to 83·6)	27·7 (15·9 to 43·5)	21·2 (13·3 to 31·8)	19·1 (11·9 to 28·8)	12·6 (7·9 to 20·2)	7·5 (4·5 to 13·6)	3 (1·8 to 4·9)	2·6 (1·2 to 5·4)	0·9 (0·4 to 1·9)	0·8 (0·3 to 1·6)	0·2 (0·1 to 0·4)	0·1 (0 to 0·3)	0 (0 to 0·1)	0 (0 to 0·1)	0 (0 to 0)	0 (0 to 0)	0 (0 to 0)	0 (0 to 0)	0 (0 to 0)	0 (0 to 0)	608
Congenital musculoskeletal and limb anomalies	230 (72 to 542)	43·3 (20·1 to 88·9)	297 (154 to 549)	299 (166 to 474)	194 (105 to 320)	164 (88·9 to 257)	141 (80·1 to 215)	80 (43·7 to 133)	66·1 (34·6 to 110)	60·3 (31·9 to 98·9)	48·2 (26 to 76·8)	39·2 (21·2 to 65·3)	32·7 (17·4 to 54·3)	26·7 (13·5 to 45·1)	21·4 (11 to 36·8)	14·3 (7·2 to 23·5)	7·6 (4 to 12·4)	3·4 (1·8 to 5·7)	1·5 (0·7 to 2·6)	0·6 (0·3 to 0·9)	0·2 (0·1 to 0·3)	0 (0 to 0·1)	0 (0 to 0)	1,770
Urogenital congenital anomalies	280 (77·6 to 745)	35·4 (9·2 to 97·7)	97·2 (21·4 to 315)	31·2 (16·3 to 58·1)	18·1 (10·5 to 28·6)	12·1 (7·4 to 18·6)	10·2 (6 to 15·9)	7·9 (4·7 to 12·3)	6·1 (3·7 to 9·7)	4·8 (3 to 7·6)	3·4 (2 to 5·1)	2·5 (1·5 to 3·9)	1·9 (1·1 to 3·1)	1·3 (0·7 to 2·2)	1 (0·5 to 1·9)	0·9 (0·4 to 2)	0·7 (0·3 to 1·5)	0 (0 to 0)	0 (0 to 0)	0 (0 to 0)	0 (0 to 0)	0 (0 to 0)	0 (0 to 0)	514
Digestive congenital anomalies	1,560 (397 to 3,540)	572 (162 to 1,260)	2,760 (382 to 7,630)	228 (69·3 to 486)	58·4 (17·4 to 127)	33·2 (10·3 to 76)	7·5 (2·8 to 15·4)	3·8 (1·5 to 7·8)	4 (1·4 to 8·7)	1·8 (0·8 to 3·7)	2 (0·7 to 3·9)	1·5 (0·6 to 3)	1·7 (0·5 to 3·9)	1 (0·3 to 2)	1·7 (0·4 to 3·8)	1·1 (0·2 to 2·4)	1·6 (0·4 to 3·3)	0 (0 to 0)	0 (0 to 0)	0 (0 to 0)	0 (0 to 0)	0 (0 to 0)	0 (0 to 0)	5,240
Other congenital birth defects	4,980 (1,530 to 12,200)	793 (243 to 1,870)	3,800 (650 to 12,100)	729 (360 to 1,480)	584 (308 to 1,200)	350 (184 to 621)	309 (162 to 526)	268 (141 to 459)	204 (96·6 to 376)	187 (91 to 329)	148 (67·9 to 272)	120 (54·7 to 216)	102 (48 to 187)	81·4 (37·9 to 154)	67·1 (31·1 to 124)	50·5 (23·8 to 89·7)	34·2 (16·7 to 61·1)	19·7 (8·5 to 37)	12·1 (5·1 to 22·3)	6·4 (2·9 to 11·9)	2·6 (1·1 to 4·7)	0·7 (0·3 to 1·3)	0·1 (0·1 to 0·3)	12,800
Urinary diseases and male infertility	0·8 (0·3 to 2)	3·4 (1·1 to 7·7)	176 (47·3 to 379)	47 (23·1 to 87·3)	76·3 (40·4 to 125)	66·6 (36·4 to 113)	76·8 (40·7 to 130)	150 (87·2 to 234)	146 (84·3 to 240)	192 (105 to 317)	194 (106 to 312)	216 (124 to 355)	258 (152 to 406)	341 (196 to 532)	445 (267 to 681)	532 (339 to 791)	515 (333 to 755)	420 (267 to 614)	291 (189 to 441)	185 (122 to 274)	74·2 (45·8 to 113)	22·2 (13·8 to 33·4)	4·9 (2·7 to 7·9)	4,430
Urolithiasis	0 (0 to 0)	0 (0 to 0)	0 (0 to 0)	1 (0·3 to 2·5)	4·7 (0·7 to 13·3)	4·5 (0·9 to 12·8)	5 (1·3 to 14·8)	7·3 (2·9 to 14·8)	18·7 (7 to 39·3)	20·3 (8·6 to 37·8)	32·3 (8·8 to 66·6)	38·4 (11·5 to 79·4)	45·9 (15·9 to 93·3)	55·4 (18·5 to 114)	43·9 (14·4 to 91·4)	54·1 (15 to 123)	45·6 (11·6 to 109)	24·4 (5·9 to 61·3)	20·4 (3·9 to 53·9)	11·8 (1·9 to 31·5)	3·6 (0·6 to 9·4)	1·1 (0·2 to 3)	0·2 (0 to 0·7)	438
Benign prostatic hyperplasia	0 (0 to 0)	0 (0 to 0)	0 (0 to 0)	0 (0 to 0)	0 (0 to 0)	0 (0 to 0)	0 (0 to 0)	0 (0 to 0)	0 (0 to 0)	0 (0 to 0)	0 (0 to 0)	4·4 (0·8 to 11·5)	29·9 (12·6 to 59·9)	80·5 (37·7 to 150)	168 (81·8 to 287)	240 (122 to 421)	265 (150 to 428)	221 (122 to 365)	145 (81·4 to 234)	71·6 (39·3 to 115)	24·2 (13·1 to 39·1)	5·3 (2·9 to 8·7)	0·8 (0·4 to 1·3)	1,260
Male infertility	0 (0 to 0)	0 (0 to 0)	0 (0 to 0)	0 (0 to 0)	0 (0 to 0)	0 (0 to 0)	0 (0 to 0)	27·8 (7·3 to 71·5)	40 (11·2 to 99·7)	42·4 (11·5 to 118)	36·9 (9·8 to 95·4)	30·3 (8 to 81·5)	4 (0·7 to 12·5)	0 (0 to 0)	0 (0 to 0)	0 (0 to 0)	0 (0 to 0)	0 (0 to 0)	0 (0 to 0)	0 (0 to 0)	0 (0 to 0)	0 (0 to 0)	0 (0 to 0)	181
Other urinary diseases	0·3 (0·1 to 0·7)	0·7 (0·2 to 2·1)	35·2 (6·1 to 107)	8·7 (2·6 to 23)	18·2 (4·5 to 42·8)	17·3 (5·3 to 39)	13 (4 to 29·9)	42·8 (13·5 to 86·7)	9·2 (4 to 18·8)	28·5 (10·6 to 57·2)	33·6 (11·8 to 68·9)	29 (12 to 61·7)	53·4 (20·8 to 112)	54·6 (23·1 to 108)	75·9 (31·5 to 154)	71 (29·3 to 140)	63·5 (25·4 to 132)	52·7 (20·8 to 105)	25·9 (9·7 to 55·9)	37·1 (15·6 to 69·3)	14·2 (5·8 to 28·2)	6·2 (2·5 to 11·8)	1 (0·3 to 2·3)	692
Gynecological diseases	0 (0 to 0)	0 (0 to 0)	0 (0 to 0)	0 (0 to 0)	0 (0 to 0)	0 (0 to 0)	0 (0 to 0)	0 (0 to 0)	0 (0 to 0)	0 (0 to 0)	0 (0 to 0)	0 (0 to 0)	0 (0 to 0)	0 (0 to 0)	0 (0 to 0)	0 (0 to 0)	0 (0 to 0)	0 (0 to 0)	0 (0 to 0)	0 (0 to 0)	0 (0 to 0)	0 (0 to 0)	0 (0 to 0)	0
Uterine fibroids	0 (0 to 0)	0 (0 to 0)	0 (0 to 0)	0 (0 to 0)	0 (0 to 0)	0 (0 to 0)	0 (0 to 0)	0 (0 to 0)	0 (0 to 0)	0 (0 to 0)	0 (0 to 0)	0 (0 to 0)	0 (0 to 0)	0 (0 to 0)	0 (0 to 0)	0 (0 to 0)	0 (0 to 0)	0 (0 to 0)	0 (0 to 0)	0 (0 to 0)	0 (0 to 0)	0 (0 to 0)	0 (0 to 0)	0
Polycystic ovarian syndrome	--	--	--	--	--	--	--	--	--	--	--	--	--	--	--	--	--	--	--	--	--	--	--	NA
Female infertility	--	--	--	--	--	--	--	--	--	--	--	--	--	--	--	--	--	--	--	--	--	--	--	NA
Endometriosis	--	--	--	--	--	--	--	--	--	--	--	--	--	--	--	--	--	--	--	--	--	--	--	NA
Genital prolapse	0 (0 to 0)	0 (0 to 0)	0 (0 to 0)	0 (0 to 0)	0 (0 to 0)	0 (0 to 0)	0 (0 to 0)	0 (0 to 0)	0 (0 to 0)	0 (0 to 0)	0 (0 to 0)	0 (0 to 0)	0 (0 to 0)	0 (0 to 0)	0 (0 to 0)	0 (0 to 0)	0 (0 to 0)	0 (0 to 0)	0 (0 to 0)	0 (0 to 0)	0 (0 to 0)	0 (0 to 0)	0 (0 to 0)	0
Premenstrual syndrome	--	--	--	--	--	--	--	--	--	--	--	--	--	--	--	--	--	--	--	--	--	--	--	NA
Other gynecological diseases	0 (0 to 0)	0 (0 to 0)	0 (0 to 0)	0 (0 to 0)	0 (0 to 0)	0 (0 to 0)	0 (0 to 0)	0 (0 to 0)	0 (0 to 0)	0 (0 to 0)	0 (0 to 0)	0 (0 to 0)	0 (0 to 0)	0 (0 to 0)	0 (0 to 0)	0 (0 to 0)	0 (0 to 0)	0 (0 to 0)	0 (0 to 0)	0 (0 to 0)	0 (0 to 0)	0 (0 to 0)	0 (0 to 0)	0
Hemoglobinopathies and hemolytic anemias	23 (13·4 to 37·5)	37·5 (22·7 to 55·2)	408 (220 to 671)	551 (289 to 899)	636 (287 to 1,110)	337 (180 to 594)	258 (145 to 439)	221 (126 to 353)	200 (109 to 353)	168 (82·7 to 309)	144 (70·3 to 273)	130 (62·6 to 235)	125 (65·2 to 223)	130 (72 to 221)	152 (90·2 to 246)	137 (82·2 to 219)	112 (68·6 to 175)	79·8 (49·1 to 119)	55·1 (33·7 to 84·3)	31·7 (18·9 to 47·2)	17 (11 to 24·6)	5·2 (3·4 to 7·5)	1·2 (0·8 to 1·9)	3,960
Thalassemias	20·7 (11 to 35)	9·1 (4·8 to 15·9)	256 (101 to 489)	156 (90·5 to 252)	161 (96 to 254)	149 (81·3 to 251)	124 (67·3 to 213)	68·7 (34·6 to 125)	28·7 (12·7 to 57·1)	12·7 (4·1 to 25·7)	7·6 (2·6 to 16·8)	5·8 (2·4 to 12)	4·5 (1·8 to 8·9)	3 (1·2 to 6·5)	1·2 (0·4 to 2·8)	0·3 (0·1 to 0·6)	0 (0 to 0·1)	0 (0 to 0)	0 (0 to 0)	0 (0 to 0)	0 (0 to 0)	0 (0 to 0)	0 (0 to 0)	1,010
Thalassemias trait	1·8 (0·7 to 3·6)	22·4 (11·9 to 34·9)	123 (60·2 to 208)	324 (147 to 570)	385 (127 to 734)	144 (35·4 to 324)	55·3 (15·2 to 139)	46·5 (11·3 to 116)	54·5 (14·5 to 131)	53 (14·7 to 131)	50 (14·4 to 120)	44·8 (11·5 to 94·3)	43·7 (14 to 96·7)	40·3 (13·1 to 85·7)	48·7 (19·9 to 92·3)	39·1 (15·4 to 70·6)	30·1 (12·3 to 56·6)	21·7 (10 to 38·4)	15·6 (7·2 to 27·7)	9 (4·3 to 14·8)	4·4 (2·4 to 7)	1·2 (0·7 to 2)	0·3 (0·1 to 0·4)	1,560
Sickle cell disorders	0 (0 to 0·1)	0 (0 to 0)	0·4 (0·1 to 0·9)	0·2 (0·1 to 0·4)	0·2 (0·1 to 0·4)	0·2 (0·1 to 0·4)	0·1 (0 to 0·3)	0·1 (0 to 0·2)	0 (0 to 0·1)	0 (0 to 0·1)	0 (0 to 0)	0 (0 to 0)	0 (0 to 0)	0 (0 to 0)	0 (0 to 0)	0 (0 to 0)	0 (0 to 0)	0 (0 to 0)	0 (0 to 0)	0 (0 to 0)	0 (0 to 0)	0 (0 to 0)	0 (0 to 0)	1·3
Sickle cell trait	0·1 (0 to 0·3)	1·3 (0·6 to 2·2)	4 (1·4 to 7·8)	10·7 (3·3 to 21·9)	12·3 (3·1 to 25·8)	4·7 (0·2 to 13)	1·8 (0·4 to 4·4)	1·5 (0·4 to 3·8)	1·8 (0·5 to 4·3)	1·7 (0·5 to 4·1)	1·6 (0·5 to 3·8)	1·5 (0·4 to 3·2)	1·4 (0·2 to 3·9)	1·3 (0·2 to 3·5)	1·6 (0·4 to 3·5)	1·3 (0·3 to 2·8)	1 (0·3 to 2·1)	0·7 (0·2 to 1·5)	0·5 (0·2 to 1·1)	0·3 (0·1 to 0·6)	0·1 (0·1 to 0·3)	0 (0 to 0·1)	0 (0 to 0)	51·3

G6PD deficiency	0·1 (0 to 0·2)	0·6 (0·3 to 1)	1·5 (0·5 to 2·9)	2·7 (0·6 to 6·7)	5·4 (2 to 10·5)	13 (5·4 to 26·7)	36·1 (14·7 to 69·5)	62·6 (28·5 to 112)
G6PD trait	0 (0 to 0)	0 (0 to 0)	0 (0 to 0)	0 (0 to 0)	0 (0 to 0)	0 (0 to 0)	0 (0 to 0)	0 (0 to 0)
Other hemoglobinopathies and hemolytic anemias	0·2 (0·1 to 0·5)	4·1 (2·1 to 6·6)	23·4 (7·7 to 47·4)	56·2 (18·2 to 119)	72 (15·8 to 175)	26·2 (5·8 to 64·4)	40 (12·1 to 101)	41·6 (16·5 to 93·1)
Endocrine, metabolic, blood, and immune disorders	60·7 (24·8 to 117)	48·1 (22·6 to 97·2)	695 (213 to 1.520)	200 (116 to 312)	196 (106 to 337)	140 (84·6 to 210)	116 (72·5 to 170)	121 (76·3 to 183)
Oral disorders	0 (0 to 0)	0 (0 to 0)	0 (0 to 0)	185 (102 to 302)	210 (114 to 364)	232 (122 to 410)	275 (150 to 471)	328 (185 to 540)
Caries of deciduous teeth	0 (0 to 0)	0 (0 to 0)	0 (0 to 0)	63·3 (23 to 131)	58·1 (22·7 to 127)	3 (0·5 to 8·3)	0 (0 to 0)	0 (0 to 0)
Caries of permanent teeth	0 (0 to 0)	0 (0 to 0)	0 (0 to 0)	0 (0 to 0)	50·1 (18·6 to 105)	122 (49·6 to 251)	116 (47·7 to 236)	117 (48·9 to 231)
Periodontal diseases	0 (0 to 0)	0 (0 to 0)	0 (0 to 0)	0 (0 to 0)	0 (0 to 0)	0 (0 to 0)	4·6 (1·1 to 12·3)	12·7 (3·6 to 33·3)
Other oral disorders	0 (0 to 0)	0 (0 to 0)	0 (0 to 0)	122 (71·4 to 193)	102 (58·7 to 158)	107 (62·1 to 170)	154 (91·1 to 242)	181 (108 to 284)
Sudden infant death syndrome	--	366 (129 to 810)	3,340 (680 to 8.590)	--	--	--	--	--
Injuries	291 (170 to 471)	234 (104 to 572)	5,110 (2.680 to 8.590)	5,330 (3.230 to 8.010)	7,460 (5.270 to 9.710)	7,130 (5.430 to 8.950)	18,600 (14.400 to 22.500)	25,400 (18.700 to 30.900)
Transport injuries	56·8 (16·9 to 140)	27·6 (9·3 to 72·1)	785 (326 to 1,510)	915 (473 to 1,570)	2,130 (1,270 to 3,180)	2,200 (1,440 to 3,240)	7,410 (4,740 to 10,400)	9,900 (6,460 to 13,700)
Road injuries	51·4 (14·9 to 127)	24·9 (8·2 to 64·1)	718 (293 to 1.400)	831 (419 to 1.430)	1,980 (1.180 to 2.960)	2,050 (1.330 to 2.990)	6,950 (4.440 to 9.710)	9,270 (5.990 to 12.900)
Pedestrian road injuries	20·6 (5·2 to 51·8)	7·1 (1·8 to 20)	205 (59·7 to 482)	286 (115 to 565)	719 (329 to 1.250)	630 (296 to 1.080)	783 (343 to 1.520)	1,040 (471 to 1.970)
Cyclist road injuries	0 (0 to 0)	0 (0 to 0)	0 (0 to 0)	83·1 (30·4 to 183)	235 (96 to 450)	241 (113 to 446)	324 (176 to 588)	383 (214 to 659)
Motorcyclist road injuries	14·8 (3·4 to 50·5)	7·9 (2 to 21·1)	269 (65·4 to 667)	215 (82·9 to 457)	439 (199 to 830)	598 (278 to 1.060)	3,550 (1.990 to 5.540)	4,890 (2.830 to 7.380)
Motor vehicle road injuries	15 (3·8 to 39·4)	9·4 (2·7 to 24·7)	232 (76·4 to 492)	231 (101 to 455)	564 (260 to 984)	551 (275 to 973)	2,190 (1.080 to 3.590)	2,830 (1.510 to 4.650)
Other road injuries	1 (0·1 to 3·7)	0·4 (0·1 to 1·6)	10·7 (2 to 31·6)	15·6 (3·9 to 40·2)	27·1 (9·7 to 62·5)	30·4 (11·9 to 64)	102 (38·7 to 224)	131 (55·9 to 262)
Other transport injuries	5·4 (1·3 to 15·6)	2·7 (0·8 to 7)	67·1 (21·9 to 165)	84·1 (33·1 to 186)	142 (59·3 to 284)	151 (72·7 to 291)	456 (202 to 924)	626 (302 to 1,170)
Unintentional injuries	174 (104 to 306)	188 (76·4 to 505)	4,130 (2.030 to 7.410)	4,160 (2.340 to 6.520)	5,020 (3.380 to 7.000)	4,140 (2.950 to 5.510)	5,910 (4.220 to 8.080)	6,730 (4.800 to 8.820)
Falls	25·7 (9·7 to 51·8)	21·6 (8·3 to 46·3)	404 (104 to 936)	522 (252 to 995)	648 (355 to 984)	636 (420 to 931)	1,110 (717 to 1,640)	1,410 (925 to 2,050)
Drowning	28·3 (9 to 64·3)	13·8 (4·4 to 38)	568 (181 to 1,400)	2,110 (899 to 4,090)	2,610 (1,380 to 4,380)	1,870 (1,060 to 2,990)	2,290 (1,180 to 3,920)	1,920 (1,010 to 3,300)
Fire, heat, and hot substances	6·6 (2·2 to 14)	4·2 (1·5 to 9·4)	381 (79·1 to 754)	319 (138 to 597)	226 (113 to 383)	200 (121 to 298)	269 (167 to 403)	380 (231 to 582)
Poisonings	4·1 (1 to 10·6)	7·3 (0·6 to 31·5)	82·6 (19·1 to 219)	149 (48 to 352)	87·6 (33·9 to 189)	61·6 (27·4 to 120)	111 (47·9 to 221)	151 (63·5 to 281)
Poisoning by carbon monoxide	0·3 (0 to 1·3)	0·5 (0 to 2·1)	2·5 (0·2 to 10·3)	9·5 (1·7 to 30·6)	5·5 (1·6 to 16)	5·4 (1·8 to 13·3)	25·4 (6·9 to 59·7)	32·6 (9·3 to 78·6)
Poisoning by other means	3·8 (0·9 to 9·8)	6·9 (0·6 to 29·9)	80·1 (18·4 to 209)	140 (44·6 to 338)	82 (31·5 to 176)	56·2 (24·6 to 111)	85·5 (35·1 to 180)	119 (47·2 to 236)
Exposure to mechanical forces	9·6 (0·5 to 46·3)	44·6 (0·4 to 353)	202 (10·7 to 1,260)	186 (80·4 to 440)	269 (147 to 433)	261 (157 to 409)	454 (274 to 728)	652 (381 to 1,010)
Unintentional firearm injuries	0·9 (0 to 5·9)	7·8 (0 to 32·4)	23·7 (0·7 to 179)	11·9 (4·2 to 36)	15·1 (6·5 to 38·6)	18·2 (8·4 to 41)	48 (17·5 to 132)	87·2 (26·6 to 231)
Other exposure to mechanical forces	8·7 (0·5 to 43·2)	36·9 (0·4 to 300)	179 (9·6 to 1.050)	174 (74·9 to 410)	254 (135 to 418)	243 (145 to 393)	406 (247 to 671)	565 (331 to 874)
Adverse effects of medical treatment	37·4 (16·9 to 68)	22·2 (7·7 to 50·9)	307 (82·9 to 662)	81·6 (37·6 to 144)	91·4 (43·1 to 161)	59·9 (33·6 to 94·2)	62·5 (31·1 to 104)	98·8 (52·7 to 162)
Animal contact	3·8 (1 to 8·5)	7·4 (1·8 to 17·7)	182 (37·3 to 421)	217 (56·4 to 489)	387 (119 to 848)	254 (72·8 to 547)	216 (74·8 to 463)	223 (76·7 to 478)
Venomous animal contact	2·6 (0·7 to 6·3)	2·5 (0·4 to 8)	93·5 (15·3 to 254)	133 (32·4 to 335)	257 (65·7 to 610)	171 (45·4 to 392)	150 (45·7 to 340)	160 (50·3 to 367)
Non-venomous animal contact	1·2 (0·2 to 3·2)	4·8 (1·1 to 12·6)	88·4 (15·2 to 211)	83·8 (15·3 to 229)	131 (29·5 to 341)	82·2 (19·9 to 233)	66·2 (21·8 to 173)	63·6 (21·6 to 158)
Foreign body	42·3 (15·7 to 121)	58·7 (23·4 to 125)	1,740 (548 to 3,760)	245 (119 to 448)	195 (103 to 341)	175 (102 to 277)	147 (91·5 to 219)	224 (134 to 345)
Pulmonary aspiration and foreign body in airway	41·9 (15·5 to 121)	58·4 (23·2 to 124)	1,710 (540 to 3.660)	226 (104 to 422)	175 (84·5 to 313)	153 (83·7 to 253)	119 (67·4 to 190)	189 (102 to 310)
Foreign body in eyes	0 (0 to 0)	0·1 (0 to 0·1)	1 (0·3 to 2·1)	4·8 (1·4 to 11)	6·8 (1·9 to 16)	8·5 (2·6 to 20·1)	11·9 (3·6 to 28·1)	16·5 (4·9 to 37·3)
Foreign body in other body part	0·4 (0 to 1·4)	0·2 (0·1 to 0·8)	29·1 (3·2 to 112)	14·2 (6·3 to 32)	13·9 (8·2 to 25·4)	13·5 (9·1 to 20)	16·1 (11·2 to 22·6)	18·4 (12·8 to 25·5)
Environmental heat and cold exposure	0·9 (0·2 to 2·4)	1·5 (0·2 to 4·9)	37·1 (4·1 to 131)	17·5 (6·7 to 39·7)	21·2 (10·9 to 41·1)	19·4 (12·3 to 31·4)	44·8 (22·8 to 82·3)	66·3 (29·9 to 127)
Exposure to forces of nature	0·6 (0·6 to 0·7)	1·4 (1·3 to 1·6)	11·7 (10·6 to 12·9)	54·1 (48·9 to 59·7)	55·6 (50·1 to 61·9)	77·1 (68·2 to 86·5)	86·3 (76·5 to 97·2)	84·1 (74·6 to 94·9)
Other unintentional injuries	14·6 (4·9 to 31·7)	5·3 (1·8 to 13·9)	211 (64·9 to 437)	263 (99 to 669)	428 (179 to 995)	532 (238 to 1,230)	1,120 (491 to 2,430)	1,520 (756 to 2,880)
Self-harm and interpersonal violence	59·8 (20·1 to 132)	18·2 (5·4 to 42·5)	190 (58·3 to 457)	251 (93·2 to 564)	319 (160 to 586)	786 (463 to 1.250)	5,270 (3.270 to 7.730)	8,800 (5.590 to 12.700)
Self-harm	0 (0 to 0)	0 (0 to 0)	0 (0 to 0)	0 (0 to 0)	0 (0 to 0)	248 (120 to 447)	2,180 (1.240 to 3.440)	3,630 (2.090 to 5.600)
Self-harm by firearm	0 (0 to 0)	0 (0 to 0)	0 (0 to 0)	0 (0 to 0)	0 (0 to 0)	3·7 (1·4 to 8·2)	44·7 (15·1 to 106)	88·4 (31 to 217)
Self-harm by other specified means	0 (0 to 0)	0 (0 to 0)	0 (0 to 0)	0 (0 to 0)	0 (0 to 0)	244 (118 to 441)	2,130 (1,230 to 3,380)	3,540 (2,040 to 5,460)
Interpersonal violence	59·8 (20·1 to 132)	18·2 (5·4 to 42·5)	190 (57·8 to 456)	247 (89·5 to 561)	314 (156 to 581)	532 (270 to 959)	3,070 (1.530 to 5.330)	5,140 (2.630 to 8.610)
Physical violence by firearm	2·4 (0·4 to 7·7)	2·8 (0·6 to 8·1)	16·6 (3·3 to 53·4)	35·7 (7·2 to 93·8)	41·8 (14·2 to 95·2)	76·4 (21·4 to 178)	817 (250 to 1.790)	1,600 (588 to 3.460)
Physical violence by sharp object	15·8 (3·9 to 42·9)	2·9 (0·6 to 8·3)	22·8 (5 to 69·8)	71·2 (17·5 to 180)	85·7 (27·5 to 190)	145 (43·3 to 318)	1,660 (618 to 3,230)	2,690 (1,030 to 5,030)
Sexual violence	0 (0 to 0)	0 (0 to 0)	0 (0 to 0)	2·1 (1·2 to 3·5)	15·2 (9 to 24·3)	30·7 (17 to 48·3)	62·6 (35 to 100)	83·7 (48·9 to 129)
Physical violence by other means	41·6 (12·9 to 92·4)	12·6 (3·6 to 30·5)	150 (41·7 to 362)	138 (45·8 to 320)	171 (77 to 345)	280 (125 to 545)	525 (225 to 1.070)	765 (349 to 1.510)
Conflict and terrorism	0 (0 to 0)	0 (0 to 0)	0 (0 to 0)	0·1 (0·1 to 0·1)	0·6 (0·4 to 1)	1·7 (0·9 to 2·9)	6 (3·6 to 10·3)	6·5 (3·9 to 10·3)

G6PD deficiency	68·4 (33·7 to 121)	55·5 (23·7 to 99)	42·1 (17·8 to 77)	37·6 (18·5 to 64)	35·9 (18·5 to 62·5)	41·1 (22·7 to 70·8)	41·5 (22·6 to 68·7)	30·8 (15·9 to 52·9)
G6PD trait	0 (0 to 0)	0 (0 to 0)	0 (0 to 0)	0 (0 to 0)	0 (0 to 0)	0 (0 to 0)	0 (0 to 0)	0 (0 to 0)
Other hemoglobinopathies and hemolytic anemias	46·4 (16·9 to 103)	44·7 (15·3 to 103)	42·5 (15 to 96·7)	39·9 (14·8 to 84·5)	39·2 (16 to 82·5)	43·7 (20·4 to 81·5)	59·1 (32·2 to 104)	65·6 (37·2 to 109)
Endocrine, metabolic, blood, and immune disorders	176 (107 to 269)	225 (135 to 346)	271 (165 to 413)	282 (162 to 437)	275 (166 to 418)	268 (167 to 406)	256 (161 to 390)	213 (138 to 313)
Oral disorders	361 (201 to 585)	361 (204 to 588)	335 (186 to 557)	315 (170 to 542)	317 (177 to 546)	336 (190 to 575)	360 (204 to 593)	349 (204 to 569)
Caries of deciduous teeth	0 (0 to 0)	0 (0 to 0)	0 (0 to 0)	0 (0 to 0)	0 (0 to 0)	0 (0 to 0)	0 (0 to 0)	0 (0 to 0)
Caries of permanent teeth	107 (42·8 to 222)	89·7 (36 to 183)	73·1 (29·9 to 152)	55·4 (22·5 to 117)	42·1 (16·6 to 89·4)	34 (13·7 to 67·4)	27·8 (10·8 to 63·1)	21·3 (7·4 to 46)
Periodontal diseases	27·8 (8·2 to 69·4)	45·7 (14·1 to 123)	62·2 (19·9 to 160)	79·4 (24·8 to 206)	94·9 (30·5 to 230)	106 (35·8 to 248)	108 (36·7 to 254)	93·4 (31·9 to 210)
Other oral disorders	194 (116 to 306)	185 (109 to 285)	156 (91·3 to 244)	130 (76·3 to 201)	109 (64·9 to 165)	91·7 (55·9 to 139)	75·3 (44·9 to 115)	55·8 (33·4 to 85·9)
Sudden infant death syndrome	--	--	--	--	--	--	--	--
Injuries	20,200 (13.900 to 25.400)	16,100 (9.630 to 20.400)	12,400 (7.400 to 16.400)	10,500 (6.750 to 14.200)	8,630 (6.340 to 11.600)	7,160 (5.420 to 9.320)	5,840 (4.460 to 7.490)	4,280 (3.320 to 5.320)
Transport injuries	7,190 (4,420 to 10,300)	5,250 (2,890 to 7,550)	3,890 (2,280 to 5,640)	3,260 (2,070 to 4,690)	2,830 (1,900 to 4,030)	2,450 (1,720 to 3,380)	2,000 (1,420 to 2,800)	1,430 (1,040 to 1,910)
Road injuries	6,700 (4.080 to 9.650)	4,890 (2.730 to 6.960)	3,630 (2.100 to 5.260)	3,050 (1.930 to 4.400)	2,640 (1.770 to 3.780)	2,290 (1.610 to 3.170)	1,880 (1.330 to 2.620)	1,330 (960 to 1.800)
Pedestrian road injuries	749 (335 to 1.380)	739 (335 to 1.360)	556 (249 to 1.010)	494 (244 to 951)	513 (262 to 923)	510 (272 to 878)	423 (231 to 709)	375 (218 to 617)
Cyclist road injuries	305 (173 to 527)	350 (191 to 603)	265 (148 to 427)	219 (134 to 351)	299 (173 to 484)	271 (161 to 432)	233 (137 to 385)	186 (110 to 288)
Motorcyclist road injuries	3,410 (2.070 to 5.400)	2,140 (1.210 to 3.310)	1,570 (912 to 2.330)	1,310 (769 to 1.920)	1,010 (642 to 1.480)	808 (530 to 1.170)	656 (430 to 953)	368 (255 to 507)
Motor vehicle road injuries	2,130 (1.140 to 3.420)	1,570 (779 to 2.530)	1,180 (571 to 1.970)	971 (516 to 1.600)	781 (448 to 1.230)	657 (389 to 1.030)	537 (330 to 868)	378 (243 to 589)
Other road injuries	101 (43·6 to 195)	82·2 (35·1 to 160)	60·3 (24 to 128)	52·7 (22·7 to 104)	44·7 (20·2 to 86·9)	46 (18·9 to 95·8)	32·1 (16 to 59·9)	24·1 (12 to 43·9)
Other transport injuries	491 (223 to 916)	364 (159 to 713)	261 (124 to 496)	212 (103 to 376)	181 (88·2 to 335)	160 (86·3 to 295)	123 (63·7 to 219)	94·8 (52·9 to 158)
Unintentional injuries	5,630 (3.990 to 7.460)	4,830 (3.120 to 6.430)	4,060 (2.610 to 5.440)	3,570 (2.470 to 4.790)	3,130 (2.270 to 4.150)	2,790 (2.100 to 3.670)	2,420 (1.830 to 3.140)	1,960 (1.480 to 2.480)
Falls	1,380 (922 to 1,920)	1,270 (815 to 1,760)	1,220 (794 to 1,690)	1,200 (821 to 1,670)	1,090 (770 to 1,500)	1,140 (794 to 1,560)	1,050 (775 to 1,440)	949 (670 to 1,310)
Drowning	1,320 (707 to 2,310)	1,040 (482 to 1,770)	802 (379 to 1,410)	653 (346 to 1,130)	527 (266 to 951)	406 (229 to 679)	315 (168 to 525)	227 (130 to 369)
Fire, heat, and hot substances	380 (236 to 583)	345 (224 to 500)	292 (195 to 414)	261 (177 to 379)	219 (153 to 316)	186 (130 to 261)	156 (108 to 222)	120 (79·1 to 174)
Poisonings	133 (62·3 to 240)	96 (42·4 to 176)	77·6 (35·7 to 149)	63·5 (32·4 to 116)	56·1 (26·5 to 104)	42·6 (22·4 to 74·1)	30·8 (14·9 to 57·1)	24·1 (12 to 43·6)
Poisoning by carbon monoxide	23·1 (6·7 to 56)	23·5 (6·5 to 54·5)	15·9 (5·3 to 35·9)	15·6 (5·3 to 35·1)	11·3 (3·4 to 26·6)	7·7 (2·6 to 17·3)	5·4 (1·7 to 12·8)	3·3 (1 to 7·2)
Poisoning by other means	110 (46·9 to 201)	72·5 (31 to 138)	61·8 (27·5 to 120)	47·9 (23·7 to 83·8)	44·7 (20·2 to 84·7)	35 (18·1 to 62·5)	25·4 (12·4 to 47·3)	20·8 (10·1 to 38·2)
Exposure to mechanical forces	609 (364 to 921)	552 (332 to 830)	477 (308 to 723)	405 (263 to 600)	371 (256 to 545)	311 (214 to 440)	272 (180 to 391)	190 (134 to 261)
Unintentional firearm injuries	61·5 (24·5 to 143)	62·7 (26·3 to 139)	59·7 (26·5 to 135)	47 (23 to 95·5)	40·7 (21·2 to 80)	30·9 (17·2 to 57)	22·5 (13·7 to 39·9)	15·3 (9·9 to 25·1)
Other exposure to mechanical forces	548 (324 to 839)	489 (298 to 731)	417 (269 to 630)	358 (230 to 530)	330 (222 to 498)	280 (194 to 408)	249 (164 to 362)	175 (124 to 244)
Adverse effects of medical treatment	81·3 (41·7 to 136)	67·3 (25·9 to 117)	69·4 (27·2 to 130)	66 (26·4 to 124)	74·4 (32·7 to 140)	60·1 (28·6 to 117)	75·4 (33·9 to 152)	70·9 (31·7 to 142)
Animal contact	191 (71·7 to 382)	147 (55·6 to 289)	153 (54·8 to 328)	130 (47 to 259)	135 (49·8 to 293)	140 (54·9 to 288)	114 (41·7 to 236)	88·4 (33·1 to 175)
Venomous animal contact	117 (37·4 to 268)	94·1 (29·2 to 200)	95·1 (26·5 to 222)	86·1 (25·8 to 188)	75·3 (21·5 to 173)	75·7 (24·3 to 172)	63 (19·6 to 140)	42·5 (13·1 to 98·5)
Non-venomous animal contact	73·9 (24·9 to 178)	53·3 (20·4 to 123)	57·6 (22·5 to 131)	43·7 (17 to 106)	60 (20·5 to 140)	64·8 (23·2 to 146)	50·7 (17·1 to 121)	45·8 (16·2 to 98·9)
Foreign body	195 (121 to 287)	157 (94·9 to 229)	119 (73·6 to 179)	104 (67·5 to 158)	89·9 (60·8 to 135)	81·7 (55·8 to 117)	72·2 (49·2 to 109)	61·7 (40·7 to 91·4)
Pulmonary aspiration and foreign body in airway	155 (83·7 to 244)	115 (56·7 to 181)	81·4 (40·6 to 143)	70·5 (37·2 to 120)	59·9 (33·3 to 105)	55·3 (31·6 to 91·4)	49·4 (29·6 to 83·6)	42·1 (23·1 to 71·7)
Foreign body in eyes	19·8 (6·6 to 42)	19·8 (7 to 41·7)	16·7 (6 to 35·3)	13·6 (5·7 to 27)	11 (5·2 to 20·2)	8·4 (4·4 to 15)	6·3 (3·6 to 10·7)	4·7 (2·7 to 7·4)
Foreign body in other body part	20·5 (14·8 to 27·5)	22·2 (15·7 to 29·6)	21·4 (15·2 to 28·5)	20·2 (14·5 to 27)	19 (13·9 to 25·5)	17·9 (13 to 24·3)	16·4 (12 to 22·1)	14·9 (10·7 to 20)
Environmental heat and cold exposure	57·3 (28·1 to 108)	57·2 (27·1 to 116)	47·6 (22·6 to 86·9)	43·2 (20·6 to 85·6)	38·6 (18·8 to 72·6)	35 (16·8 to 64·3)	29 (13·8 to 54·3)	27·1 (11·8 to 57·7)
Exposure to forces of nature	77·5 (68·3 to 87·7)	63·7 (55·4 to 72·8)	51·1 (43·9 to 59·5)	38 (32·8 to 44·5)	30·6 (26·6 to 35·7)	25 (21·7 to 28·9)	18·5 (16 to 21·4)	13·6 (11·8 to 15·8)
Other unintentional injuries	1,200 (624 to 2,230)	1,040 (514 to 2,010)	743 (367 to 1,480)	603 (317 to 1,190)	498 (271 to 941)	361 (207 to 650)	284 (164 to 509)	183 (113 to 315)
Self-harm and interpersonal violence	7,410 (4.500 to 10.700)	6,040 (3.280 to 8.840)	4,500 (2.280 to 6.850)	3,640 (1.970 to 5.650)	2,680 (1.620 to 4.030)	1,920 (1.300 to 2.810)	1,420 (940 to 2.070)	903 (620 to 1.300)
Self-harm	2,870 (1.590 to 4.400)	2,290 (1.060 to 3.710)	1,580 (753 to 2.590)	1,220 (613 to 2.020)	921 (531 to 1.530)	665 (395 to 1.070)	500 (314 to 804)	347 (213 to 556)
Self-harm by firearm	73·7 (27·4 to 175)	54·6 (19·5 to 123)	37·1 (13·1 to 92·8)	32·6 (12·5 to 82·8)	32·4 (13·2 to 80)	16·3 (6·7 to 43·1)	15·4 (6·7 to 41·1)	7·2 (3·2 to 19·5)
Self-harm by other specified means	2,800 (1,540 to 4,300)	2,240 (1,030 to 3,600)	1,550 (735 to 2,510)	1,190 (599 to 1,940)	889 (514 to 1,470)	649 (385 to 1,050)	484 (304 to 781)	340 (208 to 542)
Interpersonal violence	4,500 (2.320 to 7.450)	3,690 (1.700 to 6.110)	2,880 (1.340 to 4.960)	2,390 (1.150 to 4.150)	1,720 (956 to 2.790)	1,220 (714 to 1.980)	895 (522 to 1.440)	546 (347 to 851)
Physical violence by firearm	1,250 (436 to 2.750)	1,200 (385 to 2.460)	970 (319 to 2.090)	806 (302 to 1.670)	444 (164 to 901)	343 (139 to 682)	231 (89·8 to 468)	139 (57·8 to 264)
Physical violence by sharp object	2,460 (1,010 to 4,340)	1,820 (570 to 3,380)	1,340 (434 to 2,590)	1,080 (364 to 2,040)	876 (332 to 1,560)	538 (203 to 993)	384 (153 to 722)	213 (93·1 to 376)
Sexual violence	80·4 (44·5 to 131)	67·2 (36·7 to 109)	51·7 (29·5 to 82·7)	38·2 (22·1 to 61·9)	27·3 (15·9 to 45·6)	17·8 (10·1 to 29·1)	10·5 (6 to 16·8)	6·8 (4·2 to 10·2)
Physical violence by other means	704 (345 to 1.340)	600 (294 to 1.100)	508 (259 to 865)	466 (251 to 841)	373 (230 to 609)	322 (205 to 502)	269 (171 to 434)	187 (123 to 280)
Conflict and terrorism	10·9 (6·7 to 17·3)	33 (17·7 to 59·1)	26·7 (15·3 to 44·5)	25·6 (14 to 45·1)	28·5 (14·4 to 53·9)	28·8 (14·6 to 55·6)	17·5 (9 to 33·2)	9·2 (4·7 to 17·6)

G6PD deficiency	18·9 (9·4 to 31·4)	9 (4·2 to 16·2)	3·8 (1·7 to 6·4)	1·1 (0·5 to 2)	0·3 (0·2 to 0·6)	0·1 (0 to 0·1)	0 (0 to 0)	508
G6PD trait	0 (0 to 0)	0 (0 to 0)	0 (0 to 0)	0 (0 to 0)	0 (0 to 0)	0 (0 to 0)	0 (0 to 0)	0
Other hemoglobinopathies and hemolytic anemias	61·7 (36·4 to 97·7)	48·3 (29·8 to 72·9)	35·2 (22·1 to 52·6)	21·2 (12·8 to 31·9)	12·1 (8 to 17·4)	3·9 (2·6 to 5·5)	1 (0·6 to 1·5)	828
Endocrine, metabolic, blood, and immune disorders	150 (95·1 to 225)	104 (68·9 to 151)	66·8 (43·9 to 96·8)	41·7 (26 to 65·5)	16·8 (10·2 to 26·9)	6·3 (3·5 to 10·5)	1·5 (0·8 to 2·7)	3.930
Oral disorders	298 (171 to 481)	228 (136 to 359)	163 (99·5 to 248)	95·2 (59·3 to 144)	40·8 (24·8 to 61·6)	12·5 (7·6 to 18·9)	2·6 (1·5 to 4)	4,800
Caries of deciduous teeth	0 (0 to 0)	0 (0 to 0)	0 (0 to 0)	0 (0 to 0)	0 (0 to 0)	0 (0 to 0)	0 (0 to 0)	124
Caries of permanent teeth	14·5 (5·5 to 30·1)	9·2 (3·6 to 19·2)	5·3 (2·1 to 10·4)	2·6 (1 to 5·5)	0·9 (0·3 to 1·9)	0·2 (0·1 to 0·5)	0 (0 to 0·1)	888
Periodontal diseases	68·8 (24·6 to 154)	45·6 (16·7 to 103)	29 (11·2 to 63·6)	15·5 (6 to 34·3)	6·2 (2·2 to 13·9)	1·8 (0·6 to 3·8)	0·3 (0·1 to 0·7)	801
Other oral disorders	37·1 (22·4 to 55·9)	21·2 (12·5 to 32·6)	10·7 (6·5 to 16)	4·5 (2·8 to 6·9)	1·4 (0·8 to 2·1)	0·4 (0·2 to 0·5)	0·1 (0 to 0·1)	1,740
Sudden infant death syndrome	--	--	--	--	--	--	--	NA
Injuries	2,790 (2.200 to 3.470)	1,770 (1.410 to 2.150)	1,120 (919 to 1.340)	610 (499 to 729)	261 (212 to 316)	78 (62·6 to 94·8)	18·8 (14·7 to 23·4)	161.000
Transport injuries	938 (665 to 1,300)	576 (423 to 769)	348 (258 to 466)	179 (136 to 231)	67·1 (50·5 to 87)	16·8 (12·9 to 21·6)	4 (2·9 to 5·3)	53,800
Road injuries	877 (615 to 1.210)	539 (395 to 725)	325 (241 to 434)	166 (128 to 218)	61·6 (46·5 to 79·9)	15·2 (11·6 to 19·6)	3·5 (2·6 to 4·6)	50.300
Pedestrian road injuries	254 (144 to 420)	169 (102 to 260)	138 (88·6 to 211)	69·5 (46·4 to 100)	24·6 (16·2 to 35·8)	4·9 (3·4 to 7)	1·3 (0·8 to 2·1)	8.710
Cyclist road injuries	121 (72·4 to 194)	69·7 (42·4 to 110)	20·9 (14·8 to 28·3)	11·2 (8 to 14·9)	5·3 (3·8 to 7·2)	1·6 (1·1 to 2·2)	0·2 (0·2 to 0·3)	3,630
Motorcyclist road injuries	233 (165 to 318)	132 (95 to 178)	70·3 (49·6 to 93·5)	37 (26·2 to 50·2)	14·6 (10·5 to 19·9)	4·2 (3 to 5·8)	0·9 (0·6 to 1·2)	21.800
Motor vehicle road injuries	252 (154 to 415)	157 (98·3 to 248)	88·2 (56·8 to 141)	44·5 (29·2 to 68)	15·6 (10·4 to 23·3)	4·2 (2·8 to 6)	1 (0·6 to 1·4)	15.400
Other road injuries	16·4 (7·6 to 31·4)	10·3 (5·2 to 19)	8 (4 to 15·3)	4·2 (2 to 8)	1·5 (0·8 to 2·8)	0·3 (0·2 to 0·5)	0·1 (0 to 0·2)	802
Other transport injuries	61·5 (32·6 to 110)	36·6 (21·2 to 60·1)	23·2 (13·5 to 39)	12·1 (7·2 to 20·4)	5·4 (3·2 to 8·9)	1·6 (1 to 2·6)	0·5 (0·3 to 0·7)	3,560
Unintentional injuries	1,350 (1.040 to 1.730)	918 (706 to 1.150)	625 (504 to 768)	372 (298 to 460)	172 (137 to 216)	55·7 (43·5 to 69·4)	13·2 (9·8 to 17·2)	62.300
Falls	668 (474 to 903)	497 (360 to 664)	358 (272 to 474)	223 (163 to 300)	104 (75·1 to 139)	34·8 (25 to 47)	8 (5·1 to 11·3)	16,000
Drowning	152 (83·8 to 256)	90·8 (52·8 to 152)	52·5 (30·3 to 86·6)	23·6 (13·5 to 39)	10·8 (6 to 19·2)	2·2 (1·3 to 3·5)	0·6 (0·3 to 1)	17,000
Fire, heat, and hot substances	91 (59 to 136)	50·3 (33·3 to 71·9)	33·8 (21·8 to 49)	21·7 (13·9 to 31·3)	11·7 (7·3 to 17·5)	3·7 (2·3 to 5·4)	0·9 (0·5 to 1·3)	3.960
Poisonings	13·1 (6·2 to 24·7)	6·3 (3·4 to 11)	3·9 (1·9 to 6·7)	3·2 (1·6 to 5·8)	1·6 (0·9 to 2·8)	0·3 (0·2 to 0·6)	0·1 (0 to 0·1)	1,210
Poisoning by carbon monoxide	2·1 (0·7 to 5·2)	1·1 (0·4 to 2·5)	0·7 (0·2 to 1·7)	0·6 (0·2 to 1·4)	0·4 (0·1 to 0·9)	0 (0 to 0·1)	0 (0 to 0)	192
Poisoning by other means	11 (5·1 to 20·1)	5·2 (2·7 to 9·2)	3·2 (1·6 to 5·7)	2·6 (1·2 to 4·8)	1·2 (0·6 to 2·1)	0·3 (0·2 to 0·5)	0·1 (0 to 0·1)	1.010
Exposure to mechanical forces	126 (89·3 to 174)	83·1 (61 to 114)	49·5 (35·6 to 67·7)	26 (18·5 to 35·9)	11·3 (8·3 to 15·8)	3·6 (2·7 to 5)	0·9 (0·6 to 1·2)	5,570
Unintentional firearm injuries	10 (6·5 to 16·4)	6·9 (4·2 to 12·2)	3·3 (2·2 to 5·1)	1·8 (1·2 to 2·6)	0·8 (0·5 to 1·3)	0·3 (0·2 to 0·4)	0·1 (0 to 0·1)	576
Other exposure to mechanical forces	116 (82 to 163)	76·2 (55·5 to 106)	46·2 (33 to 63·9)	24·2 (17·3 to 33·8)	10·6 (7·8 to 14·6)	3·3 (2·5 to 4·6)	0·8 (0·6 to 1·1)	4.990
Adverse effects of medical treatment	54·7 (24·8 to 107)	35·9 (18·5 to 69·3)	23·6 (12·1 to 42·4)	14·7 (7·5 to 26·8)	6·3 (3·4 to 11·3)	2·1 (1·1 to 3·8)	0·5 (0·3 to 0·8)	1.460
Animal contact	64·4 (25·1 to 124)	41·3 (16·1 to 80)	26 (10·2 to 49·5)	16·2 (6·5 to 30)	6·4 (2·6 to 12)	1·9 (0·8 to 3·5)	0·5 (0·2 to 1)	2,750
Venomous animal contact	32·5 (10·2 to 70·9)	18·6 (5·5 to 41)	11·1 (3·2 to 26·5)	7·2 (2·1 to 16·1)	2·9 (0·9 to 6·4)	0·9 (0·3 to 2)	0·3 (0·1 to 0·6)	1.690
Non-venomous animal contact	31·9 (12 to 66)	22·7 (8·8 to 46·5)	14·9 (5·5 to 30)	8·9 (3·5 to 18·7)	3·5 (1·4 to 7·1)	1 (0·4 to 2)	0·3 (0·1 to 0·5)	1.050
Foreign body	43·1 (29·5 to 61·6)	34·5 (23·7 to 52·6)	27·1 (19 to 37·7)	17·6 (12·3 to 25·4)	8·4 (5·5 to 12·4)	3·2 (2·2 to 4·6)	0·8 (0·5 to 1·1)	3,850
Pulmonary aspiration and foreign body in airway	29·6 (17·2 to 48·6)	25 (14·7 to 42·6)	20·5 (12·9 to 31·1)	13·6 (8·5 to 20·9)	6·6 (3·9 to 10·4)	2·6 (1·6 to 4)	0·7 (0·4 to 1)	3.400
Foreign body in eyes	3·2 (2 to 4·9)	2·1 (1·4 to 3·1)	1·4 (0·9 to 2)	0·7 (0·5 to 1·1)	0·3 (0·2 to 0·4)	0·1 (0·1 to 0·1)	0 (0 to 0)	158
Foreign body in other body part	10·2 (7·3 to 13·5)	7·4 (5·2 to 10)	5·2 (3·8 to 6·9)	3·2 (2·3 to 4·3)	1·5 (1·1 to 2)	0·5 (0·4 to 0·7)	0·1 (0·1 to 0·2)	286
Environmental heat and cold exposure	16 (7·5 to 30·8)	13·1 (6 to 23·8)	9·1 (4·1 to 17·2)	5·9 (2·6 to 11·6)	3·1 (1·4 to 5·4)	1·1 (0·5 to 1·9)	0·2 (0·1 to 0·4)	592
Exposure to forces of nature	8·2 (7 to 9·6)	5·3 (4·6 to 6·2)	3·2 (2·7 to 3·7)	1·7 (1·5 to 2)	0·6 (0·5 to 0·7)	0·1 (0·1 to 0·2)	0 (0 to 0)	708
Other unintentional injuries	115 (72 to 189)	60 (38·8 to 94·5)	38·3 (25·5 to 59)	19·1 (13·5 to 27·4)	8·4 (5·8 to 12·4)	2·7 (1·8 to 3·9)	0·6 (0·4 to 0·9)	9,250
Self-harm and interpersonal violence	503 (349 to 695)	274 (197 to 374)	146 (105 to 203)	59·1 (43·7 to 79·6)	21·9 (16·5 to 30·3)	5·5 (4·1 to 7·6)	1·6 (1·1 to 2·2)	45.200
Self-harm	208 (128 to 326)	126 (78·8 to 205)	67·5 (42·7 to 104)	31·4 (19·7 to 50·9)	11·6 (7·1 to 19·8)	2·8 (1·7 to 4·9)	0·7 (0·4 to 1·2)	16.900
Self-harm by firearm	3·7 (1·7 to 9·2)	2·9 (1·3 to 7)	1·3 (0·6 to 2·9)	1·2 (0·5 to 3·2)	0·4 (0·2 to 1·1)	0·2 (0·1 to 0·5)	0 (0 to 0·1)	416
Self-harm by other specified means	204 (126 to 320)	123 (76·1 to 201)	66·2 (42 to 102)	30·3 (19·3 to 48)	11·1 (6·9 to 18·7)	2·6 (1·6 to 4·6)	0·7 (0·4 to 1·2)	16,500
Interpersonal violence	291 (184 to 448)	146 (97·3 to 228)	77·1 (50·4 to 118)	27·2 (19·7 to 36·2)	10·1 (7·3 to 13·7)	2·7 (1·9 to 3·6)	0·8 (0·5 to 1·3)	27.900
Physical violence by firearm	45·2 (17·8 to 90·4)	20·2 (7·8 to 42·1)	10·5 (4 to 22·3)	2·4 (1·1 to 4·8)	0·9 (0·4 to 1·7)	0·2 (0·1 to 0·4)	0·1 (0 to 0·3)	8.060
Physical violence by sharp object	130 (58·4 to 241)	56·8 (27·6 to 99·7)	27·5 (14·2 to 49·1)	9·8 (6·1 to 14·7)	3·4 (2·2 to 5)	0·9 (0·6 to 1·3)	0·3 (0·2 to 0·6)	13,600
Sexual violence	4·5 (2·8 to 6·7)	2·7 (1·7 to 4)	1·6 (1·1 to 2·4)	0·9 (0·6 to 1·3)	0·3 (0·2 to 0·5)	0 (0 to 0·1)	0 (0 to 0)	504
Physical violence by other means	111 (73·4 to 166)	66·3 (44 to 96·8)	37·5 (25 to 55·5)	14·1 (9·9 to 19)	5·5 (3·9 to 7·6)	1·5 (1·1 to 2·1)	0·4 (0·2 to 0·5)	5.750
Conflict and terrorism	4 (2·1 to 7·9)	1·8 (1 to 3·5)	0·8 (0·5 to 1·6)	0·4 (0·2 to 0·7)	0·1 (0·1 to 0·3)	0 (0 to 0·1)	0 (0 to 0)	203

SR Table 2a: United States of America DALYS by cause and age group for males with 95% uncertainty intervals, 2019

Cause	Early Neonatal	Late Neonatal	Post Neonatal	1 to 4	5 to 9	10 to 14	15 to 19	20 to 24	25 to 29	30 to 34	35 to 39	40 to 44	45 to 49	50 to 54	55 to 59	60 to 64	65 to 69	70 to 74	75 to 79	80 to 84	85 to 89	90 to 94	95 plus	Total
All causes	551,000 (503,000 to 605,000)	134,000 (123,000 to 147,000)	385,000 (356,000 to 416,000)	422,000 (348,000 to 513,000)	471,000 (364,000 to 601,000)	709,000 [illegible]	1,440,000 [illegible]	2,220,000 [illegible]	2,530,000 [illegible]	2,480,000 [illegible]	2,550,000 [illegible]	2,590,000 [illegible]	3,180,000 [illegible]	4,020,000 [illegible]	5,290,000 [illegible]	5,870,000 [illegible]	5,740,000 [illegible]	5,350,000 [illegible]	4,230,000 [illegible]	3,100,000 [illegible]	2,100,000 [illegible]	1,100,000 [illegible]	433,000 [illegible]	56,900,000
Communicable, maternal, neonatal, and nutritional diseases	424,000 (378,000 to 471,000)	80,300 (71,200 to 89,200)	88,100 (78,000 to 99,800)	81,000 (65,100 to 102,000)	75,300 (56,900 to 102,000)	68,100 [illegible]	64,900 [illegible]	69,900 [illegible]	85,700 [illegible]	91,900 [illegible]	100,000 [illegible]	104,000 [illegible]	121,000 [illegible]	134,000 [illegible]	157,000 [illegible]	153,000 [illegible]	141,000 [illegible]	129,000 [illegible]	115,000 [illegible]	96,800 [illegible]	77,700 [illegible]	48,600 [illegible]	22,500 [illegible]	2,530,000
HIV/AIDS and sexually transmitted infections	51·7 (44·4 to 59·8)	16·2 (13·3 to 19·3)	694 (666 to 724)	593 (570 to 620)	367 (350 to 384)	428 [illegible]	1,160 [illegible]	6,000 [illegible]	17,200 [illegible]	25,100 [illegible]	31,500 [illegible]	36,300 [illegible]	44,000 [illegible]	43,500 [illegible]	41,400 [illegible]	29,300 [illegible]	18,800 [illegible]	6,750 [illegible]	2,880 [illegible]	708 [illegible]	366 [illegible]	156 [illegible]	62 [illegible]	307,000
HIV/AIDS	0 (0 to 0·1)	0·2 (0·1 to 0·2)	672 (646 to 702)	589 (566 to 616)	367 (350 to 384)	367 [illegible]	942 [illegible]	5,580 [illegible]	16,500 [illegible]	24,200 [illegible]	30,500 [illegible]	35,400 [illegible]	43,100 [illegible]	42,600 [illegible]	40,400 [illegible]	28,500 [illegible]	18,000 [illegible]	6,120 [illegible]	2,440 [illegible]	443 [illegible]	204 [illegible]	84·2 [illegible]	32 [illegible]	297,000
HIV/AIDS - Drug-susceptible Tuberculosis	0 (0 to 0)	0 (0 to 0)	13·4 (8·4 to 20·7)	11·6 (7·3 to 17·9)	7 (4·4 to 10·9)	[illegible]	[illegible]	[illegible]	[illegible]	[illegible]	[illegible]	[illegible]	[illegible]	[illegible]	[illegible]	[illegible]	[illegible]	[illegible]	[illegible]	[illegible]	[illegible]	[illegible]	[illegible]	[illegible]

[illegible]

Gastroesophageal reflux disease	0 (0 to 0)
Appendicitis	0 (0 to 0)
Paralytic ileus and intestinal obstruction	407 (346 to 487)
Inguinal, femoral, and abdominal hernia	551 (437 to 650)
Inflammatory bowel disease	0 (0 to 0)
Vascular intestinal disorders	0 (0 to 0)
Gallbladder and biliary diseases	0 (0 to 0)
Pancreatitis	0 (0 to 0)
Other digestive diseases	0 (0 to 0)
Neurological disorders	70 (58·2 to 86·1)
Alzheimer's disease and other dementias	0 (0 to 0)
Parkinson's disease	0 (0 to 0)
Idiopathic epilepsy	14·8 (5·9 to 30·8)
Multiple sclerosis	0 (0 to 0)
Motor neuron disease	55·1 (48·5 to 62·2)
Headache disorders	0 (0 to 0)
Migraine	0 (0 to 0)
Tension-type headache	0 (0 to 0)
Other neurological disorders	0 (0 to 0)
Mental disorders	78·6 (52·3 to 112)
Schizophrenia	0 (0 to 0)
Depressive disorders	0 (0 to 0)
Major depressive disorder	0 (0 to 0)
Dysthymia	0 (0 to 0)
Bipolar disorder	0 (0 to 0)
Anxiety disorders	0 (0 to 0)
Eating disorders	0 (0 to 0)
Anorexia nervosa	0 (0 to 0)
Bulimia nervosa	0 (0 to 0)
Autism spectrum disorders	74·9 (48·6 to 107)
Attention-deficit/hyperactivity disorder	0 (0 to 0)
Conduct disorder	0 (0 to 0)
Idiopathic developmental intellectual disability	3·6 (0·1 to 10·7)
Other mental disorders	0 (0 to 0)
Substance use disorders	0·1 (0 to 0·3)
Alcohol use disorders	0·1 (0 to 0·3)
Drug use disorders	0 (0 to 0)
Opioid use disorders	0 (0 to 0)
Cocaine use disorders	0 (0 to 0)
Amphetamine use disorders	0 (0 to 0)
Cannabis use disorders	0 (0 to 0)
Other drug use disorders	0 (0 to 0)
Diabetes and kidney diseases	31·5 (24·7 to 49·5)
Diabetes mellitus	31·5 (24·6 to 49·5)
Diabetes mellitus type 1	31·5 (24·6 to 49·5)
Diabetes mellitus type 2	0 (0 to 0)
Chronic kidney disease	0 (0 to 0·1)
Chronic kidney disease due to diabetes mellitus typ[illegible]	0 (0 to 0)
Chronic kidney disease due to diabetes mellitus typ[illegible]	0 (0 to 0)
Chronic kidney disease due to hypertension	0 (0 to 0)
Chronic kidney disease due to glomerulonephritis	0 (0 to 0)
Chronic kidney disease due to other and unspecifie[illegible]	0 (0 to 0)
Acute glomerulonephritis	0 (0 to 0)
Skin and subcutaneous diseases	1·9 (1·2 to 2·8)
Dermatitis	0 (0 to 0)
Atopic dermatitis	0 (0 to 0)
Contact dermatitis	0 (0 to 0)
Seborrhoeic dermatitis	0 (0 to 0)
Psoriasis	0 (0 to 0)
Bacterial skin diseases	0·2 (0·1 to 0·3)
Cellulitis	0·2 (0·1 to 0·2)
Pyoderma	0 (0 to 0·1)
Scabies	0 (0 to 0·1)
Fungal skin diseases	0·1 (0 to 0·1)
Viral skin diseases	0 (0 to 0)
Acne vulgaris	0 (0 to 0)
Alopecia areata	0 (0 to 0)
Pruritus	0 (0 to 0)
Urticaria	1·2 (0·7 to 1·8)
Decubitus ulcer	0 (0 to 0)
Other skin and subcutaneous diseases	0·4 (0·2 to 0·7)
Sense organ diseases	5·7 (2·9 to 9·7)
Blindness and vision loss	0·7 (0·2 to 2·1)
Glaucoma	0 (0 to 0)
Cataract	0 (0 to 0)
Age-related macular degeneration	0 (0 to 0)
Refraction disorders	0 (0 to 0·1)
Near vision loss	0 (0 to 0)
Other vision loss	0·7 (0·1 to 2)
Age-related and other hearing loss	0·6 (0·1 to 1·5)
Other sense organ diseases	4·4 (2·1 to 8·1)
Musculoskeletal disorders	0 (0 to 0)
Rheumatoid arthritis	0 (0 to 0)
Osteoarthritis	0 (0 to 0)
Osteoarthritis hip	0 (0 to 0)
Osteoarthritis knee	0 (0 to 0)
Osteoarthritis hand	0 (0 to 0)
Osteoarthritis other	0 (0 to 0)
Low back pain	0 (0 to 0)
Neck pain	0 (0 to 0)
Gout	0 (0 to 0)
Other musculoskeletal disorders	0 (0 to 0)
Other non-communicable diseases	120,000 (103 000 to 156 000)
Congenital birth defects	116,000 (98 900 to 152 000)
Neural tube defects	12,200 (8 660 to 16 000)
Congenital heart anomalies	17,900 (14 400 to 27 700)
Orofacial clefts	43·6 (30 to 59·4)
Down syndrome	1,530 (1 110 to 2 140)
Turner syndrome	0 (0 to 0)
Klinefelter syndrome	0·1 (0·1 to 0·2)
Other chromosomal abnormalities	23,700 (16 100 to 34 200)
Congenital musculoskeletal and limb anomalies	5,430 (2 310 to 7 410)
Urogenital congenital anomalies	13,500 (8 010 to 19 600)
Digestive congenital anomalies	9,130 (6 700 to 12 900)
Other congenital birth defects	33,100 (27 300 to 48 700)
Urinary diseases and male infertility	325 (262 to 546)
Urolithiasis	0 (0 to 0)
Benign prostatic hyperplasia	0 (0 to 0)
Male infertility	0 (0 to 0)
Other urinary diseases	225 (165 to 443)
Gynecological diseases	0 (0 to 0)
Uterine fibroids	0 (0 to 0)
Polycystic ovarian syndrome	0 (0 to 0)
Female infertility	0 (0 to 0)
Endometriosis	0 (0 to 0)
Genital prolapse	0 (0 to 0)
Premenstrual syndrome	0 (0 to 0)
Other gynecological diseases	0 (0 to 0)
Hemoglobinopathies and hemolytic anemias	168 (143 to 201)
Thalassemias	42·7 (30 to 58·8)
Thalassemias trait	2·2 (0·4 to 6·2)
Sickle cell disorders	110 (87·6 to 135)
Sickle cell trait	1·6 (0·2 to 5·4)
G6PD deficiency	5·1 (3 to 7·7)
G6PD trait	0 (0 to 0)
Other hemoglobinopathies and hemolytic anemias	7·2 (5·3 to 9·6)
Endocrine, metabolic, blood, and immune disorders	3,220 (2 490 to 5 650)
Oral disorders	0 (0 to 0)
Caries of deciduous teeth	0 (0 to 0)
Caries of permanent teeth	0 (0 to 0)
Periodontal diseases	0 (0 to 0)
Other oral disorders	0 (0 to 0)
Sudden infant death syndrome	..
Injuries	2,760 (2 440 to 3 070)
Transport injuries	330 (292 to 372)
Road injuries	272 (239 to 306)
Pedestrian road injuries	52·3 (45·8 to 58·8)
Cyclist road injuries	0 (0 to 0)
Motorcyclist road injuries	21·6 (19 to 24·3)
Motor vehicle road injuries	174 (153 to 197)
Other road injuries	23·3 (19·5 to 27·9)
Other transport injuries	58·9 (50·4 to 69·6)
Unintentional injuries	1,510 (1 340 to 1 700)
Falls	90·1 (78·7 to 104)
Drowning	112 (98·9 to 125)
Fire, heat, and hot substances	35 (31·1 to 39·6)
Poisonings	70·9 (61·9 to 80)
Poisoning by carbon monoxide	9·3 (7·6 to 11·1)
Poisoning by other means	61·5 (53·8 to 69·7)
Exposure to mechanical forces	53 (47 to 59·5)
Unintentional firearm injuries	11·3 (8·6 to 15·5)
Other exposure to mechanical forces	41·7 (35 to 47·3)
Adverse effects of medical treatment	350 (286 to 445)
Animal contact	26·5 (22·7 to 30·6)
Venomous animal contact	1·7 (1·3 to 2·1)
Non-venomous animal contact	24·8 (21·2 to 28·6)
Foreign body	716 (638 to 795)
Pulmonary aspiration and foreign body in airway	712 (634 to 791)
Foreign body in eyes	0·9 (0·2 to 2·2)
Foreign body in other body part	3·2 (2·3 to 5·2)
Environmental heat and cold exposure	22·8 (20·2 to 26)
Exposure to forces of nature	1·7 (1·5 to 1·8)
Other unintentional injuries	30·9 (26·5 to 35·3)
Self-harm and interpersonal violence	919 (789 to 1 060)
Self-harm	0 (0 to 0)
Self-harm by firearm	0 (0 to 0)
Self-harm by other specified means	0 (0 to 0)
Interpersonal violence	916 (786 to 1 060)
Physical violence by firearm	52·5 (44·5 to 61·7)
Physical violence by sharp object	35 (29·2 to 41·1)
Sexual violence	0 (0 to 0)
Physical violence by other means	828 (709 to 958)
Conflict and terrorism	2·9 (2·6 to 3·2)

SR Table 2a: Latin America and Caribbean DALYS by cause and age group for males with 95% uncertainty intervals, 2019

Cause	Early Neonatal	Late Neonatal	Post Neonatal	1 to 4	5 to 9	10 to 14	15 to 19	20 to 24	25 to 29	30 to 34	35 to 39	40 to 44	45 to 49	50 to 54	55 to 59	60 to 64	65 to 69	70 to 74	75 to 79	80 to 84	85 to 89	90 to 94	95 plus	Total
All causes	3,600,000 (2,830,000 to 4,530,000)	1,140,000 (903,000 to 1,410,000)	3,230,000 (2,520,000 to 4,100,000)	2,050,000 (1,700,000 to 2,430,000)	1,660,000 (1,370,000 to 2,000,000)	1,950,000 (1,590,000 to 2,380,000)	3,870,000 (3,410,000 to 4,440,000)	5,300,000 (4,750,000 to 5,930,000)	5,300,000 (4,750,000 to 5,940,000)	5,230,000 (4,680,000 to 5,900,000)	5,340,000 (4,770,000 to 6,060,000)	5,390,000 (4,770,000 to 6,090,000)	5,600,000 (4,980,000 to 6,360,000)	6,160,000 (5,480,000 to 6,950,000)	6,470,000 (5,790,000 to 7,280,000)	6,370,000 (5,740,000 to 7,130,000)	5,840,000 (5,280,000 to 6,520,000)	5,120,000 (4,650,000 to 5,660,000)	4,030,000 (3,680,000 to 4,420,000)	2,920,000 (2,680,000 to 3,180,000)	1,700,000 (1,580,000 to 1,840,000)	766,000 (711,000 to 823,000)	300,000 (282,000 to 319,000)	89,300,000
Communicable, maternal, neonatal, and nutritional diseases	2,890,000 (2,240,000 to 3,650,000)	851,000 (671,000 to 1,070,000)	1,800,000 (1,400,000 to 2,280,000)	843,000 (695,000 to 1,010,000)	462,000 (359,000 to 584,000)	317,000 (253,000 to 388,000)	299,000 (250,000 to 356,000)	367,000 (321,000 to 419,000)	460,000 (411,000 to 516,000)	510,000 (462,000 to 569,000)	535,000 (490,000 to 591,000)	515,000 (471,000 to 568,000)	482,000 (441,000 to 530,000)	445,000 (405,000 to 491,000)	416,000 (378,000 to 461,000)	370,000 (337,000 to 413,000)	334,000 (303,000 to 371,000)	304,000 (276,000 to 336,000)	266,000 (240,000 to 292,000)	218,000 (192,000 to 241,000)	149,000 (127,000 to 165,000)	73,600 (60,000 to 82,700)	31,600 (24,500 to 35,900)	12,900,000
HIV/AIDS and sexually transmitted infections	52,500 (21,900 to 106,000)	18,200 (7,480 to 35,300)	47,700 (31,800 to 72,400)	24,800 (17,200 to 63,700)	13,000 (11,000 to 15,700)	11,700 (10,100 to 13,500)	25,600 (22,100 to 29,900)	77,000 (71,800 to 84,500)	159,000 (147,000 to 179,000)	202,000 (187,000 to 226,000)	212,000 (196,000 to 236,000)	191,000 (177,000 to 209,000)	158,000 (148,000 to 172,000)	108,000 (101,000 to 116,000)	75,700 (70,900 to 81,400)	42,500 (39,400 to 46,600)	27,100 (24,800 to 29,800)	7,960 (6,890 to 9,340)	3,460 (2,990 to 4,090)	1,110 (952 to 1,330)	437 (362 to 544)	149 (118 to 189)	78 (66 to 92·3)	1,460,000
HIV/AIDS	2·1 (1·4 to 3·3)	6·8 (4·5 to 10·6)	22,100 (19,700 to 31,100)	23,900 (16,500 to 62,300)	12,700 (10,700 to 15,300)	11,200 (9,620 to 12,900)	24,000 (20,600 to 28,500)	74,300 (69,300 to 80,900)	156,000 (143,000 to 175,000)	198,000 (183,000 to 221,000)	208,000 (192,000 to 232,000)	187,000 (174,000 to 206,000)	156,000 (146,000 to 169,000)	106,000 (99,400 to 113,000)	74,100 (69,600 to 79,500)	41,300 (38,400 to 45,100)	26,200 (24,000 to 28,600)	7,260 (6,310 to 8,420)	3,020 (2,620 to 3,490)	831 (737 to 931)	298 (257 to 342)	90·5 (75·2 to 108)	53·9 (47·1 to 60·6)	1,330,000
HIV/AIDS - Drug-susceptible Tuberculosis	0 (0 to 0)	0·3 (0·2 to 0·5)	2,460 (1,540 to 3,730)	2,120 (1,320 to 3,270)	1,360 (875 to 2,050)	1,100 (709 to 1,640)	2,300 (1,510 to 3,420)	7,750 (5,100 to 11,400)	16,000 (10,500 to 23,600)	19,900 (13,000 to 29,500)	20,800 (13,600 to 30,700)	18,700 (12,200 to 27,600)	15,500 (10,000 to 23,100)	10,300 (6,760 to 15,300)	7,160 (4,680 to 10,500)	4,020 (2,670 to 5,890)	2,550 (1,680 to 3,710)	674 (468 to 964)	283 (197 to 399)	79·4 (54·3 to 113)	26·7 (19 to 37·7)	8·2 (6 to 11·4)	4·7 (3·3 to 6·8)	133,000
HIV/AIDS - Multidrug-resistant Tuberculosis with	0 (0 to 0)	0 (0 to 0)	193 (60·7 to 488)	153 (52·1 to 350)	84·9 (29·3 to 200)	67·5 (23·2 to 161)	164 (59·7 to 379)	648 (237 to 1,480)	1,340 (492 to 3,100)	1,610 (582 to 3,860)	1,600 (546 to 3,970)	1,360 (458 to 3,360)	1,090 (360 to 2,710)	712 (239 to 1,730)	500 (160 to 1,250)	282 (94·5 to 686)	191 (65·6 to 466)	44·3 (16·4 to 106)	18·5 (7 to 43·7)	5·7 (2·1 to 13·6)	1·8 (0·7 to 4·2)	0·6 (0·3 to 1·4)	0·5 (0·2 to 1)	10,100
HIV/AIDS - Extensively drug-resistant Tuberculo	0 (0 to 0)	0 (0 to 0)	31·9 (9·6 to 77·4)	25·3 (8·2 to 56·8)	13·9 (4·6 to 31·8)	11 (3·6 to 25·8)	26·6 (9·1 to 62·1)	105 (35·9 to 239)	217 (74·3 to 490)	262 (87·9 to 596)	260 (85·4 to 599)	221 (71·1 to 518)	178 (56·5 to 422)	116 (37 to 272)	80·9 (25 to 192)	45·3 (14·4 to 108)	30·6 (9·8 to 72·2)	6·8 (2·3 to 15·6)	2·8 (1 to 6·4)	0·9 (0·3 to 2·1)	0·3 (0·1 to 0·6)	0·1 (0 to 0·2)	0·1 (0 to 0·2)	1,630
HIV/AIDS resulting in other diseases	2 (1·3 to 3·3)	6·4 (4·2 to 10·2)	19,400 (16,500 to 28,600)	21,600 (14,300 to 60,000)	11,200 (9,170 to 13,800)	9,980 (8,350 to 11,700)	21,500 (17,900 to 25,800)	65,800 (59,300 to 72,900)	138,000 (123,000 to 159,000)	176,000 (158,000 to 200,000)	186,000 (167,000 to 209,000)	167,000 (151,000 to 186,000)	139,000 (126,000 to 153,000)	94,500 (86,400 to 103,000)	66,400 (60,400 to 72,100)	36,900 (33,500 to 41,000)	23,400 (20,900 to 26,000)	6,540 (5,550 to 7,770)	2,720 (2,310 to 3,200)	745 (646 to 846)	269 (228 to 313)	81·6 (66·1 to 98·7)	48·6 (41·5 to 55·5)	1,190,000
Sexually transmitted infections excluding HIV	52,500 (21,900 to 106,000)	18,200 (7,470 to 35,300)	25,600 (10,700 to 50,000)	864 (337 to 1,760)	386 (144 to 778)	526 (366 to 750)	1,550 (970 to 2,370)	2,760 (1,690 to 4,300)	3,450 (2,030 to 5,720)	4,130 (2,330 to 6,840)	3,980 (2,300 to 6,770)	3,350 (1,940 to 5,900)	2,790 (1,550 to 4,960)	2,220 (1,230 to 4,170)	1,640 (886 to 3,130)	1,260 (703 to 2,360)	967 (555 to 1,750)	696 (417 to 1,230)	441 (272 to 770)	281 (178 to 467)	139 (89·2 to 226)	58·5 (37·5 to 94·4)	24·1 (15·6 to 36·1)	128,000
Syphilis	52,500 (21,900 to 106,000)	18,200 (7,470 to 35,300)	25,600 (10,700 to 50,000)	864 (337 to 1,760)	386 (144 to 778)	144 (106 to 209)	168 (114 to 263)	233 (154 to 363)	308 (206 to 475)	333 (231 to 488)	531 (371 to 833)	421 (313 to 550)	391 (289 to 531)	444 (328 to 607)	409 (309 to 550)	396 (296 to 529)	338 (256 to 464)	266 (201 to 344)	145 (109 to 182)	87·3 (65·9 to 111)	38·3 (27·7 to 51·3)	15 (10·7 to 20·2)	5·8 (3·9 to 7·8)	102,000
Chlamydial infection	0 (0 to 0)	0 (0 to 0)	0 (0 to 0)	0 (0 to 0)	0 (0 to 0)	6·3 (2·7 to 11·7)	118 (57·4 to 219)	471 (233 to 884)	860 (411 to 1,540)	961 (464 to 1,690)	841 (413 to 1,490)	664 (321 to 1,160)	429 (205 to 746)	188 (89·4 to 342)	50·5 (24·2 to 89·7)	8·8 (4·3 to 15·9)	2·3 (1·1 to 4·4)	0 (0 to 0·1)	0 (0 to 0)	0 (0 to 0)	0 (0 to 0)	0 (0 to 0)	0 (0 to 0)	4,600
Gonococcal infection	0 (0 to 0)	0 (0 to 0)	0 (0 to 0)	0 (0 to 0)	0 (0 to 0)	81·1 (42·2 to 145)	430 (236 to 715)	670 (372 to 1,090)	379 (189 to 657)	242 (122 to 413)	171 (95·6 to 290)	152 (93·7 to 249)	125 (73·4 to 202)	86·2 (50·3 to 142)	45·4 (29·1 to 71·8)	32·4 (23·4 to 45·8)	32·4 (22·6 to 48·6)	21·2 (15·4 to 29·1)	11·7 (8·2 to 15·5)	9·2 (6·3 to 12·8)	4·2 (2·4 to 6·5)	0·6 (0·3 to 0·9)	0·1 (0 to 0·1)	2,490
Trichomoniasis	0 (0 to 0)	0 (0 to 0)	0 (0 to 0)	0 (0 to 0)	0 (0 to 0)	0·4 (0·1 to 1·1)	5·7 (1·4 to 15)	31·8 (9·4 to 80·7)	85·5 (25·6 to 200)	137 (43·8 to 311)	156 (51·8 to 357)	148 (49·1 to 350)	119 (38·5 to 282)	82·4 (26·6 to 191)	45·1 (15·3 to 107)	22·1 (8·6 to 50·4)	14·3 (5·5 to 31·4)	8·9 (3·4 to 20)	4·8 (1·8 to 10·7)	2·3 (0·8 to 5)	0·9 (0·3 to 2)	0·2 (0·1 to 0·6)	0 (0 to 0·1)	864
Genital herpes	0 (0 to 0)	0 (0 to 0)	0 (0 to 0)	0 (0 to 0)	0 (0 to 0)	68·3 (22·7 to 164)	311 (103 to 742)	678 (224 to 1,660)	1,060 (353 to 2,540)	1,370 (449 to 3,390)	1,530 (491 to 3,740)	1,470 (477 to 3,650)	1,330 (418 to 3,340)	1,190 (372 to 2,960)	990 (304 to 2,440)	756 (227 to 1,880)	545 (166 to 1,350)	370 (111 to 927)	230 (68·9 to 571)	132 (38·8 to 326)	62·5 (19·1 to 156)	23·7 (7·1 to 58·5)	7·6 (2·3 to 18·6)	12,100
Other sexually transmitted infections	0 (0 to 0)	0 (0 to 0)	0 (0 to 0)	0 (0 to 0)	0 (0 to 0)	225 [illegible]	521 [illegible]	673 [illegible]	761 [illegible]	1,080 [illegible]	757 [illegible]	502 [illegible]	395 [illegible]	231 [illegible]	97·9 [illegible]	46·3 [illegible]	34·9 [illegible]	28·9 [illegible]	49·8 [illegible]	50·6 [illegible]	33·6 [illegible]	18·9 [illegible]	10·6 [illegible]	[illegible]
[illegible]	[illegible]	[illegible]	[illegible]	[illegible]	[illegible]	[illegible]	[illegible]	[illegible]	[illegible]	[illegible]	[illegible]	[illegible]	[illegible]	[illegible]	[illegible]	[illegible]	[illegible]	[illegible]	[illegible]	[illegible]	[illegible]	[illegible]	[illegible]	[illegible]

Multiple sclerosis	0 (0 to 0)	0 (0 to 0)	0 (0 to 0)	0 (0 to 0)	54·6 (5·8 to 260)
Motor neuron disease	30·4 (23·1 to 38·9)	36·2 (27·4 to 46·4)	1,920 (1,340 to 2,670)	726 (527 to 971)	408 (325 to 500)
Headache disorders	0 (0 to 0)	0 (0 to 0)	0 (0 to 0)	0 (0 to 0)	26,000 (378 to 65,800)
Migraine	0 (0 to 0)	0 (0 to 0)	0 (0 to 0)	0 (0 to 0)	23,600 (253 to 61,400)
Tension-type headache	0 (0 to 0)	0 (0 to 0)	0 (0 to 0)	0 (0 to 0)	2,390 (44·4 to 16,700)
Other neurological disorders	0 (0 to 0)	0 (0 to 0)	9,150 (6,900 to 11,800)	14,300 (9,980 to 19,800)	15,200 (9,900 to 22,600)
Mental disorders	104 (69·1 to 151)	311 (207 to 448)	5,170 (3,410 to 7,400)	26,800 (17,700 to 37,600)	119,000 (81,000 to 170,000)
Schizophrenia	0 (0 to 0)	0 (0 to 0)	0 (0 to 0)	0 (0 to 0)	0 (0 to 0)
Depressive disorders	0 (0 to 0)	0 (0 to 0)	0 (0 to 0)	49·1 (20 to 108)	5,580 (2,580 to 9,840)
Major depressive disorder	0 (0 to 0)	0 (0 to 0)	0 (0 to 0)	40·9 (14·6 to 99·8)	4,990 (2,180 to 9,220)
Dysthymia	0 (0 to 0)	0 (0 to 0)	0 (0 to 0)	8·2 (1·9 to 20·7)	588 (238 to 1,220)
Bipolar disorder	0 (0 to 0)	0 (0 to 0)	0 (0 to 0)	0 (0 to 0)	0 (0 to 0)
Anxiety disorders	0 (0 to 0)	0 (0 to 0)	0 (0 to 0)	2,130 (1,170 to 3,400)	31,100 (18,100 to 49,600)
Eating disorders	0 (0 to 0)	0 (0 to 0)	0 (0 to 0)	0 (0 to 0)	332 (125 to 665)
Anorexia nervosa	0 (0 to 0)	0 (0 to 0)	0 (0 to 0)	0 (0 to 0)	286 (88·9 to 612)
Bulimia nervosa	0 (0 to 0)	0 (0 to 0)	0 (0 to 0)	0 (0 to 0)	45·9 (13·8 to 108)
Autism spectrum disorders	96·7 (62·8 to 140)	285 (187 to 410)	4,580 (2,980 to 6,690)	19,600 (12,700 to 28,400)	23,800 (15,400 to 34,900)
Attention-deficit/hyperactivity disorder	0 (0 to 0)	0 (0 to 0)	0 (0 to 0)	1,310 (694 to 2,370)	13,800 (7,310 to 24,700)
Conduct disorder	0 (0 to 0)	0 (0 to 0)	0 (0 to 0)	0 (0 to 0)	38,800 (20,700 to 66,800)
Idiopathic developmental intellectual disability	7·8 (0·7 to 22)	26·1 (1·8 to 69·6)	584 (41·3 to 1,350)	3,760 (808 to 7,430)	5,520 (1,610 to 10,200)
Other mental disorders	0 (0 to 0)	0 (0 to 0)	0 (0 to 0)	0 (0 to 0)	0 (0 to 0)
Substance use disorders	0·1 (0 to 0·1)	0·2 (0·1 to 0·3)	3·4 (1·5 to 6·1)	22·9 (12·3 to 37·2)	29·3 (16·1 to 47·4)

SR Table 2a: Caribbean DALYS by cause and age group for males with 95% uncertainty intervals, 2019

Cause	Early Neonatal	Late Neonatal	Post Neonatal	1 to 4	5 to 9	10 to 14	15 to 19	20 to 24	25 to 29	30 to 34	35 to 39	40 to 44
All causes	585,000 (450,000 to 746,000)	182,000 (141,000 to 231,000)	470,000 (353,000 to 617,000)	431,000 (362,000 to 510,000)	211,000 (176,000 to 249,000)	188,000 (155,000 to 225,000)	268,000 (228,000 to 316,000)	351,000 (304,000 to 404,000)	379,000 (332,000 to 431,000)	403,000 (354,000 to 458,000)	404,000 (354,000 to 463,000)	436,000 (381,000 to 502,000)
Communicable, maternal, neonatal, and nutritional diseases	495,000 (373,000 to 639,000)	140,000 (106,000 to 179,000)	316,000 (225,000 to 435,000)	199,000 (151,000 to 266,000)	62,400 (49,000 to 78,900)	36,300 (28,800 to 45,900)	35,300 (27,900 to 44,700)	35,900 (29,700 to 44,000)	44,100 (36,800 to 53,400)	56,500 (47,700 to 68,700)	63,000 (51,700 to 78,700)	64,100 (53,500 to 77,600)
HIV/AIDS and sexually transmitted infections	25,900 (8,670 to 54,000)	8,900 (3,070 to 18,500)	15,200 (7,290 to 28,100)	8,960 (6,340 to 12,700)	5,090 (3,470 to 7,150)	4,500 (3,120 to 6,060)	8,060 (4,860 to 12,000)	7,920 (6,280 to 10,200)	15,200 (11,500 to 21,700)	25,800 (19,500 to 36,200)	34,300 (25,500 to 48,700)	35,500 (27,600 to 47,300)
HIV/AIDS	0·4 (0·2 to 0·6)	1·4 (0·8 to 2·1)	3,780 (2,870 to 5,150)	8,580 (5,980 to 12,300)	4,910 (3,270 to 6,890)	4,400 (3,040 to 5,950)	7,800 (4,630 to 11,700)	7,550 (5,900 to 9,870)	14,800 (11,100 to 21,200)	25,400 (19,100 to 35,700)	33,900 (25,100 to 48,100)	35,200 (27,300 to 47,000)
HIV/AIDS - Drug-susceptible Tuberculosis	0 (0 to 0)	0·1 (0 to 0·1)	295 (180 to 465)	698 (423 to 1,110)	492 (302 to 775)	381 (235 to 597)	581 (357 to 913)	536 (346 to 822)	1,050 (684 to 1,600)	1,800 (1,160 to 2,730)	2,520 (1,630 to 3,860)	2,840 (1,800 to 4,420)
HIV/AIDS - Multidrug-resistant Tuberculosis without exte	0 (0 to 0)	0 (0 to 0)	3·8 (0·6 to 12·6)	8·3 (1·1 to 31)	5·7 (0·7 to 21·1)	4·4 (0·6 to 16·2)	7 (1 to 25)	7·5 (1·8 to 23·5)	15·1 (3·8 to 50·2)	25·6 (6·1 to 81·4)	34·2 (7·7 to 110)	38·2 (8·2 to 122)
HIV/AIDS - Extensively drug-resistant Tuberculosis	0 (0 to 0)	0 (0 to 0)	0·6 (0·1 to 2·2)	1·4 (0·2 to 5·2)	0·9 (0·1 to 3·5)	0·7 (0·1 to 2·7)	1·1 (0·2 to 4·2)	1·2 (0·3 to 3·8)	2·5 (0·6 to 7·5)	4·2 (1 to 12·8)	5·6 (1·2 to 18·1)	6·3 (1·3 to 20·4)
HIV/AIDS resulting in other diseases	0·4 (0·2 to 0·6)	1·3 (0·8 to 2)	3,480 (2,530 to 4,830)	7,870 (5,270 to 11,700)	4,410 (2,770 to 6,410)	4,010 (2,630 to 5,570)	7,210 (4,000 to 11,000)	7,000 (5,380 to 9,300)	13,700 (10,100 to 20,200)	23,500 (17,300 to 33,800)	31,300 (22,600 to 45,400)	32,300 (24,500 to 44,600)
Sexually transmitted infections excluding HIV	25,900 (8,670 to 54,000)	8,900 (3,070 to 18,500)	11,400 (3,650 to 24,400)	383 (132 to 806)	173 (59·6 to 365)	97·7 (48·6 to 187)	259 (146 to 440)	373 (221 to 610)	421 (256 to 695)	462 (275 to 752)	450 (256 to 845)	324 (186 to 547)
Syphilis	25,900 (8,670 to 54,000)	8,900 (3,070 to 18,500)	11,400 (3,650 to 24,400)	383 (132 to 806)	173 (59·6 to 365)	36 (13·8 to 81·5)	60 (22·4 to 147)	62·8 (21·7 to 167)	93 (36·3 to 233)	95·7 (39·6 to 210)	163 (55·9 to 471)	76·3 (34·7 to 156)
Chlamydial infection	0 (0 to 0)	0 (0 to 0)	0 (0 to 0)	0 (0 to 0)	0 (0 to 0)	0·7 (0·3 to 1·4)	17·3 (8·2 to 32·1)	58·6 (26·4 to 111)	93·5 (42·6 to 173)	90·9 (40·2 to 162)	71·2 (31·7 to 132)	59 (26·4 to 109)
Gonococcal infection	0 (0 to 0)	0 (0 to 0)	0 (0 to 0)	0 (0 to 0)	0 (0 to 0)	13·1 (5·3 to 28·2)	73·2 (30·3 to 152)	106 (50 to 192)	47·2 (19·1 to 94·9)	32·6 (14 to 63)	25·4 (12 to 48·5)	23·9 (12·3 to 44)
Trichomoniasis	0 (0 to 0)	0 (0 to 0)	0 (0 to 0)	0 (0 to 0)	0 (0 to 0)	0 (0 to 0·1)	0·5 (0·1 to 1·4)	2·8 (0·7 to 7·3)	7·1 (2·1 to 15·7)	10·8 (3·4 to 24·6)	11·2 (3·6 to 26·3)	10·4 (3·4 to 24·7)
Genital herpes	0 (0 to 0)	0 (0 to 0)	0 (0 to 0)	0 (0 to 0)	0 (0 to 0)	4·7 (1·5 to 11·5)	21·8 (7 to 53·7)	46·7 (14·9 to 115)	76·5 (25·6 to 182)	98·7 (32·5 to 239)	102 (33·9 to 252)	102 (32·3 to 249)
Other sexually transmitted infections	0 (0 to 0)	0 (0 to 0)	0 (0 to 0)	0 (0 to 0)	0 (0 to 0)	43 (20·9 to 81)	85·9 (41·8 to 161)	96·8 (54·2 to 161)	104 (60·2 to 173)	133 (74·6 to 221)	76·8 (41·4 to 132)	53 (28·7 to 89·5)
Respiratory infections and tuberculosis	30,100 (16,100 to 53,200)	21,400 (11,300 to 36,700)	91,900 (59,300 to 138,000)	54,500 (35,400 to 78,400)	11,600 (8,540 to 15,100)	6,460 (4,830 to 8,560)	6,330 (4,660 to 8,410)	9,450 (7,070 to 12,100)	11,200 (8,650 to 14,000)	13,400 (10,700 to 17,000)	12,500 (9,870 to 15,800)	13,100 (10,300 to 16,500)
Tuberculosis	0 (0 to 0)	0 (0 to 0·1)	3,460 (1,790 to 5,890)	4,180 (2,570 to 6,670)	1,180 (804 to 1,670)	713 (497 to 1,000)	1,330 (831 to 2,000)	3,970 (2,640 to 5,660)	5,200 (3,660 to 7,210)	6,450 (4,510 to 8,840)	5,590 (3,830 to 7,710)	5,290 (3,710 to 7,240)
Latent tuberculosis infection	0 (0 to 0)	0 (0 to 0)	0 (0 to 0)	0 (0 to 0)	0 (0 to 0)	0 (0 to 0)	0 (0 to 0)	0 (0 to 0)	0 (0 to 0)	0 (0 to 0)	0 (0 to 0)	0 (0 to 0)
Drug-susceptible tuberculosis	0 (0 to 0)	0 (0 to 0·1)	3,420 (1,760 to 5,830)	4,130 (2,550 to 6,650)	1,170 (796 to 1,660)	705 (492 to 991)	1,310 (818 to 1,980)	3,920 (2,590 to 5,600)	5,140 (3,620 to 7,080)	6,370 (4,470 to 8,770)	5,530 (3,790 to 7,650)	5,230 (3,670 to 7,170)
Multidrug-resistant tuberculosis without extensive drug r	0 (0 to 0)	0 (0 to 0)	33·8 (4·3 to 135)	38·9 (5·6 to 143)	10·8 (2·1 to 37)	6·9 (1·4 to 23·7)	14·3 (2·7 to 54·5)	41·4 (8·4 to 149)	52·9 (10·9 to 185)	66·5 (13·6 to 234)	57·9 (12·5 to 212)	55·1 (12·7 to 181)
Extensively drug-resistant tuberculosis	0 (0 to 0)	0 (0 to 0)	5·6 (0·6 to 20·7)	6·4 (0·8 to 25·1)	1·7 (0·3 to 5·7)	1·1 (0·2 to 3·8)	2·4 (0·4 to 8·4)	6·7 (1·3 to 23·7)	8·5 (1·6 to 28·5)	10·8 (2 to 39·5)	9·5 (1·9 to 33·3)	9 (1·9 to 30·4)
Lower respiratory infections	30,000 (16,100 to 53,100)	21,300 (11,200 to 36,700)	87,500 (55,400 to 132,000)	46,600 (28,000 to 70,800)	7,010 (4,600 to 10,000)	3,110 (2,140 to 4,460)	2,520 (1,750 to 3,700)	3,170 (2,220 to 4,540)	3,870 (2,840 to 5,340)	5,130 (3,870 to 6,810)	5,310 (3,900 to 7,020)	6,400 (4,800 to 8,410)
Upper respiratory infections	5·8 (3·2 to 10·2)	34 (19 to 58·7)	873 (391 to 2,180)	2,810 (1,390 to 5,930)	2,230 (1,240 to 3,810)	1,820 (959 to 3,150)	1,730 (918 to 2,990)	1,610 (873 to 2,690)	1,510 (837 to 2,520)	1,390 (753 to 2,290)	1,210 (618 to 2,000)	1,070 (584 to 1,880)
Otitis media	46·9 (0·5 to 246)	60·8 (2·1 to 417)	104 (53 to 191)	860 (484 to 1,460)	1,170 (660 to 1,890)	817 (445 to 1,380)	749 (404 to 1,270)	704 (302 to 1,410)	571 (276 to 1,010)	446 (205 to 812)	389 (192 to 728)	382 (181 to 671)
Enteric infections	4,600 (1,400 to 11,900)	14,700 (4,390 to 33,900)	102,000 (39,300 to 213,000)	50,000 (19,000 to 112,000)	7,200 (4,640 to 10,800)	4,260 (2,740 to 6,190)	3,040 (2,010 to 4,300)	2,800 (1,860 to 4,290)	3,130 (2,160 to 4,730)	3,380 (2,360 to 5,030)	3,490 (2,440 to 5,130)	3,870 (2,690 to 5,890)
Diarrheal diseases	4,600 (1,400 to 11,900)	14,700 (4,380 to 33,900)	102,000 (39,200 to 213,000)	49,700 (18,900 to 112,000)	7,030 (4,440 to 10,600)	4,150 (2,620 to 6,120)	2,990 (1,960 to 4,260)	2,760 (1,810 to 4,240)	3,110 (2,140 to 4,700)	3,350 (2,330 to 4,990)	3,470 (2,410 to 5,100)	3,860 (2,660 to 5,870)
Typhoid and paratyphoid	0 (0 to 0)	0 (0 to 0)	3·8 (1·5 to 8)	73·8 (27·4 to 165)	67·2 (24·1 to 150)	46·8 (18·7 to 97)	25·9 (10·7 to 52·7)	17·2 (7·3 to 34·2)	12·4 (5·1 to 24·4)	9·8 (4·5 to 19·8)	6·7 (3 to 13·1)	5·7 (2·5 to 12·1)
Typhoid fever	0 (0 to 0)	0 (0 to 0)	2·1 (0·7 to 4·9)	38 (10·1 to 88·6)	35·4 (9·5 to 82·6)	26·3 (8·3 to 60·2)	16·4 (5·6 to 35)	11·8 (4·5 to 24·7)	9·3 (3·7 to 18·5)	7·7 (3·4 to 15·7)	5·1 (2·2 to 10·4)	4·2 (1·7 to 8·5)
Paratyphoid fever	0 (0 to 0)	0 (0 to 0)	1·6 (0·5 to 3·9)	35·8 (11·2 to 92·1)	31·7 (10·2 to 78·7)	20·4 (6·8 to 48)	9·5 (3 to 21·4)	5·4 (1·8 to 12)	3·1 (1 to 7)	2·1 (0·7 to 4·8)	1·6 (0·5 to 3·9)	1·6 (0·5 to 3·8)
Invasive Non-typhoidal Salmonella (iNTS)	0 (0 to 0)	1·8 (0·5 to 5)	25·3 (7·5 to 60·6)	74·4 (20·9 to 156)	68·6 (22 to 145)	37·9 (9·7 to 90·2)	18·5 (3·5 to 51·6)	12·4 (1·8 to 37·9)	9 (1·8 to 24·1)	7·2 (1·6 to 19·1)	6·3 (1·4 to 15·9)	5·5 (0·7 to 14·3)

Cause	45 to 49	50 to 54	55 to 59	60 to 64	65 to 69	70 to 74	75 to 79	80 to 84	85 to 89	90 to 94	95 plus	Total
All causes	506,000 (436,000 to 585,000)	591,000 (508,000 to 680,000)	628,000 (541,000 to 721,000)	605,000 (525,000 to 691,000)	572,000 (500,000 to 649,000)	532,000 (469,000 to 599,000)	414,000 (369,000 to 461,000)	298,000 (269,000 to 328,000)	179,000 (163,000 to 194,000)	83,200 (76,500 to 90,100)	34,200 (31,900 to 36,600)	8,750,000
Communicable, maternal, neonatal, and nutritional diseases	61,800 (53,300 to 72,400)	53,400 (46,900 to 61,900)	42,900 (37,300 to 49,400)	34,900 (30,100 to 40,400)	30,200 (25,900 to 35,200)	28,600 (24,300 to 33,900)	24,400 (20,700 to 28,600)	19,800 (16,800 to 23,000)	13,700 (11,500 to 15,700)	7,050 (5,750 to 8,190)	3,260 (2,580 to 3,870)	1,870,000
HIV/AIDS and sexually transmitted infections	33,100 (27,000 to 42,200)	23,600 (20,100 to 29,300)	13,700 (11,600 to 17,100)	6,930 (5,700 to 8,690)	3,730 (3,010 to 4,670)	1,400 (1,040 to 1,890)	639 (465 to 871)	185 (140 to 251)	76·8 (56·6 to 108)	23·5 (16·7 to 33·2)	7·7 (5·3 to 12·9)	279,000
HIV/AIDS	32,800 (26,700 to 41,800)	23,300 (19,800 to 29,200)	13,500 (11,300 to 16,800)	6,760 (5,520 to 8,560)	3,570 (2,850 to 4,540)	1,300 (958 to 1,800)	587 (413 to 818)	144 (105 to 202)	54·2 (38·5 to 76·8)	15·5 (11·1 to 21·2)	4·2 (3·1 to 5·5)	228,000
HIV/AIDS - Drug-susceptible Tuberculosis	2,690 (1,710 to 4,160)	1,990 (1,260 to 3,070)	1,130 (728 to 1,740)	589 (381 to 899)	277 (180 to 423)	115 (75·7 to 173)	49 (32·7 to 73·3)	11·6 (7·9 to 16·8)	4·6 (3·2 to 6·5)	1·3 (0·9 to 1·8)	0·3 (0·2 to 0·4)	18,100
HIV/AIDS - Multidrug-resistant Tuberculosis without exte	36·8 (8 to 122)	27·4 (5·9 to 89·9)	15·7 (3·5 to 51·7)	8·2 (1·8 to 26·7)	4 (0·9 to 12·6)	1·5 (0·3 to 5)	0·6 (0·1 to 2·1)	0·2 (0 to 0·5)	0·1 (0 to 0·2)	0 (0 to 0·1)	0 (0 to 0)	244
HIV/AIDS - Extensively drug-resistant Tuberculosis	6·1 (1·2 to 19·9)	4·5 (0·9 to 14·9)	2·6 (0·5 to 8·5)	1·3 (0·3 to 4·4)	0·6 (0·1 to 2)	0·2 (0 to 0·8)	0·1 (0 to 0·3)	0 (0 to 0·1)	0 (0 to 0)	0 (0 to 0)	0 (0 to 0)	40·1
HIV/AIDS resulting in other diseases	30,000 (23,900 to 38,900)	21,300 (17,500 to 27,300)	12,400 (10,100 to 15,700)	6,160 (4,890 to 8,010)	3,290 (2,560 to 4,280)	1,180 (847 to 1,670)	537 (366 to 770)	132 (92·6 to 188)	49·5 (34·1 to 71·6)	14·2 (9·8 to 19·9)	3·9 (2·9 to 5·2)	210,000
Sexually transmitted infections excluding HIV	330 (185 to 573)	288 (156 to 530)	234 (133 to 408)	167 (91·1 to 310)	159 (82·5 to 303)	96·4 (52·9 to 174)	52·5 (27·9 to 94·6)	41·3 (23 to 77·1)	22·6 (11·7 to 47·2)	8·1 (4·2 to 15·9)	3·5 (1·7 to 8·1)	50,500
Syphilis	88 (40·1 to 188)	102 (46 to 218)	105 (49·9 to 206)	83·1 (39 to 177)	87·9 (39·3 to 187)	50 (25·3 to 101)	19·5 (10·6 to 36·6)	14·2 (7·9 to 27·3)	6·1 (3·1 to 13·5)	1·8 (0·9 to 3·6)	0·7 (0·3 to 1·6)	47,900
Chlamydial infection	44·5 (18·5 to 82·6)	20·3 (6·9 to 41·2)	6 (2·9 to 10·8)	1 (0·5 to 1·8)	0·3 (0·1 to 0·5)	0 (0 to 0)	0 (0 to 0)	0 (0 to 0)	0 (0 to 0)	0 (0 to 0)	0 (0 to 0)	463
Gonococcal infection	22·8 (11·3 to 40·8)	15·9 (8 to 29·4)	10·7 (5·4 to 18·2)	8·6 (3·9 to 18·6)	11·5 (4·6 to 25·2)	5·3 (2·4 to 11·5)	2·2 (0·9 to 4·5)	2·3 (1·1 to 4·8)	1·2 (0·5 to 2·9)	0·1 (0 to 0·3)	0 (0 to 0)	402
Trichomoniasis	9·2 (2·9 to 21·9)	6·8 (2·1 to 16·6)	3·9 (1·3 to 9·1)	1·8 (0·7 to 4·3)	1·3 (0·4 to 2·9)	0·8 (0·3 to 1·9)	0·5 (0·1 to 1·1)	0·2 (0·1 to 0·5)	0·1 (0 to 0·2)	0 (0 to 0·1)	0 (0 to 0)	67·5
Genital herpes	105 (33·3 to 264)	98·3 (31·1 to 244)	82·7 (25·3 to 207)	59·8 (18 to 149)	45·8 (13·7 to 114)	33·1 (10·1 to 81·3)	21·1 (6·4 to 52)	12·3 (3·6 to 30·2)	6·1 (1·8 to 15·1)	2·4 (0·7 to 5·8)	0·8 (0·2 to 1·9)	920
Other sexually transmitted infections	59·7 (32 to 101)	45·1 (24·1 to 83·5)	25·8 (13·1 to 47·7)	12·2 (5·6 to 26·8)	12·5 (5 to 27·6)	7·3 (3·2 to 15·7)	9·2 (3·9 to 19·4)	12·4 (5·8 to 26·5)	9·2 (3·6 to 23)	3·8 (1·5 to 9·3)	2·1 (0·8 to 5·7)	791
Respiratory infections and tuberculosis	13,200 (10,300 to 16,400)	14,400 (11,400 to 17,900)	15,100 (12,100 to 18,600)	15,700 (12,700 to 19,200)	16,000 (13,100 to 19,500)	17,800 (14,600 to 21,600)	16,800 (13,800 to 20,600)	14,700 (12,000 to 17,600)	10,600 (8,740 to 12,300)	5,760 (4,610 to 6,820)	2,760 (2,130 to 3,320)	425,000
Tuberculosis	4,250 (3,000 to 6,020)	3,850 (2,740 to 5,390)	3,130 (2,200 to 4,300)	2,760 (2,020 to 3,750)	2,120 (1,570 to 2,880)	1,660 (1,210 to 2,230)	1,130 (846 to 1,540)	712 (519 to 923)	395 (287 to 525)	126 (93·4 to 164)	32·3 (23·3 to 42·2)	57,500
Latent tuberculosis infection	0 (0 to 0)	0 (0 to 0)	0 (0 to 0)	0 (0 to 0)	0 (0 to 0)	0 (0 to 0)	0 (0 to 0)	0 (0 to 0)	0 (0 to 0)	0 (0 to 0)	0 (0 to 0)	0
Drug-susceptible tuberculosis	4,190 (2,960 to 5,950)	3,800 (2,690 to 5,350)	3,080 (2,170 to 4,260)	2,730 (1,990 to 3,710)	2,090 (1,550 to 2,840)	1,640 (1,190 to 2,200)	1,110 (834 to 1,520)	701 (513 to 910)	389 (281 to 518)	124 (91·5 to 161)	31·6 (22·5 to 41·6)	56,800
Multidrug-resistant tuberculosis without extensive drug r	46·8 (11·5 to 145)	44·4 (11·7 to 134)	37·8 (9·7 to 113)	33 (8·8 to 102)	26 (6·9 to 76·5)	21·1 (5·8 to 61·4)	14·9 (4·3 to 42·3)	9·4 (2·6 to 27·3)	5·4 (1·5 to 15·6)	1·9 (0·5 to 5·3)	0·5 (0·2 to 1·4)	620
Extensively drug-resistant tuberculosis	7·7 (1·8 to 25·7)	7·2 (1·8 to 21·8)	6·2 (1·5 to 18·1)	5·4 (1·4 to 16·2)	4·3 (1·1 to 12·9)	3·5 (0·9 to 10·3)	2·4 (0·7 to 7)	1·5 (0·4 to 4·4)	0·9 (0·2 to 2·5)	0·3 (0·1 to 0·9)	0·1 (0 to 0·2)	101
Lower respiratory infections	7,520 (5,740 to 9,550)	9,310 (7,220 to 11,800)	11,000 (8,600 to 13,900)	12,200 (9,570 to 15,100)	13,300 (10,600 to 16,600)	15,800 (12,600 to 19,500)	15,400 (12,400 to 19,100)	13,800 (11,200 to 16,700)	10,100 (8,290 to 11,800)	5,600 (4,450 to 6,640)	2,710 (2,090 to 3,280)	339,000
Upper respiratory infections	1,000 (540 to 1,730)	877 (494 to 1,490)	727 (399 to 1,200)	528 (283 to 886)	423 (228 to 710)	299 (164 to 491)	191 (105 to 311)	108 (58·1 to 183)	54·2 (31·1 to 87·5)	24·2 (14·4 to 39·5)	9·2 (5·2 to 15·7)	20,600
Otitis media	394 (197 to 678)	368 (174 to 659)	300 (153 to 530)	200 (96·9 to 383)	131 (67·2 to 237)	79·2 (35·5 to 148)	54·3 (28·1 to 96)	35·8 (17 to 64·2)	19·1 (9·2 to 33·6)	8·4 (3·9 to 15·3)	3·2 (1·5 to 5·9)	7,890
Enteric infections	4,120 (2,850 to 6,050)	4,370 (3,090 to 6,350)	4,290 (3,120 to 6,350)	4,270 (3,070 to 6,320)	4,470 (3,220 to 6,530)	4,610 (3,340 to 6,520)	3,850 (2,800 to 5,480)	2,880 (2,140 to 3,990)	1,590 (1,200 to 2,210)	670 (505 to 873)	265 (194 to 353)	238,000
Diarrheal diseases	4,110 (2,840 to 6,040)	4,360 (3,080 to 6,340)	4,280 (3,110 to 6,330)	4,260 (3,060 to 6,300)	4,460 (3,210 to 6,520)	4,600 (3,340 to 6,510)	3,840 (2,800 to 5,480)	2,870 (2,140 to 3,990)	1,590 (1,200 to 2,210)	669 (505 to 873)	265 (194 to 353)	237,000
Typhoid and paratyphoid	5·2 (2·2 to 10·9)	4·9 (2·2 to 9·3)	4·4 (2·2 to 8·5)	3·9 (2 to 7·5)	3·8 (1·8 to 7·5)	2·6 (1·3 to 5·1)	1·5 (0·8 to 2·6)	1·1 (0·6 to 1·8)	0·8 (0·4 to 1·5)	0·3 (0·1 to 0·5)	0·1 (0·1 to 0·2)	298
Typhoid fever	3·6 (1·5 to 7·8)	3·3 (1·4 to 6·6)	2·7 (1·2 to 5·4)	2·4 (1·2 to 4·9)	2·7 (1·2 to 5·8)	1·9 (0·9 to 4)	1 (0·5 to 1·9)	0·7 (0·3 to 1·3)	0·5 (0·2 to 1·1)	0·2 (0·1 to 0·3)	0·1 (0 to 0·1)	175
Paratyphoid fever	1·6 (0·5 to 4)	1·6 (0·6 to 3·7)	1·7 (0·7 to 3·7)	1·5 (0·6 to 3·2)	1 (0·4 to 2·2)	0·7 (0·2 to 1·6)	0·5 (0·2 to 1·1)	0·4 (0·2 to 0·8)	0·3 (0·1 to 0·6)	0·1 (0 to 0·2)	0 (0 to 0·1)	122
Invasive Non-typhoidal Salmonella (iNTS)	5·5 (1 to 13·4)	5·6 (1 to 13·6)	5·5 (1·1 to 14)	6·6 (1·7 to 16·3)	4·9 (1·2 to 11·9)	3·2 (0·8 to 8)	2 (0·7 to 4·5)	0·8 (0·1 to 2·2)	0·4 (0·1 to 1)	0·1 (0 to 0·4)	0 (0 to 0·1)	302

Other cardiovascular and circulatory diseases	660 (297 to 1,430)
Chronic respiratory diseases	0 (0 to 0)
Chronic obstructive pulmonary disease	0 (0 to 0)
Pneumoconiosis	0 (0 to 0)
Silicosis	0 (0 to 0)
Asbestosis	0 (0 to 0)
Coal workers pneumoconiosis	0 (0 to 0)
Other pneumoconiosis	0 (0 to 0)
Asthma	0 (0 to 0)
Interstitial lung disease and pulmonary sarcoidosis	0 (0 to 0)
Other chronic respiratory diseases	0 (0 to 0)
Digestive diseases	1,350 (635 to 2,590)
Cirrhosis and other chronic liver diseases	0 (0 to 0)
Cirrhosis and other chronic liver diseases due to hepati	0 (0 to 0)
Cirrhosis and other chronic liver diseases due to hepati	0 (0 to 0)
Cirrhosis and other chronic liver diseases due to alcoho	0 (0 to 0)
Cirrhosis and other chronic liver diseases due to NAFL	0 (0 to 0)
Cirrhosis and other chronic liver diseases due to other	0 (0 to 0)
Upper digestive system diseases	0 (0 to 0)
Peptic ulcer disease	0 (0 to 0)
Gastritis and duodenitis	0 (0 to 0)
Gastroesophageal reflux disease	0 (0 to 0)
Appendicitis	0 (0 to 0)
Paralytic ileus and intestinal obstruction	750 (369 to 1,450)
Inguinal, femoral, and abdominal hernia	599 (196 to 1,360)
Inflammatory bowel disease	0 (0 to 0)
Vascular intestinal disorders	0 (0 to 0)
Gallbladder and biliary diseases	0 (0 to 0)
Pancreatitis	0 (0 to 0)
Other digestive diseases	0 (0 to 0)
Neurological disorders	6·7 (3 to 12·7)
Alzheimer's disease and other dementias	0 (0 to 0)
Parkinson's disease	0 (0 to 0)
Idiopathic epilepsy	5·8 (2·2 to 11·9)
Multiple sclerosis	0 (0 to 0)
Motor neuron disease	0·8 (0·5 to 1·3)
Headache disorders	0 (0 to 0)
Migraine	0 (0 to 0)
Tension-type headache	0 (0 to 0)
Other neurological disorders	0 (0 to 0)
Mental disorders	9·2 (5·8 to 13·5)
Schizophrenia	0 (0 to 0)
Depressive disorders	0 (0 to 0)
Major depressive disorder	0 (0 to 0)
Dysthymia	0 (0 to 0)
Bipolar disorder	0 (0 to 0)
Anxiety disorders	0 (0 to 0)
Eating disorders	0 (0 to 0)
Anorexia nervosa	0 (0 to 0)
Bulimia nervosa	0 (0 to 0)
Autism spectrum disorders	7·9 (5 to 11·6)
Attention-deficit/hyperactivity disorder	0 (0 to 0)
Conduct disorder	0 (0 to 0)
Idiopathic developmental intellectual disability	1·4 (0·1 to 3·3)
Other mental disorders	0 (0 to 0)
Substance use disorders	0 (0 to 0)
Alcohol use disorders	0 (0 to 0)
Drug use disorders	0 (0 to 0)
Opioid use disorders	0 (0 to 0)
Cocaine use disorders	0 (0 to 0)
Amphetamine use disorders	0 (0 to 0)
Cannabis use disorders	0 (0 to 0)
Other drug use disorders	0 (0 to 0)
Diabetes and kidney diseases	17·2 (11·1 to 26·4)
Diabetes mellitus	17·2 (11·1 to 26·3)
Diabetes mellitus type 1	17·2 (11·1 to 26·3)
Diabetes mellitus type 2	0 (0 to 0)
Chronic kidney disease	0 (0 to 0)
Chronic kidney disease due to diabetes mellitus type 1	0 (0 to 0)
Chronic kidney disease due to diabetes mellitus type 2	0 (0 to 0)
Chronic kidney disease due to hypertension	0 (0 to 0)
Chronic kidney disease due to glomerulonephritis	0 (0 to 0)
Chronic kidney disease due to other and unspecified ca	0 (0 to 0)
Acute glomerulonephritis	0 (0 to 0)
Skin and subcutaneous diseases	0·5 (0·3 to 0·9)
Dermatitis	0 (0 to 0)
Atopic dermatitis	0 (0 to 0)
Contact dermatitis	0 (0 to 0)
Seborrhoeic dermatitis	0 (0 to 0)
Psoriasis	0 (0 to 0)
Bacterial skin diseases	0·1 (0 to 0·1)
Cellulitis	0 (0 to 0)
Pyoderma	0 (0 to 0·1)
Scabies	0·2 (0·1 to 0·4)
Fungal skin diseases	0 (0 to 0·1)
Viral skin diseases	0 (0 to 0)
Acne vulgaris	0 (0 to 0)
Alopecia areata	0 (0 to 0)
Pruritus	0 (0 to 0)
Urticaria	0·2 (0·1 to 0·3)
Decubitus ulcer	0 (0 to 0)
Other skin and subcutaneous diseases	0 (0 to 0·1)
Sense organ diseases	1·1 (0·6 to 1·9)
Blindness and vision loss	0·4 (0·1 to 0·8)
Glaucoma	0 (0 to 0)
Cataract	0 (0 to 0)
Age-related macular degeneration	0 (0 to 0)
Refraction disorders	0 (0 to 0)
Near vision loss	0 (0 to 0)
Other vision loss	0·4 (0·1 to 0·8)
Age-related and other hearing loss	0·3 (0·1 to 0·7)
Other sense organ diseases	0·5 (0·2 to 0·9)
Musculoskeletal disorders	0 (0 to 0)
Rheumatoid arthritis	0 (0 to 0)
Osteoarthritis	0 (0 to 0)
Osteoarthritis hip	0 (0 to 0)
Osteoarthritis knee	0 (0 to 0)
Osteoarthritis hand	0 (0 to 0)
Osteoarthritis other	0 (0 to 0)
Low back pain	0 (0 to 0)
Neck pain	0 (0 to 0)
Gout	0 (0 to 0)
Other musculoskeletal disorders	0 (0 to 0)
Other non-communicable diseases	79,300 (46,300 to 131,000)
Congenital birth defects	77,600 (44,700 to 129,000)
Neural tube defects	9,430 (2,480 to 27,300)
Congenital heart anomalies	30,700 (14,700 to 54,400)
Orofacial clefts	187 (36·5 to 698)
Down syndrome	961 (367 to 2,190)
Turner syndrome	0 (0 to 0)
Klinefelter syndrome	0 (0 to 0)
Other chromosomal abnormalities	3,330 (1,140 to 8,320)
Congenital musculoskeletal and limb anomalies	1,490 (545 to 3,230)
Urogenital congenital anomalies	1,910 (823 to 4,010)
Digestive congenital anomalies	11,700 (5,010 to 20,800)
Other congenital birth defects	17,900 (6,850 to 33,000)
Urinary diseases and male infertility	106 (52·4 to 199)
Urolithiasis	0 (0 to 0)
Benign prostatic hyperplasia	0 (0 to 0)
Male infertility	0 (0 to 0)
Other urinary diseases	40·3 (16·9 to 85·8)
Gynecological diseases	0 (0 to 0)
Uterine fibroids	0 (0 to 0)
Polycystic ovarian syndrome	0 (0 to 0)
Female infertility	0 (0 to 0)
Endometriosis	0 (0 to 0)
Genital prolapse	0 (0 to 0)
Premenstrual syndrome	0 (0 to 0)
Other gynecological diseases	0 (0 to 0)
Hemoglobinopathies and hemolytic anemias	213 (132 to 329)
Thalassemias	0·2 (0·1 to 0·4)
Thalassemias trait	0·9 (0·5 to 1·7)
Sickle cell disorders	200 (119 to 315)
Sickle cell trait	9·6 (4·9 to 15·7)
G6PD deficiency	1·4 (0·7 to 2·4)
G6PD trait	0 (0 to 0)
Other hemoglobinopathies and hemolytic anemias	0·8 (0·4 to 1·3)
Endocrine, metabolic, blood, and immune disorders	1,460 (813 to 2,470)
Oral disorders	0 (0 to 0)
Caries of deciduous teeth	0 (0 to 0)
Caries of permanent teeth	0 (0 to 0)
Periodontal diseases	0 (0 to 0)
Other oral disorders	0 (0 to 0)
Sudden infant death syndrome	--
Injuries	6,340 (4,060 to 10,200)
Transport injuries	585 (244 to 1,290)
Road injuries	500 (209 to 1,120)
Pedestrian road injuries	200 (70·2 to 471)
Cyclist road injuries	0 (0 to 0)
Motorcyclist road injuries	90·4 (21·2 to 257)
Motor vehicle road injuries	200 (80·8 to 452)
Other road injuries	9 (2·4 to 28·5)

Other transport injuries	85·7 (27·4 to 250)	66·9 (25·8 to 157)	309 (126 to 630)	645 (311 to 1,480)	589 (358 to 1,140)	703 (469 to 1,290)	1,180 (851 to 1,910)	1,840 (1,370 to 2,790)
Unintentional injuries	4,600 (2,810 to 7,760)	4,630 (2,090 to 17,600)	29,000 (13,500 to 48,600)	68,000 (37,400 to 96,700)	36,000 (27,500 to 45,100)	31,800 (25,800 to 38,800)	36,900 (28,100 to 48,500)	46,500 (37,000 to 60,400)
Falls	92·6 (44·4 to 174)	62·4 (25·9 to 126)	1,110 (349 to 2,740)	2,800 (842 to 6,500)	1,850 (1,180 to 2,740)	2,210 (1,640 to 2,880)	3,190 (2,450 to 3,990)	4,180 (3,230 to 5,270)
Drowning	326 (149 to 654)	151 (61·9 to 311)	3,420 (916 to 7,680)	27,800 (9,140 to 50,100)	16,900 (10,100 to 25,100)	11,100 (7,140 to 15,500)	10,400 (6,810 to 15,700)	9,180 (6,290 to 13,600)
Fire, heat, and hot substances	122 (58 to 207)	176 (71·9 to 319)	3,150 (841 to 6,140)	11,400 (3,000 to 20,000)	4,110 (2,120 to 6,090)	2,730 (1,970 to 3,610)	2,470 (1,820 to 3,220)	2,920 (2,200 to 3,800)
Poisonings	198 (58·2 to 587)	257 (44·2 to 1,020)	1,080 (363 to 2,590)	4,790 (2,030 to 9,600)	1,030 (484 to 2,680)	559 (315 to 1,330)	465 (268 to 1,080)	703 (416 to 1,560)
Poisoning by carbon monoxide	13·1 (1·6 to 55·4)	25·4 (2·4 to 132)	27·1 (4·9 to 112)	400 (70·7 to 1,450)	166 (51·6 to 544)	147 (58·6 to 458)	179 (82·9 to 528)	289 (143 to 792)
Poisoning by other means	185 (56 to 545)	231 (40 to 949)	1,050 (351 to 2,530)	4,390 (1,800 to 8,950)	860 (400 to 2,140)	413 (237 to 949)	286 (165 to 542)	414 (240 to 834)
Exposure to mechanical forces	600 (50·2 to 3,110)	1,560 (25·9 to 15,000)	1,560 (182 to 7,220)	3,650 (1,420 to 7,140)	3,860 (1,720 to 7,660)	3,810 (1,940 to 7,040)	7,630 (3,920 to 14,900)	10,700 (6,420 to 18,700)
Unintentional firearm injuries	380 (19·5 to 2,090)	1,030 (6·5 to 11,400)	1,080 (67·4 to 5,390)	2,230 (414 to 5,190)	1,990 (498 to 5,370)	2,010 (679 to 5,100)	5,020 (1,920 to 11,900)	7,190 (3,610 to 15,600)
Other exposure to mechanical forces	219 (11·3 to 1,510)	529 (11 to 3,880)	480 (79·8 to 2,210)	1,430 (524 to 3,900)	1,870 (914 to 4,440)	1,800 (1,050 to 3,400)	2,610 (1,710 to 4,580)	3,500 (2,360 to 5,810)
Adverse effects of medical treatment	900 (525 to 1,450)	795 (389 to 1,460)	3,200 (1,100 to 6,710)	2,320 (819 to 4,400)	979 (618 to 1,510)	584 (414 to 810)	434 (301 to 603)	795 (567 to 1,090)
Animal contact	10·3 (4·3 to 21·3)	22·8 (8·6 to 45·1)	138 (43·8 to 297)	584 (185 to 1,230)	281 (171 to 441)	298 (203 to 430)	313 (221 to 433)	340 (243 to 452)
Venomous animal contact	9·5 (3·6 to 20·1)	20·4 (7·1 to 42)	101 (32·2 to 222)	447 (148 to 978)	201 (117 to 326)	212 (143 to 312)	224 (156 to 313)	241 (171 to 327)
Non-venomous animal contact	0·8 (0·5 to 1·5)	2·5 (1·1 to 5·9)	36·9 (7·7 to 94·6)	137 (27·8 to 367)	79·8 (37·2 to 148)	85·8 (47·9 to 146)	89·1 (57·2 to 136)	98·7 (62·8 to 147)
Foreign body	1,630 (885 to 2,920)	1,340 (752 to 2,230)	14,100 (5,720 to 25,900)	10,600 (4,750 to 16,900)	2,880 (1,910 to 4,160)	2,370 (1,760 to 3,090)	1,220 (888 to 1,610)	1,760 (1,260 to 2,320)
Pulmonary aspiration and foreign body in airway	1,610 (878 to 2,900)	1,330 (744 to 2,220)	13,900 (5,650 to 25,500)	10,200 (4,530 to 16,400)	2,590 (1,640 to 3,850)	2,080 (1,490 to 2,790)	920 (642 to 1,280)	1,410 (964 to 1,930)
Foreign body in eyes	0·2 (0 to 0·4)	0·5 (0·1 to 1·1)	8·8 (2·4 to 19·5)	46·7 (14 to 107)	73·9 (21·4 to 175)	90·1 (27·7 to 211)	117 (36·8 to 274)	142 (45·3 to 311)
Foreign body in other body part	11·6 (3·9 to 34·5)	8·7 (3·3 to 23·5)	140 (34·2 to 553)	357 (121 to 1,190)	216 (137 to 392)	196 (138 to 283)	184 (134 to 250)	205 (149 to 273)
Environmental heat and cold exposure	129 (20·3 to 397)	62·2 (18·2 to 164)	277 (83·1 to 733)	671 (301 to 1,640)	550 (293 to 1,210)	543 (335 to 957)	882 (524 to 1,810)	1,180 (715 to 2,400)
Exposure to forces of nature	1·1 (1 to 1·2)	2·4 (2·1 to 2·6)	19·7 (17·9 to 21·7)	102 (89·9 to 121)	597 (404 to 878)	4,540 (2,740 to 7,430)	5,700 (3,550 to 9,170)	9,220 (5,660 to 14,000)
Other unintentional injuries	598 (253 to 1,230)	204 (86·5 to 450)	1,010 (456 to 1,830)	3,200 (1,550 to 6,390)	3,010 (1,690 to 5,650)	3,050 (1,980 to 5,300)	4,190 (2,870 to 6,500)	5,510 (3,810 to 7,980)
Self-harm and interpersonal violence	1,160 (620 to 2,040)	344 (175 to 635)	2,020 (1,000 to 3,760)	6,650 (3,250 to 12,400)	5,340 (2,350 to 9,240)	9,660 (4,950 to 15,000)	51,100 (32,300 to 69,200)	91,000 (64,800 to 114,000)
Self-harm	0 (0 to 0)	0 (0 to 0)	0 (0 to 0)	0 (0 to 0)	0 (0 to 0)	1,380 (864 to 2,070)	7,770 (5,660 to 10,600)	14,700 (11,200 to 19,400)
Self-harm by firearm	0 (0 to 0)	0 (0 to 0)	0 (0 to 0)	0 (0 to 0)	0 (0 to 0)	53·8 (26·4 to 109)	805 (494 to 1,370)	1,540 (952 to 2,660)
Self-harm by other specified means	0 (0 to 0)	0 (0 to 0)	0 (0 to 0)	0 (0 to 0)	0 (0 to 0)	1,330 (828 to 1,990)	6,960 (5,040 to 9,530)	13,200 (10,000 to 17,400)
Interpersonal violence	1,160 (620 to 2,040)	344 (175 to 635)	2,010 (997 to 3,750)	6,620 (3,220 to 12,400)	5,310 (2,320 to 9,220)	8,260 (3,750 to 13,700)	43,200 (24,300 to 60,800)	76,000 (50,300 to 99,500)
Physical violence by firearm	136 (59·9 to 298)	45·1 (18·1 to 98·4)	212 (92·7 to 443)	577 (253 to 1,570)	840 (416 to 1,710)	2,260 (1,140 to 4,270)	24,800 (13,600 to 37,100)	48,800 (32,200 to 66,200)
Physical violence by sharp object	123 (38·5 to 276)	34·9 (10·6 to 94·4)	85·9 (36 to 241)	434 (180 to 1,390)	511 (228 to 1,650)	1,080 (526 to 2,650)	10,800 (5,750 to 21,900)	17,200 (10,300 to 32,400)
Sexual violence	0 (0 to 0)	0 (0 to 0)	0 (0 to 0)	9·6 (5·4 to 15·6)	74·6 (45·6 to 118)	155 (87 to 246)	330 (185 to 532)	428 (248 to 662)
Physical violence by other means	898 (485 to 1,560)	264 (127 to 485)	1,720 (792 to 3,320)	5,600 (2,510 to 10,800)	3,890 (1,420 to 7,260)	4,760 (1,560 to 9,250)	7,170 (2,130 to 14,200)	9,520 (3,370 to 18,600)
Conflict and terrorism	0 (0 to 0)	0 (0 to 0)	0 (0 to 0)	1·1 (0·6 to 1·7)	1·5 (0·8 to 2·6)	4·8 (3 to 7·4)	27·9 (15·3 to 47·7)	25·2 (14·4 to 41·2)

Other transport injuries	1,850 (1,430 to 2,840)	1,870 (1,440 to 2,770)	1,530 (1,160 to 2,290)	1,460 (1,120 to 2,210)	1,250 (985 to 1,710)	1,090 (880 to 1,450)	1,010 (806 to 1,390)	570 (451 to 772)
Unintentional injuries	44,400 (35,700 to 56,100)	43,600 (35,500 to 54,000)	39,300 (31,700 to 48,300)	36,400 (29,300 to 45,100)	33,200 (26,800 to 41,300)	31,300 (25,500 to 38,500)	27,000 (22,000 to 33,200)	21,700 (17,800 to 26,400)
Falls	4,670 (3,670 to 5,910)	5,150 (4,130 to 6,410)	5,390 (4,270 to 6,640)	5,810 (4,590 to 7,170)	6,890 (5,530 to 8,500)	8,090 (6,540 to 9,880)	7,920 (6,430 to 9,620)	7,050 (5,780 to 8,470)
Drowning	7,950 (5,800 to 11,500)	6,930 (5,220 to 9,120)	5,540 (3,980 to 7,470)	4,810 (3,610 to 6,570)	3,500 (2,640 to 4,650)	2,990 (2,340 to 3,930)	2,050 (1,600 to 2,680)	1,430 (1,120 to 1,870)
Fire, heat, and hot substances	3,260 (2,470 to 4,240)	3,620 (2,730 to 4,730)	3,240 (2,440 to 4,260)	2,990 (2,220 to 3,980)	2,890 (2,130 to 3,900)	2,580 (1,840 to 3,560)	2,190 (1,590 to 3,060)	1,700 (1,250 to 2,340)
Poisonings	903 (546 to 2,110)	899 (543 to 2,230)	737 (430 to 1,780)	558 (342 to 1,320)	476 (297 to 1,110)	359 (234 to 771)	270 (183 to 596)	199 (132 to 435)
Poisoning by carbon monoxide	346 (172 to 1,050)	350 (170 to 1,190)	298 (141 to 948)	210 (105 to 653)	141 (70·3 to 474)	82·7 (43·9 to 256)	72·9 (40·3 to 252)	48·2 (25·5 to 161)
Poisoning by other means	558 (321 to 1,210)	550 (324 to 1,170)	440 (254 to 876)	348 (210 to 711)	334 (207 to 675)	276 (180 to 532)	197 (135 to 361)	151 (101 to 271)
Exposure to mechanical forces	10,800 (6,670 to 18,700)	10,700 (7,060 to 16,600)	9,370 (6,180 to 14,800)	8,850 (5,790 to 14,200)	7,860 (5,240 to 11,800)	7,140 (4,860 to 10,300)	6,050 (4,150 to 8,760)	4,440 (3,050 to 6,310)
Unintentional firearm injuries	6,730 (3,420 to 14,600)	6,150 (3,390 to 12,100)	4,970 (2,740 to 9,930)	4,420 (2,370 to 8,910)	3,280 (1,860 to 6,570)	2,610 (1,480 to 5,060)	1,990 (1,130 to 3,920)	1,330 (812 to 2,430)
Other exposure to mechanical forces	4,100 (2,740 to 6,770)	4,580 (3,080 to 7,120)	4,400 (2,930 to 6,940)	4,420 (2,990 to 6,880)	4,580 (3,110 to 6,770)	4,540 (3,120 to 6,730)	4,060 (2,800 to 5,810)	3,110 (2,140 to 4,450)
Adverse effects of medical treatment	851 (616 to 1,160)	780 (545 to 1,100)	875 (616 to 1,270)	877 (620 to 1,270)	845 (598 to 1,170)	1,140 (830 to 1,580)	1,120 (806 to 1,520)	1,190 (888 to 1,590)
Animal contact	425 (312 to 569)	371 (270 to 488)	400 (293 to 539)	394 (289 to 531)	454 (344 to 608)	468 (357 to 626)	430 (324 to 573)	369 (280 to 495)
Venomous animal contact	296 (217 to 403)	254 (179 to 343)	285 (209 to 388)	257 (185 to 352)	282 (205 to 383)	282 (207 to 380)	254 (188 to 346)	212 (159 to 290)
Non-venomous animal contact	130 (90 to 188)	117 (78·1 to 176)	115 (78·5 to 172)	137 (93·3 to 199)	172 (120 to 244)	186 (133 to 265)	176 (125 to 248)	157 (111 to 219)
Foreign body	1,940 (1,450 to 2,560)	1,830 (1,340 to 2,370)	1,650 (1,210 to 2,170)	1,680 (1,250 to 2,200)	1,580 (1,210 to 2,030)	1,660 (1,270 to 2,150)	1,420 (1,120 to 1,790)	1,290 (982 to 1,690)
Pulmonary aspiration and foreign body in airway	1,570 (1,110 to 2,160)	1,460 (1,030 to 1,950)	1,320 (931 to 1,800)	1,340 (945 to 1,830)	1,220 (890 to 1,650)	1,330 (983 to 1,810)	1,130 (847 to 1,470)	1,060 (781 to 1,430)
Foreign body in eyes	157 (56·6 to 335)	159 (56·1 to 332)	147 (56 to 305)	142 (62·1 to 281)	142 (68·2 to 271)	124 (64·6 to 221)	99·7 (58 to 162)	77·3 (45·7 to 121)
Foreign body in other body part	218 (164 to 288)	208 (152 to 279)	191 (139 to 257)	200 (151 to 269)	217 (161 to 288)	202 (148 to 269)	191 (143 to 256)	152 (113 to 200)
Environmental heat and cold exposure	1,280 (813 to 2,340)	1,380 (904 to 2,400)	1,290 (845 to 2,210)	1,260 (826 to 2,160)	1,240 (829 to 2,020)	1,190 (783 to 1,900)	1,100 (708 to 1,800)	884 (571 to 1,500)
Exposure to forces of nature	6,150 (3,680 to 10,300)	5,900 (3,510 to 9,560)	5,580 (3,330 to 8,770)	4,450 (2,710 to 6,950)	3,270 (2,040 to 5,060)	2,410 (1,490 to 3,720)	1,840 (1,180 to 2,810)	1,410 (918 to 2,190)
Other unintentional injuries	6,120 (4,420 to 8,330)	6,040 (4,380 to 8,160)	5,180 (3,730 to 7,040)	4,710 (3,490 to 6,140)	4,170 (3,110 to 5,600)	3,310 (2,510 to 4,340)	2,630 (1,950 to 3,450)	1,770 (1,290 to 2,380)
Self-harm and interpersonal violence	89,100 (65,900 to 111,000)	76,200 (57,900 to 95,600)	58,300 (42,100 to 74,400)	45,200 (32,000 to 59,300)	35,800 (26,700 to 46,300)	27,400 (21,200 to 35,000)	20,000 (15,800 to 25,300)	13,100 (10,400 to 15,900)
Self-harm	16,300 (13,000 to 21,100)	17,000 (13,700 to 21,600)	14,700 (11,600 to 18,500)	13,600 (10,800 to 16,800)	13,000 (10,400 to 16,300)	11,900 (9,590 to 14,600)	9,640 (7,730 to 11,800)	6,810 (5,530 to 8,360)
Self-harm by firearm	1,590 (1,030 to 2,740)	1,450 (925 to 2,430)	1,120 (713 to 1,790)	1,030 (657 to 1,620)	811 (539 to 1,290)	728 (489 to 1,100)	498 (340 to 740)	403 (270 to 589)
Self-harm by other specified means	14,800 (11,800 to 18,800)	15,600 (12,600 to 19,600)	13,500 (10,800 to 17,100)	12,600 (10,000 to 15,500)	12,200 (9,730 to 15,400)	11,200 (9,010 to 13,800)	9,150 (7,310 to 11,200)	6,410 (5,210 to 7,860)
Interpersonal violence	72,300 (48,700 to 94,500)	58,800 (39,800 to 78,300)	43,400 (27,700 to 58,900)	31,500 (19,000 to 45,400)	22,500 (14,400 to 31,600)	15,300 (9,750 to 21,700)	10,200 (6,710 to 14,700)	6,190 (4,060 to 8,470)
Physical violence by firearm	44,600 (30,500 to 60,100)	33,600 (23,400 to 46,100)	22,800 (15,500 to 32,500)	14,600 (9,470 to 21,100)	9,530 (6,310 to 14,000)	5,840 (3,980 to 8,460)	3,520 (2,430 to 5,170)	2,040 (1,470 to 2,830)
Physical violence by sharp object	16,900 (10,200 to 31,000)	15,300 (9,340 to 27,500)	11,300 (6,560 to 20,200)	8,780 (5,120 to 15,900)	6,410 (4,120 to 11,000)	4,210 (2,750 to 7,280)	2,500 (1,700 to 3,960)	1,410 (952 to 2,350)
Sexual violence	388 (215 to 630)	341 (190 to 548)	279 (159 to 450)	220 (126 to 359)	185 (107 to 316)	143 (82·5 to 236)	103 (60·4 to 165)	74·3 (45·6 to 114)
Physical violence by other means	10,500 (3,910 to 19,300)	9,650 (3,460 to 17,800)	8,930 (3,150 to 16,700)	7,850 (2,800 to 15,300)	6,380 (2,590 to 11,500)	5,080 (2,360 to 9,230)	4,070 (2,000 to 7,120)	2,670 (1,330 to 4,330)
Conflict and terrorism	55·8 (32·9 to 90)	104 (60·4 to 174)	109 (63·2 to 183)	119 (72·9 to 191)	164 (103 to 261)	165 (106 to 254)	120 (78·9 to 178)	70 (46·3 to 103)

Other transport injuries	377 (301 to 484)	331 (265 to 440)	179 (144 to 229)	94·9 (76 to 118)	45·3 (36 to 57)	19·3 (15·3 to 23·7)	8·8 (6·9 to 10·9)	17,100
Unintentional injuries	17,900 (14,700 to 21,900)	15,600 (13,000 to 18,900)	12,600 (10,500 to 15,000)	10,500 (8,770 to 12,400)	7,370 (6,140 to 8,630)	4,210 (3,470 to 4,940)	1,950 (1,600 to 2,280)	605,000
Falls	6,710 (5,490 to 8,050)	7,210 (5,940 to 8,590)	6,830 (5,670 to 8,050)	6,690 (5,480 to 7,940)	5,290 (4,310 to 6,200)	3,290 (2,670 to 3,900)	1,570 (1,270 to 1,860)	104,000
Drowning	885 (700 to 1,150)	505 (407 to 623)	303 (250 to 367)	121 (96·3 to 151)	83·1 (51 to 112)	33·2 (20·5 to 43·9)	10·7 (7·8 to 13·3)	116,000
Fire, heat, and hot substances	1,290 (946 to 1,840)	1,030 (757 to 1,440)	727 (533 to 1,010)	499 (375 to 683)	309 (237 to 406)	138 (109 to 180)	54·8 (42·7 to 71·6)	53,700
Poisonings	135 (92·5 to 281)	94·4 (66·8 to 171)	62·9 (45·5 to 111)	40·6 (28·4 to 74·2)	22·8 (16·3 to 34·1)	9·8 (6·9 to 14·1)	2·8 (2·1 to 3·6)	13,800
Poisoning by carbon monoxide	30·4 (17 to 95·1)	22·3 (13·6 to 58·8)	12·3 (7·7 to 34)	10·3 (6·3 to 27·7)	4·8 (2·9 to 10·1)	1·8 (1·1 to 3·5)	0·4 (0·3 to 0·6)	2,880
Poisoning by other means	104 (72·1 to 189)	72·2 (50·9 to 113)	50·6 (36·3 to 81·4)	30·3 (21·2 to 46·4)	18 (12·9 to 25·8)	7·9 (5·7 to 11·3)	2·3 (1·7 to 3·1)	11,000
Exposure to mechanical forces	3,290 (2,300 to 4,710)	2,400 (1,650 to 3,420)	1,580 (1,090 to 2,250)	980 (678 to 1,400)	539 (371 to 761)	232 (160 to 328)	91·6 (63·9 to 129)	108,000
Unintentional firearm injuries	831 (533 to 1,450)	507 (331 to 811)	291 (189 to 449)	173 (118 to 256)	97·8 (67 to 139)	38·6 (27 to 54·2)	14·8 (10·3 to 20·9)	54,400
Other exposure to mechanical forces	2,460 (1,650 to 3,540)	1,900 (1,270 to 2,720)	1,290 (857 to 1,860)	808 (537 to 1,170)	441 (296 to 628)	194 (130 to 277)	76·8 (52·5 to 109)	53,400
Adverse effects of medical treatment	1,090 (810 to 1,440)	962 (734 to 1,240)	805 (638 to 1,030)	589 (462 to 767)	343 (267 to 439)	146 (113 to 184)	58·9 (42·9 to 73·8)	21,700
Animal contact	336 (253 to 447)	265 (203 to 349)	196 (146 to 258)	110 (82·1 to 148)	54·9 (41·2 to 71·8)	27·9 (21·6 to 35·8)	11·9 (9·1 to 15·4)	6,300
Venomous animal contact	174 (129 to 238)	124 (93·6 to 166)	96·6 (73 to 131)	56 (41·8 to 74·5)	30·2 (22·8 to 39·4)	18·1 (14·4 to 22·2)	8 (6·1 to 10·3)	4,080
Non-venomous animal contact	162 (115 to 227)	141 (103 to 195)	99·8 (70·5 to 135)	53·9 (37·9 to 76·1)	24·6 (17·4 to 34·7)	9·8 (6·8 to 13·9)	3·9 (2·7 to 5·6)	2,220
Foreign body	1,120 (877 to 1,420)	954 (755 to 1,190)	698 (570 to 840)	539 (443 to 640)	266 (220 to 316)	137 (111 to 158)	72·6 (58·7 to 85·1)	52,700
Pulmonary aspiration and foreign body in airway	919 (695 to 1,230)	774 (593 to 996)	558 (449 to 691)	433 (348 to 530)	206 (165 to 249)	103 (80·6 to 124)	56·1 (43·4 to 66·9)	47,500
Foreign body in eyes	65·1 (39·9 to 98·4)	52·7 (34·1 to 77·8)	37·9 (24·7 to 55·8)	24·5 (16·3 to 36)	13·2 (8·9 to 19·1)	5·7 (3·9 to 8·1)	2·2 (1·5 to 3·1)	1,730
Foreign body in other body part	134 (98·2 to 179)	126 (94·3 to 168)	102 (77 to 132)	81·2 (61·7 to 105)	46·9 (34·9 to 60·9)	27·7 (20·4 to 36·1)	14·3 (10·5 to 19·6)	3,430
Environmental heat and cold exposure	717 (471 to 1,160)	637 (399 to 1,130)	457 (291 to 812)	331 (209 to 592)	187 (121 to 325)	89·6 (59·7 to 152)	35·2 (25·2 to 51·6)	16,400
Exposure to forces of nature	978 (636 to 1,470)	614 (393 to 927)	331 (213 to 488)	165 (105 to 255)	61·6 (39·6 to 96·7)	18 (11·4 to 28)	3·6 (2·2 to 6·4)	53,400
Other unintentional injuries	1,340 (991 to 1,790)	953 (694 to 1,290)	641 (459 to 875)	389 (277 to 538)	217 (155 to 299)	94·3 (67·7 to 129)	37·6 (27·5 to 51·4)	58,400
Self-harm and interpersonal violence	8,510 (6,970 to 10,300)	5,960 (4,990 to 7,150)	3,750 (3,140 to 4,450)	2,110 (1,760 to 2,490)	1,060 (882 to 1,280)	324 (268 to 377)	108 (87·8 to 127)	554,000
Self-harm	4,840 (3,930 to 5,920)	3,850 (3,140 to 4,700)	2,640 (2,130 to 3,210)	1,650 (1,320 to 2,020)	845 (676 to 1,040)	245 (196 to 294)	69·9 (53·3 to 87·2)	141,000
Self-harm by firearm	248 (173 to 357)	182 (131 to 260)	105 (75·5 to 144)	61·9 (44·8 to 86·5)	27·6 (18·9 to 39·3)	6·5 (4·6 to 9·2)	2·6 (1·7 to 3·8)	10,700
Self-harm by other specified means	4,590 (3,730 to 5,630)	3,660 (2,970 to 4,500)	2,530 (2,040 to 3,100)	1,590 (1,270 to 1,950)	818 (652 to 1,020)	239 (190 to 286)	67·3 (51·1 to 83·9)	130,000
Interpersonal violence	3,620 (2,460 to 4,890)	2,090 (1,500 to 2,790)	1,100 (817 to 1,440)	452 (352 to 564)	213 (167 to 267)	77·4 (60 to 99)	37·8 (29·6 to 47)	411,000
Physical violence by firearm	1,060 (783 to 1,490)	529 (389 to 733)	203 (153 to 276)	82·2 (63·8 to 108)	37·7 (29·3 to 48·8)	12·1 (9·2 to 15·7)	6 (4·7 to 7·9)	216,000
Physical violence by sharp object	762 (557 to 1,190)	529 (400 to 751)	289 (219 to 391)	133 (101 to 176)	64·9 (49·7 to 84·1)	31·1 (23·8 to 40·1)	14·1 (10·5 to 18·4)	98,900
Sexual violence	58·4 (36·2 to 87·6)	43·7 (27·5 to 64·9)	28·8 (18·6 to 42·4)	17·5 (11·2 to 25·5)	7·2 (4·1 to 11·5)	1·2 (0·7 to 1·9)	0·1 (0 to 0·1)	2,890
Physical violence by other means	1,740 (955 to 2,790)	988 (593 to 1,510)	581 (380 to 841)	220 (151 to 295)	104 (75·8 to 136)	33 (24·3 to 43·8)	17·6 (13·4 to 22·2)	92,700
Conflict and terrorism	42·6 (28·3 to 62·8)	19·4 (13 to 28·9)	8·9 (5·9 to 13·2)	4·7 (3·1 to 6·8)	2·1 (1·4 to 3·1)	0·6 (0·4 to 0·8)	0·2 (0·1 to 0·2)	1,040

SR Table 2a: Antigua and Barbuda DALYS by cause and age group for males with 95% uncertainty intervals, 2019

Cause	Early Neonatal	Late Neonatal	Post Neonatal	1 to 4	5 to 9	10 to 14	15 to 19	20 to 24	25 to 29	30 to 34	35 to 39	40 to 44	45 to 49	50 to 54	55 to 59	60 to 64	65 to 69	70 to 74	75 to 79	80 to 84	85 to 89	90 to 94	95 plus	Total
All causes	177 (120 to 254)	97·4 (66·9 to 139)	112 (80·7 to 154)	203 (155 to 259)	196 (160 to 249)	219 (174 to 278)	355 (292 to 432)	574 (492 to 665)	619 (539 to 714)	562 (480 to 656)	636 (547 to 743)	649 (554 to 765)	783 (660 to 918)	1,020 (866 to 1,180)	1,160 (994 to 1,350)	1,220 (1,060 to 1,410)	1,000 (872 to 1,150)	928 (814 to 1,050)	752 (668 to 845)	591 (535 to 653)	296 (271 to 321)	120 (112 to 129)	35·6 (33·3 to 38·1)	12,300
Communicable, maternal, neonatal, and nutritional diseases	152 (104 to 218)	77·9 (53·2 to 112)	56·7 (39·7 to 78·5)	67·4 (49·8 to 90·3)	48·2 (32·2 to 73·5)	34·4 (24·3 to 50·9)	35·3 (27·3 to 46·1)	48·5 (40 to 58·8)	61·6 (52·8 to 73)	73 (64·7 to 84·3)	79 (70·7 to 89·9)	71 (63·2 to 82·7)	70·2 (61·5 to 80·7)	67·6 (58·6 to 79·3)	63·3 (53·1 to 75·8)	57·9 (47·3 to 71·9)	45·4 (36·8 to 55·7)	43·4 (34·5 to 54·2)	39·9 (30·5 to 52·1)	38·1 (28·5 to 48·7)	23·5 (17·7 to 29·5)	10·6 (7·9 to 13·9)	3·7 (2·8 to 4·9)	1,270
HIV/AIDS and sexually transmitted infections	0·2 (0·1 to 0·3)	0·1 (0 to 0·2)	1·6 (1·4 to 1·7)	2·4 (2·2 to 2·5)	2·1 (2 to 2·3)	1·3 (1·2 to 1·5)	3 (2·7 to 3·5)	12·2 (11·1 to 13·5)	24·4 (22·3 to 27)	39 (35·7 to 42·6)	43·6 (40·3 to 47·2)	36·6 (33·7 to 39·7)	32·6 (30·1 to 35·4)	24·4 (22·6 to 26·3)	18·3 (16·9 to 20)	10 (9 to 10·9)	6·4 (5·8 to 7)	1·7 (1·4 to 1·9)	0·9 (0·8 to 1)	0·4 (0·3 to 0·4)	0·1 (0·1 to 0·2)	0 (0 to 0)	0 (0 to 0)	261
HIV/AIDS	0 (0 to 0)	0 (0 to 0)	1·5 (1·4 to 1·6)	2·4 (2·2 to 2·5)	2·1 (2 to 2·3)	1·3 (1·2 to 1·4)	2·6 (2·4 to 2·8)	11·5 (10·5 to 12·7)	23·7 (21·6 to 26·1)	38·2 (35 to 41·8)	42·9 (39·7 to 46·4)	36 (33·2 to 39)	32 (29·6 to 34·8)	23·9 (22·2 to 25·9)	18 (16·5 to 19·6)	9·7 (8·8 to 10·7)	6·2 (5·7 to 6·7)	1·5 (1·3 to 1·7)	0·8 (0·7 to 0·9)	0·3 (0·3 to 0·3)	0·1 (0·1 to 0·1)	0 (0 to 0)	0 (0 to 0)	255
HIV/AIDS - Drug-susceptible Tuberculosis	0 (0 to 0)	0 (0 to 0)	0 (0 to 0)	0 (0 to 0)	0 (0 to 0)	0 (0 to 0)	0 (0 to 0·1)	0·2 (0·2 to 0·3)	0·5 (0·3 to 0·7)	0·6 (0·4 to 1)	0·7 (0·4 to 1)	0·6 (0·4 to 0·8)	0·5 (0·3 to 0·7)	0·4 (0·2 to 0·5)	0·3 (0·2 to 0·4)	0·2 (0·1 to 0·2)	0·1 (0·1 to 0·2)	0 (0 to 0)	0 (0 to 0)	0 (0 to 0)	0 (0 to 0)	0 (0 to 0)	0 (0 to 0)	4·1
HIV/AIDS - Multidrug-resistant Tuberculosis without extensive dru	0 (0 to 0)	0 (0 to 0)	0 (0 to 0)	0 (0 to 0)	0 (0 to 0)	0 (0 to 0)	0 (0 to 0)	0 (0 to 0)	0 (0 to 0)	0 (0 to 0)	0 (0 to 0)	0 (0 to 0)	0 (0 to 0)	0 (0 to 0)	0 (0 to 0)	0 (0 to 0)	0 (0 to 0)	0 (0 to 0)	0 (0 to 0)	0 (0 to 0)	0 (0 to 0)	0 (0 to 0)	0 (0 to 0)	0·1
HIV/AIDS - Extensively drug-resistant Tuberculosis	0 (0 to 0)	0 (0 to 0)	0 (0 to 0)	0 (0 to 0)	0 (0 to 0)	0 (0 to 0)	0 (0 to 0)	0 (0 to 0)	0 (0 to 0)	0 (0 to 0)	0 (0 to 0)	0 (0 to 0)	0 (0 to 0)	0 (0 to 0)	0 (0 to 0)	0 (0 to 0)	0 (0 to 0)	0 (0 to 0)	0 (0 to 0)	0 (0 to 0)	0 (0 to 0)	0 (0 to 0)	0 (0 to 0)	0
HIV/AIDS resulting in other diseases	0 (0 to 0)	0 (0 to 0)	1·5 (1·4 to 1·6)	2·3 (2·1 to 2·5)	2·1 (1·9 to 2·2)	1·2 (1·1 to 1·3)	2·6 (2·4 to 2·8)	11·3 (10·2 to 12·5)	23·2 (21·2 to 25·6)	37·6 (34·4 to 41·2)	42·2 (39 to 45·8)	35·4 (32·7 to 38·3)	31·6 (29·2 to 34·4)	23·6 (21·8 to 25·5)	17·7 (16·2 to 19·4)	9·6 (8·6 to 10·5)	6·1 (5·5 to 6·6)	1·5 (1·3 to 1·7)	0·8 (0·7 to 0·9)	0·3 (0·3 to 0·3)	0·1 (0·1 to 0·1)	0 (0 to 0)	0 (0 to 0)	251
Sexually transmitted infections excluding HIV	0·2 (0·1 to 0·3)	0·1 (0 to 0·2)	0·1 (0 to 0·2)	0 (0 to 0)	0 (0 to 0)	0·1 (0 to 0·1)	0·4 (0·2 to 0·8)	0·7 (0·4 to 1·1)	0·7 (0·4 to 1·2)	0·7 (0·4 to 1·3)	0·7 (0·4 to 1·2)	0·6 (0·3 to 1·1)	0·6 (0·3 to 1)	0·5 (0·2 to 0·9)	0·4 (0·2 to 0·7)	0·3 (0·1 to 0·5)	0·2 (0·1 to 0·4)	0·1 (0·1 to 0·2)	0·1 (0 to 0·1)	0·1 (0 to 0·1)	0 (0 to 0)	0 (0 to 0)	0 (0 to 0)	6·5
Syphilis	0·2 (0·1 to 0·3)	0·1 (0 to 0·2)	0·1 (0 to 0·2)	0 (0 to 0)	0 (0 to 0)	0 (0 to 0)	0·1 (0 to 0·1)	0·1 (0 to 0·1)	0·1 (0 to 0·2)	0·1 (0 to 0·2)	0·1 (0·1 to 0·2)	0·1 (0 to 0·1)	0·1 (0 to 0·1)	0·1 (0·1 to 0·2)	0·1 (0·1 to 0·2)	0·1 (0·1 to 0·2)	0·1 (0 to 0·2)	0·1 (0 to 0·1)	0 (0 to 0)	0 (0 to 0)	0 (0 to 0)	0 (0 to 0)	0 (0 to 0)	1·4
Chlamydial infection	0 (0 to 0)	0 (0 to 0)	0 (0 to 0)	0 (0 to 0)	0 (0 to 0)	0 (0 to 0)	0 (0 to 0·1)	0·1 (0 to 0·3)	0·2 (0·1 to 0·4)	0·2 (0·1 to 0·4)	0·2 (0 to 0·3)	0·1 (0 to 0·3)	0·1 (0 to 0·2)	0 (0 to 0·1)	0 (0 to 0)	0 (0 to 0)	0 (0 to 0)	0 (0 to 0)	0 (0 to 0)	0 (0 to 0)	0 (0 to 0)	0 (0 to 0)	0 (0 to 0)	1
Gonococcal infection	0 (0 to 0)	0 (0 to 0)	0 (0 to 0)	0 (0 to 0)	0 (0 to 0)	0 (0 to 0)	0·1 (0 to 0·3)	0·2 (0·1 to 0·4)	0·1 (0 to 0·2)	0·1 (0 to 0·1)	0 (0 to 0·1)	0 (0 to 0·1)	0 (0 to 0·1)	0 (0 to 0·1)	0 (0 to 0)	0 (0 to 0)	0 (0 to 0)	0 (0 to 0)	0 (0 to 0)	0 (0 to 0)	0 (0 to 0)	0 (0 to 0)	0 (0 to 0)	0·7
Trichomoniasis	0 (0 to 0)	0 (0 to 0)	0 (0 to 0)	0 (0 to 0)	0 (0 to 0)	0 (0 to 0)	0 (0 to 0)	0 (0 to 0)	0 (0 to 0)	0 (0 to 0)	0 (0 to 0·1)	0 (0 to 0·1)	0 (0 to 0)	0 (0 to 0)	0 (0 to 0)	0 (0 to 0)	0 (0 to 0)	0 (0 to 0)	0 (0 to 0)	0 (0 to 0)	0 (0 to 0)	0 (0 to 0)	0 (0 to 0)	0·1
Genital herpes	0 (0 to 0)	0 (0 to 0)	0 (0 to 0)	0 (0 to 0)	0 (0 to 0)	0 (0 to 0)	0 (0 to 0·1)	0·1 (0 to 0·2)	0·1 (0 to 0·3)	0·2 (0·1 to 0·5)	0·2 (0·1 to 0·5)	0·2 (0·1 to 0·5)	0·2 (0·1 to 0·6)	0·2 (0·1 to 0·5)	0·2 (0·1 to 0·4)	0·1 (0 to 0·3)	0·1 (0 to 0·2)	0·1 (0 to 0·2)	0 (0 to 0·1)	0 (0 to 0)	0 (0 to 0)	0 (0 to 0)	0 (0 to 0)	1·9
Other sexually transmitted infections	0 (0 to 0)	0 (0 to 0)	0 (0 to 0)	0 (0 to 0)	0 (0 to 0)	0 (0 to 0·1)	0·2 (0 to 0·3)	0·2 (0·1 to 0·3)	0·2 (0·1 to 0·3)	0·2 (0·1 to 0·4)	0·1 (0·1 to 0·3)	0·1 (0 to 0·2)	0·1 (0 to 0·2)	0·1 (0 to 0·1)	0 (0 to 0·1)	0 (0 to 0)	0 (0 to 0)	0 (0 to 0)	0 (0 to 0)	0 (0 to 0)	0 (0 to 0)	0 (0 to 0)	0 (0 to 0)	1·3
Respiratory infections and tuberculosis	5·2 (2·7 to 8·6)	7·4 (4 to 12·4)	18·7 (11·3 to 30)	19·2 (13 to 27·4)	8·4 (6·1 to 11·3)	6·2 (4·2 to 8·9)	7·3 (5·1 to 10·1)	11·3 (8·4 to 14·9)	12·3 (9·3 to 16·2)	10·6 (8 to 13·9)	12·1 (9 to 15·8)	11·5 (8·4 to 15·3)	13·6 (9·6 to 18·5)	18·5 (13·3 to 25·2)	22·4 (16·3 to 30·2)	27·9 (19·8 to 38·7)	23·9 (17·2 to 32·4)	28·5 (20·7 to 38·8)	28·8 (20·5 to 39·4)	30·4 (21·5 to 40·4)	19·5 (14·1 to 25)	9 (6·5 to 12·1)	3·2 (2·3 to 4·2)	356
Tuberculosis	0 (0 to 0)	0 (0 to 0)	0·1 (0 to 0·1)	0·2 (0·1 to 0·4)	0·3 (0·1 to 0·6)	0·1 (0 to 0·3)	0·2 (0·1 to 0·3)	0·8 (0·5 to 1·2)	0·9 (0·5 to 1·4)	0·8 (0·5 to 1·2)	0·7 (0·4 to 1·1)	0·7 (0·4 to 1·1)	0·7 (0·5 to 1·1)	0·9 (0·6 to 1·2)	0·8 (0·6 to 1·2)	0·8 (0·6 to 1·1)	0·5 (0·4 to 0·8)	0·5 (0·4 to 0·7)	0·4 (0·3 to 0·6)	0·3 (0·2 to 0·4)	0·1 (0·1 to 0·2)	0·1 (0 to 0·1)	0 (0 to 0)	10
Latent tuberculosis infection	0 (0 to 0)	0 (0 to 0)	0 (0 to 0)	0 (0 to 0)	0 (0 to 0)	0 (0 to 0)	0 (0 to 0)	0 (0 to 0)	0 (0 to 0)	0 (0 to 0)	0 (0 to 0)	0 (0 to 0)	0 (0 to 0)	0 (0 to 0)	0 (0 to 0)	0 (0 to 0)	0 (0 to 0)	0 (0 to 0)	0 (0 to 0)	0 (0 to 0)	0 (0 to 0)	0 (0 to 0)	0 (0 to 0)	0
Drug-susceptible tuberculosis	0 (0 to 0)	0 (0 to 0)	0·1 (0 to 0·1)	0·2 (0·1 to 0·4)	0·3 (0·1 to 0·6)	0·1 (0 to 0·3)	0·2 (0·1 to 0·2)	0·8 (0·5 to 1·2)	0·9 (0·5 to 1·4)	0·8 (0·5 to 1·2)	0·7 (0·4 to 1·1)	0·7 (0·4 to 1·1)	0·7 (0·5 to 1)	0·9 (0·6 to 1·2)	0·8 (0·6 to 1·1)	0·8 (0·5 to 1·1)	0·5 (0·4 to 0·8)	0·5 (0·3 to 0·7)	0·4 (0·3 to 0·5)	0·3 (0·2 to 0·4)	0·1 (0·1 to 0·2)	0·1 (0 to 0·1)	0 (0 to 0)	9·9
Multidrug-resistant tuberculosis without extensive drug resistanc	0 (0 to 0)	0 (0 to 0)	0 (0 to 0)	0 (0 to 0)	0 (0 to 0)	0 (0 to 0)	0 (0 to 0)	0 (0 to 0)	0 (0 to 0)	0 (0 to 0)	0 (0 to 0)	0 (0 to 0)	0 (0 to 0)	0 (0 to 0)	0 (0 to 0)	0 (0 to 0)	0 (0 to 0)	0 (0 to 0)	0 (0 to 0)	0 (0 to 0)	0 (0 to 0)	0 (0 to 0)	0 (0 to 0)	0·1
Extensively drug-resistant tuberculosis	0 (0 to 0)	0 (0 to 0)	0 (0 to 0)	0 (0 to 0)	0 (0 to 0)	0 (0 to 0)	0 (0 to 0)	0 (0 to 0)	0 (0 to 0)	0 (0 to 0)	0 (0 to 0)	0 (0 to 0)	0 (0 to 0)	0 (0 to 0)	0 (0 to 0)	0 (0 to 0)	0 (0 to 0)	0 (0 to 0)	0 (0 to 0)	0 (0 to 0)	0 (0 to 0)	0 (0 to 0)	0 (0 to 0)	0
Lower respiratory infections	5·2 (2·7 to 8·6)	7·4 (3·9 to 12·4)	17·9 (10·6 to 29)	15·3 (9·1 to 23·2)	3·8 (2·6 to 5·2)	2·1 (1·5 to 2·7)	3 (2·1 to 4·1)	6·5 (4·7 to 9)	7·9 (5·6 to 10·9)	6·7 (4·7 to 9·1)	8·3 (5·8 to 11·8)	7·8 (5·3 to 11·2)	10·1 (6·6 to 14·5)	15·1 (9·9 to 21·6)	19·5 (13·4 to 27)	25·6 (17·6 to 36)	22·3 (15·5 to 31)	27·3 (19·5 to 37·7)	28 (19·7 to 38·6)	29·8 (20·9 to 39·8)	19·2 (13·9 to 24·9)	8·9 (6·4 to 12)	3·1 (2·3 to 4·2)	301
Upper respiratory infections	0 (0 to 0)	0 (0 to 0·1)	0·6 (0·4 to 1)	2·7 (1·6 to 4·2)	2·9 (1·6 to 5·1)	2·9 (1·5 to 5·1)	3 (1·6 to 5·6)	3 (1·7 to 5·3)	2·8 (1·5 to 4·6)	2·6 (1·4 to 4·4)	2·5 (1·2 to 4·3)	2·4 (1·3 to 4·2)	2·1 (1·1 to 3·7)	1·9 (1 to 3·3)	1·5 (0·8 to 2·5)	1·2 (0·6 to 2)	0·9 (0·5 to 1·5)	0·6 (0·3 to 1)	0·3 (0·2 to 0·6)	0·2 (0·1 to 0·3)	0·1 (0 to 0·1)	0 (0 to 0)	0 (0 to 0)	34·3
Otitis media	0 (0 to 0)	0 (0 to 0)	0·1 (0·1 to 0·2)	1 (0·6 to 1·8)	1·4 (0·8 to 2·4)	1·1 (0·6 to 1·9)	1·1 (0·5 to 1·8)	0·9 (0·4 to 1·8)	0·7 (0·3 to 1·3)	0·6 (0·2 to 1·1)	0·6 (0·3 to 1)	0·6 (0·3 to 1·2)	0·6 (0·3 to 1·2)	0·6 (0·3 to 1·2)	0·5 (0·2 to 0·9)	0·3 (0·2 to 0·7)	0·2 (0·1 to 0·4)	0·1 (0·1 to 0·2)	0·1 (0 to 0·1)	0 (0 to 0·1)	0 (0 to 0)	0 (0 to 0)	0 (0 to 0)	10·7
Enteric infections	0·2 (0·1 to 0·4)	1·1 (0·5 to 2·3)	6·3 (4 to 9·6)	7·5 (4·9 to 10·9)	5·3 (3·2 to 8·3)	5·3 (3 to 8·6)	4·3 (2·6 to 6·5)	4 (2·5 to 5·9)	4·2 (2·8 to 6)	4·2 (2·8 to 6·1)	4·5 (3·1 to 6·5)	4·9 (3·4 to 7)	5·2 (3·6 to 7·5)	5·6 (3·9 to 7·8)	5·3 (3·7 to 7·2)	5·5 (4 to 7·5)	5·6 (4 to 7·6)	5·5 (4 to 7·3)	4·7 (3·4 to 6·2)	3·2 (2·4 to 4·2)	1·5 (1·1 to 2)	0·6 (0·5 to 0·8)	0·2 (0·1 to 0·2)	94·7
Diarrheal diseases	0·2 (0·1 to 0·4)	1·1 (0·5 to 2·3)	6·3 (4 to 9·6)	7·4 (4·9 to 10·8)	5·3 (3·2 to 8·2)	5·3 (3 to 8·5)	4·3 (2·6 to 6·5)	3·9 (2·5 to 5·9)	4·2 (2·8 to 6)	4·2 (2·8 to 6·1)	4·5 (3·1 to 6·5)	4·8 (3·3 to 6·9)	5·2 (3·6 to 7·5)	5·6 (3·9 to 7·8)	5·2 (3·7 to 7·2)	5·4 (4 to 7·4)	5·6 (4 to 7·6)	5·5 (4 to 7·3)	4·7 (3·4 to 6·2)	3·2 (2·4 to 4·2)	1·5 (1·1 to 2)	0·6 (0·5 to 0·8)	0·2 (0·1 to 0·2)	94·5
Typhoid and paratyphoid	0 (0 to 0)	0 (0 to 0)	0 (0 to 0)	0 (0 to 0)	0 (0 to 0)	0 (0 to 0)	0 (0 to 0)	0 (0 to 0)	0 (0 to 0)	0 (0 to 0)	0 (0 to 0)	0 (0 to 0)	0 (0 to 0)	0 (0 to 0)	0 (0 to 0)	0 (0 to 0)	0 (0 to 0)	0 (0 to 0)	0 (0 to 0)	0 (0 to 0)	0 (0 to 0)	0 (0 to 0)	0 (0 to 0)	0
Typhoid fever	0 (0 to 0)	0 (0 to 0)	0 (0 to 0)	0 (0 to 0)	0 (0 to 0)	0 (0 to 0)	0 (0 to 0)	0 (0 to 0)	0 (0 to 0)	0 (0 to 0)	0 (0 to 0)	0 (0 to 0)	0 (0 to 0)	0 (0 to 0)	0 (0 to 0)	0 (0 to 0)	0 (0 to 0)	0 (0 to 0)	0 (0 to 0)	0 (0 to 0)	0 (0 to 0)	0 (0 to 0)	0 (0 to 0)	0
Paratyphoid fever	0 (0 to 0)	0 (0 to 0)	0 (0 to 0)	0 (0 to 0)	0 (0 to 0)	0 (0 to 0)	0 (0 to 0)	0 (0 to 0)	0 (0 to 0)	0 (0 to 0)	0 (0 to 0)	0 (0 to 0)	0 (0 to 0)	0 (0 to 0)	0 (0 to 0)	0 (0 to 0)	0 (0 to 0)	0 (0 to 0)	0 (0 to 0)	0 (0 to 0)	0 (0 to 0)	0 (0 to 0)	0 (0 to 0)	0
Invasive Non-typhoidal Salmonella (iNTS)	0 (0 to 0)	0 (0 to 0)	0 (0 to 0)	0 (0 to 0)	0 (0 to 0)	0 (0 to 0)	0 (0 to 0)	0 (0 to 0)	0 (0 to 0)	0 (0 to 0)	0 (0 to 0)	0 (0 to 0)	0 (0 to 0)	0 (0 to 0)	0 (0 to 0)	0 (0 to 0)	0 (0 to 0)	0 (0 to 0)	0 (0 to 0)	0 (0 to 0)	0 (0 to 0)	0 (0 to 0)	0 (0 to 0)	0
Other intestinal infectious diseases	0 (0 to 0)	0 (0 to 0)	0 (0 to 0)	0 (0 to 0·1)	0 (0 to 0·1)	0 (0 to 0)	0 (0 to 0)	0 (0 to 0)	0 (0 to 0)	0 (0 to 0)	0 (0 to 0)	0 (0 to 0)	0 (0 to 0)	0 (0 to 0)	0 (0 to 0)	0 (0 to 0)	0 (0 to 0)	0 (0 to 0)	0 (0 to 0)	0 (0 to 0)	0 (0 to 0)	0 (0 to 0)	0 (0 to 0)	0·2
Neglected tropical diseases and malaria	0 (0 to 0)	0·1 (0 to 0·1)	0·6 (0·2 to 1·1)	2·1 (0·9 to 4·2)	3·4 (1·5 to 6·8)	3·3 (1·1 to 7·8)	3·4 (1·2 to 7·8)	3·5 (1·3 to 8·4)	3·5 (1·1 to 8·6)	3·4 (1·1 to 8·2)	3 (1 to 7·3)	2·9 (1 to 6·7)	2·8 (1 to 6·3)	2·6 (1 to 5·8)	2·3 (0·9 to 5)	1·8 (0·7 to 3·9)	1·1 (0·4 to 2·5)	0·8 (0·3 to 1·9)	0·5 (0·2 to 1·3)	0·3 (0·1 to 0·7)	0·2 (0·1 to 0·3)	0·1 (0 to 0·1)	0 (0 to 0)	41·5
Malaria	0 (0 to 0)	0 (0 to 0)	0 (0 to 0)	0 (0 to 0)	0 (0 to 0)	0 (0 to 0)	0 (0 to 0)	0 (0 to 0)	0 (0 to 0)	0 (0 to 0)	0 (0 to 0)	0 (0 to 0)	0 (0 to 0)	0 (0 to 0)	0 (0 to 0)	0 (0 to 0)	0 (0 to 0)	0 (0 to 0)	0 (0 to 0)	0 (0 to 0)	0 (0 to 0)	0 (0 to 0)	0 (0 to 0)	0
Chagas disease	0 (0 to 0)	0 (0 to 0)	0 (0 to 0)	0 (0 to 0)	0 (0 to 0)	0 (0 to 0)	0 (0 to 0)	0 (0 to 0)	0 (0 to 0)	0 (0 to 0)	0 (0 to 0)	0 (0 to 0)	0 (0 to 0)	0 (0 to 0)	0 (0 to 0)	0 (0 to 0)	0 (0 to 0)	0 (0 to 0)	0 (0 to 0)	0 (0 to 0)	0 (0 to 0)	0 (0 to 0)	0 (0 to 0)	0
Leishmaniasis	0 (0 to 0)	0 (0 to 0)	0 (0 to 0)	0 (0 to 0)	0 (0 to 0)	0 (0 to 0)	0 (0 to 0)	0 (0 to 0)	0 (0 to 0)	0 (0 to 0)	0 (0 to 0)	0 (0 to 0)	0 (0 to 0)	0 (0 to 0)	0 (0 to 0)	0 (0 to 0)	0 (0 to 0)	0 (0 to 0)	0 (0 to 0)	0 (0 to 0)	0 (0 to 0)	0 (0 to 0)	0 (0 to 0)	0
Visceral leishmaniasis	0 (0 to 0)	0 (0 to 0)	0 (0 to 0)	0 (0 to 0)	0 (0 to 0)	0 (0 to 0)	0 (0 to 0)	0 (0 to 0)	0 (0 to 0)	0 (0 to 0)	0 (0 to 0)	0 (0 to 0)	0 (0 to 0)	0 (0 to 0)	0 (0 to 0)	0 (0 to 0)	0 (0 to 0)	0 (0 to 0)	0 (0 to 0)	0 (0 to 0)	0 (0 to 0)	0 (0 to 0)	0 (0 to 0)	0
Cutaneous and mucocutaneous leishmaniasis	0 (0 to 0)	0 (0 to 0)	0 (0 to 0)	0 (0 to 0)	0 (0 to 0)	0 (0 to 0)	0 (0 to 0)	0 (0 to 0)	0 (0 to 0)	0 (0 to 0)	0 (0 to 0)	0 (0 to 0)	0 (0 to 0)	0 (0 to 0)	0 (0 to 0)	0 (0 to 0)	0 (0 to 0)	0 (0 to 0)	0 (0 to 0)	0 (0 to 0)	0 (0 to 0)	0 (0 to 0)	0 (0 to 0)	0
African trypanosomiasis	0 (0 to 0)	0 (0 to 0)	0 (0 to 0)	0 (0 to 0)	0 (0 to 0)	0 (0 to 0)	0 (0 to 0)	0 (0 to 0)	0 (0 to 0)	0 (0 to 0)	0 (0 to 0)	0 (0 to 0)	0 (0 to 0)	0 (0 to 0)	0 (0 to 0)	0 (0 to 0)	0 (0 to 0)	0 (0 to 0)	0 (0 to 0)	0 (0 to 0)	0 (0 to 0)	0 (0 to 0)	0 (0 to 0)	0
Schistosomiasis	0 (0 to 0)	0 (0 to 0)	0 (0 to 0)	0·1 (0 to 0·4)	1·1 (0·2 to 3·3)	2·1 (0·3 to 6·2)	2·4 (0·4 to 6·7)	2·4 (0·5 to 7·2)	2·6 (0·5 to 7·4)	2·5 (0·5 to 7·2)	2·3 (0·4 to 6·2)	2·2 (0·5 to 6)	2·1 (0·5 to 5·3)	2 (0·5 to 4·9)	1·7 (0·4 to 4·4)	1·3 (0·3 to 3·4)	0·8 (0·2 to 2·1)	0·6 (0·1 to 1·6)	0·4 (0·1 to 1)	0·2 (0 to 0·6)	0·1 (0 to 0·3)	0 (0 to 0·1)	0 (0 to 0)	26·8
Cysticercosis	0 (0 to 0)	0 (0 to 0)	0 (0 to 0)	0 (0 to 0)	0 (0 to 0)	0 (0 to 0)	0 (0 to 0·1)	0·1 (0 to 0·2)	0·1 (0 to 0·3)	0·1 (0 to 0·2)	0·1 (0 to 0·2)	0 (0 to 0·2)	0 (0 to 0·2)	0 (0 to 0·2)	0·1 (0 to 0·2)	0 (0 to 0·2)	0 (0 to 0·1)	0 (0 to 0)	0 (0 to 0)	0 (0 to 0)	0 (0 to 0)	0 (0 to 0)	0 (0 to 0)	0·6
Cystic echinococcosis	0 (0 to 0)	0 (0 to 0)	0 (0 to 0)	0 (0 to 0)	0 (0 to 0)	0 (0 to 0)	0 (0 to 0)	0 (0 to 0)	0 (0 to 0)	0 (0 to 0)	0 (0 to 0)	0 (0 to 0)	0 (0 to 0)	0 (0 to 0)	0 (0 to 0)	0 (0 to 0)	0 (0 to 0)	0 (0 to 0)	0 (0 to 0)	0 (0 to 0)	0 (0 to 0)	0 (0 to 0)	0 (0 to 0)	0
Lymphatic filariasis	0 (0 to 0)	0 (0 to 0)	0 (0 to 0)	0 (0 to 0)	0 (0 to 0)	0 (0 to 0)	0 (0 to 0)	0 (0 to 0)	0 (0 to 0)	0 (0 to 0)	0 (0 to 0)	0 (0 to 0)	0 (0 to 0)	0 (0 to 0)	0 (0 to 0)	0 (0 to 0)	0 (0 to 0)	0 (0 to 0)	0 (0 to 0)	0 (0 to 0)	0 (0 to 0)	0 (0 to 0)	0 (0 to 0)	0
Onchocerciasis	0 (0 to 0)	0 (0 to 0)	0 (0 to 0)	0 (0 to 0)	0 (0 to 0)	0 (0 to 0)	0 (0 to 0)	0 (0 to 0)	0 (0 to 0)	0 (0 to 0)	0 (0 to 0)	0 (0 to 0)	0 (0 to 0)	0 (0 to 0)	0 (0 to 0)	0 (0 to 0)	0 (0 to 0)	0 (0 to 0)	0 (0 to 0)	0 (0 to 0)	0 (0 to 0)	0 (0 to 0)	0 (0 to 0)	0
Trachoma	0 (0 to 0)	0 (0 to 0)	0 (0 to 0)	0 (0 to 0)	0 (0 to 0)	0 (0 to 0)	0 (0 to 0)	0 (0 to 0)	0 (0 to 0)	0 (0 to 0)	0 (0 to 0)	0 (0 to 0)	0 (0 to 0)	0 (0 to 0)	0 (0 to 0)	0 (0 to 0)	0 (0 to 0)	0 (0 to 0)	0 (0 to 0)	0 (0 to 0)	0 (0 to 0)	0 (0 to 0)	0 (0 to 0)	0
Dengue	0 (0 to 0)	0 (0 to 0)	0·2 (0·1 to 0·7)	0·4 (0·1 to 1·8)	0·5 (0·1 to 1·2)	0·4 (0·1 to 0·8)	0·5 (0·2 to 1·3)	0·5 (0·1 to 1·1)	0·4 (0·1 to 1)	0·4 (0·1 to 0·9)	0·3 (0·1 to 0·7)	0·3 (0·1 to 0·6)	0·3 (0·1 to 0·6)	0·3 (0·1 to 0·6)	0·2 (0·1 to 0·5)	0·3 (0·1 to 0·6)	0·2 (0·1 to 0·4)	0·1 (0 to 0·3)	0·1 (0 to 0·2)	0·1 (0 to 0·1)	0 (0 to 0·1)	0 (0 to 0)	0 (0 to 0)	5·6
Yellow fever	0 (0 to 0)	0 (0 to 0)	0 (0 to 0)	0 (0 to 0)	0 (0 to 0)	0 (0 to 0)	0 (0 to 0)	0 (0 to 0)	0 (0 to 0)	0 (0 to 0)	0 (0 to 0)	0 (0 to 0)	0 (0 to 0)	0 (0 to 0)	0 (0 to 0)	0 (0 to 0)	0 (0 to 0)	0 (0 to 0)	0 (0 to 0)	0 (0 to 0)	0 (0 to 0)	0 (0 to 0)	0 (0 to 0)	0
Rabies	0 (0 to 0)	0 (0 to 0)	0 (0 to 0)	0 (0 to 0·1)	0 (0 to 0·2)	0 (0 to 0)	0 (0 to 0)	0 (0 to 0)	0 (0 to 0)	0 (0 to 0)	0 (0 to 0)	0 (0 to 0)	0 (0 to 0)	0 (0 to 0)	0 (0 to 0)	0 (0 to 0)	0 (0 to 0)	0 (0 to 0)	0 (0 to 0)	0 (0 to 0)	0 (0 to 0)	0 (0 to 0)	0 (0 to 0)	0·2
Intestinal nematode infections	0 (0 to 0)	0 (0 to 0)	0 (0 to 0)	0 (0 to 0·2)	0·1 (0 to 0·2)	0 (0 to 0·1)	0 (0 to 0·1)	0 (0 to 0)	0 (0 to 0)	0 (0 to 0)	0 (0 to 0)	0 (0 to 0)	0 (0 to 0)	0 (0 to 0)	0 (0 to 0)	0 (0 to 0)	0 (0 to 0)	0 (0 to 0)	0 (0 to 0)	0 (0 to 0)	0 (0 to 0)	0 (0 to 0)	0 (0 to 0)	0·3
Ascariasis	0 (0 to 0)	0 (0 to 0)	0 (0 to 0)	0 (0 to 0·1)	0 (0 to 0·1)	0 (0 to 0)	0 (0 to 0)	0 (0 to 0)	0 (0 to 0)	0 (0 to 0)	0 (0 to 0)	0 (0 to 0)	0 (0 to 0)	0 (0 to 0)	0 (0 to 0)	0 (0 to 0)	0 (0 to 0)	0 (0 to 0)	0 (0 to 0)	0 (0 to 0)	0 (0 to 0)	0 (0 to 0)	0 (0 to 0)	0·1
Trichuriasis	0 (0 to 0)	0 (0 to 0)	0 (0 to 0)	0 (0 to 0)	0 (0 to 0)	0 (0 to 0)	0 (0 to 0)	0 (0 to 0)	0 (0 to 0)	0 (0 to 0)	0 (0 to 0)	0 (0 to 0)	0 (0 to 0)	0 (0 to 0)	0 (0 to 0)	0 (0 to 0)	0 (0 to 0)	0 (0 to 0)	0 (0 to 0)	0 (0 to 0)	0 (0 to 0)	0 (0 to 0)	0 (0 to 0)	0
Hookworm disease	0 (0 to 0)	0 (0 to 0)	0 (0 to 0)	0 (0 to 0·1)	0·1 (0 to 0·1)	0 (0 to 0·1)	0 (0 to 0·1)	0 (0 to 0)	0 (0 to 0)	0 (0 to 0)	0 (0 to 0)	0 (0 to 0)	0 (0 to 0)	0 (0 to 0)	0 (0 to 0)	0 (0 to 0)	0 (0 to 0)	0 (0 to 0)	0 (0 to 0)	0 (0 to 0)	0 (0 to 0)	0 (0 to 0)	0 (0 to 0)	0·3
Food-borne trematodiases	0 (0 to 0)	0 (0 to 0)	0 (0 to 0)	0 (0 to 0)	0 (0 to 0)	0 (0 to 0)	0 (0 to 0)	0 (0 to 0)	0 (0 to 0)	0 (0 to 0)	0 (0 to 0)	0 (0 to 0)	0 (0 to 0)	0 (0 to 0)	0 (0 to 0)	0 (0 to 0)	0 (0 to 0)	0 (0 to 0)	0 (0 to 0)	0 (0 to 0)	0 (0 to 0)	0 (0 to 0)	0 (0 to 0)	0
Leprosy	0 (0 to 0)	0 (0 to 0)	0 (0 to 0)	0 (0 to 0)	0 (0 to 0)	0 (0 to 0)	0 (0 to 0)	0 (0 to 0)	0 (0 to 0)	0 (0 to 0)	0 (0 to 0)	0 (0 to 0)	0 (0 to 0)	0 (0 to 0)	0 (0 to 0)	0 (0 to 0)	0 (0 to 0)	0 (0 to 0)	0 (0 to 0)	0 (0 to 0)	0 (0 to 0)	0 (0 to 0)	0 (0 to 0)	0
Ebola	0 (0 to 0)	0 (0 to 0)	0 (0 to 0)	0 (0 to 0)	0 (0 to 0)	0 (0 to 0)	0 (0 to 0)	0 (0 to 0)	0 (0 to 0)	0 (0 to 0)	0 (0 to 0)	0 (0 to 0)	0 (0 to 0)	0 (0 to 0)	0 (0 to 0)	0 (0 to 0)	0 (0 to 0)	0 (0 to 0)	0 (0 to 0)	0 (0 to 0)	0 (0 to 0)	0 (0 to 0)	0 (0 to 0)	0
Zika virus	0 (0 to 0)	0 (0 to 0)	0 (0 to 0·1)	0·1 (0 to 0·5)	0·1 (0 to 0·6)	0·1 (0 to 0·6)	0·2 (0 to 0·8)	0·2 (0 to 0·9)	0·2 (0 to 0·9)	0·2 (0 to 0·9)	0·2 (0 to 0·7)	0·1 (0 to 0·6)	0·1 (0 to 0·4)	0·1 (0 to 0·4)	0·1 (0 to 0·3)	0 (0 to 0·2)	0 (0 to 0·1)	0 (0 to 0)	0 (0 to 0)	0 (0 to 0)	0 (0 to 0)	0 (0 to 0)	0 (0 to 0)	1·9
Guinea worm disease	0 (0 to 0)	0 (0 to 0)	0 (0 to 0)	0 (0 to 0)	0 (0 to 0)	0 (0 to 0)	0 (0 to 0)	0 (0 to 0)	0 (0 to 0)	0 (0 to 0)	0 (0 to 0)	0 (0 to 0)	0 (0 to 0)	0 (0 to 0)	0 (0 to 0)	0 (0 to 0)	0 (0 to 0)	0 (0 to 0)	0 (0 to 0)	0 (0 to 0)	0 (0 to 0)	0 (0 to 0)	0 (0 to 0)	0
Other neglected tropical diseases	0 (0 to 0)	0·1 (0 to 0·1)	0·3 (0·1 to 0·7)	1·4 (0·5 to 2·9)	1·5 (0·5 to 3·5)	0·6 (0·1 to 1·6)	0·2 (0·1 to 0·5)	0·2 (0·1 to 0·5)	0·2 (0·1 to 0·4)	0·2 (0·1 to 0·4)	0·2 (0·1 to 0·4)	0·2 (0·1 to 0·4)	0·2 (0·1 to 0·5)	0·2 (0·1 to 0·4)	0·2 (0·1 to 0·5)	0·1 (0·1 to 0·3)	0·1 (0 to 0·1)	0·1 (0 to 0·2)	0·1 (0 to 0·1)	0 (0 to 0·1)	0 (0 to 0)	0 (0 to 0)	0 (0 to 0)	6·1
Other infectious diseases	0·5 (0·3 to 0·9)	1·9 (1 to 3·4)	4·6 (2·9 to 7·2)	6·2 (4 to 9·2)	4·1 (2·9 to 5·6)	2·7 (2 to 3·7)	3·3 (2·5 to 4·3)	4·1 (3·2 to 5·3)	4·2 (3·2 to 5·6)	3·6 (2·8 to 4·8)	4 (3 to 5·1)	3·7 (2·7 to 5·1)	4·5 (3·2 to 6·4)	4·8 (3·4 to 6·8)	4·3 (3 to 6·1)	4·2 (2·8 to 5·7)	2·7 (1·8 to 3·8)	2·1 (1·5 to 2·9)	1·3 (0·9 to 1·9)	0·9 (0·6 to 1·3)	0·5 (0·3 to 0·8)	0·2 (0·1 to 0·2)	0 (0 to 0·1)	68·7
Meningitis	0·4 (0·2 to 0·7)	1·6 (0·8 to 3)	2·9 (1·6 to 4·8)	3·5 (1·9 to 5·8)	1·7 (1·1 to 2·6)	1 (0·7 to 1·6)	1·2 (0·8 to 1·8)	1·4 (1 to 1·9)	1·3 (0·9 to 1·7)	1 (0·7 to 1·5)	1·1 (0·8 to 1·5)	0·9 (0·7 to 1·3)	1·1 (0·8 to 1·5)	1·3 (0·9 to 1·9)	1·2 (0·8 to 1·7)	1·3 (0·9 to 1·8)	0·8 (0·5 to 1·1)	0·6 (0·5 to 0·9)	0·5 (0·3 to 0·7)	0·3 (0·2 to 0·5)	0·2 (0·1 to 0·2)	0 (0 to 0·1)	0 (0 to 0)	25·4
Encephalitis	0 (0 to 0·1)	0·1 (0 to 0·2)	0·6 (0·3 to 1·1)	1 (0·5 to 1·7)	0·7 (0·4 to 1·1)	0·5 (0·3 to 0·7)	0·6 (0·4 to 0·9)	0·7 (0·5 to 1)	0·5 (0·4 to 0·8)	0·4 (0·3 to 0·7)	0·4 (0·3 to 0·6)	0·4 (0·3 to 0·6)	0·4 (0·3 to 0·6)	0·3 (0·2 to 0·5)	0·3 (0·2 to 0·5)	0·3 (0·2 to 0·4)	0·2 (0·1 to 0·3)	0·1 (0·1 to 0·2)	0·1 (0 to 0·1)	0·1 (0 to 0·1)	0 (0 to 0)	0 (0 to 0)	0 (0 to 0)	7·7
Diphtheria	0 (0 to 0)	0 (0 to 0)	0 (0 to 0)	0 (0 to 0)	0 (0 to 0)	0 (0 to 0)	0 (0 to 0)	0 (0 to 0)	0 (0 to 0)	0 (0 to 0)	0 (0 to 0)	0 (0 to 0)	0 (0 to 0)	0 (0 to 0)	0 (0 to 0)	0 (0 to 0)	0 (0 to 0)	0 (0 to 0)	0 (0 to 0)	0 (0 to 0)	0 (0 to 0)	0 (0 to 0)	0 (0 to 0)	0
Tetanus	0 (0 to 0)	0 (0 to 0)	0 (0 to 0)	0 (0 to 0·1)	0 (0 to 0·2)	0 (0 to 0·2)	0 (0 to 0·2)	0 (0 to 0·2)	0 (0 to 0·2)	0 (0 to 0·2)	0·1 (0 to 0·3)	0·1 (0 to 0·4)	0·1 (0 to 0·6)	0·2 (0 to 0·7)	0·2 (0·1 to 0·8)	0·1 (0 to 0·7)	0·1 (0 to 0·5)	0·1 (0 to 0·4)	0·1 (0 to 0·3)	0 (0 to 0·1)	0 (0 to 0)	0 (0 to 0)	0 (0 to 0)	1·3
Measles	0 (0 to 0)	0 (0 to 0)	0 (0 to 0)	0 (0 to 0)	0 (0 to 0)	0 (0 to 0)	0 (0 to 0)	0 (0 to 0)	0 (0 to 0)	0 (0 to 0)	0 (0 to 0)	0 (0 to 0)	0 (0 to 0)	0 (0 to 0)	0 (0 to 0)	0 (0 to 0)	0 (0 to 0)	0 (0 to 0)	0 (0 to 0)	0 (0 to 0)	0 (0 to 0)	0 (0 to 0)	0 (0 to 0)	0
Varicella and herpes zoster	0 (0 to 0)	0 (0 to 0·1)	0 (0 to 0·1)	0 (0 to 0·1)	0·1 (0 to 0·1)	0·1 (0 to 0·1)	0·1 (0 to 0·1)	0·1 (0 to 0·2)	0·1 (0 to 0·2)	0·1 (0 to 0·2)	0·1 (0 to 0·2)	0·1 (0 to 0·2)	0·1 (0·1 to 0·2)	0·1 (0·1 to 0·2)	0·1 (0·1 to 0·2)	0·1 (0·1 to 0·2)	0·1 (0 to 0·2)	0·1 (0 to 0·1)	0·1 (0 to 0·1)	0 (0 to 0)	0 (0 to 0)	0 (0 to 0)	0 (0 to 0)	1·6
Acute hepatitis	0 (0 to 0)	0 (0 to 0)	0 (0 to 0·1)	0·1 (0·1 to 0·2)	0·2 (0·1 to 0·4)	0·3 (0·2 to 0·5)	0·3 (0·2 to 0·5)	0·4 (0·2 to 0·5)	0·3 (0·2 to 0·5)	0·3 (0·2 to 0·4)	0·3 (0·2 to 0·4)	0·2 (0·1 to 0·3)	0·2 (0·1 to 0·3)	0·2 (0·1 to 0·3)	0·2 (0·1 to 0·2)	0·1 (0·1 to 0·2)	0·1 (0·1 to 0·1)	0·1 (0 to 0·1)	0 (0 to 0)	0 (0 to 0)	0 (0 to 0)	0 (0 to 0)	0 (0 to 0)	3·3
Acute hepatitis A	0 (0 to 0)	0 (0 to 0)	0 (0 to 0)	0·1 (0 to 0·1)	0·2 (0·1 to 0·3)	0·2 (0·1 to 0·4)	0·2 (0·1 to 0·4)	0·2 (0·1 to 0·4)	0·2 (0·1 to 0·3)	0·1 (0·1 to 0·2)	0·1 (0 to 0·2)	0·1 (0 to 0·1)	0·1 (0 to 0·1)	0·1 (0 to 0·1)	0 (0 to 0·1)	0 (0 to 0)	0 (0 to 0)	0 (0 to 0)	0 (0 to 0)	0 (0 to 0)	0 (0 to 0)	0 (0 to 0)	0 (0 to 0)	1·6
Acute hepatitis B	0 (0 to 0)	0 (0 to 0)	0 (0 to 0)	0 (0 to 0)	0 (0 to 0)	0 (0 to 0·1)	0 (0 to 0·1)	0·1 (0 to 0·2)	0·1 (0·1 to 0·2)	0·1 (0·1 to 0·2)	0·1 (0·1 to 0·2)	0·1 (0·1 to 0·2)	0·1 (0·1 to 0·2)	0·1 (0·1 to 0·2)	0·1 (0·1 to 0·2)	0·1 (0 to 0·1)	0·1 (0 to 0·1)	0 (0 to 0)	0 (0 to 0)	0 (0 to 0)	0 (0 to 0)	0 (0 to 0)	0 (0 to 0)	1·3
Acute hepatitis C	0 (0 to 0)	0 (0 to 0)	0 (0 to 0)	0 (0 to 0)	0 (0 to 0)	0 (0 to 0)	0 (0 to 0)	0 (0 to 0)	0 (0 to 0)	0 (0 to 0)	0 (0 to 0)	0 (0 to 0)	0 (0 to 0)	0 (0 to 0)	0 (0 to 0)	0 (0 to 0)	0 (0 to 0)	0 (0 to 0)	0 (0 to 0)	0 (0 to 0)	0 (0 to 0)	0 (0 to 0)	0 (0 to 0)	0·1
Acute hepatitis E	0 (0 to 0)	0 (0 to 0)	0 (0 to 0)	0 (0 to 0)	0 (0 to 0)	0 (0 to 0·1)	0 (0 to 0·1)	0 (0 to 0·1)	0 (0 to 0·1)	0 (0 to 0)	0 (0 to 0)	0 (0 to 0)	0 (0 to 0)	0 (0 to 0)	0 (0 to 0)	0 (0 to 0·1)	0 (0 to 0)	0 (0 to 0)	0 (0 to 0)	0 (0 to 0)	0 (0 to 0)	0 (0 to 0)	0 (0 to 0)	0·3
Other unspecified infectious diseases	0·1 (0·1 to 0·2)	0·2 (0·1 to 0·3)	0·9 (0·5 to 1·5)	1·5 (0·7 to 2·6)	1·3 (0·6 to 2·6)	0·8 (0·4 to 1·4)	1·1 (0·6 to 1·7)	1·5 (0·9 to 2·4)	2 (1·1 to 3·2)	1·8 (1·1 to 2·7)	2 (1·2 to 3·1)	2 (1·2 to 3·2)	2·7 (1·6 to 4·2)	2·6 (1·5 to 4·2)	2·3 (1·2 to 3·7)	2·2 (1·1 to 3·5)	1·5 (0·7 to 2·3)	1·1 (0·5 to 1·8)	0·6 (0·3 to 1·1)	0·4 (0·2 to 0·8)	0·3 (0·1 to 0·6)	0·1 (0 to 0·2)	0 (0 to 0)	29·1
Maternal and neonatal disorders	146 (99·3 to 209)	66·8 (44·8 to 97)	16·8 (10·7 to 25·2)	11·1 (8·4 to 14·3)	8·2 (5·9 to 10·9)	8·9 (6·4 to 11·8)	9·4 (6·7 to 12·8)	9·4 (6·7 to 12·4)	8·7 (6·2 to 11·5)	8 (5·8 to 10·5)	7·4 (5·2 to 9·8)	6·9 (5 to 9·1)	6·4 (4·7 to 8·5)	5·6 (4 to 7·5)	4 (2·9 to 5·4)	2·5 (1·8 to 3·3)	1·3 (0·9 to 1·8)	0·6 (0·4 to 0·8)	0·2 (0·2 to 0·3)	0 (0 to 0·1)	0 (0 to 0)	0 (0 to 0)	0 (0 to 0)	328
Maternal disorders	0 (0 to 0)	0 (0 to 0)	0 (0 to 0)	0 (0 to 0)	0 (0 to 0)	0 (0 to 0)	0 (0 to 0)	0 (0 to 0)	0 (0 to 0)	0 (0 to 0)	0 (0 to 0)	0 (0 to 0)	0 (0 to 0)	0 (0 to 0)	0 (0 to 0)	0 (0 to 0)	0 (0 to 0)	0 (0 to 0)	0 (0 to 0)	0 (0 to 0)	0 (0 to 0)	0 (0 to 0)	0 (0 to 0)	0
Maternal hemorrhage	0 (0 to 0)	0 (0 to 0)	0 (0 to 0)	0 (0 to 0)	0 (0 to 0)	0 (0 to 0)	0 (0 to 0)	0 (0 to 0)	0 (0 to 0)	0 (0 to 0)	0 (0 to 0)	0 (0 to 0)	0 (0 to 0)	0 (0 to 0)	0 (0 to 0)	0 (0 to 0)	0 (0 to 0)	0 (0 to 0)	0 (0 to 0)	0 (0 to 0)	0 (0 to 0)	0 (0 to 0)	0 (0 to 0)	0
Maternal sepsis and other maternal infections	0 (0 to 0)	0 (0 to 0)	0 (0 to 0)	0 (0 to 0)	0 (0 to 0)	0 (0 to 0)	0 (0 to 0)	0 (0 to 0)	0 (0 to 0)	0 (0 to 0)	0 (0 to 0)	0 (0 to 0)	0 (0 to 0)	0 (0 to 0)	0 (0 to 0)	0 (0 to 0)	0 (0 to 0)	0 (0 to 0)	0 (0 to 0)	0 (0 to 0)	0 (0 to 0)	0 (0 to 0)	0 (0 to 0)	0
Maternal hypertensive disorders	0 (0 to 0)	0 (0 to 0)	0 (0 to 0)	0 (0 to 0)	0 (0 to 0)	0 (0 to 0)	0 (0 to 0)	0 (0 to 0)	0 (0 to 0)	0 (0 to 0)	0 (0 to 0)	0 (0 to 0)	0 (0 to 0)	0 (0 to 0)	0 (0 to 0)	0 (0 to 0)	0 (0 to 0)	0 (0 to 0)	0 (0 to 0)	0 (0 to 0)	0 (0 to 0)	0 (0 to 0)	0 (0 to 0)	0
Maternal obstructed labor and uterine rupture	--	--	--	--	--	--	--	--	--	--	--	--	--	--	--	--	--	--	--	--	--	--	--	NA
Maternal abortion and miscarriage	--	--	--	--	--	--	--	--	--	--	--	--	--	--	--	--	--	--	--	--	--	--	--	NA
Ectopic pregnancy	--	--	--	--	--	--	--	--	--	--	--	--	--	--	--	--	--	--	--	--	--	--	--	NA
Indirect maternal deaths	--	--	--	--	--	--	--	--	--	--	--	--	--	--	--	--	--	--	--	--	--	--	--	NA
Late maternal deaths	--	--	--	--	--	--	--	--	--	--	--	--	--	--	--	--	--	--	--	--	--	--	--	NA
Maternal deaths aggravated by HIV/AIDS	--	--	--	--	--	--	--	--	--	--	--	--	--	--	--	--	--	--	--	--	--	--	--	NA
Neonatal disorders	146 (99·3 to 209)	66·8 (44·8 to 97)	16·8 (10·7 to 25·2)	11·1 (8·4 to 14·3)	8·2 (5·9 to 10·9)	8·9 (6·4 to 11·8)	9·4 (6·7 to 12·8)	9·4 (6·7 to 12·4)	8·7 (6·2 to 11·5)	8 (5·8 to 10·5)	7·4 (5·2 to 9·8)	6·9 (5 to 9·1)	6·4 (4·7 to 8·5)	5·6 (4 to 7·5)	4 (2·9 to 5·4)	2·5 (1·8 to 3·3)	1·3 (0·9 to 1·8)	0·6 (0·4 to 0·8)	0·2 (0·2 to 0·3)	0 (0 to 0·1)	0 (0 to 0)	0 (0 to 0)	0 (0 to 0)	328
Neonatal preterm birth	79·6 (51·9 to 117)	31·4 (19·8 to 47)	8·2 (4·9 to 12·9)	4·5 (3·2 to 5·9)	4·7 (3·3 to 6·5)	5·1 (3·5 to 6·9)	5·3 (3·6 to 7·4)	5·2 (3·5 to 7)	4·8 (3·2 to 6·6)	4·4 (3 to 6·1)	4 (2·7 to 5·4)	3·6 (2·6 to 4·9)	3·3 (2·3 to 4·5)	2·8 (2 to 3·8)	1·9 (1·3 to 2·6)	1·1 (0·8 to 1·4)	0·5 (0·4 to 0·7)	0·2 (0·2 to 0·3)	0·1 (0·1 to 0·1)	0 (0 to 0)	0 (0 to 0)	0 (0 to 0)	0 (0 to 0)	171
Neonatal encephalopathy due to birth asphyxia and trauma	26·7 (15·1 to 43·8)	7 (3·3 to 12·1)	2 (1·1 to 3·4)	1·6 (1 to 2·5)	1 (0·5 to 1·7)	1·1 (0·5 to 1·8)	1·1 (0·6 to 1·9)	1·2 (0·6 to 1·9)	1·1 (0·5 to 1·8)	1 (0·5 to 1·8)	0·9 (0·5 to 1·6)	0·9 (0·5 to 1·5)	0·9 (0·4 to 1·4)	0·8 (0·4 to 1·3)	0·6 (0·3 to 1·1)	0·4 (0·2 to 0·7)	0·2 (0·1 to 0·3)	0·1 (0 to 0·1)	0 (0 to 0)	0 (0 to 0)	0 (0 to 0)	0 (0 to 0)	0 (0 to 0)	48·5
Neonatal sepsis and other neonatal infections	17 (9·8 to 27·4)	15·9 (9·1 to 25·4)	3·6 (2 to 6)	1·9 (1·1 to 3)	2·3 (1·2 to 3·7)	2·5 (1·4 to 4·1)	2·8 (1·5 to 4·4)	2·8 (1·6 to 4·6)	2·6 (1·5 to 4·2)	2·5 (1·3 to 4·1)	2·3 (1·3 to 3·8)	2·2 (1·2 to 3·6)	2·1 (1·1 to 3·4)	1·9 (1 to 3·1)	1·4 (0·8 to 2·3)	0·9 (0·5 to 1·5)	0·5 (0·3 to 1)	0·3 (0·1 to 0·5)	0·1 (0 to 0·2)	0 (0 to 0)	0 (0 to 0)	0 (0 to 0)	0 (0 to 0)	65·8
Hemolytic disease and other neonatal jaundice	2·1 (1 to 3·8)	1·5 (0·6 to 2·9)	0·2 (0·1 to 0·5)	0·2 (0·1 to 0·3)	0·2 (0·1 to 0·2)	0·2 (0·1 to 0·2)	0·2 (0·1 to 0·2)	0·2 (0·1 to 0·2)	0·2 (0·1 to 0·2)	0·2 (0·1 to 0·2)	0·2 (0·1 to 0·2)	0·1 (0·1 to 0·2)	0·1 (0·1 to 0·2)	0·1 (0·1 to 0·2)	0·1 (0·1 to 0·1)	0·1 (0 to 0·1)	0 (0 to 0)	0 (0 to 0)	0 (0 to 0)	0 (0 to 0)	0 (0 to 0)	0 (0 to 0)	0 (0 to 0)	5·8
Other neonatal disorders	20·6 (11·9 to 33·5)	11·1 (6·6 to 17·4)	2·8 (1·7 to 4·6)	3 (2·2 to 3·8)	0 (0 to 0)	0 (0 to 0)	0 (0 to 0)	0 (0 to 0)	0 (0 to 0)	0 (0 to 0)	0 (0 to 0)	0 (0 to 0)	0 (0 to 0)	0 (0 to 0)	0 (0 to 0)	0 (0 to 0)	0 (0 to 0)	0 (0 to 0)	0 (0 to 0)	0 (0 to 0)	0 (0 to 0)	0 (0 to 0)	0 (0 to 0)	37·5
Nutritional deficiencies	0 (0 to 0·1)	0·5 (0·2 to 0·9)	8·1 (4·8 to 12·9)	18·9 (9·2 to 34·1)	16·6 (5·3 to 36·7)	6·7 (2 to 16·5)	4·6 (1·9 to 9·8)	4·2 (2·1 to 8·3)	4·3 (2·2 to 8·7)	4·3 (2·1 to 8·8)	4·4 (2·3 to 8·5)	4·5 (2·3 to 9·4)	5·1 (2·6 to 9·6)	6·1 (3·5 to 10·8)	6·7 (3·8 to 12)	6 (3·7 to 9·8)	4·3 (2·7 to 6·7)	4·1 (2·7 to 6·1)	3·4 (2·2 to 5·1)	3 (2 to 4·3)	1·7 (1·1 to 2·5)	0·8 (0·5 to 1·1)	0·3 (0·2 to 0·4)	119
Protein-energy malnutrition	0 (0 to 0)	0·1 (0 to 0·1)	5·1 (2·9 to 8·2)	6·1 (3·2 to 10·3)	1·5 (1 to 2·2)	0·7 (0·5 to 1)	0·9 (0·6 to 1·2)	1·3 (0·9 to 1·8)	1·3 (0·9 to 1·8)	1·2 (0·9 to 1·7)	1·6 (1·1 to 2·1)	1·5 (1 to 2)	1·8 (1·2 to 2·4)	2·4 (1·7 to 3·2)	2·6 (1·9 to 3·5)	3·1 (2·3 to 4·4)	2·6 (1·8 to 3·7)	2·9 (2 to 4·2)	2·5 (1·7 to 3·7)	2·4 (1·6 to 3·5)	1·4 (0·9 to 2)	0·7 (0·4 to 1)	0·3 (0·2 to 0·4)	43·9
Iodine deficiency	0 (0 to 0)	0 (0 to 0)	0 (0 to 0)	0 (0 to 0)	0 (0 to 0·1)	0·1 (0 to 0·1)	0·1 (0 to 0·2)	0·1 (0 to 0·2)	0·1 (0 to 0·3)	0·1 (0·1 to 0·3)	0·1 (0·1 to 0·2)	0·1 (0·1 to 0·2)	0·1 (0·1 to 0·2)	0·1 (0·1 to 0·2)	0·1 (0 to 0·2)	0·1 (0 to 0·1)	0 (0 to 0·1)	0 (0 to 0·1)	0 (0 to 0)	0 (0 to 0)	0 (0 to 0)	0 (0 to 0)	0 (0 to 0)	1·4
Vitamin A deficiency	0 (0 to 0)	0 (0 to 0)	0·1 (0 to 0·1)	0·3 (0·1 to 0·6)	0·4 (0·2 to 0·7)	0·2 (0·1 to 0·5)	0·1 (0 to 0·3)	0·1 (0 to 0·2)	0·1 (0 to 0·2)	0·1 (0 to 0·2)	0·1 (0 to 0·1)	0·1 (0 to 0·1)	0·1 (0 to 0·1)	0·1 (0 to 0·1)	0 (0 to 0·1)	0 (0 to 0·1)	0 (0 to 0)	0 (0 to 0)	0 (0 to 0)	0 (0 to 0)	0 (0 to 0)	0 (0 to 0)	0 (0 to 0)	1·8
Dietary iron deficiency	0 (0 to 0)	0·4 (0·2 to 0·8)	2·9 (0·8 to 6·5)	12·4 (3·5 to 27·6)	14·6 (3·6 to 34·7)	5·6 (1·1 to 15·3)	3·3 (0·7 to 8·5)	2·4 (0·5 to 6·5)	2·3 (0·5 to 6·5)	2·5 (0·5 to 7·2)	2·3 (0·5 to 6·3)	2·6 (0·5 to 7·4)	2·8 (0·6 to 7·2)	3·1 (0·8 to 7·8)	3·6 (1 to 8·7)	2·5 (0·6 to 6·1)	1·3 (0·3 to 3·6)	1 (0·2 to 2·6)	0·7 (0·2 to 1·8)	0·4 (0·1 to 1·1)	0·3 (0·1 to 0·7)	0·1 (0 to 0·3)	0 (0 to 0·1)	67·2
Other nutritional deficiencies	0 (0 to 0)	0 (0 to 0)	0 (0 to 0·1)	0·1 (0 to 0·1)	0·1 (0·1 to 0·2)	0·1 (0·1 to 0·2)	0·2 (0·1 to 0·4)	0·3 (0·2 to 0·5)	0·4 (0·2 to 0·6)	0·3 (0·1 to 0·4)	0·3 (0·2 to 0·5)	0·3 (0·2 to 0·5)	0·3 (0·2 to 0·4)	0·4 (0·2 to 0·6)	0·4 (0·2 to 0·6)	0·3 (0·2 to 0·4)	0·3 (0·2 to 0·4)	0·2 (0·1 to 0·3)	0·1 (0·1 to 0·2)	0·1 (0·1 to 0·2)	0 (0 to 0·1)	0 (0 to 0)	0 (0 to 0)	4·2
Non-communicable diseases	23·9 (14·3 to 38)	18 (10·9 to 27·8)	47·9 (32·1 to 68·4)	100 (76·2 to 130)	110 (85·4 to 139)	145 (109 to 190)	217 (166 to 278)	293 (235 to 361)	334 (271 to 408)	339 (274 to 413)	411 (346 to 490)	464 (387 to 553)	605 (504 to 716)	840 (706 to 982)	1,010 (857 to 1,160)	1,090 (940 to 1,260)	909 (789 to 1,040)	846 (741 to 966)	685 (608 to 770)	534 (481 to 589)	262 (239 to 285)	105 (97 to 113)	30·5 (28·2 to 32·8)	9,410
Neoplasms	0·4 (0·1 to 1·4)	0·6 (0·2 to 1·8)	1·9 (1·1 to 3)	15·7 (9·9 to 23·2)	14·7 (11·2 to 19·2)	9·4 (7·1 to 12·3)	11·9 (9 to 15·7)	20·3 (16·3 to 25)	22·6 (18·7 to 27)	21·3 (17·5 to 25·6)	30·9 (25·2 to 37·8)	39·1 (31·6 to 48)	66·5 (52·7 to 82·6)	125 (99·8 to 157)	184 (148 to 225)	240 (196 to 294)	203 (166 to 245)	207 (170 to 250)	181 (148 to 219)	136 (108 to 165)	62·7 (49·5 to 77)	22·7 (17·1 to 28·4)	5·6 (3·9 to 7·8)	1,620
Lip and oral cavity cancer	0 (0 to 0)	0 (0 to 0)	0 (0 to 0)	0 (0 to 0)	0 (0 to 0)	0 (0 to 0·1)	0·1 (0 to 0·1)	0·2 (0·1 to 0·3)	0·2 (0·1 to 0·4)	0·3 (0·2 to 0·5)	0·7 (0·4 to 1·1)	1·2 (0·7 to 1·9)	2·4 (1·5 to 3·8)	4·6 (3 to 6·6)	6 (4 to 8·4)	6·2 (4·3 to 8·5)	4·1 (2·7 to 5·8)	3·3 (2·2 to 4·6)	2·4 (1·7 to 3·3)	1·7 (1·1 to 2·4)	0·6 (0·4 to 0·8)	0·2 (0·1 to 0·3)	0·1 (0 to 0·1)	34·2
Nasopharynx cancer	0 (0 to 0)	0 (0 to 0)	0 (0 to 0)	0 (0 to 0)	0 (0 to 0·1)	0·1 (0 to 0·1)	0·1 (0·1 to 0·2)	0·2 (0·1 to 0·4)	0·2 (0·1 to 0·3)	0·1 (0·1 to 0·2)	0·2 (0·1 to 0·3)	0·4 (0·2 to 0·6)	0·6 (0·4 to 0·9)	1·1 (0·7 to 1·6)	1·5 (0·9 to 2·2)	1·2 (0·8 to 1·7)	0·8 (0·5 to 1·2)	0·6 (0·4 to 0·9)	0·3 (0·2 to 0·5)	0·2 (0·1 to 0·3)	0·1 (0·1 to 0·2)	0 (0 to 0·1)	0 (0 to 0)	7·9
Other pharynx cancer	0 (0 to 0)	0 (0 to 0)	0 (0 to 0)	0 (0 to 0)	0 (0 to 0)	0 (0 to 0)	0 (0 to 0)	0·1 (0·1 to 0·2)	0·1 (0·1 to 0·2)	0·1 (0·1 to 0·2)	0·4 (0·2 to 0·6)	0·7 (0·5 to 1·1)	1·9 (1·2 to 2·8)	3·4 (2·2 to 5)	4·7 (3 to 6·7)	4·6 (2·9 to 7·2)	3 (1·9 to 4·6)	2·2 (1·4 to 3·3)	1·3 (0·8 to 2)	0·8 (0·5 to 1·3)	0·3 (0·2 to 0·5)	0·1 (0·1 to 0·2)	0 (0 to 0·1)	24
Esophageal cancer	0 (0 to 0)	0 (0 to 0)	0 (0 to 0)	0 (0 to 0)	0 (0 to 0)	0 (0 to 0)	0 (0 to 0)	0·1 (0·1 to 0·1)	0·1 (0·1 to 0·2)	0·2 (0·1 to 0·3)	0·7 (0·5 to 1)	1·5 (0·9 to 2·4)	3·5 (2·3 to 5)	6·5 (4·6 to 9·3)	8·9 (6·2 to 11·8)	9·3 (6·5 to 13)	6·4 (4·6 to 8·9)	5·2 (3·7 to 7·2)	3·4 (2·4 to 4·7)	2·2 (1·5 to 3·1)	0·9 (0·6 to 1·2)	0·3 (0·2 to 0·4)	0 (0 to 0·1)	49·2
Stomach cancer	0 (0 to 0)	0 (0 to 0)	0 (0 to 0)	0 (0 to 0)	0 (0 to 0)	0 (0 to 0)	0·2 (0·1 to 0·3)	0·6 (0·4 to 0·9)	1·4 (1 to 2)	2·1 (1·4 to 3)	3·7 (2·5 to 5·4)	5·1 (3·5 to 7)	7·7 (5·3 to 10·6)	15 (10·5 to 21·1)	20·7 (14·5 to 27·8)	24·7 (18·1 to 33·5)	19·1 (14·1 to 24·9)	17·8 (13·4 to 23)	13·4 (10·4 to 16·9)	9·8 (7·4 to 12·7)	3·9 (3 to 5·1)	1·3 (0·9 to 1·7)	0·3 (0·2 to 0·4)	147
Colon and rectum cancer	0 (0 to 0)	0 (0 to 0)	0 (0 to 0)	0 (0 to 0)	0·1 (0·1 to 0·1)	0·1 (0 to 0·1)	0·3 (0·2 to 0·5)	1 (0·7 to 1·5)	1·6 (1·1 to 2·3)	2·1 (1·4 to 3·2)	3·5 (2·3 to 5·2)	4·2 (2·6 to 6·2)	6·6 (4·3 to 9·9)	11·9 (8·2 to 16·2)	17·6 (12·1 to 24·1)	22·6 (16·1 to 30·4)	17·5 (12·7 to 23·6)	16·1 (11·9 to 21·9)	12·8 (9·4 to 16·8)	9·8 (6·8 to 13·6)	3·9 (2·9 to 5·3)	1·3 (0·9 to 1·9)	0·3 (0·2 to 0·5)	133
Liver cancer	0 (0 to 0)	0 (0 to 0)	0 (0 to 0·1)	0·2 (0·1 to 0·2)	0·2 (0·1 to 0·2)	0·2 (0·1 to 0·3)	0·1 (0·1 to 0·2)	0·4 (0·2 to 0·6)	0·6 (0·4 to 1·1)	0·6 (0·3 to 1)	1·2 (0·6 to 1·9)	1·5 (0·8 to 2·5)	2·5 (1·3 to 4·2)	4·4 (2·7 to 6·7)	6·5 (4·1 to 9·8)	7·3 (4·9 to 10·3)	5·5 (3·8 to 7·8)	4·9 (3·3 to 6·7)	3·4 (2·5 to 4·6)	2 (1·5 to 2·7)	0·6 (0·4 to 0·8)	0·2 (0·1 to 0·2)	0 (0 to 0·1)	42·4
Liver cancer due to hepatitis B	0 (0 to 0)	0 (0 to 0)	0 (0 to 0)	0 (0 to 0)	0 (0 to 0)	0·1 (0 to 0·1)	0·1 (0 to 0·1)	0·3 (0·1 to 0·4)	0·4 (0·2 to 0·7)	0·4 (0·2 to 0·6)	0·7 (0·3 to 1·2)	0·8 (0·4 to 1·5)	1·2 (0·6 to 2·2)	1·8 (0·9 to 3·3)	2·5 (1·3 to 4·5)	2·5 (1·2 to 4·2)	1·5 (0·7 to 2·6)	1·1 (0·5 to 1·9)	0·7 (0·4 to 1·2)	0·4 (0·2 to 0·7)	0·1 (0·1 to 0·2)	0 (0 to 0)	0 (0 to 0)	14·6
Liver cancer due to hepatitis C	0 (0 to 0)	0 (0 to 0)	0 (0 to 0)	0 (0 to 0)	0 (0 to 0)	0 (0 to 0)	0 (0 to 0)	0 (0 to 0)	0 (0 to 0)	0 (0 to 0)	0 (0 to 0·1)	0·1 (0 to 0·2)	0·2 (0·1 to 0·4)	0·4 (0·2 to 0·9)	0·8 (0·3 to 1·7)	1 (0·4 to 2)	0·8 (0·4 to 1·4)	0·8 (0·4 to 1·4)	0·7 (0·3 to 1·2)	0·5 (0·3 to 0·9)	0·2 (0·1 to 0·3)	0·1 (0 to 0·1)	0 (0 to 0)	5·8
Liver cancer due to alcohol use	0 (0 to 0)	0 (0 to 0)	0 (0 to 0)	0 (0 to 0)	0 (0 to 0)	0 (0 to 0)	0 (0 to 0)	0 (0 to 0·1)	0·1 (0 to 0·2)	0·1 (0 to 0·2)	0·3 (0·1 to 0·6)	0·5 (0·2 to 0·9)	0·9 (0·3 to 1·7)	1·6 (0·7 to 2·9)	2·6 (1·2 to 4·3)	3·2 (1·6 to 5·2)	2·7 (1·6 to 4·3)	2·5 (1·4 to 3·8)	1·6 (1 to 2·4)	0·9 (0·5 to 1·3)	0·2 (0·1 to 0·3)	0·1 (0 to 0·1)	0 (0 to 0)	17·2
Liver cancer due to NASH	0 (0 to 0)	0 (0 to 0)	0 (0 to 0)	0 (0 to 0)	0 (0 to 0)	0 (0 to 0)	0 (0 to 0)	0 (0 to 0·1)	0 (0 to 0·1)	0 (0 to 0·1)	0·1 (0 to 0·1)	0·1 (0 to 0·1)	0·1 (0 to 0·3)	0·2 (0·1 to 0·5)	0·4 (0·2 to 0·7)	0·4 (0·2 to 0·8)	0·3 (0·2 to 0·6)	0·3 (0·2 to 0·6)	0·3 (0·2 to 0·5)	0·2 (0·1 to 0·4)	0·1 (0 to 0·1)	0 (0 to 0)	0 (0 to 0)	2·6
Liver cancer due to other causes	0 (0 to 0)	0 (0 to 0)	0 (0 to 0·1)	0·2 (0·1 to 0·2)	0·2 (0·1 to 0·2)	0·2 (0·1 to 0·2)	0 (0 to 0·1)	0·1 (0 to 0·1)	0·1 (0 to 0·1)	0 (0 to 0·1)	0·1 (0 to 0·1)	0·1 (0 to 0·2)	0·1 (0 to 0·2)	0·2 (0·1 to 0·4)	0·2 (0·1 to 0·5)	0·2 (0·1 to 0·5)	0·2 (0·1 to 0·3)	0·1 (0 to 0·3)	0·1 (0 to 0·2)	0·1 (0 to 0·1)	0 (0 to 0)	0 (0 to 0)	0 (0 to 0)	2·2
Gallbladder and biliary tract cancer	0 (0 to 0)	0 (0 to 0)	0 (0 to 0)	0 (0 to 0)	0 (0 to 0)	0 (0 to 0)	0 (0 to 0)	0·1 (0 to 0·1)	0·1 (0·1 to 0·2)	0·1 (0·1 to 0·2)	0·2 (0·2 to 0·5)	0·4 (0·2 to 0·8)	0·6 (0·4 to 1·3)	1·2 (0·8 to 2·2)	1·8 (1·1 to 2·9)	2·2 (1·4 to 3·6)	1·8 (1·1 to 3)	1·6 (1 to 2·7)	1·2 (0·8 to 2)	0·8 (0·5 to 1·3)	0·3 (0·2 to 0·6)	0·1 (0·1 to 0·2)	0 (0 to 0)	12·6
Pancreatic cancer	0 (0 to 0)	0 (0 to 0)	0 (0 to 0)	0 (0 to 0)	0 (0 to 0)	0 (0 to 0)	0·1 (0·1 to 0·2)	0·2 (0·1 to 0·3)	0·3 (0·2 to 0·6)	0·9 (0·6 to 1·2)	1·7 (1·2 to 2·3)	2·8 (1·9 to 3·8)	4·8 (3·3 to 6·8)	8·7 (5·9 to 12·5)	12·3 (8·3 to 17·1)	14 (9·6 to 19·7)	10·1 (7 to 13·9)	9·2 (6·7 to 12·2)	6·2 (4·4 to 8·5)	4·3 (3 to 6)	1·6 (1·2 to 2·2)	0·5 (0·3 to 0·7)	0·1 (0·1 to 0·2)	78
Larynx cancer	0 (0 to 0)	0 (0 to 0)	0 (0 to 0)	0 (0 to 0)	0 (0 to 0)	0 (0 to 0)	0 (0 to 0)	0·1 (0·1 to 0·2)	0·1 (0·1 to 0·2)	0·1 (0·1 to 0·2)	0·4 (0·2 to 0·7)	0·9 (0·5 to 1·6)	2·2 (1·4 to 3·5)	4·9 (3·2 to 7·2)	6·7 (4·6 to 9·2)	7·6 (5·3 to 11)	5·4 (3·7 to 7·5)	4·3 (3 to 6·1)	2·8 (2 to 4)	1·8 (1·3 to 2·5)	0·6 (0·4 to 0·9)	0·2 (0·1 to 0·3)	0 (0 to 0·1)	38·4
Tracheal, bronchus, and lung cancer	0 (0 to 0)	0 (0 to 0)	0 (0 to 0)	0 (0 to 0)	0 (0 to 0)	0 (0 to 0)	0·2 (0·1 to 0·3)	0·4 (0·3 to 0·6)	0·5 (0·4 to 0·8)	0·9 (0·6 to 1·5)	2 (1·2 to 3·1)	3·4 (2·2 to 5·1)	7·6 (5·2 to 10·3)	14·9 (10·8 to 20·1)	22·1 (15·7 to 29·6)	26·4 (19·3 to 34·4)	19·5 (14·8 to 25·3)	17·1 (13·2 to 21·8)	11·5 (8·7 to 15)	7·6 (5·5 to 10·5)	2·8 (2 to 3·9)	0·8 (0·6 to 1·1)	0·2 (0·1 to 0·2)	138
Malignant skin melanoma	0 (0 to 0)	0 (0 to 0)	0 (0 to 0·1)	0·1 (0 to 0·4)	0 (0 to 0·1)	0 (0 to 0·1)	0·1 (0 to 0·1)	0·3 (0·2 to 0·4)	0·5 (0·3 to 0·8)	0·5 (0·3 to 0·8)	0·8 (0·5 to 1·3)	0·9 (0·5 to 1·5)	1·1 (0·6 to 1·7)	1·6 (0·9 to 2·6)	2 (1·1 to 3·2)	1·7 (1 to 2·7)	1·2 (0·7 to 1·8)	1·1 (0·6 to 1·7)	0·8 (0·5 to 1·3)	0·7 (0·4 to 1·1)	0·3 (0·2 to 0·5)	0·1 (0·1 to 0·1)	0 (0 to 0·1)	13·8
Non-melanoma skin cancer	0 (0 to 0)	0 (0 to 0)	0 (0 to 0)	0 (0 to 0)	0 (0 to 0)	0 (0 to 0)	0 (0 to 0)	0·1 (0 to 0·1)	0·2 (0·1 to 0·4)	0·3 (0·1 to 0·4)	0·3 (0·2 to 0·5)	0·4 (0·3 to 0·7)	0·6 (0·4 to 1)	1·1 (0·7 to 1·6)	1·4 (0·8 to 2·1)	1·7 (1 to 2·5)	1·5 (0·9 to 2·1)	1·4 (0·8 to 2)	1·2 (0·7 to 1·8)	1·1 (0·5 to 1·6)	0·6 (0·3 to 0·9)	0·3 (0·1 to 0·5)	0·1 (0 to 0·2)	12·6
Non-melanoma skin cancer (squamous-cell carcinoma)	0 (0 to 0)	0 (0 to 0)	0 (0 to 0)	0 (0 to 0)	0 (0 to 0)	0 (0 to 0)	0 (0 to 0)	0·1 (0 to 0·1)	0·2 (0·1 to 0·4)	0·3 (0·1 to 0·4)	0·3 (0·2 to 0·5)	0·4 (0·3 to 0·7)	0·6 (0·4 to 1)	1·1 (0·7 to 1·6)	1·4 (0·8 to 2·1)	1·7 (1 to 2·5)	1·5 (0·9 to 2·1)	1·4 (0·8 to 2)	1·2 (0·7 to 1·8)	1·1 (0·5 to 1·6)	0·6 (0·3 to 0·9)	0·3 (0·1 to 0·5)	0·1 (0 to 0·2)	12·6
Non-melanoma skin cancer (basal-cell carcinoma)	0 (0 to 0)	0 (0 to 0)	0 (0 to 0)	0 (0 to 0)	0 (0 to 0)	0 (0 to 0)	0 (0 to 0)	0 (0 to 0)	0 (0 to 0)	0 (0 to 0)	0 (0 to 0)	0 (0 to 0)	0 (0 to 0)	0 (0 to 0)	0 (0 to 0)	0 (0 to 0)	0 (0 to 0)	0 (0 to 0)	0 (0 to 0)	0 (0 to 0)	0 (0 to 0)	0 (0 to 0)	0 (0 to 0)	0
Breast cancer	0 (0 to 0)	0 (0 to 0)	0 (0 to 0)	0 (0 to 0)	0 (0 to 0)	0 (0 to 0)	0 (0 to 0)	0 (0 to 0)	0 (0 to 0·1)	0·1 (0 to 0·1)	0·1 (0 to 0·2)	0·1 (0 to 0·2)	0·3 (0·1 to 0·5)	0·4 (0·2 to 0·8)	0·6 (0·3 to 0·9)	0·6 (0·3 to 1·1)	0·6 (0·3 to 0·9)	0·4 (0·2 to 0·8)	0·3 (0·2 to 0·6)	0·3 (0·1 to 0·5)	0·2 (0·1 to 0·4)	0·1 (0 to 0·2)	0 (0 to 0)	4·1
Cervical cancer	--	--	--	--	--	--	--	--	--	--	--	--	--	--	--	--	--	--	--	--	--	--	--	NA
Uterine cancer	--	--	--	--	--	--	--	--	--	--	--	--	--	--	--	--	--	--	--	--	--	--	--	NA
Ovarian cancer	--	--	--	--	--	--	--	--	--	--	--	--	--	--	--	--	--	--	--	--	--	--	--	NA
Prostate cancer	0 (0 to 0)	0 (0 to 0)	0 (0 to 0)	0 (0 to 0)	0 (0 to 0)	0 (0 to 0)	0 (0 to 0)	0·2 (0·2 to 0·3)	0·5 (0·4 to 0·7)	0·5 (0·4 to 0·7)	0·7 (0·5 to 1)	1·4 (0·9 to 2·2)	4·9 (3·1 to 7·6)	15·7 (10·4 to 23·3)	33·9 (21·5 to 51·1)	69·3 (47·7 to 98·4)	75·4 (53·1 to 107)	94·7 (67·1 to 130)	99·9 (71·9 to 132)	80·2 (54·9 to 106)	40·6 (28·4 to 53·1)	15·4 (10·3 to 20·6)	4 (2·4 to 5·9)	537
Testicular cancer	0 (0 to 0·2)	0 (0 to 0·1)	0 (0 to 0·1)	0·1 (0 to 0·3)	0·1 (0·1 to 0·2)	0·1 (0·1 to 0·2)	0·4 (0·2 to 0·7)	0·8 (0·5 to 1·3)	1 (0·6 to 1·6)	0·5 (0·3 to 0·8)	0·5 (0·3 to 0·8)	0·3 (0·2 to 0·5)	0·2 (0·1 to 0·3)	0·2 (0·1 to 0·4)	0·2 (0·1 to 0·3)	0·2 (0·1 to 0·3)	0·1 (0·1 to 0·3)	0·1 (0·1 to 0·2)	0·1 (0·1 to 0·2)	0·1 (0 to 0·1)	0 (0 to 0·1)	0 (0 to 0)	0 (0 to 0)	5·3
Kidney cancer	0·1 (0 to 0·5)	0·1 (0 to 0·4)	0·1 (0·1 to 0·2)	1·1 (0·6 to 1·7)	0·8 (0·5 to 1·2)	0·2 (0·1 to 0·4)	0·3 (0·2 to 0·5)	0·6 (0·4 to 1·1)	0·9 (0·5 to 1·3)	0·7 (0·4 to 1·1)	0·8 (0·5 to 1·2)	1·1 (0·7 to 1·6)	1·7 (1·1 to 2·6)	3·4 (2·1 to 5·2)	4·5 (2·9 to 6·7)	4·9 (3·2 to 7·2)	3·6 (2·4 to 5)	2·9 (2 to 4·2)	1·9 (1·4 to 2·6)	1·2 (0·8 to 1·6)	0·4 (0·2 to 0·5)	0·1 (0·1 to 0·2)	0 (0 to 0)	31·2
Bladder cancer	0 (0 to 0)	0 (0 to 0)	0 (0 to 0)	0 (0 to 0)	0 (0 to 0)	0 (0 to 0)	0 (0 to 0)	0·1 (0 to 0·1)	0·1 (0 to 0·1)	0·1 (0·1 to 0·2)	0·2 (0·2 to 0·3)	0·4 (0·2 to 0·6)	0·7 (0·5 to 1)	1·7 (1·1 to 2·4)	2·9 (1·9 to 4)	4·5 (3·1 to 6·4)	4 (2·7 to 5·5)	4·4 (3·1 to 6)	3·7 (2·6 to 5)	2·8 (2 to 3·8)	1·3 (0·9 to 1·8)	0·5 (0·3 to 0·7)	0·1 (0·1 to 0·2)	27·4
Brain and central nervous system cancer	0·1 (0 to 0·7)	0·1 (0 to 0·8)	0·3 (0·1 to 0·5)	1·9 (0·7 to 3·8)	2·3 (0·9 to 3·8)	1·5 (0·7 to 2·6)	1·1 (0·5 to 2)	1·5 (0·6 to 2·4)	1·7 (0·7 to 2·6)	1·6 (0·7 to 2·5)	2·1 (1 to 3·3)	2·2 (1 to 3·3)	2·5 (1·1 to 3·9)	3·4 (1·6 to 5·5)	3·9 (1·7 to 6·1)	3·5 (1·5 to 5·3)	2·4 (1 to 3·7)	1·7 (0·7 to 2·6)	0·9 (0·4 to 1·5)	0·5 (0·2 to 0·8)	0·1 (0·1 to 0·2)	0 (0 to 0·1)	0 (0 to 0)	35·3
Thyroid cancer	0 (0 to 0)	0 (0 to 0)	0 (0 to 0)	0 (0 to 0)	0 (0 to 0)	0 (0 to 0)	0 (0 to 0)	0·1 (0 to 0·1)	0·1 (0·1 to 0·2)	0·1 (0·1 to 0·2)	0·2 (0·1 to 0·3)	0·2 (0·1 to 0·3)	0·4 (0·2 to 0·6)	0·6 (0·4 to 0·9)	0·8 (0·5 to 1·1)	0·7 (0·5 to 1·1)	0·5 (0·3 to 0·7)	0·4 (0·3 to 0·6)	0·3 (0·2 to 0·4)	0·2 (0·1 to 0·3)	0·1 (0·1 to 0·1)	0 (0 to 0)	0 (0 to 0)	4·8
Mesothelioma	0 (0 to 0)	0 (0 to 0)	0 (0 to 0)	0 (0 to 0)	0 (0 to 0)	0 (0 to 0)	0 (0 to 0)	0 (0 to 0)	0 (0 to 0·1)	0 (0 to 0·1)	0·1 (0 to 0·1)	0·1 (0·1 to 0·1)	0·1 (0·1 to 0·2)	0·2 (0·2 to 0·3)	0·3 (0·2 to 0·4)	0·2 (0·1 to 0·2)	0·1 (0·1 to 0·1)	0·1 (0·1 to 0·1)	0·1 (0·1 to 0·2)	0·1 (0·1 to 0·1)	0 (0 to 0)	0 (0 to 0)	0 (0 to 0)	1·5
Hodgkin lymphoma	0 (0 to 0)	0 (0 to 0)	0 (0 to 0)	0 (0 to 0)	0 (0 to 0·1)	0 (0 to 0·1)	0·2 (0·1 to 0·2)	0·4 (0·2 to 0·6)	0·4 (0·3 to 0·6)	0·3 (0·2 to 0·4)	0·3 (0·2 to 0·4)	0·2 (0·1 to 0·3)	0·2 (0·1 to 0·3)	0·3 (0·2 to 0·4)	0·3 (0·2 to 0·4)	0·3 (0·2 to 0·4)	0·2 (0·1 to 0·3)	0·1 (0·1 to 0·2)	0·1 (0·1 to 0·1)	0·1 (0 to 0·1)	0 (0 to 0)	0 (0 to 0)	0 (0 to 0)	3·2
Non-Hodgkin lymphoma	0 (0 to 0)	0 (0 to 0)	0 (0 to 0)	1·3 (0·8 to 2·1)	1·8 (1·1 to 2·8)	1·2 (0·8 to 1·9)	1·9 (1·3 to 2·8)	3·7 (2·5 to 5·2)	3·9 (2·6 to 5·7)	3·1 (2 to 4·6)	3·5 (2·4 to 5·2)	3·1 (2·1 to 4·5)	4 (2·7 to 5·8)	5·6 (3·8 to 8·3)	7 (4·7 to 9·9)	7 (4·7 to 10)	4·9 (3·4 to 6·8)	4·2 (2·9 to 5·8)	2·8 (1·9 to 3·7)	1·6 (1·1 to 2·4)	0·5 (0·4 to 0·7)	0·1 (0·1 to 0·2)	0 (0 to 0)	61·5
Multiple myeloma	0 (0 to 0)	0 (0 to 0)	0 (0 to 0)	0 (0 to 0)	0 (0 to 0)	0 (0 to 0)	0 (0 to 0)	0·2 (0·1 to 0·2)	0·2 (0·1 to 0·2)	0·2 (0·1 to 0·3)	0·5 (0·3 to 0·7)	1 (0·7 to 1·6)	2·2 (1·5 to 3·2)	4·4 (3·1 to 6·4)	6·1 (4·2 to 8·7)	7·2 (5 to 10·1)	6·2 (4·4 to 8·3)	5 (3·6 to 7)	3·5 (2·5 to 4·8)	2·2 (1·5 to 3·1)	0·7 (0·5 to 1)	0·2 (0·1 to 0·2)	0 (0 to 0)	39·7
Leukemia	0·1 (0 to 0·5)	0·2 (0 to 1·1)	0·5 (0·2 to 0·9)	3·7 (1·9 to 6·3)	5 (3 to 7·9)	3·6 (2·2 to 5·6)	4·1 (2·5 to 6·5)	5·4 (3·4 to 8·1)	4·4 (3 to 6·5)	2·9 (1·9 to 4)	3·2 (2·2 to 4·5)	2·8 (1·8 to 4·1)	3·2 (2·2 to 4·7)	4·3 (2·9 to 6·2)	5 (3·2 to 7·2)	5·3 (3·4 to 7·7)	4·4 (2·9 to 6·4)	4 (2·7 to 5·8)	3 (2·1 to 4·3)	2·2 (1·4 to 3·2)	0·9 (0·6 to 1·4)	0·3 (0·2 to 0·4)	0·1 (0 to 0·1)	68·8
Acute lymphoid leukemia	0 (0 to 0·1)	0 (0 to 0·1)	0·1 (0 to 0·2)	1·2 (0·5 to 2·1)	1·8 (0·9 to 3·3)	1 (0·5 to 1·8)	1·2 (0·6 to 2·2)	0·9 (0·5 to 1·7)	0·4 (0·2 to 0·8)	0·3 (0·1 to 0·4)	0·2 (0·1 to 0·4)	0·2 (0·1 to 0·3)	0·1 (0·1 to 0·2)	0·2 (0·1 to 0·3)	0·2 (0·1 to 0·3)	0·1 (0·1 to 0·2)	0·1 (0·1 to 0·2)	0·1 (0·1 to 0·2)	0·1 (0 to 0·1)	0 (0 to 0·1)	0 (0 to 0)	0 (0 to 0)	0 (0 to 0)	8·4
Chronic lymphoid leukemia	0 (0 to 0)	0 (0 to 0)	0 (0 to 0)	0 (0 to 0)	0 (0 to 0)	0 (0 to 0)	0 (0 to 0)	0 (0 to 0·1)	0 (0 to 0·1)	0 (0 to 0)	0 (0 to 0)	0 (0 to 0·1)	0 (0 to 0·1)	0·1 (0 to 0·2)	0·2 (0·1 to 0·4)	0·3 (0·1 to 0·5)	0·3 (0·1 to 0·4)	0·2 (0·1 to 0·4)	0·2 (0·1 to 0·3)	0·1 (0 to 0·2)	0·1 (0 to 0·1)	0 (0 to 0)	0 (0 to 0)	1·7
Acute myeloid leukemia	0 (0 to 0·2)	0·1 (0 to 0·5)	0·2 (0·1 to 0·4)	1·5 (0·7 to 2·7)	1·5 (0·7 to 2·7)	1·4 (0·7 to 2·4)	1·7 (0·9 to 3)	2 (1·2 to 3·2)	1·9 (1·1 to 2·9)	1·1 (0·6 to 1·8)	1·2 (0·7 to 1·9)	0·9 (0·5 to 1·5)	1·1 (0·6 to 1·7)	1·4 (0·7 to 2·2)	1·4 (0·8 to 2·3)	1·3 (0·7 to 2·2)	1·1 (0·6 to 1·9)	0·9 (0·5 to 1·5)	0·6 (0·3 to 1)	0·3 (0·2 to 0·6)	0·1 (0·1 to 0·2)	0 (0 to 0·1)	0 (0 to 0)	21·7

Chronic myeloid leukemia	0 (0 to 0)	[illegible]	7·3
Other leukemia	0·1 (0 to 0·3)	[illegible]	29·8
Other malignant neoplasms	0·1 (0 to 0·3)	[illegible]	67·8
Other neoplasms	0 (0 to 0)	[illegible]	3·7
Myelodysplastic, myeloproliferative, and other hematopoietic neo	0 (0 to 0)	[illegible]	3·7
Benign and in situ intestinal neoplasms	0 (0 to 0)	[illegible]	0
Benign and in situ cervical and uterine neoplasms	0 (0 to 0)	[illegible]	0
Other benign and in situ neoplasms	0 (0 to 0)	[illegible]	0
Cardiovascular diseases	0·5 (0·3 to 0·8)	[illegible]	2,060
Rheumatic heart disease	0 (0 to 0)	[illegible]	27·2
Ischemic heart disease	0 (0 to 0)	[illegible]	810
Stroke	0·1 (0·1 to 0·2)	[illegible]	631
Ischemic stroke	0 (0 to 0)	[illegible]	203
Intracerebral hemorrhage	0 (0 to 0)	[illegible]	361
Subarachnoid hemorrhage	0·1 (0·1 to 0·2)	[illegible]	66·8
Hypertensive heart disease	0 (0 to 0)	[illegible]	242
Non-rheumatic valvular heart disease	0 (0 to 0)	[illegible]	22·6
Non-rheumatic calcific aortic valve disease	0 (0 to 0)	[illegible]	10·5
Non-rheumatic degenerative mitral valve disease	0 (0 to 0)	[illegible]	9·6
Other non-rheumatic valve diseases	0 (0 to 0)	[illegible]	2·4
Cardiomyopathy and myocarditis	0·1 (0·1 to 0·2)	[illegible]	94·8
Myocarditis	0 (0 to 0·1)	[illegible]	10·2
Alcoholic cardiomyopathy	0 (0 to 0)	[illegible]	14·8
Other cardiomyopathy	0·1 (0 to 0·2)	[illegible]	69·7
Atrial fibrillation and flutter	0 (0 to 0)	[illegible]	44·2
Aortic aneurysm	--	[illegible]	NA
Endocarditis	0 (0 to 0·1)	[illegible]	23·7
Other cardiovascular and circulatory diseases	0·2 (0·1 to 0·4)	[illegible]	120
Chronic respiratory diseases	0 (0 to 0)	[illegible]	222
Chronic obstructive pulmonary disease	0 (0 to 0)	[illegible]	97·6
Pneumoconiosis	0 (0 to 0)	[illegible]	1·3
Silicosis	0 (0 to 0)	[illegible]	0
Asbestosis	0 (0 to 0)	[illegible]	0·2
Coal workers pneumoconiosis	0 (0 to 0)	[illegible]	0
Other pneumoconiosis	0 (0 to 0)	[illegible]	1·1
Asthma	0 (0 to 0)	[illegible]	99·5
Interstitial lung disease and pulmonary sarcoidosis	0 (0 to 0)	[illegible]	5
Other chronic respiratory diseases	0 (0 to 0)	[illegible]	18·6
Digestive diseases	0·3 (0·2 to 0·5)	[illegible]	495
Cirrhosis and other chronic liver diseases	0 (0 to 0)	[illegible]	230
Cirrhosis and other chronic liver diseases due to hepatitis B	0 (0 to 0)	[illegible]	17·3
Cirrhosis and other chronic liver diseases due to hepatitis C	0 (0 to 0)	[illegible]	46·1
Cirrhosis and other chronic liver diseases due to alcohol use	0 (0 to 0)	[illegible]	109
Cirrhosis and other chronic liver diseases due to NAFLD	0 (0 to 0)	[illegible]	31·3
Cirrhosis and other chronic liver diseases due to other causes	0 (0 to 0)	[illegible]	26·4
Upper digestive system diseases	0 (0 to 0)	[illegible]	104
Peptic ulcer disease	0 (0 to 0)	[illegible]	22·3
Gastritis and duodenitis	0 (0 to 0)	[illegible]	22·4
Gastroesophageal reflux disease	0 (0 to 0)	[illegible]	59·2
Appendicitis	0 (0 to 0)	[illegible]	10·3
Paralytic ileus and intestinal obstruction	0·3 (0·2 to 0·4)	[illegible]	28·9
Inguinal, femoral, and abdominal hernia	0·1 (0 to 0·1)	[illegible]	41
Inflammatory bowel disease	0 (0 to 0)	[illegible]	13·5
Vascular intestinal disorders	0 (0 to 0)	[illegible]	4·6
Gallbladder and biliary diseases	0 (0 to 0)	[illegible]	15·7
Pancreatitis	0 (0 to 0)	[illegible]	22·7
Other digestive diseases	0 (0 to 0)	[illegible]	24·2
Neurological disorders	0 (0 to 0)	[illegible]	598
Alzheimer's disease and other dementias	0 (0 to 0)	[illegible]	107
Parkinson's disease	0 (0 to 0)	[illegible]	52·3
Idiopathic epilepsy	0 (0 to 0)	[illegible]	200
Multiple sclerosis	0 (0 to 0)	[illegible]	2·2
Motor neuron disease	0 (0 to 0)	[illegible]	8·5
Headache disorders	0 (0 to 0)	[illegible]	183
Migraine	0 (0 to 0)	[illegible]	162
Tension-type headache	0 (0 to 0)	[illegible]	21·5
Other neurological disorders	0 (0 to 0)	[illegible]	44·8
Mental disorders	0 (0 to 0)	[illegible]	670
Schizophrenia	0 (0 to 0)	[illegible]	96·7
Depressive disorders	0 (0 to 0)	[illegible]	167
Major depressive disorder	0 (0 to 0)	[illegible]	127
Dysthymia	0 (0 to 0)	[illegible]	40·1
Bipolar disorder	0 (0 to 0)	[illegible]	91·9
Anxiety disorders	0 (0 to 0)	[illegible]	144
Eating disorders	0 (0 to 0)	[illegible]	15·2
Anorexia nervosa	0 (0 to 0)	[illegible]	3·1
Bulimia nervosa	0 (0 to 0)	[illegible]	12·2
Autism spectrum disorders	0 (0 to 0)	[illegible]	33·6
Attention-deficit/hyperactivity disorder	0 (0 to 0)	[illegible]	22·1
Conduct disorder	0 (0 to 0)	[illegible]	31·5
Idiopathic developmental intellectual disability	0 (0 to 0)	[illegible]	4·3
Other mental disorders	0 (0 to 0)	[illegible]	63·6
Substance use disorders	0 (0 to 0)	[illegible]	400
Alcohol use disorders	0 (0 to 0)	[illegible]	329
Drug use disorders	0 (0 to 0)	[illegible]	70·8
Opioid use disorders	0 (0 to 0)	[illegible]	25·9
Cocaine use disorders	0 (0 to 0)	[illegible]	17·5
Amphetamine use disorders	0 (0 to 0)	[illegible]	5·3
Cannabis use disorders	0 (0 to 0)	[illegible]	9·6
Other drug use disorders	0 (0 to 0)	[illegible]	12·5
Diabetes and kidney diseases	0 (0 to 0)	[illegible]	1,510
Diabetes mellitus	0 (0 to 0)	[illegible]	973
Diabetes mellitus type 1	0 (0 to 0)	[illegible]	60
Diabetes mellitus type 2	0 (0 to 0)	[illegible]	913
Chronic kidney disease	0 (0 to 0)	[illegible]	537
Chronic kidney disease due to diabetes mellitus type 1	0 (0 to 0)	[illegible]	39
Chronic kidney disease due to diabetes mellitus type 2	0 (0 to 0)	[illegible]	128
Chronic kidney disease due to hypertension	0 (0 to 0)	[illegible]	113
Chronic kidney disease due to glomerulonephritis	0 (0 to 0)	[illegible]	124
Chronic kidney disease due to other and unspecified causes	0 (0 to 0)	[illegible]	133
Acute glomerulonephritis	0 (0 to 0)	[illegible]	0·1
Skin and subcutaneous diseases	0 (0 to 0)	[illegible]	270
Dermatitis	0 (0 to 0)	[illegible]	41·8
Atopic dermatitis	0 (0 to 0)	[illegible]	28
Contact dermatitis	0 (0 to 0)	[illegible]	12·3
Seborrhoeic dermatitis	0 (0 to 0)	[illegible]	1·6
Psoriasis	0 (0 to 0)	[illegible]	14·2
Bacterial skin diseases	0 (0 to 0)	[illegible]	29·1
Cellulitis	0 (0 to 0)	[illegible]	7·9
Pyoderma	0 (0 to 0)	[illegible]	21·2
Scabies	0 (0 to 0)	[illegible]	34
Fungal skin diseases	0 (0 to 0)	[illegible]	20·1
Viral skin diseases	0 (0 to 0)	[illegible]	17·4
Acne vulgaris	0 (0 to 0)	[illegible]	21·7
Alopecia areata	0 (0 to 0)	[illegible]	2·3
Pruritus	0 (0 to 0)	[illegible]	3·5
Urticaria	0 (0 to 0)	[illegible]	16·2
Decubitus ulcer	0 (0 to 0)	[illegible]	42·7
Other skin and subcutaneous diseases	0 (0 to 0)	[illegible]	27·3
Sense organ diseases	0 (0 to 0)	[illegible]	347
Blindness and vision loss	0 (0 to 0)	[illegible]	89·6
Glaucoma	0 (0 to 0)	[illegible]	4·8
Cataract	0 (0 to 0)	[illegible]	12·8
Age-related macular degeneration	0 (0 to 0)	[illegible]	0·6
Refraction disorders	0 (0 to 0)	[illegible]	22·5
Near vision loss	0 (0 to 0)	[illegible]	25·4
Other vision loss	0 (0 to 0)	[illegible]	23·5
Age-related and other hearing loss	0 (0 to 0)	[illegible]	242
Other sense organ diseases	0 (0 to 0)	[illegible]	15·7
Musculoskeletal disorders	0 (0 to 0)	[illegible]	655
Rheumatoid arthritis	0 (0 to 0)	[illegible]	9·5
Osteoarthritis	0 (0 to 0)	[illegible]	89·1
Osteoarthritis hip	0 (0 to 0)	[illegible]	5·6
Osteoarthritis knee	0 (0 to 0)	[illegible]	62·8
Osteoarthritis hand	0 (0 to 0)	[illegible]	7·8

Osteoarthritis other	0 (0 to 0)	0 (0 to 0)	0 (0 to 0)	0 (0 to 0)	0 (0 to 0)	0 (0 to 0)	0 (0 to 0)	0 (0 to 0)	0 (0 to 0)	0 (0 to 0)	0·1 (0 to 0·4)	0·5 (0·2 to 1·1)	0·9 (0·4 to 2·2)	1·6 (0·7 to 3·3)	2 (0·9 to 4·2)	2 (1 to 4·4)	1·9 (0·9 to 3·9)	1·5 (0·7 to 3·2)	1·1 (0·6 to 2·4)	0·7 (0·3 to 1·4)	0·3 (0·2 to 0·7)	0·1 (0·1 to 0·2)	0 (0 to 0·1)	12·8
Low back pain	0 (0 to 0)	0 (0 to 0)	0 (0 to 0)	0 (0 to 0)	1·2 (0·5 to 2·2)	6·3 (3·7 to 10·1)	12·8 (7·3 to 20·8)	16·8 (9·8 to 26·7)	19·5 (11·2 to 30·9)	21·5 (11·9 to 33·4)	24·3 (14·4 to 38·5)	26·8 (16 to 42·1)	28·3 (16·9 to 44·6)	30·3 (18·3 to 47·8)	27·9 (16·8 to 42·6)	23·8 (14·8 to 35·5)	19·3 (12·1 to 30)	14·3 (8·8 to 21·6)	9·2 (5·7 to 14·3)	5 (3·1 to 7·7)	2·1 (1·3 to 3·1)	0·7 (0·4 to 1)	0·2 (0·1 to 0·2)	290
Neck pain	0 (0 to 0)	0 (0 to 0)	0 (0 to 0)	0 (0 to 0)	0·1 (0 to 0·2)	0·5 (0·2 to 1·1)	1·2 (0·5 to 2·3)	2 (0·9 to 4·2)	3·2 (1·4 to 6·3)	4·8 (2·2 to 9·3)	6·2 (3 to 10·9)	7·7 (3·9 to 13·9)	8·6 (4·2 to 15·5)	8 (3·8 to 15·1)	6·8 (3·2 to 12·1)	5·3 (2·7 to 9·6)	3·9 (2·1 to 6·6)	2·8 (1·5 to 4·9)	1·7 (0·8 to 3)	0·8 (0·4 to 1·4)	0·3 (0·2 to 0·6)	0·1 (0·1 to 0·2)	0 (0 to 0·1)	64
Gout	0 (0 to 0)	0 (0 to 0)	0 (0 to 0)	0 (0 to 0)	0 (0 to 0)	0 (0 to 0)	0 (0 to 0)	0·1 (0 to 0·1)	0·1 (0 to 0·4)	0·3 (0·1 to 0·6)	0·4 (0·1 to 0·7)	0·5 (0·2 to 1)	0·6 (0·2 to 1)	0·6 (0·3 to 1·2)	0·6 (0·3 to 1·1)	0·5 (0·2 to 1)	0·5 (0·2 to 0·9)	0·4 (0·2 to 0·8)	0·3 (0·2 to 0·5)	0·2 (0·1 to 0·4)	0·1 (0·1 to 0·2)	0 (0 to 0·1)	0 (0 to 0)	5·3
Other musculoskeletal disorders	0 (0 to 0)	0 (0 to 0)	0 (0 to 0)	0 (0 to 0)	0 (0 to 0·1)	0·1 (0·1 to 0·4)	2·4 (0·9 to 4·6)	7 (4 to 11·1)	13·3 (7·6 to 20·7)	17·5 (10·2 to 28)	19·3 (11·5 to 30)	21·2 (12 to 33·8)	22·4 (13·4 to 35·5)	23·4 (13·6 to 37·9)	21·3 (12·8 to 32·3)	17·6 (9·7 to 27·9)	13·4 (7·9 to 20·7)	9·3 (5·1 to 14·9)	5·3 (2·9 to 8·5)	2·3 (1 to 4·1)	1·2 (0·7 to 1·8)	0·3 (0·1 to 0·5)	0 (0 to 0·1)	197
Other non-communicable diseases	22·6 (13·5 to 36·3)	16·5 (9·7 to 25·7)	35·7 (23·5 to 52·5)	29·9 (20·9 to 42·2)	18·5 (14·6 to 23·5)	14·2 (11·2 to 17·9)	16·5 (13·2 to 20·7)	24·3 (18·9 to 31·4)	26·6 (20 to 37)	26·5 (19·4 to 38·6)	29·7 (21·5 to 43·3)	29·4 (20·3 to 44·6)	30·7 (21·5 to 45)	36·4 (26·7 to 51)	39·1 (29·1 to 53·2)	41·4 (31·3 to 54·9)	35·8 (26·8 to 47·9)	32 (24·2 to 41·6)	24·3 (18·8 to 31)	16·5 (12·7 to 20·7)	8·8 (6·9 to 11)	3·4 (2·6 to 4·3)	0·9 (0·7 to 1·2)	560
Congenital birth defects	21·3 (12·1 to 34·9)	14·5 (8·1 to 23)	21·1 (12·9 to 33·4)	14·7 (9·3 to 22·5)	6·9 (5 to 9)	5·3 (3·9 to 7)	5·5 (4·2 to 7·4)	6 (4·4 to 8)	4·9 (3·6 to 6·3)	3·4 (2·5 to 4·4)	3·1 (2·4 to 4·1)	2·5 (1·8 to 3·3)	2·3 (1·8 to 3·1)	2·4 (1·8 to 3·2)	2 (1·5 to 2·7)	1·4 (1·1 to 1·8)	0·8 (0·6 to 1·1)	0·3 (0·2 to 0·5)	0·2 (0·1 to 0·3)	0·1 (0·1 to 0·1)	0 (0 to 0·1)	0 (0 to 0)	0 (0 to 0)	119
Neural tube defects	0·9 (0·4 to 1·8)	0·6 (0·2 to 1·1)	1·1 (0·4 to 2·5)	1·5 (0·6 to 3)	0·2 (0·1 to 0·3)	0·2 (0·2 to 0·3)	0·2 (0·2 to 0·3)	0·2 (0·1 to 0·3)	0·2 (0·1 to 0·3)	0·1 (0·1 to 0·2)	0·1 (0·1 to 0·1)	0·1 (0 to 0·1)	0·1 (0 to 0·1)	0 (0 to 0·1)	0 (0 to 0)	0 (0 to 0)	0 (0 to 0)	0 (0 to 0)	0 (0 to 0)	0 (0 to 0)	0 (0 to 0)	0 (0 to 0)	0 (0 to 0)	5·6
Congenital heart anomalies	9·8 (4·7 to 17·2)	8·3 (4·5 to 13·2)	10·8 (6 to 18)	6·3 (3·5 to 10·5)	2·5 (1·6 to 3·7)	1·5 (0·9 to 2·1)	1·6 (1 to 2·5)	2 (1·3 to 3)	1·6 (1 to 2·3)	1 (0·7 to 1·5)	0·9 (0·6 to 1·3)	0·6 (0·4 to 0·9)	0·5 (0·3 to 0·8)	0·5 (0·3 to 0·7)	0·3 (0·2 to 0·5)	0·3 (0·2 to 0·4)	0·2 (0·1 to 0·2)	0 (0 to 0·1)	0 (0 to 0)	0 (0 to 0)	0 (0 to 0)	0 (0 to 0)	0 (0 to 0)	48·7
Orofacial clefts	0·3 (0·1 to 0·7)	0·4 (0·1 to 0·9)	0·5 (0·1 to 1·5)	0·2 (0·1 to 0·3)	0·1 (0 to 0·2)	0·1 (0 to 0·3)	0·1 (0 to 0·2)	0·1 (0 to 0·3)	0·1 (0 to 0·2)	0·1 (0 to 0·2)	0·1 (0 to 0·2)	0·1 (0 to 0·2)	0·1 (0 to 0·2)	0·1 (0 to 0·2)	0·1 (0 to 0·2)	0·1 (0 to 0·1)	0 (0 to 0·1)	0 (0 to 0·1)	0 (0 to 0)	0 (0 to 0)	0 (0 to 0)	0 (0 to 0)	0 (0 to 0)	2·8
Down syndrome	0·3 (0·1 to 0·7)	0·3 (0·1 to 0·5)	1·1 (0·5 to 2·1)	0·9 (0·5 to 1·7)	0·3 (0·2 to 0·5)	0·4 (0·3 to 0·6)	0·6 (0·3 to 0·9)	0·6 (0·4 to 1)	0·5 (0·3 to 0·8)	0·4 (0·3 to 0·7)	0·4 (0·3 to 0·7)	0·4 (0·2 to 0·6)	0·4 (0·2 to 0·6)	0·5 (0·3 to 0·7)	0·6 (0·4 to 0·9)	0·3 (0·2 to 0·5)	0·1 (0·1 to 0·2)	0 (0 to 0)	0 (0 to 0)	0 (0 to 0)	0 (0 to 0)	0 (0 to 0)	0 (0 to 0)	8·3
Turner syndrome	0 (0 to 0)	0 (0 to 0)	0 (0 to 0)	0 (0 to 0)	0 (0 to 0)	0 (0 to 0)	0 (0 to 0)	0 (0 to 0)	0 (0 to 0)	0 (0 to 0)	0 (0 to 0)	0 (0 to 0)	0 (0 to 0)	0 (0 to 0)	0 (0 to 0)	0 (0 to 0)	0 (0 to 0)	0 (0 to 0)	0 (0 to 0)	0 (0 to 0)	0 (0 to 0)	0 (0 to 0)	0 (0 to 0)	0
Klinefelter syndrome	0 (0 to 0)	0 (0 to 0)	0 (0 to 0)	0 (0 to 0)	0 (0 to 0)	0 (0 to 0)	0 (0 to 0)	0 (0 to 0)	0 (0 to 0)	0 (0 to 0)	0 (0 to 0)	0 (0 to 0)	0 (0 to 0)	0 (0 to 0)	0 (0 to 0)	0 (0 to 0)	0 (0 to 0)	0 (0 to 0)	0 (0 to 0)	0 (0 to 0)	0 (0 to 0)	0 (0 to 0)	0 (0 to 0)	0
Other chromosomal abnormalities	1·1 (0·5 to 2)	0·6 (0·3 to 1·2)	1 (0·6 to 1·8)	0·6 (0·4 to 1)	0·4 (0·2 to 0·5)	0·3 (0·2 to 0·5)	0·4 (0·2 to 0·6)	0·3 (0·2 to 0·4)	0·3 (0·2 to 0·4)	0·1 (0·1 to 0·2)	0·1 (0·1 to 0·2)	0·1 (0 to 0·1)	0·1 (0 to 0·1)	0 (0 to 0·1)	0 (0 to 0)	0 (0 to 0)	0 (0 to 0)	0 (0 to 0)	0 (0 to 0)	0 (0 to 0)	0 (0 to 0)	0 (0 to 0)	0 (0 to 0)	5·4
Congenital musculoskeletal and limb anomalies	1·8 (0·8 to 3·5)	0·9 (0·4 to 1·8)	1·9 (1 to 3·4)	1·9 (1·1 to 2·9)	1·6 (1 to 2·4)	1·7 (1 to 2·5)	1·4 (0·8 to 2·3)	1·2 (0·7 to 1·8)	1·1 (0·7 to 1·7)	0·8 (0·4 to 1·3)	0·8 (0·5 to 1·3)	0·7 (0·4 to 1·2)	0·7 (0·4 to 1·2)	0·8 (0·5 to 1·2)	0·6 (0·4 to 1)	0·5 (0·3 to 0·7)	0·3 (0·2 to 0·5)	0·2 (0·1 to 0·3)	0·1 (0 to 0·1)	0 (0 to 0·1)	0 (0 to 0)	0 (0 to 0)	0 (0 to 0)	19·1
Urogenital congenital anomalies	0·3 (0·1 to 0·5)	0·1 (0·1 to 0·2)	0·2 (0·1 to 0·3)	0·2 (0·1 to 0·3)	0·1 (0·1 to 0·2)	0·1 (0·1 to 0·2)	0·1 (0·1 to 0·2)	0·1 (0·1 to 0·1)	0·1 (0·1 to 0·1)	0·1 (0 to 0·1)	0 (0 to 0·1)	0 (0 to 0·1)	0 (0 to 0)	0 (0 to 0)	0 (0 to 0)	0 (0 to 0)	0 (0 to 0)	0 (0 to 0)	0 (0 to 0)	0 (0 to 0)	0 (0 to 0)	0 (0 to 0)	0 (0 to 0)	1·5
Digestive congenital anomalies	1·8 (0·8 to 3·3)	1·4 (0·7 to 2·5)	1·3 (0·7 to 2·3)	0·6 (0·3 to 1)	0·2 (0·1 to 0·3)	0·1 (0·1 to 0·1)	0·1 (0 to 0·1)	0 (0 to 0·1)	0 (0 to 0·1)	0 (0 to 0)	0 (0 to 0)	0 (0 to 0)	0 (0 to 0)	0 (0 to 0)	0 (0 to 0)	0 (0 to 0)	0 (0 to 0)	0 (0 to 0)	0 (0 to 0)	0 (0 to 0)	0 (0 to 0)	0 (0 to 0)	0 (0 to 0)	5·6
Other congenital birth defects	5 (2·6 to 8·5)	2 (1·1 to 3·4)	3·3 (1·7 to 5·7)	2·5 (1·3 to 4)	1·3 (0·8 to 2)	0·9 (0·6 to 1·4)	1 (0·6 to 1·6)	1·5 (0·9 to 2·3)	1 (0·6 to 1·5)	0·7 (0·4 to 1·1)	0·6 (0·3 to 0·9)	0·5 (0·3 to 0·8)	0·4 (0·3 to 0·7)	0·5 (0·3 to 0·8)	0·3 (0·2 to 0·6)	0·2 (0·1 to 0·4)	0·2 (0·1 to 0·3)	0·1 (0 to 0·2)	0 (0 to 0·1)	0 (0 to 0·1)	0 (0 to 0)	0 (0 to 0)	0 (0 to 0)	22
Urinary diseases and male infertility	0 (0 to 0·1)	0·1 (0·1 to 0·3)	0·4 (0·2 to 0·8)	0·4 (0·2 to 0·6)	0·2 (0·1 to 0·3)	0·2 (0·1 to 0·3)	0·3 (0·2 to 0·4)	1·5 (0·9 to 2·6)	1·7 (1 to 2·9)	1·8 (1·1 to 3·3)	2·4 (1·5 to 3·8)	2·2 (1·4 to 3·7)	2·4 (1·5 to 3·4)	4·1 (2·8 to 5·6)	7·2 (4·7 to 9·8)	10·7 (7 to 14·9)	10·9 (7·2 to 15·2)	11·1 (7·3 to 15·5)	9·1 (6 to 12·6)	6·7 (4·3 to 9·1)	3·5 (2·2 to 4·9)	1·3 (0·8 to 1·9)	0·4 (0·2 to 0·6)	78·4
Urolithiasis	0 (0 to 0)	0 (0 to 0)	0 (0 to 0)	0 (0 to 0)	0 (0 to 0)	0 (0 to 0)	0 (0 to 0)	0·1 (0 to 0·1)	0·1 (0 to 0·2)	0·1 (0·1 to 0·3)	0·2 (0·1 to 0·4)	0·2 (0·1 to 0·5)	0·3 (0·1 to 0·6)	0·4 (0·2 to 0·7)	0·4 (0·2 to 0·7)	0·4 (0·2 to 0·6)	0·2 (0·1 to 0·4)	0·2 (0·1 to 0·3)	0·1 (0 to 0·2)	0 (0 to 0·1)	0 (0 to 0)	0 (0 to 0)	0 (0 to 0)	2·8
Benign prostatic hyperplasia	0 (0 to 0)	0 (0 to 0)	0 (0 to 0)	0 (0 to 0)	0 (0 to 0)	0 (0 to 0)	0 (0 to 0)	0 (0 to 0)	0 (0 to 0)	0 (0 to 0)	0 (0 to 0)	0 (0 to 0·1)	0·2 (0·1 to 0·5)	1 (0·5 to 1·8)	2·9 (1·4 to 5·1)	4·9 (2·5 to 8·3)	6 (3·4 to 9·7)	5·6 (3·1 to 9·2)	3·7 (2·1 to 6)	1·7 (1 to 2·7)	0·6 (0·3 to 1)	0·2 (0·1 to 0·3)	0 (0 to 0)	26·9
Male infertility	0 (0 to 0)	0 (0 to 0)	0 (0 to 0)	0 (0 to 0)	0 (0 to 0)	0 (0 to 0)	0 (0 to 0)	0·7 (0·2 to 1·7)	0·8 (0·2 to 2)	0·8 (0·2 to 2·3)	0·8 (0·2 to 2)	0·8 (0·2 to 2)	0·1 (0 to 0·3)	0 (0 to 0)	0 (0 to 0)	0 (0 to 0)	0 (0 to 0)	0 (0 to 0)	0 (0 to 0)	0 (0 to 0)	0 (0 to 0)	0 (0 to 0)	0 (0 to 0)	4
Other urinary diseases	0 (0 to 0)	0·1 (0 to 0·2)	0·1 (0 to 0·1)	0·1 (0 to 0·1)	0·1 (0 to 0·2)	0·1 (0 to 0·1)	0·1 (0·1 to 0·2)	0·3 (0·2 to 0·5)	0·2 (0·1 to 0·3)	0·2 (0·1 to 0·3)	0·4 (0·2 to 0·7)	0·3 (0·2 to 0·5)	0·5 (0·3 to 0·9)	0·7 (0·3 to 1·1)	0·8 (0·4 to 1·3)	1·2 (0·6 to 2·1)	1 (0·4 to 1·6)	0·8 (0·3 to 1·5)	0·8 (0·4 to 1·5)	0·8 (0·3 to 1·4)	0·3 (0·1 to 0·7)	0·2 (0·1 to 0·3)	0·1 (0 to 0·1)	9·1
Gynecological diseases	0 (0 to 0)	0 (0 to 0)	0 (0 to 0)	0 (0 to 0)	0 (0 to 0)	0 (0 to 0)	0 (0 to 0)	0 (0 to 0)	0 (0 to 0)	0 (0 to 0)	0 (0 to 0)	0 (0 to 0)	0 (0 to 0)	0 (0 to 0)	0 (0 to 0)	0 (0 to 0)	0 (0 to 0)	0 (0 to 0)	0 (0 to 0)	0 (0 to 0)	0 (0 to 0)	0 (0 to 0)	0 (0 to 0)	0
Uterine fibroids	0 (0 to 0)	0 (0 to 0)	0 (0 to 0)	0 (0 to 0)	0 (0 to 0)	0 (0 to 0)	0 (0 to 0)	0 (0 to 0)	0 (0 to 0)	0 (0 to 0)	0 (0 to 0)	0 (0 to 0)	0 (0 to 0)	0 (0 to 0)	0 (0 to 0)	0 (0 to 0)	0 (0 to 0)	0 (0 to 0)	0 (0 to 0)	0 (0 to 0)	0 (0 to 0)	0 (0 to 0)	0 (0 to 0)	0
Polycystic ovarian syndrome	--	--	--	--	--	--	--	--	--	--	--	--	--	--	--	--	--	--	--	--	--	--	--	NA
Female infertility	--	--	--	--	--	--	--	--	--	--	--	--	--	--	--	--	--	--	--	--	--	--	--	NA
Endometriosis	--	--	--	--	--	--	--	--	--	--	--	--	--	--	--	--	--	--	--	--	--	--	--	NA
Genital prolapse	0 (0 to 0)	0 (0 to 0)	0 (0 to 0)	0 (0 to 0)	0 (0 to 0)	0 (0 to 0)	0 (0 to 0)	0 (0 to 0)	0 (0 to 0)	0 (0 to 0)	0 (0 to 0)	0 (0 to 0)	0 (0 to 0)	0 (0 to 0)	0 (0 to 0)	0 (0 to 0)	0 (0 to 0)	0 (0 to 0)	0 (0 to 0)	0 (0 to 0)	0 (0 to 0)	0 (0 to 0)	0 (0 to 0)	0
Premenstrual syndrome	--	--	--	--	--	--	--	--	--	--	--	--	--	--	--	--	--	--	--	--	--	--	--	NA
Other gynecological diseases	0 (0 to 0)	0 (0 to 0)	0 (0 to 0)	0 (0 to 0)	0 (0 to 0)	0 (0 to 0)	0 (0 to 0)	0 (0 to 0)	0 (0 to 0)	0 (0 to 0)	0 (0 to 0)	0 (0 to 0)	0 (0 to 0)	0 (0 to 0)	0 (0 to 0)	0 (0 to 0)	0 (0 to 0)	0 (0 to 0)	0 (0 to 0)	0 (0 to 0)	0 (0 to 0)	0 (0 to 0)	0 (0 to 0)	0
Hemoglobinopathies and hemolytic anemias	0·1 (0·1 to 0·2)	0·2 (0·1 to 0·3)	1·3 (0·7 to 2·1)	4·8 (3·1 to 7·1)	5·6 (3·7 to 8)	3·6 (2·5 to 5·1)	4·4 (3·1 to 6·3)	6·5 (4·4 to 9·3)	5·1 (3·5 to 7)	3·6 (2·5 to 5)	3·5 (2·6 to 4·6)	3 (2·1 to 4·2)	2·8 (2 to 3·8)	2·9 (2·1 to 3·9)	3 (2 to 4·2)	2·6 (1·8 to 3·7)	1·6 (1·2 to 2·3)	1·5 (1·1 to 2)	1·1 (0·8 to 1·5)	0·8 (0·6 to 1·1)	0·7 (0·5 to 0·9)	0·3 (0·2 to 0·4)	0·1 (0·1 to 0·1)	59·2
Thalassemias	0 (0 to 0)	0 (0 to 0)	0 (0 to 0)	0 (0 to 0·1)	0 (0 to 0)	0 (0 to 0)	0 (0 to 0)	0 (0 to 0)	0 (0 to 0)	0 (0 to 0)	0 (0 to 0)	0 (0 to 0)	0 (0 to 0)	0 (0 to 0)	0 (0 to 0)	0 (0 to 0)	0 (0 to 0)	0 (0 to 0)	0 (0 to 0)	0 (0 to 0)	0 (0 to 0)	0 (0 to 0)	0 (0 to 0)	0·1
Thalassemias trait	0 (0 to 0)	0 (0 to 0)	0 (0 to 0)	0·1 (0 to 0·2)	0·1 (0 to 0·3)	0·1 (0 to 0·1)	0 (0 to 0·1)	0 (0 to 0)	0 (0 to 0)	0 (0 to 0)	0 (0 to 0)	0 (0 to 0·1)	0 (0 to 0·1)	0 (0 to 0·1)	0 (0 to 0·1)	0 (0 to 0·1)	0 (0 to 0)	0 (0 to 0)	0 (0 to 0)	0 (0 to 0)	0 (0 to 0)	0 (0 to 0)	0 (0 to 0)	0·5
Sickle cell disorders	0·1 (0·1 to 0·2)	0·1 (0·1 to 0·2)	1 (0·5 to 1·7)	3·1 (1·8 to 5)	3·5 (2·3 to 4·8)	2·6 (1·8 to 3·6)	3·1 (2·1 to 4·7)	4·2 (2·7 to 6·4)	2·8 (1·6 to 4·3)	1·8 (1 to 2·8)	1·7 (1 to 2·6)	1·3 (0·7 to 2)	1 (0·6 to 1·6)	0·7 (0·4 to 1·3)	0·4 (0·2 to 0·7)	0·1 (0 to 0·2)	0 (0 to 0)	0 (0 to 0)	0 (0 to 0)	0 (0 to 0)	0 (0 to 0)	0 (0 to 0)	0 (0 to 0)	27·5
Sickle cell trait	0 (0 to 0)	0·1 (0 to 0·1)	0·2 (0·1 to 0·3)	0·7 (0·2 to 1·5)	1 (0·3 to 2·1)	0·4 (0·1 to 1·1)	0·2 (0 to 0·5)	0·1 (0 to 0·3)	0·1 (0 to 0·4)	0·1 (0 to 0·4)	0·1 (0 to 0·4)	0·2 (0 to 0·4)	0·2 (0 to 0·4)	0·2 (0 to 0·5)	0·2 (0·1 to 0·5)	0·2 (0 to 0·4)	0·1 (0 to 0·3)	0·1 (0 to 0·2)	0·1 (0 to 0·2)	0·1 (0 to 0·1)	0 (0 to 0·1)	0 (0 to 0)	0 (0 to 0)	4·4
G6PD deficiency	0 (0 to 0)	0 (0 to 0)	0 (0 to 0·1)	0·4 (0·2 to 0·8)	0·5 (0·2 to 0·9)	0·4 (0·2 to 0·7)	0·7 (0·3 to 1·2)	1·7 (0·7 to 3·1)	1·8 (0·8 to 3·1)	1·3 (0·6 to 2·1)	1·3 (0·6 to 2·1)	1·2 (0·6 to 1·8)	1·1 (0·7 to 1·8)	1·4 (0·8 to 2)	1·5 (1 to 2·2)	1·3 (0·8 to 1·9)	0·6 (0·4 to 0·9)	0·4 (0·3 to 0·6)	0·2 (0·1 to 0·3)	0·1 (0 to 0·1)	0 (0 to 0·1)	0 (0 to 0)	0 (0 to 0)	16
G6PD trait	0 (0 to 0)	0 (0 to 0)	0 (0 to 0)	0 (0 to 0)	0 (0 to 0)	0 (0 to 0)	0 (0 to 0)	0 (0 to 0)	0 (0 to 0)	0 (0 to 0)	0 (0 to 0)	0 (0 to 0)	0 (0 to 0)	0 (0 to 0)	0 (0 to 0)	0 (0 to 0)	0 (0 to 0)	0 (0 to 0)	0 (0 to 0)	0 (0 to 0)	0 (0 to 0)	0 (0 to 0)	0 (0 to 0)	0
Other hemoglobinopathies and hemolytic anemias	0 (0 to 0)	0 (0 to 0)	0·1 (0 to 0·2)	0·5 (0·1 to 1)	0·6 (0·1 to 1·3)	0·2 (0·1 to 0·6)	0·4 (0·1 to 0·9)	0·4 (0·2 to 0·8)	0·4 (0·2 to 0·8)	0·4 (0·2 to 0·8)	0·4 (0·2 to 0·7)	0·4 (0·2 to 0·9)	0·4 (0·2 to 0·9)	0·6 (0·3 to 1·1)	0·8 (0·5 to 1·4)	1 (0·6 to 1·6)	0·8 (0·6 to 1·3)	0·9 (0·6 to 1·3)	0·8 (0·6 to 1·1)	0·7 (0·5 to 0·9)	0·6 (0·5 to 0·8)	0·3 (0·2 to 0·4)	0·1 (0·1 to 0·1)	10·8
Endocrine, metabolic, blood, and immune disorders	1·2 (0·5 to 2·5)	1·3 (0·7 to 2·5)	10·7 (5·2 to 21)	8·8 (5 to 14·8)	4·2 (2·9 to 6·2)	3·2 (2·2 to 4·9)	3·2 (2·1 to 4·7)	5·1 (3·2 to 8)	7·5 (4·9 to 13·1)	8·4 (5·6 to 14)	10·1 (6·4 to 16·6)	9·7 (6·1 to 16·7)	10·3 (6·8 to 17·9)	13·2 (8·6 to 21·7)	13·4 (9·1 to 19·8)	13·5 (9 to 20)	10·3 (6·9 to 16)	9·2 (6·3 to 13·6)	7 (4·9 to 10·2)	5·1 (3·5 to 7·3)	2·9 (1·9 to 4·2)	1·2 (0·8 to 1·8)	0·3 (0·2 to 0·4)	160
Oral disorders	0 (0 to 0)	0 (0 to 0)	0 (0 to 0)	1·2 (0·7 to 2)	1·6 (0·8 to 2·7)	1·9 (1 to 3·3)	3·1 (1·7 to 5·5)	5·2 (2·6 to 9·8)	7·4 (3·6 to 14·2)	9·2 (4·3 to 17·3)	10·6 (4·8 to 19·6)	11·9 (5·5 to 22·1)	12·8 (6·2 to 23·7)	13·8 (7 to 24·8)	13·5 (7·5 to 22·9)	13·2 (7·7 to 21·4)	12·2 (7·2 to 19·3)	10 (6·1 to 15·5)	7 (4·3 to 10·6)	3·8 (2·4 to 5·7)	1·7 (1·1 to 2·6)	0·6 (0·4 to 0·9)	0·2 (0·1 to 0·2)	141
Caries of deciduous teeth	0 (0 to 0)	0 (0 to 0)	0 (0 to 0)	0·4 (0·2 to 0·9)	0·4 (0·2 to 0·9)	0 (0 to 0·1)	0 (0 to 0)	0 (0 to 0)	0 (0 to 0)	0 (0 to 0)	0 (0 to 0)	0 (0 to 0)	0 (0 to 0)	0 (0 to 0)	0 (0 to 0)	0 (0 to 0)	0 (0 to 0)	0 (0 to 0)	0 (0 to 0)	0 (0 to 0)	0 (0 to 0)	0 (0 to 0)	0 (0 to 0)	0·9
Caries of permanent teeth	0 (0 to 0)	0 (0 to 0)	0 (0 to 0)	0 (0 to 0)	0·3 (0·1 to 0·8)	0·9 (0·4 to 2)	1·1 (0·4 to 2·3)	1·3 (0·5 to 2·6)	1·2 (0·5 to 2·5)	1·1 (0·4 to 2·2)	1 (0·4 to 2)	0·9 (0·4 to 1·9)	0·9 (0·4 to 1·9)	0·8 (0·3 to 1·7)	0·6 (0·2 to 1·4)	0·5 (0·2 to 1)	0·3 (0·1 to 0·6)	0·2 (0·1 to 0·4)	0·1 (0 to 0·2)	0·1 (0 to 0·1)	0 (0 to 0·1)	0 (0 to 0)	0 (0 to 0)	11·3
Periodontal diseases	0 (0 to 0)	0 (0 to 0)	0 (0 to 0)	0 (0 to 0)	0 (0 to 0)	0 (0 to 0)	0·5 (0·1 to 1·2)	1·9 (0·6 to 4·7)	3·8 (1·2 to 9)	5·5 (1·9 to 12·5)	6·6 (2·3 to 14·5)	7·6 (2·8 to 16)	8 (3 to 16·8)	7·9 (3·1 to 16·6)	6·4 (2·6 to 13·3)	4·6 (1·8 to 9·3)	3·1 (1·2 to 6·3)	2 (0·8 to 4·1)	1·2 (0·5 to 2·4)	0·6 (0·2 to 1·2)	0·3 (0·1 to 0·6)	0·1 (0 to 0·2)	0 (0 to 0)	60·2
Other oral disorders	0 (0 to 0)	0 (0 to 0)	0 (0 to 0)	0·8 (0·5 to 1·3)	0·8 (0·4 to 1·2)	0·9 (0·5 to 1·5)	1·5 (0·9 to 2·3)	1·9 (1·1 to 3)	2 (1·2 to 3·1)	2 (1·2 to 3·1)	2 (1·2 to 3·1)	2 (1·2 to 3·1)	2 (1·2 to 3)	1·9 (1·1 to 2·9)	1·6 (1 to 2·4)	1·2 (0·7 to 1·9)	0·9 (0·6 to 1·4)	0·6 (0·4 to 0·9)	0·3 (0·2 to 0·5)	0·1 (0·1 to 0·2)	0 (0 to 0·1)	0 (0 to 0)	0 (0 to 0)	22·6
Sudden infant death syndrome	--	0·3 (0·2 to 0·5)	2·1 (1·1 to 3·8)	--	--	--	--	--	--	--	--	--	--	--	--	--	--	--	--	--	--	--	--	NA
Injuries	1·2 (0·8 to 1·8)	1·4 (0·9 to 2·1)	7 (4·3 to 10·8)	34·7 (23·6 to 49·2)	38·6 (32·7 to 45·2)	40·1 (33·6 to 46·9)	103 (87·6 to 120)	233 (204 to 265)	224 (197 to 250)	150 (130 to 172)	146 (124 to 170)	114 (95 to 137)	107 (88·5 to 130)	108 (88·4 to 131)	94·6 (78 to 113)	76 (62·6 to 92·5)	50·3 (41·2 to 61·4)	38·3 (31·5 to 46·5)	27·7 (22·8 to 33·1)	19·7 (16·5 to 23·2)	10·2 (8·5 to 12)	4·4 (3·6 to 5·3)	1·4 (1·1 to 1·6)	1,630
Transport injuries	0·1 (0 to 0·1)	0·1 (0 to 0·1)	0·4 (0·3 to 0·7)	3·9 (2·2 to 6·2)	6·5 (4·1 to 9·2)	7·3 (5 to 10)	21·1 (15·1 to 29)	51 (37·2 to 66·5)	47·1 (33·7 to 61·2)	31·5 (23·7 to 40·6)	30·8 (23 to 40·3)	24·5 (18·9 to 30·3)	24·4 (18·7 to 31·3)	24·8 (18·8 to 32)	22 (16·7 to 27·6)	17·1 (13·2 to 21·6)	10·9 (8·4 to 13·8)	7·7 (6·1 to 9·6)	5·2 (4·1 to 6·3)	3 (2·3 to 3·8)	1·3 (1 to 1·6)	0·4 (0·3 to 0·5)	0·1 (0·1 to 0·1)	341
Road injuries	0 (0 to 0·1)	0 (0 to 0·1)	0·3 (0·2 to 0·5)	3·5 (1·9 to 5·7)	5·9 (3·6 to 8·4)	6·5 (4·5 to 9)	19·4 (13·8 to 27·1)	47 (34·1 to 61·7)	43·3 (30·7 to 56·3)	28·8 (21·7 to 36·8)	28·3 (21 to 37·1)	22·4 (17·1 to 27·9)	22·6 (17·2 to 29)	22·9 (17·6 to 29·8)	20 (15·3 to 25·1)	15·8 (12·3 to 20·1)	10·2 (7·9 to 12·9)	7 (5·6 to 8·8)	4·8 (3·8 to 5·9)	2·8 (2·2 to 3·5)	1·2 (0·9 to 1·5)	0·4 (0·3 to 0·5)	0·1 (0·1 to 0·1)	313
Pedestrian road injuries	0 (0 to 0)	0 (0 to 0)	0·1 (0 to 0·1)	1 (0·5 to 1·7)	1·7 (1 to 2·5)	1·3 (0·8 to 1·9)	1·3 (0·8 to 1·8)	2·4 (1·7 to 3·2)	2·8 (2 to 3·7)	2·3 (1·7 to 3·1)	2·7 (2 to 3·6)	2·5 (1·9 to 3·2)	2·8 (2·1 to 3·6)	3·2 (2·3 to 4·3)	3·2 (2·4 to 4·2)	2·8 (2 to 3·6)	2 (1·5 to 2·6)	1·6 (1·2 to 2·1)	1·3 (1 to 1·6)	0·9 (0·7 to 1·2)	0·4 (0·3 to 0·5)	0·1 (0·1 to 0·1)	0 (0 to 0)	36·2
Cyclist road injuries	0 (0 to 0)	0 (0 to 0)	0 (0 to 0)	0·2 (0·1 to 0·3)	0·5 (0·3 to 0·8)	1·2 (0·7 to 1·9)	2·3 (1·5 to 3·5)	3·6 (2·5 to 5·1)	3·4 (2·4 to 4·7)	3·1 (2·3 to 4·1)	3·3 (2·4 to 4·4)	3·2 (2·4 to 4·2)	3·3 (2·4 to 4·4)	3·5 (2·6 to 4·6)	3·1 (2·2 to 4)	2·5 (1·8 to 3·4)	1·8 (1·3 to 2·4)	1·2 (0·9 to 1·6)	0·8 (0·6 to 1)	0·4 (0·3 to 0·5)	0·2 (0·1 to 0·2)	0·1 (0 to 0·1)	0 (0 to 0)	37·6
Motorcyclist road injuries	0 (0 to 0)	0 (0 to 0)	0·1 (0 to 0·1)	0·4 (0·2 to 0·7)	0·7 (0·3 to 1·2)	0·7 (0·4 to 1·2)	5·2 (2·8 to 8·3)	15·6 (9·5 to 23·7)	15 (9·7 to 21·6)	9 (5·9 to 12·7)	8·3 (5·5 to 12)	5·6 (3·9 to 7·7)	5·4 (3·8 to 7·4)	4·9 (3·5 to 6·7)	4 (2·8 to 5·4)	2·6 (1·9 to 3·6)	1·7 (1·2 to 2·2)	1 (0·7 to 1·3)	0·6 (0·4 to 0·8)	0·3 (0·2 to 0·4)	0·1 (0·1 to 0·2)	0·1 (0 to 0·1)	0 (0 to 0)	81·3
Motor vehicle road injuries	0 (0 to 0)	0 (0 to 0·1)	0·2 (0·1 to 0·4)	1·9 (1 to 3·1)	3 (1·7 to 4·4)	3·2 (2 to 4·7)	10·3 (6·5 to 15·6)	24·7 (16·7 to 34·5)	21·5 (14·4 to 30)	13·8 (9·6 to 19·4)	13·3 (8·9 to 18·6)	10·4 (7·6 to 13·6)	10·4 (7·5 to 14·1)	10·5 (7·6 to 14)	9·1 (6·5 to 12·1)	7·4 (5·3 to 10·1)	4·3 (3·2 to 5·8)	3 (2·2 to 3·9)	2 (1·5 to 2·6)	1·1 (0·8 to 1·5)	0·5 (0·3 to 0·6)	0·1 (0·1 to 0·2)	0 (0 to 0·1)	151
Other road injuries	0 (0 to 0)	0 (0 to 0)	0 (0 to 0)	0·1 (0 to 0·2)	0·1 (0 to 0·2)	0·1 (0·1 to 0·2)	0·3 (0·2 to 0·5)	0·7 (0·4 to 1·2)	0·6 (0·4 to 0·9)	0·6 (0·4 to 0·9)	0·6 (0·4 to 0·9)	0·6 (0·5 to 0·9)	0·8 (0·5 to 1)	0·7 (0·5 to 1)	0·7 (0·5 to 0·9)	0·5 (0·4 to 0·7)	0·4 (0·3 to 0·5)	0·3 (0·2 to 0·4)	0·2 (0·1 to 0·3)	0·1 (0·1 to 0·1)	0 (0 to 0·1)	0 (0 to 0)	0 (0 to 0)	7·5
Other transport injuries	0 (0 to 0)	0 (0 to 0)	0·1 (0·1 to 0·2)	0·4 (0·2 to 0·7)	0·6 (0·3 to 0·9)	0·8 (0·5 to 1·2)	1·7 (1 to 2·7)	4 (2·3 to 6·2)	3·8 (2·1 to 5·8)	2·7 (1·7 to 4·2)	2·6 (1·6 to 3·8)	2·1 (1·4 to 2·9)	1·9 (1·3 to 2·7)	1·9 (1·2 to 2·7)	2 (1·3 to 2·8)	1·2 (0·8 to 1·8)	0·7 (0·5 to 1)	0·7 (0·5 to 1)	0·3 (0·2 to 0·5)	0·2 (0·1 to 0·2)	0·1 (0 to 0·1)	0 (0 to 0)	0 (0 to 0)	27·7
Unintentional injuries	0·9 (0·6 to 1·4)	1·2 (0·8 to 1·9)	6·2 (3·7 to 9·7)	29·1 (19·5 to 41·9)	30·5 (25·7 to 36·4)	29·8 (24·7 to 35·6)	58·3 (47·1 to 70·8)	115 (92·3 to 140)	109 (91·9 to 130)	77·9 (64·4 to 94)	78·8 (63·9 to 95·7)	67·3 (55 to 83·5)	65·6 (51·5 to 81·6)	68·3 (54·8 to 85·2)	61 (48·9 to 75·7)	50·6 (41 to 63·1)	34·9 (27·9 to 44·2)	27·5 (22·2 to 34·1)	20·5 (16·5 to 25·3)	15·5 (12·8 to 18·5)	8·5 (7·1 to 10·1)	3·9 (3·2 to 4·7)	1·2 (1 to 1·4)	961
Falls	0 (0 to 0)	0 (0 to 0)	0·2 (0·1 to 0·3)	1 (0·6 to 1·4)	1·3 (1 to 1·8)	2·2 (1·6 to 3)	4 (3 to 5·6)	6·5 (4·9 to 8·6)	6·9 (5·3 to 9)	6·6 (4·9 to 8·9)	7·7 (5·8 to 10)	7·8 (5·9 to 10·4)	8·5 (6·5 to 11·1)	9·8 (7·5 to 12·7)	9·4 (7·2 to 11·9)	8·9 (6·9 to 11·4)	7 (5·5 to 8·9)	6·8 (5·4 to 8·5)	6 (4·7 to 7·5)	6·2 (4·7 to 7·8)	4 (3 to 5)	2 (1·5 to 2·6)	0·7 (0·5 to 0·8)	114
Drowning	0·1 (0·1 to 0·1)	0·1 (0 to 0·1)	0·8 (0·5 to 1·3)	11·6 (6·9 to 18·5)	13·5 (10·4 to 17·4)	11 (8·3 to 13·9)	18·2 (12·8 to 25)	28·5 (20·6 to 38·8)	23·6 (17·1 to 30·9)	13·2 (10·2 to 17·2)	12·7 (9·6 to 16·5)	8·5 (6·2 to 11·6)	7·2 (4·9 to 10·2)	7·3 (5·5 to 9·3)	5·9 (4·6 to 7·5)	4·4 (3·5 to 5·5)	2·2 (1·8 to 2·8)	1·3 (1·1 to 1·7)	0·8 (0·6 to 1·1)	0·4 (0·3 to 0·5)	0·2 (0·1 to 0·2)	0·1 (0·1 to 0·1)	0 (0 to 0)	172
Fire, heat, and hot substances	0 (0 to 0·1)	0·1 (0·1 to 0·2)	0·8 (0·5 to 1·4)	4·9 (3 to 7·5)	3·4 (2·7 to 4·3)	2·8 (2·1 to 3·7)	3·4 (2·5 to 4·6)	5·6 (4·2 to 7·3)	6·2 (4·6 to 8)	6 (4·5 to 7·9)	6 (4·5 to 7·9)	5·6 (4·2 to 7·7)	5·9 (4·4 to 7·9)	5·9 (4·3 to 7·9)	5·3 (4 to 7)	4·7 (3·6 to 6·1)	3·1 (2·4 to 4·1)	2·6 (2 to 3·4)	1·9 (1·5 to 2·5)	1·5 (1·1 to 1·9)	0·8 (0·7 to 1)	0·4 (0·3 to 0·4)	0·1 (0·1 to 0·1)	77
Poisonings	0 (0 to 0)	0 (0 to 0)	0·1 (0 to 0·1)	0·5 (0·3 to 0·9)	0·3 (0·2 to 0·4)	0·3 (0·2 to 0·4)	0·3 (0·2 to 0·5)	0·6 (0·4 to 0·8)	0·8 (0·5 to 1·1)	0·6 (0·5 to 0·8)	0·6 (0·4 to 0·8)	0·4 (0·3 to 0·6)	0·4 (0·3 to 0·5)	0·4 (0·3 to 0·5)	0·3 (0·2 to 0·4)	0·2 (0·2 to 0·3)	0·2 (0·1 to 0·2)	0·1 (0·1 to 0·2)	0·1 (0·1 to 0·1)	0 (0 to 0·1)	0 (0 to 0)	0 (0 to 0)	0 (0 to 0)	6·2
Poisoning by carbon monoxide	0 (0 to 0)	0 (0 to 0)	0 (0 to 0·1)	0·4 (0·2 to 0·7)	0·1 (0·1 to 0·2)	0·1 (0·1 to 0·1)	0·1 (0·1 to 0·2)	0·4 (0·2 to 0·5)	0·5 (0·3 to 0·8)	0·4 (0·3 to 0·5)	0·4 (0·2 to 0·5)	0·2 (0·1 to 0·3)	0·2 (0·1 to 0·3)	0·2 (0·1 to 0·2)	0·1 (0·1 to 0·2)	0·1 (0·1 to 0·2)	0·1 (0 to 0·1)	0 (0 to 0·1)	0 (0 to 0)	0 (0 to 0)	0 (0 to 0)	0 (0 to 0)	0 (0 to 0)	3·4
Poisoning by other means	0 (0 to 0)	0 (0 to 0)	0 (0 to 0·1)	0·1 (0·1 to 0·2)	0·1 (0·1 to 0·2)	0·2 (0·1 to 0·3)	0·2 (0·1 to 0·3)	0·2 (0·1 to 0·4)	0·3 (0·2 to 0·4)	0·2 (0·1 to 0·4)	0·2 (0·1 to 0·3)	0·2 (0·1 to 0·3)	0·2 (0·1 to 0·3)	0·2 (0·1 to 0·3)	0·2 (0·1 to 0·3)	0·1 (0·1 to 0·2)	0·1 (0·1 to 0·1)	0·1 (0 to 0·1)	0 (0 to 0·1)	0 (0 to 0)	0 (0 to 0)	0 (0 to 0)	0 (0 to 0)	2·8
Exposure to mechanical forces	0·1 (0 to 0·1)	0·2 (0·1 to 0·3)	0·6 (0·4 to 0·9)	4·5 (2·8 to 7·1)	6·5 (4·9 to 8·6)	7·5 (5·6 to 9·8)	23·3 (16·4 to 32·3)	56·8 (40·1 to 77·4)	54·2 (38·9 to 73·1)	38·3 (28·4 to 50·9)	37·7 (28 to 50·6)	32·7 (25·1 to 43·2)	31·2 (23·1 to 40·9)	32 (23·9 to 42·7)	28·2 (21 to 37)	21·8 (16 to 28·8)	14·4 (10·6 to 19·6)	10·1 (7·3 to 13·7)	6·5 (4·6 to 9)	3·7 (2·6 to 5)	1·7 (1·3 to 2·4)	0·6 (0·5 to 0·9)	0·2 (0·1 to 0·2)	413
Unintentional firearm injuries	0 (0 to 0)	0 (0 to 0·1)	0·2 (0·1 to 0·3)	0·8 (0·4 to 1·6)	1·2 (0·7 to 2·1)	2·3 (1·3 to 3·5)	14·2 (8·8 to 21·1)	39·9 (26·1 to 57·1)	35·3 (23·6 to 49·5)	20·4 (13·7 to 29·3)	18·7 (12·8 to 26·6)	13·7 (9·8 to 18·8)	11·6 (8 to 16·1)	10·9 (7·5 to 15·5)	9 (6·4 to 12·7)	6·3 (4·5 to 8·7)	3·5 (2·6 to 4·5)	2·1 (1·6 to 2·8)	1·2 (0·9 to 1·6)	0·7 (0·5 to 0·9)	0·4 (0·3 to 0·5)	0·1 (0·1 to 0·2)	0 (0 to 0·1)	193
Other exposure to mechanical forces	0 (0 to 0·1)	0·1 (0·1 to 0·2)	0·4 (0·3 to 0·7)	3·7 (2·3 to 5·8)	5·3 (3·9 to 7)	5·3 (3·7 to 7·2)	9·1 (6·1 to 13·2)	16·9 (11·6 to 23·6)	18·9 (13·1 to 27·1)	17·8 (12·2 to 24·8)	19 (13·2 to 27·2)	19 (13·2 to 27)	19·6 (13·5 to 27·9)	21·1 (14·9 to 29·9)	19·1 (13·4 to 26·8)	15·4 (10·8 to 21·6)	10·9 (7·6 to 15·6)	8 (5·6 to 11·3)	5·3 (3·7 to 7·5)	3 (2 to 4·1)	1·4 (1 to 2)	0·5 (0·4 to 0·7)	0·1 (0·1 to 0·2)	220
Adverse effects of medical treatment	0·3 (0·1 to 0·5)	0·3 (0·2 to 0·5)	0·8 (0·3 to 1·5)	0·8 (0·4 to 1·5)	0·5 (0·4 to 0·8)	0·4 (0·3 to 0·7)	0·6 (0·3 to 0·9)	1·4 (0·9 to 2·2)	1·4 (0·9 to 2·3)	1 (0·6 to 1·5)	1·2 (0·7 to 1·9)	1·1 (0·7 to 1·8)	1·2 (0·7 to 1·9)	1·9 (1·2 to 3)	2·2 (1·4 to 3·4)	2·5 (1·6 to 3·8)	2 (1·3 to 3·1)	1·9 (1·3 to 2·8)	1·8 (1·1 to 2·7)	1·4 (1 to 2·1)	0·8 (0·5 to 1·1)	0·3 (0·2 to 0·5)	0·1 (0·1 to 0·1)	26
Animal contact	0 (0 to 0)	0 (0 to 0)	0 (0 to 0·1)	0·3 (0·2 to 0·5)	0·3 (0·2 to 0·4)	0·3 (0·2 to 0·5)	0·4 (0·3 to 0·6)	0·6 (0·4 to 0·8)	0·7 (0·5 to 1)	0·5 (0·4 to 0·7)	0·6 (0·4 to 0·9)	0·6 (0·4 to 0·8)	0·7 (0·5 to 0·9)	0·8 (0·6 to 1·1)	0·8 (0·5 to 1·1)	0·8 (0·6 to 1·1)	0·6 (0·4 to 0·8)	0·5 (0·4 to 0·7)	0·4 (0·3 to 0·5)	0·2 (0·2 to 0·3)	0·1 (0·1 to 0·1)	0 (0 to 0·1)	0 (0 to 0)	9·3
Venomous animal contact	0 (0 to 0)	0 (0 to 0)	0 (0 to 0)	0·2 (0·1 to 0·3)	0·2 (0·1 to 0·3)	0·2 (0·1 to 0·3)	0·3 (0·2 to 0·4)	0·4 (0·2 to 0·5)	0·5 (0·3 to 0·7)	0·3 (0·2 to 0·5)	0·4 (0·3 to 0·6)	0·4 (0·2 to 0·5)	0·4 (0·3 to 0·6)	0·5 (0·3 to 0·6)	0·4 (0·3 to 0·6)	0·4 (0·3 to 0·6)	0·3 (0·2 to 0·4)	0·2 (0·2 to 0·3)	0·2 (0·1 to 0·3)	0·1 (0·1 to 0·2)	0·1 (0 to 0·1)	0 (0 to 0)	0 (0 to 0)	5·6
Non-venomous animal contact	0 (0 to 0)	0 (0 to 0)	0 (0 to 0)	0·1 (0 to 0·2)	0·1 (0 to 0·1)	0·1 (0·1 to 0·2)	0·1 (0·1 to 0·2)	0·2 (0·1 to 0·3)	0·3 (0·2 to 0·4)	0·2 (0·1 to 0·3)	0·2 (0·1 to 0·3)	0·2 (0·2 to 0·4)	0·3 (0·2 to 0·4)	0·3 (0·2 to 0·5)	0·3 (0·2 to 0·4)	0·3 (0·2 to 0·5)	0·3 (0·2 to 0·4)	0·3 (0·2 to 0·4)	0·2 (0·1 to 0·3)	0·1 (0·1 to 0·1)	0 (0 to 0)	0 (0 to 0)	0 (0 to 0)	3·7
Foreign body	0·3 (0·2 to 0·6)	0·5 (0·3 to 0·8)	2·5 (1·1 to 5)	3·2 (2 to 5·1)	1·6 (1·2 to 2)	1·5 (1·2 to 1·9)	1·1 (0·8 to 1·5)	2·1 (1·6 to 2·7)	2·3 (1·7 to 2·9)	1·6 (1·3 to 2)	1·7 (1·3 to 2·2)	1·5 (1·2 to 2)	1·6 (1·2 to 2)	1·9 (1·4 to 2·4)	1·8 (1·5 to 2·2)	1·8 (1·4 to 2·2)	1·3 (1·1 to 1·7)	1·2 (0·9 to 1·5)	1 (0·7 to 1·2)	0·8 (0·7 to 1)	0·3 (0·3 to 0·4)	0·2 (0·1 to 0·2)	0·1 (0 to 0·1)	31·9
Pulmonary aspiration and foreign body in airway	0·3 (0·2 to 0·6)	0·5 (0·3 to 0·8)	2·5 (1·1 to 4·9)	2·9 (1·7 to 4·6)	1·1 (0·9 to 1·5)	1 (0·8 to 1·3)	0·7 (0·5 to 0·9)	1·4 (1·1 to 1·9)	1·4 (1 to 1·9)	0·9 (0·7 to 1·2)	1·1 (0·8 to 1·3)	0·8 (0·6 to 1)	0·8 (0·6 to 1·1)	1·2 (0·9 to 1·6)	1·1 (0·8 to 1·4)	1·2 (0·9 to 1·6)	0·9 (0·6 to 1·1)	0·8 (0·5 to 1)	0·6 (0·5 to 0·9)	0·6 (0·4 to 0·7)	0·2 (0·2 to 0·3)	0·1 (0·1 to 0·1)	0 (0 to 0·1)	22·1
Foreign body in eyes	0 (0 to 0)	0 (0 to 0)	0 (0 to 0)	0·1 (0 to 0·1)	0·1 (0 to 0·2)	0·1 (0 to 0·3)	0·2 (0·1 to 0·5)	0·3 (0·1 to 0·6)	0·3 (0·1 to 0·6)	0·3 (0·1 to 0·6)	0·3 (0·1 to 0·6)	0·3 (0·1 to 0·6)	0·3 (0·1 to 0·6)	0·3 (0·1 to 0·5)	0·2 (0·1 to 0·3)	0·2 (0·1 to 0·3)	0·1 (0·1 to 0·2)	0·1 (0·1 to 0·1)	0·1 (0 to 0·1)	0 (0 to 0)	0 (0 to 0)	0 (0 to 0)	0 (0 to 0)	3·2
Foreign body in other body part	0 (0 to 0)	0 (0 to 0)	0·1 (0 to 0·1)	0·3 (0·2 to 0·4)	0·3 (0·2 to 0·4)	0·3 (0·2 to 0·5)	0·3 (0·2 to 0·4)	0·4 (0·3 to 0·6)	0·5 (0·3 to 0·8)	0·4 (0·3 to 0·5)	0·4 (0·3 to 0·5)	0·4 (0·3 to 0·6)	0·5 (0·3 to 0·6)	0·5 (0·3 to 0·7)	0·5 (0·4 to 0·7)	0·4 (0·3 to 0·6)	0·4 (0·2 to 0·5)	0·3 (0·2 to 0·5)	0·3 (0·2 to 0·4)	0·2 (0·2 to 0·3)	0·1 (0·1 to 0·1)	0·1 (0 to 0·1)	0 (0 to 0)	6·5
Environmental heat and cold exposure	0 (0 to 0)	0 (0 to 0)	0 (0 to 0)	0·2 (0·1 to 0·3)	0·4 (0·3 to 0·6)	0·6 (0·4 to 0·9)	1·1 (0·7 to 1·6)	1·6 (1·1 to 2·2)	1·8 (1·3 to 2·5)	1·9 (1·3 to 2·7)	2 (1·4 to 2·9)	2·2 (1·5 to 3)	2·3 (1·6 to 3·2)	2·3 (1·6 to 3·2)	2·1 (1·5 to 2·8)	1·7 (1·2 to 2·4)	1·3 (0·9 to 1·8)	1·1 (0·8 to 1·4)	0·7 (0·5 to 1)	0·5 (0·4 to 0·6)	0·2 (0·2 to 0·3)	0·1 (0·1 to 0·1)	0 (0 to 0·1)	24·4
Exposure to forces of nature	0 (0 to 0)	0 (0 to 0)	0 (0 to 0)	0 (0 to 0)	0 (0 to 0)	0 (0 to 0)	0 (0 to 0)	0·1 (0·1 to 0·1)	0·1 (0·1 to 0·2)	0·2 (0·1 to 0·3)	0·2 (0·1 to 0·3)	0·3 (0·2 to 0·4)	0·3 (0·2 to 0·4)	0·3 (0·2 to 0·5)	0·3 (0·2 to 0·4)	0·2 (0·1 to 0·4)	0·2 (0·1 to 0·3)	0·2 (0·1 to 0·3)	0·2 (0·1 to 0·3)	0·1 (0·1 to 0·2)	0·1 (0 to 0·1)	0 (0 to 0)	0 (0 to 0)	2·9
Other unintentional injuries	0·1 (0 to 0·1)	0 (0 to 0·1)	0·3 (0·2 to 0·4)	2 (1·3 to 2·8)	2·7 (2 to 3·5)	3·1 (2·4 to 3·9)	5·8 (4·1 to 8·1)	10·8 (7·8 to 14·3)	11·1 (8·1 to 14·6)	8·1 (6·2 to 10·3)	8·3 (6·4 to 10·6)	6·6 (5·1 to 8·6)	6·3 (4·9 to 8·1)	5·7 (4·3 to 7·5)	4·8 (3·7 to 6·2)	3·6 (2·7 to 4·7)	2·5 (1·8 to 3·3)	1·7 (1·2 to 2·3)	1·1 (0·8 to 1·6)	0·6 (0·5 to 0·8)	0·3 (0·2 to 0·4)	0·1 (0·1 to 0·1)	0 (0 to 0)	85·5
Self-harm and interpersonal violence	0·3 (0·1 to 0·4)	0·1 (0·1 to 0·1)	0·4 (0·2 to 0·7)	1·8 (1 to 2·8)	1·6 (1·1 to 2·1)	3 (2·3 to 3·9)	23·2 (17·1 to 31)	67·4 (52·1 to 85·1)	67·5 (52·9 to 84·1)	41 (31·9 to 51)	36·3 (28·5 to 46·3)	22·1 (16·7 to 28·5)	17·4 (13·3 to 22·6)	15 (11·6 to 19·2)	11·6 (8·9 to 15·3)	8·3 (6·4 to 10·5)	4·5 (3·5 to 5·6)	3·1 (2·5 to 3·9)	2 (1·6 to 2·5)	1·2 (1 to 1·5)	0·5 (0·4 to 0·6)	0·1 (0·1 to 0·2)	0 (0 to 0·1)	328
Self-harm	0 (0 to 0)	0 (0 to 0)	0 (0 to 0)	0 (0 to 0)	0 (0 to 0)	0·5 (0·3 to 0·7)	4·8 (2·9 to 7·3)	16·6 (10·6 to 24·4)	21·8 (14·4 to 31·8)	15·1 (10·1 to 21·7)	14·8 (9·8 to 21·4)	8·7 (5·8 to 12·5)	6·4 (4·2 to 9·2)	5·8 (4 to 8·1)	4·5 (3 to 6·3)	3·4 (2·4 to 4·6)	1·9 (1·3 to 2·6)	1·5 (1·1 to 2)	1 (0·7 to 1·4)	0·7 (0·5 to 1)	0·3 (0·2 to 0·4)	0·1 (0·1 to 0·1)	0 (0 to 0)	108
Self-harm by firearm	0 (0 to 0)	0 (0 to 0)	0 (0 to 0)	0 (0 to 0)	0 (0 to 0)	0 (0 to 0)	0·1 (0·1 to 0·2)	0·4 (0·2 to 0·8)	0·4 (0·2 to 0·8)	0·3 (0·2 to 0·5)	0·3 (0·1 to 0·5)	0·2 (0·1 to 0·3)	0·2 (0·1 to 0·2)	0·2 (0·1 to 0·3)	0·1 (0·1 to 0·2)	0·1 (0·1 to 0·2)	0·1 (0 to 0·1)	0 (0 to 0·1)	0 (0 to 0)	0 (0 to 0)	0 (0 to 0)	0 (0 to 0)	0 (0 to 0)	2·5
Self-harm by other specified means	0 (0 to 0)	0 (0 to 0)	0 (0 to 0)	0 (0 to 0)	0 (0 to 0)	0·4 (0·3 to 0·6)	4·7 (2·8 to 7·1)	16·1 (10·3 to 23·7)	21·4 (14·1 to 31·2)	14·8 (9·9 to 21·3)	14·6 (9·6 to 21·1)	8·5 (5·6 to 12·3)	6·2 (4·1 to 9)	5·6 (3·9 to 7·9)	4·4 (3 to 6·1)	3·3 (2·3 to 4·5)	1·8 (1·3 to 2·6)	1·4 (1 to 2)	1 (0·7 to 1·4)	0·7 (0·5 to 0·9)	0·3 (0·2 to 0·4)	0·1 (0·1 to 0·1)	0 (0 to 0)	105
Interpersonal violence	0·3 (0·1 to 0·4)	0·1 (0·1 to 0·1)	0·4 (0·2 to 0·7)	1·7 (1 to 2·7)	1·6 (1·1 to 2·1)	2·5 (1·9 to 3·3)	18·4 (12·8 to 25·1)	50·7 (37·5 to 66·4)	45·4 (33·9 to 60·2)	25·7 (19·4 to 33·5)	21·4 (16·1 to 27·8)	13·4 (9·7 to 18·1)	10·9 (8·1 to 14·6)	9·2 (6·8 to 12·1)	7 (5·2 to 9·5)	4·8 (3·6 to 6·4)	2·6 (2 to 3·4)	1·6 (1·3 to 2)	0·9 (0·7 to 1·2)	0·4 (0·3 to 0·5)	0·2 (0·1 to 0·2)	0·1 (0 to 0·1)	0 (0 to 0)	219
Physical violence by firearm	0 (0 to 0)	0 (0 to 0)	0 (0 to 0)	0·1 (0 to 0·1)	0·1 (0·1 to 0·2)	0·4 (0·3 to 0·5)	9·2 (6·2 to 13·2)	27·1 (19·8 to 35·8)	22·8 (16·5 to 30·4)	11·4 (8·4 to 15·2)	8 (5·9 to 10·6)	4·2 (2·9 to 5·8)	2·9 (2·1 to 4)	2 (1·4 to 2·7)	1·2 (0·9 to 1·8)	0·7 (0·5 to 1)	0·3 (0·2 to 0·4)	0·2 (0·1 to 0·2)	0·1 (0 to 0·1)	0 (0 to 0)	0 (0 to 0)	0 (0 to 0)	0 (0 to 0)	90·8
Physical violence by sharp object	0 (0 to 0)	0 (0 to 0)	0 (0 to 0)	0·1 (0·1 to 0·1)	0·1 (0·1 to 0·2)	0·2 (0·2 to 0·3)	3·5 (2·4 to 4·8)	10 (7·3 to 13·1)	8·9 (6·6 to 11·9)	5·5 (4·1 to 7·1)	4·4 (3·3 to 5·7)	2·7 (2 to 3·6)	2 (1·5 to 2·7)	1·6 (1·2 to 2·1)	1 (0·8 to 1·3)	0·7 (0·5 to 0·9)	0·4 (0·3 to 0·5)	0·2 (0·2 to 0·3)	0·1 (0·1 to 0·2)	0·1 (0·1 to 0·1)	0 (0 to 0)	0 (0 to 0)	0 (0 to 0)	41·6
Sexual violence	0 (0 to 0)	0 (0 to 0)	0 (0 to 0)	0 (0 to 0)	0·1 (0·1 to 0·2)	0·3 (0·1 to 0·4)	0·6 (0·3 to 1)	0·8 (0·5 to 1·3)	0·7 (0·4 to 1·2)	0·6 (0·4 to 1)	0·6 (0·3 to 0·9)	0·5 (0·3 to 0·8)	0·4 (0·2 to 0·7)	0·3 (0·2 to 0·5)	0·2 (0·1 to 0·4)	0·2 (0·1 to 0·3)	0·1 (0·1 to 0·2)	0·1 (0·1 to 0·1)	0·1 (0 to 0·1)	0 (0 to 0)	0 (0 to 0)	0 (0 to 0)	0 (0 to 0)	5·6
Physical violence by other means	0·3 (0·1 to 0·4)	0·1 (0 to 0·1)	0·4 (0·2 to 0·7)	1·6 (0·9 to 2·5)	1·2 (0·8 to 1·6)	1·6 (1·1 to 2·2)	5 (3·5 to 7·1)	12·7 (9·2 to 16·7)	13 (9·7 to 17·1)	8·2 (6·1 to 10·7)	8·4 (6·3 to 11)	6·1 (4·4 to 8·3)	5·6 (4·2 to 7·6)	5·3 (3·9 to 7·2)	4·6 (3·3 to 6·2)	3·3 (2·4 to 4·3)	1·8 (1·4 to 2·4)	1·1 (0·8 to 1·4)	0·7 (0·5 to 0·9)	0·3 (0·2 to 0·3)	0·1 (0·1 to 0·1)	0 (0 to 0)	0 (0 to 0)	81·3
Conflict and terrorism	0 (0 to 0)	0 (0 to 0)	0 (0 to 0)	0 (0 to 0)	0 (0 to 0)	0 (0 to 0)	0 (0 to 0)	0 (0 to 0)	0 (0 to 0)	0 (0 to 0)	0 (0 to 0)	0 (0 to 0)	0 (0 to 0)	0 (0 to 0)	0 (0 to 0)	0 (0 to 0)	0 (0 to 0)	0 (0 to 0)	0·1 (0 to 0·1)	0·1 (0·1 to 0·1)	0 (0 to 0)	0 (0 to 0)	0 (0 to 0)	0·1

SR Table 2a: Bahamas DALYS by cause and age group for males with 95% uncertainty intervals, 2019

Cause	Early Neonatal	Late Neonatal	Post Neonatal	1 to 4	5 to 9	10 to 14	15 to 19
All causes	802 (631 to 1,010)	347 (207 to 578)	716 (566 to 892)	825 (669 to 1,030)	971 (788 to 1,190)	1,330 (1,100 to 1,610)	2,280 (1,940 to 2,660)
Communicable, maternal, neonatal, and nutritional diseases	670 (515 to 851)	267 (158 to 456)	332 (251 to 422)	231 (172 to 319)	225 (146 to 354)	181 (133 to 255)	179 (145 to 221)
HIV/AIDS and sexually transmitted infections	0·2 (0·1 to 0·3)	0·1 (0 to 0·1)	16·8 (15·5 to 18·3)	15·6 (14·4 to 16·9)	14·3 (13·2 to 15·5)	13·1 (12·2 to 14·3)	29·9 (27·2 to 33·7)
HIV/AIDS	0 (0 to 0)	0 (0 to 0)	16·7 (15·4 to 18·1)	15·6 (14·4 to 16·9)	14·3 (13·2 to 15·5)	12·8 (11·9 to 13·9)	28·3 (25·9 to 31·7)
HIV/AIDS - Drug-susceptible Tuberculosis	0 (0 to 0)	0 (0 to 0)	0·5 (0·3 to 0·8)	0·5 (0·3 to 0·7)	0·5 (0·3 to 0·8)	0·4 (0·2 to 0·5)	0·7 (0·5 to 1·1)
HIV/AIDS - Multidrug-resistant Tuberculosis without extensive dru	0 (0 to 0)	0 (0 to 0)	0 (0 to 0·1)	0 (0 to 0·1)	0 (0 to 0·1)	0 (0 to 0·1)	0 (0 to 0·1)
HIV/AIDS - Extensively drug-resistant Tuberculosis	0 (0 to 0)	0 (0 to 0)	0 (0 to 0)	0 (0 to 0)	0 (0 to 0)	0 (0 to 0)	0 (0 to 0)
HIV/AIDS resulting in other diseases	0 (0 to 0)	0 (0 to 0)	16·2 (14·9 to 17·6)	15·1 (13·9 to 16·4)	13·8 (12·7 to 14·9)	12·5 (11·4 to 13·6)	27·6 (25·1 to 30·7)
Sexually transmitted infections excluding HIV	0·2 (0·1 to 0·3)	0·1 (0 to 0·1)	0·1 (0 to 0·2)	0 (0 to 0)	0 (0 to 0)	0·3 (0·1 to 0·6)	1·5 (0·7 to 3)
Syphilis	0·2 (0·1 to 0·3)	0·1 (0 to 0·1)	0·1 (0 to 0·2)	0 (0 to 0)	0 (0 to 0)	0 (0 to 0)	0·1 (0 to 0·1)
Chlamydial infection	0 (0 to 0)	0 (0 to 0)	0 (0 to 0)	0 (0 to 0)	0 (0 to 0)	0 (0 to 0)	0·2 (0·1 to 0·3)
Gonococcal infection	0 (0 to 0)	0 (0 to 0)	0 (0 to 0)	0 (0 to 0)	0 (0 to 0)	0·1 (0 to 0·2)	0·5 (0·1 to 1·2)
Trichomoniasis	0 (0 to 0)	0 (0 to 0)	0 (0 to 0)	0 (0 to 0)	0 (0 to 0)	0 (0 to 0)	0 (0 to 0)
Genital herpes	0 (0 to 0)	0 (0 to 0)	0 (0 to 0)	0 (0 to 0)	0 (0 to 0)	0 (0 to 0·1)	0·2 (0·1 to 0·4)
Other sexually transmitted infections	0 (0 to 0)	0 (0 to 0)	0 (0 to 0)	0 (0 to 0)	0 (0 to 0)	0·2 (0·1 to 0·4)	0·6 (0·2 to 1·3)
Respiratory infections and tuberculosis	25·7 (17·2 to 38·5)	26·9 (14·2 to 46·4)	125 (81·4 to 176)	61·7 (42·1 to 87·8)	34·5 (24·5 to 48·1)	32·6 (22·7 to 46·1)	42·2 (30·1 to 57·8)
Tuberculosis	0 (0 to 0)	0 (0 to 0)	1·4 (0·9 to 2)	1·6 (1 to 2·5)	1·4 (0·6 to 2·4)	1·5 (1·1 to 2)	4·8 (3 to 7·4)
Latent tuberculosis infection	0 (0 to 0)	0 (0 to 0)	0 (0 to 0)	0 (0 to 0)	0 (0 to 0)	0 (0 to 0)	0 (0 to 0)
Drug-susceptible tuberculosis	0 (0 to 0)	0 (0 to 0)	1·4 (0·9 to 2)	1·6 (1 to 2·4)	1·4 (0·6 to 2·4)	1·4 (1 to 1·9)	4·6 (2·8 to 7·1)
Multidrug-resistant tuberculosis without extensive drug resistanc	0 (0 to 0)	0 (0 to 0)	0 (0 to 0·2)	0 (0 to 0·2)	0 (0 to 0·1)	0 (0 to 0·2)	0·2 (0 to 0·6)
Extensively drug-resistant tuberculosis	0 (0 to 0)	0 (0 to 0)	0 (0 to 0)	0 (0 to 0)	0 (0 to 0)	0 (0 to 0)	0 (0 to 0·1)
Lower respiratory infections	25·7 (17·2 to 38·5)	26·8 (14·1 to 46·4)	121 (77·4 to 172)	45·2 (28 to 68·6)	13 (9·1 to 18·2)	11·7 (8·1 to 16·3)	19·7 (12·4 to 30)
Upper respiratory infections	0 (0 to 0)	0·1 (0·1 to 0·2)	2·4 (1·3 to 3·9)	10·5 (5·9 to 17·3)	13·5 (7·2 to 23·3)	14·4 (7·3 to 25·3)	13·2 (6·8 to 23·7)
Otitis media	0 (0 to 0)	0 (0 to 0·1)	0·5 (0·3 to 0·9)	4·4 (2·3 to 7·7)	6·6 (3·7 to 11·2)	5·1 (2·7 to 9)	4·4 (2·2 to 7·6)
Enteric infections	0·8 (0·6 to 1·2)	3·2 (1·7 to 5·6)	30 (18·4 to 47·2)	27·1 (18·3 to 39·9)	23·7 (14·3 to 37·7)	24·9 (14·2 to 39·2)	17·7 (11·1 to 26·3)
Diarrheal diseases	0·8 (0·6 to 1·2)	3·2 (1·7 to 5·6)	29·8 (18·2 to 47)	26·8 (18·1 to 39·7)	23·2 (13·9 to 37·1)	24·6 (13·9 to 38·8)	17·6 (11·1 to 26·3)
Typhoid and paratyphoid	0 (0 to 0)	0 (0 to 0)	0 (0 to 0)	0 (0 to 0)	0 (0 to 0)	0 (0 to 0)	0 (0 to 0)
Typhoid fever	0 (0 to 0)	0 (0 to 0)	0 (0 to 0)	0 (0 to 0)	0 (0 to 0)	0 (0 to 0)	0 (0 to 0)
Paratyphoid fever	0 (0 to 0)	0 (0 to 0)	0 (0 to 0)	0 (0 to 0)	0 (0 to 0)	0 (0 to 0)	0 (0 to 0)
Invasive Non-typhoidal Salmonella (iNTS)	0 (0 to 0)	0 (0 to 0)	0·1 (0 to 0·4)	0·2 (0·1 to 0·5)	0·4 (0·1 to 0·9)	0·3 (0·1 to 0·6)	0 (0 to 0·1)
Other intestinal infectious diseases	0 (0 to 0)	0 (0 to 0)	0·1 (0 to 0·3)	0·1 (0 to 0·3)	0·1 (0 to 0·2)	0·1 (0 to 0·2)	0·1 (0 to 0·1)
Neglected tropical diseases and malaria	0 (0 to 0)	0·2 (0·1 to 0·4)	1·7 (0·7 to 3)	5·8 (2·4 to 11·5)	9·3 (3·2 to 19·4)	5·1 (1·9 to 11·1)	3 (1·5 to 5·2)
Malaria	0 (0 to 0)	0 (0 to 0)	0 (0 to 0)	0 (0 to 0)	0 (0 to 0)	0 (0 to 0)	0 (0 to 0)
Chagas disease	0 (0 to 0)	0 (0 to 0)	0 (0 to 0)	0 (0 to 0)	0 (0 to 0)	0 (0 to 0)	0 (0 to 0)
Leishmaniasis	0 (0 to 0)	0 (0 to 0)	0 (0 to 0)	0 (0 to 0)	0 (0 to 0)	0 (0 to 0)	0 (0 to 0)
Visceral leishmaniasis	0 (0 to 0)	0 (0 to 0)	0 (0 to 0)	0 (0 to 0)	0 (0 to 0)	0 (0 to 0)	0 (0 to 0)
Cutaneous and mucocutaneous leishmaniasis	0 (0 to 0)	0 (0 to 0)	0 (0 to 0)	0 (0 to 0)	0 (0 to 0)	0 (0 to 0)	0 (0 to 0)
African trypanosomiasis	0 (0 to 0)	0 (0 to 0)	0 (0 to 0)	0 (0 to 0)	0 (0 to 0)	0 (0 to 0)	0 (0 to 0)
Schistosomiasis	0 (0 to 0)	0 (0 to 0)	0 (0 to 0)	0 (0 to 0)	0 (0 to 0)	0 (0 to 0)	0 (0 to 0)
Cysticercosis	0 (0 to 0)	0 (0 to 0)	0 (0 to 0)	0 (0 to 0)	0 (0 to 0)	0·1 (0 to 0·2)	0·4 (0·1 to 1·1)
Cystic echinococcosis	0 (0 to 0)	0 (0 to 0)	0 (0 to 0)	0 (0 to 0)	0 (0 to 0)	0 (0 to 0)	0 (0 to 0)
Lymphatic filariasis	0 (0 to 0)	0 (0 to 0)	0 (0 to 0)	0 (0 to 0)	0 (0 to 0)	0 (0 to 0)	0 (0 to 0)
Onchocerciasis	0 (0 to 0)	0 (0 to 0)	0 (0 to 0)	0 (0 to 0)	0 (0 to 0)	0 (0 to 0)	0 (0 to 0)
Trachoma	0 (0 to 0)	0 (0 to 0)	0 (0 to 0)	0 (0 to 0)	0 (0 to 0)	0 (0 to 0)	0 (0 to 0)
Dengue	0 (0 to 0)	0 (0 to 0)	0·4 (0·1 to 0·9)	0·5 (0·1 to 1·2)	1·2 (0·3 to 2·8)	1·3 (0·3 to 3)	1·4 (0·4 to 3·1)
Yellow fever	0 (0 to 0)	0 (0 to 0)	0 (0 to 0)	0 (0 to 0)	0 (0 to 0)	0 (0 to 0)	0 (0 to 0)
Rabies	0 (0 to 0)	0 (0 to 0)	0 (0 to 0)	0 (0 to 0)	0 (0 to 0)	0 (0 to 0)	0 (0 to 0)
Intestinal nematode infections	0 (0 to 0)	0 (0 to 0)	0 (0 to 0·1)	0·1 (0 to 0·4)	0·3 (0·1 to 0·8)	0·2 (0 to 0·4)	0·1 (0 to 0·3)
Ascariasis	0 (0 to 0)	0 (0 to 0)	0 (0 to 0·1)	0 (0 to 0·2)	0 (0 to 0·2)	0 (0 to 0·1)	0 (0 to 0·1)
Trichuriasis	0 (0 to 0)	0 (0 to 0)	0 (0 to 0)	0 (0 to 0)	0 (0 to 0)	0 (0 to 0)	0 (0 to 0)
Hookworm disease	0 (0 to 0)	0 (0 to 0)	0 (0 to 0)	0·1 (0 to 0·2)	0·3 (0 to 0·8)	0·1 (0 to 0·4)	0·1 (0 to 0·2)
Food-borne trematodiases	0 (0 to 0)	0 (0 to 0)	0 (0 to 0)	0 (0 to 0)	0 (0 to 0)	0 (0 to 0)	0 (0 to 0)
Leprosy	0 (0 to 0)	0 (0 to 0)	0 (0 to 0)	0 (0 to 0)	0 (0 to 0)	0 (0 to 0)	0 (0 to 0)
Ebola	0 (0 to 0)	0 (0 to 0)	0 (0 to 0)	0 (0 to 0)	0 (0 to 0)	0 (0 to 0)	0 (0 to 0)
Zika virus	0 (0 to 0)	0 (0 to 0)	0 (0 to 0·1)	0·1 (0 to 0·5)	0·1 (0 to 0·5)	0·1 (0 to 0·6)	0·1 (0 to 0·6)
Guinea worm disease	0 (0 to 0)	0 (0 to 0)	0 (0 to 0)	0 (0 to 0)	0 (0 to 0)	0 (0 to 0)	0 (0 to 0)
Other neglected tropical diseases	0 (0 to 0)	0·2 (0·1 to 0·4)	1·2 (0·5 to 2·5)	5 (1·8 to 10·5)	7·6 (2·1 to 17·7)	3·4 (0·8 to 9)	0·9 (0·3 to 2·2)
Other infectious diseases	1·8 (1·2 to 2·6)	4·6 (2·4 to 8·2)	20·7 (14·3 to 29·6)	17·4 (11·6 to 25·6)	16·1 (11 to 23·4)	14 (10·2 to 18·6)	15·5 (11·4 to 20·4)
Meningitis	1 (0·6 to 1·5)	3 (1·4 to 5·8)	10·7 (6·5 to 17)	6·2 (3·7 to 9·9)	4·5 (3 to 6·2)	4·1 (2·7 to 6·1)	5·3 (3·2 to 8)
Encephalitis	0·3 (0·1 to 0·5)	0·6 (0·3 to 1·4)	5·3 (3 to 8·8)	5·2 (2·8 to 9·4)	4 (2·3 to 6·9)	4 (2·5 to 6·1)	4·7 (2·9 to 7·4)
Diphtheria	0 (0 to 0)	0 (0 to 0)	0 (0 to 0)	0 (0 to 0)	0 (0 to 0)	0 (0 to 0)	0 (0 to 0)
Tetanus	0 (0 to 0)	0 (0 to 0)	0 (0 to 0)	0 (0 to 0)	0 (0 to 0·1)	0 (0 to 0·1)	0 (0 to 0·1)
Measles	0 (0 to 0)	0 (0 to 0)	0 (0 to 0)	0 (0 to 0)	0 (0 to 0)	0 (0 to 0)	0 (0 to 0)
Varicella and herpes zoster	0·2 (0·1 to 0·6)	0·4 (0·2 to 1)	1·1 (0·4 to 2·4)	0·5 (0·2 to 1·1)	0·6 (0·3 to 1·3)	0·9 (0·4 to 1·8)	0·6 (0·3 to 1·1)
Acute hepatitis	0 (0 to 0)	0 (0 to 0)	0·1 (0·1 to 0·3)	0·3 (0·1 to 0·5)	1 (0·5 to 1·6)	1·4 (0·8 to 2·3)	1·4 (0·8 to 2·1)
Acute hepatitis A	0 (0 to 0)	0 (0 to 0)	0 (0 to 0)	0·2 (0·1 to 0·4)	0·8 (0·4 to 1·5)	1·2 (0·6 to 1·9)	1·1 (0·6 to 1·8)
Acute hepatitis B	0 (0 to 0)	0 (0 to 0)	0·1 (0 to 0·2)	0 (0 to 0·1)	0·1 (0 to 0·1)	0·1 (0 to 0·2)	0·1 (0 to 0·3)
Acute hepatitis C	0 (0 to 0)	0 (0 to 0)	0 (0 to 0)	0 (0 to 0·1)	0 (0 to 0)	0 (0 to 0·1)	0 (0 to 0·1)
Acute hepatitis E	0 (0 to 0)	0 (0 to 0)	0 (0 to 0·1)	0 (0 to 0)	0·1 (0 to 0·1)	0·1 (0 to 0·3)	0·1 (0 to 0·3)
Other unspecified infectious diseases	0·4 (0·2 to 0·7)	0·5 (0·3 to 0·9)	2·9 (1·7 to 4·7)	4·5 (2·2 to 8·5)	5·9 (2·2 to 12·8)	3·5 (1·6 to 7·3)	3·5 (1·9 to 5·5)
Maternal and neonatal disorders	642 (491 to 817)	230 (134 to 391)	108 (71·2 to 158)	45·6 (35 to 58·9)	40·4 (29·6 to 53·1)	46·3 (33·6 to 60·9)	42·3 (31·1 to 56·7)
Maternal disorders	0 (0 to 0)	0 (0 to 0)	0 (0 to 0)	0 (0 to 0)	0 (0 to 0)	0 (0 to 0)	0 (0 to 0)
Maternal hemorrhage	0 (0 to 0)	0 (0 to 0)	0 (0 to 0)	0 (0 to 0)	0 (0 to 0)	0 (0 to 0)	0 (0 to 0)
Maternal sepsis and other maternal infections	0 (0 to 0)	0 (0 to 0)	0 (0 to 0)	0 (0 to 0)	0 (0 to 0)	0 (0 to 0)	0 (0 to 0)
Maternal hypertensive disorders	0 (0 to 0)	0 (0 to 0)	0 (0 to 0)	0 (0 to 0)	0 (0 to 0)	0 (0 to 0)	0 (0 to 0)
Maternal obstructed labor and uterine rupture	--	--	--	--	--	--	--
Maternal abortion and miscarriage	--	--	--	--	--	--	--
Ectopic pregnancy	--	--	--	--	--	--	--
Indirect maternal deaths	--	--	--	--	--	--	--
Late maternal deaths	--	--	--	--	--	--	--
Maternal deaths aggravated by HIV/AIDS	--	--	--	--	--	--	--
Neonatal disorders	642 (491 to 817)	230 (134 to 391)	108 (71·2 to 158)	45·6 (35 to 58·9)	40·4 (29·6 to 53·1)	46·3 (33·6 to 60·9)	42·3 (31·1 to 56·7)
Neonatal preterm birth	249 (170 to 343)	61·6 (31·6 to 110)	34·2 (20·3 to 52·6)	17·7 (12·9 to 23·3)	23·9 (16·8 to 32·4)	27·3 (19·2 to 37·3)	24·5 (17·2 to 32·9)
Neonatal encephalopathy due to birth asphyxia and trauma	156 (90·6 to 242)	29·2 (12·5 to 54·7)	15·9 (7·8 to 27·7)	6·5 (4·1 to 9·5)	4·9 (2·4 to 8·2)	5·7 (2·8 to 9·2)	5·3 (2·6 to 8·9)
Neonatal sepsis and other neonatal infections	105 (68·6 to 151)	89·7 (46·1 to 158)	33·6 (19·2 to 54·4)	6·9 (3·9 to 11)	9 (4·8 to 15)	10·4 (5·4 to 16·6)	9·7 (5·1 to 16·4)
Hemolytic disease and other neonatal jaundice	1·3 (0·6 to 2·1)	0·8 (0·4 to 1·4)	0·6 (0·3 to 0·9)	1·8 (0·9 to 3·1)	2·6 (1·3 to 4·6)	3 (1·7 to 5·1)	2·8 (1·5 to 4·6)
Other neonatal disorders	130 (86·9 to 190)	48·8 (25·4 to 87·1)	24·3 (13·8 to 38·7)	12·7 (9·7 to 16·3)	0 (0 to 0)	0 (0 to 0)	0 (0 to 0)
Nutritional deficiencies	0·1 (0·1 to 0·2)	2 (0·9 to 3·6)	29 (18·1 to 44·4)	57·7 (24·1 to 119)	86·4 (29 to 191)	44·7 (16·3 to 106)	28·6 (14 to 53·8)
Protein-energy malnutrition	0·1 (0 to 0·1)	0·2 (0·1 to 0·3)	17·4 (11·2 to 25·1)	9·7 (5·5 to 16·2)	8·4 (5 to 13·1)	7·6 (4·3 to 12·3)	8·1 (4·8 to 12·8)
Iodine deficiency	0 (0 to 0)	0 (0 to 0)	0 (0 to 0)	0 (0 to 0)	0·1 (0 to 0·2)	0·3 (0·1 to 0·6)	0·4 (0·1 to 0·8)
Vitamin A deficiency	0 (0 to 0)	0 (0 to 0·1)	0·2 (0·1 to 0·4)	1·2 (0·6 to 2·1)	1·5 (0·7 to 3·1)	1·1 (0·4 to 2·1)	0·6 (0·2 to 1·2)
Dietary iron deficiency	0 (0 to 0·1)	1·7 (0·7 to 3·3)	10·8 (3·1 to 23·9)	46·5 (13·3 to 105)	74·8 (17·8 to 177)	33·9 (7 to 95·1)	16·2 (3·6 to 41·3)
Other nutritional deficiencies	0 (0 to 0)	0 (0 to 0)	0·7 (0·3 to 1·4)	0·4 (0·2 to 0·9)	1·6 (0·8 to 2·7)	1·9 (1 to 3·2)	3·4 (1·8 to 5·5)
Non-communicable diseases	121 (79·9 to 193)	71·2 (40·9 to 120)	303 (227 to 396)	367 (277 to 476)	465 (351 to 595)	714 (537 to 933)	981 (754 to 1,240)
Neoplasms	1 (0·7 to 1·5)	1 (0·6 to 1·8)	8·8 (6 to 12·2)	37·7 (24·4 to 55·9)	46·4 (35·4 to 58·6)	47·6 (35·6 to 62·5)	68·3 (51 to 88·4)
Lip and oral cavity cancer	0 (0 to 0)	0 (0 to 0)	0 (0 to 0)	0 (0 to 0)	0·2 (0·1 to 0·3)	0·3 (0·2 to 0·5)	0·6 (0·5 to 0·8)
Nasopharynx cancer	0 (0 to 0)	0 (0 to 0)	0 (0 to 0)	0 (0 to 0)	0·2 (0·1 to 0·3)	0·5 (0·4 to 0·7)	0·8 (0·5 to 1·1)
Other pharynx cancer	0 (0 to 0)	0 (0 to 0)	0 (0 to 0)	0 (0 to 0)	0 (0 to 0)	0 (0 to 0)	0 (0 to 0)
Esophageal cancer	0 (0 to 0)	0 (0 to 0)	0 (0 to 0)	0 (0 to 0)	0 (0 to 0)	0 (0 to 0)	0 (0 to 0)
Stomach cancer	0 (0 to 0)	0 (0 to 0)	0 (0 to 0)	0 (0 to 0)	0 (0 to 0)	0 (0 to 0)	1·1 (0·8 to 1·4)
Colon and rectum cancer	0 (0 to 0)	0 (0 to 0)	0 (0 to 0)	0 (0 to 0)	0·5 (0·3 to 0·8)	0·6 (0·4 to 0·9)	3·2 (2·2 to 4·4)
Liver cancer	0 (0 to 0·1)	0 (0 to 0)	0·2 (0·1 to 0·4)	0·5 (0·3 to 0·7)	0·6 (0·4 to 0·8)	1 (0·7 to 1·3)	0·9 (0·6 to 1·3)
Liver cancer due to hepatitis B	0 (0 to 0)	0 (0 to 0)	0 (0 to 0)	0 (0 to 0)	0 (0 to 0)	0·3 (0·2 to 0·5)	0·5 (0·3 to 0·7)
Liver cancer due to hepatitis C	0 (0 to 0)	0 (0 to 0)	0 (0 to 0)	0 (0 to 0)	0 (0 to 0)	0 (0 to 0)	0 (0 to 0)
Liver cancer due to alcohol use	0 (0 to 0)	0 (0 to 0)	0 (0 to 0)	0 (0 to 0)	0 (0 to 0)	0 (0 to 0)	0 (0 to 0·1)
Liver cancer due to NASH	0 (0 to 0)	0 (0 to 0)	0 (0 to 0)	0 (0 to 0)	0 (0 to 0)	0 (0 to 0)	0·2 (0·1 to 0·3)
Liver cancer due to other causes	0 (0 to 0·1)	0 (0 to 0)	0·2 (0·1 to 0·4)	0·5 (0·3 to 0·7)	0·6 (0·4 to 0·8)	0·6 (0·5 to 0·9)	0·2 (0·1 to 0·3)
Gallbladder and biliary tract cancer	0 (0 to 0)	0 (0 to 0)	0 (0 to 0)	0 (0 to 0)	0 (0 to 0)	0 (0 to 0)	0 (0 to 0)
Pancreatic cancer	0 (0 to 0)	0 (0 to 0)	0 (0 to 0)	0 (0 to 0)	0 (0 to 0)	0 (0 to 0)	0·5 (0·4 to 0·6)
Larynx cancer	0 (0 to 0)	0 (0 to 0)	0 (0 to 0)	0 (0 to 0)	0 (0 to 0)	0 (0 to 0)	0 (0 to 0)
Tracheal, bronchus, and lung cancer	0 (0 to 0)	0 (0 to 0)	0 (0 to 0)	0 (0 to 0)	0 (0 to 0)	0·3 (0·2 to 0·4)	2·1 (1·5 to 2·8)
Malignant skin melanoma	0 (0 to 0)	0 (0 to 0)	0·1 (0 to 0·5)	0·3 (0·1 to 2·3)	0·2 (0·1 to 0·3)	0·2 (0·1 to 0·4)	0·6 (0·3 to 0·9)
Non-melanoma skin cancer	0 (0 to 0)	0 (0 to 0)	0 (0 to 0)	0 (0 to 0)	0 (0 to 0)	0 (0 to 0)	0 (0 to 0)
Non-melanoma skin cancer (squamous-cell carcinoma)	0 (0 to 0)	0 (0 to 0)	0 (0 to 0)	0 (0 to 0)	0 (0 to 0)	0 (0 to 0)	0 (0 to 0)
Non-melanoma skin cancer (basal-cell carcinoma)	0 (0 to 0)	0 (0 to 0)	0 (0 to 0)	0 (0 to 0)	0 (0 to 0)	0 (0 to 0)	0 (0 to 0)
Breast cancer	0 (0 to 0)	0 (0 to 0)	0 (0 to 0)	0 (0 to 0)	0 (0 to 0)	0 (0 to 0)	0·1 (0·1 to 0·1)
Cervical cancer	--	--	--	--	--	--	--
Uterine cancer	--	--	--	--	--	--	--
Ovarian cancer	--	--	--	--	--	--	--
Prostate cancer	0 (0 to 0)	0 (0 to 0)	0 (0 to 0)	0 (0 to 0)	0 (0 to 0)	0 (0 to 0)	0 (0 to 0)
Testicular cancer	0 (0 to 0·1)	0 (0 to 0·1)	0 (0 to 0·1)	0·1 (0·1 to 0·3)	0·1 (0·1 to 0·2)	0·1 (0·1 to 0·2)	0·4 (0·2 to 0·6)
Kidney cancer	0·1 (0·1 to 0·1)	0 (0 to 0·1)	0·6 (0·4 to 0·9)	3·2 (2 to 5·1)	2·7 (1·8 to 3·9)	1·2 (0·9 to 1·7)	2 (1·3 to 2·8)
Bladder cancer	0 (0 to 0)	0 (0 to 0)	0 (0 to 0)	0 (0 to 0)	0 (0 to 0)	0 (0 to 0)	0·1 (0·1 to 0·1)
Brain and central nervous system cancer	0·1 (0 to 0·2)	0·1 (0 to 0·2)	1·4 (0·6 to 2·4)	5·4 (2·6 to 8·8)	8·2 (4·2 to 13·1)	7·2 (3·7 to 11·5)	5·3 (2·2 to 8·7)
Thyroid cancer	0 (0 to 0)	0 (0 to 0)	0 (0 to 0)	0 (0 to 0)	0·1 (0·1 to 0·2)	0·1 (0·1 to 0·2)	0·3 (0·2 to 0·4)
Mesothelioma	0 (0 to 0)	0 (0 to 0)	0 (0 to 0)	0 (0 to 0)	0 (0 to 0)	0 (0 to 0)	0 (0 to 0)
Hodgkin lymphoma	0 (0 to 0)	0 (0 to 0)	0 (0 to 0)	0·1 (0·1 to 0·1)	0·2 (0·1 to 0·2)	0·3 (0·2 to 0·4)	1·3 (0·8 to 1·9)
Non-Hodgkin lymphoma	0 (0 to 0)	0 (0 to 0)	0 (0 to 0)	3·6 (2·1 to 5·7)	5·8 (3·8 to 9)	6·3 (3·9 to 9·8)	10·6 (7 to 15·4)
Multiple myeloma	0 (0 to 0)	0 (0 to 0)	0 (0 to 0)	0 (0 to 0)	0 (0 to 0)	0 (0 to 0)	0 (0 to 0)
Leukemia	0·3 (0·2 to 0·4)	0·3 (0·2 to 0·5)	2·3 (1·4 to 3·6)	9·3 (5·5 to 15·3)	14·6 (9·5 to 21·7)	16·1 (10·3 to 24·7)	19·8 (13·3 to 28·2)
Acute lymphoid leukemia	0·1 (0 to 0·1)	0 (0 to 0)	0·7 (0·4 to 1·3)	4·9 (2·7 to 8·1)	9 (5·5 to 14)	7·9 (4·7 to 12·7)	9·8 (6·2 to 14·4)
Chronic lymphoid leukemia	0 (0 to 0)	0 (0 to 0)	0 (0 to 0)	0 (0 to 0)	0 (0 to 0)	0 (0 to 0)	0 (0 to 0)
Acute myeloid leukemia	0·1 (0·1 to 0·1)	0·2 (0·1 to 0·3)	1 (0·5 to 1·7)	3·1 (1·5 to 5·8)	3·6 (1·9 to 6·5)	5·4 (2·9 to 9·1)	7 (4·2 to 11·1)

Cause	20 to 24	25 to 29	30 to 34	35 to 39	40 to 44	45 to 49	50 to 54	55 to 59
All causes	3,720 (3,230 to 4,260)	4,330 (3,830 to 4,880)	4,390 (3,880 to 4,970)	4,840 (4,210 to 5,590)	5,300 (4,540 to 6,190)	5,750 (4,810 to 6,820)	6,110 (5,130 to 7,280)	5,980 (4,990 to 7,150)
Communicable, maternal, neonatal, and nutritional diseases	278 (241 to 326)	382 (336 to 435)	555 (501 to 614)	743 (677 to 822)	862 (791 to 947)	799 (724 to 886)	601 (540 to 672)	412 (354 to 479)
HIV/AIDS and sexually transmitted infections	106 (96·7 to 117)	187 (172 to 206)	353 (326 to 384)	533 (490 to 581)	646 (598 to 701)	576 (531 to 625)	374 (346 to 402)	201 (184 to 216)
HIV/AIDS	104 (94·1 to 114)	185 (169 to 203)	350 (323 to 381)	531 (487 to 579)	644 (596 to 699)	574 (529 to 622)	372 (344 to 400)	200 (183 to 215)
HIV/AIDS - Drug-susceptible Tuberculosis	2·8 (1·9 to 4·1)	5·5 (3·6 to 8·1)	11·1 (7 to 16·5)	17·4 (10·9 to 25·9)	21·6 (13·6 to 32·5)	17 (10·6 to 25·6)	11·1 (6·9 to 16·6)	6 (3·7 to 8·8)
HIV/AIDS - Multidrug-resistant Tuberculosis without extensive dru	0·1 (0 to 0·4)	0·2 (0 to 0·9)	0·4 (0·1 to 1·8)	0·7 (0·1 to 2·7)	0·8 (0·1 to 3·4)	0·7 (0·1 to 2·7)	0·4 (0·1 to 1·8)	0·2 (0 to 1)
HIV/AIDS - Extensively drug-resistant Tuberculosis	0 (0 to 0·1)	0 (0 to 0·1)	0·1 (0 to 0·3)	0·1 (0 to 0·4)	0·1 (0 to 0·6)	0·1 (0 to 0·4)	0·1 (0 to 0·3)	0 (0 to 0·2)
HIV/AIDS resulting in other diseases	101 (90·9 to 111)	179 (163 to 197)	339 (311 to 369)	513 (468 to 561)	621 (571 to 676)	557 (510 to 605)	361 (332 to 390)	193 (177 to 210)
Sexually transmitted infections excluding HIV	2·4 (1·2 to 4·4)	2·7 (1·3 to 4·8)	2·9 (1·4 to 5·1)	2·7 (1·3 to 4·6)	2·4 (1·1 to 4·5)	2·1 (1·1 to 4)	1·6 (0·7 to 3)	1·1 (0·5 to 2·1)
Syphilis	0·1 (0 to 0·2)	0·2 (0·1 to 0·3)	0·2 (0·1 to 0·3)	0·2 (0·1 to 0·4)	0·1 (0·1 to 0·2)	0·2 (0·1 to 0·3)	0·2 (0·1 to 0·3)	0·2 (0·1 to 0·2)
Chlamydial infection	0·5 (0·1 to 1·2)	0·8 (0·2 to 1·8)	0·8 (0·2 to 1·6)	0·7 (0·2 to 1·4)	0·6 (0·1 to 1·3)	0·4 (0·1 to 1)	0·2 (0 to 0·5)	0·1 (0 to 0·1)
Gonococcal infection	0·7 (0·2 to 1·5)	0·3 (0 to 0·8)	0·2 (0 to 0·6)	0·1 (0·1 to 0·3)	0·1 (0·1 to 0·3)	0·1 (0·1 to 0·2)	0·1 (0 to 0·2)	0 (0 to 0·1)
Trichomoniasis	0 (0 to 0·1)	0·1 (0 to 0·1)	0·1 (0 to 0·2)	0·1 (0 to 0·3)	0·1 (0 to 0·2)	0·1 (0 to 0·2)	0·1 (0 to 0·1)	0 (0 to 0·1)
Genital herpes	0·4 (0·1 to 0·9)	0·6 (0·2 to 1·5)	0·8 (0·3 to 2)	0·9 (0·3 to 2·2)	1 (0·3 to 2·3)	1 (0·3 to 2·4)	0·9 (0·3 to 2·1)	0·7 (0·2 to 1·8)
Other sexually transmitted infections	0·7 (0·3 to 1·4)	0·7 (0·3 to 1·3)	0·9 (0·3 to 1·7)	0·6 (0·2 to 1·2)	0·4 (0·2 to 0·9)	0·4 (0·1 to 0·7)	0·2 (0·1 to 0·4)	0·1 (0 to 0·1)
Respiratory infections and tuberculosis	68·6 (50·3 to 93·1)	87·9 (64·5 to 116)	99·3 (74·8 to 130)	107 (78 to 147)	114 (79·9 to 157)	118 (83·3 to 163)	129 (91·6 to 175)	123 (83·9 to 174)
Tuberculosis	16·3 (10·5 to 25·1)	21·3 (14·5 to 29·7)	22·9 (16·6 to 31)	21·4 (14·8 to 30·2)	21·3 (14·7 to 30·4)	21·9 (14·6 to 31·9)	21·4 (14·3 to 31·3)	17·4 (11·7 to 24·6)
Latent tuberculosis infection	0 (0 to 0)	0 (0 to 0)	0 (0 to 0)	0 (0 to 0)	0 (0 to 0)	0 (0 to 0)	0 (0 to 0)	0 (0 to 0)
Drug-susceptible tuberculosis	15·7 (10·1 to 24·4)	20·6 (13·6 to 28·9)	22·1 (15·7 to 29·7)	20·6 (14 to 29·2)	20·6 (13·8 to 29)	21·1 (14·2 to 30·8)	20·6 (13·7 to 30·6)	16·8 (11·2 to 24)
Multidrug-resistant tuberculosis without extensive drug resistanc	0·5 (0·1 to 2)	0·7 (0·1 to 2·6)	0·7 (0·1 to 2·7)	0·6 (0·1 to 2·5)	0·6 (0·1 to 2·6)	0·7 (0·1 to 2·9)	0·7 (0·1 to 2·5)	0·6 (0·1 to 2·2)
Extensively drug-resistant tuberculosis	0·1 (0 to 0·3)	0·1 (0 to 0·4)	0·1 (0 to 0·4)	0·1 (0 to 0·4)	0·1 (0 to 0·4)	0·1 (0 to 0·4)	0·1 (0 to 0·4)	0·1 (0 to 0·4)
Lower respiratory infections	35·9 (22·5 to 54·7)	51·6 (33·7 to 75·4)	63·2 (41·8 to 91·5)	73 (47·6 to 108)	80 (49·5 to 118)	84 (53·7 to 125)	97·3 (64·5 to 141)	97·3 (60·7 to 144)
Upper respiratory infections	12·8 (6·7 to 22·7)	12·1 (6·6 to 20·8)	10·8 (5·8 to 18·5)	10·4 (5·2 to 18)	9·9 (5·2 to 17·7)	9 (4·7 to 15·8)	7·7 (4·2 to 13·4)	6·1 (3·3 to 10·2)
Otitis media	3·6 (1·6 to 7·1)	3 (1·3 to 5·5)	2·4 (1 to 4·5)	2·3 (1·1 to 4·4)	2·5 (1·1 to 4·9)	2·7 (1·4 to 4·7)	2·4 (1·1 to 4·4)	1·9 (0·9 to 3·4)
Enteric infections	16 (10·3 to 23·6)	17·8 (11·7 to 25·8)	18·4 (12·6 to 25·3)	19·7 (13·7 to 26·6)	21·3 (14·9 to 29·1)	22·3 (15·9 to 30·6)	21·9 (15·3 to 30·4)	20 (14 to 27·2)
Diarrheal diseases	15·9 (10·1 to 23·4)	17·7 (11·5 to 25·6)	18·1 (12·4 to 25·2)	19·5 (13·5 to 26·5)	21·2 (14·8 to 28·9)	22·2 (15·8 to 30·4)	21·7 (15·2 to 30·2)	19·9 (13·9 to 27·1)
Typhoid and paratyphoid	0 (0 to 0)	0 (0 to 0)	0 (0 to 0)	0 (0 to 0)	0 (0 to 0)	0 (0 to 0)	0 (0 to 0)	0 (0 to 0)
Typhoid fever	0 (0 to 0)	0 (0 to 0)	0 (0 to 0)	0 (0 to 0)	0 (0 to 0)	0 (0 to 0)	0 (0 to 0)	0 (0 to 0)
Paratyphoid fever	0 (0 to 0)	0 (0 to 0)	0 (0 to 0)	0 (0 to 0)	0 (0 to 0)	0 (0 to 0)	0 (0 to 0)	0 (0 to 0)
Invasive Non-typhoidal Salmonella (iNTS)	0·1 (0 to 0·2)	0·1 (0 to 0·3)	0·1 (0 to 0·3)	0·1 (0 to 0·3)	0·1 (0 to 0·1)	0·1 (0 to 0·3)	0·1 (0 to 0·3)	0·1 (0 to 0·2)
Other intestinal infectious diseases	0·1 (0 to 0·2)	0 (0 to 0·1)	0·1 (0 to 0·2)	0 (0 to 0·1)	0 (0 to 0·1)	0 (0 to 0·1)	0 (0 to 0·1)	0 (0 to 0)
Neglected tropical diseases and malaria	4·2 (2 to 7·3)	4·9 (2·3 to 9·1)	5·3 (2·5 to 9·3)	5·6 (2·5 to 10·8)	6·6 (3·1 to 13·7)	7·8 (3·6 to 15·2)	8·4 (3·6 to 17·2)	9·2 (4·1 to 18·5)
Malaria	0 (0 to 0)	0 (0 to 0)	0 (0 to 0)	0 (0 to 0)	0 (0 to 0)	0 (0 to 0)	0 (0 to 0)	0 (0 to 0)
Chagas disease	0 (0 to 0)	0 (0 to 0)	0 (0 to 0)	0 (0 to 0)	0 (0 to 0)	0 (0 to 0)	0 (0 to 0)	0 (0 to 0)
Leishmaniasis	0 (0 to 0)	0 (0 to 0)	0 (0 to 0)	0 (0 to 0)	0 (0 to 0)	0 (0 to 0)	0 (0 to 0)	0 (0 to 0)
Visceral leishmaniasis	0 (0 to 0)	0 (0 to 0)	0 (0 to 0)	0 (0 to 0)	0 (0 to 0)	0 (0 to 0)	0 (0 to 0)	0 (0 to 0)
Cutaneous and mucocutaneous leishmaniasis	0 (0 to 0)	0 (0 to 0)	0 (0 to 0)	0 (0 to 0)	0 (0 to 0)	0 (0 to 0)	0 (0 to 0)	0 (0 to 0)
African trypanosomiasis	0 (0 to 0)	0 (0 to 0)	0 (0 to 0)	0 (0 to 0)	0 (0 to 0)	0 (0 to 0)	0 (0 to 0)	0 (0 to 0)
Schistosomiasis	0 (0 to 0)	0 (0 to 0)	0 (0 to 0)	0 (0 to 0)	0 (0 to 0)	0 (0 to 0)	0 (0 to 0)	0 (0 to 0)
Cysticercosis	1·5 (0·2 to 3·8)	2·6 (0·7 to 6·2)	3·1 (0·9 to 7)	3·6 (1 to 8·3)	4·5 (1·4 to 11·3)	5·6 (1·7 to 13·3)	6·6 (2 to 15·1)	7·5 (2·5 to 16·5)
Cystic echinococcosis	0 (0 to 0)	0 (0 to 0)	0 (0 to 0)	0 (0 to 0)	0 (0 to 0)	0 (0 to 0)	0 (0 to 0)	0 (0 to 0)
Lymphatic filariasis	0 (0 to 0)	0 (0 to 0)	0 (0 to 0)	0 (0 to 0)	0 (0 to 0)	0 (0 to 0)	0 (0 to 0)	0 (0 to 0)
Onchocerciasis	0 (0 to 0)	0 (0 to 0)	0 (0 to 0)	0 (0 to 0)	0 (0 to 0)	0 (0 to 0)	0 (0 to 0)	0 (0 to 0)
Trachoma	0 (0 to 0)	0 (0 to 0)	0 (0 to 0)	0 (0 to 0)	0 (0 to 0)	0 (0 to 0)	0 (0 to 0)	0 (0 to 0)
Dengue	1·4 (0·3 to 2·9)	1·3 (0·3 to 2·9)	1·1 (0·3 to 2·3)	1·1 (0·3 to 2·3)	1 (0·2 to 2·1)	0·9 (0·2 to 1·9)	0·8 (0·2 to 1·7)	0·6 (0·1 to 1·4)
Yellow fever	0 (0 to 0)	0 (0 to 0)	0 (0 to 0)	0 (0 to 0)	0 (0 to 0)	0 (0 to 0)	0 (0 to 0)	0 (0 to 0)
Rabies	0 (0 to 0)	0 (0 to 0)	0 (0 to 0)	0 (0 to 0)	0 (0 to 0)	0 (0 to 0)	0 (0 to 0)	0 (0 to 0)
Intestinal nematode infections	0·1 (0 to 0·2)	0·1 (0 to 0·2)	0·1 (0 to 0·1)	0·1 (0 to 0·1)	0·1 (0 to 0·1)	0·1 (0 to 0·1)	0·1 (0 to 0·1)	0·1 (0 to 0·1)
Ascariasis	0 (0 to 0·1)	0 (0 to 0·1)	0 (0 to 0·1)	0 (0 to 0·1)	0 (0 to 0)	0 (0 to 0)	0 (0 to 0)	0 (0 to 0)
Trichuriasis	0 (0 to 0)	0 (0 to 0)	0 (0 to 0)	0 (0 to 0)	0 (0 to 0)	0 (0 to 0)	0 (0 to 0)	0 (0 to 0)
Hookworm disease	0·1 (0 to 0·2)	0·1 (0 to 0·1)	0 (0 to 0·1)	0 (0 to 0·1)	0 (0 to 0·1)	0 (0 to 0·1)	0 (0 to 0·1)	0 (0 to 0·1)
Food-borne trematodiases	0 (0 to 0)	0 (0 to 0)	0 (0 to 0)	0 (0 to 0)	0 (0 to 0)	0 (0 to 0)	0 (0 to 0)	0 (0 to 0)
Leprosy	0 (0 to 0)	0 (0 to 0)	0 (0 to 0)	0 (0 to 0)	0 (0 to 0)	0 (0 to 0·1)	0 (0 to 0·1)	0 (0 to 0·1)
Ebola	0 (0 to 0)	0 (0 to 0)	0 (0 to 0)	0 (0 to 0)	0 (0 to 0)	0 (0 to 0)	0 (0 to 0)	0 (0 to 0)
Zika virus	0·1 (0 to 0·7)	0·1 (0 to 0·7)	0·2 (0 to 0·8)	0·1 (0 to 0·7)	0·1 (0 to 0·6)	0·1 (0 to 0·4)	0·1 (0 to 0·3)	0 (0 to 0·2)
Guinea worm disease	0 (0 to 0)	0 (0 to 0)	0 (0 to 0)	0 (0 to 0)	0 (0 to 0)	0 (0 to 0)	0 (0 to 0)	0 (0 to 0)
Other neglected tropical diseases	1 (0·3 to 2·1)	0·7 (0·2 to 1·8)	0·9 (0·3 to 1·8)	0·7 (0·2 to 1·7)	0·9 (0·3 to 2)	1·1 (0·4 to 2·4)	0·9 (0·3 to 2)	1 (0·3 to 2·1)
Other infectious diseases	17·4 (13 to 23·1)	19·9 (14·6 to 26·9)	20·8 (15·3 to 28·2)	22 (15·9 to 29·9)	21·9 (15·6 to 30·4)	22·3 (15·6 to 32·3)	19 (13·2 to 26)	15·5 (10·9 to 21·2)
Meningitis	5·3 (3·1 to 8·3)	6 (3·7 to 9)	6·3 (3·8 to 9·7)	6·4 (3·8 to 9·6)	6·1 (3·8 to 9·1)	6 (3·5 to 9·2)	5·6 (3·5 to 8·5)	4·4 (2·8 to 6·4)
Encephalitis	5·5 (3·3 to 8·5)	4·9 (3·1 to 7·4)	4·9 (3·1 to 7·2)	4·4 (2·8 to 6·6)	4·5 (3 to 6·7)	3·5 (2·3 to 5·6)	2·5 (1·6 to 3·7)	2·2 (1·4 to 3·3)
Diphtheria	0 (0 to 0)	0 (0 to 0)	0 (0 to 0)	0 (0 to 0)	0 (0 to 0)	0 (0 to 0)	0 (0 to 0)	0 (0 to 0)
Tetanus	0 (0 to 0·1)	0·1 (0 to 0·1)	0·1 (0 to 0·2)	0·1 (0 to 0·2)	0·1 (0 to 0·3)	0·1 (0 to 0·4)	0·1 (0 to 0·4)	0·1 (0 to 0·4)
Measles	0 (0 to 0)	0 (0 to 0)	0 (0 to 0)	0 (0 to 0)	0 (0 to 0)	0 (0 to 0)	0 (0 to 0)	0 (0 to 0)
Varicella and herpes zoster	0·8 (0·3 to 1·3)	0·9 (0·4 to 1·5)	1 (0·5 to 1·7)	1·4 (0·7 to 2·6)	1·1 (0·5 to 1·8)	1·4 (0·7 to 2·6)	1·2 (0·6 to 2)	0·9 (0·5 to 1·5)
Acute hepatitis	1·5 (1 to 2·1)	1·4 (1 to 2)	1·3 (0·8 to 1·8)	1·3 (0·8 to 1·8)	1·1 (0·8 to 1·6)	1 (0·6 to 1·5)	0·9 (0·6 to 1·2)	0·6 (0·4 to 0·8)
Acute hepatitis A	1 (0·6 to 1·6)	0·7 (0·3 to 1·2)	0·6 (0·3 to 1)	0·5 (0·2 to 0·8)	0·4 (0·2 to 0·7)	0·4 (0·2 to 0·7)	0·2 (0·1 to 0·4)	0·1 (0 to 0·3)
Acute hepatitis B	0·3 (0·1 to 0·5)	0·5 (0·3 to 0·8)	0·5 (0·3 to 0·8)	0·6 (0·4 to 0·9)	0·6 (0·3 to 0·8)	0·4 (0·2 to 0·6)	0·5 (0·3 to 0·7)	0·4 (0·2 to 0·5)
Acute hepatitis C	0·1 (0 to 0·1)	0·1 (0 to 0·2)	0·1 (0 to 0·1)	0·1 (0 to 0·1)	0·1 (0 to 0·2)	0·1 (0 to 0·3)	0·1 (0 to 0·2)	0·1 (0 to 0·1)
Acute hepatitis E	0·1 (0 to 0·3)	0·2 (0·1 to 0·3)	0·1 (0·1 to 0·2)	0·1 (0·1 to 0·2)	0·1 (0 to 0·2)	0·1 (0 to 0·2)	0 (0 to 0·1)	0 (0 to 0·1)
Other unspecified infectious diseases	4·3 (2 to 7·4)	6·6 (2·9 to 11·8)	7·2 (3·4 to 12·5)	8·4 (3·9 to 14·2)	9 (4·2 to 15·2)	10·2 (4·8 to 17·6)	8·6 (4·3 to 13·6)	7·2 (3·6 to 11·3)
Maternal and neonatal disorders	39·2 (28·2 to 51·3)	36·2 (26·3 to 47·5)	31 (22·1 to 41·1)	27·8 (20·3 to 36·6)	24·8 (17·8 to 32·6)	21·6 (15·3 to 28·7)	16·3 (11·9 to 21·6)	11·3 (8·3 to 14·7)
Maternal disorders	0 (0 to 0)	0 (0 to 0)	0 (0 to 0)	0 (0 to 0)	0 (0 to 0)	0 (0 to 0)	0 (0 to 0)	0 (0 to 0)
Maternal hemorrhage	0 (0 to 0)	0 (0 to 0)	0 (0 to 0)	0 (0 to 0)	0 (0 to 0)	0 (0 to 0)	0 (0 to 0)	0 (0 to 0)
Maternal sepsis and other maternal infections	0 (0 to 0)	0 (0 to 0)	0 (0 to 0)	0 (0 to 0)	0 (0 to 0)	0 (0 to 0)	0 (0 to 0)	0 (0 to 0)
Maternal hypertensive disorders	0 (0 to 0)	0 (0 to 0)	0 (0 to 0)	0 (0 to 0)	0 (0 to 0)	0 (0 to 0)	0 (0 to 0)	0 (0 to 0)
Maternal obstructed labor and uterine rupture	--	--	--	--	--	--	--	--
Maternal abortion and miscarriage	--	--	--	--	--	--	--	--
Ectopic pregnancy	--	--	--	--	--	--	--	--
Indirect maternal deaths	--	--	--	--	--	--	--	--
Late maternal deaths	--	--	--	--	--	--	--	--
Maternal deaths aggravated by HIV/AIDS	--	--	--	--	--	--	--	--
Neonatal disorders	39·2 (28·2 to 51·3)	36·2 (26·3 to 47·5)	31 (22·1 to 41·1)	27·8 (20·3 to 36·6)	24·8 (17·8 to 32·6)	21·6 (15·3 to 28·7)	16·3 (11·9 to 21·6)	11·3 (8·3 to 14·7)
Neonatal preterm birth	22 (15·3 to 30)	20 (13·9 to 27·3)	16·6 (11·2 to 22·9)	14·6 (10·2 to 19·9)	12·6 (8·6 to 17·4)	10·6 (7·2 to 14·5)	7·6 (5·4 to 10·2)	4·9 (3·8 to 6·2)
Neonatal encephalopathy due to birth asphyxia and trauma	5·1 (2·4 to 8·5)	4·8 (2·4 to 7·9)	4·2 (2 to 7·2)	3·9 (1·9 to 6·7)	3·5 (1·6 to 5·9)	3·2 (1·6 to 5·3)	2·6 (1·4 to 4·4)	2 (1·2 to 2·9)
Neonatal sepsis and other neonatal infections	9·2 (5 to 14·8)	8·7 (4·5 to 14·6)	7·7 (4 to 12·6)	7·1 (3·6 to 11·9)	6·5 (3·2 to 11·2)	5·9 (2·9 to 9·5)	4·6 (2·4 to 7·7)	3·3 (1·6 to 5·6)
Hemolytic disease and other neonatal jaundice	2·8 (2·1 to 3·6)	2·7 (2 to 3·5)	2·4 (1·8 to 3·1)	2·2 (1·7 to 2·9)	2·1 (1·6 to 2·7)	1·9 (1·4 to 2·5)	1·6 (1·2 to 2)	1·1 (0·8 to 1·4)
Other neonatal disorders	0 (0 to 0)	0 (0 to 0)	0 (0 to 0)	0 (0 to 0)	0 (0 to 0)	0 (0 to 0)	0 (0 to 0)	0 (0 to 0)
Nutritional deficiencies	26·3 (13·9 to 48·6)	27·6 (15·5 to 49)	27 (14·3 to 47·1)	27·5 (15·8 to 49·3)	27·6 (16·1 to 46·9)	30·8 (16·7 to 58·4)	32·2 (18·7 to 59·2)	32 (17·6 to 57·1)
Protein-energy malnutrition	8·8 (5·4 to 13·7)	9·9 (6·2 to 14·9)	10·1 (6·5 to 15)	11·3 (7·4 to 16·1)	10·8 (7·2 to 15·4)	11·8 (8 to 16·8)	12·1 (8·1 to 16·7)	11·1 (7·5 to 15·5)
Iodine deficiency	0·5 (0·1 to 0·9)	0·5 (0·2 to 1)	0·5 (0·2 to 1)	0·5 (0·2 to 1)	0·5 (0·2 to 0·9)	0·5 (0·2 to 0·8)	0·4 (0·2 to 0·7)	0·3 (0·1 to 0·6)
Vitamin A deficiency	0·5 (0·2 to 0·9)	0·4 (0·2 to 0·8)	0·4 (0·2 to 0·7)	0·3 (0·2 to 0·6)	0·3 (0·2 to 0·6)	0·3 (0·1 to 0·5)	0·3 (0·1 to 0·4)	0·2 (0·1 to 0·4)
Dietary iron deficiency	12·1 (2·5 to 33·4)	11·3 (2·4 to 31·6)	12·2 (2·7 to 32·2)	11·4 (2·5 to 30·9)	11·5 (2·5 to 29·2)	14·6 (3 to 40)	14·8 (3·9 to 39·9)	16·4 (4·3 to 39·3)
Other nutritional deficiencies	4·4 (2·6 to 6·9)	5·4 (3·2 to 8·8)	3·8 (2·4 to 5·8)	3·9 (2·4 to 6)	4·5 (2·8 to 6·5)	3·6 (2·1 to 5·4)	4·7 (3 to 6·9)	3·9 (2·5 to 5·8)
Non-communicable diseases	1,260 (997 to 1,540)	1,630 (1,330 to 1,940)	1,920 (1,610 to 2,280)	2,460 (2,070 to 2,940)	3,130 (2,580 to 3,770)	3,920 (3,200 to 4,750)	4,710 (3,860 to 5,710)	4,970 (4,110 to 6,000)
Neoplasms	105 (84·1 to 131)	148 (120 to 180)	199 (164 to 241)	283 (224 to 358)	428 (332 to 547)	617 (469 to 803)	884 (686 to 1,150)	1,080 (839 to 1,380)
Lip and oral cavity cancer	1·5 (1·1 to 2)	2 (1·4 to 2·7)	4·2 (2·7 to 6)	8·4 (5·6 to 11·9)	17·1 (11·1 to 24·9)	28·3 (19·5 to 40·2)	40·8 (27·9 to 57·1)	44·2 (30·7 to 60·3)
Nasopharynx cancer	1·2 (0·8 to 1·6)	1·5 (1 to 2)	1·5 (1 to 2)	2·1 (1·4 to 3·2)	4·3 (2·8 to 6·2)	5·9 (4 to 8·4)	8·3 (5·5 to 12·1)	8·9 (6·3 to 12·4)
Other pharynx cancer	0·8 (0·5 to 1·2)	1 (0·6 to 1·4)	1·7 (1·2 to 2·3)	4 (2·5 to 5·9)	9·1 (5·9 to 13·7)	19·6 (13·4 to 29·1)	26·9 (18·4 to 38·6)	30·2 (21·3 to 42·9)
Esophageal cancer	1·1 (0·8 to 1·5)	2 (1·4 to 2·8)	4·4 (2·9 to 6·4)	12·8 (8·5 to 18·7)	31·4 (21·6 to 44·6)	55·8 (39·3 to 78·7)	76·3 (53·6 to 106)	88·3 (63·1 to 126)
Stomach cancer	2·6 (2 to 3·5)	6·8 (4·6 to 9·4)	15 (10·7 to 20·4)	23·8 (16 to 33·9)	38·4 (26·5 to 52·7)	47·1 (33·1 to 64·2)	67·6 (48·7 to 92·4)	77·8 (55·8 to 104)
Colon and rectum cancer	8·5 (6·1 to 11·8)	16·9 (11·1 to 25·7)	32 (23·2 to 43·2)	45·7 (31·3 to 66·4)	62·7 (46·1 to 85·9)	79·3 (54·6 to 110)	115 (79·3 to 158)	137 (96·2 to 187)
Liver cancer	2·2 (1·4 to 3·3)	4·7 (2·9 to 7·3)	5·8 (3·8 to 8·5)	10·5 (6·9 to 15·4)	15·7 (10·6 to 21·9)	21 (13·8 to 30·5)	28·7 (17·9 to 43·7)	35·1 (23·9 to 48·9)
Liver cancer due to hepatitis B	1·4 (0·9 to 2·1)	3·1 (1·8 to 5·1)	3·8 (2·4 to 5·7)	6·4 (3·5 to 9·8)	8·7 (5 to 13·2)	10·3 (5·7 to 16·9)	12·4 (6·5 to 20·7)	13·8 (7·8 to 22·3)
Liver cancer due to hepatitis C	0 (0 to 0·1)	0·1 (0 to 0·2)	0·2 (0·1 to 0·4)	0·4 (0·2 to 1)	0·9 (0·4 to 1·8)	1·6 (0·7 to 3·2)	3 (1·3 to 5·8)	4·5 (2 to 8·8)
Liver cancer due to alcohol use	0·2 (0·1 to 0·4)	0·6 (0·2 to 1·4)	1·1 (0·5 to 2)	2·6 (1·1 to 5)	4·6 (2·2 to 8)	7 (3·2 to 12·2)	10·5 (5·1 to 18·9)	13·5 (7 to 22·3)
Liver cancer due to NASH	0·3 (0·1 to 0·5)	0·3 (0·1 to 0·7)	0·3 (0·1 to 0·6)	0·5 (0·2 to 1)	0·8 (0·3 to 1·4)	1·1 (0·5 to 2·1)	1·6 (0·7 to 3)	2 (1 to 3·8)
Liver cancer due to other causes	0·3 (0·2 to 0·6)	0·5 (0·2 to 0·9)	0·4 (0·2 to 0·8)	0·6 (0·3 to 1·2)	0·8 (0·4 to 1·6)	1 (0·5 to 1·9)	1·3 (0·5 to 2·5)	1·4 (0·6 to 2·5)
Gallbladder and biliary tract cancer	0·4 (0·3 to 0·6)	0·9 (0·7 to 1·3)	1·6 (1·2 to 2·4)	3·1 (2·1 to 4·6)	5·3 (3·8 to 8·1)	7·1 (4·9 to 11·1)	10·6 (7·3 to 16·4)	13·1 (9·2 to 20·9)
Pancreatic cancer	0·8 (0·6 to 1)	1·6 (1·1 to 2·2)	5·9 (4 to 8·3)	10·4 (6·7 to 14·9)	19·8 (13·5 to 28)	28·9 (19·6 to 39·8)	37·9 (27·2 to 51·6)	44·1 (30·9 to 59·1)
Larynx cancer	0·9 (0·6 to 1·3)	0·9 (0·7 to 1·2)	1·6 (1·2 to 2·1)	4·8 (3·3 to 6·7)	12·1 (8·3 to 17)	24·6 (16·3 to 37·7)	40·1 (28·4 to 55·3)	45·7 (33·4 to 63·2)
Tracheal, bronchus, and lung cancer	3·8 (2·6 to 5·3)	6 (3·9 to 9)	14·4 (9·8 to 20)	28·5 (18·3 to 42·1)	55·8 (38·1 to 75·9)	101 (67·5 to 142)	147 (101 to 208)	185 (129 to 261)
Malignant skin melanoma	1·4 (0·8 to 2·3)	3·4 (1·8 to 4·9)	4·7 (2·5 to 6·9)	7·4 (4·2 to 11·3)	9 (5·6 to 14·1)	9 (5·4 to 14)	10·7 (6·3 to 16·6)	10·8 (6·5 to 17·5)
Non-melanoma skin cancer	0·3 (0·2 to 0·5)	1·1 (0·7 to 1·6)	1·7 (1·1 to 2·5)	2·1 (1·4 to 3·2)	3·1 (2 to 4·6)	3·6 (2·4 to 5·2)	4·9 (3·3 to 7·1)	5·3 (3·6 to 7·5)
Non-melanoma skin cancer (squamous-cell carcinoma)	0·3 (0·2 to 0·5)	1·1 (0·7 to 1·6)	1·7 (1·1 to 2·5)	2·1 (1·4 to 3·2)	3·1 (2 to 4·6)	3·6 (2·4 to 5·2)	4·9 (3·3 to 7·1)	5·3 (3·6 to 7·5)
Non-melanoma skin cancer (basal-cell carcinoma)	0 (0 to 0)	0 (0 to 0)	0 (0 to 0)	0 (0 to 0)	0 (0 to 0)	0 (0 to 0)	0 (0 to 0)	0 (0 to 0)
Breast cancer	0·1 (0·1 to 0·1)	0·4 (0·3 to 0·6)	0·6 (0·5 to 0·9)	1·1 (0·8 to 1·6)	1·2 (0·8 to 1·8)	3·1 (2 to 4·8)	3·8 (2·4 to 5·7)	4·1 (2·5 to 6·2)
Cervical cancer	--	--	--	--	--	--	--	--
Uterine cancer	--	--	--	--	--	--	--	--
Ovarian cancer	--	--	--	--	--	--	--	--
Prostate cancer	1·1 (0·6 to 1·4)	2·9 (1·5 to 3·9)	3·8 (1·9 to 5·1)	4·9 (2·8 to 6·8)	11·2 (6·9 to 15·9)	31·1 (19·9 to 46·7)	78·3 (52·6 to 109)	146 (97·8 to 208)
Testicular cancer	0·7 (0·4 to 1·1)	1 (0·6 to 1·6)	0·6 (0·4 to 0·9)	0·5 (0·3 to 0·8)	0·4 (0·2 to 0·6)	0·2 (0·1 to 0·4)	0·2 (0·1 to 0·3)	0·1 (0·1 to 0·2)
Kidney cancer	3·4 (2·3 to 4·7)	5·5 (3·8 to 7·6)	6·4 (3·9 to 10·1)	6·6 (4·4 to 9·4)	10·3 (6·8 to 14·9)	14·2 (9·4 to 20·5)	20·9 (14·6 to 28·5)	22·9 (16·2 to 31·7)
Bladder cancer	0·2 (0·2 to 0·3)	0·3 (0·3 to 0·5)	0·9 (0·6 to 1·3)	1·6 (1·1 to 2·2)	3 (2·1 to 4·2)	4·8 (3·1 to 6·7)	8·3 (5·9 to 11·9)	12·1 (8·5 to 16·7)
Brain and central nervous system cancer	6·2 (2·1 to 10)	8·9 (3 to 13·8)	11·1 (4·1 to 16·7)	14·2 (5·9 to 21·3)	16·2 (7·1 to 24·3)	15·6 (6·6 to 23·7)	17·1 (7·4 to 25·9)	17 (7·8 to 25·5)
Thyroid cancer	0·6 (0·4 to 0·7)	1·1 (0·7 to 1·4)	1·3 (0·9 to 1·7)	2 (1·3 to 3)	2·4 (1·6 to 3·6)	3·9 (2·5 to 6)	5·1 (3·4 to 7·5)	5·2 (3·5 to 7·5)
Mesothelioma	0·4 (0·3 to 0·6)	0·9 (0·6 to 1·3)	0·9 (0·6 to 1·2)	1·5 (1 to 2·2)	1·6 (1·1 to 2·3)	2·8 (1·9 to 4)	3·6 (2·5 to 5)	4·2 (2·9 to 5·8)
Hodgkin lymphoma	2·8 (1·8 to 4·3)	3·7 (2·2 to 5·6)	3·2 (2·1 to 4·8)	3·1 (2 to 4·5)	2·8 (1·9 to 4·1)	2 (1·3 to 3·1)	2·1 (1·4 to 3·1)	1·7 (1·2 to 2·5)
Non-Hodgkin lymphoma	17·6 (12·3 to 24·6)	23·5 (16·6 to 32·8)	26·1 (18·9 to 35·3)	27·6 (19·9 to 37·9)	28 (19·4 to 38·8)	29·3 (20 to 42·2)	31·9 (22·6 to 44·3)	33 (22·5 to 46·6)
Multiple myeloma	1·1 (0·6 to 1·6)	1·7 (0·9 to 2·4)	2·3 (1·5 to 3·5)	5·5 (3·7 to 8)	12·7 (8·5 to 18)	23 (14·8 to 34)	36·3 (24·7 to 52·5)	42 (29 to 60·2)
Leukemia	22·5 (15·3 to 32·1)	23·4 (16·6 to 31·6)	20·9 (14·2 to 28·8)	21·7 (15 to 30·7)	21·5 (15 to 30·2)	20·7 (14·6 to 28·6)	20·8 (15 to 28·7)	20·5 (14·2 to 28)
Acute lymphoid leukemia	7·2 (4·3 to 11·1)	4·4 (2·6 to 7)	3·5 (2·1 to 5·4)	3·2 (1·9 to 5·1)	2·8 (1·7 to 4·4)	1·8 (1 to 2·8)	1·5 (0·9 to 2·5)	1·4 (0·9 to 2·3)
Chronic lymphoid leukemia	0·4 (0·2 to 0·7)	0·5 (0·2 to 0·8)	0·4 (0·1 to 0·7)	0·4 (0·2 to 0·7)	0·9 (0·5 to 1·4)	0·7 (0·4 to 1·2)	1·6 (1 to 2·7)	2·6 (1·6 to 4·2)
Acute myeloid leukemia	8·7 (5·2 to 13·3)	10·6 (6·8 to 15·5)	8·7 (5·5 to 13·2)	8·9 (5·6 to 13·5)	7·7 (4·8 to 11·3)	8·1 (5·2 to 11·8)	7·7 (4·9 to 11·2)	6·7 (4·4 to 9·9)

Cause	60 to 64	65 to 69	70 to 74	75 to 79	80 to 84	85 to 89	90 to 94	95 plus	Total
All causes	5,390 (4,540 to 6,370)	4,320 (3,680 to 5,070)	3,260 (2,800 to 3,780)	2,550 (2,200 to 2,920)	1,730 (1,520 to 1,960)	929 (835 to 1,030)	411 (377 to 446)	144 (134 to 155)	66,400
Communicable, maternal, neonatal, and nutritional diseases	283 (239 to 337)	207 (168 to 257)	149 (115 to 193)	122 (94·2 to 158)	92·6 (72 to 118)	59·1 (45·6 to 74·3)	33·1 (24·7 to 42·3)	13·3 (9·5 to 17·2)	7,670
HIV/AIDS and sexually transmitted infections	94·1 (86 to 102)	49·7 (45·7 to 53·4)	10·6 (9·4 to 11·6)	5·3 (4·7 to 5·7)	2·3 (2 to 2·5)	0·8 (0·7 to 0·9)	0·2 (0·2 to 0·2)	0·1 (0·1 to 0·1)	3,230
HIV/AIDS	93·4 (85·4 to 101)	49·2 (45·2 to 52·8)	10·4 (9·1 to 11·3)	5·1 (4·5 to 5·5)	2·2 (2 to 2·3)	0·8 (0·7 to 0·8)	0·2 (0·2 to 0·2)	0·1 (0·1 to 0·1)	3,210
HIV/AIDS - Drug-susceptible Tuberculosis	2·9 (1·9 to 4·2)	1·5 (1 to 2·2)	0·3 (0·2 to 0·4)	0·2 (0·1 to 0·2)	0·1 (0 to 0·1)	0 (0 to 0)	0 (0 to 0)	0 (0 to 0)	99·9
HIV/AIDS - Multidrug-resistant Tuberculosis without extensive dru	0·1 (0 to 0·5)	0·1 (0 to 0·2)	0 (0 to 0)	0 (0 to 0)	0 (0 to 0)	0 (0 to 0)	0 (0 to 0)	0 (0 to 0)	3·9
HIV/AIDS - Extensively drug-resistant Tuberculosis	0 (0 to 0·1)	0 (0 to 0)	0 (0 to 0)	0 (0 to 0)	0 (0 to 0)	0 (0 to 0)	0 (0 to 0)	0 (0 to 0)	0·6
HIV/AIDS resulting in other diseases	90·4 (82·4 to 97·9)	47·7 (43·5 to 51·1)	10 (8·8 to 11)	5 (4·4 to 5·4)	2·1 (1·9 to 2·3)	0·8 (0·7 to 0·8)	0·2 (0·1 to 0·2)	0·1 (0·1 to 0·1)	3,100
Sexually transmitted infections excluding HIV	0·7 (0·3 to 1·5)	0·5 (0·2 to 1)	0·3 (0·1 to 0·6)	0·2 (0·1 to 0·4)	0·1 (0·1 to 0·2)	0 (0 to 0·1)	0 (0 to 0)	0 (0 to 0)	21·8
Syphilis	0·1 (0·1 to 0·2)	0·1 (0·1 to 0·2)	0·1 (0 to 0·1)	0 (0 to 0)	0 (0 to 0)	0 (0 to 0)	0 (0 to 0)	0 (0 to 0)	2
Chlamydial infection	0 (0 to 0)	0 (0 to 0)	0 (0 to 0)	0 (0 to 0)	0 (0 to 0)	0 (0 to 0)	0 (0 to 0)	0 (0 to 0)	4·3
Gonococcal infection	0 (0 to 0)	0 (0 to 0)	0 (0 to 0)	0 (0 to 0)	0 (0 to 0)	0 (0 to 0)	0 (0 to 0)	0 (0 to 0)	2·4
Trichomoniasis	0 (0 to 0)	0 (0 to 0)	0 (0 to 0)	0 (0 to 0)	0 (0 to 0)	0 (0 to 0)	0 (0 to 0)	0 (0 to 0)	0·6
Genital herpes	0·5 (0·2 to 1·3)	0·3 (0·1 to 0·8)	0·2 (0·1 to 0·5)	0·1 (0 to 0·3)	0·1 (0 to 0·2)	0 (0 to 0·1)	0 (0 to 0)	0 (0 to 0)	7·7
Other sexually transmitted infections	0 (0 to 0)	0 (0 to 0)	0 (0 to 0)	0 (0 to 0)	0 (0 to 0)	0 (0 to 0)	0 (0 to 0)	0 (0 to 0)	4·8
Respiratory infections and tuberculosis	117 (84 to 160)	106 (73·6 to 147)	98·8 (69·3 to 136)	86·6 (62·1 to 120)	70·1 (51·7 to 92·5)	47·1 (35·4 to 61)	28·3 (20·6 to 36·6)	11·5 (8 to 15·1)	1,760
Tuberculosis	14·3 (9·8 to 20·2)	10·3 (7 to 14·7)	7·6 (5·4 to 10·3)	5·5 (4 to 7·3)	3·2 (2·3 to 4·4)	1·6 (1·2 to 2·3)	0·5 (0·4 to 0·7)	0·1 (0·1 to 0·2)	218
Latent tuberculosis infection	0 (0 to 0)	0 (0 to 0)	0 (0 to 0)	0 (0 to 0)	0 (0 to 0)	0 (0 to 0)	0 (0 to 0)	0 (0 to 0)	0
Drug-susceptible tuberculosis	13·8 (9·3 to 19·5)	9·9 (6·7 to 14·3)	7·3 (5·1 to 10)	5·3 (3·7 to 7·1)	3·1 (2·2 to 4·2)	1·6 (1·1 to 2·2)	0·5 (0·3 to 0·6)	0·1 (0·1 to 0·2)	210
Multidrug-resistant tuberculosis without extensive drug resistanc	0·5 (0 to 1·8)	0·3 (0 to 1·4)	0·2 (0 to 0·9)	0·2 (0 to 0·7)	0·1 (0 to 0·4)	0·1 (0 to 0·2)	0 (0 to 0·1)	0 (0 to 0)	6·7
Extensively drug-resistant tuberculosis	0·1 (0 to 0·3)	0·1 (0 to 0·2)	0 (0 to 0·2)	0 (0 to 0·1)	0 (0 to 0·1)	0 (0 to 0)	0 (0 to 0)	0 (0 to 0)	1·1
Lower respiratory infections	97·4 (66·1 to 139)	91·7 (60·3 to 132)	89·2 (60·5 to 126)	79·8 (55·6 to 113)	66·1 (47·8 to 88·6)	45·1 (33·6 to 59·3)	27·7 (19·9 to 36)	11·3 (7·8 to 15)	1,350
Upper respiratory infections	4·4 (2·4 to 7·5)	2·9 (1·5 to 5·1)	1·8 (0·9 to 3·1)	1·1 (0·6 to 1·8)	0·6 (0·3 to 1·1)	0·3 (0·1 to 0·4)	0·1 (0 to 0·2)	0 (0 to 0·1)	144
Otitis media	1·3 (0·6 to 2·5)	0·7 (0·3 to 1·3)	0·4 (0·2 to 0·7)	0·2 (0·1 to 0·5)	0·2 (0·1 to 0·3)	0·1 (0 to 0·1)	0 (0 to 0·1)	0 (0 to 0)	44·8
Enteric infections	18·9 (13·6 to 25·3)	17·2 (12·3 to 22·9)	14·6 (10·6 to 19·5)	12·5 (8·9 to 16·8)	8·8 (6·2 to 11·8)	4·4 (3·2 to 5·9)	1·8 (1·3 to 2·4)	0·6 (0·5 to 0·8)	364
Diarrheal diseases	18·7 (13·4 to 25)	17·1 (12·2 to 22·8)	14·6 (10·5 to 19·5)	12·4 (8·9 to 16·8)	8·8 (6·2 to 11·8)	4·4 (3·2 to 5·9)	1·8 (1·3 to 2·4)	0·6 (0·5 to 0·8)	360
Typhoid and paratyphoid	0 (0 to 0)	0 (0 to 0)	0 (0 to 0)	0 (0 to 0)	0 (0 to 0)	0 (0 to 0)	0 (0 to 0)	0 (0 to 0)	0
Typhoid fever	0 (0 to 0)	0 (0 to 0)	0 (0 to 0)	0 (0 to 0)	0 (0 to 0)	0 (0 to 0)	0 (0 to 0)	0 (0 to 0)	0
Paratyphoid fever	0 (0 to 0)	0 (0 to 0)	0 (0 to 0)	0 (0 to 0)	0 (0 to 0)	0 (0 to 0)	0 (0 to 0)	0 (0 to 0)	0
Invasive Non-typhoidal Salmonella (iNTS)	0·2 (0·1 to 0·5)	0·1 (0 to 0·2)	0 (0 to 0·1)	0·1 (0 to 0·1)	0 (0 to 0)	0 (0 to 0)	0 (0 to 0)	0 (0 to 0)	2·3
Other intestinal infectious diseases	0 (0 to 0)	0 (0 to 0)	0 (0 to 0)	0 (0 to 0)	0 (0 to 0)	0 (0 to 0)	0 (0 to 0)	0 (0 to 0)	0·8
Neglected tropical diseases and malaria	8·7 (3·6 to 17·8)	7·2 (3·1 to 14·2)	5·5 (2·4 to 10·6)	4·2 (1·7 to 8·3)	2·6 (1·1 to 5·1)	1·2 (0·5 to 2·4)	0·5 (0·2 to 0·9)	0·2 (0·1 to 0·3)	107
Malaria	0 (0 to 0)	0 (0 to 0)	0 (0 to 0)	0 (0 to 0)	0 (0 to 0)	0 (0 to 0)	0 (0 to 0)	0 (0 to 0)	0
Chagas disease	0 (0 to 0)	0 (0 to 0)	0 (0 to 0)	0 (0 to 0)	0 (0 to 0)	0 (0 to 0)	0 (0 to 0)	0 (0 to 0)	0
Leishmaniasis	0 (0 to 0)	0 (0 to 0)	0 (0 to 0)	0 (0 to 0)	0 (0 to 0)	0 (0 to 0)	0 (0 to 0)	0 (0 to 0)	0
Visceral leishmaniasis	0 (0 to 0)	0 (0 to 0)	0 (0 to 0)	0 (0 to 0)	0 (0 to 0)	0 (0 to 0)	0 (0 to 0)	0 (0 to 0)	0
Cutaneous and mucocutaneous leishmaniasis	0 (0 to 0)	0 (0 to 0)	0 (0 to 0)	0 (0 to 0)	0 (0 to 0)	0 (0 to 0)	0 (0 to 0)	0 (0 to 0)	0
African trypanosomiasis	0 (0 to 0)	0 (0 to 0)	0 (0 to 0)	0 (0 to 0)	0 (0 to 0)	0 (0 to 0)	0 (0 to 0)	0 (0 to 0)	0
Schistosomiasis	0 (0 to 0)	0 (0 to 0)	0 (0 to 0)	0 (0 to 0)	0 (0 to 0)	0 (0 to 0)	0 (0 to 0)	0 (0 to 0)	0
Cysticercosis	7·6 (2·6 to 16·4)	6·5 (2·4 to 13·5)	4·9 (1·8 to 10·1)	3·8 (1·4 to 8)	2·4 (0·9 to 4·9)	1·1 (0·4 to 2·2)	0·4 (0·1 to 0·8)	0·1 (0 to 0·3)	62·3
Cystic echinococcosis	0 (0 to 0)	0 (0 to 0)	0 (0 to 0)	0 (0 to 0)	0 (0 to 0)	0 (0 to 0)	0 (0 to 0)	0 (0 to 0)	0
Lymphatic filariasis	0 (0 to 0)	0 (0 to 0)	0 (0 to 0)	0 (0 to 0)	0 (0 to 0)	0 (0 to 0)	0 (0 to 0)	0 (0 to 0)	0
Onchocerciasis	0 (0 to 0)	0 (0 to 0)	0 (0 to 0)	0 (0 to 0)	0 (0 to 0)	0 (0 to 0)	0 (0 to 0)	0 (0 to 0)	0
Trachoma	0 (0 to 0)	0 (0 to 0)	0 (0 to 0)	0 (0 to 0)	0 (0 to 0)	0 (0 to 0)	0 (0 to 0)	0 (0 to 0)	0
Dengue	0·6 (0·1 to 1·2)	0·4 (0·1 to 0·8)	0·2 (0·1 to 0·5)	0·2 (0 to 0·4)	0·1 (0 to 0·2)	0·1 (0 to 0·1)	0 (0 to 0·1)	0 (0 to 0)	14·6
Yellow fever	0 (0 to 0)	0 (0 to 0)	0 (0 to 0)	0 (0 to 0)	0 (0 to 0)	0 (0 to 0)	0 (0 to 0)	0 (0 to 0)	0
Rabies	0 (0 to 0)	0 (0 to 0)	0 (0 to 0)	0 (0 to 0)	0 (0 to 0)	0 (0 to 0)	0 (0 to 0)	0 (0 to 0)	0
Intestinal nematode infections	0 (0 to 0·1)	0 (0 to 0·1)	0 (0 to 0)	0 (0 to 0)	0 (0 to 0)	0 (0 to 0)	0 (0 to 0)	0 (0 to 0)	1·4
Ascariasis	0 (0 to 0·1)	0 (0 to 0)	0 (0 to 0)	0 (0 to 0)	0 (0 to 0)	0 (0 to 0)	0 (0 to 0)	0 (0 to 0)	0·3
Trichuriasis	0 (0 to 0)	0 (0 to 0)	0 (0 to 0)	0 (0 to 0)	0 (0 to 0)	0 (0 to 0)	0 (0 to 0)	0 (0 to 0)	0
Hookworm disease	0 (0 to 0·1)	0 (0 to 0·1)	0 (0 to 0)	0 (0 to 0)	0 (0 to 0)	0 (0 to 0)	0 (0 to 0)	0 (0 to 0)	1·1
Food-borne trematodiases	0 (0 to 0)	0 (0 to 0)	0 (0 to 0)	0 (0 to 0)	0 (0 to 0)	0 (0 to 0)	0 (0 to 0)	0 (0 to 0)	0
Leprosy	0 (0 to 0·1)	0 (0 to 0)	0 (0 to 0)	0 (0 to 0)	0 (0 to 0)	0 (0 to 0)	0 (0 to 0)	0 (0 to 0)	0·3
Ebola	0 (0 to 0)	0 (0 to 0)	0 (0 to 0)	0 (0 to 0)	0 (0 to 0)	0 (0 to 0)	0 (0 to 0)	0 (0 to 0)	0
Zika virus	0 (0 to 0·1)	0 (0 to 0·1)	0 (0 to 0)	0 (0 to 0)	0 (0 to 0)	0 (0 to 0)	0 (0 to 0)	0 (0 to 0)	1·4
Guinea worm disease	0 (0 to 0)	0 (0 to 0)	0 (0 to 0)	0 (0 to 0)	0 (0 to 0)	0 (0 to 0)	0 (0 to 0)	0 (0 to 0)	0
Other neglected tropical diseases	0·5 (0·2 to 1)	0·3 (0·1 to 0·5)	0·3 (0·1 to 0·5)	0·2 (0·1 to 0·4)	0·1 (0 to 0·2)	0·1 (0 to 0·2)	0 (0 to 0·1)	0 (0 to 0)	27·1
Other infectious diseases	12·7 (9·1 to 17·3)	7·9 (5·7 to 10·5)	5·5 (4 to 7·4)	3·3 (2·5 to 4·3)	2 (1·4 to 2·6)	1·2 (0·9 to 1·7)	0·4 (0·3 to 0·5)	0·1 (0·1 to 0·2)	282
Meningitis	3·8 (2·3 to 5·7)	2·3 (1·4 to 3·4)	1·6 (1 to 2·4)	1·1 (0·7 to 1·7)	0·6 (0·4 to 0·9)	0·4 (0·2 to 0·5)	0·1 (0·1 to 0·1)	0 (0 to 0)	90·9
Encephalitis	1·7 (1·1 to 2·5)	1·1 (0·7 to 1·6)	0·7 (0·5 to 1·1)	0·4 (0·2 to 0·6)	0·2 (0·1 to 0·4)	0·1 (0·1 to 0·1)	0 (0 to 0)	0 (0 to 0)	60·8
Diphtheria	0 (0 to 0)	0 (0 to 0)	0 (0 to 0)	0 (0 to 0)	0 (0 to 0)	0 (0 to 0)	0 (0 to 0)	0 (0 to 0)	0
Tetanus	0·1 (0 to 0·3)	0·1 (0 to 0·2)	0 (0 to 0·2)	0 (0 to 0·1)	0 (0 to 0)	0 (0 to 0)	0 (0 to 0)	0 (0 to 0)	1·1
Measles	0 (0 to 0)	0 (0 to 0)	0 (0 to 0)	0 (0 to 0)	0 (0 to 0)	0 (0 to 0)	0 (0 to 0)	0 (0 to 0)	0
Varicella and herpes zoster	0·9 (0·5 to 1·6)	0·5 (0·3 to 0·9)	0·6 (0·3 to 1·1)	0·4 (0·2 to 0·7)	0·3 (0·1 to 0·5)	0·1 (0·1 to 0·2)	0·1 (0 to 0·1)	0 (0 to 0·1)	15·8
Acute hepatitis	0·4 (0·3 to 0·6)	0·2 (0·2 to 0·3)	0·2 (0·1 to 0·2)	0·1 (0·1 to 0·1)	0 (0 to 0·1)	0 (0 to 0)	0 (0 to 0)	0 (0 to 0)	14·3
Acute hepatitis A	0·1 (0 to 0·2)	0 (0 to 0·1)	0 (0 to 0)	0 (0 to 0)	0 (0 to 0)	0 (0 to 0)	0 (0 to 0)	0 (0 to 0)	7·4
Acute hepatitis B	0·2 (0·1 to 0·3)	0·1 (0·1 to 0·2)	0·1 (0·1 to 0·1)	0 (0 to 0·1)	0 (0 to 0)	0 (0 to 0)	0 (0 to 0)	0 (0 to 0)	4·6
Acute hepatitis C	0·1 (0 to 0·1)	0 (0 to 0·1)	0 (0 to 0)	0 (0 to 0)	0 (0 to 0)	0 (0 to 0)	0 (0 to 0)	0 (0 to 0)	0·9
Acute hepatitis E	0·1 (0 to 0·2)	0 (0 to 0)	0 (0 to 0)	0 (0 to 0)	0 (0 to 0)	0 (0 to 0)	0 (0 to 0)	0 (0 to 0)	1·4
Other unspecified infectious diseases	5·7 (2·8 to 8·9)	3·6 (1·8 to 5·7)	2·4 (1·2 to 3·5)	1·3 (0·7 to 2·1)	0·8 (0·4 to 1·3)	0·7 (0·4 to 1·1)	0·2 (0·1 to 0·3)	0·1 (0 to 0·1)	97·8
Maternal and neonatal disorders	6·4 (4·6 to 8·4)	3 (2·2 to 3·9)	1·1 (0·9 to 1·5)	0·4 (0·3 to 0·6)	0·1 (0·1 to 0·1)	0 (0 to 0)	0 (0 to 0)	0 (0 to 0)	1,370
Maternal disorders	0 (0 to 0)	0 (0 to 0)	0 (0 to 0)	0 (0 to 0)	0 (0 to 0)	0 (0 to 0)	0 (0 to 0)	0 (0 to 0)	0
Maternal hemorrhage	0 (0 to 0)	0 (0 to 0)	0 (0 to 0)	0 (0 to 0)	0 (0 to 0)	0 (0 to 0)	0 (0 to 0)	0 (0 to 0)	0
Maternal sepsis and other maternal infections	0 (0 to 0)	0 (0 to 0)	0 (0 to 0)	0 (0 to 0)	0 (0 to 0)	0 (0 to 0)	0 (0 to 0)	0 (0 to 0)	0
Maternal hypertensive disorders	0 (0 to 0)	0 (0 to 0)	0 (0 to 0)	0 (0 to 0)	0 (0 to 0)	0 (0 to 0)	0 (0 to 0)	0 (0 to 0)	0
Maternal obstructed labor and uterine rupture	--	--	--	--	--	--	--	--	NA
Maternal abortion and miscarriage	--	--	--	--	--	--	--	--	NA
Ectopic pregnancy	--	--	--	--	--	--	--	--	NA
Indirect maternal deaths	--	--	--	--	--	--	--	--	NA
Late maternal deaths	--	--	--	--	--	--	--	--	NA
Maternal deaths aggravated by HIV/AIDS	--	--	--	--	--	--	--	--	NA
Neonatal disorders	6·4 (4·6 to 8·4)	3 (2·2 to 3·9)	1·1 (0·9 to 1·5)	0·4 (0·3 to 0·6)	0·1 (0·1 to 0·1)	0 (0 to 0)	0 (0 to 0)	0 (0 to 0)	1,370
Neonatal preterm birth	2·5 (1·9 to 3·2)	1·1 (0·8 to 1·4)	0·4 (0·3 to 0·6)	0·2 (0·1 to 0·3)	0·1 (0 to 0·1)	0 (0 to 0)	0 (0 to 0)	0 (0 to 0)	552
Neonatal encephalopathy due to birth asphyxia and trauma	1·2 (0·8 to 1·8)	0·5 (0·3 to 0·8)	0·1 (0·1 to 0·2)	0 (0 to 0·1)	0 (0 to 0)	0 (0 to 0)	0 (0 to 0)	0 (0 to 0)	254
Neonatal sepsis and other neonatal infections	2 (1 to 3·4)	1 (0·5 to 1·7)	0·4 (0·3 to 0·7)	0·2 (0·1 to 0·3)	0 (0 to 0·1)	0 (0 to 0)	0 (0 to 0)	0 (0 to 0)	321
Hemolytic disease and other neonatal jaundice	0·7 (0·5 to 0·9)	0·3 (0·2 to 0·4)	0·1 (0·1 to 0·2)	0 (0 to 0·1)	0 (0 to 0)	0 (0 to 0)	0 (0 to 0)	0 (0 to 0)	31·1
Other neonatal disorders	0 (0 to 0)	0 (0 to 0)	0 (0 to 0)	0 (0 to 0)	0 (0 to 0)	0 (0 to 0)	0 (0 to 0)	0 (0 to 0)	216
Nutritional deficiencies	24·7 (14·4 to 45)	16·6 (10·3 to 27·9)	12·5 (8·1 to 19·1)	9·5 (6·3 to 14·5)	6·7 (4·3 to 10·1)	4·3 (2·8 to 6·6)	1·9 (1·3 to 2·7)	0·8 (0·6 to 1·1)	556
Protein-energy malnutrition	10 (6·9 to 13·9)	8·1 (5·5 to 10·9)	6·7 (4·4 to 9·3)	5·4 (3·9 to 7·3)	3·7 (2·5 to 5·2)	2·3 (1·6 to 3)	1·2 (0·8 to 1·5)	0·5 (0·4 to 0·7)	175
Iodine deficiency	0·2 (0·1 to 0·4)	0·2 (0·1 to 0·3)	0·1 (0 to 0·2)	0·1 (0 to 0·1)	0 (0 to 0·1)	0 (0 to 0)	0 (0 to 0)	0 (0 to 0)	5·1
Vitamin A deficiency	0·1 (0·1 to 0·2)	0·1 (0 to 0·2)	0·1 (0 to 0·1)	0 (0 to 0·1)	0 (0 to 0)	0 (0 to 0)	0 (0 to 0)	0 (0 to 0)	7·6
Dietary iron deficiency	11·3 (3 to 29·7)	5·8 (1·2 to 16)	4 (0·9 to 9·5)	2·9 (0·7 to 7·7)	2 (0·5 to 5·1)	1·6 (0·4 to 3·7)	0·5 (0·2 to 1·2)	0·2 (0 to 0·4)	317
Other nutritional deficiencies	3 (1·9 to 4·7)	2·5 (1·5 to 3·6)	1·7 (1 to 2·6)	1·1 (0·7 to 1·7)	0·9 (0·4 to 1·4)	0·4 (0·2 to 0·7)	0·2 (0·1 to 0·4)	0·1 (0 to 0·2)	52·1
Non-communicable diseases	4,700 (3,940 to 5,590)	3,860 (3,280 to 4,550)	2,950 (2,520 to 3,430)	2,310 (2,000 to 2,660)	1,570 (1,380 to 1,780)	832 (743 to 923)	361 (331 to 396)	125 (115 to 135)	43,700
Neoplasms	1,100 (883 to 1,380)	961 (764 to 1,210)	780 (632 to 966)	613 (490 to 751)	392 (316 to 485)	190 (153 to 240)	74 (57·7 to 96·2)	19·2 (13·7 to 25·7)	8,080
Lip and oral cavity cancer	37·2 (26·1 to 51·3)	25·6 (18·8 to 34·3)	16·2 (11·6 to 22)	11·2 (8·1 to 15·3)	6 (4·4 to 8·2)	2·3 (1·8 to 3)	0·9 (0·7 to 1·2)	0·3 (0·2 to 0·4)	247
Nasopharynx cancer	5·9 (4·1 to 8·1)	4 (2·9 to 5·3)	2·5 (1·8 to 3·2)	1·2 (0·9 to 1·7)	0·6 (0·5 to 0·9)	0·5 (0·3 to 0·6)	0·2 (0·1 to 0·2)	0 (0 to 0·1)	50
Other pharynx cancer	24·6 (16·7 to 34·6)	16·7 (11·3 to 23·6)	9·6 (6·5 to 13·4)	5·5 (3·8 to 7·6)	2·8 (2·1 to 3·9)	1·3 (0·9 to 1·7)	0·4 (0·3 to 0·6)	0·1 (0·1 to 0·2)	154
Esophageal cancer	72·3 (50·1 to 100)	56·3 (39·6 to 78·2)	37·1 (25·6 to 51·3)	22·7 (16 to 31·9)	11·9 (8·7 to 16·3)	5·5 (4·1 to 7·1)	1·8 (1·4 to 2·3)	0·3 (0·2 to 0·5)	480
Stomach cancer	76·4 (54·9 to 104)	61·3 (44·1 to 81·7)	45·5 (32·9 to 61·6)	32 (23·1 to 43)	18 (13·3 to 23·9)	8·5 (6·6 to 10·9)	3·1 (2·4 to 3·8)	0·7 (0·5 to 0·9)	526
Colon and rectum cancer	140 (107 to 184)	126 (87·1 to 170)	88·3 (63·9 to 117)	66 (47·7 to 88·4)	37·6 (27·7 to 49·8)	17·3 (13·5 to 22·4)	6·7 (5·2 to 8·2)	1·6 (1·2 to 2·1)	984
Liver cancer	32 (22·5 to 44·2)	25 (17·7 to 35·1)	17·9 (12·2 to 25·5)	12·1 (8·5 to 16·3)	6 (4·3 to 8·3)	1·9 (1·3 to 2·8)	0·5 (0·4 to 0·7)	0·1 (0·1 to 0·1)	222
Liver cancer due to hepatitis B	10·9 (5·6 to 17·8)	6·8 (3·5 to 11·5)	4·1 (1·9 to 7·4)	2·5 (1·4 to 4·5)	1·1 (0·5 to 2)	0·4 (0·2 to 0·6)	0·1 (0 to 0·2)	0 (0 to 0)	86·6
Liver cancer due to hepatitis C	4·6 (2 to 8·5)	3·8 (1·9 to 6·3)	3 (1·5 to 5·2)	2·5 (1·2 to 4·2)	1·6 (0·8 to 2·5)	0·6 (0·3 to 1)	0·2 (0·1 to 0·3)	0 (0 to 0·1)	27
Liver cancer due to alcohol use	13·5 (7·7 to 21·6)	12 (7·2 to 18·7)	8·8 (5·2 to 14·2)	5·5 (3·4 to 8·3)	2·5 (1·5 to 3·9)	0·7 (0·4 to 1·1)	0·2 (0·1 to 0·3)	0 (0 to 0)	83·3
Liver cancer due to NASH	1·9 (0·9 to 3·4)	1·6 (0·8 to 3·1)	1·3 (0·7 to 2·4)	1·1 (0·6 to 2)	0·6 (0·3 to 1·1)	0·2 (0·1 to 0·4)	0·1 (0 to 0·1)	0 (0 to 0)	14
Liver cancer due to other causes	1·1 (0·5 to 2·1)	0·8 (0·4 to 1·4)	0·6 (0·3 to 1)	0·4 (0·2 to 0·7)	0·2 (0·1 to 0·4)	0·1 (0 to 0·1)	0 (0 to 0)	0 (0 to 0)	11·7
Gallbladder and biliary tract cancer	13·4 (9·5 to 22·2)	11 (7·5 to 18·1)	8·3 (5·8 to 14·5)	5·7 (4·1 to 9·8)	3·3 (2·3 to 6·1)	1·6 (1·1 to 2·7)	0·6 (0·4 to 1)	0·1 (0·1 to 0·2)	86·2
Pancreatic cancer	39·6 (28·1 to 54·9)	31·3 (22·1 to 41·9)	22·6 (15·9 to 31·1)	14·6 (10·3 to 19·8)	7·8 (5·6 to 10·5)	3·4 (2·5 to 4·2)	1·1 (0·9 to 1·4)	0·3 (0·2 to 0·3)	270
Larynx cancer	42·3 (31 to 56·1)	31·8 (23·4 to 42·6)	19·8 (14·5 to 26·2)	12·7 (9·1 to 17·1)	6·4 (4·7 to 8·6)	2·5 (1·8 to 3·4)	0·8 (0·6 to 1·1)	0·2 (0·1 to 0·2)	247
Tracheal, bronchus, and lung cancer	171 (121 to 237)	133 (97·4 to 181)	90·2 (64·4 to 121)	58·9 (43·6 to 77·8)	30·6 (21·5 to 41·6)	13·1 (9·4 to 17·4)	4·2 (3·2 to 5·2)	0·9 (0·7 to 1·2)	1,040
Malignant skin melanoma	7·3 (4·5 to 11·4)	5·3 (3·2 to 8·3)	4 (2·5 to 6·4)	2·9 (1·8 to 4·5)	1·9 (1·2 to 3·1)	0·9 (0·5 to 1·4)	0·3 (0·2 to 0·5)	0·1 (0·1 to 0·2)	80·5
Non-melanoma skin cancer	5·3 (3·9 to 7·3)	4·6 (3·4 to 6·2)	3·5 (2·5 to 4·8)	2·9 (2·1 to 4)	2·3 (1·7 to 3·2)	1·4 (1 to 1·9)	0·8 (0·6 to 1·1)	0·3 (0·2 to 0·4)	43·3
Non-melanoma skin cancer (squamous-cell carcinoma)	5·3 (3·9 to 7·3)	4·6 (3·4 to 6·2)	3·5 (2·5 to 4·8)	2·9 (2·1 to 4)	2·3 (1·7 to 3·2)	1·4 (1 to 1·9)	0·8 (0·6 to 1·1)	0·3 (0·2 to 0·4)	43·3
Non-melanoma skin cancer (basal-cell carcinoma)	0 (0 to 0)	0 (0 to 0)	0 (0 to 0)	0 (0 to 0)	0 (0 to 0)	0 (0 to 0)	0 (0 to 0)	0 (0 to 0)	0
Breast cancer	3·8 (2·4 to 5·8)	3·5 (2·3 to 5·1)	2·2 (1·4 to 3·3)	1·6 (1 to 2·3)	1 (0·6 to 1·5)	0·8 (0·5 to 1·2)	0·3 (0·2 to 0·5)	0·1 (0 to 0·1)	27·9
Cervical cancer	--	--	--	--	--	--	--	--	NA
Uterine cancer	--	--	--	--	--	--	--	--	NA
Ovarian cancer	--	--	--	--	--	--	--	--	NA
Prostate cancer	252 (180 to 365)	285 (200 to 406)	312 (222 to 433)	296 (213 to 417)	219 (158 to 311)	113 (82·8 to 168)	46·6 (33·8 to 69·3)	12·7 (8·5 to 19·3)	1,820
Testicular cancer	0·1 (0·1 to 0·2)	0·1 (0·1 to 0·1)	0·1 (0 to 0·1)	0·1 (0 to 0·1)	0 (0 to 0)	0 (0 to 0)	0 (0 to 0)	0 (0 to 0)	4·9
Kidney cancer	19·9 (14·3 to 26·4)	15·3 (11 to 20·8)	10·1 (7·1 to 13·8)	6·2 (4·5 to 8·2)	3 (2·1 to 4)	1·1 (0·8 to 1·4)	0·4 (0·3 to 0·5)	0·1 (0·1 to 0·1)	156
Bladder cancer	15·1 (10·9 to 20·2)	14 (10 to 19·1)	12·1 (8·5 to 16·9)	9·6 (6·8 to 13·1)	6 (4·2 to 8·2)	3·2 (2·4 to 4·1)	1·3 (1 to 1·6)	0·3 (0·2 to 0·4)	92·8
Brain and central nervous system cancer	12·6 (5·9 to 18·6)	8·6 (4 to 12·4)	4·9 (2·3 to 7)	2·5 (1·2 to 3·6)	1·1 (0·6 to 1·7)	0·4 (0·2 to 0·6)	0·1 (0·1 to 0·2)	0 (0 to 0)	164
Thyroid cancer	4 (2·6 to 5·9)	2·9 (2 to 3·9)	2 (1·4 to 2·8)	1·3 (0·9 to 1·8)	0·8 (0·5 to 1·1)	0·3 (0·2 to 0·4)	0·1 (0·1 to 0·2)	0 (0 to 0)	33·5
Mesothelioma	1·7 (1·2 to 2·3)	1·2 (0·9 to 1·7)	1 (0·8 to 1·4)	1 (0·7 to 1·3)	0·5 (0·4 to 0·7)	0·2 (0·2 to 0·3)	0·1 (0 to 0·1)	0 (0 to 0)	21·6
Hodgkin lymphoma	1·4 (1 to 2)	1·1 (0·7 to 1·5)	0·6 (0·4 to 0·8)	0·4 (0·3 to 0·6)	0·2 (0·1 to 0·3)	0·1 (0·1 to 0·1)	0 (0 to 0)	0 (0 to 0)	27·1
Non-Hodgkin lymphoma	26·7 (18·6 to 37·3)	19·2 (13·6 to 26·7)	13·4 (9·5 to 18·7)	8·2 (5·9 to 11)	3·9 (2·8 to 5·3)	1·4 (1 to 1·8)	0·4 (0·3 to 0·6)	0·1 (0·1 to 0·1)	317
Multiple myeloma	40·7 (28·8 to 56·8)	35·2 (25·5 to 48·3)	24 (16·9 to 33·9)	15·8 (11·2 to 21·4)	8·4 (6·1 to 11·8)	3 (2·2 to 4·2)	0·8 (0·6 to 1·1)	0·2 (0·1 to 0·2)	253
Leukemia	17·5 (12·8 to 23·5)	14·9 (11 to 20·3)	11·2 (8 to 15·1)	7·7 (5·7 to 10·3)	4·5 (3·3 to 6)	2·1 (1·6 to 2·8)	0·7 (0·6 to 0·9)	0·2 (0·1 to 0·2)	294
Acute lymphoid leukemia	0·9 (0·5 to 1·4)	1 (0·6 to 1·6)	0·7 (0·4 to 1·1)	0·3 (0·2 to 0·5)	0·2 (0·1 to 0·4)	0·1 (0·1 to 0·2)	0 (0 to 0·1)	0 (0 to 0)	61·5
Chronic lymphoid leukemia	2·6 (1·6 to 4)	2·5 (1·6 to 3·8)	1·9 (1·2 to 2·8)	1·3 (0·8 to 2)	0·9 (0·6 to 1·3)	0·4 (0·3 to 0·7)	0·2 (0·1 to 0·2)	0 (0 to 0·1)	17·4
Acute myeloid leukemia	5·2 (3·4 to 7·6)	4·3 (2·9 to 6·3)	2·9 (1·9 to 4·3)	1·9 (1·2 to 2·8)	0·9 (0·6 to 1·4)	0·4 (0·2 to 0·6)	0·1 (0·1 to 0·2)	0 (0 to 0)	103

Chronic myeloid leukemia	0 (0 to 0)	0 (0 to 0)	0·1 (0 to 0·2)	0·3 (0·2 to 0·6)	0·2 (0·1 to 0·4)	0·2 (0·1 to 0·4)	0·6 (0·3 to 1)	1·9 (1 to 3·2)	3·6 (2 to 5·6)	3·7 (2·1 to 5·8)	4·4 (2·5 to 7)	3·4 (2 to 5·2)	3·5 (2·1 to 5·6)	3·7 (2·3 to 5·6)	3·6 (2·1 to 5·5)	3·4 (2·1 to 5·2)	2·7 (1·7 to 4)	1·7 (1 to 2·7)	1·3 (0·8 to 2)	0·7 (0·4 to 1·1)	0·3 (0·2 to 0·4)	0·1 (0·1 to 0·2)	0 (0 to 0)	39·5
Other leukemia	0·1 (0·1 to 0·2)	0·1 (0 to 0·2)	0·5 (0·2 to 0·8)	0·9 (0·5 to 1·8)	1·8 (0·9 to 3·2)	2·6 (1·4 to 4·4)	2·4 (1·3 to 4·1)	4·4 (2·5 to 7·2)	4·3 (2·6 to 6·8)	4·6 (2·7 to 7·3)	4·8 (2·9 to 7·3)	6·8 (4·2 to 10·4)	6·6 (4 to 10)	6·3 (4·1 to 9·2)	6·1 (3·8 to 8·8)	5·4 (3·6 to 7·5)	4·4 (3·1 to 6·3)	4 (2·6 to 5·7)	2·9 (1·9 to 4·2)	1·8 (1·2 to 2·6)	1 (0·6 to 1·4)	0·3 (0·2 to 0·5)	0·1 (0 to 0·1)	72·2
Other malignant neoplasms	0·4 (0·2 to 0·8)	0·5 (0·2 to 0·9)	4 (2·6 to 5·7)	14·2 (8·7 to 22·4)	11·7 (8·6 to 15·9)	12·2 (9·1 to 16·3)	17·6 (12·7 to 23·3)	21·5 (16·1 to 27·8)	23·6 (18 to 30·2)	24·8 (18·8 to 32)	26·6 (20·2 to 34·5)	30·7 (22·3 to 40·9)	33·4 (23·3 to 45)	37 (27 to 50·4)	38·8 (27·5 to 52·8)	30·9 (22·8 to 40·4)	23·4 (17·6 to 30·6)	16 (12·1 to 20·7)	10·9 (8·2 to 14)	6·5 (5 to 8·6)	3·4 (2·5 to 4·5)	1·3 (1 to 1·7)	0·3 (0·2 to 0·4)	390
Other neoplasms	0·1 (0 to 0·1)	0·1 (0 to 0·1)	0·2 (0·1 to 0·4)	0·9 (0·4 to 1·7)	1·2 (0·6 to 2)	1·1 (0·6 to 1·7)	1·1 (0·5 to 1·8)	0·8 (0·3 to 1·5)	2 (0·8 to 3·3)	1·4 (0·6 to 2·5)	2·4 (1·1 to 4·2)	2·2 (1·2 to 3·7)	2·5 (1·4 to 4·4)	4·3 (2·4 to 7)	5·3 (3·1 to 9·2)	5·7 (3·3 to 10)	4·9 (3·2 to 8·6)	4·7 (2·9 to 8·4)	3·6 (2·3 to 6·8)	2·1 (1·3 to 4)	1·1 (0·7 to 2)	0·4 (0·2 to 0·7)	0·1 (0 to 0·1)	48·2
Myelodysplastic, myeloproliferative, and other hematopoietic neo	0·1 (0 to 0·1)	0·1 (0 to 0·1)	0·2 (0·1 to 0·4)	0·9 (0·4 to 1·7)	1·2 (0·6 to 2)	1·1 (0·6 to 1·7)	1·1 (0·5 to 1·8)	0·8 (0·3 to 1·5)	2 (0·8 to 3·3)	1·4 (0·6 to 2·5)	2·4 (1·1 to 4·2)	2·2 (1·2 to 3·7)	2·5 (1·4 to 4·4)	4·3 (2·4 to 7)	5·3 (3·1 to 9·2)	5·7 (3·3 to 10)	4·9 (3·2 to 8·6)	4·7 (2·9 to 8·4)	3·6 (2·3 to 6·8)	2·1 (1·3 to 4)	1·1 (0·7 to 2)	0·4 (0·2 to 0·7)	0·1 (0 to 0·1)	48·2
Benign and in situ intestinal neoplasms	0 (0 to 0)	0 (0 to 0)	0 (0 to 0)	0 (0 to 0)	0 (0 to 0)	0 (0 to 0)	0 (0 to 0)	0 (0 to 0)	0 (0 to 0)	0 (0 to 0)	0 (0 to 0)	0 (0 to 0)	0 (0 to 0)	0 (0 to 0)	0 (0 to 0)	0 (0 to 0)	0 (0 to 0)	0 (0 to 0)	0 (0 to 0)	0 (0 to 0)	0 (0 to 0)	0 (0 to 0)	0 (0 to 0)	0
Benign and in situ cervical and uterine neoplasms	0 (0 to 0)	0 (0 to 0)	0 (0 to 0)	0 (0 to 0)	0 (0 to 0)	0 (0 to 0)	0 (0 to 0)	0 (0 to 0)	0 (0 to 0)	0 (0 to 0)	0 (0 to 0)	0 (0 to 0)	0 (0 to 0)	0 (0 to 0)	0 (0 to 0)	0 (0 to 0)	0 (0 to 0)	0 (0 to 0)	0 (0 to 0)	0 (0 to 0)	0 (0 to 0)	0 (0 to 0)	0 (0 to 0)	0
Other benign and in situ neoplasms	0 (0 to 0)	0 (0 to 0)	0 (0 to 0)	0 (0 to 0)	0 (0 to 0)	0 (0 to 0)	0 (0 to 0)	0 (0 to 0)	0 (0 to 0)	0 (0 to 0)	0 (0 to 0)	0 (0 to 0)	0 (0 to 0)	0 (0 to 0)	0 (0 to 0)	0 (0 to 0)	0 (0 to 0)	0 (0 to 0)	0 (0 to 0)	0 (0 to 0)	0 (0 to 0)	0 (0 to 0)	0 (0 to 0)	0
Cardiovascular diseases	2·7 (1·7 to 4)	1·7 (0·9 to 3)	9·4 (6·1 to 14·1)	7·5 (5·2 to 10·5)	12·5 (9·6 to 16·2)	23 (17·4 to 30)	60·9 (46·7 to 78·7)	110 (84·7 to 142)	195 (153 to 250)	300 (234 to 383)	514 (405 to 647)	799 (609 to 1,030)	1,140 (865 to 1,510)	1,450 (1,140 to 1,870)	1,630 (1,290 to 2,070)	1,580 (1,290 to 1,950)	1,300 (1,070 to 1,580)	983 (817 to 1,180)	793 (668 to 939)	547 (463 to 638)	308 (257 to 355)	140 (115 to 162)	50·3 (37·9 to 59·9)	12,000
Rheumatic heart disease	0 (0 to 0)	0 (0 to 0)	0 (0 to 0)	0·4 (0·2 to 0·8)	2·6 (1·4 to 4·3)	6·6 (3·7 to 11·2)	10·3 (6·6 to 15·8)	12·7 (8·4 to 18·5)	14·6 (10·1 to 20·2)	14·4 (10 to 20)	15·1 (10·6 to 20·4)	14·3 (10·3 to 19·5)	12·1 (8·5 to 17)	9·6 (6·9 to 13·1)	7·5 (5·4 to 10·2)	4·6 (3·4 to 6·3)	2·8 (2 to 3·7)	1·6 (1·2 to 2·1)	1·1 (0·8 to 1·5)	0·5 (0·4 to 0·7)	0·3 (0·2 to 0·3)	0·1 (0·1 to 0·1)	0 (0 to 0)	131
Ischemic heart disease	0 (0 to 0)	0 (0 to 0)	0 (0 to 0)	0 (0 to 0)	0 (0 to 0)	0 (0 to 0)	5·7 (3·9 to 8·1)	18·1 (12·4 to 25·5)	44·3 (30·8 to 60·9)	82·7 (59·8 to 113)	159 (117 to 210)	293 (215 to 400)	464 (341 to 627)	639 (488 to 827)	723 (561 to 927)	712 (555 to 889)	571 (464 to 696)	409 (333 to 498)	308 (247 to 368)	211 (170 to 252)	120 (96·8 to 143)	53·2 (41·9 to 64)	20·2 (14·6 to 25·3)	4,830
Stroke	0·4 (0·2 to 0·8)	0·2 (0·1 to 0·3)	0·9 (0·4 to 1·6)	0·9 (0·7 to 1·2)	2·6 (1·8 to 3·6)	4·5 (3·2 to 6·3)	13·3 (9·1 to 18·8)	28·3 (18·6 to 41·6)	41·8 (26·5 to 62·7)	66·4 (43·5 to 95)	114 (76·5 to 161)	173 (114 to 247)	236 (160 to 336)	317 (219 to 449)	345 (248 to 455)	357 (264 to 473)	309 (226 to 402)	245 (186 to 315)	222 (168 to 284)	139 (104 to 176)	73 (55·6 to 91·8)	30·4 (22·9 to 37·7)	9·6 (6·7 to 12·5)	2,730
Ischemic stroke	0 (0 to 0)	0 (0 to 0)	0·1 (0 to 0·1)	0·5 (0·4 to 0·7)	1·3 (0·7 to 2·2)	2 (1·2 to 3·3)	3·1 (2 to 4·5)	4·4 (2·9 to 6·4)	6·7 (4·4 to 9·5)	9·8 (6·5 to 14·4)	15·3 (10·1 to 22·8)	22·3 (14·9 to 31·7)	32·4 (21·9 to 46·6)	50 (34·2 to 72·1)	62·5 (43·5 to 87·8)	97 (67·4 to 133)	91·4 (64·5 to 125)	110 (78·9 to 148)	106 (77·2 to 142)	92·2 (68·6 to 120)	50·2 (38 to 63·2)	21·3 (15·7 to 26·6)	6·9 (4·8 to 9·1)	785
Intracerebral hemorrhage	0·1 (0 to 0·2)	0 (0 to 0·1)	0·4 (0·2 to 0·9)	0·1 (0·1 to 0·2)	0·6 (0·4 to 0·9)	1·1 (0·7 to 1·6)	5·7 (3·4 to 8·8)	14·6 (8·6 to 22·2)	23·1 (13·6 to 37·1)	41·1 (25·2 to 63·2)	73·3 (47 to 108)	118 (76·6 to 173)	172 (114 to 252)	234 (159 to 339)	248 (177 to 336)	238 (172 to 318)	202 (146 to 272)	127 (91·4 to 168)	109 (78·2 to 148)	42·2 (29·4 to 57·6)	20·8 (14·6 to 27·7)	8·3 (6 to 11·2)	2·4 (1·6 to 3·2)	1,680
Subarachnoid hemorrhage	0·3 (0·1 to 0·6)	0·1 (0·1 to 0·2)	0·3 (0·2 to 0·7)	0·3 (0·2 to 0·4)	0·6 (0·4 to 1)	1·4 (0·9 to 2·1)	4·5 (2·6 to 7·2)	9·3 (5·2 to 15·7)	12 (6·4 to 20·6)	15·4 (8·5 to 25·2)	25·6 (15·5 to 39·2)	32·6 (20·1 to 50·2)	31·5 (18·6 to 48·4)	32·6 (20·7 to 50)	33·9 (22·1 to 50·3)	21·2 (13·7 to 31·1)	15·5 (10·1 to 23)	9·3 (6·2 to 13·6)	6·5 (4·3 to 9·8)	4·3 (2·8 to 6·4)	2 (1·2 to 3)	0·8 (0·5 to 1·2)	0·3 (0·2 to 0·5)	261
Hypertensive heart disease	0 (0 to 0)	0 (0 to 0)	0 (0 to 0)	0 (0 to 0)	0 (0 to 0)	0 (0 to 0)	3·6 (2·1 to 5·6)	8·8 (5·2 to 14·1)	18·9 (10·2 to 30·5)	41·1 (23·9 to 62·1)	83·9 (54·6 to 123)	146 (93·8 to 208)	236 (152 to 338)	283 (186 to 396)	341 (232 to 472)	317 (221 to 435)	261 (182 to 360)	208 (152 to 275)	164 (121 to 221)	124 (91·4 to 164)	68·1 (50·1 to 89·5)	33·3 (24 to 44·3)	11·9 (7·8 to 16·3)	2,350
Non-rheumatic valvular heart disease	0 (0 to 0)	0 (0 to 0)	0 (0 to 0)	0 (0 to 0)	0 (0 to 0)	0 (0 to 0)	1·3 (0·8 to 2)	2 (1·2 to 3)	4 (2·5 to 6·3)	4·9 (3·2 to 7·3)	5·9 (3·7 to 8·7)	7·5 (4·8 to 11·3)	8 (5·1 to 11·9)	7·6 (4·9 to 11·3)	8·7 (5·8 to 12·4)	7·6 (5·2 to 10·7)	6·6 (4·6 to 9·3)	4·5 (3·2 to 6·1)	3·3 (2·4 to 4·6)	2·3 (1·7 to 3·1)	1·2 (0·8 to 1·6)	0·5 (0·4 to 0·8)	0·2 (0·1 to 0·2)	76
Non-rheumatic calcific aortic valve disease	0 (0 to 0)	0 (0 to 0)	0 (0 to 0)	0 (0 to 0)	0 (0 to 0)	0 (0 to 0)	0·9 (0·5 to 1·4)	1 (0·5 to 1·7)	2·3 (1·4 to 3·8)	2·2 (1·3 to 3·4)	3·1 (1·8 to 5·1)	4·3 (2·6 to 7)	5·1 (3·1 to 7·9)	4·7 (2·9 to 7·2)	5·6 (3·5 to 8·3)	5·1 (3·3 to 7·3)	4·8 (3·3 to 6·9)	3·3 (2·3 to 4·6)	2·5 (1·8 to 3·5)	1·8 (1·3 to 2·5)	0·9 (0·6 to 1·3)	0·4 (0·3 to 0·6)	0·1 (0·1 to 0·2)	48·2
Non-rheumatic degenerative mitral valve disease	0 (0 to 0)	0 (0 to 0)	0 (0 to 0)	0 (0 to 0)	0 (0 to 0)	0 (0 to 0)	0·3 (0·1 to 0·5)	0·5 (0·2 to 0·8)	1·5 (0·8 to 2·3)	1·9 (1 to 3·1)	2·4 (1·3 to 4)	2·9 (1·5 to 4·7)	2·7 (1·4 to 4·5)	2·5 (1·4 to 4)	2·9 (1·5 to 4·7)	2·4 (1·4 to 3·8)	1·6 (0·9 to 2·7)	1 (0·6 to 1·6)	0·7 (0·4 to 1·1)	0·4 (0·2 to 0·6)	0·2 (0·1 to 0·4)	0·1 (0·1 to 0·2)	0 (0 to 0)	23·9
Other non-rheumatic valve diseases	0 (0 to 0)	0 (0 to 0)	0 (0 to 0)	0 (0 to 0)	0 (0 to 0)	0 (0 to 0)	0·1 (0 to 0·2)	0·5 (0·2 to 0·9)	0·2 (0·1 to 0·5)	0·8 (0·4 to 1·5)	0·4 (0·2 to 0·8)	0·3 (0·1 to 0·7)	0·2 (0·1 to 0·5)	0·4 (0·2 to 0·9)	0·2 (0·1 to 0·4)	0·2 (0·1 to 0·4)	0·1 (0 to 0·3)	0·1 (0 to 0·3)	0·1 (0 to 0·2)	0·1 (0 to 0·2)	0·1 (0 to 0·1)	0 (0 to 0)	0 (0 to 0)	3·9
Cardiomyopathy and myocarditis	0·8 (0·4 to 1·4)	0·7 (0·3 to 1·3)	4·3 (2·6 to 6·7)	3·1 (1·9 to 4·8)	3·3 (2·4 to 4·5)	4·6 (3·3 to 6·4)	12·8 (8·5 to 18·4)	19·2 (11·4 to 27·1)	37·9 (22·5 to 53·7)	49·9 (30·3 to 72·3)	78·4 (48·3 to 117)	93·8 (61·7 to 134)	107 (66·7 to 155)	105 (68 to 149)	103 (68·3 to 148)	78·8 (53·1 to 112)	56·6 (38·9 to 78·6)	36·5 (24·9 to 51·1)	24·9 (17·1 to 35·3)	15·2 (10·1 to 22·3)	9·3 (6 to 13·9)	3·6 (2·4 to 5·4)	1·3 (0·8 to 2·1)	850
Myocarditis	0·1 (0 to 0·2)	0·1 (0 to 0·2)	0·6 (0·3 to 1·1)	0·5 (0·2 to 0·9)	0·4 (0·2 to 0·7)	0·4 (0·2 to 0·7)	1·1 (0·6 to 2·1)	1·1 (0·5 to 2)	2·1 (0·9 to 3·7)	2·5 (1·1 to 4·7)	3 (1·4 to 5·4)	3·1 (1·6 to 5·3)	2 (1 to 3·8)	2·3 (1·1 to 4·4)	1·7 (0·8 to 3·4)	1·3 (0·6 to 2·4)	1·1 (0·6 to 2)	0·5 (0·3 to 1)	0·4 (0·2 to 0·8)	0·3 (0·1 to 0·5)	0·2 (0·1 to 0·3)	0·1 (0 to 0·1)	0·1 (0 to 0·1)	25
Alcoholic cardiomyopathy	0 (0 to 0)	0 (0 to 0)	0 (0 to 0)	0 (0 to 0)	0 (0 to 0)	0 (0 to 0)	0·9 (0·5 to 1·7)	1·7 (0·8 to 3·3)	5·7 (2·5 to 10·6)	14·1 (6·7 to 23·8)	21·3 (10·8 to 37·2)	31·5 (18 to 51·2)	44·3 (25·5 to 69·6)	45·8 (27 to 69·6)	42·6 (25 to 67·7)	31 (17·7 to 48·3)	22·4 (13·3 to 34·1)	10·8 (6 to 17·5)	6·7 (3·3 to 11·1)	1·3 (0·5 to 2·7)	0·6 (0·2 to 1·1)	0·2 (0·1 to 0·3)	0 (0 to 0·1)	281
Other cardiomyopathy	0·7 (0·3 to 1·2)	0·6 (0·3 to 1·1)	3·7 (2·2 to 5·7)	2·6 (1·6 to 4·2)	2·8 (2 to 3·9)	4·2 (3 to 5·8)	10·7 (7·1 to 15·3)	16·3 (9·7 to 23·6)	30·1 (18·1 to 42·9)	33·4 (19·6 to 50·2)	54·1 (32·1 to 80·8)	59·2 (36·4 to 87·6)	60·2 (35·5 to 94·6)	56·9 (33·2 to 88)	58·6 (36·5 to 89·5)	46·6 (29 to 70)	33 (21·1 to 48·3)	25·1 (16·2 to 36·8)	17·8 (11·9 to 26·5)	13·7 (8·9 to 19·7)	8·6 (5·4 to 12·7)	3·4 (2·2 to 5)	1·2 (0·7 to 1·9)	544
Atrial fibrillation and flutter	0 (0 to 0)	0 (0 to 0)	0 (0 to 0)	0 (0 to 0)	0 (0 to 0)	0 (0 to 0)	0 (0 to 0)	0 (0 to 0)	0 (0 to 0)	1 (0·5 to 1·5)	1·1 (0·6 to 1·6)	4·2 (2·7 to 6·2)	6·9 (4·6 to 10·5)	10·4 (7·2 to 14·6)	15 (10·7 to 20·7)	18·3 (13·4 to 25·7)	19·2 (14·1 to 25·2)	20·3 (14·8 to 27·2)	21·4 (15·8 to 28·2)	21·4 (15·6 to 29·1)	16 (11·9 to 22·1)	10·3 (7·3 to 14·4)	3·9 (2·5 to 6·1)	169
Aortic aneurysm	--	--	--	--	--	--	0·9 (0·5 to 1·3)	0·9 (0·6 to 1·4)	2·2 (1·4 to 3·3)	3·4 (2·2 to 5)	6·5 (4·3 to 9·2)	8·4 (5·5 to 12·1)	9·9 (6·6 to 14·7)	14·3 (9·3 to 20·7)	21·8 (14·4 to 30·6)	26 (18·1 to 36)	24·4 (16·8 to 34·2)	19·8 (13·2 to 27·9)	15·6 (10·9 to 21·6)	9·3 (6·3 to 12·9)	4·6 (3·3 to 6·2)	1·6 (1·2 to 2·2)	0·4 (0·3 to 0·6)	NA
Endocarditis	0·1 (0 to 0·2)	0·1 (0·1 to 0·2)	0·6 (0·3 to 1·1)	0·7 (0·3 to 1·2)	0·5 (0·2 to 0·9)	1·2 (0·6 to 1·9)	3 (1·6 to 5)	5·3 (2·7 to 8·4)	8·3 (4·4 to 13·8)	11·7 (6 to 19·7)	14·3 (7·8 to 22·8)	17·5 (10·3 to 28)	14·5 (8·4 to 22·8)	14·1 (8·4 to 22·1)	10·8 (6·3 to 16·6)	8 (5 to 12·3)	5·5 (3·4 to 8·5)	3·6 (2·3 to 5·4)	2·1 (1·4 to 3·2)	1·3 (0·8 to 2·2)	0·7 (0·4 to 1·2)	0·2 (0·1 to 0·4)	0·1 (0·1 to 0·2)	125
Other cardiovascular and circulatory diseases	1·4 (0·7 to 2·4)	0·7 (0·3 to 1·4)	3·6 (1·8 to 6·1)	2·4 (1·4 to 3·8)	3·5 (2·1 to 5·6)	6·1 (3·9 to 9·1)	10·1 (7 to 14)	14·5 (10·1 to 19·8)	22·7 (15·3 to 31·6)	24·6 (16·9 to 34·3)	36 (24·8 to 50·3)	39·5 (28·2 to 53·9)	44·6 (31·1 to 61·9)	48·4 (32·9 to 67·4)	47 (32·5 to 65·2)	42·6 (31·7 to 56·3)	33·1 (24·1 to 43·6)	25·1 (18·5 to 33·4)	21·9 (15·9 to 28·9)	14·9 (11 to 19·8)	9·5 (6·9 to 12·6)	4·1 (3 to 5·5)	1·4 (0·9 to 1·9)	458
Chronic respiratory diseases	0 (0 to 0)	0 (0 to 0)	0 (0 to 0)	73·2 (40·4 to 125)	75·9 (44·4 to 125)	65·4 (38·9 to 103)	48·8 (31·1 to 74·6)	46·3 (32·3 to 64·3)	50·3 (37·5 to 66·9)	49·9 (37·7 to 64·6)	53·6 (40·3 to 69·6)	58·8 (45 to 75)	68·5 (52·2 to 89·1)	87·2 (65·7 to 112)	101 (75·2 to 133)	102 (77·1 to 134)	96·3 (72·5 to 126)	87·9 (66·4 to 114)	76·3 (58·8 to 99·3)	50·6 (39·2 to 65·2)	27 (20·9 to 34·1)	12·7 (9·6 to 16)	4·4 (3·2 to 5·4)	1,240
Chronic obstructive pulmonary disease	0 (0 to 0)	0 (0 to 0)	0 (0 to 0)	0·8 (0·5 to 1·3)	1 (0·7 to 1·4)	1·8 (0·6 to 3·3)	3·5 (2 to 5·4)	6·3 (3·8 to 9·4)	8·7 (5·7 to 12·3)	10·2 (6·8 to 14·1)	13·6 (9·3 to 18·7)	19·1 (13·4 to 25·4)	29·1 (20·8 to 39·2)	43·8 (31·8 to 58)	61·7 (43·9 to 82·8)	71·3 (52·6 to 95·2)	72·4 (52·7 to 95·3)	68·8 (51·4 to 89·4)	62·5 (48·1 to 81·3)	42·4 (32·7 to 54·2)	23·1 (17·9 to 29·3)	10·9 (8·2 to 13·8)	3·7 (2·7 to 4·6)	555
Pneumoconiosis	0 (0 to 0)	0 (0 to 0)	0 (0 to 0)	0 (0 to 0)	0 (0 to 0)	0 (0 to 0)	0·4 (0·2 to 0·7)	0·1 (0·1 to 0·2)	0·4 (0·2 to 0·7)	0·1 (0·1 to 0·1)	0·2 (0·1 to 0·4)	0·2 (0·1 to 0·2)	0·4 (0·2 to 0·7)	0·4 (0·2 to 0·7)	0·4 (0·2 to 0·7)	0·4 (0·2 to 0·7)	0·6 (0·4 to 1)	0·3 (0·2 to 0·5)	0·3 (0·2 to 0·4)	0·2 (0·2 to 0·4)	0·2 (0·1 to 0·3)	0·1 (0·1 to 0·1)	0 (0 to 0)	4·8
Silicosis	0 (0 to 0)	0 (0 to 0)	0 (0 to 0)	0 (0 to 0)	0 (0 to 0)	0 (0 to 0)	0·2 (0·1 to 0·3)	0·1 (0 to 0·1)	0·4 (0·2 to 0·7)	0 (0 to 0·1)	0·2 (0·1 to 0·3)	0·1 (0 to 0·1)	0·4 (0·2 to 0·6)	0·3 (0·1 to 0·5)	0·2 (0·1 to 0·4)	0·3 (0·2 to 0·6)	0·4 (0·2 to 0·6)	0·2 (0·1 to 0·3)	0·2 (0·1 to 0·3)	0·2 (0·1 to 0·3)	0·1 (0·1 to 0·2)	0 (0 to 0·1)	0 (0 to 0)	3·2
Asbestosis	0 (0 to 0)	0 (0 to 0)	0 (0 to 0)	0 (0 to 0)	0 (0 to 0)	0 (0 to 0)	0·2 (0·1 to 0·3)	0 (0 to 0)	0 (0 to 0)	0 (0 to 0)	0 (0 to 0)	0 (0 to 0)	0 (0 to 0)	0·1 (0 to 0·1)	0·1 (0 to 0·1)	0 (0 to 0·1)	0·1 (0 to 0·1)	0·1 (0 to 0·1)	0 (0 to 0)	0 (0 to 0·1)	0 (0 to 0·1)	0 (0 to 0)	0 (0 to 0)	0·7
Coal workers pneumoconiosis	0 (0 to 0)	0 (0 to 0)	0 (0 to 0)	0 (0 to 0)	0 (0 to 0)	0 (0 to 0)	0 (0 to 0)	0 (0 to 0)	0 (0 to 0)	0 (0 to 0)	0 (0 to 0)	0 (0 to 0)	0 (0 to 0)	0 (0 to 0·1)	0 (0 to 0)	0 (0 to 0·1)	0 (0 to 0)	0 (0 to 0)	0 (0 to 0)	0 (0 to 0)	0 (0 to 0)	0 (0 to 0)	0 (0 to 0)	0·1
Other pneumoconiosis	0 (0 to 0)	0 (0 to 0)	0 (0 to 0)	0 (0 to 0)	0 (0 to 0)	0 (0 to 0)	0·1 (0 to 0·2)	0 (0 to 0·1)	0 (0 to 0)	0 (0 to 0)	0 (0 to 0)	0·1 (0 to 0·1)	0·1 (0 to 0·1)	0·1 (0 to 0·1)	0·1 (0 to 0·2)	0 (0 to 0·1)	0·2 (0·1 to 0·3)	0·1 (0 to 0·1)	0·1 (0 to 0·1)	0 (0 to 0·1)	0 (0 to 0)	0 (0 to 0)	0 (0 to 0)	0·9
Asthma	0 (0 to 0)	0 (0 to 0)	0 (0 to 0)	64·6 (32·4 to 115)	72·2 (41 to 121)	59·7 (33·9 to 97·2)	40·6 (23·7 to 65·3)	33·1 (20·7 to 50·4)	31·3 (20·8 to 45·6)	28·9 (20 to 40·8)	27·1 (18·9 to 37·4)	25·3 (18·2 to 34·4)	25·1 (17·6 to 35·1)	26·4 (18·5 to 35·5)	23 (16·3 to 32·1)	20·3 (14·2 to 28·4)	14·6 (10·1 to 20·5)	10·8 (7·2 to 15·9)	7·1 (4·8 to 10·3)	4·4 (3 to 6·6)	1·9 (1·2 to 2·8)	0·9 (0·6 to 1·3)	0·3 (0·2 to 0·5)	518
Interstitial lung disease and pulmonary sarcoidosis	0 (0 to 0)	0 (0 to 0)	0 (0 to 0)	1·4 (0·7 to 2·5)	0·2 (0·1 to 0·4)	0·3 (0·2 to 0·5)	0·9 (0·5 to 1·7)	1·6 (0·7 to 2·7)	1·4 (0·6 to 2·7)	3·1 (1·5 to 5·4)	3·8 (1·9 to 6·6)	5·8 (3 to 9·7)	5·3 (2·8 to 8·7)	7·4 (4·2 to 11·9)	8 (4·8 to 12·6)	7 (4 to 11·4)	6·7 (3·7 to 10·6)	6·2 (3·6 to 10·1)	5·2 (2·9 to 8·1)	2·6 (1·4 to 4·2)	1·3 (0·7 to 2)	0·5 (0·3 to 0·8)	0·2 (0·1 to 0·4)	68·9
Other chronic respiratory diseases	0 (0 to 0)	0 (0 to 0)	0 (0 to 0)	6·3 (3·8 to 10·4)	2·4 (1·6 to 3·5)	3·7 (2 to 5·9)	3·4 (2·1 to 5·2)	5·2 (3·5 to 7·4)	8·4 (5·4 to 12·8)	7·6 (4·8 to 11·3)	8·8 (5·6 to 13·9)	8·4 (5·2 to 13·3)	8·5 (5·3 to 13·1)	9·3 (5·6 to 14·7)	8·2 (4·6 to 13·2)	2·6 (1·4 to 4·8)	2·1 (1·1 to 3·6)	1·7 (1 to 2·9)	1·3 (0·7 to 2·3)	1 (0·5 to 1·7)	0·5 (0·2 to 1)	0·2 (0·1 to 0·4)	0·1 (0·1 to 0·2)	90
Digestive diseases	1·6 (1·1 to 2·3)	1·8 (0·9 to 3·1)	15·5 (9·4 to 24·3)	24·6 (17·1 to 34·4)	23·5 (16·6 to 33)	23·3 (17 to 32)	36·2 (27·3 to 47·3)	67·4 (50·1 to 88·3)	109 (81·7 to 141)	153 (116 to 195)	228 (179 to 288)	298 (226 to 386)	358 (274 to 470)	418 (323 to 542)	348 (275 to 443)	296 (232 to 375)	207 (162 to 259)	134 (107 to 165)	92·1 (73·7 to 115)	51·7 (40·6 to 63·6)	24·3 (18·6 to 30·6)	10·2 (7·6 to 12·6)	3·3 (2·4 to 4·1)	2,920
Cirrhosis and other chronic liver diseases	0 (0 to 0)	0 (0 to 0)	0 (0 to 0)	2·5 (1·4 to 4·4)	2·4 (1·5 to 3·4)	3 (2 to 4·3)	6·7 (4 to 9·7)	14·7 (9·7 to 21·4)	30·7 (20·6 to 44·2)	57·2 (39·2 to 77·9)	105 (76·5 to 138)	157 (112 to 213)	197 (143 to 268)	252 (186 to 340)	193 (141 to 258)	159 (119 to 207)	100 (74·1 to 131)	55 (40·7 to 72·5)	32·6 (23·8 to 43·6)	16·1 (11·6 to 21·6)	6·4 (4·4 to 8·6)	2·4 (1·6 to 3·1)	0·7 (0·5 to 1)	1,390
Cirrhosis and other chronic liver diseases due to hepatitis B	0 (0 to 0)	0 (0 to 0)	0 (0 to 0)	0 (0 to 0)	0 (0 to 0)	0 (0 to 0·1)	0·1 (0 to 0·2)	0·7 (0·3 to 1·5)	2·2 (0·7 to 4·6)	5·1 (2·4 to 9)	10·6 (4·6 to 20·2)	16·1 (8 to 28·9)	19·6 (9·3 to 37·3)	23 (10·5 to 45)	14·9 (6·6 to 27·8)	10·7 (4·6 to 20·6)	6·2 (2·9 to 11·3)	3·3 (1·5 to 6·1)	2 (1 to 3·7)	1 (0·5 to 1·8)	0·4 (0·2 to 0·7)	0·1 (0·1 to 0·3)	0 (0 to 0·1)	116
Cirrhosis and other chronic liver diseases due to hepatitis C	0 (0 to 0)	0 (0 to 0)	0 (0 to 0)	0 (0 to 0)	0 (0 to 0·1)	0·1 (0·1 to 0·2)	0·4 (0·1 to 0·9)	2·5 (1·1 to 4·6)	7·7 (3·7 to 14)	15·3 (8·3 to 24·3)	27 (14 to 46·7)	38·4 (20·5 to 64·9)	44·7 (24·1 to 75)	53·2 (27·8 to 91·9)	37·8 (19·6 to 66·6)	29·4 (13·4 to 55)	18·2 (10 to 30·7)	9·8 (5·5 to 17)	5·7 (3 to 10·2)	2·9 (1·5 to 4·8)	1·2 (0·7 to 2)	0·4 (0·2 to 0·8)	0·1 (0·1 to 0·3)	295
Cirrhosis and other chronic liver diseases due to alcohol use	0 (0 to 0)	0 (0 to 0)	0 (0 to 0)	0 (0 to 0)	0 (0 to 0)	0 (0 to 0)	0 (0 to 0·1)	1·7 (0·7 to 3·5)	6·3 (2·8 to 12)	18·4 (10·5 to 29·2)	42·9 (24·4 to 66)	70·9 (43·5 to 111)	93·1 (56·9 to 138)	125 (76·4 to 187)	101 (62·1 to 143)	83·6 (54·4 to 118)	50·9 (32 to 71·7)	26·3 (16 to 37·9)	14·3 (8·6 to 21·1)	6·5 (3·8 to 9·7)	2·5 (1·5 to 3·7)	0·9 (0·5 to 1·4)	0·2 (0·1 to 0·4)	644
Cirrhosis and other chronic liver diseases due to NAFLD	0 (0 to 0)	0 (0 to 0)	0 (0 to 0)	0 (0 to 0)	0 (0 to 0)	0 (0 to 0)	0·4 (0·1 to 0·8)	1·7 (0·7 to 3·3)	4·8 (2·1 to 9)	8·5 (4·1 to 14·9)	13·3 (5·8 to 25·8)	19·3 (9 to 38)	25 (11·5 to 45·9)	32 (14·7 to 59·1)	23·7 (10·9 to 43·9)	20·4 (9·3 to 37·8)	14·6 (8·1 to 24·2)	8·9 (4·9 to 14·4)	5·9 (3 to 10·2)	3·2 (1·6 to 5·4)	1·3 (0·7 to 2·2)	0·5 (0·3 to 0·9)	0·2 (0·1 to 0·3)	184
Cirrhosis and other chronic liver diseases due to other causes	0 (0 to 0)	0 (0 to 0)	0 (0 to 0)	2·5 (1·4 to 4·3)	2·3 (1·5 to 3·3)	2·8 (1·9 to 4·1)	5·7 (3·5 to 8·4)	8·2 (4·8 to 12·9)	9·6 (4·8 to 17·1)	9·9 (4·5 to 17·4)	10·8 (4·7 to 21·2)	12·4 (5·5 to 24)	14 (6·2 to 25·9)	18·2 (8·3 to 34)	15·6 (7·2 to 28·9)	14·7 (7 to 25·6)	10·5 (4·8 to 19·2)	6·7 (3·2 to 11·4)	4·6 (2·3 to 8·4)	2·5 (1·2 to 4·4)	1·1 (0·6 to 1·8)	0·4 (0·2 to 0·7)	0·1 (0·1 to 0·3)	153
Upper digestive system diseases	0 (0 to 0)	0 (0 to 0)	0 (0 to 0)	0·9 (0·5 to 1·5)	1·2 (0·6 to 2·3)	2·4 (1·4 to 3·9)	9·2 (5·5 to 15·2)	22·7 (13·8 to 39)	33 (20·4 to 53·6)	39·3 (24·7 to 63·3)	45·7 (29·7 to 69·1)	50·6 (33·4 to 74·2)	58·4 (39·8 to 85·5)	61·7 (42·5 to 88·1)	55·5 (38·6 to 79·3)	47·4 (33·4 to 65·9)	35·8 (25·5 to 49·1)	24·7 (17·9 to 32·9)	18·4 (13·5 to 24·9)	10·3 (7·5 to 14)	5·1 (3·6 to 7)	2·2 (1·6 to 2·9)	0·6 (0·5 to 0·9)	525
Peptic ulcer disease	0 (0 to 0)	0 (0 to 0)	0 (0 to 0)	0·2 (0·1 to 0·4)	0·2 (0·1 to 0·3)	0·4 (0·3 to 0·6)	1·5 (1 to 2·4)	4·3 (2·6 to 6·3)	6·7 (4·2 to 10·1)	9·9 (6·4 to 14·9)	14·2 (9·5 to 20·7)	16·7 (10·7 to 25·4)	20·6 (13·3 to 31·1)	26·7 (17·2 to 39·9)	24·1 (15·5 to 35·8)	22·6 (14·7 to 33)	18·6 (12·3 to 26·8)	13·7 (9 to 19·4)	11·1 (7·7 to 15·8)	6·1 (4·1 to 8·7)	3·2 (2·1 to 4·6)	1·4 (1 to 2)	0·5 (0·3 to 0·6)	203
Gastritis and duodenitis	0 (0 to 0)	0 (0 to 0)	0 (0 to 0)	0·7 (0·4 to 1·3)	1 (0·4 to 2·1)	1 (0·4 to 1·9)	1·3 (0·6 to 2·5)	2·1 (1·2 to 3·6)	3·2 (1·7 to 5·3)	4·5 (2·6 to 7·4)	6·4 (3·7 to 10·1)	8·1 (4·7 to 13·2)	11·3 (6·9 to 18·4)	10·2 (5·9 to 16·1)	9·9 (6·1 to 15·3)	8 (5 to 12·3)	5·8 (3·7 to 8·6)	3·8 (2·4 to 5·7)	2·4 (1·5 to 3·7)	1·3 (0·8 to 2)	0·6 (0·4 to 1)	0·3 (0·2 to 0·5)	0·1 (0 to 0·1)	82
Gastroesophageal reflux disease	0 (0 to 0)	0 (0 to 0)	0 (0 to 0)	0 (0 to 0)	0 (0 to 0·1)	1 (0·4 to 2)	6·3 (3·1 to 11·8)	16·3 (8 to 32·4)	23·1 (11·3 to 43)	24·9 (11·6 to 48·2)	25·1 (12·1 to 47)	25·8 (12·5 to 47)	26·5 (12·8 to 48·2)	24·8 (12·1 to 45·4)	21·6 (10·1 to 42)	16·8 (8 to 31·4)	11·4 (5·9 to 20·7)	7·3 (3·7 to 12·6)	4·9 (2·4 to 8·8)	2·9 (1·4 to 5·2)	1·2 (0·6 to 2·3)	0·4 (0·2 to 0·7)	0·1 (0·1 to 0·2)	241
Appendicitis	0 (0 to 0)	0 (0 to 0)	0 (0 to 0)	1·3 (0·6 to 2·5)	1·4 (0·9 to 2·3)	2·2 (1·1 to 3·8)	3 (1·6 to 5)	3·7 (2·1 to 5·9)	3·7 (2 to 6·1)	3·5 (1·9 to 5·5)	4·4 (2·5 to 7·1)	3·9 (2·2 to 6·4)	3·7 (2·2 to 6·1)	3·6 (2 to 6·1)	2·6 (1·5 to 4·4)	2·1 (1·1 to 3·5)	2 (1·1 to 3·3)	1·4 (0·8 to 2·2)	0·7 (0·4 to 1·3)	0·4 (0·2 to 0·7)	0·2 (0·1 to 0·4)	0·1 (0·1 to 0·2)	0 (0 to 0·1)	43·8
Paralytic ileus and intestinal obstruction	1·3 (0·9 to 1·8)	1·6 (0·8 to 2·7)	13·2 (8 to 21)	5·3 (2·9 to 9)	3 (1·9 to 4·5)	2·7 (1·7 to 4·1)	3·2 (1·8 to 4·9)	4·7 (2·4 to 7·7)	6 (3 to 10·1)	7 (3·5 to 11·2)	11·4 (6·3 to 18·4)	13·1 (7·4 to 21·8)	15·3 (8·7 to 24·6)	18·6 (10·3 to 29·5)	19 (11·7 to 29·4)	17·9 (11·3 to 26·9)	16·1 (10·3 to 24·1)	14 (9·3 to 20·8)	11·4 (7·6 to 16·9)	7·7 (5·2 to 11·7)	3·8 (2·5 to 5·8)	1·6 (1 to 2·4)	0·7 (0·4 to 1)	199
Inguinal, femoral, and abdominal hernia	0·3 (0·2 to 0·6)	0·2 (0·1 to 0·5)	2·2 (1·1 to 4·2)	8·3 (4·9 to 13·1)	11·8 (6·3 to 19·8)	9·2 (4·6 to 15·9)	7·3 (3·6 to 12·7)	8 (4·1 to 13·2)	9·7 (5·4 to 16)	10·4 (5·8 to 17)	13·1 (7·1 to 21·1)	16·5 (10 to 26·6)	19·7 (11·6 to 30·8)	21·4 (13·5 to 32·6)	20 (12·8 to 30·3)	16·7 (10·8 to 24·5)	11·5 (8·1 to 16·2)	7·3 (5·2 to 9·8)	4·7 (3·5 to 6·3)	2·5 (1·8 to 3·2)	1·1 (0·8 to 1·6)	0·5 (0·3 to 0·7)	0·2 (0·1 to 0·2)	202
Inflammatory bowel disease	0 (0 to 0)	0 (0 to 0)	0 (0 to 0)	0·5 (0·2 to 0·9)	0·2 (0·1 to 0·3)	0·2 (0·1 to 0·2)	0·6 (0·4 to 0·9)	1·1 (0·7 to 1·7)	2 (1·3 to 3·2)	3 (1·9 to 4·4)	3·1 (2·1 to 4·6)	3·3 (2·1 to 4·8)	3·5 (2·2 to 5·1)	3·4 (2·2 to 5)	3·4 (2·2 to 5)	2·5 (1·6 to 3·7)	1·9 (1·2 to 2·8)	1·6 (1·1 to 2·4)	1·1 (0·7 to 1·6)	0·7 (0·4 to 1)	0·3 (0·2 to 0·4)	0·1 (0·1 to 0·2)	0 (0 to 0·1)	32·6
Vascular intestinal disorders	0 (0 to 0)	0 (0 to 0)	0 (0 to 0)	0·2 (0·1 to 0·4)	0·2 (0·2 to 0·3)	0·3 (0·1 to 0·4)	0·5 (0·3 to 0·9)	0·5 (0·3 to 0·8)	1·4 (0·9 to 2·2)	2·4 (1·5 to 3·7)	2·9 (1·8 to 4·5)	3·3 (2 to 5·2)	5·7 (3·5 to 8·9)	7·2 (4·5 to 10·9)	7·6 (4·7 to 11·6)	8·1 (5·3 to 11·6)	7·2 (4·9 to 10·1)	5·5 (3·7 to 7·9)	4·4 (2·9 to 6·2)	2·7 (1·8 to 3·8)	1·3 (0·9 to 1·8)	0·6 (0·4 to 0·9)	0·2 (0·1 to 0·2)	62·4
Gallbladder and biliary diseases	0 (0 to 0)	0 (0 to 0)	0 (0 to 0)	0·2 (0·1 to 0·3)	0·3 (0·2 to 0·5)	0·7 (0·5 to 1·1)	1·3 (0·8 to 2)	2·5 (1·4 to 4)	3·8 (2·3 to 5·8)	5 (3·1 to 7·5)	6 (3·8 to 8·8)	8·5 (5·4 to 12·4)	9·5 (6·3 to 13·6)	12 (8·1 to 17·2)	13·1 (8·7 to 18·7)	11·8 (7·9 to 16·9)	10·5 (7·4 to 14·9)	8·6 (6 to 12·4)	6·6 (4·5 to 9·4)	4 (2·8 to 6)	1·9 (1·2 to 2·7)	0·8 (0·6 to 1·2)	0·3 (0·2 to 0·4)	107
Pancreatitis	0 (0 to 0)	0 (0 to 0)	0 (0 to 0)	0·3 (0·1 to 0·4)	0 (0 to 0·1)	0·5 (0·3 to 0·9)	2·1 (1·3 to 3·2)	4·8 (3 to 7·7)	12·1 (7·3 to 18·8)	18·1 (11·1 to 28·1)	27·6 (17·8 to 41·7)	31·2 (19·1 to 46·5)	30·1 (19·2 to 45·5)	23·6 (15 to 35·6)	19·5 (12·5 to 29·4)	15·2 (9·8 to 22·2)	9·8 (6·5 to 13·8)	6·4 (4·5 to 9)	4·3 (3 to 6·2)	2·3 (1·5 to 3·2)	0·9 (0·6 to 1·3)	0·3 (0·2 to 0·5)	0·1 (0·1 to 0·2)	209
Other digestive diseases	0 (0 to 0)	0 (0 to 0)	0 (0 to 0)	5·2 (2·9 to 8·5)	2·8 (1·7 to 4·1)	2·2 (1·4 to 3·3)	2·4 (1·2 to 4)	4·5 (2 to 7·4)	6 (2·7 to 10·6)	7·3 (3·4 to 12)	9·3 (4·7 to 15·3)	10·9 (5·7 to 18·4)	15·2 (8·2 to 26·5)	15·1 (8 to 24·5)	14·8 (7·9 to 23·2)	15·9 (8·8 to 24·8)	12·1 (7·1 to 18)	9·5 (5·3 to 14·3)	7·8 (4·6 to 11·7)	5·1 (2·7 to 7·7)	3·2 (1·7 to 4·8)	1·5 (0·8 to 2·2)	0·5 (0·2 to 0·8)	151
Neurological disorders	0 (0 to 0·1)	0·1 (0 to 0·2)	8·9 (5·4 to 13·3)	20·3 (8·8 to 36·2)	39·6 (16·7 to 72·4)	106 (40·8 to 209)	137 (60·3 to 262)	151 (70·4 to 272)	151 (76·8 to 265)	137 (67·4 to 240)	140 (74·2 to 233)	141 (76·3 to 236)	138 (79·8 to 225)	126 (76·7 to 195)	114 (73·4 to 172)	103 (69·3 to 154)	95·5 (66 to 150)	95·2 (64·4 to 163)	105 (70·1 to 184)	115 (68·2 to 224)	79·8 (42·6 to 159)	44·1 (20·9 to 96·8)	21·6 (9·1 to 47·4)	2,070
Alzheimer's disease and other dementias	0 (0 to 0)	0 (0 to 0)	0 (0 to 0)	0 (0 to 0)	0 (0 to 0)	0 (0 to 0)	0 (0 to 0)	0 (0 to 0)	0 (0 to 0)	0 (0 to 0)	0 (0 to 0)	1 (0·2 to 3·3)	4·8 (1·4 to 13·5)	9 (3·2 to 22·1)	15·4 (6·1 to 35)	24·2 (9·5 to 56·9)	33·2 (12·7 to 82·4)	43·1 (18 to 105)	54·6 (23·2 to 133)	77·8 (31·2 to 188)	61 (23·3 to 143)	37·5 (14·1 to 91)	20 (7·4 to 46)	381
Parkinson's disease	0 (0 to 0)	0 (0 to 0)	0 (0 to 0)	0 (0 to 0)	0 (0 to 0)	0 (0 to 0)	0 (0 to 0)	0·2 (0·1 to 0·3)	0·3 (0·2 to 0·6)	0·3 (0·2 to 0·4)	0·7 (0·4 to 1)	2 (1·5 to 2·8)	4·2 (3·1 to 5·7)	6·9 (5·1 to 9·2)	10 (7·5 to 12·9)	14·8 (11·6 to 18·6)	21·1 (17·1 to 26·4)	28 (22·6 to 34·8)	35·2 (28·5 to 42·5)	28·9 (22·9 to 35·7)	15·2 (12·4 to 18·1)	5·3 (4·2 to 6·5)	1·3 (1 to 1·7)	175
Idiopathic epilepsy	0 (0 to 0·1)	0·1 (0 to 0·2)	2·8 (1·3 to 4·8)	13·1 (5·1 to 25)	20·7 (7·1 to 41·9)	30 (10·1 to 60·2)	38·5 (16·3 to 71·2)	48·5 (24·2 to 82·7)	51·8 (27·3 to 82·5)	45·2 (25·6 to 73·8)	45·8 (26·2 to 70)	42·4 (24·7 to 66·7)	40 (22·9 to 63·7)	31·7 (16·3 to 51·1)	26·2 (13·1 to 44·3)	19·8 (9·1 to 34)	13·8 (6 to 24)	9·1 (3·7 to 16·2)	6·8 (2·6 to 12·2)	4·3 (1·6 to 7·8)	2 (0·7 to 3·7)	0·8 (0·3 to 1·4)	0·2 (0·1 to 0·4)	494
Multiple sclerosis	0 (0 to 0)	0 (0 to 0)	0 (0 to 0)	0 (0 to 0)	0·1 (0 to 0·1)	0·1 (0 to 0·1)	0·3 (0·1 to 0·5)	1·2 (0·5 to 1·9)	1·4 (0·7 to 2)	1·9 (1·2 to 2·8)	3·2 (2 to 4·7)	3·9 (2·6 to 5·9)	3·5 (2·4 to 5·4)	2·9 (2 to 4·8)	3 (1·9 to 4·9)	2 (1·3 to 3·1)	1·1 (0·8 to 1·7)	0·6 (0·4 to 0·8)	0·3 (0·2 to 0·5)	0·1 (0·1 to 0·2)	0 (0 to 0·1)	0 (0 to 0)	0 (0 to 0)	25·5
Motor neuron disease	0 (0 to 0)	0 (0 to 0)	1·3 (0·7 to 2·4)	0·5 (0·3 to 0·8)	0·1 (0·1 to 0·1)	0·2 (0·2 to 0·3)	0·7 (0·5 to 1·1)	0·9 (0·6 to 1·4)	0·9 (0·6 to 1·3)	1·3 (0·8 to 1·8)	3 (2 to 4·3)	4·1 (2·7 to 6)	4·1 (2·6 to 6)	5·4 (3·6 to 8)	5·4 (3·5 to 7·7)	4·5 (3 to 6·2)	3·6 (2·4 to 5·2)	1·9 (1·3 to 2·8)	0·9 (0·6 to 1·2)	0·3 (0·2 to 0·4)	0·1 (0·1 to 0·1)	0 (0 to 0)	0 (0 to 0)	39·4
Headache disorders	0 (0 to 0)	0 (0 to 0)	0 (0 to 0)	0 (0 to 0)	10·3 (0·2 to 27·1)	59·2 (2·8 to 150)	77·3 (8 to 190)	82·8 (11·9 to 192)	82·5 (14·4 to 185)	76·7 (15·5 to 169)	74·5 (16·5 to 157)	74·3 (19·7 to 159)	69·3 (20·2 to 149)	57·6 (16·3 to 117)	43·3 (13·4 to 86·4)	29·7 (10·1 to 61·1)	17·6 (6·1 to 35·9)	9·3 (3·1 to 19·4)	5·1 (1·7 to 10·8)	2·6 (0·9 to 5·7)	1·1 (0·3 to 2·2)	0·3 (0·1 to 0·7)	0·1 (0 to 0·2)	774
Migraine	0 (0 to 0)	0 (0 to 0)	0 (0 to 0)	0 (0 to 0)	9·2 (0·1 to 25·4)	54·8 (1·7 to 148)	71·1 (5·3 to 184)	75·3 (8·2 to 183)	74 (9·8 to 173)	68·4 (10·7 to 154)	65·6 (11·5 to 148)	65·2 (13·5 to 149)	60·3 (14·3 to 136)	49·3 (11·3 to 106)	37·2 (9·5 to 79·4)	24·9 (7 to 53·1)	14·3 (4·2 to 29·9)	7·3 (2·3 to 15·7)	4 (1·1 to 8·9)	2·1 (0·6 to 4·7)	0·8 (0·2 to 1·8)	0·2 (0·1 to 0·5)	0 (0 to 0·1)	684
Tension-type headache	0 (0 to 0)	0 (0 to 0)	0 (0 to 0)	0 (0 to 0)	1·1 (0 to 7·7)	4·4 (0·1 to 34·2)	6·2 (0·8 to 34·3)	7·5 (1·2 to 37)	8·4 (1·4 to 38·1)	8·3 (1·6 to 34·7)	8·9 (1·8 to 35·4)	9·1 (2·2 to 34·1)	9 (2·3 to 29·7)	8·3 (2 to 28·6)	6·1 (1·6 to 18·8)	4·8 (1·2 to 15·4)	3·2 (0·8 to 11·4)	1·9 (0·4 to 7·3)	1·1 (0·2 to 4·3)	0·5 (0·1 to 2·2)	0·2 (0 to 0·9)	0·1 (0 to 0·3)	0 (0 to 0·1)	89·3
Other neurological disorders	0 (0 to 0)	0 (0 to 0)	4·8 (2·8 to 7·3)	6·6 (3·3 to 11)	8·5 (3·7 to 15·6)	16·5 (7·8 to 29·5)	20·3 (11·5 to 33·1)	17·6 (10·6 to 26·3)	14·6 (9·4 to 21)	12 (7·8 to 17·3)	12·3 (8 to 17·6)	13·2 (8·8 to 19·1)	12·3 (7·7 to 18·4)	12·4 (8·3 to 17·5)	10·7 (7·3 to 14·6)	7·9 (5·7 to 10·7)	5 (3·6 to 6·7)	3·2 (2·3 to 4·3)	2 (1·5 to 2·8)	1·2 (0·9 to 1·6)	0·4 (0·3 to 0·6)	0·2 (0·1 to 0·2)	0 (0 to 0·1)	182
Mental disorders	0 (0 to 0·1)	0·1 (0·1 to 0·2)	2 (1·3 to 3)	11·6 (7·5 to 16·8)	65·6 (43·5 to 95·5)	196 (132 to 280)	243 (166 to 338)	247 (170 to 341)	266 (187 to 366)	262 (187 to 357)	266 (189 to 355)	263 (190 to 355)	257 (186 to 341)	225 (162 to 300)	183 (131 to 242)	132 (95·6 to 174)	82·7 (59·9 to 109)	48·7 (35·4 to 64·2)	30·2 (21·7 to 39·4)	16·8 (11·7 to 22·2)	7·2 (5·1 to 9·5)	2·4 (1·7 to 3·3)	0·7 (0·5 to 1)	2,810
Schizophrenia	0 (0 to 0)	0 (0 to 0)	0 (0 to 0)	0 (0 to 0)	0 (0 to 0)	1 (0·4 to 1·9)	7·4 (2·5 to 14·5)	25·7 (12·9 to 42·9)	43·6 (24·8 to 67·5)	50·7 (30·5 to 75·6)	53·7 (33 to 79·7)	52·4 (33·5 to 73·9)	49·6 (31·9 to 70·1)	41·1 (26·4 to 56·8)	30·5 (19·8 to 42·4)	19·3 (12·5 to 27·6)	10·8 (6·8 to 15·2)	5·5 (3·5 to 8)	2·9 (1·7 to 4·3)	1·3 (0·7 to 2)	0·4 (0·2 to 0·7)	0·1 (0 to 0·2)	0 (0 to 0)	396
Depressive disorders	0 (0 to 0)	0 (0 to 0)	0 (0 to 0)	0 (0 to 0)	1·9 (0·8 to 3·7)	19·5 (9·3 to 35·7)	48 (26·9 to 76·8)	63·6 (36·3 to 99)	61·8 (36·9 to 96·3)	58·6 (34·8 to 92·8)	64·7 (39·8 to 98·1)	69·9 (43 to 109)	72·3 (46·4 to 108)	67·5 (44·2 to 98·9)	58·7 (36·9 to 87·2)	44·9 (27·3 to 67·7)	29·8 (19·5 to 43·1)	18·6 (12·3 to 26·7)	12·3 (7·7 to 18·2)	7·5 (4·6 to 11·1)	3·5 (2·3 to 5·2)	1·3 (0·8 to 2)	0·4 (0·2 to 0·7)	705
Major depressive disorder	0 (0 to 0)	0 (0 to 0)	0 (0 to 0)	0 (0 to 0)	1·6 (0·5 to 3·3)	16·8 (7·5 to 31·7)	40·3 (21·8 to 68·8)	50·1 (26·5 to 81·4)	44·9 (25·1 to 73·1)	41·2 (22·4 to 69·4)	46·9 (27·6 to 72·9)	51·6 (30 to 84·4)	54·3 (33·4 to 81·5)	51·5 (31·8 to 77·5)	45·6 (26·6 to 69·6)	35·3 (20 to 54)	23·7 (15·2 to 35·2)	15 (9·4 to 22·5)	10·1 (6 to 15·8)	6·2 (3·6 to 9·8)	3 (1·8 to 4·5)	1·1 (0·7 to 1·8)	0·4 (0·2 to 0·6)	539
Dysthymia	0 (0 to 0)	0 (0 to 0)	0 (0 to 0)	0 (0 to 0)	0·3 (0 to 0·9)	2·8 (1·1 to 5·5)	7·7 (3·6 to 13·3)	13·5 (6·5 to 23·6)	16·9 (8·1 to 30)	17·4 (9·4 to 29·1)	17·9 (9·9 to 29·9)	18·2 (9·7 to 31·8)	17·9 (9·7 to 30·9)	16 (9·2 to 26·5)	13·1 (7·5 to 21·8)	9·6 (5 to 16·1)	6·1 (3·2 to 10·5)	3·6 (2 to 6·1)	2·3 (1·2 to 3·8)	1·2 (0·7 to 2·1)	0·5 (0·3 to 0·9)	0·2 (0·1 to 0·3)	0·1 (0 to 0·1)	165
Bipolar disorder	0 (0 to 0)	0 (0 to 0)	0 (0 to 0)	0 (0 to 0)	0 (0 to 0)	9·9 (3·9 to 19·1)	34·7 (16·1 to 62·5)	43·6 (21·6 to 74·5)	42·6 (22·2 to 72·2)	37·6 (19·8 to 63·7)	36·5 (18·9 to 59·7)	37 (20 to 61·1)	36·5 (19·4 to 60·6)	31·4 (16·6 to 52·3)	24·2 (13·4 to 38·9)	16·1 (8·8 to 26·1)	9·2 (4·8 to 14·8)	4·8 (2·5 to 8)	2·5 (1·3 to 4·3)	1·1 (0·6 to 2)	0·4 (0·2 to 0·7)	0·1 (0·1 to 0·2)	0 (0 to 0·1)	368
Anxiety disorders	0 (0 to 0)	0 (0 to 0)	0 (0 to 0)	0·9 (0·2 to 2)	15·4 (7·3 to 27·1)	45·5 (24 to 77·3)	55·9 (32·7 to 87·8)	58·4 (31·9 to 97·8)	57·5 (31·8 to 95·3)	54·1 (32·2 to 85·8)	53·7 (30·7 to 82·6)	53·6 (30·1 to 84·6)	53·1 (30·5 to 84·4)	47·2 (27·6 to 70·9)	39·5 (24·5 to 59·2)	29·2 (17·2 to 46·3)	18·7 (10·6 to 29·9)	10·8 (6·3 to 17·5)	6·5 (3·8 to 10·1)	3·3 (1·9 to 5·3)	1·2 (0·7 to 1·9)	0·3 (0·2 to 0·6)	0·1 (0 to 0·1)	605
Eating disorders	0 (0 to 0)	0 (0 to 0)	0 (0 to 0)	0 (0 to 0)	0·2 (0·1 to 0·4)	3·5 (1·4 to 7)	9·5 (4·5 to 17·6)	12·3 (5·8 to 22·5)	13·2 (7 to 21·3)	13·9 (6·7 to 24·1)	10·5 (5·3 to 18·1)	4·9 (2·4 to 9)	2 (0·8 to 3·9)	0 (0 to 0)	0 (0 to 0)	0 (0 to 0)	0 (0 to 0)	0 (0 to 0)	0 (0 to 0)	0 (0 to 0)	0 (0 to 0)	0 (0 to 0)	0 (0 to 0)	70
Anorexia nervosa	0 (0 to 0)	0 (0 to 0)	0 (0 to 0)	0 (0 to 0)	0·1 (0 to 0·3)	1·3 (0·4 to 2·7)	3·2 (1·3 to 6·2)	3·2 (1·4 to 6·2)	2·4 (0·9 to 4·7)	1·4 (0·4 to 3)	1 (0·3 to 2·1)	0·8 (0·1 to 1·7)	0·7 (0·1 to 1·4)	0 (0 to 0)	0 (0 to 0)	0 (0 to 0)	0 (0 to 0)	0 (0 to 0)	0 (0 to 0)	0 (0 to 0)	0 (0 to 0)	0 (0 to 0)	0 (0 to 0)	14·2
Bulimia nervosa	0 (0 to 0)	0 (0 to 0)	0 (0 to 0)	0 (0 to 0)	0 (0 to 0·1)	2·2 (0·6 to 5·1)	6·2 (2·4 to 13·1)	9·1 (3·1 to 18·4)	10·8 (5·1 to 18·3)	12·5 (5·8 to 22·7)	9·5 (4·5 to 16·7)	4·1 (1·7 to 7·8)	1·4 (0·4 to 3)	0 (0 to 0)	0 (0 to 0)	0 (0 to 0)	0 (0 to 0)	0 (0 to 0)	0 (0 to 0)	0 (0 to 0)	0 (0 to 0)	0 (0 to 0)	0 (0 to 0)	55·8
Autism spectrum disorders	0 (0 to 0·1)	0·1 (0·1 to 0·2)	1·9 (1·2 to 2·9)	8·7 (5·4 to 12·9)	12·6 (7·9 to 19·6)	14·4 (9·1 to 21·7)	13·5 (8·7 to 20·2)	13·2 (8·2 to 20·1)	12·4 (7·7 to 18·3)	10·9 (6·7 to 16·4)	10·1 (6·4 to 15·5)	9·5 (5·8 to 14·7)	8·9 (5·6 to 13·4)	7·7 (4·7 to 12)	6·2 (3·8 to 9·2)	4·5 (2·7 to 6·8)	2·9 (1·8 to 4·4)	1·8 (1·1 to 2·8)	1·1 (0·7 to 1·7)	0·7 (0·4 to 1)	0·3 (0·2 to 0·4)	0·1 (0·1 to 0·2)	0 (0 to 0)	141
Attention-deficit/hyperactivity disorder	0 (0 to 0)	0 (0 to 0)	0 (0 to 0)	1·1 (0·5 to 2)	13·1 (7·1 to 23·6)	20·8 (11·3 to 36·9)	16·6 (9·2 to 29·4)	12·6 (6·9 to 21·9)	9·4 (5·1 to 16·3)	6·9 (3·7 to 12·3)	5·4 (2·7 to 9·4)	4·3 (2·2 to 7·6)	3·4 (1·7 to 6)	2·4 (1·2 to 4·5)	1·4 (0·7 to 2·6)	0·7 (0·3 to 1·3)	0·2 (0·1 to 0·5)	0·1 (0 to 0·2)	0 (0 to 0·1)	0 (0 to 0)	0 (0 to 0)	0 (0 to 0)	0 (0 to 0)	98·3
Conduct disorder	0 (0 to 0)	0 (0 to 0)	0 (0 to 0)	0 (0 to 0)	20·8 (10·9 to 36·6)	78 (43·3 to 126)	49·1 (25·9 to 81·2)	0 (0 to 0)	0 (0 to 0)	0 (0 to 0)	0 (0 to 0)	0 (0 to 0)	0 (0 to 0)	0 (0 to 0)	0 (0 to 0)	0 (0 to 0)	0 (0 to 0)	0 (0 to 0)	0 (0 to 0)	0 (0 to 0)	0 (0 to 0)	0 (0 to 0)	0 (0 to 0)	148
Idiopathic developmental intellectual disability	0 (0 to 0)	0 (0 to 0)	0·1 (0 to 0·3)	0·9 (0·1 to 2)	1·7 (0·3 to 3·4)	1·9 (0·3 to 3·8)	1·8 (0·3 to 3·7)	1·7 (0·3 to 3·6)	1·6 (0·3 to 3·4)	1·4 (0·3 to 2·8)	1·3 (0·2 to 2·5)	1·1 (0·2 to 2·4)	1 (0·2 to 2·1)	0·8 (0·1 to 1·8)	0·6 (0·1 to 1·3)	0·4 (0·1 to 0·9)	0·2 (0 to 0·5)	0·1 (0 to 0·3)	0·1 (0 to 0·1)	0 (0 to 0·1)	0 (0 to 0)	0 (0 to 0)	0 (0 to 0)	16·8
Other mental disorders	0 (0 to 0)	0 (0 to 0)	0 (0 to 0)	0 (0 to 0)	0 (0 to 0)	1 (0·4 to 1·8)	6·6 (3 to 11·3)	16·2 (8·1 to 27·1)	24·4 (13·9 to 38·9)	27·9 (15·6 to 43·4)	29·8 (17 to 47·3)	30·2 (18·4 to 46·8)	30 (17·8 to 46·5)	26·8 (16·3 to 40·6)	22·2 (13·5 to 34)	16·5 (9·8 to 25·1)	10·9 (6·9 to 16·8)	6·9 (4·5 to 10·2)	4·7 (3 to 7·1)	2·8 (1·8 to 4·2)	1·3 (0·8 to 1·9)	0·5 (0·3 to 0·7)	0·2 (0·1 to 0·2)	259
Substance use disorders	0 (0 to 0)	0 (0 to 0)	0 (0 to 0)	0 (0 to 0)	0 (0 to 0)	3·2 (1·6 to 5·5)	53·8 (35 to 77·2)	115 (77·7 to 159)	144 (100 to 192)	153 (111 to 206)	169 (125 to 227)	178 (130 to 240)	170 (123 to 235)	148 (108 to 197)	120 (89 to 160)	74·7 (54·3 to 97·2)	42·7 (31·4 to 57·5)	20·1 (14·7 to 26·9)	10·1 (7·5 to 13·2)	4·5 (3·3 to 6·2)	1·8 (1·3 to 2·5)	0·7 (0·5 to 1)	0·3 (0·2 to 0·4)	1,410
Alcohol use disorders	0 (0 to 0)	0 (0 to 0)	0 (0 to 0)	0 (0 to 0)	0 (0 to 0)	1·2 (0·4 to 2·6)	18·2 (10·3 to 28·3)	57·2 (34·2 to 92·1)	88·5 (58·4 to 126)	111 (76·6 to 155)	133 (95·1 to 183)	149 (104 to 205)	147 (104 to 209)	133 (94·5 to 180)	111 (80·2 to 150)	69·4 (49·2 to 91·5)	39·8 (28·9 to 53·8)	18·5 (13·4 to 24·9)	9 (6·6 to 11·8)	3·9 (2·7 to 5·4)	1·5 (1·1 to 2·1)	0·5 (0·4 to 0·8)	0·2 (0·1 to 0·3)	1,090
Drug use disorders	0 (0 to 0)	0 (0 to 0)	0 (0 to 0)	0 (0 to 0)	0 (0 to 0)	2 (0·8 to 3·6)	35·6 (21·1 to 54·5)	57·5 (38·6 to 81·6)	55·5 (37·6 to 77·5)	42·6 (29·4 to 59·4)	35·7 (25 to 48·4)	28·8 (20 to 38·9)	22·4 (15·5 to 30·9)	14·9 (9·6 to 21)	9·6 (6·1 to 13·8)	5·3 (3·1 to 8·1)	2·9 (1·7 to 4·6)	1·6 (0·9 to 2·5)	1 (0·6 to 1·7)	0·7 (0·4 to 1)	0·3 (0·2 to 0·5)	0·1 (0·1 to 0·2)	0·1 (0 to 0·1)	317
Opioid use disorders	0 (0 to 0)	0 (0 to 0)	0 (0 to 0)	0 (0 to 0)	0 (0 to 0)	0 (0 to 0)	4·5 (1·7 to 8·2)	19·7 (10·1 to 33·3)	23·5 (13·4 to 37·4)	19·5 (12 to 30·4)	16·7 (10·2 to 25·2)	13·8 (8 to 20·9)	10·9 (6·3 to 16·7)	7·8 (3·8 to 13)	5·4 (2·8 to 9·1)	3·2 (1·5 to 5·6)	1·8 (0·8 to 3)	0·9 (0·4 to 1·7)	0·6 (0·3 to 1·1)	0·4 (0·2 to 0·7)	0·2 (0·1 to 0·3)	0·1 (0 to 0·2)	0 (0 to 0·1)	129
Cocaine use disorders	0 (0 to 0)	0 (0 to 0)	0 (0 to 0)	0 (0 to 0)	0 (0 to 0)	0·2 (0·1 to 0·4)	8·5 (3·7 to 15·3)	10·4 (5·6 to 17)	9·8 (6 to 15·2)	8·2 (4·8 to 12·3)	7·5 (4·7 to 10·8)	5·8 (3·4 to 8·9)	4·2 (2·4 to 6·6)	2·3 (1·1 to 3·8)	1·4 (0·6 to 2·5)	0·8 (0·3 to 1·6)	0·4 (0·1 to 0·9)	0·3 (0·1 to 0·6)	0·2 (0 to 0·4)	0·1 (0 to 0·2)	0 (0 to 0·1)	0 (0 to 0)	0 (0 to 0)	60·2
Amphetamine use disorders	0 (0 to 0)	0 (0 to 0)	0 (0 to 0)	0 (0 to 0)	0 (0 to 0)	0 (0 to 0)	1·1 (0·1 to 2·8)	5·1 (1·9 to 9·7)	5·3 (2·2 to 9·3)	3·4 (1·6 to 6·1)	2·4 (1·1 to 4·1)	1·7 (1·1 to 2·5)	1·1 (0·7 to 1·7)	0·8 (0·5 to 1·1)	0·5 (0·3 to 0·7)	0·2 (0·2 to 0·4)	0·1 (0·1 to 0·2)	0 (0 to 0·1)	0 (0 to 0)	0 (0 to 0)	0 (0 to 0)	0 (0 to 0)	0 (0 to 0)	21·6
Cannabis use disorders	0 (0 to 0)	0 (0 to 0)	0 (0 to 0)	0 (0 to 0)	0 (0 to 0)	1·6 (0·5 to 3·2)	15·3 (7·3 to 28·4)	12·4 (5·7 to 22·5)	7·2 (3·3 to 14·2)	4·2 (1·8 to 7·8)	3 (1·3 to 5·7)	2·5 (1 to 4·6)	2 (0·8 to 3·9)	1·5 (0·6 to 2·7)	0·8 (0·3 to 1·6)	0·4 (0·1 to 0·8)	0·2 (0·1 to 0·3)	0·1 (0 to 0·1)	0 (0 to 0·1)	0 (0 to 0)	0 (0 to 0)	0 (0 to 0)	0 (0 to 0)	51·3
Other drug use disorders	0 (0 to 0)	0 (0 to 0)	0 (0 to 0)	0 (0 to 0)	0 (0 to 0)	0·2 (0·1 to 0·3)	6·2 (2·8 to 11·5)	9·9 (5·7 to 16)	9·7 (5·9 to 15·2)	7·2 (4·4 to 11·1)	6·1 (3·9 to 9·1)	5·1 (3·3 to 7·5)	4·1 (2·6 to 6)	2·6 (1·5 to 4)	1·4 (0·8 to 2·3)	0·7 (0·3 to 1·2)	0·4 (0·2 to 0·8)	0·3 (0·2 to 0·5)	0·2 (0·1 to 0·3)	0·1 (0·1 to 0·2)	0·1 (0 to 0·1)	0 (0 to 0)	0 (0 to 0)	54·2
Diabetes and kidney diseases	0 (0 to 0)	0·1 (0·1 to 0·2)	7 (4·4 to 10·5)	8·6 (5·9 to 11·8)	12·7 (9·1 to 17·3)	15·4 (11 to 21·3)	41 (30·4 to 52·8)	80·1 (62·8 to 102)	138 (108 to 171)	180 (145 to 224)	259 (208 to 324)	364 (282 to 452)	505 (396 to 636)	661 (505 to 839)	694 (537 to 861)	673 (524 to 838)	569 (453 to 702)	421 (336 to 512)	299 (241 to 360)	189 (153 to 227)	89·4 (72·4 to 106)	35·9 (29·5 to 43·2)	10·8 (8·4 to 13)	5,250
Diabetes mellitus	0 (0 to 0)	0·1 (0·1 to 0·2)	1·5 (0·7 to 2·8)	1·4 (0·8 to 2·3)	1·4 (0·9 to 2·1)	2·8 (1·8 to 4)	12·5 (9·1 to 16·8)	31·7 (23·7 to 40·9)	61·1 (46·2 to 78·8)	88·4 (66 to 116)	135 (102 to 173)	197 (148 to 257)	299 (221 to 394)	432 (321 to 560)	445 (330 to 576)	462 (349 to 594)	390 (298 to 498)	286 (219 to 367)	197 (151 to 252)	124 (95 to 156)	54·7 (42·8 to 67·3)	20·4 (16·1 to 25·4)	5·8 (4·5 to 7·2)	3,250
Diabetes mellitus type 1	0 (0 to 0)	0·1 (0·1 to 0·2)	1·5 (0·7 to 2·8)	1·4 (0·8 to 2·3)	1·4 (0·9 to 2·1)	2·8 (1·8 to 4)	5·3 (3·6 to 7·5)	12·9 (8·9 to 18·2)	25·2 (17·3 to 35·3)	18·7 (11·5 to 29·9)	27·3 (19·2 to 41·4)	37·2 (23·3 to 59·1)	12·9 (8·2 to 20·8)	16·1 (9·9 to 26·1)	16·7 (10·2 to 26·1)	13·7 (8·2 to 22·3)	11·6 (7·2 to 19·5)	6·5 (3·9 to 11·3)	4·7 (2·8 to 7·9)	2·1 (1·3 to 3·7)	1 (0·6 to 1·5)	0·4 (0·3 to 0·7)	0·1 (0·1 to 0·2)	220
Diabetes mellitus type 2	0 (0 to 0)	0 (0 to 0)	0 (0 to 0)	0 (0 to 0)	0 (0 to 0)	0 (0 to 0)	7·2 (4·6 to 10·7)	18·8 (12·7 to 26·1)	36 (24·7 to 49·9)	69·7 (52 to 93·4)	108 (78 to 143)	160 (117 to 213)	286 (212 to 381)	416 (310 to 542)	428 (317 to 555)	448 (338 to 578)	379 (290 to 482)	280 (214 to 358)	193 (147 to 247)	122 (93·2 to 153)	53·7 (41·8 to 66·4)	19·9 (15·7 to 24·9)	5·7 (4·4 to 7)	3,030
Chronic kidney disease	0 (0 to 0)	0 (0 to 0)	5·4 (3·3 to 8·3)	6·8 (4·5 to 9·6)	10·7 (7·4 to 15·1)	12·3 (8·3 to 18)	28·1 (19·3 to 38·5)	48·1 (34·9 to 65·1)	76·3 (53·8 to 104)	91·2 (67·4 to 121)	123 (88·9 to 164)	166 (121 to 222)	205 (148 to 275)	229 (162 to 317)	249 (181 to 335)	211 (151 to 285)	179 (134 to 236)	134 (102 to 171)	101 (76·9 to 130)	65·3 (50·6 to 83·9)	34·7 (27·2 to 43·3)	15·6 (12·1 to 19·3)	5 (3·7 to 6·3)	2,000
Chronic kidney disease due to diabetes mellitus type 1	0 (0 to 0)	0 (0 to 0)	0 (0 to 0·1)	0·2 (0·1 to 0·4)	0·3 (0·1 to 0·8)	0·4 (0·2 to 1)	1 (0·4 to 2·2)	2·4 (1 to 4·8)	7·6 (3·1 to 14·8)	14·2 (6·9 to 24·7)	23 (11·2 to 39·4)	30·6 (14·9 to 56·1)	31·4 (14·9 to 55·9)	25·7 (10 to 50·4)	19·1 (6·2 to 41·5)	11·7 (4·1 to 25·2)	7·8 (3 to 17·7)	4·5 (1·6 to 9)	2·2 (0·8 to 4·9)	0·7 (0·3 to 1·7)	0·1 (0 to 0·3)	0 (0 to 0)	0 (0 to 0)	183
Chronic kidney disease due to diabetes mellitus type 2	0 (0 to 0)	0 (0 to 0)	0 (0 to 0)	0 (0 to 0)	0 (0 to 0)	0 (0 to 0)	0·4 (0·1 to 0·9)	1·1 (0·4 to 2·5)	3 (1·2 to 6·5)	5·5 (2·3 to 10·9)	10·2 (4·3 to 20·3)	19·6 (8·8 to 34·9)	35·7 (18·5 to 60·2)	53·6 (29·6 to 85·9)	69·4 (38·3 to 104)	63·9 (36·8 to 95·6)	56 (35·7 to 81·4)	42·6 (27·1 to 60·1)	31·6 (20·4 to 44·8)	19·9 (12·5 to 28·3)	10 (6·2 to 14·2)	4·3 (2·5 to 6·2)	1·3 (0·7 to 2)	428
Chronic kidney disease due to hypertension	0 (0 to 0)	0 (0 to 0)	0 (0 to 0)	0 (0 to 0)	0 (0 to 0)	0 (0 to 0)	2·3 (1·1 to 4)	4·1 (1·6 to 7·6)	7 (3·2 to 12·1)	9·1 (5 to 15·2)	12·9 (7·2 to 20·5)	19·9 (12·2 to 31·2)	29·2 (18·2 to 44·7)	38·2 (23·3 to 58·9)	48·2 (29·4 to 72·8)	47·2 (28·6 to 71·9)	44·8 (28·5 to 66·3)	36·6 (24 to 52·9)	30·5 (21 to 43·1)	21·9 (15 to 30·1)	13 (9·3 to 17·7)	6·5 (4·6 to 8·7)	2·1 (1·4 to 3)	373
Chronic kidney disease due to glomerulonephritis	0 (0 to 0)	0 (0 to 0)	1·6 (0·7 to 2·9)	2·3 (1·3 to 3·7)	4·1 (2·4 to 6·2)	5·2 (3 to 8·1)	12·1 (7·1 to 18·7)	20·8 (12·7 to 30·8)	31·4 (19·3 to 47·6)	34 (20·9 to 49·3)	42·6 (25·3 to 63·6)	53·2 (31·2 to 79·9)	58·9 (36·3 to 90·6)	59·9 (34·7 to 96·9)	59·8 (32·6 to 98·4)	44·1 (24·1 to 72·5)	32·2 (16 to 56)	21 (11·3 to 36·1)	13·8 (6·7 to 23·9)	7·3 (3·3 to 13·4)	3·1 (1·4 to 5·5)	1·2 (0·5 to 2·1)	0·3 (0·1 to 0·7)	509
Chronic kidney disease due to other and unspecified causes	0 (0 to 0)	0 (0 to 0)	3·8 (2·2 to 6·1)	4·4 (2·8 to 6·4)	6·3 (3·9 to 9·5)	6·7 (3·8 to 10·6)	12·3 (7·2 to 19·2)	19·7 (11·8 to 29·5)	27·3 (15·6 to 42)	28·5 (17 to 44·4)	34·3 (19·3 to 54)	42·7 (23·3 to 68·5)	49·6 (27·8 to 79·6)	51·1 (29·7 to 81·7)	52·3 (29·7 to 84·8)	43·7 (25·1 to 73·1)	38 (21·4 to 59·8)	29·9 (18·1 to 46·6)	23·2 (13·4 to 36·2)	15·5 (9·3 to 24)	8·4 (5·2 to 12·5)	3·6 (2·4 to 5·5)	1·2 (0·7 to 1·8)	502
Acute glomerulonephritis	0 (0 to 0)	0 (0 to 0)	0 (0 to 0)	0·4 (0·1 to 0·9)	0·6 (0·2 to 1·1)	0·3 (0·1 to 0·6)	0·4 (0·2 to 0·9)	0·3 (0·1 to 0·6)	0·3 (0·1 to 0·7)	0·2 (0·1 to 0·4)	0·5 (0·2 to 1)	0·6 (0·2 to 1·4)	1 (0·4 to 1·9)	0·5 (0·2 to 0·9)	0·6 (0·3 to 1·4)	0·4 (0·2 to 0·9)	0·3 (0·1 to 0·6)	0·4 (0·1 to 0·8)	0·2 (0·1 to 0·5)	0·1 (0·1 to 0·3)	0·1 (0 to 0·2)	0 (0 to 0·1)	0 (0 to 0)	7·2
Skin and subcutaneous diseases	0 (0 to 0)	0 (0 to 0·1)	9·6 (5·2 to 15·4)	56·6 (35·8 to 83)	82 (50·2 to 120)	98·4 (63·1 to 147)	106 (68·3 to 163)	71·4 (47·2 to 108)	64 (42 to 95·2)	56·9 (37·5 to 82·8)	58·3 (39·3 to 84·4)	62 (42·3 to 90·6)	64·5 (43 to 94·8)	65·4 (43·9 to 95·8)	60·4 (40 to 88·1)	52·8 (35·7 to 79·5)	45·2 (30·5 to 67·7)	34·2 (22·5 to 53·8)	29 (19·1 to 47·7)	20·5 (13·5 to 34·2)	10·8 (7 to 19·6)	5 (3·2 to 9)	1·7 (1 to 3·6)	1,050
Dermatitis	0 (0 to 0)	0 (0 to 0)	0 (0 to 0)	18·3 (9·9 to 31·4)	25·3 (13·3 to 43·5)	20·5 (11 to 35·2)	14·8 (8·1 to 24·4)	12·7 (7 to 20·7)	11·4 (6·4 to 18·6)	10·3 (5·6 to 16·7)	9·7 (5·4 to 15·6)	9·3 (5·2 to 15·3)	9·4 (5·3 to 15·7)	9·1 (4·8 to 14·7)	8·3 (4·5 to 14·1)	6·7 (3·7 to 11·1)	5 (2·8 to 8·1)	3·3 (1·9 to 5·3)	2·2 (1·3 to 3·6)	1·3 (0·7 to 2)	0·6 (0·3 to 0·9)	0·2 (0·1 to 0·3)	0·1 (0 to 0·1)	178
Atopic dermatitis	0 (0 to 0)	0 (0 to 0)	0 (0 to 0)	18·1 (9·7 to 31·1)	24·7 (12·9 to 42·9)	18·3 (9·4 to 32·1)	11·3 (5·7 to 19·8)	8·3 (4·2 to 14·7)	6·5 (3·2 to 11·4)	5·3 (2·6 to 9·6)	4·8 (2·3 to 8·7)	4·6 (2·4 to 8·3)	4·5 (2·3 to 8·2)	4·1 (2 to 7·4)	3·7 (1·8 to 6·9)	2·9 (1·4 to 5·3)	2 (1 to 3·6)	1·3 (0·6 to 2·2)	0·9 (0·5 to 1·5)	0·6 (0·3 to 1)	0·3 (0·1 to 0·5)	0·1 (0 to 0·2)	0 (0 to 0)	122
Contact dermatitis	0 (0 to 0)	0 (0 to 0)	0 (0 to 0)	0 (0 to 0)	0·3 (0·1 to 0·7)	1·8 (0·7 to 3·9)	3·1 (1·2 to 5·9)	3·8 (1·6 to 7·4)	4·4 (1·8 to 8·4)	4·4 (1·8 to 8·4)	4·3 (1·7 to 8·6)	4·2 (1·9 to 8·5)	4·4 (1·8 to 8·6)	4·5 (1·9 to 9)	4·2 (1·6 to 8·6)	3·5 (1·5 to 6·7)	2·7 (1·2 to 5·3)	1·8 (0·8 to 3·4)	1·1 (0·5 to 2·2)	0·6 (0·3 to 1·1)	0·2 (0·1 to 0·5)	0·1 (0 to 0·1)	0 (0 to 0)	49·7
Seborrhoeic dermatitis	0 (0 to 0)	0 (0 to 0)	0 (0 to 0)	0·2 (0·1 to 0·4)	0·2 (0·1 to 0·5)	0·4 (0·2 to 0·7)	0·5 (0·2 to 0·8)	0·5 (0·2 to 0·9)	0·6 (0·3 to 1·1)	0·6 (0·3 to 1)	0·5 (0·3 to 1)	0·5 (0·3 to 1)	0·5 (0·2 to 0·9)	0·4 (0·2 to 0·8)	0·4 (0·2 to 0·7)	0·3 (0·2 to 0·5)	0·3 (0·2 to 0·5)	0·2 (0·1 to 0·4)	0·2 (0·1 to 0·3)	0·1 (0·1 to 0·2)	0 (0 to 0·1)	0 (0 to 0)	0 (0 to 0)	6·4
Psoriasis	0 (0 to 0)	0 (0 to 0)	0 (0 to 0)	0·7 (0·4 to 1·1)	2·1 (0·8 to 3·9)	3·2 (1·5 to 5·5)	3·5 (1·6 to 5·9)	3·7 (1·8 to 6·3)	4·1 (2 to 7)	4·4 (2·2 to 7·1)	4·8 (2·6 to 7·7)	5·2 (2·9 to 8·2)	5·6 (3·1 to 8·6)	5·5 (3·2 to 8·6)	5·1 (3 to 7·8)	4 (2·4 to 6·2)	2·8 (1·6 to 4·2)	1·6 (1 to 2·4)	0·9 (0·5 to 1·4)	0·4 (0·2 to 0·7)	0·2 (0·1 to 0·2)	0 (0 to 0·1)	0 (0 to 0)	58
Bacterial skin diseases	0 (0 to 0)	0 (0 to 0)	2·3 (0·7 to 5)	1·8 (0·9 to 3·2)	2·3 (1·2 to 4)	2·2 (1·1 to 4)	1·9 (1 to 3·5)	1·9 (0·9 to 3·6)	2·5 (1·1 to 5·3)	3·1 (1·4 to 6·2)	2·9 (1·3 to 6·2)	4·1 (1·7 to 9·5)	4·4 (1·9 to 9·6)	4·1 (1·6 to 8·6)	4·1 (1·4 to 9·4)	2·9 (1·1 to 6·7)	2·2 (0·8 to 5·2)	1·8 (0·6 to 4·8)	1·5 (0·4 to 4)	1 (0·2 to 2·6)	0·5 (0·1 to 1·5)	0·3 (0·1 to 0·8)	0·1 (0 to 0·3)	47·8
Cellulitis	0 (0 to 0)	0 (0 to 0)	0·4 (0·1 to 1·2)	0·4 (0·1 to 0·8)	0·6 (0·2 to 1·1)	0·5 (0·3 to 1·1)	0·5 (0·2 to 1)	0·4 (0·2 to 0·9)	0·6 (0·2 to 1·5)	0·7 (0·3 to 1·7)	0·6 (0·3 to 1·5)	0·7 (0·3 to 1·7)	0·8 (0·3 to 2)	0·7 (0·3 to 1·9)	0·6 (0·2 to 1·8)	0·5 (0·1 to 1·3)	0·3 (0·1 to 0·8)	0·2 (0·1 to 0·7)	0·2 (0 to 0·5)	0·1 (0 to 0·3)	0·1 (0 to 0·2)	0 (0 to 0·1)	0 (0 to 0)	9
Pyoderma	0 (0 to 0)	0 (0 to 0)	1·9 (0·5 to 4·2)	1·4 (0·6 to 2·6)	1·7 (0·9 to 3·2)	1·7 (0·8 to 3·2)	1·4 (0·7 to 2·7)	1·4 (0·6 to 2·9)	1·9 (0·8 to 3·9)	2·4 (1 to 4·8)	2·3 (1 to 4·8)	3·4 (1·2 to 7·9)	3·6 (1·4 to 7·5)	3·3 (1·2 to 7)	3·5 (1·1 to 8)	2·5 (0·9 to 5·7)	1·9 (0·6 to 4·6)	1·5 (0·5 to 4·1)	1·3 (0·3 to 3·5)	0·9 (0·2 to 2·3)	0·5 (0·1 to 1·3)	0·2 (0·1 to 0·7)	0·1 (0 to 0·3)	38·8
Scabies	0 (0 to 0)	0 (0 to 0)	1·4 (0·7 to 2·4)	10 (5 to 17·6)	15·3 (7·4 to 26·9)	17·5 (8·6 to 31·2)	17·6 (8·9 to 30·6)	15·9 (8 to 28·2)	11·5 (5·7 to 20·5)	8·1 (3·7 to 14·8)	7·7 (3·8 to 13·5)	8·5 (4·3 to 14·5)	8·7 (4·4 to 14·8)	7·4 (3·7 to 12·7)	5·7 (2·9 to 10)	3·8 (2 to 6·8)	2·5 (1·3 to 4·4)	1·6 (0·8 to 2·9)	1·2 (0·6 to 2·2)	0·8 (0·4 to 1·5)	0·4 (0·2 to 0·7)	0·2 (0·1 to 0·3)	0·1 (0 to 0·1)	146
Fungal skin diseases	0 (0 to 0)	0 (0 to 0)	0·2 (0·1 to 0·5)	2 (0·7 to 4·3)	3·8 (1·4 to 8·2)	4·9 (1·8 to 10·8)	4·8 (1·8 to 11)	5·1 (1·9 to 11·5)	5·3 (2 to 12)	5·1 (2 to 11·5)	5·2 (2 to 11·3)	5·2 (2 to 11·3)	5·4 (2·1 to 11·8)	5·5 (2·1 to 12·2)	5·7 (2·3 to 12·4)	5·1 (1·9 to 11)	4·7 (1·9 to 10·1)	4·4 (1·8 to 9·2)	3·7 (1·4 to 7·7)	2·9 (1·1 to 6)	1·6 (0·7 to 3·5)	0·7 (0·3 to 1·4)	0·2 (0·1 to 0·4)	81·4
Viral skin diseases	0 (0 to 0)	0 (0 to 0)	0 (0 to 0)	10·9 (6·9 to 16·6)	19·7 (12·2 to 30·8)	11·7 (7·3 to 18·3)	6·8 (4 to 10·7)	5·4 (3·2 to 8·3)	3·9 (2·3 to 6·1)	3 (1·7 to 4·8)	2·8 (1·6 to 4·4)	2·6 (1·6 to 4·2)	2·5 (1·4 to 4)	2·2 (1·3 to 3·6)	1·8 (1·1 to 2·9)	1·4 (0·8 to 2·3)	1 (0·6 to 1·6)	0·6 (0·3 to 0·9)	0·3 (0·2 to 0·5)	0·2 (0·1 to 0·3)	0·1 (0 to 0·1)	0 (0 to 0)	0 (0 to 0)	76·9
Acne vulgaris	0 (0 to 0)	0 (0 to 0)	0 (0 to 0)	0 (0 to 0)	1 (0·5 to 1·6)	25·4 (13·9 to 42·4)	43·6 (25·1 to 71·5)	11 (6·2 to 18)	4·9 (2·5 to 8·3)	3·1 (1·6 to 5·5)	2·4 (1·2 to 4·3)	1·7 (1 to 2·8)	1·2 (0·6 to 2)	0·8 (0·4 to 1·3)	0·5 (0·2 to 0·8)	0·3 (0·2 to 0·5)	0·2 (0·1 to 0·3)	0·1 (0·1 to 0·2)	0·1 (0 to 0·1)	0 (0 to 0)	0 (0 to 0)	0 (0 to 0)	0 (0 to 0)	96·3
Alopecia areata	0 (0 to 0)	0 (0 to 0)	0 (0 to 0)	0·1 (0 to 0·3)	0·2 (0·1 to 0·5)	0·4 (0·1 to 0·7)	0·6 (0·2 to 1·1)	0·9 (0·5 to 1·6)	1·3 (0·7 to 2·2)	1·2 (0·7 to 2·1)	1·1 (0·6 to 1·8)	0·9 (0·5 to 1·6)	0·8 (0·4 to 1·3)	0·6 (0·3 to 1·1)	0·5 (0·2 to 0·8)	0·4 (0·2 to 0·7)	0·2 (0·1 to 0·4)	0·1 (0·1 to 0·3)	0·1 (0 to 0·1)	0 (0 to 0·1)	0 (0 to 0)	0 (0 to 0)	0 (0 to 0)	9·6
Pruritus	0 (0 to 0)	0 (0 to 0)	0 (0 to 0·1)	0·4 (0·2 to 0·7)	0·6 (0·2 to 1·2)	0·7 (0·3 to 1·4)	0·7 (0·3 to 1·5)	0·9 (0·4 to 1·8)	1 (0·4 to 2)	1·1 (0·5 to 2·1)	1·1 (0·5 to 2·2)	1·1 (0·5 to 2·2)	1·1 (0·5 to 2·3)	1·1 (0·5 to 2·3)	1·1 (0·4 to 2·3)	1 (0·4 to 2)	0·9 (0·4 to 1·7)	0·6 (0·3 to 1·2)	0·4 (0·2 to 0·8)	0·3 (0·1 to 0·5)	0·1 (0 to 0·2)	0 (0 to 0·1)	0 (0 to 0)	14·3
Urticaria	0 (0 to 0)	0 (0 to 0)	1·2 (0·7 to 1·9)	8·4 (4·9 to 13·3)	8·5 (4·9 to 13·8)	6·7 (3·5 to 11·6)	5·5 (2·9 to 9·6)	5·2 (2·7 to 8·7)	4·8 (2·5 to 8·3)	4·6 (2·4 to 7·5)	4·5 (2·5 to 7·3)	4·2 (2·2 to 6·9)	3·9 (2 to 6·9)	3·4 (1·8 to 5·6)	2·9 (1·5 to 4·8)	2·1 (1·1 to 3·6)	1·4 (0·7 to 2·6)	0·9 (0·5 to 1·5)	0·6 (0·3 to 1)	0·3 (0·2 to 0·5)	0·1 (0 to 0·2)	0 (0 to 0·1)	0 (0 to 0)	69·4
Decubitus ulcer	0 (0 to 0)	0 (0 to 0)	0 (0 to 0)	0·6 (0·1 to 1·5)	0·5 (0·1 to 1·3)	1·4 (0·3 to 3·1)	1·8 (0·4 to 3·5)	3·9 (1 to 7·6)	6·8 (1·9 to 13·5)	6 (1·8 to 12·5)	7·5 (2·5 to 15·7)	10·7 (4 to 23·2)	11·5 (4·4 to 24·6)	13·8 (5·8 to 29·2)	14·2 (6·2 to 28·7)	15 (7 to 31·2)	15·1 (6·7 to 32)	13·4 (6·4 to 28·5)	13·5 (6·5 to 29·8)	10·7 (5·5 to 24·1)	6·1 (3·3 to 13·9)	3·1 (1·6 to 6·7)	1·1 (0·6 to 2·9)	157
Other skin and subcutaneous diseases	0 (0 to 0)	0 (0 to 0)	4·5 (1·7 to 8·6)	3·5 (1·7 to 6·2)	2·7 (1·3 to 5)	3·8 (1·8 to 6·9)	4·5 (2·4 to 8·1)	4·9 (2·5 to 8·8)	6·5 (3·5 to 11·5)	6·8 (3·7 to 11·7)	8·6 (4·6 to 14·7)	8·3 (4·4 to 14·2)	10 (5·3 to 17·1)	11·9 (6·4 to 20·5)	10·6 (5·5 to 18·1)	9·9 (5·2 to 16·8)	9·2 (5 to 15·6)	5·8 (2·9 to 9·9)	4·5 (2·3 to 8)	2·5 (1·4 to 4·4)	1·1 (0·6 to 2)	0·5 (0·3 to 0·9)	0·1 (0·1 to 0·2)	120
Sense organ diseases	0 (0 to 0)	0 (0 to 0)	0·5 (0·3 to 0·8)	5·7 (3·5 to 8·6)	14·2 (8·6 to 21·4)	20·4 (12·9 to 29·7)	23·6 (14·7 to 35·7)	30·7 (19·2 to 46·1)	39·9 (24·6 to 61·6)	48·4 (29·5 to 76)	57·9 (35 to 89·9)	74·2 (45·5 to 116)	106 (65·8 to 167)	134 (84·8 to 210)	152 (96·7 to 240)	149 (95·4 to 227)	131 (85·4 to 193)	108 (71·9 to 155)	94·1 (64 to 132)	69·8 (48·8 to 97·3)	36·9 (25·9 to 50)	14·6 (10·5 to 19·5)	4·8 (3·4 to 6·4)	1,320
Blindness and vision loss	0 (0 to 0)	0 (0 to 0)	0·1 (0 to 0·1)	1·5 (0·8 to 2·5)	3·7 (2·1 to 6·2)	4·9 (2·9 to 8·1)	5·5 (3·2 to 8·5)	6·6 (3·8 to 10·4)	7·7 (4·5 to 11·7)	9·1 (5·5 to 13·8)	12·1 (7·7 to 18·5)	17·3 (10·7 to 26·9)	24·4 (15·4 to 38·7)	30·9 (19·3 to 49·2)	36·9 (23·2 to 57·6)	38·7 (25·1 to 59·1)	37 (23·9 to 54·6)	32·5 (21·6 to 47·1)	29·2 (20 to 41·8)	22·2 (15·3 to 30·8)	12·1 (8·3 to 16·5)	5·1 (3·6 to 7)	1·9 (1·3 to 2·6)	339
Glaucoma	0 (0 to 0)	0 (0 to 0)	0 (0 to 0)	0 (0 to 0)	0 (0 to 0)	0 (0 to 0)	0 (0 to 0)	0 (0 to 0)	0 (0 to 0)	0 (0 to 0)	0 (0 to 0)	0 (0 to 0)	0·1 (0 to 0·1)	0·5 (0·1 to 1)	1·2 (0·5 to 2·1)	1·9 (1 to 3·2)	2·4 (1·3 to 3·8)	2·6 (1·6 to 4·1)	3 (1·8 to 4·8)	2·7 (1·7 to 4·2)	1·8 (1·1 to 2·7)	0·8 (0·5 to 1·2)	0·3 (0·2 to 0·5)	17·3
Cataract	0 (0 to 0)	0 (0 to 0)	0 (0 to 0)	0 (0 to 0)	0 (0 to 0)	0 (0 to 0)	0 (0 to 0)	0 (0 to 0)	0·2 (0·1 to 0·3)	0·4 (0·2 to 0·7)	0·8 (0·4 to 1·6)	1·5 (0·6 to 2·6)	2·4 (1·2 to 4·1)	3 (1·6 to 5)	3·8 (2·2 to 5·9)	4·3 (2·6 to 6·7)	5·1 (3·1 to 7·8)	6 (3·8 to 8·8)	7·1 (4·6 to 10·2)	6·3 (4·2 to 8·9)	3·7 (2·5 to 5·2)	1·5 (1 to 2·1)	0·6 (0·3 to 0·8)	46·6
Age-related macular degeneration	0 (0 to 0)	0 (0 to 0)	0 (0 to 0)	0 (0 to 0)	0 (0 to 0)	0 (0 to 0)	0 (0 to 0)	0 (0 to 0)	0 (0 to 0)	0 (0 to 0)	0 (0 to 0)	0 (0 to 0)	0 (0 to 0)	0·1 (0 to 0·2)	0·2 (0·1 to 0·4)	0·3 (0·1 to 0·6)	0·3 (0·1 to 0·6)	0·3 (0·1 to 0·5)	0·3 (0·2 to 0·5)	0·2 (0·1 to 0·4)	0·1 (0·1 to 0·2)	0 (0 to 0·1)	0 (0 to 0)	2·1
Refraction disorders	0 (0 to 0)	0 (0 to 0)	0·1 (0 to 0·1)	1·3 (0·7 to 2·2)	3·2 (1·8 to 5·5)	4·2 (2·4 to 7·1)	4·6 (2·6 to 7·3)	5·4 (2·9 to 8·6)	5·9 (3·4 to 9·3)	6 (3·4 to 9·4)	6·7 (3·9 to 10·4)	7·6 (4·3 to 12)	8·3 (5 to 12·9)	7·9 (4·7 to 12·3)	7·4 (4·5 to 11·8)	6·2 (3·8 to 9·8)	5 (2·9 to 7·6)	4 (2·4 to 6·1)	3·4 (2·1 to 5·1)	2·1 (1·3 to 3·3)	0·9 (0·6 to 1·4)	0·3 (0·2 to 0·4)	0·1 (0 to 0·1)	90·5
Near vision loss	0 (0 to 0)	0 (0 to 0)	0 (0 to 0)	0 (0 to 0)	0 (0 to 0·1)	0·1 (0 to 0·1)	0·1 (0 to 0·2)	0·2 (0·1 to 0·5)	0·4 (0·1 to 0·9)	1·1 (0·4 to 2·4)	2·1 (0·8 to 4·8)	4·3 (1·7 to 9)	7·7 (3·1 to 16·3)	11·1 (4·5 to 23·5)	13·9 (5·6 to 29·3)	14·1 (5·8 to 29·8)	12·2 (5 to 24·6)	9·4 (3·9 to 19·1)	7·4 (3·1 to 15·1)	4·9 (2·1 to 10·1)	2·4 (1 to 4·7)	0·9 (0·4 to 1·8)	0·3 (0·1 to 0·6)	92·5
Other vision loss	0 (0 to 0)	0 (0 to 0)	0 (0 to 0·1)	0·2 (0·1 to 0·4)	0·4 (0·2 to 0·8)	0·7 (0·4 to 1·1)	0·8 (0·3 to 1·5)	1 (0·4 to 2)	1·2 (0·5 to 2·3)	1·6 (0·7 to 3)	2·5 (1·2 to 4·2)	3·9 (2·1 to 6·7)	6 (3·3 to 9·5)	8·3 (4·9 to 12·4)	10·4 (6·3 to 15·6)	11·7 (7·6 to 16·9)	12 (7·8 to 17)	10·2 (6·7 to 14·5)	8 (5·1 to 11·7)	5·9 (3·8 to 8·5)	3·3 (2·1 to 4·8)	1·6 (1·1 to 2·3)	0·7 (0·4 to 1)	90·5
Age-related and other hearing loss	0 (0 to 0)	0 (0 to 0)	0·2 (0·1 to 0·3)	3 (1·5 to 5·1)	8·9 (4·8 to 14·5)	13·7 (8 to 20·7)	16·3 (9·3 to 25·2)	22 (13 to 34·1)	29·7 (17·7 to 46·5)	36 (21 to 58·7)	42 (24·1 to 67·2)	52·8 (30·9 to 86·1)	77·1 (45·1 to 126)	97·1 (59·3 to 156)	109 (66·5 to 174)	104 (63·6 to 165)	88·6 (55·5 to 134)	71·4 (46·8 to 105)	61·4 (40·8 to 88·6)	45·5 (30·4 to 63·8)	23·9 (16·4 to 33·1)	9·2 (6·5 to 12·5)	2·7 (1·9 to 3·7)	915
Other sense organ diseases	0 (0 to 0)	0 (0 to 0)	0·2 (0·1 to 0·4)	1·2 (0·6 to 2·2)	1·6 (0·7 to 2·7)	1·7 (0·8 to 3·1)	1·9 (0·8 to 3·5)	2·1 (0·8 to 4·1)	2·5 (1·1 to 4·9)	3·3 (1·4 to 6·4)	3·8 (1·7 to 7·1)	4 (1·8 to 8)	4·6 (1·9 to 8·7)	5·9 (2·8 to 11·9)	5·8 (2·6 to 11·2)	6·2 (2·8 to 11·4)	5·7 (2·6 to 10·7)	4·4 (2 to 8·1)	3·6 (1·7 to 6·5)	2·2 (1·1 to 4·2)	0·8 (0·4 to 1·5)	0·3 (0·1 to 0·5)	0·1 (0 to 0·2)	61·8
Musculoskeletal disorders	0 (0 to 0)	0 (0 to 0)	0 (0 to 0)	0 (0 to 0)	6·2 (2·8 to 11·2)	35·2 (20·8 to 56·2)	73·8 (45·8 to 111)	114 (73·2 to 165)	165 (106 to 239)	198 (125 to 280)	234 (161 to 325)	269 (185 to 373)	305 (207 to 432)	312 (213 to 445)	298 (202 to 414)	249 (169 to 353)	180 (125 to 254)	119 (80·6 to 169)	80 (53·9 to 114)	46·8 (32·1 to 67·4)	22·1 (15·5 to 31)	7·3 (5·2 to 10·3)	2·1 (1·5 to 3)	2,720
Rheumatoid arthritis	0 (0 to 0)	0 (0 to 0)	0 (0 to 0)	0 (0 to 0)	0 (0 to 0·1)	0·3 (0·1 to 0·5)	0·7 (0·3 to 1·2)	1·3 (0·6 to 2·1)	2 (1 to 3·4)	2·4 (1·2 to 4)	3·1 (1·5 to 5·4)	3·5 (1·8 to 5·7)	4·1 (2·1 to 6·6)	4·2 (2·3 to 6·8)	3·9 (2·2 to 6·2)	3·2 (1·9 to 4·9)	2·4 (1·5 to 3·5)	1·6 (0·9 to 2·3)	1·3 (0·8 to 1·9)	0·9 (0·6 to 1·3)	1·1 (0·7 to 1·8)	0·5 (0·3 to 0·8)	0·1 (0·1 to 0·2)	36·5
Osteoarthritis	0 (0 to 0)	0 (0 to 0)	0 (0 to 0)	0 (0 to 0)	0 (0 to 0)	0 (0 to 0)	0 (0 to 0)	0 (0 to 0)	0 (0 to 0)	1·1 (0·4 to 2·3)	6·3 (3 to 13·2)	17·3 (8·5 to 35·9)	33·9 (16·6 to 68·2)	49·5 (24 to 100)	58·5 (28·6 to 117)	56·3 (28·2 to 113)	44·6 (22·3 to 90·2)	31·7 (15·7 to 64·1)	22·8 (11·4 to 46·3)	14·1 (7·2 to 28·6)	6·2 (3·2 to 12·7)	2·1 (1·1 to 4·3)	0·6 (0·3 to 1·2)	345
Osteoarthritis hip	0 (0 to 0)	0 (0 to 0)	0 (0 to 0)	0 (0 to 0)	0 (0 to 0)	0 (0 to 0)	0 (0 to 0)	0 (0 to 0)	0 (0 to 0)	0·4 (0·1 to 0·9)	0·6 (0·2 to 1·6)	1·1 (0·4 to 2·5)	1·8 (0·7 to 4·1)	2·7 (1·1 to 6)	3·5 (1·5 to 7·5)	3·7 (1·6 to 7·9)	3·1 (1·3 to 6·4)	2·2 (1 to 4·7)	1·6 (0·8 to 3·4)	1 (0·5 to 2·1)	0·4 (0·2 to 0·9)	0·2 (0·1 to 0·3)	0 (0 to 0·1)	22·4
Osteoarthritis knee	0 (0 to 0)	0 (0 to 0)	0 (0 to 0)	0 (0 to 0)	0 (0 to 0)	0 (0 to 0)	0 (0 to 0)	0 (0 to 0)	0 (0 to 0)	0·5 (0·2 to 1·1)	4·5 (2 to 9·7)	12·8 (6 to 26·8)	25·1 (12 to 51·9)	36·1 (17 to 75·6)	41·9 (19·9 to 87·4)	39·8 (19·1 to 81·3)	31·2 (15·1 to 64·7)	21·9 (10·8 to 45·2)	15·4 (7·5 to 31·5)	9·2 (4·6 to 18·6)	3·9 (1·9 to 8)	1·3 (0·6 to 2·5)	0·3 (0·2 to 0·7)	244
Osteoarthritis hand	0 (0 to 0)	0 (0 to 0)	0 (0 to 0)	0 (0 to 0)	0 (0 to 0)	0 (0 to 0)	0 (0 to 0)	0 (0 to 0)	0 (0 to 0)	0·1 (0 to 0·2)	0·5 (0·1 to 1·5)	1·4 (0·5 to 3·5)	2·8 (1 to 6·6)	4·2 (1·7 to 9·2)	5 (2·1 to 11·1)	4·7 (2·1 to 10·2)	3·7 (1·7 to 7·9)	2·7 (1·3 to 5·6)	2 (1 to 4·3)	1·3 (0·6 to 2·7)	0·7 (0·3 to 1·3)	0·3 (0·1 to 0·5)	0·1 (0 to 0·2)	29·5

Osteoarthritis other	0 (0 to 0)	0 (0 to 0)	0 (0 to 0)	0 (0 to 0)	0 (0 to 0)	0 (0 to 0)	0 (0 to 0)	0 (0 to 0)	0 (0 to 0)	0·1 (0 to 0·2)	0·6 (0·1 to 1·4)	2 (0·7 to 4·8)	4·2 (1·7 to 9·6)	6·5 (2·8 to 14·1)	8·1 (3·6 to 17·4)	8·1 (3·7 to 17·2)	6·6 (3·1 to 14)	4·9 (2·4 to 10·1)	3·7 (1·8 to 7·9)	2·5 (1·3 to 5·2)	1·2 (0·6 to 2·5)	0·5 (0·2 to 0·9)	0·2 (0·1 to 0·3)	49·1
Low back pain	0 (0 to 0)	0 (0 to 0)	0 (0 to 0)	0 (0 to 0)	5·5 (2·2 to 10·5)	31·4 (17·9 to 51·4)	55·9 (32·1 to 91)	71·3 (41·1 to 115)	84·5 (48 to 132)	90·8 (48·8 to 140)	103 (61·1 to 162)	113 (67·2 to 178)	121 (74 to 193)	121 (70·3 to 191)	111 (68·6 to 169)	91·5 (57·6 to 138)	66·4 (41·4 to 103)	44·2 (27·2 to 67·4)	30·2 (18·4 to 46·6)	18·5 (11·5 to 27·7)	7·7 (4·6 to 11·3)	2·4 (1·5 to 3·7)	0·7 (0·4 to 1·1)	1,170
Neck pain	0 (0 to 0)	0 (0 to 0)	0 (0 to 0)	0 (0 to 0)	0·4 (0·1 to 1)	2·5 (0·9 to 5·4)	5·4 (2·2 to 10)	8·6 (3·6 to 17·2)	13·7 (6·1 to 27·3)	20·3 (9·5 to 39·5)	26·7 (12·7 to 47·5)	32·5 (16·1 to 59)	36·9 (18·2 to 67·7)	31·9 (15·9 to 60·7)	27·2 (13 to 47·1)	20·5 (10·3 to 35·8)	13·6 (7·2 to 22·8)	8·6 (4·5 to 15)	5·5 (2·7 to 10)	2·9 (1·4 to 5·1)	1·3 (0·7 to 2·2)	0·4 (0·2 to 0·7)	0·1 (0·1 to 0·2)	259
Gout	0 (0 to 0)	0 (0 to 0)	0 (0 to 0)	0 (0 to 0)	0 (0 to 0)	0 (0 to 0)	0 (0 to 0·1)	0·2 (0·1 to 0·4)	0·7 (0·1 to 1·6)	1·1 (0·4 to 2·4)	1·6 (0·6 to 3·1)	2·1 (0·9 to 3·9)	2·5 (1·1 to 4·5)	2·6 (1·2 to 5)	2·5 (1 to 4·6)	2·1 (0·9 to 4·1)	1·7 (0·8 to 3)	1·4 (0·6 to 2·3)	1·1 (0·5 to 1·8)	0·7 (0·3 to 1·3)	0·4 (0·2 to 0·6)	0·2 (0·1 to 0·3)	0·1 (0 to 0·1)	21·1
Other musculoskeletal disorders	0 (0 to 0)	0 (0 to 0)	0 (0 to 0)	0 (0 to 0)	0·3 (0·2 to 0·6)	1 (0·4 to 2·7)	11·8 (5·2 to 21·9)	32·9 (19·1 to 51·4)	64·2 (38·1 to 102)	82·5 (50·6 to 126)	92·8 (57 to 139)	100 (59·7 to 156)	106 (66·8 to 164)	103 (61·4 to 162)	94·6 (57·6 to 144)	75·2 (43·4 to 122)	51·3 (30·9 to 79)	31·9 (18·1 to 50·9)	19·2 (11·4 to 29·9)	9·6 (4·6 to 16·4)	5·4 (3·4 to 8·2)	1·7 (0·9 to 2·7)	0·5 (0·3 to 0·9)	884
Other non-communicable diseases	115 (75·5 to 189)	66·3 (37·8 to 112)	242 (174 to 329)	121 (85·6 to 173)	86·4 (64·3 to 112)	80·6 (62·6 to 104)	88 (67·6 to 113)	118 (88·1 to 154)	156 (119 to 206)	177 (132 to 236)	197 (147 to 272)	196 (146 to 270)	186 (133 to 259)	195 (148 to 267)	193 (145 to 265)	185 (139 to 244)	149 (113 to 195)	117 (88 to 153)	90·6 (68·9 to 117)	61·4 (47·9 to 80·1)	35·1 (27·3 to 44·7)	14·7 (11·6 to 19)	5·3 (3·8 to 7·6)	2,880
Congenital birth defects	106 (66·7 to 180)	53·9 (30·3 to 93·1)	128 (87·3 to 180)	51·9 (34·3 to 77·5)	29·6 (21·7 to 40·7)	29·6 (21·9 to 38·5)	30·7 (22·5 to 41)	29·7 (19·4 to 40·3)	28·5 (18·6 to 37·4)	23·4 (16·2 to 31·5)	21 (14·6 to 28·1)	16·7 (12·1 to 22·4)	14 (10·1 to 18·8)	12·1 (8·8 to 16·5)	9·4 (6·8 to 12·4)	5·6 (4·1 to 7·5)	3·1 (2·4 to 4·2)	0·9 (0·5 to 1·3)	0·5 (0·3 to 0·8)	0·3 (0·2 to 0·4)	0·1 (0·1 to 0·2)	0 (0 to 0·1)	0 (0 to 0)	595
Neural tube defects	9·7 (3·9 to 18·3)	3·1 (1·2 to 7·2)	6·6 (2·8 to 13·1)	3·9 (1·8 to 7·3)	1·3 (0·9 to 1·8)	1·9 (1·3 to 2·9)	2·2 (1·4 to 3·4)	1·7 (1 to 2·6)	1·9 (1·1 to 3·2)	1·2 (0·7 to 1·9)	0·6 (0·4 to 0·8)	0·6 (0·4 to 1)	0·4 (0·2 to 0·6)	0·2 (0·1 to 0·3)	0·2 (0·1 to 0·2)	0·1 (0·1 to 0·2)	0 (0 to 0·1)	0 (0 to 0)	0 (0 to 0)	0 (0 to 0)	0 (0 to 0)	0 (0 to 0)	0 (0 to 0)	35·5
Congenital heart anomalies	41·6 (20·9 to 75·9)	27 (14·4 to 47·9)	62·7 (39·2 to 94·5)	20·3 (11·4 to 33·1)	9·2 (5·5 to 14·7)	7·9 (5 to 11·7)	8·8 (5·4 to 13·2)	9·7 (5·3 to 14·6)	9·3 (5·1 to 13·9)	8·2 (4·8 to 12·6)	7·1 (4·2 to 10·6)	5 (3·1 to 7·5)	3·7 (2·3 to 5·6)	2·8 (1·7 to 4·2)	1·6 (1 to 2·4)	1·1 (0·7 to 1·8)	0·7 (0·4 to 1)	0·1 (0 to 0·2)	0·1 (0 to 0·1)	0 (0 to 0·1)	0 (0 to 0)	0 (0 to 0)	0 (0 to 0)	227
Orofacial clefts	0 (0 to 0·1)	0 (0 to 0)	0·2 (0·1 to 0·3)	0·5 (0·2 to 0·9)	0·5 (0·2 to 1)	0·6 (0·2 to 1·2)	0·6 (0·2 to 1·1)	0·5 (0·2 to 1·1)	0·5 (0·2 to 1)	0·5 (0·2 to 1)	0·5 (0·1 to 0·9)	0·5 (0·2 to 0·9)	0·4 (0·1 to 0·9)	0·4 (0·1 to 0·8)	0·3 (0·1 to 0·7)	0·2 (0·1 to 0·5)	0·2 (0 to 0·3)	0·1 (0 to 0·2)	0·1 (0 to 0·1)	0 (0 to 0·1)	0 (0 to 0)	0 (0 to 0)	0 (0 to 0)	6·7
Down syndrome	1·4 (0·5 to 2·9)	1 (0·4 to 2)	6·3 (3·3 to 10·9)	3·4 (1·8 to 6)	1·5 (1 to 2·4)	2·1 (1·3 to 3·2)	3 (1·8 to 4·6)	3 (1·7 to 5)	3·1 (1·7 to 5·4)	3·5 (1·8 to 6)	3·6 (1·9 to 6)	3·5 (2 to 5·7)	3 (1·7 to 5·2)	2·9 (1·6 to 4·8)	3·1 (1·8 to 4·7)	1·4 (0·8 to 2·3)	0·6 (0·4 to 1)	0 (0 to 0)	0 (0 to 0)	0 (0 to 0)	0 (0 to 0)	0 (0 to 0)	0 (0 to 0)	46·6
Turner syndrome	0 (0 to 0)	0 (0 to 0)	0 (0 to 0)	0 (0 to 0)	0 (0 to 0)	0 (0 to 0)	0 (0 to 0)	0 (0 to 0)	0 (0 to 0)	0 (0 to 0)	0 (0 to 0)	0 (0 to 0)	0 (0 to 0)	0 (0 to 0)	0 (0 to 0)	0 (0 to 0)	0 (0 to 0)	0 (0 to 0)	0 (0 to 0)	0 (0 to 0)	0 (0 to 0)	0 (0 to 0)	0 (0 to 0)	0
Klinefelter syndrome	0 (0 to 0)	0 (0 to 0)	0 (0 to 0)	0 (0 to 0)	0 (0 to 0)	0 (0 to 0)	0 (0 to 0)	0 (0 to 0)	0 (0 to 0)	0 (0 to 0)	0 (0 to 0)	0 (0 to 0)	0 (0 to 0)	0 (0 to 0)	0 (0 to 0)	0 (0 to 0)	0 (0 to 0)	0 (0 to 0)	0 (0 to 0)	0 (0 to 0)	0 (0 to 0)	0 (0 to 0)	0 (0 to 0)	0
Other chromosomal abnormalities	13·3 (6·7 to 25)	6·8 (3 to 14·3)	14·3 (8 to 24·8)	4·7 (2·7 to 7·7)	2·6 (1·6 to 4)	2·6 (1·6 to 3·8)	3·2 (1·9 to 5·1)	2·1 (1·3 to 3·5)	3·2 (1·7 to 5·6)	1·3 (0·7 to 2·3)	1·8 (0·9 to 3·1)	0·5 (0·3 to 0·9)	0·5 (0·3 to 1)	0·3 (0·1 to 0·4)	0·2 (0·1 to 0·4)	0·1 (0·1 to 0·2)	0 (0 to 0·1)	0 (0 to 0)	0 (0 to 0)	0 (0 to 0)	0 (0 to 0)	0 (0 to 0)	0 (0 to 0)	57·5
Congenital musculoskeletal and limb anomalies	1·4 (0·6 to 3)	0·7 (0·4 to 1·3)	3·5 (2·1 to 5·3)	5·7 (3·2 to 9·1)	6·8 (3·9 to 11)	7·9 (4·2 to 12·8)	6·4 (3·5 to 10·4)	4·1 (2·3 to 6·6)	3·6 (2 to 5·8)	3·1 (1·6 to 5·2)	3·1 (1·7 to 5·2)	2·9 (1·5 to 4·8)	2·9 (1·6 to 4·6)	2·7 (1·5 to 4·4)	2·2 (1·2 to 3·5)	1·6 (0·8 to 2·5)	0·9 (0·5 to 1·5)	0·5 (0·2 to 0·8)	0·2 (0·1 to 0·4)	0·1 (0·1 to 0·2)	0 (0 to 0·1)	0 (0 to 0)	0 (0 to 0)	60·3
Urogenital congenital anomalies	3·1 (1·2 to 10·2)	1·3 (0·6 to 2·9)	2·3 (1·2 to 4·2)	1·2 (0·7 to 1·9)	0·8 (0·5 to 1·2)	0·7 (0·4 to 1)	0·4 (0·3 to 0·7)	0·5 (0·3 to 0·8)	0·4 (0·2 to 0·6)	0·4 (0·3 to 0·7)	0·2 (0·1 to 0·3)	0·2 (0·1 to 0·3)	0·2 (0·1 to 0·3)	0·1 (0·1 to 0·1)	0·2 (0·1 to 0·3)	0·1 (0 to 0·2)	0 (0 to 0·1)	0 (0 to 0)	0 (0 to 0)	0 (0 to 0)	0 (0 to 0)	0 (0 to 0)	0 (0 to 0)	12·2
Digestive congenital anomalies	9·9 (5 to 18·4)	5·8 (2·7 to 11·7)	10·4 (6·1 to 16·2)	2·4 (1·4 to 3·9)	0·9 (0·5 to 1·5)	0·5 (0·3 to 0·7)	0·4 (0·2 to 0·6)	0·2 (0·1 to 0·3)	0·3 (0·1 to 0·4)	0·2 (0·1 to 0·2)	0·1 (0·1 to 0·2)	0·1 (0·1 to 0·1)	0·1 (0·1 to 0·2)	0·1 (0·1 to 0·2)	0·1 (0 to 0·1)	0·1 (0 to 0·1)	0·1 (0 to 0·1)	0 (0 to 0)	0 (0 to 0)	0 (0 to 0)	0 (0 to 0)	0 (0 to 0)	0 (0 to 0)	31·6
Other congenital birth defects	25·3 (14 to 44·6)	8·1 (3·9 to 15·4)	22·4 (13·6 to 33·9)	9·9 (5·5 to 16·2)	5·9 (3·8 to 9·1)	5·5 (3·5 to 8·1)	5·8 (3·6 to 8·8)	7·9 (4·3 to 12·3)	6·2 (3·4 to 9·6)	5 (2·8 to 7·7)	3·9 (2·3 to 6)	3·3 (2 to 5·1)	2·7 (1·7 to 4·2)	2·7 (1·6 to 4·2)	1·6 (1 to 2·5)	0·9 (0·5 to 1·6)	0·6 (0·4 to 1·1)	0·2 (0·1 to 0·5)	0·1 (0 to 0·3)	0·1 (0 to 0·2)	0 (0 to 0·1)	0 (0 to 0)	0 (0 to 0)	118
Urinary diseases and male infertility	0·2 (0·1 to 0·4)	0·6 (0·3 to 1·2)	3·6 (2 to 5·9)	1·4 (0·9 to 2·2)	0·9 (0·6 to 1·4)	1·1 (0·8 to 1·7)	1·9 (1·3 to 2·8)	8·1 (5·1 to 12·8)	10 (6·1 to 15·9)	13·5 (8·2 to 21)	19·3 (11·5 to 29·1)	19·1 (12 to 28·1)	20·4 (12·5 to 30·3)	27·1 (17·3 to 39·9)	38·4 (25·5 to 53·4)	48·7 (33 to 65·9)	47·2 (32·5 to 64)	41·8 (28·3 to 57·7)	35·3 (23·3 to 48·7)	23·2 (14·4 to 32·8)	13·4 (8·5 to 18·9)	5·7 (3·4 to 8·2)	1·9 (1·1 to 2·7)	383
Urolithiasis	0 (0 to 0)	0 (0 to 0)	0 (0 to 0)	0 (0 to 0)	0 (0 to 0·1)	0·1 (0 to 0·1)	0·1 (0·1 to 0·2)	0·3 (0·1 to 0·4)	0·4 (0·2 to 0·8)	0·7 (0·3 to 1·3)	1·3 (0·5 to 2·4)	1·3 (0·5 to 2·3)	2·2 (1 to 3·8)	2·2 (1·1 to 3·6)	2·4 (1·3 to 3·8)	1·8 (1·1 to 3)	1·1 (0·6 to 1·9)	0·7 (0·4 to 1·2)	0·5 (0·2 to 0·8)	0·2 (0·1 to 0·3)	0·1 (0 to 0·1)	0 (0 to 0·1)	0 (0 to 0)	15·4
Benign prostatic hyperplasia	0 (0 to 0)	0 (0 to 0)	0 (0 to 0)	0 (0 to 0)	0 (0 to 0)	0 (0 to 0)	0 (0 to 0)	0 (0 to 0)	0 (0 to 0)	0 (0 to 0)	0 (0 to 0)	0·1 (0 to 0·4)	1 (0·4 to 2·1)	4 (1·9 to 7)	11·6 (5·7 to 20·3)	18·6 (9·6 to 31·7)	20·6 (11·6 to 32·9)	17·3 (9·5 to 28·1)	11·9 (6·6 to 19·2)	6·2 (3·5 to 10)	2·2 (1·2 to 3·5)	0·6 (0·3 to 1)	0·1 (0·1 to 0·2)	94·3
Male infertility	0 (0 to 0)	0 (0 to 0)	0 (0 to 0)	0 (0 to 0)	0 (0 to 0)	0 (0 to 0)	0 (0 to 0)	2·8 (0·8 to 7·3)	3·5 (1 to 8·9)	3·6 (1 to 9·8)	3·5 (1 to 9·1)	3·2 (0·9 to 8·1)	0·5 (0·1 to 1·3)	0 (0 to 0)	0 (0 to 0)	0 (0 to 0)	0 (0 to 0)	0 (0 to 0)	0 (0 to 0)	0 (0 to 0)	0 (0 to 0)	0 (0 to 0)	0 (0 to 0)	17
Other urinary diseases	0·1 (0 to 0·1)	0·3 (0·1 to 0·6)	0·2 (0·1 to 0·5)	0·2 (0·1 to 0·4)	0·2 (0·1 to 0·5)	0·4 (0·2 to 0·7)	0·7 (0·4 to 1·1)	1·7 (0·7 to 3)	0·9 (0·4 to 1·8)	1·5 (0·7 to 2·8)	3·2 (1·4 to 5·6)	2·6 (1·3 to 4·5)	3·5 (1·7 to 6·1)	3·4 (1·6 to 6·2)	3·2 (1·6 to 5·5)	4 (1·9 to 6·9)	3·5 (1·5 to 6)	2·2 (1 to 4)	2·2 (0·9 to 4)	1·5 (0·6 to 2·9)	0·8 (0·3 to 1·5)	0·4 (0·2 to 0·8)	0·1 (0 to 0·2)	36·8
Gynecological diseases	0 (0 to 0)	0 (0 to 0)	0 (0 to 0)	0 (0 to 0)	0 (0 to 0)	0 (0 to 0)	0 (0 to 0)	0 (0 to 0)	0 (0 to 0)	0 (0 to 0)	0 (0 to 0)	0 (0 to 0)	0 (0 to 0)	0 (0 to 0)	0 (0 to 0)	0 (0 to 0)	0 (0 to 0)	0 (0 to 0)	0 (0 to 0)	0 (0 to 0)	0 (0 to 0)	0 (0 to 0)	0 (0 to 0)	0
Uterine fibroids	0 (0 to 0)	0 (0 to 0)	0 (0 to 0)	0 (0 to 0)	0 (0 to 0)	0 (0 to 0)	0 (0 to 0)	0 (0 to 0)	0 (0 to 0)	0 (0 to 0)	0 (0 to 0)	0 (0 to 0)	0 (0 to 0)	0 (0 to 0)	0 (0 to 0)	0 (0 to 0)	0 (0 to 0)	0 (0 to 0)	0 (0 to 0)	0 (0 to 0)	0 (0 to 0)	0 (0 to 0)	0 (0 to 0)	0
Polycystic ovarian syndrome	--	--	--	--	--	--	--	--	--	--	--	--	--	--	--	--	--	--	--	--	--	--	--	NA
Female infertility	--	--	--	--	--	--	--	--	--	--	--	--	--	--	--	--	--	--	--	--	--	--	--	NA
Endometriosis	--	--	--	--	--	--	--	--	--	--	--	--	--	--	--	--	--	--	--	--	--	--	--	NA
Genital prolapse	0 (0 to 0)	0 (0 to 0)	0 (0 to 0)	0 (0 to 0)	0 (0 to 0)	0 (0 to 0)	0 (0 to 0)	0 (0 to 0)	0 (0 to 0)	0 (0 to 0)	0 (0 to 0)	0 (0 to 0)	0 (0 to 0)	0 (0 to 0)	0 (0 to 0)	0 (0 to 0)	0 (0 to 0)	0 (0 to 0)	0 (0 to 0)	0 (0 to 0)	0 (0 to 0)	0 (0 to 0)	0 (0 to 0)	0
Premenstrual syndrome	--	--	--	--	--	--	--	--	--	--	--	--	--	--	--	--	--	--	--	--	--	--	--	NA
Other gynecological diseases	0 (0 to 0)	0 (0 to 0)	0 (0 to 0)	0 (0 to 0)	0 (0 to 0)	0 (0 to 0)	0 (0 to 0)	0 (0 to 0)	0 (0 to 0)	0 (0 to 0)	0 (0 to 0)	0 (0 to 0)	0 (0 to 0)	0 (0 to 0)	0 (0 to 0)	0 (0 to 0)	0 (0 to 0)	0 (0 to 0)	0 (0 to 0)	0 (0 to 0)	0 (0 to 0)	0 (0 to 0)	0 (0 to 0)	0
Hemoglobinopathies and hemolytic anemias	0·4 (0·2 to 0·6)	0·8 (0·5 to 1·3)	6 (3·6 to 9·4)	14·9 (9·1 to 23·3)	22·2 (13·3 to 34·3)	17·5 (11·1 to 27·4)	20·3 (13·3 to 30·6)	26·3 (16·6 to 40·5)	26·2 (17·3 to 37·3)	23·7 (15 to 34·3)	22 (14·7 to 30·5)	21·3 (13·7 to 31)	16·5 (11 to 23·5)	13·6 (9·5 to 19·5)	12·1 (7·7 to 17·8)	9·1 (5·7 to 13·6)	5·6 (3·7 to 8·2)	4·2 (2·8 to 5·9)	3 (2 to 4·5)	2·1 (1·4 to 3)	1·8 (1·3 to 2·6)	0·8 (0·6 to 1·1)	0·3 (0·2 to 0·5)	271
Thalassemias	0 (0 to 0)	0 (0 to 0)	0 (0 to 0)	0 (0 to 0·1)	0 (0 to 0)	0 (0 to 0)	0 (0 to 0)	0 (0 to 0)	0 (0 to 0)	0 (0 to 0)	0 (0 to 0)	0 (0 to 0)	0 (0 to 0)	0 (0 to 0)	0 (0 to 0)	0 (0 to 0)	0 (0 to 0)	0 (0 to 0)	0 (0 to 0)	0 (0 to 0)	0 (0 to 0)	0 (0 to 0)	0 (0 to 0)	0·1
Thalassemias trait	0 (0 to 0)	0 (0 to 0)	0·1 (0 to 0·2)	0·4 (0·1 to 0·8)	0·6 (0·1 to 1·4)	0·3 (0 to 0·8)	0·1 (0 to 0·3)	0·1 (0 to 0·2)	0·1 (0 to 0·2)	0·1 (0 to 0·2)	0·1 (0 to 0·2)	0·1 (0 to 0·2)	0·1 (0 to 0·3)	0·1 (0 to 0·3)	0·1 (0 to 0·3)	0·1 (0 to 0·3)	0·1 (0 to 0·2)	0 (0 to 0·1)	0 (0 to 0·1)	0 (0 to 0·1)	0 (0 to 0)	0 (0 to 0)	0 (0 to 0)	2·5
Sickle cell disorders	0·3 (0·2 to 0·5)	0·4 (0·2 to 0·8)	4·5 (2·5 to 7·7)	8·4 (5 to 12·9)	11·2 (7·4 to 16·7)	11·9 (7·7 to 18)	15·7 (9·9 to 24·2)	20 (12·2 to 31·2)	18·5 (11·1 to 28·1)	15·5 (9·4 to 23·3)	13·3 (7·8 to 19·9)	11·5 (6·4 to 18·8)	7 (3·9 to 11·3)	3·8 (1·9 to 6·6)	1·5 (0·7 to 2·8)	0·3 (0·1 to 0·7)	0 (0 to 0·1)	0 (0 to 0)	0 (0 to 0)	0 (0 to 0)	0 (0 to 0)	0 (0 to 0)	0 (0 to 0)	144
Sickle cell trait	0 (0 to 0·1)	0·3 (0·1 to 0·5)	0·9 (0·3 to 1·7)	3·9 (1·4 to 7·6)	6·7 (2 to 13·5)	3·2 (0·6 to 8)	1·2 (0·3 to 3)	0·9 (0·2 to 2·3)	0·9 (0·2 to 2·4)	0·9 (0·2 to 2·3)	0·9 (0·2 to 2·4)	0·9 (0·2 to 2·4)	1·2 (0·2 to 3)	1·2 (0·3 to 3)	1·4 (0·4 to 3·3)	1·1 (0·3 to 2·4)	0·6 (0·1 to 1·5)	0·5 (0·1 to 1)	0·4 (0·1 to 0·9)	0·3 (0·1 to 0·6)	0·2 (0·1 to 0·4)	0·1 (0 to 0·1)	0 (0 to 0)	27·7
G6PD deficiency	0 (0 to 0)	0 (0 to 0·1)	0·1 (0 to 0·1)	0·5 (0·2 to 1)	0·8 (0·3 to 1·4)	0·8 (0·3 to 1·6)	1·6 (0·7 to 3·2)	3·8 (1·4 to 7·6)	5·3 (2·2 to 9·8)	5·5 (2·2 to 10·6)	5·9 (2·7 to 10·6)	6·8 (3·1 to 11·6)	6 (3 to 9·9)	5·8 (3·2 to 9·2)	5·6 (3·4 to 8·4)	4 (2·4 to 6·2)	2 (1·1 to 3·1)	1·1 (0·6 to 1·7)	0·5 (0·2 to 0·8)	0·2 (0·1 to 0·3)	0·1 (0 to 0·2)	0 (0 to 0)	0 (0 to 0)	56·4
G6PD trait	0 (0 to 0)	0 (0 to 0)	0 (0 to 0)	0 (0 to 0)	0 (0 to 0)	0 (0 to 0)	0 (0 to 0)	0 (0 to 0)	0 (0 to 0)	0 (0 to 0)	0 (0 to 0)	0 (0 to 0)	0 (0 to 0)	0 (0 to 0)	0 (0 to 0)	0 (0 to 0)	0 (0 to 0)	0 (0 to 0)	0 (0 to 0)	0 (0 to 0)	0 (0 to 0)	0 (0 to 0)	0 (0 to 0)	0
Other hemoglobinopathies and hemolytic anemias	0 (0 to 0)	0·1 (0 to 0·1)	0·4 (0·1 to 0·9)	1·7 (0·4 to 3·8)	2·8 (0·6 to 6·8)	1·3 (0·3 to 3·6)	1·7 (0·5 to 4·2)	1·6 (0·6 to 3·6)	1·5 (0·6 to 3·5)	1·7 (0·7 to 3·5)	1·8 (0·8 to 3·8)	2 (1 to 4)	2·3 (1 to 4·8)	2·7 (1·4 to 5·1)	3·5 (1·9 to 6)	3·7 (2 to 6·3)	2·9 (1·8 to 4·6)	2·6 (1·7 to 3·7)	2·1 (1·4 to 3·1)	1·6 (1·1 to 2·2)	1·6 (1·1 to 2·1)	0·7 (0·5 to 1)	0·3 (0·2 to 0·4)	40·4
Endocrine, metabolic, blood, and immune disorders	8·9 (4·7 to 19·4)	8·7 (3·9 to 19·1)	83·7 (50·4 to 149)	48 (27·3 to 93·7)	26·5 (16·8 to 45)	23·2 (15·3 to 38·2)	21·6 (14·1 to 32·1)	31·7 (20 to 46·7)	58·4 (36 to 88·3)	77·1 (48·5 to 112)	89·1 (58·8 to 136)	88·6 (58·3 to 137)	80·7 (52·9 to 125)	87·2 (59·6 to 135)	80 (55·1 to 123)	72 (49·7 to 111)	51·9 (35·6 to 79·9)	39·7 (27·6 to 61·3)	29·6 (20·1 to 49·3)	22 (14·9 to 36·1)	13·5 (9·3 to 22·5)	6 (4 to 10·1)	2·5 (1·4 to 4·7)	1,050
Oral disorders	0 (0 to 0)	0 (0 to 0)	0 (0 to 0)	5·1 (2·9 to 8·4)	7·2 (3·9 to 12·5)	9·2 (4·8 to 16·2)	13·4 (7·3 to 23·4)	22·1 (11·4 to 42·3)	32·4 (15·6 to 63·4)	39·3 (18·3 to 74·7)	45·4 (21·3 to 85·6)	50·4 (23·7 to 93·2)	54·7 (26·9 to 100)	54·6 (28·4 to 99)	53·5 (29·3 to 91·6)	49·8 (28·9 to 82·1)	41·1 (24·4 to 65·2)	30·3 (18·6 to 46·9)	22·2 (13·8 to 34)	13·8 (8·6 to 20·9)	6·2 (3·9 to 9·3)	2·2 (1·4 to 3·3)	0·7 (0·4 to 1)	554
Caries of deciduous teeth	0 (0 to 0)	0 (0 to 0)	0 (0 to 0)	1·8 (0·7 to 3·6)	2 (0·8 to 4·6)	0·1 (0 to 0·4)	0 (0 to 0)	0 (0 to 0)	0 (0 to 0)	0 (0 to 0)	0 (0 to 0)	0 (0 to 0)	0 (0 to 0)	0 (0 to 0)	0 (0 to 0)	0 (0 to 0)	0 (0 to 0)	0 (0 to 0)	0 (0 to 0)	0 (0 to 0)	0 (0 to 0)	0 (0 to 0)	0 (0 to 0)	3·9
Caries of permanent teeth	0 (0 to 0)	0 (0 to 0)	0 (0 to 0)	0 (0 to 0)	1·6 (0·6 to 3·7)	4·5 (1·8 to 9·9)	4·7 (1·8 to 9·9)	5·4 (2·3 to 11)	5·2 (2·1 to 10·8)	4·5 (1·8 to 9·2)	4·3 (1·8 to 8·7)	4 (1·6 to 8·2)	3·7 (1·5 to 7·9)	3·2 (1·3 to 7)	2·5 (1 to 5·7)	1·7 (0·6 to 4)	1 (0·4 to 2·3)	0·6 (0·2 to 1·3)	0·4 (0·2 to 0·8)	0·2 (0·1 to 0·5)	0·1 (0 to 0·2)	0 (0 to 0·1)	0 (0 to 0)	47·7
Periodontal diseases	0 (0 to 0)	0 (0 to 0)	0 (0 to 0)	0 (0 to 0)	0 (0 to 0)	0 (0 to 0)	2·1 (0·6 to 5·6)	8 (2·4 to 19·9)	16·9 (5·4 to 40·7)	23·4 (8·1 to 53·5)	28·4 (10 to 63·5)	32·2 (11·7 to 68·5)	34·3 (13·1 to 71·6)	31·6 (12·2 to 66·6)	25·7 (10·2 to 54·4)	17·9 (7·1 to 36·4)	10·9 (4·2 to 22·7)	6·4 (2·5 to 13·1)	4·1 (1·6 to 8·2)	2·4 (0·9 to 4·6)	1 (0·4 to 2·1)	0·3 (0·1 to 0·7)	0·1 (0 to 0·2)	246
Other oral disorders	0 (0 to 0)	0 (0 to 0)	0 (0 to 0)	3·3 (2 to 5·3)	3·6 (2·1 to 5·8)	4·6 (2·6 to 7·4)	6·6 (3·9 to 10·3)	8·1 (4·8 to 12·7)	8·8 (5·2 to 13·2)	8·6 (5·1 to 13)	8·4 (5 to 13·1)	8·4 (5 to 12·9)	8·4 (5·2 to 12·9)	7·7 (4·7 to 11·5)	6·4 (3·8 to 9·7)	4·7 (2·8 to 7·2)	3·2 (1·9 to 4·8)	1·8 (1·1 to 2·8)	1 (0·6 to 1·5)	0·5 (0·3 to 0·7)	0·2 (0·1 to 0·3)	0·1 (0 to 0·1)	0 (0 to 0)	94·4
Sudden infant death syndrome	--	2·2 (1·1 to 4·1)	20 (10·3 to 34·2)	--	--	--	--	--	--	--	--	--	--	--	--	--	--	--	--	--	--	--	--	NA
Injuries	11·3 (8·1 to 15·5)	9·2 (6·1 to 13·9)	80·5 (59·3 to 109)	227 (187 to 277)	281 (254 to 314)	439 (396 to 492)	1,120 (969 to 1,310)	2,180 (1,900 to 2,550)	2,330 (2,080 to 2,680)	1,920 (1,660 to 2,200)	1,640 (1,390 to 1,950)	1,310 (1,090 to 1,580)	1,030 (854 to 1,240)	803 (668 to 960)	596 (504 to 708)	405 (346 to 474)	251 (215 to 294)	161 (138 to 188)	112 (95·2 to 131)	71·2 (60 to 83·3)	37·9 (31·7 to 44·7)	16·9 (14 to 19·6)	6·2 (5·1 to 7·5)	15,000
Transport injuries	0·5 (0·4 to 0·7)	0·4 (0·3 to 0·7)	5·2 (3·8 to 7·2)	35·9 (28·7 to 45·6)	59·5 (48·3 to 73·4)	98 (81·6 to 117)	294 (234 to 369)	558 (452 to 685)	567 (460 to 678)	499 (409 to 605)	446 (357 to 559)	384 (314 to 469)	342 (277 to 418)	273 (227 to 325)	203 (168 to 248)	131 (110 to 156)	78·2 (65·8 to 92·2)	46·5 (39·2 to 55·6)	29·7 (24·7 to 35·4)	15·7 (13·1 to 18·8)	6·8 (5·6 to 8·3)	2·2 (1·8 to 2·7)	0·7 (0·6 to 0·8)	4,080
Road injuries	0·3 (0·2 to 0·4)	0·2 (0·1 to 0·4)	2·7 (1·6 to 4·2)	14·7 (9 to 23·5)	29·1 (18·9 to 41·4)	47·7 (33·8 to 64·7)	169 (112 to 239)	354 (253 to 474)	375 (274 to 480)	321 (243 to 418)	286 (207 to 383)	228 (169 to 301)	211 (156 to 278)	168 (128 to 218)	128 (96·9 to 165)	87·6 (69·5 to 110)	54·1 (42·8 to 66·9)	31·3 (24·6 to 38·9)	21·5 (17 to 26·5)	11·8 (9·3 to 14·6)	5·4 (4·2 to 6·8)	1·8 (1·4 to 2·2)	0·6 (0·4 to 0·7)	2,550
Pedestrian road injuries	0·1 (0·1 to 0·2)	0·1 (0 to 0·1)	0·9 (0·5 to 1·3)	6·8 (3·8 to 11·7)	13·3 (8·5 to 19·1)	16·8 (11·1 to 24)	21 (13·8 to 29·7)	35·3 (22·8 to 51·1)	45·3 (32·7 to 58·2)	47·3 (35·5 to 62·1)	49·1 (35·7 to 66·5)	46·9 (33·8 to 63·8)	46·8 (33·3 to 63·1)	41·1 (30·2 to 55)	34·8 (25·6 to 46·3)	25·4 (19·5 to 32·8)	17·1 (13·1 to 21·8)	10·9 (8·3 to 13·9)	8·4 (6·6 to 10·6)	4·8 (3·8 to 6·1)	2·3 (1·8 to 3)	0·7 (0·6 to 0·9)	0·2 (0·1 to 0·2)	476
Cyclist road injuries	0 (0 to 0)	0 (0 to 0)	0 (0 to 0)	0·4 (0·2 to 0·6)	1·4 (0·8 to 2·1)	4·9 (3·1 to 7·1)	11 (6·6 to 16·8)	15·5 (10·2 to 22·6)	16·8 (11·5 to 23·3)	16·9 (11·7 to 23·3)	16·8 (11·8 to 23·2)	15·8 (11·4 to 21·3)	15·3 (11 to 21·3)	13·9 (10·2 to 18·2)	11·3 (8 to 15)	8·3 (6·2 to 11)	5·5 (4·1 to 7·2)	3·2 (2·4 to 4·3)	2·1 (1·5 to 2·8)	1·2 (0·9 to 1·7)	0·5 (0·4 to 0·7)	0·2 (0·1 to 0·3)	0·1 (0 to 0·1)	161
Motorcyclist road injuries	0 (0 to 0)	0 (0 to 0)	0·3 (0·2 to 0·4)	1·1 (0·6 to 1·7)	2·2 (1·5 to 3·1)	4·4 (2·5 to 7·3)	40·4 (26·8 to 57·1)	98·2 (62·3 to 144)	113 (81·9 to 145)	88·8 (67·7 to 115)	76·1 (55·6 to 102)	53·3 (40·1 to 69·5)	45·9 (34·4 to 59·3)	33·2 (25·7 to 41·3)	24·1 (18·4 to 31)	14·3 (11 to 18·4)	8·7 (6·5 to 11·4)	4·6 (3·4 to 6·2)	3 (2·2 to 4·1)	1·8 (1·3 to 2·5)	0·8 (0·6 to 1·2)	0·3 (0·2 to 0·4)	0·1 (0·1 to 0·1)	615
Motor vehicle road injuries	0·1 (0·1 to 0·2)	0·1 (0·1 to 0·2)	1·5 (0·9 to 2·4)	6·2 (3·3 to 10·8)	12 (7·7 to 17·6)	21·2 (14·3 to 29·8)	96·1 (62·3 to 138)	204 (138 to 279)	198 (145 to 255)	166 (123 to 217)	142 (98·6 to 194)	110 (77·3 to 151)	101 (70·5 to 136)	77·6 (57·2 to 103)	55·4 (41·1 to 74·1)	37·9 (29·1 to 49·2)	21·5 (16·7 to 27·5)	11·7 (9 to 15)	7·4 (5·8 to 9·4)	3·6 (2·8 to 4·4)	1·6 (1·2 to 2)	0·5 (0·4 to 0·7)	0·2 (0·2 to 0·3)	1,270
Other road injuries	0 (0 to 0)	0 (0 to 0)	0 (0 to 0)	0·1 (0·1 to 0·2)	0·2 (0·1 to 0·3)	0·4 (0·2 to 0·5)	0·6 (0·4 to 0·9)	1·2 (0·8 to 1·6)	1·5 (1 to 2·1)	1·7 (1·2 to 2·5)	2·1 (1·4 to 2·8)	2·3 (1·6 to 3·3)	2·6 (1·8 to 3·6)	2·5 (1·7 to 3·4)	2·1 (1·4 to 3)	1·7 (1·1 to 2·3)	1·2 (0·8 to 1·6)	0·8 (0·5 to 1·1)	0·5 (0·4 to 0·8)	0·3 (0·2 to 0·4)	0·1 (0·1 to 0·2)	0 (0 to 0·1)	0 (0 to 0)	22
Other transport injuries	0·3 (0·2 to 0·4)	0·2 (0·1 to 0·3)	2·5 (2 to 3·3)	21·2 (18·9 to 24)	30·4 (27·5 to 34·1)	50·3 (45·1 to 56·7)	125 (112 to 140)	204 (179 to 235)	192 (168 to 221)	178 (155 to 207)	161 (139 to 189)	156 (137 to 180)	131 (116 to 149)	105 (93·9 to 118)	75·6 (66·1 to 87·2)	43·3 (38·7 to 49·2)	24·2 (21·8 to 26·9)	15·2 (13·3 to 17·4)	8·2 (7·3 to 9·4)	3·9 (3·5 to 4·5)	1·4 (1·3 to 1·6)	0·4 (0·4 to 0·5)	0·1 (0·1 to 0·2)	1,530
Unintentional injuries	6·3 (4·8 to 8·2)	7·7 (5·2 to 11·4)	66·4 (48·3 to 91·1)	173 (142 to 214)	204 (182 to 231)	296 (264 to 332)	377 (326 to 437)	470 (405 to 558)	503 (431 to 592)	472 (403 to 553)	439 (377 to 513)	403 (344 to 474)	357 (299 to 426)	317 (265 to 374)	251 (211 to 299)	192 (161 to 228)	129 (110 to 154)	90·8 (76·7 to 108)	68·8 (58·3 to 81·5)	49·4 (41·6 to 58·4)	28·4 (23·7 to 33·7)	13·7 (11·3 to 16·2)	5·2 (4·1 to 6·3)	4,920
Falls	0·1 (0·1 to 0·1)	0·1 (0 to 0·2)	1·6 (0·9 to 2·5)	3·6 (2·3 to 5·6)	6 (4·3 to 8·2)	12·3 (9·1 to 16·2)	23 (17·3 to 29·9)	35·8 (27·9 to 46·1)	43·1 (33·5 to 54·2)	45·5 (35·6 to 57·7)	51·9 (40·5 to 65·5)	53·8 (42·1 to 68·8)	55·6 (42·8 to 71·5)	56·5 (43·5 to 71·3)	50·2 (38·8 to 64·2)	42·2 (33·1 to 53)	31·7 (25 to 39·7)	26·7 (20·6 to 33·5)	22·9 (17·9 to 28·7)	20·4 (16 to 25·4)	14·1 (11·2 to 17·7)	7·6 (5·9 to 9·4)	2·9 (2·2 to 3·7)	608
Drowning	0·5 (0·4 to 0·7)	0·3 (0·2 to 0·5)	6·2 (3·8 to 9·3)	38·6 (24·3 to 57·6)	50·4 (37·1 to 65·4)	64·3 (47·8 to 84·3)	103 (71·7 to 143)	129 (89·4 to 182)	150 (111 to 203)	141 (105 to 185)	118 (89·1 to 155)	96 (71·1 to 128)	67·8 (48·8 to 92·7)	52·5 (38·3 to 72·1)	36·4 (27·5 to 47·9)	22·1 (16·9 to 28·6)	11·7 (8·8 to 15)	5·6 (4·4 to 7)	3·3 (2·6 to 4·2)	1·2 (0·9 to 1·5)	0·6 (0·5 to 0·8)	0·3 (0·2 to 0·3)	0·1 (0·1 to 0·1)	1,100
Fire, heat, and hot substances	0·2 (0·2 to 0·3)	0·4 (0·2 to 0·7)	6·2 (3·9 to 9·3)	17·3 (10·8 to 26·6)	14·5 (11·3 to 18·3)	17·3 (13·2 to 22·1)	20·9 (16 to 27·4)	32·8 (25·1 to 42·9)	40·6 (30·9 to 52·1)	45·9 (35·2 to 58·6)	43·7 (33·5 to 57·3)	42 (31·7 to 54·4)	40 (30·4 to 52·9)	34 (25 to 45·1)	28·3 (21·4 to 37·2)	21·9 (16·7 to 28·8)	14·3 (10·8 to 19)	10·1 (7·8 to 13·1)	7·5 (5·8 to 9·7)	5 (3·9 to 6·4)	3 (2·2 to 3·8)	1·4 (1·1 to 1·7)	0·5 (0·4 to 0·6)	448
Poisonings	0 (0 to 0)	0 (0 to 0·1)	0·4 (0·2 to 0·7)	1·6 (1 to 2·6)	1 (0·7 to 1·5)	1·3 (0·8 to 1·9)	1·8 (1·2 to 2·5)	3·1 (2·2 to 4·1)	4·6 (3·3 to 6·3)	4·6 (3·3 to 6·2)	4 (2·9 to 5·5)	2·9 (2·1 to 3·8)	2·4 (1·7 to 3·2)	1·8 (1·3 to 2·4)	1·4 (1 to 1·9)	1 (0·7 to 1·3)	0·6 (0·4 to 0·8)	0·4 (0·2 to 0·5)	0·2 (0·2 to 0·3)	0·1 (0·1 to 0·2)	0·1 (0 to 0·1)	0 (0 to 0)	0 (0 to 0)	33·2
Poisoning by carbon monoxide	0 (0 to 0)	0 (0 to 0·1)	0·1 (0·1 to 0·3)	1·1 (0·5 to 1·9)	0·4 (0·3 to 0·7)	0·5 (0·3 to 0·7)	0·8 (0·5 to 1·3)	1·9 (1·2 to 2·9)	3·2 (2·1 to 4·8)	3·3 (2·1 to 4·8)	2·9 (1·8 to 4·2)	1·8 (1·2 to 2·6)	1·4 (0·9 to 2)	0·9 (0·6 to 1·3)	0·7 (0·4 to 1)	0·4 (0·3 to 0·6)	0·2 (0·2 to 0·3)	0·1 (0·1 to 0·2)	0·1 (0·1 to 0·1)	0·1 (0 to 0·1)	0 (0 to 0)	0 (0 to 0)	0 (0 to 0)	20·1
Poisoning by other means	0 (0 to 0)	0 (0 to 0)	0·3 (0·2 to 0·4)	0·6 (0·3 to 0·9)	0·6 (0·3 to 0·9)	0·8 (0·4 to 1·4)	0·9 (0·5 to 1·5)	1·1 (0·7 to 1·8)	1·4 (0·9 to 2)	1·2 (0·8 to 1·8)	1·1 (0·7 to 1·7)	1·1 (0·7 to 1·6)	1 (0·7 to 1·5)	0·9 (0·6 to 1·4)	0·7 (0·4 to 1·1)	0·5 (0·3 to 0·8)	0·3 (0·2 to 0·5)	0·2 (0·1 to 0·3)	0·1 (0·1 to 0·2)	0·1 (0·1 to 0·1)	0 (0 to 0·1)	0 (0 to 0)	0 (0 to 0)	13·1
Exposure to mechanical forces	0 (0 to 0)	0·1 (0 to 0·1)	0·4 (0·3 to 0·6)	2·6 (1·7 to 3·8)	6 (4 to 9)	11·1 (7·4 to 16·7)	21 (15·3 to 28·5)	34·6 (25·9 to 47·1)	38·3 (28·3 to 51·2)	37·3 (27·8 to 49·8)	36·7 (27 to 49·4)	36·3 (26·8 to 50·2)	34·6 (24·5 to 48·4)	31·5 (22·5 to 44·6)	27 (19·2 to 38·2)	20·4 (14 to 29)	13·7 (9·3 to 19·6)	8·8 (5·9 to 12·7)	6 (4·1 to 8·6)	3·7 (2·5 to 5·3)	1·7 (1·2 to 2·5)	0·7 (0·4 to 0·9)	0·2 (0·2 to 0·3)	373
Unintentional firearm injuries	0 (0 to 0)	0 (0 to 0)	0 (0 to 0·1)	0·2 (0·1 to 0·4)	0·5 (0·3 to 0·8)	0·9 (0·6 to 1·4)	2·5 (1·7 to 3·8)	5·3 (3·5 to 8·3)	5·5 (3·4 to 8)	4·8 (3·2 to 6·9)	4·7 (3·3 to 6·4)	4·6 (3·3 to 6·2)	4·3 (3·1 to 5·8)	3·8 (2·7 to 5·1)	3·2 (2·3 to 4·3)	2·4 (1·7 to 3·3)	1·6 (1·1 to 2·3)	1 (0·7 to 1·4)	0·7 (0·5 to 1)	0·4 (0·3 to 0·6)	0·2 (0·1 to 0·3)	0·1 (0·1 to 0·1)	0 (0 to 0)	46·9
Other exposure to mechanical forces	0 (0 to 0)	0·1 (0 to 0·1)	0·4 (0·3 to 0·6)	2·4 (1·6 to 3·5)	5·5 (3·6 to 8·4)	10·2 (6·7 to 15·6)	18·5 (13·2 to 25·6)	29·2 (21·8 to 40)	32·9 (23·9 to 44·7)	32·5 (23·9 to 44)	32 (23·2 to 44)	31·7 (23·1 to 44·5)	30·3 (21·3 to 42·8)	27·7 (19·5 to 39·4)	23·7 (16·7 to 34)	18 (12·1 to 25·9)	12·1 (8·1 to 17·5)	7·8 (5·2 to 11·3)	5·3 (3·5 to 7·8)	3·3 (2·2 to 4·8)	1·5 (1 to 2·2)	0·6 (0·4 to 0·8)	0·2 (0·1 to 0·3)	326
Adverse effects of medical treatment	1·5 (0·9 to 2·4)	1·5 (0·7 to 2·7)	5·3 (2·8 to 9·5)	3·2 (1·8 to 5·8)	2·7 (1·7 to 4·2)	2·8 (1·8 to 4·4)	3·6 (2·3 to 5·5)	8·6 (5·2 to 12·9)	11·3 (6·9 to 17·4)	9·4 (5·8 to 14·1)	11·5 (7·4 to 16·9)	11·6 (7·6 to 17·3)	10·5 (6·6 to 15·9)	13·5 (8·2 to 20·6)	13·1 (8·3 to 20·1)	12·5 (8 to 19·3)	10·1 (6·7 to 15·5)	7·7 (5·1 to 11·5)	6·8 (4·4 to 10)	4·8 (3·4 to 7·2)	2·8 (1·9 to 3·9)	1·3 (0·9 to 1·7)	0·5 (0·3 to 0·9)	157
Animal contact	0 (0 to 0)	0 (0 to 0·1)	0·2 (0·1 to 0·3)	0·8 (0·5 to 1·2)	0·9 (0·6 to 1·3)	1·6 (1·1 to 2·2)	2 (1·3 to 2·8)	2·4 (1·6 to 3·2)	3·6 (2·5 to 4·8)	2·7 (1·9 to 3·7)	3·5 (2·4 to 5)	3·5 (2·4 to 4·8)	3·7 (2·6 to 5)	3·5 (2·5 to 4·8)	3 (2·1 to 4·1)	2·8 (1·9 to 3·8)	2·1 (1·4 to 3)	1·5 (1 to 2)	1·1 (0·7 to 1·5)	0·5 (0·4 to 0·8)	0·2 (0·2 to 0·3)	0·1 (0·1 to 0·2)	0 (0 to 0·1)	39·8
Venomous animal contact	0 (0 to 0)	0 (0 to 0·1)	0·1 (0·1 to 0·2)	0·6 (0·4 to 0·9)	0·7 (0·4 to 1)	1·2 (0·8 to 1·7)	1·5 (1 to 2·1)	1·7 (1·1 to 2·4)	2·6 (1·7 to 3·5)	1·8 (1·2 to 2·5)	2·7 (1·8 to 3·9)	2·4 (1·6 to 3·3)	2·6 (1·8 to 3·6)	2·5 (1·7 to 3·4)	2·1 (1·4 to 2·9)	1·9 (1·3 to 2·7)	1·3 (0·9 to 1·9)	0·9 (0·6 to 1·3)	0·7 (0·5 to 1)	0·4 (0·2 to 0·5)	0·2 (0·1 to 0·2)	0·1 (0·1 to 0·1)	0 (0 to 0)	28
Non-venomous animal contact	0 (0 to 0)	0 (0 to 0)	0·1 (0 to 0·1)	0·2 (0·1 to 0·3)	0·2 (0·1 to 0·4)	0·4 (0·2 to 0·7)	0·5 (0·3 to 0·8)	0·7 (0·4 to 1·1)	1 (0·6 to 1·8)	0·9 (0·5 to 1·3)	0·8 (0·5 to 1·3)	1·1 (0·7 to 1·7)	1·1 (0·6 to 1·7)	1·1 (0·7 to 1·6)	0·9 (0·6 to 1·3)	0·9 (0·5 to 1·3)	0·8 (0·5 to 1·1)	0·6 (0·4 to 0·9)	0·3 (0·2 to 0·5)	0·2 (0·1 to 0·2)	0·1 (0 to 0·1)	0 (0 to 0)	0 (0 to 0)	11·8
Foreign body	2·9 (2 to 4)	3 (1·6 to 5·1)	27·5 (15·9 to 43·8)	17 (11 to 24·8)	9·1 (7 to 11·4)	13·3 (9·9 to 17·4)	10·1 (7·4 to 13·3)	17·6 (13·1 to 23·5)	22·3 (17·2 to 28·8)	20·1 (15·1 to 26·1)	20·1 (15·5 to 26·1)	19·3 (14·7 to 24·6)	17·7 (13·2 to 23·7)	17·6 (12·9 to 23·5)	14·4 (11·1 to 18·4)	12·4 (9·4 to 16)	9·7 (7·2 to 12·5)	6·8 (5·1 to 8·9)	5·4 (4·2 to 6·9)	3·8 (2·9 to 4·9)	1·7 (1·3 to 2·2)	0·9 (0·6 to 1·1)	0·4 (0·3 to 0·5)	273
Pulmonary aspiration and foreign body in airway	2·8 (2 to 3·9)	3 (1·6 to 5·1)	27·3 (15·7 to 43·4)	16 (10·3 to 23·5)	7·6 (5·7 to 9·7)	11·2 (8·1 to 15·1)	8·1 (5·8 to 11)	14·9 (10·8 to 20·5)	18·9 (14·2 to 25)	17·3 (12·7 to 22·9)	17·2 (13 to 22·6)	16·1 (12 to 21·3)	14·4 (10·3 to 19·9)	14·8 (10·5 to 20·5)	11·9 (8·9 to 15·5)	10·4 (7·6 to 13·9)	8·2 (5·9 to 10·9)	5·8 (4·1 to 7·7)	4·6 (3·5 to 6)	3·2 (2·3 to 4·2)	1·4 (1 to 1·9)	0·7 (0·5 to 0·9)	0·3 (0·2 to 0·4)	236
Foreign body in eyes	0 (0 to 0)	0 (0 to 0)	0 (0 to 0·1)	0·3 (0·1 to 0·6)	0·5 (0·1 to 1·2)	0·7 (0·2 to 1·7)	0·9 (0·3 to 2·1)	1·1 (0·4 to 2·5)	1·3 (0·4 to 2·7)	1·2 (0·4 to 2·6)	1·3 (0·5 to 2·6)	1·3 (0·5 to 2·6)	1·3 (0·6 to 2·4)	1·1 (0·5 to 1·9)	0·8 (0·5 to 1·4)	0·6 (0·4 to 1)	0·4 (0·3 to 0·7)	0·3 (0·2 to 0·4)	0·2 (0·1 to 0·3)	0·1 (0·1 to 0·2)	0·1 (0 to 0·1)	0 (0 to 0)	0 (0 to 0)	13·5
Foreign body in other body part	0 (0 to 0)	0 (0 to 0)	0·2 (0·1 to 0·4)	0·7 (0·4 to 1·1)	1 (0·7 to 1·4)	1·3 (0·9 to 1·9)	1·1 (0·8 to 1·6)	1·6 (1 to 2·2)	2·1 (1·4 to 3·3)	1·6 (1·1 to 2·2)	1·6 (1·1 to 2·2)	2 (1·4 to 2·7)	2 (1·4 to 2·9)	1·7 (1·2 to 2·4)	1·7 (1·2 to 2·4)	1·3 (1 to 1·9)	1 (0·7 to 1·5)	0·8 (0·5 to 1·1)	0·6 (0·5 to 0·9)	0·5 (0·4 to 0·8)	0·2 (0·2 to 0·4)	0·1 (0·1 to 0·2)	0·1 (0 to 0·1)	23·5
Environmental heat and cold exposure	0 (0 to 0)	0 (0 to 0)	0·2 (0·1 to 0·3)	0·9 (0·6 to 1·2)	1·7 (1·2 to 2·4)	2·8 (1·9 to 4)	4·9 (3·5 to 6·6)	7·8 (5·8 to 10·5)	8·9 (6·5 to 11·6)	9·3 (7 to 12·2)	9·5 (7·1 to 12·7)	9·5 (7 to 12·7)	9·7 (7 to 13)	9·1 (6·5 to 12·1)	8·3 (6·2 to 10·8)	6·4 (4·6 to 8·4)	4·4 (3·3 to 5·8)	3·5 (2·6 to 4·5)	2·6 (2 to 3·4)	2 (1·5 to 2·5)	1·1 (0·9 to 1·4)	0·6 (0·4 to 0·8)	0·2 (0·2 to 0·3)	104
Exposure to forces of nature	0·9 (0·9 to 1)	2·1 (1·9 to 2·3)	17·6 (15·9 to 19·3)	84·3 (76·5 to 92·7)	107 (96·8 to 117)	160 (145 to 176)	171 (156 to 188)	176 (160 to 193)	153 (139 to 168)	130 (118 to 143)	115 (104 to 126)	107 (97·1 to 118)	96·2 (87·4 to 106)	82·5 (74·9 to 90·9)	58·1 (52·7 to 64)	42·5 (38·6 to 46·8)	25·4 (23·1 to 28)	16·5 (15 to 18·2)	10·6 (9·6 to 11·6)	6·3 (5·7 to 7)	2·4 (2·2 to 2·6)	0·7 (0·6 to 0·8)	0·2 (0·2 to 0·3)	1,560
Other unintentional injuries	0·1 (0 to 0·2)	0·1 (0 to 0·1)	0·7 (0·5 to 1)	3 (2·1 to 4·2)	5·6 (4·1 to 7·7)	8·9 (6·6 to 12)	15·3 (11·2 to 20·2)	23·4 (17·4 to 31·2)	27·3 (21 to 35·3)	25·9 (19·3 to 33·4)	24·9 (19 to 32)	21·6 (16·4 to 28)	18·8 (13·9 to 25·2)	14·4 (10·3 to 19·2)	11·5 (8·5 to 15·6)	8 (5·6 to 11)	5·3 (3·8 to 7·4)	3·3 (2·3 to 4·6)	2·2 (1·6 to 3·2)	1·4 (0·9 to 1·9)	0·6 (0·4 to 0·9)	0·2 (0·2 to 0·3)	0·1 (0·1 to 0·1)	223
Self-harm and interpersonal violence	4·4 (2·6 to 7·5)	1·1 (0·5 to 2·1)	8·9 (5·2 to 15)	17·6 (10·5 to 29·5)	17·2 (12·2 to 25·1)	45·7 (33·4 to 63·1)	450 (347 to 584)	1,160 (930 to 1,420)	1,260 (1,050 to 1,520)	949 (751 to 1,160)	756 (592 to 974)	519 (375 to 688)	334 (238 to 454)	213 (154 to 291)	141 (101 to 190)	82·4 (62·5 to 107)	44·1 (33·9 to 58·2)	23·4 (18·3 to 30·8)	13·4 (10·5 to 17·1)	6 (4·8 to 7·4)	2·7 (2·2 to 3·3)	0·9 (0·7 to 1·1)	0·3 (0·3 to 0·4)	6,040
Self-harm	0 (0 to 0)	0 (0 to 0)	0 (0 to 0)	0 (0 to 0)	0 (0 to 0)	2·2 (1·2 to 3·7)	23·1 (14·8 to 34·1)	50·1 (34 to 72·1)	63·2 (44·2 to 90·6)	62·6 (44·1 to 85·9)	59·8 (41 to 83·6)	48·9 (33·9 to 71)	38·2 (26 to 53·5)	31·6 (21·5 to 44·8)	23 (16·1 to 32·7)	15·6 (11·2 to 21·6)	9·6 (7 to 12·9)	6·2 (4·5 to 8·4)	4 (3 to 5·4)	2·2 (1·6 to 3)	1 (0·8 to 1·4)	0·3 (0·2 to 0·4)	0·1 (0·1 to 0·1)	442
Self-harm by firearm	0 (0 to 0)	0 (0 to 0)	0 (0 to 0)	0 (0 to 0)	0 (0 to 0)	0·1 (0 to 0·2)	2·6 (1·4 to 4·3)	5·2 (2·8 to 9·1)	5·8 (3·2 to 10)	5·3 (3 to 8·6)	4·6 (2·6 to 7·8)	4 (2·3 to 6·5)	3 (1·7 to 4·9)	2·7 (1·6 to 4·3)	1·6 (1 to 2·7)	1·2 (0·7 to 1·9)	0·7 (0·4 to 1)	0·4 (0·3 to 0·6)	0·2 (0·2 to 0·4)	0·1 (0·1 to 0·2)	0 (0 to 0·1)	0 (0 to 0)	0 (0 to 0)	37·6
Self-harm by other specified means	0 (0 to 0)	0 (0 to 0)	0 (0 to 0)	0 (0 to 0)	0 (0 to 0)	2·1 (1·2 to 3·6)	20·6 (13·2 to 30·8)	44·8 (30·4 to 64·2)	57·4 (40·2 to 81·7)	57·3 (40 to 78·9)	55·2 (37·4 to 77·4)	44·9 (30·9 to 64·9)	35·2 (23·8 to 49·1)	28·9 (19·5 to 41·5)	21·4 (14·9 to 30·3)	14·4 (10·2 to 20·1)	9 (6·5 to 12)	5·8 (4·2 to 7·9)	3·8 (2·8 to 5·1)	2·1 (1·5 to 2·8)	1 (0·7 to 1·3)	0·3 (0·2 to 0·4)	0·1 (0·1 to 0·1)	404
Interpersonal violence	4·4 (2·6 to 7·5)	1·1 (0·5 to 2·1)	8·9 (5·2 to 15)	17·5 (10·4 to 29·4)	17·1 (12·1 to 25)	43·3 (31·7 to 60·5)	424 (322 to 557)	1,100 (877 to 1,360)	1,180 (981 to 1,440)	880 (692 to 1,080)	692 (531 to 898)	466 (331 to 630)	293 (201 to 406)	180 (126 to 251)	117 (80·4 to 163)	66·5 (48·6 to 90·2)	34 (25·1 to 46·5)	17·2 (12·6 to 23·4)	9·4 (7 to 12·6)	3·8 (3 to 4·9)	1·6 (1·3 to 2·1)	0·6 (0·4 to 0·7)	0·3 (0·2 to 0·4)	5,560
Physical violence by firearm	0·6 (0·2 to 1·1)	0·2 (0·1 to 0·4)	2·4 (1·4 to 3·9)	4·9 (2·8 to 8·3)	7·8 (5·2 to 11·6)	26·4 (18·7 to 37·6)	332 (250 to 441)	920 (731 to 1,150)	978 (800 to 1,200)	693 (538 to 871)	526 (393 to 690)	330 (229 to 449)	191 (127 to 271)	109 (74·9 to 157)	68·8 (45·4 to 99)	36·6 (25·7 to 51·1)	16·8 (11·9 to 24)	7·3 (5·1 to 10·6)	3 (2 to 4·4)	1 (0·8 to 1·3)	0·4 (0·3 to 0·6)	0·1 (0·1 to 0·2)	0·1 (0 to 0·1)	4,260
Physical violence by sharp object	0·4 (0·2 to 0·8)	0·1 (0 to 0·2)	0·6 (0·4 to 1)	1·9 (1·2 to 3)	2·1 (1·5 to 3)	5·5 (4 to 7·6)	71·9 (49 to 104)	139 (98 to 188)	158 (114 to 217)	143 (102 to 198)	120 (82·2 to 169)	94·5 (61·8 to 134)	64·9 (42 to 95·3)	40·8 (28·8 to 55·9)	23·4 (16·9 to 31·2)	13·7 (10·3 to 18·1)	6·9 (5·1 to 9)	4·2 (3·1 to 5·5)	2·5 (1·8 to 3·3)	1·1 (0·9 to 1·5)	0·5 (0·4 to 0·6)	0·2 (0·2 to 0·3)	0·1 (0·1 to 0·1)	895
Sexual violence	0 (0 to 0)	0 (0 to 0)	0 (0 to 0)	0·1 (0 to 0·1)	0·5 (0·3 to 0·8)	1·3 (0·7 to 2)	2·6 (1·4 to 4·2)	3·5 (2 to 5·4)	3·2 (1·8 to 5·2)	2·7 (1·5 to 4·4)	2·5 (1·4 to 4)	2·1 (1·2 to 3·4)	1·7 (1 to 2·9)	1·3 (0·7 to 2·1)	0·9 (0·5 to 1·4)	0·6 (0·4 to 1)	0·4 (0·3 to 0·6)	0·3 (0·2 to 0·4)	0·2 (0·1 to 0·3)	0·1 (0·1 to 0·1)	0 (0 to 0·1)	0 (0 to 0)	0 (0 to 0)	23·8
Physical violence by other means	3·5 (2 to 5·7)	0·8 (0·4 to 1·5)	5·9 (3·4 to 10·1)	10·7 (6·2 to 18·4)	6·7 (4·7 to 9·9)	10·2 (7·4 to 14·1)	18·2 (12·6 to 25·1)	36·1 (27·6 to 45·7)	44·7 (34·3 to 57·2)	40·7 (30·4 to 53·8)	43 (31·2 to 58)	39·7 (27·7 to 55·4)	34·6 (23·9 to 48·9)	28·9 (21·1 to 38·7)	23·7 (17 to 32·4)	15·6 (11·6 to 20·9)	9·9 (7·3 to 13·3)	5·4 (4 to 7·3)	3·8 (2·8 to 5·3)	1·6 (1·2 to 2)	0·7 (0·5 to 0·9)	0·2 (0·2 to 0·3)	0·1 (0·1 to 0·2)	385
Conflict and terrorism	0 (0 to 0)	0 (0 to 0)	0 (0 to 0)	0 (0 to 0)	0 (0 to 0)	0 (0 to 0)	0 (0 to 0)	0 (0 to 0)	0 (0 to 0)	0 (0 to 0)	0 (0 to 0·1)	1·8 (1·1 to 2·9)	1·6 (1·1 to 2·5)	0·5 (0·3 to 0·7)	1·2 (0·8 to 1·7)	0·2 (0·1 to 0·3)	0·4 (0·3 to 0·6)	0 (0 to 0)	0 (0 to 0)	0 (0 to 0)	0 (0 to 0)	0 (0 to 0)	0 (0 to 0)	5·9

SR Table 2a: Barbados DALYS by cause and age group for males with 95% uncertainty intervals, 2019

Cause	Early Neonatal	Late Neonatal	Post Neonatal	1 to 4	5 to 9	10 to 14	15 to 19	20 to 24	25 to 29	30 to 34	35 to 39	40 to 44	45 to 49	50 to 54	55 to 59	60 to 64	65 to 69	70 to 74	75 to 79	80 to 84	85 to 89	90 to 94	95 plus	Total
All causes	943 (634 to 1,370)	216 (147 to 312)	343 (246 to 476)	367 (289 to 457)	481 (375 to 631)	669 (527 to 844)	1,160 (965 to 1,390)	1,300 (1,110 to 1,550)	1,670 (1,440 to 1,940)	1,860 (1,590 to 2,160)	1,930 (1,650 to 2,260)	2,160 (1,820 to 2,550)	2,890 (2,400 to 3,410)	3,330 (2,750 to 3,960)	4,510 (3,750 to 5,320)	5,220 (4,390 to 6,070)	5,040 (4,300 to 5,800)	5,020 (4,300 to 5,740)	3,920 (3,400 to 4,430)	3,030 (2,690 to 3,360)	1,990 (1,810 to 2,160)	903 (837 to 964)	158 (148 to 168)	49,100
Communicable, maternal, neonatal, and nutritional diseases	773 (523 to 1,110)	159 (107 to 232)	154 (108 to 213)	121 (92·3 to 156)	144 (96·1 to 227)	138 (97·8 to 194)	122 (98·8 to 151)	126 (105 to 149)	196 (171 to 227)	211 (186 to 242)	247 (219 to 278)	232 (205 to 264)	289 (257 to 328)	237 (200 to 278)	259 (215 to 308)	266 (216 to 325)	243 (193 to 299)	246 (193 to 322)	215 (167 to 276)	212 (161 to 272)	159 (124 to 198)	87·1 (66 to 109)	17·2 (13 to 21·8)	4,850
HIV/AIDS and sexually transmitted infections	0·6 (0·3 to 0·9)	0·1 (0 to 0·2)	4·3 (4 to 4·7)	2·8 (2·6 to 3)	2·5 (2·3 to 2·7)	3·5 (3·1 to 3·8)	8·3 (7·2 to 9·7)	23·2 (20·9 to 26·1)	79 (72·2 to 87·4)	85·2 (77·6 to 94·1)	120 (111 to 132)	100 (92·5 to 109)	133 (123 to 144)	75·8 (69·8 to 82·4)	62·5 (57·1 to 68·5)	41·6 (37·6 to 46·2)	27·1 (24·4 to 29·9)	9·7 (8·3 to 11·2)	4·3 (3·6 to 5·1)	2·3 (2 to 2·7)	1 (0·9 to 1·2)	0·4 (0·3 to 0·4)	0·1 (0·1 to 0·1)	788
HIV/AIDS	0 (0 to 0)	0 (0 to 0)	4·2 (3·9 to 4·5)	2·8 (2·6 to 3)	2·5 (2·3 to 2·7)	3·3 (3 to 3·5)	7·1 (6·5 to 7·7)	21·3 (19·4 to 23·5)	76·7 (69·8 to 84·4)	82·5 (75·4 to 91)	118 (108 to 129)	98·3 (90·6 to 107)	131 (121 to 142)	74·2 (68·3 to 80·9)	61·2 (56·1 to 67·1)	40·7 (36·9 to 45)	26·4 (23·8 to 29)	9·2 (7·8 to 10·5)	4 (3·4 to 4·7)	2·1 (1·8 to 2·4)	0·9 (0·8 to 1·1)	0·3 (0·3 to 0·4)	0·1 (0·1 to 0·1)	767
HIV/AIDS - Drug-susceptible Tuberculosis	0 (0 to 0)	0 (0 to 0)	0·1 (0 to 0·1)	0 (0 to 0·1)	0·1 (0 to 0·1)	0 (0 to 0·1)	0·1 (0·1 to 0·2)	0·4 (0·3 to 0·6)	1·4 (0·9 to 2)	1·6 (1·1 to 2·5)	2 (1·4 to 3)	1·8 (1·2 to 2·7)	2·2 (1·5 to 3·3)	1·3 (0·9 to 1·9)	1·2 (0·8 to 1·8)	0·8 (0·6 to 1·2)	0·5 (0·4 to 0·8)	0·2 (0·1 to 0·3)	0·1 (0·1 to 0·1)	0 (0 to 0·1)	0 (0 to 0)	0 (0 to 0)	0 (0 to 0)	14·1
HIV/AIDS - Multidrug-resistant Tuberculosis without extensive dru	0 (0 to 0)	0 (0 to 0)	0 (0 to 0)	0 (0 to 0)	0 (0 to 0)	0 (0 to 0)	0 (0 to 0)	0 (0 to 0)	0 (0 to 0·1)	0 (0 to 0·1)	0 (0 to 0·1)	0 (0 to 0·1)	0 (0 to 0·1)	0 (0 to 0·1)	0 (0 to 0)	0 (0 to 0)	0 (0 to 0)	0 (0 to 0)	0 (0 to 0)	0 (0 to 0)	0 (0 to 0)	0 (0 to 0)	0 (0 to 0)	0·1
HIV/AIDS - Extensively drug-resistant Tuberculosis	0 (0 to 0)	0 (0 to 0)	0 (0 to 0)	0 (0 to 0)	0 (0 to 0)	0 (0 to 0)	0 (0 to 0)	0 (0 to 0)	0 (0 to 0)	0 (0 to 0)	0 (0 to 0)	0 (0 to 0)	0 (0 to 0)	0 (0 to 0)	0 (0 to 0)	0 (0 to 0)	0 (0 to 0)	0 (0 to 0)	0 (0 to 0)	0 (0 to 0)	0 (0 to 0)	0 (0 to 0)	0 (0 to 0)	0
HIV/AIDS resulting in other diseases	0 (0 to 0)	0 (0 to 0)	4·1 (3·8 to 4·5)	2·8 (2·5 to 3)	2·4 (2·2 to 2·6)	3·2 (3 to 3·5)	7 (6·4 to 7·6)	20·9 (19 to 23·1)	75·3 (68·4 to 83·2)	80·8 (73·7 to 89·2)	116 (106 to 127)	96·4 (89 to 105)	129 (119 to 140)	72·9 (67 to 79·5)	60 (54·9 to 66)	39·8 (36 to 44·2)	25·8 (23·3 to 28·5)	9 (7·6 to 10·3)	3·9 (3·3 to 4·6)	2 (1·8 to 2·3)	0·9 (0·7 to 1·1)	0·3 (0·3 to 0·4)	0·1 (0·1 to 0·1)	752
Sexually transmitted infections excluding HIV	0·6 (0·3 to 0·9)	0·1 (0 to 0·2)	0·1 (0 to 0·3)	0 (0 to 0)	0 (0 to 0)	0·2 (0·1 to 0·4)	1·2 (0·5 to 2·4)	2 (1 to 3·5)	2·4 (1·2 to 4)	2·7 (1·4 to 4·5)	2·5 (1·3 to 4·5)	2·2 (1·1 to 3·7)	2·1 (1·1 to 3·6)	1·6 (0·8 to 3)	1·2 (0·7 to 2·5)	0·9 (0·5 to 1·9)	0·8 (0·4 to 1·5)	0·5 (0·3 to 1·1)	0·3 (0·2 to 0·6)	0·2 (0·1 to 0·4)	0·1 (0·1 to 0·2)	0 (0 to 0·1)	0 (0 to 0)	21·7
Syphilis	0·6 (0·3 to 0·9)	0·1 (0 to 0·2)	0·1 (0 to 0·3)	0 (0 to 0)	0 (0 to 0)	0 (0 to 0)	0·1 (0 to 0·1)	0·1 (0 to 0·2)	0·1 (0·1 to 0·2)	0·2 (0·1 to 0·3)	0·2 (0·1 to 0·3)	0·1 (0·1 to 0·2)	0·2 (0·1 to 0·3)	0·2 (0·1 to 0·3)	0·2 (0·1 to 0·4)	0·2 (0·1 to 0·4)	0·2 (0·1 to 0·4)	0·2 (0·1 to 0·3)	0·1 (0·1 to 0·1)	0·1 (0 to 0·1)	0 (0 to 0)	0 (0 to 0)	0 (0 to 0)	3
Chlamydial infection	0 (0 to 0)	0 (0 to 0)	0 (0 to 0)	0 (0 to 0)	0 (0 to 0)	0 (0 to 0)	0·2 (0 to 0·4)	0·5 (0·2 to 1·1)	0·9 (0·3 to 1·7)	0·8 (0·3 to 1·6)	0·8 (0·3 to 1·6)	0·7 (0·2 to 1·4)	0·5 (0·2 to 1·1)	0·2 (0 to 0·6)	0·1 (0 to 0·1)	0 (0 to 0)	0 (0 to 0)	0 (0 to 0)	0 (0 to 0)	0 (0 to 0)	0 (0 to 0)	0 (0 to 0)	0 (0 to 0)	4·8
Gonococcal infection	0 (0 to 0)	0 (0 to 0)	0 (0 to 0)	0 (0 to 0)	0 (0 to 0)	0·1 (0 to 0·1)	0·4 (0·1 to 0·9)	0·5 (0·1 to 1·1)	0·2 (0 to 0·6)	0·2 (0 to 0·4)	0·1 (0·1 to 0·2)	0·1 (0·1 to 0·2)	0·1 (0·1 to 0·2)	0·1 (0 to 0·2)	0·1 (0 to 0·1)	0 (0 to 0)	0 (0 to 0·1)	0 (0 to 0)	0 (0 to 0)	0 (0 to 0)	0 (0 to 0)	0 (0 to 0)	0 (0 to 0)	1·9
Trichomoniasis	0 (0 to 0)	0 (0 to 0)	0 (0 to 0)	0 (0 to 0)	0 (0 to 0)	0 (0 to 0)	0 (0 to 0)	0 (0 to 0)	0 (0 to 0·1)	0·1 (0 to 0·1)	0·1 (0 to 0·2)	0·1 (0 to 0·2)	0·1 (0 to 0·2)	0·1 (0 to 0·1)	0 (0 to 0·1)	0 (0 to 0·1)	0 (0 to 0)	0 (0 to 0)	0 (0 to 0)	0 (0 to 0)	0 (0 to 0)	0 (0 to 0)	0 (0 to 0)	0·5
Genital herpes	0 (0 to 0)	0 (0 to 0)	0 (0 to 0)	0 (0 to 0)	0 (0 to 0)	0 (0 to 0·1)	0·1 (0 to 0·3)	0·2 (0·1 to 0·6)	0·4 (0·1 to 1)	0·5 (0·2 to 1·3)	0·7 (0·2 to 1·6)	0·7 (0·2 to 1·7)	0·8 (0·2 to 1·9)	0·7 (0·2 to 1·9)	0·7 (0·2 to 1·8)	0·6 (0·2 to 1·6)	0·5 (0·1 to 1·1)	0·3 (0·1 to 0·8)	0·2 (0·1 to 0·5)	0·1 (0 to 0·3)	0·1 (0 to 0·2)	0 (0 to 0·1)	0 (0 to 0)	6·7
Other sexually transmitted infections	0 (0 to 0)	0 (0 to 0)	0 (0 to 0)	0 (0 to 0)	0 (0 to 0)	0·1 (0 to 0·2)	0·5 (0·2 to 1·1)	0·6 (0·3 to 1·2)	0·7 (0·3 to 1·2)	0·9 (0·4 to 1·6)	0·7 (0·3 to 1·3)	0·5 (0·2 to 0·9)	0·4 (0·2 to 0·8)	0·2 (0·1 to 0·4)	0·1 (0·1 to 0·2)	0 (0 to 0·1)	0 (0 to 0·1)	0 (0 to 0)	0 (0 to 0·1)	0 (0 to 0·1)	0 (0 to 0·1)	0 (0 to 0)	0 (0 to 0)	4·9
Respiratory infections and tuberculosis	25·1 (14·5 to 40·9)	13·5 (7·9 to 21·6)	48·3 (28·8 to 73·4)	26·4 (18·3 to 36·7)	19·2 (13·1 to 27·8)	18 (12·4 to 26·5)	23·5 (16·2 to 32·5)	24·1 (17 to 32·9)	31·4 (22·4 to 41·5)	38·6 (28·7 to 51·2)	37·4 (27·4 to 49·4)	41·7 (30·1 to 56)	53·6 (38·3 to 72·7)	61·9 (42·5 to 86·2)	89·3 (61·6 to 124)	118 (79·3 to 164)	127 (86·7 to 175)	157 (108 to 225)	155 (109 to 212)	169 (122 to 225)	130 (97·4 to 167)	74·7 (55 to 96·2)	14·8 (10·9 to 19·2)	1,500
Tuberculosis	0 (0 to 0)	0 (0 to 0)	0·2 (0·1 to 0·3)	0·3 (0·2 to 0·4)	0·4 (0·1 to 0·9)	0·3 (0·2 to 0·4)	0·7 (0·4 to 1)	1·6 (0·9 to 2·5)	2·6 (1·7 to 3·8)	3 (2 to 4·2)	2·7 (1·8 to 4)	2·8 (1·8 to 4)	3·3 (2·2 to 4·8)	3·4 (2·2 to 4·8)	3·4 (2·3 to 4·9)	3·6 (2·5 to 5)	3 (2 to 4·5)	2·7 (2 to 3·7)	1·9 (1·4 to 2·7)	1·6 (1·1 to 2·1)	0·9 (0·7 to 1·3)	0·4 (0·3 to 0·5)	0·1 (0·1 to 0·1)	38·7
Latent tuberculosis infection	0 (0 to 0)	0 (0 to 0)	0 (0 to 0)	0 (0 to 0)	0 (0 to 0)	0 (0 to 0)	0 (0 to 0)	0 (0 to 0)	0 (0 to 0)	0 (0 to 0)	0 (0 to 0)	0 (0 to 0)	0 (0 to 0)	0 (0 to 0)	0 (0 to 0)	0 (0 to 0)	0 (0 to 0)	0 (0 to 0)	0 (0 to 0)	0 (0 to 0)	0 (0 to 0)	0 (0 to 0)	0 (0 to 0)	0
Drug-susceptible tuberculosis	0 (0 to 0)	0 (0 to 0)	0·2 (0·1 to 0·3)	0·3 (0·2 to 0·4)	0·4 (0·1 to 0·9)	0·3 (0·2 to 0·4)	0·7 (0·4 to 1)	1·6 (0·9 to 2·5)	2·6 (1·6 to 3·7)	2·9 (1·9 to 4·2)	2·7 (1·8 to 4)	2·8 (1·8 to 3·9)	3·3 (2·2 to 4·8)	3·3 (2·1 to 4·8)	3·4 (2·3 to 4·8)	3·5 (2·5 to 4·8)	3 (2 to 4·4)	2·7 (1·9 to 3·7)	1·9 (1·4 to 2·7)	1·5 (1·1 to 2)	0·9 (0·6 to 1·2)	0·4 (0·3 to 0·5)	0·1 (0 to 0·1)	38·4
Multidrug-resistant tuberculosis without extensive drug resistanc	0 (0 to 0)	0 (0 to 0)	0 (0 to 0)	0 (0 to 0)	0 (0 to 0)	0 (0 to 0)	0 (0 to 0)	0 (0 to 0)	0 (0 to 0·1)	0 (0 to 0·1)	0 (0 to 0·1)	0 (0 to 0·1)	0 (0 to 0·1)	0 (0 to 0·1)	0 (0 to 0·1)	0 (0 to 0·1)	0 (0 to 0·1)	0 (0 to 0·1)	0 (0 to 0·1)	0 (0 to 0·1)	0 (0 to 0)	0 (0 to 0)	0 (0 to 0)	0·3
Extensively drug-resistant tuberculosis	0 (0 to 0)	0 (0 to 0)	0 (0 to 0)	0 (0 to 0)	0 (0 to 0)	0 (0 to 0)	0 (0 to 0)	0 (0 to 0)	0 (0 to 0)	0 (0 to 0)	0 (0 to 0)	0 (0 to 0)	0 (0 to 0)	0 (0 to 0)	0 (0 to 0)	0 (0 to 0)	0 (0 to 0)	0 (0 to 0)	0 (0 to 0)	0 (0 to 0)	0 (0 to 0)	0 (0 to 0)	0 (0 to 0)	0
Lower respiratory infections	25·1 (14·5 to 40·9)	13·4 (7·8 to 21·5)	46·1 (26·7 to 71·2)	15·9 (10 to 24·9)	5·7 (4·1 to 8)	5·7 (4 to 7·7)	10·8 (7 to 15·9)	12 (7·6 to 18·1)	18·6 (12·2 to 27·9)	26·5 (17·5 to 38·6)	25·1 (17 to 36·3)	29·7 (19·2 to 43·2)	40·8 (27·1 to 59·9)	49·7 (31·7 to 72·7)	77·6 (49·8 to 112)	107 (70·2 to 151)	119 (78·9 to 167)	151 (102 to 219)	151 (105 to 207)	166 (119 to 222)	128 (95·9 to 165)	74·1 (54·4 to 95·7)	14·7 (10·8 to 19·1)	1,310
Upper respiratory infections	0 (0 to 0)	0·1 (0·1 to 0·2)	1·7 (1 to 2·8)	7·2 (4·1 to 11·7)	8·7 (4·7 to 14·7)	8·7 (4·7 to 15·1)	8·8 (4·5 to 15·6)	7·9 (4·3 to 13·6)	7·9 (4·4 to 13·5)	7·3 (3·9 to 12·3)	7·6 (3·8 to 12·9)	7·1 (3·8 to 12·7)	7·1 (3·7 to 12·5)	6·6 (3·6 to 11·3)	6·1 (3·4 to 10·3)	5·4 (2·9 to 8·9)	4·1 (2·2 to 7)	2·9 (1·6 to 5)	1·7 (0·9 to 2·8)	1 (0·5 to 1·8)	0·5 (0·3 to 0·9)	0·2 (0·1 to 0·4)	0 (0 to 0·1)	109
Otitis media	0 (0 to 0)	0 (0 to 0)	0·3 (0·2 to 0·6)	3 (1·6 to 5·2)	4·4 (2·4 to 7·3)	3·4 (1·8 to 5·9)	3·2 (1·7 to 5·7)	2·6 (1·1 to 5·1)	2·3 (1 to 4·3)	1·8 (0·7 to 3·6)	2 (0·9 to 3·8)	2·1 (1 to 3·9)	2·3 (1·1 to 4·2)	2·4 (1 to 4·3)	2·2 (1 to 3·8)	1·8 (0·8 to 3·4)	1·1 (0·5 to 2)	0·7 (0·3 to 1·3)	0·4 (0·2 to 0·8)	0·3 (0·1 to 0·5)	0·2 (0·1 to 0·3)	0·1 (0 to 0·1)	0 (0 to 0)	36·5
Enteric infections	0·9 (0·5 to 1·6)	2 (1·2 to 3·3)	16·5 (10·6 to 25·1)	18·3 (12·3 to 27)	17·2 (10·3 to 27·8)	17·6 (10·1 to 28·9)	13·8 (8·7 to 20·5)	10·8 (6·5 to 16·3)	12·8 (8·4 to 18·7)	13·8 (9·3 to 19·5)	15·5 (10·7 to 21·8)	16·8 (11·8 to 23·1)	19·5 (13·7 to 27·4)	21 (14·3 to 29·6)	22·8 (15·9 to 32)	26·1 (18·7 to 36·3)	27·5 (19·5 to 37·4)	27·8 (19·9 to 37·6)	22·1 (16·3 to 30)	17·1 (12·4 to 23)	10·2 (7·3 to 13·4)	4·5 (3·3 to 5·8)	0·8 (0·6 to 1)	355
Diarrheal diseases	0·9 (0·5 to 1·6)	2 (1·2 to 3·3)	16·4 (10·5 to 25·1)	18·1 (12·2 to 26·8)	17 (10·3 to 27·7)	17·5 (10 to 28·7)	13·7 (8·6 to 20·4)	10·7 (6·5 to 16·1)	12·7 (8·3 to 18·6)	13·6 (9·2 to 19·3)	15·4 (10·6 to 21·8)	16·7 (11·7 to 23)	19·4 (13·6 to 27·3)	20·9 (14·2 to 29·5)	22·7 (15·8 to 31·9)	25·9 (18·6 to 36·1)	27·4 (19·4 to 37·3)	27·7 (19·9 to 37·5)	22 (16·2 to 30)	17·1 (12·3 to 22·9)	10·2 (7·3 to 13·4)	4·5 (3·3 to 5·8)	0·8 (0·6 to 1)	353
Typhoid and paratyphoid	0 (0 to 0)	0 (0 to 0)	0 (0 to 0)	0 (0 to 0)	0 (0 to 0)	0 (0 to 0·1)	0 (0 to 0·1)	0 (0 to 0·1)	0 (0 to 0·1)	0·1 (0 to 0·2)	0 (0 to 0·1)	0 (0 to 0·1)	0 (0 to 0·1)	0 (0 to 0·1)	0·1 (0 to 0·1)	0·1 (0 to 0·1)	0 (0 to 0·1)	0 (0 to 0·1)	0 (0 to 0·1)	0 (0 to 0)	0 (0 to 0)	0 (0 to 0)	0 (0 to 0)	0·7
Typhoid fever	0 (0 to 0)	0 (0 to 0)	0 (0 to 0)	0 (0 to 0)	0 (0 to 0)	0 (0 to 0·1)	0 (0 to 0·1)	0 (0 to 0·1)	0 (0 to 0·1)	0·1 (0 to 0·2)	0 (0 to 0·1)	0 (0 to 0·1)	0 (0 to 0·1)	0 (0 to 0·1)	0 (0 to 0·1)	0 (0 to 0·1)	0 (0 to 0·1)	0 (0 to 0·1)	0 (0 to 0·1)	0 (0 to 0)	0 (0 to 0)	0 (0 to 0)	0 (0 to 0)	0·6
Paratyphoid fever	0 (0 to 0)	0 (0 to 0)	0 (0 to 0)	0 (0 to 0)	0 (0 to 0)	0 (0 to 0)	0 (0 to 0)	0 (0 to 0)	0 (0 to 0)	0 (0 to 0)	0 (0 to 0)	0 (0 to 0)	0 (0 to 0)	0 (0 to 0)	0 (0 to 0·1)	0 (0 to 0·1)	0 (0 to 0)	0 (0 to 0)	0 (0 to 0)	0 (0 to 0)	0 (0 to 0)	0 (0 to 0)	0 (0 to 0)	0·1
Invasive Non-typhoidal Salmonella (iNTS)	0 (0 to 0)	0 (0 to 0)	0 (0 to 0·1)	0 (0 to 0·1)	0·1 (0 to 0·2)	0·1 (0 to 0·2)	0 (0 to 0)	0 (0 to 0)	0 (0 to 0·1)	0 (0 to 0·1)	0 (0 to 0·1)	0 (0 to 0)	0 (0 to 0·1)	0 (0 to 0·1)	0 (0 to 0·1)	0·1 (0 to 0·3)	0·1 (0 to 0·1)	0 (0 to 0·1)	0·1 (0 to 0·1)	0 (0 to 0)	0 (0 to 0)	0 (0 to 0)	0 (0 to 0)	0·9
Other intestinal infectious diseases	0 (0 to 0)	0 (0 to 0)	0 (0 to 0·1)	0·1 (0 to 0·1)	0 (0 to 0·1)	0 (0 to 0·1)	0 (0 to 0·1)	0 (0 to 0·1)	0 (0 to 0)	0 (0 to 0·1)	0 (0 to 0·1)	0 (0 to 0·1)	0 (0 to 0)	0 (0 to 0)	0 (0 to 0)	0 (0 to 0)	0 (0 to 0·1)	0 (0 to 0)	0 (0 to 0)	0 (0 to 0)	0 (0 to 0)	0 (0 to 0)	0 (0 to 0)	0·5
Neglected tropical diseases and malaria	0 (0 to 0·1)	0·2 (0·1 to 0·4)	2·8 (0·8 to 6·7)	3·2 (1·4 to 6·5)	7·8 (3·4 to 14·8)	6·5 (3·1 to 11·5)	5·2 (2·2 to 11·4)	4·5 (2·1 to 8·3)	5·3 (2·5 to 9·8)	5·6 (2·9 to 10)	5·7 (2·8 to 9·9)	6·4 (3 to 11·1)	7·8 (4 to 14·3)	8·7 (4 to 16·3)	10·7 (5·1 to 19·4)	13·5 (6·5 to 25·3)	12·6 (5·8 to 23·6)	11·6 (5·5 to 21)	8·6 (3·9 to 15·8)	6 (2·7 to 11·2)	3·5 (1·7 to 6·5)	1·6 (0·8 to 2·9)	0·3 (0·1 to 0·5)	138
Malaria	0 (0 to 0)	0 (0 to 0)	0 (0 to 0)	0 (0 to 0)	0 (0 to 0)	0 (0 to 0)	0 (0 to 0)	0 (0 to 0)	0 (0 to 0)	0 (0 to 0)	0 (0 to 0)	0 (0 to 0)	0 (0 to 0)	0 (0 to 0)	0 (0 to 0)	0 (0 to 0)	0 (0 to 0)	0 (0 to 0)	0 (0 to 0)	0 (0 to 0)	0 (0 to 0)	0 (0 to 0)	0 (0 to 0)	0
Chagas disease	0 (0 to 0)	0 (0 to 0)	0 (0 to 0)	0 (0 to 0)	0 (0 to 0)	0 (0 to 0)	0 (0 to 0)	0 (0 to 0)	0 (0 to 0)	0 (0 to 0)	0 (0 to 0)	0 (0 to 0)	0 (0 to 0)	0 (0 to 0)	0 (0 to 0)	0 (0 to 0)	0 (0 to 0)	0 (0 to 0)	0 (0 to 0)	0 (0 to 0)	0 (0 to 0)	0 (0 to 0)	0 (0 to 0)	0
Leishmaniasis	0 (0 to 0)	0 (0 to 0)	0 (0 to 0)	0 (0 to 0)	0 (0 to 0)	0 (0 to 0)	0 (0 to 0)	0 (0 to 0)	0 (0 to 0)	0 (0 to 0)	0 (0 to 0)	0 (0 to 0)	0 (0 to 0)	0 (0 to 0)	0 (0 to 0)	0 (0 to 0)	0 (0 to 0)	0 (0 to 0)	0 (0 to 0)	0 (0 to 0)	0 (0 to 0)	0 (0 to 0)	0 (0 to 0)	0
Visceral leishmaniasis	0 (0 to 0)	0 (0 to 0)	0 (0 to 0)	0 (0 to 0)	0 (0 to 0)	0 (0 to 0)	0 (0 to 0)	0 (0 to 0)	0 (0 to 0)	0 (0 to 0)	0 (0 to 0)	0 (0 to 0)	0 (0 to 0)	0 (0 to 0)	0 (0 to 0)	0 (0 to 0)	0 (0 to 0)	0 (0 to 0)	0 (0 to 0)	0 (0 to 0)	0 (0 to 0)	0 (0 to 0)	0 (0 to 0)	0
Cutaneous and mucocutaneous leishmaniasis	0 (0 to 0)	0 (0 to 0)	0 (0 to 0)	0 (0 to 0)	0 (0 to 0)	0 (0 to 0)	0 (0 to 0)	0 (0 to 0)	0 (0 to 0)	0 (0 to 0)	0 (0 to 0)	0 (0 to 0)	0 (0 to 0)	0 (0 to 0)	0 (0 to 0)	0 (0 to 0)	0 (0 to 0)	0 (0 to 0)	0 (0 to 0)	0 (0 to 0)	0 (0 to 0)	0 (0 to 0)	0 (0 to 0)	0
African trypanosomiasis	0 (0 to 0)	0 (0 to 0)	0 (0 to 0)	0 (0 to 0)	0 (0 to 0)	0 (0 to 0)	0 (0 to 0)	0 (0 to 0)	0 (0 to 0)	0 (0 to 0)	0 (0 to 0)	0 (0 to 0)	0 (0 to 0)	0 (0 to 0)	0 (0 to 0)	0 (0 to 0)	0 (0 to 0)	0 (0 to 0)	0 (0 to 0)	0 (0 to 0)	0 (0 to 0)	0 (0 to 0)	0 (0 to 0)	0
Schistosomiasis	0 (0 to 0)	0 (0 to 0)	0 (0 to 0)	0 (0 to 0)	0 (0 to 0)	0 (0 to 0)	0 (0 to 0)	0 (0 to 0)	0 (0 to 0)	0 (0 to 0)	0 (0 to 0)	0 (0 to 0)	0 (0 to 0)	0 (0 to 0)	0 (0 to 0)	0 (0 to 0)	0 (0 to 0)	0 (0 to 0)	0 (0 to 0)	0 (0 to 0)	0 (0 to 0)	0 (0 to 0)	0 (0 to 0)	0
Cysticercosis	0 (0 to 0)	0 (0 to 0)	0 (0 to 0)	0 (0 to 0)	0 (0 to 0)	0 (0 to 0·1)	0·3 (0·1 to 0·7)	0·9 (0·1 to 2·6)	1·7 (0·4 to 3·9)	2 (0·5 to 4·6)	2·6 (0·7 to 6·2)	3·2 (0·9 to 7)	4·3 (1·2 to 9·8)	5·5 (1·8 to 12·3)	7·4 (2·6 to 15·8)	9·2 (3·2 to 20·3)	9·5 (3·4 to 19·9)	8·6 (3·2 to 17·4)	6·4 (2·3 to 13·3)	4·7 (1·8 to 9·9)	2·6 (0·9 to 5·4)	1 (0·4 to 2·2)	0·2 (0 to 0·4)	70·1
Cystic echinococcosis	0 (0 to 0)	0 (0 to 0)	0 (0 to 0)	0 (0 to 0)	0 (0 to 0)	0 (0 to 0)	0 (0 to 0)	0 (0 to 0)	0 (0 to 0)	0 (0 to 0)	0 (0 to 0)	0 (0 to 0)	0 (0 to 0)	0 (0 to 0)	0 (0 to 0)	0 (0 to 0)	0 (0 to 0)	0 (0 to 0)	0 (0 to 0)	0 (0 to 0)	0 (0 to 0)	0 (0 to 0)	0 (0 to 0)	0
Lymphatic filariasis	0 (0 to 0)	0 (0 to 0)	0 (0 to 0)	0 (0 to 0)	0 (0 to 0)	0 (0 to 0)	0 (0 to 0)	0 (0 to 0)	0 (0 to 0)	0 (0 to 0)	0 (0 to 0)	0 (0 to 0)	0 (0 to 0)	0 (0 to 0)	0 (0 to 0)	0 (0 to 0)	0 (0 to 0)	0 (0 to 0)	0 (0 to 0)	0 (0 to 0)	0 (0 to 0)	0 (0 to 0)	0 (0 to 0)	0
Onchocerciasis	0 (0 to 0)	0 (0 to 0)	0 (0 to 0)	0 (0 to 0)	0 (0 to 0)	0 (0 to 0)	0 (0 to 0)	0 (0 to 0)	0 (0 to 0)	0 (0 to 0)	0 (0 to 0)	0 (0 to 0)	0 (0 to 0)	0 (0 to 0)	0 (0 to 0)	0 (0 to 0)	0 (0 to 0)	0 (0 to 0)	0 (0 to 0)	0 (0 to 0)	0 (0 to 0)	0 (0 to 0)	0 (0 to 0)	0
Trachoma	0 (0 to 0)	0 (0 to 0)	0 (0 to 0)	0 (0 to 0)	0 (0 to 0)	0 (0 to 0)	0 (0 to 0)	0 (0 to 0)	0 (0 to 0)	0 (0 to 0)	0 (0 to 0)	0 (0 to 0)	0 (0 to 0)	0 (0 to 0)	0 (0 to 0)	0 (0 to 0)	0 (0 to 0)	0 (0 to 0)	0 (0 to 0)	0 (0 to 0)	0 (0 to 0)	0 (0 to 0)	0 (0 to 0)	0
Dengue	0 (0 to 0)	0·1 (0 to 0·3)	2·2 (0·4 to 6)	1·5 (0·4 to 3·5)	3·2 (0·8 to 7·9)	3 (0·9 to 6·9)	4·3 (1·4 to 10·5)	2·9 (0·9 to 6·4)	3·1 (1 to 6·7)	3 (1·2 to 6·6)	2·6 (1 to 5·6)	2·6 (0·8 to 5·7)	2·7 (1·1 to 6)	2·6 (1 to 5·8)	2·4 (0·8 to 5·5)	3·6 (1 to 10)	2·7 (0·8 to 7·2)	2·4 (0·8 to 5·9)	1·7 (0·6 to 4·5)	1·1 (0·4 to 2·3)	0·7 (0·3 to 1·4)	0·4 (0·2 to 0·9)	0·1 (0 to 0·1)	48·8
Yellow fever	0 (0 to 0)	0 (0 to 0)	0 (0 to 0)	0 (0 to 0)	0 (0 to 0)	0 (0 to 0)	0 (0 to 0)	0 (0 to 0)	0 (0 to 0)	0 (0 to 0)	0 (0 to 0)	0 (0 to 0)	0 (0 to 0)	0 (0 to 0)	0 (0 to 0)	0 (0 to 0)	0 (0 to 0)	0 (0 to 0)	0 (0 to 0)	0 (0 to 0)	0 (0 to 0)	0 (0 to 0)	0 (0 to 0)	0
Rabies	0 (0 to 0)	0 (0 to 0)	0 (0 to 0)	0 (0 to 0)	0 (0 to 0·1)	0 (0 to 0)	0 (0 to 0)	0 (0 to 0)	0 (0 to 0)	0 (0 to 0)	0 (0 to 0)	0 (0 to 0)	0 (0 to 0·1)	0 (0 to 0)	0 (0 to 0)	0 (0 to 0)	0 (0 to 0)	0 (0 to 0)	0 (0 to 0)	0 (0 to 0)	0 (0 to 0)	0 (0 to 0)	0 (0 to 0)	0·2
Intestinal nematode infections	0 (0 to 0)	0 (0 to 0)	0 (0 to 0)	0·1 (0 to 0·2)	0·2 (0 to 0·5)	0·2 (0 to 0·4)	0·1 (0 to 0·2)	0·1 (0 to 0·1)	0 (0 to 0·1)	0 (0 to 0·1)	0 (0 to 0·1)	0 (0 to 0·1)	0 (0 to 0·1)	0 (0 to 0·1)	0·1 (0 to 0·1)	0·1 (0 to 0·1)	0 (0 to 0·1)	0 (0 to 0·1)	0 (0 to 0·1)	0 (0 to 0)	0 (0 to 0)	0 (0 to 0)	0 (0 to 0)	1·1
Ascariasis	0 (0 to 0)	0 (0 to 0)	0 (0 to 0)	0 (0 to 0·1)	0 (0 to 0·1)	0 (0 to 0·1)	0 (0 to 0·1)	0 (0 to 0)	0 (0 to 0)	0 (0 to 0)	0 (0 to 0)	0 (0 to 0)	0 (0 to 0)	0 (0 to 0)	0 (0 to 0)	0 (0 to 0·1)	0 (0 to 0)	0 (0 to 0)	0 (0 to 0)	0 (0 to 0)	0 (0 to 0)	0 (0 to 0)	0 (0 to 0)	0·2
Trichuriasis	0 (0 to 0)	0 (0 to 0)	0 (0 to 0)	0 (0 to 0)	0 (0 to 0)	0 (0 to 0)	0 (0 to 0)	0 (0 to 0)	0 (0 to 0)	0 (0 to 0)	0 (0 to 0)	0 (0 to 0)	0 (0 to 0)	0 (0 to 0)	0 (0 to 0)	0 (0 to 0)	0 (0 to 0)	0 (0 to 0)	0 (0 to 0)	0 (0 to 0)	0 (0 to 0)	0 (0 to 0)	0 (0 to 0)	0
Hookworm disease	0 (0 to 0)	0 (0 to 0)	0 (0 to 0)	0 (0 to 0·1)	0·2 (0 to 0·5)	0·1 (0 to 0·4)	0·1 (0 to 0·2)	0·1 (0 to 0·1)	0 (0 to 0·1)	0 (0 to 0·1)	0 (0 to 0·1)	0 (0 to 0·1)	0 (0 to 0·1)	0 (0 to 0·1)	0·1 (0 to 0·1)	0 (0 to 0·1)	0 (0 to 0·1)	0 (0 to 0·1)	0 (0 to 0·1)	0 (0 to 0)	0 (0 to 0)	0 (0 to 0)	0 (0 to 0)	1
Food-borne trematodiases	0 (0 to 0)	0 (0 to 0)	0 (0 to 0)	0 (0 to 0)	0 (0 to 0)	0 (0 to 0)	0 (0 to 0)	0 (0 to 0)	0 (0 to 0)	0 (0 to 0)	0 (0 to 0)	0 (0 to 0)	0 (0 to 0)	0 (0 to 0)	0 (0 to 0)	0 (0 to 0)	0 (0 to 0)	0 (0 to 0)	0 (0 to 0)	0 (0 to 0)	0 (0 to 0)	0 (0 to 0)	0 (0 to 0)	0
Leprosy	0 (0 to 0)	0 (0 to 0)	0 (0 to 0)	0 (0 to 0)	0 (0 to 0)	0 (0 to 0)	0 (0 to 0)	0 (0 to 0)	0 (0 to 0)	0 (0 to 0)	0 (0 to 0)	0 (0 to 0)	0 (0 to 0)	0 (0 to 0)	0 (0 to 0)	0 (0 to 0)	0 (0 to 0)	0 (0 to 0)	0 (0 to 0)	0 (0 to 0)	0 (0 to 0)	0 (0 to 0)	0 (0 to 0)	0·2
Ebola	0 (0 to 0)	0 (0 to 0)	0 (0 to 0)	0 (0 to 0)	0 (0 to 0)	0 (0 to 0)	0 (0 to 0)	0 (0 to 0)	0 (0 to 0)	0 (0 to 0)	0 (0 to 0)	0 (0 to 0)	0 (0 to 0)	0 (0 to 0)	0 (0 to 0)	0 (0 to 0)	0 (0 to 0)	0 (0 to 0)	0 (0 to 0)	0 (0 to 0)	0 (0 to 0)	0 (0 to 0)	0 (0 to 0)	0
Zika virus	0 (0 to 0)	0 (0 to 0)	0·2 (0 to 0·5)	0·1 (0 to 0·5)	0·1 (0 to 0·6)	0·1 (0 to 0·7)	0·2 (0 to 0·9)	0·2 (0 to 1)	0·2 (0 to 0·9)	0·2 (0 to 1)	0·2 (0 to 0·9)	0·1 (0 to 0·7)	0·1 (0 to 0·5)	0·1 (0 to 0·4)	0·1 (0 to 0·3)	0 (0 to 0·2)	0 (0 to 0·1)	0 (0 to 0)	0 (0 to 0)	0 (0 to 0)	0 (0 to 0)	0 (0 to 0)	0 (0 to 0)	1·9
Guinea worm disease	0 (0 to 0)	0 (0 to 0)	0 (0 to 0)	0 (0 to 0)	0 (0 to 0)	0 (0 to 0)	0 (0 to 0)	0 (0 to 0)	0 (0 to 0)	0 (0 to 0)	0 (0 to 0)	0 (0 to 0)	0 (0 to 0)	0 (0 to 0)	0 (0 to 0)	0 (0 to 0)	0 (0 to 0)	0 (0 to 0)	0 (0 to 0)	0 (0 to 0)	0 (0 to 0)	0 (0 to 0)	0 (0 to 0)	0
Other neglected tropical diseases	0 (0 to 0)	0·1 (0 to 0·2)	0·4 (0·1 to 1)	1·6 (0·4 to 4)	4·3 (1·3 to 10·4)	3·2 (0·9 to 7·5)	0·3 (0·1 to 0·8)	0·4 (0·1 to 0·9)	0·3 (0·1 to 0·7)	0·3 (0·1 to 0·8)	0·3 (0·1 to 0·7)	0·4 (0·2 to 1)	0·6 (0·2 to 1·3)	0·4 (0·1 to 1)	0·7 (0·2 to 1·6)	0·5 (0·2 to 1·1)	0·3 (0·1 to 0·7)	0·5 (0·2 to 1·2)	0·4 (0·2 to 0·8)	0·2 (0·1 to 0·3)	0·2 (0·1 to 0·4)	0·1 (0 to 0·2)	0 (0 to 0)	15·6
Other infectious diseases	3 (1·9 to 4·7)	4·3 (2·7 to 6·6)	15·4 (9·2 to 23·4)	10·6 (7 to 15·8)	10·8 (7·5 to 16)	10·8 (8 to 14·8)	14·5 (10·7 to 19·7)	12·9 (9·5 to 17·4)	15·9 (11·3 to 21·9)	19·3 (13·6 to 27)	18·6 (12·9 to 26)	19·9 (13·4 to 28·4)	25·6 (16·3 to 36·5)	23·4 (15 to 34·1)	26·1 (17·1 to 37·6)	27·1 (17·9 to 37·2)	20·1 (12·9 to 27·9)	17·8 (11·8 to 24·5)	10·4 (6·8 to 14·5)	6·9 (4·5 to 9·9)	5·8 (3·3 to 8·4)	1·8 (1 to 2·7)	0·3 (0·2 to 0·5)	321
Meningitis	1·7 (0·9 to 2·7)	2·7 (1·6 to 4·4)	7·5 (3·9 to 12·6)	3·6 (2·2 to 5·7)	3·1 (2·1 to 4·3)	3 (1·9 to 4·3)	4·5 (2·9 to 6·8)	3 (2 to 4·5)	3·5 (2·4 to 5·1)	4·3 (2·8 to 6·3)	3·8 (2·6 to 5·5)	3·8 (2·5 to 5·5)	4·7 (3·1 to 6·9)	4·6 (3 to 6·6)	5·2 (3·4 to 7·3)	5·8 (3·8 to 8·4)	4·1 (2·7 to 6)	3·7 (2·5 to 5·5)	2·5 (1·6 to 3·6)	1·7 (1·1 to 2·5)	1·1 (0·7 to 1·7)	0·3 (0·2 to 0·4)	0·1 (0 to 0·1)	78·3
Encephalitis	0·4 (0·2 to 0·6)	0·6 (0·3 to 1·2)	3·7 (1·8 to 7·2)	3·3 (1·7 to 6·6)	2·7 (1·6 to 5·8)	2·8 (1·7 to 4·5)	4 (2·5 to 6·4)	3·5 (2·1 to 5·6)	3·1 (2·1 to 4·9)	3·5 (2·3 to 5·1)	2·9 (1·8 to 4·4)	3·1 (2 to 4·8)	3·1 (1·9 to 4·7)	2·4 (1·6 to 3·5)	2·8 (1·9 to 4·2)	2·8 (1·8 to 4·2)	2 (1·3 to 3·1)	1·8 (1·1 to 2·8)	0·9 (0·5 to 1·4)	0·6 (0·4 to 1)	0·3 (0·2 to 0·5)	0·1 (0·1 to 0·1)	0 (0 to 0)	50·3
Diphtheria	0 (0 to 0)	0 (0 to 0)	0 (0 to 0)	0 (0 to 0)	0 (0 to 0)	0 (0 to 0)	0 (0 to 0)	0 (0 to 0)	0 (0 to 0)	0 (0 to 0)	0 (0 to 0)	0 (0 to 0)	0 (0 to 0)	0 (0 to 0)	0 (0 to 0)	0 (0 to 0)	0 (0 to 0)	0 (0 to 0)	0 (0 to 0)	0 (0 to 0)	0 (0 to 0)	0 (0 to 0)	0 (0 to 0)	0
Tetanus	0·1 (0 to 0·1)	0·1 (0 to 0·1)	0·1 (0 to 0·2)	0 (0 to 0·2)	0·1 (0 to 0·5)	0·2 (0 to 0·5)	0·2 (0·1 to 0·7)	0·2 (0 to 0·7)	0·3 (0·1 to 0·8)	0·4 (0·1 to 1·2)	0·4 (0·1 to 1·1)	0·5 (0·1 to 1·6)	0·8 (0·2 to 2·6)	1 (0·2 to 3·2)	1·4 (0·3 to 4·7)	1·2 (0·2 to 3·8)	1 (0·2 to 3·3)	1 (0·2 to 3·2)	0·6 (0·1 to 1·9)	0·3 (0·1 to 1)	0·1 (0 to 0·4)	0·1 (0 to 0·2)	0 (0 to 0)	10
Measles	0 (0 to 0)	0 (0 to 0)	0 (0 to 0)	0 (0 to 0)	0 (0 to 0)	0 (0 to 0)	0 (0 to 0)	0 (0 to 0)	0 (0 to 0)	0 (0 to 0)	0 (0 to 0)	0 (0 to 0)	0 (0 to 0)	0 (0 to 0)	0 (0 to 0)	0 (0 to 0)	0 (0 to 0)	0 (0 to 0)	0 (0 to 0)	0 (0 to 0)	0 (0 to 0)	0 (0 to 0)	0 (0 to 0)	0
Varicella and herpes zoster	0·1 (0 to 0·3)	0·2 (0·1 to 0·4)	0·3 (0·1 to 0·7)	0·2 (0·1 to 0·3)	0·2 (0·1 to 0·4)	0·3 (0·1 to 0·6)	0·3 (0·1 to 0·5)	0·3 (0·1 to 0·5)	0·3 (0·2 to 0·6)	0·4 (0·2 to 0·7)	0·6 (0·3 to 1)	0·5 (0·2 to 0·8)	0·7 (0·4 to 1·2)	0·7 (0·3 to 1·1)	0·7 (0·4 to 1·1)	0·8 (0·4 to 1·4)	0·6 (0·3 to 1)	0·7 (0·4 to 1·2)	0·4 (0·3 to 0·8)	0·3 (0·2 to 0·6)	0·2 (0·1 to 0·3)	0·1 (0·1 to 0·2)	0 (0 to 0·1)	8·7
Acute hepatitis	0 (0 to 0)	0 (0 to 0)	0·1 (0 to 0·1)	0·2 (0·1 to 0·3)	0·6 (0·3 to 1·1)	0·9 (0·5 to 1·4)	0·9 (0·6 to 1·5)	0·8 (0·5 to 1·2)	0·8 (0·5 to 1·2)	0·8 (0·5 to 1·1)	0·7 (0·5 to 1)	0·7 (0·4 to 1)	0·7 (0·4 to 1)	0·7 (0·4 to 0·9)	0·6 (0·4 to 0·8)	0·5 (0·3 to 0·6)	0·3 (0·2 to 0·5)	0·3 (0·2 to 0·4)	0·1 (0·1 to 0·2)	0·1 (0·1 to 0·1)	0·1 (0 to 0·1)	0 (0 to 0)	0 (0 to 0)	9·8
Acute hepatitis A	0 (0 to 0)	0 (0 to 0)	0 (0 to 0)	0·1 (0 to 0·3)	0·5 (0·3 to 0·9)	0·7 (0·4 to 1·1)	0·7 (0·4 to 1·2)	0·6 (0·3 to 1)	0·5 (0·2 to 0·7)	0·4 (0·2 to 0·6)	0·3 (0·2 to 0·6)	0·3 (0·1 to 0·5)	0·3 (0·1 to 0·5)	0·2 (0·1 to 0·4)	0·1 (0 to 0·3)	0·1 (0 to 0·3)	0·1 (0 to 0·2)	0 (0 to 0·1)	0 (0 to 0)	0 (0 to 0·1)	0 (0 to 0)	0 (0 to 0)	0 (0 to 0)	5·2
Acute hepatitis B	0 (0 to 0)	0 (0 to 0)	0·1 (0 to 0·1)	0 (0 to 0·1)	0 (0 to 0·1)	0·1 (0 to 0·2)	0·1 (0 to 0·2)	0·2 (0·1 to 0·3)	0·3 (0·2 to 0·4)	0·3 (0·2 to 0·5)	0·3 (0·2 to 0·5)	0·3 (0·2 to 0·5)	0·3 (0·2 to 0·5)	0·4 (0·2 to 0·6)	0·4 (0·3 to 0·5)	0·3 (0·2 to 0·4)	0·2 (0·1 to 0·3)	0·2 (0·1 to 0·3)	0·1 (0 to 0·1)	0·1 (0 to 0·1)	0 (0 to 0·1)	0 (0 to 0)	0 (0 to 0)	3·7
Acute hepatitis C	0 (0 to 0)	0 (0 to 0)	0 (0 to 0)	0 (0 to 0)	0 (0 to 0)	0 (0 to 0)	0 (0 to 0)	0 (0 to 0)	0 (0 to 0)	0 (0 to 0)	0 (0 to 0)	0 (0 to 0)	0 (0 to 0·1)	0 (0 to 0)	0 (0 to 0)	0 (0 to 0)	0 (0 to 0)	0 (0 to 0)	0 (0 to 0)	0 (0 to 0)	0 (0 to 0)	0 (0 to 0)	0 (0 to 0)	0·2
Acute hepatitis E	0 (0 to 0)	0 (0 to 0)	0 (0 to 0)	0 (0 to 0)	0 (0 to 0·1)	0·1 (0 to 0·2)	0·1 (0 to 0·2)	0·1 (0 to 0·2)	0·1 (0 to 0·1)	0·1 (0 to 0·1)	0 (0 to 0·1)	0 (0 to 0·1)	0 (0 to 0·1)	0 (0 to 0)	0 (0 to 0)	0·1 (0 to 0·1)	0 (0 to 0)	0 (0 to 0)	0 (0 to 0·1)	0 (0 to 0)	0 (0 to 0)	0 (0 to 0)	0 (0 to 0)	0·7
Other unspecified infectious diseases	0·8 (0·3 to 1·4)	0·7 (0·4 to 1·4)	3·3 (1·6 to 5·8)	2·8 (1·5 to 5)	4 (1·8 to 8·2)	3·6 (1·9 to 6·4)	4·5 (2·3 to 7·4)	5·1 (2·7 to 8·4)	7·8 (3·8 to 13)	10 (4·7 to 17·1)	10·2 (5·2 to 16·3)	11·4 (5·7 to 18·5)	15·6 (7·7 to 25·6)	14·1 (6·9 to 23·5)	15·6 (7·7 to 24·9)	16·1 (7·7 to 24·9)	12·1 (5·7 to 18·5)	10·4 (4·8 to 16·1)	5·8 (2·5 to 9·1)	3·9 (1·6 to 6·4)	3·9 (1·6 to 6·5)	1·2 (0·4 to 2·1)	0·2 (0·1 to 0·3)	163
Maternal and neonatal disorders	743 (505 to 1,070)	138 (91·7 to 199)	53·1 (34·5 to 79·9)	41·4 (30·7 to 54)	40·6 (29·4 to 53·7)	45·9 (33·6 to 61·7)	48 (34·2 to 63·4)	43·4 (30·9 to 58)	43·5 (31·9 to 57·3)	39·9 (29·1 to 53·6)	39·7 (29 to 52·9)	36·6 (25·9 to 49·7)	36·6 (26·2 to 49·6)	32·4 (23·4 to 43·6)	28·3 (20·5 to 37·6)	21·1 (15·1 to 28·6)	12·5 (8·8 to 17·4)	6 (4·1 to 8·7)	2·1 (1·3 to 3·1)	0·5 (0·3 to 0·8)	0·1 (0 to 0·1)	0 (0 to 0)	0 (0 to 0)	1,450
Maternal disorders	0 (0 to 0)	0 (0 to 0)	0 (0 to 0)	0 (0 to 0)	0 (0 to 0)	0 (0 to 0)	0 (0 to 0)	0 (0 to 0)	0 (0 to 0)	0 (0 to 0)	0 (0 to 0)	0 (0 to 0)	0 (0 to 0)	0 (0 to 0)	0 (0 to 0)	0 (0 to 0)	0 (0 to 0)	0 (0 to 0)	0 (0 to 0)	0 (0 to 0)	0 (0 to 0)	0 (0 to 0)	0 (0 to 0)	0
Maternal hemorrhage	0 (0 to 0)	0 (0 to 0)	0 (0 to 0)	0 (0 to 0)	0 (0 to 0)	0 (0 to 0)	0 (0 to 0)	0 (0 to 0)	0 (0 to 0)	0 (0 to 0)	0 (0 to 0)	0 (0 to 0)	0 (0 to 0)	0 (0 to 0)	0 (0 to 0)	0 (0 to 0)	0 (0 to 0)	0 (0 to 0)	0 (0 to 0)	0 (0 to 0)	0 (0 to 0)	0 (0 to 0)	0 (0 to 0)	0
Maternal sepsis and other maternal infections	0 (0 to 0)	0 (0 to 0)	0 (0 to 0)	0 (0 to 0)	0 (0 to 0)	0 (0 to 0)	0 (0 to 0)	0 (0 to 0)	0 (0 to 0)	0 (0 to 0)	0 (0 to 0)	0 (0 to 0)	0 (0 to 0)	0 (0 to 0)	0 (0 to 0)	0 (0 to 0)	0 (0 to 0)	0 (0 to 0)	0 (0 to 0)	0 (0 to 0)	0 (0 to 0)	0 (0 to 0)	0 (0 to 0)	0
Maternal hypertensive disorders	0 (0 to 0)	0 (0 to 0)	0 (0 to 0)	0 (0 to 0)	0 (0 to 0)	0 (0 to 0)	0 (0 to 0)	0 (0 to 0)	0 (0 to 0)	0 (0 to 0)	0 (0 to 0)	0 (0 to 0)	0 (0 to 0)	0 (0 to 0)	0 (0 to 0)	0 (0 to 0)	0 (0 to 0)	0 (0 to 0)	0 (0 to 0)	0 (0 to 0)	0 (0 to 0)	0 (0 to 0)	0 (0 to 0)	0
Maternal obstructed labor and uterine rupture	--	--	--	--	--	--	--	--	--	--	--	--	--	--	--	--	--	--	--	--	--	--	--	NA
Maternal abortion and miscarriage	--	--	--	--	--	--	--	--	--	--	--	--	--	--	--	--	--	--	--	--	--	--	--	NA
Ectopic pregnancy	--	--	--	--	--	--	--	--	--	--	--	--	--	--	--	--	--	--	--	--	--	--	--	NA
Indirect maternal deaths	--	--	--	--	--	--	--	--	--	--	--	--	--	--	--	--	--	--	--	--	--	--	--	NA
Late maternal deaths	--	--	--	--	--	--	--	--	--	--	--	--	--	--	--	--	--	--	--	--	--	--	--	NA
Maternal deaths aggravated by HIV/AIDS	--	--	--	--	--	--	--	--	--	--	--	--	--	--	--	--	--	--	--	--	--	--	--	NA
Neonatal disorders	743 (505 to 1,070)	138 (91·7 to 199)	53·1 (34·5 to 79·9)	41·4 (30·7 to 54)	40·6 (29·4 to 53·7)	45·9 (33·6 to 61·7)	48 (34·2 to 63·4)	43·4 (30·9 to 58)	43·5 (31·9 to 57·3)	39·9 (29·1 to 53·6)	39·7 (29 to 52·9)	36·6 (25·9 to 49·7)	36·6 (26·2 to 49·6)	32·4 (23·4 to 43·6)	28·3 (20·5 to 37·6)	21·1 (15·1 to 28·6)	12·5 (8·8 to 17·4)	6 (4·1 to 8·7)	2·1 (1·3 to 3·1)	0·5 (0·3 to 0·8)	0·1 (0 to 0·1)	0 (0 to 0)	0 (0 to 0)	1,450
Neonatal preterm birth	273 (175 to 409)	34·6 (20·3 to 53·3)	16·9 (10·9 to 26·2)	12·3 (8·6 to 16·6)	17 (11·7 to 23)	19 (13·2 to 25·9)	19·5 (13·8 to 26·3)	17·2 (11·8 to 23·6)	17·2 (11·7 to 23·5)	15·5 (10·9 to 21·4)	15·1 (10·6 to 20·6)	13·5 (9·2 to 18·8)	13·1 (9·2 to 18)	11 (7·6 to 15)	9 (6 to 12·4)	5·9 (3·9 to 8·2)	3·2 (2·5 to 4·1)	1·3 (1 to 1·7)	0·4 (0·3 to 0·6)	0·1 (0 to 0·1)	0 (0 to 0)	0 (0 to 0)	0 (0 to 0)	515
Neonatal encephalopathy due to birth asphyxia and trauma	238 (136 to 375)	27·2 (13·5 to 45·9)	11 (5·3 to 19·4)	4·4 (2·7 to 6·5)	4 (2·1 to 6·6)	4·6 (2·4 to 7·6)	4·8 (2·4 to 8)	4·4 (2·4 to 7·5)	4·4 (2·2 to 7·3)	4·1 (2 to 7)	4·2 (2·1 to 7·1)	3·9 (2 to 6·4)	4 (2 to 6·5)	3·6 (1·8 to 6)	3·2 (1·5 to 5·5)	2·6 (1·2 to 4·4)	1·4 (0·7 to 2·3)	0·5 (0·3 to 0·8)	0·1 (0·1 to 0·2)	0 (0 to 0)	0 (0 to 0)	0 (0 to 0)	0 (0 to 0)	330
Neonatal sepsis and other neonatal infections	113 (65 to 183)	53·2 (31 to 85·1)	15·4 (9·8 to 24·2)	12·2 (7·6 to 18)	17·4 (11 to 25·9)	19·8 (11·9 to 29·6)	21 (12·3 to 32·5)	19·4 (11·7 to 29·2)	19·5 (11·8 to 29·7)	18 (11 to 27·5)	18·1 (11·2 to 27·9)	17·2 (10·3 to 26·2)	17·4 (10·6 to 27·1)	15·9 (9·7 to 23·8)	14·2 (8·8 to 21·4)	11·1 (6·9 to 17·2)	7 (4 to 10·9)	3·8 (2·1 to 6·1)	1·4 (0·7 to 2·4)	0·4 (0·2 to 0·7)	0·1 (0 to 0·1)	0 (0 to 0)	0 (0 to 0)	416
Hemolytic disease and other neonatal jaundice	8 (3·6 to 14·4)	1·8 (1 to 3·3)	0·7 (0·3 to 1·4)	1·6 (0·8 to 2·6)	2·1 (0·9 to 3·6)	2·5 (1·2 to 4·2)	2·6 (1·2 to 4·5)	2·4 (1·1 to 4)	2·4 (1·2 to 4·2)	2·2 (1 to 3·9)	2·3 (1 to 3·9)	2·1 (0·9 to 3·6)	2·2 (1·1 to 3·6)	2 (1 to 3·2)	1·8 (1·4 to 2·4)	1·4 (1·1 to 1·8)	0·9 (0·6 to 1·1)	0·4 (0·3 to 0·6)	0·2 (0·1 to 0·2)	0 (0 to 0·1)	0 (0 to 0)	0 (0 to 0)	0 (0 to 0)	39·7
Other neonatal disorders	111 (67·3 to 174)	21 (11·1 to 35·6)	9·1 (6·1 to 13·5)	11 (8·2 to 14·4)	0 (0 to 0)	0 (0 to 0)	0 (0 to 0)	0 (0 to 0)	0 (0 to 0)	0 (0 to 0)	0 (0 to 0)	0 (0 to 0)	0 (0 to 0)	0 (0 to 0)	0 (0 to 0)	0 (0 to 0)	0 (0 to 0)	0 (0 to 0)	0 (0 to 0)	0 (0 to 0)	0 (0 to 0)	0 (0 to 0)	0 (0 to 0)	152
Nutritional deficiencies	0·1 (0 to 0·1)	1 (0·4 to 2·1)	13·4 (8·4 to 21·4)	18·3 (7 to 41·6)	45·6 (14·2 to 109)	35·6 (10·9 to 77)	8·8 (4·2 to 19·6)	7 (3·5 to 13·2)	8·5 (4·5 to 16·2)	8·6 (4·8 to 16·1)	9·9 (5·3 to 19·2)	10·2 (5·7 to 18·7)	12·6 (6·7 to 25)	13·8 (7·7 to 25·6)	19·7 (9·9 to 39)	19 (10·9 to 34·5)	16·2 (10·1 to 26·5)	16 (10·2 to 25·2)	12·3 (8·2 to 18·7)	10·5 (7 to 16·1)	8·1 (5·3 to 12·1)	4·1 (2·8 to 5·7)	0·9 (0·7 to 1·3)	300
Protein-energy malnutrition	0·1 (0 to 0·1)	0·2 (0·1 to 0·3)	9·6 (6·1 to 15·1)	4·3 (2·3 to 7)	2·2 (1·3 to 3·4)	1·8 (1 to 2·7)	2·1 (1·3 to 3·3)	1·9 (1 to 3·1)	2·4 (1·4 to 3·9)	2·8 (1·8 to 4·1)	3·1 (2 to 4·6)	3 (1·8 to 4·5)	4·1 (2·6 to 5·9)	4·6 (2·9 to 6·6)	5·6 (3·8 to 8)	6·8 (4·5 to 9·6)	6·8 (4·5 to 9·7)	7·8 (4·9 to 11·9)	6·7 (4·5 to 9·6)	5·5 (3·5 to 8·1)	4·2 (2·7 to 6·1)	2·3 (1·4 to 3·3)	0·5 (0·3 to 0·8)	88·5
Iodine deficiency	0 (0 to 0)	0 (0 to 0)	0 (0 to 0)	0 (0 to 0·1)	0·2 (0·1 to 0·4)	0·5 (0·2 to 1)	0·8 (0·3 to 1·4)	0·8 (0·3 to 1·8)	1 (0·3 to 1·9)	1 (0·3 to 1·9)	1 (0·4 to 2)	0·9 (0·4 to 1·9)	1 (0·4 to 1·9)	0·9 (0·4 to 1·8)	0·8 (0·3 to 1·7)	0·7 (0·3 to 1·4)	0·5 (0·3 to 0·9)	0·4 (0·2 to 0·6)	0·2 (0·1 to 0·3)	0·1 (0 to 0·2)	0·1 (0 to 0·1)	0 (0 to 0)	0 (0 to 0)	11
Vitamin A deficiency	0 (0 to 0)	0 (0 to 0·1)	0·1 (0 to 0·2)	0·6 (0·2 to 1·2)	0·9 (0·3 to 2)	0·6 (0·2 to 1·4)	0·3 (0·1 to 0·4)	0·2 (0·1 to 0·4)	0·2 (0·1 to 0·3)	0·1 (0·1 to 0·3)	0·1 (0·1 to 0·2)	0·1 (0 to 0·2)	0·1 (0 to 0·2)	0·1 (0 to 0·2)	0·1 (0 to 0·2)	0·1 (0 to 0·2)	0·1 (0 to 0·1)	0 (0 to 0·1)	0 (0 to 0·1)	0 (0 to 0)	0 (0 to 0)	0 (0 to 0)	0 (0 to 0)	3·8
Dietary iron deficiency	0 (0 to 0)	0·8 (0·2 to 1·9)	3 (0·5 to 9·1)	13 (2·3 to 35·2)	41·8 (10·9 to 104)	32·1 (8·2 to 73·2)	4·6 (0·7 to 14·6)	2·9 (0·4 to 8·7)	3·5 (0·5 to 10·9)	3·4 (0·5 to 10)	4·3 (0·7 to 13)	4·6 (0·9 to 12·9)	5·9 (1 to 17·4)	6 (1 to 16·4)	10·6 (2·1 to 29·6)	8·5 (1·7 to 23·5)	5·9 (1·1 to 15·6)	4·7 (1 to 12·3)	3·3 (0·7 to 8·4)	2·5 (0·5 to 7·1)	2·3 (0·6 to 5·6)	0·8 (0·2 to 2)	0·2 (0 to 0·4)	165
Other nutritional deficiencies	0 (0 to 0)	0 (0 to 0)	0·7 (0·3 to 1·4)	0·4 (0·2 to 0·8)	0·5 (0·3 to 0·8)	0·5 (0·3 to 0·8)	1 (0·5 to 1·6)	1·2 (0·7 to 1·9)	1·4 (0·8 to 2·4)	1·3 (0·8 to 2)	1·3 (0·7 to 2)	1·6 (1 to 2·4)	1·5 (0·9 to 2·5)	2·2 (1·4 to 3·5)	2·5 (1·5 to 3·9)	3 (1·6 to 4·9)	2·9 (1·5 to 4·9)	3·1 (1·5 to 5·3)	2·1 (1 to 3·6)	2·4 (1·1 to 4·2)	1·5 (0·6 to 2·7)	0·9 (0·4 to 1·6)	0·2 (0·1 to 0·4)	32·3
Non-communicable diseases	163 (97·9 to 251)	53·6 (34·8 to 80·1)	171 (115 to 244)	208 (156 to 268)	279 (216 to 358)	427 (323 to 552)	649 (507 to 822)	681 (536 to 851)	881 (712 to 1,090)	1,070 (884 to 1,300)	1,250 (1,040 to 1,510)	1,550 (1,280 to 1,850)	2,220 (1,830 to 2,640)	2,770 (2,270 to 3,300)	3,910 (3,230 to 4,620)	4,650 (3,890 to 5,410)	4,580 (3,890 to 5,270)	4,590 (3,940 to 5,270)	3,590 (3,110 to 4,040)	2,730 (2,420 to 3,030)	1,770 (1,620 to 1,930)	790 (726 to 849)	136 (126 to 145)	39,100
Neoplasms	1·3 (0·9 to 2)	0·8 (0·5 to 1·2)	6 (3·6 to 9·4)	22·4 (14·5 to 32·8)	30·9 (23·3 to 39·9)	33 (25·6 to 42·2)	55·9 (42·4 to 73·8)	54·5 (43·8 to 71)	76·2 (61·6 to 95·3)	114 (92·8 to 142)	130 (105 to 165)	202 (159 to 258)	363 (276 to 475)	533 (410 to 682)	926 (717 to 1,170)	1,260 (992 to 1,540)	1,290 (1,040 to 1,550)	1,370 (1,120 to 1,660)	1,080 (881 to 1,310)	765 (626 to 910)	455 (370 to 549)	181 (141 to 216)	23·9 (18 to 29·8)	8,970
Lip and oral cavity cancer	0 (0 to 0)	0 (0 to 0)	0 (0 to 0)	0 (0 to 0)	0·1 (0·1 to 0·1)	0·1 (0·1 to 0·2)	0·3 (0·2 to 0·4)	0·4 (0·3 to 0·6)	0·6 (0·4 to 0·9)	1·5 (0·9 to 2·1)	2·5 (1·7 to 3·6)	5·4 (3·7 to 7·9)	11·9 (8 to 17·1)	17·5 (12·1 to 23·6)	28·1 (20·3 to 38·8)	29·2 (20·1 to 39·3)	24 (17·5 to 31·6)	20·5 (14·7 to 27·3)	14·2 (10·3 to 19·3)	9·1 (6·6 to 12)	4·3 (3·2 to 5·4)	1·7 (1·3 to 2·2)	0·3 (0·2 to 0·3)	172
Nasopharynx cancer	0 (0 to 0)	0 (0 to 0)	0 (0 to 0)	0 (0 to 0)	0·1 (0·1 to 0·2)	0·3 (0·2 to 0·5)	0·7 (0·5 to 0·9)	0·6 (0·4 to 0·9)	0·8 (0·6 to 1·2)	1 (0·7 to 1·4)	1·2 (0·8 to 1·7)	2·5 (1·6 to 3·5)	4·3 (3 to 6·1)	6·5 (4·4 to 9·4)	9·9 (7 to 13·8)	8·5 (5·9 to 11·7)	6·7 (4·8 to 8·9)	5·5 (4 to 7·3)	2·8 (2 to 4)	1·7 (1·2 to 2·3)	1·5 (1·1 to 2)	0·5 (0·4 to 0·7)	0·1 (0·1 to 0·1)	55·2
Other pharynx cancer	0 (0 to 0)	0 (0 to 0)	0 (0 to 0)	0 (0 to 0)	0 (0 to 0)	0 (0 to 0)	0 (0 to 0)	0·3 (0·2 to 0·4)	0·4 (0·3 to 0·6)	0·8 (0·6 to 1·1)	1·6 (1·1 to 2·4)	3·8 (2·4 to 5·7)	10·6 (7·1 to 15·4)	15·4 (10·6 to 21·8)	25 (17 to 35·4)	25·7 (17·1 to 36·6)	20·4 (14·2 to 28)	15·7 (10·6 to 22·1)	9·1 (6·4 to 12·7)	5·5 (4 to 7·4)	3 (2·1 to 4)	1 (0·7 to 1·3)	0·2 (0·1 to 0·2)	139
Esophageal cancer	0 (0 to 0)	0 (0 to 0)	0 (0 to 0)	0 (0 to 0)	0 (0 to 0)	0 (0 to 0)	0 (0 to 0)	0·4 (0·3 to 0·6)	0·8 (0·6 to 1·2)	2·1 (1·4 to 3·1)	5·2 (3·6 to 7·5)	13·5 (9·2 to 18·9)	31·1 (21·2 to 43·6)	43·6 (31·6 to 59·9)	68·7 (48·4 to 96·7)	74 (51·2 to 102)	61·5 (43·9 to 83·4)	53·6 (37·5 to 72·9)	34·6 (24·3 to 48·7)	21·8 (15·8 to 29·2)	13 (9·8 to 17)	4·4 (3·4 to 5·5)	0·5 (0·3 to 0·6)	429
Stomach cancer	0 (0 to 0)	0 (0 to 0)	0 (0 to 0)	0 (0 to 0)	0 (0 to 0)	0 (0 to 0)	0·9 (0·7 to 1·1)	1·3 (1 to 1·7)	3·5 (2·5 to 5)	9·1 (6·4 to 12·8)	12 (8·5 to 16·6)	20·7 (14·9 to 28·2)	32·7 (22·9 to 44·4)	46·5 (33·1 to 62·2)	72·1 (51·6 to 94·5)	92·2 (65·9 to 124)	92·7 (67 to 123)	90 (66·4 to 118)	60·8 (44·1 to 80)	41·1 (30·7 to 54·6)	25 (18·7 to 31·3)	9·3 (7·4 to 11·3)	1·2 (0·9 to 1·4)	611
Colon and rectum cancer	0 (0 to 0)	0 (0 to 0)	0 (0 to 0)	0 (0 to 0)	0·3 (0·2 to 0·4)	0·4 (0·3 to 0·6)	2·4 (1·7 to 3·2)	3·8 (2·7 to 5·5)	7·8 (5·1 to 11·7)	17·4 (12·3 to 23·7)	21·2 (14·5 to 29·8)	31·7 (23·4 to 43·1)	54·7 (38·6 to 75·4)	77·6 (55 to 106)	141 (99 to 189)	189 (135 to 256)	177 (127 to 234)	178 (134 to 227)	127 (94·5 to 167)	88·3 (66·9 to 114)	46·6 (35·6 to 59·2)	17·2 (13·5 to 21·3)	2·4 (1·8 to 2·9)	1,180
Liver cancer	0 (0 to 0)	0 (0 to 0)	0·3 (0·1 to 0·7)	0·9 (0·4 to 1·5)	0·9 (0·6 to 1·2)	0·9 (0·6 to 1·3)	0·4 (0·3 to 0·5)	0·6 (0·4 to 1)	1·4 (0·9 to 2·1)	2 (1·3 to 2·9)	3 (2 to 4·5)	4·8 (3·3 to 7·1)	8·3 (5·5 to 12·1)	12 (7·7 to 18·2)	21·9 (14·9 to 31·2)	26·3 (18·7 to 35·9)	26·4 (18·5 to 37)	25·7 (18·3 to 35·9)	17 (11·7 to 23·3)	9 (6·3 to 12·3)	3·4 (2·4 to 4·8)	1 (0·7 to 1·3)	0·1 (0·1 to 0·1)	166
Liver cancer due to hepatitis B	0 (0 to 0)	0 (0 to 0)	0 (0 to 0)	0 (0 to 0)	0 (0 to 0)	0·1 (0·1 to 0·2)	0·2 (0·1 to 0·3)	0·4 (0·2 to 0·7)	0·9 (0·5 to 1·5)	1·2 (0·7 to 2)	1·7 (1 to 2·8)	2·5 (1·4 to 3·9)	3·8 (2 to 6·3)	4·8 (2·6 to 8·2)	8 (4·3 to 12·8)	8·2 (4·2 to 13·4)	6·5 (3·3 to 11·6)	5·4 (2·4 to 9·5)	3·2 (1·7 to 5·6)	1·6 (0·7 to 2·8)	0·6 (0·3 to 1·1)	0·2 (0·1 to 0·3)	0 (0 to 0)	49·3
Liver cancer due to hepatitis C	0 (0 to 0)	0 (0 to 0)	0 (0 to 0)	0 (0 to 0)	0 (0 to 0)	0 (0 to 0)	0 (0 to 0)	0 (0 to 0)	0 (0 to 0·1)	0·1 (0 to 0·1)	0·1 (0 to 0·3)	0·3 (0·1 to 0·5)	0·6 (0·2 to 1·3)	1·1 (0·5 to 2·2)	2·5 (1 to 4·9)	3·4 (1·5 to 6·4)	3·6 (1·8 to 6·3)	4 (1·9 to 7)	3·2 (1·5 to 5·8)	2·1 (1·1 to 3·5)	1 (0·5 to 1·7)	0·3 (0·2 to 0·5)	0 (0 to 0·1)	22·4
Liver cancer due to alcohol use	0 (0 to 0)	0 (0 to 0)	0 (0 to 0)	0 (0 to 0)	0 (0 to 0)	0 (0 to 0)	0 (0 to 0)	0·1 (0 to 0·1)	0·2 (0·1 to 0·4)	0·4 (0·2 to 0·8)	0·9 (0·4 to 1·6)	1·6 (0·8 to 2·8)	3·1 (1·5 to 5·3)	4·9 (2·4 to 8·6)	9·4 (5 to 15·1)	12·3 (7 to 19·5)	13·9 (8·4 to 21·2)	13·8 (8·2 to 20·7)	8·6 (5·3 to 12·7)	4·1 (2·5 to 6·2)	1·4 (0·8 to 2·2)	0·3 (0·2 to 0·6)	0 (0 to 0·1)	75·1
Liver cancer due to NASH	0 (0 to 0)	0 (0 to 0)	0 (0 to 0)	0 (0 to 0)	0 (0 to 0)	0 (0 to 0)	0·1 (0 to 0·1)	0·1 (0 to 0·1)	0·1 (0 to 0·2)	0·1 (0 to 0·2)	0·2 (0·1 to 0·3)	0·2 (0·1 to 0·4)	0·4 (0·2 to 0·9)	0·7 (0·3 to 1·3)	1·2 (0·6 to 2·2)	1·6 (0·8 to 2·8)	1·6 (0·8 to 3·1)	1·8 (1 to 3·4)	1·5 (0·8 to 2·7)	0·9 (0·5 to 1·6)	0·4 (0·2 to 0·6)	0·1 (0·1 to 0·2)	0 (0 to 0)	11
Liver cancer due to other causes	0 (0 to 0)	0 (0 to 0)	0·3 (0·1 to 0·7)	0·9 (0·4 to 1·5)	0·9 (0·6 to 1·2)	0·8 (0·5 to 1·2)	0·1 (0 to 0·1)	0·1 (0 to 0·2)	0·1 (0·1 to 0·2)	0·1 (0·1 to 0·2)	0·2 (0·1 to 0·3)	0·2 (0·1 to 0·4)	0·4 (0·2 to 0·7)	0·5 (0·2 to 0·9)	0·8 (0·3 to 1·5)	0·8 (0·4 to 1·6)	0·7 (0·3 to 1·4)	0·7 (0·3 to 1·3)	0·5 (0·2 to 0·9)	0·3 (0·1 to 0·5)	0·1 (0·1 to 0·2)	0 (0 to 0·1)	0 (0 to 0)	8·5
Gallbladder and biliary tract cancer	0 (0 to 0)	0 (0 to 0)	0 (0 to 0)	0 (0 to 0)	0 (0 to 0)	0 (0 to 0)	0 (0 to 0)	0·1 (0·1 to 0·2)	0·3 (0·2 to 0·5)	0·5 (0·4 to 1)	0·9 (0·6 to 1·6)	1·6 (1·1 to 3·1)	2·7 (1·8 to 5)	4·3 (2·8 to 8·4)	7·5 (4·9 to 14·5)	9·8 (6·6 to 19)	9·5 (6·3 to 18)	9·4 (6·4 to 18)	6·6 (4·6 to 12·4)	4·4 (3·1 to 7·8)	2·5 (1·8 to 4·5)	0·9 (0·7 to 1·6)	0·1 (0·1 to 0·2)	61·1
Pancreatic cancer	0 (0 to 0)	0 (0 to 0)	0 (0 to 0)	0 (0 to 0)	0 (0 to 0)	0 (0 to 0)	0·5 (0·4 to 0·7)	0·5 (0·4 to 0·7)	1·1 (0·8 to 1·5)	4·8 (3·2 to 7·1)	7 (4·8 to 10)	14·2 (10 to 19·7)	26·4 (18·3 to 36)	36·4 (26·1 to 48·5)	63 (44·5 to 83)	76 (53·4 to 103)	63·5 (46 to 85)	61·2 (44·6 to 81·3)	40 (28·7 to 53·4)	27·2 (20·2 to 36·3)	13·6 (10·3 to 17·2)	4·7 (3·7 to 5·8)	0·6 (0·5 to 0·7)	441
Larynx cancer	0 (0 to 0)	0 (0 to 0)	0 (0 to 0)	0 (0 to 0)	0 (0 to 0)	0 (0 to 0)	0 (0 to 0)	0·2 (0·1 to 0·3)	0·2 (0·2 to 0·3)	0·5 (0·3 to 0·6)	1·1 (0·8 to 1·6)	3 (2·1 to 4·2)	8·1 (5·1 to 12·2)	13·5 (9·9 to 18·5)	22·5 (16·3 to 30·6)	26·3 (19·6 to 34·7)	23·5 (17·7 to 30·5)	20 (14·7 to 26·1)	12·5 (9·1 to 17)	7·5 (5·7 to 9·8)	3·5 (2·6 to 4·6)	1·1 (0·8 to 1·5)	0·1 (0·1 to 0·2)	144
Tracheal, bronchus, and lung cancer	0 (0 to 0)	0 (0 to 0)	0 (0 to 0)	0 (0 to 0)	0 (0 to 0)	0·1 (0·1 to 0·2)	0·8 (0·6 to 1·1)	0·9 (0·6 to 1·3)	1·6 (1 to 2·5)	4·4 (3 to 6·3)	7·2 (4·7 to 10·5)	15·4 (11 to 21)	36·3 (24·3 to 51·9)	56 (38·3 to 79·5)	94 (62·4 to 134)	116 (81 to 158)	114 (80 to 157)	104 (74·1 to 137)	64·6 (47·5 to 84·4)	36·9 (26·5 to 49·1)	19·8 (14·5 to 26·1)	6·5 (5·1 to 7·9)	0·8 (0·6 to 1)	678
Malignant skin melanoma	0 (0 to 0)	0 (0 to 0)	0 (0 to 0·1)	0·1 (0 to 0·6)	0·1 (0·1 to 0·2)	0·1 (0·1 to 0·2)	0·2 (0·1 to 0·3)	0·3 (0·2 to 0·5)	0·8 (0·5 to 1·2)	1·3 (0·7 to 1·9)	1·7 (1 to 2·5)	2·2 (1·3 to 3·5)	2·8 (1·6 to 4·3)	3·5 (2 to 5·4)	5·1 (3·2 to 8·3)	4·5 (2·7 to 6·8)	3·8 (2·2 to 5·6)	3·8 (2·2 to 5·9)	2·8 (1·6 to 4·1)	2·1 (1·3 to 3·3)	1·2 (0·7 to 1·8)	0·4 (0·2 to 0·6)	0·1 (0 to 0·1)	36·9
Non-melanoma skin cancer	0 (0 to 0)	0 (0 to 0)	0 (0 to 0)	0 (0 to 0)	0 (0 to 0)	0 (0 to 0)	0 (0 to 0)	0·1 (0·1 to 0·1)	0·1 (0·1 to 0·2)	0·3 (0·2 to 0·4)	0·4 (0·2 to 0·5)	0·5 (0·3 to 0·7)	0·8 (0·5 to 1·1)	1·1 (0·8 to 1·6)	1·9 (1·3 to 2·8)	3 (2·1 to 4)	3·1 (2·2 to 4·1)	3·1 (2·2 to 4·1)	2·4 (1·7 to 3·3)	1·8 (1·4 to 2·3)	1·2 (0·9 to 1·6)	0·6 (0·4 to 0·8)	0·1 (0·1 to 0·2)	20·4
Non-melanoma skin cancer (squamous-cell carcinoma)	0 (0 to 0)	0 (0 to 0)	0 (0 to 0)	0 (0 to 0)	0 (0 to 0)	0 (0 to 0)	0 (0 to 0)	0·1 (0·1 to 0·1)	0·1 (0·1 to 0·2)	0·3 (0·2 to 0·4)	0·4 (0·2 to 0·5)	0·5 (0·3 to 0·7)	0·8 (0·5 to 1·1)	1·1 (0·8 to 1·6)	1·9 (1·3 to 2·8)	3 (2·1 to 4)	3·1 (2·2 to 4·1)	3·1 (2·2 to 4·1)	2·4 (1·7 to 3·3)	1·8 (1·4 to 2·3)	1·2 (0·9 to 1·6)	0·6 (0·4 to 0·8)	0·1 (0·1 to 0·2)	20·3
Non-melanoma skin cancer (basal-cell carcinoma)	0 (0 to 0)	0 (0 to 0)	0 (0 to 0)	0 (0 to 0)	0 (0 to 0)	0 (0 to 0)	0 (0 to 0)	0 (0 to 0)	0 (0 to 0)	0 (0 to 0)	0 (0 to 0)	0 (0 to 0)	0 (0 to 0)	0 (0 to 0)	0 (0 to 0)	0 (0 to 0)	0 (0 to 0)	0 (0 to 0)	0 (0 to 0)	0 (0 to 0)	0 (0 to 0)	0 (0 to 0)	0 (0 to 0)	0
Breast cancer	0 (0 to 0)	0 (0 to 0)	0 (0 to 0)	0 (0 to 0)	0 (0 to 0)	0 (0 to 0)	0·1 (0·1 to 0·1)	0·1 (0·1 to 0·1)	0·1 (0·1 to 0·2)	0·3 (0·2 to 0·4)	0·4 (0·3 to 0·5)	0·4 (0·3 to 0·7)	1·5 (0·9 to 2·2)	1·8 (1·1 to 2·7)	2·9 (1·8 to 4·3)	3·5 (2·2 to 5·1)	3·7 (2·4 to 5·3)	3·1 (2 to 4·7)	2·3 (1·5 to 3·3)	1·7 (1·1 to 2·6)	1·6 (1 to 2·4)	0·7 (0·5 to 1)	0·1 (0·1 to 0·1)	24·2
Cervical cancer	--	--	--	--	--	--	--	--	--	--	--	--	--	--	--	--	--	--	--	--	--	--	--	NA
Uterine cancer	--	--	--	--	--	--	--	--	--	--	--	--	--	--	--	--	--	--	--	--	--	--	--	NA
Ovarian cancer	--	--	--	--	--	--	--	--	--	--	--	--	--	--	--	--	--	--	--	--	--	--	--	NA
Prostate cancer	0 (0 to 0)	0 (0 to 0)	0 (0 to 0)	0 (0 to 0)	0 (0 to 0)	0 (0 to 0)	0 (0 to 0)	0·5 (0·4 to 0·7)	1·4 (0·9 to 1·8)	2·1 (1·3 to 2·8)	2·4 (1·5 to 3·2)	5·7 (3·7 to 8·1)	20 (12·7 to 28·9)	55·4 (36·8 to 79)	147 (98·8 to 206)	333 (225 to 456)	437 (298 to 583)	569 (394 to 759)	541 (390 to 717)	417 (298 to 545)	267 (195 to 346)	114 (79·5 to 146)	15·2 (10·2 to 20·8)	2,930
Testicular cancer	0 (0 to 0·6)	0 (0 to 0·1)	0·1 (0 to 0·2)	0·2 (0 to 0·5)	0·1 (0·1 to 0·2)	0·2 (0·1 to 0·3)	0·6 (0·4 to 1)	0·9 (0·5 to 1·3)	1·5 (0·9 to 2·3)	1 (0·6 to 1·6)	0·9 (0·6 to 1·3)	0·7 (0·4 to 1)	0·5 (0·3 to 0·7)	0·4 (0·2 to 0·6)	0·3 (0·2 to 0·5)	0·4 (0·2 to 0·5)	0·3 (0·2 to 0·5)	0·3 (0·2 to 0·5)	0·3 (0·2 to 0·4)	0·2 (0·1 to 0·3)	0·1 (0·1 to 0·2)	0·1 (0 to 0·1)	0 (0 to 0)	9·1
Kidney cancer	0·1 (0·1 to 0·2)	0 (0 to 0·1)	0·4 (0·3 to 0·7)	2 (1·2 to 3)	1·9 (1·2 to 2·7)	0·9 (0·6 to 1·3)	1·8 (1·3 to 2·6)	2 (1·4 to 2·9)	3·4 (2·4 to 4·9)	4·6 (2·9 to 7)	4 (2·6 to 5·6)	6·6 (4·4 to 9·2)	11·6 (7·8 to 16)	17·7 (12·6 to 23·9)	27·8 (19·5 to 37·5)	31·6 (22·4 to 42·8)	27·7 (19·7 to 37·1)	24·5 (17·6 to 33·6)	15·5 (11·1 to 20·8)	8·7 (6·1 to 11·8)	3·7 (2·7 to 5·1)	1·4 (1 to 1·9)	0·2 (0·1 to 0·2)	198
Bladder cancer	0 (0 to 0)	0 (0 to 0)	0 (0 to 0)	0 (0 to 0)	0 (0 to 0)	0 (0 to 0)	0·1 (0·1 to 0·1)	0·1 (0·1 to 0·2)	0·2 (0·2 to 0·3)	0·6 (0·4 to 0·9)	0·9 (0·6 to 1·3)	1·8 (1·3 to 2·6)	3·7 (2·5 to 5·3)	6·8 (4·7 to 9·4)	13·9 (9·7 to 18·5)	22·8 (16·6 to 30·4)	24·5 (17·8 to 33)	28·5 (20·5 to 38·2)	22·4 (16 to 29·9)	16·7 (12·2 to 22·5)	10·8 (8 to 14·1)	4·3 (3·3 to 5·3)	0·6 (0·4 to 0·8)	159
Brain and central nervous system cancer	0·2 (0 to 0·3)	0·1 (0 to 0·2)	1·1 (0·3 to 2)	3·5 (1·2 to 5·9)	5·5 (2·2 to 9·1)	5·2 (2 to 8·4)	5 (1·6 to 8·5)	4·1 (1·4 to 6·9)	5·8 (1·9 to 9·3)	8·2 (2·7 to 13·2)	9·2 (3·2 to 14·8)	11 (3·8 to 17)	13·2 (4·3 to 20·3)	15·1 (5·3 to 23·8)	21 (7·3 to 32·6)	20·1 (7·1 to 30·8)	15·8 (5·5 to 24·3)	12·1 (4·2 to 18·2)	6·3 (2·2 to 9·7)	3·2 (1·1 to 5)	1·4 (0·4 to 2·3)	0·4 (0·1 to 0·6)	0·1 (0 to 0·1)	168
Thyroid cancer	0 (0 to 0)	0 (0 to 0)	0 (0 to 0)	0 (0 to 0)	0·1 (0·1 to 0·1)	0·1 (0·1 to 0·1)	0·1 (0·1 to 0·2)	0·2 (0·2 to 0·3)	0·4 (0·3 to 0·6)	0·6 (0·4 to 0·8)	0·8 (0·5 to 1·2)	1 (0·6 to 1·6)	2·1 (1·4 to 3·1)	2·9 (1·9 to 4·2)	4·2 (2·6 to 6·1)	4·2 (2·8 to 6·1)	3·5 (2·4 to 4·8)	3·2 (2·2 to 4·4)	2·1 (1·5 to 2·8)	1·5 (1·1 to 2)	0·7 (0·5 to 1)	0·3 (0·2 to 0·4)	0 (0 to 0)	28·1
Mesothelioma	0 (0 to 0)	0 (0 to 0)	0 (0 to 0)	0 (0 to 0)	0 (0 to 0)	0 (0 to 0)	0 (0 to 0)	0·1 (0·1 to 0·2)	0·3 (0·2 to 0·4)	0·3 (0·2 to 0·5)	0·5 (0·3 to 0·7)	0·5 (0·4 to 0·7)	1·3 (0·9 to 1·8)	1·7 (1·2 to 2·3)	2·8 (2 to 3·7)	1·5 (1·1 to 2·1)	1·2 (0·9 to 1·7)	1·4 (1 to 1·9)	1·2 (0·9 to 1·7)	0·8 (0·6 to 1)	0·5 (0·3 to 0·6)	0·1 (0·1 to 0·2)	0 (0 to 0)	14·3
Hodgkin lymphoma	0 (0 to 0)	0 (0 to 0)	0 (0 to 0)	0·1 (0 to 0·1)	0·1 (0·1 to 0·2)	0·2 (0·2 to 0·3)	1·2 (0·8 to 1·7)	1·5 (1 to 2·3)	2·2 (1·4 to 3·4)	2·2 (1·4 to 3·3)	1·7 (1·1 to 2·5)	1·7 (1·1 to 2·5)	1·6 (1 to 2·4)	1·7 (1·1 to 2·6)	2 (1·3 to 3)	2·1 (1·5 to 3·1)	1·9 (1·3 to 2·7)	1·4 (1 to 2)	1 (0·6 to 1·4)	0·5 (0·3 to 0·8)	0·3 (0·2 to 0·4)	0·1 (0·1 to 0·1)	0 (0 to 0)	23·5
Non-Hodgkin lymphoma	0 (0 to 0)	0 (0 to 0)	0 (0 to 0)	2·3 (1·4 to 3·5)	4·3 (2·8 to 6·4)	5·1 (3·2 to 7·8)	10·4 (6·9 to 15·1)	10·8 (7·3 to 15·5)	15·1 (10·6 to 21·5)	19·2 (13·7 to 26·5)	17 (12 to 23·3)	18·5 (13 to 25·3)	24·6 (16·3 to 35·8)	27·7 (19·7 to 38·4)	40·9 (27·6 to 56·3)	42·7 (30·2 to 58·2)	35·8 (26 to 47·9)	33·1 (23·6 to 44·9)	20·8 (15 to 27·8)	12·1 (8·7 to 16)	5·2 (3·8 to 6·8)	1·6 (1·2 to 2·1)	0·2 (0·1 to 0·2)	347
Multiple myeloma	0 (0 to 0)	0 (0 to 0)	0 (0 to 0)	0 (0 to 0)	0 (0 to 0)	0 (0 to 0)	0 (0 to 0)	0·5 (0·4 to 0·8)	0·8 (0·6 to 1·2)	1·3 (0·9 to 1·9)	2·7 (1·8 to 4)	6·6 (4·5 to 9·6)	15·3 (9·9 to 22·4)	25·9 (17·5 to 37·6)	42·1 (28·2 to 60·2)	50·5 (35·1 to 72)	52·9 (35·9 to 71·2)	46 (31·7 to 64·4)	29·8 (20·5 to 40·8)	17·6 (12·7 to 23·8)	8·3 (6 to 11·2)	2·3 (1·6 to 3·1)	0·3 (0·2 to 0·4)	303
Leukemia	0·5 (0·3 to 0·7)	0·3 (0·2 to 0·5)	1·7 (1 to 2·9)	6 (3·5 to 9·7)	10·4 (6·8 to 15·4)	12·5 (8·2 to 18·3)	18·9 (12·9 to 27·7)	14·2 (9·7 to 20·5)	14·9 (10·5 to 20·7)	15·3 (10·8 to 20·7)	13·4 (9·2 to 18·8)	14·3 (10·2 to 19·5)	17·7 (12·4 to 24)	18·8 (13·6 to 26)	26·1 (18·6 to 34·6)	28·9 (20·9 to 38·5)	29·1 (21·4 to 37·7)	28·5 (20·7 to 38·4)	20·1 (14·5 to 26·4)	13·9 (10·2 to 18·3)	8·1 (6 to 10·6)	2·8 (2·1 to 3·5)	0·4 (0·3 to 0·5)	317
Acute lymphoid leukemia	0·1 (0 to 0·3)	0 (0 to 0·1)	0·3 (0·1 to 0·5)	1·9 (1 to 3·5)	3·9 (2 to 6·7)	3·1 (1·5 to 5·3)	5·2 (2·9 to 8·3)	2·5 (1·2 to 4·3)	1·4 (0·7 to 2·2)	1·2 (0·6 to 2)	0·9 (0·5 to 1·6)	0·8 (0·4 to 1·4)	0·6 (0·3 to 1·1)	0·7 (0·4 to 1·2)	0·9 (0·5 to 1·5)	0·7 (0·4 to 1·1)	0·9 (0·5 to 1·4)	0·7 (0·4 to 1·1)	0·3 (0·1 to 0·5)	0·3 (0·1 to 0·4)	0·1 (0·1 to 0·2)	0 (0 to 0·1)	0 (0 to 0)	26·5
Chronic lymphoid leukemia	0 (0 to 0)	0 (0 to 0)	0 (0 to 0)	0 (0 to 0)	0 (0 to 0)	0 (0 to 0)	0 (0 to 0)	0·3 (0·1 to 0·5)	0·3 (0·2 to 0·6)	0·3 (0·1 to 0·6)	0·3 (0·1 to 0·6)	0·6 (0·3 to 1·1)	0·6 (0·3 to 1·1)	1·5 (0·9 to 2·5)	3·4 (2·1 to 5·4)	4·3 (2·5 to 6·8)	5 (2·9 to 7·5)	4·8 (2·8 to 7·8)	3·3 (1·9 to 5·5)	2·5 (1·6 to 3·7)	1·6 (0·9 to 2·5)	0·6 (0·4 to 0·9)	0·1 (0 to 0·1)	29·6
Acute myeloid leukemia	0·1 (0·1 to 0·2)	0·2 (0·1 to 0·3)	0·8 (0·4 to 1·4)	2·6 (1·4 to 4·6)	3·5 (1·9 to 6·1)	5·1 (2·9 to 8·2)	8·3 (5·1 to 13)	5·9 (3·6 to 9·2)	6·8 (4·4 to 9·7)	6·1 (4·1 to 9·1)	5·4 (3·4 to 8·1)	4·7 (2·9 to 7·2)	6·1 (3·9 to 9·2)	6·2 (4 to 9)	7·4 (4·9 to 10·6)	7·3 (4·7 to 10·6)	7·2 (4·8 to 10·4)	6·2 (4 to 8·9)	4·1 (2·5 to 6)	2·2 (1·3 to 3·5)	1·1 (0·6 to 1·7)	0·3 (0·2 to 0·5)	0 (0 to 0·1)	97·5

Chronic myeloid leukemia	0 (0 to 0)	0 (0 to 0)	0·1 (0 to 0·2)	0·2 (0·1 to 0·4)	0·2 (0·1 to 0·3)	0·2 (0·1 to 0·4)	0·7 (0·3 to 1·2)	1 (0·5 to 2·1)	2 (1 to 3·6)	2·3 (1·2 to 4)	2·3 (1·2 to 3·8)	1·7 (0·9 to 3)	2·3 (1·2 to 3·8)	2·5 (1·4 to 4·3)	3·5 (1·9 to 5·9)	4·2 (2·2 to 6·9)	4 (2·1 to 6·5)	3·2 (1·7 to 5·4)	2·5 (1·4 to 4·1)	1·6 (0·9 to 2·6)	0·7 (0·4 to 1·2)	0·3 (0·2 to 0·5)	0 (0 to 0·1)	35·5
Other leukemia	0·3 (0·1 to 0·5)	0·1 (0·1 to 0·2)	0·6 (0·3 to 1·1)	1·2 (0·6 to 2·2)	2·8 (1·3 to 4·8)	4·1 (2·3 to 6·6)	4·7 (2·8 to 7·5)	4·5 (2·8 to 7·2)	4·4 (2·7 to 6·7)	5·3 (3·4 to 8·1)	4·6 (2·9 to 7·1)	6·4 (4·3 to 9·3)	8 (5·2 to 11·7)	7·9 (5·3 to 11·5)	10·8 (7·2 to 15·3)	12·3 (8·4 to 17·2)	12·1 (8·4 to 16·8)	13·6 (9·2 to 19·5)	9·9 (6·6 to 13·7)	7·3 (5 to 10·3)	4·6 (3·2 to 6·3)	1·6 (1·1 to 2·1)	0·2 (0·1 to 0·3)	127
Other malignant neoplasms	0·4 (0·2 to 0·6)	0·3 (0·2 to 0·5)	2·3 (1·4 to 3·7)	7·3 (4·5 to 11)	6·7 (4·7 to 9·5)	6·6 (5 to 8·5)	11·2 (8·4 to 14·8)	9·5 (7·2 to 12·6)	10·3 (8·2 to 13·3)	12·5 (9·7 to 16·2)	11·2 (8·4 to 14·8)	13·5 (9·6 to 18·4)	18·6 (13 to 25·6)	21·8 (15·4 to 30)	31·9 (23 to 44)	32·1 (23·2 to 44·5)	28·7 (21·3 to 38·3)	26·2 (19·6 to 34·9)	17·8 (13·6 to 23·7)	12 (9·3 to 15·7)	7·7 (5·8 to 10·2)	3 (2·2 to 3·8)	0·4 (0·3 to 0·5)	292
Other neoplasms	0 (0 to 0·1)	0 (0 to 0)	0·1 (0 to 0·1)	0·2 (0·1 to 0·4)	0·3 (0·2 to 0·6)	0·3 (0·2 to 0·5)	0·4 (0·2 to 0·6)	0·2 (0·1 to 0·4)	0·4 (0·2 to 0·7)	0·4 (0·2 to 0·6)	0·5 (0·3 to 0·8)	0·5 (0·3 to 0·9)	0·7 (0·4 to 1·3)	1·3 (0·8 to 2·2)	2·2 (1·4 to 3·9)	3 (1·9 to 5·2)	3·1 (2 to 5·2)	3·8 (2·4 to 6·8)	3 (1·9 to 5·2)	2 (1·2 to 3·2)	1·2 (0·7 to 2)	0·4 (0·2 to 0·7)	0 (0 to 0·1)	24·2
Myelodysplastic, myeloproliferative, and other hematopoietic neo	0 (0 to 0·1)	0 (0 to 0)	0·1 (0 to 0·1)	0·2 (0·1 to 0·4)	0·3 (0·2 to 0·6)	0·3 (0·2 to 0·5)	0·4 (0·2 to 0·6)	0·2 (0·1 to 0·4)	0·4 (0·2 to 0·7)	0·4 (0·2 to 0·6)	0·5 (0·3 to 0·8)	0·5 (0·3 to 0·9)	0·7 (0·4 to 1·3)	1·3 (0·8 to 2·2)	2·2 (1·4 to 3·9)	3 (1·9 to 5·2)	3·1 (2 to 5·2)	3·8 (2·4 to 6·8)	3 (1·9 to 5·2)	2 (1·2 to 3·2)	1·2 (0·7 to 2)	0·4 (0·2 to 0·7)	0 (0 to 0·1)	24·2
Benign and in situ intestinal neoplasms	0 (0 to 0)	0 (0 to 0)	0 (0 to 0)	0 (0 to 0)	0 (0 to 0)	0 (0 to 0)	0 (0 to 0)	0 (0 to 0)	0 (0 to 0)	0 (0 to 0)	0 (0 to 0)	0 (0 to 0)	0 (0 to 0)	0 (0 to 0)	0 (0 to 0)	0 (0 to 0)	0 (0 to 0)	0 (0 to 0)	0 (0 to 0)	0 (0 to 0)	0 (0 to 0)	0 (0 to 0)	0 (0 to 0)	0
Benign and in situ cervical and uterine neoplasms	0 (0 to 0)	0 (0 to 0)	0 (0 to 0)	0 (0 to 0)	0 (0 to 0)	0 (0 to 0)	0 (0 to 0)	0 (0 to 0)	0 (0 to 0)	0 (0 to 0)	0 (0 to 0)	0 (0 to 0)	0 (0 to 0)	0 (0 to 0)	0 (0 to 0)	0 (0 to 0)	0 (0 to 0)	0 (0 to 0)	0 (0 to 0)	0 (0 to 0)	0 (0 to 0)	0 (0 to 0)	0 (0 to 0)	0
Other benign and in situ neoplasms	0 (0 to 0)	0 (0 to 0)	0 (0 to 0)	0 (0 to 0)	0 (0 to 0)	0 (0 to 0)	0 (0 to 0)	0 (0 to 0)	0 (0 to 0)	0 (0 to 0)	0 (0 to 0)	0 (0 to 0)	0 (0 to 0)	0 (0 to 0)	0 (0 to 0)	0 (0 to 0)	0 (0 to 0)	0 (0 to 0)	0 (0 to 0)	0 (0 to 0)	0 (0 to 0)	0 (0 to 0)	0 (0 to 0)	0
Cardiovascular diseases	2·4 (1·4 to 3·8)	0·8 (0·5 to 1·2)	3·4 (2·1 to 5·6)	2·7 (2 to 3·7)	6 (4·4 to 7·9)	11·5 (8·5 to 15·2)	32 (24·8 to 40·6)	38 (29·7 to 48·9)	63·3 (49·5 to 80·9)	104 (81·4 to 132)	138 (105 to 177)	224 (175 to 284)	420 (323 to 534)	567 (440 to 708)	882 (693 to 1,110)	1,070 (848 to 1,310)	1,120 (918 to 1,350)	1,180 (975 to 1,390)	933 (775 to 1,080)	731 (604 to 853)	516 (434 to 591)	249 (204 to 286)	47·1 (37·3 to 55)	8,350
Rheumatic heart disease	0 (0 to 0)	0 (0 to 0)	0 (0 to 0)	0·3 (0·1 to 0·5)	1·6 (0·8 to 2·8)	3·9 (2·2 to 6·5)	6·8 (4·3 to 10·3)	6·8 (4·3 to 10·3)	8 (5·2 to 12)	8·4 (5·6 to 12)	8·4 (5·7 to 11·8)	8·2 (5·6 to 11·5)	8·5 (5·8 to 11·6)	7·3 (5·2 to 9·9)	7·3 (5·4 to 9·7)	5·7 (4·2 to 7·4)	4 (3 to 5·2)	2·9 (2·2 to 3·8)	1·9 (1·4 to 2·5)	0·9 (0·7 to 1·2)	0·5 (0·4 to 0·7)	0·2 (0·2 to 0·3)	0 (0 to 0)	91·6
Ischemic heart disease	0 (0 to 0)	0 (0 to 0)	0 (0 to 0)	0 (0 to 0)	0 (0 to 0)	0 (0 to 0)	2·3 (1·6 to 3·2)	4·6 (3·1 to 6·6)	11·4 (8 to 15·6)	25·4 (18·6 to 33·9)	39·1 (28·5 to 52·4)	71·2 (53·6 to 93·5)	155 (119 to 202)	244 (188 to 309)	380 (297 to 474)	447 (358 to 543)	477 (392 to 572)	490 (405 to 579)	363 (300 to 421)	282 (236 to 331)	205 (173 to 240)	98·7 (80·7 to 114)	19·3 (15 to 22·7)	3,320
Stroke	0·5 (0·2 to 1)	0·1 (0 to 0·2)	0·4 (0·2 to 0·7)	0·5 (0·4 to 0·7)	1·5 (1 to 2·2)	2·6 (1·8 to 3·6)	8·1 (5·6 to 11·2)	10·9 (7·5 to 15·3)	16·9 (11·2 to 24·8)	29·9 (20·1 to 42·4)	40·4 (26·8 to 57)	67·5 (45·4 to 94·4)	134 (89·8 to 188)	161 (114 to 222)	268 (190 to 371)	349 (252 to 471)	376 (281 to 486)	412 (310 to 529)	336 (253 to 424)	253 (190 to 331)	172 (130 to 217)	80·6 (60 to 101)	13·9 (9·9 to 17·7)	2,730
Ischemic stroke	0 (0 to 0)	0 (0 to 0)	0 (0 to 0·1)	0·4 (0·3 to 0·5)	0·9 (0·5 to 1·6)	1·4 (0·8 to 2·1)	2·2 (1·4 to 3·2)	2·4 (1·6 to 3·4)	3·8 (2·5 to 5·4)	5·8 (3·9 to 8·2)	7·7 (5 to 11·1)	12·1 (8·2 to 17·3)	23·8 (16 to 33·5)	34·8 (24·5 to 48)	60·1 (42 to 81·7)	118 (82·6 to 161)	143 (102 to 192)	220 (161 to 288)	184 (138 to 239)	187 (142 to 240)	133 (100 to 171)	63·7 (46·2 to 80)	11·1 (7·9 to 14·2)	1,220
Intracerebral hemorrhage	0·1 (0 to 0·2)	0 (0 to 0)	0·1 (0 to 0·3)	0 (0 to 0·1)	0·2 (0·1 to 0·3)	0·5 (0·3 to 0·7)	2·5 (1·5 to 3·8)	3·6 (2·2 to 5·4)	6·6 (4 to 10·3)	14 (8·8 to 21·1)	19 (11·6 to 28·7)	35·4 (22·3 to 51·9)	81·5 (52·6 to 116)	97·1 (65·8 to 137)	167 (115 to 236)	199 (139 to 283)	204 (147 to 273)	170 (118 to 231)	136 (94·2 to 185)	55·7 (37·2 to 79·6)	32·3 (22·1 to 44·3)	14·3 (9·8 to 19·2)	2·3 (1·6 to 3·2)	1,240
Subarachnoid hemorrhage	0·4 (0·2 to 0·9)	0·1 (0 to 0·2)	0·2 (0·1 to 0·4)	0·1 (0·1 to 0·2)	0·3 (0·2 to 0·5)	0·8 (0·5 to 1·2)	3·4 (2 to 5·1)	4·9 (2·9 to 7·7)	6·6 (3·9 to 10·6)	10·1 (6·3 to 15·4)	13·7 (8·2 to 20·7)	20 (12·5 to 30·1)	28·9 (17·9 to 44·8)	28·8 (17·9 to 42·1)	40·9 (26 to 59·5)	33·1 (21·4 to 48·4)	29·4 (19·3 to 42·6)	22·4 (15·1 to 32·4)	15·3 (9·9 to 22·1)	10·5 (6·9 to 15·5)	6·3 (4 to 9·4)	2·6 (1·6 to 3·8)	0·5 (0·3 to 0·8)	279
Hypertensive heart disease	0 (0 to 0)	0 (0 to 0)	0 (0 to 0)	0 (0 to 0)	0 (0 to 0)	0 (0 to 0)	0·4 (0·3 to 0·6)	0·7 (0·4 to 1·2)	1·5 (0·9 to 2·5)	4 (2·5 to 6·2)	6·8 (4·3 to 10·2)	13·1 (8·6 to 20)	27·9 (17·3 to 43·2)	38·8 (25 to 57·5)	62·1 (40·7 to 89·4)	76·2 (50·7 to 111)	75·8 (51·5 to 108)	81·6 (56·7 to 115)	71·7 (49·8 to 98·8)	59·1 (42·6 to 81)	38·6 (28·4 to 52·2)	19·5 (13·7 to 26·1)	4·1 (2·8 to 5·8)	582
Non-rheumatic valvular heart disease	0 (0 to 0)	0 (0 to 0)	0 (0 to 0)	0 (0 to 0)	0 (0 to 0)	0 (0 to 0)	0·9 (0·6 to 1·3)	0·9 (0·5 to 1·3)	1·8 (1·1 to 2·8)	2·5 (1·6 to 3·7)	2·4 (1·5 to 3·6)	3·6 (2·3 to 5·4)	5·3 (3·5 to 7·8)	5·5 (3·5 to 8·1)	9·2 (6·3 to 12·7)	10 (6·9 to 14)	10·6 (7·4 to 14·7)	9·6 (6·7 to 13·3)	7·2 (5·2 to 9·8)	6·2 (4·3 to 8·5)	3·8 (2·8 to 5·2)	1·8 (1·2 to 2·5)	0·3 (0·2 to 0·4)	81·7
Non-rheumatic calcific aortic valve disease	0 (0 to 0)	0 (0 to 0)	0 (0 to 0)	0 (0 to 0)	0 (0 to 0)	0 (0 to 0)	0·7 (0·4 to 1·1)	0·6 (0·3 to 0·9)	1·2 (0·7 to 2)	1·5 (0·9 to 2·3)	1·6 (0·9 to 2·4)	2·5 (1·5 to 3·9)	4 (2·5 to 6)	4 (2·6 to 6·1)	6·9 (4·5 to 9·6)	7·7 (5·2 to 10·8)	8·7 (6 to 12·1)	8·1 (5·5 to 11·2)	6·1 (4·3 to 8·4)	5·5 (3·8 to 7·4)	3·3 (2·4 to 4·6)	1·5 (1·1 to 2·2)	0·2 (0·2 to 0·3)	64·1
Non-rheumatic degenerative mitral valve disease	0 (0 to 0)	0 (0 to 0)	0 (0 to 0)	0 (0 to 0)	0 (0 to 0)	0 (0 to 0)	0·2 (0·1 to 0·3)	0·2 (0·1 to 0·3)	0·5 (0·3 to 0·9)	0·9 (0·5 to 1·4)	0·8 (0·4 to 1·3)	1·1 (0·5 to 1·8)	1·4 (0·7 to 2·3)	1·4 (0·7 to 2·4)	2·3 (1·2 to 3·8)	2·3 (1·2 to 4)	1·8 (1 to 3)	1·6 (0·8 to 2·6)	1·1 (0·6 to 1·8)	0·7 (0·4 to 1·2)	0·5 (0·3 to 0·8)	0·2 (0·1 to 0·4)	0 (0 to 0)	16·8
Other non-rheumatic valve diseases	0 (0 to 0)	0 (0 to 0)	0 (0 to 0)	0 (0 to 0)	0 (0 to 0)	0 (0 to 0)	0 (0 to 0)	0·1 (0 to 0·2)	0 (0 to 0·1)	0·1 (0 to 0·3)	0 (0 to 0·1)	0 (0 to 0·1)	0 (0 to 0·1)	0·1 (0 to 0·2)	0 (0 to 0·1)	0 (0 to 0·1)	0 (0 to 0·1)	0 (0 to 0·1)	0 (0 to 0·1)	0 (0 to 0·1)	0 (0 to 0·1)	0 (0 to 0)	0 (0 to 0)	0·7
Cardiomyopathy and myocarditis	0·6 (0·3 to 1·1)	0·3 (0·2 to 0·5)	1·4 (0·8 to 2·4)	0·8 (0·5 to 1·3)	1·1 (0·7 to 1·5)	1·6 (1·1 to 2·3)	5·6 (3·8 to 8)	6 (4 to 8·5)	11·3 (7·5 to 16·2)	17 (11·3 to 24)	21·9 (14 to 31·2)	29·7 (20·2 to 42·6)	46·7 (31 to 65·3)	55·2 (35·3 to 77·6)	70·7 (47·3 to 101)	73·3 (49·5 to 102)	62·3 (42·8 to 85·1)	54·1 (38 to 76·1)	36·4 (25·2 to 50·8)	25 (17·3 to 35·7)	17·6 (11·3 to 25·2)	7·6 (4·8 to 11·3)	1·4 (0·9 to 2·2)	548
Myocarditis	0·1 (0 to 0·2)	0·1 (0 to 0·1)	0·3 (0·1 to 0·6)	0·2 (0·1 to 0·4)	0·2 (0·1 to 0·4)	0·2 (0·1 to 0·4)	0·8 (0·4 to 1·4)	0·7 (0·3 to 1·1)	1·1 (0·6 to 1·8)	1·4 (0·7 to 2·5)	1·4 (0·7 to 2·3)	1·6 (0·8 to 2·6)	1·4 (0·7 to 2·4)	1·8 (0·9 to 3·2)	1·8 (0·9 to 3·1)	1·8 (0·9 to 3·4)	1·9 (1 to 3·2)	1·2 (0·6 to 2·3)	1 (0·5 to 1·9)	0·7 (0·4 to 1·4)	0·5 (0·2 to 1)	0·2 (0·1 to 0·5)	0·1 (0·1 to 0·2)	20·6
Alcoholic cardiomyopathy	0 (0 to 0)	0 (0 to 0)	0 (0 to 0)	0 (0 to 0)	0 (0 to 0)	0 (0 to 0)	0·5 (0·3 to 0·8)	0·7 (0·3 to 1·2)	2·1 (1·1 to 3·8)	5·7 (3·2 to 9·2)	7·5 (4·1 to 12·3)	12·5 (7·6 to 19·2)	23·8 (14·4 to 36·2)	28·3 (17·2 to 42·6)	34·6 (21 to 52·5)	33·6 (19·6 to 51·5)	30·3 (19 to 45·2)	19·4 (10·8 to 30·8)	11·7 (6·3 to 19·1)	2·8 (1·2 to 5·2)	1·5 (0·6 to 3)	0·5 (0·2 to 1)	0·1 (0 to 0·1)	216
Other cardiomyopathy	0·5 (0·2 to 0·9)	0·2 (0·1 to 0·4)	1·1 (0·6 to 1·8)	0·6 (0·4 to 1)	0·8 (0·6 to 1·2)	1·4 (1 to 2)	4·3 (2·9 to 6·2)	4·7 (3·2 to 6·6)	8·1 (5·2 to 11·8)	9·9 (6·1 to 15·4)	12·9 (7·9 to 18·9)	15·6 (9·6 to 23·5)	21·5 (12·7 to 33·7)	25 (15 to 38)	34·3 (21·1 to 51·3)	37·9 (23·9 to 56·3)	30·1 (19·6 to 44·5)	33·4 (21·6 to 49·8)	23·7 (15·2 to 34·1)	21·6 (14·5 to 31·4)	15·6 (10 to 22·7)	6·8 (4·3 to 10·1)	1·3 (0·8 to 1·9)	311
Atrial fibrillation and flutter	0 (0 to 0)	0 (0 to 0)	0 (0 to 0)	0 (0 to 0)	0 (0 to 0)	0 (0 to 0)	0 (0 to 0)	0 (0 to 0)	0 (0 to 0)	0·3 (0·2 to 0·5)	0·4 (0·2 to 0·6)	1·5 (0·9 to 2·4)	3·4 (2·2 to 5)	5·8 (3·9 to 8·5)	11·4 (8·1 to 15·9)	17·4 (12·4 to 23·7)	21·9 (15·8 to 29·7)	29·1 (21·4 to 39·2)	29·9 (22·2 to 39·9)	34·2 (25·4 to 44·9)	30·2 (22·1 to 41·1)	20 (14·1 to 28·5)	4 (2·7 to 5·6)	210
Aortic aneurysm	--	--	--	--	--	--	0·4 (0·3 to 0·6)	0·3 (0·2 to 0·4)	0·6 (0·4 to 1)	1·2 (0·8 to 1·7)	1·8 (1·1 to 2·6)	2·7 (1·8 to 3·8)	4·4 (2·9 to 6·3)	7 (4·8 to 10·1)	15·2 (10·7 to 20·8)	23 (15·5 to 32·2)	25·3 (17 to 36·2)	27·7 (19·1 to 38·5)	21·3 (14·5 to 30)	15·4 (10·7 to 21·6)	9·4 (6·8 to 12·8)	3·6 (2·6 to 4·7)	0·5 (0·3 to 0·6)	NA
Endocarditis	0·1 (0·1 to 0·2)	0·1 (0 to 0·2)	0·4 (0·2 to 0·7)	0·3 (0·2 to 0·6)	0·3 (0·1 to 0·5)	0·7 (0·4 to 1·2)	2·4 (1·4 to 3·8)	3 (1·8 to 4·6)	4·5 (2·7 to 7)	7·3 (4·3 to 12·2)	7·3 (4·4 to 11·8)	10·3 (6·1 to 16·6)	11·8 (6·8 to 18·3)	12·5 (7·6 to 19·6)	13·5 (8·2 to 20·8)	12·6 (7·8 to 19·4)	10·6 (6·6 to 16)	9·2 (5·8 to 13·7)	5·4 (3·5 to 8·1)	4 (2·6 to 6)	2·7 (1·7 to 4)	0·9 (0·6 to 1·3)	0·2 (0·1 to 0·3)	120
Other cardiovascular and circulatory diseases	1·1 (0·5 to 2)	0·3 (0·2 to 0·5)	1·2 (0·6 to 2·1)	0·8 (0·5 to 1·2)	1·5 (0·9 to 2·5)	2·6 (1·6 to 4·1)	5·1 (3·5 to 7·2)	4·8 (3·4 to 6·7)	7·2 (4·9 to 9·9)	8·3 (5·9 to 11·5)	9·8 (6·9 to 13·7)	14·5 (10·5 to 19·6)	19·4 (13·8 to 27·1)	22·5 (16·2 to 30·7)	32 (23·3 to 43·2)	37·4 (27·3 to 48·8)	35·6 (26·8 to 46·2)	35·8 (26·9 to 46·9)	31·6 (23·4 to 41·1)	26·4 (19·4 to 35·3)	18·2 (13·4 to 23·5)	8·3 (6 to 10·7)	1·5 (1·1 to 1·9)	326
Chronic respiratory diseases	0 (0 to 0)	0 (0 to 0)	0 (0 to 0)	48·6 (25·4 to 85·2)	49·6 (28·3 to 83·8)	40·6 (24·1 to 64·4)	32·8 (20 to 50·4)	25·1 (16·2 to 37·2)	27·2 (18·8 to 38)	29·1 (21 to 39·2)	29·8 (22·2 to 40)	31·9 (24·2 to 40·8)	42·4 (32·8 to 54·4)	54·3 (41·1 to 68·7)	80·6 (60·2 to 102)	106 (80·6 to 136)	109 (83·5 to 138)	125 (94·7 to 158)	104 (82·9 to 129)	85 (67·3 to 106)	57·7 (45·6 to 72·3)	26·8 (21·1 to 33·1)	5·2 (4·1 to 6·4)	1,110
Chronic obstructive pulmonary disease	0 (0 to 0)	0 (0 to 0)	0 (0 to 0)	0·3 (0·2 to 0·4)	0·5 (0·3 to 0·7)	0·9 (0·2 to 1·8)	1·6 (0·7 to 2·8)	2·2 (1·1 to 3·5)	3·1 (1·8 to 4·9)	3·9 (2·3 to 5·9)	5 (3·1 to 7·2)	6·8 (4·6 to 9·4)	11·6 (8·1 to 15·6)	18 (12·8 to 23·8)	34·3 (24·3 to 46·3)	55·8 (40·3 to 72·9)	64·9 (48 to 84·3)	82·4 (60·8 to 106)	75 (57·3 to 95·6)	62·3 (48·6 to 79·3)	44·6 (34·9 to 56)	20·7 (16·3 to 26)	4 (3 to 4·9)	498
Pneumoconiosis	0 (0 to 0)	0 (0 to 0)	0 (0 to 0)	0 (0 to 0)	0 (0 to 0)	0 (0 to 0)	0 (0 to 0)	0 (0 to 0)	0 (0 to 0)	0 (0 to 0)	0 (0 to 0)	0 (0 to 0)	0 (0 to 0·1)	0 (0 to 0·1)	0·1 (0 to 0·1)	0·1 (0·1 to 0·2)	0·2 (0·1 to 0·2)	0·1 (0·1 to 0·2)	0·1 (0·1 to 0·1)	0·1 (0·1 to 0·1)	0·1 (0·1 to 0·1)	0 (0 to 0·1)	0 (0 to 0)	1
Silicosis	0 (0 to 0)	0 (0 to 0)	0 (0 to 0)	0 (0 to 0)	0 (0 to 0)	0 (0 to 0)	0 (0 to 0)	0 (0 to 0)	0 (0 to 0)	0 (0 to 0)	0 (0 to 0)	0 (0 to 0)	0 (0 to 0)	0 (0 to 0)	0 (0 to 0)	0 (0 to 0·1)	0 (0 to 0·1)	0 (0 to 0·1)	0 (0 to 0)	0 (0 to 0)	0 (0 to 0)	0 (0 to 0)	0 (0 to 0)	0·2
Asbestosis	0 (0 to 0)	0 (0 to 0)	0 (0 to 0)	0 (0 to 0)	0 (0 to 0)	0 (0 to 0)	0 (0 to 0)	0 (0 to 0)	0 (0 to 0)	0 (0 to 0)	0 (0 to 0)	0 (0 to 0)	0 (0 to 0)	0 (0 to 0)	0 (0 to 0)	0 (0 to 0)	0 (0 to 0)	0 (0 to 0)	0 (0 to 0)	0 (0 to 0)	0 (0 to 0)	0 (0 to 0)	0 (0 to 0)	0·1
Coal workers pneumoconiosis	0 (0 to 0)	0 (0 to 0)	0 (0 to 0)	0 (0 to 0)	0 (0 to 0)	0 (0 to 0)	0 (0 to 0)	0 (0 to 0)	0 (0 to 0)	0 (0 to 0)	0 (0 to 0)	0 (0 to 0)	0 (0 to 0)	0 (0 to 0)	0 (0 to 0)	0 (0 to 0)	0 (0 to 0)	0 (0 to 0)	0 (0 to 0)	0 (0 to 0)	0 (0 to 0)	0 (0 to 0)	0 (0 to 0)	0
Other pneumoconiosis	0 (0 to 0)	0 (0 to 0)	0 (0 to 0)	0 (0 to 0)	0 (0 to 0)	0 (0 to 0)	0 (0 to 0)	0 (0 to 0)	0 (0 to 0)	0 (0 to 0)	0 (0 to 0)	0 (0 to 0)	0 (0 to 0)	0 (0 to 0)	0 (0 to 0·1)	0 (0 to 0·1)	0·1 (0·1 to 0·2)	0·1 (0 to 0·1)	0·1 (0 to 0·1)	0·1 (0 to 0·1)	0 (0 to 0·1)	0 (0 to 0)	0 (0 to 0)	0·6
Asthma	0 (0 to 0)	0 (0 to 0)	0 (0 to 0)	45·9 (22·8 to 82·6)	48 (26·9 to 81·9)	38 (21·6 to 61·6)	29·1 (16·8 to 46·9)	20·5 (12·2 to 32·4)	20·7 (13 to 31·2)	21·2 (14·2 to 30·7)	20·4 (13·8 to 29·8)	19·8 (13·9 to 27·8)	24·1 (16·9 to 32·9)	27·6 (19·3 to 38·1)	33·9 (23·8 to 46·1)	40·5 (28·1 to 55·1)	33·6 (23·7 to 44·9)	30·1 (21 to 41·4)	19·4 (13·7 to 27·1)	15·8 (10·7 to 22·2)	8·6 (5·8 to 11·9)	4·3 (3·1 to 5·8)	0·9 (0·6 to 1·2)	502
Interstitial lung disease and pulmonary sarcoidosis	0 (0 to 0)	0 (0 to 0)	0 (0 to 0)	0·4 (0·2 to 0·8)	0·1 (0 to 0·1)	0·1 (0·1 to 0·2)	0·4 (0·2 to 0·7)	0·5 (0·3 to 0·8)	0·4 (0·3 to 0·7)	1·1 (0·6 to 1·8)	1·2 (0·7 to 1·9)	2 (1·2 to 3·1)	2·5 (1·5 to 3·9)	3·7 (2·3 to 5·7)	6 (3·6 to 9)	7·2 (4·3 to 11·1)	7·7 (4·4 to 12·1)	9·2 (5·4 to 14)	7·5 (4·5 to 11·6)	4·9 (2·7 to 8·1)	3 (1·6 to 4·8)	1·1 (0·6 to 1·9)	0·2 (0·1 to 0·4)	59·3
Other chronic respiratory diseases	0 (0 to 0)	0 (0 to 0)	0 (0 to 0)	2 (1·2 to 3·2)	1 (0·7 to 1·4)	1·6 (0·7 to 2·8)	1·6 (1 to 2·6)	1·9 (1·1 to 2·8)	2·8 (1·7 to 4·2)	2·9 (1·8 to 4·3)	3·1 (2 to 4·6)	3·2 (1·9 to 4·8)	4·2 (2·6 to 6·5)	5 (3 to 8)	6·3 (3·5 to 9·9)	2·9 (1·5 to 5·1)	2·6 (1·4 to 4·4)	2·8 (1·6 to 4·6)	2·2 (1·2 to 3·5)	2 (0·9 to 3·5)	1·5 (0·7 to 2·9)	0·6 (0·3 to 1·1)	0·2 (0·1 to 0·3)	50·3
Digestive diseases	1·5 (1 to 2·3)	1 (0·6 to 1·5)	6·5 (3·9 to 10·8)	11·1 (7·8 to 15·4)	11·9 (8 to 17·6)	11·7 (8·3 to 16·2)	20·7 (15·7 to 27·4)	29·2 (21·3 to 39·8)	47·3 (35·2 to 63·9)	70·7 (54·3 to 90·8)	90·8 (69·2 to 115)	123 (96 to 157)	180 (141 to 228)	206 (157 to 262)	259 (196 to 333)	275 (213 to 345)	220 (172 to 273)	190 (146 to 234)	124 (95·9 to 152)	87·5 (67·5 to 110)	51·3 (40·5 to 62·7)	21·1 (16·5 to 25·2)	3·5 (2·7 to 4·2)	2,040
Cirrhosis and other chronic liver diseases	0 (0 to 0)	0 (0 to 0)	0 (0 to 0)	0·7 (0·4 to 1·2)	0·9 (0·5 to 1·3)	1·3 (0·8 to 2)	3·3 (2 to 4·9)	4·3 (2·8 to 6·4)	10 (6·7 to 14·6)	22·1 (15·3 to 30·7)	32·7 (23·5 to 44·5)	55 (38·8 to 74·8)	93·9 (67·8 to 128)	110 (79 to 148)	140 (101 to 189)	145 (106 to 194)	109 (78·4 to 142)	81·8 (59·5 to 108)	42·8 (30·3 to 56·9)	27 (19·1 to 36)	13·7 (10 to 18·2)	5 (3·5 to 6·7)	0·8 (0·6 to 1·1)	899
Cirrhosis and other chronic liver diseases due to hepatitis B	0 (0 to 0)	0 (0 to 0)	0 (0 to 0)	0 (0 to 0)	0 (0 to 0)	0 (0 to 0)	0·1 (0 to 0·1)	0·2 (0·1 to 0·4)	0·7 (0·2 to 1·5)	2 (1 to 3·5)	3·3 (1·5 to 6·2)	5·7 (2·8 to 10·4)	9·4 (4·6 to 17·2)	10·2 (4·7 to 19)	11 (5 to 20·1)	9·8 (4·5 to 18·4)	6·7 (3·2 to 12·7)	5 (2·2 to 9·2)	2·7 (1·3 to 4·8)	1·7 (0·8 to 3·2)	0·9 (0·4 to 1·5)	0·3 (0·1 to 0·6)	0 (0 to 0·1)	69·7
Cirrhosis and other chronic liver diseases due to hepatitis C	0 (0 to 0)	0 (0 to 0)	0 (0 to 0)	0 (0 to 0)	0 (0 to 0)	0 (0 to 0·1)	0·2 (0·1 to 0·4)	0·7 (0·3 to 1·3)	2·4 (1·2 to 4·3)	5·6 (3·1 to 9·1)	7·9 (4 to 13·8)	12·7 (6·6 to 21·5)	20·1 (10·5 to 33·6)	21·9 (11·1 to 37·5)	25·9 (13·2 to 45·6)	25·2 (12 to 46·9)	18·5 (10·1 to 32·6)	13·7 (7·3 to 22·6)	7 (3·6 to 12)	4·5 (2·4 to 8)	2·4 (1·3 to 3·8)	0·9 (0·4 to 1·6)	0·1 (0·1 to 0·3)	170
Cirrhosis and other chronic liver diseases due to alcohol use	0 (0 to 0)	0 (0 to 0)	0 (0 to 0)	0 (0 to 0)	0 (0 to 0)	0 (0 to 0)	0 (0 to 0·1)	0·5 (0·2 to 1·1)	2·2 (1 to 4·1)	7·6 (4·2 to 12·2)	14·1 (7·7 to 22·1)	25·9 (15·8 to 39·2)	46·1 (27·9 to 68·2)	56·4 (34·1 to 83·3)	75·8 (47·3 to 107)	79·1 (50·4 to 114)	57 (36·5 to 79·6)	40·7 (24·8 to 58·4)	19·7 (12·1 to 28·6)	11·5 (6·9 to 17·1)	5·5 (3·4 to 8·1)	1·9 (1·1 to 2·9)	0·3 (0·1 to 0·5)	444
Cirrhosis and other chronic liver diseases due to NAFLD	0 (0 to 0)	0 (0 to 0)	0 (0 to 0)	0 (0 to 0)	0 (0 to 0)	0 (0 to 0)	0·2 (0·1 to 0·4)	0·5 (0·2 to 0·9)	1·6 (0·7 to 3·2)	3·3 (1·6 to 5·7)	4·2 (1·9 to 8·1)	6·8 (3·2 to 12·3)	12 (5·3 to 23·2)	14 (6·2 to 26·9)	17·3 (7·9 to 33·2)	18·7 (8·4 to 34·6)	15·7 (8·6 to 26)	13·2 (7·2 to 21·1)	7·7 (3·9 to 12·7)	5·3 (2·6 to 8·7)	2·8 (1·6 to 4·5)	1·1 (0·5 to 1·9)	0·2 (0·1 to 0·3)	124
Cirrhosis and other chronic liver diseases due to other causes	0 (0 to 0)	0 (0 to 0)	0 (0 to 0)	0·7 (0·4 to 1·2)	0·9 (0·5 to 1·3)	1·2 (0·8 to 1·8)	2·9 (1·7 to 4·2)	2·3 (1·4 to 3·8)	3 (1·5 to 5·2)	3·6 (1·8 to 7·1)	3·2 (1·4 to 5·8)	4 (1·8 to 7·8)	6·3 (2·9 to 12·4)	7·4 (3·4 to 14·1)	10·5 (5·1 to 20·4)	12·4 (6 to 22·9)	10·7 (5 to 19·3)	9·3 (4·4 to 16·7)	5·6 (2·7 to 10·1)	4 (2 to 6·9)	2·1 (1·1 to 3·5)	0·8 (0·4 to 1·4)	0·1 (0 to 0·3)	91·1
Upper digestive system diseases	0 (0 to 0)	0 (0 to 0)	0 (0 to 0)	0·4 (0·2 to 0·8)	0·7 (0·3 to 1·2)	1·4 (0·8 to 2·3)	5·8 (3·4 to 9·8)	12·3 (6·9 to 22·3)	18·9 (10·9 to 31·8)	23·1 (13·6 to 38·3)	26·2 (15·3 to 42·7)	28·9 (17·6 to 44·9)	35·4 (23 to 53·8)	39·1 (25·4 to 58·3)	45 (30·2 to 67·1)	47 (32 to 67·5)	39·4 (27·9 to 55·1)	34·1 (24·5 to 46·9)	24·9 (17·7 to 34)	17·7 (12·9 to 24·2)	10·5 (7·7 to 14)	4·7 (3·5 to 6·1)	0·7 (0·5 to 0·9)	416
Peptic ulcer disease	0 (0 to 0)	0 (0 to 0)	0 (0 to 0)	0·1 (0 to 0·1)	0·1 (0·1 to 0·1)	0·2 (0·1 to 0·3)	0·8 (0·5 to 1·2)	1·3 (0·8 to 2)	2·4 (1·5 to 3·5)	4·2 (2·7 to 6·2)	4·9 (3·2 to 7)	6·3 (4 to 9·2)	9·1 (5·7 to 13·6)	11·9 (7·5 to 17·9)	16·5 (10·2 to 24·6)	20 (12·9 to 29)	18·4 (12·1 to 25·8)	18·3 (12·1 to 26·6)	15·1 (10·2 to 21·6)	11·4 (7·6 to 16·2)	7·1 (4·8 to 10)	3·3 (2·3 to 4·5)	0·5 (0·4 to 0·7)	152
Gastritis and duodenitis	0 (0 to 0)	0 (0 to 0)	0 (0 to 0)	0·4 (0·1 to 0·7)	0·6 (0·2 to 1·1)	0·6 (0·2 to 1·1)	0·7 (0·3 to 1·4)	0·9 (0·4 to 1·7)	1·4 (0·6 to 2·5)	2·1 (1 to 3·6)	3 (1·5 to 5·5)	4 (2 to 7·4)	5·8 (3·1 to 10·5)	5·9 (3·2 to 10)	7 (4 to 11·7)	6·8 (3·9 to 11)	5·2 (3 to 8·1)	3·8 (2·3 to 5·8)	2·2 (1·4 to 3·2)	1·4 (0·9 to 2·2)	0·8 (0·5 to 1·2)	0·4 (0·2 to 0·6)	0 (0 to 0·1)	52·9
Gastroesophageal reflux disease	0 (0 to 0)	0 (0 to 0)	0 (0 to 0)	0 (0 to 0)	0 (0 to 0·1)	0·6 (0·3 to 1·2)	4·2 (2·1 to 7·9)	10·1 (5 to 20)	15·1 (7·3 to 28·2)	16·8 (8 to 31·7)	18·3 (8·7 to 33·8)	18·5 (8·9 to 34·1)	20·6 (10 to 37·5)	21·2 (10·2 to 39·2)	21·5 (9·9 to 41·6)	20·2 (9·7 to 38·2)	15·9 (8·1 to 28·8)	12 (6·1 to 21·1)	7·6 (3·8 to 13·4)	4·9 (2·4 to 8·8)	2·5 (1·3 to 4·6)	0·9 (0·5 to 1·7)	0·1 (0·1 to 0·2)	211
Appendicitis	0 (0 to 0)	0 (0 to 0)	0 (0 to 0)	0·4 (0·2 to 0·8)	0·7 (0·4 to 1)	1·1 (0·5 to 2·1)	1·8 (0·9 to 3)	1·6 (0·8 to 2·8)	1·7 (0·9 to 2·8)	1·8 (1 to 2·9)	1·8 (1 to 3·2)	1·7 (1 to 3)	1·9 (1·1 to 3·4)	2 (1·1 to 3·5)	2·1 (1·2 to 3·6)	2·1 (1·2 to 3·8)	2·2 (1·3 to 4)	2·1 (1·2 to 3·8)	1·2 (0·7 to 2·2)	0·9 (0·5 to 1·6)	0·6 (0·3 to 1·2)	0·2 (0·1 to 0·5)	0 (0 to 0·1)	28
Paralytic ileus and intestinal obstruction	1·2 (0·8 to 1·9)	0·9 (0·6 to 1·4)	5·7 (3·4 to 9·7)	2·3 (1·4 to 3·7)	1·5 (0·9 to 2·3)	1·4 (0·9 to 2·1)	2·3 (1·4 to 3·6)	2·5 (1·5 to 4)	3·2 (1·9 to 4·9)	4·3 (2·6 to 6·5)	6·1 (3·8 to 9·4)	7·5 (4·7 to 11·6)	10·1 (6·1 to 15·9)	13·3 (7·9 to 20·3)	19·5 (11·4 to 30)	23·1 (14 to 35·2)	22·8 (14·2 to 34)	27·2 (17·7 to 39·8)	22·5 (15·1 to 31·4)	17·8 (11·9 to 25·9)	11·3 (7·8 to 15·7)	4·5 (2·9 to 6·3)	0·9 (0·6 to 1·2)	212
Inguinal, femoral, and abdominal hernia	0·3 (0·2 to 0·6)	0·1 (0 to 0·2)	0·8 (0·4 to 1·5)	5 (2·8 to 8·2)	6·7 (3·6 to 11·7)	4·8 (2·3 to 8·5)	4·2 (2·1 to 7·5)	4·1 (2 to 7·2)	5·2 (2·8 to 8·8)	6 (3·2 to 10·4)	8 (4·2 to 13·4)	9·9 (5·6 to 16·4)	13·1 (7·3 to 21·3)	15·3 (8·9 to 24·4)	17·4 (10·7 to 27·5)	17·3 (10·6 to 25·9)	13·5 (9·1 to 19·8)	10·3 (7·1 to 14·1)	6·5 (4·7 to 8·7)	4·3 (3·2 to 5·8)	2·5 (1·8 to 3·4)	1·1 (0·8 to 1·5)	0·2 (0·1 to 0·2)	157
Inflammatory bowel disease	0 (0 to 0)	0 (0 to 0)	0 (0 to 0)	0·1 (0 to 0·2)	0 (0 to 0·1)	0·1 (0 to 0·1)	0·3 (0·2 to 0·4)	0·5 (0·3 to 0·7)	1 (0·5 to 1·6)	1·6 (1 to 2·3)	1·8 (1·1 to 2·9)	2·2 (1·3 to 3·3)	2·8 (1·7 to 4·2)	3 (1·8 to 4·4)	3·5 (2·2 to 5·2)	3·2 (2·1 to 4·8)	2·6 (1·8 to 3·8)	2·5 (1·7 to 3·5)	1·6 (1 to 2·2)	1·1 (0·7 to 1·6)	0·6 (0·4 to 0·8)	0·2 (0·2 to 0·3)	0 (0 to 0·1)	28·8
Vascular intestinal disorders	0 (0 to 0)	0 (0 to 0)	0 (0 to 0)	0 (0 to 0·1)	0·1 (0 to 0·1)	0·1 (0 to 0·1)	0·2 (0·1 to 0·2)	0·1 (0·1 to 0·1)	0·3 (0·2 to 0·4)	0·5 (0·3 to 0·8)	0·5 (0·3 to 0·8)	0·6 (0·4 to 1)	1·3 (0·8 to 2)	1·7 (1·1 to 2·6)	2·8 (1·7 to 4·2)	3·7 (2·3 to 5·5)	3·8 (2·4 to 5·4)	4 (2·6 to 5·8)	3·1 (2 to 4·6)	2·5 (1·6 to 3·6)	1·4 (0·9 to 2)	0·7 (0·5 to 1)	0·1 (0·1 to 0·1)	27·5
Gallbladder and biliary diseases	0 (0 to 0)	0 (0 to 0)	0 (0 to 0)	0·1 (0 to 0·1)	0·2 (0·1 to 0·3)	0·3 (0·2 to 0·5)	0·6 (0·3 to 0·9)	0·8 (0·3 to 1·4)	1·1 (0·5 to 2·1)	1·6 (0·9 to 2·5)	1·9 (1 to 3·1)	2·5 (1·5 to 3·9)	3·2 (1·8 to 4·9)	4 (2·5 to 6·1)	5·4 (3·4 to 7·8)	5·9 (4 to 8·6)	5·7 (3·8 to 7·9)	5·9 (4·2 to 8·6)	4·5 (3 to 6·4)	3·4 (2·3 to 5)	1·9 (1·3 to 2·8)	0·9 (0·6 to 1·3)	0·1 (0·1 to 0·2)	50
Pancreatitis	0 (0 to 0)	0 (0 to 0)	0 (0 to 0)	0·1 (0 to 0·1)	0 (0 to 0)	0·2 (0·1 to 0·3)	0·9 (0·6 to 1·4)	1·4 (0·8 to 2·1)	3·6 (2·1 to 5·6)	6·5 (3·9 to 10)	8·3 (5·2 to 12·3)	10·4 (6·6 to 15·2)	11·5 (7 to 17·1)	9·9 (6·1 to 14·8)	12·1 (7·5 to 18·5)	12·2 (7·6 to 18·1)	8·8 (5·7 to 13)	7·8 (5·2 to 11·3)	5·4 (3·6 to 7·7)	3·5 (2·3 to 5·1)	1·8 (1·1 to 2·6)	0·7 (0·4 to 0·9)	0·1 (0·1 to 0·2)	105
Other digestive diseases	0 (0 to 0)	0 (0 to 0)	0 (0 to 0)	1·9 (1 to 3)	1·2 (0·7 to 1·8)	1 (0·6 to 1·5)	1·3 (0·6 to 2·2)	1·7 (0·8 to 2·7)	2·3 (1·1 to 4)	3·3 (1·5 to 5·4)	3·5 (1·5 to 5·7)	4·5 (2·1 to 7·5)	7·2 (3·4 to 12·2)	7·8 (3·7 to 12·9)	11·2 (5·4 to 18·5)	15·3 (7·5 to 25)	12·9 (6·5 to 20·2)	13·9 (6·7 to 21·9)	11·4 (5·8 to 17·9)	9·1 (4·5 to 14·2)	6·9 (3·6 to 10·6)	3·2 (1·6 to 4·8)	0·5 (0·3 to 0·8)	120
Neurological disorders	0 (0 to 0·1)	0·1 (0 to 0·2)	7·5 (4·5 to 11·9)	13·6 (6·2 to 25·2)	25·4 (11·1 to 44·7)	66·1 (25·7 to 134)	98·9 (45·4 to 180)	92 (43·3 to 168)	97·6 (48·3 to 168)	95·5 (47·9 to 163)	98 (50·5 to 165)	100 (55·2 to 164)	112 (66·3 to 177)	110 (67·6 to 171)	125 (82·8 to 181)	136 (93 to 197)	141 (96·1 to 220)	171 (113 to 286)	177 (115 to 318)	202 (122 to 385)	170 (91·3 to 342)	89 (44·5 to 184)	19·7 (9·2 to 42·2)	2,150
Alzheimer's disease and other dementias	0 (0 to 0)	0 (0 to 0)	0 (0 to 0)	0 (0 to 0)	0 (0 to 0)	0 (0 to 0)	0 (0 to 0)	0 (0 to 0)	0 (0 to 0)	0 (0 to 0)	0 (0 to 0)	0·7 (0·2 to 2·1)	3·9 (1·2 to 10·7)	8 (3 to 20)	17·2 (6·7 to 42·7)	32·1 (12·2 to 78·8)	49·5 (19·4 to 122)	80·4 (31·9 to 192)	95·7 (38·4 to 233)	135 (55·2 to 323)	130 (49·6 to 306)	74·4 (29 to 170)	17·8 (7·2 to 40·6)	644
Parkinson's disease	0 (0 to 0)	0 (0 to 0)	0 (0 to 0)	0 (0 to 0)	0 (0 to 0)	0 (0 to 0)	0 (0 to 0)	0·1 (0 to 0·1)	0·1 (0·1 to 0·2)	0·1 (0·1 to 0·3)	0·3 (0·2 to 0·5)	0·9 (0·7 to 1·3)	2·2 (1·6 to 3)	3·9 (2·7 to 5·5)	7·5 (5·4 to 9·9)	13·9 (10·8 to 17·4)	23 (18·3 to 28·3)	40·2 (32·9 to 49)	51·3 (42 to 61·1)	48·1 (39·5 to 57·7)	30·8 (25·3 to 36·8)	10·9 (8·8 to 13·2)	1·4 (1 to 1·7)	235
Idiopathic epilepsy	0 (0 to 0·1)	0·1 (0 to 0·2)	1·9 (0·9 to 3·5)	8·5 (3·3 to 17·1)	13 (4·3 to 26·1)	18·2 (6·6 to 36·1)	27·4 (13·7 to 47·9)	27·9 (14·5 to 46·8)	31·7 (17·3 to 51·3)	31·1 (17·9 to 47·9)	30·1 (17·7 to 47·8)	29·1 (16·8 to 46·1)	32·5 (19·7 to 50·6)	27·4 (15·1 to 45·2)	28·9 (16·5 to 47)	26·7 (14·6 to 45·2)	21·4 (10·4 to 36·9)	17·1 (8·1 to 29·5)	12·6 (6 to 21·2)	8·9 (4·1 to 15·4)	5·1 (2·4 to 9)	2·2 (1 to 3·8)	0·3 (0·2 to 0·6)	402
Multiple sclerosis	0 (0 to 0)	0 (0 to 0)	0 (0 to 0)	0 (0 to 0)	0 (0 to 0·1)	0 (0 to 0·1)	0·2 (0·1 to 0·4)	0·7 (0·3 to 1·1)	0·7 (0·4 to 1·1)	1·2 (0·8 to 1·8)	1·8 (1·1 to 2·6)	2·2 (1·5 to 3·3)	2·5 (1·7 to 3·6)	2·2 (1·5 to 3·4)	3·2 (2 to 4·8)	2·7 (1·6 to 4)	1·8 (1·2 to 2·6)	1·2 (0·8 to 1·7)	0·6 (0·4 to 1)	0·3 (0·2 to 0·4)	0·1 (0·1 to 0·2)	0 (0 to 0·1)	0 (0 to 0)	21·6
Motor neuron disease	0 (0 to 0)	0 (0 to 0)	1·5 (0·7 to 2·8)	0·4 (0·2 to 0·7)	0·1 (0 to 0·1)	0·2 (0·1 to 0·3)	0·9 (0·6 to 1·4)	0·8 (0·5 to 1·2)	0·8 (0·5 to 1·2)	1·4 (0·8 to 2)	2·7 (1·8 to 3·8)	3·9 (2·5 to 5·7)	5·1 (3·3 to 7·5)	7 (4·6 to 10·2)	10·3 (6·7 to 14·2)	11·1 (7·5 to 15·4)	10·4 (6·7 to 15)	7·4 (4·8 to 10·8)	3·4 (2·2 to 4·9)	1·4 (0·9 to 2)	0·5 (0·4 to 0·8)	0·1 (0·1 to 0·2)	0 (0 to 0)	69·4
Headache disorders	0 (0 to 0)	0 (0 to 0)	0 (0 to 0)	0 (0 to 0)	6·5 (0·1 to 17·8)	35·8 (1·7 to 92·1)	51·6 (5·6 to 125)	51·1 (7·1 to 119)	53·9 (9·4 to 120)	51·8 (10·1 to 114)	54·3 (11·6 to 116)	53·4 (13·9 to 113)	54·2 (16·2 to 114)	49·5 (14·3 to 102)	43·4 (13·3 to 89·2)	35·9 (12·2 to 72·8)	24·5 (8·3 to 49·3)	15·3 (5·1 to 32·5)	8 (2·6 to 16·2)	4·4 (1·3 to 9·3)	2·1 (0·7 to 4·5)	0·7 (0·2 to 1·5)	0·1 (0 to 0·2)	596
Migraine	0 (0 to 0)	0 (0 to 0)	0 (0 to 0)	0 (0 to 0)	5·8 (0·1 to 16·1)	33·1 (1 to 89·6)	47·5 (3·8 to 122)	46·5 (5·2 to 115)	48·5 (6·1 to 115)	46·2 (6·9 to 105)	47·8 (8·3 to 109)	46·8 (9·7 to 107)	47·1 (11·2 to 104)	42·5 (9·8 to 92·7)	37·2 (9·4 to 80·6)	30 (8·3 to 63·5)	20 (6 to 43·4)	12·1 (3·7 to 26·3)	6·3 (1·8 to 13·4)	3·5 (1 to 7·8)	1·7 (0·5 to 3·6)	0·5 (0·2 to 1·2)	0·1 (0 to 0·1)	523
Tension-type headache	0 (0 to 0)	0 (0 to 0)	0 (0 to 0)	0 (0 to 0)	0·7 (0 to 4·5)	2·6 (0·1 to 20·8)	4·1 (0·5 to 22·5)	4·6 (0·8 to 22·6)	5·5 (1 to 25)	5·6 (1·1 to 23·2)	6·5 (1·3 to 26·5)	6·5 (1·5 to 24·2)	7 (2 to 22·8)	7 (1·6 to 24·5)	6·1 (1·7 to 18·7)	5·8 (1·5 to 18·1)	4·5 (1·1 to 15·4)	3·2 (0·7 to 12)	1·7 (0·3 to 6·7)	0·9 (0·2 to 3·7)	0·5 (0·1 to 1·8)	0·2 (0 to 0·7)	0 (0 to 0·1)	73·1
Other neurological disorders	0 (0 to 0)	0 (0 to 0)	4·1 (2·4 to 6·8)	4·7 (2·4 to 8·1)	5·8 (2·8 to 10·3)	11·9 (6·6 to 19·4)	18·7 (12·2 to 27·3)	11·5 (7·4 to 17)	10·3 (6·9 to 14·2)	9·8 (6·4 to 14)	8·8 (5·8 to 12·8)	10 (6·6 to 14·4)	11·3 (7·5 to 16·1)	12·4 (8·7 to 17·1)	14·7 (10·3 to 20·7)	14·1 (9·6 to 19·8)	10·7 (7·6 to 14·5)	9 (6·3 to 12·5)	5·7 (4 to 7·8)	3·8 (2·6 to 5·3)	1·7 (1·2 to 2·3)	0·7 (0·5 to 0·9)	0·1 (0·1 to 0·1)	180
Mental disorders	0 (0 to 0)	0·1 (0·1 to 0·1)	1·5 (0·9 to 2·2)	8·1 (5·3 to 11·6)	41·8 (28·2 to 60·5)	118 (79·9 to 168)	165 (114 to 228)	159 (111 to 217)	179 (125 to 243)	182 (129 to 248)	199 (140 to 265)	196 (141 to 264)	208 (152 to 278)	202 (144 to 269)	193 (140 to 257)	168 (122 to 224)	122 (87·4 to 163)	85·7 (61·4 to 112)	49·7 (35 to 65·8)	30 (21 to 40·7)	15·5 (11 to 20·7)	5·9 (4·1 to 8)	0·9 (0·6 to 1·3)	2,330
Schizophrenia	0 (0 to 0)	0 (0 to 0)	0 (0 to 0)	0 (0 to 0)	0 (0 to 0)	0·6 (0·2 to 1·1)	4·9 (1·7 to 9·7)	15·6 (7·7 to 26·4)	28 (15·7 to 41·8)	34·1 (20·4 to 50·5)	38·8 (24 to 57·1)	37·9 (24·3 to 53·2)	38·3 (25·7 to 53·3)	34·8 (23·9 to 49)	30 (19·7 to 42·1)	22·9 (14·5 to 32·8)	14·5 (9·2 to 20·9)	8·8 (5·2 to 13·1)	4·2 (2·4 to 6·4)	2 (1·1 to 3·1)	0·8 (0·4 to 1·2)	0·2 (0·1 to 0·4)	0 (0 to 0)	316
Depressive disorders	0 (0 to 0)	0 (0 to 0)	0 (0 to 0)	0 (0 to 0)	1·2 (0·5 to 2·5)	12·2 (6·2 to 22·1)	35·2 (20·5 to 55·2)	45·5 (26·1 to 72)	46·3 (27·1 to 71·5)	44·9 (27·2 to 68·5)	52·2 (31·9 to 77·1)	55·6 (34·9 to 84·5)	63·1 (39·7 to 91·2)	65·6 (42·3 to 97·9)	67·4 (42·2 to 101)	62·8 (39·5 to 94·9)	48·1 (31·9 to 69·5)	35·7 (23·1 to 51·7)	22·1 (13·7 to 33)	14·5 (8·9 to 22·1)	8·2 (5·3 to 12)	3·4 (2 to 5·2)	0·5 (0·3 to 0·9)	685
Major depressive disorder	0 (0 to 0)	0 (0 to 0)	0 (0 to 0)	0 (0 to 0)	1 (0·3 to 2·2)	10·6 (4·8 to 20·2)	30 (16·9 to 48·8)	37·1 (20 to 61·3)	35·3 (20·1 to 56·1)	33·2 (18·8 to 55·2)	39·2 (22·9 to 60·1)	42·5 (25·3 to 66·9)	49·1 (30·4 to 75·8)	52 (31·7 to 77·8)	54·2 (31·5 to 83·5)	51·2 (31·2 to 77·8)	39·5 (25·3 to 58·6)	29·7 (18·9 to 44·4)	18·6 (11·1 to 28·8)	12·4 (7·3 to 19·2)	7·1 (4·4 to 10·7)	3 (1·7 to 4·7)	0·5 (0·2 to 0·8)	546
Dysthymia	0 (0 to 0)	0 (0 to 0)	0 (0 to 0)	0 (0 to 0)	0·2 (0 to 0·6)	1·7 (0·6 to 3·3)	5·2 (2·4 to 9·2)	8·4 (4·1 to 14·6)	11 (5·7 to 19·3)	11·7 (6·4 to 19·9)	13 (7·4 to 22·1)	13·1 (7·1 to 22·5)	14 (7·6 to 24·1)	13·6 (7·8 to 23)	13·2 (7·4 to 21·3)	11·6 (6·3 to 19·1)	8·5 (4·3 to 14·7)	6 (3·5 to 10)	3·5 (1·9 to 5·8)	2·1 (1·1 to 3·6)	1·1 (0·6 to 1·8)	0·4 (0·2 to 0·7)	0·1 (0 to 0·1)	138
Bipolar disorder	0 (0 to 0)	0 (0 to 0)	0 (0 to 0)	0 (0 to 0)	0 (0 to 0)	6·1 (2·4 to 11·7)	23·5 (11·1 to 42·1)	27·6 (13·3 to 46·3)	28·8 (15·1 to 48·7)	26·6 (13·7 to 43·4)	27·9 (15·3 to 45·6)	28·1 (14·7 to 45·7)	29·8 (16·2 to 49·8)	28·2 (15·2 to 47·2)	25·5 (14·2 to 43·1)	20·5 (11·1 to 33·2)	13·6 (7·2 to 22·3)	8·4 (4·3 to 13·6)	4·1 (2·2 to 6·7)	2 (1 to 3·2)	0·8 (0·4 to 1·4)	0·3 (0·1 to 0·5)	0 (0 to 0·1)	302
Anxiety disorders	0 (0 to 0)	0 (0 to 0)	0 (0 to 0)	0·6 (0·1 to 1·3)	9·6 (4·8 to 16·5)	27·3 (14 to 45·5)	37·1 (21·2 to 58·5)	35·8 (19·2 to 60·4)	37·5 (20·5 to 63·9)	36·5 (21·6 to 58·4)	39·1 (23 to 60·1)	38·9 (21·6 to 64)	41·4 (23·2 to 67·5)	40·8 (24·6 to 61·7)	39·4 (24·1 to 60·1)	35·3 (20·8 to 55·6)	26·1 (15 to 42)	17·9 (10·3 to 28·9)	10 (5·8 to 16)	5·5 (3·1 to 8·6)	2·5 (1·3 to 4)	0·8 (0·4 to 1·3)	0·1 (0 to 0·2)	482
Eating disorders	0 (0 to 0)	0 (0 to 0)	0 (0 to 0)	0 (0 to 0)	0·1 (0 to 0·2)	1·9 (0·8 to 3·6)	5·5 (2·5 to 9·7)	6·5 (3·1 to 12·2)	7·2 (3·7 to 11·9)	7·8 (3·8 to 13·8)	6·3 (3 to 11·3)	2·8 (1·3 to 5·3)	1·2 (0·4 to 2·4)	0 (0 to 0)	0 (0 to 0)	0 (0 to 0)	0 (0 to 0)	0 (0 to 0)	0 (0 to 0)	0 (0 to 0)	0 (0 to 0)	0 (0 to 0)	0 (0 to 0)	39·2
Anorexia nervosa	0 (0 to 0)	0 (0 to 0)	0 (0 to 0)	0 (0 to 0)	0·1 (0 to 0·2)	0·7 (0·2 to 1·6)	1·9 (0·7 to 3·8)	1·7 (0·7 to 3·4)	1·3 (0·5 to 2·5)	0·8 (0·2 to 1·7)	0·6 (0·1 to 1·4)	0·5 (0·1 to 1·1)	0·4 (0 to 0·9)	0 (0 to 0)	0 (0 to 0)	0 (0 to 0)	0 (0 to 0)	0 (0 to 0)	0 (0 to 0)	0 (0 to 0)	0 (0 to 0)	0 (0 to 0)	0 (0 to 0)	8
Bulimia nervosa	0 (0 to 0)	0 (0 to 0)	0 (0 to 0)	0 (0 to 0)	0 (0 to 0)	1·2 (0·3 to 2·6)	3·5 (1·3 to 7·2)	4·8 (1·8 to 9·5)	5·9 (2·8 to 10·2)	7 (3·2 to 12·9)	5·7 (2·6 to 10·4)	2·4 (1 to 4·6)	0·8 (0·2 to 1·8)	0 (0 to 0)	0 (0 to 0)	0 (0 to 0)	0 (0 to 0)	0 (0 to 0)	0 (0 to 0)	0 (0 to 0)	0 (0 to 0)	0 (0 to 0)	0 (0 to 0)	31·2
Autism spectrum disorders	0 (0 to 0)	0·1 (0·1 to 0·1)	1·3 (0·8 to 2)	5·9 (3·7 to 8·8)	8 (5 to 12·1)	8·7 (5·4 to 13·1)	9·1 (5·7 to 13·7)	8·3 (5·1 to 12·6)	8·2 (5·2 to 12·4)	7·5 (4·8 to 11·1)	7·6 (4·7 to 11·6)	7 (4·4 to 10·8)	7·2 (4·5 to 10·8)	6·8 (4·2 to 10·2)	6·4 (4 to 9·6)	5·5 (3·5 to 8·4)	4·2 (2·6 to 6·3)	3 (1·9 to 4·7)	1·8 (1·1 to 2·7)	1·1 (0·7 to 1·8)	0·6 (0·4 to 0·9)	0·2 (0·1 to 0·3)	0 (0 to 0·1)	109
Attention-deficit/hyperactivity disorder	0 (0 to 0)	0 (0 to 0)	0 (0 to 0)	0·7 (0·3 to 1·3)	8·3 (4·6 to 14·8)	12·5 (6·9 to 22·2)	11·1 (6 to 19·7)	7·8 (4·3 to 13·6)	6·1 (3·4 to 10·9)	4·6 (2·4 to 8)	3·9 (2 to 6·9)	3·1 (1·5 to 5·4)	2·7 (1·3 to 4·8)	2·1 (1 to 3·8)	1·4 (0·7 to 2·6)	0·8 (0·4 to 1·5)	0·4 (0·1 to 0·7)	0·1 (0 to 0·3)	0 (0 to 0·1)	0 (0 to 0)	0 (0 to 0)	0 (0 to 0)	0 (0 to 0)	65·7
Conduct disorder	0 (0 to 0)	0 (0 to 0)	0 (0 to 0)	0 (0 to 0)	13·1 (6·9 to 23·3)	47·1 (25·6 to 76·5)	32·6 (17·3 to 52·9)	0 (0 to 0)	0 (0 to 0)	0 (0 to 0)	0 (0 to 0)	0 (0 to 0)	0 (0 to 0)	0 (0 to 0)	0 (0 to 0)	0 (0 to 0)	0 (0 to 0)	0 (0 to 0)	0 (0 to 0)	0 (0 to 0)	0 (0 to 0)	0 (0 to 0)	0 (0 to 0)	92·9
Idiopathic developmental intellectual disability	0 (0 to 0)	0 (0 to 0)	0·1 (0 to 0·3)	0·9 (0·2 to 1·8)	1·4 (0·3 to 2·7)	1·5 (0·3 to 3·1)	1·5 (0·3 to 3·1)	1·4 (0·3 to 2·7)	1·3 (0·3 to 2·8)	1·2 (0·3 to 2·4)	1·2 (0·2 to 2·4)	1·1 (0·2 to 2·2)	1·1 (0·2 to 2·1)	0·9 (0·2 to 2)	0·8 (0·1 to 1·8)	0·7 (0·1 to 1·4)	0·4 (0 to 1)	0·3 (0 to 0·6)	0·1 (0 to 0·3)	0·1 (0 to 0·2)	0 (0 to 0·1)	0 (0 to 0)	0 (0 to 0)	15·9
Other mental disorders	0 (0 to 0)	0 (0 to 0)	0 (0 to 0)	0 (0 to 0)	0 (0 to 0)	0·6 (0·3 to 1·1)	4·3 (1·9 to 7·5)	10·1 (5·2 to 17·1)	15·8 (8·8 to 25·3)	18·8 (10·7 to 29·5)	21·9 (12·9 to 34·2)	21·9 (13·4 to 34·7)	23·5 (14 to 36·8)	23 (13·8 to 35·8)	22·2 (13·5 to 34·1)	19·9 (12·4 to 30·2)	15·2 (9·6 to 23·4)	11·5 (7·4 to 17·6)	7·2 (4·7 to 10·7)	4·8 (3·1 to 7·2)	2·7 (1·7 to 3·9)	1·1 (0·7 to 1·6)	0·2 (0·1 to 0·3)	225
Substance use disorders	0 (0 to 0)	0 (0 to 0)	0 (0 to 0)	0 (0 to 0)	0 (0 to 0)	1·9 (1 to 3·2)	29·1 (18·8 to 42·1)	54·2 (36 to 76)	65·7 (45·4 to 90·8)	66·1 (47·3 to 92·6)	67·8 (49·2 to 88·1)	68·7 (50·2 to 91·4)	77 (55·8 to 104)	70·1 (51·5 to 95·5)	71·8 (52·5 to 94·4)	57·1 (41·7 to 76·9)	37·4 (27·4 to 48·5)	22 (16·1 to 29·2)	11 (8·1 to 14·5)	5·7 (4·1 to 7·7)	3 (2·1 to 4·1)	1·4 (0·9 to 2)	0·3 (0·2 to 0·5)	710
Alcohol use disorders	0 (0 to 0)	0 (0 to 0)	0 (0 to 0)	0 (0 to 0)	0 (0 to 0)	0·7 (0·2 to 1·5)	9·1 (4·9 to 14·9)	23·8 (13·4 to 37·9)	35·7 (22·1 to 54)	43·7 (29 to 64·8)	49·3 (35·1 to 67·5)	53·4 (37·8 to 74·7)	63 (43·7 to 87·7)	59·2 (42·4 to 83·2)	63 (45·1 to 85·9)	51·1 (36·7 to 70·5)	33·7 (24·3 to 44·9)	19·5 (13·8 to 26·4)	9·6 (7 to 12·8)	4·7 (3·3 to 6·5)	2·4 (1·6 to 3·5)	1·1 (0·7 to 1·7)	0·3 (0·2 to 0·4)	523
Drug use disorders	0 (0 to 0)	0 (0 to 0)	0 (0 to 0)	0 (0 to 0)	0 (0 to 0)	1·2 (0·5 to 2·2)	20 (12·5 to 30·6)	30·4 (19 to 44·2)	30 (19·5 to 43)	22·5 (15 to 32·3)	18·5 (12·4 to 26)	15·3 (9·8 to 22·4)	14 (9·1 to 20·1)	10·9 (6·7 to 16·5)	8·8 (5·2 to 13·4)	6 (3·5 to 9·3)	3·7 (2·1 to 6)	2·4 (1·4 to 3·7)	1·4 (0·8 to 2·3)	1 (0·5 to 1·5)	0·6 (0·3 to 0·9)	0·3 (0·2 to 0·4)	0·1 (0 to 0·1)	187
Opioid use disorders	0 (0 to 0)	0 (0 to 0)	0 (0 to 0)	0 (0 to 0)	0 (0 to 0)	0 (0 to 0)	3·2 (1·3 to 6)	12·3 (6·3 to 21·4)	15·2 (8·5 to 24·7)	12·4 (7 to 19·7)	10·9 (6·2 to 17·2)	9·2 (5 to 14·9)	8·5 (4·6 to 13·6)	7·1 (3·5 to 12·4)	5·9 (2·9 to 9·9)	4·2 (1·9 to 7·3)	2·6 (1·1 to 4·7)	1·7 (0·7 to 2·9)	1 (0·4 to 1·8)	0·7 (0·3 to 1·2)	0·4 (0·2 to 0·7)	0·2 (0·1 to 0·4)	0·1 (0 to 0·1)	95·7
Cocaine use disorders	0 (0 to 0)	0 (0 to 0)	0 (0 to 0)	0 (0 to 0)	0 (0 to 0)	0 (0 to 0·1)	2·1 (0·7 to 4·3)	2·4 (1·1 to 4·3)	2·5 (1·3 to 4)	2·3 (1·3 to 3·6)	2 (1·1 to 3·2)	1·6 (0·8 to 2·6)	1·5 (1 to 2·2)	0·8 (0·6 to 1·2)	0·7 (0·5 to 1)	0·4 (0·3 to 0·6)	0·3 (0·2 to 0·4)	0·2 (0·1 to 0·3)	0·1 (0·1 to 0·2)	0·1 (0 to 0·1)	0 (0 to 0)	0 (0 to 0)	0 (0 to 0)	17
Amphetamine use disorders	0 (0 to 0)	0 (0 to 0)	0 (0 to 0)	0 (0 to 0)	0 (0 to 0)	0 (0 to 0)	0·6 (0·1 to 1·6)	2·6 (0·9 to 5·2)	2·8 (1·1 to 5·3)	1·6 (0·6 to 3·1)	1 (0·3 to 2·1)	0·7 (0·4 to 1·1)	0·6 (0·3 to 0·8)	0·4 (0·3 to 0·7)	0·4 (0·2 to 0·6)	0·2 (0·1 to 0·3)	0·1 (0·1 to 0·2)	0·1 (0 to 0·1)	0 (0 to 0)	0 (0 to 0)	0 (0 to 0)	0 (0 to 0)	0 (0 to 0)	11·2
Cannabis use disorders	0 (0 to 0)	0 (0 to 0)	0 (0 to 0)	0 (0 to 0)	0 (0 to 0)	1·1 (0·5 to 2·1)	12·3 (6·5 to 21)	9·7 (5 to 16·6)	6·2 (2·9 to 10·8)	3·8 (1·8 to 6·8)	2·8 (1·3 to 4·8)	2·2 (1 to 4)	2 (0·9 to 3·7)	1·5 (0·6 to 2·8)	1·1 (0·4 to 2)	0·6 (0·2 to 1·2)	0·3 (0·1 to 0·6)	0·1 (0·1 to 0·3)	0·1 (0 to 0·1)	0 (0 to 0·1)	0 (0 to 0)	0 (0 to 0)	0 (0 to 0)	43·9
Other drug use disorders	0 (0 to 0)	0 (0 to 0)	0 (0 to 0)	0 (0 to 0)	0 (0 to 0)	0 (0 to 0·1)	1·8 (0·7 to 3·4)	3·2 (1·6 to 5·6)	3·4 (1·8 to 5·7)	2·4 (1·4 to 3·9)	1·9 (1 to 3)	1·6 (0·9 to 2·4)	1·5 (1 to 2·2)	1·1 (0·7 to 1·6)	0·8 (0·5 to 1·1)	0·5 (0·3 to 0·7)	0·4 (0·2 to 0·5)	0·4 (0·3 to 0·5)	0·2 (0·2 to 0·3)	0·2 (0·1 to 0·3)	0·1 (0·1 to 0·1)	0 (0 to 0·1)	0 (0 to 0)	19·3
Diabetes and kidney diseases	0 (0 to 0)	0·1 (0 to 0·1)	3·3 (1·9 to 5·2)	3·6 (2·4 to 5·1)	5·9 (4·2 to 8·3)	8·3 (6 to 11·6)	26·2 (20·1 to 34·3)	37·4 (29·3 to 48·4)	69·1 (55 to 86·7)	104 (83·2 to 129)	136 (109 to 167)	198 (156 to 246)	321 (251 to 398)	440 (342 to 558)	655 (517 to 808)	779 (625 to 959)	787 (625 to 953)	755 (611 to 908)	581 (473 to 698)	420 (347 to 505)	237 (198 to 279)	98·5 (80·9 to 116)	15·8 (12·6 to 18·6)	5,680
Diabetes mellitus	0 (0 to 0)	0·1 (0 to 0·1)	1 (0·4 to 1·8)	0·8 (0·5 to 1·3)	0·9 (0·6 to 1·2)	1·9 (1·1 to 2·7)	10·1 (7·5 to 13·5)	18·1 (13·6 to 23·6)	36·6 (27·8 to 48)	58·5 (44·7 to 75·8)	82·5 (63·3 to 105)	126 (97·1 to 162)	215 (161 to 282)	311 (232 to 403)	476 (365 to 610)	582 (448 to 744)	599 (470 to 744)	568 (443 to 709)	447 (352 to 556)	307 (245 to 379)	170 (137 to 206)	66·6 (53·5 to 80·5)	9·9 (7·7 to 11·9)	4,090
Diabetes mellitus type 1	0 (0 to 0)	0·1 (0 to 0·1)	1 (0·4 to 1·8)	0·8 (0·5 to 1·3)	0·9 (0·6 to 1·2)	1·9 (1·1 to 2·7)	4·8 (3·3 to 6·7)	7·6 (5·4 to 10·7)	15·9 (11·1 to 21·8)	13·5 (8·5 to 20·5)	16·7 (10·6 to 26·3)	24·2 (15·6 to 37·4)	9·8 (6·1 to 15·8)	12·6 (7·6 to 19·8)	19·8 (11·5 to 33·6)	19·6 (11·3 to 32·9)	19·8 (12 to 32·3)	14·6 (8·3 to 25)	11·2 (6·5 to 21)	5·5 (3·2 to 9·9)	3·1 (1·8 to 5·9)	1·4 (0·8 to 2·7)	0·3 (0·1 to 0·5)	205
Diabetes mellitus type 2	0 (0 to 0)	0 (0 to 0)	0 (0 to 0)	0 (0 to 0)	0 (0 to 0)	0 (0 to 0)	5·4 (3·4 to 7·9)	10·4 (7·2 to 14·8)	20·8 (14·3 to 29·6)	45 (32·4 to 60·8)	65·8 (47·4 to 87·5)	102 (75·6 to 133)	205 (153 to 269)	298 (222 to 389)	456 (349 to 588)	562 (432 to 716)	579 (455 to 721)	553 (433 to 692)	436 (344 to 542)	301 (240 to 373)	167 (135 to 203)	65·1 (52·3 to 78·9)	9·6 (7·5 to 11·6)	3,880
Chronic kidney disease	0 (0 to 0)	0 (0 to 0)	2·3 (1·3 to 3·9)	2·8 (1·7 to 4·2)	5 (3·4 to 7·4)	6·5 (4·4 to 9·4)	16 (11·1 to 22·9)	19·3 (13·4 to 26·6)	32·5 (22·6 to 45·5)	45·5 (32·8 to 60·3)	53·6 (40 to 71·3)	71·8 (51·5 to 97·7)	106 (77·4 to 142)	129 (93·3 to 173)	179 (135 to 236)	198 (146 to 258)	189 (141 to 253)	187 (144 to 241)	133 (101 to 170)	113 (86·8 to 140)	66·9 (52·9 to 83·7)	32 (24·2 to 39·5)	5·9 (4·5 to 7·4)	1,590
Chronic kidney disease due to diabetes mellitus type 1	0 (0 to 0)	0 (0 to 0)	0 (0 to 0)	0·1 (0 to 0·2)	0·2 (0·1 to 0·4)	0·2 (0·1 to 0·5)	0·6 (0·2 to 1·3)	0·9 (0·4 to 1·9)	3·1 (1·3 to 5·8)	7 (3·4 to 12·3)	9·3 (4·7 to 15·4)	12·2 (6·3 to 20·2)	15·2 (7·2 to 26·4)	13·7 (5·5 to 26·6)	13·4 (4·7 to 29·8)	10·8 (3·8 to 23·9)	8 (3 to 18·1)	6·1 (2·4 to 13)	2·8 (1 to 6·4)	1·3 (0·4 to 3)	0·3 (0·1 to 0·7)	0 (0 to 0·1)	0 (0 to 0·1)	105
Chronic kidney disease due to diabetes mellitus type 2	0 (0 to 0)	0 (0 to 0)	0 (0 to 0)	0 (0 to 0)	0 (0 to 0)	0 (0 to 0)	0·2 (0·1 to 0·6)	0·4 (0·2 to 1)	1·3 (0·5 to 2·7)	2·8 (1·2 to 5·3)	4·5 (1·9 to 8·7)	8·7 (4·1 to 15·7)	18·6 (10·1 to 30)	30·7 (17·9 to 46·7)	51 (29·5 to 75·8)	61 (38·1 to 89·5)	60·1 (38·3 to 90)	60·1 (39·7 to 83·2)	42·3 (27 to 59·4)	35·2 (22·8 to 49·3)	19·7 (12·6 to 27·6)	9 (5·5 to 13·2)	1·6 (0·9 to 2·4)	407
Chronic kidney disease due to hypertension	0 (0 to 0)	0 (0 to 0)	0 (0 to 0)	0 (0 to 0)	0 (0 to 0)	0 (0 to 0)	1·3 (0·4 to 2·7)	2 (0·7 to 3·8)	3·4 (1·4 to 6·2)	4·9 (2·4 to 8·4)	6·3 (3·4 to 10·1)	9·4 (5·5 to 15·1)	15·9 (9·9 to 24·5)	22·3 (13·7 to 35·3)	34·8 (22·5 to 52·9)	44·2 (28·4 to 65·3)	46·9 (30·5 to 71·4)	50·6 (33·9 to 71)	39·8 (27·4 to 56·8)	37·6 (26·4 to 50·7)	24·9 (17·9 to 33·2)	13 (9·2 to 17·2)	2·5 (1·8 to 3·4)	360
Chronic kidney disease due to glomerulonephritis	0 (0 to 0)	0 (0 to 0)	0·7 (0·3 to 1·3)	0·9 (0·5 to 1·5)	1·9 (1·1 to 3)	2·7 (1·6 to 4·2)	6·9 (4·1 to 10·5)	8 (4·8 to 11·8)	13 (8·1 to 19·5)	16·5 (10·4 to 24·5)	18·1 (11·3 to 26·8)	22·4 (13·3 to 33)	29·8 (18·3 to 45·6)	32·8 (19·6 to 52)	41·9 (23 to 67·1)	40·7 (21·5 to 67·2)	33·4 (16·1 to 59·5)	28·6 (14·6 to 47·2)	17·6 (8·7 to 31·3)	12·6 (5·9 to 23·1)	5·8 (2·6 to 9·9)	2·3 (1 to 4·1)	0·4 (0·1 to 0·8)	337
Chronic kidney disease due to other and unspecified causes	0 (0 to 0)	0 (0 to 0)	1·6 (0·9 to 2·9)	1·7 (1 to 2·7)	2·9 (1·8 to 4·5)	3·5 (2·1 to 5·4)	7 (4·1 to 10·7)	8 (4·5 to 12·1)	11·8 (7 to 18)	14·3 (8·4 to 22·3)	15·3 (8·6 to 24)	19·1 (11 to 30·1)	26·4 (15·7 to 41·9)	29·5 (17 to 45·9)	38·1 (22·9 to 61·2)	41·3 (24·4 to 67·5)	40·5 (23·9 to 65·1)	41·8 (26 to 63·5)	30·8 (18·5 to 46·8)	26·5 (15·8 to 40·6)	16·3 (10·3 to 24·2)	7·6 (4·8 to 11·5)	1·5 (0·9 to 2·2)	385
Acute glomerulonephritis	0 (0 to 0)	0 (0 to 0)	0 (0 to 0)	0 (0 to 0)	0 (0 to 0)	0 (0 to 0)	0 (0 to 0)	0 (0 to 0)	0 (0 to 0)	0 (0 to 0)	0 (0 to 0)	0 (0 to 0)	0 (0 to 0)	0 (0 to 0)	0 (0 to 0)	0 (0 to 0)	0 (0 to 0)	0 (0 to 0)	0 (0 to 0)	0 (0 to 0)	0 (0 to 0)	0 (0 to 0)	0 (0 to 0)	0·1
Skin and subcutaneous diseases	0 (0 to 0)	0 (0 to 0)	11·3 (5·5 to 19·8)	39 (24·9 to 57·1)	53·4 (33·3 to 78)	60·4 (39·7 to 88·6)	71·7 (47·2 to 107)	47·2 (31·6 to 68·1)	47·6 (32·4 to 67·9)	45·8 (31·2 to 66·6)	48·4 (33 to 71·1)	53·5 (36·1 to 77·4)	63·6 (43·2 to 96·6)	73·5 (49·7 to 112)	87·5 (57·3 to 138)	97·1 (63·9 to 163)	105 (68 to 189)	105 (66·1 to 196)	94·3 (59 to 193)	79·1 (48 to 153)	52·7 (30·8 to 105)	26·7 (13·7 to 52)	4·6 (2·4 to 8·9)	1,270
Dermatitis	0 (0 to 0)	0 (0 to 0)	0 (0 to 0)	12·3 (6·5 to 21·8)	16·1 (8·4 to 27·6)	12·4 (6·6 to 20·9)	9·9 (5·4 to 16·2)	7·8 (4·4 to 12·7)	7·5 (4·1 to 12·6)	7 (3·9 to 11·1)	7 (3·9 to 11·5)	6·7 (3·8 to 11)	7·3 (4 to 12·1)	7·8 (4·2 to 13)	8·3 (4·5 to 13·7)	8·1 (4·3 to 13·1)	7 (3·8 to 11·3)	5·5 (3·1 to 9)	3·5 (1·9 to 5·6)	2·2 (1·2 to 3·4)	1·1 (0·7 to 1·8)	0·4 (0·2 to 0·7)	0·1 (0 to 0·1)	138
Atopic dermatitis	0 (0 to 0)	0 (0 to 0)	0 (0 to 0)	12·2 (6·5 to 21·7)	15·7 (8·1 to 27·1)	11 (5·6 to 19·3)	7·6 (3·8 to 13)	5·2 (2·5 to 9·2)	4·3 (2·1 to 7·8)	3·6 (1·8 to 6·6)	3·5 (1·7 to 6·4)	3·3 (1·6 to 5·9)	3·5 (1·7 to 6·4)	3·5 (1·8 to 6·4)	3·7 (1·8 to 6·8)	3·5 (1·7 to 6·2)	2·8 (1·4 to 4·8)	2·1 (1·1 to 3·7)	1·4 (0·7 to 2·4)	1 (0·5 to 1·7)	0·5 (0·3 to 0·9)	0·2 (0·1 to 0·4)	0 (0 to 0·1)	88·6
Contact dermatitis	0 (0 to 0)	0 (0 to 0)	0 (0 to 0)	0 (0 to 0)	0·2 (0·1 to 0·4)	1·1 (0·4 to 2·2)	2·1 (0·8 to 4·1)	2·4 (0·9 to 4·5)	2·9 (1·2 to 5·5)	3 (1·3 to 5·6)	3·1 (1·4 to 6·1)	3 (1·3 to 6·2)	3·4 (1·5 to 7)	3·9 (1·6 to 7·3)	4·2 (1·8 to 8·2)	4·3 (1·7 to 8·3)	3·8 (1·7 to 7·3)	3 (1·3 to 5·6)	1·8 (0·8 to 3·4)	1·1 (0·5 to 2)	0·5 (0·2 to 1)	0·2 (0·1 to 0·3)	0 (0 to 0·1)	43·9
Seborrhoeic dermatitis	0 (0 to 0)	0 (0 to 0)	0 (0 to 0)	0·1 (0 to 0·2)	0·2 (0·1 to 0·3)	0·2 (0·1 to 0·4)	0·3 (0·1 to 0·5)	0·3 (0·1 to 0·6)	0·4 (0·2 to 0·7)	0·4 (0·2 to 0·7)	0·4 (0·2 to 0·7)	0·4 (0·2 to 0·7)	0·4 (0·2 to 0·7)	0·4 (0·2 to 0·7)	0·4 (0·2 to 0·7)	0·4 (0·2 to 0·6)	0·4 (0·2 to 0·7)	0·4 (0·2 to 0·6)	0·3 (0·1 to 0·4)	0·2 (0·1 to 0·3)	0·1 (0 to 0·2)	0 (0 to 0·1)	0 (0 to 0)	5·5
Psoriasis	0 (0 to 0)	0 (0 to 0)	0 (0 to 0)	0·5 (0·3 to 0·8)	1·3 (0·5 to 2·5)	1·9 (0·8 to 3·4)	2·2 (1 to 3·9)	2·2 (1·1 to 3·8)	2·6 (1·3 to 4·3)	2·9 (1·5 to 4·8)	3·4 (1·7 to 5·3)	3·6 (2 to 5·7)	4·2 (2·2 to 6·9)	4·6 (2·6 to 7·2)	4·9 (2·7 to 7·6)	4·7 (2·8 to 7·1)	3·7 (2·2 to 5·6)	2·6 (1·5 to 3·9)	1·4 (0·8 to 2·1)	0·7 (0·4 to 1·1)	0·3 (0·2 to 0·5)	0·1 (0 to 0·1)	0 (0 to 0)	48·1
Bacterial skin diseases	0 (0 to 0)	0 (0 to 0)	8·7 (3·4 to 17)	2·8 (1·4 to 5·5)	3·4 (1·8 to 6·6)	3·8 (1·7 to 7)	5·1 (2·4 to 10)	4·4 (1·8 to 9·9)	7 (2·8 to 14·3)	9·4 (4·1 to 18·8)	8·8 (3 to 18·2)	12·1 (4·2 to 25·2)	16·1 (5·2 to 34·2)	18·3 (5·7 to 38)	24·4 (8·4 to 51)	23 (7 to 52·2)	24·6 (7·5 to 62·9)	24·1 (7·4 to 61·5)	22 (6·5 to 62)	16·6 (5·3 to 43·7)	11·1 (3 to 31·6)	5·9 (1·6 to 15·9)	1·1 (0·3 to 2·9)	253
Cellulitis	0 (0 to 0)	0 (0 to 0)	3·5 (0·8 to 8·1)	1·1 (0·3 to 2·4)	1·4 (0·4 to 2·8)	1·8 (0·5 to 3·6)	2·5 (0·9 to 5·4)	1·9 (0·6 to 4·9)	3·1 (1 to 6·9)	4·1 (1·4 to 9)	3·5 (1 to 8·2)	4·1 (1·3 to 9·9)	5·7 (1·7 to 14·2)	6·5 (1·8 to 15·1)	7·6 (2·2 to 18)	7·8 (2 to 20)	7·5 (2·1 to 22)	7·5 (2·1 to 24·1)	5·5 (1·4 to 19·9)	4·6 (1·3 to 15·6)	3·3 (0·9 to 12·5)	1·7 (0·4 to 6)	0·4 (0·1 to 1·2)	85·1
Pyoderma	0 (0 to 0)	0 (0 to 0)	5·2 (1·8 to 11·2)	1·7 (0·7 to 3·5)	1·9 (1 to 4·2)	2 (0·8 to 4·4)	2·6 (1 to 5·6)	2·5 (1 to 6)	3·9 (1·3 to 8·6)	5·3 (2 to 11·1)	5·4 (1·8 to 12·2)	7·9 (2·4 to 16·8)	10·5 (3 to 24·5)	11·8 (3·1 to 25)	16·8 (5·3 to 35·5)	15·2 (4·5 to 35·1)	17·1 (4·4 to 43·5)	16·7 (4·5 to 42·1)	16·4 (4·3 to 46)	12 (3 to 31)	7·8 (2 to 21)	4·2 (1·1 to 10·8)	0·7 (0·2 to 1·8)	167
Scabies	0 (0 to 0)	0 (0 to 0)	0·9 (0·5 to 1·6)	6·7 (3·4 to 11·7)	9·6 (4·5 to 17)	10·5 (5·4 to 18)	11·6 (5·9 to 19·9)	9·7 (4·9 to 16·8)	7·5 (3·7 to 13·3)	5·5 (2·7 to 9·7)	5·6 (2·7 to 10)	6·1 (3·1 to 10·4)	6·7 (3·4 to 11·6)	6·3 (3·2 to 10·9)	5·6 (2·8 to 9·9)	4·6 (2·4 to 8)	3·5 (1·7 to 6·2)	2·7 (1·4 to 4·7)	1·9 (1 to 3·4)	1·4 (0·7 to 2·6)	0·9 (0·4 to 1·5)	0·4 (0·2 to 0·7)	0·1 (0 to 0·1)	108
Fungal skin diseases	0 (0 to 0)	0 (0 to 0)	0·2 (0·1 to 0·4)	1·3 (0·5 to 2·8)	2·3 (0·9 to 5)	2·9 (1·1 to 6·4)	3·2 (1·2 to 7·3)	3·1 (1·2 to 6·9)	3·4 (1·3 to 7·9)	3·4 (1·3 to 7·8)	3·7 (1·4 to 8·1)	3·8 (1·4 to 8·2)	4·2 (1·6 to 9·2)	4·7 (1·8 to 10·2)	5·7 (2·2 to 12·2)	6·1 (2·3 to 13·1)	6·5 (2·6 to 14·1)	7·2 (2·8 to 15·2)	5·7 (2·2 to 11·8)	4·8 (1·9 to 10·3)	3·3 (1·3 to 7·1)	1·5 (0·6 to 3·1)	0·3 (0·1 to 0·5)	77·2
Viral skin diseases	0 (0 to 0)	0 (0 to 0)	0 (0 to 0)	7·3 (4·6 to 11·2)	12·5 (7·7 to 19·5)	7 (4·3 to 11·1)	4·6 (2·8 to 7·1)	3·3 (2 to 5)	2·6 (1·5 to 4·1)	2 (1·2 to 3·2)	2 (1·2 to 3·2)	1·9 (1·1 to 3·1)	1·9 (1·1 to 3)	1·9 (1 to 3)	1·8 (1 to 2·9)	1·7 (1 to 2·8)	1·4 (0·8 to 2·2)	1 (0·6 to 1·5)	0·5 (0·3 to 0·8)	0·3 (0·2 to 0·4)	0·1 (0·1 to 0·2)	0 (0 to 0·1)	0 (0 to 0)	53·9
Acne vulgaris	0 (0 to 0)	0 (0 to 0)	0 (0 to 0)	0 (0 to 0)	0·6 (0·3 to 1)	14·2 (8 to 23·7)	26 (14·5 to 42·8)	5·9 (3·2 to 9·7)	2·8 (1·4 to 4·7)	1·9 (0·9 to 3·3)	1·6 (0·7 to 2·8)	1·1 (0·6 to 1·8)	0·9 (0·5 to 1·4)	0·6 (0·3 to 1)	0·4 (0·2 to 0·7)	0·3 (0·2 to 0·5)	0·2 (0·1 to 0·4)	0·1 (0·1 to 0·2)	0·1 (0 to 0·1)	0 (0 to 0·1)	0 (0 to 0)	0 (0 to 0·1)	0 (0 to 0)	56·7
Alopecia areata	0 (0 to 0)	0 (0 to 0)	0 (0 to 0)	0·1 (0 to 0·2)	0·1 (0 to 0·3)	0·2 (0·1 to 0·4)	0·4 (0·2 to 0·7)	0·6 (0·3 to 1)	0·8 (0·4 to 1·4)	0·8 (0·4 to 1·4)	0·8 (0·5 to 1·3)	0·7 (0·3 to 1·1)	0·6 (0·3 to 1·1)	0·5 (0·3 to 0·9)	0·5 (0·2 to 0·8)	0·5 (0·2 to 0·8)	0·3 (0·2 to 0·6)	0·2 (0·1 to 0·4)	0·1 (0·1 to 0·2)	0·1 (0 to 0·1)	0 (0 to 0·1)	0 (0 to 0)	0 (0 to 0)	7·5
Pruritus	0 (0 to 0)	0 (0 to 0)	0 (0 to 0)	0·2 (0·1 to 0·5)	0·4 (0·1 to 0·7)	0·4 (0·2 to 0·8)	0·5 (0·2 to 1)	0·5 (0·2 to 1·1)	0·7 (0·3 to 1·3)	0·7 (0·3 to 1·4)	0·8 (0·3 to 1·5)	0·8 (0·3 to 1·4)	0·9 (0·4 to 1·7)	0·9 (0·4 to 2)	1·1 (0·4 to 2·2)	1·2 (0·5 to 2·5)	1·2 (0·5 to 2·3)	1 (0·4 to 1·9)	0·6 (0·3 to 1·3)	0·4 (0·2 to 0·8)	0·2 (0·1 to 0·4)	0·1 (0 to 0·2)	0 (0 to 0)	12·6
Urticaria	0 (0 to 0)	0 (0 to 0)	0·8 (0·5 to 1·3)	5·7 (3·4 to 9·1)	5·3 (3 to 8·9)	4·1 (2·1 to 7)	3·7 (2 to 6·1)	3·2 (1·6 to 5·4)	3·1 (1·6 to 5·1)	3·1 (1·6 to 5·3)	3·3 (1·8 to 5·4)	3 (1·7 to 5)	3 (1·5 to 5·2)	2·9 (1·6 to 4·9)	2·9 (1·4 to 4·8)	2·6 (1·3 to 4·4)	2 (1 to 3·4)	1·5 (0·8 to 2·6)	0·9 (0·5 to 1·5)	0·5 (0·3 to 0·9)	0·2 (0·1 to 0·4)	0·1 (0 to 0·1)	0 (0 to 0)	52·1
Decubitus ulcer	0 (0 to 0)	0 (0 to 0)	0 (0 to 0)	0·4 (0·1 to 1·1)	0·3 (0·1 to 0·8)	1 (0·2 to 2·3)	1·8 (0·3 to 4·1)	3·8 (1 to 9·4)	6·6 (1·6 to 13·7)	5·6 (1·4 to 12·6)	7·1 (2·1 to 16·5)	9·2 (2·7 to 21·3)	12 (3·9 to 27·8)	17·7 (6·1 to 38·6)	23·3 (7·8 to 52·3)	34·6 (13·5 to 75·5)	45·1 (18·4 to 107)	51·2 (21·7 to 120)	52·4 (23·3 to 131)	48·8 (20·4 to 105)	33·7 (15·3 to 73·8)	17·6 (6·6 to 36·7)	3 (1·2 to 6·1)	375
Other skin and subcutaneous diseases	0 (0 to 0)	0 (0 to 0)	0·6 (0·3 to 1·1)	1·6 (0·8 to 3·1)	1·6 (0·8 to 3)	2·1 (1 to 4·1)	2·6 (1·3 to 4·9)	2·5 (1·2 to 4·8)	3·1 (1·5 to 5·6)	3·5 (1·6 to 6·5)	4·2 (2·1 to 7·7)	4·5 (2·2 to 8·1)	5·8 (2·9 to 11·1)	7·3 (3·6 to 13)	8·7 (4·1 to 16·3)	9·7 (4·8 to 17·6)	9·3 (4·7 to 16·6)	7·7 (3·8 to 14)	5·2 (2·7 to 9·4)	3·2 (1·6 to 5·7)	1·7 (0·8 to 2·8)	0·7 (0·3 to 1·1)	0·1 (0 to 0·2)	85·8
Sense organ diseases	0 (0 to 0)	0 (0 to 0)	0·3 (0·2 to 0·5)	3·4 (2·1 to 5·1)	7·8 (4·8 to 11·7)	10·8 (6·5 to 15·6)	13·9 (8·4 to 20·4)	16·7 (10 to 25·2)	23·4 (14·1 to 36·6)	29·6 (17·8 to 47·2)	37·9 (22·9 to 59·6)	48 (29·1 to 77·2)	75·4 (44·9 to 124)	106 (65·4 to 173)	140 (86·9 to 225)	165 (104 to 257)	164 (105 to 249)	158 (105 to 233)	126 (84·5 to 184)	101 (68·8 to 141)	63·6 (44·4 to 86·6)	27·9 (19·8 to 37·6)	4·6 (3·4 to 6·3)	1,320
Blindness and vision loss	0 (0 to 0)	0 (0 to 0)	0 (0 to 0·1)	0·6 (0·3 to 1)	1·3 (0·8 to 2·1)	1·6 (0·9 to 2·7)	2 (1·2 to 3·1)	2·1 (1·2 to 3·4)	2·6 (1·5 to 4·2)	3·3 (1·9 to 5·3)	4·8 (2·9 to 7·8)	7·2 (4·1 to 11·9)	11·5 (6·3 to 20·4)	16·9 (9·3 to 30·6)	24 (13·4 to 42·6)	30·3 (17·3 to 51·3)	32·4 (18·8 to 52·5)	32·5 (19·5 to 51·7)	26·3 (16·6 to 41·5)	21·2 (13·6 to 32·3)	13·7 (8·9 to 19·8)	6·5 (4·3 to 9·4)	1·3 (0·9 to 1·9)	242
Glaucoma	0 (0 to 0)	0 (0 to 0)	0 (0 to 0)	0 (0 to 0)	0 (0 to 0)	0 (0 to 0)	0 (0 to 0)	0 (0 to 0)	0 (0 to 0)	0 (0 to 0)	0 (0 to 0)	0 (0 to 0)	0 (0 to 0)	0·1 (0·1 to 0·2)	0·4 (0·1 to 0·9)	0·9 (0·4 to 1·7)	1·4 (0·7 to 2·3)	1·8 (1 to 3)	2 (1·2 to 3·2)	2 (1·2 to 3)	1·5 (0·9 to 2·3)	0·8 (0·5 to 1·2)	0·2 (0·1 to 0·3)	11·2
Cataract	0 (0 to 0)	0 (0 to 0)	0 (0 to 0)	0 (0 to 0)	0 (0 to 0)	0 (0 to 0)	0 (0 to 0)	0 (0 to 0)	0 (0 to 0·1)	0·1 (0 to 0·2)	0·2 (0·1 to 0·4)	0·4 (0·2 to 0·7)	0·7 (0·3 to 1·3)	1·1 (0·5 to 1·9)	1·6 (0·8 to 2·7)	2·2 (1·2 to 3·5)	3·1 (1·8 to 4·9)	4·3 (2·6 to 6·5)	4·9 (3·1 to 7·2)	4·8 (3·2 to 6·9)	3·4 (2·3 to 4·8)	1·6 (1·1 to 2·3)	0·3 (0·2 to 0·5)	28·7
Age-related macular degeneration	0 (0 to 0)	0 (0 to 0)	0 (0 to 0)	0 (0 to 0)	0 (0 to 0)	0 (0 to 0)	0 (0 to 0)	0 (0 to 0)	0 (0 to 0)	0 (0 to 0)	0 (0 to 0)	0 (0 to 0)	0 (0 to 0)	0 (0 to 0)	0·1 (0 to 0·1)	0·1 (0·1 to 0·2)	0·2 (0·1 to 0·3)	0·2 (0·1 to 0·3)	0·2 (0·1 to 0·3)	0·2 (0·1 to 0·3)	0·1 (0 to 0·2)	0 (0 to 0·1)	0 (0 to 0)	1·1
Refraction disorders	0 (0 to 0)	0 (0 to 0)	0 (0 to 0)	0·5 (0·3 to 0·9)	1·2 (0·6 to 1·9)	1·4 (0·7 to 2·4)	1·7 (0·9 to 2·8)	1·8 (1 to 2·9)	2·1 (1·1 to 3·3)	2·1 (1·2 to 3·4)	2·5 (1·5 to 4)	2·8 (1·6 to 4·5)	3·3 (1·9 to 5·1)	3·4 (2 to 5·5)	3·7 (2·1 to 5·8)	3·7 (2·1 to 5·8)	3·3 (1·9 to 5·2)	3·1 (1·8 to 4·8)	2·5 (1·5 to 3·8)	1·8 (1·1 to 2·7)	0·9 (0·6 to 1·4)	0·3 (0·2 to 0·5)	0 (0 to 0·1)	42·2
Near vision loss	0 (0 to 0)	0 (0 to 0)	0 (0 to 0)	0 (0 to 0)	0 (0 to 0)	0 (0 to 0·1)	0 (0 to 0·1)	0·1 (0 to 0·3)	0·3 (0·1 to 0·6)	0·7 (0·3 to 1·6)	1·5 (0·6 to 3·3)	3·2 (1·2 to 6·7)	6·1 (2·4 to 12·7)	9·8 (4 to 20·5)	14·4 (5·5 to 30·5)	17·7 (7·1 to 35·8)	17·5 (7·1 to 35·7)	16 (6·6 to 32·6)	11·7 (4·9 to 24·1)	8·5 (3·6 to 17·1)	5 (2·2 to 9·6)	2·1 (0·9 to 4·1)	0·3 (0·1 to 0·7)	115
Other vision loss	0 (0 to 0)	0 (0 to 0)	0 (0 to 0)	0·1 (0 to 0·2)	0·2 (0·1 to 0·3)	0·2 (0·1 to 0·4)	0·2 (0·1 to 0·4)	0·2 (0·1 to 0·4)	0·3 (0·1 to 0·5)	0·3 (0·2 to 0·6)	0·5 (0·2 to 1)	0·8 (0·3 to 1·4)	1·4 (0·6 to 2·5)	2·4 (1·3 to 3·8)	3·8 (2·2 to 6)	5·7 (3·5 to 8·7)	7 (4·5 to 10·4)	7·1 (4·5 to 10·2)	5 (3·2 to 7·4)	4 (2·6 to 5·9)	2·8 (1·8 to 4·1)	1·7 (1·1 to 2·5)	0·4 (0·3 to 0·6)	44·2
Age-related and other hearing loss	0 (0 to 0)	0 (0 to 0)	0·1 (0·1 to 0·2)	2 (1 to 3·4)	5·5 (3 to 8·8)	8·1 (4·6 to 12·3)	10·7 (6·2 to 16·2)	13·3 (7·7 to 20·7)	19·2 (11·3 to 30·1)	24·1 (14·2 to 38·5)	30·3 (17·7 to 49·4)	37·9 (21·9 to 61·7)	60·2 (35·6 to 101)	83·6 (50·3 to 136)	110 (67·1 to 175)	127 (78·2 to 198)	124 (78·8 to 191)	118 (78 to 175)	94·5 (62·5 to 138)	75·8 (50·8 to 107)	48·2 (32·8 to 66·2)	20·7 (14·5 to 28·3)	3·2 (2·2 to 4·3)	1,020
Other sense organ diseases	0 (0 to 0)	0 (0 to 0)	0·2 (0·1 to 0·3)	0·8 (0·4 to 1·4)	1 (0·4 to 1·8)	1 (0·4 to 1·9)	1·2 (0·5 to 2·2)	1·3 (0·5 to 2·4)	1·6 (0·7 to 3·2)	2·2 (0·9 to 4·4)	2·8 (1·2 to 5·1)	2·9 (1·3 to 5·6)	3·6 (1·5 to 6·8)	5·1 (2·3 to 9·8)	5·8 (2·6 to 11·1)	7·3 (3·4 to 13·7)	7·8 (3·6 to 14·5)	7·1 (3·2 to 12·8)	5·5 (2·5 to 9·9)	3·6 (1·8 to 7·1)	1·7 (0·8 to 3·1)	0·6 (0·3 to 1·1)	0·1 (0·1 to 0·2)	63·3
Musculoskeletal disorders	0 (0 to 0)	0 (0 to 0)	0 (0 to 0)	0 (0 to 0)	3·9 (1·8 to 6·8)	21·1 (12·8 to 33·9)	49·3 (30·4 to 75·2)	70·4 (44·4 to 105)	108 (69·7 to 158)	134 (85·5 to 191)	169 (114 to 237)	193 (132 to 269)	237 (163 to 336)	268 (184 to 384)	299 (201 to 420)	301 (204 to 423)	252 (173 to 358)	199 (136 to 276)	126 (85·4 to 178)	81·4 (56·2 to 114)	47·6 (34·2 to 66·4)	17·6 (12·6 to 23·9)	2·5 (1·8 to 3·5)	2,580
Rheumatoid arthritis	0 (0 to 0)	0 (0 to 0)	0 (0 to 0)	0 (0 to 0)	0 (0 to 0)	0·2 (0·1 to 0·3)	0·5 (0·2 to 0·8)	0·8 (0·4 to 1·4)	1·4 (0·7 to 2·3)	1·7 (0·9 to 2·9)	2·4 (1·1 to 4)	2·7 (1·4 to 4·6)	3·4 (1·8 to 5·4)	3·8 (2·2 to 6·2)	4·3 (2·5 to 6·5)	4·2 (2·4 to 6·4)	3·7 (2·3 to 5·5)	3 (2 to 4·4)	2·6 (1·8 to 3·6)	2·4 (1·6 to 3·2)	3·7 (2·4 to 5·5)	1·6 (0·9 to 2·5)	0·2 (0·1 to 0·3)	42·7
Osteoarthritis	0 (0 to 0)	0 (0 to 0)	0 (0 to 0)	0 (0 to 0)	0 (0 to 0)	0 (0 to 0)	0 (0 to 0)	0 (0 to 0)	0 (0 to 0)	0·7 (0·3 to 1·6)	4·6 (2·2 to 9·5)	12·5 (6·1 to 25·3)	26·5 (12·8 to 54)	42·3 (20 to 87·2)	58·5 (28·8 to 115)	68·1 (34 to 136)	62·1 (31·1 to 123)	52·6 (26·1 to 105)	35·3 (17·5 to 70·6)	23·7 (11·7 to 47·3)	12·7 (6·4 to 25·5)	4·9 (2·5 to 9·8)	0·7 (0·4 to 1·5)	405
Osteoarthritis hip	0 (0 to 0)	0 (0 to 0)	0 (0 to 0)	0 (0 to 0)	0 (0 to 0)	0 (0 to 0)	0 (0 to 0)	0 (0 to 0)	0 (0 to 0)	0·3 (0·1 to 0·6)	0·5 (0·1 to 1·1)	0·8 (0·3 to 1·8)	1·5 (0·6 to 3·3)	2·4 (1 to 5·4)	3·5 (1·5 to 7·8)	4·4 (2 to 9·6)	4·3 (1·9 to 9·1)	3·8 (1·7 to 7·9)	2·5 (1 to 5)	1·7 (0·8 to 3·6)	0·9 (0·4 to 1·9)	0·4 (0·2 to 0·7)	0·1 (0 to 0·1)	26·9
Osteoarthritis knee	0 (0 to 0)	0 (0 to 0)	0 (0 to 0)	0 (0 to 0)	0 (0 to 0)	0 (0 to 0)	0 (0 to 0)	0 (0 to 0)	0 (0 to 0)	0·3 (0·1 to 0·8)	3·3 (1·4 to 6·9)	9·3 (4·3 to 19·8)	19·5 (9·3 to 40·2)	30·9 (14·4 to 63·3)	42 (20·3 to 85·3)	48·2 (23·2 to 98·1)	43·5 (21·3 to 88·9)	36·3 (17·9 to 73·9)	23·9 (11·5 to 48·5)	15·5 (7·5 to 31·5)	8 (3·9 to 16·2)	2·9 (1·4 to 5·8)	0·4 (0·2 to 0·8)	284
Osteoarthritis hand	0 (0 to 0)	0 (0 to 0)	0 (0 to 0)	0 (0 to 0)	0 (0 to 0)	0 (0 to 0)	0 (0 to 0)	0 (0 to 0)	0 (0 to 0)	0·1 ([illegible])	0·4 ([illegible])	1·1 ([illegible])	2·2 ([illegible])	3·5 ([illegible])	4·9 ([illegible])	5·7 ([illegible])	5·2 ([illegible])	4·4 ([illegible])	3·1 ([illegible])	2·2 ([illegible])	1·3 ([illegible])	0·6 ([illegible])	0·1 ([illegible])	34·8

Osteoarthritis other	0 (0 to 0)	0 (0 to 0)	0 (0 to 0)	0 (0 to 0)	0 (0 to 0)	0 (0 to 0)	0 (0 to 0)	0 (0 to 0)
Low back pain	0 (0 to 0)	0 (0 to 0)	0 (0 to 0)	0 (0 to 0)	3·5 (1·5 to 6·5)	19 (11·2 to 30·8)	37·5 (21·8 to 59·7)	44·5 (25·6 to 72·5)
Neck pain	0 (0 to 0)	0 (0 to 0)	0 (0 to 0)	0 (0 to 0)	0·2 (0·1 to 0·6)	1·5 (0·6 to 3·1)	3·6 (1·5 to 6·8)	5·4 (2·2 to 10·6)
Gout	0 (0 to 0)	0 (0 to 0)	0 (0 to 0)	0 (0 to 0)	0 (0 to 0)	0 (0 to 0)	0 (0 to 0)	0·1 (0·1 to 0·3)
Other musculoskeletal disorders	0 (0 to 0)	0 (0 to 0)	0 (0 to 0)	0 (0 to 0)	0·1 (0·1 to 0·3)	0·4 (0·2 to 1·3)	7·7 (3·4 to 13·6)	19·6 (11·3 to 30·4)
Other non-communicable diseases	157 (93·8 to 243)	50·7 (32·7 to 76·5)	131 (84·5 to 192)	55·3 (39·9 to 75·8)	43 (32·2 to 57·3)	43·1 (33·4 to 55·9)	54·1 (42 to 69)	57·5 (43·9 to 77·2)
Congenital birth defects	151 (88·5 to 235)	44 (27·1 to 66·6)	81·7 (51·3 to 123)	28·6 (19·3 to 42)	17·6 (13·1 to 24·2)	18·1 (13·4 to 23·6)	22·8 (16·3 to 30·3)	16·7 (12·3 to 22·6)
Neural tube defects	2·7 (1·2 to 5)	0·6 (0·3 to 1·3)	1·2 (0·5 to 2·5)	0·8 (0·4 to 1·4)	0·4 (0·3 to 0·6)	0·5 (0·3 to 0·7)	0·6 (0·4 to 0·8)	0·4 (0·3 to 0·6)
Congenital heart anomalies	59·6 (31·8 to 101)	21·5 (12 to 34·5)	38·7 (22·4 to 62·9)	10·9 (5·7 to 17·7)	5·4 (3 to 9·1)	5·4 (3·5 to 8·3)	7·6 (4·7 to 11·4)	5·2 (3·1 to 8)
Orofacial clefts	0 (0 to 0)	0 (0 to 0)	0·1 (0·1 to 0·2)	0·3 (0·1 to 0·6)	0·3 (0·1 to 0·6)	0·3 (0·1 to 0·7)	0·3 (0·1 to 0·7)	0·3 (0·1 to 0·6)
Down syndrome	1·8 (0·8 to 3·3)	0·8 (0·3 to 1·4)	3·7 (1·7 to 7)	1·7 (0·8 to 3·1)	0·8 (0·5 to 1·3)	1·2 (0·7 to 1·8)	2·1 (1·2 to 3·5)	1·5 (0·9 to 2·4)
Turner syndrome	0 (0 to 0)	0 (0 to 0)	0 (0 to 0)	0 (0 to 0)	0 (0 to 0)	0 (0 to 0)	0 (0 to 0)	0 (0 to 0)
Klinefelter syndrome	0 (0 to 0)	0 (0 to 0)	0 (0 to 0)	0 (0 to 0)	0 (0 to 0)	0 (0 to 0)	0 (0 to 0)	0 (0 to 0)
Other chromosomal abnormalities	14·3 (7·1 to 25·3)	4·4 (2·1 to 8·1)	7·3 (3·7 to 13·2)	2·3 (1·4 to 4)	1·3 (0·9 to 2)	1·3 (0·8 to 2)	2·1 (1·2 to 3·4)	1 (0·6 to 1·6)
Congenital musculoskeletal and limb anomalies	5·3 (2·3 to 11)	1·5 (0·7 to 2·9)	3·8 (2·1 to 6·6)	3·6 (2·1 to 5·5)	3·9 (2·3 to 6·3)	4·3 (2·5 to 6·8)	3·9 (2·2 to 6·3)	2·7 (1·5 to 4·3)
Urogenital congenital anomalies	5·7 (2·7 to 11·6)	1·3 (0·6 to 2·5)	1·7 (0·9 to 3·3)	0·8 (0·5 to 1·3)	0·6 (0·4 to 0·9)	0·5 (0·3 to 0·8)	0·3 (0·2 to 0·5)	0·3 (0·2 to 0·5)
Digestive congenital anomalies	20·5 (10·8 to 34·6)	6·2 (3·3 to 10·7)	8·7 (4·6 to 14·9)	1·9 (1·1 to 3·1)	0·8 (0·4 to 1·3)	0·4 (0·3 to 0·7)	0·4 (0·3 to 0·7)	0·1 (0·1 to 0·2)
Other congenital birth defects	41·2 (21·6 to 69·1)	7·7 (4·2 to 12·9)	16·5 (8·9 to 27·2)	6·3 (3·5 to 10·4)	4 (2·6 to 6·3)	4·1 (2·6 to 6·1)	5·5 (3·3 to 8·3)	5·2 (3·1 to 7·7)
Urinary diseases and male infertility	0·6 (0·2 to 1)	0·8 (0·3 to 1·4)	3·6 (1·7 to 6·3)	1·2 (0·7 to 1·8)	0·8 (0·6 to 1·2)	1·1 (0·8 to 1·6)	2·3 (1·6 to 3·3)	6 (3·7 to 8·9)
Urolithiasis	0 (0 to 0)	0 (0 to 0)	0 (0 to 0)	0 (0 to 0)	0 (0 to 0·1)	0 (0 to 0·1)	0·1 (0 to 0·1)	0·2 (0·1 to 0·3)
Benign prostatic hyperplasia	0 (0 to 0)	0 (0 to 0)	0 (0 to 0)	0 (0 to 0)	0 (0 to 0)	0 (0 to 0)	0 (0 to 0)	0 (0 to 0)
Male infertility	0 (0 to 0)	0 (0 to 0)	0 (0 to 0)	0 (0 to 0)	0 (0 to 0)	0 (0 to 0)	0 (0 to 0)	1·7 (0·5 to 4·6)
Other urinary diseases	0·1 (0 to 0·3)	0·3 (0·1 to 0·5)	0·1 (0·1 to 0·3)	0·1 (0·1 to 0·2)	0·2 (0·1 to 0·3)	0·3 (0·1 to 0·5)	0·6 (0·3 to 1)	0·9 (0·4 to 1·6)
Gynecological diseases	0 (0 to 0)	0 (0 to 0)	0 (0 to 0)	0 (0 to 0)	0 (0 to 0)	0 (0 to 0)	0 (0 to 0)	0 (0 to 0)
Uterine fibroids	0 (0 to 0)	0 (0 to 0)	0 (0 to 0)	0 (0 to 0)	0 (0 to 0)	0 (0 to 0)	0 (0 to 0)	0 (0 to 0)
Polycystic ovarian syndrome	--	--	--	--	--	--	--	--
Female infertility	--	--	--	--	--	--	--	--
Endometriosis	--	--	--	--	--	--	--	--
Genital prolapse	0 (0 to 0)	0 (0 to 0)	0 (0 to 0)	0 (0 to 0)	0 (0 to 0)	0 (0 to 0)	0 (0 to 0)	0 (0 to 0)
Premenstrual syndrome	--	--	--	--	--	--	--	--
Other gynecological diseases	0 (0 to 0)	0 (0 to 0)	0 (0 to 0)	0 (0 to 0)	0 (0 to 0)	0 (0 to 0)	0 (0 to 0)	0 (0 to 0)
Hemoglobinopathies and hemolytic anemias	0·3 (0·2 to 0·5)	0·4 (0·2 to 0·6)	2·2 (1·2 to 3·7)	4·9 (2·7 to 8)	9 (5 to 15)	8·5 (5·2 to 13·2)	9·7 (6·4 to 14·3)	8·7 (5·2 to 13·3)
Thalassemias	0 (0 to 0)	0 (0 to 0)	0 (0 to 0)	0 (0 to 0·1)	0 (0 to 0)	0 (0 to 0)	0 (0 to 0)	0 (0 to 0)
Thalassemias trait	0 (0 to 0)	0 (0 to 0)	0 (0 to 0·1)	0·1 (0 to 0·4)	0·3 (0·1 to 0·7)	0·2 (0 to 0·5)	0 (0 to 0·1)	0 (0 to 0·1)
Sickle cell disorders	0·3 (0·2 to 0·5)	0·2 (0·1 to 0·4)	1·8 (0·9 to 3·2)	2·7 (1·6 to 4·5)	3·9 (2·5 to 5·8)	4·6 (2·8 to 7·1)	7·3 (4·5 to 10·9)	5·9 (3·4 to 9·6)
Sickle cell trait	0 (0 to 0)	0·1 (0 to 0·2)	0·3 (0 to 0·7)	1·1 (0·2 to 2·7)	2·5 (0·7 to 5·3)	1·8 (0·5 to 3·7)	0·3 (0 to 0·9)	0·2 (0 to 0·5)
G6PD deficiency	0 (0 to 0)	0 (0 to 0·1)	0 (0 to 0·1)	0·4 (0·2 to 0·8)	0·7 (0·3 to 1·3)	0·7 (0·3 to 1·3)	1·4 (0·6 to 2·7)	2·1 (0·8 to 4)
G6PD trait	0 (0 to 0)	0 (0 to 0)	0 (0 to 0)	0 (0 to 0)	0 (0 to 0)	0 (0 to 0)	0 (0 to 0)	0 (0 to 0)
Other hemoglobinopathies and hemolytic anemias	0 (0 to 0)	0 (0 to 0·1)	0·1 (0 to 0·3)	0·5 (0·1 to 1·3)	1·6 (0·4 to 3·8)	1·2 (0·3 to 2·8)	0·6 (0·2 to 1·5)	0·5 (0·2 to 1·1)
Endocrine, metabolic, blood, and immune disorders	5·4 (2·9 to 9·5)	4 (2 to 7·7)	32·7 (16·7 to 65·5)	17·2 (9·5 to 37·4)	11 (6·8 to 20·2)	9·8 (6·3 to 15·9)	10·3 (6·5 to 16·2)	12·7 (8 to 22·8)
Oral disorders	0 (0 to 0)	0 (0 to 0)	0 (0 to 0)	3·5 (2 to 5·7)	4·6 (2·4 to 7·9)	5·6 (2·9 to 10·1)	8·9 (4·9 to 15·7)	13·5 (6·8 to 26)
Caries of deciduous teeth	0 (0 to 0)	0 (0 to 0)	0 (0 to 0)	1·2 (0·5 to 2·5)	1·3 (0·5 to 2·8)	0·1 (0 to 0·2)	0 (0 to 0)	0 (0 to 0)
Caries of permanent teeth	0 (0 to 0)	0 (0 to 0)	0 (0 to 0)	0 (0 to 0)	1 (0·4 to 2·3)	2·8 (1·1 to 6·2)	3·2 (1·3 to 6·9)	3·5 (1·4 to 7·1)
Periodontal diseases	0 (0 to 0)	0 (0 to 0)	0 (0 to 0)	0 (0 to 0)	0 (0 to 0)	0 (0 to 0)	1·3 (0·4 to 3·5)	4·6 (1·4 to 11·7)
Other oral disorders	0 (0 to 0)	0 (0 to 0)	0 (0 to 0)	2·2 (1·4 to 3·5)	2·3 (1·3 to 3·7)	2·8 (1·6 to 4·4)	4·4 (2·6 to 6·9)	5 (3 to 7·9)
Sudden infant death syndrome	--	1·6 (0·9 to 2·7)	10·9 (5·4 to 19·6)	--	--	--	--	--
Injuries	7·4 (4·8 to 11)	2·9 (1·8 to 4·2)	18·6 (11·8 to 28·7)	37·7 (26·7 to 52)	58·5 (47·4 to 71·8)	104 (85·1 to 124)	385 (316 to 472)	497 (430 to 598)
Transport injuries	0·4 (0·2 to 0·6)	0·2 (0·1 to 0·3)	1·5 (0·9 to 2·5)	5·9 (3·5 to 9·2)	13·1 (8·7 to 18·6)	25 (16·6 to 34·4)	96·8 (63·7 to 135)	124 (84·6 to 168)
Road injuries	0·3 (0·2 to 0·5)	0·1 (0·1 to 0·2)	1·4 (0·7 to 2·3)	5·5 (3·1 to 8·8)	12·4 (8·1 to 17·8)	23·7 (15·7 to 32·8)	93·8 (61·7 to 131)	120 (81·7 to 163)
Pedestrian road injuries	0·1 (0·1 to 0·2)	0 (0 to 0)	0·3 (0·2 to 0·5)	1·7 (0·9 to 2·9)	3·8 (2·4 to 5·8)	5·7 (3·8 to 8·1)	8 (5·2 to 11·8)	8·4 (5·7 to 11·7)
Cyclist road injuries	0 (0 to 0)	0 (0 to 0)	0 (0 to 0)	0·3 (0·1 to 0·4)	1 (0·6 to 1·5)	3·2 (2 to 4·9)	7·5 (4·8 to 11·1)	7·6 (5·2 to 10·7)
Motorcyclist road injuries	0 (0 to 0·1)	0 (0 to 0)	0·1 (0·1 to 0·3)	0·4 (0·2 to 0·7)	1 (0·5 to 1·7)	2 (1·1 to 3·4)	18·4 (10·1 to 30)	28·1 (15·8 to 44·3)
Motor vehicle road injuries	0·2 (0·1 to 0·3)	0·1 (0·1 to 0·2)	0·9 (0·5 to 1·6)	3 (1·7 to 4·8)	6·5 (4 to 9·6)	12·7 (7·5 to 18)	59·5 (37·1 to 87·2)	75·5 (48·5 to 105)
Other road injuries	0 (0 to 0)	0 (0 to 0)	0 (0 to 0)	0·1 (0·1 to 0·2)	0·2 (0·1 to 0·2)	0·2 (0·2 to 0·3)	0·4 (0·3 to 0·6)	0·7 (0·5 to 1)
Other transport injuries	0 (0 to 0·1)	0 (0 to 0)	0·2 (0·1 to 0·3)	0·4 (0·3 to 0·6)	0·7 (0·5 to 1)	1·3 (0·8 to 2)	3 (1·8 to 5)	3·8 (2·3 to 6)
Unintentional injuries	3·9 (2·5 to 5·8)	2·2 (1·4 to 3·3)	14·3 (8·8 to 22·8)	27·2 (18·9 to 37·8)	40 (31·4 to 50)	62·6 (49·8 to 77·6)	124 (98·1 to 157)	122 (97 to 153)
Falls	0·1 (0 to 0·1)	0 (0 to 0·1)	0·5 (0·3 to 0·8)	1·3 (0·9 to 1·9)	3 (2·1 to 4·3)	6·2 (4·5 to 8·7)	11·7 (8·7 to 15·4)	13·6 (10 to 18·1)
Drowning	0·5 (0·3 to 0·7)	0·1 (0·1 to 0·2)	2·2 (1·3 to 3·6)	11·5 (7 to 17·3)	18·2 (13·1 to 24·5)	27·6 (20 to 36·6)	61·5 (43·4 to 83·2)	47·5 (32 to 66·9)
Fire, heat, and hot substances	0·1 (0·1 to 0·2)	0·1 (0·1 to 0·2)	1·5 (0·9 to 2·4)	3·8 (2·5 to 5·6)	4·7 (3·5 to 6·2)	6·5 (4·8 to 8·8)	8·8 (6·3 to 12·1)	9·8 (7·1 to 13·5)
Poisonings	0 (0 to 0)	0 (0 to 0)	0·2 (0·1 to 0·4)	0·7 (0·4 to 1·1)	0·6 (0·4 to 0·9)	0·8 (0·5 to 1·2)	1·2 (0·8 to 1·7)	1·4 (1 to 2)
Poisoning by carbon monoxide	0 (0 to 0)	0 (0 to 0)	0 (0 to 0)	0 (0 to 0·1)	0·1 (0 to 0·1)	0·1 (0·1 to 0·2)	0·2 (0·1 to 0·3)	0·2 (0·1 to 0·4)
Poisoning by other means	0 (0 to 0)	0 (0 to 0)	0·2 (0·1 to 0·4)	0·7 (0·4 to 1·1)	0·5 (0·3 to 0·8)	0·7 (0·5 to 1·1)	1 (0·7 to 1·4)	1·2 (0·9 to 1·7)
Exposure to mechanical forces	0·1 (0 to 0·1)	0·1 (0 to 0·1)	0·4 (0·3 to 0·6)	2·1 (1·4 to 3)	4·5 (3 to 6·5)	8 (5·7 to 11·8)	20·8 (15·1 to 27·7)	26·4 (19·9 to 35)
Unintentional firearm injuries	0 (0 to 0)	0 (0 to 0)	0·1 (0·1 to 0·1)	0·3 (0·2 to 0·4)	0·6 (0·3 to 0·8)	1·6 (1 to 2·4)	10·1 (6·7 to 15)	14 (9·6 to 20·3)
Other exposure to mechanical forces	0 (0 to 0·1)	0·1 (0 to 0·1)	0·3 (0·2 to 0·5)	1·8 (1·2 to 2·6)	3·9 (2·6 to 5·8)	6·5 (4·3 to 9·7)	10·6 (7·2 to 15·2)	12·4 (8·5 to 18·4)
Adverse effects of medical treatment	1·4 (0·8 to 2·2)	0·9 (0·5 to 1·5)	2·6 (1·2 to 5)	1·5 (0·9 to 2·6)	1·2 (0·8 to 2)	1·4 (0·8 to 2·2)	2·1 (1·4 to 3·2)	3·6 (2·2 to 5·7)
Animal contact	0 (0 to 0)	0 (0 to 0)	0 (0 to 0·1)	0·3 (0·2 to 0·4)	0·5 (0·3 to 0·7)	0·7 (0·5 to 1·1)	1 (0·6 to 1·4)	1·1 (0·7 to 1·5)
Venomous animal contact	0 (0 to 0)	0 (0 to 0)	0 (0 to 0)	0·2 (0·1 to 0·3)	0·3 (0·2 to 0·5)	0·5 (0·3 to 0·8)	0·7 (0·4 to 1·1)	0·8 (0·4 to 1·1)
Non-venomous animal contact	0 (0 to 0)	0 (0 to 0)	0 (0 to 0)	0·1 (0·1 to 0·1)	0·1 (0·1 to 0·2)	0·2 (0·1 to 0·4)	0·3 (0·2 to 0·4)	0·3 (0·2 to 0·5)
Foreign body	1·6 (1 to 2·5)	0·9 (0·5 to 1·4)	6·4 (3·6 to 10·8)	3·6 (2·4 to 5·1)	2·5 (1·9 to 3·3)	4 (3 to 5·2)	3·7 (2·8 to 4·9)	4·1 (3·1 to 5·4)
Pulmonary aspiration and foreign body in airway	1·6 (1 to 2·5)	0·9 (0·5 to 1·4)	6·3 (3·5 to 10·6)	3 (1·9 to 4·5)	1·7 (1·2 to 2·2)	2·8 (2 to 3·9)	2·4 (1·7 to 3·2)	2·6 (1·9 to 3·6)
Foreign body in eyes	0 (0 to 0)	0 (0 to 0)	0 (0 to 0·1)	0·2 (0·1 to 0·4)	0·3 (0·1 to 0·7)	0·4 (0·1 to 1)	0·6 (0·2 to 1·4)	0·7 (0·2 to 1·6)
Foreign body in other body part	0 (0 to 0)	0 (0 to 0)	0·1 (0·1 to 0·2)	0·4 (0·3 to 0·6)	0·6 (0·4 to 0·8)	0·7 (0·5 to 1·1)	0·7 (0·5 to 1)	0·8 (0·6 to 1·1)
Environmental heat and cold exposure	0 (0 to 0)	0 (0 to 0)	0·1 (0 to 0·1)	0·4 (0·3 to 0·7)	1 (0·6 to 1·4)	1·6 (1·1 to 2·3)	2·5 (1·7 to 3·7)	3·2 (2·2 to 4·6)
Exposure to forces of nature	0 (0 to 0)	0 (0 to 0)	0 (0 to 0)	0 (0 to 0)	0 (0 to 0)	0 (0 to 0)	0 (0 to 0)	0 (0 to 0)
Other unintentional injuries	0·1 (0·1 to 0·2)	0 (0 to 0·1)	0·5 (0·3 to 0·7)	2 (1·3 to 2·8)	3·7 (2·6 to 5·3)	5·7 (4·3 to 7·8)	10·7 (8·1 to 14·3)	11·5 (8·4 to 15·4)
Self-harm and interpersonal violence	3·2 (1·9 to 5·1)	0·4 (0·3 to 0·7)	2·7 (1·5 to 4·6)	4·5 (2·5 to 7·2)	5·3 (3·8 to 7·4)	16·1 (12 to 21·4)	164 (123 to 221)	251 (198 to 317)
Self-harm	0 (0 to 0)	0 (0 to 0)	0 (0 to 0)	0 (0 to 0)	0 (0 to 0)	2 (1·2 to 3·4)	21·8 (14·2 to 31·9)	35·5 (24 to 50·7)
Self-harm by firearm	0 (0 to 0)	0 (0 to 0)	0 (0 to 0)	0 (0 to 0)	0 (0 to 0)	0·1 (0 to 0·1)	1·8 (0·9 to 3·1)	2·2 (1 to 3·9)
Self-harm by other specified means	0 (0 to 0)	0 (0 to 0)	0 (0 to 0)	0 (0 to 0)	0 (0 to 0)	2 (1·1 to 3·3)	20 (12·8 to 29·3)	33·4 (22·7 to 47·3)
Interpersonal violence	3·2 (1·9 to 5·1)	0·4 (0·3 to 0·7)	2·7 (1·5 to 4·6)	4·4 (2·5 to 7)	5·3 (3·8 to 7·3)	14 (10·4 to 19)	142 (102 to 194)	215 (166 to 278)
Physical violence by firearm	0·1 (0·1 to 0·1)	0 (0 to 0)	0·2 (0·1 to 0·3)	0·4 (0·2 to 0·6)	0·8 (0·6 to 1·2)	3·8 (2·7 to 5·2)	75·5 (53·7 to 104)	120 (90·9 to 155)
Physical violence by sharp object	0·2 (0·1 to 0·3)	0 (0 to 0)	0·2 (0·1 to 0·3)	0·5 (0·3 to 0·7)	0·7 (0·5 to 1)	2·4 (1·8 to 3·1)	45·7 (32 to 63·8)	68·6 (52·9 to 89·1)
Sexual violence	0 (0 to 0)	0 (0 to 0)	0 (0 to 0)	0 (0 to 0·1)	0·3 (0·2 to 0·5)	0·8 (0·4 to 1·2)	1·7 (1 to 2·8)	2·1 (1·2 to 3·3)
Physical violence by other means	2·9 (1·7 to 4·7)	0·4 (0·2 to 0·7)	2·4 (1·3 to 4)	3·6 (1·9 to 5·8)	3·4 (2·3 to 4·9)	7·1 (5·1 to 9·7)	19·1 (13·6 to 26·1)	25·2 (19·6 to 32·4)
Conflict and terrorism	0 (0 to 0)	0 (0 to 0)	0 (0 to 0)	0 (0 to 0)	0 (0 to 0)	0 (0 to 0)	0 (0 to 0)	0 (0 to 0)

Osteoarthritis other	0 (0 to 0)	0·1 (0 to 0·1)	0·4 (0·1 to 1·2)	1·4 (0·5 to 3·4)	3·3 (1·3 to 7·4)	5·6 (2·4 to 11·8)	8·1 (3·7 to 17·6)	9·8 (4·6 to 20·9)
Low back pain	56·3 (32·5 to 88·9)	62·6 (33·8 to 99)	76 (44·6 to 119)	82·5 (48·7 to 129)	95·5 (56·8 to 154)	105 (62·9 to 169)	113 (70·1 to 175)	111 (67·7 to 167)
Neck pain	9 (3·9 to 18·1)	13·7 (6·4 to 26·8)	19·4 (9·4 to 34·2)	23·4 (11·6 to 42·6)	28·7 (13·9 to 51·1)	27·4 (13·4 to 51·7)	27·3 (13·1 to 48·1)	24·7 (12·7 to 43·9)
Gout	0·4 (0·1 to 1)	0·8 (0·2 to 1·6)	1·2 (0·5 to 2·3)	1·6 (0·7 to 2·8)	2 (0·9 to 3·6)	2·2 (0·9 to 4·1)	2·4 (0·9 to 4·6)	2·6 (1·1 to 4·8)
Other musculoskeletal disorders	40·6 (23·9 to 62·7)	54·2 (33·8 to 83·6)	65·3 (40·4 to 98·6)	70·4 (41·6 to 110)	81·2 (50·1 to 123)	87·7 (52·7 to 139)	93·8 (57·5 to 146)	90·5 (52·2 to 142)
Other non-communicable diseases	76·9 (58 to 105)	95·3 (72·4 to 131)	104 (77 to 147)	107 (77·7 to 155)	123 (89·8 to 171)	143 (108 to 196)	189 (145 to 248)	232 (176 to 299)
Congenital birth defects	16·6 (11·8 to 22·5)	15·2 (10·9 to 20·5)	12·4 (9·2 to 16·5)	10·4 (7·7 to 13·8)	10·7 (7·8 to 14·3)	9·9 (7·2 to 13·2)	10·2 (7·5 to 13·6)	7·5 (5·6 to 9·9)
Neural tube defects	0·4 (0·3 to 0·5)	0·3 (0·2 to 0·4)	0·2 (0·1 to 0·3)	0·2 (0·1 to 0·2)	0·2 (0·1 to 0·2)	0·1 (0·1 to 0·2)	0·1 (0·1 to 0·2)	0·1 (0·1 to 0·1)
Congenital heart anomalies	5·2 (3·2 to 7·8)	5·4 (3·3 to 8·2)	3·8 (2·5 to 5·6)	2·9 (1·8 to 4·4)	2·7 (1·7 to 4·1)	2·1 (1·3 to 3·3)	1·7 (1·1 to 2·6)	1·6 (1 to 2·4)
Orofacial clefts	0·3 (0·1 to 0·7)	0·3 (0·1 to 0·6)	0·3 (0·1 to 0·6)	0·3 (0·1 to 0·6)	0·3 (0·1 to 0·7)	0·3 (0·1 to 0·7)	0·3 (0·1 to 0·6)	0·3 (0·1 to 0·6)
Down syndrome	1·6 (0·9 to 2·8)	2 (1·1 to 3·4)	1·8 (1 to 3·1)	1·9 (1·1 to 3)	2·1 (1·2 to 3·3)	2 (1·1 to 3·3)	3·1 (1·8 to 4·9)	1·8 (1 to 2·9)
Turner syndrome	0 (0 to 0)	0 (0 to 0)	0 (0 to 0)	0 (0 to 0)	0 (0 to 0)	0 (0 to 0)	0 (0 to 0)	0 (0 to 0)
Klinefelter syndrome	0 (0 to 0)	0 (0 to 0)	0 (0 to 0)	0 (0 to 0)	0 (0 to 0)	0 (0 to 0)	0 (0 to 0)	0 (0 to 0)
Other chromosomal abnormalities	1·6 (0·9 to 2·6)	0·7 (0·4 to 1·2)	0·9 (0·5 to 1·4)	0·3 (0·2 to 0·5)	0·4 (0·2 to 0·6)	0·2 (0·1 to 0·3)	0·2 (0·1 to 0·4)	0·1 (0·1 to 0·3)
Congenital musculoskeletal and limb anomalies	2·8 (1·7 to 4·3)	2·2 (1·3 to 3·6)	2·5 (1·4 to 3·8)	2·2 (1·3 to 3·6)	2·4 (1·4 to 3·9)	2·5 (1·4 to 4)	2·5 (1·5 to 3·8)	2 (1·1 to 3·2)
Urogenital congenital anomalies	0·3 (0·2 to 0·4)	0·3 (0·2 to 0·5)	0·2 (0·1 to 0·2)	0·1 (0·1 to 0·2)	0·2 (0·1 to 0·3)	0·1 (0·1 to 0·1)	0·2 (0·1 to 0·3)	0·1 (0·1 to 0·2)
Digestive congenital anomalies	0·2 (0·1 to 0·3)	0·1 (0·1 to 0·2)	0·1 (0·1 to 0·1)	0·1 (0·1 to 0·1)	0·1 (0·1 to 0·2)	0·1 (0·1 to 0·2)	0·1 (0 to 0·1)	0·1 (0·1 to 0·2)
Other congenital birth defects	4·2 (2·5 to 6·6)	3·9 (2·2 to 6)	2·7 (1·7 to 4·1)	2·4 (1·5 to 3·6)	2·4 (1·4 to 3·7)	2·5 (1·6 to 3·8)	2 (1·2 to 3·2)	1·3 (0·8 to 2·2)
Urinary diseases and male infertility	7·8 (4·6 to 11·9)	11·8 (6·8 to 17·8)	15·4 (9·1 to 23)	16·3 (9·8 to 23·7)	21·9 (12 to 32·8)	31 (18·8 to 45·2)	58·5 (35·2 to 80·8)	89·5 (56·6 to 123)
Urolithiasis	0·3 (0·2 to 0·5)	0·5 (0·3 to 0·9)	1 (0·4 to 1·8)	1 (0·4 to 1·8)	2·1 (1 to 3·7)	2·2 (1·2 to 3·8)	3·3 (1·8 to 5·3)	3·4 (1·8 to 5·5)
Benign prostatic hyperplasia	0 (0 to 0)	0 (0 to 0)	0 (0 to 0)	0·1 (0 to 0·2)	0·8 (0·3 to 1·7)	3·5 (1·7 to 6·3)	11·9 (5·8 to 20·5)	22·9 (11·9 to 39·5)
Male infertility	2·3 (0·6 to 5·6)	2·4 (0·7 to 6·5)	2·5 (0·7 to 6·4)	2·3 (0·7 to 6)	0·4 (0·1 to 1·1)	0 (0 to 0)	0 (0 to 0)	0 (0 to 0)
Other urinary diseases	0·5 (0·2 to 0·9)	0·9 (0·4 to 1·6)	1·7 (0·8 to 3)	1·4 (0·7 to 2·4)	2·5 (1·1 to 4·4)	2·5 (1·2 to 4·7)	3·4 (1·5 to 6)	5·6 (2·4 to 9·9)
Gynecological diseases	0 (0 to 0)	0 (0 to 0)	0 (0 to 0)	0 (0 to 0)	0 (0 to 0)	0 (0 to 0)	0 (0 to 0)	0 (0 to 0)
Uterine fibroids	0 (0 to 0)	0 (0 to 0)	0 (0 to 0)	0 (0 to 0)	0 (0 to 0)	0 (0 to 0)	0 (0 to 0)	0 (0 to 0)
Polycystic ovarian syndrome	--	--	--	--	--	--	--	--
Female infertility	--	--	--	--	--	--	--	--
Endometriosis	--	--	--	--	--	--	--	--
Genital prolapse	0 (0 to 0)	0 (0 to 0)	0 (0 to 0)	0 (0 to 0)	0 (0 to 0)	0 (0 to 0)	0 (0 to 0)	0 (0 to 0)
Premenstrual syndrome	--	--	--	--	--	--	--	--
Other gynecological diseases	0 (0 to 0)	0 (0 to 0)	0 (0 to 0)	0 (0 to 0)	0 (0 to 0)	0 (0 to 0)	0 (0 to 0)	0 (0 to 0)
Hemoglobinopathies and hemolytic anemias	9·1 (6 to 13·4)	9 (6 to 13·3)	7·6 (5 to 11·2)	7·8 (5 to 11·7)	7·5 (4·8 to 10·8)	6·5 (4·3 to 9·3)	8·1 (5·2 to 12)	7·7 (5 to 11·7)
Thalassemias	0 (0 to 0)	0 (0 to 0)	0 (0 to 0)	0 (0 to 0)	0 (0 to 0)	0 (0 to 0)	0 (0 to 0)	0 (0 to 0)
Thalassemias trait	0 (0 to 0·1)	0 (0 to 0·1)	0 (0 to 0·1)	0 (0 to 0·1)	0 (0 to 0·1)	0 (0 to 0·1)	0·1 (0 to 0·2)	0·1 (0 to 0·2)
Sickle cell disorders	5·5 (3·2 to 8·8)	5·1 (2·9 to 7·9)	3·8 (2·1 to 6·1)	3·6 (1·9 to 5·9)	2·8 (1·4 to 4·7)	1·7 (0·8 to 2·9)	0·9 (0·4 to 1·8)	0·3 (0·1 to 0·6)
Sickle cell trait	0·2 (0 to 0·7)	0·2 (0 to 0·6)	0·3 (0 to 0·8)	0·3 (0 to 0·8)	0·4 (0·1 to 1·1)	0·4 (0·1 to 1·1)	0·7 (0·1 to 1·8)	0·6 (0·2 to 1·6)
G6PD deficiency	2·8 (1·3 to 5·2)	3·1 (1·4 to 5·6)	2·8 (1·3 to 4·8)	3·1 (1·5 to 5·2)	3·2 (1·6 to 5·1)	3·1 (1·8 to 4·9)	4 (2·5 to 6·2)	3·7 (2·2 to 5·7)
G6PD trait	0 (0 to 0)	0 (0 to 0)	0 (0 to 0)	0 (0 to 0)	0 (0 to 0)	0 (0 to 0)	0 (0 to 0)	0 (0 to 0)
Other hemoglobinopathies and hemolytic anemias	0·5 (0·2 to 1·4)	0·6 (0·3 to 1·3)	0·7 (0·3 to 1·6)	0·9 (0·4 to 1·7)	1 (0·4 to 2·2)	1·2 (0·6 to 2·3)	2·3 (1·2 to 4·3)	3 (1·7 to 5·2)
Endocrine, metabolic, blood, and immune disorders	22·6 (14·8 to 36·1)	33·1 (21·8 to 53)	35·8 (24·1 to 57·9)	36·7 (23·7 to 62·1)	40·2 (26·4 to 66)	48·1 (31·6 to 79·6)	58 (39·1 to 93·4)	64·8 (42·3 to 105)
Oral disorders	20·8 (10·3 to 41)	26·1 (12·4 to 49·3)	32·6 (15·4 to 61·5)	36·1 (17 to 66·7)	42·5 (20·7 to 77·6)	47 (24·8 to 83·5)	54·4 (30 to 90·8)	62 (36·4 to 102)
Caries of deciduous teeth	0 (0 to 0)	0 (0 to 0)	0 (0 to 0)	0 (0 to 0)	0 (0 to 0)	0 (0 to 0)	0 (0 to 0)	0 (0 to 0)
Caries of permanent teeth	3·5 (1·4 to 7·1)	3·1 (1·2 to 6·5)	3·2 (1·3 to 6·6)	3 (1·2 to 6·2)	3 (1·2 to 6·5)	3 (1·1 to 6·3)	2·7 (1 to 6·1)	2·2 (0·8 to 4·8)
Periodontal diseases	10·5 (3·3 to 25)	15·2 (5·2 to 34)	20 (7·1 to 44·8)	22·6 (8·1 to 48·9)	26·1 (9·9 to 55·7)	26·5 (10·4 to 55·4)	25·2 (10 to 52·9)	21·1 (8·3 to 43)
Other oral disorders	5·8 (3·4 to 8·8)	5·8 (3·4 to 8·8)	6·2 (3·5 to 9·5)	6·1 (3·6 to 9·3)	6·6 (4 to 9·9)	6·5 (4 to 10·1)	6·4 (3·8 to 9·6)	5·7 (3·5 to 8·6)
Sudden infant death syndrome	--	--	--	--	--	--	--	--
Injuries	592 (514 to 697)	576 (498 to 675)	433 (364 to 518)	382 (313 to 465)	380 (303 to 462)	324 (261 to 393)	342 (277 to 420)	302 (246 to 366)
Transport injuries	148 (104 to 197)	146 (103 to 194)	114 (83·2 to 150)	104 (76·8 to 136)	109 (78·6 to 145)	95·9 (72·3 to 124)	103 (76·6 to 133)	88·2 (66 to 114)
Road injuries	143 (101 to 190)	141 (99·2 to 187)	110 (79·8 to 145)	100 (73·2 to 131)	106 (75·9 to 139)	92·5 (69·7 to 119)	99 (73·3 to 127)	85·2 (63·6 to 110)
Pedestrian road injuries	12·1 (8·7 to 16·5)	14·2 (10·1 to 19·2)	12·8 (9·2 to 17·5)	13·4 (9·5 to 17·7)	15·6 (10·8 to 21·4)	15 (10·9 to 20·5)	18·2 (13·3 to 24·6)	16·6 (12·2 to 22·4)
Cyclist road injuries	9·1 (6·5 to 12·4)	10 (7·2 to 13·5)	10 (7·1 to 13·6)	10·2 (7·5 to 13·6)	11·4 (8·3 to 15·2)	11·6 (8·3 to 15·3)	11·9 (8·6 to 15·9)	10·9 (7·8 to 14·5)
Motorcyclist road injuries	35·2 (21·8 to 54·4)	32·6 (20·6 to 50)	23·5 (15·6 to 34·9)	18·4 (12·6 to 26·8)	19 (13·2 to 26·5)	15·4 (11·1 to 20·6)	15·5 (10·7 to 21·6)	11·3 (8·1 to 15·4)
Motor vehicle road injuries	86 (58·2 to 119)	82·7 (53 to 113)	61·7 (42 to 83·4)	56·6 (38·9 to 76·5)	57·5 (38 to 79·1)	48·4 (33·9 to 66·2)	51·2 (35·1 to 69·2)	44·3 (30·9 to 60·4)
Other road injuries	0·9 (0·7 to 1·3)	1·1 (0·8 to 1·6)	1·4 (1 to 2)	1·6 (1·1 to 2·3)	2 (1·4 to 2·8)	2·1 (1·4 to 3)	2·2 (1·5 to 3·1)	2·1 (1·5 to 2·9)
Other transport injuries	4·7 (2·8 to 7·1)	5·2 (3·1 to 8·1)	4·2 (2·6 to 6·4)	4·1 (2·7 to 6·1)	4 (2·5 to 6·1)	3·4 (2·4 to 4·9)	4·2 (2·7 to 6·2)	3 (2 to 4·2)
Unintentional injuries	142 (116 to 176)	148 (119 to 181)	133 (106 to 167)	130 (104 to 162)	139 (109 to 176)	135 (106 to 169)	144 (114 to 181)	137 (109 to 172)
Falls	17·2 (12·7 to 23)	19·6 (14·6 to 26·3)	22 (16·4 to 29·3)	23·6 (17·7 to 31·3)	27·9 (21·2 to 36·4)	30·3 (22·9 to 39·9)	33·5 (25·5 to 43·1)	34·3 (26·5 to 44)
Drowning	50·2 (36·2 to 69·1)	47·5 (35 to 61·6)	34 (24·7 to 45·4)	28·8 (21·5 to 38·2)	26·1 (18·9 to 34·5)	21 (15·1 to 28·4)	20·8 (15 to 28·3)	16·5 (12·4 to 21·6)
Fire, heat, and hot substances	12·3 (9·2 to 16·6)	14·9 (10·9 to 19·9)	14·3 (10·4 to 20)	14·4 (10·3 to 19·7)	16·1 (11·8 to 22·3)	15·3 (11 to 21·3)	16 (11·5 to 22·3)	15·2 (11·2 to 20·9)
Poisonings	2·1 (1·6 to 2·9)	2·4 (1·8 to 3·2)	2 (1·4 to 2·6)	1·5 (1·1 to 2)	1·6 (1·1 to 2·1)	1·3 (0·9 to 1·8)	1·3 (0·9 to 1·8)	1·2 (0·8 to 1·6)
Poisoning by carbon monoxide	0·3 (0·2 to 0·6)	0·4 (0·2 to 0·7)	0·4 (0·2 to 0·6)	0·2 (0·1 to 0·4)	0·2 (0·1 to 0·4)	0·1 (0·1 to 0·2)	0·2 (0·1 to 0·3)	0·1 (0·1 to 0·2)
Poisoning by other means	1·8 (1·4 to 2·4)	2 (1·5 to 2·5)	1·6 (1·2 to 2·1)	1·3 (0·9 to 1·7)	1·4 (1 to 1·8)	1·2 (0·8 to 1·6)	1·1 (0·8 to 1·6)	1 (0·7 to 1·4)
Exposure to mechanical forces	31·1 (23·3 to 41·4)	33·1 (25·3 to 43·2)	30·9 (23·2 to 41·7)	31·3 (22·9 to 41·6)	33·5 (24·5 to 46·3)	32·9 (23·3 to 45·7)	34·2 (24·8 to 47·2)	30·8 (22 to 42·6)
Unintentional firearm injuries	15·4 (10·6 to 21·8)	15 (10·5 to 20·6)	11·3 (8·1 to 15)	10·4 (7·3 to 14·2)	9·6 (6·9 to 12·7)	8 (6 to 10·5)	8 (6·1 to 10·4)	6·2 (4·7 to 8)
Other exposure to mechanical forces	15·7 (10·9 to 22·7)	18·1 (12·6 to 26·1)	19·6 (13·1 to 28·9)	20·9 (14·4 to 30·4)	23·9 (16·2 to 35·3)	24·9 (16·8 to 36·6)	26·2 (17·9 to 38)	24·6 (16·7 to 35·5)
Adverse effects of medical treatment	4·6 (2·9 to 7·1)	4·4 (2·8 to 6·6)	4·7 (3 to 7·2)	5 (3·2 to 7·5)	5·8 (3·6 to 9·2)	8 (4·8 to 12·2)	10·7 (6·8 to 15·9)	13·3 (8·3 to 21·1)
Animal contact	1·3 (0·8 to 1·8)	1·2 (0·8 to 1·8)	1·4 (1 to 2)	1·4 (1 to 2·1)	1·7 (1·1 to 2·4)	1·7 (1·1 to 2·4)	1·8 (1·2 to 2·6)	1·8 (1·3 to 2·5)
Venomous animal contact	0·9 (0·6 to 1·3)	0·8 (0·5 to 1·2)	1 (0·6 to 1·4)	1 (0·6 to 1·4)	1·1 (0·7 to 1·6)	1·1 (0·7 to 1·6)	1·1 (0·8 to 1·7)	1·1 (0·8 to 1·6)
Non-venomous animal contact	0·4 (0·2 to 0·6)	0·4 (0·2 to 0·6)	0·4 (0·3 to 0·7)	0·5 (0·3 to 0·7)	0·6 (0·4 to 0·9)	0·6 (0·4 to 0·9)	0·7 (0·4 to 1)	0·7 (0·5 to 1)
Foreign body	5·4 (4·1 to 7)	5·5 (4·1 to 7·1)	5 (3·7 to 6·5)	5·2 (4 to 6·6)	5·9 (4·6 to 7·6)	6 (4·5 to 8)	6·8 (5·3 to 8·6)	7·4 (5·6 to 9·4)
Pulmonary aspiration and foreign body in airway	3·5 (2·5 to 4·6)	3·8 (2·8 to 4·9)	3·1 (2·3 to 4·1)	3·1 (2·4 to 4)	3·6 (2·6 to 4·7)	3·8 (2·7 to 5·3)	4·4 (3·2 to 5·9)	5·1 (3·6 to 7)
Foreign body in eyes	0·8 (0·3 to 1·8)	0·8 (0·3 to 1·8)	0·9 (0·3 to 1·9)	0·9 (0·4 to 1·9)	1 (0·5 to 1·9)	0·9 (0·5 to 1·6)	0·8 (0·4 to 1·3)	0·7 (0·4 to 1·2)
Foreign body in other body part	1·1 (0·7 to 1·6)	0·9 (0·7 to 1·3)	1 (0·7 to 1·4)	1·2 (0·8 to 1·6)	1·4 (1 to 1·9)	1·3 (0·9 to 1·8)	1·5 (1·1 to 2·1)	1·5 (1·1 to 2·1)
Environmental heat and cold exposure	4·1 (2·7 to 5·8)	4·6 (3·1 to 6·5)	5·2 (3·5 to 7·3)	5·5 (3·7 to 7·7)	6·2 (4·3 to 8·7)	6·5 (4·5 to 9)	6·7 (4·7 to 9·3)	6·5 (4·4 to 9)
Exposure to forces of nature	0 (0 to 0·1)	0 (0 to 0)	0 (0 to 0)	0 (0 to 0·1)	0 (0 to 0·1)	0 (0 to 0·1)	0 (0 to 0)	0 (0 to 0)
Other unintentional injuries	14·2 (10·6 to 18·7)	15·1 (11·2 to 19·8)	13·8 (10·3 to 18·5)	13 (9·7 to 17·3)	13·7 (10 to 18·4)	11·9 (8·4 to 16·5)	12 (8·7 to 16·7)	10·4 (7·3 to 14·5)
Self-harm and interpersonal violence	302 (241 to 374)	282 (225 to 349)	186 (142 to 238)	147 (111 to 194)	132 (98·1 to 173)	93·6 (68·7 to 123)	94·5 (71 to 122)	76·7 (59·8 to 97·5)
Self-harm	50·3 (35·3 to 68·5)	56·6 (40·2 to 76·1)	45 (31·7 to 62·3)	39·5 (27·4 to 55·8)	40·7 (28 to 56·9)	33·2 (22·8 to 45·7)	35·7 (24·9 to 48·4)	32·1 (22·7 to 44)
Self-harm by firearm	2·8 (1·6 to 4·6)	3 (1·7 to 5)	2·1 (1·1 to 3·5)	2 (1·1 to 3·2)	2 (1·2 to 3·3)	1·9 (1 to 2·9)	1·7 (1 to 2·7)	1·6 (0·9 to 2·7)
Self-harm by other specified means	47·4 (33·3 to 65)	53·6 (38·1 to 72·2)	42·9 (30·2 to 59)	37·5 (26·2 to 52·9)	38·6 (26·6 to 54·1)	31·3 (21·5 to 43·2)	34 (23·4 to 46)	30·5 (21·5 to 41·4)
Interpersonal violence	252 (195 to 317)	225 (174 to 289)	141 (104 to 186)	106 (76·5 to 146)	89·3 (62·7 to 122)	60 (43·1 to 82·7)	57·6 (41·4 to 79·1)	43·7 (31·8 to 58·3)
Physical violence by firearm	135 (104 to 172)	116 (88·6 to 150)	62·7 (45·8 to 83·6)	41·8 (29·5 to 58·6)	31·7 (21·8 to 44·2)	18·3 (12·7 to 25·8)	16·1 (11·1 to 22·9)	10·9 (7·3 to 15·1)
Physical violence by sharp object	79·7 (61·3 to 101)	72·7 (56·1 to 93·6)	47·8 (35·3 to 63)	36·4 (26·2 to 50·2)	29·5 (20·8 to 40·5)	18·9 (13·6 to 26·1)	15·4 (11·2 to 21·1)	11·4 (8·3 to 14·9)
Sexual violence	2·1 (1·1 to 3·4)	1·8 (1 to 3)	1·8 (1 to 2·9)	1·5 (0·9 to 2·5)	1·3 (0·8 to 2·3)	1·1 (0·6 to 1·8)	0·9 (0·5 to 1·4)	0·8 (0·5 to 1·2)
Physical violence by other means	34·8 (26·7 to 44·4)	34·1 (26·4 to 43·9)	29 (21·6 to 37·9)	26·3 (19·1 to 35·7)	26·8 (19·3 to 36·5)	21·7 (15·8 to 29·9)	25·3 (18·2 to 34·6)	20·7 (15 to 27·4)
Conflict and terrorism	0 (0 to 0·1)	0·1 (0 to 0·1)	0 (0 to 0·1)	1·9 (1·1 to 3·1)	1·8 (1·1 to 2·9)	0·4 (0·2 to 0·6)	1·2 (0·8 to 2)	0·9 (0·6 to 1·3)

Osteoarthritis other	9·2 (4·2 to 19·4)	8·1 (4 to 17)	5·8 (2·9 to 11·9)	4·2 (2·1 to 8·5)	2·5 (1·2 to 4·9)	1·1 (0·5 to 2·2)	0·2 (0·1 to 0·4)	59·6
Low back pain	92·9 (57·9 to 145)	73·5 (46·2 to 113)	46·8 (28·8 to 71)	31·4 (19 to 47·9)	15·9 (9·5 to 23·4)	5·6 (3·4 to 8·6)	0·8 (0·5 to 1·3)	1,070
Neck pain	18·9 (9·9 to 32·3)	14·2 (7·5 to 24·4)	8·4 (4·1 to 15·4)	4·9 (2·4 to 8·7)	2·5 (1·3 to 4·5)	1 (0·5 to 1·6)	0·2 (0·1 to 0·3)	234
Gout	2·4 (1·1 to 4·3)	2·2 (1 to 3·8)	1·7 (0·8 to 2·7)	1·2 (0·6 to 2·2)	0·8 (0·4 to 1·3)	0·4 (0·2 to 0·6)	0·1 (0 to 0·1)	22·2
Other musculoskeletal disorders	72 (43·4 to 109)	54 (30·6 to 86·5)	31 (18·8 to 47·9)	17·8 (9·7 to 30·1)	11·9 (7·8 to 17)	4·2 (2·5 to 6·4)	0·5 (0·2 to 0·8)	803
Other non-communicable diseases	221 (172 to 287)	229 (174 to 292)	183 (137 to 234)	143 (104 to 188)	101 (73·2 to 129)	44·5 (30·8 to 57·8)	7·9 (5·5 to 10·2)	2,590
Congenital birth defects	5 (3·7 to 6·5)	1·6 (1 to 2·4)	0·9 (0·5 to 1·4)	0·5 (0·3 to 0·8)	0·3 (0·2 to 0·4)	0·1 (0·1 to 0·2)	0 (0 to 0)	482
Neural tube defects	0·1 (0 to 0·1)	0 (0 to 0)	0 (0 to 0)	0 (0 to 0)	0 (0 to 0)	0 (0 to 0)	0 (0 to 0)	8·9
Congenital heart anomalies	1·1 (0·7 to 1·6)	0·2 (0 to 0·3)	0·1 (0 to 0·2)	0·1 (0 to 0·1)	0 (0 to 0·1)	0 (0 to 0)	0 (0 to 0)	181
Orofacial clefts	0·2 (0·1 to 0·5)	0·2 (0 to 0·3)	0·1 (0 to 0·2)	0·1 (0 to 0·1)	0 (0 to 0·1)	0 (0 to 0)	0 (0 to 0)	5
Down syndrome	0·9 (0·5 to 1·4)	0 (0 to 0)	0 (0 to 0)	0 (0 to 0)	0 (0 to 0)	0 (0 to 0)	0 (0 to 0)	30·5
Turner syndrome	0 (0 to 0)	0 (0 to 0)	0 (0 to 0)	0 (0 to 0)	0 (0 to 0)	0 (0 to 0)	0 (0 to 0)	0
Klinefelter syndrome	0 (0 to 0)	0 (0 to 0)	0 (0 to 0)	0 (0 to 0)	0 (0 to 0)	0 (0 to 0)	0 (0 to 0)	0
Other chromosomal abnormalities	0·1 (0 to 0·1)	0 (0 to 0)	0 (0 to 0)	0 (0 to 0)	0 (0 to 0)	0 (0 to 0)	0 (0 to 0)	38·5
Congenital musculoskeletal and limb anomalies	1·4 (0·8 to 2·2)	0·8 (0·4 to 1·4)	0·4 (0·2 to 0·7)	0·2 (0·1 to 0·4)	0·1 (0 to 0·2)	0 (0 to 0·1)	0 (0 to 0)	51·2
Urogenital congenital anomalies	0·1 (0 to 0·1)	0 (0 to 0)	0 (0 to 0)	0 (0 to 0)	0 (0 to 0)	0 (0 to 0)	0 (0 to 0)	12·8
Digestive congenital anomalies	0·1 (0·1 to 0·2)	0 (0 to 0)	0 (0 to 0)	0 (0 to 0)	0 (0 to 0)	0 (0 to 0)	0 (0 to 0)	40·1
Other congenital birth defects	1·1 (0·7 to 1·8)	0·3 (0·1 to 0·9)	0·2 (0·1 to 0·5)	0·1 (0 to 0·4)	0·1 (0 to 0·2)	0 (0 to 0·1)	0 (0 to 0)	114
Urinary diseases and male infertility	97·2 (62·9 to 132)	117 (71 to 162)	102 (59·5 to 145)	84·7 (47 to 125)	60·5 (34 to 86·7)	27·4 (14·3 to 39·4)	4·8 (2·5 to 6·8)	762
Urolithiasis	2·5 (1·3 to 4·1)	2·2 (1·1 to 3·9)	1·6 (0·7 to 2·9)	0·7 (0·3 to 1·2)	0·4 (0·2 to 0·7)	0·2 (0·1 to 0·3)	0 (0 to 0)	21·8
Benign prostatic hyperplasia	29·3 (16·4 to 46·4)	29 (15·8 to 47·2)	18·6 (10·2 to 30)	10·5 (5·9 to 17·2)	4·5 (2·5 to 7·3)	1·3 (0·7 to 2·1)	0·2 (0·1 to 0·2)	133
Male infertility	0 (0 to 0)	0 (0 to 0)	0 (0 to 0)	0 (0 to 0)	0 (0 to 0)	0 (0 to 0)	0 (0 to 0)	11·7
Other urinary diseases	5·5 (2·3 to 9·6)	4·8 (2 to 9·1)	4·8 (1·8 to 9)	4·1 (1·4 to 8)	2·5 (0·9 to 4·9)	1·4 (0·5 to 2·9)	0·2 (0·1 to 0·4)	44·6
Gynecological diseases	0 (0 to 0)	0 (0 to 0)	0 (0 to 0)	0 (0 to 0)	0 (0 to 0)	0 (0 to 0)	0 (0 to 0)	0
Uterine fibroids	0 (0 to 0)	0 (0 to 0)	0 (0 to 0)	0 (0 to 0)	0 (0 to 0)	0 (0 to 0)	0 (0 to 0)	0
Polycystic ovarian syndrome	--	--	--	--	--	--	--	NA
Female infertility	--	--	--	--	--	--	--	NA
Endometriosis	--	--	--	--	--	--	--	NA
Genital prolapse	0 (0 to 0)	0 (0 to 0)	0 (0 to 0)	0 (0 to 0)	0 (0 to 0)	0 (0 to 0)	0 (0 to 0)	0
Premenstrual syndrome	--	--	--	--	--	--	--	NA
Other gynecological diseases	0 (0 to 0)	0 (0 to 0)	0 (0 to 0)	0 (0 to 0)	0 (0 to 0)	0 (0 to 0)	0 (0 to 0)	0
Hemoglobinopathies and hemolytic anemias	5·8 (4 to 8·2)	5·7 (3·9 to 8·1)	4·1 (2·8 to 5·9)	3·2 (2·2 to 4·5)	3·6 (2·6 to 5·1)	1·8 (1·2 to 2·5)	0·4 (0·2 to 0·6)	132
Thalassemias	0 (0 to 0)	0 (0 to 0)	0 (0 to 0)	0 (0 to 0)	0 (0 to 0)	0 (0 to 0)	0 (0 to 0)	0·1
Thalassemias trait	0·1 (0 to 0·2)	0·1 (0 to 0·1)	0 (0 to 0·1)	0 (0 to 0·1)	0 (0 to 0·1)	0 (0 to 0)	0 (0 to 0)	1·4
Sickle cell disorders	0 (0 to 0·1)	0 (0 to 0)	0 (0 to 0)	0 (0 to 0)	0 (0 to 0)	0 (0 to 0)	0 (0 to 0)	50·4
Sickle cell trait	0·5 (0·1 to 1·3)	0·4 (0·1 to 1)	0·4 (0·1 to 0·8)	0·3 (0·1 to 0·6)	0·2 (0·1 to 0·5)	0·1 (0 to 0·2)	0 (0 to 0)	11·3
G6PD deficiency	2·2 (1·3 to 3·3)	1·6 (0·9 to 2·5)	0·7 (0·4 to 1·2)	0·3 (0·2 to 0·5)	0·2 (0·1 to 0·3)	0 (0 to 0·1)	0 (0 to 0)	36·4
G6PD trait	0 (0 to 0)	0 (0 to 0)	0 (0 to 0)	0 (0 to 0)	0 (0 to 0)	0 (0 to 0)	0 (0 to 0)	0
Other hemoglobinopathies and hemolytic anemias	3 (1·9 to 4·7)	3·5 (2·3 to 5·1)	2·9 (2 to 4·2)	2·6 (1·8 to 3·5)	3·1 (2·3 to 4·4)	1·6 (1·1 to 2·3)	0·4 (0·2 to 0·6)	32
Endocrine, metabolic, blood, and immune disorders	53·8 (36·4 to 88·5)	52·6 (35·1 to 85)	40 (26·3 to 64)	30·9 (21·3 to 46·7)	23·4 (15·9 to 35·2)	10·2 (7 to 15·3)	1·9 (1·2 to 2·9)	655
Oral disorders	59·4 (35·5 to 94·2)	52·1 (31·9 to 81·4)	35·6 (22·2 to 53·2)	24 (14·7 to 35·8)	13 (7·9 to 19·4)	5·2 (3·1 to 7·7)	0·8 (0·5 to 1·2)	548
Caries of deciduous teeth	0 (0 to 0)	0 (0 to 0)	0 (0 to 0)	0 (0 to 0)	0 (0 to 0)	0 (0 to 0)	0 (0 to 0)	2·6
Caries of permanent teeth	1·5 (0·6 to 3·2)	1·1 (0·4 to 2·2)	0·6 (0·3 to 1·3)	0·4 (0·1 to 0·8)	0·2 (0·1 to 0·4)	0·1 (0 to 0·1)	0 (0 to 0)	37·9
Periodontal diseases	14·8 (5·8 to 30)	10·3 (4 to 20·7)	6·1 (2·4 to 12)	3·8 (1·5 to 7·5)	2 (0·8 to 4·1)	0·8 (0·3 to 1·6)	0·1 (0 to 0·2)	211
Other oral disorders	4·4 (2·7 to 6·7)	3 (1·8 to 4·6)	1·6 (0·9 to 2·4)	0·8 (0·5 to 1·2)	0·3 (0·2 to 0·5)	0·1 (0·1 to 0·2)	0 (0 to 0)	75·9
Sudden infant death syndrome	--	--	--	--	--	--	--	NA
Injuries	222 (181 to 267)	180 (149 to 216)	124 (103 to 149)	88·9 (74·7 to 105)	57·5 (48·2 to 67·2)	26·4 (22·2 to 30·7)	5·1 (4·2 to 6)	5,150
Transport injuries	63·2 (48·3 to 80·2)	48·8 (37·5 to 61·5)	31·5 (24 to 40·4)	19·4 (14·8 to 24·2)	10·5 (8·1 to 13·4)	3·7 (2·9 to 4·7)	0·6 (0·5 to 0·8)	1,350
Road injuries	61·1 (46·8 to 77·8)	46·7 (35·8 to 59·1)	30·5 (23·1 to 39·3)	18·9 (14·3 to 23·5)	10·2 (7·8 to 13)	3·6 (2·8 to 4·5)	0·6 (0·5 to 0·8)	1,310
Pedestrian road injuries	13·3 (9·8 to 17·4)	11·8 (8·8 to 15·5)	8·8 (6·3 to 11·9)	6·1 (4·6 to 7·9)	3·5 (2·6 to 4·5)	1·1 (0·9 to 1·4)	0·2 (0·1 to 0·2)	191
Cyclist road injuries	8·5 (6·1 to 11·2)	6·2 (4·5 to 8·2)	3·8 (2·7 to 5)	2·4 (1·7 to 3·1)	1·2 (0·8 to 1·6)	0·5 (0·3 to 0·6)	0·1 (0·1 to 0·1)	127
Motorcyclist road injuries	8·1 (5·9 to 10·8)	5·2 (3·8 to 6·9)	3·2 (2·3 to 4·3)	2·1 (1·5 to 2·8)	1·1 (0·8 to 1·6)	0·5 (0·3 to 0·6)	0·1 (0·1 to 0·1)	241
Motor vehicle road injuries	29·5 (20·9 to 39·3)	22·2 (16 to 29·1)	13·8 (9·9 to 18·3)	7·8 (5·6 to 9·9)	4·1 (3 to 5·5)	1·5 (1·1 to 1·8)	0·3 (0·2 to 0·3)	726
Other road injuries	1·7 (1·2 to 2·4)	1·4 (0·9 to 1·8)	0·9 (0·6 to 1·2)	0·6 (0·4 to 0·8)	0·3 (0·2 to 0·4)	0·1 (0·1 to 0·2)	0 (0 to 0)	20·3
Other transport injuries	2 (1·5 to 2·7)	2 (1·4 to 2·8)	1 (0·7 to 1·4)	0·6 (0·4 to 0·7)	0·3 (0·2 to 0·4)	0·1 (0·1 to 0·2)	0 (0 to 0)	48·2
Unintentional injuries	109 (86 to 138)	95·1 (76·6 to 117)	70·4 (57·7 to 85·9)	57 (46·9 to 68·6)	40·3 (33·6 to 47·6)	20·5 (17·2 to 24·1)	4·1 (3·3 to 4·9)	1,900
Falls	29·5 (23·1 to 37·1)	29·7 (23·2 to 37)	24 (19·3 to 30·3)	24·1 (19·2 to 29·7)	19·7 (15·8 to 24·1)	11·2 (9 to 13·6)	2·3 (1·8 to 2·9)	385
Drowning	10·3 (7·9 to 13·4)	6·6 (5·1 to 8·3)	3·9 (3 to 4·9)	1·7 (1·3 to 2·2)	1·1 (0·8 to 1·4)	0·5 (0·4 to 0·5)	0·1 (0·1 to 0·1)	438
Fire, heat, and hot substances	11·5 (8·5 to 15·9)	9·8 (7·3 to 13)	6·9 (5·2 to 9)	5·2 (4·1 to 6·7)	3·6 (2·9 to 4·5)	1·7 (1·4 to 2·1)	0·3 (0·3 to 0·4)	193
Poisonings	0·8 (0·6 to 1·1)	0·6 (0·4 to 0·8)	0·4 (0·3 to 0·6)	0·3 (0·2 to 0·4)	0·1 (0·1 to 0·2)	0·1 (0 to 0·1)	0 (0 to 0)	20·8
Poisoning by carbon monoxide	0·1 (0·1 to 0·2)	0·1 (0 to 0·1)	0 (0 to 0·1)	0 (0 to 0·1)	0 (0 to 0)	0 (0 to 0)	0 (0 to 0)	2·9
Poisoning by other means	0·7 (0·5 to 1)	0·5 (0·4 to 0·7)	0·4 (0·3 to 0·5)	0·2 (0·2 to 0·3)	0·1 (0·1 to 0·2)	0·1 (0 to 0·1)	0 (0 to 0)	17·8
Exposure to mechanical forces	23·4 (16·3 to 33·2)	17·9 (12·4 to 25·3)	11·2 (7·7 to 16)	7·4 (5·1 to 10·6)	4·2 (2·9 to 5·9)	1·7 (1·2 to 2·5)	0·3 (0·2 to 0·4)	386
Unintentional firearm injuries	4·2 (3·2 to 5·5)	2·9 (2·2 to 3·7)	1·6 (1·2 to 2·1)	1·1 (0·8 to 1·4)	0·6 (0·5 to 0·8)	0·3 (0·2 to 0·3)	0·1 (0 to 0·1)	121
Other exposure to mechanical forces	19·2 (12·6 to 27·8)	15 (10·1 to 21·8)	9·6 (6·5 to 14·1)	6·3 (4·2 to 9·2)	3·5 (2·4 to 5·1)	1·5 (1 to 2·1)	0·2 (0·2 to 0·4)	265
Adverse effects of medical treatment	12·4 (8·3 to 18·3)	12·8 (8·4 to 18·7)	11·5 (7·8 to 16·7)	9·2 (6·5 to 12·9)	6·5 (4·7 to 8·9)	3 (2·2 to 4·1)	0·6 (0·4 to 0·9)	127
Animal contact	1·6 (1·1 to 2·2)	1·3 (0·9 to 1·8)	0·9 (0·6 to 1·2)	0·5 (0·4 to 0·7)	0·3 (0·2 to 0·4)	0·1 (0·1 to 0·2)	0 (0 to 0)	20·6
Venomous animal contact	0·9 (0·6 to 1·3)	0·7 (0·5 to 1)	0·5 (0·3 to 0·7)	0·3 (0·2 to 0·4)	0·2 (0·1 to 0·2)	0·1 (0·1 to 0·1)	0 (0 to 0)	13·4
Non-venomous animal contact	0·7 (0·4 to 1)	0·6 (0·4 to 0·8)	0·4 (0·3 to 0·5)	0·2 (0·2 to 0·3)	0·1 (0·1 to 0·2)	0 (0 to 0·1)	0 (0 to 0)	7·2
Foreign body	6·5 (5 to 8·3)	6 (4·6 to 7·6)	4·7 (3·7 to 5·9)	3·9 (3 to 5)	2·1 (1·7 to 2·5)	1·1 (0·8 to 1·2)	0·2 (0·2 to 0·3)	98·7
Pulmonary aspiration and foreign body in airway	4·6 (3·3 to 6·3)	4·3 (3·1 to 5·8)	3·4 (2·6 to 4·5)	2·9 (2·1 to 3·8)	1·5 (1·1 to 1·9)	0·7 (0·5 to 0·9)	0·2 (0·1 to 0·2)	69·3
Foreign body in eyes	0·6 (0·4 to 0·9)	0·5 (0·3 to 0·7)	0·3 (0·2 to 0·5)	0·2 (0·1 to 0·3)	0·1 (0·1 to 0·2)	0 (0 to 0·1)	0 (0 to 0)	10·9
Foreign body in other body part	1·3 (0·9 to 1·9)	1·2 (0·8 to 1·8)	1 (0·7 to 1·4)	0·8 (0·6 to 1·2)	0·5 (0·3 to 0·7)	0·3 (0·2 to 0·4)	0·1 (0·1 to 0·1)	18·5
Environmental heat and cold exposure	5·3 (3·6 to 7·3)	4·4 (3 to 5·9)	2·9 (2 to 4)	2 (1·4 to 2·8)	1·2 (0·8 to 1·6)	0·5 (0·4 to 0·7)	0·1 (0·1 to 0·1)	70·5
Exposure to forces of nature	0 (0 to 0)	0 (0 to 0)	0 (0 to 0)	0 (0 to 0)	0 (0 to 0)	0 (0 to 0)	0 (0 to 0)	0·4
Other unintentional injuries	8 (5·6 to 11·4)	6 (4·2 to 8·6)	3·9 (2·7 to 5·6)	2·6 (1·8 to 3·7)	1·4 (1 to 2)	0·6 (0·4 to 0·8)	0·1 (0·1 to 0·1)	161
Self-harm and interpersonal violence	49·7 (39·3 to 62·2)	36·5 (29·1 to 45·5)	21·9 (17·3 to 27·6)	12·5 (10 to 15·6)	6·7 (5·3 to 8·3)	2·1 (1·7 to 2·6)	0·4 (0·3 to 0·5)	1,890
Self-harm	22·6 (16·6 to 29·9)	19·2 (14·2 to 25·8)	12·4 (9·1 to 16·9)	8·2 (6 to 10·9)	4·6 (3·4 to 5·9)	1·4 (1 to 1·7)	0·2 (0·1 to 0·2)	461
Self-harm by firearm	1·1 (0·6 to 1·7)	0·9 (0·5 to 1·4)	0·5 (0·3 to 0·8)	0·3 (0·2 to 0·5)	0·1 (0·1 to 0·2)	0 (0 to 0·1)	0 (0 to 0)	24·2
Self-harm by other specified means	21·5 (15·7 to 28·6)	18·3 (13·5 to 24·6)	11·9 (8·8 to 16·3)	7·9 (5·8 to 10·5)	4·5 (3·3 to 5·7)	1·3 (1 to 1·7)	0·2 (0·1 to 0·2)	437
Interpersonal violence	26·6 (19·8 to 35·6)	17·3 (12·9 to 22·7)	9·5 (7 to 12·5)	4·2 (3·3 to 5·2)	2·1 (1·7 to 2·7)	0·8 (0·6 to 1)	0·2 (0·2 to 0·3)	1,420
Physical violence by firearm	5·6 (3·9 to 7·8)	3 (2·1 to 4·1)	1·1 (0·8 to 1·5)	0·4 (0·3 to 0·5)	0·2 (0·2 to 0·3)	0·1 (0·1 to 0·1)	0 (0 to 0)	644
Physical violence by sharp object	6·2 (4·7 to 8·1)	4·6 (3·5 to 5·8)	2·4 (1·8 to 3·1)	1·2 (0·9 to 1·5)	0·6 (0·5 to 0·8)	0·3 (0·2 to 0·4)	0·1 (0·1 to 0·1)	445
Sexual violence	0·6 (0·4 to 0·9)	0·4 (0·3 to 0·6)	0·3 (0·2 to 0·4)	0·2 (0·1 to 0·2)	0·1 (0 to 0·1)	0 (0 to 0)	0 (0 to 0)	17·8
Physical violence by other means	14·2 (10·5 to 19·2)	9·3 (6·9 to 12·3)	5·8 (4·2 to 7·7)	2·4 (1·9 to 3·1)	1·3 (1 to 1·6)	0·4 (0·3 to 0·5)	0·1 (0·1 to 0·2)	316
Conflict and terrorism	0·5 (0·3 to 0·7)	0 (0 to 0)	0 (0 to 0)	0·1 (0·1 to 0·2)	0 (0 to 0)	0 (0 to 0)	0 (0 to 0)	6·9

SR Table 2a: Belize DALYS by cause and age group for males with 95% uncertainty intervals, 2019

Cause	Early Neonatal	Late Neonatal	Post Neonatal	1 to 4	5 to 9	10 to 14	15 to 19	20 to 24	25 to 29	30 to 34	35 to 39
All causes	2,140 (1,680 to 2,670)	1,060 (838 to 1,320)	1,760 (1,410 to 2,190)	1,420 (1,160 to 1,740)	1,390 (1,100 to 1,760)	1,770 (1,460 to 2,180)	3,550 (3,110 to 4,070)	4,670 (4,170 to 5,230)	5,120 (4,620 to 5,660)	4,870 (4,400 to 5,400)	4,250 (3,780 to 4,770)
Communicable, maternal, neonatal, and nutritional diseases	1,800 (1,400 to 2,260)	838 (659 to 1,060)	960 (754 to 1,200)	529 (409 to 683)	409 (265 to 631)	304 (222 to 442)	308 (246 to 387)	421 (359 to 496)	707 (639 to 784)	809 (732 to 890)	742 (675 to 816)
HIV/AIDS and sexually transmitted infections	0 (0 to 0)	0 (0 to 0)	34·7 (32 to 37·6)	28·4 (26·1 to 30·7)	21 (18·6 to 23·9)	14·8 (13·2 to 16·6)	35·7 (32·4 to 39·2)	147 (134 to 162)	422 (390 to 461)	518 (478 to 563)	491 (449 to 534)
HIV/AIDS	0 (0 to 0)	0 (0 to 0)	34·7 (32 to 37·6)	28·4 (26·1 to 30·7)	21 (18·6 to 23·9)	14·4 (12·9 to 16·2)	33·5 (30·8 to 36·8)	144 (131 to 158)	419 (387 to 458)	515 (475 to 560)	489 (447 to 531)
HIV/AIDS - Drug-susceptible Tuberculosis	0 (0 to 0)	0 (0 to 0)	2·5 (1·5 to 3·8)	2 (1·3 to 3)	1·5 (1 to 2·3)	1·1 (0·7 to 1·6)	2·5 (1·6 to 3·7)	10·8 (7 to 16·2)	30·8 (19·6 to 46·6)	38 (24·5 to 57·2)	35·9 (22·9 to 54·3)
HIV/AIDS - Multidrug-resistant Tuberculosis without extensive dru	0 (0 to 0)	0 (0 to 0)	0 (0 to 0·1)	0 (0 to 0·1)	0 (0 to 0·1)	0 (0 to 0·1)	0 (0 to 0·1)	0·1 (0 to 0·6)	0·4 (0 to 1·6)	0·5 (0 to 2)	0·4 (0 to 1·9)
HIV/AIDS - Extensively drug-resistant Tuberculosis	0 (0 to 0)	0 (0 to 0)	0 (0 to 0)	0 (0 to 0)	0 (0 to 0)	0 (0 to 0)	0 (0 to 0)	0 (0 to 0·1)	0·1 (0 to 0·3)	0·1 (0 to 0·3)	0·1 (0 to 0·3)
HIV/AIDS resulting in other diseases	0 (0 to 0)	0 (0 to 0)	32·2 (29·1 to 35·4)	26·4 (23·9 to 29)	19·4 (16·8 to 22·4)	13·3 (11·7 to 15·1)	31 (28 to 34·3)	133 (120 to 147)	388 (351 to 431)	476 (432 to 525)	452 (407 to 498)
Sexually transmitted infections excluding HIV	0 (0 to 0)	0 (0 to 0)	0 (0 to 0)	0 (0 to 0)	0 (0 to 0)	0·4 (0·2 to 0·9)	2·1 (0·8 to 4·4)	3 (1·3 to 5·5)	3 (1·4 to 5·4)	3·1 (1·4 to 5·4)	2·5 (1·2 to 4·4)
Syphilis	0 (0 to 0)	0 (0 to 0)	0 (0 to 0)	0 (0 to 0)	0 (0 to 0)	0 (0 to 0)	0 (0 to 0·1)	0·1 (0 to 0·1)	0·1 (0 to 0·2)	0·1 (0 to 0·2)	0·1 (0·1 to 0·1)
Chlamydial infection	0 (0 to 0)	0 (0 to 0)	0 (0 to 0)	0 (0 to 0)	0 (0 to 0)	0 (0 to 0)	0·2 (0·1 to 0·4)	0·7 (0·1 to 1·5)	1 (0·3 to 2)	0·9 (0·3 to 1·8)	0·7 (0·2 to 1·4)
Gonococcal infection	0 (0 to 0)	0 (0 to 0)	0 (0 to 0)	0 (0 to 0)	0 (0 to 0)	0·1 (0 to 0·3)	0·7 (0 to 1·8)	0·9 (0·2 to 1·9)	0·4 (0 to 1)	0·2 (0 to 0·6)	0·1 (0·1 to 0·3)
Trichomoniasis	0 (0 to 0)	0 (0 to 0)	0 (0 to 0)	0 (0 to 0)	0 (0 to 0)	0 (0 to 0)	0 (0 to 0)	0 (0 to 0·1)	0·1 (0 to 0·2)	0·1 (0 to 0·2)	0·1 (0 to 0·2)
Genital herpes	0 (0 to 0)	0 (0 to 0)	0 (0 to 0)	0 (0 to 0)	0 (0 to 0)	0·1 (0 to 0·1)	0·2 (0·1 to 0·6)	0·5 (0·1 to 1·2)	0·7 (0·2 to 1·7)	0·9 (0·3 to 2·1)	0·9 (0·3 to 2·2)
Other sexually transmitted infections	0 (0 to 0)	0 (0 to 0)	0 (0 to 0)	0 (0 to 0)	0 (0 to 0)	0·2 (0·1 to 0·5)	0·9 (0·3 to 1·9)	0·9 (0·3 to 1·8)	0·8 (0·3 to 1·5)	0·9 (0·3 to 1·8)	0·6 (0·2 to 1·2)
Respiratory infections and tuberculosis	78·6 (53 to 111)	100 (69·5 to 139)	343 (236 to 467)	141 (103 to 186)	67·3 (50·5 to 89)	61·3 (45·9 to 80·5)	93·8 (70·6 to 121)	129 (99·3 to 167)	148 (116 to 191)	165 (128 to 211)	140 (107 to 181)
Tuberculosis	0 (0 to 0)	0 (0 to 0)	8·3 (5·8 to 12)	7·3 (5·2 to 10)	4·9 (3·1 to 7·3)	4·9 (3·5 to 6·8)	17·5 (11·9 to 25)	41·2 (27·6 to 58·6)	51·9 (37·5 to 70·3)	58 (42·6 to 76·9)	45·6 (33 to 62·5)
Latent tuberculosis infection	0 (0 to 0)	0 (0 to 0)	0 (0 to 0)	0 (0 to 0)	0 (0 to 0)	0 (0 to 0)	0 (0 to 0)	0 (0 to 0)	0 (0 to 0)	0 (0 to 0)	0 (0 to 0)
Drug-susceptible tuberculosis	0 (0 to 0)	0 (0 to 0)	8·2 (5·7 to 11·9)	7·2 (5·1 to 9·9)	4·8 (3 to 7·2)	4·9 (3·5 to 6·8)	17·2 (11·7 to 24·8)	40·8 (27·2 to 58·3)	51·3 (37 to 68·8)	57·3 (42·3 to 76·6)	45·1 (32·6 to 62)
Multidrug-resistant tuberculosis without extensive drug resistanc	0 (0 to 0)	0 (0 to 0)	0·1 (0 to 0·4)	0·1 (0 to 0·3)	0 (0 to 0·2)	0 (0 to 0·2)	0·2 (0 to 0·8)	0·4 (0 to 1·6)	0·5 (0 to 2·2)	0·6 (0·1 to 2·3)	0·4 (0 to 1·8)
Extensively drug-resistant tuberculosis	0 (0 to 0)	0 (0 to 0)	0 (0 to 0·1)	0 (0 to 0·1)	0 (0 to 0)	0 (0 to 0)	0 (0 to 0·1)	0·1 (0 to 0·3)	0·1 (0 to 0·3)	0·1 (0 to 0·4)	0·1 (0 to 0·3)
Lower respiratory infections	78·5 (53 to 111)	99·7 (69·2 to 138)	327 (224 to 449)	105 (69·8 to 148)	29·1 (20·9 to 39)	26·9 (19·8 to 36·4)	48·5 (32·7 to 69)	64 (42 to 95·1)	77·3 (53·1 to 111)	91·3 (61·7 to 130)	80·3 (54·9 to 115)
Upper respiratory infections	0 (0 to 0·1)	0·3 (0·2 to 0·5)	6·5 (4·2 to 9·6)	20·8 (13 to 32·8)	21·7 (12 to 36)	20·5 (10·6 to 35·6)	19·8 (10·4 to 34·8)	16·9 (9·5 to 29·3)	14·3 (8 to 23·7)	12·1 (6·6 to 20·5)	10·7 (5·6 to 18)
Otitis media	0 (0 to 0)	0 (0 to 0)	0·9 (0·4 to 1·6)	8·1 (4·2 to 13·9)	11·7 (6·4 to 19·2)	8·9 (4·7 to 15·4)	8·1 (4·2 to 13·9)	6·5 (2·8 to 13)	4·8 (2·3 to 9·2)	3·5 (1·5 to 6·7)	3·2 (1·5 to 6·1)
Enteric infections	5·9 (3·9 to 8·6)	24·7 (14·2 to 40·3)	164 (99 to 262)	68·9 (48·1 to 95·1)	38 (24·6 to 55·2)	42·9 (25·8 to 66·3)	31·9 (21·3 to 45)	26 (17·3 to 37·2)	26·8 (18·9 to 36·5)	26·3 (18·7 to 35·6)	25 (18·2 to 33·4)
Diarrheal diseases	5·9 (3·9 to 8·6)	24·7 (14·2 to 40·3)	164 (98·6 to 262)	68·4 (47·7 to 94·6)	37·6 (24·3 to 54·8)	42·6 (25·5 to 65·9)	31·8 (21·1 to 44·9)	25·8 (17·2 to 36·9)	26·6 (18·9 to 36·4)	26·2 (18·6 to 35·5)	24·9 (18·1 to 33·2)
Typhoid and paratyphoid	0 (0 to 0)	0 (0 to 0)	0 (0 to 0)	0 (0 to 0·1)	0 (0 to 0)	0 (0 to 0)	0 (0 to 0)	0 (0 to 0)	0 (0 to 0)	0 (0 to 0)	0 (0 to 0)
Typhoid fever	0 (0 to 0)	0 (0 to 0)	0 (0 to 0)	0 (0 to 0)	0 (0 to 0)	0 (0 to 0)	0 (0 to 0)	0 (0 to 0)	0 (0 to 0)	0 (0 to 0)	0 (0 to 0)
Paratyphoid fever	0 (0 to 0)	0 (0 to 0)	0 (0 to 0)	0 (0 to 0·1)	0 (0 to 0)	0 (0 to 0)	0 (0 to 0)	0 (0 to 0)	0 (0 to 0)	0 (0 to 0)	0 (0 to 0)
Invasive Non-typhoidal Salmonella (iNTS)	0 (0 to 0)	0 (0 to 0)	0·2 (0·1 to 0·4)	0·2 (0·1 to 0·4)	0·2 (0·1 to 0·6)	0·2 (0·1 to 0·5)	0 (0 to 0·1)	0 (0 to 0·1)	0·1 (0 to 0·1)	0·1 (0 to 0·1)	0·1 (0 to 0·1)
Other intestinal infectious diseases	0 (0 to 0)	0 (0 to 0)	0·2 (0 to 0·8)	0·2 (0 to 0·8)	0·1 (0 to 0·4)	0·1 (0 to 0·4)	0·1 (0 to 0·4)	0·1 (0 to 0·4)	0·1 (0 to 0·2)	0·1 (0 to 0·4)	0 (0 to 0·1)
Neglected tropical diseases and malaria	0 (0 to 0·1)	0·4 (0·2 to 0·7)	4·3 (2 to 8)	13·5 (5·9 to 25·3)	21·5 (8·8 to 41·9)	14·2 (5·5 to 30·7)	12 (5 to 26·6)	12·6 (5 to 26·7)	13·8 (5·5 to 28·4)	13 (5·1 to 24·9)	11·6 (4·6 to 22·1)
Malaria	0 (0 to 0)	0 (0 to 0)	0 (0 to 0)	0·1 (0 to 0·4)	0·2 (0 to 0·6)	0·1 (0 to 0·2)	0 (0 to 0)	0 (0 to 0)	0 (0 to 0)	0 (0 to 0)	0 (0 to 0)
Chagas disease	0 (0 to 0)	0 (0 to 0)	0 (0 to 0)	0 (0 to 0)	0 (0 to 0)	0 (0 to 0)	0 (0 to 0)	0 (0 to 0)	0 (0 to 0·1)	0 (0 to 0·1)	0 (0 to 0·1)
Leishmaniasis	0 (0 to 0)	0 (0 to 0)	0 (0 to 0)	0 (0 to 0)	0 (0 to 0)	0 (0 to 0)	0 (0 to 0)	0 (0 to 0)	0 (0 to 0)	0 (0 to 0)	0 (0 to 0)
Visceral leishmaniasis	0 (0 to 0)	0 (0 to 0)	0 (0 to 0)	0 (0 to 0)	0 (0 to 0)	0 (0 to 0)	0 (0 to 0)	0 (0 to 0)	0 (0 to 0)	0 (0 to 0)	0 (0 to 0)
Cutaneous and mucocutaneous leishmaniasis	0 (0 to 0)	0 (0 to 0)	0 (0 to 0)	0 (0 to 0)	0 (0 to 0)	0 (0 to 0)	0 (0 to 0)	0 (0 to 0)	0 (0 to 0)	0 (0 to 0)	0 (0 to 0)
African trypanosomiasis	0 (0 to 0)	0 (0 to 0)	0 (0 to 0)	0 (0 to 0)	0 (0 to 0)	0 (0 to 0)	0 (0 to 0)	0 (0 to 0)	0 (0 to 0)	0 (0 to 0)	0 (0 to 0)
Schistosomiasis	0 (0 to 0)	0 (0 to 0)	0 (0 to 0)	0 (0 to 0)	0 (0 to 0)	0 (0 to 0)	0 (0 to 0)	0 (0 to 0)	0 (0 to 0)	0 (0 to 0)	0 (0 to 0)
Cysticercosis	0 (0 to 0)	0 (0 to 0)	0 (0 to 0)	0 (0 to 0·1)	0·2 (0 to 0·7)	0·5 (0 to 1·7)	1·8 (0 to 5)	4·3 (0·6 to 9·9)	6·7 (1·4 to 15)	7 (1·6 to 15·4)	6·6 (1·7 to 13·6)
Cystic echinococcosis	0 (0 to 0)	0 (0 to 0)	0 (0 to 0)	0 (0 to 0)	0 (0 to 0)	0 (0 to 0)	0 (0 to 0)	0 (0 to 0)	0 (0 to 0)	0 (0 to 0)	0 (0 to 0)
Lymphatic filariasis	0 (0 to 0)	0 (0 to 0)	0 (0 to 0)	0 (0 to 0)	0 (0 to 0)	0 (0 to 0)	0 (0 to 0)	0 (0 to 0)	0 (0 to 0)	0 (0 to 0)	0 (0 to 0)
Onchocerciasis	0 (0 to 0)	0 (0 to 0)	0 (0 to 0)	0 (0 to 0)	0 (0 to 0)	0 (0 to 0)	0 (0 to 0)	0 (0 to 0)	0 (0 to 0)	0 (0 to 0)	0 (0 to 0)
Trachoma	0 (0 to 0)	0 (0 to 0)	0 (0 to 0)	0 (0 to 0)	0 (0 to 0)	0 (0 to 0)	0 (0 to 0)	0 (0 to 0)	0 (0 to 0)	0 (0 to 0)	0 (0 to 0)
Dengue	0 (0 to 0)	0·1 (0 to 0·1)	1·5 (0·5 to 3·5)	2·3 (0·8 to 6·1)	5·3 (1·6 to 14·4)	6·3 (1·5 to 20·5)	6·9 (1·7 to 21·3)	5·9 (1·2 to 18·6)	5·2 (1·1 to 16·6)	4·2 (0·9 to 12·9)	3·4 (0·8 to 9·9)
Yellow fever	0 (0 to 0)	0 (0 to 0)	0 (0 to 0)	0 (0 to 0)	0 (0 to 0)	0 (0 to 0)	0 (0 to 0)	0 (0 to 0)	0 (0 to 0)	0 (0 to 0)	0 (0 to 0)
Rabies	0 (0 to 0)	0 (0 to 0)	0 (0 to 0)	0 (0 to 0)	0 (0 to 0)	0 (0 to 0)	0 (0 to 0)	0 (0 to 0)	0 (0 to 0)	0 (0 to 0)	0 (0 to 0)
Intestinal nematode infections	0 (0 to 0)	0 (0 to 0)	0·1 (0 to 0·5)	1 (0·3 to 2·2)	1·1 (0·4 to 2·2)	0·7 (0·2 to 1·4)	1·3 (0·6 to 2·5)	1 (0·4 to 1·7)	0·7 (0·3 to 1·3)	0·5 (0·2 to 1)	0·5 (0·2 to 0·9)
Ascariasis	0 (0 to 0)	0 (0 to 0)	0·1 (0 to 0·5)	0·6 (0·1 to 1·7)	0·3 (0 to 1·2)	0·1 (0 to 0·5)	0·4 (0·1 to 0·9)	0·3 (0·1 to 0·7)	0·3 (0·1 to 0·6)	0·2 (0·1 to 0·5)	0·2 (0·1 to 0·4)
Trichuriasis	0 (0 to 0)	0 (0 to 0)	0 (0 to 0)	0 (0 to 0)	0 (0 to 0)	0 (0 to 0)	0·1 (0 to 0·3)	0·1 (0 to 0·2)	0·1 (0 to 0·2)	0 (0 to 0·1)	0 (0 to 0·1)
Hookworm disease	0 (0 to 0)	0 (0 to 0)	0 (0 to 0)	0·4 (0·1 to 0·8)	0·8 (0·3 to 1·7)	0·6 (0·2 to 1·2)	0·8 (0·4 to 1·5)	0·6 (0·2 to 1·1)	0·3 (0·1 to 0·7)	0·3 (0·1 to 0·5)	0·2 (0·1 to 0·5)
Food-borne trematodiases	0 (0 to 0)	0 (0 to 0)	0 (0 to 0)	0 (0 to 0)	0 (0 to 0)	0 (0 to 0)	0 (0 to 0)	0 (0 to 0)	0 (0 to 0)	0 (0 to 0)	0 (0 to 0)
Leprosy	0 (0 to 0)	0 (0 to 0)	0 (0 to 0)	0 (0 to 0)	0 (0 to 0)	0 (0 to 0)	0 (0 to 0)	0 (0 to 0)	0 (0 to 0·1)	0 (0 to 0·1)	0·1 (0 to 0·1)
Ebola	0 (0 to 0)	0 (0 to 0)	0 (0 to 0)	0 (0 to 0)	0 (0 to 0)	0 (0 to 0)	0 (0 to 0)	0 (0 to 0)	0 (0 to 0)	0 (0 to 0)	0 (0 to 0)
Zika virus	0 (0 to 0)	0 (0 to 0)	0 (0 to 0·1)	0·1 (0 to 0·5)	0·1 (0 to 0·6)	0·1 (0 to 0·6)	0·2 (0 to 0·7)	0·2 (0 to 0·7)	0·2 (0 to 0·7)	0·2 (0 to 0·8)	0·2 (0 to 0·7)
Guinea worm disease	0 (0 to 0)	0 (0 to 0)	0 (0 to 0)	0 (0 to 0)	0 (0 to 0)	0 (0 to 0)	0 (0 to 0)	0 (0 to 0)	0 (0 to 0)	0 (0 to 0)	0 (0 to 0)
Other neglected tropical diseases	0 (0 to 0·1)	0·4 (0·2 to 0·6)	2·6 (0·9 to 5·6)	9·9 (3·5 to 20·9)	14·6 (4·5 to 31·5)	6·5 (1·5 to 15·8)	1·7 (0·4 to 4·3)	1·2 (0·3 to 3·1)	1 (0·3 to 2·5)	1 (0·3 to 2·4)	0·8 (0·2 to 2·2)
Other infectious diseases	3·7 (2·6 to 5·1)	11·1 (7·6 to 15·6)	43·7 (30·4 to 60·8)	31·8 (22·6 to 43·9)	25·5 (17·1 to 37·8)	20·8 (15·4 to 28·2)	22·1 (17 to 28·2)	19 (14·8 to 24·5)	17·3 (13·4 to 22·3)	16·7 (13·1 to 21·2)	14·3 (11·3 to 18·1)
Meningitis	2·1 (1·4 to 3)	7·6 (4·8 to 11·4)	21·4 (12·9 to 33·3)	10·2 (6·6 to 15·5)	5·5 (4 to 7·7)	5·2 (3·7 to 7·2)	6·8 (4·8 to 9·7)	5·1 (3·6 to 7·5)	4·8 (3·4 to 6·6)	4·8 (3·3 to 6·9)	3·9 (2·8 to 5·4)
Encephalitis	0·6 (0·3 to 1)	1·6 (0·9 to 2·9)	12·3 (6·8 to 20·7)	10 (5·7 to 16·4)	6·2 (4 to 10)	6·3 (4·2 to 9·4)	7·7 (4·9 to 11·6)	6·8 (4·5 to 10·3)	5·1 (3·3 to 7·5)	4·7 (3·1 to 6·7)	3·4 (2·2 to 4·9)
Diphtheria	0 (0 to 0)	0 (0 to 0)	0·1 (0 to 0·2)	0·1 (0 to 0·3)	0·1 (0 to 0·2)	0 (0 to 0)	0 (0 to 0)	0 (0 to 0)	0 (0 to 0)	0 (0 to 0)	0 (0 to 0)
Tetanus	0 (0 to 0·1)	0·1 (0 to 0·2)	0·1 (0 to 0·2)	0·1 (0 to 0·3)	0·2 (0 to 0·7)	0·2 (0 to 0·7)	0·2 (0·1 to 0·7)	0·2 (0·1 to 0·7)	0·2 (0·1 to 0·7)	0·3 (0·1 to 0·8)	0·2 (0·1 to 0·8)
Measles	0 (0 to 0)	0 (0 to 0)	0 (0 to 0)	0 (0 to 0)	0 (0 to 0)	0 (0 to 0)	0 (0 to 0)	0 (0 to 0)	0 (0 to 0)	0 (0 to 0)	0 (0 to 0)
Varicella and herpes zoster	0·6 (0·2 to 1·2)	1·1 (0·4 to 2·4)	2·7 (1·1 to 5·9)	1 (0·4 to 2)	1·1 (0·5 to 2·3)	1·2 (0·6 to 2·5)	0·9 (0·5 to 1·5)	0·9 (0·4 to 1·5)	0·9 (0·4 to 1·6)	1 (0·5 to 1·7)	1·3 (0·6 to 2·2)
Acute hepatitis	0 (0 to 0)	0 (0 to 0)	0·4 (0·2 to 0·9)	0·7 (0·4 to 1·2)	1·8 (1 to 2·9)	2·3 (1·5 to 3·5)	2·3 (1·5 to 3·5)	2·2 (1·5 to 3·1)	1·8 (1·3 to 2·5)	1·6 (1·1 to 2·1)	1·3 (0·9 to 1·8)
Acute hepatitis A	0 (0 to 0)	0 (0 to 0)	0 (0 to 0·1)	0·4 (0·2 to 0·9)	1·4 (0·7 to 2·5)	1·8 (1 to 2·9)	1·6 (0·9 to 2·5)	1·2 (0·7 to 2)	0·7 (0·4 to 1·3)	0·6 (0·3 to 1)	0·4 (0·2 to 0·7)
Acute hepatitis B	0 (0 to 0)	0 (0 to 0)	0·3 (0·1 to 0·7)	0·1 (0 to 0·3)	0·2 (0·1 to 0·3)	0·3 (0·1 to 0·6)	0·4 (0·2 to 0·8)	0·6 (0·3 to 1)	0·7 (0·4 to 1·1)	0·7 (0·4 to 1·1)	0·7 (0·4 to 1)
Acute hepatitis C	0 (0 to 0)	0 (0 to 0)	0 (0 to 0)	0·1 (0 to 0·2)	0·1 (0 to 0·1)	0·1 (0 to 0·1)	0·1 (0 to 0·2)	0·1 (0 to 0·3)	0·1 (0 to 0·4)	0·1 (0 to 0·3)	0·1 (0 to 0·2)
Acute hepatitis E	0 (0 to 0)	0 (0 to 0)	0·1 (0 to 0·2)	0 (0 to 0·1)	0·1 (0·1 to 0·2)	0·2 (0 to 0·4)	0·3 (0·1 to 0·6)	0·2 (0·1 to 0·5)	0·2 (0·1 to 0·5)	0·2 (0·1 to 0·3)	0·1 (0·1 to 0·2)
Other unspecified infectious diseases	0·4 (0·2 to 0·7)	0·7 (0·4 to 1·1)	4 (2·1 to 6·6)	7·8 (3·3 to 15·1)	10·5 (3·8 to 21·9)	5·5 (2·1 to 11·4)	4·2 (2·1 to 7·1)	3·8 (2 to 6·2)	4·3 (2·1 to 7·3)	4·4 (2·2 to 7·6)	4·1 (2·3 to 6·5)
Maternal and neonatal disorders	1,710 (1,320 to 2,140)	697 (547 to 884)	237 (161 to 337)	84·7 (64·2 to 109)	67·9 (49·9 to 90·1)	71·4 (51·3 to 94·9)	66·9 (49 to 89·4)	54·1 (39 to 71·7)	46·1 (33·2 to 60·8)	37 (26·5 to 49·2)	30·5 (22·5 to 41·1)
Maternal disorders	0 (0 to 0)	0 (0 to 0)	0 (0 to 0)	0 (0 to 0)	0 (0 to 0)	0 (0 to 0)	0 (0 to 0)	0 (0 to 0)	0 (0 to 0)	0 (0 to 0)	0 (0 to 0)
Maternal hemorrhage	0 (0 to 0)	0 (0 to 0)	0 (0 to 0)	0 (0 to 0)	0 (0 to 0)	0 (0 to 0)	0 (0 to 0)	0 (0 to 0)	0 (0 to 0)	0 (0 to 0)	0 (0 to 0)
Maternal sepsis and other maternal infections	0 (0 to 0)	0 (0 to 0)	0 (0 to 0)	0 (0 to 0)	0 (0 to 0)	0 (0 to 0)	0 (0 to 0)	0 (0 to 0)	0 (0 to 0)	0 (0 to 0)	0 (0 to 0)
Maternal hypertensive disorders	0 (0 to 0)	0 (0 to 0)	0 (0 to 0)	0 (0 to 0)	0 (0 to 0)	0 (0 to 0)	0 (0 to 0)	0 (0 to 0)	0 (0 to 0)	0 (0 to 0)	0 (0 to 0)
Maternal obstructed labor and uterine rupture	--	--	--	--	--	--	--	--	--	--	--
Maternal abortion and miscarriage	--	--	--	--	--	--	--	--	--	--	--
Ectopic pregnancy	--	--	--	--	--	--	--	--	--	--	--
Indirect maternal deaths	--	--	--	--	--	--	--	--	--	--	--
Late maternal deaths	--	--	--	--	--	--	--	--	--	--	--
Maternal deaths aggravated by HIV/AIDS	--	--	--	--	--	--	--	--	--	--	--
Neonatal disorders	1,710 (1,320 to 2,140)	697 (547 to 884)	237 (161 to 337)	84·7 (64·2 to 109)	67·9 (49·9 to 90·1)	71·4 (51·3 to 94·9)	66·9 (49 to 89·4)	54·1 (39 to 71·7)	46·1 (33·2 to 60·8)	37 (26·5 to 49·2)	30·5 (22·5 to 41·1)
Neonatal preterm birth	797 (582 to 1,050)	241 (166 to 324)	91 (55 to 138)	31·6 (22·5 to 42·4)	37·5 (25·9 to 50·6)	39·2 (27·4 to 53·4)	36·4 (25·2 to 50·2)	28·3 (19·6 to 38·8)	23·9 (16·6 to 32·7)	18·6 (12·9 to 25·5)	15 (10·4 to 20·3)
Neonatal encephalopathy due to birth asphyxia and trauma	460 (310 to 657)	104 (62·4 to 162)	41·5 (23·1 to 70·9)	13·4 (8·3 to 19·3)	8·9 (4·2 to 14·8)	9·4 (4·6 to 16)	8·9 (4 to 15·4)	7·6 (3·5 to 13)	6·5 (3·1 to 10·8)	5·4 (2·5 to 9·5)	4·5 (2·2 to 7·6)
Neonatal sepsis and other neonatal infections	217 (140 to 315)	218 (139 to 315)	62·4 (38·3 to 95)	14·1 (8·2 to 21·2)	17·2 (9·3 to 26·6)	18·2 (10·1 to 28·7)	17·3 (9·1 to 27·2)	14·5 (7·7 to 23·3)	12·5 (6·7 to 19·6)	10·3 (5·5 to 16)	8·7 (4·6 to 14)
Hemolytic disease and other neonatal jaundice	46·7 (27·1 to 76)	27·3 (14·7 to 47·1)	6·4 (2·9 to 12·7)	3·7 (1·8 to 6·5)	4·2 (2 to 7·1)	4·6 (2·2 to 7·8)	4·3 (2·4 to 7·1)	3·7 (2·1 to 6·4)	3·3 (2·4 to 4·3)	2·8 (2·1 to 3·7)	2·4 (1·8 to 3·1)
Other neonatal disorders	190 (123 to 281)	106 (66·9 to 156)	35·7 (23 to 53·8)	22 (16·6 to 28·5)	0 (0 to 0)	0 (0 to 0)	0 (0 to 0)	0 (0 to 0)	0 (0 to 0)	0 (0 to 0)	0 (0 to 0)
Nutritional deficiencies	0·2 (0·1 to 0·3)	3·9 (1·8 to 6·7)	134 (91·7 to 190)	161 (88·1 to 279)	168 (61·3 to 349)	78·1 (26·3 to 175)	45·8 (20·3 to 102)	34·4 (15·9 to 70·3)	32·8 (16·3 to 63·4)	33·5 (17·1 to 62·1)	29·8 (16·2 to 56·5)
Protein-energy malnutrition	0·1 (0 to 0·1)	0·3 (0·2 to 0·4)	106 (69·2 to 158)	57·3 (35·6 to 87)	13·5 (8·8 to 20)	7·7 (5·1 to 11·4)	10·3 (6·6 to 15·1)	9·4 (5·3 to 15·7)	11 (6·2 to 18)	11·4 (7·7 to 16)	11·6 (7·7 to 17)
Iodine deficiency	0 (0 to 0)	0 (0 to 0)	0 (0 to 0)	0 (0 to 0)	0·1 (0 to 0·3)	0·3 (0·1 to 0·6)	0·4 (0·2 to 0·8)	0·5 (0·2 to 0·8)	0·4 (0·2 to 0·8)	0·4 (0·2 to 0·7)	0·4 (0·2 to 0·6)
Vitamin A deficiency	0 (0 to 0·1)	0·3 (0·1 to 0·6)	0·9 (0·3 to 2·1)	4·5 (1·8 to 10·3)	5·1 (1·8 to 10·9)	2·9 (1·1 to 7)	0·8 (0·2 to 1·7)	0·6 (0·3 to 1·1)	0·5 (0·2 to 0·8)	0·4 (0·2 to 0·7)	0·3 (0·2 to 0·6)
Dietary iron deficiency	0·1 (0 to 0·2)	3·4 (1·5 to 6)	25·2 (8 to 54·1)	98·6 (31·7 to 212)	149 (45·3 to 330)	66·8 (16·1 to 161)	33·2 (8·4 to 89·3)	22 (4·9 to 58·1)	19·4 (4·8 to 49·2)	19·7 (4·6 to 47·9)	16·3 (4·1 to 43·6)
Other nutritional deficiencies	0 (0 to 0)	0 (0 to 0)	1·6 (0·7 to 3·1)	0·8 (0·3 to 1·7)	0·5 (0·2 to 1·1)	0·4 (0·2 to 0·9)	1·1 (0·5 to 2·3)	2 (0·8 to 4·3)	1·5 (0·6 to 3·1)	1·5 (0·7 to 3·1)	1·2 (0·6 to 2·2)
Non-communicable diseases	303 (198 to 445)	194 (136 to 261)	629 (478 to 811)	625 (495 to 780)	705 (538 to 905)	1,020 (776 to 1,330)	1,470 (1,160 to 1,850)	1,550 (1,260 to 1,890)	1,710 (1,420 to 2,050)	1,870 (1,560 to 2,230)	1,960 (1,680 to 2,320)
Neoplasms	2·2 (1·4 to 3·8)	2·3 (1·7 to 3·2)	16·8 (11·6 to 23·3)	58 (42·3 to 78·1)	62·3 (48·3 to 79·8)	65·1 (51 to 81·7)	101 (81·7 to 124)	112 (92·5 to 134)	129 (109 to 154)	159 (133 to 188)	174 (144 to 207)
Lip and oral cavity cancer	0 (0 to 0)	0 (0 to 0)	0 (0 to 0)	0 (0 to 0)	0·2 (0·1 to 0·2)	0·2 (0·1 to 0·3)	0·5 (0·3 to 0·6)	0·8 (0·6 to 1)	0·9 (0·6 to 1·2)	1·7 (1·2 to 2·6)	2·8 (2 to 3·8)
Nasopharynx cancer	0 (0 to 0)	0 (0 to 0)	0 (0 to 0)	0 (0 to 0)	0·2 (0·2 to 0·3)	0·6 (0·4 to 0·8)	1 (0·7 to 1·3)	1·1 (0·8 to 1·5)	1·1 (0·8 to 1·5)	1·1 (0·7 to 1·4)	1·2 (0·8 to 1·7)
Other pharynx cancer	0 (0 to 0)	0 (0 to 0)	0 (0 to 0)	0 (0 to 0)	0 (0 to 0)	0 (0 to 0)	0 (0 to 0)	0·4 (0·3 to 0·5)	0·4 (0·2 to 0·5)	0·6 (0·5 to 0·8)	1·2 (0·8 to 1·6)
Esophageal cancer	0 (0 to 0)	0 (0 to 0)	0 (0 to 0)	0 (0 to 0)	0 (0 to 0)	0 (0 to 0)	0 (0 to 0)	0·6 (0·5 to 0·8)	0·9 (0·6 to 1·2)	1·8 (1·2 to 2·6)	4·2 (3 to 5·7)
Stomach cancer	0 (0 to 0)	0 (0 to 0)	0 (0 to 0)	0 (0 to 0)	0 (0 to 0)	0 (0 to 0)	2·3 (1·9 to 2·8)	4 (3·1 to 5·1)	8·7 (6·1 to 11·9)	18·6 (13·2 to 25·2)	23·3 (16·3 to 31·2)
Colon and rectum cancer	0 (0 to 0)	0 (0 to 0)	0 (0 to 0)	0 (0 to 0)	0·5 (0·3 to 0·7)	0·6 (0·4 to 0·8)	3·2 (2·4 to 4·2)	6·1 (4·5 to 8·3)	10 (6·5 to 14·9)	18·3 (13·4 to 24·5)	20·9 (14·6 to 28·6)
Liver cancer	0·1 (0 to 0·1)	0 (0 to 0·1)	0·4 (0·2 to 0·6)	0·7 (0·5 to 1)	0·8 (0·6 to 1·1)	1·3 (1 to 1·6)	1·2 (0·8 to 1·6)	2·2 (1·5 to 3·2)	3·8 (2·4 to 5·8)	4·5 (2·9 to 6·3)	6·4 (4·2 to 9)
Liver cancer due to hepatitis B	0 (0 to 0)	0 (0 to 0)	0 (0 to 0)	0 (0 to 0)	0 (0 to 0)	0·4 (0·2 to 0·6)	0·6 (0·4 to 1)	1·4 (0·9 to 2·1)	2·5 (1·4 to 3·9)	2·9 (1·8 to 4·4)	3·8 (2·2 to 5·8)
Liver cancer due to hepatitis C	0 (0 to 0)	0 (0 to 0)	0 (0 to 0)	0 (0 to 0)	0 (0 to 0)	0 (0 to 0)	0 (0 to 0)	0 (0 to 0·1)	0·1 (0 to 0·2)	0·1 (0·1 to 0·3)	0·3 (0·1 to 0·6)
Liver cancer due to alcohol use	0 (0 to 0)	0 (0 to 0)	0 (0 to 0)	0 (0 to 0)	0 (0 to 0)	0 (0 to 0)	0 (0 to 0·1)	0·2 (0·1 to 0·4)	0·5 (0·2 to 1·1)	0·8 (0·4 to 1·6)	1·6 (0·7 to 3)
Liver cancer due to NASH	0 (0 to 0)	0 (0 to 0)	0 (0 to 0)	0 (0 to 0)	0 (0 to 0)	0 (0 to 0)	0·2 (0·1 to 0·4)	0·3 (0·1 to 0·5)	0·3 (0·1 to 0·6)	0·2 (0·1 to 0·5)	0·3 (0·1 to 0·6)
Liver cancer due to other causes	0·1 (0 to 0·1)	0 (0 to 0·1)	0·4 (0·2 to 0·6)	0·7 (0·5 to 1)	0·8 (0·6 to 1·1)	0·9 (0·7 to 1·1)	0·3 (0·1 to 0·5)	0·3 (0·2 to 0·6)	0·4 (0·2 to 0·7)	0·3 (0·2 to 0·6)	0·4 (0·2 to 0·7)
Gallbladder and biliary tract cancer	0 (0 to 0)	0 (0 to 0)	0 (0 to 0)	0 (0 to 0)	0 (0 to 0)	0 (0 to 0)	0 (0 to 0)	0·3 (0·2 to 0·5)	0·6 (0·4 to 0·9)	0·9 (0·6 to 1·5)	1·4 (1 to 2·1)
Pancreatic cancer	0 (0 to 0)	0 (0 to 0)	0 (0 to 0)	0 (0 to 0)	0 (0 to 0)	0 (0 to 0)	0·9 (0·7 to 1·1)	1·1 (0·8 to 1·3)	1·8 (1·2 to 2·4)	6·5 (4·4 to 9·3)	9 (6·1 to 12·5)
Larynx cancer	0 (0 to 0)	0 (0 to 0)	0 (0 to 0)	0 (0 to 0)	0 (0 to 0)	0 (0 to 0)	0 (0 to 0)	0·7 (0·5 to 1)	0·6 (0·5 to 0·8)	1·1 (0·8 to 1·4)	2·5 (1·8 to 3·3)
Tracheal, bronchus, and lung cancer	0 (0 to 0)	0 (0 to 0)	0 (0 to 0)	0 (0 to 0)	0 (0 to 0)	0·4 (0·3 to 0·6)	3·4 (2·6 to 4·2)	4·3 (3 to 5·8)	6 (3·8 to 8·9)	13·6 (9·9 to 18·7)	20·9 (14·2 to 29·5)
Malignant skin melanoma	0 (0 to 0)	0 (0 to 0)	0·1 (0 to 0·4)	0·3 (0·1 to 1·3)	0·2 (0·1 to 0·2)	0·2 (0·1 to 0·3)	0·4 (0·2 to 0·7)	0·8 (0·4 to 1·3)	1·5 (0·7 to 2·3)	2 (0·9 to 3·1)	2·4 (1·3 to 3·8)
Non-melanoma skin cancer	0 (0 to 0)	0 (0 to 0)	0 (0 to 0)	0 (0 to 0)	0 (0 to 0)	0 (0 to 0)	0 (0 to 0)	0·5 (0·3 to 0·7)	1·3 (0·7 to 1·9)	1·9 (1·1 to 2·8)	1·9 (1·2 to 2·7)
Non-melanoma skin cancer (squamous-cell carcinoma)	0 (0 to 0)	0 (0 to 0)	0 (0 to 0)	0 (0 to 0)	0 (0 to 0)	0 (0 to 0)	0 (0 to 0)	0·5 (0·3 to 0·7)	1·3 (0·7 to 1·9)	1·9 (1·1 to 2·8)	1·9 (1·2 to 2·7)
Non-melanoma skin cancer (basal-cell carcinoma)	0 (0 to 0)	0 (0 to 0)	0 (0 to 0)	0 (0 to 0)	0 (0 to 0)	0 (0 to 0)	0 (0 to 0)	0 (0 to 0)	0 (0 to 0)	0 (0 to 0)	0 (0 to 0)
Breast cancer	0 (0 to 0)	0 (0 to 0)	0 (0 to 0)	0 (0 to 0)	0 (0 to 0)	0 (0 to 0)	0·1 (0·1 to 0·2)	0·1 (0·1 to 0·1)	0·4 (0·3 to 0·6)	0·6 (0·4 to 0·8)	0·8 (0·6 to 1·1)
Cervical cancer	--	--	--	--	--	--	--	--	--	--	--
Uterine cancer	--	--	--	--	--	--	--	--	--	--	--
Ovarian cancer	--	--	--	--	--	--	--	--	--	--	--
Prostate cancer	0 (0 to 0)	0 (0 to 0)	0 (0 to 0)	0 (0 to 0)	0 (0 to 0)	0 (0 to 0)	0 (0 to 0)	0·9 (0·4 to 1·2)	1·9 (0·8 to 2·5)	2·3 (0·9 to 3·2)	2·4 (1·2 to 3·4)
Testicular cancer	0·1 (0 to 1·7)	0·1 (0 to 0·9)	0·3 (0·1 to 1·5)	0·6 (0·2 to 1·4)	0·7 (0·4 to 1·1)	0·8 (0·4 to 1·3)	2·4 (1·5 to 3·6)	3·7 (2·2 to 5·5)	5·2 (2·9 to 8)	2·9 (1·7 to 4·3)	2·1 (1·4 to 3·1)
Kidney cancer	0·2 (0·1 to 0·3)	0·1 (0·1 to 0·2)	1·2 (0·8 to 1·8)	5·4 (3·5 to 7·9)	3·7 (2·6 to 5·3)	1·7 (1·2 to 2·2)	3 (2·2 to 3·9)	3·7 (2·7 to 5)	5 (3·5 to 6·8)	5·6 (3·5 to 8·4)	4·5 (3·1 to 6·3)
Bladder cancer	0 (0 to 0)	0 (0 to 0)	0 (0 to 0)	0 (0 to 0)	0 (0 to 0)	0 (0 to 0)	0·2 (0·1 to 0·2)	0·3 (0·2 to 0·4)	0·4 (0·3 to 0·5)	0·9 (0·6 to 1·4)	1·3 (0·9 to 1·8)
Brain and central nervous system cancer	0·3 (0·1 to 0·5)	0·3 (0·1 to 0·4)	3·4 (1·3 to 5·9)	10·4 (4·8 to 16·9)	13·1 (6·4 to 21·6)	11·6 (5·5 to 18·6)	9 (3·3 to 15·2)	8·1 (2·6 to 13·3)	9·2 (2·8 to 14·5)	10·7 (3·6 to 16·4)	11 (4·4 to 16·3)
Thyroid cancer	0 (0 to 0)	0 (0 to 0)	0 (0 to 0)	0 (0 to 0)	0·2 (0·1 to 0·2)	0·2 (0·1 to 0·2)	0·3 (0·2 to 0·3)	0·4 (0·3 to 0·5)	0·6 (0·4 to 0·8)	0·7 (0·5 to 0·9)	0·9 (0·6 to 1·3)
Mesothelioma	0 (0 to 0)	0 (0 to 0)	0 (0 to 0)	0 (0 to 0)	0 (0 to 0)	0 (0 to 0)	0 (0 to 0)	1 (0·7 to 1·4)	2 (1·3 to 3)	2 (1·4 to 2·7)	2·8 (1·9 to 3·9)
Hodgkin lymphoma	0 (0 to 0)	0 (0 to 0)	0 (0 to 0)	0·2 (0·1 to 0·4)	0·6 (0·4 to 0·8)	1·1 (0·8 to 1·4)	4·7 (3·2 to 6·7)	7·2 (4·5 to 10·6)	8·2 (5 to 12·7)	6·8 (4·3 to 10·2)	5 (3·2 to 7)
Non-Hodgkin lymphoma	0 (0 to 0)	0 (0 to 0)	0 (0 to 0)	4 (2·5 to 5·9)	5·5 (3·7 to 8·2)	6 (3·8 to 8·9)	10·8 (7·5 to 15)	13·1 (9·5 to 17·5)	14·4 (10 to 19·7)	15·1 (10·6 to 20·2)	12·5 (9 to 16·7)
Multiple myeloma	0 (0 to 0)	0 (0 to 0)	0 (0 to 0)	0 (0 to 0)	0 (0 to 0)	0 (0 to 0)	0 (0 to 0)	0·3 (0·1 to 0·4)	0·3 (0·2 to 0·5)	0·4 (0·3 to 0·7)	0·8 (0·5 to 1·2)
Leukemia	0·7 (0·5 to 1)	0·8 (0·6 to 1·1)	5·2 (3·2 to 7·7)	17·2 (11·1 to 25·6)	22·2 (14·8 to 32·6)	24·2 (15·9 to 34·8)	31·6 (22 to 43·7)	26·9 (18·3 to 37)	23·2 (16·7 to 31·6)	19·5 (13·2 to 26·6)	15·9 (11·3 to 22·1)
Acute lymphoid leukemia	0·1 (0 to 0·2)	0 (0 to 0·1)	1·4 (0·7 to 2·5)	9·1 (5·4 to 14·5)	12·3 (7·7 to 19·1)	10·4 (6 to 16·2)	14·7 (9·7 to 21·4)	7·4 (4·3 to 11·4)	4·1 (2·3 to 6·6)	2·7 (1·5 to 4·4)	2 (1·1 to 3·2)
Chronic lymphoid leukemia	0 (0 to 0)	0 (0 to 0)	0 (0 to 0)	0 (0 to 0)	0 (0 to 0)	0 (0 to 0)	0 (0 to 0)	0·6 (0·2 to 1)	0·7 (0·3 to 1·2)	0·5 (0·2 to 1)	0·4 (0·2 to 0·8)
Acute myeloid leukemia	0·1 (0 to 0·4)	0·2 (0 to 0·6)	0·6 (0·3 to 1·1)	1·9 (0·9 to 3·5)	1·6 (0·7 to 3)	2·3 (1·2 to 4·2)	3·4 (1·7 to 5·7)	2·7 (1·3 to 4·8)	3 (1·6 to 5·3)	2·1 (1·1 to 3·8)	1·8 (0·9 to 3·2)

Cause	40 to 44	45 to 49	50 to 54	55 to 59	60 to 64	65 to 69	70 to 74	75 to 79	80 to 84	85 to 89	90 to 94	95 plus	Total
All causes	3,980 (3,500 to 4,520)	4,010 (3,490 to 4,570)	3,860 (3,360 to 4,400)	4,160 (3,620 to 4,730)	3,580 (3,150 to 4,060)	2,910 (2,570 to 3,290)	2,340 (2,080 to 2,630)	1,720 (1,530 to 1,920)	1,410 (1,270 to 1,550)	791 (726 to 861)	366 (338 to 394)	121 (113 to 129)	61,200
Communicable, maternal, neonatal, and nutritional diseases	665 (602 to 737)	538 (487 to 603)	396 (349 to 453)	321 (270 to 383)	244 (201 to 298)	181 (145 to 225)	151 (120 to 190)	127 (99·7 to 159)	131 (105 to 162)	85·8 (68·5 to 104)	45·6 (34·7 to 56·4)	16·5 (12·1 to 20·9)	10,700
HIV/AIDS and sexually transmitted infections	426 (392 to 466)	312 (288 to 336)	181 (167 to 198)	97·7 (90 to 105)	44·9 (41 to 48·5)	21·9 (20 to 23·8)	5·9 (5·2 to 6·5)	2·6 (2·3 to 2·9)	1·3 (1·1 to 1·4)	0·5 (0·4 to 0·6)	0·2 (0·1 to 0·2)	0·1 (0·1 to 0·1)	2,810
HIV/AIDS	424 (390 to 465)	311 (287 to 335)	180 (166 to 196)	97 (89·3 to 105)	44·4 (40·6 to 48·1)	21·6 (19·7 to 23·4)	5·7 (5·1 to 6·3)	2·5 (2·2 to 2·8)	1·2 (1·1 to 1·3)	0·5 (0·4 to 0·5)	0·2 (0·1 to 0·2)	0·1 (0·1 to 0·1)	2,790
HIV/AIDS - Drug-susceptible Tuberculosis	31·1 (19·6 to 47)	22·7 (14·4 to 34·5)	13·2 (8·5 to 20)	7·1 (4·5 to 10·7)	3·3 (2·1 to 4·9)	1·6 (1·1 to 2·3)	0·4 (0·3 to 0·6)	0·2 (0·1 to 0·3)	0·1 (0·1 to 0·1)	0 (0 to 0·1)	0 (0 to 0)	0 (0 to 0)	205
HIV/AIDS - Multidrug-resistant Tuberculosis without extensive dru	0·4 (0 to 1·6)	0·3 (0 to 1·2)	0·2 (0 to 0·7)	0·1 (0 to 0·4)	0 (0 to 0·2)	0 (0 to 0·1)	0 (0 to 0)	0 (0 to 0)	0 (0 to 0)	0 (0 to 0)	0 (0 to 0)	0 (0 to 0)	2·5
HIV/AIDS - Extensively drug-resistant Tuberculosis	0·1 (0 to 0·3)	0 (0 to 0·2)	0 (0 to 0·1)	0 (0 to 0·1)	0 (0 to 0)	0 (0 to 0)	0 (0 to 0)	0 (0 to 0)	0 (0 to 0)	0 (0 to 0)	0 (0 to 0)	0 (0 to 0)	0·4
HIV/AIDS resulting in other diseases	393 (354 to 434)	288 (260 to 313)	167 (151 to 183)	89·8 (81·8 to 97·9)	41·1 (37 to 45)	20 (17·9 to 21·9)	5·2 (4·6 to 5·9)	2·3 (2 to 2·6)	1·1 (1 to 1·2)	0·4 (0·4 to 0·5)	0·1 (0·1 to 0·2)	0·1 (0·1 to 0·1)	2,580
Sexually transmitted infections excluding HIV	2 (0·9 to 3·8)	1·6 (0·8 to 3·1)	1·1 (0·5 to 2·1)	0·7 (0·3 to 1·5)	0·4 (0·2 to 1)	0·3 (0·1 to 0·7)	0·2 (0·1 to 0·4)	0·1 (0 to 0·3)	0·1 (0 to 0·2)	0 (0 to 0·1)	0 (0 to 0)	0 (0 to 0)	20·7
Syphilis	0·1 (0 to 0·1)	0·1 (0 to 0·1)	0·1 (0 to 0·1)	0·1 (0 to 0·1)	0 (0 to 0·1)	0 (0 to 0·1)	0 (0 to 0)	0 (0 to 0)	0 (0 to 0)	0 (0 to 0)	0 (0 to 0)	0 (0 to 0)	0·8
Chlamydial infection	0·5 (0·1 to 1·1)	0·4 (0·1 to 0·8)	0·1 (0 to 0·4)	0 (0 to 0·1)	0 (0 to 0)	0 (0 to 0)	0 (0 to 0)	0 (0 to 0)	0 (0 to 0)	0 (0 to 0)	0 (0 to 0)	0 (0 to 0)	4·5
Gonococcal infection	0·1 (0 to 0·2)	0·1 (0 to 0·2)	0·1 (0 to 0·1)	0 (0 to 0·1)	0 (0 to 0)	0 (0 to 0)	0 (0 to 0)	0 (0 to 0)	0 (0 to 0)	0 (0 to 0)	0 (0 to 0)	0 (0 to 0)	2·7
Trichomoniasis	0·1 (0 to 0·2)	0·1 (0 to 0·2)	0 (0 to 0·1)	0 (0 to 0·1)	0 (0 to 0)	0 (0 to 0)	0 (0 to 0)	0 (0 to 0)	0 (0 to 0)	0 (0 to 0)	0 (0 to 0)	0 (0 to 0)	0·5
Genital herpes	0·8 (0·3 to 2·1)	0·8 (0·2 to 1·9)	0·6 (0·2 to 1·6)	0·5 (0·2 to 1·3)	0·4 (0·1 to 0·9)	0·3 (0·1 to 0·6)	0·2 (0 to 0·4)	0·1 (0 to 0·3)	0·1 (0 to 0·2)	0 (0 to 0·1)	0 (0 to 0)	0 (0 to 0)	7
Other sexually transmitted infections	0·4 (0·1 to 0·7)	0·3 (0·1 to 0·6)	0·1 (0 to 0·3)	0 (0 to 0·1)	0 (0 to 0)	0 (0 to 0)	0 (0 to 0)	0 (0 to 0)	0 (0 to 0)	0 (0 to 0)	0 (0 to 0)	0 (0 to 0)	5·1
Respiratory infections and tuberculosis	139 (106 to 181)	131 (99·7 to 171)	130 (99 to 171)	142 (105 to 187)	133 (100 to 173)	107 (77·6 to 139)	101 (75·9 to 133)	91·8 (68·4 to 119)	103 (77·1 to 133)	68·9 (54·1 to 85)	37·6 (27·9 to 47·5)	13·5 (9·5 to 17·3)	2,660
Tuberculosis	43·9 (31·7 to 59·8)	39·2 (27·7 to 53·4)	35·3 (24·9 to 50·1)	32·7 (22·5 to 45)	26·2 (18·8 to 35·4)	18·3 (12·7 to 24·7)	13·4 (9·8 to 17·5)	8·7 (6·5 to 11·7)	6·8 (5 to 8·9)	3·4 (2·4 to 4·6)	1·2 (0·8 to 1·6)	0·3 (0·2 to 0·4)	469
Latent tuberculosis infection	0 (0 to 0)	0 (0 to 0)	0 (0 to 0)	0 (0 to 0)	0 (0 to 0)	0 (0 to 0)	0 (0 to 0)	0 (0 to 0)	0 (0 to 0)	0 (0 to 0)	0 (0 to 0)	0 (0 to 0)	0
Drug-susceptible tuberculosis	43·5 (31·5 to 59·5)	38·7 (27·3 to 52·8)	34·9 (24·5 to 49·2)	32·4 (22·2 to 44·9)	25·9 (18·5 to 34·9)	18 (12·6 to 24·3)	13·3 (9·7 to 17·4)	8·6 (6·4 to 11·7)	6·8 (4·9 to 8·9)	3·4 (2·4 to 4·5)	1·2 (0·8 to 1·5)	0·3 (0·2 to 0·4)	464
Multidrug-resistant tuberculosis without extensive drug resistanc	0·4 (0 to 1·8)	0·4 (0 to 1·6)	0·3 (0 to 1·5)	0·3 (0 to 1·4)	0·3 (0 to 1·1)	0·2 (0 to 0·8)	0·1 (0 to 0·6)	0·1 (0 to 0·4)	0·1 (0 to 0·3)	0 (0 to 0·1)	0 (0 to 0·1)	0 (0 to 0)	4·6
Extensively drug-resistant tuberculosis	0·1 (0 to 0·3)	0·1 (0 to 0·3)	0·1 (0 to 0·2)	0·1 (0 to 0·2)	0 (0 to 0·2)	0 (0 to 0·1)	0 (0 to 0·1)	0 (0 to 0·1)	0 (0 to 0)	0 (0 to 0)	0 (0 to 0)	0 (0 to 0)	0·8
Lower respiratory infections	83·2 (56·2 to 119)	82·1 (56·7 to 115)	86·5 (59·4 to 122)	103 (70·7 to 144)	102 (70·9 to 140)	85·3 (57·8 to 117)	85·5 (61·2 to 117)	81·9 (58·6 to 109)	95·2 (70·4 to 126)	65·1 (50·5 to 80·9)	36·3 (26·4 to 46·2)	13·1 (9·2 to 17)	1,950
Upper respiratory infections	9 (4·9 to 15·6)	7·3 (3·9 to 12·7)	5·8 (3·2 to 9·8)	4·7 (2·6 to 7·8)	3·3 (1·8 to 5·4)	2·4 (1·3 to 4)	1·5 (0·8 to 2·5)	0·9 (0·5 to 1·5)	0·5 (0·3 to 0·9)	0·3 (0·1 to 0·4)	0·1 (0·1 to 0·2)	0 (0 to 0·1)	180
Otitis media	3 (1·4 to 5·7)	2·8 (1·3 to 5)	2·3 (1·1 to 4·3)	1·9 (0·9 to 3·3)	1·2 (0·5 to 2·4)	0·7 (0·3 to 1·3)	0·4 (0·2 to 0·8)	0·3 (0·1 to 0·5)	0·2 (0·1 to 0·3)	0·1 (0 to 0·2)	0 (0 to 0·1)	0 (0 to 0)	68·5
Enteric infections	24·9 (18·2 to 33·5)	23·9 (17·6 to 32)	22·1 (16·6 to 28·9)	21·8 (16 to 29·4)	20·2 (15 to 26·5)	19·1 (13·8 to 25·7)	17·6 (13·1 to 23·4)	14·4 (10·6 to 18·8)	11·9 (8·3 to 15·8)	6·3 (4·6 to 8·4)	2·9 (2·2 to 3·8)	1 (0·7 to 1·3)	667
Diarrheal diseases	24·8 (18·1 to 33·4)	23·8 (17·5 to 31·9)	22 (16·5 to 28·8)	21·8 (16 to 29·3)	20·1 (14·9 to 26·4)	19·1 (13·8 to 25·6)	17·6 (13 to 23·4)	14·3 (10·6 to 18·7)	11·9 (8·3 to 15·8)	6·3 (4·6 to 8·4)	2·9 (2·2 to 3·8)	1 (0·7 to 1·3)	664
Typhoid and paratyphoid	0 (0 to 0)	0 (0 to 0)	0 (0 to 0)	0 (0 to 0)	0 (0 to 0)	0 (0 to 0)	0 (0 to 0)	0 (0 to 0)	0 (0 to 0)	0 (0 to 0)	0 (0 to 0)	0 (0 to 0)	0·1
Typhoid fever	0 (0 to 0)	0 (0 to 0)	0 (0 to 0)	0 (0 to 0)	0 (0 to 0)	0 (0 to 0)	0 (0 to 0)	0 (0 to 0)	0 (0 to 0)	0 (0 to 0)	0 (0 to 0)	0 (0 to 0)	0
Paratyphoid fever	0 (0 to 0)	0 (0 to 0)	0 (0 to 0)	0 (0 to 0)	0 (0 to 0)	0 (0 to 0)	0 (0 to 0)	0 (0 to 0)	0 (0 to 0)	0 (0 to 0)	0 (0 to 0)	0 (0 to 0)	0·1
Invasive Non-typhoidal Salmonella (iNTS)	0 (0 to 0)	0·1 (0 to 0·1)	0 (0 to 0·1)	0 (0 to 0·1)	0·1 (0 to 0·2)	0 (0 to 0·1)	0 (0 to 0)	0 (0 to 0)	0 (0 to 0)	0 (0 to 0)	0 (0 to 0)	0 (0 to 0)	1·4
Other intestinal infectious diseases	0 (0 to 0·1)	0 (0 to 0·1)	0 (0 to 0·1)	0 (0 to 0·1)	0 (0 to 0)	0 (0 to 0)	0 (0 to 0)	0 (0 to 0)	0 (0 to 0)	0 (0 to 0)	0 (0 to 0)	0 (0 to 0)	1·3
Neglected tropical diseases and malaria	11 (5 to 20·5)	10·6 (4·8 to 20)	10 (4·7 to 17·5)	10·2 (4·8 to 18·6)	8·7 (4·2 to 15·3)	7·2 (3·5 to 12·4)	5·5 (2·9 to 9·5)	4 (1·9 to 7·1)	2·8 (1·3 to 5)	1·8 (0·9 to 3·2)	0·8 (0·4 to 1·4)	0·2 (0·1 to 0·4)	190
Malaria	0 (0 to 0)	0 (0 to 0)	0 (0 to 0)	0 (0 to 0)	0 (0 to 0)	0 (0 to 0)	0 (0 to 0)	0 (0 to 0)	0 (0 to 0)	0 (0 to 0)	0 (0 to 0)	0 (0 to 0)	0·5
Chagas disease	0·1 (0 to 0·2)	0·1 (0 to 0·2)	0·1 (0 to 0·2)	0·1 (0·1 to 0·3)	0·2 (0·1 to 0·5)	0·2 (0·1 to 0·4)	0·2 (0·1 to 0·4)	0 (0 to 0·1)	0 (0 to 0·1)	0·5 (0·2 to 1·4)	0·1 (0·1 to 0·5)	0 (0 to 0·1)	1·9
Leishmaniasis	0 (0 to 0)	0 (0 to 0)	0 (0 to 0)	0 (0 to 0)	0 (0 to 0)	0 (0 to 0)	0 (0 to 0)	0 (0 to 0)	0 (0 to 0)	0 (0 to 0)	0 (0 to 0)	0 (0 to 0)	0
Visceral leishmaniasis	0 (0 to 0)	0 (0 to 0)	0 (0 to 0)	0 (0 to 0)	0 (0 to 0)	0 (0 to 0)	0 (0 to 0)	0 (0 to 0)	0 (0 to 0)	0 (0 to 0)	0 (0 to 0)	0 (0 to 0)	0
Cutaneous and mucocutaneous leishmaniasis	0 (0 to 0)	0 (0 to 0)	0 (0 to 0)	0 (0 to 0)	0 (0 to 0)	0 (0 to 0)	0 (0 to 0)	0 (0 to 0)	0 (0 to 0)	0 (0 to 0)	0 (0 to 0)	0 (0 to 0)	0
African trypanosomiasis	0 (0 to 0)	0 (0 to 0)	0 (0 to 0)	0 (0 to 0)	0 (0 to 0)	0 (0 to 0)	0 (0 to 0)	0 (0 to 0)	0 (0 to 0)	0 (0 to 0)	0 (0 to 0)	0 (0 to 0)	0
Schistosomiasis	0 (0 to 0)	0 (0 to 0)	0 (0 to 0)	0 (0 to 0)	0 (0 to 0)	0 (0 to 0)	0 (0 to 0)	0 (0 to 0)	0 (0 to 0)	0 (0 to 0)	0 (0 to 0)	0 (0 to 0)	0
Cysticercosis	6·7 (1·9 to 14)	6·9 (2·1 to 14·5)	6·8 (2·4 to 13·8)	7·5 (2·7 to 15·3)	6·8 (2·7 to 13·2)	5·9 (2·3 to 10·9)	4·5 (1·9 to 8·3)	3·3 (1·4 to 6·3)	2·3 (0·9 to 4·5)	1·1 (0·5 to 2·2)	0·5 (0·2 to 1)	0·1 (0·1 to 0·3)	79·7
Cystic echinococcosis	0 (0 to 0)	0 (0 to 0)	0 (0 to 0)	0 (0 to 0)	0 (0 to 0)	0 (0 to 0)	0 (0 to 0)	0 (0 to 0)	0 (0 to 0)	0 (0 to 0)	0 (0 to 0)	0 (0 to 0)	0·1
Lymphatic filariasis	0 (0 to 0)	0 (0 to 0)	0 (0 to 0)	0 (0 to 0)	0 (0 to 0)	0 (0 to 0)	0 (0 to 0)	0 (0 to 0)	0 (0 to 0)	0 (0 to 0)	0 (0 to 0)	0 (0 to 0)	0
Onchocerciasis	0 (0 to 0)	0 (0 to 0)	0 (0 to 0)	0 (0 to 0)	0 (0 to 0)	0 (0 to 0)	0 (0 to 0)	0 (0 to 0)	0 (0 to 0)	0 (0 to 0)	0 (0 to 0)	0 (0 to 0)	0
Trachoma	0 (0 to 0)	0 (0 to 0)	0 (0 to 0)	0 (0 to 0)	0 (0 to 0)	0 (0 to 0)	0 (0 to 0)	0 (0 to 0)	0 (0 to 0)	0 (0 to 0)	0 (0 to 0)	0 (0 to 0)	0
Dengue	2·9 (0·6 to 9)	2·3 (0·5 to 7·4)	1·8 (0·5 to 5·9)	1·4 (0·3 to 4·3)	1 (0·3 to 2·8)	0·7 (0·2 to 1·8)	0·5 (0·2 to 1·4)	0·4 (0·1 to 1·1)	0·3 (0·1 to 0·7)	0·1 (0 to 0·4)	0·1 (0 to 0·2)	0 (0 to 0·1)	52·7
Yellow fever	0 (0 to 0)	0 (0 to 0)	0 (0 to 0)	0 (0 to 0)	0 (0 to 0)	0 (0 to 0)	0 (0 to 0)	0 (0 to 0)	0 (0 to 0)	0 (0 to 0)	0 (0 to 0)	0 (0 to 0)	0
Rabies	0 (0 to 0)	0 (0 to 0)	0 (0 to 0)	0 (0 to 0)	0 (0 to 0)	0 (0 to 0)	0 (0 to 0)	0 (0 to 0)	0 (0 to 0)	0 (0 to 0)	0 (0 to 0)	0 (0 to 0)	0·1
Intestinal nematode infections	0·4 (0·2 to 0·8)	0·3 (0·1 to 0·6)	0·3 (0·1 to 0·5)	0·2 (0·1 to 0·4)	0·2 (0·1 to 0·3)	0·1 (0·1 to 0·2)	0·1 (0 to 0·2)	0·1 (0 to 0·1)	0 (0 to 0·1)	0 (0 to 0)	0 (0 to 0)	0 (0 to 0)	8·6
Ascariasis	0·2 (0 to 0·3)	0·1 (0 to 0·3)	0·1 (0 to 0·2)	0·1 (0 to 0·2)	0·1 (0 to 0·2)	0 (0 to 0·1)	0 (0 to 0·1)	0 (0 to 0·1)	0 (0 to 0)	0 (0 to 0)	0 (0 to 0)	0 (0 to 0)	3·1
Trichuriasis	0 (0 to 0)	0 (0 to 0)	0 (0 to 0)	0 (0 to 0)	0 (0 to 0)	0 (0 to 0)	0 (0 to 0)	0 (0 to 0)	0 (0 to 0)	0 (0 to 0)	0 (0 to 0)	0 (0 to 0)	0·5
Hookworm disease	0·2 (0·1 to 0·4)	0·2 (0·1 to 0·4)	0·2 (0·1 to 0·3)	0·1 (0·1 to 0·3)	0·1 (0 to 0·2)	0·1 (0 to 0·2)	0·1 (0 to 0·1)	0 (0 to 0·1)	0 (0 to 0)	0 (0 to 0)	0 (0 to 0)	0 (0 to 0)	5
Food-borne trematodiases	0 (0 to 0)	0 (0 to 0)	0 (0 to 0)	0 (0 to 0)	0 (0 to 0)	0 (0 to 0)	0 (0 to 0)	0 (0 to 0)	0 (0 to 0)	0 (0 to 0)	0 (0 to 0)	0 (0 to 0)	0
Leprosy	0·1 (0 to 0·1)	0·1 (0 to 0·1)	0·1 (0 to 0·2)	0·1 (0 to 0·2)	0·1 (0 to 0·1)	0 (0 to 0·1)	0 (0 to 0·1)	0 (0 to 0·1)	0 (0 to 0)	0 (0 to 0)	0 (0 to 0)	0 (0 to 0)	0·7
Ebola	0 (0 to 0)	0 (0 to 0)	0 (0 to 0)	0 (0 to 0)	0 (0 to 0)	0 (0 to 0)	0 (0 to 0)	0 (0 to 0)	0 (0 to 0)	0 (0 to 0)	0 (0 to 0)	0 (0 to 0)	0
Zika virus	0·2 (0 to 0·7)	0·1 (0 to 0·5)	0·1 (0 to 0·4)	0·1 (0 to 0·3)	0 (0 to 0·2)	0 (0 to 0·1)	0 (0 to 0·1)	0 (0 to 0)	0 (0 to 0)	0 (0 to 0)	0 (0 to 0)	0 (0 to 0)	1·8
Guinea worm disease	0 (0 to 0)	0 (0 to 0)	0 (0 to 0)	0 (0 to 0)	0 (0 to 0)	0 (0 to 0)	0 (0 to 0)	0 (0 to 0)	0 (0 to 0)	0 (0 to 0)	0 (0 to 0)	0 (0 to 0)	0
Other neglected tropical diseases	0·8 (0·2 to 2)	0·8 (0·2 to 2)	0·8 (0·2 to 1·9)	0·8 (0·2 to 2)	0·3 (0·1 to 0·7)	0·2 (0·1 to 0·4)	0·2 (0·1 to 0·4)	0·1 (0 to 0·2)	0·1 (0 to 0·2)	0·1 (0 to 0·1)	0 (0 to 0·1)	0 (0 to 0)	43·9
Other infectious diseases	12·6 (9·9 to 16·3)	12·5 (9·4 to 16·2)	10·1 (7·8 to 13·2)	8·7 (6·8 to 11·2)	6·6 (5·1 to 8·5)	4·2 (3·3 to 5·4)	3·1 (2·4 to 4·1)	1·8 (1·4 to 2·4)	1·2 (1 to 1·7)	0·7 (0·6 to 1)	0·3 (0·2 to 0·4)	0·1 (0·1 to 0·1)	288
Meningitis	3·3 (2·4 to 4·6)	3·2 (2·2 to 4·4)	2·7 (1·9 to 3·8)	2·3 (1·7 to 3·2)	1·9 (1·3 to 2·7)	1·2 (0·8 to 1·7)	0·8 (0·6 to 1·1)	0·5 (0·4 to 0·7)	0·4 (0·3 to 0·5)	0·2 (0·1 to 0·3)	0·1 (0 to 0·1)	0 (0 to 0)	94
Encephalitis	3·2 (2·2 to 4·5)	2·6 (1·8 to 3·7)	1·7 (1·2 to 2·4)	1·6 (1 to 2·3)	1·2 (0·8 to 1·7)	0·7 (0·5 to 1·1)	0·5 (0·3 to 0·8)	0·3 (0·2 to 0·4)	0·2 (0·1 to 0·3)	0·1 (0 to 0·1)	0 (0 to 0)	0 (0 to 0)	76·6
Diphtheria	0 (0 to 0)	0 (0 to 0)	0 (0 to 0)	0 (0 to 0)	0 (0 to 0)	0 (0 to 0)	0 (0 to 0)	0 (0 to 0)	0 (0 to 0)	0 (0 to 0)	0 (0 to 0)	0 (0 to 0)	0·3
Tetanus	0·3 (0·1 to 1)	0·4 (0·1 to 1·5)	0·4 (0·1 to 1·6)	0·4 (0·1 to 1·6)	0·3 (0·1 to 1)	0·2 (0·1 to 0·9)	0·2 (0 to 0·6)	0·1 (0 to 0·4)	0·1 (0 to 0·2)	0 (0 to 0·1)	0 (0 to 0)	0 (0 to 0)	4·1
Measles	0 (0 to 0)	0 (0 to 0)	0 (0 to 0)	0 (0 to 0)	0 (0 to 0)	0 (0 to 0)	0 (0 to 0)	0 (0 to 0)	0 (0 to 0)	0 (0 to 0)	0 (0 to 0)	0 (0 to 0)	0
Varicella and herpes zoster	0·9 (0·5 to 1·5)	1·1 (0·5 to 1·8)	0·8 (0·4 to 1·4)	0·6 (0·4 to 1·1)	0·7 (0·4 to 1·1)	0·4 (0·2 to 0·7)	0·4 (0·2 to 0·8)	0·3 (0·2 to 0·6)	0·2 (0·1 to 0·4)	0·1 (0·1 to 0·2)	0·1 (0 to 0·1)	0 (0 to 0·1)	18·4
Acute hepatitis	1·1 (0·7 to 1·4)	0·9 (0·6 to 1·3)	0·8 (0·6 to 1·1)	0·5 (0·4 to 0·7)	0·4 (0·3 to 0·5)	0·2 (0·2 to 0·3)	0·1 (0·1 to 0·2)	0·1 (0 to 0·1)	0 (0 to 0·1)	0 (0 to 0)	0 (0 to 0)	0 (0 to 0)	18·7
Acute hepatitis A	0·3 (0·1 to 0·5)	0·2 (0·1 to 0·5)	0·1 (0 to 0·3)	0·1 (0 to 0·2)	0 (0 to 0·1)	0 (0 to 0·1)	0 (0 to 0)	0 (0 to 0)	0 (0 to 0)	0 (0 to 0)	0 (0 to 0)	0 (0 to 0)	9·1
Acute hepatitis B	0·6 (0·4 to 0·8)	0·4 (0·2 to 0·7)	0·5 (0·3 to 0·7)	0·3 (0·2 to 0·5)	0·2 (0·1 to 0·3)	0·1 (0·1 to 0·2)	0·1 (0·1 to 0·1)	0 (0 to 0)	0 (0 to 0)	0 (0 to 0)	0 (0 to 0)	0 (0 to 0)	6·3
Acute hepatitis C	0·1 (0 to 0·2)	0·2 (0·1 to 0·4)	0·1 (0·1 to 0·3)	0·1 (0 to 0·2)	0·1 (0 to 0·1)	0 (0 to 0·1)	0 (0 to 0)	0 (0 to 0)	0 (0 to 0)	0 (0 to 0)	0 (0 to 0)	0 (0 to 0)	1·4
Acute hepatitis E	0·1 (0 to 0·2)	0·1 (0 to 0·2)	0 (0 to 0·1)	0 (0 to 0·1)	0·1 (0 to 0·2)	0 (0 to 0)	0 (0 to 0)	0 (0 to 0)	0 (0 to 0)	0 (0 to 0)	0 (0 to 0)	0 (0 to 0)	1·9
Other unspecified infectious diseases	3·8 (2·1 to 6·2)	4·4 (2·5 to 6·9)	3·7 (2·1 to 5·6)	3·2 (1·8 to 5)	2·2 (1·2 to 3·3)	1·4 (0·8 to 2·1)	1 (0·6 to 1·5)	0·6 (0·3 to 0·8)	0·4 (0·2 to 0·6)	0·3 (0·2 to 0·5)	0·1 (0·1 to 0·2)	0 (0 to 0)	70·7
Maternal and neonatal disorders	24·1 (17·6 to 31·6)	18·9 (13·7 to 25·3)	13·7 (9·9 to 18·5)	9·7 (7·1 to 13·1)	5·6 (4·1 to 7·5)	2·8 (2 to 3·8)	1·2 (0·9 to 1·6)	0·5 (0·3 to 0·7)	0·1 (0·1 to 0·2)	0 (0 to 0·1)	0 (0 to 0)	0 (0 to 0)	3,180
Maternal disorders	0 (0 to 0)	0 (0 to 0)	0 (0 to 0)	0 (0 to 0)	0 (0 to 0)	0 (0 to 0)	0 (0 to 0)	0 (0 to 0)	0 (0 to 0)	0 (0 to 0)	0 (0 to 0)	0 (0 to 0)	0
Maternal hemorrhage	0 (0 to 0)	0 (0 to 0)	0 (0 to 0)	0 (0 to 0)	0 (0 to 0)	0 (0 to 0)	0 (0 to 0)	0 (0 to 0)	0 (0 to 0)	0 (0 to 0)	0 (0 to 0)	0 (0 to 0)	0
Maternal sepsis and other maternal infections	0 (0 to 0)	0 (0 to 0)	0 (0 to 0)	0 (0 to 0)	0 (0 to 0)	0 (0 to 0)	0 (0 to 0)	0 (0 to 0)	0 (0 to 0)	0 (0 to 0)	0 (0 to 0)	0 (0 to 0)	0
Maternal hypertensive disorders	0 (0 to 0)	0 (0 to 0)	0 (0 to 0)	0 (0 to 0)	0 (0 to 0)	0 (0 to 0)	0 (0 to 0)	0 (0 to 0)	0 (0 to 0)	0 (0 to 0)	0 (0 to 0)	0 (0 to 0)	0
Maternal obstructed labor and uterine rupture	--	--	--	--	--	--	--	--	--	--	--	--	NA
Maternal abortion and miscarriage	--	--	--	--	--	--	--	--	--	--	--	--	NA
Ectopic pregnancy	--	--	--	--	--	--	--	--	--	--	--	--	NA
Indirect maternal deaths	--	--	--	--	--	--	--	--	--	--	--	--	NA
Late maternal deaths	--	--	--	--	--	--	--	--	--	--	--	--	NA
Maternal deaths aggravated by HIV/AIDS	--	--	--	--	--	--	--	--	--	--	--	--	NA
Neonatal disorders	24·1 (17·6 to 31·6)	18·9 (13·7 to 25·3)	13·7 (9·9 to 18·5)	9·7 (7·1 to 13·1)	5·6 (4·1 to 7·5)	2·8 (2 to 3·8)	1·2 (0·9 to 1·6)	0·5 (0·3 to 0·7)	0·1 (0·1 to 0·2)	0 (0 to 0·1)	0 (0 to 0)	0 (0 to 0)	3,180
Neonatal preterm birth	11·5 (8·2 to 15·8)	8·7 (6·1 to 12·1)	6 (4·1 to 8·5)	4·1 (3 to 5·3)	2·1 (1·6 to 2·7)	1 (0·7 to 1·3)	0·4 (0·3 to 0·6)	0·2 (0·1 to 0·3)	0·1 (0 to 0·1)	0 (0 to 0)	0 (0 to 0)	0 (0 to 0)	1,390
Neonatal encephalopathy due to birth asphyxia and trauma	3·7 (1·8 to 6·4)	2·9 (1·4 to 5)	2·2 (1·1 to 3·7)	1·7 (0·8 to 3)	1·1 (0·7 to 1·6)	0·5 (0·3 to 0·8)	0·2 (0·1 to 0·2)	0 (0 to 0·1)	0 (0 to 0)	0 (0 to 0)	0 (0 to 0)	0 (0 to 0)	682
Neonatal sepsis and other neonatal infections	7 (3·7 to 11)	5·7 (3·1 to 9·3)	4·2 (2·2 to 7·2)	3·1 (1·6 to 5·1)	1·8 (0·9 to 3·1)	1 (0·5 to 1·8)	0·5 (0·3 to 0·8)	0·2 (0·1 to 0·3)	0 (0 to 0·1)	0 (0 to 0)	0 (0 to 0)	0 (0 to 0)	634
Hemolytic disease and other neonatal jaundice	2 (1·5 to 2·6)	1·6 (1·2 to 2·1)	1·2 (0·9 to 1·6)	0·9 (0·7 to 1·2)	0·5 (0·4 to 0·7)	0·3 (0·2 to 0·4)	0·1 (0·1 to 0·2)	0 (0 to 0·1)	0 (0 to 0)	0 (0 to 0)	0 (0 to 0)	0 (0 to 0)	116
Other neonatal disorders	0 (0 to 0)	0 (0 to 0)	0 (0 to 0)	0 (0 to 0)	0 (0 to 0)	0 (0 to 0)	0 (0 to 0)	0 (0 to 0)	0 (0 to 0)	0 (0 to 0)	0 (0 to 0)	0 (0 to 0)	354
Nutritional deficiencies	26·6 (14·5 to 50·4)	28·2 (15·3 to 53·3)	29 (16·4 to 51·7)	31·2 (18 to 55·7)	25·8 (15·1 to 43·1)	18·9 (12·5 to 29·1)	16·8 (10·9 to 25)	12·5 (8·3 to 18·3)	11·4 (7·4 to 16·1)	7·4 (5·1 to 10·4)	3·9 (2·7 to 5·2)	1·6 (1·2 to 2·2)	935
Protein-energy malnutrition	9·5 (6·5 to 13·4)	11 (7·1 to 16)	11·7 (7·5 to 17·6)	12·7 (8·3 to 18·5)	12·6 (8·3 to 18·2)	11·4 (7·8 to 15·6)	11 (7 to 16·4)	8·5 (5·8 to 12·1)	8·1 (5·2 to 11·8)	5 (3·4 to 7)	2·8 (1·9 to 3·8)	1·3 (0·9 to 1·8)	344
Iodine deficiency	0·3 (0·2 to 0·6)	0·3 (0·1 to 0·5)	0·2 (0·1 to 0·4)	0·2 (0·1 to 0·3)	0·1 (0·1 to 0·2)	0·1 (0 to 0·1)	0·1 (0 to 0·1)	0 (0 to 0·1)	0 (0 to 0)	0 (0 to 0)	0 (0 to 0)	0 (0 to 0)	3·9
Vitamin A deficiency	0·3 (0·1 to 0·5)	0·2 (0·1 to 0·4)	0·2 (0·1 to 0·3)	0·1 (0·1 to 0·2)	0·1 (0 to 0·2)	0·1 (0 to 0·1)	0 (0 to 0·1)	0 (0 to 0·1)	0 (0 to 0)	0 (0 to 0)	0 (0 to 0)	0 (0 to 0)	17·4
Dietary iron deficiency	14·8 (3·5 to 38·2)	15·6 (4·1 to 39·6)	15·5 (4·5 to 37·4)	16·8 (4·7 to 40·6)	11·7 (3·1 to 27·1)	6·3 (1·8 to 15·8)	4·9 (1·3 to 12·1)	3·3 (1 to 8·3)	2·5 (0·6 to 6)	2 (0·7 to 4·2)	0·8 (0·3 to 1·8)	0·3 (0·1 to 0·5)	548
Other nutritional deficiencies	1·7 (0·8 to 3·3)	1·1 (0·4 to 2·4)	1·4 (0·6 to 2·6)	1·4 (0·6 to 2·7)	1·2 (0·5 to 2·5)	1·1 (0·4 to 2)	0·9 (0·3 to 1·8)	0·5 (0·2 to 1·1)	0·7 (0·2 to 1·4)	0·4 (0·1 to 0·7)	0·2 (0·1 to 0·4)	0·1 (0 to 0·2)	21·1
Non-communicable diseases	2,180 (1,850 to 2,540)	2,580 (2,190 to 3,010)	2,830 (2,440 to 3,260)	3,330 (2,890 to 3,820)	3,010 (2,630 to 3,410)	2,520 (2,220 to 2,840)	2,050 (1,810 to 2,300)	1,500 (1,340 to 1,670)	1,210 (1,090 to 1,340)	667 (610 to 730)	302 (277 to 329)	98·6 (90·6 to 107)	34,300
Neoplasms	233 (191 to 280)	329 (268 to 400)	427 (352 to 521)	572 (473 to 686)	560 (467 to 666)	474 (398 to 557)	389 (325 to 463)	284 (237 to 339)	216 (181 to 261)	106 (84·6 to 132)	41 (30·7 to 52)	10·2 (7·3 to 13·2)	4,520
Lip and oral cavity cancer	5·1 (3·5 to 7)	8·6 (6·2 to 11·7)	11 (8 to 14·6)	13·5 (9·9 to 17·8)	10·6 (7·7 to 14·1)	7·3 (5·6 to 9·3)	4·8 (3·6 to 6·3)	3 (2·2 to 3·9)	2·1 (1·6 to 2·8)	0·8 (0·6 to 1)	0·3 (0·2 to 0·4)	0·1 (0·1 to 0·1)	74
Nasopharynx cancer	2·2 (1·5 to 3·1)	3 (2·1 to 4·1)	4 (2·8 to 5·7)	4·6 (3·4 to 6·1)	2·9 (2·1 to 4)	2 (1·4 to 2·6)	1·2 (0·9 to 1·6)	0·6 (0·4 to 0·8)	0·4 (0·3 to 0·5)	0·3 (0·2 to 0·4)	0·1 (0·1 to 0·1)	0 (0 to 0)	27·7
Other pharynx cancer	2·4 (1·6 to 3·6)	5·3 (3·7 to 7·2)	6·7 (4·8 to 9·3)	8·3 (6 to 11·2)	6·4 (4·6 to 8·9)	4·3 (3 to 5·9)	2·5 (1·8 to 3·5)	1·3 (1 to 1·8)	0·9 (0·6 to 1·1)	0·4 (0·3 to 0·5)	0·1 (0·1 to 0·2)	0 (0 to 0·1)	41·4
Esophageal cancer	9·5 (6·9 to 12·9)	17·7 (13 to 23·4)	22·9 (17·3 to 30·2)	29 (21·2 to 38)	23·4 (17·1 to 31·1)	16·7 (11·8 to 22·2)	11·2 (8·2 to 14·9)	6·2 (4·4 to 8·4)	4·1 (3 to 5·5)	1·8 (1·4 to 2·4)	0·6 (0·5 to 0·8)	0·1 (0·1 to 0·2)	151
Stomach cancer	33·8 (24·9 to 43·3)	41·5 (30·9 to 54·8)	54·7 (42·1 to 70·1)	71·3 (55·1 to 91·8)	60 (44·7 to 78·3)	48·3 (36·7 to 62·8)	38·1 (28·8 to 49·9)	25·5 (19·4 to 33·2)	18·5 (14·1 to 24·1)	8·3 (6·4 to 10·4)	3 (2·2 to 3·6)	0·7 (0·5 to 0·8)	461
Colon and rectum cancer	26·4 (20 to 34)	34·4 (25·5 to 44·7)	43·7 (32·1 to 58)	59·1 (42·7 to 78·4)	57 (43·1 to 75·6)	45·5 (33·6 to 59·3)	35·1 (26·1 to 45·4)	23·5 (17·4 to 31·6)	17·9 (13·2 to 23·1)	8 (6·1 to 10·2)	3 (2·2 to 3·7)	0·7 (0·5 to 0·9)	414
Liver cancer	8·6 (5·9 to 12·1)	11·8 (8·1 to 16·7)	15 (9·7 to 21·9)	20·5 (14·7 to 28)	19·8 (14·3 to 27)	17·6 (12·6 to 23·3)	13·8 (9·8 to 19·2)	9·6 (6·9 to 13·1)	5·9 (4·1 to 8·1)	1·8 (1·3 to 2·6)	0·5 (0·4 to 0·7)	0·1 (0·1 to 0·1)	146
Liver cancer due to hepatitis B	4·8 (2·7 to 7)	5·8 (3·3 to 9·3)	6·5 (3·4 to 11·1)	8 (4·8 to 12·5)	6·7 (3·6 to 11·1)	4·8 (2·4 to 8·2)	3·2 (1·4 to 5·8)	2 (1 to 3·5)	1·1 (0·5 to 2)	0·3 (0·2 to 0·6)	0·1 (0 to 0·2)	0 (0 to 0)	54·9
Liver cancer due to hepatitis C	0·5 (0·2 to 0·9)	0·9 (0·4 to 1·8)	1·5 (0·6 to 3)	2·5 (1·1 to 5)	2·8 (1·2 to 5·2)	2·6 (1·3 to 4·5)	2·3 (1·1 to 4·1)	2 (0·9 to 3·5)	1·5 (0·8 to 2·6)	0·6 (0·3 to 0·9)	0·2 (0·1 to 0·3)	0 (0 to 0·1)	18
Liver cancer due to alcohol use	2·5 (1·2 to 4·3)	4 (1·9 to 6·8)	5·5 (2·7 to 9·4)	7·9 (4·2 to 12·8)	8·4 (4·8 to 13·2)	8·5 (5·3 to 12·8)	6·9 (4·1 to 10·5)	4·4 (2·7 to 6·8)	2·4 (1·4 to 3·8)	0·7 (0·4 to 1)	0·2 (0·1 to 0·3)	0 (0 to 0)	54·7
Liver cancer due to NASH	0·4 (0·2 to 0·8)	0·6 (0·3 to 1·2)	0·8 (0·4 to 1·6)	1·2 (0·6 to 2·1)	1·2 (0·6 to 2·2)	1·1 (0·6 to 2·1)	1 (0·5 to 1·9)	0·9 (0·5 to 1·5)	0·6 (0·3 to 1·1)	0·2 (0·1 to 0·3)	0·1 (0 to 0·1)	0 (0 to 0)	9·4
Liver cancer due to other causes	0·4 (0·2 to 0·8)	0·6 (0·3 to 1·1)	0·7 (0·3 to 1·4)	0·8 (0·4 to 1·6)	0·7 (0·3 to 1·3)	0·6 (0·3 to 1)	0·4 (0·2 to 0·8)	0·3 (0·2 to 0·6)	0·2 (0·1 to 0·4)	0·1 (0 to 0·1)	0 (0 to 0)	0 (0 to 0)	9·4
Gallbladder and biliary tract cancer	2·1 (1·6 to 3·5)	2·9 (2·2 to 4·6)	4·1 (2·9 to 6·6)	5·4 (4 to 8·6)	5·3 (3·8 to 8·7)	4·4 (3·1 to 7·5)	3·4 (2·5 to 6·1)	2·1 (1·5 to 3·9)	1·5 (1·1 to 2·6)	0·7 (0·5 to 1·3)	0·3 (0·2 to 0·5)	0·1 (0 to 0·1)	35·6
Pancreatic cancer	15·4 (11 to 20·4)	22·5 (16·6 to 29·8)	27 (20·7 to 35·1)	35 (26·8 to 46·3)	29·9 (21·8 to 39·7)	23·1 (17 to 30·9)	17·6 (13 to 23·7)	10·6 (7·7 to 14·1)	7·3 (5·4 to 9·7)	3 (2·2 to 3·7)	1 (0·7 to 1·2)	0·2 (0·2 to 0·3)	212
Larynx cancer	5·8 (4·3 to 7·8)	11·8 (8·3 to 16·6)	17·8 (13·5 to 23·5)	22·8 (17·1 to 29·1)	19·4 (15·2 to 25)	14·4 (10·7 to 18·5)	9·4 (7·2 to 11·9)	5·3 (3·9 to 7)	3·5 (2·6 to 4·6)	1·4 (1 to 1·8)	0·4 (0·3 to 0·6)	0·1 (0·1 to 0·1)	117
Tracheal, bronchus, and lung cancer	37·8 (27·6 to 49·8)	66·3 (47·8 to 89·8)	88·4 (61·7 to 121)	124 (89·4 to 169)	120 (88·4 to 157)	89 (66·3 to 118)	66 (49·5 to 84·9)	38 (29·4 to 49·8)	23·4 (17·3 to 30·6)	9·7 (7·1 to 13)	3 (2·3 to 3·8)	0·7 (0·5 to 0·8)	715
Malignant skin melanoma	2·8 (1·6 to 4·4)	2·7 (1·5 to 4·4)	3 (1·8 to 4·7)	3·3 (2 to 5·7)	2·1 (1·3 to 3·4)	1·5 (1 to 2·5)	1·2 (0·7 to 1·9)	0·8 (0·5 to 1·3)	0·6 (0·4 to 1·1)	0·3 (0·2 to 0·5)	0·1 (0·1 to 0·2)	0 (0 to 0·1)	26·2
Non-melanoma skin cancer	2·5 (1·7 to 3·5)	3 (2·1 to 4·2)	3·8 (2·7 to 5·2)	4·3 (3 to 5·9)	4·2 (3·3 to 5·5)	3·7 (2·8 to 5)	2·9 (2·1 to 3·9)	2·2 (1·6 to 3)	2·1 (1·6 to 2·8)	1·3 (1 to 1·8)	0·8 (0·6 to 1·1)	0·3 (0·2 to 0·4)	36·8
Non-melanoma skin cancer (squamous-cell carcinoma)	2·5 (1·7 to 3·5)	3 (2·1 to 4·2)	3·8 (2·7 to 5·2)	4·3 (3 to 5·9)	4·2 (3·3 to 5·5)	3·7 (2·8 to 5)	2·9 (2·1 to 3·9)	2·2 (1·6 to 3)	2·1 (1·6 to 2·8)	1·3 (1 to 1·8)	0·8 (0·6 to 1·1)	0·3 (0·2 to 0·4)	36·8
Non-melanoma skin cancer (basal-cell carcinoma)	0 (0 to 0)	0 (0 to 0)	0 (0 to 0)	0 (0 to 0)	0 (0 to 0)	0 (0 to 0)	0 (0 to 0)	0 (0 to 0)	0 (0 to 0)	0 (0 to 0)	0 (0 to 0)	0 (0 to 0)	0
Breast cancer	0·8 (0·5 to 1·2)	2 (1·3 to 3)	2·3 (1·6 to 3·2)	2·7 (1·8 to 3·9)	2·4 (1·5 to 3·5)	2·1 (1·5 to 3)	1·4 (0·9 to 2)	0·9 (0·6 to 1·2)	0·7 (0·5 to 1·1)	0·6 (0·4 to 0·9)	0·2 (0·2 to 0·3)	0·1 (0 to 0·1)	18·3
Cervical cancer	--	--	--	--	--	--	--	--	--	--	--	--	NA
Uterine cancer	--	--	--	--	--	--	--	--	--	--	--	--	NA
Ovarian cancer	--	--	--	--	--	--	--	--	--	--	--	--	NA
Prostate cancer	4·8 (2·7 to 7)	13·9 (8·6 to 19·7)	33 (22·1 to 45·9)	64·5 (42·3 to 88)	112 (78·3 to 153)	125 (87·5 to 172)	129 (94·8 to 183)	121 (88·9 to 166)	105 (78·1 to 145)	58·2 (41·7 to 79·9)	23·9 (16·5 to 34·9)	6·2 (4 to 8·8)	803
Testicular cancer	1·4 (0·8 to 2)	0·8 (0·5 to 1·2)	0·6 (0·4 to 0·8)	0·4 (0·3 to 0·6)	0·3 (0·2 to 0·5)	0·3 (0·2 to 0·4)	0·2 (0·1 to 0·3)	0·2 (0·1 to 0·3)	0·1 (0·1 to 0·2)	0·1 (0 to 0·1)	0 (0 to 0)	0 (0 to 0)	23·4
Kidney cancer	6·4 (4·3 to 8·9)	8·7 (6 to 11·7)	11·8 (8·5 to 15·5)	14·3 (10·2 to 19)	11·9 (8·7 to 15·8)	8·9 (6·4 to 11·9)	6·1 (4·4 to 8·2)	3·4 (2·4 to 4·5)	2·1 (1·4 to 2·8)	0·7 (0·5 to 1)	0·3 (0·2 to 0·4)	0·1 (0 to 0·1)	109
Bladder cancer	2·2 (1·7 to 2·9)	3·6 (2·5 to 5)	5·8 (4·3 to 7·7)	9·2 (7 to 12)	11 (8·3 to 14·1)	10 (7·5 to 13·5)	9·2 (6·8 to 12·4)	6·7 (5·1 to 8·9)	5·2 (3·9 to 6·8)	2·7 (2 to 3·5)	1·1 (0·8 to 1·4)	0·3 (0·2 to 0·4)	70·2
Brain and central nervous system cancer	11·3 (4·9 to 16·7)	11·2 (4·9 to 16·4)	11·5 (5·3 to 16)	12·1 (5·7 to 17·3)	9·2 (4·2 to 13)	6·7 (2·9 to 9·6)	4 (1·8 to 5·7)	2·1 (0·9 to 3·1)	1·1 (0·5 to 1·7)	0·4 (0·2 to 0·6)	0·1 (0·1 to 0·2)	0 (0 to 0)	157
Thyroid cancer	1 (0·6 to 1·5)	1·6 (1·1 to 2·2)	1·9 (1·3 to 2·6)	2·1 (1·4 to 3)	1·6 (1·1 to 2·2)	1·1 (0·8 to 1·6)	0·8 (0·6 to 1·1)	0·5 (0·4 to 0·6)	0·4 (0·3 to 0·5)	0·1 (0·1 to 0·2)	0·1 (0 to 0·1)	0 (0 to 0)	14·4
Mesothelioma	2·7 (1·9 to 3·6)	4·4 (3·2 to 6)	5·2 (3·8 to 6·9)	6·9 (5·2 to 9)	2·8 (2·1 to 3·7)	1·8 (1·4 to 2·3)	1·6 (1·2 to 2)	1·4 (1 to 1·8)	1 (0·7 to 1·3)	0·4 (0·3 to 0·6)	0·1 (0·1 to 0·1)	0 (0 to 0)	35·8
Hodgkin lymphoma	4·2 (2·8 to 6)	3 (2 to 4·4)	2·9 (1·9 to 4·1)	2·6 (1·8 to 3·8)	2 (1·4 to 2·8)	1·5 (1·1 to 2·1)	0·9 (0·6 to 1·2)	0·5 (0·4 to 0·8)	0·3 (0·2 to 0·4)	0·1 (0·1 to 0·2)	0 (0 to 0)	0 (0 to 0)	51·9
Non-Hodgkin lymphoma	11·6 (8·3 to 15·8)	12·1 (8·5 to 16·8)	12·1 (8·9 to 16)	13·7 (9·7 to 18·9)	10·7 (8·1 to 14)	8·2 (6·2 to 10·7)	5·8 (4·3 to 7·5)	3·4 (2·5 to 4·5)	1·9 (1·4 to 2·6)	0·7 (0·5 to 0·8)	0·2 (0·1 to 0·3)	0 (0 to 0·1)	162
Multiple myeloma	1·7 (1·2 to 2·4)	3·2 (2·2 to 4·5)	4·8 (3·3 to 6·6)	5·9 (4·2 to 8·4)	5·5 (3·9 to 7·5)	4·8 (3·5 to 6·5)	3·3 (2·4 to 4·6)	2 (1·5 to 2·9)	1·3 (0·9 to 1·8)	0·5 (0·3 to 0·7)	0·1 (0·1 to 0·2)	0 (0 to 0)	35·1
Leukemia	14·2 (10·2 to 18·6)	14 (10·2 to 18·1)	13·3 (9·9 to 17·2)	13·9 (10·2 to 18·6)	12·2 (9·3 to 15·5)	10·7 (8 to 13·9)	7·7 (5·9 to 10·3)	5·2 (3·9 to 6·7)	3·5 (2·5 to 4·6)	1·6 (1·2 to 2·1)	0·6 (0·4 to 0·7)	0·1 (0·1 to 0·2)	284
Acute lymphoid leukemia	1·3 (0·7 to 2)	0·8 (0·5 to 1·3)	0·7 (0·4 to 1·1)	0·7 (0·4 to 1·1)	0·4 (0·2 to 0·7)	0·5 (0·3 to 0·8)	0·3 (0·2 to 0·5)	0·1 (0·1 to 0·2)	0·1 (0·1 to 0·2)	0 (0 to 0·1)	0 (0 to 0)	0 (0 to 0)	69·1
Chronic lymphoid leukemia	0·6 (0·3 to 1·1)	0·5 (0·3 to 0·8)	1·1 (0·7 to 1·8)	1·8 (1·1 to 2·9)	1·8 (1·1 to 2·9)	1·8 (1·1 to 2·8)	1·2 (0·7 to 1·9)	0·8 (0·4 to 1·3)	0·6 (0·3 to 0·9)	0·3 (0·1 to 0·4)	0·1 (0·1 to 0·2)	0 (0 to 0)	12·8
Acute myeloid leukemia	1·2 (0·6 to 2·1)	1·3 (0·7 to 2·3)	1·2 (0·6 to 2·1)	1·1 (0·6 to 1·9)	0·8 (0·4 to 1·4)	0·7 (0·4 to 1·1)	0·4 (0·2 to 0·7)	0·2 (0·1 to 0·5)	0·1 (0·1 to 0·2)	0 (0 to 0·1)	0 (0 to 0)	0 (0 to 0)	26·7

Chronic myeloid leukemia	0 (0 to 0)	0 (0 to 0)	0·2 (0·1 to 0·3)	0·4 (0·2 to 0·6)	0·2 (0·1 to 0·3)	0·2 (0·1 to 0·4)	0·6 (0·2 to 1)	1·1 (0·5 to 2·1)	1·9 (0·9 to 3·5)	1·6 (0·8 to 2·8)	1·5 (0·9 to 2·5)	0·9 (0·4 to 1·5)	0·9 (0·5 to 1·6)	0·9 (0·5 to 1·6)	0·9 (0·5 to 1·6)	0·9 (0·5 to 1·5)	0·7 (0·4 to 1·2)	0·4 (0·2 to 0·7)	0·3 (0·2 to 0·5)	0·2 (0·1 to 0·3)	0·1 (0·1 to 0·1)	0 (0 to 0)	0 (0 to 0)	13·9
Other leukemia	0·5 (0·3 to 0·8)	0·6 (0·1 to 1)	3 (1·7 to 4·7)	5·8 (3·2 to 9·3)	8·1 (4·5 to 13)	11·3 (6·7 to 17·1)	13 (8·2 to 19·4)	15·2 (9·8 to 21·4)	13·5 (9·3 to 19·1)	12·5 (8·1 to 17·6)	10·2 (6·8 to 14·3)	10·3 (7·4 to 13·9)	10·4 (7·5 to 13·7)	9·3 (6·8 to 12·4)	9·4 (6·7 to 12·6)	8·3 (6 to 10·9)	7 (5·2 to 9·4)	5·5 (4 to 7·3)	3·7 (2·7 to 5)	2·6 (1·9 to 3·4)	1·2 (0·9 to 1·7)	0·4 (0·3 to 0·6)	0·1 (0·1 to 0·1)	162
Other malignant neoplasms	0·8 (0·5 to 1·2)	0·9 (0·7 to 1·3)	6·1 (4·2 to 8·6)	18·4 (13·1 to 26)	13·4 (10·1 to 17·5)	15·6 (12·2 to 19·3)	24·8 (18·4 to 32)	23·2 (16·9 to 30)	19·6 (15 to 24·9)	17·9 (13·9 to 22·4)	15·6 (12·2 to 19·8)	15·9 (12 to 20·8)	17·9 (13·4 to 22·9)	18·7 (14·2 to 23·8)	20·7 (15·3 to 26·7)	16·2 (12·4 to 21·2)	13 (10·1 to 16·6)	9·7 (7·5 to 12·3)	6·8 (5·3 to 8·5)	4·6 (3·6 to 5·8)	2·1 (1·6 to 2·9)	0·8 (0·6 to 1·1)	0·2 (0·1 to 0·3)	283
Other neoplasms	0·1 (0 to 0·1)	0·1 (0 to 0·1)	0·2 (0·1 to 0·4)	0·8 (0·4 to 1·4)	1 (0·5 to 1·5)	0·8 (0·4 to 1·3)	0·9 (0·4 to 1·4)	0·5 (0·2 to 0·9)	0·9 (0·4 to 1·5)	0·6 (0·3 to 1·1)	0·8 (0·4 to 1·3)	0·7 (0·4 to 1·2)	0·9 (0·5 to 1·4)	1·4 (0·8 to 2·3)	1·8 (1·1 to 3)	1·8 (1·2 to 3·3)	1·7 (1·1 to 2·9)	1·6 (1·1 to 3·1)	1·2 (0·8 to 2·3)	0·8 (0·5 to 1·6)	0·4 (0·3 to 0·8)	0·2 (0·1 to 0·3)	0 (0 to 0)	19·3
Myelodysplastic, myeloproliferative, and other hematopoietic neo	0·1 (0 to 0·1)	0·1 (0 to 0·1)	0·2 (0·1 to 0·4)	0·8 (0·4 to 1·4)	1 (0·5 to 1·5)	0·8 (0·4 to 1·3)	0·9 (0·4 to 1·4)	0·5 (0·2 to 0·9)	0·9 (0·4 to 1·5)	0·6 (0·3 to 1·1)	0·8 (0·4 to 1·3)	0·7 (0·4 to 1·2)	0·9 (0·5 to 1·4)	1·4 (0·8 to 2·3)	1·8 (1·1 to 3)	1·8 (1·2 to 3·3)	1·7 (1·1 to 2·9)	1·6 (1·1 to 3·1)	1·2 (0·8 to 2·3)	0·8 (0·5 to 1·6)	0·4 (0·3 to 0·8)	0·2 (0·1 to 0·3)	0 (0 to 0)	19·3
Benign and in situ intestinal neoplasms	0 (0 to 0)	0 (0 to 0)	0 (0 to 0)	0 (0 to 0)	0 (0 to 0)	0 (0 to 0)	0 (0 to 0)	0 (0 to 0)	0 (0 to 0)	0 (0 to 0)	0 (0 to 0)	0 (0 to 0)	0 (0 to 0)	0 (0 to 0)	0 (0 to 0)	0 (0 to 0)	0 (0 to 0)	0 (0 to 0)	0 (0 to 0)	0 (0 to 0)	0 (0 to 0)	0 (0 to 0)	0 (0 to 0)	0
Benign and in situ cervical and uterine neoplasms	0 (0 to 0)	0 (0 to 0)	0 (0 to 0)	0 (0 to 0)	0 (0 to 0)	0 (0 to 0)	0 (0 to 0)	0 (0 to 0)	0 (0 to 0)	0 (0 to 0)	0 (0 to 0)	0 (0 to 0)	0 (0 to 0)	0 (0 to 0)	0 (0 to 0)	0 (0 to 0)	0 (0 to 0)	0 (0 to 0)	0 (0 to 0)	0 (0 to 0)	0 (0 to 0)	0 (0 to 0)	0 (0 to 0)	0
Other benign and in situ neoplasms	0 (0 to 0)	0 (0 to 0)	0 (0 to 0)	0 (0 to 0)	0 (0 to 0)	0 (0 to 0)	0 (0 to 0)	0 (0 to 0)	0 (0 to 0)	0 (0 to 0)	0 (0 to 0)	0 (0 to 0)	0 (0 to 0)	0 (0 to 0)	0 (0 to 0)	0 (0 to 0)	0 (0 to 0)	0 (0 to 0)	0 (0 to 0)	0 (0 to 0)	0 (0 to 0)	0 (0 to 0)	0 (0 to 0)	0
Cardiovascular diseases	5·8 (3·8 to 8·5)	4·4 (2·8 to 6·3)	18·7 (12 to 27)	12·7 (9·6 to 16·9)	18·6 (14·1 to 24·1)	33·3 (25·6 to 42·7)	90·9 (71·6 to 112)	120 (93·8 to 149)	150 (117 to 186)	218 (169 to 270)	265 (205 to 336)	367 (293 to 455)	514 (416 to 620)	618 (510 to 737)	838 (701 to 1,000)	811 (684 to 953)	720 (612 to 836)	607 (527 to 703)	471 (409 to 538)	387 (331 to 440)	237 (197 to 269)	109 (86·5 to 126)	37·4 (28 to 43·7)	6,650
Rheumatic heart disease	0 (0 to 0)	0 (0 to 0)	0 (0 to 0)	0·9 (0·5 to 1·5)	4·2 (2·4 to 6·8)	10·4 (6·6 to 16·4)	18 (12·4 to 26·5)	17·8 (12·3 to 25·5)	17·1 (11·7 to 23·5)	16·4 (11·5 to 22·5)	13·9 (9·8 to 19)	12·1 (8·6 to 16·6)	9·9 (6·9 to 13·6)	7·3 (5·2 to 9·8)	6·3 (4·5 to 8·5)	3·9 (2·8 to 5·1)	2·4 (1·8 to 3·1)	1·4 (1·1 to 1·9)	1 (0·7 to 1·2)	0·5 (0·4 to 0·6)	0·2 (0·2 to 0·3)	0·1 (0·1 to 0·1)	0 (0 to 0)	144
Ischemic heart disease	0 (0 to 0)	0 (0 to 0)	0 (0 to 0)	0 (0 to 0)	0 (0 to 0)	0 (0 to 0)	10·6 (7·6 to 14·1)	23 (16·2 to 30·5)	39·4 (28·8 to 51·4)	71·5 (52·5 to 93·4)	99·8 (74·2 to 131)	151 (113 to 199)	229 (174 to 293)	299 (240 to 368)	428 (350 to 526)	395 (332 to 465)	365 (312 to 428)	294 (246 to 343)	219 (190 to 252)	183 (156 to 212)	116 (96·9 to 134)	52·5 (42·1 to 60·5)	19·4 (14·6 to 22·7)	2,990
Stroke	1·5 (0·8 to 2·8)	0·7 (0·4 to 1·2)	2·8 (1·4 to 5)	2 (1·5 to 2·6)	4·4 (3·1 to 6·1)	7·5 (5·5 to 10·1)	24·2 (16·3 to 35)	35·5 (23·9 to 50·8)	40·4 (26·7 to 60·3)	61·1 (39·7 to 88·2)	72 (48·7 to 107)	101 (76 to 133)	141 (105 to 183)	171 (129 to 219)	235 (183 to 298)	245 (184 to 316)	206 (156 to 267)	186 (144 to 235)	151 (118 to 188)	116 (88·1 to 145)	65·3 (49 to 82·4)	28·5 (20·9 to 36·5)	8·5 (5·9 to 11·2)	1,910
Ischemic stroke	0 (0 to 0·1)	0·1 (0 to 0·1)	0·2 (0·1 to 0·3)	1 (0·7 to 1·3)	2·1 (1·2 to 3·5)	3 (1·8 to 4·8)	5·1 (3·4 to 7·6)	5·7 (3·9 to 8·1)	7·2 (4·7 to 10·6)	9·9 (6·6 to 14·1)	11·4 (7·7 to 16·6)	15 (10·6 to 21·1)	21·3 (15 to 28·6)	29·3 (21·3 to 39·5)	43·3 (31·1 to 59·4)	66·7 (47·1 to 89·8)	63·8 (47 to 86·9)	80·2 (58·7 to 106)	68·8 (51·5 to 90·4)	76·3 (57·5 to 97·2)	44·3 (32·7 to 56)	20 (14·5 to 26·2)	6·2 (4·2 to 8·2)	581
Intracerebral hemorrhage	0·3 (0·1 to 0·7)	0·2 (0·1 to 0·3)	1·6 (0·7 to 2·9)	0·3 (0·2 to 0·5)	1·2 (0·8 to 1·8)	2·1 (1·4 to 2·9)	11·7 (7·1 to 18)	19·1 (12·1 to 28·4)	23·5 (14·5 to 37·9)	39·7 (24·8 to 59)	47 (29·9 to 72·3)	70 (51·2 to 96)	103 (75·9 to 134)	125 (91·8 to 162)	173 (130 to 220)	166 (123 to 221)	133 (96·7 to 175)	99·7 (74·4 to 131)	77·1 (57·4 to 100)	36·4 (25·1 to 49·3)	19·2 (13·4 to 26·2)	7·7 (5·3 to 10·5)	2·1 (1·4 to 3)	1,160
Subarachnoid hemorrhage	1·2 (0·6 to 2·2)	0·5 (0·3 to 0·8)	1·1 (0·5 to 2)	0·7 (0·4 to 1)	1·1 (0·6 to 1·6)	2·4 (1·5 to 3·4)	7·4 (4·2 to 11·7)	10·6 (6·1 to 17)	9·6 (5·4 to 15·7)	11·5 (6·1 to 18·8)	13·7 (7·8 to 21·5)	16·2 (10·4 to 23·2)	17·2 (11·2 to 24·7)	16·7 (11·1 to 24)	19·1 (12·8 to 27·8)	12·2 (7·9 to 18·2)	9·4 (6·3 to 14·4)	6·5 (4·4 to 9·9)	4·9 (3·4 to 7·2)	3·4 (2·2 to 5)	1·8 (1·1 to 2·9)	0·8 (0·5 to 1·3)	0·2 (0·1 to 0·4)	168
Hypertensive heart disease	0 (0 to 0)	0 (0 to 0)	0 (0 to 0)	0 (0 to 0)	0 (0 to 0)	0 (0 to 0)	2·5 (1·2 to 3·6)	4·3 (2·1 to 6·9)	6·6 (3·1 to 11·1)	14 (7·1 to 21·2)	21 (12·1 to 31·3)	33·6 (19·9 to 49·6)	52·2 (33·1 to 75·3)	58·7 (38·9 to 83·9)	71·6 (48·4 to 101)	77·9 (54·3 to 107)	70·9 (50·6 to 97·6)	63 (44·5 to 84·3)	50·2 (36·4 to 68·1)	42·3 (31·5 to 57·3)	25·4 (18·1 to 33·8)	12·7 (8·7 to 17·1)	4·4 (2·9 to 6)	611
Non-rheumatic valvular heart disease	0 (0 to 0)	0 (0 to 0)	0 (0 to 0)	0 (0 to 0)	0 (0 to 0)	0 (0 to 0)	3 (2 to 4·3)	3·2 (2 to 4·6)	4·5 (2·6 to 6·8)	5·3 (3·3 to 7·7)	4·7 (2·9 to 6·9)	5·7 (3·5 to 8·8)	6·2 (4 to 8·9)	5·5 (3·7 to 7·9)	7·4 (5 to 10·5)	6·4 (4·3 to 8·8)	5·7 (4·1 to 7·9)	4 (2·9 to 5·4)	2·8 (2 to 3·8)	2·5 (1·8 to 3·4)	1·3 (0·9 to 1·8)	0·6 (0·4 to 0·8)	0·2 (0·1 to 0·2)	69
Non-rheumatic calcific aortic valve disease	0 (0 to 0)	0 (0 to 0)	0 (0 to 0)	0 (0 to 0)	0 (0 to 0)	0 (0 to 0)	1·4 (0·8 to 2·3)	1·2 (0·7 to 2)	1·5 (0·7 to 2·8)	1·4 (0·7 to 2·5)	1·3 (0·7 to 2·4)	1·8 (1 to 3·2)	2·3 (1·2 to 4)	2 (1·2 to 3·3)	2·8 (1·7 to 4·4)	2·6 (1·6 to 4)	2·8 (1·8 to 4·2)	2·1 (1·4 to 3)	1·5 (1 to 2·1)	1·5 (1·1 to 2·1)	0·8 (0·5 to 1·1)	0·3 (0·2 to 0·5)	0·1 (0·1 to 0·1)	27·5
Non-rheumatic degenerative mitral valve disease	0 (0 to 0)	0 (0 to 0)	0 (0 to 0)	0 (0 to 0)	0 (0 to 0)	0 (0 to 0)	1·6 (0·8 to 2·5)	1·8 (1 to 2·7)	2·9 (1·6 to 4·7)	3·8 (2·2 to 5·7)	3·3 (2 to 5)	3·8 (2·2 to 6·2)	3·8 (2·3 to 5·8)	3·4 (2·1 to 5·1)	4·5 (2·8 to 6·9)	3·7 (2·3 to 5·5)	2·9 (1·9 to 4·3)	2 (1·3 to 2·8)	1·3 (0·8 to 1·9)	0·9 (0·6 to 1·4)	0·5 (0·3 to 0·8)	0·3 (0·2 to 0·4)	0·1 (0 to 0·1)	40·5
Other non-rheumatic valve diseases	0 (0 to 0)	0 (0 to 0)	0 (0 to 0)	0 (0 to 0)	0 (0 to 0)	0 (0 to 0)	0·1 (0 to 0·1)	0·2 (0·1 to 0·5)	0·1 (0 to 0·1)	0·2 (0·1 to 0·4)	0·1 (0 to 0·2)	0·1 (0 to 0·1)	0 (0 to 0·1)	0·1 (0 to 0·2)	0 (0 to 0·1)	0 (0 to 0·1)	0 (0 to 0·1)	0 (0 to 0·1)	0 (0 to 0)	0 (0 to 0·1)	0 (0 to 0)	0 (0 to 0)	0 (0 to 0)	1
Cardiomyopathy and myocarditis	1·6 (1 to 2·6)	1·7 (1·1 to 2·4)	8·7 (5·7 to 12·7)	4·8 (3·2 to 7·1)	3·6 (2·6 to 4·7)	5·1 (3·7 to 6·9)	13·9 (9 to 19·3)	15·4 (9·4 to 21·9)	19·6 (10·5 to 28·5)	24·7 (13·1 to 36·6)	28·5 (17·8 to 42·6)	33·5 (20·9 to 49·1)	40·6 (24·6 to 57·2)	38·4 (24·5 to 55·1)	41·5 (27·7 to 57·9)	33 (22·2 to 46)	24·8 (16·7 to 34·2)	17·7 (12·1 to 24·8)	11·5 (7·8 to 16·4)	8·4 (5·6 to 12)	5·3 (3·5 to 7·7)	2·2 (1·4 to 3·2)	0·6 (0·4 to 1)	385
Myocarditis	0·3 (0·1 to 0·5)	0·4 (0·2 to 0·7)	2 (1 to 3·5)	1·1 (0·6 to 2)	0·8 (0·4 to 1·2)	0·7 (0·4 to 1·2)	2·1 (1 to 3·6)	1·6 (0·8 to 2·8)	2 (1 to 3·5)	2·5 (1·3 to 4·5)	2·3 (1·1 to 3·8)	2·5 (1·3 to 4·3)	1·8 (0·9 to 3·3)	2·1 (1·1 to 3·7)	1·6 (0·8 to 3)	1·2 (0·6 to 2·2)	1·2 (0·6 to 2·1)	0·6 (0·3 to 1)	0·4 (0·2 to 0·9)	0·3 (0·1 to 0·5)	0·2 (0·1 to 0·3)	0·1 (0 to 0·1)	0·1 (0 to 0·1)	27·9
Alcoholic cardiomyopathy	0 (0 to 0)	0 (0 to 0)	0 (0 to 0)	0 (0 to 0)	0 (0 to 0)	0 (0 to 0)	0·3 (0·1 to 0·5)	0·4 (0·2 to 0·8)	0·9 (0·3 to 1·9)	2·4 (0·9 to 4·8)	2·9 (1·4 to 5·3)	4·5 (2·3 to 7·8)	7·4 (3·5 to 12·1)	7·3 (3·9 to 12·2)	7·3 (3·8 to 11·9)	5·4 (2·9 to 9·4)	4·2 (2·3 to 7·1)	1·9 (0·9 to 3·5)	1·1 (0·5 to 2·1)	0·2 (0·1 to 0·4)	0·1 (0 to 0·2)	0 (0 to 0·1)	0 (0 to 0)	46·6
Other cardiomyopathy	1·4 (0·8 to 2·2)	1·3 (0·8 to 1·9)	6·7 (4·3 to 10)	3·7 (2·3 to 5·7)	2·8 (2 to 3·9)	4·4 (3·1 to 6)	11·5 (7·3 to 16·3)	13·3 (7·7 to 19)	16·6 (9 to 24·1)	19·8 (10·2 to 29·4)	23·3 (14·2 to 35)	26·6 (16·5 to 39·3)	31·3 (19·1 to 45·4)	29 (18·4 to 41·7)	32·6 (21·4 to 45·9)	26·4 (17·7 to 37)	19·3 (12·9 to 26·9)	15·2 (10·4 to 21·5)	10 (6·8 to 14·3)	7·9 (5·2 to 11·2)	5·1 (3·3 to 7·4)	2·1 (1·3 to 3)	0·6 (0·3 to 0·9)	311
Atrial fibrillation and flutter	0 (0 to 0)	0 (0 to 0)	0 (0 to 0)	0 (0 to 0)	0 (0 to 0)	0 (0 to 0)	0 (0 to 0)	0 (0 to 0)	0 (0 to 0)	0·7 (0·3 to 1·1)	0·7 (0·4 to 1)	2·3 (1·4 to 3·5)	3·9 (2·5 to 5·6)	5·7 (3·9 to 8·4)	9·1 (6·5 to 12·6)	11·1 (8·1 to 15·2)	12·3 (9·3 to 16·5)	13·7 (10·1 to 18·2)	13·6 (10·3 to 18)	15·6 (11·8 to 20·6)	12 (8·8 to 16·6)	7·9 (5·7 to 11)	2·7 (1·8 to 3·9)	111
Aortic aneurysm	--	--	--	--	--	--	0·6 (0·4 to 0·9)	0·5 (0·3 to 0·7)	0·8 (0·5 to 1·2)	1·2 (0·8 to 1·8)	1·6 (1·1 to 2·4)	2·1 (1·3 to 3)	2·5 (1·7 to 3·6)	3·4 (2·4 to 4·7)	6 (4·2 to 8·3)	7·2 (5 to 9·9)	7 (4·8 to 9·8)	6·2 (4·4 to 8·5)	4·8 (3·3 to 6·8)	3·5 (2·4 to 4·8)	1·7 (1·2 to 2·3)	0·6 (0·4 to 0·8)	0·1 (0·1 to 0·2)	NA
Endocarditis	0·1 (0·1 to 0·2)	0·2 (0·1 to 0·3)	0·7 (0·4 to 1·3)	0·6 (0·4 to 1·1)	0·4 (0·2 to 0·6)	0·8 (0·4 to 1·3)	1·9 (1·1 to 3·1)	2·5 (1·3 to 4)	2·7 (1·4 to 4·5)	3·7 (1·8 to 6)	3·3 (1·9 to 5·3)	3·9 (2·3 to 6·3)	3·4 (2 to 5·5)	3·1 (1·9 to 4·7)	2·7 (1·6 to 4)	2 (1·3 to 3·2)	1·5 (0·9 to 2·3)	1 (0·7 to 1·7)	0·6 (0·4 to 1)	0·5 (0·3 to 0·8)	0·3 (0·2 to 0·4)	0·1 (0·1 to 0·2)	0 (0 to 0·1)	36·1
Other cardiovascular and circulatory diseases	2·5 (1·3 to 4·2)	1·8 (1 to 2·8)	6·4 (3·5 to 10·4)	4·4 (2·9 to 6·3)	6 (3·5 to 9·7)	9·4 (6 to 13·9)	16·2 (11·4 to 22·2)	17·3 (12·2 to 23·1)	19·2 (13·2 to 26·5)	19·1 (12·5 to 26·4)	19·7 (13·1 to 27·4)	21 (14·6 to 29·5)	24·4 (16·9 to 33·5)	25·1 (17·7 to 34·2)	29·2 (21 to 38·9)	27 (20·5 to 34·9)	21·8 (16·9 to 27·5)	17·2 (13·2 to 22)	14·2 (10·9 to 18·1)	12·5 (9·2 to 16·4)	7·9 (5·7 to 10·4)	3·6 (2·6 to 4·6)	1·1 (0·7 to 1·5)	327
Chronic respiratory diseases	0 (0 to 0)	0 (0 to 0)	0 (0 to 0)	146 (91·7 to 233)	123 (76·8 to 194)	103 (66·4 to 156)	92·7 (64·8 to 131)	81·6 (60·1 to 109)	85 (64·5 to 110)	82·3 (63·7 to 106)	75·9 (58 to 96·6)	76·8 (60·2 to 95·7)	88·8 (68·8 to 113)	111 (87·6 to 140)	149 (114 to 191)	162 (125 to 204)	148 (117 to 185)	142 (112 to 177)	106 (83·5 to 130)	91·6 (71·9 to 113)	44·3 (34·8 to 54·9)	19·8 (15·3 to 24·6)	6·7 (4·8 to 8·3)	1,930
Chronic obstructive pulmonary disease	0 (0 to 0)	0 (0 to 0)	0 (0 to 0)	3·8 (2·5 to 5·5)	2·7 (1·7 to 4)	4 (2·1 to 6·3)	9·4 (6·1 to 13·3)	14·5 (9·5 to 20·5)	18·8 (12·8 to 26·9)	18·9 (12·5 to 26·8)	21·5 (14 to 29·8)	25·9 (18·1 to 35)	40 (28·6 to 53·8)	58·1 (43·7 to 76·2)	92·1 (69·2 to 121)	114 (85·8 to 145)	109 (83·7 to 140)	108 (84·5 to 137)	82·8 (64·5 to 104)	74·2 (58·3 to 92·9)	36·4 (28·3 to 45·8)	16·4 (12·6 to 20·5)	5·6 (4 to 6·9)	856
Pneumoconiosis	0 (0 to 0)	0 (0 to 0)	0 (0 to 0)	0 (0 to 0)	0 (0 to 0)	0 (0 to 0)	2·8 (1·7 to 4·2)	0·5 (0·2 to 0·9)	1·9 (1·1 to 3·3)	0·3 (0·2 to 0·5)	0·6 (0·4 to 1)	0·3 (0·2 to 0·5)	0·9 (0·6 to 1·4)	0·9 (0·5 to 1·4)	0·8 (0·5 to 1·4)	1 (0·6 to 1·6)	1·2 (0·8 to 2)	0·7 (0·4 to 1)	0·5 (0·3 to 0·7)	0·6 (0·4 to 0·9)	0·4 (0·2 to 0·6)	0·2 (0·1 to 0·3)	0 (0 to 0)	13·7
Silicosis	0 (0 to 0)	0 (0 to 0)	0 (0 to 0)	0 (0 to 0)	0 (0 to 0)	0 (0 to 0)	0·1 (0 to 0·1)	0 (0 to 0)	0·8 (0·4 to 1·5)	0 (0 to 0)	0 (0 to 0)	0 (0 to 0)	0·1 (0 to 0·2)	0 (0 to 0)	0 (0 to 0·1)	0 (0 to 0)	0 (0 to 0·1)	0 (0 to 0)	0 (0 to 0·1)	0 (0 to 0·1)	0 (0 to 0·1)	0 (0 to 0)	0 (0 to 0)	1·2
Asbestosis	0 (0 to 0)	0 (0 to 0)	0 (0 to 0)	0 (0 to 0)	0 (0 to 0)	0 (0 to 0)	0·3 (0·1 to 0·6)	0 (0 to 0·1)	0·1 (0 to 0·1)	0 (0 to 0)	0 (0 to 0)	0 (0 to 0)	0 (0 to 0)	0 (0 to 0)	0 (0 to 0·1)	0 (0 to 0)	0 (0 to 0)	0 (0 to 0)	0 (0 to 0)	0 (0 to 0·1)	0 (0 to 0·1)	0 (0 to 0)	0 (0 to 0)	0·5
Coal workers pneumoconiosis	0 (0 to 0)	0 (0 to 0)	0 (0 to 0)	0 (0 to 0)	0 (0 to 0)	0 (0 to 0)	2·2 (1·3 to 3·4)	0·4 (0·2 to 0·7)	0·9 (0·4 to 1·6)	0·3 (0·2 to 0·4)	0·6 (0·4 to 1)	0·3 (0·1 to 0·4)	0·7 (0·4 to 1·1)	0·8 (0·5 to 1·3)	0·7 (0·4 to 1·2)	0·9 (0·5 to 1·6)	1·1 (0·7 to 1·8)	0·6 (0·4 to 0·9)	0·4 (0·2 to 0·6)	0·5 (0·3 to 0·8)	0·3 (0·2 to 0·4)	0·2 (0·1 to 0·3)	0 (0 to 0)	10·8
Other pneumoconiosis	0 (0 to 0)	0 (0 to 0)	0 (0 to 0)	0 (0 to 0)	0 (0 to 0)	0 (0 to 0)	0·3 (0·1 to 0·6)	0·1 (0 to 0·2)	0·1 (0·1 to 0·3)	0 (0 to 0·1)	0 (0 to 0)	0·1 (0 to 0·2)	0·1 (0 to 0·2)	0 (0 to 0)	0·1 (0 to 0·2)	0 (0 to 0)	0·1 (0 to 0·2)	0 (0 to 0·1)	0·1 (0 to 0·1)	0·1 (0 to 0·1)	0 (0 to 0·1)	0 (0 to 0)	0 (0 to 0)	1·1
Asthma	0 (0 to 0)	0 (0 to 0)	0 (0 to 0)	111 (58·1 to 199)	113 (67·6 to 184)	89·7 (53·1 to 140)	65 (39·4 to 103)	47·3 (31 to 68·3)	44 (30·4 to 61·9)	36·9 (25·9 to 51·5)	28·8 (20·3 to 40·2)	22·7 (16·2 to 31)	21 (14·9 to 29)	20·2 (14·5 to 27·9)	19·9 (13·6 to 28·1)	18·3 (12·9 to 25·7)	12·3 (8·9 to 17)	9·1 (6·2 to 13·1)	5·2 (3·7 to 7·3)	4·6 (3 to 6·8)	1·8 (1·2 to 2·6)	0·9 (0·6 to 1·3)	0·3 (0·2 to 0·5)	672
Interstitial lung disease and pulmonary sarcoidosis	0 (0 to 0)	0 (0 to 0)	0 (0 to 0)	16·3 (9·7 to 25·7)	2·2 (1·2 to 3·5)	2·8 (1·5 to 4·5)	9·4 (5·2 to 14·9)	11·9 (5·8 to 19·8)	9·6 (4·4 to 17)	17·9 (9·1 to 28·9)	17·4 (10 to 27·3)	21·9 (13·3 to 32·5)	20·6 (12·6 to 30·7)	25·6 (15·8 to 37·4)	30·2 (18·1 to 46·5)	27·1 (16·4 to 41·9)	24·1 (14·5 to 34·9)	22·9 (13·6 to 32·9)	16·6 (10·2 to 25·1)	11·5 (6·5 to 18·4)	5·3 (3 to 8)	2·2 (1·2 to 3·3)	0·7 (0·4 to 1·1)	296
Other chronic respiratory diseases	0 (0 to 0)	0 (0 to 0)	0 (0 to 0)	14·7 (8·6 to 24·1)	5 (3·3 to 7·3)	7 (4 to 10·5)	6·1 (3·7 to 9·5)	7·4 (4·5 to 10·8)	10·7 (6·7 to 16·4)	8·3 (5 to 12·7)	7·6 (4·8 to 11·3)	6 (3·8 to 9)	6·3 (4 to 9·4)	6 (3·7 to 9·2)	5·6 (3·4 to 8·8)	1·8 (1 to 3·1)	1·3 (0·7 to 2·2)	1·1 (0·7 to 1·8)	0·8 (0·4 to 1·4)	0·7 (0·4 to 1·2)	0·4 (0·2 to 0·7)	0·2 (0·1 to 0·3)	0·1 (0 to 0·1)	96·9
Digestive diseases	4·4 (3 to 6·4)	5·6 (3·4 to 8·8)	38·5 (22·5 to 59·4)	49 (35·3 to 66·6)	39·3 (28·3 to 54)	37·8 (28·4 to 49·3)	63·9 (49·9 to 81·6)	92·1 (70·9 to 119)	129 (98·3 to 165)	172 (131 to 219)	207 (161 to 263)	250 (200 to 310)	316 (249 to 385)	323 (261 to 393)	330 (266 to 401)	239 (195 to 290)	160 (132 to 195)	113 (93·1 to 137)	67 (54·3 to 81·3)	50·5 (40·2 to 61·2)	23·6 (17·9 to 29·3)	9·6 (7·5 to 11·5)	2·9 (2·1 to 3·5)	2,720
Cirrhosis and other chronic liver diseases	0 (0 to 0)	0 (0 to 0)	0 (0 to 0)	7·9 (4·8 to 12·4)	6 (4·1 to 8·5)	7·8 (5·4 to 10·5)	18·8 (12·3 to 26·6)	28·1 (19·6 to 38·7)	49·6 (34·5 to 68·9)	82·2 (58·5 to 110)	115 (82·9 to 151)	158 (120 to 202)	220 (171 to 276)	230 (177 to 291)	234 (184 to 293)	161 (127 to 202)	98·8 (77·9 to 124)	63·8 (49·4 to 79·5)	32·4 (24·6 to 41·4)	22·2 (16·6 to 28·4)	8·9 (6·5 to 11·7)	3·1 (2·3 to 4·1)	1 (0·6 to 1·3)	1,550
Cirrhosis and other chronic liver diseases due to hepatitis B	0 (0 to 0)	0 (0 to 0)	0 (0 to 0)	0 (0 to 0)	0 (0 to 0·1)	0·1 (0 to 0·2)	0·3 (0·1 to 0·7)	1·4 (0·5 to 2·8)	3·7 (1·3 to 7·5)	7·7 (3·8 to 13·2)	12·2 (5·4 to 22·7)	16·9 (8·4 to 30)	23 (11·7 to 42·4)	22·1 (10·8 to 42)	19 (9 to 35·9)	11·4 (5·2 to 21·2)	6·3 (3·2 to 12)	4 (1·8 to 7·5)	2·1 (1 to 3·7)	1·5 (0·7 to 2·7)	0·6 (0·3 to 1)	0·2 (0·1 to 0·4)	0·1 (0 to 0·1)	133
Cirrhosis and other chronic liver diseases due to hepatitis C	0 (0 to 0)	0 (0 to 0)	0 (0 to 0)	0·1 (0 to 0·1)	0·1 (0 to 0·2)	0·3 (0·1 to 0·6)	1·1 (0·4 to 2·4)	4·7 (2·2 to 8·5)	12·5 (5·9 to 22·2)	22·1 (12·5 to 33·9)	29·8 (15·3 to 51·4)	38·9 (21·2 to 64·9)	51 (27·6 to 82·6)	49·7 (26·7 to 83·1)	47·1 (26·2 to 79)	30·7 (14·7 to 53·1)	18·3 (10·2 to 30·7)	11·5 (6·8 to 18·2)	5·7 (3·1 to 9·5)	4 (2·2 to 6·8)	1·6 (0·9 to 2·7)	0·6 (0·3 to 1)	0·2 (0·1 to 0·3)	330
Cirrhosis and other chronic liver diseases due to alcohol use	0 (0 to 0)	0 (0 to 0)	0 (0 to 0)	0 (0 to 0)	0 (0 to 0)	0 (0 to 0)	0·1 (0 to 0·4)	3·1 (1·2 to 6·3)	10 (4·4 to 18·3)	26·1 (15·1 to 40·9)	46·6 (25·6 to 72·9)	70 (43 to 100)	102 (64·4 to 145)	111 (72·6 to 156)	119 (77·2 to 168)	83·1 (55·7 to 115)	49·3 (32·9 to 68·2)	30·1 (18·8 to 42·3)	14 (9 to 20·1)	8·8 (5·2 to 13·3)	3·4 (2·1 to 5·1)	1·1 (0·6 to 1·7)	0·3 (0·1 to 0·6)	679
Cirrhosis and other chronic liver diseases due to NAFLD	0 (0 to 0)	0 (0 to 0)	0 (0 to 0)	0 (0 to 0)	0 (0 to 0)	0 (0 to 0)	1 (0·4 to 2·1)	3·2 (1·5 to 6)	7·7 (3·3 to 14·2)	12 (6·1 to 20·8)	14·5 (6·6 to 27·7)	19·3 (8·5 to 35)	28 (13·1 to 52·3)	29·5 (13·3 to 55·1)	28·9 (13·7 to 51)	20·8 (9·5 to 37·9)	14·4 (8 to 23·3)	10·4 (5·9 to 16)	5·9 (2·9 to 9·7)	4·4 (2·3 to 7)	1·8 (1 to 3)	0·7 (0·4 to 1·2)	0·2 (0·1 to 0·4)	203
Cirrhosis and other chronic liver diseases due to other causes	0 (0 to 0)	0 (0 to 0)	0 (0 to 0)	7·9 (4·7 to 12·3)	5·9 (4 to 8·2)	7·4 (5·2 to 10)	16·2 (10·5 to 23)	15·7 (9·4 to 23·7)	15·7 (8 to 25·7)	14·3 (6·8 to 25·1)	12 (5·4 to 22)	12·4 (5·7 to 22·5)	16 (7·7 to 29·8)	17 (8·2 to 31·1)	19·3 (8·9 to 33·6)	15 (7·6 to 26·3)	10·5 (5 to 19·5)	7·8 (3·8 to 13·4)	4·6 (2·3 to 8·2)	3·5 (1·8 to 6)	1·5 (0·8 to 2·4)	0·6 (0·3 to 1)	0·2 (0·1 to 0·3)	204
Upper digestive system diseases	0 (0 to 0)	0 (0 to 0)	0 (0 to 0)	1·9 (1 to 3·1)	2·5 (1·2 to 4·4)	4 (2·3 to 6·3)	14·1 (8·5 to 23·4)	28·8 (17·2 to 49·7)	37·2 (22·4 to 63·1)	41·4 (25·8 to 69·1)	40·9 (26 to 64·4)	39·9 (25·6 to 61·2)	40·7 (26·9 to 61·5)	39·1 (26 to 58·1)	38·5 (26·8 to 55)	30·7 (21·6 to 43·6)	23·3 (16·6 to 32·6)	17·3 (12·4 to 23·4)	11·6 (8·3 to 15·6)	8·7 (6·3 to 11·6)	4·4 (3·1 to 5·8)	1·9 (1·4 to 2·5)	0·6 (0·4 to 0·7)	427
Peptic ulcer disease	0 (0 to 0)	0 (0 to 0)	0 (0 to 0)	0·2 (0·1 to 0·4)	0·2 (0·1 to 0·4)	0·7 (0·5 to 1)	2 (1·3 to 3·1)	4·2 (2·6 to 6·2)	5·4 (3·4 to 8·4)	7·4 (4·5 to 11·3)	8 (5·3 to 12·1)	8·4 (5·4 to 12·6)	9 (5·6 to 13·6)	11·6 (7·5 to 16·7)	13 (8·3 to 18·5)	11·1 (7·4 to 15·7)	8·9 (6 to 12·8)	7·3 (4·8 to 10·5)	5·3 (3·5 to 7·6)	4·4 (2·9 to 6·3)	2·3 (1·5 to 3·3)	1 (0·6 to 1·3)	0·3 (0·2 to 0·4)	111
Gastritis and duodenitis	0 (0 to 0)	0 (0 to 0)	0 (0 to 0)	1·7 (0·8 to 2·9)	2·2 (0·9 to 4)	1·9 (0·7 to 3·5)	2·9 (1·5 to 5)	3·9 (2·4 to 6·3)	5·2 (2·9 to 8·1)	6·8 (3·9 to 10·5)	7·8 (4·7 to 12·4)	8·8 (5 to 14·3)	10·8 (6·6 to 16·9)	9·3 (5·5 to 14·5)	9·7 (6·2 to 14·6)	7·5 (4·8 to 10·9)	5·6 (3·7 to 8)	3·9 (2·5 to 5·7)	2·4 (1·6 to 3·5)	1·8 (1·2 to 2·7)	0·9 (0·6 to 1·4)	0·5 (0·3 to 0·7)	0·1 (0·1 to 0·2)	93·8
Gastroesophageal reflux disease	0 (0 to 0)	0 (0 to 0)	0 (0 to 0)	0 (0 to 0)	0 (0 to 0·1)	1·5 (0·6 to 2·9)	9·2 (4·4 to 17·8)	20·7 (10·1 to 40·6)	26·6 (12·6 to 49·2)	27·2 (13·1 to 51·8)	25 (11·7 to 46·3)	22·7 (10·8 to 41·8)	20·9 (10·2 to 38)	18·2 (8·8 to 33·4)	15·8 (7·3 to 30·5)	12·1 (5·8 to 22·7)	8·8 (4·5 to 16·2)	6 (3 to 10·5)	3·9 (2 to 6·9)	2·5 (1·2 to 4·4)	1·2 (0·6 to 2·1)	0·5 (0·2 to 0·8)	0·1 (0·1 to 0·2)	223
Appendicitis	0 (0 to 0)	0 (0 to 0)	0 (0 to 0)	2·7 (1·4 to 4·9)	2·4 (1·5 to 3·8)	3·4 (1·8 to 5·5)	4·8 (2·8 to 8)	4·7 (2·6 to 7·6)	4·1 (2·2 to 6·7)	3·5 (1·8 to 5·6)	3·3 (1·8 to 5·6)	2·6 (1·5 to 4·3)	2·3 (1·3 to 3·9)	2·1 (1·1 to 3·5)	1·8 (1 to 3)	1·3 (0·7 to 2·1)	1·2 (0·7 to 2)	0·9 (0·5 to 1·5)	0·5 (0·2 to 0·8)	0·4 (0·2 to 0·6)	0·2 (0·1 to 0·4)	0·1 (0 to 0·2)	0 (0 to 0)	42·3
Paralytic ileus and intestinal obstruction	3·3 (2·3 to 4·8)	4·6 (2·8 to 7·2)	32·3 (18·3 to 51·5)	10·6 (6·1 to 16·8)	4·8 (2·9 to 7·3)	4·4 (2·8 to 6·3)	5·1 (2·9 to 8)	5·8 (3·2 to 9·4)	5·7 (2·9 to 9·3)	5·9 (3·1 to 9·7)	7·6 (4·5 to 11·7)	7·6 (4·8 to 11·9)	8·8 (5·3 to 13·7)	10·3 (6·2 to 16·7)	11·6 (7·4 to 18)	10·2 (6·3 to 16·4)	9·3 (5·9 to 14·3)	9·2 (5·8 to 14·5)	7·1 (4·6 to 11·9)	6·4 (4·1 to 10·4)	3·4 (2·2 to 5·6)	1·5 (0·9 to 2·4)	0·5 (0·3 to 0·8)	176
Inguinal, femoral, and abdominal hernia	1·1 (0·6 to 1·8)	1 (0·4 to 1·8)	6·2 (3 to 11·5)	14·6 (8·6 to 22·2)	18·4 (9·6 to 31·6)	13 (6·9 to 22·4)	10·7 (5·6 to 18·3)	10 (5·5 to 16·6)	10·8 (6·2 to 17·8)	10·9 (6·2 to 17·9)	12·1 (6·9 to 19·4)	13 (7·8 to 20)	14·1 (8·1 to 22·6)	14·1 (8·5 to 21·7)	13·6 (8·7 to 20·2)	10·7 (6·9 to 15·7)	7·4 (4·9 to 10·5)	4·9 (3·5 to 6·8)	3 (2·2 to 4)	2·2 (1·6 to 2·9)	1·1 (0·8 to 1·5)	0·5 (0·3 to 0·6)	0·1 (0·1 to 0·2)	193
Inflammatory bowel disease	0 (0 to 0)	0 (0 to 0)	0 (0 to 0)	2·1 (1·1 to 3·7)	0·6 (0·3 to 0·9)	0·4 (0·3 to 0·7)	1·9 (1·1 to 3)	2·6 (1·6 to 4·1)	4 (2·3 to 6·7)	5·1 (3 to 8·3)	4·2 (2·6 to 6·3)	3·9 (2·5 to 5·8)	3·9 (2·4 to 5·9)	3·5 (2·2 to 5·2)	4 (2·4 to 5·9)	2·6 (1·6 to 4)	2·1 (1·4 to 3·4)	2·1 (1·4 to 3·2)	1·3 (0·8 to 2·1)	1·1 (0·7 to 1·7)	0·5 (0·3 to 0·8)	0·2 (0·2 to 0·4)	0·1 (0 to 0·1)	46
Vascular intestinal disorders	0 (0 to 0)	0 (0 to 0)	0 (0 to 0)	0·2 (0·1 to 0·3)	0·2 (0·1 to 0·2)	0·2 (0·1 to 0·3)	0·4 (0·2 to 0·6)	0·3 (0·2 to 0·4)	0·6 (0·3 to 0·8)	0·8 (0·5 to 1·3)	0·8 (0·5 to 1·2)	0·8 (0·5 to 1·1)	1·2 (0·8 to 1·8)	1·4 (0·9 to 2·1)	1·8 (1·1 to 2·7)	1·8 (1·2 to 2·5)	1·7 (1·1 to 2·3)	1·5 (1 to 2·1)	1·2 (0·8 to 1·8)	1·1 (0·8 to 1·6)	0·5 (0·3 to 0·7)	0·3 (0·2 to 0·3)	0·1 (0 to 0·1)	16·7
Gallbladder and biliary diseases	0 (0 to 0)	0 (0 to 0)	0 (0 to 0)	0·4 (0·2 to 0·5)	0·6 (0·3 to 0·9)	1·1 (0·7 to 1·7)	2·3 (1·4 to 3·3)	3·2 (1·9 to 5·1)	4·2 (2·5 to 6·4)	5·2 (3·1 to 8)	5 (3·1 to 7·4)	6·3 (4 to 9·2)	6·4 (4·2 to 9·2)	7·5 (5·1 to 10·5)	9·2 (6·3 to 13)	7·7 (5·4 to 10·7)	6·8 (4·7 to 9·4)	6·1 (4·2 to 8·5)	4·4 (3 to 6·1)	3·8 (2·6 to 5·3)	1·8 (1·2 to 2·5)	0·8 (0·5 to 1·1)	0·2 (0·2 to 0·3)	82·9
Pancreatitis	0 (0 to 0)	0 (0 to 0)	0 (0 to 0)	0·4 (0·2 to 0·6)	0·1 (0 to 0·1)	0·6 (0·4 to 0·9)	2·5 (1·5 to 4)	4·1 (2·4 to 6·3)	8·2 (4·7 to 12·8)	11·4 (6·8 to 17·7)	13 (8·4 to 20·3)	13 (8·3 to 19·5)	11·6 (7·6 to 17·8)	8·9 (5·8 to 13)	8·3 (5·4 to 12·3)	6·1 (4 to 8·8)	3·8 (2·6 to 5·5)	2·8 (1·9 to 3·9)	1·8 (1·2 to 2·5)	1·3 (0·9 to 1·8)	0·6 (0·4 to 0·8)	0·2 (0·1 to 0·3)	0·1 (0 to 0·1)	98·7
Other digestive diseases	0 (0 to 0)	0 (0 to 0)	0 (0 to 0)	8·2 (4·9 to 12·7)	3·8 (2·4 to 5·6)	2·9 (1·8 to 4·4)	3·3 (1·5 to 5·2)	4·5 (2 to 7·3)	4·8 (1·9 to 8·4)	5·3 (2·1 to 9)	5·1 (2·5 to 8·4)	5·3 (2·7 to 9)	6·8 (3·6 to 11·1)	6·5 (3·7 to 10·1)	7·4 (4·2 to 11·3)	7·3 (4·2 to 11)	5·5 (3·3 to 8·2)	4·8 (2·8 to 7·2)	3·7 (2·1 to 5·7)	3·4 (2 to 5)	2·2 (1·2 to 3·1)	1 (0·6 to 1·4)	0·3 (0·2 to 0·4)	92
Neurological disorders	0·1 (0 to 0·1)	0·2 (0 to 0·4)	17 (10·9 to 24·7)	34·9 (17 to 60·1)	60·2 (25·3 to 113)	149 (55·8 to 289)	202 (86·6 to 377)	188 (91·5 to 330)	169 (85·8 to 289)	146 (74·6 to 250)	130 (67·7 to 219)	115 (61·8 to 190)	105 (62·6 to 168)	88 (53·2 to 137)	81·5 (53·2 to 122)	72·4 (48·8 to 109)	71·3 (47·4 to 116)	76·7 (49·5 to 133)	79·9 (51·2 to 142)	101 (58·4 to 196)	74·2 (38·1 to 152)	44·4 (21 to 92·5)	17·9 (8·1 to 37·4)	2,020
Alzheimer's disease and other dementias	0 (0 to 0)	0 (0 to 0)	0 (0 to 0)	0 (0 to 0)	0 (0 to 0)	0 (0 to 0)	0 (0 to 0)	0 (0 to 0)	0 (0 to 0)	0 (0 to 0)	0 (0 to 0)	0·8 (0·2 to 2·5)	4·2 (1·2 to 11·6)	7·5 (2·5 to 19·3)	12·7 (4·5 to 30·9)	19·4 (7·6 to 46·4)	28·4 (11·1 to 69·5)	39·2 (15·9 to 93·8)	45·8 (19·2 to 109)	70·1 (28·1 to 165)	58·7 (22·7 to 136)	38·9 (15·3 to 88)	16·6 (6·7 to 36·5)	342
Parkinson's disease	0 (0 to 0)	0 (0 to 0)	0 (0 to 0)	0 (0 to 0)	0 (0 to 0)	0 (0 to 0)	0 (0 to 0)	0·2 (0·1 to 0·3)	0·4 (0·2 to 0·6)	0·3 (0·2 to 0·5)	0·6 (0·4 to 0·9)	1·5 (1·1 to 2)	3·1 (2·3 to 4)	4·6 (3·5 to 6·1)	7·1 (5·5 to 9)	10·1 (8·1 to 12·4)	14·4 (11·7 to 17·4)	20 (16·7 to 23·9)	23·6 (19·8 to 27·9)	24·6 (20·1 to 29·7)	12·4 (10·2 to 14·6)	4·3 (3·3 to 5·2)	1 (0·7 to 1·2)	128
Idiopathic epilepsy	0·1 (0 to 0·1)	0·2 (0 to 0·4)	6·2 (3·5 to 10·1)	23·9 (10·4 to 42·9)	32·2 (10·6 to 65·6)	43·5 (16·6 to 83·1)	60·5 (29·7 to 104)	63·2 (36·2 to 105)	59·8 (35·6 to 94·2)	50·7 (30·8 to 78)	43 (26·1 to 64·4)	35·2 (21·4 to 55·1)	32 (19·9 to 50·6)	22·8 (13·4 to 36·5)	19·5 (11·2 to 30·9)	13·7 (7·4 to 23)	9·7 (4·7 to 16·5)	6·6 (2·9 to 12·1)	4·7 (2 to 8·3)	3·3 (1·4 to 5·9)	1·6 (0·7 to 3)	0·7 (0·3 to 1·4)	0·2 (0·1 to 0·4)	533
Multiple sclerosis	0 (0 to 0)	0 (0 to 0)	0 (0 to 0)	0 (0 to 0)	0 (0 to 0·1)	0 (0 to 0·1)	0·2 (0·1 to 0·4)	0·7 (0·4 to 1·2)	0·7 (0·5 to 1·2)	1 (0·7 to 2)	1·4 (0·9 to 2·5)	1·5 (1 to 2·9)	1·5 (1 to 2·6)	1·2 (0·9 to 2·2)	1·3 (0·9 to 2·4)	0·8 (0·6 to 1·4)	0·5 (0·3 to 0·8)	0·3 (0·2 to 0·5)	0·2 (0·1 to 0·3)	0·1 (0·1 to 0·1)	0 (0 to 0·1)	0 (0 to 0)	0 (0 to 0)	11·6
Motor neuron disease	0 (0 to 0)	0 (0 to 0)	2·4 (1·2 to 4)	0·7 (0·4 to 1·1)	0·1 (0·1 to 0·1)	0·2 (0·2 to 0·3)	1 (0·7 to 1·4)	0·9 (0·6 to 1·3)	0·8 (0·5 to 1·1)	1 (0·7 to 1·4)	1·8 (1·3 to 2·6)	2·3 (1·6 to 3·3)	2·3 (1·6 to 3·2)	2·7 (1·8 to 3·8)	3 (2·1 to 4·1)	2·4 (1·7 to 3·2)	1·9 (1·3 to 2·6)	1 (0·7 to 1·4)	0·4 (0·3 to 0·6)	0·2 (0·1 to 0·3)	0·1 (0 to 0·1)	0 (0 to 0)	0 (0 to 0)	25·3
Headache disorders	0 (0 to 0)	0 (0 to 0)	0 (0 to 0)	0 (0 to 0)	15·9 (0·3 to 42·2)	84 (3·7 to 217)	113 (12·2 to 281)	105 (14·4 to 240)	94·6 (15·6 to 213)	83·4 (17·1 to 186)	74·6 (15·6 to 160)	65·3 (17 to 138)	54·8 (16 to 117)	42·3 (12·4 to 85·9)	31·6 (10·1 to 66·5)	21·4 (7·2 to 44·2)	13·6 (4·7 to 27·3)	7·6 (2·6 to 16)	4 (1·3 to 8·6)	2·2 (0·7 to 4·8)	1 (0·3 to 2·1)	0·3 (0·1 to 0·7)	0·1 (0 to 0·2)	814
Migraine	0 (0 to 0)	0 (0 to 0)	0 (0 to 0)	0 (0 to 0)	14·3 (0·2 to 39·1)	77·7 (2·5 to 211)	104 (7·8 to 272)	95·2 (10·2 to 232)	84·9 (10·9 to 198)	74·4 (11·2 to 169)	65·7 (11·5 to 149)	57·3 (11·8 to 130)	47·6 (11·2 to 105)	36·3 (8·4 to 79·2)	27·1 (6·8 to 59·2)	17·9 (5·2 to 38·7)	11·1 (3·3 to 23·7)	6 (1·9 to 12·7)	3·2 (0·9 to 7·1)	1·8 (0·5 to 3·8)	0·8 (0·2 to 1·8)	0·3 (0·1 to 0·6)	0·1 (0 to 0·1)	726
Tension-type headache	0 (0 to 0)	0 (0 to 0)	0 (0 to 0)	0 (0 to 0)	1·6 (0 to 11·3)	6·2 (0·2 to 49·7)	9 (1·2 to 50·7)	9·4 (1·5 to 48·2)	9·7 (1·6 to 43·3)	9 (1·6 to 38·7)	8·9 (1·8 to 37·5)	7·9 (1·8 to 30·5)	7·1 (1·9 to 24)	6 (1·5 to 20·8)	4·5 (1·3 to 14·2)	3·5 (0·9 to 10·7)	2·5 (0·6 to 8·7)	1·6 (0·3 to 5·8)	0·9 (0·1 to 3·4)	0·5 (0·1 to 1·8)	0·2 (0 to 0·8)	0·1 (0 to 0·4)	0 (0 to 0·1)	88·6
Other neurological disorders	0 (0 to 0)	0 (0 to 0)	8·4 (5·2 to 12·8)	10·3 (5·1 to 17·2)	11·9 (4·7 to 22·9)	21·5 (10·2 to 38·7)	27·3 (15·9 to 43·8)	18 (11·2 to 28·3)	12·7 (8·1 to 19)	10 (6·5 to 14·6)	8·4 (5·4 to 12·3)	8·2 (5·2 to 12·1)	7·4 (4·9 to 11·2)	6·8 (4·6 to 9·3)	6·3 (4·5 to 8·8)	4·5 (3·2 to 6·1)	2·9 (2·1 to 3·8)	2 (1·5 to 2·5)	1·2 (0·9 to 1·5)	0·8 (0·6 to 1)	0·3 (0·2 to 0·4)	0·1 (0·1 to 0·2)	0 (0 to 0)	169
Mental disorders	0·1 (0·1 to 0·1)	0·2 (0·2 to 0·4)	4 (2·5 to 6)	21·5 (14 to 31)	104 (70·1 to 149)	280 (191 to 400)	365 (253 to 509)	335 (231 to 457)	322 (225 to 439)	297 (211 to 399)	276 (198 to 370)	246 (177 to 332)	216 (153 to 284)	177 (126 to 233)	144 (104 to 193)	102 (73·3 to 136)	68·9 (49·4 to 91·4)	43·6 (31·6 to 57·7)	26 (18·3 to 34·8)	15·6 (10·7 to 21·1)	7·3 (5·2 to 9·7)	3 (2·1 to 4·1)	0·9 (0·5 to 1·3)	3,050
Schizophrenia	0 (0 to 0)	0 (0 to 0)	0 (0 to 0)	0 (0 to 0)	0 (0 to 0)	1·4 (0·6 to 2·6)	10·5 (3·8 to 19·7)	30·8 (15·7 to 52·1)	47·5 (26·8 to 73·1)	52·5 (31·7 to 78·4)	50·3 (30·9 to 74·9)	43·9 (28·5 to 61·7)	37·1 (24 to 51·9)	28·4 (18·9 to 39·5)	21 (13·6 to 29·5)	13·2 (8·4 to 18·4)	7·7 (4·9 to 11)	4·1 (2·4 to 6)	2 (1·1 to 3·1)	0·9 (0·5 to 1·4)	0·3 (0·1 to 0·5)	0·1 (0 to 0·2)	0 (0 to 0)	352
Depressive disorders	0 (0 to 0)	0 (0 to 0)	0 (0 to 0)	0 (0 to 0·1)	3 (1·3 to 5·8)	29·3 (14·4 to 52·7)	84·9 (48·6 to 135)	108 (63·9 to 172)	93·8 (56·9 to 144)	83·5 (50·3 to 127)	83·1 (52·1 to 123)	79·1 (47·6 to 120)	72·5 (44·8 to 107)	62·6 (39·8 to 91)	54·1 (34·4 to 79·7)	40·4 (25·1 to 60·2)	28·7 (18·7 to 41·6)	19·2 (12·9 to 27·4)	12·2 (7·8 to 18·4)	7·9 (4·8 to 11·9)	4 (2·6 to 5·8)	1·8 (1·1 to 2·7)	0·5 (0·3 to 0·9)	869
Major depressive disorder	0 (0 to 0)	0 (0 to 0)	0 (0 to 0)	0 (0 to 0·1)	2·5 (0·9 to 5·2)	25·4 (11·2 to 48·3)	73·6 (39·9 to 122)	91·2 (51·5 to 150)	74·4 (42·5 to 116)	64·5 (36·2 to 102)	65·2 (39·5 to 99·7)	63·1 (35·8 to 100)	58·3 (34·8 to 86·3)	51 (31·5 to 75·1)	44·5 (26·9 to 66·4)	33·5 (20·5 to 50·5)	24 (15·4 to 35·2)	16·2 (10·6 to 23·8)	10·4 (6·3 to 16·1)	6·8 (4 to 10·6)	3·5 (2·2 to 5·2)	1·6 (0·9 to 2·4)	0·5 (0·2 to 0·8)	710
Dysthymia	0 (0 to 0)	0 (0 to 0)	0 (0 to 0)	0 (0 to 0)	0·5 (0 to 1·4)	4 (1·4 to 7·8)	11·2 (5·3 to 19·6)	17·2 (8·2 to 31·1)	19·4 (9·7 to 34)	19 (10·5 to 31·7)	17·8 (10·2 to 29·6)	16 (8·3 to 27·8)	14·2 (7·6 to 24·3)	11·7 (6·6 to 20·1)	9·6 (5·5 to 15·9)	6·9 (3·6 to 11·7)	4·7 (2·5 to 8·1)	3 (1·6 to 5)	1·8 (1 to 2·9)	1·1 (0·6 to 1·8)	0·5 (0·3 to 0·8)	0·2 (0·1 to 0·4)	0·1 (0 to 0·1)	159
Bipolar disorder	0 (0 to 0)	0 (0 to 0)	0 (0 to 0)	0 (0 to 0)	0 (0 to 0)	14·2 (5·8 to 26·6)	50·9 (23·1 to 89·1)	55 (28·5 to 93·6)	48·8 (25·2 to 82·7)	40·7 (21·5 to 69·3)	36·1 (19·6 to 60)	32·9 (17·5 to 54·8)	29·1 (15·5 to 49·8)	23·3 (12·6 to 39·7)	18·1 (9·8 to 30·2)	11·8 (6·7 to 19·7)	7·3 (4 to 12·1)	4·1 (2·2 to 6·6)	2·1 (1·1 to 3·5)	1 (0·5 to 1·7)	0·4 (0·2 to 0·6)	0·1 (0·1 to 0·2)	0 (0 to 0·1)	376
Anxiety disorders	0 (0 to 0)	0 (0 to 0)	0 (0 to 0)	1·5 (0·3 to 3·2)	23·2 (11·7 to 39·2)	63·1 (34·8 to 104)	79 (47 to 125)	71·6 (39·3 to 119)	64·7 (36·8 to 109)	57·4 (33·1 to 92·9)	52·1 (29·6 to 80·7)	46·2 (26·2 to 74·9)	40·7 (22·8 to 64·5)	33·9 (21 to 51·3)	28·2 (17·3 to 42·7)	20·4 (11·9 to 32·2)	13·9 (8 to 23)	8·7 (5·1 to 14)	4·9 (2·9 to 7·7)	2·7 (1·5 to 4·3)	1·1 (0·6 to 1·8)	0·4 (0·2 to 0·6)	0·1 (0 to 0·1)	614
Eating disorders	0 (0 to 0)	0 (0 to 0)	0 (0 to 0)	0 (0 to 0)	0·2 (0·1 to 0·4)	3·7 (1·4 to 7·2)	10·1 (4·5 to 18·6)	11·2 (5 to 20·6)	10·5 (5·5 to 17·4)	10·2 (4·9 to 18·1)	7 (3·3 to 12·5)	2·7 (1·2 to 5·2)	0·9 (0·3 to 1·9)	0 (0 to 0)	0 (0 to 0)	0 (0 to 0)	0 (0 to 0)	0 (0 to 0)	0 (0 to 0)	0 (0 to 0)	0 (0 to 0)	0 (0 to 0)	0 (0 to 0)	56·5
Anorexia nervosa	0 (0 to 0)	0 (0 to 0)	0 (0 to 0)	0 (0 to 0)	0·2 (0·1 to 0·4)	1·5 (0·3 to 3·2)	3·6 (1·4 to 7·1)	3 (1·1 to 5·9)	2 (0·7 to 3·9)	1 (0·2 to 2·1)	0·7 (0·1 to 1·6)	0·5 (0 to 1·1)	0·3 (0 to 0·8)	0 (0 to 0)	0 (0 to 0)	0 (0 to 0)	0 (0 to 0)	0 (0 to 0)	0 (0 to 0)	0 (0 to 0)	0 (0 to 0)	0 (0 to 0)	0 (0 to 0)	12·7
Bulimia nervosa	0 (0 to 0)	0 (0 to 0)	0 (0 to 0)	0 (0 to 0)	0 (0 to 0·1)	2·2 (0·5 to 5·2)	6·5 (2·3 to 13·5)	8·2 (3 to 16·7)	8·6 (4 to 14·9)	9·1 (4 to 16·8)	6·3 (2·8 to 11·6)	2·3 (0·8 to 4·5)	0·6 (0·1 to 1·4)	0 (0 to 0)	0 (0 to 0)	0 (0 to 0)	0 (0 to 0)	0 (0 to 0)	0 (0 to 0)	0 (0 to 0)	0 (0 to 0)	0 (0 to 0)	0 (0 to 0)	43·8
Autism spectrum disorders	0·1 (0 to 0·1)	0·2 (0·1 to 0·3)	3·5 (2·2 to 5·2)	14·8 (9·2 to 22·6)	19·4 (12·4 to 30·6)	20·3 (13 to 30·4)	19·4 (11·8 to 29)	16·5 (10·1 to 24·7)	14·1 (8·8 to 20·9)	11·8 (7·5 to 18)	9·9 (6·1 to 15)	8·3 (5·2 to 12·4)	7 (4·2 to 10·4)	5·6 (3·4 to 8·4)	4·5 (2·8 to 6·8)	3·2 (2 to 4·8)	2·2 (1·3 to 3·4)	1·4 (0·8 to 2·2)	0·9 (0·6 to 1·4)	0·6 (0·3 to 0·8)	0·3 (0·2 to 0·4)	0·1 (0·1 to 0·2)	0 (0 to 0)	164
Attention-deficit/hyperactivity disorder	0 (0 to 0)	0 (0 to 0)	0 (0 to 0)	1·8 (0·9 to 3·5)	20·2 (11 to 36·4)	29·6 (16 to 53·4)	24·2 (13·3 to 43·5)	15·9 (8·7 to 27·5)	10·8 (5·9 to 19)	7·5 (3·9 to 12·8)	5·3 (2·8 to 9·2)	3·8 (1·9 to 6·8)	2·7 (1·4 to 4·8)	1·8 (0·9 to 3·3)	1·1 (0·5 to 1·9)	0·5 (0·2 to 0·9)	0·2 (0·1 to 0·4)	0·1 (0 to 0·2)	0 (0 to 0)	0 (0 to 0)	0 (0 to 0)	0 (0 to 0)	0 (0 to 0)	126
Conduct disorder	0 (0 to 0)	0 (0 to 0)	0 (0 to 0)	0 (0 to 0)	32·2 (16·6 to 55·4)	111 (60·9 to 179)	71·5 (37·1 to 120)	0 (0 to 0)	0 (0 to 0)	0 (0 to 0)	0 (0 to 0)	0 (0 to 0)	0 (0 to 0)	0 (0 to 0)	0 (0 to 0)	0 (0 to 0)	0 (0 to 0)	0 (0 to 0)	0 (0 to 0)	0 (0 to 0)	0 (0 to 0)	0 (0 to 0)	0 (0 to 0)	215
Idiopathic developmental intellectual disability	0 (0 to 0)	0 (0 to 0·1)	0·5 (0 to 1·3)	3·4 (0·8 to 6·9)	5·3 (1·6 to 10·2)	5·6 (1·8 to 10·8)	5·3 (1·5 to 10·2)	4·5 (1·4 to 8·6)	3·8 (1·2 to 7·2)	3·1 (1 to 5·9)	2·6 (0·9 to 4·9)	2·1 (0·6 to 3·9)	1·7 (0·5 to 3·2)	1·3 (0·4 to 2·5)	1 (0·3 to 1·9)	0·6 (0·2 to 1·3)	0·4 (0·1 to 0·8)	0·2 (0·1 to 0·5)	0·1 (0 to 0·3)	0·1 (0 to 0·2)	0 (0 to 0·1)	0 (0 to 0)	0 (0 to 0)	41·7
Other mental disorders	0 (0 to 0)	0 (0 to 0)	0 (0 to 0)	0 (0 to 0)	0 (0 to 0)	1·4 (0·7 to 2·5)	9·4 (4·2 to 16·8)	20·8 (10·4 to 35·3)	28 (15·6 to 44·9)	30·3 (18 to 47·5)	29·7 (17·9 to 46·2)	26·8 (16·1 to 41·7)	23·8 (14·4 to 36·7)	19·7 (11·5 to 30·3)	16·3 (10 to 25·5)	11·9 (7·2 to 18·4)	8·4 (5·3 to 13)	5·7 (3·6 to 8·6)	3·7 (2·4 to 5·5)	2·4 (1·5 to 3·6)	1·2 (0·8 to 1·8)	0·5 (0·4 to 0·8)	0·2 (0·1 to 0·2)	240
Substance use disorders	0 (0 to 0)	0 (0 to 0)	0 (0 to 0)	0 (0 to 0)	0 (0 to 0)	4·3 (2·2 to 7·2)	72·1 (47·3 to 105)	122 (82 to 170)	135 (93·6 to 184)	136 (99·9 to 182)	131 (96·8 to 171)	123 (91·1 to 164)	114 (84·6 to 154)	90·9 (67·6 to 120)	80·3 (59·4 to 105)	48·7 (36·2 to 64·9)	28 (20·8 to 36)	13·4 (10 to 17·9)	6·5 (4·9 to 8·4)	3·5 (2·5 to 4·6)	1·5 (1·1 to 2)	0·6 (0·4 to 0·9)	0·2 (0·2 to 0·4)	1,110
Alcohol use disorders	0 (0 to 0)	0 (0 to 0)	0 (0 to 0)	0 (0 to 0)	0 (0 to 0)	1·7 (0·6 to 3·3)	23·9 (13·6 to 37·4)	62·3 (38·8 to 96·6)	86·8 (58·4 to 126)	103 (71·8 to 143)	107 (77·7 to 144)	106 (76·6 to 145)	102 (72·5 to 142)	82·8 (60·4 to 111)	74·7 (53·9 to 99)	45·6 (33·7 to 61·6)	26·2 (19·1 to 34·1)	12·3 (9 to 16·5)	5·8 (4·3 to 7·7)	3 (2·1 to 4)	1·2 (0·8 to 1·7)	0·5 (0·3 to 0·8)	0·2 (0·1 to 0·3)	846
Drug use disorders	0 (0 to 0)	0 (0 to 0)	0 (0 to 0)	0 (0 to 0)	0 (0 to 0)	2·7 (1·2 to 4·7)	48·2 (29·4 to 73·9)	59·6 (38·3 to 86·3)	48 (31·2 to 68·1)	33·2 (21·7 to 46·9)	23·6 (15·8 to 33·6)	17 (11·2 to 23·9)	12·3 (8·1 to 17·4)	8·1 (5·1 to 11·7)	5·6 (3·4 to 8·3)	3·1 (1·9 to 4·8)	1·8 (1 to 2·9)	1·1 (0·6 to 1·8)	0·7 (0·4 to 1·1)	0·5 (0·3 to 0·7)	0·2 (0·1 to 0·3)	0·1 (0·1 to 0·2)	0 (0 to 0·1)	266
Opioid use disorders	0 (0 to 0)	0 (0 to 0)	0 (0 to 0)	0 (0 to 0)	0 (0 to 0)	0 (0 to 0)	4·3 (1·4 to 8·5)	14·9 (7 to 26·4)	16·1 (8·6 to 27·1)	12·9 (7·1 to 20·7)	10 (5·6 to 15·8)	7·5 (3·7 to 12·7)	5·4 (2·5 to 9·1)	3·8 (1·8 to 6·4)	2·8 (1·3 to 4·8)	1·6 (0·7 to 3)	1 (0·4 to 1·8)	0·5 (0·2 to 1)	0·4 (0·1 to 0·7)	0·3 (0·1 to 0·5)	0·1 (0 to 0·2)	0·1 (0 to 0·1)	0 (0 to 0)	81·8
Cocaine use disorders	0 (0 to 0)	0 (0 to 0)	0 (0 to 0)	0 (0 to 0)	0 (0 to 0)	0·3 (0·2 to 0·6)	12·4 (5·6 to 23·4)	12·5 (6·5 to 21·3)	9·5 (5·4 to 15·3)	7·3 (4·1 to 11·6)	5·4 (3·1 to 8·6)	3·7 (2 to 5·9)	2·7 (1·4 to 4·4)	1·5 (0·7 to 2·5)	1 (0·4 to 1·9)	0·5 (0·2 to 1·1)	0·3 (0·1 to 0·7)	0·2 (0 to 0·4)	0·1 (0 to 0·3)	0·1 (0 to 0·2)	0 (0 to 0·1)	0 (0 to 0)	0 (0 to 0)	57·7
Amphetamine use disorders	0 (0 to 0)	0 (0 to 0)	0 (0 to 0)	0 (0 to 0)	0 (0 to 0)	0 (0 to 0)	1·5 (0·2 to 3·8)	6·1 (2·1 to 12·4)	5·4 (2 to 10·4)	2·6 (0·9 to 5·6)	1·3 (0·4 to 2·9)	0·8 (0·5 to 1·3)	0·5 (0·3 to 0·8)	0·3 (0·2 to 0·6)	0·2 (0·1 to 0·4)	0·1 (0·1 to 0·2)	0·1 (0 to 0·1)	0 (0 to 0·1)	0 (0 to 0·1)	0 (0 to 0)	0 (0 to 0)	0 (0 to 0)	0 (0 to 0)	19·1
Cannabis use disorders	0 (0 to 0)	0 (0 to 0)	0 (0 to 0)	0 (0 to 0)	0 (0 to 0)	2·1 (0·8 to 4·1)	21·2 (10·9 to 36·1)	14·8 (7·5 to 24·9)	8 (3·6 to 14·4)	4·5 (2 to 8·7)	3 (1·2 to 5·4)	2·2 (0·9 to 4·1)	1·7 (0·7 to 3·1)	1·1 (0·4 to 2·1)	0·6 (0·2 to 1·2)	0·3 (0·1 to 0·6)	0·1 (0·1 to 0·3)	0·1 (0 to 0·1)	0 (0 to 0·1)	0 (0 to 0)	0 (0 to 0)	0 (0 to 0)	0 (0 to 0)	59·7
Other drug use disorders	0 (0 to 0)	0 (0 to 0)	0 (0 to 0)	0 (0 to 0)	0 (0 to 0)	0·2 (0·1 to 0·4)	8·8 (3·9 to 16·3)	11·4 (5·8 to 19·5)	9 (4·7 to 14·4)	5·8 (3·1 to 9·5)	3·9 (2 to 6·4)	2·7 (1·6 to 4·4)	2 (1·1 to 3·4)	1·4 (0·7 to 2·2)	0·9 (0·5 to 1·5)	0·5 (0·2 to 0·9)	0·3 (0·2 to 0·6)	0·3 (0·2 to 0·4)	0·2 (0·1 to 0·3)	0·1 (0·1 to 0·2)	0·1 (0 to 0·1)	0 (0 to 0)	0 (0 to 0)	47·7
Diabetes and kidney diseases	0·1 (0 to 0·1)	0·2 (0·1 to 0·5)	20·8 (13·9 to 30·9)	19·9 (14·3 to 26·7)	24·9 (18·1 to 34·4)	29·2 (22·1 to 38·5)	82·5 (63·8 to 107)	119 (96·5 to 147)	174 (140 to 216)	213 (171 to 261)	249 (203 to 305)	312 (252 to 376)	432 (354 to 524)	528 (432 to 643)	659 (540 to 788)	582 (473 to 691)	486 (396 to 582)	372 (309 to 446)	244 (203 to 291)	185 (156 to 217)	86·2 (71·8 to 102)	35·5 (29·1 to 41·9)	10·5 (8·1 to 12·8)	4,860
Diabetes mellitus	0·1 (0 to 0·1)	0·2 (0·1 to 0·5)	4·2 (2·2 to 7·5)	3·5 (2·2 to 5·1)	2·7 (1·7 to 3·8)	5·3 (3·5 to 7·4)	21·9 (16·9 to 28·7)	42·6 (32·9 to 54)	70·7 (54·5 to 90·4)	96·2 (73·9 to 124)	119 (92·1 to 151)	164 (129 to 208)	242 (189 to 312)	316 (249 to 401)	410 (325 to 512)	379 (302 to 465)	315 (251 to 391)	244 (192 to 302)	153 (122 to 189)	110 (86·2 to 134)	49·8 (39·7 to 61·8)	19 (15·1 to 23·3)	5·3 (4 to 6·6)	2,770
Diabetes mellitus type 1	0·1 (0 to 0·1)	0·2 (0·1 to 0·5)	4·2 (2·2 to 7·5)	3·5 (2·2 to 5·1)	2·7 (1·7 to 3·8)	5·3 (3·5 to 7·4)	10·5 (7·2 to 14·8)	19·6 (13·8 to 26·8)	32·8 (22·8 to 45·5)	21·8 (13·8 to 34·2)	24·9 (15·9 to 39·4)	31·6 (20·6 to 47·2)	10·1 (6·6 to 17)	11·4 (7·3 to 18·8)	14·2 (8·9 to 25·1)	10·5 (6·4 to 18·1)	8·7 (5·5 to 15·3)	5 (3·1 to 9·1)	3·4 (2 to 5·8)	1·8 (1·1 to 2·9)	0·8 (0·5 to 1·4)	0·4 (0·2 to 0·6)	0·1 (0·1 to 0·2)	224
Diabetes mellitus type 2	0 (0 to 0)	0 (0 to 0)	0 (0 to 0)	0 (0 to 0)	0 (0 to 0)	0 (0 to 0)	11·4 (7·5 to 16)	23 (15·7 to 31·6)	37·9 (26·7 to 52·7)	74·4 (55·6 to 97·9)	94·3 (71·5 to 122)	132 (101 to 172)	232 (180 to 300)	305 (241 to 387)	395 (312 to 494)	369 (294 to 454)	307 (243 to 382)	239 (189 to 297)	149 (119 to 185)	108 (85·1 to 131)	49 (39 to 60·9)	18·6 (14·8 to 22·8)	5·2 (3·9 to 6·5)	2,550
Chronic kidney disease	0 (0 to 0)	0 (0 to 0)	16·5 (10·5 to 24·9)	16·2 (11·1 to 22·1)	21·9 (15·2 to 31)	23·6 (17 to 32·4)	60·2 (42·9 to 82·7)	76·4 (56·9 to 99·8)	103 (75 to 138)	117 (88 to 153)	130 (97·8 to 169)	148 (113 to 191)	189 (143 to 246)	212 (161 to 274)	249 (186 to 320)	202 (152 to 261)	170 (128 to 217)	128 (99·8 to 159)	90·9 (71·3 to 115)	75·5 (59·7 to 93·5)	36·4 (28·6 to 44·8)	16·5 (12·2 to 20·4)	5·2 (3·8 to 6·6)	2,090
Chronic kidney disease due to diabetes mellitus type 1	0 (0 to 0)	0 (0 to 0)	0·1 (0·1 to 0·3)	0·4 (0·1 to 0·8)	0·7 (0·2 to 1·5)	0·8 (0·3 to 1·7)	2·3 (0·9 to 4·7)	4·3 (1·7 to 8·7)	11·2 (4·8 to 22·3)	19·6 (9·6 to 33·3)	25·4 (12·9 to 43·1)	28 (15·2 to 45·7)	30·3 (14 to 51·4)	25 (10·4 to 47·4)	20·1 (6·9 to 42·9)	11·8 (4·2 to 25·2)	7·6 (2·8 to 16·9)	4·3 (1·6 to 9)	2 (0·8 to 4·3)	0·9 (0·3 to 2)	0·2 (0 to 0·4)	0 (0 to 0)	0 (0 to 0)	195
Chronic kidney disease due to diabetes mellitus type 2	0 (0 to 0)	0 (0 to 0)	0 (0 to 0)	0 (0 to 0)	0 (0 to 0)	0 (0 to 0)	1 (0·3 to 2·3)	1·9 (0·7 to 4·3)	4·2 (1·7 to 9·1)	7·3 (2·9 to 14·1)	11·2 (5·1 to 21)	17·9 (8·6 to 31·7)	33·5 (18 to 55)	50 (28·9 to 74·8)	70·3 (40 to 103)	61·7 (36 to 89·3)	53·6 (33·9 to 78)	40·7 (27·6 to 57·3)	28·8 (18·8 to 39·6)	23·6 (15·7 to 33·3)	10·8 (7·2 to 15·4)	4·6 (2·8 to 6·9)	1·4 (0·8 to 2·1)	423
Chronic kidney disease due to hypertension	0 (0 to 0)	0 (0 to 0)	0 (0 to 0)	0 (0 to 0)	0 (0 to 0)	0 (0 to 0)	4 (1·7 to 7·9)	5·8 (2·4 to 11)	9 (4·5 to 15·5)	11·1 (6·1 to 17·7)	13·3 (7·5 to 21)	17·5 (10·9 to 27·3)	26·3 (16·8 to 39·8)	34·7 (21·6 to 51·8)	47·4 (29 to 70·4)	44·9 (27·1 to 66·4)	42·3 (27·5 to 61·6)	34·6 (23·6 to 49·3)	27·2 (18·5 to 38)	25·2 (18 to 33·9)	13·6 (9·8 to 17·8)	6·7 (4·6 to 9)	2·2 (1·5 to 3·1)	366
Chronic kidney disease due to glomerulonephritis	0 (0 to 0)	0 (0 to 0)	4·8 (2·3 to 9)	5·4 (3 to 8·6)	8·3 (4·6 to 13·1)	10·2 (6·3 to 15)	26·8 (16·2 to 41·1)	33·5 (20 to 48·8)	42·5 (26·7 to 63)	43·1 (27 to 63·3)	44·2 (27·6 to 64·6)	46·6 (28·1 to 70·6)	54 (32·8 to 80·1)	55·7 (30·8 to 87)	60 (32·8 to 94·7)	42·9 (22·3 to 70)	31 (15·8 to 53·1)	20 (10·6 to 33·3)	12·4 (6 to 22·3)	8·7 (4 to 16·2)	3·2 (1·4 to 5·7)	1·2 (0·6 to 2·3)	0·3 (0·1 to 0·7)	555
Chronic kidney disease due to other and unspecified causes	0 (0 to 0)	0 (0 to 0)	11·6 (6·7 to 17·9)	10·4 (6·7 to 15·1)	12·9 (8·3 to 19·1)	12·7 (7·3 to 19·5)	26·2 (15·6 to 39·9)	30·9 (18·1 to 45·2)	36·4 (22·2 to 53·4)	35·9 (21·3 to 56·3)	35·4 (20·1 to 55)	37·6 (21·7 to 57·4)	45·1 (25·6 to 70·9)	46·4 (27·4 to 72·8)	51 (28·7 to 84·5)	40·9 (23·6 to 66·7)	35·6 (20·5 to 56·9)	28·2 (16·9 to 43·6)	20·6 (12·4 to 31·7)	17·1 (10·4 to 26·3)	8·6 (5·3 to 12·8)	3·9 (2·6 to 5·9)	1·3 (0·8 to 1·9)	549
Acute glomerulonephritis	0 (0 to 0)	0 (0 to 0)	0 (0 to 0)	0·3 (0·1 to 0·7)	0·4 (0·2 to 0·8)	0·2 (0·1 to 0·4)	0·4 (0·2 to 0·7)	0·2 (0·1 to 0·3)	0·2 (0·1 to 0·3)	0·1 (0 to 0·2)	0·2 (0·1 to 0·4)	0·2 (0·1 to 0·4)	0·3 (0·2 to 0·6)	0·1 (0·1 to 0·3)	0·2 (0·1 to 0·4)	0·1 (0·1 to 0·3)	0·1 (0 to 0·2)	0·1 (0 to 0·3)	0·1 (0 to 0·1)	0 (0 to 0·1)	0 (0 to 0·1)	0 (0 to 0)	0 (0 to 0)	3·3
Skin and subcutaneous diseases	0 (0 to 0)	0·1 (0 to 0·1)	11·5 (7·1 to 17·1)	93·9 (58·1 to 140)	125 (77·3 to 186)	135 (86 to 204)	143 (92 to 218)	84·7 (52·5 to 128)	65·7 (42·7 to 98·9)	55·1 (35·6 to 81)	49·5 (32·3 to 72·5)	45·2 (30 to 66·5)	42·1 (28·3 to 62·8)	38·4 (25·1 to 56·8)	35·4 (23·5 to 52)	29·4 (19·8 to 43)	24·8 (16·6 to 37·3)	19·4 (12·7 to 29·3)	14·4 (9·4 to 22·6)	10·7 (7·1 to 16·7)	5·8 (3·8 to 9·2)	2·8 (1·8 to 4·5)	0·8 (0·5 to 1·4)	1,030
Dermatitis	0 (0 to 0)	0 (0 to 0)	0 (0 to 0)	31·3 (16·4 to 54)	39·3 (20·3 to 67·1)	29 (15·2 to 48·9)	21·7 (12·3 to 35·6)	16 (8·7 to 26·1)	13·2 (7·6 to 21·8)	11·3 (6·2 to 18·7)	9·6 (5·3 to 15·6)	8·2 (4·6 to 13·6)	7·4 (4 to 12·2)	6·7 (3·6 to 11·3)	6·1 (3·4 to 9·9)	4·8 (2·6 to 7·7)	3·8 (2·1 to 6·2)	2·7 (1·6 to 4·4)	1·8 (1 to 2·8)	1·1 (0·6 to 1·7)	0·5 (0·3 to 0·8)	0·2 (0·1 to 0·3)	0·1 (0 to 0·1)	215
Atopic dermatitis	0 (0 to 0)	0 (0 to 0)	0 (0 to 0)	31 (16·2 to 53·5)	38·4 (19·5 to 65·8)	25·9 (13·4 to 45)	16·5 (8·6 to 28·9)	10·5 (5·1 to 18·8)	7·5 (3·8 to 13·3)	5·8 (2·8 to 10·4)	4·8 (2·3 to 8·5)	4 (2·1 to 7·5)	3·5 (1·8 to 6·4)	3 (1·5 to 5·6)	2·7 (1·3 to 4·8)	2 (1 to 3·7)	1·5 (0·8 to 2·7)	1·1 (0·6 to 1·9)	0·7 (0·4 to 1·2)	0·5 (0·3 to 0·8)	0·2 (0·1 to 0·4)	0·1 (0·1 to 0·2)	0 (0 to 0)	160
Contact dermatitis	0 (0 to 0)	0 (0 to 0)	0 (0 to 0)	0 (0 to 0)	0·5 (0·2 to 1·2)	2·6 (0·9 to 5·4)	4·5 (1·7 to 8·7)	4·8 (2 to 9·3)	5·1 (2·3 to 9·7)	4·8 (2 to 9·1)	4·3 (1·8 to 8·5)	3·7 (1·6 to 7·4)	3·5 (1·5 to 6·8)	3·3 (1·5 to 6·5)	3·1 (1·3 to 6)	2·6 (1·1 to 4·8)	2·1 (0·9 to 4·1)	1·5 (0·7 to 2·8)	0·9 (0·4 to 1·7)	0·5 (0·2 to 1)	0·2 (0·1 to 0·4)	0·1 (0 to 0·2)	0 (0 to 0)	48·2
Seborrhoeic dermatitis	0 (0 to 0)	0 (0 to 0)	0 (0 to 0)	0·3 (0·1 to 0·6)	0·4 (0·1 to 0·7)	0·6 (0·2 to 1)	0·7 (0·3 to 1·3)	0·6 (0·3 to 1·2)	0·7 (0·3 to 1·2)	0·6 (0·3 to 1·1)	0·6 (0·3 to 1)	0·5 (0·2 to 0·8)	0·4 (0·2 to 0·7)	0·3 (0·2 to 0·6)	0·3 (0·1 to 0·5)	0·2 (0·1 to 0·4)	0·2 (0·1 to 0·4)	0·2 (0·1 to 0·3)	0·1 (0·1 to 0·2)	0·1 (0 to 0·2)	0 (0 to 0·1)	0 (0 to 0)	0 (0 to 0)	6·7
Psoriasis	0 (0 to 0)	0 (0 to 0)	0 (0 to 0)	1·4 (0·9 to 2·2)	3·9 (1·6 to 7·2)	5·4 (2·5 to 9·3)	5·8 (2·9 to 9·8)	5·6 (2·7 to 9·3)	5·6 (2·8 to 9·2)	5·7 (3 to 9·1)	5·6 (3·1 to 8·9)	5·4 (3 to 8·2)	5·2 (3 to 8·1)	4·8 (2·9 to 7·6)	4·4 (2·7 to 6·4)	3·5 (2·2 to 5·1)	2·5 (1·6 to 3·8)	1·6 (1 to 2·4)	0·9 (0·5 to 1·4)	0·5 (0·3 to 0·7)	0·2 (0·1 to 0·3)	0·1 (0 to 0·1)	0 (0 to 0)	68·2
Bacterial skin diseases	0 (0 to 0)	0 (0 to 0)	4·8 (1·7 to 9)	2·5 (1·3 to 4·2)	2·5 (1·4 to 4·1)	2·7 (1·5 to 4·3)	3 (1·7 to 4·9)	2·9 (1·5 to 5)	3·4 (1·7 to 5·9)	3·5 (1·8 to 5·7)	3·1 (1·6 to 5·3)	3·6 (1·8 to 6·8)	3·9 (1·9 to 7·7)	3·7 (1·7 to 6·8)	3·8 (1·7 to 7·6)	3·1 (1·3 to 5·9)	2·5 (0·9 to 5·3)	2·1 (0·7 to 4·8)	1·8 (0·5 to 4)	1·5 (0·4 to 3·6)	0·8 (0·2 to 2)	0·4 (0·1 to 1·1)	0·1 (0 to 0·3)	55·7
Cellulitis	0 (0 to 0)	0 (0 to 0)	1·6 (0·4 to 3·6)	0·8 (0·3 to 1·6)	0·9 (0·4 to 1·7)	1 (0·5 to 1·7)	1·2 (0·6 to 2·3)	1·1 (0·5 to 2·3)	1·3 (0·5 to 2·5)	1·4 (0·6 to 2·6)	1·1 (0·5 to 2·2)	1·2 (0·5 to 2·3)	1·2 (0·5 to 2·7)	1·2 (0·5 to 2·6)	1·1 (0·4 to 2·4)	0·9 (0·3 to 2)	0·6 (0·2 to 1·5)	0·5 (0·2 to 1·3)	0·3 (0·1 to 0·9)	0·3 (0·1 to 0·8)	0·2 (0 to 0·5)	0·1 (0 to 0·3)	0 (0 to 0·1)	18
Pyoderma	0 (0 to 0)	0 (0 to 0)	3·3 (1 to 6·3)	1·7 (0·8 to 3·1)	1·6 (0·8 to 2·7)	1·7 (0·9 to 3)	1·8 (0·9 to 3·2)	1·8 (0·9 to 3·2)	2·1 (0·9 to 3·8)	2·1 (1 to 3·6)	2 (0·9 to 3·7)	2·5 (1·1 to 4·5)	2·7 (1·2 to 5·3)	2·5 (1·1 to 4·9)	2·7 (1·1 to 5·3)	2·1 (0·9 to 4·4)	1·9 (0·6 to 4·2)	1·6 (0·5 to 3·8)	1·4 (0·4 to 3·4)	1·2 (0·3 to 2·8)	0·6 (0·2 to 1·5)	0·3 (0·1 to 0·8)	0·1 (0 to 0·3)	37·7
Scabies	0 (0 to 0)	0 (0 to 0·1)	2·5 (1·3 to 4·4)	17·2 (8·6 to 30·7)	24 (11·6 to 42·2)	25·6 (12·5 to 44·6)	26·1 (13·1 to 44·6)	20·7 (10·3 to 36·8)	13·5 (6·8 to 23)	9·1 (4·3 to 16·5)	7·9 (3·9 to 13·5)	7·7 (3·9 to 13·6)	6·9 (3·5 to 12)	5·5 (2·8 to 9·2)	4·2 (2·1 to 7·5)	2·8 (1·4 to 4·9)	2 (1 to 3·4)	1·4 (0·7 to 2·4)	1 (0·5 to 1·7)	0·7 (0·4 to 1·3)	0·4 (0·2 to 0·7)	0·2 (0·1 to 0·3)	0·1 (0 to 0·1)	179
Fungal skin diseases	0 (0 to 0)	0 (0 to 0)	0·4 (0·2 to 1)	3·4 (1·3 to 7·2)	5·7 (2·2 to 12·4)	6·9 (2·6 to 15·3)	7 (2·5 to 16·1)	6·5 (2·4 to 14·3)	6·1 (2·3 to 13·8)	5·6 (2·2 to 12·6)	5·1 (2 to 11)	4·6 (1·8 to 10)	4·2 (1·6 to 9·4)	4 (1·6 to 8·9)	4·2 (1·6 to 9·2)	3·6 (1·4 to 8)	3·6 (1·4 to 7·7)	3·6 (1·4 to 7·6)	2·9 (1·1 to 6·1)	2·4 (1 to 5·1)	1·5 (0·6 to 3·2)	0·7 (0·3 to 1·5)	0·2 (0·1 to 0·5)	82·4
Viral skin diseases	0 (0 to 0)	0 (0 to 0)	0 (0 to 0)	18·6 (11·6 to 28·3)	30·6 (19 to 47·2)	16·5 (10 to 25·6)	9·9 (6 to 15·6)	6·8 (4 to 10·5)	4·5 (2·6 to 7·2)	3·3 (1·9 to 5·2)	2·8 (1·6 to 4·6)	2·4 (1·4 to 3·7)	2 (1·1 to 3·1)	1·6 (0·9 to 2·6)	1·3 (0·8 to 2·1)	1 (0·6 to 1·6)	0·8 (0·4 to 1·2)	0·5 (0·3 to 0·8)	0·3 (0·1 to 0·4)	0·1 (0·1 to 0·2)	0·1 (0 to 0·1)	0 (0 to 0)	0 (0 to 0)	103
Acne vulgaris	0 (0 to 0)	0 (0 to 0)	0 (0 to 0)	0 (0 to 0)	1·3 (0·7 to 2·2)	32·7 (18·5 to 52·9)	54 (30·5 to 90·1)	11·5 (6 to 19·2)	4·7 (2·4 to 8·1)	3 (1·5 to 5·2)	2 (0·9 to 3·7)	1·3 (0·7 to 2·2)	0·8 (0·4 to 1·4)	0·5 (0·3 to 0·8)	0·3 (0·2 to 0·5)	0·2 (0·1 to 0·3)	0·1 (0·1 to 0·2)	0·1 (0 to 0·1)	0 (0 to 0·1)	0 (0 to 0)	0 (0 to 0)	0 (0 to 0)	0 (0 to 0)	113
Alopecia areata	0 (0 to 0)	0 (0 to 0)	0 (0 to 0)	0·2 (0·1 to 0·5)	0·4 (0·1 to 0·8)	0·5 (0·2 to 1)	0·8 (0·4 to 1·5)	1·2 (0·6 to 2)	1·4 (0·7 to 2·4)	1·3 (0·7 to 2·2)	1·1 (0·6 to 1·8)	0·8 (0·4 to 1·4)	0·6 (0·3 to 1·1)	0·5 (0·2 to 0·8)	0·4 (0·2 to 0·6)	0·3 (0·1 to 0·5)	0·2 (0·1 to 0·3)	0·1 (0·1 to 0·2)	0·1 (0 to 0·1)	0 (0 to 0·1)	0 (0 to 0)	0 (0 to 0)	0 (0 to 0)	9·9
Pruritus	0 (0 to 0)	0 (0 to 0)	0·1 (0 to 0·1)	0·6 (0·2 to 1·2)	0·9 (0·4 to 1·7)	0·9 (0·4 to 1·9)	1 (0·4 to 2·1)	1·1 (0·5 to 2·2)	1·1 (0·5 to 2·3)	1·1 (0·5 to 2·2)	1 (0·4 to 2)	0·9 (0·4 to 1·8)	0·9 (0·4 to 1·7)	0·8 (0·3 to 1·6)	0·8 (0·3 to 1·6)	0·7 (0·3 to 1·4)	0·6 (0·3 to 1·2)	0·5 (0·2 to 1)	0·3 (0·1 to 0·6)	0·2 (0·1 to 0·4)	0·1 (0 to 0·2)	0 (0 to 0·1)	0 (0 to 0)	13·7
Urticaria	0 (0 to 0)	0 (0 to 0)	2·2 (1·3 to 3·5)	14·4 (8·4 to 23)	13·1 (7·3 to 21·4)	9·6 (5 to 16·5)	8 (4·1 to 13·9)	6·5 (3·3 to 11·2)	5·5 (2·9 to 9·1)	5 (2·5 to 8·4)	4·5 (2·5 to 7·4)	3·7 (1·9 to 6·1)	3 (1·6 to 5·2)	2·5 (1·3 to 4·3)	2·1 (1·1 to 3·6)	1·6 (0·8 to 2·7)	1·1 (0·6 to 2)	0·8 (0·4 to 1·2)	0·5 (0·3 to 0·8)	0·3 (0·1 to 0·4)	0·1 (0·1 to 0·2)	0 (0 to 0·1)	0 (0 to 0)	84·4
Decubitus ulcer	0 (0 to 0)	0 (0 to 0)	0 (0 to 0)	0·1 (0 to 0·3)	0·1 (0 to 0·2)	0·2 (0·1 to 0·6)	0·4 (0·1 to 0·8)	0·9 (0·2 to 2·1)	1·2 (0·3 to 2·5)	0·9 (0·3 to 1·8)	1 (0·3 to 2·4)	1·2 (0·4 to 2·8)	1·4 (0·5 to 3·2)	1·7 (0·7 to 4·2)	1·8 (0·7 to 4·3)	2·1 (1 to 4·6)	2·5 (1 to 6)	2·3 (1 to 5·5)	2·3 (1·1 to 5·6)	2·2 (1·1 to 5)	1·4 (0·7 to 3·3)	0·7 (0·4 to 1·7)	0·2 (0·1 to 0·5)	24·7
Other skin and subcutaneous diseases	0 (0 to 0)	0 (0 to 0)	1·4 (0·7 to 2·7)	4·1 (1·9 to 7·3)	3·8 (1·7 to 7·2)	4·8 (2·2 to 9·3)	5·6 (2·7 to 10·7)	5·1 (2·4 to 9·8)	5·3 (2·6 to 9·9)	5·4 (2·6 to 10·2)	5·7 (2·8 to 10·1)	5·4 (2·6 to 10)	5·8 (2·8 to 10·6)	6·1 (3 to 11·1)	6·2 (3 to 11·4)	5·7 (2·8 to 10)	5 (2·5 to 8·9)	3·8 (1·8 to 6·8)	2·6 (1·3 to 4·6)	1·6 (0·8 to 2·9)	0·7 (0·4 to 1·3)	0·3 (0·2 to 0·5)	0·1 (0 to 0·1)	84·4
Sense organ diseases	0 (0 to 0)	0 (0 to 0·1)	0·9 (0·5 to 1·5)	10·4 (6·3 to 15·6)	23 (13·9 to 34·6)	30·6 (18·6 to 45·4)	36·2 (22·2 to 53·6)	40·1 (24·5 to 60·6)	47·6 (29·5 to 71·8)	54·7 (33·7 to 84)	59·8 (36·7 to 91·1)	68 (42·1 to 105)	88·1 (56·1 to 141)	104 (66·3 to 167)	119 (75·9 to 183)	115 (73·9 to 173)	108 (72·1 to 161)	95·2 (63·6 to 137)	79·1 (54·5 to 112)	62·9 (43·1 to 86·4)	36·6 (25·6 to 49·2)	17·5 (12·3 to 23·4)	5·5 (4 to 7·4)	1,200
Blindness and vision loss	0 (0 to 0)	0 (0 to 0)	0·2 (0·1 to 0·3)	2·8 (1·5 to 4·7)	6·1 (3·5 to 10)	7·5 (4·4 to 12·1)	8·5 (5·1 to 13·2)	8·9 (5·2 to 13·9)	9·6 (5·8 to 14·4)	10·8 (6·5 to 16·3)	13·1 (8 to 19·9)	16·6 (10·5 to 25·1)	21 (13·2 to 32·6)	24·9 (15·8 to 39·9)	29·8 (18·6 to 46·9)	30·7 (19·6 to 46·4)	31·5 (20·5 to 46·7)	29·8 (19·9 to 42·7)	25·8 (17·7 to 37)	21·1 (14·6 to 29)	12·7 (8·8 to 17·3)	6·5 (4·5 to 8·7)	2·3 (1·6 to 3·2)	320
Glaucoma	0 (0 to 0)	0 (0 to 0)	0 (0 to 0)	0 (0 to 0)	0 (0 to 0)	0 (0 to 0)	0 (0 to 0)	0 (0 to 0)	0 (0 to 0)	0 (0 to 0)	0 (0 to 0)	0 (0 to 0)	0·1 (0 to 0·1)	0·4 (0·1 to 0·9)	1·1 (0·4 to 1·9)	1·7 (0·8 to 2·8)	2·2 (1·2 to 3·5)	2·6 (1·6 to 4·1)	2·9 (1·7 to 4·6)	2·8 (1·7 to 4·2)	1·9 (1·2 to 2·9)	1 (0·6 to 1·5)	0·4 (0·2 to 0·6)	16·9
Cataract	0 (0 to 0)	0 (0 to 0)	0 (0 to 0)	0 (0 to 0)	0 (0 to 0)	0 (0 to 0)	0 (0 to 0)	0 (0 to 0·1)	0·2 (0·1 to 0·4)	0·5 (0·1 to 1·1)	1 (0·4 to 1·9)	1·5 (0·7 to 2·7)	2·2 (1·1 to 3·6)	2·6 (1·3 to 4·3)	3·1 (1·8 to 5·1)	3·5 (2·1 to 5·4)	4·5 (2·8 to 7)	5·6 (3·6 to 8)	6·3 (4·1 to 9·2)	6 (3·9 to 8·5)	3·9 (2·6 to 5·5)	2 (1·3 to 2·8)	0·7 (0·4 to 1)	43·6
Age-related macular degeneration	0 (0 to 0)	0 (0 to 0)	0 (0 to 0)	0 (0 to 0)	0 (0 to 0)	0 (0 to 0)	0 (0 to 0)	0 (0 to 0)	0 (0 to 0)	0 (0 to 0)	0 (0 to 0)	0 (0 to 0)	0 (0 to 0)	0·1 (0 to 0·2)	0·2 (0·1 to 0·3)	0·2 (0·1 to 0·5)	0·3 (0·1 to 0·5)	0·3 (0·1 to 0·5)	0·3 (0·1 to 0·4)	0·2 (0·1 to 0·3)	0·1 (0·1 to 0·2)	0·1 (0 to 0·1)	0 (0 to 0)	1·7
Refraction disorders	0 (0 to 0)	0 (0 to 0)	0·1 (0 to 0·2)	2·3 (1·2 to 4·1)	5·2 (2·8 to 8·7)	6·2 (3·5 to 10·3)	6·9 (3·8 to 11·3)	7 (3·9 to 11·3)	7 (4·2 to 10·9)	6·9 (3·9 to 10·8)	7 (4·2 to 10·8)	6·9 (4 to 11)	6·8 (4·2 to 10·4)	6 (3·5 to 9·5)	5·6 (3·4 to 8·9)	4·6 (2·7 to 7·3)	4 (2·4 to 6·1)	3·4 (2 to 5·3)	2·8 (1·7 to 4·2)	1·9 (1·1 to 2·9)	0·9 (0·6 to 1·4)	0·3 (0·2 to 0·5)	0·1 (0 to 0·1)	92·1
Near vision loss	0 (0 to 0)	0 (0 to 0)	0 (0 to 0)	0 (0 to 0)	0 (0 to 0·1)	0·1 (0 to 0·2)	0·1 (0 to 0·3)	0·3 (0·1 to 0·6)	0·5 (0·2 to 1·2)	1·2 (0·5 to 2·7)	2·3 (0·8 to 5)	4·2 (1·7 to 9·3)	6·7 (2·7 to 14·4)	9·1 (3·7 to 19·3)	11·4 (4·5 to 23·8)	11·4 (4·7 to 23·8)	10·5 (4·3 to 21·5)	8·6 (3·5 to 17·1)	6·5 (2·7 to 13·5)	4·7 (2 to 9·4)	2·5 (1·1 to 4·8)	1·1 (0·5 to 2·2)	0·3 (0·1 to 0·7)	81·6
Other vision loss	0 (0 to 0)	0 (0 to 0)	0·1 (0 to 0·1)	0·4 (0·2 to 0·8)	0·8 (0·4 to 1·5)	1·2 (0·5 to 2·3)	1·4 (0·6 to 2·7)	1·6 (0·6 to 2·9)	1·8 (0·8 to 3·3)	2·1 (1 to 3·8)	2·8 (1·4 to 4·9)	4 (2·1 to 6·7)	5·2 (2·9 to 8·4)	6·7 (4·1 to 10·6)	8·4 (5·2 to 12·7)	9·3 (6 to 13·7)	10·2 (6·5 to 14·7)	9·3 (6·1 to 13·5)	7 (4·4 to 10·5)	5·5 (3·6 to 8)	3·4 (2·2 to 5)	2 (1·4 to 2·9)	0·8 (0·5 to 1·2)	84·2
Age-related and other hearing loss	0 (0 to 0)	0 (0 to 0)	0·4 (0·2 to 0·7)	5·6 (2·6 to 10)	14·5 (7·8 to 23·2)	20·7 (11·5 to 31·3)	25 (14·2 to 38·2)	28·6 (16·6 to 43·9)	35·2 (20·7 to 54·5)	40·5 (23·5 to 63·9)	43 (25·4 to 68·1)	47·9 (27·9 to 77·5)	63·5 (37·8 to 104)	75·1 (45·7 to 120)	85·4 (52·6 to 136)	80·1 (49·6 to 123)	72·5 (46·2 to 112)	61·9 (40·2 to 91·7)	50·6 (33·8 to 72·9)	40 (26·7 to 56·2)	23·1 (15·7 to 31·8)	10·7 (7·5 to 14·7)	3·1 (2·2 to 4·2)	828
Other sense organ diseases	0 (0 to 0)	0 (0 to 0)	0·4 (0·2 to 0·7)	2 (1 to 3·6)	2·4 (1·1 to 4·1)	2·4 (1·1 to 4·5)	2·7 (1·1 to 5·1)	2·6 (1·1 to 4·9)	2·9 (1·1 to 5·7)	3·5 (1·5 to 6·9)	3·7 (1·7 to 7)	3·5 (1·5 to 6·8)	3·6 (1·5 to 6·8)	4·2 (2 to 8·6)	4·2 (1·9 to 8·3)	4·3 (1·9 to 8)	4·2 (1·9 to 8)	3·5 (1·6 to 6·5)	2·8 (1·3 to 5·1)	1·8 (0·9 to 3·4)	0·8 (0·4 to 1·4)	0·3 (0·1 to 0·5)	0·1 (0·1 to 0·2)	55·8
Musculoskeletal disorders	0 (0 to 0)	0 (0 to 0)	0 (0 to 0)	0 (0 to 0)	9·8 (4·6 to 17·9)	50·7 (29·8 to 81·6)	107 (67·3 to 162)	140 (91·8 to 208)	181 (114 to 263)	202 (127 to 285)	217 (143 to 309)	221 (150 to 311)	226 (152 to 329)	216 (146 to 316)	207 (140 to 292)	170 (114 to 244)	131 (89·6 to 189)	93 (62 to 132)	60·2 (39·8 to 87·8)	37·7 (25·1 to 54·3)	18·4 (12·7 to 26·3)	7 (4·7 to 10·2)	1·9 (1·3 to 2·8)	2,300
Rheumatoid arthritis	0 (0 to 0)	0 (0 to 0)	0 (0 to 0)	0 (0 to 0)	0 (0 to 0·1)	0·4 (0·2 to 0·7)	1 (0·4 to 1·7)	1·6 (0·8 to 2·8)	2·2 (1·1 to 3·8)	2·5 (1·3 to 4·3)	2·9 (1·3 to 5·1)	3 (1·4 to 4·9)	3 (1·6 to 5·1)	2·9 (1·6 to 4·7)	2·7 (1·5 to 4·3)	2·1 (1·2 to 3·3)	1·7 (1 to 2·5)	1·1 (0·7 to 1·7)	0·8 (0·5 to 1·2)	0·6 (0·4 to 0·8)	0·7 (0·4 to 1)	0·3 (0·2 to 0·4)	0·1 (0 to 0·1)	29·5
Osteoarthritis	0 (0 to 0)	0 (0 to 0)	0 (0 to 0)	0 (0 to 0)	0 (0 to 0)	0 (0 to 0)	0 (0 to 0)	0 (0 to 0)	0 (0 to 0)	1·2 (0·5 to 2·6)	6·4 (3 to 13·4)	15·5 (7·5 to 31·8)	27·2 (13·2 to 54·9)	36·7 (17·8 to 73·7)	43·7 (21·6 to 87·6)	41·3 (20·6 to 81·8)	35 (17·6 to 69·9)	26·6 (13·2 to 53·7)	18·4 (9·2 to 37)	12·1 (6 to 24·1)	6 (3·1 to 11·9)	2·5 (1·3 to 4·8)	0·7 (0·4 to 1·4)	273
Osteoarthritis hip	0 (0 to 0)	0 (0 to 0)	0 (0 to 0)	0 (0 to 0)	0 (0 to 0)	0 (0 to 0)	0 (0 to 0)	0 (0 to 0)	0 (0 to 0)	0·5 (0·1 to 1·1)	0·7 (0·2 to 1·6)	1 (0·3 to 2·2)	1·5 (0·6 to 3·4)	2·1 (0·8 to 5·1)	2·7 (1·2 to 5·8)	2·8 (1·2 to 6)	2·5 (1·1 to 5·3)	1·9 (0·9 to 4·1)	1·4 (0·6 to 2·8)	0·9 (0·4 to 1·8)	0·4 (0·2 to 0·9)	0·2 (0·1 to 0·4)	0·1 (0 to 0·1)	18·4
Osteoarthritis knee	0 (0 to 0)	0 (0 to 0)	0 (0 to 0)	0 (0 to 0)	0 (0 to 0)	0 (0 to 0)	0 (0 to 0)	0 (0 to 0)	0 (0 to 0)	0·6 (0·2 to 1·3)	4·6 (2 to 9·7)	11·5 (5·3 to 24·3)	20·1 (9·7 to 40·7)	26·8 (12·6 to 54·4)	31·3 (15 to 63·5)	29·2 (14·1 to 58·9)	24·5 (11·9 to 50·2)	18·4 (9 to 37·9)	12·4 (6·2 to 25·2)	8 (3·9 to 16)	3·7 (1·8 to 7·5)	1·4 (0·7 to 2·9)	0·4 (0·2 to 0·8)	193
Osteoarthritis hand	0 (0 to 0)	0 (0 to 0)	0 (0 to 0)	0 (0 to 0)	0 (0 to 0)	0 (0 to 0)	0 (0 to 0)	0 (0 to 0)	0 (0 to 0)	0·1 (0 to 0·3)	0·5 (0·1 to 1·4)	1·3 (0·4 to 2·9)	2·2 (0·9 to 4·9)	3 (1·2 to 6·9)	3·6 (1·6 to 8·2)	3·4 (1·6 to 7·4)	2·9 (1·3 to 6)	2·2 (1 to 4·7)	1·6 (0·7 to 3·3)	1·1 (0·5 to 2·4)	0·6 (0·3 to 1·3)	0·3 (0·1 to 0·6)	0·1 (0 to 0·2)	23

Osteoarthritis other	0 (0 to 0)	0 (0 to 0)	0 (0 to 0)	0 (0 to 0)	0 (0 to 0)	0 (0 to 0)	0 (0 to 0)	0 (0 to 0)	0 (0 to 0)	0·1 (0 to 0·2)	0·6 (0·2 to 1·6)	1·8 (0·6 to 4·2)	3·4 (1·4 to 7·5)	4·8 (2·1 to 10·3)	6·1 (2·7 to 13·5)	6 (2·7 to 12·4)	5·2 (2·4 to 10·8)	4·1 (2 to 8·5)	3 (1·5 to 6·2)	2·2 (1·1 to 4·5)	1·2 (0·6 to 2·4)	0·5 (0·3 to 1·1)	0·2 (0·1 to 0·3)	39
Low back pain	0 (0 to 0)	0 (0 to 0)	0 (0 to 0)	0 (0 to 0)	8·9 (3·8 to 16·8)	45·9 (25·7 to 75·7)	84·1 (49·3 to 134)	93·3 (53·8 to 150)	101 (56·3 to 162)	103 (58·7 to 156)	107 (62·7 to 167)	103 (60·9 to 160)	99·6 (60·1 to 157)	91·6 (55·4 to 147)	84·2 (51·2 to 128)	68·1 (43 to 101)	53 (32·6 to 81·3)	37·7 (23 to 56·9)	24·8 (15·2 to 37·9)	16·4 (10·4 to 25·3)	7·5 (4·5 to 11·1)	2·8 (1·7 to 4·3)	0·8 (0·5 to 1·2)	1,130
Neck pain	0 (0 to 0)	0 (0 to 0)	0 (0 to 0)	0 (0 to 0)	0·6 (0·1 to 1·4)	3·6 (1·3 to 7·5)	7·9 (3·1 to 15)	10·9 (4·5 to 22·1)	15·9 (7·1 to 32·9)	22·2 (10·3 to 42·8)	26·6 (13·3 to 46·6)	28·6 (14·2 to 51)	29 (14·3 to 51·5)	23·3 (11·4 to 43·8)	20 (9·7 to 35·6)	14·7 (7·3 to 26·6)	10·4 (5·4 to 17·8)	7·1 (3·6 to 12·4)	4·3 (2·2 to 8)	2·5 (1·2 to 4·3)	1·2 (0·6 to 2)	0·5 (0·2 to 0·8)	0·2 (0·1 to 0·3)	229
Gout	0 (0 to 0)	0 (0 to 0)	0 (0 to 0)	0 (0 to 0)	0 (0 to 0)	0 (0 to 0)	0 (0 to 0·1)	0·3 (0·1 to 0·5)	0·8 (0·1 to 1·9)	1·3 (0·4 to 2·8)	1·7 (0·7 to 3·2)	2 (0·8 to 3·7)	2·1 (1 to 3·5)	2 (0·8 to 3·7)	1·9 (0·9 to 3·5)	1·6 (0·6 to 3)	1·4 (0·7 to 2·4)	1·1 (0·6 to 2)	0·9 (0·4 to 1·5)	0·7 (0·3 to 1·2)	0·4 (0·2 to 0·7)	0·2 (0·1 to 0·3)	0·1 (0 to 0·1)	18·3
Other musculoskeletal disorders	0 (0 to 0)	0 (0 to 0)	0 (0 to 0)	0 (0 to 0)	0·3 (0·2 to 0·4)	0·8 (0·4 to 2·3)	13·8 (5·2 to 27·2)	34·1 (19 to 55·2)	60·9 (34·7 to 99·2)	71·6 (40·9 to 114)	73 (41·8 to 114)	69·5 (38·3 to 113)	64·9 (37·1 to 102)	59·1 (32·2 to 96·4)	54·4 (30·8 to 86)	42·2 (21·5 to 68·3)	30 (16·3 to 49·8)	19·4 (9·6 to 33·7)	11 (5·5 to 18·8)	5·4 (1·6 to 10·4)	2·7 (1·2 to 4·5)	0·7 (0·4 to 1·5)	0·2 (0·1 to 0·3)	614
Other non-communicable diseases	291 (187 to 430)	181 (123 to 245)	501 (364 to 667)	180 (137 to 233)	114 (82 to 151)	101 (79·4 to 130)	113 (89·7 to 142)	117 (90·8 to 151)	127 (95 to 172)	135 (101 to 185)	130 (97·3 to 180)	118 (86 to 168)	113 (82·9 to 162)	112 (83·2 to 153)	119 (89·8 to 160)	115 (87 to 151)	99·3 (74·9 to 131)	82·9 (62·7 to 109)	60·5 (45·2 to 79·6)	45·5 (34·9 to 58·3)	26·1 (20·2 to 33)	11·5 (8·9 to 14·6)	3·6 (2·7 to 4·7)	2,900
Congenital birth defects	280 (174 to 417)	157 (102 to 218)	318 (210 to 450)	99·2 (70·6 to 135)	47·2 (34·8 to 61·4)	45·3 (34·5 to 58·9)	49·6 (35·5 to 63·5)	37·8 (26 to 49·7)	30·6 (20·2 to 40·4)	23·8 (16·3 to 31·7)	17·6 (12·7 to 23·1)	12·8 (9·4 to 16·7)	10·5 (7·9 to 13·8)	8·4 (6·3 to 11·2)	6·9 (5·1 to 9)	4 (2·9 to 5·3)	2·3 (1·7 to 3·1)	0·7 (0·4 to 1·2)	0·4 (0·3 to 0·7)	0·2 (0·1 to 0·4)	0·1 (0·1 to 0·2)	0 (0 to 0·1)	0 (0 to 0)	1,150
Neural tube defects	22 (9·9 to 39·2)	8 (3·7 to 14·6)	14·8 (6·4 to 28·4)	10·6 (5·1 to 20)	2 (1·3 to 2·8)	2·6 (1·7 to 3·8)	3·2 (2 to 4·8)	1·8 (1·2 to 2·8)	1·8 (1·1 to 3)	1 (0·6 to 1·6)	0·4 (0·3 to 0·6)	0·4 (0·3 to 0·6)	0·2 (0·2 to 0·4)	0·1 (0·1 to 0·2)	0·1 (0·1 to 0·1)	0·1 (0 to 0·1)	0 (0 to 0)	0 (0 to 0)	0 (0 to 0)	0 (0 to 0)	0 (0 to 0)	0 (0 to 0)	0 (0 to 0)	69·3
Congenital heart anomalies	101 (53·9 to 162)	75·2 (46·9 to 111)	150 (94·3 to 218)	34 (21·5 to 52)	13·3 (8·2 to 19·6)	11·6 (7·4 to 17·1)	14·4 (8·7 to 20·9)	11·2 (6·5 to 17·2)	9·3 (4·9 to 13·7)	7·5 (4·3 to 11·4)	5·2 (3·1 to 7·9)	3·1 (2 to 4·7)	2·2 (1·5 to 3·4)	1·5 (0·9 to 2·3)	0·9 (0·6 to 1·4)	0·6 (0·4 to 1)	0·4 (0·2 to 0·6)	0·1 (0 to 0·1)	0 (0 to 0·1)	0 (0 to 0·1)	0 (0 to 0)	0 (0 to 0)	0 (0 to 0)	441
Orofacial clefts	0·3 (0·1 to 0·7)	0·2 (0·1 to 0·5)	0·7 (0·3 to 1·6)	0·8 (0·3 to 1·4)	0·8 (0·3 to 1·7)	0·8 (0·3 to 1·6)	0·8 (0·3 to 1·6)	0·7 (0·2 to 1·4)	0·6 (0·2 to 1·2)	0·5 (0·1 to 1)	0·5 (0·1 to 0·9)	0·4 (0·1 to 0·8)	0·4 (0·1 to 0·7)	0·3 (0·1 to 0·6)	0·2 (0·1 to 0·4)	0·2 (0·1 to 0·4)	0·1 (0 to 0·2)	0·1 (0 to 0·2)	0·1 (0 to 0·1)	0 (0 to 0·1)	0 (0 to 0)	0 (0 to 0)	0 (0 to 0)	8·5
Down syndrome	5 (1·9 to 8·9)	3·9 (2 to 6·7)	21·2 (10·6 to 35·2)	8 (4·3 to 12·9)	2·8 (1·8 to 4·2)	3·9 (2·4 to 6·1)	6·2 (3·6 to 9·9)	4·6 (2·7 to 7·1)	4·2 (2·2 to 7·3)	4·2 (2·1 to 7)	3·5 (2 to 5·6)	2·9 (1·8 to 4·5)	2·6 (1·6 to 3·9)	2·2 (1·3 to 3·4)	2·4 (1·5 to 3·6)	1·1 (0·6 to 1·7)	0·5 (0·3 to 0·7)	0 (0 to 0)	0 (0 to 0)	0 (0 to 0)	0 (0 to 0)	0 (0 to 0)	0 (0 to 0)	79·1
Turner syndrome	0 (0 to 0)	0 (0 to 0)	0 (0 to 0)	0 (0 to 0)	0 (0 to 0)	0 (0 to 0)	0 (0 to 0)	0 (0 to 0)	0 (0 to 0)	0 (0 to 0)	0 (0 to 0)	0 (0 to 0)	0 (0 to 0)	0 (0 to 0)	0 (0 to 0)	0 (0 to 0)	0 (0 to 0)	0 (0 to 0)	0 (0 to 0)	0 (0 to 0)	0 (0 to 0)	0 (0 to 0)	0 (0 to 0)	0
Klinefelter syndrome	0 (0 to 0)	0 (0 to 0)	0 (0 to 0)	0 (0 to 0)	0 (0 to 0)	0 (0 to 0)	0 (0 to 0)	0 (0 to 0)	0 (0 to 0)	0 (0 to 0)	0 (0 to 0)	0 (0 to 0)	0 (0 to 0)	0 (0 to 0)	0 (0 to 0)	0 (0 to 0)	0 (0 to 0)	0 (0 to 0)	0 (0 to 0)	0 (0 to 0)	0 (0 to 0)	0 (0 to 0)	0 (0 to 0)	0·1
Other chromosomal abnormalities	6·6 (3 to 11·7)	3·6 (1·9 to 6·2)	8·2 (4·9 to 12·9)	3·7 (2·4 to 5·4)	2·2 (1·4 to 3·3)	2·2 (1·4 to 3·3)	2·3 (1·5 to 3·5)	1·3 (0·9 to 2)	1·2 (0·8 to 1·8)	0·5 (0·3 to 0·7)	0·4 (0·3 to 0·6)	0·2 (0·1 to 0·3)	0·1 (0·1 to 0·2)	0·1 (0 to 0·1)	0·1 (0 to 0·1)	0 (0 to 0)	0 (0 to 0)	0 (0 to 0)	0 (0 to 0)	0 (0 to 0)	0 (0 to 0)	0 (0 to 0)	0 (0 to 0)	32·7
Congenital musculoskeletal and limb anomalies	7·8 (3·8 to 14·6)	4·1 (2·1 to 7·2)	12·8 (7·6 to 20·9)	12·3 (7·1 to 19)	11·4 (6·5 to 17·7)	11·8 (6·6 to 18·4)	9·4 (5·2 to 15·1)	5·4 (3·2 to 8·4)	4·5 (2·6 to 7)	3·3 (1·8 to 5·3)	3·1 (1·8 to 4·9)	2·6 (1·4 to 4·2)	2·2 (1·2 to 3·7)	1·9 (1·1 to 3)	1·6 (0·9 to 2·5)	1·1 (0·6 to 1·8)	0·7 (0·4 to 1·1)	0·4 (0·2 to 0·6)	0·2 (0·1 to 0·3)	0·1 (0 to 0·1)	0 (0 to 0·1)	0 (0 to 0)	0 (0 to 0)	96·7
Urogenital congenital anomalies	10·9 (5 to 23·5)	5·6 (2·9 to 10)	7·3 (3·8 to 13·4)	2·6 (1·6 to 4)	1·6 (1 to 2·4)	1·3 (0·8 to 1·9)	0·7 (0·4 to 1·1)	0·8 (0·4 to 1·1)	0·5 (0·3 to 0·9)	0·6 (0·3 to 0·9)	0·2 (0·1 to 0·3)	0·2 (0·1 to 0·2)	0·2 (0·1 to 0·3)	0·1 (0 to 0·1)	0·1 (0·1 to 0·2)	0·1 (0 to 0·1)	0 (0 to 0·1)	0 (0 to 0)	0 (0 to 0)	0 (0 to 0)	0 (0 to 0)	0 (0 to 0)	0 (0 to 0)	32·7
Digestive congenital anomalies	46 (24·8 to 77·9)	29·8 (16·6 to 46·5)	41·3 (23·3 to 70·4)	6·8 (4 to 10·9)	2·3 (1·3 to 3·7)	1·2 (0·7 to 1·8)	1·1 (0·6 to 1·7)	0·3 (0·2 to 0·5)	0·5 (0·3 to 0·7)	0·2 (0·1 to 0·4)	0·1 (0·1 to 0·2)	0·1 (0·1 to 0·2)	0·1 (0·1 to 0·2)	0·1 (0·1 to 0·2)	0·1 (0 to 0·1)	0·1 (0 to 0·1)	0·1 (0 to 0·1)	0 (0 to 0)	0 (0 to 0)	0 (0 to 0)	0 (0 to 0)	0 (0 to 0)	0 (0 to 0)	130
Other congenital birth defects	80 (44·9 to 128)	26·8 (15·7 to 42·2)	62·2 (35·5 to 97)	20·4 (12·9 to 31·6)	10·9 (7 to 16·2)	10 (6·6 to 14·4)	11·5 (7·2 to 17·1)	11·7 (7·1 to 17·5)	8 (4·6 to 12·2)	6 (3·5 to 9·3)	4 (2·5 to 6·2)	2·9 (1·8 to 4·6)	2·4 (1·5 to 3·8)	2·1 (1·3 to 3·4)	1·4 (0·8 to 2·3)	0·8 (0·4 to 1·4)	0·6 (0·3 to 1)	0·2 (0·1 to 0·5)	0·1 (0·1 to 0·4)	0·1 (0 to 0·2)	0 (0 to 0·1)	0 (0 to 0)	0 (0 to 0)	262
Urinary diseases and male infertility	0·7 (0·3 to 1·2)	2 (0·9 to 3·2)	9·3 (5·2 to 15·6)	2·8 (1·9 to 4·2)	1·5 (1 to 2·3)	2 (1·4 to 3)	3·7 (2·6 to 5·1)	10 (6·5 to 14·9)	10·6 (6·6 to 15·8)	13·7 (8·3 to 19·9)	16·4 (9·7 to 23·5)	14·9 (9·7 to 20·8)	16·2 (10·1 to 23·3)	20·4 (13·4 to 27·8)	31·1 (21·4 to 41·7)	37·6 (26·2 to 50·4)	36·7 (25·8 to 49·9)	34·1 (23·4 to 46·4)	25·9 (17·7 to 35·4)	20·7 (13·6 to 28·8)	12·1 (8 to 17)	5·2 (3·4 to 7·4)	1·6 (1 to 2·3)	329
Urolithiasis	0 (0 to 0)	0 (0 to 0)	0 (0 to 0)	0·1 (0 to 0·1)	0·1 (0 to 0·1)	0·1 (0·1 to 0·2)	0·2 (0·1 to 0·3)	0·4 (0·2 to 0·6)	0·6 (0·3 to 1)	0·9 (0·5 to 1·6)	1·4 (0·6 to 2·6)	1·3 (0·6 to 2·3)	2 (1 to 3·5)	1·9 (1 to 3·2)	2·4 (1·3 to 3·7)	1·8 (1·1 to 2·8)	1·1 (0·6 to 1·8)	0·8 (0·4 to 1·2)	0·5 (0·2 to 0·8)	0·2 (0·1 to 0·4)	0·1 (0 to 0·2)	0 (0 to 0·1)	0 (0 to 0)	15·8
Benign prostatic hyperplasia	0 (0 to 0)	0 (0 to 0)	0 (0 to 0)	0 (0 to 0)	0 (0 to 0)	0 (0 to 0)	0 (0 to 0)	0 (0 to 0)	0 (0 to 0)	0 (0 to 0)	0 (0 to 0)	0·1 (0 to 0·3)	0·8 (0·3 to 1·7)	3 (1·4 to 5·3)	8·7 (4·3 to 14·8)	13·6 (7 to 23·4)	16·2 (9·2 to 25·9)	14·5 (7·8 to 24)	9·6 (5·3 to 15·9)	5·3 (3 to 8·6)	2·1 (1·1 to 3·4)	0·7 (0·3 to 1·1)	0·1 (0·1 to 0·2)	74·7
Male infertility	0 (0 to 0)	0 (0 to 0)	0 (0 to 0)	0 (0 to 0)	0 (0 to 0)	0 (0 to 0)	0 (0 to 0)	2·5 (0·7 to 6·6)	2·7 (0·7 to 6·7)	2·5 (0·7 to 6·8)	2·1 (0·6 to 5·4)	1·7 (0·5 to 4·5)	0·2 (0 to 0·7)	0 (0 to 0)	0 (0 to 0)	0 (0 to 0)	0 (0 to 0)	0 (0 to 0)	0 (0 to 0)	0 (0 to 0)	0 (0 to 0)	0 (0 to 0)	0 (0 to 0)	11·7
Other urinary diseases	0·3 (0·1 to 0·5)	1·3 (0·6 to 2·1)	1·2 (0·5 to 2·5)	0·5 (0·3 to 0·9)	0·6 (0·3 to 1·2)	0·9 (0·5 to 1·5)	1·8 (1·1 to 2·8)	3·5 (1·4 to 5·5)	1·8 (0·6 to 3·3)	2·6 (1·1 to 4·5)	4·3 (2 to 7·2)	3·3 (1·7 to 5·5)	4·2 (2 to 7)	3·9 (2 to 6·8)	4·2 (2·1 to 6·7)	5·2 (2·4 to 8·4)	4·3 (2·1 to 7·2)	2·9 (1·3 to 5·2)	2·6 (1·1 to 4·6)	2·4 (1 to 4·4)	1·2 (0·5 to 2·3)	0·7 (0·3 to 1·2)	0·2 (0·1 to 0·4)	53·9
Gynecological diseases	0 (0 to 0)	0 (0 to 0)	0 (0 to 0)	0 (0 to 0)	0 (0 to 0)	0 (0 to 0)	0 (0 to 0)	0 (0 to 0)	0 (0 to 0)	0 (0 to 0)	0 (0 to 0)	0 (0 to 0)	0 (0 to 0)	0 (0 to 0)	0 (0 to 0)	0 (0 to 0)	0 (0 to 0)	0 (0 to 0)	0 (0 to 0)	0 (0 to 0)	0 (0 to 0)	0 (0 to 0)	0 (0 to 0)	0
Uterine fibroids	0 (0 to 0)	0 (0 to 0)	0 (0 to 0)	0 (0 to 0)	0 (0 to 0)	0 (0 to 0)	0 (0 to 0)	0 (0 to 0)	0 (0 to 0)	0 (0 to 0)	0 (0 to 0)	0 (0 to 0)	0 (0 to 0)	0 (0 to 0)	0 (0 to 0)	0 (0 to 0)	0 (0 to 0)	0 (0 to 0)	0 (0 to 0)	0 (0 to 0)	0 (0 to 0)	0 (0 to 0)	0 (0 to 0)	0
Polycystic ovarian syndrome	--	--	--	--	--	--	--	--	--	--	--	--	--	--	--	--	--	--	--	--	--	--	--	NA
Female infertility	--	--	--	--	--	--	--	--	--	--	--	--	--	--	--	--	--	--	--	--	--	--	--	NA
Endometriosis	--	--	--	--	--	--	--	--	--	--	--	--	--	--	--	--	--	--	--	--	--	--	--	NA
Genital prolapse	0 (0 to 0)	0 (0 to 0)	0 (0 to 0)	0 (0 to 0)	0 (0 to 0)	0 (0 to 0)	0 (0 to 0)	0 (0 to 0)	0 (0 to 0)	0 (0 to 0)	0 (0 to 0)	0 (0 to 0)	0 (0 to 0)	0 (0 to 0)	0 (0 to 0)	0 (0 to 0)	0 (0 to 0)	0 (0 to 0)	0 (0 to 0)	0 (0 to 0)	0 (0 to 0)	0 (0 to 0)	0 (0 to 0)	0
Premenstrual syndrome	--	--	--	--	--	--	--	--	--	--	--	--	--	--	--	--	--	--	--	--	--	--	--	NA
Other gynecological diseases	0 (0 to 0)	0 (0 to 0)	0 (0 to 0)	0 (0 to 0)	0 (0 to 0)	0 (0 to 0)	0 (0 to 0)	0 (0 to 0)	0 (0 to 0)	0 (0 to 0)	0 (0 to 0)	0 (0 to 0)	0 (0 to 0)	0 (0 to 0)	0 (0 to 0)	0 (0 to 0)	0 (0 to 0)	0 (0 to 0)	0 (0 to 0)	0 (0 to 0)	0 (0 to 0)	0 (0 to 0)	0 (0 to 0)	0
Hemoglobinopathies and hemolytic anemias	0·5 (0·3 to 0·7)	1·4 (0·8 to 2)	8·5 (5·2 to 12·6)	20·6 (12·1 to 32)	28·2 (15·6 to 45·3)	18·9 (11 to 30·3)	20·7 (13·8 to 30·9)	19·2 (12·4 to 28·5)	16 (10·2 to 24·1)	13·9 (8·8 to 20·8)	10·6 (7 to 16)	9·4 (6·2 to 14·1)	7·6 (5 to 12)	6·3 (4·1 to 10·1)	6·2 (3·7 to 10·2)	4·7 (2·7 to 8)	2·8 (1·7 to 4·8)	2·3 (1·4 to 3·7)	1·6 (1 to 2·7)	1·3 (0·8 to 2·1)	1·2 (0·7 to 1·8)	0·6 (0·4 to 1)	0·3 (0·1 to 0·4)	203
Thalassemias	0 (0 to 0)	0 (0 to 0)	0 (0 to 0)	0 (0 to 0·1)	0 (0 to 0)	0 (0 to 0)	0 (0 to 0)	0 (0 to 0)	0 (0 to 0)	0 (0 to 0)	0 (0 to 0)	0 (0 to 0)	0 (0 to 0)	0 (0 to 0)	0 (0 to 0)	0 (0 to 0)	0 (0 to 0)	0 (0 to 0)	0 (0 to 0)	0 (0 to 0)	0 (0 to 0)	0 (0 to 0)	0 (0 to 0)	0·1
Thalassemias trait	0 (0 to 0)	0 (0 to 0)	0·2 (0·1 to 0·3)	0·7 (0·3 to 1·5)	1·1 (0·4 to 2·2)	0·5 (0·1 to 1·3)	0·2 (0·1 to 0·6)	0·1 (0 to 0·4)	0·1 (0 to 0·3)	0·1 (0 to 0·3)	0·1 (0 to 0·3)	0·1 (0 to 0·3)	0·1 (0 to 0·3)	0·1 (0 to 0·3)	0·1 (0 to 0·3)	0·1 (0 to 0·2)	0·1 (0 to 0·1)	0 (0 to 0·1)	0 (0 to 0·1)	0 (0 to 0·1)	0 (0 to 0)	0 (0 to 0)	0 (0 to 0)	4·1
Sickle cell disorders	0·4 (0·2 to 0·6)	0·6 (0·3 to 1)	5·6 (3 to 9·1)	8·6 (5·5 to 12·9)	9·6 (6·4 to 13·7)	9·6 (6·3 to 14·1)	13·1 (8·4 to 18·9)	12·1 (7·3 to 18·7)	8·9 (5·3 to 13·7)	6·7 (3·8 to 10·6)	4·4 (2·5 to 6·8)	3·3 (1·9 to 5·3)	2 (1·1 to 3·2)	1 (0·5 to 1·8)	0·4 (0·2 to 0·8)	0·1 (0 to 0·2)	0 (0 to 0)	0 (0 to 0)	0 (0 to 0)	0 (0 to 0)	0 (0 to 0)	0 (0 to 0)	0 (0 to 0)	86·5
Sickle cell trait	0·1 (0 to 0·1)	0·5 (0·2 to 1)	1·7 (0·7 to 3·3)	7·1 (2·8 to 13·5)	11·2 (4·1 to 20·9)	5·3 (1·3 to 11·9)	2·2 (0·6 to 5·2)	1·4 (0·3 to 3·6)	1·3 (0·3 to 3·2)	1·3 (0·3 to 3·1)	1·1 (0·3 to 3)	1 (0·3 to 2·6)	1·1 (0·3 to 2·8)	1·1 (0·3 to 2·4)	1·2 (0·4 to 2·7)	0·9 (0·3 to 2)	0·6 (0·2 to 1·2)	0·4 (0·1 to 0·9)	0·3 (0·1 to 0·7)	0·3 (0·1 to 0·5)	0·2 (0·1 to 0·3)	0·1 (0 to 0·1)	0 (0 to 0)	40·4
G6PD deficiency	0 (0 to 0)	0·1 (0 to 0·1)	0·1 (0 to 0·2)	0·6 (0·3 to 1·1)	0·9 (0·4 to 1·6)	1 (0·4 to 1·8)	1·9 (0·8 to 3·6)	3·1 (1·1 to 6)	3·5 (1·6 to 6·6)	3·5 (1·6 to 6·2)	3 (1·4 to 5·2)	3 (1·6 to 4·9)	2·5 (1·5 to 3·7)	2·2 (1·3 to 3·2)	2·1 (1·3 to 3)	1·4 (0·9 to 2)	0·7 (0·4 to 1·1)	0·4 (0·2 to 0·6)	0·2 (0·1 to 0·3)	0·1 (0 to 0·1)	0 (0 to 0·1)	0 (0 to 0)	0 (0 to 0)	30·3
G6PD trait	0 (0 to 0)	0 (0 to 0)	0 (0 to 0)	0 (0 to 0)	0 (0 to 0)	0 (0 to 0)	0 (0 to 0)	0 (0 to 0)	0 (0 to 0)	0 (0 to 0)	0 (0 to 0)	0 (0 to 0)	0 (0 to 0)	0 (0 to 0)	0 (0 to 0)	0 (0 to 0)	0 (0 to 0)	0 (0 to 0)	0 (0 to 0)	0 (0 to 0)	0 (0 to 0)	0 (0 to 0)	0 (0 to 0)	0
Other hemoglobinopathies and hemolytic anemias	0 (0 to 0)	0·1 (0·1 to 0·2)	0·9 (0·3 to 2)	3·6 (1·1 to 7·8)	5·4 (1·6 to 11·8)	2·5 (0·6 to 6·1)	3·4 (0·9 to 8·6)	2·4 (0·8 to 6·3)	2·2 (0·7 to 5·1)	2·2 (0·7 to 5·1)	1·9 (0·7 to 4·5)	1·8 (0·7 to 4·2)	1·9 (0·7 to 4·2)	2 (0·8 to 4·2)	2·3 (1·1 to 4·7)	2·2 (1 to 4·3)	1·5 (0·8 to 2·8)	1·4 (0·8 to 2·2)	1 (0·6 to 1·7)	0·9 (0·6 to 1·5)	0·9 (0·6 to 1·4)	0·5 (0·3 to 0·9)	0·2 (0·1 to 0·4)	41·4
Endocrine, metabolic, blood, and immune disorders	9·7 (5·6 to 17·8)	10 (6 to 16·4)	94 (56 to 156)	48 (31·2 to 79)	26·2 (17 to 40)	21·7 (14·9 to 33·1)	19·8 (13·9 to 27·8)	23 (15·3 to 32·7)	33·8 (21·5 to 48·2)	42 (27·7 to 59·9)	41·5 (28·4 to 59·4)	37·5 (25·7 to 55·6)	35·5 (23·8 to 54·4)	36·4 (25·5 to 56·8)	34·4 (24·1 to 53·4)	30·6 (20·9 to 48·5)	23·3 (15·9 to 37·8)	18·8 (12·7 to 30·8)	13·6 (9 to 22·4)	10·6 (7·1 to 17·1)	6·5 (4·3 to 11·3)	3 (2 to 5·4)	0·9 (0·5 to 1·8)	621
Oral disorders	0 (0 to 0)	0 (0 to 0)	0 (0 to 0)	8·8 (4·9 to 14·7)	11·4 (6·1 to 19·4)	13·4 (7·2 to 23·7)	19·3 (10·4 to 34·3)	27·1 (13·9 to 49·4)	35·9 (17·6 to 69)	41·4 (19·7 to 77·5)	44·2 (20·6 to 83·6)	43·9 (21·1 to 83·3)	43 (21·6 to 80·4)	40·3 (21 to 72·7)	40·5 (22·5 to 68·9)	38·1 (22·1 to 61·9)	34·1 (20·4 to 54)	27 (16·6 to 41·9)	19 (11·7 to 28·6)	12·6 (7·8 to 19)	6·2 (3·8 to 9·4)	2·6 (1·6 to 3·9)	0·8 (0·5 to 1·2)	509
Caries of deciduous teeth	0 (0 to 0)	0 (0 to 0)	0 (0 to 0)	3·1 (1·2 to 6·3)	3·1 (1·3 to 6·9)	0·2 (0 to 0·5)	0 (0 to 0)	0 (0 to 0)	0 (0 to 0)	0 (0 to 0)	0 (0 to 0)	0 (0 to 0)	0 (0 to 0)	0 (0 to 0)	0 (0 to 0)	0 (0 to 0)	0 (0 to 0)	0 (0 to 0)	0 (0 to 0)	0 (0 to 0)	0 (0 to 0)	0 (0 to 0)	0 (0 to 0)	6·4
Caries of permanent teeth	0 (0 to 0)	0 (0 to 0)	0 (0 to 0)	0 (0 to 0)	2·7 (0·9 to 6)	6·7 (2·7 to 14·3)	7·1 (2·8 to 14·9)	7·1 (2·9 to 15·1)	6·2 (2·4 to 12·9)	5 (2 to 10·7)	4·5 (1·8 to 9)	3·7 (1·5 to 7·5)	3·1 (1·2 to 6·5)	2·5 (1 to 5·4)	2 (0·7 to 4·5)	1·3 (0·5 to 3)	0·9 (0·3 to 1·8)	0·5 (0·2 to 1·1)	0·3 (0·1 to 0·7)	0·2 (0·1 to 0·4)	0·1 (0 to 0·2)	0 (0 to 0·1)	0 (0 to 0)	53·8
Periodontal diseases	0 (0 to 0)	0 (0 to 0)	0 (0 to 0)	0 (0 to 0)	0 (0 to 0)	0 (0 to 0)	2·7 (0·8 to 7·2)	8·9 (2·7 to 22·6)	17·4 (5·3 to 41·9)	23·3 (7·8 to 53·2)	26·3 (9·4 to 59·6)	26·8 (9·5 to 60·3)	25·7 (9·6 to 55)	22 (8·7 to 47·6)	17·9 (7·3 to 38·6)	12·1 (4·8 to 25·2)	7·8 (3 to 16·2)	4·8 (1·9 to 9·9)	3 (1·2 to 5·8)	1·8 (0·7 to 3·7)	0·9 (0·3 to 1·8)	0·4 (0·1 to 0·7)	0·1 (0 to 0·2)	202
Other oral disorders	0 (0 to 0)	0 (0 to 0)	0 (0 to 0)	5·7 (3·3 to 8·9)	5·6 (3·1 to 9)	6·6 (3·8 to 10·5)	9·6 (5·8 to 14·9)	10·3 (6·2 to 16·3)	10·2 (6 to 15·4)	9·4 (5·5 to 14·4)	8·4 (5 to 13·1)	7·4 (4·4 to 11·4)	6·7 (4 to 10·1)	5·6 (3·4 to 8·5)	4·7 (2·8 to 7·2)	3·4 (2 to 5·3)	2·4 (1·5 to 3·6)	1·5 (0·9 to 2·3)	0·8 (0·5 to 1·2)	0·4 (0·2 to 0·6)	0·2 (0·1 to 0·2)	0·1 (0 to 0·1)	0 (0 to 0)	98·8
Sudden infant death syndrome	--	10·9 (6·6 to 16·7)	70·9 (39·7 to 119)	--	--	--	--	--	--	--	--	--	--	--	--	--	--	--	--	--	--	--	--	NA
Injuries	32·9 (24·2 to 45·2)	27·8 (20·7 to 36·8)	169 (120 to 232)	262 (201 to 336)	277 (236 to 324)	451 (398 to 504)	1,770 (1,570 to 1,990)	2,700 (2,420 to 3,010)	2,700 (2,430 to 2,980)	2,200 (1,960 to 2,430)	1,540 (1,330 to 1,740)	1,140 (967 to 1,330)	887 (741 to 1,040)	627 (521 to 739)	500 (423 to 582)	333 (283 to 389)	213 (182 to 248)	142 (120 to 165)	92·2 (78 to 108)	69·6 (59·5 to 80·8)	38·1 (32·1 to 44·3)	17·9 (15·1 to 21)	6 (4·9 to 7·1)	16,200
Transport injuries	1·6 (1·1 to 2·2)	1·6 (1 to 2·2)	13·8 (9·2 to 19·7)	50·2 (33·2 to 71·9)	79·6 (56 to 109)	127 (95·7 to 160)	471 (344 to 601)	701 (531 to 877)	715 (565 to 881)	571 (444 to 697)	410 (315 to 512)	334 (257 to 422)	278 (216 to 348)	197 (153 to 245)	159 (123 to 197)	104 (83 to 127)	63·8 (52·5 to 76·2)	39 (31·7 to 47·1)	23·5 (19 to 28·3)	15 (12 to 18·2)	7·1 (5·6 to 8·7)	2·6 (2·1 to 3·2)	0·8 (0·6 to 1)	4,370
Road injuries	1·2 (0·8 to 1·7)	1·1 (0·7 to 1·6)	10·8 (6·9 to 15·9)	44·8 (29·3 to 64·9)	72·5 (49·9 to 101)	112 (83·6 to 144)	432 (317 to 564)	645 (488 to 804)	659 (519 to 820)	519 (403 to 640)	374 (288 to 475)	304 (236 to 384)	255 (198 to 320)	181 (141 to 225)	143 (111 to 177)	96·2 (76·6 to 118)	59·4 (48·7 to 71·4)	35·3 (28·5 to 42·9)	21·8 (17·6 to 26·3)	14 (11·2 to 17·1)	6·7 (5·2 to 8·2)	2·4 (1·9 to 3)	0·7 (0·6 to 0·9)	3,990
Pedestrian road injuries	0·5 (0·3 to 0·7)	0·3 (0·2 to 0·4)	2·7 (1·7 to 4·1)	16·7 (10·6 to 24·1)	27 (17·4 to 38·7)	30·1 (20·6 to 41)	42 (25 to 66·3)	51·3 (34 to 74)	59·6 (39·9 to 86)	58·9 (43·4 to 78·1)	49 (35·3 to 66·5)	46·4 (34 to 61·3)	43 (30·5 to 58·2)	33·7 (25·7 to 42·4)	31·1 (23·7 to 39·2)	22·3 (17·9 to 27·8)	15·3 (12·4 to 18·7)	10·3 (8·1 to 12·9)	7·2 (5·7 to 8·8)	5·3 (4·1 to 6·6)	2·6 (2 to 3·3)	0·9 (0·7 to 1·1)	0·2 (0·2 to 0·3)	556
Cyclist road injuries	0 (0 to 0)	0 (0 to 0)	0 (0 to 0)	3 (1·6 to 5·1)	6·6 (3·9 to 10·6)	22·9 (14·6 to 34·9)	60·1 (37·6 to 91·1)	64·8 (40·3 to 94·9)	58·8 (39·5 to 81·9)	53·9 (36·1 to 76·5)	41·8 (29·4 to 56·8)	34·6 (25 to 47)	29·8 (21·9 to 39·5)	24·8 (18·3 to 32·7)	20·2 (14·4 to 27·4)	14·6 (10·8 to 19·4)	9·8 (7·4 to 12·7)	5·7 (4·2 to 7·5)	3·3 (2·5 to 4·4)	2 (1·5 to 2·7)	0·9 (0·6 to 1·2)	0·4 (0·2 to 0·5)	0·1 (0·1 to 0·1)	458
Motorcyclist road injuries	0·1 (0·1 to 0·2)	0·1 (0·1 to 0·2)	1·2 (0·8 to 1·8)	3·3 (2·2 to 4·8)	5·8 (3·2 to 9·9)	9·6 (5·5 to 15·6)	107 (74·4 to 142)	196 (149 to 247)	198 (157 to 247)	154 (114 to 203)	95·7 (70 to 127)	64·7 (49·3 to 84·2)	51 (38·4 to 65·9)	32·8 (25·8 to 40·6)	24·8 (19·3 to 30·3)	13·7 (10·8 to 17)	8·4 (6·3 to 10·8)	4·5 (3·4 to 5·9)	2·7 (2 to 3·7)	1·7 (1·2 to 2·3)	0·8 (0·6 to 1·1)	0·3 (0·2 to 0·5)	0·1 (0·1 to 0·2)	977
Motor vehicle road injuries	0·5 (0·3 to 0·8)	0·7 (0·4 to 1)	6·7 (4·3 to 9·8)	21·4 (13·8 to 31·4)	32·5 (21·9 to 46·2)	48·5 (34·6 to 65·3)	221 (157 to 286)	328 (244 to 414)	339 (258 to 421)	248 (179 to 328)	184 (133 to 243)	155 (114 to 202)	129 (91·5 to 172)	86·9 (66·3 to 110)	65·3 (49·5 to 83·4)	44·1 (34·3 to 55·2)	24·8 (19·6 to 30·3)	14·1 (11·1 to 17·6)	8·1 (6·5 to 10)	4·8 (3·7 to 5·9)	2·2 (1·7 to 2·7)	0·8 (0·6 to 1)	0·3 (0·2 to 0·4)	1,960
Other road injuries	0 (0 to 0·1)	0 (0 to 0)	0·1 (0·1 to 0·3)	0·4 (0·2 to 0·9)	0·6 (0·3 to 1)	1·1 (0·7 to 1·9)	2·6 (1·6 to 4·1)	4·7 (2·8 to 7·5)	4·3 (2·8 to 6·6)	3·9 (2·5 to 5·9)	3·5 (2·3 to 5)	3·1 (2·2 to 4·2)	3·2 (2·3 to 4·3)	2·4 (1·7 to 3·3)	2 (1·4 to 2·8)	1·4 (1 to 1·9)	1·1 (0·8 to 1·4)	0·7 (0·5 to 1)	0·5 (0·4 to 0·7)	0·3 (0·2 to 0·4)	0·1 (0·1 to 0·2)	0·1 (0 to 0·1)	0 (0 to 0)	36·2
Other transport injuries	0·4 (0·2 to 0·6)	0·5 (0·3 to 0·8)	3 (1·8 to 4·7)	5·3 (3·1 to 9·1)	7·1 (4·4 to 11·4)	14·4 (9·1 to 21·2)	38·4 (22·4 to 59·3)	56·2 (35·3 to 81·1)	55·2 (35·9 to 81·2)	52·4 (32·8 to 76·5)	36·3 (22·6 to 54)	30·5 (19·2 to 44·3)	22·4 (13·7 to 33·8)	16·2 (10·7 to 23·9)	15·9 (10·4 to 23·1)	7·9 (5·5 to 11·3)	4·3 (3·2 to 5·9)	3·8 (2·6 to 5·1)	1·7 (1·2 to 2·2)	1 (0·7 to 1·2)	0·4 (0·3 to 0·5)	0·2 (0·1 to 0·2)	0·1 (0 to 0·1)	373
Unintentional injuries	19·2 (14·1 to 26·2)	22·6 (16·5 to 29·7)	132 (89·9 to 189)	175 (129 to 234)	167 (136 to 201)	243 (202 to 284)	474 (389 to 578)	556 (451 to 671)	535 (445 to 644)	474 (393 to 565)	380 (320 to 452)	321 (262 to 390)	265 (216 to 321)	222 (182 to 274)	194 (164 to 232)	143 (117 to 172)	102 (83·9 to 122)	74·5 (62·3 to 88·7)	53·7 (44·4 to 64·4)	45·3 (38·5 to 53·4)	27·1 (22·6 to 32·1)	14 (11·7 to 16·6)	4·8 (3·8 to 5·7)	4,640
Falls	0·3 (0·2 to 0·4)	0·3 (0·2 to 0·4)	4·1 (2·5 to 6·3)	7·7 (5·3 to 11)	10·2 (7·5 to 13·8)	20·1 (15·2 to 26)	39·7 (31 to 51·2)	48·2 (37·7 to 60·5)	50·2 (38·8 to 63·7)	50·5 (39·5 to 63·5)	48·6 (38·9 to 61·9)	45·5 (35·9 to 57·1)	43·9 (34·9 to 54·8)	41·5 (32·5 to 52·3)	38·6 (30·9 to 47·7)	31·6 (25·5 to 39·1)	24·7 (19·8 to 29·9)	21·6 (17·2 to 26·6)	17·2 (13·7 to 21·3)	18·6 (14·8 to 22·6)	12·9 (10·3 to 16)	7·4 (5·8 to 8·9)	2·6 (1·9 to 3·2)	586
Drowning	1·2 (0·9 to 1·7)	0·9 (0·6 to 1·2)	13 (8·5 to 19·2)	67·4 (45·6 to 96·9)	74·4 (54·1 to 96·9)	102 (78·6 to 126)	201 (147 to 267)	195 (137 to 265)	171 (124 to 230)	138 (101 to 179)	93·3 (69·8 to 122)	68·7 (50·5 to 90·7)	46·2 (33·4 to 62·5)	33 (23·7 to 44·6)	24·7 (18 to 32·3)	14·1 (10·7 to 18·3)	7·4 (5·8 to 9·3)	3·6 (2·9 to 4·4)	1·9 (1·6 to 2·4)	0·9 (0·7 to 1·1)	0·5 (0·4 to 0·6)	0·2 (0·2 to 0·2)	0·1 (0·1 to 0·1)	1,260
Fire, heat, and hot substances	0·3 (0·2 to 0·4)	0·6 (0·4 to 0·8)	6·9 (4·6 to 9·9)	16·4 (10·9 to 23·6)	14·7 (11·2 to 18·7)	19·5 (14·7 to 25·5)	24·7 (18·3 to 32·9)	28·3 (21·5 to 36·8)	29·9 (22·9 to 38·4)	31·2 (23·8 to 40·7)	25·7 (19·7 to 33·3)	22·7 (17 to 29·7)	20·2 (15·2 to 26·7)	16 (12·1 to 21·6)	13·7 (10·4 to 18·1)	10 (7·6 to 13·5)	6·6 (5 to 9·1)	4·7 (3·6 to 6·2)	3·1 (2·3 to 4·1)	2·4 (1·9 to 3·1)	1·4 (1·1 to 1·8)	0·7 (0·5 to 0·8)	0·2 (0·2 to 0·3)	300
Poisonings	0·2 (0·1 to 0·2)	0·3 (0·2 to 0·5)	2·4 (1·5 to 3·8)	7·1 (4·3 to 11·1)	2·7 (2 to 3·6)	3·1 (2·2 to 4·1)	4·9 (3·4 to 6·7)	7·5 (5·3 to 10·2)	10·5 (7·4 to 14·6)	9·9 (6·8 to 13·5)	6·8 (4·7 to 9·4)	4·2 (2·9 to 5·9)	3·2 (2·3 to 4·4)	2·2 (1·6 to 3)	1·7 (1·2 to 2·3)	1·1 (0·8 to 1·5)	0·7 (0·5 to 0·9)	0·4 (0·3 to 0·5)	0·3 (0·2 to 0·3)	0·2 (0·1 to 0·2)	0·1 (0·1 to 0·1)	0 (0 to 0)	0 (0 to 0)	69·5
Poisoning by carbon monoxide	0 (0 to 0·1)	0·1 (0 to 0·3)	0·2 (0·1 to 0·5)	2·2 (0·8 to 4·6)	0·9 (0·4 to 1·5)	1·1 (0·6 to 1·8)	2·4 (1·3 to 4)	3·9 (2·1 to 6·5)	5·5 (3·1 to 9·1)	5·5 (3·2 to 8·6)	3·8 (2·1 to 6·3)	2·2 (1·1 to 3·6)	1·5 (0·8 to 2·5)	0·8 (0·4 to 1·3)	0·7 (0·4 to 1·2)	0·4 (0·2 to 0·7)	0·2 (0·1 to 0·4)	0·1 (0·1 to 0·2)	0·1 (0·1 to 0·1)	0·1 (0 to 0·1)	0 (0 to 0)	0 (0 to 0)	0 (0 to 0)	31·9
Poisoning by other means	0·1 (0·1 to 0·2)	0·2 (0·1 to 0·3)	2·2 (1·4 to 3·4)	4·9 (3·2 to 7)	1·8 (1·3 to 2·5)	2 (1·3 to 2·8)	2·5 (1·8 to 3·4)	3·6 (2·7 to 4·7)	4·9 (3·6 to 6·5)	4·4 (3·1 to 5·8)	2·9 (2·1 to 3·9)	2·1 (1·5 to 2·7)	1·7 (1·3 to 2·3)	1·5 (1·1 to 1·9)	1 (0·7 to 1·3)	0·7 (0·5 to 0·9)	0·4 (0·3 to 0·6)	0·3 (0·2 to 0·4)	0·2 (0·1 to 0·2)	0·1 (0·1 to 0·1)	0·1 (0 to 0·1)	0 (0 to 0)	0 (0 to 0)	37·5
Exposure to mechanical forces	0·4 (0·3 to 0·6)	1 (0·7 to 1·4)	4·4 (3·1 to 6·2)	15·1 (10·9 to 20·6)	22·7 (16·6 to 29·5)	37·9 (28·2 to 50·7)	123 (91·4 to 160)	171 (128 to 221)	157 (120 to 203)	146 (112 to 188)	121 (94·3 to 153)	106 (81·4 to 138)	85·2 (65·9 to 109)	68·4 (52·9 to 88·1)	59·2 (45·6 to 75·9)	39·6 (30·2 to 51·5)	26 (19 to 35·2)	17·1 (12·4 to 23·5)	10·8 (7·6 to 14·8)	7·1 (5·1 to 9·7)	3·6 (2·6 to 5)	1·6 (1·1 to 2·2)	0·5 (0·4 to 0·7)	1,220
Unintentional firearm injuries	0·1 (0·1 to 0·2)	0·3 (0·2 to 0·4)	1·4 (0·8 to 2·4)	3·2 (1·6 to 5·5)	4·9 (2·9 to 7·8)	13·3 (8·3 to 20·9)	81·4 (56·2 to 110)	121 (86·6 to 163)	103 (73·9 to 140)	88·1 (62·6 to 120)	66·4 (48·3 to 88·1)	54·5 (39·2 to 75·6)	38·3 (27·9 to 50·9)	26·7 (20·2 to 34·9)	21·4 (16·2 to 28)	12 (9·4 to 15·2)	6·8 (5·2 to 8·6)	3·8 (3 to 4·8)	2 (1·6 to 2·7)	1·4 (1 to 1·7)	0·7 (0·6 to 0·9)	0·3 (0·2 to 0·4)	0·1 (0·1 to 0·1)	651
Other exposure to mechanical forces	0·3 (0·2 to 0·4)	0·8 (0·5 to 1·1)	3 (2 to 4·2)	11·9 (8·5 to 16·8)	17·8 (12·6 to 23·3)	24·6 (17·8 to 33·6)	41·4 (27·8 to 58·7)	49·7 (34 to 69·4)	54·2 (37·3 to 76·8)	57·9 (40·2 to 79·7)	54·1 (38·9 to 75·1)	51·2 (35·7 to 71·9)	46·9 (32·8 to 66·4)	41·7 (29·6 to 57·4)	37·8 (27·1 to 52·1)	27·6 (19·4 to 38·1)	19·2 (13·2 to 27·2)	13·3 (9·2 to 19)	8·7 (5·9 to 12·3)	5·7 (3·9 to 8)	2·9 (2 to 4·2)	1·3 (0·9 to 1·8)	0·4 (0·3 to 0·6)	572
Adverse effects of medical treatment	8·6 (5·1 to 12·8)	9 (5·8 to 13·2)	28·9 (16 to 49·4)	14·1 (7·8 to 23·9)	9·4 (6·1 to 13·6)	10·3 (6·7 to 15·7)	13·3 (8·3 to 20)	24·8 (15 to 36·8)	26 (16·6 to 38·8)	20·6 (12·8 to 30)	20 (13·2 to 28·9)	18·2 (12·2 to 26·4)	16·9 (10·8 to 24·7)	20·6 (13·5 to 30·8)	20·9 (14 to 30)	19·3 (12·6 to 29·2)	15·7 (10·8 to 23)	12·5 (8·7 to 18·2)	10·4 (7·2 to 16·1)	8·8 (6·2 to 12·7)	5 (3·6 to 7·2)	2·4 (1·7 to 3·4)	0·7 (0·5 to 1·2)	336
Animal contact	0·2 (0·1 to 0·2)	0·6 (0·3 to 1)	2·1 (1·2 to 3·3)	5·6 (3·5 to 8·3)	4·1 (2·8 to 5·7)	6·7 (4·6 to 9·4)	9·1 (6 to 13·2)	8·7 (5·9 to 11·7)	13·2 (9·4 to 18·4)	8·6 (6 to 11·9)	10 (6·9 to 13·6)	8·7 (6·1 to 12·1)	9·2 (6·3 to 12·9)	8 (5·8 to 11)	7·6 (5·2 to 10·4)	6·8 (4·8 to 9·4)	5·4 (3·8 to 7·6)	3·9 (2·8 to 5·4)	2·7 (1·8 to 3·8)	1·6 (1·1 to 2·2)	0·7 (0·5 to 0·9)	0·4 (0·3 to 0·5)	0·1 (0·1 to 0·2)	124
Venomous animal contact	0·1 (0·1 to 0·2)	0·5 (0·2 to 0·8)	1·3 (0·7 to 2·2)	4·3 (2·7 to 6·7)	3 (2 to 4·3)	4·9 (3·2 to 7·1)	6·6 (4 to 9·9)	5·6 (3·7 to 7·8)	8·9 (5·6 to 13)	5·4 (3·5 to 7·7)	7·6 (5·1 to 10·6)	5·7 (3·7 to 8·1)	6·3 (4·2 to 9·2)	5·4 (3·7 to 7·7)	5·2 (3·6 to 7·3)	4·6 (3·2 to 6·7)	3·3 (2·1 to 4·9)	2·2 (1·4 to 3·2)	1·8 (1·2 to 2·6)	1·1 (0·7 to 1·6)	0·5 (0·4 to 0·7)	0·3 (0·2 to 0·5)	0·1 (0·1 to 0·2)	84·8
Non-venomous animal contact	0·1 (0 to 0·1)	0·1 (0·1 to 0·3)	0·8 (0·3 to 1·5)	1·2 (0·5 to 2·4)	1 (0·5 to 1·8)	1·8 (1 to 3·1)	2·5 (1·4 to 4·3)	3·1 (1·8 to 4·8)	4·3 (2·2 to 7·1)	3·2 (1·9 to 5)	2·4 (1·4 to 3·9)	3 (1·8 to 4·9)	2·9 (1·7 to 4·6)	2·6 (1·6 to 4)	2·3 (1·5 to 3·6)	2·2 (1·3 to 3·5)	2·1 (1·3 to 3·3)	1·7 (1·1 to 2·6)	0·9 (0·6 to 1·4)	0·5 (0·3 to 0·7)	0·2 (0·1 to 0·3)	0·1 (0 to 0·1)	0 (0 to 0)	39·1
Foreign body	7·8 (5·6 to 10·6)	9·7 (6·8 to 13·6)	67·8 (41·2 to 107)	33·9 (24·2 to 46·8)	15·5 (11·9 to 19·4)	22·8 (17·6 to 28·5)	18·8 (14·5 to 24·4)	23·7 (17·9 to 30·9)	26·2 (20 to 33·4)	22·3 (16·7 to 28·8)	17·5 (13·4 to 22·4)	15·7 (12 to 20·1)	13·9 (10·4 to 18·2)	12·6 (9·1 to 16·7)	11·2 (8·6 to 14·2)	9·1 (6·8 to 12·1)	6·9 (5·4 to 8·9)	5·1 (4 to 6·4)	3·7 (2·9 to 4·6)	3·3 (2·5 to 4·2)	1·5 (1·1 to 1·8)	0·7 (0·6 to 0·9)	0·3 (0·2 to 0·4)	350
Pulmonary aspiration and foreign body in airway	7·7 (5·6 to 10·6)	9·7 (6·8 to 13·6)	67·4 (40·9 to 106)	32·4 (22·5 to 45·1)	13·4 (10·1 to 17)	20·1 (15·1 to 25·9)	15·9 (11·9 to 20·8)	20·5 (15 to 27·3)	22·9 (17 to 30·2)	19·5 (14·1 to 25·8)	14·9 (11 to 19·4)	13·2 (9·8 to 17·3)	11·5 (8·3 to 15·7)	10·7 (7·4 to 14·8)	9·6 (7 to 12·6)	7·9 (5·7 to 10·8)	6 (4·5 to 7·8)	4·4 (3·3 to 5·6)	3·2 (2·4 to 4·1)	2·9 (2·2 to 3·8)	1·3 (0·9 to 1·6)	0·6 (0·5 to 0·8)	0·3 (0·2 to 0·3)	316
Foreign body in eyes	0 (0 to 0)	0 (0 to 0)	0·1 (0 to 0·2)	0·4 (0·1 to 1)	0·8 (0·2 to 1·8)	1 (0·3 to 2·4)	1·3 (0·4 to 3·1)	1·4 (0·5 to 3·2)	1·5 (0·5 to 3·1)	1·4 (0·5 to 2·9)	1·3 (0·5 to 2·6)	1·1 (0·5 to 2·3)	1 (0·5 to 1·9)	0·8 (0·4 to 1·4)	0·6 (0·3 to 1)	0·4 (0·3 to 0·7)	0·3 (0·2 to 0·5)	0·2 (0·1 to 0·4)	0·2 (0·1 to 0·2)	0·1 (0·1 to 0·2)	0·1 (0 to 0·1)	0 (0 to 0)	0 (0 to 0)	14
Foreign body in other body part	0 (0 to 0)	0 (0 to 0)	0·3 (0·2 to 0·5)	1 (0·7 to 1·5)	1·3 (0·9 to 2)	1·7 (1·1 to 2·4)	1·7 (1·2 to 2·4)	1·7 (1·2 to 2·4)	1·8 (1·3 to 2·6)	1·5 (1·1 to 2)	1·4 (1 to 1·8)	1·4 (1 to 1·8)	1·3 (1 to 1·8)	1·1 (0·8 to 1·5)	1 (0·7 to 1·4)	0·8 (0·6 to 1·1)	0·6 (0·5 to 0·8)	0·5 (0·3 to 0·6)	0·4 (0·3 to 0·5)	0·3 (0·2 to 0·4)	0·2 (0·1 to 0·2)	0·1 (0·1 to 0·1)	0 (0 to 0)	20·1
Environmental heat and cold exposure	0 (0 to 0)	0 (0 to 0)	0·2 (0·2 to 0·3)	1·2 (0·8 to 1·7)	2·5 (1·6 to 3·8)	4 (2·6 to 5·7)	6·2 (4·2 to 8·7)	7·5 (5·2 to 10·4)	8 (5·6 to 10·8)	7·9 (5·6 to 10·9)	7·5 (5·2 to 10·2)	7 (4·8 to 9·5)	6·5 (4·6 to 8·9)	5·7 (4 to 7·7)	5·1 (3·6 to 6·9)	3·9 (2·8 to 5·3)	2·9 (2 to 4)	2·2 (1·6 to 2·9)	1·5 (1·1 to 2)	1·1 (0·8 to 1·4)	0·6 (0·4 to 0·8)	0·3 (0·2 to 0·4)	0·1 (0·1 to 0·1)	81·8
Exposure to forces of nature	0 (0 to 0)	0 (0 to 0)	0 (0 to 0)	0 (0 to 0)	0 (0 to 0)	0·1 (0·1 to 0·1)	0·3 (0·2 to 0·5)	1·2 (0·8 to 2)	1·5 (0·9 to 2·4)	2 (1·3 to 3·3)	1·2 (0·8 to 2)	1·2 (0·7 to 1·8)	1·2 (0·7 to 1·8)	1 (0·6 to 1·5)	0·7 (0·4 to 1·1)	0·5 (0·3 to 0·7)	0·3 (0·2 to 0·5)	0·2 (0·2 to 0·3)	0·1 (0·1 to 0·2)	0·1 (0·1 to 0·1)	0 (0 to 0·1)	0 (0 to 0)	0 (0 to 0)	11·8
Other unintentional injuries	0·3 (0·2 to 0·6)	0·2 (0·1 to 0·4)	2·2 (1·5 to 3)	7 (5·2 to 9·3)	10·7 (8 to 14·1)	16·7 (13·2 to 21·3)	33·3 (25·2 to 43·5)	39·9 (30 to 52)	41·8 (31·4 to 54·3)	37·6 (27·8 to 49·2)	29·1 (22·7 to 37·5)	23·2 (17·6 to 30·2)	18·8 (14·3 to 24·8)	13·1 (9·8 to 17·5)	10·8 (8·2 to 14·3)	7·1 (5·2 to 9·6)	4·9 (3·6 to 6·6)	3·2 (2·3 to 4·4)	2·1 (1·5 to 2·9)	1·4 (1 to 1·9)	0·7 (0·5 to 1)	0·3 (0·2 to 0·4)	0·1 (0·1 to 0·1)	305
Self-harm and interpersonal violence	12·1 (7·6 to 20)	3·6 (2·3 to 5·5)	23·3 (14·4 to 37·1)	36·7 (23 to 58)	30·3 (21·5 to 42·1)	81·4 (62·7 to 103)	827 (663 to 1,010)	1,440 (1,220 to 1,690)	1,450 (1,250 to 1,680)	1,150 (972 to 1,350)	750 (620 to 891)	490 (381 to 608)	343 (266 to 428)	208 (162 to 262)	147 (114 to 186)	86·3 (68·5 to 108)	47·7 (37·9 to 58·3)	28 (22·6 to 34·2)	15 (12·2 to 18·2)	9·2 (7·5 to 11·2)	4 (3·2 to 4·8)	1·3 (1 to 1·6)	0·4 (0·3 to 0·5)	7,190
Self-harm	0 (0 to 0)	0 (0 to 0)	0 (0 to 0)	0 (0 to 0)	0 (0 to 0)	8·9 (5·4 to 13·8)	92·9 (64·4 to 131)	175 (126 to 237)	197 (141 to 266)	191 (138 to 260)	135 (98·5 to 177)	96·6 (70·3 to 126)	81·6 (59·2 to 106)	58·5 (43·1 to 78·8)	48·6 (35·4 to 65·1)	32·9 (24·3 to 43·6)	19·9 (15 to 25·8)	13·1 (9·8 to 17·1)	7·5 (5·5 to 9·8)	5·5 (4·2 to 7·2)	2·5 (1·8 to 3·2)	0·7 (0·5 to 0·9)	0·2 (0·1 to 0·2)	1,170
Self-harm by firearm	0 (0 to 0)	0 (0 to 0)	0 (0 to 0)	0 (0 to 0)	0 (0 to 0)	0·5 (0·2 to 1)	15·2 (8·7 to 25·8)	25 (13·8 to 41)	25·3 (14·6 to 44·6)	23 (13·2 to 38·6)	14·4 (8·4 to 23·1)	11·6 (6·7 to 18·4)	8·5 (4·9 to 13·1)	6·7 (3·9 to 10·3)	4·9 (3 to 7·7)	3·4 (2·1 to 5·2)	1·8 (1·1 to 2·7)	1·1 (0·7 to 1·7)	0·6 (0·3 to 0·9)	0·4 (0·2 to 0·6)	0·1 (0·1 to 0·2)	0 (0 to 0)	0 (0 to 0)	143
Self-harm by other specified means	0 (0 to 0)	0 (0 to 0)	0 (0 to 0)	0 (0 to 0)	0 (0 to 0)	8·4 (5·1 to 12·9)	77·6 (53·5 to 109)	150 (106 to 206)	172 (122 to 233)	168 (121 to 230)	120 (87·4 to 156)	85 (61·9 to 111)	73·1 (52·5 to 95·4)	51·7 (37·8 to 69·3)	43·7 (31·5 to 58)	29·5 (21·7 to 39·4)	18·1 (13·6 to 23·5)	12 (8·9 to 15·8)	7 (5·1 to 9·1)	5·1 (3·8 to 6·7)	2·3 (1·7 to 3)	0·7 (0·5 to 0·9)	0·2 (0·1 to 0·2)	1,020
Interpersonal violence	12·1 (7·6 to 20)	3·6 (2·3 to 5·5)	23·3 (14·4 to 37)	36·4 (22·8 to 57·7)	30·2 (21·3 to 42)	72·4 (54·2 to 94·1)	734 (570 to 916)	1,270 (1,060 to 1,500)	1,250 (1,060 to 1,460)	949 (775 to 1,130)	611 (484 to 738)	389 (296 to 496)	255 (189 to 332)	146 (107 to 190)	96·1 (69·8 to 129)	52·4 (38·6 to 70)	26·2 (19·7 to 34)	13·5 (10·1 to 17·4)	6·9 (5·1 to 8·9)	3·2 (2·5 to 4)	1·4 (1·1 to 1·8)	0·6 (0·4 to 0·7)	0·2 (0·2 to 0·3)	5,980
Physical violence by firearm	0·6 (0·4 to 1)	0·3 (0·2 to 0·5)	2·8 (1·5 to 4·8)	4·8 (2·9 to 7·8)	7·7 (5·3 to 11·1)	29·2 (21·4 to 38·9)	482 (375 to 602)	869 (723 to 1,030)	835 (697 to 984)	597 (489 to 718)	361 (271 to 455)	205 (154 to 266)	124 (90·1 to 165)	63 (45·6 to 83·2)	37·6 (26·4 to 51·9)	18·3 (12·9 to 25·2)	7·8 (5·6 to 10·4)	3·2 (2·2 to 4·6)	1·1 (0·7 to 1·6)	0·4 (0·3 to 0·6)	0·2 (0·1 to 0·2)	0·1 (0 to 0·1)	0 (0 to 0)	3,650
Physical violence by sharp object	1 (0·4 to 1·8)	0·3 (0·1 to 0·6)	1·4 (0·8 to 2·5)	3·8 (2·4 to 5·7)	4·2 (3 to 5·6)	11·9 (8·9 to 15·4)	186 (140 to 239)	290 (233 to 348)	299 (247 to 354)	252 (204 to 302)	169 (125 to 226)	120 (90·6 to 153)	76·7 (56·4 to 102)	43·4 (31·7 to 56·5)	25·7 (18·6 to 34·4)	14 (10·4 to 18·5)	6·5 (4·8 to 8·2)	3·8 (2·9 to 5)	1·9 (1·4 to 2·5)	1 (0·8 to 1·2)	0·4 (0·3 to 0·6)	0·2 (0·2 to 0·3)	0·1 (0·1 to 0·1)	1,510
Sexual violence	0 (0 to 0)	0 (0 to 0)	0 (0 to 0)	0·1 (0·1 to 0·2)	0·8 (0·5 to 1·2)	1·8 (1 to 2·8)	3·8 (2·1 to 6·1)	4·4 (2·6 to 6·8)	3·7 (2 to 6)	3 (1·7 to 4·8)	2·5 (1·4 to 4)	1·8 (1·1 to 3)	1·4 (0·8 to 2·3)	0·9 (0·5 to 1·5)	0·7 (0·4 to 1·1)	0·5 (0·3 to 0·7)	0·3 (0·2 to 0·5)	0·2 (0·1 to 0·3)	0·1 (0·1 to 0·2)	0·1 (0·1 to 0·1)	0 (0 to 0·1)	0 (0 to 0)	0 (0 to 0)	26
Physical violence by other means	10·6 (6·6 to 17·4)	3 (1·9 to 4·7)	19·1 (11·6 to 30·4)	27·8 (17·3 to 44·2)	17·6 (12·3 to 24·9)	29·4 (21·9 to 38·3)	62·8 (42·4 to 87·9)	103 (76 to 137)	111 (84·8 to 147)	96·3 (71 to 131)	77·8 (60·4 to 99·2)	63 (44·1 to 84·9)	53·1 (37·2 to 72·7)	38·6 (28·7 to 50·2)	32·1 (23·3 to 43·2)	19·7 (14·7 to 26·3)	11·7 (8·6 to 15·1)	6·2 (4·5 to 8)	3·7 (2·7 to 4·9)	1·7 (1·3 to 2·1)	0·8 (0·6 to 1)	0·3 (0·2 to 0·3)	0·1 (0·1 to 0·2)	790
Conflict and terrorism	0 (0 to 0)	0 (0 to 0)	0 (0 to 0)	0 (0 to 0)	0 (0 to 0)	0 (0 to 0)	0 (0 to 0)	0 (0 to 0)	1·9 (1·1 to 3·2)	8·5 (5·2 to 13·7)	4·2 (2·5 to 6·7)	4 (2·4 to 6·2)	6·8 (4·3 to 10·4)	3·5 (2·3 to 5·2)	1·8 (1·2 to 2·7)	1·1 (0·7 to 1·6)	1·5 (1 to 2·3)	1·4 (0·9 to 2·1)	0·6 (0·4 to 0·9)	0·5 (0·3 to 0·7)	0·1 (0 to 0·1)	0 (0 to 0)	0 (0 to 0)	35·8

SR Table 2a: Cuba DALYS by cause and age group for males with 95% uncertainty intervals, 2019

Cause	Early Neonatal	Late Neonatal	Post Neonatal	1 to 4	5 to 9	10 to 14	15 to 19	20 to 24	25 to 29	30 to 34	35 to 39	40 to 44	45 to 49	50 to 54	55 to 59	60 to 64	65 to 69	70 to 74	75 to 79	80 to 84	85 to 89	90 to 94	95 plus	Total
All causes	7,830 (6,150 to 9,740)	4,310 (3,420 to 5,300)	9,170 (7,550 to 10,900)	13,100 (10,200 to 16,700)	17,600 (13,300 to 23,600)	19,700 (15,400 to 24,700)	32,200 (25,900 to 39,300)	40,800 (33,900 to 48,700)	51,700 (43,400 to 61,100)	61,700 (51,900 to 72,900)	58,300 (49,000 to 68,800)	76,100 (63,800 to 90,300)	129,000 (108,000 to 153,000)	178,000 (147,000 to 211,000)	203,000 (167,000 to 242,000)	173,000 (145,000 to 206,000)	182,000 (152,000 to 214,000)	185,000 (156,000 to 216,000)	160,000 (136,000 to 185,000)	117,000 (101,000 to 134,000)	72,100 (63,900 to 80,400)	37,500 (33,900 to 41,300)	16,300 (15,100 to 17,600)	1,840,000
Communicable, maternal, neonatal, and nutritional diseases	6,080 (3,720 to 7,820)	2,910 (2,170 to 3,650)	3,960 (3,070 to 4,930)	3,830 (2,690 to 5,570)	4,420 (2,660 to 7,520)	2,780 (1,980 to 4,160)	2,760 (2,080 to 3,610)	3,360 (2,700 to 4,180)	4,350 (3,660 to 5,250)	5,800 (4,970 to 6,810)	5,020 (4,320 to 5,880)	5,430 (4,570 to 6,470)	7,780 (6,540 to 9,250)	8,670 (7,210 to 10,400)	8,840 (7,080 to 11,000)	7,470 (5,920 to 9,340)	7,950 (6,100 to 10,000)	9,620 (7,350 to 12,400)	10,000 (7,700 to 13,100)	8,770 (6,850 to 11,100)	6,390 (5,060 to 7,790)	3,870 (3,050 to 4,770)	2,030 (1,530 to 2,530)	132,000
HIV/AIDS and sexually transmitted infections	0·1 (0·1 to 0·2)	0·1 (0 to 0·2)	66·6 (61·5 to 71·9)	34·5 (30·3 to 41·5)	61·1 (54·5 to 69·3)	66·7 (58·6 to 78·6)	161 (135 to 200)	791 (714 to 881)	1,450 (1,310 to 1,610)	2,520 (2,310 to 2,780)	2,130 (1,930 to 2,370)	1,880 (1,710 to 2,100)	2,260 (2,050 to 2,500)	1,680 (1,530 to 1,890)	1,060 (926 to 1,260)	488 (413 to 632)	322 (266 to 430)	96·8 (70·5 to 147)	45·8 (33·8 to 66)	16·6 (12 to 25·5)	6·1 (3·6 to 10·4)	2·1 (1·1 to 3·8)	0·7 (0·4 to 1·3)	15,100
HIV/AIDS	0 (0 to 0)	0 (0 to 0·2)	66·5 (61·4 to 71·9)	34·4 (30·3 to 41·5)	61·1 (54·5 to 69·3)	61·5 (54·9 to 72·4)	129 (117 to 144)	737 (669 to 814)	1,380 (1,250 to 1,540)	2,440 (2,250 to 2,690)	2,070 (1,880 to 2,300)	1,820 (1,660 to 2,020)	2,190 (1,990 to 2,410)	1,620 (1,480 to 1,820)	1,010 (894 to 1,220)	465 (396 to 605)	303 (254 to 407)	82·3 (61·3 to 129)	36 (27·7 to 51·9)	10·8 (8·8 to 14·3)	3·1 (2 to 4·9)	0·8 (0·3 to 1·6)	0·3 (0·1 to 0·6)	14,500
HIV/AIDS - Drug-susceptible Tuberculosis	0 (0 to 0)	0 (0 to 0)	0·9 (0·5 to 1·3)	0·5 (0·3 to 0·7)	0·8 (0·5 to 1·2)	0·8 (0·5 to 1·2)	1·8 (1·2 to 2·7)	10·6 (7·1 to 15·4)	19·9 (13·3 to 29)	34·9 (23·2 to 51·1)	29·8 (20·1 to 43·5)	26·6 (18 to 38·8)	31·7 (21·4 to 46·2)	24·2 (16·5 to 34·9)	15·8 (11·2 to 22·2)	7·7 (5·6 to 10·5)	5·2 (3·8 to 7)	1·6 (1·2 to 2·2)	0·7 (0·5 to 0·9)	0·2 (0·1 to 0·2)	0·1 (0 to 0·1)	0 (0 to 0)	0 (0 to 0)	214
HIV/AIDS - Multidrug-resistant Tuberculosis without exten	0 (0 to 0)	0 (0 to 0)	0·1 (0 to 0·2)	0 (0 to 0·1)	0 (0 to 0·2)	0 (0 to 0·2)	0·1 (0 to 0·3)	0·6 (0·1 to 2)	1·1 (0·2 to 3·7)	2 (0·3 to 6·6)	1·7 (0·3 to 5·5)	1·5 (0·2 to 4·9)	1·8 (0·3 to 5·8)	1·3 (0·2 to 4·3)	0·8 (0·1 to 2·6)	0·4 (0·1 to 1·2)	0·2 (0 to 0·8)	0·1 (0 to 0·2)	0 (0 to 0·1)	0 (0 to 0)	0 (0 to 0)	0 (0 to 0)	0 (0 to 0)	12
HIV/AIDS - Extensively drug-resistant Tuberculosis	0 (0 to 0)	0 (0 to 0)	0 (0 to 0)	0 (0 to 0)	0 (0 to 0)	0 (0 to 0)	0 (0 to 0·1)	0·1 (0 to 0·3)	0·2 (0 to 0·6)	0·3 (0·1 to 1·1)	0·3 (0 to 0·9)	0·2 (0 to 0·8)	0·3 (0 to 0·9)	0·2 (0 to 0·7)	0·1 (0 to 0·4)	0·1 (0 to 0·2)	0 (0 to 0·1)	0 (0 to 0)	0 (0 to 0)	0 (0 to 0)	0 (0 to 0)	0 (0 to 0)	0 (0 to 0)	1·9
HIV/AIDS resulting in other diseases	0 (0 to 0)	0 (0 to 0·2)	65·6 (60·6 to 70·9)	33·9 (29·8 to 41·1)	60·2 (53·6 to 68·6)	60·6 (54 to 71·6)	127 (115 to 142)	725 (657 to 804)	1,360 (1,230 to 1,520)	2,410 (2,200 to 2,650)	2,040 (1,850 to 2,270)	1,790 (1,640 to 1,990)	2,160 (1,960 to 2,380)	1,600 (1,460 to 1,790)	997 (878 to 1,200)	456 (388 to 597)	298 (248 to 401)	80·6 (59·9 to 127)	35·3 (27 to 51·3)	10·6 (8·6 to 14·1)	3 (1·9 to 4·8)	0·8 (0·3 to 1·6)	0·3 (0·1 to 0·6)	14,300
Sexually transmitted infections excluding HIV	0·1 (0·1 to 0·2)	0·1 (0 to 0·1)	0·1 (0 to 0·1)	0 (0 to 0)	0 (0 to 0)	5·2 (2·1 to 11·1)	32 (12·1 to 64·7)	54·3 (23·4 to 96·7)	66 (30·6 to 116)	79·7 (38·2 to 144)	61·4 (28·3 to 109)	60·6 (27·8 to 114)	73·8 (34·6 to 140)	60·2 (24·8 to 119)	41·4 (18 to 92·6)	23·3 (8·8 to 53·7)	18·8 (7·3 to 43·5)	14·5 (5·5 to 32·1)	9·8 (3·7 to 22·4)	5·8 (2·2 to 13)	3 (1·2 to 7)	1·2 (0·5 to 2·8)	0·4 (0·2 to 1)	611
Syphilis	0·1 (0·1 to 0·2)	0·1 (0 to 0·1)	0·1 (0 to 0·1)	0 (0 to 0)	0 (0 to 0)	0·1 (0 to 0·2)	0·4 (0 to 1·2)	1 (0·3 to 2·7)	1·5 (0·6 to 3·6)	2 (0·9 to 4·2)	1·7 (0·9 to 3·4)	1·9 (1·1 to 3·4)	2·8 (1·7 to 4·6)	3·2 (1·9 to 5·3)	3·1 (1·9 to 4·8)	2·1 (1·3 to 3·2)	2 (1·2 to 3)	1·8 (1·1 to 2·6)	1·2 (0·7 to 1·8)	0·7 (0·4 to 1·1)	0·4 (0·2 to 0·6)	0·2 (0·1 to 0·3)	0·1 (0 to 0·1)	26·4
Chlamydial infection	0 (0 to 0)	0 (0 to 0)	0 (0 to 0)	0 (0 to 0)	0 (0 to 0)	0·1 (0·1 to 0·2)	3·4 (1·6 to 6·2)	12·7 (2·8 to 28·8)	21·9 (6·5 to 45·2)	22·6 (6·6 to 46·7)	16·7 (5 to 36)	16 (4·3 to 34·7)	15·9 (2·8 to 35·1)	7·8 (0·3 to 21)	2·3 (1·1 to 4·1)	0·3 (0·2 to 0·6)	0·1 (0 to 0·2)	0 (0 to 0)	0 (0 to 0)	0 (0 to 0)	0 (0 to 0)	0 (0 to 0)	0 (0 to 0)	120
Gonococcal infection	0 (0 to 0)	0 (0 to 0)	0 (0 to 0)	0 (0 to 0)	0 (0 to 0)	1·5 (0·4 to 3·6)	10·7 (1 to 26·7)	14·8 (3·1 to 33·9)	7·9 (0·2 to 21·1)	5·6 (2·1 to 11·1)	3·3 (1·4 to 6·9)	3·1 (1·2 to 6·4)	3·9 (1·6 to 8·2)	3·3 (1·2 to 7·2)	1·5 (0·5 to 3·2)	0·3 (0·1 to 0·6)	0·1 (0·1 to 0·2)	0 (0 to 0)	0 (0 to 0)	0 (0 to 0)	0 (0 to 0)	0 (0 to 0)	0 (0 to 0)	56
Trichomoniasis	0 (0 to 0)	0 (0 to 0)	0 (0 to 0)	0 (0 to 0)	0 (0 to 0)	0 (0 to 0)	0·1 (0 to 0·2)	0·5 (0 to 1·6)	1·5 (0·3 to 3·8)	2·5 (0·7 to 6)	2·5 (0·7 to 6·2)	2·6 (0·7 to 6·4)	3·1 (0·8 to 7·4)	2·6 (0·7 to 6·6)	1·5 (0·4 to 3·9)	0·6 (0·1 to 1·5)	0·4 (0·1 to 1·1)	0·3 (0·1 to 0·8)	0·2 (0 to 0·5)	0·1 (0 to 0·3)	0 (0 to 0·1)	0 (0 to 0)	0 (0 to 0)	18·6
Genital herpes	0 (0 to 0)	0 (0 to 0)	0 (0 to 0)	0 (0 to 0)	0 (0 to 0)	0·7 (0·2 to 1·8)	3·9 (1·2 to 10·2)	9 (2·6 to 22·4)	16·2 (4·7 to 40)	22·9 (7·4 to 55·2)	22·5 (7 to 54·4)	25·5 (8·1 to 62)	35·5 (11·3 to 87·5)	36·5 (11·5 to 92)	30·7 (9·6 to 77·8)	19·6 (5·9 to 48·6)	16 (4·8 to 40·3)	12·4 (3·7 to 29·9)	8·4 (2·4 to 21)	4·9 (1·4 to 12·1)	2·5 (0·8 to 6·4)	1·1 (0·3 to 2·5)	0·4 (0·1 to 0·9)	269
Other sexually transmitted infections	0 (0 to 0)	0 (0 to 0)	0 (0 to 0)	0 (0 to 0)	0 (0 to 0)	2·8 (0·9 to 6·4)	13·4 (3·9 to 29·1)	16·3 (6 to 31·2)	17 (6·5 to 32·6)	24·1 (9·1 to 45·9)	14·7 (5·6 to 29·4)	11·5 (4·2 to 23·2)	12·5 (3·8 to 25·2)	6·7 (1·6 to 14·8)	2·4 (1·2 to 4)	0·4 (0·2 to 0·6)	0·1 (0·1 to 0·2)	0 (0 to 0·1)	0 (0 to 0·1)	0 (0 to 0·1)	0 (0 to 0·1)	0 (0 to 0)	0 (0 to 0)	122
Respiratory infections and tuberculosis	394 (235 to 556)	355 (240 to 489)	1,250 (866 to 1,710)	891 (649 to 1,180)	684 (447 to 1,020)	[illegible]	[illegible]	[illegible]	[illegible]	[illegible]	[illegible]	[illegible]	[illegible]	[illegible]	[illegible]	[illegible]	[illegible]	[illegible]	[illegible]	[illegible]	[illegible]	[illegible]	[illegible]	[illegible]
[illegible]	[illegible]	[illegible]	[illegible]	[illegible]	[illegible]	[illegible]	[illegible]	[illegible]	[illegible]	[illegible]	[illegible]	[illegible]	[illegible]	[illegible]	[illegible]	[illegible]	[illegible]	[illegible]	[illegible]	[illegible]	[illegible]	[illegible]	[illegible]	[illegible]

Non-rheumatic degenerative mitral valve disease	0 (0 to 0)	0 (0 to 0)	0 (0 to 0)	0 (0 to 0)	0 (0 to 0)	0 (0 to 0)	4·7 (2·5 to 8·3)	12·1 (5·9 to 20·8)	30·3 (14·1 to 52·4)	42·7 (23·1 to 67)	40·1 (22·3 to 64)	60·5 (32·5 to 95·2)	84·6 (43·9 to 141)	125 (65·3 to 208)	154 (82·3 to 251)	125 (66·1 to 204)	123 (63·1 to 194)	91·9 (52·3 to 149)	68·4 (39·9 to 105)	41·4 (22 to 67·1)	24·5 (13·9 to 40·9)	12·1 (6·5 to 20·5)	3·6 (1·8 to 6)	1,040
Other non-rheumatic valve diseases	0 (0 to 0)	0 (0 to 0)	0 (0 to 0)	0 (0 to 0)	0 (0 to 0)	0 (0 to 0)	0·2 (0·1 to 0·4)	1·7 (0·6 to 4·2)	0·4 (0·2 to 0·8)	2 (0·7 to 4·5)	1·2 (0·2 to 3·7)	1 (0·2 to 2·9)	0·8 (0·2 to 2·2)	1·4 (0·6 to 3·3)	0·3 (0·1 to 0·7)	0·5 (0·1 to 1·3)	0·6 (0·1 to 1·5)	0·7 (0·1 to 2·1)	0·2 (0·1 to 0·4)	0·6 (0·2 to 1·2)	0·8 (0·2 to 1·9)	0·3 (0·1 to 0·7)	0 (0 to 0)	12·6
Cardiomyopathy and myocarditis	7·1 (2·3 to 11·5)	12·5 (7·6 to 18·4)	90·1 (53 to 132)	56·4 (35 to 80·6)	43·2 (31 to 56·3)	49·5 (34·4 to 67·1)	130 (79·1 to 181)	159 (95·4 to 228)	270 (170 to 375)	424 (278 to 591)	515 (337 to 718)	808 (549 to 1,130)	1,580 (1,070 to 2,310)	2,320 (1,600 to 3,220)	2,570 (1,790 to 3,620)	1,720 (1,210 to 2,610)	1,590 (1,110 to 2,250)	1,640 (1,140 to 2,210)	1,090 (749 to 1,500)	637 (409 to 892)	368 (227 to 510)	157 (93·1 to 221)	58·6 (37·7 to 87·7)	16,300
Myocarditis	1·5 (0·5 to 2·7)	3·8 (1·8 to 6·7)	28·5 (13·1 to 49·3)	16·4 (9·8 to 26·5)	9 (4·8 to 16·1)	10·2 (5·8 to 16·7)	22·3 (11·6 to 38·2)	17·1 (9·1 to 27·5)	23·1 (11·9 to 39·7)	26·9 (13·7 to 47·5)	24·7 (12·6 to 43)	26·6 (13·3 to 49·5)	23 (11·3 to 43·9)	31·3 (14·1 to 62)	23·9 (9·8 to 48·6)	16·5 (7 to 36)	19·7 (9·1 to 41·6)	14·1 (6·4 to 29·3)	14·2 (6·5 to 29·4)	13·4 (5·8 to 25·6)	8·9 (4·1 to 17)	3·9 (1·8 to 8·3)	4 (1·7 to 8·8)	383
Alcoholic cardiomyopathy	0 (0 to 0)	0 (0 to 0)	0 (0 to 0)	0 (0 to 0)	0 (0 to 0)	0 (0 to 0)	24·7 (13·6 to 41)	42·5 (22·4 to 72·1)	108 (61·3 to 170)	276 (168 to 399)	375 (227 to 533)	635 (427 to 906)	1,390 (928 to 1,980)	2,060 (1,390 to 2,820)	2,300 (1,590 to 3,200)	1,540 (1,080 to 2,170)	1,390 (946 to 1,910)	1,430 (939 to 1,940)	907 (564 to 1,270)	220 (111 to 370)	87·3 (40·4 to 158)	35·2 (17·3 to 60·7)	11·1 (6 to 18·3)	12,800
Other cardiomyopathy	5·5 (1·7 to 9·3)	8·7 (4·7 to 13·3)	61·7 (34·4 to 96·3)	40 (21·6 to 59·9)	34·2 (21 to 46·8)	39·2 (26·1 to 54·4)	83·3 (43·6 to 122)	99·1 (50·5 to 149)	139 (71·7 to 212)	121 (66·3 to 201)	115 (64·9 to 191)	146 (79·2 to 262)	166 (78·5 to 372)	227 (108 to 486)	245 (115 to 558)	162 (62·9 to 466)	180 (87·4 to 408)	196 (85·2 to 473)	172 (80·2 to 365)	403 (235 to 608)	272 (162 to 389)	118 (68·7 to 170)	43·6 (27·3 to 65·4)	3,080
Atrial fibrillation and flutter	0 (0 to 0)	0 (0 to 0)	0 (0 to 0)	0 (0 to 0)	0 (0 to 0)	0 (0 to 0)	0 (0 to 0)	0 (0 to 0)	0 (0 to 0)	8·9 (5·6 to 12·1)	9·4 (4·8 to 15·6)	45·8 (26·4 to 71·9)	128 (80·3 to 194)	255 (168 to 364)	414 (286 to 592)	481 (337 to 670)	676 (483 to 918)	947 (690 to 1,260)	1,100 (793 to 1,490)	1,200 (869 to 1,590)	993 (707 to 1,340)	747 (491 to 997)	338 (220 to 468)	7,350
Aortic aneurysm	--	--	--	--	--	--	12 (7·8 to 17·7)	14·8 (9·3 to 22·5)	35·9 (22·6 to 52·3)	41·1 (27·6 to 58·3)	58 (39·2 to 84)	100 (64·3 to 146)	217 (150 to 302)	481 (333 to 672)	865 (630 to 1,180)	1,010 (707 to 1,390)	1,440 (1,040 to 1,970)	1,730 (1,220 to 2,310)	1,590 (1,140 to 2,160)	1,060 (762 to 1,430)	554 (397 to 744)	238 (172 to 309)	73·8 (49 to 96)	NA
Endocarditis	1·8 (0·2 to 3·7)	2·9 (0·7 to 5·5)	6·7 (3 to 13·8)	4·2 (1·7 to 11·4)	3·3 (2 to 6·2)	8·1 (4·5 to 14·2)	23·1 (13·5 to 38·5)	37·7 (20 to 61·4)	54·6 (30·3 to 87·4)	79·2 (42·9 to 126)	55·7 (32·5 to 101)	102 (61·1 to 161)	146 (84·1 to 228)	196 (110 to 297)	177 (94 to 269)	116 (67·9 to 180)	109 (61·9 to 164)	98·3 (58·2 to 144)	64·3 (38·4 to 92·8)	42·5 (26·3 to 65)	24·7 (15·6 to 39·9)	9·9 (6·1 to 15·2)	4·5 (2·7 to 7)	1,370
Other cardiovascular and circulatory diseases	1 (0·2 to 4·1)	2·8 (1·1 to 7·1)	19·9 (9·3 to 40·4)	19·9 (11·3 to 32·6)	43·2 (23·7 to 73)	63·6 (38·7 to 100)	83·3 (55·4 to 121)	89·7 (61·5 to 128)	102 (69 to 144)	107 (76·4 to 145)	105 (74·8 to 145)	198 (140 to 271)	308 (218 to 422)	515 (364 to 698)	608 (434 to 829)	565 (421 to 745)	626 (459 to 829)	638 (481 to 821)	588 (438 to 771)	519 (386 to 673)	270 (201 to 349)	134 (95·2 to 172)	57·7 (39·2 to 75·7)	5,660
Chronic respiratory diseases	0 (0 to 0)	0 (0 to 0)	0·2 (0·1 to 0·3)	2,060 (1,010 to 3,730)	2,090 (1,200 to 3,530)	1,380 (794 to 2,250)	1,090 (652 to 1,760)	918 (592 to 1,390)	985 (676 to 1,430)	1,110 (785 to 1,520)	983 (719 to 1,310)	1,200 (911 to 1,540)	2,180 (1,710 to 2,750)	3,890 (3,090 to 4,800)	5,720 (4,410 to 7,190)	6,070 (4,730 to 7,680)	7,750 (6,130 to 9,770)	8,920 (7,070 to 11,200)	8,080 (6,460 to 10,100)	5,710 (4,590 to 7,050)	3,400 (2,710 to 4,160)	1,710 (1,390 to 2,070)	704 (553 to 846)	66,000
Chronic obstructive pulmonary disease	0 (0 to 0)	0 (0 to 0)	0·2 (0·1 to 0·3)	14·8 (10·4 to 20·3)	27·3 (16·4 to 43·3)	38·7 (16·2 to 67·6)	73·4 (39·5 to 115)	119 (68·7 to 180)	166 (103 to 244)	237 (151 to 336)	289 (198 to 400)	504 (364 to 662)	1,190 (889 to 1,530)	2,590 (1,990 to 3,280)	4,520 (3,450 to 5,800)	5,230 (4,040 to 6,650)	6,980 (5,460 to 8,840)	8,250 (6,490 to 10,400)	7,610 (6,050 to 9,530)	5,440 (4,370 to 6,740)	3,270 (2,610 to 4,000)	1,650 (1,340 to 2,000)	674 (528 to 810)	48,900
Pneumoconiosis	0 (0 to 0)	0 (0 to 0)	0 (0 to 0)	0 (0 to 0)	0 (0 to 0)	0 (0 to 0)	0·2 (0·1 to 0·2)	0·2 (0·1 to 0·3)	0·2 (0·1 to 0·4)	0·3 (0·1 to 0·5)	0·3 (0·2 to 0·5)	0·6 (0·4 to 0·9)	4 (2·5 to 6·2)	4 (2·5 to 6·2)	4·4 (2·8 to 7)	6·7 (4·1 to 10·8)	8·3 (5·3 to 12·7)	6·3 (4·2 to 8·9)	7·5 (5 to 10·8)	6·1 (4·1 to 8·8)	3·2 (2·2 to 4·7)	2·3 (1·6 to 3·4)	0·7 (0·5 to 1)	55·3
Silicosis	0 (0 to 0)	0 (0 to 0)	0 (0 to 0)	0 (0 to 0)	0 (0 to 0)	0 (0 to 0)	0 (0 to 0)	0 (0 to 0)	0 (0 to 0)	0 (0 to 0)	0 (0 to 0)	0·1 (0 to 0·1)	2·4 (1·3 to 4)	1·4 (0·7 to 2·7)	2·9 (1·5 to 5·1)	5·2 (2·8 to 8·9)	4·5 (2·4 to 7·6)	3·8 (2·3 to 5·7)	4·8 (2·8 to 7·3)	4·3 (2·7 to 6·6)	2·4 (1·6 to 3·7)	1·4 (0·9 to 2·1)	0·3 (0·2 to 0·4)	33·4
Asbestosis	0 (0 to 0)	0 (0 to 0)	0 (0 to 0)	0 (0 to 0)	0 (0 to 0)	0 (0 to 0)	0·1 (0 to 0·1)	0·1 (0 to 0·2)	0·1 (0 to 0·1)	0 (0 to 0·1)	0·1 (0 to 0·1)	0·1 (0 to 0·1)	0·4 (0·1 to 0·8)	0·6 (0·2 to 1·3)	0·2 (0·1 to 0·5)	0·1 (0·1 to 0·3)	0·8 (0·2 to 1·6)	0·6 (0·2 to 1·3)	0·5 (0·1 to 1·2)	0·6 (0·1 to 1·3)	0·1 (0 to 0·2)	0·2 (0·1 to 0·3)	0·1 (0 to 0·2)	4·5
Coal workers pneumoconiosis	0 (0 to 0)	0 (0 to 0)	0 (0 to 0)	0 (0 to 0)	0 (0 to 0)	0 (0 to 0)	0 (0 to 0)	0 (0 to 0)	0 (0 to 0)	0 (0 to 0)	0 (0 to 0)	0 (0 to 0)	0·1 (0 to 0·1)	0·2 (0·1 to 0·4)	0·1 (0 to 0·1)	0·2 (0·1 to 0·5)	0·2 (0·1 to 0·4)	0·1 (0·1 to 0·3)	0·1 (0 to 0·2)	0 (0 to 0·1)	0 (0 to 0)	0·1 (0 to 0·2)	0 (0 to 0)	1·3
Other pneumoconiosis	0 (0 to 0)	0 (0 to 0)	0 (0 to 0)	0 (0 to 0)	0 (0 to 0)	0 (0 to 0)	0·1 (0 to 0·1)	0·1 (0 to 0·1)	0·1 (0·1 to 0·3)	0·2 (0·1 to 0·4)	0·2 (0·1 to 0·4)	0·4 (0·2 to 0·7)	1·2 (0·6 to 2)	1·8 (0·9 to 3)	1·3 (0·7 to 2·2)	1·1 (0·6 to 2)	2·8 (1·4 to 4·4)	1·8 (1 to 3)	2·1 (1·1 to 3·3)	1·1 (0·6 to 1·8)	0·7 (0·4 to 1·2)	0·7 (0·4 to 1·1)	0·3 (0·2 to 0·5)	16·1
Asthma	0 (0 to 0)	0 (0 to 0)	0 (0 to 0)	1,980 (925 to 3,650)	2,020 (1,130 to 3,450)	1,280 (723 to 2,180)	974 (553 to 1,630)	734 (435 to 1,180)	734 (446 to 1,140)	770 (493 to 1,130)	603 (402 to 875)	579 (385 to 848)	805 (555 to 1,140)	1,030 (709 to 1,430)	917 (629 to 1,290)	712 (499 to 969)	637 (446 to 867)	531 (380 to 736)	347 (245 to 473)	197 (140 to 269)	90·7 (63·4 to 124)	43·8 (31·3 to 59·7)	21·3 (15 to 29·2)	15,000
Interstitial lung disease and pulmonary sarcoidosis	0 (0 to 0)	0 (0 to 0)	0 (0 to 0)	28·5 (7·5 to 58·2)	6·4 (2·6 to 12·2)	6·8 (2·3 to 14)	12·5 (4·8 to 23·7)	13·8 (5·4 to 25·9)	15·8 (6·4 to 31·6)	26·7 (15·2 to 43·1)	25·1 (14·5 to 41·5)	41·5 (26·2 to 64·1)	64·7 (42·3 to 96·2)	104 (65·1 to 156)	121 (74·9 to 183)	98·8 (63·3 to 151)	113 (71·1 to 174)	113 (69 to 196)	97·1 (58·3 to 173)	52·9 (29·3 to 96·1)	29·5 (16 to 52·9)	12·4 (7 to 22·8)	5 (2·4 to 9·9)	989
Other chronic respiratory diseases	0 (0 to 0)	0 (0 to 0)	0 (0 to 0)	37·7 (22 to 65·5)	42·1 (25·7 to 65·4)	47·3 (18·1 to 84·4)	30·6 (17·2 to 47·4)	51·1 (29·8 to 78·9)	69·1 (44·8 to 98·7)	75·8 (50·2 to 109)	65·8 (42·8 to 94·2)	78·5 (50·6 to 112)	119 (80·5 to 166)	158 (96·6 to 234)	152 (91·7 to 229)	27·3 (17 to 42·2)	19·4 (12·6 to 31·4)	18·4 (12·1 to 31·7)	16·9 (10·6 to 27·2)	12 (7·2 to 20·9)	7·8 (4·9 to 13·3)	4·7 (3 to 7·4)	2·4 (1·4 to 3·8)	1,040
Digestive diseases	21 (13·9 to 29·4)	29·4 (20·5 to 42·8)	165 (111 to 241)	374 (263 to 530)	411 (260 to 623)	325 (224 to 476)	590 (432 to 780)	978 (699 to 1,370)	1,600 (1,170 to 2,200)	2,310 (1,770 to 3,070)	2,780 (2,210 to 3,550)	4,960 (3,930 to 6,120)	9,480 (7,490 to 11,800)	12,900 (10,200 to 16,300)	12,900 (10,200 to 16,100)	9,260 (7,420 to 11,400)	7,830 (6,360 to 9,630)	6,880 (5,520 to 8,460)	5,210 (4,270 to 6,320)	3,250 (2,640 to 3,920)	1,750 (1,430 to 2,120)	808 (655 to 957)	311 (241 to 363)	85,100
Cirrhosis and other chronic liver diseases	0 (0 to 0)	0 (0 to 0)	0·3 (0·1 to 0·4)	49·3 (26·6 to 82·2)	48·6 (26·3 to 72·1)	47·6 (28·9 to 69·1)	101 (68 to 141)	132 (88·1 to 186)	272 (179 to 379)	605 (442 to 798)	1,060 (796 to 1,350)	2,700 (2,080 to 3,450)	5,820 (4,390 to 7,520)	8,240 (6,150 to 10,500)	8,050 (6,050 to 10,500)	5,340 (4,060 to 6,900)	3,820 (2,910 to 4,920)	2,860 (2,140 to 3,670)	1,750 (1,320 to 2,270)	826 (614 to 1,070)	385 (285 to 496)	151 (111 to 194)	53·2 (37·3 to 68·7)	42,300
Cirrhosis and other chronic liver diseases due to hepatitis	0 (0 to 0)	0 (0 to 0)	0 (0 to 0)	0·1 (0 to 0·2)	0·2 (0·1 to 0·5)	0·5 (0·1 to 1)	1·5 (0·4 to 3·3)	5·9 (2·4 to 11·6)	18·8 (7 to 38·1)	52·6 (26 to 88·5)	105 (46·1 to 200)	269 (138 to 477)	559 (282 to 1,040)	722 (343 to 1,400)	596 (275 to 1,140)	343 (156 to 660)	225 (109 to 419)	165 (76·1 to 314)	105 (51·1 to 187)	50·4 (25·2 to 91·6)	22·9 (12·6 to 38·8)	8·8 (4·2 to 15·8)	3·1 (1·1 to 6·3)	3,250
Cirrhosis and other chronic liver diseases due to hepatitis	0 (0 to 0)	0 (0 to 0)	0 (0 to 0)	0·3 (0·1 to 0·8)	1 (0·4 to 1·9)	1·8 (0·8 to 3·4)	6·1 (2·5 to 11·8)	22 (10·4 to 39·6)	67·7 (31·9 to 122)	159 (91·7 to 254)	268 (142 to 445)	646 (357 to 1,040)	1,290 (683 to 2,160)	1,680 (877 to 2,790)	1,520 (776 to 2,680)	951 (451 to 1,680)	670 (355 to 1,130)	495 (272 to 812)	298 (157 to 488)	143 (74·4 to 237)	69·2 (38·6 to 111)	27·9 (14·5 to 48·4)	10·2 (4·2 to 18·6)	8,320
Cirrhosis and other chronic liver diseases due to alcohol u	0 (0 to 0)	0 (0 to 0)	0 (0 to 0)	0 (0 to 0)	0 (0 to 0)	0 (0 to 0)	0·8 (0·2 to 2·1)	15·7 (6·9 to 31·7)	58·5 (26·7 to 109)	201 (122 to 310)	445 (252 to 674)	1,250 (805 to 1,800)	2,830 (1,730 to 4,090)	4,210 (2,620 to 5,970)	4,310 (2,740 to 6,030)	2,880 (1,840 to 4,060)	1,980 (1,270 to 2,770)	1,400 (893 to 1,970)	791 (506 to 1,150)	344 (207 to 509)	151 (95·1 to 220)	55·7 (33·5 to 85·2)	18·5 (8·9 to 30·3)	20,900
Cirrhosis and other chronic liver diseases due to NAFLD	0 (0 to 0)	0 (0 to 0)	0 (0 to 0)	0 (0 to 0)	0 (0 to 0)	0 (0 to 0)	5·9 (2·5 to 11·5)	15·4 (7·1 to 26·7)	43·8 (19·2 to 85·9)	90·8 (45·4 to 156)	136 (62·8 to 249)	334 (157 to 585)	748 (335 to 1,420)	1,070 (496 to 2,000)	1,010 (477 to 1,810)	698 (318 to 1,300)	560 (313 to 940)	468 (257 to 769)	317 (161 to 529)	163 (85·3 to 269)	80·5 (45·7 to 128)	33 (17·1 to 54·6)	12·2 (4·7 to 22·3)	5,780
Cirrhosis and other chronic liver diseases due to other cau	0 (0 to 0)	0 (0 to 0)	0·3 (0·1 to 0·4)	48·9 (26·5 to 80·8)	47·4 (25·3 to 70·4)	45·3 (27·1 to 66·2)	86·5 (56·2 to 123)	72·5 (42·8 to 111)	83·1 (43·9 to 140)	101 (49 to 181)	104 (47·3 to 186)	201 (92·3 to 375)	398 (188 to 725)	572 (265 to 1,070)	620 (283 to 1,130)	468 (225 to 835)	382 (177 to 719)	331 (153 to 584)	235 (115 to 414)	124 (62·5 to 223)	61·4 (33 to 101)	25·4 (12·8 to 46·1)	9·3 (3·8 to 18·8)	4,020
Upper digestive system diseases	0 (0 to 0)	0 (0 to 0)	0 (0 to 0)	19·2 (8·9 to 33·2)	26·8 (11·5 to 48·5)	43·4 (24 to 72·6)	186 (100 to 331)	458 (252 to 845)	716 (384 to 1,270)	904 (491 to 1,560)	842 (480 to 1,400)	1,000 (605 to 1,610)	1,570 (983 to 2,460)	1,970 (1,310 to 2,950)	1,940 (1,310 to 2,860)	1,510 (1,060 to 2,170)	1,460 (1,070 to 2,040)	1,410 (1,040 to 1,900)	1,190 (896 to 1,550)	810 (613 to 1,050)	472 (359 to 610)	240 (187 to 304)	88·3 (68·3 to 110)	16,900
Peptic ulcer disease	0 (0 to 0)	0 (0 to 0)	0 (0 to 0)	3·5 (1·7 to 5·7)	1·7 (0·4 to 2·9)	3·6 (2·1 to 5·5)	11·4 (7·5 to 16·6)	27·9 (17·4 to 41·6)	52·3 (33·5 to 77·7)	73·2 (47·5 to 105)	88·2 (61 to 125)	126 (82·3 to 188)	250 (166 to 377)	503 (334 to 754)	582 (388 to 852)	563 (389 to 813)	608 (429 to 859)	659 (469 to 919)	630 (444 to 866)	450 (319 to 605)	283 (209 to 376)	147 (108 to 191)	56·4 (41·8 to 73·6)	5,120
Gastritis and duodenitis	0 (0 to 0)	0 (0 to 0)	0 (0 to 0)	15·6 (5·5 to 29·8)	24·4 (8·8 to 45·7)	19·5 (7·2 to 40·8)	28·6 (12·6 to 53·6)	41·8 (19·5 to 75·8)	59·4 (25·5 to 107)	100 (49·4 to 182)	127 (68 to 220)	189 (104 to 325)	353 (199 to 594)	420 (243 to 682)	424 (267 to 662)	312 (197 to 474)	298 (191 to 433)	291 (184 to 429)	232 (144 to 334)	155 (92·8 to 220)	81·9 (47·6 to 115)	47·1 (26·7 to 67)	15·8 (8·7 to 22·7)	3,240
Gastroesophageal reflux disease	0 (0 to 0)	0 (0 to 0)	0 (0 to 0)	0 (0 to 0)	0·7 (0·1 to 1·8)	20·2 (8·8 to 36·7)	146 (69·8 to 282)	388 (191 to 767)	604 (288 to 1,140)	731 (345 to 1,370)	627 (296 to 1,170)	689 (329 to 1,250)	971 (469 to 1,770)	1,050 (503 to 1,900)	929 (435 to 1,800)	640 (302 to 1,210)	555 (285 to 1,020)	457 (234 to 803)	328 (165 to 581)	204 (99·9 to 368)	107 (54·4 to 194)	45·7 (23·3 to 83)	16 (7·9 to 28·4)	8,510
Appendicitis	0 (0 to 0)	0 (0 to 0)	0 (0 to 0)	18·7 (9·9 to 38·9)	26 (15·7 to 43·9)	36·2 (16·3 to 67·9)	64·7 (34·5 to 108)	73·7 (39 to 118)	74·1 (39·7 to 118)	79·3 (42·4 to 125)	80·6 (43·3 to 134)	88·5 (48·6 to 145)	136 (75·8 to 222)	171 (91·5 to 275)	159 (79·5 to 258)	127 (58·5 to 207)	151 (73·3 to 236)	141 (75 to 223)	94·4 (45·4 to 151)	63·6 (33·6 to 99·7)	38·9 (22·2 to 60·9)	17·8 (10·2 to 27·7)	7·5 (4·1 to 11·4)	1,650
Paralytic ileus and intestinal obstruction	14·2 (10·2 to 20·4)	25·6 (17·9 to 36·7)	131 (85·2 to 193)	46·5 (27·8 to 69·6)	20·1 (11·8 to 36·1)	17·1 (10 to 29·7)	33 (20·3 to 50·7)	39·8 (23 to 66·9)	64·6 (38·4 to 96·1)	86·3 (49·7 to 129)	102 (60·8 to 156)	128 (79·1 to 192)	244 (144 to 379)	381 (210 to 588)	443 (255 to 664)	380 (207 to 564)	467 (252 to 703)	531 (308 to 755)	512 (289 to 728)	409 (225 to 591)	229 (131 to 315)	102 (56·9 to 145)	45·4 (26·5 to 63·6)	4,450
Inguinal, femoral, and abdominal hernia	6·8 (1·5 to 13·7)	3·8 (1·7 to 6·8)	33·5 (17·1 to 59·3)	191 (107 to 314)	255 (134 to 431)	152 (76·4 to 271)	141 (66·4 to 259)	156 (78·5 to 276)	204 (102 to 355)	260 (139 to 448)	273 (142 to 451)	368 (207 to 602)	621 (338 to 1,030)	802 (479 to 1,240)	852 (540 to 1,310)	647 (412 to 968)	647 (451 to 916)	630 (446 to 853)	492 (369 to 648)	287 (213 to 369)	152 (113 to 193)	67 (48·8 to 88·2)	28·1 (19 to 37·6)	7,270
Inflammatory bowel disease	0 (0 to 0)	0 (0 to 0)	0 (0 to 0)	1·7 (0·4 to 6·3)	0·8 (0·4 to 2)	1·3 (0·8 to 2·1)	5·8 (3·6 to 9·5)	12·8 (8·8 to 19·4)	28 (19·1 to 40·1)	37·9 (26·7 to 50·9)	38·1 (23·6 to 57·2)	52·4 (32·5 to 77·4)	93·7 (56·8 to 137)	109 (68 to 163)	109 (68 to 160)	68·6 (43 to 102)	72·3 (44·9 to 107)	69·5 (46·7 to 98)	48·2 (32·2 to 68·7)	32 (21·7 to 45·7)	16·8 (11·4 to 24·1)	7·6 (5·1 to 11·2)	2·5 (1·6 to 4·2)	808
Vascular intestinal disorders	0 (0 to 0)	0 (0 to 0)	0 (0 to 0)	1·8 (1 to 2·8)	0·9 (0·4 to 1·6)	0·8 (0·4 to 1·4)	6·7 (3·9 to 10·9)	8 (5·1 to 12·3)	22·7 (14·3 to 34·2)	43·8 (27·8 to 67)	40·7 (25·4 to 62)	68·7 (44·7 to 101)	168 (108 to 254)	287 (187 to 423)	368 (240 to 531)	378 (265 to 524)	452 (309 to 640)	482 (342 to 658)	464 (330 to 629)	350 (252 to 473)	191 (133 to 257)	103 (70·5 to 137)	39 (25·4 to 51·6)	3,480
Gallbladder and biliary diseases	0 (0 to 0)	0 (0 to 0)	0 (0 to 0)	2·8 (1·7 to 4·4)	6·8 (3·8 to 11·1)	10·2 (6·2 to 16·4)	20·4 (11·5 to 33·3)	30·5 (12·7 to 56·2)	49 (24·8 to 82·8)	62 (33·9 to 101)	70·1 (39·3 to 111)	117 (73·1 to 173)	179 (113 to 269)	265 (174 to 379)	301 (200 to 450)	251 (177 to 357)	289 (205 to 410)	302 (215 to 453)	282 (200 to 398)	233 (167 to 314)	133 (97 to 179)	63·4 (45·6 to 85·3)	25·4 (18 to 34·1)	2,690
Pancreatitis	0 (0 to 0)	0 (0 to 0)	0 (0 to 0)	2·4 (1·2 to 4)	0·7 (0·4 to 1·2)	1·2 (0·7 to 2·8)	18·2 (11·5 to 27·5)	45 (27·9 to 70·1)	131 (77·6 to 193)	184 (115 to 271)	233 (153 to 339)	369 (229 to 546)	527 (333 to 768)	546 (340 to 779)	469 (286 to 715)	375 (242 to 536)	284 (186 to 401)	248 (167 to 355)	182 (124 to 258)	110 (74·4 to 153)	43·9 (30·2 to 62·6)	15·2 (10·4 to 22)	4·8 (3 to 7)	3,790
Other digestive diseases	0 (0 to 0)	0 (0 to 0)	0 (0 to 0)	41·3 (25·9 to 60)	25·3 (15·9 to 38·7)	15·4 (10 to 22·8)	13·1 (8·4 to 19·4)	22·5 (11 to 34·2)	36·3 (15·4 to 60·3)	45·2 (18·9 to 72·6)	46·7 (19·3 to 75·4)	63·5 (29·5 to 102)	118 (60·1 to 180)	159 (80·3 to 246)	182 (94·3 to 283)	181 (90 to 277)	188 (91·6 to 282)	208 (103 to 308)	194 (96·5 to 290)	130 (69·7 to 197)	89·3 (49·1 to 132)	42·2 (23·9 to 62·8)	16·8 (9·7 to 26)	1,820
Neurological disorders	0·5 (0·1 to 1·3)	1·5 (0·3 to 3·8)	227 (148 to 324)	443 (217 to 795)	814 (344 to 1,590)	1,850 (636 to 3,770)	2,800 (1,130 to 5,480)	2,890 (1,130 to 5,620)	3,010 (1,160 to 5,890)	3,090 (1,210 to 5,880)	2,590 (1,080 to 4,800)	2,950 (1,400 to 5,200)	4,010 (2,060 to 6,910)	4,490 (2,580 to 7,070)	4,390 (2,780 to 6,790)	3,580 (2,350 to 5,380)	4,270 (2,790 to 6,870)	5,460 (3,550 to 9,260)	6,420 (4,090 to 11,300)	7,440 (4,400 to 15,000)	6,010 (3,160 to 12,300)	3,680 (1,860 to 7,680)	1,960 (932 to 4,220)	72,400
Alzheimer's disease and other dementias	0 (0 to 0)	0 (0 to 0)	0 (0 to 0)	0 (0 to 0)	0 (0 to 0)	0 (0 to 0)	0 (0 to 0)	0 (0 to 0)	0 (0 to 0)	0 (0 to 0)	0 (0 to 0)	25 (5·7 to 76·9)	193 (57·2 to 546)	420 (140 to 1,020)	708 (277 to 1,650)	948 (384 to 2,230)	1,640 (644 to 4,060)	2,710 (1,130 to 6,390)	3,620 (1,530 to 8,490)	5,190 (2,150 to 12,700)	4,730 (1,920 to 10,900)	3,160 (1,330 to 7,170)	1,800 (767 to 4,080)	25,100
Parkinson's disease	0 (0 to 0)	0 (0 to 0)	0 (0 to 0)	0 (0 to 0)	0 (0 to 0)	0 (0 to 0)	0 (0 to 0)	2·3 (1·4 to 3·6)	4·2 (2·3 to 6·9)	5 (2·5 to 9·3)	10·4 (6·5 to 16·1)	31·2 (22·4 to 43·4)	96·8 (69·4 to 135)	200 (139 to 282)	321 (235 to 429)	461 (352 to 588)	856 (667 to 1,050)	1,460 (1,180 to 1,790)	2,020 (1,650 to 2,460)	1,810 (1,490 to 2,220)	1,070 (878 to 1,270)	443 (349 to 536)	132 (98·1 to 163)	8,930
Idiopathic epilepsy	0·5 (0·1 to 1·3)	1·5 (0·2 to 3·8)	54·7 (29·4 to 94·8)	254 (94·5 to 513)	365 (105 to 802)	384 (127 to 850)	492 (197 to 1,030)	523 (237 to 1,000)	545 (263 to 995)	533 (268 to 935)	432 (241 to 743)	473 (271 to 794)	605 (334 to 1,030)	588 (335 to 999)	541 (297 to 939)	351 (175 to 655)	298 (129 to 584)	251 (99·2 to 506)	192 (71·3 to 370)	126 (43·3 to 255)	73·1 (24·9 to 151)	35·9 (12·3 to 73·3)	14·3 (4·7 to 28·8)	7,130
Multiple sclerosis	0 (0 to 0)	0 (0 to 0)	0 (0 to 0)	0 (0 to 0)	0·1 (0 to 0·3)	0·4 (0·2 to 0·7)	3·2 (1·5 to 5·2)	14·4 (5·5 to 23·3)	17·6 (9·1 to 25·9)	31·7 (16·4 to 46)	39·6 (22·3 to 55·2)	63·4 (34·4 to 89·7)	83·8 (52·4 to 118)	88·1 (54·6 to 127)	109 (57·9 to 153)	73·5 (34·4 to 109)	54·6 (29 to 76·5)	36·7 (18·7 to 54·2)	22·5 (12·2 to 32·4)	9·4 (5·5 to 12·9)	4·1 (2·4 to 6)	1·3 (0·8 to 1·7)	0·4 (0·2 to 0·6)	654
Motor neuron disease	0 (0 to 0)	0 (0 to 0)	30·4 (16·3 to 50·8)	12·6 (7·9 to 19·1)	2 (1·3 to 2·9)	7·5 (5·2 to 10)	33 (21·9 to 48)	29·9 (20·4 to 42·3)	23·1 (15·9 to 32·5)	35 (23·2 to 50·5)	55·1 (38·1 to 77·8)	114 (77·4 to 158)	153 (103 to 214)	263 (179 to 366)	309 (220 to 420)	246 (169 to 339)	269 (184 to 382)	183 (129 to 254)	80·4 (54·1 to 116)	32 (21·1 to 47·3)	11·6 (7·7 to 16·4)	3·5 (2·5 to 4·7)	2·5 (1·9 to 3·4)	1,900
Headache disorders	0 (0 to 0)	0 (0 to 0)	0 (0 to 0)	0 (0 to 0)	254 (5 to 684)	1,160 (52·2 to 2,960)	1,800 (201 to 4,420)	1,980 (273 to 4,570)	2,160 (363 to 4,930)	2,250 (460 to 4,890)	1,870 (414 to 4,000)	1,980 (527 to 4,150)	2,530 (749 to 5,420)	2,430 (716 to 4,880)	1,870 (564 to 3,840)	1,130 (382 to 2,230)	856 (297 to 1,670)	578 (200 to 1,180)	343 (117 to 739)	184 (60·4 to 399)	90·4 (28·6 to 188)	33·6 (10·2 to 73·3)	9·3 (2 to 24·3)	23,500
Migraine	0 (0 to 0)	0 (0 to 0)	0 (0 to 0)	0 (0 to 0)	228 (3·4 to 615)	1,070 (31·5 to 2,840)	1,660 (119 to 4,370)	1,800 (185 to 4,390)	1,940 (261 to 4,610)	2,010 (293 to 4,520)	1,650 (284 to 3,810)	1,740 (369 to 3,920)	2,200 (526 to 4,830)	2,090 (496 to 4,490)	1,600 (394 to 3,570)	947 (281 to 2,020)	698 (213 to 1,470)	458 (137 to 961)	270 (83·2 to 593)	146 (42·6 to 328)	71·3 (20·5 to 159)	26·1 (7·1 to 57·3)	6·7 (1·4 to 15·4)	20,600
Tension-type headache	0 (0 to 0)	0 (0 to 0)	0 (0 to 0)	0 (0 to 0)	26·5 (0·6 to 182)	86 (2·7 to 654)	145 (18·9 to 817)	178 (29 to 887)	220 (36·9 to 999)	242 (46·7 to 1,040)	221 (44·4 to 934)	243 (61·9 to 921)	330 (87·2 to 1,080)	344 (86·1 to 1,210)	267 (69·5 to 817)	183 (47·5 to 561)	158 (38·7 to 543)	120 (23·7 to 454)	73·3 (13·5 to 284)	38 (6·4 to 150)	19·1 (3·1 to 76·1)	7·5 (1·1 to 34·1)	2·6 (0·1 to 16·1)	2,900
Other neurological disorders	0 (0 to 0)	0 (0 to 0)	141 (88·1 to 205)	176 (102 to 277)	193 (101 to 347)	306 (178 to 510)	475 (310 to 697)	343 (224 to 484)	258 (173 to 358)	231 (157 to 320)	179 (124 to 250)	258 (181 to 357)	342 (233 to 485)	495 (346 to 675)	538 (380 to 737)	370 (270 to 505)	294 (213 to 389)	235 (171 to 317)	150 (110 to 204)	87·5 (62·8 to 120)	30·1 (22·6 to 39·6)	10·5 (8 to 13·3)	4·1 (3·1 to 5·2)	5,120
Mental disorders	1·2 (0·7 to 1·8)	3·6 (2·2 to 5·3)	58·9 (36·9 to 87·7)	338 (218 to 488)	1,660 (1,110 to 2,400)	3,870 (2,590 to 5,550)	5,840 (4,030 to 8,140)	6,380 (4,400 to 8,730)	7,630 (5,330 to 10,400)	8,470 (5,920 to 11,500)	7,400 (5,380 to 9,910)	8,010 (5,730 to 10,600)	10,900 (7,890 to 14,600)	11,200 (8,050 to 15,100)	9,480 (6,890 to 12,800)	6,130 (4,440 to 8,160)	5,010 (3,620 to 6,600)	3,900 (2,810 to 5,170)	2,650 (1,850 to 3,610)	1,580 (1,070 to 2,210)	839 (595 to 1,140)	370 (251 to 516)	135 (83·7 to 198)	102,000
Schizophrenia	0 (0 to 0)	0 (0 to 0)	0 (0 to 0)	0 (0 to 0)	0 (0 to 0)	19 (7·9 to 37·5)	171 (61 to 332)	612 (306 to 1,030)	1,140 (650 to 1,750)	1,480 (880 to 2,190)	1,330 (826 to 1,940)	1,390 (916 to 1,900)	1,790 (1,170 to 2,460)	1,700 (1,130 to 2,340)	1,280 (830 to 1,770)	713 (450 to 996)	495 (318 to 718)	318 (196 to 456)	173 (100 to 264)	78·3 (42·6 to 123)	28·9 (14 to 47·3)	8·6 (3·6 to 15·3)	2 (0·5 to 4)	12,700
Depressive disorders	0 (0 to 0)	0 (0 to 0)	0 (0 to 0)	0·5 (0·2 to 1·1)	48·4 (19·7 to 94·7)	411 (199 to 744)	1,320 (770 to 2,100)	2,000 (1,200 to 3,140)	2,250 (1,350 to 3,440)	2,500 (1,550 to 3,820)	2,390 (1,500 to 3,490)	2,800 (1,740 to 4,230)	4,050 (2,670 to 5,990)	4,440 (2,950 to 6,580)	4,040 (2,580 to 6,090)	2,780 (1,750 to 4,170)	2,420 (1,590 to 3,440)	2,010 (1,310 to 2,870)	1,460 (917 to 2,240)	939 (579 to 1,420)	533 (344 to 788)	248 (154 to 379)	94·5 (50·8 to 153)	36,800
Major depressive disorder	0 (0 to 0)	0 (0 to 0)	0 (0 to 0)	0·4 (0·1 to 1)	40·2 (14·5 to 84·8)	356 (156 to 682)	1,140 (636 to 1,870)	1,680 (960 to 2,740)	1,810 (1,030 to 2,890)	1,990 (1,150 to 3,180)	1,940 (1,180 to 2,900)	2,310 (1,390 to 3,590)	3,400 (2,170 to 5,020)	3,770 (2,410 to 5,750)	3,480 (2,150 to 5,390)	2,420 (1,480 to 3,730)	2,120 (1,360 to 3,110)	1,790 (1,150 to 2,610)	1,310 (794 to 2,050)	851 (507 to 1,330)	487 (302 to 733)	228 (136 to 354)	86·8 (44·8 to 144)	31,200
Dysthymia	0 (0 to 0)	0 (0 to 0)	0 (0 to 0)	0·1 (0 to 0·3)	8·2 (0 to 22·1)	54·6 (21·2 to 110)	179 (82·5 to 308)	323 (150 to 594)	442 (226 to 764)	509 (274 to 871)	448 (253 to 755)	487 (252 to 833)	656 (359 to 1,140)	669 (381 to 1,110)	565 (328 to 933)	366 (199 to 624)	299 (155 to 506)	228 (125 to 389)	151 (84·5 to 252)	87·7 (46·9 to 149)	46 (24·7 to 78·6)	20·5 (10·8 to 34·6)	7·7 (3·8 to 13·5)	5,550
Bipolar disorder	0 (0 to 0)	0 (0 to 0)	0 (0 to 0)	0 (0 to 0)	0 (0 to 0)	194 (74·5 to 357)	820 (387 to 1,480)	1,090 (552 to 1,880)	1,190 (618 to 2,030)	1,190 (613 to 2,030)	981 (527 to 1,600)	1,070 (579 to 1,730)	1,420 (797 to 2,350)	1,420 (771 to 2,320)	1,110 (613 to 1,820)	655 (352 to 1,060)	474 (256 to 764)	320 (164 to 524)	179 (95·2 to 302)	82·6 (42·2 to 140)	34·6 (16·7 to 60)	12·3 (5·7 to 22·1)	3·8 (1·7 to 6·9)	12,200
Anxiety disorders	0 (0 to 0)	0 (0 to 0)	0 (0 to 0)	23·3 (4·7 to 49·2)	370 (178 to 639)	874 (464 to 1,440)	1,280 (748 to 2,040)	1,380 (745 to 2,280)	1,510 (812 to 2,530)	1,590 (897 to 2,520)	1,330 (780 to 2,100)	1,420 (788 to 2,310)	1,930 (1,150 to 3,030)	1,980 (1,210 to 3,020)	1,690 (1,050 to 2,540)	1,110 (667 to 1,740)	900 (531 to 1,470)	672 (418 to 1,060)	429 (259 to 661)	227 (126 to 362)	102 (55·1 to 163)	36·3 (19·3 to 60·2)	9·8 (4·7 to 16·9)	18,900
Eating disorders	0 (0 to 0)	0 (0 to 0)	0 (0 to 0)	0 (0 to 0)	3·6 (1·3 to 7·2)	52·5 (20·6 to 103)	162 (78·9 to 309)	209 (96·3 to 390)	239 (122 to 396)	274 (133 to 497)	173 (81·7 to 315)	81·5 (36·9 to 155)	41·9 (11·2 to 87)	0 (0 to 0)	0 (0 to 0)	0 (0 to 0)	0 (0 to 0)	0 (0 to 0)	0 (0 to 0)	0 (0 to 0)	0 (0 to 0)	0 (0 to 0)	0 (0 to 0)	1,240
Anorexia nervosa	0 (0 to 0)	0 (0 to 0)	0 (0 to 0)	0 (0 to 0)	3 (0·9 to 6·7)	20·5 (4·7 to 42·6)	57·7 (20·2 to 118)	56·5 (19·1 to 111)	43·6 (15·4 to 87·1)	28 (6·4 to 59·5)	17·1 (3·1 to 38·7)	14·4 (0·1 to 34·2)	13·9 (0 to 34·9)	0 (0 to 0)	0 (0 to 0)	0 (0 to 0)	0 (0 to 0)	0 (0 to 0)	0 (0 to 0)	0 (0 to 0)	0 (0 to 0)	0 (0 to 0)	0 (0 to 0)	255
Bulimia nervosa	0 (0 to 0)	0 (0 to 0)	0 (0 to 0)	0 (0 to 0)	0·6 (0·2 to 1·4)	32 (7·8 to 73·4)	104 (39·6 to 227)	152 (54·7 to 322)	195 (88·6 to 335)	246 (113 to 465)	156 (71·2 to 288)	67·2 (25·4 to 130)	28 (4·6 to 66·5)	0 (0 to 0)	0 (0 to 0)	0 (0 to 0)	0 (0 to 0)	0 (0 to 0)	0 (0 to 0)	0 (0 to 0)	0 (0 to 0)	0 (0 to 0)	0 (0 to 0)	981
Autism spectrum disorders	1 (0·6 to 1·5)	3·1 (1·8 to 4·6)	49·9 (31 to 75·6)	232 (145 to 348)	314 (201 to 476)	286 (175 to 443)	317 (200 to 475)	321 (200 to 487)	330 (202 to 503)	331 (205 to 505)	261 (160 to 395)	267 (164 to 412)	342 (218 to 508)	338 (206 to 517)	280 (172 to 419)	178 (112 to 270)	146 (90·2 to 224)	117 (74 to 176)	81·2 (51·7 to 120)	49·4 (31 to 74·6)	25·9 (15·6 to 38·8)	11·1 (7 to 16·8)	3·9 (2·5 to 6·1)	4,280
Attention-deficit/hyperactivity disorder	0 (0 to 0)	0 (0 to 0)	0 (0 to 0)	27·9 (13·5 to 51·2)	325 (178 to 585)	407 (220 to 732)	386 (214 to 688)	300 (164 to 511)	246 (132 to 426)	202 (107 to 347)	135 (66·8 to 240)	115 (58·7 to 201)	126 (62·1 to 225)	102 (47·8 to 185)	62·1 (28·5 to 115)	25·5 (11·2 to 45·8)	12·2 (4·7 to 24·3)	5 (1·3 to 11·2)	1·3 (0 to 3·6)	0·1 (0 to 0·3)	0 (0 to 0)	0 (0 to 0)	0 (0 to 0)	2,480
Conduct disorder	0 (0 to 0)	0 (0 to 0)	0 (0 to 0)	0 (0 to 0)	517 (257 to 930)	1,530 (818 to 2,500)	1,140 (615 to 1,860)	0 (0 to 0)	0 (0 to 0)	0 (0 to 0)	0 (0 to 0)	0 (0 to 0)	0 (0 to 0)	0 (0 to 0)	0 (0 to 0)	0 (0 to 0)	0 (0 to 0)	0 (0 to 0)	0 (0 to 0)	0 (0 to 0)	0 (0 to 0)	0 (0 to 0)	0 (0 to 0)	3,190
Idiopathic developmental intellectual disability	0·2 (0 to 0·4)	0·5 (0 to 1·1)	9 (1·1 to 18·6)	54·6 (16·5 to 105)	82·2 (28·3 to 159)	73·5 (22·1 to 142)	80·6 (24·2 to 160)	79·4 (21·5 to 149)	81·4 (25·5 to 161)	78·9 (22·8 to 151)	61·5 (18·2 to 121)	61 (18·5 to 122)	77 (21·7 to 146)	73·3 (20·5 to 140)	57·4 (15·2 to 113)	33·7 (8·5 to 65·3)	25·3 (5·7 to 51·1)	17·8 (3·5 to 36·6)	10·6 (1·8 to 22·2)	5·9 (1·3 to 12·9)	2·3 (0·4 to 5·3)	0·7 (0·1 to 1·7)	0·1 (0 to 0·4)	967
Other mental disorders	0 (0 to 0)	0 (0 to 0)	0 (0 to 0)	0 (0 to 0)	0 (0 to 0)	19·7 (8·4 to 35·6)	152 (66·5 to 269)	390 (185 to 674)	637 (357 to 1,030)	821 (453 to 1,300)	745 (447 to 1,170)	812 (487 to 1,270)	1,100 (661 to 1,750)	1,130 (685 to 1,740)	958 (589 to 1,460)	628 (379 to 942)	532 (328 to 813)	437 (278 to 656)	313 (194 to 470)	200 (130 to 294)	112 (72·9 to 167)	52·5 (33·2 to 76·5)	20·4 (13·4 to 29·4)	9,060
Substance use disorders	0 (0 to 0)	0 (0 to 0)	0·1 (0 to 0·1)	0·3 (0·2 to 0·5)	0·4 (0·2 to 0·7)	48·4 (21·7 to 88·8)	982 (622 to 1,470)	2,000 (1,310 to 2,840)	2,390 (1,630 to 3,320)	2,550 (1,750 to 3,560)	2,470 (1,790 to 3,240)	3,230 (2,440 to 4,130)	5,430 (3,920 to 7,200)	6,020 (4,490 to 7,920)	5,510 (4,080 to 7,150)	3,340 (2,450 to 4,360)	2,380 (1,790 to 3,070)	1,330 (980 to 1,710)	690 (517 to 890)	282 (212 to 376)	148 (109 to 204)	78 (54·3 to 113)	42·7 (28·2 to 63·9)	38,900
Alcohol use disorders	0 (0 to 0)	0 (0 to 0)	0·1 (0 to 0·1)	0·3 (0·2 to 0·5)	0·4 (0·2 to 0·7)	22·3 (8·3 to 44·9)	318 (170 to 513)	897 (496 to 1,480)	1,300 (819 to 1,980)	1,690 (1,090 to 2,530)	1,900 (1,340 to 2,580)	2,720 (2,010 to 3,590)	4,840 (3,410 to 6,550)	5,510 (4,040 to 7,290)	5,140 (3,770 to 6,760)	3,150 (2,270 to 4,120)	2,230 (1,670 to 2,880)	1,230 (884 to 1,590)	607 (448 to 795)	221 (159 to 309)	111 (77·5 to 159)	56·7 (36·1 to 90·3)	31·5 (19 to 51·7)	32,000
Drug use disorders	0 (0 to 0)	0 (0 to 0)	0 (0 to 0)	0 (0 to 0)	0 (0 to 0)	26·1 (8·7 to 58·4)	664 (369 to 1,060)	1,100 (700 to 1,650)	1,090 (703 to 1,600)	860 (545 to 1,270)	570 (372 to 822)	510 (332 to 732)	583 (373 to 835)	509 (328 to 749)	361 (219 to 542)	195 (114 to 303)	141 (81·8 to 218)	108 (58·3 to 167)	83 (48·5 to 124)	61 (37·8 to 88·8)	37·5 (23·6 to 54·8)	21·3 (13·4 to 32·9)	11·2 (6·8 to 17·4)	6,930
Opioid use disorders	0 (0 to 0)	0 (0 to 0)	0 (0 to 0)	0 (0 to 0)	0 (0 to 0)	0 (0 to 0)	89·8 (34·2 to 177)	347 (172 to 623)	450 (224 to 759)	412 (220 to 678)	293 (159 to 468)	264 (141 to 426)	298 (156 to 503)	272 (132 to 472)	203 (97·5 to 341)	114 (50 to 197)	83·5 (35·1 to 149)	61·9 (25·8 to 111)	50·6 (24·4 to 84·1)	41·4 (21·5 to 67·8)	26·5 (13·9 to 41·8)	15·7 (8·4 to 25·9)	8·9 (4·7 to 15·1)	3,030
Cocaine use disorders	0 (0 to 0)	0 (0 to 0)	0 (0 to 0)	0 (0 to 0)	0 (0 to 0)	4·2 (1·9 to 7·7)	183 (76·3 to 333)	215 (107 to 376)	176 (90·4 to 306)	143 (69·8 to 249)	99·4 (51·2 to 167)	92·1 (46·2 to 155)	106 (52·9 to 181)	83·9 (37·9 to 149)	60·3 (26·2 to 106)	33·8 (13·5 to 62·8)	23·7 (8·6 to 46·6)	19·4 (7·2 to 37·1)	15·5 (6·6 to 29·2)	6·6 (1·9 to 13·5)	4 (2·4 to 6)	1·9 (1·2 to 2·8)	0·6 (0·4 to 0·9)	1,270
Amphetamine use disorders	0 (0 to 0)	0 (0 to 0)	0 (0 to 0)	0 (0 to 0)	0 (0 to 0)	0 (0 to 0)	23·3 (2·8 to 57·1)	113 (39·5 to 223)	120 (44·6 to 246)	63·6 (17 to 138)	28·5 (5·7 to 65·8)	22·3 (13·2 to 35·9)	23·7 (14·2 to 36·2)	23·6 (14·4 to 37·2)	17·3 (10·5 to 26·7)	9·7 (6 to 14·6)	6·6 (4·2 to 9·7)	2·2 (1·1 to 4)	1·2 (0·6 to 2·2)	0·6 (0·2 to 1·2)	0·4 (0·2 to 0·8)	0·3 (0·1 to 0·5)	0·1 (0·1 to 0·3)	456
Cannabis use disorders	0 (0 to 0)	0 (0 to 0)	0 (0 to 0)	0 (0 to 0)	0 (0 to 0)	19·2 (2·5 to 50·1)	238 (88·9 to 513)	229 (101 to 440)	159 (67·4 to 309)	107 (41·2 to 196)	65·9 (27 to 124)	58·6 (23·6 to 111)	68·9 (25·1 to 132)	55·3 (19·9 to 109)	32·8 (11 to 65·1)	13·4 (3·5 to 28·7)	7·6 (3·4 to 14·5)	3·8 (1·4 to 7·4)	1·8 (0·6 to 3·4)	0·8 (0·2 to 1·6)	0·3 (0·1 to 0·6)	0·1 (0 to 0·2)	0 (0 to 0·1)	1,060
Other drug use disorders	0 (0 to 0)	0 (0 to 0)	0 (0 to 0)	0 (0 to 0)	0 (0 to 0)	2·7 (1·2 to 5·2)	129 (55·9 to 242)	200 (102 to 353)	185 (93·2 to 320)	135 (66·9 to 235)	83·3 (42·6 to 140)	73·3 (38·4 to 121)	86·1 (44·9 to 143)	74·5 (40·5 to 123)	48 (22·7 to 82·6)	24·4 (11·1 to 44·5)	19·9 (9·7 to 34·4)	20·1 (11 to 32·4)	13·9 (7·4 to 22·7)	11·7 (6·9 to 17·9)	6·2 (4·1 to 8·6)	3·3 (2·3 to 4·5)	1·4 (1 to 1·9)	1,120
Diabetes and kidney diseases	0·5 (0·1 to 0·8)	1·3 (0·5 to 2·1)	59·9 (37·7 to 88·5)	87·9 (60·7 to 125)	163 (110 to 230)	172 (115 to 256)	517 (376 to 681)	908 (681 to 1,200)	1,660 (1,250 to 2,140)	2,510 (1,900 to 3,240)	2,930 (2,250 to 3,780)	4,750 (3,520 to 6,170)	9,920 (7,430 to 13,100)	14,900 (11,000 to 19,600)	17,200 (12,900 to 22,400)	14,300 (10,700 to 18,400)	15,000 (11,400 to 19,200)	14,700 (11,300 to 18,800)	11,900 (9,390 to 15,000)	7,830 (6,240 to 9,700)	4,270 (3,380 to 5,230)	1,940 (1,540 to 2,390)	719 (568 to 898)	126,000
Diabetes mellitus	0·5 (0·1 to 0·8)	1·3 (0·5 to 2·1)	3·6 (1·3 to 10·8)	6·3 (4·1 to 14·3)	11·3 (5·2 to 20·2)	21·4 (12·5 to 34·3)	193 (123 to 283)	430 (283 to 627)	829 (567 to 1,190)	1,340 (901 to 1,890)	1,760 (1,210 to 2,490)	2,940 (2,010 to 4,160)	6,620 (4,480 to 9,600)	10,600 (7,220 to 15,100)	12,300 (8,550 to 16,800)	10,200 (7,280 to 13,800)	10,800 (7,750 to 14,600)	10,400 (7,360 to 14,000)	8,310 (6,120 to 11,000)	5,280 (3,930 to 6,870)	2,850 (2,110 to 3,700)	1,210 (883 to 1,600)	427 (303 to 568)	86,500
Diabetes mellitus type 1	0·5 (0·1 to 0·8)	1·3 (0·5 to 2·1)	3·6 (1·3 to 10·8)	6·3 (4·1 to 14·3)	11·3 (5·2 to 20·2)	21·4 (12·5 to 34·3)	47·4 (31·8 to 69)	84 (59·2 to 115)	146 (103 to 196)	127 (82·5 to 182)	143 (91·5 to 201)	198 (131 to 281)	150 (95·1 to 232)	187 (116 to 276)	192 (128 to 281)	133 (88·8 to 196)	131 (88 to 206)	100 (66·4 to 158)	76·5 (49·3 to 136)	38·7 (24·6 to 63·6)	20·1 (12·1 to 33·1)	8·7 (5·5 to 14·9)	3·2 (1·9 to 5·4)	1,830
Diabetes mellitus type 2	0 (0 to 0)	0 (0 to 0)	0 (0 to 0)	0 (0 to 0)	0 (0 to 0)	0 (0 to 0)	145 (82·9 to 229)	346 (208 to 526)	682 (433 to 1,020)	1,220 (793 to 1,740)	1,620 (1,090 to 2,320)	2,740 (1,840 to 3,940)	6,470 (4,340 to 9,390)	10,400 (7,050 to 14,800)	12,100 (8,380 to 16,500)	10,100 (7,170 to 13,600)	10,700 (7,650 to 14,500)	10,300 (7,300 to 13,900)	8,230 (6,060 to 10,900)	5,240 (3,900 to 6,830)	2,830 (2,100 to 3,680)	1,210 (877 to 1,590)	424 (301 to 564)	84,600
Chronic kidney disease	0 (0 to 0)	0 (0 to 0)	56·3 (35·3 to 83·1)	80·8 (54·3 to 117)	150 (98·9 to 214)	150 (97 to 231)	324 (219 to 461)	478 (332 to 677)	830 (575 to 1,170)	1,170 (851 to 1,590)	1,170 (833 to 1,590)	1,810 (1,300 to 2,360)	3,310 (2,460 to 4,360)	4,330 (3,200 to 5,790)	4,980 (3,680 to 6,440)	4,020 (3,030 to 5,320)	4,210 (3,150 to 5,510)	4,280 (3,290 to 5,530)	3,620 (2,780 to 4,640)	2,550 (1,980 to 3,250)	1,420 (1,110 to 1,760)	726 (561 to 908)	292 (229 to 365)	40,000
Chronic kidney disease due to diabetes mellitus type 1	0 (0 to 0)	0 (0 to 0)	0·4 (0·2 to 0·8)	2·4 (0·8 to 5·8)	5·1 (1·7 to 12·8)	4·9 (1·7 to 11·7)	10·1 (4 to 22·2)	15·2 (6·4 to 31·8)	54 (23·3 to 107)	129 (61·4 to 224)	160 (74·8 to 276)	263 (129 to 464)	416 (188 to 727)	411 (158 to 799)	325 (110 to 695)	191 (69·2 to 424)	156 (58·8 to 363)	122 (46·1 to 266)	67·4 (24·9 to 145)	23·5 (8·4 to 55·3)	4·5 (1·4 to 10·9)	0·5 (0·2 to 1·4)	0·1 (0 to 0·1)	2,360
Chronic kidney disease due to diabetes mellitus type 2	0 (0 to 0)	0 (0 to 0)	0 (0 to 0)	0 (0 to 0)	0 (0 to 0)	0 (0 to 0)	3·5 (1·2 to 7·6)	8·5 (3·4 to 18·9)	26·4 (11·7 to 52·1)	60·1 (26·6 to 116)	88 (40·3 to 158)	200 (93·5 to 346)	545 (290 to 869)	962 (553 to 1,500)	1,320 (750 to 1,980)	1,150 (682 to 1,770)	1,250 (778 to 1,830)	1,280 (801 to 1,830)	1,070 (674 to 1,550)	714 (463 to 1,040)	353 (225 to 513)	164 (99·2 to 247)	62·3 (32·8 to 97·7)	9,250
Chronic kidney disease due to hypertension	0 (0 to 0)	0 (0 to 0)	0 (0 to 0)	0 (0 to 0)	0 (0 to 0)	0 (0 to 0)	31·8 (14·8 to 57·3)	51·3 (16·9 to 105)	94·5 (40·3 to 177)	140 (65·6 to 241)	148 (75·6 to 246)	252 (142 to 398)	520 (323 to 813)	778 (476 to 1,190)	1,020 (628 to 1,530)	942 (611 to 1,400)	1,090 (698 to 1,600)	1,200 (803 to 1,720)	1,120 (769 to 1,600)	878 (619 to 1,200)	541 (400 to 724)	302 (218 to 400)	124 (86·1 to 164)	9,230
Chronic kidney disease due to glomerulonephritis	0 (0 to 0)	0 (0 to 0)	16·3 (7·7 to 28·7)	27·6 (15·5 to 45·9)	56·5 (32·1 to 90)	63·3 (35·9 to 99·1)	134 (82·4 to 203)	198 (120 to 311)	337 (213 to 500)	443 (282 to 656)	415 (256 to 622)	593 (356 to 876)	962 (591 to 1,470)	1,140 (670 to 1,730)	1,200 (678 to 1,860)	850 (458 to 1,400)	760 (399 to 1,280)	662 (350 to 1,140)	485 (234 to 844)	282 (133 to 504)	120 (57·1 to 203)	51·7 (22·3 to 91·2)	18 (6·8 to 37·4)	8,810
Chronic kidney disease due to other and unspecified caus	0 (0 to 0)	0 (0 to 0)	39·6 (23·3 to 61)	50·9 (33·7 to 74·1)	88·2 (56·4 to 129)	81·4 (43·4 to 134)	144 (79·1 to 237)	205 (113 to 331)	318 (195 to 503)	393 (226 to 610)	357 (198 to 561)	507 (287 to 762)	863 (513 to 1,310)	1,040 (628 to 1,610)	1,120 (638 to 1,790)	892 (527 to 1,410)	955 (563 to 1,460)	1,010 (617 to 1,530)	882 (546 to 1,330)	656 (391 to 980)	404 (253 to 580)	208 (137 to 301)	88·2 (56·1 to 126)	10,300
Acute glomerulonephritis	0 (0 to 0)	0 (0 to 0)	0 (0 to 0)	0·8 (0·3 to 1·6)	2·2 (1 to 4·1)	1·2 (0·5 to 2·2)	1·2 (0·6 to 2·3)	0·7 (0·4 to 1·4)	1 (0·5 to 1·8)	0·1 (0·1 to 0·2)	0·5 (0·3 to 1)	0·8 (0·4 to 1·7)	1·9 (0·9 to 3·4)	0·2 (0·1 to 0·3)	1·2 (0·5 to 2·3)	0·7 (0·4 to 1·4)	0·2 (0·1 to 0·3)	1·8 (0·7 to 3·9)	1·3 (0·5 to 2·6)	0·5 (0·2 to 1)	0·4 (0·2 to 0·7)	0·2 (0·1 to 0·4)	0 (0 to 0)	16·8
Skin and subcutaneous diseases	0·1 (0 to 0·1)	0·9 (0·5 to 1·5)	176 (84·4 to 275)	1,460 (907 to 2,180)	2,000 (1,240 to 2,970)	1,860 (1,180 to 2,790)	2,290 (1,460 to 3,480)	1,590 (1,010 to 2,370)	1,480 (958 to 2,180)	1,450 (932 to 2,170)	1,220 (781 to 1,810)	1,350 (881 to 2,020)	1,930 (1,270 to 2,920)	2,190 (1,420 to 3,270)	2,080 (1,390 to 3,110)	1,560 (1,050 to 2,300)	1,590 (1,050 to 2,410)	1,510 (991 to 2,350)	1,270 (813 to 2,070)	894 (570 to 1,460)	520 (307 to 925)	268 (168 to 468)	107 (66·8 to 189)	28,800
Dermatitis	0 (0 to 0)	0 (0 to 0)	0 (0 to 0)	484 (252 to 814)	628 (325 to 1,070)	401 (218 to 676)	344 (192 to 564)	300 (170 to 484)	301 (170 to 479)	302 (164 to 479)	242 (133 to 396)	248 (135 to 415)	345 (183 to 573)	383 (207 to 644)	355 (194 to 592)	256 (140 to 410)	241 (135 to 400)	207 (119 to 333)	149 (88·4 to 241)	91·2 (52·8 to 147)	47·3 (27·7 to 76·6)	19·9 (11·8 to 32·1)	7·2 (4 to 11·6)	5,350
Atopic dermatitis	0 (0 to 0)	0 (0 to 0)	0 (0 to 0)	480 (249 to 808)	614 (316 to 1,050)	357 (183 to 624)	262 (135 to 460)	198 (99·4 to 352)	171 (84·5 to 294)	155 (75·7 to 276)	121 (58·5 to 221)	122 (61·1 to 215)	165 (84 to 292)	173 (88·4 to 308)	157 (77·7 to 287)	108 (54·2 to 193)	96·4 (50 to 175)	80·4 (41·3 to 140)	60·5 (31·4 to 106)	39·8 (19·7 to 68·1)	22·2 (11·4 to 37·5)	9·8 (5·3 to 16·5)	3·5 (1·9 to 5·9)	3,400
Contact dermatitis	0 (0 to 0)	0 (0 to 0)	0 (0 to 0)	0 (0 to 0)	8·6 (3·2 to 17·7)	36·3 (12·7 to 73·7)	71·5 (30·1 to 138)	90·8 (36·7 to 169)	115 (49·2 to 223)	130 (54·3 to 253)	108 (44 to 216)	113 (44·6 to 229)	162 (68·3 to 320)	192 (81·3 to 370)	182 (77·8 to 359)	136 (57·3 to 255)	130 (59·3 to 256)	113 (51·5 to 216)	77·3 (35·9 to 146)	44·1 (20 to 82·1)	21·2 (9·5 to 41)	8·5 (3·8 to 15·9)	3·1 (1·2 to 6·3)	1,740
Seborrhoeic dermatitis	0 (0 to 0)	0 (0 to 0)	0 (0 to 0)	4·9 (1·8 to 9·3)	5·9 (2·4 to 11·6)	7·6 (3·1 to 13·9)	10·4 (4·6 to 20·3)	11·8 (5·5 to 22·6)	15·2 (6·9 to 26·1)	17·3 (8·6 to 30·4)	13·9 (6·5 to 25·1)	13·9 (6·1 to 25·3)	17·7 (7·7 to 33·3)	18·3 (8·4 to 34·3)	15·6 (7·4 to 29·5)	11·8 (5·4 to 20·3)	14·1 (7 to 24·1)	13·9 (6·8 to 24)	11·1 (5·7 to 19·1)	7·4 (3·8 to 12·1)	4 (2 to 6·8)	1·7 (0·8 to 2·9)	0·5 (0·3 to 0·9)	217
Psoriasis	0 (0 to 0)	0 (0 to 0)	0 (0 to 0)	21·5 (13·2 to 33)	59·5 (25·8 to 108)	71·9 (32·6 to 124)	91·1 (43·9 to 157)	102 (51·6 to 165)	122 (61·7 to 201)	145 (73·1 to 232)	134 (76·8 to 211)	156 (90·4 to 241)	230 (131 to 350)	263 (156 to 401)	248 (144 to 376)	175 (103 to 270)	153 (94·7 to 225)	117 (71·5 to 178)	72 (43 to 106)	36 (20·9 to 55·8)	14·7 (7·8 to 22·9)	4·9 (2·4 to 8·2)	1·4 (0·6 to 2·5)	2,220
Bacterial skin diseases	0 (0 to 0)	0·1 (0 to 0·2)	86 (15·6 to 171)	54·7 (18·7 to 99·7)	55·4 (22·4 to 87·5)	53·2 (21·5 to 87)	67·9 (25·3 to 118)	74·4 (29·1 to 130)	99·7 (47 to 166)	96 (53 to 175)	86·3 (48·3 to 178)	128 (70·7 to 271)	216 (118 to 500)	288 (156 to 641)	305 (161 to 692)	233 (120 to 562)	270 (140 to 711)	280 (142 to 769)	276 (127 to 852)	219 (101 to 659)	124 (52 to 416)	71·8 (31·6 to 240)	32·8 (15 to 102)	3,120
Cellulitis	0 (0 to 0)	0 (0 to 0)	35·1 (2·7 to 85·2)	19·6 (4·3 to 43·1)	25 (5·5 to 47·3)	28 (6·9 to 53·8)	28·3 (6·4 to 60·2)	26·6 (6·8 to 56·5)	35·3 (13·3 to 69)	38·1 (17·9 to 81·7)	34·3 (15·1 to 82·7)	51 (23·4 to 129)	80·8 (36·7 to 215)	117 (51·8 to 303)	99·5 (41·7 to 286)	93·3 (39·6 to 262)	86·6 (34·9 to 292)	95·6 (36·3 to 313)	87·6 (30·2 to 344)	67·8 (21·9 to 256)	47·5 (15·4 to 194)	24·8 (7·9 to 107)	11·9 (3·8 to 44·4)	1,130
Pyoderma	0 (0 to 0)	0·1 (0 to 0·1)	50·9 (9·2 to 110)	35·1 (11·8 to 65·9)	30·4 (12·6 to 52·6)	25·1 (11·1 to 47·2)	39·6 (14·8 to 74·9)	47·8 (19·5 to 89·8)	64·4 (27·5 to 118)	57·9 (28·5 to 106)	51·9 (27·3 to 109)	77 (38·9 to 153)	135 (69·9 to 290)	171 (87 to 362)	206 (101 to 425)	140 (68·9 to 323)	183 (86·9 to 457)	184 (87·4 to 467)	188 (85·9 to 537)	152 (70·8 to 432)	76·4 (32 to 250)	46·9 (21·1 to 150)	20·9 (9·2 to 61·6)	1,980
Scabies	0 (0 to 0·1)	0·4 (0·2 to 0·7)	35·6 (18·2 to 61·6)	263 (134 to 469)	376 (180 to 667)	344 (168 to 602)	407 (208 to 707)	378 (189 to 662)	302 (149 to 530)	239 (114 to 428)	194 (93·6 to 338)	229 (116 to 398)	315 (165 to 545)	313 (154 to 553)	245 (123 to 429)	146 (76·7 to 257)	122 (58·8 to 218)	103 (50·9 to 178)	83·8 (40·4 to 146)	59·7 (30·7 to 104)	37·2 (18·9 to 65·3)	18·2 (9·7 to 31·1)	6·7 (3·3 to 11·7)	4,220
Fungal skin diseases	0 (0 to 0)	0·1 (0 to 0·1)	6·2 (2·3 to 13·3)	49·6 (18·3 to 112)	86·3 (32·1 to 191)	91 (33·6 to 203)	109 (40 to 250)	120 (44·9 to 266)	137 (51·5 to 312)	148 (56·6 to 335)	127 (50·5 to 276)	138 (53·3 to 304)	193 (75·7 to 420)	228 (89 to 499)	244 (96·6 to 536)	193 (74·3 to 417)	228 (90·5 to 488)	272 (108 to 573)	248 (96·4 to 504)	201 (79·3 to 432)	140 (56·8 to 298)	72·5 (29·2 to 148)	29·3 (11·9 to 59·5)	3,060
Viral skin diseases	0 (0 to 0)	0 (0 to 0)	0 (0 to 0)	287 (180 to 433)	490 (306 to 757)	228 (140 to 352)	158 (92·8 to 245)	128 (78·9 to 204)	103 (60·8 to 159)	88·7 (50·1 to 141)	69·8 (41·3 to 112)	71·7 (42·4 to 111)	91·1 (53 to 144)	92·4 (53·3 to 150)	78 (45·7 to 124)	54 (30·5 to 86·7)	48·5 (28·2 to 76·2)	37·3 (21·3 to 59)	22·1 (12·5 to 35·7)	11·7 (6·6 to 18·8)	5·3 (3 to 8·8)	2·1 (1·1 to 3·5)	0·7 (0·4 to 1·1)	2,070
Acne vulgaris	0 (0 to 0)	0 (0 to 0)	0 (0 to 0)	0 (0 to 0)	21·1 (11·4 to 35)	450 (255 to 754)	871 (495 to 1,450)	219 (121 to 364)	108 (53·8 to 186)	80·5 (37·9 to 136)	51·6 (24·8 to 87·9)	39·9 (22·6 to 64·2)	38·5 (22·1 to 63·2)	27·5 (15·2 to 46·1)	17·9 (9·3 to 31)	9·8 (5·2 to 17)	7·3 (3·7 to 12·3)	5·4 (3·1 to 8·7)	3·3 (1·9 to 5·5)	1·7 (1 to 2·8)	0·7 (0·4 to 1·2)	0·2 (0·1 to 0·4)	0 (0 to 0·1)	1,950
Alopecia areata	0 (0 to 0)	0 (0 to 0)	0 (0 to 0)	3·8 (1 to 8·2)	5·6 (1·8 to 11·9)	6·8 (2·1 to 13·2)	13·5 (5·9 to 25·2)	22·1 (11·1 to 37·5)	33·3 (16·9 to 56·4)	35·7 (19·1 to 57·9)	27·8 (14·2 to 45·1)	25 (12·8 to 42·3)	28·5 (14·5 to 48·4)	26·2 (12·2 to 44·9)	21·5 (10·2 to 36·7)	14·6 (7 to 26·1)	11·8 (5·7 to 20·3)	9·1 (4·2 to 15·2)	5·1 (2·2 to 9·4)	2·6 (1·1 to 4·8)	1·1 (0·3 to 2·2)	0·3 (0·1 to 0·7)	0·1 (0 to 0·2)	295
Pruritus	0 (0 to 0)	0 (0 to 0)	0·7 (0·3 to 1·5)	9·1 (3·8 to 18·2)	13·7 (5·9 to 27·6)	12·3 (4·9 to 24·9)	16·2 (6·7 to 31·9)	20·5 (8·3 to 41)	25·7 (11 to 49·6)	30·1 (12·1 to 57·2)	26·2 (11·3 to 51·6)	28·1 (12·1 to 53·6)	39·8 (16·5 to 78)	45·5 (19·2 to 91)	45·3 (18·7 to 91·2)	37·2 (16·2 to 73·4)	39·2 (16·6 to 77)	35·8 (15·2 to 69·4)	26·6 (11·5 to 53·6)	16·9 (7·3 to 34)	9 (3·8 to 18·1)	3·8 (1·7 to 7·4)	1·4 (0·6 to 2·8)	483
Urticaria	0 (0 to 0)	0·3 (0·1 to 0·5)	31·4 (18 to 51·3)	223 (129 to 344)	208 (119 to 350)	131 (69·8 to 221)	127 (66·5 to 210)	123 (62 to 207)	124 (60·8 to 212)	135 (71·3 to 225)	112 (62·4 to 187)	112 (61·1 to 183)	141 (70·4 to 244)	142 (76·5 to 240)	124 (64·3 to 208)	81·4 (42·2 to 137)	70·6 (37·1 to 124)	57·3 (29·9 to 96·1)	39·8 (21·2 to 65·4)	22·6 (12 to 37·4)	10·5 (5·4 to 17·5)	4 (2 to 6·7)	1·3 (0·6 to 2·4)	2,020
Decubitus ulcer	0 (0 to 0)	0 (0 to 0)	0 (0 to 0)	0·6 (0·4 to 1)	0·9 (0·4 to 1·4)	1 (0·6 to 1·6)	1·5 (0·9 to 2·5)	3·4 (1·6 to 6)	6·1 (2·5 to 11·8)	6·6 (3·1 to 12·2)	8·4 (3·6 to 17·6)	9·5 (4·9 to 22·5)	21·4 (10·2 to 45·9)	31·7 (15·6 to 70·9)	38·5 (17·9 to 84)	49·9 (21·3 to 112)	69·2 (27·9 to 162)	85·9 (33·2 to 206)	108 (44·2 to 265)	95 (43·7 to 219)	61·9 (30·7 to 155)	37·4 (19·4 to 86·1)	15·1 (7·4 to 32·1)	652
Other skin and subcutaneous diseases	0 (0 to 0)	0·1 (0 to 0·1)	15·9 (7 to 30·9)	63·1 (30 to 118)	60·2 (27·6 to 113)	65·6 (29·6 to 124)	88·4 (41·2 to 167)	94·9 (46·1 to 179)	120 (58·4 to 222)	143 (65·4 to 279)	140 (67·3 to 248)	163 (81·8 to 289)	266 (129 to 505)	352 (173 to 627)	363 (171 to 664)	307 (151 to 553)	328 (170 to 576)	300 (149 to 533)	236 (120 to 416)	136 (68·2 to 240)	68·2 (34·5 to 118)	32·8 (17·3 to 57·4)	10·4 (5·6 to 17·7)	3,350
Sense organ diseases	0·2 (0·1 to 0·3)	0·6 (0·3 to 1)	17·6 (10·7 to 26·6)	191 (120 to 281)	414 (256 to 608)	460 (288 to 675)	623 (387 to 923)	810 (488 to 1,210)	1,150 (723 to 1,760)	1,550 (969 to 2,390)	1,590 (1,010 to 2,410)	2,190 (1,380 to 3,350)	4,320 (2,740 to 6,770)	6,270 (4,060 to 9,860)	7,320 (4,770 to 11,200)	6,390 (4,180 to 9,470)	7,250 (4,840 to 10,600)	7,810 (5,330 to 11,000)	7,270 (5,050 to 10,000)	5,660 (4,000 to 7,660)	3,670 (2,610 to 4,860)	1,860 (1,330 to 2,460)	744 (535 to 988)	67,600
Blindness and vision loss	0·1 (0 to 0·2)	0·2 (0·1 to 0·5)	6·6 (3·4 to 11·3)	77·9 (44·6 to 122)	155 (91·5 to 239)	154 (90·4 to 246)	198 (118 to 304)	238 (143 to 361)	299 (189 to 448)	386 (238 to 576)	433 (274 to 628)	650 (411 to 980)	1,230 (790 to 1,870)	1,780 (1,130 to 2,710)	2,160 (1,400 to 3,300)	2,000 (1,360 to 2,940)	2,470 (1,670 to 3,540)	2,860 (1,950 to 3,970)	2,780 (1,940 to 3,850)	2,210 (1,530 to 3,010)	1,480 (1,040 to 1,990)	787 (547 to 1,070)	344 (239 to 475)	22,700
Glaucoma	0 (0 to 0)	0 (0 to 0)	0 (0 to 0)	0 (0 to 0)	0 (0 to 0)	0 (0 to 0)	0 (0 to 0)	0 (0 to 0)	0 (0 to 0)	0 (0 to 0)	0 (0 to 0)	0 (0 to 0)	3·7 (1·5 to 7·5)	31·5 (9·3 to 64·4)	84·4 (38·9 to 148)	116 (62·9 to 189)	181 (105 to 286)	259 (158 to 404)	316 (192 to 503)	294 (180 to 454)	221 (134 to 341)	119 (71 to 182)	52·9 (30·7 to 87)	1,680
Cataract	0 (0 to 0)	0 (0 to 0)	0 (0 to 0)	0 (0 to 0)	0 (0 to 0)	0 (0 to 0)	0 (0 to 0)	1·4 (0·6 to 2·8)	9 (4 to 17·5)	23·1 (7·4 to 45·7)	39·8 (15·6 to 74·6)	72·7 (35·5 to 128)	158 (84 to 278)	228 (124 to 367)	280 (160 to 440)	280 (171 to 425)	417 (260 to 636)	624 (406 to 907)	777 (515 to 1,130)	706 (470 to 1,000)	506 (340 to 717)	262 (173 to 369)	111 (69 to 165)	4,500
Age-related macular degeneration	0 (0 to 0)	0 (0 to 0)	0 (0 to 0)	0 (0 to 0)	0 (0 to 0)	0 (0 to 0)	0 (0 to 0)	0 (0 to 0)	0 (0 to 0)	0 (0 to 0)	0 (0 to 0)	0 (0 to 0)	1·1 (0·4 to 2·4)	7 (3·3 to 12·3)	14·8 (7·7 to 24·2)	16·4 (5·4 to 30·6)	22·2 (10 to 40·5)	27·8 (13·5 to 48·3)	29·5 (16·4 to 47·7)	23·1 (12·9 to 36·2)	14·8 (8·5 to 23·3)	7 (4 to 11·2)	2·8 (1·6 to 4·6)	166
Refraction disorders	0 (0 to 0)	0 (0 to 0·1)	4 (2 to 7·1)	62·3 (34 to 105)	125 (70·8 to 200)	121 (70·4 to 199)	153 (89·5 to 246)	179 (103 to 281)	212 (123 to 332)	242 (142 to 377)	231 (134 to 346)	276 (162 to 435)	411 (251 to 634)	452 (265 to 691)	434 (263 to 667)	321 (193 to 488)	325 (194 to 491)	333 (203 to 493)	301 (186 to 449)	199 (121 to 300)	103 (64·8 to 154)	38·3 (24·3 to 58·1)	11·5 (6·4 to 18·3)	4,530
Near vision loss	0 (0 to 0)	0 (0 to 0)	0 (0 to 0)	0·2 (0·1 to 0·5)	0·7 (0 to 1·9)	1·1 (0·2 to 3)	1·7 (0·4 to 4·2)	4·9 (1·6 to 11·6)	11·5 (3·8 to 26·9)	32·4 (12 to 69·2)	55·2 (19·6 to 118)	123 (49·7 to 252)	299 (119 to 623)	501 (205 to 1,040)	644 (251 to 1,370)	579 (236 to 1,230)	636 (265 to 1,290)	634 (266 to 1,260)	531 (220 to 1,090)	372 (155 to 745)	219 (98·7 to 423)	104 (44·2 to 200)	39·6 (15·2 to 86·2)	4,790
Other vision loss	0·1 (0 to 0·1)	0·2 (0·1 to 0·4)	2·6 (0·9 to 5·3)	15·4 (6 to 28·1)	29·1 (10·2 to 55·3)	32·3 (13·1 to 59·7)	43·3 (17·2 to 77·7)	53·4 (22·9 to 97·1)	65·7 (28·5 to 119)	88·5 (44·2 to 153)	107 (56·8 to 176)	179 (101 to 294)	362 (204 to 581)	564 (349 to 867)	705 (437 to 1,060)	691 (446 to 1,000)	887 (584 to 1,280)	979 (653 to 1,430)	819 (527 to 1,220)	621 (401 to 896)	412 (264 to 613)	256 (174 to 366)	127 (80·2 to 185)	7,040
Age-related and other hearing loss	0 (0 to 0·1)	0·1 (0 to 0·2)	5·3 (2·4 to 9·7)	81·6 (40·8 to 141)	220 (118 to 350)	272 (153 to 418)	383 (219 to 586)	523 (304 to 808)	787 (463 to 1,240)	1,070 (641 to 1,730)	1,060 (635 to 1,720)	1,440 (840 to 2,280)	2,920 (1,730 to 4,760)	4,240 (2,560 to 6,820)	4,910 (3,060 to 7,710)	4,160 (2,540 to 6,430)	4,510 (2,870 to 6,880)	4,680 (3,050 to 6,820)	4,260 (2,820 to 6,110)	3,290 (2,210 to 4,570)	2,120 (1,450 to 2,870)	1,050 (739 to 1,420)	385 (279 to 516)	42,400
Other sense organ diseases	0·1 (0 to 0·1)	0·3 (0·1 to 0·5)	5·8 (2·7 to 10·1)	31 (14·9 to 54·2)	38·1 (18·1 to 67)	33·4 (14·1 to 61·6)	42·8 (18·4 to 80·4)	48·7 (19·6 to 98·6)	65·1 (27·5 to 126)	93·8 (39·4 to 182)	94·4 (40·8 to 176)	106 (46·2 to 205)	169 (75·5 to 329)	246 (110 to 463)	250 (113 to 478)	231 (104 to 429)	268 (123 to 522)	270 (124 to 501)	233 (108 to 428)	151 (73·1 to 283)	71·9 (33·7 to 132)	28·4 (13·3 to 51·5)	14·4 (6·5 to 26·1)	2,490
Musculoskeletal disorders	0 (0 to 0)	0 (0 to 0)	0 (0 to 0)	0 (0 to 0)	148 (70·6 to 260)	668 (387 to 1,060)	1,650 (1,040 to 2,470)	2,650 (1,740 to 3,860)	4,380 (2,850 to 6,370)	5,920 (3,850 to 8,330)	5,960 (4,070 to 8,290)	7,360 (5,070 to 10,200)	11,300 (7,790 to 15,700)	13,200 (9,140 to 18,400)	12,800 (8,880 to 17,600)	9,420 (6,500 to 13,300)	8,590 (5,930 to 12,100)	7,230 (4,930 to 10,200)	5,050 (3,410 to 7,190)	2,940 (1,960 to 4,290)	1,450 (964 to 2,120)	606 (405 to 886)	223 (152 to 329)	102,000
Rheumatoid arthritis	0 (0 to 0)	0 (0 to 0)	0 (0 to 0)	0 (0 to 0)	0·7 (0·2 to 1·5)	5 (2·6 to 9)	15·1 (6·2 to 28)	31·2 (15 to 53)	52·6 (26·3 to 90·2)	73·5 (36·2 to 125)	78·2 (39·1 to 134)	98·1 (47·2 to 165)	157 (82·7 to 256)	178 (100 to 280)	172 (100 to 271)	125 (77·4 to 189)	116 (70·5 to 170)	91·3 (55·3 to 135)	70·2 (44·6 to 103)	43·7 (28·9 to 62·7)	36 (23 to 54·7)	18·6 (11·9 to 28)	6·4 (4 to 9·8)	1,370
Osteoarthritis	0 (0 to 0)	0 (0 to 0)	0 (0 to 0)	0 (0 to 0)	0 (0 to 0)	0 (0 to 0)	0 (0 to 0)	0 (0 to 0)	0 (0 to 0)	29·9 (12·3 to 61·7)	153 (72·3 to 323)	450 (222 to 905)	1,200 (587 to 2,400)	2,020 (982 to 4,170)	2,450 (1,190 to 4,960)	2,090 (1,060 to 4,140)	2,110 (1,050 to 4,160)	1,940 (949 to 3,900)	1,490 (756 to 3,000)	964 (487 to 1,930)	525 (267 to 1,050)	231 (119 to 456)	83 (42·5 to 163)	15,700
Osteoarthritis hip	0 (0 to 0)	0 (0 to 0)	0 (0 to 0)	0 (0 to 0)	0 (0 to 0)	0 (0 to 0)	0 (0 to 0)	0 (0 to 0)	0 (0 to 0)	11·2 (2·6 to 25·4)	15·1 (4·7 to 34·8)	26·9 (9·2 to 61·6)	63·1 (24·5 to 146)	108 (43·8 to 249)	141 (59·9 to 313)	130 (58·1 to 279)	139 (62·4 to 299)	133 (58·1 to 276)	103 (48·8 to 219)	66·3 (31·7 to 138)	36·7 (17·3 to 78·7)	16·6 (7·8 to 34·8)	6·1 (2·8 to 12·4)	996
Osteoarthritis knee	0 (0 to 0)	0 (0 to 0)	0 (0 to 0)	0 (0 to 0)	0 (0 to 0)	0 (0 to 0)	0 (0 to 0)	0 (0 to 0)	0 (0 to 0)	14·3 (4·8 to 32)	110 (49·1 to 232)	334 (159 to 703)	890 (421 to 1,840)	1,480 (713 to 3,090)	1,760 (827 to 3,640)	1,480 (726 to 3,020)	1,480 (718 to 2,970)	1,340 (649 to 2,730)	1,010 (496 to 2,050)	633 (311 to 1,280)	329 (164 to 659)	136 (68·4 to 270)	45·6 (22·4 to 91·1)	11,000
Osteoarthritis hand	0 (0 to 0)	0 (0 to 0)	0 (0 to 0)	0 (0 to 0)	0 (0 to 0)	0 (0 to 0)	0 (0 to 0)	0 (0 to 0)	0 (0 to 0)	2·3 (0·2 to 7·3)	13·4 (2·3 to 36·8)	38·2 (11·9 to 93·2)	104 (41 to 236)	176 (71·5 to 410)	213 (97·5 to 474)	180 (81 to 403)	181 (83·2 to 382)	168 (79·2 to 347)	134 (64·3 to 282)	93·2 (44·4 to 195)	56·2 (27·2 to 118)	27·9 (13·2 to 56·8)	11·3 (5·4 to 23·2)	1,400
Osteoarthritis other	0 (0 to 0)	0 (0 to 0)	0 (0 to 0)	0 (0 to 0)	0 (0 to 0)	0 (0 to 0)	0 (0 to 0)	0 (0 to 0)	0 (0 to 0)	2·1 (0·6 to 5·5)	14·1 (3·3 to 36·3)	50·4 (17·8 to 119)	146 (56·6 to 316)	260 (114 to 555)	335 (148 to 711)	297 (138 to 662)	309 (143 to 650)	297 (148 to 605)	243 (120 to 491)	171 (87·4 to 348)	103 (53 to 210)	50·5 (25·9 to 102)	20 (10·2 to 40·2)	2,300
Low back pain	0 (0 to 0)	0 (0 to 0)	0 (0 to 0)	0 (0 to 0)	135 (60·1 to 242)	606 (338 to 995)	1,260 (738 to 2,020)	1,630 (986 to 2,540)	2,160 (1,270 to 3,330)	2,580 (1,460 to 3,920)	2,470 (1,500 to 3,750)	2,910 (1,790 to 4,410)	4,290 (2,610 to 6,470)	4,870 (3,030 to 7,500)	4,560 (2,870 to 6,700)	3,330 (2,070 to 4,910)	3,110 (1,970 to 4,680)	2,710 (1,740 to 4,150)	2,000 (1,250 to 3,040)	1,290 (799 to 1,910)	664 (395 to 978)	268 (168 to 404)	95·9 (60·8 to 145)	40,900
Neck pain	0 (0 to 0)	0 (0 to 0)	0 (0 to 0)	0 (0 to 0)	9·1 (2 to 22·7)	49·1 (18 to 103)	126 (47·9 to 252)	206 (92·7 to 401)	361 (164 to 715)	592 (278 to 1,150)	668 (338 to 1,190)	867 (425 to 1,590)	1,350 (668 to 2,460)	1,340 (644 to 2,530)	1,180 (552 to 2,090)	784 (399 to 1,390)	659 (346 to 1,120)	540 (283 to 936)	365 (183 to 661)	206 (101 to 356)	108 (55·8 to 182)	46·5 (23·7 to 77·2)	19·1 (10 to 34)	9,470
Gout	0 (0 to 0)	0 (0 to 0)	0 (0 to 0)	0 (0 to 0)	0 (0 to 0)	0 (0 to 0)	0·7 (0·2 to 1·5)	5·3 (2·5 to 9·5)	16·9 (2·8 to 41)	32·9 (7·8 to 68·7)	40·6 (14·5 to 75·4)	55·5 (22 to 104)	88·3 (41·5 to 153)	105 (46·1 to 197)	100 (40·4 to 182)	76·5 (29·6 to 146)	79·9 (33·7 to 139)	78·8 (33·6 to 137)	67·8 (31·1 to 117)	48·9 (22 to 84·1)	31·7 (16·6 to 55·2)	17 (8·5 to 28·4)	7·8 (3·7 to 13·4)	854
Other musculoskeletal disorders	0 (0 to 0)	0 (0 to 0)	0 (0 to 0)	0 (0 to 0)	2·9 (1·8 to 4·4)	8·1 (3·4 to 29·1)	249 (99·5 to 472)	781 (460 to 1,230)	1,790 (1,060 to 2,740)	2,620 (1,590 to 4,060)	2,550 (1,580 to 3,850)	2,980 (1,830 to 4,590)	4,250 (2,690 to 6,410)	4,670 (2,810 to 7,250)	4,370 (2,650 to 6,570)	3,020 (1,800 to 4,710)	2,510 (1,510 to 3,780)	1,880 (1,040 to 2,940)	1,060 (562 to 1,690)	389 (79·2 to 788)	86·4 (31·2 to 233)	24·6 (14·4 to 41·5)	11·3 (6·7 to 17·8)	33,300
Other non-communicable diseases	1,620 (985 to 3,960)	1,250 (902 to 1,910)	3,510 (2,670 to 4,690)	1,700 (1,280 to 2,500)	1,340 (985 to 1,850)	1,090 (815 to 1,450)	1,340 (1,010 to 1,780)	1,590 (1,160 to 2,200)	2,000 (1,420 to 2,910)	2,450 (1,660 to 3,710)	2,280 (1,500 to 3,530)	2,650 (1,710 to 4,040)	3,970 (2,690 to 5,930)	4,820 (3,330 to 7,030)	5,340 (3,820 to 7,430)	4,710 (3,390 to 6,430)	4,990 (3,600 to 6,840)	4,800 (3,440 to 6,430)	3,810 (2,800 to 5,060)	2,380 (1,740 to 3,170)	1,330 (995 to 1,730)	580 (431 to 766)	217 (159 to 296)	59,800
Congenital birth defects	1,580 (954 to 3,930)	1,150 (816 to 1,840)	2,770 (2,010 to 3,980)	1,090 (759 to 1,860)	659 (474 to 1,010)	573 (420 to 815)	681 (505 to 911)	623 (428 to 819)	601 (433 to 797)	588 (424 to 767)	436 (318 to 575)	415 (310 to 555)	554 (414 to 735)	595 (437 to 791)	521 (379 to 699)	275 (207 to 370)	192 (137 to 263)	64·9 (39·4 to 102)	42·8 (26·4 to 66·1)	25·1 (15 to 38·7)	12·7 (7·6 to 19·8)	5·5 (3·4 to 8·9)	1·9 (1·1 to 3·1)	13,500
Neural tube defects	35·6 (10·1 to 96·7)	38·9 (18·1 to 73·2)	113 (48·4 to 211)	226 (115 to 457)	33·8 (22·6 to 50·2)	31·6 (21·5 to 44·9)	43·2 (29·3 to 64·8)	36·1 (23·7 to 52·1)	30·1 (21·1 to 42·7)	21·4 (15 to 29·1)	14·5 (10 to 19·8)	13·9 (9·6 to 19·3)	13·2 (9·3 to 18·1)	11·6 (8·1 to 16·1)	8·7 (6·1 to 12)	5·5 (3·7 to 7·7)	3·9 (2·6 to 5·4)	2·2 (1·4 to 3·3)	1·3 (0·8 to 2)	0·7 (0·4 to 1·1)	0·3 (0·2 to 0·5)	0·1 (0·1 to 0·2)	0 (0 to 0·1)	686
Congenital heart anomalies	625 (274 to 1,660)	607 (360 to 1,050)	1,260 (813 to 1,930)	292 (164 to 577)	169 (97 to 326)	144 (86·3 to 256)	187 (119 to 291)	130 (80·7 to 206)	147 (95·4 to 220)	166 (104 to 242)	125 (78·9 to 185)	108 (73·2 to 162)	136 (87·8 to 200)	119 (74·3 to 184)	81·2 (51·1 to 127)	50·2 (29·7 to 83·2)	30·3 (17·4 to 54·2)	6·1 (1·8 to 12·6)	4·2 (1·2 to 9·4)	2·8 (0·8 to 5·9)	1·4 (0·4 to 3·1)	0·7 (0·2 to 1·4)	0·3 (0·1 to 0·6)	4,400
Orofacial clefts	0·5 (0·2 to 1)	0·3 (0·1 to 0·6)	4·5 (1·8 to 9)	12·6 (4·9 to 23·2)	12·9 (4·2 to 25·2)	11·4 (3·6 to 23·1)	12·8 (4·5 to 25·9)	13·3 (4·1 to 27·2)	13·9 (4·4 to 27·3)	14·4 (5·3 to 28·6)	11·6 (3·7 to 22·5)	12 (3·6 to 24·6)	16·5 (5·4 to 31)	16·7 (5·4 to 31·8)	14·2 (4·7 to 27·9)	9·4 (3 to 18·4)	8·1 (2·7 to 15·9)	6·6 (2·2 to 13)	4·8 (1·5 to 9·3)	3 (1 to 5·9)	1·6 (0·5 to 3·2)	0·7 (0·2 to 1·5)	0·3 (0·1 to 0·5)	202
Down syndrome	16 (3·9 to 49·2)	8·2 (3·9 to 16)	95·6 (46 to 177)	35·4 (19·4 to 73·4)	19·2 (12·6 to 28·6)	24·8 (15·4 to 38)	43·1 (27 to 68·9)	40·9 (26 to 62·9)	47·5 (29·1 to 75·1)	62·8 (36·7 to 107)	57·1 (32·5 to 93)	63·2 (37·2 to 102)	95·3 (57·7 to 152)	134 (75·1 to 213)	176 (105 to 268)	77·1 (43·6 to 121)	44·6 (27 to 72·5)	0 (0 to 0)	0 (0 to 0)	0 (0 to 0)	0 (0 to 0)	0 (0 to 0)	0 (0 to 0)	1,040
Turner syndrome	0 (0 to 0)	0 (0 to 0)	0 (0 to 0)	0 (0 to 0)	0 (0 to 0)	0 (0 to 0)	0 (0 to 0)	0 (0 to 0)	0 (0 to 0)	0 (0 to 0)	0 (0 to 0)	0 (0 to 0)	0 (0 to 0)	0 (0 to 0)	0 (0 to 0)	0 (0 to 0)	0 (0 to 0)	0 (0 to 0)	0 (0 to 0)	0 (0 to 0)	0 (0 to 0)	0 (0 to 0)	0 (0 to 0)	0
Klinefelter syndrome	0 (0 to 0)	0 (0 to 0)	0 (0 to 0)	0·1 (0 to 0·1)	0·1 (0 to 0·1)	0·1 (0 to 0·1)	0 (0 to 0·1)	0·2 (0·1 to 0·4)	0·2 (0·1 to 0·3)	0·1 (0·1 to 0·3)	0·1 (0 to 0·2)	0·1 (0 to 0·2)	0·1 (0 to 0·2)	0 (0 to 0)	0 (0 to 0)	0 (0 to 0)	0 (0 to 0)	0 (0 to 0)	0 (0 to 0)	0 (0 to 0)	0 (0 to 0)	0 (0 to 0)	0 (0 to 0)	1·1
Other chromosomal abnormalities	79 (31·5 to 173)	43·7 (24·9 to 76)	115 (64·4 to 184)	55·5 (35·7 to 84·8)	32·4 (22·1 to 46·2)	31·6 (20 to 47·8)	37·1 (23·8 to 54·2)	26·7 (15·7 to 41·2)	38·7 (18·9 to 61·8)	21·8 (11·2 to 34)	21·1 (10·1 to 36·2)	11·8 (5·6 to 18·7)	13·7 (7·8 to 20·8)	10·8 (5·4 to 17)	6·3 (4·1 to 9·5)	2·7 (1·7 to 4)	0·9 (0·5 to 1·5)	0 (0 to 0)	0 (0 to 0)	0 (0 to 0)	0 (0 to 0)	0 (0 to 0)	0 (0 to 0)	549
Congenital musculoskeletal and limb anomalies	36·9 (12·2 to 189)	19·3 (9·3 to 46·6)	97·7 (56·4 to 157)	117 (64·1 to 186)	128 (74·8 to 199)	123 (72·9 to 190)	118 (65·3 to 184)	96·6 (55·1 to 153)	98·8 (57 to 160)	90·8 (46·6 to 144)	79 (44·3 to 122)	78·9 (43 to 128)	108 (59·8 to 173)	115 (64·1 to 178)	105 (58·3 to 163)	63·6 (35·6 to 102)	49·6 (26·9 to 82·6)	34·2 (17·4 to 58·3)	21 (10·7 to 35·1)	11·3 (6·1 to 18·5)	5·3 (2·8 to 8·6)	2·1 (1·1 to 3·5)	0·7 (0·3 to 1·2)	1,600
Urogenital congenital anomalies	44·8 (14·1 to 228)	22·7 (11·2 to 53·5)	49 (24·9 to 91·2)	27·1 (16·1 to 43·3)	19·5 (11·6 to 32·4)	12·8 (7·5 to 22·1)	10·8 (7 to 16·3)	11 (6·6 to 16·4)	8·7 (5·8 to 12·5)	10·1 (6 to 15·9)	5·3 (3·4 to 8·1)	6·1 (3·4 to 9·1)	10·7 (4·8 to 18·2)	4·1 (2·8 to 6)	9·9 (4·7 to 16·6)	5·9 (2·4 to 10·6)	1·5 (0·7 to 3·6)	0 (0 to 0)	0 (0 to 0)	0 (0 to 0)	0 (0 to 0)	0 (0 to 0)	0 (0 to 0)	260
Digestive congenital anomalies	363 (163 to 953)	268 (155 to 472)	537 (321 to 882)	99·4 (55·7 to 188)	41·8 (22·9 to 79·7)	14·8 (8 to 36·1)	11·6 (6·5 to 29·4)	7 (4 to 11·2)	10·6 (6 to 17·1)	9·2 (3·9 to 15·4)	4·1 (2·3 to 6·3)	3·4 (2·2 to 5·9)	8·3 (4·8 to 16)	8·2 (4·7 to 16·5)	6·6 (3·7 to 10·6)	8 (4·3 to 13·9)	10·1 (5·4 to 16·8)	0·3 (0·1 to 0·4)	0·2 (0·1 to 0·3)	0·1 (0·1 to 0·2)	0·1 (0 to 0·1)	0 (0 to 0)	0 (0 to 0)	1,410
Other congenital birth defects	376 (165 to 887)	146 (71·3 to 351)	496 (281 to 905)	229 (126 to 438)	202 (127 to 352)	178 (115 to 286)	217 (137 to 321)	261 (136 to 384)	205 (124 to 306)	191 (113 to 284)	118 (74·8 to 180)	118 (75·5 to 176)	152 (97·4 to 222)	176 (109 to 261)	114 (67·7 to 174)	52·2 (30·5 to 87·2)	42·6 (24·5 to 71·8)	15·5 (4·7 to 37)	11·2 (3·5 to 27·5)	7·2 (2·2 to 18·1)	4 (1·2 to 9·7)	1·8 (0·5 to 4·3)	0·7 (0·2 to 1·6)	3,310
Urinary diseases and male infertility	0·2 (0·1 to 1·1)	5·3 (1·8 to 8·9)	32 (12·8 to 53·3)	16·1 (9·1 to 24·2)	14·4 (8·5 to 21·1)	15·1 (9·8 to 21·8)	22 (13·6 to 32·3)	107 (53·1 to 211)	149 (72·5 to 291)	194 (102 to 399)	195 (108 to 348)	221 (130 to 370)	338 (194 to 486)	571 (358 to 787)	999 (646 to 1,440)	1,220 (788 to 1,820)	1,520 (995 to 2,150)	1,660 (1,100 to 2,310)	1,390 (924 to 1,920)	844 (581 to 1,150)	464 (336 to 613)	185 (133 to 250)	64 (43·3 to 99·4)	10,200
Urolithiasis	0 (0 to 0)	0 (0 to 0)	0 (0 to 0)	0·4 (0·2 to 0·6)	0·8 (0·4 to 1·4)	1·2 (0·6 to 2·1)	2·5 (1·2 to 4·7)	5·3 (2·6 to 9·4)	10·3 (5·1 to 18·4)	20·6 (10·1 to 36·4)	32·2 (14·5 to 60·9)	41·3 (19·4 to 73·9)	106 (58 to 169)	136 (76 to 211)	160 (95·2 to 244)	135 (83·8 to 208)	113 (69·2 to 178)	99·2 (59·4 to 153)	80·6 (45·7 to 140)	25·6 (14·2 to 43·4)	13·6 (7·4 to 23·4)	7·2 (3·8 to 12·5)	2·1 (1 to 3·8)	994
Benign prostatic hyperplasia	0 (0 to 0)	0 (0 to 0)	0 (0 to 0)	0 (0 to 0)	0 (0 to 0)	0 (0 to 0)	0 (0 to 0)	0 (0 to 0)	0 (0 to 0)	0 (0 to 0)	0 (0 to 0)	3·1 (0 to 8·3)	36·2 (12·6 to 74·7)	161 (80 to 282)	486 (230 to 853)	685 (353 to 1,190)	962 (539 to 1,520)	1,040 (572 to 1,680)	762 (415 to 1,240)	414 (230 to 669)	180 (97·2 to 291)	59·8 (32·7 to 97·8)	16·2 (8·7 to 26·6)	4,800
Male infertility	0 (0 to 0)	0 (0 to 0)	0 (0 to 0)	0 (0 to 0)	0 (0 to 0)	0 (0 to 0)	0 (0 to 0)	68 (19·1 to 171)	92·8 (25·6 to 228)	109 (29·6 to 300)	88·5 (25·4 to 230)	85·4 (24·8 to 225)	17·3 (3·5 to 50·7)	0 (0 to 0)	0 (0 to 0)	0 (0 to 0)	0 (0 to 0)	0 (0 to 0)	0 (0 to 0)	0 (0 to 0)	0 (0 to 0)	0 (0 to 0)	0 (0 to 0)	461
Other urinary diseases	0·1 (0 to 0·6)	3·8 (1·3 to 6·4)	5·2 (1·7 to 10·1)	7·2 (3·9 to 11·3)	6·7 (3·7 to 9·6)	7·6 (4·2 to 11·2)	11·7 (5·6 to 17·4)	16·6 (7·1 to 24·9)	8·4 (5·3 to 13·7)	18·2 (10·9 to 28·6)	23·8 (13·7 to 38·4)	28 (16·2 to 44·8)	62·4 (33·7 to 96·9)	84·6 (43·6 to 136)	105 (51·1 to 168)	113 (56·6 to 190)	107 (58·3 to 193)	93·9 (50·2 to 171)	106 (53·2 to 192)	64·9 (32·8 to 126)	33·8 (16·7 to 76·4)	17·5 (7·3 to 48·7)	6·4 (2·7 to 17·8)	932
Gynecological diseases	0 (0 to 0)	0 (0 to 0)	0 (0 to 0)	0 (0 to 0)	0 (0 to 0)	0 (0 to 0)	0 (0 to 0)	0 (0 to 0)	0 (0 to 0)	0 (0 to 0)	0 (0 to 0)	0 (0 to 0)	0 (0 to 0)	0 (0 to 0)	0 (0 to 0)	0 (0 to 0)	0 (0 to 0)	0 (0 to 0)	0 (0 to 0)	0 (0 to 0)	0 (0 to 0)	0 (0 to 0)	0 (0 to 0)	0
Uterine fibroids	0 (0 to 0)	0 (0 to 0)	0 (0 to 0)	0 (0 to 0)	0 (0 to 0)	0 (0 to 0)	0 (0 to 0)	0 (0 to 0)	0 (0 to 0)	0 (0 to 0)	0 (0 to 0)	0 (0 to 0)	0 (0 to 0)	0 (0 to 0)	0 (0 to 0)	0 (0 to 0)	0 (0 to 0)	0 (0 to 0)	0 (0 to 0)	0 (0 to 0)	0 (0 to 0)	0 (0 to 0)	0 (0 to 0)	0
Polycystic ovarian syndrome	--	--	--	--	--	--	--	--	--	--	--	--	--	--	--	--	--	--	--	--	--	--	--	NA
Female infertility	--	--	--	--	--	--	--	--	--	--	--	--	--	--	--	--	--	--	--	--	--	--	--	NA
Endometriosis	--	--	--	--	--	--	--	--	--	--	--	--	--	--	--	--	--	--	--	--	--	--	--	NA
Genital prolapse	0 (0 to 0)	0 (0 to 0)	0 (0 to 0)	0 (0 to 0)	0 (0 to 0)	0 (0 to 0)	0 (0 to 0)	0 (0 to 0)	0 (0 to 0)	0 (0 to 0)	0 (0 to 0)	0 (0 to 0)	0 (0 to 0)	0 (0 to 0)	0 (0 to 0)	0 (0 to 0)	0 (0 to 0)	0 (0 to 0)	0 (0 to 0)	0 (0 to 0)	0 (0 to 0)	0 (0 to 0)	0 (0 to 0)	0
Premenstrual syndrome	--	--	--	--	--	--	--	--	--	--	--	--	--	--	--	--	--	--	--	--	--	--	--	NA
Other gynecological diseases	0 (0 to 0)	0 (0 to 0)	0 (0 to 0)	0 (0 to 0)	0 (0 to 0)	0 (0 to 0)	0 (0 to 0)	0 (0 to 0)	0 (0 to 0)	0 (0 to 0)	0 (0 to 0)	0 (0 to 0)	0 (0 to 0)	0 (0 to 0)	0 (0 to 0)	0 (0 to 0)	0 (0 to 0)	0 (0 to 0)	0 (0 to 0)	0 (0 to 0)	0 (0 to 0)	0 (0 to 0)	0 (0 to 0)	0
Hemoglobinopathies and hemolytic anemias	0·8 (0·4 to 1·4)	6·5 (3·2 to 11·4)	43·5 (23·7 to 70·5)	120 (56·8 to 221)	204 (94·2 to 382)	112 (61·3 to 196)	140 (86·4 to 222)	144 (90·7 to 222)	134 (85·7 to 203)	153 (96·7 to 235)	139 (92·7 to 208)	153 (96·1 to 229)	203 (129 to 308)	187 (120 to 306)	193 (116 to 310)	131 (75·5 to 225)	89·9 (53·2 to 143)	87·2 (51·5 to 140)	72·7 (46·3 to 123)	46·6 (25·6 to 82)	46·9 (27·5 to 79·6)	25·5 (14·5 to 41·1)	12·4 (6·7 to 21·5)	2,450
Thalassemias	0 (0 to 0)	0 (0 to 0)	0·1 (0 to 0·2)	0·6 (0·3 to 1·2)	0·5 (0·2 to 1·1)	0·2 (0·1 to 0·5)	0·2 (0·1 to 0·5)	0·2 (0 to 0·4)	0·1 (0 to 0·2)	0 (0 to 0·1)	0 (0 to 0)	0 (0 to 0)	0 (0 to 0)	0 (0 to 0)	0 (0 to 0)	0 (0 to 0)	0 (0 to 0)	0 (0 to 0)	0 (0 to 0)	0 (0 to 0)	0 (0 to 0)	0 (0 to 0)	0 (0 to 0)	2
Thalassemias trait	0 (0 to 0·1)	0·2 (0·1 to 0·4)	0·9 (0·1 to 2·2)	3·9 (0·4 to 10)	6·7 (0·9 to 16·8)	2·3 (0·4 to 6·3)	1 (0·2 to 2·9)	0·7 (0·1 to 2·1)	0·7 (0·1 to 2·1)	0·9 (0·2 to 2·6)	0·7 (0·1 to 2·2)	0·8 (0·2 to 2·4)	1·4 (0·2 to 3·6)	1·7 (0·4 to 4·4)	2 (0·5 to 5·2)	1·4 (0·3 to 3·5)	1 (0·2 to 2·4)	0·9 (0·2 to 2·4)	0·9 (0·2 to 2·2)	0·7 (0·2 to 1·7)	0·6 (0·1 to 1·4)	0·3 (0·1 to 0·6)	0·1 (0 to 0·2)	29·7
Sickle cell disorders	0·3 (0·2 to 0·5)	1·1 (0·6 to 1·7)	24·8 (13·2 to 40·9)	38·4 (24·5 to 57·8)	60·8 (39·6 to 89·2)	58·9 (37·3 to 87·6)	78·4 (46·8 to 120)	68·4 (37·9 to 114)	50 (25·7 to 85·6)	50 (24·8 to 84·5)	42·7 (21·2 to 73·2)	42·9 (21·4 to 74)	44·7 (21·8 to 76·5)	23 (10·3 to 41·4)	9·1 (3·8 to 18·1)	1·5 (0·5 to 3·1)	0·2 (0·1 to 0·5)	0 (0 to 0·1)	0 (0 to 0)	0 (0 to 0)	0 (0 to 0)	0 (0 to 0)	0 (0 to 0)	595
Sickle cell trait	0·4 (0·1 to 0·9)	3·1 (1·3 to 5·9)	9·2 (2·5 to 19·3)	39·7 (10·3 to 88·7)	69 (18·8 to 153)	23·2 (3·3 to 65·3)	10·4 (1·8 to 28·6)	7·1 (0·8 to 20·6)	7·6 (1·1 to 22·7)	8·9 (1·3 to 24·5)	7·4 (1·1 to 21·8)	8·3 (1·1 to 24·6)	13·5 (2·5 to 36)	16·7 (3·5 to 46·4)	20·2 (4·7 to 50·7)	13·5 (2·9 to 34·5)	9·3 (1·8 to 22·9)	8·8 (1·7 to 23)	8·3 (2 to 21·6)	6·6 (1·6 to 15·2)	6·2 (2 to 13·1)	2·7 (1 to 5·4)	0·9 (0·3 to 1·8)	301

G6PD deficiency	0 (0 to 0·1)	0·7 (0·2 to 1·5)	0·6 (0·3 to 1·1)	4·7 (2·4 to 7·9)	8·6 (3·9 to 14·8)	8·4 (3·9 to 15·2)	20·5 (8·5 to 37·8)	39·9 (17·2 to 71·2)	47·9 (25 to 78·3)	59 (30·8 to 99·1)	56·5 (29·4 to 93·6)	63·4 (34 to 102)	84 (46·6 to 133)	72·1 (42·7 to 110)	67·6 (38·6 to 106)	35·9 (20 to 57·7)	17·8 (9·1 to 29·3)	11·8 (5·7 to 20)	5·8 (2·7 to 10)	1·8 (0·9 to 2·9)	0·9 (0·5 to 1·5)	0·3 (0·1 to 0·4)	0·1 (0 to 0·1)	608
G6PD trait	0 (0 to 0)	0 (0 to 0)	0 (0 to 0)	0 (0 to 0)	0 (0 to 0)	0 (0 to 0)	0 (0 to 0)	0 (0 to 0)	0 (0 to 0)	0 (0 to 0)	0 (0 to 0)	0 (0 to 0)	0 (0 to 0)	0 (0 to 0)	0 (0 to 0)	0 (0 to 0)	0 (0 to 0)	0 (0 to 0)	0 (0 to 0)	0 (0 to 0)	0 (0 to 0)	0 (0 to 0)	0 (0 to 0)	0
Other hemoglobinopathies and hemolytic anemias	0 (0 to 0·1)	1·4 (0·5 to 2·9)	7·9 (1·8 to 18·3)	32·9 (8·1 to 80·7)	58·2 (13·4 to 143)	19·2 (3·4 to 55·9)	29·9 (7·8 to 77·6)	27·2 (10·4 to 66·9)	27·3 (10·1 to 65·8)	34·4 (12·7 to 80·5)	31·2 (13·8 to 71·6)	38·2 (17·8 to 79)	59·4 (28·1 to 121)	73·8 (36 to 148)	93·9 (49·5 to 171)	78·5 (41·3 to 144)	61·6 (35·9 to 101)	65·6 (39 to 104)	57·7 (37·7 to 94·1)	37·6 (21·8 to 63·8)	39·2 (23·7 to 64·7)	22·3 (13·2 to 35·5)	11·4 (6·3 to 19·5)	909
Endocrine, metabolic, blood, and immune disorders	40·4 (20·8 to 77·3)	57 (31·5 to 90·8)	476 (286 to 740)	331 (215 to 492)	278 (173 to 420)	196 (128 to 283)	181 (115 to 268)	197 (123 to 299)	286 (182 to 455)	382 (231 to 652)	385 (227 to 655)	506 (307 to 813)	837 (539 to 1,260)	1,080 (696 to 1,740)	1,140 (730 to 1,730)	940 (595 to 1,510)	926 (578 to 1,470)	870 (535 to 1,370)	692 (421 to 1,080)	445 (254 to 735)	250 (146 to 456)	117 (71·3 to 200)	46·6 (26·8 to 87)	10,700
Oral disorders	0 (0 to 0)	0 (0 to 0)	0 (0 to 0)	137 (76·6 to 227)	188 (101 to 338)	193 (101 to 345)	318 (169 to 559)	519 (269 to 965)	828 (406 to 1,590)	1,130 (547 to 2,140)	1,130 (523 to 2,140)	1,350 (642 to 2,520)	2,030 (1,030 to 3,740)	2,380 (1,270 to 4,250)	2,490 (1,400 to 4,120)	2,140 (1,300 to 3,290)	2,260 (1,420 to 3,380)	2,120 (1,360 to 3,130)	1,610 (1,050 to 2,300)	1,020 (672 to 1,460)	554 (359 to 793)	247 (161 to 351)	92·1 (59 to 132)	22,700
Caries of deciduous teeth	0 (0 to 0)	0 (0 to 0)	0 (0 to 0)	48·2 (18·7 to 97·6)	50·8 (20·8 to 113)	2·5 (0·3 to 6·9)	0 (0 to 0)	0 (0 to 0)	0 (0 to 0)	0 (0 to 0)	0 (0 to 0)	0 (0 to 0)	0 (0 to 0)	0 (0 to 0)	0 (0 to 0)	0 (0 to 0)	0 (0 to 0)	0 (0 to 0)	0 (0 to 0)	0 (0 to 0)	0 (0 to 0)	0 (0 to 0)	0 (0 to 0)	102
Caries of permanent teeth	0 (0 to 0)	0 (0 to 0)	0 (0 to 0)	0 (0 to 0)	47·1 (16·5 to 103)	100 (39·6 to 219)	122 (49·1 to 256)	142 (59·3 to 291)	151 (59·4 to 312)	149 (59·7 to 320)	125 (50·2 to 257)	125 (52 to 261)	163 (67·9 to 342)	170 (67·7 to 352)	146 (57·4 to 316)	95·4 (36·9 to 204)	71·7 (29·2 to 141)	53·2 (22 to 106)	35·5 (15 to 71·4)	20·8 (8·4 to 41·4)	10·4 (4 to 21)	4·3 (1·6 to 8·9)	1·4 (0·5 to 3)	1,730
Periodontal diseases	0 (0 to 0)	0 (0 to 0)	0 (0 to 0)	0 (0 to 0)	0 (0 to 0)	0 (0 to 0)	42·6 (12·5 to 109)	166 (51 to 433)	396 (124 to 938)	629 (210 to 1,430)	661 (233 to 1,530)	811 (296 to 1,760)	1,190 (461 to 2,590)	1,260 (499 to 2,700)	1,050 (418 to 2,190)	634 (253 to 1,330)	484 (189 to 1,000)	364 (140 to 734)	251 (97·7 to 495)	154 (59·6 to 306)	82·4 (30·8 to 167)	35·9 (13·6 to 70·7)	12·6 (4·7 to 25·4)	8,230
Other oral disorders	0 (0 to 0)	0 (0 to 0)	0 (0 to 0)	88·8 (51·5 to 139)	89·8 (51·3 to 141)	89·9 (50·9 to 145)	153 (89·4 to 239)	193 (118 to 300)	230 (135 to 351)	251 (150 to 385)	211 (123 to 325)	226 (135 to 342)	310 (183 to 467)	322 (194 to 496)	275 (166 to 412)	181 (107 to 276)	154 (93·1 to 236)	115 (69·6 to 174)	67·5 (40·2 to 104)	33·8 (20·5 to 52·2)	14·4 (8·8 to 21·5)	5·6 (3·4 to 8·4)	2·1 (1·2 to 3·2)	3,010
Sudden infant death syndrome	--	25·1 (14·7 to 37·6)	184 (100 to 303)	--	--	--	--	--	--	--	--	--	--	--	--	--	--	--	--	--	--	--	--	NA
Injuries	86·1 (48·4 to 128)	66·1 (47·5 to 87·9)	642 (449 to 894)	1,500 (1,180 to 1,840)	2,520 (2,110 to 2,970)	3,620 (3,030 to 4,290)	9,270 (7,740 to 11,100)	13,600 (11,600 to 16,000)	16,800 (14,600 to 19,200)	18,000 (15,400 to 20,900)	14,800 (12,400 to 17,600)	16,300 (13,400 to 19,700)	21,100 (17,100 to 25,900)	21,900 (17,700 to 26,800)	19,100 (15,600 to 23,600)	12,700 (10,400 to 15,500)	11,100 (9,030 to 13,300)	10,300 (8,520 to 12,600)	9,010 (7,440 to 10,800)	7,360 (6,030 to 8,830)	5,230 (4,280 to 6,170)	3,090 (2,480 to 3,690)	1,450 (1,170 to 1,720)	220,000
Transport injuries	2·8 (1·5 to 3·9)	2·9 (1·9 to 4·1)	51·4 (32·5 to 75·4)	302 (202 to 422)	657 (475 to 858)	944 (713 to 1,210)	2,160 (1,550 to 2,900)	3,430 (2,620 to 4,410)	4,580 (3,640 to 5,660)	4,640 (3,620 to 5,720)	3,730 (2,910 to 4,680)	4,110 (3,220 to 5,220)	5,120 (3,970 to 6,540)	5,150 (4,000 to 6,550)	4,500 (3,500 to 5,600)	2,750 (2,170 to 3,420)	2,300 (1,810 to 2,830)	1,810 (1,430 to 2,220)	1,320 (1,050 to 1,610)	777 (608 to 955)	395 (307 to 481)	162 (126 to 204)	61·6 (47·9 to 76·9)	48,900
Road injuries	2·6 (1·4 to 3·7)	2·4 (1·5 to 3·6)	45·9 (28·2 to 68·5)	276 (180 to 389)	610 (436 to 806)	870 (646 to 1,120)	2,020 (1,430 to 2,730)	3,210 (2,440 to 4,130)	4,320 (3,410 to 5,320)	4,340 (3,390 to 5,320)	3,490 (2,720 to 4,400)	3,810 (3,000 to 4,850)	4,770 (3,720 to 6,090)	4,800 (3,730 to 6,110)	4,160 (3,230 to 5,190)	2,600 (2,050 to 3,240)	2,190 (1,730 to 2,690)	1,690 (1,330 to 2,090)	1,250 (995 to 1,530)	742 (581 to 914)	378 (293 to 461)	155 (119 to 194)	57·9 (44·7 to 72·6)	45,800
Pedestrian road injuries	1 (0·6 to 1·4)	0·2 (0·1 to 0·3)	8·1 (4·8 to 12·2)	119 (75·6 to 178)	323 (216 to 446)	346 (234 to 487)	400 (274 to 560)	475 (347 to 622)	623 (486 to 782)	655 (495 to 838)	605 (458 to 775)	759 (583 to 987)	1,110 (839 to 1,490)	1,160 (877 to 1,540)	1,170 (872 to 1,520)	769 (595 to 1,000)	731 (553 to 933)	648 (485 to 839)	541 (408 to 690)	353 (264 to 454)	189 (142 to 238)	73·6 (55·5 to 92·3)	26·8 (20 to 32·8)	11,100
Cyclist road injuries	0 (0 to 0)	0 (0 to 0)	0 (0 to 0)	31·2 (17·1 to 52·3)	76·5 (47·8 to 115)	241 (155 to 355)	556 (361 to 834)	645 (434 to 907)	700 (488 to 979)	738 (510 to 1,020)	671 (475 to 931)	748 (548 to 998)	963 (714 to 1,270)	1,090 (789 to 1,430)	1,020 (743 to 1,370)	685 (515 to 886)	569 (421 to 744)	437 (323 to 582)	293 (221 to 393)	154 (114 to 201)	70·1 (49·9 to 93·8)	29·3 (20·3 to 39·8)	10·7 (7·4 to 14·6)	9,730
Motorcyclist road injuries	0·1 (0·1 to 0·2)	0·1 (0 to 0·1)	3·8 (2·4 to 5·5)	15·9 (10·7 to 22)	27·9 (19·8 to 37·6)	35·7 (25·8 to 47·5)	219 (155 to 303)	657 (506 to 854)	1,100 (863 to 1,370)	1,150 (905 to 1,470)	798 (615 to 1,010)	688 (541 to 876)	875 (672 to 1,110)	819 (643 to 1,040)	674 (523 to 848)	372 (287 to 472)	292 (221 to 381)	201 (148 to 269)	147 (106 to 197)	93·2 (64·6 to 128)	53·2 (37·1 to 74·2)	25·4 (17·5 to 35·8)	10·4 (7·1 to 14·6)	8,270
Motor vehicle road injuries	1·1 (0·6 to 1·6)	2·1 (1·3 to 3·1)	28·5 (17·2 to 42·9)	80·1 (51·3 to 116)	140 (87 to 211)	191 (121 to 277)	729 (477 to 1,040)	1,200 (817 to 1,640)	1,620 (1,180 to 2,100)	1,530 (1,050 to 1,980)	1,160 (823 to 1,520)	1,320 (949 to 1,750)	1,400 (997 to 1,880)	1,340 (953 to 1,780)	952 (680 to 1,240)	588 (425 to 760)	408 (298 to 519)	260 (191 to 333)	165 (124 to 208)	87·5 (64·9 to 112)	42·1 (31·2 to 54·6)	17·9 (13·4 to 23·4)	7·2 (5·3 to 9·2)	13,300
Other road injuries	0·4 (0·2 to 0·7)	0·1 (0 to 0·2)	5·5 (2·5 to 10·6)	29·8 (12 to 59·7)	42·4 (20·7 to 78·5)	56 (29·4 to 97·9)	114 (61·8 to 202)	229 (131 to 384)	269 (155 to 445)	264 (155 to 434)	254 (161 to 391)	297 (187 to 446)	419 (274 to 646)	395 (259 to 593)	339 (220 to 493)	186 (129 to 260)	186 (128 to 259)	145 (102 to 206)	104 (74·5 to 142)	54·4 (39·2 to 73·2)	23·5 (16·8 to 31·2)	8·3 (5·8 to 11·2)	2·9 (2 to 4)	3,420
Other transport injuries	0·2 (0·1 to 0·3)	0·5 (0·3 to 0·7)	5·5 (3·6 to 8·2)	25·9 (16·5 to 39·1)	46·3 (30·9 to 65·9)	74·3 (50·3 to 105)	140 (93·8 to 206)	217 (142 to 320)	268 (184 to 382)	302 (202 to 441)	237 (161 to 341)	299 (204 to 415)	341 (237 to 483)	348 (243 to 485)	346 (240 to 484)	152 (112 to 206)	111 (84 to 145)	115 (82 to 158)	64·6 (47·6 to 86·1)	35·2 (26·2 to 46·7)	17·2 (12·8 to 22·1)	7·8 (5·8 to 9·9)	3·7 (2·8 to 4·8)	3,160
Unintentional injuries	19·8 (11·9 to 28·6)	51·2 (36·4 to 69·9)	533 (362 to 757)	1,080 (837 to 1,370)	1,740 (1,390 to 2,130)	2,290 (1,820 to 2,830)	3,860 (3,070 to 4,820)	4,330 (3,420 to 5,420)	5,120 (4,140 to 6,490)	5,620 (4,550 to 7,200)	4,820 (3,770 to 6,290)	5,720 (4,510 to 7,420)	7,930 (6,200 to 10,300)	9,090 (7,110 to 11,700)	8,350 (6,520 to 10,700)	6,030 (4,730 to 7,690)	5,790 (4,570 to 7,300)	5,960 (4,790 to 7,440)	5,760 (4,640 to 7,060)	5,380 (4,310 to 6,560)	4,210 (3,390 to 5,040)	2,730 (2,170 to 3,290)	1,330 (1,060 to 1,580)	97,700
Falls	0·8 (0·4 to 1·2)	1·2 (0·8 to 1·8)	37·4 (22·7 to 59·3)	109 (75·2 to 155)	217 (159 to 287)	350 (262 to 463)	705 (524 to 911)	904 (685 to 1,170)	1,110 (847 to 1,460)	1,300 (998 to 1,690)	1,310 (1,010 to 1,720)	1,680 (1,290 to 2,160)	2,740 (2,120 to 3,470)	3,540 (2,780 to 4,410)	3,610 (2,800 to 4,550)	2,880 (2,280 to 3,540)	3,110 (2,480 to 3,850)	3,730 (2,970 to 4,610)	4,080 (3,210 to 5,010)	4,290 (3,380 to 5,300)	3,590 (2,870 to 4,340)	2,420 (1,910 to 2,930)	1,190 (932 to 1,430)	42,900
Drowning	0·8 (0·4 to 1·1)	1·1 (0·7 to 1·6)	30·9 (19·9 to 45·2)	326 (225 to 444)	629 (467 to 809)	791 (598 to 1,020)	1,220 (883 to 1,620)	1,000 (709 to 1,360)	1,070 (808 to 1,370)	956 (740 to 1,230)	670 (514 to 860)	809 (622 to 1,050)	761 (558 to 992)	816 (590 to 1,070)	554 (413 to 724)	361 (273 to 466)	239 (184 to 310)	160 (123 to 204)	105 (82·7 to 132)	37·5 (29·3 to 46·8)	18·4 (14·2 to 23·2)	8·6 (6·9 to 10·2)	4·3 (3·4 to 5·2)	10,600
Fire, heat, and hot substances	0·2 (0·1 to 0·3)	0·4 (0·3 to 0·6)	17 (11·9 to 23·2)	81·9 (57·2 to 111)	163 (115 to 232)	185 (123 to 278)	273 (173 to 410)	334 (214 to 509)	413 (266 to 627)	540 (349 to 827)	468 (297 to 719)	515 (330 to 772)	733 (473 to 1,120)	758 (489 to 1,150)	681 (446 to 1,040)	450 (290 to 685)	377 (244 to 572)	320 (212 to 481)	247 (166 to 365)	168 (115 to 238)	99 (69·6 to 141)	48·2 (34·6 to 67·1)	21 (15·7 to 28·2)	6,890
Poisonings	0·1 (0·1 to 0·2)	0·2 (0·1 to 0·4)	5·7 (3·3 to 8·8)	39·6 (24·2 to 62·5)	28·5 (17·5 to 43·6)	32·2 (19·8 to 50·5)	45·7 (28·9 to 68·4)	55·3 (37·7 to 78)	67·8 (47·7 to 94·4)	68·2 (47·8 to 94·9)	54·9 (37·7 to 77·4)	55·6 (38·2 to 77·5)	75·2 (51·4 to 104)	80·1 (55·7 to 110)	67·2 (46·9 to 93·7)	44·4 (31·1 to 60·9)	37·5 (25·6 to 51·6)	28·8 (19·9 to 39·9)	21·3 (15·1 to 29·4)	13·4 (9·4 to 18·4)	6·7 (4·6 to 9·3)	3·2 (2·2 to 4·3)	1·2 (0·8 to 1·6)	833
Poisoning by carbon monoxide	0 (0 to 0)	0 (0 to 0)	0·5 (0·2 to 0·9)	4·9 (2·3 to 9·5)	5 (3·1 to 7·7)	5·8 (3·8 to 8·8)	11·3 (7 to 16·8)	15·9 (10·4 to 22·1)	21·9 (14·9 to 30·2)	18·1 (12·3 to 25·8)	14·2 (9·3 to 20·9)	11·9 (7·9 to 17·5)	16·3 (10·8 to 23·6)	14·6 (9·6 to 21·7)	14·5 (9·3 to 21·5)	8·8 (5·9 to 12·7)	6·2 (4·1 to 9·1)	5·1 (3·4 to 7·3)	3·8 (2·7 to 5·3)	3·1 (2·2 to 4·3)	1·1 (0·8 to 1·6)	0·5 (0·3 to 0·6)	0·2 (0·1 to 0·3)	184
Poisoning by other means	0·1 (0 to 0·1)	0·2 (0·1 to 0·4)	5·2 (3 to 8·1)	34·7 (21·3 to 55·5)	23·5 (13·7 to 37·5)	26·4 (15·2 to 43)	34·3 (20·2 to 53·4)	39·4 (24 to 60·4)	45·8 (29·4 to 68)	50·1 (31·9 to 73·9)	40·7 (26·3 to 60·6)	43·7 (28·8 to 62·7)	58·9 (38·2 to 84·8)	65·5 (44·1 to 93·1)	52·7 (34 to 75·8)	35·5 (23·6 to 50·4)	31·3 (20·9 to 43·6)	23·7 (16·1 to 34·1)	17·4 (11·9 to 24·5)	10·3 (6·8 to 14·9)	5·5 (3·6 to 8)	2·7 (1·9 to 3·8)	1 (0·7 to 1·4)	649
Exposure to mechanical forces	0·4 (0·2 to 0·5)	2 (1·4 to 2·7)	15·9 (10·9 to 23·2)	113 (82·1 to 158)	238 (164 to 337)	316 (216 to 468)	591 (419 to 832)	791 (566 to 1,100)	930 (666 to 1,330)	1,090 (770 to 1,550)	971 (683 to 1,410)	1,130 (800 to 1,640)	1,580 (1,110 to 2,290)	1,760 (1,250 to 2,530)	1,580 (1,120 to 2,250)	1,050 (725 to 1,500)	899 (610 to 1,290)	742 (502 to 1,070)	534 (358 to 772)	337 (225 to 491)	190 (126 to 276)	90·4 (59·9 to 130)	36·9 (25·2 to 52·9)	15,000
Unintentional firearm injuries	0 (0 to 0·1)	0·1 (0 to 0·2)	1·8 (1·1 to 3·1)	9·8 (6·3 to 15·2)	17·3 (10·4 to 27·3)	25·4 (16·1 to 38)	74·6 (48·4 to 110)	102 (69·4 to 148)	110 (77·1 to 154)	129 (89·5 to 183)	120 (81·8 to 166)	143 (98·6 to 199)	202 (140 to 279)	226 (160 to 310)	202 (141 to 275)	136 (95·2 to 185)	117 (81·1 to 159)	93·5 (64·9 to 129)	64·8 (44·8 to 90·7)	40·6 (28 to 57·5)	23·1 (16 to 32·9)	11·5 (7·8 to 16·3)	5·2 (3·6 to 7·5)	1,850
Other exposure to mechanical forces	0·3 (0·2 to 0·4)	1·9 (1·3 to 2·6)	14·1 (9·6 to 20·4)	103 (74·7 to 144)	221 (154 to 316)	290 (197 to 440)	517 (361 to 737)	689 (487 to 980)	820 (585 to 1,190)	959 (666 to 1,390)	851 (589 to 1,250)	986 (694 to 1,450)	1,380 (962 to 2,040)	1,540 (1,080 to 2,220)	1,380 (964 to 1,980)	915 (627 to 1,340)	782 (522 to 1,140)	648 (428 to 948)	469 (308 to 692)	297 (193 to 432)	167 (110 to 244)	78·9 (51·7 to 114)	31·7 (21·4 to 45·7)	13,100
Adverse effects of medical treatment	4·8 (2·4 to 10·6)	12·3 (7·4 to 18·4)	20·7 (11·9 to 39·2)	22·5 (13·9 to 35·5)	13 (7·8 to 23·6)	10 (6·1 to 18·7)	13 (8·4 to 22·7)	21·4 (13·4 to 37·2)	28·5 (18·7 to 46·6)	29·4 (19·3 to 47·1)	31·4 (21·4 to 47·7)	34·2 (22·7 to 55)	50·9 (32·2 to 81·1)	84·3 (53·2 to 129)	84·6 (55·5 to 138)	77 (49·8 to 119)	71·5 (46·9 to 124)	79·3 (54 to 125)	79·7 (54·2 to 124)	61·5 (42·8 to 93·7)	37·4 (26·4 to 56·9)	20·2 (14·1 to 29·4)	10·2 (6·5 to 15·7)	898
Animal contact	0·1 (0 to 0·1)	0·2 (0·1 to 0·2)	2 (1·4 to 2·9)	15·6 (10·5 to 23)	28·2 (17·9 to 44·4)	39·3 (24·7 to 59)	58·7 (38·9 to 82·8)	67·4 (44·4 to 95·1)	91·6 (65·2 to 126)	91·4 (63·2 to 126)	89·1 (63·1 to 123)	104 (73·4 to 144)	161 (116 to 220)	189 (135 to 257)	175 (125 to 232)	134 (95·1 to 183)	137 (96·9 to 187)	120 (87·3 to 159)	97·5 (68·8 to 133)	54·6 (37·9 to 74·8)	28·4 (20·3 to 38·6)	15·6 (11·6 to 20·3)	7·3 (5·4 to 9·8)	1,710
Venomous animal contact	0 (0 to 0·1)	0·1 (0 to 0·1)	1·2 (0·8 to 1·9)	9 (5·3 to 14·5)	18·6 (10·7 to 31·1)	25·7 (14·9 to 39·5)	35·9 (21·5 to 53·3)	43·3 (26·4 to 64·7)	49·8 (30·9 to 74·8)	52·7 (32·9 to 79·3)	47·4 (30·5 to 69·1)	49·8 (32·5 to 72·2)	76 (50·7 to 109)	83·1 (53·8 to 118)	74·4 (49·6 to 108)	50·5 (33·9 to 73·6)	46 (30·6 to 64·9)	37·5 (25·3 to 52·4)	34 (23·2 to 48·4)	22 (14·5 to 31·6)	14 (9·9 to 19·3)	10·1 (7·5 to 13·2)	5·2 (3·7 to 7·2)	786
Non-venomous animal contact	0 (0 to 0)	0·1 (0 to 0·1)	0·7 (0·5 to 1·1)	6·7 (4·5 to 9·9)	9·6 (6 to 15·8)	13·6 (8·3 to 21·7)	22·7 (14·3 to 33·3)	24·1 (15·8 to 35·6)	41·9 (29·5 to 58·4)	38·6 (26·2 to 55·6)	41·8 (28·5 to 58·7)	54·3 (36·7 to 76·8)	85·5 (56·3 to 119)	106 (72·9 to 152)	100 (69·2 to 136)	83·9 (55·5 to 119)	90·6 (60·8 to 130)	82·5 (56·4 to 116)	63·6 (42·4 to 89·2)	32·5 (21·6 to 46·9)	14·4 (9·9 to 20·7)	5·5 (3·8 to 7·7)	2·1 (1·4 to 3)	921
Foreign body	9·9 (5·9 to 13·7)	31·2 (21·2 to 44·5)	364 (229 to 543)	205 (149 to 269)	110 (84·3 to 143)	126 (92·8 to 165)	113 (82·3 to 151)	136 (100 to 186)	171 (130 to 230)	182 (141 to 239)	158 (119 to 208)	207 (159 to 263)	308 (238 to 394)	406 (308 to 521)	375 (295 to 473)	273 (211 to 351)	267 (208 to 342)	250 (191 to 317)	202 (162 to 249)	176 (136 to 220)	89·9 (72·3 to 110)	53·9 (42·3 to 66·1)	29·7 (23·7 to 35·5)	4,240
Pulmonary aspiration and foreign body in airway	9·8 (5·8 to 13·6)	30·9 (20·9 to 44·2)	359 (225 to 537)	180 (128 to 242)	73·5 (56·2 to 93·3)	85·8 (62·1 to 114)	61·5 (45·3 to 81·2)	72·6 (52·7 to 96·4)	95·6 (72·9 to 123)	102 (77 to 133)	88 (67·4 to 114)	122 (92·9 to 158)	185 (137 to 245)	286 (202 to 387)	265 (196 to 346)	197 (145 to 266)	194 (143 to 263)	184 (134 to 242)	147 (111 to 190)	131 (97·5 to 171)	62·8 (48·2 to 80·5)	36·3 (26·5 to 46·5)	19·9 (14·7 to 24·7)	2,990
Foreign body in eyes	0 (0 to 0·1)	0·1 (0 to 0·2)	1·2 (0·3 to 2·6)	6·8 (2 to 15·7)	12·1 (3·6 to 28·7)	14·2 (4·3 to 33·2)	20·9 (6·4 to 49·5)	27·2 (8·3 to 60·2)	32·9 (11·5 to 70·7)	36·3 (12·7 to 76·3)	31·2 (11·4 to 65·6)	34·3 (14·5 to 69·3)	45·8 (21·2 to 88·6)	43·6 (21·9 to 79·3)	34·3 (19·7 to 57·5)	22·9 (13·3 to 36·7)	20·2 (12·4 to 31·6)	17·3 (10·9 to 26)	13·2 (8·5 to 19·5)	8·6 (5·6 to 12·7)	4·8 (3·2 to 6·9)	2·2 (1·5 to 3·1)	0·9 (0·6 to 1·2)	431
Foreign body in other body part	0·1 (0·1 to 0·2)	0·2 (0·1 to 0·4)	3·6 (2·1 to 6)	18·3 (12·2 to 27)	24·6 (15·8 to 36·1)	25·6 (16·7 to 36·7)	30·6 (20·5 to 44)	36 (24·7 to 51)	42·4 (30 to 60·7)	44·5 (31·2 to 62·5)	39·1 (27 to 55·6)	51·1 (36·7 to 70)	77·1 (55·2 to 105)	76·5 (53·1 to 107)	75·9 (54·1 to 104)	53·4 (38·1 to 76)	52·5 (37·3 to 73)	48·8 (35·5 to 65·8)	42·5 (30·5 to 57·8)	36·1 (24·7 to 50·7)	22·3 (15·8 to 31·8)	15·3 (10·6 to 22)	8·9 (6·1 to 12·9)	826
Environmental heat and cold exposure	0·1 (0 to 0·1)	0·1 (0·1 to 0·2)	2·3 (1·5 to 3·5)	16·1 (10·5 to 23·8)	33·7 (21·3 to 51)	46·1 (29·5 to 67)	78·1 (51·4 to 114)	110 (73·3 to 159)	144 (97·8 to 208)	174 (116 to 252)	157 (105 to 223)	180 (121 to 258)	260 (175 to 365)	284 (193 to 398)	257 (177 to 359)	181 (125 to 250)	164 (114 to 225)	147 (103 to 201)	114 (79·8 to 154)	78·7 (56·4 to 106)	47·7 (34·6 to 64·4)	27 (20 to 35·2)	13·2 (10 to 16·7)	2,510
Exposure to forces of nature	0·1 (0·1 to 0·1)	0·2 (0·2 to 0·2)	1·5 (1·4 to 1·7)	7·7 (7 to 8·5)	8·5 (7·8 to 9·4)	11·3 (10·2 to 12·5)	15·3 (13·8 to 16·9)	15·4 (13·9 to 17·1)	17·4 (15·8 to 19·3)	14·5 (13·1 to 16·2)	11·6 (10·4 to 13)	13·9 (12·4 to 15·5)	16·4 (14·6 to 18·6)	16·2 (14·3 to 18·3)	10·9 (9·6 to 12·6)	8·1 (7·1 to 9·1)	6·3 (5·6 to 7·1)	4·9 (4·3 to 5·5)	3·4 (3 to 3·8)	2 (1·8 to 2·3)	1 (0·8 to 1·1)	0·4 (0·3 to 0·4)	0·1 (0·1 to 0·1)	187
Other unintentional injuries	2·7 (1·4 to 4·4)	2·4 (1·4 to 4)	35·6 (25·2 to 48·8)	148 (109 to 196)	266 (199 to 350)	385 (301 to 495)	753 (565 to 984)	894 (673 to 1,170)	1,080 (842 to 1,380)	1,170 (918 to 1,510)	899 (699 to 1,160)	992 (766 to 1,270)	1,240 (936 to 1,620)	1,160 (853 to 1,550)	960 (699 to 1,300)	569 (409 to 784)	489 (357 to 664)	378 (269 to 530)	275 (196 to 390)	170 (118 to 243)	97 (67·8 to 137)	45·5 (31·8 to 63·4)	18·3 (13 to 25·6)	12,000
Self-harm and interpersonal violence	63·5 (34 to 102)	12 (7·4 to 18·4)	57·6 (34·2 to 87·1)	117 (80·6 to 178)	126 (98·3 to 161)	389 (295 to 500)	3,250 (2,500 to 4,150)	5,860 (4,660 to 7,330)	7,130 (5,900 to 8,600)	7,740 (6,330 to 9,370)	6,240 (4,980 to 7,640)	6,430 (5,000 to 8,280)	8,070 (6,080 to 10,600)	7,680 (5,690 to 9,870)	6,280 (4,690 to 8,210)	3,940 (3,010 to 5,160)	2,970 (2,280 to 3,780)	2,570 (1,970 to 3,310)	1,930 (1,490 to 2,490)	1,200 (914 to 1,530)	621 (484 to 794)	198 (156 to 241)	62·1 (48·9 to 75·5)	72,900
Self-harm	0 (0 to 0)	0 (0 to 0)	0 (0 to 0)	0 (0 to 0)	0 (0 to 0)	146 (84·4 to 229)	1,430 (987 to 1,940)	2,600 (1,900 to 3,430)	3,330 (2,600 to 4,240)	4,010 (3,120 to 5,060)	3,510 (2,670 to 4,470)	3,980 (2,960 to 5,320)	5,310 (3,840 to 7,160)	5,590 (3,970 to 7,410)	4,720 (3,450 to 6,280)	3,140 (2,310 to 4,230)	2,420 (1,810 to 3,200)	2,190 (1,620 to 2,870)	1,690 (1,270 to 2,240)	1,100 (822 to 1,430)	573 (439 to 744)	178 (136 to 220)	51 (39 to 63)	46,000
Self-harm by firearm	0 (0 to 0)	0 (0 to 0)	0 (0 to 0)	0 (0 to 0)	0 (0 to 0)	1·9 (0·8 to 3·6)	142 (59·2 to 232)	125 (56·1 to 209)	118 (53·4 to 189)	98·5 (52·2 to 150)	68·5 (38·3 to 106)	76·8 (44·1 to 118)	75·6 (45·6 to 117)	91·7 (56·5 to 149)	73·5 (45·8 to 116)	52·4 (33·9 to 81·9)	36·7 (24·4 to 56·5)	38·1 (26 to 59·6)	22·9 (15·6 to 35·4)	10·7 (7·5 to 17·9)	3·9 (2·8 to 5·8)	1·2 (0·9 to 1·7)	0·5 (0·4 to 0·7)	1,040
Self-harm by other specified means	0 (0 to 0)	0 (0 to 0)	0 (0 to 0)	0 (0 to 0)	0 (0 to 0)	144 (83·5 to 227)	1,290 (903 to 1,780)	2,480 (1,800 to 3,260)	3,210 (2,510 to 4,100)	3,920 (3,060 to 4,970)	3,440 (2,630 to 4,390)	3,900 (2,910 to 5,210)	5,230 (3,800 to 7,070)	5,500 (3,910 to 7,300)	4,650 (3,370 to 6,190)	3,090 (2,280 to 4,170)	2,390 (1,780 to 3,140)	2,150 (1,590 to 2,810)	1,670 (1,250 to 2,210)	1,090 (812 to 1,420)	569 (436 to 739)	176 (135 to 219)	50·5 (38·6 to 62·5)	44,900
Interpersonal violence	63·5 (34 to 102)	12 (7·4 to 18·4)	56·9 (33·7 to 86·4)	113 (76·2 to 174)	123 (95·6 to 158)	240 (183 to 312)	1,820 (1,290 to 2,430)	3,250 (2,520 to 4,160)	3,800 (2,970 to 4,840)	3,720 (2,830 to 4,750)	2,720 (2,080 to 3,480)	2,440 (1,800 to 3,210)	2,720 (2,020 to 3,670)	2,040 (1,530 to 2,680)	1,520 (1,130 to 2,020)	784 (585 to 1,020)	529 (403 to 684)	381 (292 to 490)	240 (183 to 317)	98·9 (77·7 to 122)	47·5 (37·1 to 59·5)	20·3 (15·6 to 26·1)	11·1 (8·3 to 14·2)	26,700
Physical violence by firearm	0·3 (0·1 to 0·5)	0·1 (0 to 0·1)	2·3 (1·3 to 3·7)	7 (4·9 to 10·3)	16·7 (12·4 to 22·3)	35·6 (25·8 to 48·3)	210 (130 to 320)	301 (196 to 438)	242 (159 to 351)	231 (154 to 327)	132 (88·5 to 191)	111 (74·6 to 160)	101 (68·8 to 149)	80·7 (57·5 to 114)	71·8 (49·3 to 105)	40·3 (28·2 to 55)	26·7 (19·7 to 36)	17·6 (13 to 23·1)	11·8 (8·4 to 16·1)	5·2 (3·9 to 6·6)	2·6 (2 to 3·3)	1·2 (0·9 to 1·6)	0·6 (0·4 to 0·8)	1,650
Physical violence by sharp object	3·7 (1·5 to 7·3)	2·1 (1 to 3·9)	2·6 (1·6 to 3·9)	30·3 (20·5 to 46·6)	40·3 (30 to 53·4)	90·6 (65·6 to 120)	1,360 (925 to 1,860)	2,490 (1,900 to 3,190)	2,980 (2,290 to 3,860)	2,890 (2,180 to 3,690)	2,090 (1,570 to 2,690)	1,790 (1,300 to 2,380)	1,880 (1,370 to 2,580)	1,230 (907 to 1,640)	717 (528 to 954)	345 (257 to 453)	199 (150 to 261)	142 (106 to 184)	74·3 (56·2 to 98·2)	34·6 (25·6 to 44·8)	16·7 (12·5 to 21·8)	8·9 (6·8 to 11·7)	4·3 (3·2 to 5·7)	18,400
Sexual violence	0 (0 to 0)	0 (0 to 0)	0 (0 to 0)	1·4 (0·8 to 2·3)	12·3 (7·5 to 19·5)	24·7 (13·7 to 39·1)	60·2 (33·6 to 96·8)	83·2 (48·2 to 129)	83 (45·8 to 135)	79·8 (44·4 to 128)	61·4 (34·6 to 99·3)	55·4 (31·7 to 91·1)	62·7 (36·2 to 107)	53·6 (30·7 to 88·3)	38·5 (22·5 to 62·1)	24·5 (14·9 to 37·8)	20·5 (12·6 to 30·9)	16·5 (10·3 to 24·6)	11·6 (7·4 to 17·1)	7·1 (4·5 to 10·4)	3 (1·7 to 4·9)	0·6 (0·3 to 0·9)	0 (0 to 0·1)	700
Physical violence by other means	59·6 (31·7 to 95·9)	9·8 (6·1 to 15·1)	52·1 (30·3 to 80)	73·9 (48·1 to 115)	54·1 (41 to 71·1)	89·3 (66 to 118)	186 (126 to 266)	375 (269 to 507)	489 (352 to 657)	517 (374 to 679)	441 (316 to 598)	485 (362 to 637)	676 (501 to 908)	678 (505 to 902)	689 (509 to 924)	374 (273 to 494)	282 (212 to 374)	206 (153 to 270)	142 (105 to 194)	52 (39·5 to 66·1)	25·1 (19·1 to 31·5)	9·6 (7·2 to 12·6)	6·2 (4·6 to 8·1)	5,970
Conflict and terrorism	0 (0 to 0)	0 (0 to 0)	0 (0 to 0)	0 (0 to 0)	0 (0 to 0)	0 (0 to 0)	0 (0 to 0)	0 (0 to 0·1)	0·2 (0·1 to 0·3)	0·4 (0·2 to 0·6)	2·5 (1·6 to 4·2)	11·8 (7·4 to 19)	34·2 (21·9 to 54·7)	52 (33·5 to 83·5)	41·6 (27·6 to 62·6)	20·7 (14 to 30·7)	12·4 (8·3 to 18·2)	6·3 (4·2 to 9·4)	2·4 (1·6 to 3·6)	1 (0·6 to 1·4)	0·4 (0·2 to 0·5)	0·1 (0·1 to 0·2)	0 (0 to 0·1)	186

SR Table 2a: Dominica DALYS by cause and age group for males with 95% uncertainty intervals, 2019

Cause	Early Neonatal	Late Neonatal	Post Neonatal	1 to 4	5 to 9	10 to 14	15 to 19	20 to 24	25 to 29	30 to 34	35 to 39
All causes	539 (371 to 766)	134 (92·5 to 190)	255 (182 to 354)	205 (159 to 259)	173 (143 to 212)	253 (207 to 306)	423 (351 to 506)	472 (393 to 557)	548 (462 to 640)	560 (472 to 653)	608 (509 to 713)
Communicable, maternal, neonatal, and nutritional diseases	476 (327 to 674)	108 (73 to 152)	139 (93·1 to 197)	69·1 (52·3 to 89·3)	39·1 (29·1 to 55·2)	36·8 (29·2 to 47·1)	38·1 (30·5 to 47·6)	43·7 (36·4 to 52·7)	55·4 (47·3 to 65)	69·5 (60·8 to 79·9)	66·1 (57·8 to 75·4)
HIV/AIDS and sexually transmitted infections	15·9 (4·5 to 36·6)	5·5 (1·5 to 12·9)	13·3 (6·3 to 26·2)	3·3 (3 to 3·8)	1·3 (1·2 to 1·5)	2·6 (2·3 to 2·8)	2·4 (2 to 2·9)	9 (8·1 to 10·1)	17·9 (16·3 to 19·8)	30·7 (28·3 to 33·6)	27·9 (25·7 to 30·4)
HIV/AIDS	0 (0 to 0)	0 (0 to 0)	3·8 (3·5 to 4·1)	3 (2·8 to 3·3)	1·2 (1·1 to 1·3)	2·4 (2·2 to 2·6)	1·8 (1·7 to 2)	8·3 (7·6 to 9·2)	17·2 (15·6 to 18·9)	29·9 (27·6 to 32·6)	27 (24·9 to 29·5)
HIV/AIDS - Drug-susceptible Tuberculosis	0 (0 to 0)	0 (0 to 0)	0·5 (0·3 to 0·7)	0·4 (0·2 to 0·6)	0·1 (0·1 to 0·2)	0·3 (0·2 to 0·4)	0·2 (0·1 to 0·3)	0·9 (0·6 to 1·3)	2 (1·3 to 3)	3·5 (2·3 to 5·3)	3·8 (2·4 to 5·6)
HIV/AIDS - Multidrug-resistant Tuberculosis without extensive dru	0 (0 to 0)	0 (0 to 0)	0 (0 to 0)	0 (0 to 0)	0 (0 to 0)	0 (0 to 0)	0 (0 to 0)	0 (0 to 0·1)	0 (0 to 0·1)	0·1 (0 to 0·2)	0·1 (0 to 0·3)
HIV/AIDS - Extensively drug-resistant Tuberculosis	0 (0 to 0)	0 (0 to 0)	0 (0 to 0)	0 (0 to 0)	0 (0 to 0)	0 (0 to 0)	0 (0 to 0)	0 (0 to 0)	0 (0 to 0)	0 (0 to 0)	0 (0 to 0)
HIV/AIDS resulting in other diseases	0 (0 to 0)	0 (0 to 0)	3·3 (2·9 to 3·7)	2·6 (2·3 to 2·9)	1 (0·9 to 1·1)	2·1 (1·9 to 2·3)	1·6 (1·5 to 1·8)	7·4 (6·6 to 8·3)	15·1 (13·3 to 17·1)	26·3 (23·3 to 29·3)	23·2 (20·5 to 26)
Sexually transmitted infections excluding HIV	15·9 (4·5 to 36·6)	5·5 (1·5 to 12·9)	9·6 (2·6 to 22·4)	0·3 (0·1 to 0·7)	0·1 (0 to 0·3)	0·2 (0·1 to 0·4)	0·5 (0·3 to 1)	0·7 (0·3 to 1·3)	0·8 (0·4 to 1·3)	0·8 (0·4 to 1·4)	0·8 (0·4 to 1·6)
Syphilis	15·9 (4·5 to 36·6)	5·5 (1·5 to 12·9)	9·6 (2·6 to 22·4)	0·3 (0·1 to 0·7)	0·1 (0 to 0·3)	0·1 (0 to 0·2)	0·2 (0·1 to 0·5)	0·2 (0 to 0·5)	0·2 (0·1 to 0·6)	0·2 (0·1 to 0·5)	0·4 (0·1 to 1)
Chlamydial infection	0 (0 to 0)	0 (0 to 0)	0 (0 to 0)	0 (0 to 0)	0 (0 to 0)	0 (0 to 0)	0 (0 to 0·1)	0·1 (0 to 0·2)	0·1 (0 to 0·3)	0·1 (0 to 0·3)	0·1 (0 to 0·2)
Gonococcal infection	0 (0 to 0)	0 (0 to 0)	0 (0 to 0)	0 (0 to 0)	0 (0 to 0)	0 (0 to 0)	0·1 (0 to 0·3)	0·2 (0·1 to 0·4)	0·1 (0 to 0·2)	0·1 (0 to 0·1)	0 (0 to 0·1)
Trichomoniasis	0 (0 to 0)	0 (0 to 0)	0 (0 to 0)	0 (0 to 0)	0 (0 to 0)	0 (0 to 0)	0 (0 to 0)	0 (0 to 0)	0 (0 to 0)	0 (0 to 0)	0 (0 to 0)
Genital herpes	0 (0 to 0)	0 (0 to 0)	0 (0 to 0)	0 (0 to 0)	0 (0 to 0)	0 (0 to 0)	0 (0 to 0·1)	0·1 (0 to 0·2)	0·1 (0 to 0·3)	0·1 (0 to 0·3)	0·2 (0 to 0·4)
Other sexually transmitted infections	0 (0 to 0)	0 (0 to 0)	0 (0 to 0)	0 (0 to 0)	0 (0 to 0)	0·1 (0 to 0·2)	0·1 (0·1 to 0·3)	0·2 (0·1 to 0·3)	0·2 (0·1 to 0·3)	0·2 (0·1 to 0·4)	0·1 (0·1 to 0·2)
Respiratory infections and tuberculosis	13·7 (6·8 to 25)	8·6 (4·3 to 15·6)	43 (22·8 to 72·4)	19·1 (12·3 to 29·2)	7·7 (5·5 to 10·3)	7·7 (5·4 to 10·6)	10·1 (7 to 13·8)	12·2 (8·7 to 16·7)	14·8 (10·9 to 19·7)	16·2 (11·7 to 21·6)	16·7 (12·2 to 22·1)
Tuberculosis	0 (0 to 0)	0 (0 to 0)	1 (0·5 to 1·7)	0·9 (0·5 to 1·5)	0·7 (0·4 to 1)	0·8 (0·4 to 1·1)	2·1 (1·2 to 3·7)	4·5 (2·6 to 7)	5·6 (3·4 to 8·5)	6·2 (3·9 to 9·7)	5·4 (3·4 to 7·9)
Latent tuberculosis infection	0 (0 to 0)	0 (0 to 0)	0 (0 to 0)	0 (0 to 0)	0 (0 to 0)	0 (0 to 0)	0 (0 to 0)	0 (0 to 0)	0 (0 to 0)	0 (0 to 0)	0 (0 to 0)
Drug-susceptible tuberculosis	0 (0 to 0)	0 (0 to 0)	1 (0·5 to 1·6)	0·9 (0·5 to 1·5)	0·7 (0·4 to 1)	0·7 (0·4 to 1·1)	2·1 (1·1 to 3·7)	4·4 (2·6 to 7)	5·5 (3·3 to 8·3)	6·2 (3·8 to 9·6)	5·3 (3·3 to 7·8)
Multidrug-resistant tuberculosis without extensive drug resistanc	0 (0 to 0)	0 (0 to 0)	0 (0 to 0·1)	0 (0 to 0·1)	0 (0 to 0)	0 (0 to 0)	0 (0 to 0·1)	0·1 (0 to 0·3)	0·1 (0 to 0·3)	0·1 (0 to 0·4)	0·1 (0 to 0·3)
Extensively drug-resistant tuberculosis	0 (0 to 0)	0 (0 to 0)	0 (0 to 0)	0 (0 to 0)	0 (0 to 0)	0 (0 to 0)	0 (0 to 0)	0 (0 to 0)	0 (0 to 0)	0 (0 to 0·1)	0 (0 to 0·1)
Lower respiratory infections	13·7 (6·7 to 25)	8·6 (4·2 to 15·6)	41·3 (21·6 to 70·2)	15 (8·5 to 24·7)	3·1 (2 to 4·6)	3·2 (2·1 to 4·8)	4·3 (2·7 to 6·8)	4·6 (2·7 to 7·5)	6·5 (4 to 9·7)	7·4 (4·5 to 11)	9 (5·6 to 13·4)
Upper respiratory infections	0 (0 to 0)	0 (0 to 0)	0·6 (0·4 to 1·1)	2·3 (1·3 to 3·7)	2·5 (1·4 to 4·3)	2·6 (1·3 to 4·5)	2·6 (1·4 to 4·6)	2·3 (1·2 to 3·9)	2·1 (1·1 to 3·5)	2 (1·1 to 3·4)	1·8 (0·9 to 3·1)
Otitis media	0 (0 to 0)	0 (0 to 0)	0·1 (0 to 0·2)	0·9 (0·5 to 1·6)	1·3 (0·7 to 2·2)	1·1 (0·6 to 1·9)	1 (0·5 to 1·7)	0·8 (0·3 to 1·7)	0·7 (0·3 to 1·2)	0·6 (0·2 to 1·1)	0·5 (0·2 to 0·9)
Enteric infections	0·5 (0·2 to 1)	1·3 (0·5 to 2·6)	11·1 (5 to 22·6)	7 (4·4 to 10·8)	4·6 (2·8 to 7·1)	4·8 (2·8 to 7·6)	3·8 (2·5 to 5·7)	3 (1·9 to 4·5)	3·2 (2·1 to 4·7)	3·4 (2·2 to 4·8)	3·4 (2·4 to 4·9)
Diarrheal diseases	0·5 (0·2 to 1)	1·3 (0·5 to 2·6)	11 (4·9 to 22·6)	6·9 (4·3 to 10·6)	4·5 (2·8 to 7)	4·8 (2·7 to 7·5)	3·8 (2·4 to 5·6)	3 (1·8 to 4·5)	3·2 (2·1 to 4·6)	3·3 (2·2 to 4·7)	3·4 (2·4 to 4·8)
Typhoid and paratyphoid	0 (0 to 0)	0 (0 to 0)	0 (0 to 0)	0 (0 to 0·1)	0 (0 to 0)	0 (0 to 0·1)	0 (0 to 0·1)	0 (0 to 0)	0 (0 to 0)	0 (0 to 0)	0 (0 to 0)
Typhoid fever	0 (0 to 0)	0 (0 to 0)	0 (0 to 0)	0 (0 to 0)	0 (0 to 0)	0 (0 to 0)	0 (0 to 0)	0 (0 to 0)	0 (0 to 0)	0 (0 to 0)	0 (0 to 0)
Paratyphoid fever	0 (0 to 0)	0 (0 to 0)	0 (0 to 0)	0 (0 to 0)	0 (0 to 0)	0 (0 to 0)	0 (0 to 0)	0 (0 to 0)	0 (0 to 0)	0 (0 to 0)	0 (0 to 0)
Invasive Non-typhoidal Salmonella (iNTS)	0 (0 to 0)	0 (0 to 0)	0 (0 to 0)	0·1 (0 to 0·1)	0 (0 to 0·1)	0 (0 to 0·1)	0 (0 to 0·1)	0 (0 to 0·1)	0 (0 to 0·1)	0 (0 to 0)	0 (0 to 0)
Other intestinal infectious diseases	0 (0 to 0)	0 (0 to 0)	0 (0 to 0·1)	0 (0 to 0·1)	0 (0 to 0·1)	0 (0 to 0·1)	0 (0 to 0)	0 (0 to 0)	0 (0 to 0)	0 (0 to 0)	0 (0 to 0)
Neglected tropical diseases and malaria	0 (0 to 0)	0·1 (0 to 0·2)	0·6 (0·3 to 1·1)	1·5 (0·7 to 2·8)	1·5 (0·6 to 3)	1 (0·4 to 1·9)	1 (0·4 to 1·8)	1·5 (0·7 to 2·6)	1·6 (0·8 to 3)	1·8 (0·8 to 3·2)	1·7 (0·8 to 3·1)
Malaria	0 (0 to 0)	0 (0 to 0)	0 (0 to 0)	0 (0 to 0)	0 (0 to 0)	0 (0 to 0)	0 (0 to 0)	0 (0 to 0)	0 (0 to 0)	0 (0 to 0)	0 (0 to 0)
Chagas disease	0 (0 to 0)	0 (0 to 0)	0 (0 to 0)	0 (0 to 0)	0 (0 to 0)	0 (0 to 0)	0 (0 to 0)	0 (0 to 0)	0 (0 to 0)	0 (0 to 0)	0 (0 to 0)
Leishmaniasis	0 (0 to 0)	0 (0 to 0)	0 (0 to 0)	0 (0 to 0)	0 (0 to 0)	0 (0 to 0)	0 (0 to 0)	0 (0 to 0)	0 (0 to 0)	0 (0 to 0)	0 (0 to 0)
Visceral leishmaniasis	0 (0 to 0)	0 (0 to 0)	0 (0 to 0)	0 (0 to 0)	0 (0 to 0)	0 (0 to 0)	0 (0 to 0)	0 (0 to 0)	0 (0 to 0)	0 (0 to 0)	0 (0 to 0)
Cutaneous and mucocutaneous leishmaniasis	0 (0 to 0)	0 (0 to 0)	0 (0 to 0)	0 (0 to 0)	0 (0 to 0)	0 (0 to 0)	0 (0 to 0)	0 (0 to 0)	0 (0 to 0)	0 (0 to 0)	0 (0 to 0)
African trypanosomiasis	0 (0 to 0)	0 (0 to 0)	0 (0 to 0)	0 (0 to 0)	0 (0 to 0)	0 (0 to 0)	0 (0 to 0)	0 (0 to 0)	0 (0 to 0)	0 (0 to 0)	0 (0 to 0)
Schistosomiasis	0 (0 to 0)	0 (0 to 0)	0 (0 to 0)	0 (0 to 0)	0 (0 to 0)	0 (0 to 0)	0 (0 to 0)	0 (0 to 0)	0 (0 to 0)	0 (0 to 0)	0 (0 to 0)
Cysticercosis	0 (0 to 0)	0 (0 to 0)	0 (0 to 0)	0 (0 to 0)	0 (0 to 0)	0 (0 to 0)	0·1 (0 to 0·4)	0·5 (0·1 to 1·2)	0·9 (0·3 to 1·8)	1 (0·3 to 2·3)	1·1 (0·3 to 2·4)
Cystic echinococcosis	0 (0 to 0)	0 (0 to 0)	0 (0 to 0)	0 (0 to 0)	0 (0 to 0)	0 (0 to 0)	0 (0 to 0)	0 (0 to 0)	0 (0 to 0)	0 (0 to 0)	0 (0 to 0)
Lymphatic filariasis	0 (0 to 0)	0 (0 to 0)	0 (0 to 0)	0 (0 to 0)	0 (0 to 0)	0 (0 to 0)	0 (0 to 0)	0 (0 to 0)	0 (0 to 0)	0 (0 to 0)	0 (0 to 0)
Onchocerciasis	0 (0 to 0)	0 (0 to 0)	0 (0 to 0)	0 (0 to 0)	0 (0 to 0)	0 (0 to 0)	0 (0 to 0)	0 (0 to 0)	0 (0 to 0)	0 (0 to 0)	0 (0 to 0)
Trachoma	0 (0 to 0)	0 (0 to 0)	0 (0 to 0)	0 (0 to 0)	0 (0 to 0)	0 (0 to 0)	0 (0 to 0)	0 (0 to 0)	0 (0 to 0)	0 (0 to 0)	0 (0 to 0)
Dengue	0 (0 to 0)	0 (0 to 0)	0 (0 to 0·1)	0·1 (0 to 0·2)	0·2 (0 to 0·5)	0·3 (0 to 0·6)	0·3 (0·1 to 0·7)	0·2 (0·1 to 0·6)	0·2 (0 to 0·5)	0·2 (0 to 0·5)	0·2 (0 to 0·4)
Yellow fever	0 (0 to 0)	0 (0 to 0)	0 (0 to 0)	0 (0 to 0)	0 (0 to 0)	0 (0 to 0)	0 (0 to 0)	0 (0 to 0)	0 (0 to 0)	0 (0 to 0)	0 (0 to 0)
Rabies	0 (0 to 0)	0 (0 to 0)	0 (0 to 0)	0 (0 to 0)	0 (0 to 0·1)	0 (0 to 0·1)	0 (0 to 0)	0 (0 to 0)	0 (0 to 0)	0 (0 to 0)	0 (0 to 0)
Intestinal nematode infections	0 (0 to 0)	0 (0 to 0)	0 (0 to 0)	0 (0 to 0·1)	0·1 (0 to 0·1)	0 (0 to 0·1)	0 (0 to 0·1)	0 (0 to 0)	0 (0 to 0)	0 (0 to 0)	0 (0 to 0)
Ascariasis	0 (0 to 0)	0 (0 to 0)	0 (0 to 0)	0 (0 to 0·1)	0 (0 to 0·1)	0 (0 to 0)	0 (0 to 0)	0 (0 to 0)	0 (0 to 0)	0 (0 to 0)	0 (0 to 0)
Trichuriasis	0 (0 to 0)	0 (0 to 0)	0 (0 to 0)	0 (0 to 0)	0 (0 to 0)	0 (0 to 0)	0 (0 to 0)	0 (0 to 0)	0 (0 to 0)	0 (0 to 0)	0 (0 to 0)
Hookworm disease	0 (0 to 0)	0 (0 to 0)	0 (0 to 0)	0 (0 to 0)	0 (0 to 0·1)	0 (0 to 0·1)	0 (0 to 0)	0 (0 to 0)	0 (0 to 0)	0 (0 to 0)	0 (0 to 0)
Food-borne trematodiases	0 (0 to 0)	0 (0 to 0)	0 (0 to 0)	0 (0 to 0)	0 (0 to 0)	0 (0 to 0)	0 (0 to 0)	0 (0 to 0)	0 (0 to 0)	0 (0 to 0)	0 (0 to 0)
Leprosy	0 (0 to 0)	0 (0 to 0)	0 (0 to 0)	0 (0 to 0)	0 (0 to 0)	0 (0 to 0)	0 (0 to 0)	0 (0 to 0)	0 (0 to 0)	0 (0 to 0)	0 (0 to 0)
Ebola	0 (0 to 0)	0 (0 to 0)	0 (0 to 0)	0 (0 to 0)	0 (0 to 0)	0 (0 to 0)	0 (0 to 0)	0 (0 to 0)	0 (0 to 0)	0 (0 to 0)	0 (0 to 0)
Zika virus	0 (0 to 0)	0 (0 to 0)	0 (0 to 0·1)	0·1 (0 to 0·5)	0·2 (0 to 0·7)	0·2 (0 to 0·7)	0·3 (0 to 1)	0·3 (0 to 1·1)	0·3 (0 to 1)	0·3 (0 to 1·1)	0·3 (0 to 1)
Guinea worm disease	0 (0 to 0)	0 (0 to 0)	0 (0 to 0)	0 (0 to 0)	0 (0 to 0)	0 (0 to 0)	0 (0 to 0)	0 (0 to 0)	0 (0 to 0)	0 (0 to 0)	0 (0 to 0)
Other neglected tropical diseases	0 (0 to 0)	0·1 (0 to 0·2)	0·5 (0·2 to 1·1)	1·2 (0·5 to 2·5)	1 (0·4 to 2·3)	0·5 (0·2 to 1·1)	0·2 (0·1 to 0·5)	0·4 (0·2 to 0·9)	0·2 (0·1 to 0·5)	0·3 (0·1 to 0·6)	0·2 (0·1 to 0·4)
Other infectious diseases	1·1 (0·6 to 2)	1·9 (1 to 3·6)	15·6 (7·3 to 31·2)	13·1 (6·1 to 25·6)	4·6 (3 to 6·8)	4·3 (2·9 to 6·3)	4·9 (3·3 to 7·4)	4 (2·7 to 5·9)	4·3 (2·8 to 6·4)	4·3 (2·9 to 6·8)	4·6 (3·1 to 7·3)
Meningitis	0·7 (0·3 to 1·4)	1·3 (0·5 to 2·5)	4·7 (2·4 to 8·8)	2·5 (1·2 to 4·6)	1·1 (0·7 to 1·7)	1·1 (0·7 to 1·8)	1·2 (0·7 to 1·9)	0·8 (0·5 to 1·2)	0·8 (0·5 to 1·3)	0·8 (0·5 to 1·3)	0·9 (0·5 to 1·4)
Encephalitis	0·2 (0·1 to 0·4)	0·4 (0·1 to 0·9)	3 (1 to 6·9)	3 (1·1 to 6·3)	1·7 (0·7 to 3·4)	1·8 (0·9 to 3·4)	2 (0·9 to 3·8)	1·6 (0·8 to 3)	1·3 (0·6 to 2·5)	1·3 (0·6 to 2·3)	1·2 (0·6 to 2·2)
Diphtheria	0 (0 to 0)	0 (0 to 0)	0 (0 to 0)	0 (0 to 0)	0 (0 to 0)	0 (0 to 0)	0 (0 to 0)	0 (0 to 0)	0 (0 to 0)	0 (0 to 0)	0 (0 to 0)
Tetanus	0 (0 to 0·1)	0 (0 to 0·1)	0 (0 to 0·1)	0·1 (0 to 0·3)	0·1 (0 to 0·2)	0·1 (0 to 0·2)	0·1 (0 to 0·2)	0·1 (0 to 0·2)	0·1 (0 to 0·2)	0·1 (0 to 0·2)	0·1 (0 to 0·3)
Measles	0 (0 to 0)	0 (0 to 0)	0 (0 to 0)	0 (0 to 0)	0 (0 to 0)	0 (0 to 0)	0 (0 to 0)	0 (0 to 0)	0 (0 to 0)	0 (0 to 0)	0 (0 to 0)
Varicella and herpes zoster	0 (0 to 0)	0 (0 to 0·1)	0·3 (0 to 0·9)	0·5 (0 to 1·4)	0·2 (0 to 0·5)	0·1 (0 to 0·2)	0·1 (0 to 0·2)	0·1 (0 to 0·2)	0·1 (0 to 0·2)	0·1 (0 to 0·2)	0·1 (0 to 0·2)
Acute hepatitis	0 (0 to 0)	0 (0 to 0)	0 (0 to 0·1)	0·1 (0 to 0·1)	0·2 (0·1 to 0·3)	0·3 (0·2 to 0·4)	0·3 (0·2 to 0·4)	0·2 (0·2 to 0·4)	0·2 (0·1 to 0·3)	0·2 (0·1 to 0·3)	0·2 (0·1 to 0·3)
Acute hepatitis A	0 (0 to 0)	0 (0 to 0)	0 (0 to 0)	0·1 (0 to 0·1)	0·2 (0·1 to 0·3)	0·2 (0·1 to 0·4)	0·2 (0·1 to 0·4)	0·2 (0·1 to 0·3)	0·1 (0·1 to 0·2)	0·1 (0·1 to 0·2)	0·1 (0 to 0·1)
Acute hepatitis B	0 (0 to 0)	0 (0 to 0)	0 (0 to 0·1)	0 (0 to 0)	0 (0 to 0)	0 (0 to 0·1)	0 (0 to 0·1)	0 (0 to 0·1)	0·1 (0 to 0·1)	0·1 (0 to 0·1)	0·1 (0 to 0·2)
Acute hepatitis C	0 (0 to 0)	0 (0 to 0)	0 (0 to 0)	0 (0 to 0)	0 (0 to 0)	0 (0 to 0)	0 (0 to 0)	0 (0 to 0)	0 (0 to 0)	0 (0 to 0)	0 (0 to 0)
Acute hepatitis E	0 (0 to 0)	0 (0 to 0)	0 (0 to 0)	0 (0 to 0)	0 (0 to 0)	0 (0 to 0·1)	0 (0 to 0·1)	0 (0 to 0·1)	0 (0 to 0·1)	0 (0 to 0)	0 (0 to 0)
Other unspecified infectious diseases	0·2 (0·1 to 0·5)	0·2 (0·1 to 0·5)	1·3 (0·4 to 3·4)	1·1 (0·5 to 2·4)	0·8 (0·3 to 1·7)	0·7 (0·3 to 1·4)	1·2 (0·6 to 2·3)	1·2 (0·5 to 2·5)	1·7 (0·8 to 3·5)	1·8 (0·8 to 3·7)	2·1 (1 to 4·5)
Maternal and neonatal disorders	445 (304 to 634)	90 (59·6 to 129)	44 (23·4 to 70·6)	13·5 (9·9 to 18·1)	10·7 (7·7 to 14·3)	11·8 (8·5 to 15·8)	11·7 (8·5 to 15·7)	10·1 (7·2 to 13·8)	9·3 (6·7 to 12·6)	8·6 (6·2 to 11·6)	7·4 (5·4 to 9·8)
Maternal disorders	0 (0 to 0)	0 (0 to 0)	0 (0 to 0)	0 (0 to 0)	0 (0 to 0)	0 (0 to 0)	0 (0 to 0)	0 (0 to 0)	0 (0 to 0)	0 (0 to 0)	0 (0 to 0)
Maternal hemorrhage	0 (0 to 0)	0 (0 to 0)	0 (0 to 0)	0 (0 to 0)	0 (0 to 0)	0 (0 to 0)	0 (0 to 0)	0 (0 to 0)	0 (0 to 0)	0 (0 to 0)	0 (0 to 0)
Maternal sepsis and other maternal infections	0 (0 to 0)	0 (0 to 0)	0 (0 to 0)	0 (0 to 0)	0 (0 to 0)	0 (0 to 0)	0 (0 to 0)	0 (0 to 0)	0 (0 to 0)	0 (0 to 0)	0 (0 to 0)
Maternal hypertensive disorders	0 (0 to 0)	0 (0 to 0)	0 (0 to 0)	0 (0 to 0)	0 (0 to 0)	0 (0 to 0)	0 (0 to 0)	0 (0 to 0)	0 (0 to 0)	0 (0 to 0)	0 (0 to 0)
Maternal obstructed labor and uterine rupture	--	--	--	--	--	--	--	--	--	--	--
Maternal abortion and miscarriage	--	--	--	--	--	--	--	--	--	--	--
Ectopic pregnancy	--	--	--	--	--	--	--	--	--	--	--
Indirect maternal deaths	--	--	--	--	--	--	--	--	--	--	--
Late maternal deaths	--	--	--	--	--	--	--	--	--	--	--
Maternal deaths aggravated by HIV/AIDS	--	--	--	--	--	--	--	--	--	--	--
Neonatal disorders	445 (304 to 634)	90 (59·6 to 129)	44 (23·4 to 70·6)	13·5 (9·9 to 18·1)	10·7 (7·7 to 14·3)	11·8 (8·5 to 15·8)	11·7 (8·5 to 15·7)	10·1 (7·2 to 13·8)	9·3 (6·7 to 12·6)	8·6 (6·2 to 11·6)	7·4 (5·4 to 9·8)
Neonatal preterm birth	135 (74 to 219)	20·1 (9·2 to 35·8)	11·9 (5·9 to 21·3)	3·7 (2·5 to 4·9)	4·6 (3·2 to 6·2)	5 (3·5 to 6·8)	4·9 (3·4 to 6·7)	4·1 (2·8 to 5·6)	3·7 (2·5 to 5·1)	3·3 (2·3 to 4·5)	2·8 (2 to 3·9)
Neonatal encephalopathy due to birth asphyxia and trauma	195 (115 to 296)	23·3 (10·9 to 40·4)	13·6 (5·5 to 24·2)	3 (1·7 to 5·2)	1·6 (0·8 to 2·7)	1·7 (0·9 to 3·1)	1·8 (0·9 to 3)	1·6 (0·8 to 2·7)	1·4 (0·7 to 2·4)	1·4 (0·7 to 2·4)	1·2 (0·6 to 2·1)
Neonatal sepsis and other neonatal infections	55·7 (28·3 to 101)	32·2 (17·3 to 54·7)	11·1 (5 to 20)	3·1 (1·9 to 4·7)	4·1 (2·3 to 6·3)	4·5 (2·7 to 7·3)	4·5 (2·8 to 7·2)	4 (2·3 to 6·3)	3·7 (2·2 to 5·8)	3·5 (2 to 5·6)	3 (1·8 to 4·7)
Hemolytic disease and other neonatal jaundice	6·8 (3·2 to 12·4)	1·8 (0·8 to 3·4)	0·7 (0·2 to 2·1)	0·4 (0·2 to 0·6)	0·4 (0·3 to 0·6)	0·5 (0·4 to 0·6)	0·5 (0·4 to 0·6)	0·4 (0·3 to 0·6)	0·4 (0·3 to 0·5)	0·4 (0·3 to 0·5)	0·3 (0·3 to 0·4)
Other neonatal disorders	52·3 (26·3 to 89·6)	12·5 (6·2 to 22)	6·6 (2·9 to 12·1)	3·3 (2·4 to 4·5)	0 (0 to 0)	0 (0 to 0)	0 (0 to 0)	0 (0 to 0)	0 (0 to 0)	0 (0 to 0)	0 (0 to 0)
Nutritional deficiencies	0 (0 to 0)	0·4 (0·2 to 0·6)	11·5 (6·2 to 19·4)	11·5 (6 to 19·2)	8·7 (3 to 21·1)	4·6 (1·9 to 10·7)	4·2 (1·9 to 9·2)	3·9 (1·7 to 8·2)	4·2 (2 to 8·9)	4·5 (2 to 9·6)	4·4 (2·1 to 8·2)
Protein-energy malnutrition	0 (0 to 0)	0 (0 to 0·1)	8·6 (4·2 to 15·8)	4·9 (2 to 10·2)	1·1 (0·6 to 1·8)	0·8 (0·5 to 1·2)	0·8 (0·4 to 1·2)	0·6 (0·4 to 1)	0·8 (0·5 to 1·2)	0·9 (0·5 to 1·3)	1·1 (0·7 to 1·6)
Iodine deficiency	0 (0 to 0)	0 (0 to 0)	0 (0 to 0)	0 (0 to 0)	0 (0 to 0·1)	0·1 (0 to 0·2)	0·2 (0·1 to 0·3)	0·2 (0·1 to 0·3)	0·2 (0·1 to 0·3)	0·2 (0·1 to 0·4)	0·2 (0·1 to 0·3)
Vitamin A deficiency	0 (0 to 0)	0 (0 to 0)	0 (0 to 0·1)	0·2 (0·1 to 0·4)	0·3 (0·1 to 0·6)	0·2 (0·1 to 0·3)	0·1 (0 to 0·2)	0·1 (0 to 0·2)	0·1 (0 to 0·1)	0·1 (0 to 0·1)	0·1 (0 to 0·1)
Dietary iron deficiency	0 (0 to 0)	0·3 (0·1 to 0·6)	2 (0·7 to 4·1)	5·8 (2 to 11·7)	7 (1·6 to 19)	3·3 (0·7 to 9·2)	2·8 (0·6 to 7·4)	2·5 (0·5 to 6·5)	2·6 (0·6 to 7·4)	3 (0·7 to 8)	2·5 (0·6 to 6·3)
Other nutritional deficiencies	0 (0 to 0)	0 (0 to 0)	0·9 (0·3 to 2·2)	0·6 (0·2 to 1·4)	0·2 (0·1 to 0·4)	0·2 (0·1 to 0·5)	0·4 (0·2 to 0·7)	0·5 (0·3 to 0·7)	0·5 (0·3 to 0·8)	0·4 (0·3 to 0·7)	0·5 (0·3 to 0·8)
Non-communicable diseases	59·1 (33·6 to 96·5)	23·9 (14·7 to 37·1)	96·9 (61·5 to 142)	98·6 (75·1 to 128)	99·4 (78·8 to 124)	154 (118 to 198)	224 (177 to 283)	231 (184 to 286)	277 (226 to 339)	315 (258 to 382)	381 (311 to 456)
Neoplasms	0·6 (0·3 to 1)	0·5 (0·3 to 0·9)	4·2 (2·3 to 6·9)	18·4 (11·5 to 28·6)	15·7 (11·9 to 20·9)	17·1 (12·4 to 22·7)	22 (15·7 to 30·1)	20·9 (15·2 to 27·7)	26·6 (19·8 to 34·2)	32·2 (24·6 to 41·5)	45·4 (33·9 to 58·4)
Lip and oral cavity cancer	0 (0 to 0)	0 (0 to 0)	0 (0 to 0)	0 (0 to 0)	0 (0 to 0)	0·1 (0 to 0·1)	0·1 (0·1 to 0·3)	0·2 (0·1 to 0·3)	0·2 (0·1 to 0·4)	0·5 (0·3 to 0·8)	1 (0·6 to 1·5)
Nasopharynx cancer	0 (0 to 0)	0 (0 to 0)	0 (0 to 0)	0 (0 to 0)	0·1 (0 to 0·1)	0·2 (0·1 to 0·3)	0·2 (0·1 to 0·4)	0·2 (0·1 to 0·3)	0·2 (0·1 to 0·4)	0·2 (0·1 to 0·3)	0·3 (0·2 to 0·5)
Other pharynx cancer	0 (0 to 0)	0 (0 to 0)	0 (0 to 0)	0 (0 to 0)	0 (0 to 0)	0 (0 to 0)	0 (0 to 0)	0·1 (0·1 to 0·2)	0·2 (0·1 to 0·3)	0·3 (0·1 to 0·4)	0·6 (0·4 to 1·1)
Esophageal cancer	0 (0 to 0)	0 (0 to 0)	0 (0 to 0)	0 (0 to 0)	0 (0 to 0)	0 (0 to 0)	0 (0 to 0)	0·1 (0·1 to 0·2)	0·2 (0·1 to 0·3)	0·4 (0·2 to 0·7)	1·2 (0·7 to 2)
Stomach cancer	0 (0 to 0)	0 (0 to 0)	0 (0 to 0)	0 (0 to 0)	0 (0 to 0)	0 (0 to 0)	0·5 (0·3 to 0·8)	0·7 (0·5 to 1·1)	1·8 (1·1 to 2·7)	3·6 (2·1 to 5·5)	6·2 (3·8 to 9·4)
Colon and rectum cancer	0 (0 to 0)	0 (0 to 0)	0 (0 to 0)	0 (0 to 0)	0·1 (0 to 0·1)	0·1 (0·1 to 0·2)	0·5 (0·3 to 0·9)	0·8 (0·5 to 1·4)	1·5 (0·9 to 2·3)	2·6 (1·6 to 4·1)	4·1 (2·2 to 6·5)
Liver cancer	0 (0 to 0)	0 (0 to 0)	0·1 (0 to 0·1)	0·1 (0·1 to 0·2)	0·1 (0·1 to 0·1)	0·2 (0·1 to 0·4)	0·2 (0·1 to 0·4)	0·3 (0·1 to 0·6)	0·6 (0·3 to 1)	0·7 (0·3 to 1·3)	1·3 (0·6 to 2·6)
Liver cancer due to hepatitis B	0 (0 to 0)	0 (0 to 0)	0 (0 to 0)	0 (0 to 0)	0 (0 to 0)	0·1 (0 to 0·2)	0·1 (0 to 0·2)	0·2 (0·1 to 0·4)	0·4 (0·2 to 0·7)	0·4 (0·2 to 0·8)	0·8 (0·3 to 1·6)
Liver cancer due to hepatitis C	0 (0 to 0)	0 (0 to 0)	0 (0 to 0)	0 (0 to 0)	0 (0 to 0)	0 (0 to 0)	0 (0 to 0)	0 (0 to 0)	0 (0 to 0)	0 (0 to 0)	0·1 (0 to 0·1)
Liver cancer due to alcohol use	0 (0 to 0)	0 (0 to 0)	0 (0 to 0)	0 (0 to 0)	0 (0 to 0)	0 (0 to 0)	0 (0 to 0)	0 (0 to 0·1)	0·1 (0 to 0·2)	0·1 (0·1 to 0·3)	0·4 (0·1 to 0·8)
Liver cancer due to NASH	0 (0 to 0)	0 (0 to 0)	0 (0 to 0)	0 (0 to 0)	0 (0 to 0)	0 (0 to 0)	0 (0 to 0·1)	0 (0 to 0·1)	0 (0 to 0·1)	0 (0 to 0·1)	0·1 (0 to 0·1)
Liver cancer due to other causes	0 (0 to 0)	0 (0 to 0)	0·1 (0 to 0·1)	0·1 (0·1 to 0·2)	0·1 (0·1 to 0·1)	0·1 (0·1 to 0·2)	0 (0 to 0·1)	0 (0 to 0·1)	0·1 (0 to 0·1)	0 (0 to 0·1)	0·1 (0 to 0·2)
Gallbladder and biliary tract cancer	0 (0 to 0)	0 (0 to 0)	0 (0 to 0)	0 (0 to 0)	0 (0 to 0)	0 (0 to 0)	0 (0 to 0)	0·1 (0 to 0·1)	0·1 (0·1 to 0·2)	0·2 (0·1 to 0·4)	0·4 (0·2 to 0·8)
Pancreatic cancer	0 (0 to 0)	0 (0 to 0)	0 (0 to 0)	0 (0 to 0)	0 (0 to 0)	0 (0 to 0)	0·2 (0·1 to 0·4)	0·2 (0·1 to 0·4)	0·3 (0·2 to 0·6)	1·2 (0·8 to 1·9)	2·2 (1·3 to 3·4)
Larynx cancer	0 (0 to 0)	0 (0 to 0)	0 (0 to 0)	0 (0 to 0)	0 (0 to 0)	0 (0 to 0)	0 (0 to 0)	0·1 (0·1 to 0·2)	0·1 (0·1 to 0·2)	0·2 (0·1 to 0·3)	0·5 (0·3 to 0·9)
Tracheal, bronchus, and lung cancer	0 (0 to 0)	0 (0 to 0)	0 (0 to 0)	0 (0 to 0)	0 (0 to 0)	0·1 (0 to 0·2)	0·5 (0·3 to 1)	0·6 (0·3 to 1)	0·8 (0·5 to 1·3)	1·9 (1·1 to 3·1)	3·9 (2·1 to 6·4)
Malignant skin melanoma	0 (0 to 0)	0 (0 to 0)	0 (0 to 0)	0 (0 to 0·1)	0 (0 to 0)	0 (0 to 0·1)	0·1 (0 to 0·1)	0·1 (0 to 0·2)	0·2 (0·1 to 0·3)	0·3 (0·1 to 0·4)	0·4 (0·2 to 0·7)
Non-melanoma skin cancer	0 (0 to 0)	0 (0 to 0)	0 (0 to 0)	0 (0 to 0)	0 (0 to 0)	0 (0 to 0)	0 (0 to 0)	0 (0 to 0·1)	0·1 (0·1 to 0·2)	0·2 (0·1 to 0·3)	0·2 (0·1 to 0·3)
Non-melanoma skin cancer (squamous-cell carcinoma)	0 (0 to 0)	0 (0 to 0)	0 (0 to 0)	0 (0 to 0)	0 (0 to 0)	0 (0 to 0)	0 (0 to 0)	0 (0 to 0·1)	0·1 (0·1 to 0·2)	0·2 (0·1 to 0·3)	0·2 (0·1 to 0·3)
Non-melanoma skin cancer (basal-cell carcinoma)	0 (0 to 0)	0 (0 to 0)	0 (0 to 0)	0 (0 to 0)	0 (0 to 0)	0 (0 to 0)	0 (0 to 0)	0 (0 to 0)	0 (0 to 0)	0 (0 to 0)	0 (0 to 0)
Breast cancer	0 (0 to 0)	0 (0 to 0)	0 (0 to 0)	0 (0 to 0)	0 (0 to 0)	0 (0 to 0)	0 (0 to 0)	0 (0 to 0)	0 (0 to 0·1)	0·1 (0 to 0·2)	0·1 (0 to 0·2)
Cervical cancer	--	--	--	--	--	--	--	--	--	--	--
Uterine cancer	--	--	--	--	--	--	--	--	--	--	--
Ovarian cancer	--	--	--	--	--	--	--	--	--	--	--
Prostate cancer	0 (0 to 0)	0 (0 to 0)	0 (0 to 0)	0 (0 to 0)	0 (0 to 0)	0 (0 to 0)	0 (0 to 0)	0·2 (0·1 to 0·4)	0·6 (0·4 to 0·8)	0·7 (0·5 to 1)	1 (0·7 to 1·5)
Testicular cancer	0 (0 to 0)	0 (0 to 0)	0 (0 to 0·1)	0·1 (0·1 to 0·2)	0·1 (0 to 0·1)	0·1 (0 to 0·1)	0·3 (0·1 to 0·5)	0·4 (0·2 to 0·6)	0·5 (0·3 to 0·8)	0·3 (0·2 to 0·5)	0·3 (0·2 to 0·4)
Kidney cancer	0·1 (0 to 0·2)	0 (0 to 0·1)	0·3 (0·1 to 0·5)	1·2 (0·5 to 2·3)	0·8 (0·5 to 1·3)	0·4 (0·2 to 0·8)	0·6 (0·3 to 1)	0·6 (0·3 to 1·1)	0·9 (0·5 to 1·5)	1 (0·5 to 1·7)	1·1 (0·6 to 1·8)
Bladder cancer	0 (0 to 0)	0 (0 to 0)	0 (0 to 0)	0 (0 to 0)	0 (0 to 0)	0 (0 to 0)	0 (0 to 0)	0 (0 to 0·1)	0·1 (0 to 0·1)	0·2 (0·1 to 0·3)	0·3 (0·2 to 0·5)
Brain and central nervous system cancer	0 (0 to 0·1)	0 (0 to 0·1)	0·3 (0·1 to 0·7)	1·1 (0·4 to 2·3)	1·3 (0·6 to 2·4)	1·4 (0·6 to 2·6)	1 (0·4 to 2·1)	0·9 (0·3 to 1·8)	1·3 (0·4 to 2·4)	1·5 (0·4 to 2·8)	1·7 (0·6 to 3·2)
Thyroid cancer	0 (0 to 0)	0 (0 to 0)	0 (0 to 0)	0 (0 to 0)	0 (0 to 0·1)	0 (0 to 0·1)	0·1 (0 to 0·1)	0·1 (0 to 0·2)	0·2 (0·1 to 0·3)	0·2 (0·1 to 0·3)	0·3 (0·2 to 0·5)
Mesothelioma	0 (0 to 0)	0 (0 to 0)	0 (0 to 0)	0 (0 to 0)	0 (0 to 0)	0 (0 to 0)	0 (0 to 0)	0·1 (0·1 to 0·2)	0·2 (0·1 to 0·3)	0·2 (0·1 to 0·3)	0·4 (0·2 to 0·5)
Hodgkin lymphoma	0 (0 to 0)	0 (0 to 0)	0 (0 to 0)	0 (0 to 0·1)	0·1 (0·1 to 0·3)	0·2 (0·1 to 0·4)	0·8 (0·4 to 1·4)	1 (0·6 to 1·7)	1·2 (0·7 to 2)	1 (0·6 to 1·6)	1 (0·6 to 1·7)
Non-Hodgkin lymphoma	0 (0 to 0)	0 (0 to 0)	0 (0 to 0)	2·1 (1·1 to 3·6)	2·7 (1·5 to 4·4)	3·2 (1·9 to 5·2)	4·6 (2·8 to 7·2)	4·6 (2·7 to 7·3)	5·8 (3·5 to 9·1)	6·1 (3·6 to 9·4)	6·8 (4 to 10·6)
Multiple myeloma	0 (0 to 0)	0 (0 to 0)	0 (0 to 0)	0 (0 to 0)	0 (0 to 0)	0 (0 to 0)	0 (0 to 0)	0·2 (0·1 to 0·3)	0·3 (0·1 to 0·4)	0·3 (0·2 to 0·5)	0·8 (0·5 to 1·4)
Leukemia	0·2 (0·1 to 0·5)	0·1 (0·1 to 0·3)	1·1 (0·5 to 2·1)	4·2 (2·1 to 7·1)	5·2 (3 to 8)	6·3 (3·8 to 9·9)	6·7 (3·9 to 11)	4·8 (3 to 7·5)	4·7 (3 to 7·1)	3·9 (2·5 to 5·9)	4·4 (2·7 to 6·6)
Acute lymphoid leukemia	0·1 (0 to 0·2)	0 (0 to 0·1)	0·7 (0·3 to 1·3)	3·4 (1·7 to 5·9)	4·2 (2·4 to 6·9)	4·7 (2·7 to 7·7)	5·2 (2·9 to 8·5)	2·9 (1·6 to 4·7)	2·1 (1·1 to 3·5)	1·5 (0·7 to 2·4)	1·6 (0·8 to 2·5)
Chronic lymphoid leukemia	0 (0 to 0)	0 (0 to 0)	0 (0 to 0)	0 (0 to 0)	0 (0 to 0)	0 (0 to 0)	0 (0 to 0)	0·1 (0 to 0·1)	0·1 (0 to 0·2)	0·1 (0 to 0·1)	0·1 (0 to 0·2)
Acute myeloid leukemia	0 (0 to 0·1)	0 (0 to 0·1)	0·1 (0 to 0·2)	0·2 (0·1 to 0·5)	0·2 (0·1 to 0·4)	0·3 (0·1 to 0·7)	0·4 (0·1 to 1)	0·4 (0·2 to 0·9)	0·6 (0·3 to 1·2)	0·5 (0·2 to 1)	0·5 (0·2 to 1)

Cause	40 to 44	45 to 49	50 to 54	55 to 59	60 to 64	65 to 69	70 to 74	75 to 79	80 to 84	85 to 89	90 to 94	95 plus	Total
All causes	564 (472 to 663)	697 (580 to 828)	1,100 (914 to 1,320)	1,300 (1,070 to 1,550)	1,300 (1,090 to 1,530)	1,230 (1,040 to 1,450)	1,070 (916 to 1,250)	968 (851 to 1,110)	843 (759 to 942)	415 (377 to 457)	160 (144 to 175)	28·6 (25·2 to 31·6)	13,800
Communicable, maternal, neonatal, and nutritional diseases	64·6 (56·8 to 74)	61·1 (52·4 to 71·2)	68·6 (56·9 to 82·9)	65·3 (52·8 to 80·6)	59·4 (47·4 to 75·1)	53·3 (40·9 to 69·3)	49·9 (37·3 to 65·4)	49·5 (36·4 to 66·7)	49·6 (37·5 to 65·3)	28·8 (21·4 to 37·4)	13·1 (9·5 to 17·4)	2·8 (1·9 to 3·7)	1,710
HIV/AIDS and sexually transmitted infections	30·5 (28 to 33·2)	23·4 (21·5 to 25·4)	17·8 (16·3 to 19·2)	11·6 (10·5 to 12·8)	7·5 (6·7 to 8·4)	4·3 (3·8 to 5)	1·5 (1·3 to 1·8)	0·9 (0·7 to 1·1)	0·5 (0·4 to 0·7)	0·2 (0·2 to 0·3)	0·1 (0 to 0·1)	0 (0 to 0)	228
HIV/AIDS	30 (27·6 to 32·6)	22·8 (21 to 24·8)	17·1 (15·7 to 18·4)	10·9 (10 to 11·9)	7 (6·3 to 7·8)	3·8 (3·4 to 4·3)	1·2 (1·1 to 1·4)	0·7 (0·6 to 0·8)	0·3 (0·3 to 0·4)	0·1 (0·1 to 0·1)	0 (0 to 0)	0 (0 to 0)	189
HIV/AIDS - Drug-susceptible Tuberculosis	3·4 (2·1 to 5)	2·7 (1·7 to 4)	2·4 (1·5 to 3·6)	1·6 (1 to 2·3)	0·9 (0·6 to 1·4)	0·6 (0·4 to 0·8)	0·2 (0·1 to 0·2)	0·1 (0·1 to 0·1)	0 (0 to 0·1)	0 (0 to 0)	0 (0 to 0)	0 (0 to 0)	23·5
HIV/AIDS - Multidrug-resistant Tuberculosis without extensive dru	0·1 (0 to 0·2)	0 (0 to 0·2)	0 (0 to 0·2)	0 (0 to 0·1)	0 (0 to 0·1)	0 (0 to 0)	0 (0 to 0)	0 (0 to 0)	0 (0 to 0)	0 (0 to 0)	0 (0 to 0)	0 (0 to 0)	0·4
HIV/AIDS - Extensively drug-resistant Tuberculosis	0 (0 to 0)	0 (0 to 0)	0 (0 to 0)	0 (0 to 0)	0 (0 to 0)	0 (0 to 0)	0 (0 to 0)	0 (0 to 0)	0 (0 to 0)	0 (0 to 0)	0 (0 to 0)	0 (0 to 0)	0·1
HIV/AIDS resulting in other diseases	26·5 (23·8 to 29·5)	20·1 (17·8 to 22·4)	14·6 (12·8 to 16·2)	9·4 (8·2 to 10·6)	6·1 (5·2 to 6·9)	3·3 (2·7 to 3·8)	1·1 (0·9 to 1·2)	0·6 (0·5 to 0·7)	0·3 (0·3 to 0·3)	0·1 (0·1 to 0·1)	0 (0 to 0)	0 (0 to 0)	165
Sexually transmitted infections excluding HIV	0·5 (0·3 to 0·9)	0·6 (0·3 to 1)	0·7 (0·3 to 1·4)	0·7 (0·3 to 1·4)	0·5 (0·2 to 0·9)	0·5 (0·2 to 1)	0·3 (0·1 to 0·6)	0·2 (0·1 to 0·4)	0·2 (0·1 to 0·4)	0·1 (0 to 0·2)	0 (0 to 0·1)	0 (0 to 0)	39·3
Syphilis	0·1 (0 to 0·3)	0·2 (0·1 to 0·4)	0·3 (0·1 to 0·8)	0·4 (0·1 to 0·9)	0·3 (0·1 to 0·6)	0·3 (0·1 to 0·7)	0·2 (0·1 to 0·4)	0·1 (0 to 0·2)	0·1 (0 to 0·2)	0 (0 to 0·1)	0 (0 to 0)	0 (0 to 0)	34·5
Chlamydial infection	0·1 (0 to 0·2)	0·1 (0 to 0·2)	0 (0 to 0·1)	0 (0 to 0)	0 (0 to 0)	0 (0 to 0)	0 (0 to 0)	0 (0 to 0)	0 (0 to 0)	0 (0 to 0)	0 (0 to 0)	0 (0 to 0)	0·7
Gonococcal infection	0 (0 to 0·1)	0 (0 to 0·1)	0 (0 to 0·1)	0 (0 to 0·1)	0 (0 to 0·1)	0 (0 to 0)	0 (0 to 0·1)	0 (0 to 0)	0 (0 to 0)	0 (0 to 0)	0 (0 to 0)	0 (0 to 0)	0·8
Trichomoniasis	0 (0 to 0)	0 (0 to 0)	0 (0 to 0)	0 (0 to 0)	0 (0 to 0)	0 (0 to 0)	0 (0 to 0)	0 (0 to 0)	0 (0 to 0)	0 (0 to 0)	0 (0 to 0)	0 (0 to 0)	0·1
Genital herpes	0·1 (0 to 0·4)	0·2 (0 to 0·4)	0·2 (0·1 to 0·4)	0·2 (0 to 0·4)	0·1 (0 to 0·3)	0·1 (0 to 0·2)	0·1 (0 to 0·1)	0 (0 to 0·1)	0 (0 to 0·1)	0 (0 to 0)	0 (0 to 0)	0 (0 to 0)	1·5
Other sexually transmitted infections	0·1 (0 to 0·1)	0·1 (0 to 0·2)	0·1 (0 to 0·3)	0·1 (0 to 0·2)	0 (0 to 0·1)	0 (0 to 0·1)	0 (0 to 0·1)	0 (0 to 0·1)	0·1 (0 to 0·2)	0 (0 to 0·1)	0 (0 to 0·1)	0 (0 to 0)	1·6
Respiratory infections and tuberculosis	15·6 (11·2 to 21·2)	16·6 (12 to 22·6)	25·3 (18 to 35)	28·6 (19·7 to 40·4)	29·6 (20·3 to 41·3)	31·4 (21·5 to 45·5)	34·2 (22·8 to 48·7)	35·8 (24 to 51·8)	38·3 (27 to 53·4)	22·9 (16 to 31·2)	10·9 (7·5 to 15)	2·3 (1·6 to 3·3)	461
Tuberculosis	5·6 (3·5 to 8·6)	5·2 (3·3 to 7·8)	6·6 (4·2 to 10·2)	6·3 (4 to 9·2)	5·7 (3·7 to 8·7)	4·5 (2·8 to 6·6)	4 (2·5 to 6·2)	3·4 (2·1 to 5·1)	2·7 (1·6 to 4·2)	1·4 (0·9 to 2)	0·4 (0·2 to 0·6)	0·1 (0 to 0·1)	72·9
Latent tuberculosis infection	0 (0 to 0)	0 (0 to 0)	0 (0 to 0)	0 (0 to 0)	0 (0 to 0)	0 (0 to 0)	0 (0 to 0)	0 (0 to 0)	0 (0 to 0)	0 (0 to 0)	0 (0 to 0)	0 (0 to 0)	0
Drug-susceptible tuberculosis	5·6 (3·5 to 8·4)	5·1 (3·2 to 7·7)	6·5 (4·2 to 10·1)	6·2 (3·9 to 9)	5·7 (3·6 to 8·5)	4·4 (2·8 to 6·5)	4 (2·5 to 6·1)	3·3 (2·1 to 5)	2·7 (1·6 to 4·1)	1·4 (0·9 to 2)	0·4 (0·2 to 0·6)	0·1 (0 to 0·1)	71·9
Multidrug-resistant tuberculosis without extensive drug resistanc	0·1 (0 to 0·3)	0·1 (0 to 0·3)	0·1 (0 to 0·4)	0·1 (0 to 0·4)	0·1 (0 to 0·3)	0·1 (0 to 0·3)	0·1 (0 to 0·2)	0 (0 to 0·2)	0 (0 to 0·2)	0 (0 to 0·1)	0 (0 to 0)	0 (0 to 0)	0·9
Extensively drug-resistant tuberculosis	0 (0 to 0·1)	0 (0 to 0)	0 (0 to 0·1)	0 (0 to 0·1)	0 (0 to 0·1)	0 (0 to 0)	0 (0 to 0)	0 (0 to 0)	0 (0 to 0)	0 (0 to 0)	0 (0 to 0)	0 (0 to 0)	0·1
Lower respiratory infections	7·9 (5 to 11·9)	9·3 (5·9 to 14·1)	16·5 (10·4 to 25·1)	20·4 (12·7 to 31·1)	22·3 (14 to 33·7)	25·9 (16·4 to 39)	29·6 (18·6 to 43·7)	32 (20·3 to 47·9)	35·2 (23·5 to 50·3)	21·4 (14·6 to 29·7)	10·4 (7·1 to 14·7)	2·2 (1·5 to 3·2)	350
Upper respiratory infections	1·5 (0·8 to 2·6)	1·5 (0·8 to 2·6)	1·6 (0·8 to 2·7)	1·4 (0·7 to 2·2)	1·1 (0·6 to 1·9)	0·8 (0·4 to 1·3)	0·5 (0·3 to 0·8)	0·4 (0·2 to 0·6)	0·2 (0·1 to 0·4)	0·1 (0·1 to 0·2)	0 (0 to 0·1)	0 (0 to 0)	28
Otitis media	0·5 (0·2 to 1)	0·6 (0·3 to 1)	0·6 (0·3 to 1·1)	0·5 (0·3 to 0·9)	0·4 (0·2 to 0·8)	0·2 (0·1 to 0·4)	0·1 (0 to 0·2)	0·1 (0 to 0·2)	0·1 (0 to 0·1)	0 (0 to 0·1)	0 (0 to 0)	0 (0 to 0)	10·2
Enteric infections	3·2 (2·1 to 4·4)	3·7 (2·5 to 5·3)	4·6 (3·1 to 6·6)	4·7 (3·2 to 6·7)	4·9 (3·4 to 7)	5 (3·4 to 7)	4·5 (3·1 to 6·3)	4·7 (3·2 to 6·7)	4·1 (2·8 to 5·8)	1·9 (1·3 to 2·8)	0·7 (0·5 to 1·1)	0·1 (0·1 to 0·2)	88·2
Diarrheal diseases	3·1 (2·1 to 4·4)	3·7 (2·5 to 5·3)	4·6 (3·1 to 6·6)	4·6 (3·2 to 6·6)	4·9 (3·4 to 7)	5 (3·4 to 7)	4·5 (3·1 to 6·3)	4·6 (3·2 to 6·6)	4·1 (2·8 to 5·8)	1·9 (1·3 to 2·8)	0·7 (0·5 to 1·1)	0·1 (0·1 to 0·2)	87·4
Typhoid and paratyphoid	0 (0 to 0)	0 (0 to 0)	0 (0 to 0)	0 (0 to 0)	0 (0 to 0)	0 (0 to 0)	0 (0 to 0)	0 (0 to 0)	0 (0 to 0)	0 (0 to 0)	0 (0 to 0)	0 (0 to 0)	0·2
Typhoid fever	0 (0 to 0)	0 (0 to 0)	0 (0 to 0)	0 (0 to 0)	0 (0 to 0)	0 (0 to 0)	0 (0 to 0)	0 (0 to 0)	0 (0 to 0)	0 (0 to 0)	0 (0 to 0)	0 (0 to 0)	0·1
Paratyphoid fever	0 (0 to 0)	0 (0 to 0)	0 (0 to 0)	0 (0 to 0)	0 (0 to 0)	0 (0 to 0)	0 (0 to 0)	0 (0 to 0)	0 (0 to 0)	0 (0 to 0)	0 (0 to 0)	0 (0 to 0)	0·1
Invasive Non-typhoidal Salmonella (iNTS)	0 (0 to 0)	0 (0 to 0)	0 (0 to 0·1)	0 (0 to 0·1)	0 (0 to 0·1)	0 (0 to 0·1)	0 (0 to 0)	0 (0 to 0)	0 (0 to 0)	0 (0 to 0)	0 (0 to 0)	0 (0 to 0)	0·4
Other intestinal infectious diseases	0 (0 to 0)	0 (0 to 0)	0 (0 to 0)	0 (0 to 0)	0 (0 to 0)	0 (0 to 0)	0 (0 to 0)	0 (0 to 0)	0 (0 to 0)	0 (0 to 0)	0 (0 to 0)	0 (0 to 0)	0·2
Neglected tropical diseases and malaria	1·8 (0·9 to 3·5)	2·3 (1·1 to 4·3)	2·9 (1·2 to 5·5)	3·2 (1·5 to 6)	3·4 (1·6 to 6·5)	2·9 (1·3 to 5·5)	2·4 (1·1 to 4·2)	2·2 (1·1 to 4·2)	1·6 (0·7 to 3)	0·7 (0·4 to 1·3)	0·2 (0·1 to 0·4)	0 (0 to 0·1)	36·1
Malaria	0 (0 to 0)	0 (0 to 0)	0 (0 to 0)	0 (0 to 0)	0 (0 to 0)	0 (0 to 0)	0 (0 to 0)	0 (0 to 0)	0 (0 to 0)	0 (0 to 0)	0 (0 to 0)	0 (0 to 0)	0
Chagas disease	0 (0 to 0)	0 (0 to 0)	0 (0 to 0)	0 (0 to 0)	0 (0 to 0)	0 (0 to 0)	0 (0 to 0)	0 (0 to 0)	0 (0 to 0)	0 (0 to 0)	0 (0 to 0)	0 (0 to 0)	0
Leishmaniasis	0 (0 to 0)	0 (0 to 0)	0 (0 to 0)	0 (0 to 0)	0 (0 to 0)	0 (0 to 0)	0 (0 to 0)	0 (0 to 0)	0 (0 to 0)	0 (0 to 0)	0 (0 to 0)	0 (0 to 0)	0
Visceral leishmaniasis	0 (0 to 0)	0 (0 to 0)	0 (0 to 0)	0 (0 to 0)	0 (0 to 0)	0 (0 to 0)	0 (0 to 0)	0 (0 to 0)	0 (0 to 0)	0 (0 to 0)	0 (0 to 0)	0 (0 to 0)	0
Cutaneous and mucocutaneous leishmaniasis	0 (0 to 0)	0 (0 to 0)	0 (0 to 0)	0 (0 to 0)	0 (0 to 0)	0 (0 to 0)	0 (0 to 0)	0 (0 to 0)	0 (0 to 0)	0 (0 to 0)	0 (0 to 0)	0 (0 to 0)	0
African trypanosomiasis	0 (0 to 0)	0 (0 to 0)	0 (0 to 0)	0 (0 to 0)	0 (0 to 0)	0 (0 to 0)	0 (0 to 0)	0 (0 to 0)	0 (0 to 0)	0 (0 to 0)	0 (0 to 0)	0 (0 to 0)	0
Schistosomiasis	0 (0 to 0)	0 (0 to 0)	0 (0 to 0)	0 (0 to 0)	0 (0 to 0)	0 (0 to 0)	0 (0 to 0)	0 (0 to 0)	0 (0 to 0)	0 (0 to 0)	0 (0 to 0)	0 (0 to 0)	0
Cysticercosis	1·2 (0·4 to 2·6)	1·6 (0·5 to 3·6)	2·2 (0·7 to 4·8)	2·6 (0·9 to 5·4)	2·8 (1·1 to 6)	2·5 (0·9 to 5·1)	2 (0·8 to 3·7)	1·9 (0·7 to 3·7)	1·4 (0·5 to 2·8)	0·6 (0·2 to 1·1)	0·2 (0·1 to 0·4)	0 (0 to 0·1)	22·6
Cystic echinococcosis	0 (0 to 0)	0 (0 to 0)	0 (0 to 0)	0 (0 to 0)	0 (0 to 0)	0 (0 to 0)	0 (0 to 0)	0 (0 to 0)	0 (0 to 0)	0 (0 to 0)	0 (0 to 0)	0 (0 to 0)	0
Lymphatic filariasis	0 (0 to 0)	0 (0 to 0)	0 (0 to 0)	0 (0 to 0)	0 (0 to 0)	0 (0 to 0)	0 (0 to 0)	0 (0 to 0)	0 (0 to 0)	0 (0 to 0)	0 (0 to 0)	0 (0 to 0)	0
Onchocerciasis	0 (0 to 0)	0 (0 to 0)	0 (0 to 0)	0 (0 to 0)	0 (0 to 0)	0 (0 to 0)	0 (0 to 0)	0 (0 to 0)	0 (0 to 0)	0 (0 to 0)	0 (0 to 0)	0 (0 to 0)	0
Trachoma	0 (0 to 0)	0 (0 to 0)	0 (0 to 0)	0 (0 to 0)	0 (0 to 0)	0 (0 to 0)	0 (0 to 0)	0 (0 to 0)	0 (0 to 0)	0 (0 to 0)	0 (0 to 0)	0 (0 to 0)	0
Dengue	0·1 (0 to 0·4)	0·2 (0 to 0·4)	0·2 (0 to 0·4)	0·1 (0 to 0·3)	0·1 (0 to 0·3)	0·1 (0 to 0·2)	0·1 (0 to 0·1)	0 (0 to 0·1)	0 (0 to 0·1)	0 (0 to 0)	0 (0 to 0)	0 (0 to 0)	2·7
Yellow fever	0 (0 to 0)	0 (0 to 0)	0 (0 to 0)	0 (0 to 0)	0 (0 to 0)	0 (0 to 0)	0 (0 to 0)	0 (0 to 0)	0 (0 to 0)	0 (0 to 0)	0 (0 to 0)	0 (0 to 0)	0
Rabies	0 (0 to 0)	0 (0 to 0)	0 (0 to 0)	0 (0 to 0)	0 (0 to 0)	0 (0 to 0)	0 (0 to 0)	0 (0 to 0)	0 (0 to 0)	0 (0 to 0)	0 (0 to 0)	0 (0 to 0)	0·1
Intestinal nematode infections	0 (0 to 0)	0 (0 to 0)	0 (0 to 0)	0 (0 to 0)	0 (0 to 0)	0 (0 to 0)	0 (0 to 0)	0 (0 to 0)	0 (0 to 0)	0 (0 to 0)	0 (0 to 0)	0 (0 to 0)	0·3
Ascariasis	0 (0 to 0)	0 (0 to 0)	0 (0 to 0)	0 (0 to 0)	0 (0 to 0)	0 (0 to 0)	0 (0 to 0)	0 (0 to 0)	0 (0 to 0)	0 (0 to 0)	0 (0 to 0)	0 (0 to 0)	0·1
Trichuriasis	0 (0 to 0)	0 (0 to 0)	0 (0 to 0)	0 (0 to 0)	0 (0 to 0)	0 (0 to 0)	0 (0 to 0)	0 (0 to 0)	0 (0 to 0)	0 (0 to 0)	0 (0 to 0)	0 (0 to 0)	0
Hookworm disease	0 (0 to 0)	0 (0 to 0)	0 (0 to 0)	0 (0 to 0)	0 (0 to 0)	0 (0 to 0)	0 (0 to 0)	0 (0 to 0)	0 (0 to 0)	0 (0 to 0)	0 (0 to 0)	0 (0 to 0)	0·2
Food-borne trematodiases	0 (0 to 0)	0 (0 to 0)	0 (0 to 0)	0 (0 to 0)	0 (0 to 0)	0 (0 to 0)	0 (0 to 0)	0 (0 to 0)	0 (0 to 0)	0 (0 to 0)	0 (0 to 0)	0 (0 to 0)	0
Leprosy	0 (0 to 0)	0 (0 to 0·1)	0 (0 to 0·1)	0 (0 to 0·1)	0 (0 to 0·1)	0 (0 to 0·1)	0 (0 to 0)	0 (0 to 0)	0 (0 to 0)	0 (0 to 0)	0 (0 to 0)	0 (0 to 0)	0·3
Ebola	0 (0 to 0)	0 (0 to 0)	0 (0 to 0)	0 (0 to 0)	0 (0 to 0)	0 (0 to 0)	0 (0 to 0)	0 (0 to 0)	0 (0 to 0)	0 (0 to 0)	0 (0 to 0)	0 (0 to 0)	0
Zika virus	0·2 (0 to 0·7)	0·1 (0 to 0·5)	0·1 (0 to 0·5)	0·1 (0 to 0·3)	0·1 (0 to 0·2)	0 (0 to 0·1)	0 (0 to 0·1)	0 (0 to 0)	0 (0 to 0)	0 (0 to 0)	0 (0 to 0)	0 (0 to 0)	2·7
Guinea worm disease	0 (0 to 0)	0 (0 to 0)	0 (0 to 0)	0 (0 to 0)	0 (0 to 0)	0 (0 to 0)	0 (0 to 0)	0 (0 to 0)	0 (0 to 0)	0 (0 to 0)	0 (0 to 0)	0 (0 to 0)	0
Other neglected tropical diseases	0·3 (0·1 to 0·6)	0·3 (0·1 to 0·7)	0·3 (0·1 to 0·7)	0·4 (0·1 to 0·7)	0·3 (0·1 to 0·6)	0·2 (0·1 to 0·5)	0·3 (0·1 to 0·7)	0·3 (0·1 to 0·7)	0·1 (0·1 to 0·3)	0·1 (0·1 to 0·2)	0 (0 to 0·1)	0 (0 to 0)	7·5
Other infectious diseases	3·7 (2·4 to 5·7)	4·2 (2·8 to 6·5)	5·6 (3·5 to 8·9)	5·2 (3·4 to 8·1)	4·4 (2·8 to 6·6)	3·4 (2·1 to 5·4)	2·6 (1·6 to 4·2)	1·8 (1·1 to 2·7)	1·4 (0·9 to 2·2)	0·9 (0·5 to 1·6)	0·3 (0·2 to 0·5)	0 (0 to 0·1)	96·4
Meningitis	0·7 (0·4 to 1)	0·7 (0·5 to 1·1)	1 (0·6 to 1·7)	0·9 (0·6 to 1·5)	0·9 (0·5 to 1·4)	0·6 (0·4 to 1·1)	0·5 (0·3 to 0·8)	0·4 (0·2 to 0·6)	0·3 (0·2 to 0·5)	0·1 (0·1 to 0·2)	0 (0 to 0·1)	0 (0 to 0)	22
Encephalitis	1 (0·5 to 1·7)	1 (0·5 to 1·6)	1 (0·5 to 1·7)	1 (0·5 to 1·8)	0·8 (0·4 to 1·5)	0·6 (0·3 to 1·1)	0·4 (0·2 to 0·8)	0·3 (0·1 to 0·5)	0·2 (0·1 to 0·4)	0·1 (0 to 0·1)	0 (0 to 0)	0 (0 to 0)	23·8
Diphtheria	0 (0 to 0)	0 (0 to 0)	0 (0 to 0)	0 (0 to 0)	0 (0 to 0)	0 (0 to 0)	0 (0 to 0)	0 (0 to 0)	0 (0 to 0)	0 (0 to 0)	0 (0 to 0)	0 (0 to 0)	0·1
Tetanus	0·1 (0 to 0·3)	0·2 (0·1 to 0·5)	0·3 (0·1 to 0·9)	0·3 (0·1 to 1)	0·2 (0·1 to 0·7)	0·2 (0·1 to 0·6)	0·2 (0·1 to 0·5)	0·1 (0 to 0·4)	0·1 (0 to 0·2)	0 (0 to 0·1)	0 (0 to 0)	0 (0 to 0)	2·4
Measles	0 (0 to 0)	0 (0 to 0)	0 (0 to 0)	0 (0 to 0)	0 (0 to 0)	0 (0 to 0)	0 (0 to 0)	0 (0 to 0)	0 (0 to 0)	0 (0 to 0)	0 (0 to 0)	0 (0 to 0)	0
Varicella and herpes zoster	0·1 (0 to 0·1)	0·1 (0 to 0·2)	0·1 (0·1 to 0·2)	0·1 (0·1 to 0·2)	0·1 (0·1 to 0·3)	0·1 (0·1 to 0·3)	0·1 (0 to 0·2)	0·1 (0 to 0·3)	0·2 (0 to 0·4)	0·1 (0 to 0·3)	0·1 (0 to 0·2)	0 (0 to 0·1)	2·8
Acute hepatitis	0·1 (0·1 to 0·2)	0·1 (0·1 to 0·2)	0·2 (0·1 to 0·3)	0·1 (0·1 to 0·2)	0·1 (0·1 to 0·2)	0·1 (0 to 0·1)	0 (0 to 0·1)	0 (0 to 0)	0 (0 to 0)	0 (0 to 0)	0 (0 to 0)	0 (0 to 0)	2·6
Acute hepatitis A	0·1 (0 to 0·1)	0·1 (0 to 0·1)	0·1 (0 to 0·1)	0 (0 to 0·1)	0 (0 to 0)	0 (0 to 0)	0 (0 to 0)	0 (0 to 0)	0 (0 to 0)	0 (0 to 0)	0 (0 to 0)	0 (0 to 0)	1·4
Acute hepatitis B	0·1 (0 to 0·1)	0·1 (0 to 0·1)	0·1 (0·1 to 0·2)	0·1 (0 to 0·1)	0 (0 to 0·1)	0 (0 to 0·1)	0 (0 to 0·1)	0 (0 to 0)	0 (0 to 0)	0 (0 to 0)	0 (0 to 0)	0 (0 to 0)	0·9
Acute hepatitis C	0 (0 to 0)	0 (0 to 0)	0 (0 to 0)	0 (0 to 0)	0 (0 to 0)	0 (0 to 0)	0 (0 to 0)	0 (0 to 0)	0 (0 to 0)	0 (0 to 0)	0 (0 to 0)	0 (0 to 0)	0
Acute hepatitis E	0 (0 to 0)	0 (0 to 0)	0 (0 to 0)	0 (0 to 0)	0 (0 to 0·1)	0 (0 to 0)	0 (0 to 0)	0 (0 to 0)	0 (0 to 0)	0 (0 to 0)	0 (0 to 0)	0 (0 to 0)	0·3
Other unspecified infectious diseases	1·7 (0·8 to 3·6)	2·2 (1·1 to 4·2)	3 (1·4 to 6)	2·7 (1·3 to 5·3)	2·2 (1·1 to 4·2)	1·8 (0·8 to 3·5)	1·3 (0·6 to 2·8)	0·8 (0·4 to 1·7)	0·7 (0·3 to 1·4)	0·5 (0·2 to 1·1)	0·1 (0·1 to 0·3)	0 (0 to 0)	29·4
Maternal and neonatal disorders	6 (4·4 to 8·1)	6 (4·4 to 8·3)	5·8 (4·1 to 7·8)	4·4 (3 to 6·1)	2·9 (2·1 to 4·1)	1·4 (1 to 1·9)	0·5 (0·3 to 0·8)	0·2 (0·2 to 0·3)	0 (0 to 0·1)	0 (0 to 0)	0 (0 to 0)	0 (0 to 0)	689
Maternal disorders	0 (0 to 0)	0 (0 to 0)	0 (0 to 0)	0 (0 to 0)	0 (0 to 0)	0 (0 to 0)	0 (0 to 0)	0 (0 to 0)	0 (0 to 0)	0 (0 to 0)	0 (0 to 0)	0 (0 to 0)	0
Maternal hemorrhage	0 (0 to 0)	0 (0 to 0)	0 (0 to 0)	0 (0 to 0)	0 (0 to 0)	0 (0 to 0)	0 (0 to 0)	0 (0 to 0)	0 (0 to 0)	0 (0 to 0)	0 (0 to 0)	0 (0 to 0)	0
Maternal sepsis and other maternal infections	0 (0 to 0)	0 (0 to 0)	0 (0 to 0)	0 (0 to 0)	0 (0 to 0)	0 (0 to 0)	0 (0 to 0)	0 (0 to 0)	0 (0 to 0)	0 (0 to 0)	0 (0 to 0)	0 (0 to 0)	0
Maternal hypertensive disorders	0 (0 to 0)	0 (0 to 0)	0 (0 to 0)	0 (0 to 0)	0 (0 to 0)	0 (0 to 0)	0 (0 to 0)	0 (0 to 0)	0 (0 to 0)	0 (0 to 0)	0 (0 to 0)	0 (0 to 0)	0
Maternal obstructed labor and uterine rupture	--	--	--	--	--	--	--	--	--	--	--	--	NA
Maternal abortion and miscarriage	--	--	--	--	--	--	--	--	--	--	--	--	NA
Ectopic pregnancy	--	--	--	--	--	--	--	--	--	--	--	--	NA
Indirect maternal deaths	--	--	--	--	--	--	--	--	--	--	--	--	NA
Late maternal deaths	--	--	--	--	--	--	--	--	--	--	--	--	NA
Maternal deaths aggravated by HIV/AIDS	--	--	--	--	--	--	--	--	--	--	--	--	NA
Neonatal disorders	6 (4·4 to 8·1)	6 (4·4 to 8·3)	5·8 (4·1 to 7·8)	4·4 (3 to 6·1)	2·9 (2·1 to 4·1)	1·4 (1 to 1·9)	0·5 (0·3 to 0·8)	0·2 (0·2 to 0·3)	0 (0 to 0·1)	0 (0 to 0)	0 (0 to 0)	0 (0 to 0)	689
Neonatal preterm birth	2·2 (1·5 to 3·1)	2·1 (1·4 to 2·9)	1·9 (1·3 to 2·7)	1·3 (0·9 to 1·9)	0·8 (0·6 to 1·1)	0·4 (0·3 to 0·5)	0·1 (0·1 to 0·2)	0·1 (0 to 0·1)	0 (0 to 0)	0 (0 to 0)	0 (0 to 0)	0 (0 to 0)	208
Neonatal encephalopathy due to birth asphyxia and trauma	1 (0·5 to 1·8)	1 (0·5 to 1·8)	1 (0·5 to 1·7)	0·8 (0·4 to 1·4)	0·6 (0·3 to 1)	0·3 (0·1 to 0·4)	0·1 (0 to 0·1)	0 (0 to 0)	0 (0 to 0)	0 (0 to 0)	0 (0 to 0)	0 (0 to 0)	250
Neonatal sepsis and other neonatal infections	2·5 (1·5 to 4)	2·6 (1·5 to 4·1)	2·6 (1·5 to 4·1)	2 (1·1 to 3·1)	1·4 (0·8 to 2·2)	0·7 (0·4 to 1·1)	0·3 (0·1 to 0·5)	0·1 (0·1 to 0·2)	0 (0 to 0)	0 (0 to 0)	0 (0 to 0)	0 (0 to 0)	142
Hemolytic disease and other neonatal jaundice	0·3 (0·2 to 0·4)	0·3 (0·2 to 0·4)	0·3 (0·2 to 0·4)	0·2 (0·2 to 0·3)	0·2 (0·1 to 0·2)	0·1 (0·1 to 0·1)	0 (0 to 0)	0 (0 to 0)	0 (0 to 0)	0 (0 to 0)	0 (0 to 0)	0 (0 to 0)	14·2
Other neonatal disorders	0 (0 to 0)	0 (0 to 0)	0 (0 to 0)	0 (0 to 0)	0 (0 to 0)	0 (0 to 0)	0 (0 to 0)	0 (0 to 0)	0 (0 to 0)	0 (0 to 0)	0 (0 to 0)	0 (0 to 0)	74·7
Nutritional deficiencies	3·8 (1·8 to 7·9)	4·8 (2·4 to 9·2)	6·7 (3·5 to 12·1)	7·6 (3·9 to 14·1)	6·7 (3·7 to 12·2)	4·9 (2·8 to 8·4)	4·3 (2·5 to 6·7)	4 (2·4 to 6·1)	3·7 (2·3 to 5·7)	2·2 (1·3 to 3·3)	0·9 (0·6 to 1·4)	0·2 (0·1 to 0·3)	108
Protein-energy malnutrition	0·8 (0·5 to 1·1)	1·1 (0·7 to 1·6)	1·6 (1 to 2·5)	1·8 (1·1 to 2·6)	1·9 (1·1 to 3)	1·9 (1·1 to 3·2)	1·9 (1·1 to 3·2)	2 (1·2 to 3·2)	1·8 (1 to 2·8)	1 (0·6 to 1·7)	0·5 (0·3 to 0·8)	0·1 (0·1 to 0·2)	35·8
Iodine deficiency	0·1 (0 to 0·3)	0·2 (0·1 to 0·3)	0·2 (0·1 to 0·3)	0·1 (0·1 to 0·2)	0·1 (0·1 to 0·2)	0·1 (0 to 0·1)	0 (0 to 0·1)	0 (0 to 0·1)	0 (0 to 0)	0 (0 to 0)	0 (0 to 0)	0 (0 to 0)	1·9
Vitamin A deficiency	0 (0 to 0·1)	0 (0 to 0·1)	0·1 (0 to 0·1)	0 (0 to 0·1)	0 (0 to 0·1)	0 (0 to 0)	0 (0 to 0)	0 (0 to 0)	0 (0 to 0)	0 (0 to 0)	0 (0 to 0)	0 (0 to 0)	1·4
Dietary iron deficiency	2·3 (0·5 to 6·1)	3 (0·7 to 7·2)	3·8 (1 to 9·1)	4·7 (1·3 to 10·9)	3·6 (1·1 to 8·8)	1·9 (0·5 to 4·7)	1·3 (0·3 to 3·1)	1·2 (0·3 to 2·8)	0·9 (0·2 to 2·3)	0·6 (0·2 to 1·4)	0·2 (0·1 to 0·4)	0 (0 to 0·1)	55·5
Other nutritional deficiencies	0·6 (0·3 to 0·9)	0·5 (0·3 to 0·8)	1 (0·6 to 1·7)	1 (0·6 to 1·6)	1 (0·5 to 1·7)	1 (0·5 to 2)	1 (0·5 to 1·8)	0·8 (0·4 to 1·4)	1 (0·5 to 1·7)	0·5 (0·2 to 0·9)	0·2 (0·1 to 0·5)	0·1 (0 to 0·1)	12·9
Non-communicable diseases	393 (321 to 473)	537 (443 to 644)	912 (754 to 1,090)	1,130 (928 to 1,350)	1,160 (971 to 1,370)	1,120 (942 to 1,320)	980 (836 to 1,150)	884 (774 to 1,010)	765 (689 to 861)	372 (336 to 411)	141 (126 to 156)	24·7 (21·6 to 27·5)	10,400
Neoplasms	53·3 (39·5 to 68·8)	86·8 (66·1 to 113)	191 (145 to 243)	268 (205 to 344)	301 (233 to 386)	318 (247 to 400)	298 (237 to 375)	276 (225 to 335)	239 (192 to 292)	112 (87·8 to 139)	37·7 (27·9 to 48·5)	5·6 (4 to 7·7)	2,390
Lip and oral cavity cancer	1·7 (0·9 to 2·8)	3·1 (1·8 to 5·2)	6·7 (4 to 10·5)	8·7 (5·3 to 13·6)	7·5 (4·7 to 11·4)	6·3 (4 to 9·1)	4·7 (3 to 7)	3·6 (2·5 to 5·1)	2·6 (1·7 to 4)	0·9 (0·6 to 1·4)	0·3 (0·2 to 0·5)	0·1 (0 to 0·1)	48·4
Nasopharynx cancer	0·5 (0·3 to 0·7)	0·7 (0·4 to 1·2)	1·6 (1 to 2·6)	2 (1·2 to 3·2)	1·4 (0·9 to 2·2)	1·2 (0·7 to 1·9)	0·8 (0·5 to 1·3)	0·5 (0·3 to 0·8)	0·3 (0·2 to 0·5)	0·2 (0·1 to 0·4)	0·1 (0 to 0·1)	0 (0 to 0)	10·8
Other pharynx cancer	1·1 (0·6 to 1·8)	2·8 (1·6 to 4·6)	6·1 (3·6 to 10)	7·9 (4·5 to 12·9)	6·8 (3·8 to 11·2)	5·5 (3·3 to 8·6)	3·6 (2·1 to 5·9)	2·4 (1·4 to 3·7)	1·7 (1 to 2·6)	0·7 (0·4 to 1)	0·2 (0·1 to 0·3)	0 (0 to 0·1)	40
Esophageal cancer	2·4 (1·4 to 3·9)	5 (3·1 to 7·5)	11·4 (7·4 to 17)	15·1 (9·5 to 22·9)	13·3 (8·7 to 19·5)	11·7 (7·7 to 17)	8·8 (5·7 to 13)	6 (4·1 to 8·7)	4·2 (2·8 to 6·1)	1·7 (1·1 to 2·4)	0·5 (0·3 to 0·7)	0·1 (0 to 0·1)	82·3
Stomach cancer	8 (5·1 to 11·8)	11 (7 to 16·4)	25·2 (16·1 to 36·4)	33·3 (22 to 49·2)	34·3 (23·3 to 48·4)	33·2 (22·9 to 45·8)	28·9 (20·4 to 40·2)	22·4 (16 to 30·5)	16·7 (11·6 to 23·3)	7·1 (5·2 to 9·7)	2·2 (1·5 to 3·1)	0·3 (0·2 to 0·4)	235
Colon and rectum cancer	4·6 (2·8 to 7)	6·9 (4·3 to 10·5)	14·4 (8·9 to 20·9)	20 (13·2 to 30·1)	21·6 (14·4 to 30·5)	21·3 (14·2 to 31·2)	18·5 (12·4 to 26·5)	15·4 (10·7 to 21·2)	12·1 (8 to 17·8)	5·1 (3·5 to 7·2)	1·6 (1·1 to 2·4)	0·2 (0·1 to 0·4)	152
Liver cancer	1·5 (0·8 to 2·8)	2·4 (1·2 to 4·2)	5 (2·6 to 8·6)	7·3 (4 to 12·5)	7 (3·9 to 11·4)	6·5 (4·1 to 10·4)	5·2 (3·2 to 7·9)	4 (2·7 to 5·8)	2·6 (1·7 to 3·7)	0·7 (0·4 to 1)	0·2 (0·1 to 0·3)	0 (0 to 0·1)	46·2
Liver cancer due to hepatitis B	0·8 (0·4 to 1·6)	1·1 (0·5 to 2·2)	2·1 (0·9 to 4)	2·8 (1·3 to 5·3)	2·3 (1 to 4·4)	1·7 (0·8 to 3·2)	1·1 (0·5 to 2·2)	0·8 (0·4 to 1·4)	0·5 (0·2 to 0·9)	0·1 (0·1 to 0·2)	0 (0 to 0·1)	0 (0 to 0)	15·4
Liver cancer due to hepatitis C	0·1 (0 to 0·2)	0·2 (0·1 to 0·4)	0·5 (0·2 to 1)	0·9 (0·3 to 2)	1 (0·4 to 2)	1 (0·4 to 1·8)	0·9 (0·4 to 1·6)	0·8 (0·4 to 1·4)	0·6 (0·3 to 1·1)	0·2 (0·1 to 0·3)	0·1 (0 to 0·1)	0 (0 to 0)	6·2
Liver cancer due to alcohol use	0·5 (0·2 to 1)	0·8 (0·3 to 1·9)	2 (0·8 to 3·8)	3 (1·3 to 5·7)	3·1 (1·5 to 5·7)	3·3 (1·8 to 5·5)	2·7 (1·5 to 4·5)	2 (1·1 to 3·1)	1·1 (0·6 to 1·8)	0·3 (0·1 to 0·4)	0·1 (0 to 0·1)	0 (0 to 0)	19·3
Liver cancer due to NASH	0·1 (0 to 0·2)	0·1 (0 to 0·3)	0·3 (0·1 to 0·6)	0·4 (0·2 to 0·8)	0·4 (0·2 to 0·8)	0·4 (0·2 to 0·8)	0·4 (0·2 to 0·7)	0·4 (0·2 to 0·6)	0·3 (0·1 to 0·5)	0·1 (0 to 0·1)	0 (0 to 0)	0 (0 to 0)	3
Liver cancer due to other causes	0·1 (0 to 0·2)	0·1 (0 to 0·2)	0·2 (0·1 to 0·5)	0·3 (0·1 to 0·6)	0·2 (0·1 to 0·5)	0·2 (0·1 to 0·4)	0·2 (0·1 to 0·3)	0·1 (0·1 to 0·2)	0·1 (0 to 0·2)	0 (0 to 0)	0 (0 to 0)	0 (0 to 0)	2·2
Gallbladder and biliary tract cancer	0·5 (0·3 to 1)	0·8 (0·5 to 1·6)	1·9 (1·1 to 3·8)	2·8 (1·6 to 5·5)	3 (1·7 to 5·9)	3 (1·8 to 5·9)	2·6 (1·4 to 4·7)	2 (1·2 to 3·8)	1·5 (0·9 to 2·8)	0·7 (0·4 to 1·2)	0·2 (0·1 to 0·4)	0 (0 to 0·1)	20
Pancreatic cancer	3·6 (2·2 to 5·4)	5·9 (3·6 to 9·2)	11·8 (7·4 to 18)	16 (10 to 24)	15·6 (10·5 to 23·2)	14·1 (9 to 20·9)	11·7 (7·6 to 17)	8·8 (5·9 to 12·5)	6·3 (4·1 to 9·2)	2·4 (1·6 to 3·4)	0·7 (0·5 to 1·1)	0·1 (0·1 to 0·1)	101
Larynx cancer	1·1 (0·5 to 1·8)	2·4 (1·4 to 3·8)	6 (3·6 to 9·3)	8·2 (5·1 to 12·3)	7·8 (5 to 11·5)	7 (4·4 to 10·5)	5·2 (3·2 to 7·6)	3·6 (2·3 to 5·3)	2·5 (1·6 to 3·6)	0·9 (0·6 to 1·3)	0·2 (0·2 to 0·4)	0 (0 to 0)	45·7
Tracheal, bronchus, and lung cancer	6·5 (3·8 to 10·4)	13·1 (8·4 to 19·5)	29·4 (18·9 to 42·5)	40·4 (26·7 to 56·1)	41·3 (28·3 to 58·1)	37·5 (25·8 to 51·7)	31·4 (21·9 to 42·9)	23 (16·3 to 31·7)	15·3 (10·2 to 21·7)	6·1 (4 to 8·8)	1·6 (1·1 to 2·3)	0·2 (0·1 to 0·3)	254
Malignant skin melanoma	0·4 (0·2 to 0·7)	0·5 (0·3 to 0·8)	0·9 (0·5 to 1·5)	1·1 (0·6 to 1·8)	0·8 (0·4 to 1·2)	0·7 (0·4 to 1·1)	0·6 (0·3 to 0·9)	0·5 (0·3 to 0·8)	0·4 (0·2 to 0·6)	0·2 (0·1 to 0·3)	0·1 (0 to 0·1)	0 (0 to 0)	7·2
Non-melanoma skin cancer	0·2 (0·1 to 0·4)	0·3 (0·2 to 0·5)	0·7 (0·4 to 1·1)	0·8 (0·5 to 1·2)	0·9 (0·6 to 1·4)	1 (0·6 to 1·5)	0·8 (0·5 to 1·3)	0·8 (0·5 to 1·2)	0·9 (0·5 to 1·3)	0·5 (0·3 to 0·8)	0·2 (0·1 to 0·4)	0·1 (0 to 0·1)	7·9
Non-melanoma skin cancer (squamous-cell carcinoma)	0·2 (0·1 to 0·4)	0·3 (0·2 to 0·5)	0·7 (0·4 to 1·1)	0·8 (0·5 to 1·2)	0·9 (0·6 to 1·4)	1 (0·6 to 1·5)	0·8 (0·5 to 1·3)	0·8 (0·5 to 1·2)	0·9 (0·5 to 1·3)	0·5 (0·3 to 0·8)	0·2 (0·1 to 0·4)	0·1 (0 to 0·1)	7·9
Non-melanoma skin cancer (basal-cell carcinoma)	0 (0 to 0)	0 (0 to 0)	0 (0 to 0)	0 (0 to 0)	0 (0 to 0)	0 (0 to 0)	0 (0 to 0)	0 (0 to 0)	0 (0 to 0)	0 (0 to 0)	0 (0 to 0)	0 (0 to 0)	0
Breast cancer	0·1 (0 to 0·2)	0·3 (0·1 to 0·6)	0·5 (0·2 to 1)	0·6 (0·3 to 1·2)	0·6 (0·3 to 1·2)	0·7 (0·3 to 1·3)	0·5 (0·2 to 1)	0·4 (0·2 to 0·9)	0·4 (0·1 to 0·8)	0·3 (0·1 to 0·6)	0·1 (0 to 0·2)	0 (0 to 0)	4·7
Cervical cancer	--	--	--	--	--	--	--	--	--	--	--	--	NA
Uterine cancer	--	--	--	--	--	--	--	--	--	--	--	--	NA
Ovarian cancer	--	--	--	--	--	--	--	--	--	--	--	--	NA
Prostate cancer	1·7 (1 to 2·7)	5·8 (3·7 to 8·8)	22·3 (14·4 to 33·3)	46·3 (30·3 to 68)	85·8 (56·9 to 123)	117 (82·3 to 163)	133 (93·4 to 185)	150 (109 to 196)	148 (106 to 194)	74·8 (52·6 to 99·7)	26·5 (18·3 to 36·6)	4 (2·6 to 6·1)	818
Testicular cancer	0·2 (0·1 to 0·3)	0·1 (0·1 to 0·2)	0·1 (0·1 to 0·2)	0·1 (0 to 0·2)	0·1 (0 to 0·2)	0·1 (0 to 0·2)	0·1 (0 to 0·1)	0·1 (0 to 0·2)	0 (0 to 0·1)	0 (0 to 0)	0 (0 to 0)	0 (0 to 0)	2·9
Kidney cancer	1·3 (0·7 to 2·2)	2 (1·1 to 3·3)	4·6 (2·7 to 7·4)	5·9 (3·4 to 9·5)	5·4 (3·3 to 8·1)	5·1 (3·1 to 7·9)	3·8 (2·4 to 5·9)	2·6 (1·7 to 3·9)	1·7 (1·1 to 2·6)	0·6 (0·3 to 0·8)	0·2 (0·1 to 0·3)	0 (0 to 0)	40·1
Bladder cancer	0·5 (0·3 to 0·8)	0·8 (0·5 to 1·3)	2·3 (1·4 to 3·4)	3·8 (2·4 to 5·8)	5·2 (3·3 to 7·8)	5·8 (3·7 to 8·7)	5·7 (3·8 to 8·5)	5·4 (3·6 to 7·8)	4·4 (2·9 to 6·5)	2·1 (1·4 to 3)	0·7 (0·5 to 1·1)	0·1 (0·1 to 0·2)	37·4
Brain and central nervous system cancer	1·4 (0·5 to 2·6)	1·5 (0·6 to 2·7)	2·3 (0·9 to 4)	2·6 (1·1 to 4·3)	2·1 (0·8 to 3·6)	1·6 (0·6 to 2·7)	1·1 (0·4 to 1·9)	0·7 (0·3 to 1·2)	0·4 (0·2 to 0·7)	0·1 (0·1 to 0·2)	0 (0 to 0·1)	0 (0 to 0)	24·4
Thyroid cancer	0·3 (0·2 to 0·5)	0·5 (0·3 to 0·9)	1·1 (0·6 to 1·7)	1·2 (0·7 to 2)	1 (0·6 to 1·6)	0·9 (0·5 to 1·4)	0·7 (0·4 to 1·1)	0·5 (0·3 to 0·8)	0·4 (0·2 to 0·7)	0·2 (0·1 to 0·2)	0·1 (0 to 0·1)	0 (0 to 0)	7·8
Mesothelioma	0·3 (0·2 to 0·5)	0·6 (0·4 to 1)	1·3 (0·8 to 1·9)	1·7 (1·1 to 2·4)	0·8 (0·5 to 1·1)	0·6 (0·4 to 0·9)	0·6 (0·4 to 1)	0·7 (0·5 to 1)	0·5 (0·3 to 0·7)	0·2 (0·1 to 0·3)	0 (0 to 0·1)	0 (0 to 0)	8·2
Hodgkin lymphoma	0·8 (0·4 to 1·2)	0·6 (0·4 to 1·1)	1·1 (0·6 to 1·7)	1·1 (0·6 to 1·8)	0·9 (0·5 to 1·5)	0·8 (0·5 to 1·3)	0·5 (0·3 to 0·8)	0·4 (0·2 to 0·6)	0·2 (0·1 to 0·4)	0·1 (0·1 to 0·1)	0 (0 to 0)	0 (0 to 0)	12·1
Non-Hodgkin lymphoma	5·3 (3·3 to 8)	6·5 (4·1 to 10·6)	10·9 (6·9 to 15·8)	13 (8·3 to 19·4)	11·1 (7·3 to 16·1)	9·8 (6·4 to 14·3)	7·7 (5·3 to 11·1)	5·3 (3·6 to 7·7)	3·5 (2·1 to 5·3)	1·1 (0·7 to 1·6)	0·3 (0·2 to 0·5)	0 (0 to 0·1)	111
Multiple myeloma	1·4 (0·9 to 2·2)	3·1 (1·9 to 4·6)	7·7 (4·9 to 11·3)	10·1 (6·6 to 14·7)	10·4 (6·5 to 15·2)	10·8 (6·9 to 16·1)	8·2 (5·3 to 11·9)	6·2 (4 to 9)	4·3 (2·8 to 6·5)	1·4 (0·9 to 2·4)	0·3 (0·2 to 0·5)	0 (0 to 0·1)	65·6
Leukemia	3·3 (2·1 to 5)	3·8 (2·4 to 5·7)	6·1 (3·9 to 9)	6·8 (4·2 to 10·2)	6·2 (3·7 to 9·4)	6·3 (4 to 9·3)	5·3 (3·5 to 7·9)	4·3 (2·8 to 6·5)	3·3 (2 to 5·2)	1·4 (0·9 to 2·3)	0·4 (0·3 to 0·6)	0·1 (0 to 0·1)	89
Acute lymphoid leukemia	0·9 (0·5 to 1·6)	0·8 (0·4 to 1·3)	1·1 (0·6 to 2·1)	1·2 (0·6 to 2)	0·8 (0·3 to 1·4)	1 (0·5 to 1·8)	0·7 (0·4 to 1·3)	0·4 (0·2 to 0·8)	0·4 (0·2 to 0·7)	0·1 (0·1 to 0·3)	0 (0 to 0·1)	0 (0 to 0)	33·7
Chronic lymphoid leukemia	0·1 (0·1 to 0·2)	0·1 (0·1 to 0·2)	0·5 (0·2 to 0·8)	0·8 (0·4 to 1·4)	0·9 (0·4 to 1·5)	1 (0·5 to 1·7)	0·8 (0·4 to 1·4)	0·6 (0·3 to 1·1)	0·5 (0·3 to 1)	0·2 (0·1 to 0·4)	0·1 (0 to 0·1)	0 (0 to 0)	5·8
Acute myeloid leukemia	0·3 (0·1 to 0·6)	0·4 (0·2 to 0·7)	0·5 (0·2 to 1)	0·5 (0·2 to 0·9)	0·4 (0·2 to 0·8)	0·4 (0·2 to 0·6)	0·3 (0·1 to 0·5)	0·2 (0·1 to 0·4)	0·1 (0 to 0·2)	0 (0 to 0·1)	0 (0 to 0)	0 (0 to 0)	6·5

Chronic myeloid leukemia	0 (0 to 0)	0 (0 to 0)	0·1 (0 to 0·2)	0·1 (0 to 0·3)	0·1 (0 to 0·1)	0·1 (0 to 0·2)	0·2 (0·1 to 0·4)	0·3 (0·1 to 0·7)	0·7 (0·3 to 1·5)	0·7 (0·3 to 1·2)	0·9 (0·4 to 1·7)	0·6 (0·3 to 1)	0·7 (0·3 to 1·3)	1·3 (0·6 to 2·2)	1·4 (0·7 to 2·5)	1·4 (0·6 to 2·6)	1·3 (0·6 to 2·3)	0·9 (0·4 to 1·8)	0·8 (0·4 to 1·5)	0·6 (0·2 to 1·1)	0·2 (0·1 to 0·4)	0·1 (0 to 0·1)	0 (0 to 0)	12·3
Other leukemia	0·1 (0 to 0·3)	0·1 (0 to 0·2)	0·3 (0·1 to 0·7)	0·5 (0·2 to 1·1)	0·7 (0·2 to 1·5)	1·1 (0·4 to 2·5)	1 (0·4 to 2·1)	1·2 (0·5 to 2·3)	1·1 (0·5 to 2·1)	1·2 (0·6 to 2)	1·3 (0·6 to 2·3)	1·4 (0·8 to 2·3)	1·8 (1 to 3)	2·7 (1·5 to 4·5)	2·9 (1·6 to 4·9)	2·8 (1·5 to 4·7)	2·7 (1·5 to 4·4)	2·6 (1·5 to 4·2)	2·3 (1·3 to 3·6)	1·7 (1 to 2·9)	0·8 (0·5 to 1·4)	0·2 (0·1 to 0·4)	0 (0 to 0·1)	30·7
Other malignant neoplasms	0·3 (0·1 to 0·6)	0·3 (0·1 to 0·6)	2·3 (1·2 to 4·2)	9·3 (5·1 to 16·7)	4·9 (3 to 8)	4·5 (3·1 to 6·1)	5·3 (3·7 to 7·3)	4·2 (2·9 to 5·8)	4·3 (3 to 6)	4·4 (3·1 to 6·3)	4·6 (3·2 to 6·4)	4·4 (2·9 to 6·4)	5·8 (3·7 to 8·6)	9 (5·9 to 12·9)	10·6 (6·6 to 15·2)	9·3 (6·1 to 13·1)	8 (5·3 to 11·2)	6·3 (4·2 to 8·6)	5·2 (3·5 to 7·3)	3·9 (2·8 to 5·3)	1·9 (1·3 to 2·7)	0·7 (0·5 to 0·9)	0·1 (0·1 to 0·1)	109
Other neoplasms	0 (0 to 0)	0 (0 to 0)	0·1 (0 to 0·1)	0·2 (0·1 to 0·4)	0·2 (0·1 to 0·4)	0·2 (0·1 to 0·4)	0·2 (0·1 to 0·3)	0·1 (0·1 to 0·2)	0·2 (0·1 to 0·3)	0·2 (0·1 to 0·3)	0·3 (0·2 to 0·4)	0·2 (0·1 to 0·3)	0·3 (0·2 to 0·4)	0·7 (0·4 to 1·1)	0·9 (0·6 to 1·6)	1·1 (0·7 to 1·7)	1·1 (0·7 to 1·8)	1·1 (0·7 to 1·8)	1 (0·7 to 1·6)	0·8 (0·5 to 1·2)	0·4 (0·2 to 0·5)	0·1 (0·1 to 0·2)	0 (0 to 0)	9·4
Myelodysplastic, myeloproliferative, and other hematopoietic neo	0 (0 to 0)	0 (0 to 0)	0·1 (0 to 0·1)	0·2 (0·1 to 0·4)	0·2 (0·1 to 0·4)	0·2 (0·1 to 0·4)	0·2 (0·1 to 0·3)	0·1 (0·1 to 0·2)	0·2 (0·1 to 0·3)	0·2 (0·1 to 0·3)	0·3 (0·2 to 0·4)	0·2 (0·1 to 0·3)	0·3 (0·2 to 0·4)	0·7 (0·4 to 1·1)	0·9 (0·6 to 1·6)	1·1 (0·7 to 1·7)	1·1 (0·7 to 1·8)	1·1 (0·7 to 1·8)	1 (0·7 to 1·6)	0·8 (0·5 to 1·2)	0·4 (0·2 to 0·5)	0·1 (0·1 to 0·2)	0 (0 to 0)	9·4
Benign and in situ intestinal neoplasms	0 (0 to 0)	0 (0 to 0)	0 (0 to 0)	0 (0 to 0)	0 (0 to 0)	0 (0 to 0)	0 (0 to 0)	0 (0 to 0)	0 (0 to 0)	0 (0 to 0)	0 (0 to 0)	0 (0 to 0)	0 (0 to 0)	0 (0 to 0)	0 (0 to 0)	0 (0 to 0)	0 (0 to 0)	0 (0 to 0)	0 (0 to 0)	0 (0 to 0)	0 (0 to 0)	0 (0 to 0)	0 (0 to 0)	0
Benign and in situ cervical and uterine neoplasms	0 (0 to 0)	0 (0 to 0)	0 (0 to 0)	0 (0 to 0)	0 (0 to 0)	0 (0 to 0)	0 (0 to 0)	0 (0 to 0)	0 (0 to 0)	0 (0 to 0)	0 (0 to 0)	0 (0 to 0)	0 (0 to 0)	0 (0 to 0)	0 (0 to 0)	0 (0 to 0)	0 (0 to 0)	0 (0 to 0)	0 (0 to 0)	0 (0 to 0)	0 (0 to 0)	0 (0 to 0)	0 (0 to 0)	0
Other benign and in situ neoplasms	0 (0 to 0)	0 (0 to 0)	0 (0 to 0)	0 (0 to 0)	0 (0 to 0)	0 (0 to 0)	0 (0 to 0)	0 (0 to 0)	0 (0 to 0)	0 (0 to 0)	0 (0 to 0)	0 (0 to 0)	0 (0 to 0)	0 (0 to 0)	0 (0 to 0)	0 (0 to 0)	0 (0 to 0)	0 (0 to 0)	0 (0 to 0)	0 (0 to 0)	0 (0 to 0)	0 (0 to 0)	0 (0 to 0)	0
Cardiovascular diseases	1·4 (0·6 to 2·7)	0·5 (0·2 to 1·1)	3 (1·4 to 5·8)	2·1 (1·3 to 3·6)	2·5 (1·8 to 3·4)	5·3 (3·4 to 7·8)	12·8 (8·5 to 19)	15·1 (9·4 to 23·2)	24·1 (15·9 to 35·8)	32·2 (21·2 to 46·6)	52·5 (35·1 to 73·7)	64·5 (45 to 89·4)	98·7 (70·7 to 133)	210 (158 to 274)	291 (218 to 386)	317 (244 to 408)	317 (245 to 392)	295 (235 to 372)	262 (210 to 326)	228 (180 to 280)	115 (90·5 to 143)	46·4 (35·6 to 57·4)	9·3 (7·1 to 11·6)	2,410
Rheumatic heart disease	0 (0 to 0)	0 (0 to 0)	0 (0 to 0)	0·1 (0·1 to 0·2)	0·5 (0·3 to 0·9)	1·4 (0·8 to 2·3)	2·5 (1·5 to 3·9)	2·4 (1·6 to 3·7)	2·7 (1·7 to 4)	2·7 (1·8 to 4)	2·7 (1·8 to 3·9)	2·3 (1·5 to 3·3)	2·2 (1·5 to 3·1)	2·7 (1·8 to 4)	2·6 (1·7 to 4)	1·8 (1·2 to 2·7)	1·3 (0·8 to 1·9)	0·8 (0·5 to 1·3)	0·6 (0·4 to 1)	0·3 (0·2 to 0·5)	0·1 (0·1 to 0·2)	0·1 (0 to 0·1)	0 (0 to 0)	30
Ischemic heart disease	0 (0 to 0)	0 (0 to 0)	0 (0 to 0)	0 (0 to 0)	0 (0 to 0)	0 (0 to 0)	0·7 (0·4 to 1·3)	1·5 (0·8 to 2·6)	3·4 (1·8 to 5·8)	6 (3·4 to 9·7)	12 (7 to 18·9)	17 (10·2 to 26)	28·4 (17·6 to 41·9)	66·6 (44·6 to 93·8)	97·5 (68·7 to 137)	109 (74·3 to 149)	110 (79·5 to 146)	101 (73·5 to 137)	87·3 (63·6 to 119)	74·7 (54·6 to 98·5)	39·2 (28·5 to 52·6)	16 (11·3 to 21·2)	3·4 (2·4 to 4·5)	774
Stroke	0·2 (0·1 to 0·5)	0 (0 to 0·1)	0·2 (0·1 to 0·5)	0·2 (0·1 to 0·3)	0·5 (0·3 to 0·7)	0·9 (0·6 to 1·3)	2·2 (1·4 to 3·4)	3 (1·7 to 5)	4·4 (2·6 to 7·2)	6·2 (3·7 to 9·6)	10·1 (6·1 to 16)	13·6 (8·4 to 20·4)	21·4 (14·1 to 30·9)	48·4 (32·8 to 68·6)	71 (48·2 to 102)	79·5 (55·1 to 111)	83·7 (58·6 to 114)	85·1 (60 to 120)	78·2 (54·9 to 105)	65·9 (47 to 88·7)	30·3 (21·5 to 41·8)	10·8 (7·3 to 15·2)	2 (1·3 to 2·9)	618
Ischemic stroke	0 (0 to 0)	0 (0 to 0)	0 (0 to 0)	0·1 (0·1 to 0·1)	0·2 (0·1 to 0·4)	0·4 (0·2 to 0·6)	0·6 (0·4 to 0·9)	0·6 (0·4 to 1)	0·9 (0·6 to 1·3)	1·2 (0·8 to 1·8)	1·8 (1·1 to 2·7)	2·3 (1·5 to 3·5)	3·8 (2·4 to 5·5)	9·1 (5·8 to 13·5)	14·8 (9·1 to 22·6)	26 (16·9 to 38·2)	29·8 (19·7 to 43)	41·4 (27·9 to 60)	40·4 (26·3 to 57·1)	46·1 (32·3 to 64)	21·9 (15·4 to 30·1)	8·1 (5·4 to 11·3)	1·6 (1 to 2·2)	251
Intracerebral hemorrhage	0 (0 to 0·1)	0 (0 to 0)	0·1 (0 to 0·3)	0 (0 to 0·1)	0·1 (0·1 to 0·2)	0·2 (0·1 to 0·4)	0·8 (0·4 to 1·4)	1·2 (0·6 to 2·1)	1·9 (1 to 3·3)	3 (1·7 to 5)	5·3 (3 to 8·8)	7·9 (4·5 to 12·4)	13·4 (8·2 to 20)	31·9 (20·4 to 46·4)	46·5 (29·9 to 67·7)	46·6 (31·2 to 65·7)	47·9 (32 to 68·2)	39·5 (25·8 to 57·7)	34·5 (23 to 49·2)	17·1 (10·9 to 27)	7·2 (4·5 to 10·8)	2·4 (1·5 to 3·7)	0·4 (0·2 to 0·6)	308
Subarachnoid hemorrhage	0·2 (0 to 0·4)	0 (0 to 0·1)	0·1 (0 to 0·3)	0·1 (0 to 0·1)	0·1 (0·1 to 0·2)	0·3 (0·1 to 0·6)	0·9 (0·4 to 1·5)	1·2 (0·6 to 2·2)	1·6 (0·7 to 2·8)	1·9 (1 to 3·2)	3·1 (1·6 to 5·6)	3·4 (1·8 to 5·7)	4·2 (2·3 to 7)	7·3 (4·3 to 11·9)	9·8 (5·4 to 16·6)	6·8 (3·7 to 11·8)	6 (3·4 to 10·2)	4·2 (2·5 to 7·1)	3·3 (1·8 to 5·4)	2·6 (1·5 to 4·5)	1·1 (0·6 to 1·8)	0·4 (0·2 to 0·6)	0·1 (0 to 0·1)	58·6
Hypertensive heart disease	0 (0 to 0)	0 (0 to 0)	0 (0 to 0)	0 (0 to 0)	0 (0 to 0)	0 (0 to 0)	0·4 (0·2 to 0·8)	0·7 (0·3 to 1·4)	1·4 (0·7 to 2·5)	3 (1·4 to 5·2)	6 (3 to 10·4)	8·9 (4·9 to 15)	15·4 (8·5 to 25)	34·7 (20 to 55·6)	47·4 (28·2 to 72·9)	52 (31·8 to 80·7)	52·5 (31 to 81·3)	49·3 (30 to 73·8)	43·7 (26·8 to 68·3)	40 (25·6 to 59·2)	19·9 (12·5 to 30·1)	8·7 (5·5 to 12·9)	1·9 (1·2 to 2·8)	386
Non-rheumatic valvular heart disease	0 (0 to 0)	0 (0 to 0)	0 (0 to 0)	0 (0 to 0)	0 (0 to 0)	0 (0 to 0)	0·5 (0·2 to 0·9)	0·4 (0·2 to 0·9)	0·8 (0·4 to 1·4)	0·9 (0·5 to 1·4)	1·1 (0·6 to 1·9)	1·2 (0·7 to 2)	1·6 (0·9 to 2·5)	2·6 (1·6 to 4)	3·8 (2·3 to 6)	3·7 (2·3 to 5·9)	4 (2·4 to 6)	3·2 (1·9 to 4·9)	2·6 (1·7 to 4)	2·4 (1·4 to 3·8)	1·1 (0·6 to 1·9)	0·4 (0·2 to 0·7)	0·1 (0 to 0·1)	30·6
Non-rheumatic calcific aortic valve disease	0 (0 to 0)	0 (0 to 0)	0 (0 to 0)	0 (0 to 0)	0 (0 to 0)	0 (0 to 0)	0·3 (0·1 to 0·5)	0·2 (0·1 to 0·5)	0·3 (0·2 to 0·7)	0·3 (0·2 to 0·6)	0·4 (0·2 to 0·7)	0·5 (0·3 to 0·9)	0·7 (0·4 to 1·3)	1·2 (0·7 to 2)	1·8 (1 to 3·1)	1·9 (1·1 to 3·1)	2·3 (1·3 to 3·7)	2 (1·1 to 3·1)	1·7 (1 to 2·7)	1·7 (1 to 2·8)	0·8 (0·4 to 1·3)	0·3 (0·1 to 0·5)	0 (0 to 0·1)	16·5
Non-rheumatic degenerative mitral valve disease	0 (0 to 0)	0 (0 to 0)	0 (0 to 0)	0 (0 to 0)	0 (0 to 0)	0 (0 to 0)	0·2 (0·1 to 0·4)	0·2 (0·1 to 0·5)	0·5 (0·2 to 0·9)	0·6 (0·3 to 1)	0·7 (0·4 to 1·2)	0·7 (0·4 to 1·2)	0·8 (0·5 to 1·4)	1·4 (0·8 to 2·3)	2 (1·1 to 3·3)	1·8 (1·1 to 3·1)	1·6 (0·9 to 2·7)	1·2 (0·6 to 2)	1 (0·5 to 1·6)	0·7 (0·3 to 1·3)	0·4 (0·2 to 0·7)	0·1 (0·1 to 0·3)	0 (0 to 0)	14
Other non-rheumatic valve diseases	0 (0 to 0)	0 (0 to 0)	0 (0 to 0)	0 (0 to 0)	0 (0 to 0)	0 (0 to 0)	0 (0 to 0)	0 (0 to 0)	0 (0 to 0)	0 (0 to 0)	0 (0 to 0)	0 (0 to 0)	0 (0 to 0)	0 (0 to 0)	0 (0 to 0)	0 (0 to 0)	0 (0 to 0)	0 (0 to 0)	0 (0 to 0)	0 (0 to 0)	0 (0 to 0)	0 (0 to 0)	0 (0 to 0)	0·2
Cardiomyopathy and myocarditis	0·7 (0·3 to 1·5)	0·3 (0·1 to 0·7)	2·1 (0·9 to 4)	1·3 (0·7 to 2·3)	1 (0·6 to 1·4)	1·7 (0·9 to 3·1)	4·3 (2·5 to 7·3)	4·6 (2·4 to 8·1)	8 (4·6 to 13)	9·7 (5·4 to 15·4)	15·2 (8·4 to 24·9)	15·5 (9 to 24·6)	21·9 (12·6 to 33·9)	39·2 (21·8 to 60·7)	46·5 (26·8 to 69·3)	44·5 (25·5 to 69·3)	38 (22·5 to 58·5)	28·9 (16·8 to 44·9)	22·2 (13 to 33·9)	17 (9·8 to 26·3)	8·7 (5 to 14·2)	3 (1·6 to 4·8)	0·6 (0·3 to 0·9)	335
Myocarditis	0 (0 to 0·1)	0 (0 to 0·1)	0·2 (0·1 to 0·6)	0·1 (0 to 0·3)	0·1 (0 to 0·2)	0·1 (0 to 0·2)	0·3 (0·1 to 0·7)	0·2 (0·1 to 0·5)	0·4 (0·2 to 0·8)	0·5 (0·2 to 1)	0·6 (0·3 to 1·1)	0·5 (0·3 to 1·1)	0·5 (0·2 to 1)	1 (0·5 to 1·9)	0·9 (0·4 to 1·8)	0·8 (0·3 to 1·5)	0·8 (0·4 to 1·6)	0·4 (0·2 to 0·9)	0·4 (0·2 to 0·7)	0·2 (0·1 to 0·5)	0·1 (0 to 0·2)	0 (0 to 0·1)	0 (0 to 0)	8·4
Alcoholic cardiomyopathy	0 (0 to 0)	0 (0 to 0)	0 (0 to 0)	0 (0 to 0)	0 (0 to 0)	0 (0 to 0)	0 (0 to 0·1)	0 (0 to 0·1)	0·2 (0·1 to 0·4)	0·4 (0·2 to 0·9)	0·7 (0·3 to 1·4)	0·9 (0·4 to 1·8)	1·7 (0·7 to 3·5)	3·2 (1·4 to 6·4)	3·5 (1·4 to 7)	3·1 (1·2 to 6·2)	2·7 (1·2 to 5·8)	1·4 (0·5 to 3)	0·9 (0·3 to 1·9)	0·2 (0·1 to 0·4)	0·1 (0 to 0·1)	0 (0 to 0)	0 (0 to 0)	19
Other cardiomyopathy	0·6 (0·2 to 1·4)	0·3 (0·1 to 0·6)	1·8 (0·8 to 3·6)	1·1 (0·6 to 2·1)	0·9 (0·5 to 1·3)	1·6 (0·8 to 2·9)	4 (2·2 to 6·8)	4·3 (2·3 to 7·5)	7·4 (4·2 to 12·1)	8·8 (4·9 to 14·1)	13·9 (7·6 to 22·6)	14 (8·2 to 22·2)	19·7 (11 to 30·6)	34·9 (19·7 to 55)	42·1 (24·7 to 62·9)	40·6 (23·1 to 64)	34·5 (20·3 to 53)	27·1 (15·8 to 41·7)	20·9 (12·4 to 31·5)	16·6 (9·5 to 25·7)	8·5 (4·9 to 14)	2·9 (1·6 to 4·8)	0·5 (0·3 to 0·9)	307
Atrial fibrillation and flutter	0 (0 to 0)	0 (0 to 0)	0 (0 to 0)	0 (0 to 0)	0 (0 to 0)	0 (0 to 0)	0 (0 to 0)	0 (0 to 0)	0 (0 to 0)	0·1 (0·1 to 0·2)	0·1 (0·1 to 0·2)	0·4 (0·3 to 0·7)	0·9 (0·6 to 1·2)	2 (1·4 to 2·9)	3·6 (2·6 to 4·9)	5 (3·6 to 6·8)	6 (4·5 to 7·9)	7·1 (5·3 to 9·3)	8·7 (6·5 to 11·6)	11·4 (8·5 to 15·2)	7·7 (5·6 to 10·5)	4·5 (3·1 to 6·2)	0·9 (0·6 to 1·3)	58·4
Aortic aneurysm	--	--	--	--	--	--	0·1 (0·1 to 0·3)	0·1 (0 to 0·2)	0·2 (0·1 to 0·4)	0·3 (0·2 to 0·5)	0·6 (0·3 to 0·9)	0·7 (0·4 to 1·1)	0·9 (0·5 to 1·4)	2·3 (1·4 to 3·6)	4·4 (2·8 to 6·6)	6 (4 to 8·9)	6·9 (4·7 to 9·5)	6·9 (4·8 to 9·6)	5·9 (4 to 8·1)	4·7 (3·2 to 6·5)	2 (1·4 to 2·8)	0·6 (0·4 to 0·9)	0·1 (0 to 0·1)	NA
Endocarditis	0 (0 to 0·1)	0 (0 to 0·1)	0·1 (0 to 0·4)	0·1 (0 to 0·3)	0·1 (0 to 0·2)	0·2 (0·1 to 0·4)	0·5 (0·2 to 0·9)	0·6 (0·2 to 1·1)	0·8 (0·4 to 1·5)	1 (0·5 to 1·8)	1·2 (0·6 to 2·2)	1·3 (0·6 to 2·3)	1·3 (0·7 to 2·2)	2·2 (1·2 to 3·7)	2·1 (1·2 to 3·6)	1·8 (1 to 2·8)	1·5 (0·9 to 2·4)	1·1 (0·7 to 1·7)	0·7 (0·4 to 1·1)	0·6 (0·3 to 0·9)	0·3 (0·2 to 0·5)	0·1 (0 to 0·1)	0 (0 to 0)	17·6
Other cardiovascular and circulatory diseases	0·4 (0·1 to 1)	0·1 (0 to 0·3)	0·6 (0·2 to 1·4)	0·4 (0·2 to 0·8)	0·5 (0·3 to 0·8)	1 (0·5 to 1·8)	1·5 (0·9 to 2·4)	1·7 (0·9 to 2·9)	2·4 (1·4 to 4)	2·5 (1·4 to 3·9)	3·5 (2 to 5·8)	3·5 (2·2 to 5·3)	4·5 (2·8 to 7)	8·8 (5·6 to 13)	11·1 (6·8 to 17)	11·5 (7·7 to 16·8)	11 (7·3 to 16)	9·8 (6·4 to 14·2)	9·5 (6·5 to 13·7)	8·9 (6 to 12·7)	4·8 (3·2 to 7)	1·8 (1·2 to 2·7)	0·3 (0·2 to 0·5)	100
Chronic respiratory diseases	0 (0 to 0)	0 (0 to 0)	0 (0 to 0)	17·5 (10·7 to 27·3)	15·9 (9·8 to 25·1)	16 (10·2 to 23·9)	14·5 (9·5 to 21)	11·6 (8·1 to 16·3)	11·8 (8·6 to 15·9)	11·2 (8·2 to 15·1)	11·6 (8·7 to 15·3)	10·5 (7·6 to 13·7)	13·5 (10·3 to 17·6)	24·4 (17·7 to 32·7)	33·2 (23·3 to 45·9)	39·7 (28·1 to 53·8)	42 (28·9 to 59)	42·3 (29·2 to 59·3)	39·1 (28·1 to 52·8)	37·1 (25·4 to 50·2)	18 (12·5 to 25·1)	7·3 (5·1 to 10·1)	1·4 (0·9 to 1·8)	419
Chronic obstructive pulmonary disease	0 (0 to 0)	0 (0 to 0)	0 (0 to 0)	0·3 (0·1 to 0·7)	0·2 (0·1 to 0·3)	0·4 (0·2 to 0·7)	0·8 (0·4 to 1·3)	1·1 (0·6 to 1·6)	1·5 (1 to 2·1)	1·7 (1·1 to 2·4)	2·3 (1·5 to 3·3)	2·8 (1·9 to 3·9)	4·8 (3·3 to 6·7)	10·7 (7·5 to 14·9)	18·3 (12·3 to 26·6)	25·8 (16·8 to 36·4)	29·6 (19·6 to 43·5)	31·4 (21·3 to 44·4)	30·5 (20·9 to 42·4)	29·5 (20 to 41·2)	14·8 (10·1 to 20·7)	6 (4·1 to 8·4)	1·1 (0·7 to 1·5)	214
Pneumoconiosis	0 (0 to 0)	0 (0 to 0)	0 (0 to 0)	0 (0 to 0)	0 (0 to 0)	0 (0 to 0)	0 (0 to 0·1)	0 (0 to 0)	0 (0 to 0)	0 (0 to 0)	0 (0 to 0)	0 (0 to 0)	0 (0 to 0)	0 (0 to 0·1)	0 (0 to 0·1)	0 (0 to 0·1)	0·1 (0 to 0·1)	0 (0 to 0·1)	0 (0 to 0·1)	0 (0 to 0·1)	0 (0 to 0·1)	0 (0 to 0)	0 (0 to 0)	0·4
Silicosis	0 (0 to 0)	0 (0 to 0)	0 (0 to 0)	0 (0 to 0)	0 (0 to 0)	0 (0 to 0)	0 (0 to 0)	0 (0 to 0)	0 (0 to 0)	0 (0 to 0)	0 (0 to 0)	0 (0 to 0)	0 (0 to 0)	0 (0 to 0)	0 (0 to 0)	0 (0 to 0)	0 (0 to 0)	0 (0 to 0)	0 (0 to 0)	0 (0 to 0)	0 (0 to 0)	0 (0 to 0)	0 (0 to 0)	0·1
Asbestosis	0 (0 to 0)	0 (0 to 0)	0 (0 to 0)	0 (0 to 0)	0 (0 to 0)	0 (0 to 0)	0 (0 to 0)	0 (0 to 0)	0 (0 to 0)	0 (0 to 0)	0 (0 to 0)	0 (0 to 0)	0 (0 to 0)	0 (0 to 0)	0 (0 to 0)	0 (0 to 0)	0 (0 to 0)	0 (0 to 0)	0 (0 to 0)	0 (0 to 0)	0 (0 to 0)	0 (0 to 0)	0 (0 to 0)	0·1
Coal workers pneumoconiosis	0 (0 to 0)	0 (0 to 0)	0 (0 to 0)	0 (0 to 0)	0 (0 to 0)	0 (0 to 0)	0 (0 to 0)	0 (0 to 0)	0 (0 to 0)	0 (0 to 0)	0 (0 to 0)	0 (0 to 0)	0 (0 to 0)	0 (0 to 0)	0 (0 to 0)	0 (0 to 0)	0 (0 to 0)	0 (0 to 0)	0 (0 to 0)	0 (0 to 0)	0 (0 to 0)	0 (0 to 0)	0 (0 to 0)	0
Other pneumoconiosis	0 (0 to 0)	0 (0 to 0)	0 (0 to 0)	0 (0 to 0)	0 (0 to 0)	0 (0 to 0)	0 (0 to 0)	0 (0 to 0)	0 (0 to 0)	0 (0 to 0)	0 (0 to 0)	0 (0 to 0)	0 (0 to 0)	0 (0 to 0)	0 (0 to 0)	0 (0 to 0·1)	0 (0 to 0)	0 (0 to 0)	0 (0 to 0)	0 (0 to 0)	0 (0 to 0)	0 (0 to 0)	0 (0 to 0)	0·2
Asthma	0 (0 to 0)	0 (0 to 0)	0 (0 to 0)	14·2 (8·2 to 23·2)	15 (9 to 24·2)	14·7 (9·1 to 22·8)	12·5 (7·7 to 18·8)	9·3 (6 to 13·6)	8·6 (5·7 to 12)	7·8 (5·3 to 11)	7·1 (4·9 to 9·8)	5·8 (4 to 7·9)	6·5 (4·5 to 8·9)	9·7 (6·7 to 13·9)	10·3 (6·9 to 14·8)	11·1 (7·3 to 16·4)	9·4 (6 to 14·2)	8 (4·7 to 12·7)	6 (3·6 to 9·5)	5·4 (3·2 to 8·7)	2·1 (1·3 to 3·5)	0·9 (0·5 to 1·5)	0·2 (0·1 to 0·3)	165
Interstitial lung disease and pulmonary sarcoidosis	0 (0 to 0)	0 (0 to 0)	0 (0 to 0)	0·3 (0·1 to 0·7)	0 (0 to 0·1)	0·1 (0 to 0·1)	0·2 (0·1 to 0·4)	0·2 (0·1 to 0·4)	0·2 (0·1 to 0·3)	0·3 (0·2 to 0·6)	0·5 (0·2 to 0·8)	0·6 (0·3 to 1)	0·6 (0·3 to 1·1)	1·3 (0·7 to 2·2)	1·7 (0·9 to 2·9)	1·7 (0·9 to 2·8)	1·9 (1 to 3·3)	1·9 (0·9 to 3·1)	1·8 (0·9 to 3·2)	1·3 (0·6 to 2·3)	0·6 (0·3 to 1·1)	0·2 (0·1 to 0·4)	0 (0 to 0·1)	15·3
Other chronic respiratory diseases	0 (0 to 0)	0 (0 to 0)	0 (0 to 0)	2·7 (1·1 to 5·1)	0·6 (0·4 to 0·9)	0·9 (0·5 to 1·5)	1 (0·5 to 1·8)	1 (0·5 to 1·7)	1·6 (0·8 to 2·7)	1·4 (0·8 to 2·4)	1·7 (0·9 to 3)	1·3 (0·7 to 2·2)	1·6 (0·8 to 2·8)	2·7 (1·2 to 5)	2·8 (1·2 to 5·2)	1·1 (0·5 to 2·1)	1 (0·4 to 2·1)	0·9 (0·4 to 1·8)	0·8 (0·3 to 1·6)	0·8 (0·3 to 1·7)	0·4 (0·2 to 0·9)	0·2 (0·1 to 0·3)	0 (0 to 0·1)	24·6
Digestive diseases	0·6 (0·3 to 1·3)	0·5 (0·2 to 0·9)	4·3 (1·6 to 9)	5·9 (3·1 to 10·3)	4·5 (3·1 to 6·5)	4·7 (3·1 to 6·5)	7·1 (5 to 9·8)	9·5 (6·7 to 13·1)	14 (10 to 19·5)	18·7 (13·4 to 25·7)	27·4 (19·5 to 37·5)	28·4 (20·3 to 38·2)	38·6 (27·7 to 53·2)	60·4 (43·7 to 82·4)	65·3 (47 to 86·7)	56·1 (40·1 to 76·8)	45·7 (33 to 61·7)	33·8 (23·8 to 46)	25·7 (18·3 to 34·7)	19·2 (13·7 to 26·6)	8·8 (6·4 to 11·9)	3·2 (2·3 to 4·4)	0·5 (0·3 to 0·8)	483
Cirrhosis and other chronic liver diseases	0 (0 to 0)	0 (0 to 0)	0 (0 to 0)	0·5 (0·1 to 1·1)	0·4 (0·2 to 0·7)	0·5 (0·2 to 1)	1·2 (0·6 to 2·1)	1·4 (0·7 to 2·4)	2·8 (1·6 to 4·6)	5 (2·9 to 8)	10·1 (6·2 to 15·6)	12·4 (7·9 to 18·6)	18·8 (12·4 to 28·4)	32 (21·3 to 46·8)	34·2 (22·4 to 48·7)	27·5 (17·7 to 39·7)	20·6 (13·5 to 29·2)	13·4 (8·2 to 19·7)	8·5 (5·3 to 12·6)	5·3 (3·4 to 8)	2 (1·3 to 3·1)	0·6 (0·4 to 1)	0·1 (0·1 to 0·2)	197
Cirrhosis and other chronic liver diseases due to hepatitis B	0 (0 to 0)	0 (0 to 0)	0 (0 to 0)	0 (0 to 0)	0 (0 to 0)	0 (0 to 0)	0 (0 to 0)	0·1 (0 to 0·1)	0·2 (0·1 to 0·4)	0·4 (0·2 to 0·8)	1 (0·4 to 2)	1·3 (0·6 to 2·4)	1·8 (0·8 to 3·5)	2·8 (1·3 to 5·5)	2·5 (1·1 to 4·8)	1·8 (0·8 to 3·5)	1·2 (0·5 to 2·4)	0·8 (0·3 to 1·5)	0·5 (0·2 to 1)	0·3 (0·1 to 0·7)	0·1 (0·1 to 0·2)	0 (0 to 0·1)	0 (0 to 0)	15
Cirrhosis and other chronic liver diseases due to hepatitis C	0 (0 to 0)	0 (0 to 0)	0 (0 to 0)	0 (0 to 0)	0 (0 to 0)	0 (0 to 0)	0·1 (0 to 0·2)	0·2 (0·1 to 0·5)	0·7 (0·3 to 1·4)	1·3 (0·6 to 2·4)	2·5 (1·2 to 4·6)	3 (1·5 to 5·1)	4·2 (2 to 7·4)	6·7 (3·3 to 12)	6·6 (3·2 to 12·1)	5 (2·2 to 9·3)	3·7 (1·9 to 6·4)	2·4 (1·2 to 4·2)	1·5 (0·7 to 2·7)	0·9 (0·4 to 1·8)	0·4 (0·2 to 0·7)	0·1 (0·1 to 0·2)	0 (0 to 0)	39·3
Cirrhosis and other chronic liver diseases due to alcohol use	0 (0 to 0)	0 (0 to 0)	0 (0 to 0)	0 (0 to 0)	0 (0 to 0)	0 (0 to 0)	0 (0 to 0)	0·2 (0·1 to 0·3)	0·6 (0·2 to 1·2)	1·6 (0·8 to 2·9)	4·2 (2 to 7·3)	5·7 (3 to 9·4)	9·1 (5 to 14·5)	16·1 (9·2 to 25·2)	18·2 (10·7 to 27·2)	14·8 (8·5 to 23·3)	10·7 (6·1 to 16·7)	6·5 (3·6 to 10·3)	3·8 (2 to 6·1)	2·2 (1·2 to 3·7)	0·8 (0·4 to 1·4)	0·2 (0·1 to 0·4)	0 (0 to 0·1)	94·8
Cirrhosis and other chronic liver diseases due to NAFLD	0 (0 to 0)	0 (0 to 0)	0 (0 to 0)	0 (0 to 0)	0 (0 to 0)	0 (0 to 0)	0·1 (0 to 0·2)	0·2 (0·1 to 0·3)	0·4 (0·2 to 0·9)	0·7 (0·3 to 1·3)	1·3 (0·5 to 2·6)	1·5 (0·7 to 2·9)	2·4 (1 to 4·7)	4·1 (1·7 to 7·9)	4·2 (1·9 to 8)	3·5 (1·5 to 7)	3 (1·5 to 5·2)	2·1 (1 to 3·8)	1·5 (0·7 to 2·7)	1 (0·5 to 1·8)	0·4 (0·2 to 0·7)	0·1 (0·1 to 0·3)	0 (0 to 0)	26·5
Cirrhosis and other chronic liver diseases due to other causes	0 (0 to 0)	0 (0 to 0)	0 (0 to 0)	0·5 (0·1 to 1·1)	0·4 (0·2 to 0·6)	0·5 (0·2 to 0·9)	1 (0·5 to 1·8)	0·8 (0·4 to 1·4)	0·9 (0·4 to 1·6)	0·9 (0·4 to 1·7)	1 (0·4 to 2·2)	1 (0·4 to 1·9)	1·3 (0·6 to 2·7)	2·3 (1 to 4·4)	2·7 (1·2 to 5·3)	2·5 (1·1 to 4·5)	2·1 (0·9 to 4)	1·6 (0·7 to 3·1)	1·2 (0·5 to 2·3)	0·8 (0·4 to 1·6)	0·3 (0·2 to 0·6)	0·1 (0 to 0·2)	0 (0 to 0)	21·7
Upper digestive system diseases	0 (0 to 0)	0 (0 to 0)	0 (0 to 0)	0·2 (0·1 to 0·4)	0·2 (0·1 to 0·4)	0·5 (0·3 to 0·8)	1·8 (1·1 to 3)	3·8 (2·2 to 6·6)	5·3 (3·1 to 9·1)	6·5 (3·8 to 10·9)	7·2 (4·5 to 11·3)	6·8 (4·4 to 10·5)	8·3 (5·3 to 12·5)	11·5 (7·4 to 16·9)	12 (8 to 17·7)	11 (7·2 to 16·1)	9·2 (6·3 to 13·7)	7 (4·5 to 10·3)	5·8 (3·9 to 8·4)	4·3 (2·9 to 6·3)	2 (1·4 to 2·9)	0·8 (0·5 to 1·1)	0·1 (0·1 to 0·2)	104
Peptic ulcer disease	0 (0 to 0)	0 (0 to 0)	0 (0 to 0)	0 (0 to 0·1)	0 (0 to 0·1)	0·1 (0·1 to 0·2)	0·3 (0·1 to 0·5)	0·5 (0·2 to 0·8)	0·7 (0·4 to 1·2)	1 (0·6 to 1·8)	1·6 (0·8 to 2·8)	1·6 (0·8 to 2·6)	1·8 (1 to 3·2)	3·9 (2·1 to 6·5)	4·4 (2·4 to 7·3)	4·3 (2·2 to 7·2)	4·2 (2·4 to 7·3)	3·6 (2 to 5·8)	3·1 (1·7 to 5)	2·4 (1·4 to 3·8)	1·2 (0·7 to 1·9)	0·4 (0·2 to 0·7)	0·1 (0 to 0·1)	35·3
Gastritis and duodenitis	0 (0 to 0)	0 (0 to 0)	0 (0 to 0)	0·2 (0·1 to 0·3)	0·2 (0·1 to 0·4)	0·2 (0·1 to 0·4)	0·3 (0·2 to 0·6)	0·4 (0·2 to 0·7)	0·6 (0·4 to 1)	0·9 (0·5 to 1·5)	1·3 (0·7 to 2·1)	1·4 (0·8 to 2·3)	2 (1·2 to 3·2)	2·5 (1·4 to 4·1)	2·8 (1·7 to 4·4)	2·5 (1·4 to 4)	2·1 (1·2 to 3·3)	1·6 (0·9 to 2·6)	1·2 (0·7 to 2)	0·9 (0·5 to 1·6)	0·4 (0·2 to 0·8)	0·2 (0·1 to 0·3)	0 (0 to 0·1)	21·8
Gastroesophageal reflux disease	0 (0 to 0)	0 (0 to 0)	0 (0 to 0)	0 (0 to 0)	0 (0 to 0)	0·2 (0·1 to 0·4)	1·2 (0·6 to 2·3)	2·9 (1·4 to 5·7)	4 (2 to 7·5)	4·6 (2·1 to 8·6)	4·3 (2 to 7·9)	3·9 (1·8 to 7·1)	4·4 (2·2 to 8)	5 (2·4 to 9·2)	4·7 (2·2 to 9)	4·2 (2 to 7·7)	2·9 (1·5 to 5·3)	1·9 (1 to 3·4)	1·6 (0·8 to 2·8)	1·1 (0·5 to 2)	0·4 (0·2 to 0·8)	0·1 (0·1 to 0·2)	0 (0 to 0)	47·4
Appendicitis	0 (0 to 0)	0 (0 to 0)	0 (0 to 0)	0·4 (0·1 to 1)	0·4 (0·2 to 0·6)	0·5 (0·3 to 0·9)	0·7 (0·3 to 1·2)	0·6 (0·3 to 1)	0·6 (0·3 to 1)	0·5 (0·3 to 0·9)	0·7 (0·3 to 1·2)	0·5 (0·3 to 0·9)	0·5 (0·3 to 0·9)	0·8 (0·4 to 1·4)	0·7 (0·4 to 1·2)	0·6 (0·3 to 1·1)	0·6 (0·3 to 1·1)	0·5 (0·2 to 0·9)	0·3 (0·1 to 0·5)	0·2 (0·1 to 0·4)	0·1 (0·1 to 0·2)	0 (0 to 0·1)	0 (0 to 0)	9·2
Paralytic ileus and intestinal obstruction	0·5 (0·2 to 1)	0·4 (0·2 to 0·8)	3·8 (1·4 to 8·1)	1·4 (0·5 to 3·1)	0·6 (0·3 to 1·1)	0·7 (0·3 to 1·2)	0·7 (0·3 to 1·3)	0·8 (0·4 to 1·4)	0·9 (0·5 to 1·6)	1 (0·5 to 1·9)	1·8 (1 to 3·2)	1·6 (0·8 to 2·7)	2·1 (1·1 to 3·7)	3·9 (2 to 6·3)	4·8 (2·6 to 8·1)	4·6 (2·6 to 7·9)	4·8 (2·6 to 7·9)	4·4 (2·5 to 7·2)	4 (2·4 to 6·5)	3·6 (2·1 to 5·8)	1·8 (1·1 to 2·8)	0·7 (0·4 to 1·1)	0·1 (0·1 to 0·2)	49·3
Inguinal, femoral, and abdominal hernia	0·1 (0 to 0·3)	0·1 (0 to 0·1)	0·5 (0·2 to 1·2)	1·6 (0·9 to 2·6)	2·1 (1·2 to 3·6)	1·6 (0·8 to 2·8)	1·4 (0·7 to 2·4)	1·2 (0·6 to 2·2)	1·5 (0·8 to 2·4)	1·7 (0·9 to 2·8)	2 (1·1 to 3·2)	2·1 (1·2 to 3·4)	2·7 (1·5 to 4·3)	3·6 (2·1 to 5·5)	3·6 (2·2 to 5·7)	3·2 (2 to 4·9)	2·3 (1·5 to 3·3)	1·5 (1 to 2·2)	1·1 (0·8 to 1·6)	0·8 (0·5 to 1·1)	0·4 (0·2 to 0·6)	0·1 (0·1 to 0·2)	0 (0 to 0)	35·3
Inflammatory bowel disease	0 (0 to 0)	0 (0 to 0)	0 (0 to 0)	0·4 (0·1 to 0·9)	0·1 (0 to 0·2)	0·1 (0 to 0·2)	0·3 (0·1 to 0·6)	0·4 (0·2 to 0·7)	0·7 (0·4 to 1·3)	1 (0·5 to 1·9)	1·2 (0·6 to 2·2)	0·9 (0·5 to 1·6)	1·1 (0·6 to 1·8)	1·5 (0·8 to 2·6)	1·9 (1 to 3·2)	1·4 (0·7 to 2·3)	1·3 (0·7 to 2·1)	1·3 (0·7 to 2·3)	0·9 (0·5 to 1·6)	0·8 (0·4 to 1·5)	0·4 (0·2 to 0·6)	0·1 (0·1 to 0·2)	0 (0 to 0)	15·8
Vascular intestinal disorders	0 (0 to 0)	0 (0 to 0)	0 (0 to 0)	0 (0 to 0·1)	0 (0 to 0·1)	0 (0 to 0·1)	0·1 (0 to 0·1)	0 (0 to 0·1)	0·1 (0·1 to 0·2)	0·2 (0·1 to 0·3)	0·3 (0·1 to 0·4)	0·2 (0·1 to 0·4)	0·4 (0·2 to 0·7)	0·8 (0·5 to 1·3)	1·1 (0·7 to 1·6)	1·2 (0·7 to 1·9)	1·2 (0·8 to 1·9)	1·1 (0·7 to 1·6)	1 (0·6 to 1·5)	0·8 (0·5 to 1·2)	0·4 (0·2 to 0·5)	0·2 (0·1 to 0·2)	0 (0 to 0)	9·1
Gallbladder and biliary diseases	0 (0 to 0)	0 (0 to 0)	0 (0 to 0)	0 (0 to 0)	0·1 (0 to 0·1)	0·1 (0·1 to 0·2)	0·2 (0·1 to 0·3)	0·2 (0·1 to 0·4)	0·3 (0·2 to 0·6)	0·5 (0·2 to 0·8)	0·5 (0·3 to 0·9)	0·6 (0·3 to 1)	0·7 (0·4 to 1·2)	1·2 (0·7 to 1·8)	1·4 (0·9 to 2·1)	1·3 (0·8 to 2)	1·2 (0·8 to 1·9)	1·1 (0·7 to 1·6)	0·9 (0·6 to 1·4)	0·8 (0·5 to 1·1)	0·3 (0·2 to 0·5)	0·1 (0·1 to 0·2)	0 (0 to 0)	11·6
Pancreatitis	0 (0 to 0)	0 (0 to 0)	0 (0 to 0)	0 (0 to 0·1)	0 (0 to 0)	0·1 (0 to 0·2)	0·3 (0·1 to 0·5)	0·4 (0·2 to 0·8)	1 (0·5 to 1·8)	1·5 (0·8 to 2·7)	2·6 (1·4 to 4·5)	2·3 (1·3 to 3·7)	2·4 (1·3 to 4·1)	2·9 (1·6 to 4·8)	3 (1·7 to 4·9)	2·4 (1·4 to 4)	1·8 (1 to 3)	1·3 (0·7 to 2·1)	1 (0·6 to 1·7)	0·7 (0·4 to 1·1)	0·3 (0·2 to 0·5)	0·1 (0·1 to 0·1)	0 (0 to 0)	24
Other digestive diseases	0 (0 to 0)	0 (0 to 0)	0 (0 to 0)	1·1 (0·5 to 2·5)	0·5 (0·3 to 0·9)	0·5 (0·2 to 0·8)	0·5 (0·2 to 0·8)	0·6 (0·3 to 1)	0·7 (0·3 to 1·3)	0·8 (0·4 to 1·5)	1·1 (0·5 to 1·9)	1 (0·5 to 1·8)	1·5 (0·8 to 2·6)	2·3 (1·2 to 4·1)	2·7 (1·4 to 4·5)	3 (1·6 to 5·1)	2·6 (1·3 to 4·4)	2·3 (1·2 to 4)	2·1 (1 to 3·7)	1·8 (0·9 to 3)	1·1 (0·6 to 1·9)	0·5 (0·2 to 0·8)	0·1 (0 to 0·1)	26·8
Neurological disorders	0 (0 to 0)	0 (0 to 0·1)	3·7 (2·2 to 5·8)	6·9 (3·9 to 10·6)	9·9 (4·9 to 17·4)	25·4 (12 to 43·2)	36·6 (19·3 to 60·3)	34·2 (17·9 to 55)	33·8 (18·7 to 53·1)	30·9 (16·7 to 48·5)	30·5 (18·1 to 46·7)	25·3 (14·9 to 39·1)	27·4 (16·6 to 42·9)	31·9 (20·3 to 46·9)	31 (20·5 to 44·3)	29 (19·2 to 42)	28·9 (19·8 to 44·5)	29·1 (19·5 to 47·1)	36·6 (24·2 to 62·9)	47·7 (29·2 to 91·8)	29·9 (16·1 to 60·8)	14·7 (7·1 to 32·1)	2·6 (1·2 to 5·8)	546
Alzheimer's disease and other dementias	0 (0 to 0)	0 (0 to 0)	0 (0 to 0)	0 (0 to 0)	0 (0 to 0)	0 (0 to 0)	0 (0 to 0)	0 (0 to 0)	0 (0 to 0)	0 (0 to 0)	0 (0 to 0)	0·1 (0 to 0·4)	0·8 (0·2 to 2·2)	2·1 (0·7 to 5·6)	3·9 (1·5 to 9·7)	6·3 (2·4 to 15·8)	9·8 (3·7 to 24·9)	12·5 (5 to 30·4)	18·2 (7·4 to 43·6)	31 (12·6 to 75·2)	22·2 (8·3 to 53·4)	12·3 (4·5 to 29·7)	2·3 (0·9 to 5·5)	122
Parkinson's disease	0 (0 to 0)	0 (0 to 0)	0 (0 to 0)	0 (0 to 0)	0 (0 to 0)	0 (0 to 0)	0 (0 to 0)	0 (0 to 0)	0 (0 to 0·1)	0 (0 to 0·1)	0·1 (0·1 to 0·1)	0·2 (0·2 to 0·3)	0·5 (0·4 to 0·7)	1·3 (0·9 to 1·7)	2·1 (1·5 to 2·8)	3·2 (2·5 to 4·2)	5·4 (4·2 to 6·8)	8 (6·2 to 10)	11·7 (9·1 to 14·7)	12·1 (9·7 to 15·1)	5·8 (4·4 to 7·3)	1·8 (1·3 to 2·3)	0·2 (0·1 to 0·3)	52·5
Idiopathic epilepsy	0 (0 to 0)	0 (0 to 0·1)	1·5 (0·8 to 2·7)	5 (2·6 to 7·8)	6 (2·7 to 11)	10·4 (5·1 to 18·2)	15·8 (8·4 to 26·7)	16 (9·1 to 25·2)	16·8 (9·6 to 25·5)	14·5 (8·5 to 21·6)	15 (9·7 to 22·3)	11·3 (6·9 to 16·6)	11·8 (7·4 to 17·6)	12·8 (7·5 to 19·4)	11·4 (7·1 to 17·2)	8·7 (4·9 to 13·4)	6·5 (3·7 to 10·4)	4·5 (2·4 to 7·2)	3·9 (2·1 to 6·4)	3 (1·6 to 4·9)	1·3 (0·7 to 2·1)	0·5 (0·2 to 0·7)	0·1 (0 to 0·1)	177
Multiple sclerosis	0 (0 to 0)	0 (0 to 0)	0 (0 to 0)	0 (0 to 0)	0 (0 to 0·1)	0 (0 to 0)	0 (0 to 0·1)	0·1 (0 to 0·2)	0·1 (0·1 to 0·2)	0·2 (0·1 to 0·3)	0·3 (0·2 to 0·4)	0·3 (0·2 to 0·4)	0·3 (0·2 to 0·5)	0·4 (0·3 to 0·6)	0·4 (0·3 to 0·7)	0·3 (0·2 to 0·5)	0·2 (0·1 to 0·3)	0·1 (0·1 to 0·2)	0·1 (0 to 0·1)	0 (0 to 0·1)	0 (0 to 0)	0 (0 to 0)	0 (0 to 0)	2·9
Motor neuron disease	0 (0 to 0)	0 (0 to 0)	0·6 (0·1 to 1·4)	0·2 (0·1 to 0·5)	0 (0 to 0)	0·1 (0 to 0·1)	0·2 (0·1 to 0·4)	0·1 (0·1 to 0·3)	0·1 (0·1 to 0·3)	0·2 (0·1 to 0·4)	0·5 (0·2 to 0·8)	0·5 (0·2 to 1)	0·6 (0·3 to 1·1)	1·3 (0·5 to 2·7)	1·4 (0·7 to 2·4)	1·2 (0·7 to 2·1)	1·1 (0·6 to 2)	0·7 (0·4 to 1·2)	0·4 (0·2 to 0·7)	0·2 (0·1 to 0·3)	0·1 (0 to 0·1)	0 (0 to 0)	0 (0 to 0)	9·3
Headache disorders	0 (0 to 0)	0 (0 to 0)	0 (0 to 0)	0 (0 to 0)	1·9 (0 to 5)	10·5 (0·5 to 27·1)	15·2 (1·6 to 37·8)	14·6 (2·1 to 33·6)	14·2 (2·4 to 31·9)	14 (2·8 to 31)	12·6 (2·7 to 27)	11·1 (2·9 to 23·4)	11·5 (3·3 to 24·2)	11·6 (3·4 to 23·8)	9·4 (2·9 to 19·3)	7·3 (2·5 to 14·8)	4·5 (1·5 to 9)	2·4 (0·8 to 5)	1·6 (0·5 to 3·5)	1 (0·3 to 2·1)	0·4 (0·1 to 0·8)	0·1 (0 to 0·2)	0 (0 to 0)	144
Migraine	0 (0 to 0)	0 (0 to 0)	0 (0 to 0)	0 (0 to 0)	1·7 (0 to 4·8)	9·8 (0·3 to 26·5)	14 (1·1 to 36·4)	13·3 (1·3 to 32·1)	12·8 (1·6 to 30·2)	12·5 (1·9 to 28·2)	11·1 (1·9 to 25·4)	9·8 (2·1 to 21·8)	10 (2·4 to 22·5)	9·9 (2·5 to 21·8)	8·1 (2·1 to 17·7)	6·1 (1·7 to 13·2)	3·7 (1·1 to 7·8)	1·9 (0·6 to 4)	1·3 (0·4 to 2·8)	0·8 (0·2 to 1·7)	0·3 (0·1 to 0·6)	0·1 (0 to 0·2)	0 (0 to 0)	127
Tension-type headache	0 (0 to 0)	0 (0 to 0)	0 (0 to 0)	0 (0 to 0)	0·2 (0 to 1·4)	0·8 (0 to 6·1)	1·2 (0·1 to 6·7)	1·3 (0·2 to 6·6)	1·4 (0·3 to 6·5)	1·5 (0·3 to 6·5)	1·5 (0·3 to 6·4)	1·4 (0·3 to 5·1)	1·5 (0·4 to 5)	1·6 (0·4 to 5·8)	1·4 (0·4 to 4·2)	1·2 (0·3 to 3·8)	0·8 (0·2 to 2·8)	0·5 (0·1 to 1·9)	0·3 (0·1 to 1·3)	0·2 (0 to 0·8)	0·1 (0 to 0·3)	0 (0 to 0·1)	0 (0 to 0)	17
Other neurological disorders	0 (0 to 0)	0 (0 to 0)	1·6 (0·8 to 2·7)	1·7 (0·9 to 2·8)	2 (0·8 to 3·6)	4·4 (2·2 to 7·6)	5·4 (2·8 to 8·8)	3·3 (1·7 to 5·3)	2·5 (1·4 to 3·8)	2 (1·1 to 3·1)	2 (1·2 to 3)	1·8 (1·1 to 2·8)	1·9 (1·1 to 3·1)	2·5 (1·6 to 3·6)	2·4 (1·6 to 3·4)	1·9 (1·3 to 2·6)	1·4 (1 to 1·9)	1 (0·7 to 1·4)	0·7 (0·5 to 1·1)	0·5 (0·3 to 0·8)	0·2 (0·1 to 0·3)	0·1 (0 to 0·1)	0 (0 to 0)	39·2
Mental disorders	0 (0 to 0)	0 (0 to 0)	0·4 (0·3 to 0·6)	2·4 (1·6 to 3·4)	12·3 (8·1 to 17·6)	35 (23·5 to 49·7)	47·7 (32·9 to 65·8)	44·9 (31·5 to 61·4)	46·8 (32·9 to 63·8)	48·8 (34·5 to 65·7)	45·7 (32·4 to 60·9)	40·5 (29·1 to 55)	43·9 (31·5 to 58·6)	46·4 (34 to 62·1)	41·1 (29·8 to 54·9)	33·3 (23·9 to 44·1)	21·8 (15·7 to 28·7)	13·2 (9·5 to 17·2)	9·9 (7·1 to 13·1)	6·4 (4·6 to 8·5)	2·6 (1·8 to 3·4)	0·8 (0·5 to 1·1)	0·1 (0·1 to 0·2)	544
Schizophrenia	0 (0 to 0)	0 (0 to 0)	0 (0 to 0)	0 (0 to 0)	0 (0 to 0)	0·2 (0·1 to 0·4)	1·4 (0·5 to 2·9)	4·5 (2·2 to 7·7)	7·3 (4 to 11·2)	9 (5·5 to 13·7)	8·8 (5·5 to 12·6)	7·7 (4·9 to 10·9)	8 (5·2 to 10·9)	7·9 (5·2 to 10·9)	6·3 (4·2 to 8·8)	4·5 (2·9 to 6·4)	2·6 (1·6 to 3·8)	1·3 (0·8 to 2)	0·8 (0·5 to 1·3)	0·4 (0·2 to 0·7)	0·1 (0·1 to 0·2)	0 (0 to 0)	0 (0 to 0)	70·9
Depressive disorders	0 (0 to 0)	0 (0 to 0)	0 (0 to 0)	0 (0 to 0)	0·4 (0·2 to 0·7)	3·5 (1·8 to 6·1)	10·1 (5·8 to 16·1)	12·8 (7·3 to 19·9)	12·2 (7·3 to 18·6)	12·2 (7·3 to 19·2)	12·2 (7·5 to 18)	11·6 (7·1 to 17·8)	13·2 (8·4 to 19·8)	14·8 (9·6 to 21·9)	14 (8·9 to 21·3)	12 (7·6 to 18)	8·3 (5·4 to 11·9)	5·3 (3·5 to 7·6)	4·3 (2·7 to 6·3)	3 (1·9 to 4·5)	1·3 (0·9 to 1·9)	0·4 (0·3 to 0·7)	0·1 (0 to 0·1)	152
Major depressive disorder	0 (0 to 0)	0 (0 to 0)	0 (0 to 0)	0 (0 to 0)	0·3 (0·1 to 0·6)	3 (1·4 to 5·5)	8·6 (4·7 to 14)	10·4 (5·7 to 17)	9·2 (5·1 to 14·9)	9·1 (5·1 to 14·8)	9·2 (5·4 to 13·9)	8·8 (5·2 to 14·2)	10·2 (6·3 to 15·8)	11·6 (7·2 to 17·8)	11·1 (6·7 to 17)	9·6 (5·9 to 14·9)	6·7 (4·3 to 9·7)	4·4 (2·8 to 6·4)	3·6 (2·2 to 5·5)	2·5 (1·5 to 3·9)	1·1 (0·7 to 1·7)	0·4 (0·2 to 0·6)	0·1 (0 to 0·1)	120
Dysthymia	0 (0 to 0)	0 (0 to 0)	0 (0 to 0)	0 (0 to 0)	0·1 (0 to 0·2)	0·5 (0·2 to 1)	1·5 (0·7 to 2·7)	2·4 (1·1 to 4·2)	2·9 (1·5 to 5·1)	3·2 (1·8 to 5·2)	3 (1·7 to 5·1)	2·7 (1·4 to 4·7)	3 (1·6 to 5·2)	3·2 (1·8 to 5·5)	2·9 (1·6 to 4·8)	2·4 (1·2 to 4)	1·6 (0·8 to 2·6)	1 (0·5 to 1·6)	0·7 (0·4 to 1·2)	0·5 (0·2 to 0·8)	0·2 (0·1 to 0·3)	0·1 (0 to 0·1)	0 (0 to 0)	31·7
Bipolar disorder	0 (0 to 0)	0 (0 to 0)	0 (0 to 0)	0 (0 to 0)	0 (0 to 0)	1·8 (0·7 to 3·2)	6·7 (3 to 12·2)	7·7 (3·7 to 13·2)	7·4 (3·9 to 12·4)	7 (3·6 to 11·8)	6·3 (3·4 to 10·1)	5·7 (3 to 9·3)	6·2 (3·6 to 10·3)	6·4 (3·5 to 11)	5·4 (3 to 9·1)	4·1 (2·3 to 6·6)	2·4 (1·3 to 4)	1·3 (0·7 to 2·1)	0·8 (0·4 to 1·4)	0·4 (0·2 to 0·7)	0·1 (0·1 to 0·2)	0 (0 to 0·1)	0 (0 to 0)	69·7
Anxiety disorders	0 (0 to 0)	0 (0 to 0)	0 (0 to 0)	0·2 (0 to 0·4)	2·8 (1·3 to 4·9)	8·1 (4·3 to 13·3)	10·8 (6·3 to 16·9)	10·3 (5·7 to 16·9)	9·9 (5·5 to 16·4)	9·9 (5·7 to 16·1)	9·1 (5·3 to 14)	8·1 (4·5 to 13·2)	8·9 (4·8 to 14)	9·5 (5·7 to 14·8)	8·6 (5·3 to 13·2)	7·1 (4·2 to 11·4)	4·8 (2·8 to 7·7)	2·8 (1·7 to 4·5)	2·1 (1·2 to 3·4)	1·2 (0·7 to 2)	0·4 (0·2 to 0·7)	0·1 (0·1 to 0·2)	0 (0 to 0)	115
Eating disorders	0 (0 to 0)	0 (0 to 0)	0 (0 to 0)	0 (0 to 0)	0 (0 to 0·1)	0·5 (0·2 to 1)	1·4 (0·6 to 2·6)	1·6 (0·8 to 3)	1·7 (0·8 to 2·8)	1·8 (0·9 to 3·2)	1·3 (0·6 to 2·3)	0·5 (0·2 to 0·9)	0·2 (0·1 to 0·4)	0 (0 to 0)	0 (0 to 0)	0 (0 to 0)	0 (0 to 0)	0 (0 to 0)	0 (0 to 0)	0 (0 to 0)	0 (0 to 0)	0 (0 to 0)	0 (0 to 0)	9·1
Anorexia nervosa	0 (0 to 0)	0 (0 to 0)	0 (0 to 0)	0 (0 to 0)	0 (0 to 0·1)	0·2 (0·1 to 0·4)	0·5 (0·2 to 1)	0·4 (0·2 to 0·9)	0·3 (0·1 to 0·6)	0·2 (0 to 0·4)	0·1 (0 to 0·3)	0·1 (0 to 0·2)	0·1 (0 to 0·2)	0 (0 to 0)	0 (0 to 0)	0 (0 to 0)	0 (0 to 0)	0 (0 to 0)	0 (0 to 0)	0 (0 to 0)	0 (0 to 0)	0 (0 to 0)	0 (0 to 0)	1·9
Bulimia nervosa	0 (0 to 0)	0 (0 to 0)	0 (0 to 0)	0 (0 to 0)	0 (0 to 0)	0·3 (0·1 to 0·7)	0·9 (0·3 to 2)	1·2 (0·4 to 2·5)	1·4 (0·6 to 2·4)	1·6 (0·7 to 3)	1·1 (0·5 to 2·1)	0·4 (0·2 to 0·8)	0·1 (0 to 0·3)	0 (0 to 0)	0 (0 to 0)	0 (0 to 0)	0 (0 to 0)	0 (0 to 0)	0 (0 to 0)	0 (0 to 0)	0 (0 to 0)	0 (0 to 0)	0 (0 to 0)	7·2
Autism spectrum disorders	0 (0 to 0)	0 (0 to 0)	0·4 (0·2 to 0·6)	1·7 (1·1 to 2·5)	2·3 (1·5 to 3·4)	2·6 (1·6 to 3·9)	2·6 (1·7 to 3·9)	2·3 (1·4 to 3·5)	2·1 (1·3 to 3·2)	2 (1·2 to 3·1)	1·7 (1·1 to 2·6)	1·4 (0·9 to 2·2)	1·5 (0·9 to 2·2)	1·6 (1 to 2·3)	1·4 (0·9 to 2)	1·1 (0·7 to 1·7)	0·8 (0·5 to 1·1)	0·5 (0·3 to 0·7)	0·4 (0·2 to 0·5)	0·2 (0·2 to 0·4)	0·1 (0·1 to 0·2)	0 (0 to 0)	0 (0 to 0)	26·7
Attention-deficit/hyperactivity disorder	0 (0 to 0)	0 (0 to 0)	0 (0 to 0)	0·2 (0·1 to 0·4)	2·4 (1·3 to 4·3)	3·7 (2 to 6·5)	3·2 (1·8 to 5·8)	2·2 (1·2 to 3·8)	1·6 (0·9 to 2·8)	1·3 (0·7 to 2·2)	0·9 (0·5 to 1·6)	0·6 (0·3 to 1·2)	0·6 (0·3 to 1)	0·5 (0·2 to 0·9)	0·3 (0·1 to 0·6)	0·2 (0·1 to 0·3)	0·1 (0 to 0·1)	0 (0 to 0)	0 (0 to 0)	0 (0 to 0)	0 (0 to 0)	0 (0 to 0)	0 (0 to 0)	17·9
Conduct disorder	0 (0 to 0)	0 (0 to 0)	0 (0 to 0)	0 (0 to 0)	3·8 (1·9 to 6·7)	13·9 (7·7 to 22·5)	9·6 (5 to 15·5)	0 (0 to 0)	0 (0 to 0)	0 (0 to 0)	0 (0 to 0)	0 (0 to 0)	0 (0 to 0)	0 (0 to 0)	0 (0 to 0)	0 (0 to 0)	0 (0 to 0)	0 (0 to 0)	0 (0 to 0)	0 (0 to 0)	0 (0 to 0)	0 (0 to 0)	0 (0 to 0)	27·3
Idiopathic developmental intellectual disability	0 (0 to 0)	0 (0 to 0)	0 (0 to 0·1)	0·3 (0·1 to 0·6)	0·5 (0·2 to 1)	0·6 (0·2 to 1·1)	0·6 (0·2 to 1·1)	0·5 (0·2 to 1)	0·5 (0·1 to 0·9)	0·4 (0·1 to 0·8)	0·4 (0·1 to 0·7)	0·3 (0·1 to 0·6)	0·3 (0·1 to 0·6)	0·3 (0·1 to 0·6)	0·2 (0·1 to 0·5)	0·2 (0 to 0·4)	0·1 (0 to 0·2)	0·1 (0 to 0·1)	0 (0 to 0·1)	0 (0 to 0·1)	0 (0 to 0)	0 (0 to 0)	0 (0 to 0)	5·4
Other mental disorders	0 (0 to 0)	0 (0 to 0)	0 (0 to 0)	0 (0 to 0)	0 (0 to 0)	0·2 (0·1 to 0·3)	1·3 (0·6 to 2·3)	2·9 (1·4 to 4·9)	4·2 (2·2 to 6·9)	5·1 (3 to 8)	5·1 (3 to 7·9)	4·5 (2·7 to 7·2)	5 (3 to 7·9)	5·4 (3·3 to 8·3)	4·9 (2·9 to 7·3)	4 (2·5 to 6·1)	2·8 (1·8 to 4·2)	1·8 (1·1 to 2·8)	1·5 (0·9 to 2·2)	1·1 (0·7 to 1·5)	0·5 (0·3 to 0·7)	0·2 (0·1 to 0·2)	0 (0 to 0)	50·4
Substance use disorders	0 (0 to 0)	0 (0 to 0)	0 (0 to 0)	0 (0 to 0)	0 (0 to 0)	0·6 (0·3 to 1·1)	13 (8·5 to 18·9)	22 (15·3 to 31·3)	24·5 (18·1 to 33·1)	26·4 (19·3 to 35·6)	30·1 (21·8 to 40·6)	27 (19·3 to 36·2)	28 (19·9 to 37·9)	31·8 (21·9 to 44·9)	29·2 (19·9 to 40·9)	20·1 (14 to 28·3)	13·5 (9·1 to 19·5)	6·8 (4·5 to 9·5)	3·9 (2·7 to 5·3)	2 (1·4 to 2·8)	0·8 (0·5 to 1)	0·3 (0·2 to 0·4)	0·1 (0 to 0·1)	280
Alcohol use disorders	0 (0 to 0)	0 (0 to 0)	0 (0 to 0)	0 (0 to 0)	0 (0 to 0)	0·2 (0·1 to 0·4)	4 (2·3 to 6)	10·4 (6·3 to 16·4)	14·3 (9·7 to 20·6)	18·3 (12·7 to 25·5)	23·6 (16·5 to 32·7)	22·6 (15·7 to 31·2)	24·3 (16·8 to 33·8)	28·8 (19·3 to 41·2)	27·2 (18·3 to 39)	18·9 (13 to 26·9)	12·8 (8·5 to 18·7)	6·4 (4·2 to 9·1)	3·5 (2·5 to 4·9)	1·8 (1·2 to 2·5)	0·7 (0·5 to 0·9)	0·2 (0·1 to 0·3)	0 (0 to 0·1)	218
Drug use disorders	0 (0 to 0)	0 (0 to 0)	0 (0 to 0)	0 (0 to 0)	0 (0 to 0)	0·4 (0·2 to 0·7)	9·1 (5·5 to 14·2)	11·6 (7·7 to 17·2)	10·3 (7 to 14·5)	8·1 (5·6 to 11·4)	6·5 (4·5 to 9)	4·4 (3 to 6)	3·7 (2·5 to 5·1)	3 (2 to 4·3)	2 (1·3 to 2·9)	1·2 (0·7 to 1·9)	0·7 (0·4 to 1·1)	0·4 (0·2 to 0·7)	0·3 (0·2 to 0·5)	0·2 (0·1 to 0·4)	0·1 (0·1 to 0·2)	0 (0 to 0·1)	0 (0 to 0)	62·2
Opioid use disorders	0 (0 to 0)	0 (0 to 0)	0 (0 to 0)	0 (0 to 0)	0 (0 to 0)	0 (0 to 0)	0·7 (0·2 to 1·2)	2·4 (1·2 to 4)	2·8 (1·5 to 4·3)	2·4 (1·3 to 3·8)	1·9 (1·1 to 3)	1·4 (0·8 to 2·2)	1·2 (0·6 to 2)	1·1 (0·5 to 1·8)	0·7 (0·3 to 1·3)	0·5 (0·2 to 1)	0·3 (0·1 to 0·6)	0·2 (0·1 to 0·3)	0·1 (0·1 to 0·3)	0·1 (0·1 to 0·2)	0·1 (0 to 0·1)	0 (0 to 0)	0 (0 to 0)	15·8
Cocaine use disorders	0 (0 to 0)	0 (0 to 0)	0 (0 to 0)	0 (0 to 0)	0 (0 to 0)	0·1 (0 to 0·1)	3·2 (1·6 to 5·6)	3·5 (2 to 5·7)	3·3 (2 to 5·1)	2·8 (1·7 to 4·3)	2·6 (1·6 to 4)	1·7 (1·1 to 2·5)	1·4 (0·8 to 2·1)	1 (0·6 to 1·6)	0·7 (0·4 to 1·1)	0·4 (0·2 to 0·6)	0·2 (0·1 to 0·3)	0·1 (0·1 to 0·2)	0·1 (0 to 0·2)	0 (0 to 0·1)	0 (0 to 0)	0 (0 to 0)	0 (0 to 0)	21·1
Amphetamine use disorders	0 (0 to 0)	0 (0 to 0)	0 (0 to 0)	0 (0 to 0)	0 (0 to 0)	0 (0 to 0)	0·2 (0 to 0·5)	0·9 (0·3 to 1·7)	0·8 (0·3 to 1·5)	0·4 (0·1 to 0·9)	0·2 (0·1 to 0·5)	0·1 (0·1 to 0·2)	0·1 (0·1 to 0·2)	0·1 (0·1 to 0·2)	0·1 (0 to 0·1)	0 (0 to 0·1)	0 (0 to 0)	0 (0 to 0)	0 (0 to 0)	0 (0 to 0)	0 (0 to 0)	0 (0 to 0)	0 (0 to 0)	3
Cannabis use disorders	0 (0 to 0)	0 (0 to 0)	0 (0 to 0)	0 (0 to 0)	0 (0 to 0)	0·3 (0·1 to 0·6)	3 (1·3 to 5·5)	2·3 (1 to 4·2)	1·3 (0·6 to 2·5)	0·8 (0·3 to 1·5)	0·5 (0·2 to 1)	0·4 (0·2 to 0·7)	0·4 (0·1 to 0·7)	0·3 (0·1 to 0·6)	0·2 (0·1 to 0·4)	0·1 (0 to 0·2)	0 (0 to 0·1)	0 (0 to 0)	0 (0 to 0)	0 (0 to 0)	0 (0 to 0)	0 (0 to 0)	0 (0 to 0)	9·6
Other drug use disorders	0 (0 to 0)	0 (0 to 0)	0 (0 to 0)	0 (0 to 0)	0 (0 to 0)	0 (0 to 0·1)	2 (1 to 3·8)	2·5 (1·4 to 4·3)	2·1 (1·2 to 3·5)	1·6 (0·9 to 2·6)	1·2 (0·7 to 1·9)	0·8 (0·5 to 1·3)	0·7 (0·4 to 1·1)	0·6 (0·3 to 0·9)	0·4 (0·2 to 0·6)	0·2 (0·1 to 0·4)	0·1 (0·1 to 0·2)	0·1 (0 to 0·2)	0·1 (0 to 0·1)	0·1 (0 to 0·1)	0 (0 to 0)	0 (0 to 0)	0 (0 to 0)	12·6
Diabetes and kidney diseases	0 (0 to 0)	0 (0 to 0·1)	3·3 (1·8 to 5·5)	3·2 (2 to 4·9)	3·7 (2·6 to 5·1)	4·9 (3·4 to 6·6)	12·2 (8·9 to 16·8)	16·8 (12·2 to 22·8)	27·8 (20·7 to 36·5)	36·3 (27·7 to 46·8)	53·6 (40·3 to 69·9)	62 (47·4 to 80·3)	98·2 (75·3 to 126)	180 (137 to 225)	220 (170 to 278)	216 (168 to 275)	204 (163 to 255)	163 (128 to 203)	140 (113 to 174)	112 (88·3 to 137)	48·4 (38·5 to 60·5)	17·7 (14 to 21·8)	2·9 (2·1 to 3·8)	1,630
Diabetes mellitus	0 (0 to 0)	0 (0 to 0·1)	0·6 (0·2 to 1·2)	0·5 (0·2 to 1)	0·4 (0·2 to 0·6)	0·9 (0·5 to 1·5)	3·4 (2·3 to 4·8)	6·5 (4·3 to 9·3)	12·2 (8·8 to 16·7)	18 (12·9 to 24·1)	27·9 (20·2 to 37·9)	36·2 (26 to 48·3)	62·9 (44·6 to 85·2)	118 (85·2 to 153)	143 (105 to 186)	145 (110 to 188)	138 (105 to 177)	108 (82·5 to 141)	92·7 (70·7 to 118)	70·9 (53·4 to 91·3)	28·6 (21·8 to 37·1)	9·6 (7·2 to 12·3)	1·5 (1·1 to 2)	1,030
Diabetes mellitus type 1	0 (0 to 0)	0 (0 to 0·1)	0·6 (0·2 to 1·2)	0·5 (0·2 to 1)	0·4 (0·2 to 0·6)	0·9 (0·5 to 1·5)	1·4 (0·9 to 2·2)	2·3 (1·3 to 3·7)	4·2 (2·4 to 6·5)	3 (1·7 to 4·8)	4·4 (3·1 to 6·2)	4·9 (2·8 to 8·1)	2 (1·2 to 3·2)	3·6 (2·2 to 5·7)	4·4 (2·6 to 7·3)	3·7 (2·3 to 6)	3·7 (2·3 to 6)	2·3 (1·4 to 3·8)	1·9 (1·1 to 3·1)	1·1 (0·6 to 1·7)	0·5 (0·3 to 0·7)	0·2 (0·1 to 0·3)	0 (0 to 0·1)	46
Diabetes mellitus type 2	0 (0 to 0)	0 (0 to 0)	0 (0 to 0)	0 (0 to 0)	0 (0 to 0)	0 (0 to 0)	2 (1·3 to 2·8)	4·2 (2·7 to 6·1)	8 (5·4 to 11·3)	15·1 (10·5 to 20·6)	23·5 (16·6 to 32·5)	31·2 (21·8 to 42·3)	60·9 (43·2 to 82·6)	114 (82·6 to 149)	139 (102 to 181)	142 (107 to 182)	134 (102 to 172)	106 (80·7 to 137)	90·8 (69·4 to 116)	69·8 (52·4 to 89·9)	28·1 (21·5 to 36·5)	9·4 (7·1 to 12·1)	1·5 (1 to 2)	979
Chronic kidney disease	0 (0 to 0)	0 (0 to 0)	2·7 (1·4 to 4·7)	2·7 (1·6 to 4·1)	3·3 (2·2 to 4·6)	4 (2·7 to 5·5)	8·8 (5·9 to 12·9)	10·3 (6·6 to 15·4)	15·5 (10·4 to 22·5)	18·3 (12·5 to 25·9)	25·7 (17·4 to 36·8)	25·9 (17·8 to 36·3)	35·3 (24·5 to 49·6)	62 (42·1 to 87·7)	77·2 (52·7 to 109)	71 (48·6 to 101)	66·3 (46·5 to 90·9)	54·6 (37·4 to 75·7)	47·6 (34·3 to 65)	41·2 (30·2 to 56·2)	19·8 (14·4 to 26·7)	8·1 (5·8 to 11·1)	1·4 (0·9 to 2·1)	602
Chronic kidney disease due to diabetes mellitus type 1	0 (0 to 0)	0 (0 to 0)	0 (0 to 0)	0 (0 to 0·1)	0·1 (0 to 0·2)	0·1 (0 to 0·3)	0·3 (0·1 to 0·7)	0·5 (0·2 to 1·1)	1·6 (0·6 to 3·2)	2·9 (1·3 to 5·3)	5·1 (2·4 to 9·3)	4·8 (2·4 to 8·2)	5·4 (2·3 to 9·5)	7·2 (2·7 to 15)	6·1 (2 to 13·8)	4·1 (1·3 to 9·1)	3 (1·1 to 6·9)	1·9 (0·7 to 4·2)	1·1 (0·4 to 2·4)	0·5 (0·2 to 1·1)	0·1 (0 to 0·2)	0 (0 to 0)	0 (0 to 0)	44·8
Chronic kidney disease due to diabetes mellitus type 2	0 (0 to 0)	0 (0 to 0)	0 (0 to 0)	0 (0 to 0)	0 (0 to 0)	0 (0 to 0)	0·1 (0 to 0·4)	0·3 (0·1 to 0·6)	0·6 (0·2 to 1·3)	1·1 (0·4 to 2·2)	2·2 (0·9 to 4·4)	3·1 (1·4 to 5·8)	6·2 (3·2 to 10·5)	14·7 (7·9 to 23·9)	21·8 (12 to 34·3)	21·7 (12·5 to 33·9)	20·9 (12·3 to 31·5)	17·5 (10·6 to 26·8)	15·1 (9·6 to 22·3)	12·9 (7·9 to 19·5)	6 (3·7 to 9·2)	2·3 (1·3 to 3·7)	0·4 (0·2 to 0·7)	147
Chronic kidney disease due to hypertension	0 (0 to 0)	0 (0 to 0)	0 (0 to 0)	0 (0 to 0)	0 (0 to 0)	0 (0 to 0)	0·6 (0·3 to 1·2)	0·9 (0·3 to 1·7)	1·4 (0·7 to 2·5)	1·8 (0·9 to 3)	2·6 (1·4 to 4·3)	3·1 (1·8 to 5)	5 (3·1 to 7·8)	10·2 (6 to 16·3)	14·7 (8·6 to 23·3)	15·8 (9·4 to 25)	16·5 (9·9 to 25·9)	14·8 (9·1 to 22·7)	14·3 (9·5 to 21·1)	13·8 (9·1 to 20·2)	7·5 (4·9 to 11)	3·3 (2·2 to 4·9)	0·6 (0·4 to 1)	127
Chronic kidney disease due to glomerulonephritis	0 (0 to 0)	0 (0 to 0)	0·8 (0·3 to 1·6)	0·9 (0·5 to 1·6)	1·2 (0·7 to 2)	1·8 (1 to 2·8)	3·9 (2·2 to 6·2)	4·4 (2·4 to 7·2)	6·3 (3·8 to 9·9)	6·7 (4 to 10·3)	8·8 (5 to 14)	8·2 (4·6 to 12·9)	10·1 (5·9 to 15·6)	16·3 (9 to 27·8)	18·7 (9·4 to 31·8)	15 (7·5 to 25·3)	12·2 (5·7 to 21·8)	8·7 (4·2 to 15·3)	6·6 (3·2 to 12·1)	4·8 (2·1 to 8·9)	1·8 (0·8 to 3·4)	0·6 (0·3 to 1·2)	0·1 (0 to 0·2)	138
Chronic kidney disease due to other and unspecified causes	0 (0 to 0)	0 (0 to 0)	1·9 (1 to 3·5)	1·8 (1 to 2·9)	1·9 (1·2 to 3)	2·1 (1·2 to 3·3)	3·9 (2·2 to 6·1)	4·2 (2·4 to 6·8)	5·5 (3·1 to 8·7)	5·7 (3·3 to 9)	7 (3·9 to 11·7)	6·6 (3·7 to 11)	8·6 (4·9 to 13·7)	13·6 (7·3 to 22)	15·8 (8·3 to 27·3)	14·4 (7·7 to 24·1)	13·7 (7·6 to 23·4)	11·8 (6·7 to 19·7)	10·6 (6·1 to 17·7)	9·2 (5·3 to 14·8)	4·5 (2·6 to 7)	1·8 (1·1 to 2·8)	0·3 (0·2 to 0·5)	145
Acute glomerulonephritis	0 (0 to 0)	0 (0 to 0)	0 (0 to 0)	0 (0 to 0)	0 (0 to 0)	0 (0 to 0)	0 (0 to 0)	0 (0 to 0)	0 (0 to 0)	0 (0 to 0)	0 (0 to 0)	0 (0 to 0)	0 (0 to 0)	0 (0 to 0)	0 (0 to 0)	0 (0 to 0)	0 (0 to 0)	0 (0 to 0)	0 (0 to 0)	0 (0 to 0)	0 (0 to 0)	0 (0 to 0)	0 (0 to 0)	0·2
Skin and subcutaneous diseases	0 (0 to 0)	0 (0 to 0)	3·6 (1·7 to 7·1)	11·5 (7·3 to 17·1)	15·3 (9·5 to 22·4)	17·9 (11·6 to 26·5)	20·2 (13·1 to 30·2)	12·7 (8·4 to 18·9)	11·2 (7·5 to 16·1)	10·3 (7 to 14·5)	9·9 (6·9 to 14)	9·2 (6·3 to 13·1)	10·7 (7·3 to 15·4)	14·4 (9·8 to 20·4)	14·6 (10 to 20·9)	14 (9·8 to 20·2)	14 (9·5 to 19·4)	11·2 (7·4 to 16·4)	11·7 (7·7 to 17)	10·5 (6·8 to 16·3)	5·3 (3·4 to 7·8)	2·3 (1·4 to 3·6)	0·4 (0·2 to 0·6)	231
Dermatitis	0 (0 to 0)	0 (0 to 0)	0 (0 to 0)	3·5 (1·8 to 6·1)	4·7 (2·4 to 8)	3·6 (2 to 6·1)	2·9 (1·6 to 4·8)	2·3 (1·3 to 3·7)	2 (1·1 to 3·2)	1·9 (1·1 to 3)	1·6 (0·9 to 2·6)	1·4 (0·8 to 2·2)	1·6 (0·9 to 2·6)	1·8 (1 to 3)	1·8 (1 to 3)	1·7 (0·9 to 2·7)	1·3 (0·7 to 2·1)	0·9 (0·5 to 1·4)	0·7 (0·4 to 1·2)	0·5 (0·3 to 0·8)	0·2 (0·1 to 0·3)	0·1 (0 to 0·1)	0 (0 to 0)	34·4
Atopic dermatitis	0 (0 to 0)	0 (0 to 0)	0 (0 to 0)	3·5 (1·8 to 6)	4·6 (2·3 to 7·9)	3·2 (1·7 to 5·6)	2·2 (1·1 to 3·9)	1·5 (0·7 to 2·7)	1·1 (0·5 to 2)	1 (0·5 to 1·8)	0·8 (0·4 to 1·5)	0·7 (0·3 to 1·2)	0·8 (0·4 to 1·4)	0·8 (0·4 to 1·5)	0·8 (0·4 to 1·4)	0·7 (0·4 to 1·3)	0·5 (0·3 to 0·9)	0·3 (0·2 to 0·6)	0·3 (0·1 to 0·5)	0·2 (0·1 to 0·4)	0·1 (0 to 0·2)	0 (0 to 0)	0 (0 to 0)	23·2
Contact dermatitis	0 (0 to 0)	0 (0 to 0)	0 (0 to 0)	0 (0 to 0)	0·1 (0 to 0·1)	0·3 (0·1 to 0·7)	0·6 (0·2 to 1·2)	0·7 (0·3 to 1·3)	0·8 (0·3 to 1·4)	0·8 (0·3 to 1·6)	0·7 (0·3 to 1·5)	0·6 (0·3 to 1·2)	0·7 (0·3 to 1·4)	0·9 (0·4 to 1·8)	0·9 (0·4 to 1·8)	0·9 (0·4 to 1·6)	0·7 (0·3 to 1·3)	0·5 (0·2 to 0·9)	0·4 (0·2 to 0·7)	0·2 (0·1 to 0·4)	0·1 (0 to 0·2)	0 (0 to 0)	0 (0 to 0)	10
Seborrhoeic dermatitis	0 (0 to 0)	0 (0 to 0)	0 (0 to 0)	0 (0 to 0·1)	0 (0 to 0·1)	0·1 (0 to 0·1)	0·1 (0 to 0·2)	0·1 (0 to 0·2)	0·1 (0 to 0·2)	0·1 (0 to 0·2)	0·1 (0 to 0·2)	0·1 (0 to 0·1)	0·1 (0 to 0·2)	0·1 (0 to 0·2)	0·1 (0 to 0·1)	0·1 (0 to 0·1)	0·1 (0 to 0·1)	0·1 (0 to 0·1)	0·1 (0 to 0·1)	0 (0 to 0·1)	0 (0 to 0)	0 (0 to 0)	0 (0 to 0)	1·3
Psoriasis	0 (0 to 0)	0 (0 to 0)	0 (0 to 0)	0·1 (0·1 to 0·2)	0·4 (0·2 to 0·7)	0·6 (0·3 to 1)	0·7 (0·3 to 1·2)	0·7 (0·3 to 1·1)	0·7 (0·4 to 1·2)	0·8 (0·4 to 1·3)	0·8 (0·5 to 1·3)	0·8 (0·4 to 1·2)	0·9 (0·5 to 1·5)	1·1 (0·6 to 1·7)	1·1 (0·6 to 1·8)	1 (0·6 to 1·6)	0·7 (0·4 to 1)	0·4 (0·3 to 0·7)	0·3 (0·2 to 0·5)	0·2 (0·1 to 0·3)	0·1 (0 to 0·1)	0 (0 to 0)	0 (0 to 0)	11·6
Bacterial skin diseases	0 (0 to 0)	0 (0 to 0)	2·8 (1 to 6·3)	1 (0·4 to 2·1)	0·8 (0·4 to 1·4)	1·2 (0·6 to 2·1)	1·1 (0·5 to 1·9)	0·9 (0·4 to 1·6)	1·3 (0·5 to 2·5)	1·3 (0·6 to 2·5)	1·5 (0·6 to 2·8)	1·6 (0·7 to 3)	2 (0·9 to 3·5)	3·1 (1·5 to 5·9)	3·4 (1·5 to 6·4)	2·9 (1·2 to 5·7)	3·1 (1·3 to 5·7)	2·6 (0·9 to 5·2)	2·9 (1·1 to 6·1)	2·6 (1 to 5·6)	1·3 (0·5 to 2·6)	0·6 (0·2 to 1·3)	0·1 (0 to 0·2)	38·1
Cellulitis	0 (0 to 0)	0 (0 to 0)	0·7 (0·2 to 2·1)	0·3 (0·1 to 0·7)	0·2 (0·1 to 0·6)	0·4 (0·1 to 0·9)	0·4 (0·1 to 0·9)	0·3 (0·1 to 0·6)	0·4 (0·1 to 0·9)	0·4 (0·2 to 1)	0·5 (0·1 to 1·1)	0·4 (0·1 to 1)	0·5 (0·2 to 1·1)	0·9 (0·3 to 2)	0·8 (0·3 to 1·9)	0·7 (0·2 to 1·8)	0·6 (0·2 to 1·5)	0·6 (0·1 to 1·4)	0·5 (0·1 to 1·2)	0·4 (0·1 to 1·2)	0·2 (0·1 to 0·6)	0·1 (0 to 0·3)	0 (0 to 0·1)	9·3
Pyoderma	0 (0 to 0)	0 (0 to 0)	2·1 (0·6 to 5·1)	0·8 (0·3 to 1·7)	0·5 (0·2 to 1)	0·8 (0·3 to 1·6)	0·7 (0·3 to 1·3)	0·6 (0·2 to 1·1)	0·8 (0·3 to 1·7)	0·9 (0·4 to 1·8)	1 (0·4 to 2)	1·2 (0·5 to 2·3)	1·5 (0·6 to 2·7)	2·3 (1 to 4·4)	2·6 (1·1 to 5)	2·2 (0·9 to 4·5)	2·5 (1 to 4·8)	2 (0·7 to 4·2)	2·5 (0·9 to 5·1)	2·2 (0·8 to 4·7)	1·1 (0·4 to 2·2)	0·5 (0·2 to 1·1)	0·1 (0 to 0·2)	28·8
Scabies	0 (0 to 0)	0 (0 to 0)	0·3 (0·1 to 0·5)	1·9 (1 to 3·4)	2·8 (1·4 to 5)	3·2 (1·5 to 5·6)	3·5 (1·8 to 6)	2·8 (1·4 to 5)	2 (1 to 3·6)	1·5 (0·7 to 2·6)	1·3 (0·6 to 2·3)	1·3 (0·7 to 2·3)	1·5 (0·7 to 2·5)	1·5 (0·8 to 2·6)	1·2 (0·6 to 2·2)	1 (0·5 to 1·7)	0·7 (0·3 to 1·1)	0·4 (0·2 to 0·8)	0·4 (0·2 to 0·7)	0·3 (0·2 to 0·6)	0·2 (0·1 to 0·3)	0·1 (0 to 0·1)	0 (0 to 0)	27·7
Fungal skin diseases	0 (0 to 0)	0 (0 to 0)	0·1 (0 to 0·1)	0·4 (0·1 to 0·9)	0·7 (0·3 to 1·5)	0·9 (0·3 to 2)	0·9 (0·3 to 2·2)	0·9 (0·3 to 2)	0·9 (0·4 to 2·1)	0·9 (0·4 to 2·1)	0·9 (0·3 to 1·9)	0·8 (0·3 to 1·7)	0·9 (0·4 to 2)	1·1 (0·4 to 2·4)	1·2 (0·5 to 2·7)	1·3 (0·5 to 2·8)	1·2 (0·5 to 2·6)	1·1 (0·5 to 2·4)	1·2 (0·5 to 2·4)	1·1 (0·4 to 2·2)	0·6 (0·2 to 1·2)	0·2 (0·1 to 0·4)	0 (0 to 0·1)	17·3
Viral skin diseases	0 (0 to 0)	0 (0 to 0)	0 (0 to 0)	2·1 (1·3 to 3·2)	3·6 (2·3 to 5·6)	2·1 (1·3 to 3·2)	1·3 (0·8 to 2·1)	0·9 (0·6 to 1·5)	0·7 (0·4 to 1·1)	0·6 (0·3 to 0·9)	0·5 (0·3 to 0·7)	0·4 (0·2 to 0·6)	0·4 (0·2 to 0·7)	0·4 (0·3 to 0·7)	0·4 (0·2 to 0·6)	0·4 (0·2 to 0·6)	0·3 (0·1 to 0·4)	0·2 (0·1 to 0·2)	0·1 (0·1 to 0·2)	0·1 (0 to 0·1)	0 (0 to 0)	0 (0 to 0)	0 (0 to 0)	14·4
Acne vulgaris	0 (0 to 0)	0 (0 to 0)	0 (0 to 0)	0 (0 to 0)	0·2 (0·1 to 0·3)	4·2 (2·3 to 6·8)	7·5 (4·2 to 12·4)	1·7 (0·9 to 2·8)	0·7 (0·4 to 1·2)	0·5 (0·3 to 0·9)	0·4 (0·2 to 0·6)	0·2 (0·1 to 0·4)	0·2 (0·1 to 0·3)	0·1 (0·1 to 0·2)	0·1 (0 to 0·2)	0·1 (0 to 0·1)	0 (0 to 0·1)	0 (0 to 0)	0 (0 to 0)	0 (0 to 0)	0 (0 to 0)	0 (0 to 0)	0 (0 to 0)	15·9
Alopecia areata	0 (0 to 0)	0 (0 to 0)	0 (0 to 0)	0 (0 to 0·1)	0 (0 to 0·1)	0·1 (0 to 0·1)	0·1 (0 to 0·2)	0·2 (0·1 to 0·3)	0·2 (0·1 to 0·4)	0·2 (0·1 to 0·4)	0·2 (0·1 to 0·3)	0·1 (0·1 to 0·2)	0·1 (0·1 to 0·2)	0·1 (0·1 to 0·2)	0·1 (0·1 to 0·2)	0·1 (0 to 0·2)	0·1 (0 to 0·1)	0 (0 to 0·1)	0 (0 to 0)	0 (0 to 0)	0 (0 to 0)	0 (0 to 0)	0 (0 to 0)	1·8
Pruritus	0 (0 to 0)	0 (0 to 0)	0 (0 to 0)	0·1 (0 to 0·1)	0·1 (0 to 0·2)	0·1 (0 to 0·2)	0·1 (0·1 to 0·3)	0·2 (0·1 to 0·3)	0·2 (0·1 to 0·3)	0·2 (0·1 to 0·4)	0·2 (0·1 to 0·3)	0·2 (0·1 to 0·3)	0·2 (0·1 to 0·4)	0·2 (0·1 to 0·4)	0·2 (0·1 to 0·5)	0·2 (0·1 to 0·5)	0·2 (0·1 to 0·4)	0·2 (0·1 to 0·3)	0·1 (0·1 to 0·3)	0·1 (0 to 0·2)	0 (0 to 0·1)	0 (0 to 0)	0 (0 to 0)	2·8
Urticaria	0 (0 to 0)	0 (0 to 0)	0·2 (0·1 to 0·4)	1·6 (1 to 2·7)	1·6 (0·9 to 2·7)	1·2 (0·6 to 2)	1·1 (0·6 to 1·9)	0·9 (0·5 to 1·6)	0·8 (0·4 to 1·4)	0·8 (0·4 to 1·4)	0·8 (0·4 to 1·2)	0·6 (0·3 to 1·1)	0·6 (0·3 to 1·1)	0·7 (0·4 to 1·1)	0·6 (0·3 to 1)	0·5 (0·3 to 0·9)	0·4 (0·2 to 0·6)	0·2 (0·1 to 0·4)	0·2 (0·1 to 0·3)	0·1 (0·1 to 0·2)	0 (0 to 0·1)	0 (0 to 0)	0 (0 to 0)	13·2
Decubitus ulcer	0 (0 to 0)	0 (0 to 0)	0 (0 to 0)	0·1 (0 to 0·3)	0 (0 to 0·1)	0·2 (0 to 0·6)	0·3 (0·1 to 0·7)	0·5 (0·2 to 1·1)	0·9 (0·2 to 1·9)	0·6 (0·2 to 1·3)	0·8 (0·3 to 1·7)	0·8 (0·3 to 1·7)	1 (0·4 to 2·1)	2·3 (0·9 to 4·5)	2·4 (0·8 to 4·8)	2·9 (1·3 to 5·6)	4·2 (2 to 7·6)	3·8 (1·7 to 7)	4·6 (2·2 to 8·3)	4·7 (2·4 to 8·5)	2·6 (1·3 to 4·6)	1·2 (0·6 to 2·3)	0·2 (0·1 to 0·4)	33·9
Other skin and subcutaneous diseases	0 (0 to 0)	0 (0 to 0)	0·2 (0·1 to 0·4)	0·5 (0·2 to 1)	0·4 (0·2 to 0·8)	0·6 (0·3 to 1·2)	0·8 (0·3 to 1·4)	0·7 (0·3 to 1·4)	0·8 (0·4 to 1·5)	0·9 (0·4 to 1·8)	1 (0·5 to 1·8)	0·9 (0·5 to 1·8)	1·3 (0·6 to 2·4)	1·8 (0·9 to 3·2)	1·9 (0·9 to 3·6)	2·1 (1 to 3·7)	1·9 (1 to 3·4)	1·3 (0·7 to 2·3)	1·2 (0·6 to 2·1)	0·8 (0·4 to 1·4)	0·3 (0·2 to 0·5)	0·1 (0·1 to 0·2)	0 (0 to 0)	19·6
Sense organ diseases	0 (0 to 0)	0 (0 to 0)	0·1 (0·1 to 0·2)	1·1 (0·7 to 1·7)	2·6 (1·6 to 3·9)	3·6 (2·3 to 5·3)	4·6 (2·8 to 6·8)	5·4 (3·3 to 8·2)	6·9 (4·2 to 10·4)	8·8 (5·4 to 14)	9·8 (6·1 to 15·2)	11·2 (7 to 17·5)	18 (11·2 to 28·8)	27·5 (17·5 to 43·9)	34·1 (21·6 to 52·9)	37·8 (24·4 to 57·3)	34·8 (22·8 to 51·2)	29·3 (19·6 to 41·8)	30·7 (21·3 to 43·1)	26·5 (18·5 to 36·3)	13·1 (9·2 to 17·4)	4·7 (3·4 to 6·2)	0·8 (0·6 to 1·1)	311
Blindness and vision loss	0 (0 to 0)	0 (0 to 0)	0 (0 to 0)	0·3 (0·2 to 0·5)	0·7 (0·4 to 1·1)	0·9 (0·5 to 1·4)	1·1 (0·6 to 1·7)	1·2 (0·7 to 1·9)	1·4 (0·8 to 2·1)	1·7 (1 to 2·6)	2·1 (1·3 to 3·1)	2·6 (1·7 to 4·1)	4·2 (2·6 to 6·6)	6·4 (4 to 10·3)	8·4 (5·4 to 13·5)	9·9 (6·5 to 15·8)	10 (6·5 to 14·9)	9 (6 to 13)	9·9 (6·8 to 14·2)	8·7 (6 to 12·2)	4·5 (3·1 to 6·1)	1·7 (1·2 to 2·3)	0·3 (0·2 to 0·5)	85
Glaucoma	0 (0 to 0)	0 (0 to 0)	0 (0 to 0)	0 (0 to 0)	0 (0 to 0)	0 (0 to 0)	0 (0 to 0)	0 (0 to 0)	0 (0 to 0)	0 (0 to 0)	0 (0 to 0)	0 (0 to 0)	0 (0 to 0)	0·1 (0 to 0·2)	0·3 (0·1 to 0·5)	0·5 (0·3 to 0·8)	0·7 (0·4 to 1·1)	0·8 (0·5 to 1·2)	1·1 (0·6 to 1·7)	1·1 (0·7 to 1·7)	0·7 (0·4 to 1)	0·3 (0·2 to 0·4)	0·1 (0 to 0·1)	5·5
Cataract	0 (0 to 0)	0 (0 to 0)	0 (0 to 0)	0 (0 to 0)	0 (0 to 0)	0 (0 to 0)	0 (0 to 0)	0 (0 to 0)	0 (0 to 0·1)	0·1 (0 to 0·1)	0·1 (0·1 to 0·3)	0·2 (0·1 to 0·4)	0·4 (0·2 to 0·7)	0·6 (0·3 to 1·1)	0·9 (0·5 to 1·3)	1·1 (0·7 to 1·8)	1·4 (0·8 to 2·1)	1·7 (1·1 to 2·5)	2·4 (1·6 to 3·4)	2·5 (1·6 to 3·5)	1·4 (0·9 to 1·9)	0·5 (0·3 to 0·7)	0·1 (0·1 to 0·1)	13·4
Age-related macular degeneration	0 (0 to 0)	0 (0 to 0)	0 (0 to 0)	0 (0 to 0)	0 (0 to 0)	0 (0 to 0)	0 (0 to 0)	0 (0 to 0)	0 (0 to 0)	0 (0 to 0)	0 (0 to 0)	0 (0 to 0)	0 (0 to 0)	0 (0 to 0)	0·1 (0 to 0·1)	0·1 (0 to 0·2)	0·1 (0 to 0·2)	0·1 (0 to 0·1)	0·1 (0·1 to 0·2)	0·1 (0 to 0·1)	0 (0 to 0·1)	0 (0 to 0)	0 (0 to 0)	0·6
Refraction disorders	0 (0 to 0)	0 (0 to 0)	0 (0 to 0)	0·3 (0·1 to 0·4)	0·6 (0·3 to 1)	0·8 (0·4 to 1·2)	0·9 (0·5 to 1·5)	1 (0·5 to 1·6)	1 (0·6 to 1·6)	1·1 (0·6 to 1·8)	1·2 (0·7 to 1·8)	1·2 (0·7 to 1·8)	1·4 (0·9 to 2·2)	1·6 (1 to 2·5)	1·7 (1 to 2·6)	1·6 (0·9 to 2·4)	1·3 (0·8 to 2)	1·1 (0·6 to 1·6)	1·1 (0·7 to 1·6)	0·8 (0·5 to 1·2)	0·3 (0·2 to 0·5)	0·1 (0·1 to 0·1)	0 (0 to 0)	18·9
Near vision loss	0 (0 to 0)	0 (0 to 0)	0 (0 to 0)	0 (0 to 0)	0 (0 to 0)	0 (0 to 0)	0 (0 to 0)	0 (0 to 0·1)	0·1 (0 to 0·2)	0·2 (0·1 to 0·4)	0·4 (0·1 to 0·8)	0·7 (0·3 to 1·5)	1·3 (0·5 to 2·9)	2·4 (1 to 5)	3·2 (1·3 to 7)	3·7 (1·5 to 7·8)	3·3 (1·3 to 6·6)	2·6 (1·1 to 5·3)	2·5 (1 to 5·1)	1·9 (0·8 to 3·9)	0·9 (0·4 to 1·7)	0·3 (0·1 to 0·6)	0 (0 to 0·1)	23·5
Other vision loss	0 (0 to 0)	0 (0 to 0)	0 (0 to 0)	0 (0 to 0·1)	0·1 (0 to 0·2)	0·1 (0·1 to 0·2)	0·2 (0·1 to 0·3)	0·2 (0·1 to 0·3)	0·2 (0·1 to 0·4)	0·3 (0·1 to 0·5)	0·4 (0·2 to 0·7)	0·6 (0·3 to 1)	1 (0·5 to 1·6)	1·7 (1 to 2·7)	2·3 (1·4 to 3·5)	3 (2 to 4·3)	3·2 (2·1 to 4·6)	2·8 (1·9 to 4·1)	2·7 (1·7 to 4)	2·3 (1·5 to 3·4)	1·2 (0·8 to 1·8)	0·5 (0·4 to 0·8)	0·1 (0·1 to 0·2)	23·1
Age-related and other hearing loss	0 (0 to 0)	0 (0 to 0)	0 (0 to 0·1)	0·6 (0·3 to 1)	1·6 (0·9 to 2·6)	2·4 (1·4 to 3·8)	3·1 (1·8 to 4·8)	3·8 (2·2 to 5·9)	5·1 (3 to 8)	6·5 (3·8 to 10·7)	7·1 (4·2 to 11·6)	8 (4·7 to 12·8)	13·1 (7·8 to 21·5)	19·9 (12·1 to 32)	24·4 (14·9 to 38·4)	26·4 (16·2 to 41·6)	23·4 (14·7 to 35·4)	19·1 (12·5 to 28)	19·7 (13·2 to 27·9)	17 (11·5 to 23·5)	8·4 (5·7 to 11·4)	2·9 (2·1 to 4)	0·4 (0·3 to 0·6)	213
Other sense organ diseases	0 (0 to 0)	0 (0 to 0)	0 (0 to 0·1)	0·2 (0·1 to 0·4)	0·3 (0·1 to 0·5)	0·3 (0·1 to 0·6)	0·4 (0·2 to 0·7)	0·4 (0·1 to 0·7)	0·4 (0·2 to 0·8)	0·6 (0·2 to 1·2)	0·6 (0·3 to 1·2)	0·6 (0·3 to 1·2)	0·8 (0·3 to 1·5)	1·2 (0·5 to 2·3)	1·3 (0·6 to 2·5)	1·5 (0·7 to 2·8)	1·4 (0·7 to 2·7)	1·1 (0·5 to 2·1)	1·1 (0·5 to 2)	0·8 (0·4 to 1·5)	0·3 (0·1 to 0·5)	0·1 (0 to 0·1)	0 (0 to 0)	13·5
Musculoskeletal disorders	0 (0 to 0)	0 (0 to 0)	0 (0 to 0)	0 (0 to 0)	1·2 (0·5 to 2·1)	6·4 (3·9 to 9·8)	14·3 (9·1 to 21·8)	20 (12·8 to 29·3)	28 (17·8 to 40·4)	35·5 (22·5 to 50·1)	38·7 (26·7 to 54)	39·5 (26·8 to 55·2)	49·8 (33·7 to 71·1)	61·4 (41·8 to 86·6)	63·5 (42·8 to 89·8)	59·6 (40·2 to 85·5)	44·8 (30·3 to 63·1)	30·5 (20·3 to 43·3)	24·8 (16·8 to 35·8)	17 (11·4 to 24·1)	7·5 (5·3 to 10·6)	2·2 (1·6 to 3·1)	0·3 (0·2 to 0·5)	545
Rheumatoid arthritis	0 (0 to 0)	0 (0 to 0)	0 (0 to 0)	0 (0 to 0)	0 (0 to 0)	0·1 (0 to 0·1)	0·2 (0·1 to 0·3)	0·2 (0·1 to 0·4)	0·4 (0·2 to 0·6)	0·5 (0·2 to 0·8)	0·6 (0·3 to 1·1)	0·6 (0·3 to 1)	0·8 (0·4 to 1·3)	1 (0·6 to 1·6)	1·1 (0·7 to 1·6)	0·9 (0·6 to 1·4)	0·8 (0·5 to 1·1)	0·6 (0·4 to 0·8)	0·6 (0·4 to 0·8)	0·6 (0·4 to 0·8)	0·9 (0·5 to 1·4)	0·3 (0·2 to 0·5)	0 (0 to 0·1)	10·1
Osteoarthritis	0 (0 to 0)	0 (0 to 0)	0 (0 to 0)	0 (0 to 0)	0 (0 to 0)	0 (0 to 0)	0 (0 to 0)	0 (0 to 0)	0 (0 to 0)	0·2 (0·1 to 0·4)	1 (0·5 to 2·2)	2·5 (1·2 to 5·2)	5·5 (2·6 to 11·4)	9·6 (4·7 to 19·7)	12·4 (6 to 25·1)	13·5 (6·8 to 27)	11·1 (5·5 to 22·3)	8·1 (4 to 16)	7·1 (3·5 to 14)	5·1 (2·6 to 10·2)	2·1 (1·1 to 4·2)	0·7 (0·3 to 1·3)	0·1 (0·1 to 0·2)	79·1
Osteoarthritis hip	0 (0 to 0)	0 (0 to 0)	0 (0 to 0)	0 (0 to 0)	0 (0 to 0)	0 (0 to 0)	0 (0 to 0)	0 (0 to 0)	0 (0 to 0)	0·1 (0 to 0·2)	0·1 (0 to 0·2)	0·2 (0·1 to 0·3)	0·3 (0·1 to 0·7)	0·5 (0·2 to 1·2)	0·7 (0·3 to 1·6)	0·8 (0·4 to 1·8)	0·7 (0·3 to 1·6)	0·6 (0·2 to 1·1)	0·5 (0·2 to 1)	0·3 (0·2 to 0·7)	0·1 (0·1 to 0·3)	0 (0 to 0·1)	0 (0 to 0)	5
Osteoarthritis knee	0 (0 to 0)	0 (0 to 0)	0 (0 to 0)	0 (0 to 0)	0 (0 to 0)	0 (0 to 0)	0 (0 to 0)	0 (0 to 0)	0 (0 to 0)	0·1 (0 to 0·2)	0·8 (0·3 to 1·6)	1·9 (0·9 to 3·9)	4·1 (1·9 to 8·5)	7 (3·3 to 14·3)	8·9 (4·2 to 18·6)	9·6 (4·7 to 19·6)	7·8 (3·8 to 15·9)	5·6 (2·7 to 11·4)	4·8 (2·4 to 9·7)	3·3 (1·6 to 6·8)	1·3 (0·7 to 2·7)	0·4 (0·2 to 0·8)	0·1 (0 to 0·1)	55·6
Osteoarthritis hand	0 (0 to 0)	0 (0 to 0)	0 (0 to 0)	0 (0 to 0)	0 (0 to 0)	0 (0 to 0)	0 (0 to 0)	0 (0 to 0)	0 (0 to 0)	0 (0 to 0)	0·1 (0 to 0·2)	0·2 (0·1 to 0·5)	0·5 (0·2 to 1·1)	0·8 (0·3 to 1·8)	1·1 (0·5 to 2·4)	1·2 (0·5 to 2·5)	1 (0·4 to 2)	0·7 (0·3 to 1·5)	0·6 (0·3 to 1·3)	0·5 (0·2 to 1)	0·2 (0·1 to 0·5)	0·1 (0 to 0·2)	0 (0 to 0)	7

Osteoarthritis other	0 (0 to 0)	0 (0 to 0)	0 (0 to 0)	0 (0 to 0)	0 (0 to 0)	0 (0 to 0)	0 (0 to 0)	0 (0 to 0)	0 (0 to 0)	0 (0 to 0)	0·1 (0 to 0·3)
Low back pain	0 (0 to 0)	0 (0 to 0)	0 (0 to 0)	0 (0 to 0)	1 (0·4 to 1·9)	5·7 (3·3 to 9·1)	11 (6·3 to 17·8)	12·9 (7·4 to 20·8)	15 (8·8 to 23·6)	17 (9·7 to 26·3)	17·9 (10·6 to 28·1)
Neck pain	0 (0 to 0)	0 (0 to 0)	0 (0 to 0)	0 (0 to 0)	0·1 (0 to 0·2)	0·5 (0·2 to 0·9)	1·1 (0·4 to 1·9)	1·5 (0·6 to 3)	2·4 (1 to 4·6)	3·7 (1·7 to 7·1)	4·5 (2·2 to 8·1)
Gout	0 (0 to 0)	0 (0 to 0)	0 (0 to 0)	0 (0 to 0)	0 (0 to 0)	0 (0 to 0)	0 (0 to 0)	0 (0 to 0·1)	0·1 (0 to 0·3)	0·2 (0·1 to 0·4)	0·3 (0·1 to 0·5)
Other musculoskeletal disorders	0 (0 to 0)	0 (0 to 0)	0 (0 to 0)	0 (0 to 0)	0 (0 to 0·1)	0·2 (0·1 to 0·4)	2·1 (0·9 to 3·8)	5·3 (3 to 8·2)	10·1 (5·9 to 16·3)	13·9 (8·5 to 22)	14·4 (8·7 to 22·2)
Other non-communicable diseases	56·5 (31·5 to 92·2)	22·4 (13·5 to 35·1)	74·4 (44·4 to 114)	29·6 (20·4 to 41·7)	15·9 (12·5 to 20)	17·5 (13·3 to 22·9)	19 (14·3 to 25·2)	18·2 (13·6 to 24·3)	21·8 (16·1 to 29·2)	23·8 (17·1 to 32·4)	26 (18·8 to 35·9)
Congenital birth defects	54·1 (29·7 to 89·9)	19·9 (11·7 to 31·8)	49·4 (26·7 to 80·7)	16·6 (10·4 to 25·9)	7·1 (5·1 to 9·6)	7·7 (5·4 to 10·6)	7·5 (4·9 to 11·1)	5·3 (3·5 to 7·9)	4·7 (3·2 to 7)	3·7 (2·6 to 5·3)	3·4 (2·4 to 4·8)
Neural tube defects	3·5 (0·9 to 9·1)	0·8 (0·2 to 1·7)	1·8 (0·5 to 4·7)	1·8 (0·3 to 6)	0·2 (0·1 to 0·4)	0·4 (0·2 to 0·7)	0·4 (0·2 to 0·7)	0·2 (0·1 to 0·4)	0·2 (0·1 to 0·4)	0·1 (0·1 to 0·2)	0·1 (0 to 0·1)
Congenital heart anomalies	15·9 (7·3 to 29·3)	7·7 (4 to 14)	19 (8·7 to 35·4)	5·1 (2·5 to 9·1)	1·8 (1 to 3)	1·9 (1 to 3·2)	1·9 (0·9 to 3·4)	1·3 (0·6 to 2·3)	1·2 (0·6 to 2)	1 (0·6 to 1·6)	0·9 (0·6 to 1·5)
Orofacial clefts	0·4 (0·1 to 1)	0·2 (0·1 to 0·6)	0·5 (0·1 to 1·9)	0·1 (0·1 to 0·2)	0·1 (0 to 0·2)	0·1 (0 to 0·2)	0·1 (0 to 0·2)	0·1 (0 to 0·2)	0·1 (0 to 0·2)	0·1 (0 to 0·2)	0·1 (0 to 0·2)
Down syndrome	0·4 (0·1 to 0·9)	0·2 (0·1 to 0·4)	1·4 (0·5 to 3·3)	0·7 (0·3 to 1·6)	0·3 (0·2 to 0·4)	0·4 (0·2 to 0·6)	0·6 (0·3 to 1·1)	0·4 (0·2 to 0·7)	0·3 (0·2 to 0·6)	0·4 (0·2 to 0·7)	0·4 (0·2 to 0·7)
Turner syndrome	0 (0 to 0)	0 (0 to 0)	0 (0 to 0)	0 (0 to 0)	0 (0 to 0)	0 (0 to 0)	0 (0 to 0)	0 (0 to 0)	0 (0 to 0)	0 (0 to 0)	0 (0 to 0)
Klinefelter syndrome	0 (0 to 0)	0 (0 to 0)	0 (0 to 0)	0 (0 to 0)	0 (0 to 0)	0 (0 to 0)	0 (0 to 0)	0 (0 to 0)	0 (0 to 0)	0 (0 to 0)	0 (0 to 0)
Other chromosomal abnormalities	5·5 (2 to 12·1)	2·1 (0·8 to 4·3)	4·6 (1·8 to 9·9)	1·2 (0·6 to 2·4)	0·5 (0·3 to 0·9)	0·6 (0·3 to 1·1)	0·8 (0·4 to 1·5)	0·4 (0·2 to 0·6)	0·5 (0·2 to 0·9)	0·2 (0·1 to 0·4)	0·3 (0·1 to 0·6)
Congenital musculoskeletal and limb anomalies	3·7 (1·5 to 8·1)	1·4 (0·6 to 2·8)	4 (1·8 to 8·2)	2·2 (1·3 to 3·7)	1·8 (1·1 to 2·8)	1·9 (1·1 to 2·9)	1·5 (0·9 to 2·3)	1 (0·6 to 1·5)	1 (0·6 to 1·5)	0·6 (0·4 to 1)	0·7 (0·4 to 1·1)
Urogenital congenital anomalies	0·4 (0·1 to 0·8)	0·1 (0·1 to 0·2)	0·2 (0·1 to 0·4)	0·2 (0·1 to 0·3)	0·1 (0·1 to 0·2)	0·1 (0·1 to 0·2)	0·1 (0·1 to 0·1)	0·1 (0 to 0·1)	0·1 (0 to 0·1)	0 (0 to 0·1)	0 (0 to 0·1)
Digestive congenital anomalies	6·9 (2·9 to 13·5)	2·9 (1·3 to 5·4)	5·2 (2·1 to 10·6)	0·9 (0·5 to 1·8)	0·3 (0·2 to 0·5)	0·2 (0·1 to 0·3)	0·1 (0·1 to 0·3)	0 (0 to 0·1)	0·1 (0 to 0·1)	0 (0 to 0·1)	0 (0 to 0)
Other congenital birth defects	17·5 (8 to 32)	4·4 (2·1 to 7·9)	12·6 (5·3 to 24)	4·3 (2 to 7·4)	1·9 (1·1 to 3·1)	2·1 (1·2 to 3·4)	2·1 (1·1 to 3·6)	1·9 (1 to 3·3)	1·4 (0·7 to 2·3)	1·1 (0·7 to 1·8)	0·9 (0·5 to 1·4)
Urinary diseases and male infertility	0·1 (0 to 0·1)	0·1 (0 to 0·2)	0·6 (0·3 to 1·3)	0·2 (0·1 to 0·4)	0·1 (0·1 to 0·2)	0·2 (0·1 to 0·3)	0·3 (0·2 to 0·4)	0·9 (0·5 to 1·7)	1·1 (0·6 to 2)	1·4 (0·8 to 2·6)	1·7 (1 to 2·9)
Urolithiasis	0 (0 to 0)	0 (0 to 0)	0 (0 to 0)	0 (0 to 0)	0 (0 to 0)	0 (0 to 0)	0 (0 to 0)	0 (0 to 0·1)	0·1 (0 to 0·1)	0·1 (0 to 0·2)	0·2 (0 to 0·3)
Benign prostatic hyperplasia	0 (0 to 0)	0 (0 to 0)	0 (0 to 0)	0 (0 to 0)	0 (0 to 0)	0 (0 to 0)	0 (0 to 0)	0 (0 to 0)	0 (0 to 0)	0 (0 to 0)	0 (0 to 0)
Male infertility	0 (0 to 0)	0 (0 to 0)	0 (0 to 0)	0 (0 to 0)	0 (0 to 0)	0 (0 to 0)	0 (0 to 0)	0·5 (0·1 to 1·3)	0·6 (0·2 to 1·5)	0·7 (0·2 to 1·8)	0·6 (0·2 to 1·5)
Other urinary diseases	0 (0 to 0·1)	0·1 (0 to 0·2)	0·1 (0 to 0·3)	0·1 (0 to 0·1)	0·1 (0 to 0·1)	0·1 (0 to 0·2)	0·1 (0·1 to 0·2)	0·2 (0·1 to 0·4)	0·1 (0·1 to 0·3)	0·2 (0·1 to 0·4)	0·4 (0·2 to 0·8)
Gynecological diseases	0 (0 to 0)	0 (0 to 0)	0 (0 to 0)	0 (0 to 0)	0 (0 to 0)	0 (0 to 0)	0 (0 to 0)	0 (0 to 0)	0 (0 to 0)	0 (0 to 0)	0 (0 to 0)
Uterine fibroids	0 (0 to 0)	0 (0 to 0)	0 (0 to 0)	0 (0 to 0)	0 (0 to 0)	0 (0 to 0)	0 (0 to 0)	0 (0 to 0)	0 (0 to 0)	0 (0 to 0)	0 (0 to 0)
Polycystic ovarian syndrome	--	--	--	--	--	--	--	--	--	--	--
Female infertility	--	--	--	--	--	--	--	--	--	--	--
Endometriosis	--	--	--	--	--	--	--	--	--	--	--
Genital prolapse	0 (0 to 0)	0 (0 to 0)	0 (0 to 0)	0 (0 to 0)	0 (0 to 0)	0 (0 to 0)	0 (0 to 0)	0 (0 to 0)	0 (0 to 0)	0 (0 to 0)	0 (0 to 0)
Premenstrual syndrome	--	--	--	--	--	--	--	--	--	--	--
Other gynecological diseases	0 (0 to 0)	0 (0 to 0)	0 (0 to 0)	0 (0 to 0)	0 (0 to 0)	0 (0 to 0)	0 (0 to 0)	0 (0 to 0)	0 (0 to 0)	0 (0 to 0)	0 (0 to 0)
Hemoglobinopathies and hemolytic anemias	0·2 (0·1 to 0·3)	0·2 (0·1 to 0·3)	2 (1 to 3·4)	3·8 (2·3 to 6·2)	4 (2·5 to 6)	4·4 (2·6 to 6·7)	5·2 (3·3 to 7·8)	4·5 (2·8 to 7·1)	4·3 (2·5 to 6·7)	3·7 (2·2 to 6)	3·6 (2·1 to 5·7)
Thalassemias	0 (0 to 0)	0 (0 to 0)	0 (0 to 0)	0 (0 to 0·1)	0 (0 to 0)	0 (0 to 0)	0 (0 to 0)	0 (0 to 0)	0 (0 to 0)	0 (0 to 0)	0 (0 to 0)
Thalassemias trait	0 (0 to 0)	0 (0 to 0)	0 (0 to 0)	0 (0 to 0·1)	0·1 (0 to 0·2)	0 (0 to 0·1)	0 (0 to 0)	0 (0 to 0)	0 (0 to 0)	0 (0 to 0·1)	0 (0 to 0)
Sickle cell disorders	0·2 (0·1 to 0·3)	0·2 (0·1 to 0·3)	1·7 (0·8 to 3·1)	2·9 (1·5 to 5)	2·8 (1·8 to 4·2)	3·6 (2·1 to 5·8)	3·9 (2·4 to 6·3)	3 (1·6 to 4·9)	2·4 (1·2 to 3·9)	1·8 (0·9 to 3·1)	1·7 (0·8 to 3)
Sickle cell trait	0 (0 to 0)	0 (0 to 0·1)	0·1 (0·1 to 0·2)	0·4 (0·2 to 0·8)	0·5 (0·1 to 1·2)	0·2 (0 to 0·7)	0·2 (0 to 0·4)	0·1 (0 to 0·4)	0·2 (0 to 0·4)	0·2 (0 to 0·5)	0·2 (0 to 0·4)
G6PD deficiency	0 (0 to 0)	0 (0 to 0)	0 (0 to 0·1)	0·2 (0·1 to 0·5)	0·3 (0·1 to 0·6)	0·4 (0·1 to 0·8)	0·7 (0·3 to 1·5)	1·1 (0·4 to 2)	1·4 (0·6 to 2·5)	1·3 (0·5 to 2·5)	1·4 (0·6 to 2·5)
G6PD trait	0 (0 to 0)	0 (0 to 0)	0 (0 to 0)	0 (0 to 0)	0 (0 to 0)	0 (0 to 0)	0 (0 to 0)	0 (0 to 0)	0 (0 to 0)	0 (0 to 0)	0 (0 to 0)
Other hemoglobinopathies and hemolytic anemias	0 (0 to 0)	0 (0 to 0)	0·1 (0 to 0·1)	0·2 (0·1 to 0·4)	0·3 (0·1 to 0·7)	0·1 (0 to 0·4)	0·3 (0·1 to 0·8)	0·3 (0·1 to 0·8)	0·4 (0·1 to 0·8)	0·4 (0·2 to 0·9)	0·4 (0·2 to 0·8)
Endocrine, metabolic, blood, and immune disorders	2·1 (0·9 to 4·2)	1·6 (0·7 to 3·1)	16·5 (7·7 to 30·6)	8 (4·2 to 13·9)	3·4 (2·1 to 5·2)	3·6 (2·1 to 6)	3·4 (2·1 to 5·4)	3·7 (2·2 to 6)	6·3 (3·7 to 10·6)	8 (4·9 to 12·7)	9·7 (5·9 to 15·8)
Oral disorders	0 (0 to 0)	0 (0 to 0)	0 (0 to 0)	1 (0·6 to 1·7)	1·3 (0·7 to 2·3)	1·7 (0·9 to 2·9)	2·6 (1·4 to 4·5)	3·8 (2 to 7·2)	5·4 (2·6 to 10·5)	7 (3·3 to 13)	7·6 (3·5 to 14·5)
Caries of deciduous teeth	0 (0 to 0)	0 (0 to 0)	0 (0 to 0)	0·4 (0·1 to 0·7)	0·4 (0·2 to 0·8)	0 (0 to 0·1)	0 (0 to 0)	0 (0 to 0)	0 (0 to 0)	0 (0 to 0)	0 (0 to 0)
Caries of permanent teeth	0 (0 to 0)	0 (0 to 0)	0 (0 to 0)	0 (0 to 0)	0·3 (0·1 to 0·7)	0·8 (0·3 to 1·8)	0·9 (0·4 to 2)	1 (0·4 to 2)	0·9 (0·4 to 1·9)	0·8 (0·3 to 1·8)	0·8 (0·3 to 1·6)
Periodontal diseases	0 (0 to 0)	0 (0 to 0)	0 (0 to 0)	0 (0 to 0)	0 (0 to 0)	0 (0 to 0)	0·4 (0·1 to 1)	1·3 (0·4 to 3·3)	2·6 (0·8 to 6·4)	4 (1·4 to 9)	4·5 (1·6 to 10·2)
Other oral disorders	0 (0 to 0)	0 (0 to 0)	0 (0 to 0)	0·7 (0·4 to 1)	0·7 (0·4 to 1·1)	0·8 (0·5 to 1·3)	1·3 (0·8 to 2)	1·4 (0·9 to 2·2)	1·5 (0·9 to 2·3)	1·6 (0·9 to 2·4)	1·4 (0·8 to 2·2)
Sudden infant death syndrome	--	0·6 (0·2 to 1·3)	5·9 (1·7 to 13·8)	--	--	--	--	--	--	--	--
Injuries	4·1 (2·4 to 6·6)	2·2 (1·3 to 3·6)	19·4 (11 to 31·8)	37·4 (25·3 to 54·5)	34·9 (27·5 to 42·7)	62·5 (49·8 to 76)	161 (128 to 201)	197 (159 to 245)	215 (173 to 258)	175 (140 to 213)	161 (129 to 200)
Transport injuries	0·3 (0·1 to 0·6)	0·1 (0 to 0·3)	1·6 (0·7 to 3)	6·1 (3·1 to 10·6)	9·2 (5·9 to 13·3)	18·3 (12·1 to 26·2)	50·6 (33·5 to 72·7)	61·2 (40·8 to 87·1)	65·4 (44·3 to 88·3)	54·3 (38 to 73·3)	51 (36·4 to 69·6)
Road injuries	0·2 (0·1 to 0·5)	0·1 (0 to 0·3)	1·4 (0·6 to 2·8)	5·8 (2·9 to 10·1)	8·7 (5·6 to 12·8)	17·4 (11·4 to 25·1)	48·7 (32 to 70·1)	58·9 (39·4 to 83·4)	62·8 (42·2 to 85·3)	52 (36·8 to 69·4)	48·8 (34·8 to 66·7)
Pedestrian road injuries	0·1 (0 to 0·3)	0 (0 to 0·1)	0·4 (0·1 to 0·9)	2·5 (1·1 to 4·6)	3·8 (2·1 to 6·1)	5·8 (3 to 9·7)	5·2 (2·6 to 8·8)	5·1 (2·6 to 8·6)	6·3 (3·5 to 10·3)	6·4 (3·8 to 10·1)	7·1 (4·2 to 10·9)
Cyclist road injuries	0 (0 to 0)	0 (0 to 0)	0 (0 to 0)	0·2 (0·1 to 0·5)	0·5 (0·3 to 0·8)	2·2 (1·3 to 3·8)	3·9 (2·3 to 6·4)	3·4 (2·2 to 5·1)	3·5 (2·3 to 5·2)	3·5 (2·4 to 5)	3·5 (2·4 to 4·9)
Motorcyclist road injuries	0 (0 to 0·1)	0 (0 to 0)	0·3 (0·1 to 0·6)	0·7 (0·3 to 1·5)	1·1 (0·5 to 2)	2·3 (1·1 to 4·4)	16·9 (9·5 to 28·6)	23·5 (13·5 to 36·5)	26·2 (16·1 to 39·5)	20 (12·9 to 29·5)	17·3 (11·3 to 24·9)
Motor vehicle road injuries	0·1 (0 to 0·2)	0·1 (0 to 0·1)	0·7 (0·3 to 1·4)	2·3 (1 to 4·4)	3·3 (1·8 to 5·2)	6·9 (4·1 to 10·9)	22·5 (13·3 to 35·1)	26·6 (15·8 to 40·6)	26·5 (15·6 to 39)	21·8 (13·2 to 32·5)	20·6 (13·2 to 30·6)
Other road injuries	0 (0 to 0)	0 (0 to 0)	0 (0 to 0)	0 (0 to 0·1)	0 (0 to 0·1)	0·1 (0·1 to 0·2)	0·2 (0·1 to 0·3)	0·3 (0·2 to 0·4)	0·3 (0·2 to 0·5)	0·4 (0·3 to 0·5)	0·4 (0·3 to 0·6)
Other transport injuries	0 (0 to 0·1)	0 (0 to 0·1)	0·2 (0·1 to 0·4)	0·3 (0·2 to 0·6)	0·4 (0·2 to 0·7)	0·9 (0·5 to 1·6)	1·9 (0·9 to 3·4)	2·3 (1·2 to 3·9)	2·5 (1·3 to 4·5)	2·3 (1·3 to 4·1)	2·2 (1·2 to 3·7)
Unintentional injuries	2·8 (1·6 to 4·6)	1·9 (1·1 to 3·2)	16·5 (9·1 to 27·8)	28·8 (19 to 42·6)	23·7 (17·6 to 30·1)	37·7 (28·7 to 47·5)	58·1 (42·4 to 78)	55·8 (42·2 to 72·1)	60·3 (46·8 to 76·6)	53·5 (42·1 to 66·2)	52·9 (41·7 to 65·6)
Falls	0·1 (0 to 0·1)	0 (0 to 0·1)	0·5 (0·2 to 1)	1·1 (0·6 to 2)	1·3 (0·9 to 1·8)	2·7 (1·9 to 3·8)	4·9 (3·5 to 6·7)	5·5 (4 to 7·4)	6·3 (4·7 to 8·4)	6·6 (5 to 8·8)	7·5 (5·7 to 9·7)
Drowning	0·3 (0·1 to 0·5)	0·1 (0 to 0·2)	2 (0·8 to 4·2)	11·5 (5·7 to 20·4)	11·9 (6·9 to 17·7)	18·6 (11·3 to 27)	29 (15·7 to 45·3)	21·9 (13·6 to 33·4)	20·7 (13 to 31·7)	15·4 (9·8 to 22·8)	14·4 (8·7 to 22·2)
Fire, heat, and hot substances	0·1 (0 to 0·1)	0·1 (0 to 0·2)	1·4 (0·6 to 2·8)	3·7 (1·9 to 6·4)	2·6 (1·7 to 3·8)	3·4 (2·3 to 4·8)	3·8 (2·7 to 5·2)	4·2 (3 to 5·7)	4·8 (3·5 to 6·6)	5·4 (3·9 to 7·3)	5·2 (3·7 to 7·1)
Poisonings	0·1 (0 to 0·3)	0·1 (0 to 0·4)	1·3 (0·4 to 3·3)	3·8 (1·3 to 8·6)	1·2 (0·5 to 2·6)	1·3 (0·6 to 3)	1·9 (0·8 to 4·2)	2·5 (1·2 to 6·2)	4 (1·9 to 9·4)	3·7 (1·8 to 8·6)	3·4 (1·6 to 8·2)
Poisoning by carbon monoxide	0 (0 to 0)	0 (0 to 0)	0 (0 to 0)	0·1 (0 to 0·3)	0·1 (0 to 0·1)	0·1 (0 to 0·2)	0·2 (0·1 to 0·6)	0·3 (0·1 to 0·8)	0·4 (0·1 to 1)	0·4 (0·1 to 0·9)	0·4 (0·1 to 0·9)
Poisoning by other means	0·1 (0 to 0·3)	0·1 (0 to 0·4)	1·2 (0·3 to 3·2)	3·7 (1·3 to 8·5)	1·1 (0·5 to 2·5)	1·2 (0·6 to 2·8)	1·6 (0·7 to 3·8)	2·2 (1 to 5·5)	3·6 (1·7 to 8·6)	3·3 (1·6 to 7·8)	3 (1·4 to 7·4)
Exposure to mechanical forces	0 (0 to 0·1)	0·1 (0 to 0·6)	0·3 (0·1 to 1·2)	0·9 (0·5 to 1·8)	1·5 (1 to 2·1)	2·7 (1·8 to 3·9)	6·9 (4·4 to 10·9)	8·6 (5·8 to 12·6)	9·2 (6·2 to 13·6)	8·8 (6·2 to 12·5)	8·4 (6 to 11·8)
Unintentional firearm injuries	0 (0 to 0)	0 (0 to 0·1)	0 (0 to 0·2)	0·1 (0 to 0·2)	0·1 (0·1 to 0·2)	0·3 (0·2 to 0·6)	2·2 (0·9 to 4·3)	3·2 (1·6 to 5·9)	3·1 (1·4 to 5·8)	2·4 (1·2 to 4·1)	2·1 (1·1 to 3·6)
Other exposure to mechanical forces	0 (0 to 0·1)	0·1 (0 to 0·5)	0·2 (0·1 to 1)	0·9 (0·5 to 1·7)	1·4 (0·9 to 2)	2·4 (1·6 to 3·4)	4·7 (2·9 to 7·6)	5·4 (3·5 to 8·3)	6·2 (4·1 to 9·2)	6·4 (4·3 to 9·2)	6·3 (4·5 to 8·9)
Adverse effects of medical treatment	0·7 (0·3 to 1·3)	0·5 (0·2 to 0·9)	1·9 (0·9 to 3·7)	1·1 (0·6 to 1·9)	0·7 (0·4 to 1·1)	0·9 (0·5 to 1·4)	1 (0·5 to 1·7)	1·5 (0·8 to 2·5)	1·8 (1 to 2·8)	1·4 (0·8 to 2·4)	1·9 (1 to 3·2)
Animal contact	0 (0 to 0)	0 (0 to 0)	0·1 (0 to 0·1)	0·2 (0·1 to 0·3)	0·2 (0·1 to 0·3)	0·3 (0·2 to 0·5)	0·4 (0·3 to 0·6)	0·4 (0·3 to 0·6)	0·5 (0·3 to 0·7)	0·4 (0·3 to 0·6)	0·5 (0·3 to 0·7)
Venomous animal contact	0 (0 to 0)	0 (0 to 0)	0 (0 to 0)	0·1 (0·1 to 0·2)	0·1 (0·1 to 0·2)	0·2 (0·1 to 0·3)	0·3 (0·2 to 0·4)	0·3 (0·2 to 0·4)	0·3 (0·2 to 0·5)	0·3 (0·2 to 0·4)	0·3 (0·2 to 0·5)
Non-venomous animal contact	0 (0 to 0)	0 (0 to 0)	0 (0 to 0·1)	0·1 (0 to 0·1)	0·1 (0 to 0·1)	0·1 (0·1 to 0·2)	0·1 (0·1 to 0·2)	0·1 (0·1 to 0·2)	0·2 (0·1 to 0·3)	0·2 (0·1 to 0·3)	0·2 (0·1 to 0·3)
Foreign body	1·4 (0·6 to 2·7)	0·9 (0·4 to 1·6)	8·4 (3·4 to 16·5)	4·5 (2·4 to 7·8)	1·9 (1·2 to 3)	3·2 (2 to 4·9)	2 (1·3 to 2·9)	2·1 (1·4 to 3·1)	2·6 (1·7 to 3·8)	2·2 (1·5 to 3·1)	2·3 (1·5 to 3·4)
Pulmonary aspiration and foreign body in airway	1·4 (0·6 to 2·7)	0·8 (0·4 to 1·6)	8·3 (3·4 to 16·4)	4·3 (2·2 to 7·5)	1·6 (0·9 to 2·7)	2·8 (1·7 to 4·4)	1·5 (0·9 to 2·5)	1·7 (1 to 2·6)	2·1 (1·3 to 3·2)	1·7 (1·1 to 2·6)	1·8 (1·1 to 2·9)
Foreign body in eyes	0 (0 to 0)	0 (0 to 0)	0 (0 to 0)	0·1 (0 to 0·1)	0·1 (0 to 0·2)	0·1 (0 to 0·3)	0·2 (0·1 to 0·4)	0·2 (0·1 to 0·4)	0·2 (0·1 to 0·5)	0·2 (0·1 to 0·5)	0·2 (0·1 to 0·4)
Foreign body in other body part	0 (0 to 0)	0 (0 to 0)	0·1 (0 to 0·1)	0·1 (0·1 to 0·2)	0·2 (0·1 to 0·3)	0·3 (0·2 to 0·5)	0·2 (0·2 to 0·3)	0·3 (0·2 to 0·4)	0·3 (0·2 to 0·4)	0·3 (0·2 to 0·4)	0·2 (0·2 to 0·3)
Environmental heat and cold exposure	0 (0 to 0)	0 (0 to 0)	0·1 (0 to 0·2)	0·2 (0·1 to 0·4)	0·4 (0·2 to 0·5)	0·6 (0·4 to 0·8)	1·1 (0·8 to 1·6)	1·4 (1 to 2)	1·6 (1·1 to 2·2)	1·7 (1·2 to 2·3)	1·6 (1·2 to 2·2)
Exposure to forces of nature	0 (0 to 0)	0 (0 to 0)	0 (0 to 0)	0·1 (0·1 to 0·2)	0·1 (0 to 0·1)	0·1 (0·1 to 0·2)	0·2 (0·1 to 0·4)	0·3 (0·2 to 0·5)	0·4 (0·3 to 0·6)	0·4 (0·3 to 0·5)	0·4 (0·3 to 0·6)
Other unintentional injuries	0·1 (0 to 0·3)	0 (0 to 0·1)	0·5 (0·2 to 0·9)	1·6 (0·8 to 3)	2·1 (1·1 to 3·6)	3·7 (2·1 to 6·3)	6·9 (3·8 to 12·2)	7·2 (4·3 to 11·7)	8·2 (4·7 to 13·1)	7·5 (4·5 to 11·9)	7·3 (4·5 to 12·3)
Self-harm and interpersonal violence	1 (0·4 to 2·1)	0·2 (0·1 to 0·4)	1·3 (0·5 to 2·8)	2·5 (1 to 5·2)	2 (1·1 to 3·5)	6·5 (4 to 10·4)	52·5 (32·7 to 81·3)	80·4 (53·6 to 114)	89·5 (60·9 to 122)	67·5 (46·3 to 95·8)	57 (38·1 to 82)
Self-harm	0 (0 to 0)	0 (0 to 0)	0 (0 to 0)	0 (0 to 0)	0 (0 to 0)	1 (0·5 to 1·8)	8·7 (4·9 to 14·3)	15·1 (8·8 to 23)	21·7 (13·4 to 31·3)	18·9 (11·7 to 28·1)	16·8 (10·4 to 24·9)
Self-harm by firearm	0 (0 to 0)	0 (0 to 0)	0 (0 to 0)	0 (0 to 0)	0 (0 to 0)	0 (0 to 0)	0·4 (0·2 to 0·9)	0·7 (0·3 to 1·3)	0·7 (0·3 to 1·4)	0·6 (0·3 to 1·1)	0·5 (0·2 to 1)
Self-harm by other specified means	0 (0 to 0)	0 (0 to 0)	0 (0 to 0)	0 (0 to 0)	0 (0 to 0)	1 (0·5 to 1·8)	8·2 (4·6 to 13·6)	14·5 (8·4 to 22·1)	20·9 (12·8 to 30·1)	18·3 (11·3 to 27·4)	16·2 (10 to 24)
Interpersonal violence	1 (0·4 to 2·1)	0·2 (0·1 to 0·4)	1·3 (0·5 to 2·8)	2·5 (1 to 5·2)	2 (1·1 to 3·4)	5·4 (3 to 9)	43·8 (24·6 to 71·2)	65·3 (40·5 to 98)	67·8 (42 to 100)	48·5 (29·4 to 74·2)	40·1 (24 to 62·2)
Physical violence by firearm	0 (0 to 0·1)	0 (0 to 0)	0·1 (0 to 0·2)	0·1 (0 to 0·4)	0·2 (0·1 to 0·5)	1·1 (0·4 to 2·3)	18 (7·9 to 33·1)	27·8 (13·6 to 46·8)	27·7 (13·7 to 47·2)	17·4 (8·2 to 31·1)	12·7 (5·7 to 22·7)
Physical violence by sharp object	0·1 (0 to 0·2)	0 (0 to 0)	0·1 (0 to 0·2)	0·3 (0·1 to 0·7)	0·3 (0·2 to 0·6)	1·2 (0·5 to 2·3)	18·7 (8·3 to 34·6)	28·3 (14·2 to 48·1)	29·3 (15·2 to 47·1)	22·5 (11·6 to 38·2)	18·5 (9·7 to 32·5)
Sexual violence	0 (0 to 0)	0 (0 to 0)	0 (0 to 0)	0 (0 to 0)	0·1 (0·1 to 0·1)	0·2 (0·1 to 0·4)	0·5 (0·3 to 0·8)	0·6 (0·4 to 1)	0·5 (0·3 to 0·9)	0·5 (0·3 to 0·8)	0·4 (0·2 to 0·7)
Physical violence by other means	0·9 (0·3 to 1·9)	0·1 (0·1 to 0·3)	1·2 (0·4 to 2·5)	2·1 (0·8 to 4·3)	1·4 (0·7 to 2·4)	3 (1·5 to 5·1)	6·5 (2·9 to 12·7)	8·6 (4·5 to 14·9)	10·3 (5·3 to 17·6)	8·2 (4·4 to 14·3)	8·5 (4·4 to 15·1)
Conflict and terrorism	0 (0 to 0)	0 (0 to 0)	0 (0 to 0)	0 (0 to 0)	0 (0 to 0)	0 (0 to 0)	0 (0 to 0)	0 (0 to 0)	0 (0 to 0)	0 (0 to 0)	0·1 (0·1 to 0·2)

Osteoarthritis other	0·3 (0·1 to 0·7)	0·7 (0·3 to 1·5)	1·2 (0·5 to 2·7)	1·7 (0·7 to 3·7)	1·9 (0·9 to 4)	1·6 (0·8 to 3·4)	1·2 (0·6 to 2·6)	1·2 (0·6 to 2·4)	0·9 (0·5 to 1·9)	0·4 (0·2 to 0·8)	0·1 (0·1 to 0·3)	0 (0 to 0)	11·5
Low back pain	17·4 (10·6 to 27·1)	20·8 (12·2 to 32)	24·8 (15·3 to 39·7)	24·7 (15·6 to 37·4)	22·9 (14·4 to 34·4)	17·3 (10·8 to 27)	11·9 (7·4 to 17·9)	9·8 (6·1 to 15)	7 (4·3 to 10·6)	2·7 (1·6 to 4·1)	0·8 (0·5 to 1·2)	0·1 (0·1 to 0·2)	241
Neck pain	4·9 (2·5 to 8·8)	6·2 (3·1 to 11·1)	6·4 (3·1 to 12·2)	6 (2·8 to 10·7)	5·1 (2·6 to 9)	3·5 (1·8 to 5·9)	2·2 (1·2 to 3·9)	1·7 (0·9 to 3·1)	1·1 (0·5 to 1·9)	0·4 (0·2 to 0·7)	0·1 (0·1 to 0·2)	0 (0 to 0)	51·3
Gout	0·3 (0·1 to 0·6)	0·4 (0·2 to 0·7)	0·5 (0·2 to 0·9)	0·5 (0·2 to 0·9)	0·5 (0·2 to 1)	0·4 (0·2 to 0·8)	0·3 (0·2 to 0·6)	0·3 (0·1 to 0·5)	0·3 (0·1 to 0·5)	0·1 (0·1 to 0·2)	0 (0 to 0·1)	0 (0 to 0)	4·4
Other musculoskeletal disorders	13·8 (7·8 to 22·1)	16·2 (9·8 to 25·1)	19 (11·2 to 29·6)	18·8 (11·4 to 28·6)	16·7 (9·4 to 27·2)	11·7 (6·7 to 18·1)	7·4 (4·1 to 12·2)	5·4 (2·9 to 8·6)	3 (1·2 to 5·4)	1·2 (0·6 to 2·1)	0·3 (0·1 to 0·5)	0 (0 to 0·1)	160
Other non-communicable diseases	21·3 (15·2 to 30)	23·1 (16·2 to 32·8)	32·5 (23·5 to 44·5)	36 (26·2 to 48·9)	37·4 (27·5 to 50·1)	33·1 (24·4 to 44·3)	27·1 (20·4 to 35·3)	24·3 (18·1 to 31·1)	19·7 (14·6 to 25·7)	10·2 (7·7 to 13·4)	3·7 (2·8 to 4·9)	0·6 (0·5 to 0·9)	594
Congenital birth defects	2·2 (1·6 to 3)	2·1 (1·5 to 3)	2·6 (1·8 to 3·7)	2·3 (1·6 to 3·2)	1·4 (1 to 2)	0·9 (0·7 to 1·2)	0·2 (0·1 to 0·3)	0·2 (0·1 to 0·3)	0·1 (0·1 to 0·2)	0 (0 to 0·1)	0 (0 to 0)	0 (0 to 0)	191
Neural tube defects	0·1 (0 to 0·1)	0 (0 to 0·1)	0 (0 to 0·1)	0 (0 to 0·1)	0 (0 to 0)	0 (0 to 0)	0 (0 to 0)	0 (0 to 0)	0 (0 to 0)	0 (0 to 0)	0 (0 to 0)	0 (0 to 0)	9·7
Congenital heart anomalies	0·5 (0·3 to 0·8)	0·4 (0·2 to 0·8)	0·5 (0·3 to 0·8)	0·3 (0·2 to 0·6)	0·3 (0·2 to 0·5)	0·2 (0·1 to 0·3)	0 (0 to 0)	0 (0 to 0)	0 (0 to 0)	0 (0 to 0)	0 (0 to 0)	0 (0 to 0)	60
Orofacial clefts	0·1 (0 to 0·1)	0·1 (0 to 0·2)	0·1 (0 to 0·2)	0·1 (0 to 0·1)	0·1 (0 to 0·1)	0 (0 to 0·1)	0 (0 to 0·1)	0 (0 to 0)	0 (0 to 0)	0 (0 to 0)	0 (0 to 0)	0 (0 to 0)	2·4
Down syndrome	0·3 (0·2 to 0·5)	0·3 (0·2 to 0·6)	0·4 (0·2 to 0·8)	0·5 (0·2 to 1)	0·3 (0·1 to 0·5)	0·1 (0·1 to 0·2)	0 (0 to 0)	0 (0 to 0)	0 (0 to 0)	0 (0 to 0)	0 (0 to 0)	0 (0 to 0)	7·4
Turner syndrome	0 (0 to 0)	0 (0 to 0)	0 (0 to 0)	0 (0 to 0)	0 (0 to 0)	0 (0 to 0)	0 (0 to 0)	0 (0 to 0)	0 (0 to 0)	0 (0 to 0)	0 (0 to 0)	0 (0 to 0)	0
Klinefelter syndrome	0 (0 to 0)	0 (0 to 0)	0 (0 to 0)	0 (0 to 0)	0 (0 to 0)	0 (0 to 0)	0 (0 to 0)	0 (0 to 0)	0 (0 to 0)	0 (0 to 0)	0 (0 to 0)	0 (0 to 0)	0
Other chromosomal abnormalities	0·1 (0 to 0·2)	0·1 (0 to 0·1)	0·1 (0 to 0·1)	0·1 (0 to 0·1)	0 (0 to 0·1)	0 (0 to 0)	0 (0 to 0)	0 (0 to 0)	0 (0 to 0)	0 (0 to 0)	0 (0 to 0)	0 (0 to 0)	17
Congenital musculoskeletal and limb anomalies	0·5 (0·3 to 0·8)	0·6 (0·3 to 0·8)	0·7 (0·4 to 1·1)	0·7 (0·4 to 1)	0·4 (0·3 to 0·7)	0·3 (0·2 to 0·4)	0·1 (0·1 to 0·2)	0·1 (0 to 0·1)	0 (0 to 0·1)	0 (0 to 0)	0 (0 to 0)	0 (0 to 0)	23·3
Urogenital congenital anomalies	0 (0 to 0)	0 (0 to 0)	0 (0 to 0)	0 (0 to 0)	0 (0 to 0)	0 (0 to 0)	0 (0 to 0)	0 (0 to 0)	0 (0 to 0)	0 (0 to 0)	0 (0 to 0)	0 (0 to 0)	1·5
Digestive congenital anomalies	0 (0 to 0)	0 (0 to 0)	0 (0 to 0·1)	0 (0 to 0)	0 (0 to 0)	0 (0 to 0·1)	0 (0 to 0)	0 (0 to 0)	0 (0 to 0)	0 (0 to 0)	0 (0 to 0)	0 (0 to 0)	16·8
Other congenital birth defects	0·6 (0·4 to 1)	0·6 (0·3 to 0·9)	0·8 (0·5 to 1·3)	0·5 (0·3 to 0·9)	0·3 (0·2 to 0·5)	0·2 (0·1 to 0·4)	0·1 (0 to 0·1)	0 (0 to 0·1)	0 (0 to 0·1)	0 (0 to 0)	0 (0 to 0)	0 (0 to 0)	53·4
Urinary diseases and male infertility	1·4 (0·8 to 2·4)	1·5 (0·9 to 2·2)	3·1 (2·1 to 4·7)	5·6 (3·5 to 8·4)	8·2 (5·3 to 11·8)	9 (6 to 13)	8·5 (5·8 to 11·9)	7·9 (5·2 to 11·5)	6·2 (4·1 to 9·3)	3·1 (1·9 to 4·8)	1·1 (0·7 to 1·8)	0·2 (0·1 to 0·3)	62·6
Urolithiasis	0·2 (0 to 0·3)	0·2 (0·1 to 0·5)	0·3 (0·2 to 0·6)	0·4 (0·2 to 0·7)	0·3 (0·2 to 0·6)	0·2 (0·1 to 0·4)	0·2 (0·1 to 0·3)	0·1 (0 to 0·2)	0·1 (0 to 0·1)	0 (0 to 0)	0 (0 to 0)	0 (0 to 0)	2·5
Benign prostatic hyperplasia	0 (0 to 0)	0·2 (0·1 to 0·4)	0·8 (0·4 to 1·5)	2·6 (1·2 to 4·5)	4·7 (2·4 to 8·1)	5·4 (3·1 to 8·5)	4·6 (2·5 to 7·6)	3·8 (2·1 to 6·3)	2·3 (1·3 to 3·7)	0·8 (0·4 to 1·2)	0·2 (0·1 to 0·3)	0 (0 to 0)	25·4
Male infertility	0·5 (0·1 to 1·3)	0·1 (0 to 0·2)	0 (0 to 0)	0 (0 to 0)	0 (0 to 0)	0 (0 to 0)	0 (0 to 0)	0 (0 to 0)	0 (0 to 0)	0 (0 to 0)	0 (0 to 0)	0 (0 to 0)	2·9
Other urinary diseases	0·3 (0·1 to 0·5)	0·4 (0·2 to 0·8)	0·7 (0·3 to 1·2)	0·8 (0·4 to 1·6)	1 (0·4 to 1·9)	1·1 (0·5 to 2)	0·8 (0·4 to 1·7)	0·9 (0·4 to 1·9)	0·9 (0·4 to 1·8)	0·4 (0·2 to 0·9)	0·2 (0·1 to 0·4)	0 (0 to 0·1)	9·1
Gynecological diseases	0 (0 to 0)	0 (0 to 0)	0 (0 to 0)	0 (0 to 0)	0 (0 to 0)	0 (0 to 0)	0 (0 to 0)	0 (0 to 0)	0 (0 to 0)	0 (0 to 0)	0 (0 to 0)	0 (0 to 0)	0
Uterine fibroids	0 (0 to 0)	0 (0 to 0)	0 (0 to 0)	0 (0 to 0)	0 (0 to 0)	0 (0 to 0)	0 (0 to 0)	0 (0 to 0)	0 (0 to 0)	0 (0 to 0)	0 (0 to 0)	0 (0 to 0)	0
Polycystic ovarian syndrome	--	--	--	--	--	--	--	--	--	--	--	--	NA
Female infertility	--	--	--	--	--	--	--	--	--	--	--	--	NA
Endometriosis	--	--	--	--	--	--	--	--	--	--	--	--	NA
Genital prolapse	0 (0 to 0)	0 (0 to 0)	0 (0 to 0)	0 (0 to 0)	0 (0 to 0)	0 (0 to 0)	0 (0 to 0)	0 (0 to 0)	0 (0 to 0)	0 (0 to 0)	0 (0 to 0)	0 (0 to 0)	0
Premenstrual syndrome	--	--	--	--	--	--	--	--	--	--	--	--	NA
Other gynecological diseases	0 (0 to 0)	0 (0 to 0)	0 (0 to 0)	0 (0 to 0)	0 (0 to 0)	0 (0 to 0)	0 (0 to 0)	0 (0 to 0)	0 (0 to 0)	0 (0 to 0)	0 (0 to 0)	0 (0 to 0)	0
Hemoglobinopathies and hemolytic anemias	2·8 (1·7 to 4·2)	2·5 (1·6 to 3·8)	3·1 (1·9 to 4·8)	3·1 (2 to 4·5)	2·6 (1·6 to 3·9)	1·8 (1·2 to 2·8)	1·5 (0·9 to 2·2)	1·3 (0·8 to 1·9)	1·1 (0·7 to 1·6)	0·9 (0·6 to 1·3)	0·4 (0·2 to 0·5)	0·1 (0 to 0·1)	56·8
Thalassemias	0 (0 to 0)	0 (0 to 0)	0 (0 to 0)	0 (0 to 0)	0 (0 to 0)	0 (0 to 0)	0 (0 to 0)	0 (0 to 0)	0 (0 to 0)	0 (0 to 0)	0 (0 to 0)	0 (0 to 0)	0
Thalassemias trait	0 (0 to 0)	0 (0 to 0·1)	0 (0 to 0·1)	0 (0 to 0·1)	0 (0 to 0·1)	0 (0 to 0)	0 (0 to 0)	0 (0 to 0)	0 (0 to 0)	0 (0 to 0)	0 (0 to 0)	0 (0 to 0)	0·4
Sickle cell disorders	1·1 (0·6 to 1·9)	0·8 (0·4 to 1·3)	0·6 (0·3 to 1·2)	0·3 (0·1 to 0·6)	0·1 (0 to 0·1)	0 (0 to 0)	0 (0 to 0)	0 (0 to 0)	0 (0 to 0)	0 (0 to 0)	0 (0 to 0)	0 (0 to 0)	27
Sickle cell trait	0·1 (0 to 0·4)	0·2 (0 to 0·4)	0·2 (0·1 to 0·5)	0·3 (0·1 to 0·7)	0·3 (0·1 to 0·6)	0·2 (0 to 0·4)	0·1 (0 to 0·2)	0·1 (0 to 0·3)	0·1 (0 to 0·2)	0·1 (0 to 0·1)	0 (0 to 0)	0 (0 to 0)	3·8
G6PD deficiency	1·1 (0·5 to 1·9)	1·1 (0·5 to 1·8)	1·5 (0·8 to 2·5)	1·5 (0·9 to 2·4)	1·1 (0·6 to 1·8)	0·7 (0·4 to 1·1)	0·4 (0·2 to 0·7)	0·2 (0·1 to 0·4)	0·1 (0·1 to 0·2)	0·1 (0 to 0·1)	0 (0 to 0)	0 (0 to 0)	14·6
G6PD trait	0 (0 to 0)	0 (0 to 0)	0 (0 to 0)	0 (0 to 0)	0 (0 to 0)	0 (0 to 0)	0 (0 to 0)	0 (0 to 0)	0 (0 to 0)	0 (0 to 0)	0 (0 to 0)	0 (0 to 0)	0
Other hemoglobinopathies and hemolytic anemias	0·4 (0·2 to 0·7)	0·4 (0·2 to 0·9)	0·7 (0·3 to 1·3)	0·9 (0·5 to 1·6)	1·1 (0·6 to 1·8)	1 (0·6 to 1·5)	0·9 (0·6 to 1·4)	0·9 (0·6 to 1·4)	0·9 (0·6 to 1·3)	0·8 (0·5 to 1·1)	0·3 (0·2 to 0·5)	0·1 (0 to 0·1)	10·9
Endocrine, metabolic, blood, and immune disorders	7·4 (4·8 to 11·6)	7·9 (5·1 to 12)	12·6 (7·9 to 20·2)	13 (8·2 to 20·3)	12·4 (8·1 to 18·5)	10·2 (6·6 to 15·8)	8·5 (5·5 to 12·9)	7·5 (4·8 to 11·4)	6·8 (4·1 to 10·3)	3·8 (2·3 to 6·5)	1·5 (0·9 to 2·5)	0·3 (0·1 to 0·5)	158
Oral disorders	7·5 (3·6 to 14·1)	9·1 (4·6 to 16·7)	11·1 (5·7 to 20)	12 (6·7 to 20·4)	12·9 (7·7 to 21·2)	11·2 (6·7 to 17·8)	8·4 (5·2 to 13·2)	7·5 (4·7 to 11·3)	5·4 (3·4 to 8·2)	2·3 (1·4 to 3·4)	0·7 (0·4 to 1·1)	0·1 (0·1 to 0·2)	119
Caries of deciduous teeth	0 (0 to 0)	0 (0 to 0)	0 (0 to 0)	0 (0 to 0)	0 (0 to 0)	0 (0 to 0)	0 (0 to 0)	0 (0 to 0)	0 (0 to 0)	0 (0 to 0)	0 (0 to 0)	0 (0 to 0)	0·7
Caries of permanent teeth	0·6 (0·3 to 1·3)	0·7 (0·3 to 1·4)	0·7 (0·3 to 1·4)	0·6 (0·2 to 1·3)	0·5 (0·2 to 1)	0·3 (0·1 to 0·6)	0·2 (0·1 to 0·4)	0·1 (0·1 to 0·3)	0·1 (0 to 0·2)	0 (0 to 0·1)	0 (0 to 0)	0 (0 to 0)	9·3
Periodontal diseases	4·6 (1·7 to 9·9)	5·5 (2·1 to 11·8)	6·1 (2·4 to 13)	5·4 (2·2 to 11·1)	4·2 (1·7 to 8·7)	2·6 (1 to 5·4)	1·6 (0·6 to 3·2)	1·2 (0·5 to 2·4)	0·8 (0·3 to 1·7)	0·3 (0·1 to 0·7)	0·1 (0 to 0·2)	0 (0 to 0)	45·4
Other oral disorders	1·3 (0·8 to 2)	1·4 (0·8 to 2·2)	1·5 (0·9 to 2·3)	1·4 (0·8 to 2·1)	1·2 (0·7 to 1·8)	0·8 (0·5 to 1·2)	0·5 (0·3 to 0·7)	0·3 (0·2 to 0·5)	0·2 (0·1 to 0·3)	0·1 (0 to 0·1)	0 (0 to 0)	0 (0 to 0)	18
Sudden infant death syndrome	--	--	--	--	--	--	--	--	--	--	--	--	NA
Injuries	106 (84·1 to 131)	99·1 (77·8 to 124)	118 (93·4 to 147)	103 (82·9 to 128)	77·4 (62·5 to 94·4)	57·1 (46·6 to 69·4)	40·9 (33·2 to 49·9)	33·6 (27·9 to 40·7)	28·1 (23·3 to 33·9)	14·4 (11·7 to 17·2)	5·9 (4·7 to 7·3)	1·1 (0·9 to 1·4)	1,750
Transport injuries	35·5 (25·6 to 48·1)	33·4 (24·1 to 45·5)	40·3 (28·9 to 56)	35·5 (25·4 to 49)	25·1 (19·1 to 33·3)	17·8 (13 to 24·3)	11·7 (8·6 to 15·6)	8·8 (6·6 to 11·6)	5·8 (4·5 to 7·6)	2·4 (1·8 to 3·1)	0·7 (0·6 to 0·9)	0·1 (0·1 to 0·2)	535
Road injuries	33·9 (24·3 to 45·3)	32·1 (23·2 to 43·3)	38·7 (27·7 to 53·7)	33·8 (24·2 to 46·4)	24·1 (18·4 to 32·1)	17·2 (12·6 to 23·5)	11·1 (8·2 to 14·9)	8·4 (6·3 to 11·1)	5·6 (4·3 to 7·3)	2·4 (1·8 to 3)	0·7 (0·5 to 0·9)	0·1 (0·1 to 0·2)	513
Pedestrian road injuries	5·8 (3·5 to 9·2)	6·1 (3·7 to 9·8)	8·6 (5·4 to 13·3)	8·6 (5·3 to 13·3)	6·6 (4·3 to 10·1)	5·3 (3·4 to 8·1)	4 (2·7 to 5·9)	3·4 (2·3 to 5)	2·5 (1·8 to 3·6)	1·1 (0·8 to 1·5)	0·3 (0·2 to 0·4)	0 (0 to 0·1)	95·1
Cyclist road injuries	2·8 (2 to 3·9)	2·9 (2 to 3·8)	3·7 (2·7 to 5·1)	3·3 (2·4 to 4·6)	2·6 (1·9 to 3·6)	2 (1·4 to 2·7)	1·2 (0·8 to 1·7)	0·9 (0·6 to 1·2)	0·6 (0·4 to 0·7)	0·2 (0·1 to 0·3)	0·1 (0 to 0·1)	0 (0 to 0)	40·9
Motorcyclist road injuries	10·7 (7 to 15·8)	9·4 (6·4 to 13·3)	10·1 (6·8 to 14·6)	8·2 (5·5 to 12·1)	5 (3·5 to 6·9)	3·3 (2·3 to 4·6)	1·7 (1·2 to 2·2)	1·2 (0·9 to 1·6)	0·8 (0·6 to 1·1)	0·3 (0·2 to 0·5)	0·1 (0·1 to 0·2)	0 (0 to 0)	159
Motor vehicle road injuries	14·2 (9·1 to 20·8)	13·2 (8·7 to 19·1)	15·9 (10·2 to 23·8)	13·2 (8·8 to 18·9)	9·5 (6·5 to 13·8)	6·3 (4·1 to 9·3)	4 (2·7 to 6·1)	2·8 (1·9 to 4)	1·6 (1·2 to 2·3)	0·7 (0·5 to 1)	0·2 (0·1 to 0·3)	0 (0 to 0·1)	213
Other road injuries	0·4 (0·3 to 0·5)	0·5 (0·3 to 0·6)	0·5 (0·4 to 0·7)	0·5 (0·4 to 0·7)	0·4 (0·3 to 0·6)	0·3 (0·2 to 0·4)	0·2 (0·2 to 0·3)	0·2 (0·1 to 0·2)	0·1 (0·1 to 0·2)	0 (0 to 0·1)	0 (0 to 0)	0 (0 to 0)	5
Other transport injuries	1·6 (0·9 to 2·7)	1·3 (0·8 to 2·2)	1·6 (0·9 to 2·6)	1·7 (1 to 2·8)	1 (0·6 to 1·5)	0·6 (0·4 to 1)	0·6 (0·3 to 0·9)	0·3 (0·2 to 0·5)	0·2 (0·1 to 0·3)	0·1 (0·1 to 0·1)	0 (0 to 0)	0 (0 to 0)	22·1
Unintentional injuries	39·6 (30·9 to 49·5)	39·5 (31 to 49·2)	49·2 (39 to 60·7)	45·4 (36·4 to 56·1)	37·8 (30·1 to 46·5)	29·4 (23·5 to 36·1)	22·9 (18·4 to 28·2)	20·7 (17·1 to 25·2)	19·6 (15·8 to 24·3)	10·8 (8·6 to 13·4)	4·9 (3·8 to 6·2)	1 (0·7 to 1·2)	713
Falls	6·5 (4·9 to 8·5)	7·6 (5·8 to 9·9)	10·5 (8·1 to 13·8)	10·4 (8·1 to 13·3)	9·6 (7·2 to 12·3)	8 (6·1 to 10·6)	7·4 (5·5 to 10·2)	7·5 (5·7 to 9·9)	8·9 (6·5 to 12·6)	5·6 (4 to 7·9)	2·8 (2 to 3·9)	0·5 (0·3 to 0·8)	122
Drowning	9·1 (5·6 to 14·2)	7·2 (4·3 to 11)	8·8 (5·6 to 13·2)	6·8 (4 to 10·9)	4·3 (2·8 to 6·6)	2·8 (1·7 to 4·2)	1·5 (1 to 2·3)	1 (0·6 to 1·6)	0·5 (0·3 to 0·8)	0·2 (0·1 to 0·4)	0·1 (0·1 to 0·1)	0 (0 to 0)	188
Fire, heat, and hot substances	4·3 (3·2 to 5·8)	4·6 (3·4 to 6·3)	5·3 (3·8 to 7·1)	4·8 (3·5 to 6·5)	4 (2·9 to 5·4)	2·8 (2 to 3·8)	2·1 (1·5 to 2·9)	1·8 (1·2 to 2·5)	1·5 (1 to 2·2)	0·8 (0·6 to 1·2)	0·3 (0·2 to 0·5)	0·1 (0 to 0·1)	67·2
Poisonings	1·8 (1 to 4·2)	1·6 (0·8 to 3·8)	1·7 (0·9 to 4)	1·4 (0·7 to 3·2)	1 (0·5 to 2·3)	0·6 (0·3 to 1·5)	0·4 (0·2 to 1)	0·3 (0·2 to 0·7)	0·2 (0·1 to 0·6)	0·1 (0·1 to 0·2)	0 (0 to 0·1)	0 (0 to 0)	32·6
Poisoning by carbon monoxide	0·2 (0·1 to 0·4)	0·1 (0 to 0·3)	0·1 (0 to 0·2)	0·1 (0 to 0·2)	0·1 (0 to 0·2)	0 (0 to 0·1)	0 (0 to 0·1)	0 (0 to 0)	0 (0 to 0·1)	0 (0 to 0)	0 (0 to 0)	0 (0 to 0)	2·7
Poisoning by other means	1·6 (0·8 to 3·8)	1·4 (0·7 to 3·6)	1·6 (0·8 to 3·8)	1·3 (0·7 to 3·1)	0·9 (0·5 to 2·2)	0·6 (0·3 to 1·4)	0·4 (0·2 to 0·9)	0·3 (0·2 to 0·7)	0·2 (0·1 to 0·5)	0·1 (0 to 0·2)	0 (0 to 0·1)	0 (0 to 0)	29·9
Exposure to mechanical forces	7 (5 to 9·8)	7·3 (5·3 to 10)	8·4 (6·2 to 11·7)	7·8 (5·6 to 11·1)	6·3 (4·6 to 8·7)	4·4 (3·1 to 6)	2·8 (2 to 3·9)	2·2 (1·6 to 3·2)	1·6 (1·1 to 2·2)	0·7 (0·5 to 1)	0·2 (0·2 to 0·3)	0 (0 to 0·1)	96·4
Unintentional firearm injuries	1·5 (0·9 to 2·5)	1·3 (0·8 to 2)	1·4 (0·9 to 2·2)	1·2 (0·8 to 1·8)	0·9 (0·6 to 1·2)	0·6 (0·4 to 0·8)	0·3 (0·2 to 0·4)	0·2 (0·2 to 0·3)	0·2 (0·1 to 0·2)	0·1 (0·1 to 0·1)	0 (0 to 0)	0 (0 to 0)	21·2
Other exposure to mechanical forces	5·5 (3·9 to 7·7)	6 (4·2 to 8·5)	7·1 (5 to 9·9)	6·7 (4·7 to 9·6)	5·5 (3·8 to 7·7)	3·8 (2·6 to 5·3)	2·5 (1·7 to 3·5)	2 (1·4 to 2·8)	1·4 (1 to 2)	0·6 (0·4 to 0·9)	0·2 (0·1 to 0·3)	0 (0 to 0)	75·3
Adverse effects of medical treatment	1·4 (0·8 to 2·4)	1·5 (0·9 to 2·4)	3 (1·8 to 4·8)	3·4 (2 to 5·2)	3·4 (2 to 5·3)	3·4 (2·1 to 5·3)	3 (1·8 to 4·5)	3 (1·8 to 4·6)	2·8 (1·7 to 4·3)	1·4 (0·9 to 2·2)	0·6 (0·3 to 0·9)	0·1 (0·1 to 0·2)	40·5
Animal contact	0·4 (0·3 to 0·6)	0·5 (0·3 to 0·7)	0·6 (0·4 to 0·9)	0·6 (0·4 to 0·9)	0·6 (0·4 to 0·9)	0·5 (0·3 to 0·7)	0·4 (0·2 to 0·5)	0·3 (0·2 to 0·5)	0·2 (0·1 to 0·3)	0·1 (0·1 to 0·1)	0 (0 to 0·1)	0 (0 to 0)	7·4
Venomous animal contact	0·3 (0·2 to 0·4)	0·3 (0·2 to 0·4)	0·4 (0·2 to 0·5)	0·4 (0·2 to 0·5)	0·3 (0·2 to 0·5)	0·3 (0·2 to 0·4)	0·2 (0·1 to 0·3)	0·2 (0·1 to 0·3)	0·1 (0·1 to 0·2)	0·1 (0 to 0·1)	0 (0 to 0)	0 (0 to 0)	4·4
Non-venomous animal contact	0·2 (0·1 to 0·3)	0·2 (0·1 to 0·3)	0·3 (0·2 to 0·4)	0·3 (0·2 to 0·4)	0·3 (0·2 to 0·4)	0·2 (0·1 to 0·4)	0·2 (0·1 to 0·3)	0·1 (0·1 to 0·2)	0·1 (0·1 to 0·1)	0 (0 to 0)	0 (0 to 0)	0 (0 to 0)	3
Foreign body	1·8 (1·3 to 2·6)	1·9 (1·3 to 2·7)	2·8 (1·9 to 3·9)	2·6 (1·8 to 3·8)	2·4 (1·6 to 3·5)	2·1 (1·4 to 3·2)	1·8 (1·1 to 2·7)	1·6 (1 to 2·3)	1·5 (0·9 to 2·2)	0·6 (0·4 to 0·9)	0·3 (0·2 to 0·4)	0·1 (0 to 0·1)	50·9
Pulmonary aspiration and foreign body in airway	1·4 (0·9 to 2·1)	1·4 (0·9 to 2·1)	2·2 (1·4 to 3·4)	2·1 (1·3 to 3·3)	2 (1·1 to 3·1)	1·8 (1·1 to 2·9)	1·5 (0·9 to 2·4)	1·3 (0·8 to 2)	1·3 (0·8 to 2)	0·5 (0·3 to 0·8)	0·2 (0·1 to 0·3)	0 (0 to 0·1)	44
Foreign body in eyes	0·2 (0·1 to 0·4)	0·2 (0·1 to 0·4)	0·2 (0·1 to 0·4)	0·2 (0·1 to 0·3)	0·2 (0·1 to 0·2)	0·1 (0·1 to 0·2)	0·1 (0 to 0·1)	0·1 (0 to 0·1)	0 (0 to 0·1)	0 (0 to 0)	0 (0 to 0)	0 (0 to 0)	2·6
Foreign body in other body part	0·2 (0·2 to 0·3)	0·3 (0·2 to 0·4)	0·3 (0·2 to 0·4)	0·3 (0·2 to 0·5)	0·3 (0·2 to 0·4)	0·2 (0·2 to 0·3)	0·2 (0·1 to 0·3)	0·2 (0·1 to 0·2)	0·2 (0·1 to 0·3)	0·1 (0 to 0·1)	0 (0 to 0·1)	0 (0 to 0)	4·3
Environmental heat and cold exposure	1·5 (1·1 to 2)	1·7 (1·2 to 2·3)	2 (1·4 to 2·8)	2 (1·4 to 2·8)	1·7 (1·2 to 2·4)	1·3 (0·9 to 1·7)	1·1 (0·7 to 1·5)	1 (0·7 to 1·3)	0·9 (0·6 to 1·3)	0·5 (0·3 to 0·7)	0·2 (0·2 to 0·3)	0·1 (0 to 0·1)	22·6
Exposure to forces of nature	0·4 (0·3 to 0·6)	0·6 (0·4 to 0·9)	0·9 (0·6 to 1·3)	1·1 (0·7 to 1·6)	1·3 (0·8 to 1·9)	1·2 (0·7 to 1·8)	1 (0·6 to 1·5)	0·9 (0·6 to 1·4)	0·7 (0·5 to 1·1)	0·4 (0·2 to 0·5)	0·2 (0·1 to 0·2)	0 (0 to 0)	10·9
Other unintentional injuries	5·2 (3·4 to 7·9)	5 (3·3 to 7·4)	5 (3·5 to 7·2)	4·4 (3 to 6·4)	3·2 (2·2 to 4·4)	2·3 (1·6 to 3·1)	1·4 (1 to 2·1)	1·1 (0·8 to 1·6)	0·8 (0·5 to 1·1)	0·3 (0·2 to 0·5)	0·1 (0·1 to 0·2)	0 (0 to 0)	74·2
Self-harm and interpersonal violence	31·3 (21·1 to 44·2)	26·3 (17·9 to 37·8)	28·1 (18·9 to 39·6)	21·7 (15 to 30·5)	14·5 (10 to 19·8)	9·9 (7·1 to 13·3)	6·4 (4·5 to 8·7)	4·1 (3 to 5·7)	2·7 (2 to 3·6)	1·1 (0·8 to 1·5)	0·3 (0·2 to 0·4)	0·1 (0 to 0·1)	507
Self-harm	9·6 (6·4 to 14)	8·3 (5·3 to 12·7)	10·5 (6·7 to 15·5)	8·7 (5·7 to 12·9)	6·3 (3·9 to 9·5)	4·7 (3 to 6·8)	3·4 (2·1 to 5)	2·4 (1·6 to 3·5)	1·8 (1·2 to 2·6)	0·8 (0·5 to 1·1)	0·2 (0·1 to 0·3)	0 (0 to 0)	139
Self-harm by firearm	0·3 (0·2 to 0·6)	0·3 (0·1 to 0·5)	0·4 (0·2 to 0·7)	0·3 (0·1 to 0·5)	0·2 (0·1 to 0·4)	0·1 (0·1 to 0·3)	0·1 (0·1 to 0·2)	0·1 (0 to 0·1)	0 (0 to 0·1)	0 (0 to 0)	0 (0 to 0)	0 (0 to 0)	4·8
Self-harm by other specified means	9·2 (6·1 to 13·6)	8 (5·2 to 12·3)	10·1 (6·5 to 15)	8·5 (5·5 to 12·5)	6·1 (3·7 to 9·1)	4·5 (2·9 to 6·7)	3·3 (2 to 4·9)	2·4 (1·5 to 3·5)	1·8 (1·1 to 2·6)	0·8 (0·5 to 1·1)	0·2 (0·1 to 0·3)	0 (0 to 0)	134
Interpersonal violence	21·6 (12·8 to 32·4)	17·7 (10·6 to 28·1)	17·2 (10·4 to 27)	12·5 (7·5 to 19·4)	7·7 (4·9 to 11·2)	4·6 (3 to 6·9)	2·6 (1·7 to 4·1)	1·7 (1·1 to 2·6)	0·8 (0·6 to 1·2)	0·3 (0·2 to 0·4)	0·1 (0·1 to 0·1)	0 (0 to 0)	365
Physical violence by firearm	5·8 (2·6 to 10·6)	4·3 (1·9 to 8·3)	3·6 (1·6 to 6·9)	2·4 (1·1 to 4·5)	1·2 (0·6 to 2·2)	0·6 (0·3 to 1·2)	0·3 (0·1 to 0·6)	0·1 (0·1 to 0·2)	0·1 (0 to 0·1)	0 (0 to 0)	0 (0 to 0)	0 (0 to 0)	124
Physical violence by sharp object	9·9 (5 to 16·3)	7·6 (4 to 12·7)	7 (3·6 to 12·3)	4·2 (2·3 to 7·2)	2·5 (1·5 to 4·2)	1·4 (0·8 to 2·3)	0·8 (0·5 to 1·5)	0·5 (0·3 to 0·8)	0·3 (0·2 to 0·4)	0·1 (0·1 to 0·1)	0 (0 to 0·1)	0 (0 to 0)	154
Sexual violence	0·3 (0·2 to 0·5)	0·3 (0·2 to 0·5)	0·3 (0·1 to 0·4)	0·2 (0·1 to 0·3)	0·2 (0·1 to 0·2)	0·1 (0·1 to 0·2)	0·1 (0 to 0·1)	0·1 (0 to 0·1)	0 (0 to 0·1)	0 (0 to 0)	0 (0 to 0)	0 (0 to 0)	4·4
Physical violence by other means	5·6 (3 to 9·7)	5·5 (3 to 9·3)	6·4 (3·6 to 10·5)	5·7 (3·1 to 9·6)	3·7 (2·2 to 5·9)	2·5 (1·5 to 4)	1·4 (0·9 to 2·4)	1 (0·6 to 1·6)	0·5 (0·3 to 0·7)	0·2 (0·1 to 0·3)	0 (0 to 0·1)	0 (0 to 0)	83·2
Conflict and terrorism	0·2 (0·1 to 0·3)	0·2 (0·1 to 0·4)	0·4 (0·3 to 0·6)	0·5 (0·3 to 0·7)	0·5 (0·3 to 0·7)	0·6 (0·4 to 0·9)	0·3 (0·2 to 0·5)	0 (0 to 0·1)	0 (0 to 0)	0 (0 to 0)	0 (0 to 0)	0 (0 to 0)	2·9

SR Table 2a: Dominican Republic DALYS by cause and age group for males with 95% uncertainty intervals, 2019

Cause	Early Neonatal	Late Neonatal	Post Neonatal	1 to 4	5 to 9	10 to 14	15 to 19	20 to 24	25 to 29	30 to 34	35 to 39	40 to 44	45 to 49	50 to 54	55 to 59	60 to 64	65 to 69	70 to 74	75 to 79	80 to 84	85 to 89	90 to 94	95 plus	Total
All causes	166,000 (117,000 to 229,000)	37,700 (26,700 to 51,800)	60,900 (44,900 to 81,900)	55,900 (44,300 to 68,900)	39,400 (32,800 to 46,700)	40,800 (33,600 to 49,900)	66,400 (54,600 to 80,100)	99,800 (82,100 to 118,000)	106,000 (88,800 to 122,000)	110,000 (92,400 to 128,000)	108,000 (89,500 to 127,000)	112,000 (91,200 to 134,000)	117,000 (94,000 to 144,000)	128,000 (102,000 to 159,000)	121,000 (96,300 to 151,000)	127,000 (102,000 to 157,000)	107,000 (86,300 to 130,000)	99,100 (81,800 to 119,000)	68,100 (57,600 to 80,500)	50,800 (44,300 to 58,400)	32,700 (29,100 to 36,500)	13,200 (11,800 to 14,500)	3,970 (3,640 to 4,280)	1,870,000
Communicable, maternal, neonatal, and nutritional diseases	148,000 (103,000 to 203,000)	31,400 (21,700 to 43,300)	36,200 (23,800 to 51,400)	24,000 (17,500 to 32,100)	11,400 (8,760 to 14,900)	7,870 (6,180 to 10,000)	8,140 (6,380 to 10,200)	10,300 (8,140 to 12,900)	12,200 (9,370 to 16,000)	14,600 (11,100 to 19,800)	15,100 (11,300 to 20,700)	14,600 (11,300 to 19,800)	13,400 (10,500 to 17,600)	11,200 (8,810 to 14,500)	8,310 (6,530 to 10,600)	7,010 (5,370 to 9,030)	5,360 (4,140 to 7,010)	4,970 (3,740 to 6,490)	3,460 (2,660 to 4,540)	2,690 (2,040 to 3,510)	2,020 (1,500 to 2,590)	846 (625 to 1,110)	243 (161 to 342)	393,000
HIV/AIDS and sexually transmitted infections	4,510 (1,440 to 9,930)	1,690 (503 to 3,810)	2,800 (1,020 to 5,880)	458 (197 to 1,040)	229 (151 to 313)	193 (114 to 293)	584 (316 to 1,050)	1,350 (752 to 2,340)	2,930 (1,440 to 5,930)	4,760 (2,540 to 9,160)	6,480 (3,640 to 11,500)	6,650 (4,280 to 11,100)	6,230 (4,160 to 9,970)	4,410 (3,090 to 6,580)	2,490 (1,750 to 3,770)	1,250 (844 to 1,920)	626 (393 to 982)	316 (178 to 506)	151 (84·1 to 249)	48·7 (28·3 to 85·9)	25·6 (14·3 to 47·9)	8·9 (4·7 to 17·2)	2·9 (1·3 to 7·4)	48,200
HIV/AIDS	0 (0 to 0)	0·1 (0 to 0·1)	301 (157 to 493)	375 (140 to 958)	191 (124 to 265)	156 (86·2 to 238)	490 (238 to 942)	1,210 (629 to 2,200)	2,760 (1,290 to 5,780)	4,590 (2,380 to 9,020)	6,310 (3,510 to 11,300)	6,540 (4,160 to 10,800)	6,130 (4,070 to 9,860)	4,320 (3,000 to 6,520)	2,420 (1,670 to 3,680)	1,200 (782 to 1,860)	570 (341 to 919)	283 (150 to 478)	134 (67·6 to 229)	31·7 (16 to 56·4)	14·6 (7·5 to 25·9)	5·1 (2·7 to 8·9)	1·3 (0·7 to 2·3)	38,000
HIV/AIDS - Drug-susceptible Tuberculosis	0 (0 to 0)	0 (0 to 0)	50 (31·3 to 79)	58 (36·1 to 89·2)	29·2 (19·1 to 44·8)	24 (15·9 to 36·3)	69·2 (45 to 107)	176 (115 to 270)	384 (248 to 587)	658 (424 to 1,020)	925 (600 to 1,420)	1,040 (665 to 1,600)	1,040 (670 to 1,630)	774 (498 to 1,200)	432 (276 to 670)	224 (144 to 343)	98·8 (63·1 to 150)	49·7 (32·9 to 75·3)	21·3 (14·3 to 31·9)	5·2 (3·5 to 7·5)	2·5 (1·7 to 3·6)	0·7 (0·5 to 1)	0·1 (0·1 to 0·2)	6,070
HIV/AIDS - Multidrug-resistant Tuberculosis without exten	0 (0 to 0)	0 (0 to 0)	0·7 (0·1 to 3·7)	0·8 (0·1 to 4·1)	0·4 (0 to 2)	0·3 (0 to 1·6)	1 (0·1 to 4·8)	2·5 (0·2 to 12)	5·3 (0·4 to 25·7)	9 (0·7 to 43·7)	12·6 (1 to 61·2)	14·2 (1·1 to 69·3)	14·5 (1·1 to 70·9)	10·8 (0·8 to 53·2)	6·1 (0·5 to 29·8)	3·1 (0·2 to 15·4)	1·4 (0·1 to 6·8)	0·7 (0·1 to 3·3)	0·3 (0 to 1·4)	0·1 (0 to 0·3)	0 (0 to 0·1)	0 (0 to 0)	0 (0 to 0)	83·8
HIV/AIDS - Extensively drug-resistant Tuberculosis	0 (0 to 0)	0 (0 to 0)	0·1 (0 to 0·6)	0·1 (0 to 0·7)	0·1 (0 to 0·3)	0·1 (0 to 0·3)	0·2 (0 to 0·8)	0·4 (0 to 2)	0·9 (0·1 to 4·3)	1·5 (0·1 to 7·3)	2·1 (0·2 to 10·2)	2·4 (0·2 to 11·6)	2·4 (0·2 to 11·8)	1·8 (0·1 to 8·9)	1 (0·1 to 5)	0·5 (0 to 2·6)	0·2 (0 to 1·1)	0·1 (0 to 0·5)	0 (0 to 0·2)	0 (0 to 0·1)	0 (0 to 0)	0 (0 to 0)	0 (0 to 0)	13·9
HIV/AIDS resulting in other diseases	0 (0 to 0)	0·1 (0 to 0·1)	250 (104 to 449)	316 (81·7 to 905)	161 (93·3 to 236)	131 (62·8 to 211)	420 (167 to 874)	1,030 (456 to 2,020)	2,370 (917 to 5,190)	3,930 (1,650 to 8,420)	5,370 (2,530 to 10,300)	5,490 (3,040 to 10,000)	5,070 (2,980 to 8,870)	3,530 (2,200 to 5,710)	1,980 (1,220 to 3,230)	969 (561 to 1,620)	469 (242 to 825)	232 (99 to 431)	112 (46·8 to 205)	26·5 (11·4 to 51·6)	12·1 (5·2 to 22·9)	4·4 (2 to 8·2)	1·1 (0·5 to 2·1)	31,900
Sexually transmitted infections excluding HIV	4,510 (1,440 to 9,930)	1,690 (503 to 3,810)	2,500 (740 to 5,620)	83·5 (25·4 to 191)	37·9 (12·2 to 82·8)	37 (13·5 to 106)	94 (41 to 195)	142 (63·7 to 300)	163 (77·1 to 350)	171 (80·5 to 330)	179 (76·4 to 482)	111 (53·3 to 208)	102 (47·9 to 216)	87·7 (38·3 to 202)	75·7 (34·2 to 165)	54·9 (22·1 to 127)	56·6 (20·7 to 145)	32·9 (13·6 to 70·4)	17·4 (7·2 to 38·1)	16·9 (6·6 to 41·4)	11 (3·6 to 29·6)	3·8 (1·2 to 10·9)	1·6 (0·3 to 5·9)	10,200
Syphilis	4,510 (1,440 to 9,930)	1,690 (503 to 3,810)	2,500 (740 to 5,620)	83·5 (25·4 to 191)	37·9 (12·2 to 82·8)	16 (3·7 to 55·5)	35·9 (7·3 to 118)	41·8 (8·9 to 136)	60·3 (12·5 to 197)	59·1 (13·4 to 165)	94·6 (18·8 to 357)	41·1 (11·9 to 113)	40·4 (10·8 to 113)	42·7 (11·4 to 125)	44·7 (14·5 to 116)	34·3 (10·3 to 93·1)	37·5 (11·5 to 102)	21·4 (7·2 to 51·5)	8·4 (2·9 to 20·6)	6·9 (2·6 to 18·1)	3·4 (1·1 to 9·2)	0·9 (0·3 to 2·6)	0·3 (0·1 to 1·1)	9,410
Chlamydial infection	0 (0 to 0)	0 (0 to 0)	0 (0 to 0)	0 (0 to 0)	0 (0 to 0)	0·2 (0·1 to 0·4)	4·9 (2·3 to 8·9)	16·6 (4·1 to 36·9)	26 (7·9 to 52·8)	24·7 (7 to 50·3)	20·2 (5·4 to 40·5)	15·7 (4·2 to 33·8)	10·1 (2·2 to 22·8)	4·1 (0·2 to 10·2)	1·2 (0·6 to 2·1)	0·2 (0·1 to 0·4)	0·1 (0 to 0·1)	0 (0 to 0)	0 (0 to 0)	0 (0 to 0)	0 (0 to 0)	0 (0 to 0)	0 (0 to 0)	124
Gonococcal infection	0 (0 to 0)	0 (0 to 0)	0 (0 to 0)	0 (0 to 0)	0 (0 to 0)	3·9 (1·5 to 8·6)	22·2 (6·5 to 46·4)	38·5 (13·6 to 84)	15·6 (4·2 to 34·8)	11·3 (2·5 to 26·1)	9·8 (3·9 to 25·5)	9·6 (4 to 21·6)	7·9 (3 to 18·5)	4·8 (1·9 to 11·2)	3·8 (1·5 to 8·6)	3·5 (1 to 9·8)	5 (1·4 to 14·1)	2·4 (0·7 to 6·1)	1 (0·3 to 2·8)	1·2 (0·4 to 3·4)	0·7 (0·2 to 2·1)	0·1 (0 to 0·2)	0 (0 to 0)	141
Trichomoniasis	0 (0 to 0)	0 (0 to 0)	0 (0 to 0)	0 (0 to 0)	0 (0 to 0)	0 (0 to 0)	0·1 (0 to 0·4)	0·7 (0·1 to 2·1)	1·8 (0·4 to 4·6)	2·6 (0·6 to 6·5)	2·8 (0·8 to 6·9)	2·5 (0·7 to 6·3)	1·9 (0·5 to 4·8)	1·3 (0·3 to 3·4)	0·7 (0·2 to 1·9)	0·4 (0·1 to 0·9)	0·2 (0 to 0·6)	0·1 (0 to 0·4)	0·1 (0 to 0·2)	0 (0 to 0·1)	0 (0 to 0)	0 (0 to 0)	0 (0 to 0)	15·4
Genital herpes	0 (0 to 0)	0 (0 to 0)	0 (0 to 0)	0 (0 to 0)	0 (0 to 0)	1·2 (0·3 to 3)	5·4 (1·6 to 13·9)	12 (3·4 to 29·8)	19·4 (6·2 to 45·2)	24·2 (8 to 57·8)	25·7 (8·5 to 63·3)	24·7 (7·7 to 62·4)	22·3 (7 to 56·4)	19·2 (5·9 to 47·5)	15·3 (4·7 to 38·3)	11·5 (3·5 to 29)	8·3 (2·3 to 20·2)	5·7 (1·8 to 14·3)	3·5 (1·1 to 8·8)	2·1 (0·6 to 5·2)	1·1 (0·3 to 2·6)	0·3 (0·1 to 0·9)	0·1 (0 to 0·2)	202
Other sexually transmitted infections	0 (0 to 0)	0 (0 to 0)	0 (0 to 0)	0 (0 to 0)	0 (0 to 0)	15·7 (5·7 to 41·4)	25·5 (10·1 to 49·6)	32·3 (13·9 to 61·2)	39·6 (17·2 to 83·5)	48·8 (20·4 to 96·9)	25·5 (11·1 to 50·9)	16·9 (7·4 to 30·5)	19·7 (7·9 to 44·3)	15·6 (5·6 to 39·5)	10 (3·5 to 24·6)	5 (1·4 to 14)	5·5 (1·5 to 15·4)	3·3 (0·9 to 8·2)	4·4 (1·1 to 11·9)	6·6 (2 to 18·5)	5·8 (1·4 to 16·9)	2·4 (0·5 to 7·7)	1·2 (0·2 to 4·7)	284
Respiratory infections and tuberculosis	2,420 (1,140 to 4,400)	1,310 (607 to 2,450)	7,390 (3,350 to 13,100)	4,610 (2,720 to 7,520)	2,480 (1,660 to 3,320)	1,610 (1,130 to 2,190)	1,900 (1,300 to 2,700)	3,410 (2,290 to 5,060)	3,970 (2,660 to 5,690)	4,710 (3,150 to 6,810)	3,960 (2,730 to 5,810)	3,820 (2,600 to 5,810)	3,410 (2,250 to 5,220)	3,240 (2,100 to 4,900)	2,790 (1,830 to 4,140)	2,970 (1,930 to 4,420)	2,570 (1,700 to 3,810)	2,690 (1,750 to 4,050)	1,910 (1,280 to 2,760)	1,570 (1,080 to 2,250)	1,210 (806 to 1,690)	513 (342 to 726)	119 (72·3 to 181)	64,600
Tuberculosis	0 (0 to 0)	0 (0 to 0)	452 (221 to 806)	575 (288 to 989)	324 (193 to 502)	247 (150 to 385)	631 (331 to 1,090)	2,030 (1,160 to 3,350)	2,550 (1,540 to 4,020)	3,160 (1,920 to 4,840)	2,550 (1,530 to 4,060)	2,280 (1,390 to 3,700)	1,690 (989 to 2,790)	1,460 (871 to 2,340)	1,110 (679 to 1,760)	969 (577 to 1,590)	707 (441 to 1,120)	619 (370 to 976)	406 (246 to 633)	270 (164 to 412)	167 (104 to 254)	60·6 (38·5 to 91)	14·3 (8·2 to 22·4)	22,300
Latent tuberculosis infection	0 (0 to 0)	0 (0 to 0)	0 (0 to 0)	0 (0 to 0)	0 (0 to 0)	0 (0 to 0)	0 (0 to 0)	0 (0 to 0)	0 (0 to 0)	0 (0 to 0)	0 (0 to 0)	0 (0 to 0)	0 (0 to 0)	0 (0 to 0)	0 (0 to 0)	0 (0 to 0)	0 (0 to 0)	0 (0 to 0)	0 (0 to 0)	0 (0 to 0)	0 (0 to 0)	0 (0 to 0)	0 (0 to 0)	0
Drug-susceptible tuberculosis	0 (0 to 0)	0 (0 to 0)	446 (217 to 801)	567 (284 to 975)	321 (189 to 495)	244 (148 to 382)	622 (329 to 1,070)	2,010 (1,160 to 3,260)	2,520 (1,510 to 3,980)	3,120 (1,880 to 4,780)	2,520 (1,510 to 3,970)	2,250 (1,370 to 3,660)	1,670 (983 to 2,730)	1,440 (859 to 2,310)	1,100 (667 to 1,740)	957 (567 to 1,580)	698 (429 to 1,110)	611 (365 to 969)	401 (244 to 623)	267 (162 to 407)	165 (103 to 250)	59·8 (37·9 to 89)	14·1 (8·2 to 22·2)	22,000
Multidrug-resistant tuberculosis without extensive drug res	0 (0 to 0)	0 (0 to 0)	5·2 (0·4 to 24·9)	6·3 (0·5 to 30·9)	3·4 (0·3 to 16·9)	2·6 (0·2 to 12·6)	7·3 (0·5 to 38·4)	22·3 (1·7 to 100)	27 (2·1 to 124)	34·4 (2·8 to 174)	27·6 (2·2 to 143)	24·4 (2 to 116)	18·7 (1·6 to 92·9)	15·7 (1·4 to 76·3)	12·2 (1 to 60·1)	10·6 (0·9 to 53·8)	8 (0·7 to 36·2)	6·9 (0·5 to 31·9)	4·5 (0·4 to 22·6)	3·1 (0·2 to 15·4)	1·9 (0·2 to 9·2)	0·7 (0·1 to 3·1)	0·2 (0 to 0·8)	243
Extensively drug-resistant tuberculosis	0 (0 to 0)	0 (0 to 0)	0·9 (0·1 to 4·1)	1 (0·1 to 5)	0·5 (0 to 2·6)	0·4 (0 to 2)	1·2 (0·1 to 6·6)	3·6 (0·3 to 17·4)	4·4 (0·3 to 22·2)	5·7 (0·4 to 28·7)	4·6 (0·4 to 22)	4 (0·3 to 18·8)	3·1 (0·2 to 16)	2·6 (0·2 to 12·9)	2 (0·2 to 9·8)	1·8 (0·1 to 8·9)	1·3 (0·1 to 6·6)	1·1 (0·1 to 5·7)	0·7 (0·1 to 3·8)	0·5 (0 to 2·4)	0·3 (0 to 1·5)	0·1 (0 to 0·5)	0 (0 to 0·1)	40·1
Lower respiratory infections	2,420 (1,140 to 4,400)	1,300 (600 to 2,450)	6,780 (2,890 to 12,400)	3,300 (1,620 to 5,910)	1,390 (695 to 2,010)	788 (387 to 1,180)	726 (397 to 1,160)	869 (500 to 1,440)	959 (565 to 1,540)	1,150 (674 to 1,860)	1,060 (615 to 1,740)	1,230 (715 to 2,030)	1,460 (853 to 2,420)	1,570 (905 to 2,560)	1,510 (890 to 2,480)	1,880 (1,090 to 3,030)	1,770 (1,020 to 2,810)	2,020 (1,190 to 3,300)	1,470 (903 to 2,260)	1,270 (825 to 1,910)	1,030 (653 to 1,510)	448 (283 to 665)	104 (62·6 to 164)	36,500
Upper respiratory infections	1·2 (0·6 to 2)	7·1 (3·9 to 12·2)	136 (76·8 to 228)	512 (290 to 838)	482 (264 to 820)	401 (202 to 703)	386 (194 to 687)	372 (191 to 636)	344 (188 to 573)	305 (162 to 511)	272 (131 to 477)	234 (124 to 414)	189 (99·1 to 334)	153 (81·5 to 262)	120 (63·6 to 203)	89·9 (46·9 to 152)	67·7 (34·6 to 118)	45·9 (24·4 to 76·8)	28·4 (14·8 to 48·7)	16·8 (8·8 to 28·4)	8·3 (4·6 to 14·2)	3·1 (1·7 to 5·3)	0·8 (0·4 to 1·5)	4,180
Otitis media	0·1 (0 to 0·1)	0·6 (0·3 to 1·1)	25·9 (13·4 to 46·8)	230 (126 to 395)	277 (158 to 463)	177 (94 to 311)	161 (81·1 to 278)	139 (58·4 to 271)	112 (53 to 206)	86·8 (35·9 to 170)	79·3 (35·5 to 154)	77·2 (34·5 to 144)	71 (35 to 122)	62·4 (28·2 to 113)	48 (22·8 to 84·1)	33·2 (14·6 to 65·9)	20·1 (8·8 to 37·3)	12 (5·2 to 23·7)	8 (3·8 to 15)	5·5 (2·5 to 10·3)	3 (1·4 to 5·4)	1·2 (0·5 to 2·2)	0·3 (0·1 to 0·6)	1,630
Enteric infections	361 (82·2 to 1,100)	800 (195 to 2,460)	7,480 (2,630 to 17,200)	4,370 (2,190 to 8,370)	1,610 (919 to 2,630)	1,020 (628 to 1,560)	764 (496 to 1,130)	704 (442 to 1,040)	740 (483 to 1,110)	748 (506 to 1,080)	758 (516 to 1,100)	770 (525 to 1,100)	747 (500 to 1,070)	727 (484 to 1,100)	673 (452 to 996)	717 (488 to 1,070)	701 (477 to 1,010)	749 (510 to 1,100)	600 (414 to 868)	497 (340 to 732)	332 (216 to 519)	133 (79·2 to 209)	65 (31·9 to 126)	26,100
Diarrheal diseases	361 (82·2 to 1,100)	800 (195 to 2,460)	7,460 (2,610 to 17,200)	4,330 (2,150 to 8,320)	1,590 (903 to 2,600)	1,000 (608 to 1,540)	747 (481 to 1,120)	689 (429 to 1,020)	729 (475 to 1,090)	738 (499 to 1,070)	751 (512 to 1,090)	763 (515 to 1,090)	740 (495 to 1,070)	721 (481 to 1,090)	667 (446 to 986)	712 (485 to 1,070)	697 (474 to 1,010)	746 (506 to 1,100)	599 (413 to 867)	496 (340 to 731)	331 (215 to 518)	132 (79 to 209)	64·9 (31·9 to 126)	25,900
Typhoid and paratyphoid	0 (0 to 0)	0 (0 to 0)	1·5 (0·4 to 4·1)	10·3 (2·8 to 26·5)	7·1 (1·9 to 20·4)	8·3 (2·5 to 20·4)	7·6 (2·1 to 18·6)	7·1 (1·8 to 17·9)	5·6 (1·3 to 14)	4·4 (1·2 to 11·1)	3·5 (1 to 9)	3·3 (1 to 8·5)	3·1 (1 to 8·1)	3·1 (1·1 to 6·9)	2·5 (0·9 to 5·5)	2·1 (0·7 to 4·7)	1·4 (0·5 to 3·2)	1 (0·4 to 2·3)	0·7 (0·3 to 1·6)	0·6 (0·3 to 1·2)	0·4 (0·1 to 0·8)	0·1 (0 to 0·3)	0·1 (0 to 0·1)	73·9
Typhoid fever	0 (0 to 0)	0 (0 to 0)	0·8 (0·1 to 2·4)	5·4 (1 to 14·5)	3·7 (0·8 to 11·7)	4·7 (1·1 to 12·3)	4·6 (0·9 to 12·2)	4·7 (1·1 to 12·1)	4 (0·9 to 10·7)	3·2 (0·9 to 8·4)	2·5 (0·7 to 6·7)	2·2 (0·6 to 5·6)	2 (0·5 to 5·2)	1·9 (0·6 to 4·2)	1·4 (0·5 to 3·2)	1·1 (0·3 to 2·6)	0·7 (0·2 to 1·7)	0·5 (0·1 to 1·2)	0·3 (0·1 to 0·8)	0·3 (0·1 to 0·6)	0·2 (0 to 0·4)	0·1 (0 to 0·1)	0 (0 to 0·1)	44·3
Paratyphoid fever	0 (0 to 0)	0 (0 to 0)	0·7 (0·2 to 2·1)	4·9 (1·2 to 14)	3·4 (0·8 to 9·7)	3·7 (1 to 10)	3 (0·8 to 7·7)	2·4 (0·6 to 6·6)	1·5 (0·3 to 4·3)	1·1 (0·3 to 3·3)	1 (0·3 to 2·9)	1·1 (0·3 to 2·9)	1·1 (0·3 to 3·2)	1·2 (0·4 to 3)	1·1 (0·3 to 2·6)	1 (0·3 to 2·5)	0·7 (0·3 to 1·7)	0·6 (0·2 to 1·4)	0·4 (0·2 to 0·9)	0·3 (0·1 to 0·7)	0·2 (0·1 to 0·5)	0·1 (0 to 0·2)	0 (0 to 0·1)	29·6
Invasive Non-typhoidal Salmonella (iNTS)	0 (0 to 0)	0·4 (0·1 to 1·2)	7·1 (1·4 to 19·4)	20 (4·5 to 47·9)	14·9 (4 to 33·3)	9·7 (2 to 23·9)	6·5 (1·1 to 19·2)	5·1 (0·5 to 16·2)	3·5 (0·5 to 10·5)	2·7 (0·5 to 8·1)	2·4 (0·3 to 6·2)	2·2 (0·2 to 5·9)	2·3 (0·1 to 5·9)	2·5 (0·3 to 6·3)	2·6 (0·4 to 6·6)	2·7 (0·3 to 7·1)	2·1 (0·3 to 5·5)	1·4 (0·2 to 4·1)	0·7 (0·1 to 2)	0·4 (0 to 1·2)	0·2 (0 to 0·6)	0·1 (0 to 0·2)	0 (0 to 0)	89·5
Other intestinal infectious diseases	0 (0 to 0)	0 (0 to 0)	9 (0·6 to 33)	13·3 (2·2 to 46·4)	5·3 (0·9 to 17·6)	3·7 (0·7 to 11·3)	2·4 (0·4 to 7·5)	3 (0·4 to 9·3)	1·4 (0·2 to 4·4)	3·1 (0·6 to 9·4)	1·4 (0·3 to 4·3)	1·4 (0·3 to 4·3)	0·8 (0·2 to 2·6)	0·7 (0·1 to 2·3)	0·5 (0·1 to 1·5)	0·4 (0·1 to 1·3)	0·5 (0·1 to 1·6)	0·3 (0·1 to 1·1)	0·2 (0 to 0·5)	0·2 (0 to 0·5)	0·2 (0 to 0·6)	0·1 (0 to 0·2)	0 (0 to 0·1)	47·8
Neglected tropical diseases and malaria	0·8 (0·3 to 1·8)	11·6 (5·1 to 22·5)	144 (57·1 to 348)	288 (144 to 493)	776 (404 to 1,320)	824 (398 to 1,500)	911 (445 to 1,680)	984 (509 to 1,800)	1,060 (567 to 1,840)	1,020 (539 to 1,690)	914 (492 to 1,550)	833 (483 to 1,370)	758 (442 to 1,220)	702 (405 to 1,070)	628 (361 to 977)	550 (326 to 830)	450 (268 to 679)	362 (216 to 544)	251 (147 to 385)	168 (95 to 259)	91·8 (54·8 to 145)	33·9 (19·3 to 55·1)	9·4 (5·3 to 15)	11,800
Malaria	0 (0 to 0)	0·1 (0 to 0·2)	0·2 (0 to 0·6)	0·7 (0·2 to 1·9)	1·5 (0·7 to 3·5)	2·1 (1·2 to 4·2)	19·1 (3·9 to 71·9)	28·9 (5·2 to 110)	16·9 (3·7 to 60·6)	11·5 (2·7 to 38·7)	7·5 (2 to 26)	8·9 (2 to 32·4)	7·4 (1·7 to 27·1)	6·9 (1·4 to 25·6)	3·7 (0·9 to 12·9)	4·3 (0·9 to 16·1)	3·9 (0·7 to 14·6)	4·1 (0·6 to 15·9)	1·2 (0·2 to 4·5)	0·4 (0·1 to 1·7)	0·1 (0 to 0·2)	0 (0 to 0)	0 (0 to 0)	129
Chagas disease	0 (0 to 0)	0 (0 to 0)	0 (0 to 0)	0 (0 to 0)	0 (0 to 0)	0 (0 to 0)	0 (0 to 0)	0·1 (0 to 0·1)	0·1 (0·1 to 0·2)	0·3 (0·2 to 0·5)	0·4 (0·2 to 0·6)	0·5 (0·3 to 0·7)	0·5 (0·3 to 0·8)	0·5 (0·3 to 0·8)	0·5 (0·3 to 0·8)	0·5 (0·3 to 0·7)	0·4 (0·2 to 0·6)	0·4 (0·2 to 0·6)	0·3 (0·1 to 0·5)	0·2 (0·1 to 0·5)	0·1 (0 to 0·3)	0 (0 to 0·1)	0 (0 to 0·1)	4·8
Leishmaniasis	0 (0 to 0)	0 (0 to 0)	0 (0 to 0)	0 (0 to 0·2)	0 (0 to 0·2)	0 (0 to 0·2)	0 (0 to 0·2)	0 (0 to 0·1)	0 (0 to 0·1)	0 (0 to 0·1)	0 (0 to 0·1)	0 (0 to 0·1)	0 (0 to 0·1)	0 (0 to 0·1)	0 (0 to 0·1)	0 (0 to 0·1)	0 (0 to 0)	0 (0 to 0)	0 (0 to 0)	0 (0 to 0)	0 (0 to 0)	0 (0 to 0)	0 (0 to 0)	0·3
Visceral leishmaniasis	0 (0 to 0)	0 (0 to 0)	0 (0 to 0)	0 (0 to 0)	0 (0 to 0)	0 (0 to 0)	0 (0 to 0)	0 (0 to 0)	0 (0 to 0)	0 (0 to 0)	0 (0 to 0)	0 (0 to 0)	0 (0 to 0)	0 (0 to 0)	0 (0 to 0)	0 (0 to 0)	0 (0 to 0)	0 (0 to 0)	0 (0 to 0)	0 (0 to 0)	0 (0 to 0)	0 (0 to 0)	0 (0 to 0)	0
Cutaneous and mucocutaneous leishmaniasis	0 (0 to 0)	0 (0 to 0)	0 (0 to 0)	0 (0 to 0·2)	0 (0 to 0·2)	0 (0 to 0·2)	0 (0 to 0·2)	0 (0 to 0·1)	0 (0 to 0·1)	0 (0 to 0·1)	0 (0 to 0·1)	0 (0 to 0·1)	0 (0 to 0·1)	0 (0 to 0·1)	0 (0 to 0·1)	0 (0 to 0·1)	0 (0 to 0)	0 (0 to 0)	0 (0 to 0)	0 (0 to 0)	0 (0 to 0)	0 (0 to 0)	0 (0 to 0)	0·3
African trypanosomiasis	0 (0 to 0)	0 (0 to 0)	0 (0 to 0)	0 (0 to 0)	0 (0 to 0)	0 (0 to 0)	0 (0 to 0)	0 (0 to 0)	0 (0 to 0)	0 (0 to 0)	0 (0 to 0)	0 (0 to 0)	0 (0 to 0)	0 (0 to 0)	0 (0 to 0)	0 (0 to 0)	0 (0 to 0)	0 (0 to 0)	0 (0 to 0)	0 (0 to 0)	0 (0 to 0)	0 (0 to 0)	0 (0 to 0)	0
Schistosomiasis	0 (0 to 0)	0 (0 to 0)	0 (0 to 0)	33·7 (8·2 to 87·7)	259 (75·1 to 642)	440 (131 to 1,040)	495 (151 to 1,220)	498 (157 to 1,210)	544 (184 to 1,270)	521 (180 to 1,130)	452 (170 to 987)	390 (153 to 836)	333 (133 to 687)	291 (124 to 581)	239 (93 to 485)	183 (73·8 to 359)	121 (49·9 to 250)	89·4 (36 to 181)	53·2 (19·1 to 114)	30·1 (10·4 to 64·2)	16·3 (5·9 to 36·9)	5·6 (2·1 to 12·5)	1·4 (0·5 to 3·3)	5,000
Cysticercosis	0 (0 to 0)	0 (0 to 0)	0 (0 to 0)	0·6 (0 to 2·6)	3·4 (0 to 12·5)	6·5 (0·7 to 18·8)	26·2 (0 to 69·7)	78·3 (17·1 to 187)	124 (32·9 to 261)	130 (38·2 to 269)	127 (37·8 to 276)	135 (47·1 to 298)	146 (48·3 to 299)	167 (64·1 to 332)	190 (74·8 to 358)	206 (85·3 to 392)	206 (84·7 to 386)	179 (79 to 315)	137 (57·5 to 256)	97·4 (40·8 to 180)	52·9 (22·1 to 104)	19·4 (7·8 to 39·7)	5·2 (1·8 to 10·3)	2,040
Cystic echinococcosis	0 (0 to 0)	0 (0 to 0)	0 (0 to 0)	0·1 (0 to 0·3)	0·1 (0 to 0·3)	0·1 (0 to 0·2)	0·1 (0 to 0·2)	0·1 (0 to 0·3)	0·1 (0 to 0·5)	0·1 (0 to 0·5)	0·1 (0 to 0·4)	0·1 (0 to 0·4)	0·1 (0 to 0·3)	0·1 (0 to 0·3)	0·1 (0 to 0·3)	0·1 (0 to 0·2)	0 (0 to 0·2)	0 (0 to 0·1)	0 (0 to 0·1)	0 (0 to 0)	0 (0 to 0)	0 (0 to 0)	0 (0 to 0)	1·4
Lymphatic filariasis	0 (0 to 0)	0 (0 to 0)	0 (0 to 0)	0 (0 to 0)	238 (97·2 to 503)	242 (100 to 498)	270 (119 to 557)	295 (128 to 596)	296 (133 to 598)	281 (124 to 584)	259 (112 to 534)	234 (104 to 462)	209 (92·3 to 418)	184 (80·8 to 366)	153 (68·7 to 299)	122 (54·7 to 240)	93·2 (41 to 182)	67·9 (30·5 to 134)	45·1 (20·4 to 87)	28·6 (13·4 to 54·9)	15·1 (6·8 to 29·2)	5·4 (2·5 to 10·3)	1·4 (0·7 to 2·7)	3,040
Onchocerciasis	0 (0 to 0)	0 (0 to 0)	0 (0 to 0)	0 (0 to 0)	0 (0 to 0)	0 (0 to 0)	0 (0 to 0)	0 (0 to 0)	0 (0 to 0)	0 (0 to 0)	0 (0 to 0)	0 (0 to 0)	0 (0 to 0)	0 (0 to 0)	0 (0 to 0)	0 (0 to 0)	0 (0 to 0)	0 (0 to 0)	0 (0 to 0)	0 (0 to 0)	0 (0 to 0)	0 (0 to 0)	0 (0 to 0)	0
Trachoma	0 (0 to 0)	0 (0 to 0)	0 (0 to 0)	0 (0 to 0)	0 (0 to 0)	0 (0 to 0)	0 (0 to 0)	0 (0 to 0)	0 (0 to 0)	0 (0 to 0)	0 (0 to 0)	0 (0 to 0)	0 (0 to 0)	0 (0 to 0)	0 (0 to 0)	0 (0 to 0)	0 (0 to 0)	0 (0 to 0)	0 (0 to 0)	0 (0 to 0)	0 (0 to 0)	0 (0 to 0)	0 (0 to 0)	0
Dengue	0 (0 to 0)	2·9 (0·3 to 12·1)	76·8 (12·2 to 280)	59·6 (10·5 to 156)	83·1 (17·9 to 211)	56·5 (11·7 to 127)	54·5 (11·1 to 128)	48·5 (10·4 to 109)	43·7 (8·5 to 98·9)	37·4 (7·5 to 87·1)	31·1 (6·7 to 71·3)	28·4 (5·4 to 67·6)	21·9 (3·6 to 51·1)	19·7 (3·3 to 48·5)	14·8 (2 to 36·2)	14·5 (2·7 to 35·3)	10 (1·3 to 25·2)	8·8 (1·8 to 21·6)	6·7 (1·7 to 15·9)	4·9 (1·3 to 11·8)	3 (0·8 to 7)	1·9 (0·7 to 4·3)	0·9 (0·3 to 2)	630
Yellow fever	0 (0 to 0)	0 (0 to 0)	0 (0 to 0)	0 (0 to 0)	0 (0 to 0)	0 (0 to 0)	0 (0 to 0)	0 (0 to 0)	0 (0 to 0)	0 (0 to 0)	0 (0 to 0)	0 (0 to 0)	0 (0 to 0)	0 (0 to 0)	0 (0 to 0)	0 (0 to 0)	0 (0 to 0)	0 (0 to 0)	0 (0 to 0)	0 (0 to 0)	0 (0 to 0)	0 (0 to 0)	0 (0 to 0)	0
Rabies	0 (0 to 0)	0 (0 to 0)	0·2 (0 to 0·7)	19·9 (0·4 to 71·4)	26·1 (0·7 to 93·9)	10·8 (0·1 to 36·7)	10·4 (0·2 to 30·2)	1·6 (0·1 to 5)	2 (0·1 to 6·1)	0·5 (0 to 1·6)	8 (0·1 to 24·5)	7 (0·1 to 20·3)	11·7 (0·1 to 34·1)	6·4 (0 to 18·9)	0·7 (0 to 2·4)	4·8 (0 to 16·2)	6 (0 to 17·9)	3·7 (0 to 12·6)	0·4 (0 to 1·2)	1·7 (0 to 5·1)	0·1 (0 to 0·3)	0 (0 to 0)	0 (0 to 0)	122
Intestinal nematode infections	0 (0 to 0)	0 (0 to 0)	2·6 (0·1 to 11·8)	15·5 (0·4 to 58·9)	14·2 (0·4 to 44·7)	4·4 (0·7 to 12·5)	5·1 (1·6 to 11·6)	4·1 (1·1 to 9·7)	3·1 (0·7 to 7·9)	2·8 (0·6 to 7·8)	2·5 (0·6 to 6·4)	2 (0·5 to 5)	1·7 (0·5 to 4·1)	1·6 (0·5 to 3·6)	1·4 (0·5 to 3·2)	1·6 (0·4 to 4·1)	0·9 (0·2 to 2)	0·9 (0·2 to 2·4)	0·7 (0·2 to 1·6)	0·4 (0·1 to 1)	0·3 (0·1 to 0·6)	0·1 (0 to 0·3)	0 (0 to 0)	65·8
Ascariasis	0 (0 to 0)	0 (0 to 0)	2·4 (0 to 11·7)	12·4 (0 to 57)	8 (0 to 38·1)	2·1 (0 to 9·8)	1·4 (0 to 6·1)	1·3 (0 to 6·5)	1·2 (0 to 5·5)	1·3 (0 to 5·9)	1 (0 to 4·6)	0·6 (0 to 3·2)	0·5 (0 to 2·6)	0·4 (0 to 2·3)	0·3 (0 to 1·7)	0·7 (0 to 3·2)	0·2 (0 to 1·1)	0·4 (0 to 1·8)	0·2 (0 to 1·1)	0·1 (0 to 0·6)	0·1 (0 to 0·3)	0 (0 to 0·2)	0 (0 to 0)	34·8
Trichuriasis	0 (0 to 0)	0 (0 to 0)	0 (0 to 0)	0 (0 to 0)	0 (0 to 0)	0 (0 to 0)	0 (0 to 0)	0 (0 to 0)	0 (0 to 0)	0 (0 to 0)	0 (0 to 0)	0 (0 to 0)	0 (0 to 0)	0 (0 to 0)	0 (0 to 0)	0 (0 to 0)	0 (0 to 0)	0 (0 to 0)	0 (0 to 0)	0 (0 to 0)	0 (0 to 0)	0 (0 to 0)	0 (0 to 0)	0
Hookworm disease	0 (0 to 0)	0 (0 to 0)	0·2 (0·1 to 0·3)	3·1 (0·2 to 8·9)	6·2 (0·3 to 17·2)	2·3 (0·5 to 6·3)	3·8 (1·3 to 8·3)	2·8 (1 to 6·1)	1·9 (0·6 to 4·2)	1·5 (0·5 to 3·5)	1·4 (0·5 to 3·4)	1·3 (0·4 to 3)	1·2 (0·4 to 2·8)	1·1 (0·4 to 2·5)	1·1 (0·4 to 2·4)	0·9 (0·3 to 1·9)	0·6 (0·2 to 1·4)	0·5 (0·2 to 1·1)	0·4 (0·1 to 0·8)	0·3 (0·1 to 0·6)	0·2 (0 to 0·4)	0·1 (0 to 0·2)	0 (0 to 0)	31
Food-borne trematodiases	0 (0 to 0)	0 (0 to 0)	0 (0 to 0)	0 (0 to 0)	0 (0 to 0)	0 (0 to 0)	0 (0 to 0)	0 (0 to 0)	0 (0 to 0)	0 (0 to 0)	0 (0 to 0)	0 (0 to 0)	0 (0 to 0)	0 (0 to 0)	0 (0 to 0)	0 (0 to 0)	0 (0 to 0)	0 (0 to 0)	0 (0 to 0)	0 (0 to 0)	0 (0 to 0)	0 (0 to 0)	0 (0 to 0)	0
Leprosy	0 (0 to 0)	0 (0 to 0)	0 (0 to 0)	0 (0 to 0)	0 (0 to 0)	0 (0 to 0)	0·4 (0·2 to 0·6)	0·6 (0·4 to 0·9)	0·8 (0·5 to 1·3)	1·1 (0·6 to 1·7)	1·3 (0·8 to 2)	1·4 (0·9 to 2·2)	1·6 (1 to 2·4)	1·6 (1 to 2·5)	1·5 (0 to 4·1)	1·4 (0 to 3·5)	1·1 (0 to 2·7)	1 (0 to 2·2)	0·6 (0 to 1·5)	0·5 (0·1 to 1)	0·3 (0 to 0·6)	0·1 (0 to 0·2)	0 (0 to 0·1)	15·2
Ebola	0 (0 to 0)	0 (0 to 0)	0 (0 to 0)	0 (0 to 0)	0 (0 to 0)	0 (0 to 0)	0 (0 to 0)	0 (0 to 0)	0 (0 to 0)	0 (0 to 0)	0 (0 to 0)	0 (0 to 0)	0 (0 to 0)	0 (0 to 0)	0 (0 to 0)	0 (0 to 0)	0 (0 to 0)	0 (0 to 0)	0 (0 to 0)	0 (0 to 0)	0 (0 to 0)	0 (0 to 0)	0 (0 to 0)	0
Zika virus	0·2 (0 to 0·6)	0·5 (0 to 1·7)	7·3 (0 to 26·6)	0·1 (0 to 0·5)	0·1 (0 to 0·7)	0·1 (0 to 0·7)	0·1 (0 to 0·8)	0·2 (0 to 0·8)	0·2 (0 to 0·8)	0·2 (0 to 0·9)	0·2 (0 to 0·8)	0·1 (0 to 0·7)	0·1 (0 to 0·4)	0·1 (0 to 0·4)	0·1 (0 to 0·3)	0 (0 to 0·2)	0 (0 to 0·1)	0 (0 to 0)	0 (0 to 0)	0 (0 to 0)	0 (0 to 0)	0 (0 to 0)	0 (0 to 0)	9·5
Guinea worm disease	0 (0 to 0)	0 (0 to 0)	0 (0 to 0)	0 (0 to 0)	0 (0 to 0)	0 (0 to 0)	0 (0 to 0)	0 (0 to 0)	0 (0 to 0)	0 (0 to 0)	0 (0 to 0)	0 (0 to 0)	0 (0 to 0)	0 (0 to 0)	0 (0 to 0)	0 (0 to 0)	0 (0 to 0)	0 (0 to 0)	0 (0 to 0)	0 (0 to 0)	0 (0 to 0)	0 (0 to 0)	0 (0 to 0)	0
Other neglected tropical diseases	0·7 (0·3 to 1·7)	8·2 (3·2 to 14·3)	56·8 (20·5 to 112)	158 (61·4 to 311)	150 (41·6 to 358)	61·3 (16 to 161)	31 (8·9 to 74·1)	29·2 (10·2 to 63·6)	29 (10·4 to 64·8)	30·5 (10·3 to 70·6)	25·2 (8·8 to 58·5)	25·5 (9·8 to 58·4)	24·6 (9·3 to 56·2)	22·6 (7·4 to 57·9)	22·4 (7 to 49·4)	11·8 (4·6 to 24·1)	7 (3 to 15·2)	7·3 (3·1 to 14·2)	5·4 (2·4 to 10·5)	3·5 (1·6 to 7·1)	3·7 (1·8 to 6·7)	1·4 (0·7 to 2·5)	0·4 (0·2 to 0·8)	716
Other infectious diseases	663 (294 to 1,370)	1,060 (510 to 1,980)	7,480 (2,790 to 15,100)	6,450 (2,480 to 13,300)	1,510 (937 to 2,310)	793 (536 to 1,120)	681 (457 to 999)	677 (455 to 984)	616 (424 to 869)	673 (462 to 946)	615 (419 to 877)	598 (396 to 877)	567 (370 to 870)	571 (370 to 892)	448 (292 to 670)	469 (310 to 721)	318 (202 to 494)	284 (182 to 431)	154 (102 to 228)	112 (73·8 to 167)	91·2 (56·8 to 140)	30·2 (18·5 to 46·9)	7·6 (3·9 to 14·4)	24,900
Meningitis	569 (225 to 1,230)	913 (403 to 1,830)	2,100 (846 to 4,210)	1,230 (554 to 2,360)	565 (314 to 942)	333 (190 to 545)	286 (147 to 532)	231 (125 to 392)	210 (116 to 354)	249 (133 to 405)	225 (120 to 384)	195 (106 to 336)	180 (95·6 to 319)	176 (94·7 to 311)	135 (70·7 to 239)	187 (99·4 to 333)	127 (64·2 to 232)	116 (59·2 to 207)	65·1 (34·3 to 116)	41·6 (22·3 to 73·9)	32·9 (17·4 to 56·2)	9·2 (5·2 to 15·4)	2 (1·1 to 3·3)	8,170
Encephalitis	48·5 (15·6 to 120)	74 (21·6 to 186)	686 (213 to 1,690)	721 (251 to 1,860)	281 (116 to 654)	175 (87 to 333)	136 (62·9 to 270)	129 (64·3 to 252)	101 (50 to 195)	94·3 (47·3 to 185)	89·4 (43·8 to 168)	109 (53·6 to 217)	94·6 (46·6 to 177)	61·6 (30·1 to 115)	57·9 (27·5 to 124)	60·9 (28·1 to 128)	33 (16·1 to 62·3)	28·4 (13 to 56·1)	16·7 (7·7 to 35·2)	11·6 (5·8 to 22·2)	5·7 (2·8 to 11·2)	2·6 (1 to 4·9)	0·3 (0·1 to 0·4)	3,020
Diphtheria	0 (0 to 0)	0 (0 to 0)	8·4 (0·2 to 34)	15·4 (0·6 to 62·1)	8·3 (0·2 to 31·7)	3·9 (0·1 to 13·8)	1·9 (0·1 to 7·4)	1·8 (0·1 to 6·7)	1·5 (0·1 to 5·3)	1·6 (0 to 6·2)	1·5 (0 to 5·2)	1·6 (0·1 to 5·8)	2·4 (0·1 to 8·4)	1·9 (0·1 to 7·2)	0·9 (0 to 3·5)	0 (0 to 0)	0 (0 to 0)	0 (0 to 0)	0 (0 to 0)	0 (0 to 0)	0 (0 to 0)	0 (0 to 0)	0 (0 to 0)	51·2
Tetanus	20 (4·6 to 57·9)	32·8 (5·2 to 125)	32·2 (7·6 to 94·7)	49·4 (7·4 to 301)	28·9 (7·9 to 94·3)	19·8 (5·1 to 65·1)	19·9 (5·1 to 59·6)	24·9 (6·8 to 84·1)	26 (6·8 to 83·7)	35·6 (9 to 118)	34·4 (10·3 to 118)	38·2 (11·8 to 125)	44·5 (12·9 to 157)	53·9 (15·3 to 182)	49·5 (14·6 to 170)	39·5 (11·5 to 146)	26·2 (7·2 to 88·4)	25·3 (7·9 to 86·3)	13·6 (4·2 to 48·6)	8·2 (2·5 to 25·2)	5 (1·7 to 14)	2·2 (0·7 to 6·1)	0·9 (0·2 to 2·9)	631
Measles	0 (0 to 0)	0 (0 to 0)	0 (0 to 0)	0 (0 to 0)	0 (0 to 0)	0 (0 to 0)	0 (0 to 0)	0 (0 to 0)	0 (0 to 0)	0 (0 to 0)	0 (0 to 0)	0 (0 to 0)	0 (0 to 0)	0 (0 to 0)	0 (0 to 0)	0 (0 to 0)	0 (0 to 0)	0 (0 to 0)	0 (0 to 0)	0 (0 to 0)	0 (0 to 0)	0 (0 to 0)	0 (0 to 0)	0
Varicella and herpes zoster	3·4 (0·3 to 11)	11·5 (1·1 to 36·9)	83·8 (7·4 to 267)	120 (14·3 to 392)	43 (6·1 to 131)	15·2 (3·1 to 37·6)	11·4 (3·3 to 24·2)	12·8 (4 to 27·9)	14·3 (4·5 to 29·6)	16·6 (5·9 to 34·6)	14·3 (5 to 28)	12·2 (4·3 to 24·2)	12·4 (5·2 to 24)	13·2 (5·8 to 23·4)	13·4 (5·8 to 24·6)	13·1 (5·8 to 25·3)	14·1 (5·4 to 28·7)	11·8 (5 to 23·9)	11·2 (3·9 to 25)	11·9 (3 to 32·6)	10·7 (2·3 to 29·1)	5·1 (1 to 13·7)	1·6 (0·3 to 4·3)	477
Acute hepatitis	0 (0 to 0)	0 (0 to 0·1)	78 (24·6 to 201)	74·8 (30·2 to 170)	92·3 (45·4 to 172)	108 (52·1 to 199)	111 (48·3 to 206)	161 (58·1 to 371)	144 (53·9 to 300)	147 (56·2 to 314)	133 (47·1 to 271)	120 (42·2 to 252)	98·8 (32·3 to 212)	126 (39·5 to 301)	77·8 (26·3 to 171)	68·6 (23 to 150)	42·5 (13·3 to 100)	33·8 (10·4 to 75·4)	12·7 (4·3 to 29·7)	12·5 (4·5 to 28·4)	9·7 (3·8 to 19·5)	2·8 (1·2 to 5·9)	0·5 (0·2 to 1·1)	1,650
Acute hepatitis A	0 (0 to 0)	0 (0 to 0)	11·4 (2·8 to 35·8)	41·7 (15·8 to 96·6)	62·3 (30 to 111)	58·4 (30 to 105)	63·4 (30·6 to 123)	83·5 (30·3 to 201)	35·4 (16 to 73·7)	41·4 (16·2 to 93)	25·6 (9·8 to 54·3)	17·2 (6·1 to 37·9)	18·1 (5·8 to 44·9)	17·6 (4·3 to 48·9)	4·3 (0·9 to 13·2)	4·4 (1·1 to 14·7)	6·7 (1·3 to 20·2)	1·6 (0·3 to 5·9)	0·9 (0·1 to 2·8)	0·8 (0·1 to 3·5)	0·7 (0·1 to 3)	0·2 (0 to 1)	0 (0 to 0·1)	496
Acute hepatitis B	0 (0 to 0)	0 (0 to 0)	58·5 (15·3 to 160)	14·6 (2·5 to 47·7)	16·9 (3·8 to 50·6)	32·5 (6·6 to 91·1)	21·4 (4·2 to 60·4)	29·2 (6·3 to 80·3)	64·1 (16·4 to 161)	74·7 (20·1 to 188)	66·1 (20 to 151)	43·2 (13 to 107)	17·3 (5·4 to 42·8)	30·6 (8 to 91·1)	33·5 (8·9 to 85·8)	13·6 (4·3 to 37·9)	14 (4 to 37·8)	13·4 (3·7 to 33·3)	2·3 (0·7 to 6·3)	2 (0·6 to 5)	3 (0·9 to 7·7)	0·8 (0·2 to 2)	0 (0 to 0·1)	552
Acute hepatitis C	0 (0 to 0)	0 (0 to 0·1)	2·3 (0·7 to 8)	16·3 (3·8 to 48·9)	8·5 (1·6 to 28·7)	11·8 (1·9 to 37·2)	19·5 (3·5 to 51·8)	42·3 (7·3 to 122)	35·6 (6·2 to 99·2)	27·4 (4·6 to 84·8)	37·2 (7·5 to 97·9)	52·6 (12·2 to 128)	60·3 (15·1 to 145)	75·5 (19·2 to 192)	38·5 (9·6 to 99)	40·1 (11·1 to 96·9)	21·1 (5·1 to 55·3)	18·1 (4·7 to 43·7)	6·7 (1·8 to 16·8)	9·5 (3 to 22·9)	5·4 (1·8 to 12)	1·6 (0·5 to 4)	0·4 (0·1 to 1)	531
Acute hepatitis E	0 (0 to 0)	0 (0 to 0)	5·8 (1·1 to 17·2)	2·1 (0·4 to 7·3)	4·6 (2 to 10·1)	5·1 (1·1 to 11·9)	6·4 (1·7 to 13·7)	6 (1·3 to 14·8)	8·7 (2·9 to 21·7)	3·8 (1·6 to 8)	4·4 (1·6 to 10·6)	7·2 (1·8 to 20·1)	3·1 (1 to 8·4)	1·9 (0·6 to 5·1)	1·4 (0·5 to 3·6)	10·6 (1·9 to 29·8)	0·7 (0·3 to 1·6)	0·7 (0·2 to 1·9)	2·8 (0·7 to 8)	0·3 (0·1 to 0·6)	0·4 (0·2 to 1)	0·2 (0 to 0·4)	0 (0 to 0)	76·4
Other unspecified infectious diseases	22·7 (6·4 to 56·6)	25·8 (8·7 to 60·4)	151 (46·3 to 408)	181 (75·7 to 371)	131 (53·1 to 267)	72·5 (33 to 146)	88 (44·4 to 159)	97·3 (46·4 to 178)	107 (49 to 227)	119 (50·5 to 248)	111 (49·4 to 257)	114 (49·8 to 235)	128 (60·1 to 267)	135 (60·9 to 251)	109 (52·1 to 192)	100 (49·2 to 181)	75·4 (37·3 to 136)	68·5 (30·8 to 138)	35 (16·6 to 65·2)	26·5 (12·2 to 55·1)	27·3 (11·6 to 61·1)	8·3 (3·5 to 18)	2·5 (0·6 to 8)	1,940
Maternal and neonatal disorders	140,000 (97,600 to 191,000)	26,500 (17,700 to 37,300)	6,810 (3,680 to 12,000)	3,330 (2,480 to 4,310)	2,690 (1,950 to 3,590)	2,570 (1,860 to 3,400)	2,520 (1,840 to 3,320)	2,380 (1,720 to 3,200)	2,170 (1,600 to 2,880)	1,830 (1,320 to 2,390)	1,530 (1,110 to 2,040)	1,250 (884 to 1,680)	990 (723 to 1,330)	747 (535 to 1,020)	524 (367 to 736)	332 (232 to 464)	178 (120 to 252)	82·6 (52 to 121)	30·5 (17·8 to 46·2)	8·5 (5 to 13·5)	2·1 (1·1 to 3·4)	0·5 (0·2 to 0·9)	0·1 (0 to 0·2)	196,000
Maternal disorders	0 (0 to 0)	0 (0 to 0)	0 (0 to 0)	0 (0 to 0)	0 (0 to 0)	0 (0 to 0)	0 (0 to 0)	0 (0 to 0)	0 (0 to 0)	0 (0 to 0)	0 (0 to 0)	0 (0 to 0)	0 (0 to 0)	0 (0 to 0)	0 (0 to 0)	0 (0 to 0)	0 (0 to 0)	0 (0 to 0)	0 (0 to 0)	0 (0 to 0)	0 (0 to 0)	0 (0 to 0)	0 (0 to 0)	0
Maternal hemorrhage	0 (0 to 0)	0 (0 to 0)	0 (0 to 0)	0 (0 to 0)	0 (0 to 0)	0 (0 to 0)	0 (0 to 0)	0 (0 to 0)	0 (0 to 0)	0 (0 to 0)	0 (0 to 0)	0 (0 to 0)	0 (0 to 0)	0 (0 to 0)	0 (0 to 0)	0 (0 to 0)	0 (0 to 0)	0 (0 to 0)	0 (0 to 0)	0 (0 to 0)	0 (0 to 0)	0 (0 to 0)	0 (0 to 0)	0
Maternal sepsis and other maternal infections	0 (0 to 0)	0 (0 to 0)	0 (0 to 0)	0 (0 to 0)	0 (0 to 0)	0 (0 to 0)	0 (0 to 0)	0 (0 to 0)	0 (0 to 0)	0 (0 to 0)	0 (0 to 0)	0 (0 to 0)	0 (0 to 0)	0 (0 to 0)	0 (0 to 0)	0 (0 to 0)	0 (0 to 0)	0 (0 to 0)	0 (0 to 0)	0 (0 to 0)	0 (0 to 0)	0 (0 to 0)	0 (0 to 0)	0
Maternal hypertensive disorders	0 (0 to 0)	0 (0 to 0)	0 (0 to 0)	0 (0 to 0)	0 (0 to 0)	0 (0 to 0)	0 (0 to 0)	0 (0 to 0)	0 (0 to 0)	0 (0 to 0)	0 (0 to 0)	0 (0 to 0)	0 (0 to 0)	0 (0 to 0)	0 (0 to 0)	0 (0 to 0)	0 (0 to 0)	0 (0 to 0)	0 (0 to 0)	0 (0 to 0)	0 (0 to 0)	0 (0 to 0)	0 (0 to 0)	0
Maternal obstructed labor and uterine rupture	--	--	--	--	--	--	--	--	--	--	--	--	--	--	--	--	--	--	--	--	--	--	--	NA
Maternal abortion and miscarriage	--	--	--	--	--	--	--	--	--	--	--	--	--	--	--	--	--	--	--	--	--	--	--	NA
Ectopic pregnancy	--	--	--	--	--	--	--	--	--	--	--	--	--	--	--	--	--	--	--	--	--	--	--	NA
Indirect maternal deaths	--	--	--	--	--	--	--	--	--	--	--	--	--	--	--	--	--	--	--	--	--	--	--	NA
Late maternal deaths	--	--	--	--	--	--	--	--	--	--	--	--	--	--	--	--	--	--	--	--	--	--	--	NA
Maternal deaths aggravated by HIV/AIDS	--	--	--	--	--	--	--	--	--	--	--	--	--	--	--	--	--	--	--	--	--	--	--	NA
Neonatal disorders	140,000 (97,600 to 191,000)	26,500 (17,700 to 37,300)	6,810 (3,680 to 12,000)	3,330 (2,480 to 4,310)	2,690 (1,950 to 3,590)	2,570 (1,860 to 3,400)	2,520 (1,840 to 3,320)	2,380 (1,720 to 3,200)	2,170 (1,600 to 2,880)	1,830 (1,320 to 2,390)	1,530 (1,110 to 2,040)	1,250 (884 to 1,680)	990 (723 to 1,330)	747 (535 to 1,020)	524 (367 to 736)	332 (232 to 464)	178 (120 to 252)	82·6 (52 to 121)	30·5 (17·8 to 46·2)	8·5 (5 to 13·5)	2·1 (1·1 to 3·4)	0·5 (0·2 to 0·9)	0·1 (0 to 0·2)	196,000
Neonatal preterm birth	70,600 (41,800 to 106,000)	7,690 (3,430 to 13,800)	2,200 (994 to 4,480)	1,020 (724 to 1,360)	1,130 (765 to 1,560)	1,060 (732 to 1,440)	1,010 (721 to 1,390)	927 (634 to 1,250)	833 (590 to 1,140)	678 (472 to 926)	553 (376 to 766)	437 (291 to 604)	330 (229 to 456)	235 (157 to 333)	153 (101 to 213)	91 (69·2 to 118)	44 (32·3 to 58·7)	20 (13·7 to 27·9)	8·8 (5·2 to 13·9)	2·6 (1·1 to 4·9)	1·3 (0·5 to 2·4)	0·5 (0·2 to 0·8)	0·1 (0 to 0·2)	89,000
Neonatal encephalopathy due to birth asphyxia and traum	21,700 (10,300 to 39,400)	2,010 (856 to 4,090)	608 (239 to 1,370)	231 (128 to 379)	178 (83·2 to 316)	167 (78·8 to 295)	169 (77·8 to 283)	164 (75·8 to 292)	151 (73·7 to 271)	130 (57·6 to 233)	108 (50·6 to 189)	91·4 (40·8 to 165)	73·9 (32·5 to 133)	57·1 (27·5 to 97·2)	44 (26·9 to 64·7)	29·2 (17·9 to 44·8)	13·5 (7·9 to 20·8)	4 (2·2 to 6·4)	0·8 (0·4 to 1·4)	0·1 (0 to 0·2)	0 (0 to 0)	0 (0 to 0)	0 (0 to 0)	26,000
Neonatal sepsis and other neonatal infections	33,700 (15,400 to 62,900)	14,300 (7,700 to 22,800)	3,290 (1,460 to 6,430)	1,150 (769 to 1,690)	1,310 (844 to 1,900)	1,280 (783 to 1,870)	1,260 (792 to 1,870)	1,220 (754 to 1,800)	1,120 (686 to 1,680)	959 (598 to 1,400)	816 (514 to 1,230)	677 (408 to 1,020)	551 (347 to 811)	428 (265 to 636)	307 (175 to 484)	199 (113 to 311)	113 (58·8 to 178)	55·1 (25·5 to 93·3)	19·7 (8·5 to 34·9)	5·6 (2·3 to 9·8)	0·8 (0·3 to 1·6)	0·1 (0 to 0·2)	0 (0 to 0)	62,700
Hemolytic disease and other neonatal jaundice	1,020 (574 to 1,570)	182 (77 to 357)	45·8 (19·1 to 114)	66·4 (37·3 to 105)	74·8 (43·6 to 115)	73·4 (53·1 to 96·9)	74 (54·6 to 96·2)	72·1 (53·3 to 94·7)	67 (49·5 to 86·7)	58·5 (42·9 to 75·8)	50·1 (36 to 65·1)	42·3 (30·9 to 55·4)	34·8 (25·4 to 45·5)	27·4 (19·9 to 35·7)	19·9 (14·5 to 26·1)	12·8 (9·3 to 16·7)	7·3 (5·4 to 9·6)	3·5 (2·5 to 4·7)	1·2 (0·9 to 1·6)	0·3 (0·2 to 0·4)	0 (0 to 0·1)	0 (0 to 0)	0 (0 to 0)	1,930
Other neonatal disorders	12,800 (6,420 to 22,300)	2,310 (1,020 to 4,450)	662 (347 to 1,290)	861 (638 to 1,120)	0 (0 to 0)	0 (0 to 0)	0 (0 to 0)	0 (0 to 0)	0 (0 to 0)	0 (0 to 0)	0 (0 to 0)	0 (0 to 0)	0 (0 to 0)	0 (0 to 0)	0 (0 to 0)	0 (0 to 0)	0 (0 to 0)	0 (0 to 0)	0 (0 to 0)	0 (0 to 0)	0 (0 to 0)	0 (0 to 0)	0 (0 to 0)	16,600
Nutritional deficiencies	4·1 (2·2 to 7·9)	89·6 (37·5 to 157)	4,080 (1,810 to 8,080)	4,480 (2,260 to 7,810)	2,130 (959 to 4,390)	848 (368 to 1,790)	778 (343 to 1,690)	757 (369 to 1,460)	761 (387 to 1,450)	851 (436 to 1,600)	815 (451 to 1,480)	710 (402 to 1,330)	683 (364 to 1,300)	790 (419 to 1,490)	759 (393 to 1,380)	725 (407 to 1,210)	522 (298 to 878)	484 (287 to 791)	360 (215 to 563)	289 (172 to 462)	265 (158 to 404)	127 (75·2 to 197)	38·3 (21·3 to 60·2)	21,300
Protein-energy malnutrition	2·7 (1·5 to 4·1)	9·9 (5·8 to 15·5)	3,500 (1,310 to 7,500)	2,970 (1,160 to 6,050)	611 (333 to 963)	238 (140 to 367)	180 (95 to 323)	173 (89·5 to 305)	226 (122 to 388)	298 (159 to 527)	303 (171 to 518)	199 (111 to 337)	215 (120 to 370)	265 (139 to 484)	261 (146 to 447)	318 (159 to 561)	269 (137 to 505)	276 (145 to 491)	222 (122 to 374)	190 (110 to 304)	176 (97·8 to 296)	95·1 (51·2 to 160)	29·9 (14·7 to 50·8)	11,000
Iodine deficiency	0 (0 to 0)	0 (0 to 0)	0 (0 to 0)	1·4 (0·1 to 3·3)	9·8 (2·5 to 19·6)	20·4 (6 to 39·9)	28·8 (9·5 to 55·2)	34·9 (12·4 to 64·3)	36·2 (12·4 to 78·5)	36·4 (11·9 to 78·5)	33 (11·5 to 65·3)	28·8 (9·5 to 58·2)	24·2 (8·6 to 48·5)	20·5 (6·7 to 42·3)	15·7 (5·4 to 31·9)	11·9 (5·8 to 20·1)	8·3 (4 to 14·2)	5·4 (2·6 to 9·1)	3·2 (1·5 to 5·3)	1·7 (0·7 to 3)	0·8 (0·3 to 1·4)	0·3 (0·1 to 0·5)	0·1 (0 to 0·1)	322
Vitamin A deficiency	0·5 (0·1 to 1·3)	6·1 (1·6 to 15)	22·4 (8·8 to 49·5)	88·3 (40·2 to 176)	84 (32·3 to 184)	40·5 (14 to 84·1)	19·2 (5·9 to 38·7)	15·6 (6·9 to 28·3)	14·4 (6·6 to 26·1)	12·5 (5·9 to 22·4)	11·2 (5·5 to 20·2)	9·6 (4·8 to 17·1)	8·4 (4·2 to 14·9)	7 (3·5 to 12·1)	5·5 (2·9 to 9·5)	3·9 (2 to 6·8)	2·9 (1·4 to 4·9)	2·2 (1·1 to 3·9)	1·6 (0·8 to 2·8)	1 (0·5 to 1·7)	0·5 (0·2 to 0·8)	0·1 (0·1 to 0·2)	0 (0 to 0·1)	358
Dietary iron deficiency	1 (0·1 to 3·3)	73·6 (25 to 138)	506 (166 to 1,060)	1,380 (460 to 2,890)	1,400 (328 to 3,460)	528 (114 to 1,430)	509 (108 to 1,420)	438 (98·7 to 1,100)	412 (99·5 to 1,080)	438 (104 to 1,210)	371 (84·4 to 972)	349 (85·7 to 914)	360 (89·5 to 942)	386 (94·1 to 1,020)	397 (103 to 925)	303 (86·2 to 715)	177 (47·7 to 421)	143 (37·8 to 353)	104 (27·4 to 245)	80·2 (21·6 to 195)	74·2 (24·3 to 167)	25 (8·6 to 53·9)	6·6 (2·4 to 13·8)	8,460
Other nutritional deficiencies	0 (0 to 0)	0 (0 to 0)	45·7 (14·4 to 123)	43·2 (13 to 112)	24 (10·8 to 51·1)	20·6 (9·1 to 40·5)	41·3 (19·6 to 77)	96·1 (42·3 to 195)	72·6 (34·8 to 137)	65·4 (30·3 to 127)	97·2 (44 to 190)	123 (59·2 to 233)	75·9 (31·6 to 152)	111 (47·3 to 230)	79·6 (37 to 157)	88·4 (34 to 186)	64·9 (26·6 to 146)	57·1 (22·2 to 117)	29·3 (11·5 to 61)	16·1 (6·9 to 33·1)	13·7 (5·1 to 30·9)	6·7 (2·6 to 15)	1·7 (0·5 to 3·7)	1,170
Non-communicable diseases	16,600 (8,530 to 33,400)	5,520 (3,010 to 9,970)	20,300 (12,100 to 33,000)	22,900 (16,700 to 31,500)	19,700 (15,700 to 24,700)	22,900 (17,600 to 29,500)	30,700 (23,600 to 39,000)	37,800 (29,800 to 47,300)	43,500 (34,900 to 53,200)	50,500 (40,200 to 60,600)	57,400 (46,300 to 69,500)	68,400 (55,800 to 83,500)	81,200 (65,100 to 102,000)	98,600 (78,200 to 124,000)	99,500 (78,900 to 125,000)	110,000 (87,300 to 136,000)	94,000 (76,200 to 115,000)	89,000 (73,300 to 107,000)	61,400 (52,000 to 72,800)	46,000 (40,300 to 53,100)	29,300 (26,000 to 32,800)	11,800 (10,600 to 13,000)	3,570 (3,230 to 3,880)	1,120,000
Neoplasms	207 (93·7 to 414)	111 (54·1 to 243)	636 (364 to 1,010)	2,620 (1,610 to 4,070)	2,420 (1,760 to 3,300)	1,790 (1,290 to 2,440)	1,920 (1,230 to 2,830)	2,590 (1,660 to 3,640)	3,050 (2,050 to 4,230)	4,260 (2,870 to 5,740)	5,380 (3,720 to 7,330)	7,530 (5,080 to 10,300)	9,840 (6,470 to 13,900)	14,000 (9,210 to 19,800)	15,800 (10,800 to 22,000)	19,700 (13,200 to 27,400)	17,200 (11,900 to 23,800)	16,900 (11,800 to 23,300)	11,400 (8,170 to 15,800)	7,990 (5,880 to 11,100)	4,950 (3,570 to 6,870)	1,610 (1,130 to 2,220)	246 (148 to 369)	152,000
Lip and oral cavity cancer	0 (0 to 0)	0 (0 to 0)	0 (0 to 0)	0 (0 to 0)	7·2 (3·7 to 12·9)	10·2 (4·4 to 19·6)	18·2 (8·2 to 35·9)	40·1 (18·9 to 72)	55·5 (27 to 103)	93·1 (48·4 to 161)	158 (86 to 288)	226 (113 to 404)	376 (187 to 654)	478 (252 to 828)	537 (292 to 891)	636 (339 to 1,060)	545 (304 to 871)	483 (289 to 740)	283 (178 to 436)	209 (123 to 321)	92·6 (55·7 to 141)	33·1 (19·7 to 52·9)	6·1 (3·2 to 9·8)	4,290
Nasopharynx cancer	0 (0 to 0)	0 (0 to 0)	0 (0 to 0)	0 (0 to 0)	6·9 (4 to 12·2)	15 (7·1 to 28·2)	15·5 (6 to 33·5)	18·7 (7·8 to 37)	19·9 (8·3 to 38·9)	18·9 (9·1 to 34·7)	26·6 (13·2 to 47)	40·9 (20·9 to 70·1)	56·9 (29·1 to 99·3)	80·1 (44 to 135)	99·6 (54·1 to 168)	85 (47·9 to 139)	70·3 (41 to 115)	63 (35·9 to 98·8)	29 (16 to 49·2)	20·6 (11·3 to 34·3)	19·2 (9·3 to 33)	6·3 (3·1 to 11·4)	1 (0·4 to 1·9)	693
Other pharynx cancer	0 (0 to 0)	0 (0 to 0)	0 (0 to 0)	0 (0 to 0)	0 (0 to 0)	0 (0 to 0)	0 (0 to 0)	25·4 (9·8 to 52·8)	28·8 (12·6 to 56·8)	44·3 (20·5 to 82·8)	74·2 (36·3 to 139)	126 (63·5 to 230)	252 (136 to 449)	335 (172 to 578)	388 (204 to 656)	468 (233 to 804)	381 (200 to 649)	316 (182 to 509)	154 (88·8 to 248)	118 (67·9 to 190)	68·7 (37·5 to 114)	20·8 (11 to 35·9)	3·8 (1·7 to 7·1)	2,800
Esophageal cancer	0 (0 to 0)	0 (0 to 0)	0 (0 to 0)	0 (0 to 0)	0 (0 to 0)	0 (0 to 0)	0 (0 to 0)	36·5 (10·2 to 67·3)	46·8 (14 to 92·7)	98·4 (34·8 to 183)	163 (71 to 288)	299 (134 to 519)	428 (217 to 724)	623 (310 to 1,070)	694 (379 to 1,120)	768 (414 to 1,210)	599 (340 to 920)	473 (272 to 717)	261 (151 to 398)	162 (101 to 241)	91·6 (57·4 to 139)	25 (14·8 to 36·8)	2·9 (1·6 to 4·5)	4,770
Stomach cancer	0 (0 to 0)	0 (0 to 0)	0 (0 to 0)	0 (0 to 0)	0 (0 to 0)	0 (0 to 0)	52·4 (27·7 to 90·3)	113 (65·8 to 184)	222 (132 to 357)	466 (259 to 823)	666 (382 to 1,100)	1,010 (626 to 1,610)	1,060 (639 to 1,700)	1,570 (932 to 2,550)	1,710 (1,060 to 2,680)	1,910 (1,200 to 2,950)	1,540 (1,010 to 2,380)	1,350 (876 to 2,000)	771 (511 to 1,140)	475 (334 to 683)	264 (180 to 371)	78·9 (53·2 to 115)	15·7 (9·1 to 23·7)	13,300
Colon and rectum cancer	0 (0 to 0)	0 (0 to 0)	0 (0 to 0)	0 (0 to 0)	30·5 (9·8 to 57·8)	22·6 (9·1 to 41·9)	94 (42·8 to 174)	151 (79·7 to 242)	304 (170 to 479)	591 (306 to 942)	824 (434 to 1,420)	1,020 (553 to 1,650)	1,270 (700 to 2,070)	1,690 (921 to 2,670)	1,680 (944 to 2,690)	2,070 (1,130 to 3,270)	1,600 (921 to 2,370)	1,680 (991 to 2,500)	1,050 (679 to 1,550)	665 (425 to 966)	381 (246 to 540)	117 (71·1 to 182)	19·2 (10·4 to 31·4)	15,300
Liver cancer	16·2 (3·5 to 46·6)	6·9 (1·2 to 24·2)	51·5 (17·4 to 122)	76·8 (34·3 to 146)	52·6 (31·2 to 82·9)	74·1 (38·2 to 138)	52·6 (18·4 to 118)	115 (40 to 309)	178 (65·9 to 436)	248 (89·7 to 597)	379 (138 to 907)	587 (225 to 1,450)	652 (244 to 1,540)	817 (330 to 1,840)	947 (446 to 1,980)	1,100 (495 to 2,180)	903 (447 to 1,800)	777 (405 to 1,420)	441 (230 to 779)	267 (155 to 445)	107 (63·2 to 168)	27 (15·7 to 42·4)	2·9 (0·9 to 6·4)	7,880
Liver cancer due to hepatitis B	0 (0 to 0)	0 (0 to 0)	0 (0 to 0)	0 (0 to 0)	0 (0 to 0)	31·8 (11·4 to 74)	29·7 (10·5 to 69·7)	73·4 (25·4 to 203)	120 (41·3 to 292)	161 (54·3 to 415)	227 (77·7 to 574)	321 (111 to 811)	314 (109 to 753)	349 (122 to 811)	370 (147 to 794)	368 (138 to 796)	242 (97 to 512)	175 (67·4 to 363)	91·2 (40 to 192)	50·8 (21 to 98·2)	19·7 (9·1 to 37·4)	4·7 (2 to 8·9)	0·5 (0·1 to 1·3)	2,950
Liver cancer due to hepatitis C	0 (0 to 0)	0 (0 to 0)	0 (0 to 0)	0 (0 to 0)	0 (0 to 0)	0·5 (0·1 to 1·4)	0·4 (0·1 to 1·3)	1·7 (0·4 to 5·2)	3·6 (0·8 to 12·6)	7·2 (1·7 to 21·1)	15·1 (3·6 to 41·9)	32 (9·2 to 87·4)	48·9 (13 to 124)	80·7 (24 to 202)	116 (35·8 to 278)	152 (48·3 to 395)	132 (51·3 to 302)	129 (51·9 to 274)	87·9 (34·3 to 188)	66·4 (30·1 to 126)	32·2 (16·8 to 56·4)	9·4 (4·5 to 16·7)	1·1 (0·3 to 2·8)	916
Liver cancer due to alcohol use	0 (0 to 0)	0 (0 to 0)	0 (0 to 0)	0 (0 to 0)	0 (0 to 0)	0 (0 to 0)	1·7 (0·4 to 4·4)	10·8 (3·1 to 30·1)	25·6 (6·7 to 67·1)	48·9 (14·1 to 134)	98·2 (29·9 to 263)	179 (57·2 to 474)	227 (72 to 621)	312 (100 to 754)	377 (151 to 827)	486 (195 to 1,040)	450 (204 to 902)	398 (180 to 793)	211 (97·6 to 396)	116 (57·3 to 208)	41·3 (21·6 to 72·1)	9·2 (4·2 to 16·7)	0·9 (0·2 to 2·2)	2,990
Liver cancer due to NASH	0 (0 to 0)	0 (0 to 0)	0 (0 to 0)	0 (0 to 0)	0 (0 to 0)	0 (0 to 0)	8·5 (2·5 to 21·1)	11·8 (3·5 to 34·5)	11·7 (3 to 32·4)	12·4 (3·5 to 35·4)	16·3 (4·8 to 44·9)	25 (7·6 to 68·3)	30·5 (8·7 to 87·9)	40 (12·5 to 102)	48·1 (18 to 114)	58·6 (21·5 to 134)	51·3 (20·3 to 113)	51·3 (20·4 to 110)	36·4 (15·7 to 76)	25·3 (11·6 to 50·4)	10·4 (4·8 to 19·6)	2·7 (1·2 to 5·4)	0·3 (0·1 to 0·8)	441
Liver cancer due to other causes	[illegible]	[illegible]	[illegible]	[illegible]	[illegible]	[illegible]	[illegible]	[illegible]	[illegible]	[illegible]	[illegible]	[illegible]	[illegible]	[illegible]	[illegible]	[illegible]	[illegible]	[illegible]	[illegible]	[illegible]	[illegible]	[illegible]	[illegible]	[illegible]
Gallbladder and biliary tract cancer	[illegible]	[illegible]	[illegible]	[illegible]	[illegible]	[illegible]	[illegible]	[illegible]	[illegible]	[illegible]	[illegible]	[illegible]	[illegible]	[illegible]	[illegible]	[illegible]	[illegible]	[illegible]	[illegible]	[illegible]	[illegible]	[illegible]	[illegible]	[illegible]
Pancreatic cancer	[illegible]	[illegible]	[illegible]	[illegible]	[illegible]	[illegible]	[illegible]	[illegible]	[illegible]	[illegible]	[illegible]	[illegible]	[illegible]	[illegible]	[illegible]	[illegible]	[illegible]	[illegible]	[illegible]	[illegible]	[illegible]	[illegible]	[illegible]	[illegible]
Larynx cancer	[illegible]	[illegible]	[illegible]	[illegible]	[illegible]	[illegible]	[illegible]	[illegible]	[illegible]	[illegible]	[illegible]	[illegible]	[illegible]	[illegible]	[illegible]	[illegible]	[illegible]	[illegible]	[illegible]	[illegible]	[illegible]	[illegible]	[illegible]	[illegible]
Tracheal, bronchus, and lung cancer	[illegible]	[illegible]	[illegible]	[illegible]	[illegible]	[illegible]	[illegible]	[illegible]	[illegible]	[illegible]	[illegible]	[illegible]	[illegible]	[illegible]	[illegible]	[illegible]	[illegible]	[illegible]	[illegible]	[illegible]	[illegible]	[illegible]	[illegible]	[illegible]
Malignant skin melanoma	[illegible]	[illegible]	[illegible]	[illegible]	[illegible]	[illegible]	[illegible]	[illegible]	[illegible]	[illegible]	[illegible]	[illegible]	[illegible]	[illegible]	[illegible]	[illegible]	[illegible]	[illegible]	[illegible]	[illegible]	[illegible]	[illegible]	[illegible]	[illegible]
Non-melanoma skin cancer	[illegible]	[illegible]	[illegible]	[illegible]	[illegible]	[illegible]	[illegible]	[illegible]	[illegible]	[illegible]	[illegible]	[illegible]	[illegible]	[illegible]	[illegible]	[illegible]	[illegible]	[illegible]	[illegible]	[illegible]	[illegible]	[illegible]	[illegible]	[illegible]
Non-melanoma skin cancer (squamous-cell carcinoma)	[illegible]	[illegible]	[illegible]	[illegible]	[illegible]	[illegible]	[illegible]	[illegible]	[illegible]	[illegible]	[illegible]	[illegible]	[illegible]	[illegible]	[illegible]	[illegible]	[illegible]	[illegible]	[illegible]	[illegible]	[illegible]	[illegible]	[illegible]	[illegible]
Non-melanoma skin cancer (basal-cell carcinoma)	[illegible]	[illegible]	[illegible]	[illegible]	[illegible]	[illegible]	[illegible]	[illegible]	[illegible]	[illegible]	[illegible]	[illegible]	[illegible]	[illegible]	[illegible]	[illegible]	[illegible]	[illegible]	[illegible]	[illegible]	[illegible]	[illegible]	[illegible]	[illegible]
Breast cancer	0 (0 to 0)	0 (0 to 0)	0 (0 to 0)	0 (0 to 0)	0 (0 to 0)	[illegible]	[illegible]	[illegible]	[illegible]	[illegible]	[illegible]	[illegible]	[illegible]	[illegible]	[illegible]	[illegible]	[illegible]	[illegible]	[illegible]	[illegible]	[illegible]	[illegible]	[illegible]	[illegible]
Cervical cancer	--	--	--	--	--	--	--	--	--	--	--	--	--	--	--	--	--	--	--	--	--	--	--	NA
Uterine cancer	--	--	--	--	--	--	--	--	--	--	--	--	--	--	--	--	--	--	--	--	--	--	--	NA
Ovarian cancer	--	--	--	--	--	--	--	--	--	--	--	--	--	--	--	--	--	--	--	--	--	--	--	NA
Prostate cancer	0 (0 to 0)	0 (0 to 0)	0 (0 to 0)	0 (0 to 0)	0 (0 to 0)	[illegible]	[illegible]	[illegible]	[illegible]	[illegible]	[illegible]	[illegible]	[illegible]	[illegible]	[illegible]	[illegible]	[illegible]	[illegible]	[illegible]	[illegible]	[illegible]	[illegible]	[illegible]	[illegible]
Testicular cancer	0·6 (0·3 to 1·4)	0·9 (0·3 to 1·7)	4·8 (2 to 9·4)	22·7 (10·2 to 41·8)	8·8 (4 to 24·1)	[illegible]	[illegible]	[illegible]	[illegible]	[illegible]	[illegible]	[illegible]	[illegible]	[illegible]	[illegible]	[illegible]	[illegible]	[illegible]	[illegible]	[illegible]	[illegible]	[illegible]	[illegible]	[illegible]
Kidney cancer	33·1 (7·7 to 90)	7 (1·4 to 20·9)	64·4 (26·6 to 130)	251 (105 to 496)	185 (98·4 to 310)	[illegible]	[illegible]	[illegible]	[illegible]	[illegible]	[illegible]	[illegible]	[illegible]	[illegible]	[illegible]	[illegible]	[illegible]	[illegible]	[illegible]	[illegible]	[illegible]	[illegible]	[illegible]	[illegible]
Bladder cancer	0 (0 to 0)	0 (0 to 0)	0 (0 to 0)	0 (0 to 0)	0 (0 to 0)	[illegible]	[illegible]	[illegible]	[illegible]	[illegible]	[illegible]	[illegible]	[illegible]	[illegible]	[illegible]	[illegible]	[illegible]	[illegible]	[illegible]	[illegible]	[illegible]	[illegible]	[illegible]	[illegible]
Brain and central nervous system cancer	8·8 (2·6 to 23)	5·1 (1·3 to 14·2)	48·5 (12·5 to 110)	191 (46·7 to 419)	204 (75·9 to 449)	[illegible]	[illegible]	[illegible]	[illegible]	[illegible]	[illegible]	[illegible]	[illegible]	[illegible]	[illegible]	[illegible]	[illegible]	[illegible]	[illegible]	[illegible]	[illegible]	[illegible]	[illegible]	[illegible]
Thyroid cancer	0 (0 to 0)	0 (0 to 0)	0 (0 to 0)	0 (0 to 0)	13·2 (4·7 to 30·4)	[illegible]	[illegible]	[illegible]	[illegible]	[illegible]	[illegible]	[illegible]	[illegible]	[illegible]	[illegible]	[illegible]	[illegible]	[illegible]	[illegible]	[illegible]	[illegible]	[illegible]	[illegible]	[illegible]
Mesothelioma	0 (0 to 0)	0 (0 to 0)	0 (0 to 0)	0 (0 to 0)	0 (0 to 0)	[illegible]	[illegible]	[illegible]	[illegible]	[illegible]	[illegible]	[illegible]	[illegible]	[illegible]	[illegible]	[illegible]	[illegible]	[illegible]	[illegible]	[illegible]	[illegible]	[illegible]	[illegible]	[illegible]
Hodgkin lymphoma	0 (0 to 0)	0 (0 to 0)	0 (0 to 0)	1·3 (0·3 to 3·1)	8 (3·6 to 17·5)	[illegible]	[illegible]	[illegible]	[illegible]	[illegible]	[illegible]	[illegible]	[illegible]	[illegible]	[illegible]	[illegible]	[illegible]	[illegible]	[illegible]	[illegible]	[illegible]	[illegible]	[illegible]	[illegible]
Non-Hodgkin lymphoma	0 (0 to 0)	0 (0 to 0)	0 (0 to 0)	197 (92·3 to 406)	255 (136 to 428)	[illegible]	[illegible]	[illegible]	[illegible]	[illegible]	[illegible]	[illegible]	[illegible]	[illegible]	[illegible]	[illegible]	[illegible]	[illegible]	[illegible]	[illegible]	[illegible]	[illegible]	[illegible]	[illegible]
Multiple myeloma	0 (0 to 0)	0 (0 to 0)	0 (0 to 0)	0 (0 to 0)	0 (0 to 0)	[illegible]	[illegible]	[illegible]	[illegible]	[illegible]	[illegible]	[illegible]	[illegible]	[illegible]	[illegible]	[illegible]	[illegible]	[illegible]	[illegible]	[illegible]	[illegible]	[illegible]	[illegible]	[illegible]
Leukemia	57·6 (21·2 to 135)	30·7 (11·6 to 68·2)	226 (89·9 to 444)	936 (428 to 1,720)	1,060 (596 to 1,760)	[illegible]	[illegible]	[illegible]	[illegible]	[illegible]	[illegible]	[illegible]	[illegible]	[illegible]	[illegible]	[illegible]	[illegible]	[illegible]	[illegible]	[illegible]	[illegible]	[illegible]	[illegible]	[illegible]
Acute lymphoid leukemia	17·8 (3·7 to 59·3)	6·7 (1 to 21·3)	96·3 (36·1 to 227)	487 (196 to 989)	565 (264 to 1,050)	[illegible]	[illegible]	[illegible]	[illegible]	[illegible]	[illegible]	[illegible]	[illegible]	[illegible]	[illegible]	[illegible]	[illegible]	[illegible]	[illegible]	[illegible]	[illegible]	[illegible]	[illegible]	[illegible]
Chronic lymphoid leukemia	0 (0 to 0)	0 (0 to 0)	0 (0 to 0)	0 (0 to 0)	0 (0 to 0)	[illegible]	[illegible]	[illegible]	[illegible]	[illegible]	[illegible]	[illegible]	[illegible]	[illegible]	[illegible]	[illegible]	[illegible]	[illegible]	[illegible]	[illegible]	[illegible]	[illegible]	[illegible]	[illegible]
Acute myeloid leukemia	9·6 (0·6 to 38·8)	9·7 (0·3 to 30·8)	44·9 (13·4 to 112)	240 (87·3 to 510)	192 (67·1 to 422)	[illegible]	[illegible]	[illegible]	[illegible]	[illegible]	[illegible]	[illegible]	[illegible]	[illegible]	[illegible]	[illegible]	[illegible]	[illegible]	[illegible]	[illegible]	[illegible]	[illegible]	[illegible]	[illegible]
Chronic myeloid leukemia	0·3 (0 to 1·4)	0·7 (0 to 4·5)	8·2 (1·9 to 19·8)	19·6 (5·1 to 49)	27 (8·4 to 63·3)	[illegible]	[illegible]	[illegible]	[illegible]	[illegible]	[illegible]	[illegible]	[illegible]	[illegible]	[illegible]	[illegible]	[illegible]	[illegible]	[illegible]	[illegible]	[illegible]	[illegible]	[illegible]	[illegible]
Other leukemia	30 (7·5 to 77·1)	13·6 (2·2 to 36·8)	76·6 (21·9 to 171)	189 (58·3 to 446)	272 (92·6 to 556)	[illegible]	[illegible]	[illegible]	[illegible]	[illegible]	[illegible]	[illegible]	[illegible]	[illegible]	[illegible]	[illegible]	[illegible]	[illegible]	[illegible]	[illegible]	[illegible]	[illegible]	[illegible]	[illegible]
Other malignant neoplasms	87 (26·3 to 239)	58·4 (18·6 to 172)	226 (106 to 489)	882 (486 to 1,510)	545 (373 to 789)	[illegible]	[illegible]	[illegible]	[illegible]	[illegible]	[illegible]	[illegible]	[illegible]	[illegible]	[illegible]	[illegible]	[illegible]	[illegible]	[illegible]	[illegible]	[illegible]	[illegible]	[illegible]	[illegible]
Other neoplasms	3·5 (1·5 to 6·9)	2·4 (1 to 4·8)	13·5 (4 to 38·2)	54 (18·7 to 126)	40·7 (19 to 67·6)	[illegible]	[illegible]	[illegible]	[illegible]	[illegible]	[illegible]	[illegible]	[illegible]	[illegible]	[illegible]	[illegible]	[illegible]	[illegible]	[illegible]	[illegible]	[illegible]	[illegible]	[illegible]	[illegible]
Myelodysplastic, myeloproliferative, and other hematopoie	3·5 (1·5 to 6·9)	2·4 (1 to 4·8)	13·5 (4 to 38·2)	54 (18·7 to 126)	40·7 (19 to 67·6)	[illegible]	[illegible]	[illegible]	[illegible]	[illegible]	[illegible]	[illegible]	[illegible]	[illegible]	[illegible]	[illegible]	[illegible]	[illegible]	[illegible]	[illegible]	[illegible]	[illegible]	[illegible]	[illegible]
Benign and in situ intestinal neoplasms	0 (0 to 0)	0 (0 to 0)	0 (0 to 0)	0 (0 to 0)	0 (0 to 0)	[illegible]	[illegible]	[illegible]	[illegible]	[illegible]	[illegible]	[illegible]	[illegible]	[illegible]	[illegible]	[illegible]	[illegible]	[illegible]	[illegible]	[illegible]	[illegible]	[illegible]	[illegible]	[illegible]
Benign and in situ cervical and uterine neoplasms	0 (0 to 0)	0 (0 to 0)	0 (0 to 0)	0 (0 to 0)	0 (0 to 0)	[illegible]	[illegible]	[illegible]	[illegible]	[illegible]	[illegible]	[illegible]	[illegible]	[illegible]	[illegible]	[illegible]	[illegible]	[illegible]	[illegible]	[illegible]	[illegible]	[illegible]	[illegible]	[illegible]
Other benign and in situ neoplasms	0 (0 to 0)	0 (0 to 0)	0 (0 to 0)	0 (0 to 0)	0 (0 to 0)	[illegible]	[illegible]	[illegible]	[illegible]	[illegible]	[illegible]	[illegible]	[illegible]	[illegible]	[illegible]	[illegible]	[illegible]	[illegible]	[illegible]	[illegible]	[illegible]	[illegible]	[illegible]	[illegible]
Cardiovascular diseases	445 (187 to 902)	181 (77·6 to 358)	772 (313 to 1,560)	808 (431 to 1,390)	949 (661 to 1,320)	[illegible]	[illegible]	[illegible]	[illegible]	[illegible]	[illegible]	[illegible]	[illegible]	[illegible]	[illegible]	[illegible]	[illegible]	[illegible]	[illegible]	[illegible]	[illegible]	[illegible]	[illegible]	[illegible]
Rheumatic heart disease	0 (0 to 0)	0 (0 to 0)	0 (0 to 0)	84 (41·2 to 162)	202 (118 to 314)	[illegible]	[illegible]	[illegible]	[illegible]	[illegible]	[illegible]	[illegible]	[illegible]	[illegible]	[illegible]	[illegible]	[illegible]	[illegible]	[illegible]	[illegible]	[illegible]	[illegible]	[illegible]	[illegible]
Ischemic heart disease	0 (0 to 0)	0 (0 to 0)	0 (0 to 0)	0 (0 to 0)	0 (0 to 0)	[illegible]	[illegible]	[illegible]	[illegible]	[illegible]	[illegible]	[illegible]	[illegible]	[illegible]	[illegible]	[illegible]	[illegible]	[illegible]	[illegible]	[illegible]	[illegible]	[illegible]	[illegible]	[illegible]
Stroke	239 (93·8 to 519)	60·1 (18·8 to 142)	206 (59·2 to 513)	161 (69·9 to 323)	265 (156 to 417)	[illegible]	[illegible]	[illegible]	[illegible]	[illegible]	[illegible]	[illegible]	[illegible]	[illegible]	[illegible]	[illegible]	[illegible]	[illegible]	[illegible]	[illegible]	[illegible]	[illegible]	[illegible]	[illegible]
Ischemic stroke	14·6 (3·8 to 37)	7·7 (2·2 to 19)	18·6 (5·1 to 48·9)	59·9 (29·7 to 112)	66·3 (45·1 to 92·8)	[illegible]	[illegible]	[illegible]	[illegible]	[illegible]	[illegible]	[illegible]	[illegible]	[illegible]	[illegible]	[illegible]	[illegible]	[illegible]	[illegible]	[illegible]	[illegible]	[illegible]	[illegible]	[illegible]
Intracerebral hemorrhage	127 (48·5 to 259)	20·4 (5·5 to 49·3)	99·8 (21·2 to 260)	42·7 (12·8 to 94·6)	121 (57·3 to 225)	[illegible]	[illegible]	[illegible]	[illegible]	[illegible]	[illegible]	[illegible]	[illegible]	[illegible]	[illegible]	[illegible]	[illegible]	[illegible]	[illegible]	[illegible]	[illegible]	[illegible]	[illegible]	[illegible]
Subarachnoid hemorrhage	97·2 (29·9 to 249)	32·1 (9·7 to 79·1)	87·9 (23·6 to 224)	58·7 (17·8 to 136)	77·8 (34·9 to 149)	[illegible]	[illegible]	[illegible]	[illegible]	[illegible]	[illegible]	[illegible]	[illegible]	[illegible]	[illegible]	[illegible]	[illegible]	[illegible]	[illegible]	[illegible]	[illegible]	[illegible]	[illegible]	[illegible]
Hypertensive heart disease	0 (0 to 0)	0 (0 to 0)	0 (0 to 0)	0 (0 to 0)	0 (0 to 0)	[illegible]	[illegible]	[illegible]	[illegible]	[illegible]	[illegible]	[illegible]	[illegible]	[illegible]	[illegible]	[illegible]	[illegible]	[illegible]	[illegible]	[illegible]	[illegible]	[illegible]	[illegible]	[illegible]
Non-rheumatic valvular heart disease	0 (0 to 0)	0 (0 to 0)	0 (0 to 0)	0 (0 to 0)	0 (0 to 0)	[illegible]	[illegible]	[illegible]	[illegible]	[illegible]	[illegible]	[illegible]	[illegible]	[illegible]	[illegible]	[illegible]	[illegible]	[illegible]	[illegible]	[illegible]	[illegible]	[illegible]	[illegible]	[illegible]
Non-rheumatic calcific aortic valve disease	0 (0 to 0)	0 (0 to 0)	0 (0 to 0)	0 (0 to 0)	0 (0 to 0)	[illegible]	[illegible]	[illegible]	[illegible]	[illegible]	[illegible]	[illegible]	[illegible]	[illegible]	[illegible]	[illegible]	[illegible]	[illegible]	[illegible]	[illegible]	[illegible]	[illegible]	[illegible]	[illegible]

Non-rheumatic degenerative mitral valve disease	0 (0 to 0)	0 (0 to 0)	0 (0 to 0)	0 (0 to 0)	0 (0 to 0)	0 (0 to 0)	24·9 (8 to 55·1)	14·7 (4·7 to 32·4)	40 (16·7 to 78·3)	38·7 (15·9 to 75·3)	42·8 (19·5 to 80·6)	52·5 (24·8 to 95·6)	56·5 (26·9 to 104)	61·3 (31·6 to 106)	61·9 (30·9 to 109)	55 (27·1 to 95·7)	40·1 (20·7 to 69·3)	32·7 (16·6 to 55·9)	20·3 (9·9 to 36·4)	12·9 (5·6 to 23·4)	10·9 (4·5 to 22·3)	4·1 (1·6 to 8·3)	0·9 (0·3 to 2·3)	570
Other non-rheumatic valve diseases	0 (0 to 0)	0 (0 to 0)	0 (0 to 0)	0 (0 to 0)	0 (0 to 0)	0 (0 to 0)	0·4 (0·2 to 0·7)	1·3 (0·3 to 3·6)	1·1 (0·2 to 3·4)	0·8 (0·2 to 2)	0·6 (0·2 to 1·5)	0·4 (0·1 to 0·9)	0·2 (0·1 to 0·5)	0·3 (0·1 to 0·8)	0·2 (0·1 to 0·5)	0·4 (0·1 to 1)	0·2 (0·1 to 0·4)	0·2 (0 to 0·5)	0·1 (0 to 0·3)	0·2 (0·1 to 0·7)	0·2 (0·1 to 0·6)	0·1 (0 to 0·4)	0 (0 to 0·1)	6·8
Cardiomyopathy and myocarditis	85·9 (23·8 to 220)	44·2 (14·8 to 101)	229 (70·1 to 516)	174 (68·8 to 337)	117 (57·9 to 215)	104 (45 to 200)	137 (62·8 to 275)	152 (66 to 283)	217 (97·5 to 421)	259 (112 to 481)	324 (150 to 602)	379 (183 to 666)	441 (217 to 789)	465 (237 to 785)	451 (232 to 758)	427 (216 to 705)	320 (168 to 518)	283 (151 to 461)	181 (95·5 to 287)	133 (69·5 to 219)	104 (51·8 to 177)	44·2 (18·5 to 76·6)	9·6 (4 to 18·5)	5,080
Myocarditis	8·1 (1·5 to 22·9)	6·5 (1·6 to 17·7)	32·2 (5·7 to 89·6)	19·2 (4·1 to 52·5)	15·4 (5 to 36·5)	10 (3·6 to 22·2)	17·3 (6·8 to 38·4)	16·6 (5·6 to 35·7)	20·9 (6·8 to 48·6)	20·5 (6·3 to 45·7)	23·6 (7·3 to 52·1)	18·6 (7·3 to 38·1)	10·7 (4·7 to 20·6)	12 (5·2 to 23·6)	10·4 (4·5 to 20·2)	7 (3·2 to 13·8)	6 (2·7 to 11·1)	6·2 (2·8 to 12·5)	4·4 (2·2 to 8·2)	2·9 (1·3 to 5·8)	2·5 (1 to 5)	1 (0·4 to 2·1)	0·7 (0·2 to 1·7)	273
Alcoholic cardiomyopathy	0 (0 to 0)	0 (0 to 0)	0 (0 to 0)	0 (0 to 0)	0 (0 to 0)	0 (0 to 0)	9·6 (2·9 to 22)	10·9 (3·5 to 25·3)	33·2 (10·4 to 79·1)	81·6 (30·1 to 172)	98·8 (39 to 206)	144 (59·5 to 275)	197 (85·9 to 388)	223 (105 to 409)	191 (84·8 to 346)	171 (80·4 to 320)	135 (61·9 to 246)	88·1 (34·5 to 179)	47·1 (21·5 to 92·8)	5·8 (2·2 to 12·5)	1·4 (0·5 to 3·3)	0·5 (0·2 to 1·2)	0·2 (0·1 to 0·3)	1,440
Other cardiomyopathy	77·8 (21·3 to 203)	37·7 (12·2 to 87)	197 (61·7 to 451)	155 (61·4 to 302)	102 (47·6 to 194)	93·6 (39·7 to 185)	110 (49·3 to 219)	125 (53·6 to 233)	163 (71·3 to 320)	156 (66·1 to 300)	202 (95·9 to 376)	217 (94·6 to 413)	233 (103 to 459)	230 (100 to 416)	250 (109 to 447)	248 (113 to 429)	179 (84·3 to 308)	189 (92·9 to 326)	130 (65·5 to 211)	124 (63 to 205)	100 (49·3 to 171)	42·7 (17·7 to 74·6)	8·8 (3·7 to 17)	3,370
Atrial fibrillation and flutter	0 (0 to 0)	0 (0 to 0)	0 (0 to 0)	0 (0 to 0)	0 (0 to 0)	0 (0 to 0)	0 (0 to 0)	0 (0 to 0)	0 (0 to 0)	5·3 (2·3 to 17·6)	15·1 (8·7 to 23·4)	51·9 (28·5 to 84·7)	96·7 (57·4 to 144)	163 (105 to 236)	240 (161 to 336)	336 (222 to 469)	401 (277 to 549)	508 (354 to 690)	513 (350 to 698)	572 (373 to 767)	475 (333 to 621)	261 (179 to 356)	78·6 (44·2 to 150)	3,720
Aortic aneurysm	--	--	--	--	--	--	28·6 (12·5 to 56·1)	22·6 (9·7 to 44·8)	43·7 (22·5 to 78·1)	62 (30·4 to 102)	105 (56·7 to 172)	196 (105 to 342)	190 (99·8 to 321)	275 (158 to 434)	320 (194 to 527)	420 (277 to 630)	366 (246 to 536)	330 (229 to 467)	222 (153 to 325)	159 (111 to 224)	103 (69·4 to 144)	36·6 (23·7 to 54·9)	7 (3·7 to 12)	NA
Endocarditis	15·9 (4·2 to 42·9)	12·6 (3 to 32·9)	83·6 (23 to 199)	107 (36·4 to 226)	56·5 (18·2 to 129)	80 (24·6 to 194)	82 (32·4 to 191)	101 (40·2 to 211)	122 (49·1 to 247)	147 (54·9 to 285)	146 (67·4 to 274)	157 (73·5 to 290)	131 (61·3 to 235)	144 (71·9 to 248)	109 (58·3 to 179)	88·2 (45·9 to 147)	65·1 (35 to 108)	55·2 (32·1 to 85·5)	28·5 (17 to 45·1)	20·7 (12·3 to 32·5)	16 (8·8 to 26·6)	6·9 (3·4 to 12·3)	1·4 (0·8 to 4·1)	1,780
Other cardiovascular and circulatory diseases	104 (41·1 to 215)	64·1 (23·8 to 139)	253 (98·9 to 567)	282 (129 to 501)	307 (176 to 484)	312 (163 to 519)	325 (177 to 518)	402 (194 to 711)	460 (217 to 770)	417 (175 to 712)	392 (167 to 664)	451 (232 to 717)	454 (219 to 748)	533 (271 to 874)	569 (297 to 910)	583 (311 to 905)	469 (231 to 733)	432 (242 to 664)	344 (200 to 516)	293 (186 to 420)	264 (159 to 393)	120 (67·2 to 176)	36·8 (14·2 to 88·2)	7,870
Chronic respiratory diseases	0 (0 to 0)	0 (0 to 0)	0·4 (0·2 to 0·6)	3,640 (2,230 to 5,680)	2,260 (1,460 to 3,530)	1,590 (1,050 to 2,410)	1,180 (783 to 1,760)	1,150 (793 to 1,650)	1,140 (786 to 1,620)	1,160 (804 to 1,650)	1,120 (769 to 1,560)	1,350 (939 to 2,010)	1,660 (1,130 to 2,430)	2,080 (1,380 to 3,040)	2,310 (1,520 to 3,400)	3,110 (2,030 to 4,670)	2,690 (1,790 to 4,090)	3,130 (2,040 to 4,650)	2,330 (1,540 to 3,390)	1,820 (1,250 to 2,630)	1,270 (855 to 1,810)	494 (344 to 700)	137 (82·4 to 284)	35,600
Chronic obstructive pulmonary disease	0 (0 to 0)	0 (0 to 0)	0·4 (0·2 to 0·6)	155 (72·7 to 303)	60·5 (37·1 to 94·8)	68·7 (31·3 to 118)	181 (99·6 to 295)	310 (182 to 496)	366 (222 to 551)	392 (248 to 584)	434 (276 to 667)	602 (386 to 900)	830 (531 to 1,210)	1,220 (751 to 1,840)	1,530 (980 to 2,270)	2,320 (1,480 to 3,570)	2,100 (1,360 to 3,210)	2,490 (1,610 to 3,720)	1,860 (1,230 to 2,700)	1,490 (1,010 to 2,120)	1,080 (726 to 1,530)	419 (291 to 597)	117 (69·9 to 239)	18,000
Pneumoconiosis	0 (0 to 0)	0 (0 to 0)	0 (0 to 0)	0 (0 to 0)	0 (0 to 0)	0 (0 to 0)	0·7 (0·3 to 1·6)	0·2 (0·1 to 0·4)	3 (1·1 to 7·4)	0·6 (0·3 to 1·1)	1·6 (0·7 to 3·5)	0·9 (0·5 to 1·6)	3·3 (1·3 to 7·5)	3·3 (1·5 to 7)	2·1 (1·1 to 3·7)	2·9 (1·5 to 5·7)	5·9 (2·8 to 11)	4·4 (2·1 to 8·5)	2·3 (1·3 to 4·3)	1·3 (0·8 to 2·3)	1·9 (0·9 to 3·6)	0·4 (0·2 to 0·6)	0·1 (0 to 0·1)	34·9
Silicosis	0 (0 to 0)	0 (0 to 0)	0 (0 to 0)	0 (0 to 0)	0 (0 to 0)	0 (0 to 0)	0·1 (0 to 0·2)	0 (0 to 0·1)	2·4 (0·7 to 5·9)	0·1 (0 to 0·2)	0·7 (0·2 to 1·9)	0·1 (0 to 0·4)	1·2 (0·3 to 3·1)	1·4 (0·4 to 3·7)	0·5 (0·2 to 1)	0·9 (0·3 to 2·2)	1·6 (0·4 to 3·8)	0·6 (0·3 to 2·8)	0·6 (0·2 to 1·5)	0·3 (0·1 to 0·8)	0·5 (0·2 to 1·4)	0·1 (0 to 0·2)	0 (0 to 0·1)	11·7
Asbestosis	0 (0 to 0)	0 (0 to 0)	0 (0 to 0)	0 (0 to 0)	0 (0 to 0)	0 (0 to 0)	0·3 (0·1 to 0·7)	0·1 (0 to 0·1)	0·2 (0 to 0·7)	0·1 (0 to 0·3)	0·3 (0·1 to 0·8)	0·1 (0 to 0·3)	0·6 (0·2 to 2·1)	0·6 (0·1 to 1·9)	0·5 (0·1 to 1·3)	0·9 (0·3 to 2·5)	1 (0·2 to 2·7)	1·3 (0·3 to 3·2)	0·3 (0·1 to 1)	0·2 (0·1 to 0·6)	0·5 (0·1 to 1·2)	0·1 (0 to 0·1)	0 (0 to 0)	7·2
Coal workers pneumoconiosis	0 (0 to 0)	0 (0 to 0)	0 (0 to 0)	0 (0 to 0)	0 (0 to 0)	0 (0 to 0)	0·1 (0 to 0·2)	0 (0 to 0)	0·1 (0 to 0·2)	0 (0 to 0·1)	0·2 (0 to 0·6)	0·1 (0 to 0·2)	0·3 (0 to 1)	0·4 (0·1 to 1·3)	0·1 (0 to 0·2)	0·2 (0 to 0·5)	0·2 (0 to 0·7)	0·2 (0 to 0·7)	0·2 (0 to 0·6)	0 (0 to 0·1)	0·1 (0 to 0·2)	0 (0 to 0·1)	0 (0 to 0)	2·1
Other pneumoconiosis	0 (0 to 0)	0 (0 to 0)	0 (0 to 0)	0 (0 to 0)	0 (0 to 0)	0 (0 to 0)	0·3 (0·1 to 0·6)	0·1 (0 to 0·2)	0·4 (0·1 to 1)	0·4 (0·2 to 0·7)	0·4 (0·2 to 0·8)	0·5 (0·3 to 1)	1·2 (0·4 to 3)	0·9 (0·4 to 1·8)	0·9 (0·5 to 1·7)	0·9 (0·4 to 1·7)	3·1 (1·3 to 6·2)	1·8 (0·7 to 4)	1·2 (0·6 to 2·3)	0·7 (0·4 to 1·2)	0·7 (0·3 to 1·7)	0·2 (0·1 to 0·4)	0 (0 to 0·1)	13·9
Asthma	0 (0 to 0)	0 (0 to 0)	0 (0 to 0)	2,970 (1,790 to 4,650)	2,090 (1,310 to 3,320)	1,410 (882 to 2,220)	911 (557 to 1,450)	701 (458 to 1,020)	633 (410 to 930)	625 (410 to 918)	570 (369 to 829)	603 (386 to 951)	666 (424 to 1,070)	[illegible]	627 (379 to 1,050)	680 (384 to 1,130)	495 (284 to 818)	549 (299 to 922)	395 (221 to 687)	288 (169 to 484)	163 (91·3 to 267)	66·6 (37·9 to 110)	16·5 (7·6 to 45·1)	15,200
Interstitial lung disease and pulmonary sarcoidosis	0 (0 to 0)	0 (0 to 0)	0 (0 to 0)	55·4 (25·6 to 108)	7 (3·2 to 13·4)	7·1 (3·5 to 13·5)	11·2 (4·6 to 22·3)	20·5 (9 to 43·7)	15·7 (7·7 to 32·4)	30·2 (15·5 to 58)	39·4 (17·7 to 82·9)	66·1 (30·9 to 138)	61·4 (30·1 to 115)	83·5 (40·7 to 160)	85·8 (37 to 163)	71·6 (37·8 to 131)	64·3 (33·3 to 121)	59 (31·1 to 106)	51·9 (25·2 to 98·4)	29·4 (13·6 to 56·8)	15·6 (6·9 to 29·1)	5·2 (2·4 to 10·3)	2·1 (0·5 to 5·2)	782
Other chronic respiratory diseases	0 (0 to 0)	0 (0 to 0)	0 (0 to 0)	456 (160 to 978)	98·4 (55·2 to 184)	104 (48·8 to 182)	76·8 (39·1 to 139)	114 (58·9 to 208)	119 (69·8 to 206)	114 (67·3 to 197)	74 (40 to 132)	78 (41·8 to 144)	94·7 (49·4 to 166)	78·3 (36·3 to 151)	66·9 (32·2 to 124)	38·2 (14·9 to 92)	28·3 (10·5 to 69·3)	29·7 (10·4 to 69·3)	22·3 (7·3 to 58·5)	15·3 (5·7 to 37·4)	12 (4·1 to 30·4)	3·1 (1·3 to 7·5)	1 (0·3 to 2·7)	1,620
Digestive diseases	308 (130 to 635)	234 (101 to 468)	2,510 (899 to 6,070)	3,610 (1,680 to 7,510)	1,980 (1,340 to 2,890)	1,350 (899 to 1,970)	1,720 (1,080 to 2,590)	2,630 (1,620 to 3,890)	3,410 (2,060 to 4,940)	4,490 (2,720 to 6,560)	5,770 (3,590 to 8,410)	6,670 (4,170 to 9,860)	8,050 (4,760 to 11,800)	9,330 (5,430 to 14,000)	8,610 (5,270 to 12,400)	8,500 (5,030 to 12,800)	6,300 (3,840 to 8,980)	5,390 (3,320 to 7,680)	3,190 (2,180 to 4,490)	2,140 (1,450 to 2,940)	1,360 (959 to 1,900)	475 (325 to 697)	146 (61·1 to 320)	88,200

[illegible]

G6PD deficiency	0·1 (0 to 0·3)	1·3 (0·5 to 2·5)	2·8 (1·1 to 5·5)	25·8 (11·4 to 49·9)	34·9 (13·7 to 65·5)	32·8 (12·9 to 68)	41·6 (15·2 to 87·4)
G6PD trait	0 (0 to 0)	0 (0 to 0)	0 (0 to 0)	0 (0 to 0)	0 (0 to 0)	0 (0 to 0)	0 (0 to 0)
Other hemoglobinopathies and hemolytic anemias	0·1 (0 to 0·2)	2·7 (0·9 to 5)	18·7 (5·8 to 39·5)	51·5 (16·3 to 109)	53·2 (12·2 to 131)	21·7 (5 to 58·3)	53·3 (13·4 to 148)
Endocrine, metabolic, blood, and immune disorders	610 (91·9 to 1,490)	352 (85·8 to 812)	3,270 (988 to 7,100)	1,880 (622 to 3,790)	643 (298 to 1,070)	413 (205 to 704)	389 (185 to 730)
Oral disorders	0 (0 to 0)	0 (0 to 0)	0 (0 to 0)	257 (144 to 423)	280 (150 to 493)	286 (151 to 502)	426 (230 to 749)
Caries of deciduous teeth	0 (0 to 0)	0 (0 to 0)	0 (0 to 0)	89·5 (34·6 to 184)	77·5 (31·8 to 169)	3·9 (0·4 to 10·3)	0 (0 to 0)
Caries of permanent teeth	0 (0 to 0)	0 (0 to 0)	0 (0 to 0)	0 (0 to 0)	63·6 (22 to 142)	140 (56·1 to 309)	153 (60·4 to 331)
Periodontal diseases	0 (0 to 0)	0 (0 to 0)	0 (0 to 0)	0 (0 to 0)	0 (0 to 0)	0 (0 to 0)	62·9 (17·6 to 162)
Other oral disorders	0 (0 to 0)	0 (0 to 0)	0 (0 to 0)	167 (97·6 to 268)	139 (79·8 to 218)	142 (81 to 228)	210 (125 to 324)
Sudden infant death syndrome	--	202 (84·5 to 411)	1,500 (490 to 3,270)	--	--	--	--
Injuries	1,400 (806 to 2,290)	698 (400 to 1,250)	4,410 (2,290 to 7,280)	9,060 (5,600 to 13,400)	8,310 (6,420 to 10,800)	10,100 (8,000 to 12,500)	27,600 (21,000 to 35,000)
Transport injuries	130 (38·1 to 320)	54·2 (18 to 115)	396 (138 to 958)	1,990 (856 to 4,220)	2,840 (1,690 to 4,840)	3,160 (2,070 to 5,010)	9,540 (6,080 to 13,400)
Road injuries	121 (34 to 291)	49·1 (15·5 to 104)	365 (123 to 900)	1,920 (818 to 4,100)	2,770 (1,640 to 4,760)	3,070 (2,000 to 4,860)	9,350 (5,960 to 13,200)
Pedestrian road injuries	36·4 (9 to 102)	10·7 (2·3 to 28·5)	56 (16·7 to 142)	409 (133 to 1,040)	565 (236 to 1,250)	445 (207 to 862)	417 (213 to 722)
Cyclist road injuries	0 (0 to 0)	0 (0 to 0)	0 (0 to 0)	25·8 (11·8 to 53·8)	64·9 (39·2 to 107)	135 (88·4 to 192)	266 (177 to 386)
Motorcyclist road injuries	6·9 (1·6 to 18·7)	2·2 (0·6 to 5·6)	16·1 (4·3 to 43·2)	72·2 (25·8 to 173)	131 (63·6 to 258)	193 (101 to 367)	1,360 (714 to 2,370)
Motor vehicle road injuries	76·3 (20·6 to 194)	35·9 (10·7 to 84·8)	291 (93·2 to 747)	1,400 (584 to 3,090)	2,000 (1,120 to 3,500)	2,280 (1,420 to 3,650)	7,280 (4,510 to 10,500)
Other road injuries	1·4 (0·2 to 4·9)	0·3 (0 to 0·9)	2·1 (0·5 to 5·8)	7·4 (3·2 to 16·4)	9·2 (5·7 to 14·8)	15·4 (9·9 to 23)	35·9 (23 to 54·5)
Other transport injuries	9·3 (2·2 to 26)	5·1 (1·6 to 13)	30·9 (11·5 to 76·9)	67 (31·1 to 155)	71·5 (39·6 to 133)	91·2 (55·5 to 163)	185 (98·4 to 335)
Unintentional injuries	791 (446 to 1,340)	550 (294 to 1,030)	3,310 (1,630 to 5,750)	5,610 (3,570 to 8,420)	4,390 (3,370 to 5,530)	4,920 (3,800 to 6,250)	6,630 (4,700 to 8,810)
Falls	19·7 (6·9 to 46·8)	8 (3·7 to 16·9)	109 (44·5 to 225)	243 (113 to 423)	283 (201 to 392)	415 (303 to 562)	688 (491 to 951)
Drowning	61·8 (20·8 to 145)	20·9 (7·9 to 45·3)	232 (88·1 to 525)	1,400 (601 to 2,660)	1,270 (679 to 2,170)	1,420 (810 to 2,220)	1,880 (946 to 3,210)
Fire, heat, and hot substances	24·5 (10·2 to 51·1)	25·9 (11·7 to 56·2)	269 (105 to 636)	824 (372 to 1,630)	494 (306 to 786)	454 (308 to 643)	508 (355 to 705)
Poisonings	30·6 (6·5 to 92·6)	35·2 (4·4 to 132)	152 (38·1 to 421)	394 (151 to 875)	146 (71 to 291)	121 (61·7 to 221)	111 (59 to 199)
Poisoning by carbon monoxide	0·5 (0·1 to 1·9)	1·8 (0·1 to 9)	3·6 (0·6 to 11·9)	38·7 (6·8 to 137)	24·9 (8·8 to 62·5)	27·7 (9·1 to 66·2)	40·2 (14·7 to 87)
Poisoning by other means	30·1 (6·1 to 91·8)	33·4 (4·2 to 126)	148 (37·1 to 410)	356 (133 to 792)	122 (58·6 to 236)	93 (47·8 to 171)	71·1 (38 to 129)
Exposure to mechanical forces	20·6 (1·6 to 96·7)	46·8 (1·1 to 311)	100 (17·7 to 465)	288 (139 to 512)	396 (253 to 590)	497 (343 to 687)	1,180 (724 to 1,790)
Unintentional firearm injuries	7·4 (0·3 to 35·6)	24·7 (0·1 to 172)	47·8 (3 to 221)	92·3 (26·8 to 218)	104 (49·1 to 199)	159 (85·6 to 266)	672 (324 to 1,230)
Other exposure to mechanical forces	13·2 (0·9 to 72·3)	22·1 (0·7 to 164)	52·5 (11·8 to 255)	195 (94·6 to 361)	292 (180 to 450)	338 (220 to 510)	506 (318 to 763)
Adverse effects of medical treatment	164 (66·9 to 295)	106 (47·3 to 208)	328 (137 to 673)	277 (137 to 512)	168 (84·6 to 275)	122 (64·7 to 208)	119 (54·2 to 233)
Animal contact	0·3 (0·2 to 0·8)	0·3 (0·2 to 0·9)	3·5 (2·2 to 5·6)	21·8 (14·1 to 32·1)	29·4 (18 to 47·2)	40·5 (25·4 to 62·5)	50·1 (30·5 to 73·5)
Venomous animal contact	0·1 (0 to 0·3)	0·2 (0·1 to 0·5)	2·1 (1·3 to 3·3)	15·5 (9·5 to 23·5)	20·6 (11·2 to 34·6)	28·9 (17 to 46·4)	36·2 (21 to 55·9)
Non-venomous animal contact	0·2 (0·1 to 0·6)	0·1 (0·1 to 0·4)	1·3 (0·7 to 2·5)	6·3 (3·6 to 10·4)	8·8 (5·5 to 15·1)	11·6 (6·9 to 19·4)	14 (8·2 to 23·2)
Foreign body	402 (163 to 784)	287 (137 to 519)	1,960 (716 to 4,030)	1,580 (809 to 2,860)	827 (468 to 1,350)	902 (525 to 1,430)	516 (294 to 805)
Pulmonary aspiration and foreign body in airway	401 (163 to 782)	286 (137 to 518)	1,950 (705 to 4,020)	1,530 (774 to 2,800)	765 (413 to 1,280)	835 (465 to 1,360)	438 (229 to 731)
Foreign body in eyes	0 (0 to 0·1)	0·1 (0 to 0·3)	2·5 (0·7 to 5·5)	12·9 (3·9 to 29·5)	18·9 (5·4 to 44·8)	22·3 (6·7 to 52·3)	29·1 (8·8 to 68·6)
Foreign body in other body part	1·2 (0·3 to 3·5)	0·6 (0·3 to 1)	6·8 (3·7 to 11·8)	35·6 (19·8 to 59·9)	43·5 (25·8 to 74)	44·5 (29·2 to 66·3)	48·2 (33·4 to 66·7)
Environmental heat and cold exposure	1 (0·3 to 2·5)	0·7 (0·3 to 1·4)	8·9 (4·5 to 16·4)	39·7 (25·8 to 58·6)	65·7 (42·7 to 95·1)	90 (61 to 126)	146 (100 to 200)
Exposure to forces of nature	0 (0 to 0)	0 (0 to 0)	0 (0 to 0)	0·1 (0 to 0·1)	0·3 (0·2 to 0·5)	1·6 (1 to 2·5)	10·7 (6·7 to 16·9)
Other unintentional injuries	65·5 (24·9 to 132)	20 (7 to 41·5)	155 (65·4 to 310)	545 (231 to 1,130)	712 (342 to 1,290)	853 (409 to 1,500)	1,430 (674 to 2,590)
Self-harm and interpersonal violence	474 (181 to 1,020)	93·5 (35·7 to 208)	704 (248 to 1,700)	1,470 (565 to 3,200)	1,080 (567 to 1,880)	1,980 (1,200 to 3,230)	11,400 (7,010 to 17,500)
Self-harm	0 (0 to 0)	0 (0 to 0)	0 (0 to 0)	0 (0 to 0)	0 (0 to 0)	201 (103 to 358)	1,520 (845 to 2,510)
Self-harm by firearm	0 (0 to 0)	0 (0 to 0)	0 (0 to 0)	0 (0 to 0)	0 (0 to 0)	15·4 (6·6 to 41·4)	258 (96·1 to 632)
Self-harm by other specified means	0 (0 to 0)	0 (0 to 0)	0 (0 to 0)	0 (0 to 0)	0 (0 to 0)	185 (95 to 331)	1,260 (684 to 2,110)
Interpersonal violence	474 (181 to 1,020)	93·5 (35·7 to 208)	703 (247 to 1,700)	1,460 (561 to 3,190)	1,070 (563 to 1,880)	1,780 (1,010 to 3,060)	9,870 (5,660 to 16,100)
Physical violence by firearm	59·9 (14·1 to 161)	15·2 (4 to 40·4)	106 (27·4 to 274)	203 (52·4 to 530)	270 (122 to 528)	744 (329 to 1,490)	6,820 (3,500 to 11,500)
Physical violence by sharp object	48·2 (12·1 to 131)	5·8 (1·6 to 15·8)	29·9 (9·1 to 75·8)	76·7 (27·8 to 188)	80·8 (39·2 to 167)	212 (91·9 to 414)	2,150 (934 to 4,260)
Sexual violence	0 (0 to 0)	0 (0 to 0)	0 (0 to 0)	2·7 (1·5 to 4·3)	19·1 (11·6 to 30·2)	38·8 (21·6 to 61·3)	83·2 (46·5 to 134)
Physical violence by other means	366 (141 to 782)	72·5 (27·7 to 164)	567 (185 to 1,330)	1,180 (441 to 2,670)	705 (299 to 1,290)	784 (325 to 1,450)	818 (382 to 1,580)
Conflict and terrorism	0 (0 to 0)	0 (0 to 0)	0 (0 to 0)	0 (0 to 0)	0 (0 to 0)	0 (0 to 0·1)	0·1 (0 to 0·1)

G6PD deficiency	83·6 (31·9 to 165)	95·6 (39·9 to 194)	94·1 (38·3 to 186)	76·3 (33·3 to 143)	88·5 (41·5 to 160)	71·2 (33·9 to 123)	71·7 (33·3 to 130)	60·1 (32·7 to 99·2)	53·3 (27 to 87·8)
G6PD trait	0 (0 to 0)	0 (0 to 0)	0 (0 to 0)	0 (0 to 0)	0 (0 to 0)	0 (0 to 0)	0 (0 to 0)	0 (0 to 0)	0 (0 to 0)
Other hemoglobinopathies and hemolytic anemias	51·4 (16·7 to 119)	47·6 (16·1 to 111)	51·2 (16·8 to 118)	44·4 (15·3 to 101)	46 (18·5 to 102)	44·8 (16·6 to 103)	52·6 (21·9 to 115)	58·4 (27·2 to 116)	66·9 (34·8 to 119)
Endocrine, metabolic, blood, and immune disorders	514 (222 to 1,050)	671 (309 to 1,290)	822 (375 to 1,550)	856 (420 to 1,560)	868 (436 to 1,600)	806 (441 to 1,390)	949 (501 to 1,790)	848 (462 to 1,470)	913 (481 to 1,600)
Oral disorders	697 (364 to 1,290)	1,000 (498 to 1,920)	1,220 (585 to 2,260)	1,320 (622 to 2,480)	1,330 (646 to 2,450)	1,300 (670 to 2,390)	1,270 (683 to 2,260)	1,270 (714 to 2,130)	1,280 (768 to 2,090)
Caries of deciduous teeth	0 (0 to 0)	0 (0 to 0)	0 (0 to 0)	0 (0 to 0)	0 (0 to 0)	0 (0 to 0)	0 (0 to 0)	0 (0 to 0)	0 (0 to 0)
Caries of permanent teeth	176 (73·8 to 369)	168 (66·4 to 343)	145 (59 to 297)	130 (51·2 to 263)	108 (45·4 to 230)	90·2 (35·5 to 192)	76·2 (29·9 to 162)	58 (21·8 to 133)	40·2 (13·9 to 88·5)
Periodontal diseases	235 (71 to 623)	493 (158 to 1,180)	681 (229 to 1,560)	776 (273 to 1,740)	798 (288 to 1,700)	755 (292 to 1,570)	667 (262 to 1,410)	527 (208 to 1,120)	370 (147 to 768)
Other oral disorders	259 (157 to 403)	273 (159 to 425)	264 (155 to 406)	241 (144 to 371)	217 (132 to 330)	194 (116 to 292)	167 (102 to 253)	137 (81·7 to 207)	106 (62·4 to 163)
Sudden infant death syndrome	--	--	--	--	--	--	--	--	--
Injuries	51,700 (39,800 to 63,200)	49,900 (40,300 to 59,500)	44,700 (35,900 to 54,400)	35,400 (27,800 to 44,500)	29,000 (22,100 to 37,400)	22,300 (17,000 to 29,400)	18,600 (14,000 to 24,400)	13,200 (10,100 to 17,300)	10,800 (8,210 to 14,000)
Transport injuries	19,100 (12,600 to 26,700)	18,600 (12,500 to 25,300)	17,400 (12,200 to 23,800)	13,900 (9,650 to 19,000)	11,600 (7,910 to 16,400)	9,010 (6,290 to 12,700)	7,830 (5,440 to 11,100)	5,470 (3,850 to 7,630)	4,570 (3,210 to 6,390)
Road injuries	18,800 (12,300 to 26,200)	18,300 (12,200 to 24,800)	17,100 (11,900 to 23,200)	13,600 (9,370 to 18,700)	11,300 (7,730 to 16,000)	8,800 (6,140 to 12,400)	7,660 (5,320 to 10,800)	5,330 (3,760 to 7,400)	4,460 (3,140 to 6,220)
Pedestrian road injuries	753 (396 to 1,320)	858 (478 to 1,460)	998 (556 to 1,720)	940 (566 to 1,570)	915 (549 to 1,520)	800 (473 to 1,330)	834 (489 to 1,390)	674 (418 to 1,080)	619 (367 to 983)
Cyclist road injuries	434 (295 to 653)	497 (333 to 711)	547 (380 to 777)	516 (363 to 715)	491 (339 to 699)	419 (303 to 557)	391 (280 to 524)	298 (211 to 405)	246 (172 to 334)
Motorcyclist road injuries	3,220 (1,760 to 5,430)	3,110 (1,730 to 5,090)	2,800 (1,560 to 4,710)	2,270 (1,370 to 3,590)	1,840 (1,130 to 2,990)	1,390 (894 to 2,120)	1,160 (753 to 1,710)	820 (556 to 1,180)	588 (403 to 874)
Motor vehicle road injuries	14,300 (9,050 to 20,300)	13,700 (8,800 to 19,500)	12,600 (8,540 to 17,500)	9,810 (6,570 to 13,800)	8,000 (5,200 to 11,500)	6,100 (4,020 to 8,730)	5,200 (3,390 to 7,710)	3,480 (2,300 to 5,060)	2,960 (1,970 to 4,340)
Other road injuries	72·4 (47·4 to 110)	67·7 (44·9 to 99·7)	74·4 (51·1 to 104)	83·4 (57·7 to 119)	83 (58·4 to 114)	93·7 (64·9 to 130)	78·4 (55·1 to 109)	63·8 (44 to 86·9)	54 (38·3 to 72·6)
Other transport injuries	348 (181 to 637)	348 (189 to 604)	368 (196 to 650)	304 (166 to 557)	270 (146 to 490)	202 (115 to 368)	172 (97·6 to 295)	139 (83·7 to 239)	101 (58 to 169)
Unintentional injuries	9,830 (6,880 to 13,300)	9,950 (7,050 to 13,000)	9,570 (6,910 to 12,700)	8,520 (6,190 to 11,200)	7,660 (5,650 to 10,100)	6,390 (4,720 to 8,430)	5,750 (4,370 to 7,540)	4,500 (3,420 to 5,900)	3,930 (2,980 to 5,170)
Falls	1,040 (696 to 1,490)	1,130 (779 to 1,650)	1,170 (831 to 1,670)	1,190 (859 to 1,620)	1,160 (837 to 1,570)	1,100 (804 to 1,440)	1,150 (835 to 1,600)	962 (711 to 1,300)	938 (661 to 1,290)
Drowning	2,050 (977 to 3,440)	1,630 (836 to 2,680)	1,440 (660 to 2,400)	1,120 (523 to 1,850)	870 (439 to 1,470)	609 (309 to 1,050)	503 (265 to 845)	327 (171 to 567)	277 (149 to 467)
Fire, heat, and hot substances	690 (464 to 948)	760 (543 to 1,020)	812 (574 to 1,130)	740 (542 to 1,020)	650 (460 to 909)	582 (405 to 804)	496 (351 to 689)	392 (280 to 541)	331 (234 to 458)
Poisonings	200 (95 to 379)	216 (109 to 362)	200 (97·2 to 328)	166 (84·8 to 302)	130 (69·5 to 224)	113 (61·9 to 207)	82 (45·2 to 148)	54·5 (31·5 to 91·9)	43·5 (24·7 to 76·2)
Poisoning by carbon monoxide	77·6 (26·7 to 163)	75·8 (28·4 to 150)	66·8 (21·9 to 139)	55·3 (19·1 to 113)	48·4 (18·8 to 95·1)	30·7 (11 to 69·4)	16·8 (6·2 to 37·7)	12·4 (4·6 to 26·5)	7·6 (2·8 to 17·3)
Poisoning by other means	122 (60·3 to 241)	141 (70·2 to 252)	133 (64·4 to 237)	111 (55·2 to 205)	81·5 (43 to 138)	82·2 (42·9 to 151)	65·3 (36·3 to 117)	42 (24·5 to 69·9)	35·9 (20 to 64·4)
Exposure to mechanical forces	2,080 (1,250 to 3,350)	2,090 (1,290 to 3,180)	2,050 (1,320 to 3,240)	1,840 (1,230 to 2,710)	1,690 (1,150 to 2,430)	1,420 (1,010 to 1,990)	1,250 (891 to 1,770)	1,020 (725 to 1,440)	797 (565 to 1,090)
Unintentional firearm injuries	1,350 (651 to 2,610)	1,230 (593 to 2,230)	1,100 (562 to 2,020)	906 (498 to 1,660)	786 (446 to 1,330)	568 (344 to 909)	458 (290 to 775)	327 (212 to 536)	239 (162 to 368)
Other exposure to mechanical forces	728 (471 to 1,080)	862 (567 to 1,320)	945 (629 to 1,420)	934 (621 to 1,370)	905 (602 to 1,350)	852 (581 to 1,230)	794 (536 to 1,140)	688 (467 to 991)	558 (376 to 806)
Adverse effects of medical treatment	260 (110 to 503)	278 (114 to 545)	264 (101 to 549)	280 (109 to 570)	287 (117 to 615)	262 (113 to 530)	357 (164 to 733)	307 (139 to 636)	328 (161 to 620)
Animal contact	59·3 (36·7 to 87·6)	61·7 (39·7 to 91·8)	58·9 (37·6 to 85·9)	57·4 (37·5 to 84·8)	53·7 (34·5 to 77·8)	50·5 (32·1 to 74)	47·2 (31·5 to 67·3)	41·8 (28 to 60·6)	36·7 (25 to 51·7)
Venomous animal contact	43 (25·3 to 65·8)	45·4 (28·5 to 68·7)	42·8 (26·3 to 63·8)	41·1 (26·2 to 61·9)	37·5 (23·4 to 55·8)	33·3 (20·6 to 49·6)	31·6 (20·5 to 45·6)	27·7 (18·1 to 39·4)	24·4 (16·2 to 34·4)
Non-venomous animal contact	16·3 (9·8 to 25·6)	16·3 (9·7 to 25·6)	16·2 (9·7 to 26·4)	16·3 (9·8 to 27·2)	16·2 (9·8 to 26·5)	17·2 (10·8 to 26·4)	15·7 (9·8 to 24·2)	14·1 (8·9 to 21·5)	12·4 (7·9 to 19)
Foreign body	871 (440 to 1,380)	952 (518 to 1,500)	885 (460 to 1,350)	777 (409 to 1,230)	770 (395 to 1,190)	643 (348 to 1,020)	632 (338 to 1,020)	486 (262 to 786)	496 (265 to 836)
Pulmonary aspiration and foreign body in airway	779 (368 to 1,280)	860 (442 to 1,390)	794 (379 to 1,240)	694 (339 to 1,150)	694 (329 to 1,110)	575 (286 to 947)	575 (284 to 953)	439 (216 to 735)	458 (222 to 797)
Foreign body in eyes	36·5 (11·7 to 80·8)	39·5 (14 to 84·9)	38·5 (13·6 to 80·9)	36·2 (13·4 to 75·7)	33·5 (14·4 to 67·1)	29 (13·6 to 56·4)	23·1 (11·8 to 42·3)	17·4 (9·8 to 28·9)	13·6 (8·1 to 21·7)
Foreign body in other body part	55·7 (37·7 to 77)	53·1 (37·3 to 72·4)	51·9 (37 to 73)	47·1 (34 to 64·2)	43 (31·1 to 58·7)	39 (28·6 to 53·2)	34·8 (25·4 to 46·7)	28·7 (20·9 to 38·5)	23·9 (17·1 to 32·2)
Environmental heat and cold exposure	206 (139 to 282)	233 (158 to 321)	245 (168 to 334)	234 (163 to 319)	224 (156 to 304)	206 (145 to 278)	191 (134 to 254)	163 (116 to 218)	136 (94·6 to 184)
Exposure to forces of nature	17·9 (11·4 to 27·6)	32·7 (20·5 to 51·6)	28·5 (17·9 to 44·5)	25·4 (16·6 to 38·1)	24·1 (15·3 to 36·3)	22·6 (14·5 to 34·2)	18·5 (11·9 to 27·3)	13·8 (9 to 20·2)	10·2 (6·8 to 14·9)
Other unintentional injuries	2,350 (970 to 4,210)	2,570 (1,160 to 4,720)	2,430 (1,090 to 4,490)	2,090 (970 to 3,720)	1,800 (882 to 3,030)	1,380 (712 to 2,470)	1,020 (582 to 1,610)	735 (446 to 1,150)	532 (331 to 791)
Self-harm and interpersonal violence	22,700 (14,400 to 32,900)	21,300 (14,600 to 30,000)	17,700 (11,900 to 25,600)	12,900 (8,470 to 18,700)	9,690 (5,960 to 15,000)	6,940 (4,510 to 10,700)	5,020 (3,120 to 7,700)	3,270 (2,140 to 4,980)	2,340 (1,540 to 3,460)
Self-harm	3,530 (1,860 to 5,730)	3,780 (2,130 to 5,980)	3,620 (1,950 to 5,820)	2,720 (1,500 to 4,570)	2,260 (1,310 to 3,920)	1,810 (1,070 to 2,980)	1,600 (950 to 2,680)	1,200 (718 to 2,000)	1,040 (623 to 1,630)
Self-harm by firearm	710 (267 to 1,470)	715 (299 to 1,420)	623 (236 to 1,260)	477 (192 to 972)	408 (172 to 833)	307 (136 to 609)	267 (115 to 546)	171 (78·1 to 330)	144 (63·5 to 278)
Self-harm by other specified means	2,820 (1,520 to 4,630)	3,060 (1,720 to 4,950)	3,000 (1,620 to 4,760)	2,240 (1,250 to 3,740)	1,850 (1,070 to 3,100)	1,510 (912 to 2,520)	1,330 (795 to 2,250)	1,030 (608 to 1,710)	892 (533 to 1,410)
Interpersonal violence	19,200 (11,300 to 29,200)	17,500 (11,000 to 26,400)	14,100 (8,690 to 21,700)	10,200 (6,160 to 16,100)	7,430 (4,260 to 12,200)	5,120 (3,080 to 8,420)	3,420 (1,940 to 5,580)	2,070 (1,180 to 3,450)	1,300 (762 to 2,160)
Physical violence by firearm	13,900 (7,650 to 21,700)	12,700 (7,630 to 19,700)	9,890 (5,770 to 15,700)	7,100 (3,970 to 11,500)	4,900 (2,580 to 8,390)	3,350 (1,760 to 5,870)	2,100 (1,060 to 3,700)	1,180 (566 to 2,040)	734 (371 to 1,290)
Physical violence by sharp object	4,090 (1,790 to 7,420)	3,630 (1,740 to 6,650)	3,220 (1,530 to 5,910)	2,250 (1,040 to 4,260)	1,770 (902 to 3,430)	1,150 (573 to 2,150)	785 (370 to 1,460)	501 (247 to 952)	291 (159 to 508)
Sexual violence	111 (64·4 to 172)	98·8 (54·6 to 161)	84·1 (46·8 to 135)	70·5 (39·8 to 114)	53·5 (30·7 to 88)	39·2 (22·6 to 66·9)	28 (16 to 46)	19·1 (11·2 to 30·9)	14·3 (8·6 to 21·9)
Physical violence by other means	1,100 (555 to 2,020)	1,060 (569 to 1,800)	916 (498 to 1,580)	806 (421 to 1,530)	704 (364 to 1,290)	578 (323 to 1,030)	505 (277 to 906)	370 (213 to 639)	263 (160 to 439)
Conflict and terrorism	0·2 (0·1 to 0·3)	0·5 (0·3 to 0·8)	1·2 (0·8 to 1·9)	3·5 (2·2 to 5·5)	3·6 (2·3 to 5·7)	5·3 (3·4 to 8·2)	4·1 (2·6 to 6·1)	2·2 (1·5 to 3·3)	1·6 (1·1 to 2·3)

G6PD deficiency	29·2 (15·6 to 48·8)	17·1 (8·4 to 29·1)	7·5 (3·9 to 12·6)	3·8 (1·8 to 6·6)	2·3 (1 to 4·1)	0·5 (0·2 to 0·8)	0·1 (0 to 0·1)	894
G6PD trait	0 (0 to 0)	0 (0 to 0)	0 (0 to 0)	0 (0 to 0)	0 (0 to 0)	0 (0 to 0)	0 (0 to 0)	0
Other hemoglobinopathies and hemolytic anemias	52·4 (29·2 to 86·8)	50·1 (29·4 to 80·6)	39·2 (23·7 to 61·3)	37·4 (21·8 to 59·3)	44·4 (27·8 to 66·5)	20 (12·6 to 30·5)	6·7 (3·4 to 11·8)	915
Endocrine, metabolic, blood, and immune disorders	718 (396 to 1,200)	667 (363 to 1,150)	475 (252 to 813)	361 (185 to 639)	286 (123 to 555)	116 (43·9 to 225)	35·2 (10·7 to 76·8)	17,500
Oral disorders	1,240 (744 to 1,940)	1,050 (649 to 1,640)	750 (470 to 1,130)	492 (306 to 731)	262 (162 to 385)	93·6 (57·8 to 136)	25 (15·4 to 37)	15,800
Caries of deciduous teeth	0 (0 to 0)	0 (0 to 0)	0 (0 to 0)	0 (0 to 0)	0 (0 to 0)	0 (0 to 0)	0 (0 to 0)	171
Caries of permanent teeth	27 (10·2 to 56·7)	18·4 (7·4 to 38·7)	11·6 (4·7 to 22·8)	7 (2·7 to 15)	3·4 (1·2 to 7·2)	1·1 (0·4 to 2·4)	0·2 (0·1 to 0·5)	1,420
Periodontal diseases	248 (95·7 to 501)	163 (64·1 to 327)	100 (40·4 to 199)	61·6 (23·5 to 124)	31·8 (12 to 65·9)	11 (4·1 to 22·8)	2·8 (1 to 6)	5,980
Other oral disorders	79·7 (47·6 to 121)	52·8 (31·9 to 80·9)	28·3 (17·2 to 42·4)	14·4 (8·8 to 22·1)	5·9 (3·7 to 9·1)	1·8 (1·1 to 2·8)	0·5 (0·3 to 0·8)	2,700
Sudden infant death syndrome	--	--	--	--	--	--	--	NA
Injuries	7,130 (5,570 to 9,140)	5,150 (4,050 to 6,430)	3,190 (2,590 to 3,920)	2,140 (1,750 to 2,600)	1,450 (1,130 to 1,820)	542 (416 to 685)	157 (103 to 250)	357,000
Transport injuries	2,940 (2,090 to 4,120)	1,960 (1,460 to 2,580)	1,160 (883 to 1,510)	701 (539 to 906)	461 (326 to 617)	139 (102 to 179)	33·2 (23·5 to 55)	133,000
Road injuries	2,870 (2,050 to 4,030)	1,910 (1,430 to 2,510)	1,140 (862 to 1,470)	689 (530 to 890)	455 (320 to 609)	137 (101 to 177)	32·5 (23 to 53·8)	130,000
Pedestrian road injuries	459 (280 to 735)	342 (222 to 522)	240 (156 to 356)	158 (106 to 229)	108 (69·2 to 162)	30·4 (20·7 to 44·9)	5·9 (3·8 to 10·1)	10,700
Cyclist road injuries	175 (126 to 236)	111 (80·1 to 149)	69·5 (48·8 to 94·5)	45·4 (31·3 to 61·9)	25·3 (17·4 to 34·3)	9·5 (6·5 to 12·9)	2·6 (1·8 to 3·6)	4,770
Motorcyclist road injuries	394 (279 to 556)	237 (172 to 319)	145 (105 to 196)	94·6 (67 to 128)	56·4 (40·3 to 76·8)	19·8 (13·7 to 27·5)	5·5 (3·8 to 7·7)	19,900
Motor vehicle road injuries	1,800 (1,210 to 2,690)	1,190 (840 to 1,640)	665 (488 to 896)	379 (282 to 504)	258 (169 to 366)	75·1 (51 to 103)	18 (11·4 to 35·3)	94,000
Other road injuries	41·6 (29·2 to 57·1)	28·9 (20·4 to 39·6)	19·1 (13·4 to 25·9)	11·8 (8·1 to 16·3)	6·7 (4·6 to 9·3)	2·1 (1·4 to 2·9)	0·5 (0·4 to 0·7)	853
Other transport injuries	63·9 (36·5 to 103)	52 (31·4 to 84·2)	25·9 (16·3 to 40·1)	12·1 (8·4 to 17)	6·3 (4·6 to 8·7)	2·3 (1·6 to 3·1)	0·7 (0·5 to 1·1)	2,880
Unintentional injuries	2,890 (2,200 to 3,750)	2,340 (1,810 to 2,970)	1,590 (1,240 to 1,990)	1,200 (945 to 1,490)	853 (647 to 1,110)	366 (273 to 483)	113 (68·9 to 199)	102,000
Falls	736 (533 to 1,010)	708 (504 to 972)	529 (389 to 697)	494 (363 to 667)	410 (282 to 603)	196 (133 to 303)	69 (33·7 to 153)	14,700
Drowning	162 (82·6 to 266)	103 (56·2 to 172)	60·8 (32 to 106)	32 (15·3 to 54·2)	37·7 (9·4 to 65·3)	15·2 (3·5 to 24·8)	2·8 (0·8 to 4·7)	15,500
Fire, heat, and hot substances	229 (164 to 318)	176 (122 to 254)	112 (75·7 to 165)	82·5 (56·3 to 122)	62·2 (42·2 to 93·1)	25·4 (17·4 to 37·5)	6·7 (4 to 16·1)	8,750
Poisonings	25·5 (15·4 to 41·7)	19·5 (12·1 to 31·9)	12·2 (7·5 to 19·3)	9·4 (5·2 to 16)	8 (3 to 14·1)	3·1 (1·2 to 5·5)	0·5 (0·2 to 0·9)	2,270
Poisoning by carbon monoxide	4·4 (1·8 to 9)	4·4 (1·8 to 9)	1·7 (0·7 to 3·9)	1·8 (0·7 to 4·1)	1·7 (0·5 to 3·9)	0·7 (0·2 to 1·5)	0·1 (0 to 0·2)	543
Poisoning by other means	21·1 (12·7 to 34·8)	15·1 (9·1 to 25)	10·4 (6·5 to 16·9)	7·6 (4·1 to 13·2)	6·4 (2·4 to 11·4)	2·5 (1 to 4·5)	0·4 (0·2 to 0·8)	1,730
Exposure to mechanical forces	567 (405 to 774)	409 (290 to 564)	263 (185 to 371)	173 (122 to 243)	105 (72·6 to 146)	39·9 (27·4 to 56·6)	12·2 (8 to 18·2)	18,300
Unintentional firearm injuries	147 (98·6 to 218)	93·8 (65·9 to 130)	54·3 (39 to 74·6)	35·2 (25·1 to 49)	24·8 (16·1 to 35·5)	9 (6 to 12·8)	3 (1·8 to 5)	8,440
Other exposure to mechanical forces	421 (282 to 604)	316 (213 to 455)	209 (140 to 304)	137 (91·7 to 199)	79·8 (54 to 116)	30·8 (20·7 to 44·3)	9·3 (6·1 to 13·9)	9,890
Adverse effects of medical treatment	281 (143 to 554)	242 (128 to 431)	182 (100 to 323)	131 (73·5 to 218)	85·8 (50·3 to 134)	31·6 (19·1 to 47·2)	6·6 (3·1 to 10·6)	4,870
Animal contact	29·7 (20 to 41·5)	22·6 (15·5 to 31·5)	15 (10·3 to 21·4)	10 (6·9 to 13·8)	5·7 (4 to 8)	2·3 (1·6 to 3·1)	0·6 (0·4 to 1)	699
Venomous animal contact	19·4 (12·9 to 27)	14·4 (9·6 to 20·4)	9·3 (6·3 to 13·3)	6·3 (4·2 to 8·7)	3·6 (2·5 to 5)	1·5 (1·1 to 2)	0·4 (0·3 to 0·6)	485
Non-venomous animal contact	10·3 (6·5 to 15·4)	8·2 (5·2 to 12·2)	5·7 (3·6 to 8·5)	3·8 (2·4 to 5·7)	2·1 (1·4 to 3·2)	0·8 (0·5 to 1·2)	0·2 (0·2 to 0·4)	214
Foreign body	386 (202 to 626)	326 (171 to 510)	201 (117 to 298)	135 (79·1 to 201)	58·1 (35·8 to 88·8)	20·8 (13·8 to 30)	5·5 (3·4 to 11)	14,100
Pulmonary aspiration and foreign body in airway	357 (180 to 595)	293 (142 to 474)	176 (96·1 to 268)	115 (63·4 to 177)	47·1 (26·6 to 75·4)	15·7 (9·3 to 24·2)	4·2 (2·2 to 8·8)	13,100
Foreign body in eyes	10·7 (6·5 to 16·7)	8·1 (5·2 to 12·3)	5·7 (3·6 to 8·5)	3·7 (2·5 to 5·5)	2 (1·4 to 2·9)	0·7 (0·5 to 1·1)	0·2 (0·1 to 0·3)	384
Foreign body in other body part	18·9 (13·9 to 25·7)	25·1 (14·9 to 44·8)	19·8 (12·3 to 32·2)	16·6 (10·1 to 27·1)	9 (5·6 to 15·5)	4·3 (2·5 to 7·2)	1·1 (0·6 to 1·9)	652
Environmental heat and cold exposure	105 (72·8 to 141)	84·9 (59·9 to 112)	57·9 (41 to 77·4)	41·8 (29·9 to 56·1)	25·7 (18·8 to 34·9)	10·8 (7·9 to 14·9)	3·1 (2·1 to 4·3)	2,520
Exposure to forces of nature	7·5 (5·1 to 11·1)	5·1 (3·5 to 7·4)	3·2 (2·1 to 4·5)	1·8 (1·2 to 2·6)	0·9 (0·6 to 1·3)	0·3 (0·2 to 0·4)	0·1 (0·1 to 0·1)	225
Other unintentional injuries	359 (240 to 520)	245 (163 to 360)	152 (106 to 209)	95·2 (69 to 128)	54·2 (39·1 to 75·5)	20·3 (14·8 to 27·7)	6·2 (4·2 to 9·9)	19,600
Self-harm and interpersonal violence	1,310 (878 to 1,970)	849 (579 to 1,240)	441 (298 to 617)	235 (171 to 340)	137 (97·6 to 199)	37·1 (27·5 to 52·1)	10 (6·5 to 18·3)	122,000
Self-harm	624 (379 to 1,010)	450 (281 to 712)	240 (151 to 378)	145 (90·6 to 240)	88·5 (53·8 to 150)	20·6 (13·2 to 35·6)	4 (1·9 to 12·7)	24,800
Self-harm by firearm	81·1 (37·9 to 161)	56·7 (27 to 112)	27·6 (13 to 55·6)	16·2 (7·5 to 33·3)	8·9 (4·3 to 17·1)	2·1 (0·9 to 4·2)	0·4 (0·2 to 1·4)	4,290
Self-harm by other specified means	543 (327 to 883)	394 (244 to 622)	212 (132 to 331)	128 (79 to 214)	79·6 (48·4 to 136)	18·5 (11·6 to 32·4)	3·6 (1·6 to 11·6)	20,500
Interpersonal violence	682 (428 to 1,110)	398 (245 to 638)	201 (130 to 321)	90·4 (65·4 to 126)	48·1 (35·5 to 65)	16·4 (12·1 to 21·4)	5·9 (4·1 to 8·7)	97,300
Physical violence by firearm	343 (174 to 636)	168 (82·8 to 319)	62·3 (31 to 110)	26 (15 to 42·9)	12 (7·2 to 19·4)	2·6 (1·6 to 4·1)	0·6 (0·3 to 1·3)	64,700
Physical violence by sharp object	158 (93·6 to 278)	111 (65·5 to 182)	63·1 (38·4 to 105)	30·5 (20·9 to 44·3)	18·1 (12·2 to 25·5)	8·2 (5·8 to 11)	3·6 (2·2 to 5·7)	20,700
Sexual violence	10·6 (6·5 to 15·9)	7·5 (4·7 to 11·2)	4·8 (3·1 to 7·1)	3 (1·9 to 4·4)	1·2 (0·7 to 2)	0·2 (0·1 to 0·3)	0 (0 to 0)	690
Physical violence by other means	171 (106 to 281)	111 (70·8 to 172)	70·5 (43·9 to 110)	30·9 (21·5 to 43·3)	16·8 (11·9 to 23·3)	5·4 (3·8 to 7·4)	1·8 (1·2 to 3)	11,200
Conflict and terrorism	0·7 (0·5 to 1·1)	1 (0·6 to 1·4)	0·4 (0·3 to 0·6)	0·4 (0·3 to 0·6)	0·4 (0·3 to 0·6)	0·1 (0·1 to 0·1)	0 (0 to 0)	25·4

SR Table 2a: Malaysia DALYS by cause and age group for males with 95% uncertainty intervals, 2019

Cause	Early Neonatal	Late Neonatal	Post Neonatal	1 to 4	5 to 9	10 to 14	15 to 19	20 to 24	25 to 29	30 to 34	35 to 39	40 to 44	45 to 49	50 to 54	55 to 59	60 to 64	65 to 69	70 to 74	75 to 79	80 to 84	85 to 89	90 to 94	95 plus	Total
All causes	71,700 (57,000 to 88,700)	24,500 (19,600 to 30,100)	49,200 (40,400 to 59,300)	51,700 (37,100 to 68,900)	70,600 (55,900 to 89,200)	90,800 (72,700 to 113,000)	174,000 (145,000 to 210,000)	228,000 (193,000 to 270,000)	251,000 (214,000 to 295,000)	286,000 (245,000 to 333,000)	279,000 (239,000 to 329,000)	267,000 (223,000 to 315,000)	277,000 (227,000 to 332,000)	317,000 (260,000 to 383,000)	371,000 (304,000 to 449,000)	394,000 (323,000 to 471,000)	372,000 (310,000 to 441,000)	343,000 (289,000 to 404,000)	219,000 (187,000 to 255,000)	143,000 (124,000 to 163,000)	65,800 (58,500 to 73,400)	30,700 (27,800 to 33,800)	6,760 (6,230 to 7,300)	4,380,000
Communicable, maternal, neonatal, and nutritional diseases	50,300 (37,500 to 64,500)	16,600 (12,700 to 21,200)	25,000 (18,700 to 31,400)	27,100 (18,600 to 37,300)	24,600 (17,500 to 34,700)	17,800 (13,700 to 23,000)	19,200 (14,800 to 23,800)	24,500 (19,700 to 30,600)	33,700 (27,700 to 40,700)	47,100 (39,200 to 56,100)	49,600 (41,400 to 58,400)	41,200 (33,300 to 50,500)	36,400 (28,600 to 45,900)	37,500 (28,100 to 48,100)	41,100 (29,700 to 53,900)	45,800 (31,700 to 61,100)	45,100 (31,100 to 60,300)	47,500 (31,700 to 64,900)	34,400 (22,300 to 45,600)	23,800 (16,300 to 30,700)	11,800 (8,000 to 15,100)	5,780 (4,130 to 7,340)	1,290 (887 to 1,660)	707,000
HIV/AIDS and sexually transmitted infections	455 (142 to 1,060)	161 (48 to 363)	689 (498 to 988)	488 (387 to 610)	396 (319 to 492)	427 (338 to 532)	691 (534 to 890)	1,720 (1,360 to 2,140)	5,250 (4,170 to 6,450)	13,200 (10,400 to 16,500)	17,100 (13,500 to 21,200)	11,700 (9,070 to 14,800)	7,120 (5,690 to 8,920)	4,790 (3,830 to 6,020)	3,610 (2,860 to 4,590)	1,990 (1,550 to 2,460)	670 (523 to 829)	78·4 (57·7 to 105)	29·5 (21·5 to 40·6)	8·3 (5 to 14·4)	3·7 (2·4 to 6·1)	1·2 (0·7 to 2·2)	0·4 (0·3 to 0·6)	70,500
HIV/AIDS	0·3 (0·1 to 0·4)	0·8 (0·4 to 1·3)	497 (385 to 625)	484 (381 to 607)	393 (316 to 489)	387 (301 to 476)	540 (429 to 673)	1,450 (1,130 to 1,820)	4,910 (3,900 to 6,070)	12,800 (10,100 to 16,200)	16,800 (13,300 to 20,900)	11,500 (8,930 to 14,500)	6,980 (5,580 to 8,770)	4,700 (3,720 to 5,910)	3,550 (2,810 to 4,530)	1,950 (1,500 to 2,410)	643 (500 to 801)	58·3 (44·5 to 71·1)	21·6 (16·4 to 26·5)	4 (3·1 to 5)	2 (1·6 to 2·5)	0·6 (0·4 to 0·7)	0·3 (0·2 to 0·3)	67,700
HIV/AIDS - Drug-susceptible Tuberculosis	0 (0 to 0)	0 (0 to 0)	87·4 (55·8 to 130)	85·1 (57·1 to 126)	63·7 (42·9 to 93)	58·3 (39·9 to 84·3)	95·5 (64·1 to 141)	268 (180 to 391)	911 (596 to 1,360)	2,330 (1,530 to 3,470)	3,030 (2,010 to 4,550)	2,050 (1,330 to 3,070)	1,230 (803 to 1,850)	822 (533 to 1,250)	616 (399 to 920)	338 (222 to 502)	113 (75·2 to 167)	10·8 (7·8 to 14·8)	3·9 (2·8 to 5·5)	0·7 (0·5 to 1)	0·4 (0·2 to 0·5)	0·1 (0·1 to 0·1)	0 (0 to 0·1)	12,100
HIV/AIDS - Multidrug-resistant Tuberculosis without exte	0 (0 to 0)	0 (0 to 0)	2 (0·4 to 6·3)	1·8 (0·3 to 5·7)	1·3 (0·2 to 4·2)	1·2 (0·2 to 3·7)	2 (0·4 to 6·4)	5·6 (1·1 to 17·5)	19 (3·5 to 59·9)	49 (9 to 156)	63·7 (11·6 to 203)	43·5 (7·9 to 139)	26·5 (4·8 to 84·3)	17·9 (3·3 to 57·2)	13·6 (2·5 to 43·3)	7·4 (1·4 to 23·6)	2·4 (0·4 to 7·7)	0·2 (0 to 0·6)	0·1 (0 to 0·2)	0 (0 to 0)	0 (0 to 0)	0 (0 to 0)	0 (0 to 0)	257
HIV/AIDS - Extensively drug-resistant Tuberculosis	0 (0 to 0)	0 (0 to 0)	0·4 (0·1 to 1·2)	0·3 (0·1 to 1·1)	0·2 (0 to 0·8)	0·2 (0 to 0·7)	0·4 (0·1 to 1·2)	1 (0·2 to 3·3)	3·5 (0·6 to 11·4)	9·2 (1·6 to 30)	12 (2·1 to 39·1)	8·2 (1·4 to 26·7)	5 (0·9 to 16·2)	3·4 (0·6 to 11)	2·6 (0·4 to 8·4)	1·4 (0·2 to 4·6)	0·5 (0·1 to 1·5)	0 (0 to 0·1)	0 (0 to 0)	0 (0 to 0)	0 (0 to 0)	0 (0 to 0)	0 (0 to 0)	48·4
HIV/AIDS resulting in other diseases	0·3 (0·1 to 0·4)	0·8 (0·4 to 1·3)	407 (289 to 545)	397 (283 to 521)	328 (242 to 426)	328 (238 to 420)	443 (324 to 580)	1,170 (843 to 1,550)	3,980 (2,890 to 5,170)	10,400 (7,590 to 13,900)	13,700 (9,890 to 18,100)	9,380 (6,660 to 12,600)	5,720 (4,180 to 7,440)	3,850 (2,840 to 5,090)	2,920 (2,070 to 3,970)	1,600 (1,120 to 2,080)	528 (384 to 691)	47·3 (32·8 to 60·3)	17·6 (12·3 to 22·7)	3·3 (2·3 to 4·2)	1·7 (1·2 to 2·1)	0·5 (0·3 to 0·6)	0·2 (0·2 to 0·3)	55,200
Sexually transmitted infections excluding HIV	454 (142 to 1,060)	161 (47 to 362)	192 (56·5 to 456)	4·5 (1 to 12·2)	3 (0·9 to 6·8)	39·1 (20·7 to 75·5)	151 (61·8 to 298)	270 (132 to 471)	339 (165 to 608)	370 (164 to 649)	263 (118 to 473)	190 (93·6 to 341)	140 (66·8 to 255)	96·8 (45·9 to 182)	62 (29·9 to 126)	42·5 (17·8 to 91·9)	26·7 (10·5 to 57·8)	20·1 (7·7 to 42·2)	7·9 (2·8 to 18·8)	4·3 (1·5 to 10·2)	1·7 (0·6 to 4)	0·7 (0·2 to 1·6)	0·1 (0 to 0·3)	2,840
Syphilis	454 (142 to 1,060)	161 (47 to 362)	192 (56·5 to 456)	4·5 (1 to 12·2)	3 (0·9 to 6·8)	5·5 (4·3 to 6·9)	8·6 (6·5 to 11·2)	8·8 (6·3 to 12·8)	11·1 (7·9 to 17·5)	10·8 (7·1 to 18·7)	9·7 (6·5 to 16·2)	6 (4·1 to 9·6)	4·1 (2·8 to 6·3)	4·5 (2·4 to 10)	3·9 (2·2 to 8·8)	5·9 (1·8 to 15·9)	3·3 (1·2 to 8·4)	4·4 (0·9 to 12·4)	0·4 (0·3 to 0·6)	0·2 (0·1 to 0·2)	0·1 (0 to 0·1)	0 (0 to 0)	0 (0 to 0)	902
Chlamydial infection	0 (0 to 0)	0 (0 to 0)	0 (0 to 0)	0 (0 to 0)	0 (0 to 0)	0·6 (0·2 to 1·1)	14·9 (6·9 to 27·7)	62 (16 to 137)	112 (37·9 to 223)	112 (33·5 to 228)	79·3 (22·6 to 163)	56·5 (15·7 to 113)	34·9 (8·4 to 73·7)	15·6 (0·9 to 40·2)	4·7 (2·2 to 8·4)	0·8 (0·4 to 1·5)	0·2 (0·1 to 0·4)	0 (0 to 0)	0 (0 to 0)	0 (0 to 0)	0 (0 to 0)	0 (0 to 0)	0 (0 to 0)	493
Gonococcal infection	0 (0 to 0)	0 (0 to 0)	0 (0 to 0)	0 (0 to 0)	[illegible]	[illegible]	[illegible]	[illegible]	[illegible]	[illegible]	[illegible]	[illegible]	[illegible]	[illegible]	[illegible]	[illegible]	[illegible]	[illegible]	[illegible]	[illegible]	[illegible]	[illegible]	[illegible]	[illegible]
Trichomoniasis	0 (0 to 0)	0 (0 to 0)	0 (0 to 0)	0 (0 to 0)	[illegible]	[illegible]	[illegible]	[illegible]	[illegible]	[illegible]	[illegible]	[illegible]	[illegible]	[illegible]	[illegible]	[illegible]	[illegible]	[illegible]	[illegible]	[illegible]	[illegible]	[illegible]	[illegible]	[illegible]
Genital herpes	0 (0 to 0)	0 (0 to 0)	0 (0 to 0)	0 (0 to 0)	[illegible]	[illegible]	[illegible]	[illegible]	[illegible]	[illegible]	[illegible]	[illegible]	[illegible]	[illegible]	[illegible]	[illegible]	[illegible]	[illegible]	[illegible]	[illegible]	[illegible]	[illegible]	[illegible]	[illegible]
Other sexually transmitted infections	0 (0 to 0)	0 (0 to 0)	0 (0 to 0)	0 (0 to 0)	[illegible]	[illegible]	[illegible]	[illegible]	[illegible]	[illegible]	[illegible]	[illegible]	[illegible]	[illegible]	[illegible]	[illegible]	[illegible]	[illegible]	[illegible]	[illegible]	[illegible]	[illegible]	[illegible]	[illegible]
Respiratory infections and tuberculosis	1,470 (807 to 2,530)	1,160 (652 to 2,010)	6,710 (3,910 to 10,400)	3,160 (1,990 to 4,760)	[illegible]	[illegible]	[illegible]	[illegible]	[illegible]	[illegible]	[illegible]	[illegible]	[illegible]	[illegible]	[illegible]	[illegible]	[illegible]	[illegible]	[illegible]	[illegible]	[illegible]	[illegible]	[illegible]	[illegible]
Tuberculosis	0 (0 to 0)	0 (0 to 0)	186 (108 to 297)	110 (42·2 to 202)	[illegible]	[illegible]	[illegible]	[illegible]	[illegible]	[illegible]	[illegible]	[illegible]	[illegible]	[illegible]	[illegible]	[illegible]	[illegible]	[illegible]	[illegible]	[illegible]	[illegible]	[illegible]	[illegible]	[illegible]
Latent tuberculosis infection	0 (0 to 0)	[illegible]	[illegible]	[illegible]	[illegible]	[illegible]	[illegible]	[illegible]	[illegible]	[illegible]	[illegible]	[illegible]	[illegible]	[illegible]	[illegible]	[illegible]	[illegible]	[illegible]	[illegible]	[illegible]	[illegible]	[illegible]	[illegible]	[illegible]
Drug-susceptible tuberculosis	0 (0 to 0)	[illegible]	[illegible]	[illegible]	[illegible]	[illegible]	[illegible]	[illegible]	[illegible]	[illegible]	[illegible]	[illegible]	[illegible]	[illegible]	[illegible]	[illegible]	[illegible]	[illegible]	[illegible]	[illegible]	[illegible]	[illegible]	[illegible]	[illegible]
Multidrug-resistant tuberculosis without extensive drug r	0 (0 to 0)	[illegible]	[illegible]	[illegible]	[illegible]	[illegible]	[illegible]	[illegible]	[illegible]	[illegible]	[illegible]	[illegible]	[illegible]	[illegible]	[illegible]	[illegible]	[illegible]	[illegible]	[illegible]	[illegible]	[illegible]	[illegible]	[illegible]	[illegible]
Extensively drug-resistant tuberculosis	0 (0 to 0)	[illegible]	[illegible]	[illegible]	[illegible]	[illegible]	[illegible]	[illegible]	[illegible]	[illegible]	[illegible]	[illegible]	[illegible]	[illegible]	[illegible]	[illegible]	[illegible]	[illegible]	[illegible]	[illegible]	[illegible]	[illegible]	[illegible]	[illegible]
Lower respiratory infections	[illegible]	[illegible]	[illegible]	[illegible]	[illegible]	[illegible]	[illegible]	[illegible]	[illegible]	[illegible]	[illegible]	[illegible]	[illegible]	[illegible]	[illegible]	[illegible]	[illegible]	[illegible]	[illegible]	[illegible]	[illegible]	[illegible]	[illegible]	[illegible]
Upper respiratory infections	[illegible]	[illegible]	[illegible]	[illegible]	[illegible]	[illegible]	[illegible]	[illegible]	[illegible]	[illegible]	[illegible]	[illegible]	[illegible]	[illegible]	[illegible]	[illegible]	[illegible]	[illegible]	[illegible]	[illegible]	[illegible]	[illegible]	[illegible]	[illegible]
Otitis media	[illegible]	[illegible]	[illegible]	[illegible]	[illegible]	[illegible]	[illegible]	[illegible]	[illegible]	[illegible]	[illegible]	[illegible]	[illegible]	[illegible]	[illegible]	[illegible]	[illegible]	[illegible]	[illegible]	[illegible]	[illegible]	[illegible]	[illegible]	[illegible]
Enteric infections	[illegible]	[illegible]	[illegible]	[illegible]	[illegible]	[illegible]	[illegible]	[illegible]	[illegible]	[illegible]	[illegible]	[illegible]	[illegible]	[illegible]	[illegible]	[illegible]	[illegible]	[illegible]	[illegible]	[illegible]	[illegible]	[illegible]	[illegible]	[illegible]
Diarrheal diseases	[illegible]	[illegible]	[illegible]	[illegible]	[illegible]	[illegible]	[illegible]	[illegible]	[illegible]	[illegible]	[illegible]	[illegible]	[illegible]	[illegible]	[illegible]	[illegible]	[illegible]	[illegible]	[illegible]	[illegible]	[illegible]	[illegible]	[illegible]	[illegible]
Typhoid and paratyphoid	0 (0 to 0)	[illegible]	[illegible]	[illegible]	[illegible]	[illegible]	[illegible]	[illegible]	[illegible]	[illegible]	[illegible]	[illegible]	[illegible]	[illegible]	[illegible]	[illegible]	[illegible]	[illegible]	[illegible]	[illegible]	[illegible]	[illegible]	[illegible]	[illegible]
Typhoid fever	0 (0 to 0)	[illegible]	[illegible]	[illegible]	[illegible]	[illegible]	[illegible]	[illegible]	[illegible]	[illegible]	[illegible]	[illegible]	[illegible]	[illegible]	[illegible]	[illegible]	[illegible]	[illegible]	[illegible]	[illegible]	[illegible]	[illegible]	[illegible]	[illegible]
Paratyphoid fever	0 (0 to 0)	[illegible]	[illegible]	[illegible]	[illegible]	[illegible]	[illegible]	[illegible]	[illegible]	[illegible]	[illegible]	[illegible]	[illegible]	[illegible]	[illegible]	[illegible]	[illegible]	[illegible]	[illegible]	[illegible]	[illegible]	[illegible]	[illegible]	[illegible]
Invasive Non-typhoidal Salmonella (iNTS)	0 (0 to 0)	[illegible]	[illegible]	[illegible]	[illegible]	[illegible]	[illegible]	[illegible]	[illegible]	[illegible]	[illegible]	[illegible]	[illegible]	[illegible]	[illegible]	[illegible]	[illegible]	[illegible]	[illegible]	[illegible]	[illegible]	[illegible]	[illegible]	[illegible]
Other intestinal infectious diseases	0 (0 to 0)	[illegible]	[illegible]	[illegible]	[illegible]	[illegible]	[illegible]	[illegible]	[illegible]	[illegible]	[illegible]	[illegible]	[illegible]	[illegible]	[illegible]	[illegible]	[illegible]	[illegible]	[illegible]	[illegible]	[illegible]	[illegible]	[illegible]	[illegible]
Neglected tropical diseases and malaria	[illegible]	[illegible]	[illegible]	[illegible]	[illegible]	[illegible]	[illegible]	[illegible]	[illegible]	[illegible]	[illegible]	[illegible]	[illegible]	[illegible]	[illegible]	[illegible]	[illegible]	[illegible]	[illegible]	[illegible]	[illegible]	[illegible]	[illegible]	[illegible]
Malaria	[illegible]	[illegible]	[illegible]	[illegible]	[illegible]	[illegible]	[illegible]	[illegible]	[illegible]	[illegible]	[illegible]	[illegible]	[illegible]	[illegible]	[illegible]	[illegible]	[illegible]	[illegible]	[illegible]	[illegible]	[illegible]	[illegible]	[illegible]	[illegible]
Chagas disease	0 (0 to 0)	[illegible]	[illegible]	[illegible]	[illegible]	[illegible]	[illegible]	[illegible]	[illegible]	[illegible]	[illegible]	[illegible]	[illegible]	[illegible]	[illegible]	[illegible]	[illegible]	[illegible]	[illegible]	[illegible]	[illegible]	[illegible]	[illegible]	[illegible]
Leishmaniasis	0 (0 to 0)	[illegible]	[illegible]	[illegible]	[illegible]	[illegible]	[illegible]	[illegible]	[illegible]	[illegible]	[illegible]	[illegible]	[illegible]	[illegible]	[illegible]	[illegible]	[illegible]	[illegible]	[illegible]	[illegible]	[illegible]	[illegible]	[illegible]	[illegible]
Visceral leishmaniasis	0 (0 to 0)	[illegible]	[illegible]	[illegible]	[illegible]	[illegible]	[illegible]	[illegible]	[illegible]	[illegible]	[illegible]	[illegible]	[illegible]	[illegible]	[illegible]	[illegible]	[illegible]	[illegible]	[illegible]	[illegible]	[illegible]	[illegible]	[illegible]	[illegible]
Cutaneous and mucocutaneous leishmaniasis	0 (0 to 0)	[illegible]	[illegible]	[illegible]	[illegible]	[illegible]	[illegible]	[illegible]	[illegible]	[illegible]	[illegible]	[illegible]	[illegible]	[illegible]	[illegible]	[illegible]	[illegible]	[illegible]	[illegible]	[illegible]	[illegible]	[illegible]	[illegible]	[illegible]
African trypanosomiasis	0 (0 to 0)	[illegible]	[illegible]	[illegible]	[illegible]	[illegible]	[illegible]	[illegible]	[illegible]	[illegible]	[illegible]	[illegible]	[illegible]	[illegible]	[illegible]	[illegible]	[illegible]	[illegible]	[illegible]	[illegible]	[illegible]	[illegible]	[illegible]	[illegible]
Schistosomiasis	0 (0 to 0)	[illegible]	[illegible]	[illegible]	[illegible]	[illegible]	[illegible]	[illegible]	[illegible]	[illegible]	[illegible]	[illegible]	[illegible]	[illegible]	[illegible]	[illegible]	[illegible]	[illegible]	[illegible]	[illegible]	[illegible]	[illegible]	[illegible]	[illegible]
Cysticercosis	0 (0 to 0)	[illegible]	[illegible]	[illegible]	[illegible]	[illegible]	[illegible]	[illegible]	[illegible]	[illegible]	[illegible]	[illegible]	[illegible]	[illegible]	[illegible]	[illegible]	[illegible]	[illegible]	[illegible]	[illegible]	[illegible]	[illegible]	[illegible]	[illegible]
Cystic echinococcosis	0 (0 to 0)	[illegible]	[illegible]	[illegible]	[illegible]	[illegible]	[illegible]	[illegible]	[illegible]	[illegible]	[illegible]	[illegible]	[illegible]	[illegible]	[illegible]	[illegible]	[illegible]	[illegible]	[illegible]	[illegible]	[illegible]	[illegible]	[illegible]	[illegible]
Lymphatic filariasis	0 (0 to 0)	[illegible]	[illegible]	[illegible]	[illegible]	[illegible]	[illegible]	[illegible]	[illegible]	[illegible]	[illegible]	[illegible]	[illegible]	[illegible]	[illegible]	[illegible]	[illegible]	[illegible]	[illegible]	[illegible]	[illegible]	[illegible]	[illegible]	[illegible]
Onchocerciasis	0 (0 to 0)	[illegible]	[illegible]	[illegible]	[illegible]	[illegible]	[illegible]	[illegible]	[illegible]	[illegible]	[illegible]	[illegible]	[illegible]	[illegible]	[illegible]	[illegible]	[illegible]	[illegible]	[illegible]	[illegible]	[illegible]	[illegible]	[illegible]	[illegible]
Trachoma	0 (0 to 0)	[illegible]	[illegible]	[illegible]	[illegible]	[illegible]	[illegible]	[illegible]	[illegible]	[illegible]	[illegible]	[illegible]	[illegible]	[illegible]	[illegible]	[illegible]	[illegible]	[illegible]	[illegible]	[illegible]	[illegible]	[illegible]	[illegible]	[illegible]
Dengue	0 (0 to 0)	[illegible]	[illegible]	[illegible]	[illegible]	[illegible]	[illegible]	[illegible]	[illegible]	[illegible]	[illegible]	[illegible]	[illegible]	[illegible]	[illegible]	[illegible]	[illegible]	[illegible]	[illegible]	[illegible]	[illegible]	[illegible]	[illegible]	[illegible]
Yellow fever	0 (0 to 0)	[illegible]	[illegible]	[illegible]	[illegible]	[illegible]	[illegible]	[illegible]	[illegible]	[illegible]	[illegible]	[illegible]	[illegible]	[illegible]	[illegible]	[illegible]	[illegible]	[illegible]	[illegible]	[illegible]	[illegible]	[illegible]	[illegible]	[illegible]
Rabies	0 (0 to 0)	[illegible]	[illegible]	[illegible]	[illegible]	[illegible]	[illegible]	[illegible]	[illegible]	[illegible]	[illegible]	[illegible]	[illegible]	[illegible]	[illegible]	[illegible]	[illegible]	[illegible]	[illegible]	[illegible]	[illegible]	[illegible]	[illegible]	[illegible]
Intestinal nematode infections	0 (0 to 0)	[illegible]	[illegible]	[illegible]	[illegible]	[illegible]	[illegible]	[illegible]	[illegible]	[illegible]	[illegible]	[illegible]	[illegible]	[illegible]	[illegible]	[illegible]	[illegible]	[illegible]	[illegible]	[illegible]	[illegible]	[illegible]	[illegible]	[illegible]
Ascariasis	0 (0 to 0)	[illegible]	[illegible]	[illegible]	[illegible]	[illegible]	[illegible]	[illegible]	[illegible]	[illegible]	[illegible]	[illegible]	[illegible]	[illegible]	[illegible]	[illegible]	[illegible]	[illegible]	[illegible]	[illegible]	[illegible]	[illegible]	[illegible]	[illegible]
Trichuriasis	0 (0 to 0)	[illegible]	[illegible]	[illegible]	[illegible]	[illegible]	[illegible]	[illegible]	[illegible]	[illegible]	[illegible]	[illegible]	[illegible]	[illegible]	[illegible]	[illegible]	[illegible]	[illegible]	[illegible]	[illegible]	[illegible]	[illegible]	[illegible]	[illegible]
Hookworm disease	0 (0 to 0)	[illegible]	[illegible]	[illegible]	[illegible]	[illegible]	[illegible]	[illegible]	[illegible]	[illegible]	[illegible]	[illegible]	[illegible]	[illegible]	[illegible]	[illegible]	[illegible]	[illegible]	[illegible]	[illegible]	[illegible]	[illegible]	[illegible]	[illegible]
Food-borne trematodiases	0 (0 to 0)	[illegible]	[illegible]	[illegible]	[illegible]	[illegible]	[illegible]	[illegible]	[illegible]	[illegible]	[illegible]	[illegible]	[illegible]	[illegible]	[illegible]	[illegible]	[illegible]	[illegible]	[illegible]	[illegible]	[illegible]	[illegible]	[illegible]	[illegible]
Leprosy	0 (0 to 0)	[illegible]	[illegible]	[illegible]	[illegible]	[illegible]	[illegible]	[illegible]	[illegible]	[illegible]	[illegible]	[illegible]	[illegible]	[illegible]	[illegible]	[illegible]	[illegible]	[illegible]	[illegible]	[illegible]	[illegible]	[illegible]	[illegible]	[illegible]
Ebola	0 (0 to 0)	[illegible]	[illegible]	[illegible]	[illegible]	[illegible]	[illegible]	[illegible]	[illegible]	[illegible]	[illegible]	[illegible]	[illegible]	[illegible]	[illegible]	[illegible]	[illegible]	[illegible]	[illegible]	[illegible]	[illegible]	[illegible]	[illegible]	[illegible]
Zika virus	0 (0 to 0)	[illegible]	[illegible]	[illegible]	[illegible]	[illegible]	[illegible]	[illegible]	[illegible]	[illegible]	[illegible]	[illegible]	[illegible]	[illegible]	[illegible]	[illegible]	[illegible]	[illegible]	[illegible]	[illegible]	[illegible]	[illegible]	[illegible]	[illegible]
Guinea worm disease	0 (0 to 0)	[illegible]	[illegible]	[illegible]	[illegible]	[illegible]	[illegible]	[illegible]	[illegible]	[illegible]	[illegible]	[illegible]	[illegible]	[illegible]	[illegible]	[illegible]	[illegible]	[illegible]	[illegible]	[illegible]	[illegible]	[illegible]	[illegible]	[illegible]
Other neglected tropical diseases	[illegible]	[illegible]	[illegible]	[illegible]	[illegible]	[illegible]	[illegible]	[illegible]	[illegible]	[illegible]	[illegible]	[illegible]	[illegible]	[illegible]	[illegible]	[illegible]	[illegible]	[illegible]	[illegible]	[illegible]	[illegible]	[illegible]	[illegible]	[illegible]
Other infectious diseases	[illegible]	[illegible]	[illegible]	[illegible]	[illegible]	[illegible]	[illegible]	[illegible]	[illegible]	[illegible]	[illegible]	[illegible]	[illegible]	[illegible]	[illegible]	[illegible]	[illegible]	[illegible]	[illegible]	[illegible]	[illegible]	[illegible]	[illegible]	[illegible]
Meningitis	[illegible]	[illegible]	[illegible]	[illegible]	[illegible]	[illegible]	[illegible]	[illegible]	[illegible]	[illegible]	[illegible]	[illegible]	[illegible]	[illegible]	[illegible]	[illegible]	[illegible]	[illegible]	[illegible]	[illegible]	[illegible]	[illegible]	[illegible]	[illegible]
Encephalitis	[illegible]	[illegible]	[illegible]	[illegible]	[illegible]	[illegible]	[illegible]	[illegible]	[illegible]	[illegible]	[illegible]	[illegible]	[illegible]	[illegible]	[illegible]	[illegible]	[illegible]	[illegible]	[illegible]	[illegible]	[illegible]	[illegible]	[illegible]	[illegible]
Diphtheria	0 (0 to 0)	[illegible]	[illegible]	[illegible]	[illegible]	[illegible]	[illegible]	[illegible]	[illegible]	[illegible]	[illegible]	[illegible]	[illegible]	[illegible]	[illegible]	[illegible]	[illegible]	[illegible]	[illegible]	[illegible]	[illegible]	[illegible]	[illegible]	[illegible]
Tetanus	[illegible]	[illegible]	[illegible]	[illegible]	[illegible]	[illegible]	[illegible]	[illegible]	[illegible]	[illegible]	[illegible]	[illegible]	[illegible]	[illegible]	[illegible]	[illegible]	[illegible]	[illegible]	[illegible]	[illegible]	[illegible]	[illegible]	[illegible]	[illegible]
Measles	0 (0 to 0)	[illegible]	[illegible]	[illegible]	[illegible]	[illegible]	[illegible]	[illegible]	[illegible]	[illegible]	[illegible]	[illegible]	[illegible]	[illegible]	[illegible]	[illegible]	[illegible]	[illegible]	[illegible]	[illegible]	[illegible]	[illegible]	[illegible]	[illegible]
Varicella and herpes zoster	[illegible]	[illegible]	[illegible]	[illegible]	[illegible]	[illegible]	[illegible]	[illegible]	[illegible]	[illegible]	[illegible]	[illegible]	[illegible]	[illegible]	[illegible]	[illegible]	[illegible]	[illegible]	[illegible]	[illegible]	[illegible]	[illegible]	[illegible]	[illegible]
Acute hepatitis	[illegible]	[illegible]	[illegible]	[illegible]	[illegible]	[illegible]	[illegible]	[illegible]	[illegible]	[illegible]	[illegible]	[illegible]	[illegible]	[illegible]	[illegible]	[illegible]	[illegible]	[illegible]	[illegible]	[illegible]	[illegible]	[illegible]	[illegible]	[illegible]
Acute hepatitis A	0 (0 to 0)	[illegible]	[illegible]	[illegible]	[illegible]	[illegible]	[illegible]	[illegible]	[illegible]	[illegible]	[illegible]	[illegible]	[illegible]	[illegible]	[illegible]	[illegible]	[illegible]	[illegible]	[illegible]	[illegible]	[illegible]	[illegible]	[illegible]	[illegible]
Acute hepatitis B	0 (0 to 0)	[illegible]	[illegible]	[illegible]	[illegible]	[illegible]	[illegible]	[illegible]	[illegible]	[illegible]	[illegible]	[illegible]	[illegible]	[illegible]	[illegible]	[illegible]	[illegible]	[illegible]	[illegible]	[illegible]	[illegible]	[illegible]	[illegible]	[illegible]
Acute hepatitis C	[illegible]	[illegible]	[illegible]	[illegible]	[illegible]	[illegible]	[illegible]	[illegible]	[illegible]	[illegible]	[illegible]	[illegible]	[illegible]	[illegible]	[illegible]	[illegible]	[illegible]	[illegible]	[illegible]	[illegible]	[illegible]	[illegible]	[illegible]	[illegible]
Acute hepatitis E	0 (0 to 0)	[illegible]	[illegible]	[illegible]	[illegible]	[illegible]	[illegible]	[illegible]	[illegible]	[illegible]	[illegible]	[illegible]	[illegible]	[illegible]	[illegible]	[illegible]	[illegible]	[illegible]	[illegible]	[illegible]	[illegible]	[illegible]	[illegible]	[illegible]
Other unspecified infectious diseases	[illegible]	[illegible]	[illegible]	[illegible]	[illegible]	[illegible]	[illegible]	[illegible]	[illegible]	[illegible]	[illegible]	[illegible]	[illegible]	[illegible]	[illegible]	[illegible]	[illegible]	[illegible]	[illegible]	[illegible]	[illegible]	[illegible]	[illegible]	[illegible]
Maternal and neonatal disorders	[illegible]	[illegible]	[illegible]	[illegible]	[illegible]	[illegible]	[illegible]	[illegible]	[illegible]	[illegible]	[illegible]	[illegible]	[illegible]	[illegible]	[illegible]	[illegible]	[illegible]	[illegible]	[illegible]	[illegible]	[illegible]	[illegible]	[illegible]	[illegible]
Maternal disorders	0 (0 to 0)	[illegible]	[illegible]	[illegible]	[illegible]	[illegible]	[illegible]	[illegible]	[illegible]	[illegible]	[illegible]	[illegible]	[illegible]	[illegible]	[illegible]	[illegible]	[illegible]	[illegible]	[illegible]	[illegible]	[illegible]	[illegible]	[illegible]	[illegible]
Maternal hemorrhage	0 (0 to 0)	[illegible]	[illegible]	[illegible]	[illegible]	[illegible]	[illegible]	[illegible]	[illegible]	[illegible]	[illegible]	[illegible]	[illegible]	[illegible]	[illegible]	[illegible]	[illegible]	[illegible]	[illegible]	[illegible]	[illegible]	[illegible]	[illegible]	[illegible]
Maternal sepsis and other maternal infections	0 (0 to 0)	[illegible]	[illegible]	[illegible]	[illegible]	[illegible]	[illegible]	[illegible]	[illegible]	[illegible]	[illegible]	[illegible]	[illegible]	[illegible]	[illegible]	[illegible]	[illegible]	[illegible]	[illegible]	[illegible]	[illegible]	[illegible]	[illegible]	[illegible]
Maternal hypertensive disorders	0 (0 to 0)	[illegible]	[illegible]	[illegible]	[illegible]	[illegible]	[illegible]	[illegible]	[illegible]	[illegible]	[illegible]	[illegible]	[illegible]	[illegible]	[illegible]	[illegible]	[illegible]	[illegible]	[illegible]	[illegible]	[illegible]	[illegible]	[illegible]	[illegible]
Maternal obstructed labor and uterine rupture	--	[illegible]	[illegible]	[illegible]	[illegible]	[illegible]	[illegible]	[illegible]	[illegible]	[illegible]	[illegible]	[illegible]	[illegible]	[illegible]	[illegible]	[illegible]	[illegible]	[illegible]	[illegible]	[illegible]	[illegible]	[illegible]	[illegible]	NA
Maternal abortion and miscarriage	--	[illegible]	[illegible]	[illegible]	[illegible]	[illegible]	[illegible]	[illegible]	[illegible]	[illegible]	[illegible]	[illegible]	[illegible]	[illegible]	[illegible]	[illegible]	[illegible]	[illegible]	[illegible]	[illegible]	[illegible]	[illegible]	[illegible]	NA
Ectopic pregnancy	--	[illegible]	[illegible]	[illegible]	[illegible]	[illegible]	[illegible]	[illegible]	[illegible]	[illegible]	[illegible]	[illegible]	[illegible]	[illegible]	[illegible]	[illegible]	[illegible]	[illegible]	[illegible]	[illegible]	[illegible]	[illegible]	[illegible]	NA
Indirect maternal deaths	--	[illegible]	[illegible]	[illegible]	[illegible]	[illegible]	[illegible]	[illegible]	[illegible]	[illegible]	[illegible]	[illegible]	[illegible]	[illegible]	[illegible]	[illegible]	[illegible]	[illegible]	[illegible]	[illegible]	[illegible]	[illegible]	[illegible]	NA
Late maternal deaths	--	[illegible]	[illegible]	[illegible]	[illegible]	[illegible]	[illegible]	[illegible]	[illegible]	[illegible]	[illegible]	[illegible]	[illegible]	[illegible]	[illegible]	[illegible]	[illegible]	[illegible]	[illegible]	[illegible]	[illegible]	[illegible]	[illegible]	NA
Maternal deaths aggravated by HIV/AIDS	--	[illegible]	[illegible]	[illegible]	[illegible]	[illegible]	[illegible]	[illegible]	[illegible]	[illegible]	[illegible]	[illegible]	[illegible]	[illegible]	[illegible]	[illegible]	[illegible]	[illegible]	[illegible]	[illegible]	[illegible]	[illegible]	[illegible]	NA
Neonatal disorders	[illegible]	[illegible]	[illegible]	[illegible]	[illegible]	[illegible]	[illegible]	[illegible]	[illegible]	[illegible]	[illegible]	[illegible]	[illegible]	[illegible]	[illegible]	[illegible]	[illegible]	[illegible]	[illegible]	[illegible]	[illegible]	[illegible]	[illegible]	[illegible]
Neonatal preterm birth	[illegible]	[illegible]	[illegible]	[illegible]	[illegible]	[illegible]	[illegible]	[illegible]	[illegible]	[illegible]	[illegible]	[illegible]	[illegible]	[illegible]	[illegible]	[illegible]	[illegible]	[illegible]	[illegible]	[illegible]	[illegible]	[illegible]	[illegible]	[illegible]
Neonatal encephalopathy due to birth asphyxia and trau	[illegible]	[illegible]	[illegible]	[illegible]	[illegible]	[illegible]	[illegible]	[illegible]	[illegible]	[illegible]	[illegible]	[illegible]	[illegible]	[illegible]	[illegible]	[illegible]	[illegible]	[illegible]	[illegible]	[illegible]	[illegible]	[illegible]	[illegible]	[illegible]
Neonatal sepsis and other neonatal infections	[illegible]	[illegible]	[illegible]	[illegible]	[illegible]	[illegible]	[illegible]	[illegible]	[illegible]	[illegible]	[illegible]	[illegible]	[illegible]	[illegible]	[illegible]	[illegible]	[illegible]	[illegible]	[illegible]	[illegible]	[illegible]	[illegible]	[illegible]	[illegible]
Hemolytic disease and other neonatal jaundice	[illegible]	[illegible]	[illegible]	[illegible]	[illegible]	[illegible]	[illegible]	[illegible]	[illegible]	[illegible]	[illegible]	[illegible]	[illegible]	[illegible]	[illegible]	[illegible]	[illegible]	[illegible]	[illegible]	[illegible]	[illegible]	[illegible]	[illegible]	[illegible]
Other neonatal disorders	[illegible]	[illegible]	[illegible]	[illegible]	[illegible]	[illegible]	[illegible]	[illegible]	[illegible]	[illegible]	[illegible]	[illegible]	[illegible]	[illegible]	[illegible]	[illegible]	[illegible]	[illegible]	[illegible]	[illegible]	[illegible]	[illegible]	[illegible]	[illegible]
Nutritional deficiencies	[illegible]	[illegible]	[illegible]	[illegible]	[illegible]	[illegible]	[illegible]	[illegible]	[illegible]	[illegible]	[illegible]	[illegible]	[illegible]	[illegible]	[illegible]	[illegible]	[illegible]	[illegible]	[illegible]	[illegible]	[illegible]	[illegible]	[illegible]	[illegible]
Protein-energy malnutrition	[illegible]	[illegible]	[illegible]	[illegible]	[illegible]	[illegible]	[illegible]	[illegible]	[illegible]	[illegible]	[illegible]	[illegible]	[illegible]	[illegible]	[illegible]	[illegible]	[illegible]	[illegible]	[illegible]	[illegible]	[illegible]	[illegible]	[illegible]	[illegible]
Iodine deficiency	0 (0 to 0)	[illegible]	[illegible]	[illegible]	[illegible]	[illegible]	[illegible]	[illegible]	[illegible]	[illegible]	[illegible]	[illegible]	[illegible]	[illegible]	[illegible]	[illegible]	[illegible]	[illegible]	[illegible]	[illegible]	[illegible]	[illegible]	[illegible]	[illegible]
Vitamin A deficiency	[illegible]	[illegible]	[illegible]	[illegible]	[illegible]	[illegible]	[illegible]	[illegible]	[illegible]	[illegible]	[illegible]	[illegible]	[illegible]	[illegible]	[illegible]	[illegible]	[illegible]	[illegible]	[illegible]	[illegible]	[illegible]	[illegible]	[illegible]	[illegible]
Dietary iron deficiency	[illegible]	[illegible]	[illegible]	[illegible]	[illegible]	[illegible]	[illegible]	[illegible]	[illegible]	[illegible]	[illegible]	[illegible]	[illegible]	[illegible]	[illegible]	[illegible]	[illegible]	[illegible]	[illegible]	[illegible]	[illegible]	[illegible]	[illegible]	[illegible]
Other nutritional deficiencies	0 (0 to 0)	[illegible]	[illegible]	[illegible]	[illegible]	[illegible]	[illegible]	[illegible]	[illegible]	[illegible]	[illegible]	[illegible]	[illegible]	[illegible]	[illegible]	[illegible]	[illegible]	[illegible]	[illegible]	[illegible]	[illegible]	[illegible]	[illegible]	[illegible]
Non-communicable diseases	[illegible]	[illegible]	[illegible]	[illegible]	[illegible]	[illegible]	[illegible]	[illegible]	[illegible]	[illegible]	[illegible]	[illegible]	[illegible]	[illegible]	[illegible]	[illegible]	[illegible]	[illegible]	[illegible]	[illegible]	[illegible]	[illegible]	[illegible]	[illegible]
Neoplasms	[illegible]	[illegible]	[illegible]	[illegible]	[illegible]	[illegible]	[illegible]	[illegible]	[illegible]	[illegible]	[illegible]	[illegible]	[illegible]	[illegible]	[illegible]	[illegible]	[illegible]	[illegible]	[illegible]	[illegible]	[illegible]	[illegible]	[illegible]	[illegible]
Lip and oral cavity cancer	0 (0 to 0)	[illegible]	[illegible]	[illegible]	[illegible]	[illegible]	[illegible]	[illegible]	[illegible]	[illegible]	[illegible]	[illegible]	[illegible]	[illegible]	[illegible]	[illegible]	[illegible]	[illegible]	[illegible]	[illegible]	[illegible]	[illegible]	[illegible]	[illegible]
Nasopharynx cancer	0 (0 to 0)	[illegible]	[illegible]	[illegible]	[illegible]	[illegible]	[illegible]	[illegible]	[illegible]	[illegible]	[illegible]	[illegible]	[illegible]	[illegible]	[illegible]	[illegible]	[illegible]	[illegible]	[illegible]	[illegible]	[illegible]	[illegible]	[illegible]	[illegible]
Other pharynx cancer	0 (0 to 0)	[illegible]	[illegible]	[illegible]	[illegible]	[illegible]	[illegible]	[illegible]	[illegible]	[illegible]	[illegible]	[illegible]	[illegible]	[illegible]	[illegible]	[illegible]	[illegible]	[illegible]	[illegible]	[illegible]	[illegible]	[illegible]	[illegible]	[illegible]
Esophageal cancer	0 (0 to 0)	[illegible]	[illegible]	[illegible]	[illegible]	[illegible]	[illegible]	[illegible]	[illegible]	[illegible]	[illegible]	[illegible]	[illegible]	[illegible]	[illegible]	[illegible]	[illegible]	[illegible]	[illegible]	[illegible]	[illegible]	[illegible]	[illegible]	[illegible]
Stomach cancer	0 (0 to 0)	[illegible]	[illegible]	[illegible]	[illegible]	[illegible]	[illegible]	[illegible]	[illegible]	[illegible]	[illegible]	[illegible]	[illegible]	[illegible]	[illegible]	[illegible]	[illegible]	[illegible]	[illegible]	[illegible]	[illegible]	[illegible]	[illegible]	[illegible]
Colon and rectum cancer	0 (0 to 0)	[illegible]	[illegible]	[illegible]	[illegible]	[illegible]	[illegible]	[illegible]	[illegible]	[illegible]	[illegible]	[illegible]	[illegible]	[illegible]	[illegible]	[illegible]	[illegible]	[illegible]	[illegible]	[illegible]	[illegible]	[illegible]	[illegible]	[illegible]
Liver cancer	[illegible]	[illegible]	[illegible]	[illegible]	[illegible]	[illegible]	[illegible]	[illegible]	[illegible]	[illegible]	[illegible]	[illegible]	[illegible]	[illegible]	[illegible]	[illegible]	[illegible]	[illegible]	[illegible]	[illegible]	[illegible]	[illegible]	[illegible]	[illegible]
Liver cancer due to hepatitis B	0 (0 to 0)	[illegible]	[illegible]	[illegible]	[illegible]	[illegible]	[illegible]	[illegible]	[illegible]	[illegible]	[illegible]	[illegible]	[illegible]	[illegible]	[illegible]	[illegible]	[illegible]	[illegible]	[illegible]	[illegible]	[illegible]	[illegible]	[illegible]	[illegible]
Liver cancer due to hepatitis C	0 (0 to 0)	[illegible]	[illegible]	[illegible]	[illegible]	[illegible]	[illegible]	[illegible]	[illegible]	[illegible]	[illegible]	[illegible]	[illegible]	[illegible]	[illegible]	[illegible]	[illegible]	[illegible]	[illegible]	[illegible]	[illegible]	[illegible]	[illegible]	[illegible]
Liver cancer due to alcohol use	0 (0 to 0)	[illegible]	[illegible]	[illegible]	[illegible]	[illegible]	[illegible]	[illegible]	[illegible]	[illegible]	[illegible]	[illegible]	[illegible]	[illegible]	[illegible]	[illegible]	[illegible]	[illegible]	[illegible]	[illegible]	[illegible]	[illegible]	[illegible]	[illegible]
Liver cancer due to NASH	0 (0 to 0)	[illegible]	[illegible]	[illegible]	[illegible]	[illegible]	[illegible]	[illegible]	[illegible]	[illegible]	[illegible]	[illegible]	[illegible]	[illegible]	[illegible]	[illegible]	[illegible]	[illegible]	[illegible]	[illegible]	[illegible]	[illegible]	[illegible]	[illegible]
Liver cancer due to other causes	[illegible]	[illegible]	[illegible]	[illegible]	[illegible]	[illegible]	[illegible]	[illegible]	[illegible]	[illegible]	[illegible]	[illegible]	[illegible]	[illegible]	[illegible]	[illegible]	[illegible]	[illegible]	[illegible]	[illegible]	[illegible]	[illegible]	[illegible]	[illegible]
Gallbladder and biliary tract cancer	0 (0 to 0)	[illegible]	[illegible]	[illegible]	[illegible]	[illegible]	[illegible]	[illegible]	[illegible]	[illegible]	[illegible]	[illegible]	[illegible]	[illegible]	[illegible]	[illegible]	[illegible]	[illegible]	[illegible]	[illegible]	[illegible]	[illegible]	[illegible]	[illegible]
Pancreatic cancer	0 (0 to 0)	[illegible]	[illegible]	[illegible]	[illegible]	[illegible]	[illegible]	[illegible]	[illegible]	[illegible]	[illegible]	[illegible]	[illegible]	[illegible]	[illegible]	[illegible]	[illegible]	[illegible]	[illegible]	[illegible]	[illegible]	[illegible]	[illegible]	[illegible]
Larynx cancer	0 (0 to 0)	[illegible]	[illegible]	[illegible]	[illegible]	[illegible]	[illegible]	[illegible]	[illegible]	[illegible]	[illegible]	[illegible]	[illegible]	[illegible]	[illegible]	[illegible]	[illegible]	[illegible]	[illegible]	[illegible]	[illegible]	[illegible]	[illegible]	[illegible]
Tracheal, bronchus, and lung cancer	0 (0 to 0)	[illegible]	[illegible]	[illegible]	[illegible]	[illegible]	[illegible]	[illegible]	[illegible]	[illegible]	[illegible]	[illegible]	[illegible]	[illegible]	[illegible]	[illegible]	[illegible]	[illegible]	[illegible]	[illegible]	[illegible]	[illegible]	[illegible]	[illegible]
Malignant skin melanoma	[illegible]	[illegible]	[illegible]	[illegible]	[illegible]	[illegible]	[illegible]	[illegible]	[illegible]	[illegible]	[illegible]	[illegible]	[illegible]	[illegible]	[illegible]	[illegible]	[illegible]	[illegible]	[illegible]	[illegible]	[illegible]	[illegible]	[illegible]	[illegible]
Non-melanoma skin cancer	0 (0 to 0)	[illegible]	[illegible]	[illegible]	[illegible]	[illegible]	[illegible]	[illegible]	[illegible]	[illegible]	[illegible]	[illegible]	[illegible]	[illegible]	[illegible]	[illegible]	[illegible]	[illegible]	[illegible]	[illegible]	[illegible]	[illegible]	[illegible]	[illegible]
Non-melanoma skin cancer (squamous-cell carcinoma	0 (0 to 0)	[illegible]	[illegible]	[illegible]	[illegible]	[illegible]	[illegible]	[illegible]	[illegible]	[illegible]	[illegible]	[illegible]	[illegible]	[illegible]	[illegible]	[illegible]	[illegible]	[illegible]	[illegible]	[illegible]	[illegible]	[illegible]	[illegible]	[illegible]
Non-melanoma skin cancer (basal-cell carcinoma)	0 (0 to 0)	[illegible]	[illegible]	[illegible]	[illegible]	[illegible]	[illegible]	[illegible]	[illegible]	[illegible]	[illegible]	[illegible]	[illegible]	[illegible]	[illegible]	[illegible]	[illegible]	[illegible]	[illegible]	[illegible]	[illegible]	[illegible]	[illegible]	[illegible]
Breast cancer	0 (0 to 0)	[illegible]	[illegible]	[illegible]	[illegible]	[illegible]	[illegible]	[illegible]	[illegible]	[illegible]	[illegible]	[illegible]	[illegible]	[illegible]	[illegible]	[illegible]	[illegible]	[illegible]	[illegible]	[illegible]	[illegible]	[illegible]	[illegible]	[illegible]
Cervical cancer	--	[illegible]	[illegible]	[illegible]	[illegible]	[illegible]	[illegible]	[illegible]	[illegible]	[illegible]	[illegible]	[illegible]	[illegible]	[illegible]	[illegible]	[illegible]	[illegible]	[illegible]	[illegible]	[illegible]	[illegible]	[illegible]	[illegible]	[illegible]
Uterine cancer	--	[illegible]	[illegible]	[illegible]	[illegible]	[illegible]	[illegible]	[illegible]	[illegible]	[illegible]	[illegible]	[illegible]	[illegible]	[illegible]	[illegible]	[illegible]	[illegible]	[illegible]	[illegible]	[illegible]	[illegible]	[illegible]	[illegible]	[illegible]
Ovarian cancer	--	[illegible]	[illegible]	[illegible]	[illegible]	[illegible]	[illegible]	[illegible]	[illegible]	[illegible]	[illegible]	[illegible]	[illegible]	[illegible]	[illegible]	[illegible]	[illegible]	[illegible]	[illegible]	[illegible]	[illegible]	[illegible]	[illegible]	[illegible]
Prostate cancer	0 (0 to 0)	[illegible]	[illegible]	[illegible]	[illegible]	[illegible]	[illegible]	[illegible]	[illegible]	[illegible]	[illegible]	[illegible]	[illegible]	[illegible]	[illegible]	[illegible]	[illegible]	[illegible]	[illegible]	[illegible]	[illegible]	[illegible]	[illegible]	[illegible]
Testicular cancer	[illegible]	[illegible]	[illegible]	[illegible]	[illegible]	[illegible]	[illegible]	[illegible]	[illegible]	[illegible]	[illegible]	[illegible]	[illegible]	[illegible]	[illegible]	[illegible]	[illegible]	[illegible]	[illegible]	[illegible]	[illegible]	[illegible]	[illegible]	[illegible]
Kidney cancer	[illegible]	[illegible]	[illegible]	[illegible]	[illegible]	[illegible]	[illegible]	[illegible]	[illegible]	[illegible]	[illegible]	[illegible]	[illegible]	[illegible]	[illegible]	[illegible]	[illegible]	[illegible]	[illegible]	[illegible]	[illegible]	[illegible]	[illegible]	[illegible]
Bladder cancer	0 (0 to 0)	[illegible]	[illegible]	[illegible]	[illegible]	[illegible]	[illegible]	[illegible]	[illegible]	[illegible]	[illegible]	[illegible]	[illegible]	[illegible]	[illegible]	[illegible]	[illegible]	[illegible]	[illegible]	[illegible]	[illegible]	[illegible]	[illegible]	[illegible]
Brain and central nervous system cancer	[illegible]	[illegible]	[illegible]	[illegible]	[illegible]	[illegible]	[illegible]	[illegible]	[illegible]	[illegible]	[illegible]	[illegible]	[illegible]	[illegible]	[illegible]	[illegible]	[illegible]	[illegible]	[illegible]	[illegible]	[illegible]	[illegible]	[illegible]	[illegible]
Thyroid cancer	0 (0 to 0)	[illegible]	[illegible]	[illegible]	[illegible]	[illegible]	[illegible]	[illegible]	[illegible]	[illegible]	[illegible]	[illegible]	[illegible]	[illegible]	[illegible]	[illegible]	[illegible]	[illegible]	[illegible]	[illegible]	[illegible]	[illegible]	[illegible]	[illegible]
Mesothelioma	0 (0 to 0)	[illegible]	[illegible]	[illegible]	[illegible]	[illegible]	[illegible]	[illegible]	[illegible]	[illegible]	[illegible]	[illegible]	[illegible]	[illegible]	[illegible]	[illegible]	[illegible]	[illegible]	[illegible]	[illegible]	[illegible]	[illegible]	[illegible]	[illegible]
Hodgkin lymphoma	0 (0 to 0)	[illegible]	[illegible]	[illegible]	[illegible]	[illegible]	[illegible]	[illegible]	[illegible]	[illegible]	[illegible]	[illegible]	[illegible]	[illegible]	[illegible]	[illegible]	[illegible]	[illegible]	[illegible]	[illegible]	[illegible]	[illegible]	[illegible]	[illegible]
Non-Hodgkin lymphoma	0 (0 to 0)	[illegible]	[illegible]	[illegible]	[illegible]	[illegible]	[illegible]	[illegible]	[illegible]	[illegible]	[illegible]	[illegible]	[illegible]	[illegible]	[illegible]	[illegible]	[illegible]	[illegible]	[illegible]	[illegible]	[illegible]	[illegible]	[illegible]	[illegible]
Multiple myeloma	0 (0 to 0)	[illegible]	[illegible]	[illegible]	[illegible]	[illegible]	[illegible]	[illegible]	[illegible]	[illegible]	[illegible]	[illegible]	[illegible]	[illegible]	[illegible]	[illegible]	[illegible]	[illegible]	[illegible]	[illegible]	[illegible]	[illegible]	[illegible]	[illegible]
Leukemia	[illegible]	[illegible]	[illegible]	[illegible]	[illegible]	[illegible]	[illegible]	[illegible]	[illegible]	[illegible]	[illegible]	[illegible]	[illegible]	[illegible]	[illegible]	[illegible]	[illegible]	[illegible]	[illegible]	[illegible]	[illegible]	[illegible]	[illegible]	[illegible]
Acute lymphoid leukemia	[illegible]	[illegible]	[illegible]	[illegible]	[illegible]	[illegible]	[illegible]	[illegible]	[illegible]	[illegible]	[illegible]	[illegible]	[illegible]	[illegible]	[illegible]	[illegible]	[illegible]	[illegible]	[illegible]	[illegible]	[illegible]	[illegible]	[illegible]	[illegible]
Chronic lymphoid leukemia	0 (0 to 0)	[illegible]	[illegible]	[illegible]	[illegible]	[illegible]	[illegible]	[illegible]	[illegible]	[illegible]	[illegible]	[illegible]	[illegible]	[illegible]	[illegible]	[illegible]	[illegible]	[illegible]	[illegible]	[illegible]	[illegible]	[illegible]	[illegible]	[illegible]
Acute myeloid leukemia	[illegible]	[illegible]	[illegible]	[illegible]	[illegible]	[illegible]	[illegible]	[illegible]	[illegible]	[illegible]	[illegible]	[illegible]	[illegible]	[illegible]	[illegible]	[illegible]	[illegible]	[illegible]	[illegible]	[illegible]	[illegible]	[illegible]	[illegible]	[illegible]
Chronic myeloid leukemia	[illegible]	[illegible]	[illegible]	[illegible]	[illegible]	[illegible]	[illegible]	[illegible]	[illegible]	[illegible]	[illegible]	[illegible]	[illegible]	[illegible]	[illegible]	[illegible]	[illegible]	[illegible]	[illegible]	[illegible]	[illegible]	[illegible]	[illegible]	[illegible]
Other leukemia	[illegible]	[illegible]	[illegible]	[illegible]	[illegible]	[illegible]	[illegible]	[illegible]	[illegible]	[illegible]	[illegible]	[illegible]	[illegible]	[illegible]	[illegible]	[illegible]	[illegible]	[illegible]	[illegible]	[illegible]	[illegible]	[illegible]	[illegible]	[illegible]
Other malignant neoplasms	[illegible]	[illegible]	[illegible]	[illegible]	[illegible]	[illegible]	[illegible]	[illegible]	[illegible]	[illegible]	[illegible]	[illegible]	[illegible]	[illegible]	[illegible]	[illegible]	[illegible]	[illegible]	[illegible]	[illegible]	[illegible]	[illegible]	[illegible]	[illegible]
Other neoplasms	[illegible]	[illegible]	[illegible]	[illegible]	[illegible]	[illegible]	[illegible]	[illegible]	[illegible]	[illegible]	[illegible]	[illegible]	[illegible]	[illegible]	[illegible]	[illegible]	[illegible]	[illegible]	[illegible]	[illegible]	[illegible]	[illegible]	[illegible]	[illegible]
Myelodysplastic, myeloproliferative, and other hematopo	[illegible]	[illegible]	[illegible]	[illegible]	[illegible]	[illegible]	[illegible]	[illegible]	[illegible]	[illegible]	[illegible]	[illegible]	[illegible]	[illegible]	[illegible]	[illegible]	[illegible]	[illegible]	[illegible]	[illegible]	[illegible]	[illegible]	[illegible]	[illegible]
Benign and in situ intestinal neoplasms	0 (0 to 0)	[illegible]	[illegible]	[illegible]	[illegible]	[illegible]	[illegible]	[illegible]	[illegible]	[illegible]	[illegible]	[illegible]	[illegible]	[illegible]	[illegible]	[illegible]	[illegible]	[illegible]	[illegible]	[illegible]	[illegible]	[illegible]	[illegible]	[illegible]
Benign and in situ cervical and uterine neoplasms	0 (0 to 0)	[illegible]	[illegible]	[illegible]	[illegible]	[illegible]	[illegible]	[illegible]	[illegible]	[illegible]	[illegible]	[illegible]	[illegible]	[illegible]	[illegible]	[illegible]	[illegible]	[illegible]	[illegible]	[illegible]	[illegible]	[illegible]	[illegible]	[illegible]
Other benign and in situ neoplasms	0 (0 to 0)	[illegible]	[illegible]	[illegible]	[illegible]	[illegible]	[illegible]	[illegible]	[illegible]	[illegible]	[illegible]	[illegible]	[illegible]	[illegible]	[illegible]	[illegible]	[illegible]	[illegible]	[illegible]	[illegible]	[illegible]	[illegible]	[illegible]	[illegible]
Cardiovascular diseases	[illegible]	[illegible]	[illegible]	[illegible]	[illegible]	[illegible]	[illegible]	[illegible]	[illegible]	[illegible]	[illegible]	[illegible]	[illegible]	[illegible]	[illegible]	[illegible]	[illegible]	[illegible]	[illegible]	[illegible]	[illegible]	[illegible]	[illegible]	[illegible]
Rheumatic heart disease	0 (0 to 0)	[illegible]	[illegible]	[illegible]	[illegible]	[illegible]	[illegible]	[illegible]	[illegible]	[illegible]	[illegible]	[illegible]	[illegible]	[illegible]	[illegible]	[illegible]	[illegible]	[illegible]	[illegible]	[illegible]	[illegible]	[illegible]	[illegible]	[illegible]
Ischemic heart disease	0 (0 to 0)	[illegible]	[illegible]	[illegible]	[illegible]	[illegible]	[illegible]	[illegible]	[illegible]	[illegible]	[illegible]	[illegible]	[illegible]	[illegible]	[illegible]	[illegible]	[illegible]	[illegible]	[illegible]	[illegible]	[illegible]	[illegible]	[illegible]	[illegible]
Stroke	[illegible]	[illegible]	[illegible]	[illegible]	[illegible]	[illegible]	[illegible]	[illegible]	[illegible]	[illegible]	[illegible]	[illegible]	[illegible]	[illegible]	[illegible]	[illegible]	[illegible]	[illegible]	[illegible]	[illegible]	[illegible]	[illegible]	[illegible]	[illegible]
Ischemic stroke	[illegible]	[illegible]	[illegible]	[illegible]	[illegible]	[illegible]	[illegible]	[illegible]	[illegible]	[illegible]	[illegible]	[illegible]	[illegible]	[illegible]	[illegible]	[illegible]	[illegible]	[illegible]	[illegible]	[illegible]	[illegible]	[illegible]	[illegible]	[illegible]
Intracerebral hemorrhage	[illegible]	[illegible]	[illegible]	[illegible]	[illegible]	[illegible]	[illegible]	[illegible]	[illegible]	[illegible]	[illegible]	[illegible]	[illegible]	[illegible]	[illegible]	[illegible]	[illegible]	[illegible]	[illegible]	[illegible]	[illegible]	[illegible]	[illegible]	[illegible]
Subarachnoid hemorrhage	[illegible]	[illegible]	[illegible]	[illegible]	[illegible]	[illegible]	[illegible]	[illegible]	[illegible]	[illegible]	[illegible]	[illegible]	[illegible]	[illegible]	[illegible]	[illegible]	[illegible]	[illegible]	[illegible]	[illegible]	[illegible]	[illegible]	[illegible]	[illegible]
Hypertensive heart disease	0 (0 to 0)	[illegible]	[illegible]	[illegible]	[illegible]	[illegible]	[illegible]	[illegible]	[illegible]	[illegible]	[illegible]	[illegible]	[illegible]	[illegible]	[illegible]	[illegible]	[illegible]	[illegible]	[illegible]	[illegible]	[illegible]	[illegible]	[illegible]	[illegible]
Non-rheumatic valvular heart disease	0 (0 to 0)	[illegible]	[illegible]	[illegible]	[illegible]	[illegible]	[illegible]	[illegible]	[illegible]	[illegible]	[illegible]	[illegible]	[illegible]	[illegible]	[illegible]	[illegible]	[illegible]	[illegible]	[illegible]	[illegible]	[illegible]	[illegible]	[illegible]	[illegible]
Non-rheumatic calcific aortic valve disease	0 (0 to 0)	[illegible]	[illegible]	[illegible]	[illegible]	[illegible]	[illegible]	[illegible]	[illegible]	[illegible]	[illegible]	[illegible]	[illegible]	[illegible]	[illegible]	[illegible]	[illegible]	[illegible]	[illegible]	[illegible]	[illegible]	[illegible]	[illegible]	[illegible]
Non-rheumatic degenerative mitral valve disease	0 (0 to 0)	[illegible]	[illegible]	[illegible]	[illegible]	[illegible]	[illegible]	[illegible]	[illegible]	[illegible]	[illegible]	[illegible]	[illegible]	[illegible]	[illegible]	[illegible]	[illegible]	[illegible]	[illegible]	[illegible]	[illegible]	[illegible]	[illegible]	[illegible]
Other non-rheumatic valve diseases	0 (0 to 0)	[illegible]	[illegible]	[illegible]	[illegible]	[illegible]	[illegible]	[illegible]	[illegible]	[illegible]	[illegible]	[illegible]	[illegible]	[illegible]	[illegible]	[illegible]	[illegible]	[illegible]	[illegible]	[illegible]	[illegible]	[illegible]	[illegible]	[illegible]
Cardiomyopathy and myocarditis	[illegible]	[illegible]	[illegible]	[illegible]	[illegible]	[illegible]	[illegible]	[illegible]	[illegible]	[illegible]	[illegible]	[illegible]	[illegible]	[illegible]	[illegible]	[illegible]	[illegible]	[illegible]	[illegible]	[illegible]	[illegible]	[illegible]	[illegible]	[illegible]
Myocarditis	[illegible]	[illegible]	[illegible]	[illegible]	[illegible]	[illegible]	[illegible]	[illegible]	[illegible]	[illegible]	[illegible]	[illegible]	[illegible]	[illegible]	[illegible]	[illegible]	[illegible]	[illegible]	[illegible]	[illegible]	[illegible]	[illegible]	[illegible]	[illegible]
Alcoholic cardiomyopathy	0 (0 to 0)	[illegible]	[illegible]	[illegible]	[illegible]	[illegible]	[illegible]	[illegible]	[illegible]	[illegible]	[illegible]	[illegible]	[illegible]	[illegible]	[illegible]	[illegible]	[illegible]	[illegible]	[illegible]	[illegible]	[illegible]	[illegible]	[illegible]	[illegible]
Other cardiomyopathy	[illegible]	[illegible]	[illegible]	[illegible]	[illegible]	[illegible]	[illegible]	[illegible]	[illegible]	[illegible]	[illegible]	[illegible]	[illegible]	[illegible]	[illegible]	[illegible]	[illegible]	[illegible]	[illegible]	[illegible]	[illegible]	[illegible]	[illegible]	[illegible]
Atrial fibrillation and flutter	0 (0 to 0)	[illegible]	[illegible]	[illegible]	[illegible]	[illegible]	[illegible]	[illegible]	[illegible]	[illegible]	[illegible]	[illegible]	[illegible]	[illegible]	[illegible]	[illegible]	[illegible]	[illegible]	[illegible]	[illegible]	[illegible]	[illegible]	[illegible]	[illegible]
Aortic aneurysm	--	[illegible]	[illegible]	[illegible]	[illegible]	[illegible]	[illegible]	[illegible]	[illegible]	[illegible]	[illegible]	[illegible]	[illegible]	[illegible]	[illegible]	[illegible]	[illegible]	[illegible]	[illegible]	[illegible]	[illegible]	[illegible]	[illegible]	[illegible]
Endocarditis	[illegible]	[illegible]	[illegible]	[illegible]	[illegible]	[illegible]	[illegible]	[illegible]	[illegible]	[illegible]	[illegible]	[illegible]	[illegible]	[illegible]	[illegible]	[illegible]	[illegible]	[illegible]	[illegible]	[illegible]	[illegible]	[illegible]	[illegible]	[illegible]

Other cardiovascular and circulatory diseases	16 (4 to 64·8)	26·1 (9·7 to 56·3)	125 (44·8 to 284)	120 (54·7 to 224)	394 (222 to 656)	380 (213 to 619)	611 (364 to 953)	975 (588 to 1,460)	1,190 (728 to 1,810)	1,330 (828 to 2,020)	1,310 (821 to 1,990)	1,550 (1,050 to 2,190)	1,490 (1,010 to 2,170)	1,610 (1,110 to 2,280)	1,890 (1,250 to 2,770)	2,000 (1,400 to 2,770)	1,870 (1,250 to 2,690)	1,610 (1,090 to 2,230)	1,010 (677 to 1,420)	650 (435 to 900)	258 (165 to 371)	125 (77·8 to 181)	25·6 (13 to 40)	20,600
Chronic respiratory diseases	0 (0 to 0)	0 (0 to 0)	1·9 (0·7 to 3)	2,110 (1,110 to 3,700)	3,020 (1,790 to 4,990)	2,690 (1,700 to 4,210)	2,560 (1,750 to 3,670)	3,320 (2,440 to 4,360)	4,210 (3,200 to 5,380)	5,150 (3,880 to 6,580)	5,350 (4,110 to 6,840)	5,320 (4,120 to 6,720)	6,720 (5,250 to 8,470)	9,350 (7,420 to 12,000)	12,400 (9,520 to 16,100)	16,100 (12,000 to 21,200)	18,500 (13,800 to 24,900)	21,100 (15,500 to 28,900)	15,000 (11,100 to 19,900)	10,400 (7,680 to 13,800)	5,060 (3,850 to 6,530)	2,390 (1,790 to 3,110)	518 (355 to 681)	151,000
Chronic obstructive pulmonary disease	0 (0 to 0)	0 (0 to 0)	1·9 (0·7 to 3)	63·5 (30·3 to 109)	160 (56·2 to 306)	262 (118 to 441)	600 (359 to 901)	1,130 (756 to 1,610)	1,650 (1,180 to 2,170)	2,310 (1,680 to 3,050)	2,700 (2,000 to 3,590)	2,910 (2,170 to 3,710)	4,380 (3,390 to 5,550)	6,880 (5,360 to 8,850)	9,380 (7,270 to 12,200)	13,800 (10,300 to 18,000)	16,400 (12,100 to 22,100)	19,100 (14,000 to 26,100)	13,800 (10,300 to 18,500)	9,750 (7,200 to 12,700)	4,720 (3,610 to 6,080)	2,240 (1,680 to 2,930)	488 (338 to 635)	113,000
Pneumoconiosis	0 (0 to 0)	0 (0 to 0)	0 (0 to 0)	0 (0 to 0)	0 (0 to 0)	0 (0 to 0)	0·2 (0·1 to 0·3)	0·5 (0·2 to 0·9)	1 (0·5 to 1·8)	2·3 (1·2 to 4·1)	7 (3·5 to 12)	12 (5·8 to 21·5)	17·8 (9·9 to 28·8)	27·7 (15·9 to 44·5)	35·8 (21·7 to 54·3)	32·7 (9·1 to 73·3)	33·8 (12·7 to 66·2)	26·8 (10·5 to 52)	15·8 (6·1 to 29·8)	9·2 (3·3 to 17·6)	4·2 (1·7 to 8·2)	1·8 (0·7 to 3·4)	0·3 (0·1 to 0·6)	229
Silicosis	0 (0 to 0)	0 (0 to 0)	0 (0 to 0)	0 (0 to 0)	0 (0 to 0)	0 (0 to 0)	0 (0 to 0·1)	0·2 (0·1 to 0·5)	0·6 (0·2 to 1·2)	1·8 (0·9 to 3·4)	5·9 (2·6 to 10·7)	11·3 (5·2 to 20·5)	17 (9·1 to 27·9)	25 (14·1 to 41·1)	31·7 (18·6 to 49·8)	29·9 (6·6 to 70·1)	29·7 (9·3 to 62·2)	24·9 (9 to 49·7)	13·4 (4·2 to 27·4)	8·2 (2·5 to 16·4)	3·6 (1·2 to 7·5)	1·6 (0·5 to 3·1)	0·3 (0·1 to 0·5)	205
Asbestosis	0 (0 to 0)	0 (0 to 0)	0 (0 to 0)	0 (0 to 0)	0 (0 to 0)	0 (0 to 0)	0·1 (0 to 0·2)	0·2 (0 to 0·5)	0·2 (0·1 to 0·6)	0·2 (0·1 to 0·7)	0·3 (0·1 to 0·7)	0·2 (0·1 to 0·5)	0·2 (0·1 to 0·5)	1 (0·3 to 2·7)	1·1 (0·3 to 3)	1 (0·3 to 2·7)	1 (0·3 to 2·8)	0·5 (0·1 to 1·6)	0·3 (0·1 to 0·9)	0·1 (0 to 0·2)	0·1 (0 to 0·2)	0·1 (0 to 0·1)	0 (0 to 0)	6·8
Coal workers pneumoconiosis	0 (0 to 0)	0 (0 to 0)	0 (0 to 0)	0 (0 to 0)	0 (0 to 0)	0 (0 to 0)	0 (0 to 0)	0 (0 to 0·1)	0 (0 to 0·1)	0·1 (0 to 0·1)	0·3 (0·1 to 0·8)	0·1 (0 to 0·3)	0·1 (0·1 to 0·4)	0·6 (0·1 to 2)	0·6 (0·2 to 2)	0·2 (0·1 to 0·7)	0·3 (0·1 to 1·1)	0·2 (0 to 0·4)	0·3 (0·1 to 0·9)	0·1 (0 to 0·2)	0 (0 to 0·1)	0 (0 to 0)	0 (0 to 0)	3
Other pneumoconiosis	0 (0 to 0)	0 (0 to 0)	0 (0 to 0)	0 (0 to 0)	0 (0 to 0)	0 (0 to 0)	0 (0 to 0·1)	0 (0 to 0·1)	0·1 (0 to 0·2)	0·1 (0·1 to 0·3)	0·5 (0·2 to 1)	0·3 (0·2 to 0·6)	0·4 (0·2 to 0·8)	1 (0·3 to 2·5)	2·3 (0·8 to 5·1)	1·6 (0·6 to 3·5)	2·8 (1 to 6·9)	1·3 (0·4 to 3·1)	1·7 (0·7 to 3·4)	0·8 (0·4 to 1·5)	0·5 (0·2 to 0·8)	0·2 (0·1 to 0·4)	0 (0 to 0·1)	13·8
Asthma	0 (0 to 0)	0 (0 to 0)	0 (0 to 0)	1,900 (945 to 3,440)	2,600 (1,420 to 4,610)	2,040 (1,170 to 3,400)	1,630 (987 to 2,590)	1,600 (1,030 to 2,350)	1,890 (1,270 to 2,680)	2,040 (1,370 to 2,850)	1,890 (1,270 to 2,670)	1,640 (1,100 to 2,260)	1,580 (1,090 to 2,260)	1,650 (1,070 to 2,370)	1,820 (1,110 to 2,760)	1,780 (1,060 to 2,870)	1,570 (955 to 2,580)	1,430 (835 to 2,440)	856 (479 to 1,450)	522 (290 to 914)	240 (138 to 401)	103 (57·5 to 168)	23·1 (10 to 39·2)	28,800
Interstitial lung disease and pulmonary sarcoidosis	0 (0 to 0)	0 (0 to 0)	0 (0 to 0)	10·5 (3·7 to 25·1)	10 (5·2 to 17·9)	13·7 (7·2 to 24·9)	29·9 (12·8 to 63·1)	46·9 (19·7 to 93·2)	90·5 (43·7 to 180)	120 (58·4 to 218)	106 (42·6 to 213)	213 (110 to 358)	227 (125 to 371)	335 (187 to 557)	484 (279 to 790)	412 (226 to 686)	425 (222 to 779)	505 (271 to 919)	296 (151 to 576)	136 (65·6 to 250)	90 (42·3 to 162)	28·2 (13·6 to 52·2)	4·7 (2 to 9·1)	3,580
Other chronic respiratory diseases	0 (0 to 0)	0 (0 to 0)	0 (0 to 0)	136 (57·3 to 275)	250 (95·6 to 472)	380 (190 to 623)	304 (174 to 505)	545 (334 to 813)	569 (373 to 832)	682 (451 to 988)	649 (417 to 990)	556 (360 to 870)	520 (336 to 828)	461 (275 to 795)	647 (360 to 1,200)	79·6 (51·6 to 135)	73·8 (44·1 to 125)	48·5 (29·8 to 82·9)	27·3 (17·1 to 51·1)	26·1 (15·1 to 48·2)	8·9 (6·4 to 14·1)	7·5 (4·1 to 13·9)	1·5 (1 to 2·3)	5,970
Digestive diseases	103 (65·6 to 166)	168 (110 to 263)	1,180 (580 to 2,200)	794 (435 to 1,490)	1,050 (708 to 1,460)	1,040 (702 to 1,450)	2,020 (1,350 to 2,880)	3,190 (2,290 to 4,430)	5,430 (3,890 to 7,230)	9,500 (6,690 to 12,700)	12,000 (8,450 to 16,200)	14,000 (10,100 to 19,100)	16,100 (11,300 to 22,100)	18,800 (13,400 to 25,800)	22,500 (15,900 to 30,900)	22,000 (15,700 to 30,100)	19,400 (13,500 to 27,300)	17,600 (12,400 to 24,100)	10,500 (7,230 to 14,300)	6,460 (4,580 to 8,800)	3,010 (2,120 to 4,100)	1,450 (1,000 to 1,920)	312 (188 to 445)	189,000
Cirrhosis and other chronic liver diseases	0 (0 to 0)	0 (0 to 0)	1·2 (0·6 to 2·1)	130 (37·1 to 371)	184 (102 to 312)	216 (118 to 355)	596 (318 to 983)	1,090 (658 to 1,660)	2,020 (1,230 to 3,060)	4,910 (3,060 to 7,120)	7,370 (4,880 to 10,800)	9,240 (6,230 to 13,100)	11,100 (7,350 to 15,800)	12,400 (8,290 to 17,700)	14,200 (9,510 to 20,600)	12,500 (8,390 to 17,800)	9,330 (6,110 to 14,000)	7,330 (4,540 to 10,800)	3,400 (2,030 to 5,140)	1,820 (1,140 to 2,710)	748 (445 to 1,160)	324 (195 to 494)	65 (33·2 to 105)	99,000
Cirrhosis and other chronic liver diseases due to hepati	0 (0 to 0)	0 (0 to 0)	0 (0 to 0)	1·9 (0·4 to 5·8)	7·2 (3 to 15·4)	15·7 (6·4 to 30·8)	67·3 (25·9 to 134)	373 (182 to 641)	925 (479 to 1,530)	2,390 (1,400 to 3,710)	3,630 (2,230 to 5,590)	4,460 (2,810 to 6,600)	5,110 (3,140 to 7,820)	5,480 (3,270 to 8,370)	5,910 (3,480 to 9,240)	4,980 (2,930 to 8,140)	3,630 (1,980 to 6,010)	2,880 (1,520 to 4,650)	1,390 (727 to 2,270)	752 (413 to 1,230)	305 (163 to 498)	130 (69·7 to 215)	25·4 (10·7 to 46·4)	42,500
Cirrhosis and other chronic liver diseases due to hepati	0 (0 to 0)	0 (0 to 0)	0 (0 to 0)	1·3 (0·3 to 4·3)	5·2 (2·1 to 11·8)	11·2 (4·7 to 23)	48·4 (17·8 to 106)	210 (96·2 to 401)	500 (218 to 937)	1,270 (650 to 2,140)	1,890 (974 to 3,190)	2,350 (1,250 to 3,990)	2,780 (1,450 to 4,690)	3,030 (1,590 to 5,030)	3,340 (1,740 to 5,690)	2,880 (1,390 to 5,180)	2,200 (1,140 to 3,900)	1,740 (890 to 2,980)	793 (398 to 1,420)	429 (213 to 758)	180 (90·3 to 316)	80 (38·9 to 144)	16·4 (5·8 to 33·1)	23,800
Cirrhosis and other chronic liver diseases due to alcoho	0 (0 to 0)	0 (0 to 0)	0 (0 to 0)	0 (0 to 0)	0 (0 to 0)	0 (0 to 0)	3·3 (0·8 to 9·8)	48·5 (19·3 to 109)	132 (49·3 to 296)	515 (265 to 900)	1,070 (490 to 1,930)	1,550 (822 to 2,690)	2,080 (1,020 to 3,700)	2,580 (1,260 to 4,490)	3,250 (1,490 to 5,950)	2,920 (1,440 to 5,240)	2,030 (936 to 3,720)	1,410 (666 to 2,560)	548 (257 to 974)	254 (119 to 471)	96·7 (46 to 176)	39·1 (17·6 to 78·6)	7·4 (2·5 to 16·4)	18,500
Cirrhosis and other chronic liver diseases due to NAFLD	0 (0 to 0)	0 (0 to 0)	0 (0 to 0)	0 (0 to 0)	0 (0 to 0)	0 (0 to 0)	22·2 (8·2 to 50·8)	62·1 (25·9 to 118)	131 (49·5 to 270)	285 (134 to 528)	368 (151 to 744)	461 (198 to 869)	616 (254 to 1,250)	743 (308 to 1,490)	885 (366 to 1,710)	853 (355 to 1,710)	717 (350 to 1,330)	623 (318 to 1,120)	311 (135 to 593)	179 (83·7 to 323)	78·4 (37·2 to 147)	35·7 (15·5 to 68·2)	7·6 (2·4 to 17·5)	6,380
Cirrhosis and other chronic liver diseases due to other c	0 (0 to 0)	0 (0 to 0)	1·2 (0·6 to 2·1)	127 (36·1 to 363)	172 (95·2 to 289)	189 (102 to 310)	455 (242 to 746)	393 (209 to 656)	334 (154 to 625)	445 (215 to 842)	412 (192 to 776)	414 (200 to 766)	496 (228 to 953)	606 (287 to 1,180)	829 (389 to 1,550)	870 (413 to 1,620)	753 (334 to 1,420)	679 (302 to 1,270)	353 (151 to 729)	203 (93·7 to 379)	87·5 (40·6 to 168)	39·3 (17·4 to 75·6)	8·2 (2·6 to 18·9)	7,870
Upper digestive system diseases	0 (0 to 0)	0 (0 to 0)	0 (0 to 0)	120 (48·8 to 228)	148 (68·1 to 277)	162 (90·2 to 273)	421 (253 to 649)	822 (495 to 1,370)	1,200 (720 to 1,930)	1,570 (954 to 2,410)	1,520 (948 to 2,410)	1,490 (956 to 2,200)	1,550 (1,040 to 2,230)	1,790 (1,170 to 2,560)	2,090 (1,370 to 3,100)	2,450 (1,620 to 3,530)	2,720 (1,730 to 4,000)	2,820 (1,820 to 4,360)	2,020 (1,240 to 3,070)	1,360 (830 to 2,060)	687 (420 to 1,060)	319 (194 to 488)	76·7 (40 to 121)	25,300
Peptic ulcer disease	0 (0 to 0)	[illegible]	[illegible]	[illegible]	19 ([illegible] to 35·5)	37 (19·7 to 65·1)	122 (67·8 to 219)	196 (102 to 330)	330 (172 to 563)	567 (293 to 1,010)	565 (324 to 924)	613 (346 to 1,050)	737 (426 to 1,190)	1,020 (593 to 1,640)	1,400 (815 to 2,230)	1,860 (1,120 to 2,840)	2,270 (1,310 to 3,490)	2,470 (1,510 to 3,910)	1,830 (1,070 to 2,890)	1,250 (732 to 1,930)	650 (388 to 1,010)	304 (182 to 469)	73·5 (37·1 to 118)	16,300
Gastritis and duodenitis	0 (0 to 0)	[illegible]	[illegible]	[illegible]	127 ([illegible] to 248)	84·2 (27·5 to 175)	74·6 (23·7 to 161)	91·3 (35·7 to 180)	128 (49·1 to 240)	178 (67·8 to 354)	227 (103 to 416)	247 (108 to 473)	258 (130 to 499)	258 (135 to 455)	242 (126 to 437)	192 (99·1 to 333)	135 (76·1 to 228)	108 (59 to 185)	46·6 (24·7 to 83)	28·3 (14·4 to 49·3)	6·1 (3·3 to 11)	2·7 (1·4 to 5·3)	0·7 (0·3 to 1·6)	2,550
Gastroesophageal reflux disease	0 (0 to 0)	[illegible]	[illegible]	[illegible]	[illegible]	[illegible]	[illegible]	[illegible]	[illegible]	[illegible]	[illegible]	[illegible]	[illegible]	[illegible]	[illegible]	[illegible]	[illegible]	[illegible]	[illegible]	[illegible]	[illegible]	[illegible]	[illegible]	[illegible]
Appendicitis	0 (0 to 0)	[illegible]	[illegible]	[illegible]	[illegible]	[illegible]	[illegible]	[illegible]	[illegible]	[illegible]	[illegible]	[illegible]	[illegible]	[illegible]	[illegible]	[illegible]	[illegible]	[illegible]	[illegible]	[illegible]	[illegible]	[illegible]	[illegible]	[illegible]
Paralytic ileus and intestinal obstruction	92·2 (59 to 141)	[illegible]	[illegible]	[illegible]	[illegible]	[illegible]	[illegible]	[illegible]	[illegible]	[illegible]	[illegible]	[illegible]	[illegible]	[illegible]	[illegible]	[illegible]	[illegible]	[illegible]	[illegible]	[illegible]	[illegible]	[illegible]	[illegible]	[illegible]
Inguinal, femoral, and abdominal hernia	11·3 (2·3 to 30·5)	[illegible]	[illegible]	[illegible]	[illegible]	[illegible]	[illegible]	[illegible]	[illegible]	[illegible]	[illegible]	[illegible]	[illegible]	[illegible]	[illegible]	[illegible]	[illegible]	[illegible]	[illegible]	[illegible]	[illegible]	[illegible]	[illegible]	[illegible]
Inflammatory bowel disease	0 (0 to 0)	[illegible]	[illegible]	[illegible]	[illegible]	[illegible]	[illegible]	[illegible]	[illegible]	[illegible]	[illegible]	[illegible]	[illegible]	[illegible]	[illegible]	[illegible]	[illegible]	[illegible]	[illegible]	[illegible]	[illegible]	[illegible]	[illegible]	[illegible]
Vascular intestinal disorders	0 (0 to 0)	[illegible]	[illegible]	[illegible]	[illegible]	[illegible]	[illegible]	[illegible]	[illegible]	[illegible]	[illegible]	[illegible]	[illegible]	[illegible]	[illegible]	[illegible]	[illegible]	[illegible]	[illegible]	[illegible]	[illegible]	[illegible]	[illegible]	[illegible]
Gallbladder and biliary diseases	0 (0 to 0)	[illegible]	[illegible]	[illegible]	[illegible]	[illegible]	[illegible]	[illegible]	[illegible]	[illegible]	[illegible]	[illegible]	[illegible]	[illegible]	[illegible]	[illegible]	[illegible]	[illegible]	[illegible]	[illegible]	[illegible]	[illegible]	[illegible]	[illegible]
Pancreatitis	0 (0 to 0)	[illegible]	[illegible]	[illegible]	[illegible]	[illegible]	[illegible]	[illegible]	[illegible]	[illegible]	[illegible]	[illegible]	[illegible]	[illegible]	[illegible]	[illegible]	[illegible]	[illegible]	[illegible]	[illegible]	[illegible]	[illegible]	[illegible]	[illegible]
Other digestive diseases	0 (0 to 0)	[illegible]	[illegible]	[illegible]	[illegible]	[illegible]	[illegible]	[illegible]	[illegible]	[illegible]	[illegible]	[illegible]	[illegible]	[illegible]	[illegible]	[illegible]	[illegible]	[illegible]	[illegible]	[illegible]	[illegible]	[illegible]	[illegible]	[illegible]
Neurological disorders	3·7 (0·7 to 9)	[illegible]	[illegible]	[illegible]	[illegible]	[illegible]	[illegible]	[illegible]	[illegible]	[illegible]	[illegible]	[illegible]	[illegible]	[illegible]	[illegible]	[illegible]	[illegible]	[illegible]	[illegible]	[illegible]	[illegible]	[illegible]	[illegible]	[illegible]
Alzheimer's disease and other dementias	0 (0 to 0)	[illegible]	[illegible]	[illegible]	[illegible]	[illegible]	[illegible]	[illegible]	[illegible]	[illegible]	[illegible]	[illegible]	[illegible]	[illegible]	[illegible]	[illegible]	[illegible]	[illegible]	[illegible]	[illegible]	[illegible]	[illegible]	[illegible]	[illegible]
Parkinson's disease	0 (0 to 0)	[illegible]	[illegible]	[illegible]	[illegible]	[illegible]	[illegible]	[illegible]	[illegible]	[illegible]	[illegible]	[illegible]	[illegible]	[illegible]	[illegible]	[illegible]	[illegible]	[illegible]	[illegible]	[illegible]	[illegible]	[illegible]	[illegible]	[illegible]
Idiopathic epilepsy	3·7 (0·7 to 9)	[illegible]	[illegible]	[illegible]	[illegible]	[illegible]	[illegible]	[illegible]	[illegible]	[illegible]	[illegible]	[illegible]	[illegible]	[illegible]	[illegible]	[illegible]	[illegible]	[illegible]	[illegible]	[illegible]	[illegible]	[illegible]	[illegible]	[illegible]
Multiple sclerosis	0 (0 to 0)	[illegible]	[illegible]	[illegible]	[illegible]	[illegible]	[illegible]	[illegible]	[illegible]	[illegible]	[illegible]	[illegible]	[illegible]	[illegible]	[illegible]	[illegible]	[illegible]	[illegible]	[illegible]	[illegible]	[illegible]	[illegible]	[illegible]	[illegible]
Motor neuron disease	0 (0 to 0)	[illegible]	[illegible]	[illegible]	[illegible]	[illegible]	[illegible]	[illegible]	[illegible]	[illegible]	[illegible]	[illegible]	[illegible]	[illegible]	[illegible]	[illegible]	[illegible]	[illegible]	[illegible]	[illegible]	[illegible]	[illegible]	[illegible]	[illegible]
Headache disorders	0 (0 to 0)	[illegible]	[illegible]	[illegible]	[illegible]	[illegible]	[illegible]	[illegible]	[illegible]	[illegible]	[illegible]	[illegible]	[illegible]	[illegible]	[illegible]	[illegible]	[illegible]	[illegible]	[illegible]	[illegible]	[illegible]	[illegible]	[illegible]	[illegible]
Migraine	0 (0 to 0)	[illegible]	[illegible]	[illegible]	[illegible]	[illegible]	[illegible]	[illegible]	[illegible]	[illegible]	[illegible]	[illegible]	[illegible]	[illegible]	[illegible]	[illegible]	[illegible]	[illegible]	[illegible]	[illegible]	[illegible]	[illegible]	[illegible]	[illegible]
Tension-type headache	0 (0 to 0)	[illegible]	[illegible]	[illegible]	[illegible]	[illegible]	[illegible]	[illegible]	[illegible]	[illegible]	[illegible]	[illegible]	[illegible]	[illegible]	[illegible]	[illegible]	[illegible]	[illegible]	[illegible]	[illegible]	[illegible]	[illegible]	[illegible]	[illegible]
Other neurological disorders	0 (0 to 0)	[illegible]	[illegible]	[illegible]	[illegible]	[illegible]	[illegible]	[illegible]	[illegible]	[illegible]	[illegible]	[illegible]	[illegible]	[illegible]	[illegible]	[illegible]	[illegible]	[illegible]	[illegible]	[illegible]	[illegible]	[illegible]	[illegible]	[illegible]
Mental disorders	5·2 (3·3 to 7·7)	[illegible]	[illegible]	[illegible]	[illegible]	[illegible]	[illegible]	[illegible]	[illegible]	[illegible]	[illegible]	[illegible]	[illegible]	[illegible]	[illegible]	[illegible]	[illegible]	[illegible]	[illegible]	[illegible]	[illegible]	[illegible]	[illegible]	[illegible]
Schizophrenia	0 (0 to 0)	[illegible]	[illegible]	[illegible]	[illegible]	[illegible]	[illegible]	[illegible]	[illegible]	[illegible]	[illegible]	[illegible]	[illegible]	[illegible]	[illegible]	[illegible]	[illegible]	[illegible]	[illegible]	[illegible]	[illegible]	[illegible]	[illegible]	[illegible]
Depressive disorders	0 (0 to 0)	[illegible]	[illegible]	[illegible]	[illegible]	[illegible]	[illegible]	[illegible]	[illegible]	[illegible]	[illegible]	[illegible]	[illegible]	[illegible]	[illegible]	[illegible]	[illegible]	[illegible]	[illegible]	[illegible]	[illegible]	[illegible]	[illegible]	[illegible]
Major depressive disorder	0 (0 to 0)	[illegible]	[illegible]	[illegible]	[illegible]	[illegible]	[illegible]	[illegible]	[illegible]	[illegible]	[illegible]	[illegible]	[illegible]	[illegible]	[illegible]	[illegible]	[illegible]	[illegible]	[illegible]	[illegible]	[illegible]	[illegible]	[illegible]	[illegible]
Dysthymia	0 (0 to 0)	[illegible]	[illegible]	[illegible]	[illegible]	[illegible]	[illegible]	[illegible]	[illegible]	[illegible]	[illegible]	[illegible]	[illegible]	[illegible]	[illegible]	[illegible]	[illegible]	[illegible]	[illegible]	[illegible]	[illegible]	[illegible]	[illegible]	[illegible]
Bipolar disorder	0 (0 to 0)	[illegible]	[illegible]	[illegible]	[illegible]	[illegible]	[illegible]	[illegible]	[illegible]	[illegible]	[illegible]	[illegible]	[illegible]	[illegible]	[illegible]	[illegible]	[illegible]	[illegible]	[illegible]	[illegible]	[illegible]	[illegible]	[illegible]	[illegible]
Anxiety disorders	0 (0 to 0)	[illegible]	[illegible]	[illegible]	[illegible]	[illegible]	[illegible]	[illegible]	[illegible]	[illegible]	[illegible]	[illegible]	[illegible]	[illegible]	[illegible]	[illegible]	[illegible]	[illegible]	[illegible]	[illegible]	[illegible]	[illegible]	[illegible]	[illegible]
Eating disorders	0 (0 to 0)	[illegible]	[illegible]	[illegible]	[illegible]	[illegible]	[illegible]	[illegible]	[illegible]	[illegible]	[illegible]	[illegible]	[illegible]	[illegible]	[illegible]	[illegible]	[illegible]	[illegible]	[illegible]	[illegible]	[illegible]	[illegible]	[illegible]	[illegible]
Anorexia nervosa	0 (0 to 0)	[illegible]	[illegible]	[illegible]	[illegible]	[illegible]	[illegible]	[illegible]	[illegible]	[illegible]	[illegible]	[illegible]	[illegible]	[illegible]	[illegible]	[illegible]	[illegible]	[illegible]	[illegible]	[illegible]	[illegible]	[illegible]	[illegible]	[illegible]
Bulimia nervosa	0 (0 to 0)	[illegible]	[illegible]	[illegible]	[illegible]	[illegible]	[illegible]	[illegible]	[illegible]	[illegible]	[illegible]	[illegible]	[illegible]	[illegible]	[illegible]	[illegible]	[illegible]	[illegible]	[illegible]	[illegible]	[illegible]	[illegible]	[illegible]	[illegible]
Autism spectrum disorders	4·9 (3·1 to 7·4)	[illegible]	[illegible]	[illegible]	[illegible]	[illegible]	[illegible]	[illegible]	[illegible]	[illegible]	[illegible]	[illegible]	[illegible]	[illegible]	[illegible]	[illegible]	[illegible]	[illegible]	[illegible]	[illegible]	[illegible]	[illegible]	[illegible]	[illegible]
Attention-deficit/hyperactivity disorder	0 (0 to 0)	[illegible]	[illegible]	[illegible]	[illegible]	[illegible]	[illegible]	[illegible]	[illegible]	[illegible]	[illegible]	[illegible]	[illegible]	[illegible]	[illegible]	[illegible]	[illegible]	[illegible]	[illegible]	[illegible]	[illegible]	[illegible]	[illegible]	[illegible]
Conduct disorder	0 (0 to 0)	[illegible]	[illegible]	[illegible]	[illegible]	[illegible]	[illegible]	[illegible]	[illegible]	[illegible]	[illegible]	[illegible]	[illegible]	[illegible]	[illegible]	[illegible]	[illegible]	[illegible]	[illegible]	[illegible]	[illegible]	[illegible]	[illegible]	[illegible]
Idiopathic developmental intellectual disability	0·2 (0 to 0·9)	[illegible]	[illegible]	[illegible]	[illegible]	[illegible]	[illegible]	[illegible]	[illegible]	[illegible]	[illegible]	[illegible]	[illegible]	[illegible]	[illegible]	[illegible]	[illegible]	[illegible]	[illegible]	[illegible]	[illegible]	[illegible]	[illegible]	[illegible]
Other mental disorders	0 (0 to 0)	[illegible]	[illegible]	[illegible]	[illegible]	[illegible]	[illegible]	[illegible]	[illegible]	[illegible]	[illegible]	[illegible]	[illegible]	[illegible]	[illegible]	[illegible]	[illegible]	[illegible]	[illegible]	[illegible]	[illegible]	[illegible]	[illegible]	[illegible]
Substance use disorders	0 (0 to 0)	[illegible]	[illegible]	[illegible]	[illegible]	[illegible]	[illegible]	[illegible]	[illegible]	[illegible]	[illegible]	[illegible]	[illegible]	[illegible]	[illegible]	[illegible]	[illegible]	[illegible]	[illegible]	[illegible]	[illegible]	[illegible]	[illegible]	[illegible]
Alcohol use disorders	0 (0 to 0)	[illegible]	[illegible]	[illegible]	[illegible]	[illegible]	[illegible]	[illegible]	[illegible]	[illegible]	[illegible]	[illegible]	[illegible]	[illegible]	[illegible]	[illegible]	[illegible]	[illegible]	[illegible]	[illegible]	[illegible]	[illegible]	[illegible]	[illegible]
Drug use disorders	0 (0 to 0)	[illegible]	[illegible]	[illegible]	[illegible]	[illegible]	[illegible]	[illegible]	[illegible]	[illegible]	[illegible]	[illegible]	[illegible]	[illegible]	[illegible]	[illegible]	[illegible]	[illegible]	[illegible]	[illegible]	[illegible]	[illegible]	[illegible]	[illegible]
Opioid use disorders	0 (0 to 0)	[illegible]	[illegible]	[illegible]	[illegible]	[illegible]	[illegible]	[illegible]	[illegible]	[illegible]	[illegible]	[illegible]	[illegible]	[illegible]	[illegible]	[illegible]	[illegible]	[illegible]	[illegible]	[illegible]	[illegible]	[illegible]	[illegible]	[illegible]
Cocaine use disorders	0 (0 to 0)	[illegible]	[illegible]	[illegible]	[illegible]	[illegible]	[illegible]	[illegible]	[illegible]	[illegible]	[illegible]	[illegible]	[illegible]	[illegible]	[illegible]	[illegible]	[illegible]	[illegible]	[illegible]	[illegible]	[illegible]	[illegible]	[illegible]	[illegible]
Amphetamine use disorders	0 (0 to 0)	[illegible]	[illegible]	[illegible]	[illegible]	[illegible]	[illegible]	[illegible]	[illegible]	[illegible]	[illegible]	[illegible]	[illegible]	[illegible]	[illegible]	[illegible]	[illegible]	[illegible]	[illegible]	[illegible]	[illegible]	[illegible]	[illegible]	[illegible]
Cannabis use disorders	0 (0 to 0)	[illegible]	[illegible]	[illegible]	[illegible]	[illegible]	[illegible]	[illegible]	[illegible]	[illegible]	[illegible]	[illegible]	[illegible]	[illegible]	[illegible]	[illegible]	[illegible]	[illegible]	[illegible]	[illegible]	[illegible]	[illegible]	[illegible]	[illegible]
Other drug use disorders	0 (0 to 0)	[illegible]	[illegible]	[illegible]	[illegible]	[illegible]	[illegible]	[illegible]	[illegible]	[illegible]	[illegible]	[illegible]	[illegible]	[illegible]	[illegible]	[illegible]	[illegible]	[illegible]	[illegible]	[illegible]	[illegible]	[illegible]	[illegible]	[illegible]
Diabetes and kidney diseases	3·1 (1·8 to 4·9)	[illegible]	[illegible]	[illegible]	[illegible]	[illegible]	[illegible]	[illegible]	[illegible]	[illegible]	[illegible]	[illegible]	[illegible]	[illegible]	[illegible]	[illegible]	[illegible]	[illegible]	[illegible]	[illegible]	[illegible]	[illegible]	[illegible]	[illegible]
Diabetes mellitus	3·1 (1·8 to 4·9)	[illegible]	[illegible]	[illegible]	[illegible]	[illegible]	[illegible]	[illegible]	[illegible]	[illegible]	[illegible]	[illegible]	[illegible]	[illegible]	[illegible]	[illegible]	[illegible]	[illegible]	[illegible]	[illegible]	[illegible]	[illegible]	[illegible]	[illegible]
Diabetes mellitus type 1	3·1 (1·8 to 4·9)	[illegible]	[illegible]	[illegible]	[illegible]	[illegible]	[illegible]	[illegible]	[illegible]	[illegible]	[illegible]	[illegible]	[illegible]	[illegible]	[illegible]	[illegible]	[illegible]	[illegible]	[illegible]	[illegible]	[illegible]	[illegible]	[illegible]	[illegible]
Diabetes mellitus type 2	0 (0 to 0)	[illegible]	[illegible]	[illegible]	[illegible]	[illegible]	[illegible]	[illegible]	[illegible]	[illegible]	[illegible]	[illegible]	[illegible]	[illegible]	[illegible]	[illegible]	[illegible]	[illegible]	[illegible]	[illegible]	[illegible]	[illegible]	[illegible]	[illegible]
Chronic kidney disease	0 (0 to 0)	[illegible]	[illegible]	[illegible]	[illegible]	[illegible]	[illegible]	[illegible]	[illegible]	[illegible]	[illegible]	[illegible]	[illegible]	[illegible]	[illegible]	[illegible]	[illegible]	[illegible]	[illegible]	[illegible]	[illegible]	[illegible]	[illegible]	[illegible]
Chronic kidney disease due to diabetes mellitus type 1	0 (0 to 0)	[illegible]	[illegible]	[illegible]	[illegible]	[illegible]	[illegible]	[illegible]	[illegible]	[illegible]	[illegible]	[illegible]	[illegible]	[illegible]	[illegible]	[illegible]	[illegible]	[illegible]	[illegible]	[illegible]	[illegible]	[illegible]	[illegible]	[illegible]
Chronic kidney disease due to diabetes mellitus type 2	0 (0 to 0)	[illegible]	[illegible]	[illegible]	[illegible]	[illegible]	[illegible]	[illegible]	[illegible]	[illegible]	[illegible]	[illegible]	[illegible]	[illegible]	[illegible]	[illegible]	[illegible]	[illegible]	[illegible]	[illegible]	[illegible]	[illegible]	[illegible]	[illegible]
Chronic kidney disease due to hypertension	0 (0 to 0)	[illegible]	[illegible]	[illegible]	[illegible]	[illegible]	[illegible]	[illegible]	[illegible]	[illegible]	[illegible]	[illegible]	[illegible]	[illegible]	[illegible]	[illegible]	[illegible]	[illegible]	[illegible]	[illegible]	[illegible]	[illegible]	[illegible]	[illegible]
Chronic kidney disease due to glomerulonephritis	0 (0 to 0)	[illegible]	[illegible]	[illegible]	[illegible]	[illegible]	[illegible]	[illegible]	[illegible]	[illegible]	[illegible]	[illegible]	[illegible]	[illegible]	[illegible]	[illegible]	[illegible]	[illegible]	[illegible]	[illegible]	[illegible]	[illegible]	[illegible]	[illegible]
Chronic kidney disease due to other and unspecified ca	0 (0 to 0)	[illegible]	[illegible]	[illegible]	[illegible]	[illegible]	[illegible]	[illegible]	[illegible]	[illegible]	[illegible]	[illegible]	[illegible]	[illegible]	[illegible]	[illegible]	[illegible]	[illegible]	[illegible]	[illegible]	[illegible]	[illegible]	[illegible]	[illegible]
Acute glomerulonephritis	0 (0 to 0)	[illegible]	[illegible]	[illegible]	[illegible]	[illegible]	[illegible]	[illegible]	[illegible]	[illegible]	[illegible]	[illegible]	[illegible]	[illegible]	[illegible]	[illegible]	[illegible]	[illegible]	[illegible]	[illegible]	[illegible]	[illegible]	[illegible]	[illegible]
Skin and subcutaneous diseases	0·5 (0·2 to 0·8)	[illegible]	[illegible]	[illegible]	[illegible]	[illegible]	[illegible]	[illegible]	[illegible]	[illegible]	[illegible]	[illegible]	[illegible]	[illegible]	[illegible]	[illegible]	[illegible]	[illegible]	[illegible]	[illegible]	[illegible]	[illegible]	[illegible]	[illegible]
Dermatitis	0 (0 to 0)	[illegible]	[illegible]	[illegible]	[illegible]	[illegible]	[illegible]	[illegible]	[illegible]	[illegible]	[illegible]	[illegible]	[illegible]	[illegible]	[illegible]	[illegible]	[illegible]	[illegible]	[illegible]	[illegible]	[illegible]	[illegible]	[illegible]	[illegible]
Atopic dermatitis	0 (0 to 0)	[illegible]	[illegible]	[illegible]	[illegible]	[illegible]	[illegible]	[illegible]	[illegible]	[illegible]	[illegible]	[illegible]	[illegible]	[illegible]	[illegible]	[illegible]	[illegible]	[illegible]	[illegible]	[illegible]	[illegible]	[illegible]	[illegible]	[illegible]
Contact dermatitis	0 (0 to 0)	[illegible]	[illegible]	[illegible]	[illegible]	[illegible]	[illegible]	[illegible]	[illegible]	[illegible]	[illegible]	[illegible]	[illegible]	[illegible]	[illegible]	[illegible]	[illegible]	[illegible]	[illegible]	[illegible]	[illegible]	[illegible]	[illegible]	[illegible]
Seborrhoeic dermatitis	0 (0 to 0)	[illegible]	[illegible]	[illegible]	[illegible]	[illegible]	[illegible]	[illegible]	[illegible]	[illegible]	[illegible]	[illegible]	[illegible]	[illegible]	[illegible]	[illegible]	[illegible]	[illegible]	[illegible]	[illegible]	[illegible]	[illegible]	[illegible]	[illegible]
Psoriasis	0 (0 to 0)	[illegible]	[illegible]	[illegible]	[illegible]	[illegible]	[illegible]	[illegible]	[illegible]	[illegible]	[illegible]	[illegible]	[illegible]	[illegible]	[illegible]	[illegible]	[illegible]	[illegible]	[illegible]	[illegible]	[illegible]	[illegible]	[illegible]	[illegible]
Bacterial skin diseases	0·1 (0 to 0·1)	[illegible]	[illegible]	[illegible]	[illegible]	[illegible]	[illegible]	[illegible]	[illegible]	[illegible]	[illegible]	[illegible]	[illegible]	[illegible]	[illegible]	[illegible]	[illegible]	[illegible]	[illegible]	[illegible]	[illegible]	[illegible]	[illegible]	[illegible]
Cellulitis	0 (0 to 0)	[illegible]	[illegible]	[illegible]	[illegible]	[illegible]	[illegible]	[illegible]	[illegible]	[illegible]	[illegible]	[illegible]	[illegible]	[illegible]	[illegible]	[illegible]	[illegible]	[illegible]	[illegible]	[illegible]	[illegible]	[illegible]	[illegible]	[illegible]
Pyoderma	0 (0 to 0·1)	[illegible]	[illegible]	[illegible]	[illegible]	[illegible]	[illegible]	[illegible]	[illegible]	[illegible]	[illegible]	[illegible]	[illegible]	[illegible]	[illegible]	[illegible]	[illegible]	[illegible]	[illegible]	[illegible]	[illegible]	[illegible]	[illegible]	[illegible]
Scabies	0·3 (0·1 to 0·5)	[illegible]	[illegible]	[illegible]	[illegible]	[illegible]	[illegible]	[illegible]	[illegible]	[illegible]	[illegible]	[illegible]	[illegible]	[illegible]	[illegible]	[illegible]	[illegible]	[illegible]	[illegible]	[illegible]	[illegible]	[illegible]	[illegible]	[illegible]
Fungal skin diseases	0 (0 to 0·1)	[illegible]	[illegible]	[illegible]	[illegible]	[illegible]	[illegible]	[illegible]	[illegible]	[illegible]	[illegible]	[illegible]	[illegible]	[illegible]	[illegible]	[illegible]	[illegible]	[illegible]	[illegible]	[illegible]	[illegible]	[illegible]	[illegible]	[illegible]
Viral skin diseases	0 (0 to 0)	[illegible]	[illegible]	[illegible]	[illegible]	[illegible]	[illegible]	[illegible]	[illegible]	[illegible]	[illegible]	[illegible]	[illegible]	[illegible]	[illegible]	[illegible]	[illegible]	[illegible]	[illegible]	[illegible]	[illegible]	[illegible]	[illegible]	[illegible]
Acne vulgaris	0 (0 to 0)	[illegible]	[illegible]	[illegible]	[illegible]	[illegible]	[illegible]	[illegible]	[illegible]	[illegible]	[illegible]	[illegible]	[illegible]	[illegible]	[illegible]	[illegible]	[illegible]	[illegible]	[illegible]	[illegible]	[illegible]	[illegible]	[illegible]	[illegible]
Alopecia areata	0 (0 to 0)	[illegible]	[illegible]	[illegible]	[illegible]	[illegible]	[illegible]	[illegible]	[illegible]	[illegible]	[illegible]	[illegible]	[illegible]	[illegible]	[illegible]	[illegible]	[illegible]	[illegible]	[illegible]	[illegible]	[illegible]	[illegible]	[illegible]	[illegible]
Pruritus	0 (0 to 0)	[illegible]	[illegible]	[illegible]	[illegible]	[illegible]	[illegible]	[illegible]	[illegible]	[illegible]	[illegible]	[illegible]	[illegible]	[illegible]	[illegible]	[illegible]	[illegible]	[illegible]	[illegible]	[illegible]	[illegible]	[illegible]	[illegible]	[illegible]
Urticaria	0 (0 to 0·1)	[illegible]	[illegible]	[illegible]	[illegible]	[illegible]	[illegible]	[illegible]	[illegible]	[illegible]	[illegible]	[illegible]	[illegible]	[illegible]	[illegible]	[illegible]	[illegible]	[illegible]	[illegible]	[illegible]	[illegible]	[illegible]	[illegible]	[illegible]
Decubitus ulcer	0 (0 to 0)	[illegible]	[illegible]	[illegible]	[illegible]	[illegible]	[illegible]	[illegible]	[illegible]	[illegible]	[illegible]	[illegible]	[illegible]	[illegible]	[illegible]	[illegible]	[illegible]	[illegible]	[illegible]	[illegible]	[illegible]	[illegible]	[illegible]	[illegible]
Other skin and subcutaneous diseases	0·1 (0 to 0·1)	[illegible]	[illegible]	[illegible]	[illegible]	[illegible]	[illegible]	[illegible]	[illegible]	[illegible]	[illegible]	[illegible]	[illegible]	[illegible]	[illegible]	[illegible]	[illegible]	[illegible]	[illegible]	[illegible]	[illegible]	[illegible]	[illegible]	[illegible]
Sense organ diseases	1·8 (1 to 2·8)	[illegible]	[illegible]	[illegible]	[illegible]	[illegible]	[illegible]	[illegible]	[illegible]	[illegible]	[illegible]	[illegible]	[illegible]	[illegible]	[illegible]	[illegible]	[illegible]	[illegible]	[illegible]	[illegible]	[illegible]	[illegible]	[illegible]	[illegible]
Blindness and vision loss	0·7 (0·3 to 1·3)	[illegible]	[illegible]	[illegible]	[illegible]	[illegible]	[illegible]	[illegible]	[illegible]	[illegible]	[illegible]	[illegible]	[illegible]	[illegible]	[illegible]	[illegible]	[illegible]	[illegible]	[illegible]	[illegible]	[illegible]	[illegible]	[illegible]	[illegible]
Glaucoma	0 (0 to 0)	[illegible]	[illegible]	[illegible]	[illegible]	[illegible]	[illegible]	[illegible]	[illegible]	[illegible]	[illegible]	[illegible]	[illegible]	[illegible]	[illegible]	[illegible]	[illegible]	[illegible]	[illegible]	[illegible]	[illegible]	[illegible]	[illegible]	[illegible]
Cataract	0 (0 to 0)	[illegible]	[illegible]	[illegible]	[illegible]	[illegible]	[illegible]	[illegible]	[illegible]	[illegible]	[illegible]	[illegible]	[illegible]	[illegible]	[illegible]	[illegible]	[illegible]	[illegible]	[illegible]	[illegible]	[illegible]	[illegible]	[illegible]	[illegible]
Age-related macular degeneration	0 (0 to 0)	[illegible]	[illegible]	[illegible]	[illegible]	[illegible]	[illegible]	[illegible]	[illegible]	[illegible]	[illegible]	[illegible]	[illegible]	[illegible]	[illegible]	[illegible]	[illegible]	[illegible]	[illegible]	[illegible]	[illegible]	[illegible]	[illegible]	[illegible]
Refraction disorders	0·1 (0 to 0·1)	[illegible]	[illegible]	[illegible]	[illegible]	[illegible]	[illegible]	[illegible]	[illegible]	[illegible]	[illegible]	[illegible]	[illegible]	[illegible]	[illegible]	[illegible]	[illegible]	[illegible]	[illegible]	[illegible]	[illegible]	[illegible]	[illegible]	[illegible]
Near vision loss	0 (0 to 0)	[illegible]	[illegible]	[illegible]	[illegible]	[illegible]	[illegible]	[illegible]	[illegible]	[illegible]	[illegible]	[illegible]	[illegible]	[illegible]	[illegible]	[illegible]	[illegible]	[illegible]	[illegible]	[illegible]	[illegible]	[illegible]	[illegible]	[illegible]
Other vision loss	0·6 (0·3 to 1·2)	[illegible]	[illegible]	[illegible]	[illegible]	[illegible]	[illegible]	[illegible]	[illegible]	[illegible]	[illegible]	[illegible]	[illegible]	[illegible]	[illegible]	[illegible]	[illegible]	[illegible]	[illegible]	[illegible]	[illegible]	[illegible]	[illegible]	[illegible]
Age-related and other hearing loss	0·7 (0·3 to 1·5)	[illegible]	[illegible]	[illegible]	[illegible]	[illegible]	[illegible]	[illegible]	[illegible]	[illegible]	[illegible]	[illegible]	[illegible]	[illegible]	[illegible]	[illegible]	[illegible]	[illegible]	[illegible]	[illegible]	[illegible]	[illegible]	[illegible]	[illegible]
Other sense organ diseases	0·3 (0·1 to 0·7)	[illegible]	[illegible]	[illegible]	[illegible]	[illegible]	[illegible]	[illegible]	[illegible]	[illegible]	[illegible]	[illegible]	[illegible]	[illegible]	[illegible]	[illegible]	[illegible]	[illegible]	[illegible]	[illegible]	[illegible]	[illegible]	[illegible]	[illegible]
Musculoskeletal disorders	0 (0 to 0)	[illegible]	[illegible]	[illegible]	[illegible]	[illegible]	[illegible]	[illegible]	[illegible]	[illegible]	[illegible]	[illegible]	[illegible]	[illegible]	[illegible]	[illegible]	[illegible]	[illegible]	[illegible]	[illegible]	[illegible]	[illegible]	[illegible]	[illegible]
Rheumatoid arthritis	0 (0 to 0)	[illegible]	[illegible]	[illegible]	[illegible]	[illegible]	[illegible]	[illegible]	[illegible]	[illegible]	[illegible]	[illegible]	[illegible]	[illegible]	[illegible]	[illegible]	[illegible]	[illegible]	[illegible]	[illegible]	[illegible]	[illegible]	[illegible]	[illegible]
Osteoarthritis	0 (0 to 0)	[illegible]	[illegible]	[illegible]	[illegible]	[illegible]	[illegible]	[illegible]	[illegible]	[illegible]	[illegible]	[illegible]	[illegible]	[illegible]	[illegible]	[illegible]	[illegible]	[illegible]	[illegible]	[illegible]	[illegible]	[illegible]	[illegible]	[illegible]
Osteoarthritis hip	0 (0 to 0)	[illegible]	[illegible]	[illegible]	[illegible]	[illegible]	[illegible]	[illegible]	[illegible]	[illegible]	[illegible]	[illegible]	[illegible]	[illegible]	[illegible]	[illegible]	[illegible]	[illegible]	[illegible]	[illegible]	[illegible]	[illegible]	[illegible]	[illegible]
Osteoarthritis knee	0 (0 to 0)	[illegible]	[illegible]	[illegible]	[illegible]	[illegible]	[illegible]	[illegible]	[illegible]	[illegible]	[illegible]	[illegible]	[illegible]	[illegible]	[illegible]	[illegible]	[illegible]	[illegible]	[illegible]	[illegible]	[illegible]	[illegible]	[illegible]	[illegible]
Osteoarthritis hand	0 (0 to 0)	[illegible]	[illegible]	[illegible]	[illegible]	[illegible]	[illegible]	[illegible]	[illegible]	[illegible]	[illegible]	[illegible]	[illegible]	[illegible]	[illegible]	[illegible]	[illegible]	[illegible]	[illegible]	[illegible]	[illegible]	[illegible]	[illegible]	[illegible]
Osteoarthritis other	0 (0 to 0)	[illegible]	[illegible]	[illegible]	[illegible]	[illegible]	[illegible]	[illegible]	[illegible]	[illegible]	[illegible]	[illegible]	[illegible]	[illegible]	[illegible]	[illegible]	[illegible]	[illegible]	[illegible]	[illegible]	[illegible]	[illegible]	[illegible]	[illegible]
Low back pain	0 (0 to 0)	[illegible]	[illegible]	[illegible]	[illegible]	[illegible]	[illegible]	[illegible]	[illegible]	[illegible]	[illegible]	[illegible]	[illegible]	[illegible]	[illegible]	[illegible]	[illegible]	[illegible]	[illegible]	[illegible]	[illegible]	[illegible]	[illegible]	[illegible]
Neck pain	0 (0 to 0)	[illegible]	[illegible]	[illegible]	[illegible]	[illegible]	[illegible]	[illegible]	[illegible]	[illegible]	[illegible]	[illegible]	[illegible]	[illegible]	[illegible]	[illegible]	[illegible]	[illegible]	[illegible]	[illegible]	[illegible]	[illegible]	[illegible]	[illegible]
Gout	0 (0 to 0)	[illegible]	[illegible]	[illegible]	[illegible]	[illegible]	[illegible]	[illegible]	[illegible]	[illegible]	[illegible]	[illegible]	[illegible]	[illegible]	[illegible]	[illegible]	[illegible]	[illegible]	[illegible]	[illegible]	[illegible]	[illegible]	[illegible]	[illegible]
Other musculoskeletal disorders	0 (0 to 0)	[illegible]	[illegible]	[illegible]	[illegible]	[illegible]	[illegible]	[illegible]	[illegible]	[illegible]	[illegible]	[illegible]	[illegible]	[illegible]	[illegible]	[illegible]	[illegible]	[illegible]	[illegible]	[illegible]	[illegible]	[illegible]	[illegible]	[illegible]
Other non-communicable diseases	20,800 (12,500 to 31,600)	[illegible]	[illegible]	[illegible]	[illegible]	[illegible]	[illegible]	[illegible]	[illegible]	[illegible]	[illegible]	[illegible]	[illegible]	[illegible]	[illegible]	[illegible]	[illegible]	[illegible]	[illegible]	[illegible]	[illegible]	[illegible]	[illegible]	[illegible]
Congenital birth defects	20,700 (12,400 to 31,400)	[illegible]	[illegible]	[illegible]	[illegible]	[illegible]	[illegible]	[illegible]	[illegible]	[illegible]	[illegible]	[illegible]	[illegible]	[illegible]	[illegible]	[illegible]	[illegible]	[illegible]	[illegible]	[illegible]	[illegible]	[illegible]	[illegible]	[illegible]
Neural tube defects	2,350 (824 to 5,200)	[illegible]	[illegible]	[illegible]	[illegible]	[illegible]	[illegible]	[illegible]	[illegible]	[illegible]	[illegible]	[illegible]	[illegible]	[illegible]	[illegible]	[illegible]	[illegible]	[illegible]	[illegible]	[illegible]	[illegible]	[illegible]	[illegible]	[illegible]
Congenital heart anomalies	5,000 (2,740 to 8,430)	[illegible]	[illegible]	[illegible]	[illegible]	[illegible]	[illegible]	[illegible]	[illegible]	[illegible]	[illegible]	[illegible]	[illegible]	[illegible]	[illegible]	[illegible]	[illegible]	[illegible]	[illegible]	[illegible]	[illegible]	[illegible]	[illegible]	[illegible]
Orofacial clefts	96·9 (16·1 to 369)	[illegible]	[illegible]	[illegible]	[illegible]	[illegible]	[illegible]	[illegible]	[illegible]	[illegible]	[illegible]	[illegible]	[illegible]	[illegible]	[illegible]	[illegible]	[illegible]	[illegible]	[illegible]	[illegible]	[illegible]	[illegible]	[illegible]	[illegible]
Down syndrome	108 (44·4 to 232)	[illegible]	[illegible]	[illegible]	[illegible]	[illegible]	[illegible]	[illegible]	[illegible]	[illegible]	[illegible]	[illegible]	[illegible]	[illegible]	[illegible]	[illegible]	[illegible]	[illegible]	[illegible]	[illegible]	[illegible]	[illegible]	[illegible]	[illegible]
Turner syndrome	0 (0 to 0)	[illegible]	[illegible]	[illegible]	[illegible]	[illegible]	[illegible]	[illegible]	[illegible]	[illegible]	[illegible]	[illegible]	[illegible]	[illegible]	[illegible]	[illegible]	[illegible]	[illegible]	[illegible]	[illegible]	[illegible]	[illegible]	[illegible]	[illegible]
Klinefelter syndrome	0 (0 to 0)	[illegible]	[illegible]	[illegible]	[illegible]	[illegible]	[illegible]	[illegible]	[illegible]	[illegible]	[illegible]	[illegible]	[illegible]	[illegible]	[illegible]	[illegible]	[illegible]	[illegible]	[illegible]	[illegible]	[illegible]	[illegible]	[illegible]	[illegible]
Other chromosomal abnormalities	3,530 (1,230 to 6,750)	[illegible]	[illegible]	[illegible]	[illegible]	[illegible]	[illegible]	[illegible]	[illegible]	[illegible]	[illegible]	[illegible]	[illegible]	[illegible]	[illegible]	[illegible]	[illegible]	[illegible]	[illegible]	[illegible]	[illegible]	[illegible]	[illegible]	[illegible]
Congenital musculoskeletal and limb anomalies	1,720 (752 to 3,180)	[illegible]	[illegible]	[illegible]	[illegible]	[illegible]	[illegible]	[illegible]	[illegible]	[illegible]	[illegible]	[illegible]	[illegible]	[illegible]	[illegible]	[illegible]	[illegible]	[illegible]	[illegible]	[illegible]	[illegible]	[illegible]	[illegible]	[illegible]
Urogenital congenital anomalies	538 (91·6 to 1,150)	[illegible]	[illegible]	[illegible]	[illegible]	[illegible]	[illegible]	[illegible]	[illegible]	[illegible]	[illegible]	[illegible]	[illegible]	[illegible]	[illegible]	[illegible]	[illegible]	[illegible]	[illegible]	[illegible]	[illegible]	[illegible]	[illegible]	[illegible]
Digestive congenital anomalies	1,230 (457 to 2,610)	[illegible]	[illegible]	[illegible]	[illegible]	[illegible]	[illegible]	[illegible]	[illegible]	[illegible]	[illegible]	[illegible]	[illegible]	[illegible]	[illegible]	[illegible]	[illegible]	[illegible]	[illegible]	[illegible]	[illegible]	[illegible]	[illegible]	[illegible]
Other congenital birth defects	6,110 (2,770 to 10,500)	[illegible]	[illegible]	[illegible]	[illegible]	[illegible]	[illegible]	[illegible]	[illegible]	[illegible]	[illegible]	[illegible]	[illegible]	[illegible]	[illegible]	[illegible]	[illegible]	[illegible]	[illegible]	[illegible]	[illegible]	[illegible]	[illegible]	[illegible]
Urinary diseases and male infertility	0·8 (0·3 to 1·9)	[illegible]	[illegible]	[illegible]	[illegible]	[illegible]	[illegible]	[illegible]	[illegible]	[illegible]	[illegible]	[illegible]	[illegible]	[illegible]	[illegible]	[illegible]	[illegible]	[illegible]	[illegible]	[illegible]	[illegible]	[illegible]	[illegible]	[illegible]
Urolithiasis	0 (0 to 0)	[illegible]	[illegible]	[illegible]	[illegible]	[illegible]	[illegible]	[illegible]	[illegible]	[illegible]	[illegible]	[illegible]	[illegible]	[illegible]	[illegible]	[illegible]	[illegible]	[illegible]	[illegible]	[illegible]	[illegible]	[illegible]	[illegible]	[illegible]
Benign prostatic hyperplasia	0 (0 to 0)	[illegible]	[illegible]	[illegible]	[illegible]	[illegible]	[illegible]	[illegible]	[illegible]	[illegible]	[illegible]	[illegible]	[illegible]	[illegible]	[illegible]	[illegible]	[illegible]	[illegible]	[illegible]	[illegible]	[illegible]	[illegible]	[illegible]	[illegible]
Male infertility	0 (0 to 0)	[illegible]	[illegible]	[illegible]	[illegible]	[illegible]	[illegible]	[illegible]	[illegible]	[illegible]	[illegible]	[illegible]	[illegible]	[illegible]	[illegible]	[illegible]	[illegible]	[illegible]	[illegible]	[illegible]	[illegible]	[illegible]	[illegible]	[illegible]
Other urinary diseases	0·4 (0·2 to 1·1)	[illegible]	[illegible]	[illegible]	[illegible]	[illegible]	[illegible]	[illegible]	[illegible]	[illegible]	[illegible]	[illegible]	[illegible]	[illegible]	[illegible]	[illegible]	[illegible]	[illegible]	[illegible]	[illegible]	[illegible]	[illegible]	[illegible]	[illegible]
Gynecological diseases	0 (0 to 0)	[illegible]	[illegible]	[illegible]	[illegible]	[illegible]	[illegible]	[illegible]	[illegible]	[illegible]	[illegible]	[illegible]	[illegible]	[illegible]	[illegible]	[illegible]	[illegible]	[illegible]	[illegible]	[illegible]	[illegible]	[illegible]	[illegible]	[illegible]
Uterine fibroids	0 (0 to 0)	[illegible]	[illegible]	[illegible]	[illegible]	[illegible]	[illegible]	[illegible]	[illegible]	[illegible]	[illegible]	[illegible]	[illegible]	[illegible]	[illegible]	[illegible]	[illegible]	[illegible]	[illegible]	[illegible]	[illegible]	[illegible]	[illegible]	[illegible]
Polycystic ovarian syndrome	--	[illegible]	[illegible]	[illegible]	[illegible]	[illegible]	[illegible]	[illegible]	[illegible]	[illegible]	[illegible]	[illegible]	[illegible]	[illegible]	[illegible]	[illegible]	[illegible]	[illegible]	[illegible]	[illegible]	[illegible]	[illegible]	[illegible]	[illegible]
Female infertility	--	[illegible]	[illegible]	[illegible]	[illegible]	[illegible]	[illegible]	[illegible]	[illegible]	[illegible]	[illegible]	[illegible]	[illegible]	[illegible]	[illegible]	[illegible]	[illegible]	[illegible]	[illegible]	[illegible]	[illegible]	[illegible]	[illegible]	[illegible]
Endometriosis	--	[illegible]	[illegible]	[illegible]	[illegible]	[illegible]	[illegible]	[illegible]	[illegible]	[illegible]	[illegible]	[illegible]	[illegible]	[illegible]	[illegible]	[illegible]	[illegible]	[illegible]	[illegible]	[illegible]	[illegible]	[illegible]	[illegible]	[illegible]
Genital prolapse	0 (0 to 0)	[illegible]	[illegible]	[illegible]	[illegible]	[illegible]	[illegible]	[illegible]	[illegible]	[illegible]	[illegible]	[illegible]	[illegible]	[illegible]	[illegible]	[illegible]	[illegible]	[illegible]	[illegible]	[illegible]	[illegible]	[illegible]	[illegible]	[illegible]
Premenstrual syndrome	--	[illegible]	[illegible]	[illegible]	[illegible]	[illegible]	[illegible]	[illegible]	[illegible]	[illegible]	[illegible]	[illegible]	[illegible]	[illegible]	[illegible]	[illegible]	[illegible]	[illegible]	[illegible]	[illegible]	[illegible]	[illegible]	[illegible]	[illegible]
Other gynecological diseases	0 (0 to 0)	[illegible]	[illegible]	[illegible]	[illegible]	[illegible]	[illegible]	[illegible]	[illegible]	[illegible]	[illegible]	[illegible]	[illegible]	[illegible]	[illegible]	[illegible]	[illegible]	[illegible]	[illegible]	[illegible]	[illegible]	[illegible]	[illegible]	[illegible]
Hemoglobinopathies and hemolytic anemias	15·6 (8·4 to 31·9)	[illegible]	[illegible]	[illegible]	[illegible]	[illegible]	[illegible]	[illegible]	[illegible]	[illegible]	[illegible]	[illegible]	[illegible]	[illegible]	[illegible]	[illegible]	[illegible]	[illegible]	[illegible]	[illegible]	[illegible]	[illegible]	[illegible]	[illegible]
Thalassemias	11·1 (5·4 to 25·4)	[illegible]	[illegible]	[illegible]	[illegible]	[illegible]	[illegible]	[illegible]	[illegible]	[illegible]	[illegible]	[illegible]	[illegible]	[illegible]	[illegible]	[illegible]	[illegible]	[illegible]	[illegible]	[illegible]	[illegible]	[illegible]	[illegible]	[illegible]
Thalassemias trait	2·3 (1·1 to 5·7)	[illegible]	[illegible]	[illegible]	[illegible]	[illegible]	[illegible]	[illegible]	[illegible]	[illegible]	[illegible]	[illegible]	[illegible]	[illegible]	[illegible]	[illegible]	[illegible]	[illegible]	[illegible]	[illegible]	[illegible]	[illegible]	[illegible]	[illegible]
Sickle cell disorders	0·2 (0 to 0·4)	[illegible]	[illegible]	[illegible]	[illegible]	[illegible]	[illegible]	[illegible]	[illegible]	[illegible]	[illegible]	[illegible]	[illegible]	[illegible]	[illegible]	[illegible]	[illegible]	[illegible]	[illegible]	[illegible]	[illegible]	[illegible]	[illegible]	[illegible]
Sickle cell trait	0·3 (0·1 to 0·6)	[illegible]	[illegible]	[illegible]	[illegible]	[illegible]	[illegible]	[illegible]	[illegible]	[illegible]	[illegible]	[illegible]	[illegible]	[illegible]	[illegible]	[illegible]	[illegible]	[illegible]	[illegible]	[illegible]	[illegible]	[illegible]	[illegible]	[illegible]
G6PD deficiency	0·6 (0·2 to 1·5)	[illegible]	[illegible]	[illegible]	[illegible]	[illegible]	[illegible]	[illegible]	[illegible]	[illegible]	[illegible]	[illegible]	[illegible]	[illegible]	[illegible]	[illegible]	[illegible]	[illegible]	[illegible]	[illegible]	[illegible]	[illegible]	[illegible]	[illegible]
G6PD trait	0 (0 to 0)	[illegible]	[illegible]	[illegible]	[illegible]	[illegible]	[illegible]	[illegible]	[illegible]	[illegible]	[illegible]	[illegible]	[illegible]	[illegible]	[illegible]	[illegible]	[illegible]	[illegible]	[illegible]	[illegible]	[illegible]	[illegible]	[illegible]	[illegible]
Other hemoglobinopathies and hemolytic anemias	1·1 (0·5 to 2·3)	[illegible]	[illegible]	[illegible]	[illegible]	[illegible]	[illegible]	[illegible]	[illegible]	[illegible]	[illegible]	[illegible]	[illegible]	[illegible]	[illegible]	[illegible]	[illegible]	[illegible]	[illegible]	[illegible]	[illegible]	[illegible]	[illegible]	[illegible]
Endocrine, metabolic, blood, and immune disorders	95·6 (45·4 to 172)	[illegible]	[illegible]	[illegible]	[illegible]	[illegible]	[illegible]	[illegible]	[illegible]	[illegible]	[illegible]	[illegible]	[illegible]	[illegible]	[illegible]	[illegible]	[illegible]	[illegible]	[illegible]	[illegible]	[illegible]	[illegible]	[illegible]	[illegible]
Oral disorders	0 (0 to 0)	[illegible]	[illegible]	[illegible]	[illegible]	[illegible]	[illegible]	[illegible]	[illegible]	[illegible]	[illegible]	[illegible]	[illegible]	[illegible]	[illegible]	[illegible]	[illegible]	[illegible]	[illegible]	[illegible]	[illegible]	[illegible]	[illegible]	[illegible]
Caries of deciduous teeth	0 (0 to 0)	[illegible]	[illegible]	[illegible]	[illegible]	[illegible]	[illegible]	[illegible]	[illegible]	[illegible]	[illegible]	[illegible]	[illegible]	[illegible]	[illegible]	[illegible]	[illegible]	[illegible]	[illegible]	[illegible]	[illegible]	[illegible]	[illegible]	[illegible]
Caries of permanent teeth	0 (0 to 0)	[illegible]	[illegible]	[illegible]	[illegible]	[illegible]	[illegible]	[illegible]	[illegible]	[illegible]	[illegible]	[illegible]	[illegible]	[illegible]	[illegible]	[illegible]	[illegible]	[illegible]	[illegible]	[illegible]	[illegible]	[illegible]	[illegible]	[illegible]
Periodontal diseases	0 (0 to 0)	[illegible]	[illegible]	[illegible]	[illegible]	[illegible]	[illegible]	[illegible]	[illegible]	[illegible]	[illegible]	[illegible]	[illegible]	[illegible]	[illegible]	[illegible]	[illegible]	[illegible]	[illegible]	[illegible]	[illegible]	[illegible]	[illegible]	[illegible]
Other oral disorders	0 (0 to 0)	[illegible]	[illegible]	[illegible]	[illegible]	[illegible]	[illegible]	[illegible]	[illegible]	[illegible]	[illegible]	[illegible]	[illegible]	[illegible]	[illegible]	[illegible]	[illegible]	[illegible]	[illegible]	[illegible]	[illegible]	[illegible]	[illegible]	[illegible]
Sudden infant death syndrome	--	[illegible]	[illegible]	[illegible]	[illegible]	[illegible]	[illegible]	[illegible]	[illegible]	[illegible]	[illegible]	[illegible]	[illegible]	[illegible]	[illegible]	[illegible]	[illegible]	[illegible]	[illegible]	[illegible]	[illegible]	[illegible]	[illegible]	[illegible]
Injuries	296 (164 to 493)	[illegible]	[illegible]	[illegible]	[illegible]	[illegible]	[illegible]	[illegible]	[illegible]	[illegible]	[illegible]	[illegible]	[illegible]	[illegible]	[illegible]	[illegible]	[illegible]	[illegible]	[illegible]	[illegible]	[illegible]	[illegible]	[illegible]	[illegible]
Transport injuries	31·4 (8·3 to 82·1)	[illegible]	[illegible]	[illegible]	[illegible]	[illegible]	[illegible]	[illegible]	[illegible]	[illegible]	[illegible]	[illegible]	[illegible]	[illegible]	[illegible]	[illegible]	[illegible]	[illegible]	[illegible]	[illegible]	[illegible]	[illegible]	[illegible]	[illegible]
Road injuries	28·2 (7·2 to 74·1)	[illegible]	[illegible]	[illegible]	[illegible]	[illegible]	[illegible]	[illegible]	[illegible]	[illegible]	[illegible]	[illegible]	[illegible]	[illegible]	[illegible]	[illegible]	[illegible]	[illegible]	[illegible]	[illegible]	[illegible]	[illegible]	[illegible]	[illegible]
Pedestrian road injuries	12·6 (3 to 32)	[illegible]	[illegible]	[illegible]	[illegible]	[illegible]	[illegible]	[illegible]	[illegible]	[illegible]	[illegible]	[illegible]	[illegible]	[illegible]	[illegible]	[illegible]	[illegible]	[illegible]	[illegible]	[illegible]	[illegible]	[illegible]	[illegible]	[illegible]
Cyclist road injuries	0 (0 to 0)	[illegible]	[illegible]	[illegible]	[illegible]	[illegible]	[illegible]	[illegible]	[illegible]	[illegible]	[illegible]	[illegible]	[illegible]	[illegible]	[illegible]	[illegible]	[illegible]	[illegible]	[illegible]	[illegible]	[illegible]	[illegible]	[illegible]	[illegible]
Motorcyclist road injuries	3·6 (0·7 to 9·7)	[illegible]	[illegible]	[illegible]	[illegible]	[illegible]	[illegible]	[illegible]	[illegible]	[illegible]	[illegible]	[illegible]	[illegible]	[illegible]	[illegible]	[illegible]	[illegible]	[illegible]	[illegible]	[illegible]	[illegible]	[illegible]	[illegible]	[illegible]
Motor vehicle road injuries	11·6 (2·3 to 37·3)	[illegible]	[illegible]	[illegible]	[illegible]	[illegible]	[illegible]	[illegible]	[illegible]	[illegible]	[illegible]	[illegible]	[illegible]	[illegible]	[illegible]	[illegible]	[illegible]	[illegible]	[illegible]	[illegible]	[illegible]	[illegible]	[illegible]	[illegible]
Other road injuries	0·4 (0·1 to 1·4)	[illegible]	[illegible]	[illegible]	[illegible]	[illegible]	[illegible]	[illegible]	[illegible]	[illegible]	[illegible]	[illegible]	[illegible]	[illegible]	[illegible]	[illegible]	[illegible]	[illegible]	[illegible]	[illegible]	[illegible]	[illegible]	[illegible]	[illegible]

Other transport injuries	3·2 (0·8 to 10·1)	3·1 (1·2 to 6·9)	45 (21 to 90·1)	111 (57·1 to 208)	356 (180 to 615)	745 (440 to 1,250)	4,350 (2,420 to 7,490)	6,000 (3,520 to 9,330)
Unintentional injuries	237 (124 to 409)	305 (178 to 503)	2,550 (1,440 to 4,080)	2,430 (1,310 to 4,250)	5,820 (4,340 to 7,600)	7,870 (5,950 to 10,200)	14,100 (10,300 to 19,000)	17,400 (13,200 to 22,200)
Falls	12·3 (3·6 to 36·2)	10·7 (3·3 to 28·3)	126 (58·2 to 246)	303 (136 to 761)	767 (529 to 1,090)	1,200 (854 to 1,600)	3,000 (2,040 to 4,250)	4,250 (3,100 to 5,790)
Drowning	15·1 (6·3 to 31·8)	5·7 (2·2 to 12·2)	185 (71·7 to 389)	995 (353 to 2,170)	3,000 (1,600 to 4,630)	3,850 (2,260 to 5,770)	5,400 (2,970 to 8,920)	4,760 (2,800 to 7,280)
Fire, heat, and hot substances	4·5 (2 to 9·1)	2·3 (1·1 to 4·2)	99·1 (48·1 to 178)	161 (71·1 to 351)	296 (190 to 458)	407 (264 to 629)	690 (419 to 1,070)	1,020 (617 to 1,600)
Poisonings	5·2 (1·4 to 12·8)	2·6 (0·2 to 11·5)	14·2 (3·4 to 54·5)	45·8 (14·8 to 138)	68·6 (31·3 to 157)	107 (52·8 to 230)	468 (223 to 879)	857 (415 to 1,610)
Poisoning by carbon monoxide	0·3 (0 to 1·1)	0·9 (0·1 to 3·9)	3 (0·6 to 10·8)	17·7 (4·8 to 52·3)	19·9 (8·1 to 49)	18·9 (8·1 to 41·1)	68·3 (20·5 to 196)	106 (34·2 to 240)
Poisoning by other means	5 (1·4 to 12·3)	1·8 (0·1 to 8·8)	11·2 (2·5 to 46·5)	28·1 (7·4 to 111)	48·6 (19·5 to 130)	88·4 (42·6 to 200)	400 (190 to 772)	751 (363 to 1,400)
Exposure to mechanical forces	9·6 (1·4 to 43·9)	12·8 (1·3 to 62·2)	73·5 (24·8 to 230)	188 (112 to 302)	383 (251 to 551)	526 (360 to 737)	1,050 (677 to 1,550)	1,550 (1,010 to 2,270)
Unintentional firearm injuries	0·4 (0·1 to 1·9)	2·5 (0·1 to 10·2)	10·1 (2·4 to 40·6)	26·8 (14 to 52)	33 (21 to 49)	41·3 (27·1 to 60·8)	66·9 (40·8 to 122)	142 (76·6 to 259)
Other exposure to mechanical forces	9·2 (1·3 to 40·4)	10·3 (1·1 to 56)	63·4 (21·5 to 214)	161 (94·6 to 261)	350 (224 to 511)	485 (325 to 684)	979 (632 to 1,460)	1,410 (900 to 2,080)
Adverse effects of medical treatment	16·4 (5 to 46·4)	17·6 (7·1 to 38·1)	143 (73·9 to 243)	78·4 (40·4 to 168)	106 (61·2 to 159)	107 (62 to 163)	167 (84·6 to 281)	273 (154 to 449)
Animal contact	1·3 (0·6 to 2·6)	1·1 (0·5 to 2·9)	19·2 (10·2 to 36·7)	50·2 (25·2 to 99)	162 (91·9 to 271)	172 (92·7 to 295)	230 (131 to 393)	272 (157 to 445)
Venomous animal contact	1·1 (0·5 to 2·2)	0·6 (0·2 to 1·8)	14·1 (6·5 to 29·1)	33·6 (13·7 to 75·3)	129 (67·9 to 227)	135 (64·6 to 250)	191 (98·7 to 334)	227 (120 to 389)
Non-venomous animal contact	0·2 (0·1 to 0·5)	0·5 (0·2 to 1·1)	5·1 (2·7 to 9·4)	16·6 (9·2 to 29·6)	32·1 (18·4 to 56·3)	37·2 (20·3 to 62·3)	39·2 (23·9 to 61·4)	44·7 (27·1 to 67·3)
Foreign body	165 (75·8 to 314)	247 (139 to 416)	1,800 (860 to 3,220)	337 (150 to 650)	466 (284 to 741)	665 (413 to 1,000)	611 (400 to 962)	989 (624 to 1,550)
Pulmonary aspiration and foreign body in airway	165 (75·5 to 313)	246 (138 to 415)	1,790 (853 to 3,200)	294 (115 to 600)	404 (234 to 679)	589 (344 to 922)	510 (305 to 854)	849 (492 to 1,420)
Foreign body in eyes	0·1 (0 to 0·1)	0·2 (0 to 0·4)	3·2 (0·8 to 6·9)	15·4 (4·4 to 35·5)	24 (6·6 to 56·5)	30·9 (9·3 to 72·8)	46·4 (13·8 to 110)	70·6 (21 to 160)
Foreign body in other body part	0·3 (0·1 to 0·6)	0·5 (0·3 to 0·8)	6·9 (3·7 to 12·7)	27·5 (15·9 to 45·5)	38·3 (25·1 to 55·6)	45·1 (29·9 to 66·7)	54·3 (37·4 to 77·6)	68·8 (46·8 to 98)
Environmental heat and cold exposure	0·3 (0·1 to 0·6)	0·4 (0·2 to 0·7)	5·7 (3·3 to 10·8)	20·7 (13·7 to 29·5)	43·6 (29·5 to 62·1)	53·7 (36·7 to 75·2)	85·4 (58·6 to 118)	117 (81·7 to 161)
Exposure to forces of nature	0 (0 to 0)	0·1 (0·1 to 0·1)	0·5 (0·4 to 0·5)	2·4 (2·2 to 2·6)	3·3 (2·8 to 3·9)	5·3 (4·4 to 6·6)	10 (7·8 to 13·1)	14·9 (11·4 to 19·9)
Other unintentional injuries	6·6 (2·3 to 25·1)	4·5 (2·4 to 8)	89·7 (50·7 to 146)	251 (148 to 395)	524 (322 to 829)	777 (470 to 1,280)	2,360 (1,200 to 4,100)	3,270 (1,760 to 5,230)
Self-harm and interpersonal violence	27·6 (10·8 to 66·3)	7·8 (3·3 to 17·2)	79·4 (36·4 to 174)	158 (70·8 to 348)	285 (201 to 423)	845 (577 to 1,290)	8,410 (5,230 to 14,300)	14,800 (9,770 to 23,200)
Self-harm	0 (0 to 0)	0 (0 to 0)	0 (0 to 0)	0 (0 to 0)	0 (0 to 0)	243 (111 to 623)	5,010 (2,740 to 10,400)	8,770 (5,100 to 15,600)
Self-harm by firearm	0 (0 to 0)	0 (0 to 0)	0 (0 to 0)	0 (0 to 0)	0 (0 to 0)	10·2 (3·7 to 28·1)	157 (50·8 to 437)	296 (129 to 629)
Self-harm by other specified means	0 (0 to 0)	0 (0 to 0)	0 (0 to 0)	0 (0 to 0)	0 (0 to 0)	233 (106 to 602)	4,850 (2,640 to 9,970)	8,470 (4,920 to 15,100)
Interpersonal violence	27·5 (10·8 to 66·3)	7·7 (3·2 to 17·2)	77·3 (34·6 to 172)	152 (65·9 to 337)	273 (189 to 410)	590 (399 to 862)	3,390 (1,870 to 5,880)	5,960 (3,510 to 9,900)
Physical violence by firearm	0·5 (0·1 to 1·2)	0·3 (0·1 to 0·9)	2 (0·8 to 4·9)	6·5 (2·8 to 15·1)	9·6 (5·9 to 15·5)	23·5 (10·9 to 50·7)	708 (257 to 1,510)	1,390 (562 to 2,830)
Physical violence by sharp object	5·6 (1·4 to 14)	0·6 (0·3 to 1·5)	5·9 (3·2 to 10·4)	22·5 (12·6 to 39·6)	36·2 (22·8 to 55·1)	65·7 (39·2 to 105)	889 (375 to 1,880)	1,600 (743 to 3,240)
Sexual violence	0 (0 to 0)	0 (0 to 0)	0 (0 to 0)	6·9 (3·9 to 11·4)	53·6 (31·5 to 85·6)	112 (61·9 to 176)	245 (137 to 392)	362 (211 to 557)
Physical violence by other means	21·5 (8·2 to 53)	6·8 (2·8 to 15·5)	69·5 (28·6 to 157)	116 (40·5 to 280)	174 (106 to 298)	389 (236 to 620)	1,540 (749 to 2,910)	2,620 (1,460 to 4,470)
Conflict and terrorism	0 (0 to 0)	0 (0 to 0)	0·1 (0·1 to 0·1)	0·7 (0·6 to 0·8)	2·2 (1·5 to 3·4)	2·4 (1·5 to 4·1)	4·6 (3·2 to 6·8)	9·5 (6·8 to 13·8)

Other transport injuries	4,800 (2,880 to 7,450)	4,410 (2,440 to 7,440)	2,940 (1,730 to 4,920)	2,190 (1,220 to 3,690)	1,900 (1,090 to 3,170)	1,890 (1,130 to 3,060)	1,670 (1,000 to 2,680)	1,590 (900 to 2,570)
Unintentional injuries	17,700 (13,900 to 21,700)	17,600 (13,900 to 21,800)	14,700 (11,700 to 18,300)	12,100 (9,560 to 15,000)	10,800 (8,400 to 13,700)	10,500 (8,080 to 13,200)	10,500 (8,220 to 13,200)	10,200 (7,900 to 13,000)
Falls	4,670 (3,490 to 6,170)	4,850 (3,670 to 6,270)	4,530 (3,330 to 6,000)	4,140 (3,030 to 5,530)	3,800 (2,870 to 5,030)	4,410 (3,250 to 5,760)	4,670 (3,480 to 6,080)	5,080 (3,570 to 6,910)
Drowning	3,910 (2,450 to 5,850)	3,380 (2,160 to 5,070)	2,460 (1,540 to 3,700)	1,720 (1,120 to 2,620)	1,410 (873 to 2,200)	1,100 (664 to 1,680)	902 (538 to 1,420)	730 (472 to 1,080)
Fire, heat, and hot substances	1,140 (737 to 1,730)	1,250 (841 to 1,760)	1,050 (731 to 1,460)	889 (632 to 1,220)	758 (537 to 1,060)	742 (517 to 1,030)	733 (525 to 1,040)	719 (472 to 1,050)
Poisonings	1,080 (619 to 1,840)	1,130 (592 to 1,960)	858 (480 to 1,490)	667 (381 to 1,110)	491 (263 to 852)	403 (225 to 659)	346 (181 to 611)	322 (166 to 562)
Poisoning by carbon monoxide	112 (42 to 238)	182 (62·6 to 407)	134 (48·7 to 286)	137 (50·4 to 298)	85·2 (30·8 to 195)	57·8 (21 to 125)	46·2 (16·2 to 100)	31·8 (11 to 74·8)
Poisoning by other means	965 (541 to 1,650)	950 (474 to 1,630)	723 (397 to 1,280)	530 (301 to 876)	406 (216 to 715)	345 (193 to 593)	300 (156 to 525)	290 (147 to 517)
Exposure to mechanical forces	1,730 (1,180 to 2,410)	1,870 (1,280 to 2,610)	1,670 (1,190 to 2,340)	1,440 (1,050 to 1,940)	1,410 (995 to 1,990)	1,270 (923 to 1,740)	1,250 (875 to 1,770)	1,030 (728 to 1,410)
Unintentional firearm injuries	156 (94 to 272)	192 (116 to 328)	212 (121 to 370)	151 (98·9 to 228)	145 (92·2 to 232)	127 (84·9 to 193)	109 (73·3 to 163)	78·7 (56·2 to 107)
Other exposure to mechanical forces	1,570 (1,060 to 2,250)	1,680 (1,160 to 2,330)	1,460 (1,020 to 2,070)	1,280 (925 to 1,750)	1,270 (881 to 1,790)	1,150 (819 to 1,590)	1,140 (791 to 1,640)	950 (668 to 1,330)
Adverse effects of medical treatment	283 (160 to 448)	289 (159 to 469)	309 (170 to 514)	284 (161 to 467)	315 (182 to 523)	258 (156 to 403)	347 (197 to 548)	355 (209 to 568)
Animal contact	284 (176 to 459)	234 (146 to 374)	231 (141 to 365)	185 (110 to 302)	177 (105 to 304)	221 (136 to 350)	232 (132 to 380)	235 (130 to 390)
Venomous animal contact	221 (129 to 377)	163 (90·8 to 280)	162 (88·6 to 274)	135 (70·6 to 247)	124 (64·2 to 239)	168 (92·5 to 288)	185 (97·8 to 327)	190 (99·1 to 334)
Non-venomous animal contact	62·4 (36·5 to 106)	71 (40·3 to 115)	68·5 (39·4 to 110)	49·3 (29·6 to 80)	53·4 (31·3 to 87)	53·5 (33·1 to 82)	47·4 (28·7 to 76·5)	44·5 (27·2 to 72·7)
Foreign body	1,150 (790 to 1,700)	1,270 (870 to 1,750)	964 (651 to 1,380)	745 (511 to 1,050)	645 (434 to 957)	641 (445 to 922)	692 (467 to 992)	642 (428 to 956)
Pulmonary aspiration and foreign body in airway	980 (640 to 1,480)	1,080 (692 to 1,550)	786 (489 to 1,180)	591 (368 to 907)	510 (311 to 824)	515 (317 to 790)	578 (365 to 861)	538 (323 to 850)
Foreign body in eyes	91·1 (30·3 to 194)	98·7 (33·9 to 209)	85 (31·1 to 181)	68·7 (28·2 to 137)	55·1 (25·5 to 103)	45 (23 to 80·8)	36 (20·4 to 60·6)	29 (17·1 to 46)
Foreign body in other body part	82·3 (58·4 to 115)	95·4 (66·5 to 132)	92·8 (65·7 to 126)	84·4 (59·9 to 116)	79·5 (55·5 to 110)	80·5 (57·4 to 111)	78·3 (56 to 108)	74·4 (52·7 to 101)
Environmental heat and cold exposure	129 (89·7 to 179)	142 (98·8 to 199)	118 (82·7 to 162)	101 (70·8 to 140)	89·8 (62·7 to 124)	83·1 (58 to 115)	74·1 (51·9 to 102)	67·2 (46 to 96·4)
Exposure to forces of nature	27·3 (19·7 to 38·2)	31·5 (22·5 to 44·9)	35·3 (24·8 to 51·3)	24·2 (16·9 to 34·1)	20·2 (14·3 to 27·8)	18 (12·4 to 25·1)	14·6 (10·2 to 20·5)	11·2 (8·1 to 15·2)
Other unintentional injuries	3,240 (1,890 to 4,850)	3,170 (1,920 to 5,060)	2,490 (1,560 to 3,970)	1,900 (1,240 to 2,760)	1,650 (1,070 to 2,470)	1,360 (907 to 1,990)	1,290 (809 to 1,960)	996 (644 to 1,500)
Self-harm and interpersonal violence	16,300 (11,200 to 24,200)	16,000 (11,100 to 23,700)	11,400 (8,070 to 16,000)	8,520 (6,140 to 11,400)	6,360 (4,460 to 8,940)	5,350 (3,720 to 7,460)	4,750 (3,360 to 6,360)	3,660 (2,560 to 5,120)
Self-harm	10,300 (6,550 to 16,700)	10,800 (6,670 to 17,000)	7,530 (4,990 to 11,300)	5,690 (3,750 to 8,160)	4,320 (2,750 to 6,530)	3,750 (2,360 to 5,540)	3,390 (2,130 to 4,900)	2,730 (1,640 to 4,090)
Self-harm by firearm	337 (136 to 646)	311 (116 to 615)	230 (86·3 to 413)	188 (68·4 to 346)	182 (68·8 to 332)	103 (33·1 to 200)	108 (37·9 to 198)	45·3 (19·6 to 84·2)
Self-harm by other specified means	9,940 (6,290 to 16,100)	10,400 (6,440 to 16,700)	7,300 (4,830 to 11,100)	5,500 (3,610 to 7,900)	4,130 (2,650 to 6,240)	3,640 (2,290 to 5,410)	3,290 (2,040 to 4,770)	2,690 (1,630 to 4,030)
Interpersonal violence	5,900 (3,610 to 9,550)	5,110 (3,190 to 8,250)	3,730 (2,350 to 5,880)	2,760 (1,860 to 4,110)	2,000 (1,370 to 2,980)	1,580 (1,090 to 2,220)	1,330 (914 to 1,920)	921 (663 to 1,250)
Physical violence by firearm	1,190 (526 to 2,390)	1,030 (440 to 1,990)	732 (335 to 1,390)	543 (250 to 1,080)	308 (137 to 635)	217 (101 to 414)	175 (79·4 to 339)	95·3 (44 to 179)
Physical violence by sharp object	1,660 (825 to 3,250)	1,300 (610 to 2,660)	863 (451 to 1,700)	603 (332 to 1,130)	454 (276 to 806)	300 (187 to 508)	260 (161 to 442)	174 (113 to 272)
Sexual violence	372 (206 to 608)	338 (185 to 550)	265 (152 to 424)	195 (113 to 316)	139 (81·3 to 233)	97·1 (55·2 to 159)	61 (35·2 to 98)	42·9 (26·4 to 64·9)
Physical violence by other means	2,670 (1,520 to 4,670)	2,440 (1,430 to 4,070)	1,870 (1,140 to 3,120)	1,420 (927 to 2,170)	1,100 (737 to 1,670)	963 (660 to 1,350)	836 (573 to 1,180)	608 (430 to 837)
Conflict and terrorism	11·7 (8·1 to 18·3)	11·1 (7·6 to 16·8)	10·5 (6·8 to 17·4)	11·1 (5·3 to 29·3)	7·7 (3·8 to 18·9)	9·2 (4·1 to 25·9)	10·6 (3·9 to 34·6)	9·1 (2·9 to 33·7)

Other transport injuries	1,250 (708 to 2,090)	997 (572 to 1,620)	577 (323 to 955)	293 (177 to 472)	104 (64·8 to 166)	47·2 (29 to 74·2)	12·4 (7 to 20·3)	36,300
Unintentional injuries	8,590 (6,590 to 11,200)	7,380 (5,480 to 9,670)	4,610 (3,420 to 6,050)	2,890 (2,140 to 3,810)	1,400 (1,030 to 1,830)	694 (507 to 907)	165 (120 to 221)	180,000
Falls	4,460 (3,080 to 6,260)	4,210 (2,710 to 6,010)	2,740 (1,780 to 3,960)	1,810 (1,190 to 2,610)	873 (570 to 1,230)	445 (289 to 634)	105 (69·9 to 155)	60,400
Drowning	555 (335 to 867)	441 (267 to 675)	222 (133 to 336)	99·4 (59·1 to 158)	46·1 (24·8 to 75·4)	15·5 (9·5 to 23·9)	3·9 (2·3 to 6·3)	35,200
Fire, heat, and hot substances	641 (429 to 922)	440 (286 to 655)	275 (164 to 421)	169 (104 to 250)	90·8 (57·1 to 132)	40·4 (26·2 to 59·9)	9·2 (5·7 to 13·4)	11,600
Poisonings	218 (111 to 401)	135 (61·5 to 253)	75·4 (34 to 137)	47·9 (24·1 to 87·5)	25·7 (14·1 to 44·2)	9·3 (4·7 to 17)	2·2 (1·1 to 4·2)	7,380
Poisoning by carbon monoxide	29·7 (8·9 to 70·7)	23 (5·7 to 52·3)	12·5 (3·2 to 31·1)	6·5 (2·1 to 15·3)	4·8 (1·7 to 10·5)	1·2 (0·3 to 2·8)	0·2 (0·1 to 0·6)	1,100
Poisoning by other means	188 (91·7 to 351)	112 (52·6 to 211)	62·9 (29·3 to 117)	41·4 (20·6 to 76·9)	20·9 (11·3 to 36·6)	8·1 (4 to 14·6)	2 (1 to 3·8)	6,280
Exposure to mechanical forces	772 (540 to 1,090)	575 (410 to 796)	310 (222 to 417)	172 (123 to 236)	75·3 (54·4 to 103)	34·4 (24·9 to 46·2)	7·8 (5·6 to 10·5)	17,400
Unintentional firearm injuries	61·6 (43·4 to 86·5)	43·6 (30·5 to 61)	21·2 (15 to 29·2)	12·7 (8·9 to 17·3)	5·7 (3·9 to 7·8)	2·6 (1·8 to 3·6)	0·6 (0·4 to 0·8)	1,640
Other exposure to mechanical forces	711 (492 to 1,010)	532 (376 to 742)	289 (205 to 393)	160 (113 to 220)	69·6 (50·1 to 95·7)	31·7 (22·8 to 42·8)	7·2 (5·2 to 9·8)	15,800
Adverse effects of medical treatment	334 (195 to 539)	266 (150 to 406)	149 (85·4 to 235)	91·7 (53·5 to 143)	40·2 (24·2 to 60·6)	19·1 (11·1 to 29·5)	4·2 (2·5 to 6·6)	4,250
Animal contact	213 (118 to 354)	200 (104 to 333)	105 (55·5 to 174)	64·3 (34·5 to 106)	28·5 (15·8 to 46·7)	11·9 (7 to 18)	3 (1·7 to 4·7)	3,330
Venomous animal contact	175 (90 to 305)	159 (75·6 to 273)	79·4 (37·7 to 140)	49·6 (24·3 to 85·3)	22·6 (11·4 to 38·4)	9·7 (5·3 to 15·3)	2·5 (1·3 to 4·1)	2,580
Non-venomous animal contact	38·6 (22·5 to 64·2)	40·7 (21·3 to 76·9)	25·8 (13·9 to 45·9)	14·7 (8·3 to 26·3)	5·9 (3·5 to 9·8)	2·2 (1·5 to 3·3)	0·5 (0·3 to 0·8)	754
Foreign body	550 (363 to 777)	591 (377 to 891)	433 (282 to 629)	274 (176 to 405)	138 (85·2 to 221)	80·9 (50·2 to 117)	21 (12·9 to 30·7)	14,100
Pulmonary aspiration and foreign body in airway	470 (285 to 697)	530 (315 to 826)	395 (247 to 592)	249 (154 to 380)	126 (72·8 to 208)	74·7 (43·7 to 111)	19·5 (11·4 to 29)	12,300
Foreign body in eyes	21·7 (13·2 to 32·9)	15·8 (10·1 to 23·5)	9·1 (5·9 to 13·3)	5·5 (3·6 to 8·1)	2·3 (1·5 to 3·4)	1 (0·7 to 1·4)	0·2 (0·1 to 0·3)	755
Foreign body in other body part	58·7 (40·8 to 79·5)	45·9 (31·7 to 62·4)	28·2 (20 to 38·9)	19·4 (13·8 to 26·7)	9·8 (7 to 13·6)	5·2 (3·6 to 7·2)	1·3 (0·9 to 1·8)	1,080
Environmental heat and cold exposure	49·7 (34·7 to 68·8)	40·7 (28·4 to 58·3)	24·7 (17·2 to 33·8)	16 (11·4 to 22·7)	8·7 (5·9 to 12·3)	4·2 (2·9 to 6·1)	0·8 (0·6 to 1·2)	1,270
Exposure to forces of nature	8·1 (5·8 to 11·1)	5·8 (4·2 to 8·2)	3·2 (2·3 to 4·4)	1·8 (1·3 to 2·5)	0·8 (0·5 to 1·1)	0·3 (0·2 to 0·4)	0·1 (0 to 0·1)	239
Other unintentional injuries	786 (498 to 1,170)	473 (304 to 720)	277 (182 to 409)	149 (102 to 211)	70 (47·4 to 98·9)	33·5 (22·9 to 47)	7·9 (5·3 to 11·2)	25,200
Self-harm and interpersonal violence	2,560 (1,780 to 3,630)	1,890 (1,270 to 2,640)	926 (633 to 1,270)	434 (297 to 594)	184 (124 to 252)	74·1 (48 to 102)	17·9 (11·2 to 24·9)	103,000
Self-harm	1,940 (1,200 to 2,910)	1,490 (894 to 2,220)	725 (429 to 1,070)	345 (209 to 509)	149 (89·8 to 217)	59·2 (33·9 to 86·8)	14·1 (7·7 to 21·4)	67,200
Self-harm by firearm	24·3 (11·6 to 43·5)	16·7 (8·5 to 31·3)	6·8 (3·6 to 12·3)	5·8 (2·7 to 11·8)	1·7 (0·8 to 3·6)	1·1 (0·5 to 2·4)	0·2 (0·1 to 0·5)	2,020
Self-harm by other specified means	1,910 (1,190 to 2,870)	1,470 (881 to 2,190)	718 (425 to 1,050)	339 (206 to 501)	147 (87·9 to 214)	58 (32·8 to 85·2)	13·9 (7·5 to 21·1)	65,200
Interpersonal violence	612 (449 to 836)	393 (282 to 537)	200 (144 to 274)	88·4 (66·5 to 116)	35·6 (26·6 to 46·3)	14·8 (10·8 to 19·8)	3·7 (2·7 to 5·1)	35,200
Physical violence by firearm	45·2 (22·9 to 84·7)	23 (11·6 to 45·6)	11 (5·4 to 22·4)	2·6 (1·8 to 3·8)	1·3 (0·9 to 1·9)	0·7 (0·4 to 0·9)	0·2 (0·1 to 0·5)	6,510
Physical violence by sharp object	119 (79·7 to 180)	69·6 (46·8 to 102)	33·2 (22·6 to 48·5)	16·6 (11·8 to 23·3)	6·8 (4·9 to 9·4)	3·1 (2·2 to 4·2)	0·9 (0·6 to 1·4)	8,490
Sexual violence	30·6 (18·9 to 46·3)	21·3 (13·5 to 31·7)	11·4 (7·4 to 16·8)	6·6 (4·3 to 9·9)	2·3 (1·3 to 3·7)	0·4 (0·3 to 0·7)	0 (0 to 0)	2,360
Physical violence by other means	417 (300 to 581)	279 (196 to 391)	144 (103 to 203)	62·5 (45 to 83·9)	25·2 (18·1 to 34)	10·6 (7·6 to 14·7)	2·6 (1·8 to 3·5)	17,800
Conflict and terrorism	4·9 (1·6 to 17·9)	2·5 (0·8 to 8·2)	1·2 (0·5 to 3·3)	0·5 (0·2 to 1·4)	0·2 (0·1 to 0·5)	0·1 (0 to 0·2)	0 (0 to 0)	110

SR Table 2a: Grenada DALYS by cause and age group for males with 95% uncertainty intervals, 2019

Cause	Early Neonatal	Late Neonatal	Post Neonatal	1 to 4	5 to 9	10 to 14	15 to 19
All causes	508 (347 to 728)	84·9 (58·8 to 120)	256 (184 to 352)	228 (175 to 293)	268 (214 to 349)	299 (239 to 369)	515 (436 to 603)
Communicable, maternal, neonatal, and nutritional diseases	444 (304 to 626)	65·8 (44·8 to 94·9)	131 (92·2 to 185)	85·1 (61·3 to 118)	77·9 (50·1 to 122)	48·8 (36·4 to 69·4)	48·3 (38·9 to 59·4)
HIV/AIDS and sexually transmitted infections	0·1 (0·1 to 0·2)	0 (0 to 0·1)	1·4 (1·3 to 1·6)	1·4 (1·2 to 1·4)	1·3 (1·2 to 1·4)	1·2 (1·1 to 1·3)	1·6 (1·3 to 2·1)
HIV/AIDS	0 (0 to 0)	0 (0 to 0)	1·4 (1·3 to 1·5)	1·3 (1·2 to 1·4)	1·3 (1·2 to 1·4)	1·1 (1 to 1·1)	1·1 (1 to 1·1)
HIV/AIDS - Drug-susceptible Tuberculosis	0 (0 to 0)	0 (0 to 0)	0 (0 to 0)	0 (0 to 0)	0 (0 to 0)	0 (0 to 0)	0 (0 to 0)
HIV/AIDS - Multidrug-resistant Tuberculosis without extensive dru	0 (0 to 0)	0 (0 to 0)	0 (0 to 0)	0 (0 to 0)	0 (0 to 0)	0 (0 to 0)	0 (0 to 0)
HIV/AIDS - Extensively drug-resistant Tuberculosis	0 (0 to 0)	0 (0 to 0)	0 (0 to 0)	0 (0 to 0)	0 (0 to 0)	0 (0 to 0)	0 (0 to 0)
HIV/AIDS resulting in other diseases	0 (0 to 0)	0 (0 to 0)	1·3 (1·2 to 1·4)	1·3 (1·2 to 1·4)	1·3 (1·2 to 1·4)	1 (1 to 1·1)	1 (1 to 1·1)
Sexually transmitted infections excluding HIV	0·1 (0·1 to 0·2)	0 (0 to 0·1)	0·1 (0 to 0·1)	0 (0 to 0)	0 (0 to 0)	0·1 (0 to 0·2)	0·5 (0·2 to 1·1)
Syphilis	0·1 (0·1 to 0·2)	0 (0 to 0·1)	0·1 (0 to 0·1)	0 (0 to 0)	0 (0 to 0)	0 (0 to 0)	0·1 (0 to 0·1)
Chlamydial infection	0 (0 to 0)	0 (0 to 0)	0 (0 to 0)	0 (0 to 0)	0 (0 to 0)	0 (0 to 0)	0 (0 to 0·1)
Gonococcal infection	0 (0 to 0)	0 (0 to 0)	0 (0 to 0)	0 (0 to 0)	0 (0 to 0)	0 (0 to 0·1)	0·2 (0 to 0·4)
Trichomoniasis	0 (0 to 0)	0 (0 to 0)	0 (0 to 0)	0 (0 to 0)	0 (0 to 0)	0 (0 to 0)	0 (0 to 0)
Genital herpes	0 (0 to 0)	0 (0 to 0)	0 (0 to 0)	0 (0 to 0)	0 (0 to 0)	0 (0 to 0)	0·1 (0 to 0·1)
Other sexually transmitted infections	0 (0 to 0)	0 (0 to 0)	0 (0 to 0)	0 (0 to 0)	0 (0 to 0)	0 (0 to 0·1)	0·2 (0·1 to 0·4)
Respiratory infections and tuberculosis	19·1 (11·7 to 30)	7·9 (4·7 to 11·9)	55·6 (34·8 to 84·5)	21·1 (14·3 to 30·2)	12·1 (8·8 to 16·2)	9·6 (7 to 13·3)	13 (9·4 to 17·8)
Tuberculosis	0 (0 to 0)	0 (0 to 0)	0·1 (0·1 to 0·2)	0·2 (0·1 to 0·4)	0·3 (0·1 to 0·6)	0·2 (0·1 to 0·3)	0·4 (0·2 to 0·5)
Latent tuberculosis infection	0 (0 to 0)	0 (0 to 0)	0 (0 to 0)	0 (0 to 0)	0 (0 to 0)	0 (0 to 0)	0 (0 to 0)
Drug-susceptible tuberculosis	0 (0 to 0)	0 (0 to 0)	0·1 (0·1 to 0·2)	0·2 (0·1 to 0·4)	0·3 (0·1 to 0·6)	0·2 (0·1 to 0·3)	0·3 (0·2 to 0·5)
Multidrug-resistant tuberculosis without extensive drug resistanc	0 (0 to 0)	0 (0 to 0)	0 (0 to 0)	0 (0 to 0)	0 (0 to 0)	0 (0 to 0)	0 (0 to 0)
Extensively drug-resistant tuberculosis	0 (0 to 0)	0 (0 to 0)	0 (0 to 0)	0 (0 to 0)	0 (0 to 0)	0 (0 to 0)	0 (0 to 0)
Lower respiratory infections	19·1 (11·7 to 30)	7·8 (4·7 to 11·9)	54·3 (33·5 to 83)	15·7 (9·1 to 24·5)	5·8 (4·1 to 8·1)	4·1 (3 to 5·5)	7·1 (5 to 10)
Upper respiratory infections	0 (0 to 0)	0·1 (0 to 0·1)	1 (0·7 to 1·6)	3·7 (2·2 to 5·8)	4 (2·2 to 6·7)	3·8 (2 to 6·7)	4 (2·1 to 7·2)
Otitis media	0 (0 to 0)	0 (0 to 0)	0·2 (0·1 to 0·3)	1·5 (0·8 to 2·5)	2 (1·1 to 3·4)	1·5 (0·8 to 2·7)	1·5 (0·8 to 2·6)
Enteric infections	0·4 (0·2 to 0·7)	0·8 (0·5 to 1·3)	10·3 (6·3 to 16·4)	9·5 (6·3 to 14)	8·7 (5·1 to 13·8)	8·8 (4·9 to 14·3)	7·4 (4·7 to 10·9)
Diarrheal diseases	0·4 (0·2 to 0·7)	0·8 (0·5 to 1·3)	10·3 (6·3 to 16·3)	9·5 (6·3 to 13·9)	8·7 (5·1 to 13·8)	8·8 (4·9 to 14·3)	7·3 (4·7 to 10·9)
Typhoid and paratyphoid	0 (0 to 0)	0 (0 to 0)	0 (0 to 0)	0 (0 to 0)	0 (0 to 0)	0 (0 to 0)	0 (0 to 0)
Typhoid fever	0 (0 to 0)	0 (0 to 0)	0 (0 to 0)	0 (0 to 0)	0 (0 to 0)	0 (0 to 0)	0 (0 to 0)
Paratyphoid fever	0 (0 to 0)	0 (0 to 0)	0 (0 to 0)	0 (0 to 0)	0 (0 to 0)	0 (0 to 0)	0 (0 to 0)
Invasive Non-typhoidal Salmonella (iNTS)	0 (0 to 0)	0 (0 to 0)	0 (0 to 0)	0 (0 to 0)	0 (0 to 0)	0 (0 to 0)	0 (0 to 0)
Other intestinal infectious diseases	0 (0 to 0)	0 (0 to 0)	0 (0 to 0·1)	0 (0 to 0·1)	0 (0 to 0·1)	0 (0 to 0·1)	0 (0 to 0·1)
Neglected tropical diseases and malaria	0 (0 to 0·1)	0·1 (0·1 to 0·3)	1·1 (0·5 to 1·9)	3·2 (1·5 to 5·9)	4·3 (1·8 to 7·9)	1·8 (0·8 to 3·6)	1·5 (0·6 to 3·2)
Malaria	0 (0 to 0)	0 (0 to 0)	0 (0 to 0)	0 (0 to 0)	0 (0 to 0)	0 (0 to 0)	0 (0 to 0)
Chagas disease	0 (0 to 0)	0 (0 to 0)	0 (0 to 0)	0 (0 to 0)	0 (0 to 0)	0 (0 to 0)	0 (0 to 0)
Leishmaniasis	0 (0 to 0)	0 (0 to 0)	0 (0 to 0)	0 (0 to 0)	0 (0 to 0)	0 (0 to 0)	0 (0 to 0)
Visceral leishmaniasis	0 (0 to 0)	0 (0 to 0)	0 (0 to 0)	0 (0 to 0)	0 (0 to 0)	0 (0 to 0)	0 (0 to 0)
Cutaneous and mucocutaneous leishmaniasis	0 (0 to 0)	0 (0 to 0)	0 (0 to 0)	0 (0 to 0)	0 (0 to 0)	0 (0 to 0)	0 (0 to 0)
African trypanosomiasis	0 (0 to 0)	0 (0 to 0)	0 (0 to 0)	0 (0 to 0)	0 (0 to 0)	0 (0 to 0)	0 (0 to 0)
Schistosomiasis	0 (0 to 0)	0 (0 to 0)	0 (0 to 0)	0 (0 to 0)	0 (0 to 0)	0 (0 to 0)	0 (0 to 0)
Cysticercosis	0 (0 to 0)	0 (0 to 0)	0 (0 to 0)	0 (0 to 0)	0 (0 to 0)	0 (0 to 0·1)	0·2 (0 to 0·5)
Cystic echinococcosis	0 (0 to 0)	0 (0 to 0)	0 (0 to 0)	0 (0 to 0)	0 (0 to 0)	0 (0 to 0)	0 (0 to 0)
Lymphatic filariasis	0 (0 to 0)	0 (0 to 0)	0 (0 to 0)	0 (0 to 0)	0 (0 to 0)	0 (0 to 0)	0 (0 to 0)
Onchocerciasis	0 (0 to 0)	0 (0 to 0)	0 (0 to 0)	0 (0 to 0)	0 (0 to 0)	0 (0 to 0)	0 (0 to 0)
Trachoma	0 (0 to 0)	0 (0 to 0)	0 (0 to 0)	0 (0 to 0)	0 (0 to 0)	0 (0 to 0)	0 (0 to 0)
Dengue	0 (0 to 0)	0 (0 to 0)	0·1 (0 to 0·2)	0·3 (0·1 to 0·7)	0·7 (0·2 to 1·9)	0·7 (0·2 to 1·6)	0·8 (0·2 to 2·4)
Yellow fever	0 (0 to 0)	0 (0 to 0)	0 (0 to 0)	0 (0 to 0)	0 (0 to 0)	0 (0 to 0)	0 (0 to 0)
Rabies	0 (0 to 0)	0 (0 to 0)	0 (0 to 0)	0 (0 to 0)	0 (0 to 0)	0 (0 to 0)	0 (0 to 0)
Intestinal nematode infections	0 (0 to 0)	0 (0 to 0)	0 (0 to 0)	0·1 (0·1 to 0·3)	0·3 (0·1 to 0·5)	0·2 (0·1 to 0·4)	0·2 (0·1 to 0·3)
Ascariasis	0 (0 to 0)	0 (0 to 0)	0 (0 to 0)	0·1 (0 to 0·2)	0·1 (0 to 0·2)	0 (0 to 0·1)	0 (0 to 0·1)
Trichuriasis	0 (0 to 0)	0 (0 to 0)	0 (0 to 0)	0 (0 to 0)	0 (0 to 0)	0 (0 to 0)	0 (0 to 0)
Hookworm disease	0 (0 to 0)	0 (0 to 0)	0 (0 to 0)	0·1 (0 to 0·2)	0·2 (0·1 to 0·4)	0·2 (0·1 to 0·3)	0·1 (0·1 to 0·3)
Food-borne trematodiases	0 (0 to 0)	0 (0 to 0)	0 (0 to 0)	0 (0 to 0)	0 (0 to 0)	0 (0 to 0)	0 (0 to 0)
Leprosy	0 (0 to 0)	0 (0 to 0)	0 (0 to 0)	0 (0 to 0)	0 (0 to 0)	0 (0 to 0)	0 (0 to 0)
Ebola	0 (0 to 0)	0 (0 to 0)	0 (0 to 0)	0 (0 to 0)	0 (0 to 0)	0 (0 to 0)	0 (0 to 0)
Zika virus	0 (0 to 0)	0 (0 to 0)	0·2 (0 to 0·6)	0·1 (0 to 0·5)	0·1 (0 to 0·6)	0·1 (0 to 0·6)	0·2 (0 to 0·9)
Guinea worm disease	0 (0 to 0)	0 (0 to 0)	0 (0 to 0)	0 (0 to 0)	0 (0 to 0)	0 (0 to 0)	0 (0 to 0)
Other neglected tropical diseases	0 (0 to 0·1)	0·1 (0·1 to 0·3)	0·8 (0·3 to 1·5)	2·6 (1·1 to 5·4)	3·1 (1·1 to 6·6)	0·8 (0·2 to 2·2)	0·2 (0 to 0·5)
Other infectious diseases	0·7 (0·4 to 1·1)	0·9 (0·6 to 1·5)	6·1 (3·9 to 9·3)	4·9 (3·3 to 7·3)	4·6 (3 to 7·1)	3 (2·2 to 4·2)	4·2 (3·2 to 5·9)
Meningitis	0·3 (0·1 to 0·4)	0·4 (0·2 to 0·6)	1·9 (1·1 to 3·1)	0·9 (0·6 to 1·4)	0·8 (0·5 to 1·1)	0·6 (0·4 to 0·8)	0·8 (0·6 to 1·1)
Encephalitis	0·1 (0 to 0·2)	0·1 (0·1 to 0·3)	1·5 (0·8 to 3·3)	1·2 (0·6 to 2·5)	0·9 (0·6 to 1·6)	0·8 (0·5 to 1·3)	1·1 (0·7 to 1·8)
Diphtheria	0 (0 to 0)	0 (0 to 0)	0 (0 to 0)	0 (0 to 0)	0 (0 to 0)	0 (0 to 0)	0 (0 to 0)
Tetanus	0 (0 to 0)	0 (0 to 0)	0 (0 to 0)	0 (0 to 0·1)	0 (0 to 0·3)	0 (0 to 0·2)	0·1 (0 to 0·3)
Measles	0 (0 to 0)	0 (0 to 0)	0 (0 to 0)	0 (0 to 0)	0 (0 to 0)	0 (0 to 0)	0 (0 to 0)
Varicella and herpes zoster	0·1 (0 to 0·2)	0·1 (0 to 0·2)	0·2 (0·1 to 0·6)	0·1 (0 to 0·2)	0·1 (0·1 to 0·3)	0·1 (0·1 to 0·4)	0·1 (0 to 0·2)
Acute hepatitis	0 (0 to 0)	0 (0 to 0)	0·1 (0 to 0·1)	0·1 (0·1 to 0·2)	0·3 (0·2 to 0·5)	0·4 (0·2 to 0·6)	0·4 (0·3 to 0·7)
Acute hepatitis A	0 (0 to 0)	0 (0 to 0)	0 (0 to 0)	0·1 (0 to 0·1)	0·2 (0·1 to 0·4)	0·3 (0·2 to 0·5)	0·3 (0·2 to 0·5)
Acute hepatitis B	0 (0 to 0)	0 (0 to 0)	0·1 (0 to 0·1)	0 (0 to 0·1)	0 (0 to 0·1)	0·1 (0 to 0·1)	0·1 (0 to 0·1)
Acute hepatitis C	0 (0 to 0)	0 (0 to 0)	0 (0 to 0)	0 (0 to 0)	0 (0 to 0)	0 (0 to 0)	0 (0 to 0)
Acute hepatitis E	0 (0 to 0)	0 (0 to 0)	0 (0 to 0)	0 (0 to 0)	0 (0 to 0)	0 (0 to 0·1)	0 (0 to 0·1)
Other unspecified infectious diseases	0·3 (0·1 to 0·5)	0·3 (0·1 to 0·5)	2·1 (1·1 to 3·7)	2·4 (1·3 to 4)	2·4 (1·1 to 4·7)	1·1 (0·6 to 2)	1·8 (1·1 to 2·8)
Maternal and neonatal disorders	423 (289 to 599)	55·1 (37·7 to 80·1)	41 (25·2 to 63·7)	17·2 (13 to 22·4)	14·5 (10·4 to 18·9)	15·5 (11·3 to 20·8)	16·3 (11·9 to 21·3)
Maternal disorders	0 (0 to 0)	0 (0 to 0)	0 (0 to 0)	0 (0 to 0)	0 (0 to 0)	0 (0 to 0)	0 (0 to 0)
Maternal hemorrhage	0 (0 to 0)	0 (0 to 0)	0 (0 to 0)	0 (0 to 0)	0 (0 to 0)	0 (0 to 0)	0 (0 to 0)
Maternal sepsis and other maternal infections	0 (0 to 0)	0 (0 to 0)	0 (0 to 0)	0 (0 to 0)	0 (0 to 0)	0 (0 to 0)	0 (0 to 0)
Maternal hypertensive disorders	0 (0 to 0)	0 (0 to 0)	0 (0 to 0)	0 (0 to 0)	0 (0 to 0)	0 (0 to 0)	0 (0 to 0)
Maternal obstructed labor and uterine rupture	--	--	--	--	--	--	--
Maternal abortion and miscarriage	--	--	--	--	--	--	--
Ectopic pregnancy	--	--	--	--	--	--	--
Indirect maternal deaths	--	--	--	--	--	--	--
Late maternal deaths	--	--	--	--	--	--	--
Maternal deaths aggravated by HIV/AIDS	--	--	--	--	--	--	--
Neonatal disorders	423 (289 to 599)	55·1 (37·7 to 80·1)	41 (25·2 to 63·7)	17·2 (13 to 22·4)	14·5 (10·4 to 18·9)	15·5 (11·3 to 20·8)	16·3 (11·9 to 21·3)
Neonatal preterm birth	165 (106 to 253)	14 (8·1 to 22·8)	13·1 (7·9 to 20·6)	5·3 (3·7 to 7·1)	6·4 (4·5 to 8·8)	6·8 (4·6 to 9·4)	7·1 (5 to 9·7)
Neonatal encephalopathy due to birth asphyxia and trauma	159 (103 to 238)	12·6 (6·9 to 21·6)	10·3 (5·2 to 18·7)	2·9 (1·8 to 4·2)	2·1 (1·1 to 3·4)	2·2 (1·1 to 3·7)	2·4 (1·2 to 4)
Neonatal sepsis and other neonatal infections	57·3 (34·5 to 91·4)	22·5 (13·6 to 34·2)	12·6 (7·5 to 20·2)	4·1 (2·5 to 6·3)	5·2 (3·2 to 8·1)	5·6 (3·3 to 8·6)	5·9 (3·5 to 9·1)
Hemolytic disease and other neonatal jaundice	0·2 (0·1 to 0·3)	0·1 (0·1 to 0·2)	0·1 (0·1 to 0·2)	0·6 (0·3 to 1)	0·7 (0·3 to 1·3)	0·8 (0·4 to 1·3)	0·9 (0·5 to 1·5)
Other neonatal disorders	42 (25·8 to 65·4)	5·9 (3·6 to 8·8)	4·8 (3·1 to 7·4)	4·4 (3·3 to 5·7)	0 (0 to 0)	0 (0 to 0)	0 (0 to 0)
Nutritional deficiencies	0 (0 to 0·1)	0·8 (0·4 to 1·4)	15·5 (9·4 to 23·5)	27·8 (12·8 to 53·5)	32·5 (11·5 to 68·2)	8·9 (2·8 to 23·7)	4·3 (2·2 to 8·7)
Protein-energy malnutrition	0 (0 to 0)	0·1 (0 to 0·1)	9·6 (5·8 to 14·7)	4·2 (2·4 to 7)	1·7 (1·1 to 2·5)	1 (0·6 to 1·5)	1·4 (0·9 to 1·9)
Iodine deficiency	0 (0 to 0)	0 (0 to 0)	0 (0 to 0)	0 (0 to 0)	0 (0 to 0·1)	0·1 (0 to 0·2)	0·2 (0·1 to 0·4)
Vitamin A deficiency	0 (0 to 0)	0 (0 to 0·1)	0·1 (0·1 to 0·3)	0·7 (0·3 to 1·5)	0·8 (0·3 to 1·7)	0·4 (0·1 to 0·9)	0·2 (0 to 0·4)
Dietary iron deficiency	0 (0 to 0·1)	0·7 (0·3 to 1·2)	5·6 (2 to 11·8)	22·8 (8 to 47·5)	29·7 (9·3 to 64·6)	7·3 (1·5 to 21·5)	2·3 (0·4 to 6·6)
Other nutritional deficiencies	0 (0 to 0)	0 (0 to 0)	0·1 (0·1 to 0·3)	0·1 (0 to 0·1)	0·1 (0·1 to 0·2)	0·1 (0·1 to 0·3)	0·3 (0·2 to 0·5)
Non-communicable diseases	59·8 (36·7 to 92·9)	17·4 (11·3 to 26·8)	103 (69·3 to 148)	112 (83·4 to 148)	143 (111 to 183)	191 (143 to 247)	301 (236 to 375)
Neoplasms	0·5 (0·3 to 0·8)	0·2 (0·2 to 0·4)	2·8 (1·8 to 4·3)	10·4 (6·5 to 15·5)	16·2 (12·5 to 20·4)	14 (11·1 to 17·4)	22·7 (18·3 to 27·5)
Lip and oral cavity cancer	0 (0 to 0)	0 (0 to 0)	0 (0 to 0)	0 (0 to 0)	0 (0 to 0·1)	0·1 (0 to 0·1)	0·2 (0·1 to 0·3)
Nasopharynx cancer	0 (0 to 0)	0 (0 to 0)	0 (0 to 0)	0 (0 to 0)	0·1 (0 to 0·1)	0·1 (0·1 to 0·2)	0·2 (0·1 to 0·4)
Other pharynx cancer	0 (0 to 0)	0 (0 to 0)	0 (0 to 0)	0 (0 to 0)	0 (0 to 0)	0 (0 to 0)	0 (0 to 0)
Esophageal cancer	0 (0 to 0)	0 (0 to 0)	0 (0 to 0)	0 (0 to 0)	0 (0 to 0)	0 (0 to 0)	0 (0 to 0)
Stomach cancer	0 (0 to 0)	0 (0 to 0)	0 (0 to 0)	0 (0 to 0)	0 (0 to 0)	0 (0 to 0)	0·3 (0·2 to 0·4)
Colon and rectum cancer	0 (0 to 0)	0 (0 to 0)	0 (0 to 0)	0 (0 to 0)	0·2 (0·1 to 0·2)	0·1 (0·1 to 0·2)	0·8 (0·5 to 1·2)
Liver cancer	0 (0 to 0)	0 (0 to 0)	0·1 (0 to 0·1)	0·2 (0·1 to 0·2)	0·2 (0·1 to 0·2)	0·2 (0·2 to 0·3)	0·2 (0·1 to 0·3)
Liver cancer due to hepatitis B	0 (0 to 0)	0 (0 to 0)	0 (0 to 0)	0 (0 to 0)	0 (0 to 0)	0·1 (0 to 0·1)	0·1 (0·1 to 0·2)
Liver cancer due to hepatitis C	0 (0 to 0)	0 (0 to 0)	0 (0 to 0)	0 (0 to 0)	0 (0 to 0)	0 (0 to 0)	0 (0 to 0)
Liver cancer due to alcohol use	0 (0 to 0)	0 (0 to 0)	0 (0 to 0)	0 (0 to 0)	0 (0 to 0)	0 (0 to 0)	0 (0 to 0)
Liver cancer due to NASH	0 (0 to 0)	0 (0 to 0)	0 (0 to 0)	0 (0 to 0)	0 (0 to 0)	0 (0 to 0)	0 (0 to 0·1)
Liver cancer due to other causes	0 (0 to 0)	0 (0 to 0)	0·1 (0 to 0·1)	0·2 (0·1 to 0·2)	0·2 (0·1 to 0·2)	0·2 (0·1 to 0·2)	0 (0 to 0·1)
Gallbladder and biliary tract cancer	0 (0 to 0)	0 (0 to 0)	0 (0 to 0)	0 (0 to 0)	0 (0 to 0)	0 (0 to 0)	0 (0 to 0)
Pancreatic cancer	0 (0 to 0)	0 (0 to 0)	0 (0 to 0)	0 (0 to 0)	0 (0 to 0)	0 (0 to 0)	0·2 (0·1 to 0·4)
Larynx cancer	0 (0 to 0)	0 (0 to 0)	0 (0 to 0)	0 (0 to 0)	0 (0 to 0)	0 (0 to 0)	0 (0 to 0)
Tracheal, bronchus, and lung cancer	0 (0 to 0)	0 (0 to 0)	0 (0 to 0)	0 (0 to 0)	0 (0 to 0)	0·1 (0 to 0·1)	0·4 (0·3 to 0·6)
Malignant skin melanoma	0 (0 to 0)	0 (0 to 0)	0 (0 to 0·1)	0·1 (0 to 0·3)	0 (0 to 0·1)	0 (0 to 0·1)	0·1 (0·1 to 0·2)
Non-melanoma skin cancer	0 (0 to 0)	0 (0 to 0)	0 (0 to 0)	0 (0 to 0)	0 (0 to 0)	0 (0 to 0)	0 (0 to 0)
Non-melanoma skin cancer (squamous-cell carcinoma)	0 (0 to 0)	0 (0 to 0)	0 (0 to 0)	0 (0 to 0)	0 (0 to 0)	0 (0 to 0)	0 (0 to 0)
Non-melanoma skin cancer (basal-cell carcinoma)	0 (0 to 0)	0 (0 to 0)	0 (0 to 0)	0 (0 to 0)	0 (0 to 0)	0 (0 to 0)	0 (0 to 0)
Breast cancer	0 (0 to 0)	0 (0 to 0)	0 (0 to 0)	0 (0 to 0)	0 (0 to 0)	0 (0 to 0)	0 (0 to 0)
Cervical cancer	--	--	--	--	--	--	--
Uterine cancer	--	--	--	--	--	--	--
Ovarian cancer	--	--	--	--	--	--	--
Prostate cancer	0 (0 to 0)	0 (0 to 0)	0 (0 to 0)	0 (0 to 0)	0 (0 to 0)	0 (0 to 0)	0 (0 to 0)
Testicular cancer	0 (0 to 0·3)	0 (0 to 0)	0 (0 to 0·2)	0 (0 to 0·1)	0·1 (0 to 0·1)	0·1 (0 to 0·1)	0·2 (0·2 to 0·4)
Kidney cancer	0 (0 to 0·1)	0 (0 to 0)	0·2 (0·1 to 0·3)	0·8 (0·5 to 1·2)	0·8 (0·5 to 1·2)	0·3 (0·2 to 0·4)	0·5 (0·4 to 0·7)
Bladder cancer	0 (0 to 0)	0 (0 to 0)	0 (0 to 0)	0 (0 to 0)	0 (0 to 0)	0 (0 to 0)	0 (0 to 0)
Brain and central nervous system cancer	0·1 (0 to 0·1)	0 (0 to 0·1)	0·7 (0·3 to 1·2)	2·1 (0·9 to 3·6)	3·1 (1·6 to 5)	2·3 (1·1 to 3·7)	2 (1 to 3·3)
Thyroid cancer	0 (0 to 0)	0 (0 to 0)	0 (0 to 0)	0 (0 to 0)	0·1 (0 to 0·1)	0 (0 to 0·1)	0·1 (0·1 to 0·2)
Mesothelioma	0 (0 to 0)	0 (0 to 0)	0 (0 to 0)	0 (0 to 0)	0 (0 to 0)	0 (0 to 0)	0 (0 to 0)
Hodgkin lymphoma	0 (0 to 0)	0 (0 to 0)	0 (0 to 0)	0 (0 to 0)	0·1 (0 to 0·2)	0·1 (0·1 to 0·2)	0·5 (0·3 to 0·9)
Non-Hodgkin lymphoma	0 (0 to 0)	0 (0 to 0)	0 (0 to 0)	2 (1·2 to 3·4)	4 (2·6 to 5·8)	3·4 (2·3 to 4·9)	6·2 (4·5 to 8·5)
Multiple myeloma	0 (0 to 0)	0 (0 to 0)	0 (0 to 0)	0 (0 to 0)	0 (0 to 0)	0 (0 to 0)	0 (0 to 0)
Leukemia	0·1 (0·1 to 0·2)	0·1 (0 to 0·1)	0·8 (0·4 to 1·3)	2·4 (1·4 to 4)	4·3 (2·8 to 6·2)	3·8 (2·5 to 5·5)	5 (3·6 to 6·8)
Acute lymphoid leukemia	0 (0 to 0·1)	0 (0 to 0)	0·1 (0·1 to 0·2)	0·7 (0·3 to 1·3)	1·4 (0·6 to 2·4)	1 (0·5 to 1·8)	1·5 (0·8 to 2·4)
Chronic lymphoid leukemia	0 (0 to 0)	0 (0 to 0)	0 (0 to 0)	0 (0 to 0)	0 (0 to 0)	0 (0 to 0)	0 (0 to 0)
Acute myeloid leukemia	0 (0 to 0·1)	0 (0 to 0·1)	0·2 (0·1 to 0·4)	0·8 (0·4 to 1·6)	1 (0·5 to 1·7)	1 (0·5 to 1·7)	1·6 (0·9 to 2·6)

Cause	20 to 24	25 to 29	30 to 34	35 to 39	40 to 44	45 to 49	50 to 54	55 to 59
All causes	730 (627 to 842)	735 (626 to 859)	737 (636 to 852)	721 (626 to 825)	867 (750 to 989)	1,240 (1,080 to 1,410)	1,600 (1,410 to 1,800)	1,850 (1,660 to 2,050)
Communicable, maternal, neonatal, and nutritional diseases	59·8 (48·5 to 73)	76·2 (64·7 to 89)	75·6 (64·5 to 88·3)	78·2 (68·5 to 89·4)	80·8 (69·7 to 93·4)	93·2 (80·1 to 109)	100 (85·6 to 117)	96 (79·5 to 119)
HIV/AIDS and sexually transmitted infections	4·8 (4·1 to 5·6)	22·1 (20·2 to 24·8)	24·6 (22·3 to 27·2)	32·1 (29·4 to 35·3)	29·1 (26·7 to 31·7)	27·3 (25 to 29·6)	24·9 (22·8 to 27·1)	15·2 (13·7 to 16·7)
HIV/AIDS	3·9 (3·5 to 4·4)	21·2 (19·4 to 23·6)	23·7 (21·5 to 26·3)	31·4 (28·7 to 34·5)	28·5 (26·1 to 31·1)	26·6 (24·4 to 28·9)	24·3 (22·2 to 26·4)	14·8 (13·4 to 16·2)
HIV/AIDS - Drug-susceptible Tuberculosis	0·1 (0·1 to 0·1)	0·5 (0·4 to 0·7)	0·6 (0·4 to 0·9)	0·7 (0·4 to 1)	0·5 (0·4 to 0·8)	0·5 (0·4 to 0·8)	0·6 (0·4 to 0·8)	0·4 (0·3 to 0·6)
HIV/AIDS - Multidrug-resistant Tuberculosis without extensive dru	0 (0 to 0)	0 (0 to 0)	0 (0 to 0)	0 (0 to 0·1)	0 (0 to 0)	0 (0 to 0)	0 (0 to 0)	0 (0 to 0)
HIV/AIDS - Extensively drug-resistant Tuberculosis	0 (0 to 0)	0 (0 to 0)	0 (0 to 0)	0 (0 to 0)	0 (0 to 0)	0 (0 to 0)	0 (0 to 0)	0 (0 to 0)
HIV/AIDS resulting in other diseases	3·8 (3·4 to 4·3)	20·7 (18·8 to 23·1)	23·1 (20·9 to 25·6)	30·8 (27·9 to 33·8)	27·9 (25·6 to 30·5)	26 (23·9 to 28·3)	23·8 (21·6 to 25·8)	14·3 (13 to 15·7)
Sexually transmitted infections excluding HIV	0·9 (0·5 to 1·6)	0·9 (0·5 to 1·6)	0·9 (0·5 to 1·5)	0·7 (0·4 to 1·2)	0·6 (0·3 to 1·1)	0·7 (0·4 to 1·2)	0·6 (0·3 to 1·1)	0·5 (0·3 to 0·9)
Syphilis	0·1 (0 to 0·1)	0·1 (0·1 to 0·2)	0·1 (0 to 0·2)	0·1 (0·1 to 0·2)	0·1 (0 to 0·1)	0·1 (0·1 to 0·2)	0·1 (0·1 to 0·2)	0·2 (0·1 to 0·3)
Chlamydial infection	0·2 (0 to 0·4)	0·3 (0·1 to 0·6)	0·2 (0·1 to 0·5)	0·2 (0 to 0·3)	0·1 (0 to 0·3)	0·1 (0 to 0·3)	0·1 (0 to 0·2)	0 (0 to 0)
Gonococcal infection	0·3 (0·1 to 0·5)	0·1 (0 to 0·3)	0·1 (0 to 0·2)	0 (0 to 0·1)	0 (0 to 0·1)	0 (0 to 0·1)	0 (0 to 0·1)	0 (0 to 0)
Trichomoniasis	0 (0 to 0)	0 (0 to 0)	0 (0 to 0·1)	0 (0 to 0·1)	0 (0 to 0·1)	0 (0 to 0·1)	0 (0 to 0)	0 (0 to 0)
Genital herpes	0·1 (0 to 0·3)	0·2 (0·1 to 0·5)	0·2 (0·1 to 0·5)	0·2 (0·1 to 0·5)	0·2 (0·1 to 0·6)	0·3 (0·1 to 0·7)	0·3 (0·1 to 0·7)	0·2 (0·1 to 0·5)
Other sexually transmitted infections	0·3 (0·1 to 0·5)	0·2 (0·1 to 0·4)	0·3 (0·1 to 0·5)	0·2 (0·1 to 0·3)	0·1 (0 to 0·2)	0·1 (0·1 to 0·2)	0·1 (0 to 0·2)	0 (0 to 0·1)
Respiratory infections and tuberculosis	18 (13 to 24·7)	17·8 (13 to 23·9)	20·1 (14·6 to 27·5)	19·3 (14·1 to 25·8)	23·4 (16·9 to 31·5)	32·6 (23·6 to 43·6)	39·6 (29·4 to 52·8)	47·1 (34·4 to 63·9)
Tuberculosis	1·1 (0·7 to 1·8)	1·4 (0·9 to 2)	1·3 (0·9 to 1·8)	1·2 (0·9 to 1·8)	1·6 (1·1 to 2·2)	1·7 (1·2 to 2·3)	1·6 (1·1 to 2·2)	1·3 (0·9 to 1·8)
Latent tuberculosis infection	0 (0 to 0)	0 (0 to 0)	0 (0 to 0)	0 (0 to 0)	0 (0 to 0)	0 (0 to 0)	0 (0 to 0)	0 (0 to 0)
Drug-susceptible tuberculosis	1·1 (0·7 to 1·8)	1·3 (0·8 to 2)	1·3 (0·9 to 1·8)	1·2 (0·8 to 1·7)	1·5 (1 to 2·1)	1·7 (1·2 to 2·3)	1·6 (1·1 to 2·1)	1·3 (0·9 to 1·8)
Multidrug-resistant tuberculosis without extensive drug resistanc	0 (0 to 0·1)	0 (0 to 0·1)	0 (0 to 0·1)	0 (0 to 0·1)	0 (0 to 0·1)	0 (0 to 0·1)	0 (0 to 0·1)	0 (0 to 0·1)
Extensively drug-resistant tuberculosis	0 (0 to 0)	0 (0 to 0)	0 (0 to 0)	0 (0 to 0)	0 (0 to 0)	0 (0 to 0)	0 (0 to 0)	0 (0 to 0)
Lower respiratory infections	11·2 (7·2 to 16·6)	11·5 (7·7 to 16·8)	15·1 (9·9 to 22·1)	14·9 (10·1 to 21)	18·6 (12·7 to 26·5)	27·5 (18·9 to 38·3)	34·6 (24·9 to 47·6)	43·1 (30·6 to 59·6)
Upper respiratory infections	4·2 (2·3 to 7·5)	3·8 (2·1 to 6·2)	3 (1·6 to 5·2)	2·5 (1·3 to 4·1)	2·5 (1·3 to 4·3)	2·5 (1·4 to 4·5)	2·5 (1·3 to 4·3)	2 (1·1 to 3·3)
Otitis media	1·4 (0·6 to 2·7)	1·1 (0·5 to 2·1)	0·8 (0·3 to 1·5)	0·7 (0·3 to 1·2)	0·7 (0·3 to 1·3)	0·9 (0·4 to 1·5)	0·9 (0·4 to 1·7)	0·7 (0·3 to 1·2)
Enteric infections	7 (4·3 to 10·3)	7 (4·5 to 10·2)	6·1 (4·1 to 8·6)	5·5 (3·7 to 7·6)	6 (4·2 to 8·4)	7·2 (4·9 to 10·1)	7·9 (5·4 to 11·2)	7·3 (5·1 to 10·2)
Diarrheal diseases	7 (4·3 to 10·3)	6·9 (4·5 to 10·2)	6·1 (4·1 to 8·6)	5·5 (3·7 to 7·6)	6 (4·2 to 8·4)	7·2 (4·9 to 10·1)	7·9 (5·4 to 11·2)	7·2 (5 to 10·1)
Typhoid and paratyphoid	0 (0 to 0)	0 (0 to 0)	0 (0 to 0)	0 (0 to 0)	0 (0 to 0)	0 (0 to 0)	0 (0 to 0)	0 (0 to 0)
Typhoid fever	0 (0 to 0)	0 (0 to 0)	0 (0 to 0)	0 (0 to 0)	0 (0 to 0)	0 (0 to 0)	0 (0 to 0)	0 (0 to 0)
Paratyphoid fever	0 (0 to 0)	0 (0 to 0)	0 (0 to 0)	0 (0 to 0)	0 (0 to 0)	0 (0 to 0)	0 (0 to 0)	0 (0 to 0)
Invasive Non-typhoidal Salmonella (iNTS)	0 (0 to 0)	0 (0 to 0)	0 (0 to 0)	0 (0 to 0)	0 (0 to 0)	0 (0 to 0)	0 (0 to 0)	0 (0 to 0)
Other intestinal infectious diseases	0 (0 to 0·1)	0 (0 to 0)	0 (0 to 0·1)	0 (0 to 0)	0 (0 to 0)	0 (0 to 0)	0 (0 to 0)	0 (0 to 0)
Neglected tropical diseases and malaria	2·4 (1·1 to 4·7)	2·5 (1·2 to 4·4)	2·1 (1 to 3·9)	2 (0·9 to 3·6)	2·5 (1·2 to 4·5)	3·2 (1·6 to 6·3)	3·8 (1·7 to 7·3)	4·3 (1·9 to 7·8)
Malaria	0 (0 to 0)	0 (0 to 0)	0 (0 to 0)	0 (0 to 0)	0 (0 to 0)	0 (0 to 0)	0 (0 to 0)	0 (0 to 0)
Chagas disease	0 (0 to 0)	0 (0 to 0)	0 (0 to 0)	0 (0 to 0)	0 (0 to 0)	0 (0 to 0)	0 (0 to 0)	0 (0 to 0)
Leishmaniasis	0 (0 to 0)	0 (0 to 0)	0 (0 to 0)	0 (0 to 0)	0 (0 to 0)	0 (0 to 0)	0 (0 to 0)	0 (0 to 0)
Visceral leishmaniasis	0 (0 to 0)	0 (0 to 0)	0 (0 to 0)	0 (0 to 0)	0 (0 to 0)	0 (0 to 0)	0 (0 to 0)	0 (0 to 0)
Cutaneous and mucocutaneous leishmaniasis	0 (0 to 0)	0 (0 to 0)	0 (0 to 0)	0 (0 to 0)	0 (0 to 0)	0 (0 to 0)	0 (0 to 0)	0 (0 to 0)
African trypanosomiasis	0 (0 to 0)	0 (0 to 0)	0 (0 to 0)	0 (0 to 0)	0 (0 to 0)	0 (0 to 0)	0 (0 to 0)	0 (0 to 0)
Schistosomiasis	0 (0 to 0)	0 (0 to 0)	0 (0 to 0)	0 (0 to 0)	0 (0 to 0)	0 (0 to 0)	0 (0 to 0)	0 (0 to 0)
Cysticercosis	0·6 (0·1 to 1·6)	1·1 (0·3 to 2·4)	1·1 (0·3 to 2·6)	1·2 (0·3 to 2·7)	1·6 (0·5 to 3·6)	2·3 (0·8 to 5·5)	3 (0·9 to 6·3)	3·3 (1·2 to 6·8)
Cystic echinococcosis	0 (0 to 0)	0 (0 to 0)	0 (0 to 0)	0 (0 to 0)	0 (0 to 0)	0 (0 to 0)	0 (0 to 0)	0 (0 to 0)
Lymphatic filariasis	0 (0 to 0)	0 (0 to 0)	0 (0 to 0)	0 (0 to 0)	0 (0 to 0)	0 (0 to 0)	0 (0 to 0)	0 (0 to 0)
Onchocerciasis	0 (0 to 0)	0 (0 to 0)	0 (0 to 0)	0 (0 to 0)	0 (0 to 0)	0 (0 to 0)	0 (0 to 0)	0 (0 to 0)
Trachoma	0 (0 to 0)	0 (0 to 0)	0 (0 to 0)	0 (0 to 0)	0 (0 to 0)	0 (0 to 0)	0 (0 to 0)	0 (0 to 0)
Dengue	0·9 (0·2 to 2·6)	0·8 (0·2 to 2·3)	0·5 (0·1 to 1·2)	0·3 (0·1 to 0·8)	0·3 (0·1 to 0·7)	0·3 (0·1 to 0·8)	0·4 (0·1 to 0·9)	0·5 (0·1 to 1·4)
Yellow fever	0 (0 to 0)	0 (0 to 0)	0 (0 to 0)	0 (0 to 0)	0 (0 to 0)	0 (0 to 0)	0 (0 to 0)	0 (0 to 0)
Rabies	0 (0 to 0)	0 (0 to 0)	0 (0 to 0)	0 (0 to 0)	0 (0 to 0)	0 (0 to 0)	0 (0 to 0)	0 (0 to 0)
Intestinal nematode infections	0·2 (0·1 to 0·3)	0·1 (0 to 0·2)	0·1 (0 to 0·1)	0·1 (0 to 0·1)	0·1 (0 to 0·1)	0·1 (0 to 0·1)	0·1 (0 to 0·1)	0·1 (0 to 0·1)
Ascariasis	0 (0 to 0·1)	0 (0 to 0·1)	0 (0 to 0·1)	0 (0 to 0·1)	0 (0 to 0)	0 (0 to 0)	0 (0 to 0)	0 (0 to 0)
Trichuriasis	0 (0 to 0)	0 (0 to 0)	0 (0 to 0)	0 (0 to 0)	0 (0 to 0)	0 (0 to 0)	0 (0 to 0)	0 (0 to 0)
Hookworm disease	0·1 (0 to 0·2)	0·1 (0 to 0·2)	0·1 (0 to 0·1)	0 (0 to 0·1)	0 (0 to 0·1)	0·1 (0 to 0·1)	0·1 (0 to 0·1)	0 (0 to 0·1)
Food-borne trematodiases	0 (0 to 0)	0 (0 to 0)	0 (0 to 0)	0 (0 to 0)	0 (0 to 0)	0 (0 to 0)	0 (0 to 0)	0 (0 to 0)
Leprosy	0 (0 to 0)	0 (0 to 0)	0 (0 to 0)	0 (0 to 0)	0 (0 to 0·1)	0 (0 to 0·1)	0 (0 to 0·1)	0 (0 to 0·1)
Ebola	0 (0 to 0)	0 (0 to 0)	0 (0 to 0)	0 (0 to 0)	0 (0 to 0)	0 (0 to 0)	0 (0 to 0)	0 (0 to 0)
Zika virus	0·2 (0 to 1)	0·2 (0 to 1)	0·2 (0 to 1)	0·2 (0 to 0·8)	0·1 (0 to 0·7)	0·1 (0 to 0·5)	0·1 (0 to 0·4)	0·1 (0 to 0·2)
Guinea worm disease	0 (0 to 0)	0 (0 to 0)	0 (0 to 0)	0 (0 to 0)	0 (0 to 0)	0 (0 to 0)	0 (0 to 0)	0 (0 to 0)
Other neglected tropical diseases	0·4 (0·1 to 1·7)	0·2 (0·1 to 0·6)	0·2 (0·1 to 0·7)	0·2 (0 to 0·4)	0·3 (0·1 to 0·7)	0·3 (0·1 to 1)	0·3 (0·1 to 0·7)	0·3 (0·1 to 0·8)
Other infectious diseases	5·2 (3·7 to 7·7)	5·1 (3·5 to 7·9)	5·2 (3·6 to 7·7)	4·6 (3·2 to 6·6)	4·8 (3·4 to 6·8)	7 (4·8 to 10·2)	7·3 (5·2 to 10·6)	6·9 (4·8 to 10·1)
Meningitis	0·7 (0·5 to 1)	0·7 (0·5 to 0·9)	0·7 (0·5 to 1)	0·6 (0·4 to 0·8)	0·6 (0·4 to 0·9)	0·8 (0·5 to 1·1)	0·8 (0·6 to 1·1)	0·7 (0·5 to 1)
Encephalitis	1·1 (0·7 to 2·2)	0·8 (0·5 to 1·5)	0·8 (0·5 to 1·4)	0·6 (0·4 to 0·9)	0·7 (0·5 to 1)	0·7 (0·5 to 1·1)	0·6 (0·4 to 1)	0·6 (0·4 to 1)
Diphtheria	0 (0 to 0)	0 (0 to 0)	0 (0 to 0)	0 (0 to 0)	0 (0 to 0)	0 (0 to 0)	0 (0 to 0)	0 (0 to 0)
Tetanus	0·1 (0 to 0·4)	0·1 (0 to 0·3)	0·1 (0 to 0·4)	0·1 (0 to 0·3)	0·1 (0 to 0·5)	0·2 (0·1 to 0·9)	0·3 (0·1 to 1·2)	0·4 (0·1 to 1·6)
Measles	0 (0 to 0)	0 (0 to 0)	0 (0 to 0)	0 (0 to 0)	0 (0 to 0)	0 (0 to 0)	0 (0 to 0)	0 (0 to 0)
Varicella and herpes zoster	0·1 (0 to 0·3)	0·1 (0·1 to 0·3)	0·1 (0·1 to 0·3)	0·2 (0·1 to 0·4)	0·2 (0·1 to 0·3)	0·2 (0·1 to 0·5)	0·3 (0·2 to 0·5)	0·2 (0·1 to 0·4)
Acute hepatitis	0·5 (0·3 to 0·7)	0·4 (0·3 to 0·6)	0·4 (0·2 to 0·5)	0·3 (0·2 to 0·4)	0·3 (0·2 to 0·4)	0·3 (0·2 to 0·5)	0·4 (0·3 to 0·5)	0·3 (0·2 to 0·3)
Acute hepatitis A	0·3 (0·2 to 0·5)	0·2 (0·1 to 0·4)	0·2 (0·1 to 0·3)	0·1 (0·1 to 0·2)	0·1 (0 to 0·2)	0·1 (0 to 0·2)	0·1 (0 to 0·1)	0 (0 to 0·1)
Acute hepatitis B	0·1 (0·1 to 0·2)	0·2 (0·1 to 0·2)	0·1 (0·1 to 0·2)	0·2 (0·1 to 0·2)	0·2 (0·1 to 0·2)	0·2 (0·1 to 0·3)	0·2 (0·2 to 0·3)	0·2 (0·1 to 0·3)
Acute hepatitis C	0 (0 to 0)	0 (0 to 0)	0 (0 to 0)	0 (0 to 0)	0 (0 to 0)	0 (0 to 0)	0 (0 to 0)	0 (0 to 0)
Acute hepatitis E	0 (0 to 0·1)	0 (0 to 0·1)	0 (0 to 0·1)	0 (0 to 0·1)	0 (0 to 0·1)	0 (0 to 0·1)	0 (0 to 0)	0 (0 to 0)
Other unspecified infectious diseases	2·5 (1·5 to 4·5)	3·1 (1·7 to 5·6)	3·1 (1·7 to 5·3)	2·8 (1·6 to 4·4)	3 (1·5 to 4·7)	4·7 (2·6 to 7·6)	5 (2·9 to 7·6)	4·7 (2·8 to 7·6)
Maternal and neonatal disorders	17·4 (12·4 to 23)	15·9 (11·4 to 21·3)	12·3 (9·2 to 16·2)	9·8 (7·1 to 13)	9·4 (6·8 to 12·5)	9·4 (6·9 to 12·5)	8·4 (6·2 to 11·1)	6 (4·3 to 8·1)
Maternal disorders	0 (0 to 0)	0 (0 to 0)	0 (0 to 0)	0 (0 to 0)	0 (0 to 0)	0 (0 to 0)	0 (0 to 0)	0 (0 to 0)
Maternal hemorrhage	0 (0 to 0)	0 (0 to 0)	0 (0 to 0)	0 (0 to 0)	0 (0 to 0)	0 (0 to 0)	0 (0 to 0)	0 (0 to 0)
Maternal sepsis and other maternal infections	0 (0 to 0)	0 (0 to 0)	0 (0 to 0)	0 (0 to 0)	0 (0 to 0)	0 (0 to 0)	0 (0 to 0)	0 (0 to 0)
Maternal hypertensive disorders	0 (0 to 0)	0 (0 to 0)	0 (0 to 0)	0 (0 to 0)	0 (0 to 0)	0 (0 to 0)	0 (0 to 0)	0 (0 to 0)
Maternal obstructed labor and uterine rupture	--	--	--	--	--	--	--	--
Maternal abortion and miscarriage	--	--	--	--	--	--	--	--
Ectopic pregnancy	--	--	--	--	--	--	--	--
Indirect maternal deaths	--	--	--	--	--	--	--	--
Late maternal deaths	--	--	--	--	--	--	--	--
Maternal deaths aggravated by HIV/AIDS	--	--	--	--	--	--	--	--
Neonatal disorders	17·4 (12·4 to 23)	15·9 (11·4 to 21·3)	12·3 (9·2 to 16·2)	9·8 (7·1 to 13)	9·4 (6·8 to 12·5)	9·4 (6·9 to 12·5)	8·4 (6·2 to 11·1)	6 (4·3 to 8·1)
Neonatal preterm birth	7·3 (5·1 to 10·1)	6·7 (4·7 to 9·2)	5·1 (3·6 to 6·9)	4 (2·8 to 5·4)	3·7 (2·6 to 5)	3·5 (2·4 to 4·8)	3 (2 to 4)	1·9 (1·3 to 2·7)
Neonatal encephalopathy due to birth asphyxia and trauma	2·6 (1·3 to 4·3)	2·4 (1·1 to 3·9)	1·9 (0·9 to 3·1)	1·5 (0·8 to 2·5)	1·5 (0·8 to 2·5)	1·5 (0·8 to 2·5)	1·4 (0·7 to 2·4)	1 (0·5 to 1·8)
Neonatal sepsis and other neonatal infections	6·5 (3·8 to 9·8)	6 (3·5 to 9·3)	4·7 (2·8 to 7·3)	3·8 (2·2 to 5·8)	3·7 (2·3 to 5·7)	3·8 (2·3 to 6)	3·5 (2·2 to 5·4)	2·6 (1·5 to 4·1)
Hemolytic disease and other neonatal jaundice	0·9 (0·7 to 1·2)	0·9 (0·6 to 1·1)	0·7 (0·5 to 0·9)	0·6 (0·4 to 0·7)	0·5 (0·4 to 0·7)	0·6 (0·4 to 0·7)	0·5 (0·4 to 0·7)	0·4 (0·3 to 0·5)
Other neonatal disorders	0 (0 to 0)	0 (0 to 0)	0 (0 to 0)	0 (0 to 0)	0 (0 to 0)	0 (0 to 0)	0 (0 to 0)	0 (0 to 0)
Nutritional deficiencies	5·2 (2·6 to 10·5)	5·8 (3 to 11·3)	5·2 (2·7 to 10)	5 (2·7 to 9·2)	5·4 (2·9 to 9·9)	6·5 (3·9 to 11·2)	8 (4·9 to 13·8)	9·3 (5·5 to 16·3)
Protein-energy malnutrition	1·7 (1·1 to 2·4)	1·7 (1·1 to 2·5)	1·8 (1·3 to 2·5)	1·9 (1·3 to 2·6)	2 (1·5 to 2·8)	3 (2·1 to 4)	3·7 (2·7 to 5·1)	3·9 (2·8 to 5·3)
Iodine deficiency	0·2 (0·1 to 0·5)	0·3 (0·1 to 0·5)	0·2 (0·1 to 0·4)	0·2 (0·1 to 0·3)	0·2 (0·1 to 0·3)	0·2 (0·1 to 0·4)	0·2 (0·1 to 0·4)	0·2 (0·1 to 0·3)
Vitamin A deficiency	0·2 (0·1 to 0·3)	0·1 (0·1 to 0·2)	0·1 (0 to 0·2)	0·1 (0 to 0·1)	0·1 (0 to 0·1)	0·1 (0 to 0·1)	0·1 (0 to 0·1)	0·1 (0 to 0·1)
Dietary iron deficiency	2·6 (0·5 to 7·7)	3·1 (0·7 to 8·5)	2·6 (0·5 to 7·2)	2·4 (0·5 to 6·3)	2·6 (0·6 to 7·1)	2·7 (0·6 to 7·4)	3·2 (0·7 to 8·4)	4·5 (1·1 to 11·6)
Other nutritional deficiencies	0·5 (0·3 to 0·8)	0·6 (0·3 to 1·1)	0·4 (0·2 to 0·7)	0·4 (0·2 to 0·6)	0·5 (0·3 to 0·8)	0·5 (0·3 to 0·8)	0·8 (0·5 to 1·2)	0·7 (0·4 to 1·1)
Non-communicable diseases	393 (318 to 480)	425 (341 to 514)	446 (370 to 528)	469 (396 to 547)	623 (534 to 720)	965 (838 to 1,110)	1,320 (1,160 to 1,500)	1,600 (1,440 to 1,780)
Neoplasms	30·5 (23·6 to 37·4)	30 (22·9 to 38·1)	40·1 (32·2 to 49·4)	46·5 (37·4 to 56·9)	74·9 (60·3 to 92·1)	146 (121 to 176)	236 (200 to 275)	335 (285 to 387)
Lip and oral cavity cancer	0·3 (0·2 to 0·5)	0·3 (0·2 to 0·5)	0·7 (0·4 to 1·1)	1·1 (0·7 to 1·7)	2·7 (1·6 to 4·2)	6·2 (4 to 9·5)	9·9 (6·8 to 14·1)	12·7 (8·8 to 17·9)
Nasopharynx cancer	0·3 (0·1 to 0·5)	0·2 (0·1 to 0·4)	0·3 (0·1 to 0·4)	0·3 (0·2 to 0·5)	0·7 (0·4 to 1)	1·3 (0·9 to 2)	2·2 (1·5 to 3·2)	2·8 (1·8 to 4)
Other pharynx cancer	0·1 (0·1 to 0·2)	0·1 (0·1 to 0·2)	0·2 (0·1 to 0·3)	0·4 (0·3 to 0·7)	1·1 (0·7 to 1·6)	3·5 (2·3 to 4·9)	5·4 (3·8 to 7·6)	7·2 (4·7 to 10·7)
Esophageal cancer	0·3 (0·2 to 0·4)	0·4 (0·2 to 0·6)	0·8 (0·5 to 1·2)	2·1 (1·3 to 3)	5·9 (3·5 to 8·6)	15·2 (10·5 to 21·2)	24·2 (17·8 to 32·7)	31·8 (22·9 to 43·4)
Stomach cancer	0·5 (0·4 to 0·7)	1 (0·6 to 1·4)	2·3 (1·5 to 3·3)	3·1 (2·1 to 4·5)	5·7 (4·1 to 7·8)	10 (7 to 13·7)	16·5 (12·1 to 22·4)	21·6 (15·9 to 28·9)
Colon and rectum cancer	1·7 (1·1 to 2·4)	2·3 (1·5 to 3·2)	4·7 (3 to 6·8)	6 (3·9 to 8·8)	9·4 (6·2 to 13·9)	16·9 (11·4 to 23·7)	26 (18 to 35·9)	36·3 (27 to 48·1)
Liver cancer	0·4 (0·2 to 0·6)	0·6 (0·3 to 1)	0·8 (0·4 to 1·2)	1·2 (0·7 to 2·1)	2 (1·1 to 3·4)	3·8 (2·1 to 6)	5·9 (3·6 to 9·2)	8·7 (5·8 to 12·8)
Liver cancer due to hepatitis B	0·3 (0·1 to 0·4)	0·4 (0·2 to 0·7)	0·5 (0·3 to 0·8)	0·7 (0·4 to 1·2)	1·1 (0·5 to 1·9)	1·8 (0·9 to 3·2)	2·4 (1·2 to 4·2)	3·2 (1·7 to 5·6)
Liver cancer due to hepatitis C	0 (0 to 0)	0 (0 to 0)	0 (0 to 0)	0 (0 to 0·1)	0·1 (0 to 0·2)	0·3 (0·1 to 0·6)	0·6 (0·2 to 1·1)	1 (0·4 to 2)
Liver cancer due to alcohol use	0 (0 to 0·1)	0·1 (0 to 0·2)	0·2 (0·1 to 0·3)	0·3 (0·1 to 0·7)	0·7 (0·3 to 1·3)	1·4 (0·6 to 2·6)	2·4 (1·1 to 4·2)	3·7 (1·9 to 6·2)
Liver cancer due to NASH	0 (0 to 0·1)	0 (0 to 0·1)	0 (0 to 0·1)	0·1 (0 to 0·1)	0·1 (0 to 0·2)	0·2 (0·1 to 0·3)	0·3 (0·1 to 0·5)	0·4 (0·2 to 0·8)
Liver cancer due to other causes	0·1 (0 to 0·1)	0·1 (0 to 0·1)	0·1 (0 to 0·1)	0·1 (0 to 0·2)	0·1 (0 to 0·2)	0·2 (0·1 to 0·3)	0·2 (0·1 to 0·5)	0·3 (0·1 to 0·6)
Gallbladder and biliary tract cancer	0·1 (0·1 to 0·2)	0·1 (0·1 to 0·3)	0·2 (0·1 to 0·4)	0·4 (0·2 to 0·6)	0·7 (0·4 to 1·1)	1·3 (0·8 to 2·1)	2·2 (1·4 to 3·9)	3·2 (2·1 to 5·9)
Pancreatic cancer	0·3 (0·2 to 0·5)	0·5 (0·3 to 0·7)	1·8 (1·3 to 2·5)	2·8 (1·9 to 3·8)	5·9 (4·1 to 8·2)	12·1 (8·2 to 17·1)	18 (12·9 to 24·9)	24·2 (17·4 to 32·7)
Larynx cancer	0·1 (0·1 to 0·1)	0·1 (0 to 0·1)	0·1 (0·1 to 0·2)	0·3 (0·2 to 0·5)	0·8 (0·4 to 1·4)	2·3 (1·4 to 3·6)	4·1 (2·8 to 5·8)	5·4 (3·9 to 7·6)
Tracheal, bronchus, and lung cancer	0·7 (0·4 to 1)	0·8 (0·5 to 1·1)	2 (1·2 to 3·2)	3·5 (2·1 to 5·7)	7·9 (5·2 to 11·5)	19·9 (14·2 to 27·2)	33·5 (25·1 to 43·4)	48·6 (35·7 to 63·5)
Malignant skin melanoma	0·2 (0·1 to 0·4)	0·3 (0·2 to 0·6)	0·5 (0·3 to 0·9)	0·6 (0·4 to 1·2)	0·8 (0·5 to 1·6)	1·1 (0·6 to 2·2)	1·6 (0·9 to 3·1)	1·8 (1·1 to 4)
Non-melanoma skin cancer	0·1 (0·1 to 0·1)	0·2 (0·1 to 0·3)	0·3 (0·2 to 0·5)	0·3 (0·2 to 0·5)	0·5 (0·3 to 0·8)	0·9 (0·6 to 1·2)	1·4 (0·9 to 1·8)	1·7 (1·2 to 2·4)
Non-melanoma skin cancer (squamous-cell carcinoma)	0·1 (0·1 to 0·1)	0·2 (0·1 to 0·3)	0·3 (0·2 to 0·5)	0·3 (0·2 to 0·5)	0·5 (0·3 to 0·8)	0·9 (0·6 to 1·2)	1·4 (0·9 to 1·8)	1·7 (1·2 to 2·4)
Non-melanoma skin cancer (basal-cell carcinoma)	0 (0 to 0)	0 (0 to 0)	0 (0 to 0)	0 (0 to 0)	0 (0 to 0)	0 (0 to 0)	0 (0 to 0)	0 (0 to 0)
Breast cancer	0 (0 to 0·1)	0 (0 to 0·1)	0·1 (0 to 0·1)	0·1 (0 to 0·2)	0·1 (0·1 to 0·3)	0·4 (0·2 to 0·8)	0·6 (0·3 to 1·2)	0·8 (0·5 to 1·3)
Cervical cancer	--	--	--	--	--	--	--	--
Uterine cancer	--	--	--	--	--	--	--	--
Ovarian cancer	--	--	--	--	--	--	--	--
Prostate cancer	0·3 (0·2 to 0·5)	0·6 (0·4 to 0·9)	0·8 (0·6 to 1·1)	0·9 (0·6 to 1·2)	2·2 (1·3 to 3·2)	8·5 (5·5 to 12·3)	25·9 (17·4 to 36·2)	55·5 (37 to 81·2)
Testicular cancer	0·4 (0·3 to 0·7)	0·5 (0·3 to 0·8)	0·3 (0·2 to 0·5)	0·3 (0·2 to 0·4)	0·4 (0·2 to 0·5)	0·2 (0·1 to 0·3)	0·2 (0·1 to 0·3)	0·1 (0·1 to 0·2)
Kidney cancer	0·7 (0·5 to 1)	0·9 (0·6 to 1·2)	1·1 (0·7 to 1·6)	0·9 (0·7 to 1·3)	1·6 (1·1 to 2·2)	3·1 (2·3 to 4)	5·2 (3·9 to 6·8)	6·7 (5·1 to 8·8)
Bladder cancer	0·1 (0 to 0·1)	0·1 (0 to 0·1)	0·2 (0·1 to 0·4)	0·3 (0·2 to 0·5)	0·7 (0·5 to 1·1)	1·6 (1·1 to 2·2)	3·2 (2·2 to 4·4)	5·3 (3·8 to 7·4)
Brain and central nervous system cancer	2·2 (1·1 to 3·3)	2·3 (1·1 to 3·6)	2·9 (1·4 to 4·5)	3·1 (1·4 to 4·6)	3·8 (1·5 to 6)	5 (2·1 to 7·8)	6·4 (3 to 9·6)	7·3 (3·5 to 11·2)
Thyroid cancer	0·2 (0·1 to 0·3)	0·2 (0·1 to 0·4)	0·3 (0·2 to 0·5)	0·5 (0·3 to 0·7)	0·6 (0·4 to 0·9)	1·4 (0·8 to 2)	2 (1·3 to 3)	2·5 (1·7 to 3·7)
Mesothelioma	0·1 (0·1 to 0·1)	0·1 (0·1 to 0·2)	0·1 (0·1 to 0·2)	0·2 (0·1 to 0·3)	0·2 (0·2 to 0·3)	0·5 (0·4 to 0·7)	0·8 (0·6 to 1·1)	1 (0·7 to 1·3)
Hodgkin lymphoma	1 (0·6 to 1·5)	0·9 (0·5 to 1·4)	0·8 (0·5 to 1·2)	0·7 (0·4 to 1)	0·7 (0·4 to 1)	0·7 (0·4 to 1)	0·8 (0·5 to 1·2)	0·8 (0·5 to 1·2)
Non-Hodgkin lymphoma	8·8 (6·3 to 12·2)	8·5 (5·8 to 11·9)	9·9 (7·2 to 13·5)	9 (6·6 to 12·2)	10·2 (7·4 to 14·2)	14·7 (10·5 to 20·1)	18·1 (13·5 to 23·6)	21·7 (15·9 to 29·4)
Multiple myeloma	0·1 (0·1 to 0·2)	0·2 (0·1 to 0·3)	0·2 (0·1 to 0·4)	0·5 (0·3 to 0·8)	1·2 (0·8 to 1·7)	2·9 (2 to 4·2)	5·2 (3·5 to 7·8)	7·1 (4·8 to 10·3)
Leukemia	5 (3·4 to 7·3)	3·8 (2·6 to 5·2)	3·5 (2·4 to 4·8)	3·1 (2·1 to 4·3)	3·4 (2·4 to 4·6)	4·6 (3·5 to 5·9)	5·4 (4·2 to 6·9)	6·1 (4·6 to 8)
Acute lymphoid leukemia	1 (0·5 to 1·7)	0·5 (0·2 to 0·8)	0·4 (0·2 to 0·6)	0·3 (0·2 to 0·5)	0·2 (0·1 to 0·4)	0·2 (0·1 to 0·4)	0·3 (0·2 to 0·5)	0·3 (0·2 to 0·5)
Chronic lymphoid leukemia	0·1 (0 to 0·2)	0·1 (0 to 0·1)	0 (0 to 0·1)	0 (0 to 0)	0·1 (0 to 0·1)	0·1 (0 to 0·1)	0·2 (0·1 to 0·4)	0·4 (0·2 to 0·7)
Acute myeloid leukemia	1·4 (0·8 to 2·3)	1·3 (0·8 to 2·1)	1·1 (0·6 to 1·6)	0·9 (0·5 to 1·4)	0·7 (0·4 to 1·2)	1·1 (0·6 to 1·6)	1·2 (0·7 to 1·9)	1·2 (0·7 to 1·9)

Cause	60 to 64	65 to 69	70 to 74	75 to 79	80 to 84	85 to 89	90 to 94	95 plus	Total
All causes	1,760 (1,610 to 1,920)	1,730 (1,610 to 1,880)	1,520 (1,420 to 1,630)	1,200 (1,130 to 1,280)	480 (450 to 508)	155 (146 to 164)	52·1 (49·2 to 55·4)	12·4 (11·8 to 13·2)	17,600
Communicable, maternal, neonatal, and nutritional diseases	83·9 (70·7 to 97·8)	81·7 (65·8 to 103)	81 (62·5 to 105)	71·4 (55 to 91·5)	32·9 (26·1 to 41·4)	14·1 (11·3 to 17·1)	5·6 (4·5 to 6·9)	1·6 (1·3 to 2)	1,930
HIV/AIDS and sexually transmitted infections	7·8 (7 to 8·7)	4·2 (3·6 to 4·7)	1·3 (1·1 to 1·6)	0·5 (0·4 to 0·6)	0·1 (0·1 to 0·2)	0 (0 to 0)	0 (0 to 0)	0 (0 to 0)	201
HIV/AIDS	7·5 (6·7 to 8·4)	3·9 (3·4 to 4·4)	1·1 (0·9 to 1·4)	0·4 (0·3 to 0·5)	0·1 (0·1 to 0·1)	0 (0 to 0)	0 (0 to 0)	0 (0 to 0)	194
HIV/AIDS - Drug-susceptible Tuberculosis	0·2 (0·1 to 0·3)	0·1 (0·1 to 0·1)	0 (0 to 0)	0 (0 to 0)	0 (0 to 0)	0 (0 to 0)	0 (0 to 0)	0 (0 to 0)	4·4
HIV/AIDS - Multidrug-resistant Tuberculosis without extensive dru	0 (0 to 0)	0 (0 to 0)	0 (0 to 0)	0 (0 to 0)	0 (0 to 0)	0 (0 to 0)	0 (0 to 0)	0 (0 to 0)	0·1
HIV/AIDS - Extensively drug-resistant Tuberculosis	0 (0 to 0)	0 (0 to 0)	0 (0 to 0)	0 (0 to 0)	0 (0 to 0)	0 (0 to 0)	0 (0 to 0)	0 (0 to 0)	0
HIV/AIDS resulting in other diseases	7·3 (6·5 to 8·1)	3·8 (3·3 to 4·3)	1·1 (0·9 to 1·3)	0·4 (0·3 to 0·5)	0·1 (0·1 to 0·1)	0 (0 to 0)	0 (0 to 0)	0 (0 to 0)	189
Sexually transmitted infections excluding HIV	0·3 (0·2 to 0·5)	0·3 (0·2 to 0·5)	0·2 (0·1 to 0·3)	0·1 (0·1 to 0·2)	0 (0 to 0·1)	0 (0 to 0)	0 (0 to 0)	0 (0 to 0)	7·5
Syphilis	0·1 (0·1 to 0·2)	0·1 (0·1 to 0·2)	0·1 (0 to 0·1)	0 (0 to 0)	0 (0 to 0)	0 (0 to 0)	0 (0 to 0)	0 (0 to 0)	1·5
Chlamydial infection	0 (0 to 0)	0 (0 to 0)	0 (0 to 0)	0 (0 to 0)	0 (0 to 0)	0 (0 to 0)	0 (0 to 0)	0 (0 to 0)	1·2
Gonococcal infection	0 (0 to 0)	0 (0 to 0)	0 (0 to 0)	0 (0 to 0)	0 (0 to 0)	0 (0 to 0)	0 (0 to 0)	0 (0 to 0)	0·8
Trichomoniasis	0 (0 to 0)	0 (0 to 0)	0 (0 to 0)	0 (0 to 0)	0 (0 to 0)	0 (0 to 0)	0 (0 to 0)	0 (0 to 0)	0·2
Genital herpes	0·2 (0 to 0·4)	0·1 (0 to 0·3)	0·1 (0 to 0·2)	0 (0 to 0·1)	0 (0 to 0)	0 (0 to 0)	0 (0 to 0)	0 (0 to 0)	2·2
Other sexually transmitted infections	0 (0 to 0)	0 (0 to 0)	0 (0 to 0)	0 (0 to 0)	0 (0 to 0)	0 (0 to 0)	0 (0 to 0)	0 (0 to 0)	1·6
Respiratory infections and tuberculosis	49 (38·3 to 61·1)	55·1 (40·2 to 74·9)	59·9 (42·3 to 82·2)	55·8 (41·2 to 75·4)	26·8 (20·4 to 34·5)	12 (9·3 to 14·9)	4·9 (3·8 to 6·2)	1·4 (1·1 to 1·8)	621
Tuberculosis	1·2 (0·9 to 1·6)	1·2 (0·9 to 1·7)	1·2 (0·9 to 1·6)	0·8 (0·6 to 1)	0·3 (0·2 to 0·3)	0·1 (0·1 to 0·1)	0 (0 to 0)	0 (0 to 0)	17·1
Latent tuberculosis infection	0 (0 to 0)	0 (0 to 0)	0 (0 to 0)	0 (0 to 0)	0 (0 to 0)	0 (0 to 0)	0 (0 to 0)	0 (0 to 0)	0
Drug-susceptible tuberculosis	1·2 (0·9 to 1·5)	1·2 (0·9 to 1·7)	1·2 (0·9 to 1·6)	0·8 (0·6 to 1)	0·3 (0·2 to 0·3)	0·1 (0·1 to 0·1)	0 (0 to 0)	0 (0 to 0)	16·9
Multidrug-resistant tuberculosis without extensive drug resistanc	0 (0 to 0·1)	0 (0 to 0·1)	0 (0 to 0·1)	0 (0 to 0·1)	0 (0 to 0)	0 (0 to 0)	0 (0 to 0)	0 (0 to 0)	0·2
Extensively drug-resistant tuberculosis	0 (0 to 0)	0 (0 to 0)	0 (0 to 0)	0 (0 to 0)	0 (0 to 0)	0 (0 to 0)	0 (0 to 0)	0 (0 to 0)	0
Lower respiratory infections	46 (35·3 to 58)	52·6 (37·9 to 72·1)	57·8 (40·3 to 79·8)	54·4 (39·8 to 74)	26·4 (20 to 34·1)	11·9 (9·2 to 14·8)	4·8 (3·8 to 6·1)	1·4 (1·1 to 1·8)	546
Upper respiratory infections	1·4 (0·7 to 2·3)	1 (0·5 to 1·6)	0·7 (0·4 to 1·1)	0·4 (0·2 to 0·7)	0·1 (0·1 to 0·2)	0 (0 to 0·1)	0 (0 to 0)	0 (0 to 0)	43·3
Otitis media	0·5 (0·2 to 0·9)	0·3 (0·1 to 0·5)	0·2 (0·1 to 0·3)	0·1 (0·1 to 0·2)	0 (0 to 0·1)	0 (0 to 0)	0 (0 to 0)	0 (0 to 0)	14·9
Enteric infections	6·6 (4·7 to 9)	6·6 (4·9 to 8·8)	6·5 (4·8 to 8·6)	5·6 (4·1 to 7·3)	2·3 (1·7 to 3)	0·7 (0·5 to 0·9)	0·2 (0·2 to 0·3)	0·1 (0 to 0·1)	129
Diarrheal diseases	6·6 (4·7 to 8·9)	6·6 (4·9 to 8·8)	6·5 (4·8 to 8·6)	5·6 (4·1 to 7·3)	2·3 (1·7 to 3)	0·7 (0·5 to 0·9)	0·2 (0·2 to 0·3)	0·1 (0 to 0·1)	128
Typhoid and paratyphoid	0 (0 to 0)	0 (0 to 0)	0 (0 to 0)	0 (0 to 0)	0 (0 to 0)	0 (0 to 0)	0 (0 to 0)	0 (0 to 0)	0
Typhoid fever	0 (0 to 0)	0 (0 to 0)	0 (0 to 0)	0 (0 to 0)	0 (0 to 0)	0 (0 to 0)	0 (0 to 0)	0 (0 to 0)	0
Paratyphoid fever	0 (0 to 0)	0 (0 to 0)	0 (0 to 0)	0 (0 to 0)	0 (0 to 0)	0 (0 to 0)	0 (0 to 0)	0 (0 to 0)	0
Invasive Non-typhoidal Salmonella (iNTS)	0 (0 to 0)	0 (0 to 0)	0 (0 to 0)	0 (0 to 0)	0 (0 to 0)	0 (0 to 0)	0 (0 to 0)	0 (0 to 0)	0
Other intestinal infectious diseases	0 (0 to 0)	0 (0 to 0)	0 (0 to 0)	0 (0 to 0)	0 (0 to 0)	0 (0 to 0)	0 (0 to 0)	0 (0 to 0)	0·3
Neglected tropical diseases and malaria	3·9 (1·8 to 7·2)	3·5 (1·6 to 6·3)	3·3 (1·6 to 5·8)	2·5 (1·2 to 4·5)	0·9 (0·4 to 1·6)	0·3 (0·1 to 0·5)	0·1 (0 to 0·2)	0 (0 to 0)	49·4
Malaria	0 (0 to 0)	0 (0 to 0)	0 (0 to 0)	0 (0 to 0)	0 (0 to 0)	0 (0 to 0)	0 (0 to 0)	0 (0 to 0)	0
Chagas disease	0 (0 to 0)	0 (0 to 0)	0 (0 to 0)	0 (0 to 0)	0 (0 to 0)	0 (0 to 0)	0 (0 to 0)	0 (0 to 0)	0·1
Leishmaniasis	0 (0 to 0)	0 (0 to 0)	0 (0 to 0)	0 (0 to 0)	0 (0 to 0)	0 (0 to 0)	0 (0 to 0)	0 (0 to 0)	0
Visceral leishmaniasis	0 (0 to 0)	0 (0 to 0)	0 (0 to 0)	0 (0 to 0)	0 (0 to 0)	0 (0 to 0)	0 (0 to 0)	0 (0 to 0)	0
Cutaneous and mucocutaneous leishmaniasis	0 (0 to 0)	0 (0 to 0)	0 (0 to 0)	0 (0 to 0)	0 (0 to 0)	0 (0 to 0)	0 (0 to 0)	0 (0 to 0)	0
African trypanosomiasis	0 (0 to 0)	0 (0 to 0)	0 (0 to 0)	0 (0 to 0)	0 (0 to 0)	0 (0 to 0)	0 (0 to 0)	0 (0 to 0)	0
Schistosomiasis	0 (0 to 0)	0 (0 to 0)	0 (0 to 0)	0 (0 to 0)	0 (0 to 0)	0 (0 to 0)	0 (0 to 0)	0 (0 to 0)	0
Cysticercosis	3·2 (1·2 to 6·5)	3 (1·1 to 5·8)	2·7 (1·1 to 5·2)	2·2 (0·8 to 4·1)	0·8 (0·3 to 1·6)	0·2 (0·1 to 0·5)	0·1 (0 to 0·1)	0 (0 to 0)	26·6
Cystic echinococcosis	0 (0 to 0)	0 (0 to 0)	0 (0 to 0)	0 (0 to 0)	0 (0 to 0)	0 (0 to 0)	0 (0 to 0)	0 (0 to 0)	0
Lymphatic filariasis	0 (0 to 0)	0 (0 to 0)	0 (0 to 0)	0 (0 to 0)	0 (0 to 0)	0 (0 to 0)	0 (0 to 0)	0 (0 to 0)	0
Onchocerciasis	0 (0 to 0)	0 (0 to 0)	0 (0 to 0)	0 (0 to 0)	0 (0 to 0)	0 (0 to 0)	0 (0 to 0)	0 (0 to 0)	0
Trachoma	0 (0 to 0)	0 (0 to 0)	0 (0 to 0)	0 (0 to 0)	0 (0 to 0)	0 (0 to 0)	0 (0 to 0)	0 (0 to 0)	0
Dengue	0·3 (0·1 to 0·7)	0·2 (0 to 0·4)	0·1 (0 to 0·3)	0·1 (0 to 0·2)	0 (0 to 0·1)	0 (0 to 0)	0 (0 to 0)	0 (0 to 0)	7·5
Yellow fever	0 (0 to 0)	0 (0 to 0)	0 (0 to 0)	0 (0 to 0)	0 (0 to 0)	0 (0 to 0)	0 (0 to 0)	0 (0 to 0)	0
Rabies	0 (0 to 0)	0 (0 to 0)	0 (0 to 0)	0 (0 to 0)	0 (0 to 0)	0 (0 to 0)	0 (0 to 0)	0 (0 to 0)	0·1
Intestinal nematode infections	0·1 (0 to 0·1)	0 (0 to 0·1)	0 (0 to 0·1)	0 (0 to 0)	0 (0 to 0)	0 (0 to 0)	0 (0 to 0)	0 (0 to 0)	1·7
Ascariasis	0 (0 to 0)	0 (0 to 0)	0 (0 to 0)	0 (0 to 0)	0 (0 to 0)	0 (0 to 0)	0 (0 to 0)	0 (0 to 0)	0·5
Trichuriasis	0 (0 to 0)	0 (0 to 0)	0 (0 to 0)	0 (0 to 0)	0 (0 to 0)	0 (0 to 0)	0 (0 to 0)	0 (0 to 0)	0
Hookworm disease	0 (0 to 0·1)	0 (0 to 0·1)	0 (0 to 0)	0 (0 to 0)	0 (0 to 0)	0 (0 to 0)	0 (0 to 0)	0 (0 to 0)	1·2
Food-borne trematodiases	0 (0 to 0)	0 (0 to 0)	0 (0 to 0)	0 (0 to 0)	0 (0 to 0)	0 (0 to 0)	0 (0 to 0)	0 (0 to 0)	0
Leprosy	0 (0 to 0·1)	0 (0 to 0·1)	0 (0 to 0·1)	0 (0 to 0)	0 (0 to 0)	0 (0 to 0)	0 (0 to 0)	0 (0 to 0)	0·4
Ebola	0 (0 to 0)	0 (0 to 0)	0 (0 to 0)	0 (0 to 0)	0 (0 to 0)	0 (0 to 0)	0 (0 to 0)	0 (0 to 0)	0
Zika virus	0 (0 to 0·2)	0 (0 to 0·1)	0 (0 to 0)	0 (0 to 0)	0 (0 to 0)	0 (0 to 0)	0 (0 to 0)	0 (0 to 0)	2·1
Guinea worm disease	0 (0 to 0)	0 (0 to 0)	0 (0 to 0)	0 (0 to 0)	0 (0 to 0)	0 (0 to 0)	0 (0 to 0)	0 (0 to 0)	0
Other neglected tropical diseases	0·2 (0·1 to 0·7)	0·2 (0·1 to 0·4)	0·3 (0·2 to 0·7)	0·2 (0·1 to 0·5)	0·1 (0 to 0·1)	0 (0 to 0·1)	0 (0 to 0)	0 (0 to 0)	11
Other infectious diseases	5·5 (3·8 to 7·7)	3·9 (2·4 to 5·4)	2·9 (1·8 to 4)	1·7 (1·1 to 2·4)	0·6 (0·4 to 0·9)	0·3 (0·1 to 0·4)	0·1 (0 to 0·1)	0 (0 to 0)	85·6
Meningitis	0·6 (0·4 to 0·9)	0·5 (0·3 to 0·7)	0·4 (0·3 to 0·5)	0·3 (0·2 to 0·4)	0·1 (0·1 to 0·1)	0 (0 to 0)	0 (0 to 0)	0 (0 to 0)	13·3
Encephalitis	0·5 (0·3 to 0·8)	0·3 (0·2 to 0·5)	0·3 (0·2 to 0·4)	0·1 (0·1 to 0·2)	0·1 (0 to 0·1)	0 (0 to 0)	0 (0 to 0)	0 (0 to 0)	12·8
Diphtheria	0 (0 to 0)	0 (0 to 0)	0 (0 to 0)	0 (0 to 0)	0 (0 to 0)	0 (0 to 0)	0 (0 to 0)	0 (0 to 0)	0
Tetanus	0·2 (0·1 to 1·1)	0·1 (0 to 0·9)	0·1 (0 to 0·6)	0·1 (0 to 0·4)	0 (0 to 0·1)	0 (0 to 0)	0 (0 to 0)	0 (0 to 0)	2·1
Measles	0 (0 to 0)	0 (0 to 0)	0 (0 to 0)	0 (0 to 0)	0 (0 to 0)	0 (0 to 0)	0 (0 to 0)	0 (0 to 0)	0
Varicella and herpes zoster	0·2 (0·1 to 0·4)	0·1 (0·1 to 0·2)	0·2 (0·1 to 0·4)	0·1 (0·1 to 0·2)	0 (0 to 0·1)	0 (0 to 0)	0 (0 to 0)	0 (0 to 0)	3
Acute hepatitis	0·2 (0·1 to 0·2)	0·1 (0·1 to 0·2)	0·1 (0·1 to 0·1)	0 (0 to 0·1)	0 (0 to 0)	0 (0 to 0)	0 (0 to 0)	0 (0 to 0)	4·6
Acute hepatitis A	0 (0 to 0·1)	0 (0 to 0·1)	0 (0 to 0)	0 (0 to 0)	0 (0 to 0)	0 (0 to 0)	0 (0 to 0)	0 (0 to 0)	2·1
Acute hepatitis B	0·1 (0·1 to 0·1)	0·1 (0·1 to 0·1)	0·1 (0 to 0·1)	0 (0 to 0)	0 (0 to 0)	0 (0 to 0)	0 (0 to 0)	0 (0 to 0)	1·9
Acute hepatitis C	0 (0 to 0)	0 (0 to 0)	0 (0 to 0)	0 (0 to 0)	0 (0 to 0)	0 (0 to 0)	0 (0 to 0)	0 (0 to 0)	0·1
Acute hepatitis E	0 (0 to 0·1)	0 (0 to 0)	0 (0 to 0)	0 (0 to 0)	0 (0 to 0)	0 (0 to 0)	0 (0 to 0)	0 (0 to 0)	0·5
Other unspecified infectious diseases	3·8 (2 to 5·6)	2·6 (1·2 to 3·9)	1·9 (0·8 to 2·9)	1·1 (0·4 to 1·7)	0·4 (0·2 to 0·6)	0·2 (0·1 to 0·3)	0 (0 to 0·1)	0 (0 to 0)	49·2
Maternal and neonatal disorders	3·4 (2·4 to 4·7)	1·6 (1·1 to 2·2)	0·6 (0·4 to 0·8)	0·2 (0·1 to 0·3)	0 (0 to 0)	0 (0 to 0)	0 (0 to 0)	0 (0 to 0)	677
Maternal disorders	0 (0 to 0)	0 (0 to 0)	0 (0 to 0)	0 (0 to 0)	0 (0 to 0)	0 (0 to 0)	0 (0 to 0)	0 (0 to 0)	0
Maternal hemorrhage	0 (0 to 0)	0 (0 to 0)	0 (0 to 0)	0 (0 to 0)	0 (0 to 0)	0 (0 to 0)	0 (0 to 0)	0 (0 to 0)	0
Maternal sepsis and other maternal infections	0 (0 to 0)	0 (0 to 0)	0 (0 to 0)	0 (0 to 0)	0 (0 to 0)	0 (0 to 0)	0 (0 to 0)	0 (0 to 0)	0
Maternal hypertensive disorders	0 (0 to 0)	0 (0 to 0)	0 (0 to 0)	0 (0 to 0)	0 (0 to 0)	0 (0 to 0)	0 (0 to 0)	0 (0 to 0)	0
Maternal obstructed labor and uterine rupture	--	--	--	--	--	--	--	--	NA
Maternal abortion and miscarriage	--	--	--	--	--	--	--	--	NA
Ectopic pregnancy	--	--	--	--	--	--	--	--	NA
Indirect maternal deaths	--	--	--	--	--	--	--	--	NA
Late maternal deaths	--	--	--	--	--	--	--	--	NA
Maternal deaths aggravated by HIV/AIDS	--	--	--	--	--	--	--	--	NA
Neonatal disorders	3·4 (2·4 to 4·7)	1·6 (1·1 to 2·2)	0·6 (0·4 to 0·8)	0·2 (0·1 to 0·3)	0 (0 to 0)	0 (0 to 0)	0 (0 to 0)	0 (0 to 0)	677
Neonatal preterm birth	1 (0·8 to 1·3)	0·4 (0·3 to 0·5)	0·2 (0·1 to 0·2)	0·1 (0 to 0·1)	0 (0 to 0)	0 (0 to 0)	0 (0 to 0)	0 (0 to 0)	255
Neonatal encephalopathy due to birth asphyxia and trauma	0·6 (0·3 to 1·1)	0·3 (0·2 to 0·4)	0·1 (0 to 0·1)	0 (0 to 0)	0 (0 to 0)	0 (0 to 0)	0 (0 to 0)	0 (0 to 0)	206
Neonatal sepsis and other neonatal infections	1·5 (0·8 to 2·4)	0·7 (0·4 to 1·2)	0·3 (0·1 to 0·5)	0·1 (0 to 0·1)	0 (0 to 0)	0 (0 to 0)	0 (0 to 0)	0 (0 to 0)	151
Hemolytic disease and other neonatal jaundice	0·2 (0·2 to 0·3)	0·1 (0·1 to 0·2)	0·1 (0 to 0·1)	0 (0 to 0)	0 (0 to 0)	0 (0 to 0)	0 (0 to 0)	0 (0 to 0)	8·9
Other neonatal disorders	0 (0 to 0)	0 (0 to 0)	0 (0 to 0)	0 (0 to 0)	0 (0 to 0)	0 (0 to 0)	0 (0 to 0)	0 (0 to 0)	57
Nutritional deficiencies	7·7 (4·8 to 12·7)	6·9 (4·7 to 10·3)	6·5 (4·4 to 9·4)	5·1 (3·6 to 7·4)	2·2 (1·6 to 3·1)	0·9 (0·6 to 1·2)	0·3 (0·2 to 0·4)	0·1 (0·1 to 0·1)	170
Protein-energy malnutrition	4 (2·9 to 5·4)	4·3 (3·1 to 5·6)	4·4 (3 to 6·3)	3·7 (2·6 to 4·8)	1·6 (1·1 to 2·3)	0·6 (0·4 to 0·8)	0·2 (0·2 to 0·3)	0·1 (0·1 to 0·1)	56·7
Iodine deficiency	0·1 (0·1 to 0·2)	0·1 (0 to 0·1)	0 (0 to 0·1)	0 (0 to 0·1)	0 (0 to 0)	0 (0 to 0)	0 (0 to 0)	0 (0 to 0)	2·3
Vitamin A deficiency	0 (0 to 0·1)	0 (0 to 0)	0 (0 to 0)	0 (0 to 0)	0 (0 to 0)	0 (0 to 0)	0 (0 to 0)	0 (0 to 0)	3·1
Dietary iron deficiency	3 (0·7 to 7·6)	2 (0·5 to 5·2)	1·6 (0·4 to 4)	1·1 (0·3 to 3)	0·4 (0·1 to 1·1)	0·2 (0·1 to 0·5)	0·1 (0 to 0·1)	0 (0 to 0)	100
Other nutritional deficiencies	0·5 (0·3 to 0·8)	0·5 (0·3 to 0·8)	0·4 (0·3 to 0·7)	0·3 (0·2 to 0·5)	0·1 (0·1 to 0·3)	0 (0 to 0·1)	0 (0 to 0)	0 (0 to 0)	7·1
Non-communicable diseases	1,570 (1,430 to 1,710)	1,570 (1,450 to 1,700)	1,380 (1,280 to 1,480)	1,080 (1,010 to 1,160)	427 (401 to 453)	134 (126 to 142)	44 (41·3 to 47)	10·1 (9·4 to 10·9)	13,400
Neoplasms	371 (320 to 428)	387 (338 to 447)	375 (308 to 429)	295 (236 to 341)	106 (85·5 to 123)	31·9 (25·9 to 37·6)	9·8 (7·5 to 11·7)	2 (1·5 to 2·6)	2,580
Lip and oral cavity cancer	11 (7·8 to 14·5)	9·8 (7·2 to 13·4)	7·2 (5·1 to 9·6)	4·9 (3·5 to 6·6)	1·6 (1·1 to 2·2)	0·3 (0·2 to 0·5)	0·1 (0·1 to 0·1)	0 (0 to 0)	69·3
Nasopharynx cancer	1·9 (1·3 to 2·7)	1·6 (1 to 2·3)	1·1 (0·7 to 1·7)	0·6 (0·4 to 0·8)	0·2 (0·1 to 0·3)	0·1 (0 to 0·1)	0 (0 to 0)	0 (0 to 0)	13·9
Other pharynx cancer	6·1 (3·9 to 9)	5·2 (3·3 to 7·5)	3·4 (2·2 to 5)	1·9 (1·2 to 2·8)	0·6 (0·4 to 0·9)	0·2 (0·1 to 0·2)	0 (0 to 0·1)	0 (0 to 0)	35·5
Esophageal cancer	28·8 (21 to 37·8)	25·5 (18·6 to 33·3)	19·9 (14·6 to 26·6)	11·7 (8·4 to 15·8)	3·6 (2·6 to 4·9)	1 (0·7 to 1·2)	0·2 (0·2 to 0·3)	0 (0 to 0)	171
Stomach cancer	22·5 (17·2 to 28·8)	23·3 (18·3 to 29·7)	19·9 (15·3 to 25·2)	13·7 (11 to 16·9)	4·6 (3·5 to 5·9)	1·2 (1 to 1·6)	0·3 (0·2 to 0·4)	0·1 (0 to 0·1)	146
Colon and rectum cancer	41·4 (31·1 to 54·6)	42·2 (31·2 to 54·3)	34·8 (26·3 to 45·4)	23·9 (18·4 to 30·1)	8·6 (6·3 to 11·7)	2·4 (1·8 to 3·1)	0·7 (0·5 to 0·9)	0·1 (0·1 to 0·2)	258
Liver cancer	8·3 (5·7 to 11·5)	8·1 (5·8 to 11·3)	6·8 (4·9 to 9·2)	4·7 (3·5 to 6·2)	1·4 (1·1 to 1·9)	0·3 (0·2 to 0·4)	0·1 (0 to 0·1)	0 (0 to 0)	53·9
Liver cancer due to hepatitis B	2·6 (1·4 to 4·6)	2 (1 to 3·5)	1·4 (0·7 to 2·5)	0·9 (0·5 to 1·6)	0·3 (0·1 to 0·4)	0·1 (0 to 0·1)	0 (0 to 0)	0 (0 to 0)	17·8
Liver cancer due to hepatitis C	1·1 (0·5 to 2·1)	1·1 (0·6 to 1·9)	1·1 (0·5 to 1·8)	0·9 (0·4 to 1·6)	0·3 (0·2 to 0·6)	0·1 (0·1 to 0·1)	0 (0 to 0)	0 (0 to 0)	6·8
Liver cancer due to alcohol use	3·9 (2·2 to 6)	4·2 (2·6 to 6·3)	3·6 (2·3 to 5·4)	2·4 (1·6 to 3·4)	0·7 (0·4 to 0·9)	0·1 (0·1 to 0·2)	0 (0 to 0)	0 (0 to 0)	23·7
Liver cancer due to NASH	0·4 (0·2 to 0·8)	0·4 (0·2 to 0·8)	0·4 (0·2 to 0·8)	0·4 (0·2 to 0·7)	0·1 (0·1 to 0·2)	0 (0 to 0)	0 (0 to 0)	0 (0 to 0)	2·9
Liver cancer due to other causes	0·3 (0·1 to 0·5)	0·2 (0·1 to 0·4)	0·2 (0·1 to 0·3)	0·1 (0·1 to 0·3)	0 (0 to 0·1)	0 (0 to 0)	0 (0 to 0)	0 (0 to 0)	2·6
Gallbladder and biliary tract cancer	3·5 (2·3 to 5·9)	3·4 (2·3 to 5·4)	2·8 (1·9 to 4·5)	2 (1·3 to 3·2)	0·7 (0·4 to 1·1)	0·2 (0·1 to 0·3)	0·1 (0 to 0·1)	0 (0 to 0)	20·7
Pancreatic cancer	23·8 (17·4 to 31·9)	23·7 (17·6 to 31·8)	18·9 (13·6 to 25·1)	12·4 (9·3 to 16·6)	4 (2·8 to 5·5)	1 (0·7 to 1·3)	0·2 (0·2 to 0·3)	0 (0 to 0·1)	150
Larynx cancer	5·5 (3·8 to 7·6)	5·3 (3·7 to 7·3)	4 (2·9 to 5·5)	2·6 (1·9 to 3·6)	0·8 (0·6 to 1·1)	0·2 (0·1 to 0·3)	0 (0 to 0·1)	0 (0 to 0)	31·7
Tracheal, bronchus, and lung cancer	46 (35·6 to 57·8)	43·7 (34·8 to 54·1)	36·4 (28·6 to 45)	23·3 (18·1 to 29·9)	6·8 (5 to 9)	1·7 (1·3 to 2·3)	0·4 (0·3 to 0·5)	0·1 (0·1 to 0·1)	276
Malignant skin melanoma	1·3 (0·7 to 2·6)	1·1 (0·7 to 2·3)	1 (0·6 to 2)	0·7 (0·4 to 1·4)	0·3 (0·2 to 0·6)	0·1 (0 to 0·2)	0 (0 to 0)	0 (0 to 0)	11·6
Non-melanoma skin cancer	1·9 (1·4 to 2·4)	2 (1·2 to 2·6)	1·7 (0·9 to 2·2)	1·5 (0·8 to 2)	0·7 (0·4 to 0·9)	0·3 (0·2 to 0·3)	0·1 (0·1 to 0·1)	0 (0 to 0)	13·5
Non-melanoma skin cancer (squamous-cell carcinoma)	1·9 (1·4 to 2·4)	2 (1·2 to 2·6)	1·7 (0·9 to 2·2)	1·5 (0·8 to 2)	0·7 (0·4 to 0·9)	0·3 (0·2 to 0·3)	0·1 (0·1 to 0·1)	0 (0 to 0)	13·5
Non-melanoma skin cancer (basal-cell carcinoma)	0 (0 to 0)	0 (0 to 0)	0 (0 to 0)	0 (0 to 0)	0 (0 to 0)	0 (0 to 0)	0 (0 to 0)	0 (0 to 0)	0
Breast cancer	0·8 (0·5 to 1·4)	0·9 (0·5 to 1·5)	0·7 (0·3 to 1·2)	0·5 (0·2 to 0·8)	0·2 (0·1 to 0·3)	0·1 (0 to 0·1)	0 (0 to 0)	0 (0 to 0)	5·5
Cervical cancer	--	--	--	--	--	--	--	--	NA
Uterine cancer	--	--	--	--	--	--	--	--	NA
Ovarian cancer	--	--	--	--	--	--	--	--	NA
Prostate cancer	102 (71·4 to 141)	128 (87·1 to 174)	165 (92·5 to 216)	156 (89·1 to 200)	60·8 (38·5 to 77·1)	20·1 (13·9 to 25·8)	6·7 (4·6 to 8·6)	1·4 (0·9 to 2)	735
Testicular cancer	0·1 (0·1 to 0·2)	0·1 (0·1 to 0·2)	0·1 (0·1 to 0·2)	0·1 (0·1 to 0·2)	0 (0 to 0·1)	0 (0 to 0)	0 (0 to 0)	0 (0 to 0)	3·5
Kidney cancer	6·3 (4·8 to 8)	5·9 (4·5 to 7·6)	4·5 (3·5 to 5·9)	2·7 (2 to 3·5)	0·8 (0·6 to 1·1)	0·2 (0·1 to 0·2)	0 (0 to 0·1)	0 (0 to 0)	43·2
Bladder cancer	7·2 (5·1 to 9·7)	8 (5·8 to 11)	8·1 (5·8 to 11)	6·5 (4·7 to 8·7)	2·4 (1·7 to 3·1)	0·7 (0·5 to 1)	0·2 (0·2 to 0·3)	0 (0 to 0·1)	44·8
Brain and central nervous system cancer	5·7 (2·7 to 8·7)	4·6 (1·8 to 7)	3 (1 to 4·8)	1·6 (0·5 to 2·5)	0·4 (0·1 to 0·7)	0·1 (0 to 0·1)	0 (0 to 0)	0 (0 to 0)	58·9
Thyroid cancer	2·1 (1·4 to 3)	1·9 (1·2 to 2·7)	1·5 (1 to 2·2)	1 (0·7 to 1·4)	0·4 (0·2 to 0·5)	0·1 (0·1 to 0·1)	0 (0 to 0)	0 (0 to 0)	14·8
Mesothelioma	0·4 (0·3 to 0·5)	0·3 (0·2 to 0·4)	0·3 (0·2 to 0·4)	0·2 (0·2 to 0·4)	0·1 (0·1 to 0·1)	0 (0 to 0)	0 (0 to 0)	0 (0 to 0)	4·4
Hodgkin lymphoma	0·7 (0·5 to 1)	0·7 (0·4 to 0·9)	0·4 (0·3 to 0·6)	0·3 (0·2 to 0·4)	0·1 (0 to 0·1)	0 (0 to 0)	0 (0 to 0)	0 (0 to 0)	9·4
Non-Hodgkin lymphoma	18·7 (14·5 to 24)	17·2 (13·2 to 22·4)	13·4 (10·2 to 17·7)	8·1 (6 to 10·5)	2·3 (1·7 to 3)	0·5 (0·4 to 0·6)	0·1 (0·1 to 0·1)	0 (0 to 0)	177
Multiple myeloma	7·2 (5 to 10·3)	7·3 (5 to 10·3)	5·5 (3·8 to 7·8)	3·6 (2·4 to 5)	1·1 (0·7 to 1·6)	0·2 (0·2 to 0·3)	0 (0 to 0·1)	0 (0 to 0)	42·3
Leukemia	5·6 (4·4 to 7)	5·8 (4·5 to 7·4)	5 (3·8 to 6·4)	3·5 (2·7 to 4·5)	1·2 (0·9 to 1·5)	0·3 (0·3 to 0·4)	0·1 (0·1 to 0·1)	0 (0 to 0)	72·8
Acute lymphoid leukemia	0·2 (0·1 to 0·3)	0·2 (0·1 to 0·4)	0·2 (0·1 to 0·3)	0·1 (0 to 0·1)	0 (0 to 0)	0 (0 to 0)	0 (0 to 0)	0 (0 to 0)	8·6
Chronic lymphoid leukemia	0·4 (0·2 to 0·7)	0·5 (0·3 to 0·8)	0·4 (0·2 to 0·6)	0·3 (0·1 to 0·5)	0·1 (0·1 to 0·2)	0 (0 to 0)	0 (0 to 0)	0 (0 to 0)	2·6
Acute myeloid leukemia	0·9 (0·5 to 1·5)	0·8 (0·4 to 1·4)	0·6 (0·3 to 1·1)	0·3 (0·2 to 0·7)	0·1 (0 to 0·2)	0 (0 to 0)	0 (0 to 0)	0 (0 to 0)	16·1

Chronic myeloid leukemia	0 (0 to 0)	0 (0 to 0)	0·1 (0 to 0·2)	0·2 (0·1 to 0·3)	0·1 (0·1 to 0·2)	0·1 (0·1 to 0·2)	0·2 (0·1 to 0·4)	0·4 (0·2 to 0·8)	0·5 (0·2 to 0·9)	0·5 (0·3 to 0·9)	0·5 (0·3 to 0·9)	0·4 (0·2 to 0·8)	0·6 (0·4 to 1)	0·7 (0·4 to 1·2)	0·9 (0·5 to 1·4)	0·8 (0·4 to 1·3)	0·8 (0·5 to 1·3)	0·6 (0·3 to 0·9)	0·4 (0·2 to 0·7)	0·1 (0·1 to 0·2)	0 (0 to 0)	0 (0 to 0)	0 (0 to 0)	8
Other leukemia	0·1 (0 to 0·2)	0 (0 to 0·1)	0·4 (0·2 to 0·6)	0·7 (0·3 to 1·3)	1·8 (0·9 to 3)	1·7 (1 to 2·8)	1·8 (1 to 2·8)	2·1 (1·2 to 3·3)	1·4 (0·9 to 2·2)	1·5 (1 to 2·3)	1·4 (0·9 to 2)	1·9 (1·3 to 2·7)	2·6 (1·8 to 3·5)	2·9 (2·1 to 4)	3·3 (2·4 to 4·5)	3·3 (2·4 to 4·4)	3·5 (2·5 to 4·5)	3·3 (2·4 to 4·4)	2·4 (1·8 to 3·2)	0·9 (0·6 to 1·1)	0·3 (0·2 to 0·3)	0·1 (0 to 0·1)	0 (0 to 0)	37·5
Other malignant neoplasms	0·2 (0·1 to 0·3)	0·1 (0·1 to 0·2)	1 (0·6 to 1·6)	2·5 (1·5 to 4·2)	2·8 (2·1 to 3·8)	3 (2·3 to 3·9)	5·3 (4 to 6·9)	6·1 (4·5 to 8·4)	4·8 (3·4 to 6·8)	4·8 (3·6 to 6·4)	4·3 (3·3 to 5·5)	5·2 (3·7 to 7)	7·8 (5·7 to 10·4)	9·9 (6·9 to 13·8)	12 (8·6 to 16·9)	10·2 (7·5 to 13·7)	9·2 (6·8 to 12·3)	7·3 (5·5 to 9·8)	5·2 (3·9 to 6·8)	1·8 (1·4 to 2·4)	0·5 (0·4 to 0·7)	0·2 (0·1 to 0·2)	0 (0 to 0)	104
Other neoplasms	0 (0 to 0·1)	0 (0 to 0)	0·1 (0 to 0·2)	0·3 (0·1 to 0·5)	0·4 (0·2 to 0·6)	0·3 (0·1 to 0·4)	0·3 (0·2 to 0·5)	0·2 (0·1 to 0·4)	0·4 (0·2 to 0·7)	0·3 (0·2 to 0·5)	0·4 (0·2 to 0·6)	0·4 (0·2 to 0·6)	0·6 (0·4 to 1)	1·2 (0·7 to 2)	1·8 (1·1 to 3)	1·9 (1·1 to 3·2)	2 (1 to 2·9)	2·1 (1 to 3·2)	1·6 (0·8 to 2·5)	0·6 (0·3 to 0·9)	0·2 (0·1 to 0·3)	0 (0 to 0·1)	0 (0 to 0)	15·3
Myelodysplastic, myeloproliferative, and other hematopoietic neo	0 (0 to 0·1)	0 (0 to 0)	0·1 (0 to 0·2)	0·3 (0·1 to 0·5)	0·4 (0·2 to 0·6)	0·3 (0·1 to 0·4)	0·3 (0·2 to 0·5)	0·2 (0·1 to 0·4)	0·4 (0·2 to 0·7)	0·3 (0·2 to 0·5)	0·4 (0·2 to 0·6)	0·4 (0·2 to 0·6)	0·6 (0·4 to 1)	1·2 (0·7 to 2)	1·8 (1·1 to 3)	1·9 (1·1 to 3·2)	2 (1 to 2·9)	2·1 (1 to 3·2)	1·6 (0·8 to 2·5)	0·6 (0·3 to 0·9)	0·2 (0·1 to 0·3)	0 (0 to 0·1)	0 (0 to 0)	15·3
Benign and in situ intestinal neoplasms	0 (0 to 0)	0 (0 to 0)	0 (0 to 0)	0 (0 to 0)	0 (0 to 0)	0 (0 to 0)	0 (0 to 0)	0 (0 to 0)	0 (0 to 0)	0 (0 to 0)	0 (0 to 0)	0 (0 to 0)	0 (0 to 0)	0 (0 to 0)	0 (0 to 0)	0 (0 to 0)	0 (0 to 0)	0 (0 to 0)	0 (0 to 0)	0 (0 to 0)	0 (0 to 0)	0 (0 to 0)	0 (0 to 0)	0
Benign and in situ cervical and uterine neoplasms	0 (0 to 0)	0 (0 to 0)	0 (0 to 0)	0 (0 to 0)	0 (0 to 0)	0 (0 to 0)	0 (0 to 0)	0 (0 to 0)	0 (0 to 0)	0 (0 to 0)	0 (0 to 0)	0 (0 to 0)	0 (0 to 0)	0 (0 to 0)	0 (0 to 0)	0 (0 to 0)	0 (0 to 0)	0 (0 to 0)	0 (0 to 0)	0 (0 to 0)	0 (0 to 0)	0 (0 to 0)	0 (0 to 0)	0
Other benign and in situ neoplasms	0 (0 to 0)	0 (0 to 0)	0 (0 to 0)	0 (0 to 0)	0 (0 to 0)	0 (0 to 0)	0 (0 to 0)	0 (0 to 0)	0 (0 to 0)	0 (0 to 0)	0 (0 to 0)	0 (0 to 0)	0 (0 to 0)	0 (0 to 0)	0 (0 to 0)	0 (0 to 0)	0 (0 to 0)	0 (0 to 0)	0 (0 to 0)	0 (0 to 0)	0 (0 to 0)	0 (0 to 0)	0 (0 to 0)	0
Cardiovascular diseases	1·4 (0·8 to 2·1)	0·4 (0·2 to 0·6)	3·1 (1·9 to 4·9)	1·9 (1·4 to 2·8)	3·7 (2·9 to 4·6)	5·8 (4·5 to 7·3)	16·5 (13·3 to 19·7)	27 (21·5 to 32·4)	32·6 (25·5 to 40)	47·1 (37·5 to 57·3)	64·1 (51·8 to 79·1)	110 (89·2 to 131)	203 (170 to 241)	317 (273 to 367)	448 (396 to 504)	457 (411 to 505)	494 (446 to 546)	431 (385 to 472)	343 (307 to 381)	147 (131 to 162)	47·7 (42·6 to 53·1)	15·7 (13·2 to 17·6)	4 (3·1 to 4·6)	3,220
Rheumatic heart disease	0 (0 to 0)	0 (0 to 0)	0 (0 to 0)	0·2 (0·1 to 0·3)	1 (0·6 to 1·6)	2·3 (1·5 to 3·4)	4·5 (3·1 to 6·2)	5·7 (4 to 7·7)	5·6 (3·9 to 7·6)	5·4 (3·9 to 7·2)	5 (3·6 to 6·6)	5·5 (3·9 to 7·4)	6·2 (4·8 to 7·7)	6 (4·4 to 8·2)	5·8 (4·3 to 7·8)	3·8 (2·8 to 5)	2·9 (2·2 to 3·8)	1·8 (1·4 to 2·4)	1·3 (1 to 1·7)	0·3 (0·2 to 0·4)	0·1 (0·1 to 0·1)	0 (0 to 0)	0 (0 to 0)	63·5
Ischemic heart disease	0 (0 to 0)	0 (0 to 0)	0 (0 to 0)	0 (0 to 0)	0 (0 to 0)	0 (0 to 0)	1·2 (0·9 to 1·6)	3·4 (2·4 to 4·5)	5·8 (4·2 to 7·9)	11·7 (8·7 to 15·5)	19·4 (15 to 25)	38·3 (30·5 to 47·4)	79·6 (64·8 to 96·6)	133 (111 to 157)	204 (177 to 234)	206 (182 to 230)	232 (209 to 256)	198 (177 to 221)	147 (131 to 164)	61·1 (54·2 to 67·5)	20·4 (17·8 to 22·9)	6·7 (5·6 to 7·6)	1·8 (1·4 to 2·1)	1,370
Stroke	0·5 (0·2 to 0·8)	0·1 (0 to 0·1)	0·4 (0·2 to 0·8)	0·3 (0·2 to 0·4)	0·9 (0·6 to 1·2)	1·3 (0·9 to 1·7)	3·8 (2·7 to 5)	7·3 (5·1 to 9·8)	8·1 (5·4 to 11·3)	12·4 (8·5 to 17·1)	17·6 (12·5 to 24·6)	31·2 (22·1 to 41·6)	58·2 (40·5 to 78·2)	94·1 (71 to 120)	129 (99 to 162)	140 (112 to 172)	152 (119 to 188)	137 (110 to 168)	119 (94·2 to 145)	50·7 (40·5 to 62·5)	15·2 (12 to 18·8)	4·5 (3·5 to 5·6)	1·1 (0·8 to 1·4)	983
Ischemic stroke	0 (0 to 0)	0 (0 to 0)	0 (0 to 0·1)	0·2 (0·1 to 0·3)	0·4 (0·2 to 0·7)	0·6 (0·4 to 1)	1·1 (0·7 to 1·5)	1·5 (1 to 2·1)	1·9 (1·3 to 2·8)	2·6 (1·8 to 3·7)	3·5 (2·4 to 5)	5·9 (4·1 to 8·2)	11·4 (7·7 to 15·8)	20·9 (15·2 to 27·2)	31·8 (23·1 to 42·6)	51·3 (38·2 to 66·1)	60·2 (44·6 to 77·6)	75·3 (57·7 to 95·8)	69 (52·6 to 86·3)	38·6 (30·6 to 48·6)	11·8 (9·2 to 14·6)	3·5 (2·7 to 4·4)	0·9 (0·7 to 1·1)	393
Intracerebral hemorrhage	0·1 (0 to 0·2)	0 (0 to 0)	0·2 (0·1 to 0·4)	0 (0 to 0·1)	0·2 (0·1 to 0·3)	0·3 (0·2 to 0·4)	1·4 (0·9 to 1·9)	3 (1·9 to 4·4)	3·4 (2·1 to 5·2)	6·4 (4 to 9·6)	9·6 (6·4 to 13·8)	18·6 (12·4 to 25·6)	37·7 (26·1 to 51·6)	61·4 (45·8 to 79·3)	81·9 (61·5 to 105)	79·3 (61 to 101)	83·9 (61·9 to 108)	56·3 (41·4 to 74·2)	46·2 (33·4 to 60·6)	10·8 (7·9 to 14·5)	3 (2·2 to 4·1)	0·9 (0·6 to 1·2)	0·2 (0·1 to 0·3)	505
Subarachnoid hemorrhage	0·4 (0·2 to 0·6)	0 (0 to 0·1)	0·2 (0·1 to 0·4)	0·1 (0 to 0·2)	0·2 (0·1 to 0·4)	0·4 (0·2 to 0·6)	1·4 (0·9 to 2·1)	2·8 (1·7 to 4·3)	2·7 (1·6 to 4·2)	3·4 (2·1 to 5·1)	4·5 (2·8 to 6·8)	6·7 (4·2 to 9·7)	9·1 (5·7 to 13·5)	11·8 (8 to 16·7)	15·1 (10·5 to 21·8)	9·2 (6·3 to 12·8)	7·7 (5 to 10·9)	4·9 (3·3 to 6·9)	3·4 (2·2 to 4·9)	1·3 (0·8 to 1·9)	0·4 (0·2 to 0·6)	0·1 (0·1 to 0·2)	0 (0 to 0)	85·7
Hypertensive heart disease	0 (0 to 0)	0 (0 to 0)	0 (0 to 0)	0 (0 to 0)	0 (0 to 0)	0 (0 to 0)	0·3 (0·2 to 0·4)	0·7 (0·4 to 1·1)	1 (0·6 to 1·7)	2·3 (1·4 to 3·4)	3·9 (2·5 to 5·8)	7·8 (5 to 11·5)	16·9 (11 to 23·9)	26·5 (18·5 to 37)	37·6 (26·3 to 52·6)	38·8 (26·4 to 52·5)	38·6 (25·9 to 53·1)	35·1 (23·8 to 47·5)	28·3 (19·1 to 38·6)	13·3 (8·8 to 17·5)	4·4 (3 to 5·7)	1·6 (1·1 to 2·1)	0·4 (0·3 to 0·6)	257
Non-rheumatic valvular heart disease	0 (0 to 0)	0 (0 to 0)	0 (0 to 0)	0 (0 to 0)	0 (0 to 0)	0 (0 to 0)	0·8 (0·5 to 1·1)	1·1 (0·7 to 1·6)	1·5 (0·9 to 2·3)	2 (1·3 to 2·8)	2 (1·3 to 2·9)	3·1 (2·1 to 4·5)	4·7 (3·2 to 6·6)	5·6 (4 to 7·6)	7·7 (5·4 to 10·5)	7·1 (5·2 to 9·5)	7·6 (5·6 to 10·1)	5·9 (4·3 to 7·9)	4·3 (3·1 to 5·8)	1·9 (1·4 to 2·5)	0·6 (0·4 to 0·8)	0·2 (0·1 to 0·3)	0 (0 to 0·1)	56·1
Non-rheumatic calcific aortic valve disease	0 (0 to 0)	0 (0 to 0)	0 (0 to 0)	0 (0 to 0)	0 (0 to 0)	0 (0 to 0)	0·4 (0·2 to 0·6)	0·5 (0·3 to 0·7)	0·5 (0·3 to 1)	0·5 (0·3 to 0·9)	0·6 (0·3 to 1)	1·1 (0·6 to 1·8)	1·8 (1 to 2·9)	2·2 (1·4 to 3·4)	3·1 (1·8 to 4·8)	3 (1·9 to 4·6)	3·9 (2·6 to 5·6)	3·2 (2·1 to 4·6)	2·4 (1·6 to 3·6)	1·2 (0·8 to 1·7)	0·3 (0·2 to 0·5)	0·1 (0·1 to 0·2)	0 (0 to 0)	24·9
Non-rheumatic degenerative mitral valve disease	0 (0 to 0)	0 (0 to 0)	0 (0 to 0)	0 (0 to 0)	0 (0 to 0)	0 (0 to 0)	0·4 (0·2 to 0·6)	0·6 (0·4 to 1)	1 (0·6 to 1·6)	1·4 (0·9 to 2·1)	1·4 (0·9 to 2)	2·1 (1·3 to 3)	2·8 (1·7 to 4·3)	3·4 (2·1 to 5)	4·6 (3 to 6·7)	4 (2·6 to 5·7)	3·7 (2·4 to 5·3)	2·7 (1·6 to 3·9)	1·9 (1·1 to 2·9)	0·7 (0·4 to 1·1)	0·2 (0·1 to 0·3)	0·1 (0 to 0·1)	0 (0 to 0)	31·2
Other non-rheumatic valve diseases	0 (0 to 0)	0 (0 to 0)	0 (0 to 0)	0 (0 to 0)	0 (0 to 0)	0 (0 to 0)	0 (0 to 0)	0 (0 to 0)	0 (0 to 0)	0 (0 to 0)	0 (0 to 0)	0 (0 to 0)	0 (0 to 0)	0 (0 to 0)	0 (0 to 0)	0 (0 to 0)	0 (0 to 0)	0 (0 to 0)	0 (0 to 0)	0 (0 to 0)	0 (0 to 0)	0 (0 to 0)	0 (0 to 0)	0
Cardiomyopathy and myocarditis	0·5 (0·3 to 0·8)	0·2 (0·1 to 0·3)	1·6 (0·9 to 2·6)	0·8 (0·5 to 1·2)	0·8 (0·6 to 1·1)	0·9 (0·6 to 1·2)	2·7 (1·8 to 3·7)	4 (2·7 to 5·6)	5·2 (3·2 to 7·4)	6·8 (4·3 to 9·5)	8·8 (5·4 to 12·6)	12·2 (7·9 to 17·1)	20·1 (12·5 to 28·2)	26·2 (16·7 to 36·1)	29·8 (19·1 to 41·3)	24·4 (16·2 to 33·2)	21·6 (13·2 to 29·9)	16·7 (9 to 23·5)	11·2 (6·7 to 15·4)	4·3 (2·5 to 6·2)	1·5 (0·9 to 2·2)	0·5 (0·3 to 0·7)	0·1 (0 to 0·1)	201
Myocarditis	0·1 (0 to 0·2)	0 (0 to 0·1)	0·4 (0·2 to 0·8)	0·2 (0·1 to 0·4)	0·2 (0·1 to 0·3)	0·1 (0·1 to 0·2)	0·4 (0·2 to 0·9)	0·5 (0·2 to 1·1)	0·6 (0·3 to 1·2)	0·7 (0·3 to 1·3)	0·6 (0·3 to 1·1)	0·7 (0·4 to 1·3)	0·7 (0·3 to 1·2)	1·1 (0·5 to 1·9)	0·9 (0·4 to 1·8)	0·7 (0·3 to 1·2)	0·7 (0·4 to 1·2)	0·4 (0·2 to 0·7)	0·3 (0·2 to 0·6)	0·1 (0·1 to 0·2)	0 (0 to 0·1)	0 (0 to 0)	0 (0 to 0)	9·6
Alcoholic cardiomyopathy	0 (0 to 0)	0 (0 to 0)	0 (0 to 0)	0 (0 to 0)	0 (0 to 0)	0 (0 to 0)	0·2 (0·1 to 0·3)	0·3 (0·1 to 0·6)	0·7 (0·3 to 1·3)	1·7 (0·8 to 2·9)	2·3 (1·1 to 3·7)	4 (2·1 to 6·4)	8·3 (4·4 to 13·1)	11·1 (6·1 to 16·7)	11·9 (6·6 to 18·5)	9·4 (5·1 to 14·1)	8·4 (4·3 to 13·1)	4·7 (2·2 to 8)	2·9 (1·4 to 5)	0·3 (0·1 to 0·7)	0·1 (0 to 0·2)	0 (0 to 0)	0 (0 to 0)	66·3
Other cardiomyopathy	0·4 (0·2 to 0·7)	0·1 (0·1 to 0·2)	1·2 (0·7 to 2)	0·6 (0·3 to 1)	0·6 (0·4 to 0·9)	0·7 (0·5 to 1)	2·1 (1·4 to 3)	3·2 (2·1 to 4·5)	3·9 (2·4 to 5·7)	4·4 (2·7 to 6·4)	5·9 (3·6 to 8·6)	7·5 (4·5 to 11·2)	11·1 (6·6 to 16·2)	14·1 (9 to 20·7)	16·9 (10·6 to 24)	14·4 (9·1 to 21)	12·5 (7·4 to 18·2)	11·6 (6·2 to 16·9)	8·1 (4·8 to 11·3)	3·8 (2·2 to 5·6)	1·4 (0·8 to 2·1)	0·4 (0·2 to 0·6)	0·1 (0 to 0·1)	125
Atrial fibrillation and flutter	0 (0 to 0)	0 (0 to 0)	0 (0 to 0)	0 (0 to 0)	0 (0 to 0)	0 (0 to 0)	0 (0 to 0)	0 (0 to 0)	0 (0 to 0)	0·1 (0·1 to 0·2)	0·1 (0·1 to 0·2)	0·6 (0·4 to 0·9)	1·3 (0·9 to 2)	2·5 (1·8 to 3·7)	4·2 (3·1 to 6)	5·2 (3·9 to 7·3)	6·3 (4·9 to 8·6)	7·7 (5·8 to 10·7)	8·1 (6·1 to 11·3)	5 (3·8 to 7·1)	2·2 (1·7 to 3·2)	1·1 (0·8 to 1·6)	0·3 (0·2 to 0·4)	44·8
Aortic aneurysm	--	--	--	--	--	--	0·3 (0·2 to 0·3)	0·2 (0·2 to 0·4)	0·4 (0·3 to 0·6)	0·7 (0·4 to 1)	1 (0·7 to 1·5)	1·6 (1·1 to 2·3)	2·7 (2 to 3·7)	4·9 (3·6 to 6·7)	9·2 (6·8 to 11·9)	11·7 (8·7 to 15·4)	14 (10·1 to 18·2)	13·2 (9·6 to 17·8)	10·3 (7·5 to 13·8)	3·8 (2·8 to 5·1)	1·1 (0·8 to 1·4)	0·3 (0·2 to 0·4)	0 (0 to 0·1)	NA
Endocarditis	0·1 (0 to 0·1)	0 (0 to 0·1)	0·3 (0·1 to 0·6)	0·2 (0·1 to 0·4)	0·2 (0·1 to 0·3)	0·3 (0·2 to 0·5)	0·9 (0·5 to 1·5)	1·6 (0·9 to 2·9)	1·7 (0·9 to 3·3)	2·4 (1·3 to 4·3)	2·3 (1·4 to 3·8)	3·3 (1·9 to 5·5)	3·9 (2·4 to 6·2)	4·9 (3·1 to 7·9)	4·5 (2·9 to 7·9)	3·5 (2·1 to 5·7)	2·9 (1·9 to 4·5)	2·1 (1·3 to 3·3)	1·2 (0·8 to 2)	0·5 (0·3 to 0·8)	0·2 (0·1 to 0·3)	0 (0 to 0·1)	0 (0 to 0)	37·2
Other cardiovascular and circulatory diseases	0·3 (0·2 to 0·6)	0·1 (0 to 0·2)	0·8 (0·4 to 1·4)	0·4 (0·3 to 0·7)	0·8 (0·5 to 1·3)	1·1 (0·7 to 1·7)	2·1 (1·5 to 2·8)	3 (2·1 to 4)	3·2 (2·2 to 4·4)	3·3 (2·3 to 4·3)	3·9 (2·8 to 5·3)	5·7 (4·1 to 7·4)	8·2 (6·1 to 11)	11·1 (8·1 to 15)	13·5 (10·2 to 17·5)	13·1 (10·2 to 16·3)	13 (9·8 to 16·9)	11·1 (8·6 to 14·1)	9·6 (7·4 to 12·5)	4·4 (3·3 to 5·7)	1·5 (1·1 to 2)	0·5 (0·4 to 0·6)	0·1 (0·1 to 0·2)	111
Chronic respiratory diseases	0 (0 to 0)	0 (0 to 0)	0 (0 to 0)	27·3 (14·8 to 48·4)	25 (14·6 to 41·8)	18·8 (11·3 to 29·9)	15·2 (9·8 to 23·7)	14·3 (9·7 to 20·7)	13·3 (9·2 to 19·1)	12·5 (9·2 to 16·9)	11·5 (8·6 to 15·1)	13·6 (10·4 to 17·2)	20·3 (15·8 to 25·3)	29·1 (23·3 to 36·5)	37·8 (30·2 to 47·2)	40·6 (32·3 to 50·2)	44 (35·2 to 54·8)	43·3 (34·9 to 53·6)	35·8 (28·2 to 44·3)	15·1 (11·8 to 18·3)	4·8 (3·7 to 5·9)	1·8 (1·4 to 2·2)	0·5 (0·4 to 0·6)	425
Chronic obstructive pulmonary disease	0 (0 to 0)	0 (0 to 0)	0 (0 to 0)	0·2 (0·1 to 0·3)	0·3 (0·2 to 0·5)	0·5 (0·2 to 0·8)	1·1 (0·6 to 1·6)	1·9 (1·2 to 2·8)	2·2 (1·4 to 3·2)	2·5 (1·6 to 3·4)	2·9 (2 to 3·8)	4·4 (3·2 to 5·8)	8·7 (6·5 to 11·2)	14·9 (11·2 to 19)	23·6 (18·3 to 30·1)	30·5 (23·7 to 38·7)	34·9 (27·6 to 43·9)	35·3 (28·1 to 44·1)	30·1 (23·2 to 37·6)	12·8 (10 to 15·9)	4·1 (3·2 to 5·1)	1·5 (1·2 to 1·9)	0·4 (0·3 to 0·5)	213
Pneumoconiosis	0 (0 to 0)	0 (0 to 0)	0 (0 to 0)	0 (0 to 0)	0 (0 to 0)	0 (0 to 0)	0·1 (0·1 to 0·2)	0 (0 to 0·1)	0·1 (0 to 0·2)	0 (0 to 0)	0·1 (0 to 0·1)	0·1 (0 to 0·1)	0·2 (0·1 to 0·3)	0·3 (0·2 to 0·5)	0·3 (0·2 to 0·5)	0·4 (0·2 to 0·7)	0·6 (0·4 to 1)	0·4 (0·3 to 0·6)	0·3 (0·2 to 0·5)	0·2 (0·1 to 0·3)	0·1 (0 to 0·1)	0 (0 to 0)	0 (0 to 0)	3·2
Silicosis	0 (0 to 0)	0 (0 to 0)	0 (0 to 0)	0 (0 to 0)	0 (0 to 0)	0 (0 to 0)	0 (0 to 0)	0 (0 to 0)	0 (0 to 0·1)	0 (0 to 0)	0 (0 to 0)	0 (0 to 0)	0 (0 to 0)	0 (0 to 0)	0 (0 to 0)	0 (0 to 0)	0 (0 to 0)	0 (0 to 0)	0 (0 to 0)	0 (0 to 0)	0 (0 to 0)	0 (0 to 0)	0 (0 to 0)	0·1
Asbestosis	0 (0 to 0)	0 (0 to 0)	0 (0 to 0)	0 (0 to 0)	0 (0 to 0)	0 (0 to 0)	0 (0 to 0)	0 (0 to 0)	0 (0 to 0)	0 (0 to 0)	0 (0 to 0)	0 (0 to 0)	0 (0 to 0)	0 (0 to 0)	0 (0 to 0)	0 (0 to 0)	0 (0 to 0)	0 (0 to 0)	0 (0 to 0)	0 (0 to 0)	0 (0 to 0)	0 (0 to 0)	0 (0 to 0)	0·1
Coal workers pneumoconiosis	0 (0 to 0)	0 (0 to 0)	0 (0 to 0)	0 (0 to 0)	0 (0 to 0)	0 (0 to 0)	0·1 (0 to 0·1)	0 (0 to 0)	0 (0 to 0·1)	0 (0 to 0)	0·1 (0 to 0·1)	0 (0 to 0·1)	0·2 (0·1 to 0·3)	0·3 (0·1 to 0·4)	0·2 (0·1 to 0·4)	0·4 (0·2 to 0·6)	0·5 (0·3 to 0·8)	0·3 (0·2 to 0·5)	0·2 (0·1 to 0·3)	0·2 (0·1 to 0·2)	0·1 (0 to 0·1)	0 (0 to 0)	0 (0 to 0)	2·7
Other pneumoconiosis	0 (0 to 0)	0 (0 to 0)	0 (0 to 0)	0 (0 to 0)	0 (0 to 0)	0 (0 to 0)	0 (0 to 0)	0 (0 to 0)	0 (0 to 0)	0 (0 to 0)	0 (0 to 0)	0 (0 to 0)	0 (0 to 0)	0 (0 to 0)	0 (0 to 0)	0 (0 to 0)	0·1 (0 to 0·2)	0 (0 to 0·1)	0·1 (0 to 0·1)	0 (0 to 0·1)	0 (0 to 0)	0 (0 to 0)	0 (0 to 0)	0·3
Asthma	0 (0 to 0)	0 (0 to 0)	0 (0 to 0)	24 (11·8 to 44·4)	23·3 (13 to 40·1)	16·8 (9·3 to 28)	12·1 (6·6 to 20·2)	9·4 (5·2 to 15·8)	7·7 (4·3 to 12·8)	6·5 (3·8 to 10·2)	5·2 (3·2 to 8·1)	5·1 (3·2 to 7·8)	5·9 (3·9 to 8·6)	6·8 (4·5 to 9·7)	6·4 (4·4 to 9·1)	5·6 (3·8 to 7·8)	4·7 (3·3 to 6·5)	3·9 (2·7 to 5·3)	2·5 (1·7 to 3·5)	1 (0·7 to 1·3)	0·3 (0·2 to 0·4)	0·1 (0·1 to 0·1)	0 (0 to 0)	147
Interstitial lung disease and pulmonary sarcoidosis	0 (0 to 0)	0 (0 to 0)	0 (0 to 0)	0·2 (0·1 to 0·4)	0·1 (0 to 0·1)	0·1 (0 to 0·1)	0·2 (0·1 to 0·4)	0·3 (0·2 to 0·7)	0·2 (0·1 to 0·4)	0·5 (0·3 to 0·8)	0·5 (0·3 to 0·8)	0·8 (0·5 to 1·3)	1·1 (0·7 to 1·6)	1·6 (1 to 2·5)	2 (1·2 to 3·2)	2 (1·2 to 3)	2 (1·2 to 3·1)	2 (1·1 to 3·2)	1·6 (0·9 to 2·5)	0·5 (0·3 to 0·8)	0·1 (0·1 to 0·2)	0 (0 to 0·1)	0 (0 to 0)	15·7
Other chronic respiratory diseases	0 (0 to 0)	0 (0 to 0)	0 (0 to 0)	2·9 (1·8 to 4·5)	1·3 (0·9 to 1·8)	1·5 (1 to 2·1)	1·8 (1·2 to 2·5)	2·6 (1·7 to 3·9)	3 (2 to 4·4)	3 (1·9 to 4·3)	2·9 (1·9 to 4·1)	3·2 (2·2 to 4·6)	4·5 (3 to 6·6)	5·5 (3·6 to 8·3)	5·5 (3·2 to 8·8)	2·1 (1·1 to 3·5)	1·8 (1 to 3·1)	1·7 (1 to 2·7)	1·3 (0·7 to 2·2)	0·6 (0·3 to 1)	0·2 (0·1 to 0·3)	0·1 (0 to 0·1)	0 (0 to 0)	45·6
Digestive diseases	0·9 (0·6 to 1·4)	0·4 (0·3 to 0·7)	5·9 (3·4 to 9·6)	7·3 (5 to 10·3)	7·4 (5·5 to 10·1)	6·2 (4·7 to 8·4)	10·8 (8·3 to 13·6)	19·2 (14·9 to 25·2)	24·4 (18·7 to 32·2)	33·1 (26·1 to 41·4)	41·6 (33·6 to 51·6)	57·8 (46 to 70·1)	91 (73·9 to 110)	119 (98·1 to 141)	121 (101 to 142)	105 (89 to 122)	87·4 (73·5 to 103)	67·9 (57·1 to 79·5)	46·8 (38·7 to 55·5)	15·8 (13 to 19·1)	4·5 (3·7 to 5·3)	1·4 (1·1 to 1·6)	0·3 (0·2 to 0·4)	875
Cirrhosis and other chronic liver diseases	0 (0 to 0)	0 (0 to 0)	0 (0 to 0)	0·6 (0·3 to 1)	0·9 (0·6 to 1·3)	0·9 (0·6 to 1·2)	2 (1·4 to 2·7)	3·5 (2·4 to 5)	5·5 (3·7 to 7·6)	11·4 (8·3 to 14·8)	18·6 (14 to 24·3)	30·2 (22·7 to 38·5)	51·8 (40·1 to 66·1)	69·6 (54 to 87·6)	69·5 (55·1 to 84·5)	58 (47 to 70·4)	45 (34·8 to 56·8)	29·8 (23 to 37·7)	17·7 (13·4 to 23·1)	5 (3·8 to 6·6)	1·2 (0·9 to 1·5)	0·3 (0·2 to 0·4)	0·1 (0 to 0·1)	422
Cirrhosis and other chronic liver diseases due to hepatitis B	0 (0 to 0)	0 (0 to 0)	0 (0 to 0)	0 (0 to 0)	0 (0 to 0)	0 (0 to 0)	0 (0 to 0·1)	0·2 (0·1 to 0·3)	0·4 (0·1 to 0·8)	1 (0·5 to 1·7)	1·8 (0·9 to 3·5)	3 (1·5 to 5·3)	5 (2·5 to 8·9)	6·2 (2·9 to 11·4)	5·2 (2·5 to 9·2)	3·7 (1·7 to 7·1)	2·6 (1·3 to 4·9)	1·7 (0·8 to 3·2)	1·1 (0·5 to 1·9)	0·3 (0·1 to 0·5)	0·1 (0 to 0·1)	0 (0 to 0)	0 (0 to 0)	32·3
Cirrhosis and other chronic liver diseases due to hepatitis C	0 (0 to 0)	0 (0 to 0)	0 (0 to 0)	0 (0 to 0)	0 (0 to 0)	0 (0 to 0·1)	0·1 (0 to 0·2)	0·6 (0·3 to 1·1)	1·4 (0·6 to 2·4)	3 (1·6 to 4·8)	4·7 (2·4 to 8·3)	7·3 (3·9 to 12·1)	11·6 (6·3 to 18·9)	14·5 (8 to 23·8)	13·5 (7·2 to 22·6)	10·6 (5·1 to 18·8)	8 (4·4 to 13·2)	5·2 (2·9 to 8·7)	3 (1·6 to 5·2)	0·9 (0·5 to 1·5)	0·2 (0·1 to 0·4)	0·1 (0 to 0·1)	0 (0 to 0)	84·7
Cirrhosis and other chronic liver diseases due to alcohol use	0 (0 to 0)	0 (0 to 0)	0 (0 to 0)	0 (0 to 0)	0 (0 to 0)	0 (0 to 0)	0 (0 to 0)	0·4 (0·2 to 0·9)	1·2 (0·5 to 2·3)	3·8 (2·3 to 5·8)	7·9 (4·6 to 11·9)	14·1 (8·9 to 20·2)	25·2 (16 to 36)	35·3 (23 to 48)	37 (25 to 49·9)	31·2 (21·4 to 41·5)	23·4 (15·6 to 32·4)	14·7 (9·5 to 21)	8·1 (5·3 to 11·5)	2·1 (1·3 to 3·3)	0·5 (0·3 to 0·7)	0·1 (0·1 to 0·2)	0 (0 to 0)	205
Cirrhosis and other chronic liver diseases due to NAFLD	0 (0 to 0)	0 (0 to 0)	0 (0 to 0)	0 (0 to 0)	0 (0 to 0)	0 (0 to 0)	0·1 (0 to 0·2)	0·4 (0·2 to 0·8)	0·8 (0·4 to 1·6)	1·6 (0·8 to 2·7)	2·3 (1 to 4·3)	3·6 (1·7 to 6·6)	6·4 (2·9 to 11·5)	8·7 (4·1 to 15·7)	8·4 (4 to 15·3)	7·2 (3·6 to 13·2)	6·3 (3·5 to 10·5)	4·6 (2·6 to 7·3)	3·1 (1·6 to 5·2)	1 (0·5 to 1·6)	0·2 (0·1 to 0·4)	0·1 (0 to 0·1)	0 (0 to 0)	54·9
Cirrhosis and other chronic liver diseases due to other causes	0 (0 to 0)	0 (0 to 0)	0 (0 to 0)	0·6 (0·3 to 1)	0·8 (0·5 to 1·2)	0·8 (0·6 to 1·1)	1·7 (1·2 to 2·4)	2 (1·2 to 3)	1·7 (0·9 to 2·8)	1·9 (0·9 to 3·4)	1·9 (0·9 to 3·4)	2·3 (1·1 to 4·3)	3·6 (1·8 to 6·5)	4·9 (2·5 to 8·6)	5·4 (2·5 to 9·8)	5·2 (2·6 to 9·2)	4·6 (2·2 to 8·5)	3·5 (1·7 to 6·5)	2·4 (1·2 to 4·4)	0·8 (0·4 to 1·4)	0·2 (0·1 to 0·3)	0·1 (0 to 0·1)	0 (0 to 0)	44·4
Upper digestive system diseases	0 (0 to 0)	0 (0 to 0)	0 (0 to 0)	0·3 (0·2 to 0·5)	0·4 (0·2 to 0·7)	0·7 (0·4 to 1·1)	2·8 (1·7 to 4·7)	7·2 (4·2 to 12·5)	9·5 (5·5 to 15·8)	10·3 (6·4 to 16·8)	10·3 (6·7 to 15·6)	12·1 (8 to 18·1)	16·6 (11·6 to 23·7)	20·5 (14·6 to 28·8)	20 (14·5 to 27·9)	17·8 (13·1 to 24)	15·9 (12 to 20·9)	13·2 (9·9 to 17·4)	9·9 (7·4 to 12·9)	3·4 (2·6 to 4·4)	1 (0·8 to 1·3)	0·3 (0·2 to 0·4)	0·1 (0·1 to 0·1)	172
Peptic ulcer disease	0 (0 to 0)	0 (0 to 0)	0 (0 to 0)	0 (0 to 0·1)	0·1 (0 to 0·1)	0·1 (0·1 to 0·2)	0·4 (0·3 to 0·6)	1 (0·6 to 1·4)	1·1 (0·7 to 1·7)	1·8 (1·2 to 2·7)	2·3 (1·5 to 3·3)	3 (1·9 to 4·5)	4·5 (3 to 6·4)	7·5 (4·9 to 10·7)	7·9 (5·3 to 11·5)	8·1 (5·5 to 11·3)	8 (5·6 to 11·2)	7 (4·8 to 9·7)	5·7 (4 to 7·8)	2 (1·3 to 2·7)	0·6 (0·4 to 0·8)	0·2 (0·1 to 0·2)	0·1 (0 to 0·1)	61·2
Gastritis and duodenitis	0 (0 to 0)	0 (0 to 0)	0 (0 to 0)	0·3 (0·1 to 0·5)	0·4 (0·2 to 0·7)	0·3 (0·1 to 0·5)	0·5 (0·3 to 0·9)	0·9 (0·5 to 1·4)	1·2 (0·7 to 1·9)	1·7 (1 to 2·6)	2·1 (1·3 to 3·1)	2·9 (1·8 to 4·3)	4·9 (3·2 to 7·3)	5·1 (3·3 to 7·6)	5·4 (3·6 to 7·9)	4·7 (3·1 to 6·8)	4·4 (3 to 6·2)	3·6 (2·4 to 5·3)	2·5 (1·6 to 3·6)	0·9 (0·6 to 1·3)	0·3 (0·2 to 0·4)	0·1 (0·1 to 0·1)	0 (0 to 0)	41·9
Gastroesophageal reflux disease	0 (0 to 0)	0 (0 to 0)	0 (0 to 0)	0 (0 to 0)	0 (0 to 0)	0·3 (0·1 to 0·5)	1·9 (0·9 to 3·6)	5·3 (2·6 to 10·6)	7·2 (3·5 to 13·3)	6·8 (3·2 to 13)	5·8 (2·8 to 11)	6·2 (3 to 11·5)	7·2 (3·6 to 13·1)	7·8 (3·8 to 14·4)	6·8 (3·1 to 13)	5 (2·4 to 9·5)	3·6 (1·8 to 6·4)	2·6 (1·4 to 4·7)	1·8 (0·9 to 3·2)	0·6 (0·3 to 1·1)	0·2 (0·1 to 0·3)	0 (0 to 0)	0 (0 to 0)	69·2
Appendicitis	0 (0 to 0)	0 (0 to 0)	0 (0 to 0)	0·2 (0·1 to 0·4)	0·3 (0·2 to 0·5)	0·4 (0·2 to 0·8)	0·6 (0·3 to 1·1)	0·7 (0·4 to 1·3)	0·6 (0·3 to 1)	0·5 (0·3 to 0·8)	0·5 (0·3 to 0·8)	0·5 (0·3 to 0·8)	0·6 (0·3 to 0·9)	0·6 (0·4 to 1·1)	0·5 (0·3 to 0·9)	0·5 (0·3 to 0·7)	0·5 (0·3 to 0·8)	0·4 (0·2 to 0·6)	0·2 (0·1 to 0·4)	0·1 (0 to 0·1)	0 (0 to 0)	0 (0 to 0)	0 (0 to 0)	7·8
Paralytic ileus and intestinal obstruction	0·7 (0·4 to 1)	0·4 (0·2 to 0·7)	5·3 (2·9 to 8·6)	1·7 (0·9 to 2·8)	1·1 (0·7 to 1·6)	0·9 (0·6 to 1·3)	1·3 (0·8 to 1·8)	1·8 (1·1 to 2·9)	1·8 (1 to 2·9)	2 (1·2 to 3·2)	2·7 (1·7 to 4·1)	3 (1·9 to 4·6)	4·8 (3 to 7·2)	7·3 (4·5 to 11·2)	8·7 (5·5 to 13·7)	8·2 (5·4 to 12·2)	7·7 (4·9 to 11·1)	7·7 (4·7 to 10·7)	6·3 (3·8 to 8·7)	2·6 (1·6 to 3·6)	0·8 (0·5 to 1·1)	0·3 (0·2 to 0·3)	0·1 (0 to 0·1)	77
Inguinal, femoral, and abdominal hernia	0·2 (0·1 to 0·4)	0 (0 to 0·1)	0·7 (0·3 to 1·2)	2·5 (1·5 to 4)	3·3 (1·9 to 5·6)	2·3 (1·2 to 4)	2·1 (1 to 3·6)	2·4 (1·1 to 4·1)	2·6 (1·4 to 4·3)	2·6 (1·4 to 4·3)	2·9 (1·6 to 4·7)	3·8 (2·3 to 6)	5·2 (3·1 to 8·3)	6·4 (4 to 9·8)	6·2 (3·9 to 9·5)	5·3 (3·5 to 7·7)	4·5 (3·2 to 6·2)	3·8 (2·8 to 5·1)	2·5 (1·9 to 3·2)	0·8 (0·6 to 1)	0·2 (0·2 to 0·3)	0·1 (0 to 0·1)	0 (0 to 0)	60·6
Inflammatory bowel disease	0 (0 to 0)	0 (0 to 0)	0 (0 to 0)	0·4 (0·2 to 0·8)	0·2 (0·1 to 0·3)	0·1 (0·1 to 0·2)	0·5 (0·3 to 0·8)	0·9 (0·6 to 1·5)	1·3 (0·7 to 2·3)	1·9 (1·1 to 3)	1·6 (1·1 to 2·4)	1·7 (1·2 to 2·5)	2·4 (1·6 to 3·4)	2·7 (1·8 to 4)	3·2 (2 to 5)	2·3 (1·5 to 3·5)	2·1 (1·4 to 3·1)	2·2 (1·5 to 3·2)	1·5 (1 to 2·2)	0·6 (0·4 to 0·8)	0·2 (0·1 to 0·2)	0·1 (0 to 0·1)	0 (0 to 0)	25·9
Vascular intestinal disorders	0 (0 to 0)	0 (0 to 0)	0 (0 to 0)	0 (0 to 0)	0 (0 to 0)	0 (0 to 0)	0·1 (0 to 0·1)	0 (0 to 0·1)	0·1 (0·1 to 0·1)	0·1 (0·1 to 0·2)	0·2 (0·1 to 0·2)	0·2 (0·1 to 0·3)	0·4 (0·3 to 0·6)	0·6 (0·4 to 0·9)	0·8 (0·5 to 1·2)	0·9 (0·6 to 1·2)	1·1 (0·8 to 1·5)	1·1 (0·8 to 1·5)	0·9 (0·6 to 1·3)	0·4 (0·3 to 0·5)	0·1 (0·1 to 0·1)	0 (0 to 0·1)	0 (0 to 0)	7·2
Gallbladder and biliary diseases	0 (0 to 0)	0 (0 to 0)	0 (0 to 0)	0 (0 to 0·1)	0·1 (0·1 to 0·2)	0·2 (0·1 to 0·3)	0·3 (0·2 to 0·5)	0·6 (0·3 to 1)	0·7 (0·4 to 1·2)	0·9 (0·6 to 1·4)	0·9 (0·6 to 1·4)	1·4 (0·9 to 2·1)	1·9 (1·2 to 2·8)	2·8 (1·8 to 4·1)	3·3 (2·2 to 5·3)	3·1 (2·1 to 4·9)	3·2 (2·2 to 5)	3·1 (2·1 to 5·1)	2·4 (1·7 to 3·9)	0·9 (0·6 to 1·5)	0·3 (0·2 to 0·4)	0·1 (0·1 to 0·1)	0 (0 to 0)	26·3
Pancreatitis	0 (0 to 0)	0 (0 to 0)	0 (0 to 0)	0 (0 to 0)	0 (0 to 0)	0·1 (0 to 0·1)	0·3 (0·2 to 0·4)	0·5 (0·3 to 0·8)	0·9 (0·5 to 1·4)	1·5 (0·9 to 2·2)	2 (1·3 to 2·8)	2·4 (1·5 to 3·4)	2·9 (2 to 4·2)	2·9 (1·9 to 4·2)	2·7 (1·7 to 3·9)	2·2 (1·6 to 3·1)	1·6 (1·1 to 2·3)	1·3 (0·9 to 1·8)	0·9 (0·6 to 1·2)	0·3 (0·2 to 0·4)	0·1 (0 to 0·1)	0 (0 to 0)	0 (0 to 0)	22·5
Other digestive diseases	0 (0 to 0)	0 (0 to 0)	0 (0 to 0)	1·5 (0·8 to 2·5)	1 (0·6 to 1·5)	0·7 (0·4 to 1)	0·9 (0·5 to 1·3)	1·5 (0·7 to 2·2)	1·5 (0·8 to 2·4)	1·9 (0·9 to 3)	2 (1 to 3·2)	2·5 (1·2 to 4)	4·4 (2·2 to 7·1)	5·4 (2·9 to 8·3)	6·2 (3·4 to 9·6)	6·8 (3·7 to 10·1)	5·8 (2·9 to 8·6)	5·3 (2·5 to 7·7)	4·3 (2·1 to 6·5)	1·7 (0·8 to 2·6)	0·6 (0·4 to 0·9)	0·2 (0·1 to 0·3)	0 (0 to 0·1)	54·3
Neurological disorders	0 (0 to 0)	0 (0 to 0·1)	3·9 (2·4 to 5·9)	6·6 (3 to 11·7)	12·1 (5·6 to 20·8)	28·6 (11·2 to 55·5)	42·8 (20·4 to 77·9)	48·9 (23·4 to 87·5)	44·4 (21·8 to 79)	36·9 (18·7 to 62·9)	32·6 (17·9 to 54·4)	34·9 (19·9 to 57·4)	41·1 (24·5 to 64·7)	42·1 (26·6 to 63·5)	40·2 (27·4 to 58)	34·8 (24·2 to 51)	34 (24·4 to 50·5)	35·7 (25·8 to 54·8)	37·4 (26·7 to 58·6)	22·4 (14·3 to 38·9)	9·8 (5·5 to 19·2)	4·3 (2·1 to 9)	0·9 (0·5 to 1·9)	594
Alzheimer's disease and other dementias	0 (0 to 0)	0 (0 to 0)	0 (0 to 0)	0 (0 to 0)	0 (0 to 0)	0 (0 to 0)	0 (0 to 0)	0 (0 to 0)	0 (0 to 0)	0 (0 to 0)	0 (0 to 0)	0·2 (0 to 0·5)	1·3 (0·4 to 3·5)	3·1 (1 to 7·7)	5·7 (2 to 13·9)	8·1 (3·1 to 19·9)	10·4 (4·2 to 26)	13·8 (6·2 to 31·5)	16·8 (7·5 to 38·3)	13·6 (5·8 to 30·7)	7·2 (2·9 to 16·8)	3·6 (1·4 to 8·4)	0·8 (0·4 to 1·8)	84·6
Parkinson's disease	0 (0 to 0)	0 (0 to 0)	0 (0 to 0)	0 (0 to 0)	0 (0 to 0)	0 (0 to 0)	0 (0 to 0)	0 (0 to 0)	0·1 (0 to 0·1)	0·1 (0 to 0·1)	0·1 (0·1 to 0·2)	0·3 (0·2 to 0·4)	0·8 (0·6 to 1·1)	1·5 (1·1 to 2·1)	2·5 (1·9 to 3·1)	3·8 (3·1 to 4·5)	6·6 (5·5 to 7·9)	10·4 (8·8 to 12·2)	13·3 (11·4 to 15·5)	6·3 (5·3 to 7·4)	1·9 (1·6 to 2·2)	0·5 (0·4 to 0·6)	0·1 (0·1 to 0·1)	48·3
Idiopathic epilepsy	0 (0 to 0)	0 (0 to 0·1)	1·2 (0·7 to 2)	4·4 (1·8 to 8·3)	6·4 (2·3 to 12·5)	8·5 (3·1 to 16·1)	12·9 (6·2 to 22·7)	16·3 (8·4 to 27·6)	14·8 (7·5 to 25·2)	12·6 (7 to 20·1)	11·3 (6·6 to 18·1)	11·6 (6·6 to 18·5)	13·9 (8·3 to 21·5)	12·1 (7·2 to 18·9)	10·8 (6·3 to 16·5)	8 (4·5 to 12·8)	6·2 (3·3 to 10·1)	4·8 (2·2 to 8·1)	3·6 (1·7 to 6·2)	1·3 (0·6 to 2·3)	0·4 (0·2 to 0·7)	0·1 (0·1 to 0·2)	0 (0 to 0)	161
Multiple sclerosis	0 (0 to 0)	0 (0 to 0)	0 (0 to 0)	0 (0 to 0)	0 (0 to 0)	0 (0 to 0)	0·1 (0 to 0·1)	0·3 (0·2 to 0·5)	0·3 (0·2 to 0·5)	0·4 (0·3 to 0·7)	0·6 (0·4 to 0·9)	0·8 (0·5 to 1·2)	1 (0·7 to 1·5)	0·9 (0·6 to 1·6)	1·1 (0·7 to 2)	0·7 (0·5 to 1·3)	0·5 (0·3 to 0·8)	0·3 (0·2 to 0·5)	0·2 (0·1 to 0·3)	0 (0 to 0·1)	0 (0 to 0)	0 (0 to 0)	0 (0 to 0)	6·9
Motor neuron disease	0 (0 to 0)	0 (0 to 0)	0·7 (0·4 to 1·4)	0·2 (0·1 to 0·3)	0 (0 to 0)	0·1 (0 to 0·1)	0·3 (0·2 to 0·4)	0·3 (0·2 to 0·5)	0·2 (0·2 to 0·4)	0·4 (0·2 to 0·5)	0·8 (0·5 to 1·1)	1·2 (0·8 to 1·6)	1·6 (1·1 to 2·3)	2·4 (1·7 to 3·3)	2·8 (2 to 3·6)	2·4 (1·8 to 3·2)	2·4 (1·8 to 3·2)	1·5 (1·1 to 2)	0·7 (0·4 to 0·9)	0·1 (0·1 to 0·2)	0 (0 to 0)	0 (0 to 0)	0 (0 to 0)	18·1
Headache disorders	0 (0 to 0)	0 (0 to 0)	0 (0 to 0)	0 (0 to 0)	2·9 (0·1 to 7·7)	15·5 (0·8 to 39·9)	23·2 (2·3 to 56·2)	26·9 (3·6 to 62·2)	25·7 (4·4 to 58·6)	20·8 (4·2 to 46·2)	17·4 (3·8 to 36·9)	17·9 (4·7 to 38·2)	18·9 (5·7 to 39·7)	18·2 (5·4 to 38)	13·6 (4·2 to 28·3)	8·8 (3 to 18·1)	5·5 (1·9 to 11)	3·4 (1·1 to 7)	1·8 (0·6 to 4)	0·6 (0·2 to 1·2)	0·1 (0 to 0·3)	0 (0 to 0·1)	0 (0 to 0)	221
Migraine	0 (0 to 0)	0 (0 to 0)	0 (0 to 0)	0 (0 to 0)	2·6 (0 to 7·3)	14·3 (0·5 to 38·4)	21·3 (1·5 to 54·9)	24·5 (2·5 to 59·8)	23·1 (2·9 to 55)	18·5 (2·9 to 42·2)	15·3 (2·8 to 34·5)	15·8 (3·3 to 35·5)	16·5 (3·7 to 36·3)	15·6 (3·6 to 33·9)	11·6 (2·9 to 25·6)	7·4 (2·1 to 15·9)	4·5 (1·3 to 9·4)	2·6 (0·8 to 5·5)	1·5 (0·4 to 3·2)	0·4 (0·1 to 1)	0·1 (0 to 0·2)	0 (0 to 0·1)	0 (0 to 0)	196
Tension-type headache	0 (0 to 0)	0 (0 to 0)	0 (0 to 0)	0 (0 to 0)	0·3 (0 to 2·2)	1·1 (0 to 8·6)	1·8 (0·3 to 10·3)	2·4 (0·4 to 11·9)	2·6 (0·4 to 11·8)	2·2 (0·4 to 9·9)	2·1 (0·4 to 8·5)	2·2 (0·5 to 8·3)	2·5 (0·7 to 8·3)	2·6 (0·6 to 9)	1·9 (0·5 to 5·9)	1·4 (0·4 to 4·5)	1 (0·2 to 3·6)	0·7 (0·1 to 2·6)	0·4 (0·1 to 1·5)	0·1 (0 to 0·4)	0 (0 to 0·1)	0 (0 to 0)	0 (0 to 0)	25·5
Other neurological disorders	0 (0 to 0)	0 (0 to 0)	1·9 (1·1 to 3·2)	2 (1 to 3·5)	2·7 (1·2 to 4·6)	4·6 (2·2 to 7·9)	6·3 (3·7 to 9·7)	5 (3·1 to 7·6)	3·3 (2 to 5·1)	2·7 (1·7 to 4·1)	2·4 (1·5 to 3·6)	3 (1·8 to 4·3)	3·5 (2·3 to 5·1)	4 (2·8 to 5·5)	3·9 (2·8 to 5·1)	3 (2·1 to 4)	2·3 (1·7 to 3)	1·7 (1·2 to 2·3)	1·1 (0·8 to 1·4)	0·4 (0·3 to 0·5)	0·1 (0·1 to 0·1)	0 (0 to 0)	0 (0 to 0)	53·9
Mental disorders	0 (0 to 0)	0 (0 to 0·1)	0·7 (0·4 to 1·1)	3·9 (2·5 to 5·5)	18·9 (12·7 to 27·1)	51·7 (35·1 to 74·1)	75·5 (52·6 to 104)	87·8 (61 to 122)	89·3 (62 to 121)	75·5 (52·6 to 102)	65·6 (47 to 88·8)	67·8 (48·4 to 91)	74·9 (54·2 to 99·7)	75·7 (55·1 to 100)	61·5 (44·3 to 82·5)	42·1 (30 to 55·8)	27·7 (20 to 36·2)	19 (13·8 to 25·2)	11·8 (8·4 to 15·7)	3·8 (2·6 to 5)	1 (0·7 to 1·4)	0·3 (0·2 to 0·4)	0·1 (0 to 0·1)	854
Schizophrenia	0 (0 to 0)	0 (0 to 0)	0 (0 to 0)	0 (0 to 0)	0 (0 to 0)	0·3 (0·1 to 0·5)	2·1 (0·7 to 4)	8·2 (4·2 to 13·9)	13·5 (7·6 to 20·7)	13·6 (8·1 to 20)	12·3 (7·8 to 18)	12·5 (8 to 17·7)	13·3 (8·8 to 18·3)	12·5 (8·2 to 17·6)	9·2 (5·9 to 12·8)	5·6 (3·5 to 7·9)	3·2 (2 to 4·6)	1·9 (1·1 to 2·7)	1 (0·5 to 1·4)	0·2 (0·1 to 0·4)	0 (0 to 0·1)	0 (0 to 0)	0 (0 to 0)	110
Depressive disorders	0 (0 to 0)	0 (0 to 0)	0 (0 to 0)	0 (0 to 0)	0·6 (0·2 to 1·1)	5·5 (2·7 to 9·7)	17·6 (10·2 to 28·1)	28·2 (16·5 to 45·2)	25·5 (15·3 to 39)	20·5 (12·4 to 30·8)	18·9 (12·2 to 27·8)	20·9 (12·6 to 31·5)	24·3 (15·6 to 36·3)	26·1 (17·2 to 38·1)	22·5 (14·4 to 33·2)	16·3 (10·5 to 24)	11·2 (7·3 to 16)	8·2 (5·4 to 11·6)	5·3 (3·3 to 7·9)	1·8 (1·1 to 2·7)	0·6 (0·3 to 0·8)	0·2 (0·1 to 0·3)	0 (0 to 0·1)	254
Major depressive disorder	0 (0 to 0)	0 (0 to 0)	0 (0 to 0)	0 (0 to 0)	0·5 (0·2 to 0·9)	4·8 (2·1 to 8·8)	15·3 (8·4 to 25·1)	23·8 (12·7 to 40·2)	20·2 (11·3 to 31·5)	15·8 (8·9 to 25)	14·8 (9·2 to 22·1)	16·5 (9·6 to 25·4)	19·4 (11·8 to 29·5)	21·1 (13·2 to 31·2)	18·4 (11·4 to 28)	13·5 (8·1 to 20·6)	9·3 (5·9 to 13·4)	6·8 (4·4 to 10)	4·5 (2·6 to 6·8)	1·6 (0·9 to 2·4)	0·5 (0·3 to 0·7)	0·2 (0·1 to 0·2)	0 (0 to 0·1)	207
Dysthymia	0 (0 to 0)	0 (0 to 0)	0 (0 to 0)	0 (0 to 0)	0·1 (0 to 0·3)	0·7 (0·3 to 1·5)	2·3 (1·1 to 4·1)	4·4 (2·2 to 8)	5·3 (2·7 to 9·2)	4·7 (2·6 to 7·5)	4·2 (2·3 to 6·9)	4·4 (2·3 to 7·7)	4·9 (2·6 to 8·4)	5 (2·8 to 8·5)	4·1 (2·3 to 6·8)	2·9 (1·5 to 4·8)	1·9 (1 to 3·2)	1·3 (0·7 to 2·2)	0·8 (0·5 to 1·3)	0·3 (0·1 to 0·4)	0·1 (0 to 0·1)	0 (0 to 0)	0 (0 to 0)	47·4
Bipolar disorder	0 (0 to 0)	0 (0 to 0)	0 (0 to 0)	0 (0 to 0)	0 (0 to 0)	2·6 (1 to 5)	10·5 (4·9 to 18·6)	14·6 (7·2 to 25·2)	13·9 (7·2 to 24)	10·8 (5·7 to 18)	8·9 (4·7 to 14·4)	9·3 (5 to 15·3)	10·3 (5·7 to 17·3)	10·1 (5·5 to 17)	7·8 (4·4 to 13)	4·9 (2·8 to 7·9)	2·9 (1·6 to 4·8)	1·8 (1 to 3)	0·9 (0·5 to 1·5)	0·2 (0·1 to 0·4)	0·1 (0 to 0·1)	0 (0 to 0)	0 (0 to 0)	110
Anxiety disorders	0 (0 to 0)	0 (0 to 0)	0 (0 to 0)	0·3 (0·1 to 0·6)	4·3 (2·2 to 7·4)	11·9 (6·4 to 19·4)	16·7 (9·8 to 26·2)	19 (10·2 to 31·3)	18·1 (9·9 to 30·8)	14·7 (8·6 to 23·4)	12·5 (7·3 to 19·4)	13 (7·2 to 21)	14·4 (8 to 23)	14·9 (9 to 22·9)	12·3 (7·5 to 18·5)	8·7 (5·1 to 13·6)	5·8 (3·4 to 9·3)	3·9 (2·4 to 6·3)	2·3 (1·4 to 3·6)	0·7 (0·4 to 1·1)	0·2 (0·1 to 0·3)	0 (0 to 0·1)	0 (0 to 0)	174
Eating disorders	0 (0 to 0)	0 (0 to 0)	0 (0 to 0)	0 (0 to 0)	0 (0 to 0·1)	0·8 (0·3 to 1·5)	2·4 (1·1 to 4·5)	3·3 (1·5 to 6·1)	3·3 (1·7 to 5·5)	3 (1·4 to 5·3)	1·9 (1 to 3·4)	0·9 (0·4 to 1·6)	0·4 (0·1 to 0·8)	0 (0 to 0)	0 (0 to 0)	0 (0 to 0)	0 (0 to 0)	0 (0 to 0)	0 (0 to 0)	0 (0 to 0)	0 (0 to 0)	0 (0 to 0)	0 (0 to 0)	15·9
Anorexia nervosa	0 (0 to 0)	0 (0 to 0)	0 (0 to 0)	0 (0 to 0)	0 (0 to 0·1)	0·3 (0·1 to 0·7)	0·8 (0·3 to 1·6)	0·9 (0·3 to 1·7)	0·6 (0·2 to 1·1)	0·3 (0·1 to 0·7)	0·2 (0 to 0·4)	0·1 (0 to 0·4)	0·1 (0 to 0·3)	0 (0 to 0)	0 (0 to 0)	0 (0 to 0)	0 (0 to 0)	0 (0 to 0)	0 (0 to 0)	0 (0 to 0)	0 (0 to 0)	0 (0 to 0)	0 (0 to 0)	3·4
Bulimia nervosa	0 (0 to 0)	0 (0 to 0)	0 (0 to 0)	0 (0 to 0)	0 (0 to 0)	0·5 (0·1 to 1·1)	1·5 (0·6 to 3·3)	2·4 (0·9 to 4·9)	2·7 (1·3 to 4·8)	2·6 (1·2 to 4·8)	1·7 (0·8 to 3·1)	0·8 (0·3 to 1·4)	0·3 (0·1 to 0·6)	0 (0 to 0)	0 (0 to 0)	0 (0 to 0)	0 (0 to 0)	0 (0 to 0)	0 (0 to 0)	0 (0 to 0)	0 (0 to 0)	0 (0 to 0)	0 (0 to 0)	12·5
Autism spectrum disorders	0 (0 to 0)	0 (0 to 0·1)	0·6 (0·4 to 1)	2·8 (1·8 to 4·1)	3·6 (2·3 to 5·3)	3·8 (2·4 to 5·6)	4 (2·5 to 6)	4·3 (2·7 to 6·6)	3·9 (2·3 to 5·8)	3 (1·8 to 4·5)	2·4 (1·5 to 3·6)	2·4 (1·4 to 3·6)	2·5 (1·5 to 3·7)	2·5 (1·5 to 3·6)	2 (1·2 to 3·1)	1·4 (0·8 to 2)	0·9 (0·6 to 1·4)	0·7 (0·4 to 1)	0·4 (0·3 to 0·6)	0·1 (0·1 to 0·2)	0 (0 to 0·1)	0 (0 to 0)	0 (0 to 0)	41·3
Attention-deficit/hyperactivity disorder	0 (0 to 0)	0 (0 to 0)	0 (0 to 0)	0·3 (0·2 to 0·6)	3·7 (2 to 6·8)	5·5 (3 to 9·7)	4·9 (2·7 to 8·7)	4·1 (2·2 to 6·9)	2·9 (1·6 to 5·1)	1·8 (1 to 3·2)	1·2 (0·6 to 2·1)	1·1 (0·5 to 1·9)	0·9 (0·5 to 1·7)	0·8 (0·4 to 1·4)	0·4 (0·2 to 0·8)	0·2 (0·1 to 0·4)	0·1 (0 to 0·2)	0 (0 to 0·1)	0 (0 to 0)	0 (0 to 0)	0 (0 to 0)	0 (0 to 0)	0 (0 to 0)	28·1
Conduct disorder	0 (0 to 0)	0 (0 to 0)	0 (0 to 0)	0 (0 to 0)	5·9 (3·1 to 10·4)	20·5 (11·5 to 33·3)	14·6 (7·8 to 24·5)	0 (0 to 0)	0 (0 to 0)	0 (0 to 0)	0 (0 to 0)	0 (0 to 0)	0 (0 to 0)	0 (0 to 0)	0 (0 to 0)	0 (0 to 0)	0 (0 to 0)	0 (0 to 0)	0 (0 to 0)	0 (0 to 0)	0 (0 to 0)	0 (0 to 0)	0 (0 to 0)	41
Idiopathic developmental intellectual disability	0 (0 to 0)	0 (0 to 0)	0·1 (0 to 0·2)	0·4 (0·1 to 0·9)	0·7 (0·2 to 1·4)	0·7 (0·2 to 1·5)	0·8 (0·2 to 1·5)	0·8 (0·2 to 1·6)	0·7 (0·2 to 1·4)	0·6 (0·1 to 1·1)	0·4 (0·1 to 0·9)	0·4 (0·1 to 0·8)	0·4 (0·1 to 0·9)	0·4 (0·1 to 0·8)	0·3 (0·1 to 0·6)	0·2 (0 to 0·4)	0·1 (0 to 0·3)	0·1 (0 to 0·2)	0 (0 to 0·1)	0 (0 to 0)	0 (0 to 0)	0 (0 to 0)	0 (0 to 0)	7·2
Other mental disorders	0 (0 to 0)	0 (0 to 0)	0 (0 to 0)	0 (0 to 0)	0 (0 to 0)	0·3 (0·1 to 0·5)	1·9 (0·8 to 3·4)	5·3 (2·6 to 8·9)	7·6 (4·1 to 12)	7·5 (4·3 to 11·8)	7 (4 to 11·1)	7·4 (4·4 to 11·2)	8·2 (5 to 12·8)	8·4 (5·1 to 12·9)	7 (4·3 to 10·6)	4·9 (3 to 7·5)	3·4 (2·1 to 5·1)	2·5 (1·6 to 3·8)	1·7 (1·1 to 2·5)	0·6 (0·4 to 0·9)	0·2 (0·1 to 0·3)	0·1 (0 to 0·1)	0 (0 to 0)	73·9
Substance use disorders	0 (0 to 0)	0 (0 to 0)	0 (0 to 0)	0 (0 to 0)	0 (0 to 0)	0·8 (0·4 to 1·4)	17·6 (11·6 to 25·3)	35·9 (25·3 to 49·5)	38·7 (28 to 51·7)	36 (26·6 to 47·3)	33·4 (25·7 to 43)	36·6 (27·3 to 47·1)	44·3 (33·3 to 57·5)	41·5 (31·9 to 53·6)	35·7 (27·4 to 45·3)	22·3 (16·5 to 29·1)	14·3 (10·8 to 18·2)	7·4 (5·6 to 9·7)	3·7 (2·8 to 4·7)	0·9 (0·7 to 1·2)	0·2 (0·2 to 0·3)	0·1 (0·1 to 0·1)	0 (0 to 0)	369
Alcohol use disorders	0 (0 to 0)	0 (0 to 0)	0 (0 to 0)	0 (0 to 0)	0 (0 to 0)	0·3 (0·1 to 0·6)	4·3 (2·4 to 7·1)	13·6 (7·8 to 21·9)	18·4 (11·8 to 27·5)	20·2 (13·7 to 29·4)	21·5 (15·6 to 29·1)	26·5 (19 to 35·6)	34·9 (25 to 47·5)	35·2 (26·1 to 46·4)	31·7 (23·5 to 41)	20·2 (14·5 to 26·9)	13·1 (9·7 to 17)	6·7 (5 to 8·9)	3·2 (2·4 to 4·2)	0·7 (0·5 to 1)	0·2 (0·1 to 0·3)	0·1 (0 to 0·1)	0 (0 to 0)	251
Drug use disorders	0 (0 to 0)	0 (0 to 0)	0 (0 to 0)	0 (0 to 0)	0 (0 to 0)	0·5 (0·2 to 0·9)	13·2 (8·2 to 20·3)	22·3 (15·3 to 32·3)	20·3 (14·4 to 27·7)	15·8 (11·3 to 21)	11·9 (8·6 to 15·7)	10·1 (7·3 to 13·6)	9·3 (6·7 to 12·3)	6·4 (4·6 to 8·6)	4·1 (2·8 to 5·6)	2 (1·3 to 3)	1·1 (0·7 to 1·7)	0·7 (0·5 to 1)	0·5 (0·3 to 0·7)	0·2 (0·1 to 0·2)	0 (0 to 0·1)	0 (0 to 0)	0 (0 to 0)	119
Opioid use disorders	0 (0 to 0)	0 (0 to 0)	0 (0 to 0)	0 (0 to 0)	0 (0 to 0)	0 (0 to 0)	1·1 (0·3 to 2·1)	5 (2·6 to 8·6)	5·9 (3·4 to 9·3)	5 (3·2 to 7·5)	3·9 (2·5 to 5·8)	3·3 (2·1 to 5·1)	3 (1·8 to 4·4)	2·4 (1·3 to 3·7)	1·6 (0·9 to 2·6)	0·9 (0·4 to 1·5)	0·5 (0·2 to 0·8)	0·3 (0·1 to 0·5)	0·2 (0·1 to 0·4)	0·1 (0 to 0·1)	0 (0 to 0)	0 (0 to 0)	0 (0 to 0)	33
Cocaine use disorders	0 (0 to 0)	0 (0 to 0)	0 (0 to 0)	0 (0 to 0)	0 (0 to 0)	0·1 (0·1 to 0·2)	5 (2·6 to 8·4)	7·4 (4·7 to 11·5)	6·7 (4·6 to 9·7)	5·8 (4 to 8·2)	4·7 (3·3 to 6·5)	4 (2·7 to 5·6)	3·7 (2·5 to 5·2)	2·1 (1·4 to 3)	1·3 (0·8 to 2)	0·6 (0·4 to 1)	0·4 (0·2 to 0·6)	0·2 (0·1 to 0·4)	0·2 (0·1 to 0·2)	0 (0 to 0·1)	0 (0 to 0)	0 (0 to 0)	0 (0 to 0)	42·4
Amphetamine use disorders	0 (0 to 0)	0 (0 to 0)	0 (0 to 0)	0 (0 to 0)	0 (0 to 0)	0 (0 to 0)	0·3 (0·1 to 0·8)	1·7 (0·7 to 3·2)	1·7 (0·7 to 3)	1·1 (0·6 to 1·8)	0·7 (0·4 to 1·2)	0·6 (0·4 to 0·8)	0·5 (0·3 to 0·7)	0·4 (0·3 to 0·6)	0·3 (0·2 to 0·4)	0·1 (0·1 to 0·2)	0·1 (0 to 0·1)	0 (0 to 0)	0 (0 to 0)	0 (0 to 0)	0 (0 to 0)	0 (0 to 0)	0 (0 to 0)	7·6
Cannabis use disorders	0 (0 to 0)	0 (0 to 0)	0 (0 to 0)	0 (0 to 0)	0 (0 to 0)	0·3 (0·1 to 0·7)	3·7 (1·6 to 7·2)	3·3 (1·5 to 5·9)	1·8 (0·8 to 3·6)	1 (0·4 to 1·8)	0·6 (0·2 to 1·1)	0·5 (0·2 to 0·9)	0·5 (0·2 to 0·9)	0·4 (0·1 to 0·8)	0·2 (0·1 to 0·4)	0·1 (0 to 0·2)	0 (0 to 0·1)	0 (0 to 0)	0 (0 to 0)	0 (0 to 0)	0 (0 to 0)	0 (0 to 0)	0 (0 to 0)	12·4
Other drug use disorders	0 (0 to 0)	0 (0 to 0)	0 (0 to 0)	0 (0 to 0)	0 (0 to 0)	0·1 (0 to 0·1)	3·2 (1·6 to 5·7)	4·9 (2·8 to 8·1)	4·1 (2·5 to 6·5)	2·8 (1·8 to 4·3)	2 (1·3 to 3)	1·7 (1·1 to 2·6)	1·6 (1 to 2·4)	1·1 (0·7 to 1·7)	0·7 (0·4 to 1·1)	0·3 (0·2 to 0·5)	0·2 (0·1 to 0·3)	0·2 (0·1 to 0·3)	0·1 (0·1 to 0·2)	0·1 (0 to 0·1)	0 (0 to 0)	0 (0 to 0)	0 (0 to 0)	23·1
Diabetes and kidney diseases	0 (0 to 0)	0 (0 to 0·1)	3·7 (2·2 to 5·8)	3·2 (2·1 to 4·6)	5·3 (3·9 to 7)	5·3 (3·9 to 7·2)	15·6 (12·1 to 19·7)	27·7 (21·7 to 34·3)	37·6 (28·8 to 48·2)	50·6 (40·7 to 62·4)	63·8 (50·6 to 77·6)	98·7 (79·4 to 120)	176 (142 to 211)	254 (207 to 307)	309 (255 to 372)	306 (256 to 362)	315 (267 to 368)	255 (216 to 298)	196 (166 to 230)	72·4 (62·1 to 83)	19·4 (16·4 to 22·5)	5·9 (5 to 7)	1·3 (1·1 to 1·6)	2,220
Diabetes mellitus	0 (0 to 0)	0 (0 to 0·1)	0·7 (0·4 to 1·4)	0·5 (0·3 to 0·9)	0·5 (0·3 to 0·8)	0·9 (0·6 to 1·3)	4·3 (3·3 to 5·5)	10·3 (7·8 to 13·2)	15·9 (11·8 to 20·8)	23·2 (17·5 to 30)	31·6 (24·1 to 39·9)	53·3 (39·9 to 67·7)	103 (78·4 to 130)	163 (125 to 210)	200 (157 to 252)	197 (155 to 245)	207 (166 to 252)	169 (136 to 206)	130 (105 to 158)	45 (36·3 to 53·9)	11·8 (9·6 to 14·2)	3·3 (2·6 to 4)	0·7 (0·5 to 0·8)	1,370
Diabetes mellitus type 1	0 (0 to 0)	0 (0 to 0·1)	0·7 (0·4 to 1·4)	0·5 (0·3 to 0·9)	0·5 (0·3 to 0·8)	0·9 (0·6 to 1·3)	1·8 (1·3 to 2·5)	4 (2·8 to 5·5)	5·6 (3·8 to 7·8)	4·3 (2·7 to 6·6)	5·3 (3·5 to 8·4)	8·2 (5·4 to 12·3)	3·5 (2·2 to 5·5)	5 (3·1 to 7·9)	6·2 (3·9 to 9·7)	5 (3 to 8)	5·3 (3·4 to 8·9)	3·3 (2 to 5·8)	2·5 (1·5 to 4·7)	0·6 (0·4 to 1·1)	0·2 (0·1 to 0·3)	0·1 (0 to 0·1)	0 (0 to 0)	63·7
Diabetes mellitus type 2	0 (0 to 0)	0 (0 to 0)	0 (0 to 0)	0 (0 to 0)	0 (0 to 0)	0 (0 to 0)	2·5 (1·7 to 3·5)	6·3 (4·4 to 8·8)	10·3 (7 to 14·7)	18·9 (13·8 to 25·2)	26·3 (19·5 to 34·1)	45·2 (32·9 to 59·2)	99·3 (75·9 to 126)	159 (121 to 204)	194 (152 to 244)	192 (152 to 239)	202 (162 to 247)	166 (134 to 203)	128 (103 to 156)	44·3 (35·8 to 53·1)	11·6 (9·4 to 13·9)	3·2 (2·6 to 3·9)	0·7 (0·5 to 0·8)	1,310
Chronic kidney disease	0 (0 to 0)	0 (0 to 0)	3 (1·7 to 4·7)	2·7 (1·8 to 3·8)	4·8 (3·4 to 6·5)	4·3 (3·1 to 6·1)	11·2 (8 to 15·1)	17·5 (13·1 to 22·8)	21·7 (15 to 29·6)	27·4 (19·9 to 36·1)	32·2 (24 to 42·1)	45·3 (33·6 to 58·1)	72·8 (54·7 to 93)	90 (68·6 to 115)	109 (84·5 to 140)	109 (85·8 to 136)	108 (84·2 to 134)	86 (69·2 to 106)	65·9 (51·8 to 82·2)	27·5 (22·2 to 33·5)	7·6 (6·3 to 9·3)	2·6 (2·1 to 3·3)	0·7 (0·5 to 0·8)	849
Chronic kidney disease due to diabetes mellitus type 1	0 (0 to 0)	0 (0 to 0)	0 (0 to 0·1)	0·1 (0 to 0·2)	0·1 (0·1 to 0·3)	0·1 (0·1 to 0·3)	0·4 (0·2 to 0·9)	1 (0·4 to 2·1)	2·2 (1 to 4·2)	4·6 (2·4 to 7·9)	6·3 (3·3 to 10·4)	8·4 (4·3 to 13·7)	11·6 (5·4 to 19·7)	10·5 (4·3 to 20·1)	8·8 (3 to 18·8)	6·4 (2·2 to 13·6)	5 (1·9 to 10·9)	3 (1·1 to 6·1)	1·5 (0·6 to 3·4)	0·3 (0·1 to 0·7)	0 (0 to 0·1)	0 (0 to 0)	0 (0 to 0)	70·5
Chronic kidney disease due to diabetes mellitus type 2	0 (0 to 0)	0 (0 to 0)	0 (0 to 0)	0 (0 to 0)	0 (0 to 0)	0 (0 to 0)	0·2 (0·1 to 0·5)	0·5 (0·2 to 1)	0·9 (0·4 to 1·8)	1·8 (0·7 to 3·4)	2·8 (1·2 to 5·5)	5·6 (2·7 to 10)	13·1 (6·9 to 21)	21·8 (12·5 to 32·9)	31·5 (18·6 to 46·2)	33·8 (21 to 48·5)	34·6 (22 to 48·2)	27·8 (18·7 to 37·3)	21·2 (14·1 to 29·2)	8·7 (5·9 to 12·1)	2·3 (1·5 to 3·2)	0·8 (0·5 to 1·1)	0·2 (0·1 to 0·3)	207
Chronic kidney disease due to hypertension	0 (0 to 0)	0 (0 to 0)	0 (0 to 0)	0 (0 to 0)	0 (0 to 0)	0 (0 to 0)	0·8 (0·3 to 1·6)	1·5 (0·7 to 2·8)	2·1 (1 to 3·8)	2·7 (1·3 to 4·4)	3·4 (1·9 to 5·5)	5·5 (3·3 to 8·4)	10·2 (6·6 to 15·2)	14·8 (9·3 to 21·7)	20·7 (13·1 to 30·7)	24 (15·5 to 34·4)	26·8 (17·5 to 38·2)	23·2 (16 to 32)	19·6 (13·8 to 27·1)	9·2 (6·4 to 12·2)	2·8 (2·1 to 3·8)	1·1 (0·8 to 1·4)	0·3 (0·2 to 0·4)	169
Chronic kidney disease due to glomerulonephritis	0 (0 to 0)	0 (0 to 0)	0·8 (0·4 to 1·6)	0·9 (0·5 to 1·5)	1·8 (1 to 2·8)	1·9 (1·1 to 2·9)	4·9 (3·1 to 7·4)	7·5 (4·7 to 10·7)	8·6 (5·3 to 13·1)	9·9 (6·2 to 14·2)	10·9 (6·9 to 16·1)	14·2 (8·1 to 20·8)	20·7 (13 to 30·5)	23·3 (13·6 to 35·5)	26 (14·9 to 40·3)	23 (12·5 to 36·7)	19·8 (10·1 to 33·9)	13·4 (7·1 to 22·8)	9 (4·4 to 15·4)	3·2 (1·4 to 5·8)	0·7 (0·3 to 1·2)	0·2 (0·1 to 0·4)	0 (0 to 0·1)	201
Chronic kidney disease due to other and unspecified causes	0 (0 to 0)	0 (0 to 0)	2·1 (1·1 to 3·5)	1·7 (1·1 to 2·6)	2·8 (1·9 to 4·1)	2·3 (1·4 to 3·6)	4·9 (2·9 to 7·3)	7·1 (4·3 to 10·3)	7·8 (4·6 to 11·9)	8·4 (5·2 to 13·3)	8·8 (5·1 to 14·1)	11·6 (6·7 to 17·3)	17·3 (10·1 to 26·9)	19·6 (11·4 to 30·8)	22·1 (12·5 to 35·6)	21·4 (11·8 to 34·9)	22·1 (12·3 to 35·1)	18·7 (11·2 to 28·5)	14·6 (8·6 to 22·5)	6·1 (3·7 to 9·3)	1·8 (1·1 to 2·6)	0·6 (0·4 to 0·9)	0·2 (0·1 to 0·2)	202
Acute glomerulonephritis	0 (0 to 0)	0 (0 to 0)	0 (0 to 0)	0 (0 to 0)	0 (0 to 0)	0 (0 to 0)	0 (0 to 0)	0 (0 to 0)	0 (0 to 0)	0 (0 to 0)	0 (0 to 0)	0 (0 to 0)	0 (0 to 0)	0 (0 to 0)	0 (0 to 0)	0 (0 to 0)	0 (0 to 0)	0 (0 to 0)	0 (0 to 0)	0 (0 to 0)	0 (0 to 0)	0 (0 to 0)	0 (0 to 0)	0·1
Skin and subcutaneous diseases	0 (0 to 0)	0 (0 to 0)	5·4 (2·8 to 10·5)	18·4 (11·8 to 27·2)	24 (15·2 to 34·7)	25·9 (16·8 to 38·4)	31·3 (20·5 to 46·6)	23·8 (15·6 to 35·1)	20·2 (13·4 to 29·4)	16·1 (10·6 to 24)	14 (9·6 to 20·4)	15·8 (10·9 to 23)	19·9 (13·5 to 29·2)	24·3 (16·6 to 37·3)	23·9 (15·9 to 37·8)	21·1 (14·1 to 33·2)	20·9 (13·6 to 32·3)	18·3 (11·1 to 28·2)	16·1 (9·2 to 27·5)	6·8 (3·9 to 11·1)	2·3 (1·3 to 4·1)	0·9 (0·5 to 1·5)	0·2 (0·1 to 0·3)	350
Dermatitis	0 (0 to 0)	0 (0 to 0)	0 (0 to 0)	5·9 (3·2 to 10)	7·2 (3·8 to 12·7)	5·4 (2·9 to 9·4)	4·4 (2·5 to 7·3)	4·1 (2·3 to 6·7)	3·6 (2 to 5·8)	2·8 (1·5 to 4·6)	2·3 (1·3 to 3·6)	2·3 (1·3 to 3·8)	2·6 (1·4 to 4·3)	2·9 (1·5 to 4·8)	2·6 (1·4 to 4·2)	2 (1·1 to 3·3)	1·6 (0·9 to 2·5)	1·2 (0·7 to 2)	0·8 (0·5 to 1·3)	0·3 (0·2 to 0·4)	0·1 (0 to 0·1)	0 (0 to 0)	0 (0 to 0)	51·9
Atopic dermatitis	0 (0 to 0)	0 (0 to 0)	0 (0 to 0)	5·8 (3·1 to 9·9)	7 (3·7 to 12·4)	4·8 (2·5 to 8·5)	3·4 (1·8 to 6·2)	2·7 (1·4 to 4·8)	2 (1 to 3·7)	1·4 (0·7 to 2·6)	1·1 (0·6 to 2)	1·1 (0·6 to 2)	1·2 (0·6 to 2·2)	1·3 (0·6 to 2·4)	1·1 (0·6 to 2·1)	0·9 (0·4 to 1·5)	0·6 (0·3 to 1·1)	0·5 (0·2 to 0·8)	0·3 (0·2 to 0·6)	0·1 (0·1 to 0·2)	0 (0 to 0·1)	0 (0 to 0)	0 (0 to 0)	35·6
Contact dermatitis	0 (0 to 0)	0 (0 to 0)	0 (0 to 0)	0 (0 to 0)	0·1 (0 to 0·2)	0·5 (0·2 to 1)	0·9 (0·4 to 1·8)	1·2 (0·5 to 2·3)	1·4 (0·6 to 2·6)	1·2 (0·5 to 2·3)	1 (0·4 to 2)	1 (0·4 to 2)	1·2 (0·5 to 2·3)	1·4 (0·6 to 2·7)	1·3 (0·6 to 2·6)	1·1 (0·4 to 2)	0·8 (0·4 to 1·6)	0·7 (0·3 to 1·2)	0·4 (0·2 to 0·8)	0·1 (0·1 to 0·2)	0 (0 to 0·1)	0 (0 to 0)	0 (0 to 0)	14·4
Seborrhoeic dermatitis	0 (0 to 0)	0 (0 to 0)	0 (0 to 0)	0·1 (0 to 0·1)	0·1 (0 to 0·1)	0·1 (0 to 0·2)	0·1 (0·1 to 0·2)	0·2 (0·1 to 0·3)	0·2 (0·1 to 0·3)	0·2 (0·1 to 0·3)	0·1 (0·1 to 0·2)	0·1 (0·1 to 0·2)	0·1 (0·1 to 0·2)	0·1 (0·1 to 0·2)	0·1 (0·1 to 0·2)	0·1 (0 to 0·2)	0·1 (0 to 0·2)	0·1 (0 to 0·1)	0·1 (0 to 0·1)	0 (0 to 0)	0 (0 to 0)	0 (0 to 0)	0 (0 to 0)	1·9
Psoriasis	0 (0 to 0)	0 (0 to 0)	0 (0 to 0)	0·3 (0·2 to 0·4)	0·7 (0·3 to 1·2)	0·9 (0·4 to 1·6)	1·1 (0·5 to 1·9)	1·3 (0·6 to 2·2)	1·4 (0·7 to 2·3)	1·3 (0·7 to 2·1)	1·2 (0·7 to 1·9)	1·3 (0·8 to 2)	1·6 (0·9 to 2·5)	1·8 (1·1 to 2·8)	1·7 (1 to 2·5)	1·3 (0·8 to 1·9)	0·9 (0·5 to 1·3)	0·6 (0·4 to 0·9)	0·4 (0·2 to 0·6)	0·1 (0·1 to 0·2)	0 (0 to 0)	0 (0 to 0)	0 (0 to 0)	17·9
Bacterial skin diseases	0 (0 to 0)	0 (0 to 0)	4·2 (1·7 to 9·1)	1·2 (0·6 to 2·3)	1·4 (0·7 to 2·4)	1·4 (0·7 to 2·5)	1·8 (0·9 to 3·7)	2·1 (1 to 4·9)	2·5 (1·1 to 5·9)	2·8 (1·4 to 6·2)	2·5 (1·1 to 5·3)	3·6 (1·6 to 7·3)	5·3 (2·1 to 10·7)	6·9 (2·6 to 14·4)	7·6 (2·9 to 16·6)	6·5 (2·5 to 13·7)	6·9 (2·4 to 14·2)	6·1 (1·9 to 13·2)	5·5 (1·7 to 12·8)	2·2 (0·7 to 4·9)	0·8 (0·2 to 1·7)	0·3 (0·1 to 0·7)	0·1 (0 to 0·1)	71·7
Cellulitis	0 (0 to 0)	0 (0 to 0)	0·4 (0·1 to 1·2)	0·2 (0·1 to 0·3)	0·2 (0·1 to 0·5)	0·2 (0·1 to 0·4)	0·3 (0·1 to 0·8)	0·3 (0·1 to 1·1)	0·4 (0·1 to 1·2)	0·4 (0·2 to 1·3)	0·4 (0·1 to 1·1)	0·5 (0·2 to 1·4)	0·7 (0·3 to 2·2)	0·9 (0·3 to 2·5)	0·8 (0·3 to 2·8)	0·8 (0·3 to 2·5)	0·7 (0·2 to 2·4)	0·7 (0·2 to 2·4)	0·4 (0·1 to 2)	0·2 (0·1 to 0·8)	0·1 (0 to 0·3)	0 (0 to 0·1)	0 (0 to 0)	8·6
Pyoderma	0 (0 to 0)	0 (0 to 0)	3·8 (1·4 to 8·4)	1 (0·5 to 2·1)	1·2 (0·6 to 2)	1·2 (0·5 to 2·2)	1·5 (0·7 to 3·1)	1·8 (0·8 to 4·3)	2·1 (0·9 to 5·2)	2·4 (1 to 4·9)	2·1 (0·9 to 4·4)	3·1 (1·2 to 5·9)	4·6 (1·7 to 9·1)	6 (1·9 to 12·4)	6·8 (2·4 to 14·7)	5·7 (2 to 11·9)	6·2 (1·9 to 12·8)	5·4 (1·6 to 11·1)	5·1 (1·5 to 11·4)	2 (0·6 to 4·2)	0·7 (0·2 to 1·4)	0·3 (0·1 to 0·6)	0·1 (0 to 0·1)	63
Scabies	0 (0 to 0)	0 (0 to 0)	0·5 (0·2 to 0·8)	3·2 (1·6 to 5·7)	4·4 (2·1 to 7·7)	4·6 (2·2 to 8·2)	5·2 (2·7 to 9·1)	5·2 (2·6 to 9)	3·6 (1·8 to 6·3)	2·2 (1·1 to 4·1)	1·8 (0·9 to 3·1)	2·1 (1·1 to 3·6)	2·4 (1·2 to 4·1)	2·3 (1·2 to 4)	1·8 (0·9 to 3·1)	1·1 (0·6 to 2)	0·8 (0·4 to 1·4)	0·6 (0·3 to 1·1)	0·5 (0·2 to 0·8)	0·2 (0·1 to 0·3)	0·1 (0 to 0·1)	0 (0 to 0)	0 (0 to 0)	42·5
Fungal skin diseases	0 (0 to 0)	0 (0 to 0)	0·1 (0 to 0·2)	0·6 (0·2 to 1·4)	1·1 (0·4 to 2·3)	1·3 (0·5 to 2·8)	1·4 (0·5 to 3·2)	1·7 (0·6 to 3·6)	1·6 (0·6 to 3·8)	1·4 (0·5 to 3·1)	1·2 (0·5 to 2·6)	1·3 (0·5 to 2·8)	1·5 (0·6 to 3·2)	1·7 (0·7 to 3·8)	1·8 (0·7 to 4)	1·5 (0·6 to 3·3)	1·5 (0·6 to 3·2)	1·6 (0·6 to 3·4)	1·4 (0·5 to 2·8)	0·6 (0·2 to 1·3)	0·2 (0·1 to 0·5)	0·1 (0 to 0·2)	0 (0 to 0)	23·4
Viral skin diseases	0 (0 to 0)	0 (0 to 0)	0 (0 to 0)	3·5 (2·2 to 5·3)	5·6 (3·5 to 8·7)	3 (1·9 to 4·8)	2 (1·3 to 3·2)	1·7 (1 to 2·7)	1·2 (0·7 to 1·9)	0·8 (0·5 to 1·3)	0·6 (0·4 to 1·1)	0·6 (0·4 to 1·1)	0·7 (0·4 to 1·1)	0·7 (0·4 to 1·1)	0·6 (0·3 to 0·9)	0·4 (0·2 to 0·7)	0·3 (0·2 to 0·5)	0·2 (0·1 to 0·3)	0·1 (0·1 to 0·2)	0 (0 to 0·1)	0 (0 to 0)	0 (0 to 0)	0 (0 to 0)	22·3
Acne vulgaris	0 (0 to 0)	0 (0 to 0)	0 (0 to 0)	0 (0 to 0)	0·3 (0·1 to 0·4)	6·2 (3·5 to 10·4)	11·8 (6·8 to 19·9)	3·2 (1·7 to 5·2)	1·4 (0·7 to 2·3)	0·8 (0·4 to 1·3)	0·5 (0·2 to 0·9)	0·4 (0·2 to 0·6)	0·3 (0·2 to 0·5)	0·2 (0·1 to 0·4)	0·1 (0·1 to 0·2)	0·1 (0 to 0·1)	0 (0 to 0·1)	0 (0 to 0·1)	0 (0 to 0)	0 (0 to 0)	0 (0 to 0)	0 (0 to 0)	0 (0 to 0)	25·3
Alopecia areata	0 (0 to 0)	0 (0 to 0)	0 (0 to 0)	0 (0 to 0·1)	0·1 (0 to 0·1)	0·1 (0 to 0·2)	0·2 (0·1 to 0·3)	0·3 (0·1 to 0·5)	0·4 (0·2 to 0·7)	0·3 (0·2 to 0·6)	0·3 (0·1 to 0·4)	0·2 (0·1 to 0·4)	0·2 (0·1 to 0·4)	0·2 (0·1 to 0·3)	0·2 (0·1 to 0·3)	0·1 (0·1 to 0·2)	0·1 (0 to 0·1)	0·1 (0 to 0·1)	0 (0 to 0·1)	0 (0 to 0)	0 (0 to 0)	0 (0 to 0)	0 (0 to 0)	2·7
Pruritus	0 (0 to 0)	0 (0 to 0)	0 (0 to 0)	0·1 (0 to 0·2)	0·2 (0·1 to 0·3)	0·2 (0·1 to 0·3)	0·2 (0·1 to 0·4)	0·3 (0·1 to 0·6)	0·3 (0·1 to 0·6)	0·3 (0·1 to 0·6)	0·2 (0·1 to 0·5)	0·3 (0·1 to 0·5)	0·3 (0·1 to 0·6)	0·4 (0·1 to 0·7)	0·3 (0·1 to 0·7)	0·3 (0·1 to 0·6)	0·3 (0·1 to 0·5)	0·2 (0·1 to 0·4)	0·1 (0·1 to 0·3)	0·1 (0 to 0·1)	0 (0 to 0)	0 (0 to 0)	0 (0 to 0)	4
Urticaria	0 (0 to 0)	0 (0 to 0)	0·4 (0·2 to 0·7)	2·7 (1·6 to 4·3)	2·4 (1·3 to 4)	1·8 (0·9 to 3)	1·6 (0·9 to 2·8)	1·7 (0·9 to 2·9)	1·5 (0·8 to 2·5)	1·2 (0·6 to 2·1)	1·1 (0·6 to 1·8)	1 (0·5 to 1·7)	1·1 (0·5 to 1·8)	1·1 (0·6 to 1·8)	0·9 (0·4 to 1·5)	0·6 (0·3 to 1·1)	0·4 (0·2 to 0·7)	0·3 (0·2 to 0·6)	0·2 (0·1 to 0·4)	0·1 (0 to 0·1)	0 (0 to 0)	0 (0 to 0)	0 (0 to 0)	20·2
Decubitus ulcer	0 (0 to 0)	0 (0 to 0)	0 (0 to 0)	0·1 (0 to 0·2)	0 (0 to 0·1)	0·1 (0 to 0·3)	0·3 (0 to 0·7)	0·9 (0·2 to 2·8)	1·2 (0·3 to 3·4)	0·8 (0·2 to 2·4)	1 (0·2 to 2·4)	1·2 (0·3 to 2·9)	1·9 (0·6 to 5·1)	3·2 (1 to 8·5)	3·5 (1·3 to 9·9)	4·6 (1·4 to 10·2)	5·6 (1·7 to 12·1)	5·4 (1·6 to 10·6)	5·6 (1·7 to 11·5)	2·7 (0·9 to 5·4)	1 (0·3 to 2·1)	0·4 (0·1 to 0·8)	0·1 (0 to 0·1)	39·7
Other skin and subcutaneous diseases	0 (0 to 0)	0 (0 to 0)	0·2 (0·1 to 0·5)	0·8 (0·4 to 1·5)	0·7 (0·3 to 1·3)	0·9 (0·4 to 1·8)	1·2 (0·6 to 2·2)	1·4 (0·7 to 2·5)	1·6 (0·8 to 2·9)	1·4 (0·7 to 2·5)	1·4 (0·7 to 2·4)	1·6 (0·8 to 2·8)	2·1 (1·1 to 3·9)	2·8 (1·4 to 5)	2·8 (1·4 to 5·2)	2·6 (1·3 to 4·6)	2·5 (1·4 to 4·5)	1·9 (1 to 3·4)	1·5 (0·8 to 2·5)	0·5 (0·3 to 0·8)	0·1 (0·1 to 0·2)	0 (0 to 0·1)	0 (0 to 0)	28·1
Sense organ diseases	0 (0 to 0)	0 (0 to 0)	0·2 (0·1 to 0·3)	1·9 (1·1 to 2·8)	4·1 (2·6 to 6·3)	5·5 (3·4 to 8·1)	7·3 (4·6 to 10·9)	10·2 (6·2 to 15·2)	12·7 (7·8 to 19·4)	13·4 (8·3 to 20·4)	13·7 (8·4 to 21·2)	18·3 (11·3 to 28·5)	29·8 (18·7 to 47·7)	43·7 (27·4 to 69·5)	49·6 (31·7 to 76·8)	46·5 (29·7 to 70·4)	42·8 (28·2 to 63·7)	41·2 (27·9 to 58·6)	35·6 (24·6 to 49·9)	15·2 (10·4 to 20·9)	5·2 (3·6 to 6·9)	1·7 (1·2 to 2·3)	0·4 (0·3 to 0·5)	399
Blindness and vision loss	0 (0 to 0)	0 (0 to 0)	0 (0 to 0·1)	0·5 (0·3 to 0·8)	1·1 (0·6 to 1·8)	1·3 (0·8 to 2·1)	1·7 (1 to 2·6)	2·2 (1·3 to 3·4)	2·5 (1·5 to 3·7)	2·5 (1·5 to 3·8)	2·9 (1·8 to 4·4)	4·3 (2·6 to 6·6)	6·8 (4·1 to 10·8)	10·1 (6·1 to 16·5)	12·1 (7·3 to 19·1)	12·1 (7·9 to 18·8)	12·2 (8 to 18·1)	12·6 (8·4 to 18·2)	11·3 (7·8 to 16·1)	5 (3·4 to 6·9)	1·8 (1·2 to 2·4)	0·6 (0·4 to 0·9)	0·2 (0·1 to 0·2)	104
Glaucoma	0 (0 to 0)	0 (0 to 0)	0 (0 to 0)	0 (0 to 0)	0 (0 to 0)	0 (0 to 0)	0 (0 to 0)	0 (0 to 0)	0 (0 to 0)	0 (0 to 0)	0 (0 to 0)	0 (0 to 0)	0 (0 to 0)	0·1 (0 to 0·3)	0·4 (0·2 to 0·7)	0·6 (0·3 to 1)	0·8 (0·4 to 1·3)	1·1 (0·6 to 1·7)	1·2 (0·7 to 1·9)	0·6 (0·4 to 1)	0·3 (0·2 to 0·4)	0·1 (0·1 to 0·1)	0 (0 to 0)	5·2
Cataract	0 (0 to 0)	0 (0 to 0)	0 (0 to 0)	0 (0 to 0)	0 (0 to 0)	0 (0 to 0)	0 (0 to 0)	0 (0 to 0)	0 (0 to 0·1)	0·1 (0·1 to 0·2)	0·2 (0·1 to 0·4)	0·4 (0·2 to 0·6)	0·6 (0·3 to 1·1)	1 (0·5 to 1·6)	1·2 (0·7 to 2)	1·3 (0·8 to 2)	1·7 (1 to 2·5)	2·3 (1·5 to 3·4)	2·7 (1·8 to 3·9)	1·4 (0·9 to 2)	0·5 (0·4 to 0·7)	0·2 (0·1 to 0·3)	0 (0 to 0·1)	13·8
Age-related macular degeneration	0 (0 to 0)	0 (0 to 0)	0 (0 to 0)	0 (0 to 0)	0 (0 to 0)	0 (0 to 0)	0 (0 to 0)	0 (0 to 0)	0 (0 to 0)	0 (0 to 0)	0 (0 to 0)	0 (0 to 0)	0 (0 to 0)	0 (0 to 0·1)	0·1 (0 to 0·1)	0·1 (0·1 to 0·2)	0·1 (0 to 0·2)	0·1 (0·1 to 0·2)	0·1 (0·1 to 0·2)	0·1 (0 to 0·1)	0 (0 to 0)	0 (0 to 0)	0 (0 to 0)	0·6
Refraction disorders	0 (0 to 0)	0 (0 to 0)	0 (0 to 0)	0·4 (0·2 to 0·7)	1 (0·5 to 1·6)	1·1 (0·6 to 1·9)	1·4 (0·8 to 2·3)	1·8 (1 to 2·8)	1·9 (1·1 to 2·9)	1·7 (1 to 2·6)	1·6 (1 to 2·5)	1·9 (1·1 to 3)	2·3 (1·4 to 3·5)	2·5 (1·5 to 3·9)	2·4 (1·4 to 3·7)	1·9 (1·1 to 2·9)	1·6 (1 to 2·4)	1·5 (0·9 to 2·3)	1·3 (0·8 to 1·9)	0·5 (0·3 to 0·7)	0·1 (0·1 to 0·2)	0 (0 to 0)	0 (0 to 0)	26·8
Near vision loss	0 (0 to 0)	0 (0 to 0)	0 (0 to 0)	0 (0 to 0)	0 (0 to 0)	0 (0 to 0)	0 (0 to 0·1)	0·1 (0 to 0·2)	0·1 (0 to 0·3)	0·3 (0·1 to 0·7)	0·5 (0·2 to 1·2)	1·1 (0·4 to 2·5)	2·3 (0·9 to 4·8)	3·8 (1·6 to 7·9)	4·8 (1·9 to 10)	4·6 (1·9 to 9·7)	4·2 (1·7 to 8·3)	3·8 (1·6 to 7·4)	2·9 (1·2 to 6)	1·1 (0·5 to 2·3)	0·4 (0·2 to 0·7)	0·1 (0 to 0·2)	0 (0 to 0·1)	30·3
Other vision loss	0 (0 to 0)	0 (0 to 0)	0 (0 to 0)	0·1 (0 to 0·1)	0·1 (0·1 to 0·2)	0·2 (0·1 to 0·3)	0·3 (0·1 to 0·5)	0·3 (0·1 to 0·7)	0·4 (0·2 to 0·7)	0·4 (0·2 to 0·8)	0·6 (0·3 to 1)	0·9 (0·5 to 1·6)	1·5 (0·8 to 2·5)	2·5 (1·5 to 3·8)	3·2 (2 to 4·9)	3·6 (2·3 to 5·1)	3·9 (2·6 to 5·6)	3·9 (2·5 to 5·6)	3·1 (2 to 4·6)	1·3 (0·8 to 1·9)	0·5 (0·3 to 0·7)	0·2 (0·1 to 0·3)	0·1 (0 to 0·1)	27·1
Age-related and other hearing loss	0 (0 to 0)	0 (0 to 0)	0·1 (0 to 0·1)	1 (0·5 to 1·7)	2·6 (1·4 to 4·3)	3·7 (2·2 to 5·7)	5 (2·9 to 7·8)	7·3 (4·2 to 11·5)	9·4 (5·6 to 14·6)	10 (6 to 15·9)	10 (5·8 to 15·9)	13·1 (7·7 to 21)	21·7 (12·7 to 35·9)	31·8 (19 to 51·4)	35·7 (22·1 to 56·3)	32·6 (20·2 to 51)	28·8 (18·4 to 44·1)	27 (17·5 to 39·1)	23 (15·5 to 32·8)	9·8 (6·6 to 13·6)	3·3 (2·2 to 4·5)	1·1 (0·8 to 1·4)	0·2 (0·2 to 0·3)	277
Other sense organ diseases	0 (0 to 0)	0 (0 to 0)	0·1 (0 to 0·1)	0·4 (0·2 to 0·7)	0·4 (0·2 to 0·8)	0·4 (0·2 to 0·8)	0·6 (0·2 to 1)	0·7 (0·3 to 1·3)	0·8 (0·3 to 1·5)	0·9 (0·4 to 1·7)	0·9 (0·4 to 1·6)	1 (0·4 to 1·8)	1·2 (0·6 to 2·4)	1·8 (0·8 to 3·6)	1·8 (0·8 to 3·4)	1·8 (0·8 to 3·3)	1·7 (0·8 to 3·2)	1·6 (0·7 to 2·9)	1·3 (0·6 to 2·3)	0·4 (0·2 to 0·8)	0·1 (0·1 to 0·2)	0 (0 to 0·1)	0 (0 to 0)	17·9
Musculoskeletal disorders	0 (0 to 0)	0 (0 to 0)	0 (0 to 0)	0 (0 to 0)	1·8 (0·8 to 3·2)	9·2 (5·3 to 14·4)	21·6 (13·5 to 33·2)	35·9 (22·5 to 53·1)	49·3 (31·1 to 72·4)	51 (32·3 to 73·2)	50·5 (33·6 to 71·1)	61·3 (40·9 to 86·4)	78·5 (53·2 to 111)	92·4 (61·6 to 134)	87·3 (57·5 to 123)	69·5 (46·6 to 98·8)	52·7 (36·3 to 74·8)	40·5 (27·1 to 57·1)	26·7 (17·8 to 38·6)	8·8 (6 to 12·7)	2·5 (1·7 to 3·6)	0·7 (0·5 to 1)	0·1 (0·1 to 0·2)	740
Rheumatoid arthritis	0 (0 to 0)	0 (0 to 0)	0 (0 to 0)	0 (0 to 0)	0 (0 to 0)	0·1 (0 to 0·1)	0·2 (0·1 to 0·3)	0·4 (0·2 to 0·6)	0·5 (0·2 to 0·9)	0·6 (0·3 to 0·9)	0·6 (0·2 to 1)	0·7 (0·3 to 1·2)	0·9 (0·4 to 1·6)	1·1 (0·5 to 1·8)	1 (0·6 to 1·6)	0·7 (0·4 to 1·1)	0·5 (0·3 to 0·9)	0·4 (0·2 to 0·6)	0·3 (0·2 to 0·4)	0·1 (0·1 to 0·1)	0 (0 to 0·1)	0 (0 to 0)	0 (0 to 0)	8
Osteoarthritis	0 (0 to 0)	0 (0 to 0)	0 (0 to 0)	0 (0 to 0)	0 (0 to 0)	0 (0 to 0)	0 (0 to 0)	0 (0 to 0)	0 (0 to 0)	0·3 (0·1 to 0·6)	1·4 (0·6 to 3)	4 (1·9 to 8·1)	8·8 (4·2 to 18·2)	14·7 (7 to 30·9)	17·4 (8·5 to 35)	15·9 (7·9 to 32·1)	13·2 (6·6 to 26·5)	11 (5·5 to 21·7)	7·9 (4 to 16)	2·8 (1·4 to 5·7)	0·8 (0·4 to 1·6)	0·2 (0·1 to 0·5)	0 (0 to 0·1)	98·5
Osteoarthritis hip	0 (0 to 0)	0 (0 to 0)	0 (0 to 0)	0 (0 to 0)	0 (0 to 0)	0 (0 to 0)	0 (0 to 0)	0 (0 to 0)	0 (0 to 0)	0·1 (0 to 0·2)	0·1 (0 to 0·3)	0·2 (0·1 to 0·6)	0·4 (0·2 to 1)	0·8 (0·3 to 1·8)	1 (0·4 to 2·1)	1 (0·4 to 2·1)	0·8 (0·4 to 1·9)	0·7 (0·3 to 1·5)	0·5 (0·2 to 1·1)	0·2 (0·1 to 0·4)	0·1 (0 to 0·1)	0 (0 to 0)	0 (0 to 0)	5·9
Osteoarthritis knee	0 (0 to 0)	0 (0 to 0)	0 (0 to 0)	0 (0 to 0)	0 (0 to 0)	0 (0 to 0)	0 (0 to 0)	0 (0 to 0)	0 (0 to 0)	0·1 (0 to 0·3)	1 (0·4 to 2·2)	3 (1·3 to 6·1)	6·5 (3 to 13·6)	10·8 (5 to 22·8)	12·5 (5·8 to 25·5)	11·3 (5·5 to 22·9)	9·3 (4·5 to 18·9)	7·6 (3·7 to 15·6)	5·3 (2·6 to 10·6)	1·8 (0·9 to 3·7)	0·5 (0·3 to 1)	0·1 (0·1 to 0·3)	0 (0 to 0·1)	69·9
Osteoarthritis hand	0 (0 to 0)	0 (0 to 0)	0 (0 to 0)	0 (0 to 0)	0 (0 to 0)	0 (0 to 0)	0 (0 to 0)	0 (0 to 0)	0 (0 to 0)	0 (0 to 0·1)	0·1 (0 to 0·3)	0·4 (0·1 to 0·9)	0·8 (0·3 to 1·8)	1·3 (0·5 to 3·1)	1·6 (0·7 to 3·5)	1·4 (0·6 to 3·1)	1·2 (0·5 to 2·4)	1 (0·4 to 2)	0·7 (0·3 to 1·5)	0·3 (0·1 to 0·6)	0·1 (0 to 0·2)	0 (0 to 0·1)	0 (0 to 0)	8·8

Osteoarthritis other	0 (0 to 0)	0 (0 to 0)	0 (0 to 0)	0 (0 to 0)	0 (0 to 0)	0 (0 to 0)	0 (0 to 0)	0 (0 to 0)	0 (0 to 0)	0 (0 to 0·1)	0·1 (0 to 0·3)	0·5 (0·2 to 1·1)	1·1 (0·4 to 2·5)	1·9 (0·8 to 4)	2·4 (1·1 to 5·2)	2·3 (1·1 to 4·7)	2 (0·9 to 4)	1·7 (0·8 to 3·4)	1·3 (0·7 to 2·7)	0·5 (0·3 to 1)	0·2 (0·1 to 0·3)	0·1 (0 to 0·1)	0 (0 to 0)	13·9
Low back pain	0 (0 to 0)	0 (0 to 0)	0 (0 to 0)	0 (0 to 0)	1·6 (0·7 to 3)	8·3 (4·6 to 13·6)	17 (10 to 27·6)	23·7 (13·7 to 38·6)	27 (15·5 to 42·2)	25·1 (14·2 to 39·2)	24·4 (14·6 to 37·8)	27·9 (17·3 to 43·5)	33·7 (20·3 to 53·6)	38·7 (22·9 to 61·1)	35·2 (21·6 to 53·7)	27·6 (17·5 to 42·2)	21 (12·9 to 32·6)	16·3 (9·8 to 24·6)	11 (6·8 to 16·8)	3·9 (2·4 to 5·9)	1·1 (0·6 to 1·5)	0·3 (0·2 to 0·4)	0·1 (0 to 0·1)	344
Neck pain	0 (0 to 0)	0 (0 to 0)	0 (0 to 0)	0 (0 to 0)	0·1 (0 to 0·3)	0·7 (0·2 to 1·4)	1·6 (0·7 to 3·1)	2·8 (1·2 to 5·5)	4·3 (2 to 8·6)	5·5 (2·6 to 10·6)	6·2 (3 to 11)	7·9 (3·9 to 14·3)	10·1 (5 to 18·3)	10 (5 to 19)	8·5 (4·1 to 15·3)	6·1 (3·1 to 10·9)	4·2 (2·2 to 7·2)	3·1 (1·6 to 5·4)	2 (1 to 3·6)	0·6 (0·3 to 1·1)	0·2 (0·1 to 0·3)	0 (0 to 0·1)	0 (0 to 0)	73·9
Gout	0 (0 to 0)	0 (0 to 0)	0 (0 to 0)	0 (0 to 0)	0 (0 to 0)	0 (0 to 0)	0 (0 to 0)	0·1 (0 to 0·1)	0·2 (0 to 0·5)	0·3 (0·1 to 0·6)	0·4 (0·2 to 0·7)	0·5 (0·2 to 1)	0·6 (0·3 to 1·2)	0·8 (0·3 to 1·4)	0·7 (0·3 to 1·4)	0·6 (0·2 to 1·1)	0·5 (0·2 to 0·9)	0·5 (0·2 to 0·8)	0·4 (0·2 to 0·6)	0·1 (0·1 to 0·3)	0 (0 to 0·1)	0 (0 to 0)	0 (0 to 0)	5·7
Other musculoskeletal disorders	0 (0 to 0)	0 (0 to 0)	0 (0 to 0)	0 (0 to 0)	0·1 (0 to 0·1)	0·1 (0·1 to 0·4)	2·8 (1·1 to 5·4)	8·9 (5 to 14·2)	17·3 (10·2 to 27·9)	19·4 (11·4 to 30·7)	17·6 (10·5 to 27)	20·4 (11·4 to 33·1)	24·3 (14·6 to 38·5)	27·2 (15·7 to 44·1)	24·5 (14 to 37·9)	18·6 (10·4 to 30·7)	13·2 (7·7 to 20·9)	9·3 (4·7 to 16·3)	5·2 (2·6 to 8·6)	1·2 (0·3 to 2·4)	0·3 (0·2 to 0·6)	0·1 (0 to 0·1)	0 (0 to 0)	210
Other non-communicable diseases	57 (35 to 88·9)	16·2 (10·4 to 25·3)	77·2 (50·1 to 117)	30·8 (21·8 to 43)	24·4 (18·6 to 32·2)	18·8 (14·5 to 24·3)	23·8 (19·1 to 30·5)	31·3 (23·9 to 41·4)	32·1 (23·3 to 46)	33·3 (24·6 to 47·3)	31·1 (22·9 to 43)	33·4 (24·4 to 47·1)	40·7 (29·9 to 57)	49·3 (36·7 to 67·7)	54·2 (42 to 72·3)	52 (39·9 to 66·4)	47·4 (36·7 to 61·2)	43·5 (33·2 to 55)	34·2 (26·4 to 43·3)	13·3 (10 to 16·7)	4·6 (3·5 to 5·7)	1·5 (1·2 to 1·8)	0·3 (0·2 to 0·4)	751
Congenital birth defects	55·2 (33·6 to 85·7)	14·3 (8·9 to 22·7)	53·6 (32·8 to 84·1)	15·6 (10·5 to 23·1)	9·5 (7·1 to 12·5)	8 (6·1 to 10·3)	9·4 (7·1 to 12·1)	9 (6·6 to 11·8)	6·7 (4·9 to 9·1)	5·4 (4 to 7·1)	4 (3 to 5·2)	3·5 (2·6 to 4·6)	3·9 (2·9 to 5·1)	3·9 (2·9 to 5)	3·3 (2·5 to 4·4)	2 (1·5 to 2·5)	1·2 (0·9 to 1·6)	0·3 (0·2 to 0·5)	0·2 (0·1 to 0·3)	0·1 (0 to 0·1)	0 (0 to 0)	0 (0 to 0)	0 (0 to 0)	209
Neural tube defects	3·4 (1·7 to 6·3)	0·7 (0·3 to 1·6)	2·6 (1·1 to 5·3)	2·1 (1 to 4·2)	0·3 (0·2 to 0·5)	0·4 (0·3 to 0·6)	0·4 (0·3 to 0·6)	0·3 (0·2 to 0·5)	0·3 (0·2 to 0·4)	0·2 (0·1 to 0·3)	0·1 (0·1 to 0·1)	0·1 (0·1 to 0·2)	0·1 (0·1 to 0·1)	0·1 (0 to 0·1)	0 (0 to 0·1)	0 (0 to 0)	0 (0 to 0)	0 (0 to 0)	0 (0 to 0)	0 (0 to 0)	0 (0 to 0)	0 (0 to 0)	0 (0 to 0)	11·4
Congenital heart anomalies	33·8 (20·1 to 52·9)	9·1 (5·2 to 14·8)	35·6 (20·7 to 55·7)	7·1 (4 to 12)	4·5 (2·9 to 6·5)	3·2 (2·1 to 4·5)	4·1 (2·6 to 5·8)	4 (2·5 to 6)	2·7 (1·7 to 4·1)	2·4 (1·6 to 3·5)	1·7 (1·1 to 2·4)	1·3 (0·8 to 1·8)	1·3 (0·9 to 1·9)	1·1 (0·7 to 1·7)	0·7 (0·5 to 1·1)	0·5 (0·3 to 0·8)	0·4 (0·2 to 0·5)	0 (0 to 0·1)	0 (0 to 0)	0 (0 to 0)	0 (0 to 0)	0 (0 to 0)	0 (0 to 0)	113
Orofacial clefts	0 (0 to 0)	0 (0 to 0)	0·1 (0 to 0·1)	0·1 (0·1 to 0·3)	0·2 (0·1 to 0·3)	0·2 (0·1 to 0·3)	0·2 (0·1 to 0·3)	0·2 (0·1 to 0·4)	0·2 (0·1 to 0·3)	0·1 (0 to 0·2)	0·1 (0 to 0·2)	0·1 (0 to 0·2)	0·1 (0 to 0·2)	0·1 (0 to 0·2)	0·1 (0 to 0·2)	0·1 (0 to 0·1)	0 (0 to 0·1)	0 (0 to 0·1)	0 (0 to 0)	0 (0 to 0)	0 (0 to 0)	0 (0 to 0)	0 (0 to 0)	1·9
Down syndrome	0·8 (0·4 to 1·5)	0·4 (0·1 to 0·7)	2·6 (1·3 to 4·8)	1 (0·5 to 1·8)	0·5 (0·3 to 0·8)	0·6 (0·4 to 0·9)	1 (0·6 to 1·5)	1 (0·6 to 1·5)	0·8 (0·4 to 1·3)	0·8 (0·4 to 1·3)	0·7 (0·4 to 1)	0·7 (0·4 to 1·1)	0·9 (0·5 to 1·4)	1 (0·6 to 1·5)	1·2 (0·8 to 1·8)	0·5 (0·3 to 0·8)	0·3 (0·2 to 0·4)	0 (0 to 0)	0 (0 to 0)	0 (0 to 0)	0 (0 to 0)	0 (0 to 0)	0 (0 to 0)	14·8
Turner syndrome	0 (0 to 0)	0 (0 to 0)	0 (0 to 0)	0 (0 to 0)	0 (0 to 0)	0 (0 to 0)	0 (0 to 0)	0 (0 to 0)	0 (0 to 0)	0 (0 to 0)	0 (0 to 0)	0 (0 to 0)	0 (0 to 0)	0 (0 to 0)	0 (0 to 0)	0 (0 to 0)	0 (0 to 0)	0 (0 to 0)	0 (0 to 0)	0 (0 to 0)	0 (0 to 0)	0 (0 to 0)	0 (0 to 0)	0
Klinefelter syndrome	0 (0 to 0)	0 (0 to 0)	0 (0 to 0)	0 (0 to 0)	0 (0 to 0)	0 (0 to 0)	0 (0 to 0)	0 (0 to 0)	0 (0 to 0)	0 (0 to 0)	0 (0 to 0)	0 (0 to 0)	0 (0 to 0)	0 (0 to 0)	0 (0 to 0)	0 (0 to 0)	0 (0 to 0)	0 (0 to 0)	0 (0 to 0)	0 (0 to 0)	0 (0 to 0)	0 (0 to 0)	0 (0 to 0)	0
Other chromosomal abnormalities	2·5 (1·2 to 5·1)	0·7 (0·3 to 1·5)	2·2 (1·2 to 4)	0·8 (0·5 to 1·2)	0·5 (0·3 to 0·7)	0·5 (0·3 to 0·7)	0·6 (0·4 to 0·9)	0·4 (0·3 to 0·6)	0·4 (0·2 to 0·6)	0·2 (0·1 to 0·2)	0·1 (0·1 to 0·2)	0·1 (0 to 0·1)	0·1 (0 to 0·1)	0 (0 to 0·1)	0 (0 to 0·1)	0 (0 to 0)	0 (0 to 0)	0 (0 to 0)	0 (0 to 0)	0 (0 to 0)	0 (0 to 0)	0 (0 to 0)	0 (0 to 0)	9·1
Congenital musculoskeletal and limb anomalies	1·4 (0·7 to 3)	0·4 (0·2 to 0·8)	1·9 (1·1 to 3·4)	1·9 (1·1 to 3)	1·9 (1 to 3)	1·9 (1·1 to 3·1)	1·8 (1 to 3)	1·4 (0·8 to 2·2)	1·2 (0·7 to 1·9)	0·9 (0·5 to 1·5)	0·8 (0·4 to 1·2)	0·8 (0·4 to 1·2)	0·8 (0·5 to 1·4)	0·9 (0·5 to 1·4)	0·8 (0·4 to 1·2)	0·5 (0·3 to 0·8)	0·3 (0·2 to 0·5)	0·2 (0·1 to 0·3)	0·1 (0 to 0·2)	0 (0 to 0)	0 (0 to 0)	0 (0 to 0)	0 (0 to 0)	19·9
Urogenital congenital anomalies	0·6 (0·3 to 1·2)	0·1 (0 to 0·2)	0·3 (0·2 to 0·5)	0·3 (0·1 to 0·4)	0·2 (0·1 to 0·3)	0·1 (0·1 to 0·2)	0·1 (0·1 to 0·2)	0·1 (0·1 to 0·2)	0·1 (0·1 to 0·2)	0·1 (0 to 0·1)	0 (0 to 0·1)	0 (0 to 0·1)	0 (0 to 0·1)	0 (0 to 0)	0 (0 to 0)	0 (0 to 0)	0 (0 to 0)	0 (0 to 0)	0 (0 to 0)	0 (0 to 0)	0 (0 to 0)	0 (0 to 0)	0 (0 to 0)	2·2
Digestive congenital anomalies	5·9 (3 to 11·5)	1·6 (0·8 to 2·9)	3·9 (2 to 7·1)	0·7 (0·4 to 1·1)	0·3 (0·2 to 0·5)	0·1 (0·1 to 0·2)	0·1 (0·1 to 0·2)	0·1 (0·1 to 0·1)	0·1 (0 to 0·1)	0 (0 to 0·1)	0 (0 to 0)	0 (0 to 0)	0 (0 to 0)	0 (0 to 0)	0 (0 to 0)	0 (0 to 0)	0 (0 to 0)	0 (0 to 0)	0 (0 to 0)	0 (0 to 0)	0 (0 to 0)	0 (0 to 0)	0 (0 to 0)	13
Other congenital birth defects	6·7 (3·4 to 12·1)	1·2 (0·6 to 2)	4·5 (2·3 to 7·8)	1·6 (0·9 to 2·6)	1·2 (0·8 to 1·8)	1 (0·6 to 1·5)	1·2 (0·7 to 1·8)	1·4 (0·9 to 2·2)	1 (0·6 to 1·5)	0·7 (0·4 to 1·2)	0·5 (0·3 to 0·9)	0·5 (0·3 to 0·8)	0·5 (0·3 to 0·9)	0·6 (0·3 to 1)	0·4 (0·2 to 0·8)	0·3 (0·1 to 0·5)	0·2 (0·1 to 0·4)	0·1 (0 to 0·2)	0·1 (0 to 0·2)	0 (0 to 0·1)	0 (0 to 0)	0 (0 to 0)	0 (0 to 0)	23·6
Urinary diseases and male infertility	0·1 (0·1 to 0·2)	0·1 (0·1 to 0·3)	1·4 (0·7 to 2·4)	0·4 (0·2 to 0·6)	0·3 (0·2 to 0·4)	0·3 (0·2 to 0·5)	0·6 (0·4 to 0·8)	2·4 (1·4 to 4)	2·4 (1·4 to 4·2)	3 (1·9 to 4·9)	3·6 (2·2 to 5·2)	4 (2·4 to 5·9)	5·5 (3·3 to 7·9)	8·5 (5·4 to 11·7)	13·7 (9 to 18·2)	17·4 (11·6 to 23·1)	19·2 (13·1 to 25·7)	19·9 (12·7 to 26·1)	16·6 (11 to 22·3)	6·6 (4 to 9·2)	2·3 (1·5 to 3·2)	0·7 (0·4 to 1)	0·2 (0·1 to 0·3)	129
Urolithiasis	0 (0 to 0)	0 (0 to 0)	0 (0 to 0)	0 (0 to 0)	0 (0 to 0)	0 (0 to 0)	0 (0 to 0·1)	0·1 (0·1 to 0·2)	0·2 (0·1 to 0·3)	0·3 (0·1 to 0·4)	0·5 (0·2 to 0·8)	0·5 (0·2 to 0·8)	1·2 (0·6 to 1·9)	1·4 (0·8 to 2·1)	1·8 (1 to 2·8)	1·6 (1 to 2·5)	1·3 (0·8 to 2·1)	1·2 (0·6 to 1·9)	0·8 (0·4 to 1·4)	0·2 (0·1 to 0·3)	0 (0 to 0·1)	0 (0 to 0)	0 (0 to 0)	11
Benign prostatic hyperplasia	0 (0 to 0)	0 (0 to 0)	0 (0 to 0)	0 (0 to 0)	0 (0 to 0)	0 (0 to 0)	0 (0 to 0)	0 (0 to 0)	0 (0 to 0)	0 (0 to 0)	0 (0 to 0)	0 (0 to 0·1)	0·3 (0·1 to 0·6)	1·3 (0·6 to 2·3)	3·8 (1·8 to 6·5)	5·7 (2·9 to 9·8)	6·6 (3·7 to 10·4)	6·5 (3·5 to 10·4)	4·5 (2·5 to 7)	1·3 (0·7 to 2·1)	0·3 (0·2 to 0·5)	0·1 (0 to 0·1)	0 (0 to 0)	30·3
Male infertility	0 (0 to 0)	0 (0 to 0)	0 (0 to 0)	0 (0 to 0)	0 (0 to 0)	0 (0 to 0)	0 (0 to 0)	0·9 (0·3 to 2·3)	1·1 (0·3 to 2·7)	1 (0·3 to 2·6)	0·8 (0·2 to 2)	0·8 (0·2 to 2)	0·1 (0 to 0·4)	0 (0 to 0)	0 (0 to 0)	0 (0 to 0)	0 (0 to 0)	0 (0 to 0)	0 (0 to 0)	0 (0 to 0)	0 (0 to 0)	0 (0 to 0)	0 (0 to 0)	4·7
Other urinary diseases	0·1 (0 to 0·1)	0·1 (0 to 0·2)	0·2 (0·1 to 0·4)	0·1 (0 to 0·2)	0·1 (0·1 to 0·2)	0·1 (0·1 to 0·2)	0·3 (0·2 to 0·4)	0·6 (0·3 to 1)	0·3 (0·1 to 0·5)	0·5 (0·2 to 0·7)	0·8 (0·4 to 1·3)	0·8 (0·4 to 1·3)	1·3 (0·6 to 2·2)	1·6 (0·8 to 2·6)	1·8 (0·8 to 2·9)	2·4 (1·1 to 3·9)	2·7 (1·2 to 4·7)	2·1 (0·9 to 3·8)	2 (0·8 to 3·7)	0·9 (0·3 to 1·6)	0·3 (0·1 to 0·5)	0·1 (0 to 0·2)	0 (0 to 0·1)	19·1
Gynecological diseases	0 (0 to 0)	0 (0 to 0)	0 (0 to 0)	0 (0 to 0)	0 (0 to 0)	0 (0 to 0)	0 (0 to 0)	0 (0 to 0)	0 (0 to 0)	0 (0 to 0)	0 (0 to 0)	0 (0 to 0)	0 (0 to 0)	0 (0 to 0)	0 (0 to 0)	0 (0 to 0)	0 (0 to 0)	0 (0 to 0)	0 (0 to 0)	0 (0 to 0)	0 (0 to 0)	0 (0 to 0)	0 (0 to 0)	0
Uterine fibroids	0 (0 to 0)	0 (0 to 0)	0 (0 to 0)	0 (0 to 0)	0 (0 to 0)	0 (0 to 0)	0 (0 to 0)	0 (0 to 0)	0 (0 to 0)	0 (0 to 0)	0 (0 to 0)	0 (0 to 0)	0 (0 to 0)	0 (0 to 0)	0 (0 to 0)	0 (0 to 0)	0 (0 to 0)	0 (0 to 0)	0 (0 to 0)	0 (0 to 0)	0 (0 to 0)	0 (0 to 0)	0 (0 to 0)	0
Polycystic ovarian syndrome	--	--	--	--	--	--	--	--	--	--	--	--	--	--	--	--	--	--	--	--	--	--	--	NA
Female infertility	--	--	--	--	--	--	--	--	--	--	--	--	--	--	--	--	--	--	--	--	--	--	--	NA
Endometriosis	--	--	--	--	--	--	--	--	--	--	--	--	--	--	--	--	--	--	--	--	--	--	--	NA
Genital prolapse	0 (0 to 0)	0 (0 to 0)	0 (0 to 0)	0 (0 to 0)	0 (0 to 0)	0 (0 to 0)	0 (0 to 0)	0 (0 to 0)	0 (0 to 0)	0 (0 to 0)	0 (0 to 0)	0 (0 to 0)	0 (0 to 0)	0 (0 to 0)	0 (0 to 0)	0 (0 to 0)	0 (0 to 0)	0 (0 to 0)	0 (0 to 0)	0 (0 to 0)	0 (0 to 0)	0 (0 to 0)	0 (0 to 0)	0
Premenstrual syndrome	--	--	--	--	--	--	--	--	--	--	--	--	--	--	--	--	--	--	--	--	--	--	--	NA
Other gynecological diseases	0 (0 to 0)	0 (0 to 0)	0 (0 to 0)	0 (0 to 0)	0 (0 to 0)	0 (0 to 0)	0 (0 to 0)	0 (0 to 0)	0 (0 to 0)	0 (0 to 0)	0 (0 to 0)	0 (0 to 0)	0 (0 to 0)	0 (0 to 0)	0 (0 to 0)	0 (0 to 0)	0 (0 to 0)	0 (0 to 0)	0 (0 to 0)	0 (0 to 0)	0 (0 to 0)	0 (0 to 0)	0 (0 to 0)	0
Hemoglobinopathies and hemolytic anemias	0·2 (0·1 to 0·3)	0·3 (0·2 to 0·4)	2·5 (1·5 to 4·2)	5·4 (3·3 to 8·1)	7·7 (4·9 to 11·6)	4·8 (3·1 to 6·9)	6·2 (4·2 to 8·8)	7·6 (5 to 11·2)	5·7 (3·7 to 8·1)	5·2 (3·4 to 7·7)	4·2 (2·9 to 5·9)	4·5 (3 to 6·2)	4·6 (3·2 to 6·4)	4·3 (3 to 5·8)	4·4 (3 to 6·1)	3·4 (2·4 to 4·8)	2·6 (1·8 to 3·6)	2·3 (1·6 to 3·1)	1·7 (1·2 to 2·2)	0·6 (0·5 to 0·9)	0·4 (0·3 to 0·5)	0·1 (0·1 to 0·2)	0 (0 to 0·1)	78·5
Thalassemias	0 (0 to 0)	0 (0 to 0)	0 (0 to 0)	0 (0 to 0)	0 (0 to 0)	0 (0 to 0)	0 (0 to 0)	0 (0 to 0)	0 (0 to 0)	0 (0 to 0)	0 (0 to 0)	0 (0 to 0)	0 (0 to 0)	0 (0 to 0)	0 (0 to 0)	0 (0 to 0)	0 (0 to 0)	0 (0 to 0)	0 (0 to 0)	0 (0 to 0)	0 (0 to 0)	0 (0 to 0)	0 (0 to 0)	0
Thalassemias trait	0 (0 to 0)	0 (0 to 0)	0 (0 to 0·1)	0·1 (0·1 to 0·3)	0·2 (0·1 to 0·4)	0·1 (0 to 0·2)	0 (0 to 0·1)	0 (0 to 0·1)	0 (0 to 0·1)	0 (0 to 0·1)	0 (0 to 0)	0 (0 to 0·1)	0 (0 to 0·1)	0 (0 to 0·1)	0 (0 to 0·1)	0 (0 to 0·1)	0 (0 to 0·1)	0 (0 to 0)	0 (0 to 0)	0 (0 to 0)	0 (0 to 0)	0 (0 to 0)	0 (0 to 0)	0·7
Sickle cell disorders	0·2 (0·1 to 0·3)	0·1 (0·1 to 0·2)	1·9 (1 to 3·4)	2·8 (1·6 to 4·7)	4·1 (2·6 to 6)	3·4 (2·2 to 5)	4·8 (3·1 to 7·1)	5·1 (3·1 to 8)	3·3 (2 to 5·3)	2·9 (1·7 to 4·8)	2·2 (1·3 to 3·3)	2·1 (1·1 to 3·3)	1·8 (1 to 2·8)	1 (0·5 to 1·8)	0·4 (0·2 to 0·8)	0·1 (0 to 0·2)	0 (0 to 0)	0 (0 to 0)	0 (0 to 0)	0 (0 to 0)	0 (0 to 0)	0 (0 to 0)	0 (0 to 0)	36·3
Sickle cell trait	0 (0 to 0)	0·1 (0 to 0·2)	0·3 (0·1 to 0·6)	1·3 (0·6 to 2·4)	1·8 (0·7 to 3·5)	0·6 (0·1 to 1·6)	0·2 (0 to 0·5)	0·2 (0 to 0·5)	0·2 (0 to 0·6)	0·2 (0 to 0·5)	0·2 (0 to 0·4)	0·2 (0 to 0·5)	0·2 (0 to 0·5)	0·2 (0·1 to 0·6)	0·3 (0·1 to 0·8)	0·2 (0·1 to 0·6)	0·2 (0 to 0·4)	0·2 (0·1 to 0·4)	0·1 (0 to 0·3)	0·1 (0 to 0·1)	0 (0 to 0)	0 (0 to 0)	0 (0 to 0)	6·8
G6PD deficiency	0 (0 to 0)	0 (0 to 0)	0 (0 to 0·1)	0·2 (0·1 to 0·5)	0·4 (0·2 to 0·8)	0·4 (0·2 to 0·7)	0·9 (0·4 to 1·8)	1·8 (0·7 to 3·4)	1·7 (0·7 to 3)	1·7 (0·8 to 3)	1·5 (0·7 to 2·5)	1·7 (0·9 to 2·9)	2·1 (1·1 to 3·2)	2·2 (1·3 to 3·3)	2·4 (1·6 to 3·6)	1·8 (1·1 to 2·6)	1·1 (0·6 to 1·6)	0·7 (0·4 to 1·1)	0·3 (0·2 to 0·5)	0·1 (0 to 0·1)	0 (0 to 0)	0 (0 to 0)	0 (0 to 0)	21
G6PD trait	0 (0 to 0)	0 (0 to 0)	0 (0 to 0)	0 (0 to 0)	0 (0 to 0)	0 (0 to 0)	0 (0 to 0)	0 (0 to 0)	0 (0 to 0)	0 (0 to 0)	0 (0 to 0)	0 (0 to 0)	0 (0 to 0)	0 (0 to 0)	0 (0 to 0)	0 (0 to 0)	0 (0 to 0)	0 (0 to 0)	0 (0 to 0)	0 (0 to 0)	0 (0 to 0)	0 (0 to 0)	0 (0 to 0)	0
Other hemoglobinopathies and hemolytic anemias	0 (0 to 0)	0 (0 to 0)	0·2 (0·1 to 0·4)	0·8 (0·3 to 1·7)	1·1 (0·3 to 2·4)	0·3 (0·1 to 0·8)	0·3 (0·1 to 0·8)	0·4 (0·2 to 0·9)	0·4 (0·2 to 1)	0·4 (0·2 to 0·8)	0·4 (0·2 to 0·8)	0·5 (0·2 to 0·9)	0·6 (0·3 to 1)	0·8 (0·4 to 1·3)	1·2 (0·7 to 2)	1·3 (0·8 to 2·1)	1·3 (0·9 to 2)	1·4 (1 to 2)	1·2 (0·8 to 1·6)	0·5 (0·4 to 0·7)	0·3 (0·2 to 0·4)	0·1 (0·1 to 0·2)	0 (0 to 0)	13·6
Endocrine, metabolic, blood, and immune disorders	1·5 (0·8 to 2·9)	1·2 (0·6 to 2·8)	15·7 (8·5 to 31·8)	7·8 (4·5 to 14·2)	4·8 (3·1 to 7·5)	3·3 (2·1 to 5·1)	3·6 (2·3 to 6)	5·4 (3·3 to 10·6)	7·4 (4·5 to 14·9)	9·3 (6 to 16·6)	8·8 (6·1 to 13·3)	9·3 (6·3 to 13·7)	11·7 (8 to 18·1)	15·3 (10·2 to 25·2)	15·7 (11 to 25·3)	13·9 (9·5 to 21·2)	11 (7·7 to 16)	9·4 (6·5 to 13·1)	7·3 (5·1 to 10·1)	2·9 (2 to 4)	1·1 (0·8 to 1·5)	0·4 (0·3 to 0·5)	0·1 (0 to 0·1)	167
Oral disorders	0 (0 to 0)	0 (0 to 0)	0 (0 to 0)	1·7 (0·9 to 2·7)	2·1 (1·1 to 3·6)	2·4 (1·3 to 4·3)	4 (2·2 to 6·9)	7·1 (3·5 to 13·4)	9·9 (4·8 to 19·3)	10·4 (5 to 19·7)	10·4 (4·9 to 19·9)	12·2 (5·7 to 22·7)	15 (7·4 to 27·6)	17·3 (9·1 to 31·2)	17·1 (9·4 to 29·2)	15·4 (9·1 to 25)	13·4 (8 to 21)	11·6 (7·2 to 18)	8·5 (5·2 to 12·8)	3 (1·9 to 4·5)	0·9 (0·5 to 1·3)	0·3 (0·2 to 0·4)	0·1 (0 to 0·1)	163
Caries of deciduous teeth	0 (0 to 0)	0 (0 to 0)	0 (0 to 0)	0·6 (0·2 to 1·2)	0·6 (0·2 to 1·3)	0 (0 to 0·1)	0 (0 to 0)	0 (0 to 0)	0 (0 to 0)	0 (0 to 0)	0 (0 to 0)	0 (0 to 0)	0 (0 to 0)	0 (0 to 0)	0 (0 to 0)	0 (0 to 0)	0 (0 to 0)	0 (0 to 0)	0 (0 to 0)	0 (0 to 0)	0 (0 to 0)	0 (0 to 0)	0 (0 to 0)	1·2
Caries of permanent teeth	0 (0 to 0)	0 (0 to 0)	0 (0 to 0)	0 (0 to 0)	0·5 (0·2 to 1·1)	1·2 (0·5 to 2·6)	1·4 (0·6 to 3)	1·8 (0·7 to 3·7)	1·6 (0·7 to 3·4)	1·2 (0·5 to 2·5)	1 (0·4 to 2)	1 (0·4 to 2)	1·1 (0·4 to 2·2)	1·1 (0·4 to 2·2)	0·8 (0·3 to 1·8)	0·5 (0·2 to 1·2)	0·3 (0·1 to 0·7)	0·2 (0·1 to 0·5)	0·2 (0·1 to 0·3)	0 (0 to 0·1)	0 (0 to 0)	0 (0 to 0)	0 (0 to 0)	14·1
Periodontal diseases	0 (0 to 0)	0 (0 to 0)	0 (0 to 0)	0 (0 to 0)	0 (0 to 0)	0 (0 to 0)	0·6 (0·2 to 1·5)	2·4 (0·7 to 6·2)	4·9 (1·5 to 11·8)	6 (2·1 to 13·6)	6·4 (2·3 to 14·2)	7·6 (2·8 to 16·4)	9·1 (3·5 to 19·4)	9·7 (3·8 to 20·9)	7·8 (3·2 to 16·5)	5·2 (2·1 to 10·6)	3·3 (1·3 to 6·7)	2·2 (0·9 to 4·5)	1·4 (0·6 to 2·8)	0·5 (0·2 to 0·9)	0·1 (0·1 to 0·3)	0 (0 to 0·1)	0 (0 to 0)	67·2
Other oral disorders	0 (0 to 0)	0 (0 to 0)	0 (0 to 0)	1·1 (0·6 to 1·7)	1 (0·6 to 1·6)	1·2 (0·7 to 1·9)	2 (1·2 to 3·1)	2·7 (1·6 to 4·1)	2·7 (1·6 to 4·2)	2·3 (1·4 to 3·5)	2 (1·2 to 3)	2 (1·2 to 3·1)	2·3 (1·4 to 3·5)	2·4 (1·5 to 3·7)	2 (1·2 to 3)	1·4 (0·9 to 2·2)	1 (0·6 to 1·5)	0·7 (0·4 to 1)	0·4 (0·2 to 0·6)	0·1 (0·1 to 0·2)	0 (0 to 0)	0 (0 to 0)	0 (0 to 0)	27·2
Sudden infant death syndrome	--	0·3 (0·2 to 0·5)	3·8 (1·9 to 7·2)	--	--	--	--	--	--	--	--	--	--	--	--	--	--	--	--	--	--	--	--	NA
Injuries	4·3 (2·8 to 6·3)	1·7 (1·1 to 2·7)	22·5 (13·6 to 34·7)	30·7 (20·7 to 45)	47·2 (39·7 to 55·8)	59·4 (51·2 to 67·9)	166 (145 to 186)	277 (233 to 317)	234 (195 to 273)	216 (186 to 249)	174 (150 to 201)	163 (139 to 190)	179 (152 to 207)	173 (149 to 201)	152 (131 to 175)	111 (96·5 to 127)	84·7 (74·3 to 97)	66·2 (58 to 76·3)	49·1 (42·6 to 56·9)	20 (17·2 to 22·9)	7 (6 to 8)	2·6 (2·2 to 3)	0·7 (0·6 to 0·8)	2,240
Transport injuries	0·2 (0·1 to 0·3)	0·1 (0 to 0·1)	1·1 (0·7 to 1·8)	3·6 (2·1 to 5·7)	8·9 (6·1 to 12·1)	11·9 (8·7 to 15·7)	40·7 (28·7 to 54·5)	72 (51·3 to 95·2)	60·1 (45·1 to 76·6)	56·3 (43·1 to 72·1)	43·9 (33·2 to 56·3)	41·8 (32·3 to 53·5)	46·9 (36·7 to 58·5)	43·2 (34·4 to 53·6)	37·6 (30·8 to 45·4)	25·7 (21·1 to 30·4)	18·3 (14·8 to 21·8)	12·4 (10·2 to 15)	7·9 (6·5 to 9·7)	2·5 (2 to 2·9)	0·7 (0·5 to 0·8)	0·2 (0·1 to 0·2)	0 (0 to 0·1)	536
Road injuries	0·1 (0·1 to 0·2)	0 (0 to 0·1)	0·8 (0·5 to 1·3)	3·1 (1·7 to 5)	7·9 (5·2 to 11)	10·4 (7·5 to 13·9)	36·7 (25·8 to 49·8)	64·8 (45·1 to 86·8)	54·1 (40 to 69·9)	50·2 (38·2 to 65·1)	39·2 (29·1 to 51)	37·1 (28·2 to 47·8)	42·2 (32·6 to 53)	38·9 (31 to 48·7)	33·1 (26·8 to 40·3)	23·3 (18·9 to 27·6)	16·8 (13·6 to 20·1)	10·9 (8·9 to 13·3)	7·2 (5·9 to 8·9)	2·3 (1·8 to 2·7)	0·6 (0·5 to 0·8)	0·2 (0·1 to 0·2)	0 (0 to 0)	480
Pedestrian road injuries	0 (0 to 0·1)	0 (0 to 0)	0·2 (0·1 to 0·3)	1 (0·5 to 1·6)	2·6 (1·7 to 3·8)	2·4 (1·6 to 3·3)	2·9 (2·1 to 4)	4·2 (3·1 to 5·6)	4·3 (3·2 to 5·5)	4·8 (3·6 to 6·2)	4·3 (3·3 to 5·6)	4·8 (3·6 to 6·2)	6 (4·6 to 7·7)	6·3 (4·9 to 7·9)	6·1 (4·8 to 7·7)	4·7 (3·7 to 5·7)	3·9 (3·2 to 4·7)	2·9 (2·4 to 3·6)	2·3 (1·8 to 2·8)	0·8 (0·6 to 1)	0·2 (0·2 to 0·3)	0·1 (0 to 0·1)	0 (0 to 0)	64·9
Cyclist road injuries	0 (0 to 0)	0 (0 to 0)	0 (0 to 0)	0·2 (0·1 to 0·4)	0·8 (0·4 to 1·2)	2·2 (1·4 to 3·4)	5·3 (3·2 to 8·2)	6·8 (4·5 to 10)	5·8 (4·1 to 8·3)	5·6 (4 to 8·1)	4·8 (3·4 to 6·7)	4·9 (3·5 to 6·4)	5·5 (4·1 to 7·5)	5·9 (4·4 to 7·6)	5·1 (3·8 to 6·7)	3·8 (2·8 to 4·9)	2·8 (2·1 to 3·6)	1·7 (1·3 to 2·3)	1·1 (0·8 to 1·4)	0·3 (0·2 to 0·4)	0·1 (0·1 to 0·1)	0 (0 to 0)	0 (0 to 0)	62·8
Motorcyclist road injuries	0 (0 to 0)	0 (0 to 0)	0·1 (0·1 to 0·2)	0·2 (0·1 to 0·4)	0·6 (0·4 to 0·9)	0·9 (0·7 to 1·2)	8 (5·5 to 11)	17·3 (12·2 to 23)	15·1 (11·2 to 19·6)	13·1 (9·9 to 17·2)	9·6 (7·1 to 12·4)	7·9 (6·1 to 10·1)	8·5 (6·7 to 10·5)	7·2 (5·7 to 9)	5·8 (4·6 to 7)	3·4 (2·6 to 4·2)	2·2 (1·8 to 2·8)	1·3 (1 to 1·7)	0·8 (0·6 to 1·1)	0·3 (0·2 to 0·4)	0·1 (0·1 to 0·1)	0 (0 to 0)	0 (0 to 0)	102
Motor vehicle road injuries	0·1 (0 to 0·1)	0 (0 to 0)	0·5 (0·3 to 0·9)	1·6 (0·9 to 2·5)	3·8 (2·4 to 5·3)	4·7 (3·2 to 6·6)	20·3 (13·9 to 27·9)	36 (24·4 to 48·8)	28·4 (20·3 to 38)	26·1 (19·3 to 34·9)	19·9 (14·2 to 26·7)	19 (13·9 to 25·6)	21·4 (15·8 to 28)	18·8 (14·4 to 23·8)	15·4 (12 to 19·3)	11 (8·6 to 13·3)	7·4 (5·9 to 9·2)	4·7 (3·7 to 5·9)	2·9 (2·3 to 3·7)	0·8 (0·7 to 1)	0·2 (0·2 to 0·3)	0·1 (0 to 0·1)	0 (0 to 0)	243
Other road injuries	0 (0 to 0)	0 (0 to 0)	0 (0 to 0)	0 (0 to 0·1)	0·1 (0 to 0·1)	0·1 (0·1 to 0·2)	0·2 (0·2 to 0·3)	0·5 (0·3 to 0·7)	0·5 (0·4 to 0·7)	0·5 (0·4 to 0·7)	0·5 (0·4 to 0·7)	0·6 (0·4 to 0·8)	0·7 (0·5 to 1)	0·8 (0·6 to 1·1)	0·7 (0·5 to 1)	0·5 (0·4 to 0·7)	0·4 (0·3 to 0·5)	0·3 (0·2 to 0·4)	0·2 (0·1 to 0·2)	0·1 (0 to 0·1)	0 (0 to 0)	0 (0 to 0)	0 (0 to 0)	6·7
Other transport injuries	0 (0 to 0·1)	0 (0 to 0)	0·3 (0·2 to 0·5)	0·5 (0·3 to 0·9)	1 (0·6 to 1·5)	1·5 (1 to 2·2)	4 (2·4 to 6·2)	7·1 (4·4 to 10·9)	6 (3·8 to 8·7)	6·1 (3·9 to 8·9)	4·7 (3 to 7·1)	4·7 (3·2 to 6·6)	4·7 (3·2 to 6·8)	4·3 (3·1 to 5·9)	4·5 (3·2 to 6·2)	2·4 (1·7 to 3·2)	1·5 (1·1 to 2)	1·5 (1·1 to 2)	0·7 (0·5 to 1)	0·2 (0·1 to 0·3)	0 (0 to 0·1)	0 (0 to 0)	0 (0 to 0)	55·8
Unintentional injuries	3·5 (2·3 to 5·2)	1·6 (1 to 2·5)	20·4 (12·1 to 32·2)	25·6 (17·2 to 38·3)	36·4 (29·4 to 44·3)	43·1 (35·5 to 51)	86·2 (69 to 103)	122 (96·6 to 148)	104 (82·8 to 127)	97·6 (79·8 to 119)	82·9 (67·3 to 99·8)	84·6 (69·7 to 102)	95·4 (78·8 to 116)	98·2 (81·7 to 119)	88·3 (73·5 to 104)	67·8 (57·6 to 81·5)	54·2 (46·3 to 64·3)	45·4 (38·8 to 53·7)	36 (30·6 to 42·5)	15·9 (13·5 to 18·5)	5·8 (5 to 6·8)	2·3 (1·9 to 2·7)	0·6 (0·5 to 0·7)	1,220
Falls	0·1 (0·1 to 0·1)	0 (0 to 0)	0·9 (0·5 to 1·5)	1·5 (1 to 2·4)	2·5 (1·8 to 3·3)	4·2 (3·1 to 5·4)	8·7 (6·7 to 11·2)	12·7 (9·6 to 16·1)	12·5 (9·5 to 16·4)	12·7 (10·1 to 16·1)	12·8 (9·9 to 16·4)	14·8 (11·6 to 18·5)	19·2 (15·3 to 23·8)	22·8 (18·1 to 28·7)	21·9 (17·6 to 26·9)	18·8 (15·2 to 22·8)	16·7 (13·6 to 20·3)	17·2 (14 to 21·1)	15·5 (12·4 to 19·2)	8·4 (6·7 to 10·2)	3·5 (2·8 to 4·2)	1·5 (1·2 to 1·8)	0·4 (0·3 to 0·5)	229
Drowning	0·3 (0·2 to 0·4)	0·1 (0 to 0·1)	2 (1·1 to 3·1)	9 (5·6 to 14·5)	16 (12 to 20·4)	16·9 (13·3 to 20·6)	33·8 (25·4 to 41·9)	37·4 (27·1 to 48·3)	26·2 (19·1 to 34·1)	22·9 (17·5 to 29)	17·4 (13·6 to 22·1)	15·4 (12·1 to 19)	14·8 (11·5 to 18·7)	13 (10·1 to 16·3)	10·2 (8·2 to 12·4)	6·6 (5·5 to 7·9)	4·4 (3·5 to 5·3)	2·4 (2 to 2·9)	1·4 (1·1 to 1·7)	0·3 (0·2 to 0·4)	0·1 (0·1 to 0·1)	0 (0 to 0)	0 (0 to 0)	251
Fire, heat, and hot substances	0·1 (0·1 to 0·1)	0·1 (0 to 0·1)	1·7 (1 to 2·6)	3·6 (2·3 to 5·5)	4·1 (3·2 to 5·1)	4·2 (3·2 to 5·4)	5·5 (4·2 to 7·3)	7·9 (6 to 10·3)	8·1 (6·2 to 10·5)	8·5 (6·6 to 11·1)	7·2 (5·5 to 9·2)	7·6 (5·7 to 9·8)	8·9 (6·8 to 11·5)	8·7 (6·7 to 11·2)	7·7 (5·9 to 9·9)	5·9 (4·6 to 7·6)	4·3 (3·4 to 5·5)	3·5 (2·7 to 4·4)	2·5 (2 to 3·2)	1 (0·8 to 1·3)	0·4 (0·3 to 0·5)	0·1 (0·1 to 0·2)	0 (0 to 0)	102
Poisonings	0 (0 to 0)	0 (0 to 0)	0·1 (0·1 to 0·2)	0·4 (0·2 to 0·7)	0·3 (0·2 to 0·5)	0·4 (0·3 to 0·6)	0·6 (0·4 to 0·8)	1 (0·7 to 1·3)	1·1 (0·8 to 1·5)	1·1 (0·8 to 1·4)	0·9 (0·6 to 1·1)	0·7 (0·5 to 0·9)	0·7 (0·5 to 0·9)	0·6 (0·5 to 0·8)	0·6 (0·4 to 0·7)	0·4 (0·3 to 0·5)	0·2 (0·2 to 0·3)	0·2 (0·1 to 0·2)	0·1 (0·1 to 0·1)	0 (0 to 0·1)	0 (0 to 0)	0 (0 to 0)	0 (0 to 0)	9·4
Poisoning by carbon monoxide	0 (0 to 0)	0 (0 to 0)	0 (0 to 0)	0·1 (0 to 0·1)	0·1 (0 to 0·1)	0·1 (0·1 to 0·1)	0·2 (0·1 to 0·3)	0·4 (0·2 to 0·6)	0·4 (0·2 to 0·7)	0·4 (0·2 to 0·7)	0·3 (0·2 to 0·5)	0·2 (0·1 to 0·3)	0·2 (0·1 to 0·3)	0·1 (0·1 to 0·2)	0·1 (0·1 to 0·2)	0·1 (0 to 0·1)	0 (0 to 0·1)	0 (0 to 0)	0 (0 to 0)	0 (0 to 0)	0 (0 to 0)	0 (0 to 0)	0 (0 to 0)	2·8
Poisoning by other means	0 (0 to 0)	0 (0 to 0)	0·1 (0·1 to 0·2)	0·4 (0·2 to 0·5)	0·2 (0·2 to 0·4)	0·3 (0·2 to 0·5)	0·4 (0·3 to 0·6)	0·6 (0·4 to 0·8)	0·7 (0·5 to 0·9)	0·7 (0·5 to 0·9)	0·5 (0·4 to 0·7)	0·5 (0·3 to 0·6)	0·5 (0·4 to 0·7)	0·5 (0·4 to 0·7)	0·5 (0·4 to 0·6)	0·3 (0·2 to 0·4)	0·2 (0·1 to 0·3)	0·1 (0·1 to 0·2)	0·1 (0·1 to 0·1)	0 (0 to 0)	0 (0 to 0)	0 (0 to 0)	0 (0 to 0)	6·6
Exposure to mechanical forces	0·1 (0 to 0·1)	0·1 (0 to 0·1)	0·7 (0·5 to 1)	2·2 (1·6 to 3)	4·4 (3·5 to 5·6)	6·3 (4·9 to 8·2)	21 (16·4 to 25·7)	37·3 (29·4 to 45·8)	31·9 (24·5 to 40·4)	30 (23·7 to 37·5)	24·8 (19·9 to 31·3)	25·8 (20·3 to 32·8)	27·8 (21·8 to 36)	28 (21·7 to 36·5)	24·2 (18·6 to 31·6)	16·6 (12·7 to 22·1)	11·3 (8·5 to 15·2)	8·1 (5·8 to 11)	5·2 (3·7 to 7·2)	1·8 (1·3 to 2·5)	0·6 (0·4 to 0·8)	0·2 (0·1 to 0·2)	0 (0 to 0·1)	308
Unintentional firearm injuries	0 (0 to 0)	0 (0 to 0)	0·1 (0 to 0·1)	0·1 (0·1 to 0·2)	0·3 (0·2 to 0·5)	0·8 (0·5 to 1·2)	7·8 (5·3 to 10·9)	16·8 (11·6 to 23·4)	11·9 (8·3 to 16·2)	10 (6·7 to 13·8)	7·2 (5·1 to 9·6)	6·7 (4·7 to 9·1)	6·1 (4·4 to 8·5)	5 (3·7 to 6·6)	4 (3 to 5·3)	2·4 (1·8 to 3·1)	1·5 (1·1 to 2)	0·8 (0·6 to 1·1)	0·5 (0·3 to 0·6)	0·2 (0·1 to 0·2)	0·1 (0 to 0·1)	0 (0 to 0)	0 (0 to 0)	82·2
Other exposure to mechanical forces	0·1 (0 to 0·1)	0·1 (0 to 0·1)	0·6 (0·4 to 0·9)	2·1 (1·4 to 2·8)	4·1 (3·3 to 5·2)	5·5 (4·2 to 7·4)	13·2 (9·4 to 17·3)	20·5 (14·7 to 27)	19·9 (14·5 to 26·8)	20 (14·9 to 26·2)	17·5 (13·2 to 23·3)	19·1 (14·2 to 25·7)	21·7 (16·3 to 29·3)	23 (17 to 31)	20·3 (15 to 27·3)	14·2 (10·4 to 19·3)	9·8 (7·1 to 13·3)	7·2 (5·2 to 9·9)	4·8 (3·4 to 6·6)	1·7 (1·2 to 2·3)	0·5 (0·3 to 0·7)	0·2 (0·1 to 0·2)	0 (0 to 0·1)	226
Adverse effects of medical treatment	1 (0·6 to 1·5)	0·6 (0·3 to 1·1)	2·8 (1·3 to 5·7)	1·4 (0·7 to 2·6)	1·1 (0·7 to 1·7)	1 (0·7 to 1·5)	1·5 (0·9 to 2·2)	3·2 (1·9 to 5·5)	3·1 (1·9 to 5)	2·6 (1·6 to 4)	2·7 (1·8 to 4)	3 (2 to 4·3)	3·8 (2·4 to 5·7)	5·7 (3·8 to 8·6)	6·5 (4·3 to 9·6)	6·3 (4·3 to 9·9)	6·1 (4·2 to 8·4)	5·4 (3·9 to 7·6)	4·9 (3·4 to 6·8)	2 (1·5 to 2·8)	0·7 (0·5 to 0·9)	0·2 (0·2 to 0·3)	0·1 (0 to 0·1)	65·6
Animal contact	0 (0 to 0)	0 (0 to 0)	0·2 (0·1 to 0·4)	0·5 (0·3 to 0·9)	0·6 (0·4 to 0·9)	0·8 (0·6 to 1·1)	1·1 (0·8 to 1·6)	1·3 (0·9 to 1·7)	1·7 (1·1 to 2·2)	1·2 (0·8 to 1·6)	1·4 (1 to 1·9)	1·5 (1 to 2)	2·1 (1·4 to 2·8)	2·2 (1·6 to 3)	2·2 (1·6 to 3)	2·2 (1·5 to 3)	2·1 (1·4 to 3)	1·7 (1·2 to 2·3)	1·2 (0·8 to 1·8)	0·3 (0·2 to 0·5)	0·1 (0·1 to 0·1)	0 (0 to 0)	0 (0 to 0)	24·6
Venomous animal contact	0 (0 to 0)	0 (0 to 0)	0 (0 to 0)	0·1 (0·1 to 0·2)	0·2 (0·1 to 0·3)	0·3 (0·2 to 0·4)	0·4 (0·2 to 0·6)	0·5 (0·3 to 0·7)	0·5 (0·3 to 0·7)	0·4 (0·3 to 0·6)	0·5 (0·3 to 0·6)	0·4 (0·3 to 0·6)	0·5 (0·4 to 0·7)	0·6 (0·4 to 0·8)	0·5 (0·4 to 0·7)	0·4 (0·3 to 0·6)	0·3 (0·2 to 0·4)	0·2 (0·2 to 0·3)	0·2 (0·1 to 0·3)	0·1 (0 to 0·1)	0 (0 to 0)	0 (0 to 0)	0 (0 to 0)	6·2
Non-venomous animal contact	0 (0 to 0)	0 (0 to 0)	0·2 (0·1 to 0·3)	0·4 (0·2 to 0·7)	0·4 (0·3 to 0·6)	0·5 (0·3 to 0·8)	0·7 (0·5 to 1·1)	0·8 (0·6 to 1·2)	1·1 (0·7 to 1·6)	0·8 (0·5 to 1·2)	1 (0·6 to 1·4)	1·1 (0·7 to 1·5)	1·6 (1 to 2·2)	1·7 (1·2 to 2·3)	1·7 (1·2 to 2·4)	1·7 (1·1 to 2·5)	1·8 (1·1 to 2·6)	1·5 (1 to 2·1)	1 (0·6 to 1·5)	0·3 (0·2 to 0·4)	0·1 (0 to 0·1)	0 (0 to 0)	0 (0 to 0)	18·4
Foreign body	1·8 (1·2 to 2·7)	0·7 (0·4 to 1)	11·4 (6·3 to 18·8)	5 (3·2 to 7·7)	3·5 (2·8 to 4·4)	4 (3·1 to 5)	3·2 (2·5 to 3·9)	4·8 (3·7 to 6)	4·6 (3·5 to 5·7)	4·2 (3·2 to 5·2)	3·6 (2·9 to 4·6)	3·9 (3·2 to 4·8)	4·9 (3·9 to 6·1)	5·5 (4·3 to 6·9)	5·1 (4·2 to 6)	4·5 (3·6 to 5·5)	4·3 (3·4 to 5·4)	3·5 (2·8 to 4·3)	2·8 (2·2 to 3·4)	1·1 (0·9 to 1·5)	0·3 (0·2 to 0·4)	0·1 (0·1 to 0·1)	0 (0 to 0)	82·8
Pulmonary aspiration and foreign body in airway	1·8 (1·2 to 2·7)	0·7 (0·4 to 1)	11·3 (6·2 to 18·7)	4·7 (3 to 7·4)	3·2 (2·5 to 4)	3·5 (2·7 to 4·5)	2·6 (2 to 3·2)	4 (3 to 5)	3·8 (2·8 to 4·8)	3·5 (2·6 to 4·5)	3 (2·4 to 3·9)	3·3 (2·6 to 4)	4·2 (3·2 to 5·3)	4·7 (3·5 to 6·1)	4·4 (3·6 to 5·3)	4 (3·2 to 5·1)	4 (3 to 5)	3·2 (2·5 to 4)	2·5 (2 to 3·1)	1·1 (0·8 to 1·4)	0·3 (0·2 to 0·3)	0·1 (0·1 to 0·1)	0 (0 to 0)	74·1
Foreign body in eyes	0 (0 to 0)	0 (0 to 0)	0 (0 to 0)	0·1 (0 to 0·2)	0·1 (0 to 0·3)	0·2 (0·1 to 0·4)	0·3 (0·1 to 0·6)	0·4 (0·1 to 0·8)	0·4 (0·1 to 0·8)	0·3 (0·1 to 0·7)	0·3 (0·1 to 0·6)	0·3 (0·1 to 0·6)	0·3 (0·2 to 0·7)	0·3 (0·2 to 0·6)	0·3 (0·1 to 0·4)	0·2 (0·1 to 0·3)	0·1 (0·1 to 0·2)	0·1 (0·1 to 0·2)	0·1 (0 to 0·1)	0 (0 to 0)	0 (0 to 0)	0 (0 to 0)	0 (0 to 0)	3·9
Foreign body in other body part	0 (0 to 0)	0 (0 to 0)	0 (0 to 0·1)	0·2 (0·1 to 0·3)	0·2 (0·1 to 0·3)	0·3 (0·2 to 0·4)	0·3 (0·2 to 0·5)	0·4 (0·3 to 0·6)	0·4 (0·3 to 0·6)	0·3 (0·2 to 0·5)	0·3 (0·2 to 0·4)	0·3 (0·2 to 0·4)	0·4 (0·3 to 0·5)	0·4 (0·3 to 0·6)	0·4 (0·3 to 0·5)	0·3 (0·2 to 0·4)	0·2 (0·2 to 0·3)	0·2 (0·1 to 0·2)	0·1 (0·1 to 0·2)	0·1 (0 to 0·1)	0 (0 to 0)	0 (0 to 0)	0 (0 to 0)	4·9
Environmental heat and cold exposure	0 (0 to 0)	0 (0 to 0)	0 (0 to 0·1)	0·2 (0·2 to 0·4)	0·5 (0·3 to 0·8)	0·8 (0·5 to 1·2)	1·4 (0·9 to 2)	2·1 (1·4 to 2·9)	2·3 (1·6 to 3·2)	2·2 (1·5 to 3·1)	2 (1·4 to 2·7)	2·2 (1·5 to 3)	2·6 (1·8 to 3·6)	2·8 (2 to 3·8)	2·5 (1·8 to 3·4)	1·9 (1·3 to 2·5)	1·4 (1 to 1·8)	1·1 (0·8 to 1·4)	0·7 (0·5 to 1)	0·3 (0·2 to 0·4)	0·1 (0·1 to 0·1)	0 (0 to 0)	0 (0 to 0)	27·1
Exposure to forces of nature	0 (0 to 0)	0 (0 to 0)	0 (0 to 0)	0 (0 to 0)	0 (0 to 0)	0 (0 to 0)	0·1 (0·1 to 0·2)	0·2 (0·1 to 0·3)	0·4 (0·3 to 0·7)	0·2 (0·1 to 0·4)	0·2 (0·1 to 0·3)	0·2 (0·1 to 0·4)	0·3 (0·2 to 0·4)	0·2 (0·1 to 0·4)	0·2 (0·1 to 0·3)	0·1 (0·1 to 0·2)	0·1 (0·1 to 0·1)	0·1 (0 to 0·1)	0 (0 to 0·1)	0 (0 to 0)	0 (0 to 0)	0 (0 to 0)	0 (0 to 0)	2·4
Other unintentional injuries	0·1 (0·1 to 0·3)	0 (0 to 0·1)	0·6 (0·4 to 1)	1·8 (1·2 to 2·5)	3·3 (2·6 to 4·2)	4·4 (3·5 to 5·5)	9·4 (7·3 to 11·7)	13·8 (10·6 to 17·4)	12·6 (9·5 to 16)	12 (9·3 to 15·2)	10 (7·8 to 12·5)	9·5 (7·5 to 11·9)	10·3 (7·9 to 13·2)	8·6 (6·7 to 10·9)	7·2 (5·6 to 9·2)	4·7 (3·6 to 6·1)	3·4 (2·6 to 4·4)	2·3 (1·7 to 3·1)	1·6 (1·2 to 2·1)	0·5 (0·4 to 0·7)	0·2 (0·1 to 0·2)	0 (0 to 0·1)	0 (0 to 0)	116
Self-harm and interpersonal violence	0·6 (0·4 to 1)	0·1 (0 to 0·1)	1 (0·5 to 1·6)	1·5 (0·9 to 2·5)	1·9 (1·4 to 2·5)	4·3 (3·3 to 5·6)	39·2 (29·3 to 50·5)	83·8 (63·8 to 106)	69·9 (51·9 to 89·4)	62·4 (49 to 78·5)	47·2 (36·7 to 58·9)	37·1 (29·2 to 47·3)	37·1 (29 to 46·4)	31·2 (25·2 to 38·5)	26·4 (21·4 to 32·1)	17·4 (14·2 to 21·3)	12·2 (10·1 to 14·8)	8·4 (6·8 to 10·3)	5·2 (4·2 to 6·2)	1·6 (1·3 to 2)	0·5 (0·4 to 0·6)	0·1 (0·1 to 0·1)	0 (0 to 0)	489
Self-harm	0 (0 to 0)	0 (0 to 0)	0 (0 to 0)	0 (0 to 0)	0 (0 to 0)	0·9 (0·5 to 1·4)	9·4 (6·4 to 13·2)	21·7 (15·1 to 30·2)	20·5 (14·1 to 28·6)	21·5 (15·2 to 29·6)	17·6 (12·6 to 23·5)	15·7 (11·3 to 21·4)	17 (12·3 to 22·6)	15·7 (11·5 to 20·8)	13·7 (10·1 to 17·6)	9·8 (7·3 to 12·8)	7·5 (5·8 to 9·6)	5·6 (4·2 to 7·3)	3·5 (2·7 to 4·6)	1·2 (0·9 to 1·5)	0·3 (0·2 to 0·4)	0·1 (0·1 to 0·1)	0 (0 to 0)	182
Self-harm by firearm	0 (0 to 0)	0 (0 to 0)	0 (0 to 0)	0 (0 to 0)	0 (0 to 0)	0 (0 to 0)	0·1 (0 to 0·2)	0·2 (0·1 to 0·4)	0·2 (0·1 to 0·3)	0·3 (0·1 to 0·4)	0·2 (0·1 to 0·3)	0·2 (0·1 to 0·2)	0·2 (0·1 to 0·2)	0·2 (0·1 to 0·2)	0·1 (0·1 to 0·2)	0·1 (0·1 to 0·1)	0·1 (0·1 to 0·1)	0·1 (0 to 0·1)	0 (0 to 0)	0 (0 to 0)	0 (0 to 0)	0 (0 to 0)	0 (0 to 0)	1·8
Self-harm by other specified means	0 (0 to 0)	0 (0 to 0)	0 (0 to 0)	0 (0 to 0)	0 (0 to 0)	0·9 (0·5 to 1·4)	9·3 (6·4 to 13)	21·5 (14·9 to 29·9)	20·3 (14 to 28·3)	21·2 (15 to 29·2)	17·4 (12·5 to 23·3)	15·6 (11·2 to 21·2)	16·9 (12·2 to 22·4)	15·6 (11·3 to 20·6)	13·5 (10 to 17·5)	9·7 (7·2 to 12·7)	7·4 (5·7 to 9·6)	5·5 (4·2 to 7·2)	3·5 (2·7 to 4·6)	1·2 (0·9 to 1·5)	0·3 (0·2 to 0·4)	0·1 (0·1 to 0·1)	0 (0 to 0)	180
Interpersonal violence	0·6 (0·4 to 1)	0·1 (0 to 0·1)	1 (0·5 to 1·6)	1·5 (0·9 to 2·5)	1·9 (1·4 to 2·5)	3·4 (2·6 to 4·5)	29·7 (20·8 to 40·7)	62·1 (45·5 to 80·4)	49·4 (35·4 to 65·7)	40·5 (29·6 to 52·2)	26·9 (19·8 to 35·4)	20·5 (15·2 to 27·4)	19·2 (14·3 to 25·9)	14·3 (10·9 to 18·5)	10·7 (8·2 to 14·1)	6·4 (5 to 8·3)	4·1 (3·1 to 5·2)	2·4 (1·8 to 3·1)	1·3 (1 to 1·6)	0·3 (0·2 to 0·4)	0·1 (0·1 to 0·1)	0 (0 to 0)	0 (0 to 0)	296
Physical violence by firearm	0 (0 to 0)	0 (0 to 0)	0 (0 to 0)	0 (0 to 0·1)	0·1 (0 to 0·1)	0·1 (0·1 to 0·2)	4·4 (3·1 to 6·1)	10·3 (7·4 to 13·5)	7·1 (4·9 to 9·6)	4·9 (3·5 to 6·4)	2·4 (1·7 to 3·1)	1·4 (1 to 1·9)	1 (0·7 to 1·4)	0·6 (0·4 to 0·8)	0·3 (0·3 to 0·4)	0·2 (0·1 to 0·2)	0·1 (0·1 to 0·1)	0·1 (0 to 0·1)	0 (0 to 0)	0 (0 to 0)	0 (0 to 0)	0 (0 to 0)	0 (0 to 0)	33
Physical violence by sharp object	0 (0 to 0)	0 (0 to 0)	0 (0 to 0)	0·1 (0 to 0·1)	0·1 (0·1 to 0·2)	0·2 (0·1 to 0·3)	3·3 (2·3 to 4·7)	7·5 (5·4 to 9·8)	5·5 (4 to 7·3)	4·6 (3·4 to 6)	2·6 (2 to 3·4)	1·8 (1·3 to 2·4)	1·4 (1·1 to 1·9)	1 (0·8 to 1·3)	0·7 (0·5 to 0·8)	0·4 (0·3 to 0·5)	0·2 (0·2 to 0·3)	0·2 (0·1 to 0·2)	0·1 (0·1 to 0·1)	0 (0 to 0)	0 (0 to 0)	0 (0 to 0)	0 (0 to 0)	29·8
Sexual violence	0 (0 to 0)	0 (0 to 0)	0 (0 to 0)	0 (0 to 0)	0·1 (0·1 to 0·2)	0·3 (0·2 to 0·5)	0·8 (0·4 to 1·2)	1·1 (0·7 to 1·8)	1 (0·5 to 1·6)	0·7 (0·4 to 1·2)	0·6 (0·3 to 0·9)	0·5 (0·3 to 0·8)	0·5 (0·3 to 0·8)	0·4 (0·2 to 0·7)	0·3 (0·2 to 0·5)	0·2 (0·1 to 0·3)	0·1 (0·1 to 0·2)	0·1 (0·1 to 0·1)	0·1 (0 to 0·1)	0 (0 to 0)	0 (0 to 0)	0 (0 to 0)	0 (0 to 0)	6·9
Physical violence by other means	0·6 (0·4 to 1)	0·1 (0 to 0·1)	0·9 (0·5 to 1·6)	1·4 (0·8 to 2·4)	1·6 (1·1 to 2·2)	2·8 (2 to 3·7)	21·2 (14·5 to 29)	43·1 (31·4 to 56·3)	35·8 (25·5 to 48)	30·3 (21·9 to 39·4)	21·4 (15·4 to 28·3)	16·8 (12·3 to 22·8)	16·2 (11·8 to 21·9)	12·3 (9·3 to 16·1)	9·4 (7·1 to 12·6)	5·6 (4·2 to 7·3)	3·6 (2·7 to 4·7)	2 (1·5 to 2·7)	1·1 (0·8 to 1·4)	0·2 (0·2 to 0·3)	0·1 (0 to 0·1)	0 (0 to 0)	0 (0 to 0)	226
Conflict and terrorism	0 (0 to 0)	0 (0 to 0)	0 (0 to 0)	0 (0 to 0)	0 (0 to 0)	0 (0 to 0)	0 (0 to 0)	0 (0 to 0)	0 (0 to 0)	0·4 (0·2 to 0·7)	2·7 (1·3 to 4·8)	0·8 (0·4 to 1·6)	0·9 (0·4 to 1·6)	1·2 (0·6 to 2·1)	2 (1·2 to 3·2)	1·2 (0·7 to 2)	0·6 (0·3 to 1·1)	0·4 (0·2 to 0·7)	0·3 (0·2 to 0·6)	0·1 (0·1 to 0·2)	0·1 (0 to 0·1)	0 (0 to 0)	0 (0 to 0)	10·7

SR Table 2a: Guyana DALYS by cause and age group for males with 95% uncertainty intervals, 2019

Cause	Early Neonatal	Late Neonatal	Post Neonatal	1 to 4	5 to 9	10 to 14	15 to 19	20 to 24	25 to 29	30 to 34	35 to 39	40 to 44	45 to 49	50 to 54	55 to 59	60 to 64	65 to 69	70 to 74	75 to 79	80 to 84	85 to 89	90 to 94	95 plus	Total
All causes	9,600 (6,680 to 13,500)	1,690 (1,180 to 2,350)	3,820 (2,760 to 5,250)	2,910 (2,250 to 3,650)	3,320 (2,700 to 4,060)	3,730 (3,050 to 4,580)	6,520 (5,370 to 7,800)	11,600 (9,760 to 13,700)	11,800 (10,200 to 13,500)	10,300 (8,890 to 11,900)	10,400 (8,720 to 12,200)	11,900 (9,750 to 14,300)	12,800 (10,300 to 15,800)	14,000 (11,200 to 17,400)	15,000 (12,000 to 18,600)	13,700 (11,100 to 16,700)	11,600 (9,560 to 14,000)	7,820 (6,510 to 9,310)	5,260 (4,440 to 6,170)	3,170 (2,730 to 3,630)	1,410 (1,250 to 1,560)	633 (578 to 684)	181 (168 to 191)	173,000
Communicable, maternal, neonatal, and nutritional diseases	8,590 (5,930 to 12,000)	1,400 (968 to 1,960)	2,390 (1,690 to 3,290)	1,250 (956 to 1,580)	1,160 (799 to 1,610)	857 (638 to 1,170)	943 (718 to 1,210)	1,450 (1,170 to 1,780)	1,850 (1,610 to 2,130)	1,890 (1,660 to 2,120)	1,910 (1,680 to 2,160)	1,900 (1,650 to 2,190)	1,720 (1,450 to 2,040)	1,440 (1,170 to 1,760)	1,210 (976 to 1,480)	982 (800 to 1,190)	777 (615 to 976)	544 (434 to 682)	389 (306 to 493)	282 (217 to 368)	145 (112 to 184)	69·7 (54·6 to 87·6)	22·8 (17·3 to 28·9)	33,200
HIV/AIDS and sexually transmitted infections	6·5 (2·7 to 12·3)	1·1 (0·5 to 2·3)	69·3 (64·1 to 75)	48·1 (44·3 to 52·3)	40·2 (33·7 to 53·1)	27·8 (23·9 to 34·2)	48·5 (43·9 to 54·3)	241 (219 to 265)	744 (682 to 814)	907 (835 to 987)	965 (895 to 1,040)	840 (772 to 912)	682 (631 to 741)	403 (370 to 437)	245 (222 to 271)	137 (123 to 156)	79 (68·2 to 93·2)	18·1 (13·6 to 24·6)	6·9 (5·3 to 9·4)	2·5 (2 to 3·3)	0·9 (0·7 to 1·2)	0·3 (0·2 to 0·4)	0·1 (0·1 to 0·1)	5,510
HIV/AIDS	0 (0 to 0)	0 (0 to 0·1)	67·5 (62·4 to 73·3)	48·1 (44·2 to 52·2)	40·1 (33·7 to 53)	26·7 (23 to 33)	43·9 (40·3 to 47·6)	233 (211 to 256)	737 (676 to 806)	901 (829 to 979)	959 (889 to 1,040)	835 (768 to 906)	677 (626 to 737)	400 (366 to 434)	242 (219 to 267)	136 (122 to 154)	77·5 (67 to 91·1)	17·3 (12·9 to 23·5)	6·5 (4·9 to 9)	2·3 (1·8 to 3)	0·8 (0·6 to 1·1)	0·2 (0·2 to 0·4)	0·1 (0·1 to 0·1)	5,450
HIV/AIDS - Drug-susceptible Tuberculosis	0 (0 to 0)	0 (0 to 0)	8·4 (5·1 to 12·9)	5·8 (3·6 to 8·8)	4·7 (3 to 6·9)	3 (1·9 to 4·5)	5·2 (3·2 to 7·8)	29 (18 to 43·8)	93·6 (58·2 to 143)	114 (71·5 to 171)	120 (74·4 to 181)	103 (63·9 to 157)	82·7 (51·9 to 126)	48·3 (30·2 to 72·3)	28·7 (18·3 to 43·8)	15·8 (10·2 to 23·7)	8·9 (5·8 to 13·1)	1·8 (1·2 to 2·7)	0·7 (0·4 to 1)	0·3 (0·2 to 0·4)	0·1 (0·1 to 0·1)	0 (0 to 0)	0 (0 to 0)	674
HIV/AIDS - Multidrug-resistant Tuberculosis without extensive	0 (0 to 0)	0 (0 to 0)	0·2 (0 to 0·9)	0·1 (0 to 0·6)	0·1 (0 to 0·5)	0·1 (0 to 0·3)	0·1 (0 to 0·6)	0·6 (0 to 3·1)	1·8 (0·1 to 9·9)	2·1 (0·1 to 12)	2·2 (0·1 to 12·6)	1·9 (0·1 to 10·9)	1·6 (0·1 to 8·8)	0·9 (0·1 to 5·1)	0·5 (0 to 3)	0·3 (0 to 1·6)	0·2 (0 to 0·9)	0 (0 to 0·2)	0 (0 to 0·1)	0 (0 to 0)	0 (0 to 0)	0 (0 to 0)	0 (0 to 0)	12·6
HIV/AIDS - Extensively drug-resistant Tuberculosis	0 (0 to 0)	0 (0 to 0)	0 (0 to 0·2)	0 (0 to 0·1)	0 (0 to 0·1)	0 (0 to 0·1)	0 (0 to 0·1)	0·1 (0 to 0·5)	0·3 (0 to 1·6)	0·3 (0 to 2)	0·4 (0 to 2·1)	0·3 (0 to 1·8)	0·3 (0 to 1·4)	0·1 (0 to 0·8)	0·1 (0 to 0·5)	0 (0 to 0·3)	0 (0 to 0·1)	0 (0 to 0)	0 (0 to 0)	0 (0 to 0)	0 (0 to 0)	0 (0 to 0)	0 (0 to 0)	2·1
HIV/AIDS resulting in other diseases	0 (0 to 0)	0 (0 to 0·1)	58·9 (52·6 to 65·7)	42·1 (37·2 to 47·2)	35·4 (28·5 to 47·9)	23·6 (19·5 to 29·9)	38·6 (34·7 to 42·6)	203 (179 to 228)	641 (566 to 717)	784 (693 to 880)	836 (739 to 929)	730 (644 to 811)	593 (528 to 660)	350 (308 to 390)	212 (186 to 239)	119 (102 to 138)	68·4 (56·9 to 82·6)	15·5 (10·9 to 21·6)	5·8 (4·2 to 8·2)	2 (1·5 to 2·8)	0·7 (0·5 to 1)	0·2 (0·1 to 0·3)	0·1 (0·1 to 0·1)	4,760
Sexually transmitted infections excluding HIV	6·5 (2·6 to 12·2)	1 (0·4 to 2·2)	1·8 (0·6 to 4·6)	0·1 (0 to 0·2)	0·1 (0 to 0·1)	1·1 (0·6 to 1·9)	4·6 (2·1 to 8·6)	7·8 (4·2 to 12·9)	7·5 (4 to 12·5)	6·6 (3·7 to 11·1)	5·9 (3·3 to 9·4)	5 (2·8 to 8·5)	4·5 (2·5 to 7·7)	3·6 (2·1 to 6·3)	2·8 (1·6 to 5)	1·8 (1 to 3·3)	1·5 (0·9 to 2·6)	0·8 (0·4 to 1·4)	0·4 (0·2 to 0·7)	0·2 (0·1 to 0·4)	0·1 (0·1 to 0·2)	0 (0 to 0·1)	0 (0 to 0)	63·8
Syphilis	6·5 (2·6 to 12·2)	1 (0·4 to 2·2)	1·8 (0·6 to 4·6)	0·1 (0 to 0·2)	0·1 (0 to 0·1)	0·2 (0·1 to 0·4)	0·7 (0·2 to 1·8)	1 (0·5 to 1·9)	1·3 (0·6 to 2·4)	1·1 (0·5 to 2·4)	1·5 (0·7 to 2·7)	0·8 (0·4 to 1·5)	1 (0·5 to 1·7)	1·1 (0·5 to 1·9)	1·1 (0·6 to 1·9)	0·8 (0·4 to 1·3)	0·7 (0·4 to 1·2)	0·3 (0·2 to 0·7)	0·1 (0·1 to 0·2)	0·1 (0 to 0·1)	0 (0 to 0)	0 (0 to 0)	0 (0 to 0)	21·3
Chlamydial infection	0 (0 to 0)	0 (0 to 0)	0 (0 to 0)	0 (0 to 0)	0 (0 to 0)	0 (0 to 0)	0·4 (0·2 to 0·7)	1·4 (0·3 to 3·2)	1·9 (0·5 to 4)	1·5 (0·4 to 3)	1·2 (0·4 to 2·5)	1·1 (0·3 to 2·2)	0·7 (0·1 to 1·6)	0·3 (0 to 0·9)	0·1 (0 to 0·2)	0 (0 to 0)	0 (0 to 0)	0 (0 to 0)	0 (0 to 0)	0 (0 to 0)	0 (0 to 0)	0 (0 to 0)	0 (0 to 0)	8·6
Gonococcal infection	0 (0 to 0)	0 (0 to 0)	0 (0 to 0)	0 (0 to 0)	0 (0 to 0)	0·2 (0·1 to 0·5)	1·4 (0·3 to 3·2)	2·2 (0·8 to 4·5)	0·9 (0·1 to 2·1)	0·5 (0·1 to 1·1)	0·3 (0·2 to 0·6)	0·3 (0·2 to 0·6)	0·3 (0·2 to 0·5)	0·2 (0·1 to 0·4)	0·1 (0·1 to 0·2)	0·1 (0 to 0·1)	0·1 (0 to 0·2)	0 (0 to 0·1)	0 (0 to 0)	0 (0 to 0)	0 (0 to 0)	0 (0 to 0)	0 (0 to 0)	6·7
Trichomoniasis	0 (0 to 0)	0 (0 to 0)	0 (0 to 0)	0 (0 to 0)	0 (0 to 0)	0 (0 to 0)	0 (0 to 0)	0·1 (0 to 0·2)	0·1 (0 to 0·3)	0·2 (0 to 0·4)	0·2 (0 to 0·4)	0·2 (0 to 0·4)	0·1 (0 to 0·3)	0·1 (0 to 0·2)	0·1 (0 to 0·1)	0 (0 to 0·1)	0 (0 to 0)	0 (0 to 0)	0 (0 to 0)	0 (0 to 0)	0 (0 to 0)	0 (0 to 0)	0 (0 to 0)	1
Genital herpes	0 (0 to 0)	0 (0 to 0)	0 (0 to 0)	0 (0 to 0)	0 (0 to 0)	0·1 (0 to 0·2)	0·4 (0·1 to 1)	1 (0·3 to 2·5)	1·4 (0·5 to 3·3)	1·4 (0·4 to 3·5)	1·6 (0·5 to 3·6)	1·7 (0·5 to 4)	1·6 (0·5 to 3·9)	1·4 (0·4 to 3·4)	1·2 (0·4 to 2·9)	0·9 (0·2 to 2·1)	0·6 (0·2 to 1·5)	0·3 (0·1 to 0·8)	0·2 (0·1 to 0·5)	0·1 (0 to 0·2)	0 (0 to 0·1)	0 (0 to 0)	0 (0 to 0)	13·8
Other sexually transmitted infections	0 (0 to 0)	[illegible]	[illegible]	[illegible]	[illegible]	[illegible]	[illegible]	[illegible]	[illegible]	[illegible]	[illegible]	[illegible]	[illegible]	[illegible]	[illegible]	[illegible]	[illegible]	[illegible]	[illegible]	[illegible]	[illegible]	[illegible]	[illegible]	[illegible]
Respiratory infections and tuberculosis	315 (167 to 526)	[illegible]	[illegible]	[illegible]	[illegible]	[illegible]	[illegible]	[illegible]	[illegible]	[illegible]	[illegible]	[illegible]	[illegible]	[illegible]	[illegible]	[illegible]	[illegible]	[illegible]	[illegible]	[illegible]	[illegible]	[illegible]	[illegible]	[illegible]
Tuberculosis	0 (0 to 0)	[illegible]	[illegible]	[illegible]	[illegible]	[illegible]	[illegible]	[illegible]	[illegible]	[illegible]	[illegible]	[illegible]	[illegible]	[illegible]	[illegible]	[illegible]	[illegible]	[illegible]	[illegible]	[illegible]	[illegible]	[illegible]	[illegible]	[illegible]
Latent tuberculosis infection	0 (0 to 0)	[illegible]	[illegible]	[illegible]	[illegible]	[illegible]	[illegible]	[illegible]	[illegible]	[illegible]	[illegible]	[illegible]	[illegible]	[illegible]	[illegible]	[illegible]	[illegible]	[illegible]	[illegible]	[illegible]	[illegible]	[illegible]	[illegible]	[illegible]
Drug-susceptible tuberculosis	0 (0 to 0)	[illegible]	[illegible]	[illegible]	[illegible]	[illegible]	[illegible]	[illegible]	[illegible]	[illegible]	[illegible]	[illegible]	[illegible]	[illegible]	[illegible]	[illegible]	[illegible]	[illegible]	[illegible]	[illegible]	[illegible]	[illegible]	[illegible]	[illegible]
Multidrug-resistant tuberculosis without extensive drug resista	0 (0 to 0)	[illegible]	[illegible]	[illegible]	[illegible]	[illegible]	[illegible]	[illegible]	[illegible]	[illegible]	[illegible]	[illegible]	[illegible]	[illegible]	[illegible]	[illegible]	[illegible]	[illegible]	[illegible]	[illegible]	[illegible]	[illegible]	[illegible]	[illegible]
Extensively drug-resistant tuberculosis	0 (0 to 0)	[illegible]	[illegible]	[illegible]	[illegible]	[illegible]	[illegible]	[illegible]	[illegible]	[illegible]	[illegible]	[illegible]	[illegible]	[illegible]	[illegible]	[illegible]	[illegible]	[illegible]	[illegible]	[illegible]	[illegible]	[illegible]	[illegible]	[illegible]
Lower respiratory infections	315 (167 to 526)	[illegible]	[illegible]	[illegible]	[illegible]	[illegible]	[illegible]	[illegible]	[illegible]	[illegible]	[illegible]	[illegible]	[illegible]	[illegible]	[illegible]	[illegible]	[illegible]	[illegible]	[illegible]	[illegible]	[illegible]	[illegible]	[illegible]	[illegible]
Upper respiratory infections	0·1 (0 to 0·1)	[illegible]	[illegible]	[illegible]	[illegible]	[illegible]	[illegible]	[illegible]	[illegible]	[illegible]	[illegible]	[illegible]	[illegible]	[illegible]	[illegible]	[illegible]	[illegible]	[illegible]	[illegible]	[illegible]	[illegible]	[illegible]	[illegible]	[illegible]
Otitis media	0 (0 to 0·1)	[illegible]	[illegible]	[illegible]	[illegible]	[illegible]	[illegible]	[illegible]	[illegible]	[illegible]	[illegible]	[illegible]	[illegible]	[illegible]	[illegible]	[illegible]	[illegible]	[illegible]	[illegible]	[illegible]	[illegible]	[illegible]	[illegible]	[illegible]
Enteric infections	42·5 (20·1 to 78·8)	[illegible]	[illegible]	[illegible]	[illegible]	[illegible]	[illegible]	[illegible]	[illegible]	[illegible]	[illegible]	[illegible]	[illegible]	[illegible]	[illegible]	[illegible]	[illegible]	[illegible]	[illegible]	[illegible]	[illegible]	[illegible]	[illegible]	[illegible]
Diarrheal diseases	42·5 (20·1 to 78·8)	[illegible]	[illegible]	[illegible]	[illegible]	[illegible]	[illegible]	[illegible]	[illegible]	[illegible]	[illegible]	[illegible]	[illegible]	[illegible]	[illegible]	[illegible]	[illegible]	[illegible]	[illegible]	[illegible]	[illegible]	[illegible]	[illegible]	[illegible]
Typhoid and paratyphoid	0 (0 to 0)	[illegible]	[illegible]	[illegible]	[illegible]	[illegible]	[illegible]	[illegible]	[illegible]	[illegible]	[illegible]	[illegible]	[illegible]	[illegible]	[illegible]	[illegible]	[illegible]	[illegible]	[illegible]	[illegible]	[illegible]	[illegible]	[illegible]	[illegible]
Typhoid fever	0 (0 to 0)	[illegible]	[illegible]	[illegible]	[illegible]	[illegible]	[illegible]	[illegible]	[illegible]	[illegible]	[illegible]	[illegible]	[illegible]	[illegible]	[illegible]	[illegible]	[illegible]	[illegible]	[illegible]	[illegible]	[illegible]	[illegible]	[illegible]	[illegible]
Paratyphoid fever	0 (0 to 0)	[illegible]	[illegible]	[illegible]	[illegible]	[illegible]	[illegible]	[illegible]	[illegible]	[illegible]	[illegible]	[illegible]	[illegible]	[illegible]	[illegible]	[illegible]	[illegible]	[illegible]	[illegible]	[illegible]	[illegible]	[illegible]	[illegible]	[illegible]
Invasive Non-typhoidal Salmonella (iNTS)	0 (0 to 0)	[illegible]	[illegible]	[illegible]	[illegible]	[illegible]	[illegible]	[illegible]	[illegible]	[illegible]	[illegible]	[illegible]	[illegible]	[illegible]	[illegible]	[illegible]	[illegible]	[illegible]	[illegible]	[illegible]	[illegible]	[illegible]	[illegible]	[illegible]
Other intestinal infectious diseases	0 (0 to 0)	[illegible]	[illegible]	[illegible]	[illegible]	[illegible]	[illegible]	[illegible]	[illegible]	[illegible]	[illegible]	[illegible]	[illegible]	[illegible]	[illegible]	[illegible]	[illegible]	[illegible]	[illegible]	[illegible]	[illegible]	[illegible]	[illegible]	[illegible]
Neglected tropical diseases and malaria	0·5 (0·2 to 1·2)	[illegible]	[illegible]	[illegible]	[illegible]	[illegible]	[illegible]	[illegible]	[illegible]	[illegible]	[illegible]	[illegible]	[illegible]	[illegible]	[illegible]	[illegible]	[illegible]	[illegible]	[illegible]	[illegible]	[illegible]	[illegible]	[illegible]	[illegible]
Malaria	0 (0 to 0·1)	[illegible]	[illegible]	[illegible]	[illegible]	[illegible]	[illegible]	[illegible]	[illegible]	[illegible]	[illegible]	[illegible]	[illegible]	[illegible]	[illegible]	[illegible]	[illegible]	[illegible]	[illegible]	[illegible]	[illegible]	[illegible]	[illegible]	[illegible]
Chagas disease	0 (0 to 0)	[illegible]	[illegible]	[illegible]	[illegible]	[illegible]	[illegible]	[illegible]	[illegible]	[illegible]	[illegible]	[illegible]	[illegible]	[illegible]	[illegible]	[illegible]	[illegible]	[illegible]	[illegible]	[illegible]	[illegible]	[illegible]	[illegible]	[illegible]
Leishmaniasis	0 (0 to 0)	[illegible]	[illegible]	[illegible]	[illegible]	[illegible]	[illegible]	[illegible]	[illegible]	[illegible]	[illegible]	[illegible]	[illegible]	[illegible]	[illegible]	[illegible]	[illegible]	[illegible]	[illegible]	[illegible]	[illegible]	[illegible]	[illegible]	[illegible]
Visceral leishmaniasis	0 (0 to 0)	[illegible]	[illegible]	[illegible]	[illegible]	[illegible]	[illegible]	[illegible]	[illegible]	[illegible]	[illegible]	[illegible]	[illegible]	[illegible]	[illegible]	[illegible]	[illegible]	[illegible]	[illegible]	[illegible]	[illegible]	[illegible]	[illegible]	[illegible]
Cutaneous and mucocutaneous leishmaniasis	0 (0 to 0)	[illegible]	[illegible]	[illegible]	[illegible]	[illegible]	[illegible]	[illegible]	[illegible]	[illegible]	[illegible]	[illegible]	[illegible]	[illegible]	[illegible]	[illegible]	[illegible]	[illegible]	[illegible]	[illegible]	[illegible]	[illegible]	[illegible]	[illegible]
African trypanosomiasis	0 (0 to 0)	[illegible]	[illegible]	[illegible]	[illegible]	[illegible]	[illegible]	[illegible]	[illegible]	[illegible]	[illegible]	[illegible]	[illegible]	[illegible]	[illegible]	[illegible]	[illegible]	[illegible]	[illegible]	[illegible]	[illegible]	[illegible]	[illegible]	[illegible]
Schistosomiasis	0 (0 to 0)	[illegible]	[illegible]	[illegible]	[illegible]	[illegible]	[illegible]	[illegible]	[illegible]	[illegible]	[illegible]	[illegible]	[illegible]	[illegible]	[illegible]	[illegible]	[illegible]	[illegible]	[illegible]	[illegible]	[illegible]	[illegible]	[illegible]	[illegible]
Cysticercosis	0 (0 to 0)	[illegible]	[illegible]	[illegible]	[illegible]	[illegible]	[illegible]	[illegible]	[illegible]	[illegible]	[illegible]	[illegible]	[illegible]	[illegible]	[illegible]	[illegible]	[illegible]	[illegible]	[illegible]	[illegible]	[illegible]	[illegible]	[illegible]	[illegible]
Cystic echinococcosis	0 (0 to 0)	[illegible]	[illegible]	[illegible]	[illegible]	[illegible]	[illegible]	[illegible]	[illegible]	[illegible]	[illegible]	[illegible]	[illegible]	[illegible]	[illegible]	[illegible]	[illegible]	[illegible]	[illegible]	[illegible]	[illegible]	[illegible]	[illegible]	[illegible]
Lymphatic filariasis	0 (0 to 0)	[illegible]	[illegible]	[illegible]	[illegible]	[illegible]	[illegible]	[illegible]	[illegible]	[illegible]	[illegible]	[illegible]	[illegible]	[illegible]	[illegible]	[illegible]	[illegible]	[illegible]	[illegible]	[illegible]	[illegible]	[illegible]	[illegible]	[illegible]
Onchocerciasis	0 (0 to 0)	[illegible]	[illegible]	[illegible]	[illegible]	[illegible]	[illegible]	[illegible]	[illegible]	[illegible]	[illegible]	[illegible]	[illegible]	[illegible]	[illegible]	[illegible]	[illegible]	[illegible]	[illegible]	[illegible]	[illegible]	[illegible]	[illegible]	[illegible]
Trachoma	0 (0 to 0)	[illegible]	[illegible]	[illegible]	[illegible]	[illegible]	[illegible]	[illegible]	[illegible]	[illegible]	[illegible]	[illegible]	[illegible]	[illegible]	[illegible]	[illegible]	[illegible]	[illegible]	[illegible]	[illegible]	[illegible]	[illegible]	[illegible]	[illegible]
Dengue	0 (0 to 0)	[illegible]	[illegible]	[illegible]	[illegible]	[illegible]	[illegible]	[illegible]	[illegible]	[illegible]	[illegible]	[illegible]	[illegible]	[illegible]	[illegible]	[illegible]	[illegible]	[illegible]	[illegible]	[illegible]	[illegible]	[illegible]	[illegible]	[illegible]
Yellow fever	0 (0 to 0)	[illegible]	[illegible]	[illegible]	[illegible]	[illegible]	[illegible]	[illegible]	[illegible]	[illegible]	[illegible]	[illegible]	[illegible]	[illegible]	[illegible]	[illegible]	[illegible]	[illegible]	[illegible]	[illegible]	[illegible]	[illegible]	[illegible]	[illegible]
Rabies	0 (0 to 0)	[illegible]	[illegible]	[illegible]	[illegible]	[illegible]	[illegible]	[illegible]	[illegible]	[illegible]	[illegible]	[illegible]	[illegible]	[illegible]	[illegible]	[illegible]	[illegible]	[illegible]	[illegible]	[illegible]	[illegible]	[illegible]	[illegible]	[illegible]
Intestinal nematode infections	0 (0 to 0)	[illegible]	[illegible]	[illegible]	[illegible]	[illegible]	[illegible]	[illegible]	[illegible]	[illegible]	[illegible]	[illegible]	[illegible]	[illegible]	[illegible]	[illegible]	[illegible]	[illegible]	[illegible]	[illegible]	[illegible]	[illegible]	[illegible]	[illegible]
Ascariasis	0 (0 to 0)	[illegible]	[illegible]	[illegible]	[illegible]	[illegible]	[illegible]	[illegible]	[illegible]	[illegible]	[illegible]	[illegible]	[illegible]	[illegible]	[illegible]	[illegible]	[illegible]	[illegible]	[illegible]	[illegible]	[illegible]	[illegible]	[illegible]	[illegible]
Trichuriasis	0 (0 to 0)	[illegible]	[illegible]	[illegible]	[illegible]	[illegible]	[illegible]	[illegible]	[illegible]	[illegible]	[illegible]	[illegible]	[illegible]	[illegible]	[illegible]	[illegible]	[illegible]	[illegible]	[illegible]	[illegible]	[illegible]	[illegible]	[illegible]	[illegible]
Hookworm disease	0 (0 to 0)	[illegible]	[illegible]	[illegible]	[illegible]	[illegible]	[illegible]	[illegible]	[illegible]	[illegible]	[illegible]	[illegible]	[illegible]	[illegible]	[illegible]	[illegible]	[illegible]	[illegible]	[illegible]	[illegible]	[illegible]	[illegible]	[illegible]	[illegible]
Food-borne trematodiases	0 (0 to 0)	[illegible]	[illegible]	[illegible]	[illegible]	[illegible]	[illegible]	[illegible]	[illegible]	[illegible]	[illegible]	[illegible]	[illegible]	[illegible]	[illegible]	[illegible]	[illegible]	[illegible]	[illegible]	[illegible]	[illegible]	[illegible]	[illegible]	[illegible]
Leprosy	0 (0 to 0)	[illegible]	[illegible]	[illegible]	[illegible]	[illegible]	[illegible]	[illegible]	[illegible]	[illegible]	[illegible]	[illegible]	[illegible]	[illegible]	[illegible]	[illegible]	[illegible]	[illegible]	[illegible]	[illegible]	[illegible]	[illegible]	[illegible]	[illegible]
Ebola	0 (0 to 0)	[illegible]	[illegible]	[illegible]	[illegible]	[illegible]	[illegible]	[illegible]	[illegible]	[illegible]	[illegible]	[illegible]	[illegible]	[illegible]	[illegible]	[illegible]	[illegible]	[illegible]	[illegible]	[illegible]	[illegible]	[illegible]	[illegible]	[illegible]
Zika virus	0 (0 to 0)	[illegible]	[illegible]	[illegible]	[illegible]	[illegible]	[illegible]	[illegible]	[illegible]	[illegible]	[illegible]	[illegible]	[illegible]	[illegible]	[illegible]	[illegible]	[illegible]	[illegible]	[illegible]	[illegible]	[illegible]	[illegible]	[illegible]	[illegible]
Guinea worm disease	0 (0 to 0)	[illegible]	[illegible]	[illegible]	[illegible]	[illegible]	[illegible]	[illegible]	[illegible]	[illegible]	[illegible]	[illegible]	[illegible]	[illegible]	[illegible]	[illegible]	[illegible]	[illegible]	[illegible]	[illegible]	[illegible]	[illegible]	[illegible]	[illegible]
Other neglected tropical diseases	0·5 (0·2 to 1·2)	[illegible]	[illegible]	[illegible]	[illegible]	[illegible]	[illegible]	[illegible]	[illegible]	[illegible]	[illegible]	[illegible]	[illegible]	[illegible]	[illegible]	[illegible]	[illegible]	[illegible]	[illegible]	[illegible]	[illegible]	[illegible]	[illegible]	[illegible]
Other infectious diseases	14·8 (8·9 to 23·7)	[illegible]	[illegible]	[illegible]	[illegible]	[illegible]	[illegible]	[illegible]	[illegible]	[illegible]	[illegible]	[illegible]	[illegible]	[illegible]	[illegible]	[illegible]	[illegible]	[illegible]	[illegible]	[illegible]	[illegible]	[illegible]	[illegible]	[illegible]
Meningitis	7·3 (3·7 to 12·9)	[illegible]	[illegible]	[illegible]	[illegible]	[illegible]	[illegible]	[illegible]	[illegible]	[illegible]	[illegible]	[illegible]	[illegible]	[illegible]	[illegible]	[illegible]	[illegible]	[illegible]	[illegible]	[illegible]	[illegible]	[illegible]	[illegible]	[illegible]
Encephalitis	3·4 (1·8 to 6·1)	[illegible]	[illegible]	[illegible]	[illegible]	[illegible]	[illegible]	[illegible]	[illegible]	[illegible]	[illegible]	[illegible]	[illegible]	[illegible]	[illegible]	[illegible]	[illegible]	[illegible]	[illegible]	[illegible]	[illegible]	[illegible]	[illegible]	[illegible]
Diphtheria	0 (0 to 0)	[illegible]	[illegible]	[illegible]	[illegible]	[illegible]	[illegible]	[illegible]	[illegible]	[illegible]	[illegible]	[illegible]	[illegible]	[illegible]	[illegible]	[illegible]	[illegible]	[illegible]	[illegible]	[illegible]	[illegible]	[illegible]	[illegible]	[illegible]
Tetanus	0·2 (0·1 to 0·4)	[illegible]	[illegible]	[illegible]	[illegible]	[illegible]	[illegible]	[illegible]	[illegible]	[illegible]	[illegible]	[illegible]	[illegible]	[illegible]	[illegible]	[illegible]	[illegible]	[illegible]	[illegible]	[illegible]	[illegible]	[illegible]	[illegible]	[illegible]
Measles	0 (0 to 0)	[illegible]	[illegible]	[illegible]	[illegible]	[illegible]	[illegible]	[illegible]	[illegible]	[illegible]	[illegible]	[illegible]	[illegible]	[illegible]	[illegible]	[illegible]	[illegible]	[illegible]	[illegible]	[illegible]	[illegible]	[illegible]	[illegible]	[illegible]
Varicella and herpes zoster	0·4 (0·1 to 0·8)	[illegible]	[illegible]	[illegible]	[illegible]	[illegible]	[illegible]	[illegible]	[illegible]	[illegible]	[illegible]	[illegible]	[illegible]	[illegible]	[illegible]	[illegible]	[illegible]	[illegible]	[illegible]	[illegible]	[illegible]	[illegible]	[illegible]	[illegible]
Acute hepatitis	0 (0 to 0)	[illegible]	[illegible]	[illegible]	[illegible]	[illegible]	[illegible]	[illegible]	[illegible]	[illegible]	[illegible]	[illegible]	[illegible]	[illegible]	[illegible]	[illegible]	[illegible]	[illegible]	[illegible]	[illegible]	[illegible]	[illegible]	[illegible]	[illegible]
Acute hepatitis A	0 (0 to 0)	[illegible]	[illegible]	[illegible]	[illegible]	[illegible]	[illegible]	[illegible]	[illegible]	[illegible]	[illegible]	[illegible]	[illegible]	[illegible]	[illegible]	[illegible]	[illegible]	[illegible]	[illegible]	[illegible]	[illegible]	[illegible]	[illegible]	[illegible]
Acute hepatitis B	0 (0 to 0)	[illegible]	[illegible]	[illegible]	[illegible]	[illegible]	[illegible]	[illegible]	[illegible]	[illegible]	[illegible]	[illegible]	[illegible]	[illegible]	[illegible]	[illegible]	[illegible]	[illegible]	[illegible]	[illegible]	[illegible]	[illegible]	[illegible]	[illegible]
Acute hepatitis C	0 (0 to 0)	[illegible]	[illegible]	[illegible]	[illegible]	[illegible]	[illegible]	[illegible]	[illegible]	[illegible]	[illegible]	[illegible]	[illegible]	[illegible]	[illegible]	[illegible]	[illegible]	[illegible]	[illegible]	[illegible]	[illegible]	[illegible]	[illegible]	[illegible]
Acute hepatitis E	0 (0 to 0)	[illegible]	[illegible]	[illegible]	[illegible]	[illegible]	[illegible]	[illegible]	[illegible]	[illegible]	[illegible]	[illegible]	[illegible]	[illegible]	[illegible]	[illegible]	[illegible]	[illegible]	[illegible]	[illegible]	[illegible]	[illegible]	[illegible]	[illegible]
Other unspecified infectious diseases	3·4 (1·4 to 6·7)	[illegible]	[illegible]	[illegible]	[illegible]	[illegible]	[illegible]	[illegible]	[illegible]	[illegible]	[illegible]	[illegible]	[illegible]	[illegible]	[illegible]	[illegible]	[illegible]	[illegible]	[illegible]	[illegible]	[illegible]	[illegible]	[illegible]	[illegible]
Maternal and neonatal disorders	8,210 (5,670 to 11,400)	[illegible]	[illegible]	[illegible]	[illegible]	[illegible]	[illegible]	[illegible]	[illegible]	[illegible]	[illegible]	[illegible]	[illegible]	[illegible]	[illegible]	[illegible]	[illegible]	[illegible]	[illegible]	[illegible]	[illegible]	[illegible]	[illegible]	[illegible]
Maternal disorders	0 (0 to 0)	[illegible]	[illegible]	[illegible]	[illegible]	[illegible]	[illegible]	[illegible]	[illegible]	[illegible]	[illegible]	[illegible]	[illegible]	[illegible]	[illegible]	[illegible]	[illegible]	[illegible]	[illegible]	[illegible]	[illegible]	[illegible]	[illegible]	[illegible]
Maternal hemorrhage	0 (0 to 0)	[illegible]	[illegible]	[illegible]	[illegible]	[illegible]	[illegible]	[illegible]	[illegible]	[illegible]	[illegible]	[illegible]	[illegible]	[illegible]	[illegible]	[illegible]	[illegible]	[illegible]	[illegible]	[illegible]	[illegible]	[illegible]	[illegible]	[illegible]
Maternal sepsis and other maternal infections	0 (0 to 0)	[illegible]	[illegible]	[illegible]	[illegible]	[illegible]	[illegible]	[illegible]	[illegible]	[illegible]	[illegible]	[illegible]	[illegible]	[illegible]	[illegible]	[illegible]	[illegible]	[illegible]	[illegible]	[illegible]	[illegible]	[illegible]	[illegible]	[illegible]
Maternal hypertensive disorders	0 (0 to 0)	[illegible]	[illegible]	[illegible]	[illegible]	[illegible]	[illegible]	[illegible]	[illegible]	[illegible]	[illegible]	[illegible]	[illegible]	[illegible]	[illegible]	[illegible]	[illegible]	[illegible]	[illegible]	[illegible]	[illegible]	[illegible]	[illegible]	[illegible]
Maternal obstructed labor and uterine rupture	--	[illegible]	[illegible]	[illegible]	[illegible]	[illegible]	[illegible]	[illegible]	[illegible]	[illegible]	[illegible]	[illegible]	[illegible]	[illegible]	[illegible]	[illegible]	[illegible]	[illegible]	[illegible]	[illegible]	[illegible]	[illegible]	[illegible]	[illegible]
Maternal abortion and miscarriage	--	[illegible]	[illegible]	[illegible]	[illegible]	[illegible]	[illegible]	[illegible]	[illegible]	[illegible]	[illegible]	[illegible]	[illegible]	[illegible]	[illegible]	[illegible]	[illegible]	[illegible]	[illegible]	[illegible]	[illegible]	[illegible]	[illegible]	[illegible]
Ectopic pregnancy	--	[illegible]	[illegible]	[illegible]	[illegible]	[illegible]	[illegible]	[illegible]	[illegible]	[illegible]	[illegible]	[illegible]	[illegible]	[illegible]	[illegible]	[illegible]	[illegible]	[illegible]	[illegible]	[illegible]	[illegible]	[illegible]	[illegible]	[illegible]
Indirect maternal deaths	--	[illegible]	[illegible]	[illegible]	[illegible]	[illegible]	[illegible]	[illegible]	[illegible]	[illegible]	[illegible]	[illegible]	[illegible]	[illegible]	[illegible]	[illegible]	[illegible]	[illegible]	[illegible]	[illegible]	[illegible]	[illegible]	[illegible]	[illegible]
Late maternal deaths	--	[illegible]	[illegible]	[illegible]	[illegible]	[illegible]	[illegible]	[illegible]	[illegible]	[illegible]	[illegible]	[illegible]	[illegible]	[illegible]	[illegible]	[illegible]	[illegible]	[illegible]	[illegible]	[illegible]	[illegible]	[illegible]	[illegible]	[illegible]
Maternal deaths aggravated by HIV/AIDS	--	[illegible]	[illegible]	[illegible]	[illegible]	[illegible]	[illegible]	[illegible]	[illegible]	[illegible]	[illegible]	[illegible]	[illegible]	[illegible]	[illegible]	[illegible]	[illegible]	[illegible]	[illegible]	[illegible]	[illegible]	[illegible]	[illegible]	[illegible]
Neonatal disorders	8,210 (5,670 to 11,400)	[illegible]	[illegible]	[illegible]	[illegible]	[illegible]	[illegible]	[illegible]	[illegible]	[illegible]	[illegible]	[illegible]	[illegible]	[illegible]	[illegible]	[illegible]	[illegible]	[illegible]	[illegible]	[illegible]	[illegible]	[illegible]	[illegible]	[illegible]
Neonatal preterm birth	3,740 (2,420 to 5,520)	[illegible]	[illegible]	[illegible]	[illegible]	[illegible]	[illegible]	[illegible]	[illegible]	[illegible]	[illegible]	[illegible]	[illegible]	[illegible]	[illegible]	[illegible]	[illegible]	[illegible]	[illegible]	[illegible]	[illegible]	[illegible]	[illegible]	[illegible]
Neonatal encephalopathy due to birth asphyxia and trauma	2,030 (1,200 to 3,020)	[illegible]	[illegible]	[illegible]	[illegible]	[illegible]	[illegible]	[illegible]	[illegible]	[illegible]	[illegible]	[illegible]	[illegible]	[illegible]	[illegible]	[illegible]	[illegible]	[illegible]	[illegible]	[illegible]	[illegible]	[illegible]	[illegible]	[illegible]
Neonatal sepsis and other neonatal infections	1,240 (751 to 1,910)	[illegible]	[illegible]	[illegible]	[illegible]	[illegible]	[illegible]	[illegible]	[illegible]	[illegible]	[illegible]	[illegible]	[illegible]	[illegible]	[illegible]	[illegible]	[illegible]	[illegible]	[illegible]	[illegible]	[illegible]	[illegible]	[illegible]	[illegible]
Hemolytic disease and other neonatal jaundice	100 (46·4 to 177)	[illegible]	[illegible]	[illegible]	[illegible]	[illegible]	[illegible]	[illegible]	[illegible]	[illegible]	[illegible]	[illegible]	[illegible]	[illegible]	[illegible]	[illegible]	[illegible]	[illegible]	[illegible]	[illegible]	[illegible]	[illegible]	[illegible]	[illegible]
Other neonatal disorders	1,100 (644 to 1,730)	[illegible]	[illegible]	[illegible]	[illegible]	[illegible]	[illegible]	[illegible]	[illegible]	[illegible]	[illegible]	[illegible]	[illegible]	[illegible]	[illegible]	[illegible]	[illegible]	[illegible]	[illegible]	[illegible]	[illegible]	[illegible]	[illegible]	[illegible]
Nutritional deficiencies	0·9 (0·5 to 1·4)	[illegible]	[illegible]	[illegible]	[illegible]	[illegible]	[illegible]	[illegible]	[illegible]	[illegible]	[illegible]	[illegible]	[illegible]	[illegible]	[illegible]	[illegible]	[illegible]	[illegible]	[illegible]	[illegible]	[illegible]	[illegible]	[illegible]	[illegible]
Protein-energy malnutrition	0·7 (0·4 to 1)	[illegible]	[illegible]	[illegible]	[illegible]	[illegible]	[illegible]	[illegible]	[illegible]	[illegible]	[illegible]	[illegible]	[illegible]	[illegible]	[illegible]	[illegible]	[illegible]	[illegible]	[illegible]	[illegible]	[illegible]	[illegible]	[illegible]	[illegible]
Iodine deficiency	0 (0 to 0)	[illegible]	[illegible]	[illegible]	[illegible]	[illegible]	[illegible]	[illegible]	[illegible]	[illegible]	[illegible]	[illegible]	[illegible]	[illegible]	[illegible]	[illegible]	[illegible]	[illegible]	[illegible]	[illegible]	[illegible]	[illegible]	[illegible]	[illegible]
Vitamin A deficiency	0·1 (0 to 0·2)	[illegible]	[illegible]	[illegible]	[illegible]	[illegible]	[illegible]	[illegible]	[illegible]	[illegible]	[illegible]	[illegible]	[illegible]	[illegible]	[illegible]	[illegible]	[illegible]	[illegible]	[illegible]	[illegible]	[illegible]	[illegible]	[illegible]	[illegible]
Dietary iron deficiency	0·2 (0 to 0·5)	[illegible]	[illegible]	[illegible]	[illegible]	[illegible]	[illegible]	[illegible]	[illegible]	[illegible]	[illegible]	[illegible]	[illegible]	[illegible]	[illegible]	[illegible]	[illegible]	[illegible]	[illegible]	[illegible]	[illegible]	[illegible]	[illegible]	[illegible]
Other nutritional deficiencies	0 (0 to 0)	[illegible]	[illegible]	[illegible]	[illegible]	[illegible]	[illegible]	[illegible]	[illegible]	[illegible]	[illegible]	[illegible]	[illegible]	[illegible]	[illegible]	[illegible]	[illegible]	[illegible]	[illegible]	[illegible]	[illegible]	[illegible]	[illegible]	[illegible]
Non-communicable diseases	909 (557 to 1,430)	[illegible]	[illegible]	[illegible]	[illegible]	[illegible]	[illegible]	[illegible]	[illegible]	[illegible]	[illegible]	[illegible]	[illegible]	[illegible]	[illegible]	[illegible]	[illegible]	[illegible]	[illegible]	[illegible]	[illegible]	[illegible]	[illegible]	[illegible]
Neoplasms	15·3 (2·8 to 61·6)	[illegible]	[illegible]	[illegible]	[illegible]	[illegible]	[illegible]	[illegible]	[illegible]	[illegible]	[illegible]	[illegible]	[illegible]	[illegible]	[illegible]	[illegible]	[illegible]	[illegible]	[illegible]	[illegible]	[illegible]	[illegible]	[illegible]	[illegible]
Lip and oral cavity cancer	0 (0 to 0)	[illegible]	[illegible]	[illegible]	[illegible]	[illegible]	[illegible]	[illegible]	[illegible]	[illegible]	[illegible]	[illegible]	[illegible]	[illegible]	[illegible]	[illegible]	[illegible]	[illegible]	[illegible]	[illegible]	[illegible]	[illegible]	[illegible]	[illegible]
Nasopharynx cancer	0 (0 to 0)	[illegible]	[illegible]	[illegible]	[illegible]	[illegible]	[illegible]	[illegible]	[illegible]	[illegible]	[illegible]	[illegible]	[illegible]	[illegible]	[illegible]	[illegible]	[illegible]	[illegible]	[illegible]	[illegible]	[illegible]	[illegible]	[illegible]	[illegible]
Other pharynx cancer	0 (0 to 0)	[illegible]	[illegible]	[illegible]	[illegible]	[illegible]	[illegible]	[illegible]	[illegible]	[illegible]	[illegible]	[illegible]	[illegible]	[illegible]	[illegible]	[illegible]	[illegible]	[illegible]	[illegible]	[illegible]	[illegible]	[illegible]	[illegible]	[illegible]
Esophageal cancer	0 (0 to 0)	[illegible]	[illegible]	[illegible]	[illegible]	[illegible]	[illegible]	[illegible]	[illegible]	[illegible]	[illegible]	[illegible]	[illegible]	[illegible]	[illegible]	[illegible]	[illegible]	[illegible]	[illegible]	[illegible]	[illegible]	[illegible]	[illegible]	[illegible]
Stomach cancer	0 (0 to 0)	[illegible]	[illegible]	[illegible]	[illegible]	[illegible]	[illegible]	[illegible]	[illegible]	[illegible]	[illegible]	[illegible]	[illegible]	[illegible]	[illegible]	[illegible]	[illegible]	[illegible]	[illegible]	[illegible]	[illegible]	[illegible]	[illegible]	[illegible]
Colon and rectum cancer	0 (0 to 0)	[illegible]	[illegible]	[illegible]	[illegible]	[illegible]	[illegible]	[illegible]	[illegible]	[illegible]	[illegible]	[illegible]	[illegible]	[illegible]	[illegible]	[illegible]	[illegible]	[illegible]	[illegible]	[illegible]	[illegible]	[illegible]	[illegible]	[illegible]
Liver cancer	0·1 (0·1 to 0·3)	[illegible]	[illegible]	[illegible]	[illegible]	[illegible]	[illegible]	[illegible]	[illegible]	[illegible]	[illegible]	[illegible]	[illegible]	[illegible]	[illegible]	[illegible]	[illegible]	[illegible]	[illegible]	[illegible]	[illegible]	[illegible]	[illegible]	[illegible]
Liver cancer due to hepatitis B	0 (0 to 0)	[illegible]	[illegible]	[illegible]	[illegible]	[illegible]	[illegible]	[illegible]	[illegible]	[illegible]	[illegible]	[illegible]	[illegible]	[illegible]	[illegible]	[illegible]	[illegible]	[illegible]	[illegible]	[illegible]	[illegible]	[illegible]	[illegible]	[illegible]
Liver cancer due to hepatitis C	0 (0 to 0)	[illegible]	[illegible]	[illegible]	[illegible]	[illegible]	[illegible]	[illegible]	[illegible]	[illegible]	[illegible]	[illegible]	[illegible]	[illegible]	[illegible]	[illegible]	[illegible]	[illegible]	[illegible]	[illegible]	[illegible]	[illegible]	[illegible]	[illegible]
Liver cancer due to alcohol use	0 (0 to 0)	[illegible]	[illegible]	[illegible]	[illegible]	[illegible]	[illegible]	[illegible]	[illegible]	[illegible]	[illegible]	[illegible]	[illegible]	[illegible]	[illegible]	[illegible]	[illegible]	[illegible]	[illegible]	[illegible]	[illegible]	[illegible]	[illegible]	[illegible]
Liver cancer due to NASH	0 (0 to 0)	[illegible]	[illegible]	[illegible]	[illegible]	[illegible]	[illegible]	[illegible]	[illegible]	[illegible]	[illegible]	[illegible]	[illegible]	[illegible]	[illegible]	[illegible]	[illegible]	[illegible]	[illegible]	[illegible]	[illegible]	[illegible]	[illegible]	[illegible]
Liver cancer due to other causes	0·1 (0·1 to 0·3)	[illegible]	[illegible]	[illegible]	[illegible]	[illegible]	[illegible]	[illegible]	[illegible]	[illegible]	[illegible]	[illegible]	[illegible]	[illegible]	[illegible]	[illegible]	[illegible]	[illegible]	[illegible]	[illegible]	[illegible]	[illegible]	[illegible]	[illegible]
Gallbladder and biliary tract cancer	0 (0 to 0)	[illegible]	[illegible]	[illegible]	[illegible]	[illegible]	[illegible]	[illegible]	[illegible]	[illegible]	[illegible]	[illegible]	[illegible]	[illegible]	[illegible]	[illegible]	[illegible]	[illegible]	[illegible]	[illegible]	[illegible]	[illegible]	[illegible]	[illegible]
Pancreatic cancer	0 (0 to 0)	[illegible]	[illegible]	[illegible]	[illegible]	[illegible]	[illegible]	[illegible]	[illegible]	[illegible]	[illegible]	[illegible]	[illegible]	[illegible]	[illegible]	[illegible]	[illegible]	[illegible]	[illegible]	[illegible]	[illegible]	[illegible]	[illegible]	[illegible]
Larynx cancer	0 (0 to 0)	[illegible]	[illegible]	[illegible]	[illegible]	[illegible]	[illegible]	[illegible]	[illegible]	[illegible]	[illegible]	[illegible]	[illegible]	[illegible]	[illegible]	[illegible]	[illegible]	[illegible]	[illegible]	[illegible]	[illegible]	[illegible]	[illegible]	[illegible]
Tracheal, bronchus, and lung cancer	0 (0 to 0)	[illegible]	[illegible]	[illegible]	[illegible]	[illegible]	[illegible]	[illegible]	[illegible]	[illegible]	[illegible]	[illegible]	[illegible]	[illegible]	[illegible]	[illegible]	[illegible]	[illegible]	[illegible]	[illegible]	[illegible]	[illegible]	[illegible]	[illegible]
Malignant skin melanoma	0 (0 to 0)	[illegible]	[illegible]	[illegible]	[illegible]	[illegible]	[illegible]	[illegible]	[illegible]	[illegible]	[illegible]	[illegible]	[illegible]	[illegible]	[illegible]	[illegible]	[illegible]	[illegible]	[illegible]	[illegible]	[illegible]	[illegible]	[illegible]	[illegible]
Non-melanoma skin cancer	0 (0 to 0)	[illegible]	[illegible]	[illegible]	[illegible]	[illegible]	[illegible]	[illegible]	[illegible]	[illegible]	[illegible]	[illegible]	[illegible]	[illegible]	[illegible]	[illegible]	[illegible]	[illegible]	[illegible]	[illegible]	[illegible]	[illegible]	[illegible]	[illegible]
Non-melanoma skin cancer (squamous-cell carcinoma)	0 (0 to 0)	[illegible]	[illegible]	[illegible]	[illegible]	[illegible]	[illegible]	[illegible]	[illegible]	[illegible]	[illegible]	[illegible]	[illegible]	[illegible]	[illegible]	[illegible]	[illegible]	[illegible]	[illegible]	[illegible]	[illegible]	[illegible]	[illegible]	[illegible]
Non-melanoma skin cancer (basal-cell carcinoma)	0 (0 to 0)	[illegible]	[illegible]	[illegible]	[illegible]	[illegible]	[illegible]	[illegible]	[illegible]	[illegible]	[illegible]	[illegible]	[illegible]	[illegible]	[illegible]	[illegible]	[illegible]	[illegible]	[illegible]	[illegible]	[illegible]	[illegible]	[illegible]	[illegible]
Breast cancer	0 (0 to 0)	[illegible]	[illegible]	[illegible]	[illegible]	[illegible]	[illegible]	[illegible]	[illegible]	[illegible]	[illegible]	[illegible]	[illegible]	[illegible]	[illegible]	[illegible]	[illegible]	[illegible]	[illegible]	[illegible]	[illegible]	[illegible]	[illegible]	[illegible]
Cervical cancer	--	[illegible]	[illegible]	[illegible]	[illegible]	[illegible]	[illegible]	[illegible]	[illegible]	[illegible]	[illegible]	[illegible]	[illegible]	[illegible]	[illegible]	[illegible]	[illegible]	[illegible]	[illegible]	[illegible]	[illegible]	[illegible]	[illegible]	[illegible]
Uterine cancer	--	[illegible]	[illegible]	[illegible]	[illegible]	[illegible]	[illegible]	[illegible]	[illegible]	[illegible]	[illegible]	[illegible]	[illegible]	[illegible]	[illegible]	[illegible]	[illegible]	[illegible]	[illegible]	[illegible]	[illegible]	[illegible]	[illegible]	[illegible]
Ovarian cancer	--	[illegible]	[illegible]	[illegible]	[illegible]	[illegible]	[illegible]	[illegible]	[illegible]	[illegible]	[illegible]	[illegible]	[illegible]	[illegible]	[illegible]	[illegible]	[illegible]	[illegible]	[illegible]	[illegible]	[illegible]	[illegible]	[illegible]	[illegible]
Prostate cancer	0 (0 to 0)	[illegible]	[illegible]	[illegible]	[illegible]	[illegible]	[illegible]	[illegible]	[illegible]	[illegible]	[illegible]	[illegible]	[illegible]	[illegible]	[illegible]	[illegible]	[illegible]	[illegible]	[illegible]	[illegible]	[illegible]	[illegible]	[illegible]	[illegible]
Testicular cancer	0·7 (0 to 7·2)	[illegible]	[illegible]	[illegible]	[illegible]	[illegible]	[illegible]	[illegible]	[illegible]	[illegible]	[illegible]	[illegible]	[illegible]	[illegible]	[illegible]	[illegible]	[illegible]	[illegible]	[illegible]	[illegible]	[illegible]	[illegible]	[illegible]	[illegible]
Kidney cancer	4·7 (0 to 25·3)	[illegible]	[illegible]	[illegible]	[illegible]	[illegible]	[illegible]	[illegible]	[illegible]	[illegible]	[illegible]	[illegible]	[illegible]	[illegible]	[illegible]	[illegible]	[illegible]	[illegible]	[illegible]	[illegible]	[illegible]	[illegible]	[illegible]	[illegible]
Bladder cancer	0 (0 to 0)	[illegible]	[illegible]	[illegible]	[illegible]	[illegible]	[illegible]	[illegible]	[illegible]	[illegible]	[illegible]	[illegible]	[illegible]	[illegible]	[illegible]	[illegible]	[illegible]	[illegible]	[illegible]	[illegible]	[illegible]	[illegible]	[illegible]	[illegible]
Brain and central nervous system cancer	2 (0 to 13·3)	[illegible]	[illegible]	[illegible]	[illegible]	[illegible]	[illegible]	[illegible]	[illegible]	[illegible]	[illegible]	[illegible]	[illegible]	[illegible]	[illegible]	[illegible]	[illegible]	[illegible]	[illegible]	[illegible]	[illegible]	[illegible]	[illegible]	[illegible]
Thyroid cancer	0 (0 to 0)	[illegible]	[illegible]	[illegible]	[illegible]	[illegible]	[illegible]	[illegible]	[illegible]	[illegible]	[illegible]	[illegible]	[illegible]	[illegible]	[illegible]	[illegible]	[illegible]	[illegible]	[illegible]	[illegible]	[illegible]	[illegible]	[illegible]	[illegible]
Mesothelioma	0 (0 to 0)	[illegible]	[illegible]	[illegible]	[illegible]	[illegible]	[illegible]	[illegible]	[illegible]	[illegible]	[illegible]	[illegible]	[illegible]	[illegible]	[illegible]	[illegible]	[illegible]	[illegible]	[illegible]	[illegible]	[illegible]	[illegible]	[illegible]	[illegible]
Hodgkin lymphoma	0 (0 to 0)	[illegible]	[illegible]	[illegible]	[illegible]	[illegible]	[illegible]	[illegible]	[illegible]	[illegible]	[illegible]	[illegible]	[illegible]	[illegible]	[illegible]	[illegible]	[illegible]	[illegible]	[illegible]	[illegible]	[illegible]	[illegible]	[illegible]	[illegible]
Non-Hodgkin lymphoma	0 (0 to 0)	[illegible]	[illegible]	[illegible]	[illegible]	[illegible]	[illegible]	[illegible]	[illegible]	[illegible]	[illegible]	[illegible]	[illegible]	[illegible]	[illegible]	[illegible]	[illegible]	[illegible]	[illegible]	[illegible]	[illegible]	[illegible]	[illegible]	[illegible]
Multiple myeloma	0 (0 to 0)	[illegible]	[illegible]	[illegible]	[illegible]	[illegible]	[illegible]	[illegible]	[illegible]	[illegible]	[illegible]	[illegible]	[illegible]	[illegible]	[illegible]	[illegible]	[illegible]	[illegible]	[illegible]	[illegible]	[illegible]	[illegible]	[illegible]	[illegible]
Leukemia	4·5 (0·2 to 21·7)	[illegible]	[illegible]	[illegible]	[illegible]	[illegible]	[illegible]	[illegible]	[illegible]	[illegible]	[illegible]	[illegible]	[illegible]	[illegible]	[illegible]	[illegible]	[illegible]	[illegible]	[illegible]	[illegible]	[illegible]	[illegible]	[illegible]	[illegible]
Acute lymphoid leukemia	2 (0·1 to 10·6)	[illegible]	[illegible]	[illegible]	[illegible]	[illegible]	[illegible]	[illegible]	[illegible]	[illegible]	[illegible]	[illegible]	[illegible]	[illegible]	[illegible]	[illegible]	[illegible]	[illegible]	[illegible]	[illegible]	[illegible]	[illegible]	[illegible]	[illegible]
Chronic lymphoid leukemia	0 (0 to 0)	[illegible]	[illegible]	[illegible]	[illegible]	[illegible]	[illegible]	[illegible]	[illegible]	[illegible]	[illegible]	[illegible]	[illegible]	[illegible]	[illegible]	[illegible]	[illegible]	[illegible]	[illegible]	[illegible]	[illegible]	[illegible]	[illegible]	[illegible]
Acute myeloid leukemia	0·5 (0 to 3·6)	[illegible]	[illegible]	[illegible]	[illegible]	[illegible]	[illegible]	[illegible]	[illegible]	[illegible]	[illegible]	[illegible]	[illegible]	[illegible]	[illegible]	[illegible]	[illegible]	[illegible]	[illegible]	[illegible]	[illegible]	[illegible]	[illegible]	[illegible]
Chronic myeloid leukemia	0 (0 to 0)	[illegible]	[illegible]	[illegible]	[illegible]	[illegible]	[illegible]	[illegible]	[illegible]	[illegible]	[illegible]	[illegible]	[illegible]	[illegible]	[illegible]	[illegible]	[illegible]	[illegible]	[illegible]	[illegible]	[illegible]	[illegible]	[illegible]	[illegible]
Other leukemia	1·9 (0 to 9·6)	[illegible]	[illegible]	[illegible]	[illegible]	[illegible]	[illegible]	[illegible]	[illegible]	[illegible]	[illegible]	[illegible]	[illegible]	[illegible]	[illegible]	[illegible]	[illegible]	[illegible]	[illegible]	[illegible]	[illegible]	[illegible]	[illegible]	[illegible]
Other malignant neoplasms	3·1 (0·6 to 9·8)	[illegible]	[illegible]	[illegible]	[illegible]	[illegible]	[illegible]	[illegible]	[illegible]	[illegible]	[illegible]	[illegible]	[illegible]	[illegible]	[illegible]	[illegible]	[illegible]	[illegible]	[illegible]	[illegible]	[illegible]	[illegible]	[illegible]	[illegible]

Other neoplasms	0·2 (0·1 to 0·4)	0·1 (0 to 0·2)	0·4 (0·1 to 0·7)	[illegible]	[illegible]	[illegible]	[illegible]	[illegible]	[illegible]	[illegible]	[illegible]	[illegible]	[illegible]	[illegible]	[illegible]	[illegible]	[illegible]	[illegible]	[illegible]	[illegible]	[illegible]	[illegible]	[illegible]	44·2
Myelodysplastic, myeloproliferative, and other hematopoietic r	0·2 (0·1 to 0·4)	0·1 (0 to 0·2)	0·4 (0·1 to 0·7)	[illegible]	[illegible]	[illegible]	[illegible]	[illegible]	[illegible]	[illegible]	[illegible]	[illegible]	[illegible]	[illegible]	[illegible]	[illegible]	[illegible]	[illegible]	[illegible]	[illegible]	[illegible]	[illegible]	[illegible]	44·2
Benign and in situ intestinal neoplasms	0 (0 to 0)	0 (0 to 0)	0 (0 to 0)	[illegible]	[illegible]	[illegible]	[illegible]	[illegible]	[illegible]	[illegible]	[illegible]	[illegible]	[illegible]	[illegible]	[illegible]	[illegible]	[illegible]	[illegible]	[illegible]	[illegible]	[illegible]	[illegible]	[illegible]	0
Benign and in situ cervical and uterine neoplasms	0 (0 to 0)	0 (0 to 0)	0 (0 to 0)	[illegible]	[illegible]	[illegible]	[illegible]	[illegible]	[illegible]	[illegible]	[illegible]	[illegible]	[illegible]	[illegible]	[illegible]	[illegible]	[illegible]	[illegible]	[illegible]	[illegible]	[illegible]	[illegible]	[illegible]	0
Other benign and in situ neoplasms	0 (0 to 0)	0 (0 to 0)	0 (0 to 0)	[illegible]	[illegible]	[illegible]	[illegible]	[illegible]	[illegible]	[illegible]	[illegible]	[illegible]	[illegible]	[illegible]	[illegible]	[illegible]	[illegible]	[illegible]	[illegible]	[illegible]	[illegible]	[illegible]	[illegible]	0
Cardiovascular diseases	36·7 (20·9 to 60·7)	10·3 (5·4 to 18·1)	60·2 (32·9 to 105)	[illegible]	[illegible]	[illegible]	[illegible]	[illegible]	[illegible]	[illegible]	[illegible]	[illegible]	[illegible]	[illegible]	[illegible]	[illegible]	[illegible]	[illegible]	[illegible]	[illegible]	[illegible]	[illegible]	[illegible]	34,000
Rheumatic heart disease	0 (0 to 0)	0 (0 to 0)	0 (0 to 0)	[illegible]	[illegible]	[illegible]	[illegible]	[illegible]	[illegible]	[illegible]	[illegible]	[illegible]	[illegible]	[illegible]	[illegible]	[illegible]	[illegible]	[illegible]	[illegible]	[illegible]	[illegible]	[illegible]	[illegible]	421
Ischemic heart disease	0 (0 to 0)	0 (0 to 0)	0 (0 to 0)	[illegible]	[illegible]	[illegible]	[illegible]	[illegible]	[illegible]	[illegible]	[illegible]	[illegible]	[illegible]	[illegible]	[illegible]	[illegible]	[illegible]	[illegible]	[illegible]	[illegible]	[illegible]	[illegible]	[illegible]	15,400
Stroke	14·5 (6·6 to 26·6)	2·3 (0·9 to 4·8)	12·3 (5·4 to 23·4)	[illegible]	[illegible]	[illegible]	[illegible]	[illegible]	[illegible]	[illegible]	[illegible]	[illegible]	[illegible]	[illegible]	[illegible]	[illegible]	[illegible]	[illegible]	[illegible]	[illegible]	[illegible]	[illegible]	[illegible]	10,600
Ischemic stroke	0·3 (0·1 to 0·5)	0·2 (0·1 to 0·3)	0·9 (0·4 to 1·8)	[illegible]	[illegible]	[illegible]	[illegible]	[illegible]	[illegible]	[illegible]	[illegible]	[illegible]	[illegible]	[illegible]	[illegible]	[illegible]	[illegible]	[illegible]	[illegible]	[illegible]	[illegible]	[illegible]	[illegible]	3,680
Intracerebral hemorrhage	3·8 (1·3 to 8)	0·4 (0·1 to 0·8)	6·5 (2·6 to 13·3)	[illegible]	[illegible]	[illegible]	[illegible]	[illegible]	[illegible]	[illegible]	[illegible]	[illegible]	[illegible]	[illegible]	[illegible]	[illegible]	[illegible]	[illegible]	[illegible]	[illegible]	[illegible]	[illegible]	[illegible]	6,110
Subarachnoid hemorrhage	10·5 (4·5 to 19·4)	1·8 (0·7 to 3·6)	4·9 (1·9 to 9·7)	[illegible]	[illegible]	[illegible]	[illegible]	[illegible]	[illegible]	[illegible]	[illegible]	[illegible]	[illegible]	[illegible]	[illegible]	[illegible]	[illegible]	[illegible]	[illegible]	[illegible]	[illegible]	[illegible]	[illegible]	793
Hypertensive heart disease	0 (0 to 0)	0 (0 to 0)	0 (0 to 0)	[illegible]	[illegible]	[illegible]	[illegible]	[illegible]	[illegible]	[illegible]	[illegible]	[illegible]	[illegible]	[illegible]	[illegible]	[illegible]	[illegible]	[illegible]	[illegible]	[illegible]	[illegible]	[illegible]	[illegible]	3,780
Non-rheumatic valvular heart disease	0 (0 to 0)	0 (0 to 0)	0 (0 to 0)	[illegible]	[illegible]	[illegible]	[illegible]	[illegible]	[illegible]	[illegible]	[illegible]	[illegible]	[illegible]	[illegible]	[illegible]	[illegible]	[illegible]	[illegible]	[illegible]	[illegible]	[illegible]	[illegible]	[illegible]	396
Non-rheumatic calcific aortic valve disease	0 (0 to 0)	0 (0 to 0)	0 (0 to 0)	[illegible]	[illegible]	[illegible]	[illegible]	[illegible]	[illegible]	[illegible]	[illegible]	[illegible]	[illegible]	[illegible]	[illegible]	[illegible]	[illegible]	[illegible]	[illegible]	[illegible]	[illegible]	[illegible]	[illegible]	238
Non-rheumatic degenerative mitral valve disease	0 (0 to 0)	0 (0 to 0)	0 (0 to 0)	[illegible]	[illegible]	[illegible]	[illegible]	[illegible]	[illegible]	[illegible]	[illegible]	[illegible]	[illegible]	[illegible]	[illegible]	[illegible]	[illegible]	[illegible]	[illegible]	[illegible]	[illegible]	[illegible]	[illegible]	150
Other non-rheumatic valve diseases	0 (0 to 0)	0 (0 to 0)	0 (0 to 0)	[illegible]	[illegible]	[illegible]	[illegible]	[illegible]	[illegible]	[illegible]	[illegible]	[illegible]	[illegible]	[illegible]	[illegible]	[illegible]	[illegible]	[illegible]	[illegible]	[illegible]	[illegible]	[illegible]	[illegible]	7·5
Cardiomyopathy and myocarditis	9·8 (5 to 16·6)	4·3 (2·2 to 7·7)	28·5 (14·3 to 51·8)	[illegible]	[illegible]	[illegible]	[illegible]	[illegible]	[illegible]	[illegible]	[illegible]	[illegible]	[illegible]	[illegible]	[illegible]	[illegible]	[illegible]	[illegible]	[illegible]	[illegible]	[illegible]	[illegible]	[illegible]	1,680
Myocarditis	4·5 (1·7 to 9·2)	2·6 (1·3 to 5·2)	16·8 (7 to 34)	[illegible]	[illegible]	[illegible]	[illegible]	[illegible]	[illegible]	[illegible]	[illegible]	[illegible]	[illegible]	[illegible]	[illegible]	[illegible]	[illegible]	[illegible]	[illegible]	[illegible]	[illegible]	[illegible]	[illegible]	357
Alcoholic cardiomyopathy	0 (0 to 0)	0 (0 to 0)	0 (0 to 0)	[illegible]	[illegible]	[illegible]	[illegible]	[illegible]	[illegible]	[illegible]	[illegible]	[illegible]	[illegible]	[illegible]	[illegible]	[illegible]	[illegible]	[illegible]	[illegible]	[illegible]	[illegible]	[illegible]	[illegible]	442
Other cardiomyopathy	5·2 (2·5 to 9·6)	1·7 (0·7 to 3·1)	11·7 (5·2 to 23·1)	[illegible]	[illegible]	[illegible]	[illegible]	[illegible]	[illegible]	[illegible]	[illegible]	[illegible]	[illegible]	[illegible]	[illegible]	[illegible]	[illegible]	[illegible]	[illegible]	[illegible]	[illegible]	[illegible]	[illegible]	878
Atrial fibrillation and flutter	0 (0 to 0)	0 (0 to 0)	0 (0 to 0)	[illegible]	[illegible]	[illegible]	[illegible]	[illegible]	[illegible]	[illegible]	[illegible]	[illegible]	[illegible]	[illegible]	[illegible]	[illegible]	[illegible]	[illegible]	[illegible]	[illegible]	[illegible]	[illegible]	[illegible]	272
Aortic aneurysm	--	--	--	[illegible]	[illegible]	[illegible]	[illegible]	[illegible]	[illegible]	[illegible]	[illegible]	[illegible]	[illegible]	[illegible]	[illegible]	[illegible]	[illegible]	[illegible]	[illegible]	[illegible]	[illegible]	[illegible]	[illegible]	NA
Endocarditis	1·1 (0·5 to 2)	0·8 (0·4 to 1·6)	4·4 (1·8 to 9·2)	[illegible]	[illegible]	[illegible]	[illegible]	[illegible]	[illegible]	[illegible]	[illegible]	[illegible]	[illegible]	[illegible]	[illegible]	[illegible]	[illegible]	[illegible]	[illegible]	[illegible]	[illegible]	[illegible]	[illegible]	269
Other cardiovascular and circulatory diseases	11·3 (4·5 to 22·6)	2·9 (1·3 to 5·7)	15 (6·1 to 31·7)	[illegible]	[illegible]	[illegible]	[illegible]	[illegible]	[illegible]	[illegible]	[illegible]	[illegible]	[illegible]	[illegible]	[illegible]	[illegible]	[illegible]	[illegible]	[illegible]	[illegible]	[illegible]	[illegible]	[illegible]	745
Chronic respiratory diseases	0 (0 to 0)	0 (0 to 0)	0 (0 to 0)	[illegible]	[illegible]	[illegible]	[illegible]	[illegible]	[illegible]	[illegible]	[illegible]	[illegible]	[illegible]	[illegible]	[illegible]	[illegible]	[illegible]	[illegible]	[illegible]	[illegible]	[illegible]	[illegible]	[illegible]	3,220
Chronic obstructive pulmonary disease	0 (0 to 0)	0 (0 to 0)	0 (0 to 0)	[illegible]	[illegible]	[illegible]	[illegible]	[illegible]	[illegible]	[illegible]	[illegible]	[illegible]	[illegible]	[illegible]	[illegible]	[illegible]	[illegible]	[illegible]	[illegible]	[illegible]	[illegible]	[illegible]	[illegible]	1,370
Pneumoconiosis	0 (0 to 0)	0 (0 to 0)	0 (0 to 0)	[illegible]	[illegible]	[illegible]	[illegible]	[illegible]	[illegible]	[illegible]	[illegible]	[illegible]	[illegible]	[illegible]	[illegible]	[illegible]	[illegible]	[illegible]	[illegible]	[illegible]	[illegible]	[illegible]	[illegible]	6
Silicosis	0 (0 to 0)	0 (0 to 0)	0 (0 to 0)	[illegible]	[illegible]	[illegible]	[illegible]	[illegible]	[illegible]	[illegible]	[illegible]	[illegible]	[illegible]	[illegible]	[illegible]	[illegible]	[illegible]	[illegible]	[illegible]	[illegible]	[illegible]	[illegible]	[illegible]	3·2
Asbestosis	0 (0 to 0)	0 (0 to 0)	0 (0 to 0)	[illegible]	[illegible]	[illegible]	[illegible]	[illegible]	[illegible]	[illegible]	[illegible]	[illegible]	[illegible]	[illegible]	[illegible]	[illegible]	[illegible]	[illegible]	[illegible]	[illegible]	[illegible]	[illegible]	[illegible]	1
Coal workers pneumoconiosis	0 (0 to 0)	0 (0 to 0)	0 (0 to 0)	[illegible]	[illegible]	[illegible]	[illegible]	[illegible]	[illegible]	[illegible]	[illegible]	[illegible]	[illegible]	[illegible]	[illegible]	[illegible]	[illegible]	[illegible]	[illegible]	[illegible]	[illegible]	[illegible]	[illegible]	0·2
Other pneumoconiosis	0 (0 to 0)	0 (0 to 0)	0 (0 to 0)	[illegible]	[illegible]	[illegible]	[illegible]	[illegible]	[illegible]	[illegible]	[illegible]	[illegible]	[illegible]	[illegible]	[illegible]	[illegible]	[illegible]	[illegible]	[illegible]	[illegible]	[illegible]	[illegible]	[illegible]	1·4
Asthma	0 (0 to 0)	0 (0 to 0)	0 (0 to 0)	[illegible]	[illegible]	[illegible]	[illegible]	[illegible]	[illegible]	[illegible]	[illegible]	[illegible]	[illegible]	[illegible]	[illegible]	[illegible]	[illegible]	[illegible]	[illegible]	[illegible]	[illegible]	[illegible]	[illegible]	1,570
Interstitial lung disease and pulmonary sarcoidosis	0 (0 to 0)	0 (0 to 0)	0 (0 to 0)	[illegible]	[illegible]	[illegible]	[illegible]	[illegible]	[illegible]	[illegible]	[illegible]	[illegible]	[illegible]	[illegible]	[illegible]	[illegible]	[illegible]	[illegible]	[illegible]	[illegible]	[illegible]	[illegible]	[illegible]	112
Other chronic respiratory diseases	0 (0 to 0)	0 (0 to 0)	0 (0 to 0)	[illegible]	[illegible]	[illegible]	[illegible]	[illegible]	[illegible]	[illegible]	[illegible]	[illegible]	[illegible]	[illegible]	[illegible]	[illegible]	[illegible]	[illegible]	[illegible]	[illegible]	[illegible]	[illegible]	[illegible]	164
Digestive diseases	23·5 (13·5 to 37·4)	11·1 (5·9 to 19·6)	99·7 (57·8 to 172)	[illegible]	[illegible]	[illegible]	[illegible]	[illegible]	[illegible]	[illegible]	[illegible]	[illegible]	[illegible]	[illegible]	[illegible]	[illegible]	[illegible]	[illegible]	[illegible]	[illegible]	[illegible]	[illegible]	[illegible]	10,500
Cirrhosis and other chronic liver diseases	0 (0 to 0)	0 (0 to 0)	0 (0 to 0·1)	[illegible]	[illegible]	[illegible]	[illegible]	[illegible]	[illegible]	[illegible]	[illegible]	[illegible]	[illegible]	[illegible]	[illegible]	[illegible]	[illegible]	[illegible]	[illegible]	[illegible]	[illegible]	[illegible]	[illegible]	6,610
Cirrhosis and other chronic liver diseases due to hepatitis B	0 (0 to 0)	0 (0 to 0)	0 (0 to 0)	[illegible]	[illegible]	[illegible]	[illegible]	[illegible]	[illegible]	[illegible]	[illegible]	[illegible]	[illegible]	[illegible]	[illegible]	[illegible]	[illegible]	[illegible]	[illegible]	[illegible]	[illegible]	[illegible]	[illegible]	542
Cirrhosis and other chronic liver diseases due to hepatitis C	0 (0 to 0)	0 (0 to 0)	0 (0 to 0)	[illegible]	[illegible]	[illegible]	[illegible]	[illegible]	[illegible]	[illegible]	[illegible]	[illegible]	[illegible]	[illegible]	[illegible]	[illegible]	[illegible]	[illegible]	[illegible]	[illegible]	[illegible]	[illegible]	[illegible]	1,410
Cirrhosis and other chronic liver diseases due to alcohol use	0 (0 to 0)	0 (0 to 0)	0 (0 to 0)	[illegible]	[illegible]	[illegible]	[illegible]	[illegible]	[illegible]	[illegible]	[illegible]	[illegible]	[illegible]	[illegible]	[illegible]	[illegible]	[illegible]	[illegible]	[illegible]	[illegible]	[illegible]	[illegible]	[illegible]	3,030
Cirrhosis and other chronic liver diseases due to NAFLD	0 (0 to 0)	0 (0 to 0)	0 (0 to 0)	[illegible]	[illegible]	[illegible]	[illegible]	[illegible]	[illegible]	[illegible]	[illegible]	[illegible]	[illegible]	[illegible]	[illegible]	[illegible]	[illegible]	[illegible]	[illegible]	[illegible]	[illegible]	[illegible]	[illegible]	847
Cirrhosis and other chronic liver diseases due to other causes	0 (0 to 0)	0 (0 to 0)	0 (0 to 0·1)	[illegible]	[illegible]	[illegible]	[illegible]	[illegible]	[illegible]	[illegible]	[illegible]	[illegible]	[illegible]	[illegible]	[illegible]	[illegible]	[illegible]	[illegible]	[illegible]	[illegible]	[illegible]	[illegible]	[illegible]	785
Upper digestive system diseases	0 (0 to 0)	0 (0 to 0)	0 (0 to 0)	[illegible]	[illegible]	[illegible]	[illegible]	[illegible]	[illegible]	[illegible]	[illegible]	[illegible]	[illegible]	[illegible]	[illegible]	[illegible]	[illegible]	[illegible]	[illegible]	[illegible]	[illegible]	[illegible]	[illegible]	1,470
Peptic ulcer disease	0 (0 to 0)	0 (0 to 0)	0 (0 to 0)	[illegible]	[illegible]	[illegible]	[illegible]	[illegible]	[illegible]	[illegible]	[illegible]	[illegible]	[illegible]	[illegible]	[illegible]	[illegible]	[illegible]	[illegible]	[illegible]	[illegible]	[illegible]	[illegible]	[illegible]	816
Gastritis and duodenitis	0 (0 to 0)	0 (0 to 0)	0 (0 to 0)	[illegible]	[illegible]	[illegible]	[illegible]	[illegible]	[illegible]	[illegible]	[illegible]	[illegible]	[illegible]	[illegible]	[illegible]	[illegible]	[illegible]	[illegible]	[illegible]	[illegible]	[illegible]	[illegible]	[illegible]	213
Gastroesophageal reflux disease	0 (0 to 0)	0 (0 to 0)	0 (0 to 0)	[illegible]	[illegible]	[illegible]	[illegible]	[illegible]	[illegible]	[illegible]	[illegible]	[illegible]	[illegible]	[illegible]	[illegible]	[illegible]	[illegible]	[illegible]	[illegible]	[illegible]	[illegible]	[illegible]	[illegible]	437
Appendicitis	0 (0 to 0)	0 (0 to 0)	0 (0 to 0)	[illegible]	[illegible]	[illegible]	[illegible]	[illegible]	[illegible]	[illegible]	[illegible]	[illegible]	[illegible]	[illegible]	[illegible]	[illegible]	[illegible]	[illegible]	[illegible]	[illegible]	[illegible]	[illegible]	[illegible]	256
Paralytic ileus and intestinal obstruction	14·4 (7·9 to 24·7)	9 (4·8 to 16)	80·6 (45·6 to 136)	[illegible]	[illegible]	[illegible]	[illegible]	[illegible]	[illegible]	[illegible]	[illegible]	[illegible]	[illegible]	[illegible]	[illegible]	[illegible]	[illegible]	[illegible]	[illegible]	[illegible]	[illegible]	[illegible]	[illegible]	463
Inguinal, femoral, and abdominal hernia	9·1 (4·9 to 16)	2·1 (0·9 to 4·3)	19·1 (8·9 to 37·6)	[illegible]	[illegible]	[illegible]	[illegible]	[illegible]	[illegible]	[illegible]	[illegible]	[illegible]	[illegible]	[illegible]	[illegible]	[illegible]	[illegible]	[illegible]	[illegible]	[illegible]	[illegible]	[illegible]	[illegible]	524
Inflammatory bowel disease	0 (0 to 0)	0 (0 to 0)	0 (0 to 0)	[illegible]	[illegible]	[illegible]	[illegible]	[illegible]	[illegible]	[illegible]	[illegible]	[illegible]	[illegible]	[illegible]	[illegible]	[illegible]	[illegible]	[illegible]	[illegible]	[illegible]	[illegible]	[illegible]	[illegible]	149
Vascular intestinal disorders	0 (0 to 0)	0 (0 to 0)	0 (0 to 0)	[illegible]	[illegible]	[illegible]	[illegible]	[illegible]	[illegible]	[illegible]	[illegible]	[illegible]	[illegible]	[illegible]	[illegible]	[illegible]	[illegible]	[illegible]	[illegible]	[illegible]	[illegible]	[illegible]	[illegible]	91·8
Gallbladder and biliary diseases	0 (0 to 0)	0 (0 to 0)	0 (0 to 0)	[illegible]	[illegible]	[illegible]	[illegible]	[illegible]	[illegible]	[illegible]	[illegible]	[illegible]	[illegible]	[illegible]	[illegible]	[illegible]	[illegible]	[illegible]	[illegible]	[illegible]	[illegible]	[illegible]	[illegible]	193
Pancreatitis	0 (0 to 0)	0 (0 to 0)	0 (0 to 0)	[illegible]	[illegible]	[illegible]	[illegible]	[illegible]	[illegible]	[illegible]	[illegible]	[illegible]	[illegible]	[illegible]	[illegible]	[illegible]	[illegible]	[illegible]	[illegible]	[illegible]	[illegible]	[illegible]	[illegible]	395
Other digestive diseases	0 (0 to 0)	0 (0 to 0)	0 (0 to 0)	[illegible]	[illegible]	[illegible]	[illegible]	[illegible]	[illegible]	[illegible]	[illegible]	[illegible]	[illegible]	[illegible]	[illegible]	[illegible]	[illegible]	[illegible]	[illegible]	[illegible]	[illegible]	[illegible]	[illegible]	304
Neurological disorders	0·1 (0 to 0·2)	0·3 (0·1 to 0·6)	36·7 (22·9 to 54·7)	[illegible]	[illegible]	[illegible]	[illegible]	[illegible]	[illegible]	[illegible]	[illegible]	[illegible]	[illegible]	[illegible]	[illegible]	[illegible]	[illegible]	[illegible]	[illegible]	[illegible]	[illegible]	[illegible]	[illegible]	4,460
Alzheimer's disease and other dementias	0 (0 to 0)	0 (0 to 0)	0 (0 to 0)	[illegible]	[illegible]	[illegible]	[illegible]	[illegible]	[illegible]	[illegible]	[illegible]	[illegible]	[illegible]	[illegible]	[illegible]	[illegible]	[illegible]	[illegible]	[illegible]	[illegible]	[illegible]	[illegible]	[illegible]	546
Parkinson's disease	0 (0 to 0)	0 (0 to 0)	0 (0 to 0)	[illegible]	[illegible]	[illegible]	[illegible]	[illegible]	[illegible]	[illegible]	[illegible]	[illegible]	[illegible]	[illegible]	[illegible]	[illegible]	[illegible]	[illegible]	[illegible]	[illegible]	[illegible]	[illegible]	[illegible]	279
Idiopathic epilepsy	0·1 (0 to 0·2)	0·3 (0·1 to 0·6)	15·8 (9·1 to 25·4)	[illegible]	[illegible]	[illegible]	[illegible]	[illegible]	[illegible]	[illegible]	[illegible]	[illegible]	[illegible]	[illegible]	[illegible]	[illegible]	[illegible]	[illegible]	[illegible]	[illegible]	[illegible]	[illegible]	[illegible]	1,620
Multiple sclerosis	0 (0 to 0)	0 (0 to 0)	0 (0 to 0)	[illegible]	[illegible]	[illegible]	[illegible]	[illegible]	[illegible]	[illegible]	[illegible]	[illegible]	[illegible]	[illegible]	[illegible]	[illegible]	[illegible]	[illegible]	[illegible]	[illegible]	[illegible]	[illegible]	[illegible]	21·4
Motor neuron disease	0 (0 to 0)	0 (0 to 0)	3·8 (2 to 6·8)	[illegible]	[illegible]	[illegible]	[illegible]	[illegible]	[illegible]	[illegible]	[illegible]	[illegible]	[illegible]	[illegible]	[illegible]	[illegible]	[illegible]	[illegible]	[illegible]	[illegible]	[illegible]	[illegible]	[illegible]	58·9
Headache disorders	0 (0 to 0)	0 (0 to 0)	0 (0 to 0)	[illegible]	[illegible]	[illegible]	[illegible]	[illegible]	[illegible]	[illegible]	[illegible]	[illegible]	[illegible]	[illegible]	[illegible]	[illegible]	[illegible]	[illegible]	[illegible]	[illegible]	[illegible]	[illegible]	[illegible]	1,520
Migraine	0 (0 to 0)	0 (0 to 0)	0 (0 to 0)	[illegible]	[illegible]	[illegible]	[illegible]	[illegible]	[illegible]	[illegible]	[illegible]	[illegible]	[illegible]	[illegible]	[illegible]	[illegible]	[illegible]	[illegible]	[illegible]	[illegible]	[illegible]	[illegible]	[illegible]	1,350
Tension-type headache	0 (0 to 0)	0 (0 to 0)	0 (0 to 0)	[illegible]	[illegible]	[illegible]	[illegible]	[illegible]	[illegible]	[illegible]	[illegible]	[illegible]	[illegible]	[illegible]	[illegible]	[illegible]	[illegible]	[illegible]	[illegible]	[illegible]	[illegible]	[illegible]	[illegible]	168
Other neurological disorders	0 (0 to 0)	0 (0 to 0)	17·2 (10 to 27·4)	[illegible]	[illegible]	[illegible]	[illegible]	[illegible]	[illegible]	[illegible]	[illegible]	[illegible]	[illegible]	[illegible]	[illegible]	[illegible]	[illegible]	[illegible]	[illegible]	[illegible]	[illegible]	[illegible]	[illegible]	417
Mental disorders	0·2 (0·1 to 0·2)	0·5 (0·3 to 0·7)	7·6 (4·8 to 11·4)	[illegible]	[illegible]	[illegible]	[illegible]	[illegible]	[illegible]	[illegible]	[illegible]	[illegible]	[illegible]	[illegible]	[illegible]	[illegible]	[illegible]	[illegible]	[illegible]	[illegible]	[illegible]	[illegible]	[illegible]	6,720
Schizophrenia	0 (0 to 0)	0 (0 to 0)	0 (0 to 0)	[illegible]	[illegible]	[illegible]	[illegible]	[illegible]	[illegible]	[illegible]	[illegible]	[illegible]	[illegible]	[illegible]	[illegible]	[illegible]	[illegible]	[illegible]	[illegible]	[illegible]	[illegible]	[illegible]	[illegible]	660
Depressive disorders	0 (0 to 0)	0 (0 to 0)	0 (0 to 0)	[illegible]	[illegible]	[illegible]	[illegible]	[illegible]	[illegible]	[illegible]	[illegible]	[illegible]	[illegible]	[illegible]	[illegible]	[illegible]	[illegible]	[illegible]	[illegible]	[illegible]	[illegible]	[illegible]	[illegible]	2,670
Major depressive disorder	0 (0 to 0)	0 (0 to 0)	0 (0 to 0)	[illegible]	[illegible]	[illegible]	[illegible]	[illegible]	[illegible]	[illegible]	[illegible]	[illegible]	[illegible]	[illegible]	[illegible]	[illegible]	[illegible]	[illegible]	[illegible]	[illegible]	[illegible]	[illegible]	[illegible]	2,360
Dysthymia	0 (0 to 0)	0 (0 to 0)	0 (0 to 0)	[illegible]	[illegible]	[illegible]	[illegible]	[illegible]	[illegible]	[illegible]	[illegible]	[illegible]	[illegible]	[illegible]	[illegible]	[illegible]	[illegible]	[illegible]	[illegible]	[illegible]	[illegible]	[illegible]	[illegible]	307
Bipolar disorder	0 (0 to 0)	0 (0 to 0)	0 (0 to 0)	[illegible]	[illegible]	[illegible]	[illegible]	[illegible]	[illegible]	[illegible]	[illegible]	[illegible]	[illegible]	[illegible]	[illegible]	[illegible]	[illegible]	[illegible]	[illegible]	[illegible]	[illegible]	[illegible]	[illegible]	692
Anxiety disorders	0 (0 to 0)	0 (0 to 0)	0 (0 to 0)	[illegible]	[illegible]	[illegible]	[illegible]	[illegible]	[illegible]	[illegible]	[illegible]	[illegible]	[illegible]	[illegible]	[illegible]	[illegible]	[illegible]	[illegible]	[illegible]	[illegible]	[illegible]	[illegible]	[illegible]	1,200
Eating disorders	0 (0 to 0)	0 (0 to 0)	0 (0 to 0)	[illegible]	[illegible]	[illegible]	[illegible]	[illegible]	[illegible]	[illegible]	[illegible]	[illegible]	[illegible]	[illegible]	[illegible]	[illegible]	[illegible]	[illegible]	[illegible]	[illegible]	[illegible]	[illegible]	[illegible]	97·9
Anorexia nervosa	0 (0 to 0)	0 (0 to 0)	0 (0 to 0)	[illegible]	[illegible]	[illegible]	[illegible]	[illegible]	[illegible]	[illegible]	[illegible]	[illegible]	[illegible]	[illegible]	[illegible]	[illegible]	[illegible]	[illegible]	[illegible]	[illegible]	[illegible]	[illegible]	[illegible]	22·1
Bulimia nervosa	0 (0 to 0)	0 (0 to 0)	0 (0 to 0)	[illegible]	[illegible]	[illegible]	[illegible]	[illegible]	[illegible]	[illegible]	[illegible]	[illegible]	[illegible]	[illegible]	[illegible]	[illegible]	[illegible]	[illegible]	[illegible]	[illegible]	[illegible]	[illegible]	[illegible]	75·8
Autism spectrum disorders	0·1 (0·1 to 0·2)	0·4 (0·3 to 0·6)	6·5 (4 to 9·9)	[illegible]	[illegible]	[illegible]	[illegible]	[illegible]	[illegible]	[illegible]	[illegible]	[illegible]	[illegible]	[illegible]	[illegible]	[illegible]	[illegible]	[illegible]	[illegible]	[illegible]	[illegible]	[illegible]	[illegible]	300
Attention-deficit/hyperactivity disorder	0 (0 to 0)	0 (0 to 0)	0 (0 to 0)	[illegible]	[illegible]	[illegible]	[illegible]	[illegible]	[illegible]	[illegible]	[illegible]	[illegible]	[illegible]	[illegible]	[illegible]	[illegible]	[illegible]	[illegible]	[illegible]	[illegible]	[illegible]	[illegible]	[illegible]	220
Conduct disorder	0 (0 to 0)	0 (0 to 0)	0 (0 to 0)	[illegible]	[illegible]	[illegible]	[illegible]	[illegible]	[illegible]	[illegible]	[illegible]	[illegible]	[illegible]	[illegible]	[illegible]	[illegible]	[illegible]	[illegible]	[illegible]	[illegible]	[illegible]	[illegible]	[illegible]	345
Idiopathic developmental intellectual disability	0 (0 to 0)	0·1 (0 to 0·1)	1·1 (0·1 to 2·5)	[illegible]	[illegible]	[illegible]	[illegible]	[illegible]	[illegible]	[illegible]	[illegible]	[illegible]	[illegible]	[illegible]	[illegible]	[illegible]	[illegible]	[illegible]	[illegible]	[illegible]	[illegible]	[illegible]	[illegible]	76·7
Other mental disorders	0 (0 to 0)	0 (0 to 0)	0 (0 to 0)	[illegible]	[illegible]	[illegible]	[illegible]	[illegible]	[illegible]	[illegible]	[illegible]	[illegible]	[illegible]	[illegible]	[illegible]	[illegible]	[illegible]	[illegible]	[illegible]	[illegible]	[illegible]	[illegible]	[illegible]	467
Substance use disorders	0 (0 to 0)	0 (0 to 0)	0 (0 to 0)	[illegible]	[illegible]	[illegible]	[illegible]	[illegible]	[illegible]	[illegible]	[illegible]	[illegible]	[illegible]	[illegible]	[illegible]	[illegible]	[illegible]	[illegible]	[illegible]	[illegible]	[illegible]	[illegible]	[illegible]	2,340
Alcohol use disorders	0 (0 to 0)	0 (0 to 0)	0 (0 to 0)	[illegible]	[illegible]	[illegible]	[illegible]	[illegible]	[illegible]	[illegible]	[illegible]	[illegible]	[illegible]	[illegible]	[illegible]	[illegible]	[illegible]	[illegible]	[illegible]	[illegible]	[illegible]	[illegible]	[illegible]	1,780
Drug use disorders	0 (0 to 0)	0 (0 to 0)	0 (0 to 0)	[illegible]	[illegible]	[illegible]	[illegible]	[illegible]	[illegible]	[illegible]	[illegible]	[illegible]	[illegible]	[illegible]	[illegible]	[illegible]	[illegible]	[illegible]	[illegible]	[illegible]	[illegible]	[illegible]	[illegible]	563
Opioid use disorders	0 (0 to 0)	0 (0 to 0)	0 (0 to 0)	[illegible]	[illegible]	[illegible]	[illegible]	[illegible]	[illegible]	[illegible]	[illegible]	[illegible]	[illegible]	[illegible]	[illegible]	[illegible]	[illegible]	[illegible]	[illegible]	[illegible]	[illegible]	[illegible]	[illegible]	267
Cocaine use disorders	0 (0 to 0)	0 (0 to 0)	0 (0 to 0)	[illegible]	[illegible]	[illegible]	[illegible]	[illegible]	[illegible]	[illegible]	[illegible]	[illegible]	[illegible]	[illegible]	[illegible]	[illegible]	[illegible]	[illegible]	[illegible]	[illegible]	[illegible]	[illegible]	[illegible]	97·5
Amphetamine use disorders	0 (0 to 0)	0 (0 to 0)	0 (0 to 0)	[illegible]	[illegible]	[illegible]	[illegible]	[illegible]	[illegible]	[illegible]	[illegible]	[illegible]	[illegible]	[illegible]	[illegible]	[illegible]	[illegible]	[illegible]	[illegible]	[illegible]	[illegible]	[illegible]	[illegible]	39·7
Cannabis use disorders	0 (0 to 0)	0 (0 to 0)	0 (0 to 0)	[illegible]	[illegible]	[illegible]	[illegible]	[illegible]	[illegible]	[illegible]	[illegible]	[illegible]	[illegible]	[illegible]	[illegible]	[illegible]	[illegible]	[illegible]	[illegible]	[illegible]	[illegible]	[illegible]	[illegible]	70·9
Other drug use disorders	0 (0 to 0)	0 (0 to 0)	0 (0 to 0)	[illegible]	[illegible]	[illegible]	[illegible]	[illegible]	[illegible]	[illegible]	[illegible]	[illegible]	[illegible]	[illegible]	[illegible]	[illegible]	[illegible]	[illegible]	[illegible]	[illegible]	[illegible]	[illegible]	[illegible]	87·4
Diabetes and kidney diseases	0·2 (0·1 to 0·4)	0·5 (0·2 to 1)	36·1 (23·1 to 53·8)	[illegible]	[illegible]	[illegible]	[illegible]	[illegible]	[illegible]	[illegible]	[illegible]	[illegible]	[illegible]	[illegible]	[illegible]	[illegible]	[illegible]	[illegible]	[illegible]	[illegible]	[illegible]	[illegible]	[illegible]	15,300
Diabetes mellitus	0·2 (0·1 to 0·4)	0·5 (0·2 to 1)	10·2 (5·4 to 18·5)	[illegible]	[illegible]	[illegible]	[illegible]	[illegible]	[illegible]	[illegible]	[illegible]	[illegible]	[illegible]	[illegible]	[illegible]	[illegible]	[illegible]	[illegible]	[illegible]	[illegible]	[illegible]	[illegible]	[illegible]	10,200
Diabetes mellitus type 1	0·2 (0·1 to 0·4)	0·5 (0·2 to 1)	10·2 (5·4 to 18·5)	[illegible]	[illegible]	[illegible]	[illegible]	[illegible]	[illegible]	[illegible]	[illegible]	[illegible]	[illegible]	[illegible]	[illegible]	[illegible]	[illegible]	[illegible]	[illegible]	[illegible]	[illegible]	[illegible]	[illegible]	654
Diabetes mellitus type 2	0 (0 to 0)	0 (0 to 0)	0 (0 to 0)	[illegible]	[illegible]	[illegible]	[illegible]	[illegible]	[illegible]	[illegible]	[illegible]	[illegible]	[illegible]	[illegible]	[illegible]	[illegible]	[illegible]	[illegible]	[illegible]	[illegible]	[illegible]	[illegible]	[illegible]	9,590
Chronic kidney disease	0 (0 to 0)	0 (0 to 0)	25·8 (15·9 to 39·3)	[illegible]	[illegible]	[illegible]	[illegible]	[illegible]	[illegible]	[illegible]	[illegible]	[illegible]	[illegible]	[illegible]	[illegible]	[illegible]	[illegible]	[illegible]	[illegible]	[illegible]	[illegible]	[illegible]	[illegible]	5,070
Chronic kidney disease due to diabetes mellitus type 1	0 (0 to 0)	0 (0 to 0)	0·2 (0·1 to 0·5)	[illegible]	[illegible]	[illegible]	[illegible]	[illegible]	[illegible]	[illegible]	[illegible]	[illegible]	[illegible]	[illegible]	[illegible]	[illegible]	[illegible]	[illegible]	[illegible]	[illegible]	[illegible]	[illegible]	[illegible]	480
Chronic kidney disease due to diabetes mellitus type 2	0 (0 to 0)	0 (0 to 0)	0 (0 to 0)	[illegible]	[illegible]	[illegible]	[illegible]	[illegible]	[illegible]	[illegible]	[illegible]	[illegible]	[illegible]	[illegible]	[illegible]	[illegible]	[illegible]	[illegible]	[illegible]	[illegible]	[illegible]	[illegible]	[illegible]	1,120
Chronic kidney disease due to hypertension	0 (0 to 0)	0 (0 to 0)	0 (0 to 0)	[illegible]	[illegible]	[illegible]	[illegible]	[illegible]	[illegible]	[illegible]	[illegible]	[illegible]	[illegible]	[illegible]	[illegible]	[illegible]	[illegible]	[illegible]	[illegible]	[illegible]	[illegible]	[illegible]	[illegible]	904
Chronic kidney disease due to glomerulonephritis	0 (0 to 0)	0 (0 to 0)	7·4 (3·5 to 13·6)	[illegible]	[illegible]	[illegible]	[illegible]	[illegible]	[illegible]	[illegible]	[illegible]	[illegible]	[illegible]	[illegible]	[illegible]	[illegible]	[illegible]	[illegible]	[illegible]	[illegible]	[illegible]	[illegible]	[illegible]	1,300
Chronic kidney disease due to other and unspecified causes	0 (0 to 0)	0 (0 to 0)	18·2 (10·2 to 29·3)	[illegible]	[illegible]	[illegible]	[illegible]	[illegible]	[illegible]	[illegible]	[illegible]	[illegible]	[illegible]	[illegible]	[illegible]	[illegible]	[illegible]	[illegible]	[illegible]	[illegible]	[illegible]	[illegible]	[illegible]	1,260
Acute glomerulonephritis	0 (0 to 0)	0 (0 to 0)	0 (0 to 0·1)	[illegible]	[illegible]	[illegible]	[illegible]	[illegible]	[illegible]	[illegible]	[illegible]	[illegible]	[illegible]	[illegible]	[illegible]	[illegible]	[illegible]	[illegible]	[illegible]	[illegible]	[illegible]	[illegible]	[illegible]	7·9
Skin and subcutaneous diseases	0 (0 to 0)	0·1 (0·1 to 0·2)	18·8 (11·5 to 29·7)	[illegible]	[illegible]	[illegible]	[illegible]	[illegible]	[illegible]	[illegible]	[illegible]	[illegible]	[illegible]	[illegible]	[illegible]	[illegible]	[illegible]	[illegible]	[illegible]	[illegible]	[illegible]	[illegible]	[illegible]	1,870
Dermatitis	0 (0 to 0)	0 (0 to 0)	0 (0 to 0)	[illegible]	[illegible]	[illegible]	[illegible]	[illegible]	[illegible]	[illegible]	[illegible]	[illegible]	[illegible]	[illegible]	[illegible]	[illegible]	[illegible]	[illegible]	[illegible]	[illegible]	[illegible]	[illegible]	[illegible]	391
Atopic dermatitis	0 (0 to 0)	0 (0 to 0)	0 (0 to 0)	[illegible]	[illegible]	[illegible]	[illegible]	[illegible]	[illegible]	[illegible]	[illegible]	[illegible]	[illegible]	[illegible]	[illegible]	[illegible]	[illegible]	[illegible]	[illegible]	[illegible]	[illegible]	[illegible]	[illegible]	286
Contact dermatitis	0 (0 to 0)	0 (0 to 0)	0 (0 to 0)	[illegible]	[illegible]	[illegible]	[illegible]	[illegible]	[illegible]	[illegible]	[illegible]	[illegible]	[illegible]	[illegible]	[illegible]	[illegible]	[illegible]	[illegible]	[illegible]	[illegible]	[illegible]	[illegible]	[illegible]	92·7
Seborrhoeic dermatitis	0 (0 to 0)	0 (0 to 0)	0 (0 to 0)	[illegible]	[illegible]	[illegible]	[illegible]	[illegible]	[illegible]	[illegible]	[illegible]	[illegible]	[illegible]	[illegible]	[illegible]	[illegible]	[illegible]	[illegible]	[illegible]	[illegible]	[illegible]	[illegible]	[illegible]	12·6
Psoriasis	0 (0 to 0)	0 (0 to 0)	0 (0 to 0)	[illegible]	[illegible]	[illegible]	[illegible]	[illegible]	[illegible]	[illegible]	[illegible]	[illegible]	[illegible]	[illegible]	[illegible]	[illegible]	[illegible]	[illegible]	[illegible]	[illegible]	[illegible]	[illegible]	[illegible]	116
Bacterial skin diseases	0 (0 to 0)	0 (0 to 0)	5·7 (1·9 to 12·2)	[illegible]	[illegible]	[illegible]	[illegible]	[illegible]	[illegible]	[illegible]	[illegible]	[illegible]	[illegible]	[illegible]	[illegible]	[illegible]	[illegible]	[illegible]	[illegible]	[illegible]	[illegible]	[illegible]	[illegible]	90·6
Cellulitis	0 (0 to 0)	0 (0 to 0)	1·2 (0·3 to 3·2)	[illegible]	[illegible]	[illegible]	[illegible]	[illegible]	[illegible]	[illegible]	[illegible]	[illegible]	[illegible]	[illegible]	[illegible]	[illegible]	[illegible]	[illegible]	[illegible]	[illegible]	[illegible]	[illegible]	[illegible]	20·7
Pyoderma	0 (0 to 0)	0 (0 to 0)	4·5 (1·4 to 10·1)	[illegible]	[illegible]	[illegible]	[illegible]	[illegible]	[illegible]	[illegible]	[illegible]	[illegible]	[illegible]	[illegible]	[illegible]	[illegible]	[illegible]	[illegible]	[illegible]	[illegible]	[illegible]	[illegible]	[illegible]	69·9
Scabies	0 (0 to 0)	0·1 (0 to 0·1)	4·8 (2·5 to 8·4)	[illegible]	[illegible]	[illegible]	[illegible]	[illegible]	[illegible]	[illegible]	[illegible]	[illegible]	[illegible]	[illegible]	[illegible]	[illegible]	[illegible]	[illegible]	[illegible]	[illegible]	[illegible]	[illegible]	[illegible]	326
Fungal skin diseases	0 (0 to 0)	0 (0 to 0)	0·9 (0·3 to 1·9)	[illegible]	[illegible]	[illegible]	[illegible]	[illegible]	[illegible]	[illegible]	[illegible]	[illegible]	[illegible]	[illegible]	[illegible]	[illegible]	[illegible]	[illegible]	[illegible]	[illegible]	[illegible]	[illegible]	[illegible]	157
Viral skin diseases	0 (0 to 0)	0 (0 to 0)	0 (0 to 0)	[illegible]	[illegible]	[illegible]	[illegible]	[illegible]	[illegible]	[illegible]	[illegible]	[illegible]	[illegible]	[illegible]	[illegible]	[illegible]	[illegible]	[illegible]	[illegible]	[illegible]	[illegible]	[illegible]	[illegible]	183
Acne vulgaris	0 (0 to 0)	0 (0 to 0)	0 (0 to 0)	[illegible]	[illegible]	[illegible]	[illegible]	[illegible]	[illegible]	[illegible]	[illegible]	[illegible]	[illegible]	[illegible]	[illegible]	[illegible]	[illegible]	[illegible]	[illegible]	[illegible]	[illegible]	[illegible]	[illegible]	193
Alopecia areata	0 (0 to 0)	0 (0 to 0)	0 (0 to 0)	[illegible]	[illegible]	[illegible]	[illegible]	[illegible]	[illegible]	[illegible]	[illegible]	[illegible]	[illegible]	[illegible]	[illegible]	[illegible]	[illegible]	[illegible]	[illegible]	[illegible]	[illegible]	[illegible]	[illegible]	18·8
Pruritus	0 (0 to 0)	0 (0 to 0)	0·1 (0 to 0·2)	[illegible]	[illegible]	[illegible]	[illegible]	[illegible]	[illegible]	[illegible]	[illegible]	[illegible]	[illegible]	[illegible]	[illegible]	[illegible]	[illegible]	[illegible]	[illegible]	[illegible]	[illegible]	[illegible]	[illegible]	25·9
Urticaria	0 (0 to 0)	0 (0 to 0·1)	4·1 (2·3 to 6·6)	[illegible]	[illegible]	[illegible]	[illegible]	[illegible]	[illegible]	[illegible]	[illegible]	[illegible]	[illegible]	[illegible]	[illegible]	[illegible]	[illegible]	[illegible]	[illegible]	[illegible]	[illegible]	[illegible]	[illegible]	155
Decubitus ulcer	0 (0 to 0)	0 (0 to 0)	0 (0 to 0)	[illegible]	[illegible]	[illegible]	[illegible]	[illegible]	[illegible]	[illegible]	[illegible]	[illegible]	[illegible]	[illegible]	[illegible]	[illegible]	[illegible]	[illegible]	[illegible]	[illegible]	[illegible]	[illegible]	[illegible]	47·6
Other skin and subcutaneous diseases	0 (0 to 0)	0 (0 to 0)	3·3 (1·4 to 7·2)	[illegible]	[illegible]	[illegible]	[illegible]	[illegible]	[illegible]	[illegible]	[illegible]	[illegible]	[illegible]	[illegible]	[illegible]	[illegible]	[illegible]	[illegible]	[illegible]	[illegible]	[illegible]	[illegible]	[illegible]	168
Sense organ diseases	0 (0 to 0)	0·1 (0 to 0·1)	1·8 (1 to 2·8)	[illegible]	[illegible]	[illegible]	[illegible]	[illegible]	[illegible]	[illegible]	[illegible]	[illegible]	[illegible]	[illegible]	[illegible]	[illegible]	[illegible]	[illegible]	[illegible]	[illegible]	[illegible]	[illegible]	[illegible]	2,370
Blindness and vision loss	0 (0 to 0)	0 (0 to 0)	0·3 (0·1 to 0·5)	[illegible]	[illegible]	[illegible]	[illegible]	[illegible]	[illegible]	[illegible]	[illegible]	[illegible]	[illegible]	[illegible]	[illegible]	[illegible]	[illegible]	[illegible]	[illegible]	[illegible]	[illegible]	[illegible]	[illegible]	634
Glaucoma	0 (0 to 0)	0 (0 to 0)	0 (0 to 0)	[illegible]	[illegible]	[illegible]	[illegible]	[illegible]	[illegible]	[illegible]	[illegible]	[illegible]	[illegible]	[illegible]	[illegible]	[illegible]	[illegible]	[illegible]	[illegible]	[illegible]	[illegible]	[illegible]	[illegible]	33·3
Cataract	0 (0 to 0)	0 (0 to 0)	0 (0 to 0)	[illegible]	[illegible]	[illegible]	[illegible]	[illegible]	[illegible]	[illegible]	[illegible]	[illegible]	[illegible]	[illegible]	[illegible]	[illegible]	[illegible]	[illegible]	[illegible]	[illegible]	[illegible]	[illegible]	[illegible]	84·1
Age-related macular degeneration	0 (0 to 0)	0 (0 to 0)	0 (0 to 0)	[illegible]	[illegible]	[illegible]	[illegible]	[illegible]	[illegible]	[illegible]	[illegible]	[illegible]	[illegible]	[illegible]	[illegible]	[illegible]	[illegible]	[illegible]	[illegible]	[illegible]	[illegible]	[illegible]	[illegible]	3·5
Refraction disorders	0 (0 to 0)	0 (0 to 0)	0·2 (0·1 to 0·4)	[illegible]	[illegible]	[illegible]	[illegible]	[illegible]	[illegible]	[illegible]	[illegible]	[illegible]	[illegible]	[illegible]	[illegible]	[illegible]	[illegible]	[illegible]	[illegible]	[illegible]	[illegible]	[illegible]	[illegible]	175
Near vision loss	0 (0 to 0)	0 (0 to 0)	0 (0 to 0)	[illegible]	[illegible]	[illegible]	[illegible]	[illegible]	[illegible]	[illegible]	[illegible]	[illegible]	[illegible]	[illegible]	[illegible]	[illegible]	[illegible]	[illegible]	[illegible]	[illegible]	[illegible]	[illegible]	[illegible]	168
Other vision loss	0 (0 to 0)	0 (0 to 0)	0·1 (0 to 0·2)	[illegible]	[illegible]	[illegible]	[illegible]	[illegible]	[illegible]	[illegible]	[illegible]	[illegible]	[illegible]	[illegible]	[illegible]	[illegible]	[illegible]	[illegible]	[illegible]	[illegible]	[illegible]	[illegible]	[illegible]	171
Age-related and other hearing loss	0 (0 to 0)	0 (0 to 0)	0·7 (0·3 to 1·4)	[illegible]	[illegible]	[illegible]	[illegible]	[illegible]	[illegible]	[illegible]	[illegible]	[illegible]	[illegible]	[illegible]	[illegible]	[illegible]	[illegible]	[illegible]	[illegible]	[illegible]	[illegible]	[illegible]	[illegible]	1,630
Other sense organ diseases	0 (0 to 0)	0 (0 to 0·1)	0·8 (0·4 to 1·3)	[illegible]	[illegible]	[illegible]	[illegible]	[illegible]	[illegible]	[illegible]	[illegible]	[illegible]	[illegible]	[illegible]	[illegible]	[illegible]	[illegible]	[illegible]	[illegible]	[illegible]	[illegible]	[illegible]	[illegible]	109
Musculoskeletal disorders	0 (0 to 0)	0 (0 to 0)	0 (0 to 0)	[illegible]	[illegible]	[illegible]	[illegible]	[illegible]	[illegible]	[illegible]	[illegible]	[illegible]	[illegible]	[illegible]	[illegible]	[illegible]	[illegible]	[illegible]	[illegible]	[illegible]	[illegible]	[illegible]	[illegible]	4,440
Rheumatoid arthritis	0 (0 to 0)	0 (0 to 0)	0 (0 to 0)	[illegible]	[illegible]	[illegible]	[illegible]	[illegible]	[illegible]	[illegible]	[illegible]	[illegible]	[illegible]	[illegible]	[illegible]	[illegible]	[illegible]	[illegible]	[illegible]	[illegible]	[illegible]	[illegible]	[illegible]	49·9
Osteoarthritis	0 (0 to 0)	0 (0 to 0)	0 (0 to 0)	[illegible]	[illegible]	[illegible]	[illegible]	[illegible]	[illegible]	[illegible]	[illegible]	[illegible]	[illegible]	[illegible]	[illegible]	[illegible]	[illegible]	[illegible]	[illegible]	[illegible]	[illegible]	[illegible]	[illegible]	537
Osteoarthritis hip	0 (0 to 0)	0 (0 to 0)	0 (0 to 0)	[illegible]	[illegible]	[illegible]	[illegible]	[illegible]	[illegible]	[illegible]	[illegible]	[illegible]	[illegible]	[illegible]	[illegible]	[illegible]	[illegible]	[illegible]	[illegible]	[illegible]	[illegible]	[illegible]	[illegible]	32·7
Osteoarthritis knee	0 (0 to 0)	0 (0 to 0)	0 (0 to 0)	[illegible]	[illegible]	[illegible]	[illegible]	[illegible]	[illegible]	[illegible]	[illegible]	[illegible]	[illegible]	[illegible]	[illegible]	[illegible]	[illegible]	[illegible]	[illegible]	[illegible]	[illegible]	[illegible]	[illegible]	381
Osteoarthritis hand	0 (0 to 0)	0 (0 to 0)	0 (0 to 0)	[illegible]	[illegible]	[illegible]	[illegible]	[illegible]	[illegible]	[illegible]	[illegible]	[illegible]	[illegible]	[illegible]	[illegible]	[illegible]	[illegible]	[illegible]	[illegible]	[illegible]	[illegible]	[illegible]	[illegible]	47·8
Osteoarthritis other	0 (0 to 0)	0 (0 to 0)	0 (0 to 0)	[illegible]	[illegible]	[illegible]	[illegible]	[illegible]	[illegible]	[illegible]	[illegible]	[illegible]	[illegible]	[illegible]	[illegible]	[illegible]	[illegible]	[illegible]	[illegible]	[illegible]	[illegible]	[illegible]	[illegible]	75·5
Low back pain	0 (0 to 0)	0 (0 to 0)	0 (0 to 0)	[illegible]	[illegible]	[illegible]	[illegible]	[illegible]	[illegible]	[illegible]	[illegible]	[illegible]	[illegible]	[illegible]	[illegible]	[illegible]	[illegible]	[illegible]	[illegible]	[illegible]	[illegible]	[illegible]	[illegible]	2,160
Neck pain	0 (0 to 0)	0 (0 to 0)	0 (0 to 0)	[illegible]	[illegible]	[illegible]	[illegible]	[illegible]	[illegible]	[illegible]	[illegible]	[illegible]	[illegible]	[illegible]	[illegible]	[illegible]	[illegible]	[illegible]	[illegible]	[illegible]	[illegible]	[illegible]	[illegible]	453
Gout	0 (0 to 0)	0 (0 to 0)	0 (0 to 0)	[illegible]	[illegible]	[illegible]	[illegible]	[illegible]	[illegible]	[illegible]	[illegible]	[illegible]	[illegible]	[illegible]	[illegible]	[illegible]	[illegible]	[illegible]	[illegible]	[illegible]	[illegible]	[illegible]	[illegible]	33·1
Other musculoskeletal disorders	0 (0 to 0)	0 (0 to 0)	0 (0 to 0)	[illegible]	[illegible]	[illegible]	[illegible]	[illegible]	[illegible]	[illegible]	[illegible]	[illegible]	[illegible]	[illegible]	[illegible]	[illegible]	[illegible]	[illegible]	[illegible]	[illegible]	[illegible]	[illegible]	[illegible]	1,210
Other non-communicable diseases	833 (490 to 1,310)	225 (137 to 363)	802 (502 to 1,240)	[illegible]	[illegible]	[illegible]	[illegible]	[illegible]	[illegible]	[illegible]	[illegible]	[illegible]	[illegible]	[illegible]	[illegible]	[illegible]	[illegible]	[illegible]	[illegible]	[illegible]	[illegible]	[illegible]	[illegible]	6,330

Congenital birth defects	798 (466 to 1,270)	197 (114 to 324)	543 (305 to 886)	173 (113 to 274)	103 (76·5 to 138)	85·6 (62·6 to 114)	81·2 (57·2 to 113)	78·9 (56 to 108)	60·8 (43·2 to 85)
Neural tube defects	44·4 (20·7 to 83·9)	7·2 (2·8 to 15·4)	17·5 (6·9 to 37·2)	9·6 (4·4 to 19·6)	3·2 (2·2 to 4·6)	4 (2·6 to 5·8)	4·2 (2·6 to 6·4)	3·1 (2·1 to 4·7)	2·9 (1·8 to 4·5)
Congenital heart anomalies	322 (164 to 529)	97·3 (51·3 to 168)	276 (141 to 470)	67·6 (34·4 to 127)	36·2 (22·3 to 56·4)	27·7 (16·6 to 41·8)	27·7 (16·1 to 45·2)	27·8 (16·8 to 42·1)	23·5 (14·5 to 36·8)
Orofacial clefts	3·6 (1·4 to 7·7)	1 (0·3 to 2·8)	3·4 (1 to 11·5)	1·8 (0·8 to 3·3)	1·4 (0·5 to 2·6)	1·2 (0·4 to 2·5)	1·3 (0·4 to 2·5)	1·4 (0·5 to 2·8)	1·1 (0·3 to 2·3)
Down syndrome	2·5 (1·1 to 5)	1 (0·4 to 2·1)	7·2 (2·9 to 14)	4·2 (2·3 to 8·1)	2·7 (1·7 to 3·9)	2·9 (1·9 to 4·3)	3·6 (2·3 to 5·5)	3·3 (2·1 to 4·9)	2·6 (1·6 to 4)
Turner syndrome	0 (0 to 0)	0 (0 to 0)	0 (0 to 0)	0 (0 to 0)	0 (0 to 0)	0 (0 to 0)	0 (0 to 0)	0 (0 to 0)	0 (0 to 0)
Klinefelter syndrome	0 (0 to 0)	0 (0 to 0)	0 (0 to 0)	0 (0 to 0)	0 (0 to 0)	0 (0 to 0)	0 (0 to 0)	0 (0 to 0)	0 (0 to 0)
Other chromosomal abnormalities	5·9 (2·6 to 10·9)	2·7 (1·2 to 5)	8·7 (5·1 to 13·7)	5·9 (3·7 to 8·9)	3·3 (2 to 4·8)	3 (1·8 to 4·5)	2·9 (1·8 to 4·5)	2·1 (1·4 to 3)	1·4 (0·9 to 2)
Congenital musculoskeletal and limb anomalies	9·5 (4·2 to 18·1)	3·1 (1·7 to 5·8)	18·1 (10·9 to 28·3)	25·7 (15 to 41·8)	21·8 (12 to 34·4)	20·4 (11·2 to 32·2)	17·2 (9·6 to 27·8)	11 (6·2 to 18·2)	8·1 (4·4 to 13)
Urogenital congenital anomalies	43·9 (19·8 to 83·5)	8·6 (3·6 to 20·3)	16·4 (7·3 to 33·2)	5·6 (3 to 9·9)	3·6 (2·3 to 5·4)	2·8 (1·6 to 4·5)	1·3 (0·8 to 2·1)	2 (1·1 to 3·1)	1·4 (0·8 to 2·2)
Digestive congenital anomalies	160 (84·2 to 282)	40·9 (20·5 to 76·4)	84·2 (42 to 151)	13·7 (6·7 to 26·2)	5·8 (3·3 to 9·5)	2·7 (1·6 to 4·6)	2·1 (1·2 to 3·5)	0·8 (0·5 to 1·3)	1·2 (0·7 to 2)
Other congenital birth defects	207 (104 to 364)	35·1 (17·6 to 61·1)	112 (56·4 to 200)	38·9 (20·3 to 66·4)	25·4 (16·4 to 38·4)	20·9 (13 to 31·7)	20·9 (12·2 to 32·5)	27·3 (16·4 to 41·4)	18·6 (11·1 to 29·3)
Urinary diseases and male infertility	2·1 (0·8 to 3·6)	2·3 (1 to 4·6)	15 (7·6 to 25·9)	4·6 (2·6 to 7·3)	3·6 (2·3 to 5·6)	3·9 (2·4 to 6·1)	5·5 (3·7 to 8·1)	24·9 (15 to 40·5)	25·4 (15·3 to 41·8)
Urolithiasis	0 (0 to 0)	0 (0 to 0)	0 (0 to 0)	0·2 (0·1 to 0·3)	0·2 (0·1 to 0·4)	0·2 (0·1 to 0·3)	0·3 (0·1 to 0·5)	0·9 (0·5 to 1·4)	1·2 (0·7 to 2)
Benign prostatic hyperplasia	0 (0 to 0)	0 (0 to 0)	0 (0 to 0)	0 (0 to 0)	0 (0 to 0)	0 (0 to 0)	0 (0 to 0)	0 (0 to 0)	0 (0 to 0)
Male infertility	0 (0 to 0)	0 (0 to 0)	0 (0 to 0)	0 (0 to 0)	0 (0 to 0)	0 (0 to 0)	0 (0 to 0)	9·4 (2·5 to 24·9)	10·1 (2·7 to 24·7)
Other urinary diseases	0·8 (0·3 to 1·5)	1·3 (0·5 to 2·7)	1·5 (0·6 to 3·1)	0·7 (0·4 to 1·3)	1·3 (0·7 to 2·5)	1·5 (0·8 to 2·8)	2·3 (1·3 to 3·7)	6 (2·7 to 9·8)	2·7 (1·1 to 4·7)
Gynecological diseases	0 (0 to 0)	0 (0 to 0)	0 (0 to 0)	0 (0 to 0)	0 (0 to 0)	0 (0 to 0)	0 (0 to 0)	0 (0 to 0)	0 (0 to 0)
Uterine fibroids	0 (0 to 0)	0 (0 to 0)	0 (0 to 0)	0 (0 to 0)	0 (0 to 0)	0 (0 to 0)	0 (0 to 0)	0 (0 to 0)	0 (0 to 0)
Polycystic ovarian syndrome	--	--	--	--	--	--	--	--	--
Female infertility	--	--	--	--	--	--	--	--	--
Endometriosis	--	--	--	--	--	--	--	--	--
Genital prolapse	0 (0 to 0)	0 (0 to 0)	0 (0 to 0)	0 (0 to 0)	0 (0 to 0)	0 (0 to 0)	0 (0 to 0)	0 (0 to 0)	0 (0 to 0)
Premenstrual syndrome	--	--	--	--	--	--	--	--	--
Other gynecological diseases	0 (0 to 0)	0 (0 to 0)	0 (0 to 0)	0 (0 to 0)	0 (0 to 0)	0 (0 to 0)	0 (0 to 0)	0 (0 to 0)	0 (0 to 0)
Hemoglobinopathies and hemolytic anemias	3·2 (1·9 to 5·1)	3·5 (2·1 to 5·3)	29·1 (16·7 to 46·8)	55·5 (36·5 to 81)	94·8 (66 to 129)	67·5 (46·7 to 96)	67·6 (46·3 to 96·4)	91·7 (60·9 to 131)	71·7 (47·8 to 98·7)
Thalassemias	0 (0 to 0)	0 (0 to 0)	0·1 (0 to 0·1)	0·2 (0·1 to 0·5)	0·1 (0·1 to 0·3)	0 (0 to 0·1)	0 (0 to 0·1)	0 (0 to 0)	0 (0 to 0)
Thalassemias trait	0 (0 to 0)	0·1 (0 to 0·1)	0·4 (0·2 to 0·7)	1·3 (0·5 to 2·4)	2·5 (1 to 4·6)	1·1 (0·3 to 2·5)	0·4 (0·1 to 1·1)	0·5 (0·2 to 0·9)	0·4 (0·1 to 0·8)
Sickle cell disorders	3 (1·8 to 4·9)	2 (1 to 3·6)	22·5 (11·2 to 40·5)	33·2 (19·5 to 54·6)	51 (36·6 to 70·1)	45 (29·7 to 66·1)	47·1 (30·2 to 70·7)	58·9 (35·9 to 93·4)	41·5 (24·9 to 61·2)
Sickle cell trait	0·1 (0 to 0·3)	1·1 (0·5 to 1·9)	3·6 (1·7 to 6·1)	11·7 (6·1 to 19·4)	22·8 (9·5 to 39·1)	10·2 (3·3 to 21)	3·9 (1·2 to 8·3)	4 (1·4 to 8·4)	3·2 (1 to 6·5)
G6PD deficiency	0 (0 to 0)	0·1 (0·1 to 0·3)	0·4 (0·2 to 0·8)	2·6 (1·1 to 5·2)	5·1 (2·3 to 9·2)	5·3 (2·4 to 9·8)	8·8 (3·6 to 16·2)	19·2 (7·9 to 35·8)	19·8 (8·8 to 35)
G6PD trait	0 (0 to 0)	0 (0 to 0)	0 (0 to 0)	0 (0 to 0)	0 (0 to 0)	0 (0 to 0)	0 (0 to 0)	0 (0 to 0)	0 (0 to 0)
Other hemoglobinopathies and hemolytic anemias	0 (0 to 0)	0·3 (0·1 to 0·5)	2·2 (0·8 to 4·1)	6·6 (2·9 to 11·8)	13·2 (4·3 to 24·4)	5·8 (1·7 to 13·2)	7·3 (2·5 to 15·4)	9·1 (4·1 to 17·5)	6·8 (2·9 to 13·5)
Endocrine, metabolic, blood, and immune disorders	29·3 (14·2 to 55·3)	18·7 (9·2 to 39)	183 (89·4 to 342)	101 (56·5 to 182)	64·1 (41·6 to 93)	46·1 (31·4 to 66·8)	39·9 (25·6 to 58·6)	59·3 (37·3 to 91·7)	82·7 (51·9 to 125)
Oral disorders	0 (0 to 0)	0 (0 to 0)	0 (0 to 0)	16·7 (9·2 to 27·1)	19·4 (10·3 to 34)	20·9 (11 to 36·3)	32·2 (17·4 to 56·8)	55·7 (29·4 to 104)	68·7 (33·9 to 135)
Caries of deciduous teeth	0 (0 to 0)	0 (0 to 0)	0 (0 to 0)	5·9 (2·2 to 12·1)	5·4 (2·2 to 11·8)	0·3 (0 to 0·8)	0 (0 to 0)	0 (0 to 0)	0 (0 to 0)
Caries of permanent teeth	0 (0 to 0)	0 (0 to 0)	0 (0 to 0)	0 (0 to 0)	4·6 (1·6 to 10·2)	10·4 (4·1 to 22·7)	11·9 (4·7 to 24·9)	14·8 (6·2 to 30·7)	12·1 (5 to 24·9)
Periodontal diseases	0 (0 to 0)	0 (0 to 0)	0 (0 to 0)	0 (0 to 0)	0 (0 to 0)	0 (0 to 0)	4·4 (1·2 to 11·1)	17·9 (5·3 to 44·9)	33 (10·1 to 77·3)
Other oral disorders	0 (0 to 0)	0 (0 to 0)	0 (0 to 0)	10·8 (6·3 to 17)	9·5 (5·5 to 14·8)	10·2 (5·9 to 16)	15·9 (9·6 to 25)	21·3 (12·4 to 33·9)	19·6 (11·4 to 30·2)
Sudden infant death syndrome	--	3·8 (2 to 6·6)	32·2 (15·6 to 58·5)	--	--	--	--	--	--
Injuries	99 (64·3 to 146)	36 (22·9 to 55·5)	343 (193 to 566)	476 (325 to 660)	717 (609 to 835)	1,040 (847 to 1,270)	2,910 (2,270 to 3,660)	6,370 (5,140 to 7,780)	5,860 (4,920 to 6,900)
Transport injuries	4 (2·4 to 6·5)	1·6 (0·9 to 2·6)	19·5 (11·4 to 32)	68·1 (39·2 to 115)	177 (120 to 245)	261 (182 to 358)	698 (460 to 966)	1,510 (1,050 to 2,020)	1,310 (957 to 1,710)
Road injuries	2·8 (1·6 to 4·6)	0·9 (0·5 to 1·6)	13·8 (7·7 to 22·8)	57·6 (32·3 to 97·1)	156 (104 to 220)	226 (156 to 315)	626 (414 to 876)	1,360 (932 to 1,830)	1,180 (841 to 1,550)
Pedestrian road injuries	1·3 (0·8 to 2·2)	0·3 (0·1 to 0·4)	4 (2·2 to 6·8)	24 (13·4 to 39·9)	67·6 (44·3 to 96·2)	71·3 (47·2 to 102)	66·8 (42·7 to 96·3)	118 (80·6 to 160)	125 (90·8 to 165)
Cyclist road injuries	0 (0 to 0)	0 (0 to 0)	0 (0 to 0)	3·5 (1·6 to 7·1)	12 (6·4 to 21·6)	41·1 (22 to 74·1)	80·2 (43·5 to 135)	119 (68·1 to 192)	98 (60·6 to 150)
Motorcyclist road injuries	0·3 (0·2 to 0·5)	0·1 (0 to 0·1)	1·4 (0·8 to 2·3)	3·8 (2·1 to 6)	10·7 (6·8 to 15·2)	16·7 (11·1 to 23·6)	129 (83·6 to 184)	366 (250 to 500)	316 (230 to 412)
Motor vehicle road injuries	1·2 (0·7 to 1·9)	0·6 (0·3 to 1)	8·3 (4·6 to 13·8)	25·8 (13·8 to 44·8)	65·2 (42·6 to 92·7)	95 (62·9 to 136)	346 (221 to 493)	747 (500 to 1,030)	631 (448 to 839)
Other road injuries	0 (0 to 0·1)	0 (0 to 0)	0·1 (0 to 0·3)	0·5 (0·2 to 1)	0·8 (0·4 to 1·4)	1·5 (0·8 to 2·6)	2·9 (1·7 to 4·7)	6·7 (3·8 to 12·5)	5·8 (3·7 to 8·9)
Other transport injuries	1·3 (0·7 to 2·1)	0·6 (0·4 to 1·1)	5·7 (3·1 to 9·6)	10·5 (6 to 19·3)	20·3 (12·7 to 31)	35·4 (21·3 to 53·8)	72·6 (41·8 to 112)	150 (87·1 to 236)	137 (86·3 to 201)
Unintentional injuries	65·8 (41·4 to 101)	31·1 (19 to 49·2)	294 (159 to 506)	360 (239 to 517)	484 (401 to 586)	600 (473 to 762)	884 (655 to 1,190)	1,430 (1,070 to 1,920)	1,300 (1,000 to 1,630)
Falls	2·3 (1·4 to 3·6)	0·9 (0·5 to 1·4)	17·4 (10·3 to 29·2)	29·7 (18·5 to 47·5)	44·2 (33·4 to 58·6)	70·3 (51·7 to 92·4)	126 (88·5 to 174)	214 (156 to 287)	207 (159 to 266)
Drowning	4·4 (2·8 to 6·6)	1·1 (0·7 to 1·7)	23·8 (14·6 to 36)	126 (73·2 to 198)	211 (163 to 268)	247 (178 to 329)	368 (243 to 525)	554 (380 to 781)	427 (298 to 579)
Fire, heat, and hot substances	1·3 (0·8 to 1·9)	1 (0·6 to 1·6)	16·8 (10 to 27)	38·8 (25·1 to 57·9)	45·6 (36·4 to 57·1)	47·1 (36 to 60·8)	53·3 (40·1 to 69·8)	80·3 (61·2 to 104)	79·2 (62·3 to 99·1)
Poisonings	0·2 (0·1 to 0·4)	0·2 (0·1 to 0·3)	2 (1·1 to 3·5)	6·1 (3·4 to 10·1)	3·9 (2·8 to 5·3)	3·9 (2·7 to 5·3)	5 (3·4 to 7)	9·3 (6·5 to 13·1)	11·5 (8 to 15·8)
Poisoning by carbon monoxide	0 (0 to 0·1)	0·1 (0 to 0·1)	0·2 (0·1 to 0·4)	1·6 (0·6 to 3·2)	1·1 (0·5 to 1·8)	1·2 (0·7 to 1·9)	1·9 (1 to 3·3)	4·2 (2·4 to 6·2)	5·3 (2·5 to 8·9)
Poisoning by other means	0·2 (0·1 to 0·3)	0·1 (0·1 to 0·2)	1·9 (1 to 3·2)	4·5 (2·4 to 7·7)	2·8 (2 to 3·8)	2·7 (1·8 to 4·1)	3·1 (2·1 to 4·5)	5·2 (3·5 to 7·3)	6·2 (4·6 to 8·1)
Exposure to mechanical forces	0·5 (0·3 to 0·8)	0·5 (0·3 to 0·8)	3·4 (2·2 to 4·9)	13·1 (9·2 to 18·5)	27·3 (20·9 to 35·9)	39·3 (29·8 to 52·3)	97·9 (67·7 to 140)	188 (129 to 266)	166 (119 to 224)
Unintentional firearm injuries	0 (0 to 0)	0 (0 to 0)	0·2 (0·1 to 0·4)	0·8 (0·5 to 1·2)	1·5 (1 to 2·3)	3·5 (2·4 to 5·3)	23·6 (12·2 to 41·3)	58·3 (30·4 to 101)	45·3 (23·7 to 75·8)
Other exposure to mechanical forces	0·5 (0·3 to 0·8)	0·5 (0·3 to 0·7)	3·1 (2 to 4·6)	12·4 (8·6 to 17·4)	25·8 (19·6 to 34·1)	35·7 (27·1 to 48·4)	74·2 (51·2 to 106)	129 (86·2 to 187)	120 (84·8 to 167)
Adverse effects of medical treatment	15·2 (8·3 to 27·2)	8·4 (4·3 to 14·6)	32·9 (15·5 to 69·1)	16·6 (7·6 to 33·1)	15·1 (9·2 to 23·3)	13·7 (8·1 to 21·6)	14·6 (8·4 to 24·2)	35 (21 to 55·6)	35·9 (22·3 to 59)
Animal contact	1·1 (0·6 to 1·7)	1·4 (0·8 to 2·4)	7·2 (4·1 to 13)	18·4 (9·9 to 32·8)	18·8 (11·6 to 30)	24·7 (15·8 to 36·3)	26·5 (17·2 to 40·8)	33·9 (21·6 to 51)	50·6 (32·9 to 73·6)
Venomous animal contact	1 (0·6 to 1·6)	1·4 (0·8 to 2·3)	6·7 (3·8 to 12·3)	17·6 (9·4 to 31·6)	17·8 (11 to 28·8)	23·2 (14·6 to 34·4)	24·7 (15·8 to 38·3)	31 (19·5 to 46·8)	47·1 (30·3 to 68·8)
Non-venomous animal contact	0·1 (0 to 0·1)	0 (0 to 0·1)	0·4 (0·2 to 1)	0·8 (0·4 to 1·5)	1 (0·6 to 1·7)	1·5 (0·9 to 2·4)	1·8 (1·1 to 3)	3 (1·7 to 5·1)	3·4 (1·9 to 5·9)
Foreign body	37 (19·8 to 63·2)	16·5 (9·1 to 27·4)	178 (77·8 to 343)	75·6 (46·9 to 112)	53·6 (42·9 to 67·3)	65·2 (50·2 to 83·5)	40·4 (29·1 to 54·1)	70·4 (51 to 94·2)	73·3 (55·8 to 94·5)
Pulmonary aspiration and foreign body in airway	36·6 (19·6 to 62·5)	16·3 (8·9 to 27)	175 (74·5 to 337)	68·5 (41·6 to 103)	45·3 (34·7 to 57·6)	55·2 (41 to 72·9)	33·2 (22·6 to 45·9)	58·5 (40·9 to 80·7)	59·7 (42·8 to 78·3)
Foreign body in eyes	0 (0 to 0)	0 (0 to 0)	0·2 (0 to 0·3)	0·8 (0·3 to 1·9)	1·3 (0·4 to 3·1)	1·6 (0·5 to 3·8)	2·2 (0·7 to 5·2)	3 (1 to 6·7)	2·8 (1 to 6)
Foreign body in other body part	0·4 (0·2 to 0·9)	0·3 (0·1 to 0·5)	3 (1·1 to 8·2)	6·3 (3·3 to 12·2)	7·1 (4·6 to 10·6)	8·4 (5 to 14·2)	5 (3·3 to 7·3)	8·8 (5·7 to 14)	10·8 (6·7 to 18)
Environmental heat and cold exposure	0·2 (0·1 to 0·3)	0·1 (0·1 to 0·2)	1·4 (0·9 to 2)	4·4 (3·1 to 5·9)	7·7 (5·6 to 10·4)	10·4 (7·3 to 13·9)	19·4 (14·7 to 25·5)	33·1 (25·5 to 42)	31·2 (23·7 to 40·3)
Exposure to forces of nature	0 (0 to 0)	0 (0 to 0)	0 (0 to 0)	0 (0 to 0)	0 (0 to 0)	0 (0 to 0·1)	0·4 (0·2 to 0·7)	0·6 (0·4 to 0·9)	1 (0·6 to 1·6)
Other unintentional injuries	3·5 (2·3 to 5·2)	0·9 (0·6 to 1·3)	11·3 (7·4 to 16·5)	30·8 (21·7 to 42·3)	56·3 (44·8 to 70·8)	78·3 (58 to 103)	132 (92 to 182)	214 (149 to 303)	213 (152 to 288)
Self-harm and interpersonal violence	29·1 (17·3 to 47·2)	3·4 (2 to 5·7)	29·5 (16·4 to 50·3)	47·7 (27·2 to 78·2)	57·3 (40·5 to 79·2)	180 (130 to 242)	1,330 (971 to 1,820)	3,430 (2,630 to 4,340)	3,250 (2,600 to 4,000)
Self-harm	0 (0 to 0)	0 (0 to 0)	0 (0 to 0)	0 (0 to 0)	0 (0 to 0)	71·3 (43·8 to 106)	651 (431 to 983)	1,680 (1,140 to 2,340)	1,580 (1,120 to 2,130)
Self-harm by firearm	0 (0 to 0)	0 (0 to 0)	0 (0 to 0)	0 (0 to 0)	0 (0 to 0)	0·5 (0·2 to 1)	11·1 (4·7 to 23·1)	26·6 (11·7 to 55·5)	22·2 (11·5 to 40·5)
Self-harm by other specified means	0 (0 to 0)	0 (0 to 0)	0 (0 to 0)	0 (0 to 0)	0 (0 to 0)	70·8 (43·6 to 105)	640 (422 to 972)	1,660 (1,120 to 2,300)	1,560 (1,100 to 2,100)
Interpersonal violence	29·1 (17·3 to 47·2)	3·4 (2 to 5·7)	29·4 (16·3 to 50·2)	47·1 (26·8 to 77·5)	56·9 (40·2 to 78·8)	107 (74·1 to 150)	675 (443 to 964)	1,740 (1,220 to 2,320)	1,660 (1,200 to 2,200)
Physical violence by firearm	1·2 (0·7 to 1·9)	0·2 (0·1 to 0·4)	3 (1·6 to 5·4)	5·5 (3 to 10·8)	12·7 (8·6 to 18·5)	36·7 (24·6 to 53·2)	346 (221 to 510)	1,030 (725 to 1,370)	961 (698 to 1,280)
Physical violence by sharp object	1·4 (0·8 to 2·3)	0·2 (0·1 to 0·3)	1·2 (0·7 to 1·9)	3·4 (2·1 to 5·4)	5·5 (4·1 to 7·6)	14 (9·6 to 19·4)	214 (137 to 311)	495 (338 to 668)	467 (330 to 619)
Sexual violence	0 (0 to 0)	0 (0 to 0)	0 (0 to 0)	0·2 (0·1 to 0·3)	1·3 (0·8 to 2·1)	2·8 (1·6 to 4·4)	6·3 (3·5 to 10·2)	9·2 (5·3 to 14·2)	7·1 (3·9 to 11·6)
Physical violence by other means	26·6 (15·8 to 43)	3 (1·7 to 5·1)	25·2 (13·8 to 43·4)	38·1 (21·5 to 63·2)	37·4 (25·9 to 52·9)	53·7 (36·8 to 75)	109 (72 to 155)	209 (147 to 279)	224 (165 to 294)
Conflict and terrorism	0 (0 to 0)	0 (0 to 0)	0 (0 to 0)	0 (0 to 0)	0 (0 to 0)	1·4 (0·8 to 2·4)	2 (1·1 to 3·3)	2·5 (1·4 to 4·1)	3·5 (2·1 to 5·7)

Congenital birth defects	42 (29·7 to 57·8)	34·6 (24·6 to 47·3)	29·6 (21 to 40·2)	24·5 (17·4 to 33·4)	21·2 (14·7 to 30·3)	16·7 (11·8 to 22·7)	9·9 (7·2 to 12·9)	5·7 (4·1 to 7·6)
Neural tube defects	1·5 (1 to 2·3)	0·7 (0·5 to 1)	0·9 (0·5 to 1·4)	0·5 (0·3 to 0·8)	0·2 (0·2 to 0·4)	0·3 (0·2 to 0·4)	0·2 (0·1 to 0·3)	0·1 (0 to 0·1)
Congenital heart anomalies	17·1 (10·5 to 26·2)	14·9 (9 to 22·6)	11·2 (7 to 17·4)	8·3 (5·3 to 12·3)	6·4 (3·6 to 10·8)	4·2 (2·5 to 6·6)	2·8 (1·8 to 4·1)	1·5 (0·9 to 2·2)
Orofacial clefts	0·8 (0·3 to 1·7)	0·8 (0·2 to 1·5)	0·7 (0·3 to 1·5)	0·7 (0·2 to 1·4)	0·6 (0·2 to 1·3)	0·5 (0·2 to 1·1)	0·4 (0·1 to 0·8)	0·3 (0·1 to 0·6)
Down syndrome	2 (1·1 to 3·4)	2 (1·1 to 3·5)	2·1 (1·1 to 3·6)	1·9 (1 to 3·1)	1·8 (0·8 to 3·2)	2·3 (1·1 to 3·9)	1 (0·5 to 1·7)	0·4 (0·2 to 0·6)
Turner syndrome	0 (0 to 0)	0 (0 to 0)	0 (0 to 0)	0 (0 to 0)	0 (0 to 0)	0 (0 to 0)	0 (0 to 0)	0 (0 to 0)
Klinefelter syndrome	0 (0 to 0)	0 (0 to 0)	0 (0 to 0)	0 (0 to 0)	0 (0 to 0)	0 (0 to 0)	0 (0 to 0)	0 (0 to 0)
Other chromosomal abnormalities	0·6 (0·4 to 0·8)	0·4 (0·3 to 0·6)	0·2 (0·1 to 0·3)	0·2 (0·1 to 0·2)	0·1 (0·1 to 0·1)	0·1 (0 to 0·1)	0 (0 to 0)	0 (0 to 0)
Congenital musculoskeletal and limb anomalies	5·5 (2·9 to 9)	5·3 (3·1 to 8·6)	5 (2·7 to 8·4)	4·6 (2·5 to 7·5)	4·2 (2·3 to 6·8)	3·6 (2 to 6)	2·5 (1·3 to 4)	1·4 (0·7 to 2·3)
Urogenital congenital anomalies	1·4 (0·8 to 2·4)	0·6 (0·4 to 0·9)	0·5 (0·3 to 0·9)	0·8 (0·4 to 1·3)	0·2 (0·1 to 0·4)	0·8 (0·4 to 1·4)	0·4 (0·2 to 0·7)	0·1 (0·1 to 0·2)
Digestive congenital anomalies	0·6 (0·3 to 1)	0·3 (0·2 to 0·5)	0·4 (0·2 to 0·6)	0·5 (0·3 to 0·9)	0·5 (0·3 to 0·8)	0·2 (0·1 to 0·4)	0·3 (0·2 to 0·5)	0·3 (0·2 to 0·5)
Other congenital birth defects	12·4 (7·3 to 19·5)	9·7 (5·9 to 14·7)	8·6 (5·2 to 13·1)	7 (4·2 to 10·7)	7·2 (4·3 to 11·1)	4·8 (3 to 7·3)	2·4 (1·4 to 3·8)	1·6 (0·9 to 2·8)
Urinary diseases and male infertility	28·6 (17·4 to 44·7)	36·9 (22·4 to 57·5)	39·4 (24·4 to 60·2)	40·3 (23·8 to 61·1)	53·3 (33·6 to 79·6)	76·2 (49·9 to 109)	94·4 (61·4 to 131)	93·9 (62·2 to 132)
Urolithiasis	1·8 (1 to 3)	3·5 (1·6 to 5·9)	3·6 (1·9 to 6·1)	6·8 (3·6 to 11·7)	6·7 (3·8 to 10·9)	8·3 (4·6 to 13·4)	6·7 (3·9 to 10·6)	4·4 (2·2 to 7·3)
Benign prostatic hyperplasia	0 (0 to 0)	0 (0 to 0)	0·2 (0 to 0·6)	1·7 (0·6 to 3·6)	6·6 (3·2 to 11·8)	19·8 (9·4 to 34·8)	32·1 (16·6 to 54·8)	38·4 (21·5 to 61·5)
Male infertility	8·5 (2·3 to 22·7)	7·8 (2·2 to 19·9)	7 (2 to 18·3)	1 (0·2 to 2·8)	0 (0 to 0)	0 (0 to 0)	0 (0 to 0)	0 (0 to 0)
Other urinary diseases	3·7 (1·7 to 6·5)	7 (3·4 to 11·8)	6·5 (3·2 to 12·1)	8·2 (3·6 to 14·4)	8·3 (3·8 to 14·9)	8·5 (3·8 to 15·5)	10·7 (5 to 19·1)	9·2 (4·2 to 17)
Gynecological diseases	0 (0 to 0)	0 (0 to 0)	0 (0 to 0)	0 (0 to 0)	0 (0 to 0)	0 (0 to 0)	0 (0 to 0)	0 (0 to 0)
Uterine fibroids	0 (0 to 0)	0 (0 to 0)	0 (0 to 0)	0 (0 to 0)	0 (0 to 0)	0 (0 to 0)	0 (0 to 0)	0 (0 to 0)
Polycystic ovarian syndrome	--	--	--	--	--	--	--	--
Female infertility	--	--	--	--	--	--	--	--
Endometriosis	--	--	--	--	--	--	--	--
Genital prolapse	0 (0 to 0)	0 (0 to 0)	0 (0 to 0)	0 (0 to 0)	0 (0 to 0)	0 (0 to 0)	0 (0 to 0)	0 (0 to 0)
Premenstrual syndrome	--	--	--	--	--	--	--	--
Other gynecological diseases	0 (0 to 0)	0 (0 to 0)	0 (0 to 0)	0 (0 to 0)	0 (0 to 0)	0 (0 to 0)	0 (0 to 0)	0 (0 to 0)
Hemoglobinopathies and hemolytic anemias	56·4 (38 to 80)	45·4 (31·4 to 60·7)	49·2 (33·3 to 68·7)	38·7 (27·1 to 53·4)	34·8 (24·7 to 50·5)	31·3 (21·4 to 44·6)	24·2 (15·9 to 35)	16·6 (11 to 24·4)
Thalassemias	0 (0 to 0)	0 (0 to 0)	0 (0 to 0)	0 (0 to 0)	0 (0 to 0)	0 (0 to 0)	0 (0 to 0)	0 (0 to 0)
Thalassemias trait	0·3 (0·1 to 0·7)	0·2 (0·1 to 0·5)	0·3 (0·1 to 0·6)	0·3 (0·1 to 0·7)	0·4 (0·1 to 0·9)	0·4 (0·1 to 0·9)	0·3 (0·1 to 0·7)	0·2 (0·1 to 0·5)
Sickle cell disorders	29·3 (17·3 to 45)	21·6 (12·4 to 32·3)	19·6 (10·5 to 32·1)	11·5 (6·1 to 19·5)	5·6 (2·6 to 10)	2 (0·9 to 4)	0·4 (0·1 to 0·8)	0 (0 to 0·1)
Sickle cell trait	2·6 (0·8 to 5·6)	1·9 (0·6 to 4·5)	2·4 (0·9 to 4·8)	2·5 (0·9 to 4·9)	3·5 (1·3 to 6·8)	3·7 (1·5 to 7)	2·9 (1·1 to 5·4)	2·2 (0·9 to 4·2)
G6PD deficiency	18 (8·2 to 31·4)	17·2 (8·4 to 28·6)	20·6 (10·1 to 33·2)	17·8 (9·6 to 27·2)	16 (9·6 to 24·5)	14·6 (9 to 21·4)	10 (6·1 to 14·7)	5·1 (3·1 to 7·8)
G6PD trait	0 (0 to 0)	0 (0 to 0)	0 (0 to 0)	0 (0 to 0)	0 (0 to 0)	0 (0 to 0)	0 (0 to 0)	0 (0 to 0)
Other hemoglobinopathies and hemolytic anemias	6·2 (2·9 to 12)	4·5 (2·3 to 8·8)	6·4 (3·5 to 11·5)	6·5 (3·3 to 11·7)	9·2 (4·7 to 16·2)	10·6 (5·8 to 17·3)	10·7 (6·1 to 17·6)	9 (5·5 to 14·3)
Endocrine, metabolic, blood, and immune disorders	89·8 (57·2 to 136)	93·3 (56·6 to 150)	99·7 (59·8 to 152)	91·5 (60 to 141)	97·6 (61·9 to 150)	95·5 (61·8 to 140)	86·2 (57·2 to 131)	65·6 (44·7 to 98·7)
Oral disorders	67·9 (32·6 to 129)	75·9 (36 to 145)	85·8 (40·3 to 163)	87·9 (44·7 to 161)	88·6 (46·3 to 158)	90·3 (50·2 to 153)	87·3 (52 to 143)	79·6 (47·6 to 125)
Caries of deciduous teeth	0 (0 to 0)	0 (0 to 0)	0 (0 to 0)	0 (0 to 0)	0 (0 to 0)	0 (0 to 0)	0 (0 to 0)	0 (0 to 0)
Caries of permanent teeth	8·5 (3·5 to 18)	7·8 (3·2 to 16·2)	7·4 (3 to 14·8)	6·5 (2·7 to 13·8)	5·7 (2·3 to 12·2)	4·5 (1·8 to 10)	3·1 (1·1 to 6·7)	2·1 (0·8 to 4·4)
Periodontal diseases	38 (12·7 to 86)	45 (16 to 103)	52·1 (18·8 to 116)	52·3 (19·7 to 113)	48·1 (19·2 to 106)	39·8 (15·7 to 83·4)	27·7 (11·1 to 56·6)	18·1 (7·1 to 36·1)
Other oral disorders	15·4 (9·2 to 23·4)	14·5 (8·5 to 22·6)	14·6 (8·6 to 22·2)	13·7 (8·3 to 20·9)	12·3 (7·6 to 18·3)	10·5 (6·4 to 16·1)	7·9 (4·8 to 11·9)	5·7 (3·5 to 8·6)
Sudden infant death syndrome	--	--	--	--	--	--	--	--
Injuries	4,400 (3,590 to 5,250)	3,630 (2,860 to 4,470)	3,310 (2,550 to 4,190)	2,730 (2,070 to 3,570)	2,100 (1,600 to 2,700)	1,610 (1,240 to 2,030)	1,090 (862 to 1,360)	709 (572 to 860)
Transport injuries	924 (649 to 1,240)	724 (512 to 990)	673 (471 to 920)	613 (427 to 833)	504 (353 to 703)	402 (283 to 546)	246 (179 to 333)	146 (105 to 194)
Road injuries	805 (564 to 1,100)	637 (445 to 882)	586 (404 to 808)	546 (381 to 748)	451 (315 to 622)	352 (247 to 474)	221 (161 to 300)	133 (94·5 to 176)
Pedestrian road injuries	109 (77·4 to 149)	98·2 (67·8 to 138)	108 (73·3 to 152)	111 (75·3 to 158)	104 (70·8 to 148)	91·5 (62·4 to 128)	60·7 (42·8 to 85·4)	40·7 (28·3 to 56)
Cyclist road injuries	77·8 (48·2 to 120)	65·2 (42·5 to 98·8)	64·9 (43·1 to 102)	58·3 (39·2 to 87·7)	54·8 (36·4 to 81·8)	42·2 (28 to 64·2)	29 (19·2 to 42·6)	19·1 (13·1 to 27·2)
Motorcyclist road injuries	194 (137 to 260)	155 (111 to 213)	127 (90·7 to 173)	107 (77·7 to 145)	77·3 (55·5 to 104)	54·8 (40·6 to 72·9)	28·7 (22·2 to 36·9)	17·3 (13·2 to 22·4)
Motor vehicle road injuries	419 (277 to 581)	314 (212 to 441)	281 (185 to 399)	265 (175 to 373)	211 (140 to 299)	159 (106 to 226)	100 (67·4 to 139)	53·4 (35·8 to 73·3)
Other road injuries	4·7 (3 to 7·3)	4·8 (3·2 to 7·1)	5 (3·5 to 7·4)	5·3 (3·7 to 7·4)	4·4 (3·1 to 6·2)	3·7 (2·6 to 5)	2·7 (1·9 to 3·6)	2 (1·4 to 2·7)
Other transport injuries	119 (73·1 to 181)	86·3 (55 to 130)	87·1 (54·9 to 129)	66·4 (41·8 to 100)	53·2 (34·7 to 80·1)	50·3 (32·5 to 72·9)	25 (16·8 to 35·9)	13·7 (9·2 to 20·1)
Unintentional injuries	1,020 (812 to 1,300)	912 (716 to 1,120)	918 (709 to 1,160)	787 (618 to 1,000)	704 (557 to 889)	590 (469 to 737)	461 (371 to 566)	334 (272 to 406)
Falls	182 (140 to 238)	192 (145 to 247)	209 (159 to 266)	209 (158 to 277)	223 (166 to 288)	201 (151 to 257)	175 (134 to 224)	134 (104 to 170)
Drowning	312 (227 to 416)	245 (176 to 324)	227 (158 to 312)	157 (106 to 221)	119 (82·7 to 164)	79·4 (57·7 to 108)	46·6 (34·3 to 60·6)	24·1 (17·8 to 32)
Fire, heat, and hot substances	72·3 (56·4 to 92·8)	63·6 (49·5 to 81·6)	64·8 (50·9 to 83·9)	58·4 (45 to 74·8)	47·7 (37·1 to 62·5)	39·6 (30·6 to 51·6)	29·9 (22·7 to 39·9)	19·8 (15·1 to 26·3)
Poisonings	10 (6·5 to 14)	7·9 (5·4 to 10·9)	6·1 (4·2 to 8·3)	4·8 (3·3 to 6·7)	3·6 (2·5 to 4·9)	2·7 (2 to 3·6)	1·9 (1·3 to 2·6)	1·2 (0·8 to 1·6)
Poisoning by carbon monoxide	5 (2·6 to 8·6)	4 (2·1 to 6·5)	2·7 (1·5 to 4·2)	1·9 (1 to 3·4)	1 (0·5 to 1·8)	1 (0·5 to 1·6)	0·6 (0·4 to 1)	0·3 (0·2 to 0·5)
Poisoning by other means	4·9 (3·2 to 6·5)	3·9 (2·8 to 5·3)	3·4 (2·3 to 4·8)	2·9 (2 to 3·8)	2·5 (1·8 to 3·3)	1·8 (1·3 to 2·4)	1·3 (0·9 to 1·8)	0·8 (0·6 to 1·2)
Exposure to mechanical forces	134 (98·1 to 180)	113 (85·2 to 150)	114 (85·4 to 147)	96 (71·4 to 127)	83 (62·1 to 112)	69·9 (52·4 to 93·2)	48·8 (35·8 to 66)	32·8 (23·7 to 45·2)
Unintentional firearm injuries	31·1 (17·5 to 51·6)	23 (12·7 to 37·1)	21·1 (12·7 to 33·9)	14·9 (9 to 23·4)	10·5 (6·8 to 16·3)	7·8 (5·3 to 11·8)	4·9 (3·4 to 6·9)	3 (2·2 to 4)
Other exposure to mechanical forces	103 (72·4 to 141)	90·3 (65·1 to 122)	92·6 (68·3 to 123)	81·1 (59·9 to 109)	72·5 (53·7 to 97·7)	62·1 (45·8 to 83·6)	44 (32 to 59·8)	29·8 (21·1 to 41·3)
Adverse effects of medical treatment	25·2 (14·3 to 40·4)	29 (16·9 to 46)	31·1 (18·5 to 48·5)	29·1 (16·5 to 47·6)	36·8 (21·9 to 58·1)	35·9 (20·8 to 56·1)	33·3 (19·6 to 54)	28·2 (16·8 to 43·7)
Animal contact	29 (19·4 to 43)	39·9 (25·2 to 61·5)	41·5 (25·6 to 65·5)	44·8 (28·6 to 70·5)	41·3 (25·3 to 64·5)	37·6 (23·8 to 55·4)	35·6 (22·8 to 53·4)	28·9 (18·6 to 42·8)
Venomous animal contact	26·4 (17·4 to 39·4)	37·8 (23·7 to 58)	38·3 (23·3 to 61·3)	41·8 (26·5 to 65·8)	38·3 (23·1 to 60·3)	35 (22 to 52·1)	33·1 (21·2 to 49·4)	26·5 (16·8 to 39·7)
Non-venomous animal contact	2·6 (1·6 to 4·3)	2 (1·2 to 3·3)	3·1 (1·8 to 5·4)	3 (1·9 to 5·2)	2·9 (1·8 to 4·5)	2·6 (1·6 to 4·3)	2·5 (1·5 to 4)	2·4 (1·5 to 3·8)
Foreign body	57 (43·5 to 74·6)	50·6 (37·9 to 65·9)	53·7 (39·1 to 71·4)	48·2 (35·2 to 64·2)	47·9 (34·7 to 64·3)	40·1 (29·9 to 53·1)	35 (25·6 to 45·7)	27·3 (19·8 to 37·3)
Pulmonary aspiration and foreign body in airway	48·8 (35·4 to 65·2)	43 (30·7 to 57)	43·7 (30·2 to 60·1)	38·6 (26·4 to 54·1)	40·3 (28·2 to 55·3)	32·5 (23·3 to 43·9)	29·1 (20·1 to 39·4)	22·5 (15·7 to 31·1)
Foreign body in eyes	2·3 (0·8 to 4·8)	2·2 (0·8 to 4·6)	2·3 (0·9 to 4·5)	2·1 (1 to 4)	1·7 (0·9 to 3·1)	1·4 (0·8 to 2·2)	1 (0·6 to 1·6)	0·8 (0·5 to 1·2)
Foreign body in other body part	6 (4 to 8·8)	5·4 (3·8 to 7·9)	7·7 (5 to 12)	7·6 (4·9 to 11·5)	5·8 (3·9 to 8·3)	6·2 (4 to 9·4)	4·9 (3·1 to 7·8)	4·1 (2·7 to 6·1)
Environmental heat and cold exposure	26·9 (20·6 to 34·7)	25·4 (19·2 to 32·6)	26·2 (19·9 to 33·4)	25 (18·9 to 32)	23 (17·4 to 29·7)	21·6 (16·5 to 28·3)	16·4 (12·2 to 21·7)	11·9 (8·8 to 15·5)
Exposure to forces of nature	0·4 (0·3 to 0·7)	0·4 (0·2 to 0·7)	0·5 (0·3 to 0·8)	0·4 (0·3 to 0·7)	0·3 (0·2 to 0·5)	0·3 (0·2 to 0·4)	0·2 (0·1 to 0·3)	0·1 (0·1 to 0·2)
Other unintentional injuries	174 (129 to 236)	145 (108 to 191)	145 (107 to 190)	114 (84 to 149)	78·7 (59·5 to 105)	62·2 (47·3 to 80·9)	38·5 (29·4 to 49·1)	25·5 (19·4 to 33·3)
Self-harm and interpersonal violence	2,450 (1,910 to 3,080)	1,990 (1,440 to 2,630)	1,720 (1,240 to 2,330)	1,330 (912 to 1,880)	892 (613 to 1,250)	614 (436 to 847)	385 (270 to 535)	229 (164 to 312)
Self-harm	1,310 (931 to 1,810)	1,140 (752 to 1,580)	1,030 (693 to 1,460)	844 (549 to 1,250)	577 (377 to 848)	416 (276 to 603)	282 (186 to 409)	173 (117 to 246)
Self-harm by firearm	17·7 (9·4 to 34·7)	14·5 (7·8 to 26·8)	15·2 (8·3 to 26·4)	11·6 (6·1 to 20·7)	10·3 (6 to 17·3)	6·8 (3·7 to 11·7)	5 (2·9 to 8)	2·8 (1·6 to 4·3)
Self-harm by other specified means	1,290 (919 to 1,780)	1,130 (743 to 1,560)	1,020 (682 to 1,440)	832 (542 to 1,230)	567 (370 to 831)	409 (271 to 593)	277 (182 to 401)	170 (115 to 243)
Interpersonal violence	1,130 (813 to 1,490)	840 (589 to 1,140)	680 (475 to 951)	480 (319 to 693)	312 (204 to 441)	195 (131 to 286)	101 (68·9 to 143)	54·4 (38·2 to 73·8)
Physical violence by firearm	639 (459 to 835)	427 (296 to 589)	345 (238 to 483)	233 (152 to 340)	133 (84·4 to 192)	75 (49·2 to 112)	33·8 (22 to 49·3)	15·6 (10·3 to 22)
Physical violence by sharp object	328 (229 to 437)	247 (172 to 340)	186 (127 to 268)	119 (79·7 to 175)	74·7 (48·6 to 107)	40·2 (27·9 to 59·1)	20·5 (14·4 to 28·1)	9·9 (7·3 to 13·4)
Sexual violence	4·9 (2·7 to 7·9)	4·3 (2·4 to 6·9)	3·6 (2·1 to 6)	2·8 (1·6 to 4·8)	2·1 (1·2 to 3·4)	1·5 (0·9 to 2·4)	1·1 (0·7 to 1·7)	0·8 (0·5 to 1·2)
Physical violence by other means	159 (116 to 206)	161 (113 to 222)	145 (104 to 202)	124 (84·2 to 175)	102 (68 to 145)	78·1 (53 to 113)	45·5 (31·6 to 64·1)	28·1 (19·8 to 38·4)
Conflict and terrorism	4·4 (2·8 to 6·8)	5·7 (3·7 to 8·4)	3·1 (2 to 4·8)	4·8 (3·1 to 7·1)	3 (2 to 4·3)	2·9 (1·9 to 4·2)	2·4 (1·6 to 3·4)	1·7 (1·1 to 2·4)

Congenital birth defects	1·4 (0·8 to 2·2)	0·7 (0·4 to 1·2)	0·4 (0·2 to 0·6)	0·1 (0·1 to 0·2)	0 (0 to 0·1)	0 (0 to 0)	2,310
Neural tube defects	0 (0 to 0)	0 (0 to 0)	0 (0 to 0)	0 (0 to 0)	0 (0 to 0)	0 (0 to 0)	101
Congenital heart anomalies	0·1 (0 to 0·2)	0·1 (0 to 0·2)	0 (0 to 0·1)	0 (0 to 0)	0 (0 to 0)	0 (0 to 0)	972
Orofacial clefts	0·2 (0 to 0·3)	0·1 (0 to 0·2)	0·1 (0 to 0·1)	0 (0 to 0)	0 (0 to 0)	0 (0 to 0)	21·4
Down syndrome	0 (0 to 0)	0 (0 to 0)	0 (0 to 0)	0 (0 to 0)	0 (0 to 0)	0 (0 to 0)	43·5
Turner syndrome	0 (0 to 0)	0 (0 to 0)	0 (0 to 0)	0 (0 to 0)	0 (0 to 0)	0 (0 to 0)	0
Klinefelter syndrome	0 (0 to 0)	0 (0 to 0)	0 (0 to 0)	0 (0 to 0)	0 (0 to 0)	0 (0 to 0)	0·1
Other chromosomal abnormalities	0 (0 to 0)	0 (0 to 0)	0 (0 to 0)	0 (0 to 0)	0 (0 to 0)	0 (0 to 0)	37·4
Congenital musculoskeletal and limb anomalies	0·6 (0·3 to 1)	0·3 (0·1 to 0·5)	0·1 (0·1 to 0·2)	0 (0 to 0·1)	0 (0 to 0)	0 (0 to 0)	168
Urogenital congenital anomalies	0 (0 to 0)	0 (0 to 0)	0 (0 to 0)	0 (0 to 0)	0 (0 to 0)	0 (0 to 0)	90·4
Digestive congenital anomalies	0 (0 to 0)	0 (0 to 0)	0 (0 to 0)	0 (0 to 0)	0 (0 to 0)	0 (0 to 0)	314
Other congenital birth defects	0·5 (0·2 to 1·1)	0·3 (0·1 to 0·7)	0·2 (0·1 to 0·4)	0·1 (0 to 0·2)	0 (0 to 0·1)	0 (0 to 0)	560
Urinary diseases and male infertility	73·8 (49·1 to 103)	53·5 (35·3 to 77·3)	31·4 (20·9 to 44·5)	15·6 (10 to 22·7)	6·5 (4 to 10)	1·8 (1·1 to 2·7)	733
Urolithiasis	2·7 (1·4 to 4·8)	1·6 (0·7 to 3)	0·5 (0·2 to 0·9)	0·2 (0·1 to 0·4)	0·1 (0 to 0·2)	0 (0 to 0)	49·9
Benign prostatic hyperplasia	28·9 (16 to 46·3)	18·2 (10·1 to 29·4)	8·6 (4·9 to 13·8)	2·8 (1·6 to 4·6)	0·8 (0·4 to 1·3)	0·1 (0·1 to 0·2)	158
Male infertility	0 (0 to 0)	0 (0 to 0)	0 (0 to 0)	0 (0 to 0)	0 (0 to 0)	0 (0 to 0)	43·8
Other urinary diseases	5·3 (2·5 to 9·3)	4·5 (1·7 to 8·9)	2·8 (1·2 to 5·3)	1·2 (0·5 to 2·2)	0·7 (0·2 to 1·3)	0·2 (0·1 to 0·4)	94·9
Gynecological diseases	0 (0 to 0)	0 (0 to 0)	0 (0 to 0)	0 (0 to 0)	0 (0 to 0)	0 (0 to 0)	0
Uterine fibroids	0 (0 to 0)	0 (0 to 0)	0 (0 to 0)	0 (0 to 0)	0 (0 to 0)	0 (0 to 0)	0
Polycystic ovarian syndrome	--	--	--	--	--	--	NA
Female infertility	--	--	--	--	--	--	NA
Endometriosis	--	--	--	--	--	--	NA
Genital prolapse	0 (0 to 0)	0 (0 to 0)	0 (0 to 0)	0 (0 to 0)	0 (0 to 0)	0 (0 to 0)	0
Premenstrual syndrome	--	--	--	--	--	--	NA
Other gynecological diseases	0 (0 to 0)	0 (0 to 0)	0 (0 to 0)	0 (0 to 0)	0 (0 to 0)	0 (0 to 0)	0
Hemoglobinopathies and hemolytic anemias	10·5 (7 to 15·2)	6 (4·2 to 8·7)	3·4 (2·3 to 4·9)	2·6 (1·8 to 3·5)	1·2 (0·8 to 1·7)	0·4 (0·2 to 0·6)	805
Thalassemias	0 (0 to 0)	0 (0 to 0)	0 (0 to 0)	0 (0 to 0)	0 (0 to 0)	0 (0 to 0)	0·5
Thalassemias trait	0·1 (0 to 0·3)	0·1 (0 to 0·2)	0 (0 to 0·1)	0 (0 to 0·1)	0 (0 to 0)	0 (0 to 0)	9·4
Sickle cell disorders	0 (0 to 0)	0 (0 to 0)	0 (0 to 0)	0 (0 to 0)	0 (0 to 0)	0 (0 to 0)	394
Sickle cell trait	1·3 (0·5 to 2·4)	0·7 (0·3 to 1·4)	0·4 (0·1 to 0·8)	0·2 (0·1 to 0·4)	0·1 (0 to 0·1)	0 (0 to 0)	85
G6PD deficiency	2·5 (1·4 to 3·8)	1 (0·5 to 1·5)	0·3 (0·2 to 0·5)	0·1 (0·1 to 0·2)	0 (0 to 0·1)	0 (0 to 0)	185
G6PD trait	0 (0 to 0)	0 (0 to 0)	0 (0 to 0)	0 (0 to 0)	0 (0 to 0)	0 (0 to 0)	0
Other hemoglobinopathies and hemolytic anemias	6·6 (4·1 to 9·8)	4·2 (2·9 to 6·1)	2·6 (1·8 to 3·7)	2·2 (1·6 to 3)	1·1 (0·8 to 1·5)	0·4 (0·2 to 0·6)	132
Endocrine, metabolic, blood, and immune disorders	44·4 (30·2 to 69·9)	29·7 (19·7 to 47·9)	18·6 (12 to 31·3)	9·8 (6·3 to 18)	4·2 (2·5 to 8)	1 (0·6 to 1·8)	1,450
Oral disorders	53·2 (32·9 to 82·6)	35·8 (22·5 to 54)	20·1 (12·5 to 30·1)	8·4 (5·2 to 12·6)	3·3 (2 to 4·8)	0·8 (0·5 to 1·2)	999
Caries of deciduous teeth	0 (0 to 0)	0 (0 to 0)	0 (0 to 0)	0 (0 to 0)	0 (0 to 0)	0 (0 to 0)	11·5
Caries of permanent teeth	1·1 (0·4 to 2·3)	0·7 (0·3 to 1·3)	0·3 (0·1 to 0·7)	0·1 (0 to 0·3)	0 (0 to 0·1)	0 (0 to 0)	102
Periodontal diseases	9·4 (3·8 to 19·3)	5·5 (2·2 to 10·9)	2·9 (1·1 to 5·8)	1·2 (0·4 to 2·4)	0·4 (0·2 to 0·9)	0·1 (0 to 0·2)	396
Other oral disorders	3 (1·8 to 4·5)	1·5 (0·9 to 2·3)	0·6 (0·4 to 1)	0·2 (0·1 to 0·3)	0·1 (0 to 0·1)	0 (0 to 0)	187
Sudden infant death syndrome	--	--	--	--	--	--	NA
Injuries	420 (343 to 513)	257 (209 to 311)	147 (120 to 176)	72·9 (60·3 to 88)	35·3 (28·8 to 42)	11 (8·9 to 13·1)	38,400
Transport injuries	76·2 (56·3 to 97·9)	42·1 (31·9 to 53·7)	19·6 (15·3 to 24)	7·8 (6·1 to 9·7)	2·7 (2·1 to 3·4)	0·7 (0·6 to 0·9)	8,430
Road injuries	66 (48·6 to 85·4)	37·8 (28·6 to 48·4)	17·9 (14 to 22)	7·2 (5·7 to 9·1)	2·5 (2 to 3·1)	0·6 (0·5 to 0·8)	7,480
Pedestrian road injuries	22·9 (16 to 31·1)	14·6 (10·5 to 19·6)	7·3 (5·6 to 9·5)	3 (2·3 to 3·9)	1 (0·7 to 1·2)	0·2 (0·2 to 0·3)	1,250
Cyclist road injuries	9 (6·4 to 12·9)	4·8 (3·5 to 6·6)	2·3 (1·7 to 3·1)	0·9 (0·6 to 1·2)	0·3 (0·2 to 0·4)	0·1 (0·1 to 0·1)	783
Motorcyclist road injuries	7·7 (5·5 to 10·1)	4·5 (3·2 to 5·9)	2·4 (1·7 to 3·2)	1 (0·7 to 1·4)	0·4 (0·3 to 0·6)	0·1 (0·1 to 0·2)	1,620
Motor vehicle road injuries	25·3 (17·6 to 33·6)	13·3 (9·6 to 17·7)	5·5 (4·2 to 6·9)	2·1 (1·6 to 2·7)	0·7 (0·6 to 0·9)	0·2 (0·2 to 0·3)	3,770
Other road injuries	1·1 (0·8 to 1·6)	0·7 (0·5 to 1)	0·4 (0·3 to 0·5)	0·1 (0·1 to 0·2)	0·1 (0 to 0·1)	0 (0 to 0)	53·4
Other transport injuries	10·2 (7 to 14·5)	4·2 (3 to 5·8)	1·7 (1·3 to 2·3)	0·6 (0·5 to 0·8)	0·2 (0·2 to 0·3)	0·1 (0·1 to 0·1)	951
Unintentional injuries	220 (179 to 269)	151 (122 to 184)	96·4 (78·9 to 116)	53·2 (43·5 to 65·4)	28·9 (23 to 35·1)	9·5 (7·6 to 11·3)	11,700
Falls	102 (79·4 to 133)	74·5 (57·2 to 96·5)	54·1 (41 to 71·4)	34·5 (26·6 to 45·4)	20·2 (15 to 25·6)	6·7 (4·9 to 8·5)	2,530
Drowning	10·2 (7·7 to 13·4)	5·1 (3·8 to 6·7)	1·7 (1·3 to 2·2)	0·7 (0·6 to 0·9)	0·3 (0·2 to 0·4)	0·1 (0·1 to 0·1)	3,190
Fire, heat, and hot substances	12·6 (9·6 to 16·6)	8·2 (6·3 to 10·9)	4·9 (3·9 to 6·4)	2·5 (1·9 to 3·1)	1·1 (0·9 to 1·4)	0·3 (0·3 to 0·5)	789
Poisonings	0·6 (0·4 to 0·8)	0·4 (0·3 to 0·5)	0·2 (0·1 to 0·3)	0·1 (0·1 to 0·1)	0 (0 to 0)	0 (0 to 0)	81·6
Poisoning by carbon monoxide	0·2 (0·1 to 0·3)	0·1 (0·1 to 0·2)	0·1 (0 to 0·1)	0 (0 to 0)	0 (0 to 0)	0 (0 to 0)	32·4
Poisoning by other means	0·4 (0·3 to 0·6)	0·3 (0·2 to 0·4)	0·1 (0·1 to 0·2)	0·1 (0 to 0·1)	0 (0 to 0)	0 (0 to 0)	49·2
Exposure to mechanical forces	18·3 (13 to 25·4)	11 (7·8 to 15·3)	6 (4·2 to 8·3)	2·6 (1·8 to 3·6)	1·1 (0·7 to 1·5)	0·3 (0·2 to 0·4)	1,270
Unintentional firearm injuries	1·5 (1·1 to 2)	0·8 (0·6 to 1·1)	0·5 (0·3 to 0·6)	0·2 (0·2 to 0·3)	0·1 (0·1 to 0·1)	0 (0 to 0)	253
Other exposure to mechanical forces	16·9 (11·9 to 23·6)	10·2 (7·1 to 14·2)	5·6 (3·8 to 7·8)	2·4 (1·7 to 3·4)	1 (0·7 to 1·4)	0·3 (0·2 to 0·4)	1,010
Adverse effects of medical treatment	18·8 (11·6 to 28·9)	14·5 (8·8 to 22·8)	8·9 (5·9 to 13·8)	4·3 (2·8 to 7·1)	2 (1·2 to 3·1)	0·5 (0·3 to 0·8)	485
Animal contact	17·6 (12 to 25·4)	11·4 (7·7 to 17)	4·8 (3·3 to 6·8)	1·8 (1·3 to 2·5)	1 (0·7 to 1·3)	0·3 (0·2 to 0·4)	518
Venomous animal contact	15·9 (10·8 to 22·9)	10·6 (7·1 to 15·9)	4·4 (3 to 6·3)	1·6 (1·1 to 2·3)	0·9 (0·6 to 1·2)	0·3 (0·2 to 0·4)	482
Non-venomous animal contact	1·7 (1·1 to 2·8)	0·8 (0·5 to 1·2)	0·4 (0·3 to 0·6)	0·1 (0·1 to 0·2)	0·1 (0 to 0·1)	0 (0 to 0)	36·4
Foreign body	17·6 (13·4 to 23)	12 (9·2 to 15·5)	7·5 (6 to 9·5)	2·8 (2·1 to 3·7)	1·4 (1·1 to 1·8)	0·5 (0·4 to 0·7)	1,010
Pulmonary aspiration and foreign body in airway	14·2 (10·5 to 19)	9·6 (7 to 12·7)	5·9 (4·4 to 7·6)	2·1 (1·5 to 2·8)	1 (0·7 to 1·3)	0·4 (0·3 to 0·6)	880
Foreign body in eyes	0·5 (0·3 to 0·7)	0·3 (0·2 to 0·4)	0·2 (0·1 to 0·2)	0·1 (0 to 0·1)	0 (0 to 0)	0 (0 to 0)	26·7
Foreign body in other body part	2·9 (1·8 to 4·5)	2·1 (1·4 to 3·4)	1·5 (1 to 2·2)	0·7 (0·4 to 1)	0·4 (0·2 to 0·6)	0·1 (0·1 to 0·2)	106
Environmental heat and cold exposure	8·7 (6·5 to 10·9)	6 (4·5 to 8)	4·1 (3·1 to 5·5)	2·1 (1·6 to 2·7)	1·2 (0·9 to 1·5)	0·4 (0·3 to 0·6)	307
Exposure to forces of nature	0·1 (0 to 0·1)	0 (0 to 0·1)	0 (0 to 0)	0 (0 to 0)	0 (0 to 0)	0 (0 to 0)	4·9
Other unintentional injuries	13·3 (10·1 to 17·5)	8 (6 to 10·5)	4 (3 to 5·4)	1·7 (1·3 to 2·3)	0·7 (0·5 to 1)	0·2 (0·2 to 0·3)	1,550
Self-harm and interpersonal violence	124 (88·8 to 173)	63·6 (46·4 to 87·8)	31·1 (22·6 to 42·3)	12 (8·9 to 15·4)	3·6 (2·7 to 5)	0·8 (0·6 to 1·1)	18,200
Self-harm	99·7 (66·4 to 144)	52·2 (36·1 to 75·4)	27·3 (19·1 to 37·9)	10·4 (7·5 to 13·9)	3·1 (2·2 to 4·3)	0·6 (0·4 to 0·9)	9,950
Self-harm by firearm	1·5 (1 to 2·4)	0·8 (0·4 to 1·3)	0·4 (0·2 to 0·6)	0·1 (0·1 to 0·2)	0 (0 to 0)	0 (0 to 0)	147
Self-harm by other specified means	98·2 (65·3 to 142)	51·5 (35·6 to 74)	26·9 (18·8 to 37·5)	10·3 (7·5 to 13·7)	3·1 (2·2 to 4·3)	0·6 (0·4 to 0·9)	9,810
Interpersonal violence	23·8 (17·3 to 32)	11 (7·8 to 14·9)	3·7 (2·9 to 4·6)	1·4 (1·1 to 1·8)	0·5 (0·4 to 0·6)	0·2 (0·2 to 0·3)	8,180
Physical violence by firearm	5·6 (3·8 to 7·9)	1·7 (1·1 to 2·4)	0·5 (0·3 to 0·6)	0·2 (0·1 to 0·2)	0·1 (0 to 0·1)	0 (0 to 0)	4,300
Physical violence by sharp object	5·1 (3·7 to 6·8)	2·4 (1·7 to 3·1)	1 (0·7 to 1·2)	0·4 (0·3 to 0·5)	0·2 (0·1 to 0·2)	0·1 (0 to 0·1)	2,240
Sexual violence	0·4 (0·3 to 0·6)	0·3 (0·2 to 0·4)	0·1 (0·1 to 0·2)	0 (0 to 0·1)	0 (0 to 0)	0 (0 to 0)	48·8
Physical violence by other means	12·6 (9·1 to 17)	6·8 (4·8 to 9·3)	2·2 (1·7 to 2·7)	0·8 (0·6 to 1·1)	0·3 (0·2 to 0·3)	0·1 (0·1 to 0·2)	1,590
Conflict and terrorism	0·7 (0·5 to 1)	0·3 (0·2 to 0·4)	0 (0 to 0·1)	0·1 (0·1 to 0·2)	0 (0 to 0·1)	0 (0 to 0)	38·4

SR Table 2a: Haiti DALYS by cause and age group for males with 95% uncertainty intervals, 2019

Cause	Early Neonatal	Late Neonatal	Post Neonatal	1 to 4	5 to 9	10 to 14	15 to 19	20 to 24	25 to 29	30 to 34	35 to 39	40 to 44	45 to 49	50 to 54	55 to 59	60 to 64	65 to 69	70 to 74	75 to 79	80 to 84	85 to 89	90 to 94	95 plus	Total
All causes	342,000 (266,000 to 429,000)	119,000 (92,700 to 149,000)	364,000 (263,000 to 497,000)	327,000 (274,000 to 388,000)	124,000 (102,000 to 144,000)	91,300 (75,900 to 107,000)	109,000 (91,000 to 129,000)	120,000 (99,100 to 141,000)	123,000 (105,000 to 144,000)	131,000 (111,000 to 153,000)	134,000 (112,000 to 161,000)	137,000 (112,000 to 166,000)	130,000 (103,000 to 165,000)	129,000 (102,000 to 163,000)	124,000 (97,500 to 161,000)	122,000 (97,600 to 155,000)	109,000 (88,200 to 137,000)	82,200 (68,800 to 99,000)	58,100 (49,800 to 68,800)	37,100 (32,400 to 42,100)	17,700 (15,300 to 20,400)	5,780 (5,110 to 6,540)	1,200 (1,090 to 1,310)	2,940,000
Communicable, maternal, neonatal, and nutritional diseases	281,000 (209,000 to 360,000)	88,800 (65,900 to 115,000)	255,000 (172,000 to 365,000)	157,000 (113,000 to 222,000)	37,700 (28,400 to 49,000)	19,200 (14,100 to 26,300)	18,000 (12,700 to 24,900)	14,300 (10,100 to 21,100)	16,700 (11,900 to 24,000)	22,600 (16,000 to 32,500)	28,100 (19,400 to 40,900)	29,400 (21,700 to 41,200)	26,600 (20,400 to 36,000)	21,200 (16,700 to 27,600)	15,200 (11,600 to 20,100)	11,700 (8,590 to 15,800)	9,300 (6,500 to 12,500)	7,120 (4,710 to 9,790)	5,200 (3,350 to 7,320)	3,630 (2,260 to 5,140)	1,990 (1,190 to 2,840)	699 (412 to 996)	158 (101 to 217)	1,070,000
HIV/AIDS and sexually transmitted infections	20,300 (6,830 to 43,400)	6,840 (2,390 to 14,500)	11,300 (5,330 to 21,400)	7,800 (5,220 to 11,400)	4,370 (2,800 to 6,370)	3,910 (2,570 to 5,420)	6,620 (3,510 to 10,300)	4,000 (2,620 to 5,880)	6,350 (3,460 to 11,500)	11,400 (6,220 to 20,300)	17,300 (9,970 to 29,400)	18,700 (12,100 to 29,000)	17,100 (12,200 to 25,300)	12,100 (9,060 to 17,000)	6,870 (5,040 to 9,830)	3,430 (2,390 to 5,050)	1,650 (1,070 to 2,500)	689 (409 to 1,110)	316 (177 to 529)	73·3 (41·5 to 126)	27·8 (15·9 to 46·8)	7 (4 to 11·7)	1·4 (0·8 to 2·3)	161,000
HIV/AIDS	0·3 (0·2 to 0·6)	1·1 (0·6 to 1·8)	2,950 (2,040 to 4,280)	7,510 (4,940 to 11,100)	4,240 (2,660 to 6,120)	3,860 (2,550 to 5,360)	6,540 (3,440 to 10,300)	3,910 (2,530 to 5,760)	6,250 (3,370 to 11,400)	11,300 (6,160 to 20,300)	17,200 (9,820 to 29,400)	18,700 (12,000 to 29,000)	17,100 (12,100 to 25,300)	12,100 (8,960 to 16,900)	6,800 (4,990 to 9,760)	3,380 (2,360 to 4,980)	1,590 (1,020 to 2,470)	662 (391 to 1,080)	303 (168 to 509)	64·1 (33·8 to 113)	23·8 (12·8 to 41·2)	5·9 (3·2 to 9·9)	1·1 (0·6 to 1·9)	124,000
HIV/AIDS - Drug-susceptible Tuberculosis	0 (0 to 0)	0·1 (0 to 0·1)	216 (130 to 344)	600 (363 to 954)	434 (266 to 681)	335 (207 to 524)	475 (290 to 748)	259 (161 to 401)	404 (249 to 638)	745 (462 to 1,160)	1,140 (725 to 1,780)	1,370 (842 to 2,130)	1,260 (784 to 1,980)	937 (577 to 1,460)	533 (329 to 832)	277 (172 to 436)	127 (79·8 to 197)	51·3 (32·3 to 79·8)	21·8 (13·7 to 34·3)	4·5 (3 to 6·8)	1·5 (1 to 2·2)	0·4 (0·2 to 0·5)	0·1 (0 to 0·1)	9,190
HIV/AIDS - Multidrug-resistant Tuberculosis without exte	0 (0 to 0)	0 (0 to 0)	2·5 (0·2 to 9·9)	6·8 (0·5 to 27·5)	4·8 (0·4 to 19·4)	3·7 (0·3 to 14·9)	5·3 (0·4 to 21·5)	2·8 (0·2 to 11·4)	4·3 (0·3 to 17·5)	8 (0·6 to 32·3)	12·2 (0·9 to 49·3)	14·7 (1·1 to 59·5)	13·5 (1 to 54·8)	10·2 (0·8 to 41·2)	5·8 (0·4 to 23·5)	3 (0·2 to 12·2)	1·4 (0·1 to 5·6)	0·6 (0 to 2·2)	0·2 (0 to 0·9)	0 (0 to 0·2)	0 (0 to 0·1)	0 (0 to 0)	0 (0 to 0)	100
HIV/AIDS - Extensively drug-resistant Tuberculosis	0 (0 to 0)	0 (0 to 0)	0·4 (0 to 1·6)	1·1 (0·1 to 4·5)	0·8 (0·1 to 3·2)	0·6 (0 to 2·4)	0·9 (0·1 to 3·5)	0·5 (0 to 1·9)	0·7 (0·1 to 2·8)	1·3 (0·1 to 5·2)	2 (0·1 to 8)	2·4 (0·2 to 9·7)	2·2 (0·2 to 8·9)	1·7 (0·1 to 6·7)	1 (0·1 to 3·8)	0·5 (0 to 2)	0·2 (0 to 0·9)	0·1 (0 to 0·4)	0 (0 to 0·2)	0 (0 to 0)	0 (0 to 0)	0 (0 to 0)	0 (0 to 0)	16·4
HIV/AIDS resulting in other diseases	0·3 (0·2 to 0·6)	1 (0·5 to 1·7)	2,730 (1,810 to 4,060)	6,900 (4,290 to 10,500)	3,800 (2,220 to 5,720)	3,520 (2,190 to 5,040)	6,060 (2,950 to 9,740)	3,650 (2,270 to 5,510)	5,840 (2,910 to 10,900)	10,600 (5,410 to 19,400)	16,000 (8,600 to 28,000)	17,300 (10,700 to 28,000)	15,800 (10,800 to 24,000)	11,100 (8,080 to 16,000)	6,260 (4,450 to 9,240)	3,100 (2,070 to 4,750)	1,460 (897 to 2,350)	610 (334 to 1,040)	281 (146 to 491)	59·5 (29·2 to 109)	22·3 (11·4 to 39·9)	5·5 (2·8 to 9·6)	1 (0·5 to 1·8)	115,000
Sexually transmitted infections excluding HIV	20,300 (6,830 to 43,400)	6,840 (2,390 to 14,500)	8,380 (2,580 to 18,400)	283 (93·9 to 603)	128 (45 to 265)	43·2 (14·6 to 105)	79·1 (33·2 to 151)	94·4 (46 to 175)	103 (51·5 to 185)	114 (57·4 to 207)	121 (54·9 to 288)	78·5 (40 to 148)	81·2 (35·5 to 181)	79·2 (27·1 to 215)	68·6 (23 to 168)	52·6 (17·3 to 138)	53·5 (14·6 to 156)	27·5 (8 to 72·5)	12·8 (3·9 to 35·9)	9·2 (2·9 to 27·8)	4 (1·1 to 12·7)	1·1 (0·3 to 3·5)	0·3 (0·1 to 0·8)	37,000
Syphilis	20,300 (6,830 to 43,400)	6,840 (2,390 to 14,500)	8,380 (2,580 to 18,400)	283 (93·9 to 603)	128 (45 to 265)	17·7 (3·8 to 49·9)	18·8 (3·8 to 55·3)	14·5 (3·3 to 48)	23·1 (5·8 to 65·4)	26·5 (7·3 to 70·1)	53·6 (9·7 to 186)	25·3 (7·5 to 66·4)	33·6 (7·7 to 102)	42 (8·1 to 143)	42·7 (8·6 to 119)	34·7 (6·8 to 105)	36·6 (7·2 to 118)	18·6 (4 to 54·1)	6·3 (1·6 to 19·3)	3·8 (1 to 11·9)	1·2 (0·3 to 4)	0·3 (0·1 to 0·8)	0·1 (0 to 0·2)	36,300

Other cardiovascular and circulatory diseases	475 (150 to 1,190)	332 (105 to 798)	1,770 (411 to 4,490)	1,810 (508 to 4,040)	630 (292 to 1,140)	426 (191 to 791)	327 (165 to 566)	368 (167 to 775)	440 (208 to 829)	534 (255 to 969)	698 (353 to 1,310)	827 (462 to 1,490)	801 (409 to 1,610)	869 (459 to 1,730)	902 (447 to 1,920)	943 (528 to 1,890)	854 (471 to 1,740)	641 (347 to 1,310)	519 (292 to 1,060)	362 (203 to 760)	186 (100 to 415)	58·1 (30·9 to 129)	11·4 (6·1 to 25·2)	14,800
Chronic respiratory diseases	0 (0 to 0)	0 (0 to 0)	0·7 (0·4 to 0·9)	23,200 (7,970 to 38,700)	6,960 (4,430 to 9,950)	3,900 (2,630 to 5,620)	2,400 (1,510 to 3,500)	1,860 (1,140 to 2,810)	2,010 (1,230 to 3,110)	2,190 (1,330 to 3,190)	2,310 (1,320 to 3,460)	2,600 (1,520 to 3,960)	2,810 (1,550 to 4,410)	3,490 (1,920 to 5,360)	4,250 (2,280 to 6,900)	5,460 (2,800 to 8,560)	5,710 (3,040 to 8,910)	5,040 (2,680 to 7,790)	3,900 (2,080 to 5,990)	2,540 (1,350 to 3,870)	1,190 (643 to 1,780)	386 (214 to 566)	77·7 (43·5 to 112)	82,300
Chronic obstructive pulmonary disease	0 (0 to 0)	0 (0 to 0)	0·7 (0·4 to 0·9)	1,870 (213 to 5,430)	227 (98·2 to 470)	146 (70·1 to 264)	244 (110 to 484)	324 (160 to 676)	417 (216 to 814)	508 (277 to 960)	668 (333 to 1,270)	955 (479 to 1,700)	1,300 (658 to 2,240)	1,910 (986 to 3,120)	2,790 (1,420 to 4,620)	4,000 (2,040 to 6,350)	4,420 (2,340 to 6,980)	4,030 (2,140 to 6,130)	3,230 (1,710 to 4,950)	2,110 (1,120 to 3,170)	1,020 (552 to 1,530)	329 (179 to 488)	66·6 (37·4 to 94·8)	30,600
Pneumoconiosis	0 (0 to 0)	0 (0 to 0)	0 (0 to 0)	0 (0 to 0)	0 (0 to 0)	0 (0 to 0)	33·9 (6·4 to 85·4)	5 (0·8 to 15·6)	18·2 (3 to 52·4)	3·9 (0·8 to 12·1)	9·3 (2·1 to 25)	6·6 (1·6 to 17·4)	17·7 (3·2 to 54·3)	18·7 (4·1 to 49·2)	18·3 (3·5 to 46·2)	24·6 (5 to 65·3)	38·8 (7·7 to 108)	21·5 (4·7 to 54·4)	14·6 (3·4 to 36·4)	11·8 (2·4 to 29·2)	7·6 (1·6 to 21·4)	2·3 (0·5 to 5·7)	0·2 (0 to 0·4)	253
Silicosis	0 (0 to 0)	0 (0 to 0)	0 (0 to 0)	0 (0 to 0)	0 (0 to 0)	0 (0 to 0)	4·7 (0·3 to 17·8)	0·9 (0 to 3·6)	11·6 (1·2 to 39·7)	0·6 (0 to 2·9)	3·2 (0·2 to 10·8)	0·9 (0·1 to 4·2)	8·3 (0·6 to 32·2)	4·8 (0·2 to 17·5)	5·6 (0·3 to 20)	9·2 (0·5 to 31·3)	10·1 (0·6 to 36·8)	6·8 (0·5 to 23·1)	5·5 (0·5 to 17·8)	4·2 (0·4 to 13·2)	3 (0·3 to 10·5)	0·5 (0 to 1·7)	0 (0 to 0·1)	80
Asbestosis	0 (0 to 0)	0 (0 to 0)	0 (0 to 0)	0 (0 to 0)	0 (0 to 0)	0 (0 to 0)	11·6 (1·5 to 37)	0·7 (0·1 to 3·1)	1·4 (0·2 to 5·3)	0·5 (0·1 to 1·6)	0·5 (0·1 to 1·6)	0·3 (0·1 to 1·1)	1 (0·1 to 3·5)	2·7 (0·2 to 10·2)	3·2 (0·4 to 11·3)	3·1 (0·3 to 12·8)	3·8 (0·4 to 13·4)	4·1 (0·5 to 14·3)	1·3 (0·1 to 4·9)	1·8 (0·2 to 7)	1·5 (0·2 to 5·2)	0·5 (0·1 to 1·6)	0 (0 to 0·1)	37·9
Coal workers pneumoconiosis	0 (0 to 0)	0 (0 to 0)	0 (0 to 0)	0 (0 to 0)	0 (0 to 0)	0 (0 to 0)	2·6 (0·3 to 10·1)	0·3 (0 to 1·5)	0·5 (0 to 2·6)	0·5 (0 to 2)	2·7 (0·3 to 9·9)	0·4 (0 to 1·9)	1·2 (0·1 to 4·9)	6·5 (0·6 to 21)	1·5 (0·1 to 5·8)	7·1 (0·8 to 23·1)	4·9 (0·3 to 17·9)	2·7 (0·2 to 9·9)	1·2 (0·1 to 5)	1·1 (0·1 to 4)	0·4 (0 to 1·8)	0·3 (0 to 1·4)	0 (0 to 0·1)	34·2
Other pneumoconiosis	0 (0 to 0)	0 (0 to 0)	0 (0 to 0)	0 (0 to 0)	0 (0 to 0)	0 (0 to 0)	15 (2·1 to 44·3)	3·1 (0·3 to 11)	4·7 (0·4 to 18·1)	2·3 (0·4 to 8·2)	2·9 (0·5 to 9·4)	4·9 (1·1 to 12·9)	7·2 (1 to 23·4)	4·7 (0·7 to 16·2)	8·1 (1·4 to 22·5)	5·1 (0·7 to 18·4)	20 (3·6 to 57·3)	7·9 (1·3 to 27·8)	6·5 (1·3 to 17·2)	4·7 (0·8 to 13·9)	2·6 (0·5 to 8)	1·1 (0·2 to 3)	0·1 (0 to 0·2)	101
Asthma	0 (0 to 0)	0 (0 to 0)	0 (0 to 0)	17,100 (6,530 to 31,200)	6,290 (3,930 to 9,160)	3,440 (2,260 to 5,150)	1,890 (1,160 to 2,840)	1,290 (780 to 1,910)	1,270 (766 to 1,920)	1,320 (791 to 1,960)	1,240 (715 to 1,910)	1,210 (687 to 1,990)	1,100 (583 to 1,900)	1,130 (575 to 1,960)	1,020 (474 to 1,940)	1,140 (530 to 2,150)	959 (451 to 1,890)	739 (336 to 1,550)	461 (200 to 908)	322 (130 to 645)	118 (45·5 to 237)	40 (18 to 88·8)	8·4 (3·5 to 16·9)	42,100
Interstitial lung disease and pulmonary sarcoidosis	0 (0 to 0)	0 (0 to 0)	0 (0 to 0)	547 (82·7 to 2,130)	36·3 (11·7 to 98·1)	25·3 (8·6 to 58·1)	47·4 (12·3 to 120)	50·8 (15 to 116)	39·2 (13·1 to 79·5)	93·7 (33·3 to 208)	111 (38·6 to 242)	171 (65·2 to 361)	143 (52·5 to 298)	182 (67·1 to 373)	206 (70·1 to 437)	204 (63·5 to 429)	217 (73·9 to 474)	189 (64·3 to 386)	150 (45·4 to 320)	75·4 (23 to 176)	31·6 (9·1 to 72·6)	9·3 (2·4 to 21)	1·6 (0·4 to 4)	2,530
Other chronic respiratory diseases	0 (0 to 0)	0 (0 to 0)	0 (0 to 0)	3,710 (560 to 11,200)	400 (141 to 988)	294 (122 to 594)	184 (72·2 to 368)	191 (84·8 to 360)	270 (108 to 505)	267 (111 to 522)	275 (104 to 572)	261 (102 to 523)	254 (88 to 554)	242 (79·5 to 509)	221 (70·9 to 512)	87·7 (25·8 to 224)	74·6 (21·5 to 175)	62·7 (18·8 to 150)	45·8 (12·3 to 117)	28·9 (8·1 to 70·2)	15·1 (4·1 to 38·9)	4·7 (1·2 to 13·1)	0·9 (0·2 to 2·2)	6,890
Digestive diseases	868 (238 to 2,050)	743 (163 to 1,800)	7,240 (801 to 22,100)	9,680 (1,880 to 25,600)	3,630 (1,890 to 5,890)	2,160 (1,250 to 3,160)	1,890 (1,200 to 2,870)	2,350 (1,460 to 3,600)	3,360 (2,070 to 5,270)	4,990 (3,060 to 7,540)	6,880 (3,870 to 10,200)	9,250 (5,550 to 14,100)	10,200 (6,000 to 15,400)	10,800 (6,500 to 15,800)	10,400 (5,920 to 16,100)	9,330 (5,350 to 14,100)	7,190 (4,180 to 11,200)	4,680 (2,880 to 7,070)	2,880 (1,820 to 4,520)	1,580 (981 to 2,400)	653 (408 to 975)	189 (114 to 285)	33·3 (20·9 to 49·7)	111,000
Cirrhosis and other chronic liver diseases	0 (0 to 0)	0 (0 to 0)	1 (0·5 to 1·7)	1,780 (84·9 to 5,930)	613 (137 to 1,360)	385 (132 to 737)	354 (130 to 693)	424 (167 to 841)	839 (298 to 1,680)	1,770 (722 to 3,130)	3,090 (1,260 to 5,280)	5,060 (2,370 to 8,540)	6,090 (2,990 to 10,000)	6,620 (3,430 to 10,400)	6,210 (3,200 to 10,500)	5,320 (2,520 to 8,430)	3,700 (1,850 to 6,090)	2,100 (1,070 to 3,260)	1,080 (567 to 1,840)	505 (279 to 837)	173 (86·4 to 304)	42·4 (20·4 to 73·8)	7·3 (3·5 to 12·7)	46,200
Cirrhosis and other chronic liver diseases due to hepati	0 (0 to 0)	0 (0 to 0)	0 (0 to 0)	5·4 (0·2 to 19·8)	5·1 (0·9 to 13·8)	6·2 (1·4 to 15·4)	9·4 (2 to 25·5)	35·2 (10 to 87·1)	105 (26·9 to 254)	279 (96·6 to 567)	549 (180 to 1,150)	912 (363 to 1,840)	1,070 (415 to 2,190)	1,070 (402 to 2,130)	860 (338 to 1,710)	643 (234 to 1,260)	410 (156 to 808)	227 (92·4 to 463)	122 (50·7 to 253)	57·3 (23·8 to 116)	19·4 (8·1 to 37·1)	4·7 (1·7 to 9·8)	0·8 (0·2 to 1·8)	6,380
Cirrhosis and other chronic liver diseases due to hepati	0 (0 to 0)	0 (0 to 0)	0 (0 to 0)	12·2 (0·5 to 45·5)	11·8 (2 to 33·1)	14·4 (3·7 to 36·1)	21·4 (5·3 to 52·5)	70·9 (21·2 to 160)	205 (61·5 to 503)	455 (169 to 956)	755 (255 to 1,580)	1,180 (489 to 2,270)	1,330 (551 to 2,520)	1,360 (569 to 2,610)	1,190 (485 to 2,400)	970 (365 to 1,970)	664 (264 to 1,260)	369 (168 to 679)	187 (80·6 to 373)	87·9 (37·9 to 175)	31·2 (12·7 to 60·8)	7·9 (3·1 to 15·8)	1·4 (0·5 to 3)	8,930
Cirrhosis and other chronic liver diseases due to alcoho	0 (0 to 0)	0 (0 to 0)	0 (0 to 0)	0 (0 to 0)	0 (0 to 0)	0 (0 to 0)	2·4 (0·4 to 7·5)	45·7 (11·9 to 117)	161 (44·3 to 386)	520 (190 to 1,030)	1,150 (420 to 2,280)	2,070 (849 to 3,830)	2,620 (1,090 to 4,720)	3,010 (1,310 to 5,050)	3,010 (1,370 to 5,310)	2,630 (1,160 to 4,420)	1,770 (816 to 3,060)	953 (450 to 1,650)	452 (214 to 855)	194 (87·2 to 364)	62·9 (28·7 to 120)	14·5 (5·7 to 29·3)	2·4 (0·9 to 4·5)	18,700
Cirrhosis and other chronic liver diseases due to NAFL[	0 (0 to 0)	0 (0 to 0)	0 (0 to 0)	0 (0 to 0)	0 (0 to 0)	0 (0 to 0)	17·6 (4·7 to 43)	42·7 (12 to 102)	113 (29·9 to 270)	222 (70·6 to 471)	334 (93·5 to 768)	526 (184 to 1,070)	656 (247 to 1,400)	718 (286 to 1,470)	663 (248 to 1,380)	600 (209 to 1,240)	473 (190 to 937)	300 (127 to 571)	171 (68·9 to 353)	87·1 (36·9 to 173)	31·2 (12·9 to 61·5)	8 (3·4 to 16·7)	1·4 (0·5 to 3·2)	4,960
Cirrhosis and other chronic liver diseases due to other c	0 (0 to 0)	0 (0 to 0)	1 (0·5 to 1·7)	1,770 (84 to 5,880)	596 (133 to 1,320)	364 (123 to 704)	303 (109 to 603)	230 (82·1 to 476)	254 (80·5 to 584)	292 (97·2 to 648)	302 (91·3 to 648)	375 (133 to 802)	415 (142 to 847)	460 (176 to 924)	487 (192 to 997)	479 (191 to 1,030)	382 (130 to 757)	250 (98·3 to 462)	150 (59·4 to 299)	78·1 (30·4 to 155)	28·4 (11·2 to 54·9)	7·3 (2·8 to 14·7)	1·3 (0·4 to 2·8)	7,220
Upper digestive system diseases	0 (0 to 0)	0 (0 to 0)	0 (0 to 0)	825 (190 to 2,140)	314 (153 to 572)	234 (135 to 371)	467 (279 to 770)	890 (514 to 1,460)	1,230 (703 to 1,960)	1,550 (925 to 2,360)	1,700 (1,040 to 2,670)	1,830 (1,150 to 2,700)	1,830 (1,110 to 2,740)	1,930 (1,200 to 3,070)	1,850 (1,080 to 2,970)	1,780 (1,050 to 2,840)	1,580 (920 to 2,550)	1,110 (658 to 1,840)	800 (445 to 1,330)	469 (282 to 787)	209 (120 to 331)	63·1 (36·1 to 104)	11·8 (6·7 to 19·1)	20,700
Peptic ulcer disease	0 (0 to 0)	0 (0 to 0)	0 (0 to 0)	531 (84·9 to 1,510)	174 (56·4 to 385)	121 (50·8 to 235)	118 (45·4 to 237)	206 (84 to 416)	298 (126 to 569)	479 (223 to 889)	658 (303 to 1,150)	864 (419 to 1,510)	891 (413 to 1,640)	1,150 (546 to 2,030)	1,120 (521 to 2,050)	1,150 (551 to 2,080)	1,060 (490 to 1,870)	777 (378 to 1,370)	591 (287 to 1,070)	351 (186 to 613)	157 (80·9 to 269)	45·9 (24·7 to 76·3)	9·2 (4·8 to 15·6)	10,800
Gastritis and duodenitis	0 (0 to 0)	0 (0 to 0)	0 (0 to 0)	294 (67·8 to 843)	138 (62 to 243)	69·4 (28·4 to 135)	98·6 (45·6 to 186)	113 (52·3 to 202)	156 (76·5 to 281)	232 (113 to 415)	299 (144 to 536)	338 (166 to 594)	424 (208 to 766)	352 (185 to 607)	369 (190 to 692)	342 (167 to 638)	296 (149 to 584)	194 (90·3 to 389)	124 (52·8 to 260)	72·7 (30·7 to 153)	32·5 (12·3 to 69·6)	11·8 (4·2 to 27)	1·6 (0·5 to 3·8)	3,960
Gastroesophageal reflux disease	0 (0 to 0)	0 (0 to 0)	0 (0 to 0)	0 (0 to 0)	1·7 (0·3 to 4·3)	44·2 (18·5 to 85)	251 (120 to 475)	571 (281 to 1,130)	780 (374 to 1,450)	840 (400 to 1,610)	740 (349 to 1,370)	623 (306 to 1,140)	512 (250 to 929)	429 (206 to 777)	359 (165 to 685)	290 (138 to 544)	220 (113 to 396)	137 (69·7 to 239)	84·9 (42·7 to 151)	46·1 (22·9 to 83·1)	19·2 (9·7 to 34·9)	5·3 (2·7 to 9·6)	0·9 (0·5 to 1·7)	5,950
Appendicitis	0 (0 to 0)	[illegible]	[illegible]	[illegible]	[illegible]	[illegible]	[illegible]	[illegible]	[illegible]	[illegible]	[illegible]	[illegible]	[illegible]	[illegible]	[illegible]	[illegible]	[illegible]	[illegible]	[illegible]	[illegible]	[illegible]	[illegible]	[illegible]	5,700
Paralytic ileus and intestinal obstruction	429 (101 to 1,070)	[illegible]	[illegible]	[illegible]	[illegible]	[illegible]	[illegible]	[illegible]	[illegible]	[illegible]	[illegible]	[illegible]	[illegible]	[illegible]	[illegible]	[illegible]	[illegible]	[illegible]	[illegible]	[illegible]	[illegible]	[illegible]	[illegible]	11,900
Inguinal, femoral, and abdominal hernia	439 (80·1 to 1,160)	[illegible]	[illegible]	[illegible]	[illegible]	[illegible]	[illegible]	[illegible]	[illegible]	[illegible]	[illegible]	[illegible]	[illegible]	[illegible]	[illegible]	[illegible]	[illegible]	[illegible]	[illegible]	[illegible]	[illegible]	[illegible]	[illegible]	12,700
Inflammatory bowel disease	0 (0 to 0)	[illegible]	[illegible]	[illegible]	[illegible]	[illegible]	[illegible]	[illegible]	[illegible]	[illegible]	[illegible]	[illegible]	[illegible]	[illegible]	[illegible]	[illegible]	[illegible]	[illegible]	[illegible]	[illegible]	[illegible]	[illegible]	[illegible]	1,740
Vascular intestinal disorders	0 (0 to 0)	[illegible]	[illegible]	[illegible]	[illegible]	[illegible]	[illegible]	[illegible]	[illegible]	[illegible]	[illegible]	[illegible]	[illegible]	[illegible]	[illegible]	[illegible]	[illegible]	[illegible]	[illegible]	[illegible]	[illegible]	[illegible]	[illegible]	659
Gallbladder and biliary diseases	0 (0 to 0)	[illegible]	[illegible]	[illegible]	[illegible]	[illegible]	[illegible]	[illegible]	[illegible]	[illegible]	[illegible]	[illegible]	[illegible]	[illegible]	[illegible]	[illegible]	[illegible]	[illegible]	[illegible]	[illegible]	[illegible]	[illegible]	[illegible]	2,610
Pancreatitis	0 (0 to 0)	[illegible]	[illegible]	[illegible]	[illegible]	[illegible]	[illegible]	[illegible]	[illegible]	[illegible]	[illegible]	[illegible]	[illegible]	[illegible]	[illegible]	[illegible]	[illegible]	[illegible]	[illegible]	[illegible]	[illegible]	[illegible]	[illegible]	3,920
Other digestive diseases	0 (0 to 0)	[illegible]	[illegible]	[illegible]	[illegible]	[illegible]	[illegible]	[illegible]	[illegible]	[illegible]	[illegible]	[illegible]	[illegible]	[illegible]	[illegible]	[illegible]	[illegible]	[illegible]	[illegible]	[illegible]	[illegible]	[illegible]	[illegible]	4,790
Neurological disorders	2·9 (0·7 to 6·9)	[illegible]	[illegible]	[illegible]	[illegible]	[illegible]	[illegible]	[illegible]	[illegible]	[illegible]	[illegible]	[illegible]	[illegible]	[illegible]	[illegible]	[illegible]	[illegible]	[illegible]	[illegible]	[illegible]	[illegible]	[illegible]	[illegible]	60,800
Alzheimer's disease and other dementias	0 (0 to 0)	[illegible]	[illegible]	[illegible]	[illegible]	[illegible]	[illegible]	[illegible]	[illegible]	[illegible]	[illegible]	[illegible]	[illegible]	[illegible]	[illegible]	[illegible]	[illegible]	[illegible]	[illegible]	[illegible]	[illegible]	[illegible]	[illegible]	6,690
Parkinson's disease	0 (0 to 0)	[illegible]	[illegible]	[illegible]	[illegible]	[illegible]	[illegible]	[illegible]	[illegible]	[illegible]	[illegible]	[illegible]	[illegible]	[illegible]	[illegible]	[illegible]	[illegible]	[illegible]	[illegible]	[illegible]	[illegible]	[illegible]	[illegible]	2,960
Idiopathic epilepsy	2·6 (0·4 to 6·8)	[illegible]	[illegible]	[illegible]	[illegible]	[illegible]	[illegible]	[illegible]	[illegible]	[illegible]	[illegible]	[illegible]	[illegible]	[illegible]	[illegible]	[illegible]	[illegible]	[illegible]	[illegible]	[illegible]	[illegible]	[illegible]	[illegible]	21,400
Multiple sclerosis	0 (0 to 0)	[illegible]	[illegible]	[illegible]	[illegible]	[illegible]	[illegible]	[illegible]	[illegible]	[illegible]	[illegible]	[illegible]	[illegible]	[illegible]	[illegible]	[illegible]	[illegible]	[illegible]	[illegible]	[illegible]	[illegible]	[illegible]	[illegible]	366
Motor neuron disease	0·3 (0·1 to 0·6)	[illegible]	[illegible]	[illegible]	[illegible]	[illegible]	[illegible]	[illegible]	[illegible]	[illegible]	[illegible]	[illegible]	[illegible]	[illegible]	[illegible]	[illegible]	[illegible]	[illegible]	[illegible]	[illegible]	[illegible]	[illegible]	[illegible]	723
Headache disorders	0 (0 to 0)	[illegible]	[illegible]	[illegible]	[illegible]	[illegible]	[illegible]	[illegible]	[illegible]	[illegible]	[illegible]	[illegible]	[illegible]	[illegible]	[illegible]	[illegible]	[illegible]	[illegible]	[illegible]	[illegible]	[illegible]	[illegible]	[illegible]	22,600
Migraine	0 (0 to 0)	[illegible]	[illegible]	[illegible]	[illegible]	[illegible]	[illegible]	[illegible]	[illegible]	[illegible]	[illegible]	[illegible]	[illegible]	[illegible]	[illegible]	[illegible]	[illegible]	[illegible]	[illegible]	[illegible]	[illegible]	[illegible]	[illegible]	20,100
Tension-type headache	0 (0 to 0)	[illegible]	[illegible]	[illegible]	[illegible]	[illegible]	[illegible]	[illegible]	[illegible]	[illegible]	[illegible]	[illegible]	[illegible]	[illegible]	[illegible]	[illegible]	[illegible]	[illegible]	[illegible]	[illegible]	[illegible]	[illegible]	[illegible]	2,410
Other neurological disorders	0 (0 to 0)	[illegible]	[illegible]	[illegible]	[illegible]	[illegible]	[illegible]	[illegible]	[illegible]	[illegible]	[illegible]	[illegible]	[illegible]	[illegible]	[illegible]	[illegible]	[illegible]	[illegible]	[illegible]	[illegible]	[illegible]	[illegible]	[illegible]	6,090
Mental disorders	4·1 (2·5 to 6·2)	[illegible]	[illegible]	[illegible]	[illegible]	[illegible]	[illegible]	[illegible]	[illegible]	[illegible]	[illegible]	[illegible]	[illegible]	[illegible]	[illegible]	[illegible]	[illegible]	[illegible]	[illegible]	[illegible]	[illegible]	[illegible]	[illegible]	85,700
Schizophrenia	0 (0 to 0)	[illegible]	[illegible]	[illegible]	[illegible]	[illegible]	[illegible]	[illegible]	[illegible]	[illegible]	[illegible]	[illegible]	[illegible]	[illegible]	[illegible]	[illegible]	[illegible]	[illegible]	[illegible]	[illegible]	[illegible]	[illegible]	[illegible]	8,740
Depressive disorders	0 (0 to 0)	[illegible]	[illegible]	[illegible]	[illegible]	[illegible]	[illegible]	[illegible]	[illegible]	[illegible]	[illegible]	[illegible]	[illegible]	[illegible]	[illegible]	[illegible]	[illegible]	[illegible]	[illegible]	[illegible]	[illegible]	[illegible]	[illegible]	23,300
Major depressive disorder	0 (0 to 0)	[illegible]	[illegible]	[illegible]	[illegible]	[illegible]	[illegible]	[illegible]	[illegible]	[illegible]	[illegible]	[illegible]	[illegible]	[illegible]	[illegible]	[illegible]	[illegible]	[illegible]	[illegible]	[illegible]	[illegible]	[illegible]	[illegible]	19,100
Dysthymia	0 (0 to 0)	[illegible]	[illegible]	[illegible]	[illegible]	[illegible]	[illegible]	[illegible]	[illegible]	[illegible]	[illegible]	[illegible]	[illegible]	[illegible]	[illegible]	[illegible]	[illegible]	[illegible]	[illegible]	[illegible]	[illegible]	[illegible]	[illegible]	4,300
Bipolar disorder	0 (0 to 0)	[illegible]	[illegible]	[illegible]	[illegible]	[illegible]	[illegible]	[illegible]	[illegible]	[illegible]	[illegible]	[illegible]	[illegible]	[illegible]	[illegible]	[illegible]	[illegible]	[illegible]	[illegible]	[illegible]	[illegible]	[illegible]	[illegible]	9,910
Anxiety disorders	0 (0 to 0)	[illegible]	[illegible]	[illegible]	[illegible]	[illegible]	[illegible]	[illegible]	[illegible]	[illegible]	[illegible]	[illegible]	[illegible]	[illegible]	[illegible]	[illegible]	[illegible]	[illegible]	[illegible]	[illegible]	[illegible]	[illegible]	[illegible]	18,500
Eating disorders	0 (0 to 0)	[illegible]	[illegible]	[illegible]	[illegible]	[illegible]	[illegible]	[illegible]	[illegible]	[illegible]	[illegible]	[illegible]	[illegible]	[illegible]	[illegible]	[illegible]	[illegible]	[illegible]	[illegible]	[illegible]	[illegible]	[illegible]	[illegible]	1,040
Anorexia nervosa	0 (0 to 0)	[illegible]	[illegible]	[illegible]	[illegible]	[illegible]	[illegible]	[illegible]	[illegible]	[illegible]	[illegible]	[illegible]	[illegible]	[illegible]	[illegible]	[illegible]	[illegible]	[illegible]	[illegible]	[illegible]	[illegible]	[illegible]	[illegible]	249
Bulimia nervosa	0 (0 to 0)	[illegible]	[illegible]	[illegible]	[illegible]	[illegible]	[illegible]	[illegible]	[illegible]	[illegible]	[illegible]	[illegible]	[illegible]	[illegible]	[illegible]	[illegible]	[illegible]	[illegible]	[illegible]	[illegible]	[illegible]	[illegible]	[illegible]	792
Autism spectrum disorders	3·2 (2 to 4·8)	[illegible]	[illegible]	[illegible]	[illegible]	[illegible]	[illegible]	[illegible]	[illegible]	[illegible]	[illegible]	[illegible]	[illegible]	[illegible]	[illegible]	[illegible]	[illegible]	[illegible]	[illegible]	[illegible]	[illegible]	[illegible]	[illegible]	4,870
Attention-deficit/hyperactivity disorder	0 (0 to 0)	[illegible]	[illegible]	[illegible]	[illegible]	[illegible]	[illegible]	[illegible]	[illegible]	[illegible]	[illegible]	[illegible]	[illegible]	[illegible]	[illegible]	[illegible]	[illegible]	[illegible]	[illegible]	[illegible]	[illegible]	[illegible]	[illegible]	3,720
Conduct disorder	0 (0 to 0)	[illegible]	[illegible]	[illegible]	[illegible]	[illegible]	[illegible]	[illegible]	[illegible]	[illegible]	[illegible]	[illegible]	[illegible]	[illegible]	[illegible]	[illegible]	[illegible]	[illegible]	[illegible]	[illegible]	[illegible]	[illegible]	[illegible]	6,370
Idiopathic developmental intellectual disability	0·9 (0·1 to 2·1)	[illegible]	[illegible]	[illegible]	[illegible]	[illegible]	[illegible]	[illegible]	[illegible]	[illegible]	[illegible]	[illegible]	[illegible]	[illegible]	[illegible]	[illegible]	[illegible]	[illegible]	[illegible]	[illegible]	[illegible]	[illegible]	[illegible]	2,840
Other mental disorders	0 (0 to 0)	[illegible]	[illegible]	[illegible]	[illegible]	[illegible]	[illegible]	[illegible]	[illegible]	[illegible]	[illegible]	[illegible]	[illegible]	[illegible]	[illegible]	[illegible]	[illegible]	[illegible]	[illegible]	[illegible]	[illegible]	[illegible]	[illegible]	6,430
Substance use disorders	0 (0 to 0)	[illegible]	[illegible]	[illegible]	[illegible]	[illegible]	[illegible]	[illegible]	[illegible]	[illegible]	[illegible]	[illegible]	[illegible]	[illegible]	[illegible]	[illegible]	[illegible]	[illegible]	[illegible]	[illegible]	[illegible]	[illegible]	[illegible]	29,900
Alcohol use disorders	0 (0 to 0)	[illegible]	[illegible]	[illegible]	[illegible]	[illegible]	[illegible]	[illegible]	[illegible]	[illegible]	[illegible]	[illegible]	[illegible]	[illegible]	[illegible]	[illegible]	[illegible]	[illegible]	[illegible]	[illegible]	[illegible]	[illegible]	[illegible]	22,500
Drug use disorders	0 (0 to 0)	[illegible]	[illegible]	[illegible]	[illegible]	[illegible]	[illegible]	[illegible]	[illegible]	[illegible]	[illegible]	[illegible]	[illegible]	[illegible]	[illegible]	[illegible]	[illegible]	[illegible]	[illegible]	[illegible]	[illegible]	[illegible]	[illegible]	7,430
Opioid use disorders	0 (0 to 0)	[illegible]	[illegible]	[illegible]	[illegible]	[illegible]	[illegible]	[illegible]	[illegible]	[illegible]	[illegible]	[illegible]	[illegible]	[illegible]	[illegible]	[illegible]	[illegible]	[illegible]	[illegible]	[illegible]	[illegible]	[illegible]	[illegible]	1,760
Cocaine use disorders	0 (0 to 0)	[illegible]	[illegible]	[illegible]	[illegible]	[illegible]	[illegible]	[illegible]	[illegible]	[illegible]	[illegible]	[illegible]	[illegible]	[illegible]	[illegible]	[illegible]	[illegible]	[illegible]	[illegible]	[illegible]	[illegible]	[illegible]	[illegible]	2,350
Amphetamine use disorders	0 (0 to 0)	[illegible]	[illegible]	[illegible]	[illegible]	[illegible]	[illegible]	[illegible]	[illegible]	[illegible]	[illegible]	[illegible]	[illegible]	[illegible]	[illegible]	[illegible]	[illegible]	[illegible]	[illegible]	[illegible]	[illegible]	[illegible]	[illegible]	572
Cannabis use disorders	0 (0 to 0)	[illegible]	[illegible]	[illegible]	[illegible]	[illegible]	[illegible]	[illegible]	[illegible]	[illegible]	[illegible]	[illegible]	[illegible]	[illegible]	[illegible]	[illegible]	[illegible]	[illegible]	[illegible]	[illegible]	[illegible]	[illegible]	[illegible]	900
Other drug use disorders	0 (0 to 0)	[illegible]	[illegible]	[illegible]	[illegible]	[illegible]	[illegible]	[illegible]	[illegible]	[illegible]	[illegible]	[illegible]	[illegible]	[illegible]	[illegible]	[illegible]	[illegible]	[illegible]	[illegible]	[illegible]	[illegible]	[illegible]	[illegible]	1,850
Diabetes and kidney diseases	12·2 (6·9 to 19·8)	[illegible]	[illegible]	[illegible]	[illegible]	[illegible]	[illegible]	[illegible]	[illegible]	[illegible]	[illegible]	[illegible]	[illegible]	[illegible]	[illegible]	[illegible]	[illegible]	[illegible]	[illegible]	[illegible]	[illegible]	[illegible]	[illegible]	105,000
Diabetes mellitus	12·2 (6·9 to 19·8)	[illegible]	[illegible]	[illegible]	[illegible]	[illegible]	[illegible]	[illegible]	[illegible]	[illegible]	[illegible]	[illegible]	[illegible]	[illegible]	[illegible]	[illegible]	[illegible]	[illegible]	[illegible]	[illegible]	[illegible]	[illegible]	[illegible]	60,700
Diabetes mellitus type 1	12·2 (6·9 to 19·8)	[illegible]	[illegible]	[illegible]	[illegible]	[illegible]	[illegible]	[illegible]	[illegible]	[illegible]	[illegible]	[illegible]	[illegible]	[illegible]	[illegible]	[illegible]	[illegible]	[illegible]	[illegible]	[illegible]	[illegible]	[illegible]	[illegible]	7,490
Diabetes mellitus type 2	0 (0 to 0)	[illegible]	[illegible]	[illegible]	[illegible]	[illegible]	[illegible]	[illegible]	[illegible]	[illegible]	[illegible]	[illegible]	[illegible]	[illegible]	[illegible]	[illegible]	[illegible]	[illegible]	[illegible]	[illegible]	[illegible]	[illegible]	[illegible]	53,200
Chronic kidney disease	0 (0 to 0)	[illegible]	[illegible]	[illegible]	[illegible]	[illegible]	[illegible]	[illegible]	[illegible]	[illegible]	[illegible]	[illegible]	[illegible]	[illegible]	[illegible]	[illegible]	[illegible]	[illegible]	[illegible]	[illegible]	[illegible]	[illegible]	[illegible]	44,200
Chronic kidney disease due to diabetes mellitus type 1	0 (0 to 0)	[illegible]	[illegible]	[illegible]	[illegible]	[illegible]	[illegible]	[illegible]	[illegible]	[illegible]	[illegible]	[illegible]	[illegible]	[illegible]	[illegible]	[illegible]	[illegible]	[illegible]	[illegible]	[illegible]	[illegible]	[illegible]	[illegible]	3,560
Chronic kidney disease due to diabetes mellitus type 2	0 (0 to 0)	[illegible]	[illegible]	[illegible]	[illegible]	[illegible]	[illegible]	[illegible]	[illegible]	[illegible]	[illegible]	[illegible]	[illegible]	[illegible]	[illegible]	[illegible]	[illegible]	[illegible]	[illegible]	[illegible]	[illegible]	[illegible]	[illegible]	8,030
Chronic kidney disease due to hypertension	0 (0 to 0)	[illegible]	[illegible]	[illegible]	[illegible]	[illegible]	[illegible]	[illegible]	[illegible]	[illegible]	[illegible]	[illegible]	[illegible]	[illegible]	[illegible]	[illegible]	[illegible]	[illegible]	[illegible]	[illegible]	[illegible]	[illegible]	[illegible]	7,140
Chronic kidney disease due to glomerulonephritis	0 (0 to 0)	[illegible]	[illegible]	[illegible]	[illegible]	[illegible]	[illegible]	[illegible]	[illegible]	[illegible]	[illegible]	[illegible]	[illegible]	[illegible]	[illegible]	[illegible]	[illegible]	[illegible]	[illegible]	[illegible]	[illegible]	[illegible]	[illegible]	12,000
Chronic kidney disease due to other and unspecified ca	0 (0 to 0)	[illegible]	[illegible]	[illegible]	[illegible]	[illegible]	[illegible]	[illegible]	[illegible]	[illegible]	[illegible]	[illegible]	[illegible]	[illegible]	[illegible]	[illegible]	[illegible]	[illegible]	[illegible]	[illegible]	[illegible]	[illegible]	[illegible]	13,400
Acute glomerulonephritis	0 (0 to 0)	[illegible]	[illegible]	[illegible]	[illegible]	[illegible]	[illegible]	[illegible]	[illegible]	[illegible]	[illegible]	[illegible]	[illegible]	[illegible]	[illegible]	[illegible]	[illegible]	[illegible]	[illegible]	[illegible]	[illegible]	[illegible]	[illegible]	289
Skin and subcutaneous diseases	0·2 (0·1 to 0·4)	[illegible]	[illegible]	[illegible]	[illegible]	[illegible]	[illegible]	[illegible]	[illegible]	[illegible]	[illegible]	[illegible]	[illegible]	[illegible]	[illegible]	[illegible]	[illegible]	[illegible]	[illegible]	[illegible]	[illegible]	[illegible]	[illegible]	35,500
Dermatitis	0 (0 to 0)	[illegible]	[illegible]	[illegible]	[illegible]	[illegible]	[illegible]	[illegible]	[illegible]	[illegible]	[illegible]	[illegible]	[illegible]	[illegible]	[illegible]	[illegible]	[illegible]	[illegible]	[illegible]	[illegible]	[illegible]	[illegible]	[illegible]	6,550
Atopic dermatitis	0 (0 to 0)	[illegible]	[illegible]	[illegible]	[illegible]	[illegible]	[illegible]	[illegible]	[illegible]	[illegible]	[illegible]	[illegible]	[illegible]	[illegible]	[illegible]	[illegible]	[illegible]	[illegible]	[illegible]	[illegible]	[illegible]	[illegible]	[illegible]	5,070
Contact dermatitis	0 (0 to 0)	[illegible]	[illegible]	[illegible]	[illegible]	[illegible]	[illegible]	[illegible]	[illegible]	[illegible]	[illegible]	[illegible]	[illegible]	[illegible]	[illegible]	[illegible]	[illegible]	[illegible]	[illegible]	[illegible]	[illegible]	[illegible]	[illegible]	1,290
Seborrhoeic dermatitis	0 (0 to 0)	[illegible]	[illegible]	[illegible]	[illegible]	[illegible]	[illegible]	[illegible]	[illegible]	[illegible]	[illegible]	[illegible]	[illegible]	[illegible]	[illegible]	[illegible]	[illegible]	[illegible]	[illegible]	[illegible]	[illegible]	[illegible]	[illegible]	189
Psoriasis	0 (0 to 0)	[illegible]	[illegible]	[illegible]	[illegible]	[illegible]	[illegible]	[illegible]	[illegible]	[illegible]	[illegible]	[illegible]	[illegible]	[illegible]	[illegible]	[illegible]	[illegible]	[illegible]	[illegible]	[illegible]	[illegible]	[illegible]	[illegible]	2,290
Bacterial skin diseases	0 (0 to 0·1)	[illegible]	[illegible]	[illegible]	[illegible]	[illegible]	[illegible]	[illegible]	[illegible]	[illegible]	[illegible]	[illegible]	[illegible]	[illegible]	[illegible]	[illegible]	[illegible]	[illegible]	[illegible]	[illegible]	[illegible]	[illegible]	[illegible]	4,590
Cellulitis	0 (0 to 0)	[illegible]	[illegible]	[illegible]	[illegible]	[illegible]	[illegible]	[illegible]	[illegible]	[illegible]	[illegible]	[illegible]	[illegible]	[illegible]	[illegible]	[illegible]	[illegible]	[illegible]	[illegible]	[illegible]	[illegible]	[illegible]	[illegible]	1,330
Pyoderma	0 (0 to 0)	[illegible]	[illegible]	[illegible]	[illegible]	[illegible]	[illegible]	[illegible]	[illegible]	[illegible]	[illegible]	[illegible]	[illegible]	[illegible]	[illegible]	[illegible]	[illegible]	[illegible]	[illegible]	[illegible]	[illegible]	[illegible]	[illegible]	3,260
Scabies	0·1 (0 to 0·2)	[illegible]	[illegible]	[illegible]	[illegible]	[illegible]	[illegible]	[illegible]	[illegible]	[illegible]	[illegible]	[illegible]	[illegible]	[illegible]	[illegible]	[illegible]	[illegible]	[illegible]	[illegible]	[illegible]	[illegible]	[illegible]	[illegible]	5,380
Fungal skin diseases	0 (0 to 0)	[illegible]	[illegible]	[illegible]	[illegible]	[illegible]	[illegible]	[illegible]	[illegible]	[illegible]	[illegible]	[illegible]	[illegible]	[illegible]	[illegible]	[illegible]	[illegible]	[illegible]	[illegible]	[illegible]	[illegible]	[illegible]	[illegible]	2,320
Viral skin diseases	0 (0 to 0)	[illegible]	[illegible]	[illegible]	[illegible]	[illegible]	[illegible]	[illegible]	[illegible]	[illegible]	[illegible]	[illegible]	[illegible]	[illegible]	[illegible]	[illegible]	[illegible]	[illegible]	[illegible]	[illegible]	[illegible]	[illegible]	[illegible]	3,300
Acne vulgaris	0 (0 to 0)	[illegible]	[illegible]	[illegible]	[illegible]	[illegible]	[illegible]	[illegible]	[illegible]	[illegible]	[illegible]	[illegible]	[illegible]	[illegible]	[illegible]	[illegible]	[illegible]	[illegible]	[illegible]	[illegible]	[illegible]	[illegible]	[illegible]	3,230
Alopecia areata	0 (0 to 0)	[illegible]	[illegible]	[illegible]	[illegible]	[illegible]	[illegible]	[illegible]	[illegible]	[illegible]	[illegible]	[illegible]	[illegible]	[illegible]	[illegible]	[illegible]	[illegible]	[illegible]	[illegible]	[illegible]	[illegible]	[illegible]	[illegible]	282
Pruritus	0 (0 to 0)	[illegible]	[illegible]	[illegible]	[illegible]	[illegible]	[illegible]	[illegible]	[illegible]	[illegible]	[illegible]	[illegible]	[illegible]	[illegible]	[illegible]	[illegible]	[illegible]	[illegible]	[illegible]	[illegible]	[illegible]	[illegible]	[illegible]	353
Urticaria	0·1 (0 to 0·1)	[illegible]	[illegible]	[illegible]	[illegible]	[illegible]	[illegible]	[illegible]	[illegible]	[illegible]	[illegible]	[illegible]	[illegible]	[illegible]	[illegible]	[illegible]	[illegible]	[illegible]	[illegible]	[illegible]	[illegible]	[illegible]	[illegible]	2,630
Decubitus ulcer	0 (0 to 0)	[illegible]	[illegible]	[illegible]	[illegible]	[illegible]	[illegible]	[illegible]	[illegible]	[illegible]	[illegible]	[illegible]	[illegible]	[illegible]	[illegible]	[illegible]	[illegible]	[illegible]	[illegible]	[illegible]	[illegible]	[illegible]	[illegible]	2,070
Other skin and subcutaneous diseases	0 (0 to 0)	[illegible]	[illegible]	[illegible]	[illegible]	[illegible]	[illegible]	[illegible]	[illegible]	[illegible]	[illegible]	[illegible]	[illegible]	[illegible]	[illegible]	[illegible]	[illegible]	[illegible]	[illegible]	[illegible]	[illegible]	[illegible]	[illegible]	2,490
Sense organ diseases	0·4 (0·2 to 0·7)	[illegible]	[illegible]	[illegible]	[illegible]	[illegible]	[illegible]	[illegible]	[illegible]	[illegible]	[illegible]	[illegible]	[illegible]	[illegible]	[illegible]	[illegible]	[illegible]	[illegible]	[illegible]	[illegible]	[illegible]	[illegible]	[illegible]	31,900
Blindness and vision loss	0·1 (0 to 0·3)	[illegible]	[illegible]	[illegible]	[illegible]	[illegible]	[illegible]	[illegible]	[illegible]	[illegible]	[illegible]	[illegible]	[illegible]	[illegible]	[illegible]	[illegible]	[illegible]	[illegible]	[illegible]	[illegible]	[illegible]	[illegible]	[illegible]	9,440
Glaucoma	0 (0 to 0)	[illegible]	[illegible]	[illegible]	[illegible]	[illegible]	[illegible]	[illegible]	[illegible]	[illegible]	[illegible]	[illegible]	[illegible]	[illegible]	[illegible]	[illegible]	[illegible]	[illegible]	[illegible]	[illegible]	[illegible]	[illegible]	[illegible]	532
Cataract	0 (0 to 0)	[illegible]	[illegible]	[illegible]	[illegible]	[illegible]	[illegible]	[illegible]	[illegible]	[illegible]	[illegible]	[illegible]	[illegible]	[illegible]	[illegible]	[illegible]	[illegible]	[illegible]	[illegible]	[illegible]	[illegible]	[illegible]	[illegible]	1,340
Age-related macular degeneration	0 (0 to 0)	[illegible]	[illegible]	[illegible]	[illegible]	[illegible]	[illegible]	[illegible]	[illegible]	[illegible]	[illegible]	[illegible]	[illegible]	[illegible]	[illegible]	[illegible]	[illegible]	[illegible]	[illegible]	[illegible]	[illegible]	[illegible]	[illegible]	45·8
Refraction disorders	0 (0 to 0)	[illegible]	[illegible]	[illegible]	[illegible]	[illegible]	[illegible]	[illegible]	[illegible]	[illegible]	[illegible]	[illegible]	[illegible]	[illegible]	[illegible]	[illegible]	[illegible]	[illegible]	[illegible]	[illegible]	[illegible]	[illegible]	[illegible]	2,590
Near vision loss	0 (0 to 0)	[illegible]	[illegible]	[illegible]	[illegible]	[illegible]	[illegible]	[illegible]	[illegible]	[illegible]	[illegible]	[illegible]	[illegible]	[illegible]	[illegible]	[illegible]	[illegible]	[illegible]	[illegible]	[illegible]	[illegible]	[illegible]	[illegible]	2,090
Other vision loss	0·1 (0 to 0·2)	[illegible]	[illegible]	[illegible]	[illegible]	[illegible]	[illegible]	[illegible]	[illegible]	[illegible]	[illegible]	[illegible]	[illegible]	[illegible]	[illegible]	[illegible]	[illegible]	[illegible]	[illegible]	[illegible]	[illegible]	[illegible]	[illegible]	2,840
Age-related and other hearing loss	0·1 (0 to 0·3)	[illegible]	[illegible]	[illegible]	[illegible]	[illegible]	[illegible]	[illegible]	[illegible]	[illegible]	[illegible]	[illegible]	[illegible]	[illegible]	[illegible]	[illegible]	[illegible]	[illegible]	[illegible]	[illegible]	[illegible]	[illegible]	[illegible]	21,000
Other sense organ diseases	0·2 (0·1 to 0·4)	[illegible]	[illegible]	[illegible]	[illegible]	[illegible]	[illegible]	[illegible]	[illegible]	[illegible]	[illegible]	[illegible]	[illegible]	[illegible]	[illegible]	[illegible]	[illegible]	[illegible]	[illegible]	[illegible]	[illegible]	[illegible]	[illegible]	1,440
Musculoskeletal disorders	0 (0 to 0)	[illegible]	[illegible]	[illegible]	[illegible]	[illegible]	[illegible]	[illegible]	[illegible]	[illegible]	[illegible]	[illegible]	[illegible]	[illegible]	[illegible]	[illegible]	[illegible]	[illegible]	[illegible]	[illegible]	[illegible]	[illegible]	[illegible]	50,400
Rheumatoid arthritis	0 (0 to 0)	[illegible]	[illegible]	[illegible]	[illegible]	[illegible]	[illegible]	[illegible]	[illegible]	[illegible]	[illegible]	[illegible]	[illegible]	[illegible]	[illegible]	[illegible]	[illegible]	[illegible]	[illegible]	[illegible]	[illegible]	[illegible]	[illegible]	625
Osteoarthritis	0 (0 to 0)	[illegible]	[illegible]	[illegible]	[illegible]	[illegible]	[illegible]	[illegible]	[illegible]	[illegible]	[illegible]	[illegible]	[illegible]	[illegible]	[illegible]	[illegible]	[illegible]	[illegible]	[illegible]	[illegible]	[illegible]	[illegible]	[illegible]	5,400
Osteoarthritis hip	0 (0 to 0)	[illegible]	[illegible]	[illegible]	[illegible]	[illegible]	[illegible]	[illegible]	[illegible]	[illegible]	[illegible]	[illegible]	[illegible]	[illegible]	[illegible]	[illegible]	[illegible]	[illegible]	[illegible]	[illegible]	[illegible]	[illegible]	[illegible]	287
Osteoarthritis knee	0 (0 to 0)	[illegible]	[illegible]	[illegible]	[illegible]	[illegible]	[illegible]	[illegible]	[illegible]	[illegible]	[illegible]	[illegible]	[illegible]	[illegible]	[illegible]	[illegible]	[illegible]	[illegible]	[illegible]	[illegible]	[illegible]	[illegible]	[illegible]	3,810
Osteoarthritis hand	0 (0 to 0)	[illegible]	[illegible]	[illegible]	[illegible]	[illegible]	[illegible]	[illegible]	[illegible]	[illegible]	[illegible]	[illegible]	[illegible]	[illegible]	[illegible]	[illegible]	[illegible]	[illegible]	[illegible]	[illegible]	[illegible]	[illegible]	[illegible]	534
Osteoarthritis other	0 (0 to 0)	[illegible]	[illegible]	[illegible]	[illegible]	[illegible]	[illegible]	[illegible]	[illegible]	[illegible]	[illegible]	[illegible]	[illegible]	[illegible]	[illegible]	[illegible]	[illegible]	[illegible]	[illegible]	[illegible]	[illegible]	[illegible]	[illegible]	764
Low back pain	0 (0 to 0)	[illegible]	[illegible]	[illegible]	[illegible]	[illegible]	[illegible]	[illegible]	[illegible]	[illegible]	[illegible]	[illegible]	[illegible]	[illegible]	[illegible]	[illegible]	[illegible]	[illegible]	[illegible]	[illegible]	[illegible]	[illegible]	[illegible]	29,500
Neck pain	0 (0 to 0)	[illegible]	[illegible]	[illegible]	[illegible]	[illegible]	[illegible]	[illegible]	[illegible]	[illegible]	[illegible]	[illegible]	[illegible]	[illegible]	[illegible]	[illegible]	[illegible]	[illegible]	[illegible]	[illegible]	[illegible]	[illegible]	[illegible]	6,000
Gout	0 (0 to 0)	[illegible]	[illegible]	[illegible]	[illegible]	[illegible]	[illegible]	[illegible]	[illegible]	[illegible]	[illegible]	[illegible]	[illegible]	[illegible]	[illegible]	[illegible]	[illegible]	[illegible]	[illegible]	[illegible]	[illegible]	[illegible]	[illegible]	368
Other musculoskeletal disorders	0 (0 to 0)	[illegible]	[illegible]	[illegible]	[illegible]	[illegible]	[illegible]	[illegible]	[illegible]	[illegible]	[illegible]	[illegible]	[illegible]	[illegible]	[illegible]	[illegible]	[illegible]	[illegible]	[illegible]	[illegible]	[illegible]	[illegible]	[illegible]	8,460
Other non-communicable diseases	53,400 (28,200 to 92,300)	[illegible]	[illegible]	[illegible]	[illegible]	[illegible]	[illegible]	[illegible]	[illegible]	[illegible]	[illegible]	[illegible]	[illegible]	[illegible]	[illegible]	[illegible]	[illegible]	[illegible]	[illegible]	[illegible]	[illegible]	[illegible]	[illegible]	238,000
Congenital birth defects	52,600 (27,400 to 91,400)	[illegible]	[illegible]	[illegible]	[illegible]	[illegible]	[illegible]	[illegible]	[illegible]	[illegible]	[illegible]	[illegible]	[illegible]	[illegible]	[illegible]	[illegible]	[illegible]	[illegible]	[illegible]	[illegible]	[illegible]	[illegible]	[illegible]	168,000
Neural tube defects	7,900 (1,370 to 24,700)	[illegible]	[illegible]	[illegible]	[illegible]	[illegible]	[illegible]	[illegible]	[illegible]	[illegible]	[illegible]	[illegible]	[illegible]	[illegible]	[illegible]	[illegible]	[illegible]	[illegible]	[illegible]	[illegible]	[illegible]	[illegible]	[illegible]	21,200
Congenital heart anomalies	19,700 (7,260 to 40,800)	[illegible]	[illegible]	[illegible]	[illegible]	[illegible]	[illegible]	[illegible]	[illegible]	[illegible]	[illegible]	[illegible]	[illegible]	[illegible]	[illegible]	[illegible]	[illegible]	[illegible]	[illegible]	[illegible]	[illegible]	[illegible]	[illegible]	69,500
Orofacial clefts	154 (15 to 646)	[illegible]	[illegible]	[illegible]	[illegible]	[illegible]	[illegible]	[illegible]	[illegible]	[illegible]	[illegible]	[illegible]	[illegible]	[illegible]	[illegible]	[illegible]	[illegible]	[illegible]	[illegible]	[illegible]	[illegible]	[illegible]	[illegible]	537
Down syndrome	531 (112 to 1,690)	[illegible]	[illegible]	[illegible]	[illegible]	[illegible]	[illegible]	[illegible]	[illegible]	[illegible]	[illegible]	[illegible]	[illegible]	[illegible]	[illegible]	[illegible]	[illegible]	[illegible]	[illegible]	[illegible]	[illegible]	[illegible]	[illegible]	6,070
Turner syndrome	0 (0 to 0)	[illegible]	[illegible]	[illegible]	[illegible]	[illegible]	[illegible]	[illegible]	[illegible]	[illegible]	[illegible]	[illegible]	[illegible]	[illegible]	[illegible]	[illegible]	[illegible]	[illegible]	[illegible]	[illegible]	[illegible]	[illegible]	[illegible]	0
Klinefelter syndrome	0 (0 to 0)	[illegible]	[illegible]	[illegible]	[illegible]	[illegible]	[illegible]	[illegible]	[illegible]	[illegible]	[illegible]	[illegible]	[illegible]	[illegible]	[illegible]	[illegible]	[illegible]	[illegible]	[illegible]	[illegible]	[illegible]	[illegible]	[illegible]	1·8
Other chromosomal abnormalities	2,630 (600 to 7,340)	[illegible]	[illegible]	[illegible]	[illegible]	[illegible]	[illegible]	[illegible]	[illegible]	[illegible]	[illegible]	[illegible]	[illegible]	[illegible]	[illegible]	[illegible]	[illegible]	[illegible]	[illegible]	[illegible]	[illegible]	[illegible]	[illegible]	7,610
Congenital musculoskeletal and limb anomalies	1,170 (328 to 2,770)	[illegible]	[illegible]	[illegible]	[illegible]	[illegible]	[illegible]	[illegible]	[illegible]	[illegible]	[illegible]	[illegible]	[illegible]	[illegible]	[illegible]	[illegible]	[illegible]	[illegible]	[illegible]	[illegible]	[illegible]	[illegible]	[illegible]	5,940
Urogenital congenital anomalies	1,300 (375 to 3,350)	[illegible]	[illegible]	[illegible]	[illegible]	[illegible]	[illegible]	[illegible]	[illegible]	[illegible]	[illegible]	[illegible]	[illegible]	[illegible]	[illegible]	[illegible]	[illegible]	[illegible]	[illegible]	[illegible]	[illegible]	[illegible]	[illegible]	3,110
Digestive congenital anomalies	7,060 (2,230 to 14,800)	[illegible]	[illegible]	[illegible]	[illegible]	[illegible]	[illegible]	[illegible]	[illegible]	[illegible]	[illegible]	[illegible]	[illegible]	[illegible]	[illegible]	[illegible]	[illegible]	[illegible]	[illegible]	[illegible]	[illegible]	[illegible]	[illegible]	17,800
Other congenital birth defects	12,200 (3,780 to 26,000)	[illegible]	[illegible]	[illegible]	[illegible]	[illegible]	[illegible]	[illegible]	[illegible]	[illegible]	[illegible]	[illegible]	[illegible]	[illegible]	[illegible]	[illegible]	[illegible]	[illegible]	[illegible]	[illegible]	[illegible]	[illegible]	[illegible]	35,800
Urinary diseases and male infertility	80·3 (33·8 to 168)	[illegible]	[illegible]	[illegible]	[illegible]	[illegible]	[illegible]	[illegible]	[illegible]	[illegible]	[illegible]	[illegible]	[illegible]	[illegible]	[illegible]	[illegible]	[illegible]	[illegible]	[illegible]	[illegible]	[illegible]	[illegible]	[illegible]	9,280
Urolithiasis	0 (0 to 0)	[illegible]	[illegible]	[illegible]	[illegible]	[illegible]	[illegible]	[illegible]	[illegible]	[illegible]	[illegible]	[illegible]	[illegible]	[illegible]	[illegible]	[illegible]	[illegible]	[illegible]	[illegible]	[illegible]	[illegible]	[illegible]	[illegible]	577
Benign prostatic hyperplasia	0 (0 to 0)	[illegible]	[illegible]	[illegible]	[illegible]	[illegible]	[illegible]	[illegible]	[illegible]	[illegible]	[illegible]	[illegible]	[illegible]	[illegible]	[illegible]	[illegible]	[illegible]	[illegible]	[illegible]	[illegible]	[illegible]	[illegible]	[illegible]	1,660
Male infertility	0 (0 to 0)	[illegible]	[illegible]	[illegible]	[illegible]	[illegible]	[illegible]	[illegible]	[illegible]	[illegible]	[illegible]	[illegible]	[illegible]	[illegible]	[illegible]	[illegible]	[illegible]	[illegible]	[illegible]	[illegible]	[illegible]	[illegible]	[illegible]	549
Other urinary diseases	31·6 (10·1 to 74·1)	[illegible]	[illegible]	[illegible]	[illegible]	[illegible]	[illegible]	[illegible]	[illegible]	[illegible]	[illegible]	[illegible]	[illegible]	[illegible]	[illegible]	[illegible]	[illegible]	[illegible]	[illegible]	[illegible]	[illegible]	[illegible]	[illegible]	1,230
Gynecological diseases	0 (0 to 0)	[illegible]	[illegible]	[illegible]	[illegible]	[illegible]	[illegible]	[illegible]	[illegible]	[illegible]	[illegible]	[illegible]	[illegible]	[illegible]	[illegible]	[illegible]	[illegible]	[illegible]	[illegible]	[illegible]	[illegible]	[illegible]	[illegible]	0
Uterine fibroids	0 (0 to 0)	[illegible]	[illegible]	[illegible]	[illegible]	[illegible]	[illegible]	[illegible]	[illegible]	[illegible]	[illegible]	[illegible]	[illegible]	[illegible]	[illegible]	[illegible]	[illegible]	[illegible]	[illegible]	[illegible]	[illegible]	[illegible]	[illegible]	0
Polycystic ovarian syndrome	--	[illegible]	[illegible]	[illegible]	[illegible]	[illegible]	[illegible]	[illegible]	[illegible]	[illegible]	[illegible]	[illegible]	[illegible]	[illegible]	[illegible]	[illegible]	[illegible]	[illegible]	[illegible]	[illegible]	[illegible]	[illegible]	[illegible]	NA
Female infertility	--	[illegible]	[illegible]	[illegible]	[illegible]	[illegible]	[illegible]	[illegible]	[illegible]	[illegible]	[illegible]	[illegible]	[illegible]	[illegible]	[illegible]	[illegible]	[illegible]	[illegible]	[illegible]	[illegible]	[illegible]	[illegible]	[illegible]	NA
Endometriosis	--	[illegible]	[illegible]	[illegible]	[illegible]	[illegible]	[illegible]	[illegible]	[illegible]	[illegible]	[illegible]	[illegible]	[illegible]	[illegible]	[illegible]	[illegible]	[illegible]	[illegible]	[illegible]	[illegible]	[illegible]	[illegible]	[illegible]	NA
Genital prolapse	0 (0 to 0)	[illegible]	[illegible]	[illegible]	[illegible]	[illegible]	[illegible]	[illegible]	[illegible]	[illegible]	[illegible]	[illegible]	[illegible]	[illegible]	[illegible]	[illegible]	[illegible]	[illegible]	[illegible]	[illegible]	[illegible]	[illegible]	[illegible]	0
Premenstrual syndrome	--	[illegible]	[illegible]	[illegible]	[illegible]	[illegible]	[illegible]	[illegible]	[illegible]	[illegible]	[illegible]	[illegible]	[illegible]	[illegible]	[illegible]	[illegible]	[illegible]	[illegible]	[illegible]	[illegible]	[illegible]	[illegible]	[illegible]	NA
Other gynecological diseases	0 (0 to 0)	[illegible]	[illegible]	[illegible]	[illegible]	[illegible]	[illegible]	[illegible]	[illegible]	[illegible]	[illegible]	[illegible]	[illegible]	[illegible]	[illegible]	[illegible]	[illegible]	[illegible]	[illegible]	[illegible]	[illegible]	[illegible]	[illegible]	0
Hemoglobinopathies and hemolytic anemias	153 (85·3 to 259)	[illegible] (107 to 352)	[illegible]	[illegible]	[illegible]	[illegible]	[illegible]	[illegible]	[illegible]	[illegible]	[illegible]	[illegible]	[illegible]	[illegible]	[illegible]	[illegible]	[illegible]	[illegible]	[illegible]	[illegible]	[illegible]	[illegible]	[illegible]	20,600
Thalassemias	0·2 (0·1 to 0·3)	0·2 (0·1 to 0·4)	[illegible]	[illegible]	[illegible]	[illegible]	[illegible]	[illegible]	[illegible]	[illegible]	[illegible]	[illegible]	[illegible]	[illegible]	[illegible]	[illegible]	[illegible]	[illegible]	[illegible]	[illegible]	[illegible]	[illegible]	[illegible]	45·7
Thalassemias trait	0·6 (0·3 to 1·2)	3·1 (1·7 to 5·2)	[illegible]	[illegible]	[illegible]	[illegible]	[illegible]	[illegible]	[illegible]	[illegible]	[illegible]	[illegible]	[illegible]	[illegible]	[illegible]	[illegible]	[illegible]	[illegible]	[illegible]	[illegible]	[illegible]	[illegible]	[illegible]	562
Sickle cell disorders	144 (77 to 250)	154 (56 to 294)	[illegible]	[illegible]	[illegible]	[illegible]	[illegible]	[illegible]	[illegible]	[illegible]	[illegible]	[illegible]	[illegible]	[illegible]	[illegible]	[illegible]	[illegible]	[illegible]	[illegible]	[illegible]	[illegible]	[illegible]	[illegible]	12,300
Sickle cell trait	6·4 (3·1 to 10·7)	42·1 (22·3 to 71·2)	[illegible]	[illegible]	[illegible]	[illegible]	[illegible]	[illegible]	[illegible]	[illegible]	[illegible]	[illegible]	[illegible]	[illegible]	[illegible]	[illegible]	[illegible]	[illegible]	[illegible]	[illegible]	[illegible]	[illegible]	[illegible]	2,390
G6PD deficiency	1·1 (0·5 to 2)	5·6 (2·8 to 9·2)	[illegible]	[illegible]	[illegible]	[illegible]	[illegible]	[illegible]	[illegible]	[illegible]	[illegible]	[illegible]	[illegible]	[illegible]	[illegible]	[illegible]	[illegible]	[illegible]	[illegible]	[illegible]	[illegible]	[illegible]	[illegible]	3,080
G6PD trait	0 (0 to 0)	0 (0 to 0)	[illegible]	[illegible]	[illegible]	[illegible]	[illegible]	[illegible]	[illegible]	[illegible]	[illegible]	[illegible]	[illegible]	[illegible]	[illegible]	[illegible]	[illegible]	[illegible]	[illegible]	[illegible]	[illegible]	[illegible]	[illegible]	0
Other hemoglobinopathies and hemolytic anemias	0·5 (0·3 to 1)	6·7 (3·6 to 10·7)	[illegible]	[illegible]	[illegible]	[illegible]	[illegible]	[illegible]	[illegible]	[illegible]	[illegible]	[illegible]	[illegible]	[illegible]	[illegible]	[illegible]	[illegible]	[illegible]	[illegible]	[illegible]	[illegible]	[illegible]	[illegible]	2,210
Endocrine, metabolic, blood, and immune disorders	604 (257 to 1,140)	571 (246 to 1,110)	[illegible]	[illegible]	[illegible]	[illegible]	[illegible]	[illegible]	[illegible]	[illegible]	[illegible]	[illegible]	[illegible]	[illegible]	[illegible]	[illegible]	[illegible]	[illegible]	[illegible]	[illegible]	[illegible]	[illegible]	[illegible]	21,700
Oral disorders	0 (0 to 0)	0 (0 to 0)	[illegible]	[illegible]	[illegible]	[illegible]	[illegible]	[illegible]	[illegible]	[illegible]	[illegible]	[illegible]	[illegible]	[illegible]	[illegible]	[illegible]	[illegible]	[illegible]	[illegible]	[illegible]	[illegible]	[illegible]	[illegible]	13,300
Caries of deciduous teeth	0 (0 to 0)	0 (0 to 0)	[illegible]	[illegible]	[illegible]	[illegible]	[illegible]	[illegible]	[illegible]	[illegible]	[illegible]	[illegible]	[illegible]	[illegible]	[illegible]	[illegible]	[illegible]	[illegible]	[illegible]	[illegible]	[illegible]	[illegible]	[illegible]	242
Caries of permanent teeth	0 (0 to 0)	0 (0 to 0)	[illegible]	[illegible]	[illegible]	[illegible]	[illegible]	[illegible]	[illegible]	[illegible]	[illegible]	[illegible]	[illegible]	[illegible]	[illegible]	[illegible]	[illegible]	[illegible]	[illegible]	[illegible]	[illegible]	[illegible]	[illegible]	1,640
Periodontal diseases	0 (0 to 0)	0 (0 to 0)	[illegible]	[illegible]	[illegible]	[illegible]	[illegible]	[illegible]	[illegible]	[illegible]	[illegible]	[illegible]	[illegible]	[illegible]	[illegible]	[illegible]	[illegible]	[illegible]	[illegible]	[illegible]	[illegible]	[illegible]	[illegible]	4,690
Other oral disorders	0 (0 to 0)	0 (0 to 0)	[illegible]	[illegible]	[illegible]	[illegible]	[illegible]	[illegible]	[illegible]	[illegible]	[illegible]	[illegible]	[illegible]	[illegible]	[illegible]	[illegible]	[illegible]	[illegible]	[illegible]	[illegible]	[illegible]	[illegible]	[illegible]	2,810
Sudden infant death syndrome	--	480 (132 to 1,420)	[illegible]	[illegible]	[illegible]	[illegible]	[illegible]	[illegible]	[illegible]	[illegible]	[illegible]	[illegible]	[illegible]	[illegible]	[illegible]	[illegible]	[illegible]	[illegible]	[illegible]	[illegible]	[illegible]	[illegible]	[illegible]	NA
Injuries	4,250 (2,400 to 7,380)	4,150 (1,710 to [illegible])	[illegible]	[illegible]	[illegible]	[illegible]	[illegible]	[illegible]	[illegible]	[illegible]	[illegible]	[illegible]	[illegible]	[illegible]	[illegible]	[illegible]	[illegible]	[illegible]	[illegible]	[illegible]	[illegible]	[illegible]	[illegible]	573,000
Transport injuries	402 (115 to 1,120)	253 (86·6 to 616)	[illegible]	[illegible]	[illegible]	[illegible]	[illegible]	[illegible]	[illegible]	[illegible]	[illegible]	[illegible]	[illegible]	[illegible]	[illegible]	[illegible]	[illegible]	[illegible]	[illegible]	[illegible]	[illegible]	[illegible]	[illegible]	70,400
Road injuries	333 (94·6 to 965)	198 (63·6 to 490)	[illegible]	[illegible]	[illegible]	[illegible]	[illegible]	[illegible]	[illegible]	[illegible]	[illegible]	[illegible]	[illegible]	[illegible]	[illegible]	[illegible]	[illegible]	[illegible]	[illegible]	[illegible]	[illegible]	[illegible]	[illegible]	65,100
Pedestrian road injuries	143 (32·7 to 403)	53·7 (11·6 to 166)	[illegible]	[illegible]	[illegible]	[illegible]	[illegible]	[illegible]	[illegible]	[illegible]	[illegible]	[illegible]	[illegible]	[illegible]	[illegible]	[illegible]	[illegible]	[illegible]	[illegible]	[illegible]	[illegible]	[illegible]	[illegible]	13,000
Cyclist road injuries	0 (0 to 0)	0 (0 to 0)	[illegible]	[illegible]	[illegible]	[illegible]	[illegible]	[illegible]	[illegible]	[illegible]	[illegible]	[illegible]	[illegible]	[illegible]	[illegible]	[illegible]	[illegible]	[illegible]	[illegible]	[illegible]	[illegible]	[illegible]	[illegible]	8,220
Motorcyclist road injuries	77·8 (13·1 to 241)	45·2 (8·7 to 132)	[illegible]	[illegible]	[illegible]	[illegible]	[illegible]	[illegible]	[illegible]	[illegible]	[illegible]	[illegible]	[illegible]	[illegible]	[illegible]	[illegible]	[illegible]	[illegible]	[illegible]	[illegible]	[illegible]	[illegible]	[illegible]	20,100
Motor vehicle road injuries	106 (24·8 to 330)	97·6 (26·9 to 262)	[illegible]	[illegible]	[illegible]	[illegible]	[illegible]	[illegible]	[illegible]	[illegible]	[illegible]	[illegible]	[illegible]	[illegible]	[illegible]	[illegible]	[illegible]	[illegible]	[illegible]	[illegible]	[illegible]	[illegible]	[illegible]	23,300
Other road injuries	6·4 (0·8 to 23·6)	1·7 (0·2 to 5·5)	[illegible]	[illegible]	[illegible]	[illegible]	[illegible]	[illegible]	[illegible]	[illegible]	[illegible]	[illegible]	[illegible]	[illegible]	[illegible]	[illegible]	[illegible]	[illegible]	[illegible]	[illegible]	[illegible]	[illegible]	[illegible]	466

Other transport injuries	68·6 (15·8 to 225)	54·7 (16·6 to 143)	239 (72·6 to 546)	468 (168 to 1,240)	355 (148 to 878)	344 (147 to 834)	419 (178 to 1,070)	531 (234 to 1,430)	530 (236 to 1,430)	544 (247 to 1,450)	436 (208 to 1,140)	389 (186 to 1,100)	269 (137 to 715)	202 (111 to 516)	182 (93·6 to 497)	113 (63·6 to 291)	72·4 (44 to 161)	53·3 (30·8 to 138)	27·4 (17·1 to 66·6)	13 (8·6 to 26·7)	5·4 (3·7 to 10·4)	1·7 (1·2 to 2·9)	0·4 (0·3 to 0·7)	5,320
Unintentional injuries	3,370 (1,840 to 6,480)	3,700 (1,340 to 16,300)	23,000 (7,680 to 41,000)	57,200 (27,400 to 83,300)	26,600 (18,700 to 34,800)	20,500 (15,700 to 26,600)	20,300 (12,900 to 29,800)	23,800 (15,700 to 36,400)	20,800 (13,500 to 31,600)	20,200 (14,100 to 28,700)	18,100 (12,600 to 25,400)	15,600 (10,900 to 21,900)	11,700 (8,020 to 16,700)	9,300 (6,440 to 13,400)	7,400 (5,120 to 10,400)	5,830 (4,250 to 8,040)	4,200 (3,100 to 5,660)	2,850 (2,150 to 3,760)	1,880 (1,410 to 2,460)	1,230 (940 to 1,650)	625 (466 to 835)	229 (169 to 314)	48·9 (36·5 to 68·1)	298,000
Falls	60·5 (21 to 136)	43·7 (9·9 to 106)	854 (115 to 2,480)	2,200 (324 to 5,850)	1,060 (467 to 1,870)	970 (560 to 1,500)	989 (611 to 1,440)	1,070 (672 to 1,620)	1,150 (762 to 1,600)	1,260 (865 to 1,740)	1,310 (892 to 1,840)	1,270 (874 to 1,760)	1,140 (786 to 1,610)	1,130 (773 to 1,610)	991 (672 to 1,420)	932 (643 to 1,310)	767 (531 to 1,090)	655 (459 to 915)	516 (361 to 740)	445 (306 to 653)	268 (179 to 384)	109 (71·5 to 162)	23·2 (14·4 to 33·7)	19,200
Drowning	239 (78·8 to 551)	118 (37·2 to 271)	2,960 (520 to 7,160)	24,600 (6,790 to 45,500)	13,700 (7,210 to 21,300)	7,640 (4,080 to 11,600)	5,410 (2,280 to 10,500)	4,050 (1,730 to 8,180)	3,470 (1,620 to 6,790)	3,060 (1,530 to 5,310)	2,560 (1,280 to 4,320)	2,110 (1,130 to 3,800)	1,360 (692 to 2,460)	1,020 (532 to 1,880)	689 (346 to 1,290)	476 (250 to 915)	287 (155 to 552)	134 (73·5 to 251)	71·5 (39·9 to 137)	25·1 (14·2 to 49·3)	11·6 (6·2 to 21·3)	3·4 (2 to 6·3)	0·8 (0·4 to 1·5)	74,100
Fire, heat, and hot substances	88 (30·5 to 167)	138 (31·1 to 275)	2,670 (319 to 5,450)	9,910 (1,410 to 18,100)	3,060 (1,100 to 4,920)	1,690 (1,030 to 2,400)	1,230 (819 to 1,650)	1,290 (857 to 1,800)	1,420 (988 to 1,890)	1,540 (1,090 to 2,040)	1,340 (936 to 1,760)	1,150 (810 to 1,520)	905 (619 to 1,230)	682 (472 to 937)	531 (362 to 720)	421 (270 to 598)	296 (198 to 419)	200 (123 to 297)	132 (83·1 to 198)	84·8 (52 to 126)	45·3 (27·4 to 67·9)	15·3 (8·9 to 22·4)	3·2 (1·9 to 4·8)	28,800
Poisonings	159 (40·2 to 518)	211 (21·6 to 947)	876 (225 to 2,370)	4,150 (1,480 to 8,850)	783 (285 to 2,420)	348 (138 to 1,110)	242 (84·4 to 840)	343 (120 to 1,230)	483 (173 to 1,760)	499 (185 to 1,860)	406 (152 to 1,450)	288 (113 to 1,060)	212 (77·8 to 857)	137 (52·4 to 543)	98·6 (38·2 to 417)	72·5 (26·9 to 321)	44·7 (18·1 to 188)	24·8 (10 to 96·5)	15·4 (6·3 to 61·2)	8·8 (3·3 to 40·5)	3·3 (1·4 to 12·9)	1·1 (0·5 to 4·7)	0·2 (0·1 to 0·6)	9,400
Poisoning by carbon monoxide	11·9 (1 to 52·9)	22·4 (1 to 126)	20·9 (1·3 to 105)	333 (29·3 to 1,350)	122 (17·1 to 485)	99·3 (20 to 415)	106 (22·5 to 455)	153 (37·4 to 676)	195 (43·5 to 916)	210 (52·2 to 1,040)	181 (47·1 to 824)	119 (28·8 to 570)	73·9 (15·4 to 423)	38·3 (7·6 to 217)	34·4 (7·1 to 210)	23·5 (4·9 to 134)	14·3 (2·9 to 79·2)	8·5 (1·8 to 46·1)	4·3 (0·9 to 26·1)	3·5 (0·8 to 21·2)	1·1 (0·3 to 6·4)	0·3 (0·1 to 2)	0 (0 to 0·2)	1,780
Poisoning by other means	147 (37·6 to 491)	188 (19·2 to 874)	855 (217 to 2,310)	3,810 (1,370 to 8,150)	661 (228 to 1,880)	248 (97·3 to 754)	136 (52 to 398)	190 (64·8 to 577)	288 (103 to 927)	290 (103 to 967)	225 (86·6 to 673)	169 (66 to 517)	138 (50·3 to 468)	99 (37·6 to 352)	64·2 (25·6 to 235)	49 (19·7 to 176)	30·4 (11·8 to 106)	16·4 (7·1 to 52·6)	11·1 (4·8 to 38·6)	5·3 (2·1 to 19)	2·2 (1 to 7·3)	0·8 (0·3 to 3·1)	0·1 (0·1 to 0·4)	7,620
Exposure to mechanical forces	554 (30·4 to 2,990)	1,450 (7·9 to 14,400)	1,360 (73·4 to 6,820)	3,000 (895 to 6,230)	2,860 (876 to 6,460)	2,480 (836 to 5,490)	4,570 (1,220 to 11,400)	5,580 (1,670 to 13,500)	5,490 (1,870 to 12,900)	5,290 (2,130 to 11,100)	4,470 (1,910 to 9,520)	4,080 (1,750 to 8,870)	3,010 (1,340 to 6,400)	2,430 (1,090 to 5,120)	1,920 (897 to 4,050)	1,360 (697 to 2,530)	889 (498 to 1,530)	522 (300 to 866)	307 (184 to 495)	167 (104 to 262)	77·5 (47·2 to 118)	23·8 (15 to 36·8)	4·9 (3·1 to 7·3)	51,900
Unintentional firearm injuries	359 (10·1 to 2,020)	972 (1·9 to 11,000)	991 (27·3 to 5,180)	2,030 (291 to 4,860)	1,780 (359 to 5,030)	1,680 (398 to 4,670)	3,610 (668 to 10,300)	4,390 (905 to 12,700)	4,090 (936 to 11,500)	3,720 (1,020 to 9,760)	2,980 (889 to 7,910)	2,690 (784 to 7,110)	1,840 (560 to 4,970)	1,420 (426 to 3,800)	1,060 (330 to 2,940)	685 (243 to 1,740)	395 (156 to 975)	207 (88·1 to 497)	108 (48·9 to 253)	56·9 (27·6 to 128)	27·8 (12·9 to 63·6)	8·2 (4·2 to 18·4)	1·8 (1 to 3·6)	35,100
Other exposure to mechanical forces	195 (2·4 to 1,430)	481 (1·2 to 3,730)	372 (17·9 to 2,060)	973 (169 to 3,360)	1,080 (258 to 3,610)	798 (316 to 2,320)	966 (465 to 2,830)	1,190 (608 to 3,280)	1,400 (755 to 3,490)	1,560 (874 to 3,450)	1,490 (843 to 3,330)	1,380 (778 to 3,240)	1,170 (678 to 2,500)	1,010 (577 to 2,200)	861 (492 to 1,810)	680 (407 to 1,230)	494 (308 to 855)	315 (196 to 509)	199 (125 to 325)	110 (71 to 173)	49·7 (31·9 to 77·6)	15·7 (9·9 to 24·7)	3·1 (2 to 4·8)	16,800
Adverse effects of medical treatment	634 (306 to 1,140)	603 (232 to 1,220)	2,550 (582 to 6,020)	1,840 (437 to 3,800)	678 (356 to 1,170)	338 (192 to 528)	184 (79·6 to 330)	275 (114 to 498)	303 (136 to 545)	278 (126 to 475)	321 (149 to 576)	323 (152 to 583)	277 (131 to 518)	345 (164 to 667)	336 (152 to 665)	364 (163 to 739)	333 (161 to 632)	250 (127 to 480)	202 (99·3 to 383)	134 (65·6 to 264)	63 (31·5 to 123)	21·3 (10·4 to 42·9)	4·6 (2·4 to 9)	10,600
Animal contact	7·9 (2·1 to 18·2)	18·3 (4·2 to 39·8)	112 (19·3 to 267)	481 (92·4 to 1,090)	169 (79·4 to 316)	140 (78·2 to 237)	115 (74·2 to 181)	111 (73·3 to 162)	134 (84·2 to 216)	118 (76 to 173)	126 (77·4 to 206)	113 (68·7 to 193)	105 (60·9 to 195)	94·1 (55·6 to 180)	82·3 (47·7 to 158)	77·8 (44·2 to 158)	67·6 (37·2 to 136)	44·6 (24·8 to 87·3)	29·4 (15·7 to 65)	14·8 (8·5 to 31)	6 (3·6 to 11·4)	2·2 (1·3 to 4·7)	0·5 (0·3 to 1)	2,170
Venomous animal contact	7·6 (2·1 to 17·6)	16·8 (3·9 to 37·8)	81·9 (14·8 to 197)	368 (72·5 to 879)	119 (51·2 to 235)	97·2 (53·5 to 173)	82·8 (51·2 to 134)	77·5 (50·1 to 118)	93·8 (57·8 to 155)	82 (52·4 to 125)	95·7 (58·2 to 166)	78·2 (46·1 to 135)	71·5 (40·1 to 143)	66·3 (38·5 to 137)	57·4 (32·5 to 120)	52·8 (28·7 to 125)	43·2 (23 to 104)	26·8 (14·4 to 62·9)	19·7 (10·3 to 48·3)	10·2 (5·5 to 24·7)	4·3 (2·4 to 9·2)	1·8 (1 to 4·2)	0·4 (0·2 to 0·8)	1,550
Non-venomous animal contact	0·3 (0·1 to 0·9)	1·5 (0·2 to 4·8)	30·2 (2 to 86·5)	113 (8·3 to 335)	50 (13·4 to 113)	42·7 (16·2 to 90·3)	32·4 (16·8 to 58·7)	33·7 (17·5 to 58·3)	40·5 (19·9 to 76·8)	35·9 (18·9 to 60·6)	30·7 (16·6 to 53·1)	34·8 (17·1 to 70·5)	33·5 (15·8 to 67·2)	27·8 (14·4 to 52)	24·9 (12·3 to 51·4)	25 (11·9 to 54)	24·4 (10·7 to 49)	17·8 (7·6 to 37·8)	9·7 (4·7 to 20·4)	4·6 (2·5 to 8·2)	1·7 (1 to 3·1)	0·5 (0·3 to 0·7)	0·1 (0·1 to 0·1)	616
Foreign body	1,010 (415 to 2,110)	883 (338 to 1,680)	10,600 (2,240 to 22,300)	8,130 (2,250 to 14,000)	1,630 (818 to 2,790)	983 (585 to 1,550)	346 (208 to 561)	379 (223 to 640)	407 (255 to 634)	395 (258 to 577)	367 (236 to 552)	353 (238 to 530)	279 (181 to 427)	248 (156 to 377)	201 (125 to 325)	185 (111 to 291)	154 (94·7 to 245)	104 (63·1 to 166)	73·5 (46·5 to 110)	48·5 (31·5 to 74)	19·5 (12·3 to 30·8)	6·8 (4·5 to 10)	1·7 (1·1 to 2·6)	26,800
Pulmonary aspiration and foreign body in airway	1,000 (409 to 2,070)	876 (336 to 1,670)	10,500 (2,210 to 21,600)	7,850 (2,190 to 13,800)	1,500 (716 to 2,580)	877 (468 to 1,430)	251 (128 to 447)	282 (144 to 536)	301 (166 to 528)	294 (172 to 475)	277 (156 to 461)	271 (158 to 450)	213 (122 to 346)	196 (110 to 322)	159 (89·9 to 279)	150 (81·1 to 252)	126 (70·1 to 216)	84·2 (46 to 142)	59·3 (34·8 to 93)	39·4 (23·3 to 63·1)	15·2 (8·9 to 25·1)	5·2 (3·1 to 8)	1·3 (0·8 to 2·1)	25,300
Foreign body in eyes	0·1 (0 to 0·2)	0·2 (0·1 to 0·5)	3·5 (1 to 7·7)	17·7 (5·3 to 40·6)	26·4 (7·8 to 62·6)	31·3 (9·7 to 73·3)	36·5 (11·7 to 85·8)	40·5 (13 to 88·5)	43·2 (15·5 to 92)	42·5 (15 to 88·9)	37·6 (14·3 to 78·3)	31·9 (13·6 to 63·4)	25 (11·9 to 48·1)	18·6 (9·5 to 33·1)	13·8 (7·9 to 22·8)	10·9 (6·5 to 17·3)	8·4 (5·1 to 12·9)	5·5 (3·5 to 8·2)	3·6 (2·3 to 5·3)	2 (1·3 to 3)	0·9 (0·6 to 1·3)	0·3 (0·2 to 0·4)	0·1 (0 to 0·1)	400
Foreign body in other body part	8·3 (1·6 to 28·8)	6·6 (1·7 to 20·9)	115 (16·2 to 510)	263 (48·9 to 1,050)	102 (51·1 to 257)	75 (47·2 to 130)	58·4 (42·3 to 79·9)	57·2 (41 to 78)	62·1 (43·9 to 87·3)	58·5 (41·8 to 79·4)	52·8 (37·7 to 71·5)	49·8 (35·3 to 69)	41·4 (28·5 to 59·9)	32·8 (23·5 to 45·2)	28·6 (20·1 to 41·3)	24 (16·3 to 35·6)	19·1 (13·1 to 28·3)	14·4 (9·3 to 24·8)	10·6 (6·8 to 16·9)	7 (4·5 to 11·3)	3·3 (2·2 to 5·5)	1·3 (0·8 to 2·2)	0·4 (0·2 to 0·6)	1,090
Environmental heat and cold exposure	123 (18·5 to 382)	58·9 (16·5 to 157)	252 (64·5 to 690)	570 (221 to 1,490)	383 (165 to 991)	313 (168 to 675)	508 (244 to 1,420)	651 (307 to 1,760)	671 (349 to 1,590)	713 (382 to 1,610)	648 (353 to 1,450)	602 (316 to 1,350)	514 (267 to 1,220)	454 (226 to 1,060)	422 (192 to 1,070)	343 (159 to 888)	257 (125 to 624)	237 (93·1 to 651)	166 (65·2 to 471)	125 (45·8 to 352)	62·6 (24·6 to 184)	26·2 (9·5 to 75·6)	6·4 (2·3 to 19·1)	8,100
Exposure to forces of nature	0 (0 to 0)	0 (0 to 0)	0 (0 to 0)	3·7 (2·2 to 6·2)	455 (276 to 723)	4,190 (2,490 to 6,960)	5,290 (3,240 to 8,530)	8,680 (5,280 to 13,300)	5,710 (3,380 to 9,620)	5,500 (3,200 to 9,010)	5,210 (3,050 to 8,210)	4,120 (2,470 to 6,520)	3,000 (1,820 to 4,710)	2,180 (1,300 to 3,410)	1,670 (1,050 to 2,590)	1,280 (814 to 2,010)	882 (564 to 1,320)	548 (342 to 827)	290 (182 to 424)	141 (87·2 to 221)	50·6 (31·7 to 76·3)	14·1 (8·9 to 21·3)	2·2 (1·4 to 3·3)	49,200
Other unintentional injuries	498 (162 to 1,090)	171 (64 to 397)	745 (239 to 1,520)	2,280 (817 to 5,110)	1,730 (583 to 4,410)	1,420 (535 to 3,520)	1,380 (516 to 3,910)	1,370 (582 to 3,380)	1,530 (635 to 3,860)	1,530 (685 to 3,570)	1,360 (616 to 3,190)	1,160 (538 to 2,630)	865 (392 to 2,080)	582 (289 to 1,330)	454 (221 to 1,050)	312 (162 to 725)	220 (120 to 451)	126 (71·5 to 259)	76·2 (45·6 to 142)	39·7 (25·1 to 67·2)	17·9 (11·4 to 28·9)	5·6 (3·7 to 8·9)	1·2 (0·8 to 1·9)	17,900
Self-harm and interpersonal violence	470 (167 to 1,040)	202 (69 to 437)	1,020 (330 to 2,370)	4,440 (1,620 to 9,710)	3,540 (950 to 7,310)	5,840 (1,770 to 11,000)	24,300 (8,510 to 39,900)	34,800 (13,200 to 52,600)	33,300 (12,900 to 50,500)	28,100 (11,000 to 43,200)	21,900 (7,640 to 36,300)	16,600 (5,630 to 29,200)	11,000 (3,520 to 19,600)	7,380 (2,540 to 13,200)	4,910 (1,740 to 8,860)	3,210 (1,230 to 5,490)	1,900 (790 to 3,210)	999 (467 to 1,660)	513 (260 to 839)	216 (122 to 319)	83·5 (48·8 to 122)	18·6 (11·8 to 25·8)	4·5 (2·4 to 7·2)	205,000
Self-harm	0 (0 to 0)	0 (0 to 0)	0 (0 to 0)	0 (0 to 0)	0 (0 to 0)	756 (283 to 1,370)	2,580 (1,000 to 4,770)	3,610 (1,590 to 7,070)	3,790 (1,770 to 6,810)	4,070 (2,000 to 7,080)	3,730 (1,940 to 6,310)	3,240 (1,610 to 5,100)	2,300 (1,110 to 3,790)	1,770 (882 to 2,860)	1,250 (644 to 2,020)	949 (488 to 1,530)	652 (341 to 1,050)	395 (206 to 621)	223 (111 to 344)	118 (61·6 to 187)	46·8 (26·8 to 72·4)	9·3 (5·1 to 14·3)	1·4 (0·7 to 2·2)	29,500
Self-harm by firearm	0 (0 to 0)	0 (0 to 0)	0 (0 to 0)	0 (0 to 0)	0 (0 to 0)	27·8 (6·9 to 73·7)	250 (75·9 to 600)	361 (106 to 909)	370 (108 to 937)	349 (128 to 817)	277 (113 to 580)	249 (102 to 515)	162 (59·4 to 341)	133 (54·4 to 298)	80·2 (31·4 to 184)	64 (26·7 to 147)	40·2 (15·3 to 93·8)	23·5 (9·1 to 53·2)	12·4 (4·7 to 28·7)	6·1 (2·3 to 14)	1·9 (0·8 to 4·3)	0·3 (0·1 to 0·7)	0·1 (0 to 0·1)	2,410
Self-harm by other specified means	0 (0 to 0)	0 (0 to 0)	0 (0 to 0)	0 (0 to 0)	0 (0 to 0)	728 (265 to 1,320)	2,330 (852 to 4,360)	3,250 (1,440 to 6,320)	3,420 (1,610 to 6,120)	3,720 (1,800 to 6,460)	3,450 (1,750 to 5,820)	2,990 (1,440 to 4,710)	2,140 (1,000 to 3,550)	1,630 (789 to 2,630)	1,170 (586 to 1,900)	885 (445 to 1,430)	612 (320 to 991)	372 (187 to 575)	211 (101 to 327)	112 (58·6 to 179)	44·9 (25·2 to 68·6)	9 (4·9 to 13·8)	1·4 (0·7 to 2·1)	27,100
Interpersonal violence	470 (167 to 1,040)	202 (68·9 to 437)	1,010 (328 to 2,360)	4,430 (1,600 to 9,700)	3,530 (937 to 7,300)	5,070 (1,120 to 10,300)	21,600 (5,470 to 37,300)	31,100 (8,700 to 49,600)	29,400 (7,920 to 47,400)	23,900 (6,370 to 40,100)	18,100 (3,930 to 32,000)	13,300 (2,760 to 25,900)	8,600 (1,610 to 16,700)	5,560 (1,050 to 10,900)	3,620 (682 to 7,540)	2,240 (461 to 4,300)	1,240 (269 to 2,420)	599 (146 to 1,210)	288 (74·8 to 587)	96·4 (35·2 to 165)	36·3 (14·9 to 62·1)	9·2 (4·7 to 14·6)	3 (1·2 to 5·7)	174,000
Physical violence by firearm	54·8 (11 to 164)	23·5 (4·2 to 68·5)	56·6 (9·6 to 234)	239 (30·1 to 1,180)	364 (50·4 to 1,220)	915 (95·3 to 2,600)	10,300 (1,410 to 21,400)	16,100 (2,340 to 29,400)	14,000 (2,080 to 26,300)	9,910 (1,320 to 19,900)	6,320 (736 to 13,500)	3,800 (381 to 8,660)	2,120 (189 to 5,260)	1,100 (104 to 2,870)	604 (60·1 to 1,680)	316 (36·2 to 776)	142 (18·9 to 395)	54·1 (8·3 to 144)	16·7 (3·8 to 48·7)	5·5 (1·6 to 14·4)	2 (0·7 to 5·1)	0·6 (0·2 to 1·3)	0·1 (0 to 0·4)	66,300
Physical violence by sharp object	60·2 (8·7 to 177)	24·2 (3·6 to 83·2)	42·1 (9·3 to 187)	268 (50·3 to 1,200)	312 (59·7 to 1,420)	605 (122 to 2,160)	5,810 (1,420 to 15,800)	8,020 (2,010 to 22,200)	7,650 (1,860 to 21,700)	6,840 (1,770 to 18,200)	5,160 (1,050 to 14,300)	3,770 (736 to 10,200)	2,250 (413 to 6,580)	1,350 (246 to 4,250)	713 (157 to 2,150)	422 (103 to 1,340)	201 (60·7 to 626)	112 (35·7 to 315)	52·1 (19·3 to 144)	20·9 (9·9 to 49)	8 (4·3 to 17)	3·3 (1·7 to 6·1)	0·8 (0·4 to 2)	43,700
Sexual violence	0 (0 to 0)	0 (0 to 0)	0 (0 to 0)	3·7 (2·1 to 6)	26·7 (16·3 to 42·3)	54·2 (30·2 to 85·8)	104 (58·1 to 167)	123 (71·1 to 190)	108 (59·4 to 175)	92·1 (51·2 to 148)	72·8 (41 to 118)	50·4 (29 to 82·9)	33·4 (19·3 to 56·9)	22·1 (12·7 to 36·4)	15 (8·8 to 24·1)	11·2 (6·8 to 17·2)	8·2 (5 to 12·3)	5 (3·1 to 7·4)	3 (1·9 to 4·4)	1·6 (1 to 2·4)	0·5 (0·3 to 0·9)	0·1 (0 to 0·1)	0 (0 to 0)	734
Physical violence by other means	355 (111 to 809)	154 (47·6 to 346)	916 (279 to 2,230)	3,920 (1,320 to 8,940)	2,830 (703 to 6,070)	3,490 (674 to 7,710)	5,460 (737 to 12,500)	6,900 (927 to 15,400)	7,700 (1,180 to 16,300)	7,040 (1,070 to 15,000)	6,540 (1,030 to 14,300)	5,620 (792 to 12,600)	4,190 (578 to 9,150)	3,080 (472 to 6,980)	2,290 (356 to 5,050)	1,490 (243 to 3,070)	886 (158 to 1,910)	428 (83·2 to 917)	216 (45·8 to 470)	68·4 (20·6 to 131)	25·8 (9 to 48·6)	5·2 (2·6 to 8·7)	2·1 (0·7 to 4·5)	63,600
Conflict and terrorism	0 (0 to 0)	0 (0 to 0)	0 (0 to 0)	1 (0·6 to 1·6)	1·4 (0·8 to 2·4)	3·1 (1·9 to 4·8)	24·8 (13·2 to 42·8)	21 (11·7 to 35·7)	46 (26·6 to 76·1)	80·6 (44·8 to 138)	80·1 (44 to 138)	72·1 (40·7 to 121)	67·9 (38·2 to 115)	50·7 (29·3 to 86·3)	29·2 (16·7 to 49·6)	15·8 (9·1 to 27)	8·3 (4·8 to 14·3)	3·9 (2·3 to 6·6)	1·9 (1·1 to 3·3)	0·9 (0·5 to 1·6)	0·4 (0·2 to 0·6)	0·1 (0·1 to 0·2)	0 (0 to 0)	509

SR Table 2a: Jamaica DALYS by cause and age group for males with 95% uncertainty intervals, 2019

Cause	Early Neonatal	Late Neonatal	Post Neonatal	1 to 4	5 to 9	10 to 14	15 to 19	20 to 24	25 to 29	30 to 34	35 to 39	40 to 44	45 to 49	50 to 54	55 to 59	60 to 64	65 to 69	70 to 74	75 to 79	80 to 84	85 to 89	90 to 94	95 plus	Total
All causes	17,300 (12,000 to 24,100)	4,650 (3,240 to 6,490)	3,210 (2,100 to 4,840)	5,770 (4,540 to 7,430)	6,360 (4,970 to 8,020)	7,770 (6,140 to 9,850)	11,500 (9,280 to 14,100)	16,700 (14,000 to 19,900)	18,600 (15,700 to 21,900)	18,800 (16,100 to 22,200)	18,700 (15,900 to 22,100)	19,100 (15,800 to 22,700)	23,500 (19,100 to 28,200)	27,900 (22,700 to 33,600)	32,200 (26,300 to 38,900)	32,400 (26,700 to 38,700)	30,600 (25,500 to 36,300)	31,300 (26,200 to 37,000)	23,400 (19,900 to 27,400)	16,900 (14,600 to 19,400)	10,600 (9,380 to 11,900)	5,450 (4,870 to 6,040)	3,030 (2,780 to 3,280)	386,000
Communicable, maternal, neonatal, and nutritional diseases	15,200 (10,500 to 21,300)	3,840 (2,680 to 5,360)	1,750 (1,150 to 2,660)	2,340 (1,720 to 3,260)	1,810 (1,220 to 2,740)	1,370 (1,010 to 1,840)	1,420 (1,110 to 1,790)	1,770 (1,440 to 2,190)	2,460 (2,110 to 2,890)	2,990 (2,650 to 3,400)	3,160 (2,850 to 3,540)	2,920 (2,610 to 3,290)	2,730 (2,390 to 3,160)	2,280 (2,010 to 2,600)	1,970 (1,680 to 2,350)	1,390 (1,160 to 1,670)	1,170 (968 to 1,420)	979 (771 to 1,220)	748 (579 to 950)	602 (464 to 764)	432 (344 to 541)	234 (179 to 298)	152 (112 to 193)	53,800
HIV/AIDS and sexually transmitted infections	3·9 (2·3 to 6·6)	2·9 (1·4 to 5·1)	109 (99·9 to 118)	151 (139 to 162)	100 (92·2 to 109)	58·9 (54·6 to 63·8)	169 (153 to 188)	395 (360 to 439)	1,110 (1,010 to 1,220)	1,740 (1,590 to 1,910)	2,030 (1,870 to 2,210)	1,880 (1,730 to 2,050)	1,570 (1,440 to 1,690)	1,160 (1,070 to 1,250)	782 (723 to 845)	349 (317 to 380)	296 (271 to 320)	64 (55·2 to 73)	23·4 (19·9 to 26·7)	3·8 (2·9 to 5·2)	1·9 (1·4 to 2·6)	0·4 (0·2 to 0·7)	0·2 (0·1 to 0·3)	12,000
HIV/AIDS	0 (0 to 0)	0 (0 to 0)	108 (99·2 to 117)	151 (139 to 162)	100 (92·2 to 109)	56·6 (52·6 to 61·3)	156 (143 to 172)	374 (342 to 413)	1,090 (994 to 1,190)	1,720 (1,570 to 1,890)	2,010 (1,860 to 2,190)	1,860 (1,720 to 2,020)	1,550 (1,430 to 1,670)	1,150 (1,060 to 1,230)	773 (715 to 837)	342 (311 to 374)	291 (267 to 315)	59·9 (51·8 to 68)	21·4 (18·4 to 24·4)	2·3 (1·9 to 2·7)	1·1 (0·9 to 1·4)	0·2 (0·1 to 0·3)	0·1 (0 to 0·1)	11,800
HIV/AIDS - Drug-susceptible Tuberculosis	0 (0 to 0)	0 (0 to 0)	1·4 (0·9 to 2·2)	2 (1·2 to 3·1)	1·5 (1 to 2·3)	0·8 (0·5 to 1·3)	1·8 (1·2 to 2·6)	4·6 (3·2 to 6·6)	13·1 (8·9 to 19)	22·3 (14·8 to 32·7)	27·5 (18·3 to 40·6)	26 (17·2 to 38·4)	23·6 (15·7 to 34·9)	17·8 (11·8 to 26·2)	12·5 (8·5 to 18·1)	6·1 (4·3 to 8·6)	5 (3·5 to 7·1)	1·3 (1 to 1·8)	0·5 (0·3 to 0·6)	0 (0 to 0·1)	0 (0 to 0)	0 (0 to 0)	0 (0 to 0)	168
HIV/AIDS - Multidrug-resistant Tuberculosis without extensive	0 (0 to 0)	0 (0 to 0)	0·1 (0 to 0·2)	0·1 (0 to 0·3)	0·1 (0 to 0·2)	0 (0 to 0·1)	0·1 (0 to 0·3)	0·2 (0 to 0·6)	0·5 (0·1 to 1·7)	0·8 (0·1 to 3)	1 (0·1 to 3·6)	0·9 (0·1 to 3·4)	0·8 (0·1 to 3·2)	0·6 (0·1 to 2·4)	0·4 (0·1 to 1·6)	0·2 (0 to 0·7)	0·2 (0 to 0·6)	0 (0 to 0·1)	0 (0 to 0)	0 (0 to 0)	0 (0 to 0)	0 (0 to 0)	0 (0 to 0)	6
HIV/AIDS - Extensively drug-resistant Tuberculosis	0 (0 to 0)	0 (0 to 0)	0 (0 to 0)	0 (0 to 0·1)	0 (0 to 0)	0 (0 to 0)	0 (0 to 0)	0 (0 to 0·1)	0·1 (0 to 0·3)	0·1 (0 to 0·5)	0·2 (0 to 0·6)	0·1 (0 to 0·5)	0·1 (0 to 0·5)	0·1 (0 to 0·4)	0·1 (0 to 0·3)	0 (0 to 0·1)	0 (0 to 0·1)	0 (0 to 0)	0 (0 to 0)	0 (0 to 0)	0 (0 to 0)	0 (0 to 0)	0 (0 to 0)	1
HIV/AIDS resulting in other diseases	0 (0 to 0)	0 (0 to 0)	106 (97·9 to 115)	148 (137 to 160)	98·4 (90·8 to 107)	55·8 (51·6 to 60·6)	154 (141 to 170)	369 (337 to 408)	1,070 (978 to 1,180)	1,700 (1,550 to 1,860)	1,990 (1,830 to 2,160)	1,840 (1,690 to 2,000)	1,530 (1,400 to 1,640)	1,130 (1,040 to 1,210)	760 (701 to 824)	336 (305 to 368)	286 (261 to 309)	58·6 (50·8 to 66·5)	20·9 (17·9 to 23·9)	2·2 (1·8 to 2·6)	1·1 (0·9 to 1·3)	0·2 (0·1 to 0·3)	0·1 (0 to 0·1)	11,600
Sexually transmitted infections excluding HIV	3·9 (2·3 to 6·6)	2·9 (1·4 to 5·1)	0·8 (0·3 to 2)	0 (0 to 0)	0 (0 to 0)	2·2 (1 to 4·6)	12·7 (4·8 to 25·6)	21·1 (10·1 to 37·5)	22·4 (10·2 to 41·5)	22·2 (10·5 to 40·1)	18 (9·3 to 31·5)	15·5 (8 to 27·3)	16·4 (8·7 to 27·1)	13·3 (6·8 to 23·4)	9·9 (5·2 to 17·7)	7·3 (3·6 to 13·3)	5 (2·6 to 9)	4·1 (2·2 to 7·1)	2 (1·1 to 3·7)	1·6 (0·8 to 2·9)	0·8 (0·4 to 1·4)	0·3 (0·1 to 0·5)	0·1 (0·1 to 0·2)	183
Syphilis	3·9 (2·3 to 6·6)	2·9 (1·4 to 5·1)	0·8 (0·3 to 2)	0 (0 to 0)	0 (0 to 0)	0·1 (0·1 to 0·2)	0·4 (0·2 to 0·9)	0·5 (0·2 to 1·1)	0·6 (0·3 to 1·3)	0·7 (0·3 to 1·3)	1·2 (0·7 to 2)	1·5 (0·8 to 2·5)	3 (1·1 to 5·5)	3·4 (1·2 to 6·8)	3·1 (1·3 to 5·4)	2·8 (1·1 to 5·2)	1·9 (1 to 3·4)	1·7 (0·8 to 3·2)	0·6 (0·4 to 0·9)	0·5 (0·3 to 0·8)	0·2 (0·1 to 0·3)	0·1 (0 to 0·1)	0 (0 to 0)	29·8
Chlamydial infection	0 (0 to 0)	[illegible]	[illegible]	[illegible]	[illegible]	[illegible]	[illegible]	[illegible]	[illegible]	[illegible]	[illegible]	[illegible]	[illegible]	[illegible]	[illegible]	[illegible]	[illegible]	[illegible]	[illegible]	[illegible]	[illegible]	[illegible]	[illegible]	32·5
Gonococcal infection	0 (0 to 0)	[illegible]	[illegible]	[illegible]	[illegible]	[illegible]	[illegible]	[illegible]	[illegible]	[illegible]	[illegible]	[illegible]	[illegible]	[illegible]	[illegible]	[illegible]	[illegible]	[illegible]	[illegible]	[illegible]	[illegible]	[illegible]	[illegible]	20·3
Trichomoniasis	0 (0 to 0)	[illegible]	[illegible]	[illegible]	[illegible]	[illegible]	[illegible]	[illegible]	[illegible]	[illegible]	[illegible]	[illegible]	[illegible]	[illegible]	[illegible]	[illegible]	[illegible]	[illegible]	[illegible]	[illegible]	[illegible]	[illegible]	[illegible]	4·1
Genital herpes	0 (0 to 0)	[illegible]	[illegible]	[illegible]	[illegible]	[illegible]	[illegible]	[illegible]	[illegible]	[illegible]	[illegible]	[illegible]	[illegible]	[illegible]	[illegible]	[illegible]	[illegible]	[illegible]	[illegible]	[illegible]	[illegible]	[illegible]	[illegible]	56·2
Other sexually transmitted infections	0 (0 to 0)	[illegible]	[illegible]	[illegible]	[illegible]	[illegible]	[illegible]	[illegible]	[illegible]	[illegible]	[illegible]	[illegible]	[illegible]	[illegible]	[illegible]	[illegible]	[illegible]	[illegible]	[illegible]	[illegible]	[illegible]	[illegible]	[illegible]	39·7
Respiratory infections and tuberculosis	260 (155 to 404)	[illegible]	[illegible]	[illegible]	[illegible]	[illegible]	[illegible]	[illegible]	[illegible]	[illegible]	[illegible]	[illegible]	[illegible]	[illegible]	[illegible]	[illegible]	[illegible]	[illegible]	[illegible]	[illegible]	[illegible]	[illegible]	[illegible]	6,900
Tuberculosis	0 (0 to 0)	[illegible]	[illegible]	[illegible]	[illegible]	[illegible]	[illegible]	[illegible]	[illegible]	[illegible]	[illegible]	[illegible]	[illegible]	[illegible]	[illegible]	[illegible]	[illegible]	[illegible]	[illegible]	[illegible]	[illegible]	[illegible]	[illegible]	317
Latent tuberculosis infection	0 (0 to 0)	[illegible]	[illegible]	[illegible]	[illegible]	[illegible]	[illegible]	[illegible]	[illegible]	[illegible]	[illegible]	[illegible]	[illegible]	[illegible]	[illegible]	[illegible]	[illegible]	[illegible]	[illegible]	[illegible]	[illegible]	[illegible]	[illegible]	0
Drug-susceptible tuberculosis	0 (0 to 0)	[illegible]	[illegible]	[illegible]	[illegible]	[illegible]	[illegible]	[illegible]	[illegible]	[illegible]	[illegible]	[illegible]	[illegible]	[illegible]	[illegible]	[illegible]	[illegible]	[illegible]	[illegible]	[illegible]	[illegible]	[illegible]	[illegible]	306
Multidrug-resistant tuberculosis without extensive drug resistar	0 (0 to 0)	[illegible]	[illegible]	[illegible]	[illegible]	[illegible]	[illegible]	[illegible]	[illegible]	[illegible]	[illegible]	[illegible]	[illegible]	[illegible]	[illegible]	[illegible]	[illegible]	[illegible]	[illegible]	[illegible]	[illegible]	[illegible]	[illegible]	8·9
Extensively drug-resistant tuberculosis	0 (0 to 0)	[illegible]	[illegible]	[illegible]	[illegible]	[illegible]	[illegible]	[illegible]	[illegible]	[illegible]	[illegible]	[illegible]	[illegible]	[illegible]	[illegible]	[illegible]	[illegible]	[illegible]	[illegible]	[illegible]	[illegible]	[illegible]	[illegible]	1·4
Lower respiratory infections	260 (155 to 404)	[illegible]	[illegible]	[illegible]	[illegible]	[illegible]	[illegible]	[illegible]	[illegible]	[illegible]	[illegible]	[illegible]	[illegible]	[illegible]	[illegible]	[illegible]	[illegible]	[illegible]	[illegible]	[illegible]	[illegible]	[illegible]	[illegible]	5,030
Upper respiratory infections	0·2 (0·1 to 0·3)	[illegible]	[illegible]	[illegible]	[illegible]	[illegible]	[illegible]	[illegible]	[illegible]	[illegible]	[illegible]	[illegible]	[illegible]	[illegible]	[illegible]	[illegible]	[illegible]	[illegible]	[illegible]	[illegible]	[illegible]	[illegible]	[illegible]	1,110
Otitis media	0 (0 to 0)	[illegible]	[illegible]	[illegible]	[illegible]	[illegible]	[illegible]	[illegible]	[illegible]	[illegible]	[illegible]	[illegible]	[illegible]	[illegible]	[illegible]	[illegible]	[illegible]	[illegible]	[illegible]	[illegible]	[illegible]	[illegible]	[illegible]	437
Enteric infections	33·1 (18·2 to 55·5)	[illegible]	[illegible]	[illegible]	[illegible]	[illegible]	[illegible]	[illegible]	[illegible]	[illegible]	[illegible]	[illegible]	[illegible]	[illegible]	[illegible]	[illegible]	[illegible]	[illegible]	[illegible]	[illegible]	[illegible]	[illegible]	[illegible]	2,720
Diarrheal diseases	33·1 (18·2 to 55·5)	[illegible]	[illegible]	[illegible]	[illegible]	[illegible]	[illegible]	[illegible]	[illegible]	[illegible]	[illegible]	[illegible]	[illegible]	[illegible]	[illegible]	[illegible]	[illegible]	[illegible]	[illegible]	[illegible]	[illegible]	[illegible]	[illegible]	2,720
Typhoid and paratyphoid	0 (0 to 0)	[illegible]	[illegible]	[illegible]	[illegible]	[illegible]	[illegible]	[illegible]	[illegible]	[illegible]	[illegible]	[illegible]	[illegible]	[illegible]	[illegible]	[illegible]	[illegible]	[illegible]	[illegible]	[illegible]	[illegible]	[illegible]	[illegible]	0·3
Typhoid fever	0 (0 to 0)	[illegible]	[illegible]	[illegible]	[illegible]	[illegible]	[illegible]	[illegible]	[illegible]	[illegible]	[illegible]	[illegible]	[illegible]	[illegible]	[illegible]	[illegible]	[illegible]	[illegible]	[illegible]	[illegible]	[illegible]	[illegible]	[illegible]	0·1
Paratyphoid fever	0 (0 to 0)	[illegible]	[illegible]	[illegible]	[illegible]	[illegible]	[illegible]	[illegible]	[illegible]	[illegible]	[illegible]	[illegible]	[illegible]	[illegible]	[illegible]	[illegible]	[illegible]	[illegible]	[illegible]	[illegible]	[illegible]	[illegible]	[illegible]	0·2
Invasive Non-typhoidal Salmonella (iNTS)	0 (0 to 0)	[illegible]	[illegible]	[illegible]	[illegible]	[illegible]	[illegible]	[illegible]	[illegible]	[illegible]	[illegible]	[illegible]	[illegible]	[illegible]	[illegible]	[illegible]	[illegible]	[illegible]	[illegible]	[illegible]	[illegible]	[illegible]	[illegible]	1·2
Other intestinal infectious diseases	0 (0 to 0)	[illegible]	[illegible]	[illegible]	[illegible]	[illegible]	[illegible]	[illegible]	[illegible]	[illegible]	[illegible]	[illegible]	[illegible]	[illegible]	[illegible]	[illegible]	[illegible]	[illegible]	[illegible]	[illegible]	[illegible]	[illegible]	[illegible]	4·6
Neglected tropical diseases and malaria	0·2 (0·1 to 0·3)	[illegible]	[illegible]	[illegible]	[illegible]	[illegible]	[illegible]	[illegible]	[illegible]	[illegible]	[illegible]	[illegible]	[illegible]	[illegible]	[illegible]	[illegible]	[illegible]	[illegible]	[illegible]	[illegible]	[illegible]	[illegible]	[illegible]	1,160
Malaria	0 (0 to 0)	[illegible]	[illegible]	[illegible]	[illegible]	[illegible]	[illegible]	[illegible]	[illegible]	[illegible]	[illegible]	[illegible]	[illegible]	[illegible]	[illegible]	[illegible]	[illegible]	[illegible]	[illegible]	[illegible]	[illegible]	[illegible]	[illegible]	0
Chagas disease	0 (0 to 0)	[illegible]	[illegible]	[illegible]	[illegible]	[illegible]	[illegible]	[illegible]	[illegible]	[illegible]	[illegible]	[illegible]	[illegible]	[illegible]	[illegible]	[illegible]	[illegible]	[illegible]	[illegible]	[illegible]	[illegible]	[illegible]	[illegible]	0
Leishmaniasis	0 (0 to 0)	[illegible]	[illegible]	[illegible]	[illegible]	[illegible]	[illegible]	[illegible]	[illegible]	[illegible]	[illegible]	[illegible]	[illegible]	[illegible]	[illegible]	[illegible]	[illegible]	[illegible]	[illegible]	[illegible]	[illegible]	[illegible]	[illegible]	0
Visceral leishmaniasis	0 (0 to 0)	[illegible]	[illegible]	[illegible]	[illegible]	[illegible]	[illegible]	[illegible]	[illegible]	[illegible]	[illegible]	[illegible]	[illegible]	[illegible]	[illegible]	[illegible]	[illegible]	[illegible]	[illegible]	[illegible]	[illegible]	[illegible]	[illegible]	0
Cutaneous and mucocutaneous leishmaniasis	0 (0 to 0)	[illegible]	[illegible]	[illegible]	[illegible]	[illegible]	[illegible]	[illegible]	[illegible]	[illegible]	[illegible]	[illegible]	[illegible]	[illegible]	[illegible]	[illegible]	[illegible]	[illegible]	[illegible]	[illegible]	[illegible]	[illegible]	[illegible]	0
African trypanosomiasis	0 (0 to 0)	[illegible]	[illegible]	[illegible]	[illegible]	[illegible]	[illegible]	[illegible]	[illegible]	[illegible]	[illegible]	[illegible]	[illegible]	[illegible]	[illegible]	[illegible]	[illegible]	[illegible]	[illegible]	[illegible]	[illegible]	[illegible]	[illegible]	0
Schistosomiasis	0 (0 to 0)	[illegible]	[illegible]	[illegible]	[illegible]	[illegible]	[illegible]	[illegible]	[illegible]	[illegible]	[illegible]	[illegible]	[illegible]	[illegible]	[illegible]	[illegible]	[illegible]	[illegible]	[illegible]	[illegible]	[illegible]	[illegible]	[illegible]	0
Cysticercosis	0 (0 to 0)	[illegible]	[illegible]	[illegible]	[illegible]	[illegible]	[illegible]	[illegible]	[illegible]	[illegible]	[illegible]	[illegible]	[illegible]	[illegible]	[illegible]	[illegible]	[illegible]	[illegible]	[illegible]	[illegible]	[illegible]	[illegible]	[illegible]	511
Cystic echinococcosis	0 (0 to 0)	[illegible]	[illegible]	[illegible]	[illegible]	[illegible]	[illegible]	[illegible]	[illegible]	[illegible]	[illegible]	[illegible]	[illegible]	[illegible]	[illegible]	[illegible]	[illegible]	[illegible]	[illegible]	[illegible]	[illegible]	[illegible]	[illegible]	0·2
Lymphatic filariasis	0 (0 to 0)	[illegible]	[illegible]	[illegible]	[illegible]	[illegible]	[illegible]	[illegible]	[illegible]	[illegible]	[illegible]	[illegible]	[illegible]	[illegible]	[illegible]	[illegible]	[illegible]	[illegible]	[illegible]	[illegible]	[illegible]	[illegible]	[illegible]	0
Onchocerciasis	0 (0 to 0)	[illegible]	[illegible]	[illegible]	[illegible]	[illegible]	[illegible]	[illegible]	[illegible]	[illegible]	[illegible]	[illegible]	[illegible]	[illegible]	[illegible]	[illegible]	[illegible]	[illegible]	[illegible]	[illegible]	[illegible]	[illegible]	[illegible]	0
Trachoma	0 (0 to 0)	[illegible]	[illegible]	[illegible]	[illegible]	[illegible]	[illegible]	[illegible]	[illegible]	[illegible]	[illegible]	[illegible]	[illegible]	[illegible]	[illegible]	[illegible]	[illegible]	[illegible]	[illegible]	[illegible]	[illegible]	[illegible]	[illegible]	0
Dengue	0 (0 to 0)	[illegible]	[illegible]	[illegible]	[illegible]	[illegible]	[illegible]	[illegible]	[illegible]	[illegible]	[illegible]	[illegible]	[illegible]	[illegible]	[illegible]	[illegible]	[illegible]	[illegible]	[illegible]	[illegible]	[illegible]	[illegible]	[illegible]	133
Yellow fever	0 (0 to 0)	[illegible]	[illegible]	[illegible]	[illegible]	[illegible]	[illegible]	[illegible]	[illegible]	[illegible]	[illegible]	[illegible]	[illegible]	[illegible]	[illegible]	[illegible]	[illegible]	[illegible]	[illegible]	[illegible]	[illegible]	[illegible]	[illegible]	0
Rabies	0 (0 to 0)	[illegible]	[illegible]	[illegible]	[illegible]	[illegible]	[illegible]	[illegible]	[illegible]	[illegible]	[illegible]	[illegible]	[illegible]	[illegible]	[illegible]	[illegible]	[illegible]	[illegible]	[illegible]	[illegible]	[illegible]	[illegible]	[illegible]	0·1
Intestinal nematode infections	0 (0 to 0)	[illegible]	[illegible]	[illegible]	[illegible]	[illegible]	[illegible]	[illegible]	[illegible]	[illegible]	[illegible]	[illegible]	[illegible]	[illegible]	[illegible]	[illegible]	[illegible]	[illegible]	[illegible]	[illegible]	[illegible]	[illegible]	[illegible]	308
Ascariasis	0 (0 to 0)	[illegible]	[illegible]	[illegible]	[illegible]	[illegible]	[illegible]	[illegible]	[illegible]	[illegible]	[illegible]	[illegible]	[illegible]	[illegible]	[illegible]	[illegible]	[illegible]	[illegible]	[illegible]	[illegible]	[illegible]	[illegible]	[illegible]	19·5
Trichuriasis	0 (0 to 0)	[illegible]	[illegible]	[illegible]	[illegible]	[illegible]	[illegible]	[illegible]	[illegible]	[illegible]	[illegible]	[illegible]	[illegible]	[illegible]	[illegible]	[illegible]	[illegible]	[illegible]	[illegible]	[illegible]	[illegible]	[illegible]	[illegible]	244
Hookworm disease	0 (0 to 0)	[illegible]	[illegible]	[illegible]	[illegible]	[illegible]	[illegible]	[illegible]	[illegible]	[illegible]	[illegible]	[illegible]	[illegible]	[illegible]	[illegible]	[illegible]	[illegible]	[illegible]	[illegible]	[illegible]	[illegible]	[illegible]	[illegible]	44·8
Food-borne trematodiases	0 (0 to 0)	[illegible]	[illegible]	[illegible]	[illegible]	[illegible]	[illegible]	[illegible]	[illegible]	[illegible]	[illegible]	[illegible]	[illegible]	[illegible]	[illegible]	[illegible]	[illegible]	[illegible]	[illegible]	[illegible]	[illegible]	[illegible]	[illegible]	0
Leprosy	0 (0 to 0)	[illegible]	[illegible]	[illegible]	[illegible]	[illegible]	[illegible]	[illegible]	[illegible]	[illegible]	[illegible]	[illegible]	[illegible]	[illegible]	[illegible]	[illegible]	[illegible]	[illegible]	[illegible]	[illegible]	[illegible]	[illegible]	[illegible]	2
Ebola	0 (0 to 0)	[illegible]	[illegible]	[illegible]	[illegible]	[illegible]	[illegible]	[illegible]	[illegible]	[illegible]	[illegible]	[illegible]	[illegible]	[illegible]	[illegible]	[illegible]	[illegible]	[illegible]	[illegible]	[illegible]	[illegible]	[illegible]	[illegible]	0
Zika virus	0 (0 to 0)	[illegible]	[illegible]	[illegible]	[illegible]	[illegible]	[illegible]	[illegible]	[illegible]	[illegible]	[illegible]	[illegible]	[illegible]	[illegible]	[illegible]	[illegible]	[illegible]	[illegible]	[illegible]	[illegible]	[illegible]	[illegible]	[illegible]	2·1
Guinea worm disease	0 (0 to 0)	[illegible]	[illegible]	[illegible]	[illegible]	[illegible]	[illegible]	[illegible]	[illegible]	[illegible]	[illegible]	[illegible]	[illegible]	[illegible]	[illegible]	[illegible]	[illegible]	[illegible]	[illegible]	[illegible]	[illegible]	[illegible]	[illegible]	0
Other neglected tropical diseases	0·2 (0·1 to 0·3)	[illegible]	[illegible]	[illegible]	[illegible]	[illegible]	[illegible]	[illegible]	[illegible]	[illegible]	[illegible]	[illegible]	[illegible]	[illegible]	[illegible]	[illegible]	[illegible]	[illegible]	[illegible]	[illegible]	[illegible]	[illegible]	[illegible]	202
Other infectious diseases	41·8 (26·1 to 63·5)	[illegible]	[illegible]	[illegible]	[illegible]	[illegible]	[illegible]	[illegible]	[illegible]	[illegible]	[illegible]	[illegible]	[illegible]	[illegible]	[illegible]	[illegible]	[illegible]	[illegible]	[illegible]	[illegible]	[illegible]	[illegible]	[illegible]	2,470
Meningitis	26·2 (15·9 to 41·4)	[illegible]	[illegible]	[illegible]	[illegible]	[illegible]	[illegible]	[illegible]	[illegible]	[illegible]	[illegible]	[illegible]	[illegible]	[illegible]	[illegible]	[illegible]	[illegible]	[illegible]	[illegible]	[illegible]	[illegible]	[illegible]	[illegible]	621
Encephalitis	10 (4·6 to 17·1)	[illegible]	[illegible]	[illegible]	[illegible]	[illegible]	[illegible]	[illegible]	[illegible]	[illegible]	[illegible]	[illegible]	[illegible]	[illegible]	[illegible]	[illegible]	[illegible]	[illegible]	[illegible]	[illegible]	[illegible]	[illegible]	[illegible]	436
Diphtheria	0 (0 to 0)	[illegible]	[illegible]	[illegible]	[illegible]	[illegible]	[illegible]	[illegible]	[illegible]	[illegible]	[illegible]	[illegible]	[illegible]	[illegible]	[illegible]	[illegible]	[illegible]	[illegible]	[illegible]	[illegible]	[illegible]	[illegible]	[illegible]	0·2
Tetanus	0 (0 to 0·1)	[illegible]	[illegible]	[illegible]	[illegible]	[illegible]	[illegible]	[illegible]	[illegible]	[illegible]	[illegible]	[illegible]	[illegible]	[illegible]	[illegible]	[illegible]	[illegible]	[illegible]	[illegible]	[illegible]	[illegible]	[illegible]	[illegible]	7
Measles	0 (0 to 0)	[illegible]	[illegible]	[illegible]	[illegible]	[illegible]	[illegible]	[illegible]	[illegible]	[illegible]	[illegible]	[illegible]	[illegible]	[illegible]	[illegible]	[illegible]	[illegible]	[illegible]	[illegible]	[illegible]	[illegible]	[illegible]	[illegible]	0
Varicella and herpes zoster	0·3 (0·1 to 0·7)	[illegible]	[illegible]	[illegible]	[illegible]	[illegible]	[illegible]	[illegible]	[illegible]	[illegible]	[illegible]	[illegible]	[illegible]	[illegible]	[illegible]	[illegible]	[illegible]	[illegible]	[illegible]	[illegible]	[illegible]	[illegible]	[illegible]	41·3
Acute hepatitis	0 (0 to 0)	[illegible]	[illegible]	[illegible]	[illegible]	[illegible]	[illegible]	[illegible]	[illegible]	[illegible]	[illegible]	[illegible]	[illegible]	[illegible]	[illegible]	[illegible]	[illegible]	[illegible]	[illegible]	[illegible]	[illegible]	[illegible]	[illegible]	91·6
Acute hepatitis A	0 (0 to 0)	[illegible]	[illegible]	[illegible]	[illegible]	[illegible]	[illegible]	[illegible]	[illegible]	[illegible]	[illegible]	[illegible]	[illegible]	[illegible]	[illegible]	[illegible]	[illegible]	[illegible]	[illegible]	[illegible]	[illegible]	[illegible]	[illegible]	56·7
Acute hepatitis B	0 (0 to 0)	[illegible]	[illegible]	[illegible]	[illegible]	[illegible]	[illegible]	[illegible]	[illegible]	[illegible]	[illegible]	[illegible]	[illegible]	[illegible]	[illegible]	[illegible]	[illegible]	[illegible]	[illegible]	[illegible]	[illegible]	[illegible]	[illegible]	24·6
Acute hepatitis C	0 (0 to 0)	[illegible]	[illegible]	[illegible]	[illegible]	[illegible]	[illegible]	[illegible]	[illegible]	[illegible]	[illegible]	[illegible]	[illegible]	[illegible]	[illegible]	[illegible]	[illegible]	[illegible]	[illegible]	[illegible]	[illegible]	[illegible]	[illegible]	3·8
Acute hepatitis E	0 (0 to 0)	[illegible]	[illegible]	[illegible]	[illegible]	[illegible]	[illegible]	[illegible]	[illegible]	[illegible]	[illegible]	[illegible]	[illegible]	[illegible]	[illegible]	[illegible]	[illegible]	[illegible]	[illegible]	[illegible]	[illegible]	[illegible]	[illegible]	6·4
Other unspecified infectious diseases	5·3 (1·9 to 9·6)	[illegible]	[illegible]	[illegible]	[illegible]	[illegible]	[illegible]	[illegible]	[illegible]	[illegible]	[illegible]	[illegible]	[illegible]	[illegible]	[illegible]	[illegible]	[illegible]	[illegible]	[illegible]	[illegible]	[illegible]	[illegible]	[illegible]	1,270
Maternal and neonatal disorders	14,900 (10,300 to 20,900)	[illegible]	[illegible]	[illegible]	[illegible]	[illegible]	[illegible]	[illegible]	[illegible]	[illegible]	[illegible]	[illegible]	[illegible]	[illegible]	[illegible]	[illegible]	[illegible]	[illegible]	[illegible]	[illegible]	[illegible]	[illegible]	[illegible]	24,500
Maternal disorders	0 (0 to 0)	[illegible]	[illegible]	[illegible]	[illegible]	[illegible]	[illegible]	[illegible]	[illegible]	[illegible]	[illegible]	[illegible]	[illegible]	[illegible]	[illegible]	[illegible]	[illegible]	[illegible]	[illegible]	[illegible]	[illegible]	[illegible]	[illegible]	0
Maternal hemorrhage	0 (0 to 0)	[illegible]	[illegible]	[illegible]	[illegible]	[illegible]	[illegible]	[illegible]	[illegible]	[illegible]	[illegible]	[illegible]	[illegible]	[illegible]	[illegible]	[illegible]	[illegible]	[illegible]	[illegible]	[illegible]	[illegible]	[illegible]	[illegible]	0
Maternal sepsis and other maternal infections	0 (0 to 0)	[illegible]	[illegible]	[illegible]	[illegible]	[illegible]	[illegible]	[illegible]	[illegible]	[illegible]	[illegible]	[illegible]	[illegible]	[illegible]	[illegible]	[illegible]	[illegible]	[illegible]	[illegible]	[illegible]	[illegible]	[illegible]	[illegible]	0
Maternal hypertensive disorders	0 (0 to 0)	[illegible]	[illegible]	[illegible]	[illegible]	[illegible]	[illegible]	[illegible]	[illegible]	[illegible]	[illegible]	[illegible]	[illegible]	[illegible]	[illegible]	[illegible]	[illegible]	[illegible]	[illegible]	[illegible]	[illegible]	[illegible]	[illegible]	0
Maternal obstructed labor and uterine rupture	--	[illegible]	[illegible]	[illegible]	[illegible]	[illegible]	[illegible]	[illegible]	[illegible]	[illegible]	[illegible]	[illegible]	[illegible]	[illegible]	[illegible]	[illegible]	[illegible]	[illegible]	[illegible]	[illegible]	[illegible]	[illegible]	[illegible]	NA
Maternal abortion and miscarriage	--	[illegible]	[illegible]	[illegible]	[illegible]	[illegible]	[illegible]	[illegible]	[illegible]	[illegible]	[illegible]	[illegible]	[illegible]	[illegible]	[illegible]	[illegible]	[illegible]	[illegible]	[illegible]	[illegible]	[illegible]	[illegible]	[illegible]	NA
Ectopic pregnancy	--	[illegible]	[illegible]	[illegible]	[illegible]	[illegible]	[illegible]	[illegible]	[illegible]	[illegible]	[illegible]	[illegible]	[illegible]	[illegible]	[illegible]	[illegible]	[illegible]	[illegible]	[illegible]	[illegible]	[illegible]	[illegible]	[illegible]	NA
Indirect maternal deaths	--	[illegible]	[illegible]	[illegible]	[illegible]	[illegible]	[illegible]	[illegible]	[illegible]	[illegible]	[illegible]	[illegible]	[illegible]	[illegible]	[illegible]	[illegible]	[illegible]	[illegible]	[illegible]	[illegible]	[illegible]	[illegible]	[illegible]	NA
Late maternal deaths	--	[illegible]	[illegible]	[illegible]	[illegible]	[illegible]	[illegible]	[illegible]	[illegible]	[illegible]	[illegible]	[illegible]	[illegible]	[illegible]	[illegible]	[illegible]	[illegible]	[illegible]	[illegible]	[illegible]	[illegible]	[illegible]	[illegible]	NA
Maternal deaths aggravated by HIV/AIDS	--	[illegible]	[illegible]	[illegible]	[illegible]	[illegible]	[illegible]	[illegible]	[illegible]	[illegible]	[illegible]	[illegible]	[illegible]	[illegible]	[illegible]	[illegible]	[illegible]	[illegible]	[illegible]	[illegible]	[illegible]	[illegible]	[illegible]	NA
Neonatal disorders	14,900 (10,300 to 20,900)	[illegible]	[illegible]	[illegible]	[illegible]	[illegible]	[illegible]	[illegible]	[illegible]	[illegible]	[illegible]	[illegible]	[illegible]	[illegible]	[illegible]	[illegible]	[illegible]	[illegible]	[illegible]	[illegible]	[illegible]	[illegible]	[illegible]	24,500
Neonatal preterm birth	8,760 (5,940 to 12,500)	[illegible]	[illegible]	[illegible]	[illegible]	[illegible]	[illegible]	[illegible]	[illegible]	[illegible]	[illegible]	[illegible]	[illegible]	[illegible]	[illegible]	[illegible]	[illegible]	[illegible]	[illegible]	[illegible]	[illegible]	[illegible]	[illegible]	12,700
Neonatal encephalopathy due to birth asphyxia and trauma	3,320 (1,950 to 5,150)	[illegible]	[illegible]	[illegible]	[illegible]	[illegible]	[illegible]	[illegible]	[illegible]	[illegible]	[illegible]	[illegible]	[illegible]	[illegible]	[illegible]	[illegible]	[illegible]	[illegible]	[illegible]	[illegible]	[illegible]	[illegible]	[illegible]	4,460
Neonatal sepsis and other neonatal infections	1,690 (1,000 to 2,710)	[illegible]	[illegible]	[illegible]	[illegible]	[illegible]	[illegible]	[illegible]	[illegible]	[illegible]	[illegible]	[illegible]	[illegible]	[illegible]	[illegible]	[illegible]	[illegible]	[illegible]	[illegible]	[illegible]	[illegible]	[illegible]	[illegible]	5,300
Hemolytic disease and other neonatal jaundice	49·9 (22·5 to 91·3)	[illegible]	[illegible]	[illegible]	[illegible]	[illegible]	[illegible]	[illegible]	[illegible]	[illegible]	[illegible]	[illegible]	[illegible]	[illegible]	[illegible]	[illegible]	[illegible]	[illegible]	[illegible]	[illegible]	[illegible]	[illegible]	[illegible]	292
Other neonatal disorders	1,080 (656 to 1,670)	[illegible]	[illegible]	[illegible]	[illegible]	[illegible]	[illegible]	[illegible]	[illegible]	[illegible]	[illegible]	[illegible]	[illegible]	[illegible]	[illegible]	[illegible]	[illegible]	[illegible]	[illegible]	[illegible]	[illegible]	[illegible]	[illegible]	1,720
Nutritional deficiencies	1·1 (0·6 to 2·1)	[illegible]	[illegible]	[illegible]	[illegible]	[illegible]	[illegible]	[illegible]	[illegible]	[illegible]	[illegible]	[illegible]	[illegible]	[illegible]	[illegible]	[illegible]	[illegible]	[illegible]	[illegible]	[illegible]	[illegible]	[illegible]	[illegible]	4,010
Protein-energy malnutrition	0·7 (0·4 to 1·1)	[illegible]	[illegible]	[illegible]	[illegible]	[illegible]	[illegible]	[illegible]	[illegible]	[illegible]	[illegible]	[illegible]	[illegible]	[illegible]	[illegible]	[illegible]	[illegible]	[illegible]	[illegible]	[illegible]	[illegible]	[illegible]	[illegible]	1,110
Iodine deficiency	0 (0 to 0)	[illegible]	[illegible]	[illegible]	[illegible]	[illegible]	[illegible]	[illegible]	[illegible]	[illegible]	[illegible]	[illegible]	[illegible]	[illegible]	[illegible]	[illegible]	[illegible]	[illegible]	[illegible]	[illegible]	[illegible]	[illegible]	[illegible]	89·4
Vitamin A deficiency	0·1 (0 to 0·2)	[illegible]	[illegible]	[illegible]	[illegible]	[illegible]	[illegible]	[illegible]	[illegible]	[illegible]	[illegible]	[illegible]	[illegible]	[illegible]	[illegible]	[illegible]	[illegible]	[illegible]	[illegible]	[illegible]	[illegible]	[illegible]	[illegible]	65·6
Dietary iron deficiency	0·4 (0·1 to 1·3)	[illegible]	[illegible]	[illegible]	[illegible]	[illegible]	[illegible]	[illegible]	[illegible]	[illegible]	[illegible]	[illegible]	[illegible]	[illegible]	[illegible]	[illegible]	[illegible]	[illegible]	[illegible]	[illegible]	[illegible]	[illegible]	[illegible]	2,550
Other nutritional deficiencies	0 (0 to 0)	[illegible]	[illegible]	[illegible]	[illegible]	[illegible]	[illegible]	[illegible]	[illegible]	[illegible]	[illegible]	[illegible]	[illegible]	[illegible]	[illegible]	[illegible]	[illegible]	[illegible]	[illegible]	[illegible]	[illegible]	[illegible]	[illegible]	190
Non-communicable diseases	1,930 (1,080 to 2,970)	[illegible]	[illegible]	[illegible]	[illegible]	[illegible]	[illegible]	[illegible]	[illegible]	[illegible]	[illegible]	[illegible]	[illegible]	[illegible]	[illegible]	[illegible]	[illegible]	[illegible]	[illegible]	[illegible]	[illegible]	[illegible]	[illegible]	286,000
Neoplasms	11·3 (6·8 to 25·8)	[illegible]	[illegible]	[illegible]	[illegible]	[illegible]	[illegible]	[illegible]	[illegible]	[illegible]	[illegible]	[illegible]	[illegible]	[illegible]	[illegible]	[illegible]	[illegible]	[illegible]	[illegible]	[illegible]	[illegible]	[illegible]	[illegible]	55,000
Lip and oral cavity cancer	0 (0 to 0)	[illegible]	[illegible]	[illegible]	[illegible]	[illegible]	[illegible]	[illegible]	[illegible]	[illegible]	[illegible]	[illegible]	[illegible]	[illegible]	[illegible]	[illegible]	[illegible]	[illegible]	[illegible]	[illegible]	[illegible]	[illegible]	[illegible]	456
Nasopharynx cancer	0 (0 to 0)	[illegible]	[illegible]	[illegible]	[illegible]	[illegible]	[illegible]	[illegible]	[illegible]	[illegible]	[illegible]	[illegible]	[illegible]	[illegible]	[illegible]	[illegible]	[illegible]	[illegible]	[illegible]	[illegible]	[illegible]	[illegible]	[illegible]	319
Other pharynx cancer	0 (0 to 0)	[illegible]	[illegible]	[illegible]	[illegible]	[illegible]	[illegible]	[illegible]	[illegible]	[illegible]	[illegible]	[illegible]	[illegible]	[illegible]	[illegible]	[illegible]	[illegible]	[illegible]	[illegible]	[illegible]	[illegible]	[illegible]	[illegible]	370
Esophageal cancer	0 (0 to 0)	[illegible]	[illegible]	[illegible]	[illegible]	[illegible]	[illegible]	[illegible]	[illegible]	[illegible]	[illegible]	[illegible]	[illegible]	[illegible]	[illegible]	[illegible]	[illegible]	[illegible]	[illegible]	[illegible]	[illegible]	[illegible]	[illegible]	1,320
Stomach cancer	0 (0 to 0)	[illegible]	[illegible]	[illegible]	[illegible]	[illegible]	[illegible]	[illegible]	[illegible]	[illegible]	[illegible]	[illegible]	[illegible]	[illegible]	[illegible]	[illegible]	[illegible]	[illegible]	[illegible]	[illegible]	[illegible]	[illegible]	[illegible]	4,230
Colon and rectum cancer	0 (0 to 0)	[illegible]	[illegible]	[illegible]	[illegible]	[illegible]	[illegible]	[illegible]	[illegible]	[illegible]	[illegible]	[illegible]	[illegible]	[illegible]	[illegible]	[illegible]	[illegible]	[illegible]	[illegible]	[illegible]	[illegible]	[illegible]	[illegible]	6,130
Liver cancer	0·8 (0·3 to 1·7)	[illegible]	[illegible]	[illegible]	[illegible]	[illegible]	[illegible]	[illegible]	[illegible]	[illegible]	[illegible]	[illegible]	[illegible]	[illegible]	[illegible]	[illegible]	[illegible]	[illegible]	[illegible]	[illegible]	[illegible]	[illegible]	[illegible]	1,200
Liver cancer due to hepatitis B	0 (0 to 0)	[illegible]	[illegible]	[illegible]	[illegible]	[illegible]	[illegible]	[illegible]	[illegible]	[illegible]	[illegible]	[illegible]	[illegible]	[illegible]	[illegible]	[illegible]	[illegible]	[illegible]	[illegible]	[illegible]	[illegible]	[illegible]	[illegible]	416
Liver cancer due to hepatitis C	0 (0 to 0)	[illegible]	[illegible]	[illegible]	[illegible]	[illegible]	[illegible]	[illegible]	[illegible]	[illegible]	[illegible]	[illegible]	[illegible]	[illegible]	[illegible]	[illegible]	[illegible]	[illegible]	[illegible]	[illegible]	[illegible]	[illegible]	[illegible]	172
Liver cancer due to alcohol use	0 (0 to 0)	[illegible]	[illegible]	[illegible]	[illegible]	[illegible]	[illegible]	[illegible]	[illegible]	[illegible]	[illegible]	[illegible]	[illegible]	[illegible]	[illegible]	[illegible]	[illegible]	[illegible]	[illegible]	[illegible]	[illegible]	[illegible]	[illegible]	461
Liver cancer due to NASH	0 (0 to 0)	[illegible]	[illegible]	[illegible]	[illegible]	[illegible]	[illegible]	[illegible]	[illegible]	[illegible]	[illegible]	[illegible]	[illegible]	[illegible]	[illegible]	[illegible]	[illegible]	[illegible]	[illegible]	[illegible]	[illegible]	[illegible]	[illegible]	79·9
Liver cancer due to other causes	0·8 (0·3 to 1·7)	[illegible]	[illegible]	[illegible]	[illegible]	[illegible]	[illegible]	[illegible]	[illegible]	[illegible]	[illegible]	[illegible]	[illegible]	[illegible]	[illegible]	[illegible]	[illegible]	[illegible]	[illegible]	[illegible]	[illegible]	[illegible]	[illegible]	68·4
Gallbladder and biliary tract cancer	0 (0 to 0)	[illegible]	[illegible]	[illegible]	[illegible]	[illegible]	[illegible]	[illegible]	[illegible]	[illegible]	[illegible]	[illegible]	[illegible]	[illegible]	[illegible]	[illegible]	[illegible]	[illegible]	[illegible]	[illegible]	[illegible]	[illegible]	[illegible]	420
Pancreatic cancer	0 (0 to 0)	[illegible]	[illegible]	[illegible]	[illegible]	[illegible]	[illegible]	[illegible]	[illegible]	[illegible]	[illegible]	[illegible]	[illegible]	[illegible]	[illegible]	[illegible]	[illegible]	[illegible]	[illegible]	[illegible]	[illegible]	[illegible]	[illegible]	1,920
Larynx cancer	0 (0 to 0)	[illegible]	[illegible]	[illegible]	[illegible]	[illegible]	[illegible]	[illegible]	[illegible]	[illegible]	[illegible]	[illegible]	[illegible]	[illegible]	[illegible]	[illegible]	[illegible]	[illegible]	[illegible]	[illegible]	[illegible]	[illegible]	[illegible]	848
Tracheal, bronchus, and lung cancer	0 (0 to 0)	[illegible]	[illegible]	[illegible]	[illegible]	[illegible]	[illegible]	[illegible]	[illegible]	[illegible]	[illegible]	[illegible]	[illegible]	[illegible]	[illegible]	[illegible]	[illegible]	[illegible]	[illegible]	[illegible]	[illegible]	[illegible]	[illegible]	10,900
Malignant skin melanoma	0·1 (0 to 0·2)	[illegible]	[illegible]	[illegible]	[illegible]	[illegible]	[illegible]	[illegible]	[illegible]	[illegible]	[illegible]	[illegible]	[illegible]	[illegible]	[illegible]	[illegible]	[illegible]	[illegible]	[illegible]	[illegible]	[illegible]	[illegible]	[illegible]	197
Non-melanoma skin cancer	0 (0 to 0)	[illegible]	[illegible]	[illegible]	[illegible]	[illegible]	[illegible]	[illegible]	[illegible]	[illegible]	[illegible]	[illegible]	[illegible]	[illegible]	[illegible]	[illegible]	[illegible]	[illegible]	[illegible]	[illegible]	[illegible]	[illegible]	[illegible]	189
Non-melanoma skin cancer (squamous-cell carcinoma)	0 (0 to 0)	[illegible]	[illegible]	[illegible]	[illegible]	[illegible]	[illegible]	[illegible]	[illegible]	[illegible]	[illegible]	[illegible]	[illegible]	[illegible]	[illegible]	[illegible]	[illegible]	[illegible]	[illegible]	[illegible]	[illegible]	[illegible]	[illegible]	189
Non-melanoma skin cancer (basal-cell carcinoma)	0 (0 to 0)	[illegible]	[illegible]	[illegible]	[illegible]	[illegible]	[illegible]	[illegible]	[illegible]	[illegible]	[illegible]	[illegible]	[illegible]	[illegible]	[illegible]	[illegible]	[illegible]	[illegible]	[illegible]	[illegible]	[illegible]	[illegible]	[illegible]	0·1
Breast cancer	0 (0 to 0)	[illegible]	[illegible]	[illegible]	[illegible]	[illegible]	[illegible]	[illegible]	[illegible]	[illegible]	[illegible]	[illegible]	[illegible]	[illegible]	[illegible]	[illegible]	[illegible]	[illegible]	[illegible]	[illegible]	[illegible]	[illegible]	[illegible]	176
Cervical cancer	--	[illegible]	[illegible]	[illegible]	[illegible]	[illegible]	[illegible]	[illegible]	[illegible]	[illegible]	[illegible]	[illegible]	[illegible]	[illegible]	[illegible]	[illegible]	[illegible]	[illegible]	[illegible]	[illegible]	[illegible]	[illegible]	[illegible]	NA
Uterine cancer	--	[illegible]	[illegible]	[illegible]	[illegible]	[illegible]	[illegible]	[illegible]	[illegible]	[illegible]	[illegible]	[illegible]	[illegible]	[illegible]	[illegible]	[illegible]	[illegible]	[illegible]	[illegible]	[illegible]	[illegible]	[illegible]	[illegible]	NA
Ovarian cancer	--	[illegible]	[illegible]	[illegible]	[illegible]	[illegible]	[illegible]	[illegible]	[illegible]	[illegible]	[illegible]	[illegible]	[illegible]	[illegible]	[illegible]	[illegible]	[illegible]	[illegible]	[illegible]	[illegible]	[illegible]	[illegible]	[illegible]	NA
Prostate cancer	0 (0 to 0)	[illegible]	[illegible]	[illegible]	[illegible]	[illegible]	[illegible]	[illegible]	[illegible]	[illegible]	[illegible]	[illegible]	[illegible]	[illegible]	[illegible]	[illegible]	[illegible]	[illegible]	[illegible]	[illegible]	[illegible]	[illegible]	[illegible]	14,100
Testicular cancer	1·3 (0 to 14)	[illegible]	[illegible]	[illegible]	[illegible]	[illegible]	[illegible]	[illegible]	[illegible]	[illegible]	[illegible]	[illegible]	[illegible]	[illegible]	[illegible]	[illegible]	[illegible]	[illegible]	[illegible]	[illegible]	[illegible]	[illegible]	[illegible]	85·1
Kidney cancer	0·3 (0·2 to 0·4)	[illegible]	[illegible]	[illegible]	[illegible]	[illegible]	[illegible]	[illegible]	[illegible]	[illegible]	[illegible]	[illegible]	[illegible]	[illegible]	[illegible]	[illegible]	[illegible]	[illegible]	[illegible]	[illegible]	[illegible]	[illegible]	[illegible]	593
Bladder cancer	0 (0 to 0)	[illegible]	[illegible]	[illegible]	[illegible]	[illegible]	[illegible]	[illegible]	[illegible]	[illegible]	[illegible]	[illegible]	[illegible]	[illegible]	[illegible]	[illegible]	[illegible]	[illegible]	[illegible]	[illegible]	[illegible]	[illegible]	[illegible]	879
Brain and central nervous system cancer	0·8 (0·4 to 1·3)	[illegible]	[illegible]	[illegible]	[illegible]	[illegible]	[illegible]	[illegible]	[illegible]	[illegible]	[illegible]	[illegible]	[illegible]	[illegible]	[illegible]	[illegible]	[illegible]	[illegible]	[illegible]	[illegible]	[illegible]	[illegible]	[illegible]	929
Thyroid cancer	0 (0 to 0)	[illegible]	[illegible]	[illegible]	[illegible]	[illegible]	[illegible]	[illegible]	[illegible]	[illegible]	[illegible]	[illegible]	[illegible]	[illegible]	[illegible]	[illegible]	[illegible]	[illegible]	[illegible]	[illegible]	[illegible]	[illegible]	[illegible]	158
Mesothelioma	0 (0 to 0)	[illegible]	[illegible]	[illegible]	[illegible]	[illegible]	[illegible]	[illegible]	[illegible]	[illegible]	[illegible]	[illegible]	[illegible]	[illegible]	[illegible]	[illegible]	[illegible]	[illegible]	[illegible]	[illegible]	[illegible]	[illegible]	[illegible]	104
Hodgkin lymphoma	0 (0 to 0)	[illegible]	[illegible]	[illegible]	[illegible]	[illegible]	[illegible]	[illegible]	[illegible]	[illegible]	[illegible]	[illegible]	[illegible]	[illegible]	[illegible]	[illegible]	[illegible]	[illegible]	[illegible]	[illegible]	[illegible]	[illegible]	[illegible]	142
Non-Hodgkin lymphoma	0 (0 to 0)	[illegible]	[illegible]	[illegible]	[illegible]	[illegible]	[illegible]	[illegible]	[illegible]	[illegible]	[illegible]	[illegible]	[illegible]	[illegible]	[illegible]	[illegible]	[illegible]	[illegible]	[illegible]	[illegible]	[illegible]	[illegible]	[illegible]	2,940
Multiple myeloma	0 (0 to 0)	[illegible]	[illegible]	[illegible]	[illegible]	[illegible]	[illegible]	[illegible]	[illegible]	[illegible]	[illegible]	[illegible]	[illegible]	[illegible]	[illegible]	[illegible]	[illegible]	[illegible]	[illegible]	[illegible]	[illegible]	[illegible]	[illegible]	1,480
Leukemia	3·4 (2·2 to 5)	[illegible]	[illegible]	[illegible]	[illegible]	[illegible]	[illegible]	[illegible]	[illegible]	[illegible]	[illegible]	[illegible]	[illegible]	[illegible]	[illegible]	[illegible]	[illegible]	[illegible]	[illegible]	[illegible]	[illegible]	[illegible]	[illegible]	2,430
Acute lymphoid leukemia	0·8 (0·4 to 1·3)	[illegible]	[illegible]	[illegible]	[illegible]	[illegible]	[illegible]	[illegible]	[illegible]	[illegible]	[illegible]	[illegible]	[illegible]	[illegible]	[illegible]	[illegible]	[illegible]	[illegible]	[illegible]	[illegible]	[illegible]	[illegible]	[illegible]	304
Chronic lymphoid leukemia	0 (0 to 0)	[illegible]	[illegible]	[illegible]	[illegible]	[illegible]	[illegible]	[illegible]	[illegible]	[illegible]	[illegible]	[illegible]	[illegible]	[illegible]	[illegible]	[illegible]	[illegible]	[illegible]	[illegible]	[illegible]	[illegible]	[illegible]	[illegible]	108
Acute myeloid leukemia	1·4 (0·8 to 2·2)	[illegible]	[illegible]	[illegible]	[illegible]	[illegible]	[illegible]	[illegible]	[illegible]	[illegible]	[illegible]	[illegible]	[illegible]	[illegible]	[illegible]	[illegible]	[illegible]	[illegible]	[illegible]	[illegible]	[illegible]	[illegible]	[illegible]	559
Chronic myeloid leukemia	0 (0 to 0·1)	[illegible]	[illegible]	[illegible]	[illegible]	[illegible]	[illegible]	[illegible]	[illegible]	[illegible]	[illegible]	[illegible]	[illegible]	[illegible]	[illegible]	[illegible]	[illegible]	[illegible]	[illegible]	[illegible]	[illegible]	[illegible]	[illegible]	224
Other leukemia	1·1 (0·6 to 1·9)	[illegible]	[illegible]	[illegible]	[illegible]	[illegible]	[illegible]	[illegible]	[illegible]	[illegible]	[illegible]	[illegible]	[illegible]	[illegible]	[illegible]	[illegible]	[illegible]	[illegible]	[illegible]	[illegible]	[illegible]	[illegible]	[illegible]	1,240
Other malignant neoplasms	4·5 (2·7 to 7·3)	[illegible]	[illegible]	[illegible]	[illegible]	[illegible]	[illegible]	[illegible]	[illegible]	[illegible]	[illegible]	[illegible]	[illegible]	[illegible]	[illegible]	[illegible]	[illegible]	[illegible]	[illegible]	[illegible]	[illegible]	[illegible]	[illegible]	2,170
Other neoplasms	0·1 (0 to 0·8)	[illegible]	[illegible]	[illegible]	[illegible]	[illegible]	[illegible]	[illegible]	[illegible]	[illegible]	[illegible]	[illegible]	[illegible]	[illegible]	[illegible]	[illegible]	[illegible]	[illegible]	[illegible]	[illegible]	[illegible]	[illegible]	[illegible]	307
Myelodysplastic, myeloproliferative, and other hematopoietic n	0·1 (0 to 0·8)	[illegible]	[illegible]	[illegible]	[illegible]	[illegible]	[illegible]	[illegible]	[illegible]	[illegible]	[illegible]	[illegible]	[illegible]	[illegible]	[illegible]	[illegible]	[illegible]	[illegible]	[illegible]	[illegible]	[illegible]	[illegible]	[illegible]	307
Benign and in situ intestinal neoplasms	0 (0 to 0)	[illegible]	[illegible]	[illegible]	[illegible]	[illegible]	[illegible]	[illegible]	[illegible]	[illegible]	[illegible]	[illegible]	[illegible]	[illegible]	[illegible]	[illegible]	[illegible]	[illegible]	[illegible]	[illegible]	[illegible]	[illegible]	[illegible]	0
Benign and in situ cervical and uterine neoplasms	0 (0 to 0)	[illegible]	[illegible]	[illegible]	[illegible]	[illegible]	[illegible]	[illegible]	[illegible]	[illegible]	[illegible]	[illegible]	[illegible]	[illegible]	[illegible]	[illegible]	[illegible]	[illegible]	[illegible]	[illegible]	[illegible]	[illegible]	[illegible]	0
Other benign and in situ neoplasms	0 (0 to 0)	[illegible]	[illegible]	[illegible]	[illegible]	[illegible]	[illegible]	[illegible]	[illegible]	[illegible]	[illegible]	[illegible]	[illegible]	[illegible]	[illegible]	[illegible]	[illegible]	[illegible]	[illegible]	[illegible]	[illegible]	[illegible]	[illegible]	0
Cardiovascular diseases	82·6 (51·2 to 125)	[illegible]	[illegible]	[illegible]	[illegible]	[illegible]	[illegible]	[illegible]	[illegible]	[illegible]	[illegible]	[illegible]	[illegible]	[illegible]	[illegible]	[illegible]	[illegible]	[illegible]	[illegible]	[illegible]	[illegible]	[illegible]	[illegible]	61,500
Rheumatic heart disease	0 (0 to 0)	[illegible]	[illegible]	[illegible]	[illegible]	[illegible]	[illegible]	[illegible]	[illegible]	[illegible]	[illegible]	[illegible]	[illegible]	[illegible]	[illegible]	[illegible]	[illegible]	[illegible]	[illegible]	[illegible]	[illegible]	[illegible]	[illegible]	1,060

Ischemic heart disease	0 (0 to 0)	0 (0 to 0)	0 (0 to 0)	0 (0 to 0)	0 (0 to 0)	0 (0 to 0)	28·9 (19·3 to 41·2)	66·2 (45·2 to 95·1)	107 (77·4 to 145)	214 (154 to 283)	350 (256 to 474)	618 (442 to 826)	1,160 (837 to 1,540)	1,810 (1,350 to 2,370)	2,460 (1,880 to 3,190)	2,640 (2,040 to 3,330)	2,620 (2,050 to 3,280)	2,880 (2,300 to 3,550)	2,090 (1,710 to 2,550)	1,640 (1,350 to 1,960)	1,110 (912 to 1,320)	664 (520 to 795)	447 (336 to 527)	20,900
Stroke	59·2 (35 to 92·3)	9·9 (5·6 to 16·4)	10·1 (4·4 to 19·9)	15·2 (9·8 to 22·2)	44·5 (29·7 to 63·3)	59·2 (41·2 to 82·2)	107 (70·6 to 154)	186 (127 to 262)	224 (156 to 323)	291 (199 to 406)	454 (306 to 654)	721 (482 to 1,060)	1,150 (763 to 1,630)	1,740 (1,190 to 2,440)	2,410 (1,710 to 3,320)	2,830 (2,070 to 3,740)	3,190 (2,360 to 4,210)	3,910 (2,930 to 5,020)	3,150 (2,400 to 3,990)	2,290 (1,800 to 2,920)	1,340 (1,020 to 1,740)	597 (434 to 753)	325 (234 to 415)	25,100
Ischemic stroke	0·8 (0·4 to 1·6)	0·5 (0·3 to 1)	0·4 (0·2 to 0·5)	5·6 (3·8 to 7·6)	11·9 (6·6 to 19·9)	17·1 (10·1 to 26·8)	23·1 (14·2 to 35·1)	38·2 (25·2 to 54·3)	56·9 (39·9 to 81·7)	54·4 (37·8 to 77)	61·6 (41 to 87·9)	90·6 (61 to 125)	146 (101 to 205)	247 (171 to 356)	341 (237 to 478)	707 (498 to 983)	898 (629 to 1,250)	1,810 (1,320 to 2,430)	1,630 (1,220 to 2,100)	1,770 (1,390 to 2,270)	1,050 (791 to 1,350)	466 (342 to 589)	257 (179 to 331)	9,690
Intracerebral hemorrhage	13·8 (6·2 to 29·7)	2·8 (1·4 to 4·9)	5 (2 to 10·5)	2·6 (1·3 to 4·4)	14·2 (8·1 to 22·8)	21·4 (12·8 to 33·6)	48 (28·6 to 74·4)	78·3 (48·2 to 117)	93·5 (59·8 to 140)	160 (103 to 238)	268 (168 to 401)	467 (305 to 697)	801 (524 to 1,150)	1,250 (825 to 1,790)	1,770 (1,250 to 2,440)	1,950 (1,380 to 2,640)	2,130 (1,530 to 2,860)	1,960 (1,390 to 2,610)	1,430 (1,030 to 1,900)	466 (327 to 632)	251 (181 to 340)	116 (78·6 to 153)	60·7 (41·9 to 83·4)	13,400
Subarachnoid hemorrhage	44·7 (23·8 to 72)	6·6 (3·1 to 11)	4·6 (1·8 to 10·3)	7 (3·9 to 11·7)	18·5 (10·5 to 29·2)	20·8 (11·3 to 35·1)	36 (19·8 to 59·1)	69 (40·8 to 106)	74 (44·4 to 119)	75·9 (44·3 to 115)	124 (76·2 to 193)	163 (99·3 to 251)	204 (125 to 321)	244 (155 to 380)	299 (184 to 441)	170 (111 to 245)	155 (97·2 to 225)	130 (86·5 to 185)	84·3 (56·2 to 122)	56·8 (38·8 to 81·1)	37·3 (22·6 to 54·8)	14·4 (9·1 to 21·5)	6·5 (4·2 to 9·9)	2,050
Hypertensive heart disease	0 (0 to 0)	0 (0 to 0)	0 (0 to 0)	0 (0 to 0)	0 (0 to 0)	0 (0 to 0)	11·9 (6·8 to 18·5)	18·8 (10·4 to 31·3)	26·5 (14·4 to 46)	44·2 (25·8 to 82·9)	84·9 (50·7 to 171)	165 (100 to 280)	369 (232 to 570)	506 (337 to 753)	636 (420 to 976)	846 (573 to 1,200)	915 (637 to 1,260)	941 (672 to 1,330)	810 (570 to 1,110)	659 (482 to 888)	445 (329 to 587)	227 (165 to 306)	150 (105 to 206)	6,860
Non-rheumatic valvular heart disease	0 (0 to 0)	0 (0 to 0)	0 (0 to 0)	0 (0 to 0)	0 (0 to 0)	0 (0 to 0)	4·9 (2·9 to 7·5)	11·5 (7 to 16·7)	10 (5·9 to 15·3)	8·6 (5·4 to 12·5)	11·8 (7·1 to 18)	15·4 (9·8 to 24)	22·1 (13·6 to 33·2)	17·9 (11·3 to 28·1)	31·2 (20 to 45·1)	27·4 (18·6 to 38·9)	25·9 (17·5 to 37·1)	24 (16·6 to 34)	16·7 (11·6 to 23·8)	11·5 (8·2 to 15·9)	7·8 (5·5 to 10·9)	4·8 (3·3 to 6·6)	1·5 (0·9 to 2)	253
Non-rheumatic calcific aortic valve disease	0 (0 to 0)	0 (0 to 0)	0 (0 to 0)	0 (0 to 0)	0 (0 to 0)	0 (0 to 0)	3·8 (2·2 to 5·8)	6·5 (3·5 to 10·5)	4·6 (2·4 to 7·8)	2·2 (1·1 to 4·1)	3·3 (1·6 to 6·1)	5·2 (2·6 to 9·4)	11·5 (6·5 to 18·5)	12·4 (6·1 to 21·1)	18·5 (10·9 to 27·9)	14 (8·4 to 21·2)	16·1 (10·4 to 24·1)	14·5 (9·4 to 21)	9·9 (6·6 to 14·6)	6·9 (4·7 to 9·8)	4·6 (3 to 6·8)	2·3 (1·4 to 3·8)	0·9 (0·5 to 1·3)	137
Non-rheumatic degenerative mitral valve disease	0 (0 to 0)	0 (0 to 0)	0 (0 to 0)	0 (0 to 0)	0 (0 to 0)	0 (0 to 0)	1 (0·5 to 2·2)	4·7 (2·2 to 8)	5·3 (2·9 to 8·7)	6·2 (3·7 to 9·3)	8·4 (4·9 to 13·2)	10·1 (5·8 to 16·5)	10·5 (5·7 to 16·8)	5·4 (2·3 to 12·7)	12·7 (7·4 to 20·3)	13·4 (8·1 to 20·8)	9·8 (5·5 to 15·9)	9·5 (5·7 to 14·7)	6·8 (4·2 to 10·8)	4·5 (2·7 to 6·9)	3·1 (1·8 to 4·8)	2·4 (1·2 to 3·6)	0·5 (0·3 to 0·8)	114
Other non-rheumatic valve diseases	0 (0 to 0)	0 (0 to 0)	0 (0 to 0)	0 (0 to 0)	0 (0 to 0)	0 (0 to 0)	0·1 (0 to 0·3)	0·3 (0·1 to 0·6)	0·1 (0 to 0·2)	0·2 (0·1 to 0·3)	0·1 (0 to 0·2)	0·1 (0 to 0·3)	0·1 (0 to 0·4)	0·1 (0 to 0·2)	0 (0 to 0·1)	0 (0 to 0·1)	0 (0 to 0·1)	0 (0 to 0)	0 (0 to 0)	0·1 (0 to 0·3)	0·1 (0 to 0·2)	0 (0 to 0·1)	0 (0 to 0)	1·3
Cardiomyopathy and myocarditis	11·4 (6·2 to 17·9)	6·4 (3·9 to 10·3)	12·4 (5·2 to 24·5)	16·6 (9·3 to 28·6)	22·9 (14·6 to 33·8)	21·7 (13·5 to 33·1)	33·4 (21·3 to 49·6)	54·1 (34·5 to 79·6)	76·2 (48·5 to 109)	83·6 (52·4 to 125)	150 (83·2 to 225)	162 (101 to 244)	222 (132 to 330)	223 (136 to 337)	237 (154 to 347)	218 (140 to 315)	191 (128 to 272)	162 (108 to 231)	96·5 (65·5 to 144)	64·9 (40·9 to 94·8)	42·3 (28 to 61·9)	20·7 (14 to 29·3)	11·6 (7 to 17·2)	2,140
Myocarditis	2·7 (0·6 to 5·7)	1·8 (0·5 to 3·2)	1·9 (0·6 to 4·3)	3·1 (1·4 to 6·2)	3·3 (1·7 to 5·7)	2·1 (1·1 to 3·8)	4·5 (2·1 to 8·7)	3·6 (1·9 to 6·1)	3·5 (1·6 to 6·6)	3·3 (1·4 to 7·2)	4·8 (2·5 to 8·8)	5·5 (2·7 to 9·6)	3 (1·5 to 5·4)	4·3 (2·1 to 8·1)	4 (2 to 7·3)	2·4 (1·1 to 4·5)	3·7 (1·9 to 6·5)	3 (1·5 to 5·5)	2·2 (1·1 to 4·1)	1·4 (0·7 to 2·6)	0·7 (0·4 to 1·4)	0·4 (0·2 to 0·8)	0·4 (0·1 to 0·9)	65·7
Alcoholic cardiomyopathy	0 (0 to 0)	0 (0 to 0)	0 (0 to 0)	0 (0 to 0)	0 (0 to 0)	0 (0 to 0)	1·7 (0·8 to 3·1)	3·3 (1·6 to 6)	3 (1·3 to 5·9)	8·1 (3·8 to 15·4)	16·6 (7·6 to 32·1)	26·7 (12·9 to 50·6)	81·2 (43·7 to 128)	75·5 (42·4 to 124)	61·5 (32·2 to 109)	96·5 (55·2 to 155)	71·7 (42·4 to 111)	14·6 (7 to 26·6)	5·3 (2·5 to 10·4)	2·5 (1·1 to 5·3)	3·1 (1·3 to 6·7)	0·3 (0·1 to 0·6)	0·2 (0·1 to 0·3)	472
Other cardiomyopathy	8·7 (5 to 14·5)	4·6 (2·6 to 7·9)	10·5 (4·2 to 21·2)	13·5 (7·3 to 23·7)	19·6 (12·4 to 28·5)	19·6 (11·9 to 30·1)	27·2 (17 to 41)	47·3 (29·4 to 70·8)	69·7 (43·7 to 100)	72·2 (45 to 109)	128 (65·1 to 198)	129 (72·1 to 201)	138 (69·5 to 229)	143 (82 to 228)	171 (106 to 256)	119 (71·5 to 183)	115 (71·8 to 171)	144 (96·5 to 205)	89 (60·9 to 132)	61 (37·7 to 90·1)	38·4 (24·6 to 57·4)	20 (13·5 to 28·3)	11·1 (6·8 to 16·2)	1,600
Atrial fibrillation and flutter	0 (0 to 0)	0 (0 to 0)	0 (0 to 0)	0 (0 to 0)	0 (0 to 0)	0 (0 to 0)	0 (0 to 0)	0 (0 to 0)	0 (0 to 0)	6·8 (2·6 to 10·8)	7·2 (3·2 to 13)	19·1 (11·5 to 28·2)	38·2 (24·7 to 54·5)	55·6 (36·9 to 81)	91·4 (63·6 to 130)	114 (78·5 to 172)	140 (98·6 to 209)	185 (129 to 297)	183 (131 to 303)	198 (140 to 331)	176 (127 to 288)	138 (100 to 210)	85·2 (53 to 131)	1,440
Aortic aneurysm	--	--	--	--	--	--	11·6 (7·4 to 17·2)	8 (4·8 to 12·1)	7·3 (4·5 to 10·9)	17·3 (11·6 to 25·3)	28·2 (18·1 to 42)	28·1 (18·1 to 42·1)	44·4 (29 to 63·8)	57·8 (38 to 87·1)	104 (68·3 to 147)	158 (107 to 228)	137 (91·9 to 197)	135 (91·7 to 188)	88·9 (59·8 to 127)	54·5 (36·2 to 77·5)	31·9 (22·3 to 43·2)	11 (7·6 to 14·7)	5·1 (3·1 to 7)	NA
Endocarditis	1·1 (0·5 to 2·7)	0·6 (0·1 to 2·7)	3·6 (1·5 to 7·9)	6·2 (3 to 12·8)	2·6 (1·2 to 6·7)	15·3 (5·9 to 28·3)	24·2 (8·1 to 44·4)	27·9 (13 to 47·3)	23·3 (13·4 to 37·4)	21 (10 to 41·4)	37·6 (22 to 59)	54·1 (29·2 to 87·7)	56·1 (28·2 to 93·9)	55·3 (25·9 to 91·9)	57·4 (22·1 to 94·1)	45·2 (19·3 to 76·5)	32·3 (17·1 to 48·7)	20·8 (10·3 to 32·1)	15·9 (7·4 to 25·2)	10·6 (4·7 to 17·4)	4·7 (1·9 to 7·8)	2·5 (1·1 to 4·1)	582	
Other cardiovascular and circulatory diseases	10·8 (6·5 to 18·4)	5·2 (2·8 to 9·2)	5·3 (2·2 to 11)	10·9 (6 to 18·5)	29 (17·9 to 45·4)	29·4 (18·5 to 44·9)	34 (22·2 to 50·4)	53·8 (37·6 to 76)	52·6 (36·5 to 73·7)	53·9 (38·9 to 73·2)	89·1 (60·8 to 124)	91·4 (64·1 to 130)	95·2 (65·5 to 132)	130 (89·7 to 179)	128 (90 to 175)	113 (82·6 to 150)	101 (72·4 to 135)	120 (87·8 to 156)	110 (79·3 to 146)	89·2 (64·8 to 118)	54 (39·3 to 71·9)	27·4 (19·6 to 36·3)	15·9 (10·6 to 21·2)	1,450
Chronic respiratory diseases	0 (0 to 0)	0 (0 to 0)	0·1 (0 to 0·1)	657 (373 to 1,100)	660 (402 to 1,060)	540 (342 to 838)	393 (253 to 597)	363 (250 to 512)	339 (250 to 461)	321 (242 to 425)	345 (261 to 446)	358 (272 to 457)	502 (385 to 645)	799 (601 to 1,020)	1,160 (851 to 1,510)	1,330 (978 to 1,720)	1,350 (1,030 to 1,740)	1,690 (1,280 to 2,160)	1,280 (988 to 1,610)	893 (683 to 1,120)	495 (386 to 615)	243 (185 to 302)	126 (91·9 to 158)	13,800
Chronic obstructive pulmonary disease	0 (0 to 0)	0 (0 to 0)	0·1 (0 to 0·1)	21·9 (13·8 to 34)	19·3 (12·6 to 28·2)	15·3 (6·3 to 26·6)	28·4 (15·6 to 44·1)	72·9 (47·7 to 103)	89·2 (59·9 to 124)	95·9 (66·6 to 131)	126 (88·1 to 169)	168 (122 to 225)	314 (231 to 412)	572 (417 to 744)	942 (672 to 1,250)	1,100 (802 to 1,440)	1,190 (877 to 1,530)	1,500 (1,140 to 1,940)	1,170 (902 to 1,470)	805 (613 to 1,000)	456 (351 to 568)	224 (170 to 280)	118 (85·4 to 149)	9,030
Pneumoconiosis	0 (0 to 0)	0 (0 to 0)	0 (0 to 0)	0 (0 to 0)	0 (0 to 0)	0 (0 to 0)	1 (0·5 to 1·7)	0·2 (0·1 to 0·3)	0·3 (0·2 to 0·5)	0·1 (0·1 to 0·2)	0·1 (0·1 to 0·2)	0·1 (0·1 to 0·2)	0·2 (0·1 to 0·3)	0·3 (0·2 to 0·5)	1·1 (0·6 to 1·8)	1·2 (0·8 to 1·8)	2·2 (1·4 to 3·5)	1·6 (1 to 2·4)	1·3 (0·8 to 1·9)	1·2 (0·8 to 1·8)	0·7 (0·4 to 1)	0·3 (0·2 to 0·5)	0·1 (0 to 0·1)	12
Silicosis	0 (0 to 0)	0 (0 to 0)	0 (0 to 0)	0 (0 to 0)	0 (0 to 0)	0 (0 to 0)	0·1 (0 to 0·2)	0 (0 to 0·1)	0·1 (0 to 0·1)	0 (0 to 0)	0 (0 to 0)	0 (0 to 0)	0 (0 to 0·1)	0 (0 to 0·1)	0·1 (0·1 to 0·3)	0·2 (0·1 to 0·5)	0·4 (0·2 to 0·9)	0·3 (0·1 to 0·5)	0·2 (0·1 to 0·4)	0·2 (0·1 to 0·3)	0·1 (0 to 0·2)	0 (0 to 0·1)	0 (0 to 0)	1·8
Asbestosis	0 (0 to 0)	0 (0 to 0)	0 (0 to 0)	0 (0 to 0)	0 (0 to 0)	0 (0 to 0)	0·8 (0·4 to 1·3)	0·1 (0 to 0·1)	0·1 (0·1 to 0·2)	0 (0 to 0·1)	0 (0 to 0)	0 (0 to 0)	0 (0 to 0·1)	0·1 (0 to 0·2)	0·5 (0·2 to 1)	0·5 (0·3 to 1)	0·8 (0·4 to 1·5)	0·7 (0·4 to 1·3)	0·4 (0·2 to 0·8)	0·6 (0·3 to 1)	0·3 (0·2 to 0·6)	0·1 (0·1 to 0·2)	0 (0 to 0)	5·3
Coal workers pneumoconiosis	0 (0 to 0)	0 (0 to 0)	0 (0 to 0)	0 (0 to 0)	0 (0 to 0)	0 (0 to 0)	0 (0 to 0·1)	0 (0 to 0)	0 (0 to 0)	0 (0 to 0)	0 (0 to 0)	0 (0 to 0)	0 (0 to 0)	0 (0 to 0·1)	0 (0 to 0)	0 (0 to 0·1)	0 (0 to 0·1)	0 (0 to 0·1)	0·1 (0 to 0·2)	0 (0 to 0)	0 (0 to 0)	0 (0 to 0)	0 (0 to 0)	0·3
Other pneumoconiosis	0 (0 to 0)	0 (0 to 0)	0 (0 to 0)	0 (0 to 0)	0 (0 to 0)	0 (0 to 0)	0·2 (0·1 to 0·4)	0·1 (0 to 0·2)	0·1 (0·1 to 0·2)	0·1 (0 to 0·1)	0·1 (0 to 0·1)	0·1 (0·1 to 0·2)	0·1 (0·1 to 0·2)	0·2 (0·1 to 0·3)	0·4 (0·2 to 0·7)	0·4 (0·2 to 0·7)	0·9 (0·5 to 1·8)	0·5 (0·3 to 0·9)	0·5 (0·3 to 0·9)	0·5 (0·2 to 0·8)	0·2 (0·1 to 0·4)	0·1 (0·1 to 0·2)	0 (0 to 0·1)	4·6
Asthma	0 (0 to 0)	0 (0 to 0)	0 (0 to 0)	573 (293 to 1,020)	623 (369 to 1,010)	491 (298 to 773)	333 (201 to 535)	248 (152 to 391)	201 (129 to 308)	184 (121 to 266)	162 (111 to 232)	148 (104 to 205)	144 (101 to 201)	161 (111 to 226)	157 (105 to 222)	175 (120 to 247)	121 (83·3 to 171)	132 (86·4 to 199)	78·3 (53·2 to 115)	64·6 (43·8 to 91·4)	25·8 (17·5 to 36·6)	13·8 (9·5 to 19·7)	5·2 (3·3 to 7·3)	4,040
Interstitial lung disease and pulmonary sarcoidosis	0 (0 to 0)	0 (0 to 0)	0 (0 to 0)	11·3 (5·6 to 19·7)	1·7 (1 to 2·9)	1·7 (1 to 2·9)	3·1 (1·5 to 6·2)	10·1 (3·8 to 20·3)	6·1 (2·9 to 11·4)	12·1 (6 to 21)	14·4 (7·4 to 24·4)	12·1 (6 to 21·2)	14·1 (7·7 to 24·6)	29·7 (16·1 to 50·4)	39·8 (20 to 70·9)	38·1 (19·2 to 68)	31·4 (18 to 51·7)	42 (22·2 to 70·4)	23·5 (14 to 38·9)	14·2 (8·1 to 23·5)	8·3 (4·8 to 13·8)	4 (2·3 to 6·2)	2 (1·1 to 3·4)	320
Other chronic respiratory diseases	0 (0 to 0)	0 (0 to 0)	0 (0 to 0)	50·2 (28·3 to 81)	15·7 (9·8 to 24·7)	32·1 (17·8 to 49·5)	27·3 (15·3 to 41·4)	31·4 (19 to 45·8)	42·4 (27·4 to 60·5)	29·1 (19·3 to 42·6)	42·4 (26·3 to 63·7)	29·3 (18·1 to 45·2)	29·8 (19·3 to 44·3)	36·2 (23 to 55·2)	21·7 (13·7 to 39·1)	10 (5·7 to 17·5)	10·1 (5·8 to 17·4)	11·4 (6·4 to 18·4)	8 (4·5 to 13·4)	7·7 (3·7 to 13·9)	3·7 (1·9 to 6·6)	0·8 (0·4 to 2·1)	0·4 (0·2 to 1·3)	440
Digestive diseases	29·5 (18·9 to 43·3)	24·7 (14·3 to 40·7)	57·2 (31·7 to 99·2)	171 (118 to 242)	193 (135 to 271)	180 (130 to 247)	220 (161 to 293)	409 (296 to 557)	523 (378 to 724)	589 (439 to 792)	659 (501 to 857)	718 (536 to 930)	945 (725 to 1,240)	1,200 (901 to 1,540)	1,360 (1,020 to 1,760)	1,250 (965 to 1,570)	944 (718 to 1,190)	896 (699 to 1,120)	581 (452 to 721)	401 (316 to 494)	230 (183 to 284)	109 (83·5 to 132)	52·5 (36·8 to 63·4)	11,700
Cirrhosis and other chronic liver diseases	0 (0 to 0)	0 (0 to 0)	0·1 (0 to 0·2)	12·5 (6·8 to 22·2)	18·1 (9·2 to 33·7)	21·2 (10·3 to 39·8)	31·2 (17·6 to 50·1)	48·3 (29·5 to 73·9)	76·2 (48·1 to 116)	108 (71·1 to 159)	145 (97·7 to 206)	177 (118 to 253)	277 (182 to 407)	418 (275 to 597)	499 (325 to 716)	440 (298 to 608)	313 (214 to 422)	287 (202 to 392)	166 (118 to 227)	104 (74·5 to 137)	49·7 (35·7 to 65·7)	17·7 (12·2 to 23·6)	9·8 (6 to 13·3)	3,220
Cirrhosis and other chronic liver diseases due to hepatitis B	0 (0 to 0)	0 (0 to 0)	0 (0 to 0)	0 (0 to 0)	0·1 (0 to 0·2)	0·2 (0·1 to 0·5)	0·5 (0·1 to 1·1)	2·2 (0·8 to 4·5)	5·3 (1·7 to 11·2)	9·5 (4·5 to 17·3)	14·6 (6·3 to 29·5)	17·9 (8 to 34·8)	27 (12·3 to 53)	37·4 (16·4 to 73·1)	37·8 (16·2 to 71·4)	29·2 (13·4 to 55·9)	18·9 (8·7 to 35·7)	16·9 (7·5 to 31·1)	10·2 (4·8 to 19)	6·4 (3·1 to 11·9)	3 (1·5 to 5·3)	1·1 (0·5 to 1·9)	0·6 (0·2 to 1·2)	239
Cirrhosis and other chronic liver diseases due to hepatitis C	0 (0 to 0)	0 (0 to 0)	0 (0 to 0)	0·1 (0 to 0·2)	0·4 (0·1 to 0·8)	0·8 (0·3 to 1·8)	1·9 (0·7 to 4)	8·2 (3·5 to 15·9)	19·4 (9·3 to 36·3)	29·2 (16·2 to 47·7)	37·7 (18·5 to 67·9)	43·6 (21·8 to 74·6)	63·8 (32 to 112)	89·8 (44·9 to 158)	99·6 (48·7 to 172)	83 (38·1 to 154)	57·7 (30·7 to 100)	51·9 (28 to 87·2)	29·4 (15·3 to 50·7)	18·5 (10 to 31·4)	9·2 (4·9 to 14·7)	3·4 (1·7 to 5·9)	1·9 (0·8 to 3·7)	649
Cirrhosis and other chronic liver diseases due to alcohol use	0 (0 to 0)	0 (0 to 0)	0 (0 to 0)	0 (0 to 0)	0 (0 to 0)	0 (0 to 0)	0·2 (0·1 to 0·6)	5·3 (2·1 to 11·4)	15·4 (6·6 to 30·9)	34·5 (18·9 to 55·8)	59·2 (31·9 to 93·7)	79·3 (45·2 to 126)	130 (72·8 to 211)	206 (118 to 318)	258 (149 to 404)	230 (135 to 350)	158 (93·6 to 236)	136 (81·7 to 208)	72·2 (43·7 to 108)	41·5 (24·4 to 63)	18·8 (11 to 28·3)	6·3 (3·5 to 10)	3·3 (1·5 to 5·6)	1,450
Cirrhosis and other chronic liver diseases due to NAFLD	0 (0 to 0)	0 (0 to 0)	0 (0 to 0)	0 (0 to 0)	0 (0 to 0)	0 (0 to 0)	1·7 (0·7 to 3·7)	5·6 (2·3 to 10·9)	12·2 (5 to 24·6)	16·3 (7·9 to 30·5)	18·8 (8·1 to 37·7)	22·1 (9·6 to 43·2)	36 (14·9 to 70)	54·5 (23·1 to 111)	62·6 (28·1 to 118)	57·5 (24·7 to 109)	46·1 (24·2 to 77·7)	47·3 (25·1 to 76·6)	30·3 (15·3 to 50·5)	20·7 (10·8 to 34·3)	10·4 (6 to 17·1)	3·9 (2 to 6·7)	2·2 (0·8 to 4·3)	448
Cirrhosis and other chronic liver diseases due to other causes	0 (0 to 0)	0 (0 to 0)	0·1 (0 to 0·2)	12·4 (6·8 to 22·1)	17·7 (8·9 to 33·3)	20·2 (9·7 to 38·2)	26·9 (15·4 to 42·7)	27·1 (14·9 to 43·5)	24·1 (11·7 to 42·6)	18·9 (8·6 to 36·2)	15·1 (6·8 to 30·2)	13·9 (6 to 28·1)	20 (8·9 to 39·4)	30·7 (13·1 to 59·2)	40·7 (18 to 78·2)	40·8 (18·1 to 75·5)	33·1 (14·9 to 63·9)	35·1 (16·2 to 64·1)	23·6 (11·5 to 42·1)	16·4 (8·1 to 29·5)	8·3 (4·4 to 13·6)	3·1 (1·5 to 5·5)	1·8 (0·6 to 3·5)	430
Upper digestive system diseases	0 (0 to 0)	0 (0 to 0)	0 (0 to 0)	6·6 (3 to 11·5)	10·2 (4·8 to 17·8)	17·7 (9·6 to 29·9)	70·7 (40·5 to 121)	195 (116 to 341)	262 (155 to 440)	285 (178 to 467)	280 (174 to 434)	296 (196 to 441)	349 (240 to 513)	391 (268 to 558)	400 (277 to 572)	361 (258 to 502)	291 (211 to 397)	267 (200 to 358)	184 (134 to 242)	125 (90·9 to 163)	72 (53·1 to 93·8)	35·5 (26 to 45·7)	18·3 (13·2 to 23·5)	3,920
Peptic ulcer disease	0 (0 to 0)	0 (0 to 0)	0 (0 to 0)	0·8 (0·4 to 1·5)	1·2 (0·6 to 2·8)	2·5 (1·4 to 4·7)	6·9 (4·2 to 11·4)	36·9 (22·6 to 56·3)	38·6 (24·7 to 56·7)	59·6 (39 to 86·5)	68·7 (45·8 to 98·6)	94·4 (60·3 to 142)	126 (80·6 to 191)	180 (114 to 270)	193 (126 to 289)	183 (124 to 262)	154 (105 to 216)	151 (102 to 215)	120 (82·6 to 169)	78·2 (53·1 to 107)	43·9 (31 to 59·8)	21·4 (14·7 to 28·4)	12·5 (8·6 to 16·3)	1,570
Gastritis and duodenitis	0 (0 to 0)	0 (0 to 0)	0 (0 to 0)	5·7 (2·2 to 10·8)	8·7 (3·6 to 16·3)	7·6 (2·7 to 15·3)	12·1 (5·6 to 21·6)	13·7 (5·5 to 26·3)	26·5 (14 to 43·5)	31·1 (16·4 to 53·8)	38·3 (21 to 63·3)	42·6 (22·6 to 74·3)	55·5 (29·4 to 97·8)	47·4 (23·6 to 84·4)	58·8 (34·2 to 94·2)	56·7 (34·3 to 85·9)	45·7 (29·6 to 67·2)	45·6 (26·8 to 68)	19·6 (12·5 to 29·1)	18·4 (9·8 to 27·7)	11·9 (5·2 to 18)	6·5 (2·5 to 9·6)	2·2 (0·9 to 3·4)	554
Gastroesophageal reflux disease	0 (0 to 0)	0 (0 to 0)	0 (0 to 0)	0 (0 to 0)	0·2 (0·1 to 0·6)	7·6 (3·3 to 15·1)	51·7 (25·4 to 98·2)	144 (69·8 to 285)	196 (94·7 to 367)	195 (92·1 to 373)	173 (81·9 to 323)	159 (75·1 to 289)	167 (81·9 to 303)	164 (78·1 to 300)	147 (69 to 285)	121 (58 to 226)	91·8 (46·5 to 167)	70·4 (36·2 to 122)	44·1 (22·2 to 79·5)	28·2 (13·9 to 50·9)	16·2 (8·2 to 29·4)	7·7 (3·8 to 13·8)	3·6 (1·7 to 6·4)	1,790
Appendicitis	0 (0 to 0)	0 (0 to 0)	0 (0 to 0)	7·7 (3·5 to 18)	13·7 (7·9 to 25·9)	16·9 (8·2 to 30·7)	20·1 (9·7 to 34·5)	24·8 (13·6 to 39·7)	22·7 (10·8 to 37·9)	15·7 (8·1 to 25·9)	17·4 (9·3 to 29·2)	13·9 (8·3 to 23·5)	18 (9·4 to 29·4)	18·8 (10·3 to 32·5)	9·8 (4·3 to 28·9)	8·6 (4 to 24·1)	10·4 (5·3 to 24·7)	13·2 (7·4 to 25·5)	6·6 (3·6 to 12·9)	4 (2·2 to 9·1)	2·8 (1·6 to 6·1)	1·5 (0·9 to 2·7)	0·5 (0·2 to 1·3)	247
Paralytic ileus and intestinal obstruction	19 (10·9 to 30·2)	16·5 (9·4 to 26·6)	46·6 (24·5 to 83·1)	29·9 (15·5 to 49·9)	26·4 (15·6 to 41·6)	27 (15·6 to 42·1)	18·2 (10·1 to 29·3)	25·8 (14·1 to 43·3)	19·6 (12 to 29·3)	21·2 (12·7 to 32·8)	27·7 (16·5 to 44·4)	30·5 (18 to 49·5)	41·3 (23·3 to 72·7)	56·1 (30·9 to 97·5)	93·5 (55·5 to 143)	99·3 (60·9 to 152)	74·7 (47·4 to 115)	90·1 (59·6 to 138)	63·7 (41·1 to 105)	52·7 (32·9 to 85·3)	29·5 (18·4 to 51·9)	14·3 (8·7 to 24·9)	5·8 (3·1 to 11·6)	929
Inguinal, femoral, and abdominal hernia	10·5 (5·4 to 19·4)	8·2 (3·8 to 15·2)	10·4 (5·2 to 18·9)	64·6 (36·2 to 105)	89·3 (47·3 to 154)	62 (30·9 to 114)	54·8 (27 to 96·8)	65 (34 to 109)	74·1 (39·9 to 119)	76·1 (41·4 to 125)	88·5 (51·7 to 143)	101 (61·2 to 155)	123 (73·8 to 194)	151 (93·1 to 227)	147 (93·7 to 215)	125 (82·5 to 180)	83·5 (56·7 to 118)	63·7 (45·3 to 88·5)	34·9 (25·5 to 46·6)	22·9 (17 to 30·1)	13·7 (9·8 to 18)	6·3 (4·4 to 8·4)	3 (1·8 to 4·1)	1,480
Inflammatory bowel disease	0 (0 to 0)	0 (0 to 0)	0 (0 to 0)	5·5 (2·6 to 10·3)	2·1 (1·1 to 3·9)	2·3 (1·1 to 4·7)	2·2 (1·2 to 5)	10·6 (6·1 to 16·6)	9·4 (6·2 to 14·1)	15·1 (10 to 21·4)	15·8 (10 to 23·5)	12·5 (7·6 to 19·4)	16·8 (10·5 to 25·3)	19·4 (12·7 to 27·5)	22·1 (14 to 32·3)	15·9 (9·8 to 24·1)	13·2 (8·2 to 20·1)	12·4 (7·5 to 21·9)	9·7 (6·3 to 14·7)	7·6 (4·9 to 11)	3·2 (2 to 5·7)	1·7 (1·1 to 2·7)	0·9 (0·6 to 1·4)	198
Vascular intestinal disorders	0 (0 to 0)	0 (0 to 0)	0 (0 to 0)	1·7 (0·9 to 3)	0·5 (0·3 to 0·8)	2·6 (1·2 to 5·4)	0·5 (0·3 to 0·8)	0·5 (0·3 to 0·8)	0·8 (0·5 to 1·2)	1 (0·6 to 1·5)	3·2 (1·9 to 5·1)	4·5 (2·6 to 7·1)	14·3 (8·5 to 22)	16·2 (10·1 to 24·4)	20·4 (12·1 to 31·1)	27 (17·5 to 39·2)	25·9 (17·2 to 37·3)	27·1 (17·9 to 38·8)	17·3 (11·2 to 24·3)	13 (8·4 to 18·3)	7·4 (4·9 to 10·5)	4·5 (3 to 6·1)	1·9 (1·1 to 2·7)	190
Gallbladder and biliary diseases	0 (0 to 0)	0 (0 to 0)	0 (0 to 0)	0·9 (0·5 to 1·5)	2·4 (1·4 to 3·8)	4·1 (2·4 to 6·5)	6·3 (3·2 to 11·1)	10·9 (4·5 to 20·3)	15·8 (7·9 to 27·4)	19·2 (10·5 to 31·1)	17·3 (8·6 to 29·6)	21·7 (12·4 to 34·2)	24·5 (13·8 to 38·4)	32·7 (19·6 to 48·9)	37·7 (23·1 to 56·9)	34·3 (22·2 to 53·4)	28·7 (18 to 49·5)	30·9 (19·7 to 57·5)	23·9 (15·2 to 41·5)	18·3 (12 to 30·2)	12·1 (8·5 to 17·1)	6·6 (4·7 to 9·2)	2·9 (1·8 to 4·2)	351
Pancreatitis	0 (0 to 0)	0 (0 to 0)	0 (0 to 0)	3 (0·8 to 5·3)	0·4 (0·2 to 0·6)	4 (1·5 to 7·3)	1·3 (0·7 to 3·7)	2·5 (1·4 to 8·2)	16·3 (9·9 to 26·1)	21·8 (12·8 to 33·6)	32·5 (20·4 to 48·4)	30·2 (18·7 to 47·2)	29·6 (18·5 to 45·2)	23·2 (14·2 to 39·5)	38·7 (23·7 to 61·2)	39·8 (24·7 to 60·1)	24·2 (15·3 to 36·1)	16·8 (11 to 24·3)	12·1 (7·9 to 18)	8·3 (5·8 to 11·5)	4·8 (3·1 to 6·8)	2·2 (1·5 to 3·1)	0·8 (0·5 to 1·2)	312
Other digestive diseases	0 (0 to 0)	0 (0 to 0)	0 (0 to 0)	38·8 (20·8 to 61·8)	29·4 (14·9 to 46)	22·3 (10·1 to 35·8)	14·8 (6·8 to 24·4)	25·6 (11·7 to 42·4)	26·4 (11·2 to 43·9)	25·6 (12·5 to 41·5)	30·9 (15·4 to 49·5)	31 (16·9 to 51·4)	51 (27·2 to 82·6)	76·1 (34·9 to 127)	94·1 (41·9 to 159)	94·2 (50 to 149)	78·5 (41·4 to 123)	87·3 (45·8 to 136)	63·5 (35·2 to 94)	45·5 (26·2 to 67·6)	35·1 (20·4 to 50·4)	18·4 (10·2 to 26·3)	8·5 (4·4 to 12·8)	897
Neurological disorders	0·6 (0·2 to 1·1)	1·3 (0·4 to 2·8)	60·8 (29·4 to 105)	179 (77·8 to 342)	329 (144 to 600)	760 (276 to 1,530)	1,020 (384 to 1,990)	1,130 (431 to 2,180)	1,080 (459 to 2,010)	917 (391 to 1,680)	789 (363 to 1,390)	746 (370 to 1,270)	801 (449 to 1,330)	778 (458 to 1,240)	745 (468 to 1,140)	700 (450 to 1,060)	736 (483 to 1,170)	902 (583 to 1,570)	941 (596 to 1,670)	1,140 (658 to 2,270)	1,020 (539 to 2,100)	689 (337 to 1,390)	417 (205 to 844)	15,900
Alzheimer's disease and other dementias	0 (0 to 0)	0 (0 to 0)	0 (0 to 0)	0 (0 to 0)	0 (0 to 0)	0 (0 to 0)	0 (0 to 0)	0 (0 to 0)	0 (0 to 0)	0 (0 to 0)	0 (0 to 0)	5·5 (1·2 to 17·7)	35·2 (10·1 to 100)	68·7 (23·7 to 174)	118 (44·6 to 284)	192 (75·5 to 475)	285 (111 to 707)	458 (188 to 1,120)	535 (219 to 1,250)	797 (316 to 1,920)	806 (319 to 1,890)	592 (243 to 1,310)	379 (165 to 812)	4,270
Parkinson's disease	0 (0 to 0)	0 (0 to 0)	0 (0 to 0)	0 (0 to 0)	0 (0 to 0)	0 (0 to 0)	0 (0 to 0)	0·2 (0·1 to 0·3)	0·7 (0·3 to 1·3)	1·1 (0·5 to 2·2)	2·5 (1·5 to 4·1)	6·8 (4·6 to 9·7)	18·7 (13·1 to 25·4)	35 (24·6 to 47·7)	55·4 (40·1 to 74·5)	89·1 (67·2 to 115)	128 (98·7 to 164)	215 (168 to 264)	263 (211 to 322)	250 (200 to 312)	164 (131 to 202)	71·3 (54 to 87·6)	27·2 (19 to 34·8)	1,330
Idiopathic epilepsy	0·4 (0·1 to 0·8)	1 (0·2 to 2·5)	19·6 (5·1 to 45·5)	128 (51·8 to 247)	186 (72·5 to 362)	221 (84·7 to 441)	270 (117 to 507)	319 (147 to 581)	313 (159 to 538)	271 (150 to 448)	214 (117 to 357)	213 (122 to 334)	214 (121 to 328)	180 (99·3 to 285)	162 (89·9 to 266)	139 (71·2 to 238)	128 (68·3 to 209)	105 (53·6 to 178)	75·5 (36·7 to 128)	56·2 (28·9 to 93·9)	30·5 (13·8 to 54·3)	15·3 (6·7 to 27·3)	6·2 (2·1 to 12·3)	3,270
Multiple sclerosis	0 (0 to 0)	0 (0 to 0)	0 (0 to 0)	0 (0 to 0)	0 (0 to 0·1)	0·1 (0·1 to 0·2)	0·6 (0·4 to 0·9)	1·8 (1·2 to 3·3)	3·3 (2·2 to 4·8)	5 (3·5 to 7·1)	7 (4·7 to 10)	8·8 (5·9 to 12·6)	11·5 (7 to 16·5)	11·3 (6·4 to 16·5)	11·8 (6·6 to 16·6)	8·2 (4·9 to 11·8)	5·1 (3·4 to 7·4)	4·2 (2·3 to 6)	2 (1·2 to 2·9)	0·9 (0·6 to 1·3)	0·5 (0·3 to 0·7)	0·2 (0·1 to 0·3)	0·1 (0 to 0·1)	82·3
Motor neuron disease	0·2 (0·1 to 0·3)	0·3 (0·2 to 0·4)	20·4 (8·6 to 42·6)	7·6 (4·3 to 12·6)	0·7 (0·4 to 0·9)	0·9 (0·6 to 1·3)	4·2 (2·9 to 6·1)	6·1 (4·1 to 8·9)	8·6 (5·7 to 12·5)	7·4 (4·7 to 11·2)	18·3 (11·5 to 27)	13·2 (8·2 to 19·6)	27·1 (16·7 to 40·9)	36·2 (22·4 to 54·4)	44·4 (28·4 to 64·4)	20·3 (13·2 to 28·1)	20·6 (13·4 to 29·9)	5·8 (3·8 to 8·2)	1·6 (1 to 2·3)	0·3 (0·2 to 0·4)	0·5 (0·3 to 0·7)	0·1 (0 to 0·1)	0·3 (0·2 to 0·5)	245
Headache disorders	0 (0 to 0)	0 (0 to 0)	0 (0 to 0)	0 (0 to 0)	81·7 (1·6 to 223)	435 (21 to 1,120)	639 (68 to 1,600)	730 (106 to 1,660)	703 (120 to 1,590)	598 (114 to 1,310)	514 (117 to 1,100)	457 (120 to 953)	438 (128 to 938)	382 (113 to 783)	296 (96·6 to 598)	214 (72·2 to 431)	141 (48·4 to 283)	89·3 (30·5 to 187)	46 (15·4 to 96)	25·4 (8·6 to 53·7)	13·7 (4·5 to 28·5)	5·7 (1·7 to 12·1)	2·1 (0·4 to 5·3)	5,810
Migraine	0 (0 to 0)	0 (0 to 0)	0 (0 to 0)	0 (0 to 0)	73·4 (1·1 to 205)	403 (13·7 to 1,090)	588 (45·9 to 1,500)	664 (71·6 to 1,590)	631 (80·2 to 1,480)	533 (75·6 to 1,200)	453 (79·1 to 1,030)	401 (83·2 to 895)	381 (86·7 to 852)	327 (76·8 to 708)	254 (64·3 to 546)	179 (52·4 to 383)	115 (33·9 to 239)	70·7 (22·3 to 152)	36·2 (11·2 to 78·7)	20·1 (5·9 to 43·8)	10·8 (3·1 to 23·7)	4·4 (1·2 to 9·8)	1·5 (0·3 to 3·4)	5,150
Tension-type headache	0 (0 to 0)	0 (0 to 0)	0 (0 to 0)	0 (0 to 0)	8·4 (0·2 to 57·6)	31·9 (0 to 254)	50·8 (5·7 to 281)	66·2 (10·5 to 334)	71·8 (12·2 to 324)	64·5 (11·4 to 280)	61·2 (12·7 to 256)	56·2 (12·1 to 207)	56·9 (14·7 to 184)	54·3 (13·4 to 187)	42·4 (11 to 129)	34·5 (8·7 to 110)	26 (6·1 to 92·3)	18·6 (3·6 to 69·2)	9·8 (1·8 to 37·1)	5·2 (0·9 to 20·9)	2·9 (0·5 to 11·7)	1·3 (0·2 to 5·7)	0·6 (0 to 3·7)	664
Other neurological disorders	0 (0 to 0)	0 (0 to 0)	20·7 (9·7 to 37·6)	43·9 (16·4 to 85·8)	60·1 (23·5 to 120)	103 (45·3 to 193)	105 (52·2 to 189)	73·4 (37·1 to 132)	50·6 (26 to 86·9)	35·1 (17·1 to 61·9)	33·2 (17·3 to 58·1)	41·8 (24·3 to 66·6)	56·9 (35·3 to 83·5)	65·3 (43·7 to 91·1)	57·8 (38·7 to 77·9)	37·3 (25·8 to 52·1)	27·8 (19·7 to 37·1)	25·4 (17·8 to 33·9)	17·8 (12·7 to 23·6)	14·4 (10·2 to 19·9)	5 (3·7 to 6·6)	4·7 (3·5 to 6·1)	2·2 (1·4 to 2·9)	882
Mental disorders	0·4 (0·2 to 0·6)	1·2 (0·7 to 1·7)	19·4 (12·1 to 28·9)	107 (69·2 to 154)	527 (352 to 753)	1,430 (972 to 2,060)	1,990 (1,390 to 2,740)	2,190 (1,550 to 3,020)	2,270 (1,590 to 3,090)	2,030 (1,440 to 2,710)	1,810 (1,290 to 2,410)	1,620 (1,160 to 2,170)	1,620 (1,170 to 2,170)	1,490 (1,080 to 1,970)	1,260 (910 to 1,670)	953 (697 to 1,270)	667 (483 to 876)	470 (343 to 622)	270 (195 to 357)	160 (113 to 213)	91·8 (64·6 to 123)	44·6 (30·6 to 60·6)	21·6 (14·1 to 30·8)	21,000
Schizophrenia	0 (0 to 0)	0 (0 to 0)	0 (0 to 0)	0 (0 to 0)	0 (0 to 0)	7·2 (3 to 13·6)	61·1 (22·3 to 119)	225 (122 to 370)	369 (198 to 563)	393 (238 to 593)	364 (221 to 529)	322 (213 to 451)	307 (196 to 428)	263 (174 to 368)	200 (128 to 286)	133 (83·7 to 186)	81·2 (51·5 to 118)	48·5 (28·6 to 71·1)	23·2 (13·2 to 34·8)	10·6 (5·7 to 16·9)	4·4 (2·1 to 7·3)	1·4 (0·6 to 2·5)	0·4 (0·1 to 0·9)	2,810
Depressive disorders	0 (0 to 0)	0 (0 to 0)	0 (0 to 0)	0·2 (0·1 to 0·4)	15·7 (6·6 to 30·7)	148 (74 to 266)	412 (236 to 652)	597 (350 to 932)	548 (329 to 855)	469 (276 to 720)	445 (279 to 668)	430 (265 to 651)	460 (298 to 691)	454 (295 to 680)	415 (257 to 625)	335 (213 to 502)	247 (161 to 359)	185 (121 to 264)	114 (70·2 to 169)	73·7 (44·5 to 112)	46·6 (29·9 to 68·1)	24·6 (15·1 to 37·3)	12·8 (6·8 to 20·8)	5,430
Major depressive disorder	0 (0 to 0)	0 (0 to 0)	0 (0 to 0)	0·1 (0 to 0·3)	13 (5 to 26·7)	128 (58·6 to 240)	349 (193 to 575)	477 (264 to 779)	404 (231 to 662)	334 (184 to 544)	322 (187 to 502)	317 (181 to 508)	347 (215 to 527)	349 (217 to 522)	326 (191 to 510)	266 (161 to 415)	198 (123 to 294)	150 (95·3 to 221)	93·5 (54·9 to 144)	61·6 (34·9 to 96·2)	39·6 (24 to 60·1)	21·1 (12·2 to 32·9)	11 (5·5 to 18·7)	4,210
Dysthymia	0 (0 to 0)	0 (0 to 0)	0 (0 to 0)	0 (0 to 0·1)	2·6 (0 to 7·7)	20·6 (7·8 to 42·8)	63·1 (29·7 to 110)	120 (60·6 to 212)	144 (71·5 to 250)	135 (73·7 to 218)	123 (69·1 to 212)	113 (59 to 194)	113 (60 to 199)	105 (59·5 to 175)	89·2 (51·2 to 147)	68·7 (35·9 to 114)	49·3 (25·7 to 84·2)	35·2 (19·6 to 59·3)	20·3 (11·4 to 33·7)	12·1 (6·4 to 20·8)	7 (3·7 to 11·8)	3·4 (1·9 to 5·8)	1·7 (0·9 to 3·1)	1,230
Bipolar disorder	0 (0 to 0)	0 (0 to 0)	0 (0 to 0)	0 (0 to 0)	0 (0 to 0)	75·3 (30·6 to 140)	297 (140 to 535)	406 (199 to 682)	390 (200 to 650)	315 (162 to 519)	270 (149 to 447)	245 (136 to 393)	246 (135 to 409)	221 (122 to 370)	177 (100 to 290)	123 (67·6 to 204)	78·7 (43·2 to 129)	49 (26·5 to 80·7)	23·9 (12·7 to 38·6)	11·4 (6 to 18·9)	5·3 (2·5 to 9·1)	2·1 (1 to 3·8)	0·9 (0·4 to 1·5)	2,940
Anxiety disorders	0 (0 to 0)	0 (0 to 0)	0 (0 to 0)	7 (1·2 to 15·7)	114 (57·2 to 206)	315 (169 to 533)	433 (250 to 691)	486 (271 to 787)	465 (259 to 784)	400 (231 to 643)	348 (201 to 533)	313 (172 to 513)	314 (175 to 490)	296 (180 to 450)	256 (161 to 389)	200 (119 to 323)	142 (84·1 to 233)	99·1 (58·7 to 163)	54·6 (31·6 to 85·5)	29·7 (16·7 to 46·6)	14·6 (7·8 to 23·3)	5·8 (3 to 9·5)	2·1 (1 to 3·6)	4,300
Eating disorders	0 (0 to 0)	0 (0 to 0)	0 (0 to 0)	0 (0 to 0)	1·1 (0·4 to 2·4)	19·1 (7·2 to 36·4)	56·7 (26·4 to 104)	77·6 (35·4 to 143)	76·9 (40·5 to 134)	72·5 (35·7 to 130)	47·7 (24·2 to 86)	19·1 (8·5 to 35·6)	7·3 (2·1 to 15·1)	0 (0 to 0)	0 (0 to 0)	0 (0 to 0)	0 (0 to 0)	0 (0 to 0)	0 (0 to 0)	0 (0 to 0)	0 (0 to 0)	0 (0 to 0)	0 (0 to 0)	378
Anorexia nervosa	0 (0 to 0)	0 (0 to 0)	0 (0 to 0)	0 (0 to 0)	1 (0·3 to 2·2)	7·6 (1·5 to 16)	20·4 (7·8 to 39·4)	21 (7·8 to 43·1)	14·1 (5 to 27·8)	7·2 (1·7 to 15·7)	4·6 (0·8 to 10·8)	3·4 (0 to 7·9)	2·5 (0·1 to 6·3)	0 (0 to 0)	0 (0 to 0)	0 (0 to 0)	0 (0 to 0)	0 (0 to 0)	0 (0 to 0)	0 (0 to 0)	0 (0 to 0)	0 (0 to 0)	0 (0 to 0)	81·7
Bulimia nervosa	0 (0 to 0)	0 (0 to 0)	0 (0 to 0)	0 (0 to 0)	0·2 (0·1 to 0·5)	11·5 (2·4 to 27·2)	36·3 (13·6 to 75·3)	56·7 (20·1 to 119)	62·9 (30·3 to 114)	65·2 (29·5 to 118)	43·2 (20·7 to 81·8)	15·6 (6 to 31·1)	4·8 (0·7 to 11·6)	0 (0 to 0)	0 (0 to 0)	0 (0 to 0)	0 (0 to 0)	0 (0 to 0)	0 (0 to 0)	0 (0 to 0)	0 (0 to 0)	0 (0 to 0)	0 (0 to 0)	296
Autism spectrum disorders	0·4 (0·2 to 0·5)	1 (0·6 to 1·6)	16·6 (10·7 to 24·7)	74·2 (45·6 to 110)	101 (62·4 to 152)	106 (66·5 to 162)	112 (71·5 to 167)	118 (76 to 176)	108 (64·4 to 163)	87 (53·2 to 131)	71·4 (44·6 to 108)	60·5 (37·9 to 91·4)	58·2 (34·8 to 88·4)	52·2 (32·1 to 79·7)	43·7 (27·4 to 66·1)	33·2 (20·4 to 48·8)	23·9 (14·7 to 35·3)	17·7 (10·8 to 26·9)	10·7 (6·5 to 16·1)	6·6 (4·1 to 9·9)	3·8 (2·3 to 5·7)	1·8 (1·2 to 2·8)	0·9 (0·5 to 1·3)	1,110
Attention-deficit/hyperactivity disorder	0 (0 to 0)	0 (0 to 0)	0 (0 to 0)	9 (4·3 to 16·6)	104 (57·1 to 189)	153 (82·9 to 272)	136 (75·7 to 238)	111 (59·7 to 192)	79·8 (43·2 to 139)	53·4 (28·3 to 92·1)	37 (19·1 to 65·9)	26·7 (13·4 to 47)	21·6 (10·7 to 38·9)	16 (7·5 to 29)	9·8 (4·6 to 17·4)	4·9 (2·1 to 8·7)	2 (0·7 to 4)	0·8 (0·2 to 1·8)	0·2 (0 to 0·5)	0 (0 to 0)	0 (0 to 0)	0 (0 to 0)	0 (0 to 0)	766
Conduct disorder	0 (0 to 0)	0 (0 to 0)	0 (0 to 0)	0 (0 to 0)	165 (83·1 to 286)	575 (324 to 928)	404 (213 to 662)	0 (0 to 0)	0 (0 to 0)	0 (0 to 0)	0 (0 to 0)	0 (0 to 0)	0 (0 to 0)	0 (0 to 0)	0 (0 to 0)	0 (0 to 0)	0 (0 to 0)	0 (0 to 0)	0 (0 to 0)	0 (0 to 0)	0 (0 to 0)	0 (0 to 0)	0 (0 to 0)	1,140
Idiopathic developmental intellectual disability	0 (0 to 0·1)	0·1 (0 to 0·3)	2·7 (0·2 to 6·1)	16·5 (3·5 to 32·5)	25·5 (7·6 to 48·9)	26·6 (7·5 to 51·4)	27·3 (8·4 to 51·8)	28·6 (8·3 to 56·1)	25·6 (7·1 to 51·5)	20·5 (4·7 to 39·8)	16·3 (4·3 to 31·5)	13·5 (3·8 to 26·5)	12·8 (3·7 to 24·8)	11·1 (2·9 to 21·3)	8·9 (2·4 to 17·8)	6·2 (1·6 to 12·4)	4 (0·8 to 8·1)	2·5 (0·5 to 5·3)	1·3 (0·2 to 2·8)	0·7 (0·2 to 1·6)	0·3 (0·1 to 0·8)	0·1 (0 to 0·3)	0 (0 to 0·1)	251
Other mental disorders	0 (0 to 0)	0 (0 to 0)	0 (0 to 0)	0 (0 to 0)	0 (0 to 0)	7·4 (3·2 to 13)	53·8 (24·3 to 93·5)	145 (73·6 to 247)	207 (114 to 335)	218 (126 to 337)	206 (120 to 325)	188 (112 to 294)	190 (115 to 298)	177 (106 to 275)	152 (92·9 to 230)	118 (73·3 to 180)	88·1 (54·4 to 131)	66·8 (40·8 to 99·5)	42·3 (27 to 61·7)	27·5 (17·5 to 40·5)	16·9 (10·8 to 24·9)	8·8 (5·7 to 12·8)	4·6 (2·9 to 6·7)	1,920
Substance use disorders	0 (0 to 0)	0 (0 to 0)	0 (0 to 0)	0·1 (0·1 to 0·2)	0·1 (0·1 to 0·2)	24·8 (13·5 to 40·9)	464 (279 to 689)	823 (533 to 1,170)	776 (493 to 1,080)	579 (371 to 840)	434 (290 to 608)	342 (233 to 486)	311 (218 to 434)	260 (178 to 365)	221 (158 to 298)	155 (108 to 215)	104 (72·2 to 142)	67·2 (46·1 to 93)	37·4 (26·4 to 52)	21·2 (14·7 to 29·7)	11·8 (8 to 16·6)	6·1 (4 to 9)	3·6 (2·3 to 5·6)	4,640
Alcohol use disorders	0 (0 to 0)	0 (0 to 0)	0 (0 to 0)	0·1 (0·1 to 0·2)	0·1 (0·1 to 0·2)	8·1 (3 to 16·3)	99·4 (47·1 to 177)	298 (155 to 526)	351 (187 to 562)	309 (167 to 515)	251 (152 to 387)	207 (126 to 323)	194 (124 to 290)	163 (102 to 248)	148 (98 to 210)	107 (70·2 to 158)	74·8 (50·6 to 107)	48·1 (32·1 to 70·4)	26·4 (17·9 to 38·5)	13·8 (8·7 to 21)	7·6 (4·6 to 11·6)	3·9 (2·3 to 6·3)	2·4 (1·3 to 4·2)	2,310
Drug use disorders	0 (0 to 0)	0 (0 to 0)	0 (0 to 0)	0 (0 to 0)	0 (0 to 0)	16·7 (8·3 to 29·2)	365 (209 to 574)	525 (326 to 790)	425 (276 to 605)	269 (170 to 389)	183 (121 to 265)	136 (86·2 to 198)	117 (74·8 to 170)	96·9 (60·9 to 140)	72·9 (47 to 106)	47·9 (29·9 to 69·6)	29·2 (17·1 to 43·6)	19·1 (11·6 to 29·7)	11 (6·5 to 16·8)	7·4 (4·5 to 11)	4·2 (2·4 to 6·4)	2·2 (1·3 to 3·4)	1·2 (0·7 to 1·9)	2,330
Opioid use disorders	0 (0 to 0)	0 (0 to 0)	0 (0 to 0)	0 (0 to 0)	0 (0 to 0)	0 (0 to 0)	24·9 (7·6 to 50·8)	113 (52·5 to 197)	128 (64·8 to 212)	96·4 (49·6 to 156)	73·1 (41·4 to 116)	57·4 (31·6 to 90·6)	49·5 (26·2 to 82·7)	41·5 (20·3 to 68·7)	33·1 (17·4 to 53·4)	22·6 (11·4 to 37)	12·9 (5·6 to 22·6)	7·5 (3·1 to 13·9)	4·9 (2·2 to 8·4)	3·6 (1·8 to 6)	2 (0·9 to 3·5)	1·1 (0·5 to 2)	0·7 (0·3 to 1·2)	673
Cocaine use disorders	0 (0 to 0)	0 (0 to 0)	0 (0 to 0)	0 (0 to 0)	0 (0 to 0)	3·1 (1·6 to 5·6)	124 (59·6 to 227)	144 (70·9 to 248)	104 (51·9 to 179)	67·4 (32·1 to 115)	45 (20·5 to 78·6)	31·8 (15·3 to 56·4)	27·1 (13·2 to 47·4)	23·3 (11·9 to 39·5)	17·2 (8·4 to 29·1)	11·6 (5·3 to 19·8)	7·1 (3·2 to 12·8)	6 (2·7 to 10·3)	2·8 (1·1 to 5·3)	1·4 (0·4 to 2·8)	0·9 (0·3 to 1·8)	0·4 (0·1 to 0·8)	0·2 (0 to 0·4)	617
Amphetamine use disorders	0 (0 to 0)	0 (0 to 0)	0 (0 to 0)	0 (0 to 0)	0 (0 to 0)	0 (0 to 0)	7·9 (0·7 to 19·6)	40·8 (13·7 to 81·1)	39·1 (14·9 to 75·8)	16·3 (3·9 to 35·2)	7·5 (1·3 to 17·2)	4·6 (2·6 to 7·7)	3·8 (2·3 to 6)	4·1 (2·6 to 6·4)	3·7 (2·3 to 5·7)	2·6 (1·7 to 3·9)	1·2 (0·7 to 1·8)	0·4 (0·2 to 0·6)	0·2 (0·1 to 0·3)	0·1 (0 to 0·2)	0·1 (0 to 0·1)	0 (0 to 0·1)	0 (0 to 0·1)	132
Cannabis use disorders	0 (0 to 0)	0 (0 to 0)	0 (0 to 0)	0 (0 to 0)	0 (0 to 0)	11·6 (3·9 to 23·5)	124 (64·1 to 213)	108 (52·4 to 196)	60·9 (27·4 to 118)	32·9 (13·6 to 63·1)	20·2 (8·7 to 37·6)	15·2 (6 to 28)	13·1 (5·2 to 26)	9·5 (3·6 to 19)	5·7 (1·9 to 10·9)	2·8 (0·9 to 6)	1·4 (0·7 to 2·6)	0·7 (0·3 to 1·2)	0·3 (0·1 to 0·5)	0·1 (0 to 0·3)	0·1 (0 to 0·1)	0 (0 to 0)	0 (0 to 0)	407
Other drug use disorders	0 (0 to 0)	0 (0 to 0)	0 (0 to 0)	0 (0 to 0)	0 (0 to 0)	2 (1 to 3·8)	84·5 (38·7 to 155)	119 (59·2 to 203)	93·1 (49·3 to 156)	56·4 (28·9 to 94·9)	37·1 (18·4 to 63·7)	26·9 (14·3 to 45·1)	23·5 (13·3 to 38)	18·6 (9·5 to 29·9)	13·2 (7·2 to 20·8)	8·3 (4·1 to 14·4)	6·7 (3·6 to 10·8)	4·5 (2·3 to 7·5)	2·8 (1·5 to 4·6)	2·1 (1·2 to 3·4)	1·2 (0·7 to 1·9)	0·6 (0·4 to 1)	0·3 (0·2 to 0·5)	500
Diabetes and kidney diseases	0·4 (0·2 to 0·6)	1 (0·6 to 1·8)	24·4 (12·2 to 42·4)	58·8 (39·5 to 81·1)	93·8 (67·9 to 125)	99·8 (69·2 to 142)	252 (189 to 336)	440 (334 to 567)	665 (513 to 850)	892 (707 to 1,120)	1,240 (979 to 1,540)	1,710 (1,310 to 2,150)	2,980 (2,250 to 3,770)	4,450 (3,480 to 5,630)	5,730 (4,490 to 7,150)	5,640 (4,410 to 7,070)	5,480 (4,310 to 6,770)	5,680 (4,520 to 7,060)	4,300 (3,470 to 5,260)	2,840 (2,300 to 3,420)	1,670 (1,380 to 2,000)	794 (639 to 945)	405 (320 to 485)	45,500
Diabetes mellitus	0·4 (0·2 to 0·6)	1 (0·6 to 1·8)	4·5 (1·5 to 13)	12·4 (7·2 to 22)	12·8 (8·2 to 18·7)	19·5 (13·7 to 27·5)	84 (59·8 to 116)	177 (127 to 242)	286 (206 to 385)	423 (313 to 556)	621 (471 to 808)	955 (697 to 1,260)	1,780 (1,320 to 2,330)	2,910 (2,200 to 3,770)	3,910 (3,000 to 4,970)	4,050 (3,120 to 5,120)	4,030 (3,080 to 5,030)	4,210 (3,250 to 5,330)	3,230 (2,520 to 4,070)	2,080 (1,630 to 2,560)	1,210 (975 to 1,470)	535 (422 to 652)	258 (199 to 316)	30,800
Diabetes mellitus type 1	0·4 (0·2 to 0·6)	1 (0·6 to 1·8)	4·5 (1·5 to 13)	12·4 (7·2 to 22)	12·8 (8·2 to 18·7)	19·5 (13·7 to 27·5)	27·6 (18·6 to 40·6)	48·7 (32·6 to 70·2)	73 (50·2 to 109)	59·2 (38·1 to 93·8)	83·3 (55·7 to 137)	117 (74·1 to 192)	57·3 (36·3 to 93·4)	74·4 (46·4 to 130)	92·3 (57 to 182)	77 (46 to 145)	75·4 (45·2 to 151)	62 (37·3 to 128)	48·2 (27·8 to 108)	22·2 (13·4 to 48·4)	12·8 (7·6 to 28·7)	6·5 (4·1 to 14·2)	3·8 (2·2 to 8·1)	991
Diabetes mellitus type 2	0 (0 to 0)	0 (0 to 0)	0 (0 to 0)	0 (0 to 0)	0 (0 to 0)	0 (0 to 0)	56·4 (34·8 to 83·9)	128 (86·5 to 186)	213 (144 to 306)	364 (261 to 487)	537 (399 to 709)	837 (602 to 1,120)	1,730 (1,270 to 2,260)	2,840 (2,140 to 3,690)	3,820 (2,920 to 4,860)	3,970 (3,050 to 5,020)	3,950 (3,010 to 4,910)	4,150 (3,200 to 5,260)	3,180 (2,480 to 4,000)	2,050 (1,610 to 2,520)	1,200 (963 to 1,460)	529 (417 to 645)	254 (196 to 312)	29,800
Chronic kidney disease	0 (0 to 0)	0 (0 to 0)	19·9 (10 to 34)	46·4 (31 to 64·5)	80·8 (56·5 to 110)	80·1 (53·1 to 121)	168 (113 to 241)	263 (184 to 359)	379 (266 to 521)	469 (333 to 634)	621 (452 to 836)	753 (546 to 1,030)	1,190 (847 to 1,620)	1,540 (1,100 to 2,090)	1,820 (1,340 to 2,420)	1,590 (1,140 to 2,140)	1,450 (1,080 to 1,910)	1,470 (1,110 to 1,890)	1,070 (813 to 1,400)	762 (585 to 966)	463 (366 to 572)	258 (195 to 321)	148 (112 to 185)	14,600
Chronic kidney disease due to diabetes mellitus type 1	0 (0 to 0)	0 (0 to 0)	0·2 (0·1 to 0·4)	1·2 (0·4 to 2·8)	2·4 (0·9 to 5·6)	2·8 (1·1 to 6·2)	6·9 (2·8 to 14·8)	14·1 (5·7 to 29·3)	38·3 (17·4 to 74)	75·2 (35·7 to 129)	121 (64·2 to 206)	139 (73·9 to 231)	190 (89·9 to 331)	182 (73·6 to 350)	147 (49·7 to 316)	92·5 (32·5 to 212)	65·1 (23·9 to 148)	50·6 (19·3 to 106)	24·4 (8·3 to 53·8)	8·9 (3·1 to 20·9)	2 (0·6 to 5)	0·3 (0·1 to 0·6)	0 (0 to 0·1)	1,160
Chronic kidney disease due to diabetes mellitus type 2	0 (0 to 0)	0 (0 to 0)	0 (0 to 0)	0 (0 to 0)	0 (0 to 0)	0 (0 to 0)	2·7 (0·9 to 6·5)	6·6 (2·7 to 14·6)	15·7 (6·3 to 31·7)	29·7 (12·7 to 55·8)	54·8 (24·5 to 103)	92·6 (44·5 to 163)	214 (112 to 357)	372 (209 to 569)	525 (310 to 780)	497 (297 to 734)	468 (297 to 687)	478 (319 to 679)	346 (219 to 490)	243 (158 to 342)	142 (93·9 to 201)	75·6 (45·5 to 107)	41·2 (23·3 to 59·5)	3,610
Chronic kidney disease due to hypertension	0 (0 to 0)	0 (0 to 0)	0 (0 to 0)	0 (0 to 0)	0 (0 to 0)	0 (0 to 0)	14·5 (6·9 to 27·4)	25·3 (9·6 to 50·9)	39 (15·8 to 71·8)	50·1 (24·4 to 85·2)	66·8 (35·4 to 108)	92·5 (56·6 to 145)	168 (105 to 258)	251 (157 to 384)	344 (217 to 528)	350 (213 to 531)	356 (229 to 515)	395 (266 to 557)	318 (215 to 450)	252 (176 to 348)	169 (122 to 220)	103 (71·8 to 138)	61·3 (42·3 to 83·7)	3,060
Chronic kidney disease due to glomerulonephritis	0 (0 to 0)	0 (0 to 0)	5·7 (2·4 to 11·2)	15·5 (8·5 to 24·6)	30·9 (18·2 to 46·9)	34·3 (18·6 to 56)	71 (42·1 to 111)	109 (63·6 to 162)	149 (91·1 to 220)	168 (106 to 251)	208 (126 to 312)	235 (132 to 359)	337 (202 to 519)	397 (227 to 628)	429 (238 to 714)	329 (174 to 560)	259 (126 to 447)	229 (113 to 396)	145 (68·9 to 255)	85·6 (38·6 to 154)	39·8 (17·3 to 70·5)	18·6 (8·1 to 33·8)	9·5 (3·6 to 20·5)	3,300
Chronic kidney disease due to other and unspecified causes	0 (0 to 0)	0 (0 to 0)	14 (6·8 to 25·2)	29·7 (19·5 to 43·7)	47·5 (29·9 to 69·6)	43·1 (24·8 to 72·3)	72·9 (43·2 to 112)	108 (62·9 to 164)	136 (78·9 to 207)	146 (86·9 to 225)	170 (98 to 266)	194 (109 to 303)	285 (161 to 465)	337 (190 to 545)	372 (213 to 604)	322 (180 to 530)	303 (180 to 485)	320 (194 to 508)	238 (142 to 377)	173 (106 to 263)	109 (68·6 to 157)	60·6 (38·8 to 89·3)	35·5 (21·6 to 53·7)	3,520
Acute glomerulonephritis	0 (0 to 0)	0 (0 to 0)	0 (0 to 0)	0 (0 to 0·1)	0·2 (0·1 to 0·4)	0·2 (0·1 to 0·4)	0·4 (0·2 to 0·7)	0·3 (0·1 to 0·6)	0·1 (0 to 0·1)	0 (0 to 0·1)	0 (0 to 0·1)	0 (0 to 0)	0 (0 to 0)	0 (0 to 0)	0 (0 to 0)	0 (0 to 0)	0 (0 to 0)	0 (0 to 0)	0·1 (0 to 0·1)	0 (0 to 0)	0 (0 to 0)	0 (0 to 0)	0 (0 to 0)	1·5
Skin and subcutaneous diseases	0 (0 to 0)	0·3 (0·2 to 0·5)	41·6 (18·4 to 94·5)	466 (287 to 691)	646 (400 to 951)	707 (460 to 1,050)	824 (534 to 1,250)	597 (386 to 892)	503 (328 to 735)	412 (267 to 611)	366 (244 to 533)	350 (239 to 506)	375 (258 to 545)	405 (272 to 586)	384 (260 to 559)	368 (246 to 521)	347 (232 to 498)	331 (217 to 490)	283 (168 to 415)	210 (126 to 314)	138 (82·8 to 208)	73·2 (44·1 to 115)	36·7 (22·7 to 56·8)	7,860
Dermatitis	0 (0 to 0)	0 (0 to 0)	0 (0 to 0)	155 (80·8 to 272)	200 (106 to 349)	151 (80 to 254)	123 (68·6 to 202)	111 (61·4 to 182)	97·7 (54·1 to 160)	80·2 (44 to 131)	66·7 (37·8 to 106)	57·5 (32·9 to 94·9)	59·3 (32 to 98·4)	60·2 (32·9 to 102)	56·5 (30·6 to 98·8)	48·3 (26·6 to 77·4)	39·9 (22·1 to 65·6)	32·1 (17·8 to 52·7)	20 (11·6 to 32·5)	12·6 (7·3 to 19·9)	7·1 (4·2 to 11·6)	3·3 (1·9 to 5·3)	1·6 (0·9 to 2·6)	1,380
Atopic dermatitis	[illegible]	[illegible]	[illegible]	[illegible]	[illegible]	[illegible]	[illegible]	[illegible]	[illegible]	[illegible]	[illegible]	[illegible]	[illegible]	[illegible]	[illegible]	[illegible]	[illegible]	[illegible]	[illegible]	[illegible]	[illegible]	[illegible]	[illegible]	[illegible]
Contact dermatitis	[illegible]	[illegible]	[illegible]	[illegible]	[illegible]	[illegible]	[illegible]	[illegible]	[illegible]	[illegible]	[illegible]	[illegible]	[illegible]	[illegible]	[illegible]	[illegible]	[illegible]	[illegible]	[illegible]	[illegible]	[illegible]	[illegible]	[illegible]	[illegible]
Seborrhoeic dermatitis	[illegible]	[illegible]	[illegible]	[illegible]	[illegible]	[illegible]	[illegible]	[illegible]	[illegible]	[illegible]	[illegible]	[illegible]	[illegible]	[illegible]	[illegible]	[illegible]	[illegible]	[illegible]	[illegible]	[illegible]	[illegible]	[illegible]	[illegible]	[illegible]
Psoriasis	[illegible]	[illegible]	[illegible]	[illegible]	[illegible]	[illegible]	[illegible]	[illegible]	[illegible]	[illegible]	[illegible]	[illegible]	[illegible]	[illegible]	[illegible]	[illegible]	[illegible]	[illegible]	[illegible]	[illegible]	[illegible]	[illegible]	[illegible]	[illegible]
Bacterial skin diseases	[illegible]	[illegible]	[illegible]	[illegible]	[illegible]	[illegible]	[illegible]	[illegible]	[illegible]	[illegible]	[illegible]	[illegible]	[illegible]	[illegible]	[illegible]	[illegible]	[illegible]	[illegible]	[illegible]	[illegible]	[illegible]	[illegible]	[illegible]	[illegible]
Cellulitis	[illegible]	[illegible]	[illegible]	[illegible]	[illegible]	[illegible]	[illegible]	[illegible]	[illegible]	[illegible]	[illegible]	[illegible]	[illegible]	[illegible]	[illegible]	[illegible]	[illegible]	[illegible]	[illegible]	[illegible]	[illegible]	[illegible]	[illegible]	[illegible]
Pyoderma	[illegible]	[illegible]	[illegible]	[illegible]	[illegible]	[illegible]	[illegible]	[illegible]	[illegible]	[illegible]	[illegible]	[illegible]	[illegible]	[illegible]	[illegible]	[illegible]	[illegible]	[illegible]	[illegible]	[illegible]	[illegible]	[illegible]	[illegible]	[illegible]
Scabies	[illegible]	[illegible]	[illegible]	[illegible]	[illegible]	[illegible]	[illegible]	[illegible]	[illegible]	[illegible]	[illegible]	[illegible]	[illegible]	[illegible]	[illegible]	[illegible]	[illegible]	[illegible]	[illegible]	[illegible]	[illegible]	[illegible]	[illegible]	[illegible]
Fungal skin diseases	[illegible]	[illegible]	[illegible]	[illegible]	[illegible]	[illegible]	[illegible]	[illegible]	[illegible]	[illegible]	[illegible]	[illegible]	[illegible]	[illegible]	[illegible]	[illegible]	[illegible]	[illegible]	[illegible]	[illegible]	[illegible]	[illegible]	[illegible]	[illegible]
Viral skin diseases	[illegible]	[illegible]	[illegible]	[illegible]	[illegible]	[illegible]	[illegible]	[illegible]	[illegible]	[illegible]	[illegible]	[illegible]	[illegible]	[illegible]	[illegible]	[illegible]	[illegible]	[illegible]	[illegible]	[illegible]	[illegible]	[illegible]	[illegible]	[illegible]
Acne vulgaris	[illegible]	[illegible]	[illegible]	[illegible]	[illegible]	[illegible]	[illegible]	[illegible]	[illegible]	[illegible]	[illegible]	[illegible]	[illegible]	[illegible]	[illegible]	[illegible]	[illegible]	[illegible]	[illegible]	[illegible]	[illegible]	[illegible]	[illegible]	[illegible]
Alopecia areata	[illegible]	[illegible]	[illegible]	[illegible]	[illegible]	[illegible]	[illegible]	[illegible]	[illegible]	[illegible]	[illegible]	[illegible]	[illegible]	[illegible]	[illegible]	[illegible]	[illegible]	[illegible]	[illegible]	[illegible]	[illegible]	[illegible]	[illegible]	[illegible]
Pruritus	[illegible]	[illegible]	[illegible]	[illegible]	[illegible]	[illegible]	[illegible]	[illegible]	[illegible]	[illegible]	[illegible]	[illegible]	[illegible]	[illegible]	[illegible]	[illegible]	[illegible]	[illegible]	[illegible]	[illegible]	[illegible]	[illegible]	[illegible]	[illegible]
Urticaria	[illegible]	[illegible]	[illegible]	[illegible]	[illegible]	[illegible]	[illegible]	[illegible]	[illegible]	[illegible]	[illegible]	[illegible]	[illegible]	[illegible]	[illegible]	[illegible]	[illegible]	[illegible]	[illegible]	[illegible]	[illegible]	[illegible]	[illegible]	[illegible]
Decubitus ulcer	[illegible]	[illegible]	[illegible]	[illegible]	[illegible]	[illegible]	[illegible]	[illegible]	[illegible]	[illegible]	[illegible]	[illegible]	[illegible]	[illegible]	[illegible]	[illegible]	[illegible]	[illegible]	[illegible]	[illegible]	[illegible]	[illegible]	[illegible]	[illegible]
Other skin and subcutaneous diseases	[illegible]	[illegible]	[illegible]	[illegible]	[illegible]	[illegible]	[illegible]	[illegible]	[illegible]	[illegible]	[illegible]	[illegible]	[illegible]	[illegible]	[illegible]	[illegible]	[illegible]	[illegible]	[illegible]	[illegible]	[illegible]	[illegible]	[illegible]	[illegible]
Sense organ diseases	[illegible]	[illegible]	[illegible]	[illegible]	[illegible]	[illegible]	[illegible]	[illegible]	[illegible]	[illegible]	[illegible]	[illegible]	[illegible]	[illegible]	[illegible]	[illegible]	[illegible]	[illegible]	[illegible]	[illegible]	[illegible]	[illegible]	[illegible]	[illegible]
Blindness and vision loss	[illegible]	[illegible]	[illegible]	[illegible]	[illegible]	[illegible]	[illegible]	[illegible]	[illegible]	[illegible]	[illegible]	[illegible]	[illegible]	[illegible]	[illegible]	[illegible]	[illegible]	[illegible]	[illegible]	[illegible]	[illegible]	[illegible]	[illegible]	[illegible]
Glaucoma	[illegible]	[illegible]	[illegible]	[illegible]	[illegible]	[illegible]	[illegible]	[illegible]	[illegible]	[illegible]	[illegible]	[illegible]	[illegible]	[illegible]	[illegible]	[illegible]	[illegible]	[illegible]	[illegible]	[illegible]	[illegible]	[illegible]	[illegible]	[illegible]
Cataract	[illegible]	[illegible]	[illegible]	[illegible]	[illegible]	[illegible]	[illegible]	[illegible]	[illegible]	[illegible]	[illegible]	[illegible]	[illegible]	[illegible]	[illegible]	[illegible]	[illegible]	[illegible]	[illegible]	[illegible]	[illegible]	[illegible]	[illegible]	[illegible]
Age-related macular degeneration	[illegible]	[illegible]	[illegible]	[illegible]	[illegible]	[illegible]	[illegible]	[illegible]	[illegible]	[illegible]	[illegible]	[illegible]	[illegible]	[illegible]	[illegible]	[illegible]	[illegible]	[illegible]	[illegible]	[illegible]	[illegible]	[illegible]	[illegible]	[illegible]
Refraction disorders	[illegible]	[illegible]	[illegible]	[illegible]	[illegible]	[illegible]	[illegible]	[illegible]	[illegible]	[illegible]	[illegible]	[illegible]	[illegible]	[illegible]	[illegible]	[illegible]	[illegible]	[illegible]	[illegible]	[illegible]	[illegible]	[illegible]	[illegible]	[illegible]
Near vision loss	[illegible]	[illegible]	[illegible]	[illegible]	[illegible]	[illegible]	[illegible]	[illegible]	[illegible]	[illegible]	[illegible]	[illegible]	[illegible]	[illegible]	[illegible]	[illegible]	[illegible]	[illegible]	[illegible]	[illegible]	[illegible]	[illegible]	[illegible]	[illegible]
Other vision loss	[illegible]	[illegible]	[illegible]	[illegible]	[illegible]	[illegible]	[illegible]	[illegible]	[illegible]	[illegible]	[illegible]	[illegible]	[illegible]	[illegible]	[illegible]	[illegible]	[illegible]	[illegible]	[illegible]	[illegible]	[illegible]	[illegible]	[illegible]	[illegible]
Age-related and other hearing loss	[illegible]	[illegible]	[illegible]	[illegible]	[illegible]	[illegible]	[illegible]	[illegible]	[illegible]	[illegible]	[illegible]	[illegible]	[illegible]	[illegible]	[illegible]	[illegible]	[illegible]	[illegible]	[illegible]	[illegible]	[illegible]	[illegible]	[illegible]	[illegible]
Other sense organ diseases	[illegible]	[illegible]	[illegible]	[illegible]	[illegible]	[illegible]	[illegible]	[illegible]	[illegible]	[illegible]	[illegible]	[illegible]	[illegible]	[illegible]	[illegible]	[illegible]	[illegible]	[illegible]	[illegible]	[illegible]	[illegible]	[illegible]	[illegible]	[illegible]
Musculoskeletal disorders	[illegible]	[illegible]	[illegible]	[illegible]	[illegible]	[illegible]	[illegible]	[illegible]	[illegible]	[illegible]	[illegible]	[illegible]	[illegible]	[illegible]	[illegible]	[illegible]	[illegible]	[illegible]	[illegible]	[illegible]	[illegible]	[illegible]	[illegible]	[illegible]
Rheumatoid arthritis	[illegible]	[illegible]	[illegible]	[illegible]	[illegible]	[illegible]	[illegible]	[illegible]	[illegible]	[illegible]	[illegible]	[illegible]	[illegible]	[illegible]	[illegible]	[illegible]	[illegible]	[illegible]	[illegible]	[illegible]	[illegible]	[illegible]	[illegible]	[illegible]
Osteoarthritis	[illegible]	[illegible]	[illegible]	[illegible]	[illegible]	[illegible]	[illegible]	[illegible]	[illegible]	[illegible]	[illegible]	[illegible]	[illegible]	[illegible]	[illegible]	[illegible]	[illegible]	[illegible]	[illegible]	[illegible]	[illegible]	[illegible]	[illegible]	[illegible]
Osteoarthritis hip	[illegible]	[illegible]	[illegible]	[illegible]	[illegible]	[illegible]	[illegible]	[illegible]	[illegible]	[illegible]	[illegible]	[illegible]	[illegible]	[illegible]	[illegible]	[illegible]	[illegible]	[illegible]	[illegible]	[illegible]	[illegible]	[illegible]	[illegible]	[illegible]
Osteoarthritis knee	[illegible]	[illegible]	[illegible]	[illegible]	[illegible]	[illegible]	[illegible]	[illegible]	[illegible]	[illegible]	[illegible]	[illegible]	[illegible]	[illegible]	[illegible]	[illegible]	[illegible]	[illegible]	[illegible]	[illegible]	[illegible]	[illegible]	[illegible]	[illegible]
Osteoarthritis hand	[illegible]	[illegible]	[illegible]	[illegible]	[illegible]	[illegible]	[illegible]	[illegible]	[illegible]	[illegible]	[illegible]	[illegible]	[illegible]	[illegible]	[illegible]	[illegible]	[illegible]	[illegible]	[illegible]	[illegible]	[illegible]	[illegible]	[illegible]	[illegible]
Osteoarthritis other	[illegible]	[illegible]	[illegible]	[illegible]	[illegible]	[illegible]	[illegible]	[illegible]	[illegible]	[illegible]	[illegible]	[illegible]	[illegible]	[illegible]	[illegible]	[illegible]	[illegible]	[illegible]	[illegible]	[illegible]	[illegible]	[illegible]	[illegible]	[illegible]
Low back pain	[illegible]	[illegible]	[illegible]	[illegible]	[illegible]	[illegible]	[illegible]	[illegible]	[illegible]	[illegible]	[illegible]	[illegible]	[illegible]	[illegible]	[illegible]	[illegible]	[illegible]	[illegible]	[illegible]	[illegible]	[illegible]	[illegible]	[illegible]	[illegible]
Neck pain	[illegible]	[illegible]	[illegible]	[illegible]	[illegible]	[illegible]	[illegible]	[illegible]	[illegible]	[illegible]	[illegible]	[illegible]	[illegible]	[illegible]	[illegible]	[illegible]	[illegible]	[illegible]	[illegible]	[illegible]	[illegible]	[illegible]	[illegible]	[illegible]
Gout	[illegible]	[illegible]	[illegible]	[illegible]	[illegible]	[illegible]	[illegible]	[illegible]	[illegible]	[illegible]	[illegible]	[illegible]	[illegible]	[illegible]	[illegible]	[illegible]	[illegible]	[illegible]	[illegible]	[illegible]	[illegible]	[illegible]	[illegible]	[illegible]
Other musculoskeletal disorders	[illegible]	[illegible]	[illegible]	[illegible]	[illegible]	[illegible]	[illegible]	[illegible]	[illegible]	[illegible]	[illegible]	[illegible]	[illegible]	[illegible]	[illegible]	[illegible]	[illegible]	[illegible]	[illegible]	[illegible]	[illegible]	[illegible]	[illegible]	[illegible]
Other non-communicable diseases	[illegible]	[illegible]	[illegible]	[illegible]	[illegible]	[illegible]	[illegible]	[illegible]	[illegible]	[illegible]	[illegible]	[illegible]	[illegible]	[illegible]	[illegible]	[illegible]	[illegible]	[illegible]	[illegible]	[illegible]	[illegible]	[illegible]	[illegible]	[illegible]
Congenital birth defects	[illegible]	[illegible]	[illegible]	[illegible]	[illegible]	[illegible]	[illegible]	[illegible]	[illegible]	[illegible]	[illegible]	[illegible]	[illegible]	[illegible]	[illegible]	[illegible]	[illegible]	[illegible]	[illegible]	[illegible]	[illegible]	[illegible]	[illegible]	[illegible]
Neural tube defects	[illegible]	[illegible]	[illegible]	[illegible]	[illegible]	[illegible]	[illegible]	[illegible]	[illegible]	[illegible]	[illegible]	[illegible]	[illegible]	[illegible]	[illegible]	[illegible]	[illegible]	[illegible]	[illegible]	[illegible]	[illegible]	[illegible]	[illegible]	[illegible]
Congenital heart anomalies	[illegible]	[illegible]	[illegible]	[illegible]	[illegible]	[illegible]	[illegible]	[illegible]	[illegible]	[illegible]	[illegible]	[illegible]	[illegible]	[illegible]	[illegible]	[illegible]	[illegible]	[illegible]	[illegible]	[illegible]	[illegible]	[illegible]	[illegible]	[illegible]
Orofacial clefts	[illegible]	[illegible]	[illegible]	[illegible]	[illegible]	[illegible]	[illegible]	[illegible]	[illegible]	[illegible]	[illegible]	[illegible]	[illegible]	[illegible]	[illegible]	[illegible]	[illegible]	[illegible]	[illegible]	[illegible]	[illegible]	[illegible]	[illegible]	[illegible]
Down syndrome	[illegible]	[illegible]	[illegible]	[illegible]	[illegible]	[illegible]	[illegible]	[illegible]	[illegible]	[illegible]	[illegible]	[illegible]	[illegible]	[illegible]	[illegible]	[illegible]	[illegible]	[illegible]	[illegible]	[illegible]	[illegible]	[illegible]	[illegible]	[illegible]
Turner syndrome	[illegible]	[illegible]	[illegible]	[illegible]	[illegible]	[illegible]	[illegible]	[illegible]	[illegible]	[illegible]	[illegible]	[illegible]	[illegible]	[illegible]	[illegible]	[illegible]	[illegible]	[illegible]	[illegible]	[illegible]	[illegible]	[illegible]	[illegible]	[illegible]
Klinefelter syndrome	[illegible]	[illegible]	[illegible]	[illegible]	[illegible]	[illegible]	[illegible]	[illegible]	[illegible]	[illegible]	[illegible]	[illegible]	[illegible]	[illegible]	[illegible]	[illegible]	[illegible]	[illegible]	[illegible]	[illegible]	[illegible]	[illegible]	[illegible]	[illegible]
Other chromosomal abnormalities	[illegible]	[illegible]	[illegible]	[illegible]	[illegible]	[illegible]	[illegible]	[illegible]	[illegible]	[illegible]	[illegible]	[illegible]	[illegible]	[illegible]	[illegible]	[illegible]	[illegible]	[illegible]	[illegible]	[illegible]	[illegible]	[illegible]	[illegible]	[illegible]
Congenital musculoskeletal and limb anomalies	[illegible]	[illegible]	[illegible]	[illegible]	[illegible]	[illegible]	[illegible]	[illegible]	[illegible]	[illegible]	[illegible]	[illegible]	[illegible]	[illegible]	[illegible]	[illegible]	[illegible]	[illegible]	[illegible]	[illegible]	[illegible]	[illegible]	[illegible]	[illegible]
Urogenital congenital anomalies	[illegible]	[illegible]	[illegible]	[illegible]	[illegible]	[illegible]	[illegible]	[illegible]	[illegible]	[illegible]	[illegible]	[illegible]	[illegible]	[illegible]	[illegible]	[illegible]	[illegible]	[illegible]	[illegible]	[illegible]	[illegible]	[illegible]	[illegible]	[illegible]
Digestive congenital anomalies	[illegible]	[illegible]	[illegible]	[illegible]	[illegible]	[illegible]	[illegible]	[illegible]	[illegible]	[illegible]	[illegible]	[illegible]	[illegible]	[illegible]	[illegible]	[illegible]	[illegible]	[illegible]	[illegible]	[illegible]	[illegible]	[illegible]	[illegible]	[illegible]
Other congenital birth defects	[illegible]	[illegible]	[illegible]	[illegible]	[illegible]	[illegible]	[illegible]	[illegible]	[illegible]	[illegible]	[illegible]	[illegible]	[illegible]	[illegible]	[illegible]	[illegible]	[illegible]	[illegible]	[illegible]	[illegible]	[illegible]	[illegible]	[illegible]	[illegible]
Urinary diseases and male infertility	[illegible]	[illegible]	[illegible]	[illegible]	[illegible]	[illegible]	[illegible]	[illegible]	[illegible]	[illegible]	[illegible]	[illegible]	[illegible]	[illegible]	[illegible]	[illegible]	[illegible]	[illegible]	[illegible]	[illegible]	[illegible]	[illegible]	[illegible]	[illegible]
Urolithiasis	[illegible]	[illegible]	[illegible]	[illegible]	[illegible]	[illegible]	[illegible]	[illegible]	[illegible]	[illegible]	[illegible]	[illegible]	[illegible]	[illegible]	[illegible]	[illegible]	[illegible]	[illegible]	[illegible]	[illegible]	[illegible]	[illegible]	[illegible]	[illegible]

Benign prostatic hyperplasia	0 (0 to 0)	0 (0 to 0)	0 (0 to 0)	0 (0 to 0)	0 (0 to 0)	0 (0 to 0)	0 (0 to 0)	0 (0 to 0)	0 (0 to 0)	0 (0 to 0)	0 (0 to 0)	0·8 (0 to 2·1)	6·5 (2·4 to 13·9)	27·3 (12·7 to 49·4)	83·4 (39·5 to 147)	140 (72·2 to 241)	173 (97·1 to 277)	173 (96·4 to 280)	111 (61·7 to 179)	61·9 (34·4 to 98·6)	29·5 (15·6 to 48·1)	10·8 (5·9 to 17·8)	4 (2·1 to 6·5)	821
Male infertility	0 (0 to 0)	0 (0 to 0)	0 (0 to 0)	0 (0 to 0)	0 (0 to 0)	0 (0 to 0)	0 (0 to 0)	29·4 (7·8 to 74·9)	34·3 (9·8 to 85·1)	31·8 (8·9 to 86·6)	27·4 (7·7 to 70·3)	23 (6·8 to 59)	3·5 (0·8 to 10·4)	0 (0 to 0)	0 (0 to 0)	0 (0 to 0)	0 (0 to 0)	0 (0 to 0)	0 (0 to 0)	0 (0 to 0)	0 (0 to 0)	0 (0 to 0)	0 (0 to 0)	149
Other urinary diseases	1 (0·3 to 1·9)	0·6 (0·1 to 3)	0·3 (0·1 to 1)	2 (1·1 to 3·5)	2·1 (1·3 to 3·6)	2·5 (1·5 to 4·1)	3·6 (1·7 to 5·6)	2·9 (1·6 to 5·4)	2·2 (1·4 to 3·2)	2·9 (1·5 to 5·3)	12·4 (4·4 to 24·3)	12·4 (4·1 to 22·4)	13·3 (6·1 to 22·9)	12·1 (6 to 20·4)	14·8 (7 to 27)	34·4 (8·9 to 65·5)	28·6 (8·8 to 52·9)	21·9 (7 to 42·7)	29 (6·2 to 56·4)	24 (4·5 to 48·9)	13 (2·6 to 26·1)	13·2 (1·6 to 26)	9·9 (0·9 to 19·3)	259
Gynecological diseases	0 (0 to 0)	0 (0 to 0)	0 (0 to 0)	0 (0 to 0)	0 (0 to 0)	0 (0 to 0)	0 (0 to 0)	0 (0 to 0)	0 (0 to 0)	0 (0 to 0)	0 (0 to 0)	0 (0 to 0)	0 (0 to 0)	0 (0 to 0)	0 (0 to 0)	0 (0 to 0)	0 (0 to 0)	0 (0 to 0)	0 (0 to 0)	0 (0 to 0)	0 (0 to 0)	0 (0 to 0)	0 (0 to 0)	0
Uterine fibroids	0 (0 to 0)	0 (0 to 0)	0 (0 to 0)	0 (0 to 0)	0 (0 to 0)	0 (0 to 0)	0 (0 to 0)	0 (0 to 0)	0 (0 to 0)	0 (0 to 0)	0 (0 to 0)	0 (0 to 0)	0 (0 to 0)	0 (0 to 0)	0 (0 to 0)	0 (0 to 0)	0 (0 to 0)	0 (0 to 0)	0 (0 to 0)	0 (0 to 0)	0 (0 to 0)	0 (0 to 0)	0 (0 to 0)	0
Polycystic ovarian syndrome	--	--	--	--	--	--	--	--	--	--	--	--	--	--	--	--	--	--	--	--	--	--	--	NA
Female infertility	--	--	--	--	--	--	--	--	--	--	--	--	--	--	--	--	--	--	--	--	--	--	--	NA
Endometriosis	--	--	--	--	--	--	--	--	--	--	--	--	--	--	--	--	--	--	--	--	--	--	--	NA
Genital prolapse	0 (0 to 0)	0 (0 to 0)	0 (0 to 0)	0 (0 to 0)	0 (0 to 0)	0 (0 to 0)	0 (0 to 0)	0 (0 to 0)	0 (0 to 0)	0 (0 to 0)	0 (0 to 0)	0 (0 to 0)	0 (0 to 0)	0 (0 to 0)	0 (0 to 0)	0 (0 to 0)	0 (0 to 0)	0 (0 to 0)	0 (0 to 0)	0 (0 to 0)	0 (0 to 0)	0 (0 to 0)	0 (0 to 0)	0
Premenstrual syndrome	--	--	--	--	--	--	--	--	--	--	--	--	--	--	--	--	--	--	--	--	--	--	--	NA
Other gynecological diseases	0 (0 to 0)	0 (0 to 0)	0 (0 to 0)	0 (0 to 0)	0 (0 to 0)	0 (0 to 0)	0 (0 to 0)	0 (0 to 0)	0 (0 to 0)	0 (0 to 0)	0 (0 to 0)	0 (0 to 0)	0 (0 to 0)	0 (0 to 0)	0 (0 to 0)	0 (0 to 0)	0 (0 to 0)	0 (0 to 0)	0 (0 to 0)	0 (0 to 0)	0 (0 to 0)	0 (0 to 0)	0 (0 to 0)	0
Hemoglobinopathies and hemolytic anemias	8 (5 to 12·9)	9·3 (6 to 13·7)	52·7 (29·6 to 89·5)	172 (109 to 261)	203 (133 to 298)	171 (112 to 247)	157 (103 to 236)	208 (133 to 313)	177 (116 to 258)	165 (108 to 243)	135 (89·2 to 200)	122 (79·7 to 182)	126 (80·9 to 187)	122 (83·4 to 172)	121 (78·2 to 177)	83·5 (54·7 to 123)	57·9 (38·3 to 82·3)	41·2 (27·1 to 60·7)	30·8 (20·6 to 44)	18·2 (12·2 to 27·8)	16·1 (10·6 to 24·3)	11·5 (7·3 to 17·7)	8 (4·5 to 13·3)	2,220
Thalassemias	0 (0 to 0)	0 (0 to 0)	0 (0 to 0·1)	0·5 (0·2 to 0·9)	0·3 (0·1 to 0·6)	0·1 (0 to 0·3)	0·1 (0 to 0·2)	0·1 (0 to 0·1)	0 (0 to 0·1)	0 (0 to 0)	0 (0 to 0)	0 (0 to 0)	0 (0 to 0)	0 (0 to 0)	0 (0 to 0)	0 (0 to 0)	0 (0 to 0)	0 (0 to 0)	0 (0 to 0)	0 (0 to 0)	0 (0 to 0)	0 (0 to 0)	0 (0 to 0)	1·2
Thalassemias trait	0 (0 to 0)	0·1 (0·1 to 0·2)	1·1 (0·4 to 2·1)	4·9 (1·8 to 9·8)	5·3 (1·2 to 12·3)	2 (0·2 to 5·7)	1·1 (0·2 to 2·9)	0·9 (0·2 to 2·3)	0·9 (0·2 to 2·6)	0·9 (0·2 to 2·4)	0·8 (0·2 to 2)	0·7 (0·2 to 2·2)	0·9 (0·2 to 2·2)	1 (0·2 to 2·5)	1·2 (0·3 to 2·9)	0·9 (0·2 to 2·4)	0·6 (0·1 to 1·6)	0·6 (0·1 to 1·3)	0·4 (0·1 to 1)	0·3 (0·1 to 0·7)	0·3 (0·1 to 0·6)	0·1 (0 to 0·3)	0·1 (0 to 0·1)	25·1
Sickle cell disorders	7·6 (4·5 to 12·3)	5·3 (2·9 to 9·3)	37·1 (16·7 to 73·2)	97·6 (58·3 to 151)	125 (84·2 to 177)	134 (84·5 to 198)	119 (75·6 to 185)	152 (91·7 to 239)	117 (71·7 to 179)	105 (63·6 to 162)	82·8 (48·6 to 129)	69·8 (40·7 to 108)	64·3 (37·6 to 104)	47·8 (26·4 to 76·5)	26·2 (12·3 to 47·1)	5·7 (2·3 to 11·6)	0·9 (0·3 to 1·9)	0·1 (0 to 0·3)	0 (0 to 0)	0 (0 to 0)	0 (0 to 0)	0 (0 to 0)	0 (0 to 0)	1,200
Sickle cell trait	0·4 (0·1 to 0·7)	2·9 (1·2 to 5)	9·3 (4 to 17·3)	41·8 (18·3 to 74·2)	45·1 (13·7 to 95·2)	16·8 (2·7 to 46·5)	9 (2 to 24·5)	7·6 (1·6 to 21·6)	8·1 (1·7 to 21·5)	7·5 (1·5 to 19·7)	6·5 (1·3 to 17·1)	6·3 (1·4 to 17·5)	7·5 (1·8 to 17·8)	8·4 (2·3 to 19·2)	10 (2·7 to 21·7)	7·9 (2·2 to 18·5)	5·2 (1·3 to 12)	4·7 (1·3 to 10·2)	3·6 (1 to 7·8)	2·6 (0·9 to 5·5)	2·3 (0·9 to 4·3)	1·1 (0·5 to 2·1)	0·5 (0·2 to 0·9)	215
G6PD deficiency	0 (0 to 0·1)	0·4 (0·1 to 0·6)	0·4 (0·2 to 0·8)	5·8 (2·8 to 10·6)	10·1 (4·3 to 18·8)	11·2 (5 to 21·8)	15·1 (6·3 to 28·2)	34·4 (12·9 to 66·5)	37·4 (16 to 68·9)	37·9 (16·4 to 70·1)	33·4 (14·5 to 60)	32·7 (13·9 to 58·7)	38·8 (17·9 to 68·5)	45·6 (22·9 to 75·1)	54·8 (30·8 to 87·1)	38·1 (22·2 to 61·1)	22·7 (12·9 to 35·4)	11·1 (6·2 to 17·7)	5·5 (2·8 to 8·6)	1·6 (0·8 to 2·8)	0·7 (0·3 to 1·1)	0·3 (0·1 to 0·5)	0·1 (0 to 0·1)	438
G6PD trait	0 (0 to 0)	0 (0 to 0)	0 (0 to 0)	0 (0 to 0)	0 (0 to 0)	0 (0 to 0)	0 (0 to 0)	0 (0 to 0)	0 (0 to 0)	0 (0 to 0)	0 (0 to 0)	0 (0 to 0)	0 (0 to 0)	0 (0 to 0)	0 (0 to 0)	0 (0 to 0)	0 (0 to 0)	0 (0 to 0)	0 (0 to 0)	0 (0 to 0)	0 (0 to 0)	0 (0 to 0)	0 (0 to 0)	0
Other hemoglobinopathies and hemolytic anemias	0 (0 to 0·1)	0·6 (0·3 to 1·1)	4·7 (1·6 to 9·5)	21·2 (7·3 to 43·2)	17·3 (4·3 to 42·4)	6·7 (1·5 to 18·4)	12·8 (3·4 to 34·7)	13·5 (5·4 to 32·2)	13·4 (4·7 to 33·8)	13·3 (4·9 to 31·5)	11·9 (4·5 to 26·8)	12·1 (5·3 to 27·4)	14·7 (6·8 to 29·7)	19·4 (10 to 35·3)	28·6 (15·6 to 48·3)	30·8 (17·7 to 49·6)	28·5 (17·4 to 42·4)	24·7 (15·8 to 37·4)	21·3 (14·2 to 30·6)	13·6 (9·4 to 19·7)	12·8 (8·6 to 19·4)	10 (6·4 to 15·4)	7·4 (4·1 to 12·5)	339
Endocrine, metabolic, blood, and immune disorders	30 (15·7 to 51·2)	22·2 (12·3 to 37·8)	123 (53·8 to 305)	172 (108 to 294)	98·4 (64·1 to 148)	77·2 (50·2 to 116)	59·9 (36·7 to 91)	78·5 (50·5 to 117)	111 (72 to 165)	136 (88·8 to 210)	148 (98·7 to 221)	157 (104 to 228)	207 (141 to 301)	231 (161 to 333)	239 (160 to 345)	246 (167 to 350)	210 (143 to 303)	206 (139 to 290)	153 (103 to 214)	107 (73·2 to 147)	64·1 (45·2 to 92·8)	37·6 (26·2 to 51·6)	16·8 (11·6 to 24·7)	2,930
Oral disorders	0 (0 to 0)	0 (0 to 0)	0 (0 to 0)	43·9 (24·4 to 73)	58·4 (31·2 to 102)	69·3 (35·8 to 122)	109 (58·7 to 192)	190 (98·5 to 352)	266 (131 to 503)	297 (141 to 554)	307 (145 to 587)	309 (148 to 573)	345 (174 to 637)	365 (193 to 651)	380 (211 to 634)	382 (224 to 631)	358 (213 to 573)	319 (197 to 501)	218 (136 to 329)	145 (89·8 to 216)	87·1 (53·5 to 129)	43·7 (26·7 to 64·7)	22·1 (13·4 to 33·1)	4,310
Caries of deciduous teeth	0 (0 to 0)	0 (0 to 0)	0 (0 to 0)	15·5 (6·1 to 32·1)	16·1 (6·6 to 35·8)	1 (0·1 to 2·6)	0 (0 to 0)	0 (0 to 0)	0 (0 to 0)	0 (0 to 0)	0 (0 to 0)	0 (0 to 0)	0 (0 to 0)	0 (0 to 0)	0 (0 to 0)	0 (0 to 0)	0 (0 to 0)	0 (0 to 0)	0 (0 to 0)	0 (0 to 0)	0 (0 to 0)	0 (0 to 0)	0 (0 to 0)	32·5
Caries of permanent teeth	0 (0 to 0)	0 (0 to 0)	0 (0 to 0)	0 (0 to 0)	13·5 (4·8 to 30·3)	34·2 (13·6 to 74)	39·8 (15·4 to 83)	49·6 (20·5 to 104)	45·9 (18·1 to 92)	36·4 (14·5 to 76·6)	31·1 (12·7 to 63)	25·9 (10·5 to 53·2)	24·9 (9·8 to 52·5)	23·1 (9·2 to 48·8)	18·7 (7 to 42·9)	13·4 (4·7 to 29·6)	8·9 (3·4 to 18·8)	6·4 (2·5 to 13·7)	3·8 (1·6 to 7·8)	2·4 (0·9 to 5)	1·3 (0·5 to 2·8)	0·6 (0·2 to 1·3)	0·2 (0·1 to 0·5)	380
Periodontal diseases	0 (0 to 0)	0 (0 to 0)	0 (0 to 0)	0 (0 to 0)	0 (0 to 0)	0 (0 to 0)	15·1 (4·2 to 38·8)	61·9 (18·2 to 162)	129 (40·9 to 311)	167 (56·5 to 371)	183 (64 to 423)	188 (68·9 to 417)	206 (78·1 to 440)	199 (77·9 to 432)	168 (66 to 357)	121 (48·7 to 249)	81·7 (32·1 to 168)	56·8 (22·4 to 116)	33·6 (13·2 to 66·7)	20·8 (8 to 41·6)	12·1 (4·5 to 25·2)	5·8 (2·1 to 12·2)	2·7 (1 to 5·8)	1,650
Other oral disorders	0 (0 to 0)	0 (0 to 0)	0 (0 to 0)	28·4 (16·7 to 45·3)	28·8 (16·6 to 45·1)	34·1 (19·3 to 54·6)	53·9 (32·3 to 85·8)	72·2 (43·5 to 112)	75·1 (44·9 to 115)	66·7 (39·2 to 103)	58·2 (34·2 to 90·2)	52·2 (31·1 to 80·4)	53·4 (32 to 82·5)	50·2 (30·3 to 76·2)	43·6 (26·2 to 66·6)	34·1 (20·5 to 51·3)	25·4 (14·9 to 38·8)	17·7 (10·7 to 27·4)	9·1 (5·5 to 13·9)	4·7 (2·8 to 7·1)	2·2 (1·3 to 3·3)	0·9 (0·6 to 1·4)	0·5 (0·3 to 0·7)	711
Sudden infant death syndrome	--	13 (7 to 21·9)	35·1 (14·5 to 69·4)	--	--	--	--	--	--	--	--	--	--	--	--	--	--	--	--	--	--	--	--	NA
Injuries	95·3 (64·2 to 137)	37·1 (24·9 to 54·2)	88·8 (54·6 to 140)	405 (295 to 562)	676 (549 to 815)	1,050 (858 to 1,270)	2,770 (2,190 to 3,420)	5,800 (4,820 to 6,970)	6,030 (5,060 to 7,040)	5,550 (4,630 to 6,670)	4,660 (3,740 to 5,750)	3,830 (3,030 to 4,760)	3,700 (2,870 to 4,690)	3,110 (2,460 to 3,920)	2,610 (2,050 to 3,220)	1,880 (1,530 to 2,300)	1,350 (1,090 to 1,640)	1,060 (864 to 1,280)	680 (558 to 816)	448 (365 to 538)	271 (220 to 328)	142 (114 to 173)	86·6 (71 to 104)	46,300
Transport injuries	13·4 (8·4 to 20·6)	6·1 (3·6 to 9·6)	9·1 (4·8 to 15·7)	88·3 (55·9 to 142)	199 (134 to 282)	254 (176 to 354)	503 (345 to 713)	952 (684 to 1,280)	888 (651 to 1,140)	702 (526 to 920)	664 (491 to 895)	564 (414 to 731)	615 (455 to 823)	592 (442 to 764)	563 (429 to 725)	397 (310 to 494)	284 (221 to 353)	216 (169 to 264)	139 (111 to 170)	84·2 (66·5 to 104)	46·8 (37·2 to 57·8)	21 (16·6 to 26)	11·3 (8·7 to 14·1)	7,810
Road injuries	12·4 (7·7 to 19)	5·2 (3 to 8·1)	7·3 (3·6 to 12·9)	81·1 (50 to 133)	186 (124 to 266)	236 (164 to 333)	477 (323 to 674)	901 (650 to 1,220)	838 (614 to 1,070)	656 (495 to 856)	628 (465 to 852)	532 (390 to 689)	579 (427 to 774)	560 (419 to 729)	530 (403 to 681)	375 (293 to 466)	269 (211 to 337)	201 (157 to 245)	130 (104 to 160)	78·4 (62 to 96·7)	43·7 (34·6 to 53·9)	19·3 (15·1 to 24)	10·2 (7·9 to 12·8)	7,360
Pedestrian road injuries	6·2 (3·8 to 9·5)	2·2 (1·3 to 3·4)	2·6 (1·3 to 4·7)	42·9 (25·7 to 70·6)	98·8 (65·2 to 145)	84·5 (53·2 to 125)	56·5 (32·2 to 92)	66·3 (42·6 to 100)	68·3 (48 to 93·8)	65·7 (47·5 to 89)	76·4 (51·9 to 112)	74 (53·4 to 96·8)	97·5 (68·2 to 139)	121 (88 to 163)	122 (87·1 to 165)	103 (78·6 to 134)	73·4 (55·4 to 95·1)	68·7 (52·1 to 87·3)	45·9 (35·6 to 58·5)	27·8 (20·9 to 36·7)	15·6 (12·1 to 19·7)	6·2 (5 to 7·8)	3·3 (2·6 to 4·2)	1,330
Cyclist road injuries	0 (0 to 0)	0 (0 to 0)	0 (0 to 0)	6·3 (3·3 to 11)	13·4 (8·2 to 21·2)	39·2 (24·1 to 57·5)	64·8 (42·4 to 97·6)	89·1 (61·4 to 125)	86·2 (62·3 to 114)	82·8 (60·8 to 112)	91·6 (66·5 to 122)	85·7 (63·3 to 113)	99·7 (69·5 to 136)	98 (72·6 to 128)	85·5 (62·9 to 114)	65·3 (49·3 to 85·3)	51·3 (39 to 67·7)	31·4 (23·1 to 41)	21·6 (16 to 28)	13·7 (9·9 to 17·7)	7·7 (5·6 to 10·3)	3·6 (2·6 to 4·9)	1·8 (1·2 to 2·4)	1,040
Motorcyclist road injuries	1·1 (0·7 to 1·6)	0·5 (0·3 to 0·8)	0·8 (0·4 to 1·5)	5·1 (3·2 to 8)	13·8 (9·3 to 19·6)	22·1 (14 to 32·9)	98·6 (64·4 to 142)	235 (167 to 322)	237 (169 to 314)	155 (106 to 221)	142 (105 to 195)	107 (77·9 to 139)	104 (75·8 to 146)	92·2 (70 to 118)	77·3 (56·9 to 102)	42·4 (32·9 to 52·9)	28·9 (21·9 to 36·9)	18·9 (13·9 to 24·6)	12·2 (8·9 to 15·9)	8·2 (5·9 to 10·9)	5·2 (3·7 to 7)	2·8 (2 to 3·8)	1·5 (1·1 to 2·1)	1,410
Motor vehicle road injuries	5 (3 to 7·5)	2·5 (1·4 to 3·8)	3·7 (1·8 to 6·6)	25·2 (14·9 to 40·9)	58·5 (37·8 to 83·7)	87·8 (54·5 to 133)	253 (163 to 364)	504 (348 to 702)	437 (307 to 577)	344 (238 to 475)	308 (216 to 441)	254 (176 to 343)	265 (182 to 381)	235 (170 to 317)	233 (166 to 318)	153 (115 to 200)	107 (79·7 to 138)	75·4 (57·9 to 94·3)	46·1 (36·1 to 58·2)	25·9 (19·4 to 34·2)	13·5 (10·5 to 17·1)	5·8 (4·5 to 7·3)	3·2 (2·3 to 4)	3,440
Other road injuries	0·2 (0·1 to 0·3)	0·1 (0 to 0·1)	0·2 (0·1 to 0·3)	1·6 (0·9 to 2·8)	1·9 (1·2 to 2·8)	2·8 (1·9 to 3·9)	4·4 (3 to 6·2)	7·2 (5 to 10)	8·8 (6·1 to 12·2)	9·5 (6·5 to 13·4)	10·3 (7·1 to 14·2)	11 (7·5 to 15·6)	13 (8·8 to 18·1)	13·7 (9·3 to 19·2)	12·7 (8·7 to 17·6)	10·5 (7·2 to 14·8)	8·6 (6 to 11·8)	6·8 (4·7 to 9·3)	4·5 (3·2 to 6·2)	2·9 (2 to 4)	1·7 (1·2 to 2·3)	0·8 (0·6 to 1·1)	0·4 (0·3 to 0·5)	134
Other transport injuries	1 (0·5 to 1·7)	0·9 (0·5 to 1·7)	1·8 (1·1 to 3)	7·2 (4·3 to 11·4)	12·7 (8·1 to 19·6)	17·3 (11·5 to 26·9)	26·4 (16·4 to 42)	50·7 (31·2 to 82·1)	50 (31·9 to 74)	45·1 (29·5 to 69·4)	35·5 (23·5 to 52·5)	32·5 (21·7 to 46·5)	35·6 (23·1 to 54·3)	31·8 (22 to 46·1)	32·9 (22·8 to 46)	22·3 (15·9 to 31·2)	15·1 (10·9 to 20·1)	14·6 (10·4 to 20·6)	9·2 (6·3 to 12·9)	5·8 (4·1 to 8·1)	3·1 (2·3 to 4·2)	1·7 (1·2 to 2·1)	1·1 (0·8 to 1·4)	454
Unintentional injuries	65 (42·5 to 95·9)	27·1 (17·9 to 39·5)	61·6 (37·2 to 98·4)	214 (156 to 285)	352 (274 to 441)	508 (396 to 643)	827 (637 to 1,050)	1,510 (1,180 to 1,900)	1,670 (1,320 to 2,080)	1,620 (1,290 to 2,020)	1,470 (1,150 to 1,830)	1,310 (1,020 to 1,670)	1,340 (1,040 to 1,730)	1,240 (956 to 1,590)	1,140 (890 to 1,470)	938 (729 to 1,190)	719 (557 to 917)	637 (503 to 794)	435 (347 to 537)	318 (254 to 389)	200 (159 to 245)	111 (88·4 to 135)	69·2 (56·3 to 83·9)	16,800
Falls	1·9 (1·2 to 2·7)	1·7 (1 to 2·7)	3·9 (2·1 to 6·7)	28·7 (19·4 to 42·1)	54·7 (39·9 to 73·8)	91·3 (65·8 to 120)	135 (98·9 to 182)	207 (154 to 272)	235 (176 to 313)	236 (180 to 309)	245 (185 to 320)	233 (180 to 298)	254 (190 to 324)	286 (221 to 370)	271 (210 to 345)	246 (189 to 309)	201 (157 to 248)	203 (161 to 255)	145 (115 to 182)	122 (95·7 to 149)	82·1 (64·3 to 101)	49·6 (39·2 to 61·2)	34·2 (26 to 41·9)	3,370
Drowning	0·3 (0·2 to 0·4)	0·2 (0·1 to 0·3)	2·3 (1·1 to 4·1)	21·2 (12·2 to 32·8)	49·3 (31·8 to 72·7)	51 (35 to 71·1)	64·3 (41 to 92·1)	86·4 (57·3 to 127)	78·1 (55·8 to 108)	46·1 (34 to 62·9)	36·5 (26·4 to 49·5)	31·4 (22·6 to 43·8)	22·4 (15·9 to 30·4)	22·8 (16·2 to 31·5)	26·9 (19·2 to 37)	17·4 (12·9 to 22·7)	12 (8·9 to 15·6)	8·7 (6·7 to 11)	4·8 (3·7 to 6)	2·6 (2·1 to 3·2)	1·6 (1·3 to 2·1)	0·8 (0·7 to 1)	0·6 (0·5 to 0·8)	588
Fire, heat, and hot substances	0·5 (0·3 to 0·7)	0·6 (0·4 to 0·9)	5 (2·8 to 8·5)	35·3 (22·8 to 51·7)	50·6 (36·1 to 70·4)	72 (49·9 to 99·6)	97·5 (66·1 to 140)	131 (92·3 to 185)	145 (102 to 200)	133 (91·3 to 188)	124 (84·9 to 175)	114 (79·9 to 159)	119 (82·6 to 167)	110 (74·8 to 155)	97·4 (68 to 138)	79·2 (55·5 to 111)	57·3 (40·8 to 81·2)	49·7 (36·7 to 68·4)	31·2 (22·6 to 43·5)	23·6 (17·6 to 31·3)	15·5 (11·7 to 20·6)	8·3 (6·4 to 10·8)	4·5 (3·6 to 5·9)	1,500
Poisonings	0·6 (0·3 to 1)	0·5 (0·3 to 1)	1·2 (0·6 to 2·3)	6·2 (3·5 to 10·1)	9·6 (6·3 to 14)	11·4 (7·3 to 16·8)	11·8 (7·5 to 17·8)	16·4 (11·2 to 23·5)	19·8 (14·1 to 27·2)	19·5 (13·7 to 26·8)	13·6 (9·5 to 19·1)	12·9 (9 to 17·4)	13·4 (9·4 to 18·6)	10·1 (7 to 14)	8 (5·5 to 11·3)	5·9 (4·1 to 8·3)	4·1 (2·8 to 5·8)	3·8 (2·6 to 5·1)	2·2 (1·5 to 3)	1·5 (1 to 2)	0·8 (0·5 to 1·1)	0·4 (0·3 to 0·6)	0·2 (0·2 to 0·3)	174
Poisoning by carbon monoxide	0·1 (0 to 0·2)	0 (0 to 0·1)	0·4 (0·2 to 0·7)	1·1 (0·5 to 2·3)	2·1 (1·2 to 3·3)	2·8 (1·7 to 4·2)	3·1 (2 to 4·6)	5·4 (3·4 to 7·7)	7·3 (4·5 to 10·7)	7 (4·2 to 11·1)	4·5 (2·7 to 6·9)	2·4 (1·4 to 3·6)	1·8 (1·1 to 2·8)	1·1 (0·7 to 1·7)	1·1 (0·7 to 1·6)	0·6 (0·4 to 0·9)	0·5 (0·3 to 0·8)	0·8 (0·5 to 1·2)	0·3 (0·2 to 0·5)	0·2 (0·2 to 0·4)	0·1 (0·1 to 0·2)	0·1 (0 to 0·1)	0 (0 to 0)	43
Poisoning by other means	0·5 (0·3 to 0·9)	0·5 (0·2 to 0·9)	0·8 (0·4 to 1·6)	5 (2·8 to 8·1)	7·4 (4·7 to 11·2)	8·7 (5·3 to 13·5)	8·7 (4·9 to 14)	11 (6·6 to 17·1)	12·6 (8 to 18·7)	12·4 (8·4 to 17·9)	9·1 (5·9 to 13·6)	10·5 (7 to 14·5)	11·6 (7·9 to 16·4)	8·9 (6·2 to 12·6)	6·9 (4·7 to 9·9)	5·3 (3·6 to 7·5)	3·5 (2·4 to 5·1)	2·9 (1·9 to 4·2)	1·8 (1·2 to 2·5)	1·2 (0·8 to 1·7)	0·7 (0·5 to 1)	0·3 (0·2 to 0·5)	0·2 (0·1 to 0·3)	131
Exposure to mechanical forces	1·2 (0·7 to 1·8)	1·6 (1 to 2·4)	4·1 (2·7 to 5·9)	26·3 (17·5 to 37·3)	57·7 (38·8 to 82·6)	105 (75·9 to 152)	317 (235 to 413)	755 (566 to 973)	871 (670 to 1,120)	884 (694 to 1,120)	769 (599 to 978)	678 (516 to 868)	662 (498 to 874)	555 (422 to 727)	476 (357 to 642)	362 (267 to 492)	254 (185 to 351)	187 (132 to 261)	114 (80·3 to 162)	72·4 (50·2 to 102)	43·3 (29·8 to 61·1)	22·4 (15·4 to 31·9)	12·2 (8·4 to 17·1)	7,230
Unintentional firearm injuries	0·4 (0·2 to 1)	0·5 (0·3 to 0·9)	0·9 (0·5 to 1·6)	5·4 (3·3 to 9·3)	9·4 (6·1 to 15·4)	28 (19·6 to 39·4)	188 (129 to 262)	546 (386 to 745)	614 (453 to 827)	610 (462 to 806)	490 (368 to 637)	402 (291 to 541)	359 (255 to 490)	252 (190 to 327)	192 (145 to 245)	123 (93·9 to 155)	73·2 (54·7 to 95·2)	44·8 (32·7 to 58·5)	23·8 (17 to 32)	14·2 (10 to 19·3)	8 (5·5 to 11·1)	4 (2·7 to 5·6)	2·1 (1·5 to 3)	3,990
Other exposure to mechanical forces	0·8 (0·3 to 1·2)	1·1 (0·6 to 1·7)	3·2 (2 to 4·7)	20·9 (13·4 to 31·1)	48·3 (31·8 to 71·7)	77·4 (50·4 to 123)	128 (82·8 to 194)	208 (137 to 307)	257 (173 to 388)	274 (182 to 412)	279 (187 to 423)	276 (186 to 414)	303 (203 to 452)	303 (202 to 453)	284 (193 to 414)	239 (163 to 346)	181 (124 to 263)	143 (96 to 206)	90·4 (60·9 to 132)	58·3 (38·9 to 84)	35·3 (23·6 to 50·9)	18·4 (12·5 to 26·3)	10 (6·9 to 14·3)	3,240
Adverse effects of medical treatment	13·1 (7·8 to 21·4)	7·5 (4·5 to 11·8)	18·8 (8·8 to 37)	24·4 (14·2 to 41·3)	24·9 (15·9 to 36·3)	28·3 (15·3 to 45)	28·5 (13·3 to 44)	63·1 (27·2 to 99·7)	47·6 (27·7 to 72·8)	45·8 (24·9 to 69·5)	51·1 (28·3 to 78·8)	35 (21·8 to 54·4)	55·5 (31·8 to 86·8)	66·2 (39·4 to 102)	86·6 (49·1 to 132)	81·7 (49·1 to 128)	76·4 (48 to 113)	90·4 (52·2 to 131)	74·3 (46·2 to 110)	51·8 (34·2 to 72·9)	29·7 (20·2 to 40·7)	14·3 (9·6 to 19·4)	8 (4·9 to 11·9)	1,020
Animal contact	0 (0 to 0·1)	0·2 (0·1 to 0·3)	0·8 (0·4 to 1·3)	4·1 (2·9 to 5·7)	7 (4·5 to 10·8)	10·9 (6·9 to 16·3)	13·3 (8·1 to 19·4)	17·2 (10·9 to 25·6)	19·5 (12·9 to 27·7)	15·4 (9·9 to 22·6)	14·4 (9·4 to 21·1)	13·4 (9 to 19·9)	14·5 (9·7 to 21)	14·5 (9·8 to 21·2)	13·3 (9 to 19·3)	11·4 (7·8 to 16·2)	10·6 (7·5 to 15)	7·5 (5·2 to 10·6)	5 (3·5 to 7)	4·1 (2·9 to 5·5)	2·1 (1·5 to 2·9)	1·1 (0·8 to 1·6)	0·6 (0·5 to 0·8)	201
Venomous animal contact	0 (0 to 0)	0 (0 to 0)	0·4 (0·2 to 0·6)	2·7 (1·8 to 4)	4·9 (2·9 to 7·6)	7·6 (4·4 to 11·8)	9·7 (5·7 to 14·6)	12·5 (7·5 to 19)	13 (7·9 to 19·3)	11 (6·8 to 16·7)	10·1 (6·4 to 15·1)	9·2 (6 to 13·7)	9·7 (6·2 to 14·1)	9·5 (6·3 to 14·1)	8·5 (5·6 to 12·3)	6·9 (4·6 to 10)	6·3 (4·3 to 8·8)	4·4 (3 to 6·1)	2·9 (2 to 4)	1·9 (1·3 to 2·7)	1·2 (0·8 to 1·6)	0·6 (0·5 to 0·9)	0·3 (0·2 to 0·5)	133
Non-venomous animal contact	0 (0 to 0)	0·1 (0·1 to 0·2)	0·4 (0·2 to 0·7)	1·4 (0·9 to 2)	2·2 (1·4 to 3·5)	3·2 (1·9 to 5·3)	3·7 (2·2 to 6·1)	4·7 (2·7 to 7·4)	6·5 (4·3 to 9·4)	4·4 (2·7 to 7·3)	4·3 (2·6 to 7·2)	4·2 (2·6 to 6·6)	4·8 (3 to 7·3)	5·1 (3·2 to 7·6)	4·9 (3·1 to 7·4)	4·4 (2·8 to 6·5)	4·3 (2·9 to 6·3)	3·1 (2 to 4·6)	2·1 (1·4 to 3·1)	2·1 (1·5 to 3)	0·9 (0·6 to 1·3)	0·5 (0·3 to 0·7)	0·3 (0·2 to 0·4)	67·5
Foreign body	44·5 (28·9 to 65·4)	13·6 (8·6 to 20·6)	20·9 (10·8 to 37·2)	38·6 (26·4 to 53·4)	38·3 (28·7 to 49·9)	51·5 (38·4 to 67·7)	35·1 (25·6 to 48·4)	49·6 (36·8 to 65·6)	56·4 (43·1 to 74·2)	46·8 (35·1 to 61·5)	41·1 (30·4 to 53·9)	36·6 (27·2 to 48·3)	37 (27·9 to 48·9)	34·8 (26·1 to 45·3)	30·1 (23·5 to 38)	28·3 (21·8 to 35·7)	21·4 (16·7 to 27·2)	18·8 (14·8 to 23·3)	14·2 (11·2 to 17·7)	10 (7·9 to 12·3)	5·5 (4·5 to 6·7)	3·1 (2·5 to 3·8)	2·1 (1·6 to 2·5)	678
Pulmonary aspiration and foreign body in airway	44·3 (28·8 to 65·1)	13·4 (8·4 to 20·3)	19·1 (9·3 to 34·9)	29·8 (18·7 to 43·5)	24·7 (18 to 33·3)	35·9 (26·1 to 48·5)	17·3 (12·9 to 22·8)	26·9 (20 to 35·3)	33·7 (25·9 to 44·3)	26·1 (19·9 to 34·3)	22·4 (16·9 to 29·6)	19·3 (14·5 to 25·6)	19·2 (14·1 to 25·4)	18·1 (12·9 to 25·1)	16 (12 to 21·4)	16·7 (12·1 to 22·2)	12·4 (8·9 to 16·4)	11·5 (8·5 to 15·2)	8·9 (6·7 to 11·6)	6·2 (4·7 to 8·2)	3·1 (2·4 to 3·9)	1·8 (1·3 to 2·2)	1·2 (0·9 to 1·6)	428
Foreign body in eyes	0 (0 to 0)	0 (0 to 0·1)	0·4 (0·1 to 0·9)	2·2 (0·7 to 5)	3·9 (1·1 to 9·2)	5·3 (1·6 to 12·5)	7·4 (2·3 to 17·4)	10·1 (3·2 to 22·3)	10·8 (3·8 to 23·2)	9·7 (3·4 to 20·6)	8·7 (3·3 to 18·1)	8 (3·4 to 16·1)	8 (3·7 to 15·3)	6·9 (3·5 to 12·4)	5·5 (3·1 to 9·2)	4·4 (2·5 to 7)	3·4 (2·1 to 5·3)	2·7 (1·7 to 4·1)	1·8 (1·2 to 2·7)	1·2 (0·8 to 1·8)	0·7 (0·5 to 1·1)	0·4 (0·3 to 0·5)	0·2 (0·1 to 0·3)	102
Foreign body in other body part	0·2 (0·1 to 0·4)	0·2 (0·1 to 0·3)	1·4 (0·8 to 2·3)	6·6 (4·4 to 9·6)	9·7 (6·8 to 13·6)	10·3 (7·2 to 14·5)	10·4 (7·3 to 14·8)	12·5 (8·6 to 17·6)	11·9 (8·4 to 16·9)	11 (7·8 to 15·2)	10 (7·2 to 13·8)	9·3 (6·5 to 12·8)	9·9 (7·1 to 13·6)	9·7 (7 to 13·3)	8·6 (6·1 to 11·8)	7·3 (5·2 to 10)	5·6 (4 to 7·7)	4·6 (3·2 to 6·3)	3·5 (2·5 to 4·7)	2·6 (1·9 to 3·5)	1·6 (1·2 to 2·3)	1 (0·7 to 1·3)	0·7 (0·5 to 0·9)	149
Environmental heat and cold exposure	0·1 (0 to 0·1)	0·1 (0·1 to 0·2)	0·8 (0·6 to 1·3)	5·8 (3·9 to 8·3)	12·9 (8·4 to 18·6)	20·4 (13·4 to 29·4)	32·3 (21·6 to 45·8)	50 (34·2 to 70·2)	54·8 (37 to 77·5)	54 (36·3 to 77·3)	50·7 (34·6 to 71·3)	50 (34·1 to 69·1)	53·6 (37·8 to 73·9)	52 (36·4 to 70·9)	47·5 (33·5 to 65·4)	39·5 (27·7 to 54·2)	31·2 (21·9 to 42·5)	25·6 (18·4 to 34·5)	16·9 (12 to 23)	11·9 (8·4 to 15·9)	7·7 (5·6 to 10·3)	4·2 (3·1 to 5·7)	2·5 (1·8 to 3·3)	625
Exposure to forces of nature	0 (0 to 0)	0 (0 to 0)	0 (0 to 0)	0 (0 to 0)	0 (0 to 0)	0·2 (0·1 to 0·3)	0·6 (0·3 to 0·9)	1·1 (0·7 to 1·7)	1·5 (0·9 to 2·3)	1·7 (1·1 to 2·5)	2·2 (1·5 to 3·1)	3 (2 to 4·3)	3 (2 to 4·4)	2·4 (1·6 to 3·3)	2·2 (1·5 to 3·1)	1·8 (1·2 to 2·5)	1·2 (0·8 to 1·7)	0·8 (0·6 to 1·2)	0·5 (0·3 to 0·7)	0·3 (0·2 to 0·4)	0·2 (0·1 to 0·2)	0·1 (0·1 to 0·1)	0 (0 to 0·1)	22·6
Other unintentional injuries	2·8 (1·5 to 5)	1·1 (0·5 to 1·9)	3·8 (2·3 to 5·6)	23·1 (15 to 33·6)	47·1 (31·5 to 69·5)	65·4 (46 to 92·6)	92·5 (63·8 to 132)	129 (92·3 to 182)	139 (101 to 189)	136 (99·5 to 187)	118 (85·9 to 161)	107 (76·5 to 147)	103 (71·3 to 145)	90·9 (61·4 to 131)	80·5 (53·6 to 115)	65·5 (44·3 to 94·3)	49·2 (33·6 to 71·8)	40·7 (28 to 57·7)	27·6 (19·8 to 38·8)	17·9 (12·7 to 25·1)	11·2 (8 to 15·8)	6·6 (4·8 to 8·9)	4·2 (3·1 to 5·5)	1,360
Self-harm and interpersonal violence	16·9 (9·8 to 26·8)	3·8 (2·2 to 6·1)	18·1 (8·8 to 32·9)	103 (60·1 to 170)	124 (87 to 169)	284 (213 to 381)	1,430 (1,040 to 1,930)	3,340 (2,650 to 4,220)	3,470 (2,750 to 4,300)	3,230 (2,480 to 4,100)	2,530 (1,870 to 3,310)	1,950 (1,400 to 2,620)	1,750 (1,220 to 2,420)	1,270 (912 to 1,740)	906 (636 to 1,230)	542 (406 to 706)	343 (256 to 449)	210 (160 to 271)	105 (81·6 to 134)	45·4 (36·6 to 56·1)	24·8 (19·9 to 30·5)	10·2 (8·2 to 12·4)	6·2 (4·9 to 7·7)	21,700
Self-harm	0 (0 to 0)	0 (0 to 0)	0 (0 to 0)	0 (0 to 0)	0 (0 to 0)	31·5 (18 to 51·2)	177 (114 to 257)	395 (270 to 564)	436 (295 to 609)	420 (289 to 582)	361 (248 to 503)	299 (202 to 434)	286 (190 to 411)	227 (148 to 326)	180 (118 to 251)	117 (80 to 165)	76·3 (54·2 to 104)	56·8 (40·2 to 78·4)	29·3 (21·2 to 39·6)	14·3 (10·5 to 19·3)	8·3 (6 to 11·2)	3·7 (2·6 to 4·7)	2 (1·3 to 2·6)	3,120
Self-harm by firearm	0 (0 to 0)	0 (0 to 0)	0 (0 to 0)	0 (0 to 0)	0 (0 to 0)	1·1 (0·5 to 2·3)	24·5 (12 to 45)	85·1 (47·7 to 145)	106 (63 to 174)	111 (65·8 to 177)	73·3 (42·3 to 119)	62·3 (34·9 to 103)	70·5 (38·4 to 112)	54·8 (30·8 to 89·3)	34·8 (19·6 to 58)	22·5 (13·7 to 36·6)	12·1 (7·1 to 18·9)	5·7 (3·6 to 8·9)	2·1 (1·3 to 3·2)	1·3 (0·8 to 2)	0·8 (0·5 to 1·3)	0·4 (0·3 to 0·6)	0·3 (0·2 to 0·4)	668
Self-harm by other specified means	0 (0 to 0)	0 (0 to 0)	0 (0 to 0)	0 (0 to 0)	0 (0 to 0)	30·4 (17·3 to 49·5)	152 (96·7 to 224)	310 (203 to 458)	330 (220 to 467)	309 (214 to 441)	288 (197 to 407)	237 (159 to 346)	215 (143 to 317)	173 (111 to 252)	145 (94·1 to 205)	94·6 (63·7 to 134)	64·2 (45·1 to 89·7)	51·1 (35·9 to 70·9)	27·2 (19·5 to 37)	13 (9·2 to 17·7)	7·5 (5·3 to 10·1)	3·2 (2·3 to 4·2)	1·7 (1·1 to 2·2)	2,450
Interpersonal violence	16·9 (9·8 to 26·8)	3·8 (2·2 to 6·1)	17·9 (8·7 to 32·7)	101 (59 to 169)	123 (85·9 to 168)	252 (181 to 340)	1,260 (905 to 1,720)	2,940 (2,280 to 3,740)	3,030 (2,340 to 3,790)	2,800 (2,120 to 3,610)	2,160 (1,570 to 2,900)	1,650 (1,170 to 2,230)	1,460 (993 to 2,060)	1,030 (720 to 1,440)	714 (489 to 989)	415 (297 to 569)	261 (184 to 351)	151 (108 to 205)	74·8 (55·3 to 101)	30·5 (24·1 to 38·8)	16·2 (12·6 to 20·7)	6·5 (4·9 to 8·3)	4·2 (3·1 to 5·4)	18,500
Physical violence by firearm	8·9 (5·1 to 14·1)	1·9 (1·1 to 3·1)	9·1 (4·2 to 17·1)	54·7 (30·9 to 92·3)	66·9 (44·8 to 92·5)	159 (112 to 221)	971 (686 to 1,340)	2,440 (1,880 to 3,120)	2,550 (1,940 to 3,190)	2,340 (1,750 to 3,020)	1,720 (1,240 to 2,330)	1,260 (878 to 1,730)	1,080 (717 to 1,550)	723 (491 to 1,030)	469 (308 to 665)	248 (168 to 350)	141 (95 to 197)	56·4 (38·6 to 79·4)	22·3 (15·4 to 31·7)	6·5 (5 to 8·7)	1·8 (1·4 to 2·3)	0·8 (0·6 to 1)	0·9 (0·6 to 1·3)	14,300
Physical violence by sharp object	1·7 (1 to 2·8)	0·4 (0·2 to 0·6)	1·4 (0·7 to 2·4)	11·5 (6·8 to 18·7)	19·5 (13·7 to 26·6)	48·1 (34·5 to 64·8)	224 (157 to 306)	384 (298 to 487)	349 (273 to 434)	344 (263 to 442)	329 (242 to 438)	295 (210 to 398)	267 (185 to 370)	214 (153 to 295)	157 (110 to 215)	89·4 (65·3 to 118)	56·4 (40·8 to 73·9)	57·1 (40·5 to 77·7)	33·6 (24·2 to 46·3)	16 (12·4 to 21·1)	8·9 (6·7 to 11·7)	3·4 (2·5 to 4·3)	1·9 (1·4 to 2·6)	2,910
Sexual violence	0 (0 to 0)	0 (0 to 0)	0 (0 to 0)	0·5 (0·3 to 0·7)	4 (2·4 to 6·2)	9·3 (5·2 to 14·7)	21·3 (11·9 to 34·2)	30·9 (17·9 to 47·8)	27 (14·9 to 44)	21·2 (11·8 to 34·1)	16·9 (9·5 to 27·4)	12·8 (7·3 to 21·1)	10·8 (6·2 to 18·4)	8·4 (4·8 to 13·8)	6·1 (3·6 to 9·8)	4·6 (2·8 to 7·1)	3·4 (2·1 to 5·1)	2·5 (1·6 to 3·8)	1·6 (1 to 2·3)	1 (0·6 to 1·4)	0·5 (0·3 to 0·7)	0·1 (0·1 to 0·1)	0 (0 to 0)	183
Physical violence by other means	6·3 (3·6 to 9·9)	1·5 (0·9 to 2·4)	7·5 (3·7 to 13·5)	34·7 (19·9 to 58)	32·9 (22·8 to 45·7)	35·3 (26·1 to 46)	40 (30·6 to 52·3)	90 (72·4 to 111)	110 (88·2 to 134)	102 (79·5 to 126)	95·9 (74·2 to 123)	83·7 (63·7 to 107)	103 (76·1 to 136)	87·5 (66·2 to 113)	81·9 (61·1 to 106)	73·2 (55·6 to 94·8)	60·2 (45·1 to 78·3)	34·9 (26·5 to 45·6)	17·3 (13·1 to 22·3)	7 (5·1 to 9·5)	5·1 (3·8 to 6·6)	2·2 (1·6 to 3)	1·3 (1 to 1·8)	1,110
Conflict and terrorism	0 (0 to 0)	0 (0 to 0)	0 (0 to 0)	0 (0 to 0)	0 (0 to 0)	0 (0 to 0)	0 (0 to 0)	0·2 (0·1 to 0·3)	1 (0·6 to 1·8)	3·1 (1·9 to 5·1)	2·5 (1·5 to 4)	4 (2·4 to 6·3)	6·5 (4·1 to 10·4)	11·6 (7·4 to 17·9)	11·8 (7·9 to 17·9)	10·4 (6·8 to 15·3)	6·2 (4·1 to 9·1)	1·7 (1·1 to 2·5)	0·8 (0·5 to 1·2)	0·5 (0·3 to 0·7)	0·3 (0·2 to 0·4)	0·1 (0 to 0·1)	0 (0 to 0)	60·6

SR Table 2a: Saint Lucia DALYS by cause and age group for males with 95% uncertainty intervals, 2019

Cause	Early Neonatal	Late Neonatal	Post Neonatal	1 to 4	5 to 9	10 to 14	15 to 19	20 to 24	25 to 29	30 to 34	35 to 39	40 to 44
All causes	755 (506 to 1,100)	105 (70·6 to 152)	212 (151 to 296)	290 (221 to 376)	353 (279 to 458)	463 (373 to 578)	893 (751 to 1,060)	1,320 (1,150 to 1,520)	1,540 (1,350 to 1,750)	1,480 (1,300 to 1,690)	1,600 (1,390 to 1,840)	1,900 (1,640 to 2,190)
Communicable, maternal, neonatal, and nutritional diseases	656 (438 to 942)	80·5 (54·1 to 118)	110 (76·7 to 156)	102 (74·3 to 139)	97·9 (63·5 to 158)	75·9 (55·1 to 107)	77·6 (60·7 to 102)	93 (77 to 113)	121 (102 to 143)	132 (114 to 153)	138 (120 to 160)	150 (128 to 172)
HIV/AIDS and sexually transmitted infections	0·9 (0·5 to 1·6)	0·1 (0·1 to 0·2)	1 (0·9 to 1·2)	0·7 (0·7 to 0·8)	0·9 (0·8 to 1)	1·1 (0·9 to 1·2)	2·4 (1·9 to 3·1)	10·5 (9·3 to 12)	28·9 (26 to 32·9)	39·6 (36·2 to 44·7)	40 (36·5 to 43·8)	41·1 (37·7 to 44·9)
HIV/AIDS	0 (0 to 0)	0 (0 to 0)	0·8 (0·8 to 0·9)	0·7 (0·6 to 0·8)	0·9 (0·8 to 1)	0·9 (0·8 to 1)	1·6 (1·4 to 1·7)	9·2 (8·2 to 10·3)	27·4 (24·7 to 31·1)	38·1 (34·8 to 43)	38·4 (35·2 to 42)	39·7 (36·6 to 43·4)
HIV/AIDS - Drug-susceptible Tuberculosis	0 (0 to 0)	0 (0 to 0)	0·1 (0 to 0·1)	0·1 (0 to 0·1)	0·1 (0·1 to 0·1)	0·1 (0 to 0·1)	0·1 (0·1 to 0·2)	0·9 (0·6 to 1·3)	2·5 (1·7 to 3·7)	3·7 (2·4 to 5·3)	3·5 (2·4 to 5·2)	3·9 (2·6 to 5·8)
HIV/AIDS - Multidrug-resistant Tuberculosis without extensive dru	0 (0 to 0)	0 (0 to 0)	0 (0 to 0)	0 (0 to 0)	0 (0 to 0)	0 (0 to 0)	0 (0 to 0)	0 (0 to 0)	0 (0 to 0·1)	0 (0 to 0·2)	0 (0 to 0·2)	0 (0 to 0·2)
HIV/AIDS - Extensively drug-resistant Tuberculosis	0 (0 to 0)	0 (0 to 0)	0 (0 to 0)	0 (0 to 0)	0 (0 to 0)	0 (0 to 0)	0 (0 to 0)	0 (0 to 0)	0 (0 to 0)	0 (0 to 0)	0 (0 to 0)	0 (0 to 0)
HIV/AIDS resulting in other diseases	0 (0 to 0)	0 (0 to 0)	0·7 (0·7 to 0·8)	0·6 (0·6 to 0·7)	0·8 (0·7 to 0·9)	0·8 (0·7 to 0·9)	1·4 (1·3 to 1·5)	8·3 (7·2 to 9·2)	24·9 (22·1 to 28·5)	34·4 (30·6 to 39·3)	34·9 (31·2 to 38·8)	35·8 (32·1 to 39·8)
Sexually transmitted infections excluding HIV	0·9 (0·5 to 1·6)	0·1 (0·1 to 0·2)	0·2 (0·1 to 0·4)	0 (0 to 0)	0 (0 to 0)	0·2 (0·1 to 0·3)	0·8 (0·4 to 1·6)	1·3 (0·7 to 2·2)	1·5 (0·9 to 2·4)	1·6 (0·8 to 2·6)	1·5 (0·9 to 2·4)	1·3 (0·7 to 2·2)
Syphilis	0·9 (0·5 to 1·6)	0·1 (0·1 to 0·2)	0·2 (0·1 to 0·4)	0 (0 to 0)	0 (0 to 0)	0 (0 to 0)	0·1 (0·1 to 0·2)	0·2 (0·1 to 0·3)	0·2 (0·1 to 0·4)	0·2 (0·1 to 0·4)	0·3 (0·2 to 0·6)	0·2 (0·1 to 0·3)
Chlamydial infection	0 (0 to 0)	0 (0 to 0)	0 (0 to 0)	0 (0 to 0)	0 (0 to 0)	0 (0 to 0)	0·1 (0 to 0·1)	0·2 (0·1 to 0·6)	0·4 (0·1 to 0·8)	0·4 (0·1 to 0·8)	0·3 (0·1 to 0·7)	0·3 (0·1 to 0·6)
Gonococcal infection	0 (0 to 0)	0 (0 to 0)	0 (0 to 0)	0 (0 to 0)	0 (0 to 0)	0 (0 to 0·1)	0·3 (0·1 to 0·6)	0·4 (0·1 to 0·8)	0·2 (0 to 0·4)	0·1 (0 to 0·3)	0·1 (0 to 0·2)	0·1 (0 to 0·2)
Trichomoniasis	0 (0 to 0)	0 (0 to 0)	0 (0 to 0)	0 (0 to 0)	0 (0 to 0)	0 (0 to 0)	0 (0 to 0)	0 (0 to 0)	0 (0 to 0·1)	0 (0 to 0·1)	0 (0 to 0·1)	0 (0 to 0·1)
Genital herpes	0 (0 to 0)	0 (0 to 0)	0 (0 to 0)	0 (0 to 0)	0 (0 to 0)	0 (0 to 0)	0·1 (0 to 0·2)	0·2 (0 to 0·4)	0·3 (0·1 to 0·7)	0·4 (0·1 to 0·9)	0·4 (0·1 to 1·1)	0·5 (0·1 to 1·1)
Other sexually transmitted infections	0 (0 to 0)	0 (0 to 0)	0 (0 to 0)	0 (0 to 0)	0 (0 to 0)	0·1 (0 to 0·2)	0·3 (0·1 to 0·6)	0·4 (0·2 to 0·7)	0·4 (0·2 to 0·7)	0·5 (0·2 to 0·9)	0·3 (0·1 to 0·6)	0·2 (0·1 to 0·5)
Respiratory infections and tuberculosis	18·9 (11·1 to 29·9)	6·5 (4·1 to 10·2)	31·8 (19·1 to 49·3)	22 (15·6 to 30·7)	14·3 (10·3 to 20·1)	13 (9·1 to 18·8)	17·6 (12·6 to 23·8)	26 (19·7 to 33·5)	32·3 (25 to 41·7)	35 (27 to 45·4)	37·6 (29·3 to 48·5)	44·8 (34·6 to 58·9)
Tuberculosis	0 (0 to 0)	0 (0 to 0)	0·5 (0·3 to 0·8)	0·6 (0·3 to 0·9)	0·7 (0·4 to 1·1)	0·7 (0·6 to 1)	2 (1·3 to 2·9)	6·3 (4·2 to 9)	9 (6·5 to 12)	9·8 (7 to 13)	10 (7·2 to 13·5)	11·2 (8 to 15·2)
Latent tuberculosis infection	0 (0 to 0)	0 (0 to 0)	0 (0 to 0)	0 (0 to 0)	0 (0 to 0)	0 (0 to 0)	0 (0 to 0)	0 (0 to 0)	0 (0 to 0)	0 (0 to 0)	0 (0 to 0)	0 (0 to 0)
Drug-susceptible tuberculosis	0 (0 to 0)	0 (0 to 0)	0·5 (0·3 to 0·8)	0·6 (0·3 to 0·9)	0·7 (0·4 to 1·1)	0·7 (0·5 to 1)	2 (1·3 to 2·8)	6·2 (4·2 to 8·8)	8·9 (6·5 to 11·9)	9·7 (7 to 12·8)	9·8 (7 to 13·4)	11 (8 to 15)
Multidrug-resistant tuberculosis without extensive drug resistanc	0 (0 to 0)	0 (0 to 0)	0 (0 to 0)	0 (0 to 0)	0 (0 to 0)	0 (0 to 0)	0 (0 to 0·1)	0·1 (0 to 0·2)	0·1 (0 to 0·3)	0·1 (0 to 0·4)	0·1 (0 to 0·4)	0·1 (0 to 0·5)
Extensively drug-resistant tuberculosis	0 (0 to 0)	0 (0 to 0)	0 (0 to 0)	0 (0 to 0)	0 (0 to 0)	0 (0 to 0)	0 (0 to 0)	0 (0 to 0)	0 (0 to 0·1)	0 (0 to 0·1)	0 (0 to 0·1)	0 (0 to 0·1)
Lower respiratory infections	18·9 (11·1 to 29·9)	6·4 (4 to 10·1)	29·6 (17·1 to 47)	14·1 (8·8 to 21·5)	5 (3·6 to 6·7)	4·3 (3·1 to 6·1)	7·2 (4·6 to 10·4)	11·6 (7·5 to 17·3)	15·7 (10·1 to 22·7)	18·7 (12·6 to 26·5)	21 (14·4 to 29·5)	27·1 (17·8 to 39·2)
Upper respiratory infections	0 (0 to 0)	0·1 (0 to 0·1)	1·4 (0·9 to 2·2)	5·3 (3·2 to 8·3)	5·8 (3·3 to 9·5)	5·6 (2·9 to 9·6)	6·1 (3·1 to 10·6)	6·1 (3·4 to 10·3)	5·8 (3·2 to 9·3)	5·1 (2·8 to 8·4)	5·2 (2·7 to 8·7)	5 (2·8 to 8·7)
Otitis media	0 (0 to 0)	0 (0 to 0)	0·2 (0·1 to 0·4)	2 (1·1 to 3·4)	2·9 (1·6 to 4·8)	2·3 (1·2 to 4)	2·3 (1·2 to 4)	2·1 (0·9 to 4·1)	1·7 (0·8 to 3·4)	1·3 (0·5 to 2·5)	1·4 (0·6 to 2·7)	1·5 (0·7 to 2·8)
Enteric infections	1·4 (0·7 to 2·3)	1·9 (1·1 to 3·1)	17·2 (10·4 to 27·2)	15·6 (10·6 to 22·1)	13 (7·8 to 20·4)	13·6 (7·7 to 21·8)	11·4 (7·2 to 16·4)	10·3 (6·5 to 15)	11·6 (7·8 to 16·3)	11·6 (8·1 to 15·9)	13 (9 to 17·6)	14·7 (10·5 to 20)
Diarrheal diseases	1·4 (0·7 to 2·3)	1·9 (1·1 to 3·1)	17·2 (10·3 to 27·1)	15·6 (10·6 to 22)	13 (7·8 to 20·3)	13·5 (7·7 to 21·8)	11·4 (7·2 to 16·4)	10·3 (6·5 to 14·9)	11·6 (7·7 to 16·3)	11·5 (8 to 15·9)	12·9 (9 to 17·5)	14·7 (10·4 to 19·9)
Typhoid and paratyphoid	0 (0 to 0)	0 (0 to 0)	0 (0 to 0)	0 (0 to 0)	0 (0 to 0)	0 (0 to 0)	0 (0 to 0)	0 (0 to 0)	0 (0 to 0)	0 (0 to 0)	0 (0 to 0)	0 (0 to 0)
Typhoid fever	0 (0 to 0)	0 (0 to 0)	0 (0 to 0)	0 (0 to 0)	0 (0 to 0)	0 (0 to 0)	0 (0 to 0)	0 (0 to 0)	0 (0 to 0)	0 (0 to 0)	0 (0 to 0)	0 (0 to 0)
Paratyphoid fever	0 (0 to 0)	0 (0 to 0)	0 (0 to 0)	0 (0 to 0)	0 (0 to 0)	0 (0 to 0)	0 (0 to 0)	0 (0 to 0)	0 (0 to 0)	0 (0 to 0)	0 (0 to 0)	0 (0 to 0)
Invasive Non-typhoidal Salmonella (iNTS)	0 (0 to 0)	0 (0 to 0)	0 (0 to 0)	0 (0 to 0)	0 (0 to 0)	0 (0 to 0)	0 (0 to 0)	0 (0 to 0)	0 (0 to 0)	0 (0 to 0)	0 (0 to 0)	0 (0 to 0)
Other intestinal infectious diseases	0 (0 to 0)	0 (0 to 0)	0 (0 to 0·1)	0 (0 to 0·1)	0 (0 to 0·1)	0 (0 to 0·1)	0 (0 to 0·1)	0 (0 to 0·1)	0 (0 to 0·1)	0 (0 to 0·1)	0 (0 to 0·1)	0 (0 to 0·1)
Neglected tropical diseases and malaria	0 (0 to 0·1)	0·1 (0·1 to 0·2)	1·2 (0·6 to 2·2)	3·6 (1·8 to 6·6)	5·7 (2·8 to 10·6)	3·4 (1·7 to 6)	2·9 (1·5 to 4·8)	3·9 (2·2 to 6·3)	4·4 (2·3 to 7·4)	4·3 (2·3 to 7·2)	4·8 (2·6 to 8·6)	5·7 (2·9 to 10·5)
Malaria	0 (0 to 0)	0 (0 to 0)	0 (0 to 0)	0 (0 to 0)	0 (0 to 0)	0 (0 to 0)	0 (0 to 0)	0 (0 to 0)	0 (0 to 0)	0 (0 to 0)	0 (0 to 0)	0 (0 to 0)
Chagas disease	0 (0 to 0)	0 (0 to 0)	0 (0 to 0)	0 (0 to 0)	0 (0 to 0)	0 (0 to 0)	0 (0 to 0)	0 (0 to 0)	0 (0 to 0)	0 (0 to 0)	0 (0 to 0)	0 (0 to 0)
Leishmaniasis	0 (0 to 0)	0 (0 to 0)	0 (0 to 0)	0 (0 to 0)	0 (0 to 0)	0 (0 to 0)	0 (0 to 0)	0 (0 to 0)	0 (0 to 0)	0 (0 to 0)	0 (0 to 0)	0 (0 to 0)
Visceral leishmaniasis	0 (0 to 0)	0 (0 to 0)	0 (0 to 0)	0 (0 to 0)	0 (0 to 0)	0 (0 to 0)	0 (0 to 0)	0 (0 to 0)	0 (0 to 0)	0 (0 to 0)	0 (0 to 0)	0 (0 to 0)
Cutaneous and mucocutaneous leishmaniasis	0 (0 to 0)	0 (0 to 0)	0 (0 to 0)	0 (0 to 0)	0 (0 to 0)	0 (0 to 0)	0 (0 to 0)	0 (0 to 0)	0 (0 to 0)	0 (0 to 0)	0 (0 to 0)	0 (0 to 0)
African trypanosomiasis	0 (0 to 0)	0 (0 to 0)	0 (0 to 0)	0 (0 to 0)	0 (0 to 0)	0 (0 to 0)	0 (0 to 0)	0 (0 to 0)	0 (0 to 0)	0 (0 to 0)	0 (0 to 0)	0 (0 to 0)
Schistosomiasis	0 (0 to 0)	0 (0 to 0)	0 (0 to 0)	0 (0 to 0)	0 (0 to 0)	0 (0 to 0)	0 (0 to 0·1)	0 (0 to 0·1)	0 (0 to 0·2)	0 (0 to 0·2)	0 (0 to 0·2)	0·1 (0 to 0·3)
Cysticercosis	0 (0 to 0)	0 (0 to 0)	0 (0 to 0)	0 (0 to 0)	0 (0 to 0)	0 (0 to 0·1)	0·3 (0 to 0·8)	1 (0·2 to 2·6)	1·9 (0·5 to 4·5)	2·1 (0·7 to 4·7)	2·8 (0·9 to 6·5)	3·5 (1·1 to 8·2)
Cystic echinococcosis	0 (0 to 0)	0 (0 to 0)	0 (0 to 0)	0 (0 to 0)	0 (0 to 0)	0 (0 to 0)	0 (0 to 0)	0 (0 to 0)	0 (0 to 0)	0 (0 to 0)	0 (0 to 0)	0 (0 to 0)
Lymphatic filariasis	0 (0 to 0)	0 (0 to 0)	0 (0 to 0)	0 (0 to 0)	0 (0 to 0)	0 (0 to 0)	0 (0 to 0)	0 (0 to 0)	0 (0 to 0)	0 (0 to 0)	0 (0 to 0)	0 (0 to 0)
Onchocerciasis	0 (0 to 0)	0 (0 to 0)	0 (0 to 0)	0 (0 to 0)	0 (0 to 0)	0 (0 to 0)	0 (0 to 0)	0 (0 to 0)	0 (0 to 0)	0 (0 to 0)	0 (0 to 0)	0 (0 to 0)
Trachoma	0 (0 to 0)	0 (0 to 0)	0 (0 to 0)	0 (0 to 0)	0 (0 to 0)	0 (0 to 0)	0 (0 to 0)	0 (0 to 0)	0 (0 to 0)	0 (0 to 0)	0 (0 to 0)	0 (0 to 0)
Dengue	0 (0 to 0)	0 (0 to 0)	0·5 (0·1 to 1·3)	0·5 (0·1 to 1·2)	1·2 (0·4 to 2·6)	1·1 (0·4 to 2·5)	1·4 (0·5 to 2·9)	1·3 (0·4 to 2·7)	1·4 (0·4 to 2·9)	1·1 (0·4 to 2·1)	1 (0·4 to 2·1)	1 (0·3 to 2)
Yellow fever	0 (0 to 0)	0 (0 to 0)	0 (0 to 0)	0 (0 to 0)	0 (0 to 0)	0 (0 to 0)	0 (0 to 0)	0 (0 to 0)	0 (0 to 0)	0 (0 to 0)	0 (0 to 0)	0 (0 to 0)
Rabies	0 (0 to 0)	0 (0 to 0)	0 (0 to 0)	0 (0 to 0)	0 (0 to 0·1)	0 (0 to 0·1)	0 (0 to 0·1)	0 (0 to 0)	0 (0 to 0)	0 (0 to 0)	0 (0 to 0)	0 (0 to 0)
Intestinal nematode infections	0 (0 to 0)	0 (0 to 0)	0 (0 to 0)	0·2 (0·1 to 0·4)	0·5 (0·3 to 1)	0·4 (0·2 to 0·8)	0·5 (0·2 to 0·8)	0·4 (0·2 to 0·7)	0·3 (0·2 to 0·6)	0·3 (0·1 to 0·5)	0·3 (0·1 to 0·5)	0·2 (0·1 to 0·5)
Ascariasis	0 (0 to 0)	0 (0 to 0)	0 (0 to 0)	0·1 (0 to 0·2)	0·2 (0·1 to 0·4)	0·1 (0·1 to 0·3)	0·1 (0·1 to 0·3)	0·1 (0 to 0·3)	0·1 (0 to 0·3)	0·1 (0 to 0·2)	0·1 (0 to 0·2)	0·1 (0 to 0·2)
Trichuriasis	0 (0 to 0)	0 (0 to 0)	0 (0 to 0)	0 (0 to 0)	0·1 (0 to 0·1)	0·1 (0 to 0·2)	0·1 (0 to 0·3)	0·1 (0 to 0·3)	0·1 (0 to 0·2)	0·1 (0 to 0·1)	0·1 (0 to 0·1)	0 (0 to 0·1)
Hookworm disease	0 (0 to 0)	0 (0 to 0)	0 (0 to 0)	0·1 (0 to 0·2)	0·3 (0·1 to 0·6)	0·2 (0·1 to 0·5)	0·2 (0·1 to 0·4)	0·2 (0·1 to 0·3)	0·1 (0 to 0·2)	0·1 (0 to 0·2)	0·1 (0 to 0·2)	0·1 (0 to 0·2)
Food-borne trematodiases	0 (0 to 0)	0 (0 to 0)	0 (0 to 0)	0 (0 to 0)	0 (0 to 0)	0 (0 to 0)	0 (0 to 0)	0 (0 to 0)	0 (0 to 0)	0 (0 to 0)	0 (0 to 0)	0 (0 to 0)
Leprosy	0 (0 to 0)	0 (0 to 0)	0 (0 to 0)	0 (0 to 0)	0 (0 to 0)	0 (0 to 0)	0 (0 to 0)	0 (0 to 0)	0 (0 to 0)	0 (0 to 0)	0 (0 to 0·1)	0 (0 to 0·1)
Ebola	0 (0 to 0)	0 (0 to 0)	0 (0 to 0)	0 (0 to 0)	0 (0 to 0)	0 (0 to 0)	0 (0 to 0)	0 (0 to 0)	0 (0 to 0)	0 (0 to 0)	0 (0 to 0)	0 (0 to 0)
Zika virus	0 (0 to 0)	0 (0 to 0)	0 (0 to 0·1)	0·1 (0 to 0·5)	0·2 (0 to 0·7)	0·2 (0 to 0·7)	0·2 (0 to 0·8)	0·2 (0 to 0·8)	0·2 (0 to 0·8)	0·2 (0 to 0·9)	0·2 (0 to 0·8)	0·2 (0 to 0·7)
Guinea worm disease	0 (0 to 0)	0 (0 to 0)	0 (0 to 0)	0 (0 to 0)	0 (0 to 0)	0 (0 to 0)	0 (0 to 0)	0 (0 to 0)	0 (0 to 0)	0 (0 to 0)	0 (0 to 0)	0 (0 to 0)
Other neglected tropical diseases	0 (0 to 0·1)	0·1 (0·1 to 0·2)	0·7 (0·3 to 1·4)	2·7 (1·1 to 5·6)	3·7 (1·1 to 8·2)	1·6 (0·4 to 3·6)	0·5 (0·2 to 1·3)	0·9 (0·4 to 1·9)	0·5 (0·2 to 1·1)	0·6 (0·2 to 1·2)	0·5 (0·2 to 1)	0·7 (0·3 to 1·4)
Other infectious diseases	2·4 (1·4 to 3·5)	2·3 (1·4 to 3·7)	10·3 (6·4 to 15·9)	9·9 (6·6 to 14·4)	8·4 (6 to 11·9)	7·3 (5·5 to 9·8)	9·8 (7·4 to 13)	11 (8 to 14·7)	12·3 (8·8 to 17)	12·7 (9 to 17·5)	13·7 (9·7 to 18·3)	15·6 (10·9 to 21·4)
Meningitis	1·3 (0·8 to 2·1)	1·4 (0·8 to 2·2)	4·7 (2·5 to 8·3)	3·4 (2·1 to 5·2)	2·3 (1·6 to 3·3)	2·1 (1·4 to 3)	2·8 (1·9 to 4·1)	2·6 (1·8 to 3·9)	2·8 (1·9 to 3·9)	2·8 (1·9 to 3·9)	2·8 (1·9 to 4)	3·1 (2·1 to 4·3)
Encephalitis	0·2 (0·1 to 0·4)	0·3 (0·1 to 0·6)	2·1 (1·1 to 4·2)	2·8 (1·5 to 5·6)	2 (1·2 to 3·5)	2 (1·3 to 3)	2·7 (1·7 to 4)	3 (1·8 to 4·6)	2·4 (1·6 to 3·6)	2·4 (1·6 to 3·6)	2·1 (1·4 to 3·1)	2·4 (1·7 to 3·5)
Diphtheria	0 (0 to 0)	0 (0 to 0)	0 (0 to 0)	0 (0 to 0)	0 (0 to 0)	0 (0 to 0)	0 (0 to 0)	0 (0 to 0)	0 (0 to 0)	0 (0 to 0)	0 (0 to 0)	0 (0 to 0)
Tetanus	0 (0 to 0·1)	0 (0 to 0)	0 (0 to 0·1)	0 (0 to 0·1)	0·1 (0 to 0·3)	0·1 (0 to 0·3)	0·1 (0 to 0·4)	0·2 (0 to 0·5)	0·2 (0 to 0·5)	0·2 (0·1 to 0·6)	0·2 (0·1 to 0·7)	0·3 (0·1 to 1)
Measles	0 (0 to 0)	0 (0 to 0)	0 (0 to 0)	0 (0 to 0)	0 (0 to 0)	0 (0 to 0)	0 (0 to 0)	0 (0 to 0)	0 (0 to 0)	0 (0 to 0)	0 (0 to 0)	0 (0 to 0)
Varicella and herpes zoster	0·3 (0·1 to 0·6)	0·2 (0·1 to 0·6)	0·6 (0·2 to 1·4)	0·3 (0·1 to 0·6)	0·5 (0·2 to 1)	0·6 (0·2 to 1·2)	0·4 (0·2 to 0·6)	0·4 (0·2 to 0·7)	0·5 (0·2 to 0·9)	0·6 (0·3 to 1·1)	0·9 (0·4 to 1·6)	0·7 (0·3 to 1·2)
Acute hepatitis	0 (0 to 0)	0 (0 to 0)	0 (0 to 0·1)	0·1 (0·1 to 0·2)	0·4 (0·2 to 0·7)	0·5 (0·3 to 0·9)	0·6 (0·4 to 0·9)	0·6 (0·4 to 0·9)	0·6 (0·4 to 0·8)	0·5 (0·3 to 0·7)	0·5 (0·3 to 0·7)	0·4 (0·3 to 0·6)
Acute hepatitis A	0 (0 to 0)	0 (0 to 0)	0 (0 to 0)	0·1 (0 to 0·2)	0·3 (0·2 to 0·6)	0·5 (0·2 to 0·8)	0·5 (0·3 to 0·7)	0·4 (0·2 to 0·7)	0·3 (0·2 to 0·5)	0·2 (0·1 to 0·4)	0·2 (0·1 to 0·4)	0·2 (0·1 to 0·3)
Acute hepatitis B	0 (0 to 0)	0 (0 to 0)	0 (0 to 0)	0 (0 to 0)	0 (0 to 0)	0 (0 to 0·1)	0·1 (0 to 0·1)	0·1 (0·1 to 0·2)	0·2 (0·1 to 0·2)	0·2 (0·1 to 0·2)	0·2 (0·1 to 0·3)	0·2 (0·1 to 0·3)
Acute hepatitis C	0 (0 to 0)	0 (0 to 0)	0 (0 to 0)	0 (0 to 0)	0 (0 to 0)	0 (0 to 0)	0 (0 to 0)	0 (0 to 0)	0 (0 to 0)	0 (0 to 0)	0 (0 to 0)	0 (0 to 0)
Acute hepatitis E	0 (0 to 0)	0 (0 to 0)	0 (0 to 0)	0 (0 to 0)	0 (0 to 0·1)	0 (0 to 0·1)	0·1 (0 to 0·1)	0·1 (0 to 0·1)	0·1 (0 to 0·1)	0 (0 to 0·1)	0 (0 to 0·1)	0 (0 to 0·1)
Other unspecified infectious diseases	0·5 (0·2 to 0·9)	0·4 (0·2 to 0·8)	2·1 (1·1 to 3·8)	3 (1·6 to 5·1)	3 (1·3 to 6)	2 (1·1 to 3·5)	3·2 (1·8 to 5)	4·2 (2·2 to 6·8)	5·8 (2·8 to 9·7)	6·2 (3·1 to 10·3)	7·2 (3·8 to 11·3)	8·5 (4·3 to 13·8)
Maternal and neonatal disorders	633 (422 to 909)	68·7 (45·4 to 101)	33·6 (21·6 to 50·7)	21·4 (16·2 to 27·8)	18·6 (13·4 to 24·5)	20·8 (15·1 to 27·1)	22·2 (15·9 to 29·7)	21·4 (15·5 to 28·2)	20·7 (14·8 to 27·5)	17·6 (12·7 to 23·4)	17·3 (12·8 to 22·6)	15·8 (11·5 to 21·3)
Maternal disorders	0 (0 to 0)	0 (0 to 0)	0 (0 to 0)	0 (0 to 0)	0 (0 to 0)	0 (0 to 0)	0 (0 to 0)	0 (0 to 0)	0 (0 to 0)	0 (0 to 0)	0 (0 to 0)	0 (0 to 0)
Maternal hemorrhage	0 (0 to 0)	0 (0 to 0)	0 (0 to 0)	0 (0 to 0)	0 (0 to 0)	0 (0 to 0)	0 (0 to 0)	0 (0 to 0)	0 (0 to 0)	0 (0 to 0)	0 (0 to 0)	0 (0 to 0)
Maternal sepsis and other maternal infections	0 (0 to 0)	0 (0 to 0)	0 (0 to 0)	0 (0 to 0)	0 (0 to 0)	0 (0 to 0)	0 (0 to 0)	0 (0 to 0)	0 (0 to 0)	0 (0 to 0)	0 (0 to 0)	0 (0 to 0)
Maternal hypertensive disorders	0 (0 to 0)	0 (0 to 0)	0 (0 to 0)	0 (0 to 0)	0 (0 to 0)	0 (0 to 0)	0 (0 to 0)	0 (0 to 0)	0 (0 to 0)	0 (0 to 0)	0 (0 to 0)	0 (0 to 0)
Maternal obstructed labor and uterine rupture	--	--	--	--	--	--	--	--	--	--	--	--
Maternal abortion and miscarriage	--	--	--	--	--	--	--	--	--	--	--	--
Ectopic pregnancy	--	--	--	--	--	--	--	--	--	--	--	--
Indirect maternal deaths	--	--	--	--	--	--	--	--	--	--	--	--
Late maternal deaths	--	--	--	--	--	--	--	--	--	--	--	--
Maternal deaths aggravated by HIV/AIDS	--	--	--	--	--	--	--	--	--	--	--	--
Neonatal disorders	633 (422 to 909)	68·7 (45·4 to 101)	33·6 (21·6 to 50·7)	21·4 (16·2 to 27·8)	18·6 (13·4 to 24·5)	20·8 (15·1 to 27·1)	22·2 (15·9 to 29·7)	21·4 (15·5 to 28·2)	20·7 (14·8 to 27·5)	17·6 (12·7 to 23·4)	17·3 (12·8 to 22·6)	15·8 (11·5 to 21·3)
Neonatal preterm birth	310 (199 to 455)	23·1 (13·3 to 36·6)	13·7 (8·6 to 21·3)	7·8 (5·5 to 10·5)	10 (6·9 to 13·7)	11 (7·5 to 15·1)	11·6 (7·9 to 16·6)	10·9 (7·8 to 14·9)	10·4 (7·2 to 14·4)	8·7 (6·1 to 11·9)	8·3 (5·8 to 11·5)	7·5 (5·2 to 10·1)
Neonatal encephalopathy due to birth asphyxia and trauma	159 (89·8 to 248)	12·5 (6·3 to 21·8)	6·1 (3·1 to 10·5)	3·1 (2 to 4·7)	2·5 (1·3 to 4·1)	2·8 (1·4 to 4·6)	3 (1·6 to 5)	3 (1·6 to 5)	2·9 (1·5 to 4·8)	2·5 (1·2 to 4·3)	2·5 (1·3 to 4·2)	2·4 (1·3 to 3·9)
Neonatal sepsis and other neonatal infections	51·3 (29·3 to 86·8)	17·5 (10·4 to 27·6)	6·1 (3·8 to 9·5)	3·9 (2·3 to 5·8)	5·2 (3 to 8·1)	5·8 (3·4 to 9)	6·2 (3·5 to 9·8)	6·2 (3·5 to 9·8)	6·1 (3·6 to 9·9)	5·3 (3 to 8·4)	5·4 (2·9 to 8·2)	5 (2·8 to 7·9)
Hemolytic disease and other neonatal jaundice	2·2 (1 to 3·9)	0·4 (0·2 to 0·6)	0·2 (0·2 to 0·4)	0·8 (0·4 to 1·4)	1 (0·5 to 1·7)	1·1 (0·6 to 1·8)	1·3 (0·9 to 1·6)	1·2 (0·9 to 1·6)	1·2 (0·9 to 1·6)	1·1 (0·8 to 1·4)	1·1 (0·8 to 1·4)	1 (0·8 to 1·3)
Other neonatal disorders	111 (65·6 to 174)	15·3 (9·2 to 23·8)	7·4 (4·7 to 11·4)	5·8 (4·4 to 7·5)	0 (0 to 0)	0 (0 to 0)	0 (0 to 0)	0 (0 to 0)	0 (0 to 0)	0 (0 to 0)	0 (0 to 0)	0 (0 to 0)
Nutritional deficiencies	0·1 (0 to 0·1)	0·9 (0·4 to 1·5)	15·3 (9·6 to 24·2)	28·5 (11·9 to 55·8)	37 (11·4 to 85·6)	16·9 (5·6 to 38·1)	11·3 (4·8 to 27·7)	9·9 (4·7 to 22)	10·5 (5·1 to 20·7)	10·6 (5·2 to 20·6)	11·6 (6 to 22·4)	12 (6·6 to 22·5)
Protein-energy malnutrition	0 (0 to 0·1)	0·1 (0·1 to 0·2)	10 (6·2 to 15·8)	6·8 (3·6 to 11·1)	2·6 (1·7 to 3·8)	1·7 (1·1 to 2·4)	2·1 (1·4 to 3)	2·5 (1·6 to 3·8)	3 (1·9 to 4·6)	3·3 (2·4 to 4·4)	4·1 (2·8 to 5·5)	4·3 (3·1 to 6)
Iodine deficiency	0 (0 to 0)	0 (0 to 0)	0 (0 to 0)	0 (0 to 0)	0 (0 to 0·1)	0·1 (0 to 0·2)	0·2 (0·1 to 0·3)	0·2 (0·1 to 0·4)	0·2 (0·1 to 0·4)	0·2 (0·1 to 0·4)	0·2 (0·1 to 0·4)	0·2 (0·1 to 0·4)
Vitamin A deficiency	0 (0 to 0)	0 (0 to 0·1)	0·1 (0 to 0·3)	0·7 (0·3 to 1·4)	0·9 (0·4 to 2·1)	0·6 (0·2 to 1·3)	0·2 (0·1 to 0·5)	0·2 (0·1 to 0·4)	0·2 (0·1 to 0·4)	0·2 (0·1 to 0·3)	0·2 (0·1 to 0·3)	0·1 (0·1 to 0·3)
Dietary iron deficiency	0 (0 to 0)	0·7 (0·3 to 1·3)	5·1 (1·5 to 11·4)	20·9 (6 to 47·9)	33·2 (8·1 to 80·8)	14·3 (3·4 to 35·2)	8·3 (2 to 24·4)	6·2 (1·4 to 18·3)	6·2 (1·4 to 16·3)	6·3 (1·4 to 16·1)	6·5 (1·4 to 16·8)	6·3 (1·4 to 16·4)
Other nutritional deficiencies	0 (0 to 0)	0 (0 to 0)	0·2 (0·1 to 0·3)	0·1 (0 to 0·2)	0·2 (0·1 to 0·4)	0·2 (0·1 to 0·4)	0·5 (0·2 to 0·8)	0·7 (0·4 to 1·2)	0·9 (0·5 to 1·4)	0·6 (0·4 to 1)	0·7 (0·4 to 1·1)	1 (0·6 to 1·6)
Non-communicable diseases	92 (57·1 to 143)	22·2 (13·9 to 33·7)	86·5 (57·9 to 124)	147 (110 to 193)	193 (149 to 247)	290 (220 to 373)	466 (365 to 584)	586 (471 to 713)	716 (592 to 863)	788 (658 to 935)	981 (828 to 1,160)	1,310 (1,110 to 1,530)
Neoplasms	0·9 (0·5 to 2·1)	0·4 (0·2 to 0·6)	2·8 (1·7 to 4·3)	15·7 (9·9 to 24)	21·4 (16·8 to 26·4)	21·7 (16·8 to 27·7)	35·8 (27·8 to 45·7)	50 (40·1 to 62·3)	62·4 (51·9 to 75·6)	75·3 (62·6 to 90·2)	99·8 (82·4 to 119)	168 (137 to 205)
Lip and oral cavity cancer	0 (0 to 0)	0 (0 to 0)	0 (0 to 0)	0 (0 to 0)	0·1 (0 to 0·1)	0·1 (0·1 to 0·2)	0·3 (0·2 to 0·3)	0·6 (0·4 to 0·7)	0·7 (0·5 to 0·9)	1·4 (0·9 to 2)	2·8 (2 to 3·9)	6·7 (4·6 to 9·3)
Nasopharynx cancer	0 (0 to 0)	0 (0 to 0)	0 (0 to 0)	0 (0 to 0)	0·1 (0·1 to 0·2)	0·3 (0·2 to 0·4)	0·5 (0·3 to 0·7)	0·6 (0·4 to 0·9)	0·7 (0·5 to 1)	0·7 (0·5 to 1)	1 (0·7 to 1·4)	2·3 (1·6 to 3·3)
Other pharynx cancer	0 (0 to 0)	0 (0 to 0)	0 (0 to 0)	0 (0 to 0)	0 (0 to 0)	0 (0 to 0)	0 (0 to 0)	0·2 (0·1 to 0·3)	0·2 (0·2 to 0·4)	0·4 (0·3 to 0·6)	1 (0·7 to 1·4)	2·6 (1·7 to 3·6)
Esophageal cancer	0 (0 to 0)	0 (0 to 0)	0 (0 to 0)	0 (0 to 0)	0 (0 to 0)	0 (0 to 0)	0 (0 to 0)	0·4 (0·3 to 0·5)	0·6 (0·4 to 0·8)	1·2 (0·8 to 1·8)	3·6 (2·5 to 4·9)	10·2 (7·3 to 13·6)
Stomach cancer	0 (0 to 0)	0 (0 to 0)	0 (0 to 0)	0 (0 to 0)	0 (0 to 0)	0 (0 to 0)	0·7 (0·5 to 0·8)	1·4 (1·1 to 1·8)	3·4 (2·4 to 4·9)	7·3 (5·3 to 10)	11·6 (8·4 to 15·5)	22 (16·1 to 29·5)
Colon and rectum cancer	0 (0 to 0)	0 (0 to 0)	0 (0 to 0)	0 (0 to 0)	0·1 (0·1 to 0·2)	0·2 (0·1 to 0·2)	0·8 (0·6 to 1·1)	2 (1·4 to 2·7)	3·5 (2·3 to 5·3)	6·6 (4·7 to 8·8)	9·5 (6·7 to 13)	15·6 (11·5 to 20·5)
Liver cancer	0 (0 to 0)	0 (0 to 0)	0·1 (0 to 0·1)	0·2 (0·1 to 0·4)	0·2 (0·1 to 0·3)	0·3 (0·2 to 0·4)	0·2 (0·1 to 0·3)	0·5 (0·3 to 0·7)	1 (0·6 to 1·5)	1·2 (0·8 to 1·7)	2·1 (1·4 to 2·9)	3·6 (2·5 to 5·1)
Liver cancer due to hepatitis B	0 (0 to 0)	0 (0 to 0)	0 (0 to 0)	0 (0 to 0)	0 (0 to 0)	0·1 (0 to 0·1)	0·1 (0·1 to 0·2)	0·3 (0·2 to 0·5)	0·6 (0·3 to 1)	0·7 (0·4 to 1·1)	1·2 (0·7 to 1·8)	1·9 (1·1 to 2·9)
Liver cancer due to hepatitis C	0 (0 to 0)	0 (0 to 0)	0 (0 to 0)	0 (0 to 0)	0 (0 to 0)	0 (0 to 0)	0 (0 to 0)	0 (0 to 0)	0 (0 to 0·1)	0 (0 to 0·1)	0·1 (0 to 0·2)	0·2 (0·1 to 0·4)
Liver cancer due to alcohol use	0 (0 to 0)	0 (0 to 0)	0 (0 to 0)	0 (0 to 0)	0 (0 to 0)	0 (0 to 0)	0 (0 to 0)	0·1 (0 to 0·1)	0·2 (0·1 to 0·3)	0·3 (0·1 to 0·5)	0·6 (0·3 to 1·1)	1·2 (0·6 to 2)
Liver cancer due to NASH	0 (0 to 0)	0 (0 to 0)	0 (0 to 0)	0 (0 to 0)	0 (0 to 0)	0 (0 to 0)	0 (0 to 0·1)	0·1 (0 to 0·1)	0·1 (0 to 0·1)	0·1 (0 to 0·1)	0·1 (0 to 0·2)	0·2 (0·1 to 0·3)
Liver cancer due to other causes	0 (0 to 0)	0 (0 to 0)	0·1 (0 to 0·1)	0·2 (0·1 to 0·4)	0·2 (0·1 to 0·3)	0·2 (0·1 to 0·3)	0 (0 to 0·1)	0·1 (0 to 0·1)	0·1 (0 to 0·2)	0·1 (0 to 0·1)	0·1 (0·1 to 0·2)	0·2 (0·1 to 0·3)
Gallbladder and biliary tract cancer	0 (0 to 0)	0 (0 to 0)	0 (0 to 0)	0 (0 to 0)	0 (0 to 0)	0 (0 to 0)	0 (0 to 0)	0·1 (0·1 to 0·2)	0·2 (0·1 to 0·3)	0·4 (0·2 to 0·6)	0·6 (0·4 to 1·1)	1·3 (0·9 to 2·2)
Pancreatic cancer	0 (0 to 0)	0 (0 to 0)	0 (0 to 0)	0 (0 to 0)	0 (0 to 0)	0 (0 to 0)	0·3 (0·2 to 0·4)	0·4 (0·3 to 0·5)	0·8 (0·5 to 1·1)	2·9 (1·9 to 4·1)	5 (3·5 to 7)	11·2 (8·1 to 15·5)
Larynx cancer	0 (0 to 0)	0 (0 to 0)	0 (0 to 0)	0 (0 to 0)	0 (0 to 0)	0 (0 to 0)	0 (0 to 0)	0·3 (0·2 to 0·4)	0·3 (0·2 to 0·4)	0·5 (0·3 to 0·6)	1·4 (1 to 1·8)	4 (2·9 to 5·3)
Tracheal, bronchus, and lung cancer	0 (0 to 0)	0 (0 to 0)	0 (0 to 0)	0 (0 to 0)	0 (0 to 0)	0·1 (0·1 to 0·1)	0·7 (0·5 to 0·9)	1·1 (0·8 to 1·5)	1·6 (1 to 2·4)	3·7 (2·7 to 5·2)	7·4 (5·1 to 10·8)	17·3 (12·7 to 23)
Malignant skin melanoma	0 (0 to 0)	0 (0 to 0)	0 (0 to 0·1)	0·1 (0 to 0·5)	0·1 (0 to 0·1)	0·1 (0 to 0·1)	0·1 (0·1 to 0·2)	0·3 (0·2 to 0·6)	0·7 (0·4 to 1·2)	1 (0·6 to 1·5)	1·6 (1 to 2·5)	2·2 (1·4 to 3·6)
Non-melanoma skin cancer	0 (0 to 0)	0 (0 to 0)	0 (0 to 0)	0 (0 to 0)	0 (0 to 0)	0 (0 to 0)	0 (0 to 0)	0·1 (0·1 to 0·1)	0·2 (0·2 to 0·4)	0·4 (0·2 to 0·5)	0·4 (0·3 to 0·6)	0·7 (0·5 to 1·1)
Non-melanoma skin cancer (squamous-cell carcinoma)	0 (0 to 0)	0 (0 to 0)	0 (0 to 0)	0 (0 to 0)	0 (0 to 0)	0 (0 to 0)	0 (0 to 0)	0·1 (0·1 to 0·1)	0·2 (0·2 to 0·4)	0·4 (0·2 to 0·5)	0·4 (0·3 to 0·6)	0·7 (0·5 to 1·1)
Non-melanoma skin cancer (basal-cell carcinoma)	0 (0 to 0)	0 (0 to 0)	0 (0 to 0)	0 (0 to 0)	0 (0 to 0)	0 (0 to 0)	0 (0 to 0)	0 (0 to 0)	0 (0 to 0)	0 (0 to 0)	0 (0 to 0)	0 (0 to 0)
Breast cancer	0 (0 to 0)	0 (0 to 0)	0 (0 to 0)	0 (0 to 0)	0 (0 to 0)	0 (0 to 0)	0 (0 to 0·1)	0·1 (0 to 0·1)	0·2 (0·1 to 0·3)	0·3 (0·2 to 0·4)	0·5 (0·3 to 0·6)	0·6 (0·4 to 0·9)
Cervical cancer	--	--	--	--	--	--	--	--	--	--	--	--
Uterine cancer	--	--	--	--	--	--	--	--	--	--	--	--
Ovarian cancer	--	--	--	--	--	--	--	--	--	--	--	--
Prostate cancer	0 (0 to 0)	0 (0 to 0)	0 (0 to 0)	0 (0 to 0)	0 (0 to 0)	0 (0 to 0)	0 (0 to 0)	0·5 (0·3 to 0·6)	1·1 (0·7 to 1·5)	1·5 (0·8 to 1·9)	1·8 (1·2 to 2·4)	4·7 (3·3 to 6·5)
Testicular cancer	0·1 (0 to 1·3)	0 (0 to 0·2)	0·1 (0 to 0·4)	0·3 (0·1 to 0·8)	0·3 (0·2 to 0·5)	0·4 (0·2 to 0·6)	1·3 (0·8 to 2)	2·5 (1·5 to 3·5)	3·8 (2·2 to 5·8)	2·3 (1·4 to 3·5)	2 (1·4 to 2·9)	1·6 (1·1 to 2·5)
Kidney cancer	0·1 (0 to 0·1)	0 (0 to 0)	0·2 (0·1 to 0·2)	1·1 (0·7 to 1·7)	1 (0·7 to 1·4)	0·4 (0·3 to 0·6)	0·8 (0·5 to 1·1)	1·2 (0·8 to 1·6)	1·8 (1·2 to 2·5)	2 (1·2 to 3)	2 (1·4 to 2·8)	3·6 (2·4 to 5·3)
Bladder cancer	0 (0 to 0)	0 (0 to 0)	0 (0 to 0)	0 (0 to 0)	0 (0 to 0)	0 (0 to 0)	0·1 (0 to 0·1)	0·1 (0·1 to 0·2)	0·2 (0·2 to 0·3)	0·5 (0·4 to 0·8)	0·9 (0·6 to 1·2)	2 (1·5 to 2·7)
Brain and central nervous system cancer	0·1 (0 to 0·2)	0 (0 to 0·1)	0·5 (0·2 to 0·9)	2·3 (0·9 to 4·2)	3·3 (1·6 to 5·2)	2·8 (1·3 to 4·7)	2·5 (1 to 4)	2·7 (1 to 4·4)	3·5 (1·2 to 5·5)	4·2 (1·6 to 6·5)	5·2 (2·1 to 7·7)	6·8 (2·7 to 9·9)
Thyroid cancer	0 (0 to 0)	0 (0 to 0)	0 (0 to 0)	0 (0 to 0)	0 (0 to 0·1)	0 (0 to 0·1)	0·1 (0·1 to 0·1)	0·1 (0·1 to 0·2)	0·3 (0·2 to 0·3)	0·3 (0·2 to 0·4)	0·5 (0·3 to 0·7)	0·7 (0·4 to 1)
Mesothelioma	0 (0 to 0)	0 (0 to 0)	0 (0 to 0)	0 (0 to 0)	0 (0 to 0)	0 (0 to 0)	0 (0 to 0)	0·1 (0·1 to 0·1)	0·2 (0·1 to 0·2)	0·1 (0·1 to 0·2)	0·2 (0·2 to 0·3)	0·3 (0·2 to 0·4)
Hodgkin lymphoma	0 (0 to 0)	0 (0 to 0)	0 (0 to 0)	0·1 (0 to 0·1)	0·2 (0·1 to 0·3)	0·4 (0·3 to 0·5)	1·6 (1·1 to 2·4)	3·1 (1·9 to 4·7)	3·8 (2·3 to 5·8)	3·2 (2·1 to 4·8)	3 (2 to 4·3)	3·2 (2·1 to 4·6)
Non-Hodgkin lymphoma	0 (0 to 0)	0 (0 to 0)	0 (0 to 0)	2·1 (1·2 to 3·5)	3·8 (2·6 to 5·6)	4 (2·6 to 6)	7·1 (4·8 to 10·2)	10·7 (7·3 to 14·8)	13·3 (9·2 to 18·1)	14·2 (10·3 to 18·9)	14·7 (10·7 to 19·4)	17·3 (12·6 to 23)
Multiple myeloma	0 (0 to 0)	0 (0 to 0)	0 (0 to 0)	0 (0 to 0)	0 (0 to 0)	0 (0 to 0)	0 (0 to 0)	0·4 (0·3 to 0·6)	0·5 (0·3 to 0·7)	0·7 (0·5 to 1)	1·6 (1·1 to 2·3)	4·3 (2·9 to 6)
Leukemia	0·3 (0·2 to 0·4)	0·1 (0·1 to 0·2)	0·8 (0·5 to 1·4)	4·3 (2·3 to 7·3)	7·1 (4·8 to 10·4)	7·5 (4·5 to 10·7)	10·2 (6·9 to 14·1)	10·8 (7·3 to 15·1)	10 (7·3 to 13·1)	8·7 (6·1 to 11·9)	8·8 (6·3 to 12·6)	10·1 (7·3 to 13·4)
Acute lymphoid leukemia	0·1 (0 to 0·2)	0 (0 to 0)	0·3 (0·1 to 0·5)	2·5 (1·3 to 4·3)	4·4 (2·7 to 6·8)	3·7 (2 to 5·8)	5·5 (3·4 to 8·3)	3·8 (2·2 to 6·1)	2·2 (1·3 to 3·4)	1·6 (0·9 to 2·6)	1·4 (0·8 to 2·2)	1·3 (0·8 to 2·1)
Chronic lymphoid leukemia	0 (0 to 0)	0 (0 to 0)	0 (0 to 0)	0 (0 to 0)	0 (0 to 0)	0 (0 to 0)	0 (0 to 0)	0·3 (0·2 to 0·6)	0·4 (0·2 to 0·7)	0·3 (0·1 to 0·6)	0·3 (0·2 to 0·6)	0·8 (0·4 to 1·4)
Acute myeloid leukemia	0 (0 to 0·1)	0·1 (0 to 0·1)	0·2 (0·1 to 0·5)	1 (0·5 to 2)	1·2 (0·6 to 2·2)	1·7 (0·9 to 2·8)	2·6 (1·4 to 4·2)	2·8 (1·6 to 4·5)	3·3 (2·1 to 4·7)	2·6 (1·6 to 3·8)	2·6 (1·6 to 4)	2·3 (1·5 to 3·5)

Cause	45 to 49	50 to 54	55 to 59	60 to 64	65 to 69	70 to 74	75 to 79	80 to 84	85 to 89	90 to 94	95 plus	Total
All causes	2,300 (1,970 to 2,670)	2,650 (2,250 to 3,070)	2,730 (2,330 to 3,180)	2,660 (2,300 to 3,070)	2,140 (1,860 to 2,450)	2,410 (2,110 to 2,740)	1,750 (1,550 to 1,970)	1,330 (1,190 to 1,470)	787 (722 to 852)	357 (334 to 379)	63·1 (58·9 to 66·9)	30,100
Communicable, maternal, neonatal, and nutritional diseases	151 (128 to 181)	147 (121 to 178)	131 (104 to 161)	116 (94·3 to 143)	91·2 (72·3 to 114)	109 (85·9 to 139)	86·4 (67·8 to 110)	75·9 (59·5 to 98·3)	55·4 (44·6 to 69·1)	27·4 (21·3 to 34·1)	6·4 (5 to 7·8)	2,830
HIV/AIDS and sexually transmitted infections	30·1 (27·6 to 32·6)	22·4 (20·5 to 24·4)	14·8 (13·5 to 16·3)	7 (6·2 to 7·9)	3·8 (3·3 to 4·3)	1·6 (1·3 to 1·9)	0·7 (0·6 to 0·9)	0·4 (0·3 to 0·5)	0·2 (0·1 to 0·2)	0 (0 to 0·1)	0 (0 to 0)	248
HIV/AIDS	28·8 (26·5 to 31·1)	21·3 (19·6 to 23·1)	14 (12·8 to 15·3)	6·4 (5·8 to 7·2)	3·3 (2·9 to 3·7)	1·2 (1 to 1·4)	0·5 (0·5 to 0·6)	0·2 (0·2 to 0·3)	0·1 (0·1 to 0·1)	0 (0 to 0)	0 (0 to 0)	234
HIV/AIDS - Drug-susceptible Tuberculosis	2·8 (1·9 to 4·2)	2·1 (1·4 to 3·2)	1·3 (0·9 to 1·9)	0·6 (0·4 to 0·9)	0·3 (0·2 to 0·5)	0·1 (0·1 to 0·2)	0 (0 to 0·1)	0 (0 to 0)	0 (0 to 0)	0 (0 to 0)	0 (0 to 0)	22·3
HIV/AIDS - Multidrug-resistant Tuberculosis without extensive dru	0 (0 to 0·1)	0 (0 to 0·1)	0 (0 to 0·1)	0 (0 to 0)	0 (0 to 0)	0 (0 to 0)	0 (0 to 0)	0 (0 to 0)	0 (0 to 0)	0 (0 to 0)	0 (0 to 0)	0·3
HIV/AIDS - Extensively drug-resistant Tuberculosis	0 (0 to 0)	0 (0 to 0)	0 (0 to 0)	0 (0 to 0)	0 (0 to 0)	0 (0 to 0)	0 (0 to 0)	0 (0 to 0)	0 (0 to 0)	0 (0 to 0)	0 (0 to 0)	0
HIV/AIDS resulting in other diseases	25·9 (23·2 to 28·3)	19·1 (17·2 to 21)	12·7 (11·3 to 14)	5·8 (5·1 to 6·6)	3 (2·6 to 3·4)	1·1 (0·9 to 1·3)	0·5 (0·4 to 0·6)	0·2 (0·2 to 0·2)	0·1 (0·1 to 0·1)	0 (0 to 0)	0 (0 to 0)	211
Sexually transmitted infections excluding HIV	1·3 (0·7 to 2·2)	1·1 (0·6 to 1·9)	0·8 (0·5 to 1·4)	0·5 (0·3 to 1)	0·4 (0·2 to 0·7)	0·3 (0·2 to 0·6)	0·2 (0·1 to 0·3)	0·1 (0·1 to 0·2)	0·1 (0 to 0·1)	0 (0 to 0)	0 (0 to 0)	14·4
Syphilis	0·3 (0·1 to 0·5)	0·3 (0·1 to 0·5)	0·3 (0·2 to 0·5)	0·2 (0·1 to 0·4)	0·2 (0·1 to 0·4)	0·2 (0·1 to 0·3)	0·1 (0 to 0·1)	0 (0 to 0·1)	0 (0 to 0)	0 (0 to 0)	0 (0 to 0)	4
Chlamydial infection	0·2 (0 to 0·5)	0·1 (0 to 0·3)	0 (0 to 0·1)	0 (0 to 0)	0 (0 to 0)	0 (0 to 0)	0 (0 to 0)	0 (0 to 0)	0 (0 to 0)	0 (0 to 0)	0 (0 to 0)	2
Gonococcal infection	0·1 (0 to 0·2)	0·1 (0 to 0·1)	0 (0 to 0·1)	0 (0 to 0)	0 (0 to 0)	0 (0 to 0)	0 (0 to 0)	0 (0 to 0)	0 (0 to 0)	0 (0 to 0)	0 (0 to 0)	1·4
Trichomoniasis	0 (0 to 0·1)	0 (0 to 0·1)	0 (0 to 0)	0 (0 to 0)	0 (0 to 0)	0 (0 to 0)	0 (0 to 0)	0 (0 to 0)	0 (0 to 0)	0 (0 to 0)	0 (0 to 0)	0·3
Genital herpes	0·5 (0·2 to 1·2)	0·5 (0·1 to 1·1)	0·4 (0·1 to 0·9)	0·3 (0·1 to 0·7)	0·2 (0·1 to 0·4)	0·1 (0 to 0·3)	0·1 (0 to 0·2)	0 (0 to 0·1)	0 (0 to 0·1)	0 (0 to 0)	0 (0 to 0)	3·9
Other sexually transmitted infections	0·2 (0·1 to 0·4)	0·2 (0·1 to 0·3)	0·1 (0 to 0·1)	0 (0 to 0·1)	0 (0 to 0·1)	0 (0 to 0)	0 (0 to 0)	0 (0 to 0·1)	0 (0 to 0·1)	0 (0 to 0)	0 (0 to 0)	2·8
Respiratory infections and tuberculosis	50·6 (37·8 to 67·2)	55 (40·2 to 73·7)	55·5 (39·9 to 76)	56·9 (41·6 to 75·1)	48·3 (34·6 to 65·2)	65·6 (47·2 to 90)	56 (39·9 to 76)	53·7 (40·1 to 72·3)	41·9 (32·5 to 53·5)	21·4 (16·2 to 27·4)	5·1 (3·9 to 6·6)	810
Tuberculosis	11·6 (8 to 16·2)	11·1 (7·8 to 15·4)	10 (6·9 to 13·9)	8·5 (6·1 to 11·5)	5·8 (4 to 8·3)	6·4 (4·6 to 8·5)	4·3 (3·1 to 5·9)	3 (2·2 to 4·1)	1·6 (1·2 to 2·3)	0·6 (0·4 to 0·8)	0·1 (0·1 to 0·1)	114
Latent tuberculosis infection	0 (0 to 0)	0 (0 to 0)	0 (0 to 0)	0 (0 to 0)	0 (0 to 0)	0 (0 to 0)	0 (0 to 0)	0 (0 to 0)	0 (0 to 0)	0 (0 to 0)	0 (0 to 0)	0
Drug-susceptible tuberculosis	11·4 (7·9 to 15·9)	11 (7·7 to 15·4)	9·9 (6·8 to 13·7)	8·4 (6 to 11·4)	5·8 (4 to 8·2)	6·3 (4·5 to 8·5)	4·3 (3 to 5·8)	3 (2·2 to 4)	1·6 (1·2 to 2·3)	0·6 (0·4 to 0·8)	0·1 (0·1 to 0·1)	113
Multidrug-resistant tuberculosis without extensive drug resistanc	0·1 (0 to 0·4)	0·1 (0 to 0·5)	0·1 (0 to 0·4)	0·1 (0 to 0·3)	0·1 (0 to 0·2)	0·1 (0 to 0·3)	0 (0 to 0·2)	0 (0 to 0·1)	0 (0 to 0·1)	0 (0 to 0)	0 (0 to 0)	1·1
Extensively drug-resistant tuberculosis	0 (0 to 0·1)	0 (0 to 0·1)	0 (0 to 0·1)	0 (0 to 0·1)	0 (0 to 0)	0 (0 to 0)	0 (0 to 0)	0 (0 to 0)	0 (0 to 0)	0 (0 to 0)	0 (0 to 0)	0·2
Lower respiratory infections	32·8 (21·8 to 46·4)	38·2 (24·9 to 55·2)	41 (26·5 to 59·8)	45·2 (30·8 to 63·2)	40·3 (27·1 to 57·6)	57·5 (39·3 to 80·7)	50·7 (35 to 70·5)	50·1 (36·7 to 68·6)	40 (30·6 to 51·3)	20·7 (15·6 to 26·7)	5 (3·8 to 6·4)	601
Upper respiratory infections	4·6 (2·5 to 7·8)	4·1 (2·3 to 6·9)	3·3 (1·8 to 5·4)	2·5 (1·3 to 4)	1·7 (0·9 to 2·9)	1·3 (0·8 to 2·2)	0·8 (0·4 to 1·3)	0·4 (0·2 to 0·7)	0·2 (0·1 to 0·3)	0·1 (0·1 to 0·1)	0 (0 to 0)	70·6
Otitis media	1·6 (0·7 to 2·8)	1·6 (0·7 to 2·8)	1·2 (0·6 to 2·1)	0·8 (0·4 to 1·6)	0·5 (0·2 to 0·9)	0·3 (0·1 to 0·6)	0·2 (0·1 to 0·4)	0·1 (0·1 to 0·2)	0·1 (0 to 0·1)	0 (0 to 0)	0 (0 to 0)	24·1
Enteric infections	16 (11·7 to 21·7)	16·8 (11·8 to 23)	15·2 (10·8 to 20·7)	14·7 (10·9 to 19·7)	13·8 (10·1 to 18·4)	16·3 (11·9 to 21)	12·7 (9·5 to 16·7)	9·5 (7 to 12·8)	5·1 (3·7 to 6·8)	2·1 (1·6 to 2·8)	0·4 (0·3 to 0·5)	258
Diarrheal diseases	16 (11·6 to 21·7)	16·7 (11·8 to 22·9)	15·2 (10·7 to 20·7)	14·6 (10·8 to 19·7)	13·8 (10·1 to 18·3)	16·2 (11·9 to 20·9)	12·6 (9·5 to 16·6)	9·5 (7 to 12·8)	5·1 (3·7 to 6·8)	2·1 (1·6 to 2·8)	0·4 (0·3 to 0·5)	257
Typhoid and paratyphoid	0 (0 to 0)	0 (0 to 0)	0 (0 to 0·1)	0 (0 to 0)	0 (0 to 0)	0 (0 to 0)	0 (0 to 0)	0 (0 to 0)	0 (0 to 0)	0 (0 to 0)	0 (0 to 0)	0·1
Typhoid fever	0 (0 to 0)	0 (0 to 0)	0 (0 to 0)	0 (0 to 0)	0 (0 to 0)	0 (0 to 0)	0 (0 to 0)	0 (0 to 0)	0 (0 to 0)	0 (0 to 0)	0 (0 to 0)	0
Paratyphoid fever	0 (0 to 0)	0 (0 to 0)	0 (0 to 0·1)	0 (0 to 0)	0 (0 to 0)	0 (0 to 0)	0 (0 to 0)	0 (0 to 0)	0 (0 to 0)	0 (0 to 0)	0 (0 to 0)	0·1
Invasive Non-typhoidal Salmonella (iNTS)	0 (0 to 0)	0 (0 to 0)	0 (0 to 0)	0 (0 to 0)	0 (0 to 0)	0 (0 to 0)	0 (0 to 0)	0 (0 to 0)	0 (0 to 0)	0 (0 to 0)	0 (0 to 0)	0·1
Other intestinal infectious diseases	0 (0 to 0)	0 (0 to 0)	0 (0 to 0)	0 (0 to 0)	0 (0 to 0)	0 (0 to 0)	0 (0 to 0)	0 (0 to 0)	0 (0 to 0)	0 (0 to 0)	0 (0 to 0)	0·4
Neglected tropical diseases and malaria	6·7 (3·5 to 12·4)	7·2 (3·5 to 13·3)	7·3 (3·5 to 13·5)	7·1 (3·4 to 13·1)	5·9 (2·8 to 10·9)	6 (2·9 to 10·8)	4·3 (2 to 7·7)	2·8 (1·3 to 5)	1·4 (0·7 to 2·5)	0·6 (0·3 to 1)	0·1 (0 to 0·2)	89·6
Malaria	0 (0 to 0)	0 (0 to 0)	0 (0 to 0)	0 (0 to 0)	0 (0 to 0)	0 (0 to 0)	0 (0 to 0)	0 (0 to 0)	0 (0 to 0)	0 (0 to 0)	0 (0 to 0)	0
Chagas disease	0 (0 to 0)	0 (0 to 0)	0 (0 to 0)	0 (0 to 0)	0 (0 to 0)	0 (0 to 0)	0 (0 to 0)	0 (0 to 0)	0 (0 to 0)	0 (0 to 0)	0 (0 to 0)	0
Leishmaniasis	0 (0 to 0)	0 (0 to 0)	0 (0 to 0)	0 (0 to 0)	0 (0 to 0)	0 (0 to 0)	0 (0 to 0)	0 (0 to 0)	0 (0 to 0)	0 (0 to 0)	0 (0 to 0)	0
Visceral leishmaniasis	0 (0 to 0)	0 (0 to 0)	0 (0 to 0)	0 (0 to 0)	0 (0 to 0)	0 (0 to 0)	0 (0 to 0)	0 (0 to 0)	0 (0 to 0)	0 (0 to 0)	0 (0 to 0)	0
Cutaneous and mucocutaneous leishmaniasis	0 (0 to 0)	0 (0 to 0)	0 (0 to 0)	0 (0 to 0)	0 (0 to 0)	0 (0 to 0)	0 (0 to 0)	0 (0 to 0)	0 (0 to 0)	0 (0 to 0)	0 (0 to 0)	0
African trypanosomiasis	0 (0 to 0)	0 (0 to 0)	0 (0 to 0)	0 (0 to 0)	0 (0 to 0)	0 (0 to 0)	0 (0 to 0)	0 (0 to 0)	0 (0 to 0)	0 (0 to 0)	0 (0 to 0)	0
Schistosomiasis	0·1 (0 to 0·5)	0·1 (0 to 0·5)	0·1 (0 to 0·6)	0·1 (0 to 0·6)	0·1 (0 to 0·3)	0·1 (0 to 0·4)	0 (0 to 0·3)	0 (0 to 0·2)	0 (0 to 0·1)	0 (0 to 0)	0 (0 to 0)	0·7
Cysticercosis	4·3 (1·4 to 9·6)	5·1 (1·5 to 11·1)	5·5 (2 to 11·7)	5·6 (2 to 11·5)	5 (1·8 to 9·8)	4·9 (1·9 to 9·7)	3·5 (1·3 to 6·9)	2·4 (0·9 to 4·5)	1·1 (0·5 to 2·2)	0·4 (0·2 to 0·8)	0·1 (0 to 0·1)	49·5
Cystic echinococcosis	0 (0 to 0)	0 (0 to 0)	0 (0 to 0)	0 (0 to 0)	0 (0 to 0)	0 (0 to 0)	0 (0 to 0)	0 (0 to 0)	0 (0 to 0)	0 (0 to 0)	0 (0 to 0)	0
Lymphatic filariasis	0 (0 to 0)	0 (0 to 0)	0 (0 to 0)	0 (0 to 0)	0 (0 to 0)	0 (0 to 0)	0 (0 to 0)	0 (0 to 0)	0 (0 to 0)	0 (0 to 0)	0 (0 to 0)	0
Onchocerciasis	0 (0 to 0)	0 (0 to 0)	0 (0 to 0)	0 (0 to 0)	0 (0 to 0)	0 (0 to 0)	0 (0 to 0)	0 (0 to 0)	0 (0 to 0)	0 (0 to 0)	0 (0 to 0)	0
Trachoma	0 (0 to 0)	0 (0 to 0)	0 (0 to 0)	0 (0 to 0)	0 (0 to 0)	0 (0 to 0)	0 (0 to 0)	0 (0 to 0)	0 (0 to 0)	0 (0 to 0)	0 (0 to 0)	0
Dengue	1 (0·4 to 1·9)	0·9 (0·4 to 1·9)	0·7 (0·2 to 1·4)	0·8 (0·2 to 1·8)	0·5 (0·1 to 1·1)	0·4 (0·1 to 0·9)	0·3 (0·1 to 0·7)	0·2 (0·1 to 0·3)	0·1 (0 to 0·2)	0·1 (0 to 0·1)	0 (0 to 0)	15·6
Yellow fever	0 (0 to 0)	0 (0 to 0)	0 (0 to 0)	0 (0 to 0)	0 (0 to 0)	0 (0 to 0)	0 (0 to 0)	0 (0 to 0)	0 (0 to 0)	0 (0 to 0)	0 (0 to 0)	0
Rabies	0 (0 to 0·1)	0 (0 to 0)	0 (0 to 0)	0 (0 to 0)	0 (0 to 0)	0 (0 to 0)	0 (0 to 0)	0 (0 to 0)	0 (0 to 0)	0 (0 to 0)	0 (0 to 0)	0·2
Intestinal nematode infections	0·2 (0·1 to 0·4)	0·2 (0·1 to 0·4)	0·2 (0·1 to 0·3)	0·1 (0·1 to 0·3)	0·1 (0 to 0·2)	0·1 (0 to 0·1)	0·1 (0 to 0·1)	0 (0 to 0·1)	0 (0 to 0)	0 (0 to 0)	0 (0 to 0)	4·2
Ascariasis	0·1 (0 to 0·2)	0·1 (0 to 0·2)	0·1 (0 to 0·1)	0·1 (0 to 0·1)	0 (0 to 0·1)	0 (0 to 0·1)	0 (0 to 0)	0 (0 to 0)	0 (0 to 0)	0 (0 to 0)	0 (0 to 0)	1·6
Trichuriasis	0 (0 to 0·1)	0 (0 to 0·1)	0 (0 to 0·1)	0 (0 to 0)	0 (0 to 0)	0 (0 to 0)	0 (0 to 0)	0 (0 to 0)	0 (0 to 0)	0 (0 to 0)	0 (0 to 0)	0·8
Hookworm disease	0·1 (0 to 0·2)	0·1 (0 to 0·2)	0·1 (0 to 0·2)	0·1 (0 to 0·1)	0 (0 to 0·1)	0 (0 to 0·1)	0 (0 to 0·1)	0 (0 to 0)	0 (0 to 0)	0 (0 to 0)	0 (0 to 0)	1·8
Food-borne trematodiases	0 (0 to 0)	0 (0 to 0)	0 (0 to 0)	0 (0 to 0)	0 (0 to 0)	0 (0 to 0)	0 (0 to 0)	0 (0 to 0)	0 (0 to 0)	0 (0 to 0)	0 (0 to 0)	0
Leprosy	0·1 (0 to 0·2)	0·1 (0 to 0·2)	0·1 (0 to 0·2)	0·1 (0 to 0·1)	0·1 (0 to 0·1)	0·1 (0 to 0·1)	0 (0 to 0·1)	0 (0 to 0)	0 (0 to 0)	0 (0 to 0)	0 (0 to 0)	0·6
Ebola	0 (0 to 0)	0 (0 to 0)	0 (0 to 0)	0 (0 to 0)	0 (0 to 0)	0 (0 to 0)	0 (0 to 0)	0 (0 to 0)	0 (0 to 0)	0 (0 to 0)	0 (0 to 0)	0
Zika virus	0·1 (0 to 0·5)	0·1 (0 to 0·4)	0·1 (0 to 0·3)	0 (0 to 0·2)	0 (0 to 0·1)	0 (0 to 0)	0 (0 to 0)	0 (0 to 0)	0 (0 to 0)	0 (0 to 0)	0 (0 to 0)	2
Guinea worm disease	0 (0 to 0)	0 (0 to 0)	0 (0 to 0)	0 (0 to 0)	0 (0 to 0)	0 (0 to 0)	0 (0 to 0)	0 (0 to 0)	0 (0 to 0)	0 (0 to 0)	0 (0 to 0)	0
Other neglected tropical diseases	0·9 (0·4 to 2)	0·7 (0·3 to 1·5)	0·7 (0·2 to 1·4)	0·5 (0·2 to 0·9)	0·3 (0·1 to 0·5)	0·5 (0·2 to 1·1)	0·3 (0·1 to 0·7)	0·1 (0·1 to 0·3)	0·2 (0·1 to 0·3)	0·1 (0 to 0·1)	0 (0 to 0)	16·8
Other infectious diseases	19 (12·8 to 27)	16·7 (11·4 to 23·2)	12·9 (9·1 to 18)	11·1 (7·7 to 15·3)	7·1 (4·9 to 9·6)	7 (4·9 to 9·7)	3·9 (2·7 to 5·3)	2·5 (1·8 to 3·4)	1·9 (1·2 to 2·7)	0·6 (0·4 to 0·9)	0·1 (0·1 to 0·2)	199
Meningitis	3·4 (2·3 to 5)	3·3 (2·2 to 4·7)	2·6 (1·7 to 3·6)	2·4 (1·6 to 3·4)	1·4 (0·9 to 2)	1·4 (0·9 to 2·1)	0·9 (0·6 to 1·3)	0·6 (0·4 to 0·8)	0·4 (0·2 to 0·5)	0·1 (0·1 to 0·1)	0 (0 to 0)	48·5
Encephalitis	2·2 (1·5 to 3·3)	1·6 (1·1 to 2·5)	1·4 (0·9 to 2·1)	1·1 (0·8 to 1·7)	0·7 (0·5 to 1·1)	0·7 (0·4 to 1·1)	0·3 (0·2 to 0·5)	0·2 (0·1 to 0·4)	0·1 (0·1 to 0·2)	0 (0 to 0)	0 (0 to 0)	32·9
Diphtheria	0 (0 to 0)	0 (0 to 0)	0 (0 to 0)	0 (0 to 0)	0 (0 to 0)	0 (0 to 0)	0 (0 to 0)	0 (0 to 0)	0 (0 to 0)	0 (0 to 0)	0 (0 to 0)	0
Tetanus	0·5 (0·1 to 1·6)	0·6 (0·1 to 2·2)	0·6 (0·1 to 1·9)	0·4 (0·1 to 1·4)	0·3 (0·1 to 1·1)	0·3 (0·1 to 1·1)	0·2 (0·1 to 0·6)	0·1 (0 to 0·3)	0 (0 to 0·1)	0 (0 to 0·1)	0 (0 to 0)	4·4
Measles	0 (0 to 0)	0 (0 to 0)	0 (0 to 0)	0 (0 to 0)	0 (0 to 0)	0 (0 to 0)	0 (0 to 0)	0 (0 to 0)	0 (0 to 0)	0 (0 to 0)	0 (0 to 0)	0
Varicella and herpes zoster	1 (0·5 to 1·9)	0·8 (0·4 to 1·5)	0·6 (0·3 to 1·1)	0·7 (0·4 to 1·2)	0·3 (0·2 to 0·6)	0·6 (0·3 to 1·2)	0·4 (0·2 to 0·8)	0·3 (0·1 to 0·6)	0·1 (0·1 to 0·3)	0·1 (0 to 0·2)	0 (0 to 0·1)	10·8
Acute hepatitis	0·4 (0·3 to 0·6)	0·4 (0·3 to 0·5)	0·2 (0·2 to 0·3)	0·2 (0·1 to 0·2)	0·1 (0·1 to 0·1)	0·1 (0·1 to 0·1)	0 (0 to 0·1)	0 (0 to 0)	0 (0 to 0)	0 (0 to 0)	0 (0 to 0)	5·6
Acute hepatitis A	0·2 (0·1 to 0·3)	0·1 (0 to 0·2)	0·1 (0 to 0·1)	0 (0 to 0·1)	0 (0 to 0)	0 (0 to 0)	0 (0 to 0)	0 (0 to 0)	0 (0 to 0)	0 (0 to 0)	0 (0 to 0)	3·1
Acute hepatitis B	0·2 (0·1 to 0·3)	0·2 (0·1 to 0·3)	0·1 (0·1 to 0·2)	0·1 (0 to 0·1)	0·1 (0 to 0·1)	0·1 (0 to 0·1)	0 (0 to 0)	0 (0 to 0)	0 (0 to 0)	0 (0 to 0)	0 (0 to 0)	1·7
Acute hepatitis C	0 (0 to 0·1)	0 (0 to 0·1)	0 (0 to 0)	0 (0 to 0)	0 (0 to 0)	0 (0 to 0)	0 (0 to 0)	0 (0 to 0)	0 (0 to 0)	0 (0 to 0)	0 (0 to 0)	0·2
Acute hepatitis E	0 (0 to 0·1)	0 (0 to 0)	0 (0 to 0)	0 (0 to 0·1)	0 (0 to 0)	0 (0 to 0)	0 (0 to 0)	0 (0 to 0)	0 (0 to 0)	0 (0 to 0)	0 (0 to 0)	0·6
Other unspecified infectious diseases	11·5 (6·1 to 18·9)	10 (5·7 to 15·8)	7·5 (4·2 to 11·6)	6·4 (3·5 to 9·9)	4·3 (2·3 to 6·4)	3·9 (2·1 to 5·9)	2 (1 to 3·1)	1·3 (0·6 to 2)	1·3 (0·6 to 2)	0·4 (0·2 to 0·7)	0·1 (0 to 0·1)	94·9
Maternal and neonatal disorders	14·4 (10·4 to 19·1)	12·2 (8·8 to 16·2)	8·5 (6·1 to 11·2)	5 (3·6 to 6·7)	2·4 (1·8 to 3·3)	1·2 (0·9 to 1·7)	0·4 (0·3 to 0·6)	0·1 (0·1 to 0·1)	0 (0 to 0)	0 (0 to 0)	0 (0 to 0)	955
Maternal disorders	0 (0 to 0)	0 (0 to 0)	0 (0 to 0)	0 (0 to 0)	0 (0 to 0)	0 (0 to 0)	0 (0 to 0)	0 (0 to 0)	0 (0 to 0)	0 (0 to 0)	0 (0 to 0)	0
Maternal hemorrhage	0 (0 to 0)	0 (0 to 0)	0 (0 to 0)	0 (0 to 0)	0 (0 to 0)	0 (0 to 0)	0 (0 to 0)	0 (0 to 0)	0 (0 to 0)	0 (0 to 0)	0 (0 to 0)	0
Maternal sepsis and other maternal infections	0 (0 to 0)	0 (0 to 0)	0 (0 to 0)	0 (0 to 0)	0 (0 to 0)	0 (0 to 0)	0 (0 to 0)	0 (0 to 0)	0 (0 to 0)	0 (0 to 0)	0 (0 to 0)	0
Maternal hypertensive disorders	0 (0 to 0)	0 (0 to 0)	0 (0 to 0)	0 (0 to 0)	0 (0 to 0)	0 (0 to 0)	0 (0 to 0)	0 (0 to 0)	0 (0 to 0)	0 (0 to 0)	0 (0 to 0)	0
Maternal obstructed labor and uterine rupture	--	--	--	--	--	--	--	--	--	--	--	NA
Maternal abortion and miscarriage	--	--	--	--	--	--	--	--	--	--	--	NA
Ectopic pregnancy	--	--	--	--	--	--	--	--	--	--	--	NA
Indirect maternal deaths	--	--	--	--	--	--	--	--	--	--	--	NA
Late maternal deaths	--	--	--	--	--	--	--	--	--	--	--	NA
Maternal deaths aggravated by HIV/AIDS	--	--	--	--	--	--	--	--	--	--	--	NA
Neonatal disorders	14·4 (10·4 to 19·1)	12·2 (8·8 to 16·2)	8·5 (6·1 to 11·2)	5 (3·6 to 6·7)	2·4 (1·8 to 3·3)	1·2 (0·9 to 1·7)	0·4 (0·3 to 0·6)	0·1 (0·1 to 0·1)	0 (0 to 0)	0 (0 to 0)	0 (0 to 0)	955
Neonatal preterm birth	6·5 (4·4 to 8·9)	5·3 (3·6 to 7·4)	3·5 (2·4 to 4·8)	1·9 (1·4 to 2·4)	0·8 (0·6 to 1·1)	0·4 (0·3 to 0·6)	0·2 (0·1 to 0·2)	0 (0 to 0·1)	0 (0 to 0)	0 (0 to 0)	0 (0 to 0)	451
Neonatal encephalopathy due to birth asphyxia and trauma	2·2 (1·1 to 3·9)	1·9 (0·9 to 3·3)	1·4 (0·7 to 2·5)	0·9 (0·5 to 1·6)	0·5 (0·3 to 0·7)	0·2 (0·1 to 0·3)	0 (0 to 0·1)	0 (0 to 0)	0 (0 to 0)	0 (0 to 0)	0 (0 to 0)	209
Neonatal sepsis and other neonatal infections	4·7 (2·7 to 7·4)	4·1 (2·3 to 6·7)	3 (1·6 to 4·8)	1·8 (1 to 3)	1 (0·5 to 1·6)	0·6 (0·3 to 1)	0·2 (0·1 to 0·3)	0 (0 to 0·1)	0 (0 to 0)	0 (0 to 0)	0 (0 to 0)	139
Hemolytic disease and other neonatal jaundice	1 (0·7 to 1·3)	0·9 (0·6 to 1·1)	0·6 (0·5 to 0·8)	0·4 (0·3 to 0·5)	0·2 (0·2 to 0·3)	0·1 (0·1 to 0·2)	0 (0 to 0·1)	0 (0 to 0)	0 (0 to 0)	0 (0 to 0)	0 (0 to 0)	15·8
Other neonatal disorders	0 (0 to 0)	0 (0 to 0)	0 (0 to 0)	0 (0 to 0)	0 (0 to 0)	0 (0 to 0)	0 (0 to 0)	0 (0 to 0)	0 (0 to 0)	0 (0 to 0)	0 (0 to 0)	139
Nutritional deficiencies	14·4 (7·7 to 27·3)	16·8 (9·1 to 30·1)	16·7 (9·1 to 29·7)	14·3 (8·5 to 24·4)	9·9 (6·3 to 15·8)	11·4 (7·2 to 16·9)	8·4 (5·8 to 12)	6·8 (4·6 to 9·8)	4·9 (3·3 to 6·9)	2·5 (1·8 to 3·3)	0·6 (0·4 to 0·8)	271
Protein-energy malnutrition	5·5 (3·7 to 7·7)	6·3 (4·3 to 9)	6 (4·2 to 8·4)	6·4 (4·5 to 8·9)	5·6 (4 to 7·7)	7·5 (4·8 to 11·3)	5·8 (4 to 8·2)	4·9 (3·2 to 7·1)	3·5 (2·4 to 4·9)	2 (1·4 to 2·7)	0·5 (0·4 to 0·7)	94·6
Iodine deficiency	0·2 (0·1 to 0·4)	0·2 (0·1 to 0·4)	0·2 (0·1 to 0·3)	0·1 (0·1 to 0·2)	0·1 (0 to 0·1)	0·1 (0 to 0·1)	0 (0 to 0·1)	0 (0 to 0)	0 (0 to 0)	0 (0 to 0)	0 (0 to 0)	2·3
Vitamin A deficiency	0·1 (0·1 to 0·3)	0·1 (0·1 to 0·2)	0·1 (0·1 to 0·2)	0·1 (0 to 0·1)	0 (0 to 0·1)	0 (0 to 0·1)	0 (0 to 0·1)	0 (0 to 0)	0 (0 to 0)	0 (0 to 0)	0 (0 to 0)	4·1
Dietary iron deficiency	7·7 (1·8 to 20·3)	9 (2 to 22·1)	9·4 (2·6 to 23·3)	6·8 (1·8 to 16·8)	3·5 (0·9 to 9·2)	3 (0·8 to 7·1)	2·1 (0·5 to 4·9)	1·4 (0·4 to 3·6)	1·1 (0·3 to 2·5)	0·4 (0·1 to 0·8)	0·1 (0 to 0·1)	159
Other nutritional deficiencies	0·8 (0·5 to 1·3)	1·2 (0·7 to 1·8)	1 (0·6 to 1·6)	0·9 (0·5 to 1·4)	0·7 (0·4 to 1·1)	0·8 (0·4 to 1·4)	0·5 (0·3 to 0·8)	0·5 (0·2 to 0·9)	0·3 (0·1 to 0·5)	0·2 (0·1 to 0·3)	0 (0 to 0·1)	11·8
Non-communicable diseases	1,740 (1,480 to 2,030)	2,170 (1,840 to 2,530)	2,350 (2,000 to 2,730)	2,370 (2,040 to 2,740)	1,940 (1,690 to 2,220)	2,200 (1,920 to 2,510)	1,600 (1,410 to 1,800)	1,210 (1,080 to 1,340)	703 (642 to 765)	316 (295 to 338)	54·2 (50·4 to 57·8)	22,300
Neoplasms	280 (223 to 351)	398 (322 to 492)	481 (387 to 594)	526 (422 to 638)	450 (367 to 549)	543 (446 to 668)	399 (327 to 489)	283 (230 to 345)	168 (139 to 205)	65 (51·9 to 77·9)	9·4 (7·3 to 11·5)	4,160
Lip and oral cavity cancer	13·3 (9·4 to 18·9)	18·8 (13·3 to 25·5)	20·7 (14·8 to 27·9)	18·5 (12·7 to 25·5)	12·3 (9·1 to 16·4)	12·1 (8·7 to 16·1)	7·6 (5·4 to 10·2)	4·8 (3·6 to 6·4)	2 (1·6 to 2·6)	0·8 (0·7 to 1·1)	0·1 (0·1 to 0·2)	124
Nasopharynx cancer	3·7 (2·6 to 5·1)	5·3 (3·7 to 7·4)	5·7 (4·1 to 7·8)	4 (2·8 to 5·5)	2·6 (2 to 3·5)	2·5 (1·8 to 3·3)	1·1 (0·8 to 1·6)	0·7 (0·5 to 0·9)	0·6 (0·4 to 0·7)	0·2 (0·2 to 0·3)	0 (0 to 0)	32·7
Other pharynx cancer	6·7 (4·7 to 9·3)	9·2 (6·5 to 12·9)	10·3 (7·3 to 14·1)	8·9 (6 to 12·2)	5·8 (4·1 to 8·1)	5 (3·5 to 7)	2·7 (1·9 to 3·6)	1·6 (1·1 to 2·1)	0·8 (0·6 to 1·1)	0·3 (0·2 to 0·4)	0·1 (0 to 0·1)	55·8
Esophageal cancer	22·6 (16·5 to 30·1)	31·3 (23·2 to 41·7)	36·2 (25·7 to 49)	33 (23·4 to 43·6)	22·5 (16·1 to 30·2)	21·8 (15·7 to 29·5)	12·3 (8·8 to 16·6)	7·5 (5·4 to 10·3)	3·9 (2·8 to 5)	1·3 (1 to 1·7)	0·1 (0·1 to 0·2)	208
Stomach cancer	31·9 (23·2 to 42·8)	46·2 (33·8 to 60·4)	55 (41·1 to 72)	56·3 (41·7 to 74·1)	43·1 (31·8 to 57·9)	49·2 (36·2 to 64·4)	31·9 (23·5 to 42·5)	20·2 (15·1 to 26·6)	10·9 (8·5 to 13·9)	4 (3·2 to 4·9)	0·5 (0·4 to 0·6)	396
Colon and rectum cancer	23·8 (17·5 to 31·7)	32·3 (23·6 to 43·8)	39·8 (28·7 to 54·3)	43·5 (31·8 to 58·2)	34·8 (25·3 to 47·1)	39·8 (29·2 to 53)	27·5 (20·3 to 36·8)	18·3 (13·6 to 24·5)	9·6 (7·4 to 12·2)	3·6 (2·8 to 4·5)	0·5 (0·4 to 0·6)	312
Liver cancer	5·7 (3·9 to 8·2)	8·1 (5·2 to 11·7)	10·3 (7·3 to 14·4)	11·6 (8·2 to 15·8)	9·8 (6·9 to 13·7)	11 (7·7 to 15·4)	7 (5 to 9·7)	3·7 (2·6 to 5·1)	1·4 (1 to 2)	0·4 (0·3 to 0·6)	0·1 (0·1 to 0·1)	78·4
Liver cancer due to hepatitis B	2·6 (1·4 to 4·2)	3·3 (1·7 to 5·5)	3·8 (2·1 to 6)	3·6 (1·9 to 6·1)	2·5 (1·2 to 4·2)	2·3 (1·1 to 4·1)	1·3 (0·7 to 2·4)	0·7 (0·3 to 1·1)	0·2 (0·1 to 0·4)	0·1 (0 to 0·1)	0 (0 to 0)	25·3
Liver cancer due to hepatitis C	0·4 (0·2 to 0·9)	0·8 (0·3 to 1·5)	1·2 (0·5 to 2·5)	1·5 (0·7 to 2·9)	1·4 (0·7 to 2·4)	1·7 (0·8 to 3·1)	1·3 (0·6 to 2·4)	0·9 (0·5 to 1·5)	0·4 (0·2 to 0·7)	0·1 (0·1 to 0·3)	0 (0 to 0)	10·2
Liver cancer due to alcohol use	2·1 (1 to 3·7)	3·3 (1·7 to 5·2)	4·4 (2·3 to 7·1)	5·4 (3·1 to 8·3)	5·2 (3·2 to 7·6)	5·9 (3·5 to 9)	3·5 (2·2 to 5·1)	1·7 (1 to 2·5)	0·6 (0·3 to 0·9)	0·2 (0·1 to 0·2)	0 (0 to 0)	34·5
Liver cancer due to NASH	0·3 (0·1 to 0·6)	0·4 (0·2 to 0·8)	0·5 (0·3 to 1)	0·6 (0·3 to 1·2)	0·6 (0·3 to 1·1)	0·7 (0·4 to 1·4)	0·6 (0·3 to 1)	0·4 (0·2 to 0·6)	0·1 (0·1 to 0·2)	0 (0 to 0·1)	0 (0 to 0)	4·8
Liver cancer due to other causes	0·3 (0·1 to 0·5)	0·3 (0·1 to 0·6)	0·4 (0·2 to 0·7)	0·4 (0·2 to 0·7)	0·3 (0·1 to 0·5)	0·3 (0·1 to 0·6)	0·2 (0·1 to 0·4)	0·1 (0·1 to 0·2)	0 (0 to 0·1)	0 (0 to 0)	0 (0 to 0)	3·6
Gallbladder and biliary tract cancer	2 (1·4 to 3·5)	3 (2·1 to 5·6)	3·7 (2·6 to 6·7)	4 (2·8 to 7·1)	3·3 (2·3 to 6·1)	3·6 (2·5 to 6·5)	2·3 (1·6 to 4·2)	1·5 (1 to 2·6)	0·8 (0·6 to 1·4)	0·3 (0·2 to 0·5)	0 (0 to 0·1)	27·2
Pancreatic cancer	19·2 (13·9 to 26·3)	25·2 (18·6 to 33)	30 (21·9 to 39·5)	29·3 (21 to 39·7)	22 (16 to 29·4)	23·7 (17 to 31·4)	13·9 (9·9 to 18·7)	8·8 (6·5 to 11·8)	4·2 (3·3 to 5·4)	1·5 (1·2 to 1·9)	0·2 (0·2 to 0·2)	199
Larynx cancer	9·8 (6·6 to 14·1)	16 (11·7 to 21·3)	18·3 (13·4 to 24·4)	17·7 (13·3 to 22·4)	12·9 (9·7 to 16·7)	12·4 (9·4 to 16·1)	7·2 (5·4 to 9·6)	4·2 (3·2 to 5·5)	1·9 (1·4 to 2·4)	0·6 (0·5 to 0·8)	0·1 (0·1 to 0·1)	107
Tracheal, bronchus, and lung cancer	37·8 (27 to 53)	56·4 (40·1 to 78·4)	67·2 (46·8 to 92·1)	67·7 (48·5 to 90·3)	51·8 (37·5 to 68·6)	56·2 (42·1 to 74·9)	32·5 (24·6 to 42·5)	19·3 (14·2 to 25·7)	9·2 (6·7 to 12)	3 (2·4 to 3·6)	0·4 (0·3 to 0·4)	433
Malignant skin melanoma	2·6 (1·5 to 4·3)	3·1 (2 to 5)	3·2 (1·9 to 5·3)	2·3 (1·4 to 3·6)	1·6 (1 to 2·5)	1·8 (1·1 to 2·9)	1·2 (0·8 to 1·9)	0·9 (0·6 to 1·5)	0·5 (0·3 to 0·8)	0·2 (0·1 to 0·3)	0 (0 to 0·1)	23·7
Non-melanoma skin cancer	1 (0·7 to 1·4)	1·4 (1 to 1·8)	1·4 (1 to 2)	1·6 (1·1 to 2)	1·4 (1 to 1·8)	1·5 (1·1 to 2)	1·2 (0·9 to 1·6)	1·1 (0·8 to 1·4)	0·7 (0·6 to 1)	0·4 (0·3 to 0·6)	0·1 (0·1 to 0·1)	13·6
Non-melanoma skin cancer (squamous-cell carcinoma)	1 (0·7 to 1·4)	1·4 (0·9 to 1·8)	1·4 (1 to 2)	1·6 (1·1 to 2)	1·4 (1 to 1·8)	1·5 (1·1 to 2)	1·2 (0·9 to 1·6)	1·1 (0·8 to 1·4)	0·7 (0·6 to 1)	0·4 (0·3 to 0·6)	0·1 (0·1 to 0·1)	13·6
Non-melanoma skin cancer (basal-cell carcinoma)	0 (0 to 0)	0 (0 to 0)	0 (0 to 0)	0 (0 to 0)	0 (0 to 0)	0 (0 to 0)	0 (0 to 0)	0 (0 to 0)	0 (0 to 0)	0 (0 to 0)	0 (0 to 0)	0
Breast cancer	1·8 (1·2 to 2·6)	2·2 (1·4 to 3·2)	2·4 (1·5 to 3·6)	2·4 (1·5 to 3·5)	2·1 (1·4 to 3)	2 (1·3 to 3)	1·3 (0·9 to 1·9)	0·9 (0·6 to 1·4)	0·9 (0·6 to 1·3)	0·4 (0·3 to 0·5)	0 (0 to 0·1)	18
Cervical cancer	--	--	--	--	--	--	--	--	--	--	--	NA
Uterine cancer	--	--	--	--	--	--	--	--	--	--	--	NA
Ovarian cancer	--	--	--	--	--	--	--	--	--	--	--	NA
Prostate cancer	15·4 (10·4 to 21·7)	40·4 (28·9 to 56·9)	72 (50·4 to 105)	128 (91·8 to 184)	147 (102 to 215)	218 (157 to 309)	196 (143 to 275)	157 (115 to 214)	104 (77 to 138)	41·8 (30·3 to 53·3)	6·2 (4·2 to 8·3)	1,140
Testicular cancer	1 (0·6 to 1·6)	0·8 (0·5 to 1·1)	0·6 (0·4 to 0·9)	0·5 (0·3 to 0·7)	0·4 (0·2 to 0·6)	0·4 (0·3 to 0·6)	0·4 (0·2 to 0·5)	0·2 (0·1 to 0·3)	0·1 (0·1 to 0·2)	0·1 (0 to 0·1)	0 (0 to 0)	19·3
Kidney cancer	5·9 (4·2 to 8·2)	8·6 (6·3 to 11·4)	9·5 (6·9 to 12·8)	8·9 (6·5 to 11·8)	6·5 (4·6 to 8·9)	6·5 (4·7 to 8·8)	3·7 (2·7 to 5·1)	2·1 (1·5 to 2·8)	0·8 (0·6 to 1·1)	0·3 (0·2 to 0·4)	0 (0 to 0·1)	67
Bladder cancer	3·8 (2·6 to 5·3)	6·5 (4·7 to 8·7)	9·4 (6·8 to 12·5)	12·7 (9·3 to 16·6)	11·4 (8·4 to 15·4)	14·9 (10·6 to 19·5)	11·1 (8·1 to 15)	7·9 (5·7 to 10·6)	4·8 (3·6 to 6·2)	2 (1·5 to 2·4)	0·3 (0·2 to 0·4)	88·7
Brain and central nervous system cancer	7·7 (3·1 to 11·3)	8·4 (3·7 to 12·4)	8·2 (3·4 to 12·2)	6·3 (2·8 to 9·2)	4·3 (1·8 to 6·2)	3·6 (1·5 to 5·2)	1·8 (0·8 to 2·6)	0·8 (0·4 to 1·3)	0·3 (0·1 to 0·5)	0·1 (0 to 0·2)	0 (0 to 0)	75·4
Thyroid cancer	1·2 (0·8 to 1·8)	1·6 (1·1 to 2·4)	1·7 (1·1 to 2·4)	1·4 (0·9 to 2)	1 (0·7 to 1·3)	1 (0·7 to 1·4)	0·6 (0·4 to 0·8)	0·4 (0·3 to 0·6)	0·2 (0·1 to 0·3)	0·1 (0·1 to 0·1)	0 (0 to 0)	11·2
Mesothelioma	0·6 (0·4 to 0·9)	0·8 (0·6 to 1·1)	0·9 (0·7 to 1·2)	0·4 (0·3 to 0·5)	0·3 (0·2 to 0·4)	0·3 (0·2 to 0·5)	0·3 (0·2 to 0·4)	0·2 (0·1 to 0·2)	0·1 (0·1 to 0·1)	0 (0 to 0)	0 (0 to 0)	5
Hodgkin lymphoma	2·7 (1·7 to 4)	2·8 (1·8 to 4·2)	2·4 (1·5 to 3·5)	2 (1·4 to 2·8)	1·5 (1 to 2·1)	1·3 (0·9 to 1·8)	0·8 (0·5 to 1·1)	0·4 (0·3 to 0·6)	0·2 (0·1 to 0·3)	0·1 (0 to 0·1)	0 (0 to 0)	32·8
Non-Hodgkin lymphoma	21·4 (14·6 to 29·5)	22·9 (16·4 to 30·2)	23·5 (16·6 to 31·9)	20·2 (14·5 to 26·6)	14·4 (10·5 to 19)	15·1 (10·8 to 19·8)	8·5 (6·3 to 11·3)	4·7 (3·4 to 6·3)	1·9 (1·4 to 2·5)	0·6 (0·5 to 0·8)	0·1 (0 to 0·1)	221
Multiple myeloma	9·4 (6·4 to 13·6)	14·8 (10·3 to 20·7)	16·7 (11·7 to 23·7)	17 (12 to 23·8)	14·8 (10·7 to 20·2)	14·5 (10·2 to 19·7)	8·9 (6·5 to 12·1)	5·2 (3·7 to 7·3)	2·2 (1·6 to 3)	0·6 (0·4 to 0·8)	0·1 (0 to 0·1)	112
Leukemia	11·5 (8·4 to 15·4)	11·7 (8·6 to 15·6)	11·3 (8·3 to 15)	10·3 (7·5 to 13·4)	8·7 (6·3 to 11·5)	9·6 (7 to 13)	6·3 (4·6 to 8·2)	4·1 (3 to 5·4)	2·3 (1·6 to 3)	0·8 (0·6 to 1)	0·1 (0·1 to 0·1)	155
Acute lymphoid leukemia	1 (0·6 to 1·6)	0·9 (0·5 to 1·4)	0·7 (0·4 to 1·2)	0·4 (0·2 to 0·7)	0·5 (0·3 to 0·8)	0·5 (0·3 to 0·8)	0·2 (0·1 to 0·4)	0·2 (0·1 to 0·3)	0·1 (0 to 0·1)	0 (0 to 0)	0 (0 to 0)	31·4
Chronic lymphoid leukemia	0·7 (0·4 to 1·2)	1·6 (0·9 to 2·6)	2·4 (1·4 to 3·8)	2·5 (1·5 to 3·9)	2·4 (1·4 to 3·6)	2·5 (1·5 to 4)	1·6 (0·9 to 2·5)	1·1 (0·7 to 1·8)	0·6 (0·4 to 1)	0·2 (0·1 to 0·4)	0 (0 to 0)	18
Acute myeloid leukemia	3 (1·9 to 4·4)	2·8 (1·8 to 4·2)	2·2 (1·5 to 3·2)	1·8 (1·1 to 2·7)	1·4 (0·9 to 2·2)	1·4 (0·9 to 2·1)	0·8 (0·5 to 1·3)	0·4 (0·3 to 0·7)	0·2 (0·1 to 0·3)	0·1 (0 to 0·1)	0 (0 to 0)	34·7

Chronic myeloid leukemia	0 (0 to 0)	0 (0 to 0)	0 (0 to 0·1)	0·1 (0 to 0·2)	0·1 (0 to 0·1)	0·1 (0 to 0·1)	0·2 (0·1 to 0·4)	0·7 (0·3 to 1·2)	1·2 (0·6 to 2)	1·2 (0·6 to 2)	1·4 (0·8 to 2·3)	1·1 (0·7 to 2)	1·4 (0·8 to 2·3)	1·5 (0·9 to 2·5)	1·4 (0·7 to 2·3)	1·4 (0·7 to 2·3)	1 (0·6 to 1·6)	0·9 (0·5 to 1·6)	0·7 (0·4 to 1·2)	0·4 (0·2 to 0·7)	0·2 (0·1 to 0·3)	0·1 (0 to 0·1)	0 (0 to 0)	15
Other leukemia	0·2 (0 to 0·3)	0 (0 to 0·1)	0·3 (0·1 to 0·5)	0·6 (0·3 to 1·2)	1·4 (0·7 to 2·6)	1·9 (1 to 3·1)	1·9 (1·1 to 3·1)	3·1 (1·8 to 4·7)	2·9 (1·8 to 4·3)	2·9 (1·8 to 4·5)	3·1 (2 to 4·6)	4·5 (3 to 6·3)	5·3 (3·5 to 7·6)	4·9 (3·4 to 6·9)	4·6 (3·1 to 6·4)	4·2 (2·8 to 5·9)	3·3 (2·2 to 4·7)	4·3 (2·9 to 6·1)	2·9 (1·9 to 4)	2 (1·3 to 2·8)	1·2 (0·8 to 1·6)	0·4 (0·3 to 0·5)	0·1 (0 to 0·1)	56·1
Other malignant neoplasms	0·3 (0·2 to 0·5)	0·2 (0·1 to 0·3)	1·1 (0·7 to 1·8)	4·8 (2·9 to 7·7)	4·4 (3·3 to 5·9)	4·8 (3·6 to 6·2)	8·1 (6·1 to 10·6)	9·4 (7·3 to 12)	9·1 (7·3 to 11·3)	9·1 (7·1 to 11·5)	9·5 (7·5 to 12·3)	12·4 (9·2 to 16·2)	16 (11·8 to 21·1)	18·2 (13·3 to 24·5)	18·3 (13·3 to 24·8)	15·4 (11·6 to 20·6)	11·7 (8·9 to 15·7)	11·8 (9 to 15·6)	7·4 (5·7 to 10)	4·8 (3·8 to 6·3)	2·9 (2·2 to 3·9)	1·1 (0·9 to 1·4)	0·2 (0·1 to 0·2)	181
Other neoplasms	0·1 (0 to 0·1)	0 (0 to 0·1)	0·1 (0 to 0·1)	0·4 (0·2 to 0·7)	0·5 (0·2 to 0·8)	0·4 (0·2 to 0·7)	0·5 (0·2 to 0·8)	0·4 (0·2 to 0·6)	0·8 (0·3 to 1·2)	0·5 (0·2 to 1)	0·9 (0·4 to 1·4)	0·9 (0·5 to 1·5)	1·2 (0·7 to 2·1)	2·1 (1·2 to 3·5)	2·6 (1·5 to 4·3)	2·8 (1·7 to 4·8)	2·5 (1·6 to 4·2)	3·4 (2·2 to 6)	2·5 (1·6 to 4·3)	1·6 (1 to 2·7)	0·9 (0·5 to 1·5)	0·3 (0·2 to 0·5)	0 (0 to 0)	25·5
Myelodysplastic, myeloproliferative, and other hematopoietic neo	0·1 (0 to 0·1)	0 (0 to 0·1)	0·1 (0 to 0·1)	0·4 (0·2 to 0·7)	0·5 (0·2 to 0·8)	0·4 (0·2 to 0·7)	0·5 (0·2 to 0·8)	0·4 (0·2 to 0·6)	0·8 (0·3 to 1·2)	0·5 (0·2 to 1)	0·9 (0·4 to 1·4)	0·9 (0·5 to 1·5)	1·2 (0·7 to 2·1)	2·1 (1·2 to 3·5)	2·6 (1·5 to 4·3)	2·8 (1·7 to 4·8)	2·5 (1·6 to 4·2)	3·4 (2·2 to 6)	2·5 (1·6 to 4·3)	1·6 (1 to 2·7)	0·9 (0·5 to 1·5)	0·3 (0·2 to 0·5)	0 (0 to 0)	25·5
Benign and in situ intestinal neoplasms	0 (0 to 0)	0 (0 to 0)	0 (0 to 0)	0 (0 to 0)	0 (0 to 0)	0 (0 to 0)	0 (0 to 0)	0 (0 to 0)	0 (0 to 0)	0 (0 to 0)	0 (0 to 0)	0 (0 to 0)	0 (0 to 0)	0 (0 to 0)	0 (0 to 0)	0 (0 to 0)	0 (0 to 0)	0 (0 to 0)	0 (0 to 0)	0 (0 to 0)	0 (0 to 0)	0 (0 to 0)	0 (0 to 0)	0
Benign and in situ cervical and uterine neoplasms	0 (0 to 0)	0 (0 to 0)	0 (0 to 0)	0 (0 to 0)	0 (0 to 0)	0 (0 to 0)	0 (0 to 0)	0 (0 to 0)	0 (0 to 0)	0 (0 to 0)	0 (0 to 0)	0 (0 to 0)	0 (0 to 0)	0 (0 to 0)	0 (0 to 0)	0 (0 to 0)	0 (0 to 0)	0 (0 to 0)	0 (0 to 0)	0 (0 to 0)	0 (0 to 0)	0 (0 to 0)	0 (0 to 0)	0
Other benign and in situ neoplasms	0 (0 to 0)	0 (0 to 0)	0 (0 to 0)	0 (0 to 0)	0 (0 to 0)	0 (0 to 0)	0 (0 to 0)	0 (0 to 0)	0 (0 to 0)	0 (0 to 0)	0 (0 to 0)	0 (0 to 0)	0 (0 to 0)	0 (0 to 0)	0 (0 to 0)	0 (0 to 0)	0 (0 to 0)	0 (0 to 0)	0 (0 to 0)	0 (0 to 0)	0 (0 to 0)	0 (0 to 0)	0 (0 to 0)	0
Cardiovascular diseases	2 (1·2 to 3·2)	0·4 (0·3 to 0·7)	2·4 (1·4 to 3·8)	2·5 (1·8 to 3·6)	5 (3·8 to 6·5)	8·9 (6·7 to 11·5)	24·4 (19 to 30·4)	39·4 (30·6 to 49)	59·3 (46·9 to 73)	84·8 (66·1 to 105)	125 (100 to 153)	224 (180 to 277)	350 (277 to 433)	484 (387 to 595)	578 (474 to 707)	634 (519 to 764)	541 (452 to 635)	639 (536 to 760)	472 (398 to 559)	388 (330 to 447)	227 (191 to 261)	114 (94·5 to 129)	21·4 (17·7 to 24·2)	5,030
Rheumatic heart disease	0 (0 to 0)	0 (0 to 0)	0 (0 to 0)	0·2 (0·1 to 0·3)	1·1 (0·7 to 1·9)	2·8 (1·7 to 4·5)	5·3 (3·4 to 7·9)	6·5 (4·3 to 9·3)	7·4 (5·2 to 10·2)	7·2 (5·1 to 9·9)	7·5 (5·4 to 10·2)	8·4 (6 to 11·2)	8 (5·6 to 10·9)	7 (5 to 9·6)	5·7 (4 to 7·7)	3·8 (2·7 to 5)	2·3 (1·7 to 3)	1·9 (1·4 to 2·5)	1·2 (0·9 to 1·6)	0·6 (0·4 to 0·7)	0·3 (0·2 to 0·4)	0·1 (0·1 to 0·2)	0 (0 to 0)	77·3
Ischemic heart disease	0 (0 to 0)	0 (0 to 0)	0 (0 to 0)	0 (0 to 0)	0 (0 to 0)	0 (0 to 0)	1·3 (0·9 to 1·8)	3·6 (2·5 to 5)	8·2 (5·7 to 11·2)	15·7 (11·6 to 20·6)	27·9 (20·9 to 36·3)	58·1 (43·8 to 75·3)	101 (76·6 to 128)	162 (128 to 205)	192 (153 to 236)	226 (185 to 271)	196 (164 to 235)	216 (181 to 255)	149 (125 to 174)	127 (106 to 149)	71·7 (59·3 to 83·6)	38 (30·1 to 45)	7·5 (6·1 to 8·9)	1,600
Stroke	0·5 (0·2 to 0·9)	0 (0 to 0·1)	0·2 (0·1 to 0·5)	0·4 (0·3 to 0·5)	1·1 (0·8 to 1·6)	1·9 (1·3 to 2·6)	5·8 (4 to 8)	11·1 (7·5 to 15·9)	15·4 (10·1 to 22·6)	23·5 (15·4 to 33·2)	36 (24·3 to 50·2)	66·4 (46·7 to 91·2)	110 (77·6 to 152)	152 (110 to 206)	206 (154 to 268)	232 (174 to 301)	199 (149 to 251)	250 (191 to 319)	193 (149 to 242)	152 (121 to 187)	84·3 (64·7 to 106)	38·1 (30·7 to 45·8)	6·9 (5·4 to 8·3)	1,790
Ischemic stroke	0 (0 to 0)	0 (0 to 0)	0 (0 to 0)	0·2 (0·2 to 0·3)	0·6 (0·3 to 1)	0·9 (0·5 to 1·4)	1·6 (1 to 2·2)	2·1 (1·4 to 3)	3·1 (2·1 to 4·4)	4·3 (2·9 to 6·1)	6·3 (4·2 to 8·8)	10·9 (7·6 to 15·7)	18·1 (12·6 to 25·4)	28·9 (20 to 40·6)	41·1 (29·1 to 56)	67·6 (47·7 to 90·3)	64·8 (47·5 to 86·6)	117 (86·4 to 156)	98·3 (71·9 to 127)	109 (83·6 to 137)	61·6 (46·7 to 78·6)	28·2 (22·2 to 34·3)	5·3 (4·1 to 6·4)	670
Intracerebral hemorrhage	0·1 (0 to 0·2)	0 (0 to 0)	0·1 (0·1 to 0·2)	0 (0 to 0·1)	0·3 (0·2 to 0·4)	0·5 (0·3 to 0·7)	2·4 (1·5 to 3·7)	5·4 (3·3 to 8)	8·2 (5 to 12·4)	14·1 (8·8 to 21)	22·2 (14·7 to 32·4)	43·6 (29·4 to 61·2)	78 (53·5 to 109)	108 (76·7 to 146)	147 (109 to 197)	153 (111 to 200)	125 (90·5 to 164)	126 (91·8 to 168)	90 (66·8 to 118)	40·3 (30 to 54)	20·8 (14·7 to 28·2)	9·2 (6·8 to 11·9)	1·5 (1·1 to 2)	996
Subarachnoid hemorrhage	0·3 (0·1 to 0·6)	0 (0 to 0·1)	0·1 (0 to 0·2)	0·1 (0 to 0·1)	0·2 (0·1 to 0·3)	0·5 (0·3 to 0·8)	1·8 (1·1 to 2·9)	3·6 (2·1 to 5·5)	4·1 (2·4 to 6·8)	5·1 (3·1 to 8)	7·5 (4·5 to 11·2)	11·9 (7·3 to 18·3)	13·9 (8·6 to 21·1)	15·3 (9·6 to 23·2)	17·3 (11·4 to 24·9)	11·5 (7·6 to 16·8)	8·6 (5·8 to 12·7)	7·5 (5·1 to 10·5)	4·7 (3·2 to 6·8)	3·3 (2·2 to 4·8)	1·8 (1·2 to 2·7)	0·8 (0·5 to 1·2)	0·1 (0·1 to 0·2)	120
Hypertensive heart disease	0 (0 to 0)	0 (0 to 0)	0 (0 to 0)	0 (0 to 0)	0 (0 to 0)	0 (0 to 0)	0·4 (0·3 to 0·6)	1 (0·6 to 1·7)	2 (1·1 to 3·2)	4·5 (2·8 to 6·7)	8·4 (5·2 to 12·3)	18·3 (11·9 to 26·4)	33·9 (22·6 to 50·2)	46·6 (31·1 to 66·9)	53·3 (37·1 to 74·8)	55·4 (37·4 to 79·4)	45·5 (31·9 to 65·6)	58 (40·9 to 80·6)	46·5 (33·1 to 63·7)	37·7 (27 to 51·1)	23·4 (17·1 to 30·9)	12·6 (8·8 to 17·1)	2·5 (1·7 to 3·5)	450
Non-rheumatic valvular heart disease	0 (0 to 0)	0 (0 to 0)	0 (0 to 0)	0 (0 to 0)	0 (0 to 0)	0 (0 to 0)	0·4 (0·3 to 0·6)	0·6 (0·4 to 0·9)	1·1 (0·7 to 1·7)	1·4 (0·9 to 2)	1·5 (0·9 to 2·2)	2·6 (1·6 to 3·7)	3·3 (2·1 to 4·8)	3·4 (2·2 to 5)	4·1 (2·8 to 5·7)	3·9 (2·6 to 5·5)	3·4 (2·4 to 4·7)	3·5 (2·4 to 4·7)	2·4 (1·7 to 3·2)	2 (1·4 to 2·7)	1·1 (0·8 to 1·6)	0·6 (0·4 to 0·8)	0·1 (0·1 to 0·1)	35·4
Non-rheumatic calcific aortic valve disease	0 (0 to 0)	0 (0 to 0)	0 (0 to 0)	0 (0 to 0)	0 (0 to 0)	0 (0 to 0)	0·3 (0·2 to 0·5)	0·4 (0·3 to 0·7)	0·8 (0·5 to 1·2)	0·8 (0·5 to 1·2)	1 (0·6 to 1·5)	1·8 (1·1 to 2·6)	2·4 (1·5 to 3·5)	2·5 (1·6 to 3·7)	3 (2 to 4·3)	3 (2 to 4·3)	2·8 (2 to 3·8)	2·9 (2 to 4)	2 (1·4 to 2·8)	1·8 (1·2 to 2·4)	1 (0·7 to 1·3)	0·5 (0·3 to 0·7)	0·1 (0·1 to 0·1)	27
Non-rheumatic degenerative mitral valve disease	0 (0 to 0)	[illegible]	[illegible]	[illegible]	[illegible]	[illegible]	[illegible]	[illegible]	[illegible]	[illegible]	[illegible]	[illegible]	[illegible]	[illegible]	[illegible]	[illegible]	[illegible]	[illegible]	[illegible]	[illegible]	[illegible]	[illegible]	[illegible]	8
Other non-rheumatic valve diseases	0 (0 to 0)	[illegible]	[illegible]	[illegible]	[illegible]	[illegible]	[illegible]	[illegible]	[illegible]	[illegible]	[illegible]	[illegible]	[illegible]	[illegible]	[illegible]	[illegible]	[illegible]	[illegible]	[illegible]	[illegible]	[illegible]	[illegible]	[illegible]	0·3
Cardiomyopathy and myocarditis	0·6 (0·3 to 1·1)	[illegible]	[illegible]	[illegible]	[illegible]	[illegible]	[illegible]	[illegible]	[illegible]	[illegible]	[illegible]	[illegible]	[illegible]	[illegible]	[illegible]	[illegible]	[illegible]	[illegible]	[illegible]	[illegible]	[illegible]	[illegible]	[illegible]	443
Myocarditis	0·2 (0·1 to 0·3)	[illegible]	[illegible]	[illegible]	[illegible]	[illegible]	[illegible]	[illegible]	[illegible]	[illegible]	[illegible]	[illegible]	[illegible]	[illegible]	[illegible]	[illegible]	[illegible]	[illegible]	[illegible]	[illegible]	[illegible]	[illegible]	[illegible]	31·1
Alcoholic cardiomyopathy	0 (0 to 0)	[illegible]	[illegible]	[illegible]	[illegible]	[illegible]	[illegible]	[illegible]	[illegible]	[illegible]	[illegible]	[illegible]	[illegible]	[illegible]	[illegible]	[illegible]	[illegible]	[illegible]	[illegible]	[illegible]	[illegible]	[illegible]	[illegible]	164
Other cardiomyopathy	0·5 (0·3 to 0·8)	[illegible]	[illegible]	[illegible]	[illegible]	[illegible]	[illegible]	[illegible]	[illegible]	[illegible]	[illegible]	[illegible]	[illegible]	[illegible]	[illegible]	[illegible]	[illegible]	[illegible]	[illegible]	[illegible]	[illegible]	[illegible]	[illegible]	247
Atrial fibrillation and flutter	0 (0 to 0)	[illegible]	[illegible]	[illegible]	[illegible]	[illegible]	[illegible]	[illegible]	[illegible]	[illegible]	[illegible]	[illegible]	[illegible]	[illegible]	[illegible]	[illegible]	[illegible]	[illegible]	[illegible]	[illegible]	[illegible]	[illegible]	[illegible]	109
Aortic aneurysm	--	[illegible]	[illegible]	[illegible]	[illegible]	[illegible]	[illegible]	[illegible]	[illegible]	[illegible]	[illegible]	[illegible]	[illegible]	[illegible]	[illegible]	[illegible]	[illegible]	[illegible]	[illegible]	[illegible]	[illegible]	[illegible]	[illegible]	NA
Endocarditis	0·1 (0 to 0·2)	[illegible]	[illegible]	[illegible]	[illegible]	[illegible]	[illegible]	[illegible]	[illegible]	[illegible]	[illegible]	[illegible]	[illegible]	[illegible]	[illegible]	[illegible]	[illegible]	[illegible]	[illegible]	[illegible]	[illegible]	[illegible]	[illegible]	89·2
Other cardiovascular and circulatory diseases	0·8 (0·4 to 1·4)	[illegible]	[illegible]	[illegible]	[illegible]	[illegible]	[illegible]	[illegible]	[illegible]	[illegible]	[illegible]	[illegible]	[illegible]	[illegible]	[illegible]	[illegible]	[illegible]	[illegible]	[illegible]	[illegible]	[illegible]	[illegible]	[illegible]	258
Chronic respiratory diseases	0 (0 to 0)	[illegible]	[illegible]	[illegible]	[illegible]	[illegible]	[illegible]	[illegible]	[illegible]	[illegible]	[illegible]	[illegible]	[illegible]	[illegible]	[illegible]	[illegible]	[illegible]	[illegible]	[illegible]	[illegible]	[illegible]	[illegible]	[illegible]	1,210
Chronic obstructive pulmonary disease	0 (0 to 0)	[illegible]	[illegible]	[illegible]	[illegible]	[illegible]	[illegible]	[illegible]	[illegible]	[illegible]	[illegible]	[illegible]	[illegible]	[illegible]	[illegible]	[illegible]	[illegible]	[illegible]	[illegible]	[illegible]	[illegible]	[illegible]	[illegible]	701
Pneumoconiosis	0 (0 to 0)	[illegible]	[illegible]	[illegible]	[illegible]	[illegible]	[illegible]	[illegible]	[illegible]	[illegible]	[illegible]	[illegible]	[illegible]	[illegible]	[illegible]	[illegible]	[illegible]	[illegible]	[illegible]	[illegible]	[illegible]	[illegible]	[illegible]	1
Silicosis	0 (0 to 0)	[illegible]	[illegible]	[illegible]	[illegible]	[illegible]	[illegible]	[illegible]	[illegible]	[illegible]	[illegible]	[illegible]	[illegible]	[illegible]	[illegible]	[illegible]	[illegible]	[illegible]	[illegible]	[illegible]	[illegible]	[illegible]	[illegible]	0·2
Asbestosis	0 (0 to 0)	[illegible]	[illegible]	[illegible]	[illegible]	[illegible]	[illegible]	[illegible]	[illegible]	[illegible]	[illegible]	[illegible]	[illegible]	[illegible]	[illegible]	[illegible]	[illegible]	[illegible]	[illegible]	[illegible]	[illegible]	[illegible]	[illegible]	0·3
Coal workers pneumoconiosis	0 (0 to 0)	[illegible]	[illegible]	[illegible]	[illegible]	[illegible]	[illegible]	[illegible]	[illegible]	[illegible]	[illegible]	[illegible]	[illegible]	[illegible]	[illegible]	[illegible]	[illegible]	[illegible]	[illegible]	[illegible]	[illegible]	[illegible]	[illegible]	0·1
Other pneumoconiosis	0 (0 to 0)	[illegible]	[illegible]	[illegible]	[illegible]	[illegible]	[illegible]	[illegible]	[illegible]	[illegible]	[illegible]	[illegible]	[illegible]	[illegible]	[illegible]	[illegible]	[illegible]	[illegible]	[illegible]	[illegible]	[illegible]	[illegible]	[illegible]	0·4
Asthma	0 (0 to 0)	[illegible]	[illegible]	[illegible]	[illegible]	[illegible]	[illegible]	[illegible]	[illegible]	[illegible]	[illegible]	[illegible]	[illegible]	[illegible]	[illegible]	[illegible]	[illegible]	[illegible]	[illegible]	[illegible]	[illegible]	[illegible]	[illegible]	390
Interstitial lung disease and pulmonary sarcoidosis	0 (0 to 0)	[illegible]	[illegible]	[illegible]	[illegible]	[illegible]	[illegible]	[illegible]	[illegible]	[illegible]	[illegible]	[illegible]	[illegible]	[illegible]	[illegible]	[illegible]	[illegible]	[illegible]	[illegible]	[illegible]	[illegible]	[illegible]	[illegible]	58·2
Other chronic respiratory diseases	0 (0 to 0)	[illegible]	[illegible]	[illegible]	[illegible]	[illegible]	[illegible]	[illegible]	[illegible]	[illegible]	[illegible]	[illegible]	[illegible]	[illegible]	[illegible]	[illegible]	[illegible]	[illegible]	[illegible]	[illegible]	[illegible]	[illegible]	[illegible]	59·8
Digestive diseases	1·2 (0·7 to 1·8)	[illegible]	[illegible]	[illegible]	[illegible]	[illegible]	[illegible]	[illegible]	[illegible]	[illegible]	[illegible]	[illegible]	[illegible]	[illegible]	[illegible]	[illegible]	[illegible]	[illegible]	[illegible]	[illegible]	[illegible]	[illegible]	[illegible]	1,260
Cirrhosis and other chronic liver diseases	0 (0 to 0)	[illegible]	[illegible]	[illegible]	[illegible]	[illegible]	[illegible]	[illegible]	[illegible]	[illegible]	[illegible]	[illegible]	[illegible]	[illegible]	[illegible]	[illegible]	[illegible]	[illegible]	[illegible]	[illegible]	[illegible]	[illegible]	[illegible]	654
Cirrhosis and other chronic liver diseases due to hepatitis B	0 (0 to 0)	[illegible]	[illegible]	[illegible]	[illegible]	[illegible]	[illegible]	[illegible]	[illegible]	[illegible]	[illegible]	[illegible]	[illegible]	[illegible]	[illegible]	[illegible]	[illegible]	[illegible]	[illegible]	[illegible]	[illegible]	[illegible]	[illegible]	51·7
Cirrhosis and other chronic liver diseases due to hepatitis C	0 (0 to 0)	[illegible]	[illegible]	[illegible]	[illegible]	[illegible]	[illegible]	[illegible]	[illegible]	[illegible]	[illegible]	[illegible]	[illegible]	[illegible]	[illegible]	[illegible]	[illegible]	[illegible]	[illegible]	[illegible]	[illegible]	[illegible]	[illegible]	132
Cirrhosis and other chronic liver diseases due to alcohol use	0 (0 to 0)	[illegible]	[illegible]	[illegible]	[illegible]	[illegible]	[illegible]	[illegible]	[illegible]	[illegible]	[illegible]	[illegible]	[illegible]	[illegible]	[illegible]	[illegible]	[illegible]	[illegible]	[illegible]	[illegible]	[illegible]	[illegible]	[illegible]	316
Cirrhosis and other chronic liver diseases due to NAFLD	0 (0 to 0)	[illegible]	[illegible]	[illegible]	[illegible]	[illegible]	[illegible]	[illegible]	[illegible]	[illegible]	[illegible]	[illegible]	[illegible]	[illegible]	[illegible]	[illegible]	[illegible]	[illegible]	[illegible]	[illegible]	[illegible]	[illegible]	[illegible]	86
Cirrhosis and other chronic liver diseases due to other causes	0 (0 to 0)	[illegible]	[illegible]	[illegible]	[illegible]	[illegible]	[illegible]	[illegible]	[illegible]	[illegible]	[illegible]	[illegible]	[illegible]	[illegible]	[illegible]	[illegible]	[illegible]	[illegible]	[illegible]	[illegible]	[illegible]	[illegible]	[illegible]	68
Upper digestive system diseases	0 (0 to 0)	[illegible]	[illegible]	[illegible]	[illegible]	[illegible]	[illegible]	[illegible]	[illegible]	[illegible]	[illegible]	[illegible]	[illegible]	[illegible]	[illegible]	[illegible]	[illegible]	[illegible]	[illegible]	[illegible]	[illegible]	[illegible]	[illegible]	243
Peptic ulcer disease	0 (0 to 0)	[illegible]	[illegible]	[illegible]	[illegible]	[illegible]	[illegible]	[illegible]	[illegible]	[illegible]	[illegible]	[illegible]	[illegible]	[illegible]	[illegible]	[illegible]	[illegible]	[illegible]	[illegible]	[illegible]	[illegible]	[illegible]	[illegible]	79·6
Gastritis and duodenitis	0 (0 to 0)	[illegible]	[illegible]	[illegible]	[illegible]	[illegible]	[illegible]	[illegible]	[illegible]	[illegible]	[illegible]	[illegible]	[illegible]	[illegible]	[illegible]	[illegible]	[illegible]	[illegible]	[illegible]	[illegible]	[illegible]	[illegible]	[illegible]	42·4
Gastroesophageal reflux disease	0 (0 to 0)	[illegible]	[illegible]	[illegible]	[illegible]	[illegible]	[illegible]	[illegible]	[illegible]	[illegible]	[illegible]	[illegible]	[illegible]	[illegible]	[illegible]	[illegible]	[illegible]	[illegible]	[illegible]	[illegible]	[illegible]	[illegible]	[illegible]	121
Appendicitis	0 (0 to 0)	[illegible]	[illegible]	[illegible]	[illegible]	[illegible]	[illegible]	[illegible]	[illegible]	[illegible]	[illegible]	[illegible]	[illegible]	[illegible]	[illegible]	[illegible]	[illegible]	[illegible]	[illegible]	[illegible]	[illegible]	[illegible]	[illegible]	20·2
Paralytic ileus and intestinal obstruction	0·8 (0·5 to 1·3)	[illegible]	[illegible]	[illegible]	[illegible]	[illegible]	[illegible]	[illegible]	[illegible]	[illegible]	[illegible]	[illegible]	[illegible]	[illegible]	[illegible]	[illegible]	[illegible]	[illegible]	[illegible]	[illegible]	[illegible]	[illegible]	[illegible]	74·1
Inguinal, femoral, and abdominal hernia	0·3 (0·2 to 0·6)	[illegible]	[illegible]	[illegible]	[illegible]	[illegible]	[illegible]	[illegible]	[illegible]	[illegible]	[illegible]	[illegible]	[illegible]	[illegible]	[illegible]	[illegible]	[illegible]	[illegible]	[illegible]	[illegible]	[illegible]	[illegible]	[illegible]	89·8
Inflammatory bowel disease	0 (0 to 0)	[illegible]	[illegible]	[illegible]	[illegible]	[illegible]	[illegible]	[illegible]	[illegible]	[illegible]	[illegible]	[illegible]	[illegible]	[illegible]	[illegible]	[illegible]	[illegible]	[illegible]	[illegible]	[illegible]	[illegible]	[illegible]	[illegible]	21·3
Vascular intestinal disorders	0 (0 to 0)	[illegible]	[illegible]	[illegible]	[illegible]	[illegible]	[illegible]	[illegible]	[illegible]	[illegible]	[illegible]	[illegible]	[illegible]	[illegible]	[illegible]	[illegible]	[illegible]	[illegible]	[illegible]	[illegible]	[illegible]	[illegible]	[illegible]	23·3
Gallbladder and biliary diseases	0 (0 to 0)	[illegible]	[illegible]	[illegible]	[illegible]	[illegible]	[illegible]	[illegible]	[illegible]	[illegible]	[illegible]	[illegible]	[illegible]	[illegible]	[illegible]	[illegible]	[illegible]	[illegible]	[illegible]	[illegible]	[illegible]	[illegible]	[illegible]	41·9
Pancreatitis	0 (0 to 0)	[illegible]	[illegible]	[illegible]	[illegible]	[illegible]	[illegible]	[illegible]	[illegible]	[illegible]	[illegible]	[illegible]	[illegible]	[illegible]	[illegible]	[illegible]	[illegible]	[illegible]	[illegible]	[illegible]	[illegible]	[illegible]	[illegible]	29·3
Other digestive diseases	0 (0 to 0)	[illegible]	[illegible]	[illegible]	[illegible]	[illegible]	[illegible]	[illegible]	[illegible]	[illegible]	[illegible]	[illegible]	[illegible]	[illegible]	[illegible]	[illegible]	[illegible]	[illegible]	[illegible]	[illegible]	[illegible]	[illegible]	[illegible]	60·3
Neurological disorders	0 (0 to 0)	[illegible]	[illegible]	[illegible]	[illegible]	[illegible]	[illegible]	[illegible]	[illegible]	[illegible]	[illegible]	[illegible]	[illegible]	[illegible]	[illegible]	[illegible]	[illegible]	[illegible]	[illegible]	[illegible]	[illegible]	[illegible]	[illegible]	1,180
Alzheimer's disease and other dementias	0 (0 to 0)	[illegible]	[illegible]	[illegible]	[illegible]	[illegible]	[illegible]	[illegible]	[illegible]	[illegible]	[illegible]	[illegible]	[illegible]	[illegible]	[illegible]	[illegible]	[illegible]	[illegible]	[illegible]	[illegible]	[illegible]	[illegible]	[illegible]	250
Parkinson's disease	0 (0 to 0)	[illegible]	[illegible]	[illegible]	[illegible]	[illegible]	[illegible]	[illegible]	[illegible]	[illegible]	[illegible]	[illegible]	[illegible]	[illegible]	[illegible]	[illegible]	[illegible]	[illegible]	[illegible]	[illegible]	[illegible]	[illegible]	[illegible]	117
Idiopathic epilepsy	0 (0 to 0)	[illegible]	[illegible]	[illegible]	[illegible]	[illegible]	[illegible]	[illegible]	[illegible]	[illegible]	[illegible]	[illegible]	[illegible]	[illegible]	[illegible]	[illegible]	[illegible]	[illegible]	[illegible]	[illegible]	[illegible]	[illegible]	[illegible]	326
Multiple sclerosis	0 (0 to 0)	[illegible]	[illegible]	[illegible]	[illegible]	[illegible]	[illegible]	[illegible]	[illegible]	[illegible]	[illegible]	[illegible]	[illegible]	[illegible]	[illegible]	[illegible]	[illegible]	[illegible]	[illegible]	[illegible]	[illegible]	[illegible]	[illegible]	9·6
Motor neuron disease	0 (0 to 0)	[illegible]	[illegible]	[illegible]	[illegible]	[illegible]	[illegible]	[illegible]	[illegible]	[illegible]	[illegible]	[illegible]	[illegible]	[illegible]	[illegible]	[illegible]	[illegible]	[illegible]	[illegible]	[illegible]	[illegible]	[illegible]	[illegible]	26·1
Headache disorders	0 (0 to 0)	[illegible]	[illegible]	[illegible]	[illegible]	[illegible]	[illegible]	[illegible]	[illegible]	[illegible]	[illegible]	[illegible]	[illegible]	[illegible]	[illegible]	[illegible]	[illegible]	[illegible]	[illegible]	[illegible]	[illegible]	[illegible]	[illegible]	370
Migraine	0 (0 to 0)	[illegible]	[illegible]	[illegible]	[illegible]	[illegible]	[illegible]	[illegible]	[illegible]	[illegible]	[illegible]	[illegible]	[illegible]	[illegible]	[illegible]	[illegible]	[illegible]	[illegible]	[illegible]	[illegible]	[illegible]	[illegible]	[illegible]	326
Tension-type headache	0 (0 to 0)	[illegible]	[illegible]	[illegible]	[illegible]	[illegible]	[illegible]	[illegible]	[illegible]	[illegible]	[illegible]	[illegible]	[illegible]	[illegible]	[illegible]	[illegible]	[illegible]	[illegible]	[illegible]	[illegible]	[illegible]	[illegible]	[illegible]	43·6
Other neurological disorders	0 (0 to 0)	[illegible]	[illegible]	[illegible]	[illegible]	[illegible]	[illegible]	[illegible]	[illegible]	[illegible]	[illegible]	[illegible]	[illegible]	[illegible]	[illegible]	[illegible]	[illegible]	[illegible]	[illegible]	[illegible]	[illegible]	[illegible]	[illegible]	85·3
Mental disorders	0 (0 to 0)	[illegible]	[illegible]	[illegible]	[illegible]	[illegible]	[illegible]	[illegible]	[illegible]	[illegible]	[illegible]	[illegible]	[illegible]	[illegible]	[illegible]	[illegible]	[illegible]	[illegible]	[illegible]	[illegible]	[illegible]	[illegible]	[illegible]	1,460
Schizophrenia	0 (0 to 0)	[illegible]	[illegible]	[illegible]	[illegible]	[illegible]	[illegible]	[illegible]	[illegible]	[illegible]	[illegible]	[illegible]	[illegible]	[illegible]	[illegible]	[illegible]	[illegible]	[illegible]	[illegible]	[illegible]	[illegible]	[illegible]	[illegible]	188
Depressive disorders	0 (0 to 0)	[illegible]	[illegible]	[illegible]	[illegible]	[illegible]	[illegible]	[illegible]	[illegible]	[illegible]	[illegible]	[illegible]	[illegible]	[illegible]	[illegible]	[illegible]	[illegible]	[illegible]	[illegible]	[illegible]	[illegible]	[illegible]	[illegible]	454
Major depressive disorder	0 (0 to 0)	[illegible]	[illegible]	[illegible]	[illegible]	[illegible]	[illegible]	[illegible]	[illegible]	[illegible]	[illegible]	[illegible]	[illegible]	[illegible]	[illegible]	[illegible]	[illegible]	[illegible]	[illegible]	[illegible]	[illegible]	[illegible]	[illegible]	372
Dysthymia	0 (0 to 0)	[illegible]	[illegible]	[illegible]	[illegible]	[illegible]	[illegible]	[illegible]	[illegible]	[illegible]	[illegible]	[illegible]	[illegible]	[illegible]	[illegible]	[illegible]	[illegible]	[illegible]	[illegible]	[illegible]	[illegible]	[illegible]	[illegible]	81·9
Bipolar disorder	0 (0 to 0)	[illegible]	[illegible]	[illegible]	[illegible]	[illegible]	[illegible]	[illegible]	[illegible]	[illegible]	[illegible]	[illegible]	[illegible]	[illegible]	[illegible]	[illegible]	[illegible]	[illegible]	[illegible]	[illegible]	[illegible]	[illegible]	[illegible]	182
Anxiety disorders	0 (0 to 0)	[illegible]	[illegible]	[illegible]	[illegible]	[illegible]	[illegible]	[illegible]	[illegible]	[illegible]	[illegible]	[illegible]	[illegible]	[illegible]	[illegible]	[illegible]	[illegible]	[illegible]	[illegible]	[illegible]	[illegible]	[illegible]	[illegible]	292
Eating disorders	0 (0 to 0)	[illegible]	[illegible]	[illegible]	[illegible]	[illegible]	[illegible]	[illegible]	[illegible]	[illegible]	[illegible]	[illegible]	[illegible]	[illegible]	[illegible]	[illegible]	[illegible]	[illegible]	[illegible]	[illegible]	[illegible]	[illegible]	[illegible]	24·9
Anorexia nervosa	0 (0 to 0)	[illegible]	[illegible]	[illegible]	[illegible]	[illegible]	[illegible]	[illegible]	[illegible]	[illegible]	[illegible]	[illegible]	[illegible]	[illegible]	[illegible]	[illegible]	[illegible]	[illegible]	[illegible]	[illegible]	[illegible]	[illegible]	[illegible]	5·2
Bulimia nervosa	0 (0 to 0)	[illegible]	[illegible]	[illegible]	[illegible]	[illegible]	[illegible]	[illegible]	[illegible]	[illegible]	[illegible]	[illegible]	[illegible]	[illegible]	[illegible]	[illegible]	[illegible]	[illegible]	[illegible]	[illegible]	[illegible]	[illegible]	[illegible]	19·8
Autism spectrum disorders	0 (0 to 0)	[illegible]	[illegible]	[illegible]	[illegible]	[illegible]	[illegible]	[illegible]	[illegible]	[illegible]	[illegible]	[illegible]	[illegible]	[illegible]	[illegible]	[illegible]	[illegible]	[illegible]	[illegible]	[illegible]	[illegible]	[illegible]	[illegible]	66·3
Attention-deficit/hyperactivity disorder	0 (0 to 0)	[illegible]	[illegible]	[illegible]	[illegible]	[illegible]	[illegible]	[illegible]	[illegible]	[illegible]	[illegible]	[illegible]	[illegible]	[illegible]	[illegible]	[illegible]	[illegible]	[illegible]	[illegible]	[illegible]	[illegible]	[illegible]	[illegible]	43·5
Conduct disorder	0 (0 to 0)	[illegible]	[illegible]	[illegible]	[illegible]	[illegible]	[illegible]	[illegible]	[illegible]	[illegible]	[illegible]	[illegible]	[illegible]	[illegible]	[illegible]	[illegible]	[illegible]	[illegible]	[illegible]	[illegible]	[illegible]	[illegible]	[illegible]	61
Idiopathic developmental intellectual disability	0 (0 to 0)	[illegible]	[illegible]	[illegible]	[illegible]	[illegible]	[illegible]	[illegible]	[illegible]	[illegible]	[illegible]	[illegible]	[illegible]	[illegible]	[illegible]	[illegible]	[illegible]	[illegible]	[illegible]	[illegible]	[illegible]	[illegible]	[illegible]	12·9
Other mental disorders	0 (0 to 0)	[illegible]	[illegible]	[illegible]	[illegible]	[illegible]	[illegible]	[illegible]	[illegible]	[illegible]	[illegible]	[illegible]	[illegible]	[illegible]	[illegible]	[illegible]	[illegible]	[illegible]	[illegible]	[illegible]	[illegible]	[illegible]	[illegible]	130
Substance use disorders	0 (0 to 0)	[illegible]	[illegible]	[illegible]	[illegible]	[illegible]	[illegible]	[illegible]	[illegible]	[illegible]	[illegible]	[illegible]	[illegible]	[illegible]	[illegible]	[illegible]	[illegible]	[illegible]	[illegible]	[illegible]	[illegible]	[illegible]	[illegible]	625
Alcohol use disorders	0 (0 to 0)	[illegible]	[illegible]	[illegible]	[illegible]	[illegible]	[illegible]	[illegible]	[illegible]	[illegible]	[illegible]	[illegible]	[illegible]	[illegible]	[illegible]	[illegible]	[illegible]	[illegible]	[illegible]	[illegible]	[illegible]	[illegible]	[illegible]	471
Drug use disorders	0 (0 to 0)	[illegible]	[illegible]	[illegible]	[illegible]	[illegible]	[illegible]	[illegible]	[illegible]	[illegible]	[illegible]	[illegible]	[illegible]	[illegible]	[illegible]	[illegible]	[illegible]	[illegible]	[illegible]	[illegible]	[illegible]	[illegible]	[illegible]	153
Opioid use disorders	0 (0 to 0)	[illegible]	[illegible]	[illegible]	[illegible]	[illegible]	[illegible]	[illegible]	[illegible]	[illegible]	[illegible]	[illegible]	[illegible]	[illegible]	[illegible]	[illegible]	[illegible]	[illegible]	[illegible]	[illegible]	[illegible]	[illegible]	[illegible]	54·8
Cocaine use disorders	0 (0 to 0)	[illegible]	[illegible]	[illegible]	[illegible]	[illegible]	[illegible]	[illegible]	[illegible]	[illegible]	[illegible]	[illegible]	[illegible]	[illegible]	[illegible]	[illegible]	[illegible]	[illegible]	[illegible]	[illegible]	[illegible]	[illegible]	[illegible]	33·1
Amphetamine use disorders	0 (0 to 0)	[illegible]	[illegible]	[illegible]	[illegible]	[illegible]	[illegible]	[illegible]	[illegible]	[illegible]	[illegible]	[illegible]	[illegible]	[illegible]	[illegible]	[illegible]	[illegible]	[illegible]	[illegible]	[illegible]	[illegible]	[illegible]	[illegible]	9·6
Cannabis use disorders	0 (0 to 0)	[illegible]	[illegible]	[illegible]	[illegible]	[illegible]	[illegible]	[illegible]	[illegible]	[illegible]	[illegible]	[illegible]	[illegible]	[illegible]	[illegible]	[illegible]	[illegible]	[illegible]	[illegible]	[illegible]	[illegible]	[illegible]	[illegible]	31·7
Other drug use disorders	0 (0 to 0)	[illegible]	[illegible]	[illegible]	[illegible]	[illegible]	[illegible]	[illegible]	[illegible]	[illegible]	[illegible]	[illegible]	[illegible]	[illegible]	[illegible]	[illegible]	[illegible]	[illegible]	[illegible]	[illegible]	[illegible]	[illegible]	[illegible]	24·1
Diabetes and kidney diseases	0 (0 to 0)	[illegible]	[illegible]	[illegible]	[illegible]	[illegible]	[illegible]	[illegible]	[illegible]	[illegible]	[illegible]	[illegible]	[illegible]	[illegible]	[illegible]	[illegible]	[illegible]	[illegible]	[illegible]	[illegible]	[illegible]	[illegible]	[illegible]	3,580
Diabetes mellitus	0 (0 to 0)	[illegible]	[illegible]	[illegible]	[illegible]	[illegible]	[illegible]	[illegible]	[illegible]	[illegible]	[illegible]	[illegible]	[illegible]	[illegible]	[illegible]	[illegible]	[illegible]	[illegible]	[illegible]	[illegible]	[illegible]	[illegible]	[illegible]	2,400
Diabetes mellitus type 1	0 (0 to 0)	[illegible]	[illegible]	[illegible]	[illegible]	[illegible]	[illegible]	[illegible]	[illegible]	[illegible]	[illegible]	[illegible]	[illegible]	[illegible]	[illegible]	[illegible]	[illegible]	[illegible]	[illegible]	[illegible]	[illegible]	[illegible]	[illegible]	114
Diabetes mellitus type 2	0 (0 to 0)	[illegible]	[illegible]	[illegible]	[illegible]	[illegible]	[illegible]	[illegible]	[illegible]	[illegible]	[illegible]	[illegible]	[illegible]	[illegible]	[illegible]	[illegible]	[illegible]	[illegible]	[illegible]	[illegible]	[illegible]	[illegible]	[illegible]	2,290
Chronic kidney disease	0 (0 to 0)	[illegible]	[illegible]	[illegible]	[illegible]	[illegible]	[illegible]	[illegible]	[illegible]	[illegible]	[illegible]	[illegible]	[illegible]	[illegible]	[illegible]	[illegible]	[illegible]	[illegible]	[illegible]	[illegible]	[illegible]	[illegible]	[illegible]	1,180
Chronic kidney disease due to diabetes mellitus type 1	0 (0 to 0)	[illegible]	[illegible]	[illegible]	[illegible]	[illegible]	[illegible]	[illegible]	[illegible]	[illegible]	[illegible]	[illegible]	[illegible]	[illegible]	[illegible]	[illegible]	[illegible]	[illegible]	[illegible]	[illegible]	[illegible]	[illegible]	[illegible]	97·6
Chronic kidney disease due to diabetes mellitus type 2	0 (0 to 0)	[illegible]	[illegible]	[illegible]	[illegible]	[illegible]	[illegible]	[illegible]	[illegible]	[illegible]	[illegible]	[illegible]	[illegible]	[illegible]	[illegible]	[illegible]	[illegible]	[illegible]	[illegible]	[illegible]	[illegible]	[illegible]	[illegible]	274
Chronic kidney disease due to hypertension	0 (0 to 0)	[illegible]	[illegible]	[illegible]	[illegible]	[illegible]	[illegible]	[illegible]	[illegible]	[illegible]	[illegible]	[illegible]	[illegible]	[illegible]	[illegible]	[illegible]	[illegible]	[illegible]	[illegible]	[illegible]	[illegible]	[illegible]	[illegible]	240
Chronic kidney disease due to glomerulonephritis	0 (0 to 0)	[illegible]	[illegible]	[illegible]	[illegible]	[illegible]	[illegible]	[illegible]	[illegible]	[illegible]	[illegible]	[illegible]	[illegible]	[illegible]	[illegible]	[illegible]	[illegible]	[illegible]	[illegible]	[illegible]	[illegible]	[illegible]	[illegible]	282
Chronic kidney disease due to other and unspecified causes	0 (0 to 0)	[illegible]	[illegible]	[illegible]	[illegible]	[illegible]	[illegible]	[illegible]	[illegible]	[illegible]	[illegible]	[illegible]	[illegible]	[illegible]	[illegible]	[illegible]	[illegible]	[illegible]	[illegible]	[illegible]	[illegible]	[illegible]	[illegible]	289
Acute glomerulonephritis	0 (0 to 0)	[illegible]	[illegible]	[illegible]	[illegible]	[illegible]	[illegible]	[illegible]	[illegible]	[illegible]	[illegible]	[illegible]	[illegible]	[illegible]	[illegible]	[illegible]	[illegible]	[illegible]	[illegible]	[illegible]	[illegible]	[illegible]	[illegible]	0·2
Skin and subcutaneous diseases	0 (0 to 0)	[illegible]	[illegible]	[illegible]	[illegible]	[illegible]	[illegible]	[illegible]	[illegible]	[illegible]	[illegible]	[illegible]	[illegible]	[illegible]	[illegible]	[illegible]	[illegible]	[illegible]	[illegible]	[illegible]	[illegible]	[illegible]	[illegible]	515
Dermatitis	0 (0 to 0)	[illegible]	[illegible]	[illegible]	[illegible]	[illegible]	[illegible]	[illegible]	[illegible]	[illegible]	[illegible]	[illegible]	[illegible]	[illegible]	[illegible]	[illegible]	[illegible]	[illegible]	[illegible]	[illegible]	[illegible]	[illegible]	[illegible]	82·9
Atopic dermatitis	0 (0 to 0)	[illegible]	[illegible]	[illegible]	[illegible]	[illegible]	[illegible]	[illegible]	[illegible]	[illegible]	[illegible]	[illegible]	[illegible]	[illegible]	[illegible]	[illegible]	[illegible]	[illegible]	[illegible]	[illegible]	[illegible]	[illegible]	[illegible]	54·8
Contact dermatitis	0 (0 to 0)	[illegible]	[illegible]	[illegible]	[illegible]	[illegible]	[illegible]	[illegible]	[illegible]	[illegible]	[illegible]	[illegible]	[illegible]	[illegible]	[illegible]	[illegible]	[illegible]	[illegible]	[illegible]	[illegible]	[illegible]	[illegible]	[illegible]	24·9
Seborrhoeic dermatitis	0 (0 to 0)	[illegible]	[illegible]	[illegible]	[illegible]	[illegible]	[illegible]	[illegible]	[illegible]	[illegible]	[illegible]	[illegible]	[illegible]	[illegible]	[illegible]	[illegible]	[illegible]	[illegible]	[illegible]	[illegible]	[illegible]	[illegible]	[illegible]	3·2
Psoriasis	0 (0 to 0)	[illegible]	[illegible]	[illegible]	[illegible]	[illegible]	[illegible]	[illegible]	[illegible]	[illegible]	[illegible]	[illegible]	[illegible]	[illegible]	[illegible]	[illegible]	[illegible]	[illegible]	[illegible]	[illegible]	[illegible]	[illegible]	[illegible]	30·5
Bacterial skin diseases	0 (0 to 0)	[illegible]	[illegible]	[illegible]	[illegible]	[illegible]	[illegible]	[illegible]	[illegible]	[illegible]	[illegible]	[illegible]	[illegible]	[illegible]	[illegible]	[illegible]	[illegible]	[illegible]	[illegible]	[illegible]	[illegible]	[illegible]	[illegible]	66
Cellulitis	0 (0 to 0)	[illegible]	[illegible]	[illegible]	[illegible]	[illegible]	[illegible]	[illegible]	[illegible]	[illegible]	[illegible]	[illegible]	[illegible]	[illegible]	[illegible]	[illegible]	[illegible]	[illegible]	[illegible]	[illegible]	[illegible]	[illegible]	[illegible]	19·2
Pyoderma	0 (0 to 0)	[illegible]	[illegible]	[illegible]	[illegible]	[illegible]	[illegible]	[illegible]	[illegible]	[illegible]	[illegible]	[illegible]	[illegible]	[illegible]	[illegible]	[illegible]	[illegible]	[illegible]	[illegible]	[illegible]	[illegible]	[illegible]	[illegible]	46·8
Scabies	0 (0 to 0)	[illegible]	[illegible]	[illegible]	[illegible]	[illegible]	[illegible]	[illegible]	[illegible]	[illegible]	[illegible]	[illegible]	[illegible]	[illegible]	[illegible]	[illegible]	[illegible]	[illegible]	[illegible]	[illegible]	[illegible]	[illegible]	[illegible]	67·4
Fungal skin diseases	0 (0 to 0)	[illegible]	[illegible]	[illegible]	[illegible]	[illegible]	[illegible]	[illegible]	[illegible]	[illegible]	[illegible]	[illegible]	[illegible]	[illegible]	[illegible]	[illegible]	[illegible]	[illegible]	[illegible]	[illegible]	[illegible]	[illegible]	[illegible]	41·4
Viral skin diseases	0 (0 to 0)	[illegible]	[illegible]	[illegible]	[illegible]	[illegible]	[illegible]	[illegible]	[illegible]	[illegible]	[illegible]	[illegible]	[illegible]	[illegible]	[illegible]	[illegible]	[illegible]	[illegible]	[illegible]	[illegible]	[illegible]	[illegible]	[illegible]	33·9
Acne vulgaris	0 (0 to 0)	[illegible]	[illegible]	[illegible]	[illegible]	[illegible]	[illegible]	[illegible]	[illegible]	[illegible]	[illegible]	[illegible]	[illegible]	[illegible]	[illegible]	[illegible]	[illegible]	[illegible]	[illegible]	[illegible]	[illegible]	[illegible]	[illegible]	38·7
Alopecia areata	0 (0 to 0)	[illegible]	[illegible]	[illegible]	[illegible]	[illegible]	[illegible]	[illegible]	[illegible]	[illegible]	[illegible]	[illegible]	[illegible]	[illegible]	[illegible]	[illegible]	[illegible]	[illegible]	[illegible]	[illegible]	[illegible]	[illegible]	[illegible]	4·6
Pruritus	0 (0 to 0)	[illegible]	[illegible]	[illegible]	[illegible]	[illegible]	[illegible]	[illegible]	[illegible]	[illegible]	[illegible]	[illegible]	[illegible]	[illegible]	[illegible]	[illegible]	[illegible]	[illegible]	[illegible]	[illegible]	[illegible]	[illegible]	[illegible]	7
Urticaria	0 (0 to 0)	[illegible]	[illegible]	[illegible]	[illegible]	[illegible]	[illegible]	[illegible]	[illegible]	[illegible]	[illegible]	[illegible]	[illegible]	[illegible]	[illegible]	[illegible]	[illegible]	[illegible]	[illegible]	[illegible]	[illegible]	[illegible]	[illegible]	31·9
Decubitus ulcer	0 (0 to 0)	[illegible]	[illegible]	[illegible]	[illegible]	[illegible]	[illegible]	[illegible]	[illegible]	[illegible]	[illegible]	[illegible]	[illegible]	[illegible]	[illegible]	[illegible]	[illegible]	[illegible]	[illegible]	[illegible]	[illegible]	[illegible]	[illegible]	61·4
Other skin and subcutaneous diseases	0 (0 to 0)	[illegible]	[illegible]	[illegible]	[illegible]	[illegible]	[illegible]	[illegible]	[illegible]	[illegible]	[illegible]	[illegible]	[illegible]	[illegible]	[illegible]	[illegible]	[illegible]	[illegible]	[illegible]	[illegible]	[illegible]	[illegible]	[illegible]	49·8
Sense organ diseases	0 (0 to 0)	[illegible]	[illegible]	[illegible]	[illegible]	[illegible]	[illegible]	[illegible]	[illegible]	[illegible]	[illegible]	[illegible]	[illegible]	[illegible]	[illegible]	[illegible]	[illegible]	[illegible]	[illegible]	[illegible]	[illegible]	[illegible]	[illegible]	745
Blindness and vision loss	0 (0 to 0)	[illegible]	[illegible]	[illegible]	[illegible]	[illegible]	[illegible]	[illegible]	[illegible]	[illegible]	[illegible]	[illegible]	[illegible]	[illegible]	[illegible]	[illegible]	[illegible]	[illegible]	[illegible]	[illegible]	[illegible]	[illegible]	[illegible]	199
Glaucoma	0 (0 to 0)	[illegible]	[illegible]	[illegible]	[illegible]	[illegible]	[illegible]	[illegible]	[illegible]	[illegible]	[illegible]	[illegible]	[illegible]	[illegible]	[illegible]	[illegible]	[illegible]	[illegible]	[illegible]	[illegible]	[illegible]	[illegible]	[illegible]	11·6
Cataract	0 (0 to 0)	[illegible]	[illegible]	[illegible]	[illegible]	[illegible]	[illegible]	[illegible]	[illegible]	[illegible]	[illegible]	[illegible]	[illegible]	[illegible]	[illegible]	[illegible]	[illegible]	[illegible]	[illegible]	[illegible]	[illegible]	[illegible]	[illegible]	30
Age-related macular degeneration	0 (0 to 0)	[illegible]	[illegible]	[illegible]	[illegible]	[illegible]	[illegible]	[illegible]	[illegible]	[illegible]	[illegible]	[illegible]	[illegible]	[illegible]	[illegible]	[illegible]	[illegible]	[illegible]	[illegible]	[illegible]	[illegible]	[illegible]	[illegible]	1·3
Refraction disorders	0 (0 to 0)	[illegible]	[illegible]	[illegible]	[illegible]	[illegible]	[illegible]	[illegible]	[illegible]	[illegible]	[illegible]	[illegible]	[illegible]	[illegible]	[illegible]	[illegible]	[illegible]	[illegible]	[illegible]	[illegible]	[illegible]	[illegible]	[illegible]	47
Near vision loss	0 (0 to 0)	[illegible]	[illegible]	[illegible]	[illegible]	[illegible]	[illegible]	[illegible]	[illegible]	[illegible]	[illegible]	[illegible]	[illegible]	[illegible]	[illegible]	[illegible]	[illegible]	[illegible]	[illegible]	[illegible]	[illegible]	[illegible]	[illegible]	56·2
Other vision loss	0 (0 to 0)	[illegible]	[illegible]	[illegible]	[illegible]	[illegible]	[illegible]	[illegible]	[illegible]	[illegible]	[illegible]	[illegible]	[illegible]	[illegible]	[illegible]	[illegible]	[illegible]	[illegible]	[illegible]	[illegible]	[illegible]	[illegible]	[illegible]	53·1
Age-related and other hearing loss	0 (0 to 0)	[illegible]	[illegible]	[illegible]	[illegible]	[illegible]	[illegible]	[illegible]	[illegible]	[illegible]	[illegible]	[illegible]	[illegible]	[illegible]	[illegible]	[illegible]	[illegible]	[illegible]	[illegible]	[illegible]	[illegible]	[illegible]	[illegible]	514
Other sense organ diseases	0 (0 to 0)	[illegible]	[illegible]	[illegible]	[illegible]	[illegible]	[illegible]	[illegible]	[illegible]	[illegible]	[illegible]	[illegible]	[illegible]	[illegible]	[illegible]	[illegible]	[illegible]	[illegible]	[illegible]	[illegible]	[illegible]	[illegible]	[illegible]	32·2
Musculoskeletal disorders	0 (0 to 0)	[illegible]	[illegible]	[illegible]	[illegible]	[illegible]	[illegible]	[illegible]	[illegible]	[illegible]	[illegible]	[illegible]	[illegible]	[illegible]	[illegible]	[illegible]	[illegible]	[illegible]	[illegible]	[illegible]	[illegible]	[illegible]	[illegible]	1,380
Rheumatoid arthritis	0 (0 to 0)	[illegible]	[illegible]	[illegible]	[illegible]	[illegible]	[illegible]	[illegible]	[illegible]	[illegible]	[illegible]	[illegible]	[illegible]	[illegible]	[illegible]	[illegible]	[illegible]	[illegible]	[illegible]	[illegible]	[illegible]	[illegible]	[illegible]	20·6
Osteoarthritis	0 (0 to 0)	[illegible]	[illegible]	[illegible]	[illegible]	[illegible]	[illegible]	[illegible]	[illegible]	[illegible]	[illegible]	[illegible]	[illegible]	[illegible]	[illegible]	[illegible]	[illegible]	[illegible]	[illegible]	[illegible]	[illegible]	[illegible]	[illegible]	188
Osteoarthritis hip	0 (0 to 0)	[illegible]	[illegible]	[illegible]	[illegible]	[illegible]	[illegible]	[illegible]	[illegible]	[illegible]	[illegible]	[illegible]	[illegible]	[illegible]	[illegible]	[illegible]	[illegible]	[illegible]	[illegible]	[illegible]	[illegible]	[illegible]	[illegible]	11·9
Osteoarthritis knee	0 (0 to 0)	[illegible]	[illegible]	[illegible]	[illegible]	[illegible]	[illegible]	[illegible]	[illegible]	[illegible]	[illegible]	[illegible]	[illegible]	[illegible]	[illegible]	[illegible]	[illegible]	[illegible]	[illegible]	[illegible]	[illegible]	[illegible]	[illegible]	132
Osteoarthritis hand	0 (0 to 0)	[illegible]	[illegible]	[illegible]	[illegible]	[illegible]	[illegible]	[illegible]	[illegible]	[illegible]	[illegible]	[illegible]	[illegible]	[illegible]	[illegible]	[illegible]	[illegible]	[illegible]	[illegible]	[illegible]	[illegible]	[illegible]	[illegible]	16·4

Osteoarthritis other	0 (0 to 0)	0 (0 to 0)	0 (0 to 0)	0 (0 to 0)	0 (0 to 0)	0 (0 to 0)	0 (0 to 0)	0 (0 to 0)	0 (0 to 0)	0 (0 to 0·1)	0·3 (0·1 to 0·7)	0·9 (0·4 to 2·3)	2 (0·8 to 4·5)	3·4 (1·5 to 7·4)	4·1 (1·8 to 8·7)	4·1 (1·9 to 8·7)	3·5 (1·7 to 7·5)	3·4 (1·7 to 7·2)	2·4 (1·2 to 4·9)	1·7 (0·8 to 3·3)	0·8 (0·4 to 1·7)	0·3 (0·2 to 0·6)	0·1 (0 to 0·1)	27·1
Low back pain	0 (0 to 0)	0 (0 to 0)	0 (0 to 0)	0 (0 to 0)	2·3 (1 to 4·4)	12·7 (7·2 to 20·9)	26·3 (15·2 to 42·9)	33·8 (20 to 54·4)	41·2 (23·8 to 65·6)	42·9 (24 to 67)	52·3 (30·3 to 81·4)	57·4 (34·4 to 88·4)	61·8 (36·6 to 97·4)	66·3 (39·2 to 107)	59 (35·9 to 90·2)	49 (31 to 73·7)	37·3 (23·4 to 58·3)	32·8 (20·2 to 50·2)	20·7 (12·4 to 32·2)	13 (7·9 to 19·9)	5·6 (3·4 to 8·4)	1·8 (1·1 to 2·6)	0·2 (0·2 to 0·4)	617
Neck pain	0 (0 to 0)	0 (0 to 0)	0 (0 to 0)	0 (0 to 0)	0·1 (0 to 0·4)	1 (0·4 to 2)	2·4 (1 to 4·6)	4 (1·7 to 8)	6·4 (2·8 to 12·9)	9·2 (4·1 to 17·8)	12·9 (6·3 to 22·6)	15·8 (7·8 to 28·9)	18 (8·9 to 32·3)	16·9 (8·5 to 32·2)	14·1 (6·8 to 25·3)	10·7 (5·2 to 19·4)	7·4 (3·9 to 12·5)	6·2 (3·2 to 10·6)	3·6 (1·8 to 6·7)	2 (1 to 3·5)	0·9 (0·5 to 1·5)	0·3 (0·1 to 0·5)	0 (0 to 0·1)	132
Gout	0 (0 to 0)	0 (0 to 0)	0 (0 to 0)	0 (0 to 0)	0 (0 to 0)	0 (0 to 0)	0 (0 to 0)	0·1 (0 to 0·2)	0·3 (0 to 0·7)	0·5 (0·1 to 1·1)	0·8 (0·3 to 1·5)	1 (0·4 to 1·9)	1·2 (0·5 to 2·1)	1·3 (0·6 to 2·4)	1·2 (0·5 to 2·3)	1·1 (0·4 to 2·1)	0·9 (0·4 to 1·6)	0·9 (0·4 to 1·6)	0·7 (0·3 to 1·1)	0·5 (0·2 to 0·9)	0·3 (0·1 to 0·5)	0·1 (0·1 to 0·2)	0 (0 to 0)	11
Other musculoskeletal disorders	0 (0 to 0)	0 (0 to 0)	0 (0 to 0)	0 (0 to 0)	0·1 (0·1 to 0·2)	0·3 (0·2 to 0·8)	4·8 (2 to 8·7)	13·6 (8·1 to 21·3)	27·3 (15·9 to 43·3)	34·3 (20·9 to 53·3)	40·4 (25·4 to 60·3)	44·4 (25·9 to 70·3)	47 (28·8 to 71·9)	48·7 (28·3 to 77·7)	43·7 (26 to 66·4)	35·2 (20·7 to 55·8)	25·1 (14·6 to 39·2)	20·8 (11·9 to 33·8)	11·3 (6·4 to 17·9)	5·8 (2·6 to 10·2)	3·8 (2·4 to 5·5)	1·2 (0·7 to 1·9)	0·2 (0·1 to 0·3)	408
Other non-communicable diseases	87·9 (53·9 to 138)	20·7 (12·7 to 31·9)	66·2 (41·9 to 98·9)	39·3 (27·4 to 55·4)	31·1 (23·4 to 41·2)	28·4 (21·9 to 36·6)	36·4 (28·4 to 46·2)	46·5 (36 to 60)	53·6 (41 to 72·8)	56·8 (42·9 to 76·9)	64 (47·5 to 87·9)	69·2 (50·6 to 97·8)	73·2 (54·1 to 102)	80·5 (59·7 to 110)	80·9 (60·9 to 109)	82 (61·3 to 108)	69·2 (52·7 to 90·6)	76 (58 to 98·4)	55·2 (42·3 to 70·8)	38·6 (29·1 to 48·6)	23·9 (18·5 to 29·8)	10·2 (7·7 to 13)	1·8 (1·2 to 2·3)	1,190
Congenital birth defects	85·3 (52·1 to 135)	17·7 (10·4 to 28·9)	41 (25·2 to 62·6)	20·6 (13·6 to 31·9)	12·1 (9·2 to 16)	11·7 (8·6 to 15·3)	13·5 (9·9 to 17·5)	12·7 (9·2 to 17·1)	11·3 (8·1 to 15)	9·1 (6·7 to 11·9)	8·2 (6 to 11)	7·3 (5·4 to 9·4)	6·9 (5·2 to 9·2)	6·2 (4·6 to 8·1)	4·8 (3·6 to 6·3)	3 (2·2 to 3·9)	1·7 (1·2 to 2·3)	0·7 (0·4 to 1)	0·4 (0·2 to 0·6)	0·2 (0·1 to 0·3)	0·1 (0·1 to 0·1)	0 (0 to 0)	0 (0 to 0)	275
Neural tube defects	0·6 (0·3 to 1)	0·3 (0·1 to 0·7)	0·6 (0·2 to 1·2)	0·5 (0·3 to 0·9)	0·3 (0·2 to 0·4)	0·3 (0·2 to 0·4)	0·3 (0·2 to 0·4)	0·3 (0·2 to 0·4)	0·2 (0·1 to 0·3)	0·2 (0·1 to 0·2)	0·1 (0·1 to 0·2)	0·1 (0·1 to 0·1)	0·1 (0·1 to 0·1)	0·1 (0 to 0·1)	0 (0 to 0·1)	0 (0 to 0)	0 (0 to 0)	0 (0 to 0)	0 (0 to 0)	0 (0 to 0)	0 (0 to 0)	0 (0 to 0)	0 (0 to 0)	3·9
Congenital heart anomalies	35·1 (19·4 to 59·9)	8·8 (4·7 to 15)	19·3 (10·9 to 31)	7·5 (4 to 13·1)	3·6 (2·1 to 5·4)	3·1 (1·9 to 4·7)	3·8 (2·4 to 5·7)	3·5 (2·1 to 5·4)	3·3 (2 to 5)	2·7 (1·6 to 3·9)	2·3 (1·4 to 3·5)	1·8 (1·1 to 2·6)	1·5 (0·9 to 2·3)	1·1 (0·7 to 1·6)	0·6 (0·4 to 1)	0·5 (0·3 to 0·8)	0·3 (0·2 to 0·4)	0·1 (0 to 0·1)	0 (0 to 0·1)	0 (0 to 0·1)	0 (0 to 0)	0 (0 to 0)	0 (0 to 0)	98·9
Orofacial clefts	0 (0 to 0·1)	0 (0 to 0)	0·1 (0 to 0·2)	0·2 (0·1 to 0·4)	0·2 (0·1 to 0·4)	0·2 (0·1 to 0·4)	0·2 (0·1 to 0·5)	0·2 (0·1 to 0·5)	0·2 (0·1 to 0·5)	0·2 (0·1 to 0·4)	0·2 (0·1 to 0·5)	0·2 (0·1 to 0·4)	0·2 (0·1 to 0·4)	0·2 (0·1 to 0·4)	0·2 (0 to 0·3)	0·1 (0 to 0·3)	0·1 (0 to 0·2)	0·1 (0 to 0·1)	0 (0 to 0·1)	0 (0 to 0·1)	0 (0 to 0)	0 (0 to 0)	0 (0 to 0)	3
Down syndrome	1·6 (0·7 to 3)	0·5 (0·2 to 1·1)	2·8 (1·4 to 5·3)	1·8 (0·9 to 3·6)	0·7 (0·4 to 1·1)	1 (0·6 to 1·6)	1·7 (1·1 to 2·7)	1·5 (0·9 to 2·5)	1·6 (0·9 to 2·7)	1·6 (0·9 to 2·6)	1·6 (0·9 to 2·6)	1·7 (1·1 to 2·6)	1·7 (1·1 to 2·7)	1·6 (0·9 to 2·5)	1·6 (1 to 2·4)	0·8 (0·5 to 1·2)	0·3 (0·2 to 0·5)	0 (0 to 0)	0 (0 to 0)	0 (0 to 0)	0 (0 to 0)	0 (0 to 0)	0 (0 to 0)	24·3
Turner syndrome	0 (0 to 0)	0 (0 to 0)	0 (0 to 0)	0 (0 to 0)	0 (0 to 0)	0 (0 to 0)	0 (0 to 0)	0 (0 to 0)	0 (0 to 0)	0 (0 to 0)	0 (0 to 0)	0 (0 to 0)	0 (0 to 0)	0 (0 to 0)	0 (0 to 0)	0 (0 to 0)	0 (0 to 0)	0 (0 to 0)	0 (0 to 0)	0 (0 to 0)	0 (0 to 0)	0 (0 to 0)	0 (0 to 0)	0
Klinefelter syndrome	0 (0 to 0)	0 (0 to 0)	0 (0 to 0)	0 (0 to 0)	0 (0 to 0)	0 (0 to 0)	0 (0 to 0)	0 (0 to 0)	0 (0 to 0)	0 (0 to 0)	0 (0 to 0)	0 (0 to 0)	0 (0 to 0)	0 (0 to 0)	0 (0 to 0)	0 (0 to 0)	0 (0 to 0)	0 (0 to 0)	0 (0 to 0)	0 (0 to 0)	0 (0 to 0)	0 (0 to 0)	0 (0 to 0)	0
Other chromosomal abnormalities	0·9 (0·4 to 1·8)	0·4 (0·2 to 0·7)	1·1 (0·6 to 1·7)	0·8 (0·5 to 1·2)	0·5 (0·3 to 0·8)	0·5 (0·3 to 0·8)	0·6 (0·4 to 0·9)	0·4 (0·3 to 0·6)	0·3 (0·2 to 0·5)	0·2 (0·1 to 0·2)	0·1 (0·1 to 0·2)	0·1 (0·1 to 0·1)	0·1 (0 to 0·1)	0 (0 to 0·1)	0 (0 to 0)	0 (0 to 0)	0 (0 to 0)	0 (0 to 0)	0 (0 to 0)	0 (0 to 0)	0 (0 to 0)	0 (0 to 0)	0 (0 to 0)	6·2
Congenital musculoskeletal and limb anomalies	4·3 (1·9 to 8·9)	1 (0·5 to 2)	2·8 (1·6 to 5)	3 (1·7 to 4·6)	2·9 (1·7 to 4·6)	3·2 (1·8 to 5·1)	2·9 (1·7 to 4·5)	2·1 (1·3 to 3·2)	2·1 (1·3 to 3·2)	1·5 (0·8 to 2·4)	1·7 (0·9 to 2·6)	1·5 (0·8 to 2·4)	1·5 (0·9 to 2·4)	1·6 (0·9 to 2·4)	1·2 (0·7 to 2)	0·9 (0·5 to 1·4)	0·5 (0·3 to 0·9)	0·3 (0·2 to 0·6)	0·2 (0·1 to 0·3)	0·1 (0 to 0·1)	0 (0 to 0·1)	0 (0 to 0)	0 (0 to 0)	35·2
Urogenital congenital anomalies	5·4 (2·5 to 10·8)	0·8 (0·3 to 2)	1·4 (0·6 to 2·7)	0·7 (0·4 to 1·2)	0·5 (0·3 to 0·7)	0·4 (0·2 to 0·6)	0·2 (0·1 to 0·4)	0·3 (0·2 to 0·4)	0·2 (0·1 to 0·4)	0·3 (0·1 to 0·4)	0·1 (0·1 to 0·2)	0·1 (0·1 to 0·2)	0·2 (0·1 to 0·2)	0·1 (0 to 0·1)	0·1 (0·1 to 0·2)	0·1 (0 to 0·1)	0 (0 to 0)	0 (0 to 0)	0 (0 to 0)	0 (0 to 0)	0 (0 to 0)	0 (0 to 0)	0 (0 to 0)	10·8
Digestive congenital anomalies	8 (4·1 to 14·6)	1·7 (0·8 to 3·2)	2·9 (1·4 to 5·2)	0·8 (0·5 to 1·5)	0·4 (0·2 to 0·6)	0·2 (0·1 to 0·3)	0·2 (0·1 to 0·3)	0·1 (0·1 to 0·1)	0·1 (0·1 to 0·1)	0·1 (0 to 0·1)	0 (0 to 0·1)	0 (0 to 0·1)	0·1 (0 to 0·1)	0 (0 to 0·1)	0 (0 to 0)	0 (0 to 0)	0 (0 to 0)	0 (0 to 0)	0 (0 to 0)	0 (0 to 0)	0 (0 to 0)	0 (0 to 0)	0 (0 to 0)	14·7
Other congenital birth defects	29·3 (16·2 to 48·4)	4·1 (2·2 to 6·8)	10·1 (5·6 to 16·6)	5·3 (3 to 9)	3·1 (2 to 4·7)	2·9 (1·8 to 4·3)	3·6 (2·2 to 5·3)	4·3 (2·7 to 6·5)	3·3 (2 to 4·9)	2·5 (1·5 to 3·8)	2 (1·2 to 3)	1·7 (1·1 to 2·6)	1·6 (1 to 2·5)	1·6 (1 to 2·4)	0·9 (0·6 to 1·5)	0·6 (0·3 to 1)	0·4 (0·2 to 0·7)	0·2 (0·1 to 0·4)	0·1 (0 to 0·3)	0·1 (0 to 0·2)	0 (0 to 0·1)	0 (0 to 0)	0 (0 to 0)	77·7
Urinary diseases and male infertility	0·2 (0·1 to 0·4)	0·2 (0·1 to 0·4)	1·2 (0·6 to 2·2)	0·6 (0·3 to 1)	0·4 (0·3 to 0·7)	0·5 (0·3 to 0·8)	0·9 (0·7 to 1·3)	3·6 (2·3 to 5·7)	4·4 (2·6 to 6·9)	5·6 (3·4 to 8·8)	7·9 (5 to 11·7)	8·8 (5·6 to 13)	10·5 (6·2 to 14·8)	14·6 (9·3 to 20·7)	20·4 (13·3 to 27·9)	27·2 (17·8 to 37·5)	26·2 (17·8 to 35·5)	33·6 (22·4 to 45·2)	26·2 (16·7 to 36)	19·2 (11·7 to 26·9)	12·5 (7·8 to 17·9)	5·6 (3·4 to 8·1)	1 (0·6 to 1·5)	231
Urolithiasis	0 (0 to 0)	0 (0 to 0)	0 (0 to 0)	0 (0 to 0)	0 (0 to 0)	0 (0 to 0)	0 (0 to 0·1)	0·1 (0·1 to 0·2)	0·2 (0·1 to 0·3)	0·3 (0·1 to 0·5)	0·4 (0·1 to 0·9)	0·5 (0·1 to 1)	0·7 (0·3 to 1·2)	0·8 (0·3 to 1·4)	0·8 (0·3 to 1·4)	0·6 (0·3 to 1·1)	0·4 (0·2 to 0·7)	0·3 (0·1 to 0·5)	0·2 (0·1 to 0·3)	0·1 (0 to 0·1)	0 (0 to 0)	0 (0 to 0)	0 (0 to 0)	5·2
Benign prostatic hyperplasia	0 (0 to 0)	0 (0 to 0)	0 (0 to 0)	0 (0 to 0)	0 (0 to 0)	0 (0 to 0)	0 (0 to 0)	0 (0 to 0)	0 (0 to 0)	0 (0 to 0)	0 (0 to 0)	0·1 (0 to 0·2)	0·5 (0·2 to 1)	2·1 (1 to 3·7)	6·1 (2·8 to 10·5)	9·8 (5 to 16·9)	11·3 (6·4 to 17·9)	12·5 (6·9 to 20·5)	7·9 (4·4 to 12·7)	4·2 (2·4 to 6·7)	1·5 (0·8 to 2·5)	0·4 (0·2 to 0·7)	0 (0 to 0·1)	56·3
Male infertility	0 (0 to 0)	0 (0 to 0)	0 (0 to 0)	0 (0 to 0)	0 (0 to 0)	0 (0 to 0)	0 (0 to 0)	1·3 (0·3 to 3·3)	1·6 (0·5 to 4)	1·6 (0·5 to 4·6)	1·7 (0·5 to 4·4)	1·6 (0·4 to 4)	0·2 (0 to 0·7)	0 (0 to 0)	0 (0 to 0)	0 (0 to 0)	0 (0 to 0)	0 (0 to 0)	0 (0 to 0)	0 (0 to 0)	0 (0 to 0)	0 (0 to 0)	0 (0 to 0)	8·1
Other urinary diseases	0·1 (0 to 0·2)	0·1 (0·1 to 0·2)	0·2 (0·1 to 0·3)	0·1 (0·1 to 0·2)	0·2 (0·1 to 0·3)	0·2 (0·1 to 0·4)	0·5 (0·3 to 0·7)	1·1 (0·5 to 1·7)	0·6 (0·3 to 1·1)	1 (0·5 to 1·6)	2 (1 to 3·3)	1·9 (1 to 3·2)	3 (1·5 to 5·1)	3·1 (1·6 to 5·4)	3 (1·4 to 5)	4 (2 to 6·6)	3·3 (1·6 to 5·5)	3·5 (1·6 to 6·1)	3·2 (1·3 to 5·5)	2·5 (1 to 4·6)	1·5 (0·6 to 2·8)	0·9 (0·4 to 1·5)	0·2 (0·1 to 0·3)	36·2
Gynecological diseases	0 (0 to 0)	0 (0 to 0)	0 (0 to 0)	0 (0 to 0)	0 (0 to 0)	0 (0 to 0)	0 (0 to 0)	0 (0 to 0)	0 (0 to 0)	0 (0 to 0)	0 (0 to 0)	0 (0 to 0)	0 (0 to 0)	0 (0 to 0)	0 (0 to 0)	0 (0 to 0)	0 (0 to 0)	0 (0 to 0)	0 (0 to 0)	0 (0 to 0)	0 (0 to 0)	0 (0 to 0)	0 (0 to 0)	0
Uterine fibroids	0 (0 to 0)	0 (0 to 0)	0 (0 to 0)	0 (0 to 0)	0 (0 to 0)	0 (0 to 0)	0 (0 to 0)	0 (0 to 0)	0 (0 to 0)	0 (0 to 0)	0 (0 to 0)	0 (0 to 0)	0 (0 to 0)	0 (0 to 0)	0 (0 to 0)	0 (0 to 0)	0 (0 to 0)	0 (0 to 0)	0 (0 to 0)	0 (0 to 0)	0 (0 to 0)	0 (0 to 0)	0 (0 to 0)	0
Polycystic ovarian syndrome	--	--	--	--	--	--	--	--	--	--	--	--	--	--	--	--	--	--	--	--	--	--	--	NA
Female infertility	--	--	--	--	--	--	--	--	--	--	--	--	--	--	--	--	--	--	--	--	--	--	--	NA
Endometriosis	--	--	--	--	--	--	--	--	--	--	--	--	--	--	--	--	--	--	--	--	--	--	--	NA
Genital prolapse	0 (0 to 0)	0 (0 to 0)	0 (0 to 0)	0 (0 to 0)	0 (0 to 0)	0 (0 to 0)	0 (0 to 0)	0 (0 to 0)	0 (0 to 0)	0 (0 to 0)	0 (0 to 0)	0 (0 to 0)	0 (0 to 0)	0 (0 to 0)	0 (0 to 0)	0 (0 to 0)	0 (0 to 0)	0 (0 to 0)	0 (0 to 0)	0 (0 to 0)	0 (0 to 0)	0 (0 to 0)	0 (0 to 0)	0
Premenstrual syndrome	--	--	--	--	--	--	--	--	--	--	--	--	--	--	--	--	--	--	--	--	--	--	--	NA
Other gynecological diseases	0 (0 to 0)	0 (0 to 0)	0 (0 to 0)	0 (0 to 0)	0 (0 to 0)	0 (0 to 0)	0 (0 to 0)	0 (0 to 0)	0 (0 to 0)	0 (0 to 0)	0 (0 to 0)	0 (0 to 0)	0 (0 to 0)	0 (0 to 0)	0 (0 to 0)	0 (0 to 0)	0 (0 to 0)	0 (0 to 0)	0 (0 to 0)	0 (0 to 0)	0 (0 to 0)	0 (0 to 0)	0 (0 to 0)	0
Hemoglobinopathies and hemolytic anemias	0·4 (0·2 to 0·6)	0·3 (0·2 to 0·5)	2·4 (1·4 to 3·9)	6·9 (4·1 to 10·7)	10 (6·4 to 15·1)	8 (5·1 to 11·6)	11·1 (7·3 to 16·8)	13·7 (8·8 to 20·9)	12·5 (8·5 to 18·2)	11 (7·3 to 15·4)	10·1 (6·9 to 14·4)	10·9 (7·2 to 15·6)	9·7 (6·7 to 13·8)	8·2 (5·6 to 11·7)	7 (4·7 to 10·2)	5·6 (3·6 to 8·3)	3·4 (2·3 to 5)	3·7 (2·6 to 5·2)	2·5 (1·8 to 3·5)	1·8 (1·3 to 2·5)	1·9 (1·3 to 2·5)	0·9 (0·6 to 1·2)	0·2 (0·1 to 0·2)	142
Thalassemias	0 (0 to 0)	0 (0 to 0)	0 (0 to 0)	0 (0 to 0·1)	0 (0 to 0)	0 (0 to 0)	0 (0 to 0)	0 (0 to 0)	0 (0 to 0)	0 (0 to 0)	0 (0 to 0)	0 (0 to 0)	0 (0 to 0)	0 (0 to 0)	0 (0 to 0)	0 (0 to 0)	0 (0 to 0)	0 (0 to 0)	0 (0 to 0)	0 (0 to 0)	0 (0 to 0)	0 (0 to 0)	0 (0 to 0)	0·1
Thalassemias trait	0 (0 to 0)	0 (0 to 0)	0 (0 to 0·1)	0·2 (0 to 0·3)	0·3 (0·1 to 0·6)	0·1 (0 to 0·3)	0·1 (0 to 0·2)	0 (0 to 0·1)	0 (0 to 0·1)	0 (0 to 0·1)	0 (0 to 0·1)	0 (0 to 0·1)	0·1 (0 to 0·1)	0·1 (0 to 0·2)	0·1 (0 to 0·2)	0·1 (0 to 0·1)	0 (0 to 0·1)	0 (0 to 0·1)	0 (0 to 0·1)	0 (0 to 0)	0 (0 to 0)	0 (0 to 0)	0 (0 to 0)	1·2
Sickle cell disorders	0·3 (0·2 to 0·5)	0·2 (0·1 to 0·4)	1·8 (0·9 to 3·3)	4·1 (2·4 to 7·1)	5·5 (3·6 to 7·9)	5·6 (3·5 to 8·2)	8·3 (5·3 to 12·9)	9·6 (5·4 to 15·3)	7·7 (4·7 to 12·3)	6·2 (3·6 to 9·7)	5·1 (3 to 8·1)	5 (2·7 to 7·8)	3·5 (1·9 to 5·9)	1·9 (0·9 to 3·5)	0·7 (0·3 to 1·4)	0·1 (0·1 to 0·3)	0 (0 to 0)	0 (0 to 0)	0 (0 to 0)	0 (0 to 0)	0 (0 to 0)	0 (0 to 0)	0 (0 to 0)	65·7
Sickle cell trait	0 (0 to 0)	0·1 (0 to 0·2)	0·3 (0·1 to 0·7)	1·4 (0·5 to 2·8)	2·4 (0·7 to 5)	1·1 (0·2 to 2·6)	0·5 (0·1 to 1·4)	0·4 (0·1 to 1·1)	0·4 (0·1 to 1)	0·4 (0·1 to 1)	0·4 (0·1 to 1)	0·4 (0·1 to 1·1)	0·5 (0·1 to 1·2)	0·6 (0·1 to 1·4)	0·7 (0·2 to 1·5)	0·5 (0·2 to 1·1)	0·3 (0·1 to 0·7)	0·3 (0·1 to 0·6)	0·2 (0·1 to 0·5)	0·2 (0 to 0·3)	0·1 (0 to 0·2)	0 (0 to 0·1)	0 (0 to 0)	11·3
G6PD deficiency	0 (0 to 0)	0 (0 to 0)	0 (0 to 0·1)	0·4 (0·2 to 0·7)	0·6 (0·2 to 1·1)	0·6 (0·3 to 1·2)	1·4 (0·6 to 2·6)	2·9 (1·1 to 5·8)	3·5 (1·6 to 6·3)	3·5 (1·6 to 6)	3·5 (1·7 to 6·2)	4·3 (2·1 to 7·2)	4·3 (2·2 to 6·7)	4 (2·3 to 5·9)	3·6 (2·3 to 5·3)	2·6 (1·6 to 4·1)	1·3 (0·8 to 2)	1·1 (0·6 to 1·7)	0·5 (0·2 to 0·7)	0·2 (0·1 to 0·3)	0·1 (0·1 to 0·2)	0 (0 to 0)	0 (0 to 0)	38·2
G6PD trait	0 (0 to 0)	0 (0 to 0)	0 (0 to 0)	0 (0 to 0)	0 (0 to 0)	0 (0 to 0)	0 (0 to 0)	0 (0 to 0)	0 (0 to 0)	0 (0 to 0)	0 (0 to 0)	0 (0 to 0)	0 (0 to 0)	0 (0 to 0)	0 (0 to 0)	0 (0 to 0)	0 (0 to 0)	0 (0 to 0)	0 (0 to 0)	0 (0 to 0)	0 (0 to 0)	0 (0 to 0)	0 (0 to 0)	0
Other hemoglobinopathies and hemolytic anemias	0 (0 to 0)	0 (0 to 0)	0·2 (0·1 to 0·4)	0·8 (0·2 to 1·8)	1·2 (0·3 to 3)	0·6 (0·1 to 1·4)	0·9 (0·3 to 2·5)	0·9 (0·3 to 2)	0·8 (0·4 to 1·8)	0·9 (0·4 to 1·9)	1 (0·4 to 2)	1·2 (0·6 to 2·2)	1·4 (0·7 to 2·6)	1·7 (0·9 to 3·1)	2·1 (1·1 to 3·7)	2·3 (1·3 to 3·8)	1·8 (1·1 to 2·7)	2·3 (1·5 to 3·3)	1·8 (1·3 to 2·5)	1·5 (1·1 to 2)	1·7 (1·2 to 2·3)	0·8 (0·6 to 1·1)	0·2 (0·1 to 0·2)	25·9
Endocrine, metabolic, blood, and immune disorders	2 (1·1 to 3·6)	1·2 (0·6 to 2·8)	10·7 (5·4 to 22·3)	9·1 (5 to 18)	5·6 (3·3 to 9·2)	4·5 (2·9 to 7·1)	4·9 (3·3 to 7·2)	6·6 (4·4 to 9·6)	10·7 (6·9 to 15·3)	13·7 (9·2 to 20·1)	16·2 (11·2 to 23·8)	17·9 (12·3 to 26·6)	19·3 (12·7 to 30)	22·3 (15 to 35·6)	20·4 (13·8 to 30·6)	19·1 (13 to 28·7)	14·3 (9·7 to 22·7)	14·9 (10·3 to 23)	10·6 (6·9 to 16·3)	7·5 (5·1 to 11·2)	4·8 (3·3 to 7·5)	2·1 (1·5 to 3)	0·4 (0·2 to 0·5)	239
Oral disorders	0 (0 to 0)	0 (0 to 0)	0 (0 to 0)	2·2 (1·2 to 3·6)	3 (1·6 to 5·2)	3·6 (1·9 to 6·4)	6 (3·2 to 10·8)	9·9 (5·1 to 18·4)	14·8 (7·2 to 28·9)	17·4 (8·1 to 32·1)	21·6 (10·1 to 40·9)	24·3 (11·5 to 45·8)	26·8 (13·4 to 48·8)	29·1 (15·1 to 51·6)	28·3 (15·8 to 48·2)	27·1 (16 to 44)	23·6 (13·9 to 37·2)	23·1 (14·2 to 36·7)	15·5 (9·6 to 23·5)	9·8 (6·1 to 14·7)	4·6 (2·8 to 6·9)	1·6 (1 to 2·4)	0·2 (0·1 to 0·4)	293
Caries of deciduous teeth	0 (0 to 0)	0 (0 to 0)	0 (0 to 0)	0·8 (0·3 to 1·6)	0·8 (0·3 to 1·8)	0·1 (0 to 0·1)	0 (0 to 0)	0 (0 to 0)	0 (0 to 0)	0 (0 to 0)	0 (0 to 0)	0 (0 to 0)	0 (0 to 0)	0 (0 to 0)	0 (0 to 0)	0 (0 to 0)	0 (0 to 0)	0 (0 to 0)	0 (0 to 0)	0 (0 to 0)	0 (0 to 0)	0 (0 to 0)	0 (0 to 0)	1·6
Caries of permanent teeth	0 (0 to 0)	0 (0 to 0)	0 (0 to 0)	0 (0 to 0)	0·7 (0·2 to 1·5)	1·8 (0·7 to 3·8)	2·2 (0·9 to 4·7)	2·5 (1·1 to 5·2)	2·5 (1 to 5)	2·1 (0·8 to 4·2)	2·2 (0·9 to 4·3)	2 (0·8 to 4·1)	1·9 (0·8 to 4)	1·8 (0·7 to 3·8)	1·4 (0·5 to 3·2)	1 (0·3 to 2·1)	0·6 (0·2 to 1·3)	0·5 (0·2 to 1)	0·3 (0·1 to 0·5)	0·2 (0·1 to 0·3)	0·1 (0 to 0·1)	0 (0 to 0)	0 (0 to 0)	23·7
Periodontal diseases	0 (0 to 0)	0 (0 to 0)	0 (0 to 0)	0 (0 to 0)	0 (0 to 0)	0 (0 to 0)	0·9 (0·2 to 2·3)	3·3 (1 to 8·5)	7·3 (2·3 to 17·9)	10 (3·4 to 23·1)	13·1 (4·6 to 29·7)	15 (5·4 to 32·4)	16·2 (6 to 34·4)	16·2 (6·3 to 34·6)	12·8 (5·1 to 27)	8·9 (3·5 to 18·5)	5·6 (2·2 to 11·6)	4·4 (1·7 to 8·8)	2·5 (1 to 5·1)	1·5 (0·6 to 3)	0·7 (0·3 to 1·4)	0·2 (0·1 to 0·5)	0 (0 to 0·1)	119
Other oral disorders	0 (0 to 0)	0 (0 to 0)	0 (0 to 0)	1·4 (0·8 to 2·2)	1·5 (0·9 to 2·3)	1·8 (1 to 2·9)	3 (1·7 to 4·7)	3·7 (2·2 to 5·6)	4·1 (2·4 to 6·2)	3·9 (2·3 to 6)	4·1 (2·4 to 6·4)	4·1 (2·5 to 6·3)	4·2 (2·5 to 6·4)	4 (2·5 to 6·1)	3·3 (2 to 5·1)	2·5 (1·5 to 3·8)	1·7 (1 to 2·7)	1·3 (0·8 to 2)	0·7 (0·4 to 1)	0·3 (0·2 to 0·5)	0·1 (0·1 to 0·2)	0 (0 to 0·1)	0 (0 to 0)	45·7
Sudden infant death syndrome	--	1·3 (0·7 to 2·3)	10·9 (5·3 to 20·6)	--	--	--	--	--	--	--	--	--	--	--	--	--	--	--	--	--	--	--	--	NA
Injuries	7·2 (4·5 to 10·5)	1·9 (1·2 to 3)	15·5 (9·8 to 23·9)	41·1 (28·3 to 58·5)	62 (52·1 to 73·1)	97 (81·7 to 114)	348 (293 to 411)	645 (560 to 735)	699 (623 to 781)	564 (499 to 637)	483 (416 to 561)	444 (377 to 517)	409 (342 to 489)	332 (278 to 393)	250 (208 to 297)	180 (150 to 211)	113 (95·6 to 134)	104 (87·9 to 122)	67·7 (57·1 to 79·2)	47·4 (40·5 to 55·3)	28·6 (24·2 to 33·1)	13·2 (11·2 to 15·2)	2·6 (2·2 to 3)	4,950
Transport injuries	0·4 (0·2 to 0·6)	0·1 (0·1 to 0·2)	1·3 (0·7 to 2·1)	6·8 (4 to 11·4)	15·2 (10·3 to 21·5)	24·5 (17·7 to 33·9)	83·2 (58·5 to 113)	161 (116 to 211)	164 (126 to 208)	141 (111 to 179)	127 (99·5 to 163)	123 (96·5 to 155)	117 (89·5 to 156)	95·5 (74·3 to 120)	74·2 (58·8 to 92)	51·9 (41·4 to 62·8)	31·6 (25·8 to 38·7)	27·2 (21·9 to 33·3)	16·5 (13·2 to 20·3)	9·6 (7·8 to 11·7)	4·7 (3·7 to 5·9)	1·6 (1·3 to 1·9)	0·3 (0·2 to 0·3)	1,280
Road injuries	0·3 (0·2 to 0·4)	0·1 (0 to 0·1)	1 (0·5 to 1·6)	6 (3·5 to 10·3)	13·8 (9·1 to 19·8)	21·7 (15·6 to 30·8)	76 (52·9 to 104)	147 (105 to 195)	150 (114 to 193)	128 (99·2 to 165)	116 (89·3 to 149)	111 (85·9 to 140)	107 (80·1 to 142)	87·3 (67·5 to 111)	66·8 (52·6 to 83·7)	47·8 (37·9 to 58·3)	29·4 (23·9 to 36·2)	24·5 (19·6 to 30·4)	15·3 (12·1 to 18·9)	9 (7·3 to 11·1)	4·4 (3·5 to 5·5)	1·5 (1·2 to 1·8)	0·2 (0·2 to 0·3)	1,160
Pedestrian road injuries	0·1 (0·1 to 0·2)	0 (0 to 0)	0·2 (0·1 to 0·4)	2 (1·2 to 3·4)	4·9 (3·2 to 7·1)	5·5 (3·8 to 8)	6·3 (4·4 to 8·5)	9·7 (6·9 to 12·8)	12·1 (9·3 to 15·4)	12·5 (9·7 to 16·4)	13·4 (10·2 to 17·4)	15·1 (11·4 to 19·8)	16·2 (12 to 21·9)	15 (11·3 to 19·5)	13·1 (10·1 to 17·1)	10·2 (7·8 to 12·9)	7 (5·5 to 8·8)	6·8 (5·3 to 8·8)	4·9 (3·8 to 6·2)	3·3 (2·6 to 4·2)	1·7 (1·3 to 2·2)	0·5 (0·4 to 0·7)	0·1 (0·1 to 0·1)	161
Cyclist road injuries	0 (0 to 0)	0 (0 to 0)	0 (0 to 0)	0·3 (0·2 to 0·6)	0·9 (0·5 to 1·5)	3·3 (1·9 to 5·3)	7·6 (4·4 to 12·5)	10·2 (6·4 to 15·9)	10·2 (6·9 to 14·4)	9·8 (6·7 to 14)	9·9 (7 to 13·9)	10·1 (7·3 to 13·5)	10·1 (7·1 to 13·9)	9·8 (7·2 to 12·9)	7·8 (5·8 to 10·6)	5·9 (4·3 to 7·8)	4 (3 to 5·3)	3·2 (2·3 to 4·2)	1·8 (1·3 to 2·5)	1·1 (0·8 to 1·4)	0·5 (0·3 to 0·6)	0·2 (0·1 to 0·2)	0 (0 to 0)	107
Motorcyclist road injuries	0 (0 to 0·1)	0 (0 to 0)	0·1 (0·1 to 0·2)	0·5 (0·3 to 0·8)	1·2 (0·8 to 1·7)	2 (1·4 to 2·8)	17·8 (12 to 24·6)	41·2 (28·9 to 54·7)	43·8 (33·1 to 57·2)	34·9 (26·9 to 45·7)	29·6 (22·7 to 38·6)	24·3 (18·9 to 31·4)	22·3 (16·7 to 29·3)	16·7 (12·8 to 21·5)	12·2 (9·4 to 15·4)	7·3 (5·8 to 9·1)	4·5 (3·5 to 5·7)	3·1 (2·3 to 4·1)	[illegible]	[illegible]	[illegible]	[illegible]	0 (0 to 0)	265
Motor vehicle road injuries	0·1 (0·1 to 0·2)	0·1 (0 to 0·1)	0·6 (0·3 to 1·1)	3·1 (1·7 to 5·6)	6·7 (4·4 to 9·7)	10·6 (7·3 to 14·9)	43·5 (30·1 to 59·7)	84·5 (59·3 to 113)	81·8 (61·8 to 106)	68·8 (53·1 to 89·5)	61 (46 to 81·1)	59·3 (44·7 to 76·7)	56 (41 to 75·8)	44 (33·3 to 57·3)	32·2 (24·9 to 41)	23·3 (18 to 29)	13·1 (10·6 to 16·3)	10·7 (8·5 to 13·5)	[illegible]	[illegible]	[illegible]	[illegible]	0·1 (0·1 to 0·1)	612
Other road injuries	0 (0 to 0)	0 (0 to 0)	0 (0 to 0)	0·1 (0 to 0·2)	0·2 (0·1 to 0·3)	0·3 (0·2 to 0·5)	0·7 (0·4 to 1·3)	1·7 (1 to 2·8)	1·7 (1 to 2·7)	1·6 (1 to 2·7)	1·7 (1·1 to 2·6)	1·8 (1·2 to 2·6)	2·1 (1·4 to 2·9)	1·8 (1·3 to 2·5)	1·5 (1 to 2)	1·1 (0·8 to 1·5)	0·8 (0·6 to 1·1)	0·7 (0·5 to 0·9)	[illegible]	[illegible]	[illegible]	[illegible]	0 (0 to 0)	18·6
Other transport injuries	0·1 (0 to 0·1)	0 (0 to 0·1)	0·3 (0·2 to 0·5)	0·8 (0·5 to 1·4)	1·4 (0·9 to 2·2)	2·7 (1·7 to 4·3)	7·2 (4 to 11·4)	13·6 (8·3 to 21·7)	14·2 (8·6 to 22·6)	13·5 (8·4 to 20·1)	11·9 (7·4 to 18·8)	12·2 (8·2 to 18)	10·4 (6·6 to 16·1)	8·3 (5·4 to 12)	7·4 (5·1 to 10·5)	4·1 (2·8 to 5·9)	2·2 (1·6 to 3)	2·7 (1·8 to 3·8)	[illegible]	[illegible]	[illegible]	[illegible]	0 (0 to 0)	115
Unintentional injuries	4 (2·5 to 6)	1·5 (1 to 2·4)	12·2 (7·4 to 19·5)	29·2 (19·9 to 42)	41 (32·7 to 50·3)	56·6 (45·5 to 69·7)	106 (81 to 134)	149 (115 to 190)	154 (123 to 190)	140 (115 to 169)	136 (111 to 164)	139 (114 to 169)	134 (107 to 162)	124 (100 to 153)	100 (81·3 to 122)	79 (63·9 to 96·1)	54·2 (43·7 to 66·2)	53·8 (43·9 to 65·9)	[illegible]	[illegible]	[illegible]	[illegible]	2·2 (1·8 to 2·5)	1,620
Falls	0·1 (0 to 0·1)	0 (0 to 0)	0·4 (0·3 to 0·7)	1·4 (0·9 to 2·1)	2·5 (1·8 to 3·3)	4·9 (3·6 to 6·5)	9·7 (7·4 to 12·6)	14 (10·6 to 17·8)	16·2 (12·6 to 20·6)	16·9 (13·3 to 21·4)	19·7 (15·3 to 25·2)	22·2 (17·5 to 28)	24·6 (19·1 to 30·4)	26·2 (20·6 to 32·8)	23·1 (18·1 to 28·7)	20·1 (15·6 to 24·8)	15·1 (12 to 18·7)	17·8 (14·3 to 22)	[illegible]	[illegible]	[illegible]	[illegible]	1·3 (1 to 1·6)	282
Drowning	0·4 (0·2 to 0·7)	0·1 (0 to 0·1)	1·6 (0·9 to 2·5)	12·3 (7·7 to 18·5)	20·5 (15·1 to 26·8)	26·1 (19·6 to 33·7)	49·4 (34·6 to 66·5)	59·3 (42 to 81·6)	53·2 (39·5 to 69·8)	43·4 (32·6 to 55·6)	37·1 (28·7 to 46·8)	34·7 (27 to 44)	28·2 (21·7 to 36·7)	22·3 (16·6 to 29)	14·8 (11·4 to 19·5)	9·6 (7·5 to 12·4)	4·9 (3·8 to 6·1)	3·6 (2·8 to 4·5)	[illegible]	[illegible]	[illegible]	[illegible]	0 (0 to 0·1)	425
Fire, heat, and hot substances	0·1 (0·1 to 0·2)	0·1 (0 to 0·1)	1·2 (0·7 to 1·8)	4·2 (2·7 to 6·4)	4·7 (3·6 to 5·9)	5·7 (4·3 to 7·4)	7·4 (5·5 to 9·9)	10·2 (7·8 to 13·2)	12 (9·2 to 15·4)	13·1 (10·1 to 16·8)	13·1 (10 to 17·2)	13·7 (10·4 to 17·8)	14·1 (10·7 to 18·2)	12·8 (9·6 to 16·9)	10·5 (7·9 to 13·9)	8·3 (6·2 to 11)	5·4 (4 to 7·2)	5·2 (4 to 6·8)	[illegible]	[illegible]	[illegible]	[illegible]	0·2 (0·1 to 0·2)	150
Poisonings	0 (0 to 0)	0 (0 to 0)	0·1 (0·1 to 0·2)	0·6 (0·4 to 1)	0·4 (0·3 to 0·6)	0·6 (0·4 to 0·8)	0·8 (0·6 to 1·1)	1·3 (1 to 1·8)	1·8 (1·3 to 2·4)	1·8 (1·3 to 2·3)	1·6 (1 to 2)	1·3 (1 to 1·7)	1·2 (0·8 to 1·6)	0·9 (0·7 to 1·3)	0·7 (0·5 to 1)	0·5 (0·4 to 0·7)	0·3 (0·2 to 0·4)	0·3 (0·2 to 0·4)	[illegible]	[illegible]	[illegible]	[illegible]	0 (0 to 0)	14·6
Poisoning by carbon monoxide	0 (0 to 0)	0 (0 to 0)	0 (0 to 0·1)	0·4 (0·2 to 0·7)	0·2 (0·1 to 0·3)	0·2 (0·1 to 0·3)	0·4 (0·2 to 0·5)	0·8 (0·5 to 1·1)	1·2 (0·8 to 1·7)	1·2 (0·8 to 1·7)	1 (0·7 to 1·4)	0·8 (0·5 to 1·1)	0·6 (0·4 to 0·9)	0·4 (0·3 to 0·6)	0·3 (0·2 to 0·5)	0·2 (0·2 to 0·3)	0·1 (0·1 to 0·2)	0·1 (0·1 to 0·2)	[illegible]	[illegible]	[illegible]	[illegible]	0 (0 to 0)	8·2
Poisoning by other means	0 (0 to 0)	0 (0 to 0)	0·1 (0·1 to 0·1)	0·2 (0·1 to 0·4)	0·2 (0·1 to 0·4)	0·3 (0·2 to 0·6)	0·4 (0·3 to 0·7)	0·5 (0·3 to 0·9)	0·6 (0·4 to 0·9)	0·6 (0·4 to 0·8)	0·6 (0·4 to 0·8)	0·5 (0·3 to 0·8)	0·5 (0·3 to 0·8)	0·5 (0·3 to 0·8)	0·4 (0·2 to 0·6)	0·3 (0·2 to 0·5)	0·2 (0·1 to 0·3)	0·2 (0·1 to 0·3)	[illegible]	[illegible]	[illegible]	[illegible]	0 (0 to 0)	6·5
Exposure to mechanical forces	0·1 (0 to 0·1)	0·1 (0 to 0·1)	0·4 (0·3 to 0·5)	2 (1·5 to 2·8)	4·1 (3·1 to 5·4)	6·9 (5·1 to 9·2)	20·4 (15·1 to 26·1)	36·1 (27·3 to 46·2)	37·8 (28·9 to 47·3)	35·1 (27·9 to 43·9)	34 (26·9 to 43)	35·4 (27·5 to 44·6)	33·5 (25·5 to 43·3)	31·1 (23·6 to 41)	25·3 (19·2 to 33·9)	18·5 (13·7 to 25·1)	12·2 (8·7 to 16·8)	10·3 (7·4 to 14·3)	[illegible]	[illegible]	[illegible]	[illegible]	0·1 (0·1 to 0·2)	356
Unintentional firearm injuries	0 (0 to 0)	0 (0 to 0)	0 (0 to 0·1)	0·2 (0·1 to 0·3)	0·4 (0·2 to 0·6)	1·1 (0·7 to 1·7)	8·8 (5·5 to 12·5)	18·7 (12·6 to 26·4)	17·5 (12 to 24·1)	14·1 (9·8 to 19·1)	11·8 (8·4 to 15·6)	11·4 (8·1 to 15·4)	9 (6·5 to 12·1)	6·9 (5 to 9)	4·9 (3·7 to 6·3)	3·1 (2·3 to 3·9)	1·8 (1·4 to 2·3)	1·3 (1 to 1·7)	[illegible]	[illegible]	[illegible]	[illegible]	0 (0 to 0)	112
Other exposure to mechanical forces	0·1 (0 to 0·1)	0·1 (0 to 0·1)	0·3 (0·2 to 0·5)	1·9 (1·3 to 2·5)	3·7 (2·8 to 5)	5·8 (4·2 to 8)	11·6 (8·2 to 16·4)	17·4 (12·3 to 23·9)	20·3 (14·4 to 27)	21 (15·2 to 28·4)	22·3 (16·2 to 30·7)	24 (17·5 to 32·6)	24·4 (17·5 to 33·8)	24·3 (17·7 to 33·7)	20·4 (14·9 to 28·4)	15·4 (10·9 to 21·5)	10·4 (7·2 to 14·7)	9 (6·3 to 12·7)	[illegible]	[illegible]	[illegible]	[illegible]	0·1 (0·1 to 0·1)	244
Adverse effects of medical treatment	1·1 (0·7 to 1·8)	0·6 (0·3 to 1)	1·8 (0·9 to 3·6)	1·5 (0·8 to 2·8)	1·1 (0·7 to 1·8)	1·1 (0·7 to 1·8)	1·7 (1·1 to 2·6)	4 (2·4 to 6)	4·7 (2·9 to 7)	3·7 (2·3 to 5·6)	4·3 (2·8 to 6·4)	5 (3·2 to 7·5)	5·3 (3·4 to 8·3)	7 (4·5 to 10·6)	6·5 (4·2 to 9·6)	6·5 (4·1 to 10·2)	5·3 (3·5 to 8)	5·9 (3·8 to 8·7)	[illegible]	[illegible]	[illegible]	[illegible]	0·3 (0·2 to 0·4)	80·2
Animal contact	0 (0 to 0)	0 (0 to 0)	0·1 (0 to 0·1)	0·4 (0·2 to 0·6)	0·5 (0·3 to 0·7)	0·8 (0·5 to 1·1)	1·1 (0·7 to 1·5)	1·3 (0·9 to 1·7)	1·8 (1·3 to 2·4)	1·4 (1 to 1·8)	1·8 (1·3 to 2·3)	1·9 (1·3 to 2·5)	2·2 (1·6 to 3)	2·1 (1·6 to 2·8)	1·9 (1·4 to 2·5)	1·8 (1·2 to 2·4)	1·3 (1 to 1·8)	1·3 (1 to 1·8)	[illegible]	[illegible]	[illegible]	[illegible]	0 (0 to 0)	23·3
Venomous animal contact	0 (0 to 0)	0 (0 to 0)	0·1 (0 to 0·1)	0·3 (0·2 to 0·5)	0·4 (0·2 to 0·6)	0·6 (0·4 to 0·9)	0·8 (0·6 to 1·2)	1 (0·7 to 1·3)	1·4 (1 to 1·9)	1 (0·7 to 1·4)	1·4 (1 to 1·9)	1·4 (1 to 1·9)	1·7 (1·2 to 2·4)	1·6 (1·1 to 2·2)	1·4 (1 to 2)	1·3 (0·9 to 1·8)	1 (0·7 to 1·4)	0·9 (0·7 to 1·3)	[illegible]	[illegible]	[illegible]	[illegible]	0 (0 to 0)	17·8
Non-venomous animal contact	0 (0 to 0)	0 (0 to 0)	0 (0 to 0)	0·1 (0 to 0·1)	0·1 (0·1 to 0·2)	0·2 (0·1 to 0·3)	0·2 (0·1 to 0·4)	0·3 (0·2 to 0·5)	0·4 (0·2 to 0·6)	0·3 (0·2 to 0·5)	0·3 (0·2 to 0·5)	0·5 (0·3 to 0·7)	0·5 (0·3 to 0·8)	0·5 (0·3 to 0·7)	0·4 (0·3 to 0·7)	0·4 (0·3 to 0·6)	0·4 (0·2 to 0·5)	0·4 (0·3 to 0·6)	[illegible]	[illegible]	[illegible]	[illegible]	0 (0 to 0)	5·5
Foreign body	2 (1·2 to 3)	0·7 (0·4 to 1·1)	6·2 (3·5 to 10·3)	4·7 (3·1 to 6·9)	3·1 (2·4 to 3·9)	4·4 (3·3 to 5·7)	3·5 (2·7 to 4·6)	5·5 (4·2 to 7·1)	6·4 (4·9 to 8·1)	5·7 (4·5 to 7·1)	5·7 (4·5 to 7·1)	6·4 (5·1 to 7·9)	6·6 (5·2 to 8·3)	6·6 (5 to 8·7)	5·4 (4·3 to 6·8)	4·9 (3·8 to 6·3)	3·6 (2·8 to 4·5)	3·9 (3 to 5)	[illegible]	[illegible]	[illegible]	[illegible]	0·1 (0·1 to 0·2)	92·2
Pulmonary aspiration and foreign body in airway	2 (1·2 to 3)	0·7 (0·4 to 1·1)	6·1 (3·4 to 10·2)	4·3 (2·8 to 6·5)	2·6 (2 to 3·3)	3·7 (2·7 to 4·9)	2·6 (1·9 to 3·5)	4·4 (3·2 to 5·8)	5·2 (4 to 6·7)	4·6 (3·4 to 5·9)	4·5 (3·4 to 5·7)	5·1 (4 to 6·4)	5·3 (4·1 to 6·9)	5·4 (3·9 to 7·2)	4·4 (3·4 to 5·8)	4·1 (3 to 5·4)	3 (2·2 to 3·9)	3·3 (2·5 to 4·4)	[illegible]	[illegible]	[illegible]	[illegible]	0·1 (0·1 to 0·2)	77·3
Foreign body in eyes	0 (0 to 0)	0 (0 to 0)	0 (0 to 0)	0·1 (0 to 0·3)	0·2 (0·1 to 0·5)	0·3 (0·1 to 0·7)	0·4 (0·1 to 1)	0·5 (0·2 to 1·2)	0·6 (0·2 to 1·3)	0·6 (0·2 to 1·2)	0·6 (0·2 to 1·3)	0·6 (0·3 to 1·3)	0·6 (0·3 to 1·2)	0·6 (0·3 to 1)	0·4 (0·2 to 0·7)	0·3 (0·2 to 0·5)	0·2 (0·1 to 0·4)	0·2 (0·1 to 0·3)	[illegible]	[illegible]	[illegible]	[illegible]	0 (0 to 0)	6·5
Foreign body in other body part	0 (0 to 0)	0 (0 to 0)	0 (0 to 0·1)	0·2 (0·2 to 0·4)	0·3 (0·2 to 0·5)	0·4 (0·3 to 0·6)	0·5 (0·3 to 0·7)	0·6 (0·4 to 0·8)	0·6 (0·4 to 0·8)	0·6 (0·4 to 0·8)	0·6 (0·4 to 0·8)	0·7 (0·5 to 0·9)	0·7 (0·5 to 0·9)	0·7 (0·5 to 0·9)	0·6 (0·4 to 0·8)	0·5 (0·4 to 0·7)	0·4 (0·3 to 0·5)	0·4 (0·3 to 0·5)	[illegible]	[illegible]	[illegible]	[illegible]	0 (0 to 0)	8·3
Environmental heat and cold exposure	0 (0 to 0)	0 (0 to 0)	0 (0 to 0·1)	0·3 (0·2 to 0·4)	0·6 (0·4 to 0·9)	1 (0·7 to 1·5)	1·9 (1·3 to 2·6)	2·7 (1·9 to 3·7)	3·2 (2·3 to 4·3)	3·3 (2·3 to 4·5)	3·6 (2·5 to 5)	3·9 (2·7 to 5·3)	4·1 (2·9 to 5·5)	4·2 (3 to 5·7)	3·6 (2·6 to 4·8)	2·9 (2·1 to 3·8)	2·1 (1·5 to 2·8)	2 (1·5 to 2·6)	[illegible]	[illegible]	[illegible]	[illegible]	0·1 (0 to 0·1)	42·7
Exposure to forces of nature	0 (0 to 0)	0 (0 to 0)	0 (0 to 0)	0 (0 to 0)	0 (0 to 0·1)	0·1 (0·1 to 0·2)	0·3 (0·2 to 0·4)	0·4 (0·2 to 0·6)	0·3 (0·2 to 0·5)	0·3 (0·2 to 0·5)	0·5 (0·3 to 0·7)	0·5 (0·3 to 0·8)	0·5 (0·3 to 0·7)	0·6 (0·4 to 0·9)	0·4 (0·3 to 0·6)	0·3 (0·2 to 0·4)	0·2 (0·2 to 0·3)	0·2 (0·1 to 0·2)	[illegible]	[illegible]	[illegible]	[illegible]	0 (0 to 0)	5
Other unintentional injuries	0·1 (0·1 to 0·3)	0 (0 to 0·1)	0·4 (0·3 to 0·6)	1·8 (1·3 to 2·5)	3·3 (2·5 to 4·4)	5 (3·9 to 6·5)	9·8 (7·4 to 12·8)	14·4 (10·9 to 19)	16·3 (12·6 to 20·8)	15·1 (11·7 to 19·5)	14·7 (11·4 to 18·5)	14·3 (11·2 to 18)	13·3 (10·1 to 17·3)	10·4 (7·8 to 13·9)	8·1 (6·2 to 10·8)	5·6 (4·1 to 7·6)	3·8 (2·7 to 5·2)	3·1 (2·3 to 4·3)	[illegible]	[illegible]	[illegible]	[illegible]	0 (0 to 0·1)	144
Self-harm and interpersonal violence	2·8 (1·6 to 4·5)	0·3 (0·1 to 0·4)	2 (1·1 to 3·3)	5 (2·7 to 9·1)	5·8 (4·1 to 8)	15·8 (12 to 20·7)	159 (121 to 204)	335 (270 to 405)	381 (318 to 450)	283 (230 to 343)	220 (173 to 278)	182 (141 to 231)	158 (122 to 206)	112 (86 to 142)	75·2 (57·8 to 97)	49 (38·2 to 62·1)	27·5 (21·7 to 34·1)	22·7 (18·2 to 28·6)	[illegible]	[illegible]	[illegible]	[illegible]	0·2 (0·2 to 0·2)	2,060
Self-harm	0 (0 to 0)	0 (0 to 0)	0 (0 to 0)	0 (0 to 0)	0 (0 to 0)	2·2 (1·2 to 3·5)	22 (14·4 to 32·4)	52·6 (36·6 to 74·2)	62·9 (45·2 to 84·4)	61·1 (44·3 to 82·1)	57·6 (41·3 to 76·8)	56·1 (40·6 to 75·2)	50·7 (35·7 to 70·4)	41·9 (30·1 to 56·2)	31 (22·5 to 41·2)	22·9 (16·4 to 30·1)	13·5 (9·8 to 18)	13·1 (9·7 to 17·5)	[illegible]	[illegible]	[illegible]	[illegible]	0·1 (0·1 to 0·1)	504
Self-harm by firearm	0 (0 to 0)	0 (0 to 0)	0 (0 to 0)	0 (0 to 0)	0 (0 to 0)	0·1 (0 to 0·1)	1·4 (0·7 to 2·5)	2·7 (1·4 to 5·1)	2·9 (1·5 to 4·9)	2·5 (1·4 to 4·4)	2·3 (1·2 to 4)	2·4 (1·3 to 4)	2·1 (1·1 to 3·7)	2 (1 to 3·2)	1·2 (0·7 to 2·1)	1 (0·6 to 1·7)	0·5 (0·3 to 0·9)	0·5 (0·3 to 0·8)	[illegible]	[illegible]	[illegible]	[illegible]	0 (0 to 0)	22·2
Self-harm by other specified means	0 (0 to 0)	0 (0 to 0)	0 (0 to 0)	0 (0 to 0)	0 (0 to 0)	2·1 (1·2 to 3·4)	20·7 (13·6 to 30·5)	49·9 (34·4 to 70·1)	60 (43·4 to 80·7)	58·5 (42·1 to 79·2)	55·3 (39·6 to 73·4)	53·7 (38·9 to 71·7)	48·6 (34·1 to 67·4)	40 (28·7 to 53·8)	29·8 (21·6 to 39·7)	21·9 (15·6 to 29)	13 (9·4 to 17·2)	12·6 (9·3 to 16·9)	[illegible]	[illegible]	[illegible]	[illegible]	0·1 (0·1 to 0·1)	482
Interpersonal violence	2·8 (1·6 to 4·5)	0·3 (0·1 to 0·4)	2 (1·1 to 3·3)	5 (2·7 to 9)	5·7 (4·1 to 8)	13·6 (10 to 17·9)	137 (102 to 178)	280 (221 to 345)	316 (257 to 380)	221 (174 to 275)	161 (121 to 208)	125 (92·6 to 166)	106 (78 to 143)	67·7 (49·3 to 90·5)	43 (31·7 to 57·6)	25·8 (19·1 to 34·7)	13·3 (9·9 to 17·8)	9·6 (7·3 to 12·4)	[illegible]	[illegible]	[illegible]	[illegible]	0·1 (0·1 to 0·1)	1,540
Physical violence by firearm	0·2 (0·1 to 0·3)	0 (0 to 0·1)	0·4 (0·2 to 0·6)	0·9 (0·5 to 1·7)	1·9 (1·3 to 2·7)	6·5 (4·6 to 8·7)	89·6 (65·6 to 118)	183 (141 to 227)	206 (166 to 249)	132 (101 to 165)	89·9 (66·1 to 119)	64·2 (46·5 to 86)	53·3 (38·1 to 72·4)	30·8 (21·5 to 42)	18·7 (13·1 to 25·7)	10·4 (7·1 to 14·5)	4·7 (3·3 to 6·6)	2·8 (2 to 3·8)	[illegible]	[illegible]	[illegible]	[illegible]	0 (0 to 0)	896
Physical violence by sharp object	0·4 (0·2 to 0·6)	0 (0 to 0·1)	0·2 (0·1 to 0·3)	0·7 (0·4 to 1·3)	1 (0·7 to 1·4)	2·7 (2 to 3·7)	38·6 (28·3 to 52·2)	81·3 (63·7 to 101)	92 (74·3 to 113)	73 (56·8 to 91·5)	55·2 (41·6 to 72·3)	45·1 (33·4 to 60·2)	37·3 (27·1 to 50·4)	23·7 (17·4 to 31·4)	13·5 (10·1 to 18·2)	8·1 (6 to 10·7)	4 (3 to 5·2)	3·3 (2·5 to 4·3)	[illegible]	[illegible]	[illegible]	[illegible]	0 (0 to 0·1)	483
Sexual violence	0 (0 to 0)	0 (0 to 0)	0 (0 to 0)	0 (0 to 0)	0·2 (0·1 to 0·3)	0·5 (0·3 to 0·8)	1·2 (0·7 to 1·9)	1·6 (0·9 to 2·5)	1·5 (0·8 to 2·4)	1·2 (0·7 to 2)	1·2 (0·7 to 1·9)	1 (0·6 to 1·7)	0·8 (0·5 to 1·4)	0·7 (0·4 to 1·1)	0·5 (0·3 to 0·7)	0·3 (0·2 to 0·5)	0·2 (0·1 to 0·3)	0·2 (0·1 to 0·3)	[illegible]	[illegible]	[illegible]	[illegible]	0 (0 to 0)	11·4
Physical violence by other means	2·3 (1·3 to 3·6)	0·2 (0·1 to 0·3)	1·4 (0·8 to 2·4)	3·3 (1·7 to 6)	2·6 (1·8 to 3·8)	4 (3 to 5·3)	7·3 (5·5 to 9·4)	14·1 (11·2 to 17·4)	17·1 (13·9 to 20·5)	14·4 (11·4 to 17·8)	14·8 (11·2 to 18·9)	14·6 (11 to 18·7)	14·8 (11·1 to 19·7)	12·5 (9·3 to 16·4)	10·3 (7·8 to 13·9)	7 (5·2 to 9·3)	4·4 (3·3 to 5·9)	3·3 (2·5 to 4·3)	[illegible]	[illegible]	[illegible]	[illegible]	0 (0 to 0·1)	152
Conflict and terrorism	0 (0 to 0)	0 (0 to 0)	0 (0 to 0)	0 (0 to 0)	0 (0 to 0)	0 (0 to 0)	0 (0 to 0)	0·1 (0 to 0·1)	0 (0 to 0·1)	0·1 (0 to 0·1)	0·1 (0·1 to 0·2)	0·2 (0·1 to 0·3)	1·1 (0·7 to 1·7)	2·3 (1·4 to 3·3)	1·1 (0·7 to 1·7)	0·2 (0·2 to 0·3)	0·6 (0·4 to 0·9)	0·1 (0 to 0·1)	[illegible]	[illegible]	[illegible]	[illegible]	0 (0 to 0)	6·4

SR Table 2a: Saint Vincent and the Grenadines DALYS by cause and age group for males with 95% uncertainty intervals, 2019

Cause	Early Neonatal	Late Neonatal	Post Neonatal	1 to 4	5 to 9	10 to 14	15 to 19	20 to 24	25 to 29	30 to 34	35 to 39	40 to 44	45 to 49	50 to 54	55 to 59	60 to 64	65 to 69	70 to 74	75 to 79	80 to 84	85 to 89	90 to 94	95 plus	Total
All causes	585 (403 to 831)	149 (104 to 211)	264 (192 to 363)	285 (218 to 368)	262 (201 to 346)	364 (296 to 450)	[illegible]	[illegible]	[illegible]	[illegible]	[illegible]	[illegible]	[illegible]	[illegible]	[illegible]	[illegible]	[illegible]	[illegible]	[illegible]	[illegible]	[illegible]	[illegible]	25·5 (24·4 to 26·7)	21,500
Communicable, maternal, neonatal, and nutritional diseases	498 (339 to 701)	115 (79·1 to 162)	136 (95·1 to 190)	106 (77·7 to 144)	83·1 (52·8 to 133)	63·4 (47·2 to 88·3)	[illegible]	[illegible]	[illegible]	[illegible]	[illegible]	[illegible]	[illegible]	[illegible]	[illegible]	[illegible]	[illegible]	[illegible]	[illegible]	[illegible]	[illegible]	[illegible]	2·4 (1·8 to 3)	2,720
HIV/AIDS and sexually transmitted infections	1·4 (0·8 to 2·4)	0·3 (0·2 to 0·6)	4·8 (4·3 to 5·4)	3·5 (3·2 to 3·8)	4·6 (4·3 to 5)	3·4 (3·2 to 3·7)	[illegible]	[illegible]	[illegible]	[illegible]	[illegible]	[illegible]	[illegible]	[illegible]	[illegible]	[illegible]	[illegible]	[illegible]	[illegible]	[illegible]	[illegible]	[illegible]	0 (0 to 0)	655
HIV/AIDS	0 (0 to 0)	0 (0 to 0)	4·3 (4 to 4·7)	3·5 (3·2 to 3·7)	4·6 (4·3 to 5)	3·2 (3 to 3·5)	[illegible]	[illegible]	[illegible]	[illegible]	[illegible]	[illegible]	[illegible]	[illegible]	[illegible]	[illegible]	[illegible]	[illegible]	[illegible]	[illegible]	[illegible]	[illegible]	0 (0 to 0)	641
HIV/AIDS - Drug-susceptible Tuberculosis	0 (0 to 0)	0 (0 to 0)	0·2 (0·1 to 0·3)	0·2 (0·1 to 0·3)	0·2 (0·1 to 0·3)	0·2 (0·1 to 0·2)	[illegible]	[illegible]	[illegible]	[illegible]	[illegible]	[illegible]	[illegible]	[illegible]	[illegible]	[illegible]	[illegible]	[illegible]	[illegible]	[illegible]	[illegible]	[illegible]	0 (0 to 0)	31·6
HIV/AIDS - Multidrug-resistant Tuberculosis without extensive dru	0 (0 to 0)	0 (0 to 0)	0 (0 to 0)	0 (0 to 0)	0 (0 to 0)	0 (0 to 0)	[illegible]	[illegible]	[illegible]	[illegible]	[illegible]	[illegible]	[illegible]	[illegible]	[illegible]	[illegible]	[illegible]	[illegible]	[illegible]	[illegible]	[illegible]	[illegible]	0 (0 to 0)	0·4
HIV/AIDS - Extensively drug-resistant Tuberculosis	0 (0 to 0)	0 (0 to 0)	0 (0 to 0)	0 (0 to 0)	0 (0 to 0)	0 (0 to 0)	[illegible]	[illegible]	[illegible]	[illegible]	[illegible]	[illegible]	[illegible]	[illegible]	[illegible]	[illegible]	[illegible]	[illegible]	[illegible]	[illegible]	[illegible]	[illegible]	0 (0 to 0)	0·1
HIV/AIDS resulting in other diseases	0 (0 to 0)	0 (0 to 0)	4·1 (3·7 to 4·5)	3·3 (3 to 3·6)	4·4 (4·1 to 4·8)	3·1 (2·8 to 3·4)	[illegible]	[illegible]	[illegible]	[illegible]	[illegible]	[illegible]	[illegible]	[illegible]	[illegible]	[illegible]	[illegible]	[illegible]	[illegible]	[illegible]	[illegible]	[illegible]	0 (0 to 0)	609
Sexually transmitted infections excluding HIV	1·4 (0·8 to 2·4)	0·3 (0·2 to 0·6)	0·4 (0·2 to 1)	0 (0 to 0·1)	0 (0 to 0)	0·2 (0·1 to 0·3)	[illegible]	[illegible]	[illegible]	[illegible]	[illegible]	[illegible]	[illegible]	[illegible]	[illegible]	[illegible]	[illegible]	[illegible]	[illegible]	[illegible]	[illegible]	[illegible]	0 (0 to 0)	14·2
Syphilis	1·4 (0·8 to 2·4)	0·3 (0·2 to 0·6)	0·4 (0·2 to 1)	0 (0 to 0·1)	0 (0 to 0)	0·1 (0 to 0·1)	[illegible]	[illegible]	[illegible]	[illegible]	[illegible]	[illegible]	[illegible]	[illegible]	[illegible]	[illegible]	[illegible]	[illegible]	[illegible]	[illegible]	[illegible]	[illegible]	0 (0 to 0)	6·6
Chlamydial infection	0 (0 to 0)	0 (0 to 0)	0 (0 to 0)	0 (0 to 0)	0 (0 to 0)	0 (0 to 0)	[illegible]	[illegible]	[illegible]	[illegible]	[illegible]	[illegible]	[illegible]	[illegible]	[illegible]	[illegible]	[illegible]	[illegible]	[illegible]	[illegible]	[illegible]	[illegible]	0 (0 to 0)	1·3
Gonococcal infection	0 (0 to 0)	0 (0 to 0)	0 (0 to 0)	0 (0 to 0)	0 (0 to 0)	0 (0 to 0·1)	[illegible]	[illegible]	[illegible]	[illegible]	[illegible]	[illegible]	[illegible]	[illegible]	[illegible]	[illegible]	[illegible]	[illegible]	[illegible]	[illegible]	[illegible]	[illegible]	0 (0 to 0)	1·2
Trichomoniasis	0 (0 to 0)	0 (0 to 0)	0 (0 to 0)	0 (0 to 0)	0 (0 to 0)	0 (0 to 0)	[illegible]	[illegible]	[illegible]	[illegible]	[illegible]	[illegible]	[illegible]	[illegible]	[illegible]	[illegible]	[illegible]	[illegible]	[illegible]	[illegible]	[illegible]	[illegible]	0 (0 to 0)	0·2
Genital herpes	0 (0 to 0)	0 (0 to 0)	0 (0 to 0)	0 (0 to 0)	0 (0 to 0)	0 (0 to 0)	[illegible]	[illegible]	[illegible]	[illegible]	[illegible]	[illegible]	[illegible]	[illegible]	[illegible]	[illegible]	[illegible]	[illegible]	[illegible]	[illegible]	[illegible]	[illegible]	0 (0 to 0)	2·5
Other sexually transmitted infections	0 (0 to 0)	0 (0 to 0)	0 (0 to 0)	0 (0 to 0)	0 (0 to 0)	0·1 (0 to 0·2)	[illegible]	[illegible]	[illegible]	[illegible]	[illegible]	[illegible]	[illegible]	[illegible]	[illegible]	[illegible]	[illegible]	[illegible]	[illegible]	[illegible]	[illegible]	[illegible]	0 (0 to 0)	2·5
Respiratory infections and tuberculosis	14·4 (8·9 to 23·1)	9·2 (5·8 to 13·7)	35·9 (22·9 to 54·3)	22·3 (15·5 to 32·1)	10·5 (7·4 to 14·8)	10·3 (7·3 to 14·3)	[illegible]	[illegible]	[illegible]	[illegible]	[illegible]	[illegible]	[illegible]	[illegible]	[illegible]	[illegible]	[illegible]	[illegible]	[illegible]	[illegible]	[illegible]	[illegible]	1·8 (1·3 to 2·3)	618
Tuberculosis	0 (0 to 0)	0 (0 to 0)	0·5 (0·3 to 0·8)	0·7 (0·4 to 1·1)	0·6 (0·3 to 0·9)	0·6 (0·4 to 0·7)	[illegible]	[illegible]	[illegible]	[illegible]	[illegible]	[illegible]	[illegible]	[illegible]	[illegible]	[illegible]	[illegible]	[illegible]	[illegible]	[illegible]	[illegible]	[illegible]	0 (0 to 0)	78·5
Latent tuberculosis infection	0 (0 to 0)	0 (0 to 0)	0 (0 to 0)	0 (0 to 0)	0 (0 to 0)	0 (0 to 0)	[illegible]	[illegible]	[illegible]	[illegible]	[illegible]	[illegible]	[illegible]	[illegible]	[illegible]	[illegible]	[illegible]	[illegible]	[illegible]	[illegible]	[illegible]	[illegible]	0 (0 to 0)	0
Drug-susceptible tuberculosis	[illegible]	[illegible]	[illegible]	[illegible]	[illegible]	[illegible]	[illegible]	[illegible]	[illegible]	[illegible]	[illegible]	[illegible]	[illegible]	[illegible]	[illegible]	[illegible]	[illegible]	[illegible]	[illegible]	[illegible]	[illegible]	[illegible]	0 (0 to 0)	77·5
Multidrug-resistant tuberculosis without extensive drug resistance	[illegible]	[illegible]	[illegible]	[illegible]	[illegible]	[illegible]	[illegible]	[illegible]	[illegible]	[illegible]	[illegible]	[illegible]	[illegible]	[illegible]	[illegible]	[illegible]	[illegible]	[illegible]	[illegible]	[illegible]	[illegible]	[illegible]	0 (0 to 0)	0·9
Extensively drug-resistant tuberculosis	[illegible]	[illegible]	[illegible]	[illegible]	[illegible]	[illegible]	[illegible]	[illegible]	[illegible]	[illegible]	[illegible]	[illegible]	[illegible]	[illegible]	[illegible]	[illegible]	[illegible]	[illegible]	[illegible]	[illegible]	[illegible]	[illegible]	0 (0 to 0)	0·1
Lower respiratory infections	[illegible]	[illegible]	[illegible]	[illegible]	[illegible]	[illegible]	[illegible]	[illegible]	[illegible]	[illegible]	[illegible]	[illegible]	[illegible]	[illegible]	[illegible]	[illegible]	[illegible]	[illegible]	[illegible]	[illegible]	[illegible]	[illegible]	1·7 (1·2 to 2·3)	473
Upper respiratory infections	[illegible]	[illegible]	[illegible]	[illegible]	[illegible]	[illegible]	[illegible]	[illegible]	[illegible]	[illegible]	[illegible]	[illegible]	[illegible]	[illegible]	[illegible]	[illegible]	[illegible]	[illegible]	[illegible]	[illegible]	[illegible]	[illegible]	0 (0 to 0)	49·1
Otitis media	[illegible]	[illegible]	[illegible]	[illegible]	[illegible]	[illegible]	[illegible]	[illegible]	[illegible]	[illegible]	[illegible]	[illegible]	[illegible]	[illegible]	[illegible]	[illegible]	[illegible]	[illegible]	[illegible]	[illegible]	[illegible]	[illegible]	0 (0 to 0)	16·8
Enteric infections	[illegible]	[illegible]	[illegible]	[illegible]	[illegible]	[illegible]	[illegible]	[illegible]	[illegible]	[illegible]	[illegible]	[illegible]	[illegible]	[illegible]	[illegible]	[illegible]	[illegible]	[illegible]	[illegible]	[illegible]	[illegible]	[illegible]	0·2 (0·1 to 0·2)	169
Diarrheal diseases	[illegible]	[illegible]	[illegible]	[illegible]	[illegible]	[illegible]	[illegible]	[illegible]	[illegible]	[illegible]	[illegible]	[illegible]	[illegible]	[illegible]	[illegible]	[illegible]	[illegible]	[illegible]	[illegible]	[illegible]	[illegible]	[illegible]	0·2 (0·1 to 0·2)	168
Typhoid and paratyphoid	[illegible]	[illegible]	[illegible]	[illegible]	[illegible]	[illegible]	[illegible]	[illegible]	[illegible]	[illegible]	[illegible]	[illegible]	[illegible]	[illegible]	[illegible]	[illegible]	[illegible]	[illegible]	[illegible]	[illegible]	[illegible]	[illegible]	0 (0 to 0)	0·1
Typhoid fever	[illegible]	[illegible]	[illegible]	[illegible]	[illegible]	[illegible]	[illegible]	[illegible]	[illegible]	[illegible]	[illegible]	[illegible]	[illegible]	[illegible]	[illegible]	[illegible]	[illegible]	[illegible]	[illegible]	[illegible]	[illegible]	[illegible]	0 (0 to 0)	0
Paratyphoid fever	[illegible]	[illegible]	[illegible]	[illegible]	[illegible]	[illegible]	[illegible]	[illegible]	[illegible]	[illegible]	[illegible]	[illegible]	[illegible]	[illegible]	[illegible]	[illegible]	[illegible]	[illegible]	[illegible]	[illegible]	[illegible]	[illegible]	0 (0 to 0)	0
Invasive Non-typhoidal Salmonella (iNTS)	[illegible]	[illegible]	[illegible]	[illegible]	[illegible]	[illegible]	[illegible]	[illegible]	[illegible]	[illegible]	[illegible]	[illegible]	[illegible]	[illegible]	[illegible]	[illegible]	[illegible]	[illegible]	[illegible]	[illegible]	[illegible]	[illegible]	0 (0 to 0)	0·2
Other intestinal infectious diseases	[illegible]	[illegible]	[illegible]	[illegible]	[illegible]	[illegible]	[illegible]	[illegible]	[illegible]	[illegible]	[illegible]	[illegible]	[illegible]	[illegible]	[illegible]	[illegible]	[illegible]	[illegible]	[illegible]	[illegible]	[illegible]	[illegible]	0 (0 to 0)	0·3
Neglected tropical diseases and malaria	[illegible]	[illegible]	[illegible]	[illegible]	[illegible]	[illegible]	[illegible]	[illegible]	[illegible]	[illegible]	[illegible]	[illegible]	[illegible]	[illegible]	[illegible]	[illegible]	[illegible]	[illegible]	[illegible]	[illegible]	[illegible]	[illegible]	0 (0 to 0·1)	58·5
Malaria	[illegible]	[illegible]	[illegible]	[illegible]	[illegible]	[illegible]	[illegible]	[illegible]	[illegible]	[illegible]	[illegible]	[illegible]	[illegible]	[illegible]	[illegible]	[illegible]	[illegible]	[illegible]	[illegible]	[illegible]	[illegible]	[illegible]	0 (0 to 0)	0
Chagas disease	[illegible]	[illegible]	[illegible]	[illegible]	[illegible]	[illegible]	[illegible]	[illegible]	[illegible]	[illegible]	[illegible]	[illegible]	[illegible]	[illegible]	[illegible]	[illegible]	[illegible]	[illegible]	[illegible]	[illegible]	[illegible]	[illegible]	0 (0 to 0)	0
Leishmaniasis	[illegible]	[illegible]	[illegible]	[illegible]	[illegible]	[illegible]	[illegible]	[illegible]	[illegible]	[illegible]	[illegible]	[illegible]	[illegible]	[illegible]	[illegible]	[illegible]	[illegible]	[illegible]	[illegible]	[illegible]	[illegible]	[illegible]	0 (0 to 0)	0
Visceral leishmaniasis	[illegible]	[illegible]	[illegible]	[illegible]	[illegible]	[illegible]	[illegible]	[illegible]	[illegible]	[illegible]	[illegible]	[illegible]	[illegible]	[illegible]	[illegible]	[illegible]	[illegible]	[illegible]	[illegible]	[illegible]	[illegible]	[illegible]	0 (0 to 0)	0
Cutaneous and mucocutaneous leishmaniasis	[illegible]	[illegible]	[illegible]	[illegible]	[illegible]	[illegible]	[illegible]	[illegible]	[illegible]	[illegible]	[illegible]	[illegible]	[illegible]	[illegible]	[illegible]	[illegible]	[illegible]	[illegible]	[illegible]	[illegible]	[illegible]	[illegible]	0 (0 to 0)	0
African trypanosomiasis	[illegible]	[illegible]	[illegible]	[illegible]	[illegible]	[illegible]	[illegible]	[illegible]	[illegible]	[illegible]	[illegible]	[illegible]	[illegible]	[illegible]	[illegible]	[illegible]	[illegible]	[illegible]	[illegible]	[illegible]	[illegible]	[illegible]	0 (0 to 0)	0
Schistosomiasis	[illegible]	[illegible]	[illegible]	[illegible]	[illegible]	[illegible]	[illegible]	[illegible]	[illegible]	[illegible]	[illegible]	[illegible]	[illegible]	[illegible]	[illegible]	[illegible]	[illegible]	[illegible]	[illegible]	[illegible]	[illegible]	[illegible]	0 (0 to 0)	0
Cysticercosis	[illegible]	[illegible]	[illegible]	[illegible]	[illegible]	[illegible]	[illegible]	[illegible]	[illegible]	[illegible]	[illegible]	[illegible]	[illegible]	[illegible]	[illegible]	[illegible]	[illegible]	[illegible]	[illegible]	[illegible]	[illegible]	[illegible]	0 (0 to 0)	35·6
Cystic echinococcosis	[illegible]	[illegible]	[illegible]	[illegible]	[illegible]	[illegible]	[illegible]	[illegible]	[illegible]	[illegible]	[illegible]	[illegible]	[illegible]	[illegible]	[illegible]	[illegible]	[illegible]	[illegible]	[illegible]	[illegible]	[illegible]	[illegible]	0 (0 to 0)	0
Lymphatic filariasis	[illegible]	[illegible]	[illegible]	[illegible]	[illegible]	[illegible]	[illegible]	[illegible]	[illegible]	[illegible]	[illegible]	[illegible]	[illegible]	[illegible]	[illegible]	[illegible]	[illegible]	[illegible]	[illegible]	[illegible]	[illegible]	[illegible]	0 (0 to 0)	0
Onchocerciasis	[illegible]	[illegible]	[illegible]	[illegible]	[illegible]	[illegible]	[illegible]	[illegible]	[illegible]	[illegible]	[illegible]	[illegible]	[illegible]	[illegible]	[illegible]	[illegible]	[illegible]	[illegible]	[illegible]	[illegible]	[illegible]	[illegible]	0 (0 to 0)	0
Trachoma	[illegible]	[illegible]	[illegible]	[illegible]	[illegible]	[illegible]	[illegible]	[illegible]	[illegible]	[illegible]	[illegible]	[illegible]	[illegible]	[illegible]	[illegible]	[illegible]	[illegible]	[illegible]	[illegible]	[illegible]	[illegible]	[illegible]	0 (0 to 0)	0
Dengue	[illegible]	[illegible]	[illegible]	[illegible]	[illegible]	[illegible]	[illegible]	[illegible]	[illegible]	[illegible]	[illegible]	[illegible]	[illegible]	[illegible]	[illegible]	[illegible]	[illegible]	[illegible]	[illegible]	[illegible]	[illegible]	[illegible]	0 (0 to 0)	5·3
Yellow fever	[illegible]	[illegible]	[illegible]	[illegible]	[illegible]	[illegible]	[illegible]	[illegible]	[illegible]	[illegible]	[illegible]	[illegible]	[illegible]	[illegible]	[illegible]	[illegible]	[illegible]	[illegible]	[illegible]	[illegible]	[illegible]	[illegible]	0 (0 to 0)	0
Rabies	[illegible]	[illegible]	[illegible]	[illegible]	[illegible]	[illegible]	[illegible]	[illegible]	[illegible]	[illegible]	[illegible]	[illegible]	[illegible]	[illegible]	[illegible]	[illegible]	[illegible]	[illegible]	[illegible]	[illegible]	[illegible]	[illegible]	0 (0 to 0)	0·3
Intestinal nematode infections	[illegible]	[illegible]	[illegible]	[illegible]	[illegible]	[illegible]	[illegible]	[illegible]	[illegible]	[illegible]	[illegible]	[illegible]	[illegible]	[illegible]	[illegible]	[illegible]	[illegible]	[illegible]	[illegible]	[illegible]	[illegible]	[illegible]	0 (0 to 0)	2·2
Ascariasis	[illegible]	[illegible]	[illegible]	[illegible]	[illegible]	[illegible]	[illegible]	[illegible]	[illegible]	[illegible]	[illegible]	[illegible]	[illegible]	[illegible]	[illegible]	[illegible]	[illegible]	[illegible]	[illegible]	[illegible]	[illegible]	[illegible]	0 (0 to 0)	0·5
Trichuriasis	[illegible]	[illegible]	[illegible]	[illegible]	[illegible]	[illegible]	[illegible]	[illegible]	[illegible]	[illegible]	[illegible]	[illegible]	[illegible]	[illegible]	[illegible]	[illegible]	[illegible]	[illegible]	[illegible]	[illegible]	[illegible]	[illegible]	0 (0 to 0)	0
Hookworm disease	[illegible]	[illegible]	[illegible]	[illegible]	[illegible]	[illegible]	[illegible]	[illegible]	[illegible]	[illegible]	[illegible]	[illegible]	[illegible]	[illegible]	[illegible]	[illegible]	[illegible]	[illegible]	[illegible]	[illegible]	[illegible]	[illegible]	0 (0 to 0)	1·6
Food-borne trematodiases	[illegible]	[illegible]	[illegible]	[illegible]	[illegible]	[illegible]	[illegible]	[illegible]	[illegible]	[illegible]	[illegible]	[illegible]	[illegible]	[illegible]	[illegible]	[illegible]	[illegible]	[illegible]	[illegible]	[illegible]	[illegible]	[illegible]	0 (0 to 0)	0
Leprosy	[illegible]	[illegible]	[illegible]	[illegible]	[illegible]	[illegible]	[illegible]	[illegible]	[illegible]	[illegible]	[illegible]	[illegible]	[illegible]	[illegible]	[illegible]	[illegible]	[illegible]	[illegible]	[illegible]	[illegible]	[illegible]	[illegible]	0 (0 to 0)	0·3
Ebola	[illegible]	[illegible]	[illegible]	[illegible]	[illegible]	[illegible]	[illegible]	[illegible]	[illegible]	[illegible]	[illegible]	[illegible]	[illegible]	[illegible]	[illegible]	[illegible]	[illegible]	[illegible]	[illegible]	[illegible]	[illegible]	[illegible]	0 (0 to 0)	0
Zika virus	[illegible]	[illegible]	[illegible]	[illegible]	[illegible]	[illegible]	[illegible]	[illegible]	[illegible]	[illegible]	[illegible]	[illegible]	[illegible]	[illegible]	[illegible]	[illegible]	[illegible]	[illegible]	[illegible]	[illegible]	[illegible]	[illegible]	0 (0 to 0)	2
Guinea worm disease	[illegible]	[illegible]	[illegible]	[illegible]	[illegible]	[illegible]	[illegible]	[illegible]	[illegible]	[illegible]	[illegible]	[illegible]	[illegible]	[illegible]	[illegible]	[illegible]	[illegible]	[illegible]	[illegible]	[illegible]	[illegible]	[illegible]	0 (0 to 0)	0
Other neglected tropical diseases	[illegible]	[illegible]	[illegible]	[illegible]	[illegible]	[illegible]	[illegible]	[illegible]	[illegible]	[illegible]	[illegible]	[illegible]	[illegible]	[illegible]	[illegible]	[illegible]	[illegible]	[illegible]	[illegible]	[illegible]	[illegible]	[illegible]	0 (0 to 0)	12·9
Other infectious diseases	[illegible]	[illegible]	[illegible]	[illegible]	[illegible]	[illegible]	[illegible]	[illegible]	[illegible]	[illegible]	[illegible]	[illegible]	[illegible]	[illegible]	[illegible]	[illegible]	[illegible]	[illegible]	[illegible]	[illegible]	[illegible]	[illegible]	0 (0 to 0·1)	154
Meningitis	[illegible]	[illegible]	[illegible]	[illegible]	[illegible]	[illegible]	[illegible]	[illegible]	[illegible]	[illegible]	[illegible]	[illegible]	[illegible]	[illegible]	[illegible]	[illegible]	[illegible]	[illegible]	[illegible]	[illegible]	[illegible]	[illegible]	0 (0 to 0)	40·7
Encephalitis	[illegible]	[illegible]	[illegible]	[illegible]	[illegible]	[illegible]	[illegible]	[illegible]	[illegible]	[illegible]	[illegible]	[illegible]	[illegible]	[illegible]	[illegible]	[illegible]	[illegible]	[illegible]	[illegible]	[illegible]	[illegible]	[illegible]	0 (0 to 0)	20·5
Diphtheria	[illegible]	[illegible]	[illegible]	[illegible]	[illegible]	[illegible]	[illegible]	[illegible]	[illegible]	[illegible]	[illegible]	[illegible]	[illegible]	[illegible]	[illegible]	[illegible]	[illegible]	[illegible]	[illegible]	[illegible]	[illegible]	[illegible]	0 (0 to 0)	0
Tetanus	[illegible]	[illegible]	[illegible]	[illegible]	[illegible]	[illegible]	[illegible]	[illegible]	[illegible]	[illegible]	[illegible]	[illegible]	[illegible]	[illegible]	[illegible]	[illegible]	[illegible]	[illegible]	[illegible]	[illegible]	[illegible]	[illegible]	0 (0 to 0)	0·4
Measles	[illegible]	[illegible]	[illegible]	[illegible]	[illegible]	[illegible]	[illegible]	[illegible]	[illegible]	[illegible]	[illegible]	[illegible]	[illegible]	[illegible]	[illegible]	[illegible]	[illegible]	[illegible]	[illegible]	[illegible]	[illegible]	[illegible]	0 (0 to 0)	0
Varicella and herpes zoster	[illegible]	[illegible]	[illegible]	[illegible]	[illegible]	[illegible]	[illegible]	[illegible]	[illegible]	[illegible]	[illegible]	[illegible]	[illegible]	[illegible]	[illegible]	[illegible]	[illegible]	[illegible]	[illegible]	[illegible]	[illegible]	[illegible]	0 (0 to 0)	3·3
Acute hepatitis	[illegible]	[illegible]	[illegible]	[illegible]	[illegible]	[illegible]	[illegible]	[illegible]	[illegible]	[illegible]	[illegible]	[illegible]	[illegible]	[illegible]	[illegible]	[illegible]	[illegible]	[illegible]	[illegible]	[illegible]	[illegible]	[illegible]	0 (0 to 0)	3·4
Acute hepatitis A	[illegible]	[illegible]	[illegible]	[illegible]	[illegible]	[illegible]	[illegible]	[illegible]	[illegible]	[illegible]	[illegible]	[illegible]	[illegible]	[illegible]	[illegible]	[illegible]	[illegible]	[illegible]	[illegible]	[illegible]	[illegible]	[illegible]	0 (0 to 0)	2
Acute hepatitis B	[illegible]	[illegible]	[illegible]	[illegible]	[illegible]	[illegible]	[illegible]	[illegible]	[illegible]	[illegible]	[illegible]	[illegible]	[illegible]	[illegible]	[illegible]	[illegible]	[illegible]	[illegible]	[illegible]	[illegible]	[illegible]	[illegible]	0 (0 to 0)	0·9
Acute hepatitis C	[illegible]	[illegible]	[illegible]	[illegible]	[illegible]	[illegible]	[illegible]	[illegible]	[illegible]	[illegible]	[illegible]	[illegible]	[illegible]	[illegible]	[illegible]	[illegible]	[illegible]	[illegible]	[illegible]	[illegible]	[illegible]	[illegible]	0 (0 to 0)	0·1
Acute hepatitis E	[illegible]	[illegible]	[illegible]	[illegible]	[illegible]	[illegible]	[illegible]	[illegible]	[illegible]	[illegible]	[illegible]	[illegible]	[illegible]	[illegible]	[illegible]	[illegible]	[illegible]	[illegible]	[illegible]	[illegible]	[illegible]	[illegible]	0 (0 to 0)	0·4
Other unspecified infectious diseases	[illegible]	[illegible]	[illegible]	[illegible]	[illegible]	[illegible]	[illegible]	[illegible]	[illegible]	[illegible]	[illegible]	[illegible]	[illegible]	[illegible]	[illegible]	[illegible]	[illegible]	[illegible]	[illegible]	[illegible]	[illegible]	[illegible]	0 (0 to 0·1)	85
Maternal and neonatal disorders	[illegible]	[illegible]	[illegible]	[illegible]	[illegible]	[illegible]	[illegible]	[illegible]	[illegible]	[illegible]	[illegible]	[illegible]	[illegible]	[illegible]	[illegible]	[illegible]	[illegible]	[illegible]	[illegible]	[illegible]	[illegible]	[illegible]	0 (0 to 0)	774
Maternal disorders	[illegible]	[illegible]	[illegible]	[illegible]	[illegible]	[illegible]	[illegible]	[illegible]	[illegible]	[illegible]	[illegible]	[illegible]	[illegible]	[illegible]	[illegible]	[illegible]	[illegible]	[illegible]	[illegible]	[illegible]	[illegible]	[illegible]	0 (0 to 0)	0
Maternal hemorrhage	[illegible]	[illegible]	[illegible]	[illegible]	[illegible]	[illegible]	[illegible]	[illegible]	[illegible]	[illegible]	[illegible]	[illegible]	[illegible]	[illegible]	[illegible]	[illegible]	[illegible]	[illegible]	[illegible]	[illegible]	[illegible]	[illegible]	0 (0 to 0)	0
Maternal sepsis and other maternal infections	[illegible]	[illegible]	[illegible]	[illegible]	[illegible]	[illegible]	[illegible]	[illegible]	[illegible]	[illegible]	[illegible]	[illegible]	[illegible]	[illegible]	[illegible]	[illegible]	[illegible]	[illegible]	[illegible]	[illegible]	[illegible]	[illegible]	0 (0 to 0)	0
Maternal hypertensive disorders	[illegible]	[illegible]	[illegible]	[illegible]	[illegible]	[illegible]	[illegible]	[illegible]	[illegible]	[illegible]	[illegible]	[illegible]	[illegible]	[illegible]	[illegible]	[illegible]	[illegible]	[illegible]	[illegible]	[illegible]	[illegible]	[illegible]	0 (0 to 0)	0
Maternal obstructed labor and uterine rupture	--	--	--	--	--	--	--	--	--	--	--	--	--	--	--	--	--	--	--	--	--	--	--	NA
Maternal abortion and miscarriage	--	--	--	--	--	--	--	--	--	--	--	--	--	--	--	--	--	--	--	--	--	--	--	NA
Ectopic pregnancy	--	--	--	--	--	--	--	--	--	--	--	--	--	--	--	--	--	--	--	--	--	--	--	NA
Indirect maternal deaths	--	--	--	--	--	--	--	--	--	--	--	--	--	--	--	--	--	--	--	--	--	--	--	NA
Late maternal deaths	--	--	--	--	--	--	--	--	--	--	--	--	--	--	--	--	--	--	--	--	--	--	--	NA
Maternal deaths aggravated by HIV/AIDS	--	--	--	--	--	--	--	--	--	--	--	--	--	--	--	--	--	--	--	--	--	--	--	NA
Neonatal disorders	[illegible]	[illegible]	[illegible]	[illegible]	[illegible]	[illegible]	[illegible]	[illegible]	[illegible]	[illegible]	[illegible]	[illegible]	[illegible]	[illegible]	[illegible]	[illegible]	[illegible]	[illegible]	[illegible]	[illegible]	[illegible]	[illegible]	0 (0 to 0)	774
Neonatal preterm birth	[illegible]	[illegible]	[illegible]	[illegible]	[illegible]	[illegible]	[illegible]	[illegible]	[illegible]	[illegible]	[illegible]	[illegible]	[illegible]	[illegible]	[illegible]	[illegible]	[illegible]	[illegible]	[illegible]	[illegible]	[illegible]	[illegible]	0 (0 to 0)	356
Neonatal encephalopathy due to birth asphyxia and trauma	[illegible]	[illegible]	[illegible]	[illegible]	[illegible]	[illegible]	[illegible]	[illegible]	[illegible]	[illegible]	[illegible]	[illegible]	[illegible]	[illegible]	[illegible]	[illegible]	[illegible]	[illegible]	[illegible]	[illegible]	[illegible]	[illegible]	0 (0 to 0)	136
Neonatal sepsis and other neonatal infections	[illegible]	[illegible]	[illegible]	[illegible]	[illegible]	[illegible]	[illegible]	[illegible]	[illegible]	[illegible]	[illegible]	[illegible]	[illegible]	[illegible]	[illegible]	[illegible]	[illegible]	[illegible]	[illegible]	[illegible]	[illegible]	[illegible]	0 (0 to 0)	173
Hemolytic disease and other neonatal jaundice	[illegible]	[illegible]	[illegible]	[illegible]	[illegible]	[illegible]	[illegible]	[illegible]	[illegible]	[illegible]	[illegible]	[illegible]	[illegible]	[illegible]	[illegible]	[illegible]	[illegible]	[illegible]	[illegible]	[illegible]	[illegible]	[illegible]	0 (0 to 0)	12·4
Other neonatal disorders	[illegible]	[illegible]	[illegible]	[illegible]	[illegible]	[illegible]	[illegible]	[illegible]	[illegible]	[illegible]	[illegible]	[illegible]	[illegible]	[illegible]	[illegible]	[illegible]	[illegible]	[illegible]	[illegible]	[illegible]	[illegible]	[illegible]	0 (0 to 0)	97
Nutritional deficiencies	[illegible]	[illegible]	[illegible]	[illegible]	[illegible]	[illegible]	[illegible]	[illegible]	[illegible]	[illegible]	[illegible]	[illegible]	[illegible]	[illegible]	[illegible]	[illegible]	[illegible]	[illegible]	[illegible]	[illegible]	[illegible]	[illegible]	0·4 (0·3 to 0·6)	289
Protein-energy malnutrition	[illegible]	[illegible]	[illegible]	[illegible]	[illegible]	[illegible]	[illegible]	[illegible]	[illegible]	[illegible]	[illegible]	[illegible]	[illegible]	[illegible]	[illegible]	[illegible]	[illegible]	[illegible]	[illegible]	[illegible]	[illegible]	[illegible]	0·3 (0·2 to 0·5)	128
Iodine deficiency	[illegible]	[illegible]	[illegible]	[illegible]	[illegible]	[illegible]	[illegible]	[illegible]	[illegible]	[illegible]	[illegible]	[illegible]	[illegible]	[illegible]	[illegible]	[illegible]	[illegible]	[illegible]	[illegible]	[illegible]	[illegible]	[illegible]	0 (0 to 0)	2·9
Vitamin A deficiency	[illegible]	[illegible]	[illegible]	[illegible]	[illegible]	[illegible]	[illegible]	[illegible]	[illegible]	[illegible]	[illegible]	[illegible]	[illegible]	[illegible]	[illegible]	[illegible]	[illegible]	[illegible]	[illegible]	[illegible]	[illegible]	[illegible]	0 (0 to 0)	3·5
Dietary iron deficiency	[illegible]	[illegible]	[illegible]	[illegible]	[illegible]	[illegible]	[illegible]	[illegible]	[illegible]	[illegible]	[illegible]	[illegible]	[illegible]	[illegible]	[illegible]	[illegible]	[illegible]	[illegible]	[illegible]	[illegible]	[illegible]	[illegible]	0 (0 to 0·1)	134
Other nutritional deficiencies	[illegible]	[illegible]	[illegible]	[illegible]	[illegible]	[illegible]	[illegible]	[illegible]	[illegible]	[illegible]	[illegible]	[illegible]	[illegible]	[illegible]	[illegible]	[illegible]	[illegible]	[illegible]	[illegible]	[illegible]	[illegible]	[illegible]	0·1 (0 to 0·1)	20·3
Non-communicable diseases	[illegible]	[illegible]	[illegible]	[illegible]	[illegible]	[illegible]	[illegible]	[illegible]	[illegible]	[illegible]	[illegible]	[illegible]	[illegible]	[illegible]	[illegible]	[illegible]	[illegible]	[illegible]	[illegible]	[illegible]	[illegible]	[illegible]	21·8 (20·6 to 23)	15,700
Neoplasms	[illegible]	[illegible]	[illegible]	[illegible]	[illegible]	[illegible]	[illegible]	[illegible]	[illegible]	[illegible]	[illegible]	[illegible]	[illegible]	[illegible]	[illegible]	[illegible]	[illegible]	[illegible]	[illegible]	[illegible]	[illegible]	[illegible]	3·3 (2·4 to 4·2)	2,740
Lip and oral cavity cancer	[illegible]	[illegible]	[illegible]	[illegible]	[illegible]	[illegible]	[illegible]	[illegible]	[illegible]	[illegible]	[illegible]	[illegible]	[illegible]	[illegible]	[illegible]	[illegible]	[illegible]	[illegible]	[illegible]	[illegible]	[illegible]	[illegible]	0·1 (0 to 0·1)	110
Nasopharynx cancer	[illegible]	[illegible]	[illegible]	[illegible]	[illegible]	[illegible]	[illegible]	[illegible]	[illegible]	[illegible]	[illegible]	[illegible]	[illegible]	[illegible]	[illegible]	[illegible]	[illegible]	[illegible]	[illegible]	[illegible]	[illegible]	[illegible]	0 (0 to 0)	16·2
Other pharynx cancer	[illegible]	[illegible]	[illegible]	[illegible]	[illegible]	[illegible]	[illegible]	[illegible]	[illegible]	[illegible]	[illegible]	[illegible]	[illegible]	[illegible]	[illegible]	[illegible]	[illegible]	[illegible]	[illegible]	[illegible]	[illegible]	[illegible]	0 (0 to 0)	45·3
Esophageal cancer	[illegible]	[illegible]	[illegible]	[illegible]	[illegible]	[illegible]	[illegible]	[illegible]	[illegible]	[illegible]	[illegible]	[illegible]	[illegible]	[illegible]	[illegible]	[illegible]	[illegible]	[illegible]	[illegible]	[illegible]	[illegible]	[illegible]	0 (0 to 0)	74·9
Stomach cancer	[illegible]	[illegible]	[illegible]	[illegible]	[illegible]	[illegible]	[illegible]	[illegible]	[illegible]	[illegible]	[illegible]	[illegible]	[illegible]	[illegible]	[illegible]	[illegible]	[illegible]	[illegible]	[illegible]	[illegible]	[illegible]	[illegible]	0·1 (0·1 to 0·2)	226
Colon and rectum cancer	[illegible]	[illegible]	[illegible]	[illegible]	[illegible]	[illegible]	[illegible]	[illegible]	[illegible]	[illegible]	[illegible]	[illegible]	[illegible]	[illegible]	[illegible]	[illegible]	[illegible]	[illegible]	[illegible]	[illegible]	[illegible]	[illegible]	0·2 (0·1 to 0·2)	236
Liver cancer	[illegible]	[illegible]	[illegible]	[illegible]	[illegible]	[illegible]	[illegible]	[illegible]	[illegible]	[illegible]	[illegible]	[illegible]	[illegible]	[illegible]	[illegible]	[illegible]	[illegible]	[illegible]	[illegible]	[illegible]	[illegible]	[illegible]	0 (0 to 0)	75
Liver cancer due to hepatitis B	[illegible]	[illegible]	[illegible]	[illegible]	[illegible]	[illegible]	[illegible]	[illegible]	[illegible]	[illegible]	[illegible]	[illegible]	[illegible]	[illegible]	[illegible]	[illegible]	[illegible]	[illegible]	[illegible]	[illegible]	[illegible]	[illegible]	0 (0 to 0)	25·8
Liver cancer due to hepatitis C	[illegible]	[illegible]	[illegible]	[illegible]	[illegible]	[illegible]	[illegible]	[illegible]	[illegible]	[illegible]	[illegible]	[illegible]	[illegible]	[illegible]	[illegible]	[illegible]	[illegible]	[illegible]	[illegible]	[illegible]	[illegible]	[illegible]	0 (0 to 0)	9·6
Liver cancer due to alcohol use	[illegible]	[illegible]	[illegible]	[illegible]	[illegible]	[illegible]	[illegible]	[illegible]	[illegible]	[illegible]	[illegible]	[illegible]	[illegible]	[illegible]	[illegible]	[illegible]	[illegible]	[illegible]	[illegible]	[illegible]	[illegible]	[illegible]	0 (0 to 0)	31·6
Liver cancer due to NASH	[illegible]	[illegible]	[illegible]	[illegible]	[illegible]	[illegible]	[illegible]	[illegible]	[illegible]	[illegible]	[illegible]	[illegible]	[illegible]	[illegible]	[illegible]	[illegible]	[illegible]	[illegible]	[illegible]	[illegible]	[illegible]	[illegible]	0 (0 to 0)	4·6
Liver cancer due to other causes	[illegible]	[illegible]	[illegible]	[illegible]	[illegible]	[illegible]	[illegible]	[illegible]	[illegible]	[illegible]	[illegible]	[illegible]	[illegible]	[illegible]	[illegible]	[illegible]	[illegible]	[illegible]	[illegible]	[illegible]	[illegible]	[illegible]	0 (0 to 0)	3·5
[truncated: 2,157,363 more chars]
